# Exploring the intestinal ecosystem: from gut microbiota to associations with subtypes of inflammatory bowel disease

**DOI:** 10.3389/fcimb.2023.1304858

**Published:** 2024-01-04

**Authors:** Fan Li, Chanjiao Yu, Qi Zhao, Zhaodi Wang, Zhi Wang, Yu Chang, Zifeng Xu, Xiaoping Han, Hongyan Li, Yue Liu, Sileng Hu, Shiyu Chang, Tongyu Tang, Yuqin Li

**Affiliations:** Department of Gastroenterology, The First Hospital of Jilin University, Changchun, China

**Keywords:** inflammatory bowel disease, gut microbiota, Mendelian randomization, stratification, extraintestinal manifestations

## Abstract

**Objective:**

Significant differences have been discovered between subtypes of Crohn’s disease (CD) and ulcerative colitis (UC). The role of gut microbiota in promoting the onset of UC and CD is established, but conclusions regarding subtype-specific analyses remain limited.

**Methods:**

This study aims to explore the influence of gut microbiota on subtypes of UC and CD, offering novel insights into the pathogenesis and treatment of UC and CD.Two-sample Mendelian randomization (MR) analysis was employed to examine the causal relationship between subtypes of UC and CD and gut microbiota composition. Gut microbiota data were sourced from the International Consortium MiBioGen, while UC and CD data were obtained from FINNGEN. Eligible single nucleotide polymorphisms (SNPs) were selected as instrumental variables. Multiple analytical approaches such as inverse variance-weighted (IVW), MR-Egger regression, weighted median, weighted mode, and MR-RAPS were utilized. Sensitivity analyses including MR-Egger intercept test, Cochran’s Q test, and leave-one-out analysis were conducted for quality control. Subsequently, we employed multivariable IVW, MR-Egger, weighted median, and LASSO regression methods to identify independently significant genera or families and conducted sensitivity analyses.

**Results:**

We have determined that *Hungatella*, *Acidaminococcaceae*, and 15 other microbial taxa act as protective factors for various CD and UC subtypes, while *Terrisporobacter*, *Anaerostipes*, and 23 other microbial taxa are associated with increased risk for different CD and UC subtypes. Furthermore, through multivariable MR analysis, we have identified significant genera or families with independent effects.

**Conclusion:**

Our study confirms a causal relationship between dysbiosis of gut microbiota and the occurrence of CD and UC subtypes. Furthermore, it validates etiological distinctions among different subtypes of CD and UC. A novel approach to adjunctive therapy involving distinct UC or CD subtypes may involve the use of probiotics and represents a potential avenue for future treatments.

## 1 Introduction

Inflammatory bowel disease (IBD) comprises a group of chronic immune-mediated disorders affecting the gastrointestinal tract, encompassing two main subtypes: ulcerative colitis (UC) and Crohn’s disease (CD) (Nóbrega et al., 2018). Prominent symptoms include diarrhea, abdominal pain, rectal bleeding, and weight loss. Controversies persist regarding variations among subtypes of UC and CD involving different anatomical sites. Due to significant differences (Pierre et al., 2021), Dulai et al. (2019) proposed distinguishing ileal-dominant CD from isolated colonic CD. Given the correlation between the anatomical site of onset and the natural history of UC, and response to therapeutic interventions, the renowned Montreal classification segregates UC into static severity levels based on colonic involvement (Satsangi et al., 2006). CD-associated spondyloarthritis (CD-SpA) is an extension of the gut-specific inflammatory process in Crohn’s disease (CD), facilitated by the ectopic expression of gut-specific chemokine CCL25, adhesion molecules (MAdCAM, ICAM, E-cadherin), and integrins (α4β7, αEβ7). This phenomenon leads to the immune response extending from the intestines to the joints (Kumar et al., 2020).

Primary sclerosing cholangitis associated with ulcerative colitis (PSC-UC) exhibits distinct clinical and histopathological characteristics compared to intestinal UC, suggesting that PSC-UC represents a unique form of inflammatory bowel disease (Aranake-Chrisinger et al., 2020). In recent years, disruption of gut ecosystem (pathological alterations in gut microbiota composition) has been extensively studied in patients with IBD (Roda et al., 2020). Research has confirmed significant differences in gut microbiota composition between individuals with IBD and healthy individuals (Mosca et al., 2016; Oligschlaeger et al., 2019). Notable features of IBD patients’ gut microbiota include decreased diversity and abundance of Firmicutes and increased that of Bacteroidetes (Ott et al., 2004; Machiels et al., 2014; Nishino et al., 2018).. The microbiota is proposed as an early potential triggering factor for CD-SpA (Kumar et al., 2020). However, due to variations among CD and UC subtypes, the relationship between gut microbiota and distinct subtypes remains elusive.

Mendelian randomization (MR) experiments are a statistical method employed to explore causal relationships between exposure factors and outcomes. It employs genetic variants identified through genome-wide association studies (GWAS) as instrumental variables to assess the relationship between instrumental variables and outcomes (Emdin et al., 2017). Given the resource-intensive nature of randomized controlled trials (RCTs), and to account for confounding and reverse causation, we conducted a two-sample Mendelian randomization (MR) analysis based on summary data from genome-wide association studies (GWAS). We examined the causal relationships between exposure factors from 150 genera or families and 10 subtypes of inflammatory bowel disease (IBD), including Crohn’s disease of the small intestine, Crohn’s disease of the colon, Crohn’s disease of the ileocolon, Ulcerative pancolitis, Ulcerative proctitis, Ulcerative rectosigmoiditis, Left-sided ulcerative colitis, Arthropathy in Crohn’s disease, Ulcerative colitis with primary sclerosing cholangitis (PSC), and Arthropathy in ulcerative colitis.

## 2 Methods

### 2.1 Data source

We acquired summary data of single nucleotide polymorphisms (SNPs) associated with individual gut microbiota from the MiBioGen database, an international consortium. This consortium conducted genome-wide association studies (GWAS) on 18,340 participants from 24 countries including the United States, Canada, Germany,and more, utilizing 16S rRNA gene sequencing profiles and genotype data (see **Supplementary Table 8**). We included data from European ancestry participants for exposure in our Mendelian randomization study (Kurilshikov et al., 2021). FinnGen aims to collect and analyze genomic and health data from 500,000 participants in the Finnish Biobank, offering novel medical and therapeutic insights (Kurki et al., 2023). We obtained GWAS datasets for stratified diagnoses of UC and CD from FINNGEN, comprising a total of 373,819 individuals, with 15,779 individuals diagnosed with UC or CD. Given variations due to race and population stratification, exposure and outcome data were sourced from European populations, encompassing both male and female individuals to mitigate bias from population stratification (Emdin et al., 2017).

### 2.2 Study design

This study employed a two-sample MR design to investigate the causal relationships between gut microbiota and subtypes of UC and CD, as well as extraintestinal manifestations, elucidating the pathogenesis of UC and CD with distinct anatomical distributions. We stratified inflammatory CD and UC based on both Montreal classification and ICD-10 coding, as detailed in **Table 1** (Verstockt et al., 2022). We utilized single nucleotide polymorphisms (SNPs) as instrumental variables. After screening for qualified SNPs, we employed various analysis methods including inverse variance-weighted (IVW), MR-Egger regression, weighted median method, weighted mode method, and MR-Robust Adjusted Profile Score (MR-RAPS). We conducted sensitivity analyses such as MR-Egger intercept test, Cochran’s Q test, and leave-one-out analysis to ensure robust results.

**Table 1:** Inclusion information and stratification details of the dataset.

Datasets	ICD-10 encoding	Montreal classification	Case	Sample Size	Year	Consortium	Gender	Population	NSNP
Abundance of 150 Gut microbiota	–	–	14306	14306	2021	MiBioGen	Males and Females	European	5486191
Crohn’s disease of small intestine	K500	L1+part of L4	2004	361931	2023	FinnGen	Males and Females	European	20167370
Crohn’s disease of colon	K501	L2	1581	361508	2023	FinnGen	Males and Females	European	20167370
Crohn’s disease of ileocolon	K502	L3	2098	362025	2023	FinnGen	Males and Females	European	20167370
Arthropathy in Crohn disease	M07.4*K50.9		273	373819	2023	FinnGen	Males and Females	European	20167370
Ulcerative proctitis	k512	E1	1773	361700	2023	FinnGen	Males and Females	European	20167370
Ulcerative rectosigmoiditis	K513		2487	362414	2023	FinnGen	Males and Females	European	20167370
Left-sided ulcerative colitis	K515	E2	4085	364012	2023	FinnGen	Males and Females	European	20167370
Ulcerative pancolitis	k510	E3	933	360860	2023	FinnGen	Males and Females	European	20167370
Ulcerative colitis with PSC	K83.0*K51		209	364784	2023	FinnGen	Males and Females	European	20167370
Arthropathy in ulcerative colitis	M07.5*K51.9		336	373882	2023	FinnGen	Males and Females	European	20167370

ICD, International Classification of Diseases; NSNP, Number of Single Nucleotide Polymorphisms.

### 2.3 Instrumental variable selection

**Figure 1:**
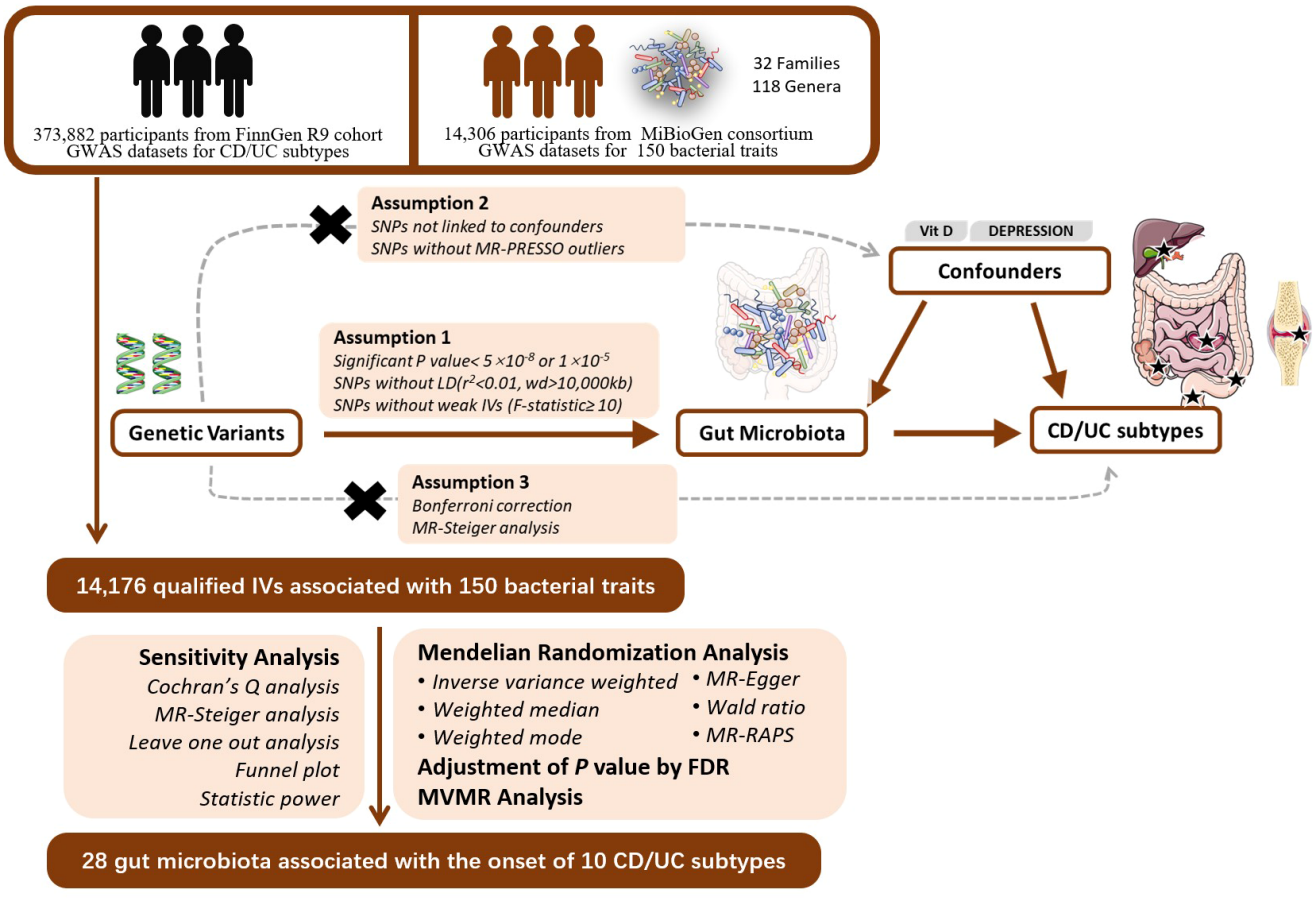
The study design of the MR study of the associations of Gut Microbiota and IBD subtypes. GWAS, Genome-wide association study; LD, linkage disequilibrium; MR-PRESSO, Mendelian Randomization Pleiotropy RESidual Sum and Outlier, a method test the pleiotropic biases in the SNPs and correct the pleiotropic effects; MR, Mendelian randomization; SNP, single nucleotide polymorphism, as instrumental variables for the exposures and outcomes; CD, Crohn’s disease; UC, ulcerative colitis; FDR, false discovery rate; MVMR, multivariable mendelian randomization.

Mendelian randomization is based on three assumptions: the relevance assumption, the independence assumption, and the exclusion assumption. To fulfill the relevance assumption, we selected SNPs significantly associated with exposure at a genome-wide significance level (alpha=5×10^-8^). In cases where such SNPs could not be identified, we lowered the threshold to alpha=1×10^-5^ to ensure a sufficient number of SNPs. To ensure the independence assumption and minimize multicollinearity risks, we established r^2^<0.01 and kb=10000 as criteria to remove linkage disequilibrium (LD). We calculated the F-statistic for each SNP. SNPs with F<10 were considered weak instrumental variables and were excluded (Burgess et al., 2017). To satisfy the independence assumption, we queried each SNP in the PhenoScanner database to exclude SNPs related to confounders such as serum vitamin D levels and depression (Staley et al., 2016). We applied the MR-PRESSO test to identify and exclude outlier SNPs due to horizontal pleiotropy. To satisfy the exclusion assumption, we employed MR-Steiger analysis to determine the causal direction of all SNPs and removed SNPs with incorrect directions (Li F. et al., 2022). Finally, we employed Bonferroni correction (P<0.05/n, where n represents the number of remaining SNPs) to remove SNPs directly associated with outcomes. Thus, we have completed instrumental variable selection in accordance with the MR assumptions. The flowchart and directed acyclic graphs representing the MR study hypotheses are shown in **Figure 1**.

### 2.4 UVMR analysis

We employed the inverse variance-weighted method (IVW), MR-Egger regression, weighted median, weighted mode, and MR-RAPS to compute the causal relationships between gut microbiota and subtypes of UC or CD. The inverse variance-weighted method is a weighted linear regression without an intercept term, where the slope parameter represents the causal estimate and the weights are the reciprocal of the genetic association variance with the outcome. IVW estimation using first-stage weights is equivalent to the 2-stage least squares (2SLS) method (Burgess et al., 2013). Following Mendelian randomization guidelines, in the absence of horizontal pleiotropy and heterogeneity, we chose the IVW method based on the multiplicative random effects model as the primary analysis (Burgess et al., 2019). The weighted median and weighted mode methods calculate causal effects based on the majority valid assumption and plurality valid assumption, respectively (Bowden et al., 2016a; Burgess et al., 2019). As long as over 50% of genetic variants are valid instruments, the median of the weighted median ratio estimate tends toward the true causal effect. In cases where less than 50% of variants are valid instruments, the weighted mode can identify the true causal effect as long as no larger group of invalid instruments with the same ratio estimation exists. When there is heterogeneity among SNPs, significant conclusions require support from both the weighted median and IVW methods. MR-Egger method resembles the IVW method but includes an intercept term in the regression model (Burgess and Thompson, 2017). MR-Egger method provides consistent causal effect estimates under the InSIDE assumption and the NOME hypothesis (Bowden et al., 2016b; Burgess and Thompson, 2017). The intercept term in MR-Egger also offers a test for horizontal pleiotropy between instruments. In cases of horizontal pleiotropy among SNPs, this study employed MR-Egger method as the primary analysis. MR-RAPS method is a common modeling approach that estimates causal effects using the probability profile likelihood function based on the assumption of multivariate normal distribution with zero-centered pleiotropy and heteroskedasticity. Finally, a False Discovery Rate (FDR) correction was applied to MR results. Causal relationships were inferred based on corrected P-values < 0.05.

### 2.5 Sensitivity analysis

Cochran’s Q test evaluates heterogeneity among IVs by calculating the weighted sum of squared distances between specific estimates and the overall estimate, considering SNPs with Q test P-value < 0.05 as heterogeneous. Additionally, we employed the MR-Steiger model to verify the overall direction of estimates for robustness (Hemani et al., 2017). To ascertain the influence of individual SNPs, we conducted leave-one-out sensitivity tests (Burgess and Thompson, 2017). The InSIDE assumption and the NOME hypothesis need to be satisfied for MR-Egger regression. We generated a funnel plot and calculated the I^2^ statistic to validate these assumptions. If I^2^ is < 90% and the primary analytical method is MR-Egger, a correction for causal estimates is necessary (Bowden et al., 2016b; Burgess and Thompson, 2017).

### 2.6 MVMR

Following univariate MR, we performed multivariate MR analyses on the significantly associated gut microbial taxa using the same parameters. We employed IVW, MR-Egger, weighted median, and LASSO regression methods to identify independently significant genera or families. Sensitivity analyses were conducted to assess heterogeneity and pleiotropy. Outcomes with fewer than 2 microbial taxa or a smaller number of available SNPs than the number of predictors were excluded from the study.

### 2.7 Results visualization

For each batch of MR analysis, this study created scatterplots and regression curve plots, along with forest plots depicting SNP effects, which will be presented in the results. Circular heatmaps and overall forest plots were generated for the results. Several figures were partly generated using Servier Medical Art (smart.servier.com), provided by Servier, licensed under a Creative Commons Attribution 3.0 unported license.

### 2.8 Statistical analysis software

This study conducted statistical analysis and visualization using R (version 4.1.2), and employed packages such as “TwoSampleMR,” “MR-PRESSO,” “mr.raps,” “forestploter,” and several foundational R packages.

## 3 Results

### 3.1 Instrumental variable selection

Initially, we identified a total of 17,957 SNPs associated with gut microbial taxa. No weak instrument bias was detected. Among these, 904 SNPs were removed due to missing data in the outcome database, 2,749 SNPs were eliminated as ambiguous or palindromic SNPs during dataset merging, and 29 SNPs, identified through PhenoScanner, were found to be associated with confounding factors such as serum vitamin D levels and depression, and were consequently excluded. The MR-PRESSO test identified 44 SNPs exhibiting horizontal pleiotropy. After Bonferroni correction, 55 SNPs directly related to the outcome were removed. In the end, 14,176 eligible SNPs were included in the study.

### 3.2 Changes in gut microbiota abundance impact subtypes of UC and CD

**Figure 2:**
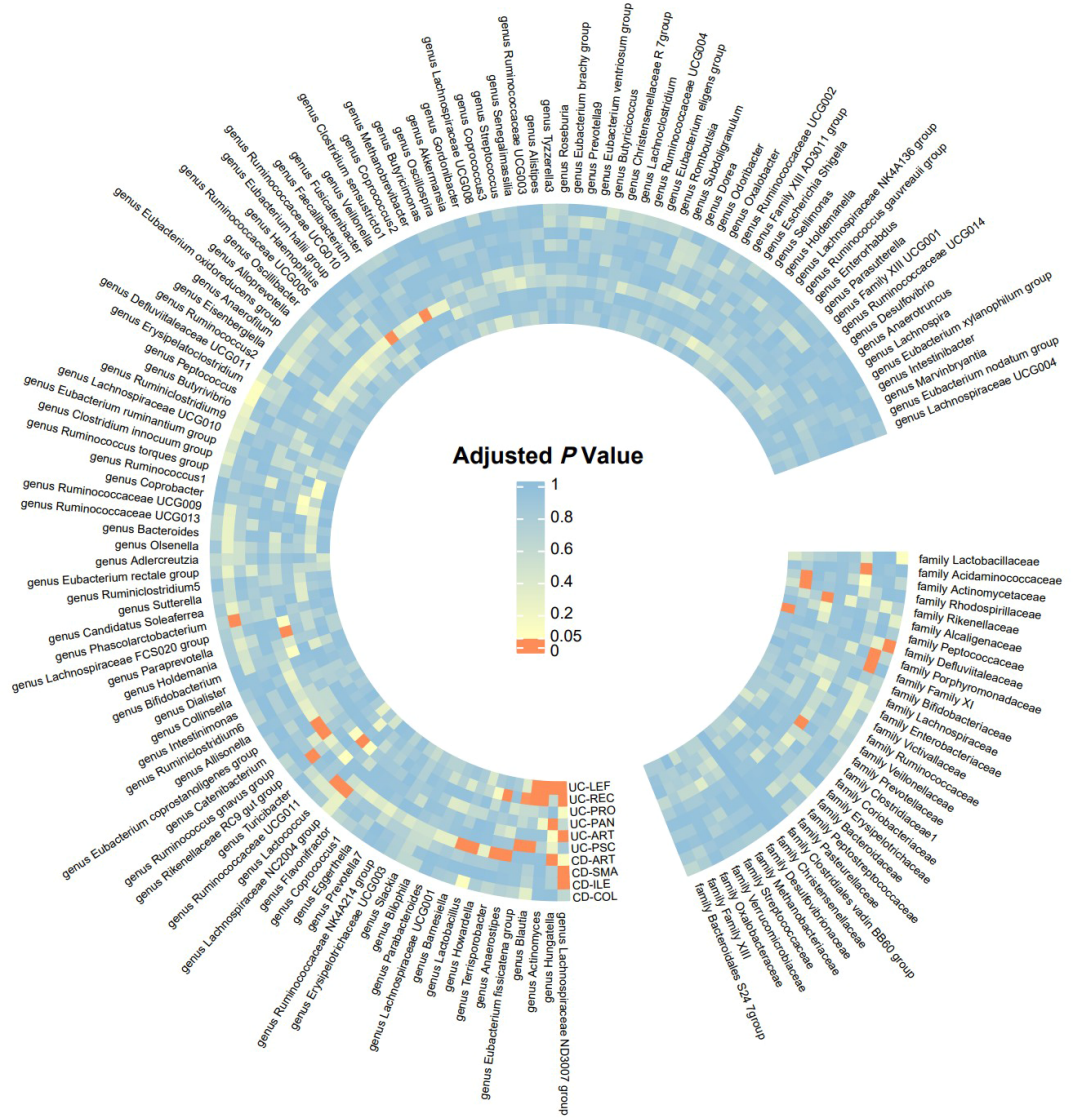
Significance Heatmap of MR Analysis. CD-SMA, Crohn’s disease of small intestine; CD-COL, Crohn’s disease of colon; CD-ILE, Crohn’s disease of ileocolon; UC-PAN, Ulcerative pancolitis; UC-PRO, Ulcerative proctitis; UC-REC, Ulcerative rectosigmoiditis; UC-LEF, Left-sided ulcerative colitis; CD-ART, Arthropathy in Crohn disease; UC-PSC, Ulcerative colitis with PSC; UC-ART, Arthropathy in ulcerative colitis.

**Figure 3:**
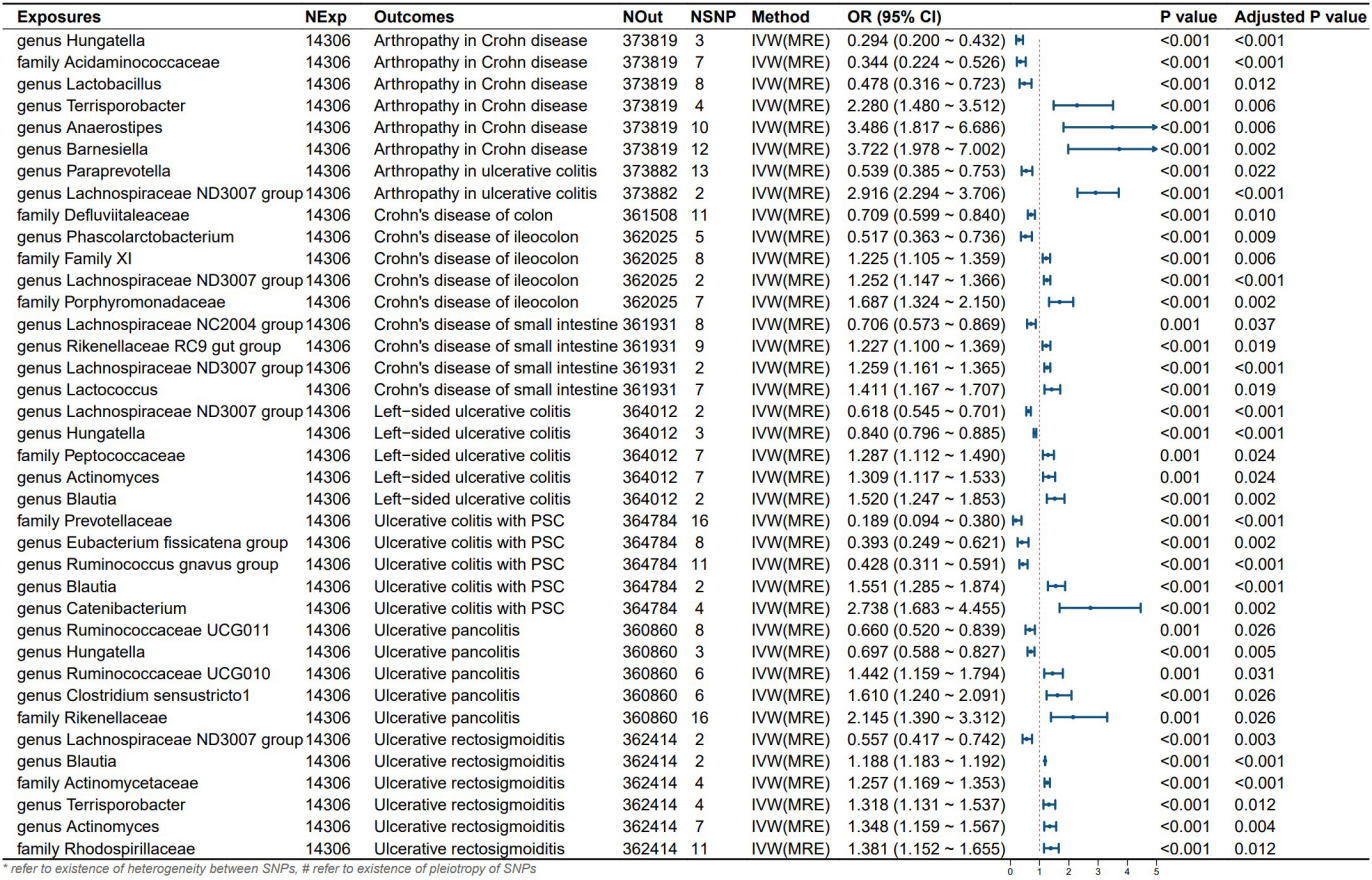
Results and forest polt of the significant UVMR analysis. IVW(MRE), inverse variance weighted (multiplicative random effects model); CI, confidence interval; NExp, sample size of exposure dataset; NOut, sample size of outcome dataset; NSNP, number of SNP included in MR analysis; *refer to existence of heterogeneity between SNPs, #refer to existence of pleiotropy of SNPs.

Detailed information of the MR study is summarized in the **Supplementary Material**. In this study, we included genera or families of 150 gut microbiota species in the analysis. The number of SNPs for each type of gut microbiota ranged from 1 to 18. **Supplementary Table** provides detailed information on the IVs of the 150 gut microbiota species. Through UVMR analysis, we identified 6 gut microbial taxa that exert a protective effect on CD subtypes, while 9 gut microbial taxa promote the onset of CD subtypes. Additionally, 9 gut microbial taxa exhibited protective effects on UC subtypes and extraintestinal manifestations, while 14 gut microbial taxa contributed to the onset of UC subtypes and extraintestinal manifestations. The results of the UVMR analysis are presented in the heatmap (**Figure 2**) and forest plot (**Figure 3**).

### 3.3 Positive findings

Six gut microbial taxa exhibit a protective effect on CD subtypes and extraintestinal manifestations. Specifically, the family *Defluviitaleaceae* reduced the risk of Crohn’s disease of the colon. Genus *Lachnospiraceae NC2004 group* decreased the risk of Crohn’s disease of the small intestine.Genus *Phascolarctobacterium* reduced the risk of Crohn’s disease of the ileocolon. Genus *Hungatella*, family *Acidaminococcaceae*, and genus *Lactobacillus* decreased the risk of Arthropathy in Crohn’s disease patients.UVMR analysis revealed that 9 gut microbial taxa had protective effects on UC subtypes and extraintestinal manifestations. Genus *Lachnospiraceae ND3007 group* decreased the risk of Ulcerative rectosigmoiditis. Genus *Ruminococcaceae UCG011* and genus *Hungatella* reduced the risk of Ulcerative pancolitis. Genus *Lachnospiraceae ND3007 group* and genus *Hungatella* decreased the risk of Left-sided ulcerative colitis. Family *Prevotellaceae*, genus *Eubacterium fissicatena group*, and genus *Ruminococcus gnavus group* reduced the risk of primary sclerosing cholangitis (PSC) in ulcerative colitis patients.

Additionally, we identified 9 gut microbial taxa that promote the onset of CD subtypes and extraintestinal manifestations, as well as 14 gut microbial taxa that contribute to the onset of UC subtypes and extraintestinal manifestations (see **Figure 3**). According to Cochran’s Q test and MR-Egger intercept, there was no evidence suggesting that potential heterogeneity or pleiotropy introduced bias in our findings (all P-values > 0.05; **Table 2**).

**Table 2:** Sensitivity analysis of the significant MR analysis.

Exposures	Outcomes	Pval of pleotropy	I^2^ for MR-Egger	Q from IVW	Q from MR-Egger	Pval_Q from IVW	Pval_Q from MR-Egger	Dirction from MR-Steiger
genus *Hungatella*	Arthropathy in Crohn disease	0.964	0.000	0.313	0.310	0.855	0.578	TRUE
family *Acidaminococcaceae*	Arthropathy in Crohn disease	0.549	0.943	1.251	0.838	0.974	0.975	TRUE
genus *Lactobacillus*	Arthropathy in Crohn disease	0.445	0.958	2.887	2.219	0.895	0.899	TRUE
genus *Terrisporobacter*	Arthropathy in Crohn disease	0.893	0.709	0.484	0.461	0.922	0.794	TRUE
genus *Anaerostipes*	Arthropathy in Crohn disease	0.940	0.959	3.257	3.251	0.953	0.918	TRUE
genus *Barnesiella*	Arthropathy in Crohn disease	0.734	0.958	5.702	5.581	0.892	0.849	TRUE
genus *Paraprevotella*	Arthropathy in ulcerative colitis	0.537	0.957	5.444	5.039	0.941	0.929	TRUE
genus *Lachnospiraceae ND3007 group*	Arthropathy in ulcerative colitis	–	0.000	–	–	–	–	TRUE
family *Defluviitaleaceae*	Crohn’s disease of colon	0.954	0.959	3.192	3.189	0.977	0.956	TRUE
genus *Phascolarctobacterium*	Crohn’s disease of ileocolon	0.901	0.965	2.861	2.842	0.581	0.417	TRUE
family *Family XI*	Crohn’s disease of ileocolon	0.711	0.914	2.311	2.160	0.941	0.904	TRUE
genus *Lachnospiraceae ND3007 group*	Crohn’s disease of ileocolon	–	0.000	–	–	–	–	TRUE
family *Porphyromonadaceae*	Crohn’s disease of ileocolon	0.725	0.957	1.425	1.286	0.964	0.936	TRUE
genus *Lachnospiraceae NC2004 group*	Crohn’s disease of small intestine	0.562	0.955	4.415	4.038	0.731	0.671	TRUE
genus *Rikenellaceae RC9 gut group*	Crohn’s disease of small intestine	0.603	0.958	2.845	2.549	0.944	0.923	TRUE
genus *Lachnospiraceae ND3007 group*	Crohn’s disease of small intestine	–	0.000	–	–	–	–	TRUE
genus *Lactococcus*	Crohn’s disease of small intestine	0.976	0.950	3.754	3.753	0.710	0.586	TRUE
genus *Lachnospiraceae ND3007 group*	Left-sided ulcerative colitis	–	0.000	–	–	–	–	TRUE
genus *Hungatella*	Left-sided ulcerative colitis	0.929	0.000	0.087	0.075	0.957	0.785	TRUE
family *Peptococcaceae*	Left-sided ulcerative colitis	0.595	0.962	2.348	2.027	0.885	0.845	TRUE
genus *Actinomyces*	Left-sided ulcerative colitis	0.488	0.958	3.623	3.063	0.728	0.690	TRUE
genus *Blautia*	Left-sided ulcerative colitis	–	0.970	–	–	–	–	TRUE
family *Prevotellaceae*	Ulcerative colitis with PSC	0.052	0.956	9.910	5.394	0.825	0.980	TRUE
genus *Eubacterium fissicatena group*	Ulcerative colitis with PSC	0.391	0.947	3.229	2.374	0.863	0.882	TRUE
genus *Ruminococcus gnavus group*	Ulcerative colitis with PSC	0.689	0.956	2.163	1.992	0.995	0.992	TRUE
genus *Blautia*	Ulcerative colitis with PSC	–	0.970	–	–	–	–	TRUE
genus *Catenibacterium*	Ulcerative colitis with PSC	0.437	0.000	0.989	0.060	0.804	0.971	TRUE
genus *Ruminococcaceae UCG011*	Ulcerative pancolitis	0.488	0.960	5.042	4.496	0.655	0.610	TRUE
genus *Hungatella*	Ulcerative pancolitis	0.906	0.000	0.212	0.190	0.900	0.663	TRUE
genus *Ruminococcaceae* UCG010	Ulcerative pancolitis	0.900	0.959	0.705	0.687	0.983	0.953	TRUE
genus *Clostridium sensustricto1*	Ulcerative pancolitis	0.712	0.958	1.154	0.996	0.949	0.910	TRUE
family *Rikenellaceae*	Ulcerative pancolitis	0.717	0.956	15.001	14.856	0.451	0.388	TRUE
genus *Lachnospiraceae ND3007 group*	Ulcerative rectosigmoiditis	–	0.000	–	–	–	–	TRUE
genus *Blautia*	Ulcerative rectosigmoiditis	–	0.970	–	–	–	–	TRUE
family *Actinomycetaceae*	Ulcerative rectosigmoiditis	0.792	0.960	0.141	0.051	0.987	0.975	TRUE
genus *Terrisporobacter*	Ulcerative rectosigmoiditis	0.656	0.709	0.552	0.284	0.907	0.868	TRUE
genus *Actinomyces*	Ulcerative rectosigmoiditis	0.768	0.958	2.015	1.918	0.918	0.860	TRUE
family *Rhodospirillaceae*	Ulcerative rectosigmoiditis	0.699	0.956	6.742	6.582	0.750	0.681	TRUE

MR analysis with less than 3 SNPs are not avaliable for Cochran’s Q test. IVW refer to inverse variance weighted method.

### 3.4 Multivariable MR results

**Figure 4:**
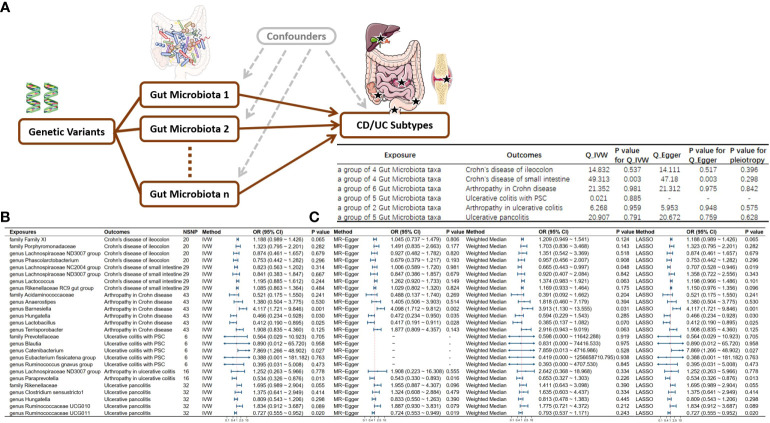
Directed Acyclic Graph and Results of Multivariable Mendelian Randomization. **(A)** Directed acyclic graph for multivariable MR; **(B)** Multivariable MR results; **(C)** Sensitivity analysis results for multivariable MR; NSNP, Number of Single Nucleotide Polymorphisms included in the study. IVW, Inverse variance weighted; OR, Odds ratio; CI, Confidence interval; MR, Mendelian randomization; CD, Crohn’s disease; UC, Ulcerative colitis.

Following multivariable MR analysis, we identified 6 independent influencing factors. Specifically, genus *Ruminococcaceae UCG011* reduced the risk of ulcerative pancolitis onset. Genus *Paraprevotella* lowered the risk of Arthropathy in UC. Genus *Catenibacterium* promoted the onset of UC-PSC. Genus *Hungatella* and genus *Lactobacillus* reduced the risk of Arthropathy in CD while genus *Barnesiella* promoted its onset. Additionally, due to the existence of pleiotropy, Genus *lachnospiraceae NC2004 group* may serve as a protective factor for CD in the small intestine (see **Figure 4**). Due to the extraction of only 2 eligible instrumental variables in the analysis of left-sided colitis and proctosigmoiditis outcomes, it is not feasible to conduct a multivariable Mendelian randomization study. Consequently, the conclusions for these outcomes primarily rely on univariable Mendelian randomization results.

## 4 Discussions

In this Mendelian randomization study of European ancestry, through summary GWAS datasets of gut microbiota and CD/UC subtypes and extraintestinal manifestations, we identified *Hungatella*, *Acidaminococcaceae*, and other microbial taxa as protective factors for different CD and UC subtypes, while *Terrisporobacter*, *Anaerostipes*, and other taxa were identified as risk factors. Furthermore, through multivariable MR analysis, we identified significant genera or species independently influencing the outcomes. This suggests a causal relationship between gut microbiota dysbiosis and the occurrence of CD and UC subtypes, confirming etiological differences among various CD and UC subtypes. Many perspectives posit that the subtypes of UC and CD, along with extraintestinal manifestations, may exhibit heterogeneity. At the 2005 World Congress of Gastroenterology in Montreal, researchers classified ulcerative colitis into proctitis, left-sided colitis, or pancolitis based on the involved anatomical extent, as the natural history of UC and its response to medications are related to the affected site​​ (Satsangi et al., 2006). Multiple omics analyses revealed significant differences between colonic and ileal CD, indicating pathologic site specificity​​ (Gonzalez et al., 2022)and disparities in serum analytes​ (Boucher et al., 2022). Given this, researchers propose a subdivision of CD into ileum-dominant and isolated colonic disease​​ (Dulai et al., 2019). As a significant extraintestinal manifestation of UC (Conrad et al., 2014), primary sclerosing cholangitis (PSC) is associated with gut microbiota-induced bacteremia (O'Toole et al., 2012)and elevated endotoxin levels (Sasatomi et al., 1998). Previous studies have employed Mendelian randomization to explore the causal relationship between gut microbiota and UC and CD. However, considering the heterogeneity among different UC and CD subtypes, to comprehensively investigate their pathogenic mechanisms and formulate tailored therapeutic strategies for specific subtypes, this study stratifies according to CD and UC subtypes, offering novel evidence in the field of IBD and gut microbiota research.

The human intestines harbor a vast array of bacteria that have co-evolved with the human host, exerting crucial influence on our physiology and metabolism (Bäckhed, 2011). Consistent with our findings, previous clinical and murine model studies have linked the gut microbiota to the pathogenesis of inflammatory bowel disease (IBD), revealing the roles of both beneficial and pathogenic bacteria in IBD (Zheng et al., 2022). The intestinal microbiota can produce various bioactive metabolites, such as short-chain fatty acids (Franzosa et al., 2019), medium-chain fatty acids (De Preter et al., 2015), which can be absorbed into the host circulation through the hepatic portal system (Wu et al., 2021). Recent research on the characteristics of CD and UC subtypes has inspired us to link them with the gut microbiota. A study on the gut microbiota of different subtypes of ulcerative colitis (UC) confirmed that, compared to the total colitis group (E3), the partial colitis group (E1 or E2) exhibited higher abundance of the family *Actinomycetaceae*​ *(* He et al., 2021). Interestingly, our study revealed that the genus *Actinomyces*, as a representative genus of the family *Actinomycetaceae*, is a shared risk factor for left-sided colitis and ulcerative proctosigmoiditis. This facultative anaerobic bacterium, with pathogenic fimbriae, can elevate local mucosal levels of IL-6 and IL-8, promoting mucosal inflammation and neutrophil-mediated tissue damage​​ (Kövér et al., 2023). Multiple studies on patients with concurrent inflammatory bowel disease (IBD) and primary sclerosing cholangitis (PSC) have confirmed a significant increase in the genus *Blautia* (Quraishi et al., 2017)and a notable decrease in the genus Prevotella in the IBD-PSC population​​ (Quraishi et al., 2017; Quraishi et al., 2020). Our study arrived at the same conclusion, identifying the genus *Blautia* as a risk factor for the onset of IBD-PSC, while an increased abundance of the family *Prevotellaceae* was associated with a reduced risk of IBD-PSC onset. Lastly, our research additionally revealed that *Ruminococcus gnavus* acts as a protective factor in ulcerative colitis accompanied by primary sclerosing cholangitis. Despite the current lack of direct research on *R. gnavus* and PSC, Ahn et al. discovered that oral administration of *R. gnavus* increased the number of Treg cells in the lymphatic circulation of mice (Ahn et al., 2022). In liver biopsies of PSC patients, the predominant cell type involved in portal vein inflammation is T cells (Whiteside et al., 1985; Ponsioen et al., 1999)​​. Given the negative regulatory role of Treg cells on T cells, this indirect pathway highlights the need for further exploration in this direction.

Short-chain fatty acids (SCFAs), primarily consisting of acetic acid, propionic acid, and butyric acid, are crucial microbial metabolites generated through bacterial fermentation of dietary fiber, serving as a primary energy source for colonic cells​​ (Shin et al., 2023). In this study, certain intestinal microbial communities associated with UC/CD subtypes were identified as SCFA-producing bacteria, including *Lachnospiraceae*​ *(* Zhang et al., 2021), *Acidaminococcaceae*​​ *(* Smith et al., 2013), *Lactococcus*​​ *(* He et al., 2022), *Ruminococcaceae (* Vital et al., 2017)​​, *paraprevotella (* Liu et al., 2022)​​, and *Lactobacillus (* Lee et al., 2020)​​. SCFAs play a crucial role in host metabolic regulation, contributing to the reduction of the risk of inflammatory bowel diseases​​ (Hu et al., 2022). Previous studies indicate that intestinal SCFA levels may decrease with the activity of IBD and recover during remission​​ (Huda-Faujan et al., 2010; Kumari et al., 2013).SCFAs produced by intestinal microbiota can effectively promote polarization of anti-inflammatory M2 macrophages, thereby ameliorating intestinal inflammation in an IBD mouse model​​ (Ji et al., 2016). SCFAs can also exert anti-inflammatory effects by inhibiting IL-6 signaling transduction and the activator of transcription 3 (STAT3) pathway, promoting forkhead box protein P3 (Foxp3)​​. SCFAs can inhibit histone deacetylase activity, acting as ligands for GPR41 and GPR43 receptors. These dual actions enhance the anti-inflammatory properties of epithelial cells​​ (Cani et al., 2013; Siddiqui and Cresci, 2021). SCFAs also exhibit broad effects outside the intestinal tract. Research suggests that the combined anti-inflammatory and immunomodulatory properties of SCFAs, coupled with the direct inhibition of osteoclast activity, may be particularly beneficial in controlling human inflammatory arthritis​​ (Luu and Visekruna, 2019). Furthermore, SCFA-producing bacteria and purified SCFAs can reduce liver fibrosis levels in patients with primary sclerosing cholangitis (PSC) (Luu and Visekruna, 2019).

The maintenance of intestinal barrier function relies on the balance between pathogenic and probiotic bacteria​​ (Li P. et al., 2022). The classical cause of IBD onset is dysbiosis in the intestinal microbiota, disrupting the balance between beneficial and harmful bacteria, resulting in compromised intestinal microbial barrier​​ (Chelakkot et al., 2018). The intestinal microbiota can produce various metabolites to resist invasion by pathogenic bacteria, promoting intestinal homeostasis​​ (Deleu et al., 2021). Bacteriocins play the role of antimicrobial agents among the metabolites. Lactobacillin produced by intestinal *Lactobacillus* inhibits infection by Listeria monocytogenes​​ (Rolhion et al., 2019). Enterotoxins produced by certain bacteria, by increasing intestinal epithelial permeability, inhibit the absorption of vitamin C (Subramenium et al., 2019)​. Roy et al. demonstrated that imbalances in the intestinal microbiota result from damage to the intestinal barrier​​ (Roy et al., 2017). Glycosylation changes in intestinal epithelial cells alter the expression of terminal polysaccharides, leading to mucosal layer damage, a decline in mucosal immunity, and ultimately the onset of IBD​ (Kudelka et al., 2020).

In the course of our research, we also identified some intriguing findings. Firstly, our univariate MR analysis suggested that Anaerostipe poses a risk in multiple subtypes, while multivariate MR analysis confirmed that *Anaerostipes* may not be an independent risk factor in Arthropathy in Crohn disease. Retrospective analysis revealed the depletion of *Anaerostipes* bacteria producing SCFA in both pediatric and adult CD patients (Wang et al., 2018; Lo Presti et al., 2019), with a more significant decline observed in late-stage Crohn’s disease patients compared to early-stage patients. In Crohn’s disease patients undergoing ADA therapy, there was a significant increase in the abundance of *Anaerostipes*, a change that can reverse microbial ecosystem disruption, generate more short-chain fatty acids, and correct immune imbalance (Meehan and Beiko, 2014). The impact of Anaerostipe on IBD requires further investigation.Secondly, in the comparison between ulcerative proctosigmoiditis and left-sided colitis, the genus *Lachnospiraceae ND3007 group* shared protective factors, whereas *Hungatella* did not. We hypothesize that *Hungatella* is associated with the onset of inflammation in descending colon. Experimental evidence suggests an increased presence of the *Hungatella* genus at Crohn’s disease surgical sites, indicating its potential role in common postoperative complications. If the intestinal microbiota plays a beneficial role in the pathogenesis of postoperative infections, targeted therapeutic or preventive interventions aimed at the intestinal microbiota may be considered to reduce the risk of postoperative complications in CD patients and enhance the primary anastomosis success rate (Sun et al., 2021).

Our research findings provide evidence for revealing the causal relationship between gut microbiota and subtypes of UC and CD. The gut microbiota is involved in crucial activities related to both disease and health (Guo et al., 2021). Currently, the primary therapeutic approaches for IBD involve anti-inflammatory and immune-modulating agents, such as 5-aminosalicylic acid, corticosteroids, monoclonal antibody compounds, etc. Although they can suppress intestinal inflammation and alleviate symptoms, the high recurrence rate and associated side effects, coupled with poor tolerance in some patients, fail to address the underlying issues troubling patients. This study, by revealing the causal relationship between gut microbiota and IBD, offers novel perspectives for the clinical treatment of this disease. Prebiotics and probiotics play a crucial role in maintaining the balance of gut microbiota​ (Quigley, 2019) and are key targets for treating IBD. A meta-analysis systematically reviewed and synthesized 38 randomized controlled trials, revealing positive outcomes of probiotics and prebiotics in alleviating IBD and reducing its disease activity index. Shen et al.​ (Shen et al., 2014)further observed that, in addition to the mentioned advantages, probiotics also demonstrate significant benefits in preventing IBD relapse. In conclusion, compared to traditional medications, probiotics and prebiotics present safer and more economical advantages, showcasing tremendous therapeutic potential.

This study, through stratified research, may pave the way for novel therapeutic approaches tailored to distinct subtypes of UC or CD. Mendelian randomization analysis, with consideration of confounding factors, is an effective method for exploring causal relationships between exposure and outcomes. Our investigation, in contrast to prior studies, is more comprehensive; we examined the relationship between bacteria from 150 genera or families and 10 subtypes of UC or CD. This provides an opportunity to assess common gut microbiota causally linked to multiple UC or CD subtypes, and offers a theoretical foundation for subsequent investigations.

Additionally, leveraging publicly available large-scale GWAS summary statistics provides a cost-effective and reliable selection for mining genetic information. However, our study has limitations. Firstly, bacterial taxonomic groups were analyzed only at the genus level, without more specific levels such as species or strains. Although, UC incidence is slightly higher in males (60%), while CD has a higher incidence of 20%-30% in females, however, our study did not stratify the analysis based on gender (Molinié et al., 2004; Bernstein et al., 2006). Furthermore, multivariable MR studies with outcomes such as left-sided colitis and rectal sigmoid colonitis are constrained by the number of valid instrumental variables. Some outcomes are not amenable to multivariable MR analysis, limiting a thorough exploration of the independent effects of these microbial communities on the diseases.

Overall, we assessed the causal relationships between gut microbiota and various subtypes of UC and CD. Through two-sample MR analysis using publicly available GWAS summary statistics, we identified the specific bacterial groups that influence different subtypes of UC and CD. This study contributes to providing insights into the pathogenic mechanisms of different subtypes of UC and CD, opening up new avenues for clinical treatment based on probiotics and prebiotics.

## 5 Conclusion

We have identified *Hungatella*, *Acidaminococcaceae*, and 13 other microbial taxa as protective factors for various CD and UC subtypes, while *Terrisporobacter*, *Anaerostipes*, and 21 other microbial taxa are associated with increased risk for different CD and UC subtypes. Furthermore, through multivariable MR analysis, we have identified significant genera or families with independent effects. Our study confirms a causal relationship between dysbiosis of the gut microbiota and the occurrence of subtypes of CD and UC. Additionally, we establish the etiological distinctions among different subtypes of CD and UC. The potential of novel probiotics for adjunctive therapy targeting different subtypes of UC or CD represents a novel avenue for future treatments.

## Data availability statement

Publicly available datasets were analyzed in this study. This data can be found here: https://www.finngen.fi/ https://mibiogen.gcc.rug.nl/.

## Ethics statement

The studies involving humans were approved by https://mibiogen.gcc.rug.nl/ https://www.finngen.fi/. The studies were conducted in accordance with the local legislation and institutional requirements. The participants provided their written informed consent to participate in this study. Written informed consent was obtained from the individual(s) for the publication of any potentially identifiable images or data included in this article.

## Author contributions

FL: Writing – original draft. CY: Writing – original draft. QZ: Writing – original draft. ZhaW: Writing – original draft. ZhiW: Writing – original draft. YC: Writing – original draft. ZX: Writing – original draft. XH: Writing – original draft. HL: Writing – original draft. YueL: Writing – original draft. SH: Writing – original draft. SC: Writing – original draft. TT: Writing – review & editing. YuqL: Writing – review & editing.
