# Supplementary material for: Exploring the intestinal ecosystem: from gut microbiota to associations with subtypes of inflammatory bowel disease
Source: Front Cell Infect Microbiol. 2024 Jan 4;13:1304858. doi: 10.3389/fcimb.2023.1304858 (PMC10794348; doi:10.3389/fcimb.2023.1304858)
Supplement: Supplementary file 2 [file Image_2.pdf]

Batch 1 : Gut microbiota abundance (family Acidaminococcaceae id.2166) on Crohn's disease of large intestine

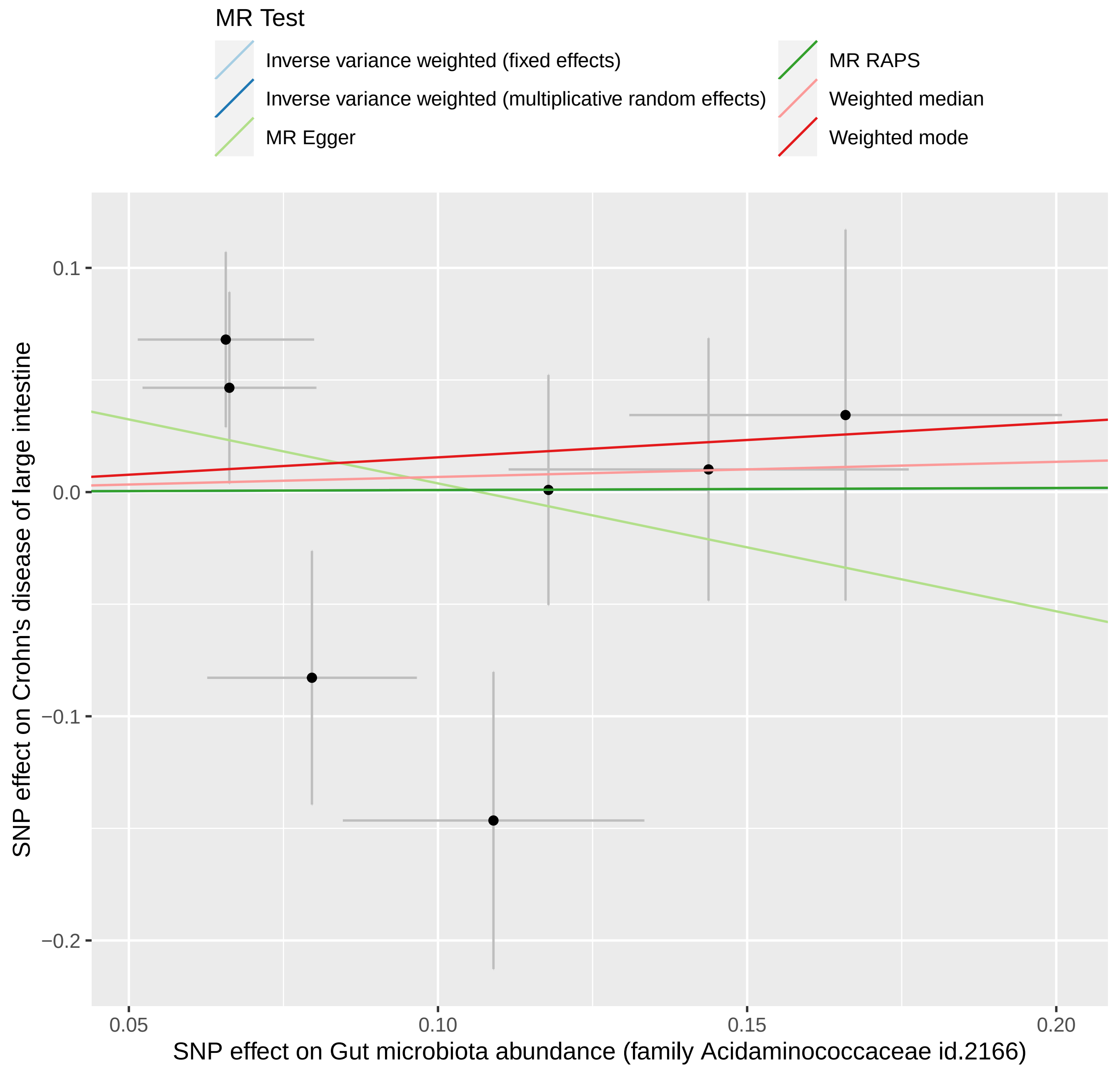

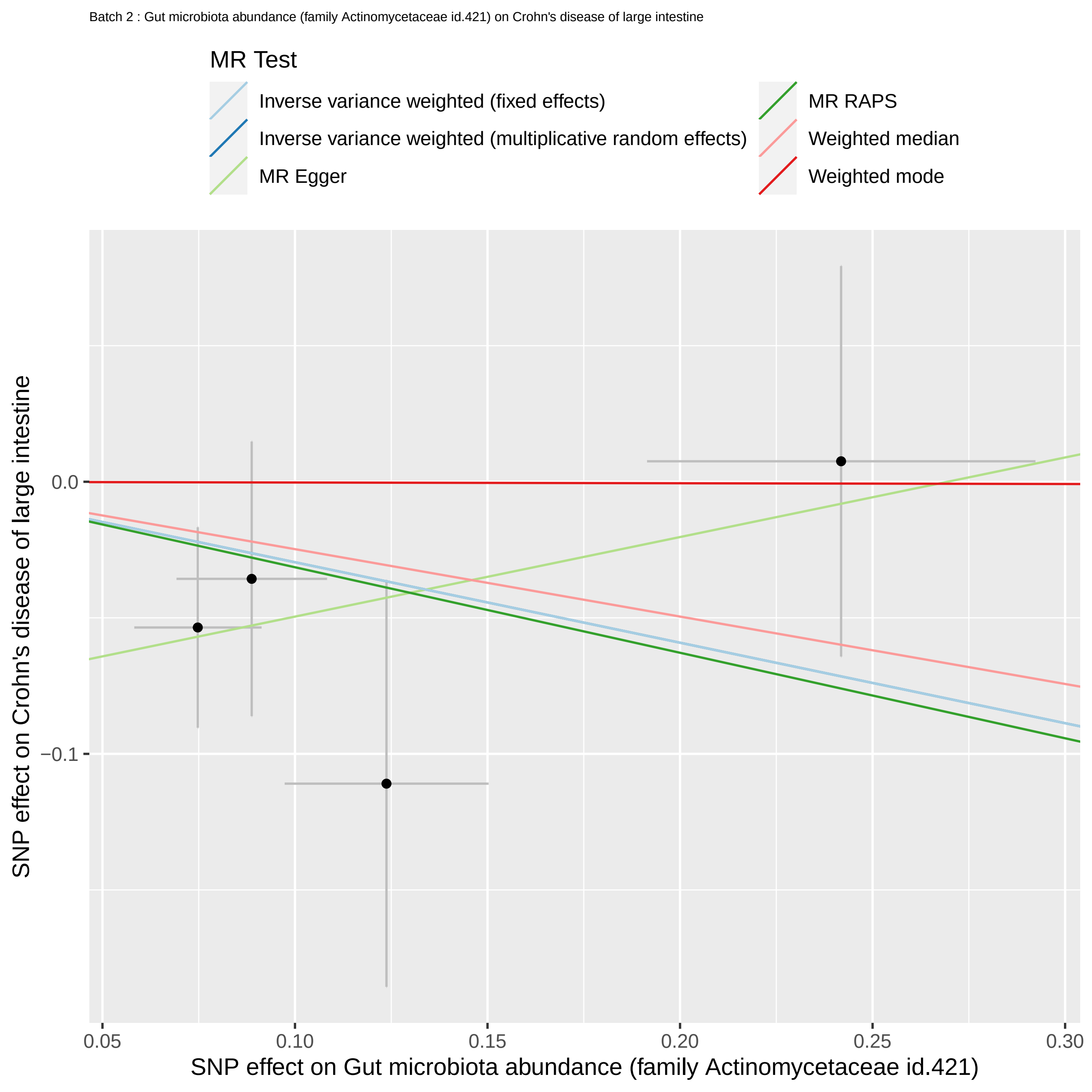

Batch 2 : Gut microbiota abundance (family Actinomycetaceae id.421) on Crohn's disease of large intestine
MR Test
Inverse variance weighted (fixed effects)
Inverse variance weighted (multiplicative random effects)
MR Egger
MR RAPS
Weighted median
Weighted mode
0.0
−0.1
0.05
0.10
0.15
0.20
0.25
0.30
SNP effect on Gut microbiota abundance (family Actinomycetaceae id.421)
SNP effect on Crohn's disease of large intestine

Batch 3 : Gut microbiota abundance (family Alcaligenaceae id.2875) on Crohn's disease of large intestine

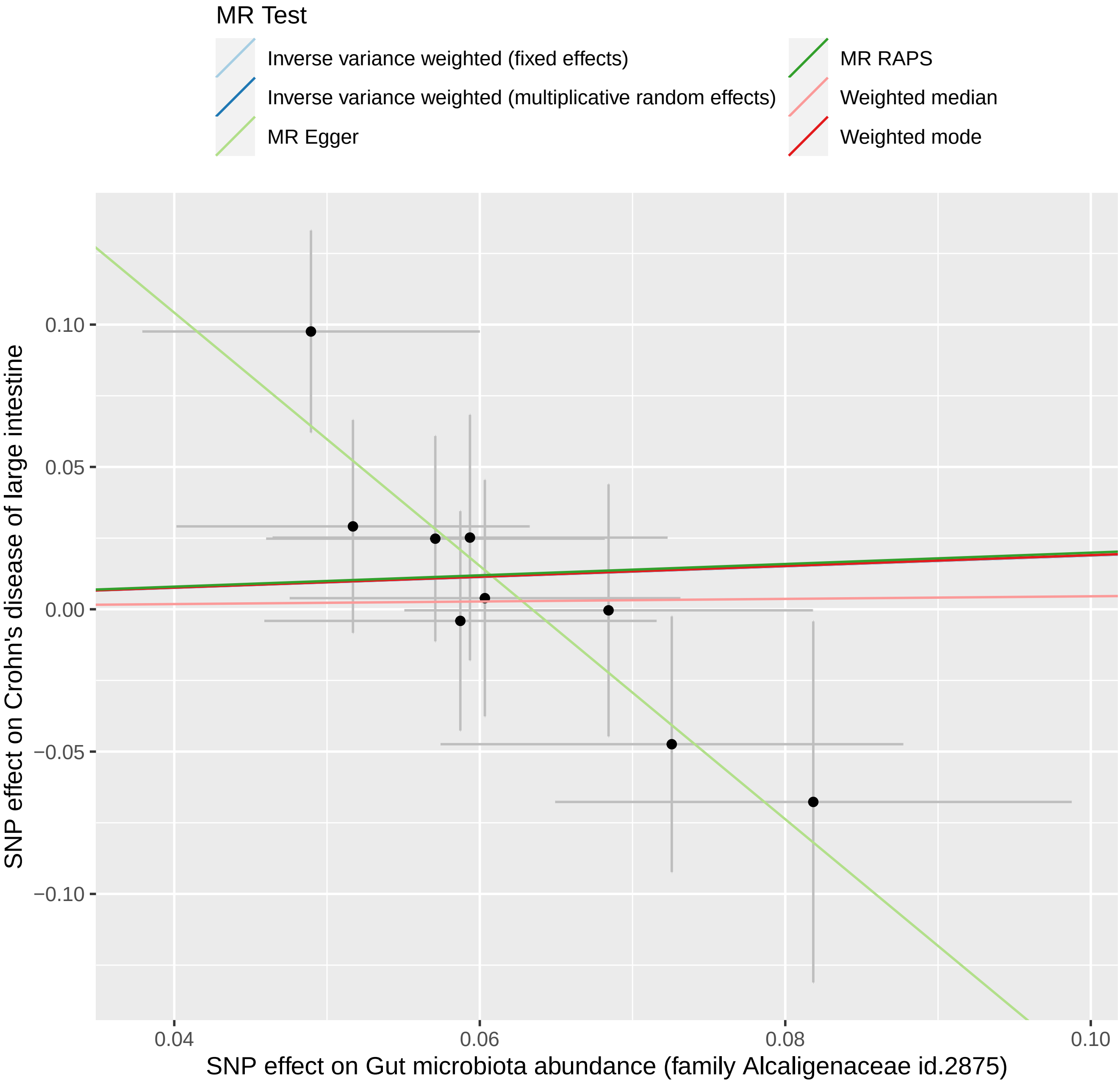

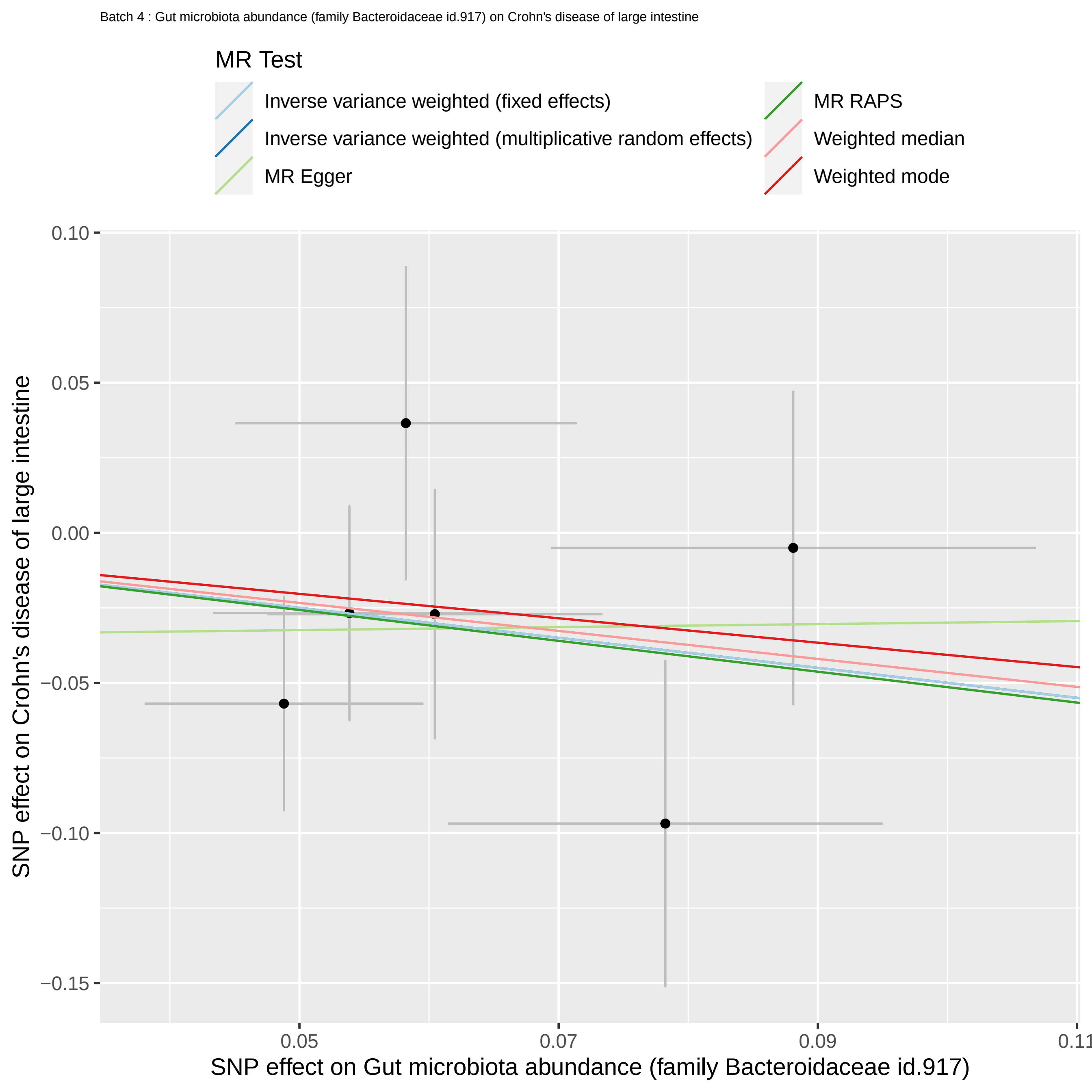
Batch 4 : Gut microbiota abundance (family Bacteroidaceae id.917) on Crohn's disease of large intestine
MR Test
Inverse variance weighted (fixed effects)
Inverse variance weighted (multiplicative random effects)
MR Egger
MR RAPS
Weighted median
Weighted mode
0.10
0.05
0.00
−0.05
−0.10
−0.15
0.05
0.07
0.09
0.11
SNP effect on Crohn's disease of large intestine
SNP effect on Gut microbiota abundance (family Bacteroidaceae id.917)

Batch 5 : Gut microbiota abundance (family Bacteroidales S24 7group id.11173) on Crohn's disease of large intestine

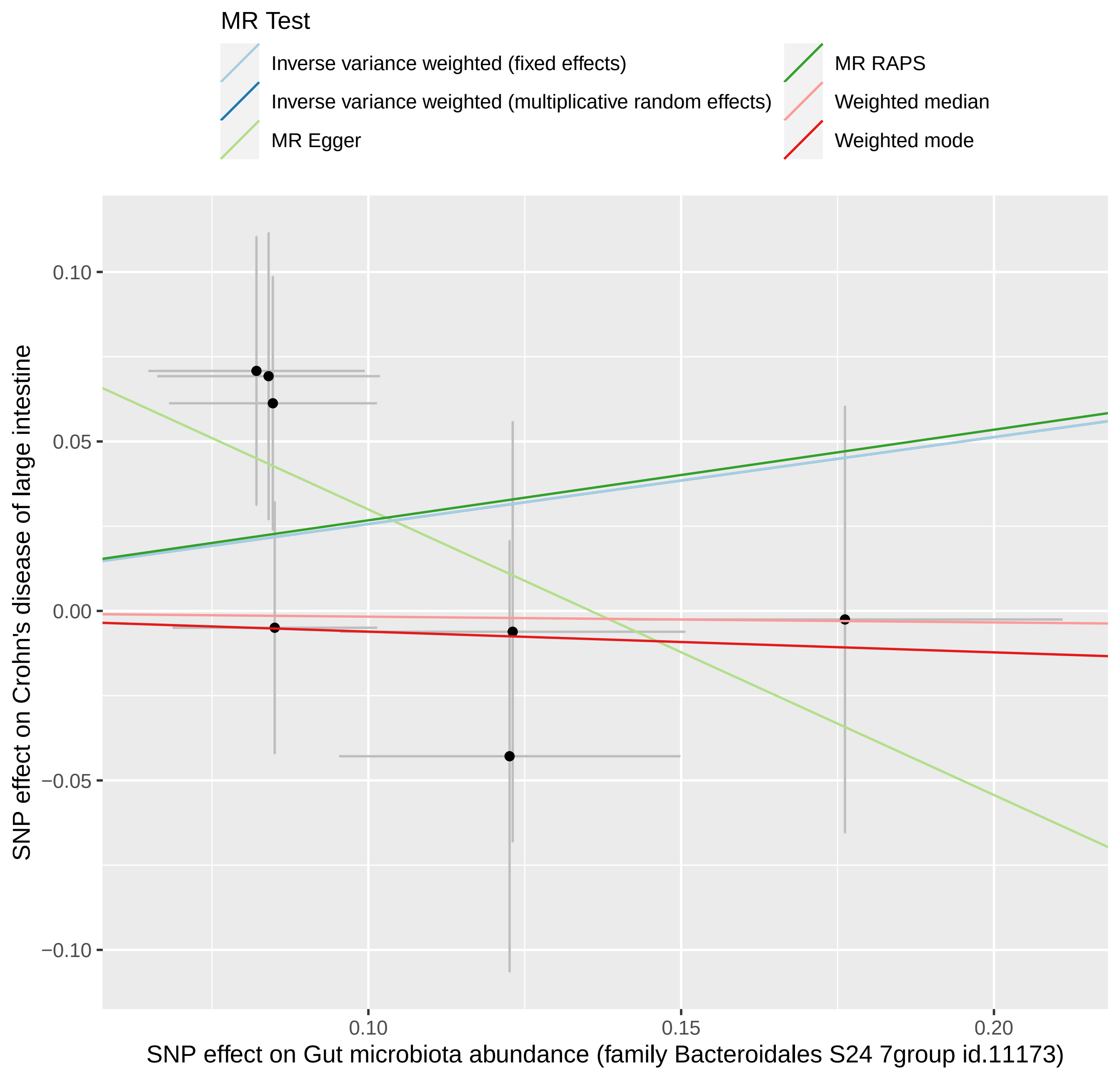

Batch 6 : Gut microbiota abundance (family Bifidobacteriaceae id.433) on Crohn's disease of large intestine

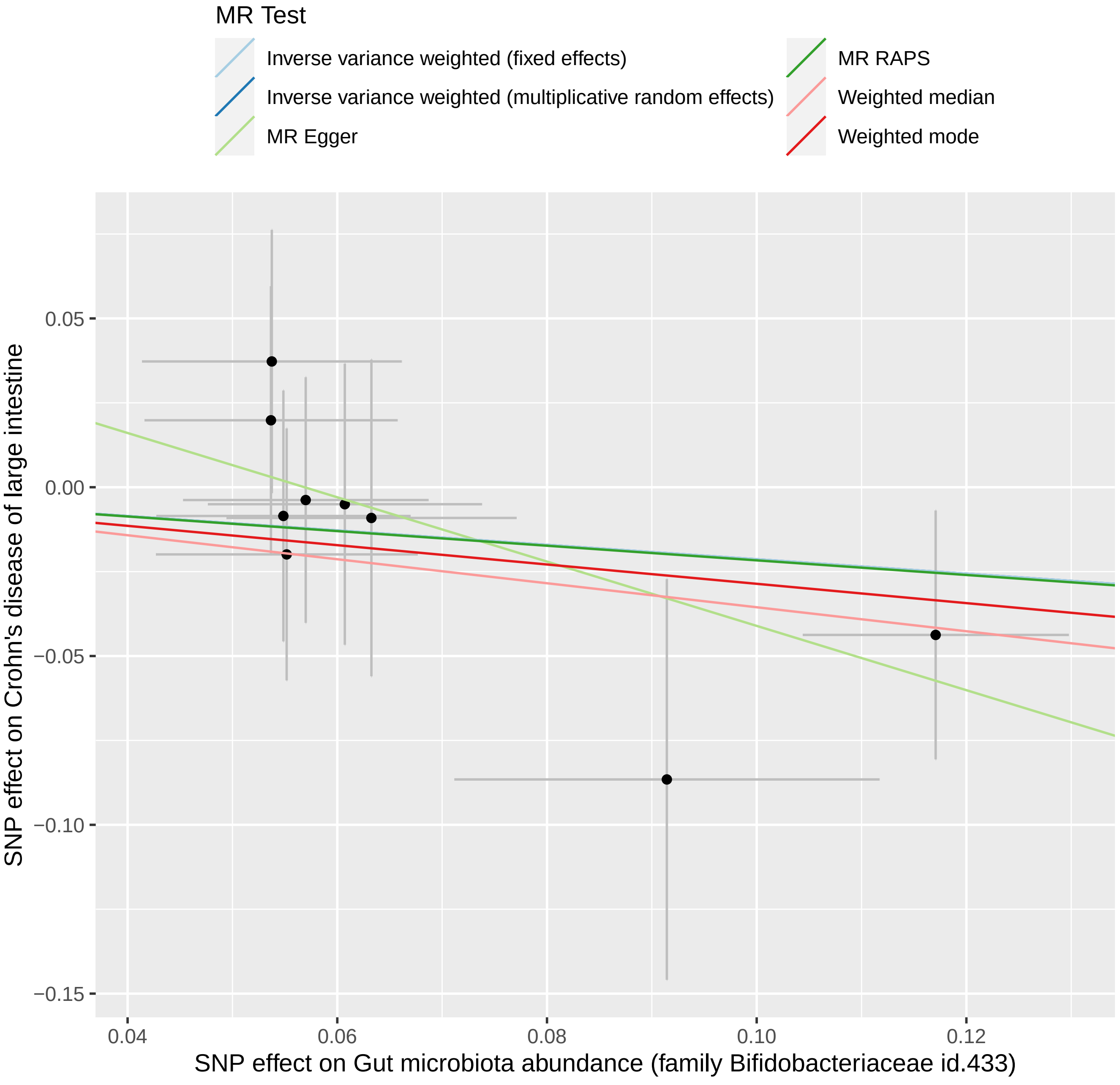

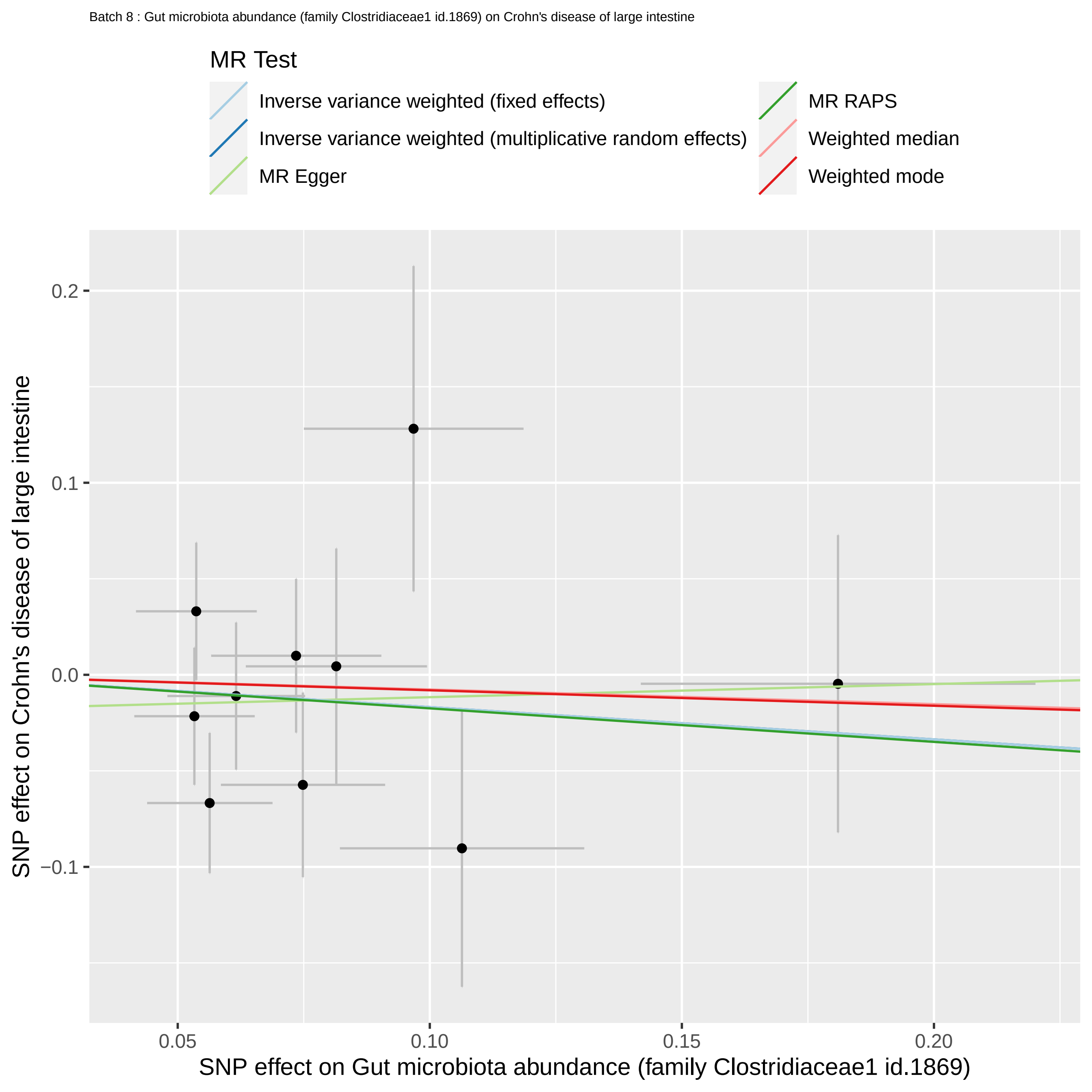
Batch 8 : Gut microbiota abundance (family Clostridiaceae1 id.1869) on Crohn's disease of large intestine
MR Test
Inverse variance weighted (fixed effects)
Inverse variance weighted (multiplicative random effects)
MR Egger
MR RAPS
Weighted median
Weighted mode
SNP effect on Crohn's disease of large intestine
0.2
0.1
0.0
−0.1
0.05
0.10
0.15
0.20
SNP effect on Gut microbiota abundance (family Clostridiaceae1 id.1869)

Batch 9 : Gut microbiota abundance (family Clostridiales vadin BB60 group id.11286) on Crohn's disease of large intestine

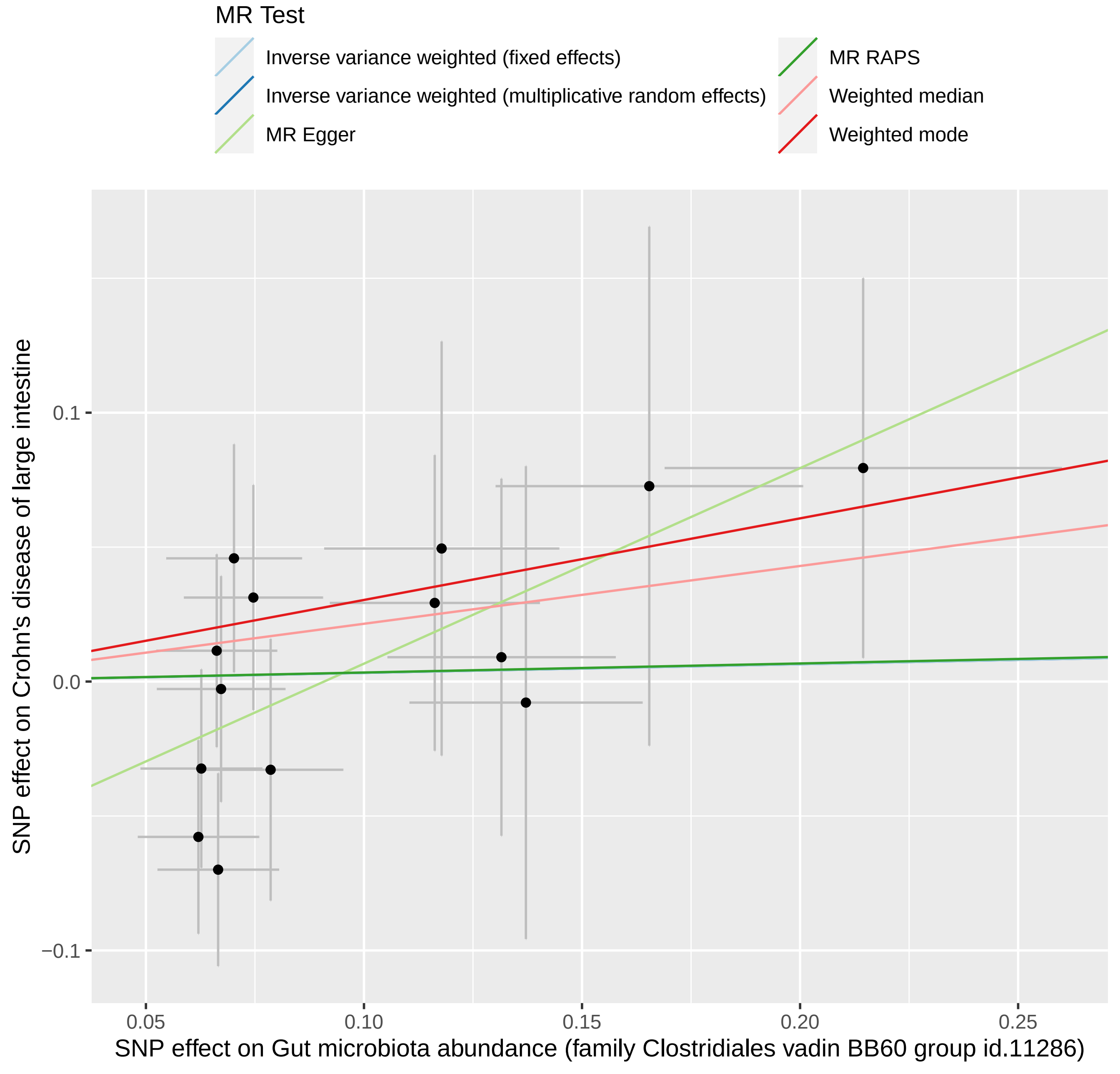

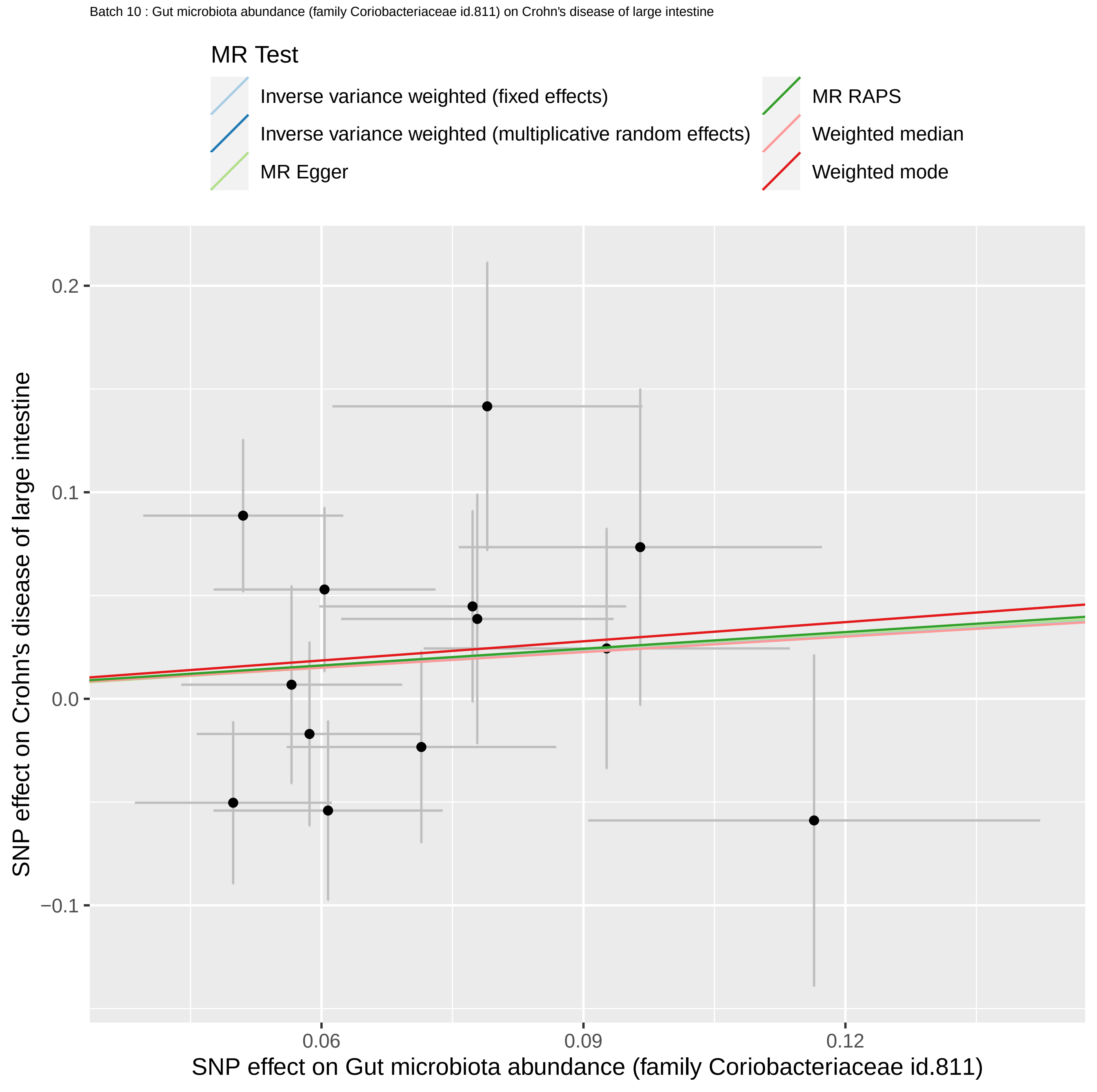

Batch 10 : Gut microbiota abundance (family Coriobacteriaceae id.811) on Crohn's disease of large intestine
MR Test
Inverse variance weighted (fixed effects)
Inverse variance weighted (multiplicative random effects)
MR Egger
MR RAPS
Weighted median
Weighted mode
0.2
0.1
0.0
−0.1
0.06
0.09
0.12
SNP effect on Crohn's disease of large intestine
SNP effect on Gut microbiota abundance (family Coriobacteriaceae id.811)

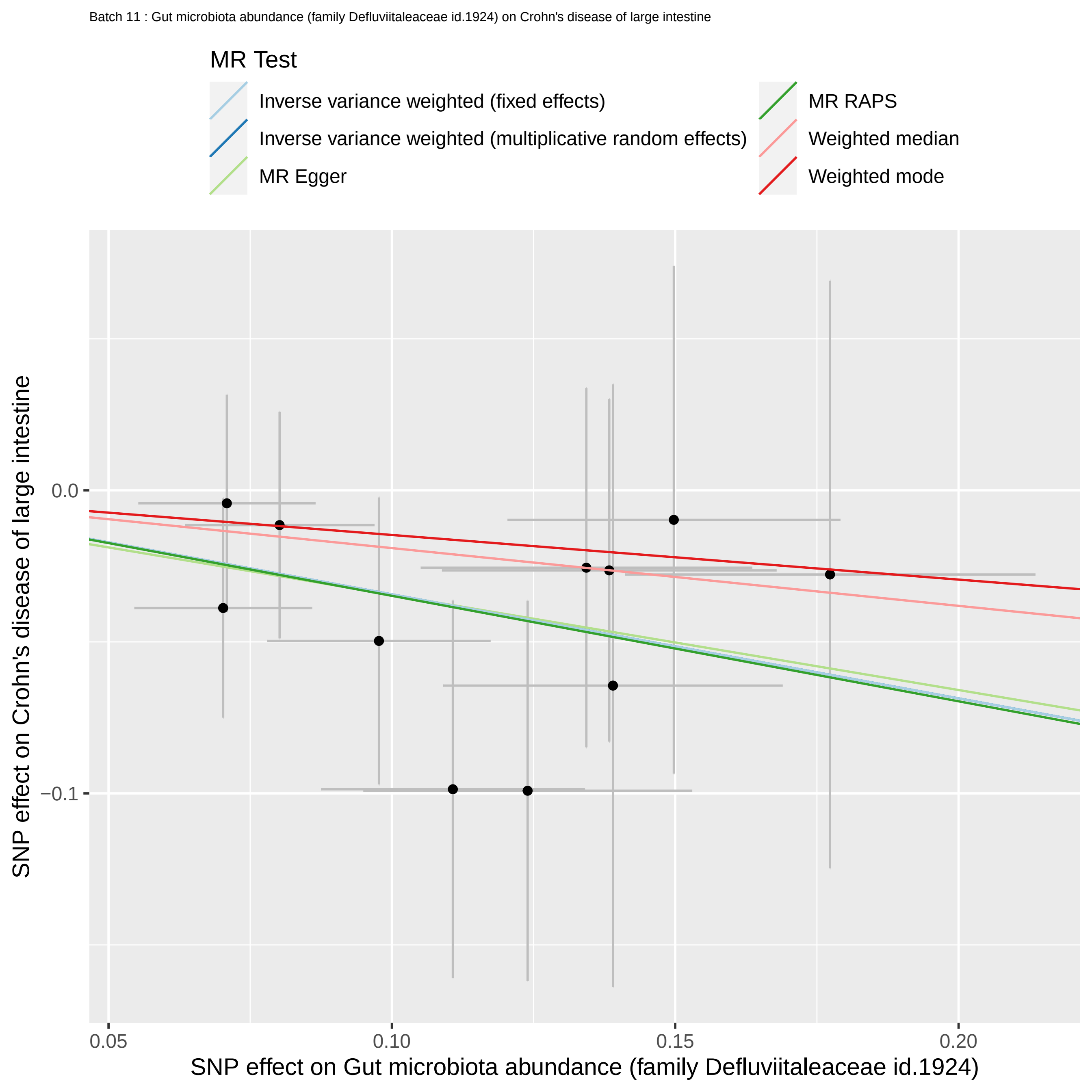

Batch 11 : Gut microbiota abundance (family Defluviitaleaceae id.1924) on Crohn's disease of large intestine
MR Test
Inverse variance weighted (fixed effects)
Inverse variance weighted (multiplicative random effects)
MR Egger
MR RAPS
Weighted median
Weighted mode
SNP effect on Crohn's disease of large intestine
0.0
−0.1
0.05
0.10
0.15
0.20
SNP effect on Gut microbiota abundance (family Defluviitaleaceae id.1924)

Batch 12 : Gut microbiota abundance (family Desulfovibrionaceae id.3169) on Crohn's disease of large intestine

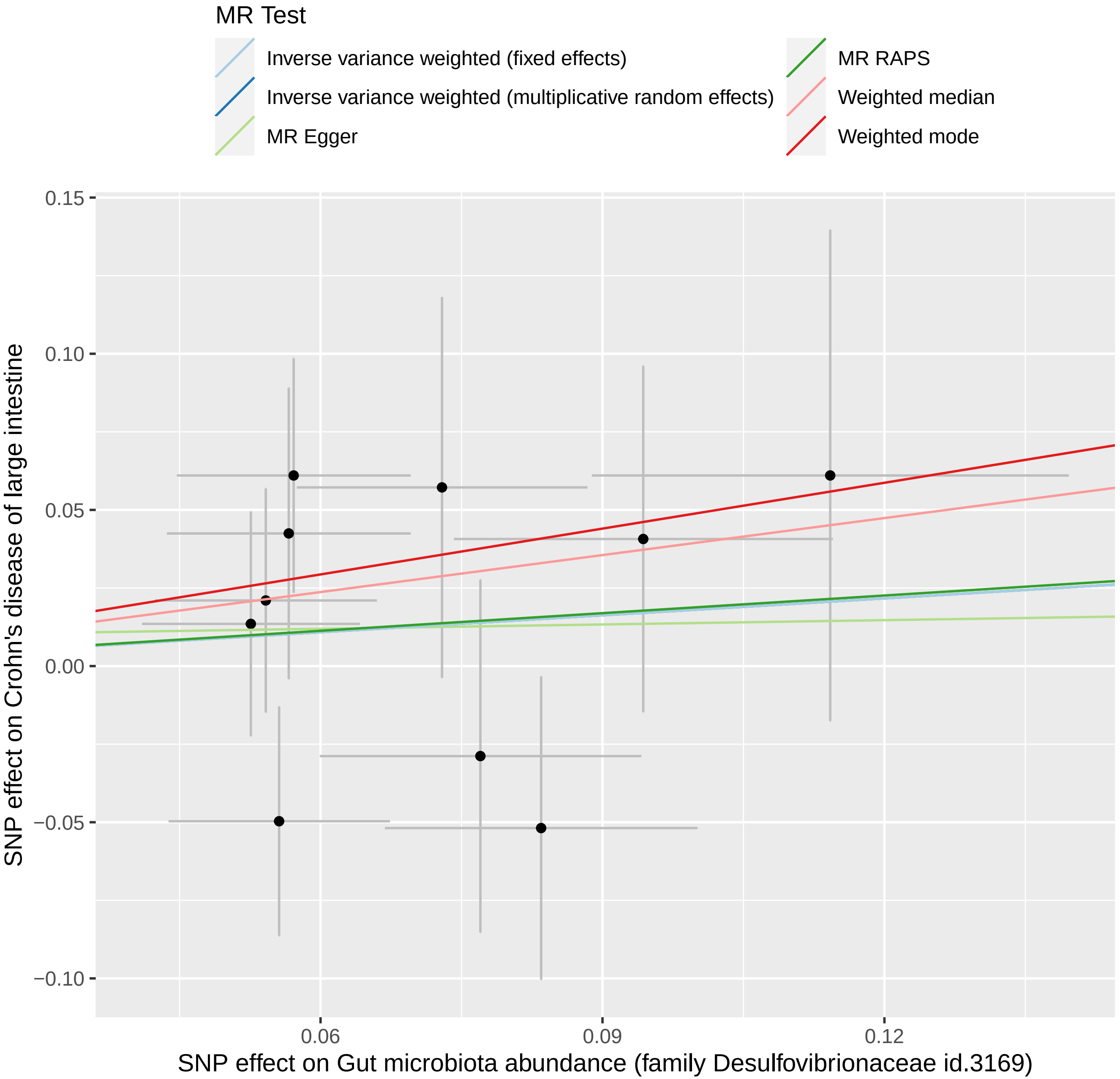

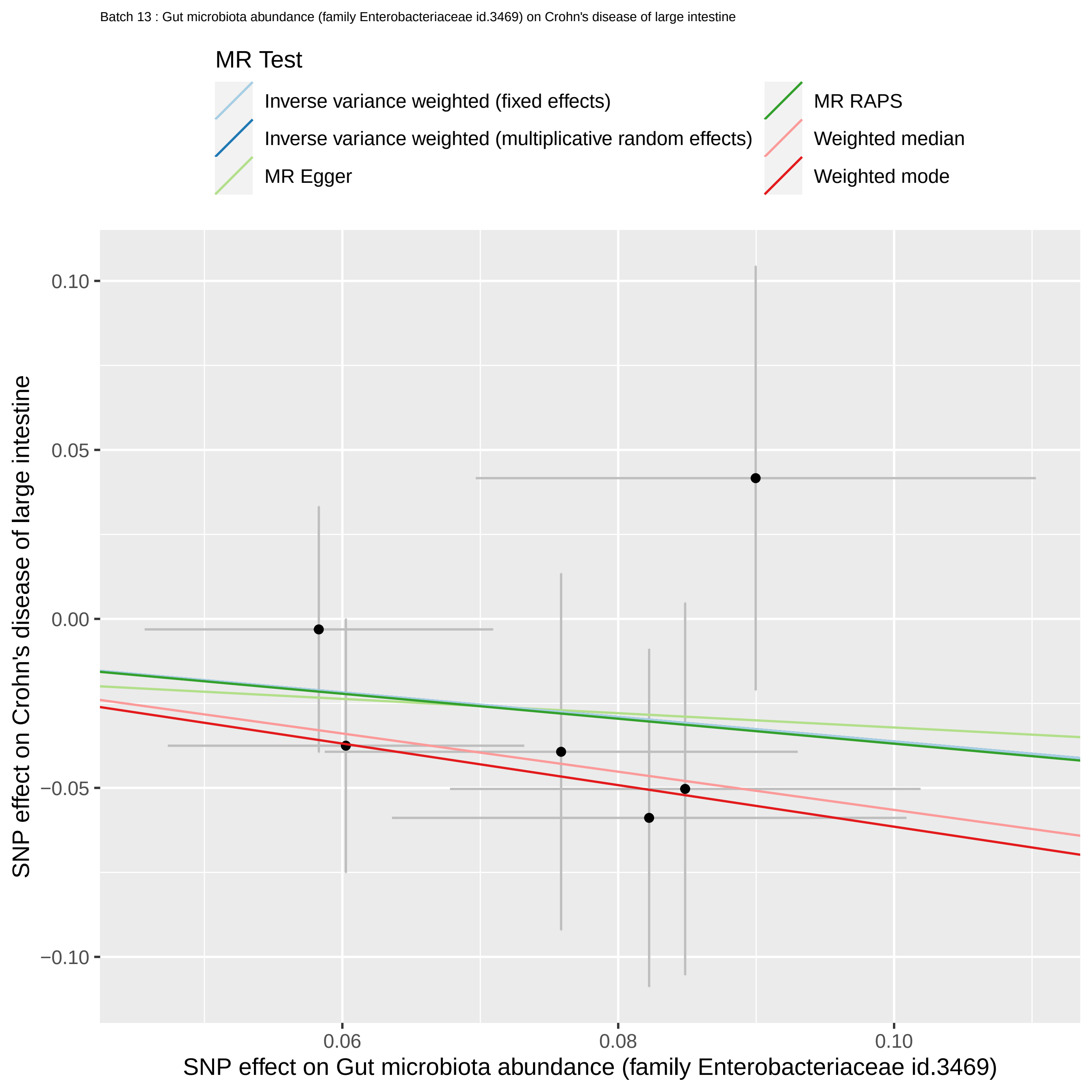

Batch 13 : Gut microbiota abundance (family Enterobacteriaceae id.3469) on Crohn's disease of large intestine
MR Test
Inverse variance weighted (fixed effects)
Inverse variance weighted (multiplicative random effects)
MR Egger
MR RAPS
Weighted median
Weighted mode
0.10
0.05
0.00
−0.05
−0.10
0.06
0.08
0.10
SNP effect on Crohn's disease of large intestine
SNP effect on Gut microbiota abundance (family Enterobacteriaceae id.3469)

Batch 14 : Gut microbiota abundance (family Erysipelotrichaceae id.2149) on Crohn's disease of large intestine

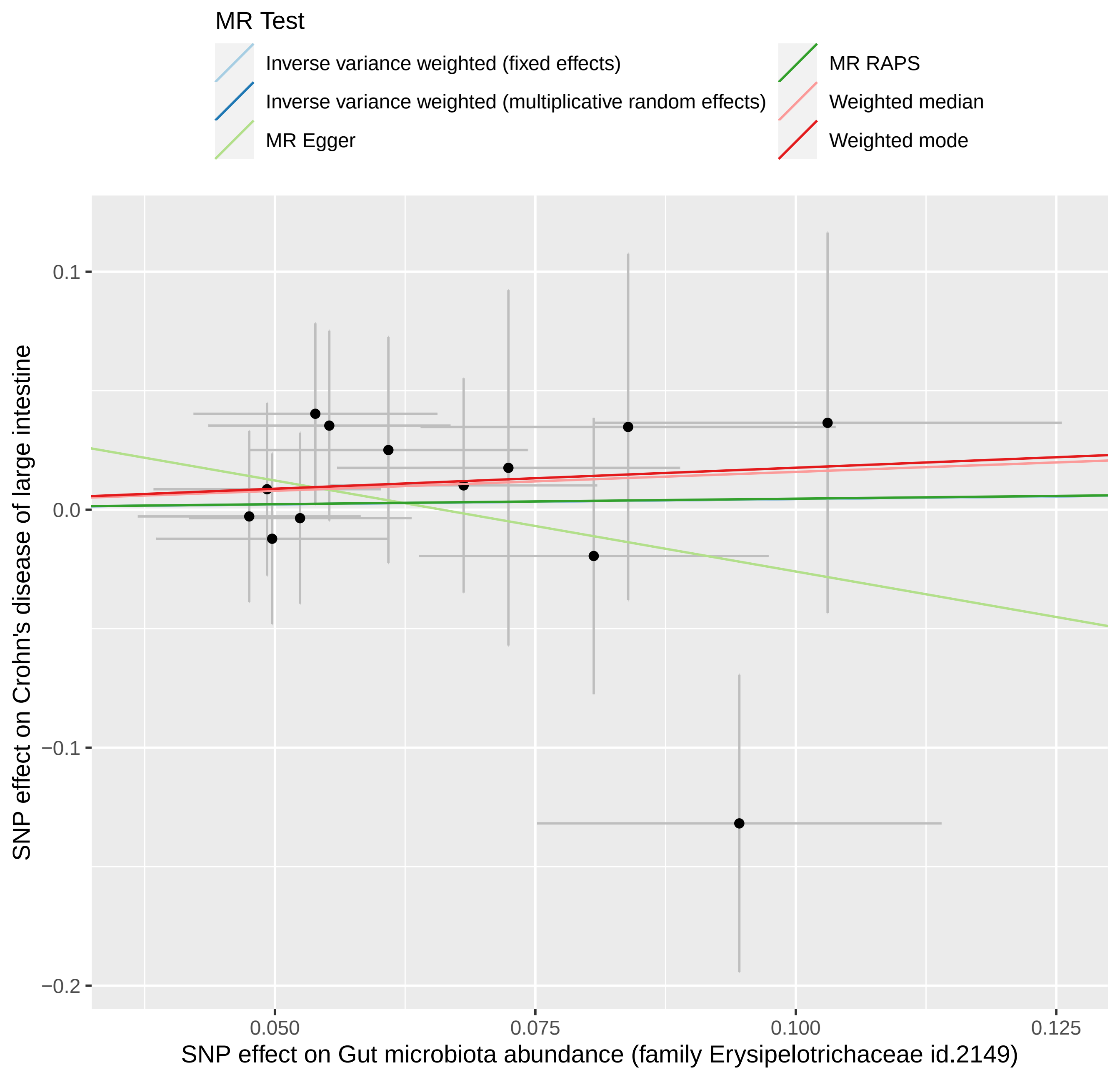

Batch 15 : Gut microbiota abundance (family Family XI id.1936) on Crohn's disease of large intestine

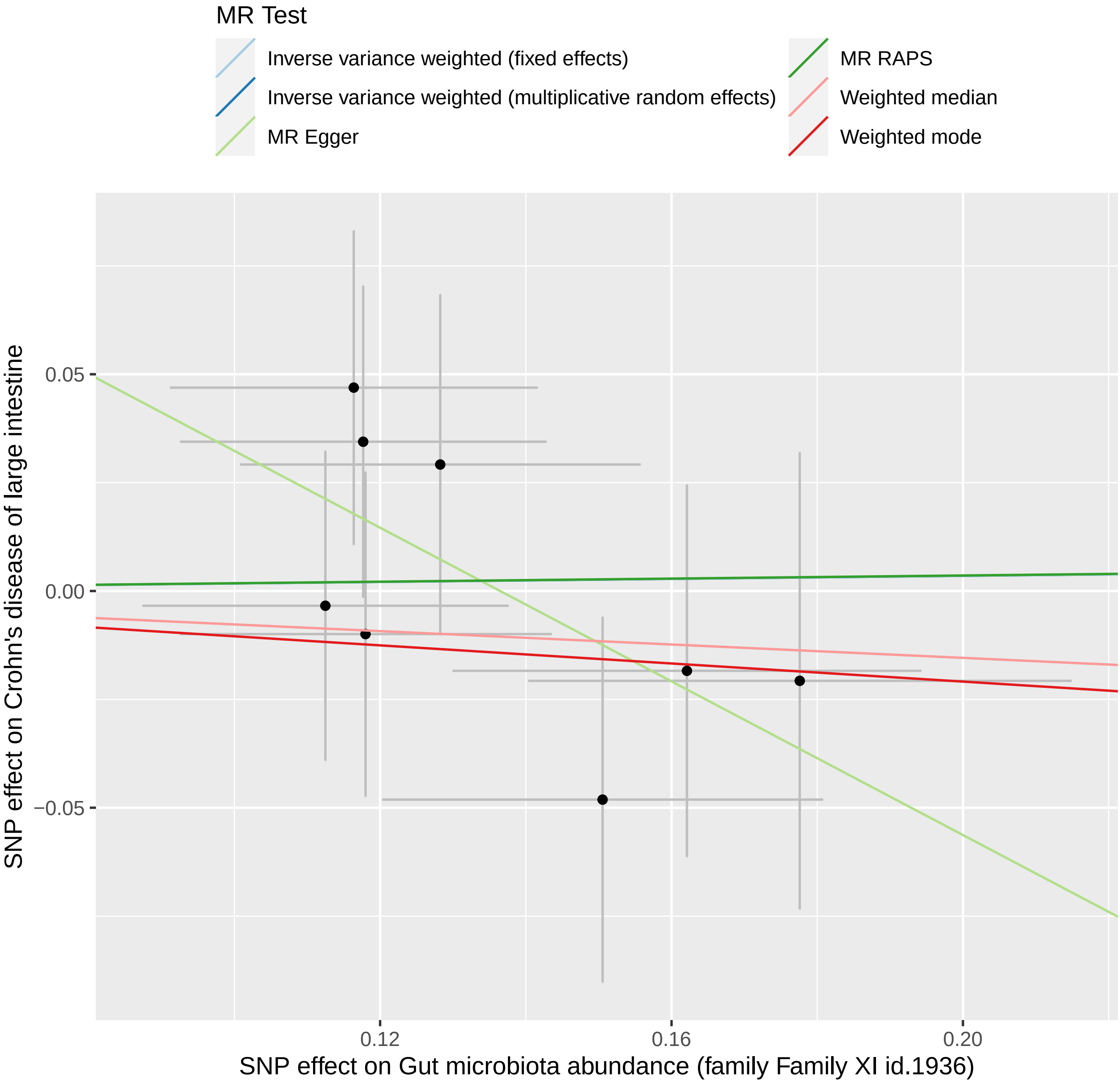

Batch 16 : Gut microbiota abundance (family Family XIII id.1957) on Crohn's disease of large intestine

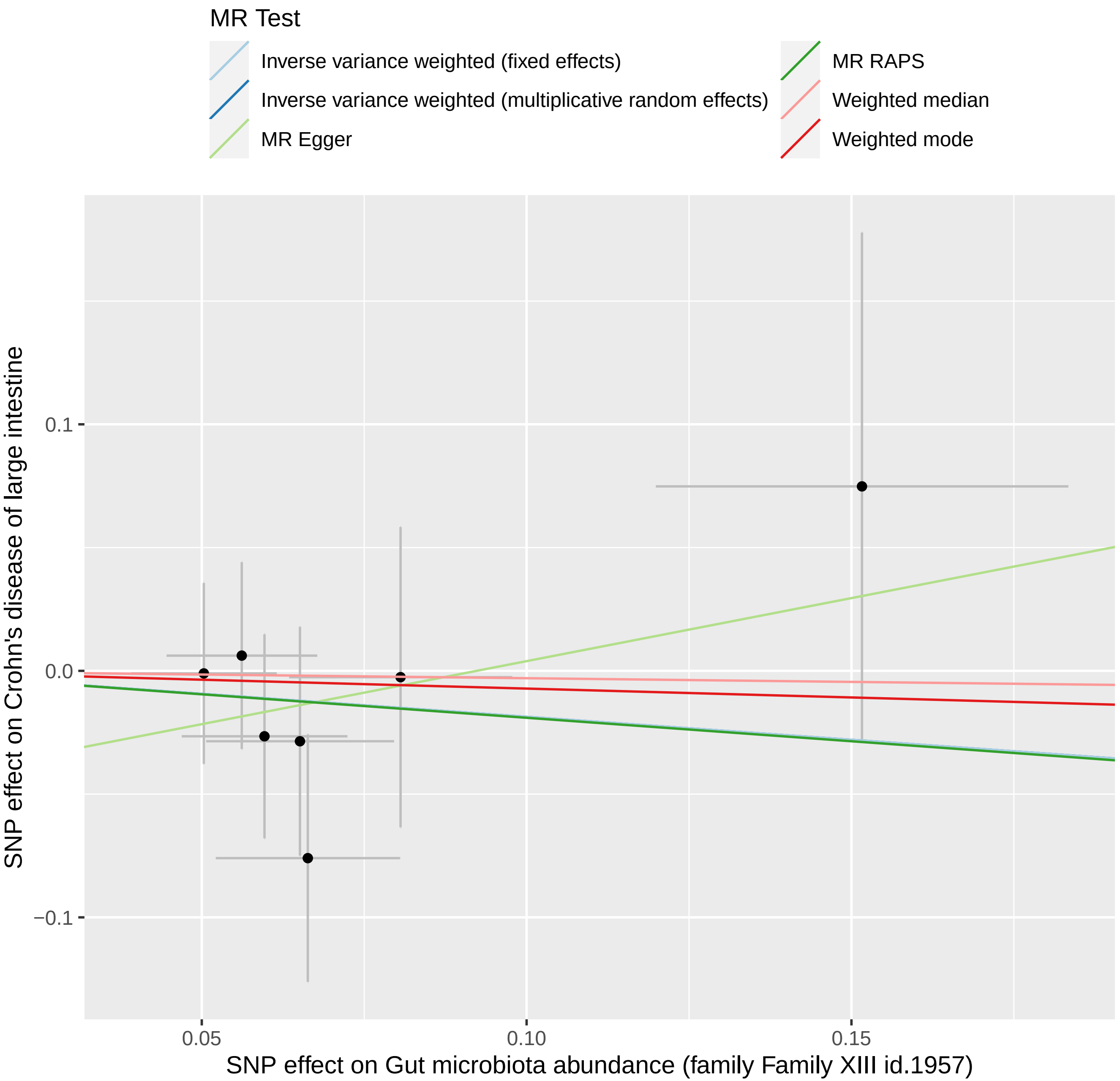

Batch 17 : Gut microbiota abundance (family Lachnospiraceae id.1987) on Crohn's disease of large intestine

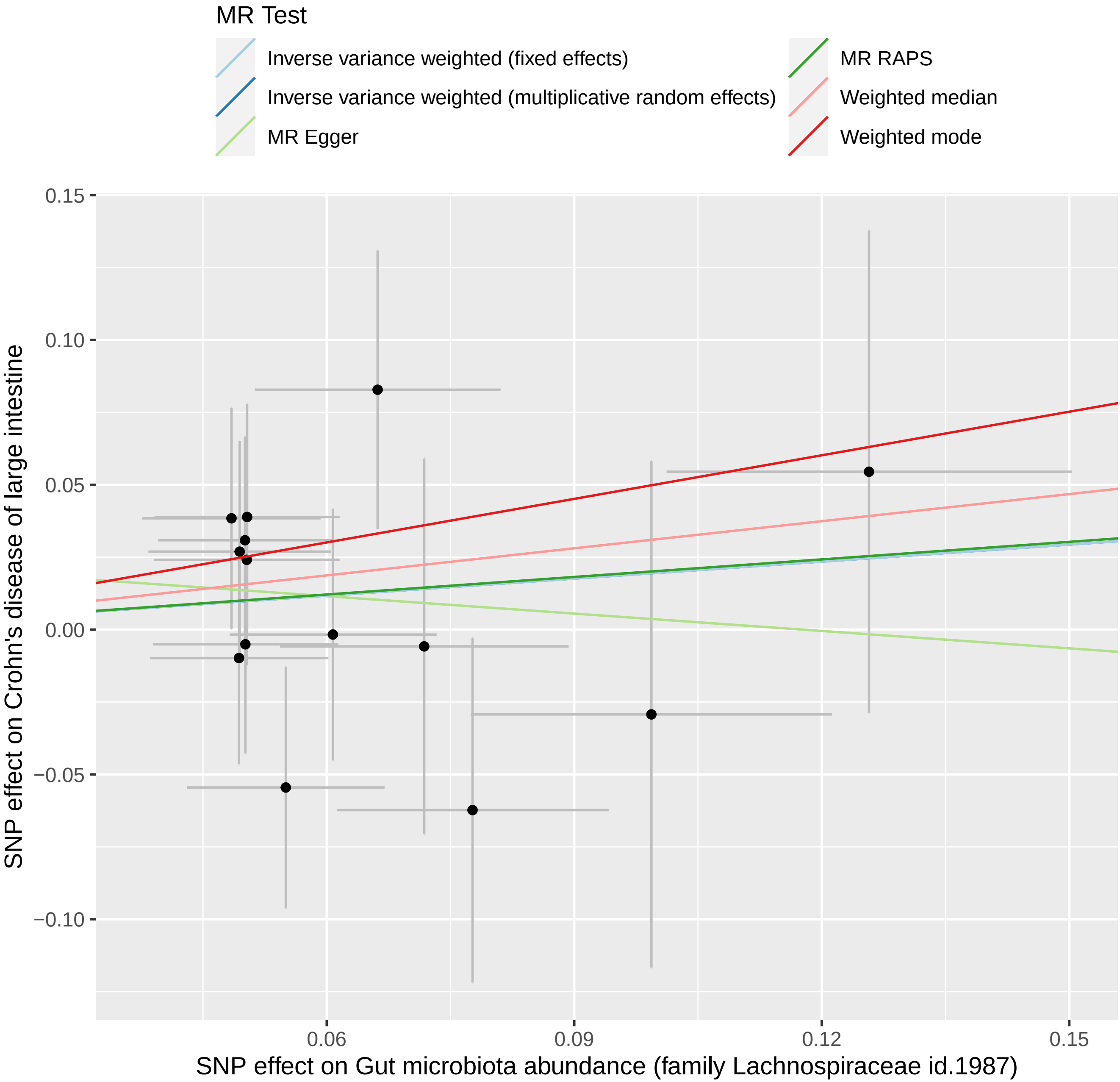

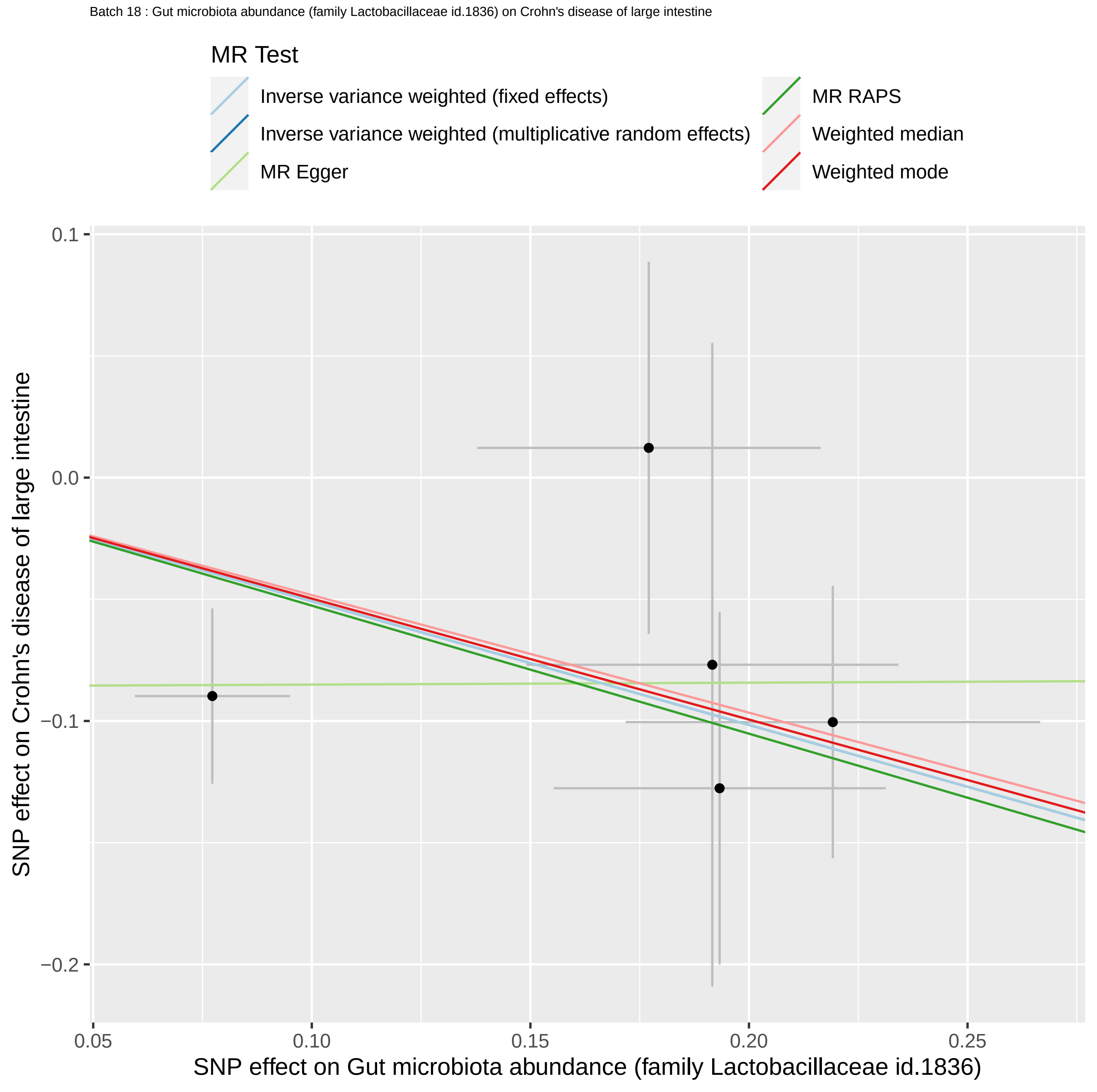
Batch 18 : Gut microbiota abundance (family Lactobacillaceae id.1836) on Crohn's disease of large intestine
MR Test
Inverse variance weighted (fixed effects)
Inverse variance weighted (multiplicative random effects)
MR Egger
MR RAPS
Weighted median
Weighted mode
0.1
0.0
−0.1
−0.2
0.05
0.10
0.15
0.20
0.25
SNP effect on Crohn's disease of large intestine
SNP effect on Gut microbiota abundance (family Lactobacillaceae id.1836)

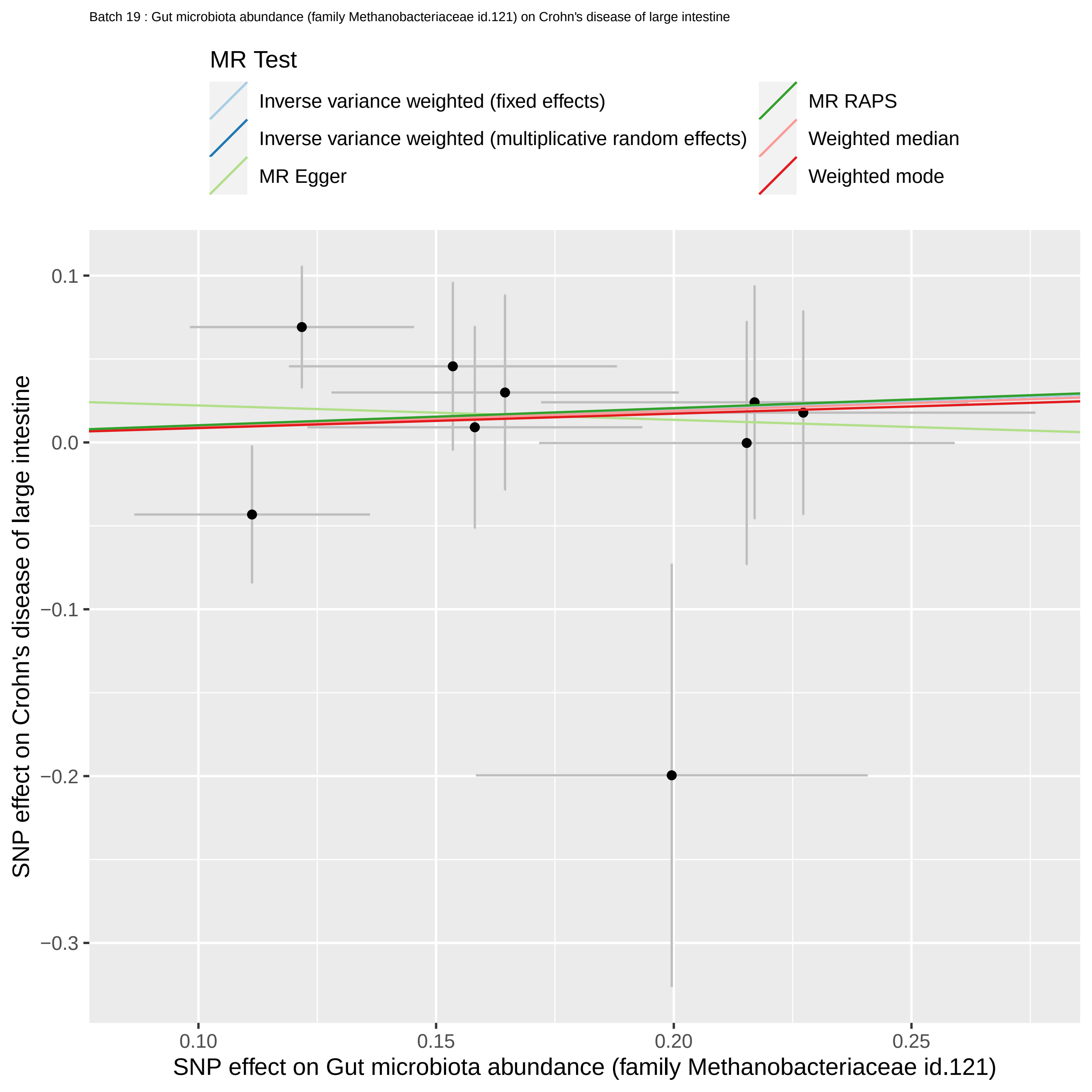

Batch 19 : Gut microbiota abundance (family Methanobacteriaceae id.121) on Crohn's disease of large intestine
MR Test
Inverse variance weighted (fixed effects)
Inverse variance weighted (multiplicative random effects)
MR Egger
MR RAPS
Weighted median
Weighted mode
0.1
0.0
−0.1
−0.2
−0.3
0.10
0.15
0.20
0.25
SNP effect on Crohn's disease of large intestine
SNP effect on Gut microbiota abundance (family Methanobacteriaceae id.121)

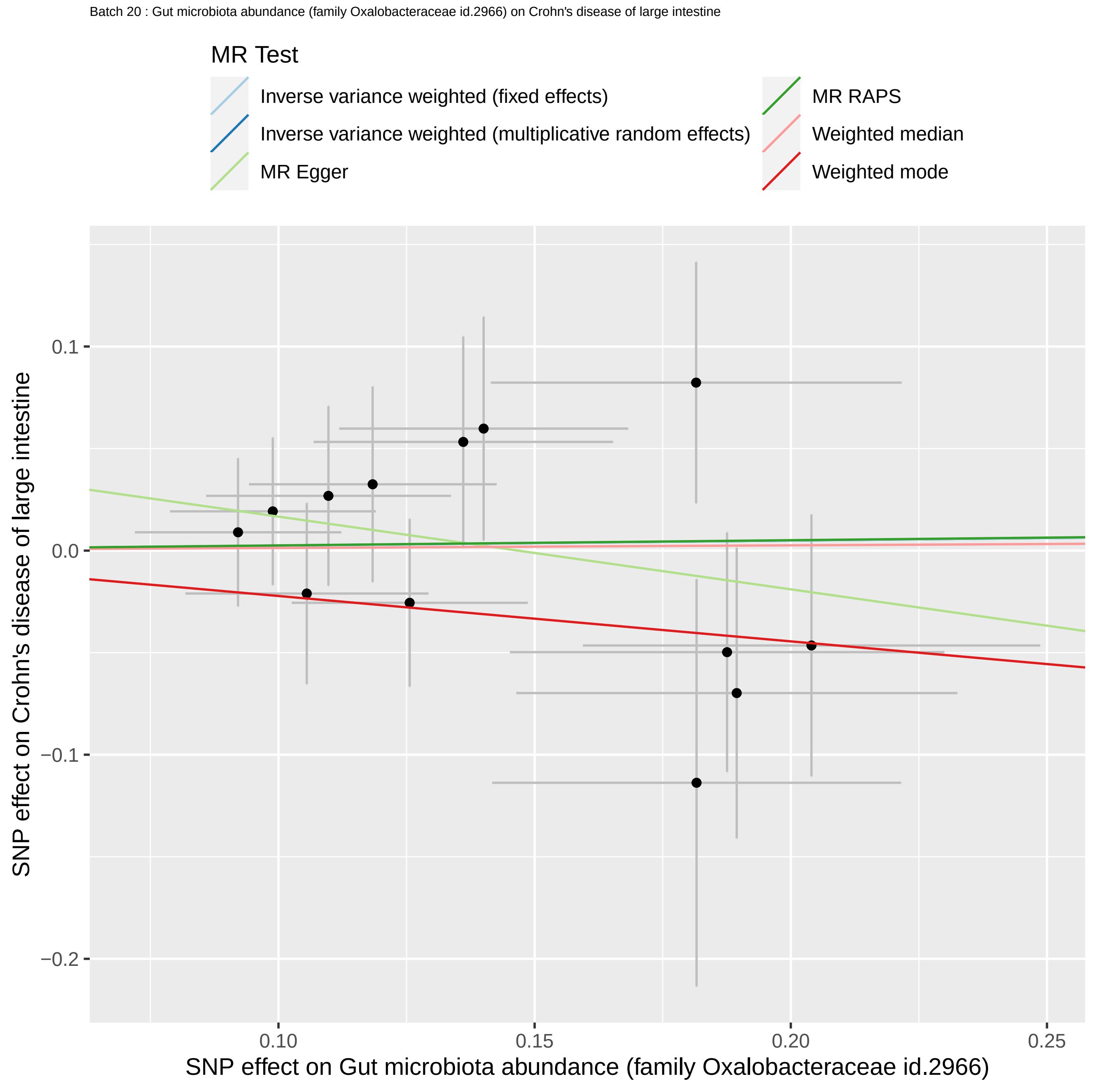
Batch 20 : Gut microbiota abundance (family Oxalobacteraceae id.2966) on Crohn's disease of large intestine
MR Test
Inverse variance weighted (fixed effects)
Inverse variance weighted (multiplicative random effects)
MR Egger
MR RAPS
Weighted median
Weighted mode
0.1
0.0
−0.1
−0.2
0.10
0.15
0.20
0.25
SNP effect on Crohn's disease of large intestine
SNP effect on Gut microbiota abundance (family Oxalobacteraceae id.2966)

Batch 21 : Gut microbiota abundance (family Pasteurellaceae id.3689) on Crohn's disease of large intestine

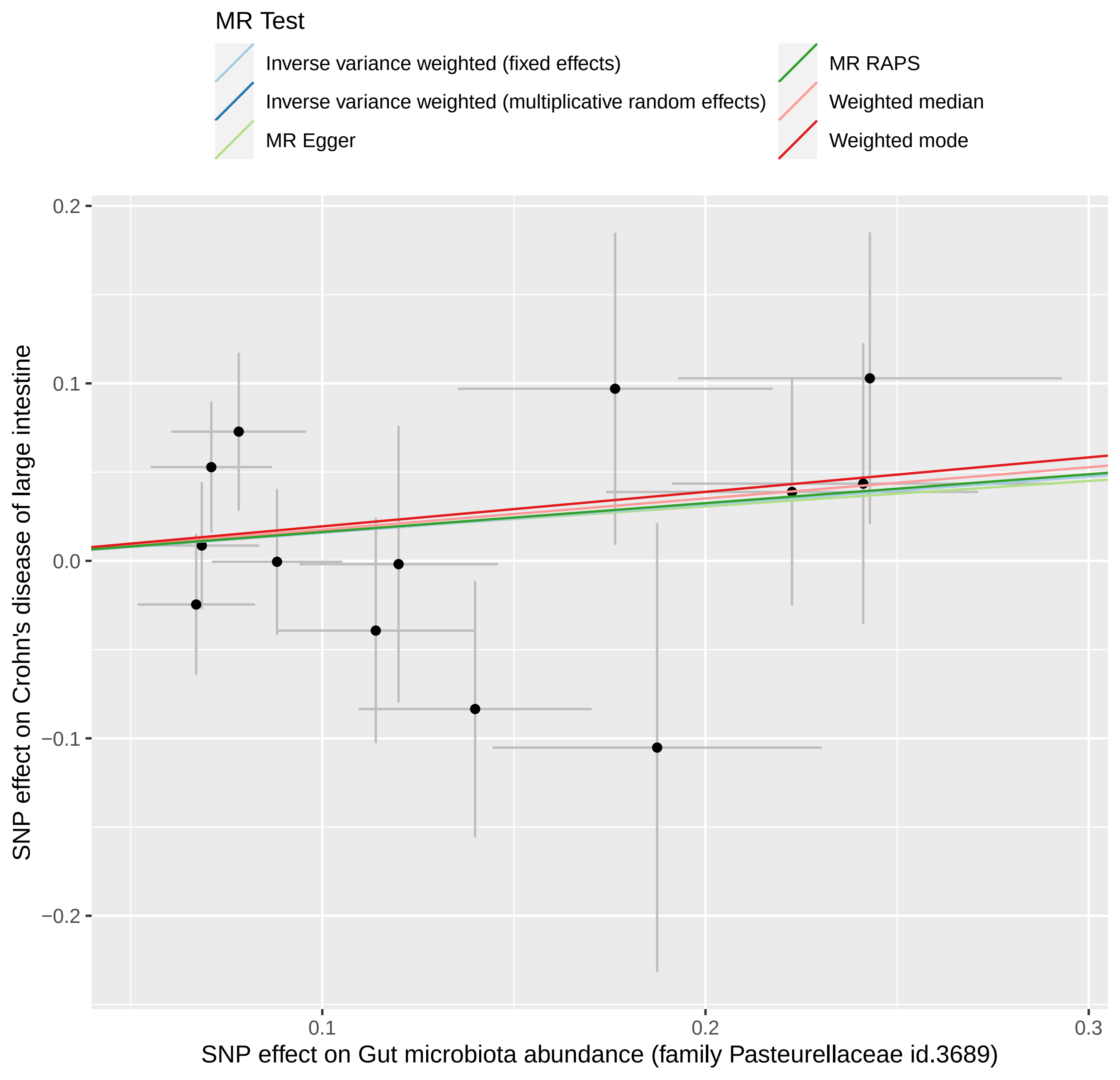

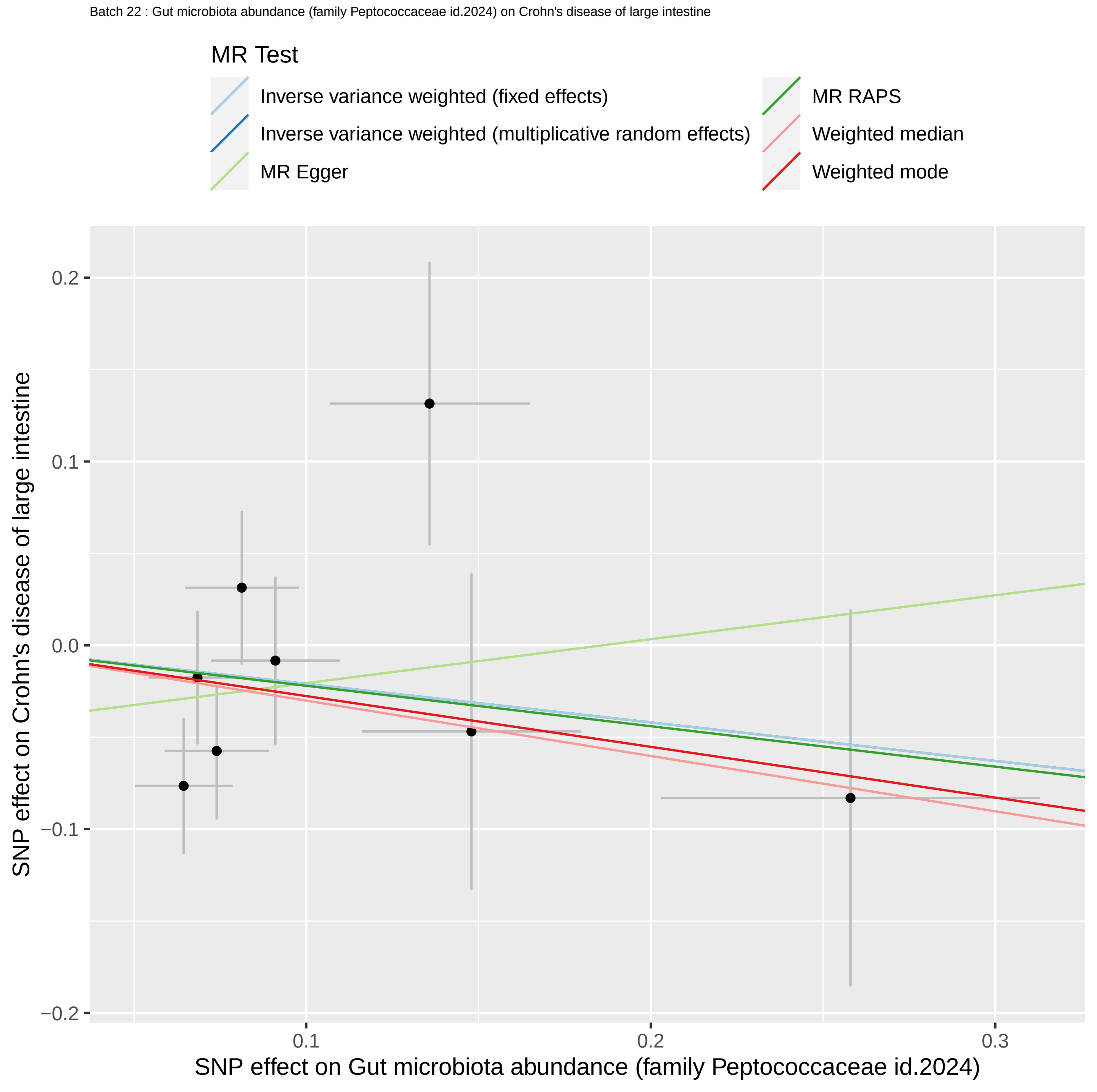
Batch 22 : Gut microbiota abundance (family Peptococcaceae id.2024) on Crohn's disease of large intestine
MR Test
Inverse variance weighted (fixed effects)
Inverse variance weighted (multiplicative random effects)
MR Egger
MR RAPS
Weighted median
Weighted mode
0.2
0.1
0.0
−0.1
−0.2
0.1
0.2
0.3
SNP effect on Crohn's disease of large intestine
SNP effect on Gut microbiota abundance (family Peptococcaceae id.2024)

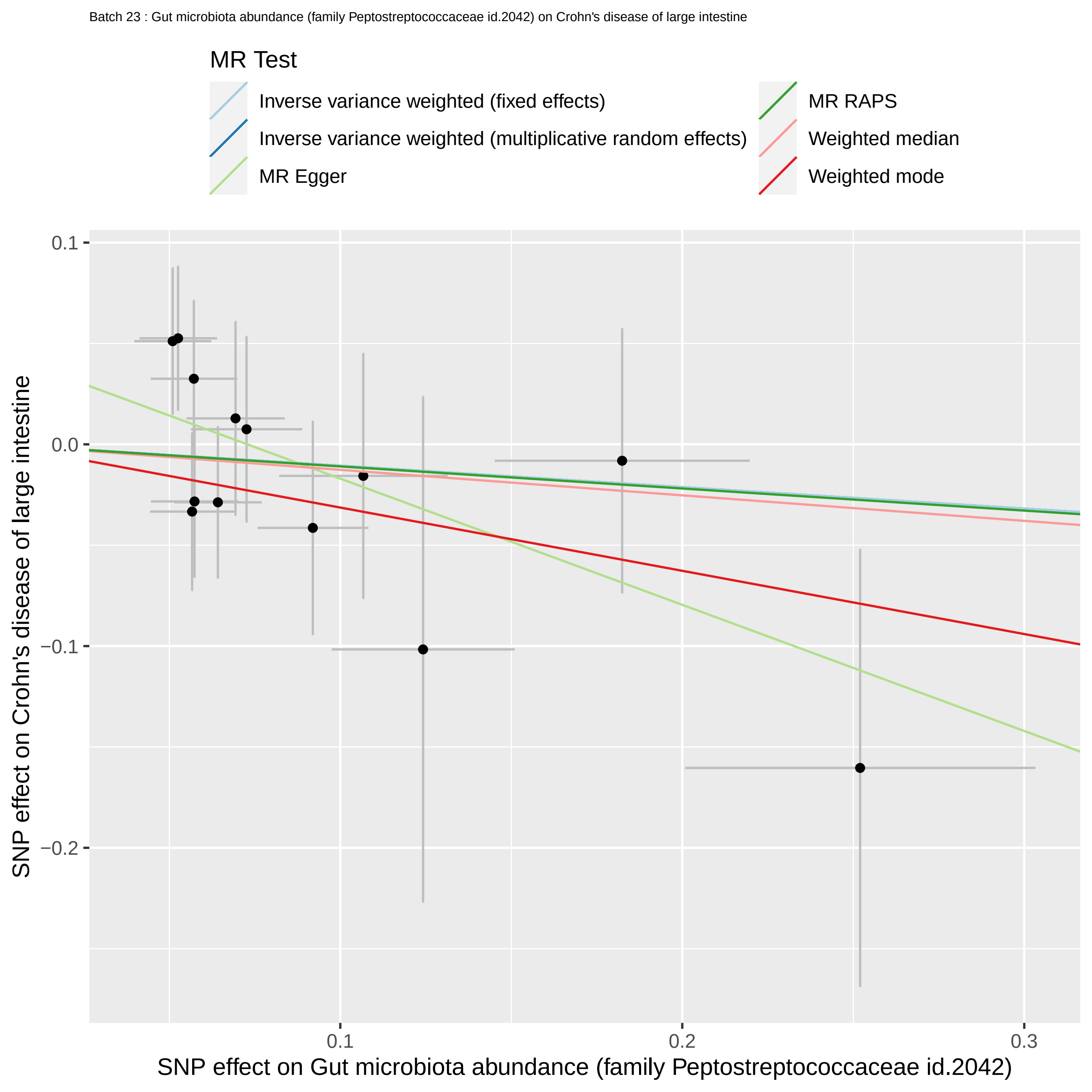

Batch 23 : Gut microbiota abundance (family Peptostreptococcaceae id.2042) on Crohn's disease of large intestine
MR Test
Inverse variance weighted (fixed effects)
Inverse variance weighted (multiplicative random effects)
MR Egger
MR RAPS
Weighted median
Weighted mode
0.1
0.0
−0.1
−0.2
0.1
0.2
0.3
SNP effect on Crohn's disease of large intestine
SNP effect on Gut microbiota abundance (family Peptostreptococcaceae id.2042)

Batch 24 : Gut microbiota abundance (family Porphyromonadaceae id.943) on Crohn's disease of large intestine

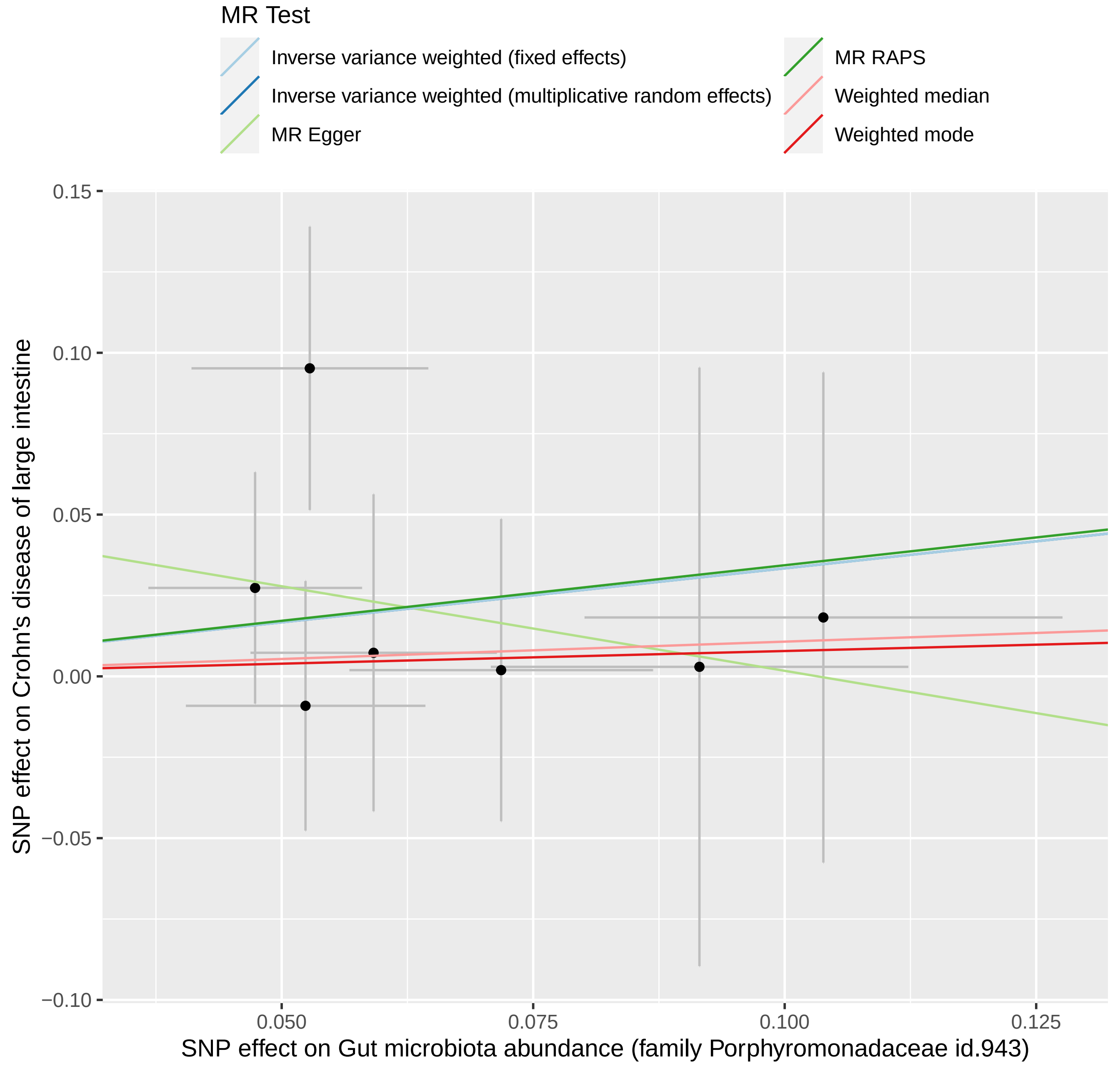

Batch 25 : Gut microbiota abundance (family Prevotellaceae id.960) on Crohn's disease of large intestine

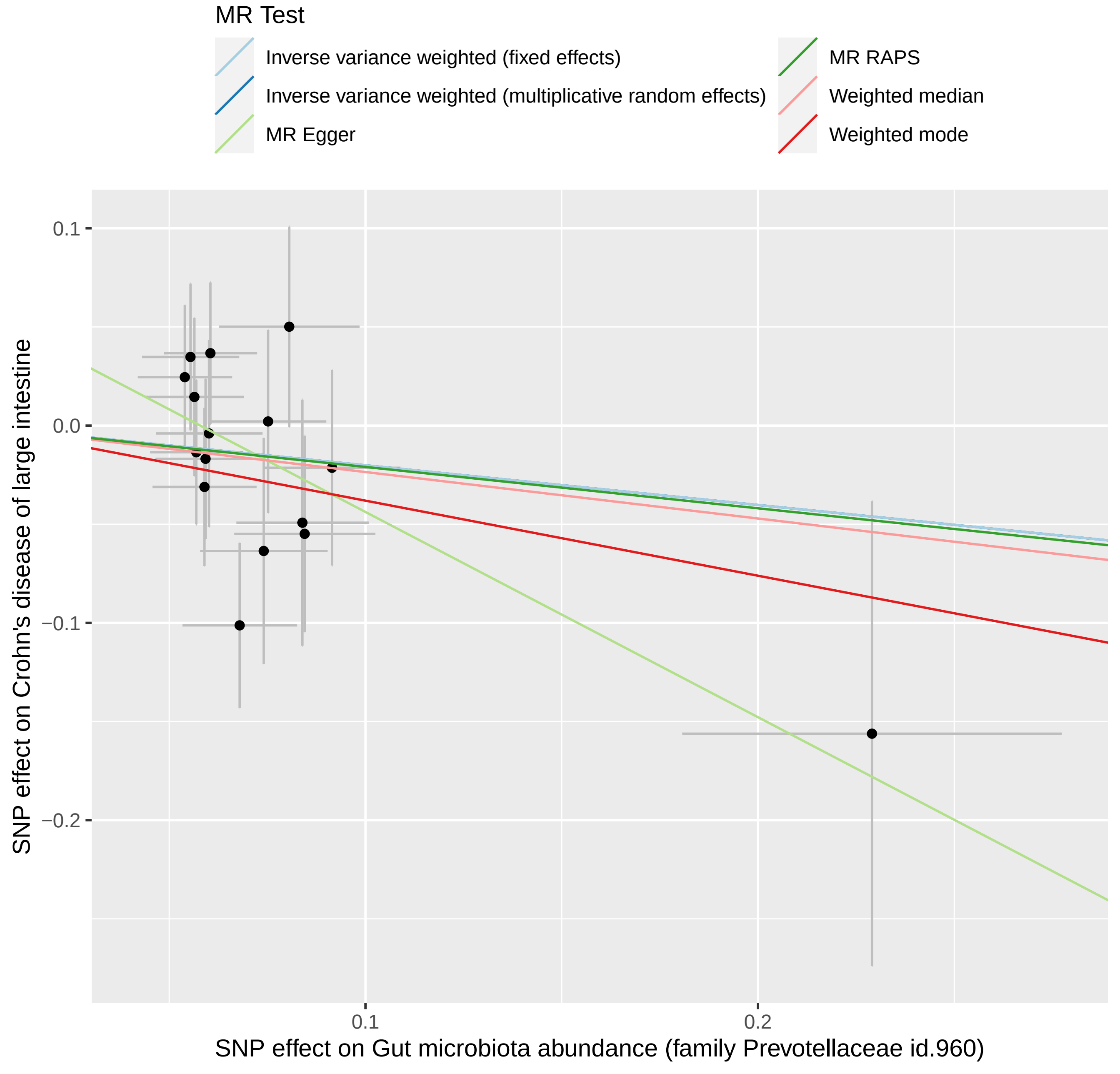

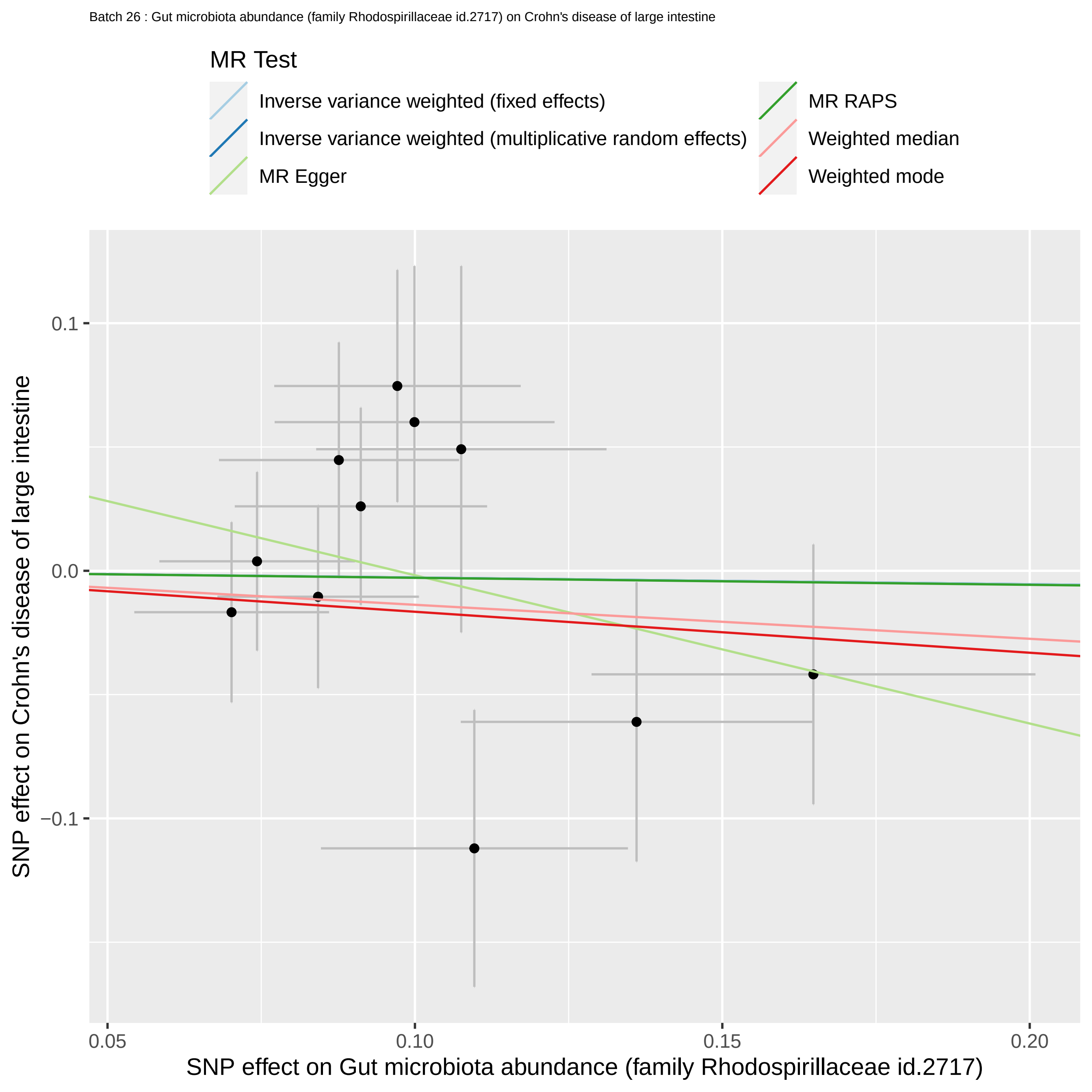

Batch 26 : Gut microbiota abundance (family Rhodospirillaceae id.2717) on Crohn's disease of large intestine
MR Test
Inverse variance weighted (fixed effects)
Inverse variance weighted (multiplicative random effects)
MR Egger
MR RAPS
Weighted median
Weighted mode
0.1
0.0
−0.1
0.05
0.10
0.15
0.20
SNP effect on Crohn's disease of large intestine
SNP effect on Gut microbiota abundance (family Rhodospirillaceae id.2717)

Batch 27 : Gut microbiota abundance (family Rikenellaceae id.967) on Crohn's disease of large intestine

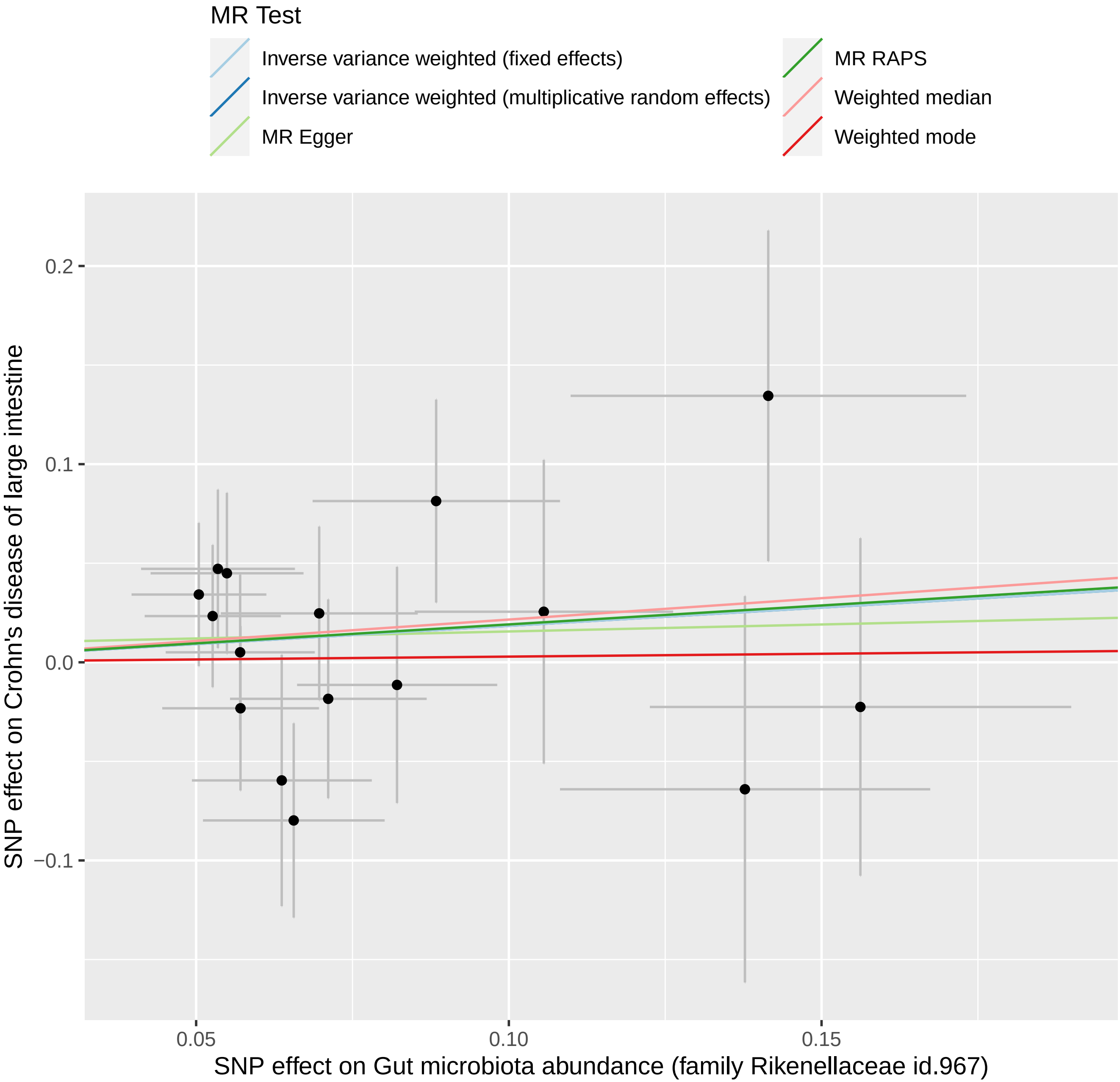

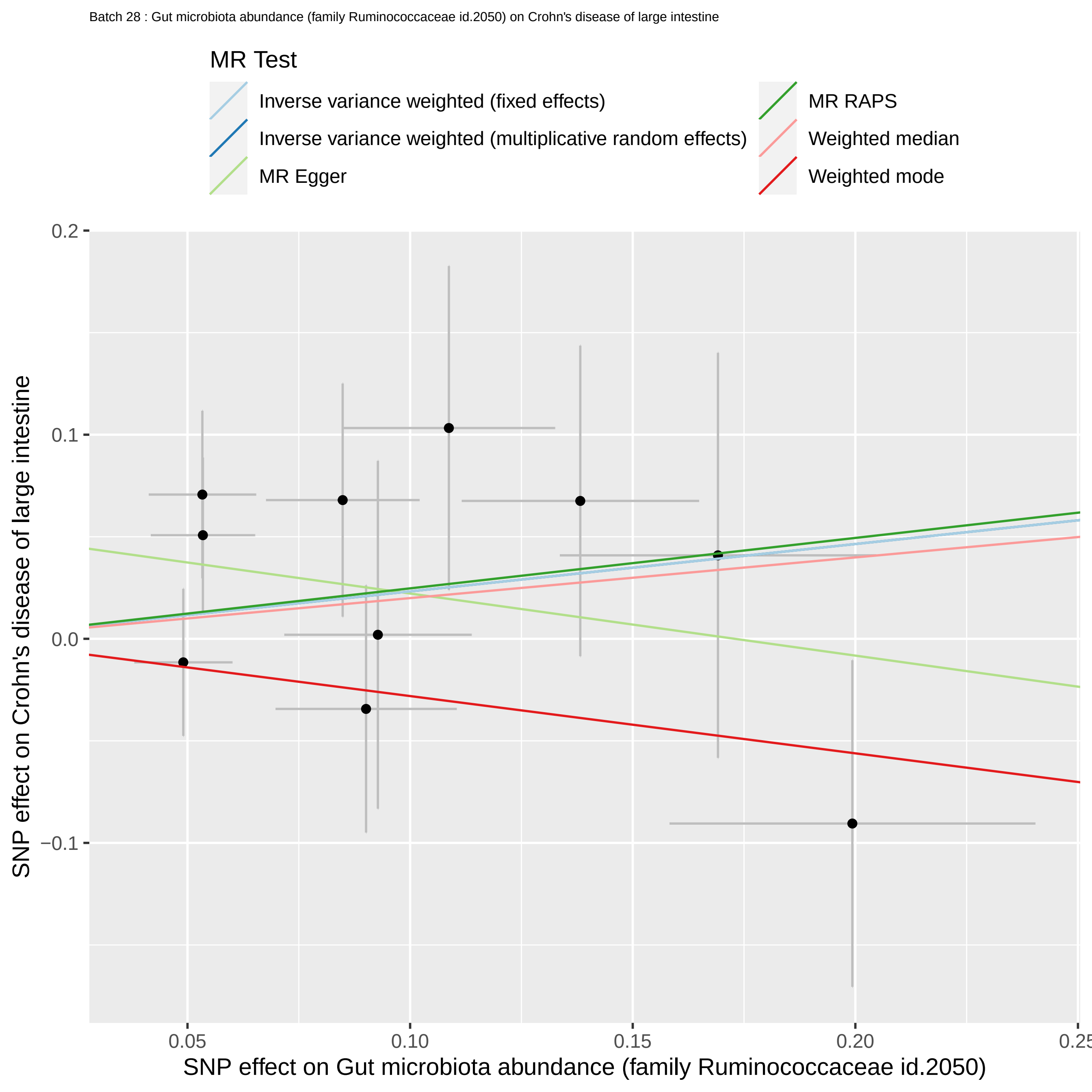
Batch 28 : Gut microbiota abundance (family Ruminococcaceae id.2050) on Crohn's disease of large intestine
MR Test
Inverse variance weighted (fixed effects)
Inverse variance weighted (multiplicative random effects)
MR Egger
MR RAPS
Weighted median
Weighted mode
0.2
0.1
0.0
−0.1
0.05
0.10
0.15
0.20
SNP effect on Crohn's disease of large intestine
SNP effect on Gut microbiota abundance (family Ruminococcaceae id.2050)

Batch 29 : Gut microbiota abundance (family Streptococcaceae id.1850) on Crohn's disease of large intestine

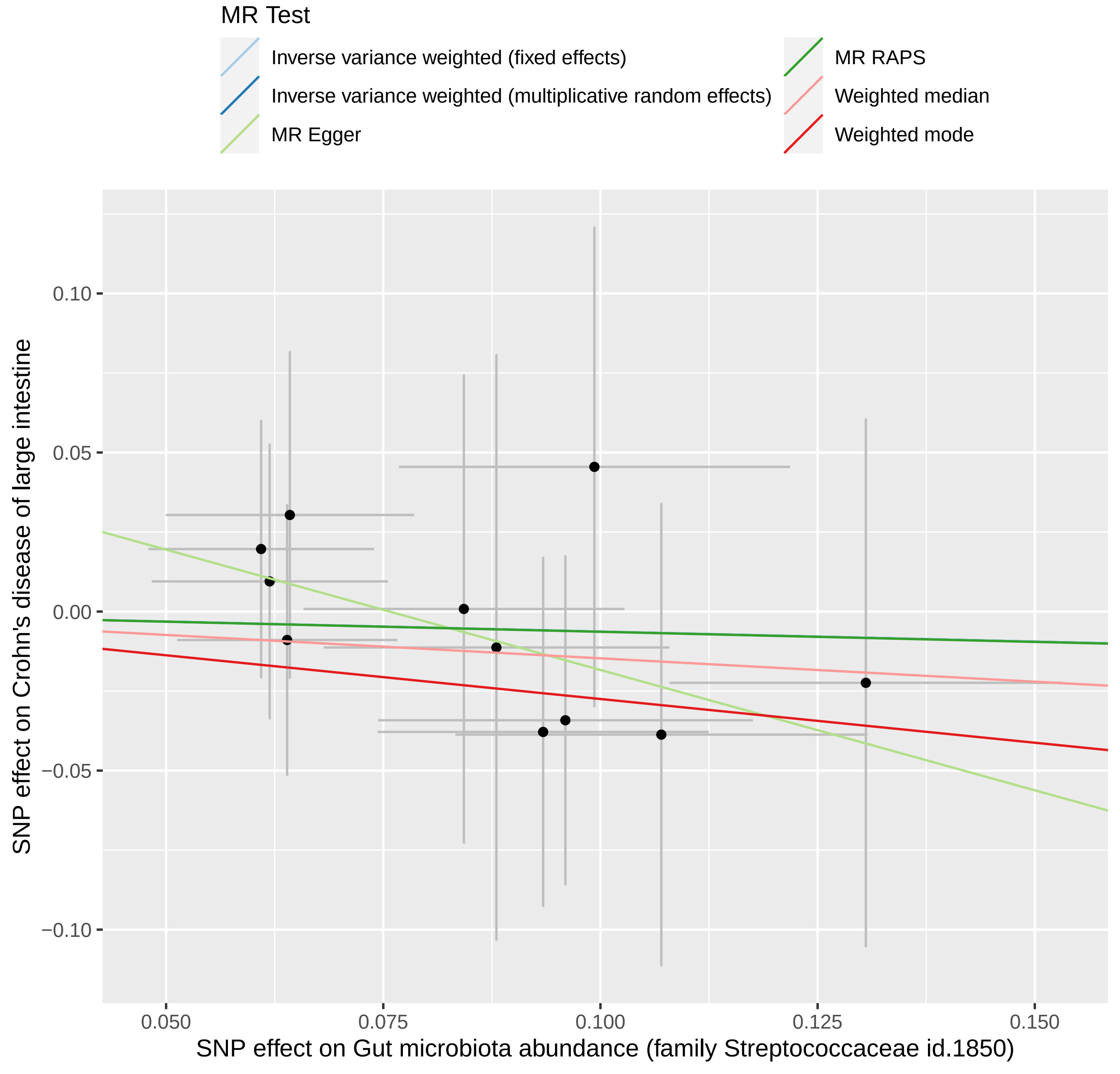

Batch 30 : Gut microbiota abundance (family Veillonellaceae id.2172) on Crohn's disease of large intestine

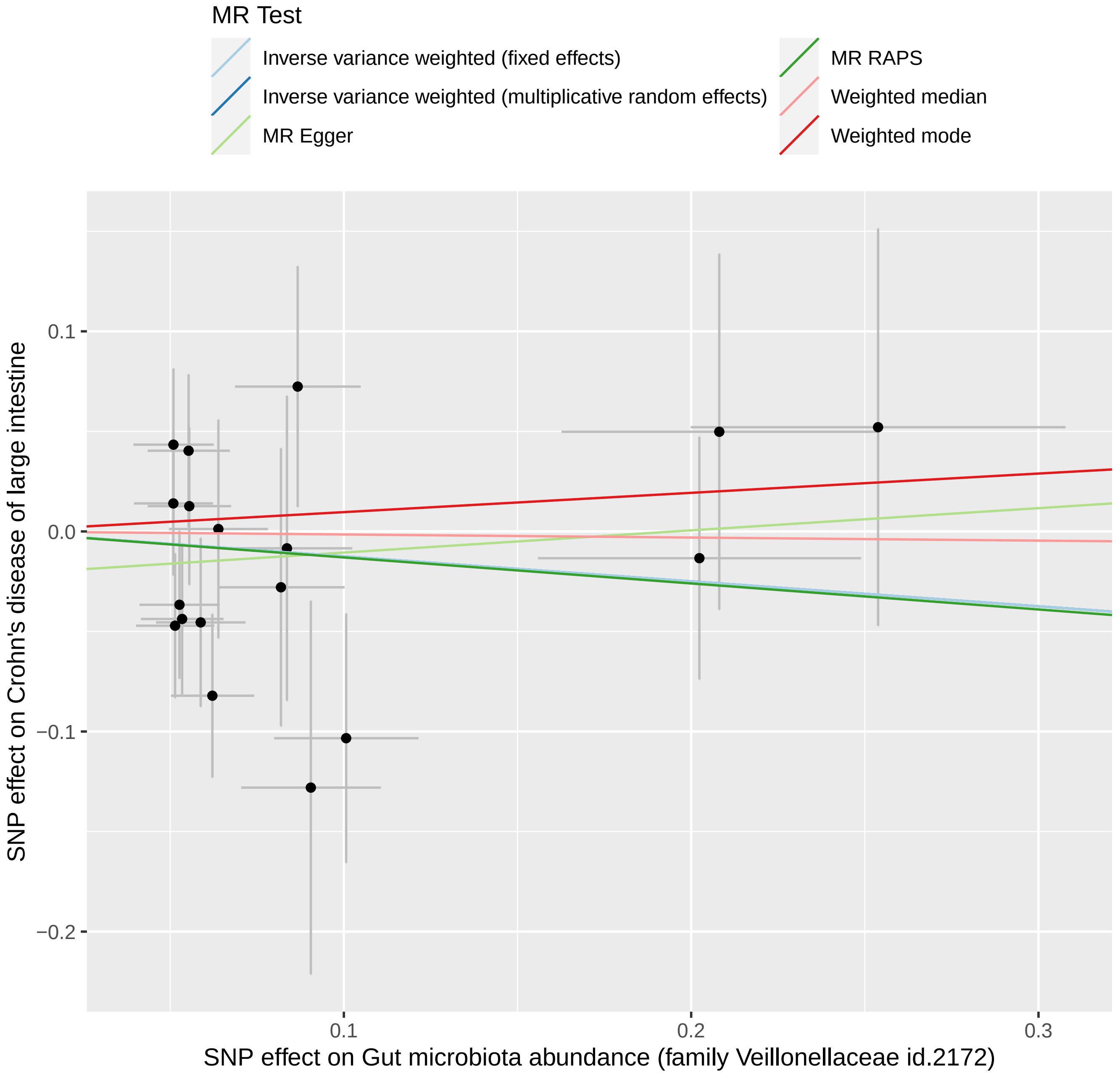

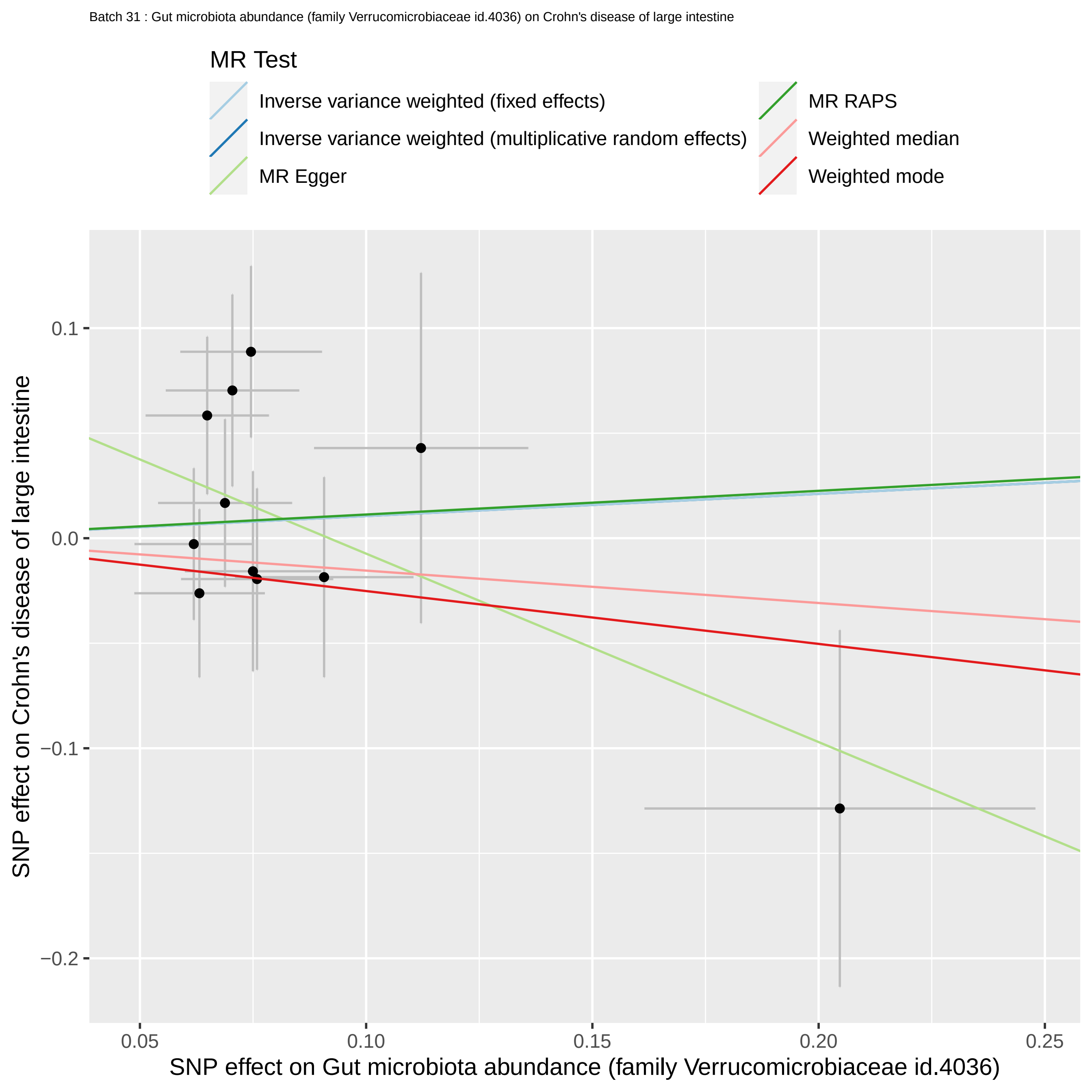
Batch 31 : Gut microbiota abundance (family Verrucomicrobiaceae id.4036) on Crohn's disease of large intestine
MR Test
Inverse variance weighted (fixed effects)
Inverse variance weighted (multiplicative random effects)
MR Egger
MR RAPS
Weighted median
Weighted mode
0.1
0.0
−0.1
−0.2
0.05
0.10
0.15
0.20
0.25
SNP effect on Crohn's disease of large intestine
SNP effect on Gut microbiota abundance (family Verrucomicrobiaceae id.4036)

Batch 32 : Gut microbiota abundance (family Victivallaceae id.2255) on Crohn's disease of large intestine

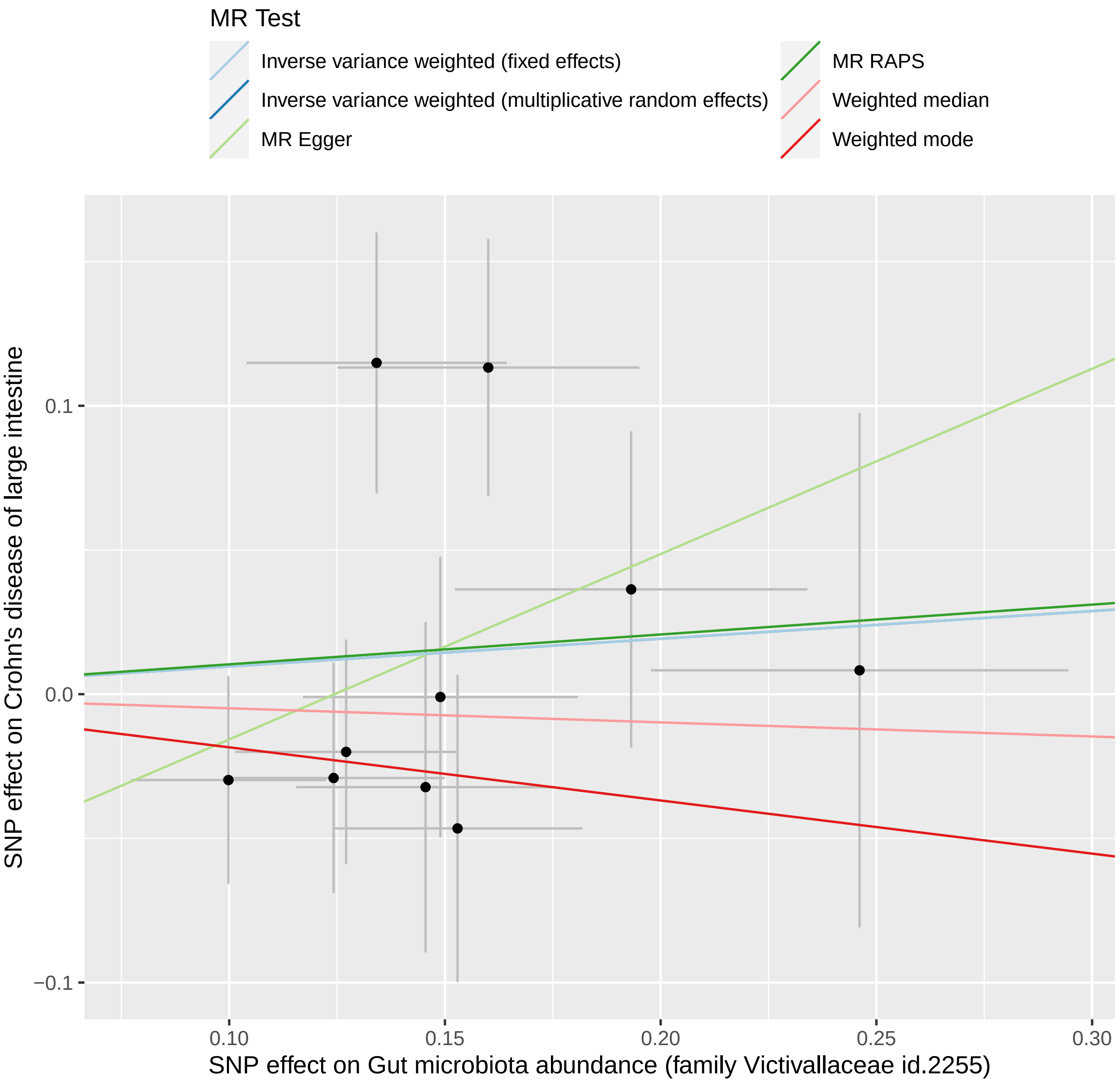

Batch 33 : Gut microbiota abundance (genus Actinomyces id.423) on Crohn's disease of large intestine

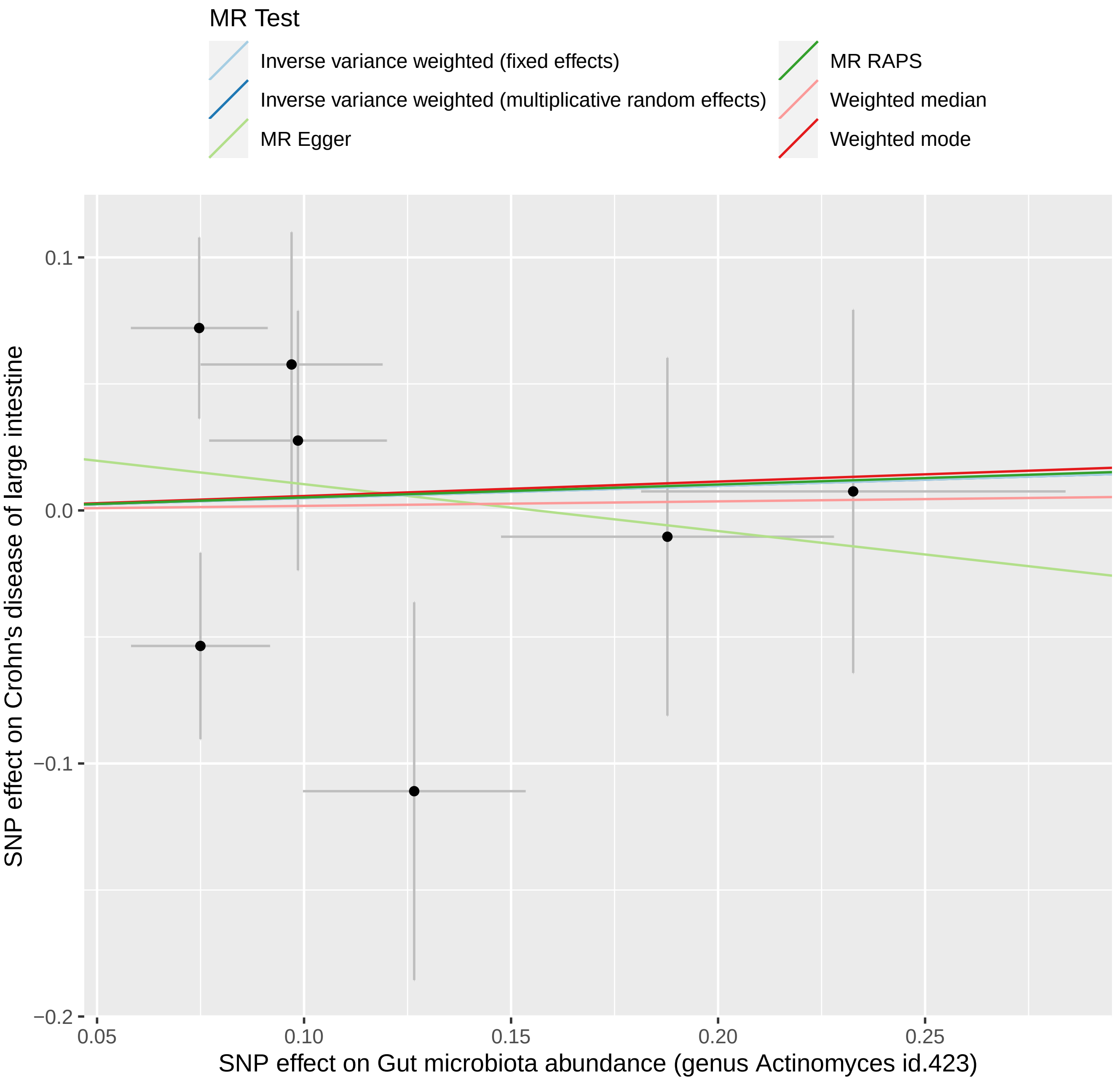

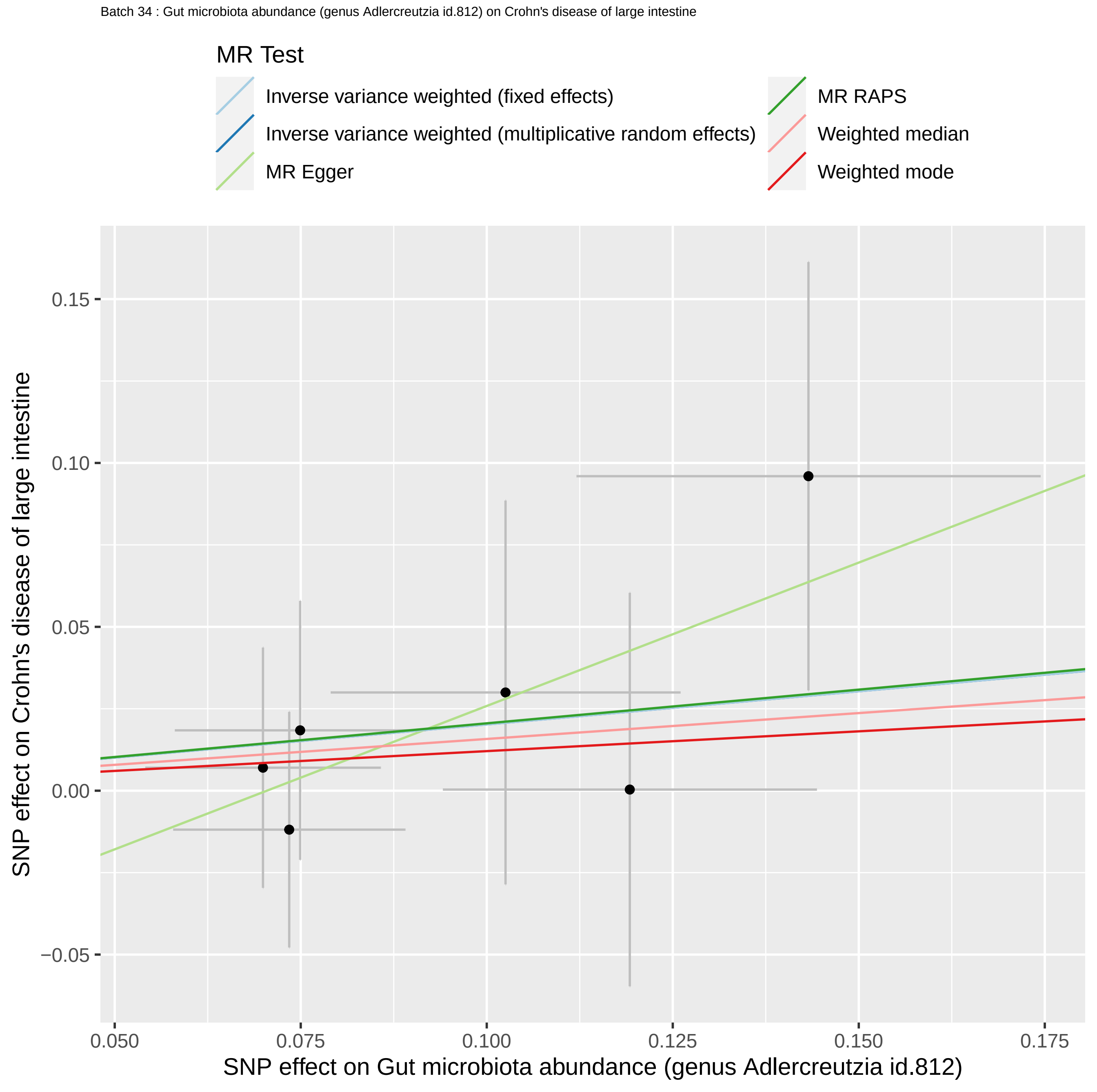
Batch 34 : Gut microbiota abundance (genus Adlercreutzia id.812) on Crohn's disease of large intestine
MR Test
Inverse variance weighted (fixed effects)
Inverse variance weighted (multiplicative random effects)
MR Egger
MR RAPS
Weighted median
Weighted mode
SNP effect on Crohn's disease of large intestine
0.15
0.10
0.05
0.00
−0.05
0.050
0.075
0.100
0.125
0.150
0.175
SNP effect on Gut microbiota abundance (genus Adlercreutzia id.812)

Batch 35 : Gut microbiota abundance (genus Akkermansia id.4037) on Crohn's disease of large intestine

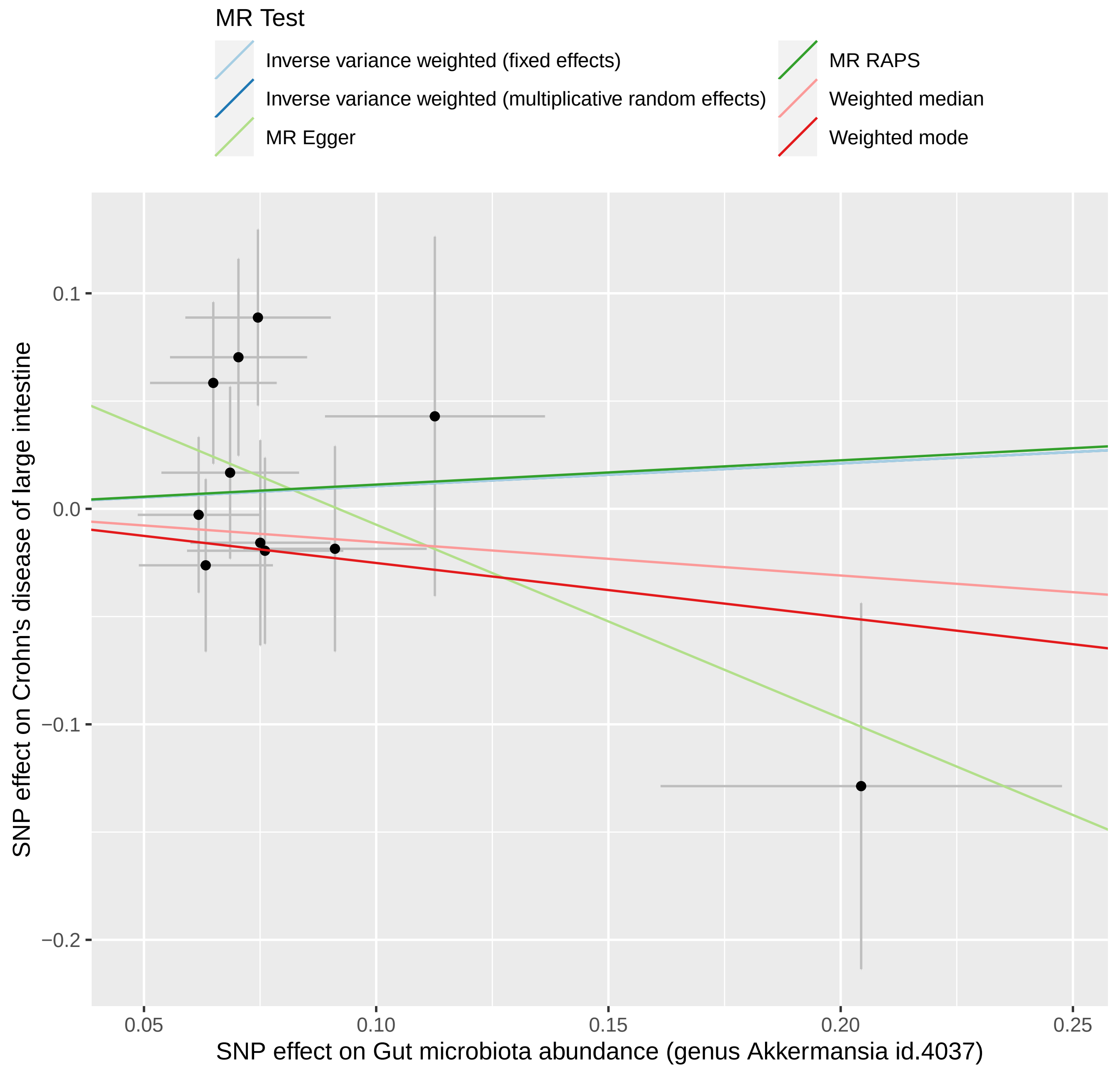

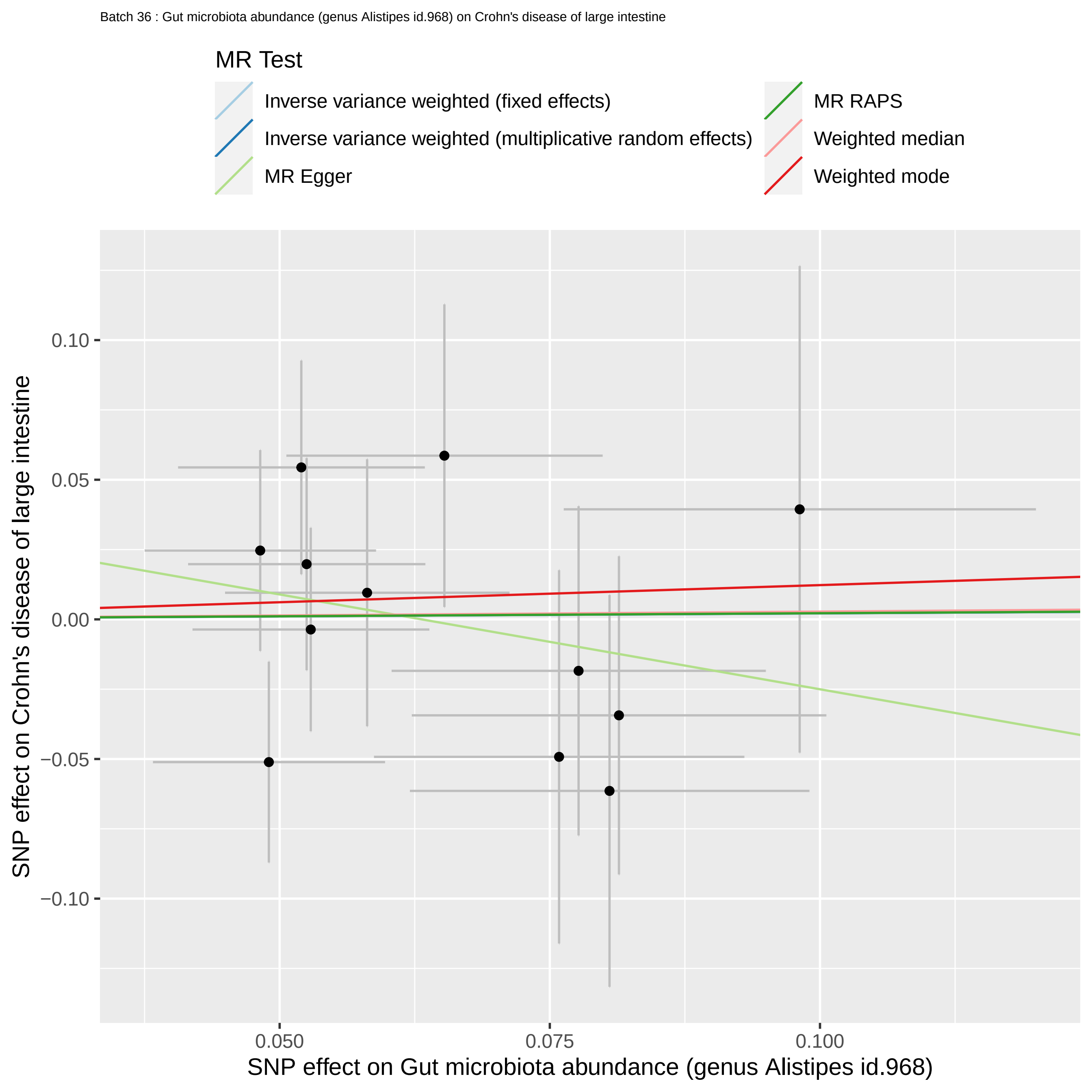

Batch 36 : Gut microbiota abundance (genus Alistipes id.968) on Crohn's disease of large intestine
MR Test
Inverse variance weighted (fixed effects)
Inverse variance weighted (multiplicative random effects)
MR Egger
MR RAPS
Weighted median
Weighted mode
0.10
0.05
0.00
−0.05
−0.10
0.050
0.075
0.100
SNP effect on Crohn's disease of large intestine
SNP effect on Gut microbiota abundance (genus Alistipes id.968)

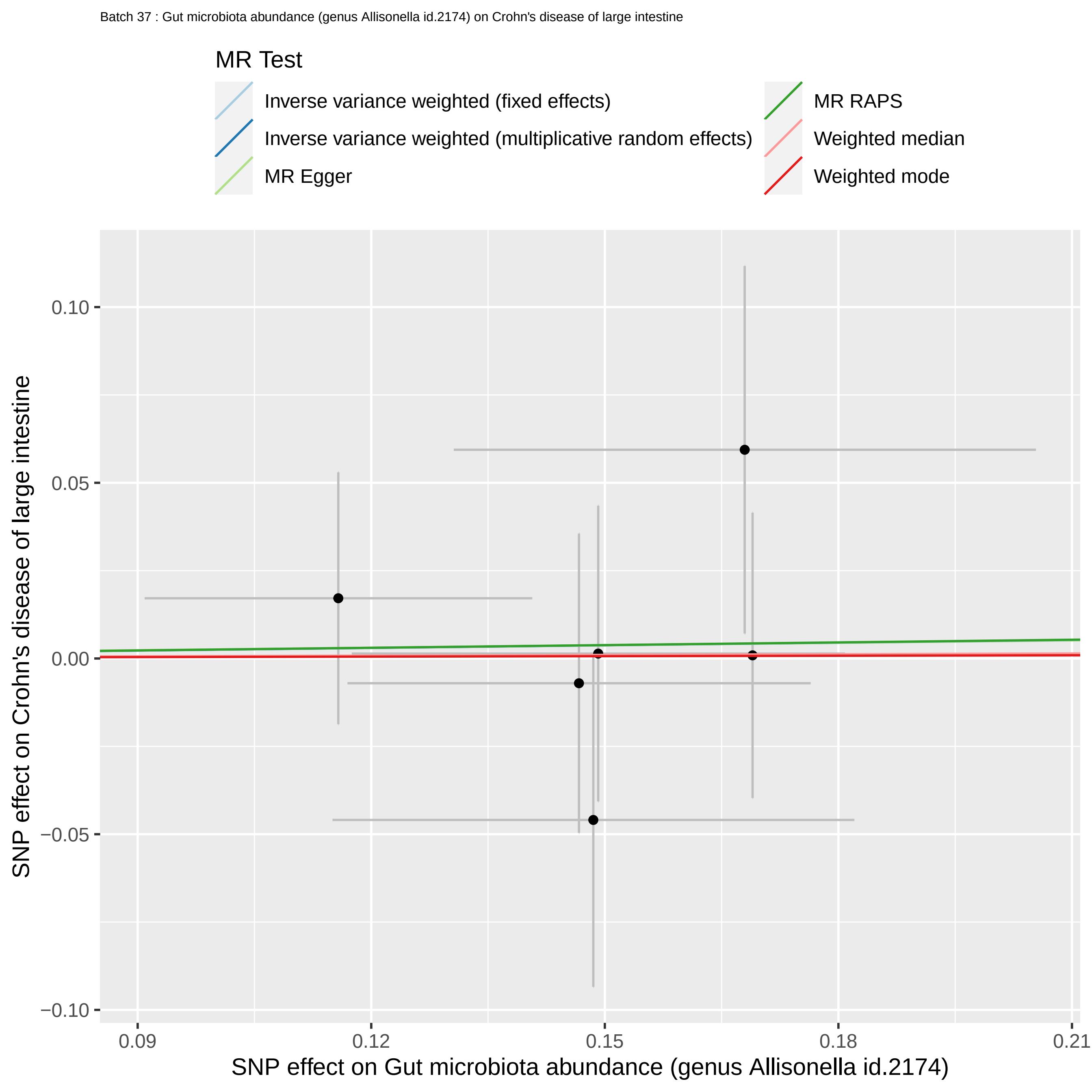
Batch 37 : Gut microbiota abundance (genus Allisonella id.2174) on Crohn's disease of large intestine
MR Test
Inverse variance weighted (fixed effects)
Inverse variance weighted (multiplicative random effects)
MR Egger
MR RAPS
Weighted median
Weighted mode
0.10
0.05
0.00
−0.05
−0.10
0.09
0.12
0.15
0.18
0.21
SNP effect on Crohn's disease of large intestine
SNP effect on Gut microbiota abundance (genus Allisonella id.2174)

Batch 38 : Gut microbiota abundance (genus Alloprevotella id.961) on Crohn's disease of large intestine

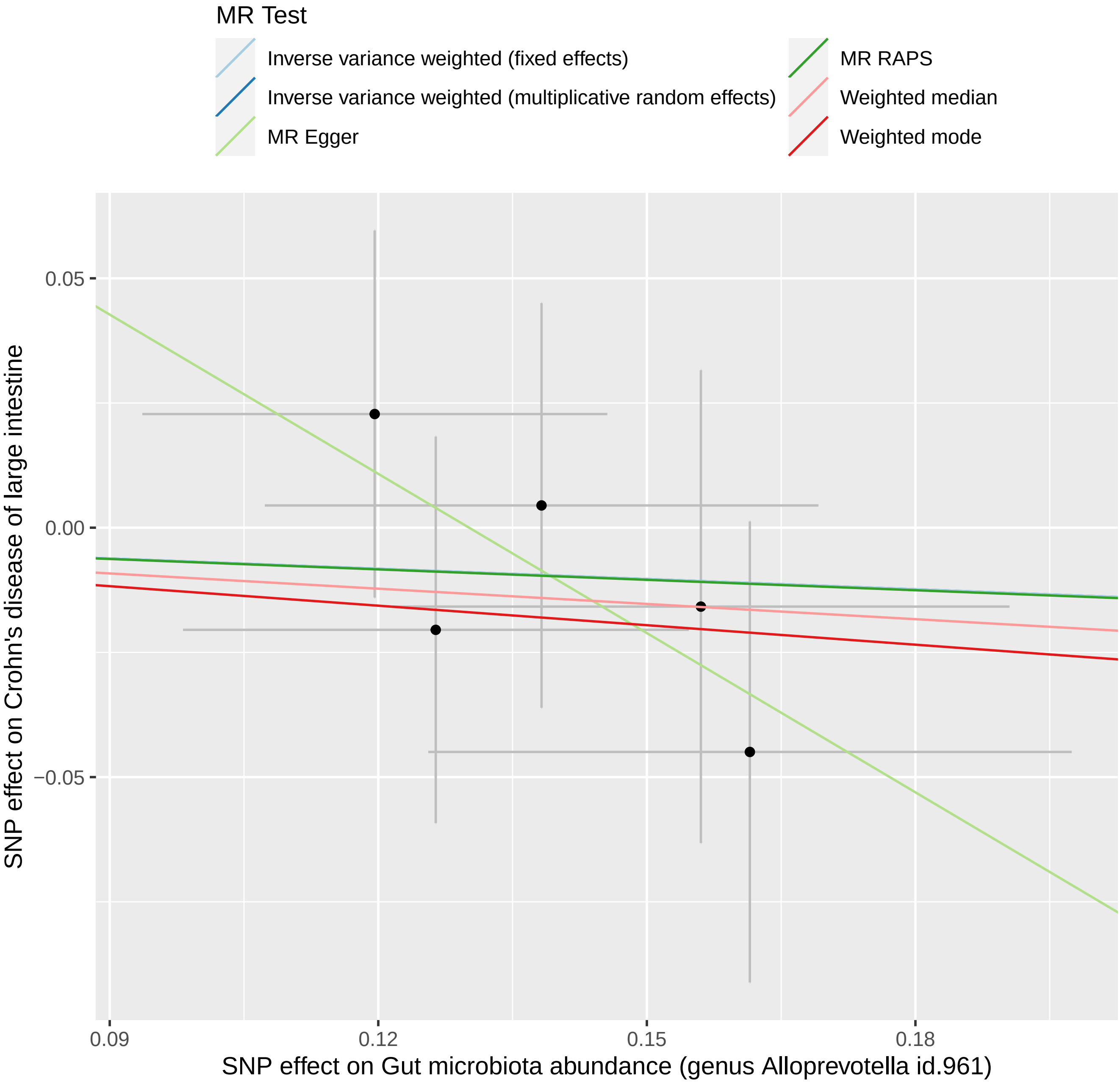

Batch 39 : Gut microbiota abundance (genus Anaerofilum id.2053) on Crohn's disease of large intestine

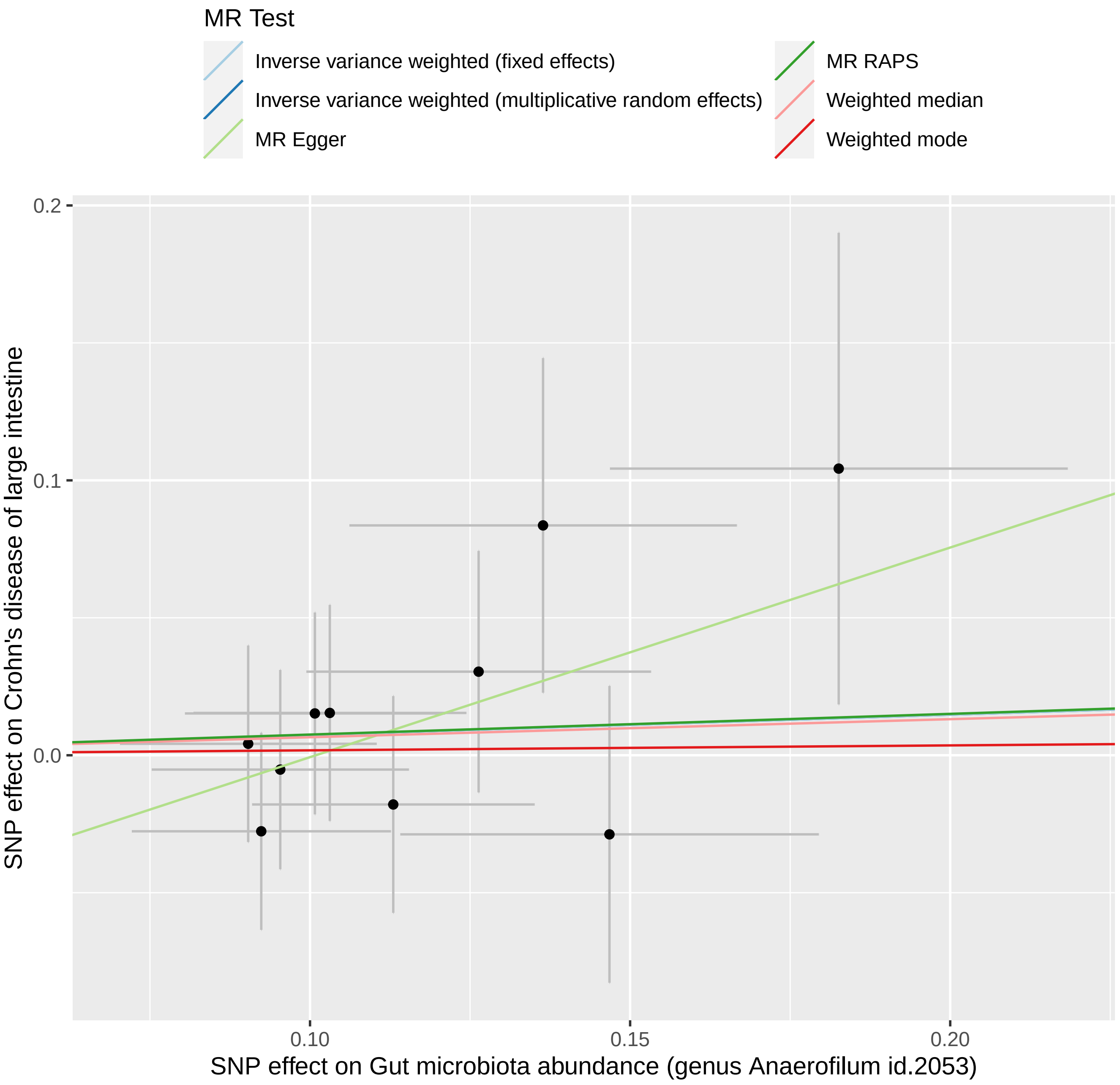

Batch 40 : Gut microbiota abundance (genus Anaerostipes id.1991) on Crohn's disease of large intestine

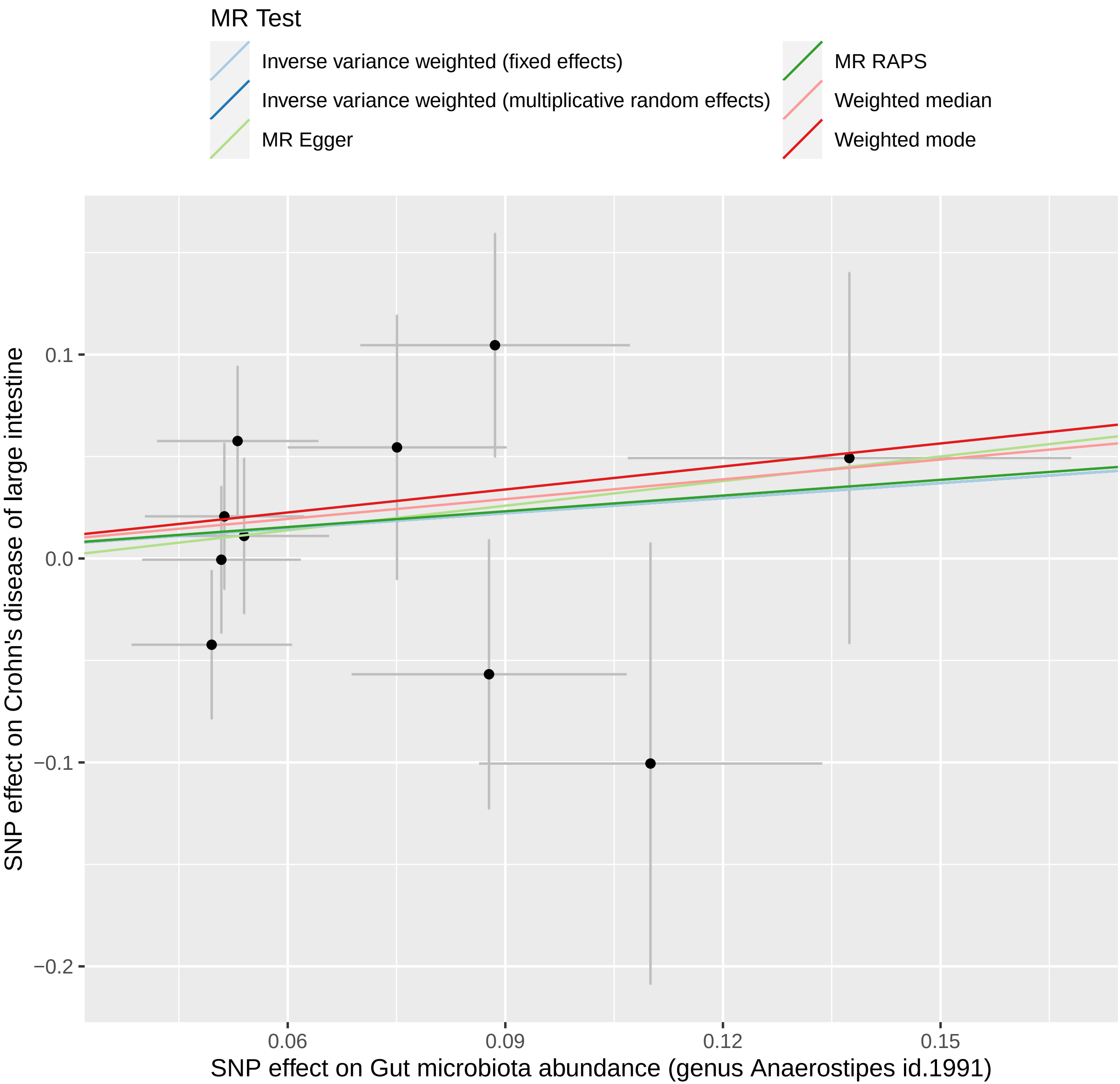

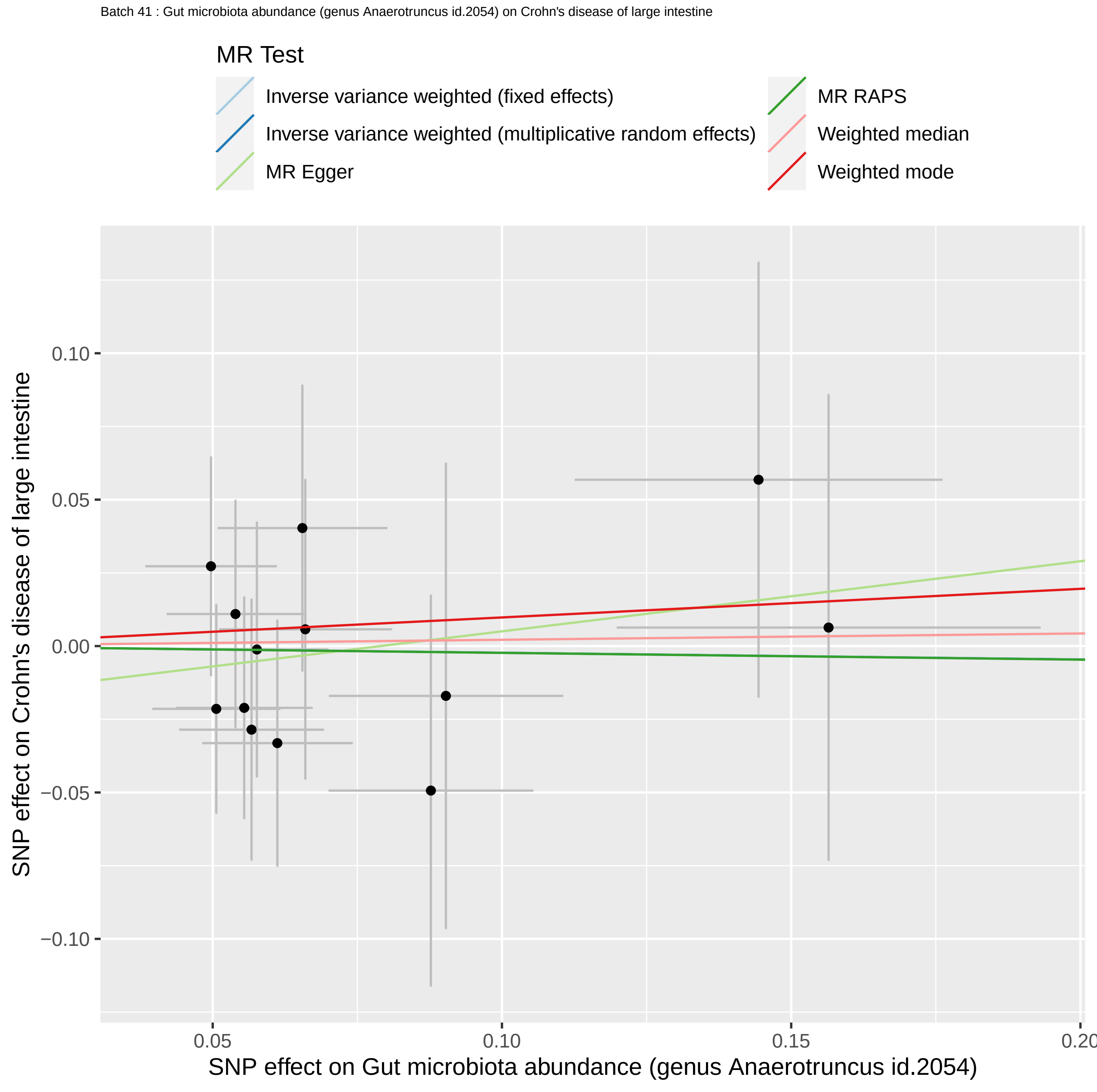

Batch 41 : Gut microbiota abundance (genus Anaerotruncus id.2054) on Crohn's disease of large intestine
MR Test
Inverse variance weighted (fixed effects)
Inverse variance weighted (multiplicative random effects)
MR Egger
MR RAPS
Weighted median
Weighted mode
0.10
0.05
0.00
−0.05
−0.10
SNP effect on Crohn's disease of large intestine
0.05
0.10
0.15
0.20
SNP effect on Gut microbiota abundance (genus Anaerotruncus id.2054)

Batch 42 : Gut microbiota abundance (genus Bacteroides id.918) on Crohn's disease of large intestine

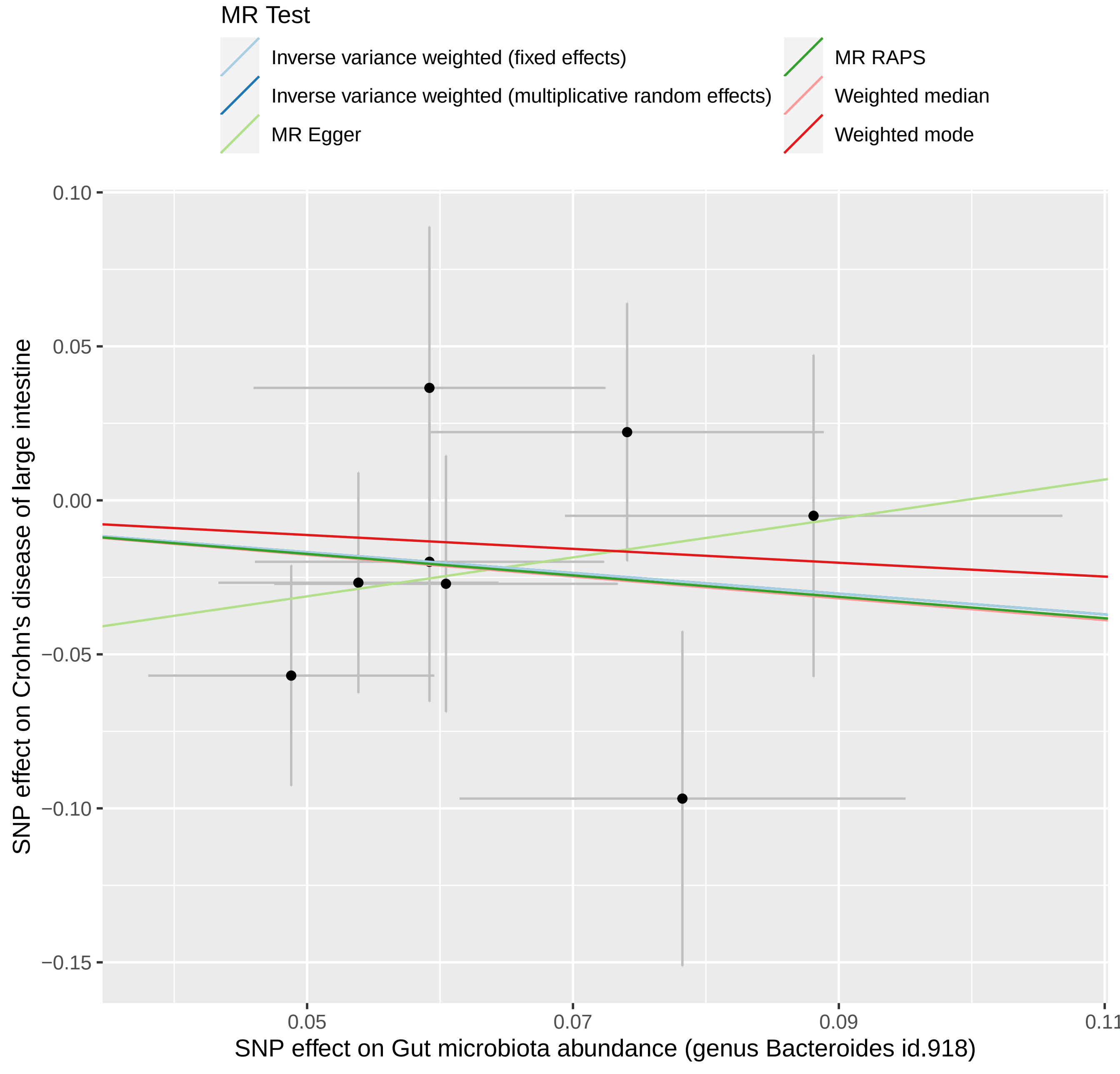

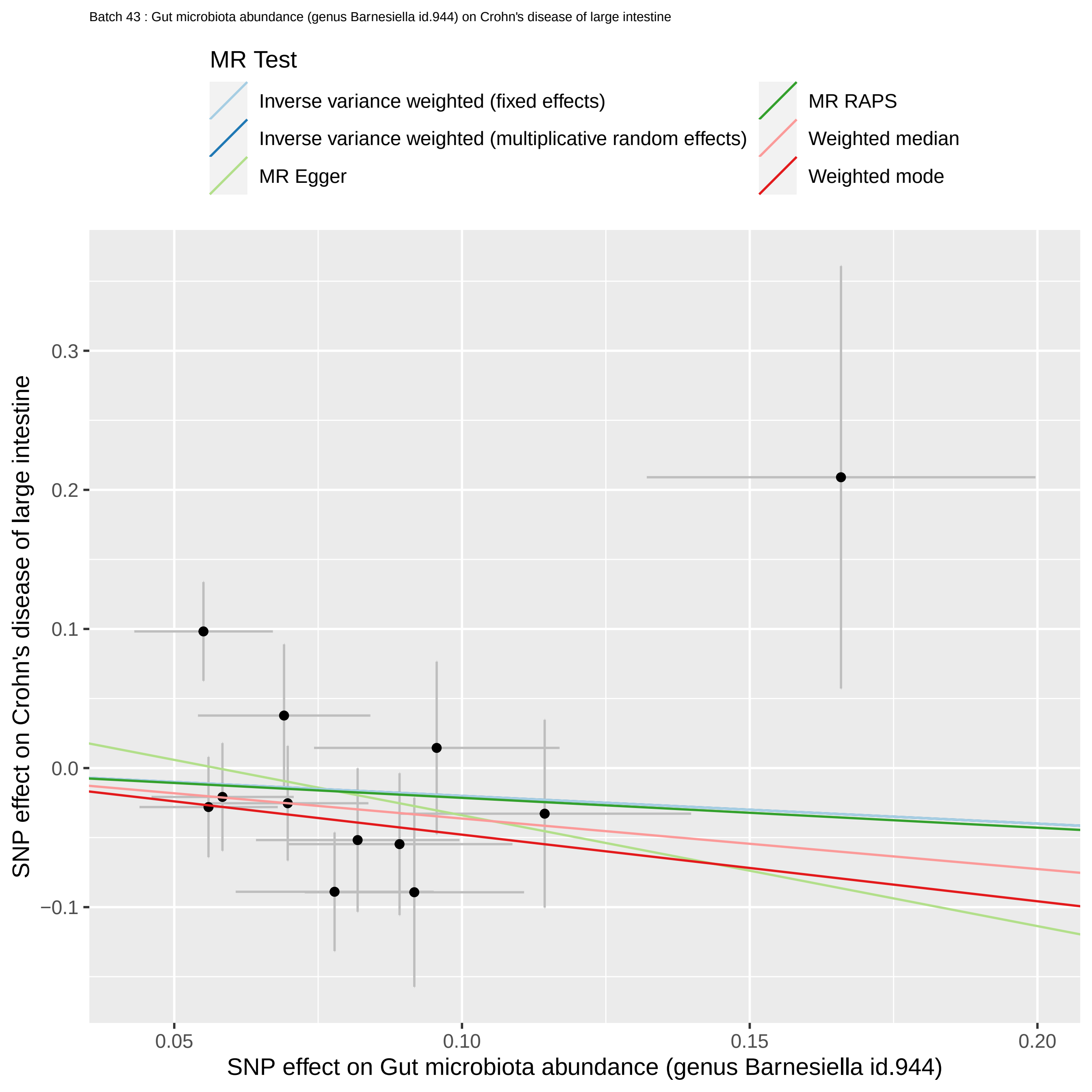

Batch 43 : Gut microbiota abundance (genus Barnesiella id.944) on Crohn's disease of large intestine
MR Test
Inverse variance weighted (fixed effects)
Inverse variance weighted (multiplicative random effects)
MR Egger
MR RAPS
Weighted median
Weighted mode
0.3
0.2
0.1
0.0
−0.1
0.05
0.10
0.15
0.20
SNP effect on Crohn's disease of large intestine
SNP effect on Gut microbiota abundance (genus Barnesiella id.944)

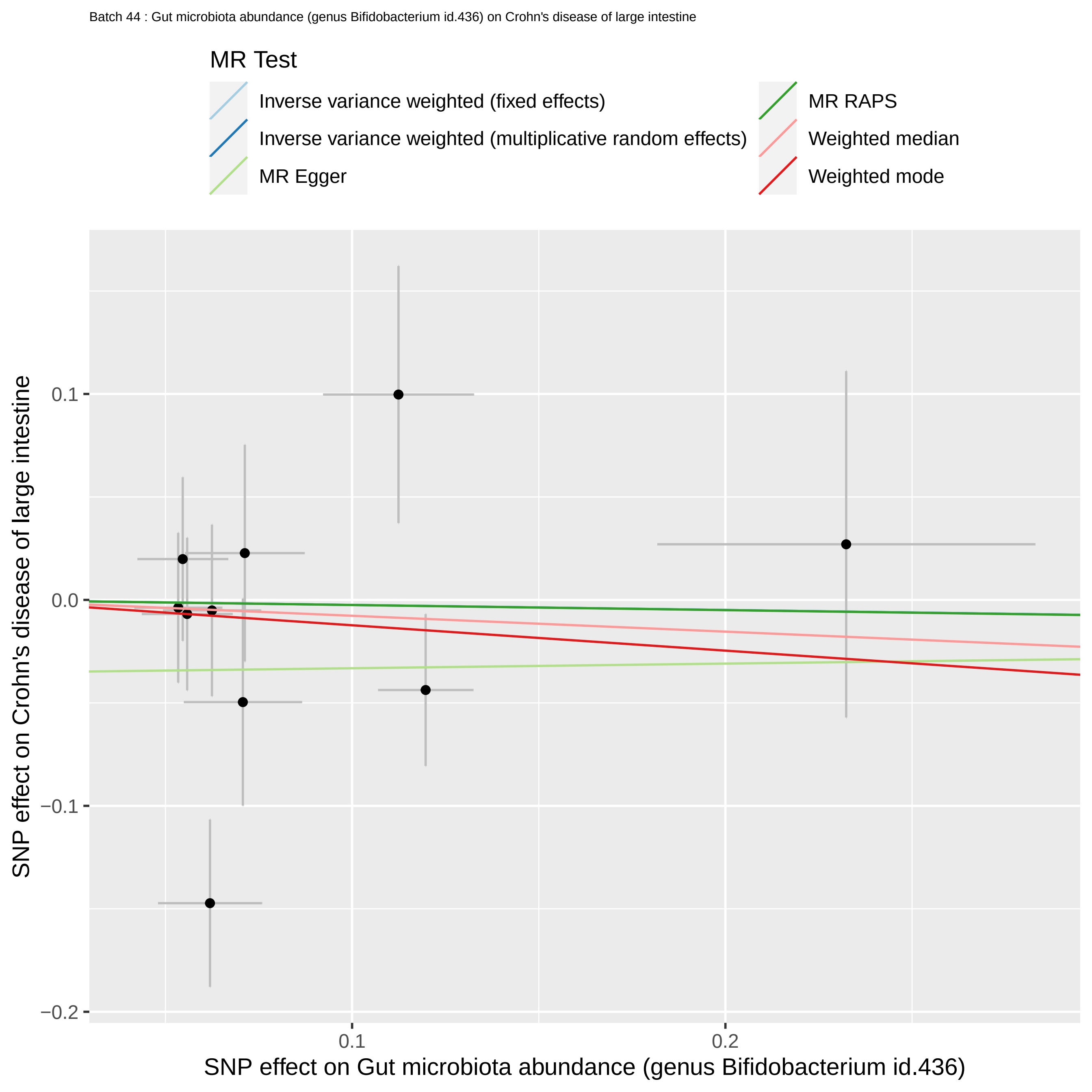
Batch 44 : Gut microbiota abundance (genus Bifidobacterium id.436) on Crohn's disease of large intestine
MR Test
Inverse variance weighted (fixed effects)
Inverse variance weighted (multiplicative random effects)
MR Egger
MR RAPS
Weighted median
Weighted mode
0.1
0.0
−0.1
−0.2
0.1
0.2
SNP effect on Crohn's disease of large intestine
SNP effect on Gut microbiota abundance (genus Bifidobacterium id.436)

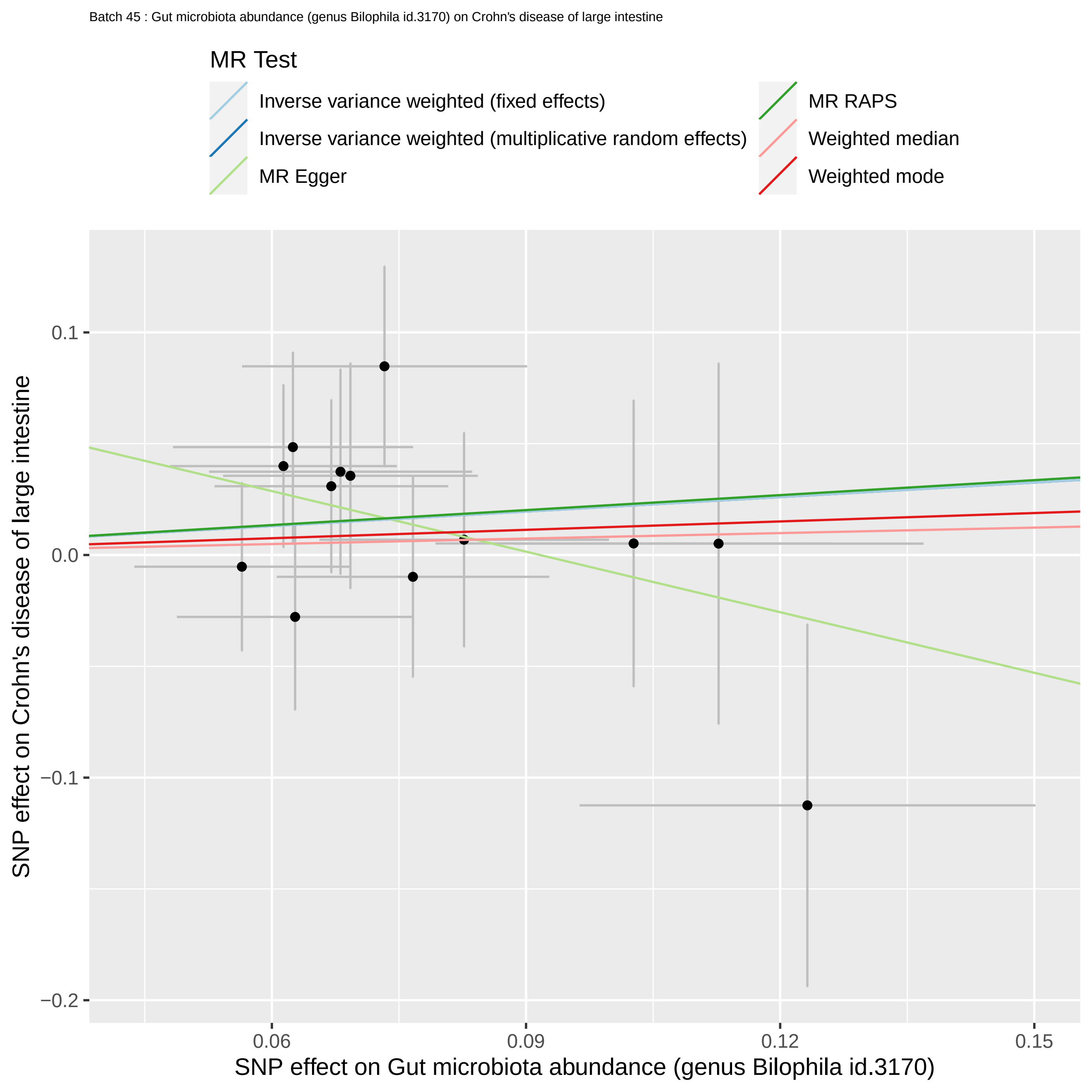
Batch 45 : Gut microbiota abundance (genus Bilophila id.3170) on Crohn's disease of large intestine
MR Test
Inverse variance weighted (fixed effects)
Inverse variance weighted (multiplicative random effects)
MR Egger
MR RAPS
Weighted median
Weighted mode
0.1
0.0
−0.1
−0.2
0.06
0.09
0.12
0.15
SNP effect on Crohn's disease of large intestine
SNP effect on Gut microbiota abundance (genus Bilophila id.3170)

Batch 46 : Gut microbiota abundance (genus Blautia id.1992) on Crohn's disease of large intestine

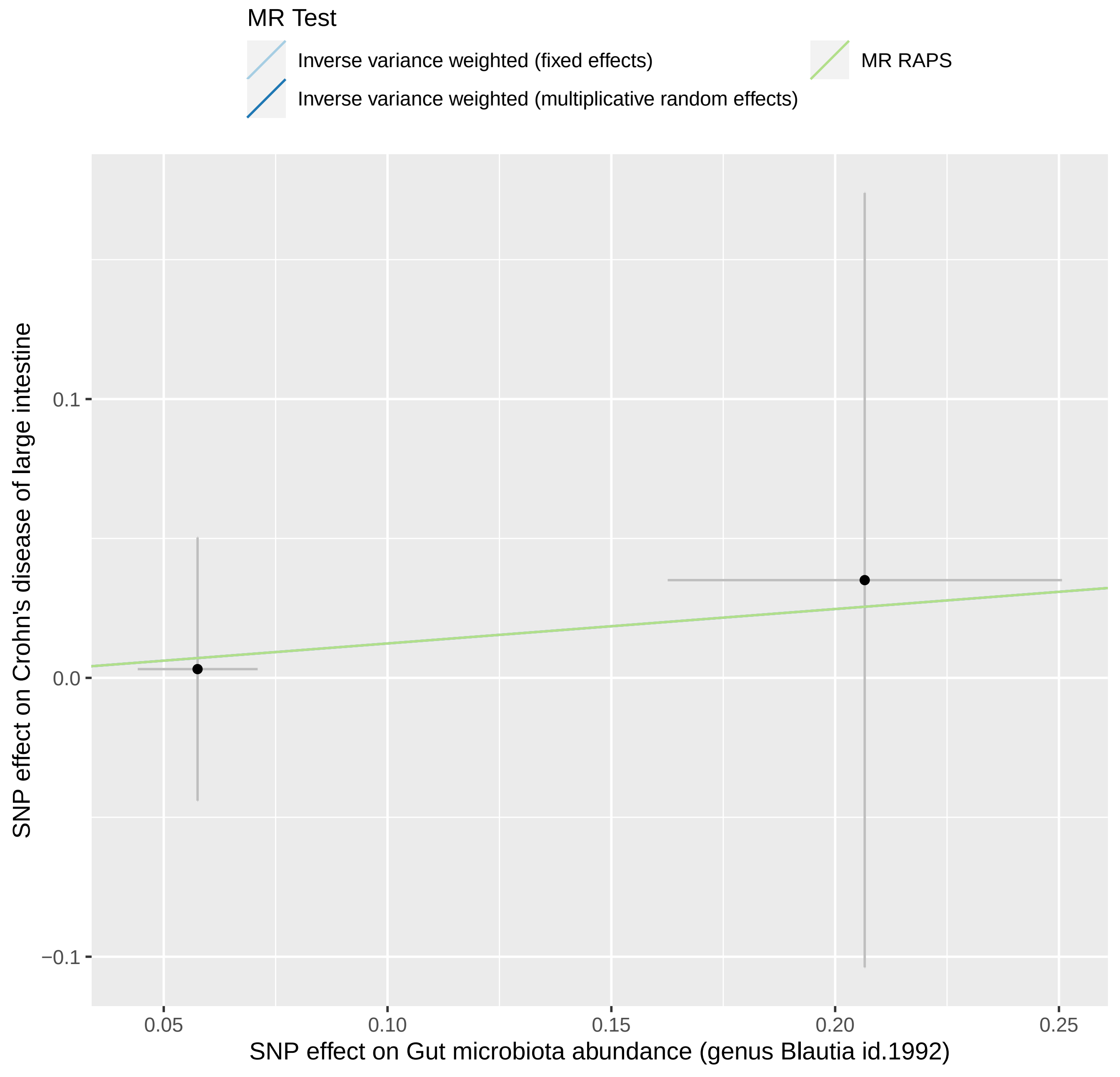

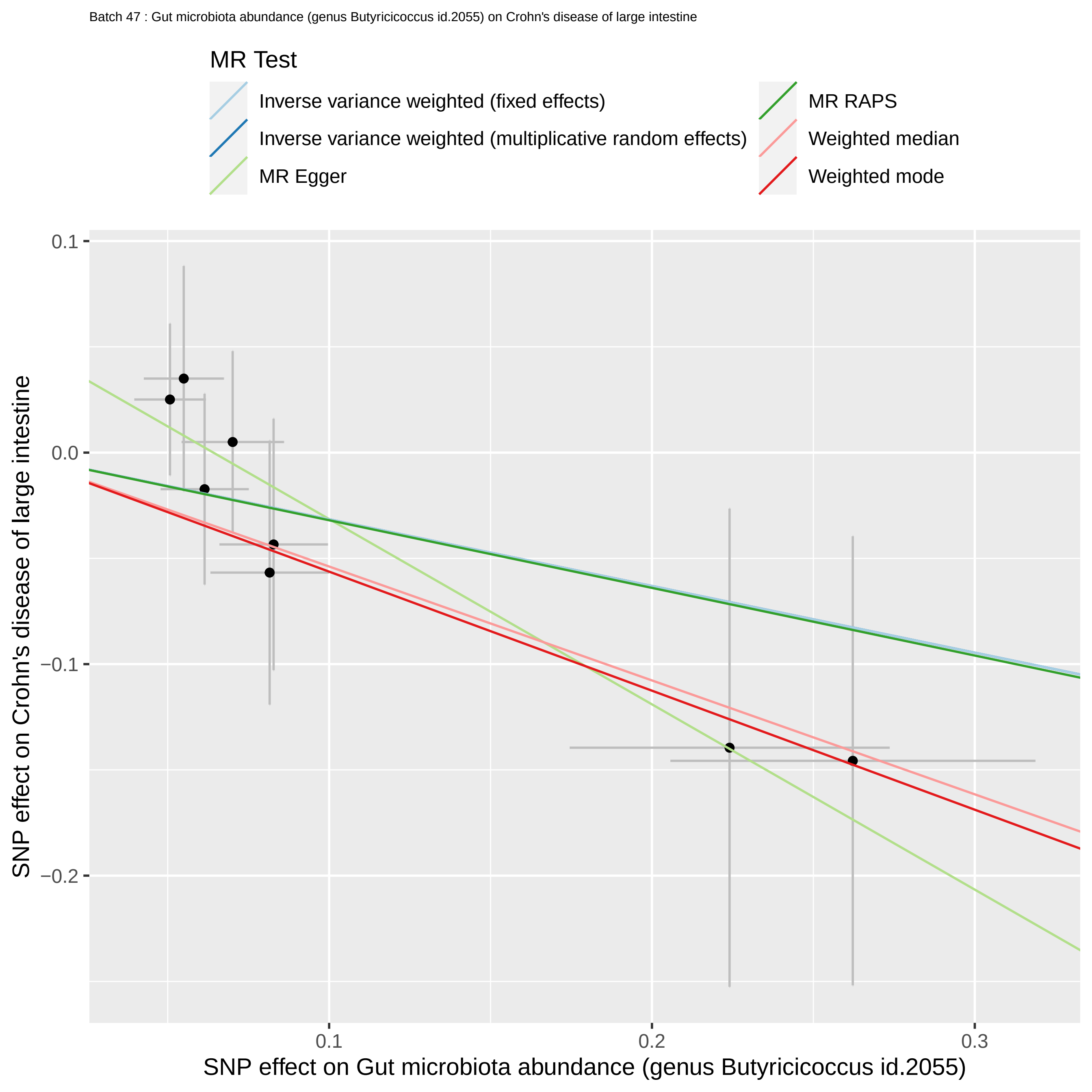
Batch 47 : Gut microbiota abundance (genus Butyricicoccus id.2055) on Crohn's disease of large intestine
MR Test
Inverse variance weighted (fixed effects)
Inverse variance weighted (multiplicative random effects)
MR Egger
MR RAPS
Weighted median
Weighted mode
0.1
0.0
−0.1
−0.2
0.1
0.2
0.3
SNP effect on Crohn's disease of large intestine
SNP effect on Gut microbiota abundance (genus Butyricicoccus id.2055)

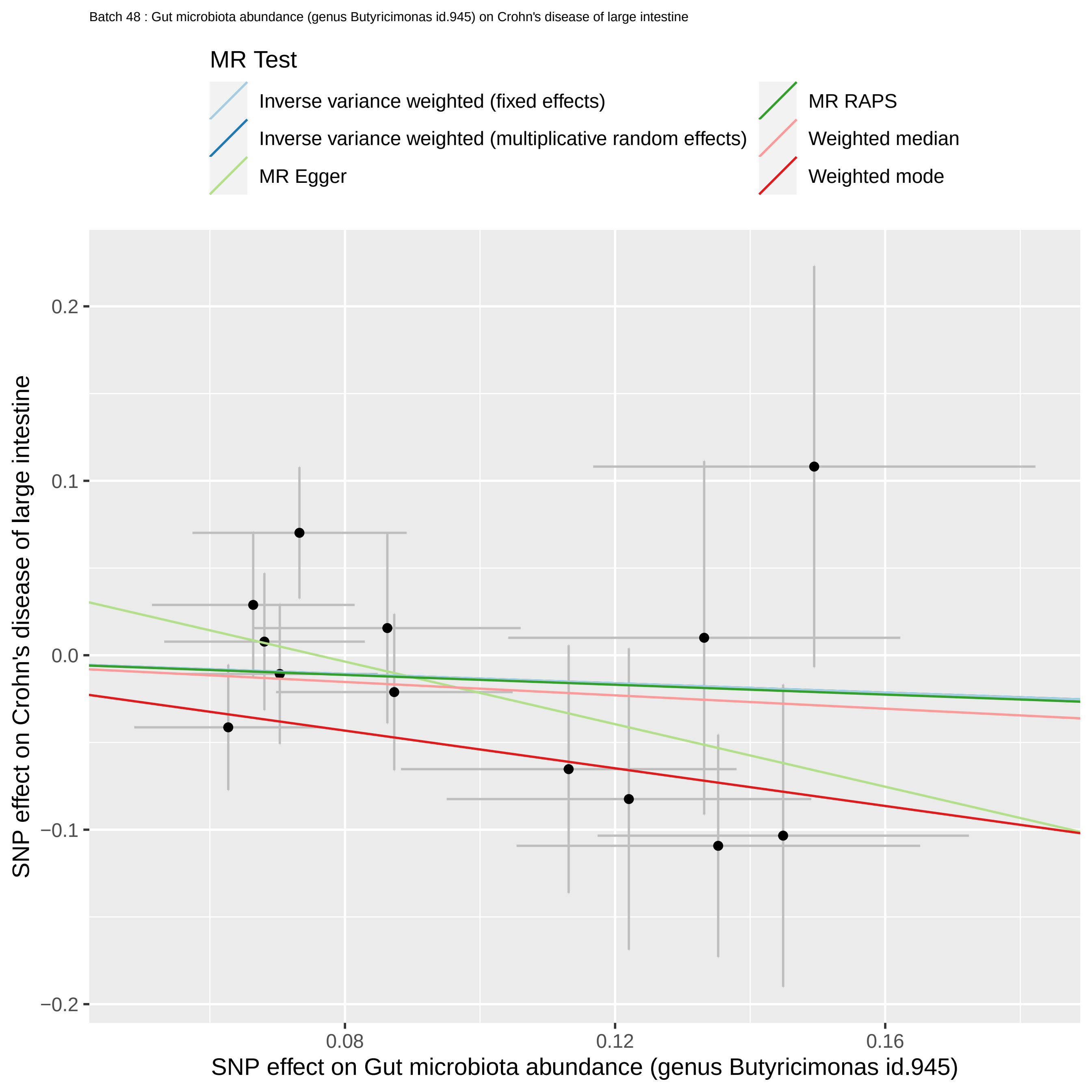
Batch 48 : Gut microbiota abundance (genus Butyricimonas id.945) on Crohn's disease of large intestine
MR Test
Inverse variance weighted (fixed effects)
Inverse variance weighted (multiplicative random effects)
MR Egger
MR RAPS
Weighted median
Weighted mode
SNP effect on Crohn's disease of large intestine
0.2
0.1
0.0
−0.1
−0.2
0.08
0.12
0.16
SNP effect on Gut microbiota abundance (genus Butyricimonas id.945)

Batch 49 : Gut microbiota abundance (genus Butyrivibrio id.1993) on Crohn's disease of large intestine

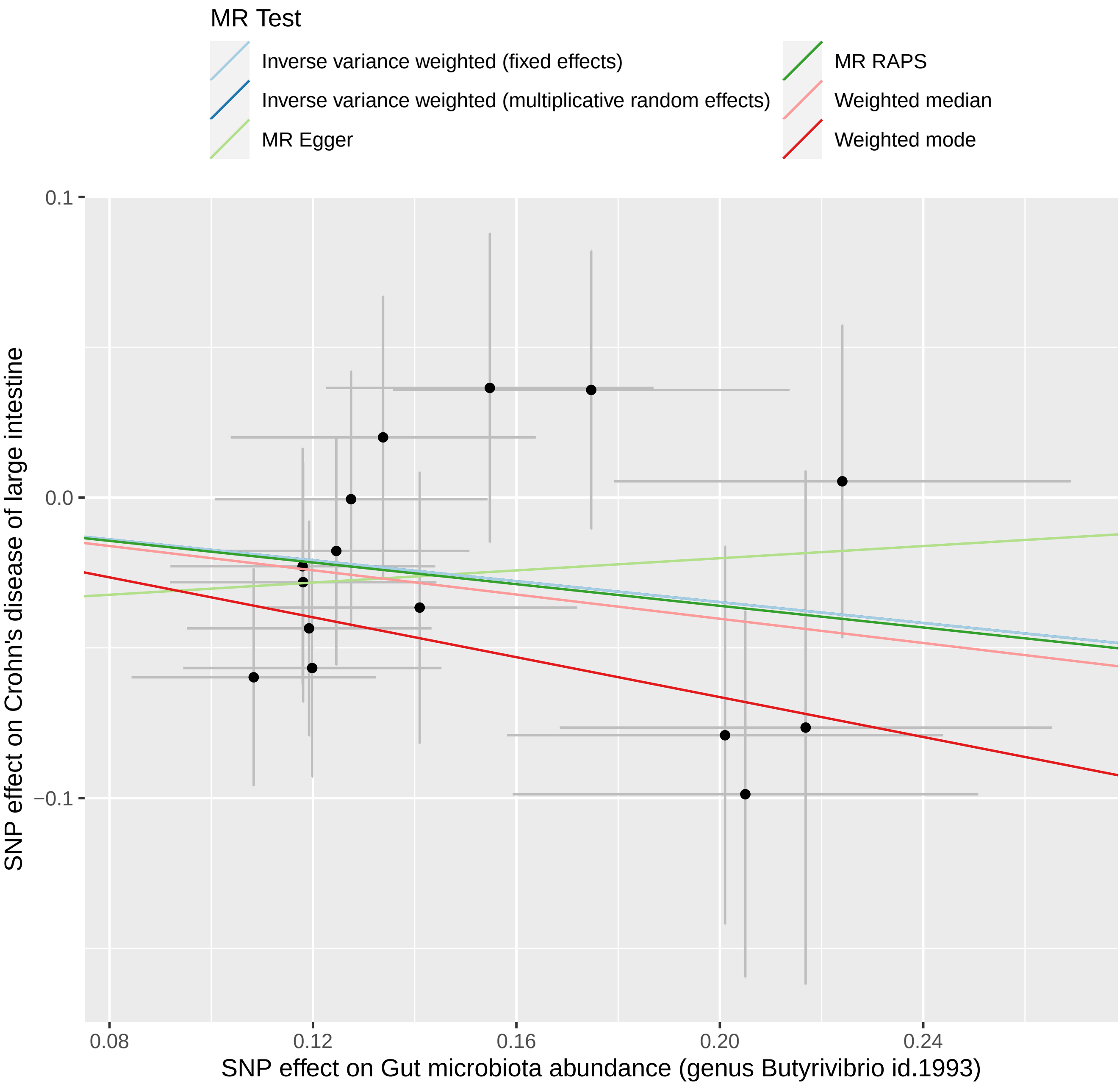

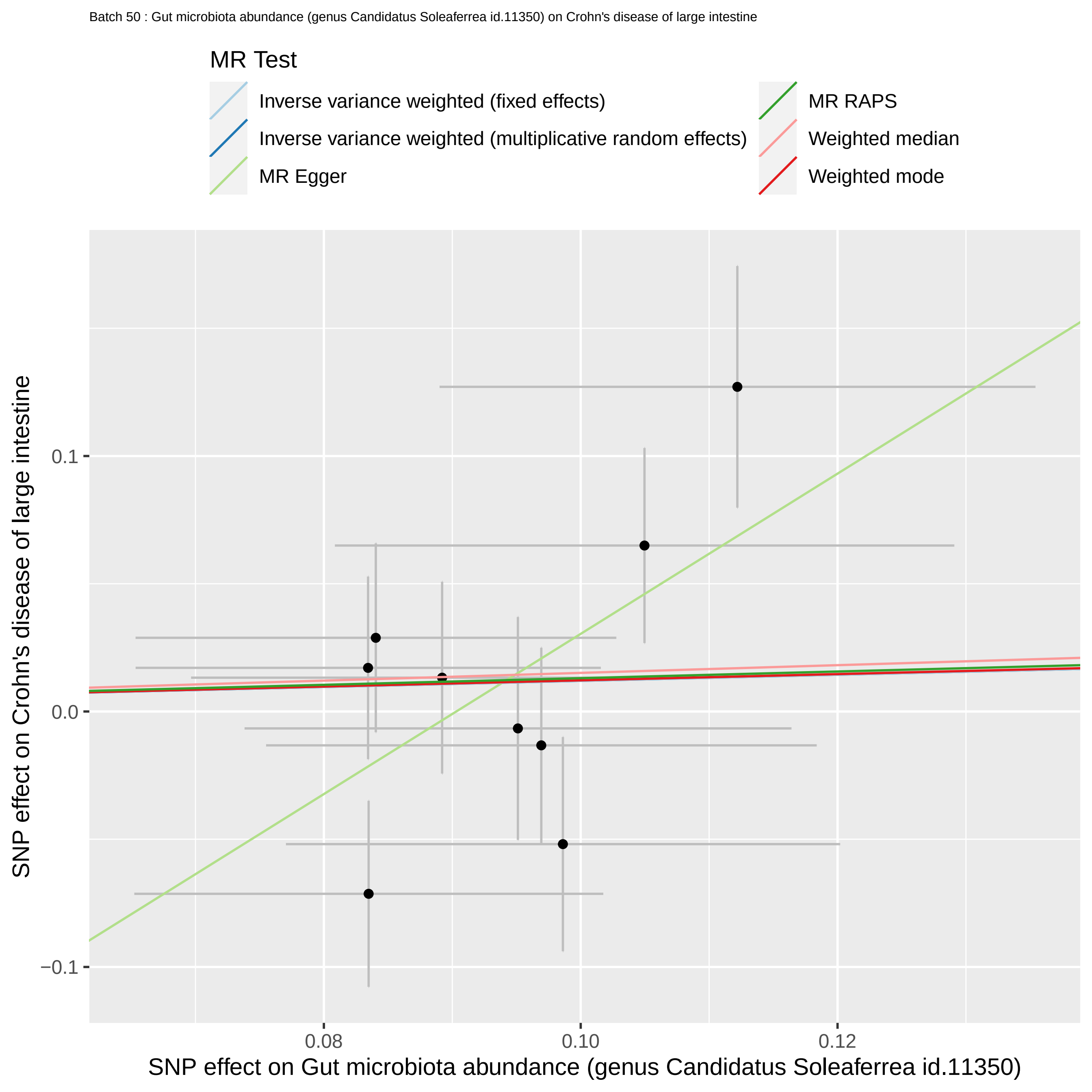
Batch 50 : Gut microbiota abundance (genus Candidatus Soleaferrea id.11350) on Crohn's disease of large intestine
MR Test
Inverse variance weighted (fixed effects)
Inverse variance weighted (multiplicative random effects)
MR Egger
MR RAPS
Weighted median
Weighted mode
SNP effect on Crohn's disease of large intestine
0.1
0.0
−0.1
0.08
0.10
0.12
SNP effect on Gut microbiota abundance (genus Candidatus Soleaferrea id.11350)

Batch 51 : Gut microbiota abundance (genus Catenibacterium id.2153) on Crohn's disease of large intestine

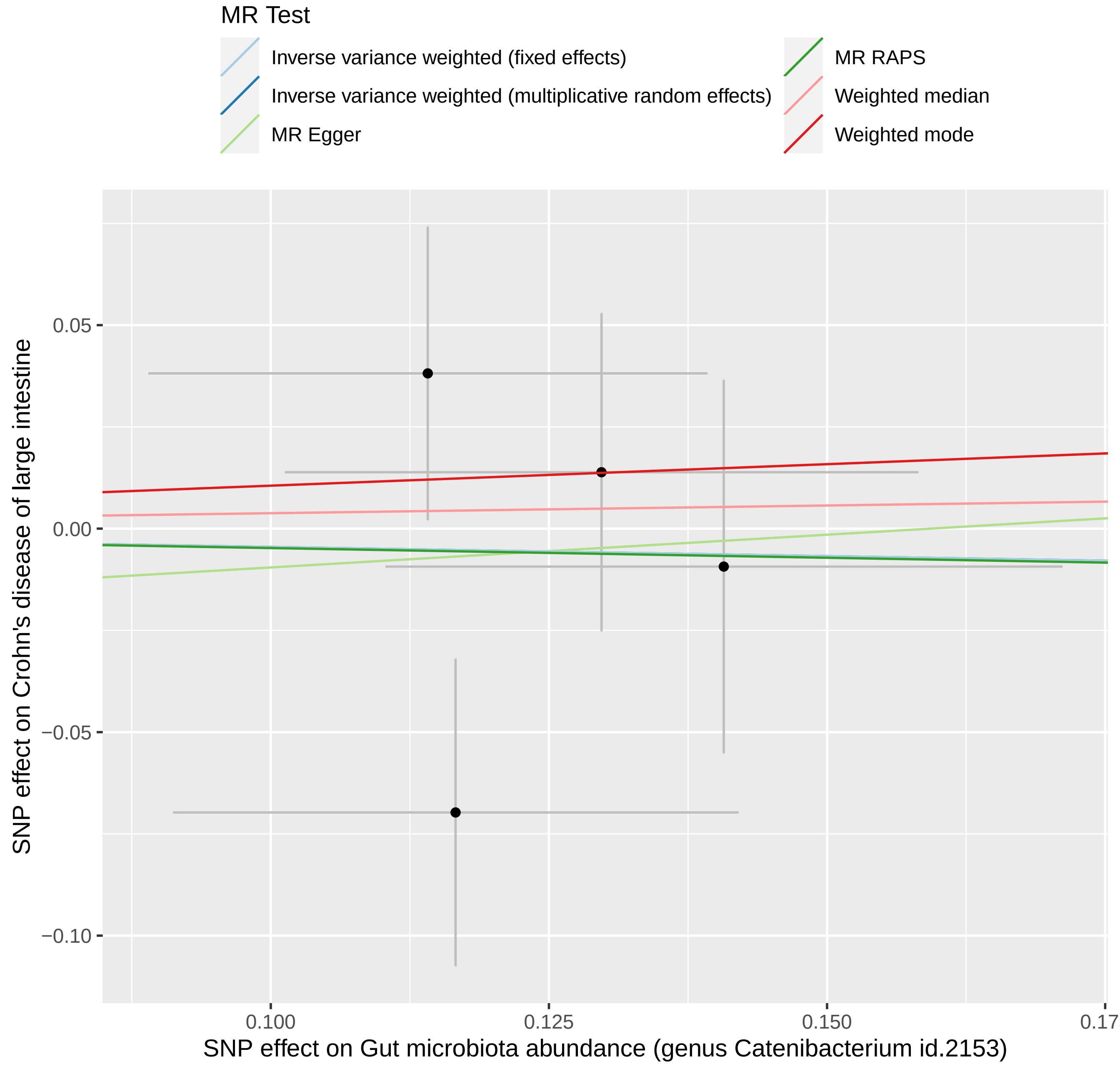

Batch 52 : Gut microbiota abundance (genus Christensenellaceae R 7group id.11283) on Crohn's disease of large intestine

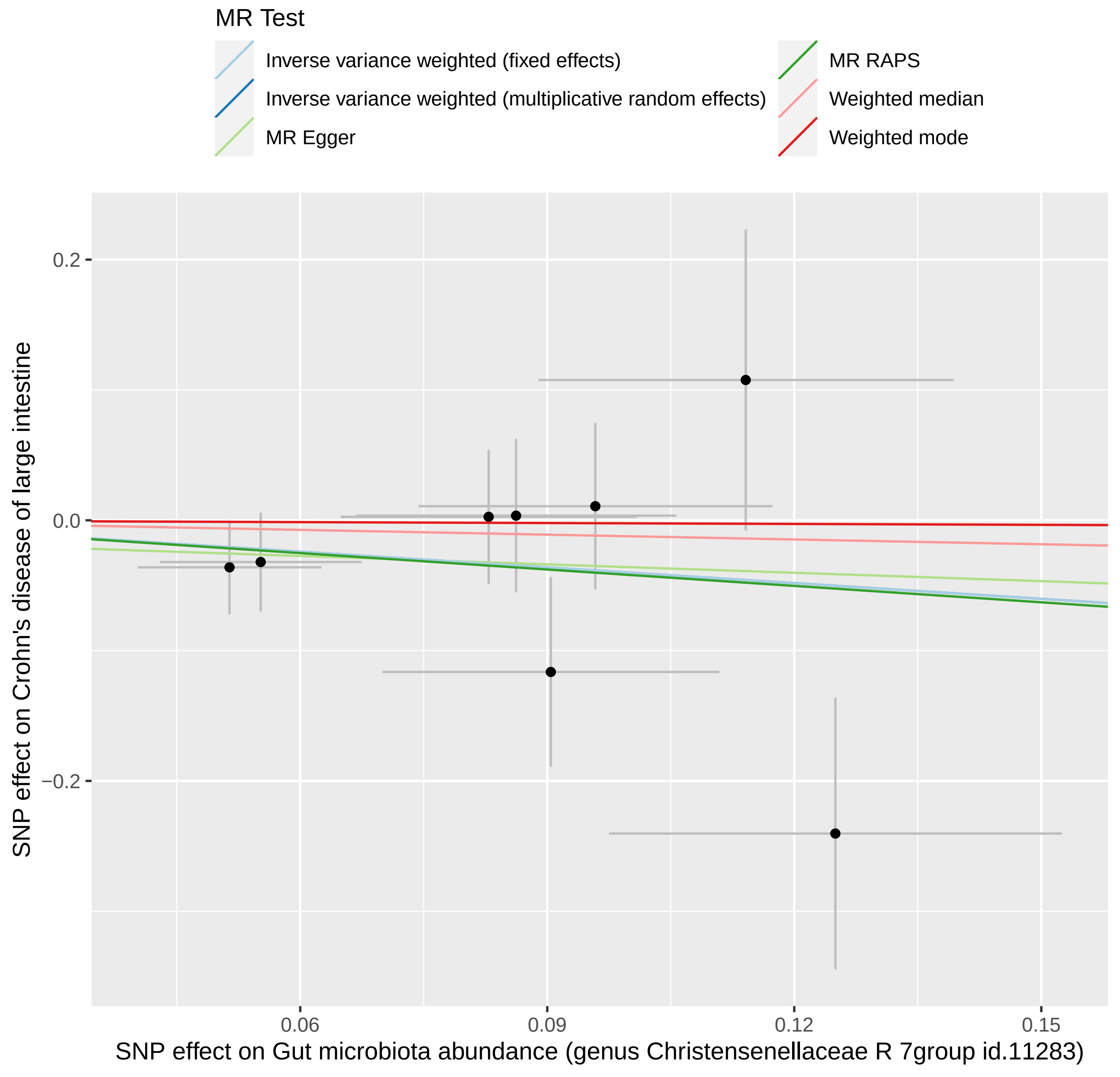

Batch 53 : Gut microbiota abundance (genus Clostridium innocuum group id.14397) on Crohn's disease of large intestine

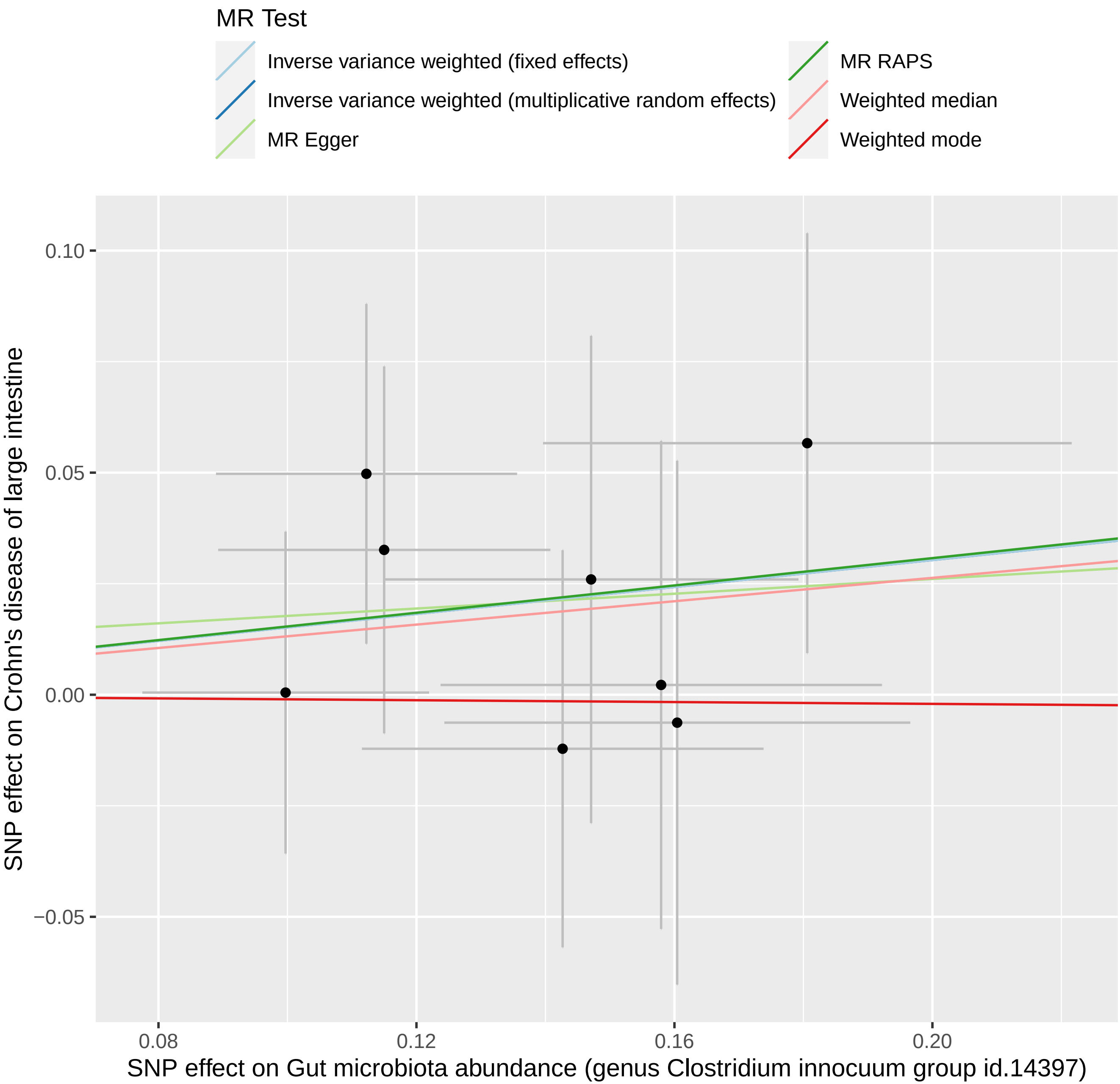

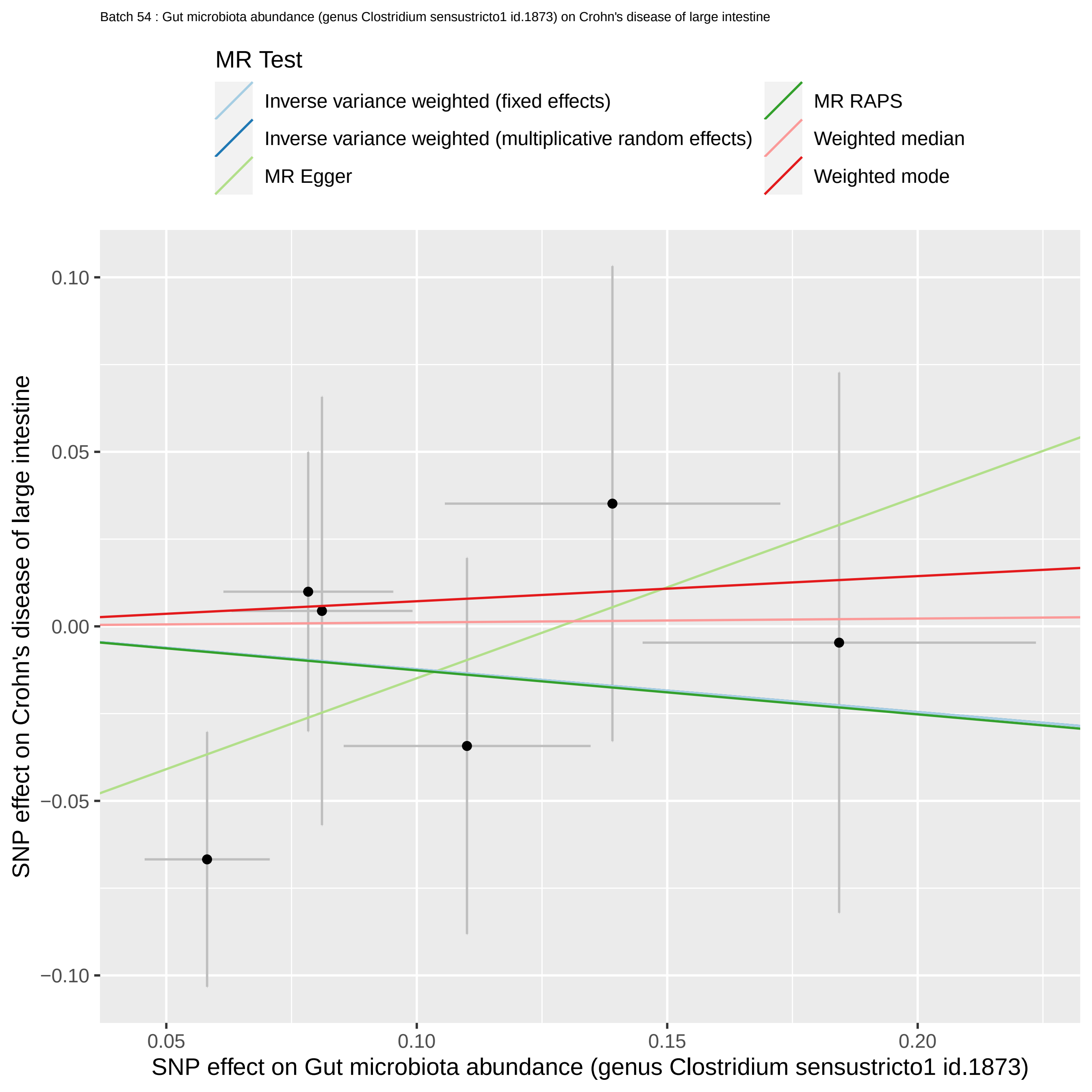

Batch 54 : Gut microbiota abundance (genus Clostridium sensustricto1 id.1873) on Crohn's disease of large intestine
MR Test
Inverse variance weighted (fixed effects)
Inverse variance weighted (multiplicative random effects)
MR Egger
MR RAPS
Weighted median
Weighted mode
0.10
0.05
0.00
−0.05
−0.10
0.05
0.10
0.15
0.20
SNP effect on Crohn's disease of large intestine
SNP effect on Gut microbiota abundance (genus Clostridium sensustricto1 id.1873)

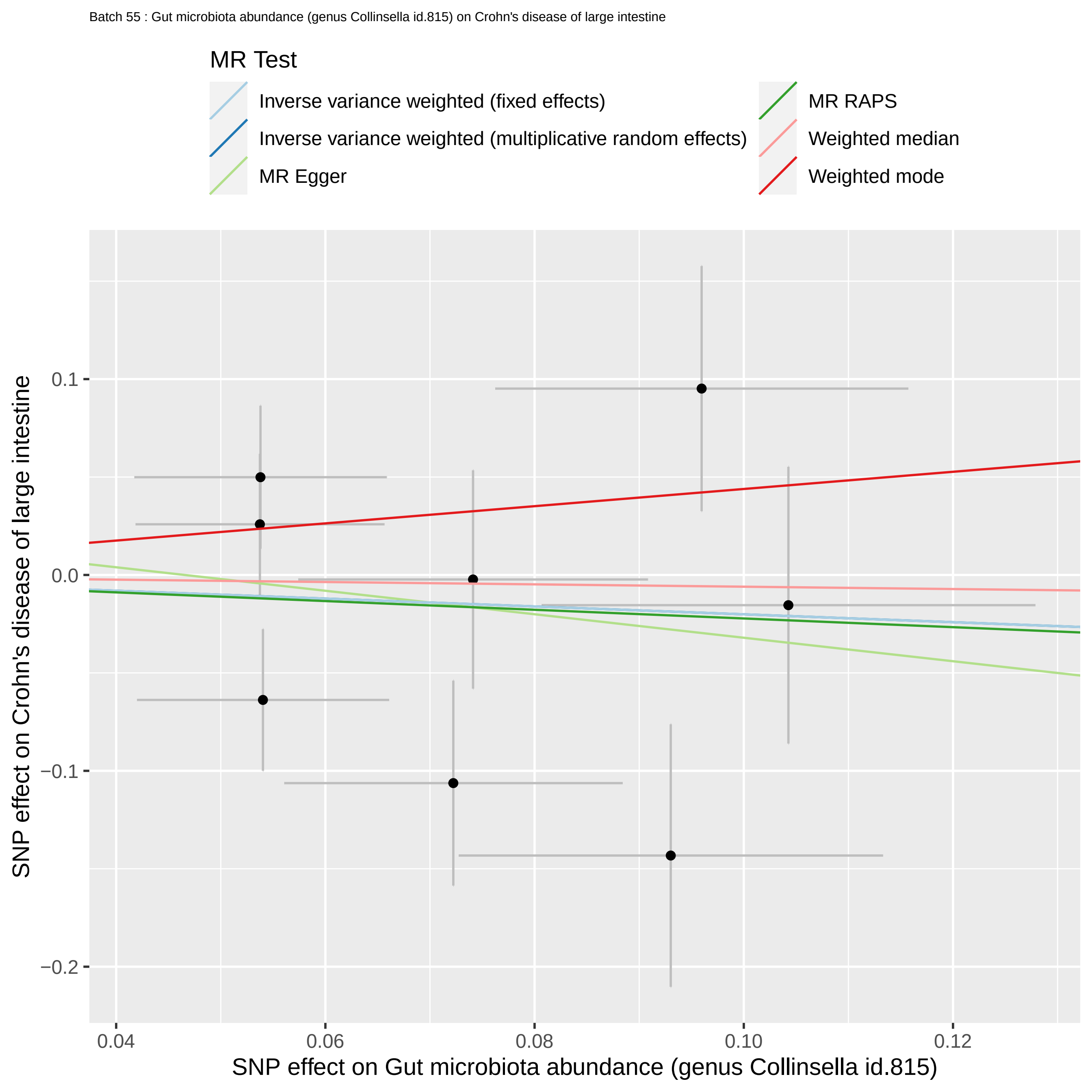

Batch 55 : Gut microbiota abundance (genus Collinsella id.815) on Crohn's disease of large intestine
MR Test
Inverse variance weighted (fixed effects)
Inverse variance weighted (multiplicative random effects)
MR Egger
MR RAPS
Weighted median
Weighted mode
0.1
0.0
−0.1
−0.2
0.04
0.06
0.08
0.10
0.12
SNP effect on Crohn's disease of large intestine
SNP effect on Gut microbiota abundance (genus Collinsella id.815)

Batch 56 : Gut microbiota abundance (genus Coprobacter id.949) on Crohn's disease of large intestine

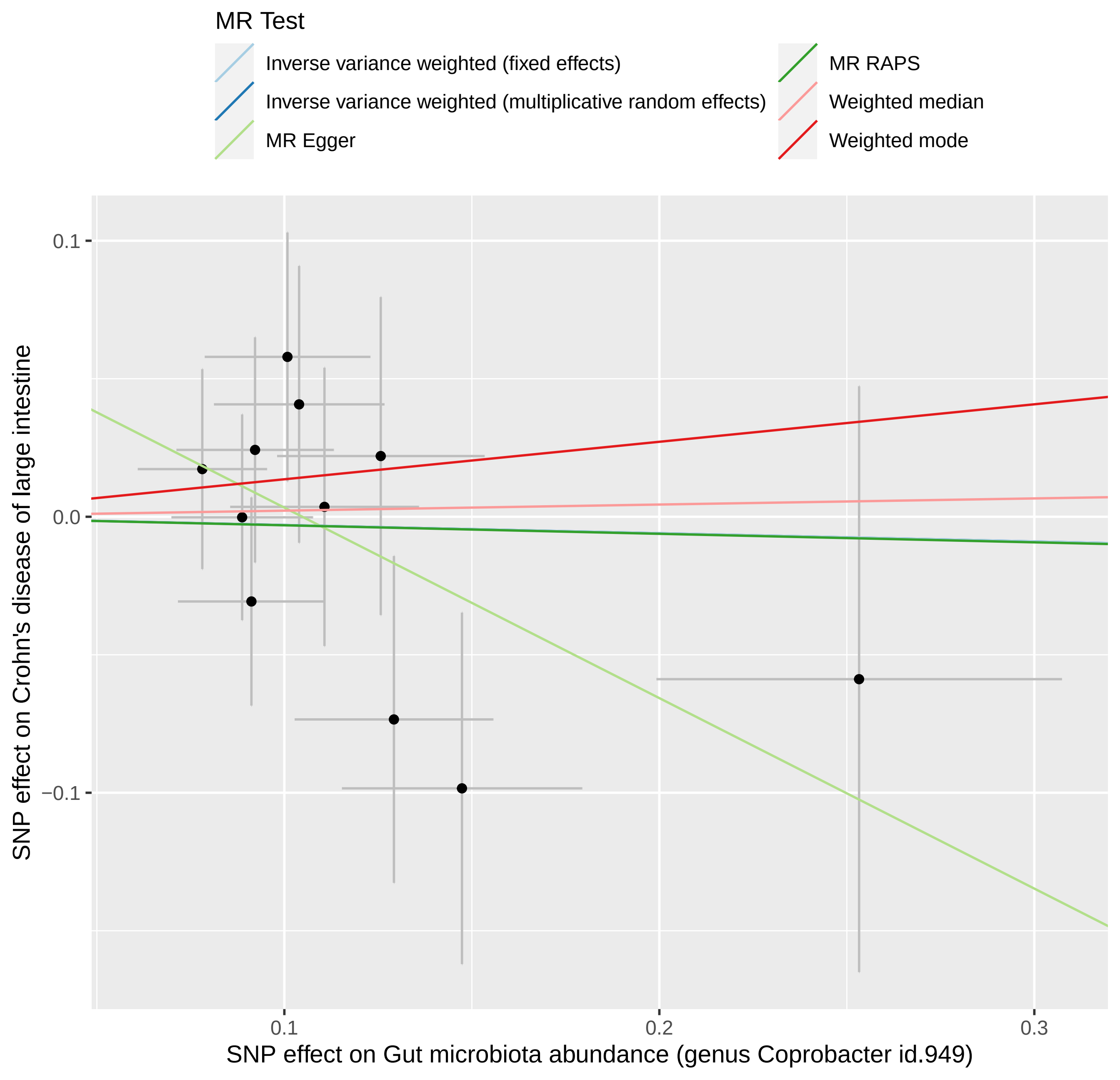

Batch 57 : Gut microbiota abundance (genus Coprococcus1 id.11301) on Crohn's disease of large intestine

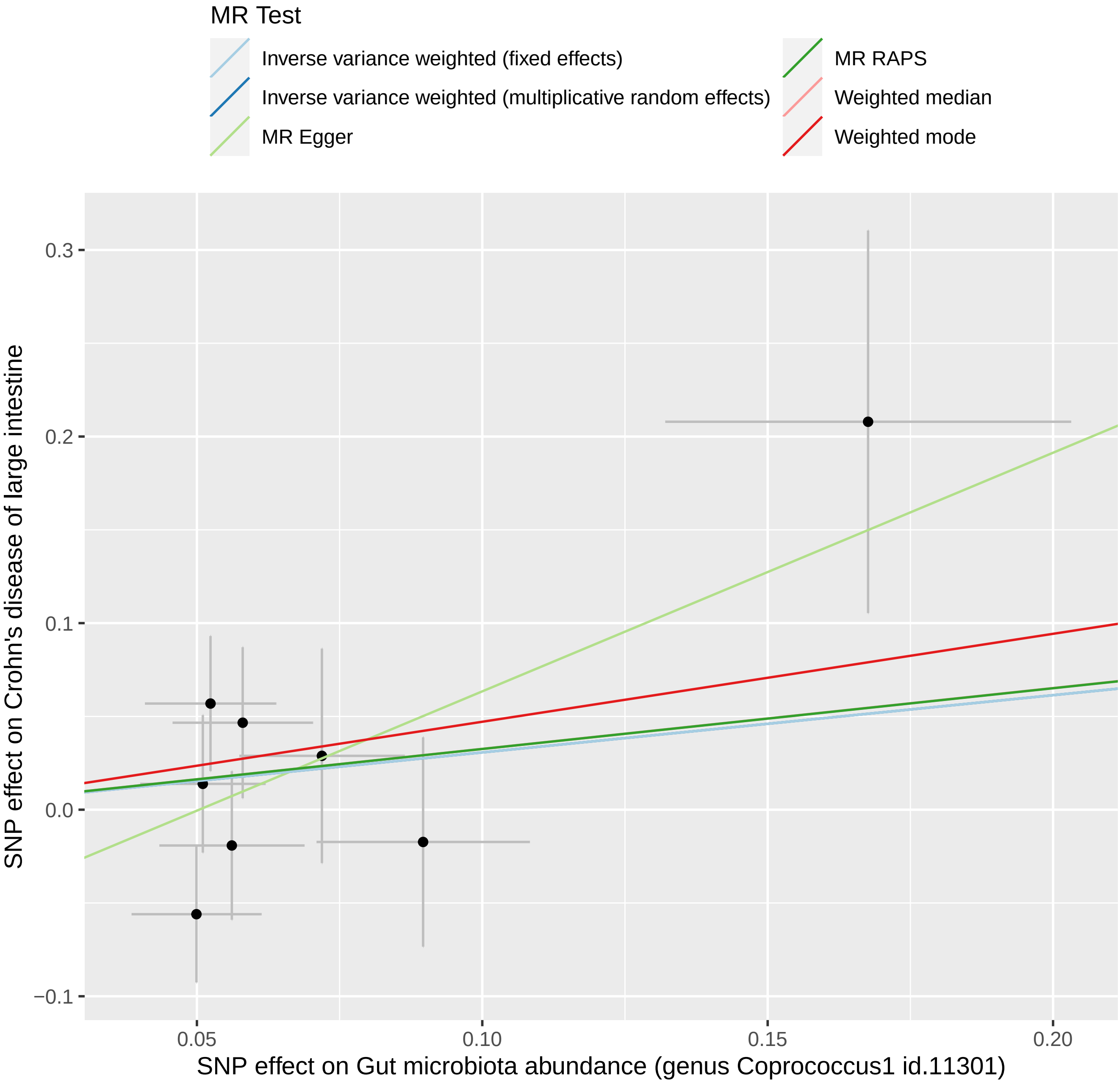

Batch 58 : Gut microbiota abundance (genus Coprococcus2 id.11302) on Crohn's disease of large intestine

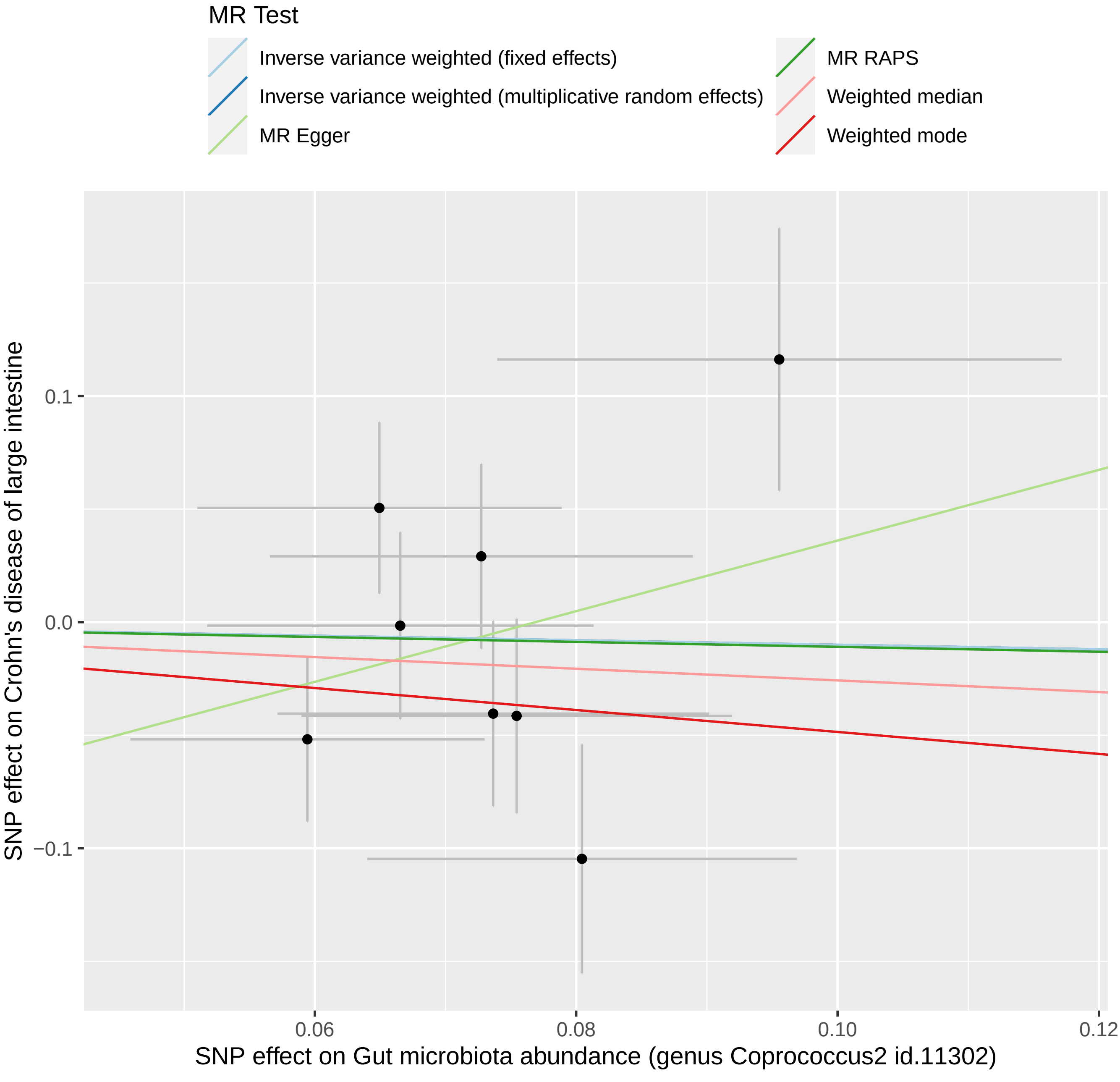

Batch 59 : Gut microbiota abundance (genus Coprococcus3 id.11303) on Crohn's disease of large intestine

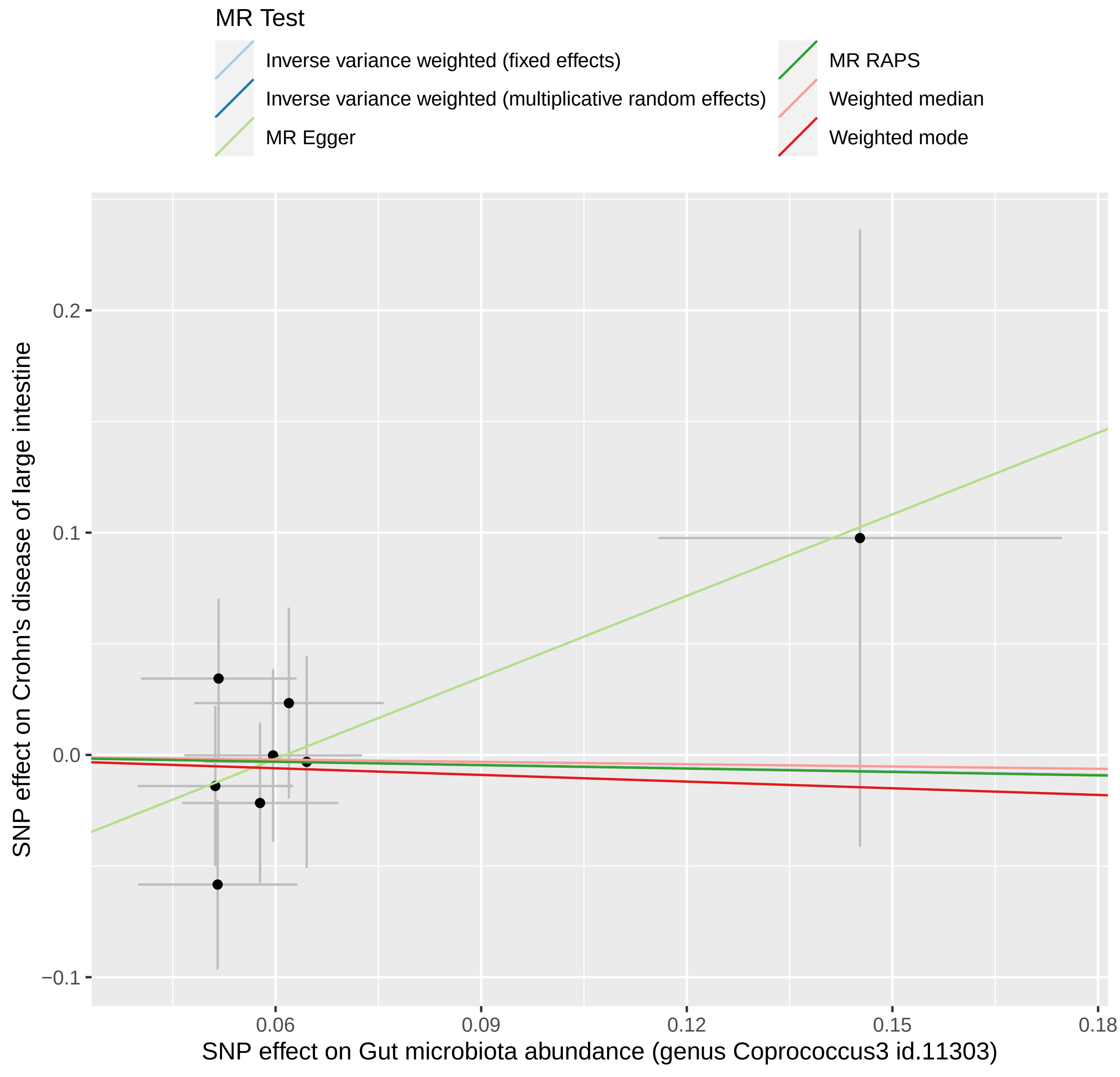

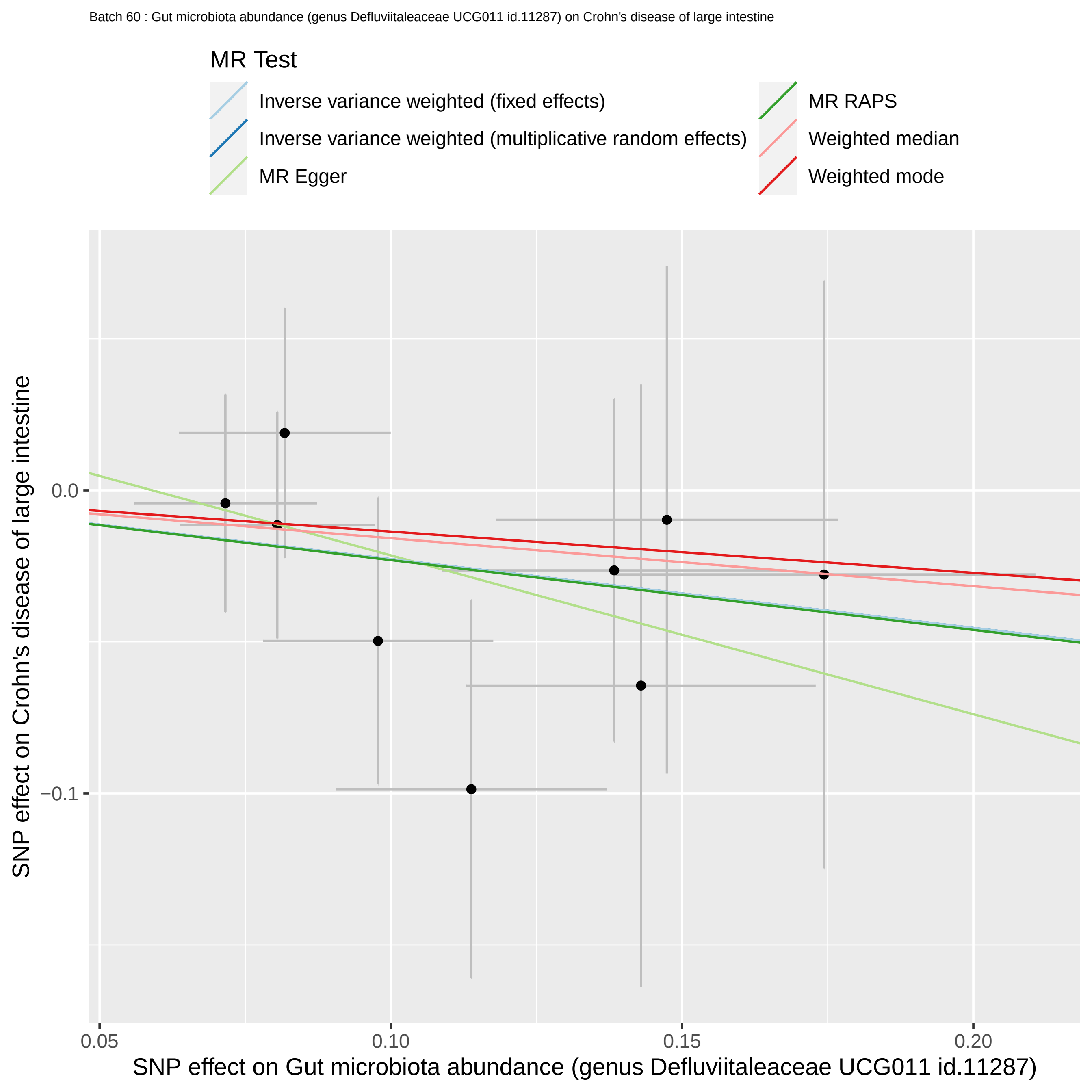

Batch 60 : Gut microbiota abundance (genus Defluviitaleaceae UCG011 id.11287) on Crohn's disease of large intestine
MR Test
Inverse variance weighted (fixed effects)
Inverse variance weighted (multiplicative random effects)
MR Egger
MR RAPS
Weighted median
Weighted mode
SNP effect on Crohn's disease of large intestine
0.0
−0.1
0.05
0.10
0.15
0.20
SNP effect on Gut microbiota abundance (genus Defluviitaleaceae UCG011 id.11287)

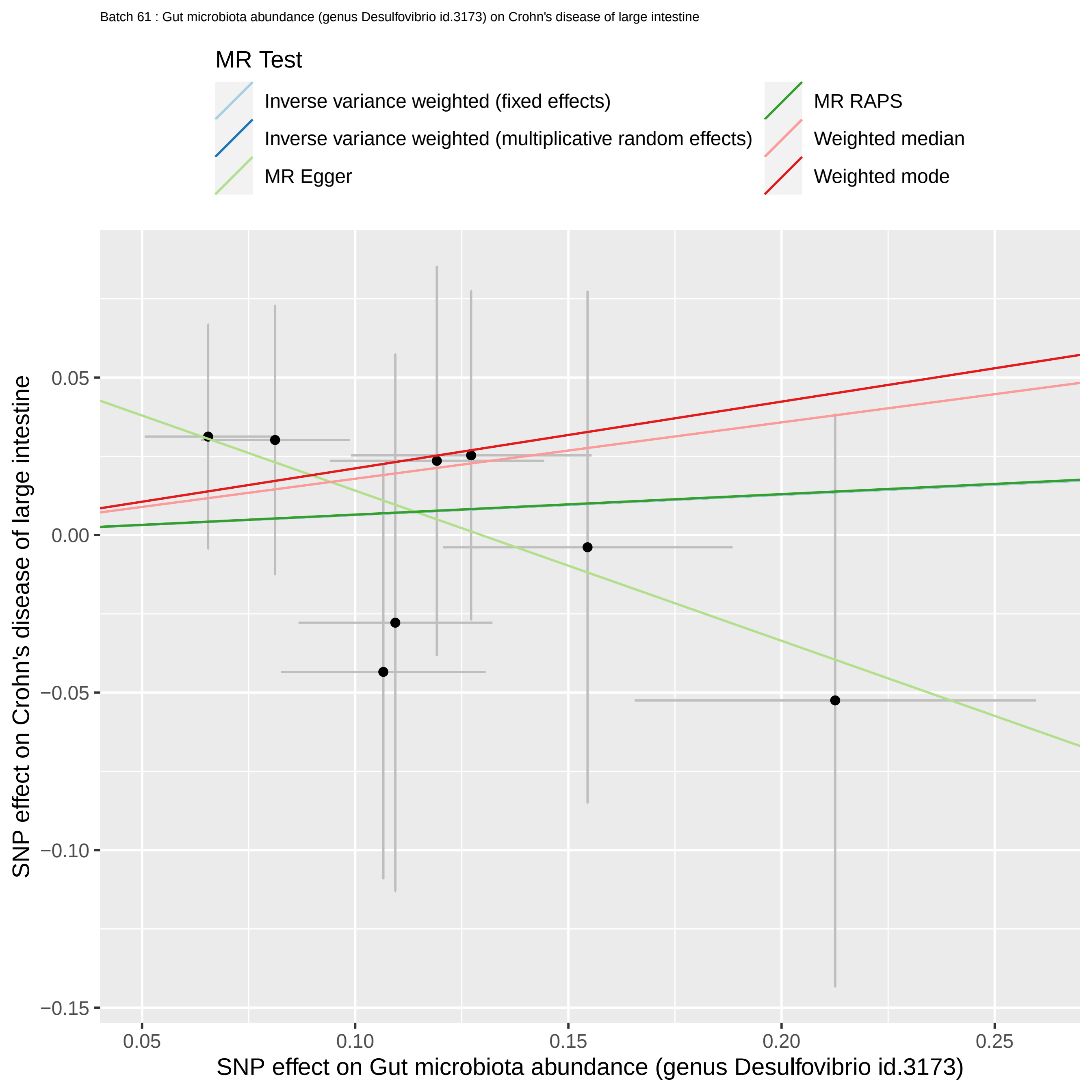

Batch 61 : Gut microbiota abundance (genus Desulfovibrio id.3173) on Crohn's disease of large intestine
MR Test
Inverse variance weighted (fixed effects)
Inverse variance weighted (multiplicative random effects)
MR Egger
MR RAPS
Weighted median
Weighted mode
0.05
0.00
−0.05
−0.10
−0.15
0.05
0.10
0.15
0.20
0.25
SNP effect on Crohn's disease of large intestine
SNP effect on Gut microbiota abundance (genus Desulfovibrio id.3173)

Batch 62 : Gut microbiota abundance (genus Dialister id.2183) on Crohn's disease of large intestine

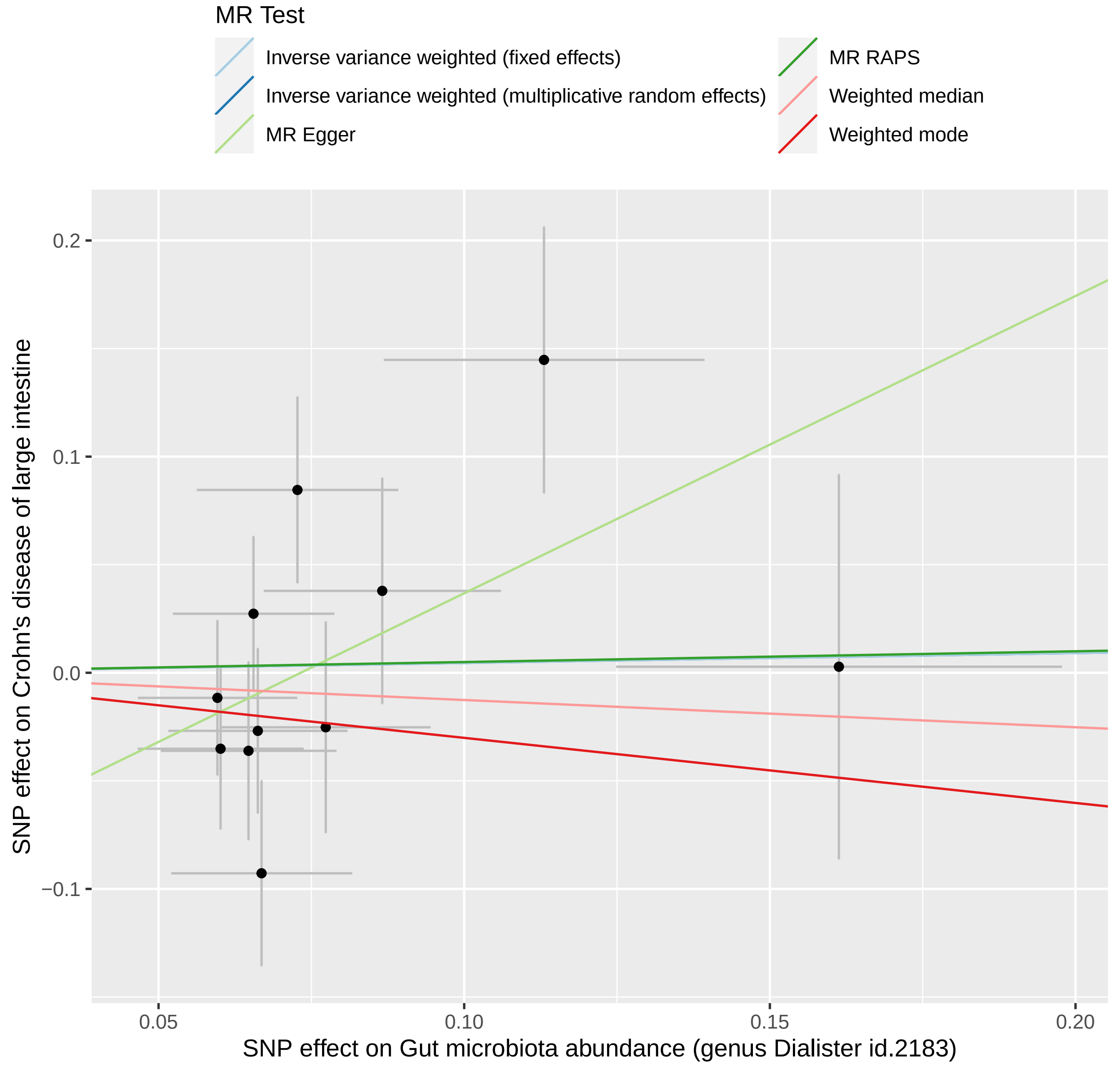

Batch 63 : Gut microbiota abundance (genus Dorea id.1997) on Crohn's disease of large intestine

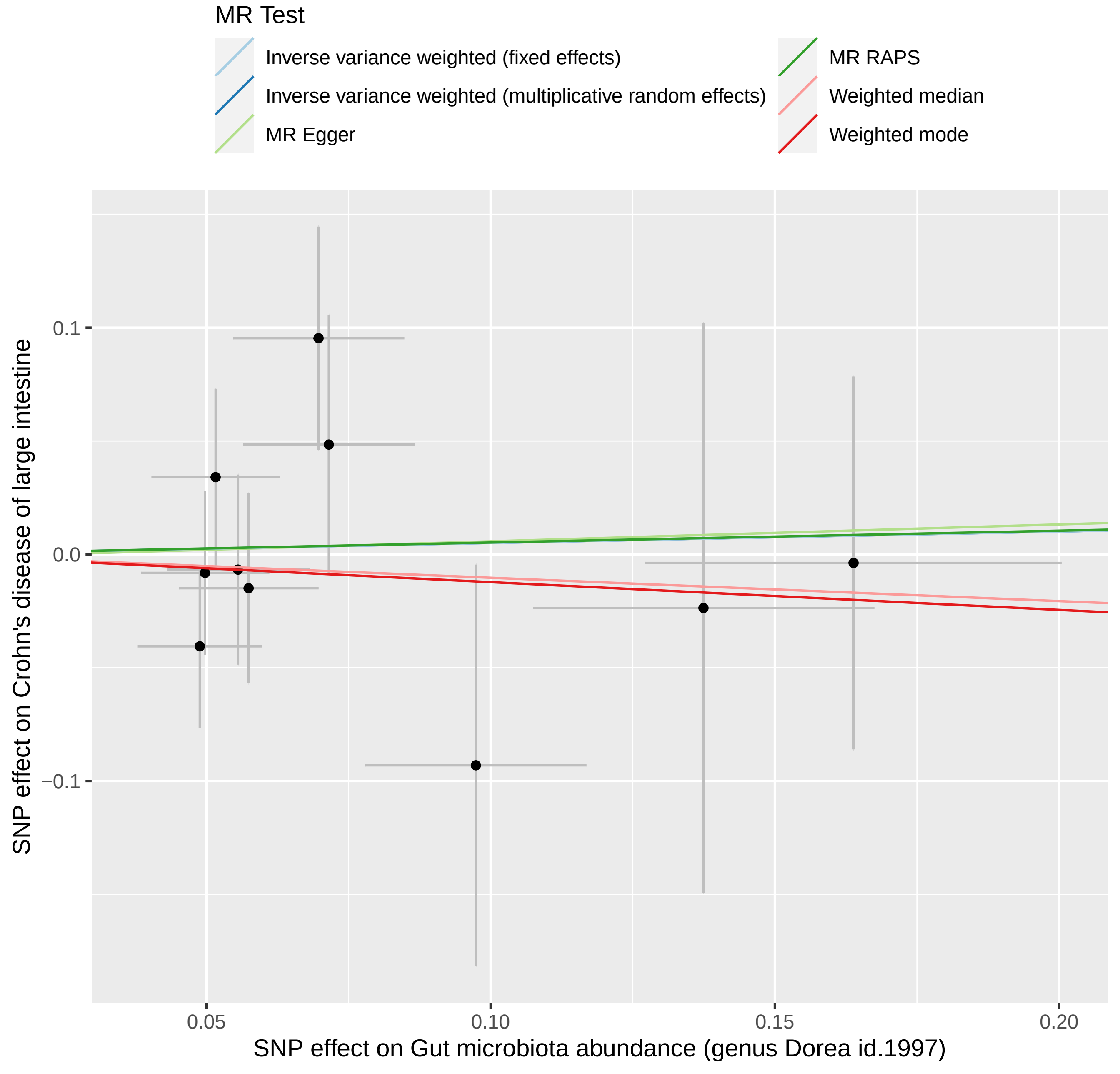

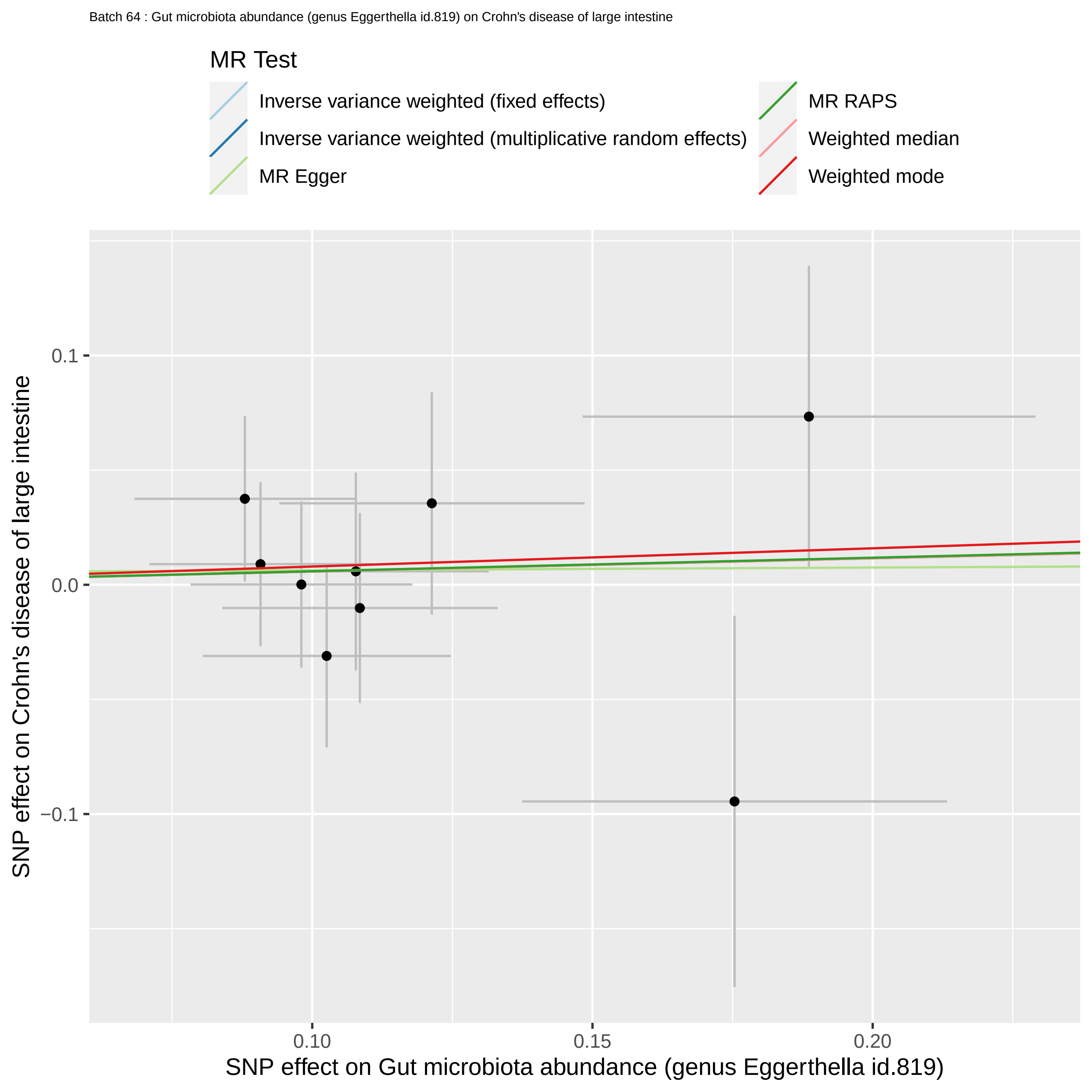

Batch 64 : Gut microbiota abundance (genus Eggerthella id.819) on Crohn's disease of large intestine
MR Test
Inverse variance weighted (fixed effects)
Inverse variance weighted (multiplicative random effects)
MR Egger
MR RAPS
Weighted median
Weighted mode
0.1
0.0
−0.1
0.10
0.15
0.20
SNP effect on Crohn's disease of large intestine
SNP effect on Gut microbiota abundance (genus Eggerthella id.819)

Batch 65 : Gut microbiota abundance (genus Eisenbergiella id.11304) on Crohn's disease of large intestine

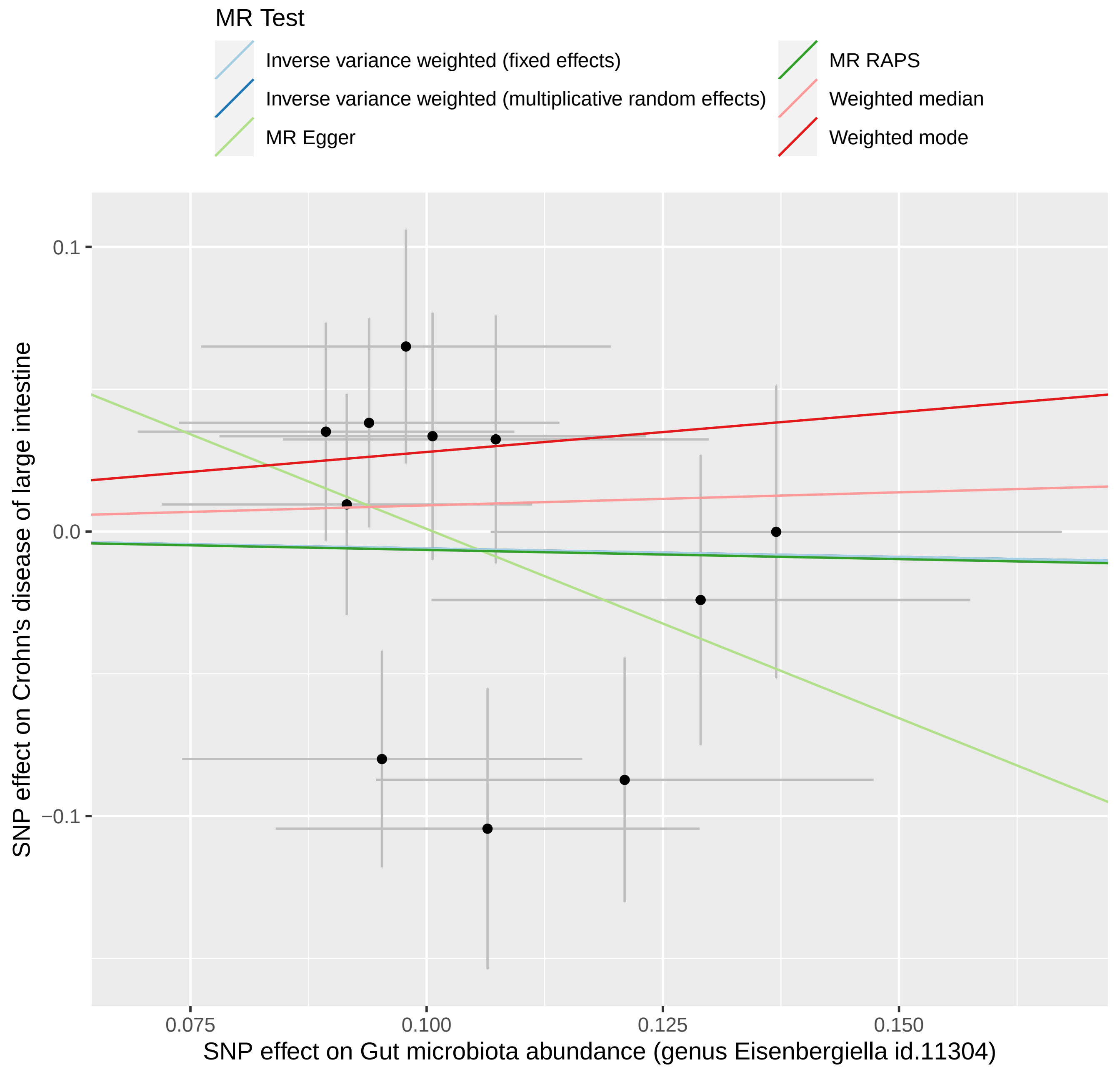

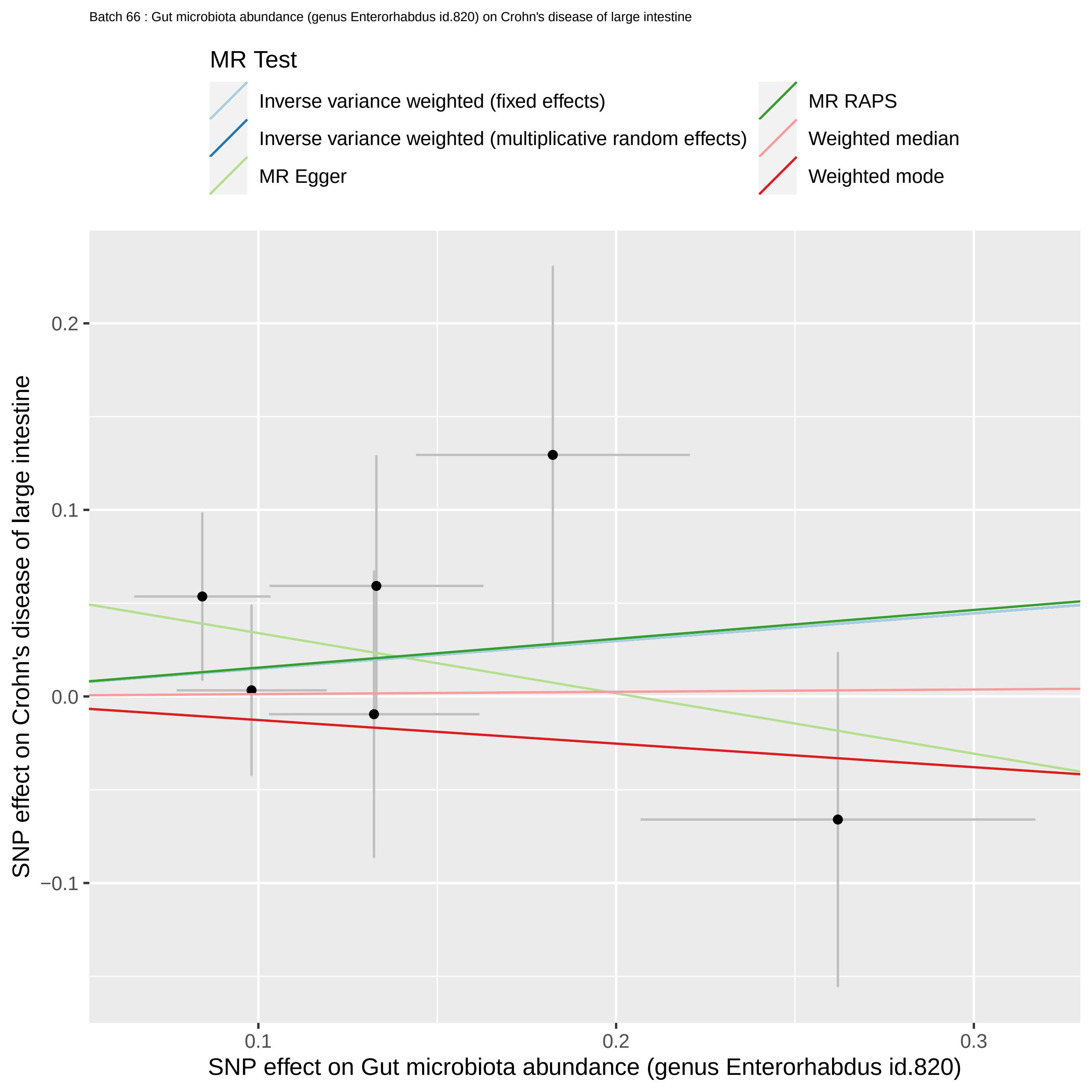
Batch 66 : Gut microbiota abundance (genus Enterorhabdus id.820) on Crohn's disease of large intestine
MR Test
Inverse variance weighted (fixed effects)
Inverse variance weighted (multiplicative random effects)
MR Egger
MR RAPS
Weighted median
Weighted mode
0.2
0.1
0.0
−0.1
0.1
0.2
0.3
SNP effect on Crohn's disease of large intestine
SNP effect on Gut microbiota abundance (genus Enterorhabdus id.820)

Batch 67 : Gut microbiota abundance (genus Erysipelatoclostridium id.11381) on Crohn's disease of large intestine

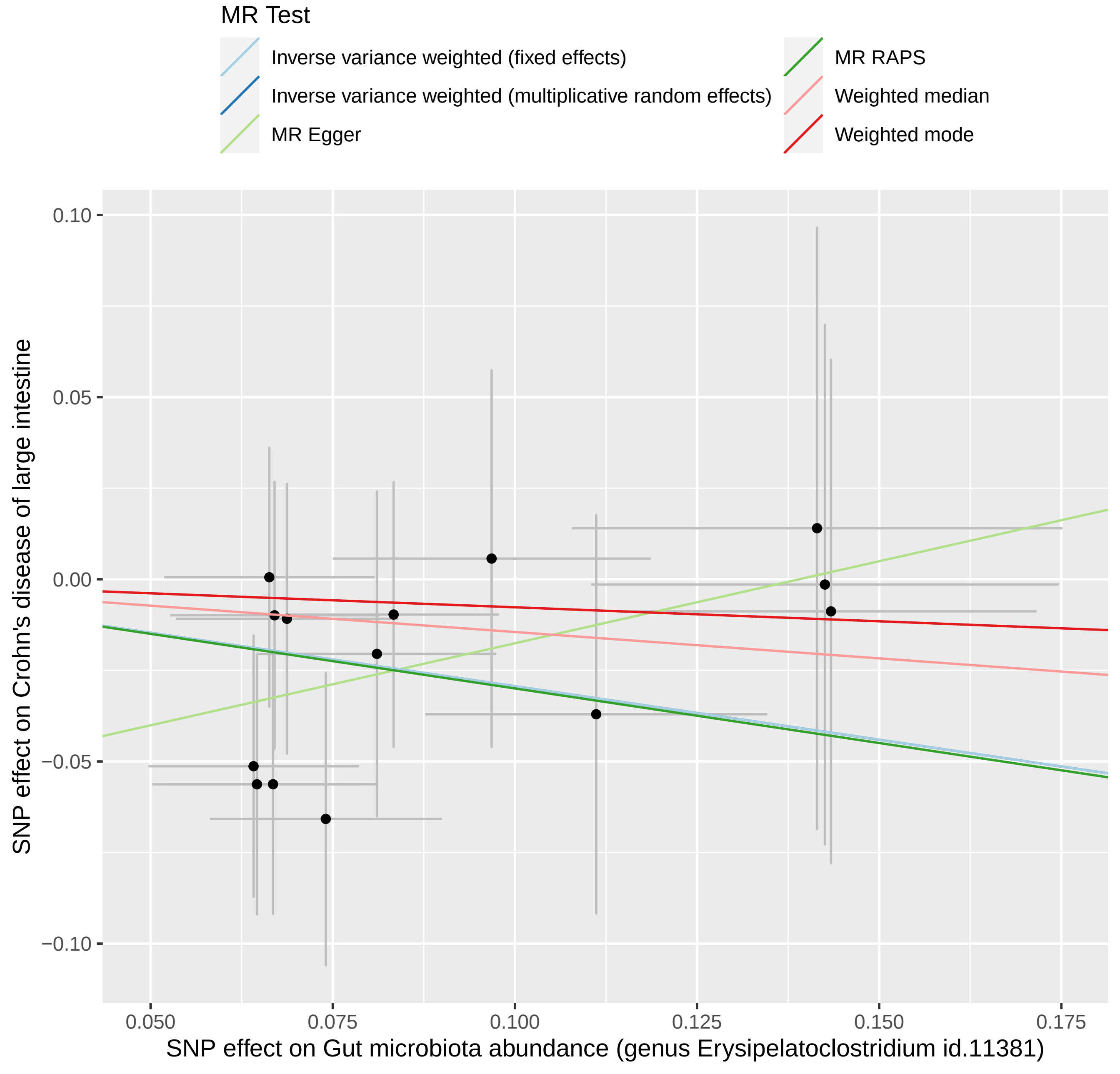

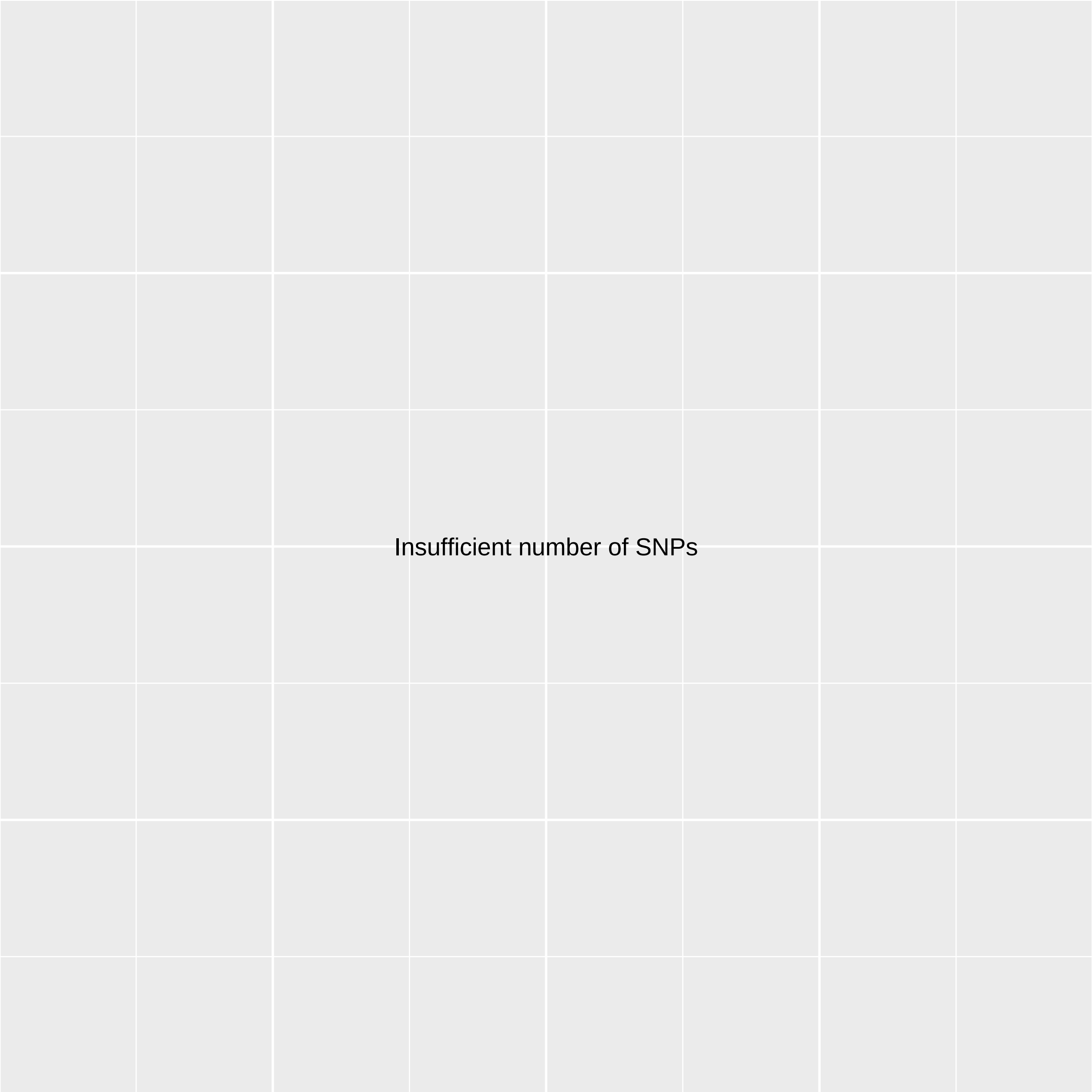
Insufficient number of SNPs

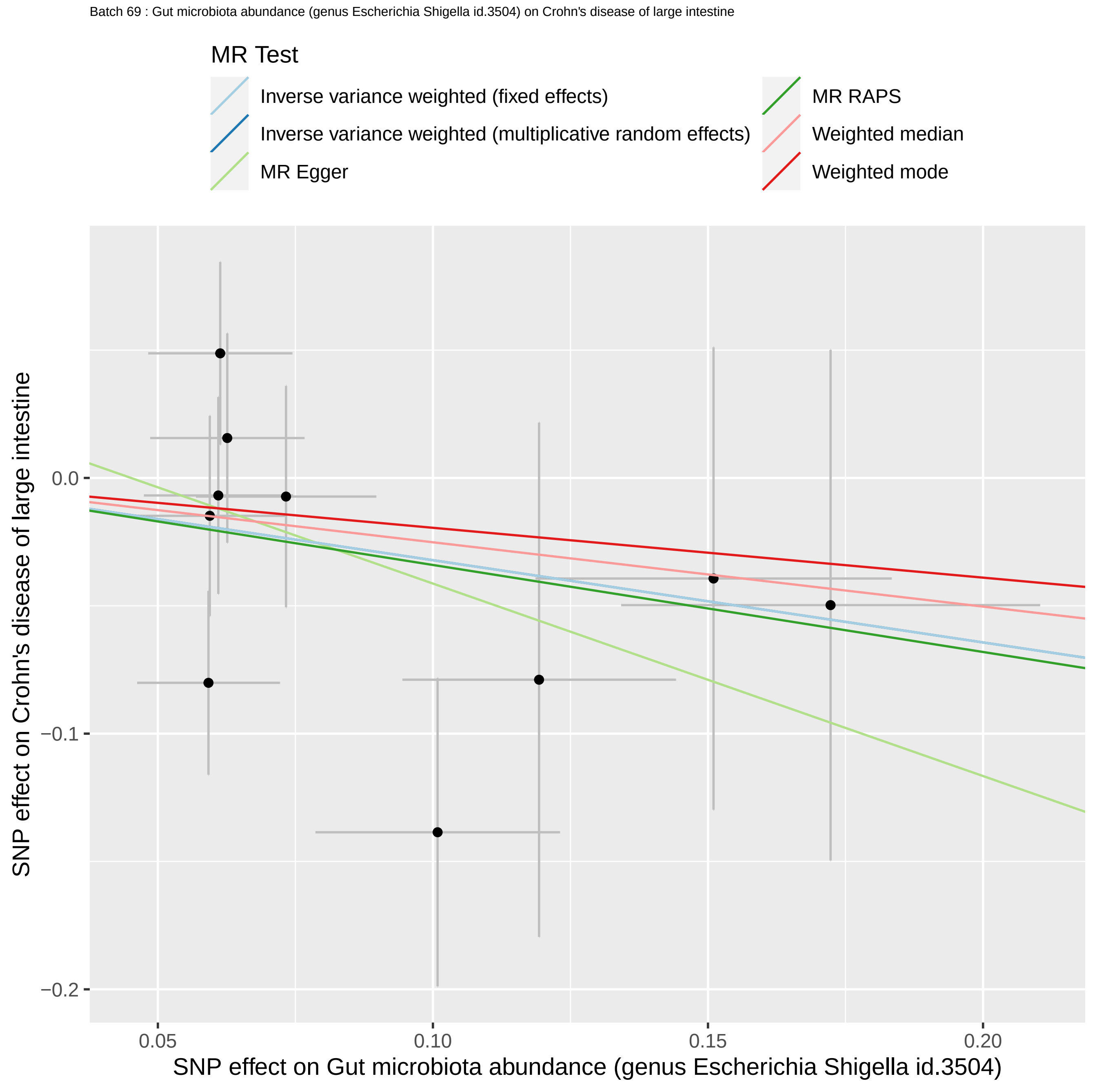
Batch 69 : Gut microbiota abundance (genus Escherichia Shigella id.3504) on Crohn's disease of large intestine
MR Test
Inverse variance weighted (fixed effects)
Inverse variance weighted (multiplicative random effects)
MR Egger
MR RAPS
Weighted median
Weighted mode
SNP effect on Crohn's disease of large intestine
0.0
−0.1
−0.2
0.05
0.10
0.15
0.20
SNP effect on Gut microbiota abundance (genus Escherichia Shigella id.3504)

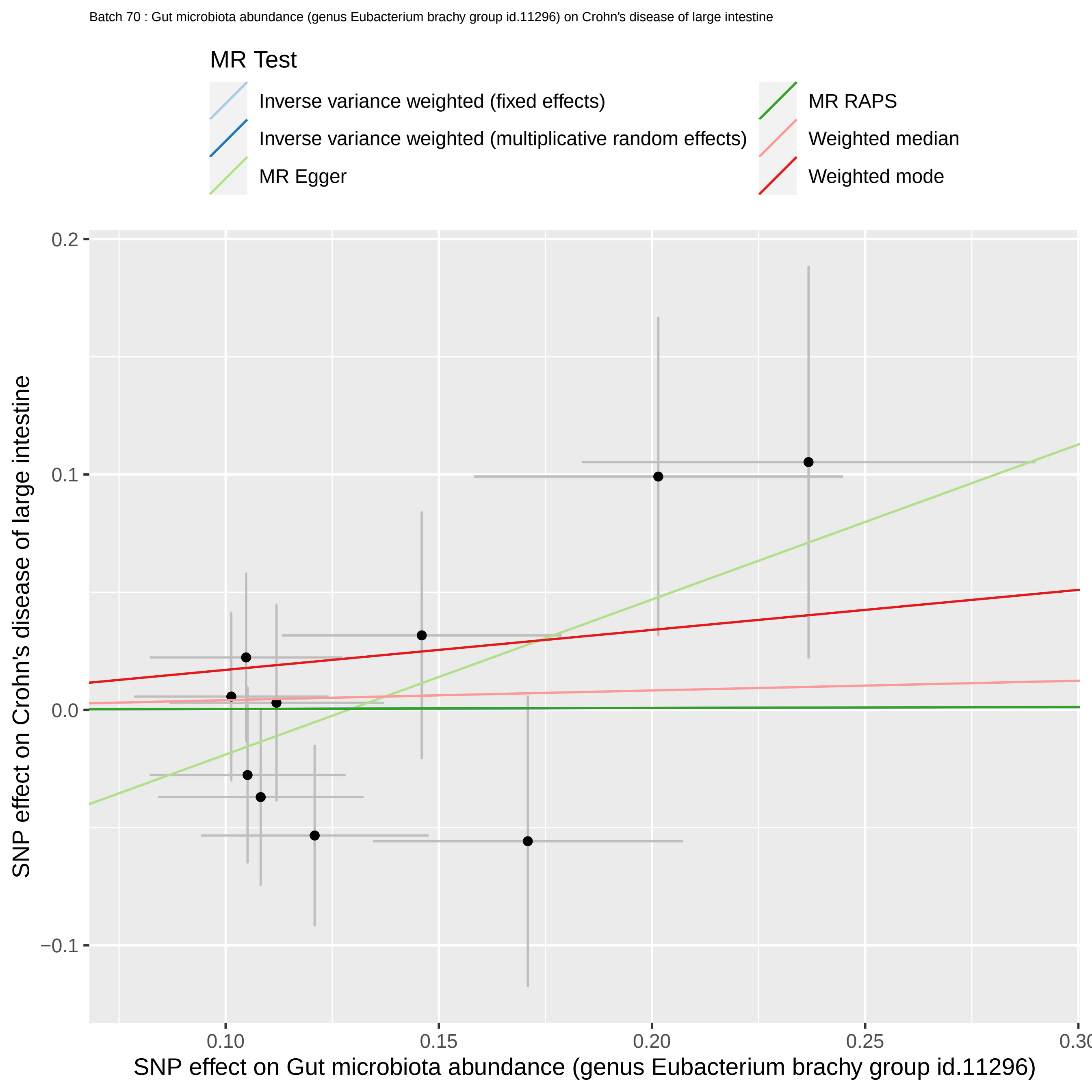

Batch 70 : Gut microbiota abundance (genus Eubacterium brachy group id.11296) on Crohn's disease of large intestine
MR Test
Inverse variance weighted (fixed effects)
Inverse variance weighted (multiplicative random effects)
MR Egger
MR RAPS
Weighted median
Weighted mode
0.2
0.1
0.0
−0.1
0.10
0.15
0.20
0.25
0.30
SNP effect on Crohn's disease of large intestine
SNP effect on Gut microbiota abundance (genus Eubacterium brachy group id.11296)

Batch 71 : Gut microbiota abundance (genus Eubacterium coprostanoligenes group id.11375) on Crohn's disease of large intestine

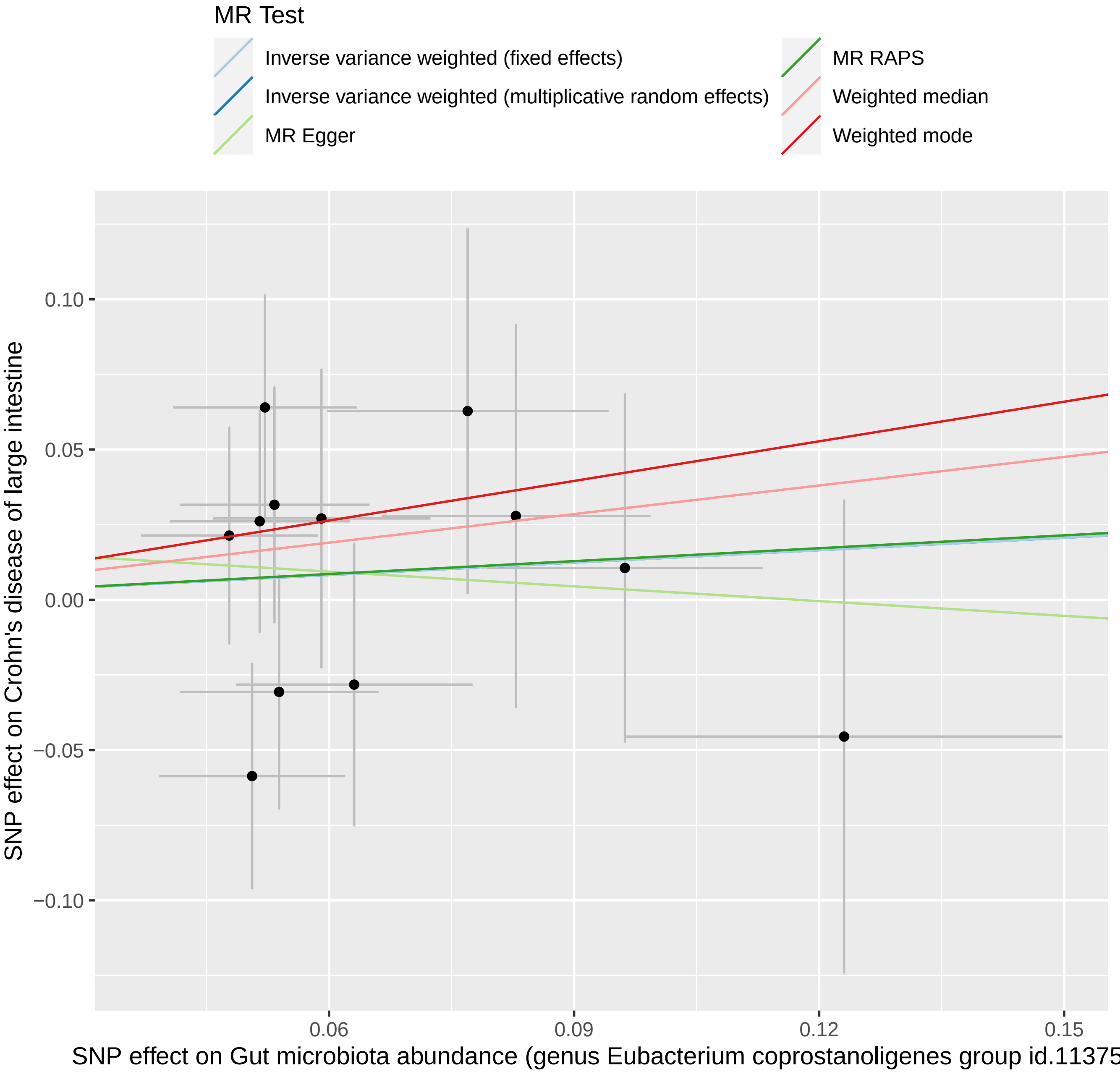

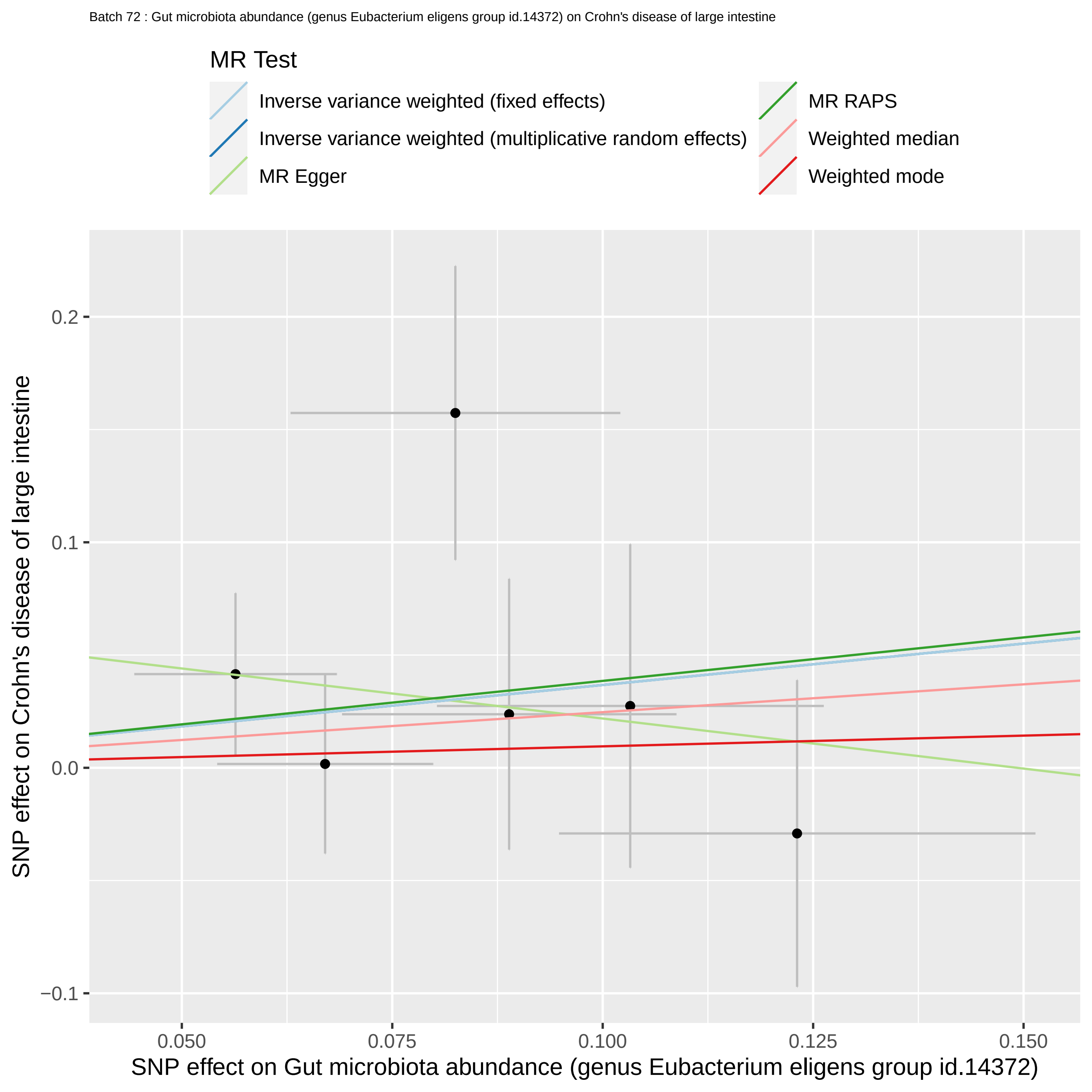
Batch 72 : Gut microbiota abundance (genus Eubacterium eligens group id.14372) on Crohn's disease of large intestine
MR Test
Inverse variance weighted (fixed effects)
Inverse variance weighted (multiplicative random effects)
MR Egger
MR RAPS
Weighted median
Weighted mode
0.2
0.1
0.0
−0.1
0.050
0.075
0.100
0.125
0.150
SNP effect on Crohn's disease of large intestine
SNP effect on Gut microbiota abundance (genus Eubacterium eligens group id.14372)

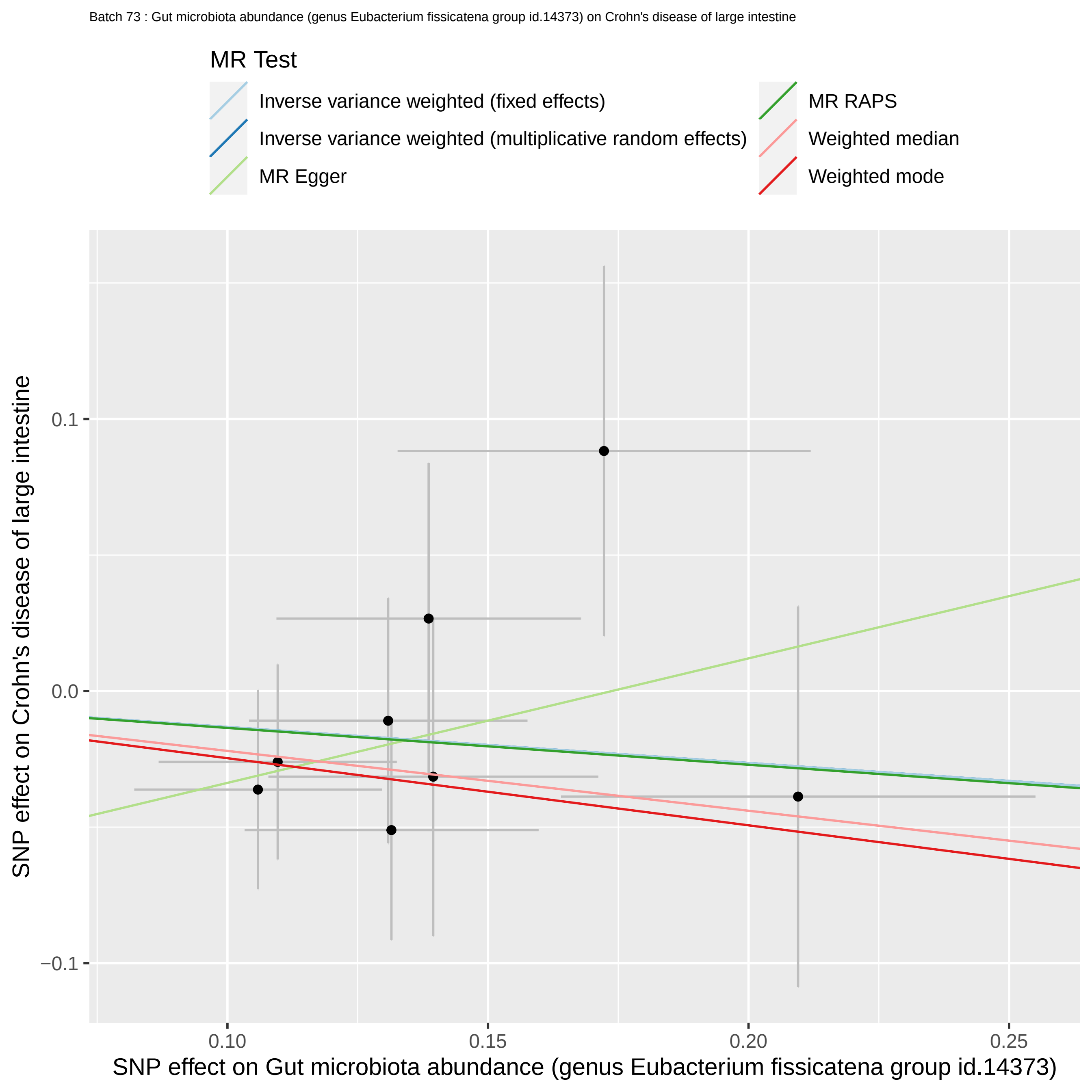

Batch 73 : Gut microbiota abundance (genus Eubacterium fissicatena group id.14373) on Crohn's disease of large intestine
MR Test
Inverse variance weighted (fixed effects)
Inverse variance weighted (multiplicative random effects)
MR Egger
MR RAPS
Weighted median
Weighted mode
SNP effect on Crohn's disease of large intestine
0.1
0.0
−0.1
0.10
0.15
0.20
0.25
SNP effect on Gut microbiota abundance (genus Eubacterium fissicatena group id.14373)

Batch 74 : Gut microbiota abundance (genus Eubacterium hallii group id.11338) on Crohn's disease of large intestine

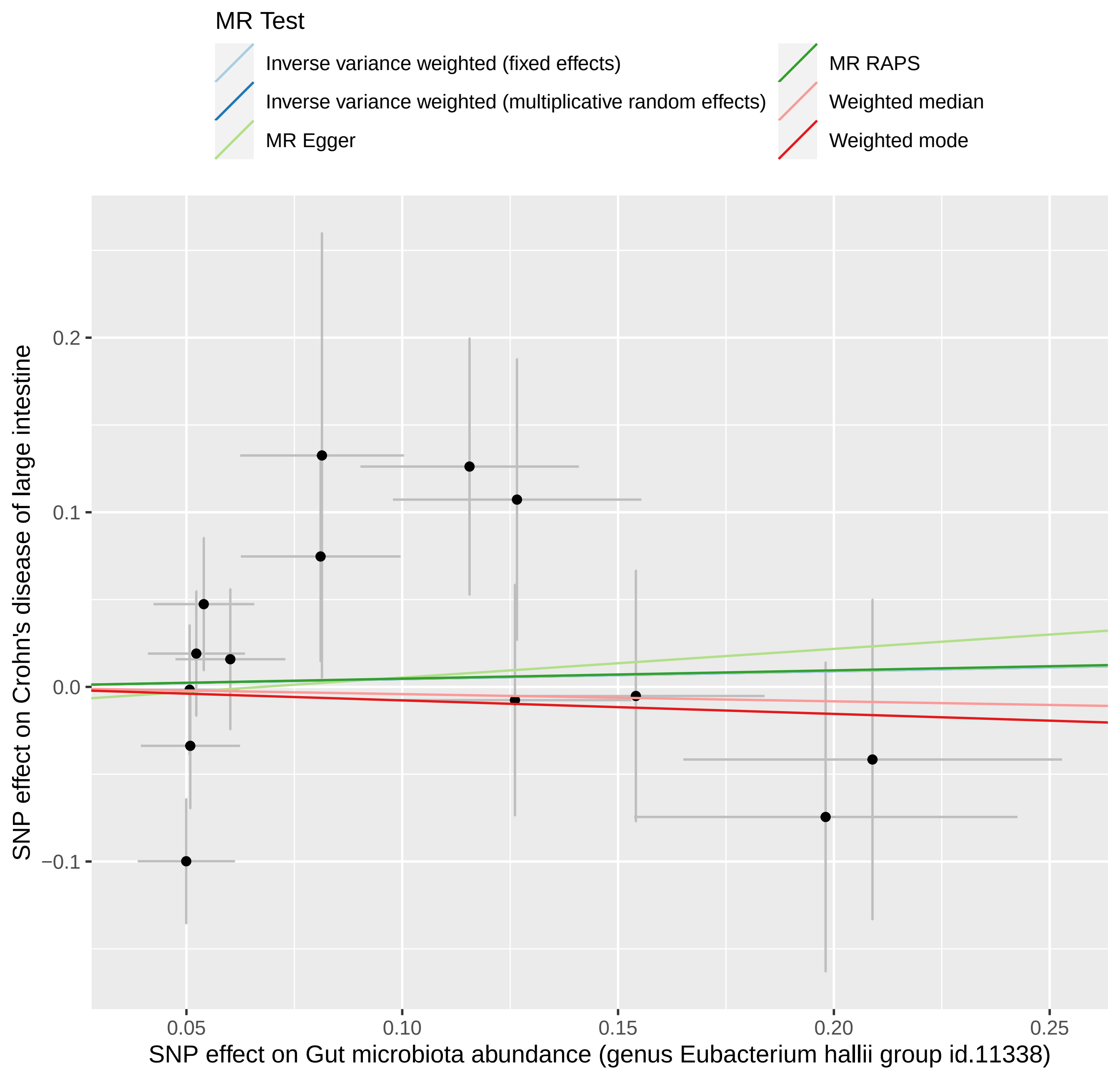

Batch 75 : Gut microbiota abundance (genus Eubacterium nodatum group id.11297) on Crohn's disease of large intestine

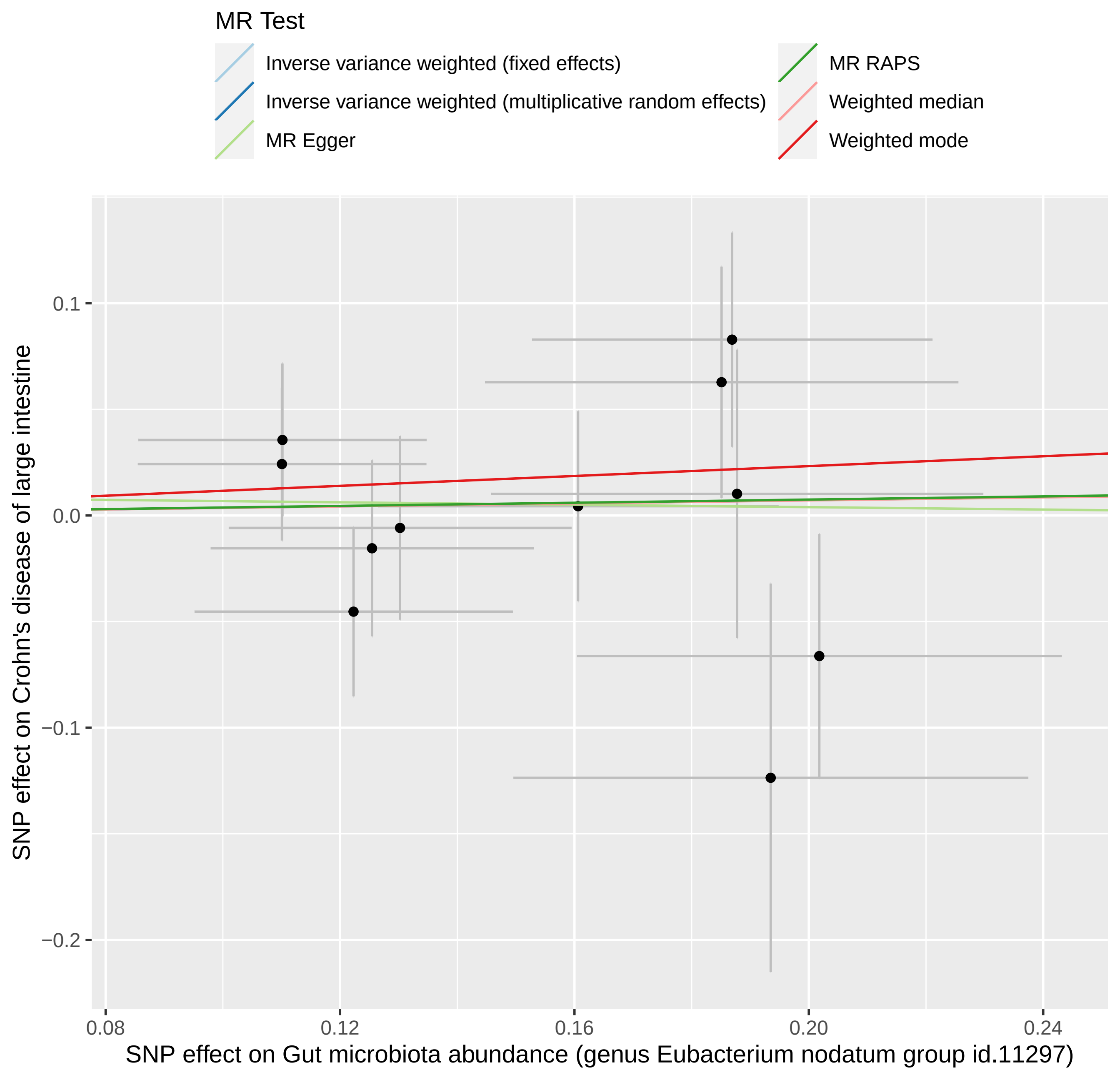

Batch 76 : Gut microbiota abundance (genus Eubacterium oxidoreducens group id.11339) on Crohn's disease of large intestine

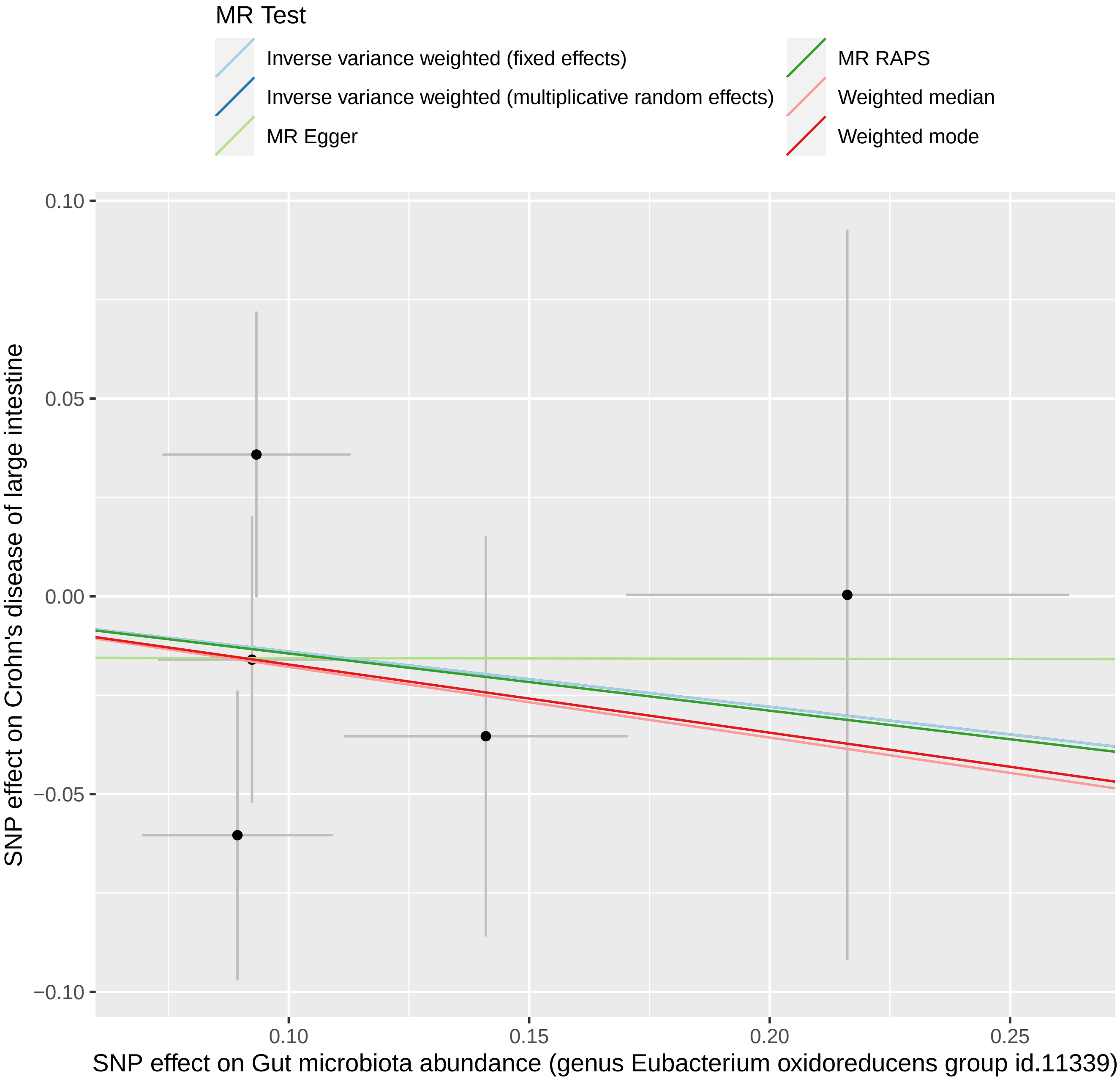

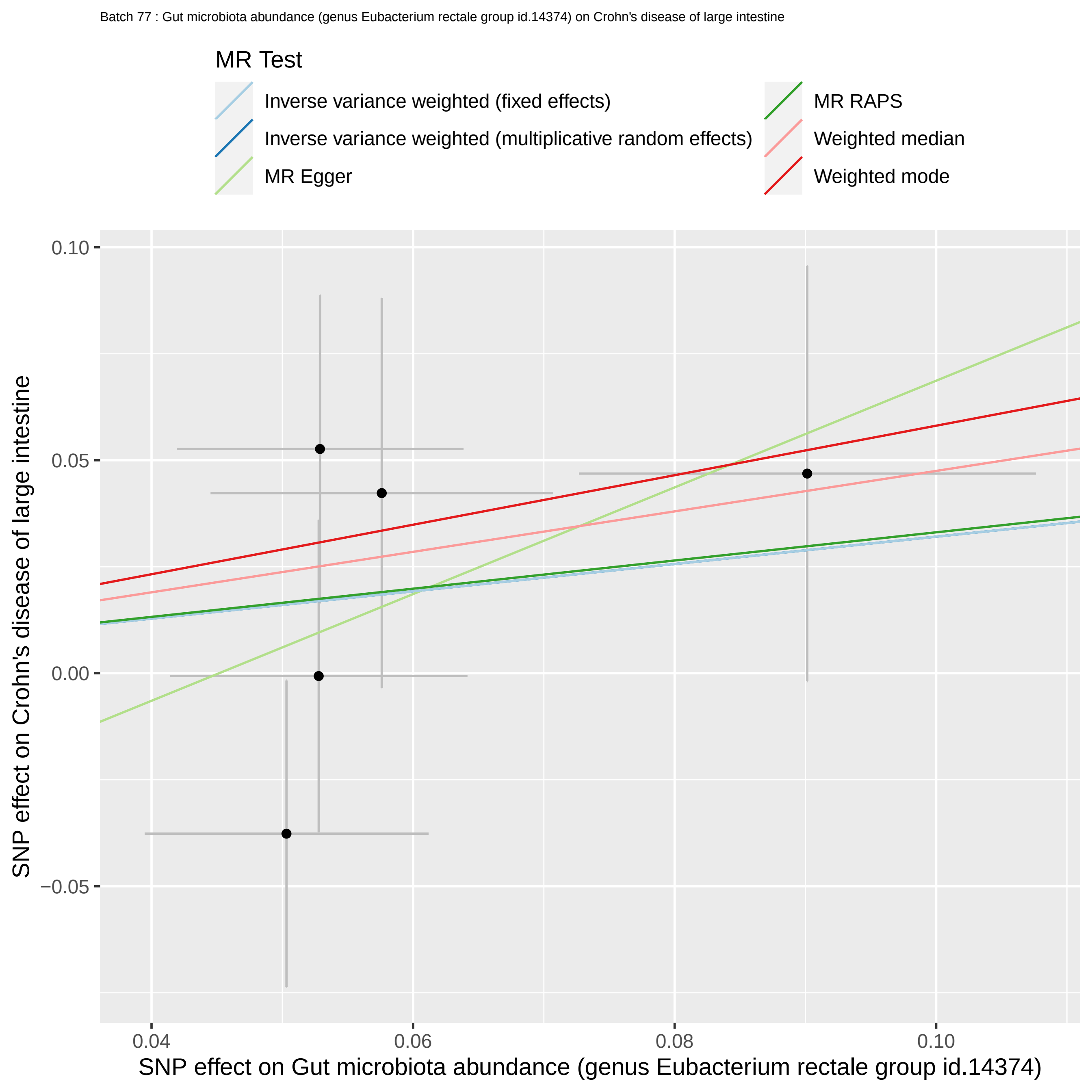

Batch 77 : Gut microbiota abundance (genus Eubacterium rectale group id.14374) on Crohn's disease of large intestine
MR Test
Inverse variance weighted (fixed effects)
Inverse variance weighted (multiplicative random effects)
MR Egger
MR RAPS
Weighted median
Weighted mode
SNP effect on Crohn's disease of large intestine
0.10
0.05
0.00
−0.05
0.04
0.06
0.08
0.10
SNP effect on Gut microbiota abundance (genus Eubacterium rectale group id.14374)

Batch 78 : Gut microbiota abundance (genus Eubacterium ruminantium group id.11340) on Crohn's disease of large intestine

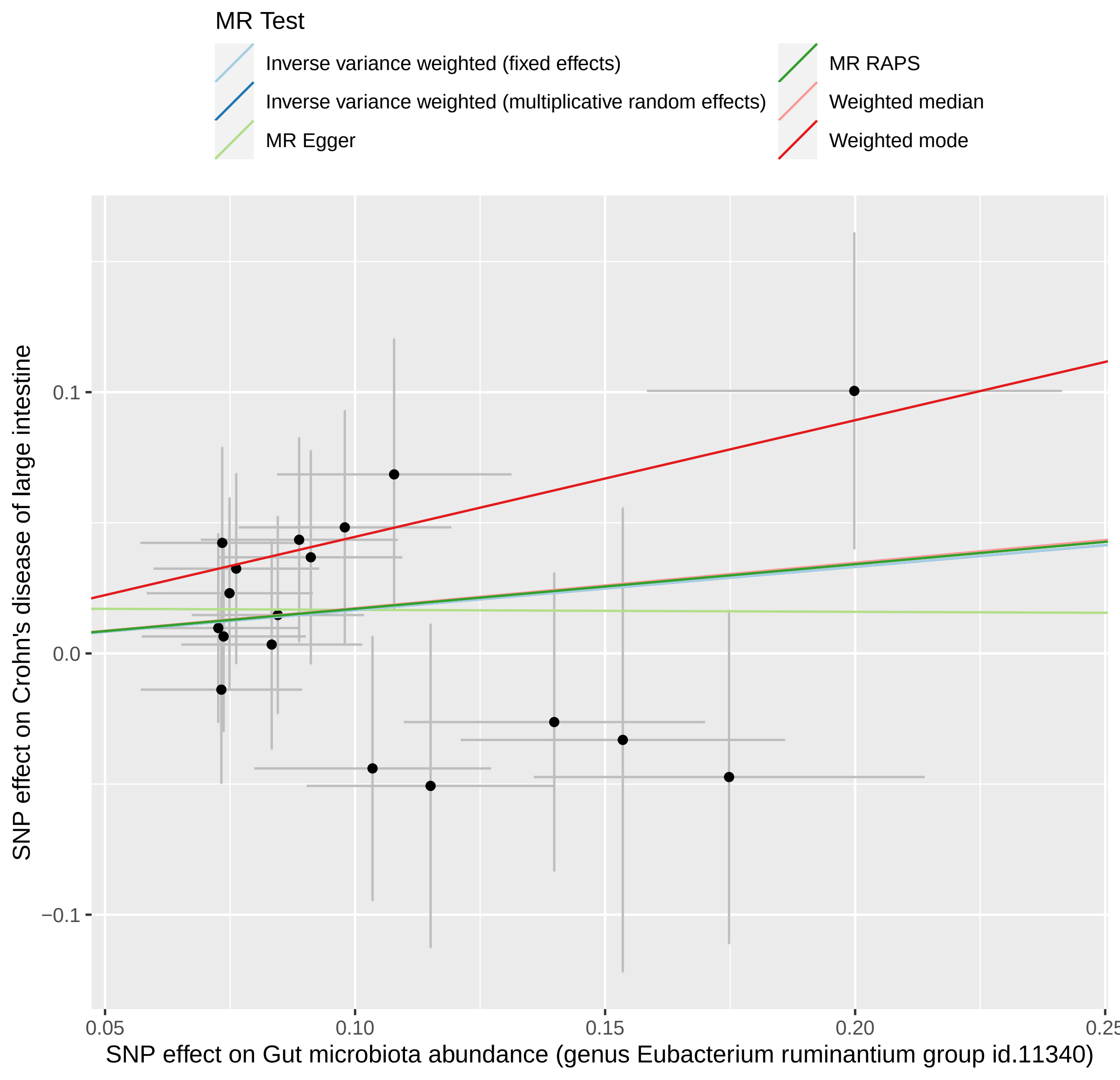

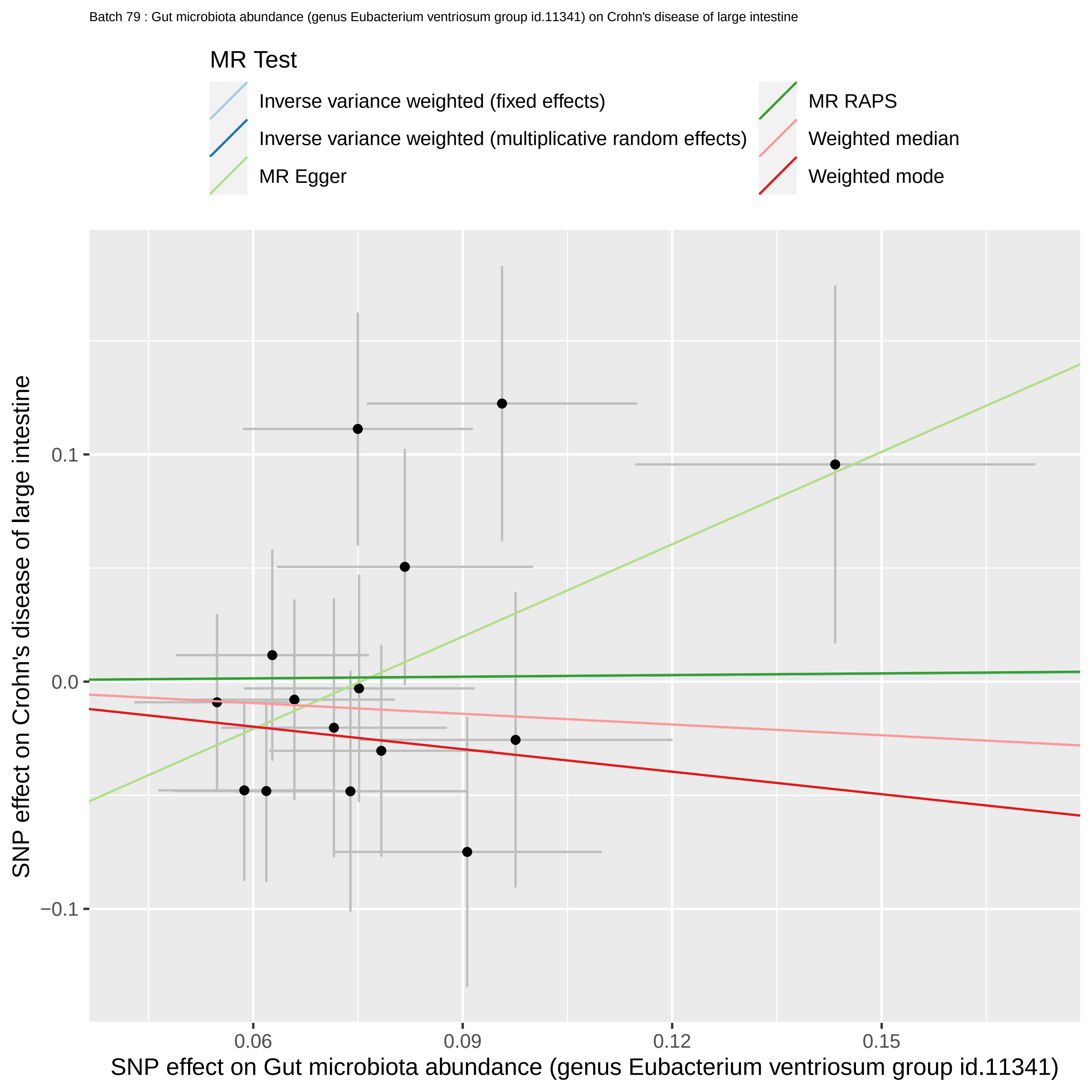
Batch 79 : Gut microbiota abundance (genus Eubacterium ventriosum group id.11341) on Crohn's disease of large intestine
MR Test
Inverse variance weighted (fixed effects)
Inverse variance weighted (multiplicative random effects)
MR Egger
MR RAPS
Weighted median
Weighted mode
0.1
0.0
−0.1
0.06
0.09
0.12
0.15
SNP effect on Crohn's disease of large intestine
SNP effect on Gut microbiota abundance (genus Eubacterium ventriosum group id.11341)

Batch 80 : Gut microbiota abundance (genus Eubacterium xylanophilum group id.14375) on Crohn's disease of large intestine

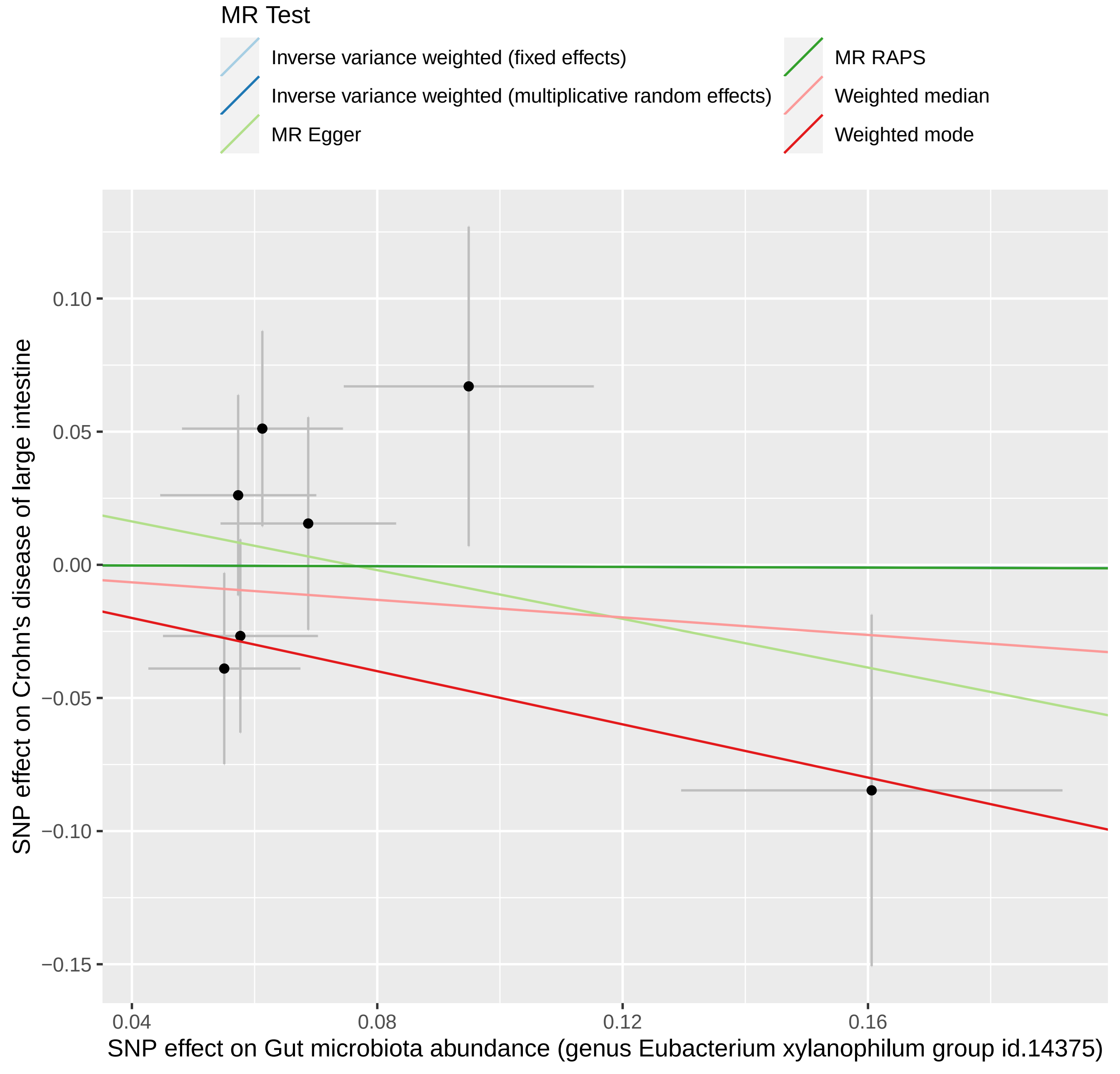

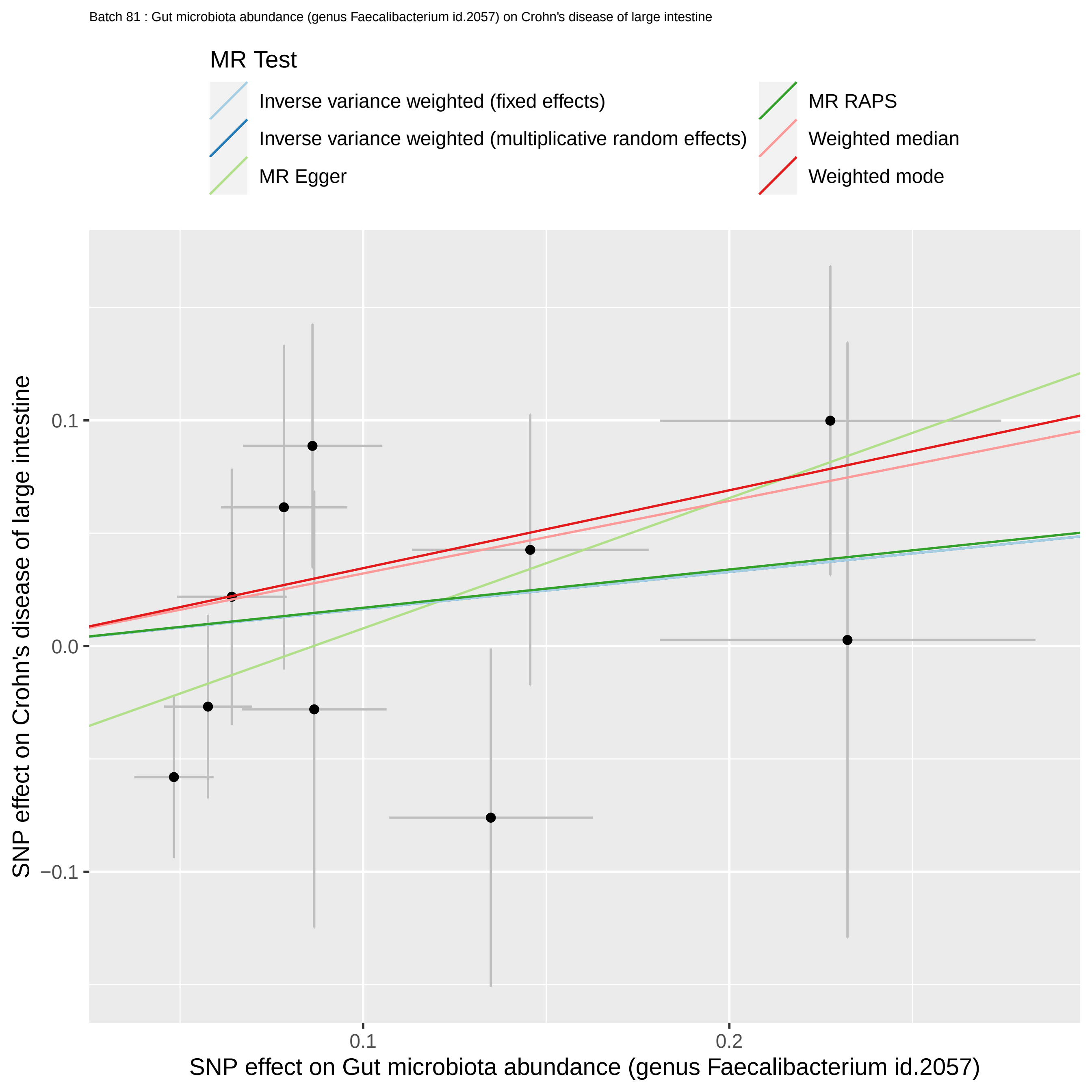
Batch 81 : Gut microbiota abundance (genus Faecalibacterium id.2057) on Crohn's disease of large intestine
MR Test
Inverse variance weighted (fixed effects)
Inverse variance weighted (multiplicative random effects)
MR Egger
MR RAPS
Weighted median
Weighted mode
0.1
0.0
−0.1
0.1
0.2
SNP effect on Crohn's disease of large intestine
SNP effect on Gut microbiota abundance (genus Faecalibacterium id.2057)

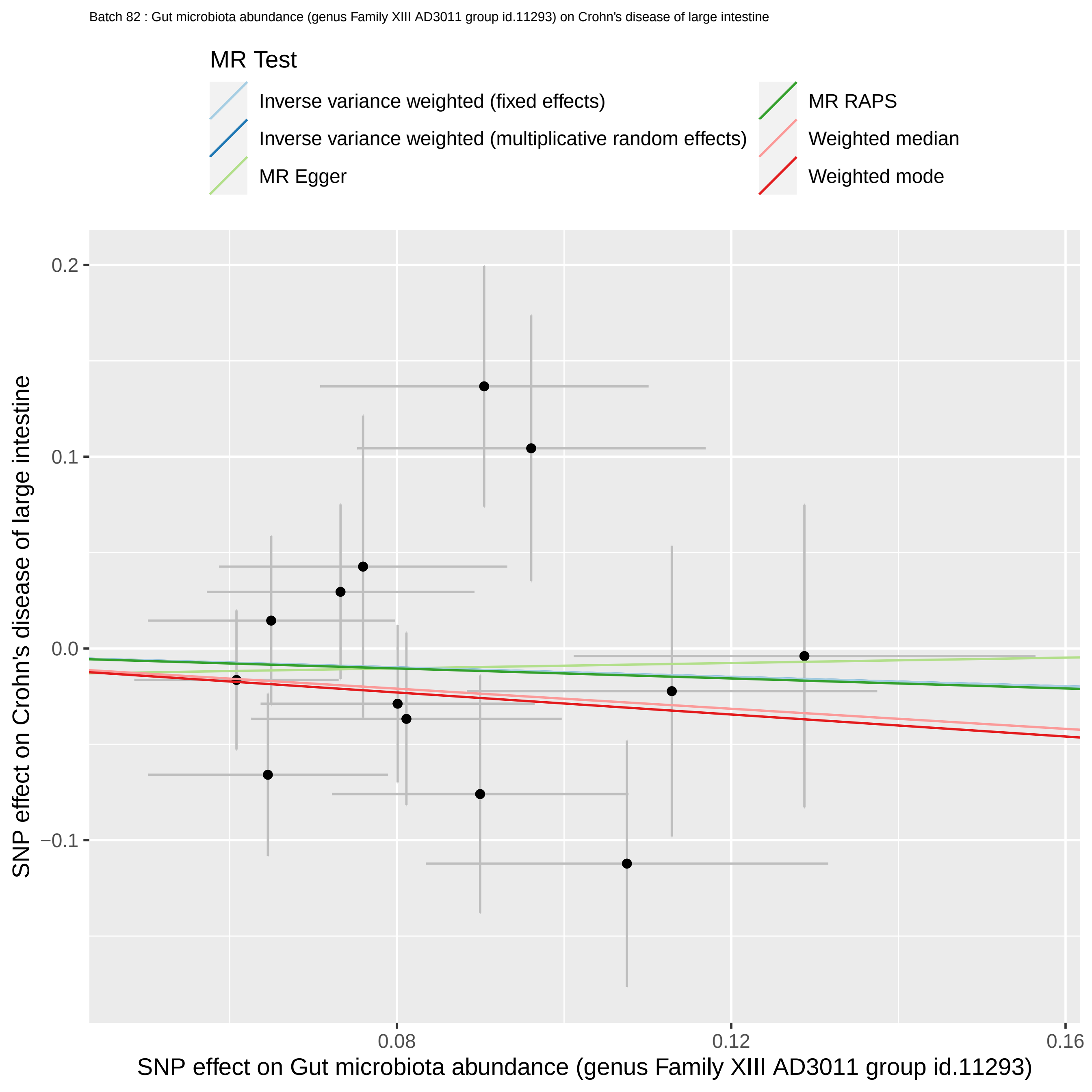

Batch 82 : Gut microbiota abundance (genus Family XIII AD3011 group id.11293) on Crohn's disease of large intestine
MR Test
Inverse variance weighted (fixed effects)
Inverse variance weighted (multiplicative random effects)
MR Egger
MR RAPS
Weighted median
Weighted mode
0.2
0.1
0.0
−0.1
0.08
0.12
0.16
SNP effect on Crohn's disease of large intestine
SNP effect on Gut microbiota abundance (genus Family XIII AD3011 group id.11293)

Batch 83 : Gut microbiota abundance (genus Family XIII UCG001 id.11294) on Crohn's disease of large intestine

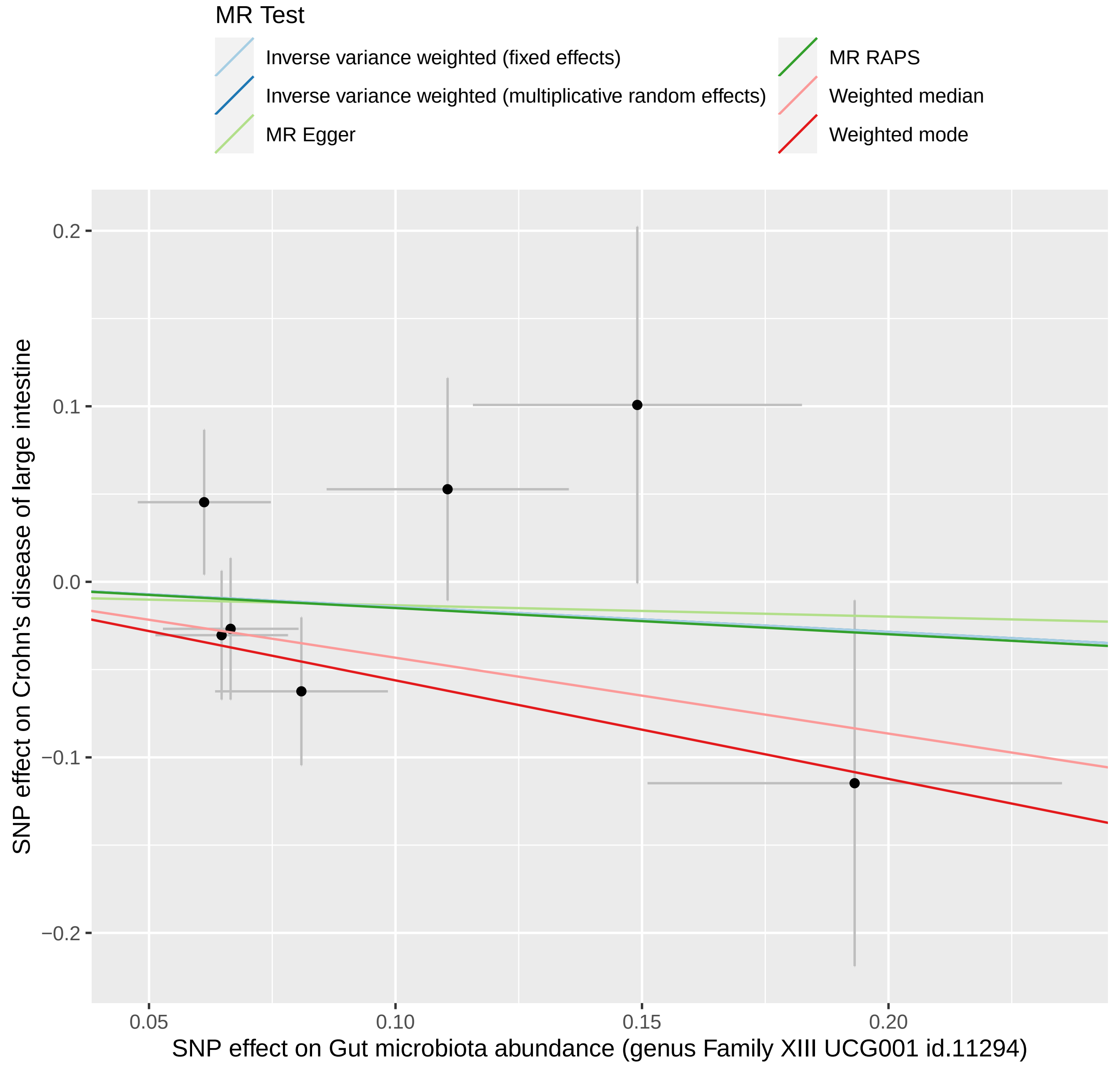

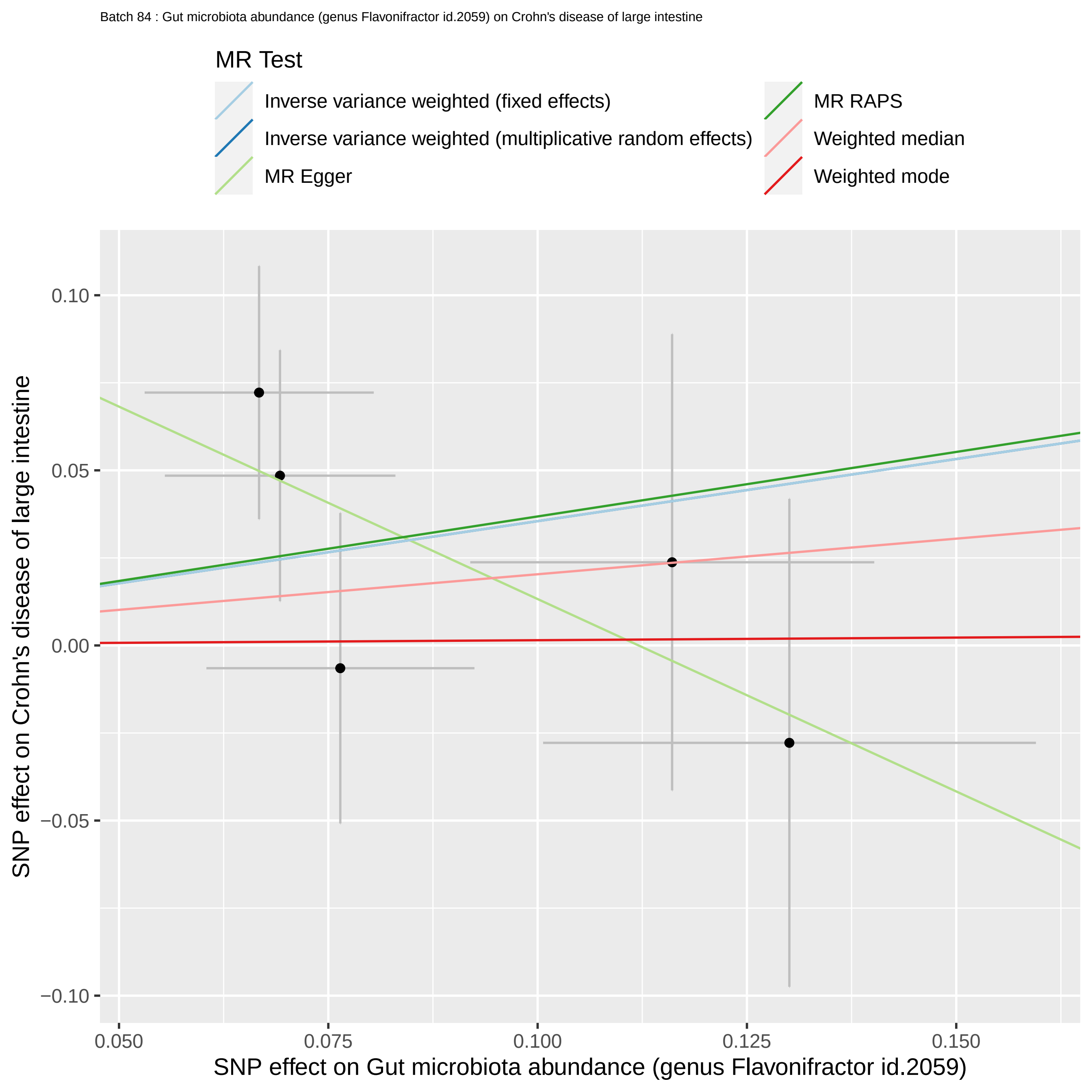

Batch 84 : Gut microbiota abundance (genus Flavonifractor id.2059) on Crohn's disease of large intestine
MR Test
Inverse variance weighted (fixed effects)
Inverse variance weighted (multiplicative random effects)
MR Egger
MR RAPS
Weighted median
Weighted mode
0.10
0.05
0.00
−0.05
−0.10
0.050
0.075
0.100
0.125
0.150
SNP effect on Crohn's disease of large intestine
SNP effect on Gut microbiota abundance (genus Flavonifractor id.2059)

Batch 85 : Gut microbiota abundance (genus Fusicatenibacter id.11305) on Crohn's disease of large intestine

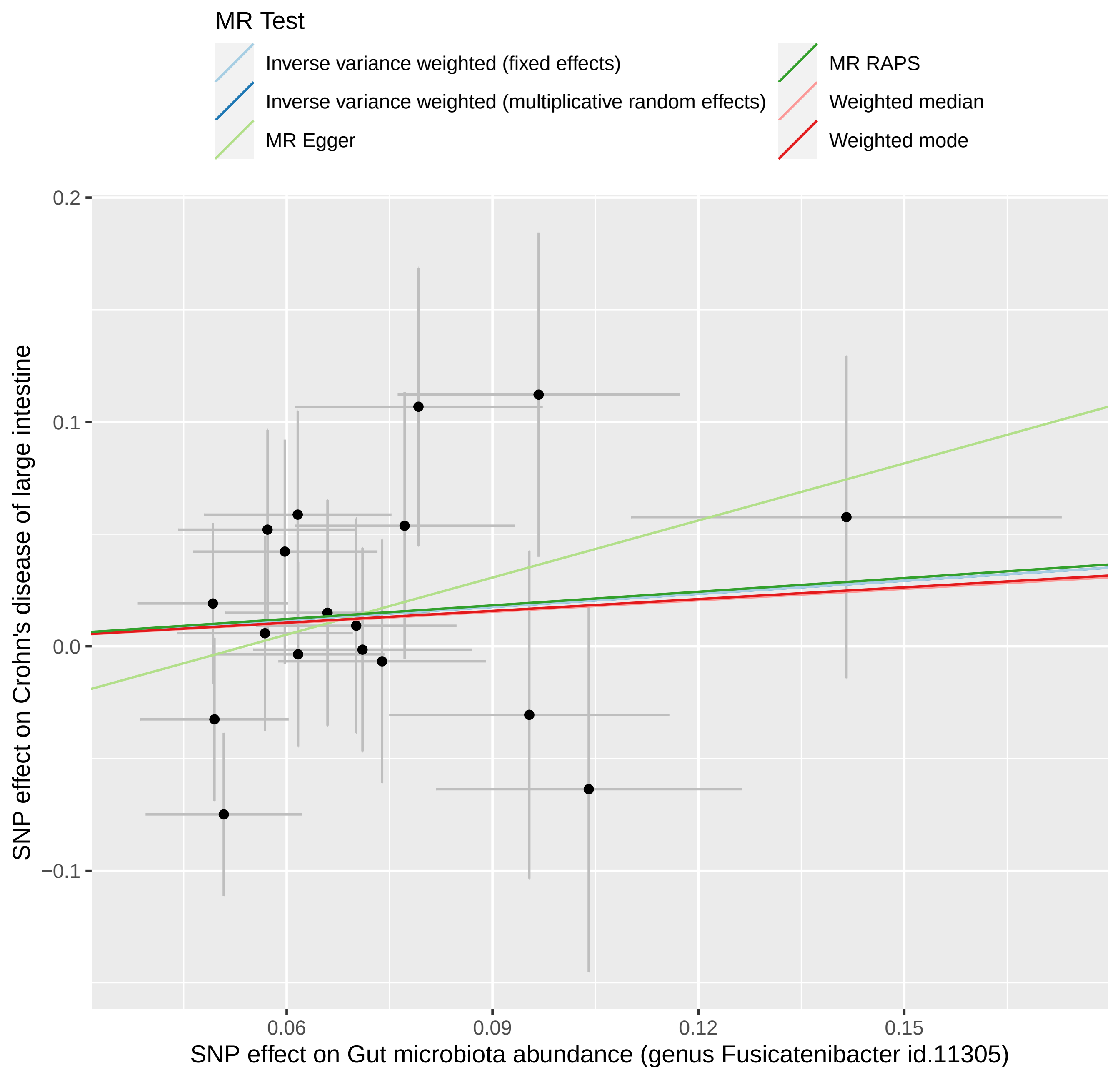

Batch 86 : Gut microbiota abundance (genus Gordonibacter id.821) on Crohn's disease of large intestine

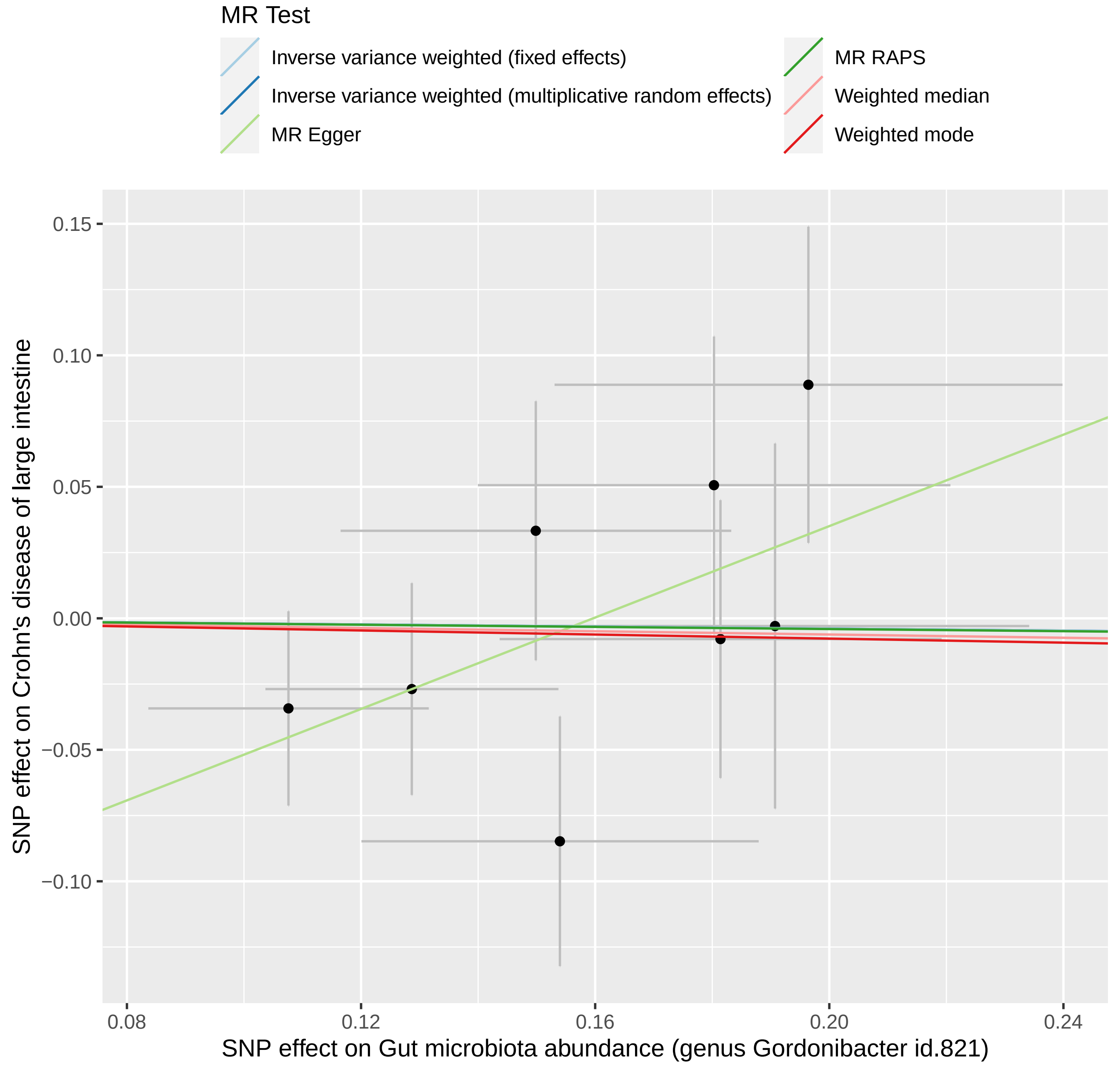

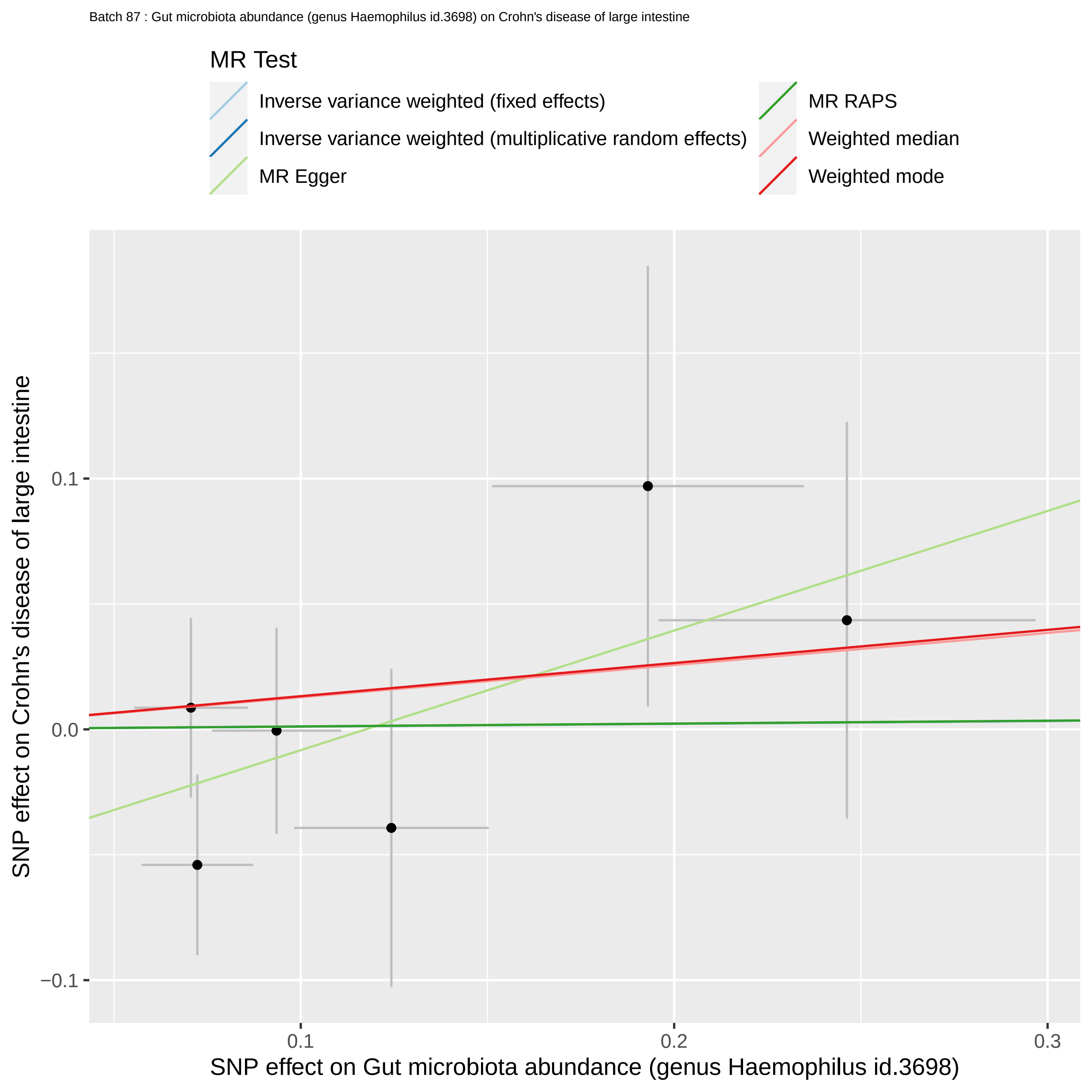
Batch 87 : Gut microbiota abundance (genus Haemophilus id.3698) on Crohn's disease of large intestine
MR Test
Inverse variance weighted (fixed effects)
Inverse variance weighted (multiplicative random effects)
MR Egger
MR RAPS
Weighted median
Weighted mode
0.1
0.0
−0.1
0.1
0.2
0.3
SNP effect on Crohn's disease of large intestine
SNP effect on Gut microbiota abundance (genus Haemophilus id.3698)

Batch 88 : Gut microbiota abundance (genus Holdemanella id.11393) on Crohn's disease of large intestine

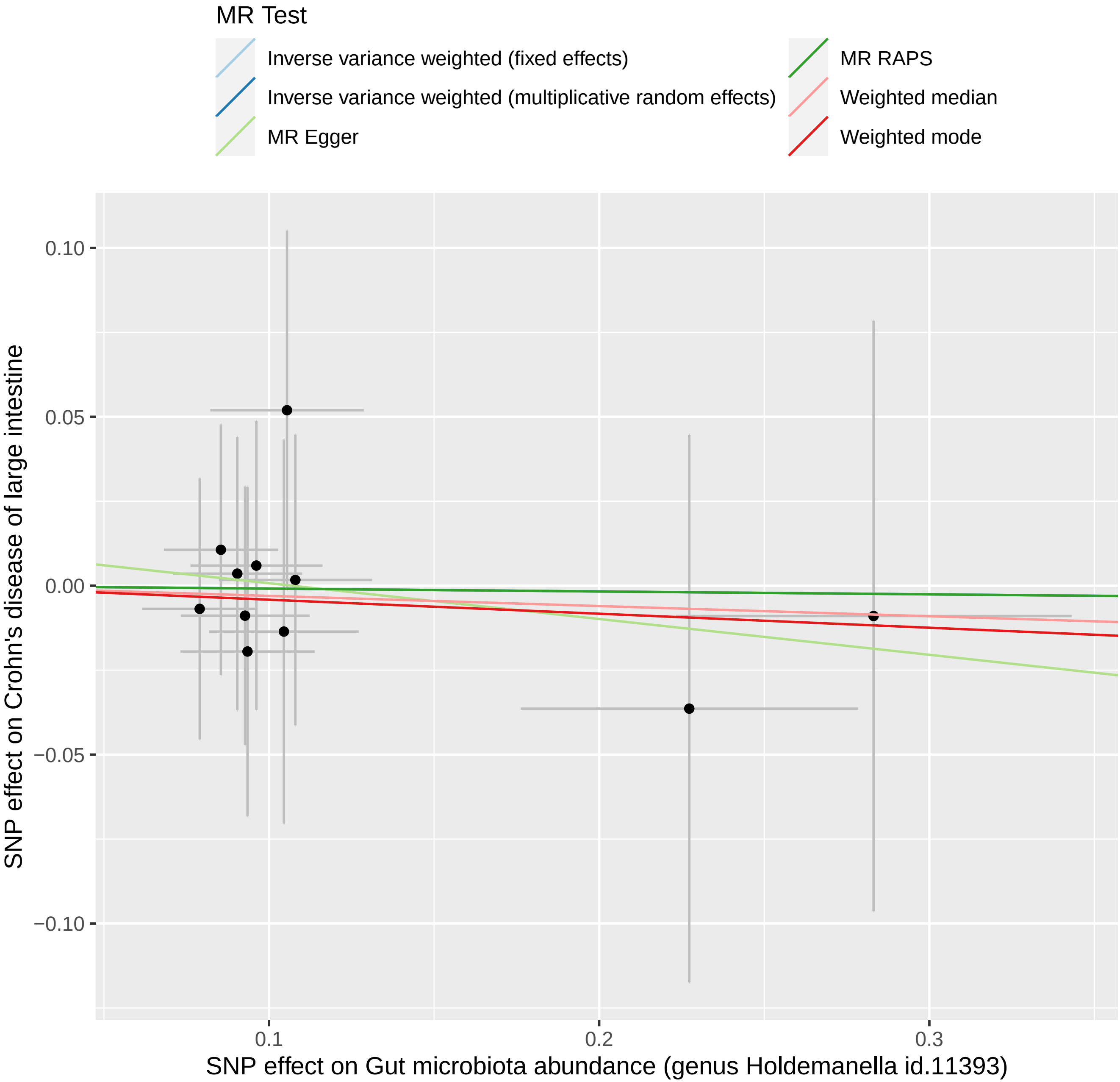

Batch 89 : Gut microbiota abundance (genus Holdemania id.2157) on Crohn's disease of large intestine

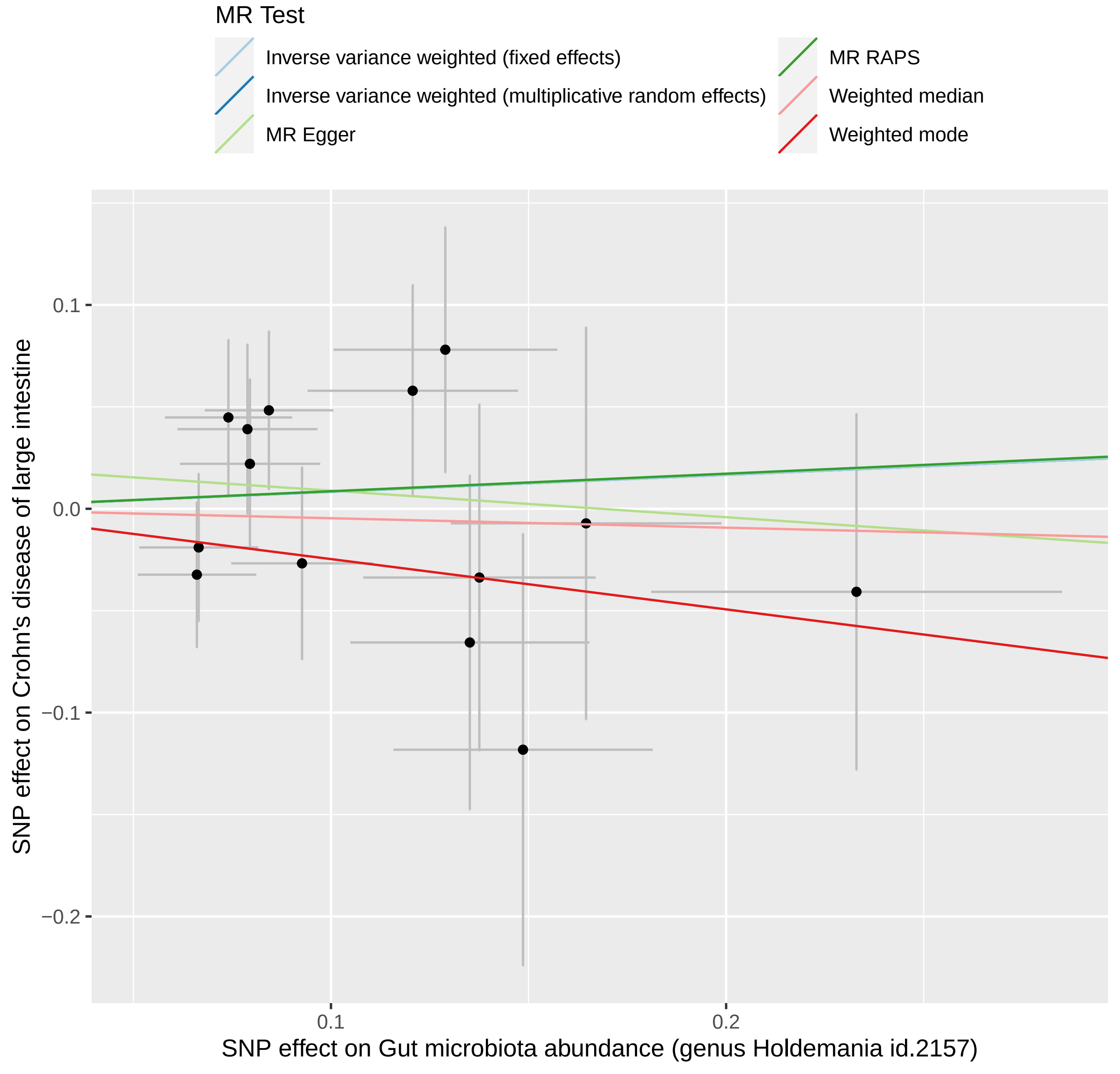

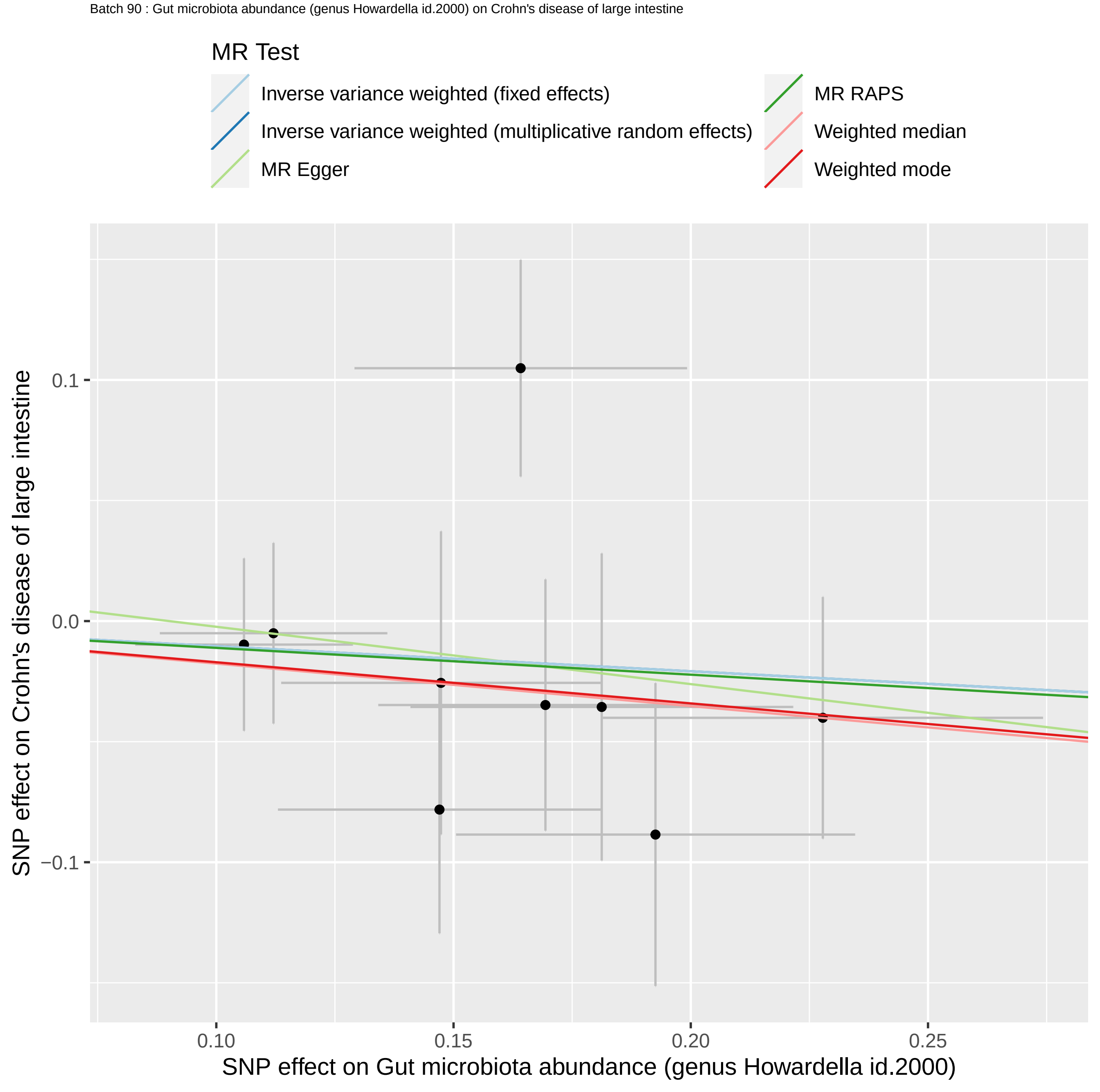
Batch 90 : Gut microbiota abundance (genus Howardella id.2000) on Crohn's disease of large intestine
MR Test
Inverse variance weighted (fixed effects)
Inverse variance weighted (multiplicative random effects)
MR Egger
MR RAPS
Weighted median
Weighted mode
0.1
0.0
−0.1
0.10
0.15
0.20
0.25
SNP effect on Crohn's disease of large intestine
SNP effect on Gut microbiota abundance (genus Howardella id.2000)

Batch 91 : Gut microbiota abundance (genus Hungatella id.11306) on Crohn's disease of large intestine

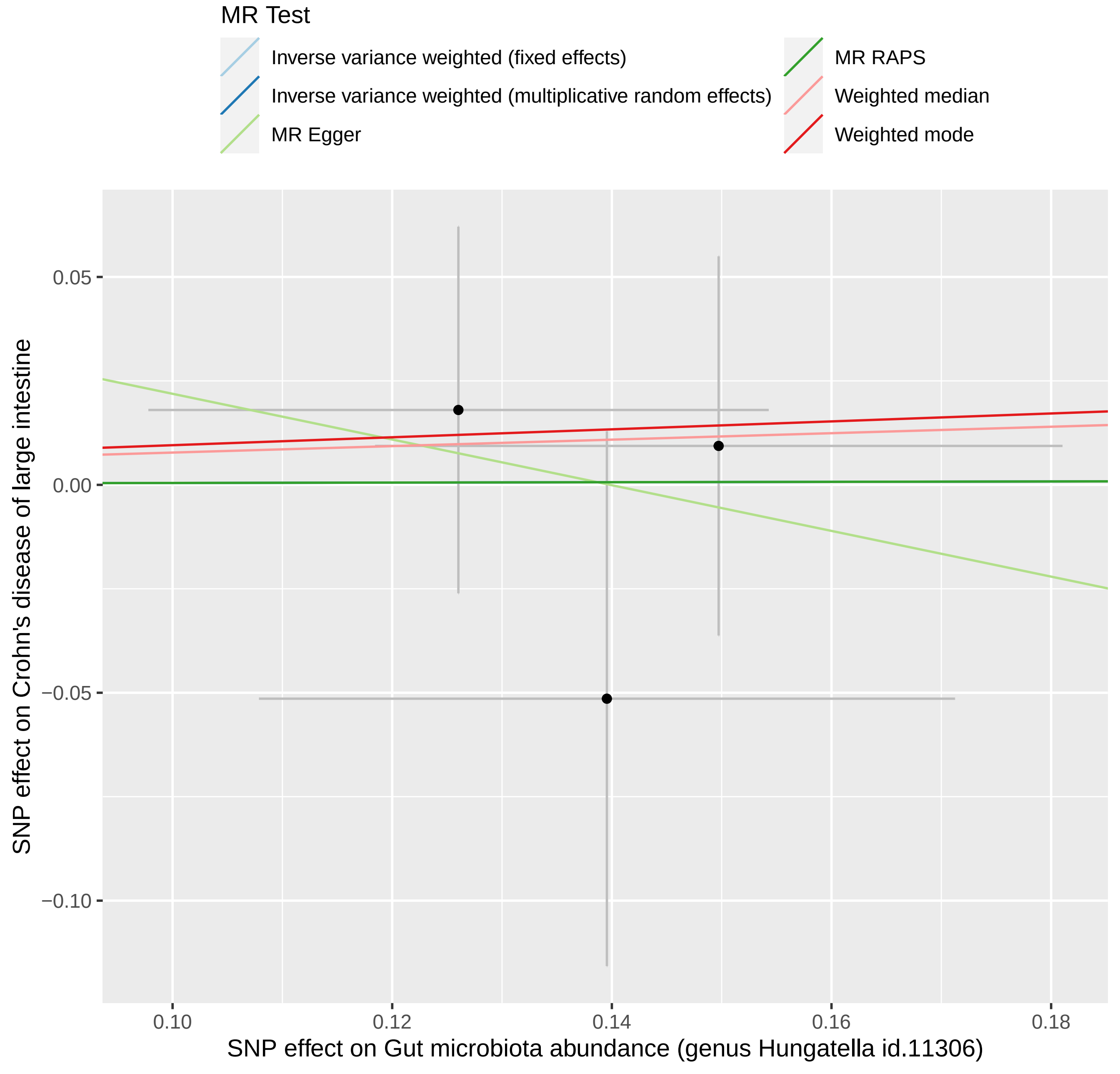

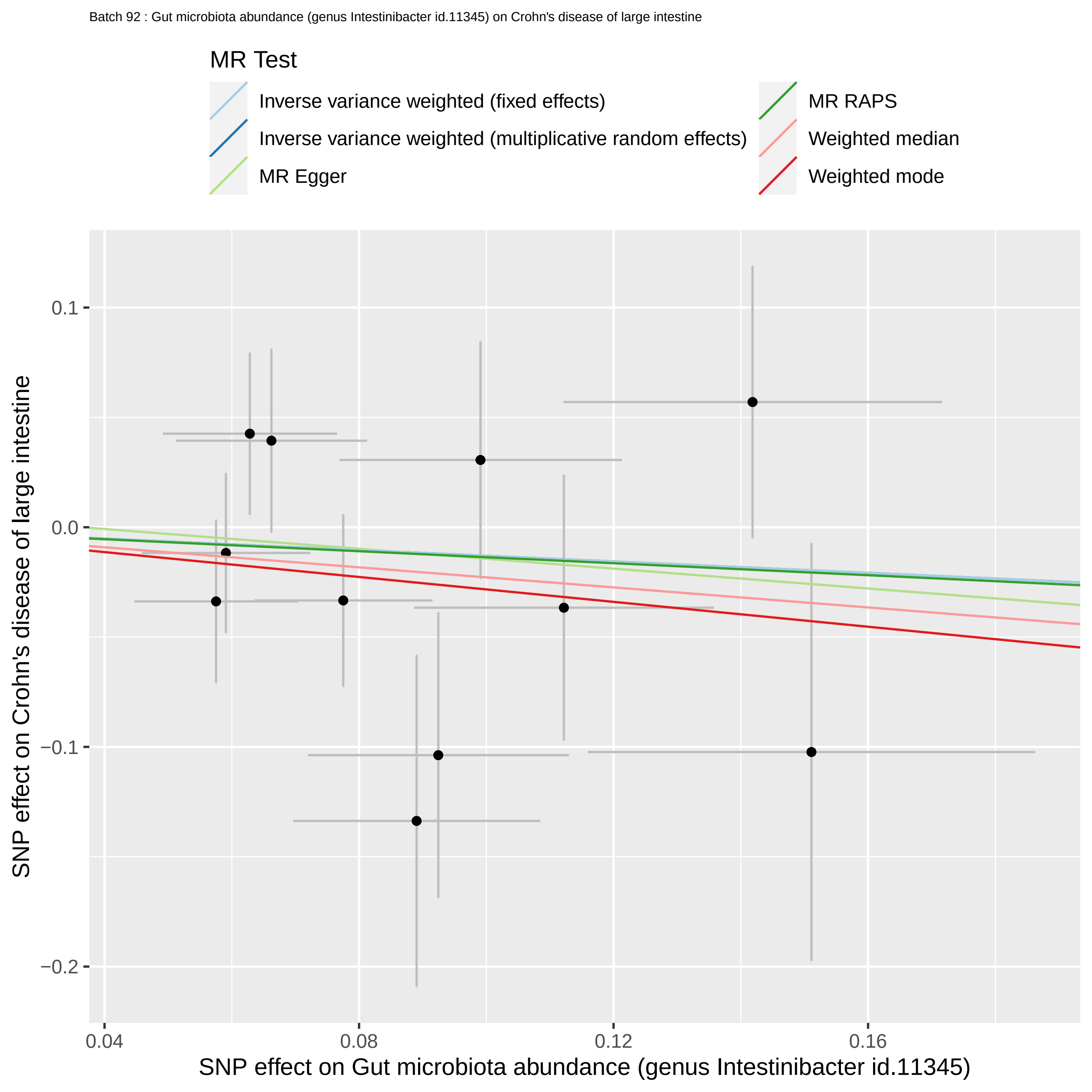
Batch 92 : Gut microbiota abundance (genus Intestinibacter id.11345) on Crohn's disease of large intestine
MR Test
Inverse variance weighted (fixed effects)
Inverse variance weighted (multiplicative random effects)
MR Egger
MR RAPS
Weighted median
Weighted mode
SNP effect on Crohn's disease of large intestine
0.1
0.0
−0.1
−0.2
0.04
0.08
0.12
0.16
SNP effect on Gut microbiota abundance (genus Intestinibacter id.11345)

Batch 93 : Gut microbiota abundance (genus Intestinimonas id.2062) on Crohn's disease of large intestine

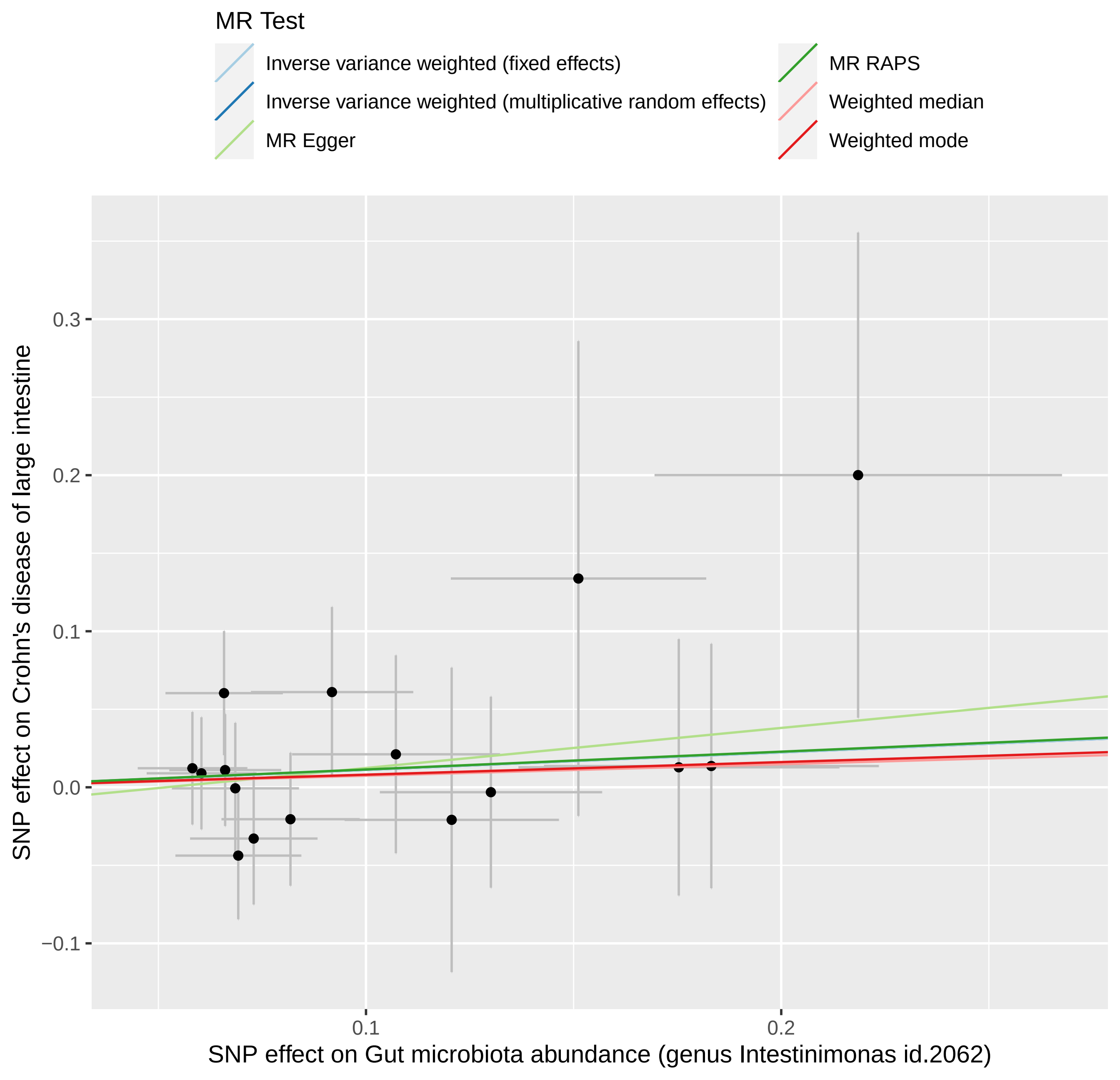

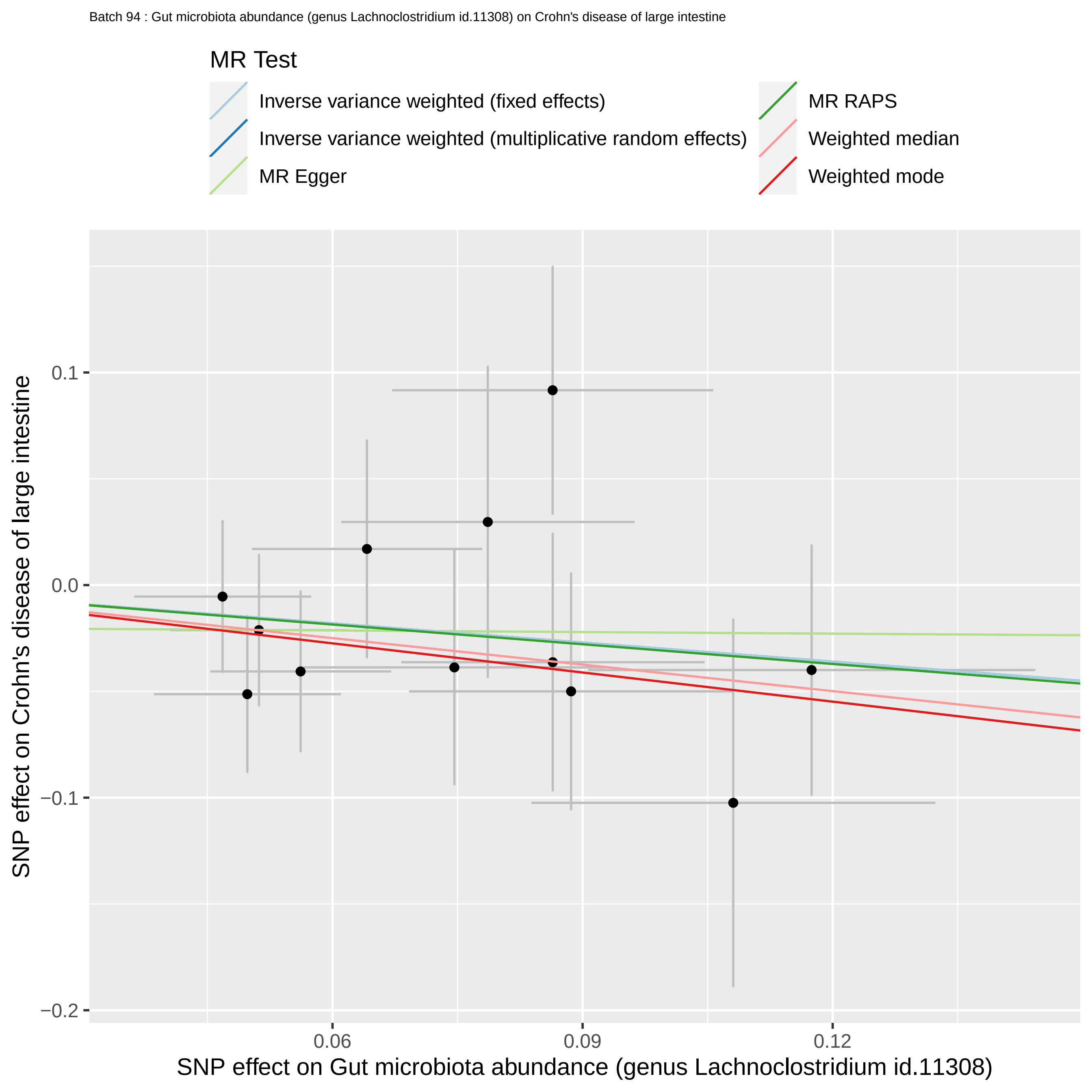

Batch 94 : Gut microbiota abundance (genus Lachnoclostridium id.11308) on Crohn's disease of large intestine
MR Test
Inverse variance weighted (fixed effects)
Inverse variance weighted (multiplicative random effects)
MR Egger
MR RAPS
Weighted median
Weighted mode
0.1
0.0
−0.1
−0.2
0.06
0.09
0.12
SNP effect on Crohn's disease of large intestine
SNP effect on Gut microbiota abundance (genus Lachnoclostridium id.11308)

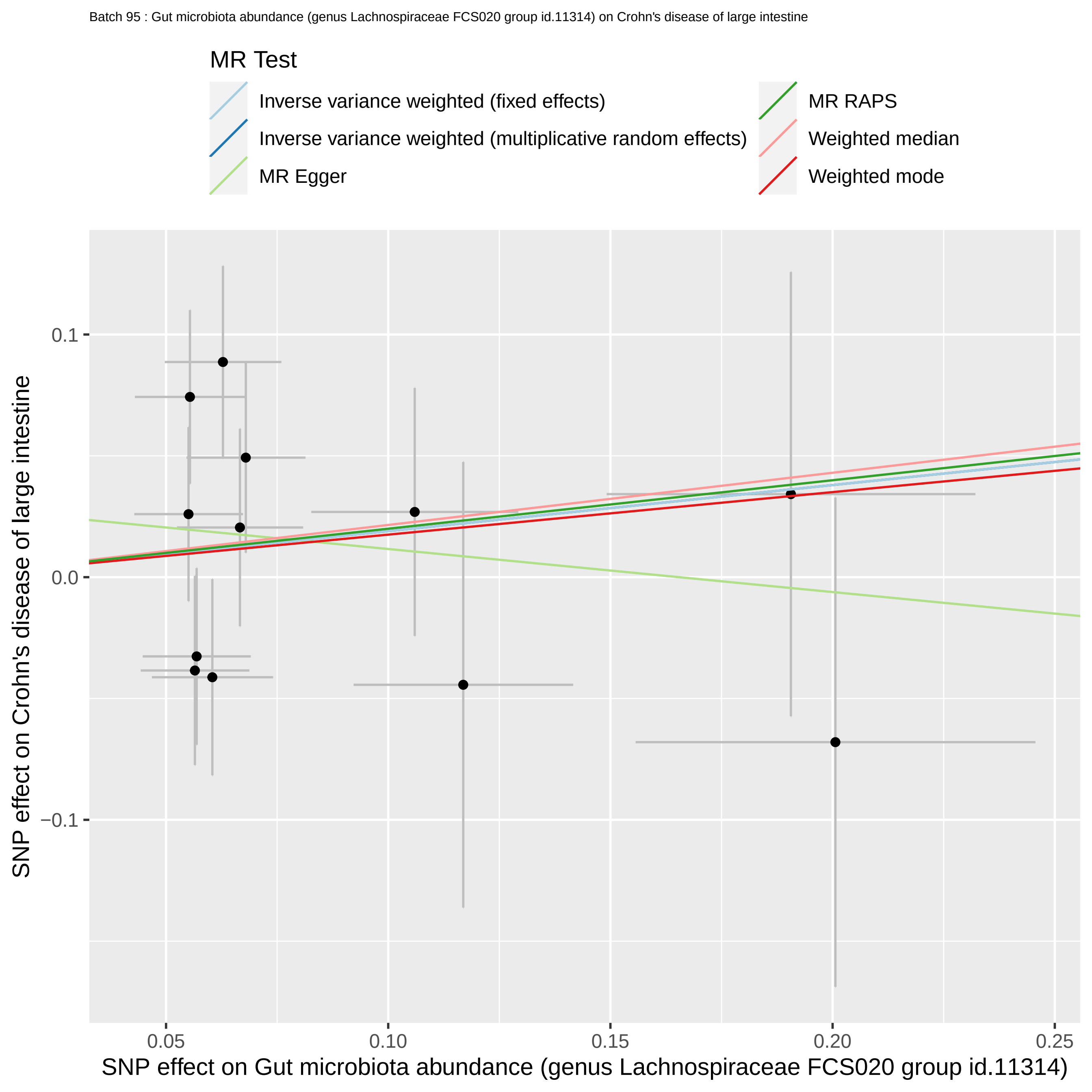

Batch 95 : Gut microbiota abundance (genus Lachnospiraceae FCS020 group id.11314) on Crohn's disease of large intestine
MR Test
Inverse variance weighted (fixed effects)
Inverse variance weighted (multiplicative random effects)
MR Egger
MR RAPS
Weighted median
Weighted mode
0.1
0.0
−0.1
0.05
0.10
0.15
0.20
0.25
SNP effect on Crohn's disease of large intestine
SNP effect on Gut microbiota abundance (genus Lachnospiraceae FCS020 group id.11314)

Batch 96 : Gut microbiota abundance (genus Lachnospiraceae NC2004 group id.11316) on Crohn's disease of large intestine

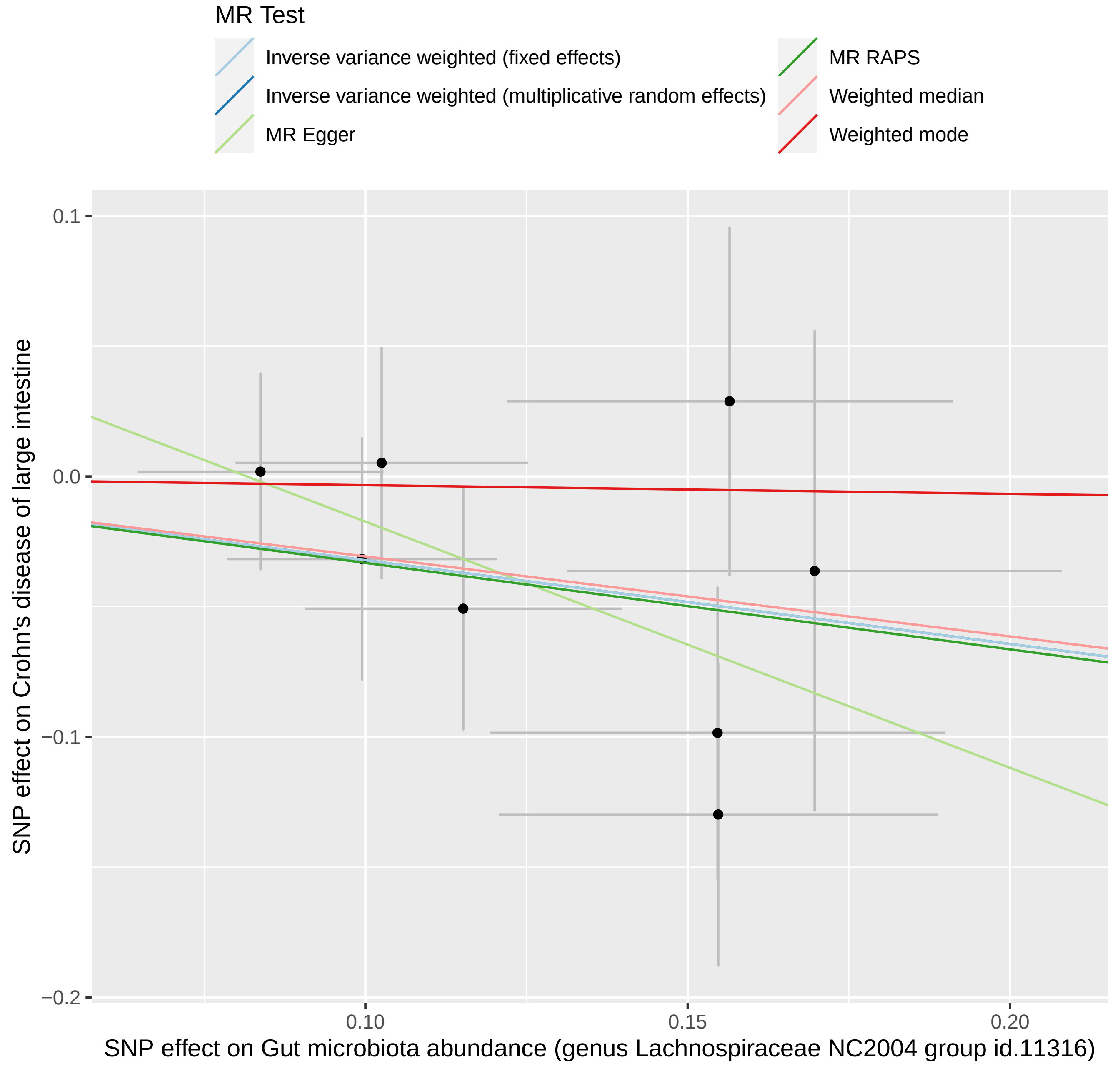

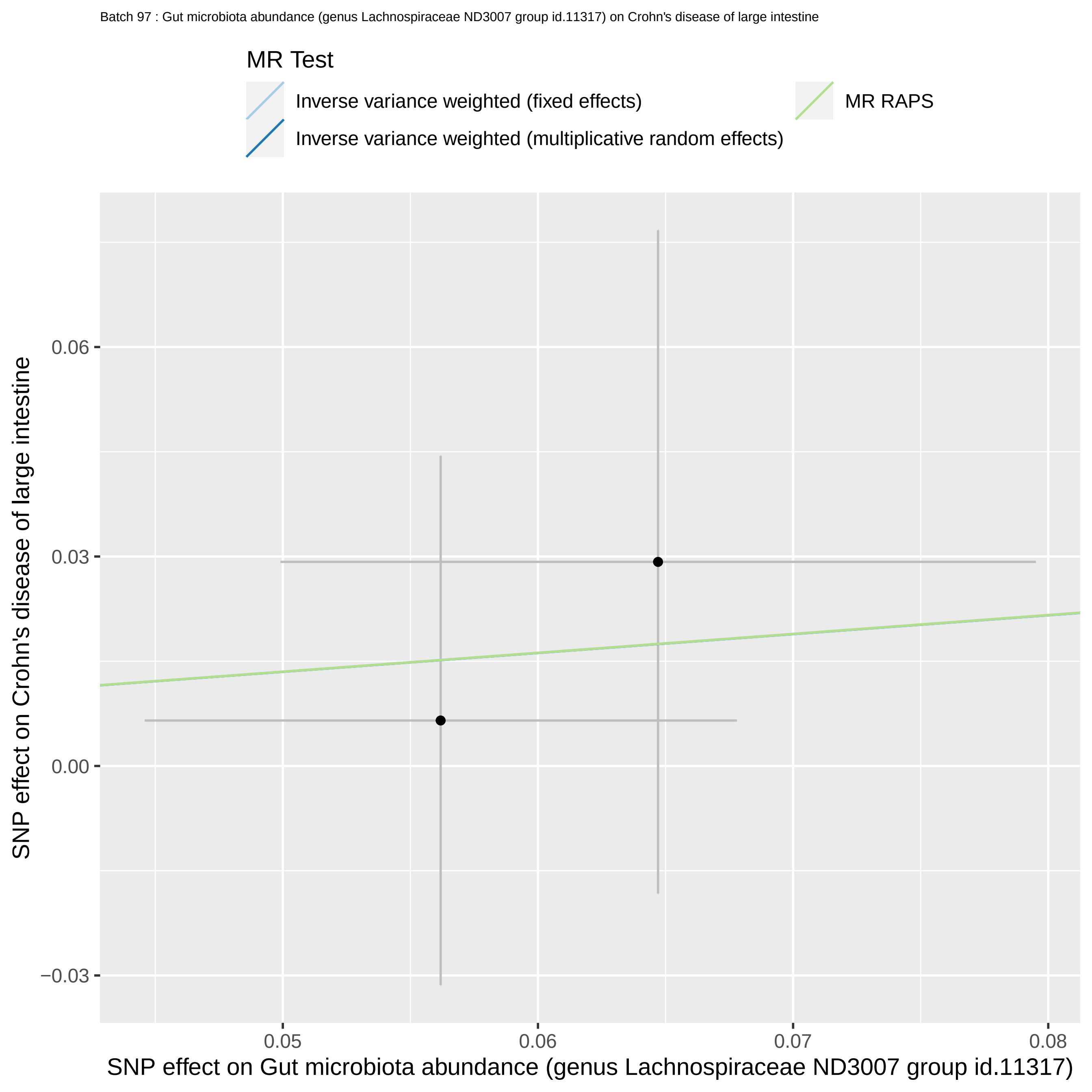
Batch 97 : Gut microbiota abundance (genus Lachnospiraceae ND3007 group id.11317) on Crohn's disease of large intestine
MR Test
Inverse variance weighted (fixed effects)
Inverse variance weighted (multiplicative random effects)
MR RAPS
0.06
0.03
0.00
−0.03
0.05
0.06
0.07
0.08
SNP effect on Crohn's disease of large intestine
SNP effect on Gut microbiota abundance (genus Lachnospiraceae ND3007 group id.11317)

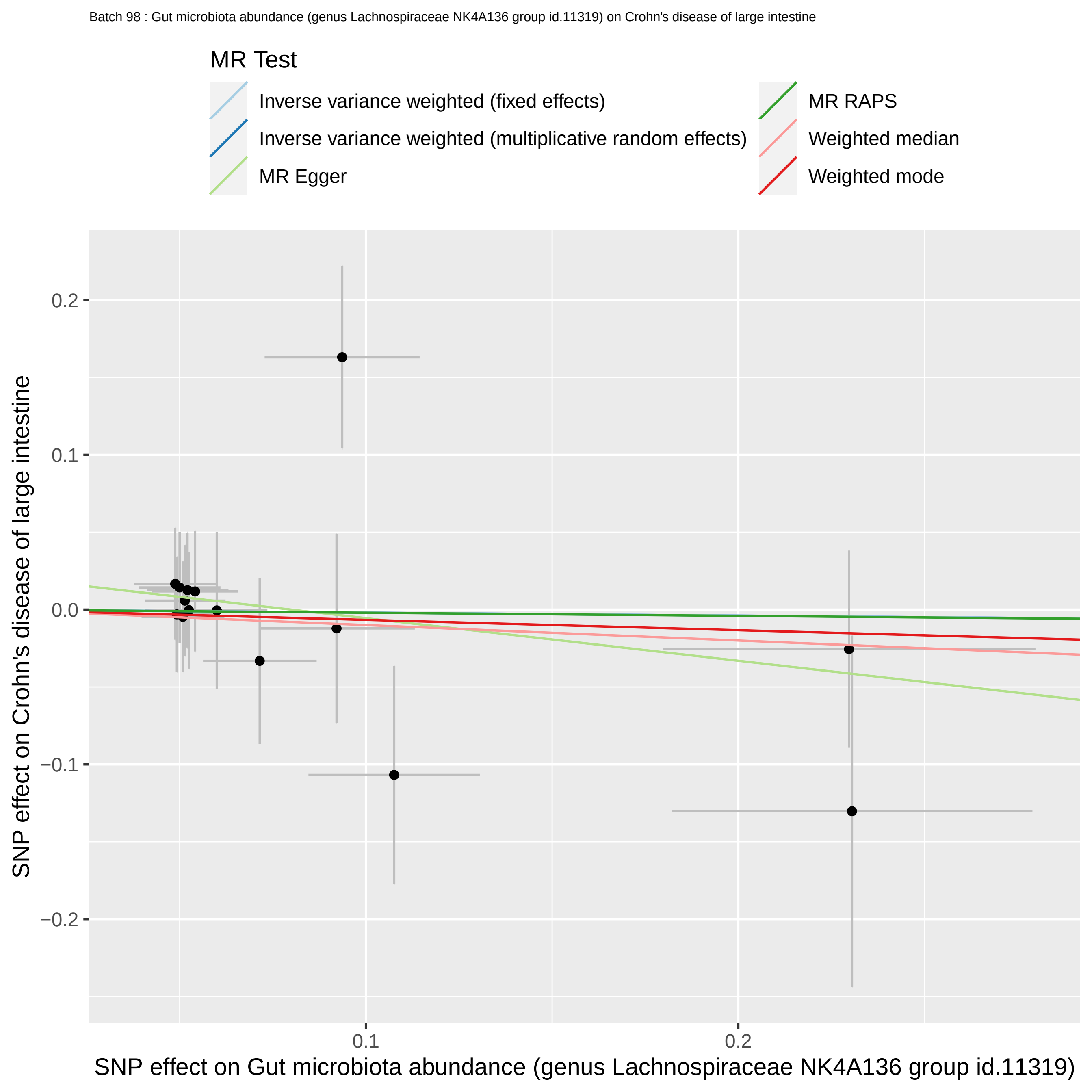

Batch 98 : Gut microbiota abundance (genus Lachnospiraceae NK4A136 group id.11319) on Crohn's disease of large intestine
MR Test
Inverse variance weighted (fixed effects)
Inverse variance weighted (multiplicative random effects)
MR Egger
MR RAPS
Weighted median
Weighted mode
0.2
0.1
0.0
−0.1
−0.2
0.1
0.2
SNP effect on Crohn's disease of large intestine
SNP effect on Gut microbiota abundance (genus Lachnospiraceae NK4A136 group id.11319)

Batch 99 : Gut microbiota abundance (genus Lachnospiraceae UCG001 id.11321) on Crohn's disease of large intestine

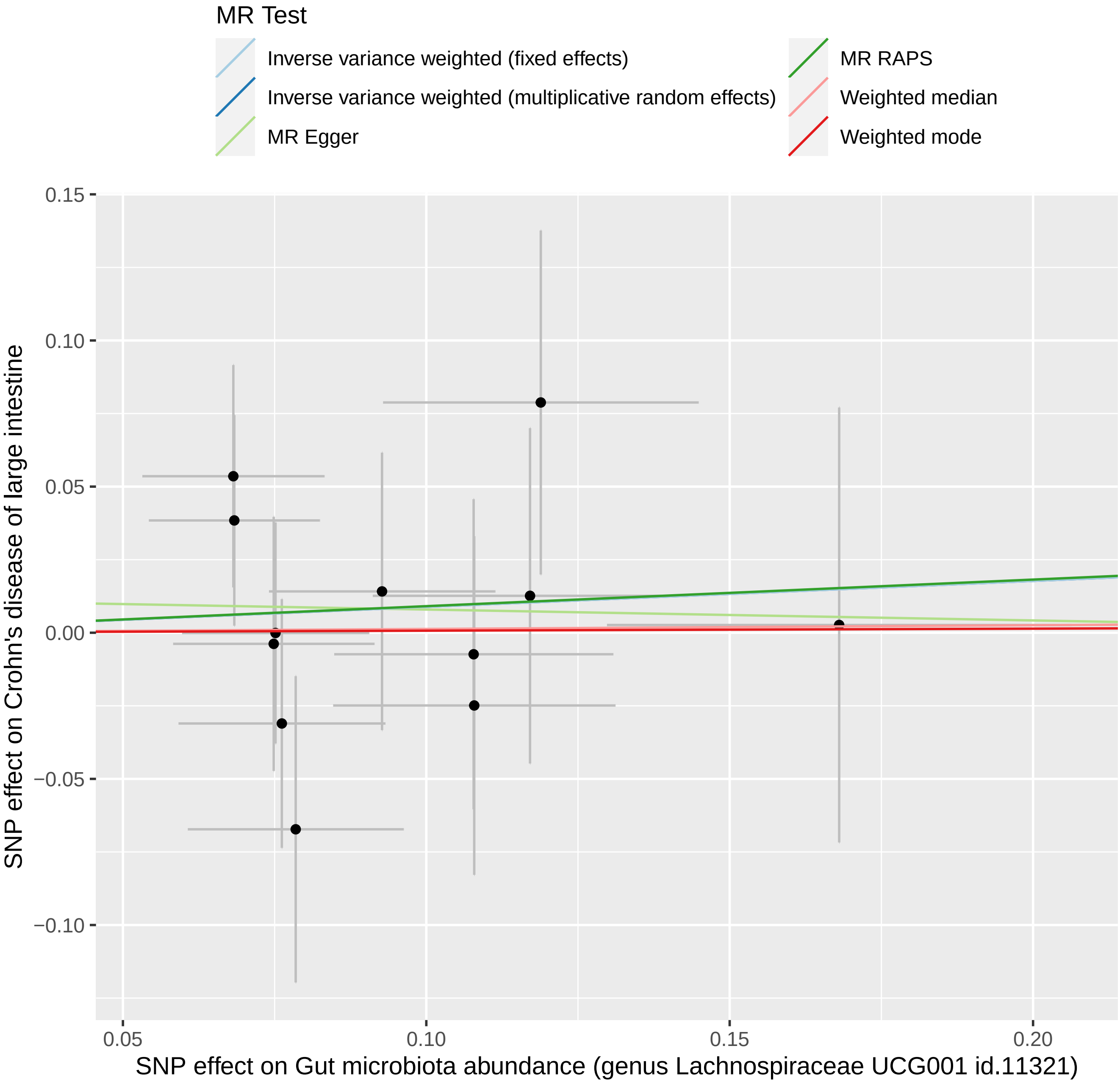

Batch 100 : Gut microbiota abundance (genus Lachnospiraceae UCG004 id.11324) on Crohn's disease of large intestine

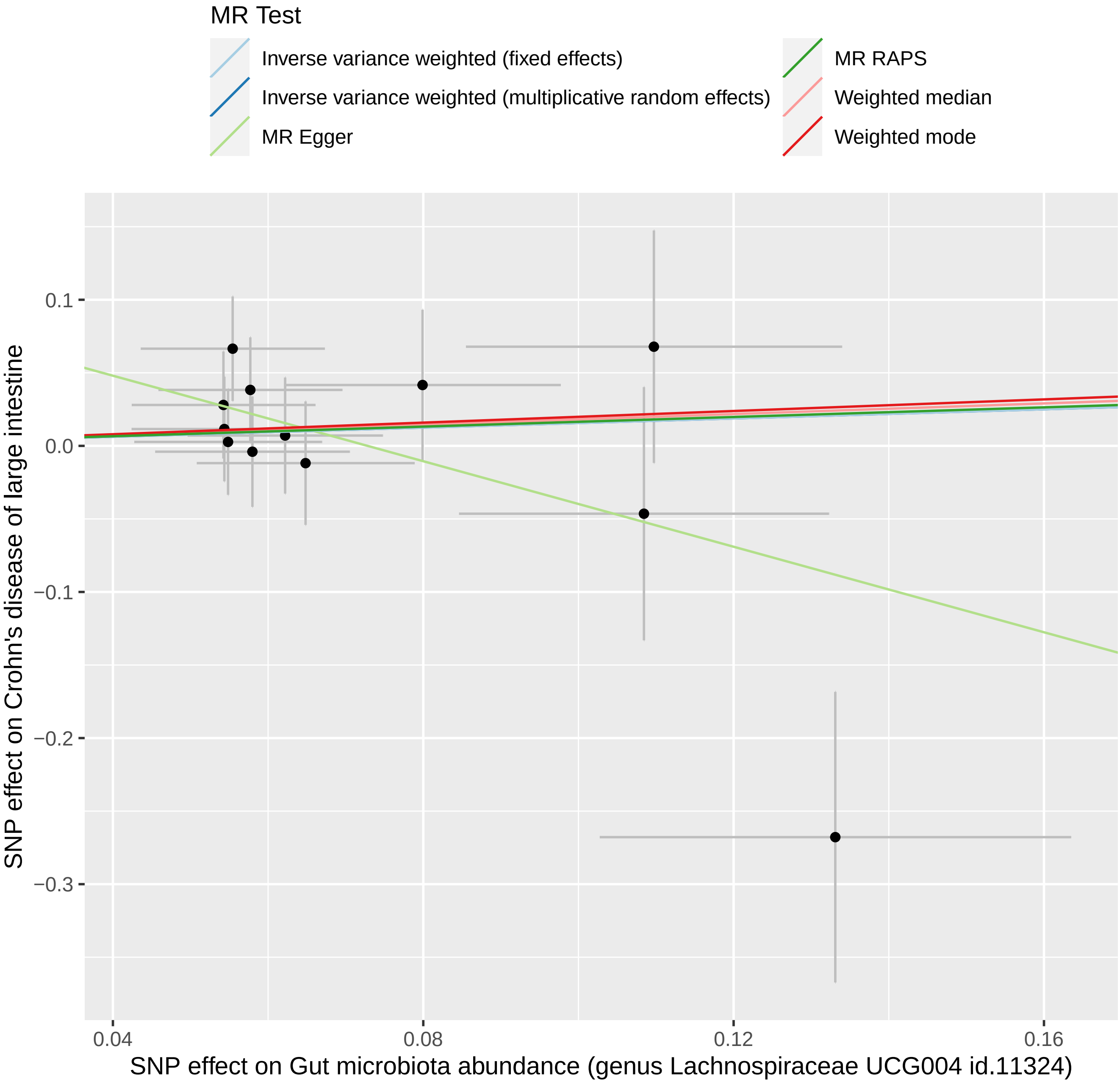

Batch 101 : Gut microbiota abundance (genus Lachnospiraceae UCG008 id.11328) on Crohn's disease of large intestine

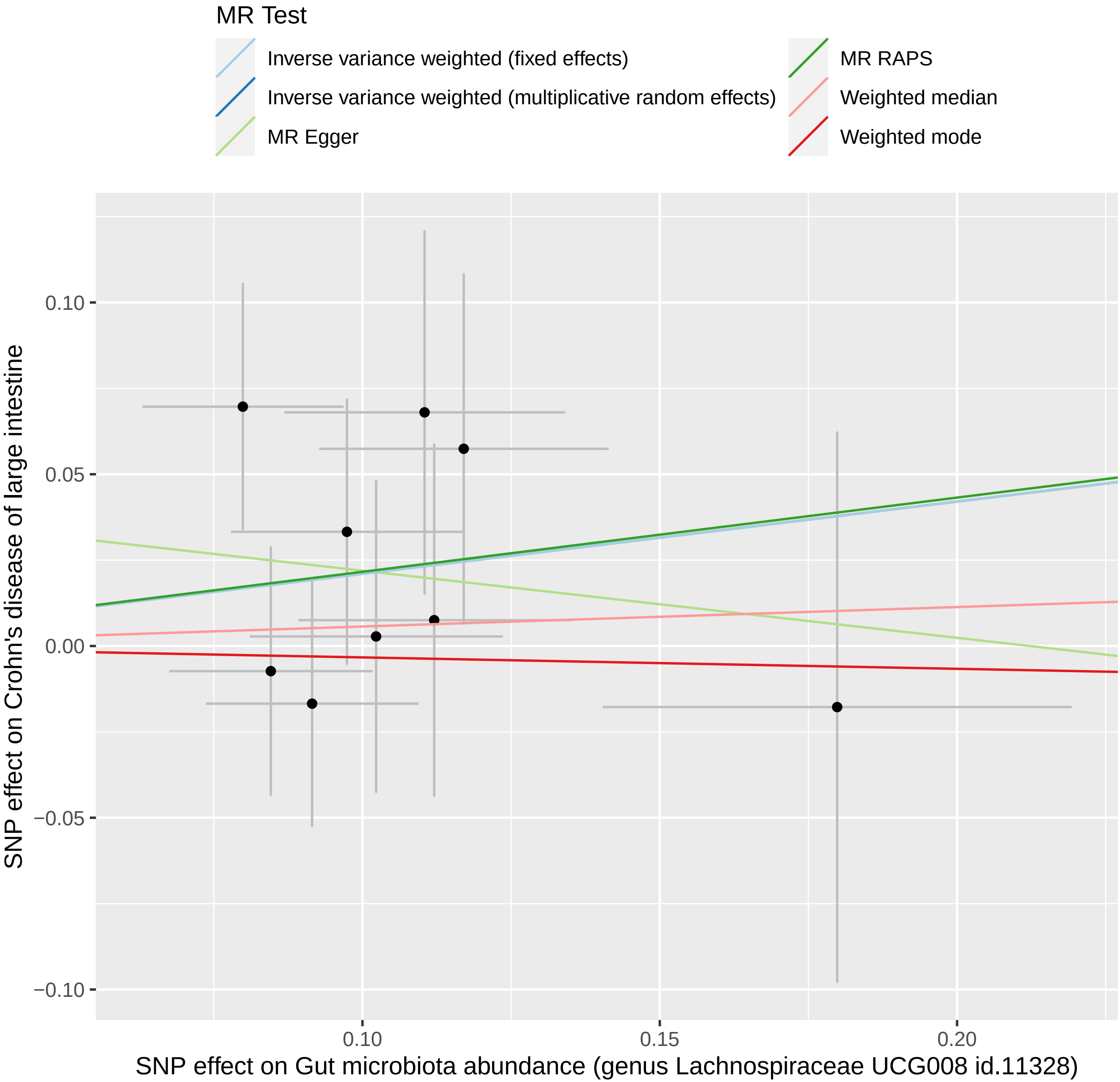

Batch 102 : Gut microbiota abundance (genus Lachnospiraceae UCG010 id.11330) on Crohn's disease of large intestine

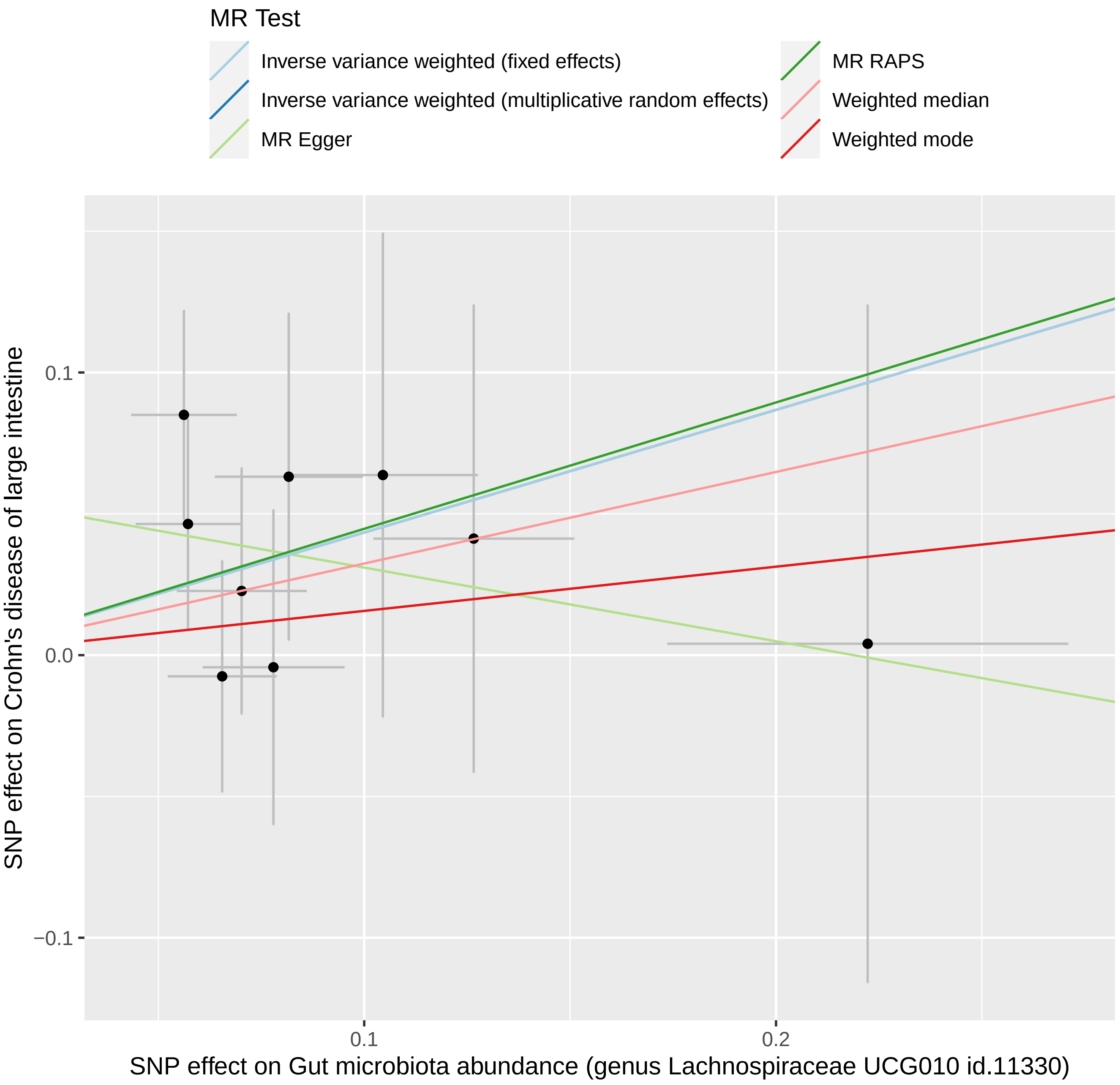

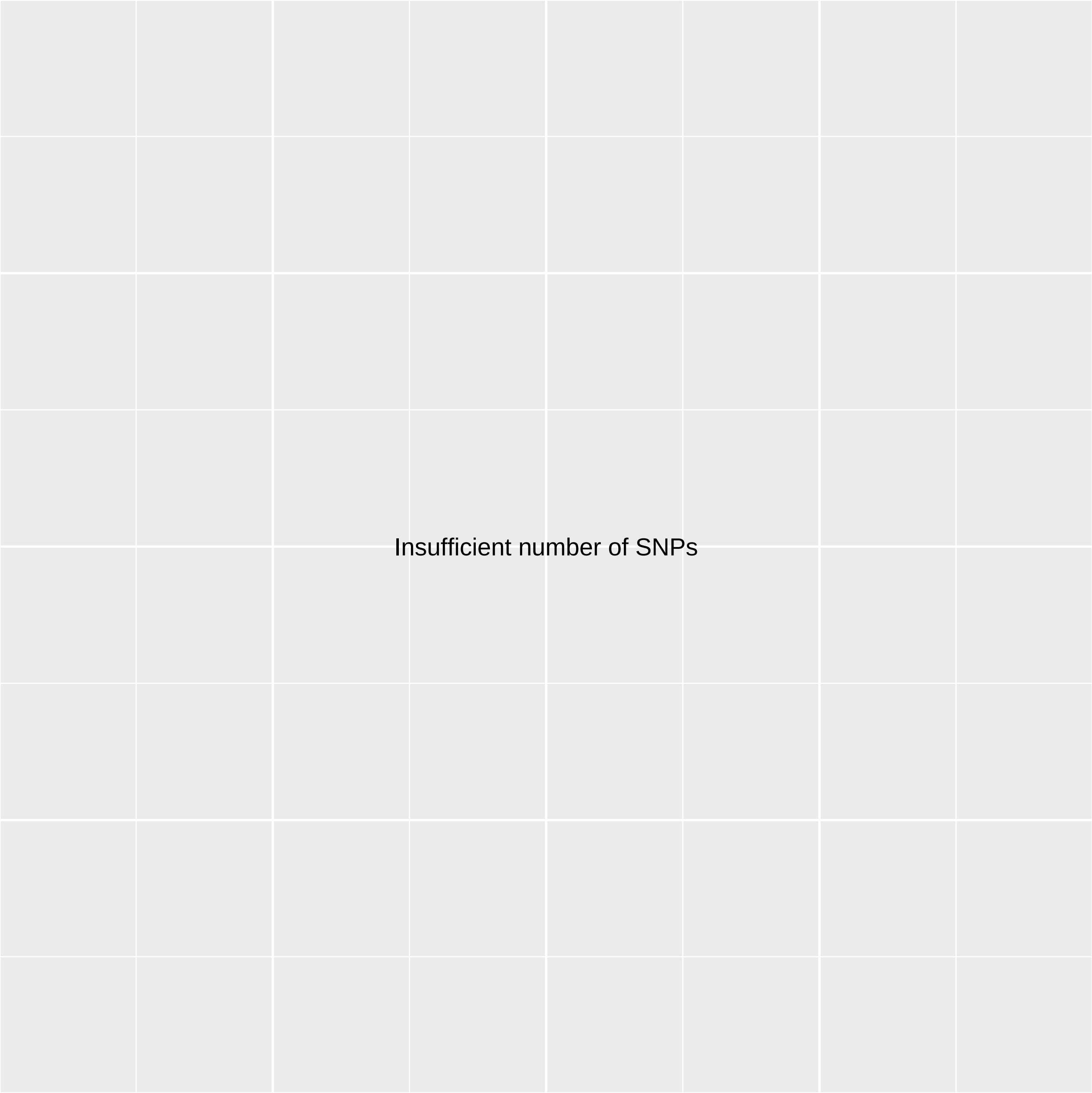
Insufficient number of SNPs

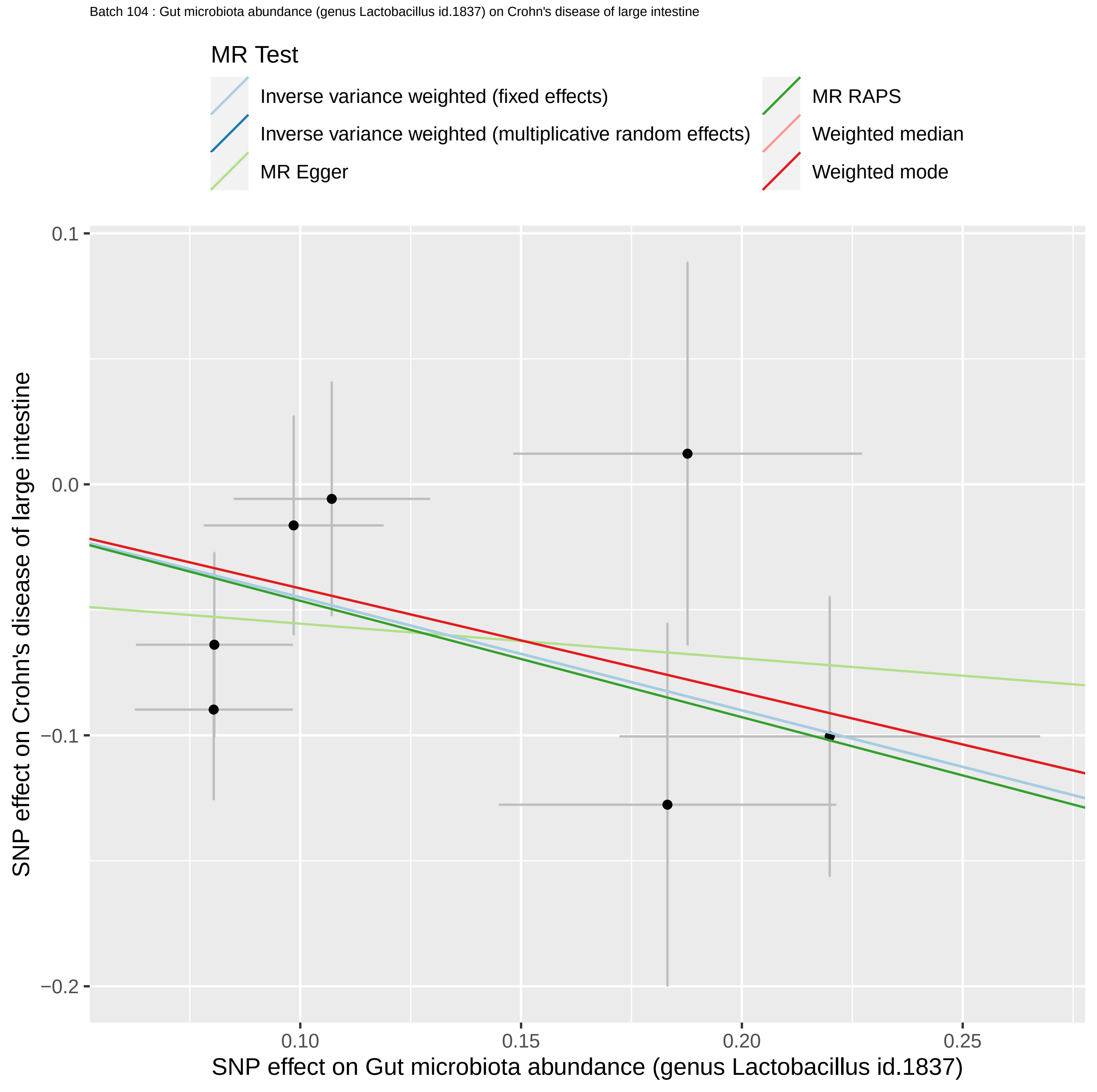

Batch 104 : Gut microbiota abundance (genus Lactobacillus id.1837) on Crohn's disease of large intestine
MR Test
Inverse variance weighted (fixed effects)
Inverse variance weighted (multiplicative random effects)
MR Egger
MR RAPS
Weighted median
Weighted mode
0.1
0.0
−0.1
−0.2
0.10
0.15
0.20
0.25
SNP effect on Crohn's disease of large intestine
SNP effect on Gut microbiota abundance (genus Lactobacillus id.1837)

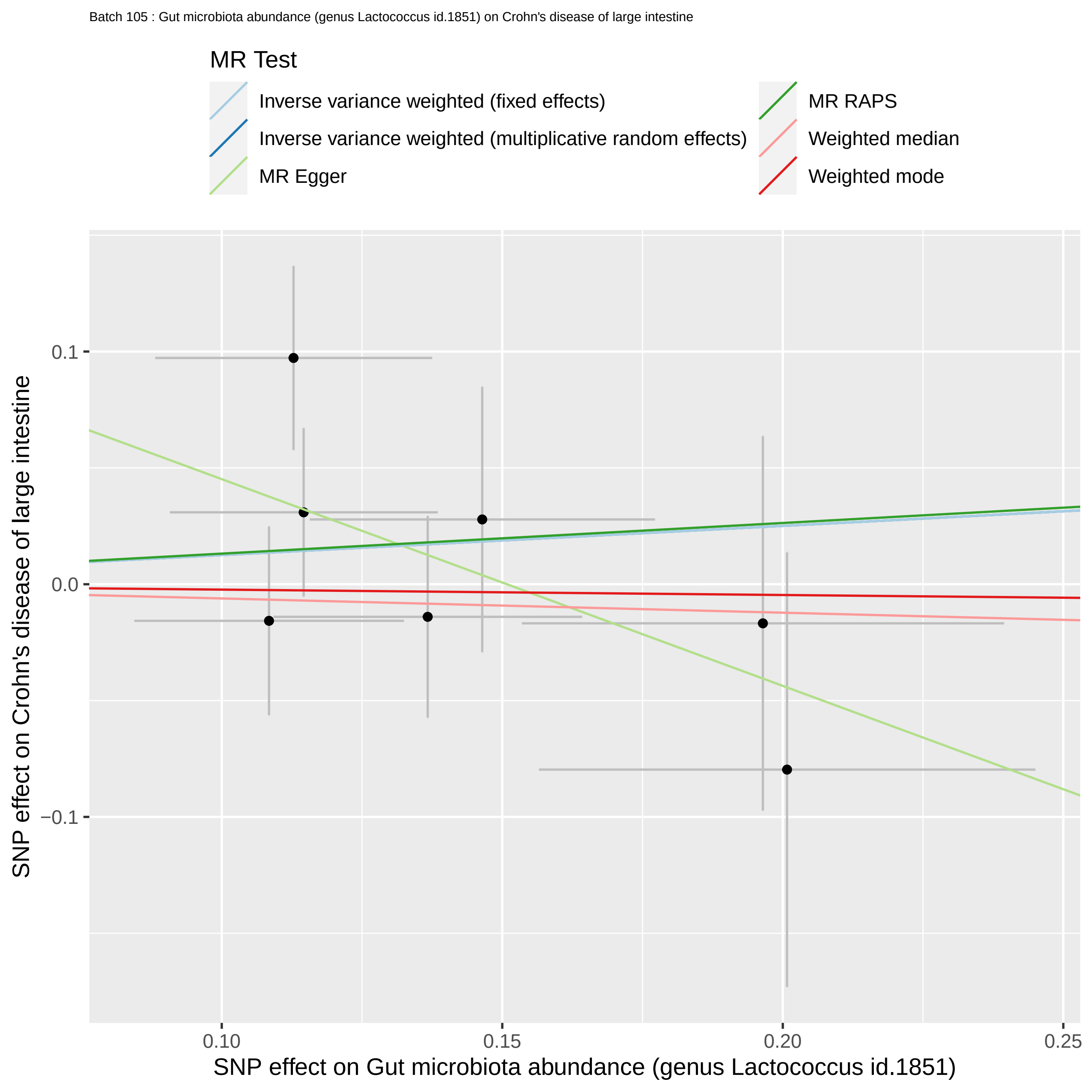
Batch 105 : Gut microbiota abundance (genus Lactococcus id.1851) on Crohn's disease of large intestine
MR Test
Inverse variance weighted (fixed effects)
Inverse variance weighted (multiplicative random effects)
MR Egger
MR RAPS
Weighted median
Weighted mode
0.1
0.0
−0.1
0.10
0.15
0.20
0.25
SNP effect on Gut microbiota abundance (genus Lactococcus id.1851)
SNP effect on Crohn's disease of large intestine

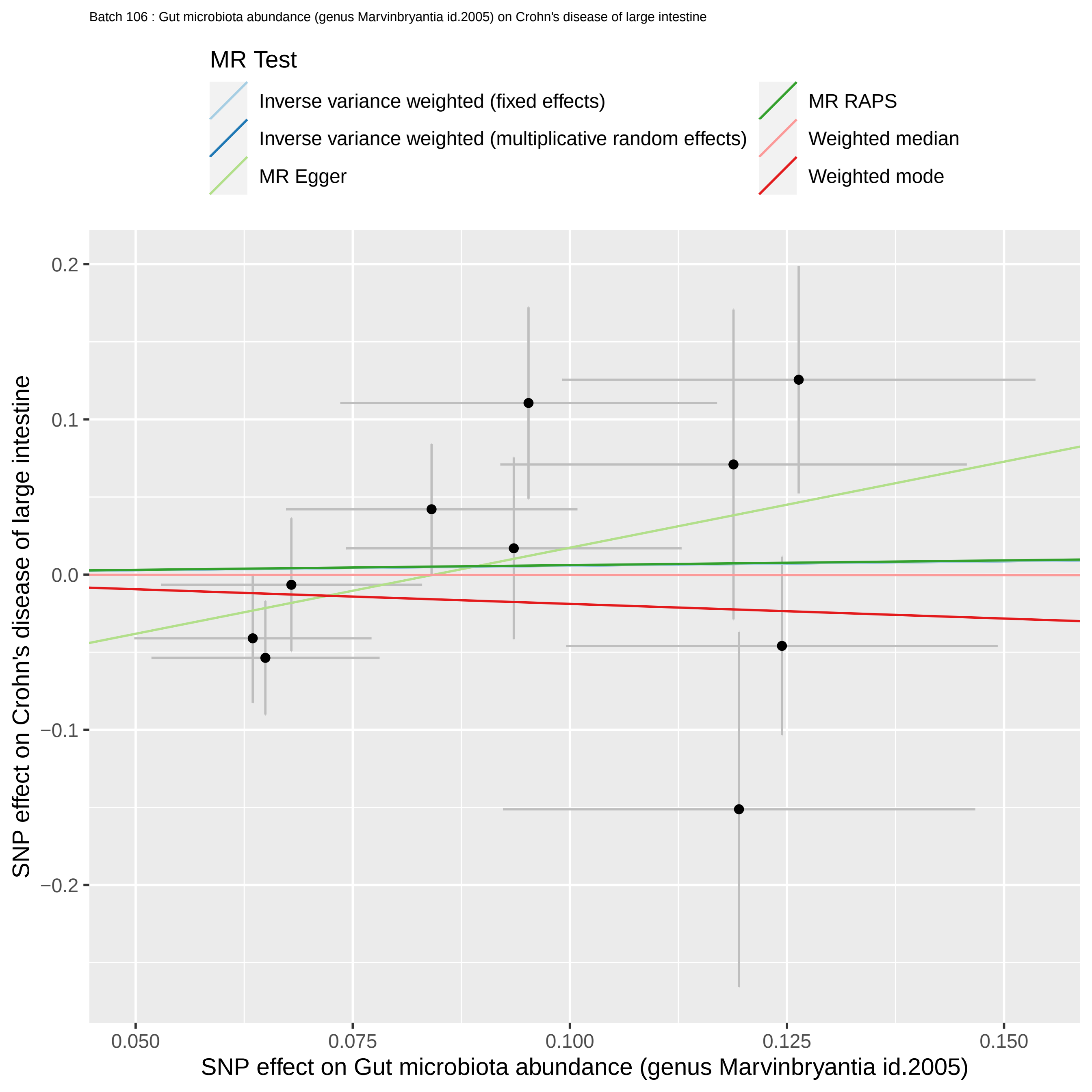
Batch 106 : Gut microbiota abundance (genus Marvinbryantia id.2005) on Crohn's disease of large intestine
MR Test
Inverse variance weighted (fixed effects)
Inverse variance weighted (multiplicative random effects)
MR Egger
MR RAPS
Weighted median
Weighted mode
0.2
0.1
0.0
−0.1
−0.2
0.050
0.075
0.100
0.125
0.150
SNP effect on Crohn's disease of large intestine
SNP effect on Gut microbiota abundance (genus Marvinbryantia id.2005)

Batch 107 : Gut microbiota abundance (genus Methanobrevibacter id.123) on Crohn's disease of large intestine

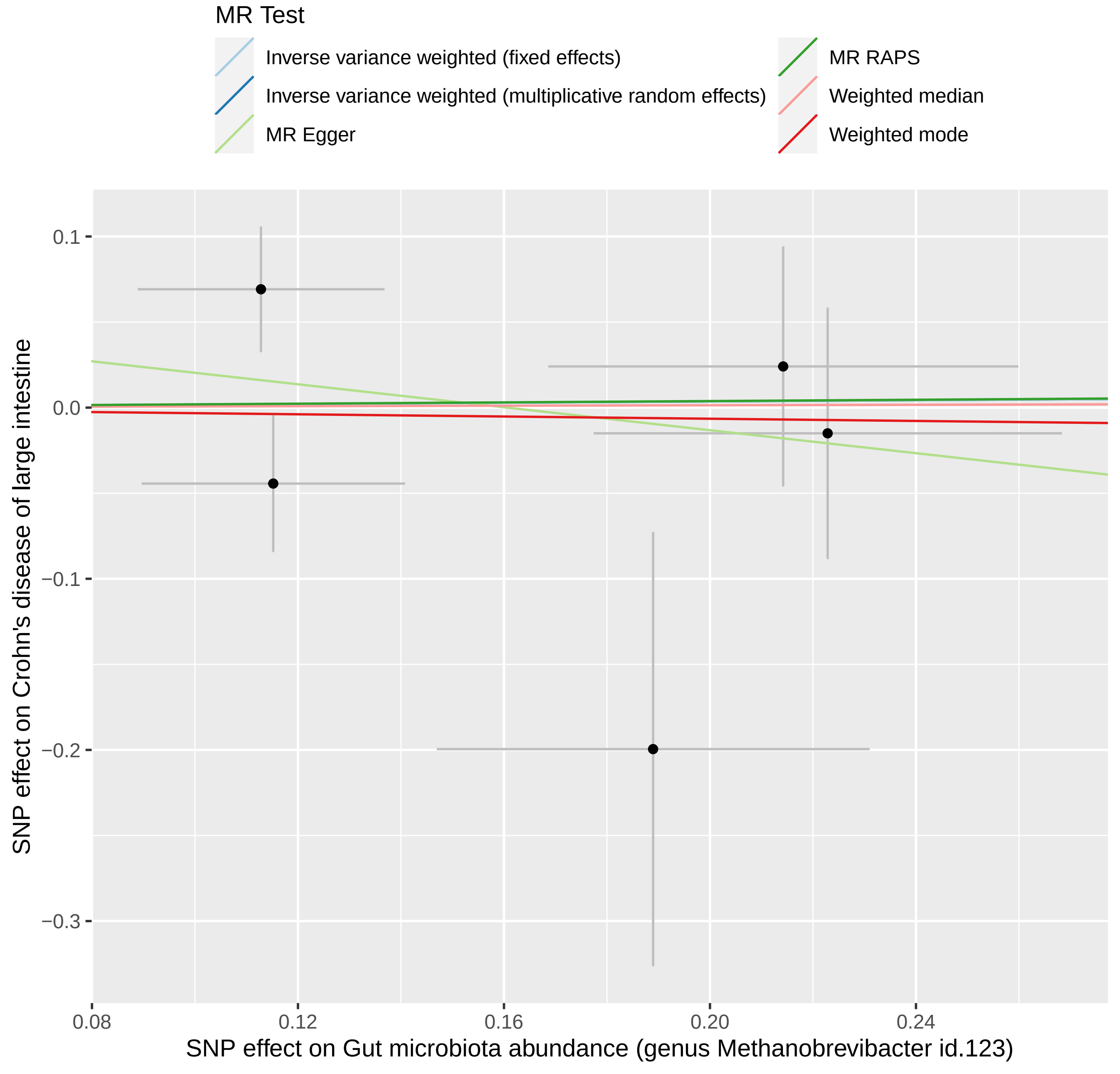

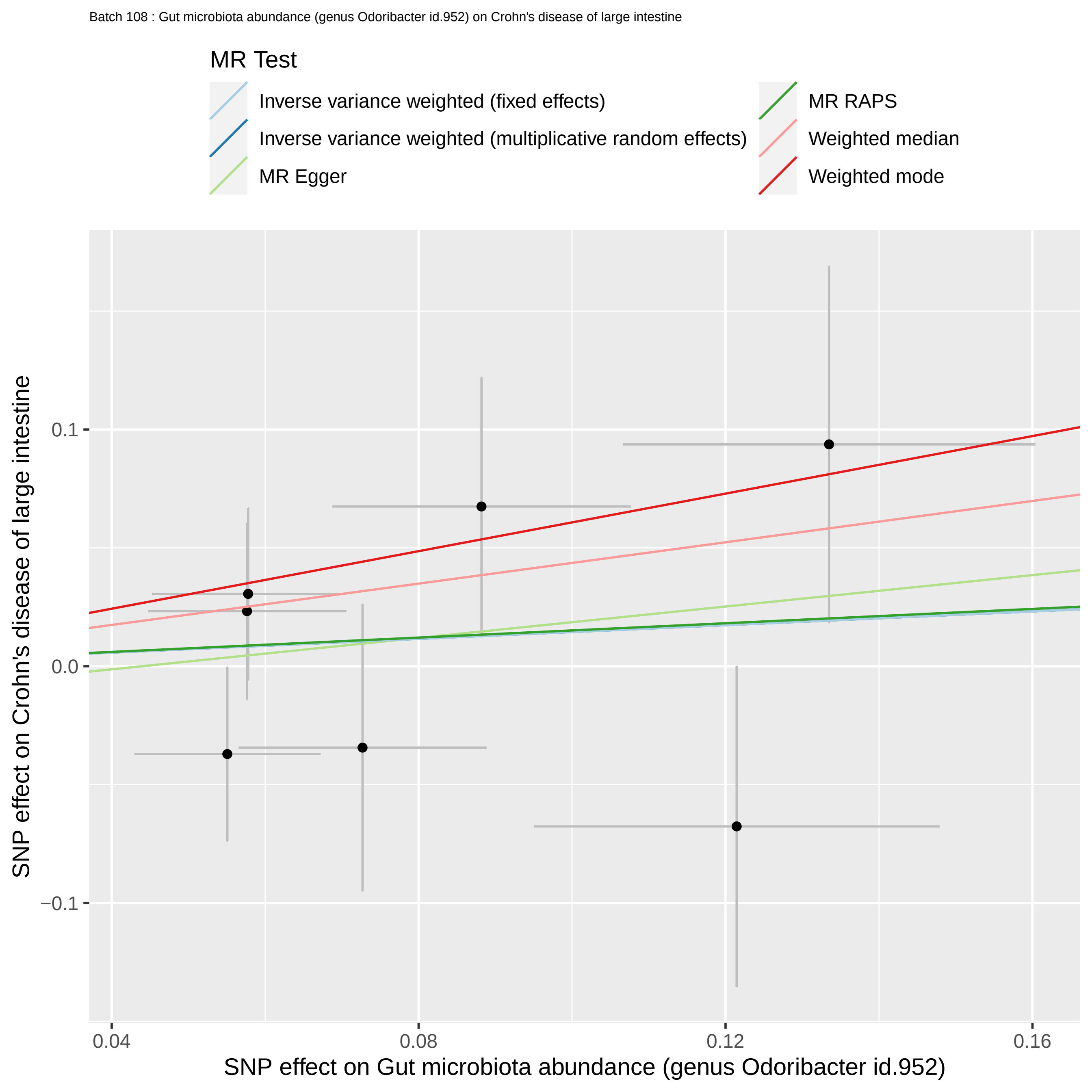
Batch 108 : Gut microbiota abundance (genus Odoribacter id.952) on Crohn's disease of large intestine
MR Test
Inverse variance weighted (fixed effects)
Inverse variance weighted (multiplicative random effects)
MR Egger
MR RAPS
Weighted median
Weighted mode
SNP effect on Crohn's disease of large intestine
0.1
0.0
−0.1
0.04
0.08
0.12
0.16
SNP effect on Gut microbiota abundance (genus Odoribacter id.952)

Batch 109 : Gut microbiota abundance (genus Olsenella id.822) on Crohn's disease of large intestine

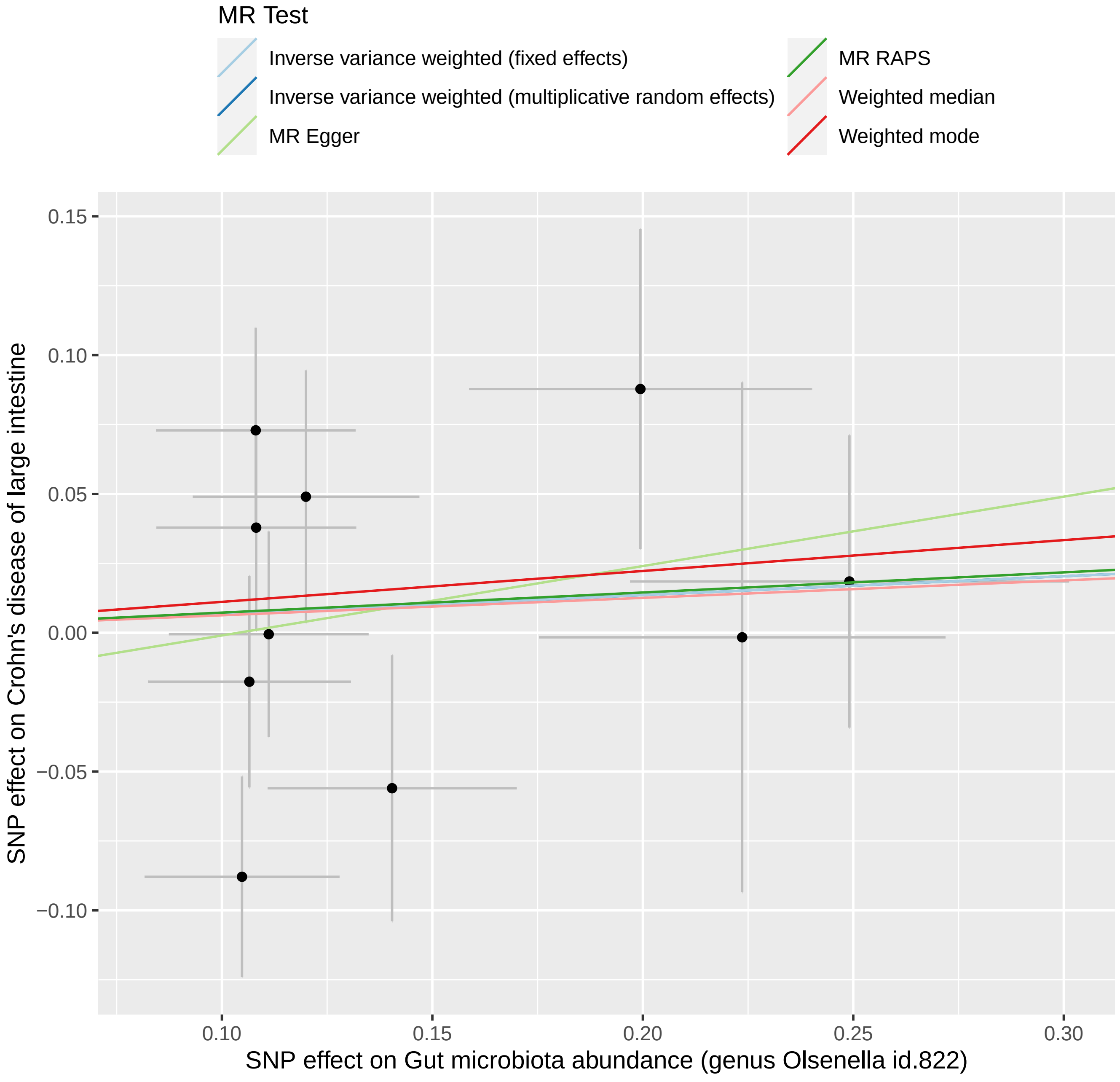

Batch 110 : Gut microbiota abundance (genus Oscillibacter id.2063) on Crohn's disease of large intestine

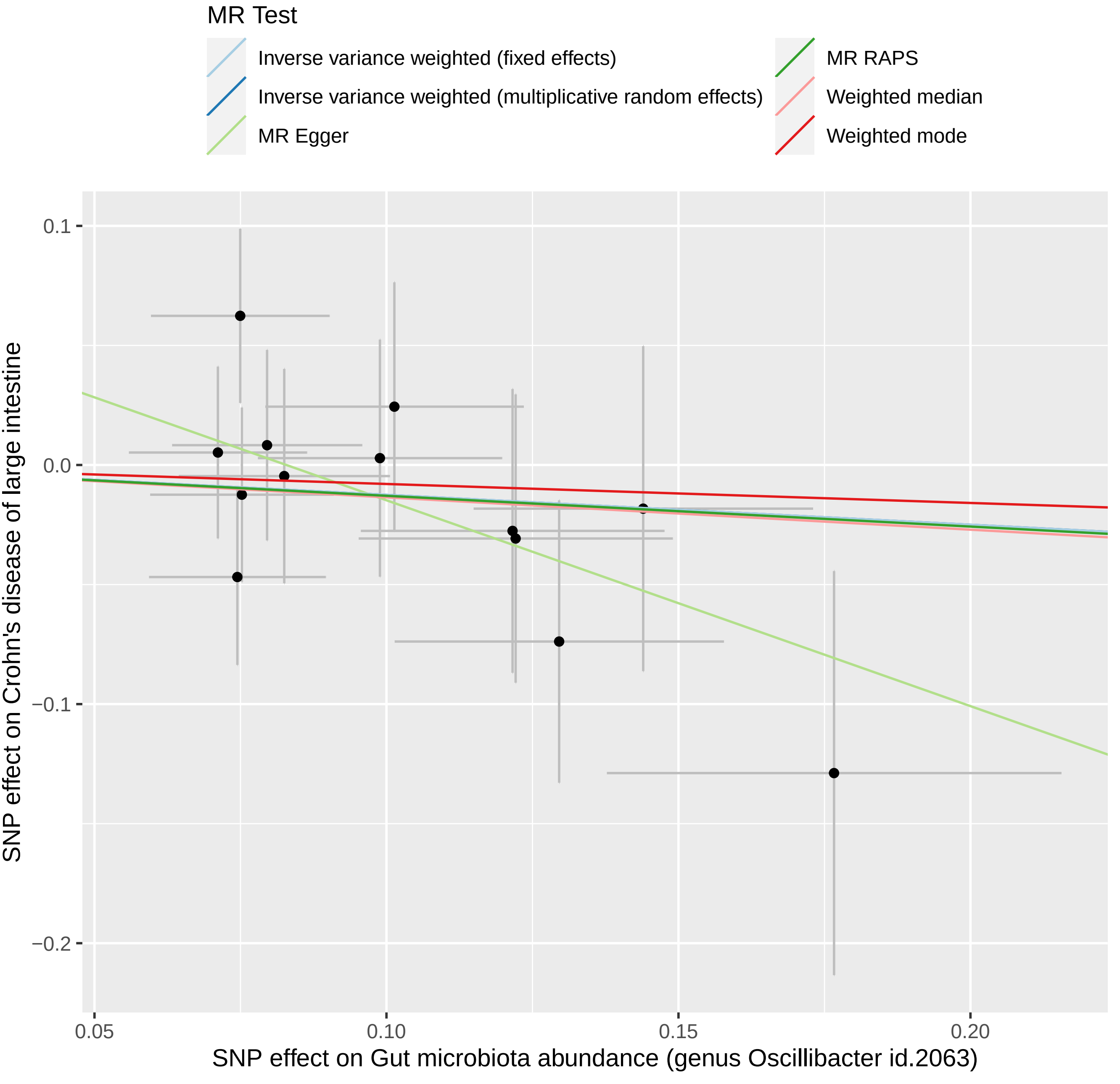

Batch 111 : Gut microbiota abundance (genus Oscillospira id.2064) on Crohn's disease of large intestine

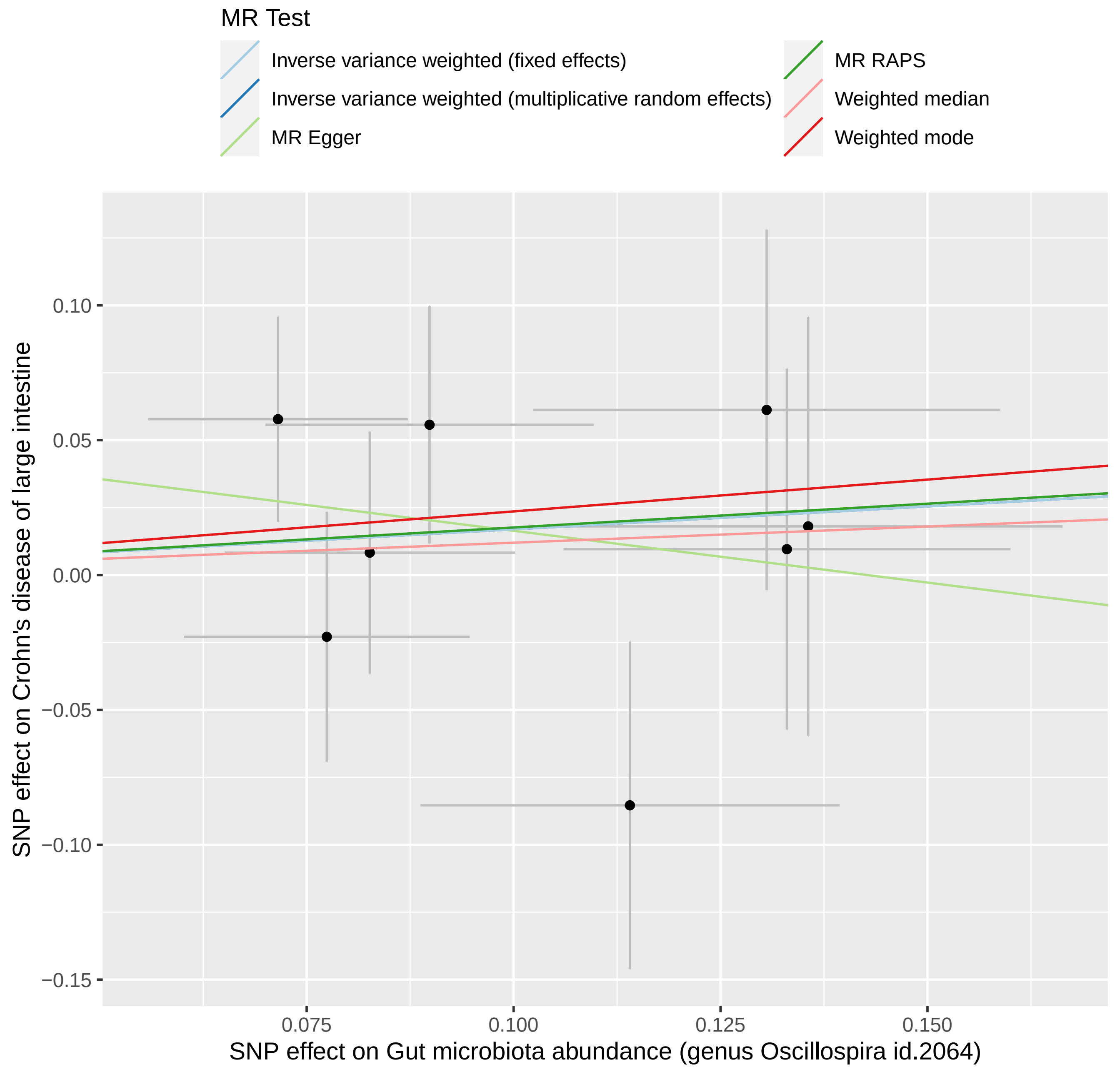

Batch 112 : Gut microbiota abundance (genus Oxalobacter id.2978) on Crohn's disease of large intestine

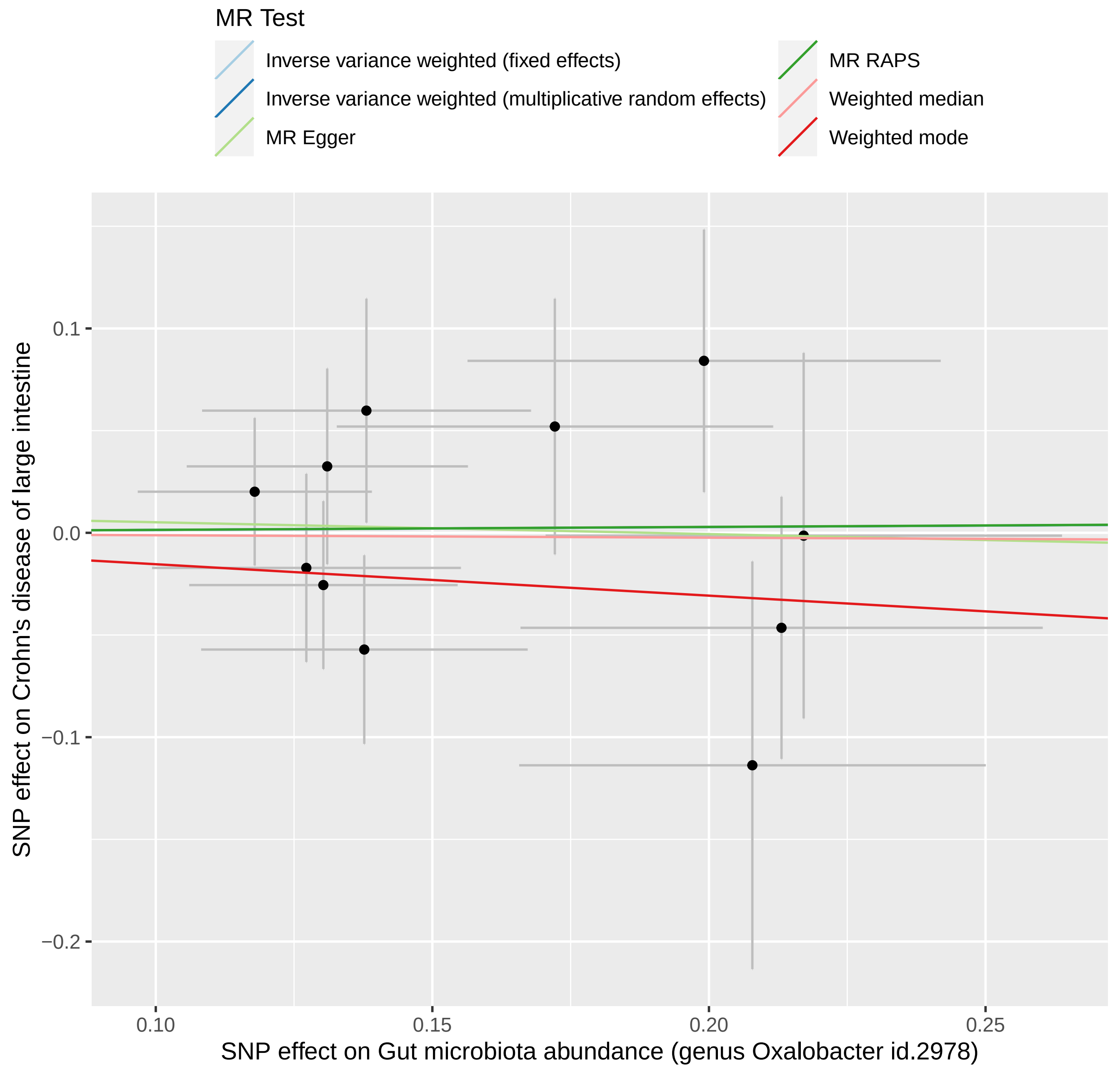

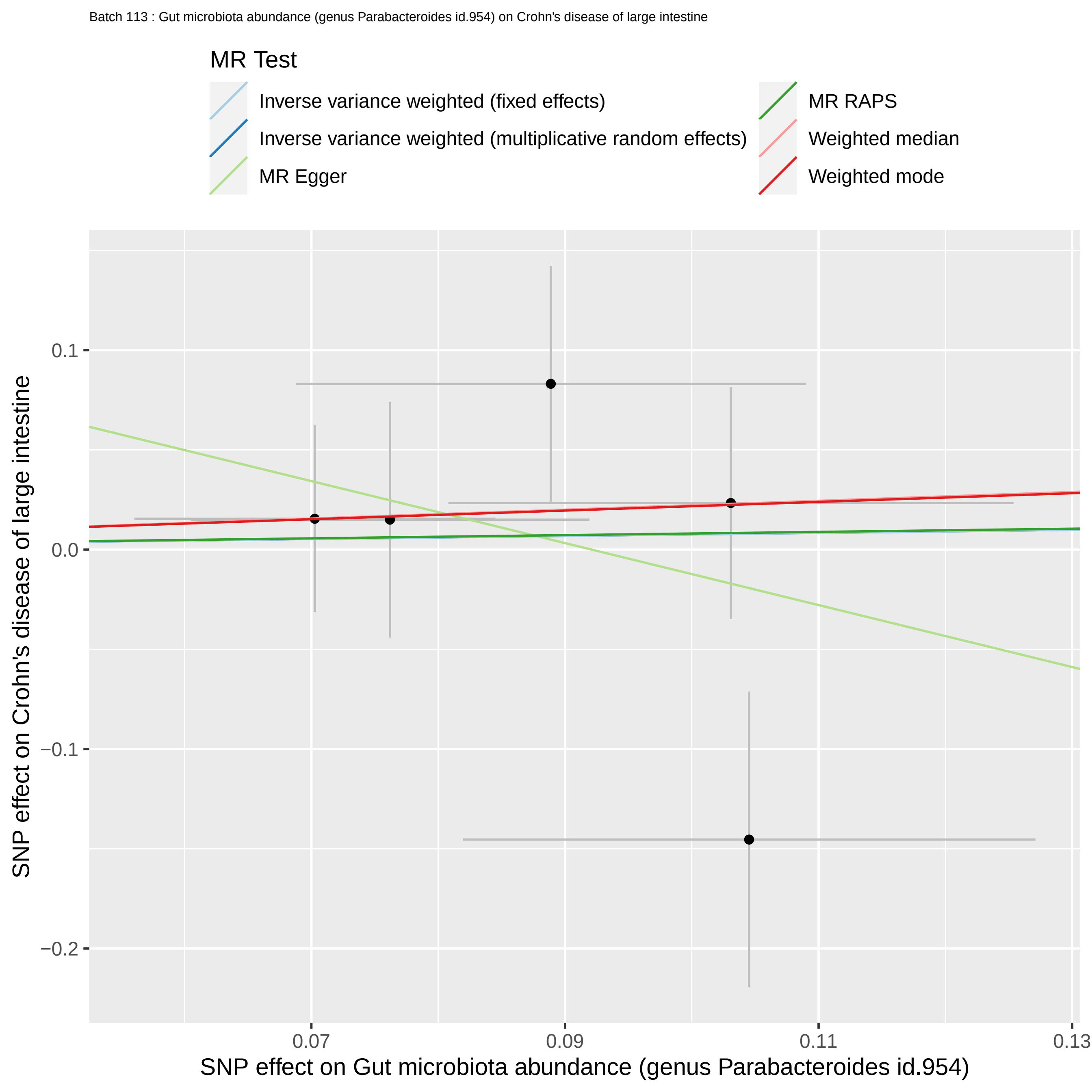
Batch 113 : Gut microbiota abundance (genus Parabacteroides id.954) on Crohn's disease of large intestine
MR Test
Inverse variance weighted (fixed effects)
Inverse variance weighted (multiplicative random effects)
MR Egger
MR RAPS
Weighted median
Weighted mode
0.1
0.0
−0.1
−0.2
0.07
0.09
0.11
0.13
SNP effect on Crohn's disease of large intestine
SNP effect on Gut microbiota abundance (genus Parabacteroides id.954)

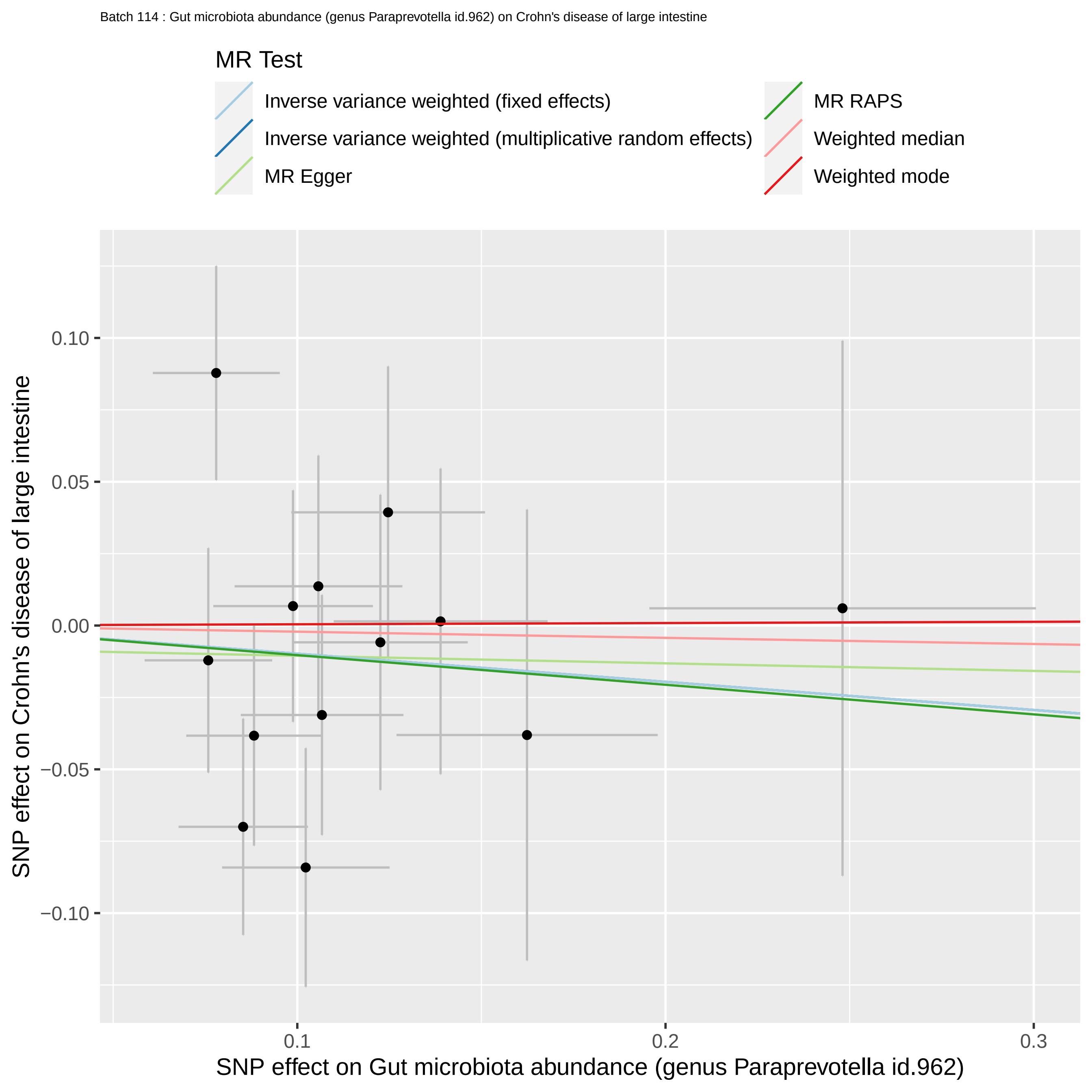
Batch 114 : Gut microbiota abundance (genus Paraprevotella id.962) on Crohn's disease of large intestine
MR Test
Inverse variance weighted (fixed effects)
Inverse variance weighted (multiplicative random effects)
MR Egger
MR RAPS
Weighted median
Weighted mode
SNP effect on Crohn's disease of large intestine
0.10
0.05
0.00
−0.05
−0.10
0.1
0.2
0.3
SNP effect on Gut microbiota abundance (genus Paraprevotella id.962)

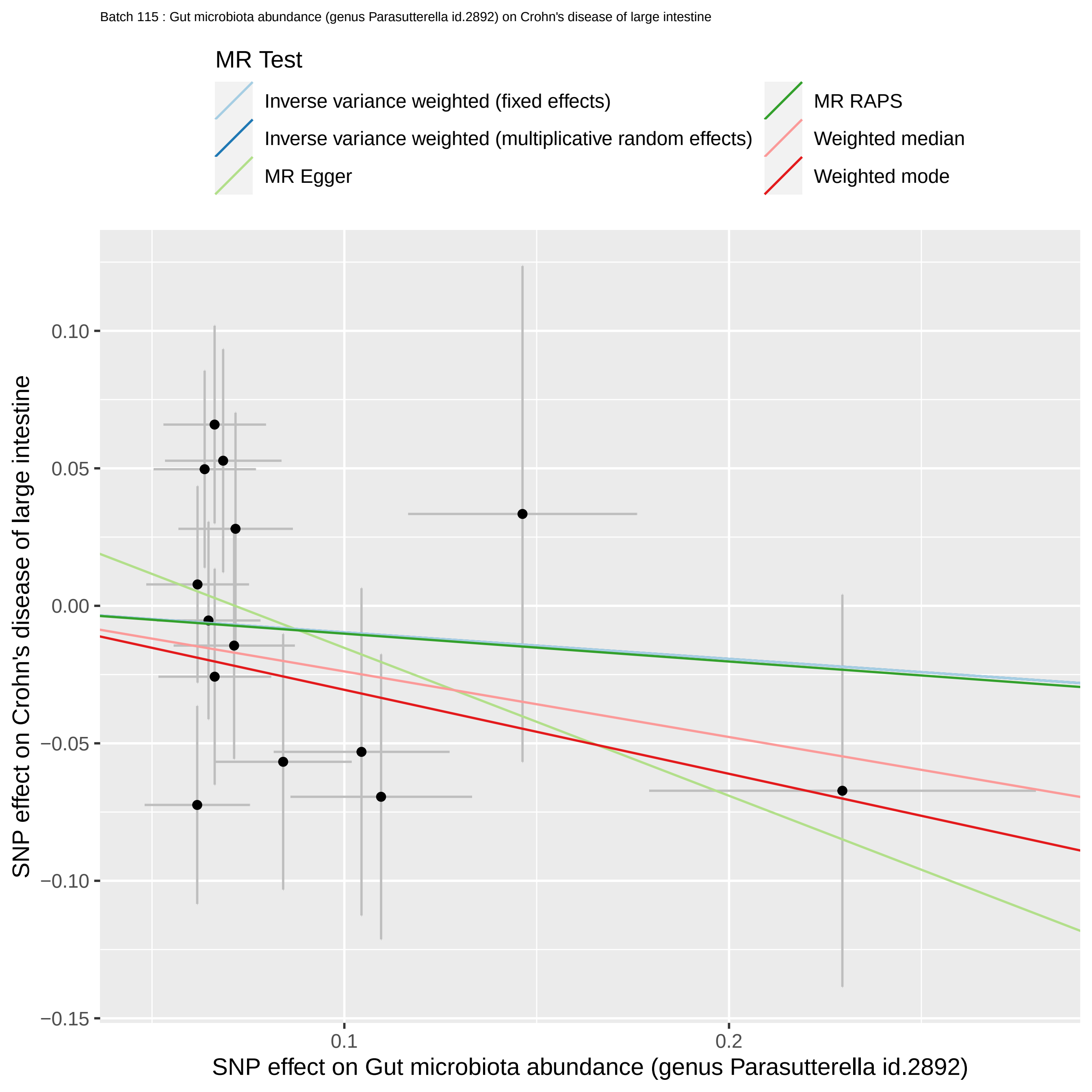

Batch 115 : Gut microbiota abundance (genus Parasutterella id.2892) on Crohn's disease of large intestine
MR Test
Inverse variance weighted (fixed effects)
Inverse variance weighted (multiplicative random effects)
MR Egger
MR RAPS
Weighted median
Weighted mode
0.10
0.05
0.00
−0.05
−0.10
−0.15
0.1
0.2
SNP effect on Crohn's disease of large intestine
SNP effect on Gut microbiota abundance (genus Parasutterella id.2892)

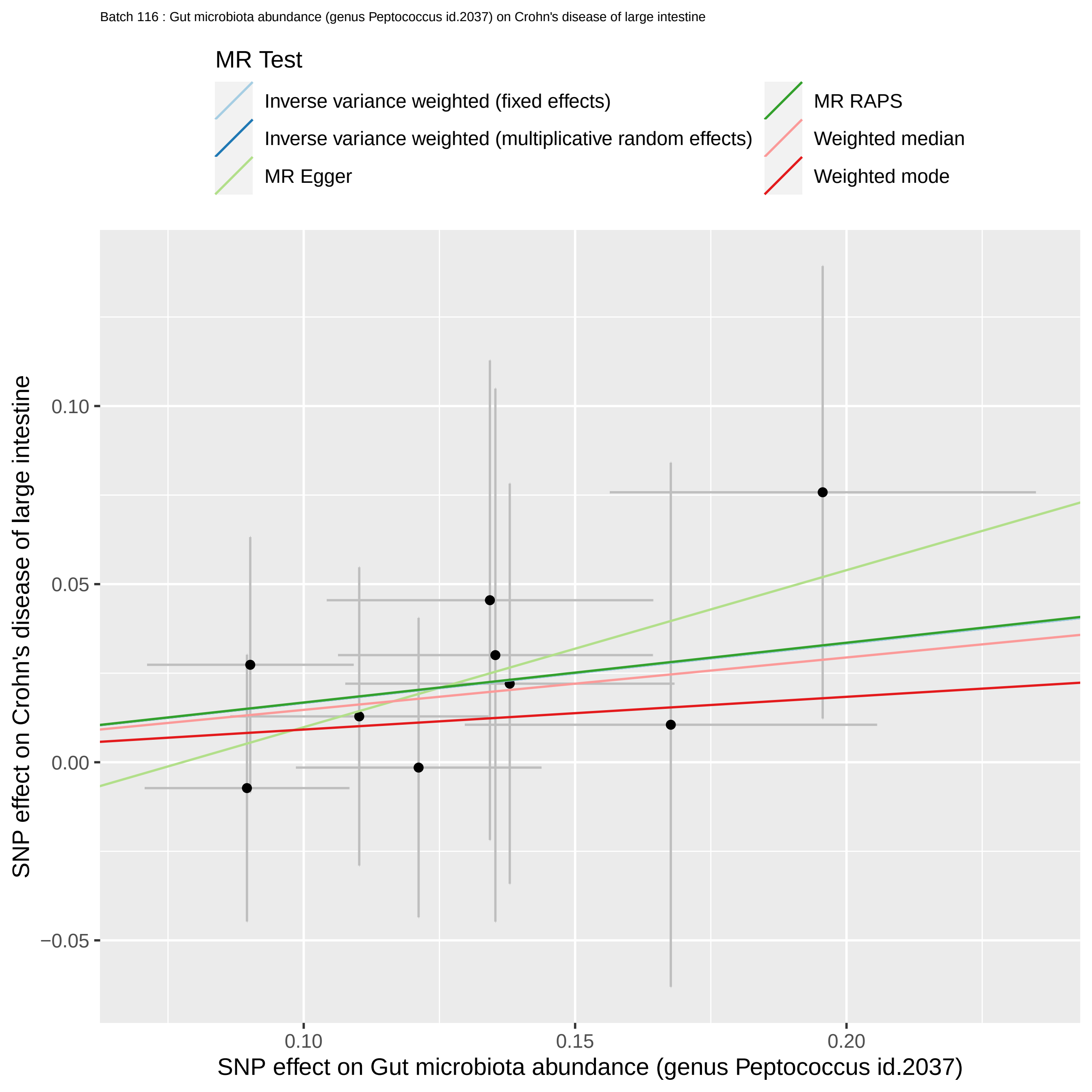

Batch 116 : Gut microbiota abundance (genus Peptococcus id.2037) on Crohn's disease of large intestine
MR Test
Inverse variance weighted (fixed effects)
Inverse variance weighted (multiplicative random effects)
MR Egger
MR RAPS
Weighted median
Weighted mode
SNP effect on Crohn's disease of large intestine
0.10
0.05
0.00
−0.05
0.10
0.15
0.20
SNP effect on Gut microbiota abundance (genus Peptococcus id.2037)

Batch 117 : Gut microbiota abundance (genus Phascolarctobacterium id.2168) on Crohn's disease of large intestine

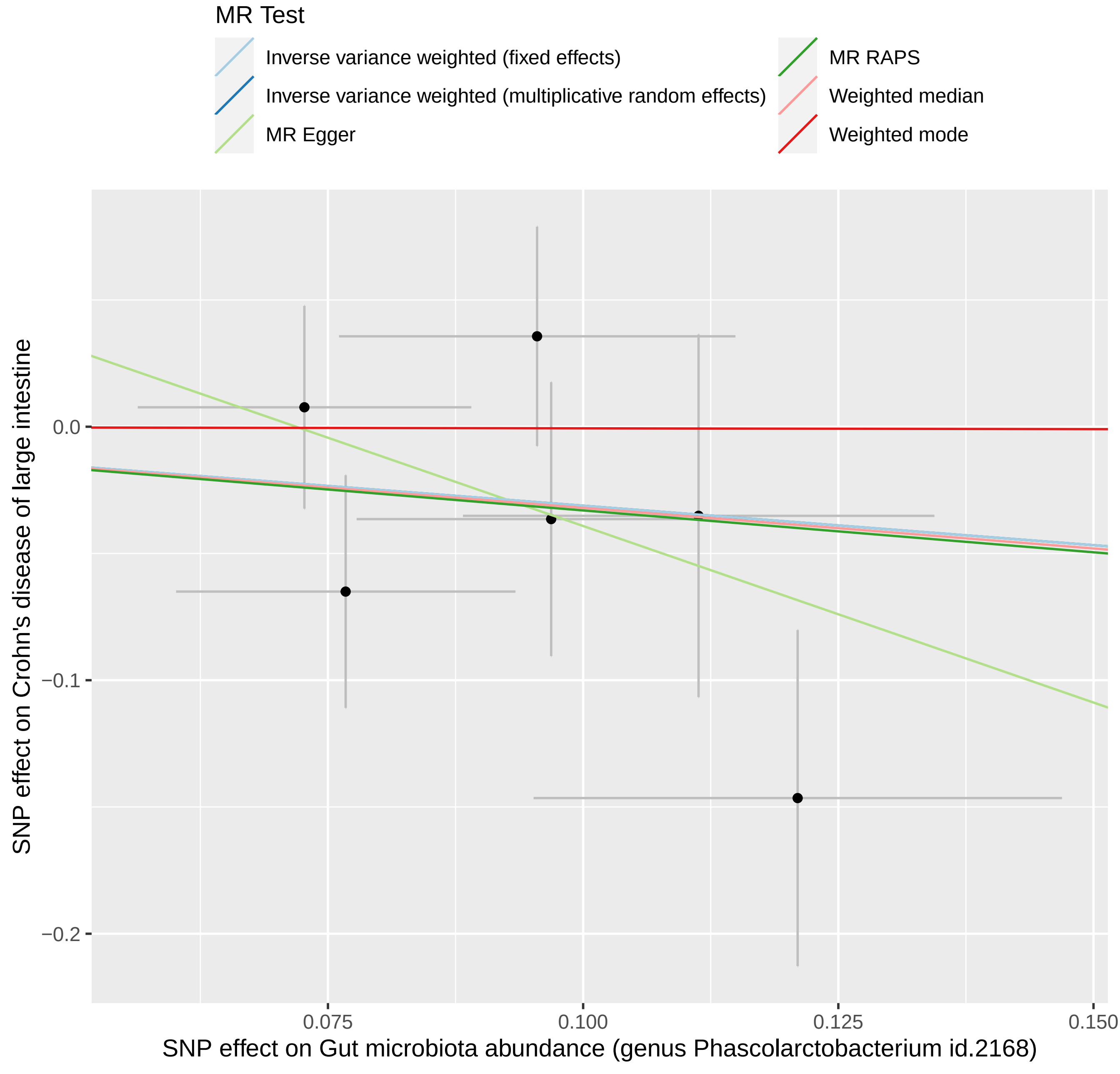

Batch 118 : Gut microbiota abundance (genus Prevotella7 id.11182) on Crohn's disease of large intestine

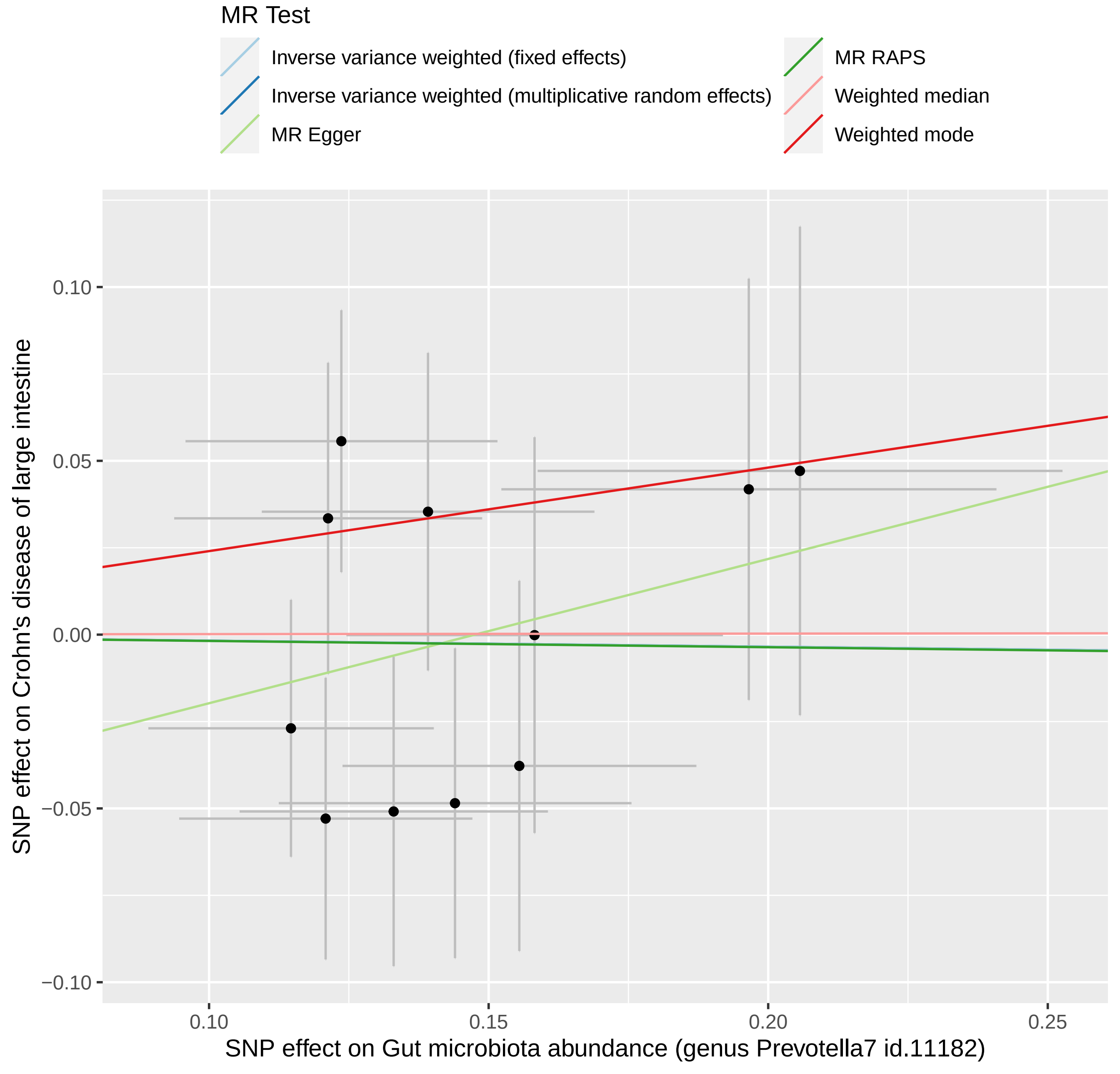

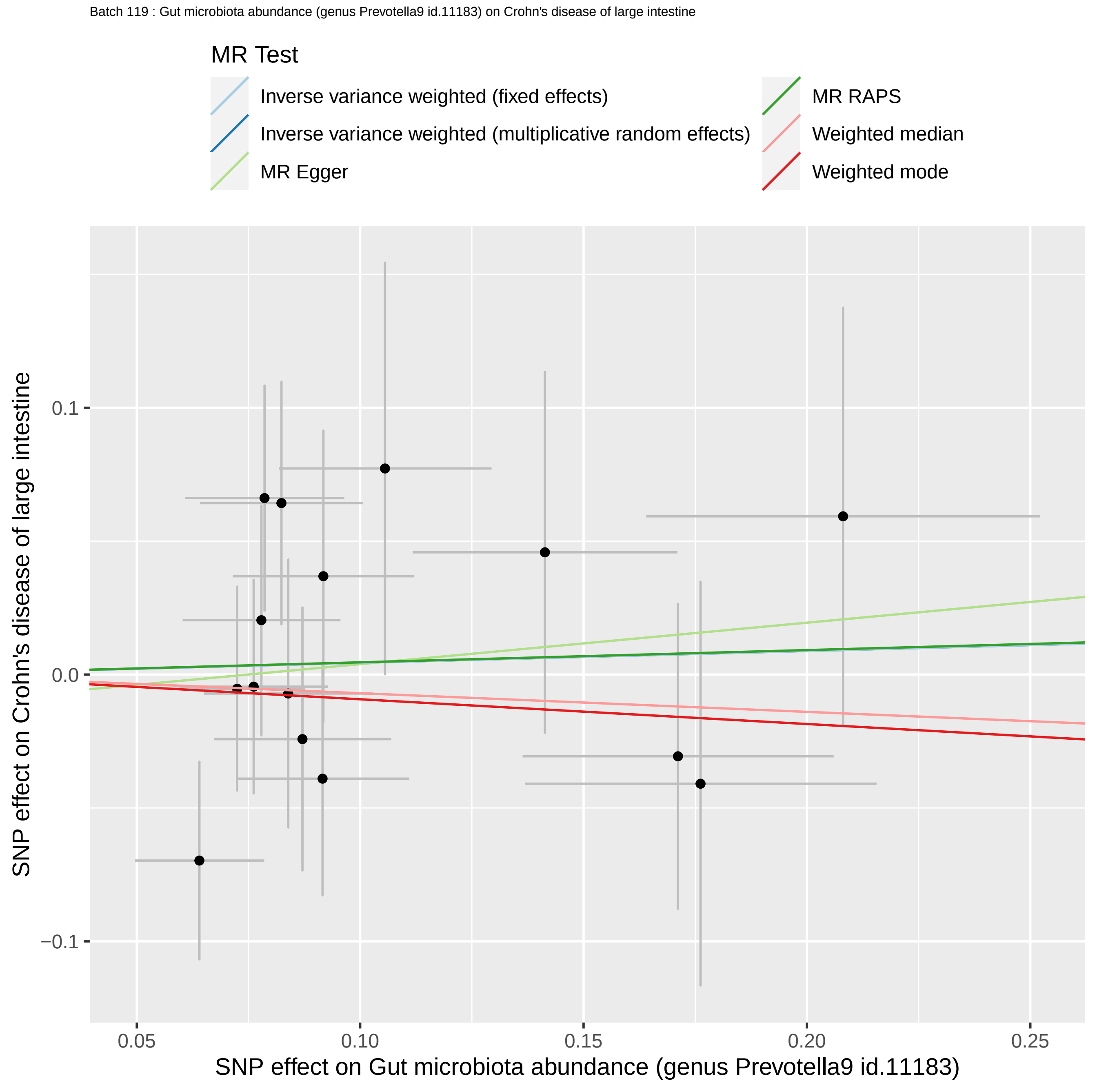

Batch 119 : Gut microbiota abundance (genus Prevotella9 id.11183) on Crohn's disease of large intestine
MR Test
Inverse variance weighted (fixed effects)
Inverse variance weighted (multiplicative random effects)
MR Egger
MR RAPS
Weighted median
Weighted mode
SNP effect on Crohn's disease of large intestine
0.1
0.0
−0.1
0.05
0.10
0.15
0.20
0.25
SNP effect on Gut microbiota abundance (genus Prevotella9 id.11183)

Batch 120 : Gut microbiota abundance (genus Rikenellaceae RC9 gut group id.11191) on Crohn's disease of large intestine

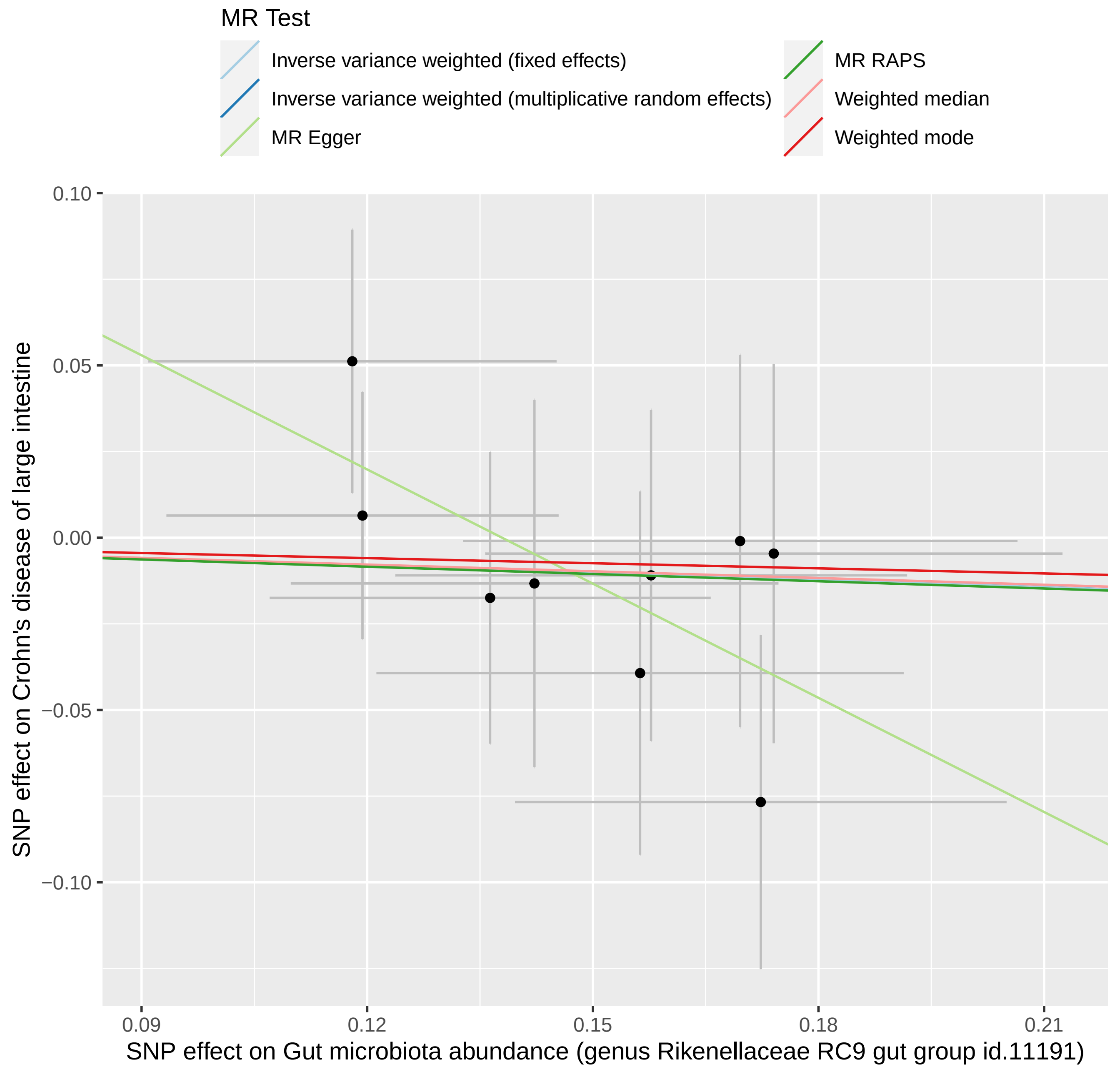

Batch 121 : Gut microbiota abundance (genus Romboutsia id.11347) on Crohn's disease of large intestine

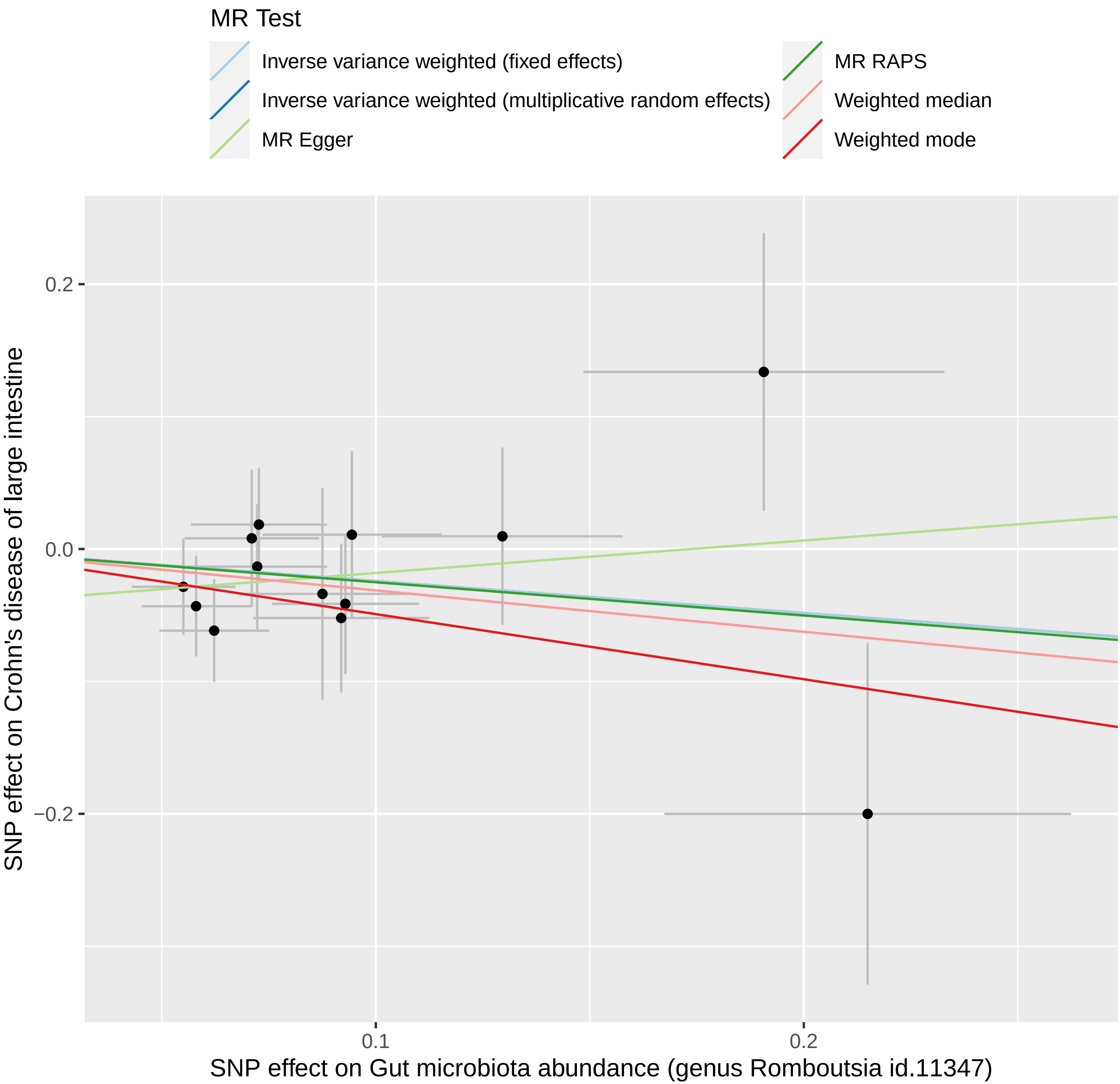

Batch 122 : Gut microbiota abundance (genus Roseburia id.2012) on Crohn's disease of large intestine

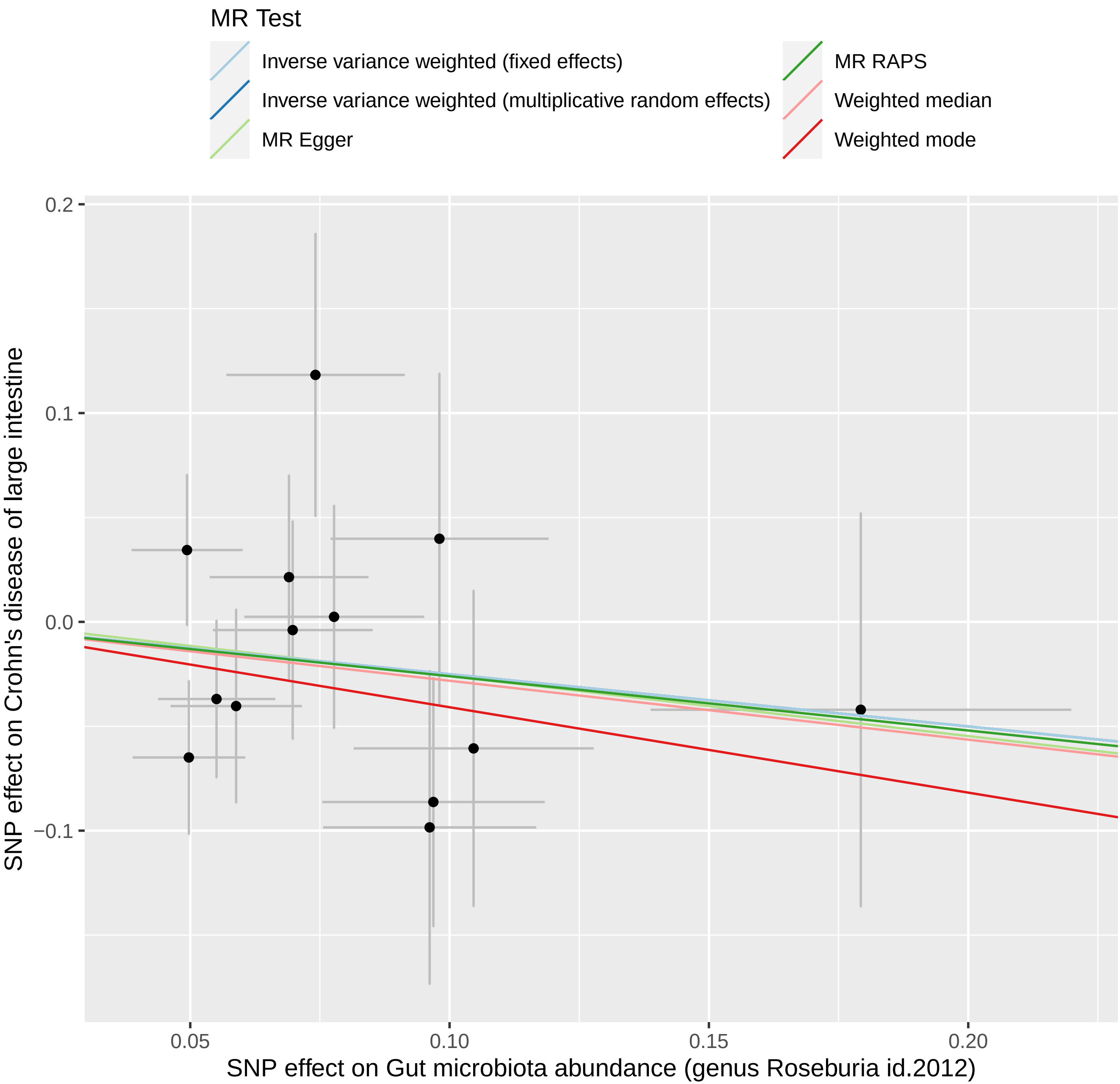

Batch 123 : Gut microbiota abundance (genus Ruminiclostridium5 id.11355) on Crohn's disease of large intestine

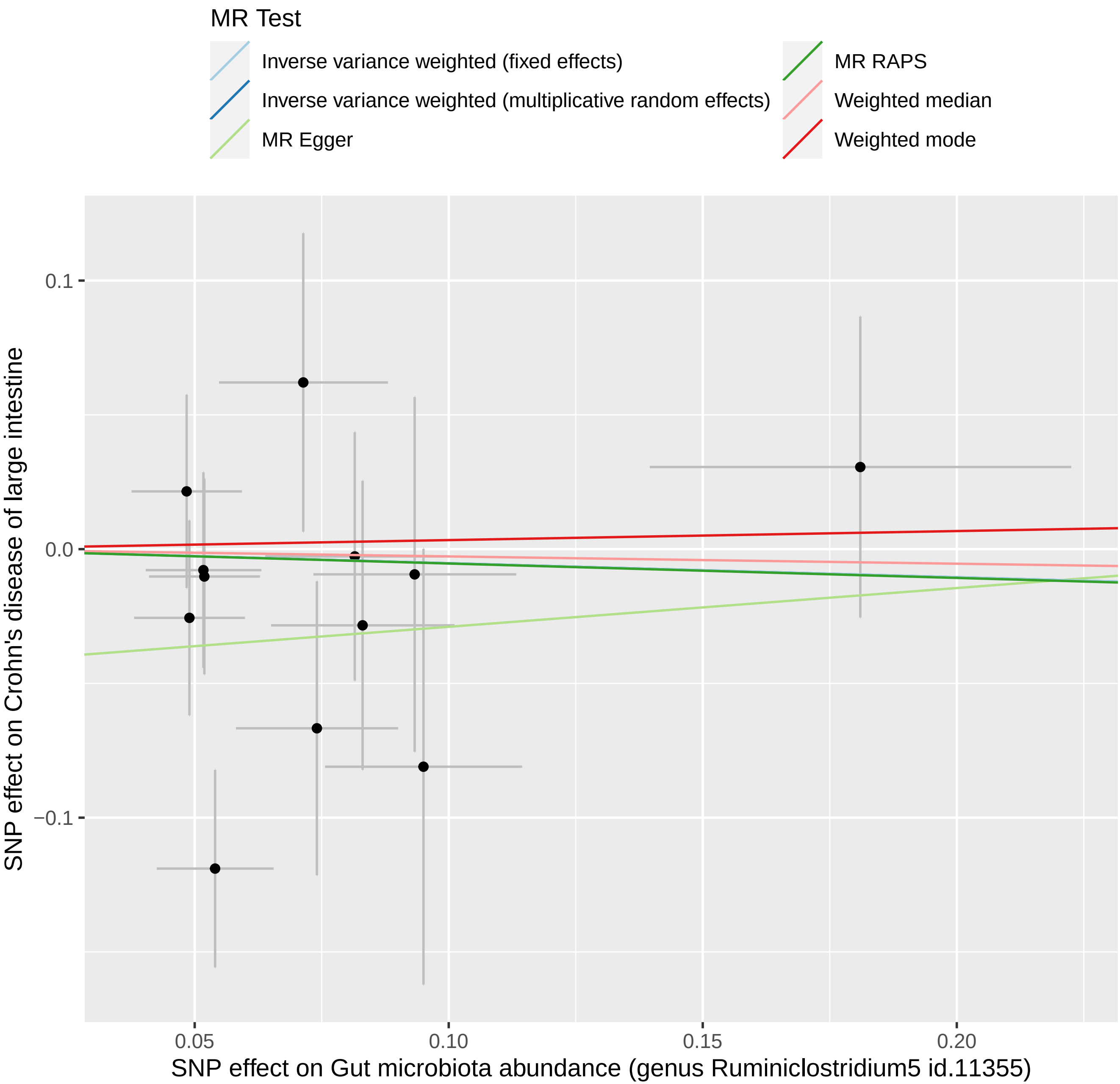

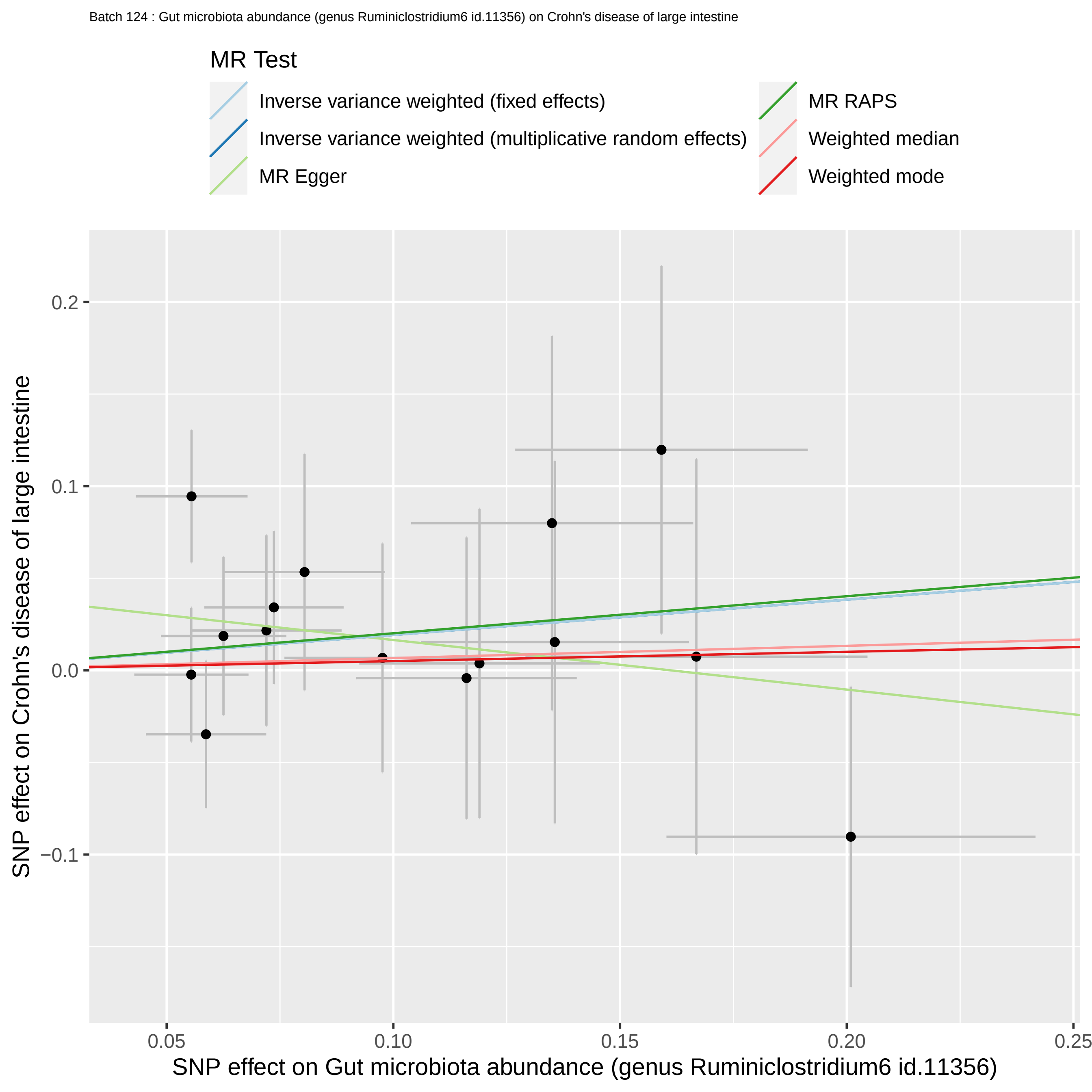
Batch 124 : Gut microbiota abundance (genus Ruminiclostridium6 id.11356) on Crohn's disease of large intestine
MR Test
Inverse variance weighted (fixed effects)
Inverse variance weighted (multiplicative random effects)
MR Egger
MR RAPS
Weighted median
Weighted mode
0.2
0.1
0.0
−0.1
0.05
0.10
0.15
0.20
0.25
SNP effect on Crohn's disease of large intestine
SNP effect on Gut microbiota abundance (genus Ruminiclostridium6 id.11356)

Batch 125 : Gut microbiota abundance (genus Ruminiclostridium9 id.11357) on Crohn's disease of large intestine

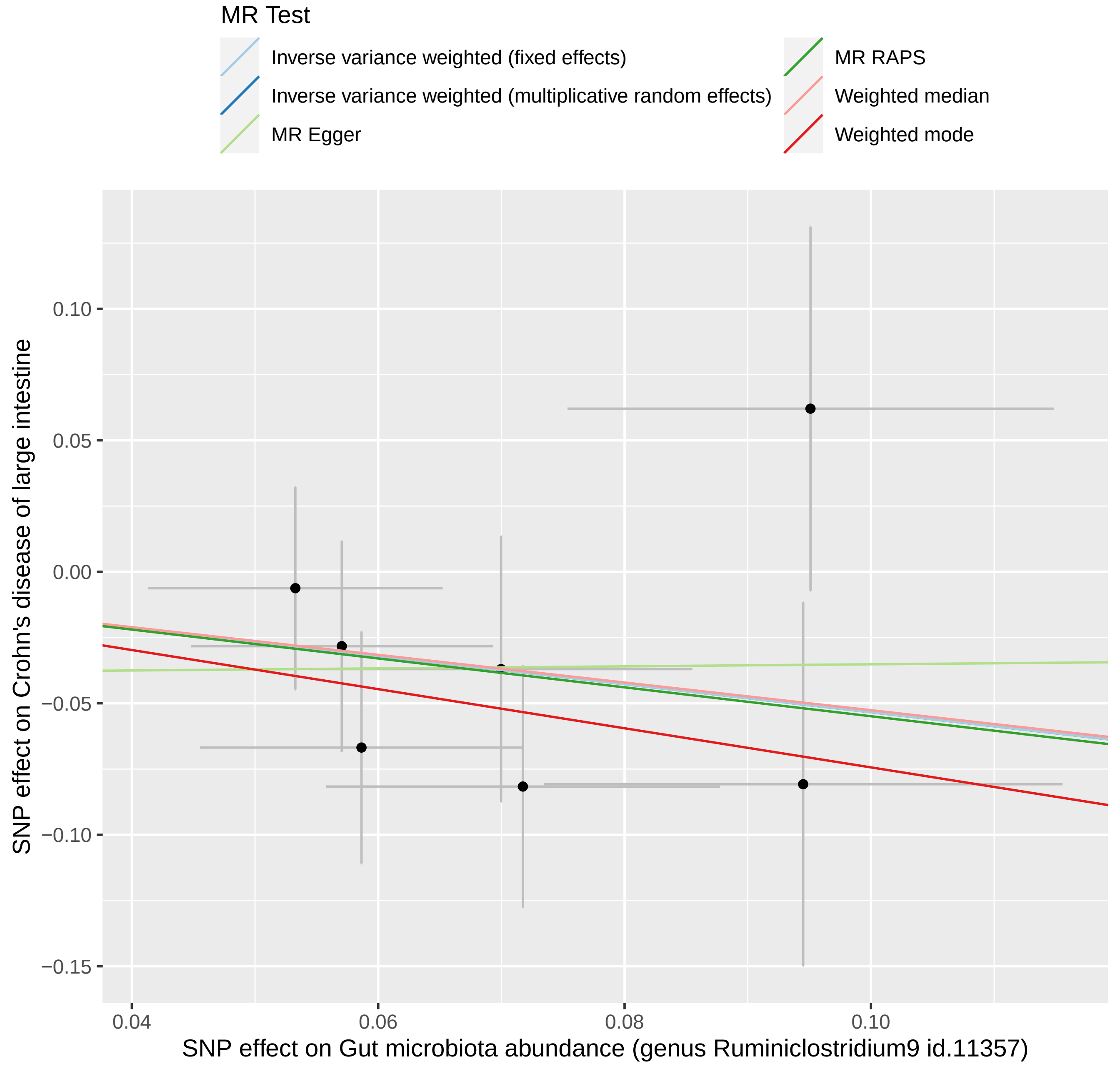

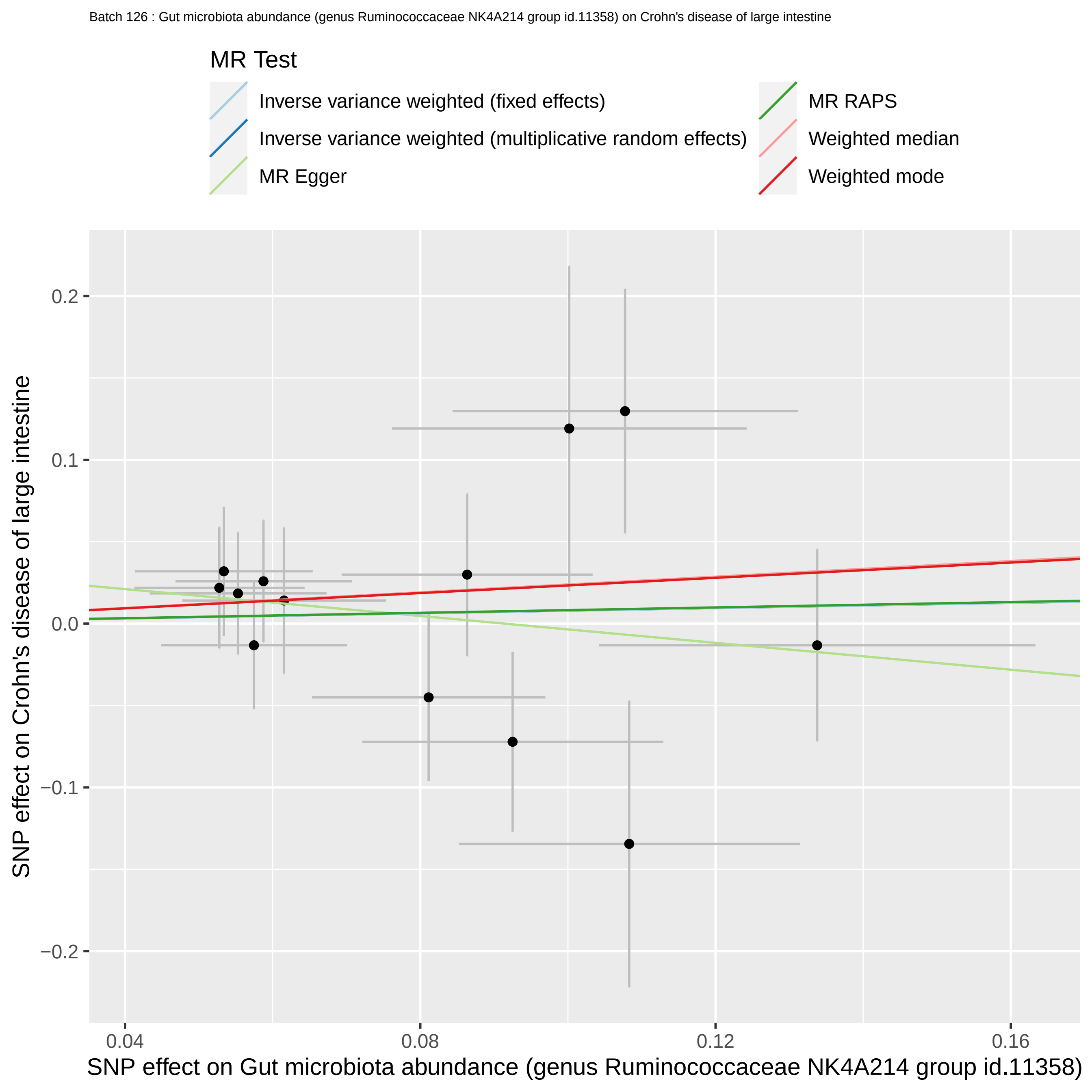

Batch 126 : Gut microbiota abundance (genus Ruminococcaceae NK4A214 group id.11358) on Crohn's disease of large intestine
MR Test
Inverse variance weighted (fixed effects)
Inverse variance weighted (multiplicative random effects)
MR Egger
MR RAPS
Weighted median
Weighted mode
0.2
0.1
0.0
−0.1
−0.2
0.04
0.08
0.12
0.16
SNP effect on Crohn's disease of large intestine
SNP effect on Gut microbiota abundance (genus Ruminococcaceae NK4A214 group id.11358)

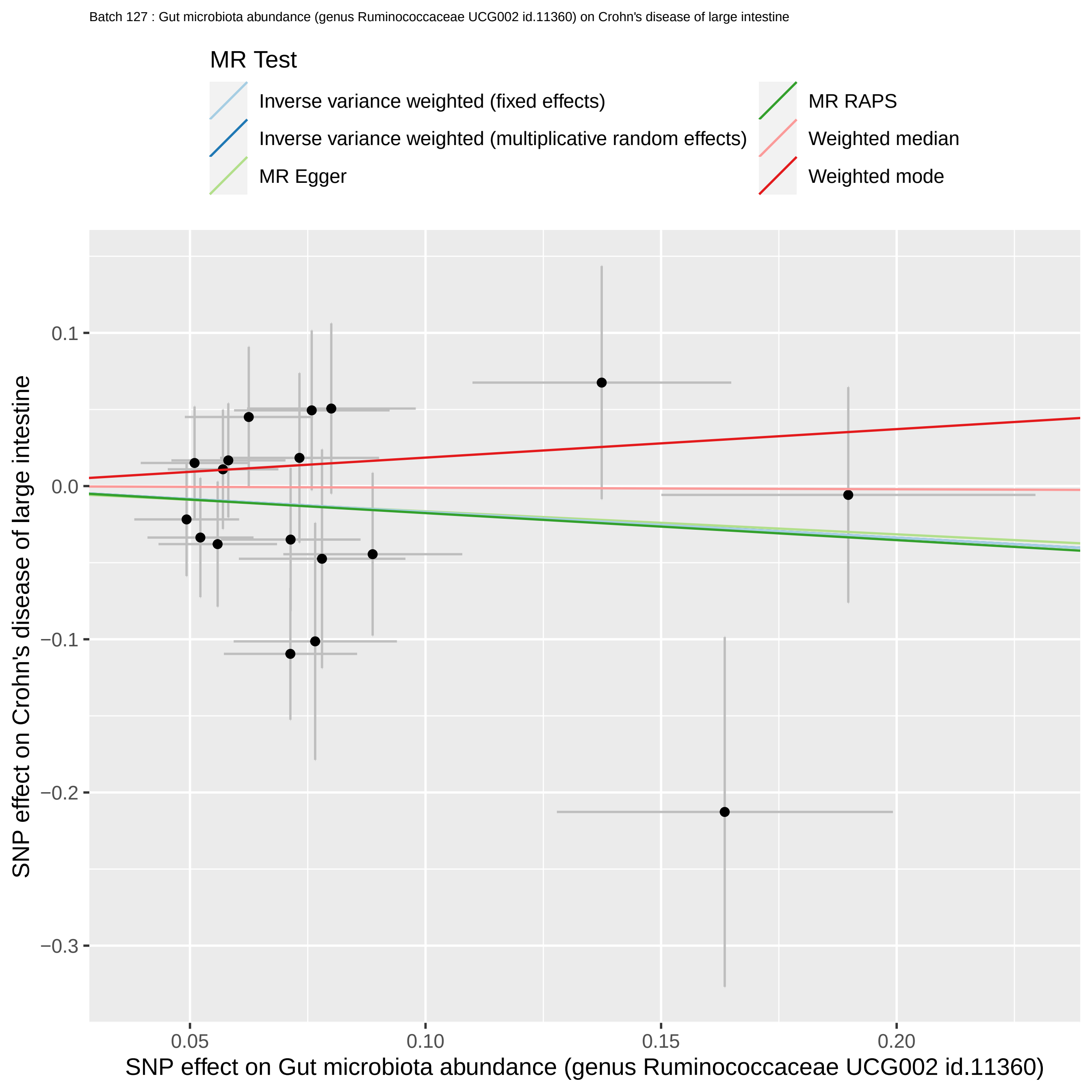
Batch 127 : Gut microbiota abundance (genus Ruminococcaceae UCG002 id.11360) on Crohn's disease of large intestine
MR Test
Inverse variance weighted (fixed effects)
Inverse variance weighted (multiplicative random effects)
MR Egger
MR RAPS
Weighted median
Weighted mode
0.1
0.0
−0.1
−0.2
−0.3
0.05
0.10
0.15
0.20
SNP effect on Crohn's disease of large intestine
SNP effect on Gut microbiota abundance (genus Ruminococcaceae UCG002 id.11360)

Batch 128 : Gut microbiota abundance (genus Ruminococcaceae UCG003 id.11361) on Crohn's disease of large intestine

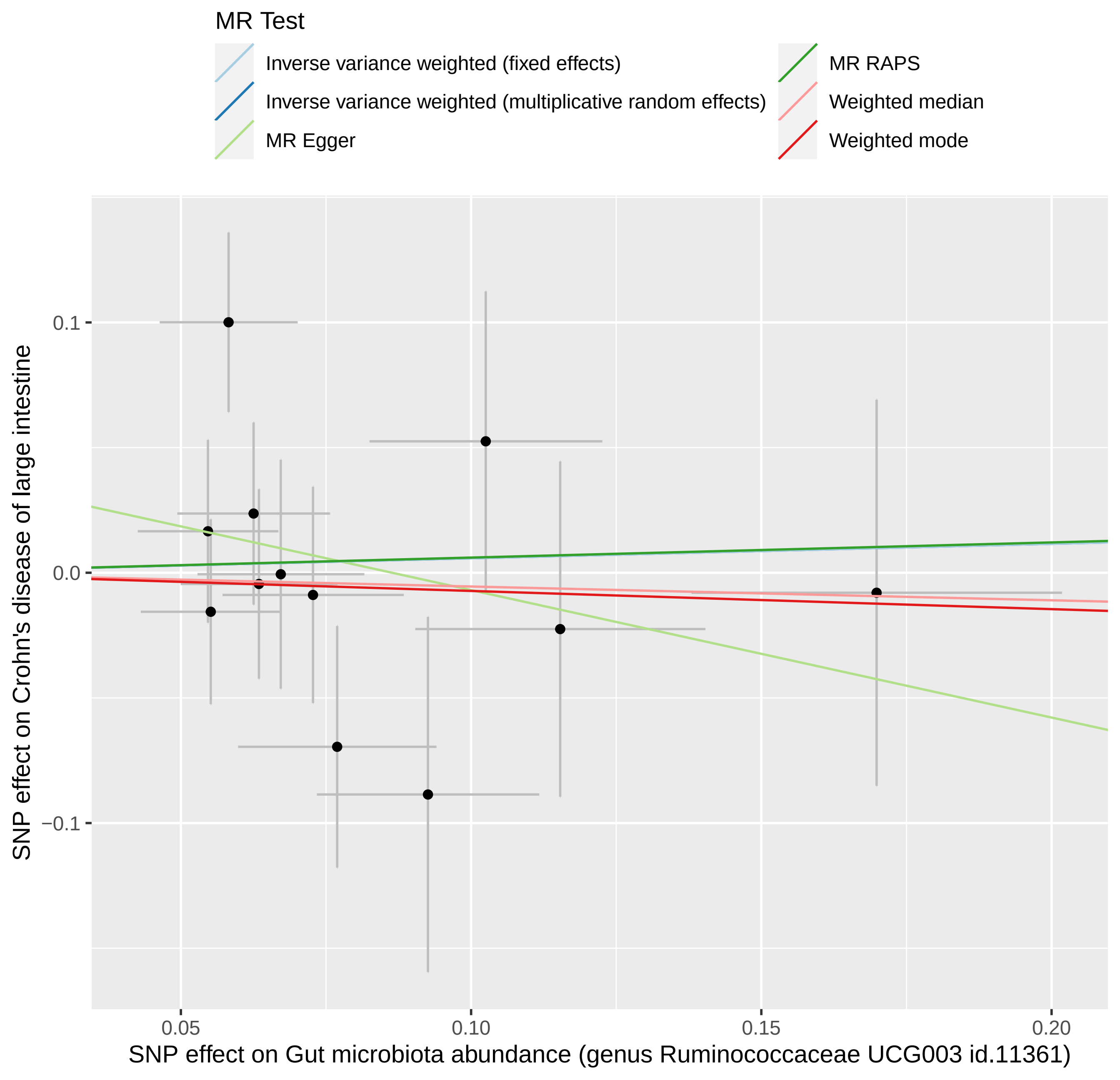

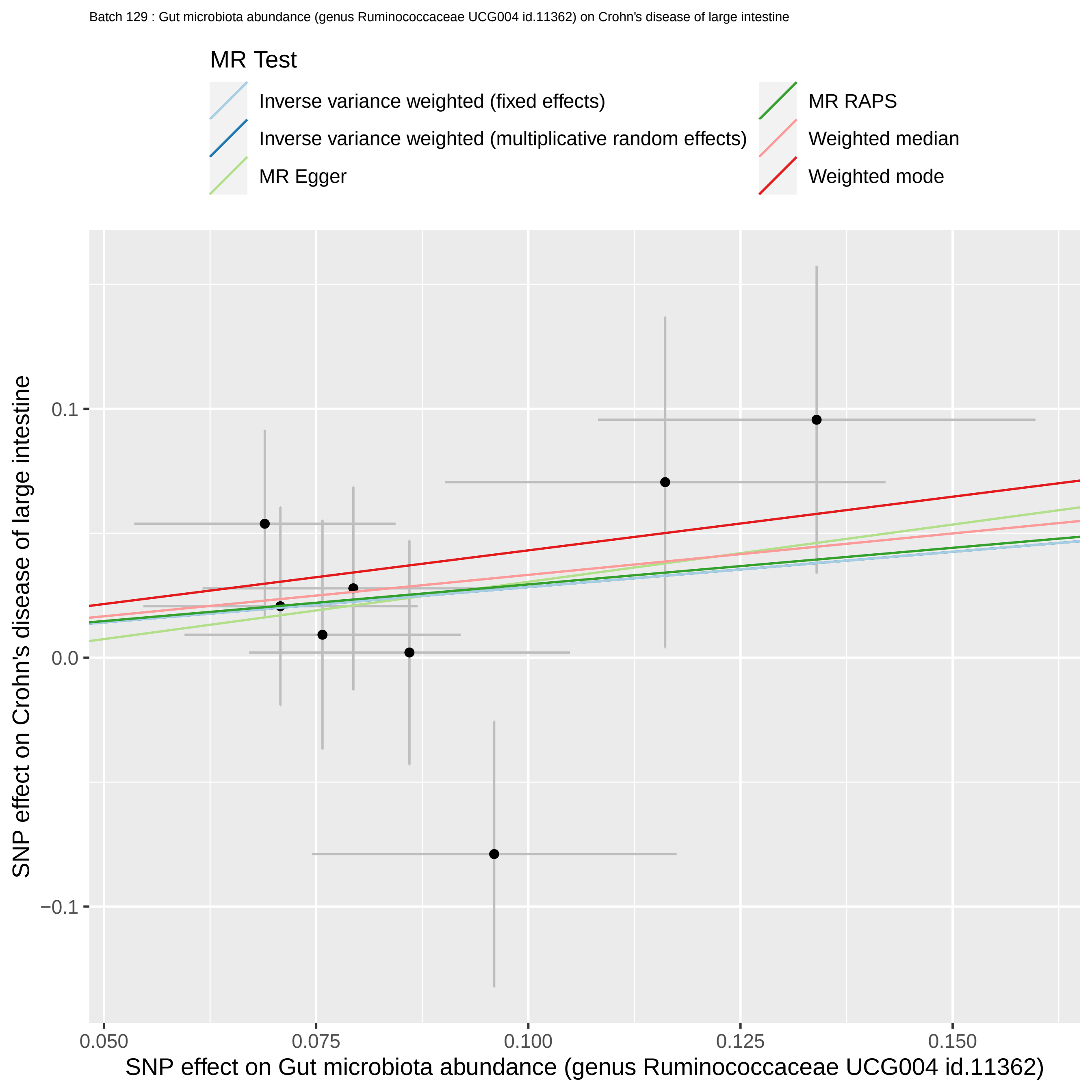

Batch 129 : Gut microbiota abundance (genus Ruminococcaceae UCG004 id.11362) on Crohn's disease of large intestine
MR Test
Inverse variance weighted (fixed effects)
Inverse variance weighted (multiplicative random effects)
MR Egger
MR RAPS
Weighted median
Weighted mode
SNP effect on Crohn's disease of large intestine
0.1
0.0
−0.1
0.050
0.075
0.100
0.125
0.150
SNP effect on Gut microbiota abundance (genus Ruminococcaceae UCG004 id.11362)

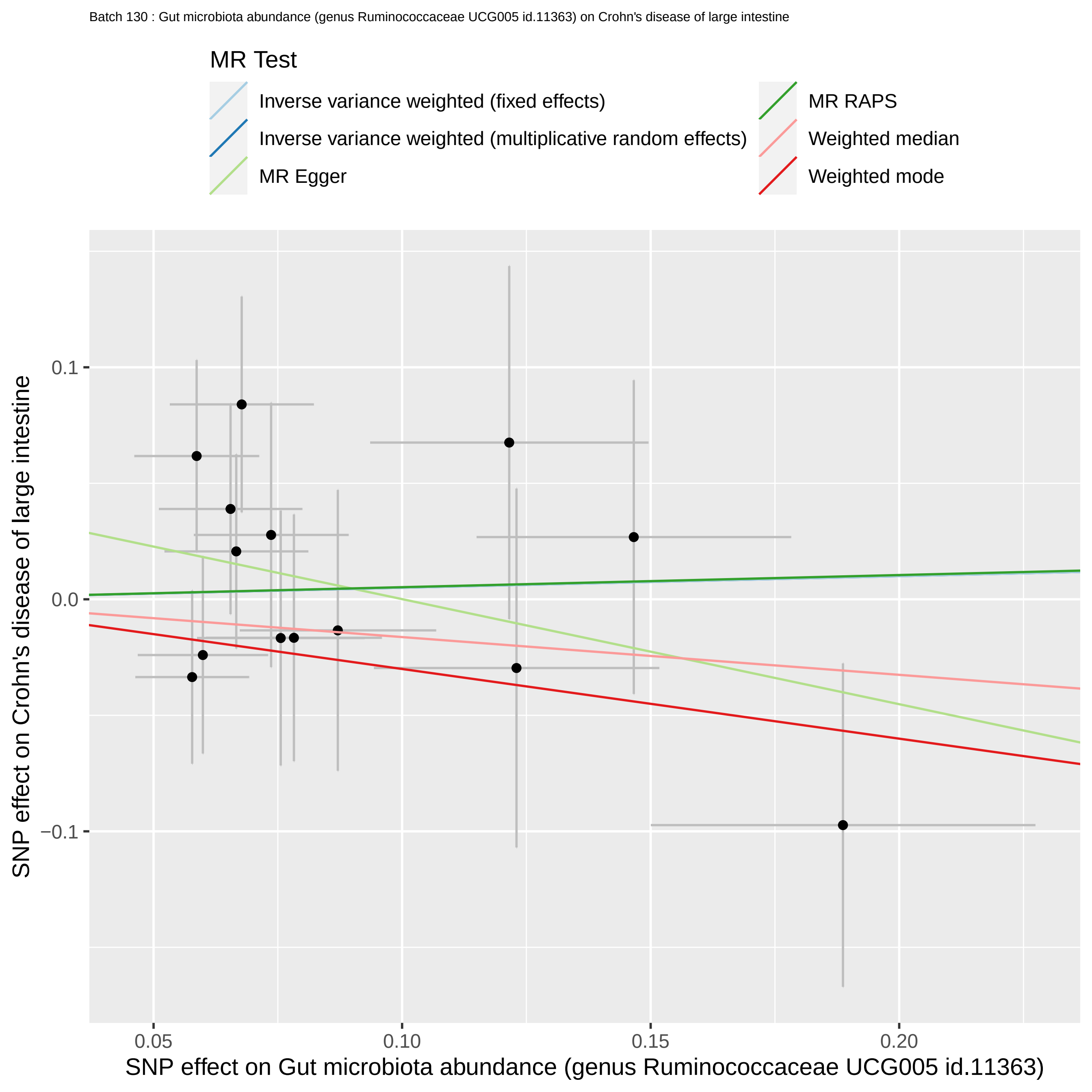
Batch 130 : Gut microbiota abundance (genus Ruminococcaceae UCG005 id.11363) on Crohn's disease of large intestine
MR Test
Inverse variance weighted (fixed effects)
Inverse variance weighted (multiplicative random effects)
MR Egger
MR RAPS
Weighted median
Weighted mode
SNP effect on Crohn's disease of large intestine
0.1
0.0
−0.1
0.05
0.10
0.15
0.20
SNP effect on Gut microbiota abundance (genus Ruminococcaceae UCG005 id.11363)

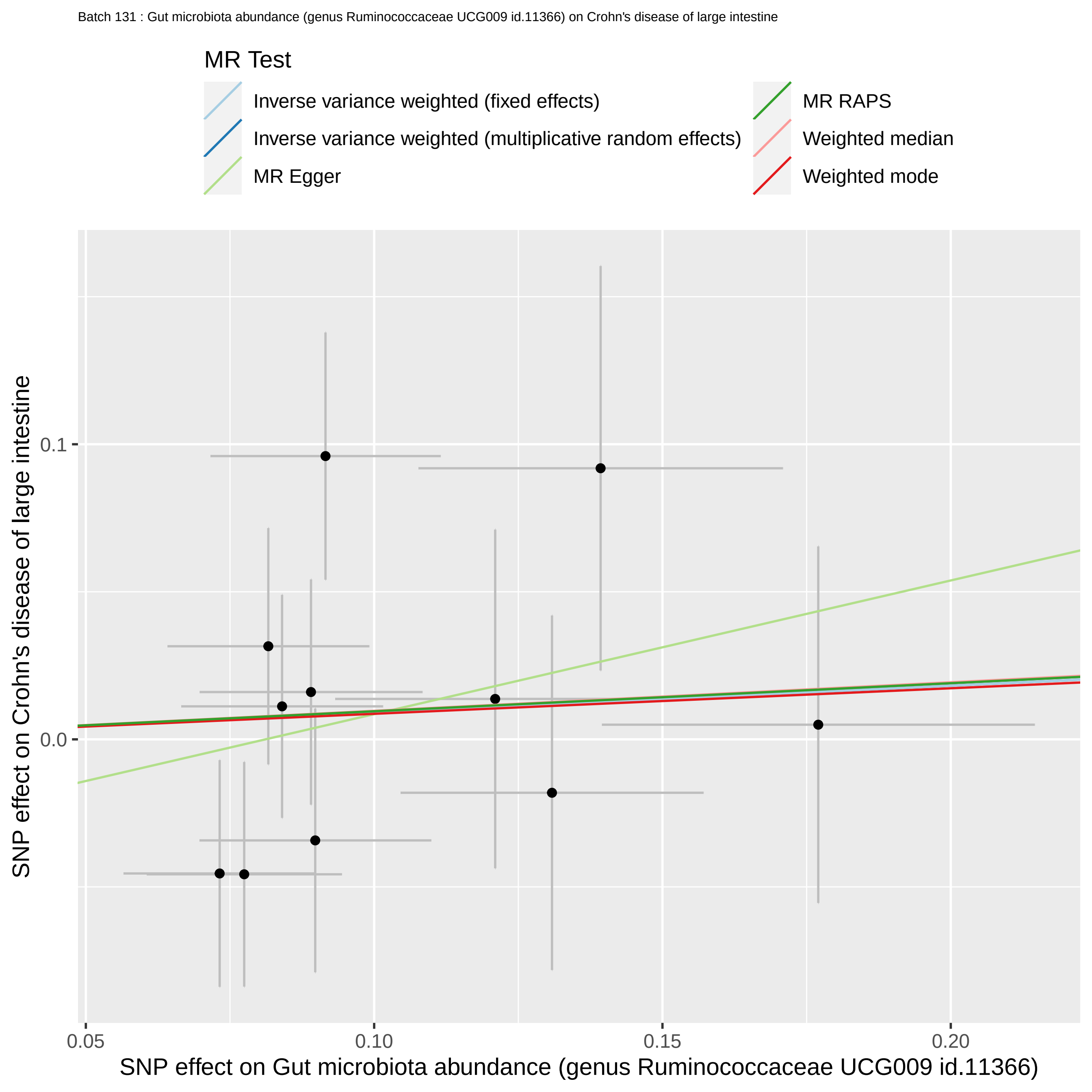

Batch 131 : Gut microbiota abundance (genus Ruminococcaceae UCG009 id.11366) on Crohn's disease of large intestine
MR Test
Inverse variance weighted (fixed effects)
Inverse variance weighted (multiplicative random effects)
MR Egger
MR RAPS
Weighted median
Weighted mode
SNP effect on Crohn's disease of large intestine
0.1
0.0
0.05
0.10
0.15
0.20
SNP effect on Gut microbiota abundance (genus Ruminococcaceae UCG009 id.11366)

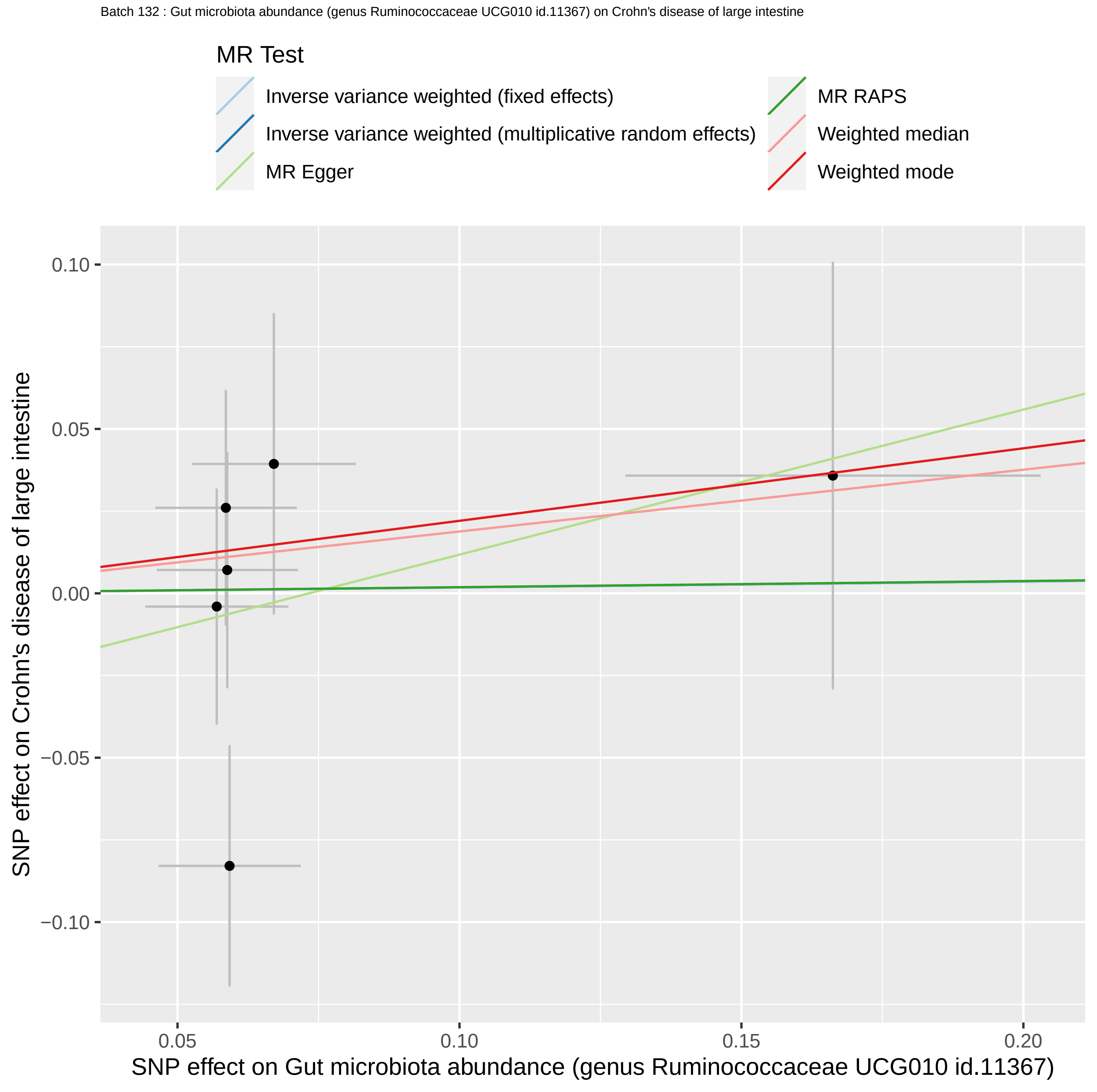

Batch 132 : Gut microbiota abundance (genus Ruminococcaceae UCG010 id.11367) on Crohn's disease of large intestine
MR Test
Inverse variance weighted (fixed effects)
Inverse variance weighted (multiplicative random effects)
MR Egger
MR RAPS
Weighted median
Weighted mode
0.10
0.05
0.00
−0.05
−0.10
0.05
0.10
0.15
0.20
SNP effect on Crohn's disease of large intestine
SNP effect on Gut microbiota abundance (genus Ruminococcaceae UCG010 id.11367)

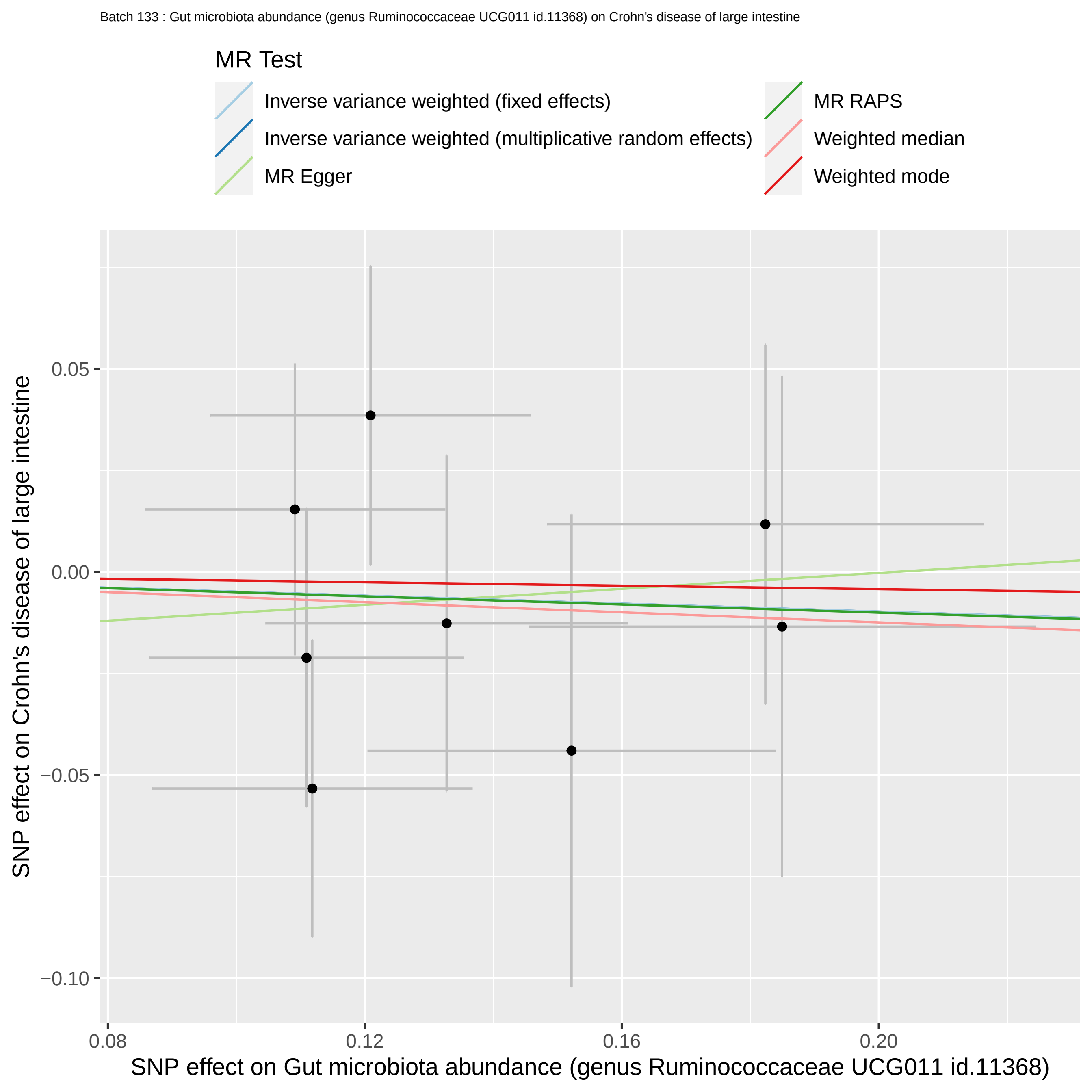

Batch 133 : Gut microbiota abundance (genus Ruminococcaceae UCG011 id.11368) on Crohn's disease of large intestine
MR Test
Inverse variance weighted (fixed effects)
Inverse variance weighted (multiplicative random effects)
MR Egger
MR RAPS
Weighted median
Weighted mode
SNP effect on Crohn's disease of large intestine
0.05
0.00
−0.05
−0.10
0.08
0.12
0.16
0.20
SNP effect on Gut microbiota abundance (genus Ruminococcaceae UCG011 id.11368)

Batch 134 : Gut microbiota abundance (genus Ruminococcaceae UCG013 id.11370) on Crohn's disease of large intestine

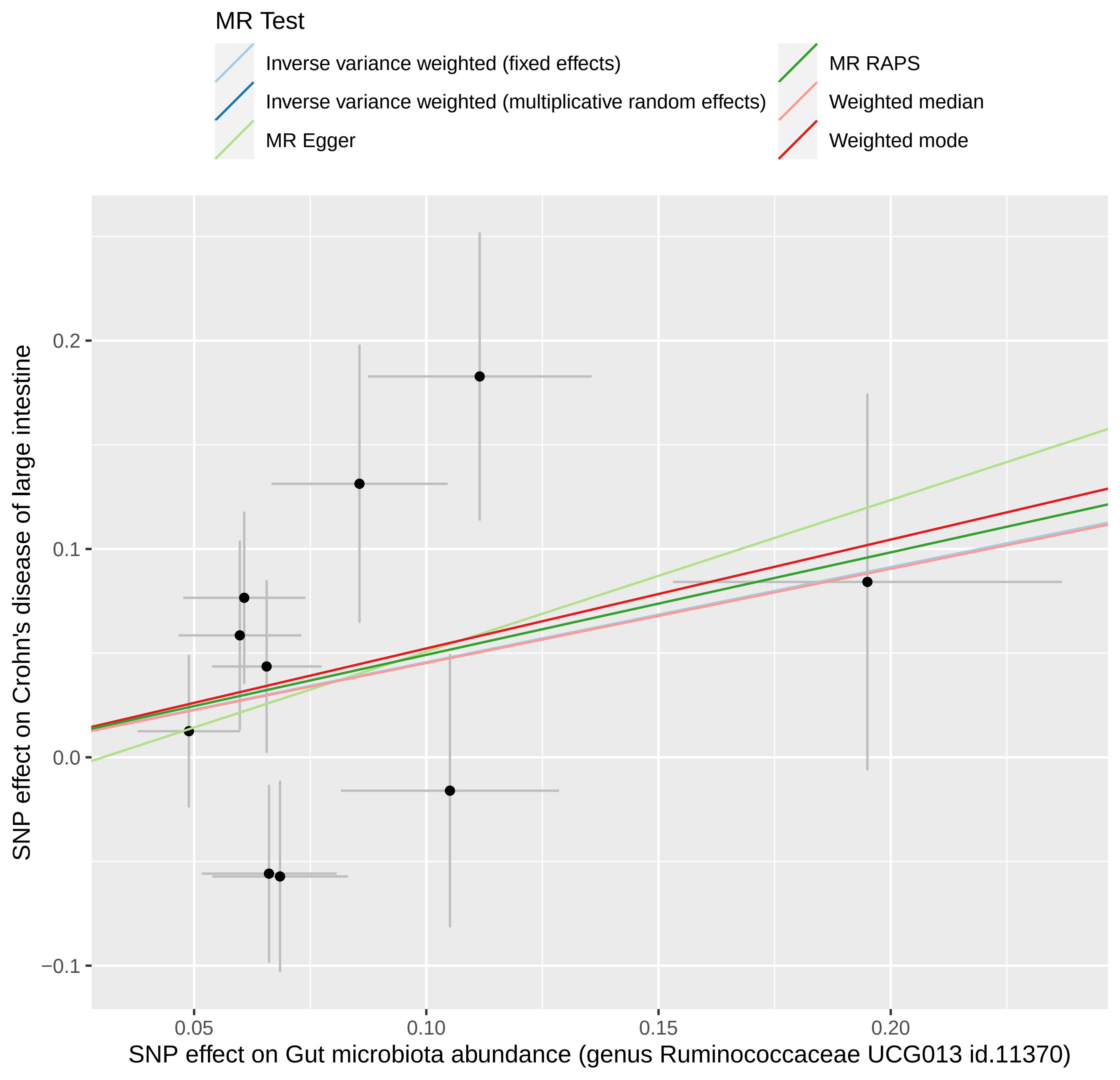

Batch 135 : Gut microbiota abundance (genus Ruminococcaceae UCG014 id.11371) on Crohn's disease of large intestine

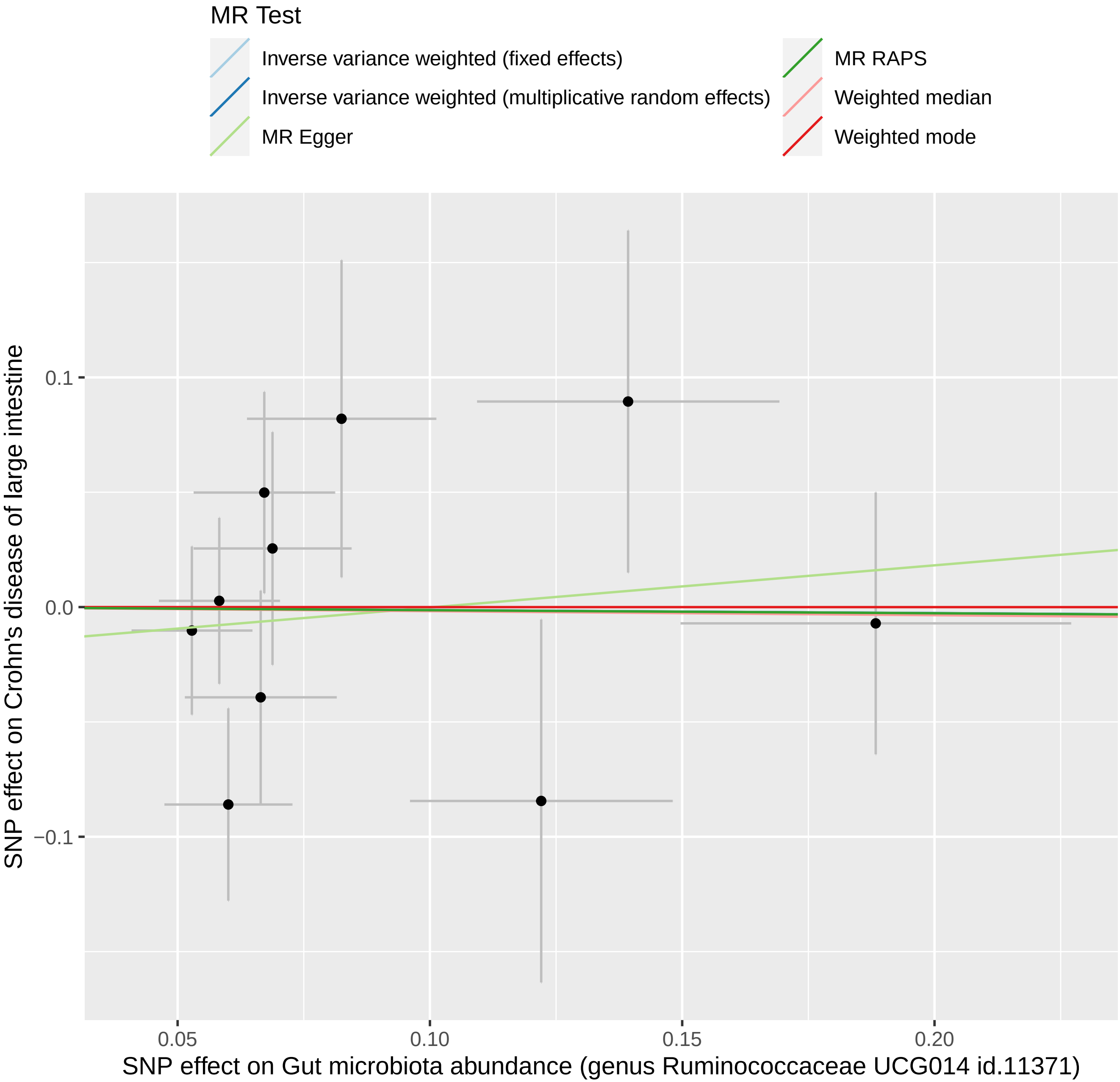

Batch 136 : Gut microbiota abundance (genus Ruminococcus1 id.11373) on Crohn's disease of large intestine

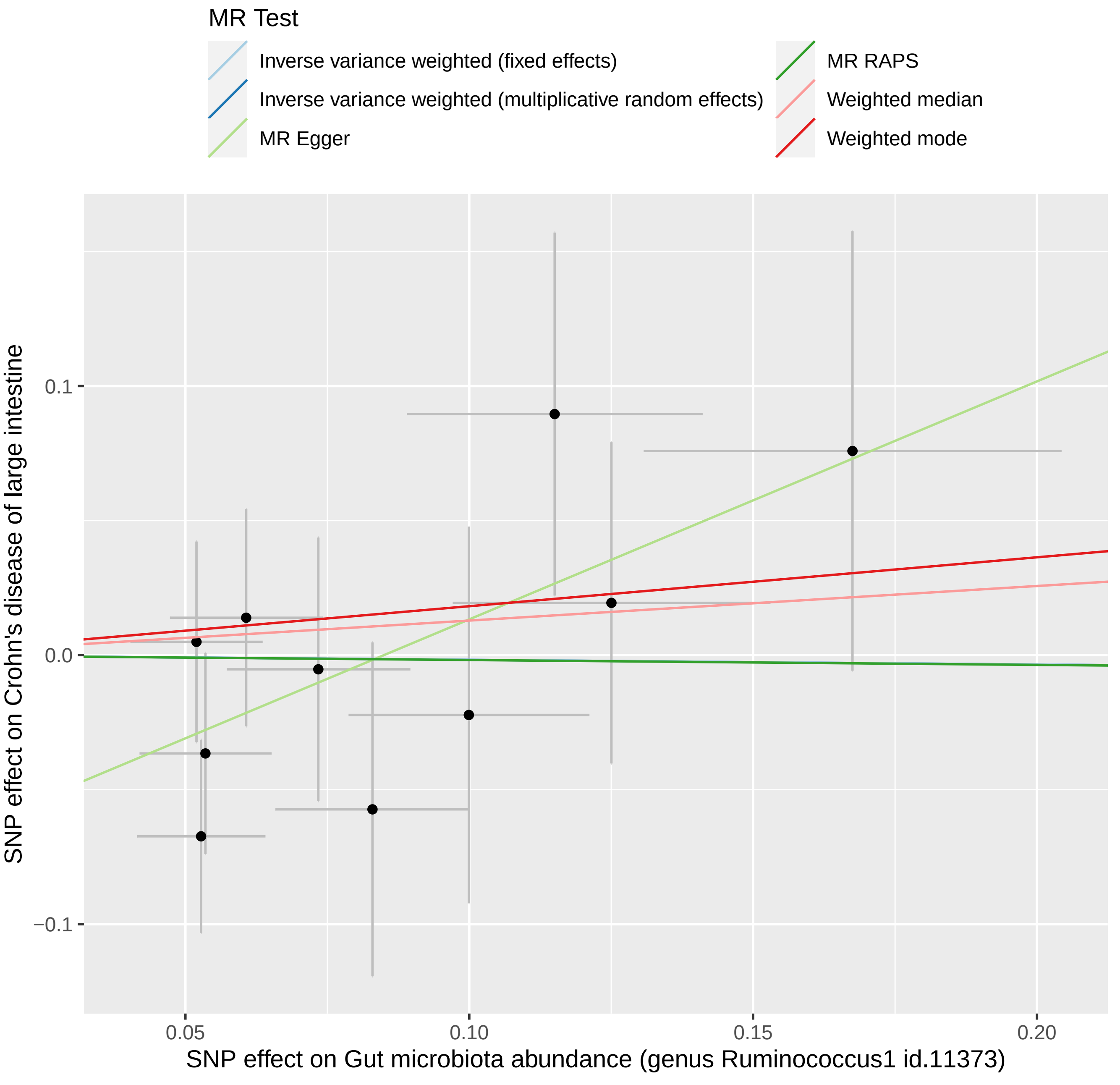

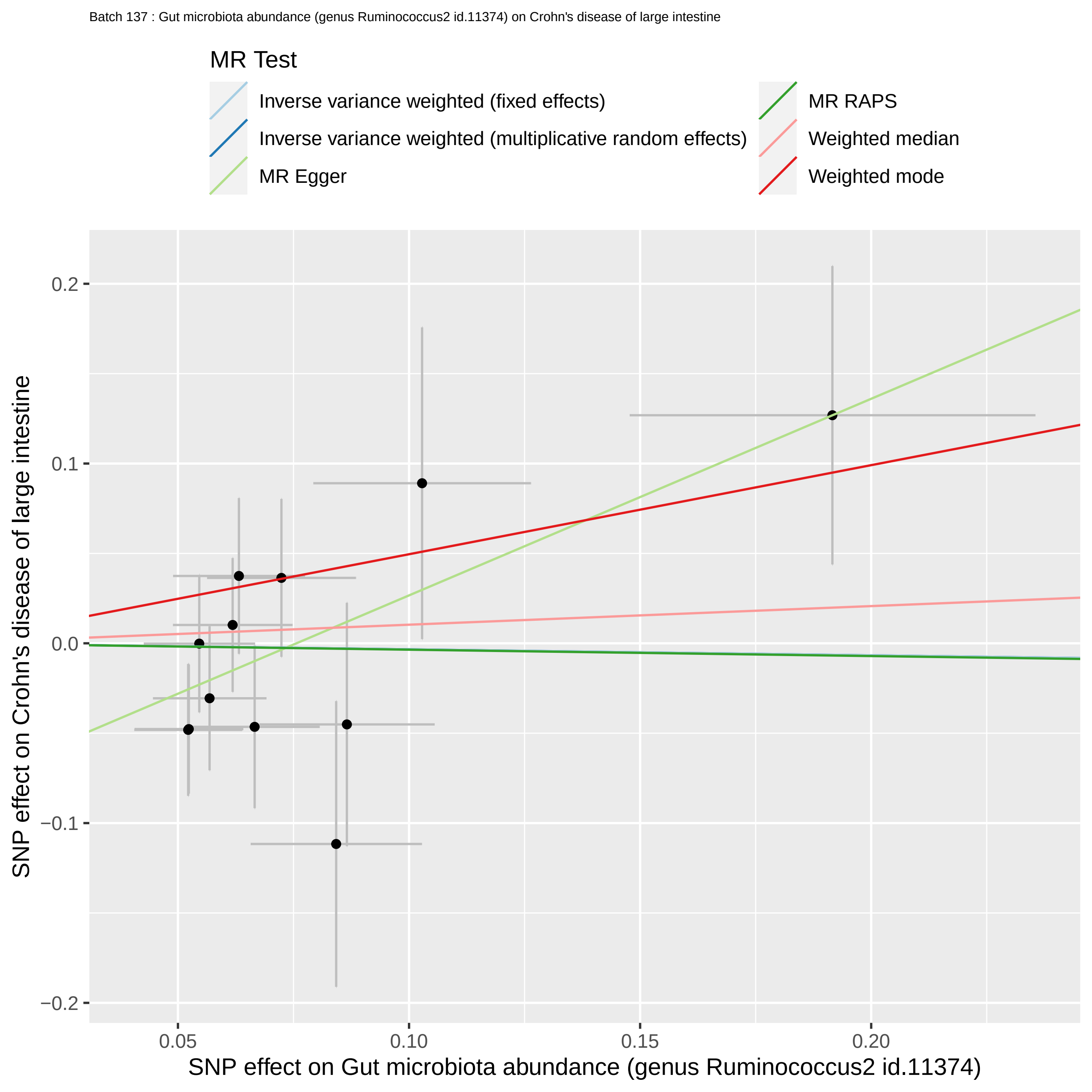
Batch 137 : Gut microbiota abundance (genus Ruminococcus2 id.11374) on Crohn's disease of large intestine
MR Test
Inverse variance weighted (fixed effects)
Inverse variance weighted (multiplicative random effects)
MR Egger
MR RAPS
Weighted median
Weighted mode
0.2
0.1
0.0
−0.1
−0.2
0.05
0.10
0.15
0.20
SNP effect on Crohn's disease of large intestine
SNP effect on Gut microbiota abundance (genus Ruminococcus2 id.11374)

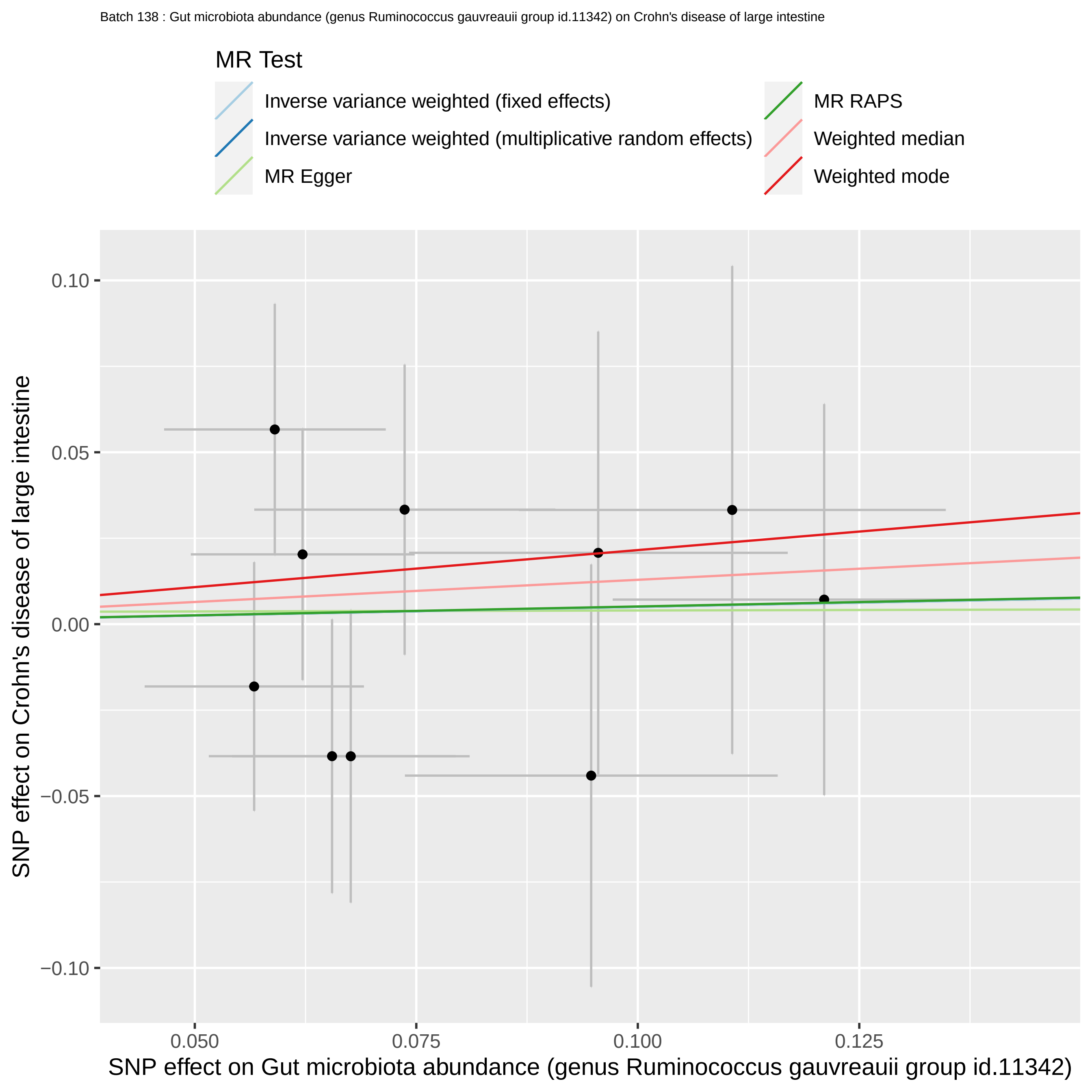
Batch 138 : Gut microbiota abundance (genus Ruminococcus gauvreauii group id.11342) on Crohn's disease of large intestine
MR Test
Inverse variance weighted (fixed effects)
Inverse variance weighted (multiplicative random effects)
MR Egger
MR RAPS
Weighted median
Weighted mode
0.10
0.05
0.00
−0.05
−0.10
0.050
0.075
0.100
0.125
SNP effect on Crohn's disease of large intestine
SNP effect on Gut microbiota abundance (genus Ruminococcus gauvreauii group id.11342)

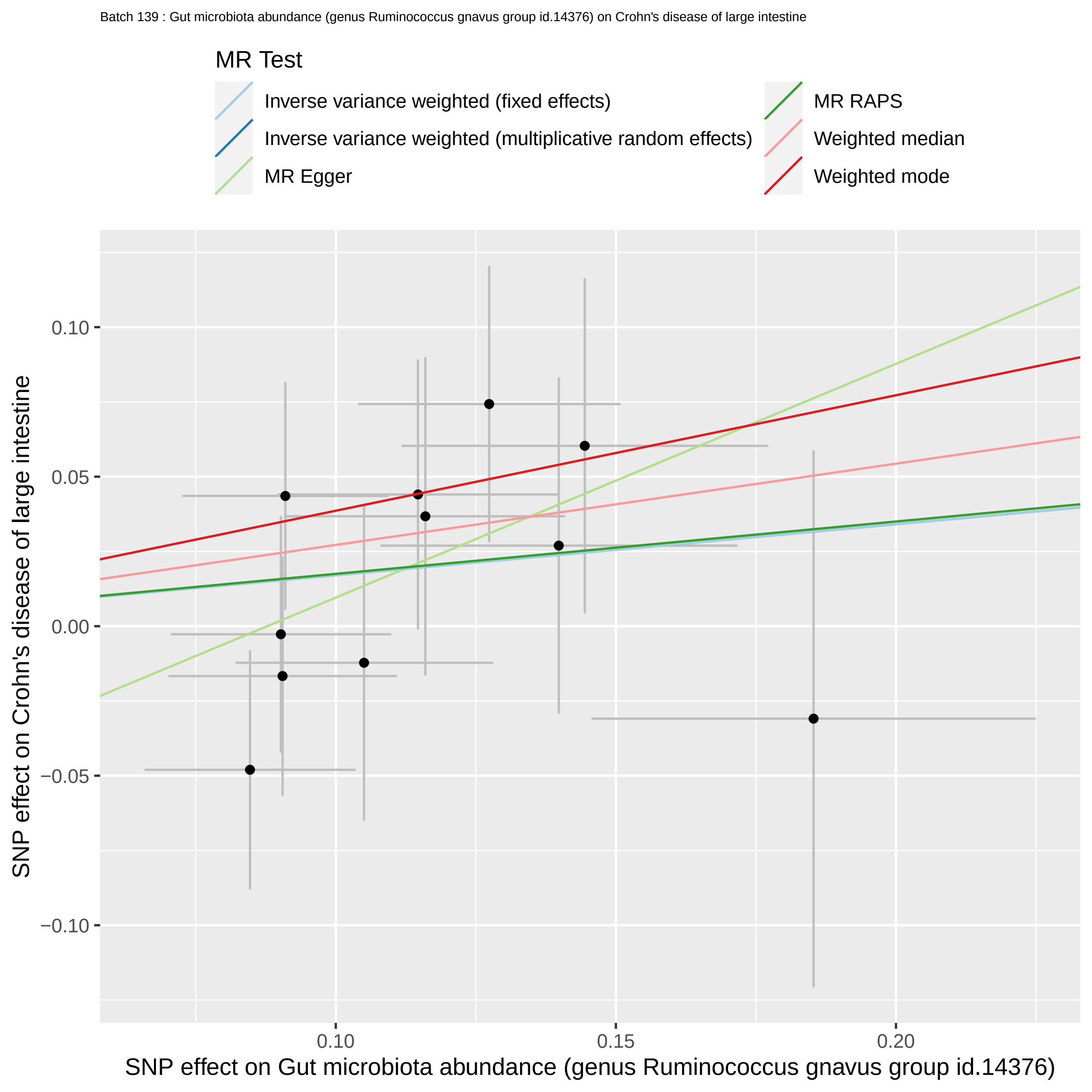
Batch 139 : Gut microbiota abundance (genus Ruminococcus gnavus group id.14376) on Crohn's disease of large intestine
MR Test
Inverse variance weighted (fixed effects)
Inverse variance weighted (multiplicative random effects)
MR Egger
MR RAPS
Weighted median
Weighted mode
0.10
0.05
0.00
−0.05
−0.10
0.10
0.15
0.20
SNP effect on Crohn's disease of large intestine
SNP effect on Gut microbiota abundance (genus Ruminococcus gnavus group id.14376)

Batch 140 : Gut microbiota abundance (genus Ruminococcus torques group id.14377) on Crohn's disease of large intestine

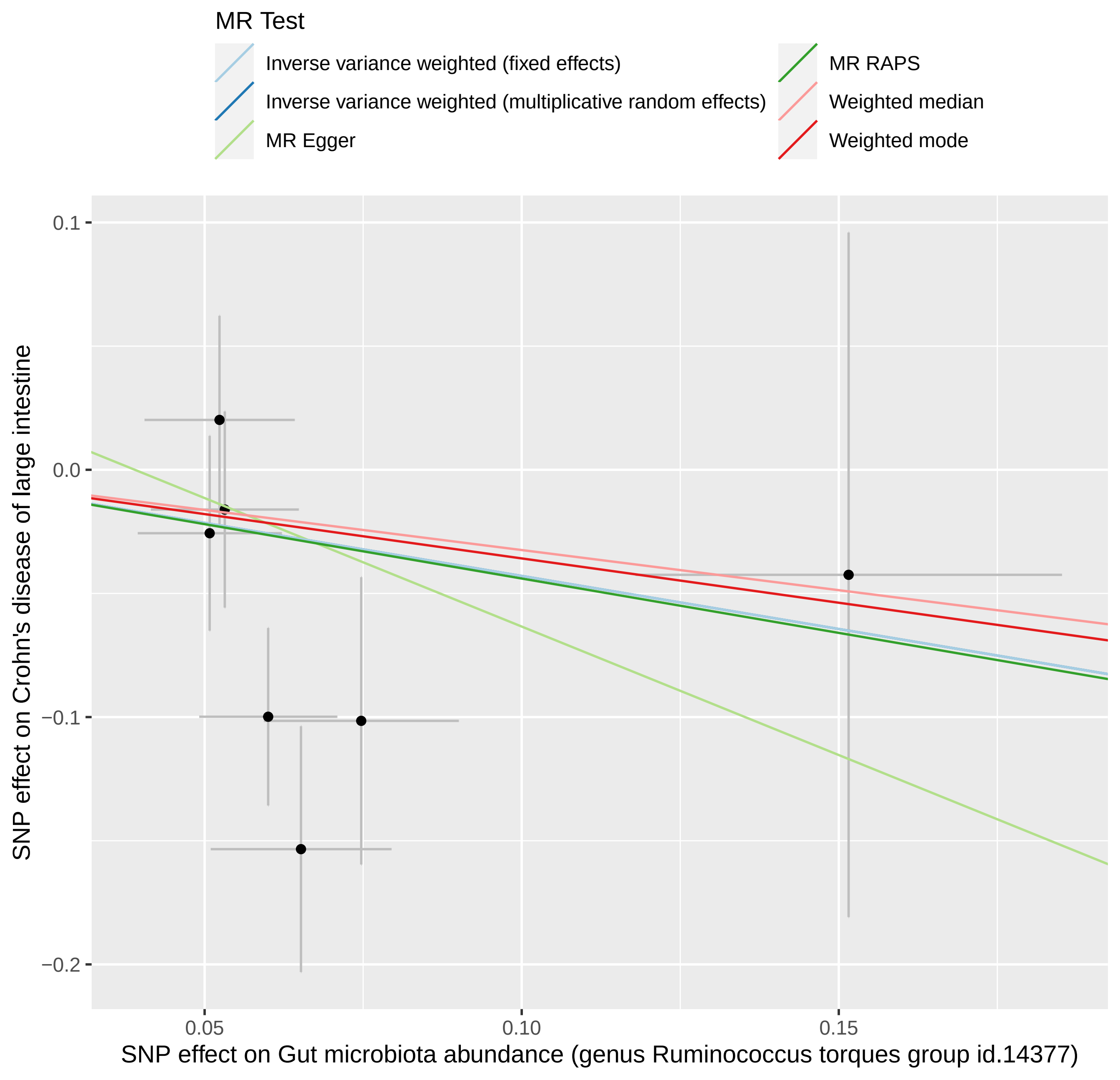

Batch 141 : Gut microbiota abundance (genus Sellimonas id.14369) on Crohn's disease of large intestine

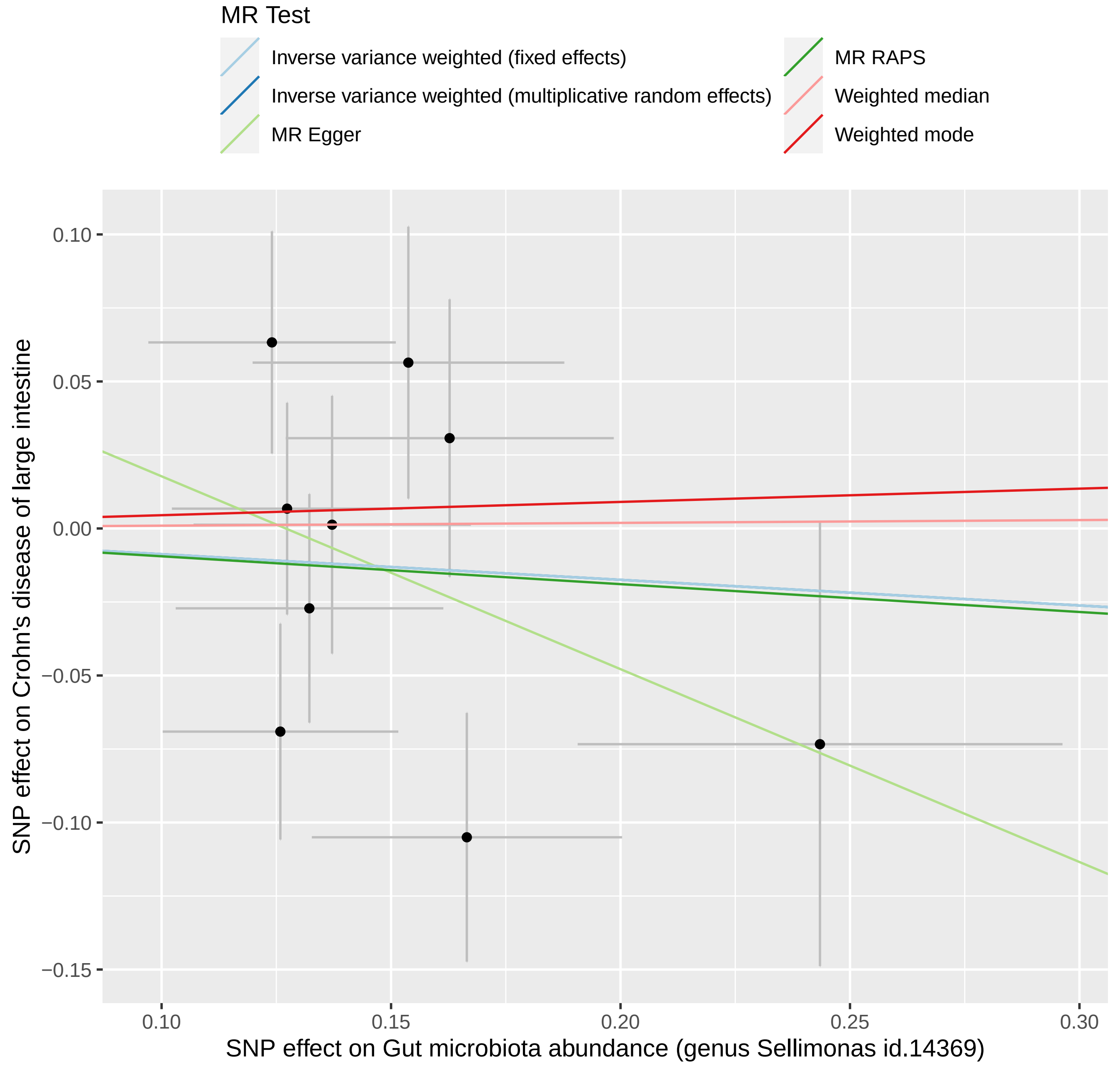

Batch 142 : Gut microbiota abundance (genus Senegalimassilia id.11160) on Crohn's disease of large intestine

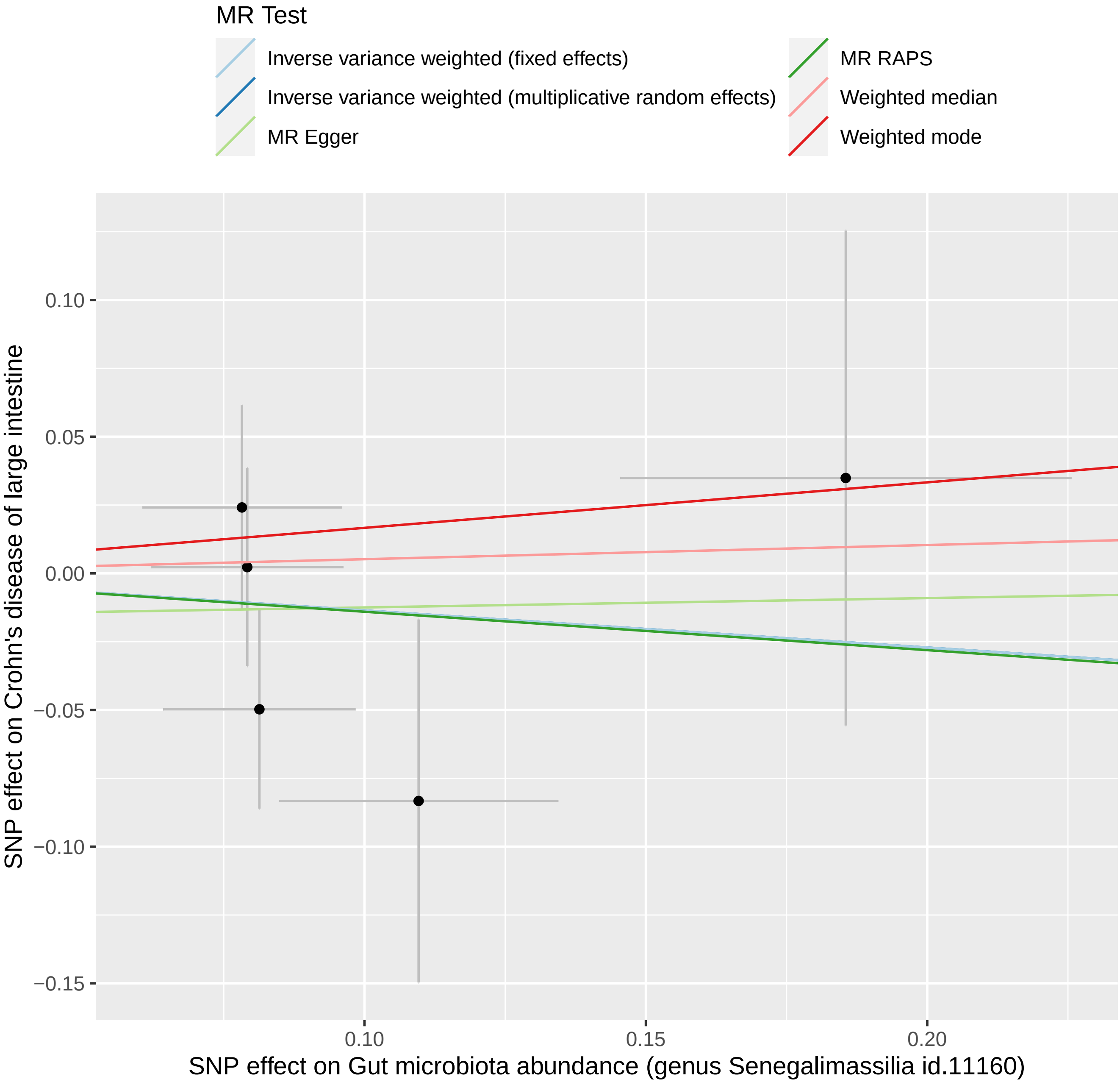

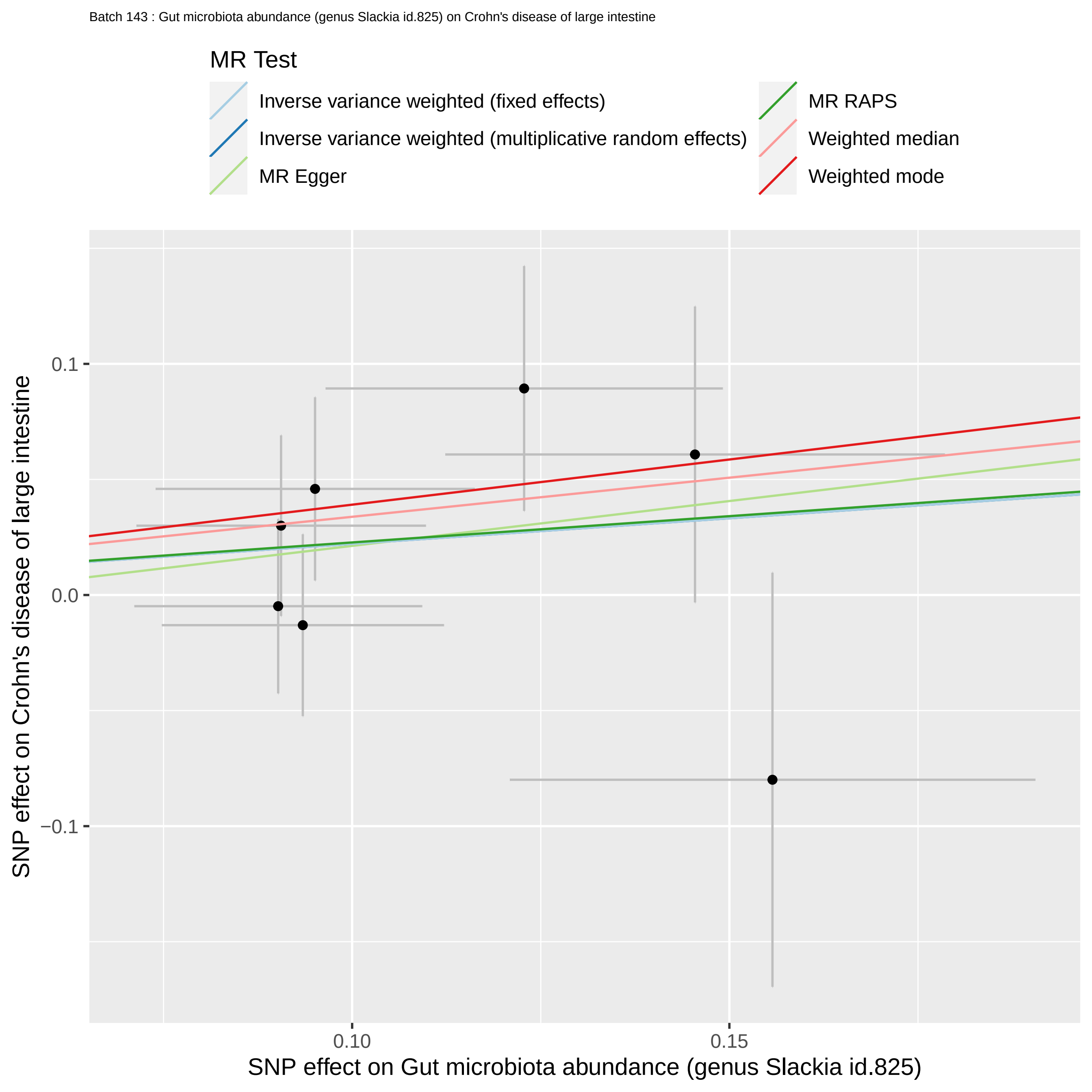

Batch 143 : Gut microbiota abundance (genus Slackia id.825) on Crohn's disease of large intestine
MR Test
Inverse variance weighted (fixed effects)
Inverse variance weighted (multiplicative random effects)
MR Egger
MR RAPS
Weighted median
Weighted mode
0.1
0.0
−0.1
0.10
0.15
SNP effect on Gut microbiota abundance (genus Slackia id.825)
SNP effect on Crohn's disease of large intestine

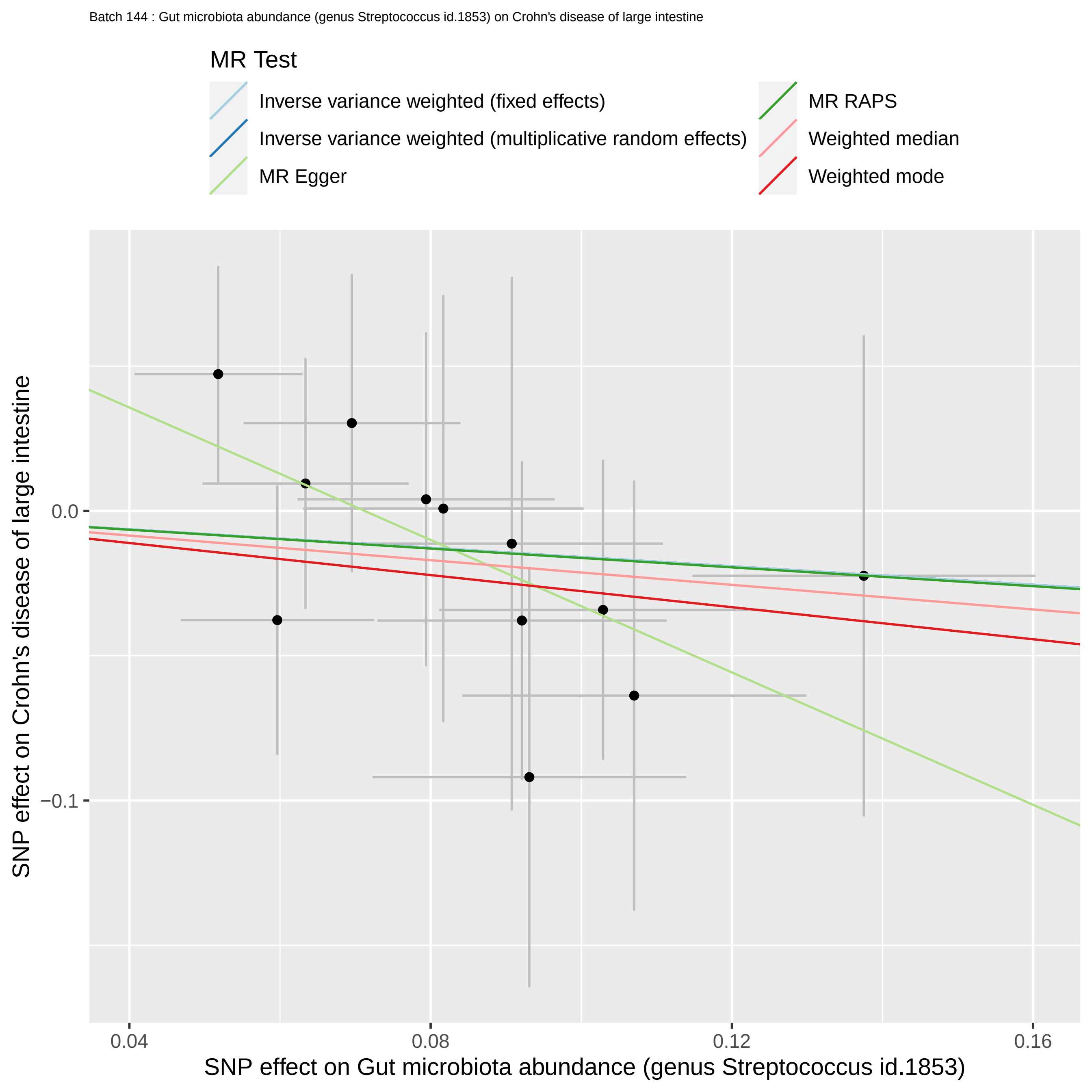
Batch 144 : Gut microbiota abundance (genus Streptococcus id.1853) on Crohn's disease of large intestine
MR Test
Inverse variance weighted (fixed effects)
Inverse variance weighted (multiplicative random effects)
MR Egger
MR RAPS
Weighted median
Weighted mode
0.0
−0.1
0.04
0.08
0.12
0.16
SNP effect on Gut microbiota abundance (genus Streptococcus id.1853)
SNP effect on Crohn's disease of large intestine

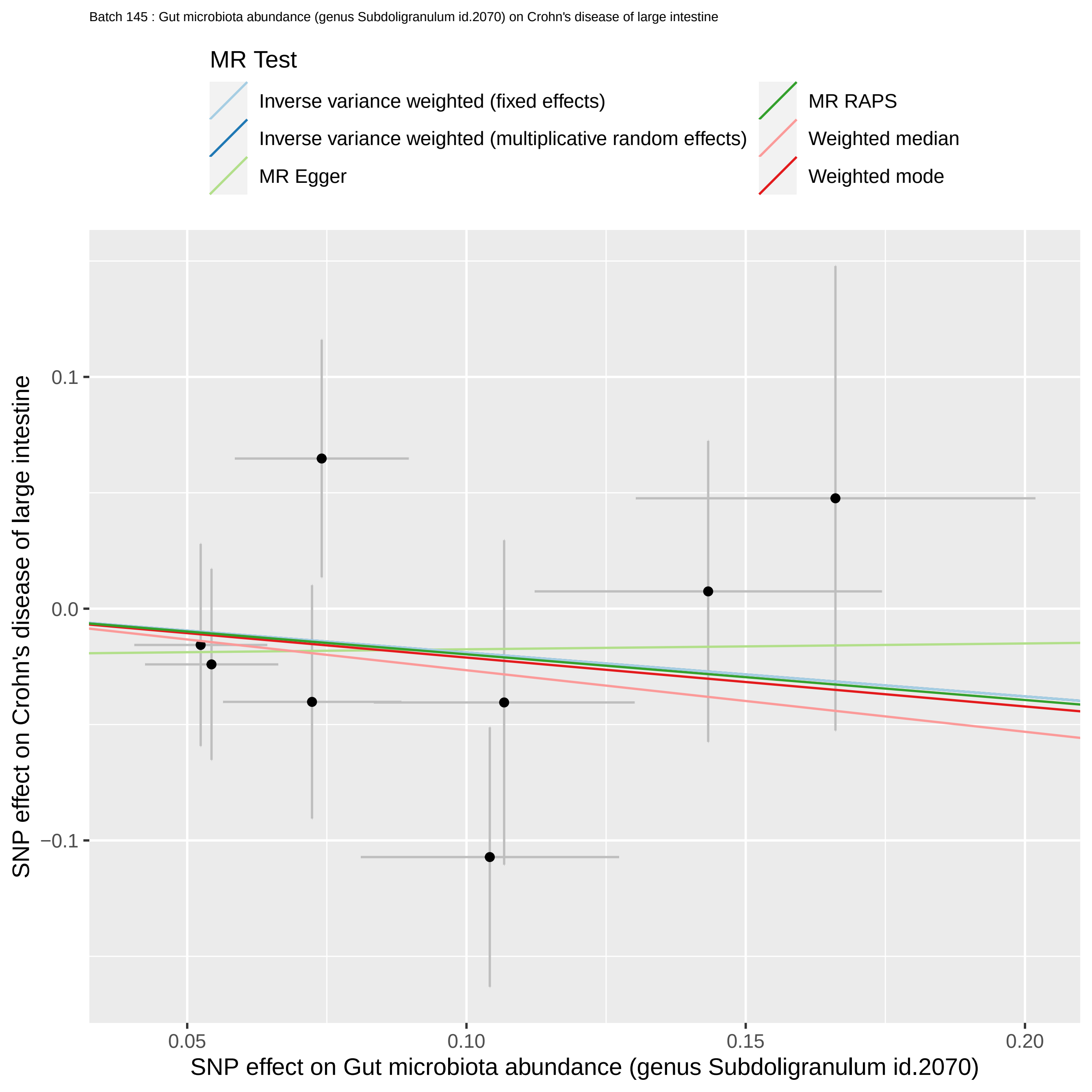
Batch 145 : Gut microbiota abundance (genus Subdoligranulum id.2070) on Crohn's disease of large intestine
MR Test
Inverse variance weighted (fixed effects)
Inverse variance weighted (multiplicative random effects)
MR Egger
MR RAPS
Weighted median
Weighted mode
0.1
0.0
−0.1
0.05
0.10
0.15
0.20
SNP effect on Crohn's disease of large intestine
SNP effect on Gut microbiota abundance (genus Subdoligranulum id.2070)

Batch 146 : Gut microbiota abundance (genus Sutterella id.2896) on Crohn's disease of large intestine

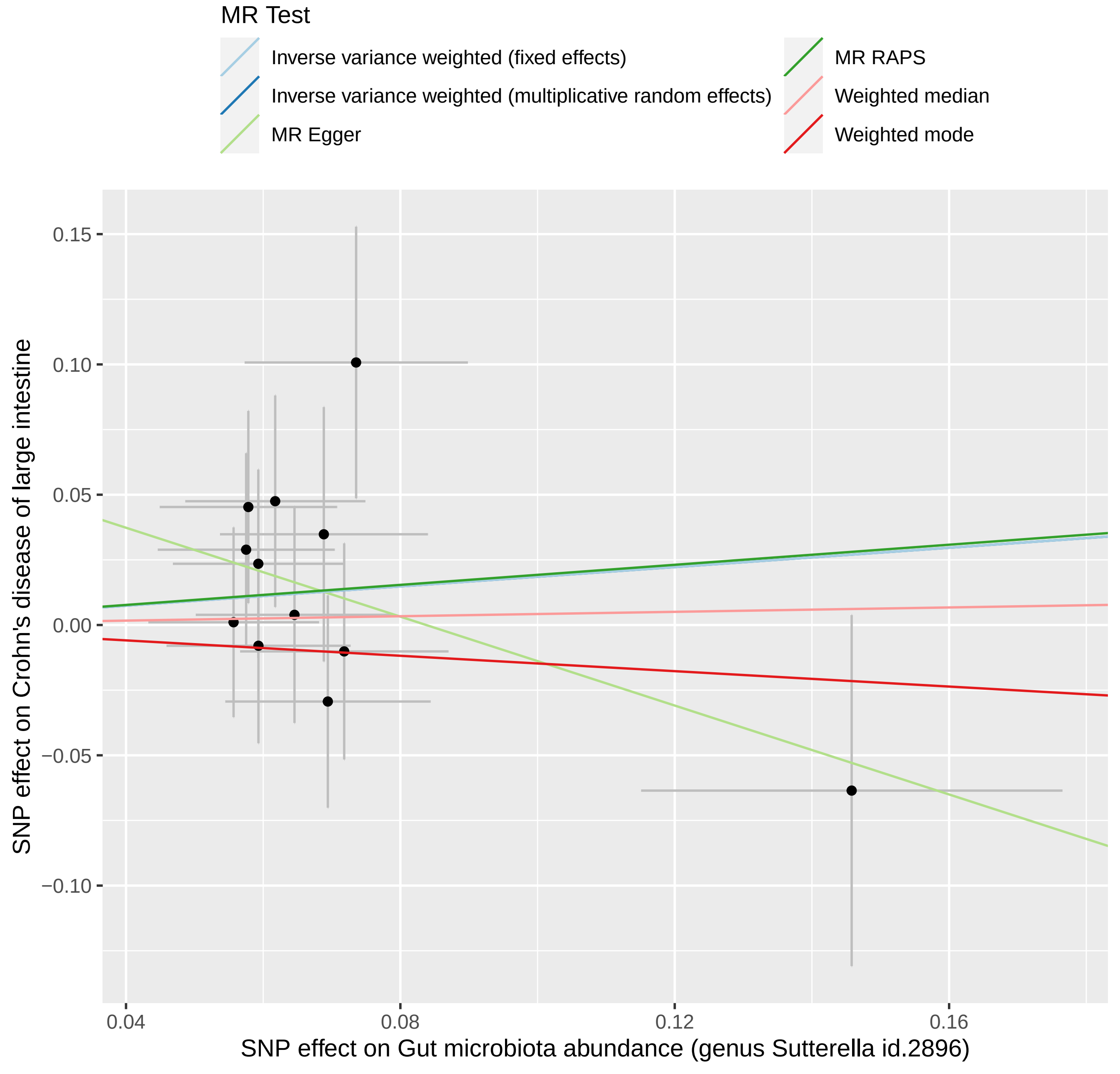

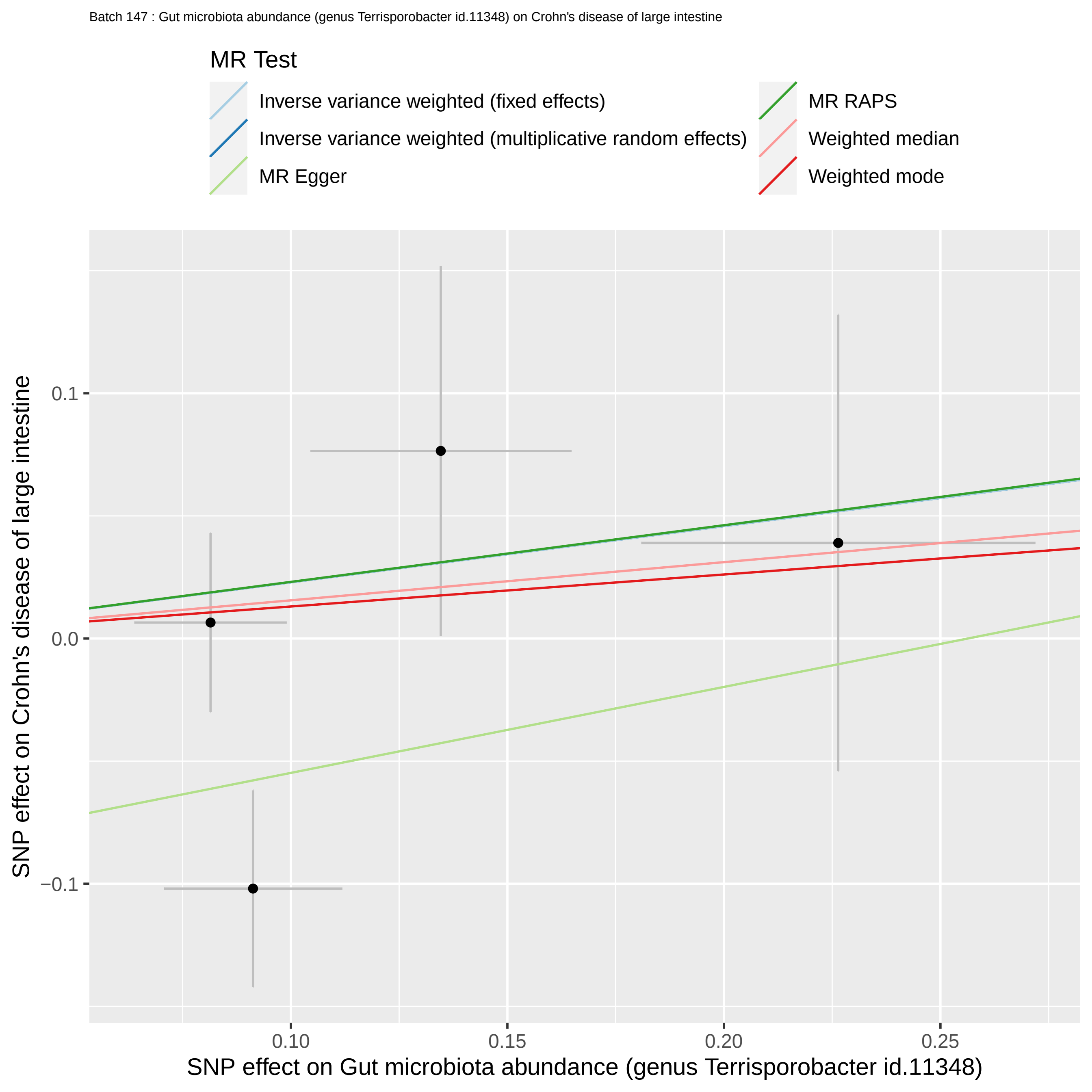

Batch 147 : Gut microbiota abundance (genus Terrisporobacter id.11348) on Crohn's disease of large intestine
MR Test
Inverse variance weighted (fixed effects)
Inverse variance weighted (multiplicative random effects)
MR Egger
MR RAPS
Weighted median
Weighted mode
0.1
0.0
−0.1
0.10
0.15
0.20
0.25
SNP effect on Crohn's disease of large intestine
SNP effect on Gut microbiota abundance (genus Terrisporobacter id.11348)

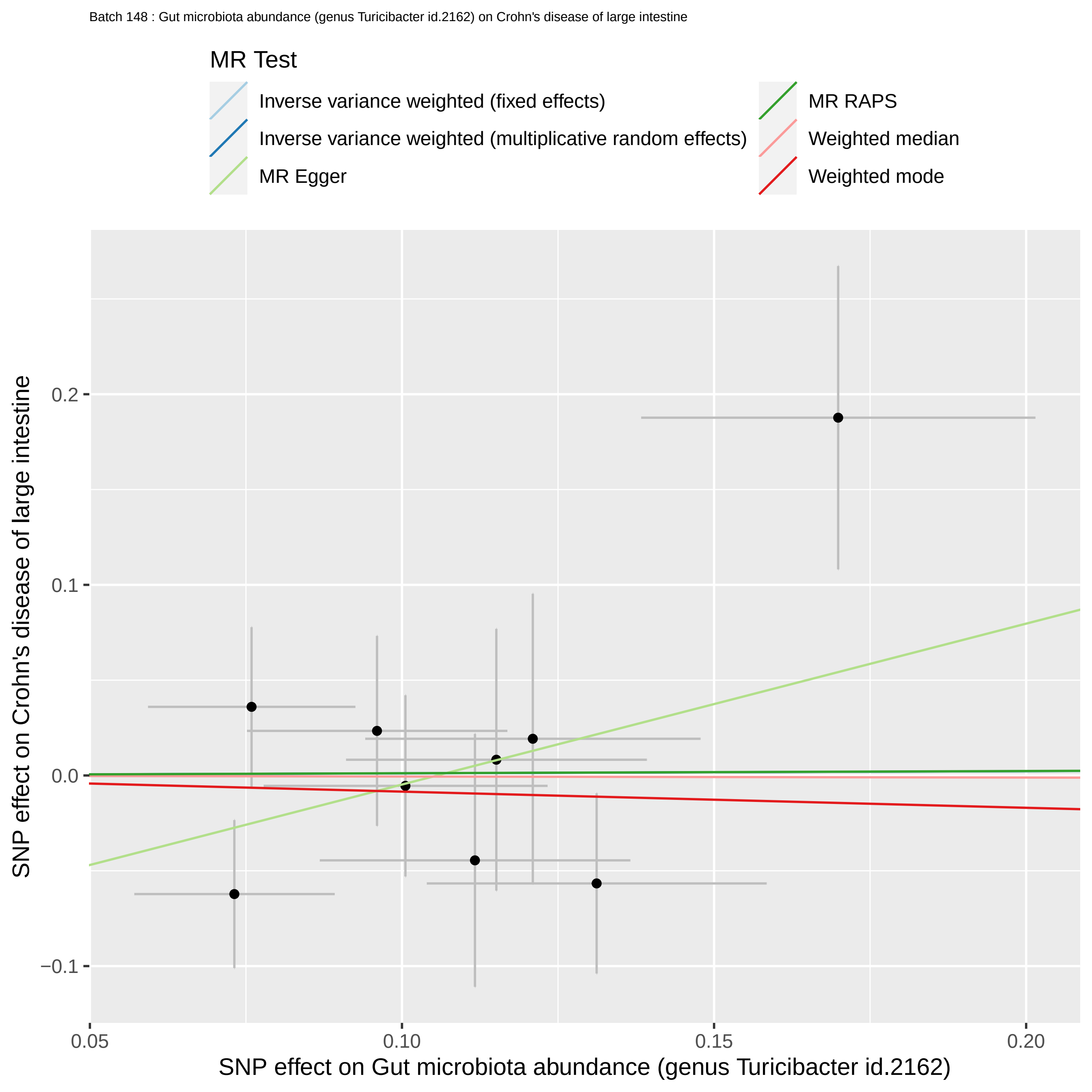
Batch 148 : Gut microbiota abundance (genus Turicibacter id.2162) on Crohn's disease of large intestine
MR Test
Inverse variance weighted (fixed effects)
Inverse variance weighted (multiplicative random effects)
MR Egger
MR RAPS
Weighted median
Weighted mode
SNP effect on Crohn's disease of large intestine
0.2
0.1
0.0
−0.1
0.05
0.10
0.15
0.20
SNP effect on Gut microbiota abundance (genus Turicibacter id.2162)

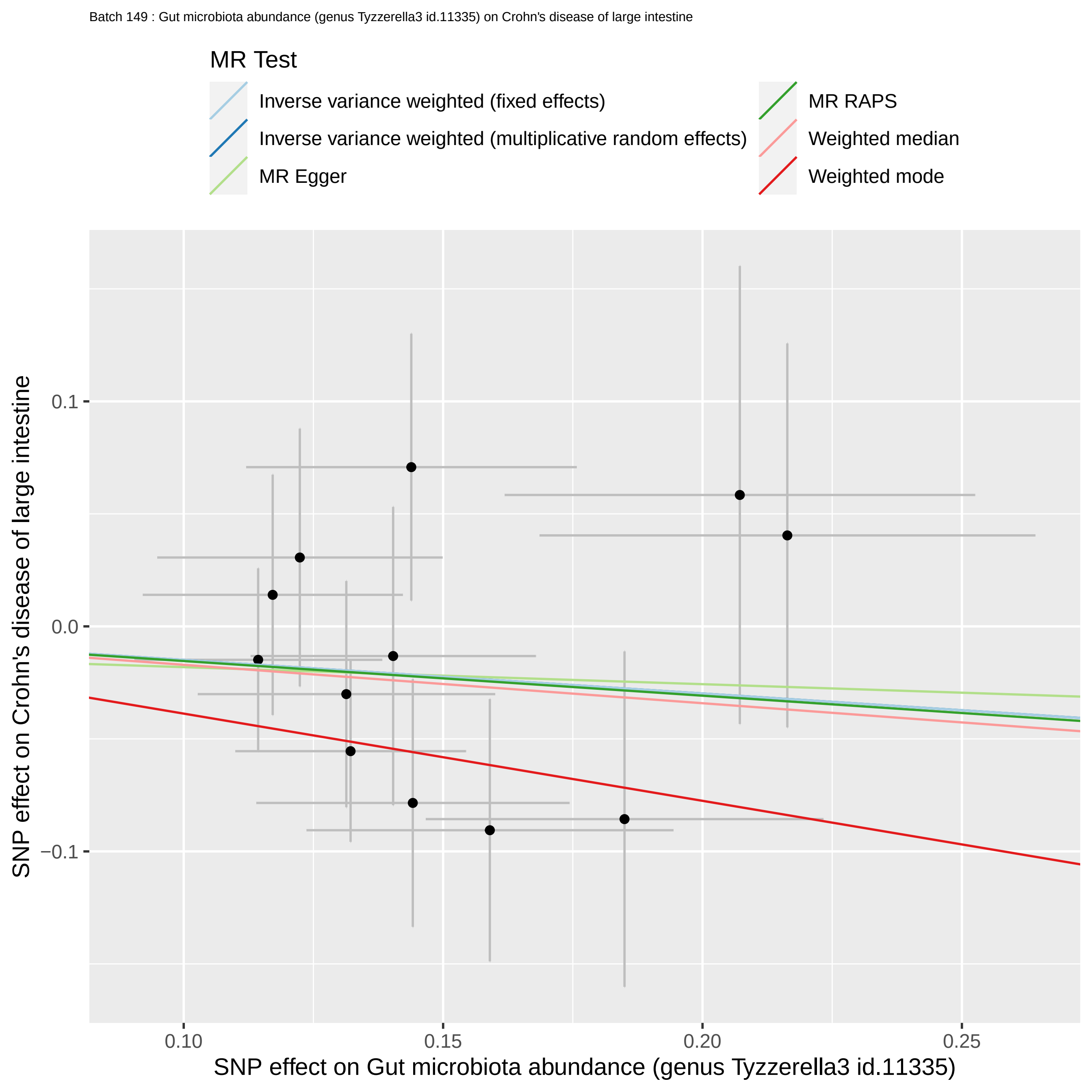
Batch 149 : Gut microbiota abundance (genus Tyzzerella3 id.11335) on Crohn's disease of large intestine
MR Test
Inverse variance weighted (fixed effects)
Inverse variance weighted (multiplicative random effects)
MR Egger
MR RAPS
Weighted median
Weighted mode
SNP effect on Crohn's disease of large intestine
0.1
0.0
−0.1
0.10
0.15
0.20
0.25
SNP effect on Gut microbiota abundance (genus Tyzzerella3 id.11335)

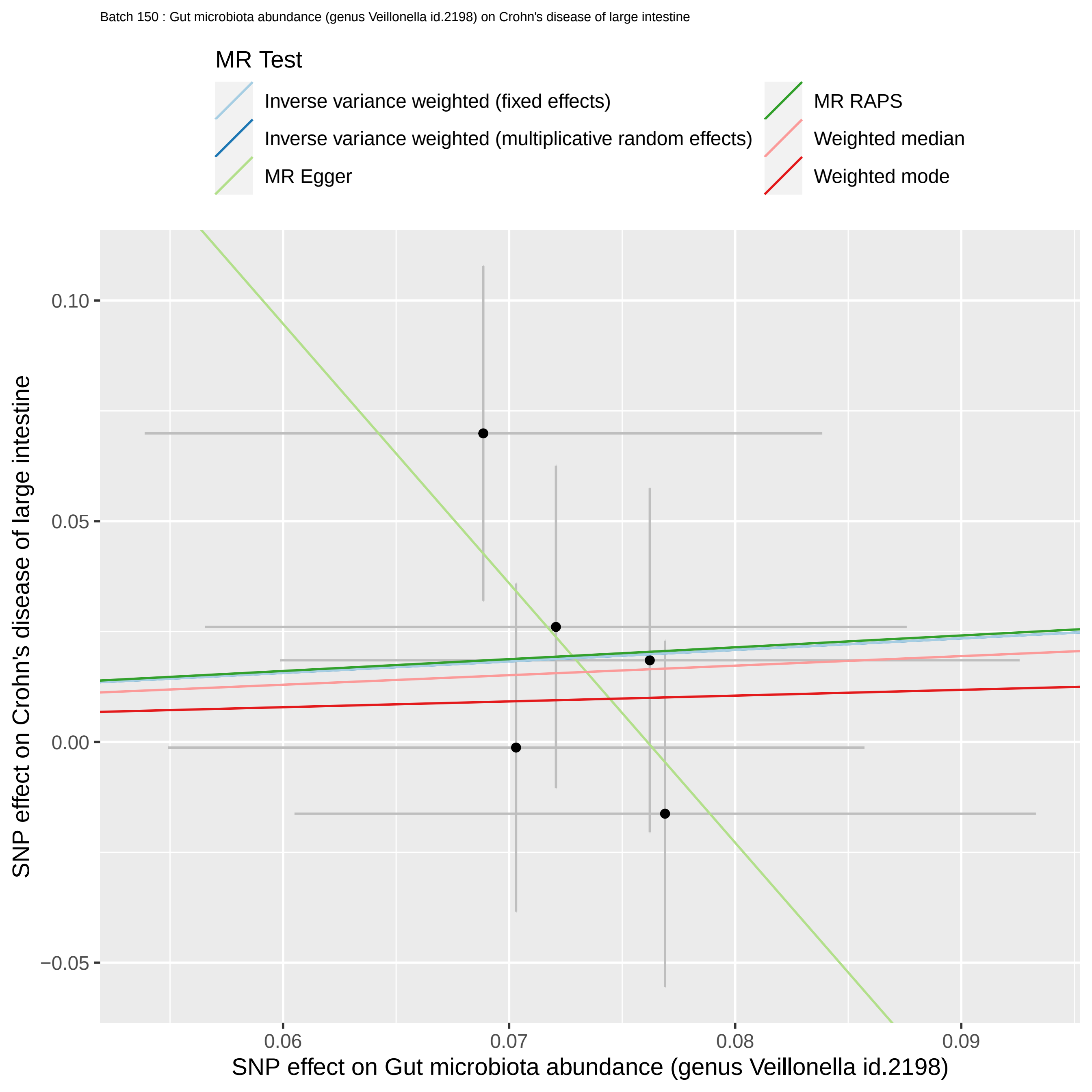

Batch 150 : Gut microbiota abundance (genus Veillonella id.2198) on Crohn's disease of large intestine
MR Test
Inverse variance weighted (fixed effects)
Inverse variance weighted (multiplicative random effects)
MR Egger
MR RAPS
Weighted median
Weighted mode
SNP effect on Crohn's disease of large intestine
0.10
0.05
0.00
−0.05
0.06
0.07
0.08
0.09
SNP effect on Gut microbiota abundance (genus Veillonella id.2198)

Batch 151 : Gut microbiota abundance (family Acidaminococcaceae id.2166) on Other Crohn's disease

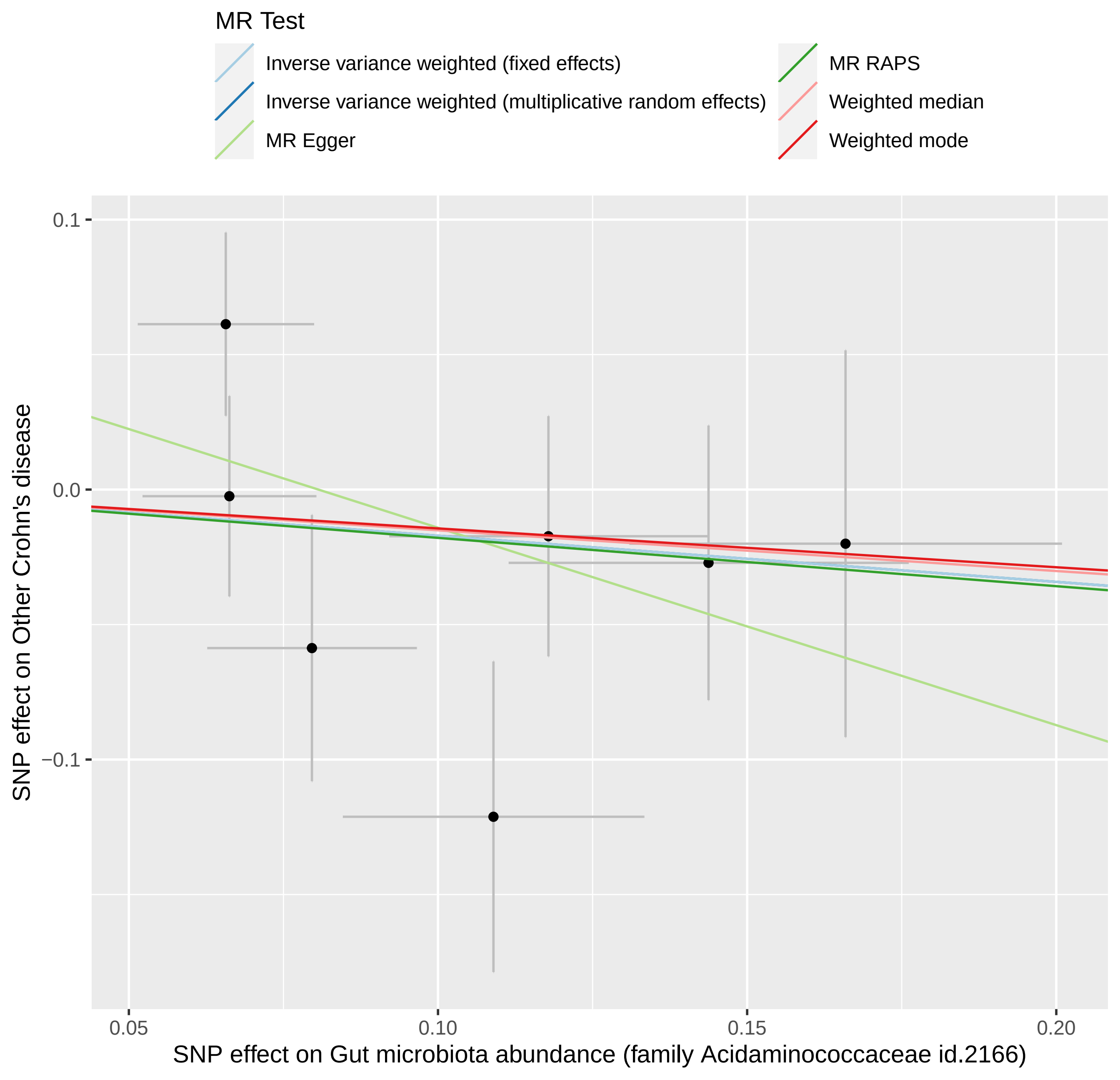

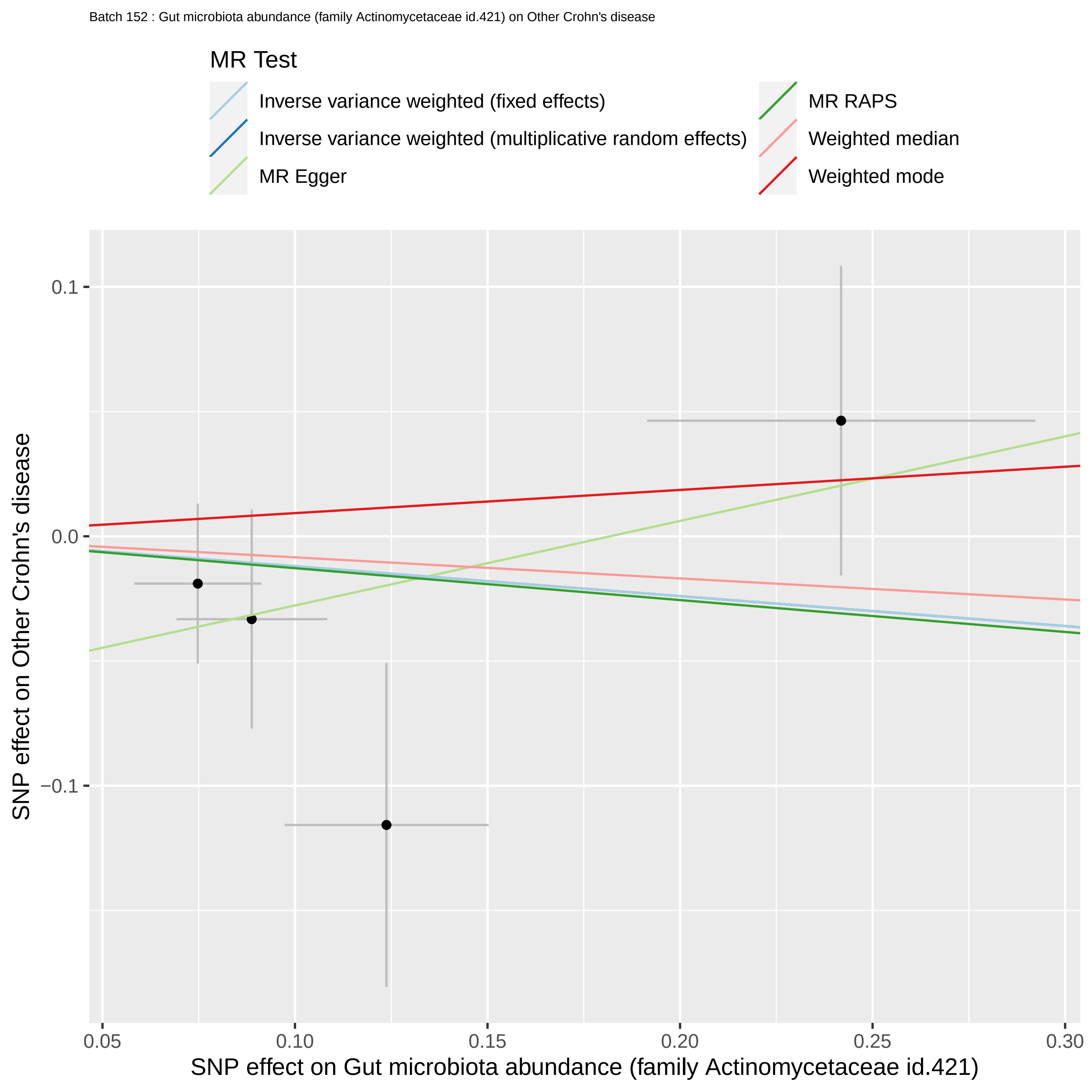
Batch 152 : Gut microbiota abundance (family Actinomycetaceae id.421) on Other Crohn's disease
MR Test
Inverse variance weighted (fixed effects)
Inverse variance weighted (multiplicative random effects)
MR Egger
MR RAPS
Weighted median
Weighted mode
0.1
0.0
−0.1
0.05
0.10
0.15
0.20
0.25
0.30
SNP effect on Other Crohn's disease
SNP effect on Gut microbiota abundance (family Actinomycetaceae id.421)

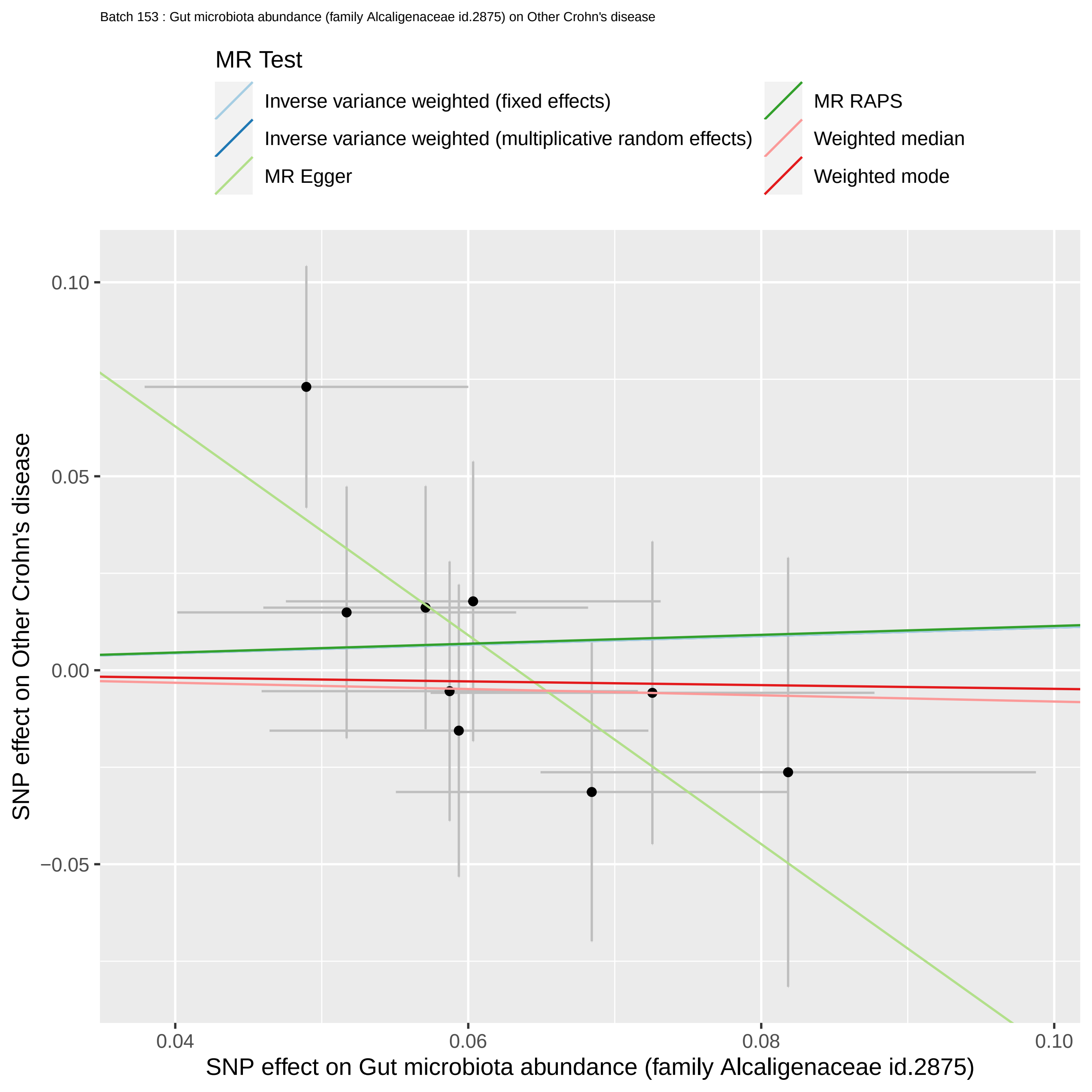

Batch 153 : Gut microbiota abundance (family Alcaligenaceae id.2875) on Other Crohn's disease
MR Test
Inverse variance weighted (fixed effects)
Inverse variance weighted (multiplicative random effects)
MR Egger
MR RAPS
Weighted median
Weighted mode
0.10
0.05
0.00
−0.05
0.04
0.06
0.08
0.10
SNP effect on Other Crohn's disease
SNP effect on Gut microbiota abundance (family Alcaligenaceae id.2875)

Batch 154 : Gut microbiota abundance (family Bacteroidaceae id.917) on Other Crohn's disease

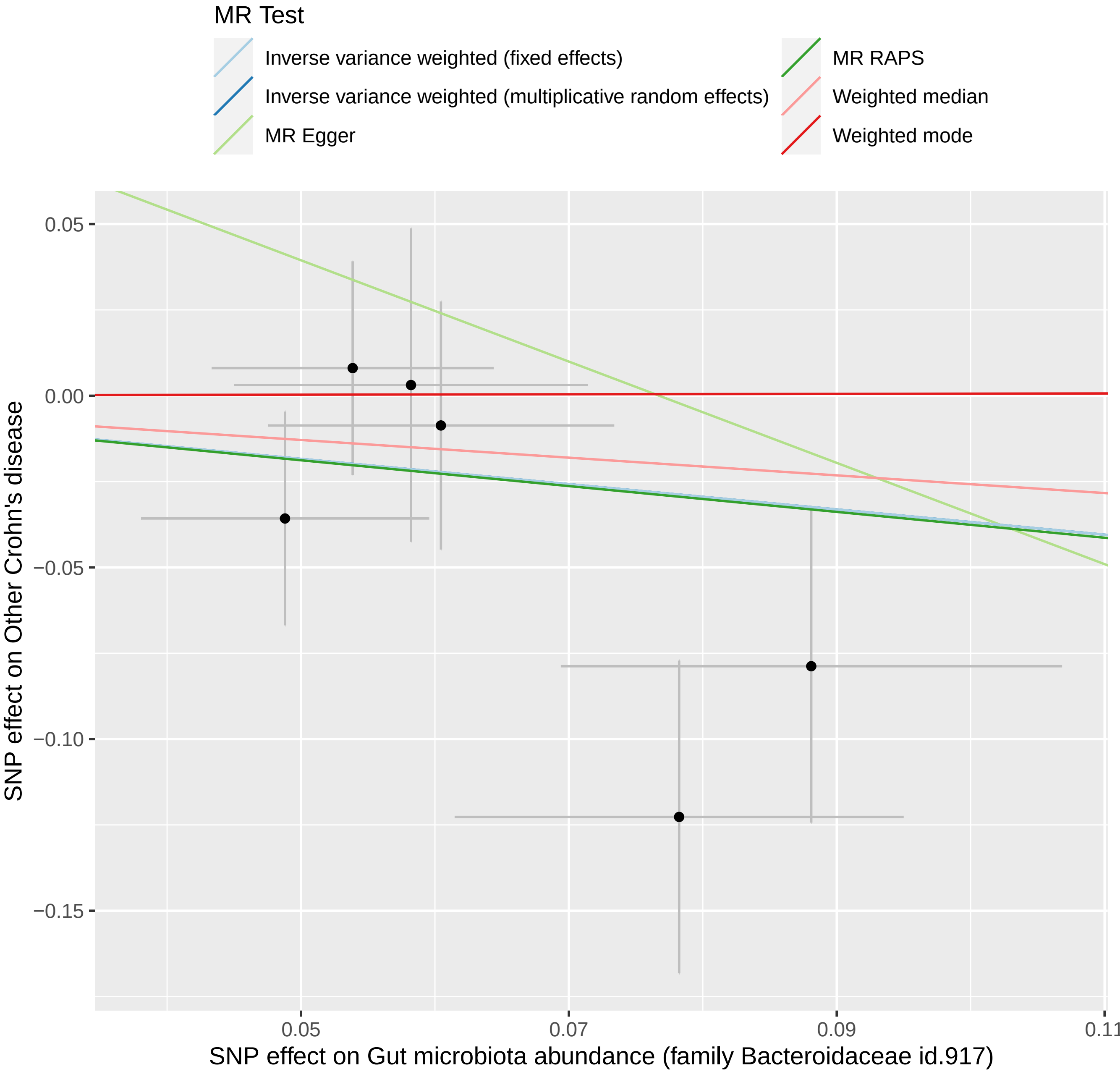

Batch 155 : Gut microbiota abundance (family Bacteroidales S24 7group id.11173) on Other Crohn's disease

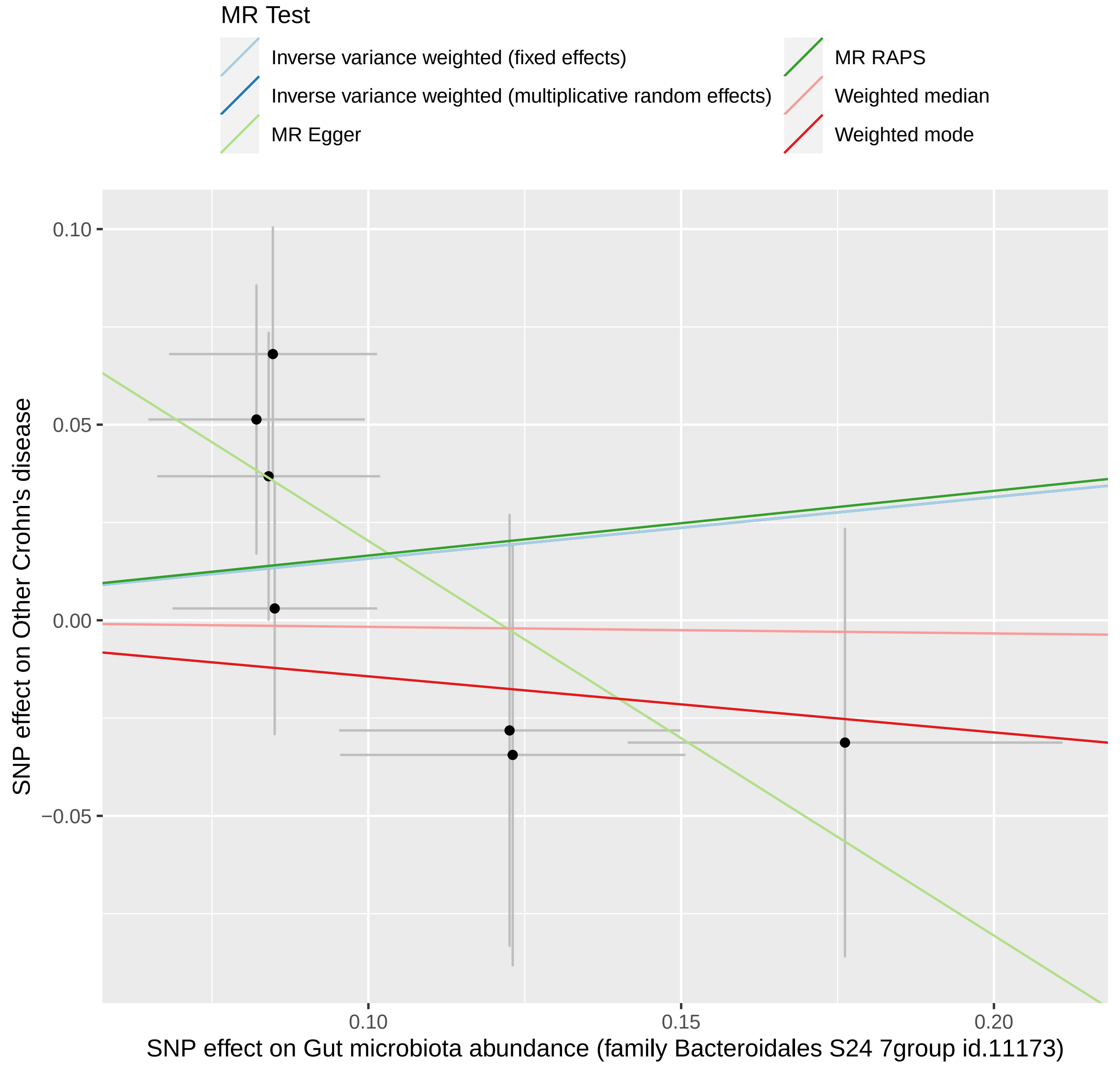

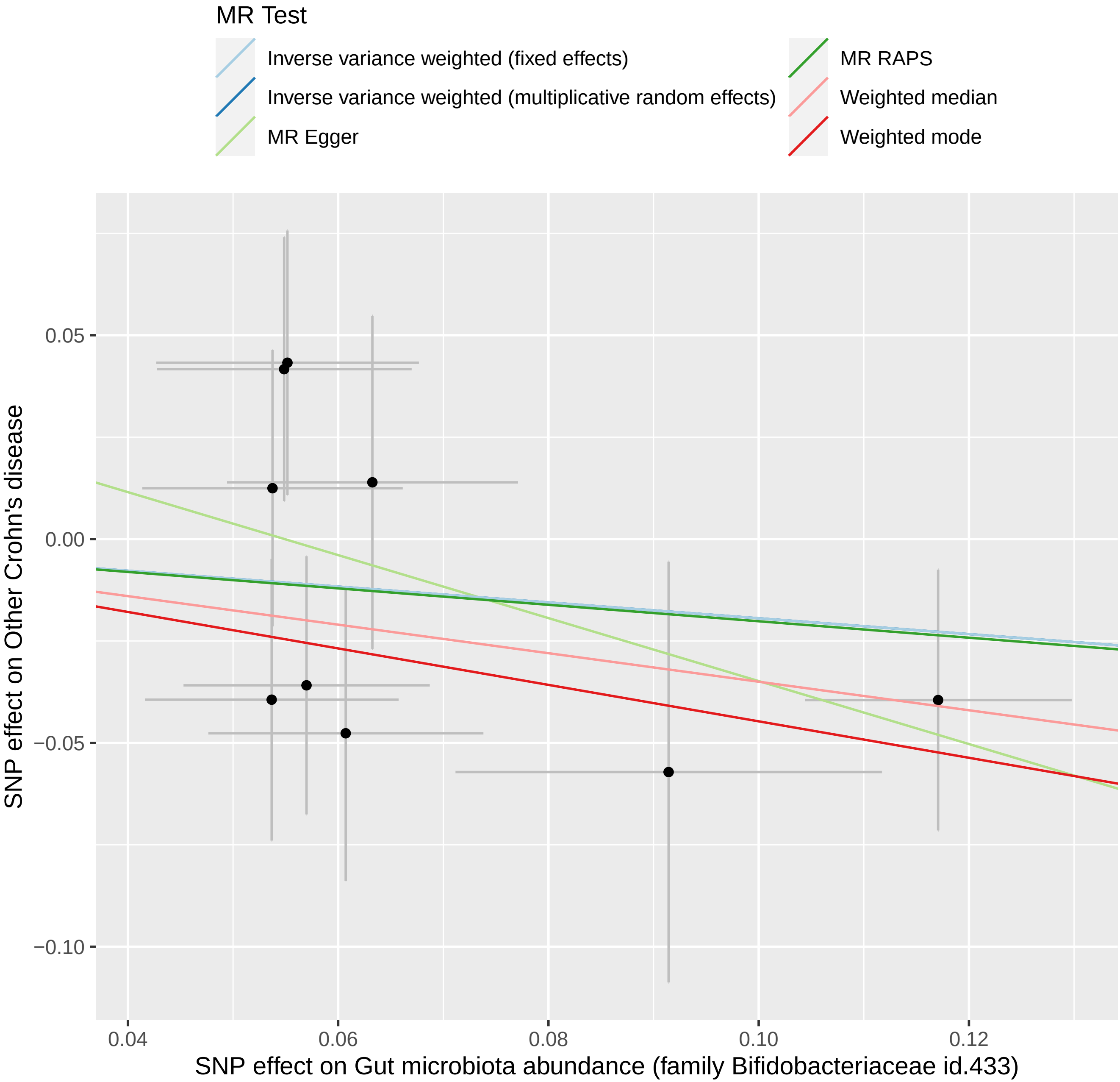
MR Test
Inverse variance weighted (fixed effects)
Inverse variance weighted (multiplicative random effects)
MR Egger
MR RAPS
Weighted median
Weighted mode
0.05
0.00
−0.05
−0.10
0.04
0.06
0.08
0.10
0.12
SNP effect on Other Crohn's disease
SNP effect on Gut microbiota abundance (family Bifidobacteriaceae id.433)

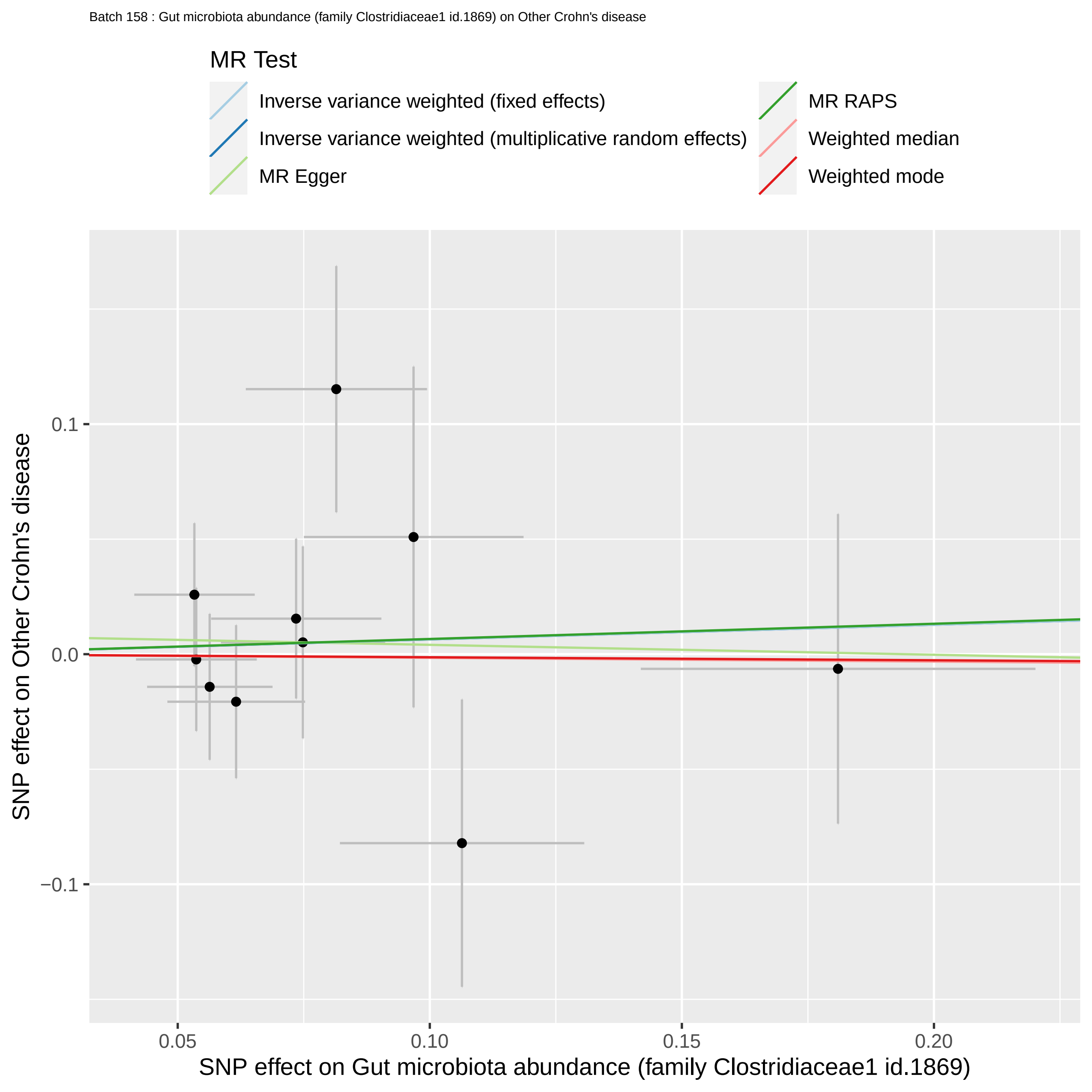

Batch 158 : Gut microbiota abundance (family Clostridiaceae1 id.1869) on Other Crohn's disease
MR Test
Inverse variance weighted (fixed effects)
Inverse variance weighted (multiplicative random effects)
MR Egger
MR RAPS
Weighted median
Weighted mode
SNP effect on Other Crohn's disease
0.1
0.0
−0.1
0.05
0.10
0.15
0.20
SNP effect on Gut microbiota abundance (family Clostridiaceae1 id.1869)

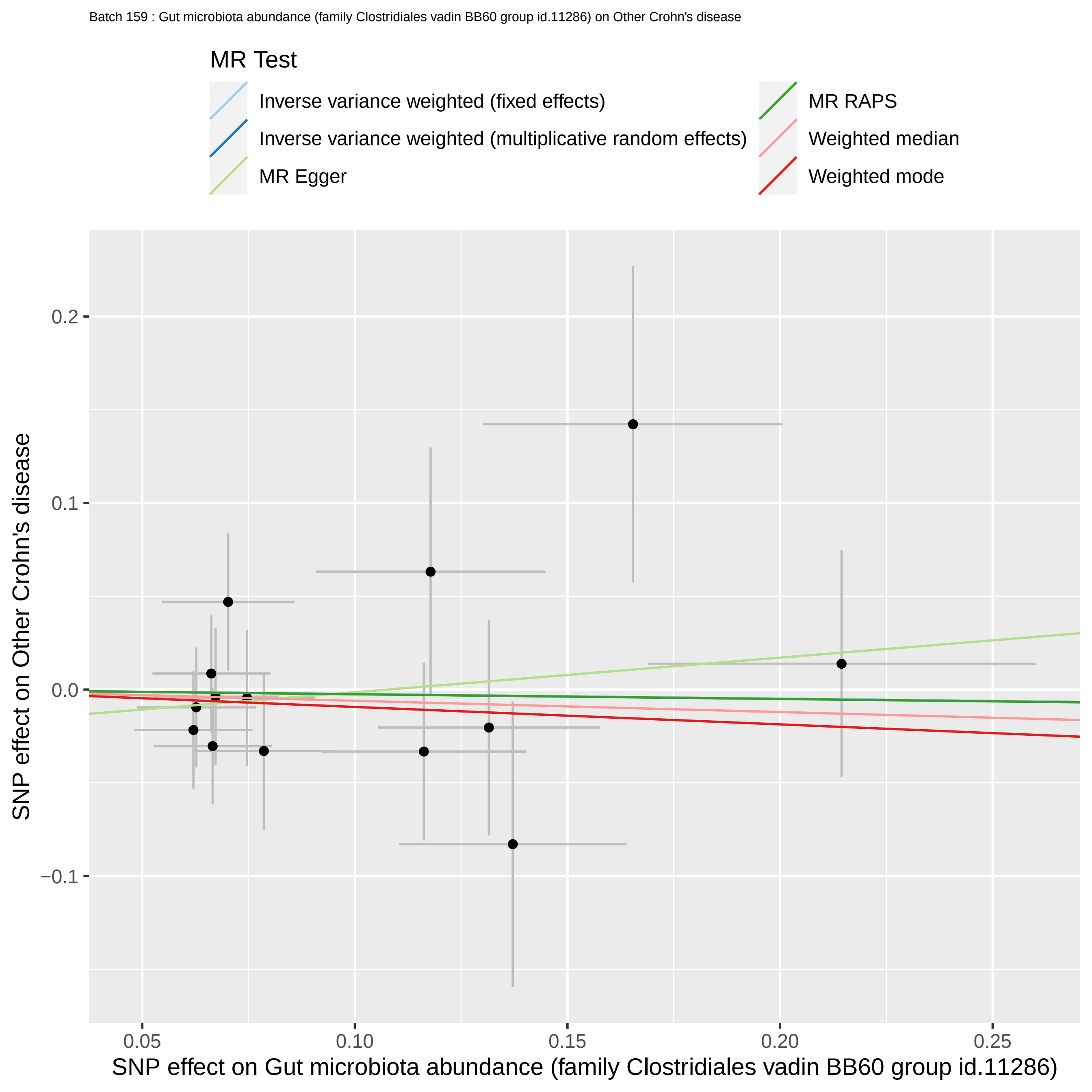
Batch 159 : Gut microbiota abundance (family Clostridiales vadin BB60 group id.11286) on Other Crohn's disease
MR Test
Inverse variance weighted (fixed effects)
Inverse variance weighted (multiplicative random effects)
MR Egger
MR RAPS
Weighted median
Weighted mode
0.2
0.1
0.0
−0.1
0.05
0.10
0.15
0.20
0.25
SNP effect on Other Crohn's disease
SNP effect on Gut microbiota abundance (family Clostridiales vadin BB60 group id.11286)

Batch 160 : Gut microbiota abundance (family Coriobacteriaceae id.811) on Other Crohn's disease

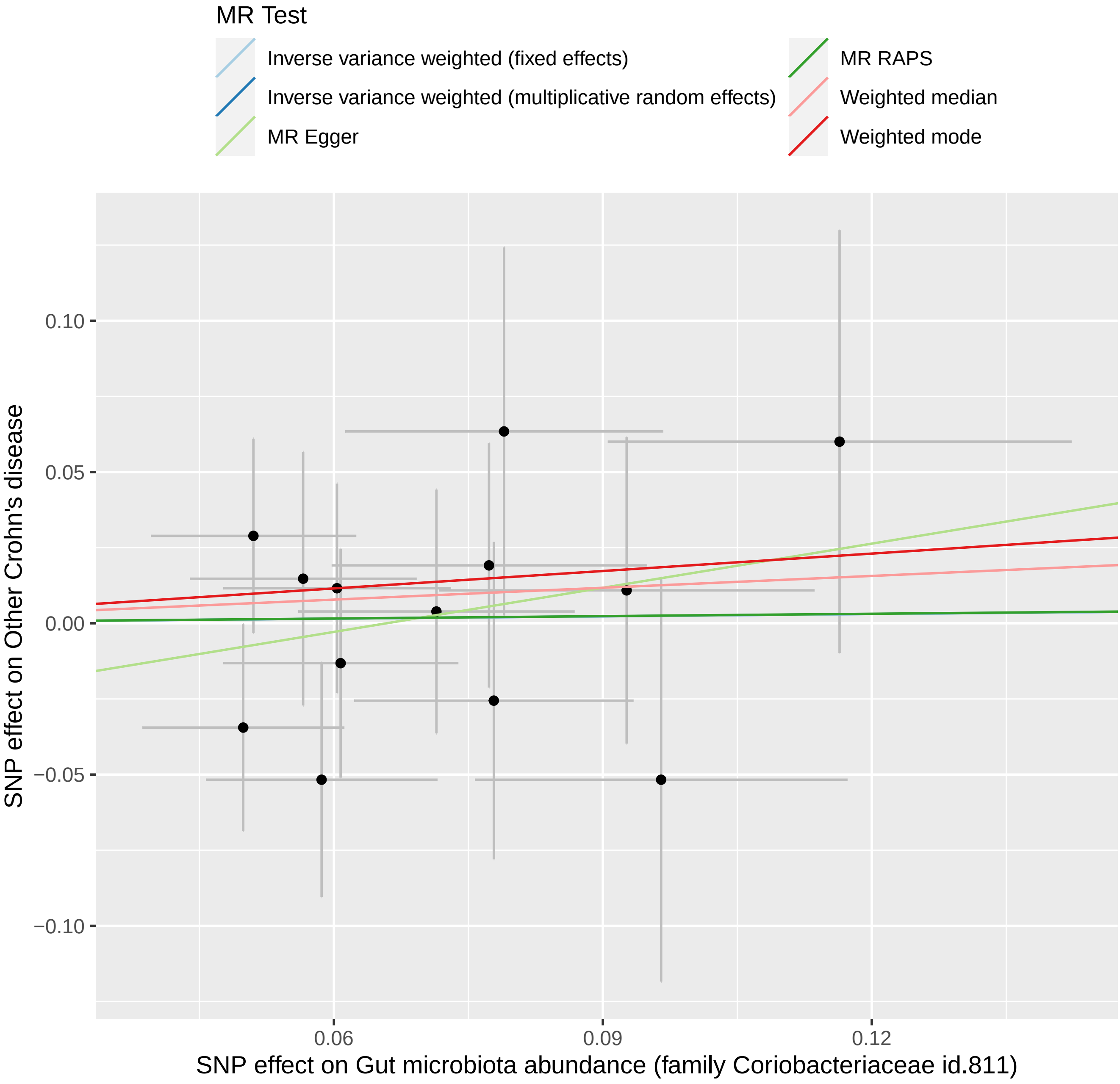

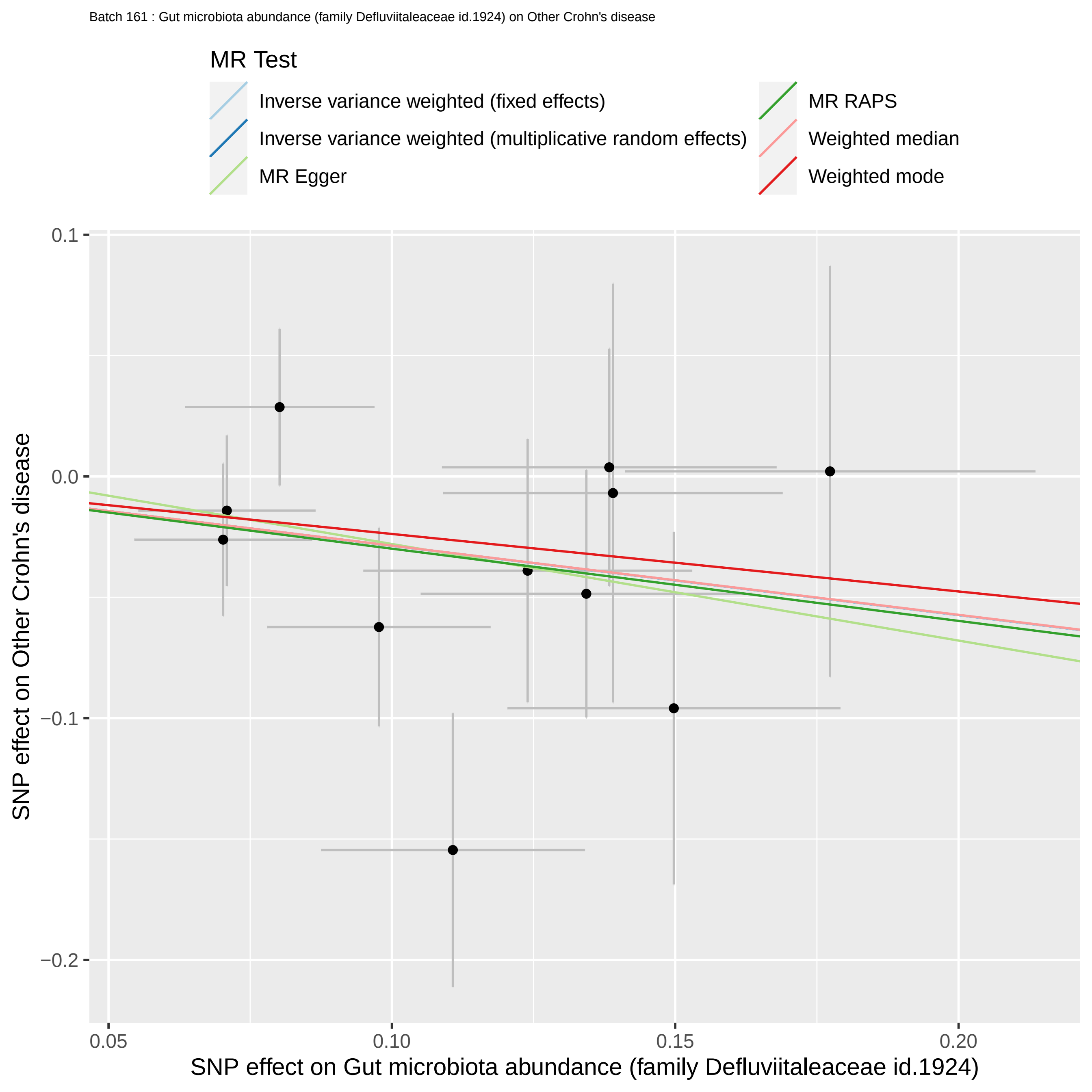

Batch 161 : Gut microbiota abundance (family Defluviitaleaceae id.1924) on Other Crohn's disease
MR Test
Inverse variance weighted (fixed effects)
Inverse variance weighted (multiplicative random effects)
MR Egger
MR RAPS
Weighted median
Weighted mode
0.1
0.0
−0.1
−0.2
0.05
0.10
0.15
0.20
SNP effect on Other Crohn's disease
SNP effect on Gut microbiota abundance (family Defluviitaleaceae id.1924)

Batch 162 : Gut microbiota abundance (family Desulfovibrionaceae id.3169) on Other Crohn's disease

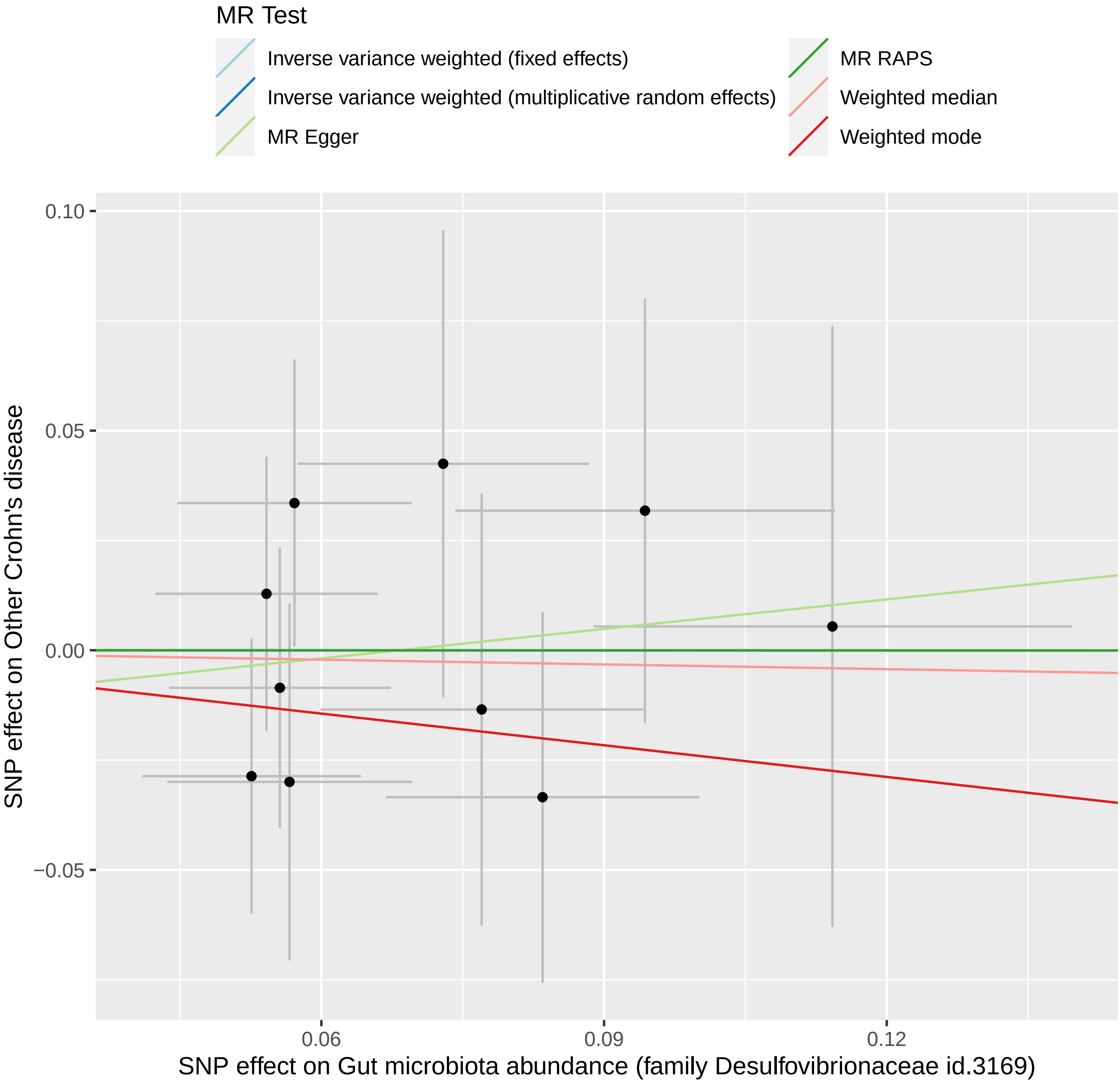

Batch 163 : Gut microbiota abundance (family Enterobacteriaceae id.3469) on Other Crohn's disease

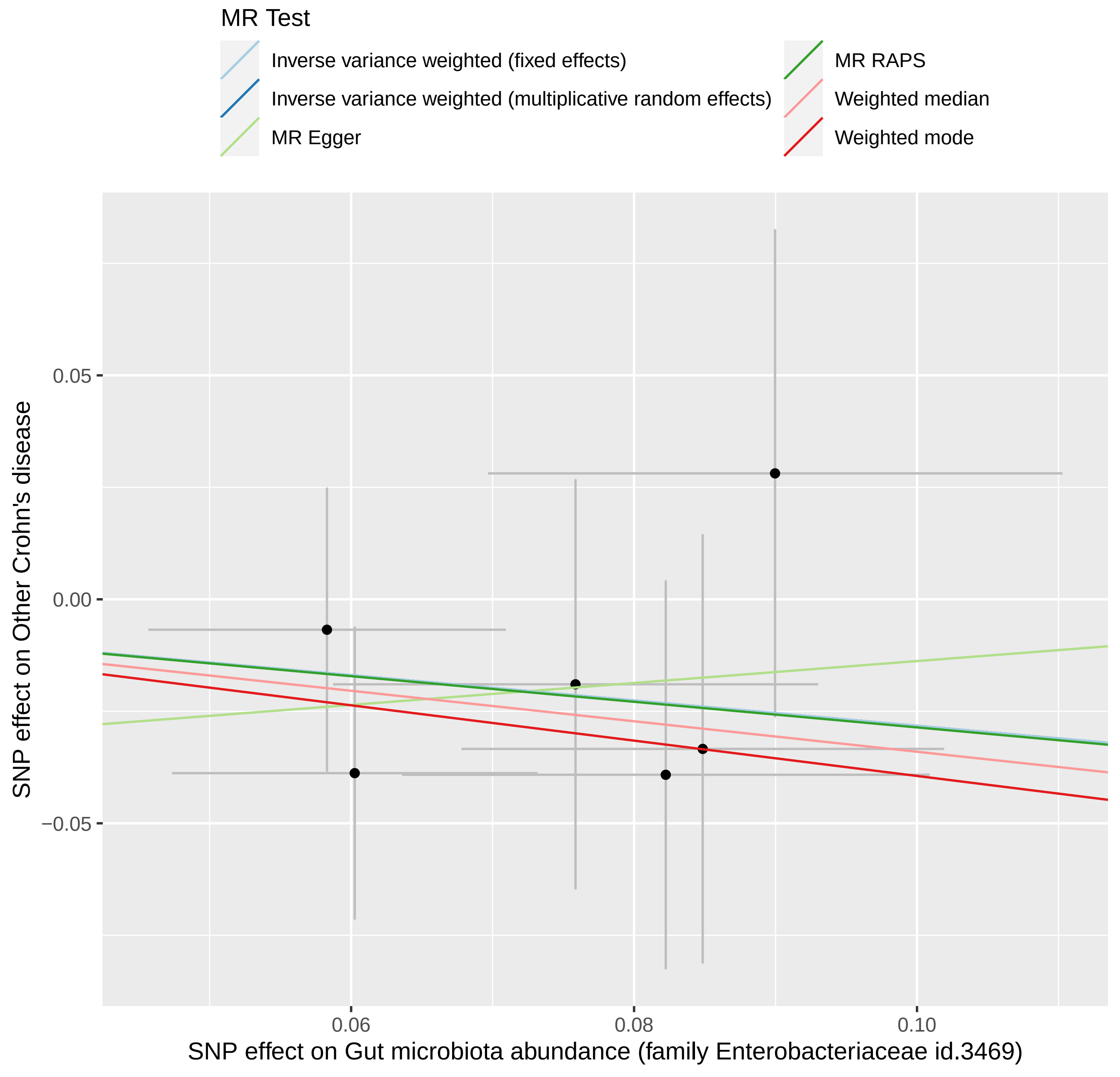

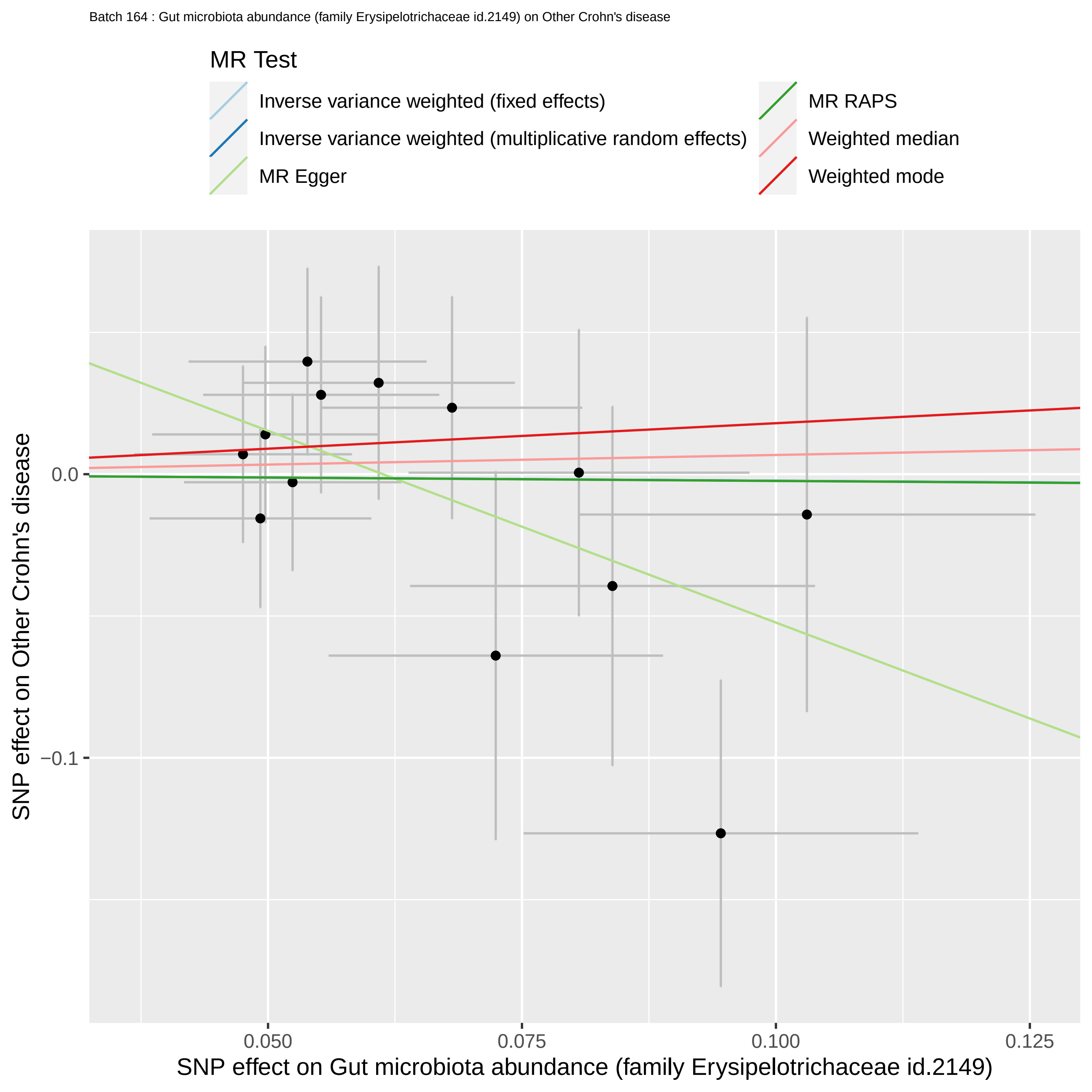
Batch 164 : Gut microbiota abundance (family Erysipelotrichaceae id.2149) on Other Crohn's disease
MR Test
Inverse variance weighted (fixed effects)
Inverse variance weighted (multiplicative random effects)
MR Egger
MR RAPS
Weighted median
Weighted mode
SNP effect on Other Crohn's disease
0.0
−0.1
0.050
0.075
0.100
0.125
SNP effect on Gut microbiota abundance (family Erysipelotrichaceae id.2149)

Batch 165 : Gut microbiota abundance (family Family XI id.1936) on Other Crohn's disease

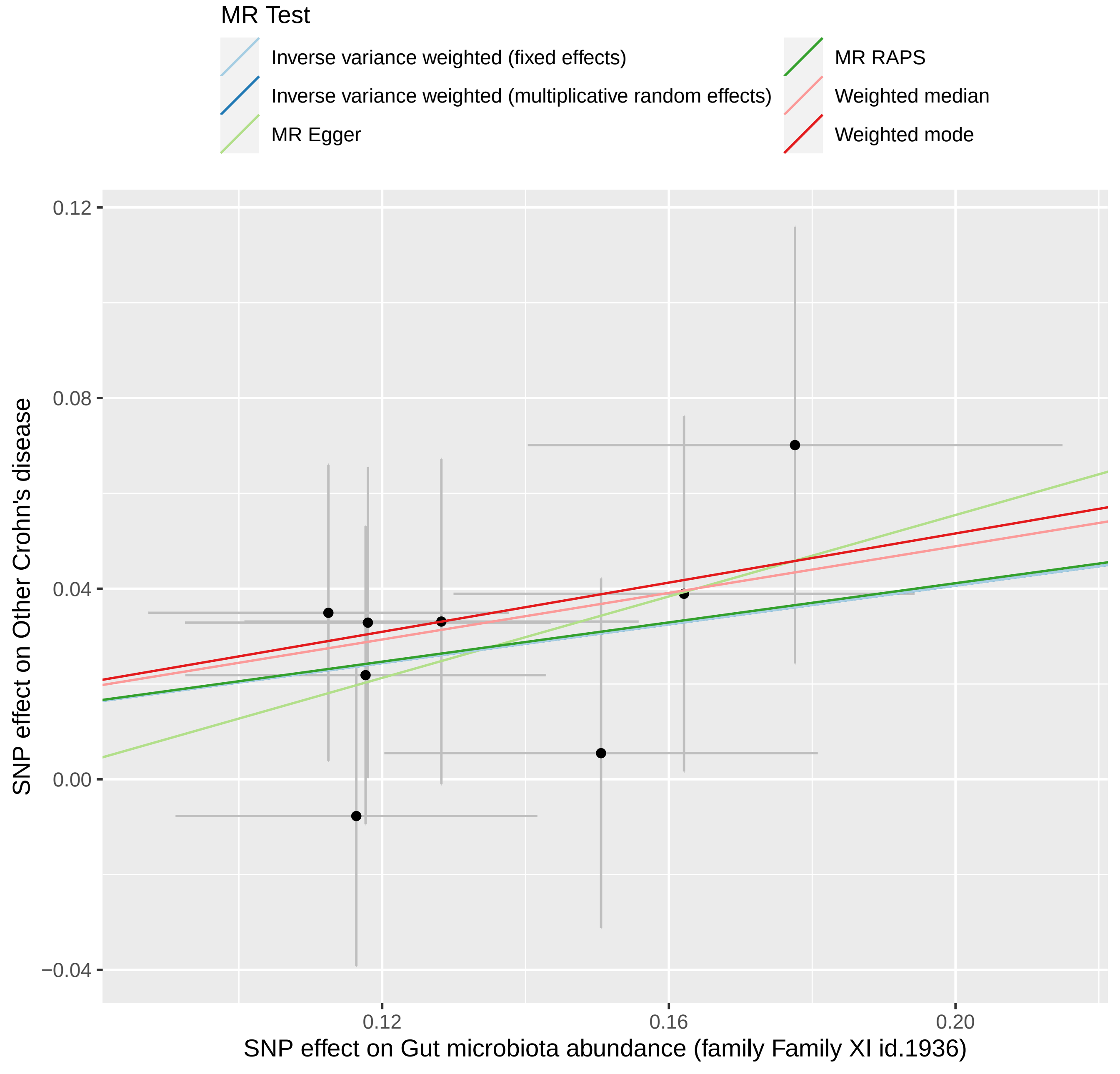

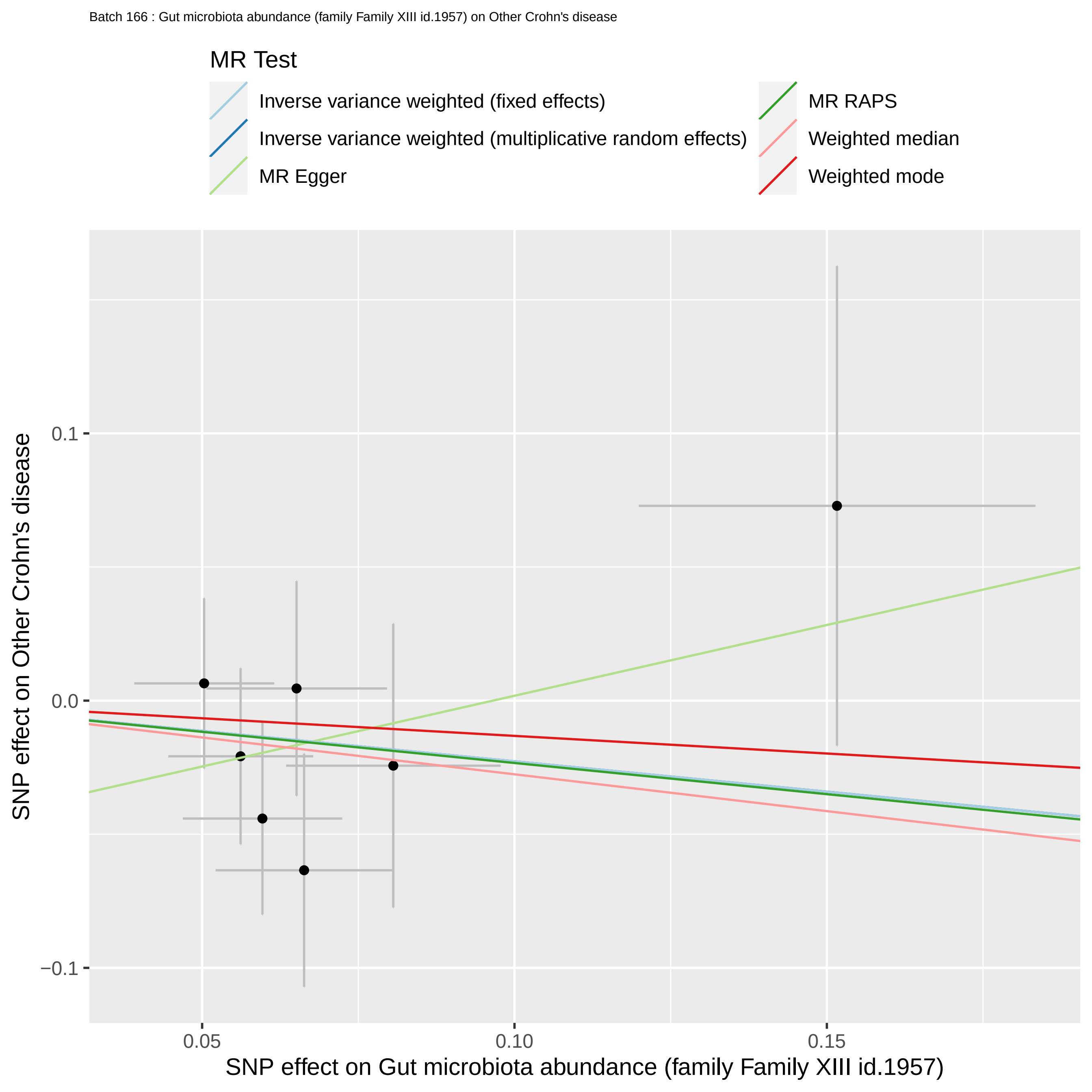

Batch 166 : Gut microbiota abundance (family Family XIII id.1957) on Other Crohn's disease
MR Test
Inverse variance weighted (fixed effects)
Inverse variance weighted (multiplicative random effects)
MR Egger
MR RAPS
Weighted median
Weighted mode
SNP effect on Other Crohn's disease
0.1
0.0
−0.1
0.05
0.10
0.15
SNP effect on Gut microbiota abundance (family Family XIII id.1957)

Batch 167 : Gut microbiota abundance (family Lachnospiraceae id.1987) on Other Crohn's disease

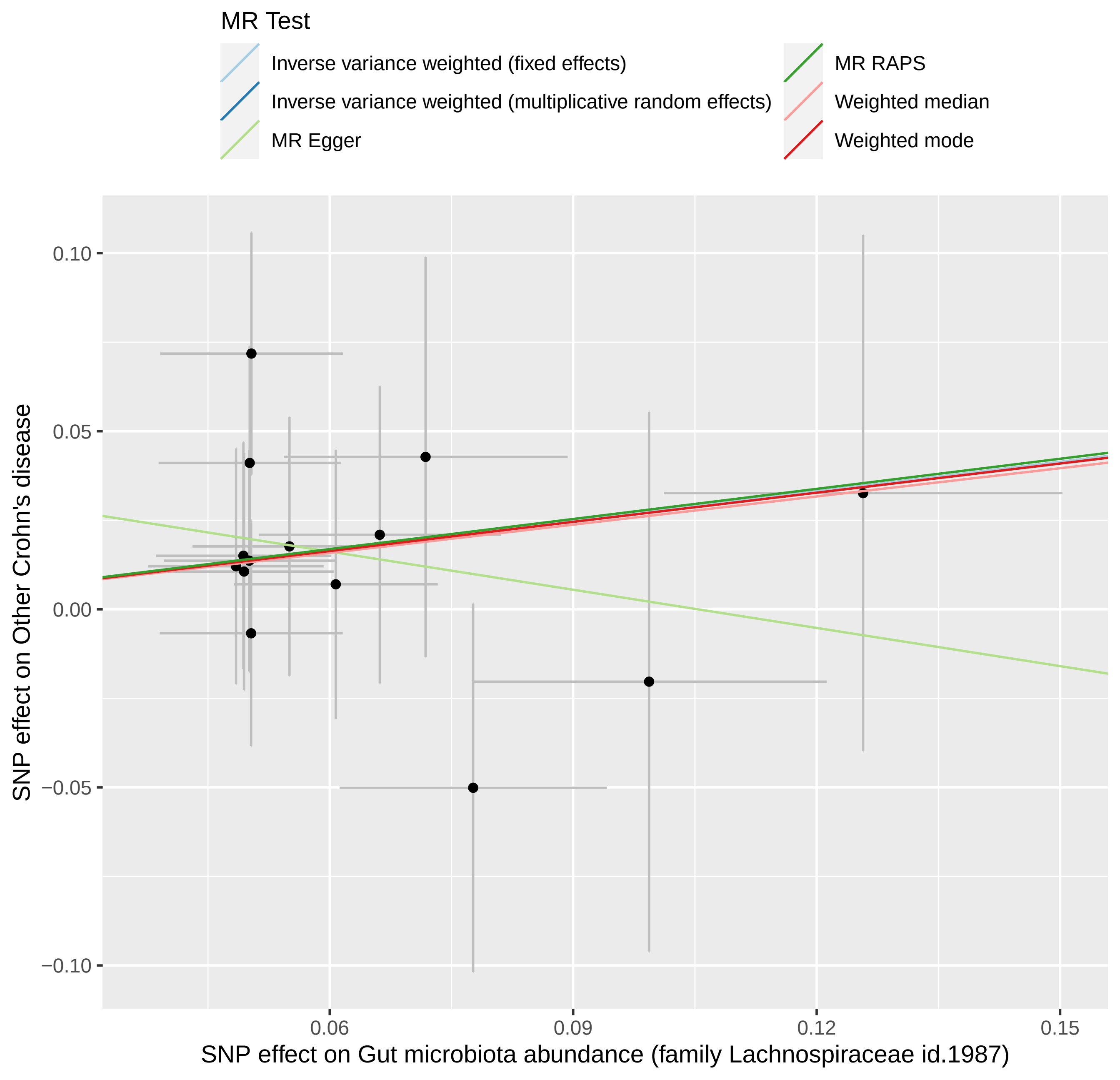

Batch 168 : Gut microbiota abundance (family Lactobacillaceae id.1836) on Other Crohn's disease

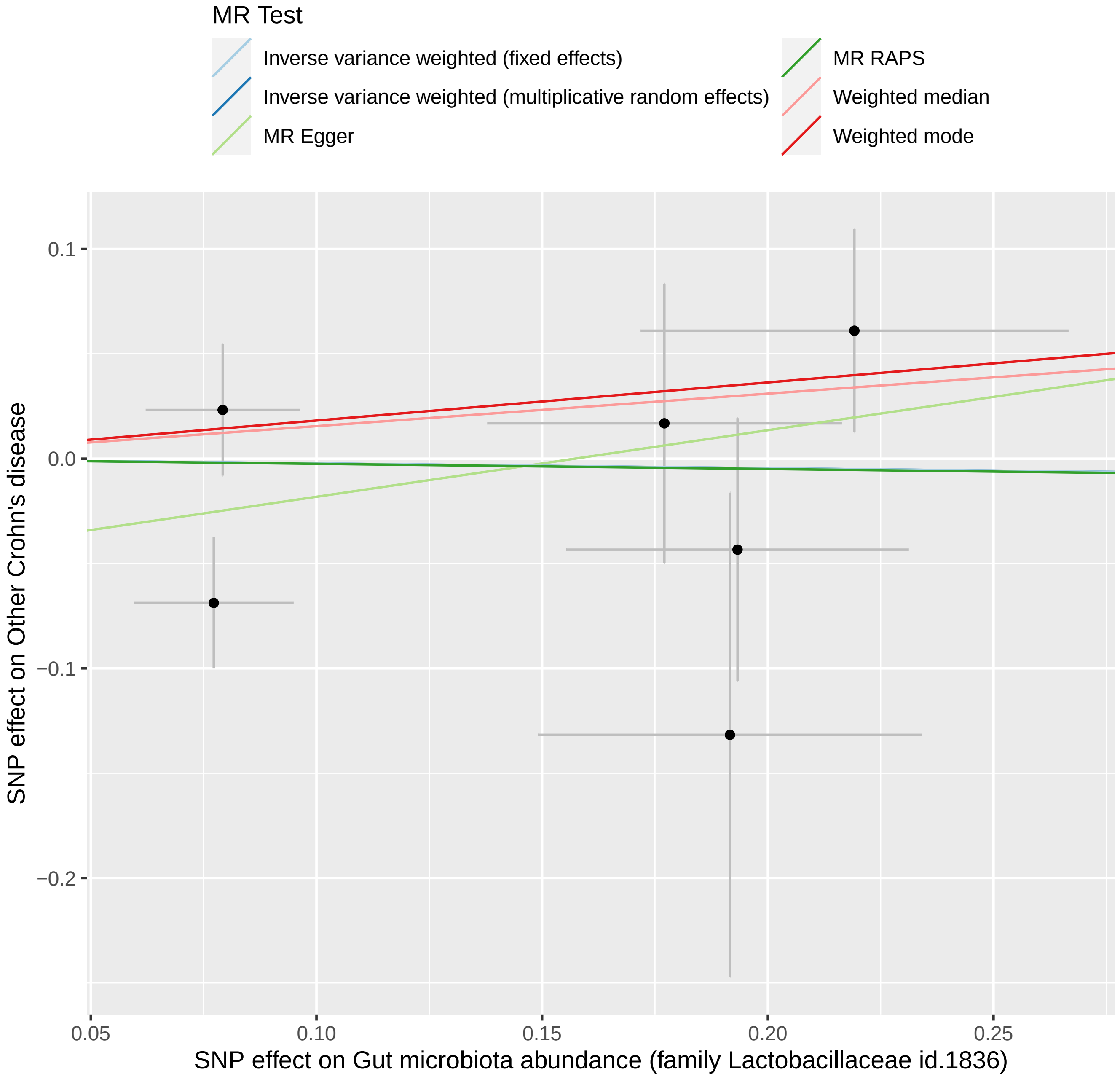

Batch 169 : Gut microbiota abundance (family Methanobacteriaceae id.121) on Other Crohn's disease

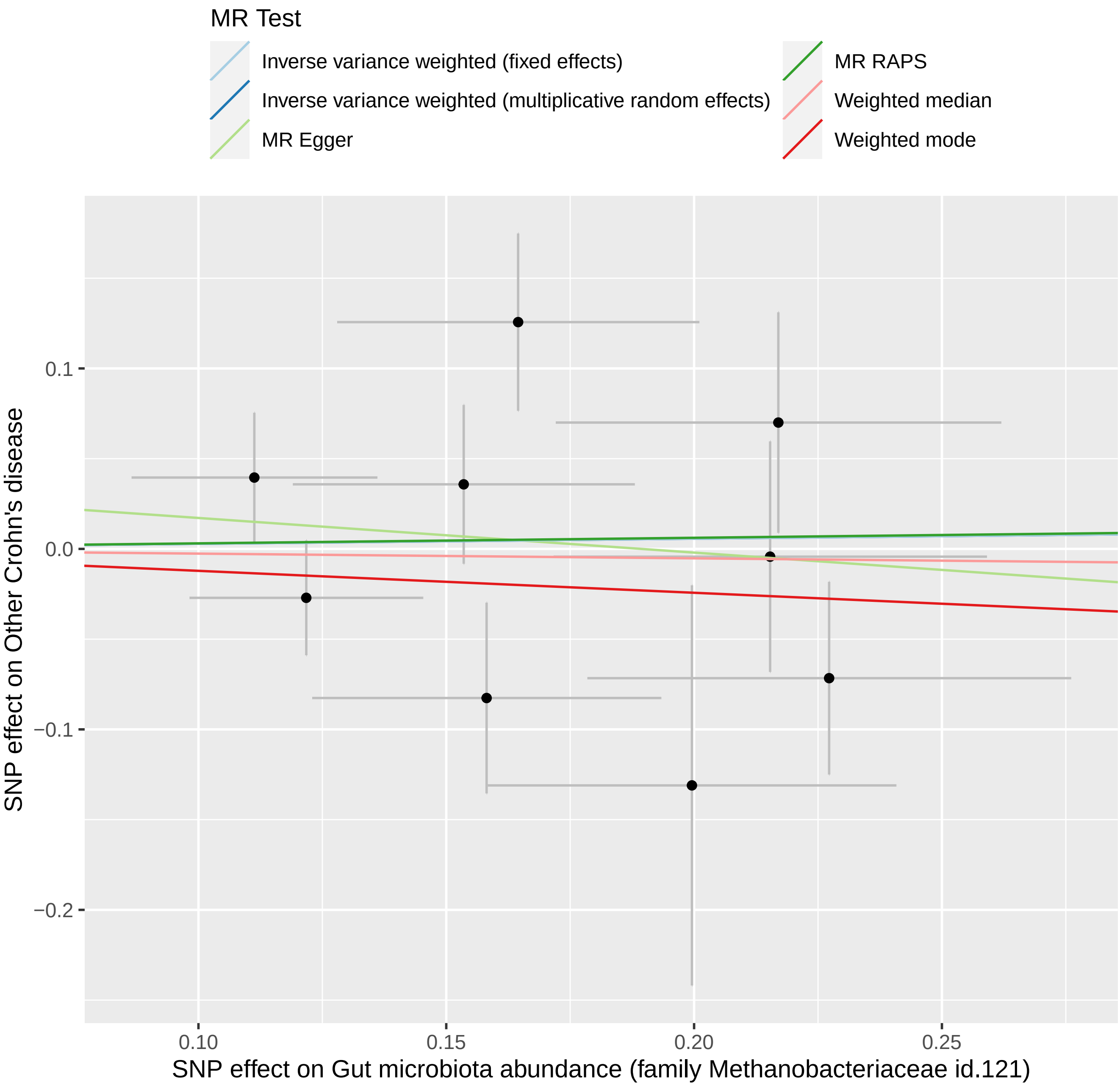

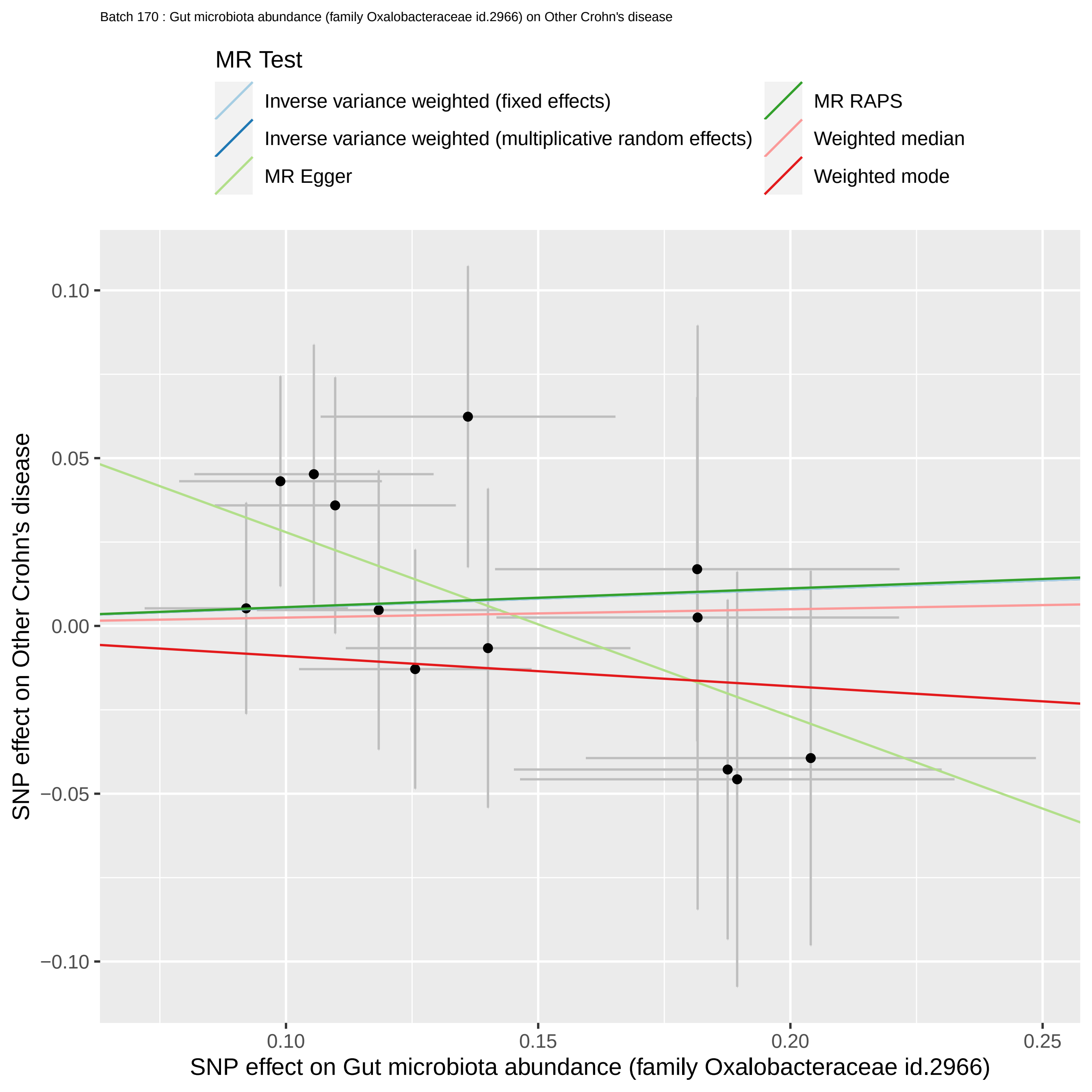
Batch 170 : Gut microbiota abundance (family Oxalobacteraceae id.2966) on Other Crohn's disease
MR Test
Inverse variance weighted (fixed effects)
Inverse variance weighted (multiplicative random effects)
MR Egger
MR RAPS
Weighted median
Weighted mode
0.10
0.05
0.00
−0.05
−0.10
SNP effect on Other Crohn's disease
0.10
0.15
0.20
0.25
SNP effect on Gut microbiota abundance (family Oxalobacteraceae id.2966)

Batch 171 : Gut microbiota abundance (family Pasteurellaceae id.3689) on Other Crohn's disease

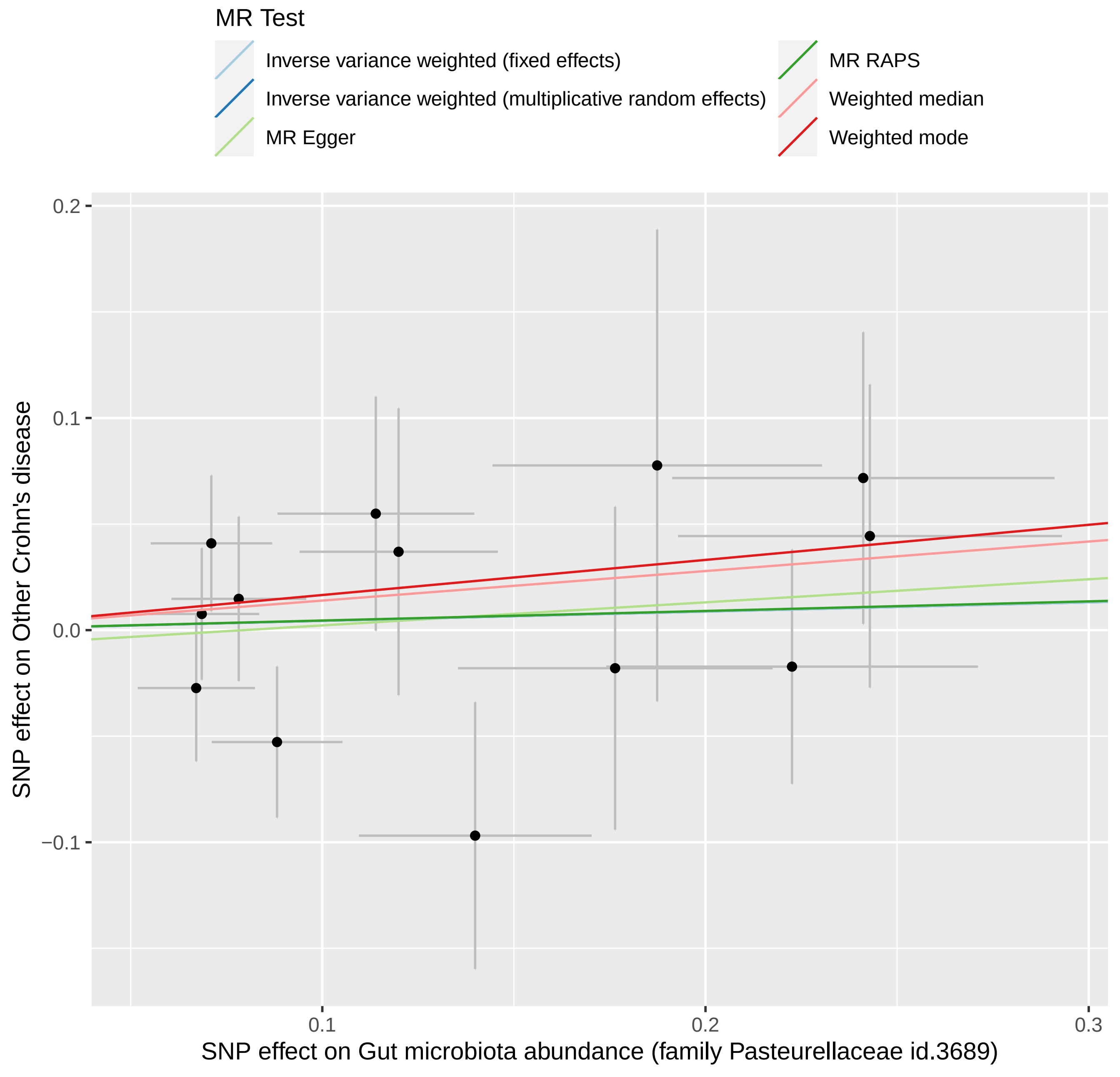

Batch 172 : Gut microbiota abundance (family Peptococcaceae id.2024) on Other Crohn's disease

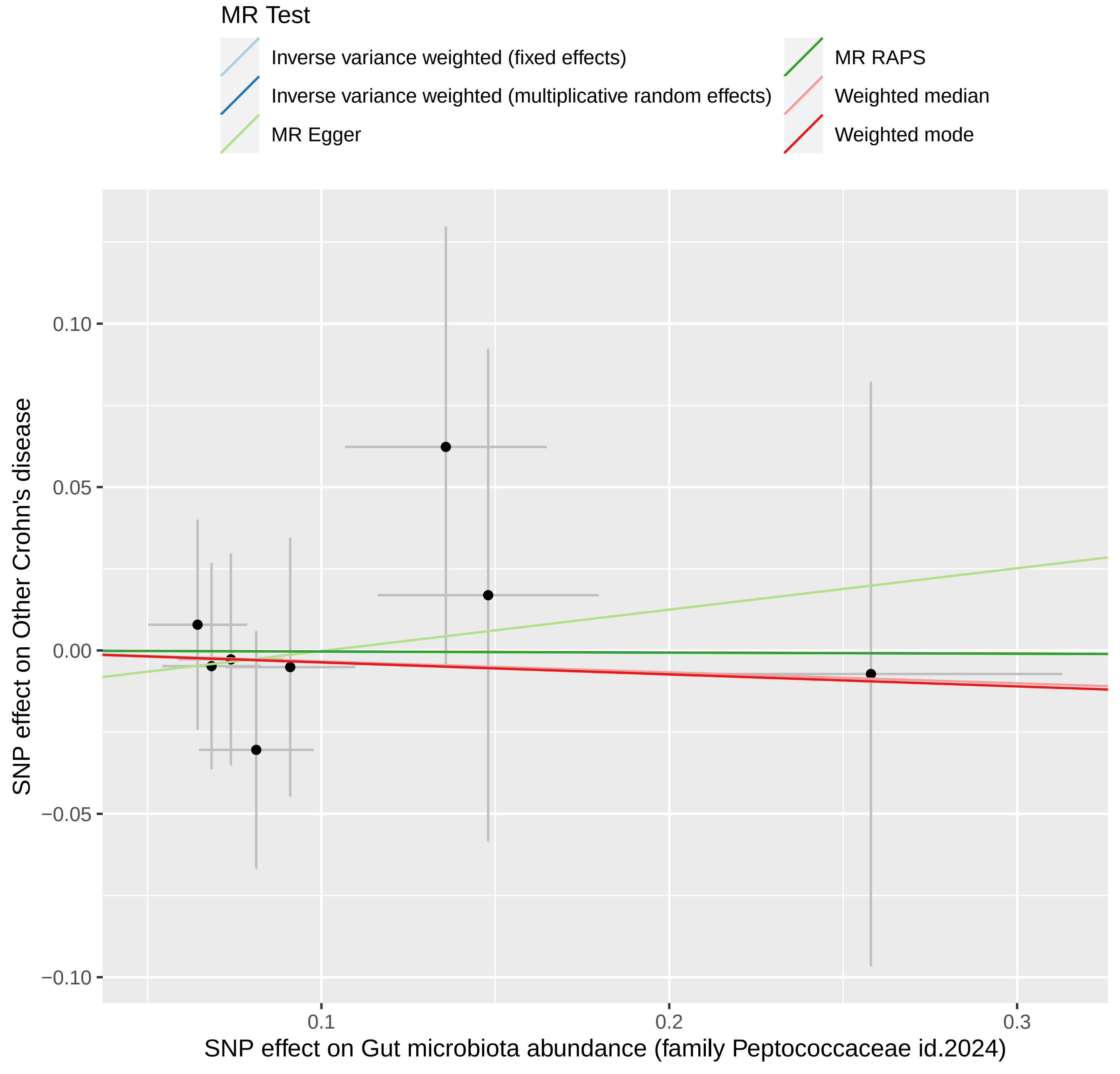

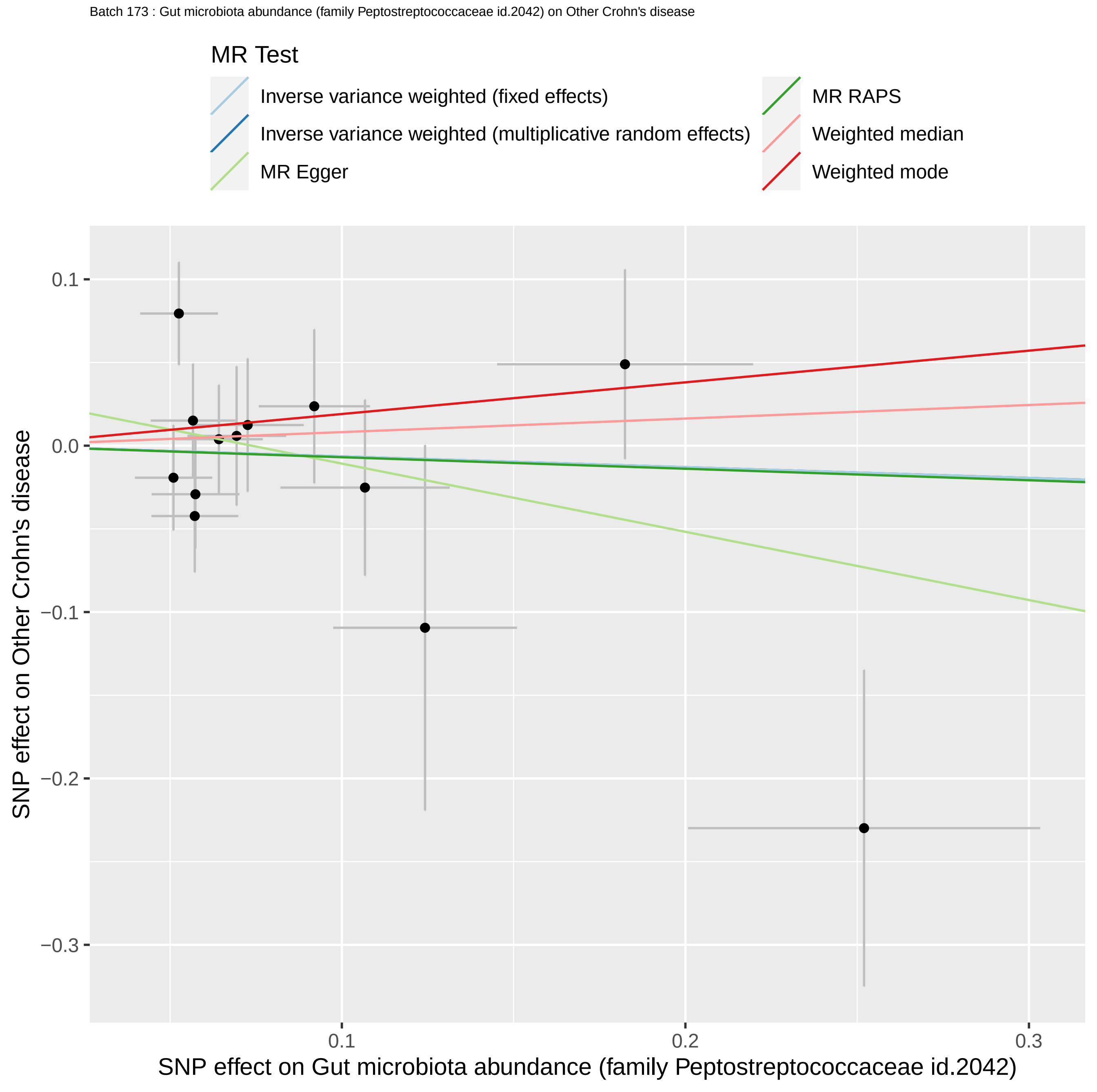
Batch 173 : Gut microbiota abundance (family Peptostreptococcaceae id.2042) on Other Crohn's disease
MR Test
Inverse variance weighted (fixed effects)
Inverse variance weighted (multiplicative random effects)
MR Egger
MR RAPS
Weighted median
Weighted mode
0.1
0.0
−0.1
−0.2
−0.3
0.1
0.2
0.3
SNP effect on Other Crohn's disease
SNP effect on Gut microbiota abundance (family Peptostreptococcaceae id.2042)

Batch 174 : Gut microbiota abundance (family Porphyromonadaceae id.943) on Other Crohn's disease

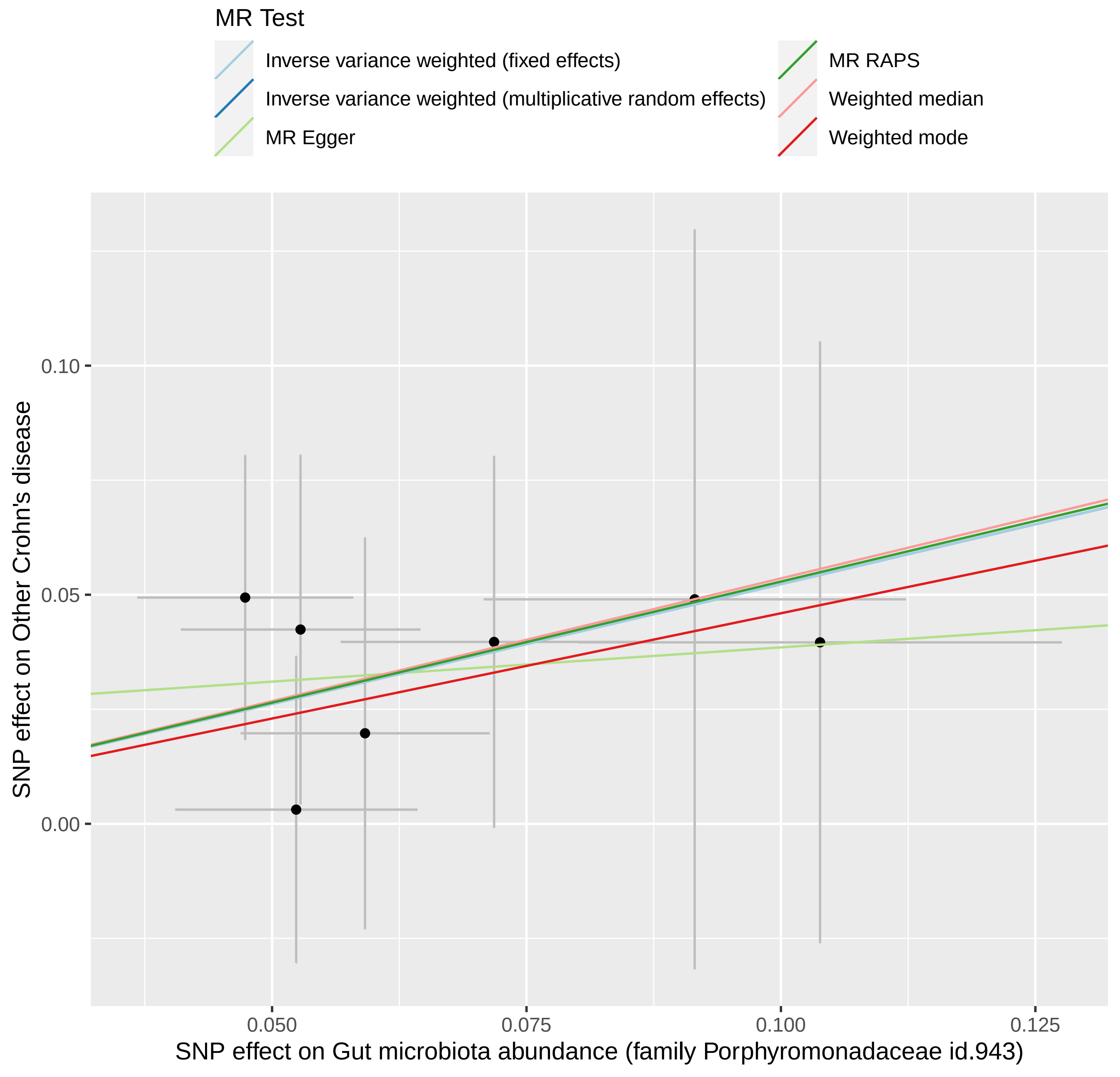

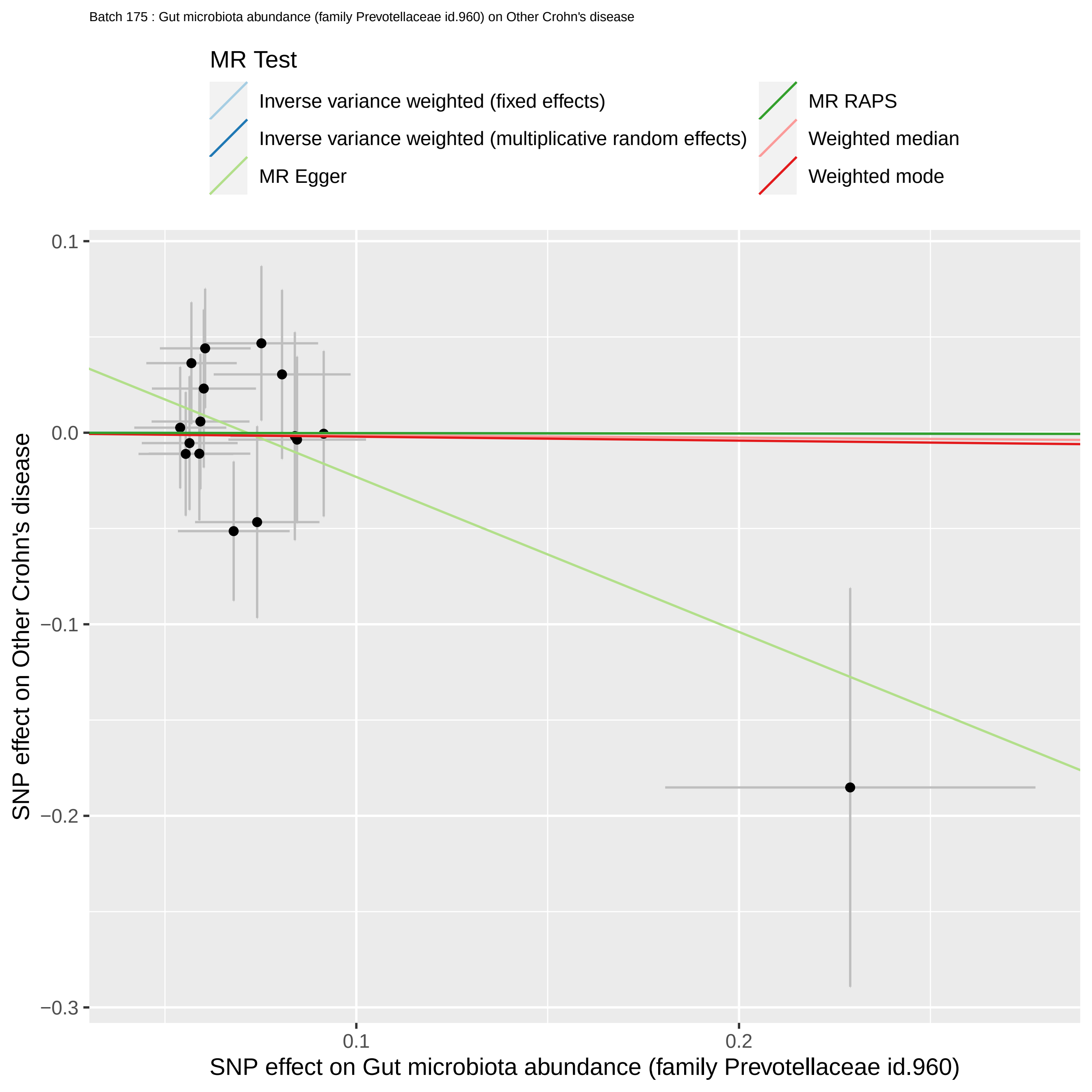

Batch 175 : Gut microbiota abundance (family Prevotellaceae id.960) on Other Crohn's disease
MR Test
Inverse variance weighted (fixed effects)
Inverse variance weighted (multiplicative random effects)
MR Egger
MR RAPS
Weighted median
Weighted mode
0.1
0.0
−0.1
−0.2
−0.3
0.1
0.2
SNP effect on Other Crohn's disease
SNP effect on Gut microbiota abundance (family Prevotellaceae id.960)

Batch 176 : Gut microbiota abundance (family Rhodospirillaceae id.2717) on Other Crohn's disease

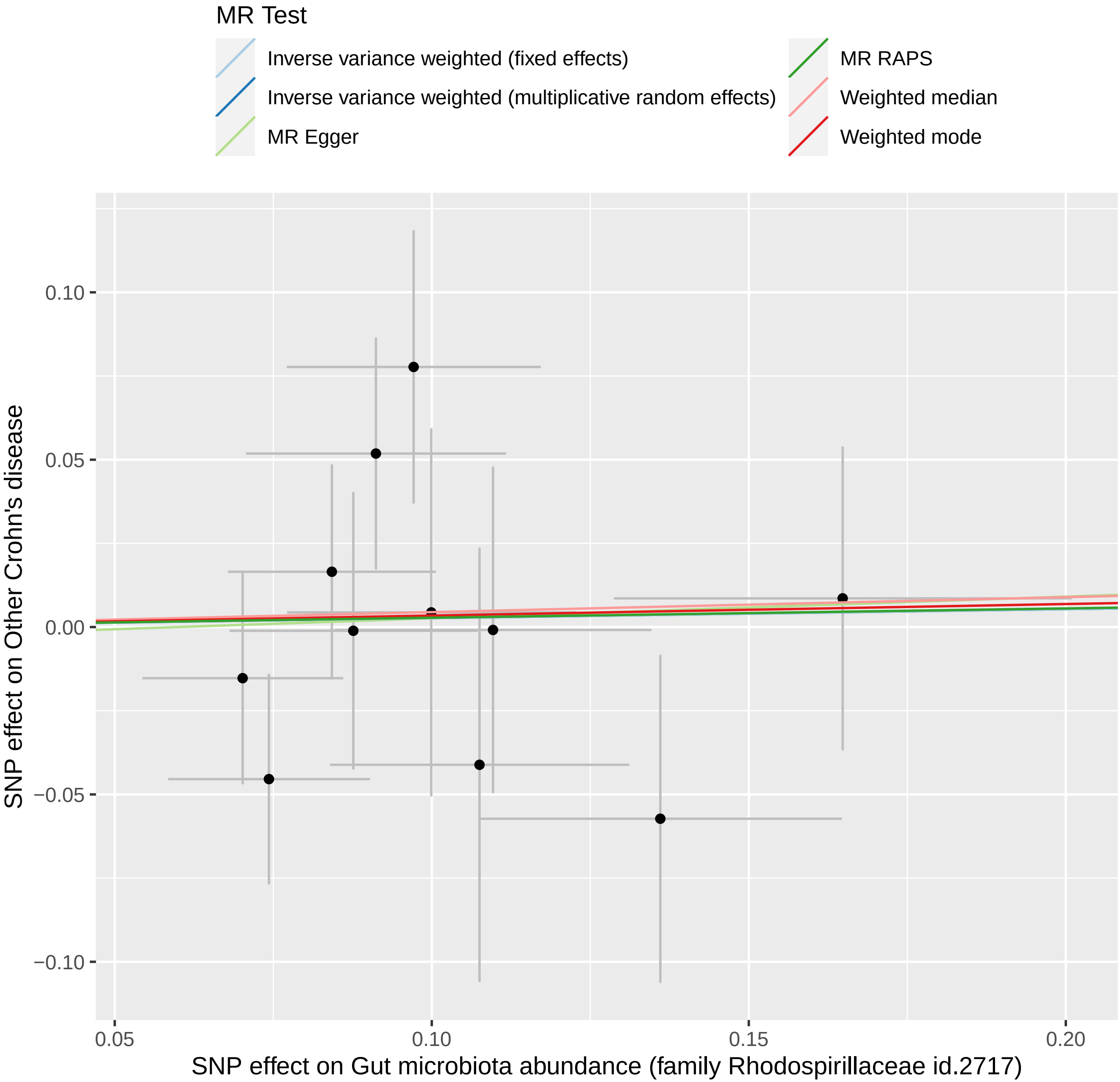

Batch 177 : Gut microbiota abundance (family Rikenellaceae id.967) on Other Crohn's disease

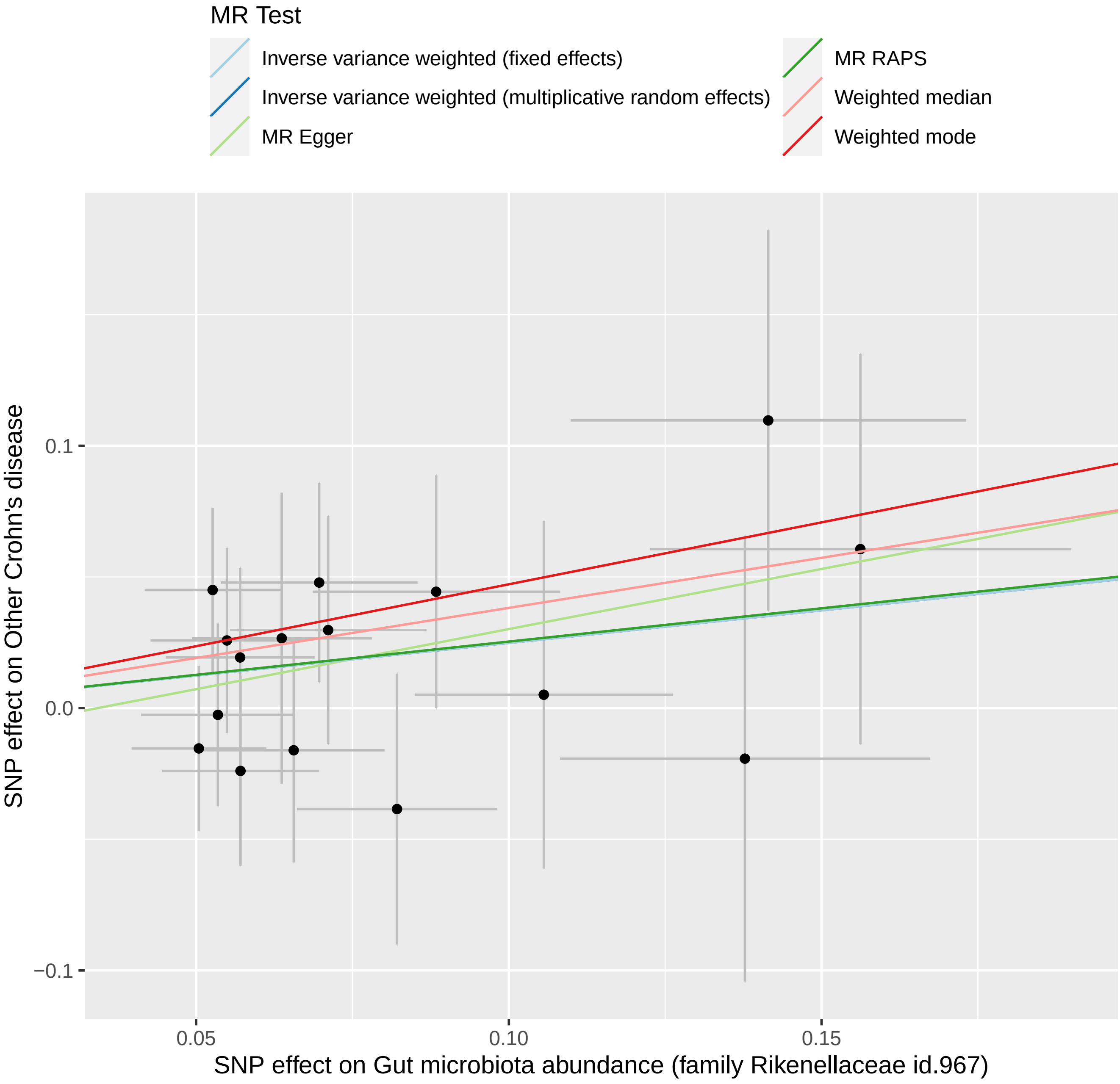

Batch 178 : Gut microbiota abundance (family Ruminococcaceae id.2050) on Other Crohn's disease

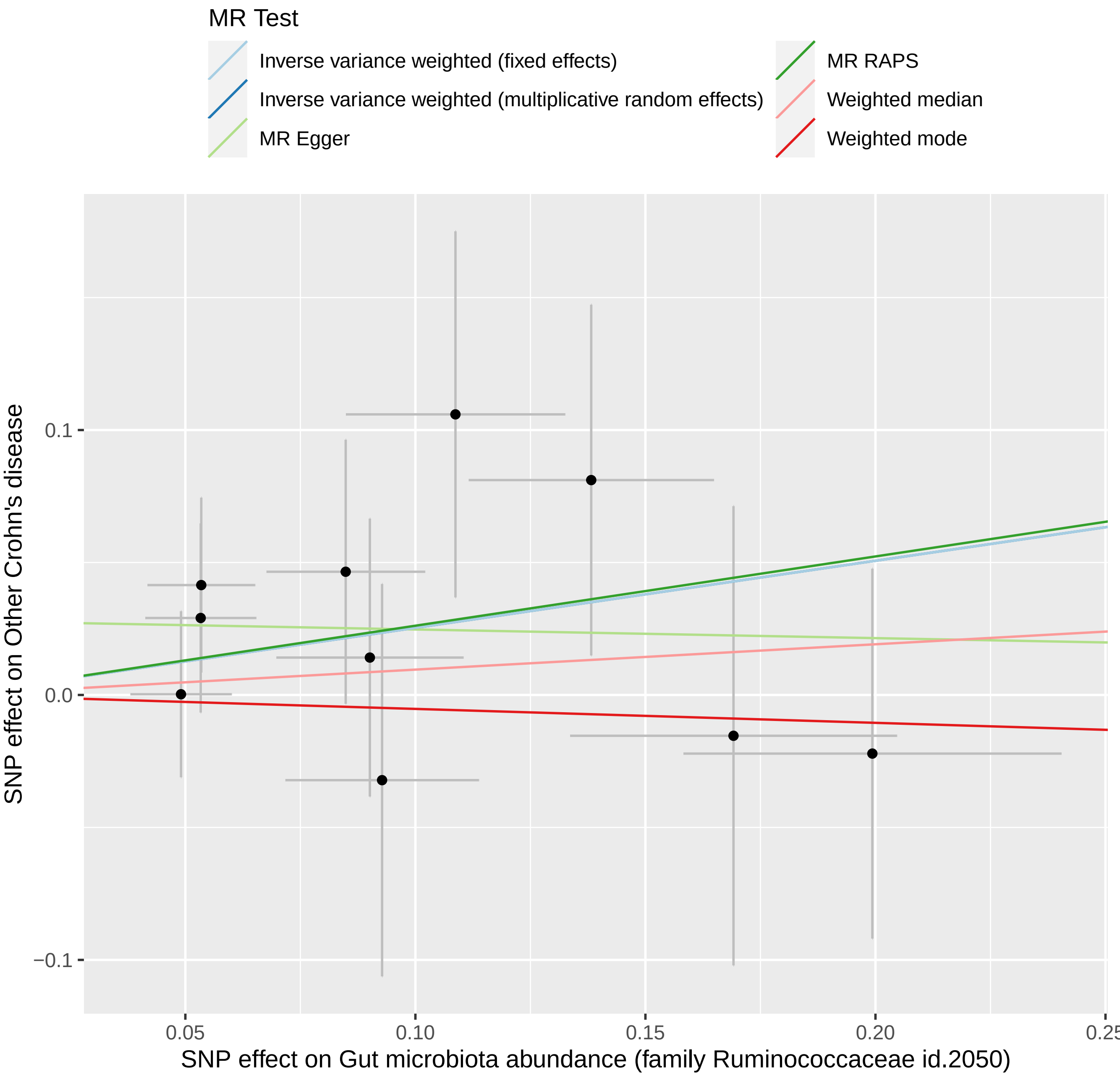

Batch 179 : Gut microbiota abundance (family Streptococcaceae id.1850) on Other Crohn's disease

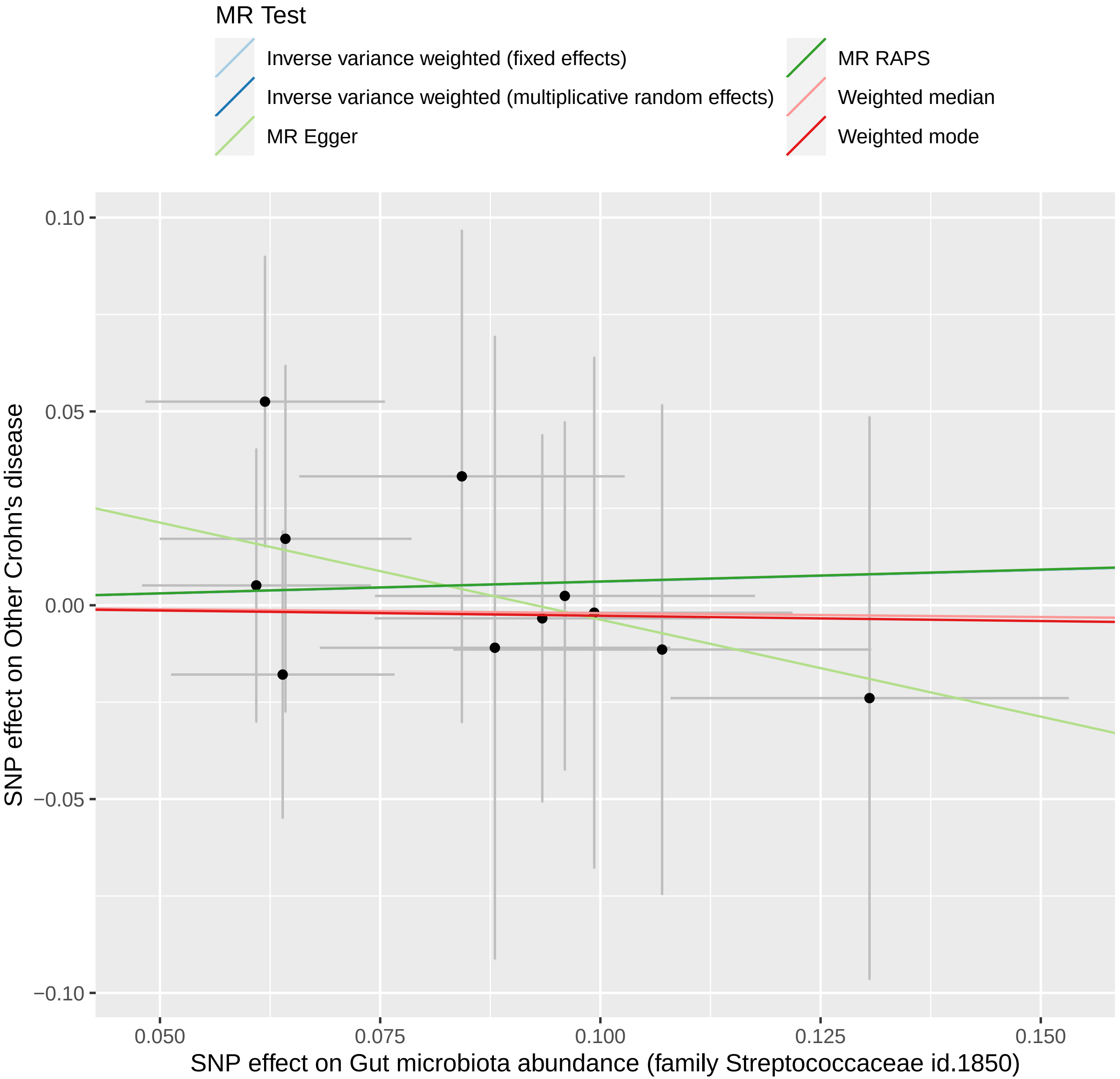

Batch 180 : Gut microbiota abundance (family Veillonellaceae id.2172) on Other Crohn's disease

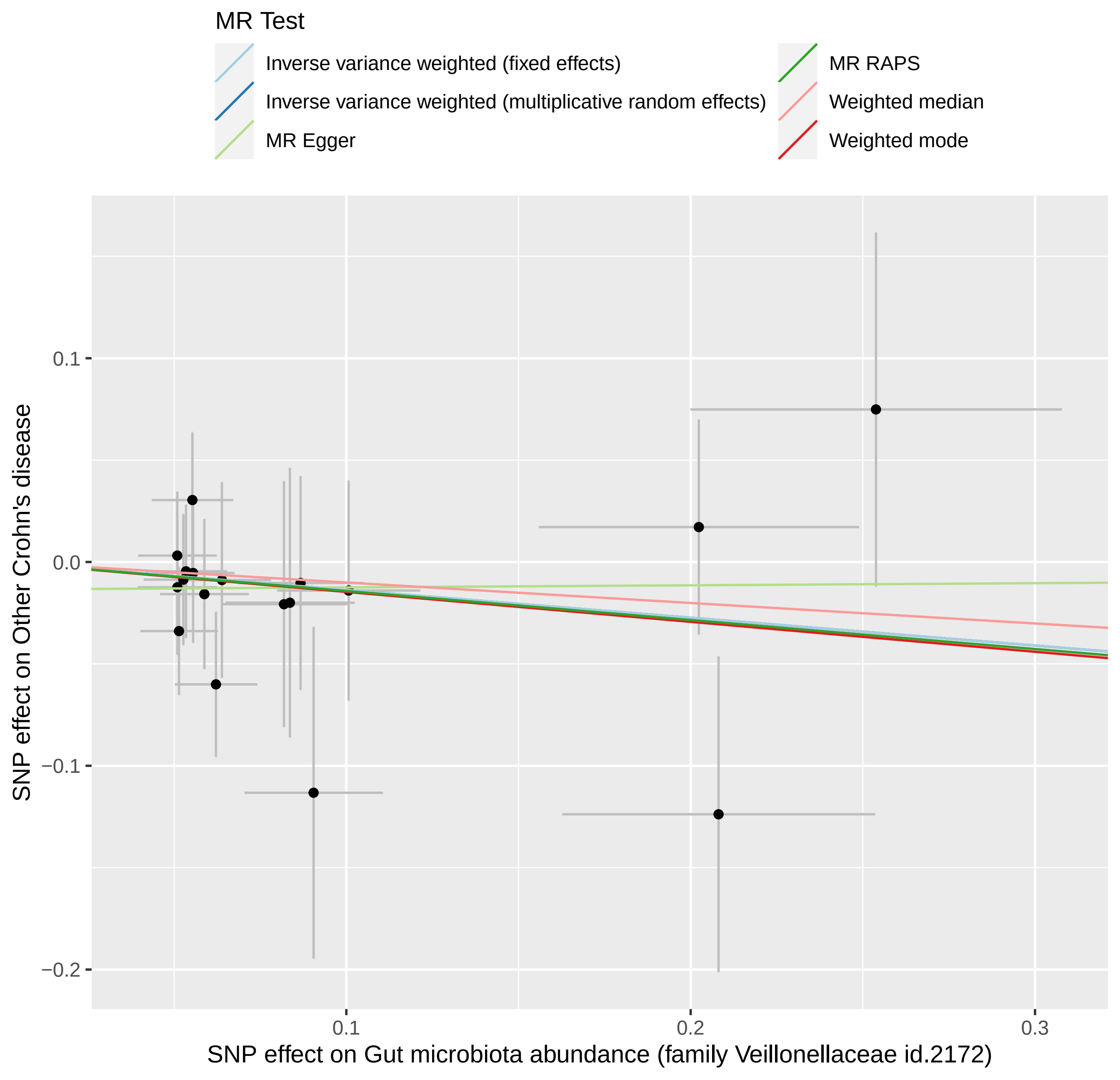

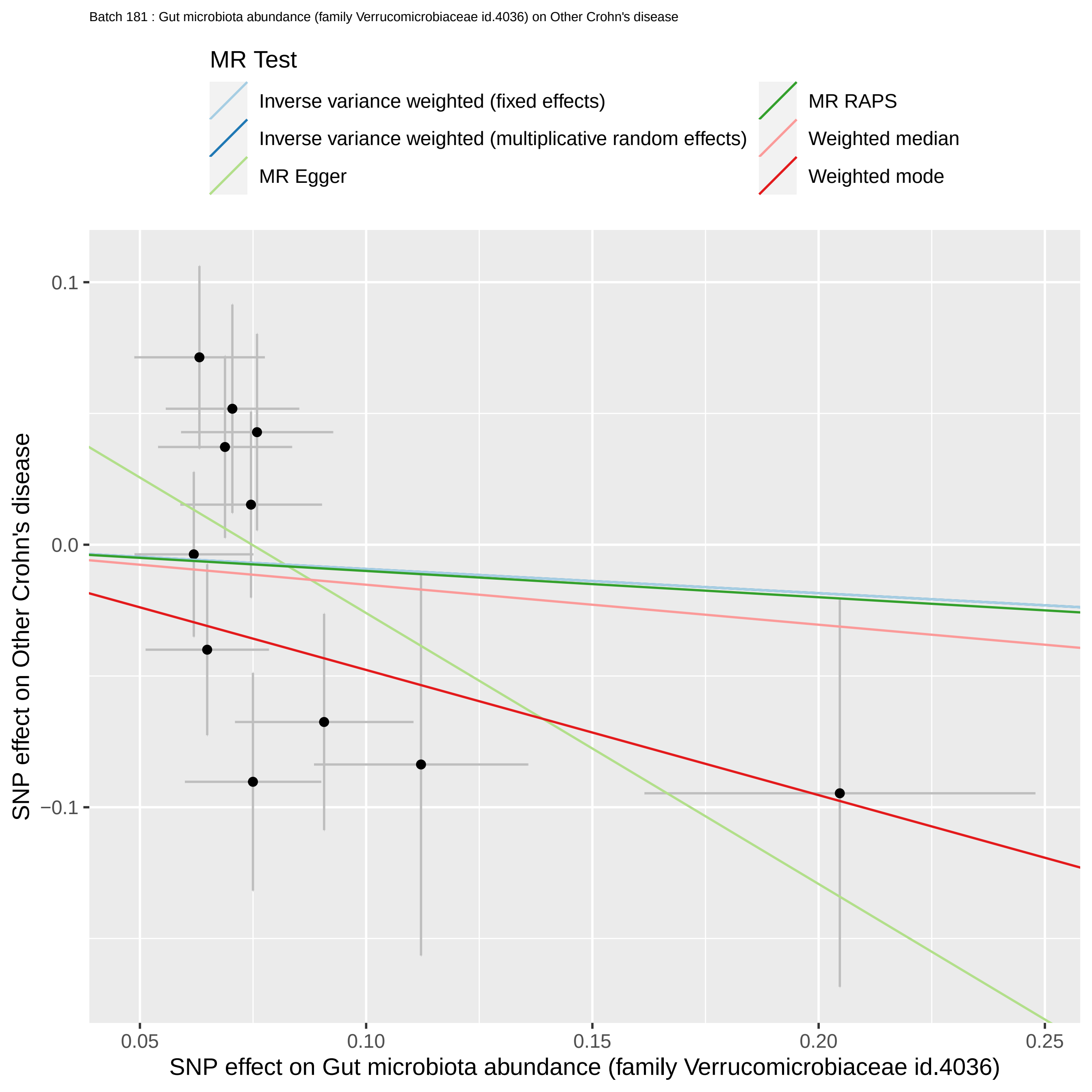

Batch 181 : Gut microbiota abundance (family Verrucomicrobiaceae id.4036) on Other Crohn's disease
MR Test
Inverse variance weighted (fixed effects)
Inverse variance weighted (multiplicative random effects)
MR Egger
MR RAPS
Weighted median
Weighted mode
SNP effect on Other Crohn's disease
0.1
0.0
−0.1
0.05
0.10
0.15
0.20
0.25
SNP effect on Gut microbiota abundance (family Verrucomicrobiaceae id.4036)

Batch 182 : Gut microbiota abundance (family Victivallaceae id.2255) on Other Crohn's disease

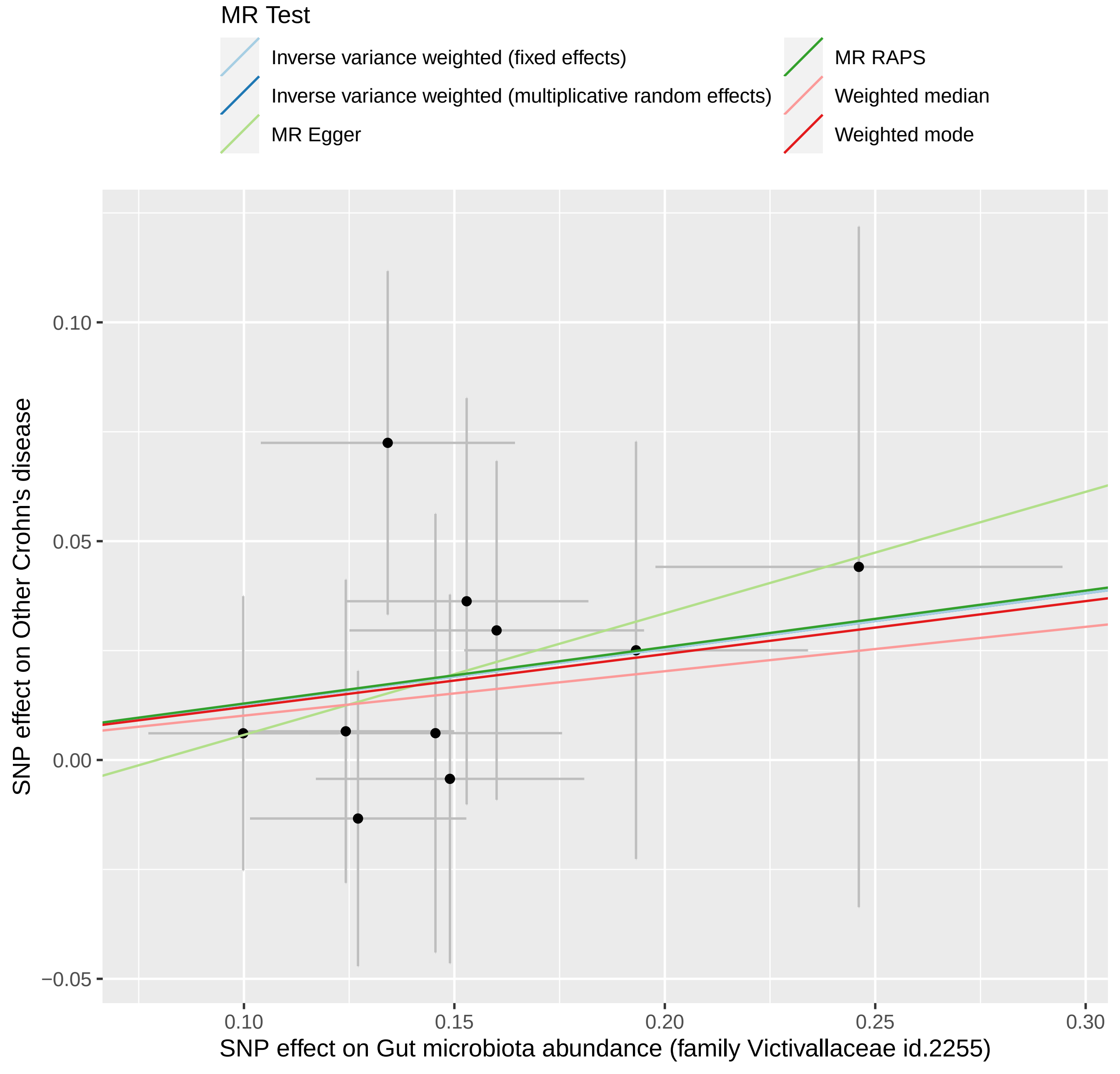

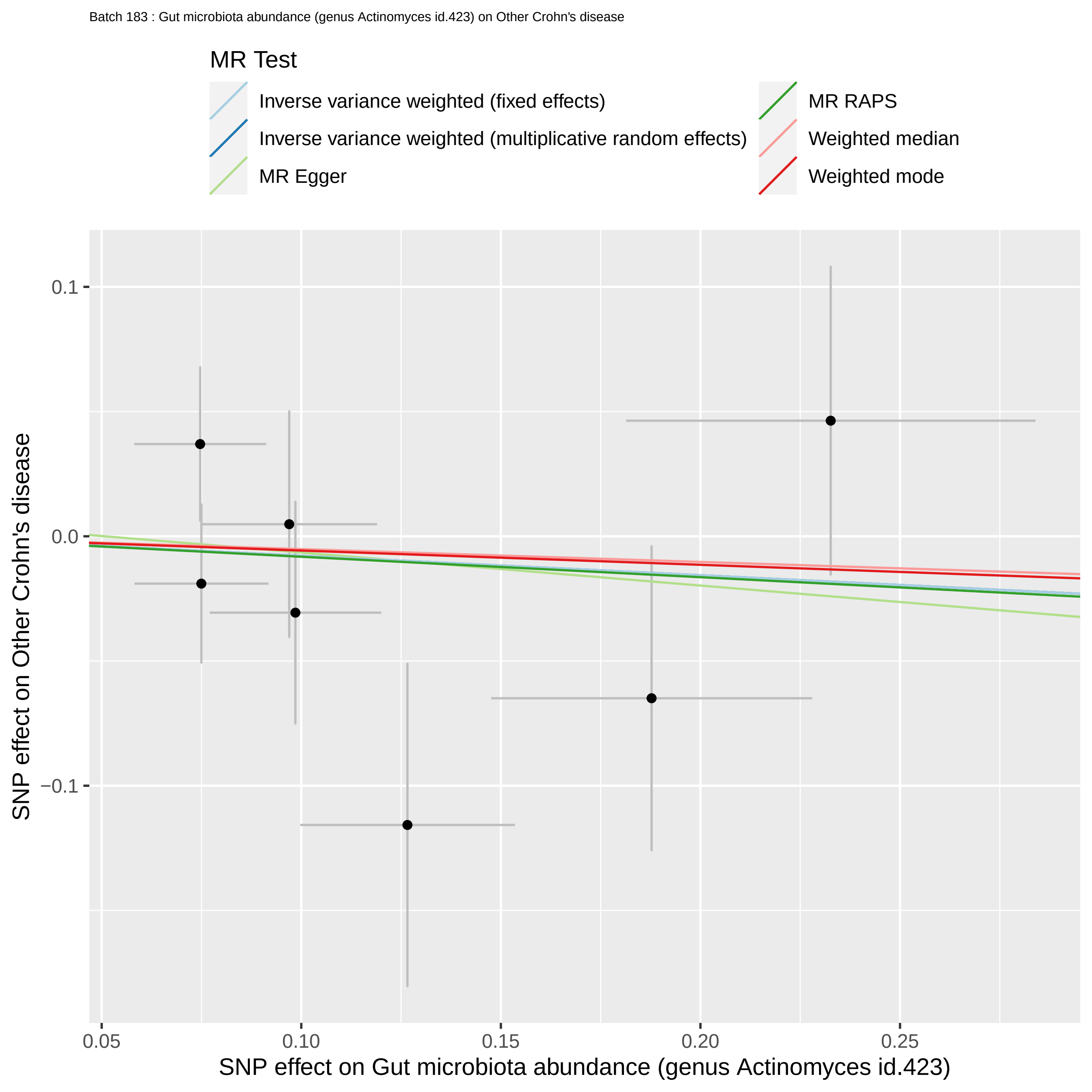

Batch 183 : Gut microbiota abundance (genus Actinomyces id.423) on Other Crohn's disease
MR Test
Inverse variance weighted (fixed effects)
Inverse variance weighted (multiplicative random effects)
MR Egger
MR RAPS
Weighted median
Weighted mode
SNP effect on Other Crohn's disease
0.1
0.0
−0.1
0.05
0.10
0.15
0.20
0.25
SNP effect on Gut microbiota abundance (genus Actinomyces id.423)

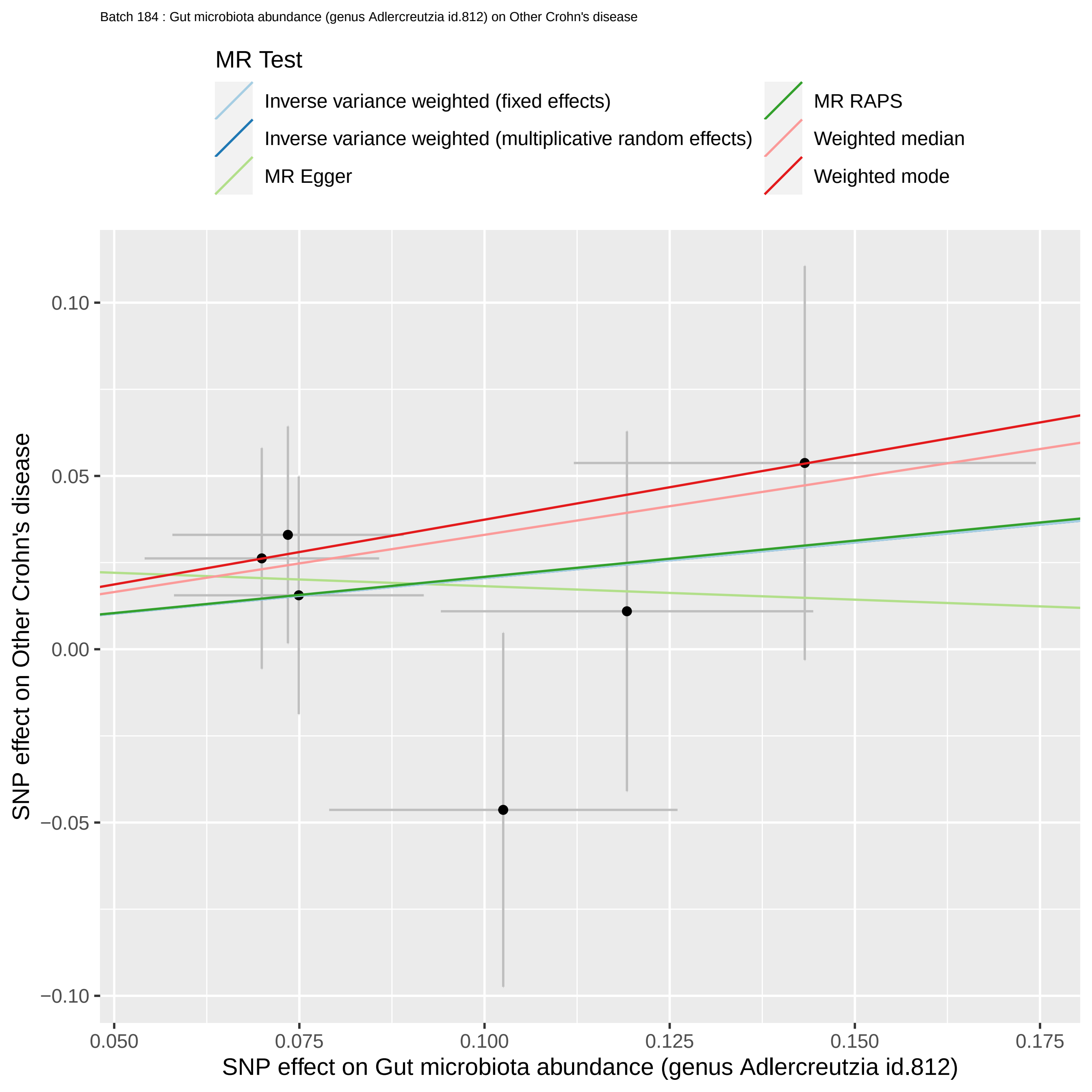
Batch 184 : Gut microbiota abundance (genus Adlercreutzia id.812) on Other Crohn's disease
MR Test
Inverse variance weighted (fixed effects)
Inverse variance weighted (multiplicative random effects)
MR Egger
MR RAPS
Weighted median
Weighted mode
0.10
0.05
0.00
−0.05
−0.10
0.050
0.075
0.100
0.125
0.150
0.175
SNP effect on Other Crohn's disease
SNP effect on Gut microbiota abundance (genus Adlercreutzia id.812)

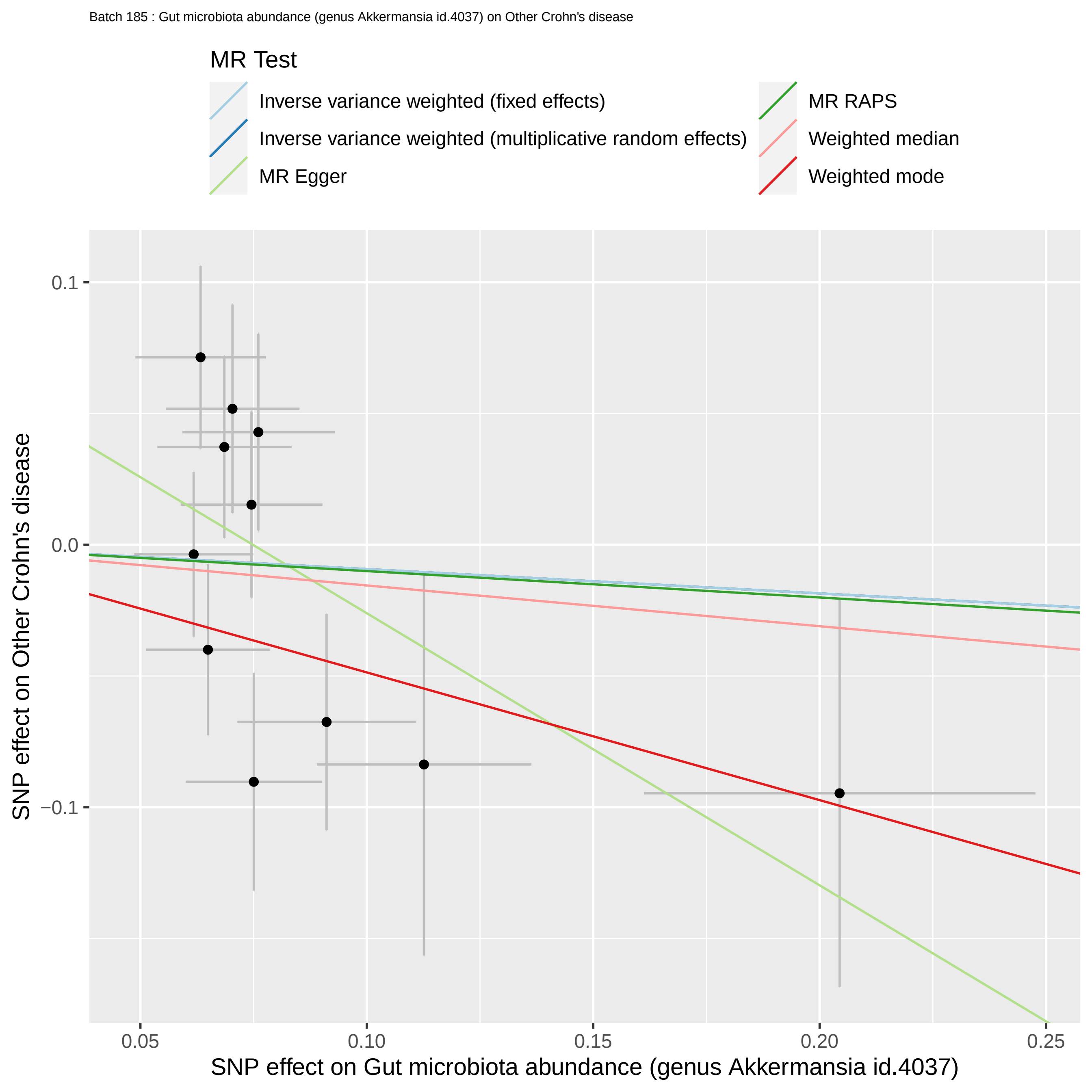

Batch 185 : Gut microbiota abundance (genus Akkermansia id.4037) on Other Crohn's disease
MR Test
Inverse variance weighted (fixed effects)
Inverse variance weighted (multiplicative random effects)
MR Egger
MR RAPS
Weighted median
Weighted mode
0.1
0.0
−0.1
0.05
0.10
0.15
0.20
0.25
SNP effect on Other Crohn's disease
SNP effect on Gut microbiota abundance (genus Akkermansia id.4037)

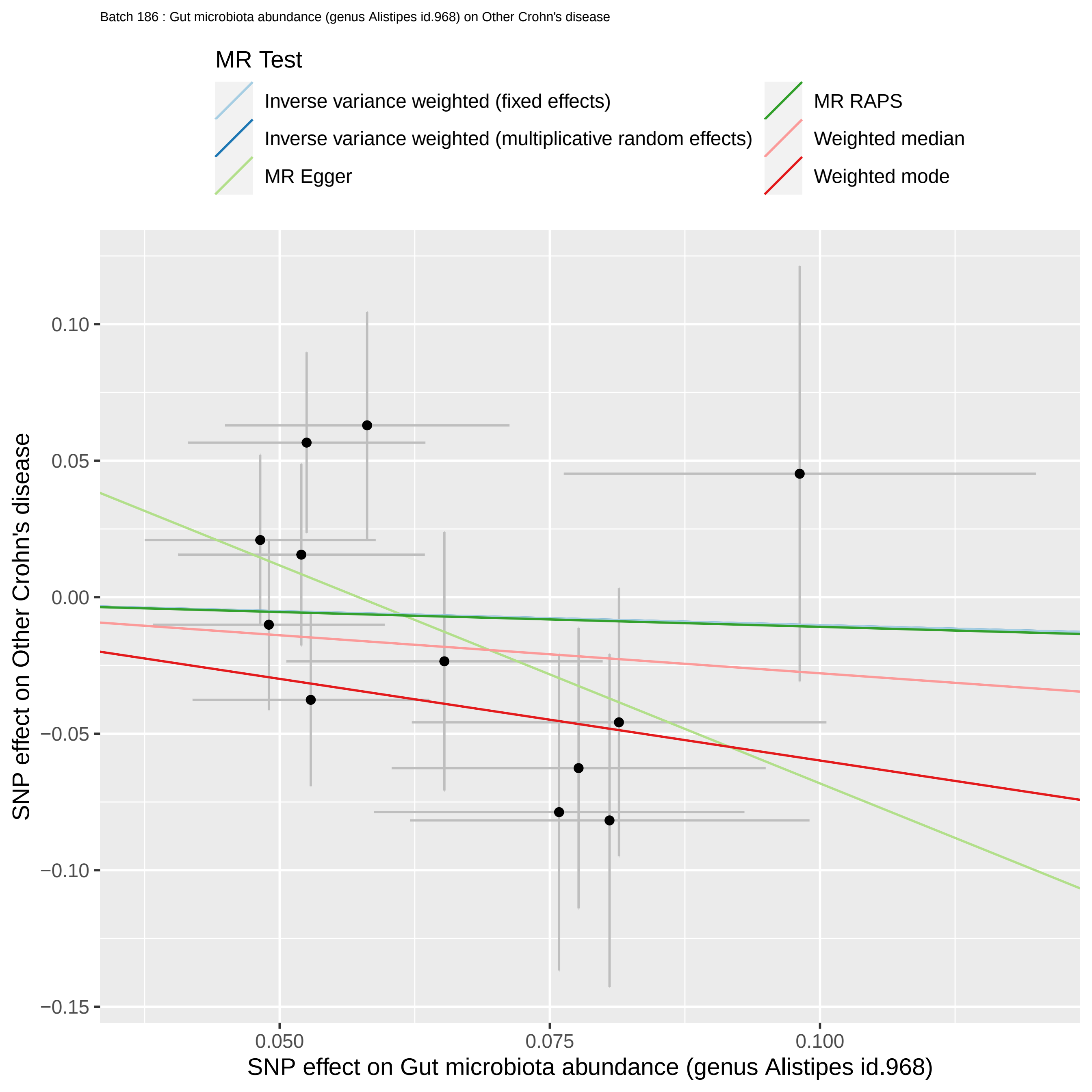

Batch 186 : Gut microbiota abundance (genus Alistipes id.968) on Other Crohn's disease
MR Test
Inverse variance weighted (fixed effects)
Inverse variance weighted (multiplicative random effects)
MR Egger
MR RAPS
Weighted median
Weighted mode
0.10
0.05
0.00
−0.05
−0.10
−0.15
0.050
0.075
0.100
SNP effect on Other Crohn's disease
SNP effect on Gut microbiota abundance (genus Alistipes id.968)

Batch 187 : Gut microbiota abundance (genus Allisonella id.2174) on Other Crohn's disease

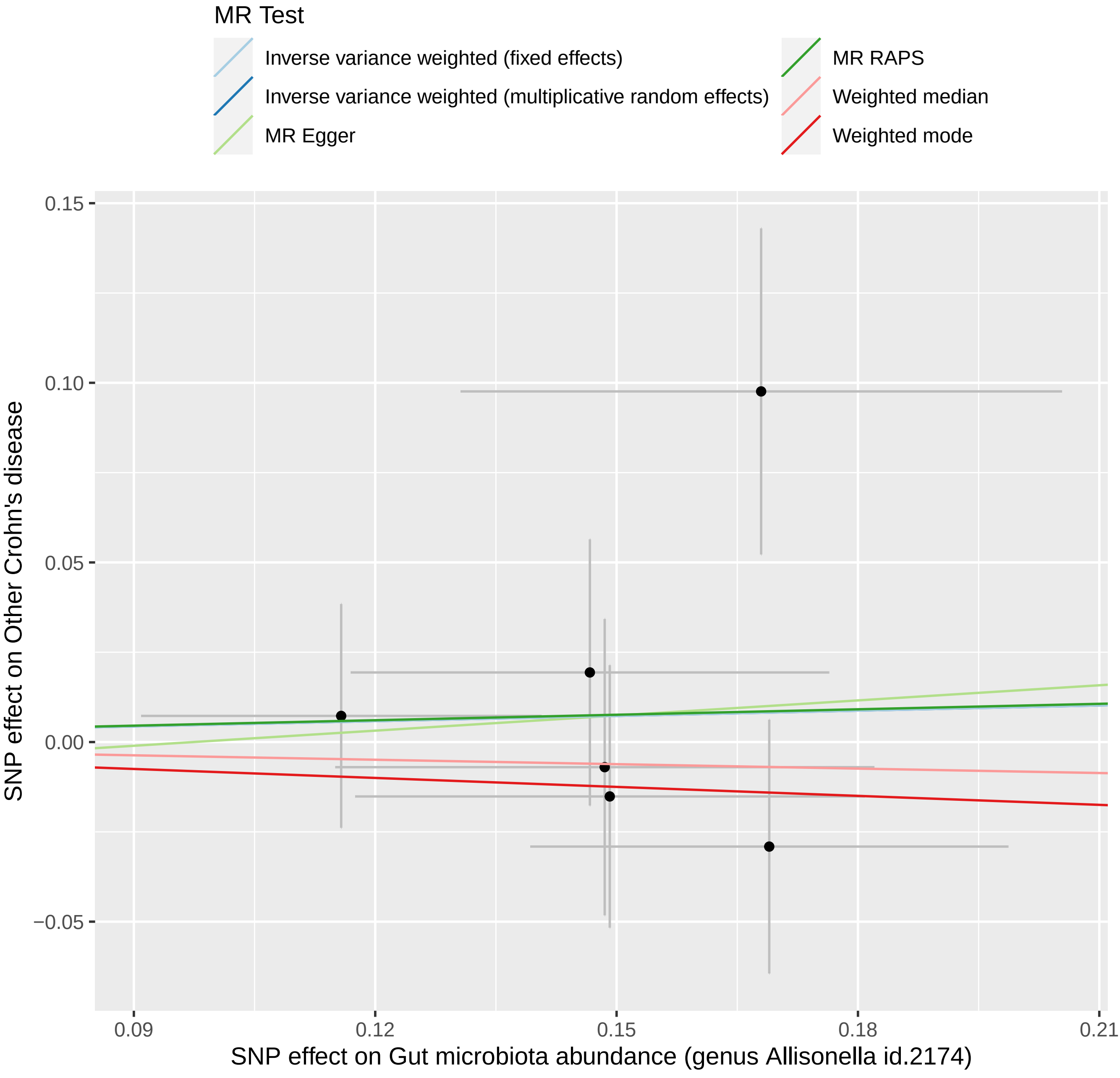

Batch 188 : Gut microbiota abundance (genus Alloprevotella id.961) on Other Crohn's disease

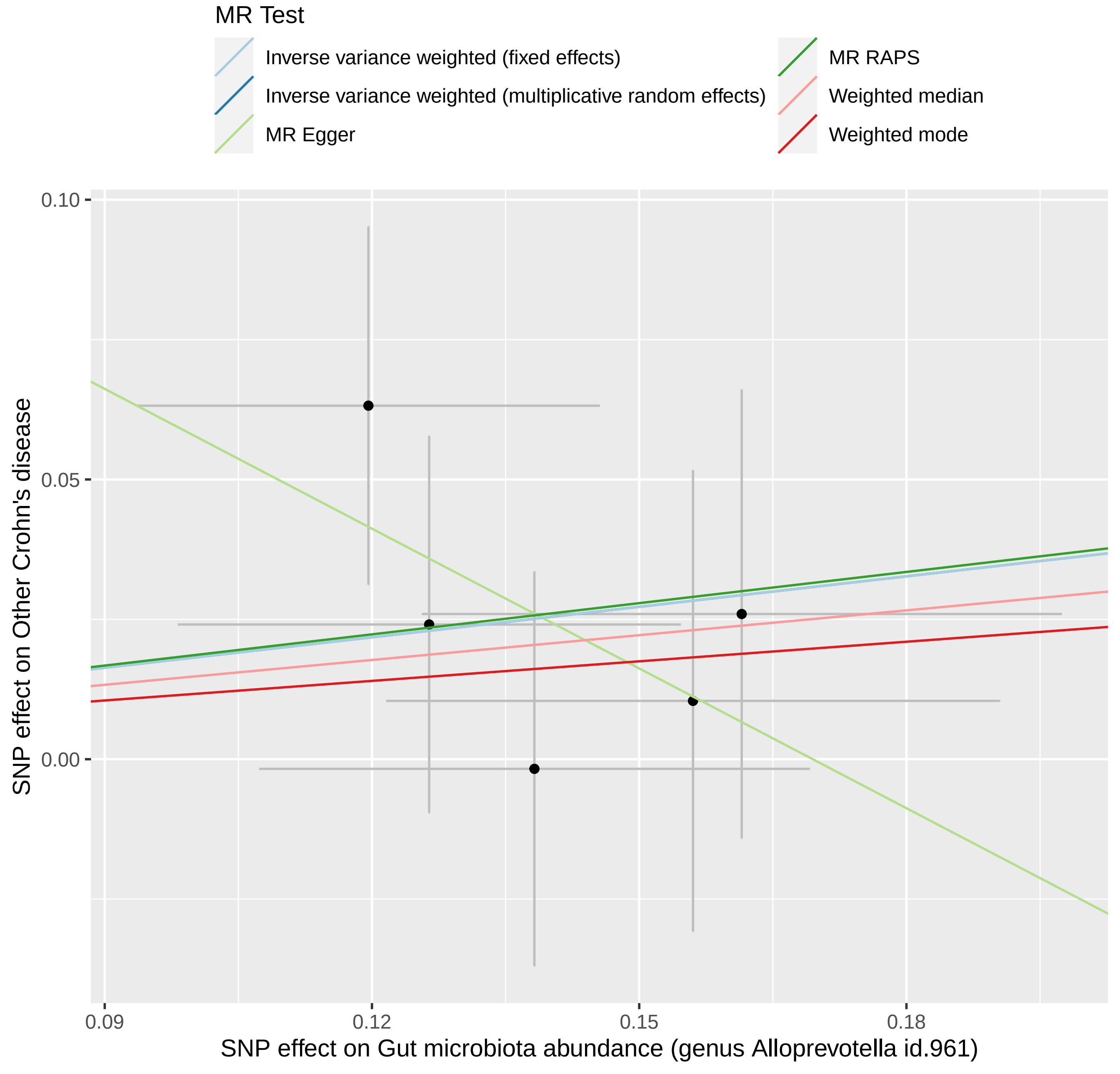

Batch 189 : Gut microbiota abundance (genus Anaerofilum id.2053) on Other Crohn's disease

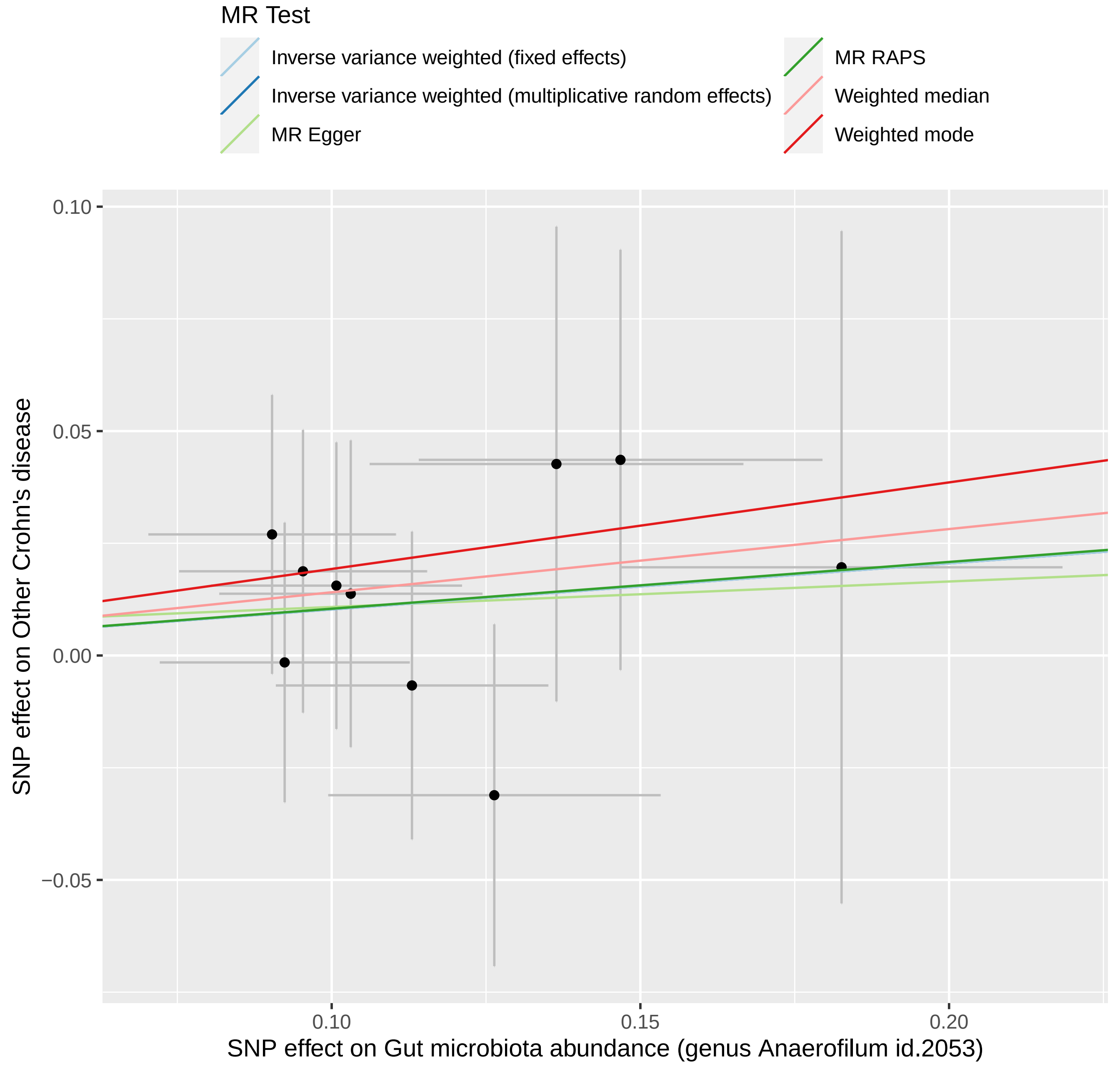

Batch 190 : Gut microbiota abundance (genus Anaerostipes id.1991) on Other Crohn's disease

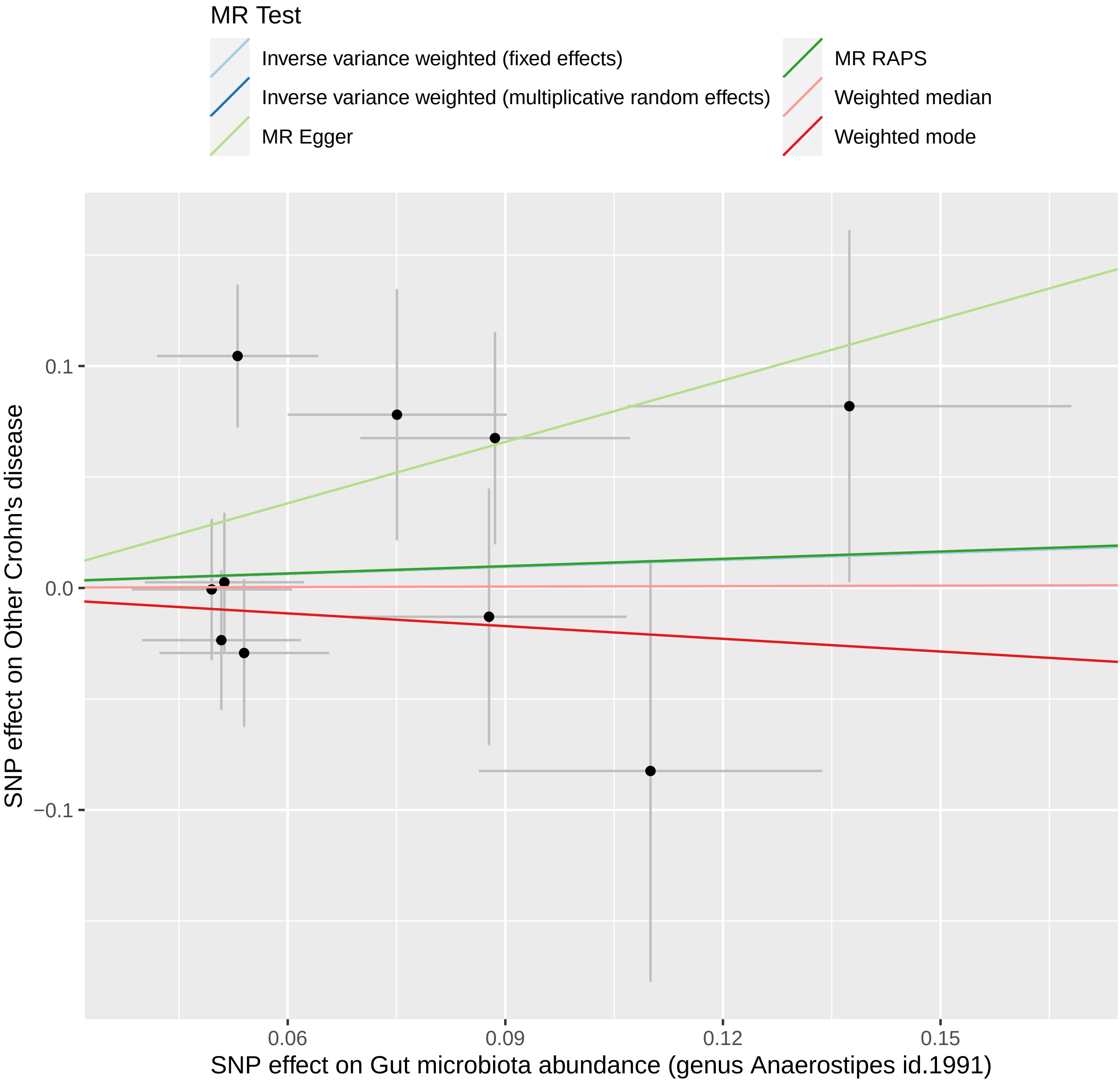

Batch 191 : Gut microbiota abundance (genus Anaerotruncus id.2054) on Other Crohn's disease

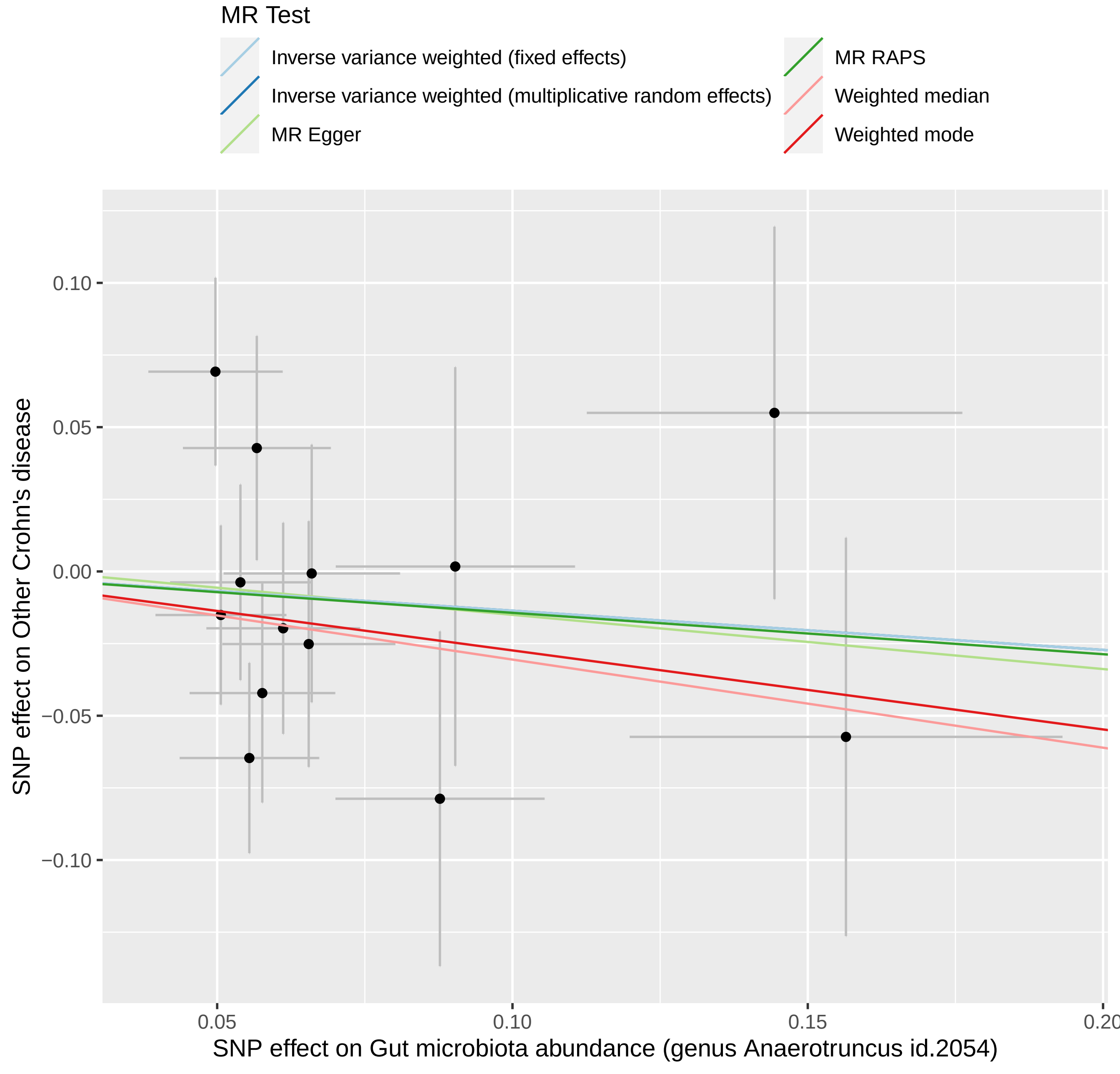

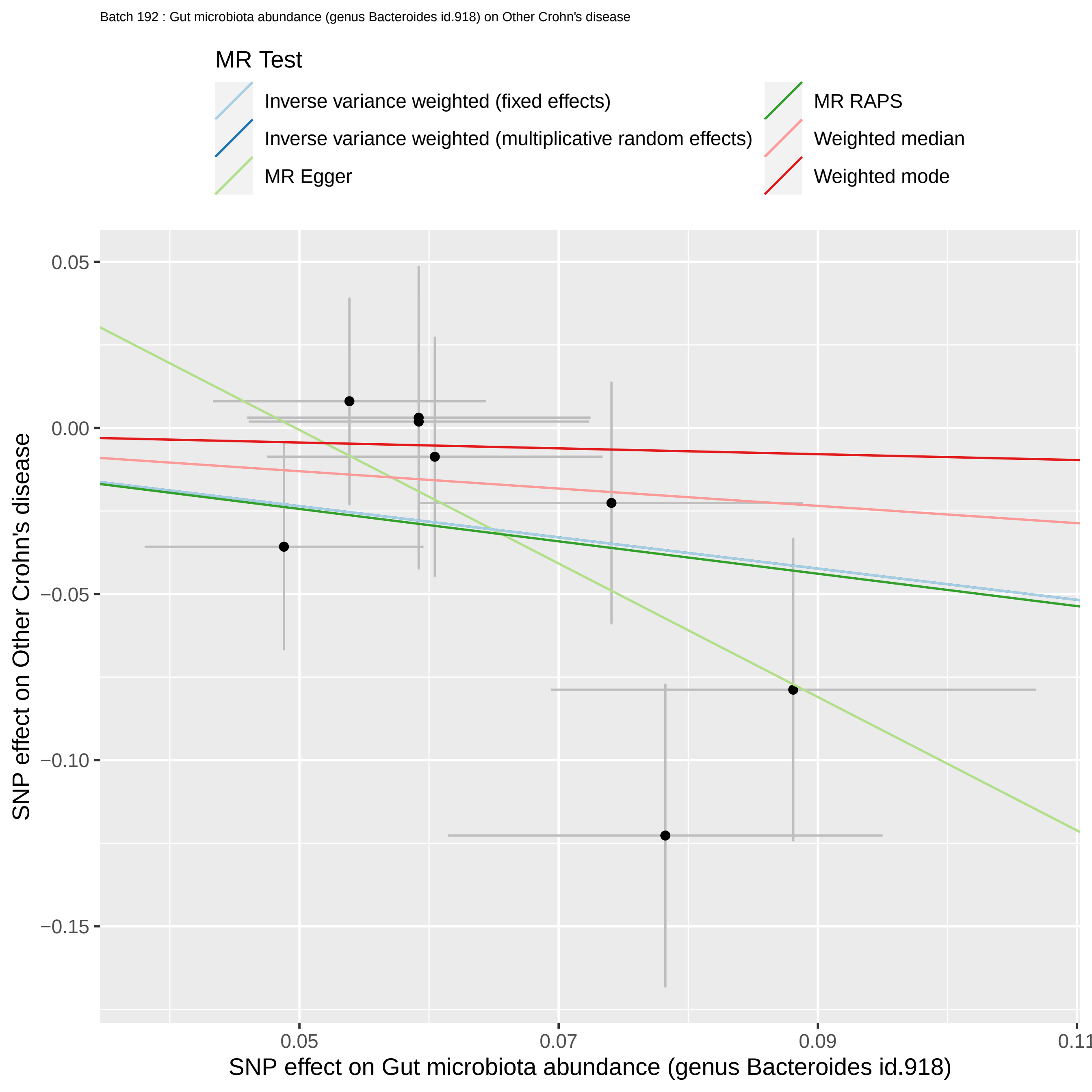

Batch 192 : Gut microbiota abundance (genus Bacteroides id.918) on Other Crohn's disease
MR Test
Inverse variance weighted (fixed effects)
Inverse variance weighted (multiplicative random effects)
MR Egger
MR RAPS
Weighted median
Weighted mode
0.05
0.00
−0.05
−0.10
−0.15
SNP effect on Other Crohn's disease
0.05
0.07
0.09
0.11
SNP effect on Gut microbiota abundance (genus Bacteroides id.918)

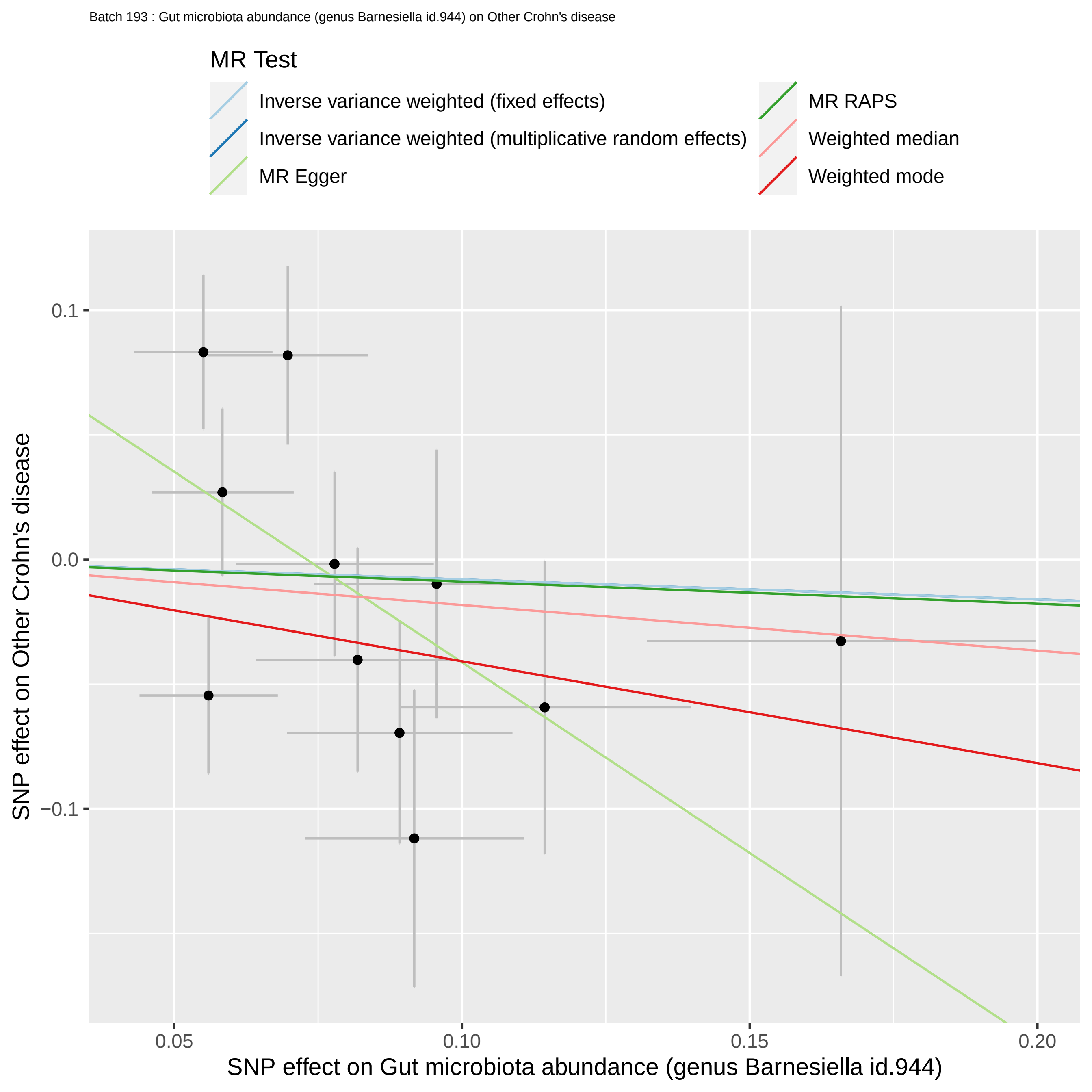

Batch 193 : Gut microbiota abundance (genus Barnesiella id.944) on Other Crohn's disease
MR Test
Inverse variance weighted (fixed effects)
Inverse variance weighted (multiplicative random effects)
MR Egger
MR RAPS
Weighted median
Weighted mode
0.1
0.0
−0.1
0.05
0.10
0.15
0.20
SNP effect on Other Crohn's disease
SNP effect on Gut microbiota abundance (genus Barnesiella id.944)

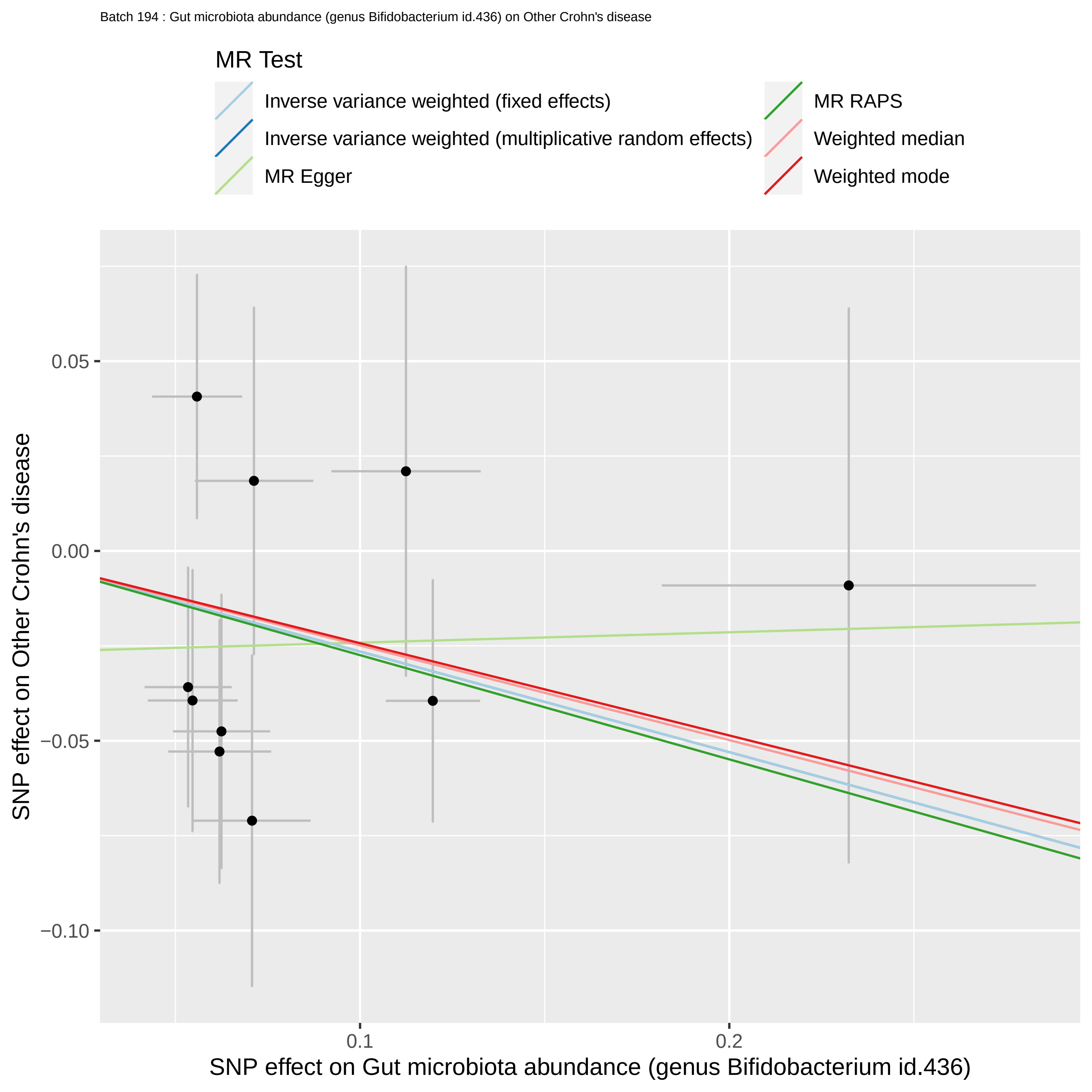
Batch 194 : Gut microbiota abundance (genus Bifidobacterium id.436) on Other Crohn's disease
MR Test
Inverse variance weighted (fixed effects)
Inverse variance weighted (multiplicative random effects)
MR Egger
MR RAPS
Weighted median
Weighted mode
0.05
0.00
−0.05
−0.10
SNP effect on Other Crohn's disease
0.1
0.2
SNP effect on Gut microbiota abundance (genus Bifidobacterium id.436)

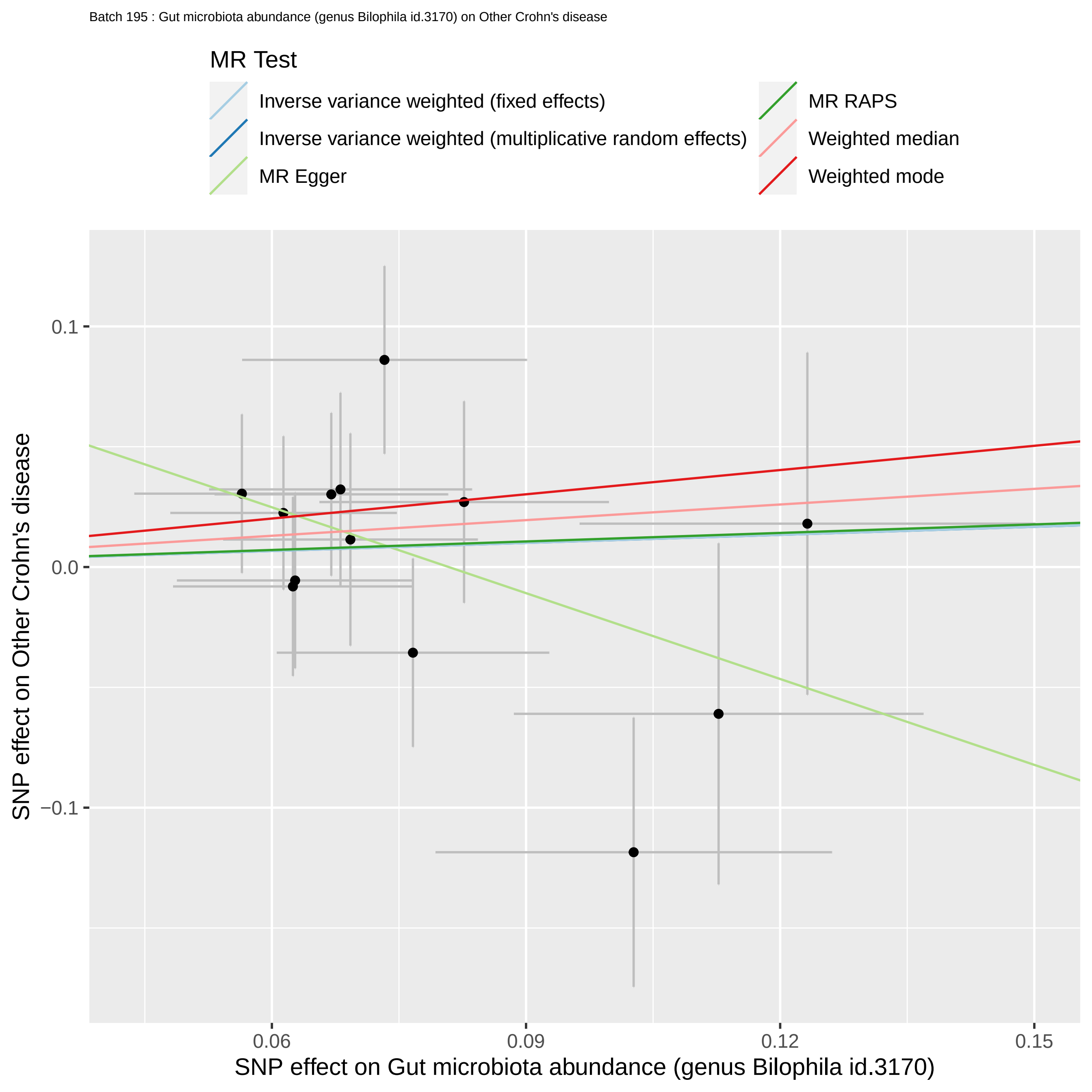

Batch 195 : Gut microbiota abundance (genus Bilophila id.3170) on Other Crohn's disease
MR Test
Inverse variance weighted (fixed effects)
Inverse variance weighted (multiplicative random effects)
MR Egger
MR RAPS
Weighted median
Weighted mode
0.1
0.0
−0.1
0.06
0.09
0.12
0.15
SNP effect on Other Crohn's disease
SNP effect on Gut microbiota abundance (genus Bilophila id.3170)

Batch 196 : Gut microbiota abundance (genus Blautia id.1992) on Other Crohn's disease

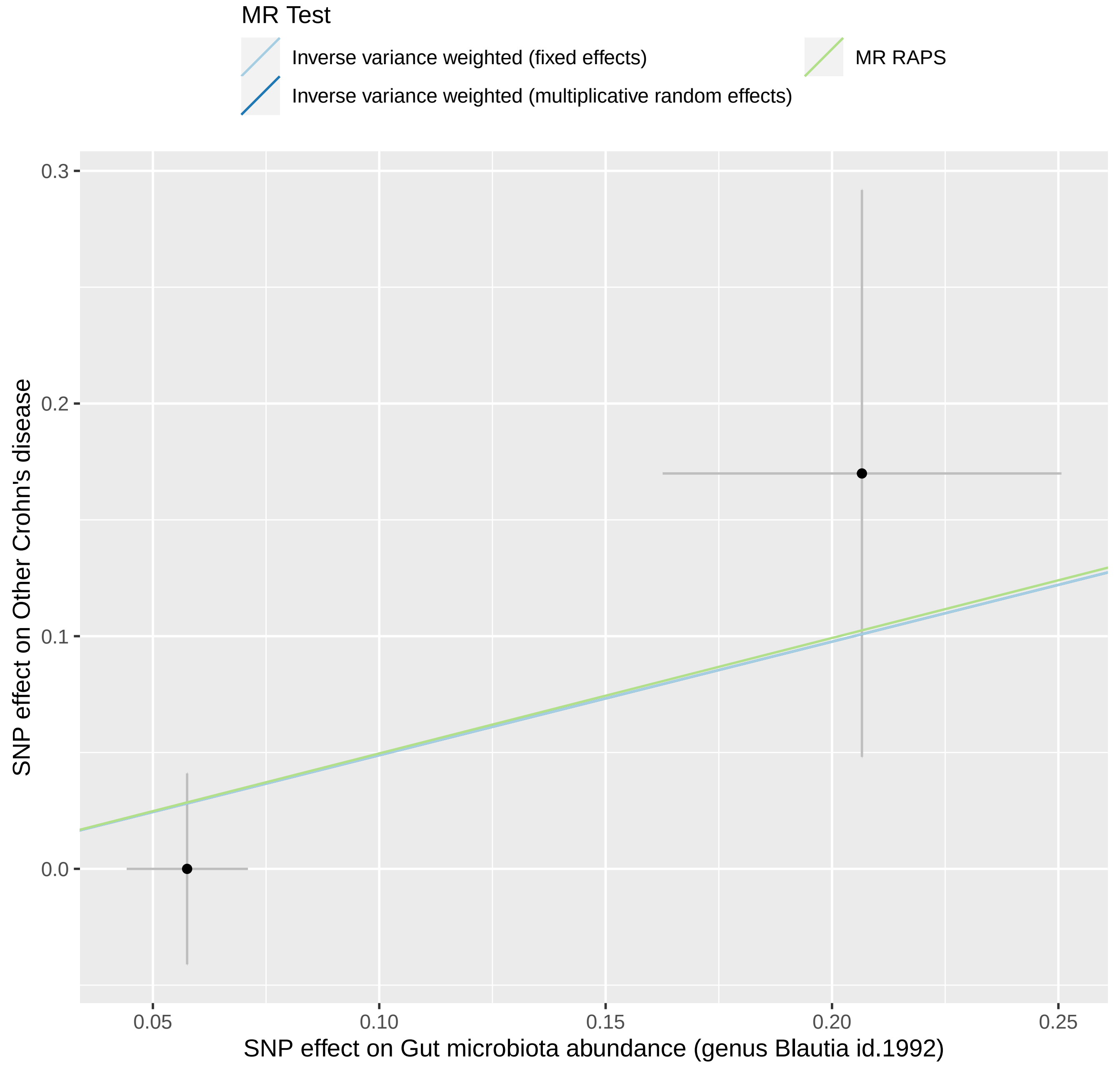

Batch 197 : Gut microbiota abundance (genus Butyricicoccus id.2055) on Other Crohn's disease

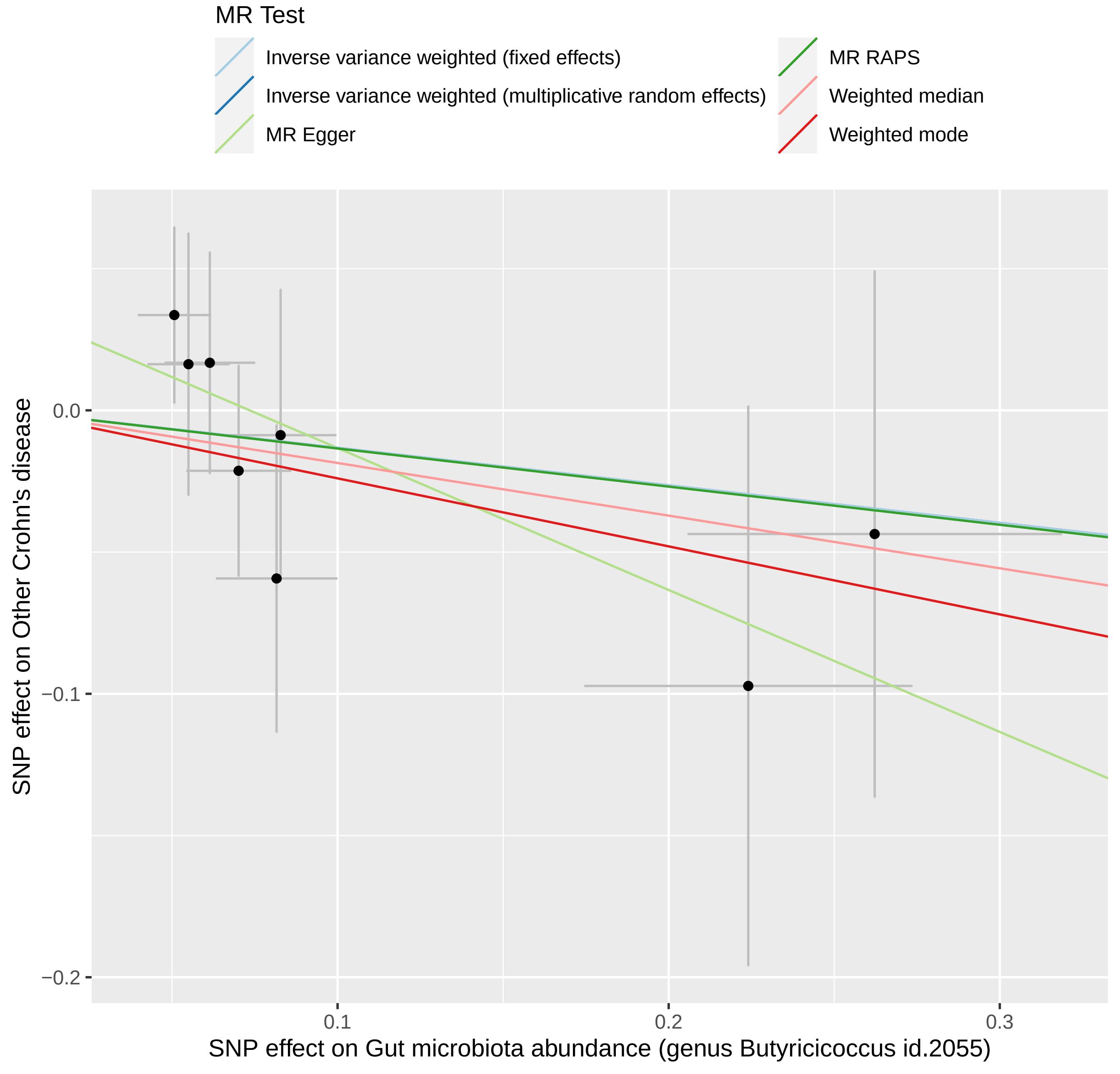

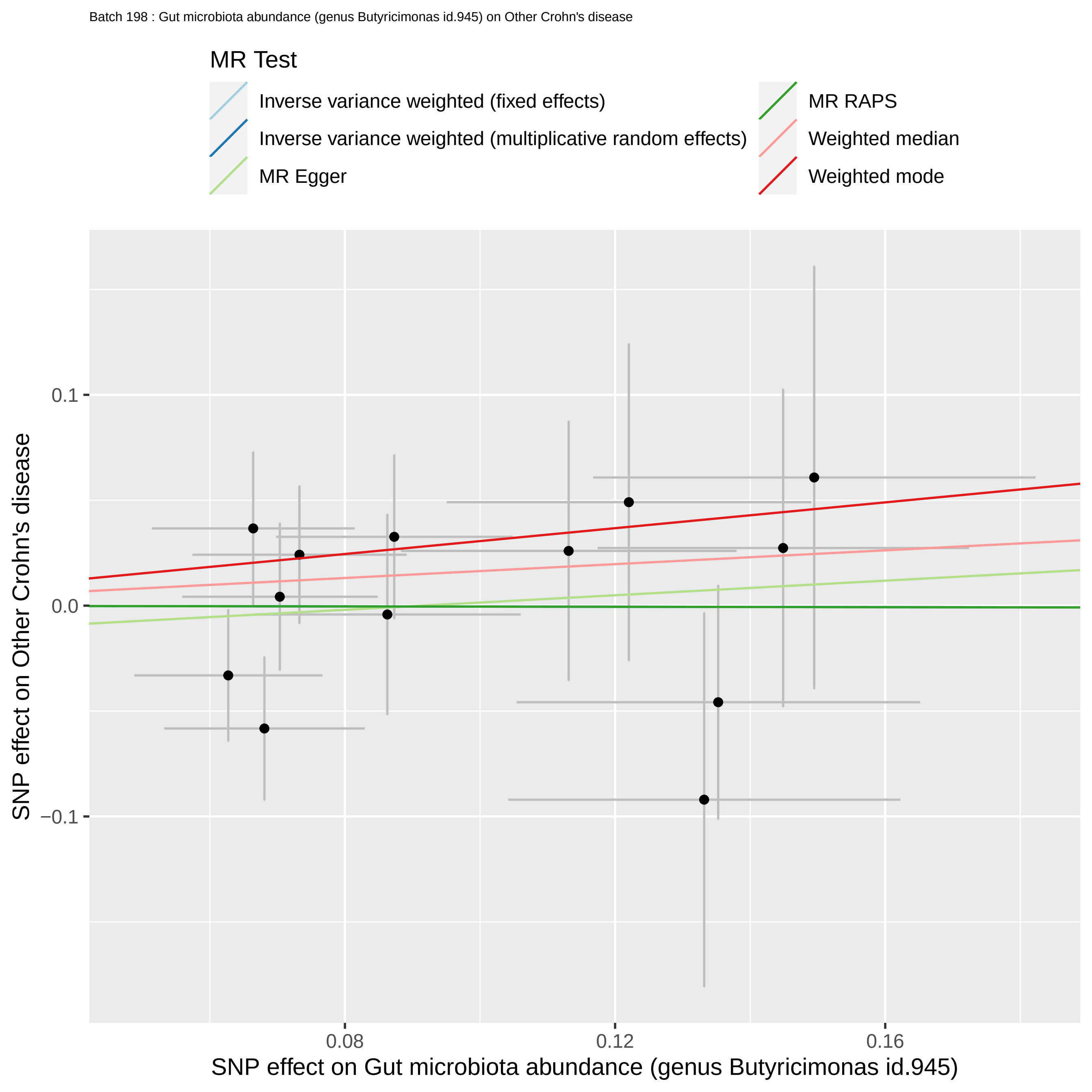
Batch 198 : Gut microbiota abundance (genus Butyricimonas id.945) on Other Crohn's disease
MR Test
Inverse variance weighted (fixed effects)
Inverse variance weighted (multiplicative random effects)
MR Egger
MR RAPS
Weighted median
Weighted mode
SNP effect on Other Crohn's disease
0.1
0.0
−0.1
0.08
0.12
0.16
SNP effect on Gut microbiota abundance (genus Butyricimonas id.945)

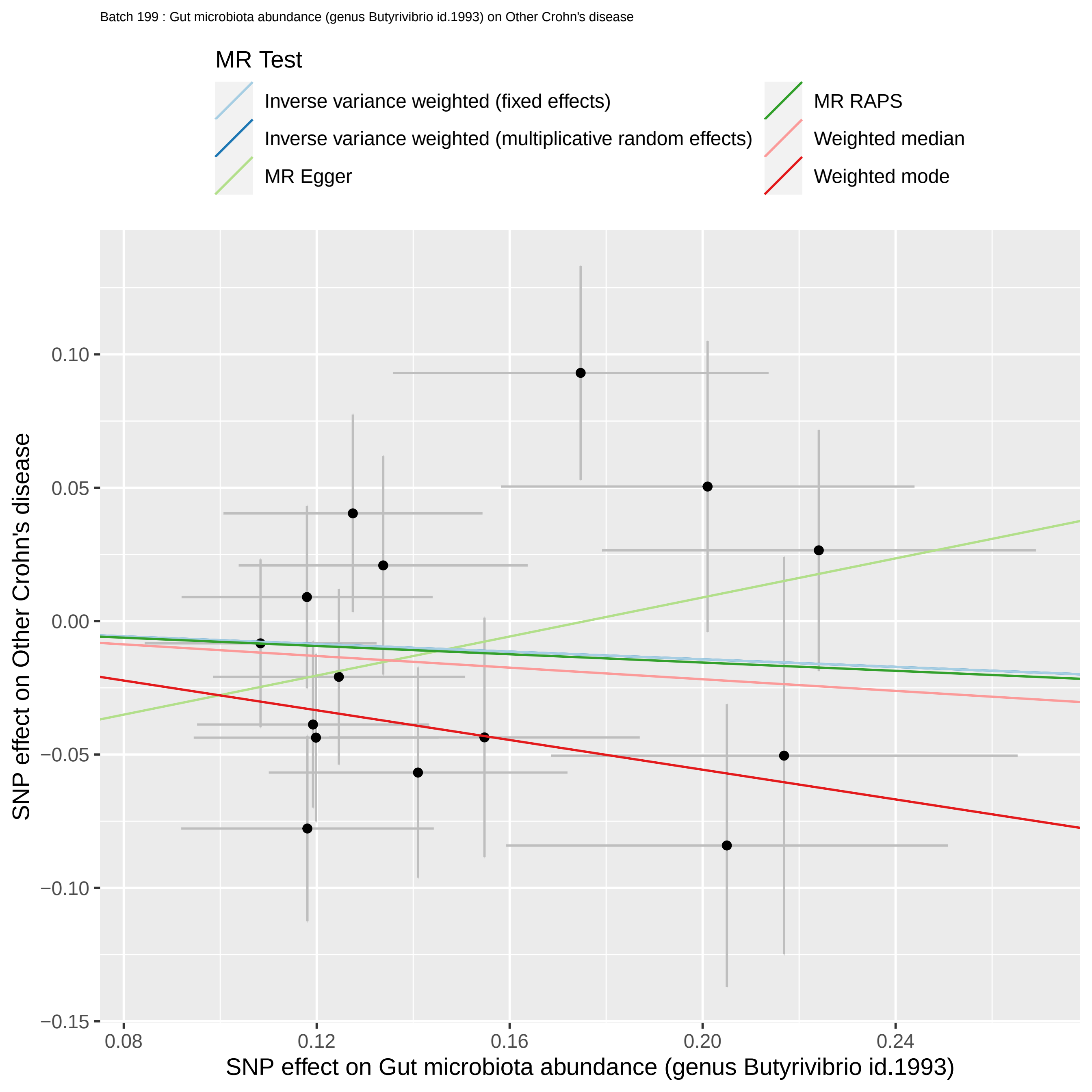

Batch 199 : Gut microbiota abundance (genus Butyrivibrio id.1993) on Other Crohn's disease
MR Test
Inverse variance weighted (fixed effects)
Inverse variance weighted (multiplicative random effects)
MR Egger
MR RAPS
Weighted median
Weighted mode
SNP effect on Other Crohn's disease
0.10
0.05
0.00
−0.05
−0.10
−0.15
0.08
0.12
0.16
0.20
0.24
SNP effect on Gut microbiota abundance (genus Butyrivibrio id.1993)

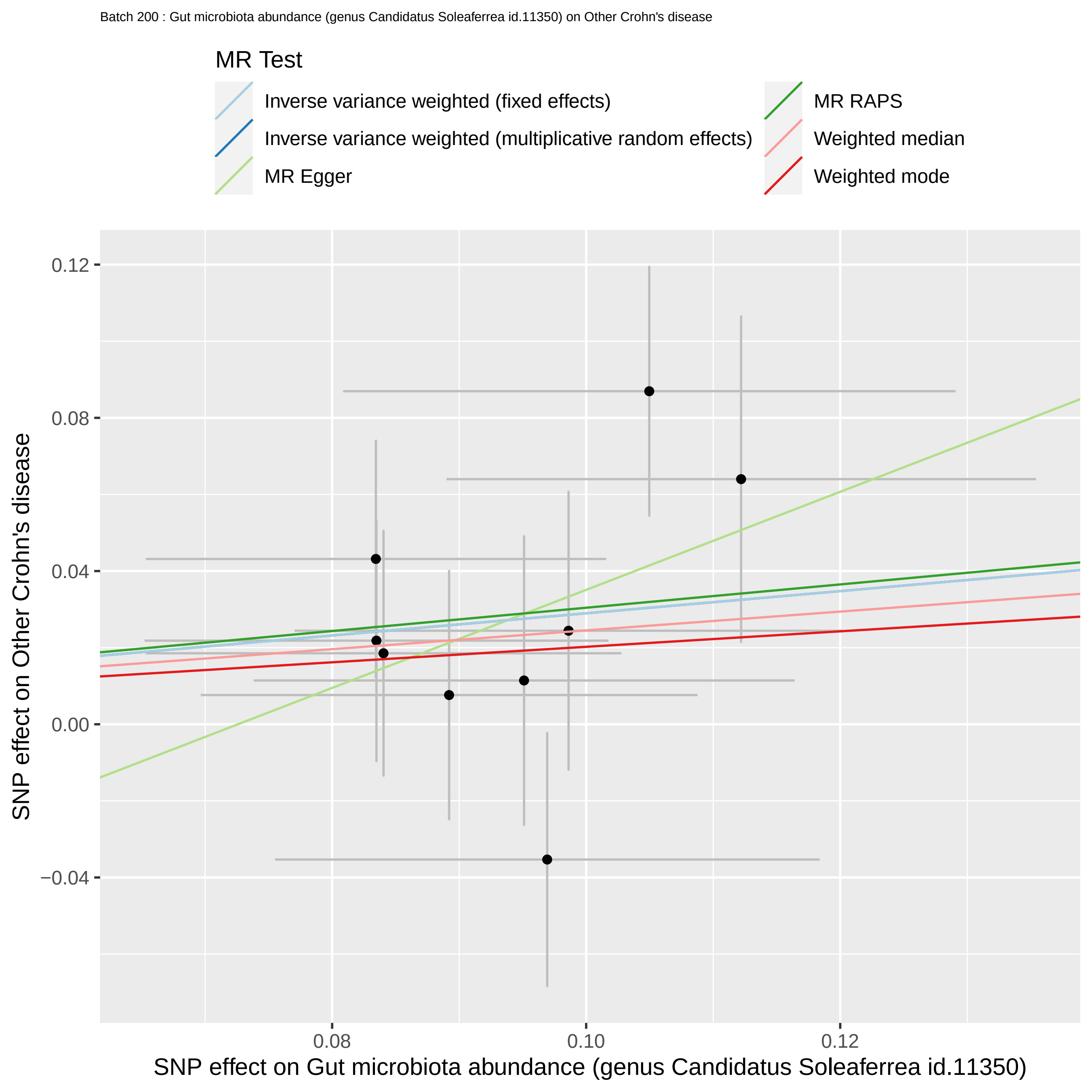

Batch 200 : Gut microbiota abundance (genus Candidatus Soleaferrea id.11350) on Other Crohn's disease
MR Test
Inverse variance weighted (fixed effects)
Inverse variance weighted (multiplicative random effects)
MR Egger
MR RAPS
Weighted median
Weighted mode
0.12
0.08
0.04
0.00
−0.04
SNP effect on Other Crohn's disease
0.08
0.10
0.12
SNP effect on Gut microbiota abundance (genus Candidatus Soleaferrea id.11350)

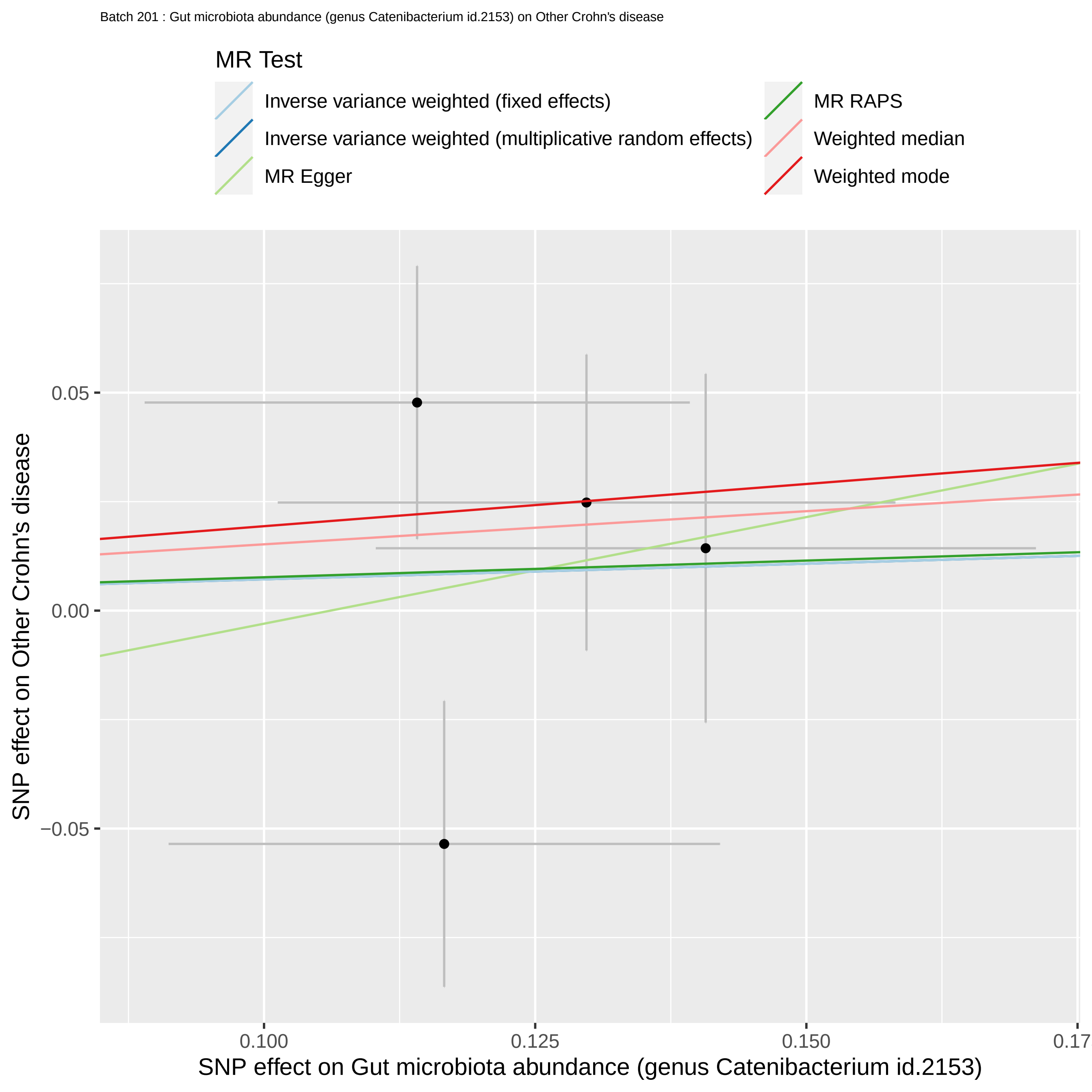
Batch 201 : Gut microbiota abundance (genus Catenibacterium id.2153) on Other Crohn's disease
MR Test
Inverse variance weighted (fixed effects)
Inverse variance weighted (multiplicative random effects)
MR Egger
MR RAPS
Weighted median
Weighted mode
0.05
0.00
−0.05
0.100
0.125
0.150
0.17
SNP effect on Other Crohn's disease
SNP effect on Gut microbiota abundance (genus Catenibacterium id.2153)

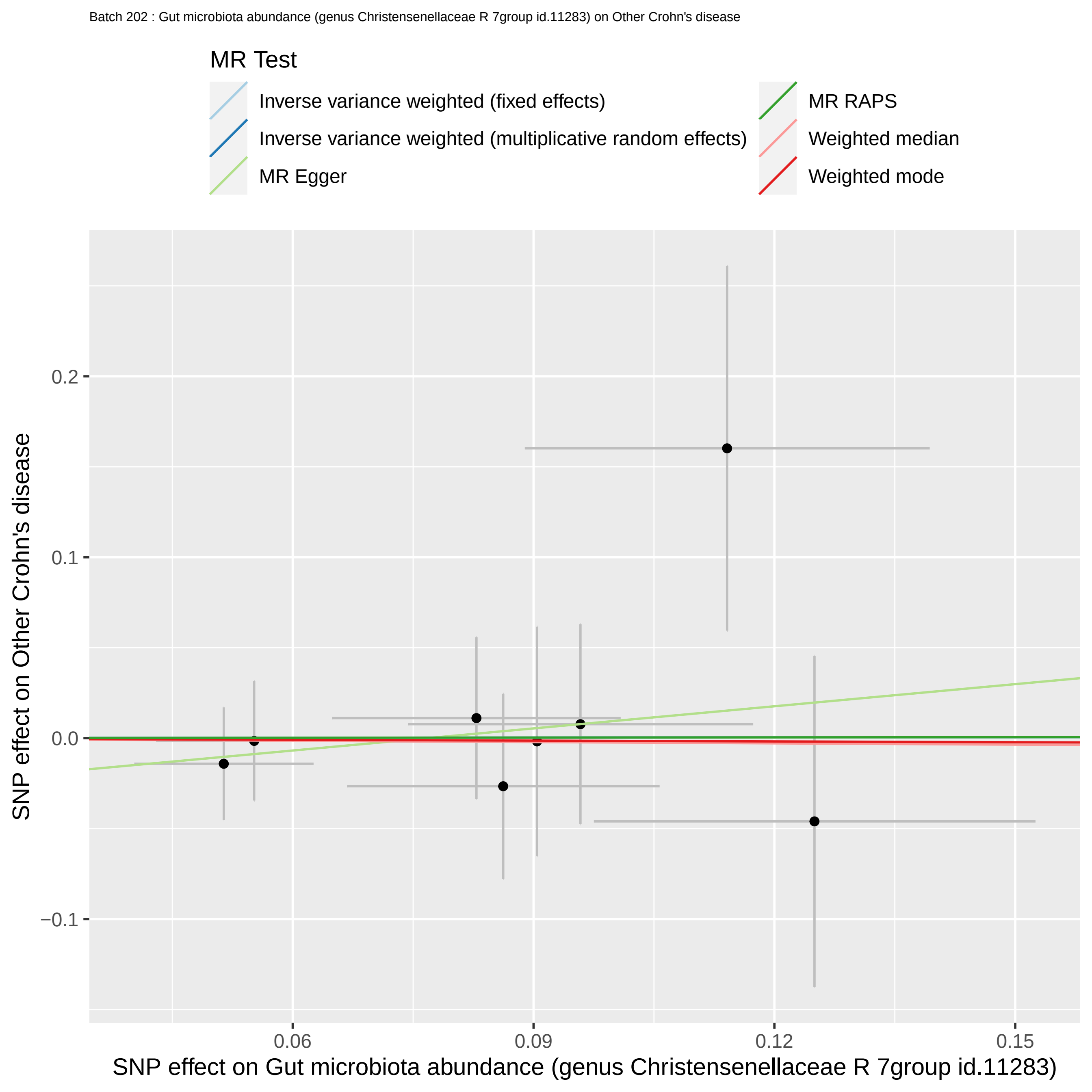
Batch 202 : Gut microbiota abundance (genus Christensenellaceae R 7group id.11283) on Other Crohn's disease
MR Test
Inverse variance weighted (fixed effects)
Inverse variance weighted (multiplicative random effects)
MR Egger
MR RAPS
Weighted median
Weighted mode
0.2
0.1
0.0
−0.1
0.06
0.09
0.12
0.15
SNP effect on Other Crohn's disease
SNP effect on Gut microbiota abundance (genus Christensenellaceae R 7group id.11283)

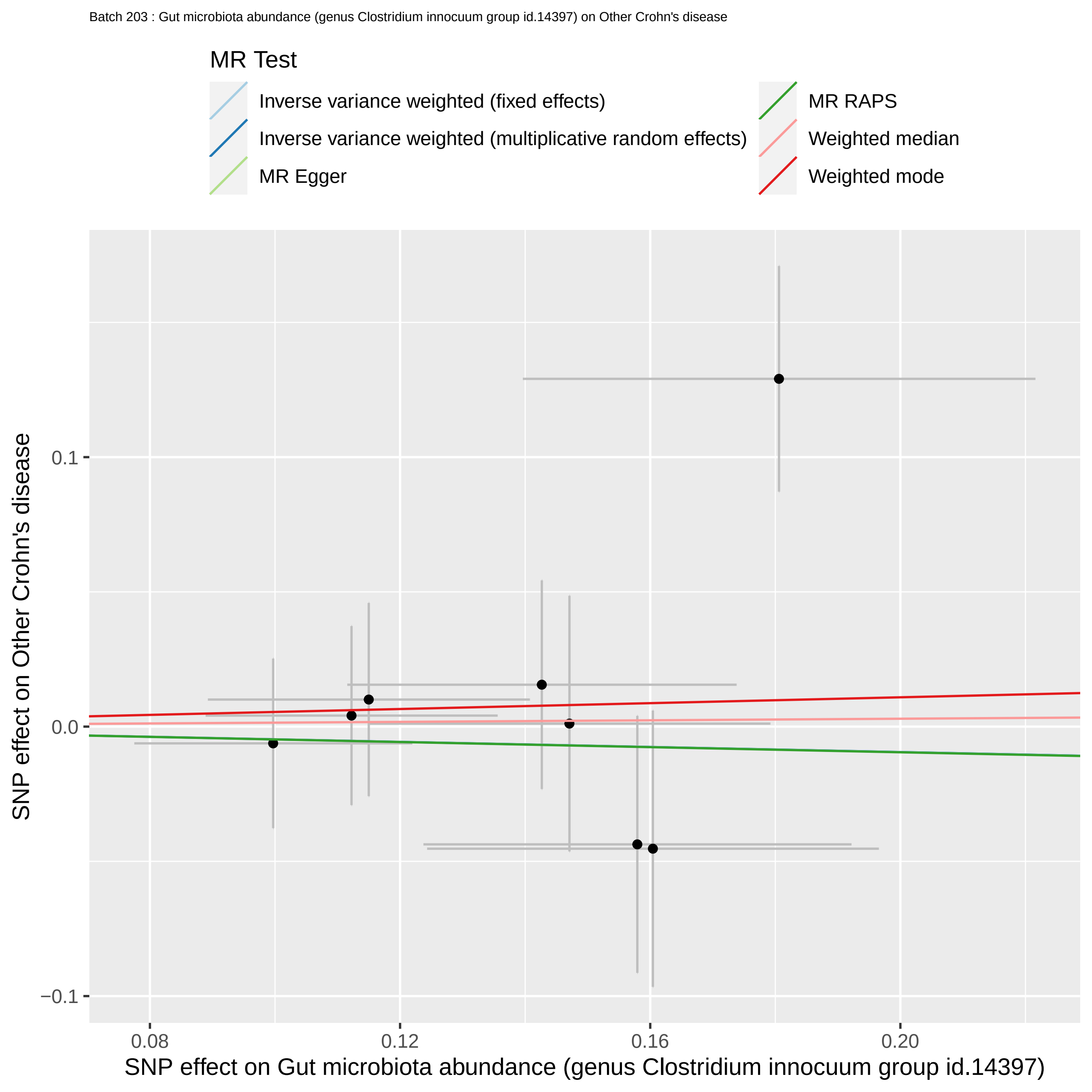
Batch 203 : Gut microbiota abundance (genus Clostridium innocuum group id.14397) on Other Crohn's disease
MR Test
Inverse variance weighted (fixed effects)
Inverse variance weighted (multiplicative random effects)
MR Egger
MR RAPS
Weighted median
Weighted mode
0.1
0.0
−0.1
0.08
0.12
0.16
0.20
SNP effect on Other Crohn's disease
SNP effect on Gut microbiota abundance (genus Clostridium innocuum group id.14397)

Batch 204 : Gut microbiota abundance (genus Clostridium sensustricto1 id.1873) on Other Crohn's disease

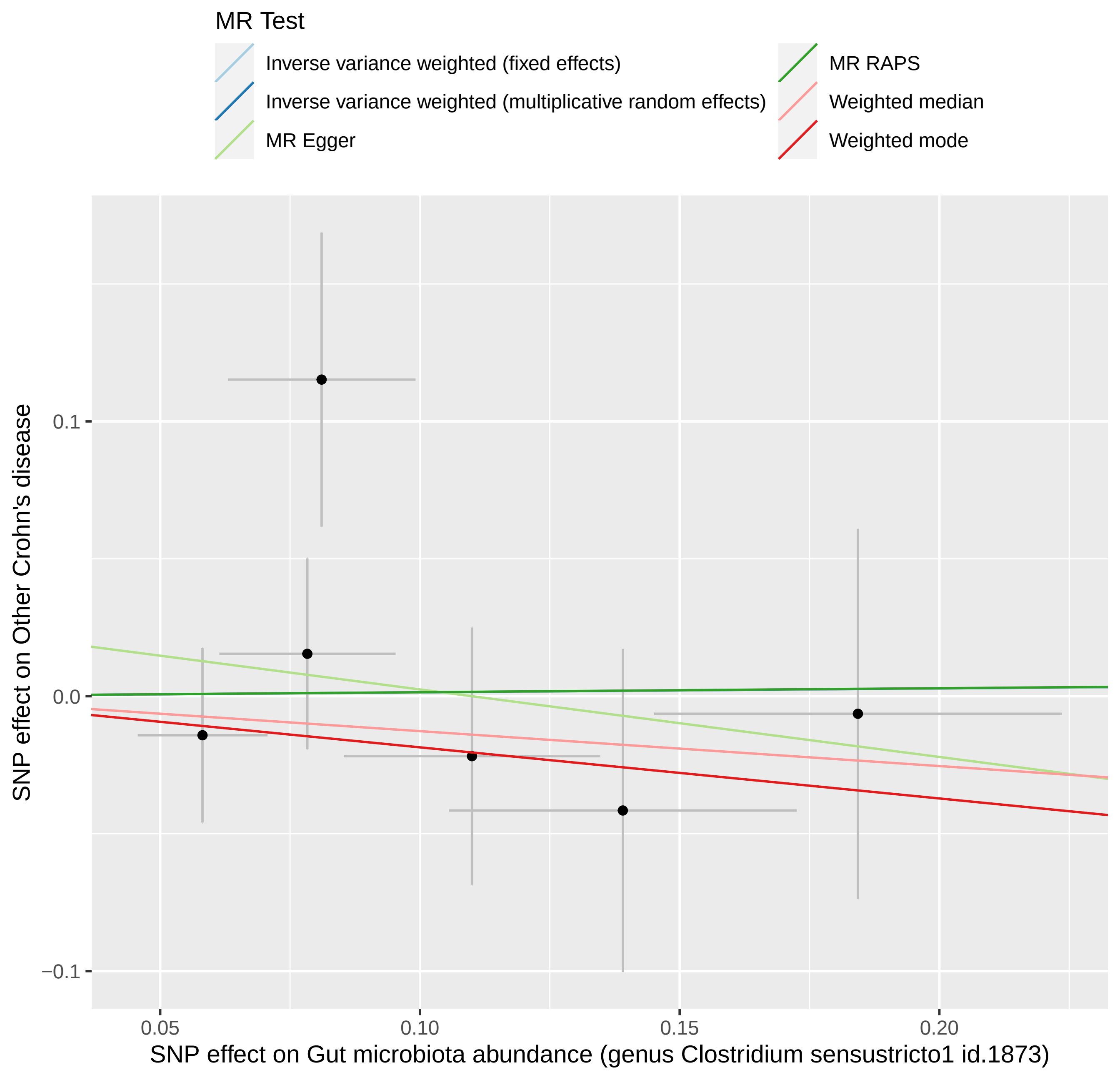

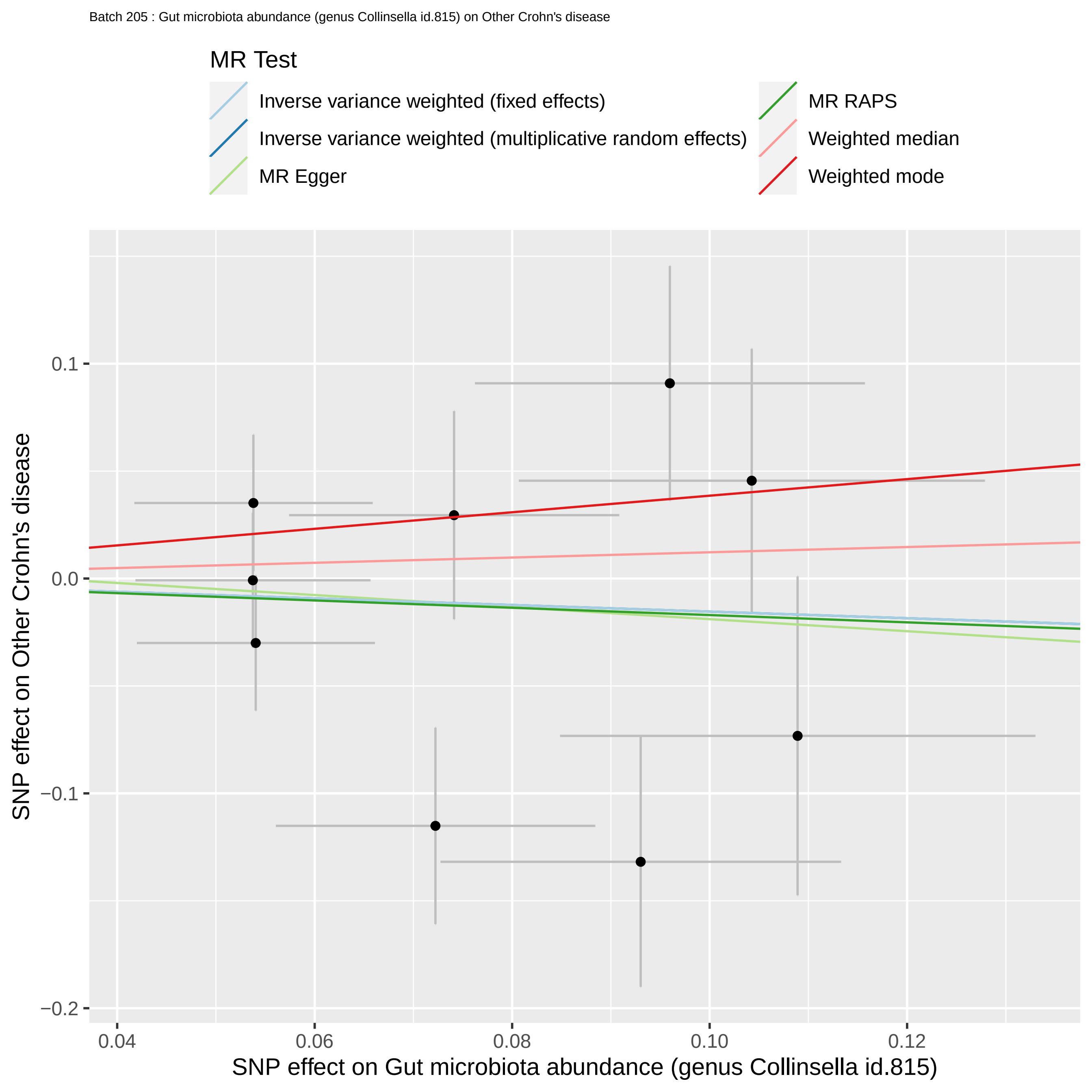
Batch 205 : Gut microbiota abundance (genus Collinsella id.815) on Other Crohn's disease
MR Test
Inverse variance weighted (fixed effects)
Inverse variance weighted (multiplicative random effects)
MR Egger
MR RAPS
Weighted median
Weighted mode
SNP effect on Other Crohn's disease
0.1
0.0
−0.1
−0.2
0.04
0.06
0.08
0.10
0.12
SNP effect on Gut microbiota abundance (genus Collinsella id.815)

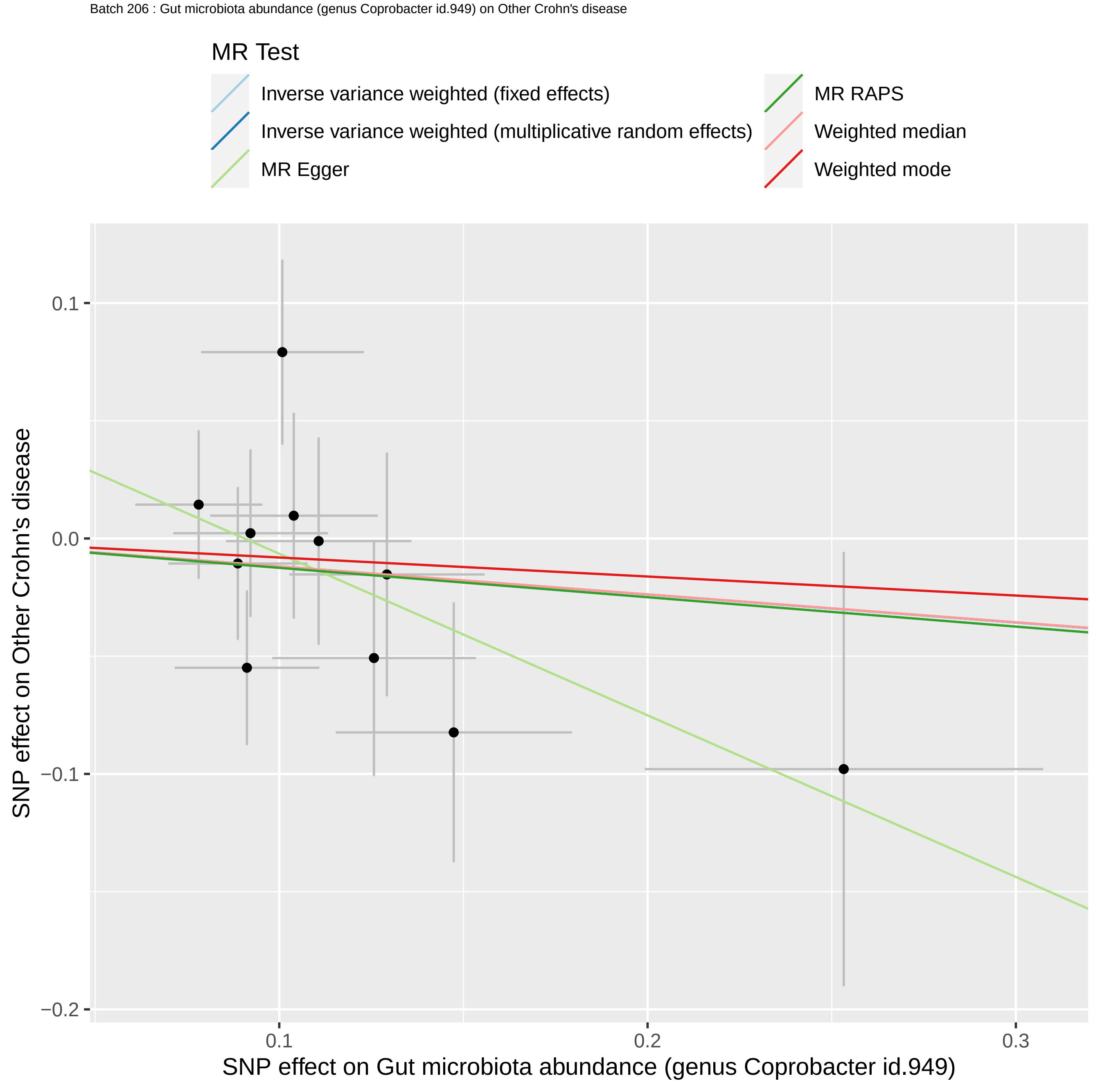

Batch 206 : Gut microbiota abundance (genus Coprobacter id.949) on Other Crohn's disease
MR Test
Inverse variance weighted (fixed effects)
Inverse variance weighted (multiplicative random effects)
MR Egger
MR RAPS
Weighted median
Weighted mode
0.1
0.0
−0.1
−0.2
0.1
0.2
0.3
SNP effect on Other Crohn's disease
SNP effect on Gut microbiota abundance (genus Coprobacter id.949)

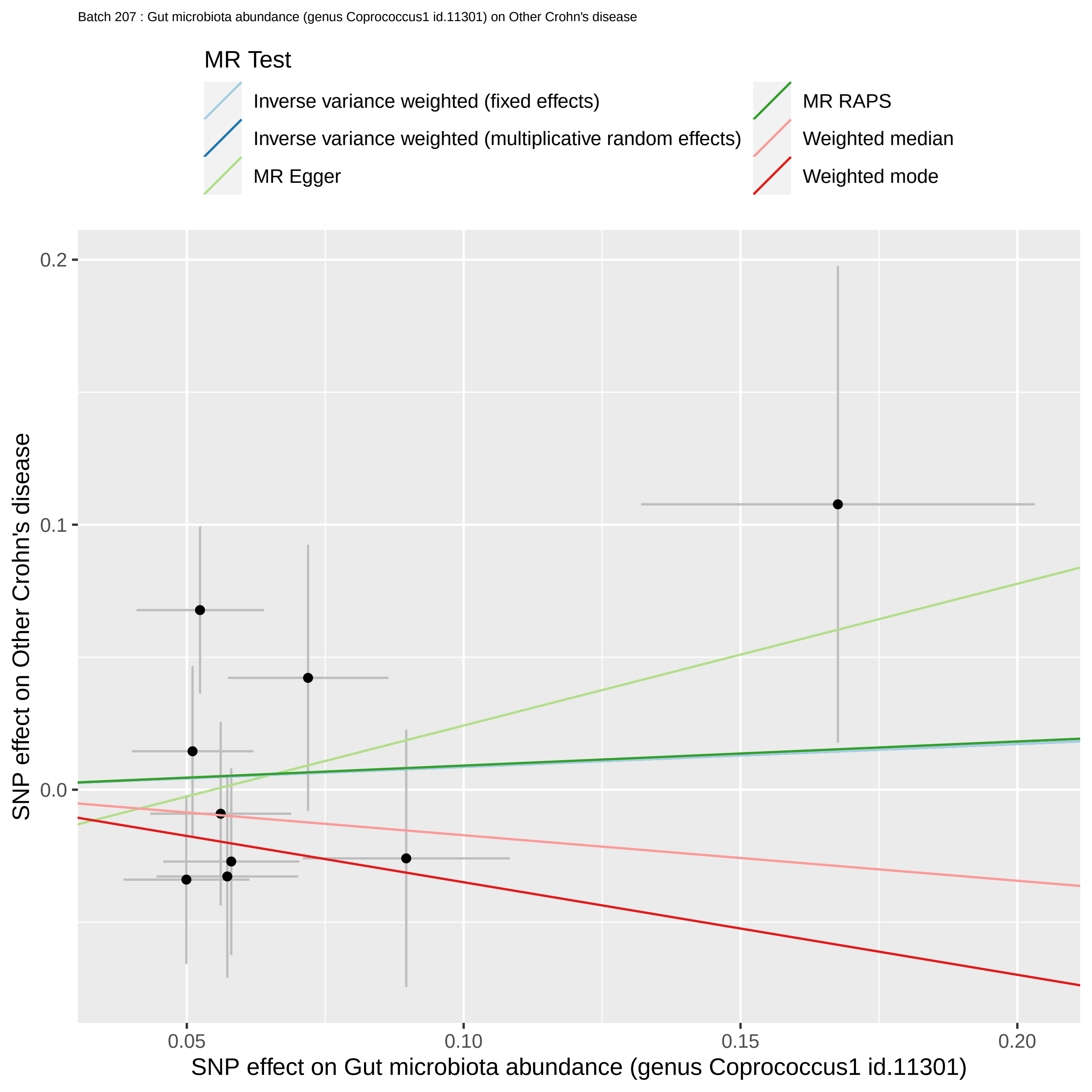
Batch 207 : Gut microbiota abundance (genus Coprococcus1 id.11301) on Other Crohn's disease
MR Test
Inverse variance weighted (fixed effects)
Inverse variance weighted (multiplicative random effects)
MR Egger
MR RAPS
Weighted median
Weighted mode
0.2
0.1
0.0
0.05
0.10
0.15
0.20
SNP effect on Other Crohn's disease
SNP effect on Gut microbiota abundance (genus Coprococcus1 id.11301)

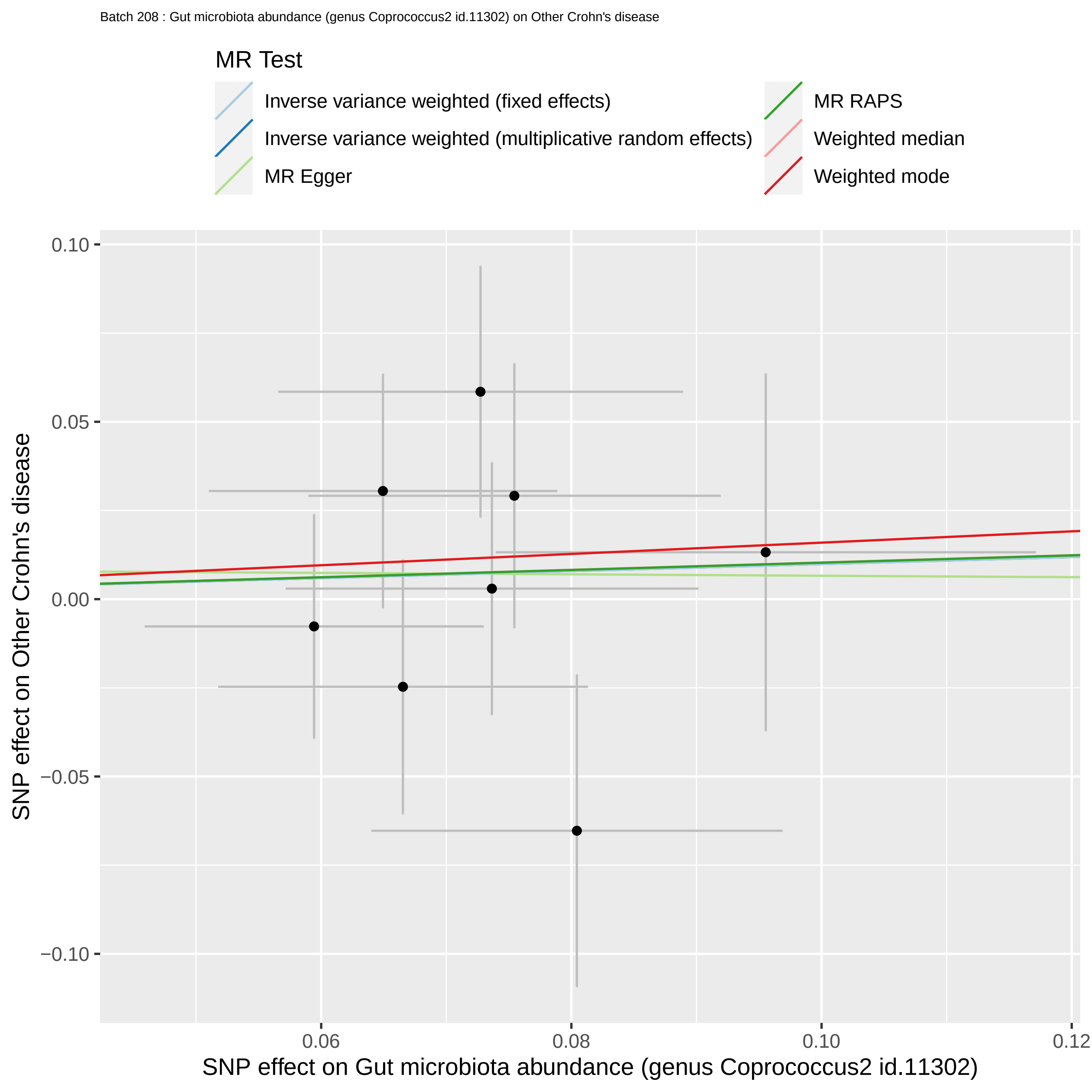

Batch 208 : Gut microbiota abundance (genus Coprococcus2 id.11302) on Other Crohn's disease
MR Test
Inverse variance weighted (fixed effects)
Inverse variance weighted (multiplicative random effects)
MR Egger
MR RAPS
Weighted median
Weighted mode
0.10
0.05
0.00
−0.05
−0.10
0.06
0.08
0.10
0.12
SNP effect on Other Crohn's disease
SNP effect on Gut microbiota abundance (genus Coprococcus2 id.11302)

Batch 209 : Gut microbiota abundance (genus Coprococcus3 id.11303) on Other Crohn's disease

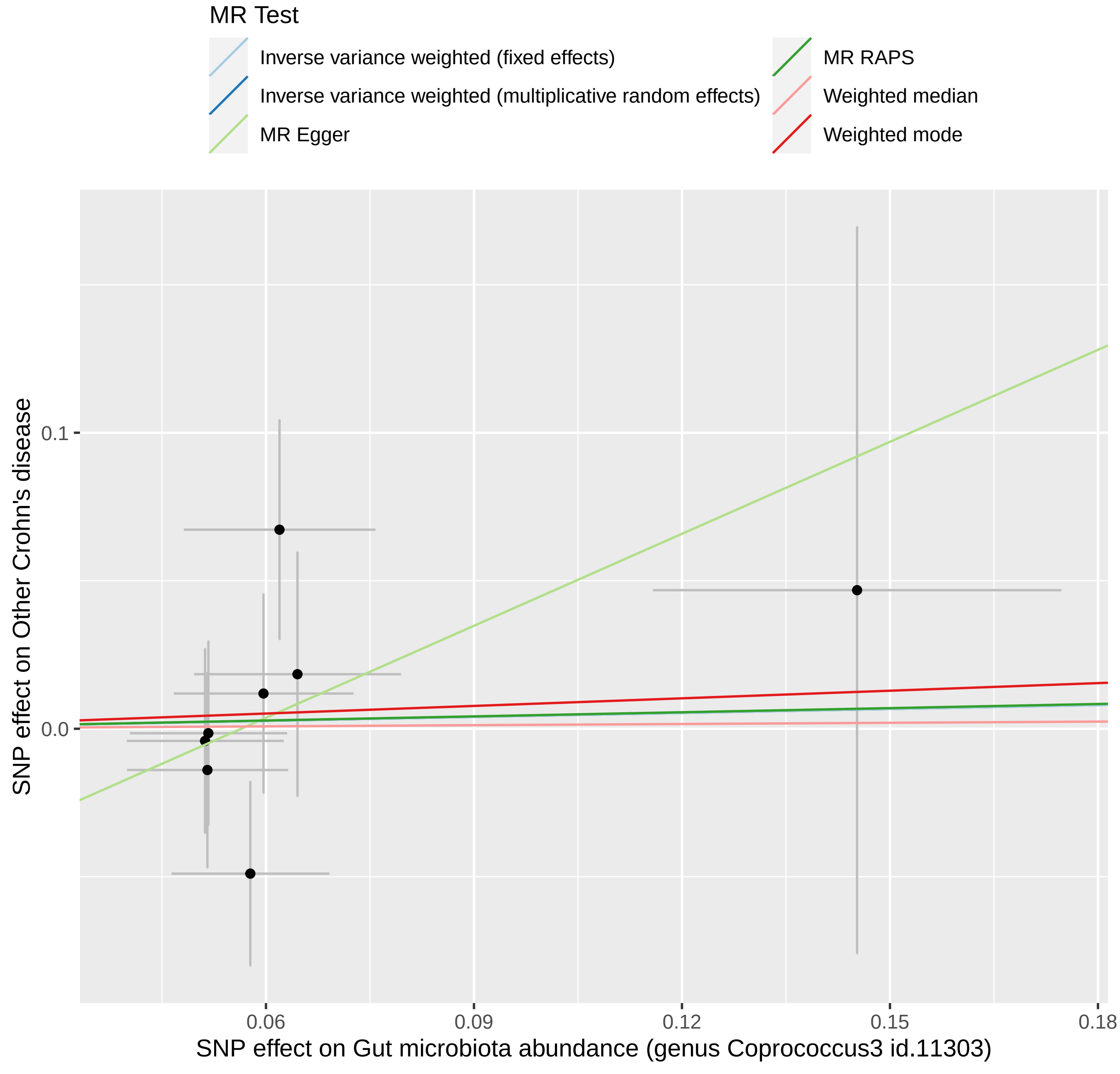

Batch 210 : Gut microbiota abundance (genus Defluviitaleaceae UCG011 id.11287) on Other Crohn's disease

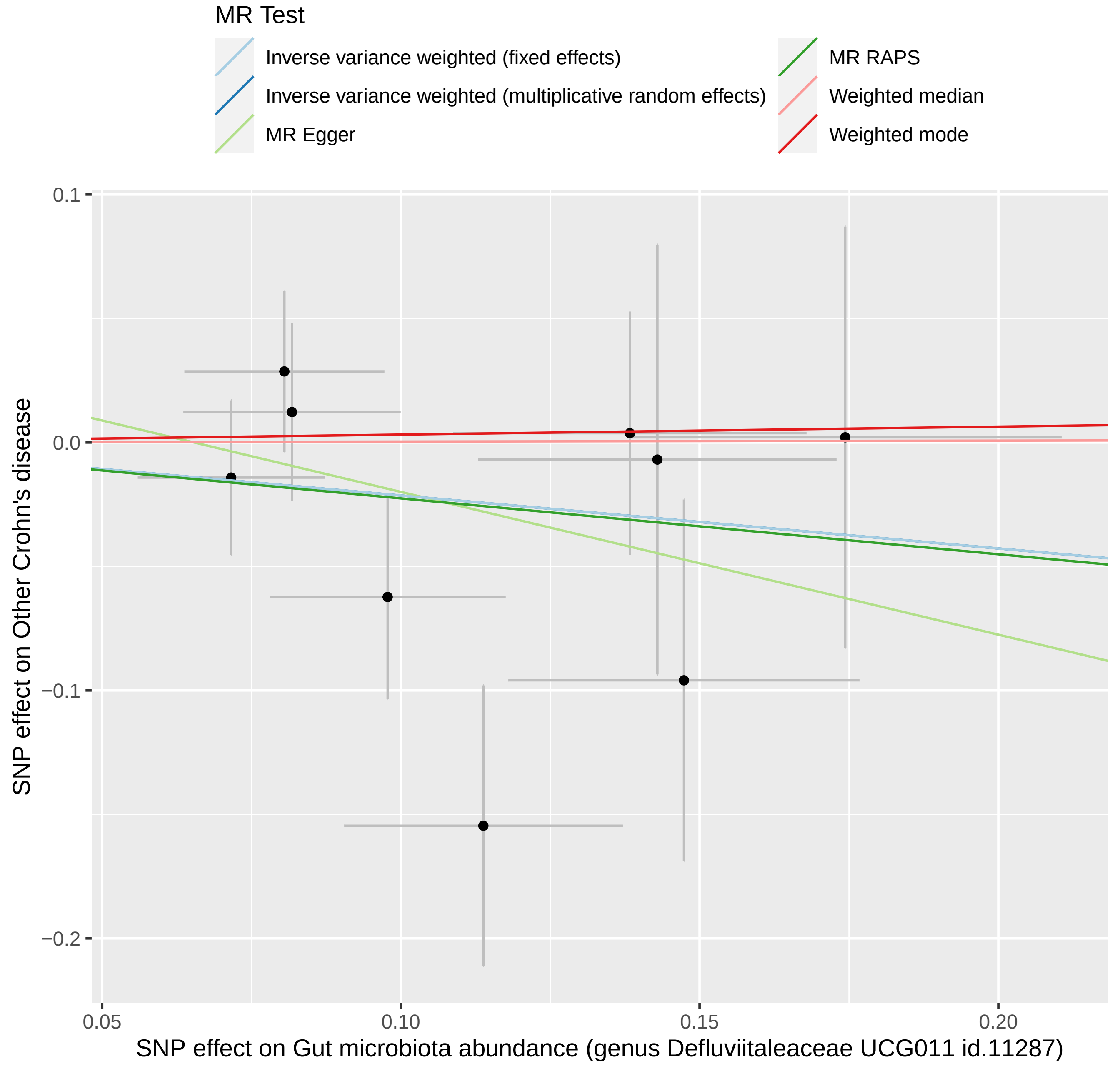

Batch 211 : Gut microbiota abundance (genus Desulfovibrio id.3173) on Other Crohn's disease

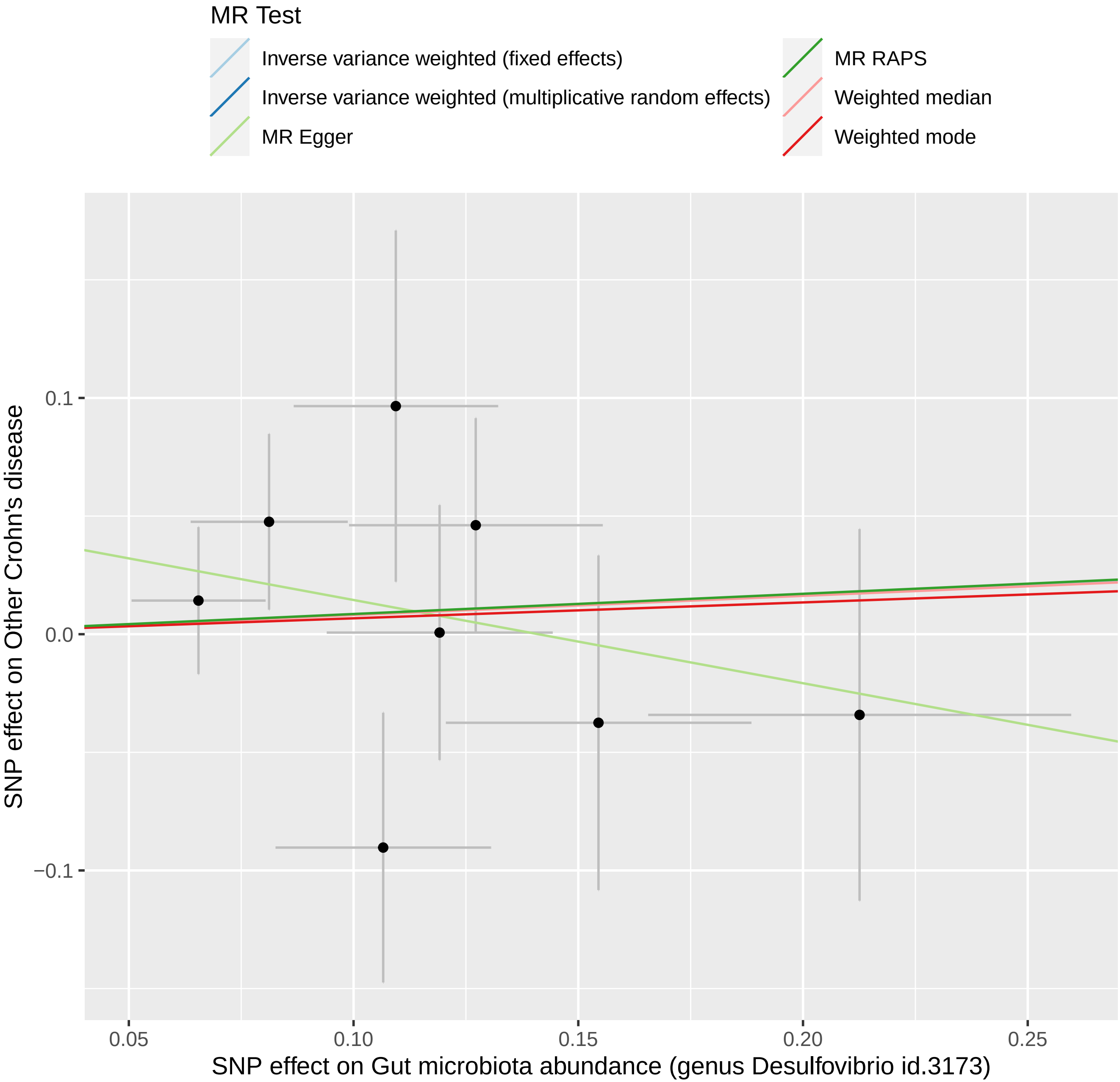

Batch 212 : Gut microbiota abundance (genus Dialister id.2183) on Other Crohn's disease

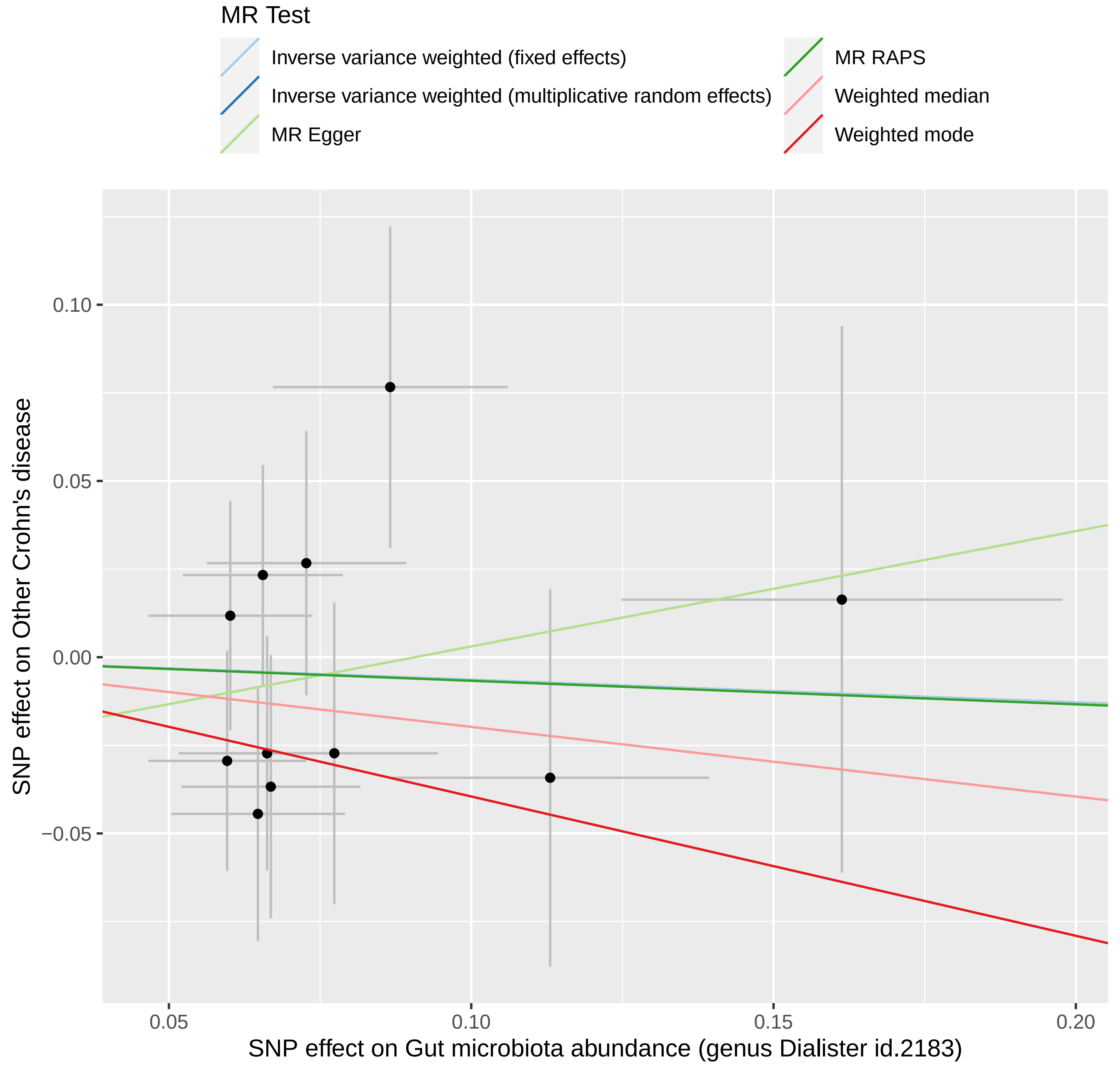

Batch 213 : Gut microbiota abundance (genus Dorea id.1997) on Other Crohn's disease

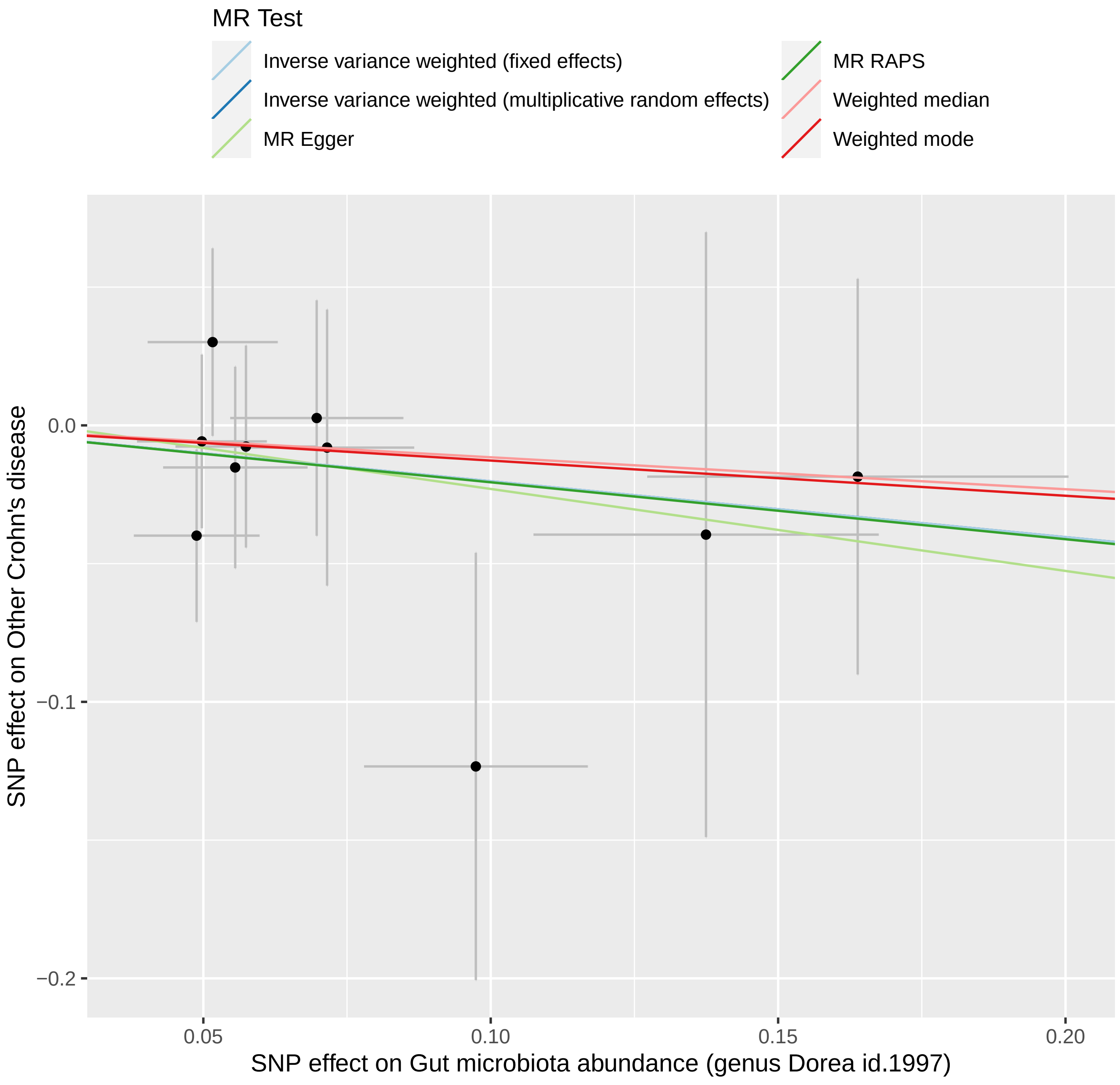

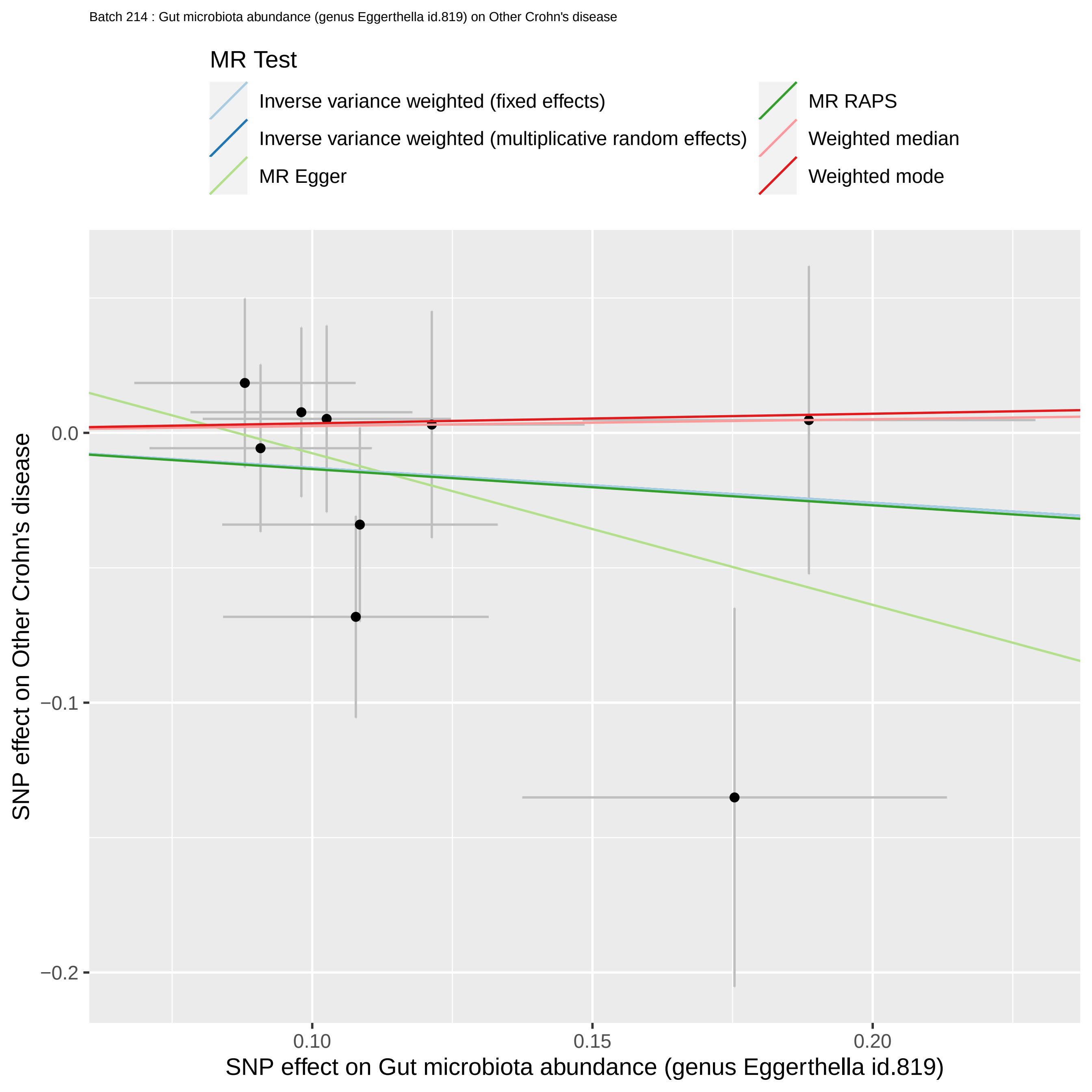
Batch 214 : Gut microbiota abundance (genus Eggerthella id.819) on Other Crohn's disease
MR Test
Inverse variance weighted (fixed effects)
Inverse variance weighted (multiplicative random effects)
MR Egger
MR RAPS
Weighted median
Weighted mode
0.0
−0.1
−0.2
0.10
0.15
0.20
SNP effect on Other Crohn's disease
SNP effect on Gut microbiota abundance (genus Eggerthella id.819)

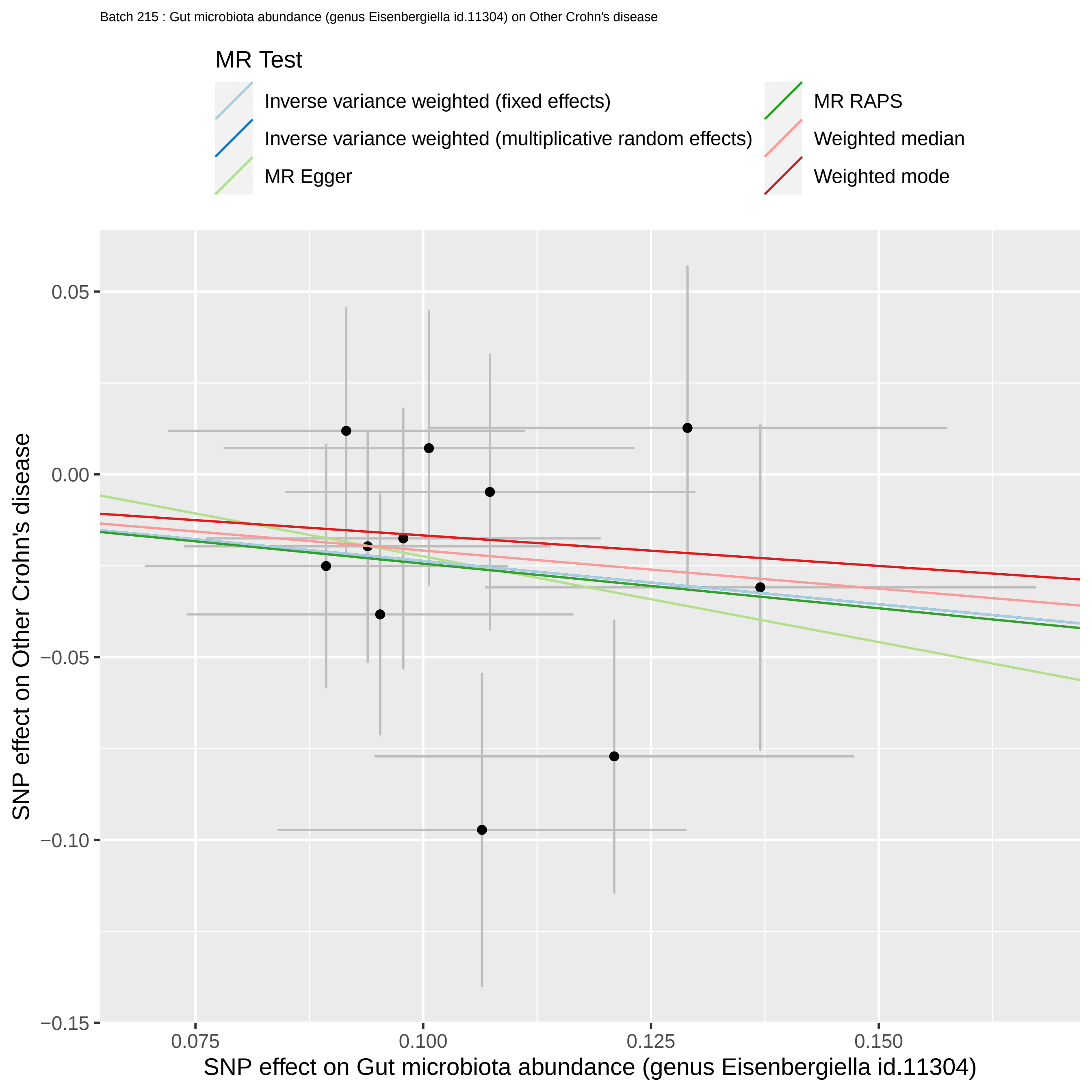
Batch 215 : Gut microbiota abundance (genus Eisenbergiella id.11304) on Other Crohn's disease
MR Test
Inverse variance weighted (fixed effects)
Inverse variance weighted (multiplicative random effects)
MR Egger
MR RAPS
Weighted median
Weighted mode
0.05
0.00
−0.05
−0.10
−0.15
0.075
0.100
0.125
0.150
SNP effect on Other Crohn's disease
SNP effect on Gut microbiota abundance (genus Eisenbergiella id.11304)

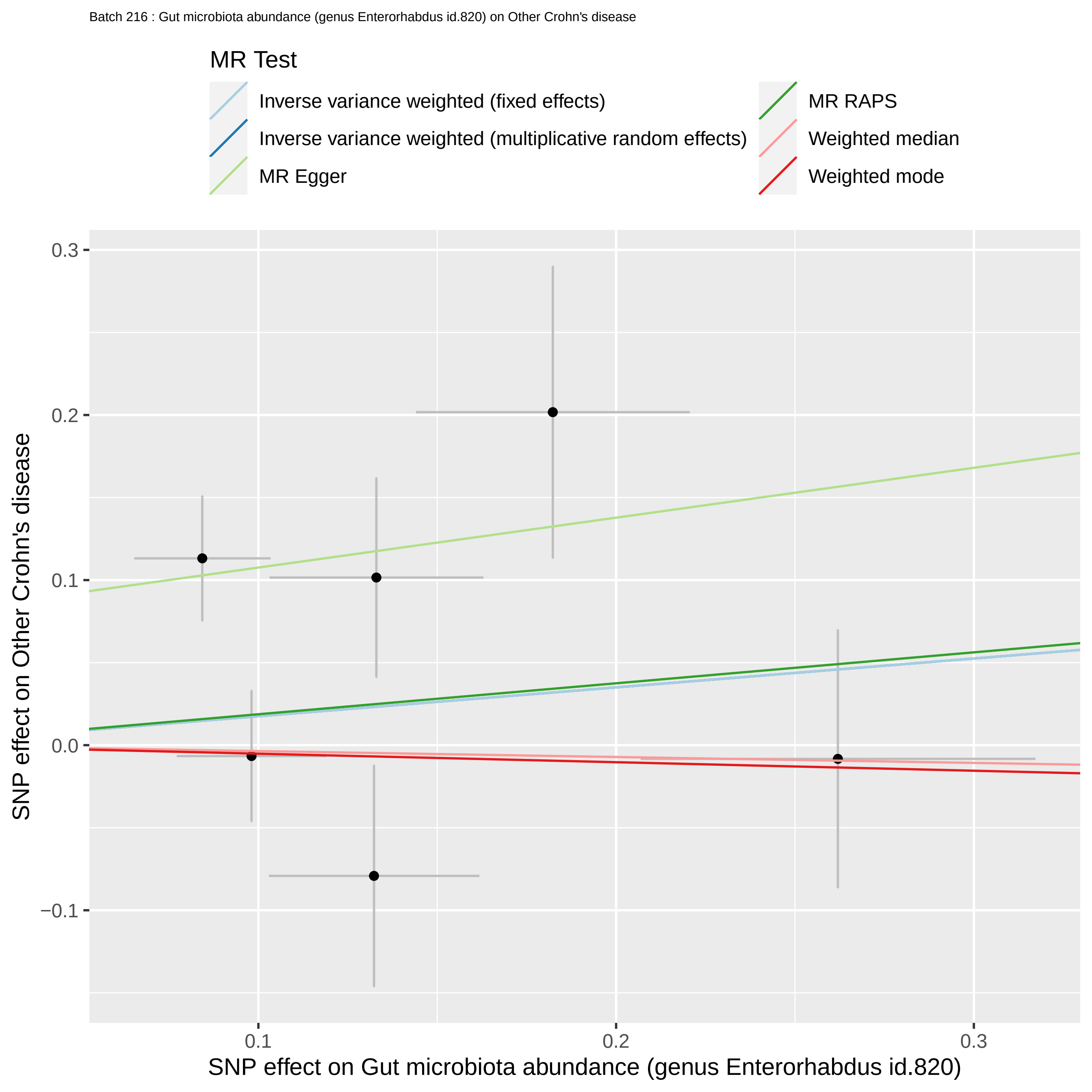

Batch 216 : Gut microbiota abundance (genus Enterorhabdus id.820) on Other Crohn's disease
MR Test
Inverse variance weighted (fixed effects)
Inverse variance weighted (multiplicative random effects)
MR Egger
MR RAPS
Weighted median
Weighted mode
0.3
0.2
0.1
0.0
−0.1
0.1
0.2
0.3
SNP effect on Other Crohn's disease
SNP effect on Gut microbiota abundance (genus Enterorhabdus id.820)

Batch 217 : Gut microbiota abundance (genus Erysipelatoclostridium id.11381) on Other Crohn's disease

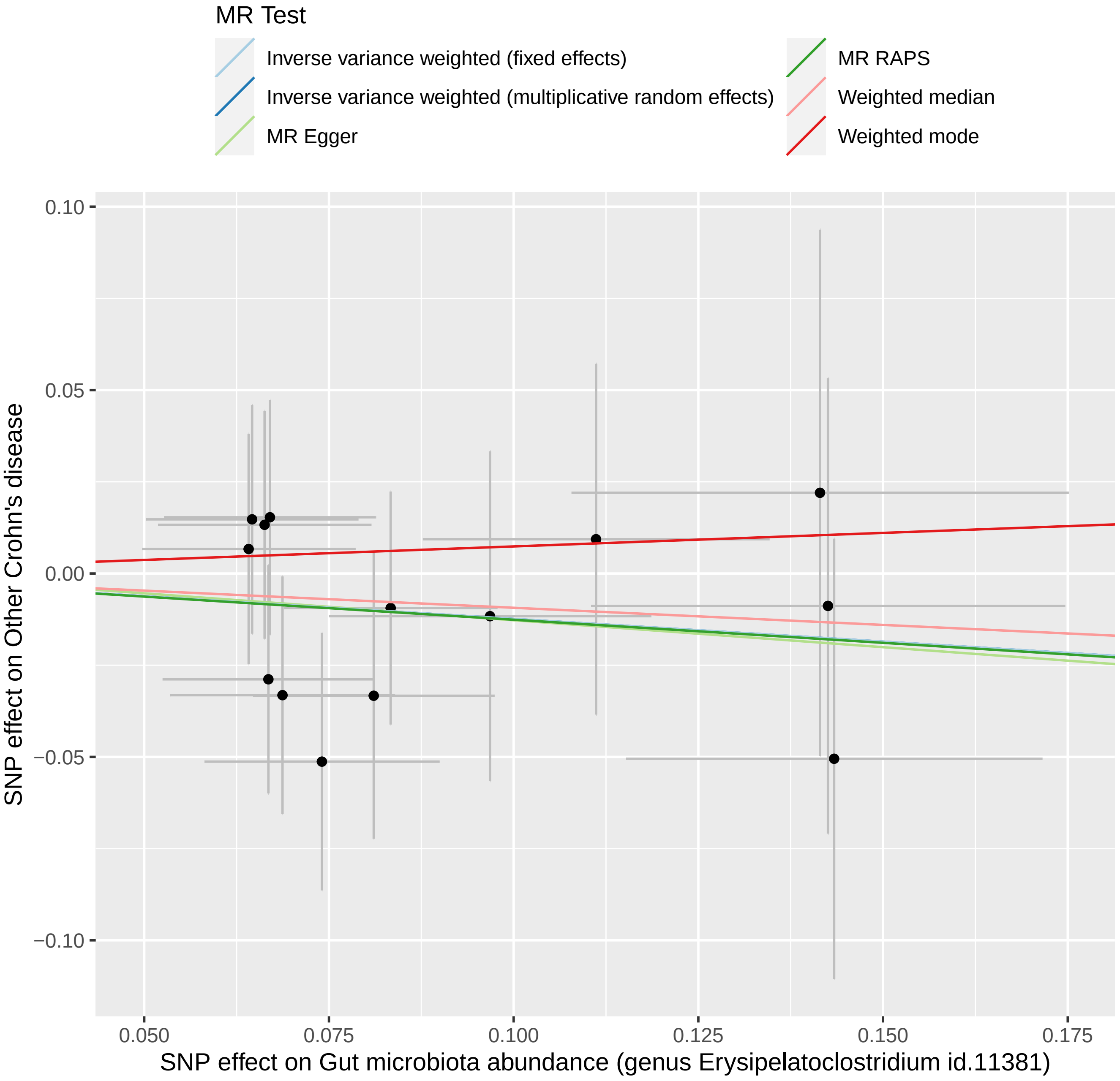

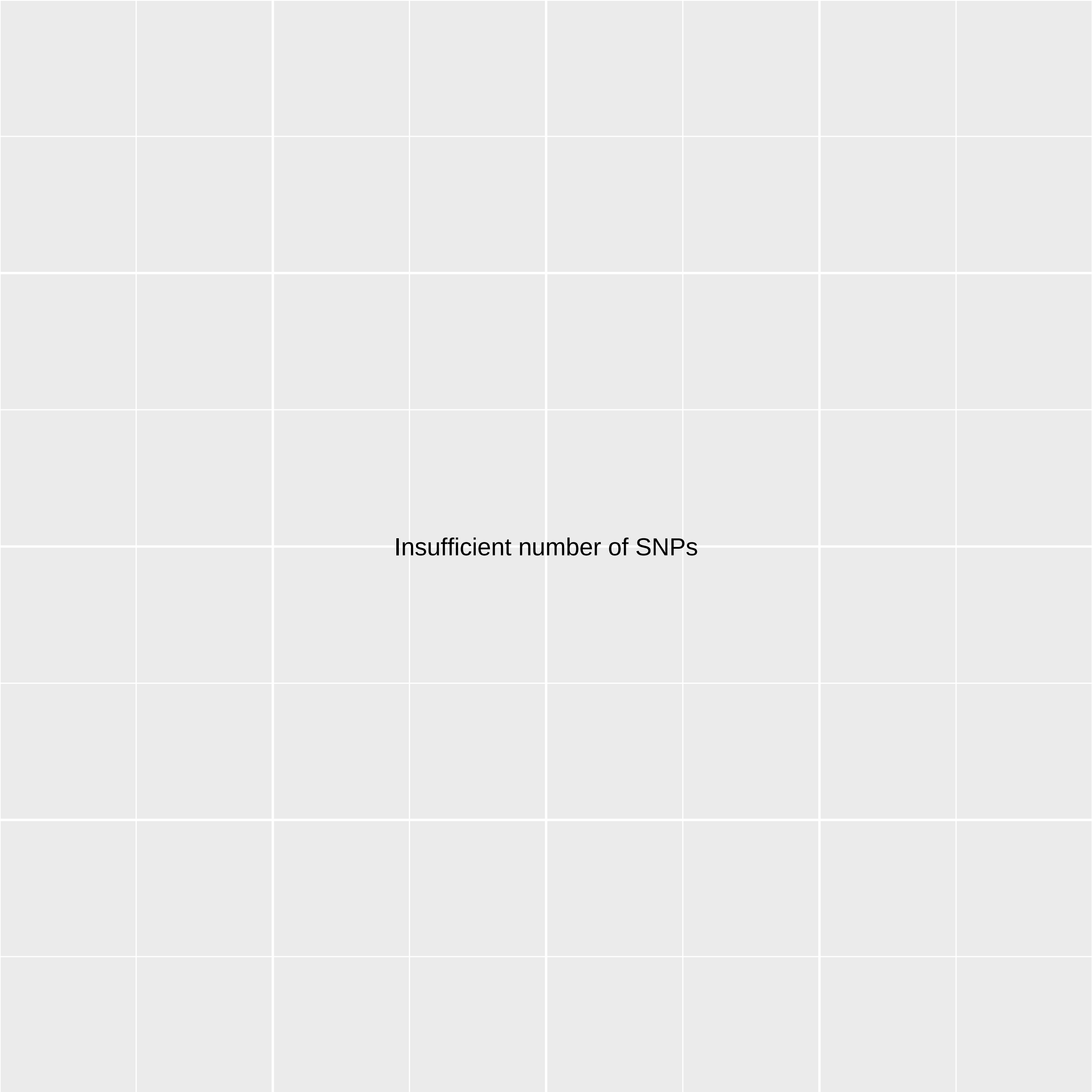
Insufficient number of SNPs

Batch 219 : Gut microbiota abundance (genus Escherichia Shigella id.3504) on Other Crohn's disease

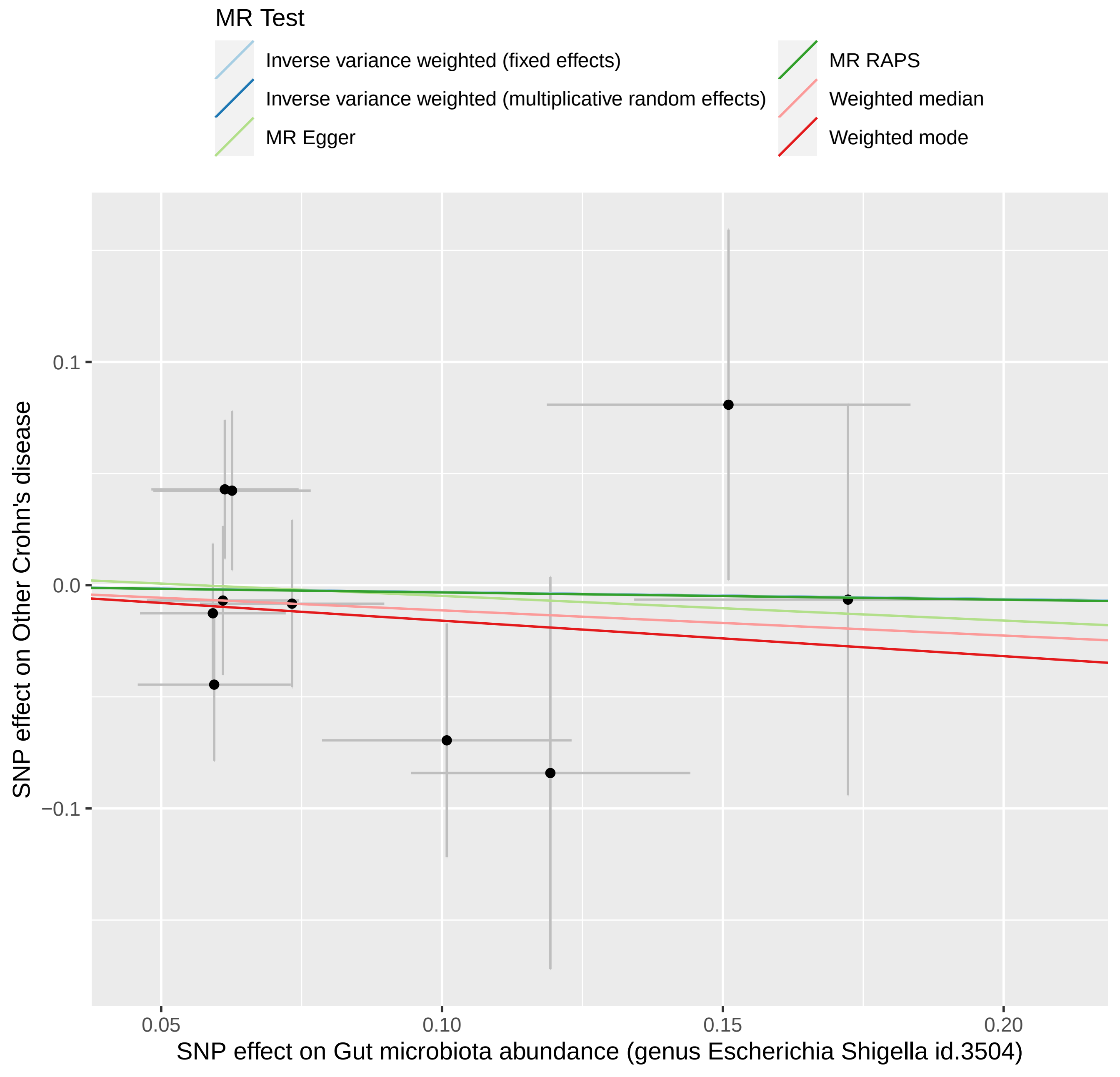

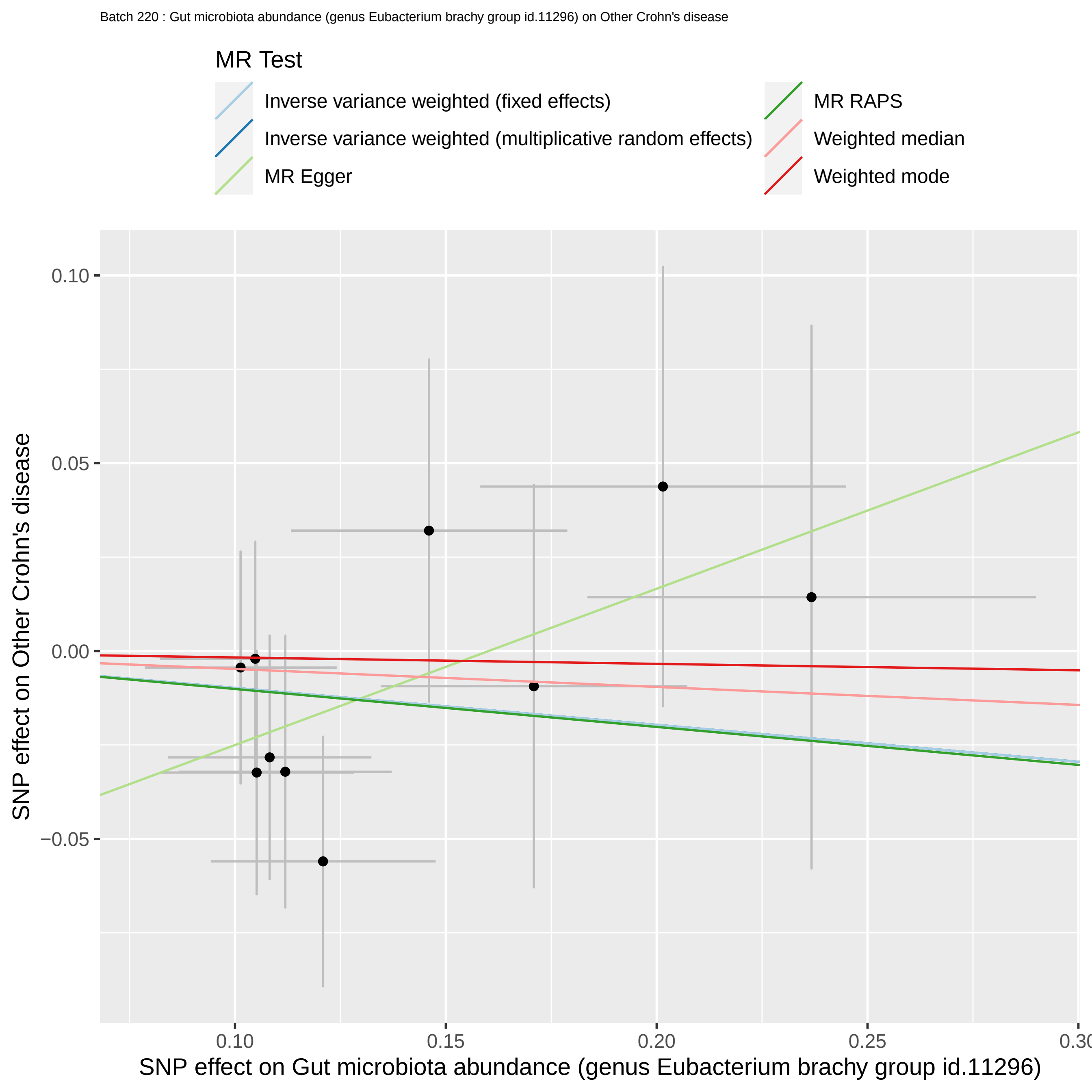
Batch 220 : Gut microbiota abundance (genus Eubacterium brachy group id.11296) on Other Crohn's disease
MR Test
Inverse variance weighted (fixed effects)
Inverse variance weighted (multiplicative random effects)
MR Egger
MR RAPS
Weighted median
Weighted mode
0.10
0.05
0.00
−0.05
0.10
0.15
0.20
0.25
0.30
SNP effect on Other Crohn's disease
SNP effect on Gut microbiota abundance (genus Eubacterium brachy group id.11296)

Batch 221 : Gut microbiota abundance (genus Eubacterium coprostanoligenes group id.11375) on Other Crohn's disease

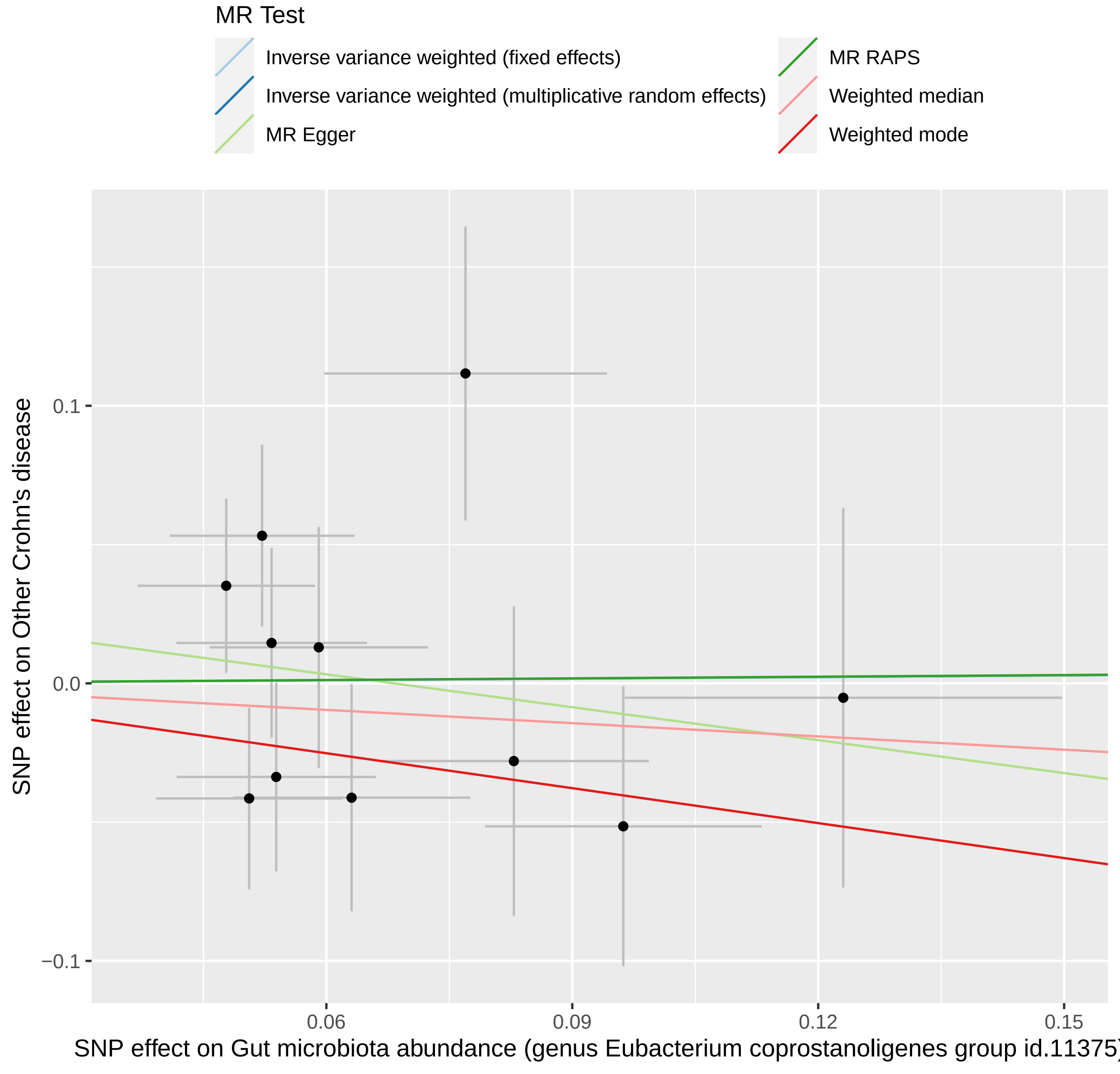

Batch 222 : Gut microbiota abundance (genus Eubacterium eligens group id.14372) on Other Crohn's disease

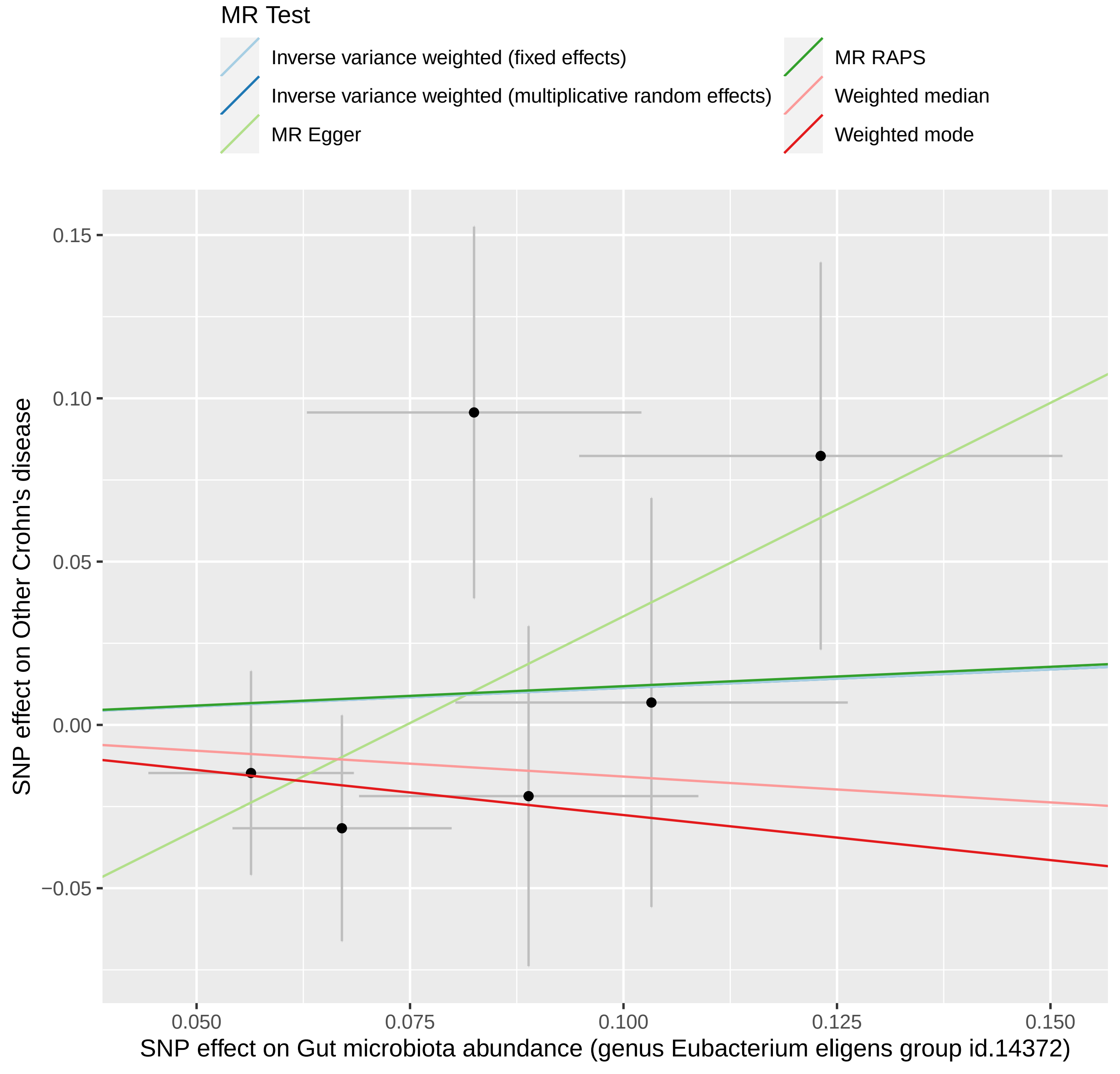

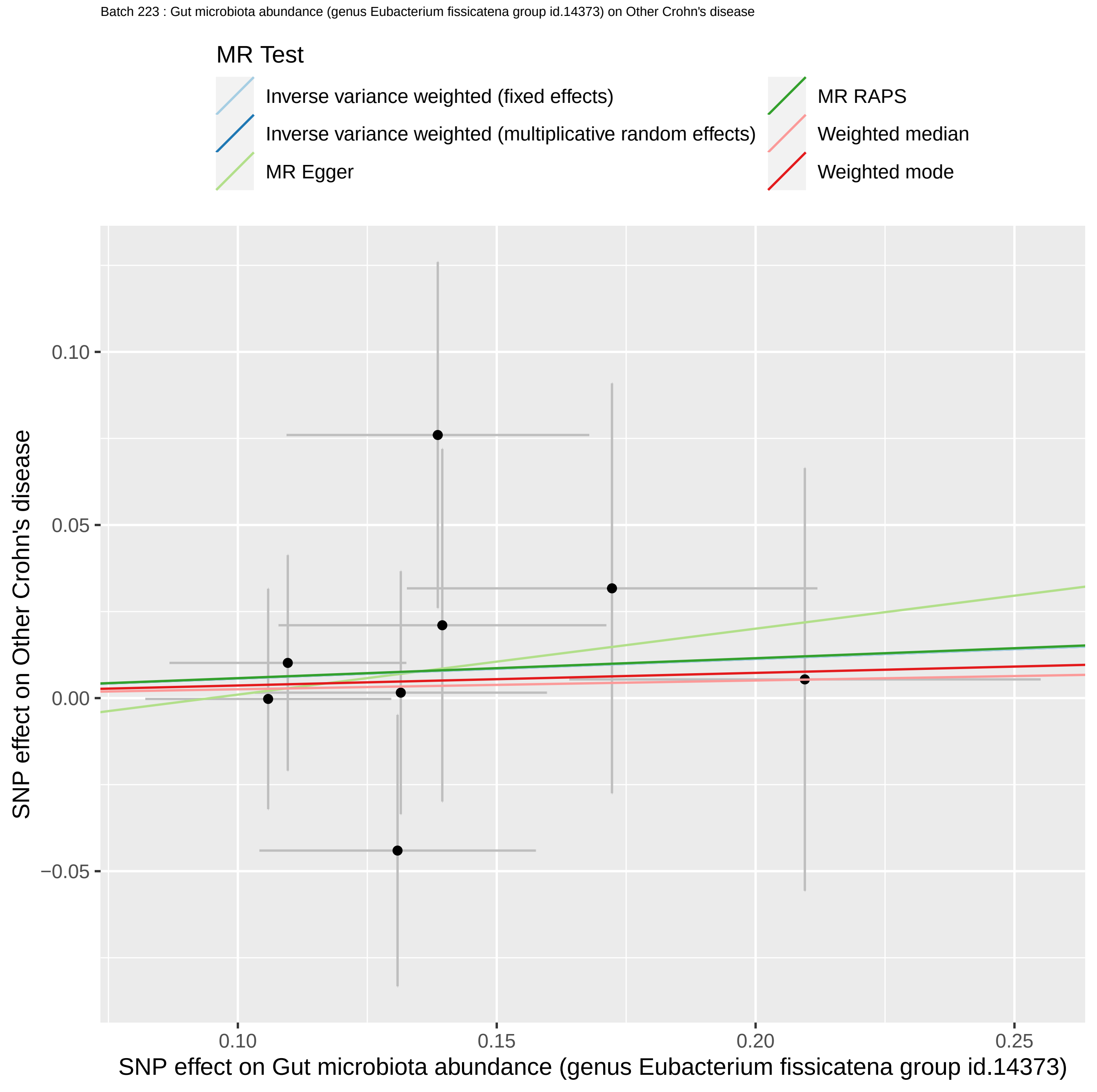

Batch 223 : Gut microbiota abundance (genus Eubacterium fissicatena group id.14373) on Other Crohn's disease
MR Test
Inverse variance weighted (fixed effects)
Inverse variance weighted (multiplicative random effects)
MR Egger
MR RAPS
Weighted median
Weighted mode
0.10
0.05
0.00
−0.05
0.10
0.15
0.20
0.25
SNP effect on Other Crohn's disease
SNP effect on Gut microbiota abundance (genus Eubacterium fissicatena group id.14373)

Batch 224 : Gut microbiota abundance (genus Eubacterium hallii group id.11338) on Other Crohn's disease

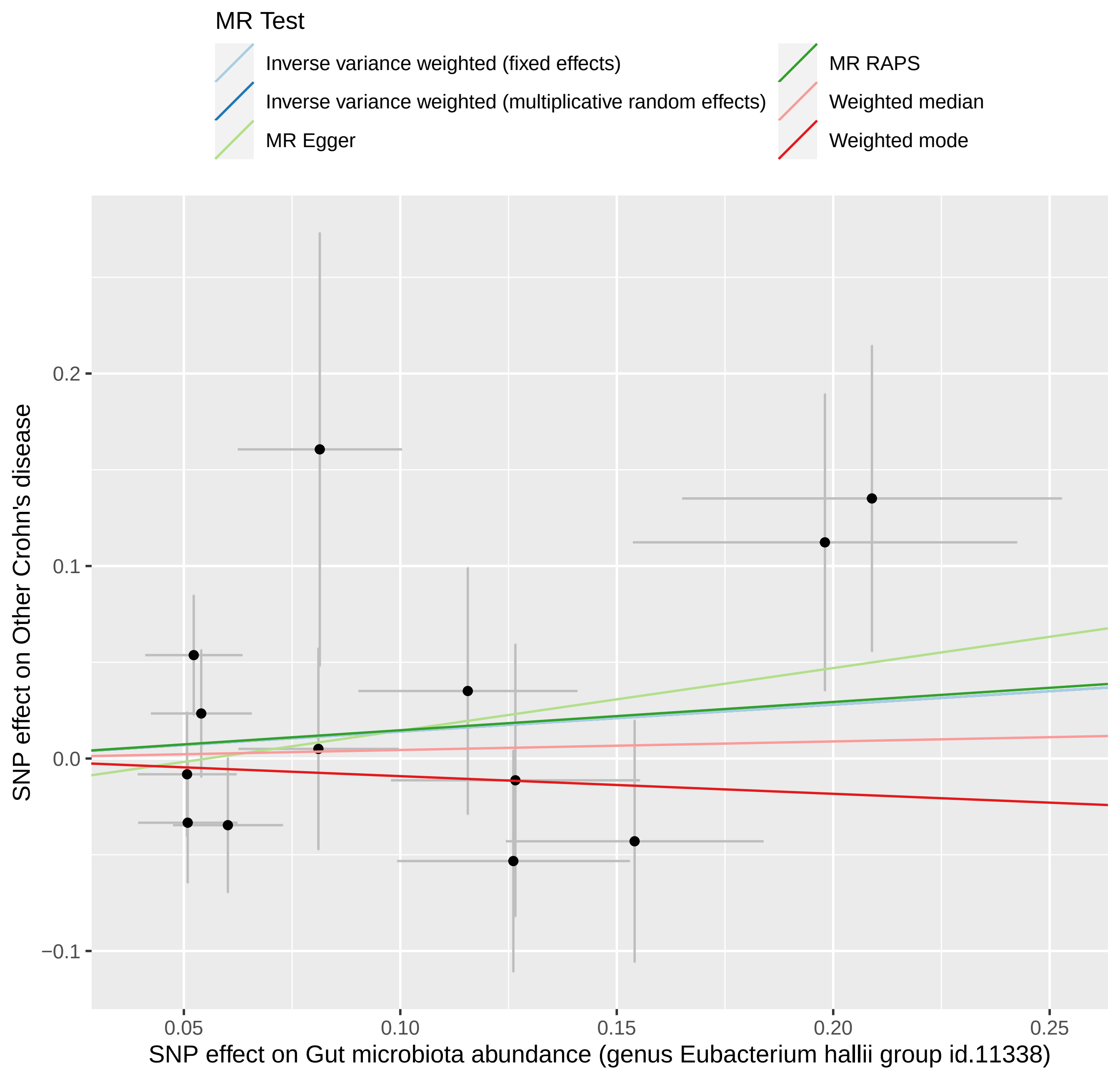

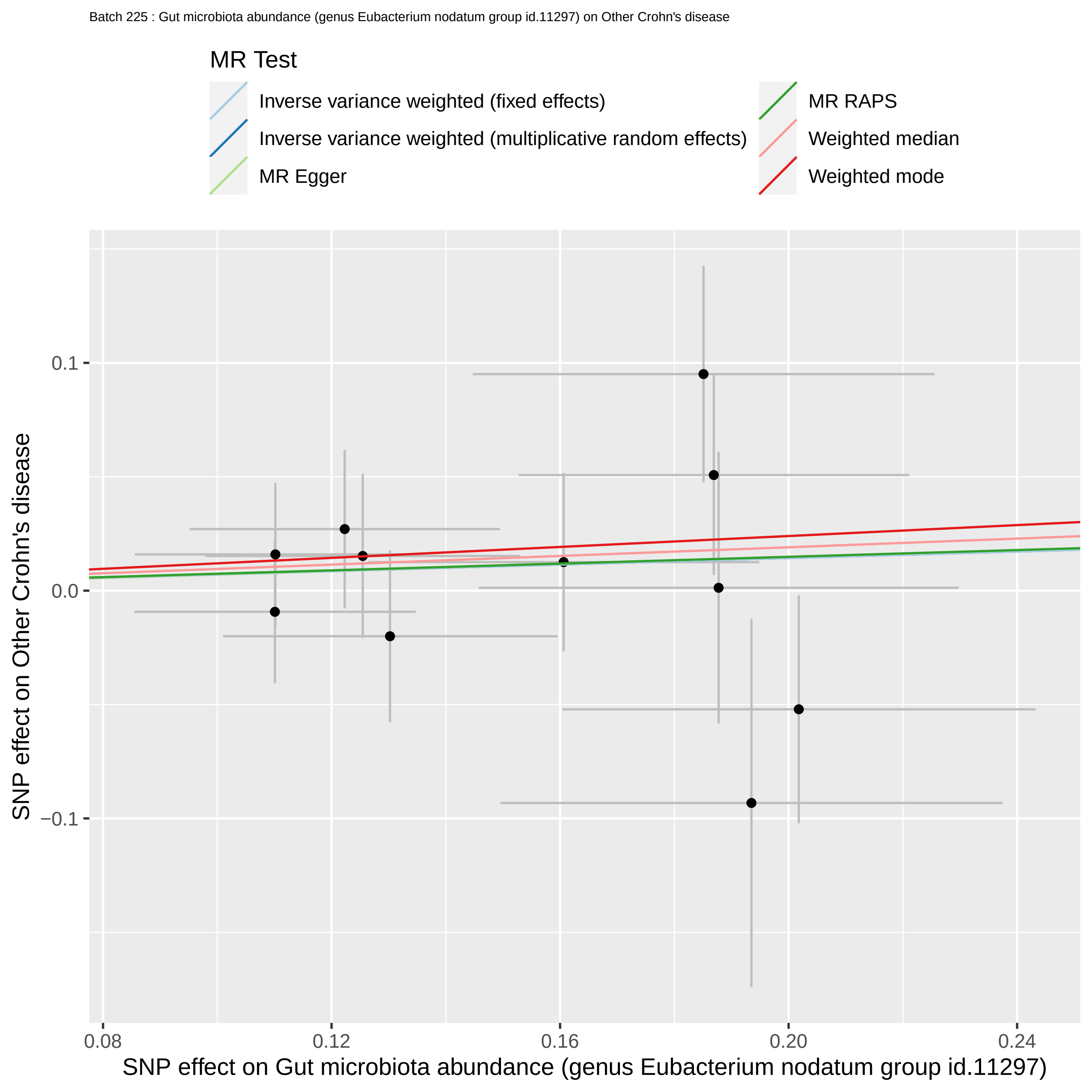

Batch 225 : Gut microbiota abundance (genus Eubacterium nodatum group id.11297) on Other Crohn's disease
MR Test
Inverse variance weighted (fixed effects)
Inverse variance weighted (multiplicative random effects)
MR Egger
MR RAPS
Weighted median
Weighted mode
0.1
0.0
−0.1
0.08
0.12
0.16
0.20
0.24
SNP effect on Other Crohn's disease
SNP effect on Gut microbiota abundance (genus Eubacterium nodatum group id.11297)

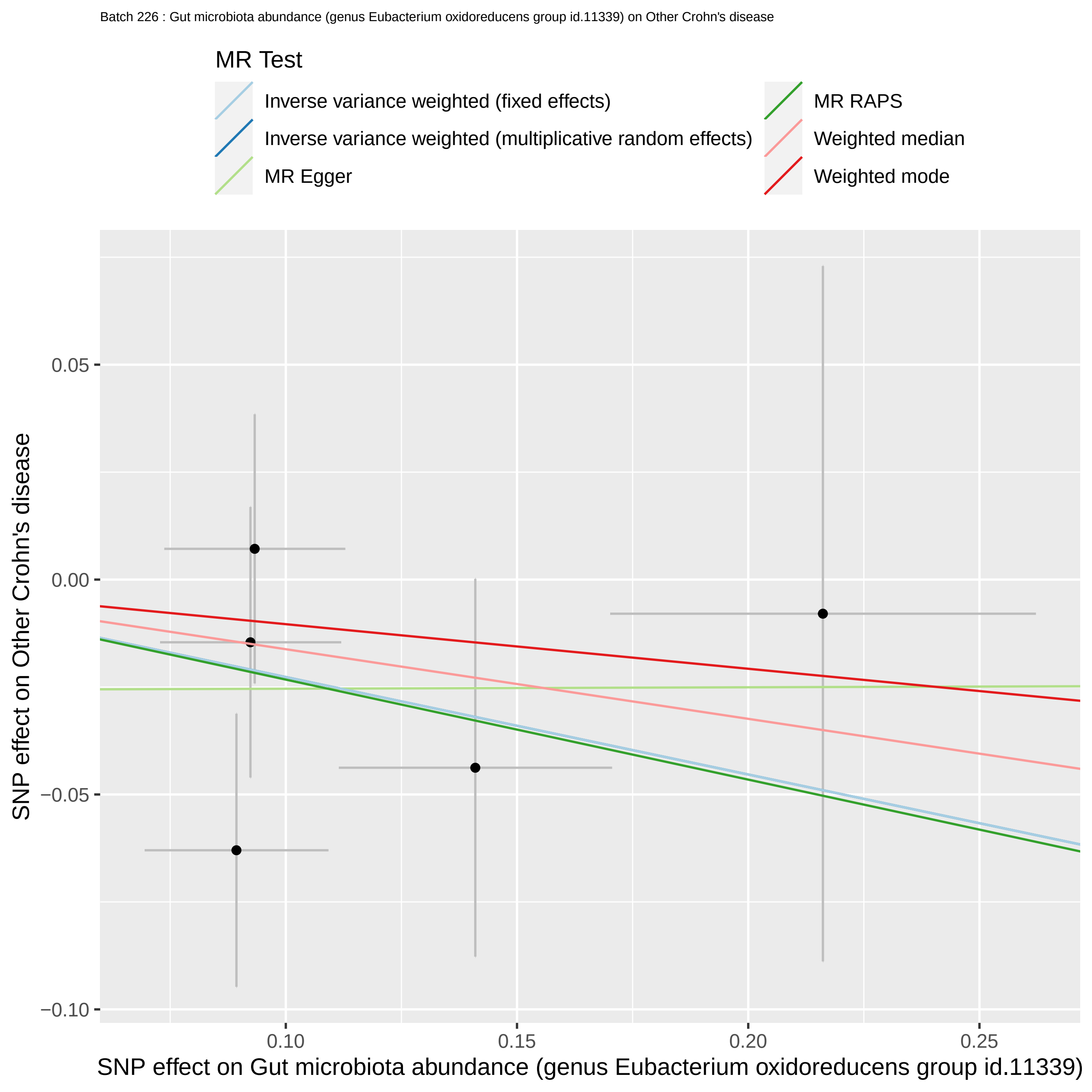
Batch 226 : Gut microbiota abundance (genus Eubacterium oxidoreducens group id.11339) on Other Crohn's disease
MR Test
Inverse variance weighted (fixed effects)
Inverse variance weighted (multiplicative random effects)
MR Egger
MR RAPS
Weighted median
Weighted mode
0.05
0.00
−0.05
−0.10
0.10
0.15
0.20
0.25
SNP effect on Other Crohn's disease
SNP effect on Gut microbiota abundance (genus Eubacterium oxidoreducens group id.11339)

Batch 227 : Gut microbiota abundance (genus Eubacterium rectale group id.14374) on Other Crohn's disease

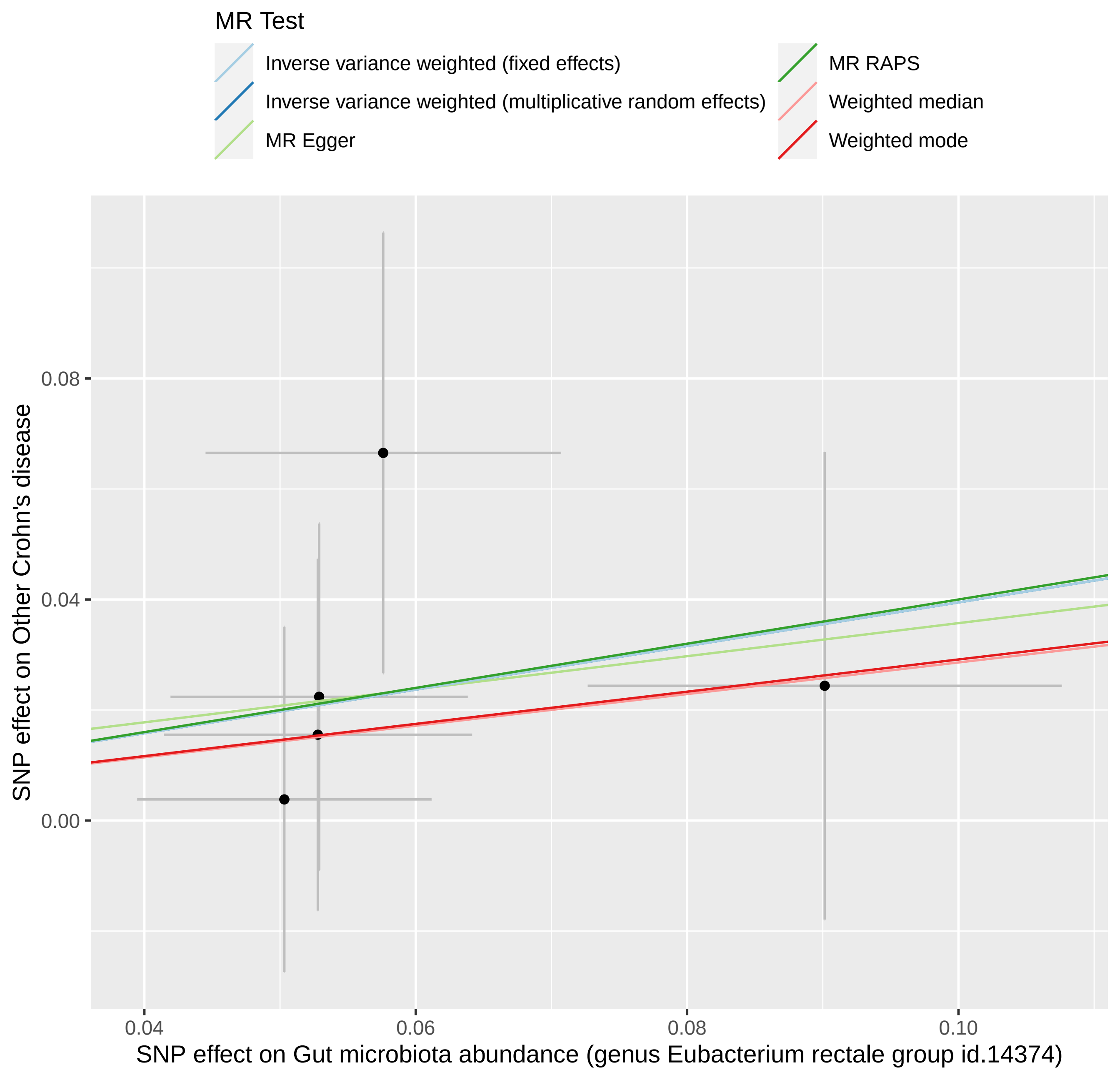

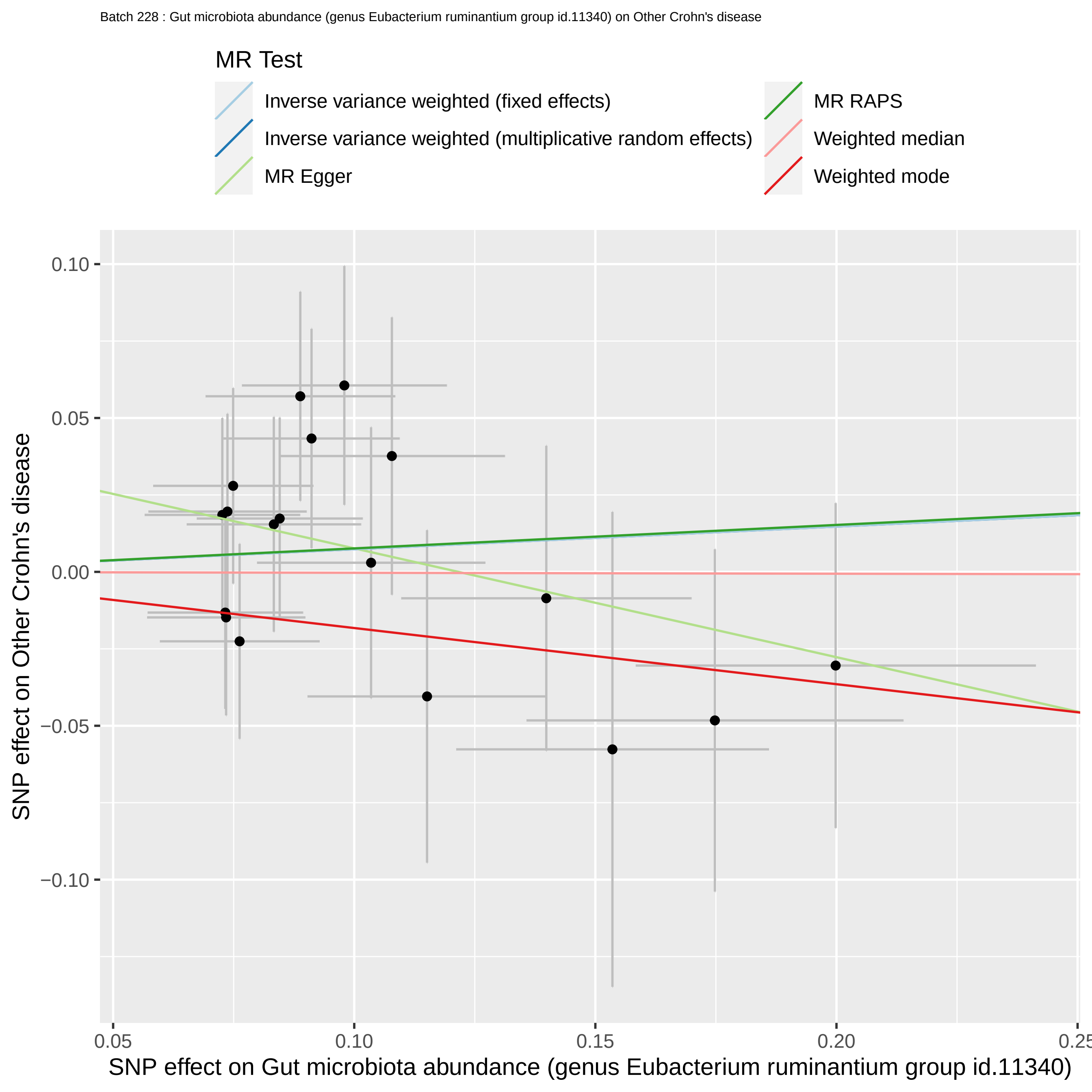

Batch 228 : Gut microbiota abundance (genus Eubacterium ruminantium group id.11340) on Other Crohn's disease
MR Test
Inverse variance weighted (fixed effects)
Inverse variance weighted (multiplicative random effects)
MR Egger
MR RAPS
Weighted median
Weighted mode
0.10
0.05
0.00
−0.05
−0.10
SNP effect on Other Crohn's disease
0.05
0.10
0.15
0.20
0.25
SNP effect on Gut microbiota abundance (genus Eubacterium ruminantium group id.11340)

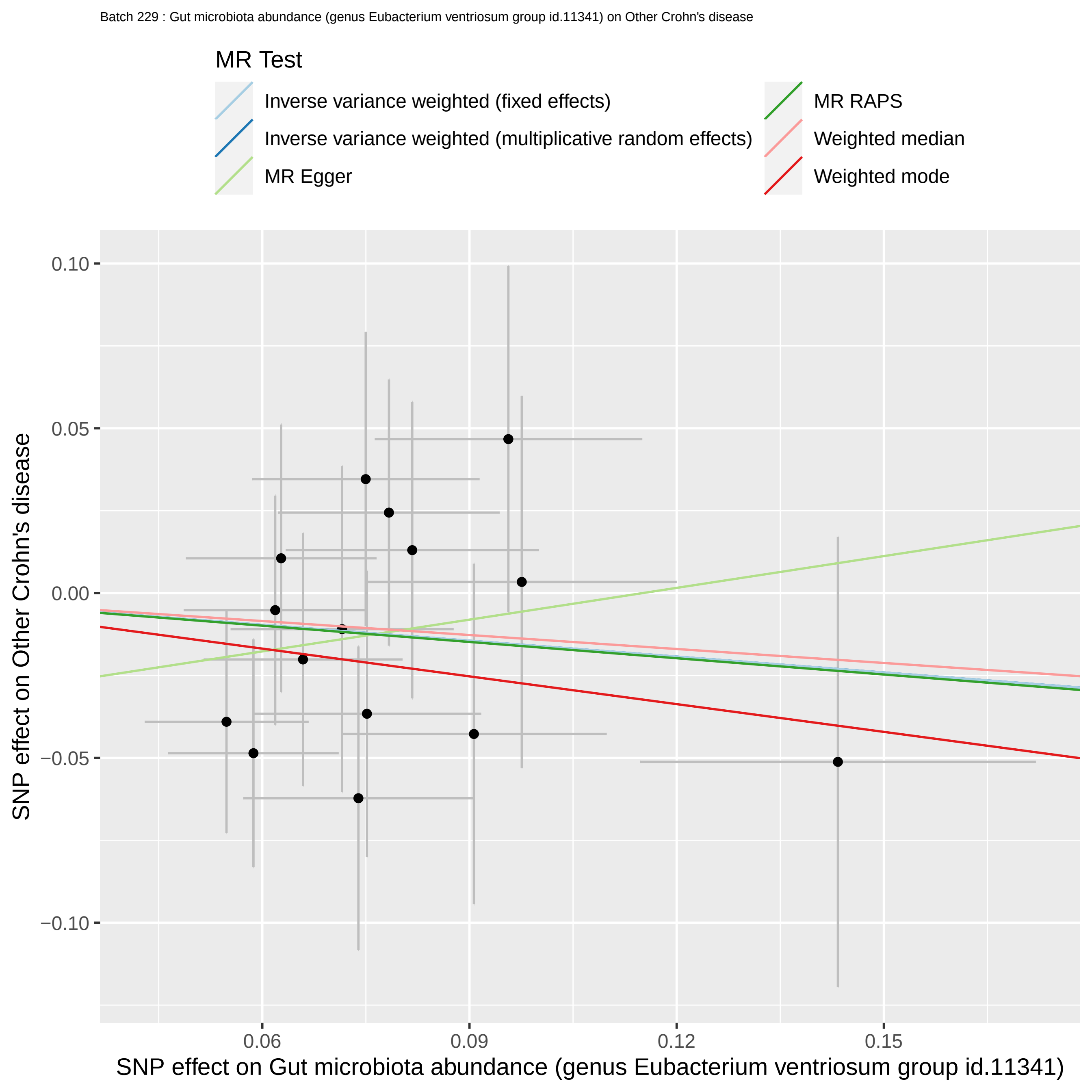
Batch 229 : Gut microbiota abundance (genus Eubacterium ventriosum group id.11341) on Other Crohn's disease
MR Test
Inverse variance weighted (fixed effects)
Inverse variance weighted (multiplicative random effects)
MR Egger
MR RAPS
Weighted median
Weighted mode
0.10
0.05
0.00
−0.05
−0.10
0.06
0.09
0.12
0.15
SNP effect on Other Crohn's disease
SNP effect on Gut microbiota abundance (genus Eubacterium ventriosum group id.11341)

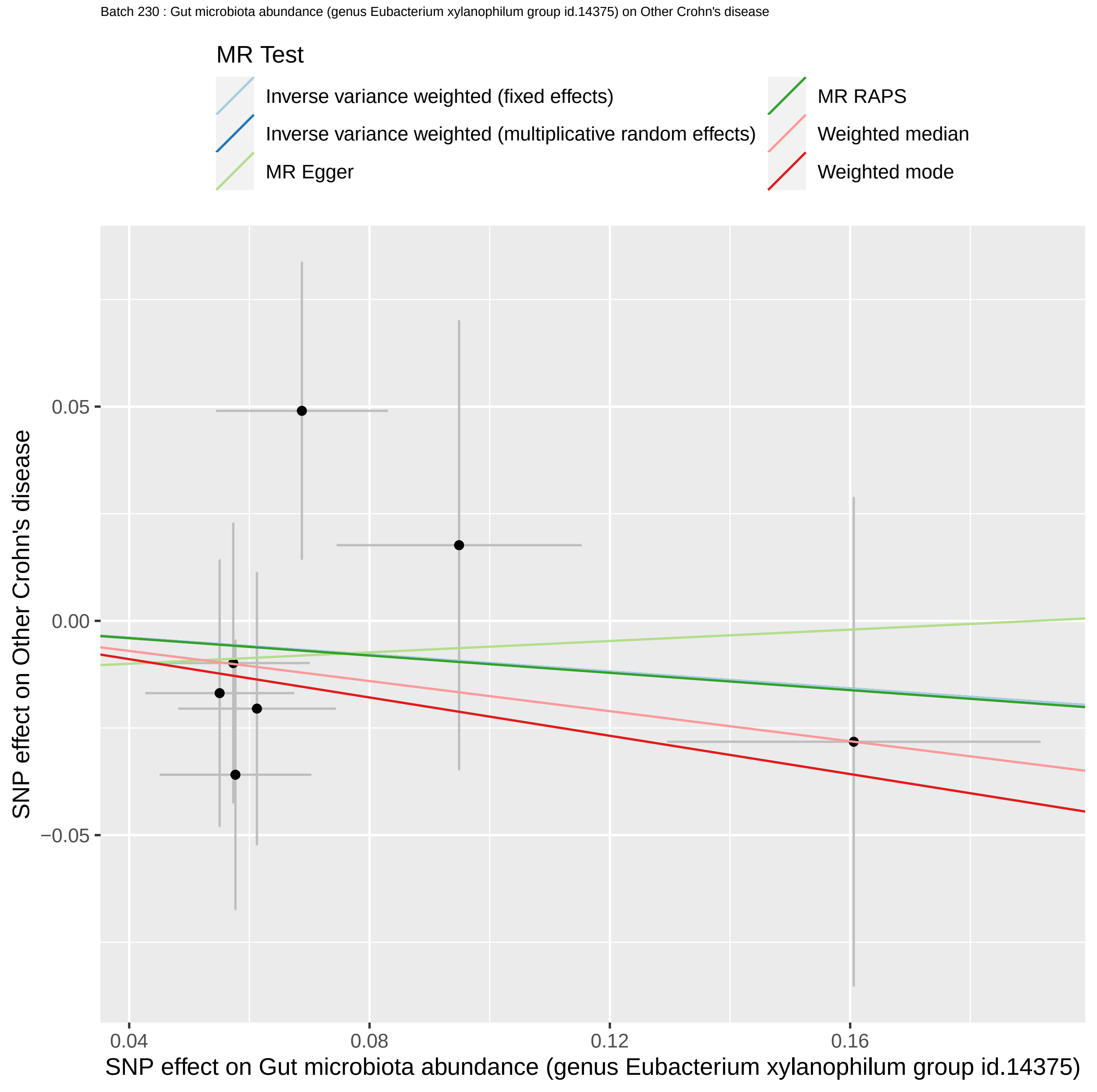

Batch 230 : Gut microbiota abundance (genus Eubacterium xylanophilum group id.14375) on Other Crohn's disease
MR Test
Inverse variance weighted (fixed effects)
Inverse variance weighted (multiplicative random effects)
MR Egger
MR RAPS
Weighted median
Weighted mode
0.05
0.00
−0.05
0.04
0.08
0.12
0.16
SNP effect on Other Crohn's disease
SNP effect on Gut microbiota abundance (genus Eubacterium xylanophilum group id.14375)

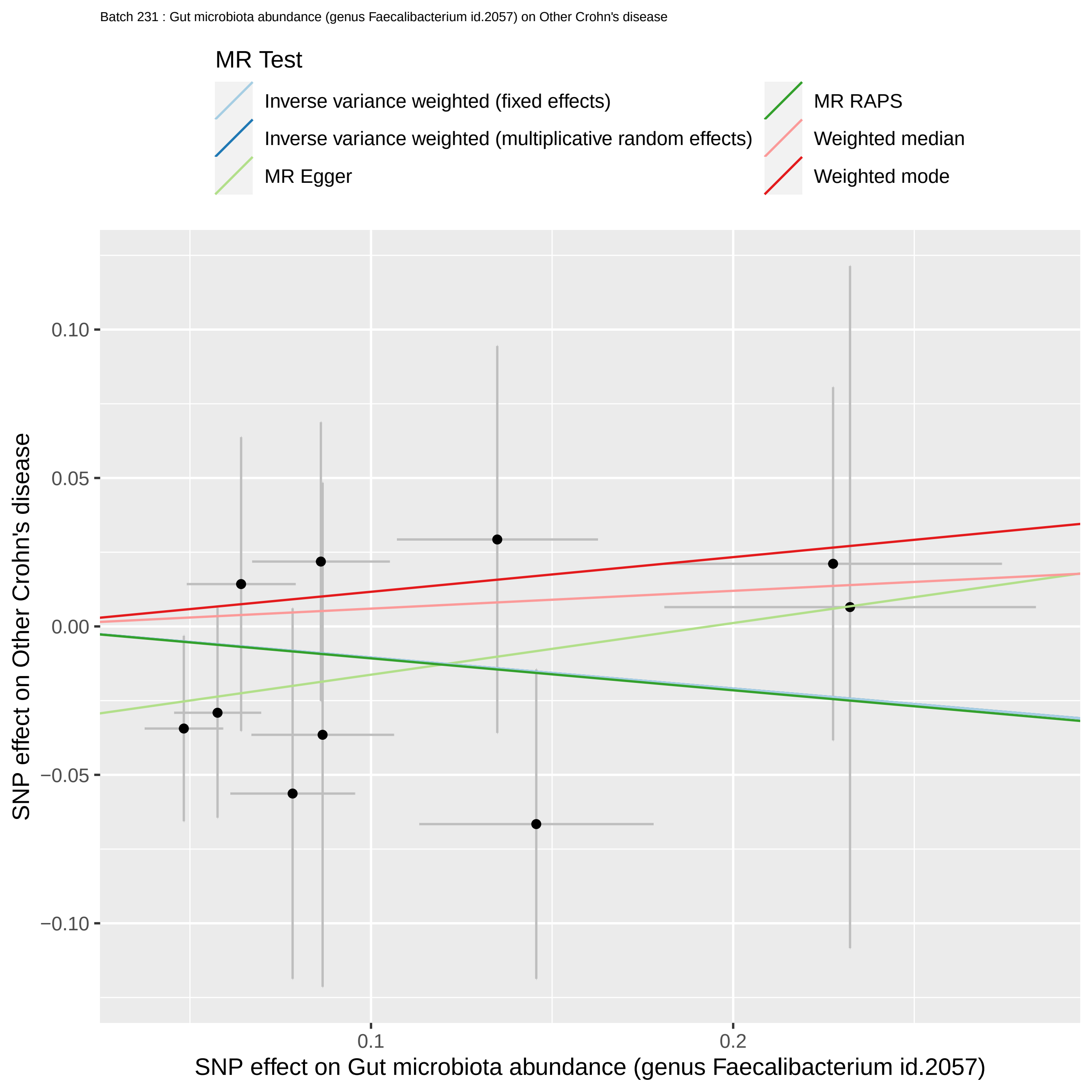

Batch 231 : Gut microbiota abundance (genus Faecalibacterium id.2057) on Other Crohn's disease
MR Test
Inverse variance weighted (fixed effects)
Inverse variance weighted (multiplicative random effects)
MR Egger
MR RAPS
Weighted median
Weighted mode
0.10
0.05
0.00
−0.05
−0.10
0.1
0.2
SNP effect on Other Crohn's disease
SNP effect on Gut microbiota abundance (genus Faecalibacterium id.2057)

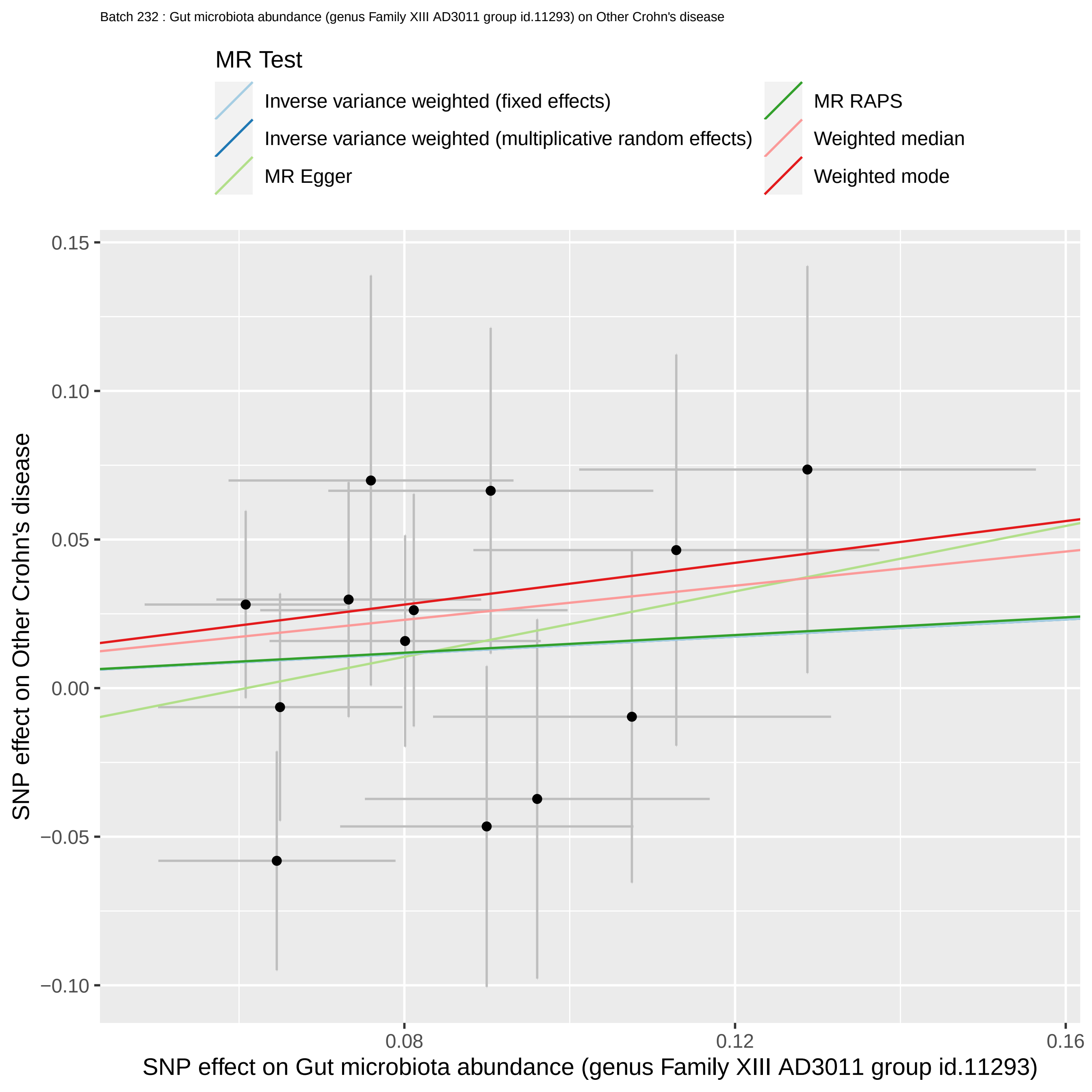
Batch 232 : Gut microbiota abundance (genus Family XIII AD3011 group id.11293) on Other Crohn's disease
MR Test
Inverse variance weighted (fixed effects)
Inverse variance weighted (multiplicative random effects)
MR Egger
MR RAPS
Weighted median
Weighted mode
0.15
0.10
0.05
0.00
−0.05
−0.10
0.08
0.12
0.16
SNP effect on Other Crohn's disease
SNP effect on Gut microbiota abundance (genus Family XIII AD3011 group id.11293)

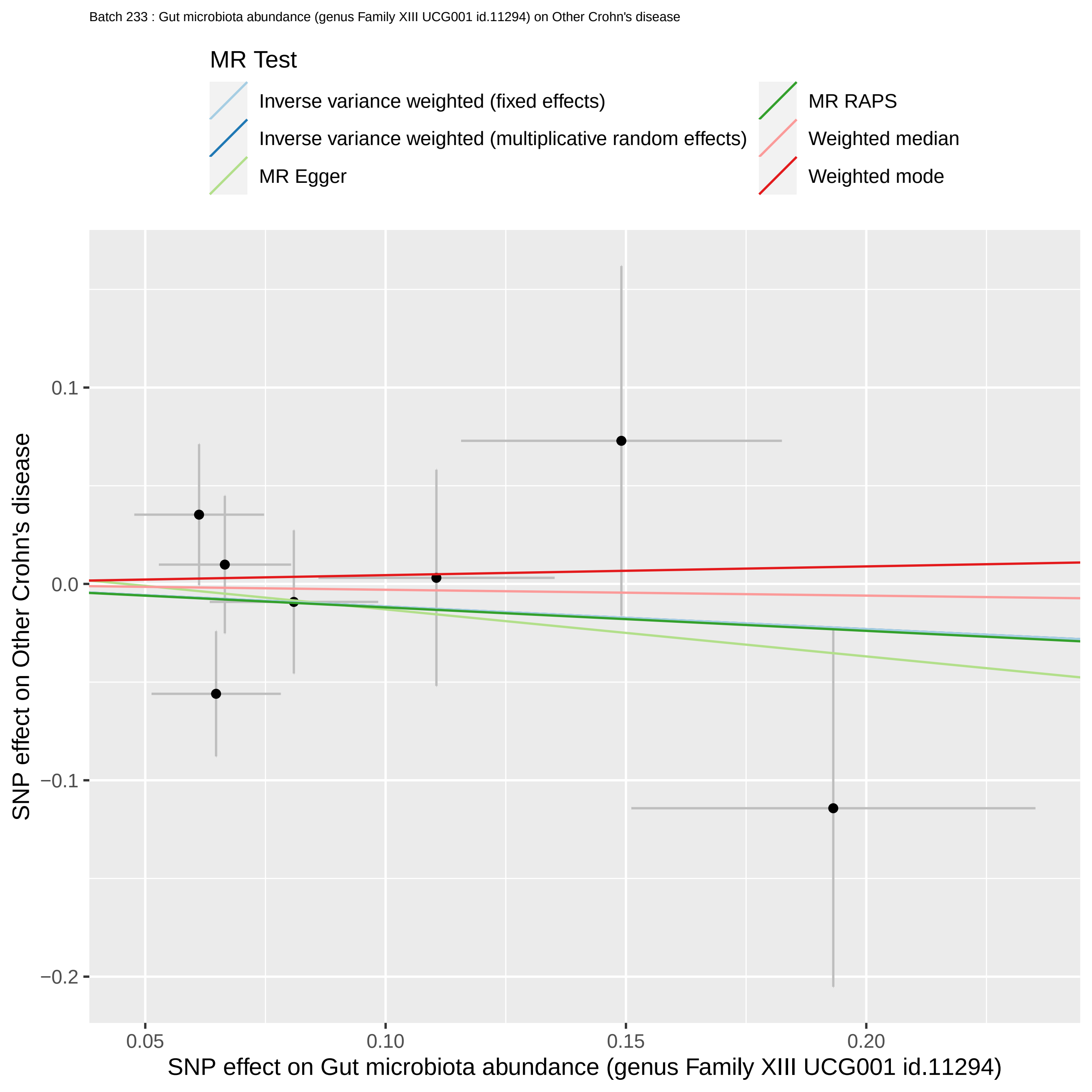

Batch 233 : Gut microbiota abundance (genus Family XIII UCG001 id.11294) on Other Crohn's disease
MR Test
Inverse variance weighted (fixed effects)
Inverse variance weighted (multiplicative random effects)
MR Egger
MR RAPS
Weighted median
Weighted mode
SNP effect on Other Crohn's disease
0.1
0.0
−0.1
−0.2
0.05
0.10
0.15
0.20
SNP effect on Gut microbiota abundance (genus Family XIII UCG001 id.11294)

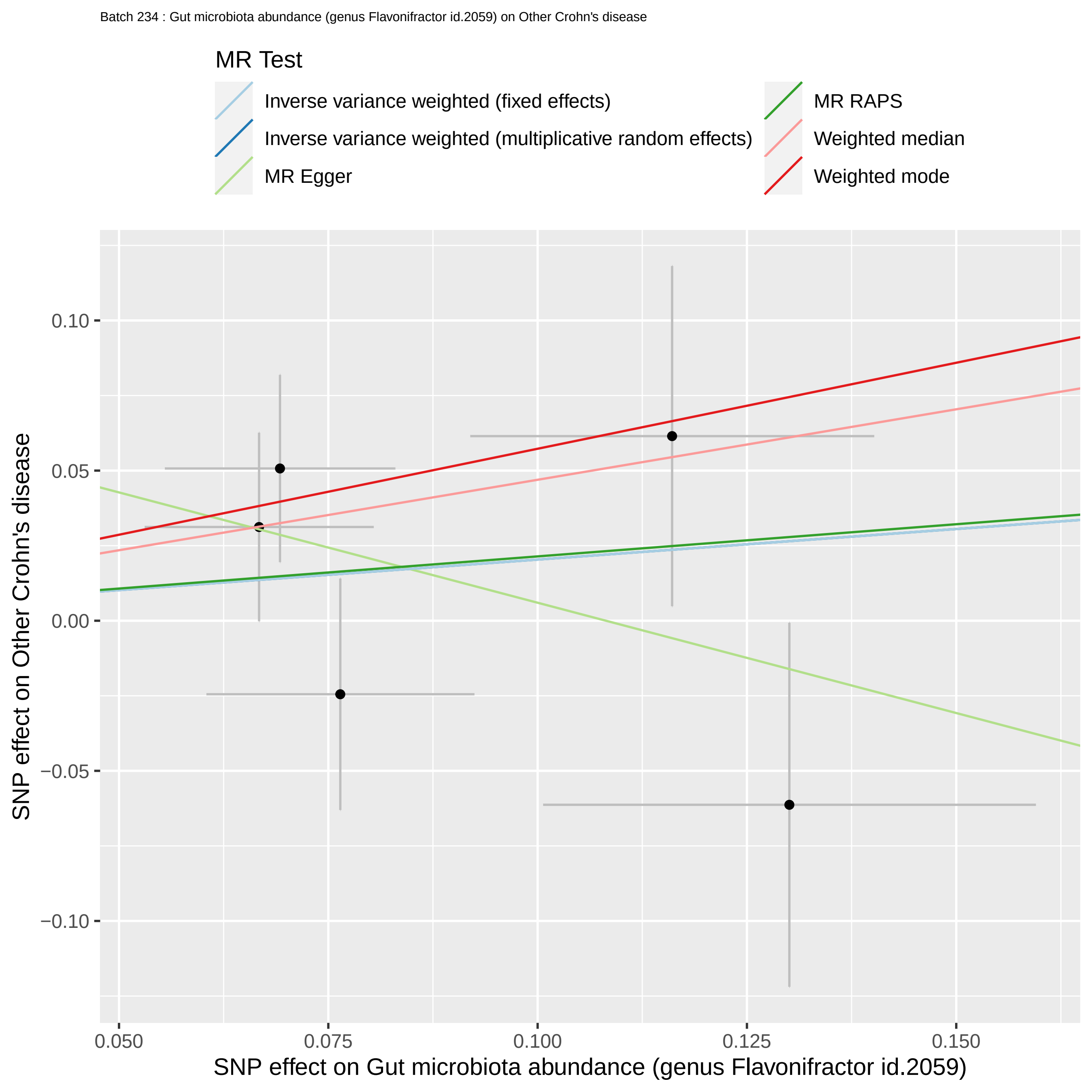
Batch 234 : Gut microbiota abundance (genus Flavonifractor id.2059) on Other Crohn's disease
MR Test
Inverse variance weighted (fixed effects)
Inverse variance weighted (multiplicative random effects)
MR Egger
MR RAPS
Weighted median
Weighted mode
0.10
0.05
0.00
−0.05
−0.10
0.050
0.075
0.100
0.125
0.150
SNP effect on Other Crohn's disease
SNP effect on Gut microbiota abundance (genus Flavonifractor id.2059)

Batch 235 : Gut microbiota abundance (genus Fusicatenibacter id.11305) on Other Crohn's disease

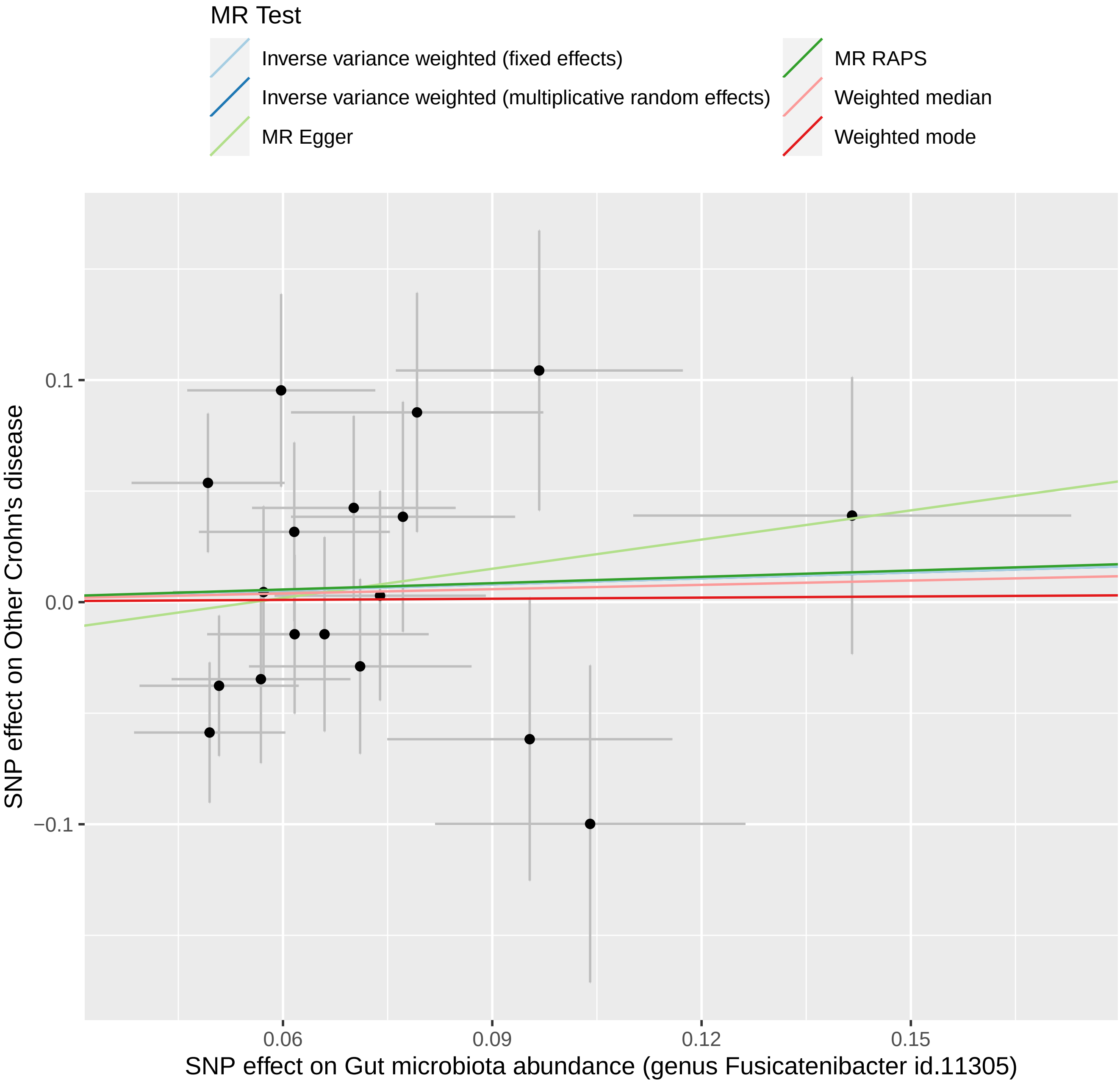

Batch 236 : Gut microbiota abundance (genus Gordonibacter id.821) on Other Crohn's disease

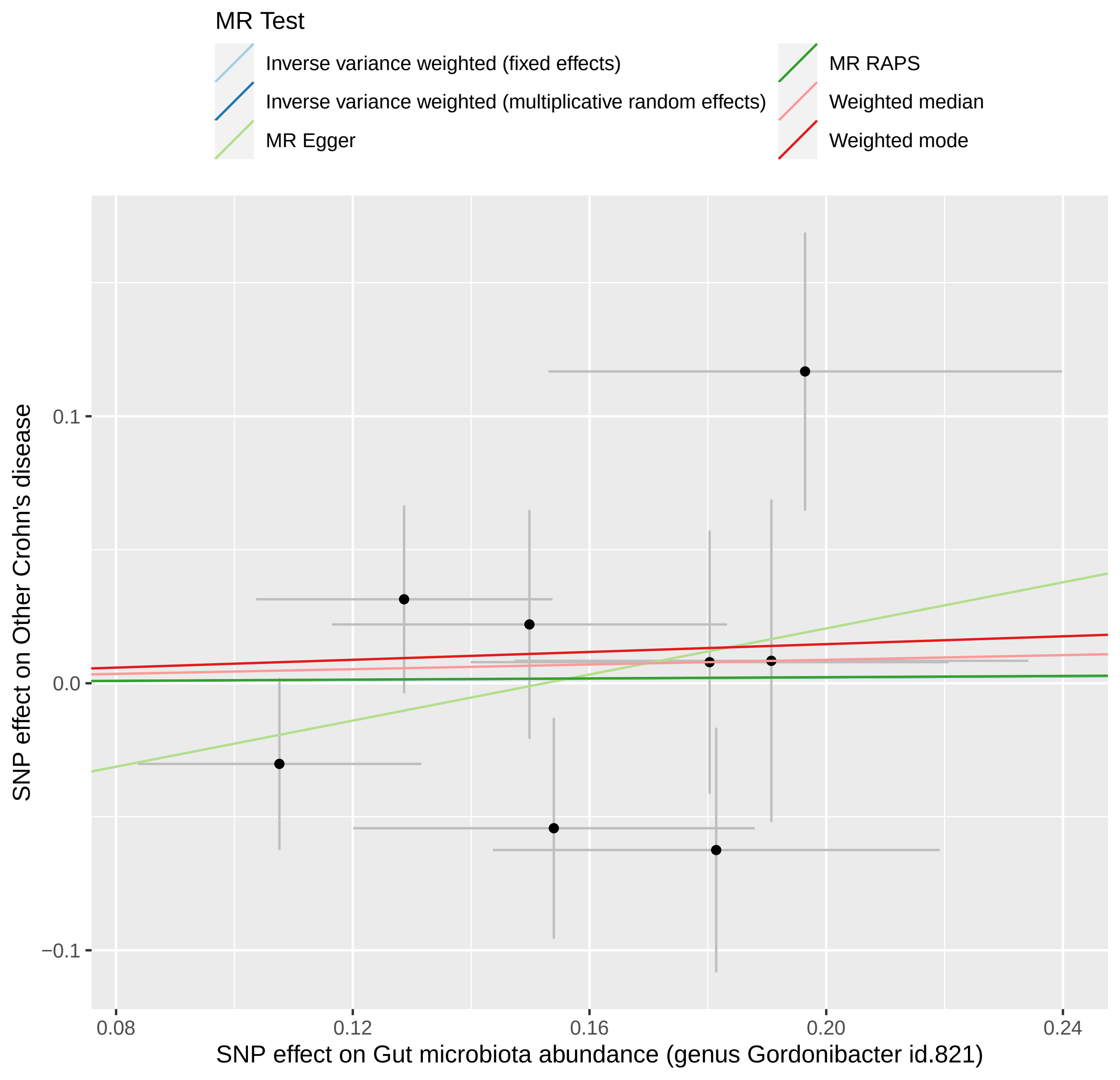

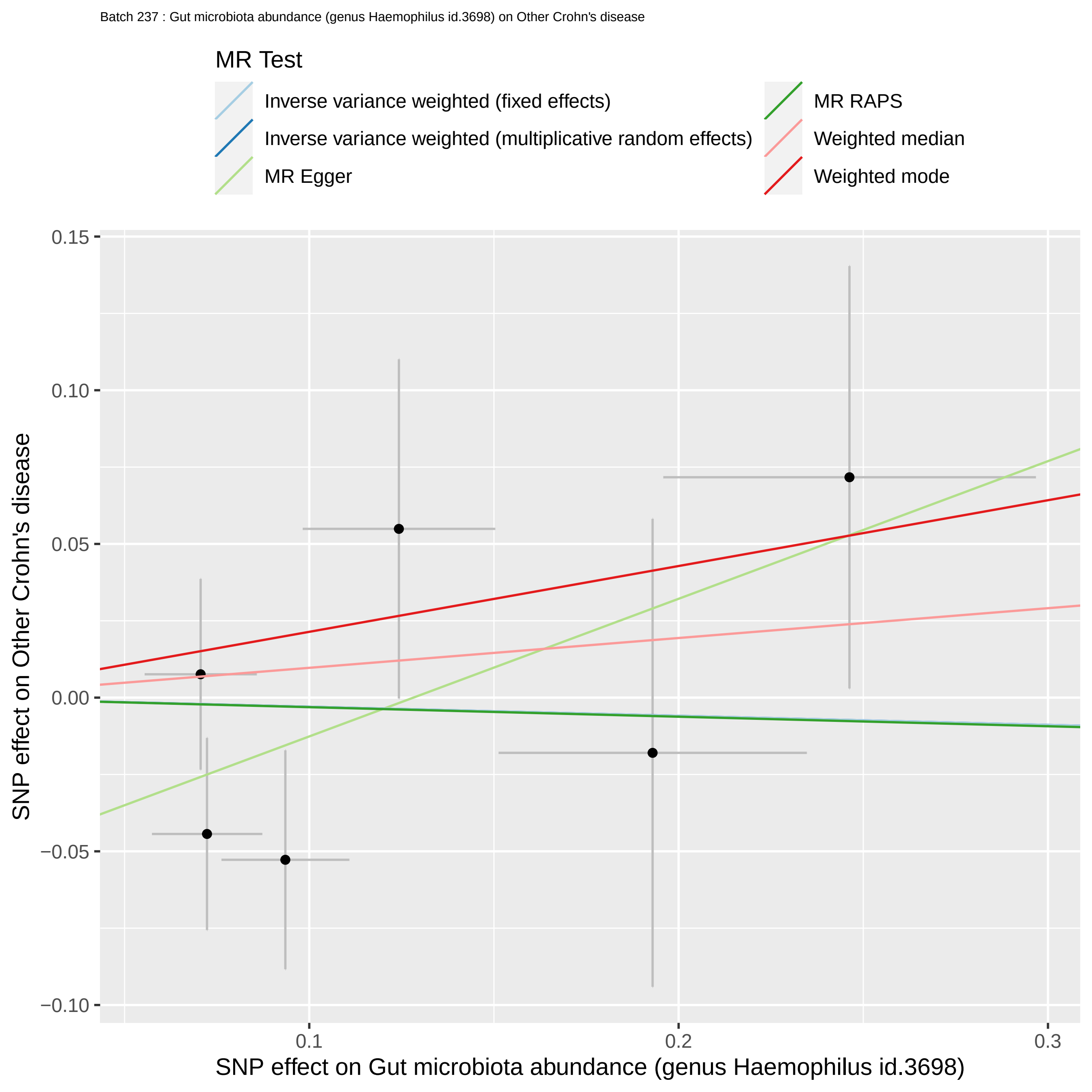
Batch 237 : Gut microbiota abundance (genus Haemophilus id.3698) on Other Crohn's disease
MR Test
Inverse variance weighted (fixed effects)
Inverse variance weighted (multiplicative random effects)
MR Egger
MR RAPS
Weighted median
Weighted mode
0.15
0.10
0.05
0.00
−0.05
−0.10
0.1
0.2
0.3
SNP effect on Other Crohn's disease
SNP effect on Gut microbiota abundance (genus Haemophilus id.3698)

Batch 238 : Gut microbiota abundance (genus Holdemanella id.11393) on Other Crohn's disease

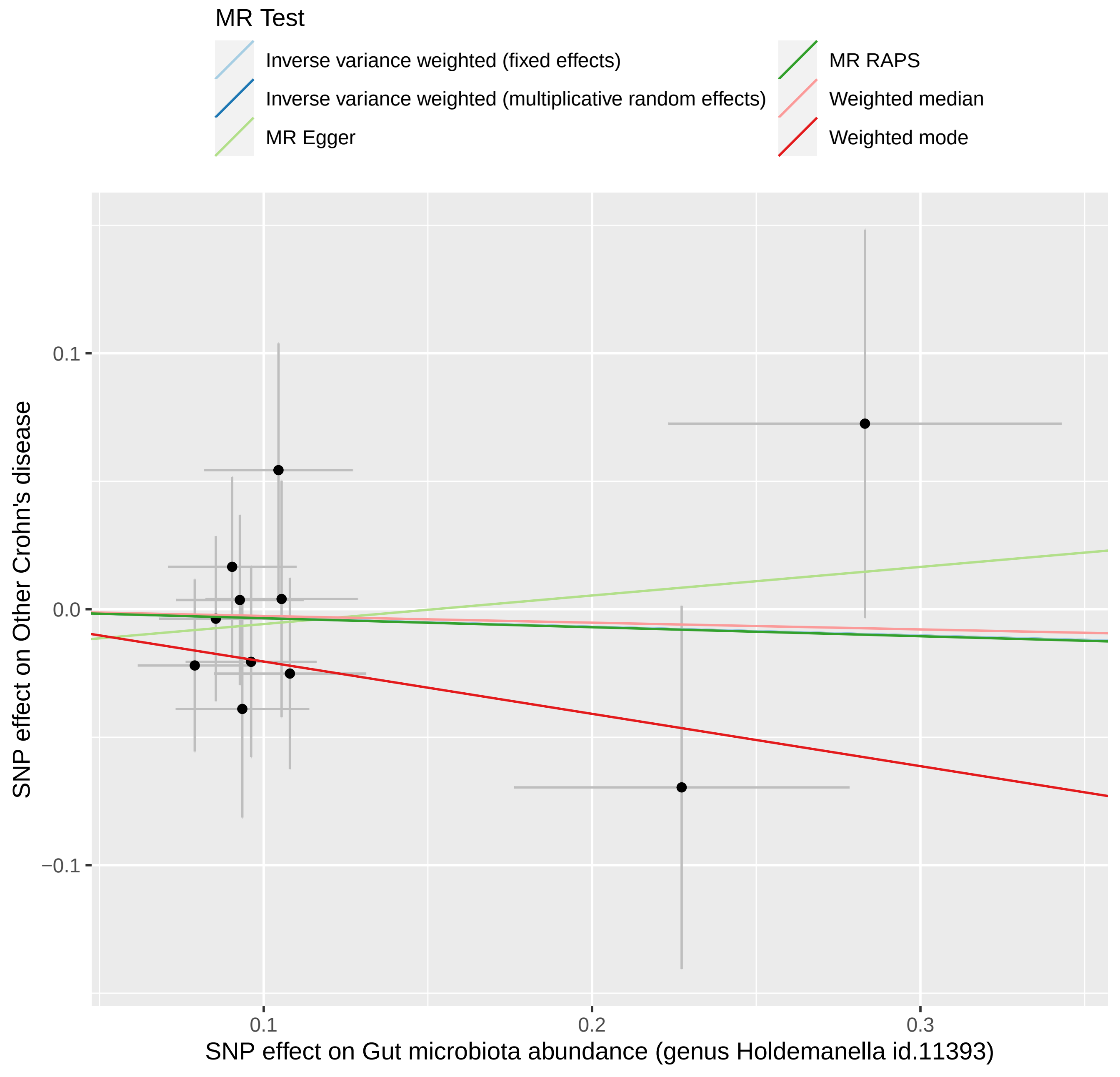

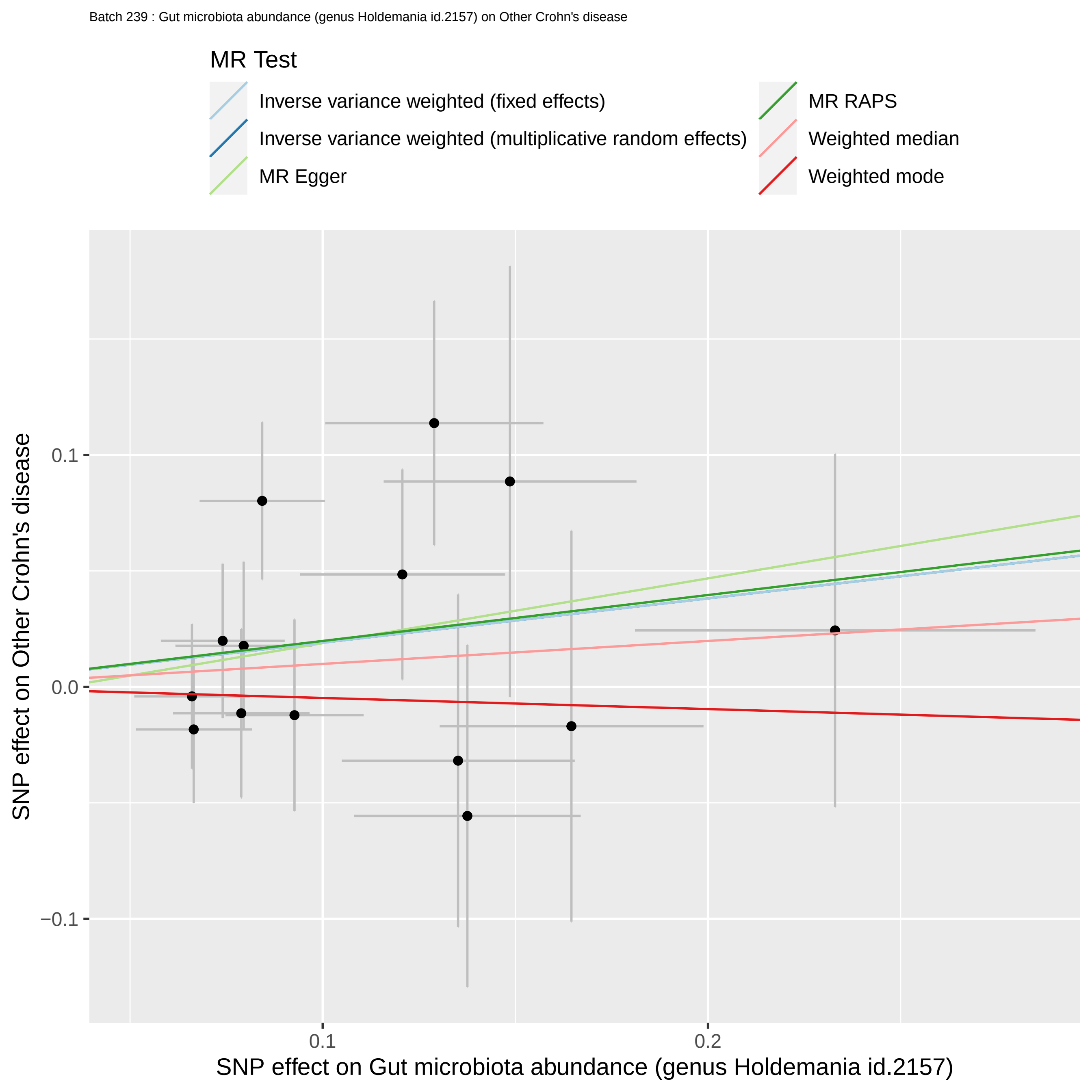
Batch 239 : Gut microbiota abundance (genus Holdemania id.2157) on Other Crohn's disease
MR Test
Inverse variance weighted (fixed effects)
Inverse variance weighted (multiplicative random effects)
MR Egger
MR RAPS
Weighted median
Weighted mode
SNP effect on Other Crohn's disease
0.1
0.0
−0.1
0.1
0.2
SNP effect on Gut microbiota abundance (genus Holdemania id.2157)

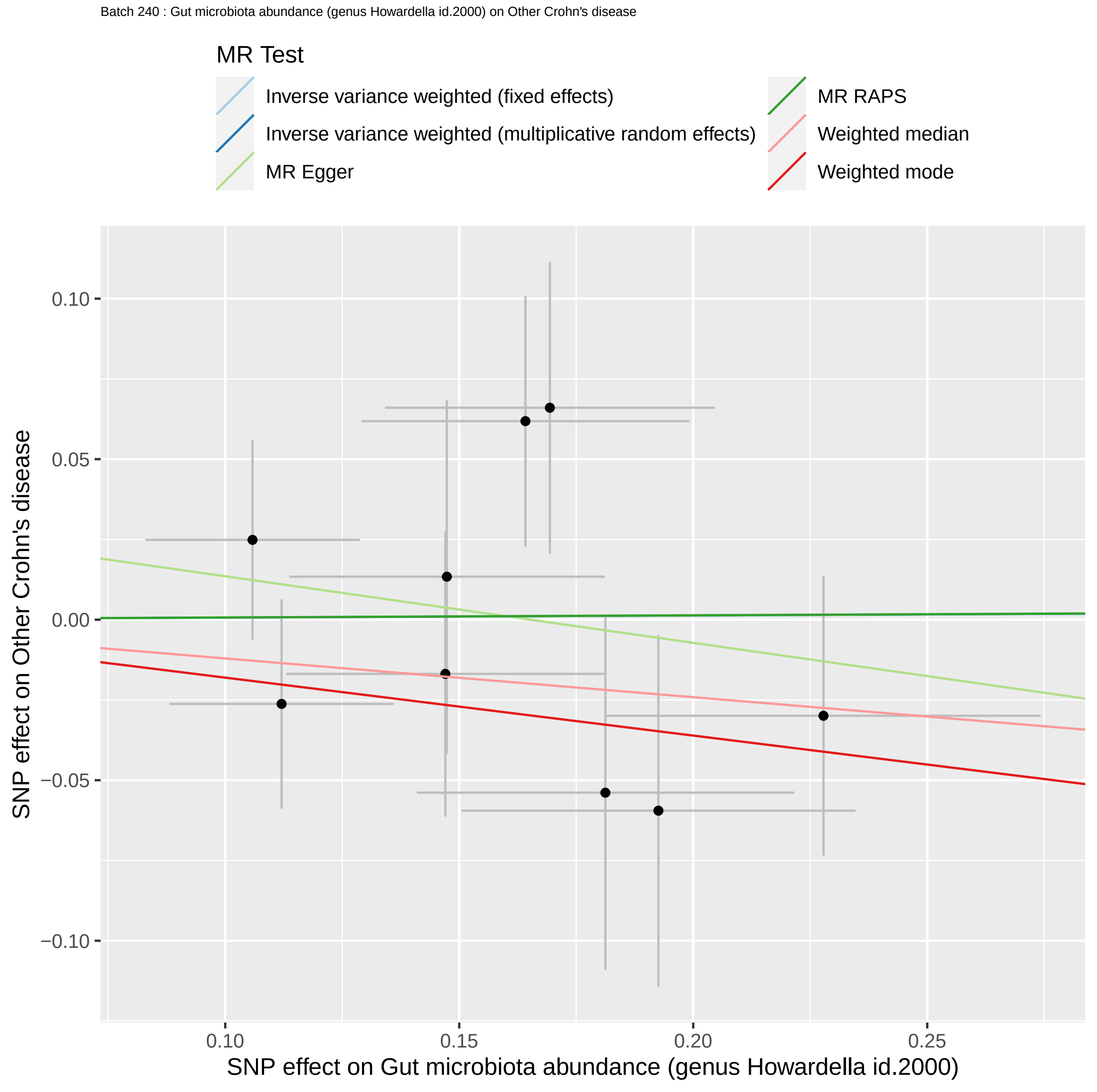

Batch 240 : Gut microbiota abundance (genus Howardella id.2000) on Other Crohn's disease
MR Test
Inverse variance weighted (fixed effects)
Inverse variance weighted (multiplicative random effects)
MR Egger
MR RAPS
Weighted median
Weighted mode
0.10
0.05
0.00
−0.05
−0.10
0.10
0.15
0.20
0.25
SNP effect on Other Crohn's disease
SNP effect on Gut microbiota abundance (genus Howardella id.2000)

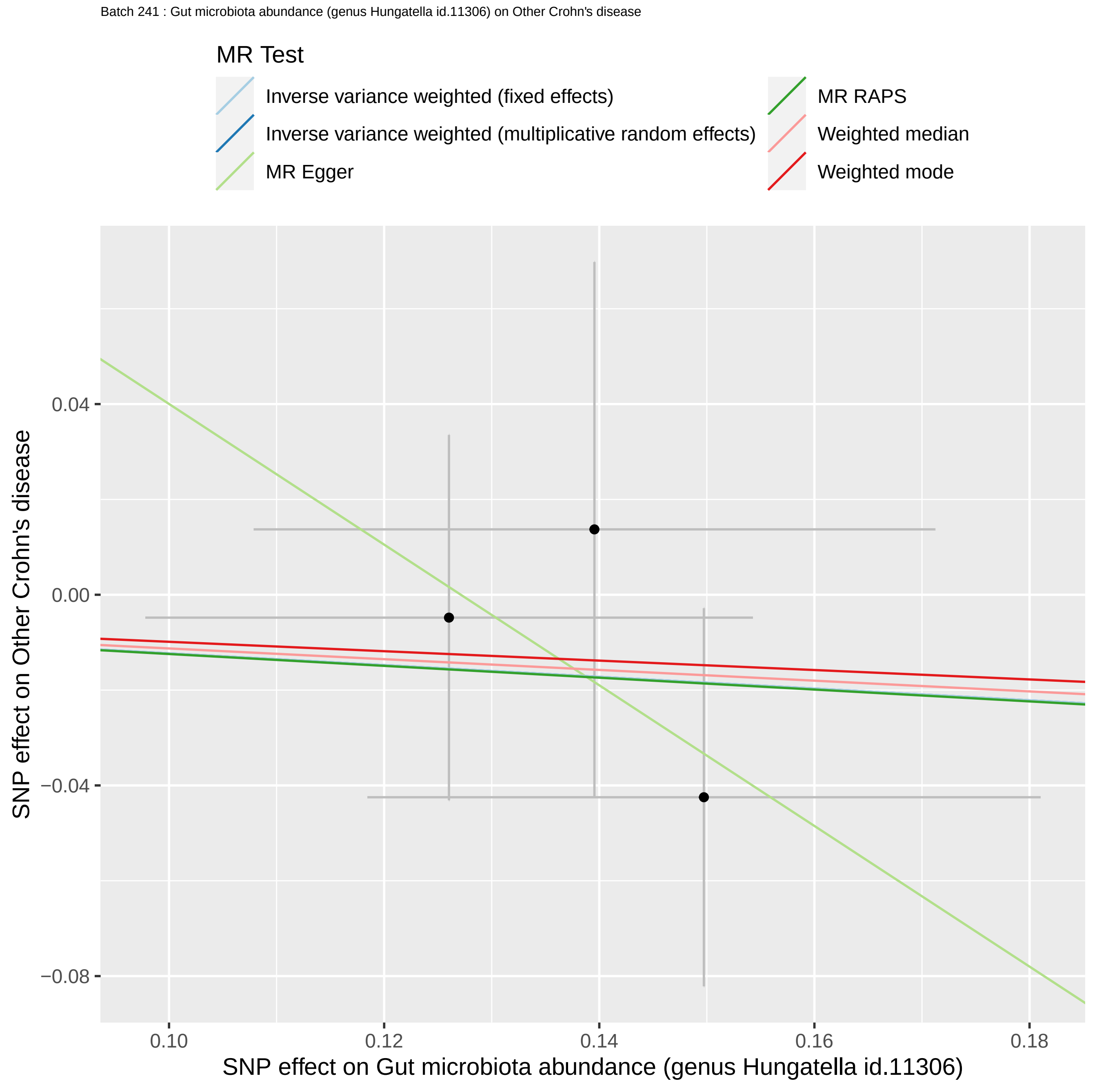

Batch 241 : Gut microbiota abundance (genus Hungatella id.11306) on Other Crohn's disease
MR Test
Inverse variance weighted (fixed effects)
Inverse variance weighted (multiplicative random effects)
MR Egger
MR RAPS
Weighted median
Weighted mode
0.04
0.00
−0.04
−0.08
0.10
0.12
0.14
0.16
0.18
SNP effect on Other Crohn's disease
SNP effect on Gut microbiota abundance (genus Hungatella id.11306)

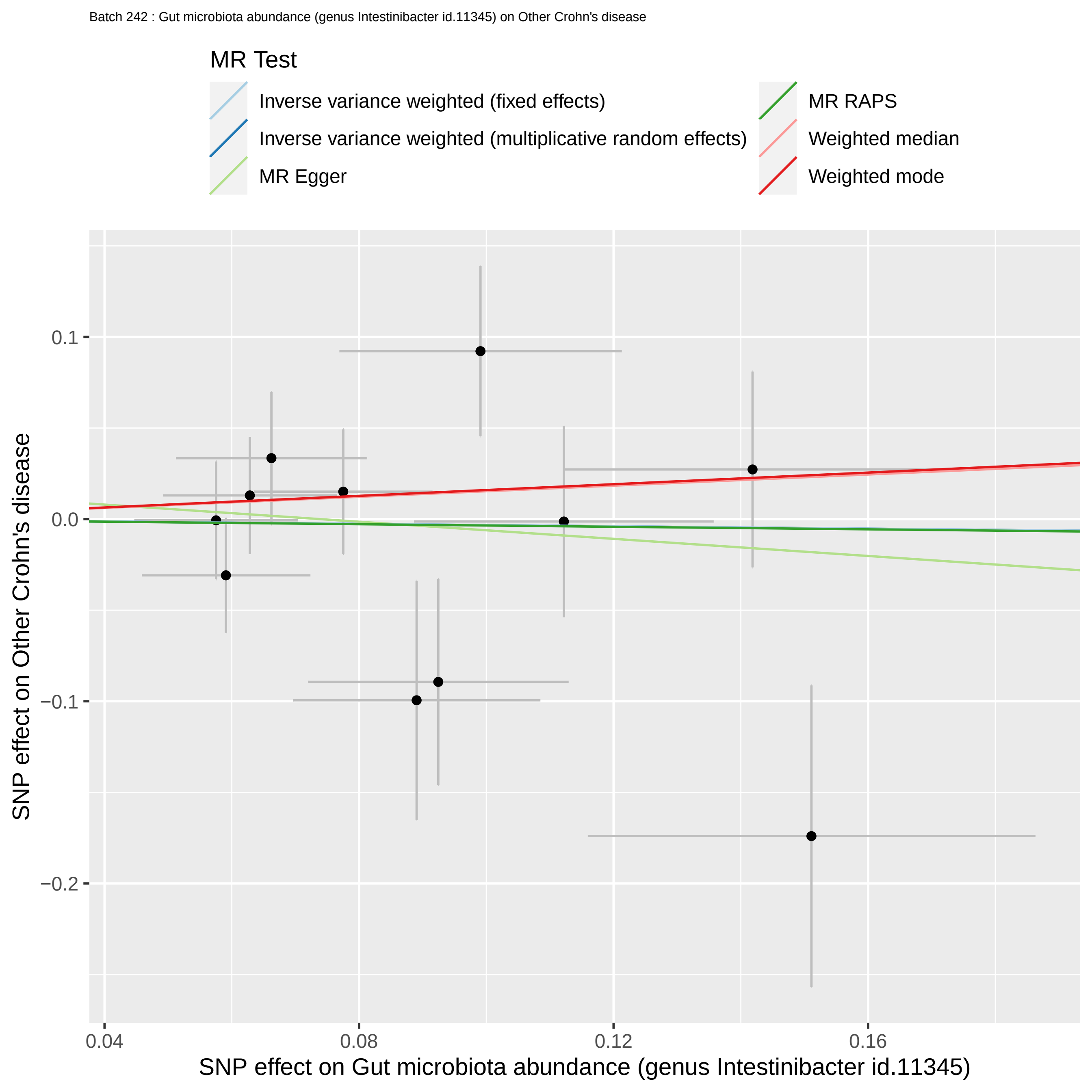

Batch 242 : Gut microbiota abundance (genus Intestinibacter id.11345) on Other Crohn's disease
MR Test
Inverse variance weighted (fixed effects)
Inverse variance weighted (multiplicative random effects)
MR Egger
MR RAPS
Weighted median
Weighted mode
0.1
0.0
−0.1
−0.2
0.04
0.08
0.12
0.16
SNP effect on Other Crohn's disease
SNP effect on Gut microbiota abundance (genus Intestinibacter id.11345)

Batch 243 : Gut microbiota abundance (genus Intestinimonas id.2062) on Other Crohn's disease

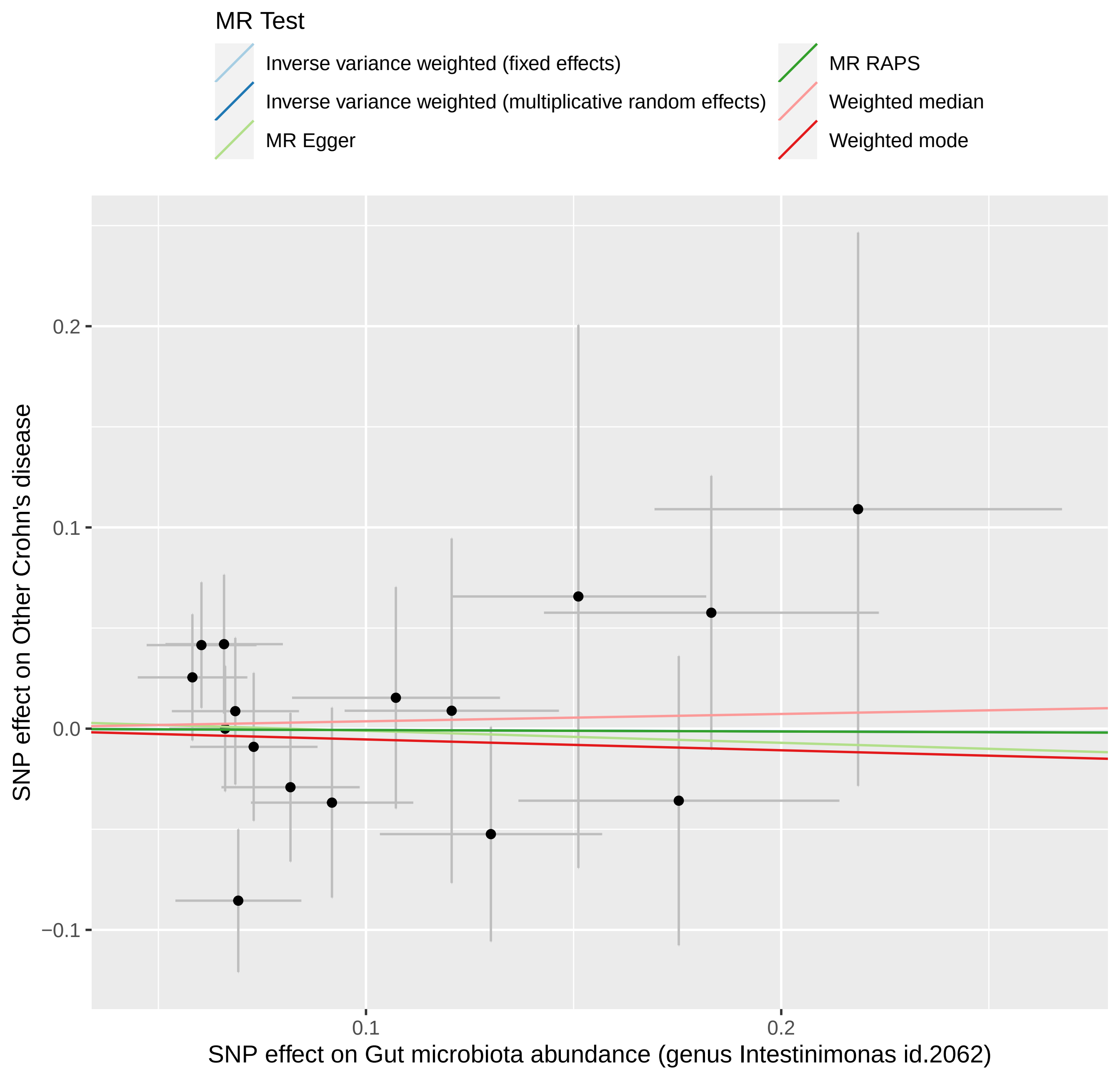

Batch 244 : Gut microbiota abundance (genus Lachnoclostridium id.11308) on Other Crohn's disease

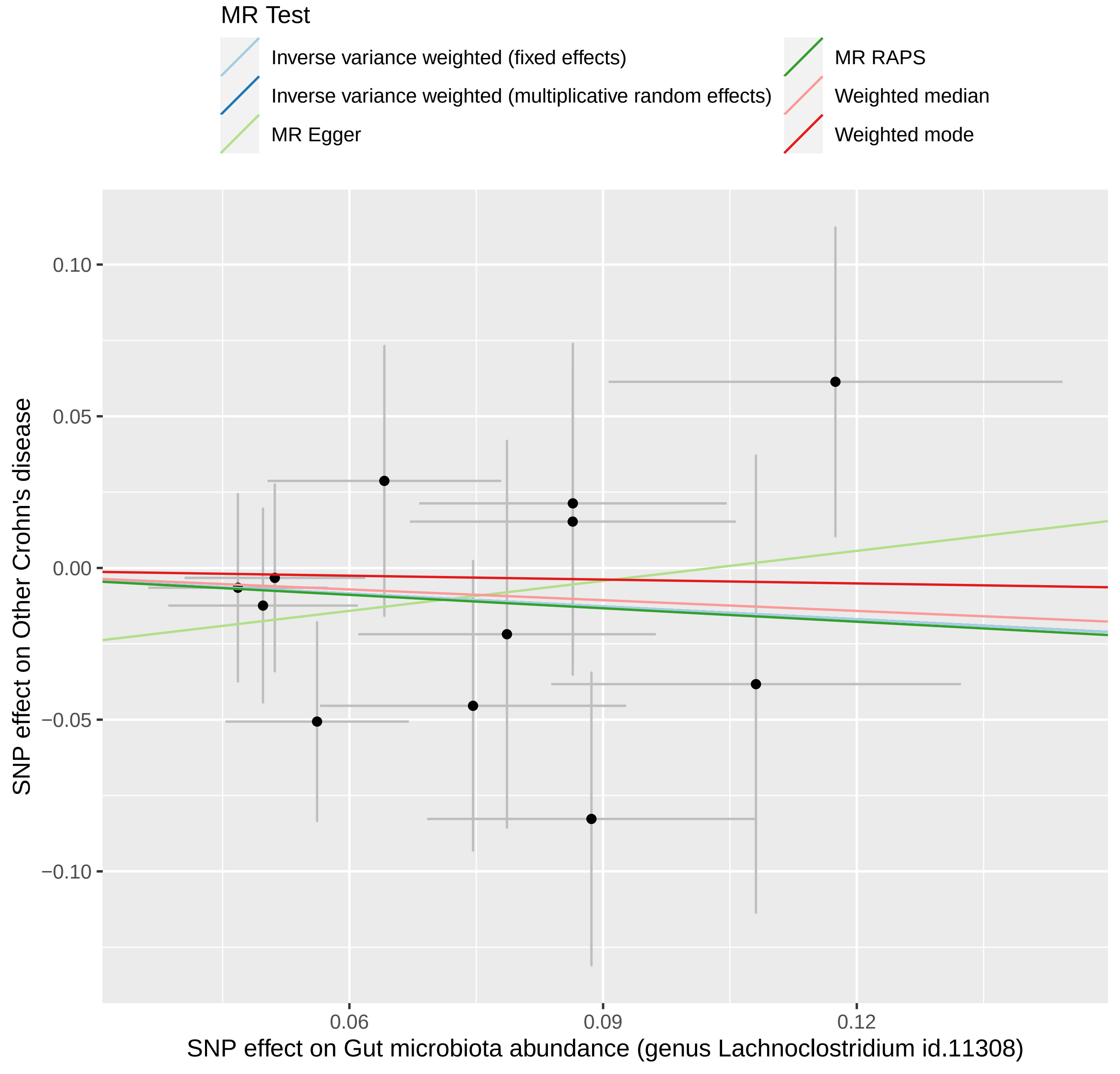

Batch 245 : Gut microbiota abundance (genus Lachnospiraceae FCS020 group id.11314) on Other Crohn's disease

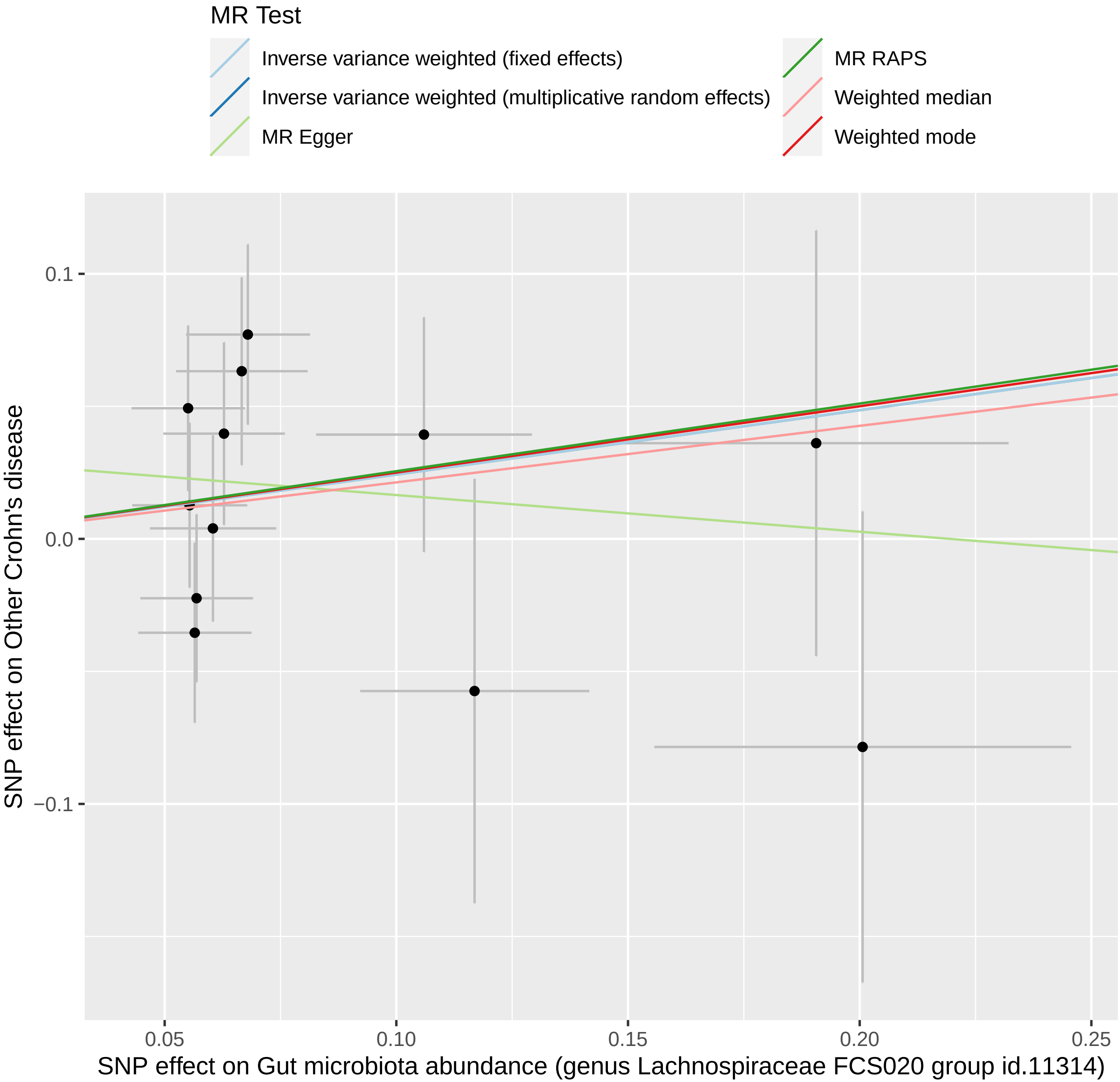

Batch 246 : Gut microbiota abundance (genus Lachnospiraceae NC2004 group id.11316) on Other Crohn's disease

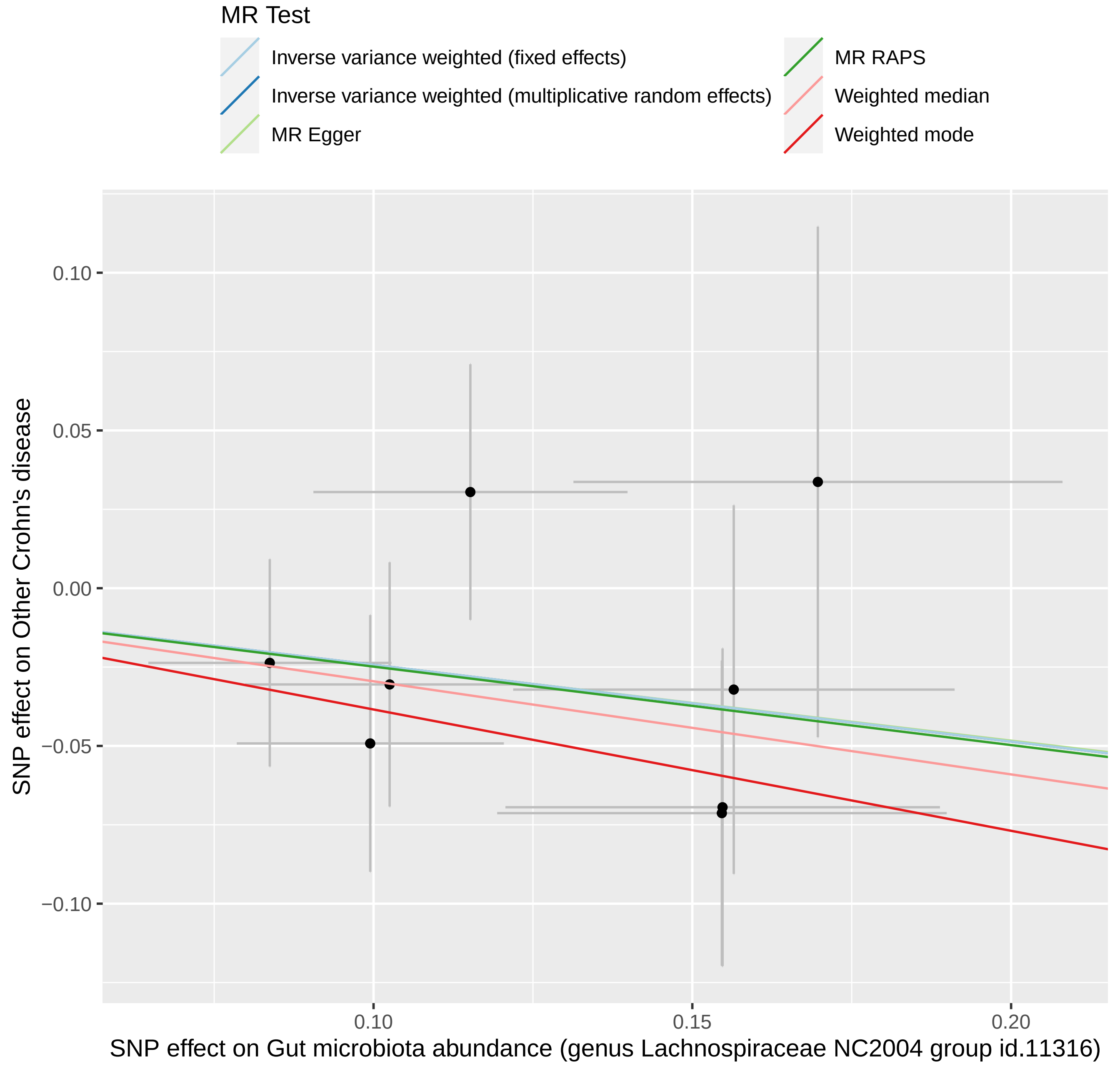

Batch 247 : Gut microbiota abundance (genus Lachnospiraceae ND3007 group id.11317) on Other Crohn's disease

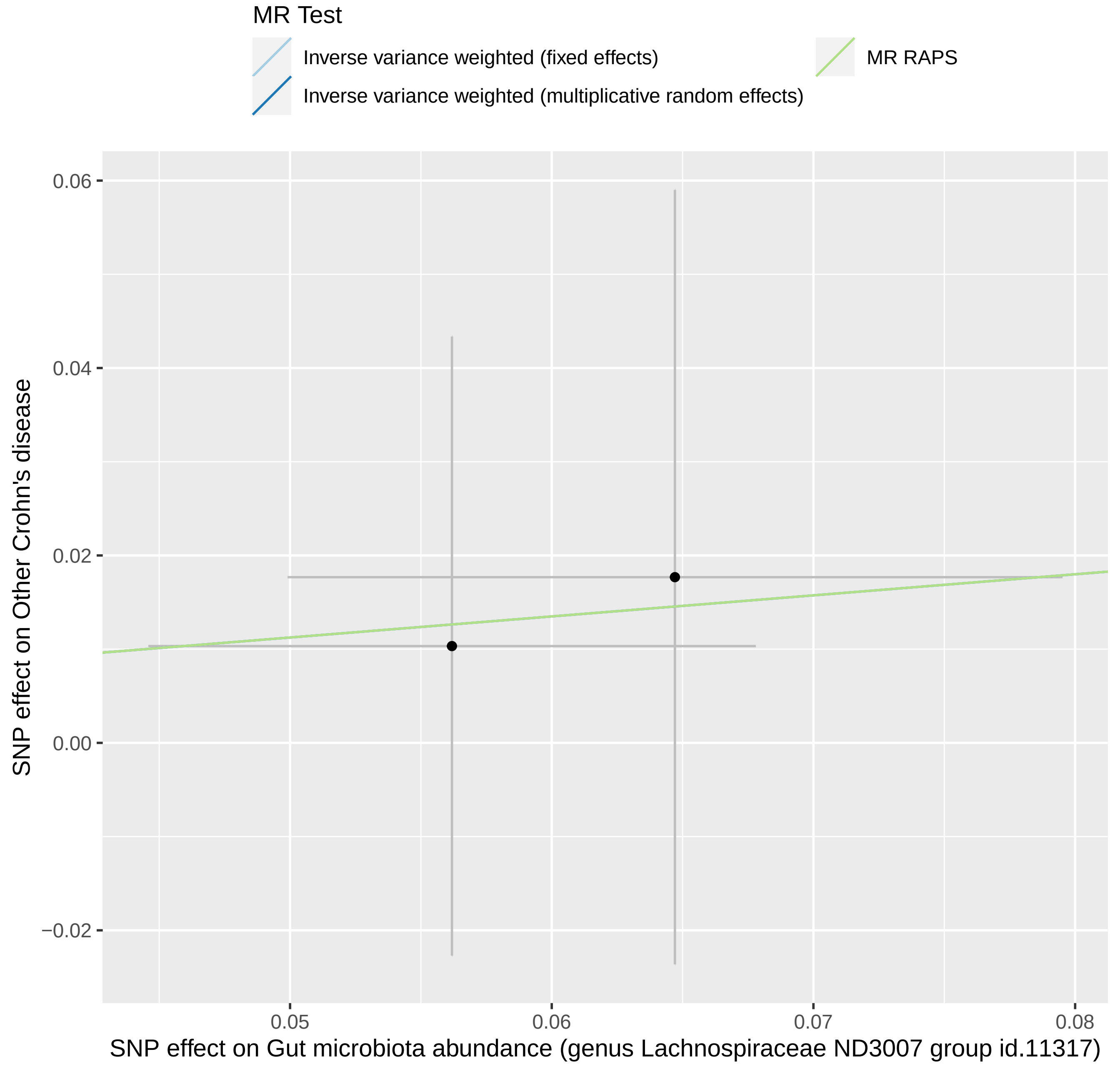

Batch 248 : Gut microbiota abundance (genus Lachnospiraceae NK4A136 group id.11319) on Other Crohn's disease

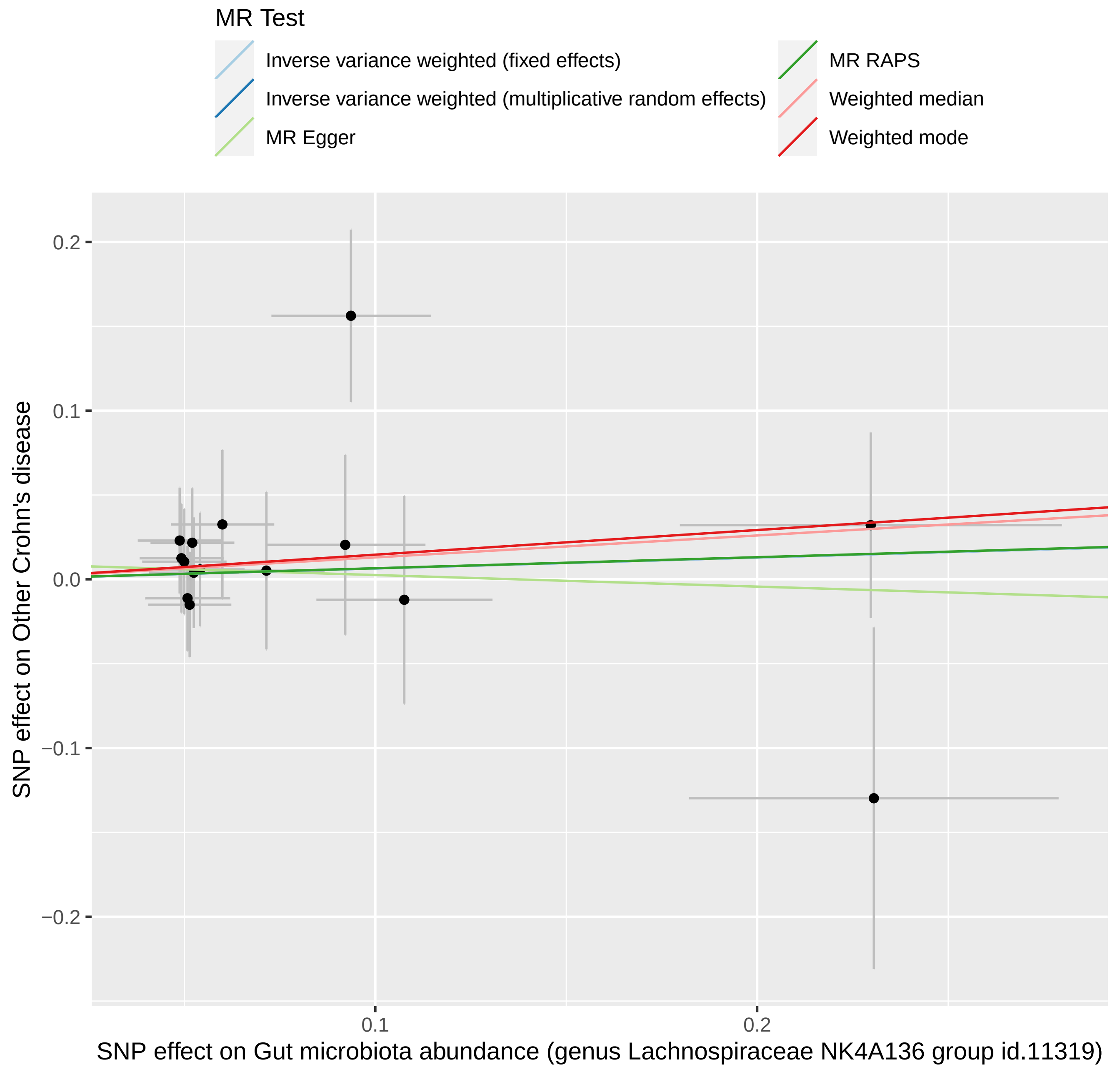

Batch 249 : Gut microbiota abundance (genus Lachnospiraceae UCG001 id.11321) on Other Crohn's disease

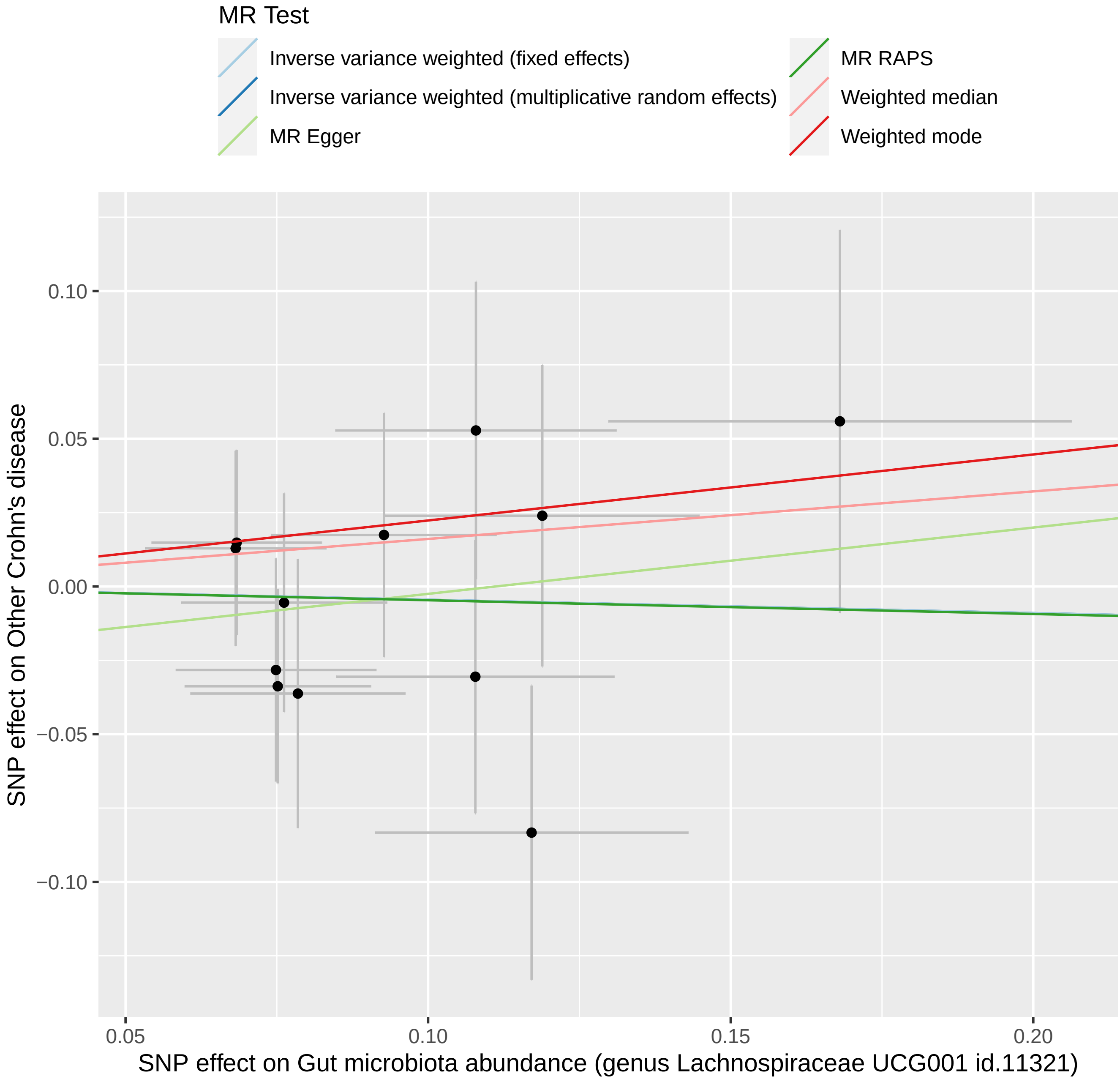

Batch 250 : Gut microbiota abundance (genus Lachnospiraceae UCG004 id.11324) on Other Crohn's disease

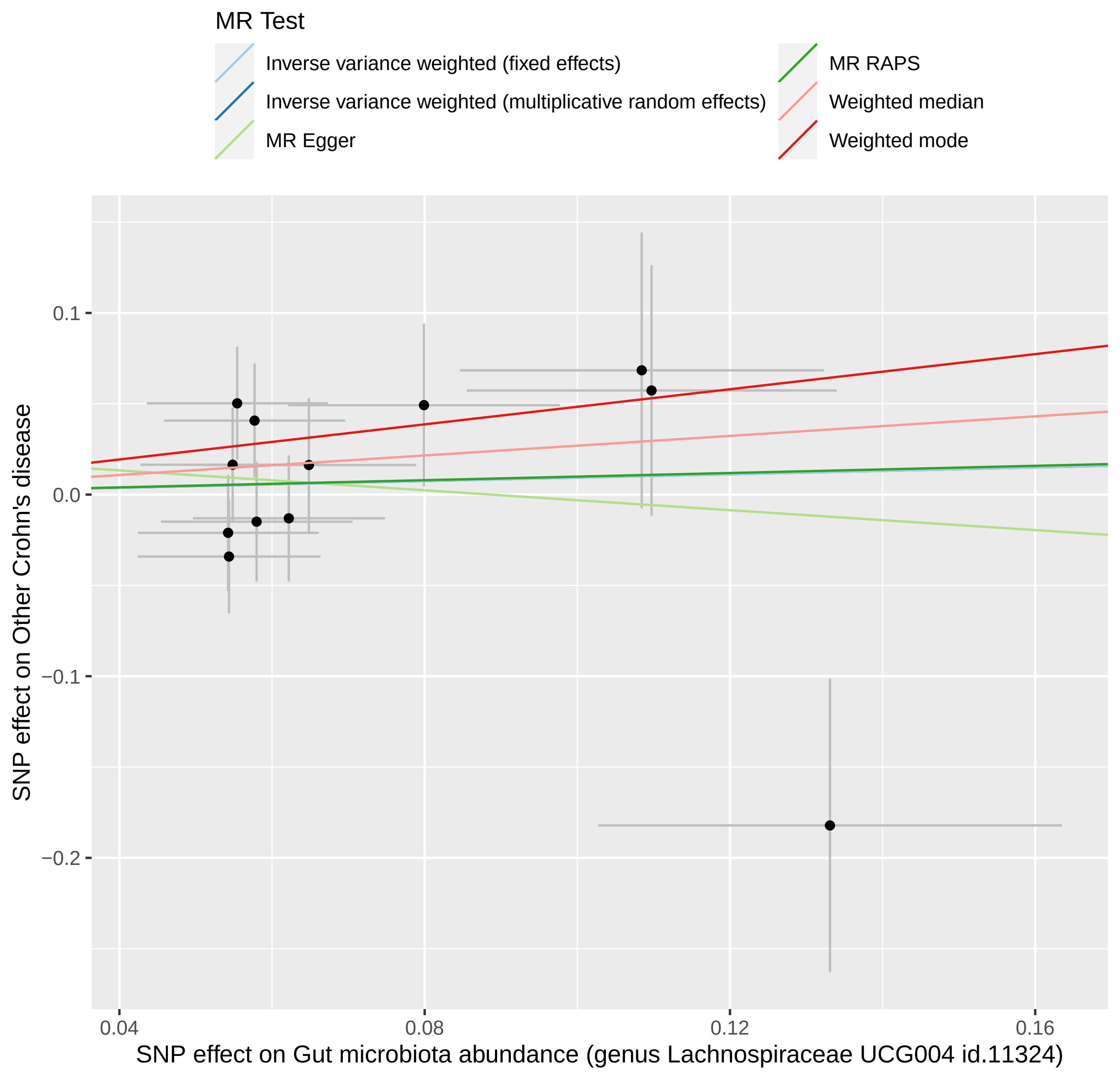

Batch 251 : Gut microbiota abundance (genus Lachnospiraceae UCG008 id.11328) on Other Crohn's disease

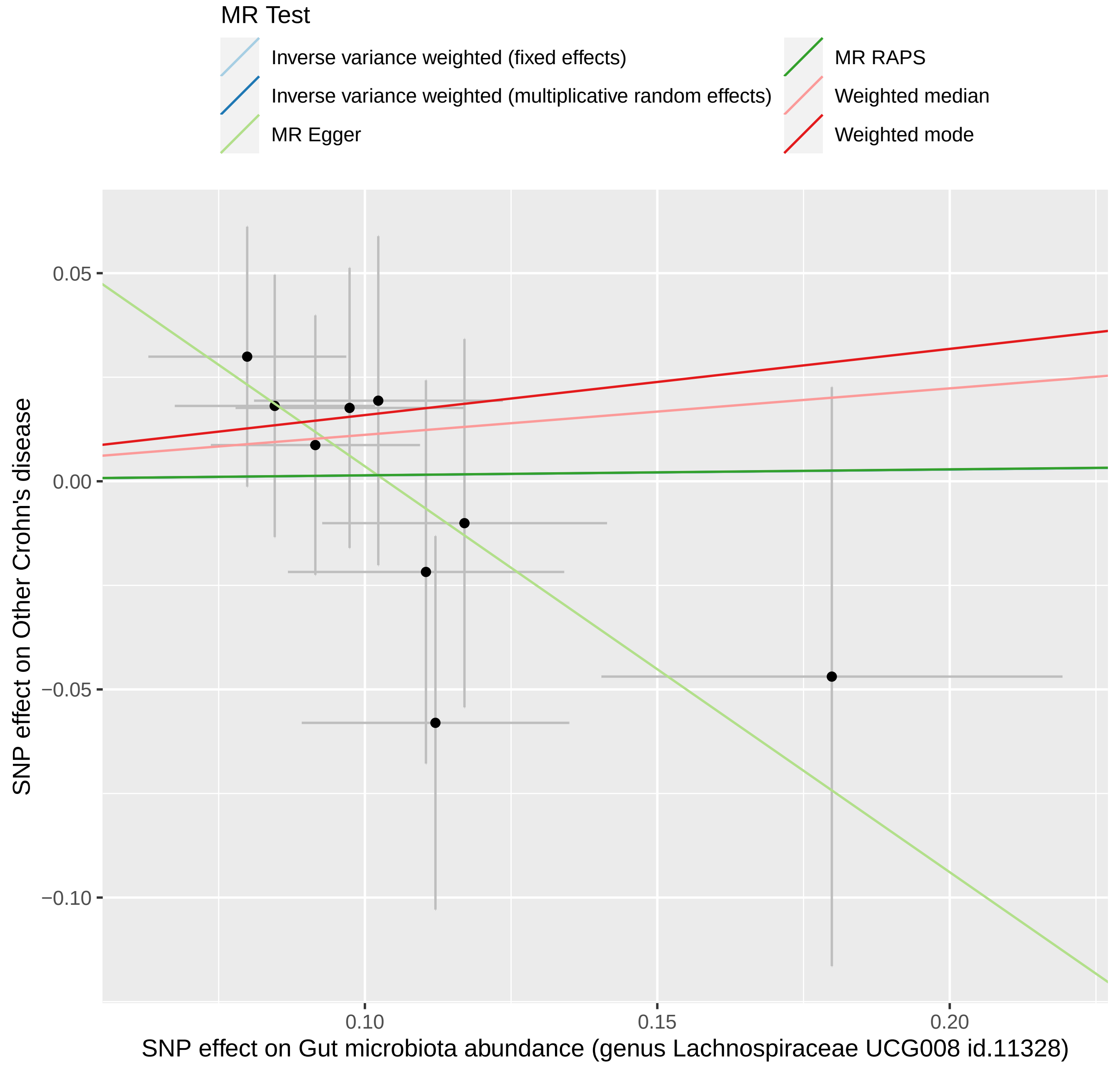

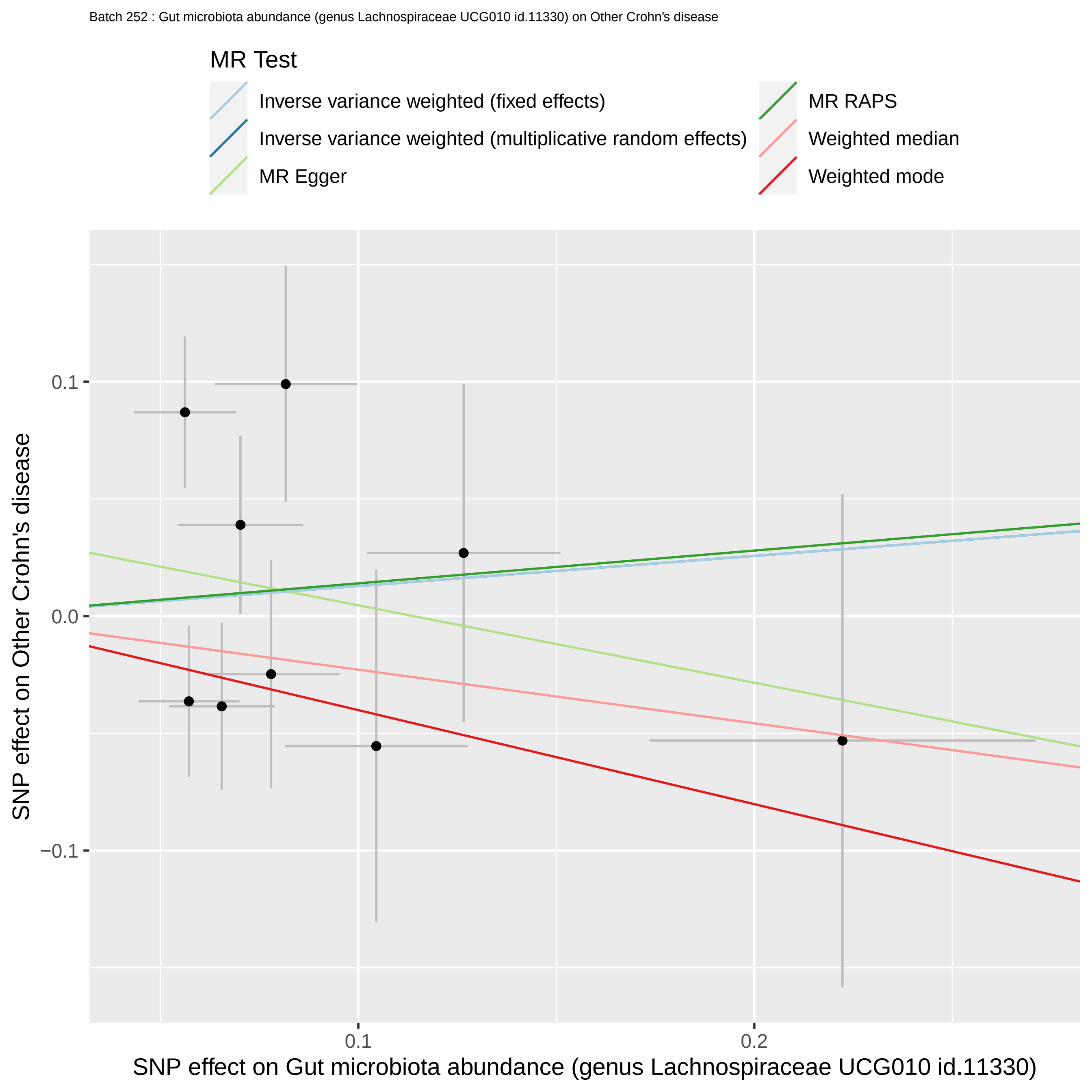
Batch 252 : Gut microbiota abundance (genus Lachnospiraceae UCG010 id.11330) on Other Crohn's disease
MR Test
Inverse variance weighted (fixed effects)
Inverse variance weighted (multiplicative random effects)
MR Egger
MR RAPS
Weighted median
Weighted mode
0.1
0.0
−0.1
0.1
0.2
SNP effect on Other Crohn's disease
SNP effect on Gut microbiota abundance (genus Lachnospiraceae UCG010 id.11330)

Insufficient number of SNPs

Batch 254 : Gut microbiota abundance (genus Lactobacillus id.1837) on Other Crohn's disease

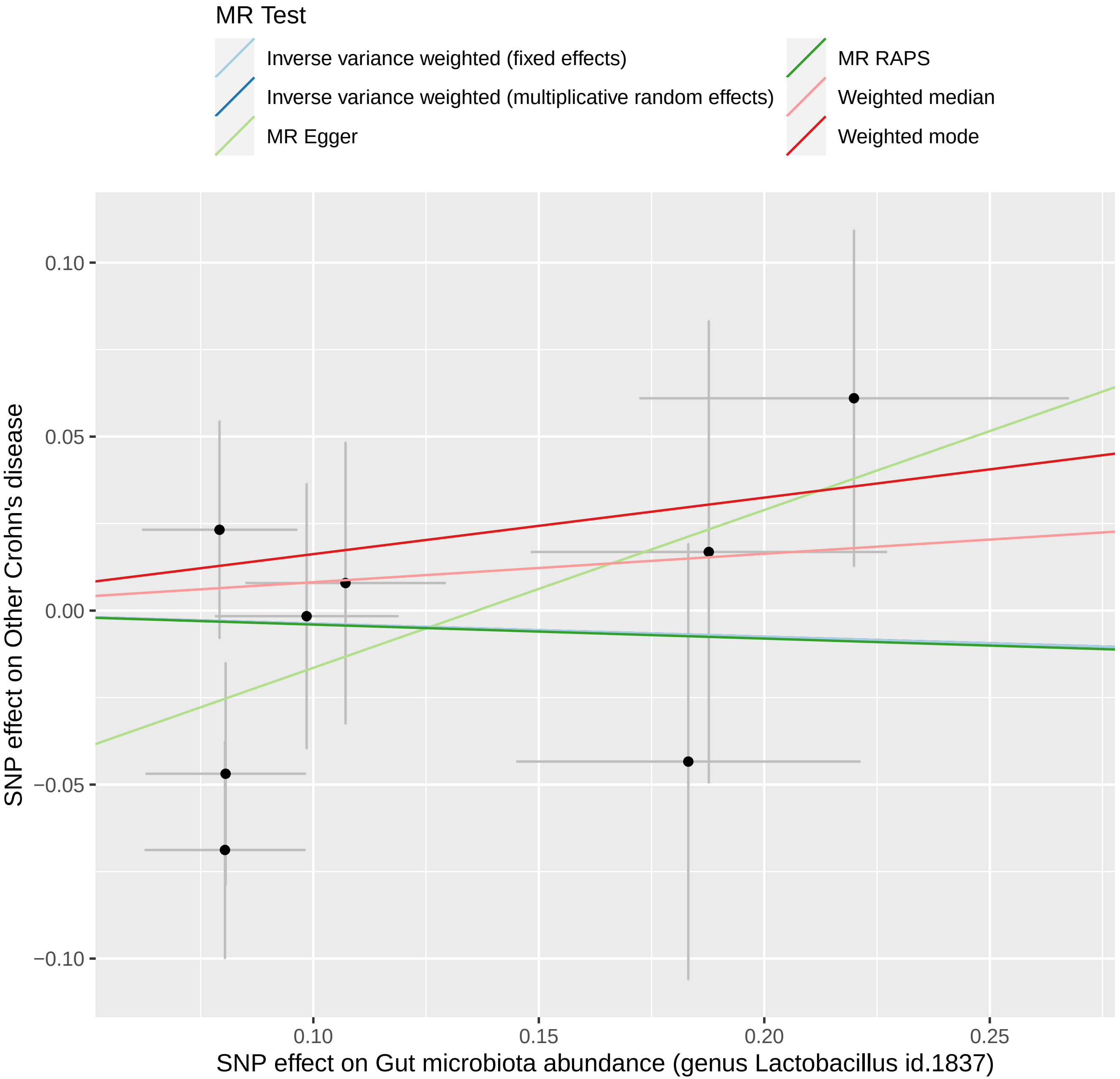

Batch 255 : Gut microbiota abundance (genus Lactococcus id.1851) on Other Crohn's disease

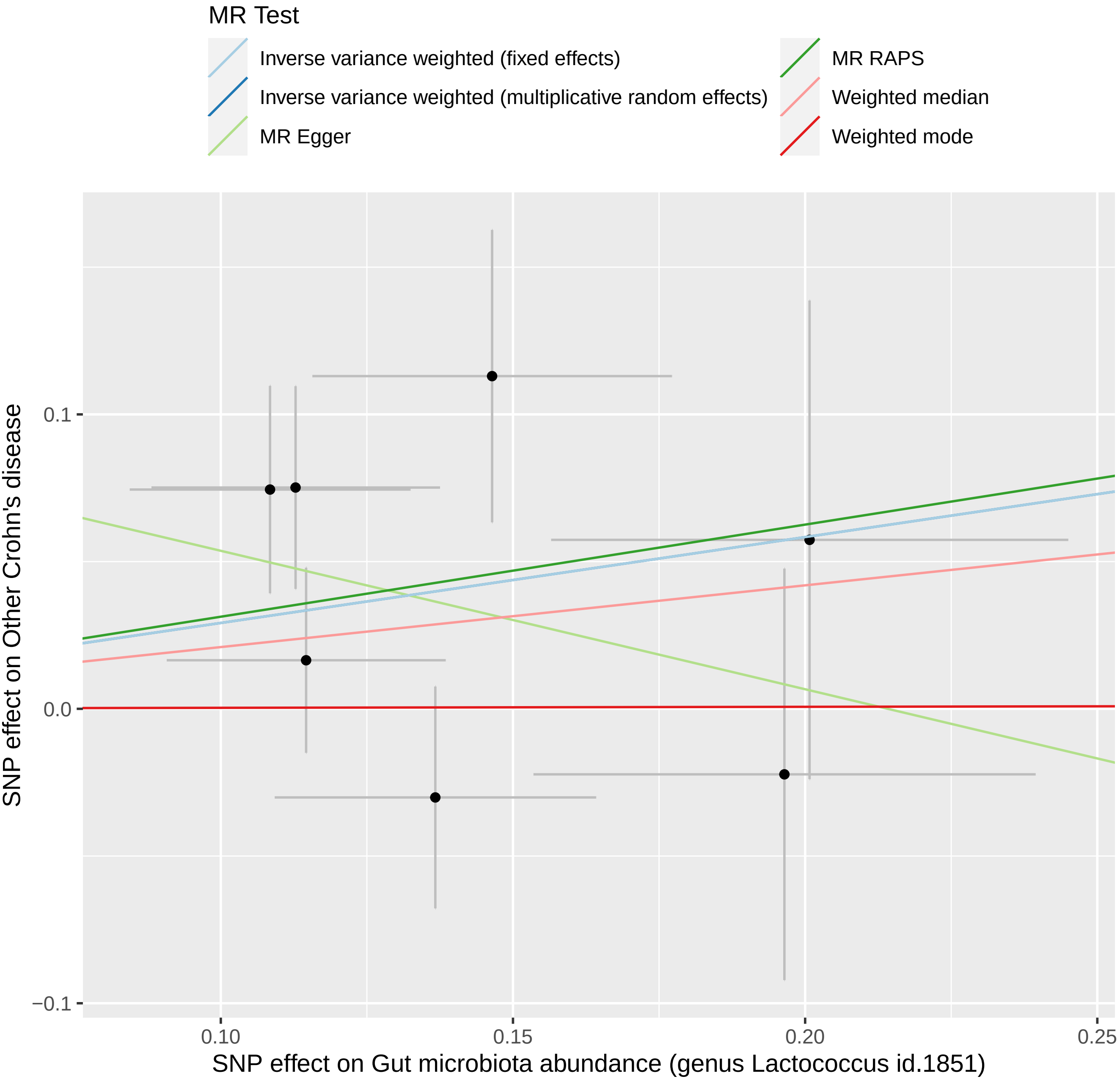

Batch 256 : Gut microbiota abundance (genus Marvinbryantia id.2005) on Other Crohn's disease

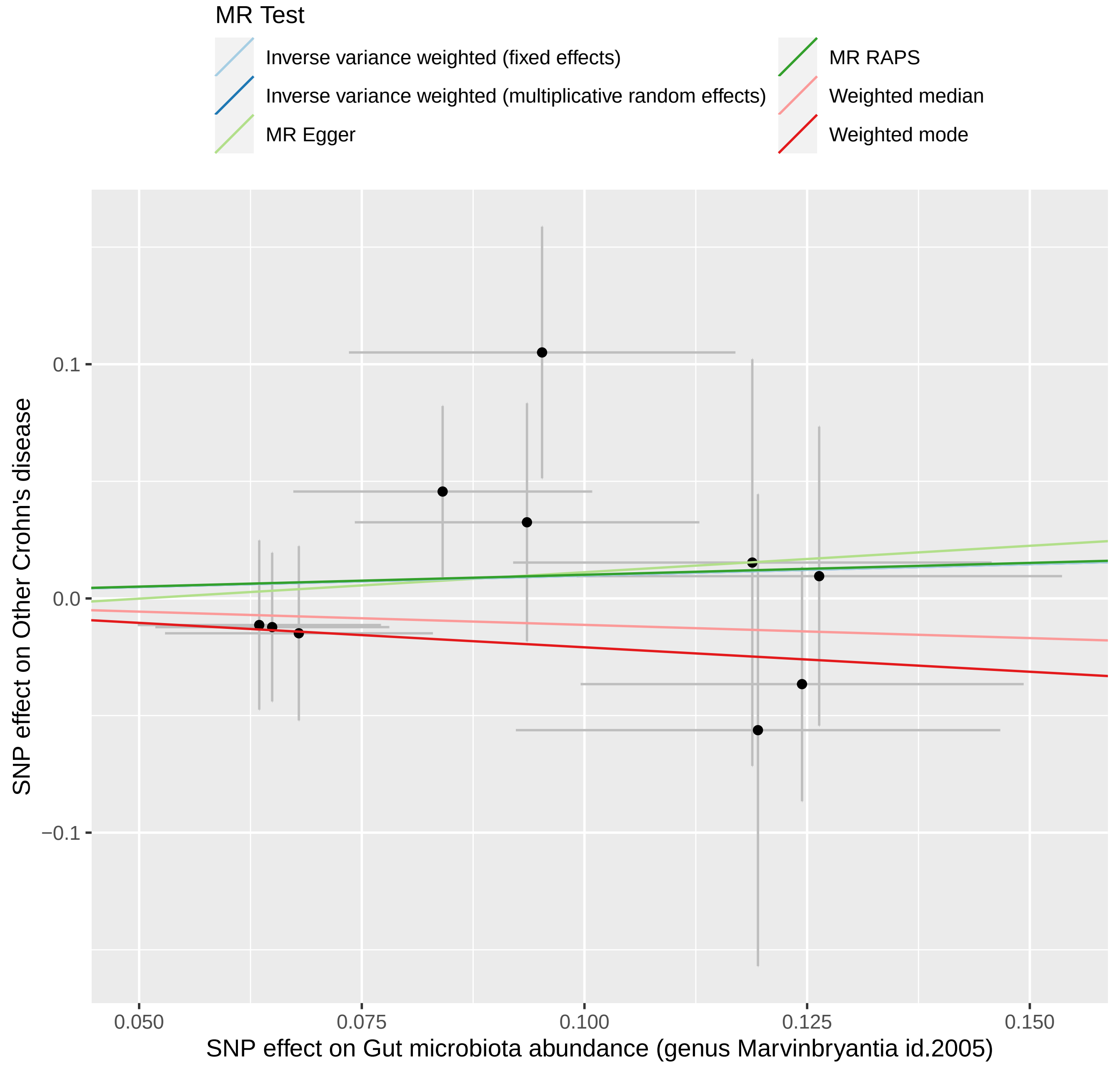

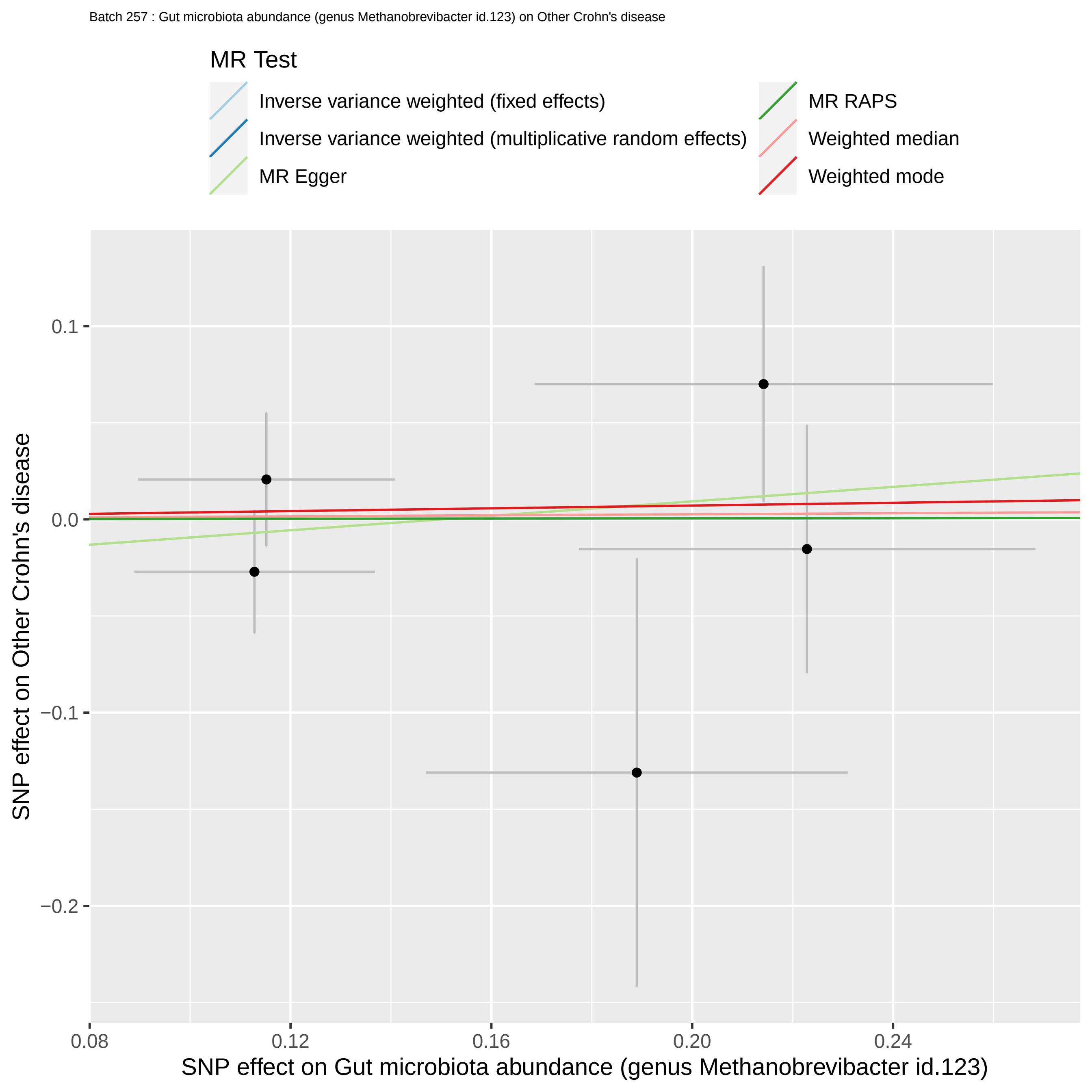
Batch 257 : Gut microbiota abundance (genus Methanobrevibacter id.123) on Other Crohn's disease
MR Test
Inverse variance weighted (fixed effects)
Inverse variance weighted (multiplicative random effects)
MR Egger
MR RAPS
Weighted median
Weighted mode
0.1
0.0
−0.1
−0.2
0.08
0.12
0.16
0.20
0.24
SNP effect on Other Crohn's disease
SNP effect on Gut microbiota abundance (genus Methanobrevibacter id.123)

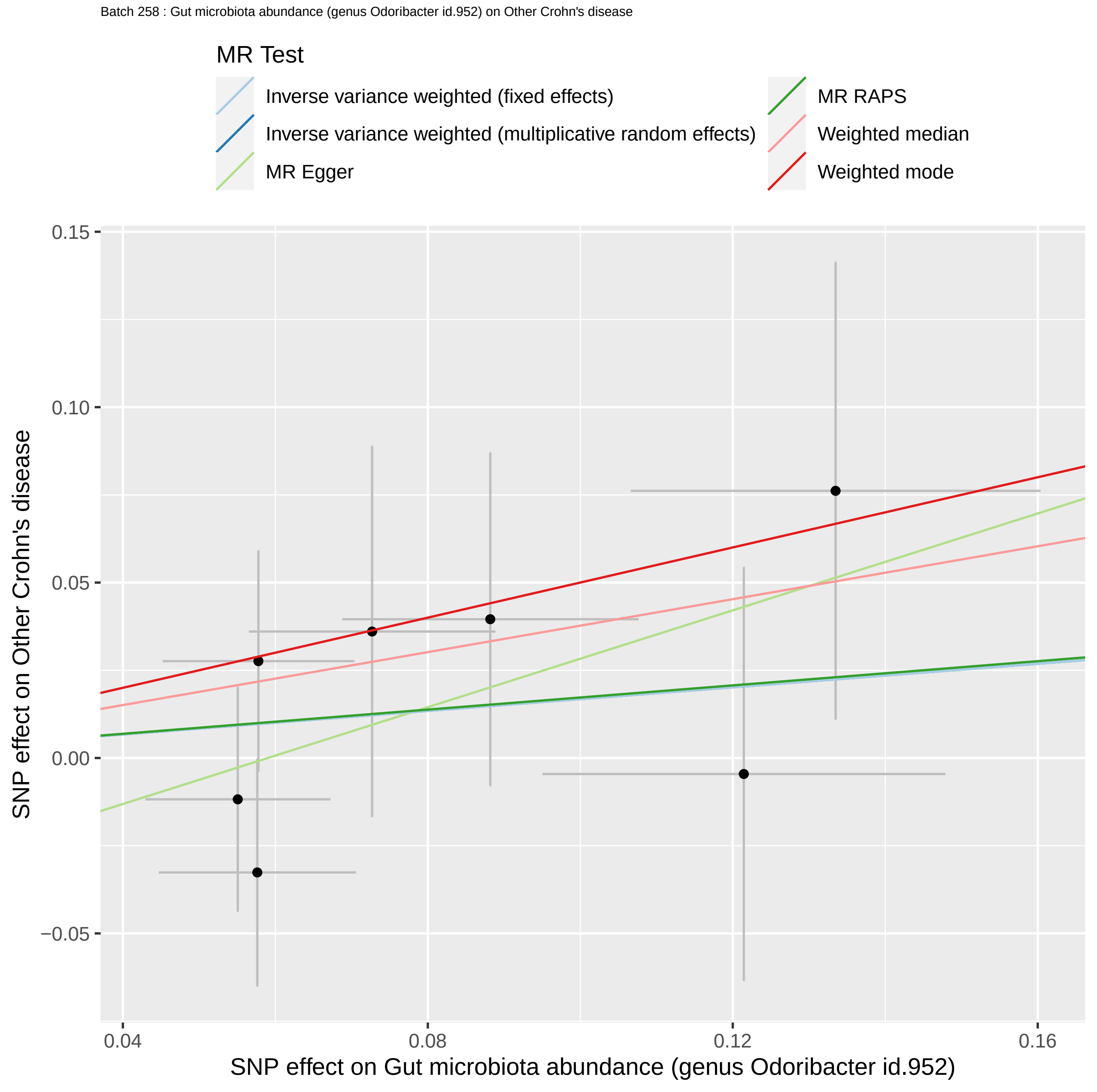

Batch 258 : Gut microbiota abundance (genus Odoribacter id.952) on Other Crohn's disease
MR Test
Inverse variance weighted (fixed effects)
Inverse variance weighted (multiplicative random effects)
MR Egger
MR RAPS
Weighted median
Weighted mode
0.15
0.10
0.05
0.00
−0.05
0.04
0.08
0.12
0.16
SNP effect on Other Crohn's disease
SNP effect on Gut microbiota abundance (genus Odoribacter id.952)

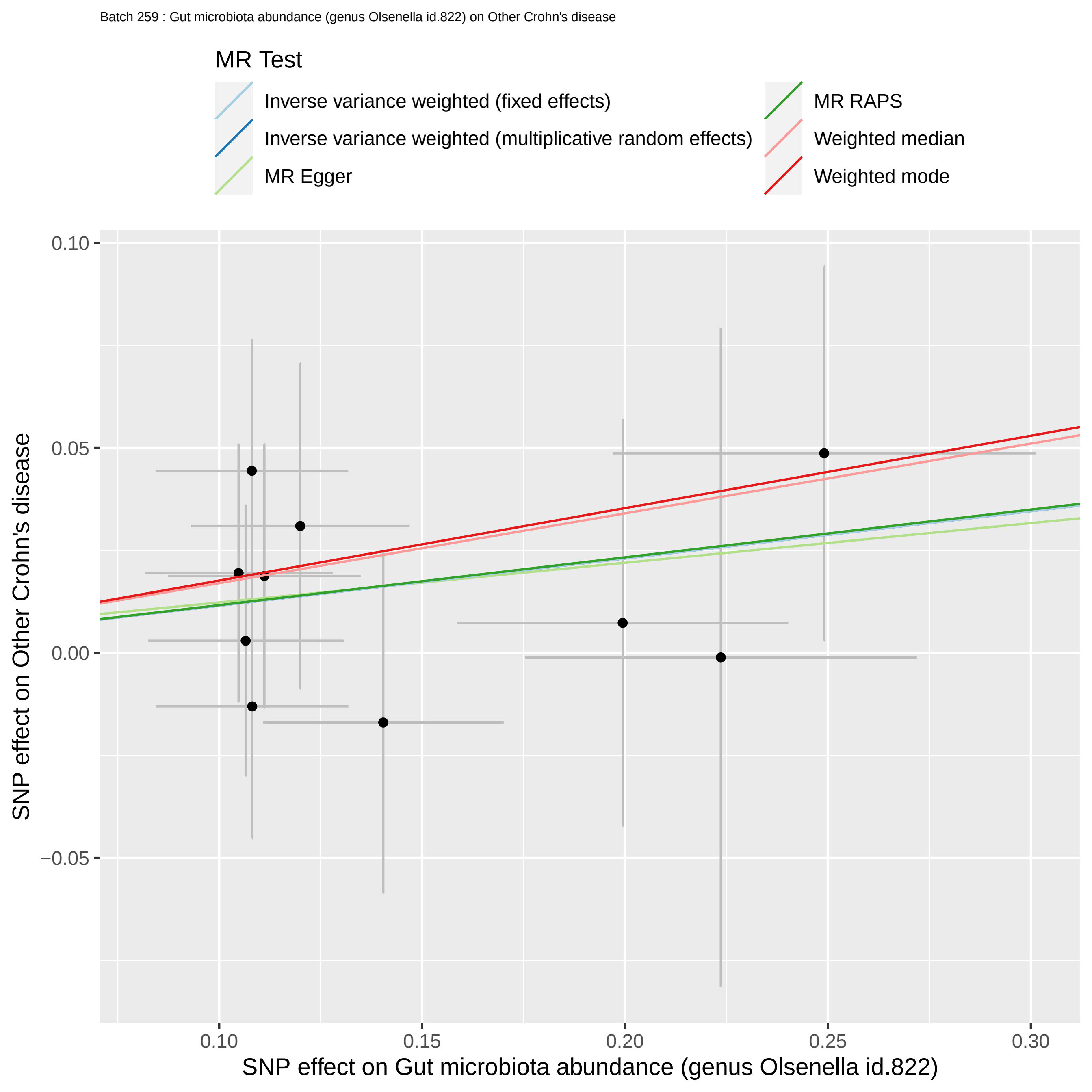
Batch 259 : Gut microbiota abundance (genus Olsenella id.822) on Other Crohn's disease
MR Test
Inverse variance weighted (fixed effects)
Inverse variance weighted (multiplicative random effects)
MR Egger
MR RAPS
Weighted median
Weighted mode
0.10
0.05
0.00
−0.05
SNP effect on Other Crohn's disease
0.10
0.15
0.20
0.25
0.30
SNP effect on Gut microbiota abundance (genus Olsenella id.822)

Batch 260 : Gut microbiota abundance (genus Oscillibacter id.2063) on Other Crohn's disease

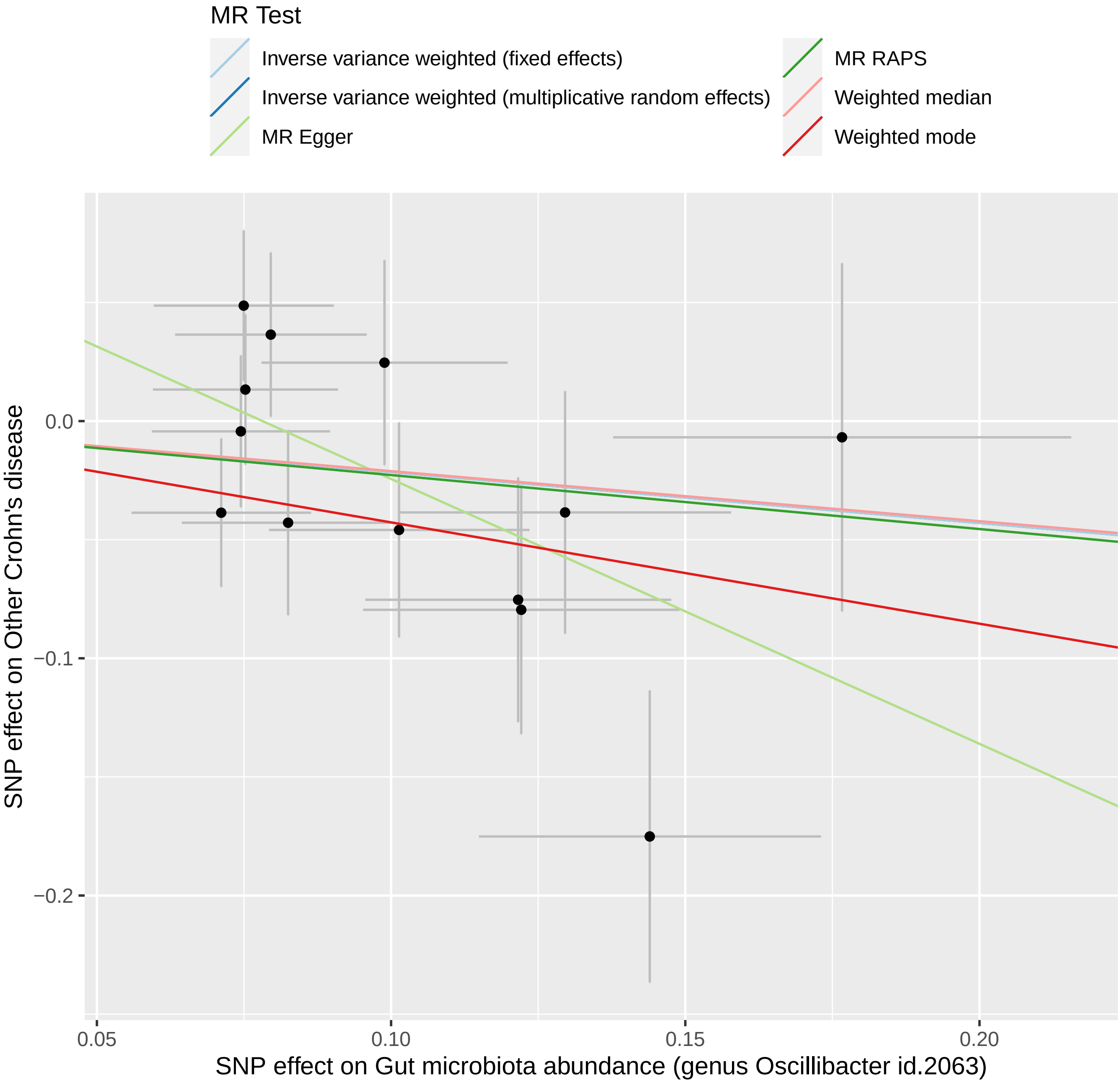

Batch 261 : Gut microbiota abundance (genus Oscillospira id.2064) on Other Crohn's disease

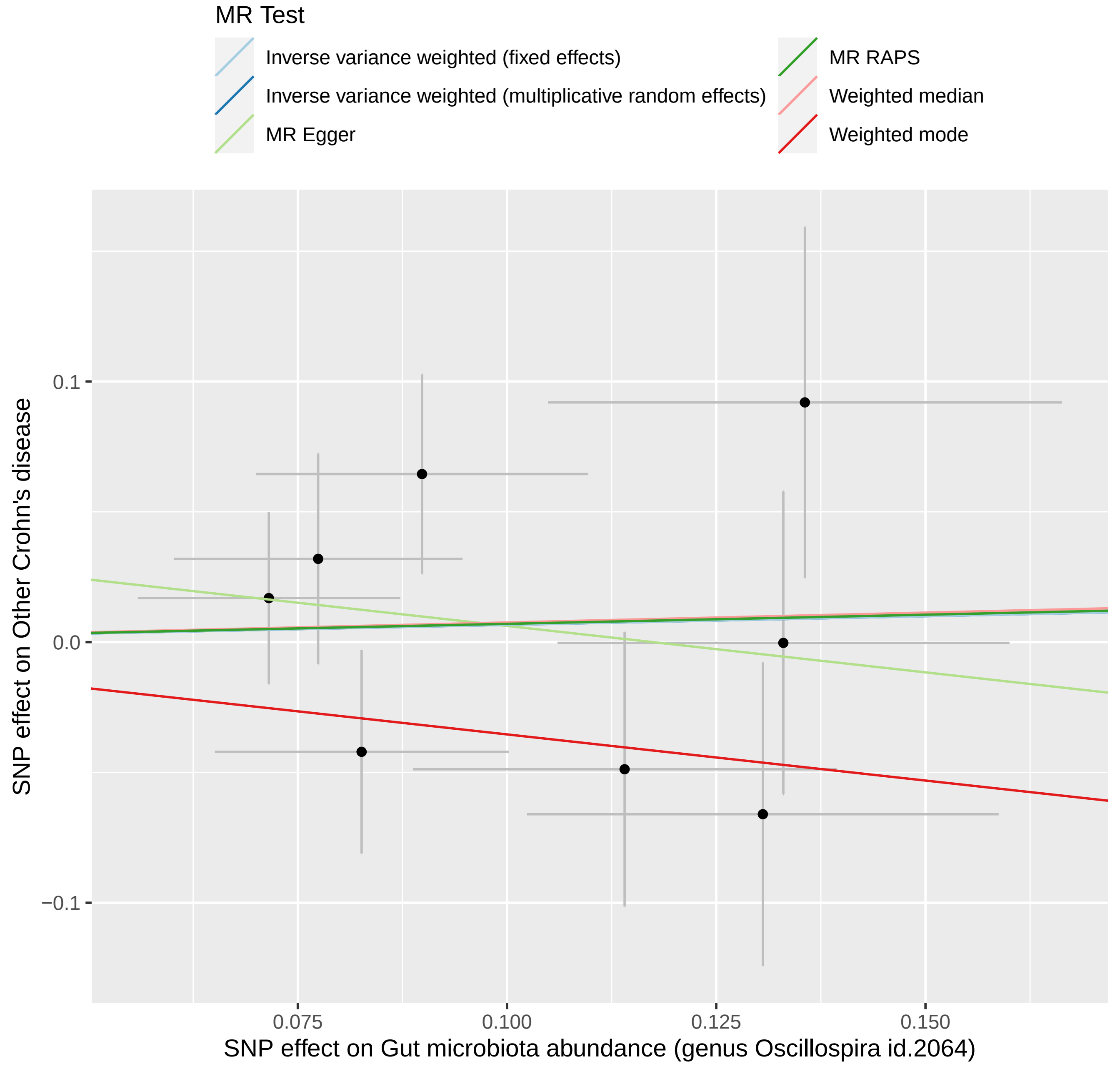

Batch 262 : Gut microbiota abundance (genus Oxalobacter id.2978) on Other Crohn's disease

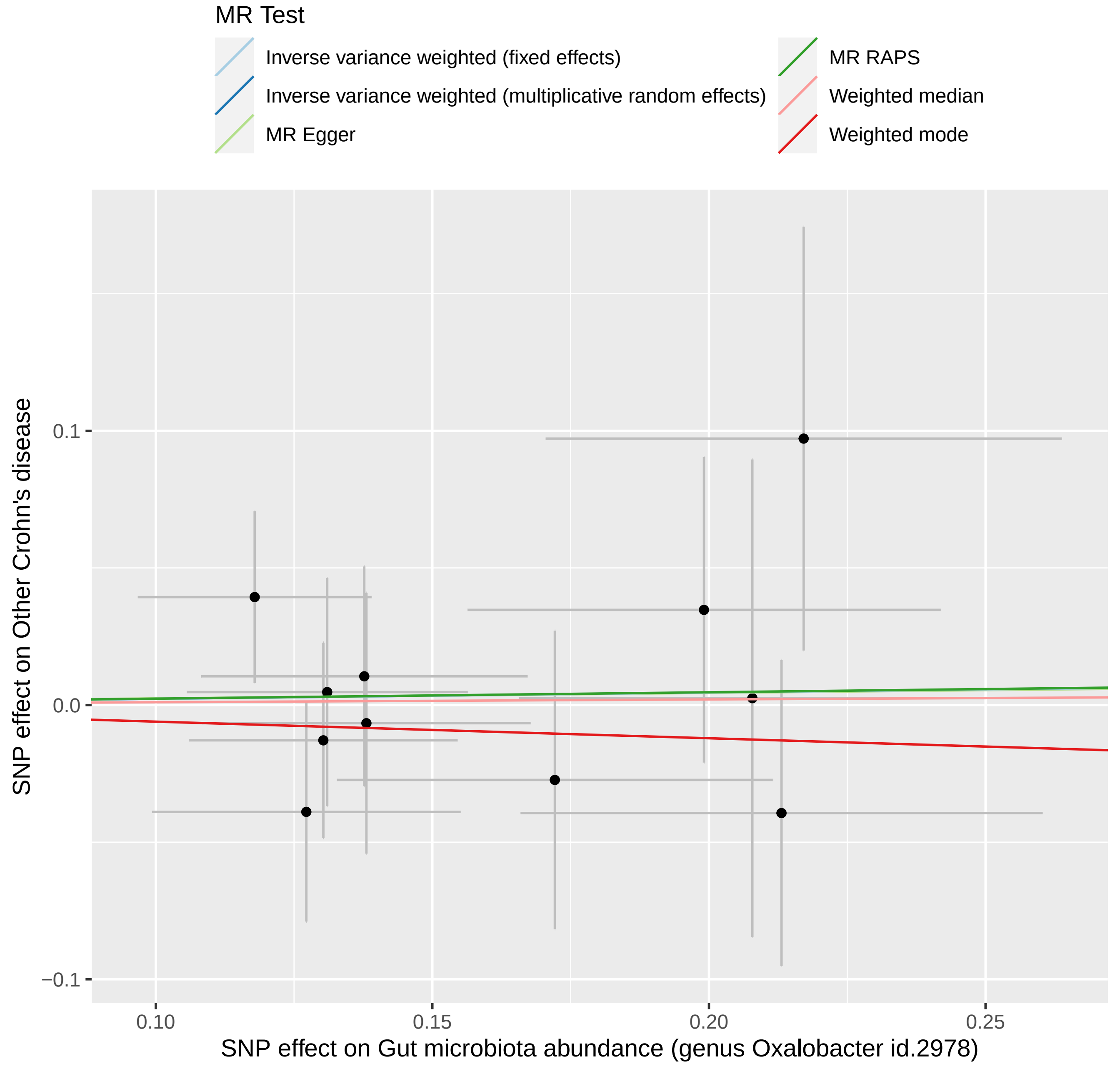

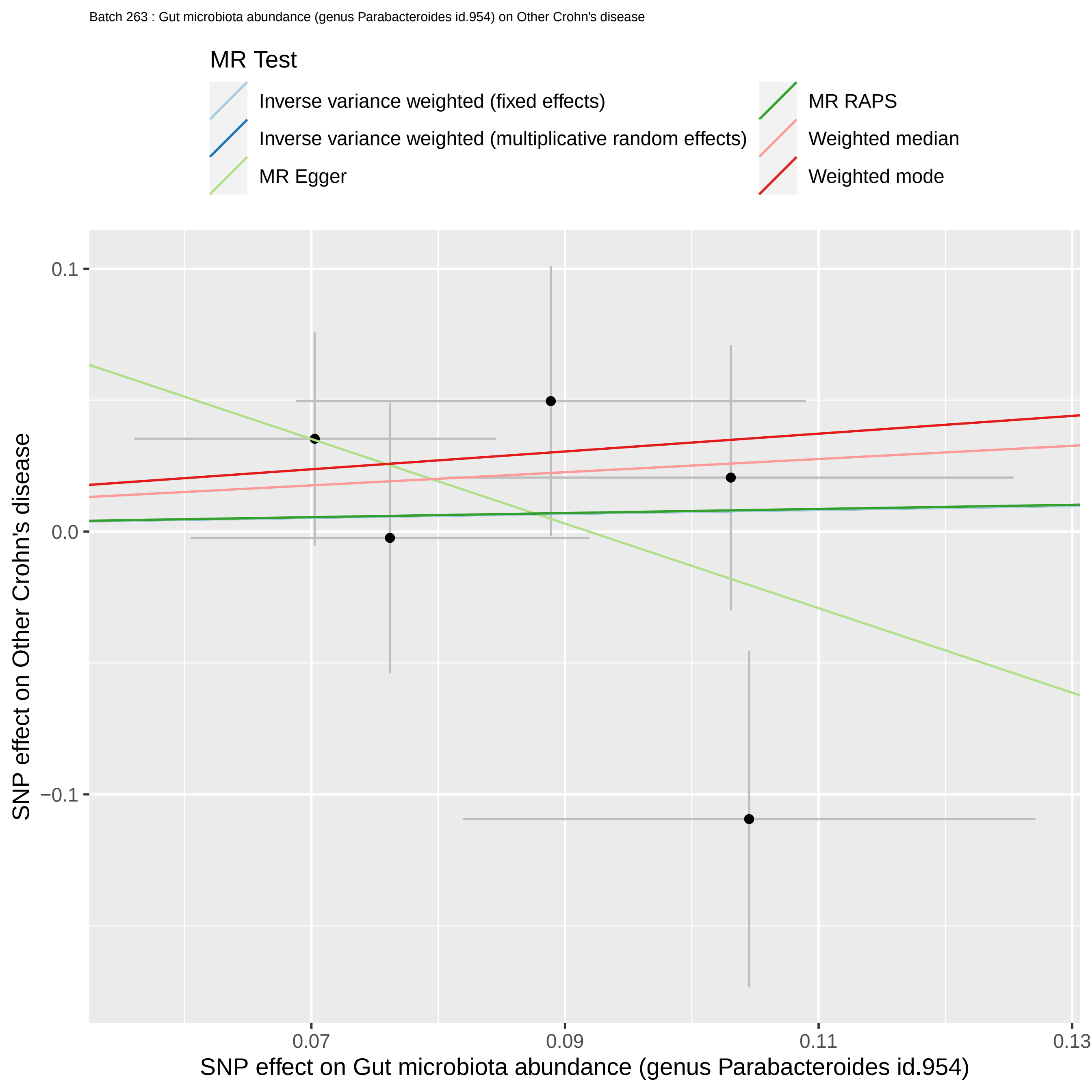
Batch 263 : Gut microbiota abundance (genus Parabacteroides id.954) on Other Crohn's disease
MR Test
Inverse variance weighted (fixed effects)
Inverse variance weighted (multiplicative random effects)
MR Egger
MR RAPS
Weighted median
Weighted mode
0.1
0.0
−0.1
SNP effect on Other Crohn's disease
0.07
0.09
0.11
0.13
SNP effect on Gut microbiota abundance (genus Parabacteroides id.954)

Batch 264 : Gut microbiota abundance (genus Paraprevotella id.962) on Other Crohn's disease

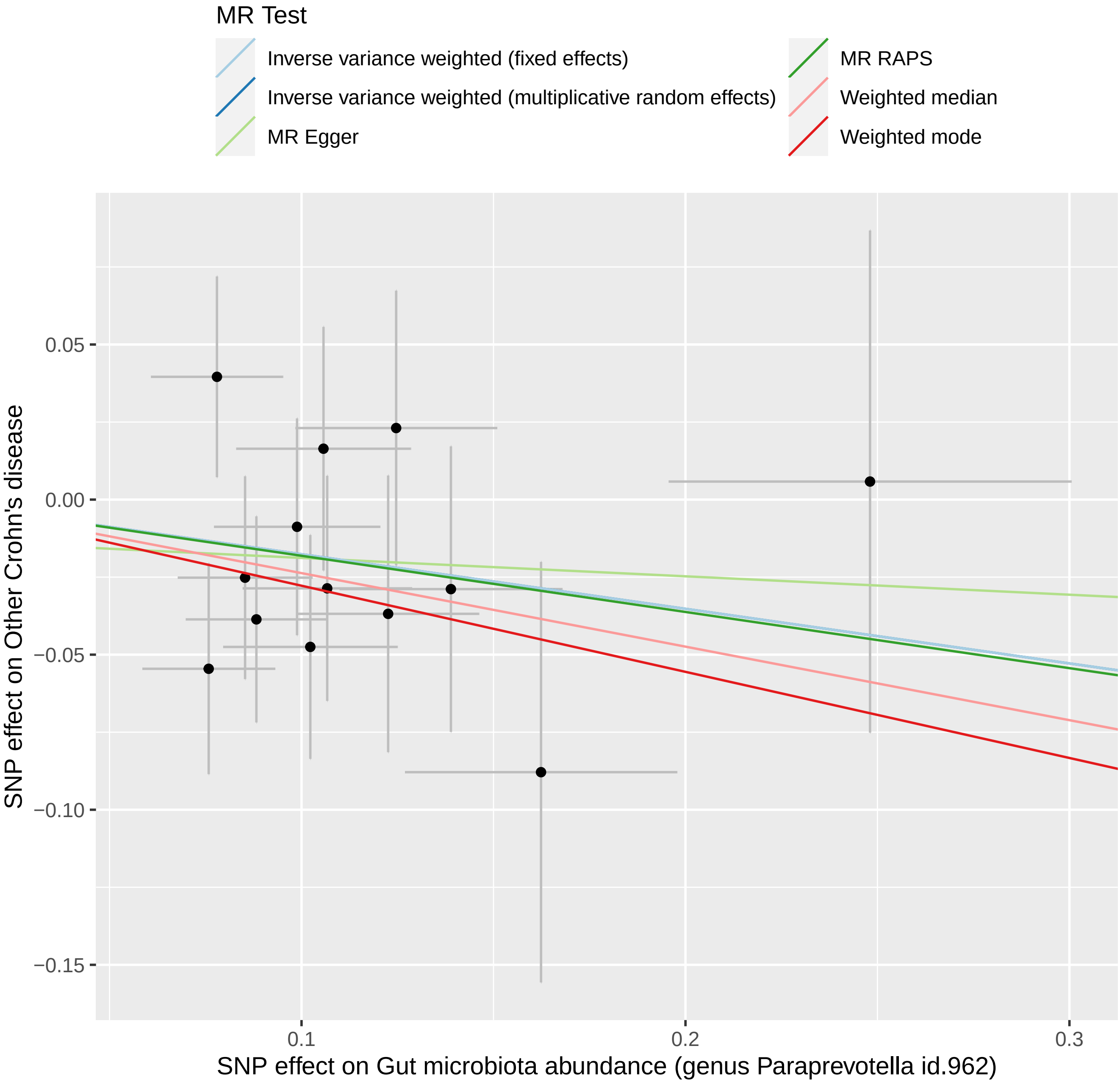

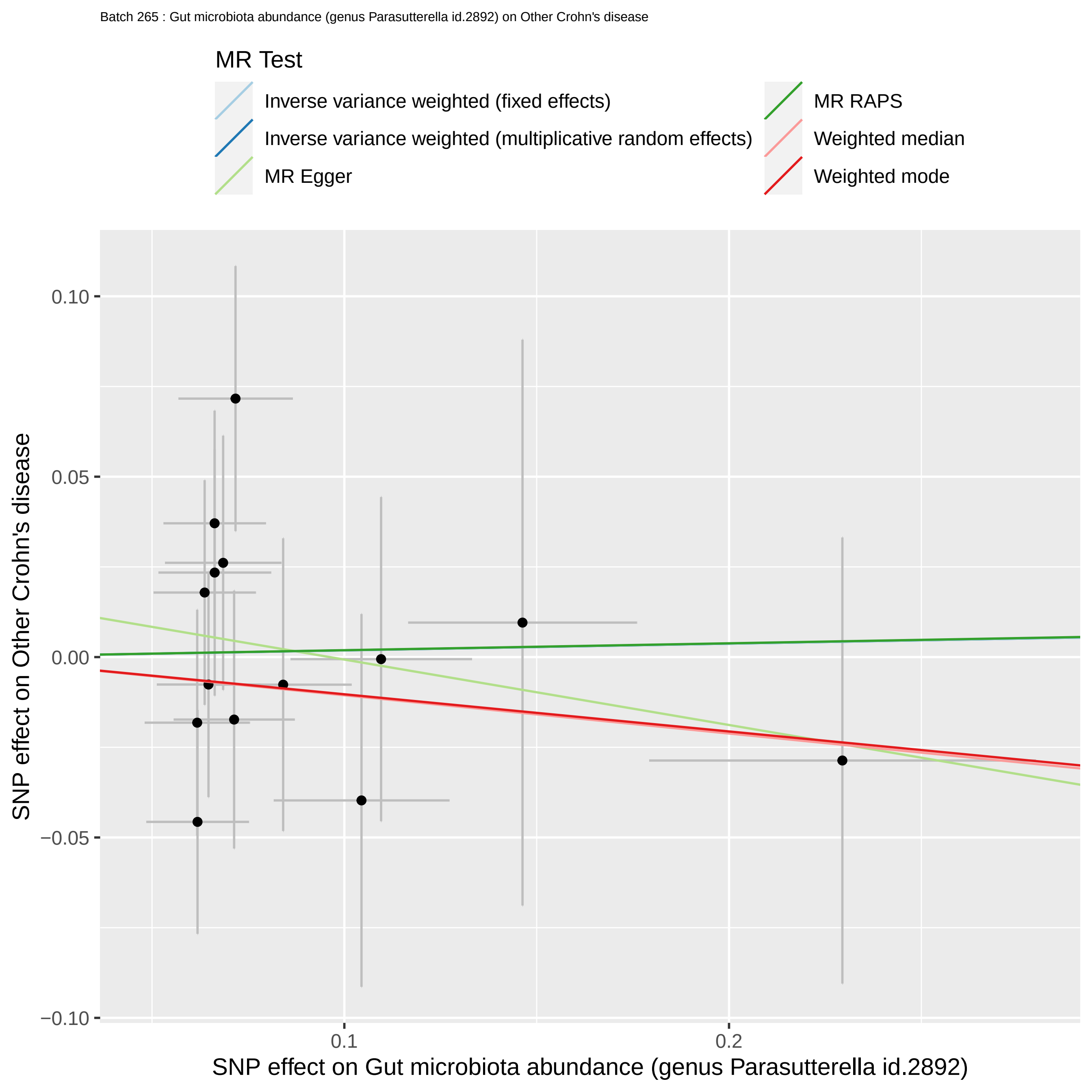
Batch 265 : Gut microbiota abundance (genus Parasutterella id.2892) on Other Crohn's disease
MR Test
Inverse variance weighted (fixed effects)
Inverse variance weighted (multiplicative random effects)
MR Egger
MR RAPS
Weighted median
Weighted mode
0.10
0.05
0.00
−0.05
−0.10
0.1
0.2
SNP effect on Other Crohn's disease
SNP effect on Gut microbiota abundance (genus Parasutterella id.2892)

Batch 266 : Gut microbiota abundance (genus Peptococcus id.2037) on Other Crohn's disease

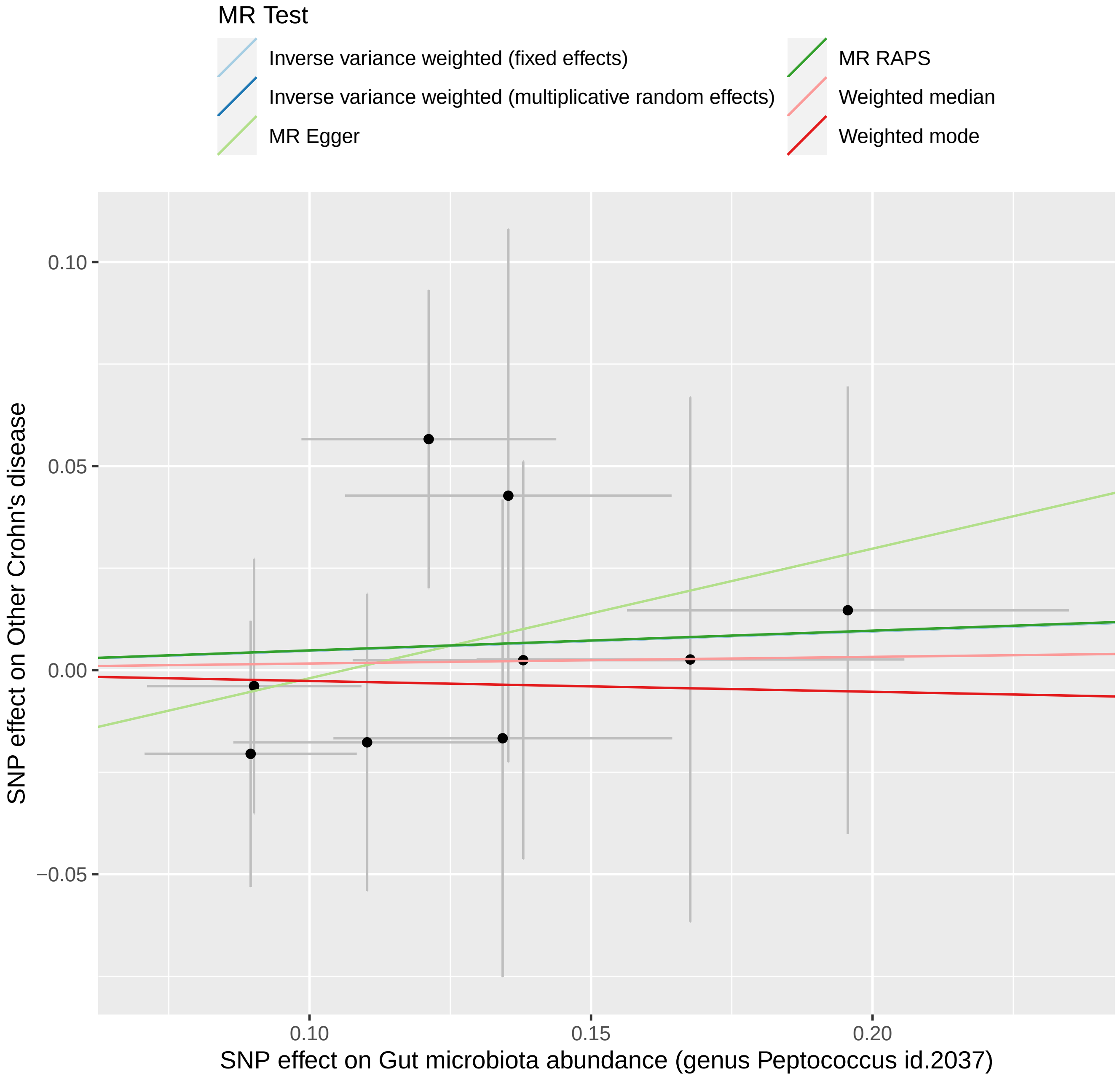

Batch 267 : Gut microbiota abundance (genus Phascolarctobacterium id.2168) on Other Crohn's disease

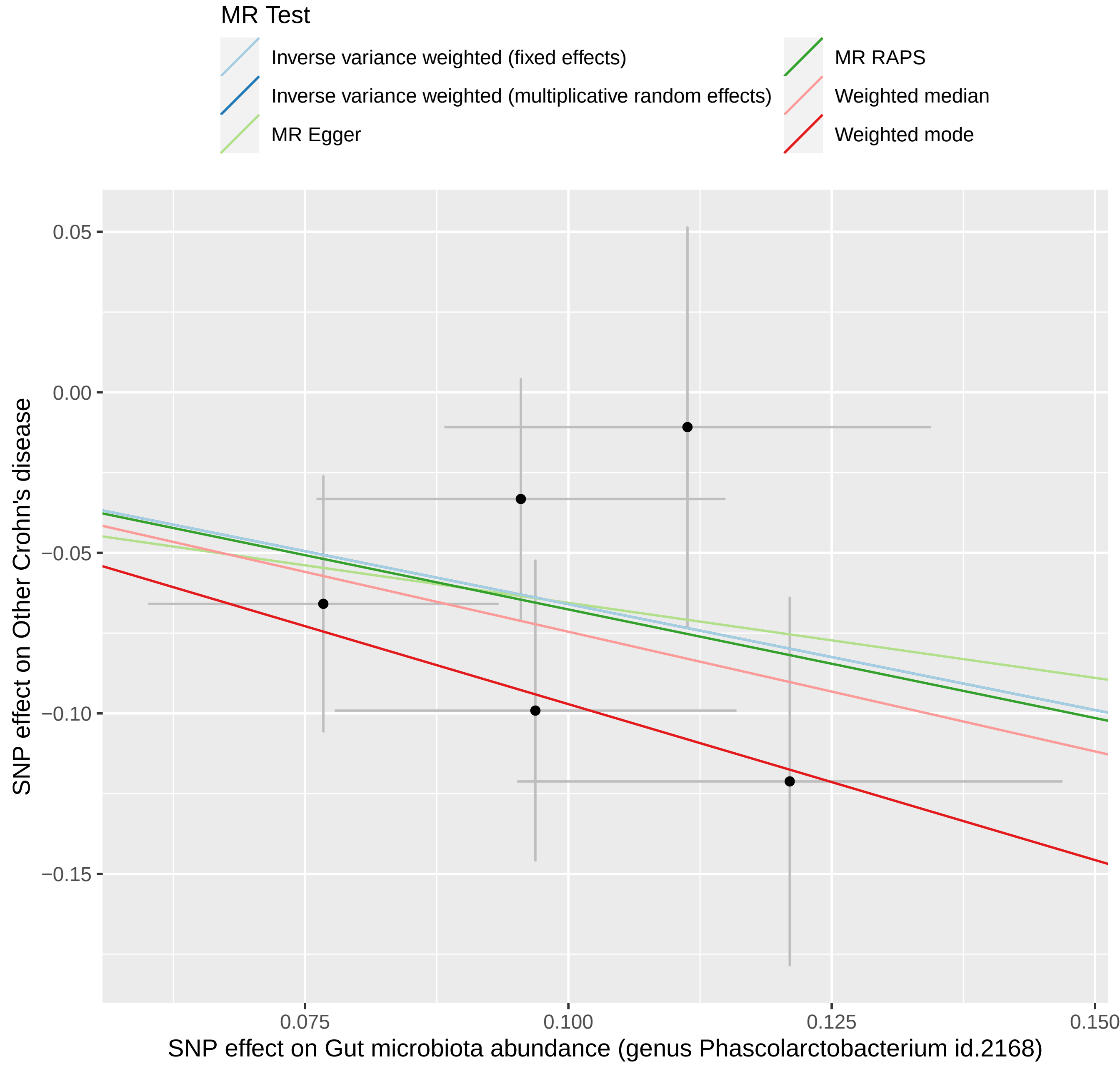

Batch 268 : Gut microbiota abundance (genus Prevotella7 id.11182) on Other Crohn's disease

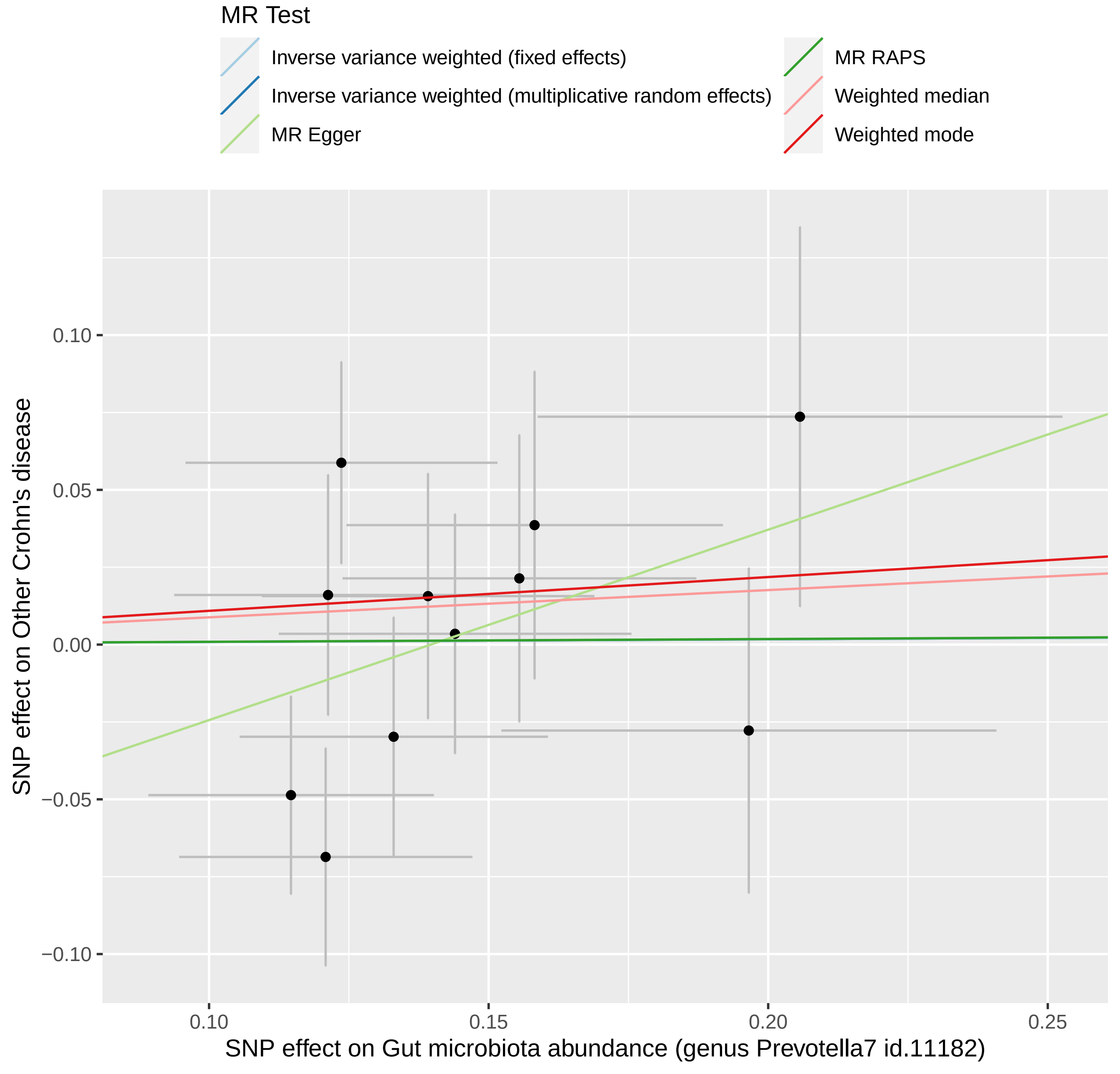

Batch 269 : Gut microbiota abundance (genus Prevotella9 id.11183) on Other Crohn's disease

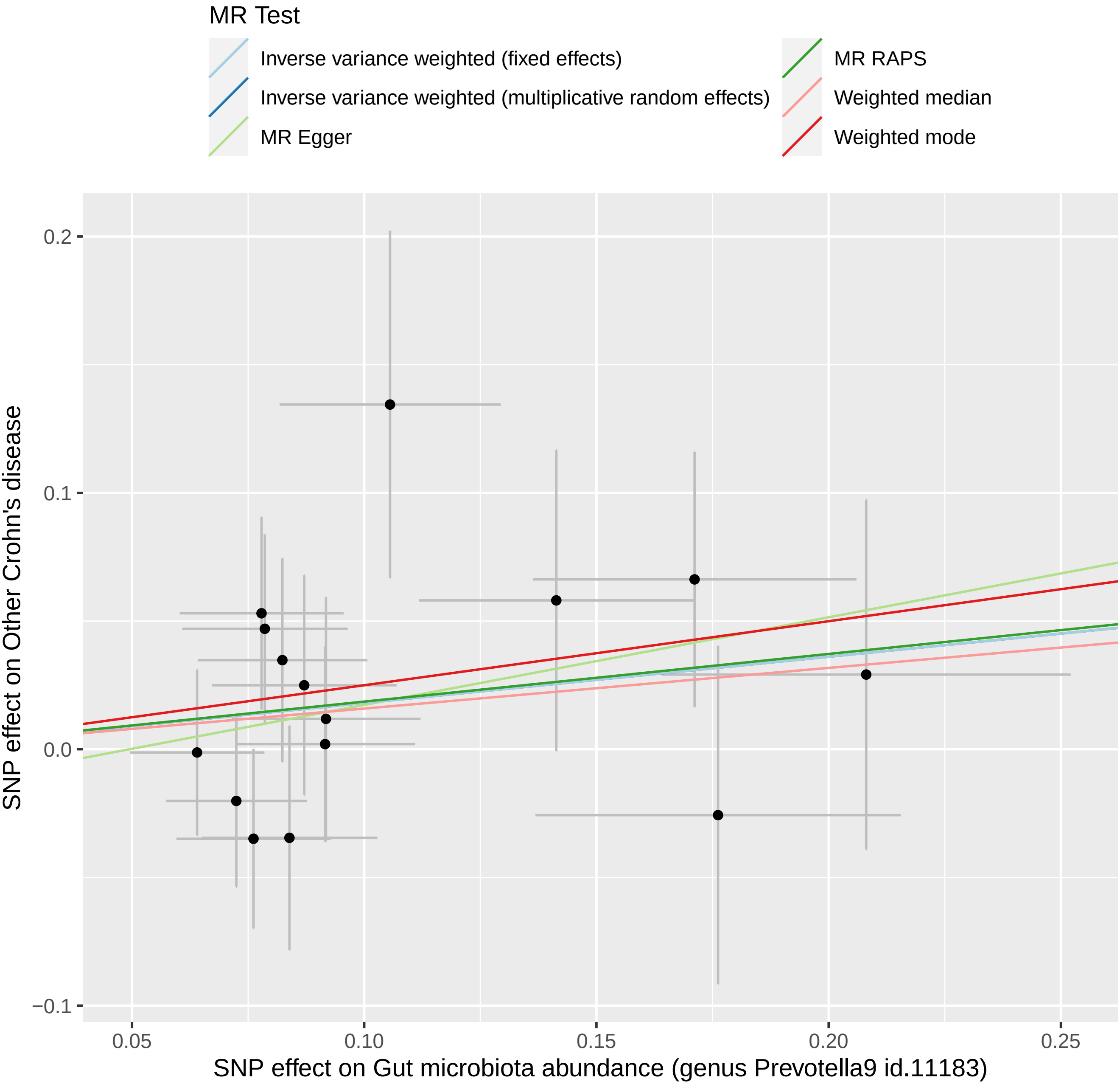

Batch 270 : Gut microbiota abundance (genus Rikenellaceae RC9 gut group id.11191) on Other Crohn's disease

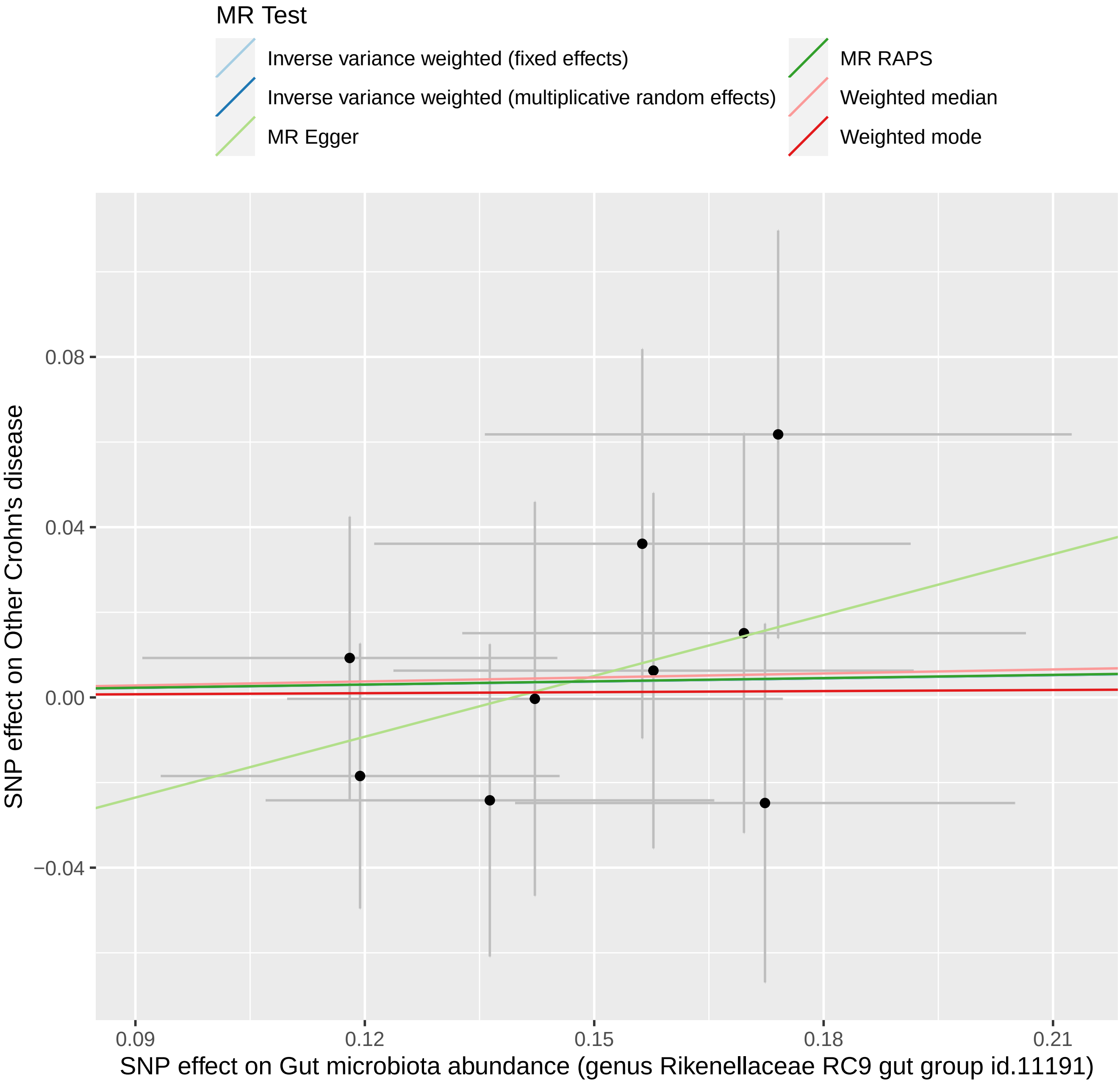

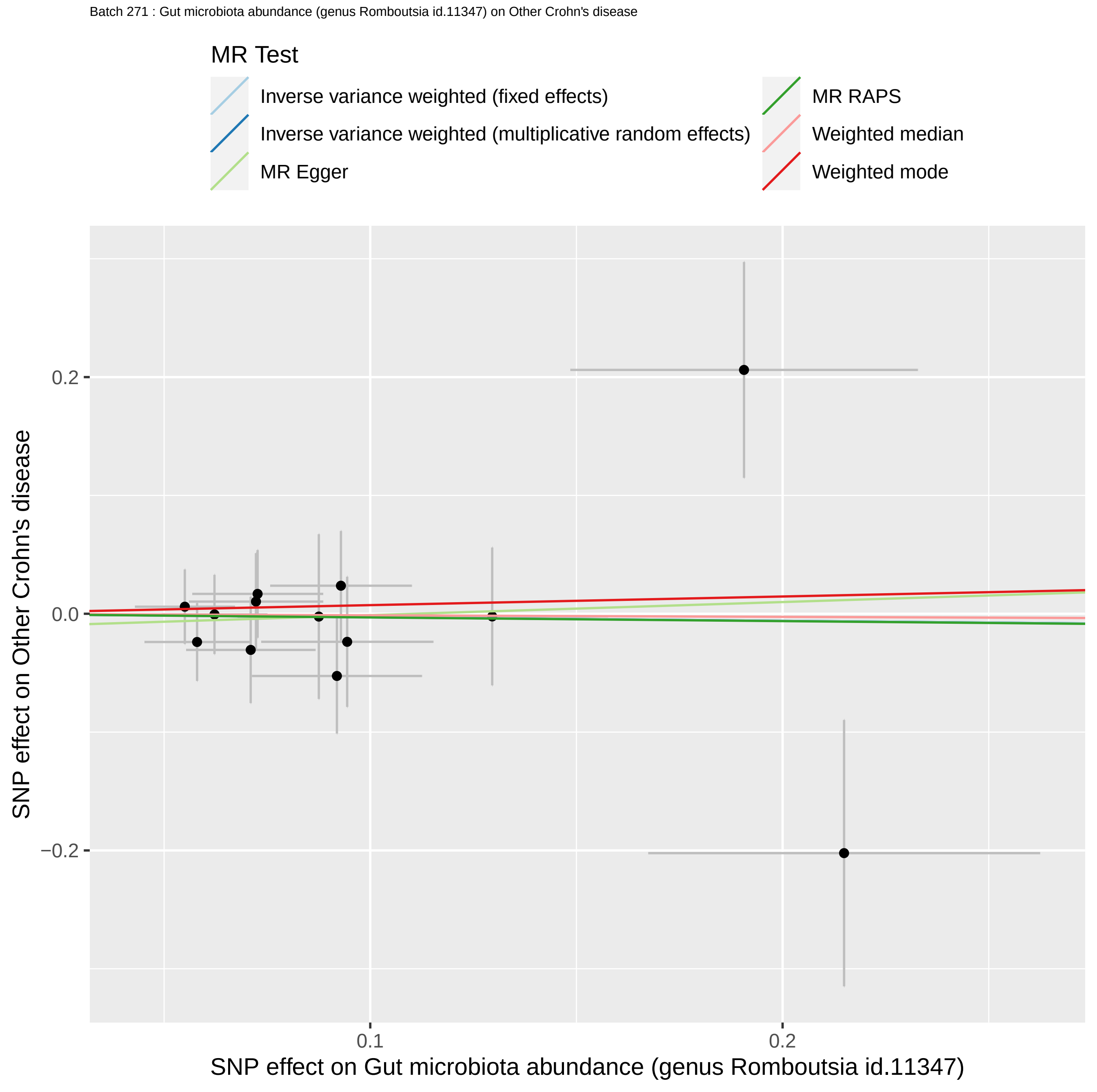
Batch 271 : Gut microbiota abundance (genus Romboutsia id.11347) on Other Crohn's disease
MR Test
Inverse variance weighted (fixed effects)
Inverse variance weighted (multiplicative random effects)
MR Egger
MR RAPS
Weighted median
Weighted mode
0.2
0.0
−0.2
0.1
0.2
SNP effect on Other Crohn's disease
SNP effect on Gut microbiota abundance (genus Romboutsia id.11347)

Batch 272 : Gut microbiota abundance (genus Roseburia id.2012) on Other Crohn's disease

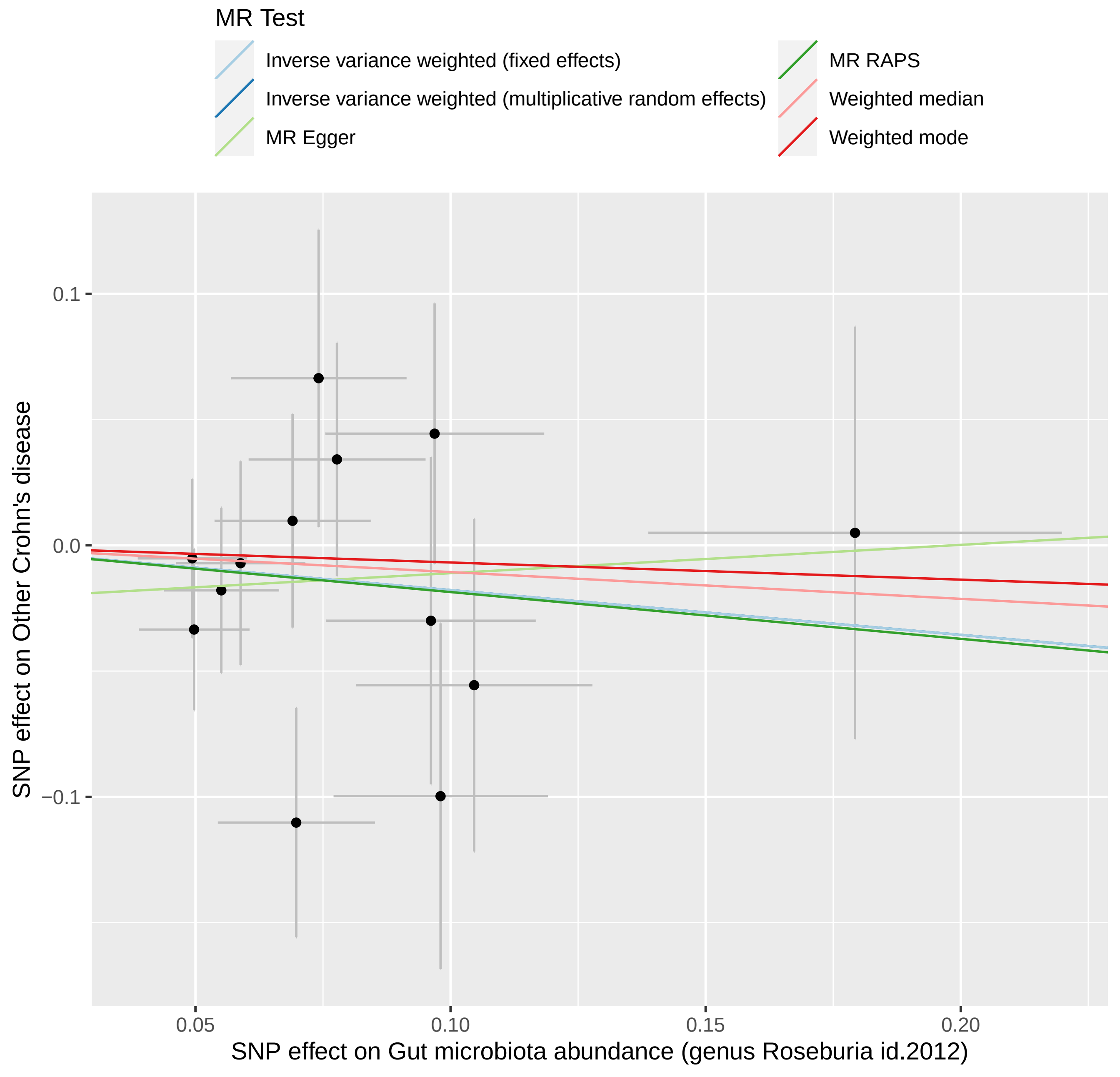

Batch 273 : Gut microbiota abundance (genus Ruminiclostridium5 id.11355) on Other Crohn's disease

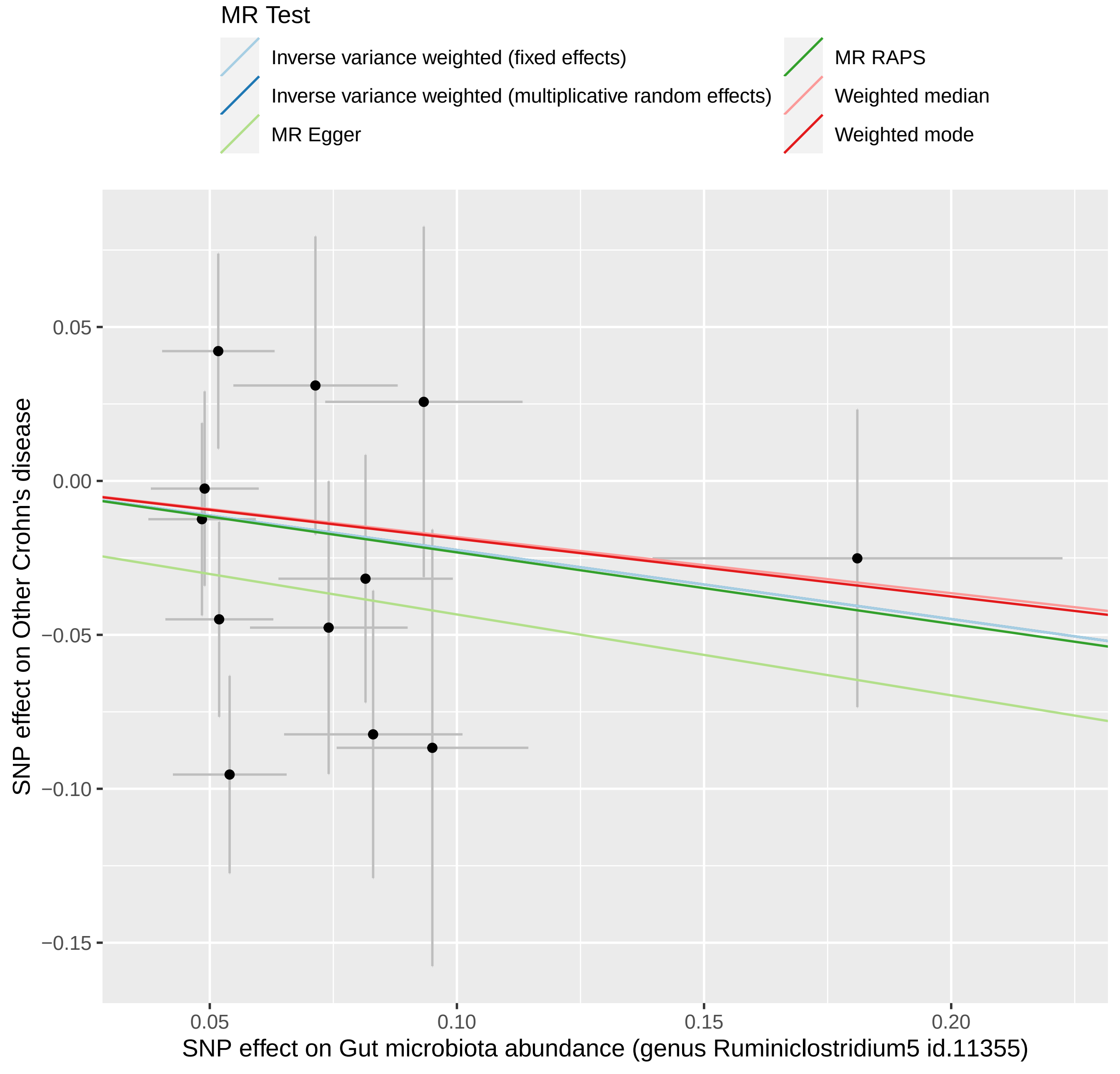

Batch 274 : Gut microbiota abundance (genus Ruminiclostridium6 id.11356) on Other Crohn's disease

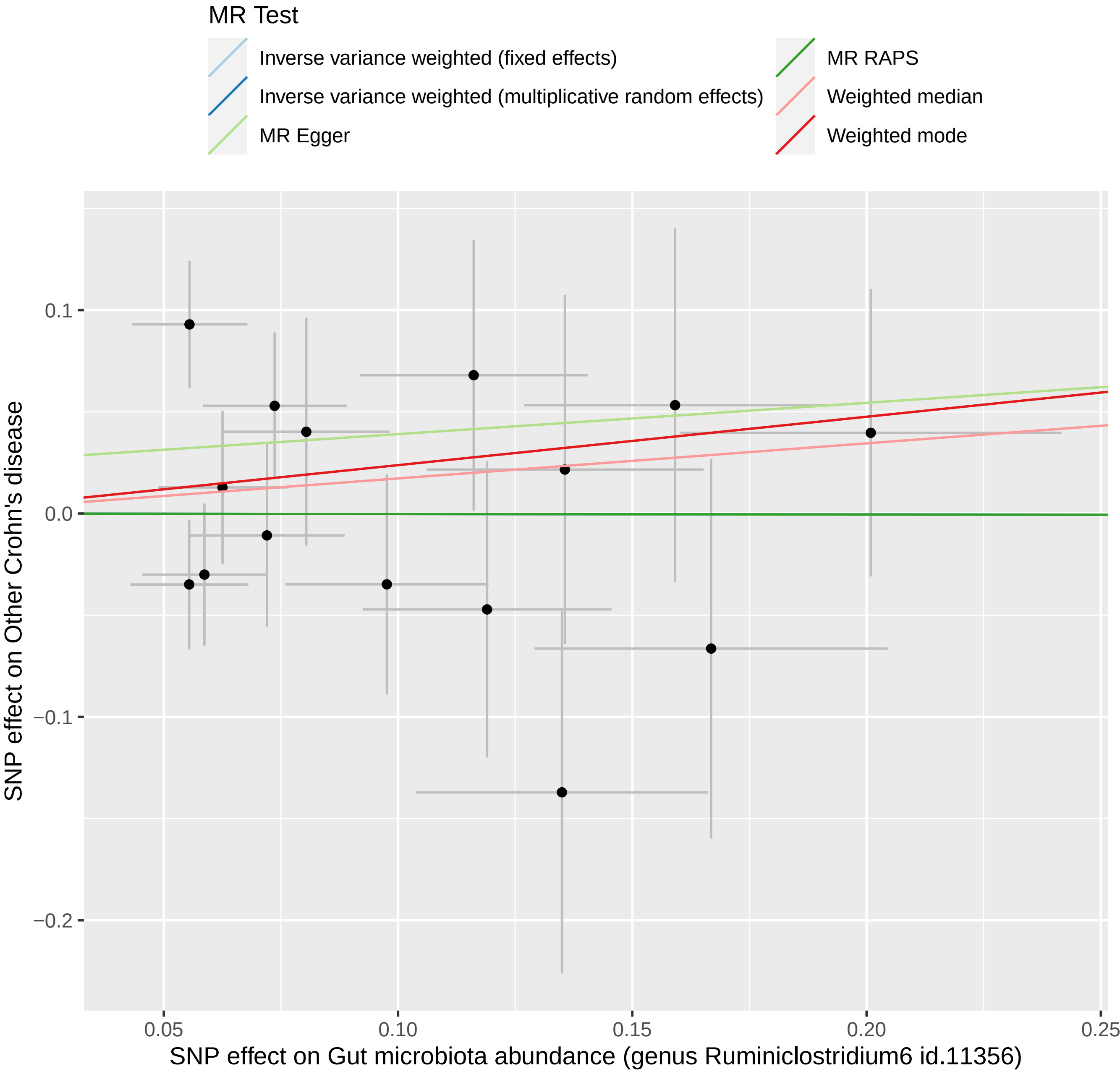

Batch 275 : Gut microbiota abundance (genus Ruminiclostridium9 id.11357) on Other Crohn's disease

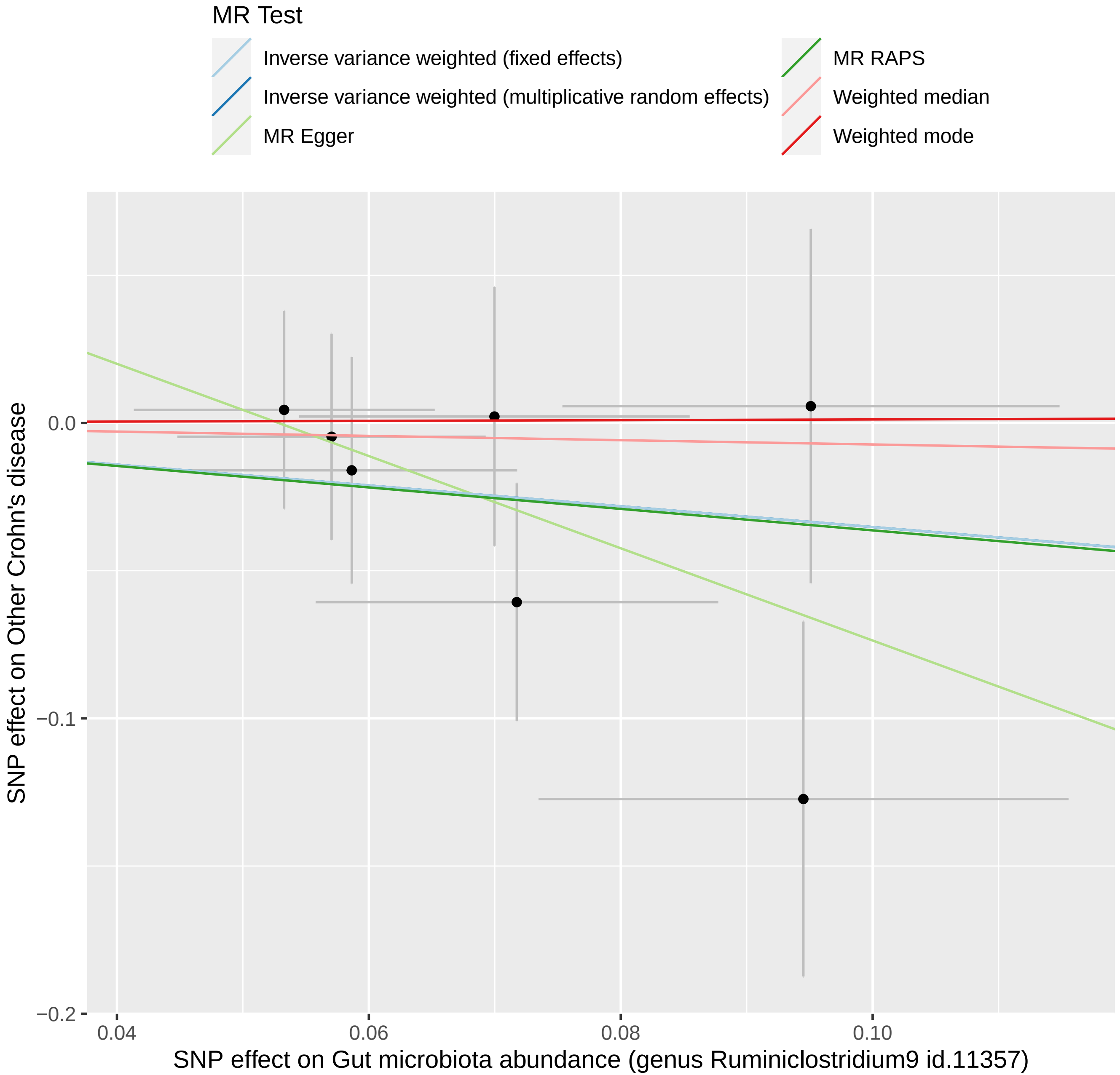

Batch 276 : Gut microbiota abundance (genus Ruminococcaceae NK4A214 group id.11358) on Other Crohn's disease

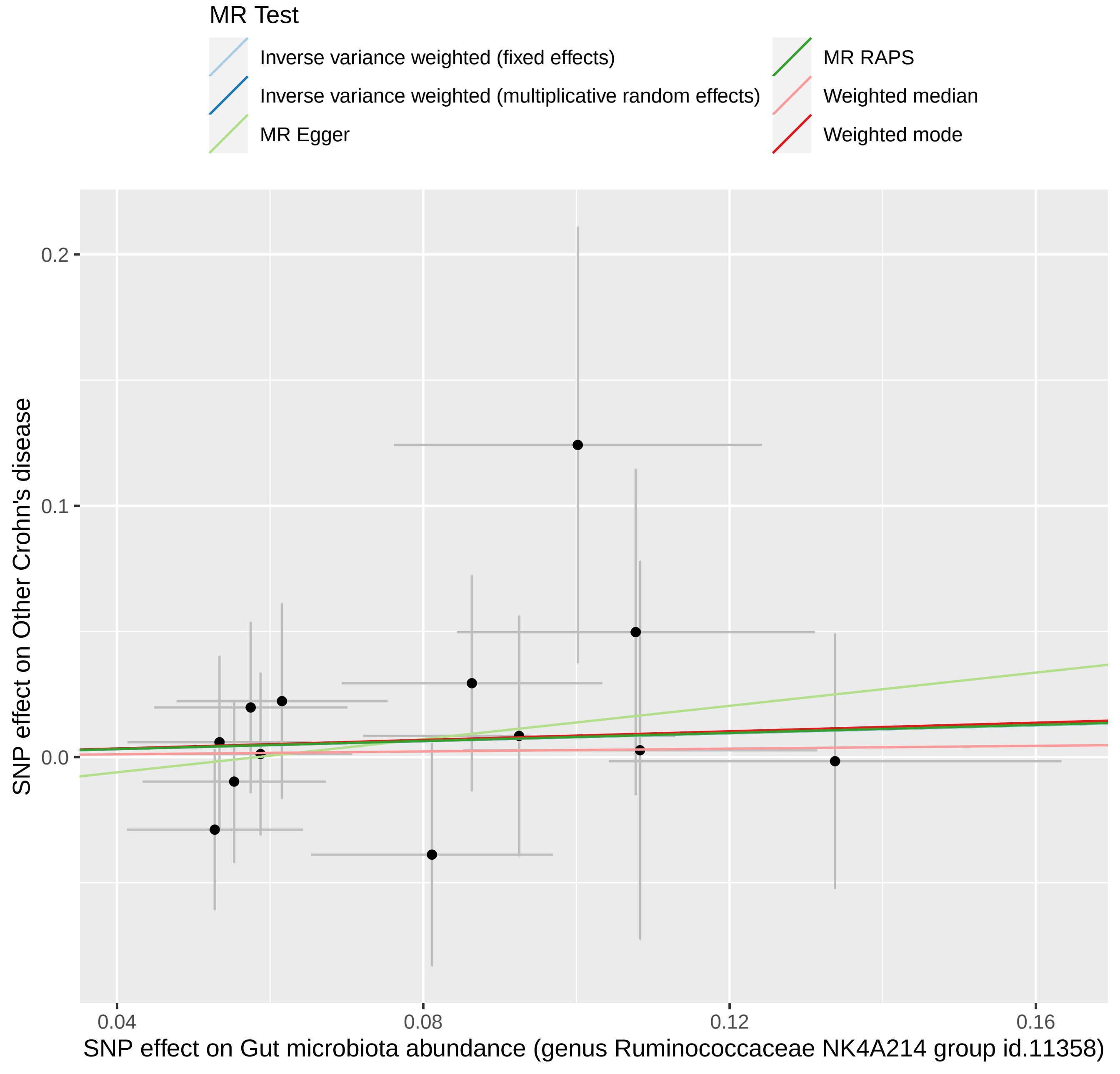

Batch 277 : Gut microbiota abundance (genus Ruminococcaceae UCG002 id.11360) on Other Crohn's disease

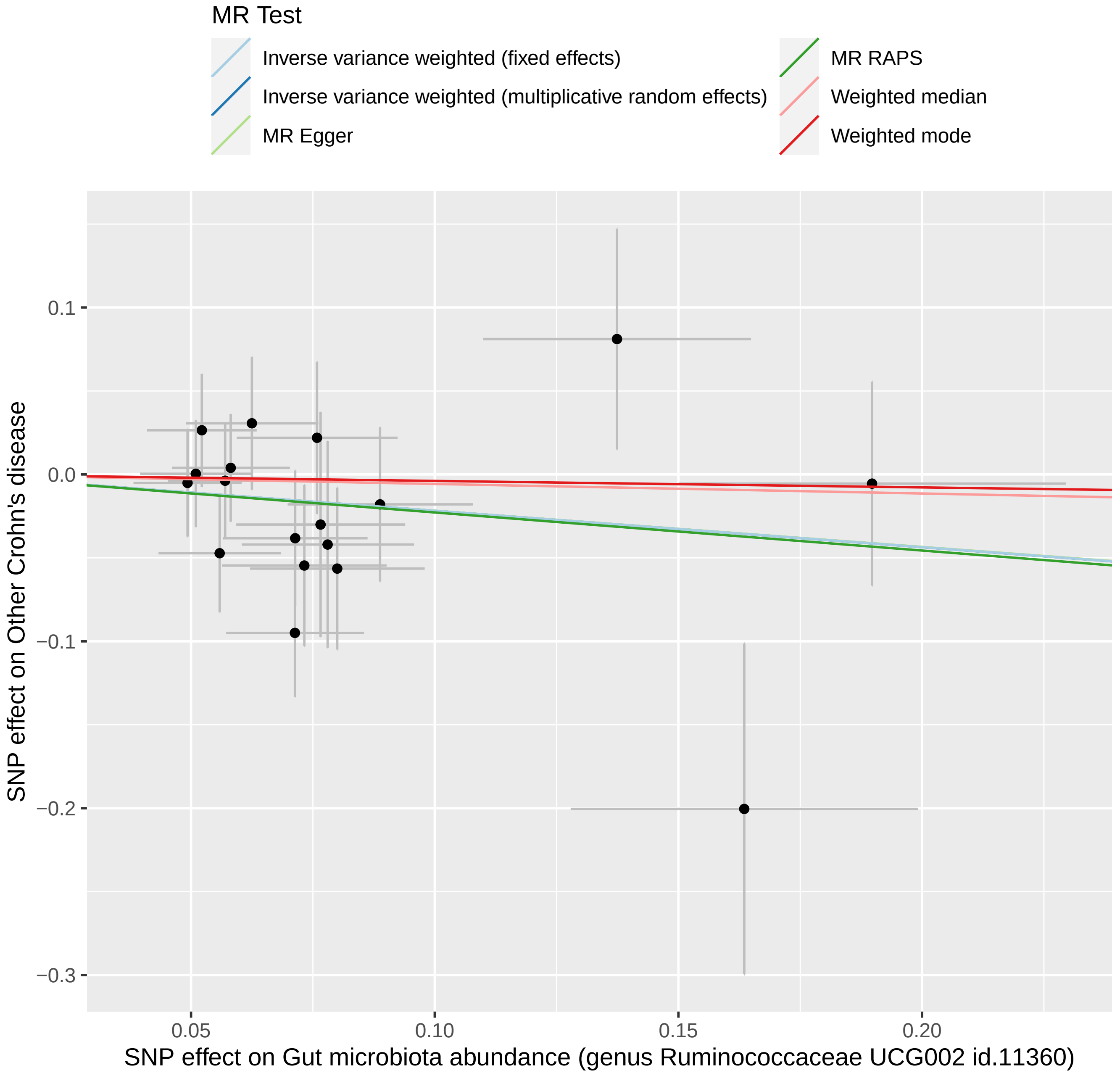

Batch 278 : Gut microbiota abundance (genus Ruminococcaceae UCG003 id.11361) on Other Crohn's disease

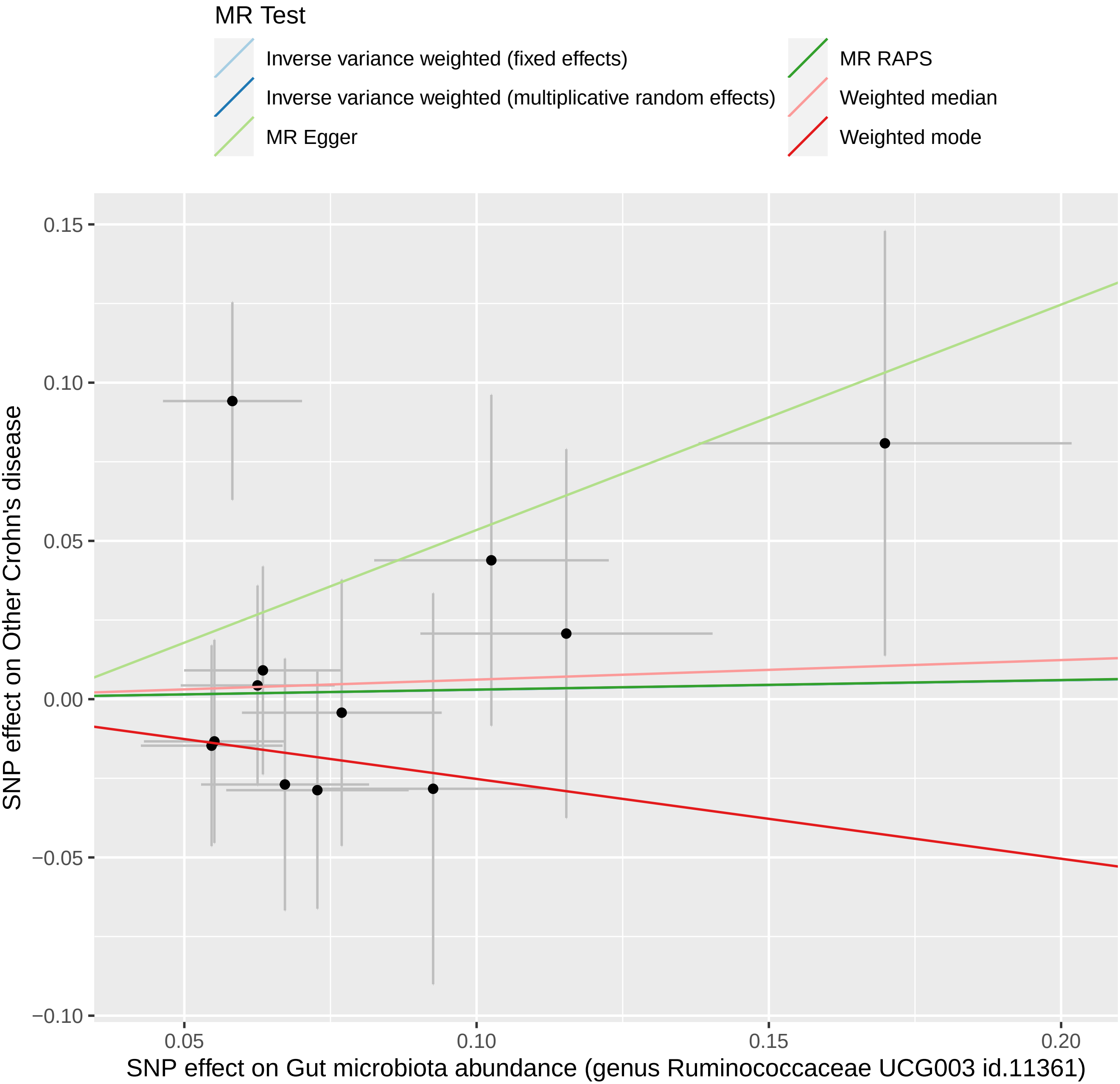

Batch 279 : Gut microbiota abundance (genus Ruminococcaceae UCG004 id.11362) on Other Crohn's disease

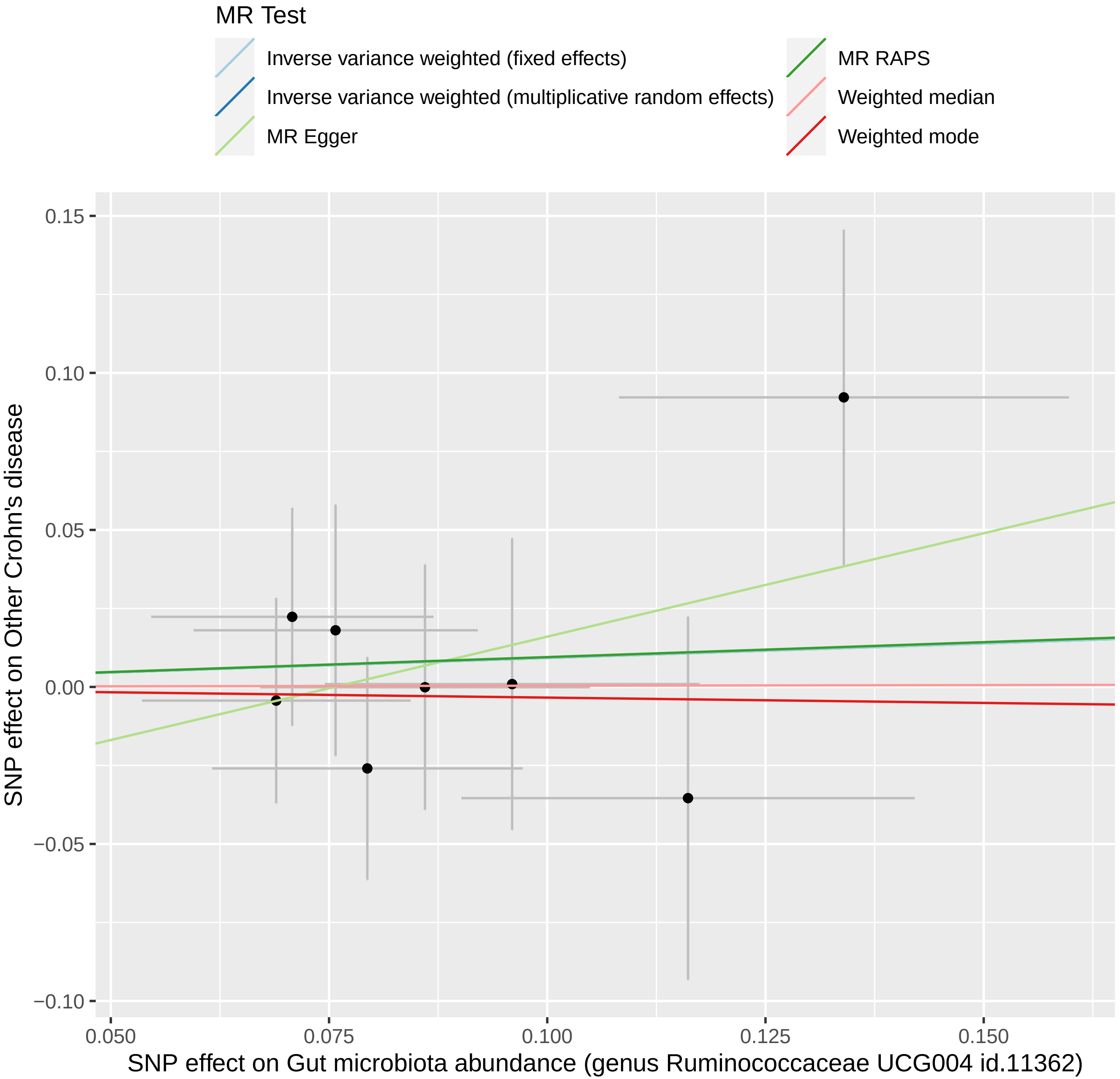

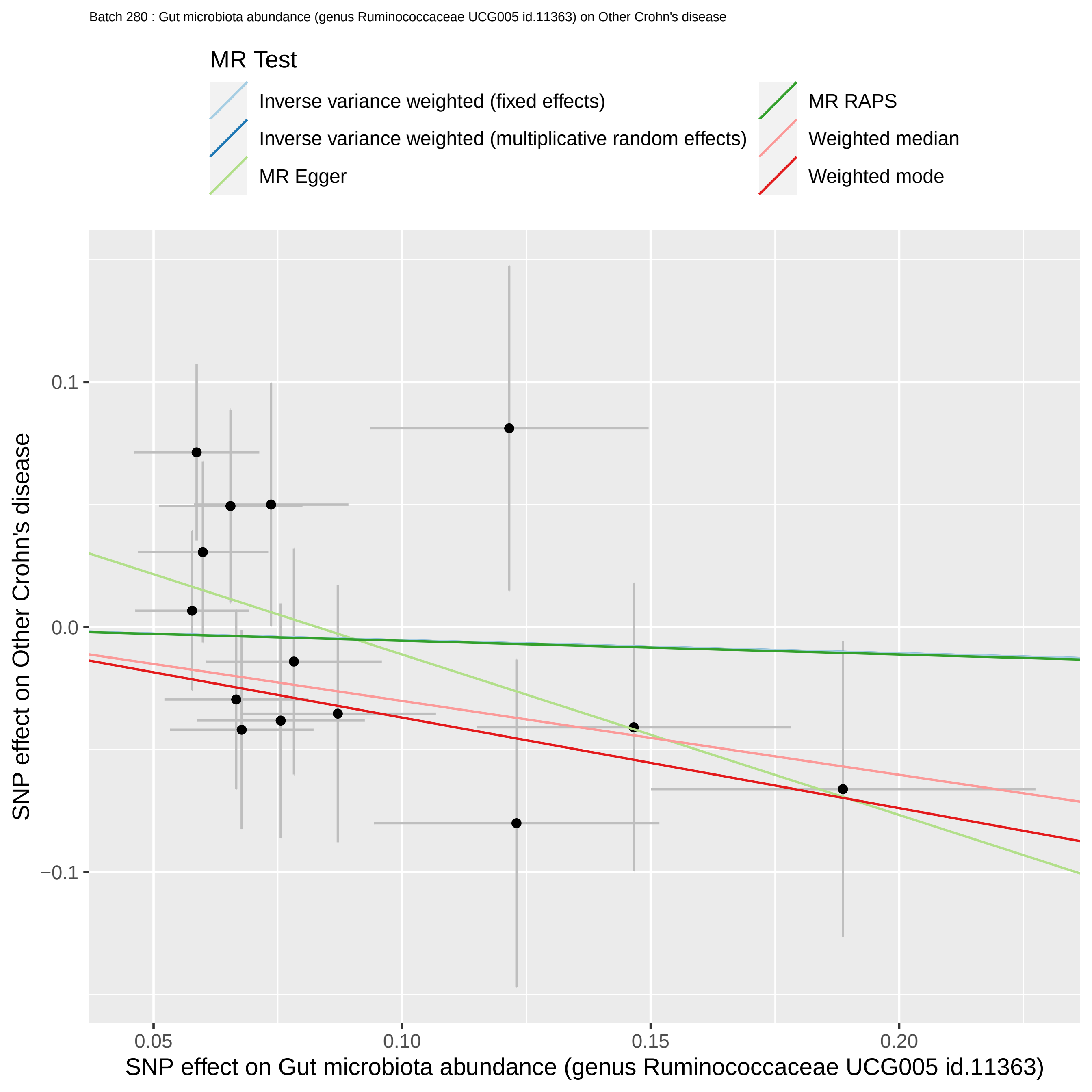

Batch 280 : Gut microbiota abundance (genus Ruminococcaceae UCG005 id.11363) on Other Crohn's disease
MR Test
Inverse variance weighted (fixed effects)
Inverse variance weighted (multiplicative random effects)
MR Egger
MR RAPS
Weighted median
Weighted mode
0.1
0.0
−0.1
0.05
0.10
0.15
0.20
SNP effect on Other Crohn's disease
SNP effect on Gut microbiota abundance (genus Ruminococcaceae UCG005 id.11363)

Batch 281 : Gut microbiota abundance (genus Ruminococcaceae UCG009 id.11366) on Other Crohn's disease

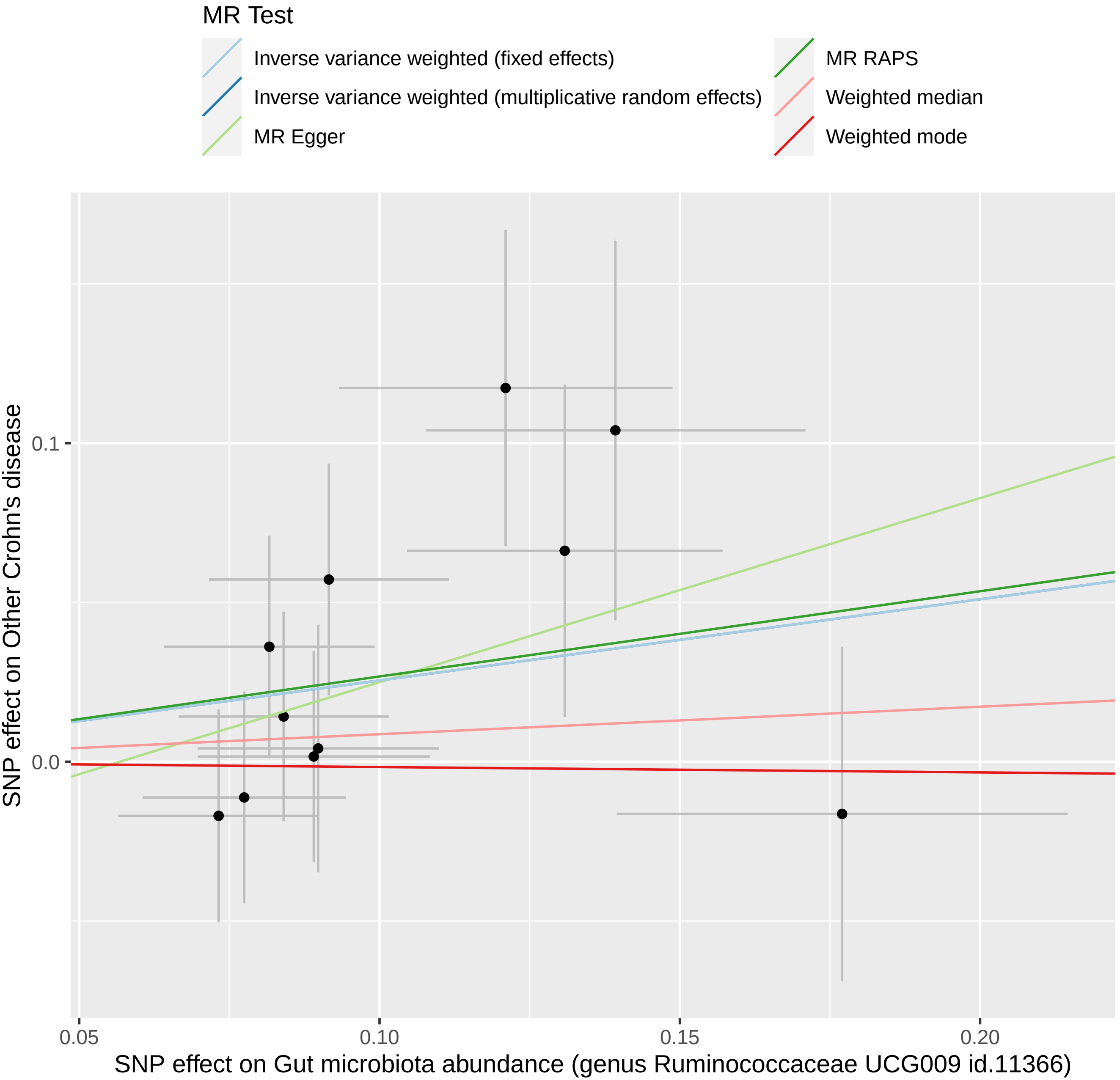

Batch 282 : Gut microbiota abundance (genus Ruminococcaceae UCG010 id.11367) on Other Crohn's disease

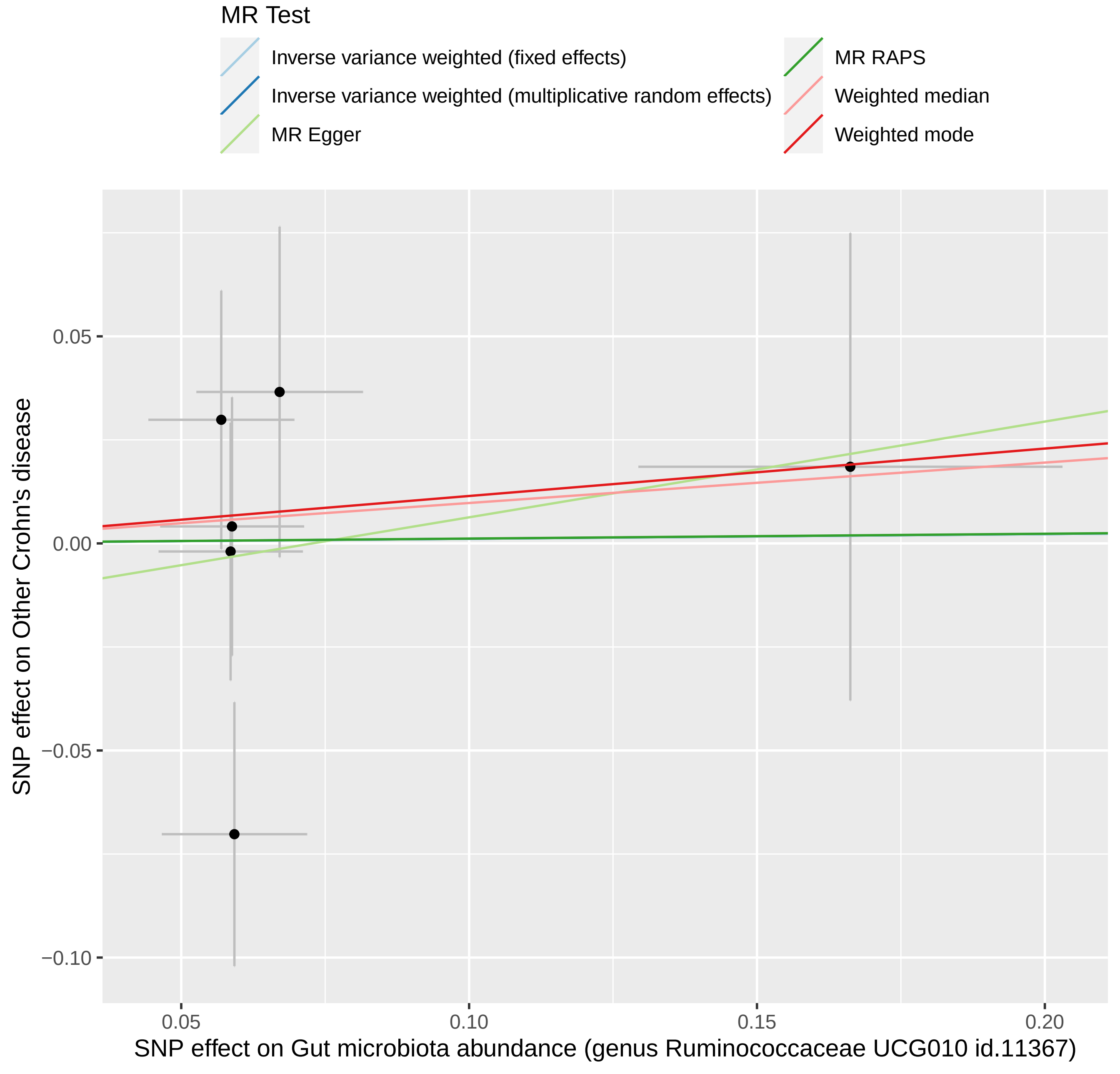

Batch 283 : Gut microbiota abundance (genus Ruminococcaceae UCG011 id.11368) on Other Crohn's disease

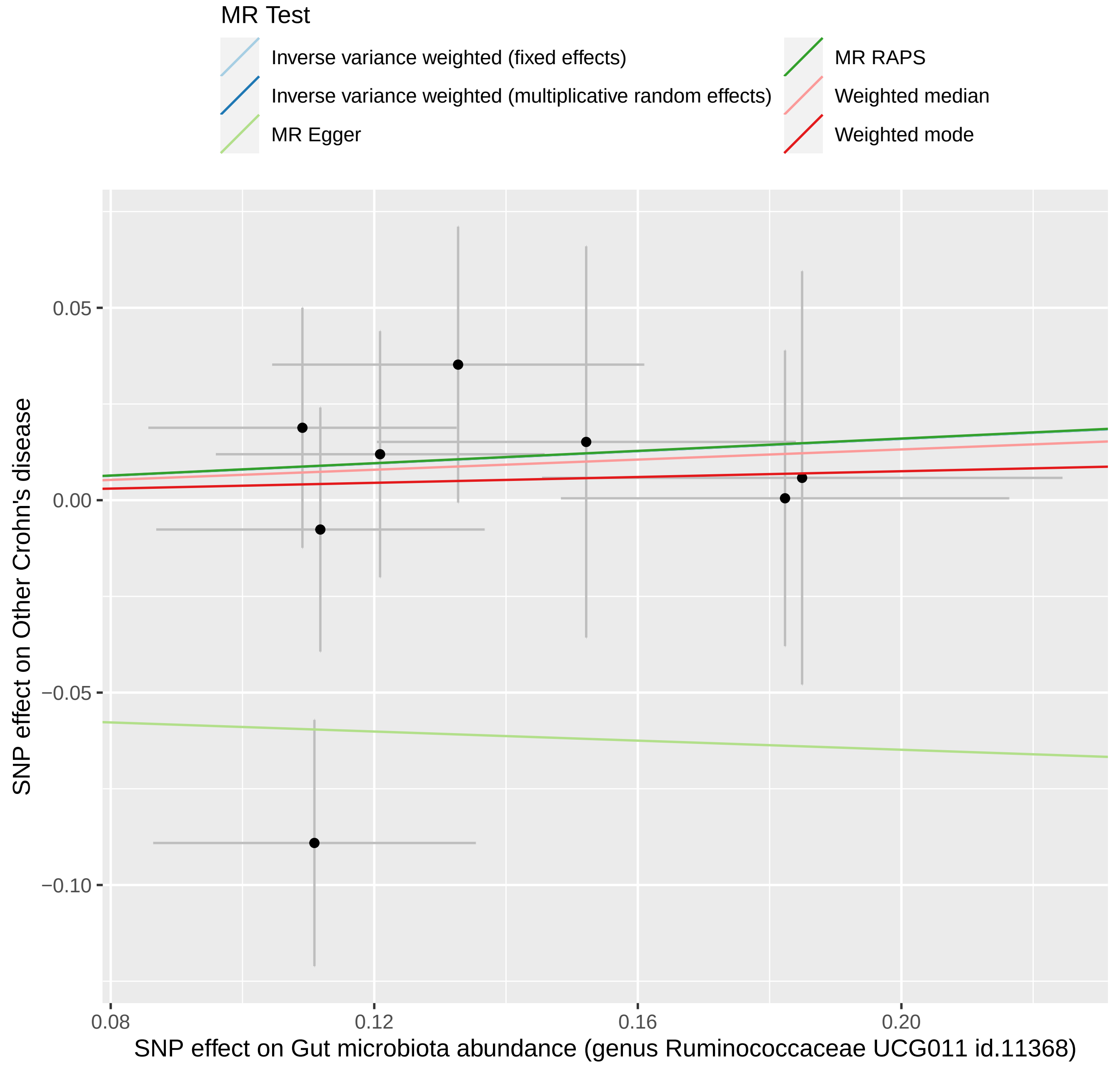

Batch 284 : Gut microbiota abundance (genus Ruminococcaceae UCG013 id.11370) on Other Crohn's disease

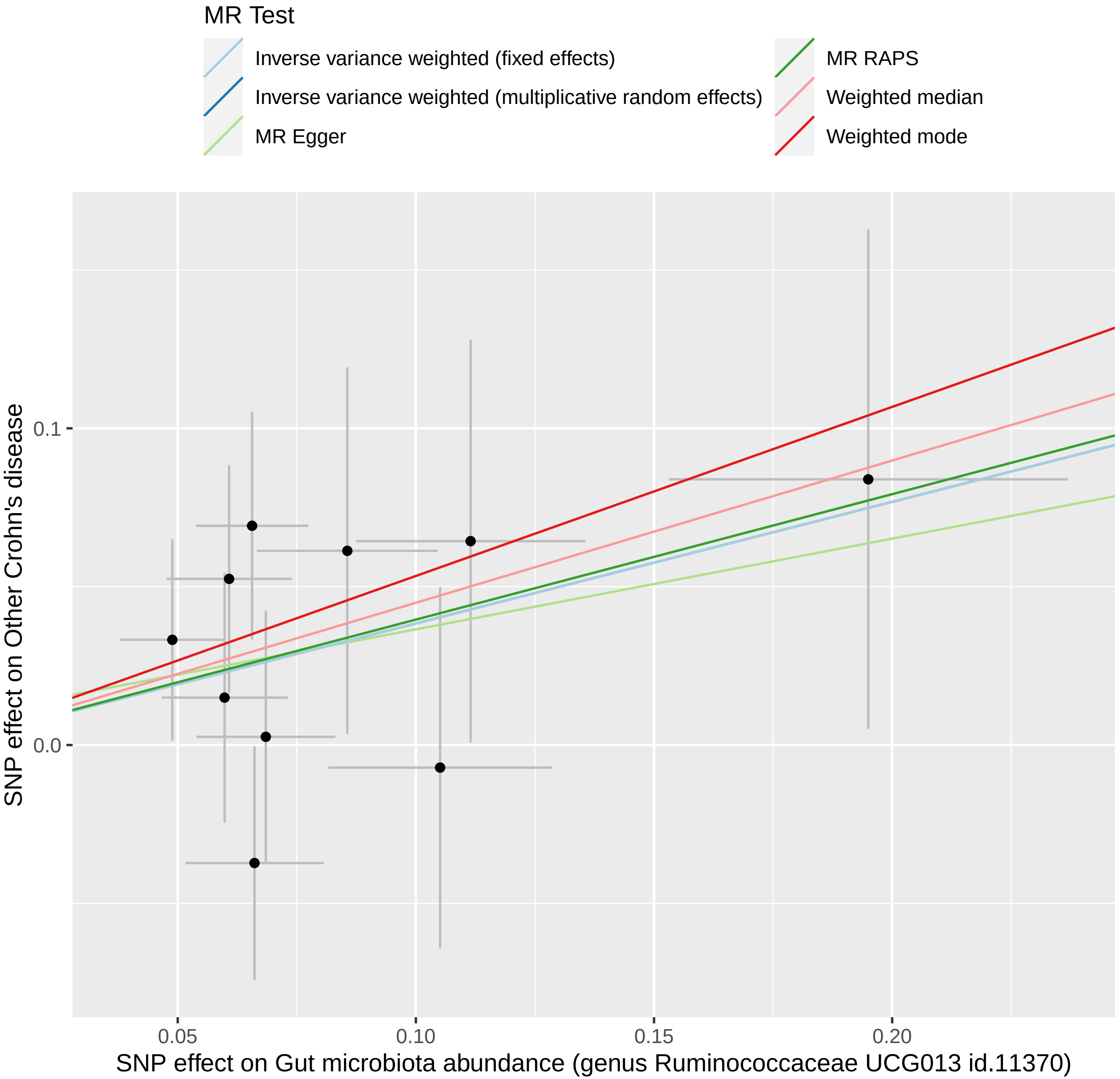

Batch 285 : Gut microbiota abundance (genus Ruminococcaceae UCG014 id.11371) on Other Crohn's disease

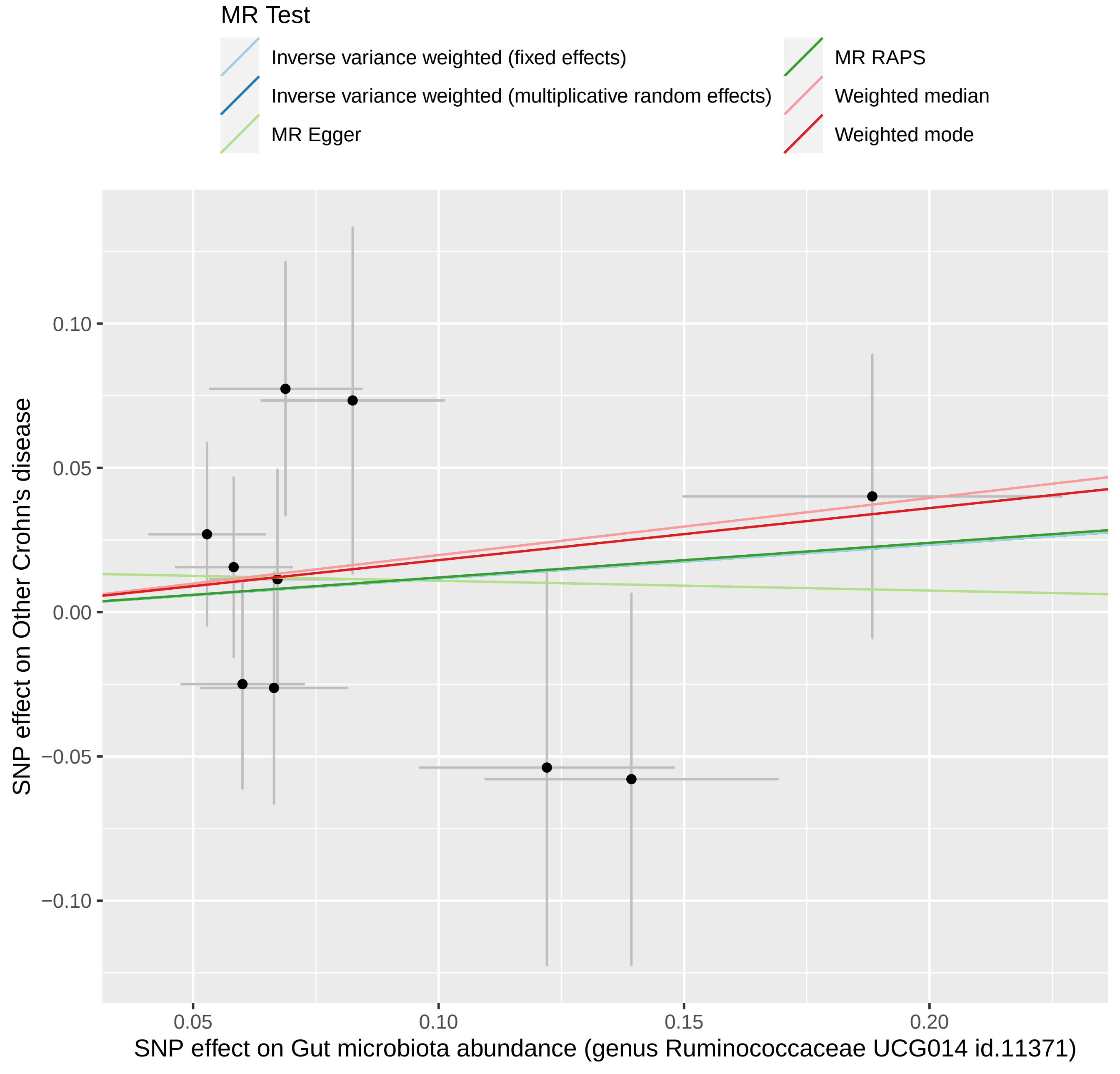

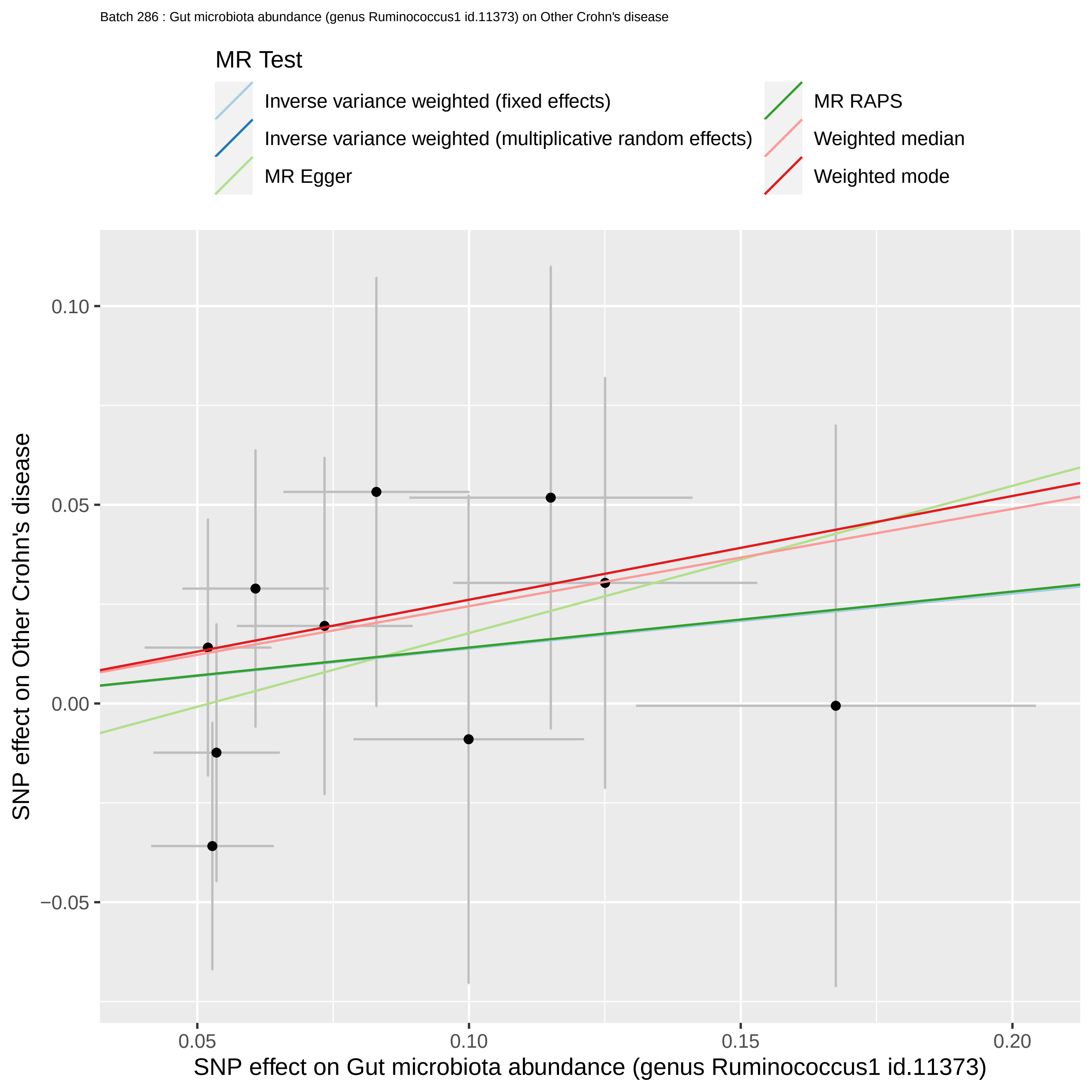

Batch 286 : Gut microbiota abundance (genus Ruminococcus1 id.11373) on Other Crohn's disease
MR Test
Inverse variance weighted (fixed effects)
Inverse variance weighted (multiplicative random effects)
MR Egger
MR RAPS
Weighted median
Weighted mode
0.10
0.05
0.00
−0.05
SNP effect on Other Crohn's disease
0.05
0.10
0.15
0.20
SNP effect on Gut microbiota abundance (genus Ruminococcus1 id.11373)

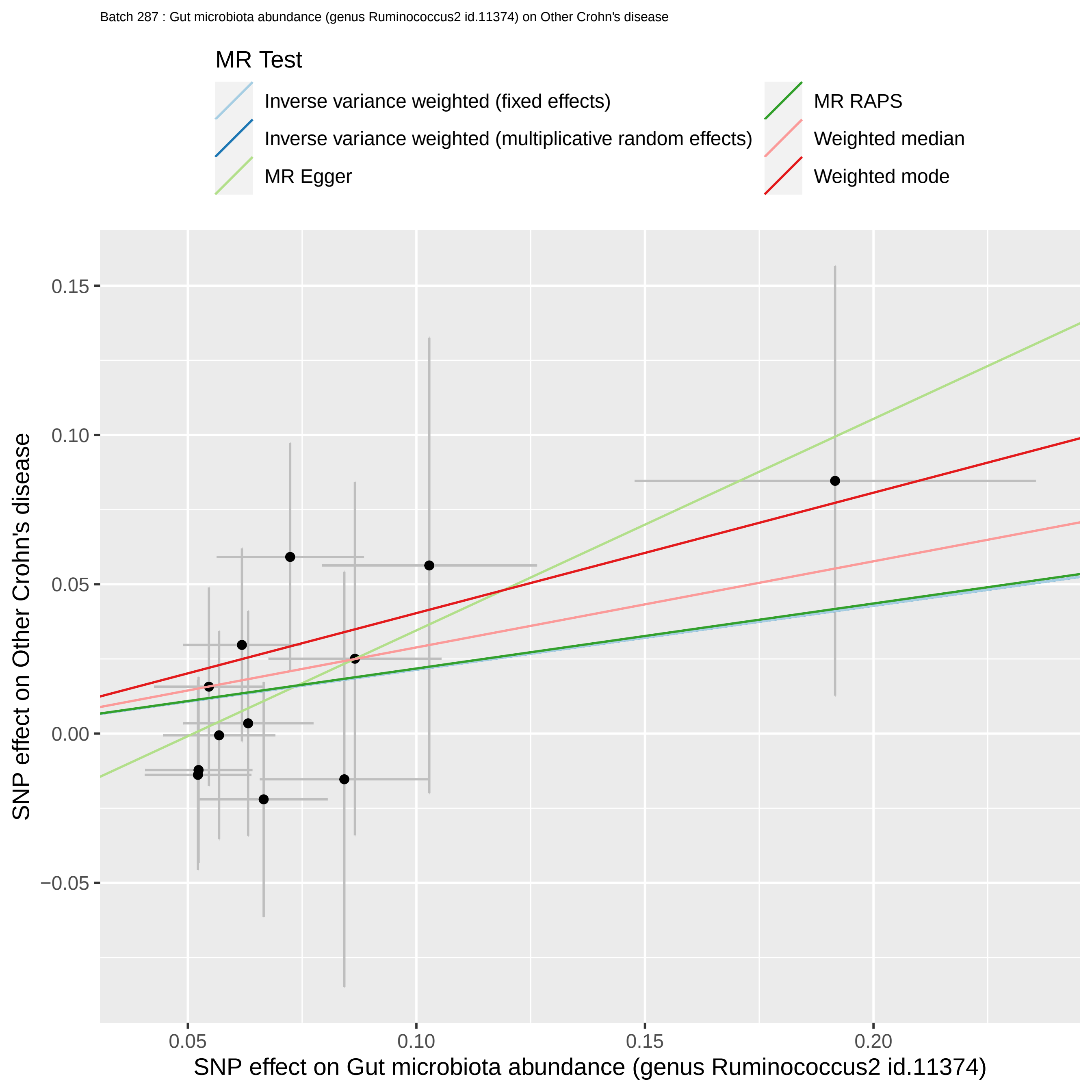

Batch 287 : Gut microbiota abundance (genus Ruminococcus2 id.11374) on Other Crohn's disease
MR Test
Inverse variance weighted (fixed effects)
Inverse variance weighted (multiplicative random effects)
MR Egger
MR RAPS
Weighted median
Weighted mode
0.15
0.10
0.05
0.00
−0.05
0.05
0.10
0.15
0.20
SNP effect on Other Crohn's disease
SNP effect on Gut microbiota abundance (genus Ruminococcus2 id.11374)

Batch 288 : Gut microbiota abundance (genus Ruminococcus gauvreauii group id.11342) on Other Crohn's disease

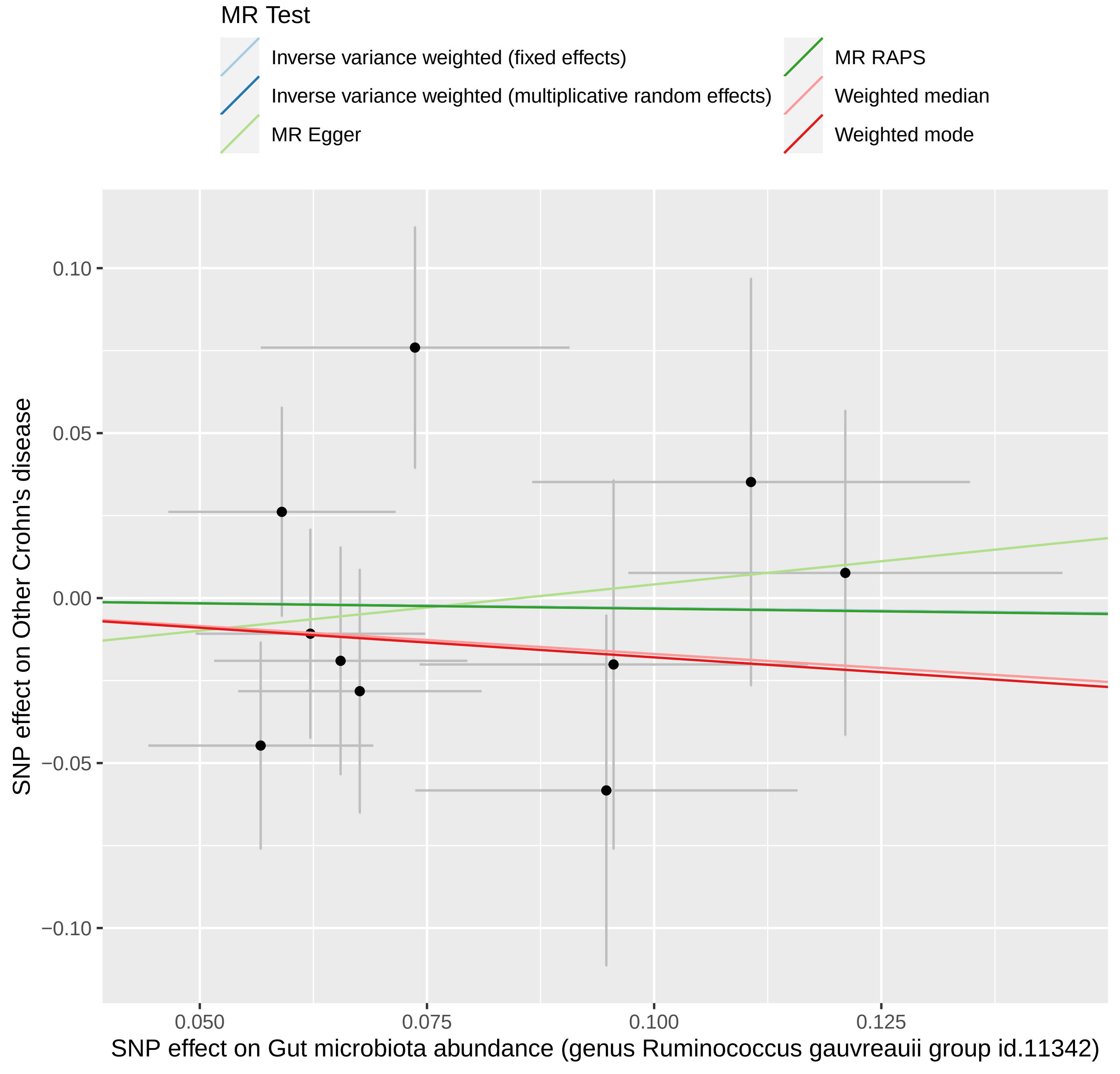

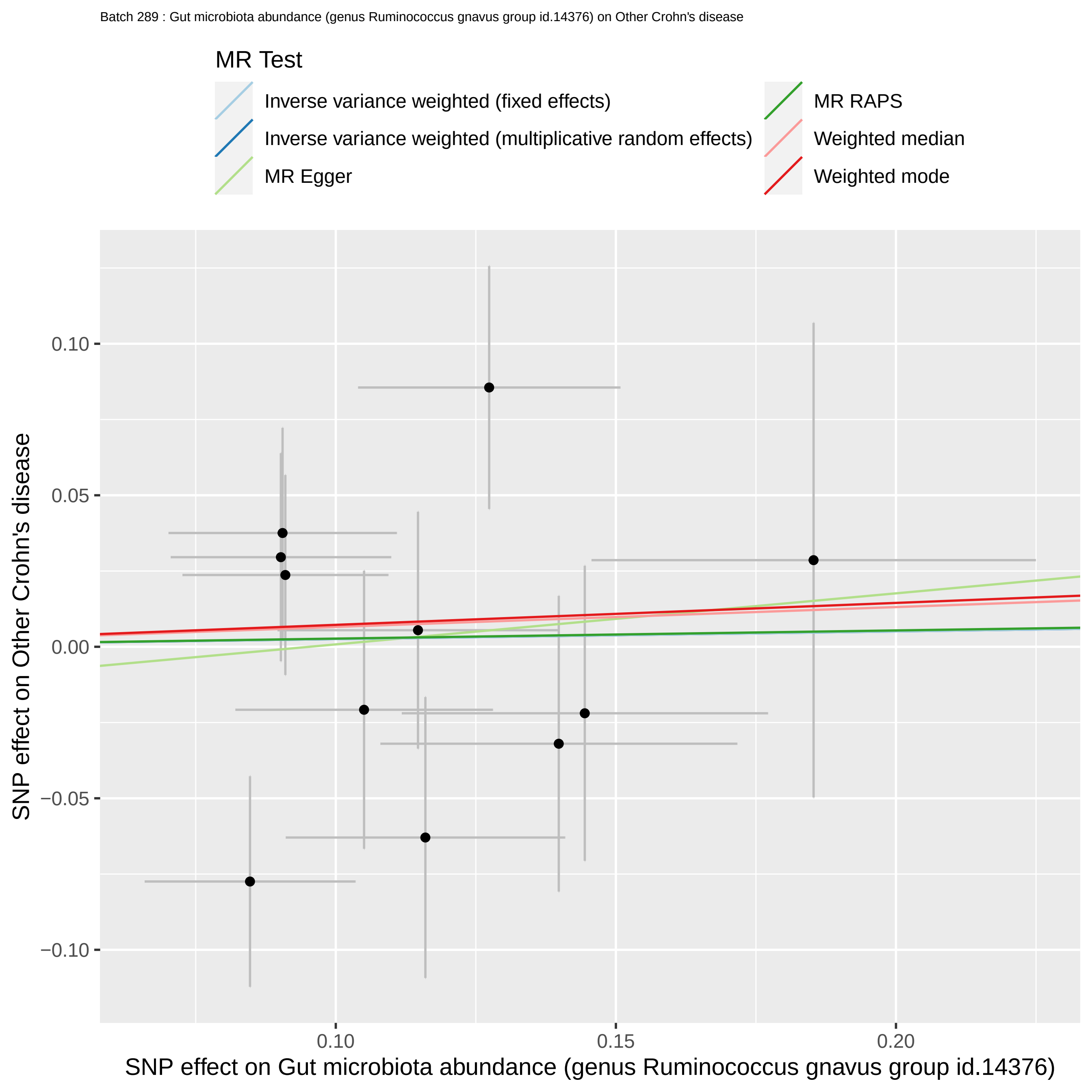
Batch 289 : Gut microbiota abundance (genus Ruminococcus gnavus group id.14376) on Other Crohn's disease
MR Test
Inverse variance weighted (fixed effects)
Inverse variance weighted (multiplicative random effects)
MR Egger
MR RAPS
Weighted median
Weighted mode
0.10
0.05
0.00
−0.05
−0.10
SNP effect on Other Crohn's disease
0.10
0.15
0.20
SNP effect on Gut microbiota abundance (genus Ruminococcus gnavus group id.14376)

Batch 290 : Gut microbiota abundance (genus Ruminococcus torques group id.14377) on Other Crohn's disease

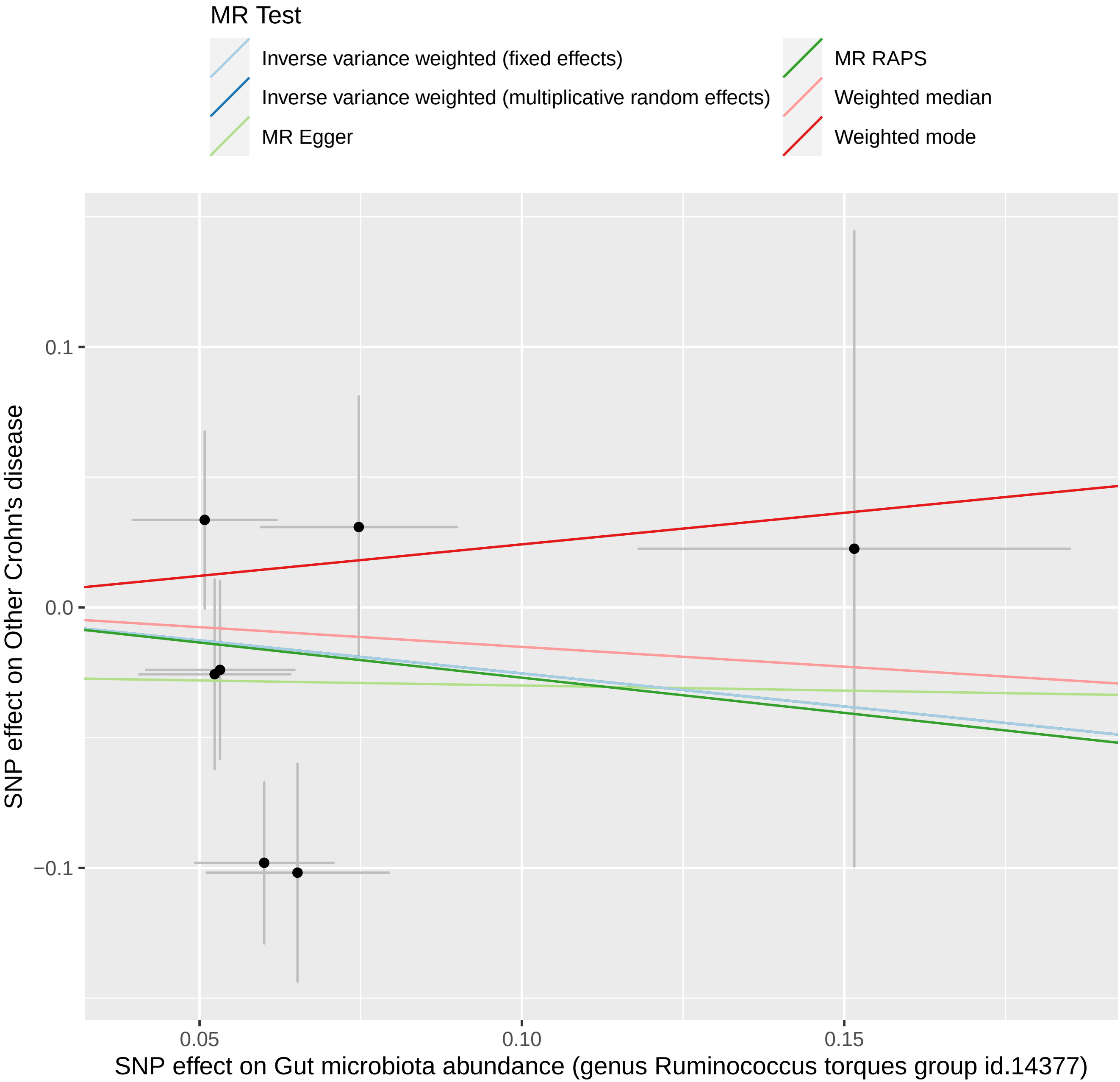

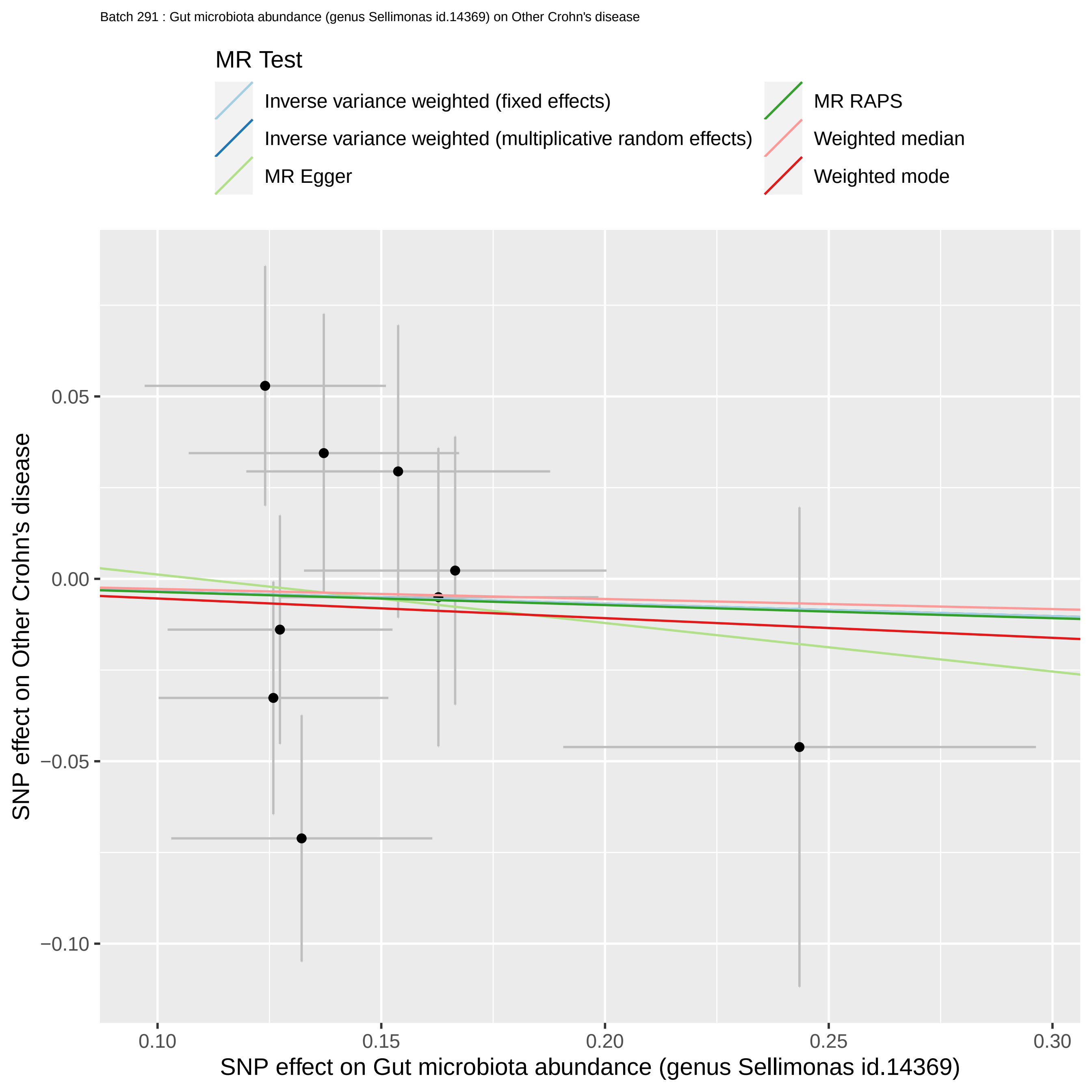
Batch 291 : Gut microbiota abundance (genus Sellimonas id.14369) on Other Crohn's disease
MR Test
Inverse variance weighted (fixed effects)
Inverse variance weighted (multiplicative random effects)
MR Egger
MR RAPS
Weighted median
Weighted mode
SNP effect on Other Crohn's disease
0.05
0.00
−0.05
−0.10
0.10
0.15
0.20
0.25
0.30
SNP effect on Gut microbiota abundance (genus Sellimonas id.14369)

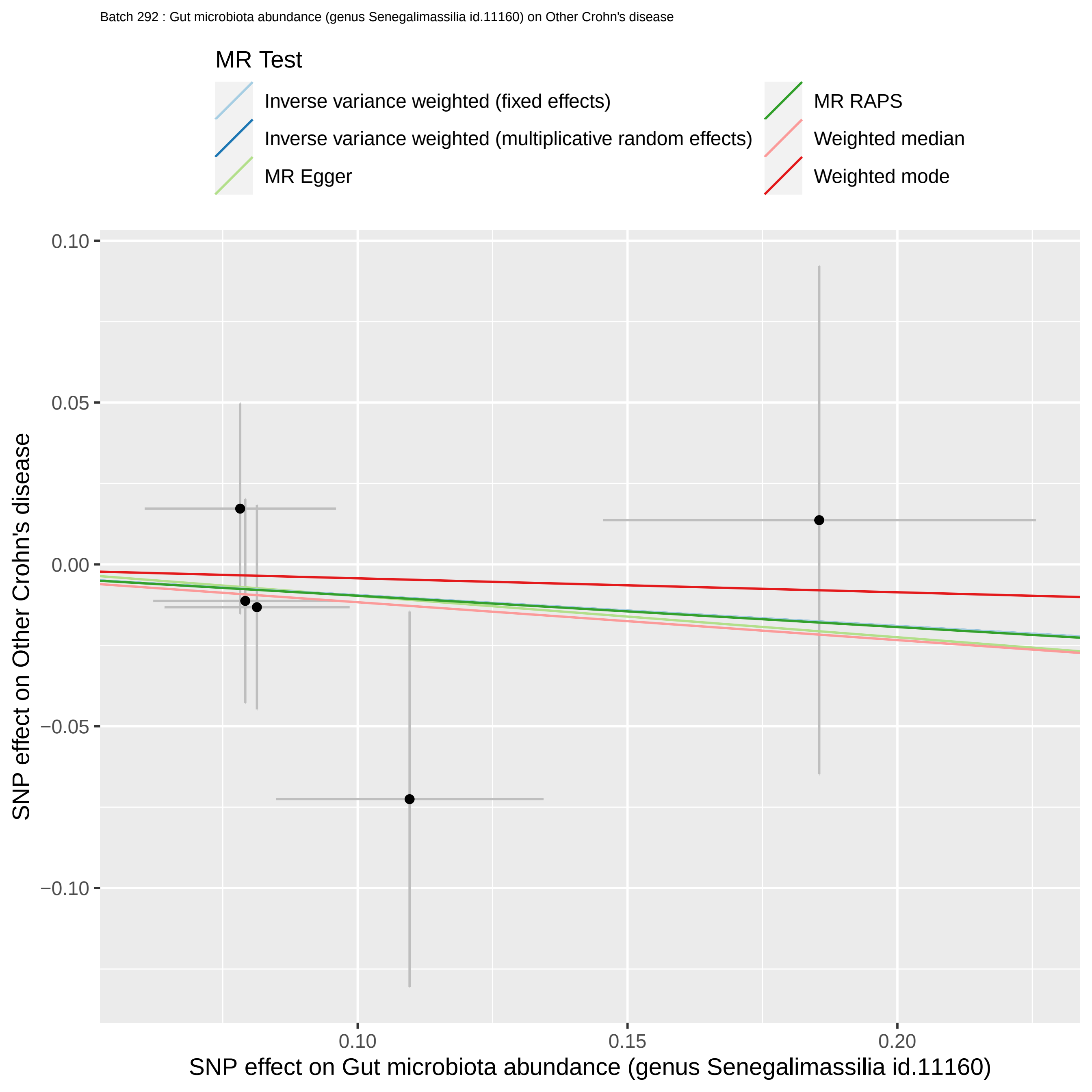

Batch 292 : Gut microbiota abundance (genus Senegalimassilia id.11160) on Other Crohn's disease
MR Test
Inverse variance weighted (fixed effects)
Inverse variance weighted (multiplicative random effects)
MR Egger
MR RAPS
Weighted median
Weighted mode
0.10
0.05
0.00
−0.05
−0.10
SNP effect on Other Crohn's disease
0.10
0.15
0.20
SNP effect on Gut microbiota abundance (genus Senegalimassilia id.11160)

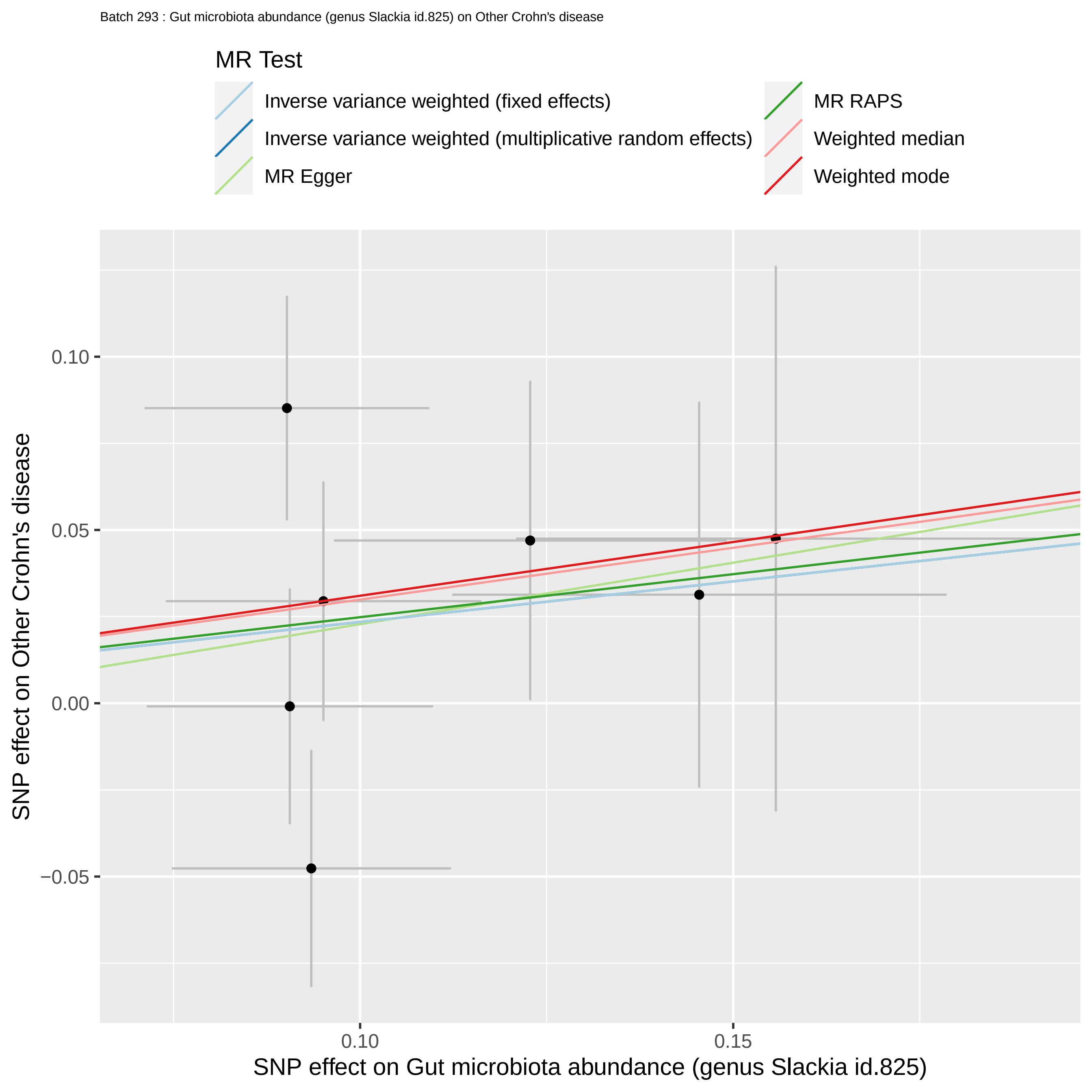
Batch 293 : Gut microbiota abundance (genus Slackia id.825) on Other Crohn's disease
MR Test
Inverse variance weighted (fixed effects)
Inverse variance weighted (multiplicative random effects)
MR Egger
MR RAPS
Weighted median
Weighted mode
0.10
0.05
0.00
−0.05
0.10
0.15
SNP effect on Other Crohn's disease
SNP effect on Gut microbiota abundance (genus Slackia id.825)

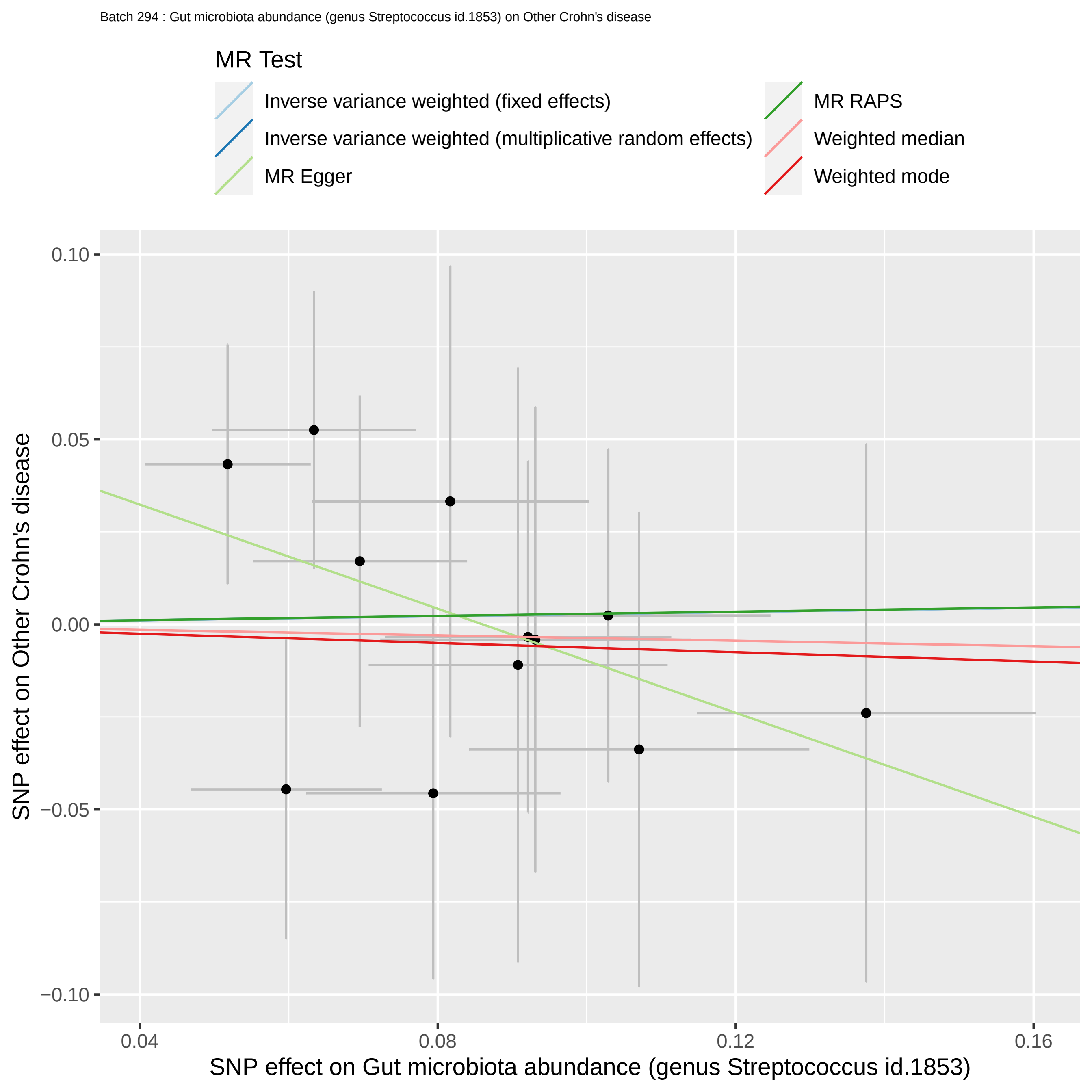

Batch 294 : Gut microbiota abundance (genus Streptococcus id.1853) on Other Crohn's disease
MR Test
Inverse variance weighted (fixed effects)
Inverse variance weighted (multiplicative random effects)
MR Egger
MR RAPS
Weighted median
Weighted mode
SNP effect on Other Crohn's disease
0.10
0.05
0.00
−0.05
−0.10
0.04
0.08
0.12
0.16
SNP effect on Gut microbiota abundance (genus Streptococcus id.1853)

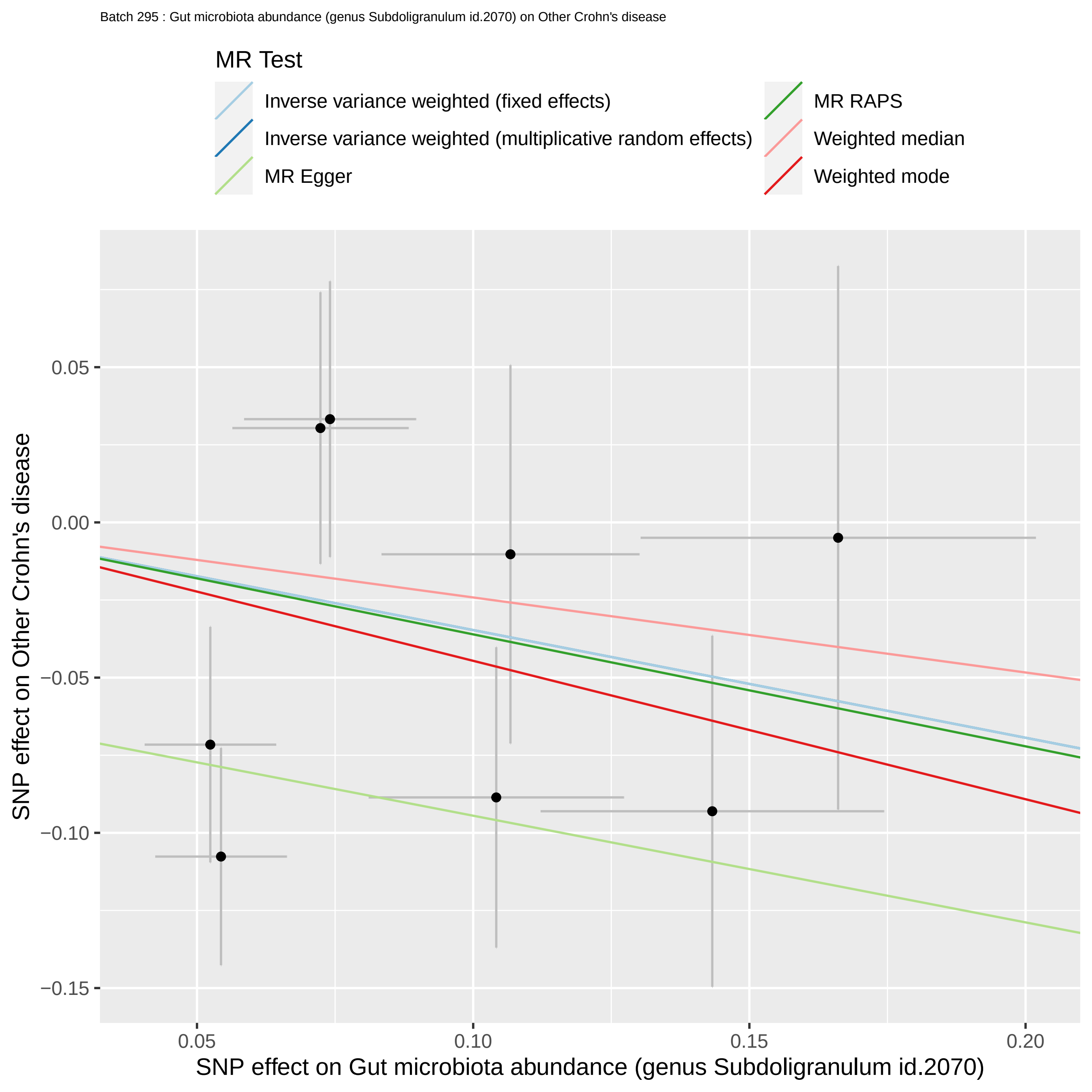

Batch 295 : Gut microbiota abundance (genus Subdoligranulum id.2070) on Other Crohn's disease
MR Test
Inverse variance weighted (fixed effects)
Inverse variance weighted (multiplicative random effects)
MR Egger
MR RAPS
Weighted median
Weighted mode
SNP effect on Other Crohn's disease
0.05
0.00
−0.05
−0.10
−0.15
0.05
0.10
0.15
0.20
SNP effect on Gut microbiota abundance (genus Subdoligranulum id.2070)

Batch 296 : Gut microbiota abundance (genus Sutterella id.2896) on Other Crohn's disease

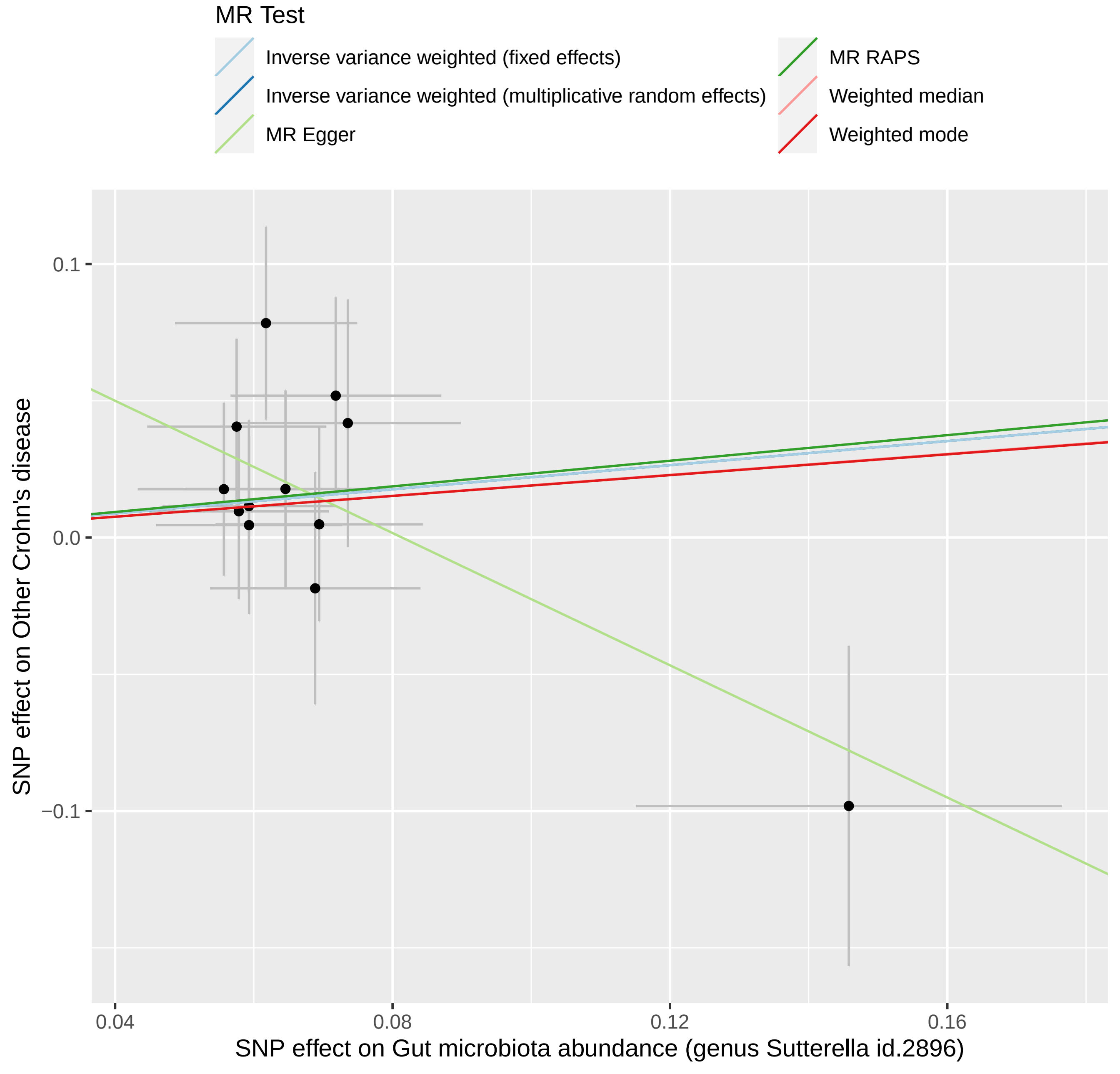

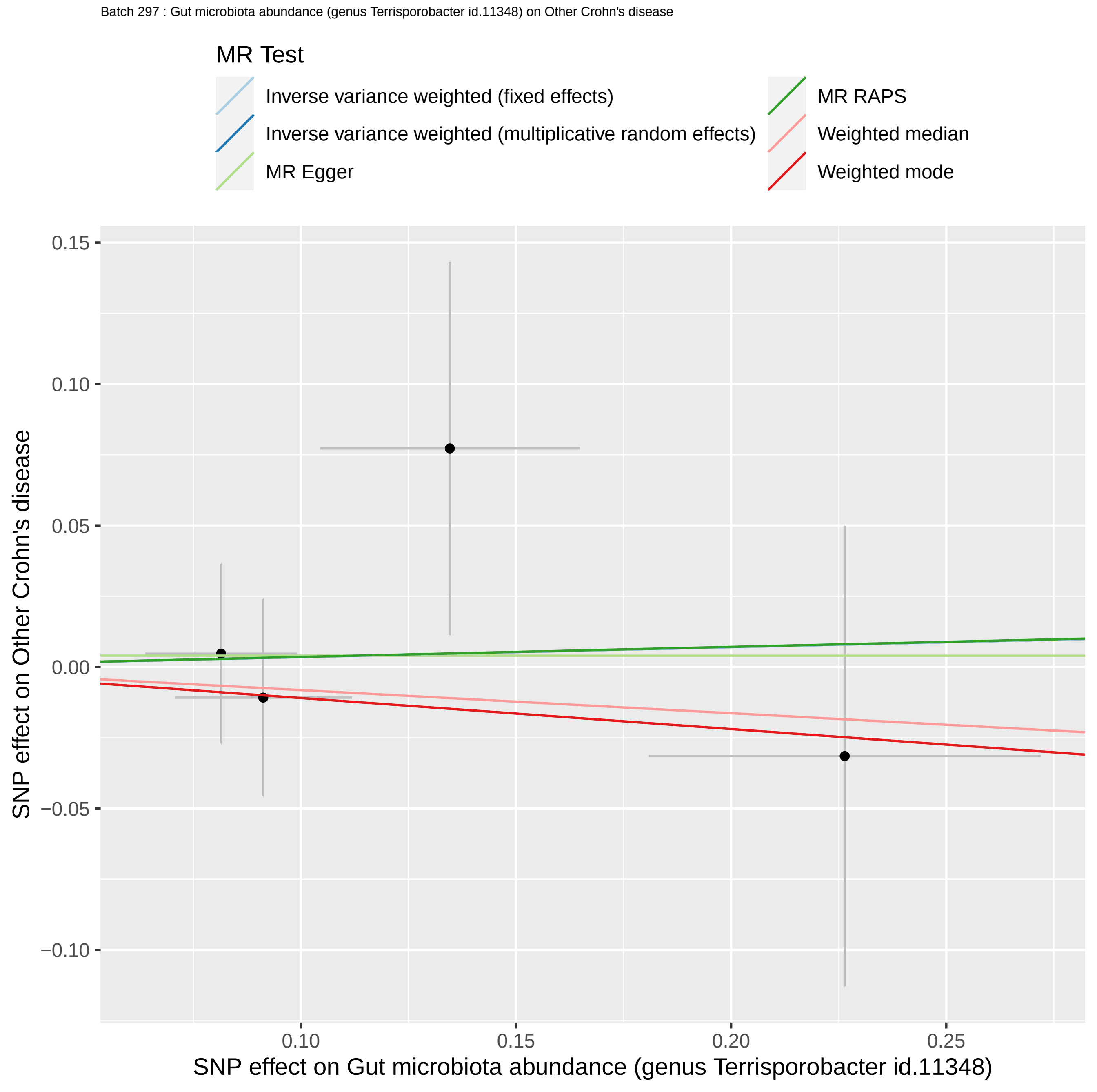
Batch 297 : Gut microbiota abundance (genus Terrisporobacter id.11348) on Other Crohn's disease
MR Test
Inverse variance weighted (fixed effects)
Inverse variance weighted (multiplicative random effects)
MR Egger
MR RAPS
Weighted median
Weighted mode
SNP effect on Other Crohn's disease
0.15
0.10
0.05
0.00
−0.05
−0.10
0.10
0.15
0.20
0.25
SNP effect on Gut microbiota abundance (genus Terrisporobacter id.11348)

Batch 298 : Gut microbiota abundance (genus Turicibacter id.2162) on Other Crohn's disease

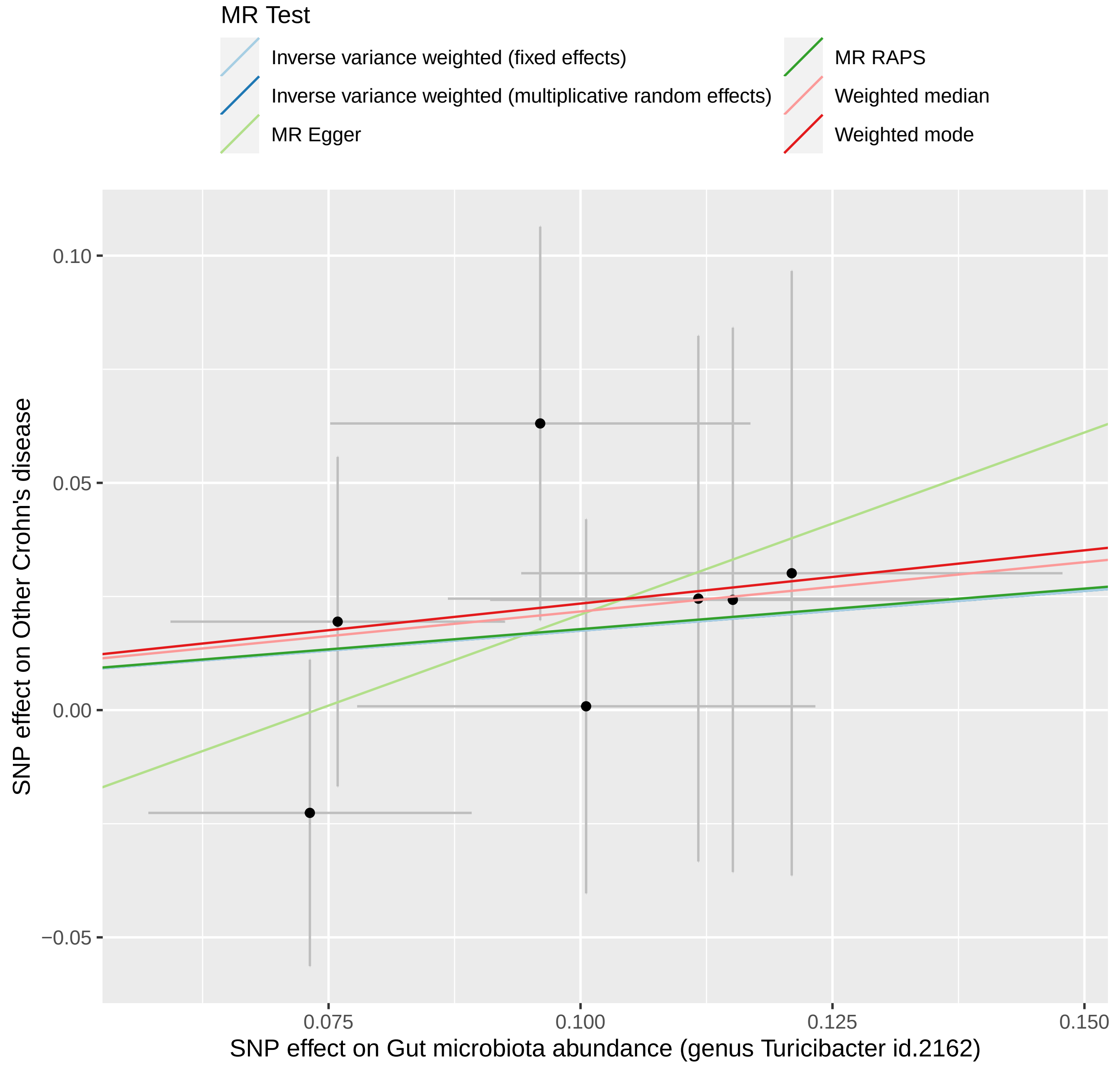

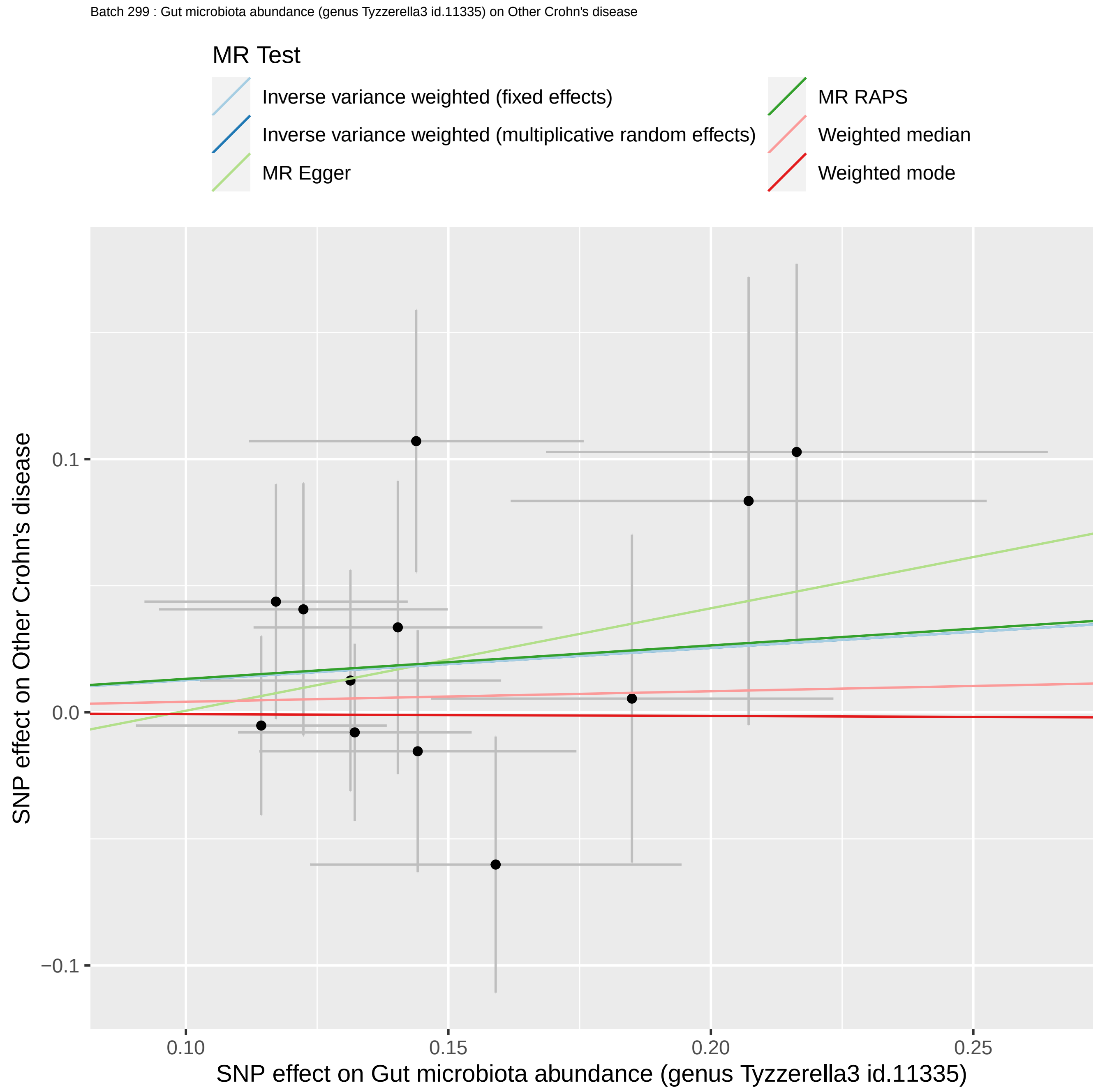
Batch 299 : Gut microbiota abundance (genus Tyzzerella3 id.11335) on Other Crohn's disease
MR Test
Inverse variance weighted (fixed effects)
Inverse variance weighted (multiplicative random effects)
MR Egger
MR RAPS
Weighted median
Weighted mode
SNP effect on Other Crohn's disease
0.1
0.0
−0.1
0.10
0.15
0.20
0.25
SNP effect on Gut microbiota abundance (genus Tyzzerella3 id.11335)

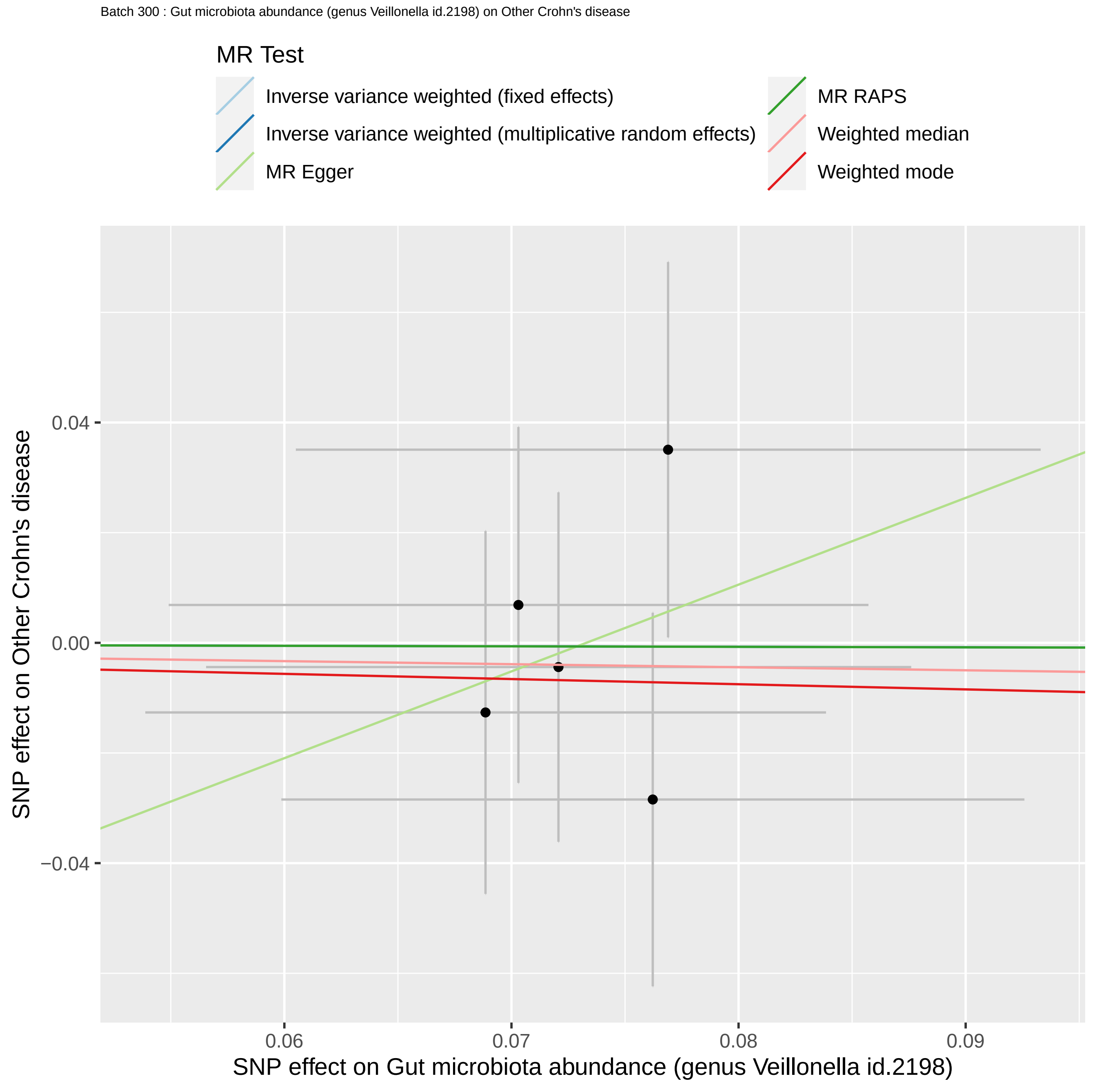

Batch 300 : Gut microbiota abundance (genus Veillonella id.2198) on Other Crohn's disease
MR Test
Inverse variance weighted (fixed effects)
Inverse variance weighted (multiplicative random effects)
MR Egger
MR RAPS
Weighted median
Weighted mode
0.04
0.00
−0.04
SNP effect on Other Crohn's disease
0.06
0.07
0.08
0.09
SNP effect on Gut microbiota abundance (genus Veillonella id.2198)

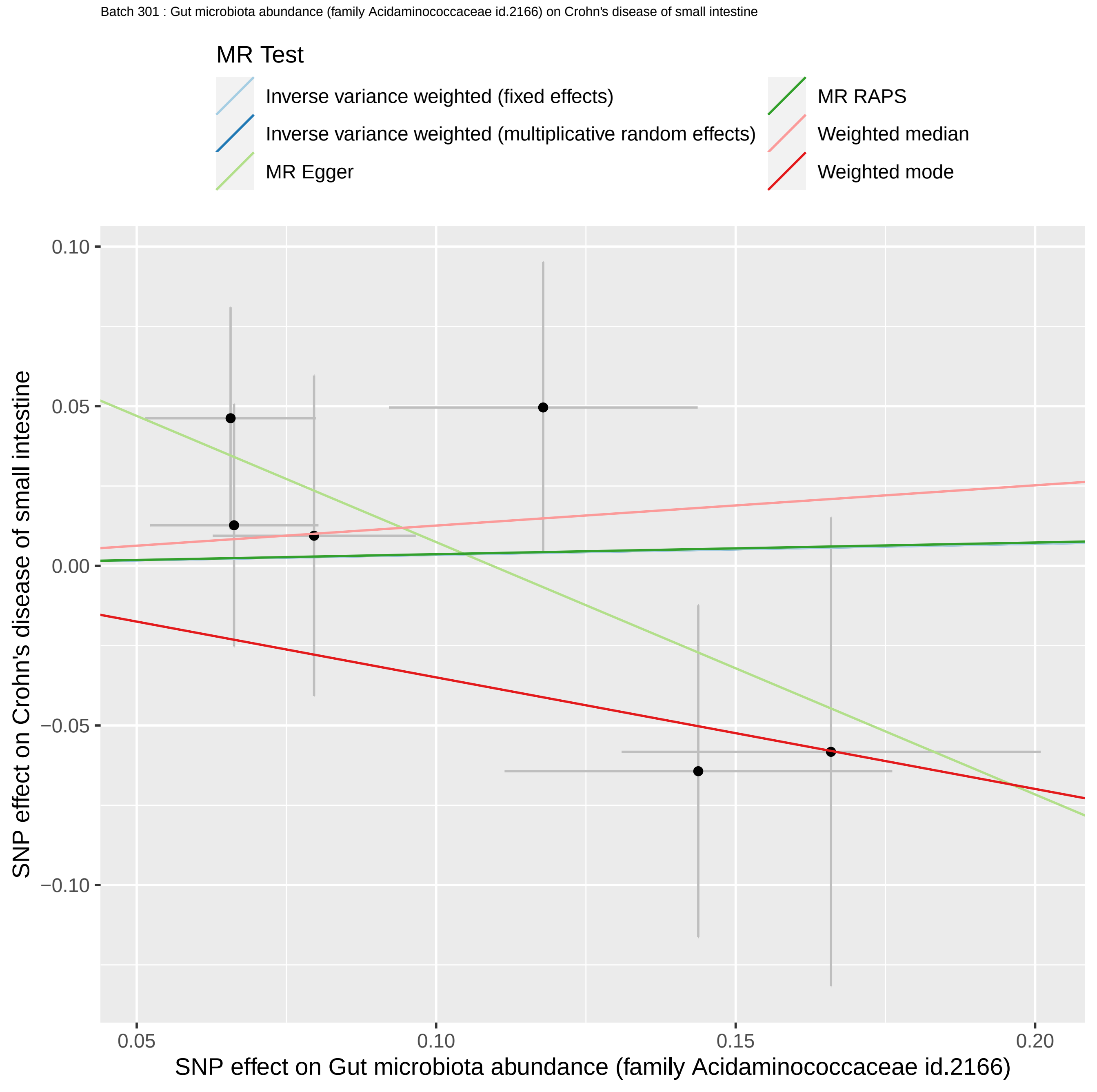
Batch 301 : Gut microbiota abundance (family Acidaminococcaceae id.2166) on Crohn's disease of small intestine
MR Test
Inverse variance weighted (fixed effects)
Inverse variance weighted (multiplicative random effects)
MR Egger
MR RAPS
Weighted median
Weighted mode
0.10
0.05
0.00
−0.05
−0.10
0.05
0.10
0.15
0.20
SNP effect on Crohn's disease of small intestine
SNP effect on Gut microbiota abundance (family Acidaminococcaceae id.2166)

Batch 302 : Gut microbiota abundance (family Actinomycetaceae id.421) on Crohn's disease of small intestine

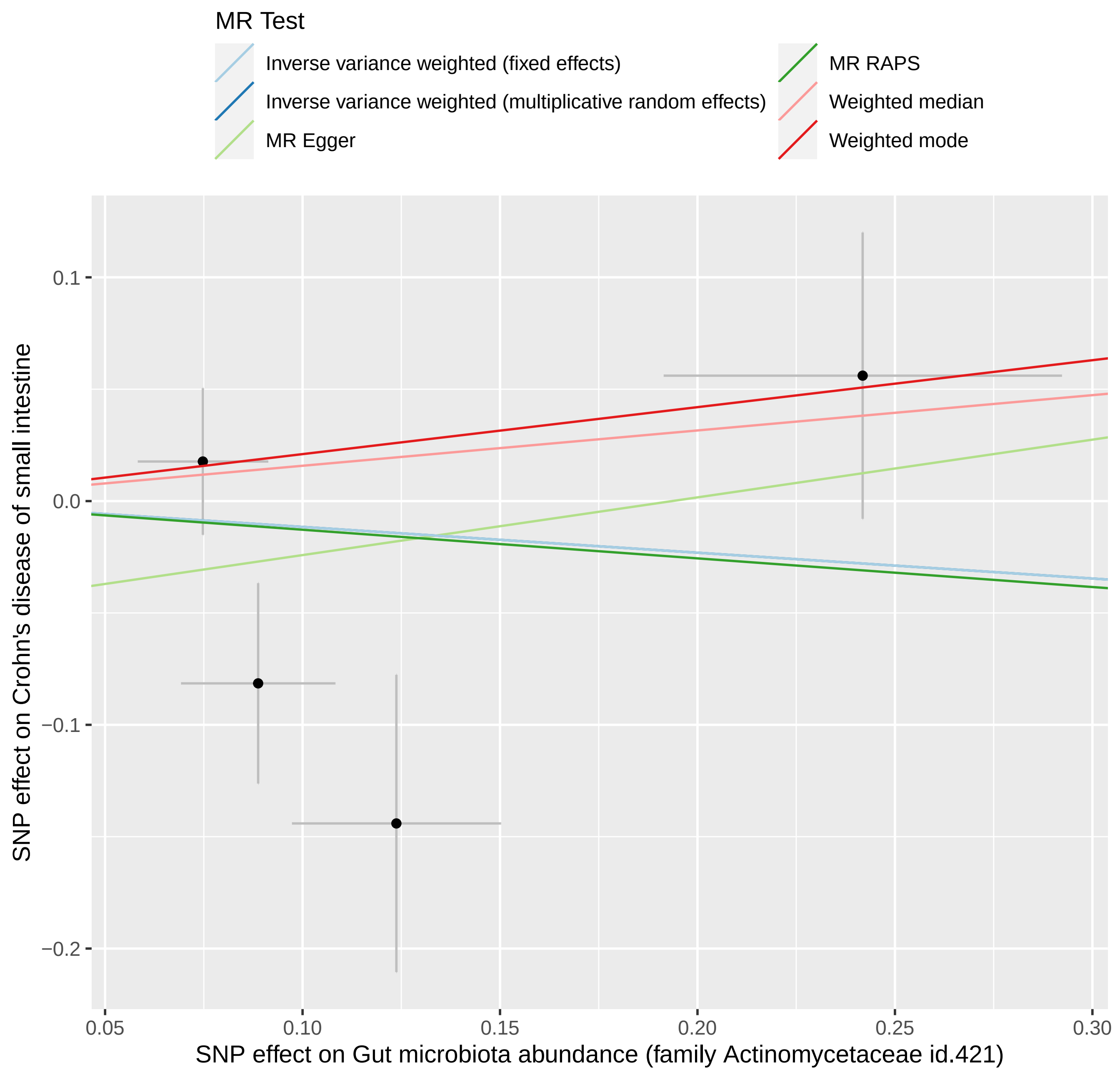

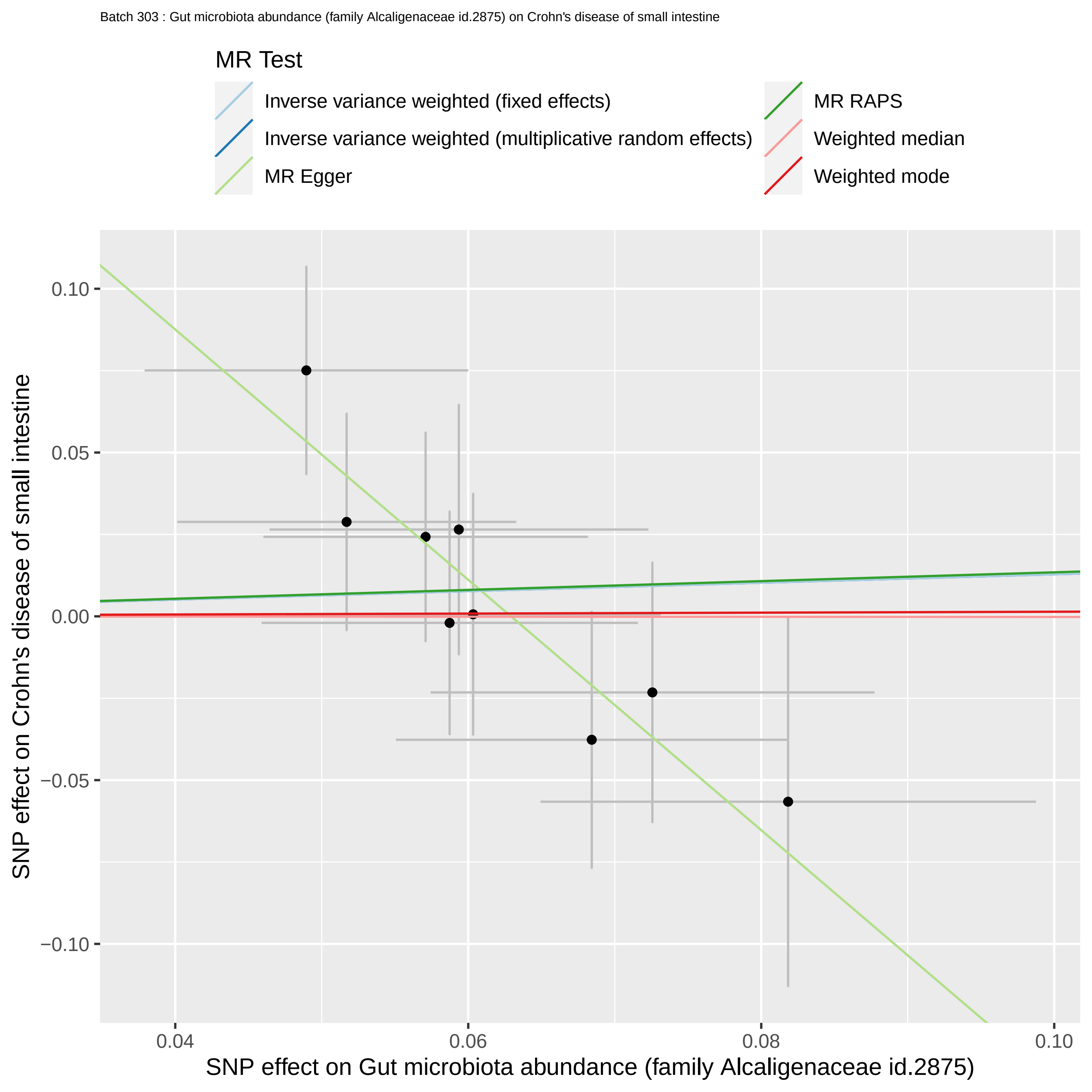

Batch 303 : Gut microbiota abundance (family Alcaligenaceae id.2875) on Crohn's disease of small intestine
MR Test
Inverse variance weighted (fixed effects)
Inverse variance weighted (multiplicative random effects)
MR Egger
MR RAPS
Weighted median
Weighted mode
0.10
0.05
0.00
−0.05
−0.10
0.04
0.06
0.08
0.10
SNP effect on Crohn's disease of small intestine
SNP effect on Gut microbiota abundance (family Alcaligenaceae id.2875)

Batch 304 : Gut microbiota abundance (family Bacteroidaceae id.917) on Crohn's disease of small intestine

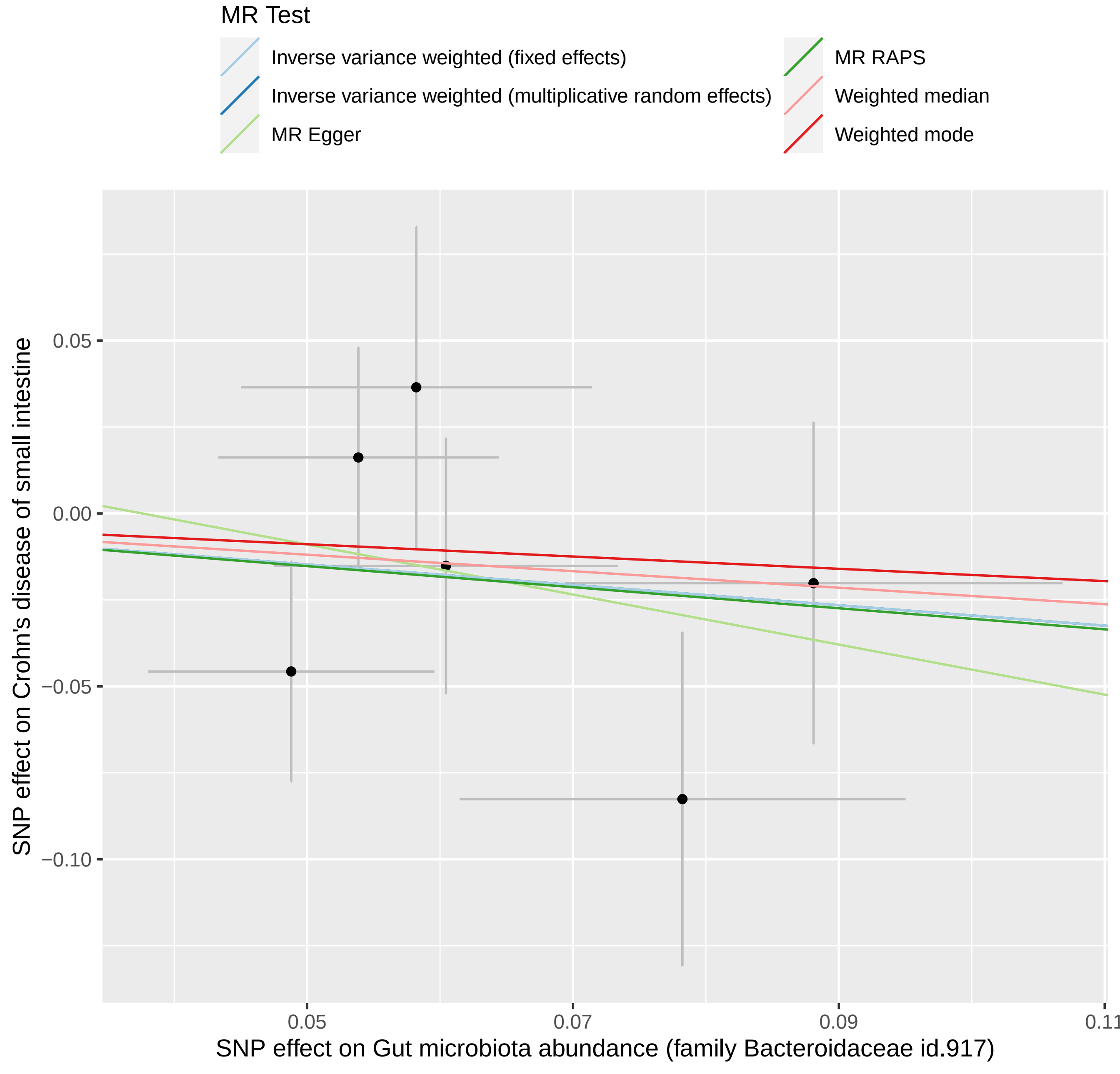

Batch 305 : Gut microbiota abundance (family Bacteroidales S24 7group id.11173) on Crohn's disease of small intestine

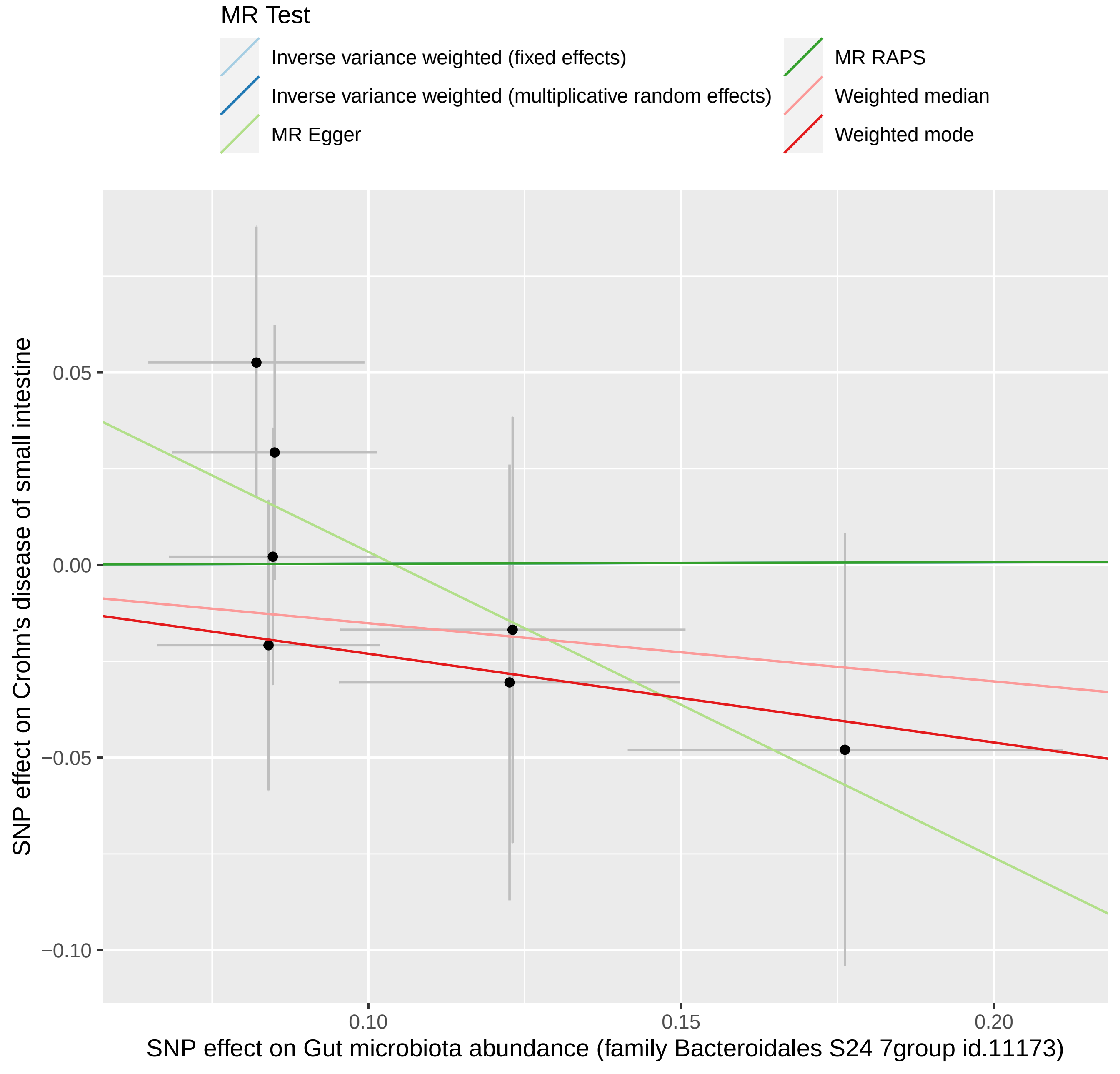

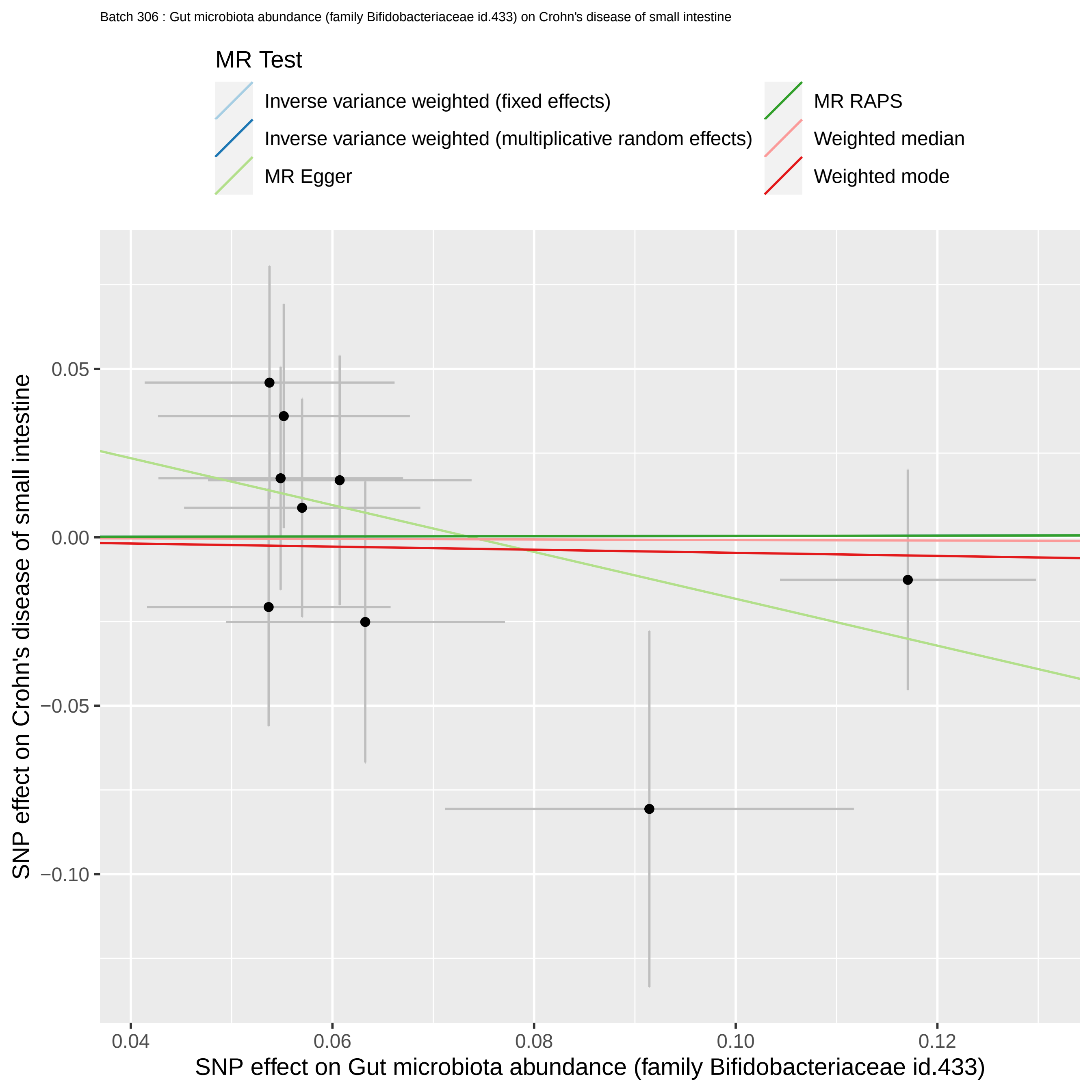

Batch 306 : Gut microbiota abundance (family Bifidobacteriaceae id.433) on Crohn's disease of small intestine
MR Test
Inverse variance weighted (fixed effects)
Inverse variance weighted (multiplicative random effects)
MR Egger
MR RAPS
Weighted median
Weighted mode
0.05
0.00
−0.05
−0.10
0.04
0.06
0.08
0.10
0.12
SNP effect on Crohn's disease of small intestine
SNP effect on Gut microbiota abundance (family Bifidobacteriaceae id.433)

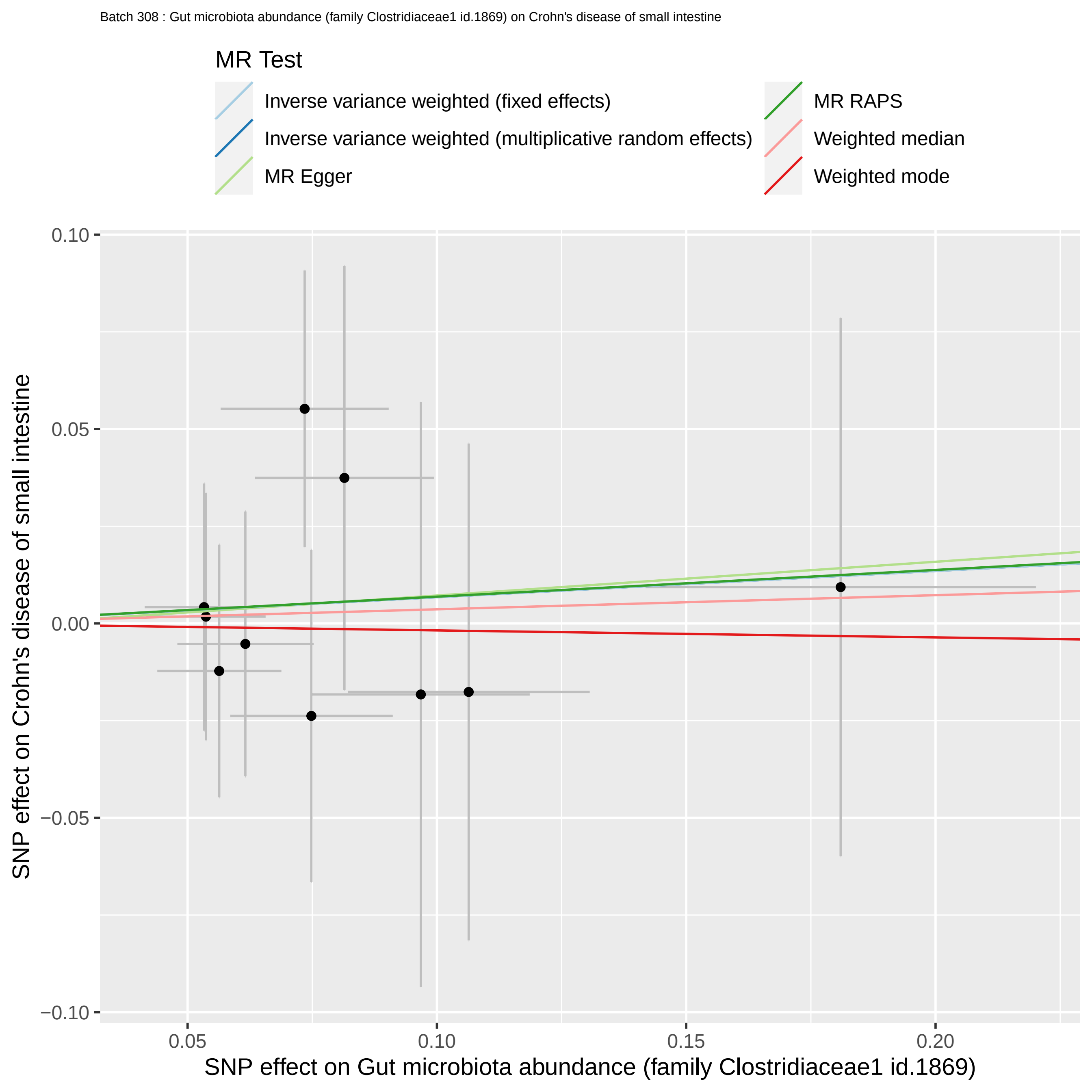

Batch 308 : Gut microbiota abundance (family Clostridiaceae1 id.1869) on Crohn's disease of small intestine
MR Test
Inverse variance weighted (fixed effects)
Inverse variance weighted (multiplicative random effects)
MR Egger
MR RAPS
Weighted median
Weighted mode
SNP effect on Crohn's disease of small intestine
0.10
0.05
0.00
−0.05
−0.10
0.05
0.10
0.15
0.20
SNP effect on Gut microbiota abundance (family Clostridiaceae1 id.1869)

Batch 309 : Gut microbiota abundance (family Clostridiales vadin BB60 group id.11286) on Crohn's disease of small intestine

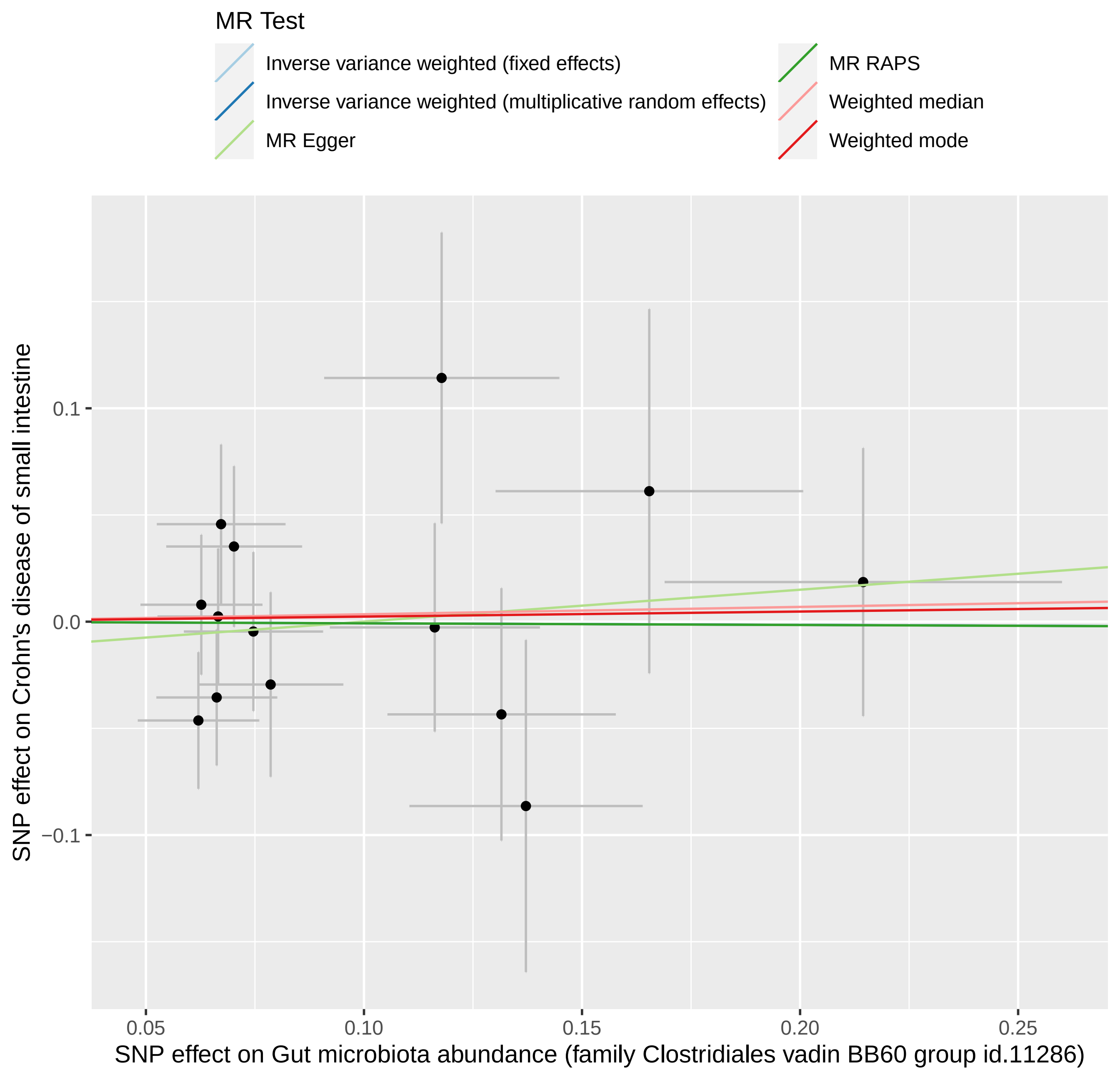

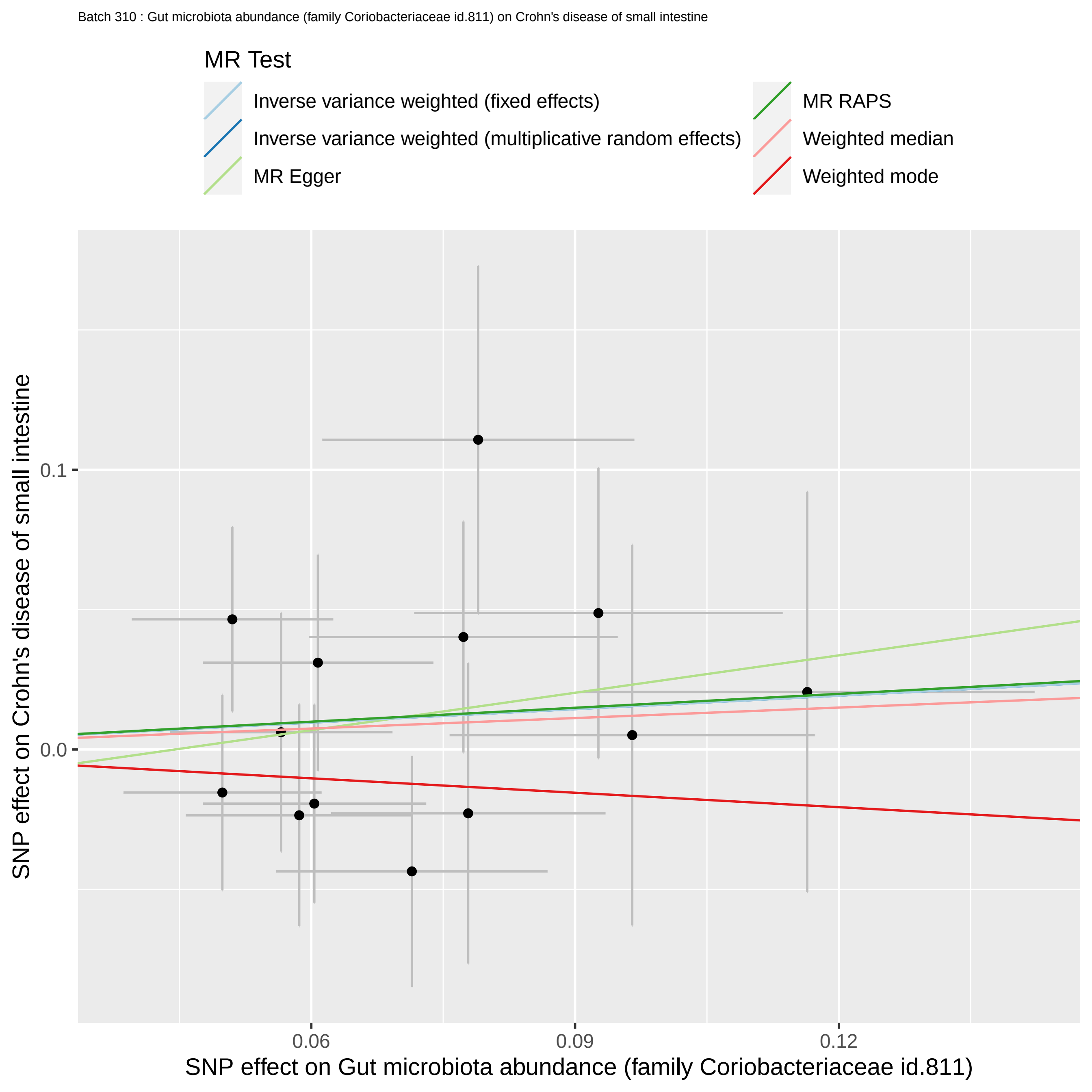

Batch 310 : Gut microbiota abundance (family Coriobacteriaceae id.811) on Crohn's disease of small intestine
MR Test
Inverse variance weighted (fixed effects)
Inverse variance weighted (multiplicative random effects)
MR Egger
MR RAPS
Weighted median
Weighted mode
SNP effect on Crohn's disease of small intestine
0.1
0.0
0.06
0.09
0.12
SNP effect on Gut microbiota abundance (family Coriobacteriaceae id.811)

Batch 311 : Gut microbiota abundance (family Defluviitaleaceae id.1924) on Crohn's disease of small intestine

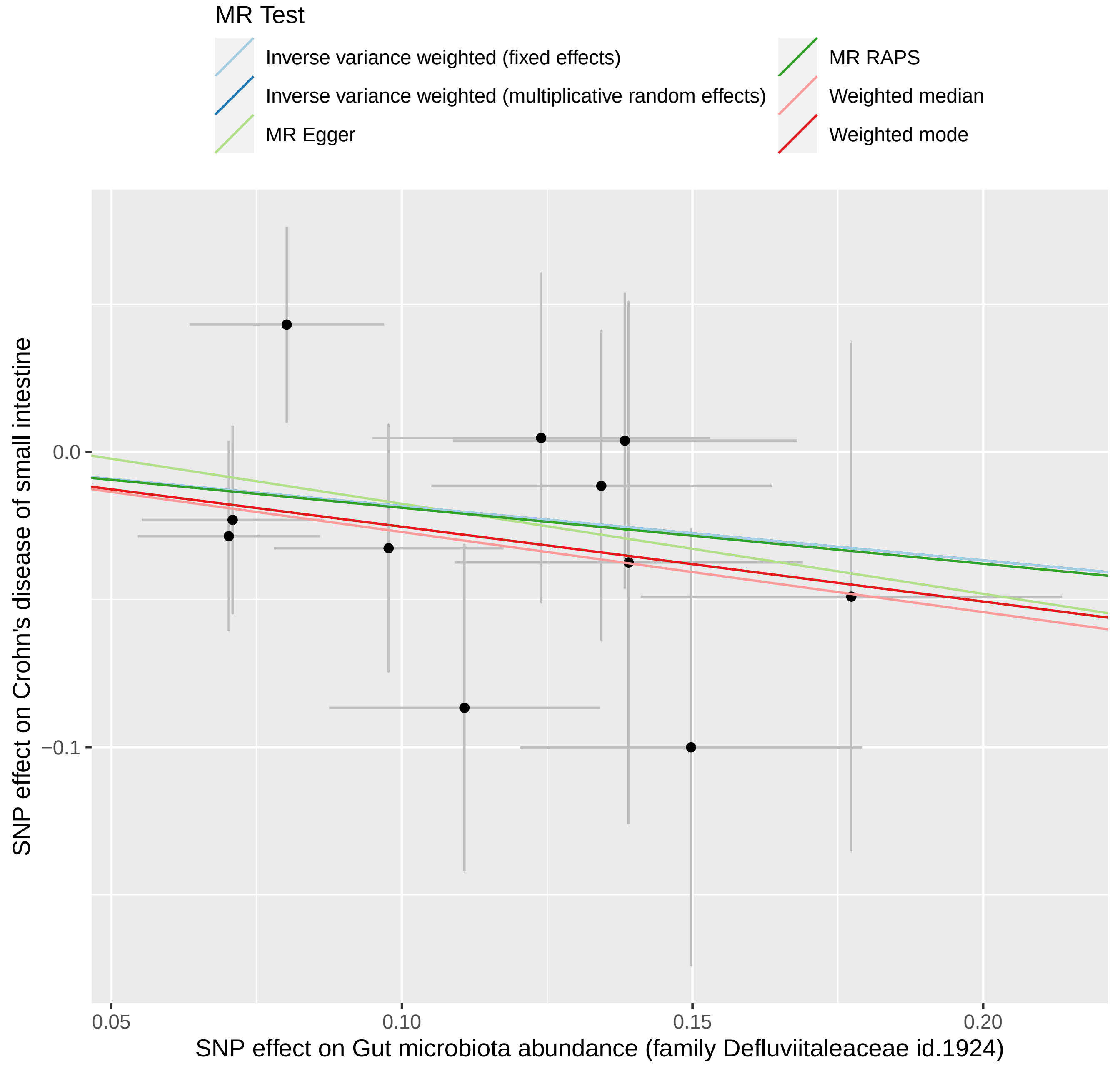

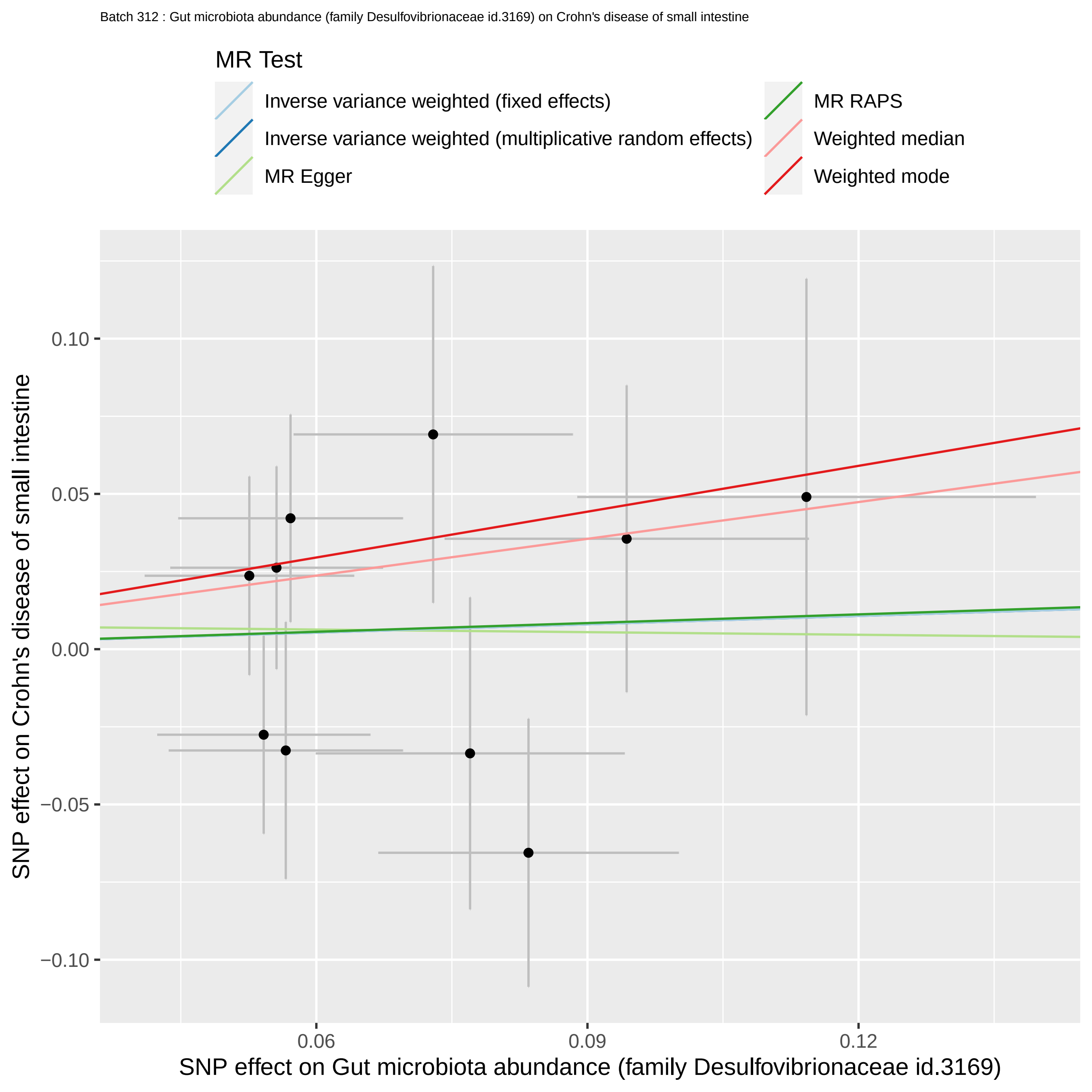

Batch 312 : Gut microbiota abundance (family Desulfovibrionaceae id.3169) on Crohn's disease of small intestine
MR Test
Inverse variance weighted (fixed effects)
Inverse variance weighted (multiplicative random effects)
MR Egger
MR RAPS
Weighted median
Weighted mode
SNP effect on Crohn's disease of small intestine
0.10
0.05
0.00
−0.05
−0.10
0.06
0.09
0.12
SNP effect on Gut microbiota abundance (family Desulfovibrionaceae id.3169)

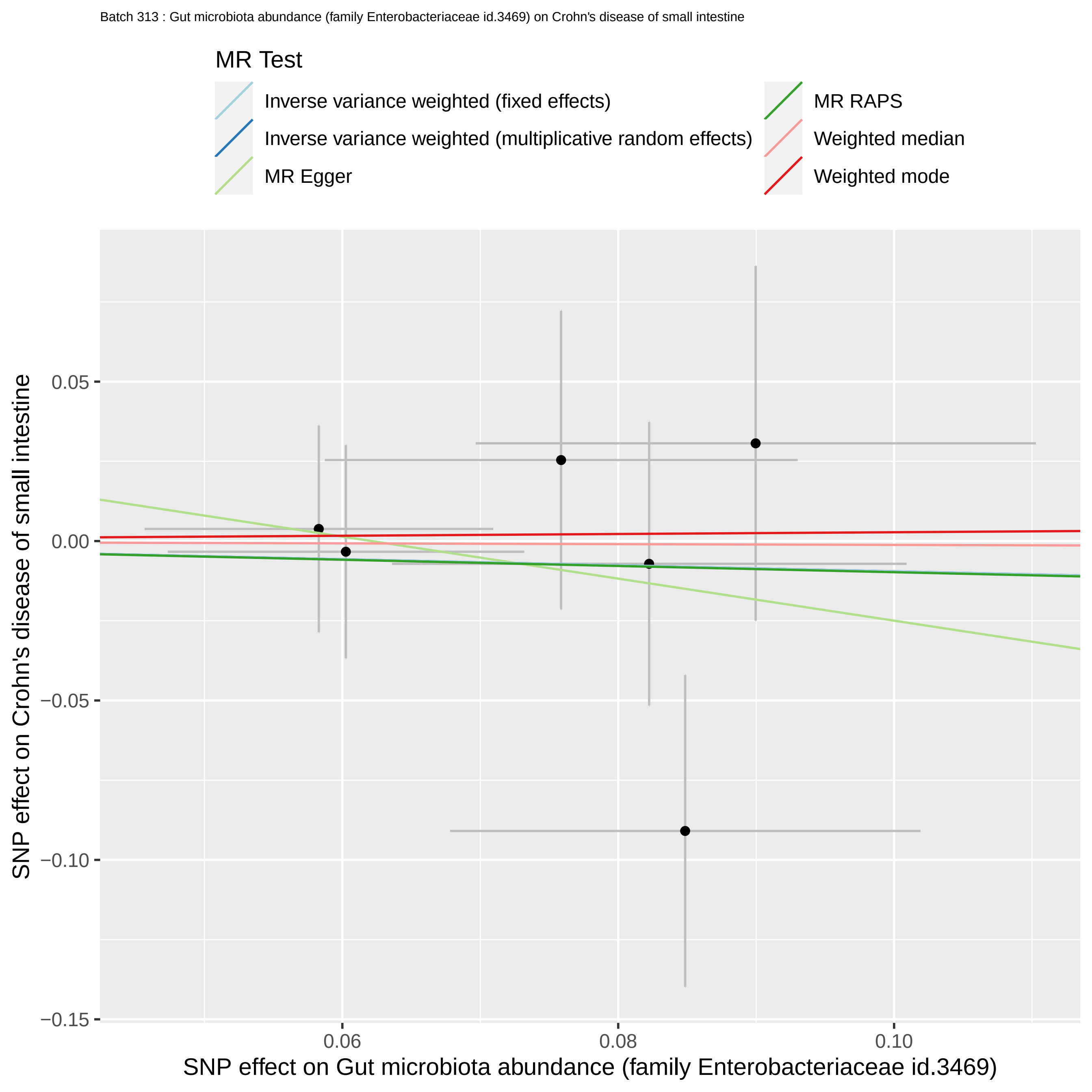

Batch 313 : Gut microbiota abundance (family Enterobacteriaceae id.3469) on Crohn's disease of small intestine
MR Test
Inverse variance weighted (fixed effects)
Inverse variance weighted (multiplicative random effects)
MR Egger
MR RAPS
Weighted median
Weighted mode
0.05
0.00
−0.05
−0.10
−0.15
0.06
0.08
0.10
SNP effect on Crohn's disease of small intestine
SNP effect on Gut microbiota abundance (family Enterobacteriaceae id.3469)

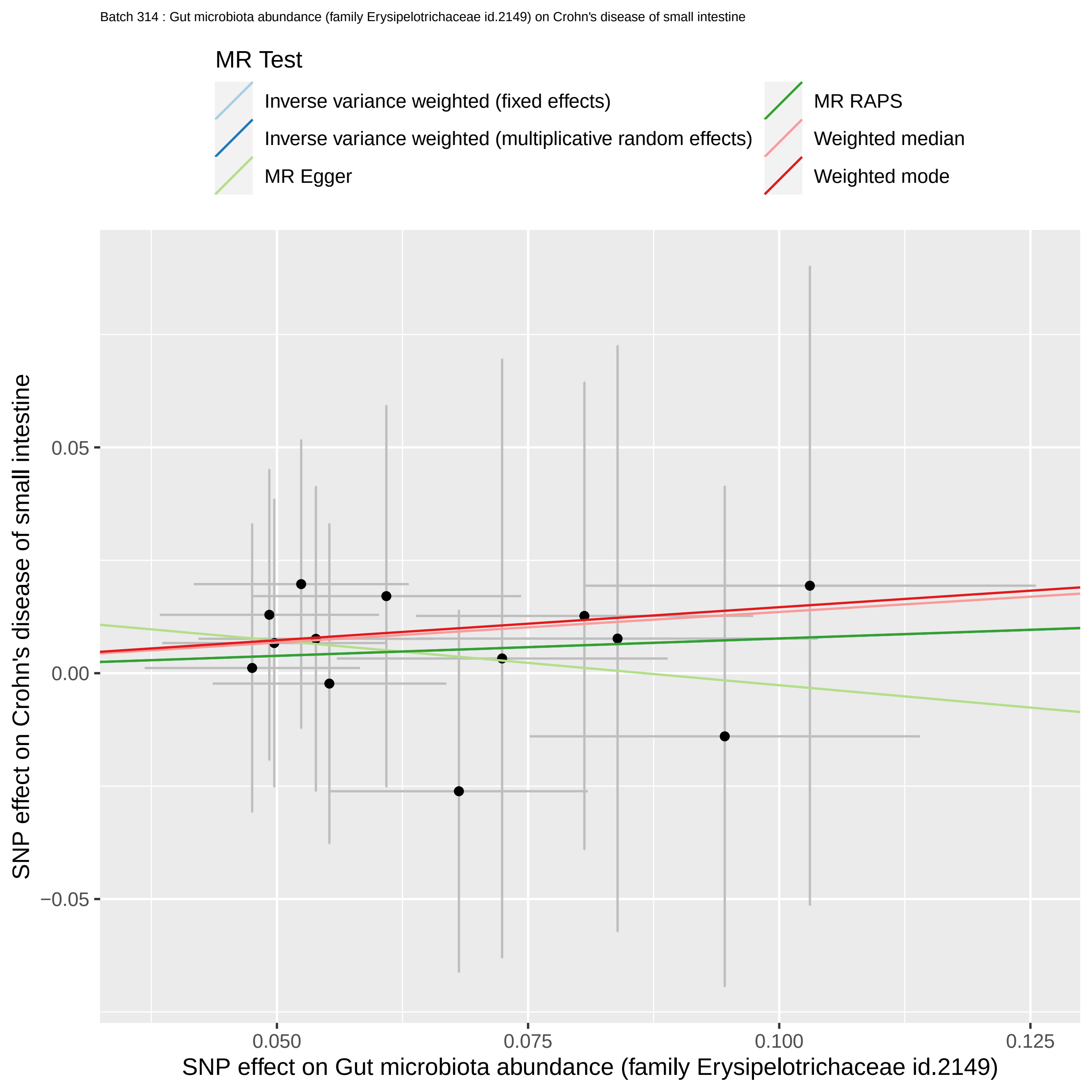

Batch 314 : Gut microbiota abundance (family Erysipelotrichaceae id.2149) on Crohn's disease of small intestine
MR Test
Inverse variance weighted (fixed effects)
Inverse variance weighted (multiplicative random effects)
MR Egger
MR RAPS
Weighted median
Weighted mode
SNP effect on Crohn's disease of small intestine
0.05
0.00
−0.05
0.050
0.075
0.100
0.125
SNP effect on Gut microbiota abundance (family Erysipelotrichaceae id.2149)

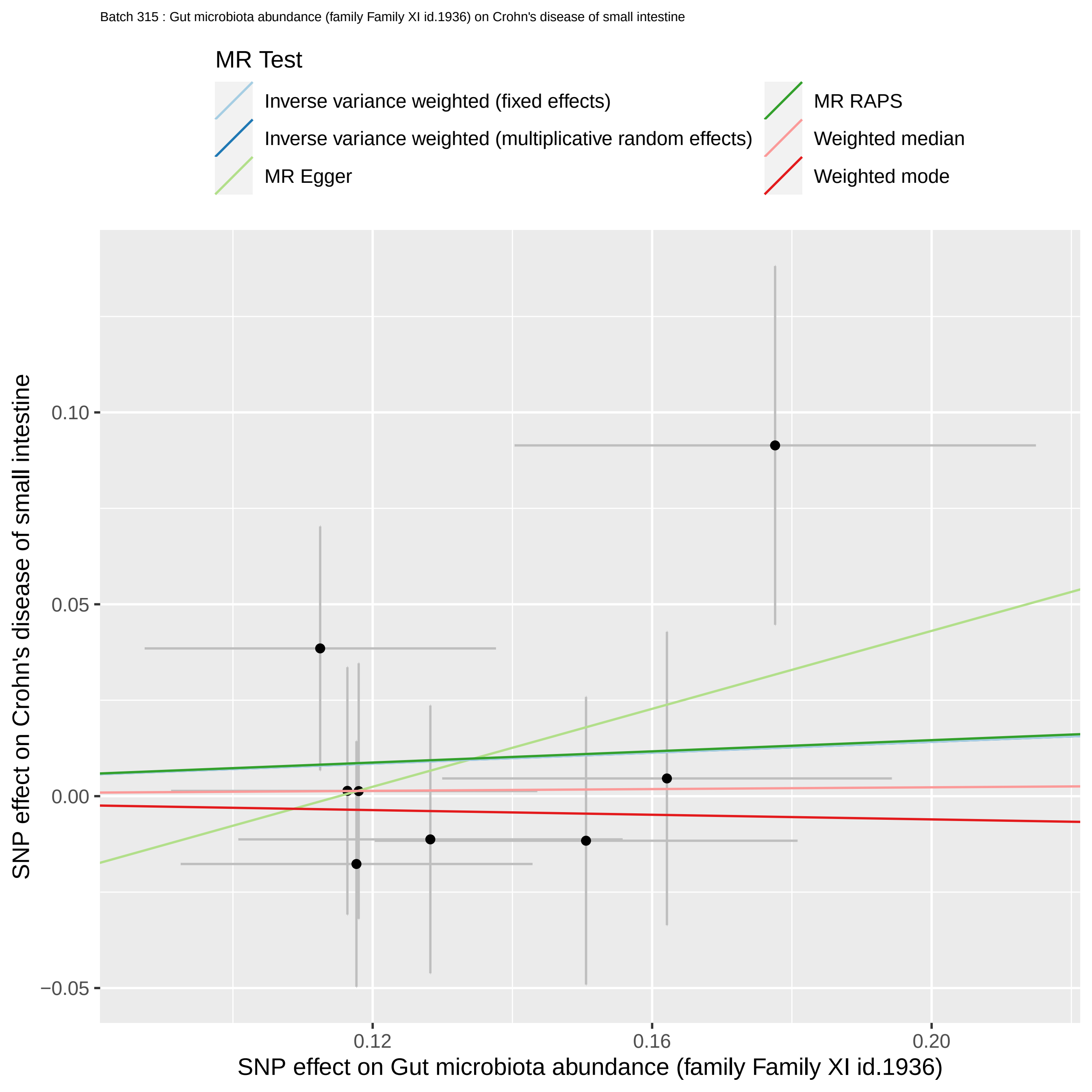
Batch 315 : Gut microbiota abundance (family Family XI id.1936) on Crohn's disease of small intestine
MR Test
Inverse variance weighted (fixed effects)
Inverse variance weighted (multiplicative random effects)
MR Egger
MR RAPS
Weighted median
Weighted mode
0.10
0.05
0.00
−0.05
0.12
0.16
0.20
SNP effect on Crohn's disease of small intestine
SNP effect on Gut microbiota abundance (family Family XI id.1936)

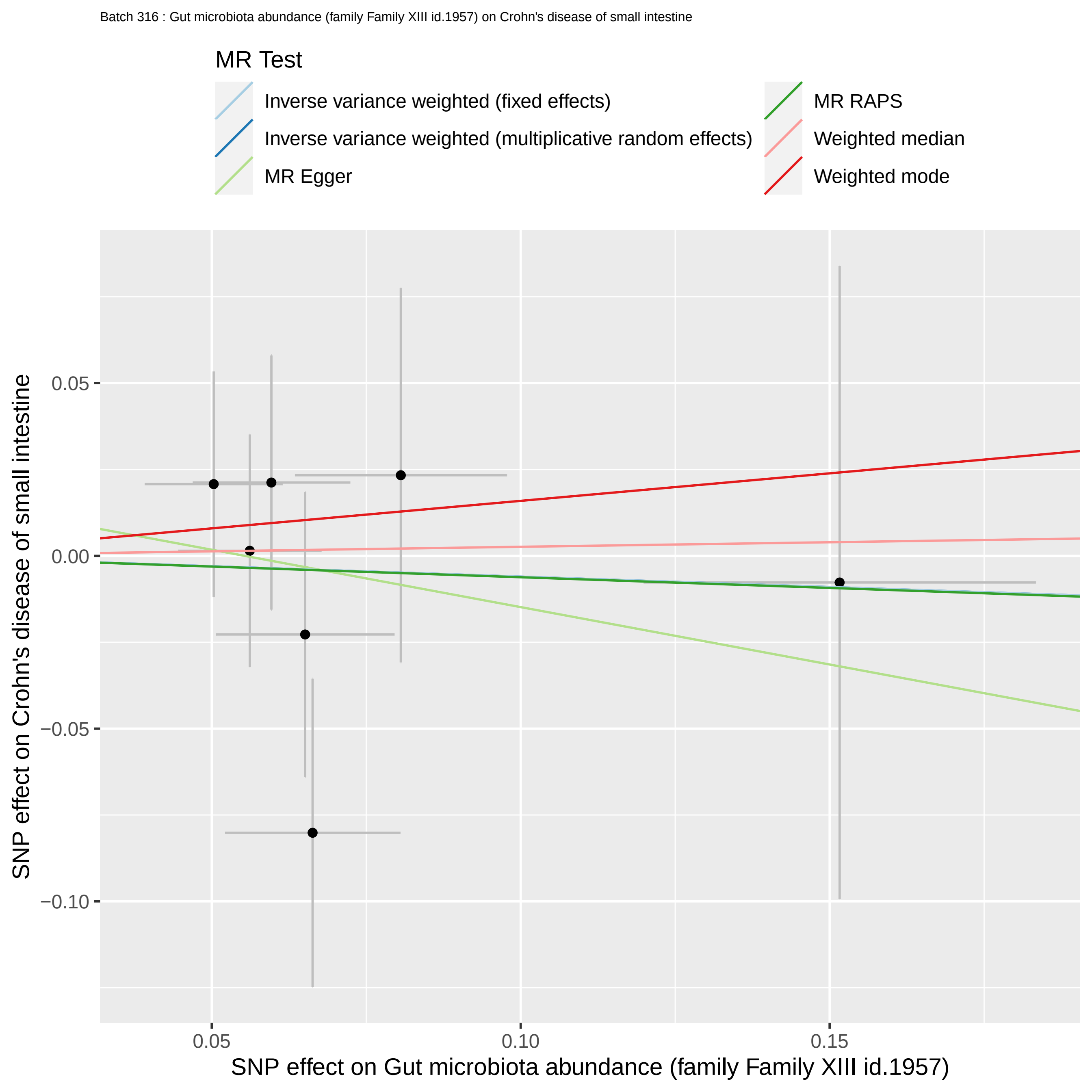
Batch 316 : Gut microbiota abundance (family Family XIII id.1957) on Crohn's disease of small intestine
MR Test
Inverse variance weighted (fixed effects)
Inverse variance weighted (multiplicative random effects)
MR Egger
MR RAPS
Weighted median
Weighted mode
0.05
0.00
−0.05
−0.10
0.05
0.10
0.15
SNP effect on Crohn's disease of small intestine
SNP effect on Gut microbiota abundance (family Family XIII id.1957)

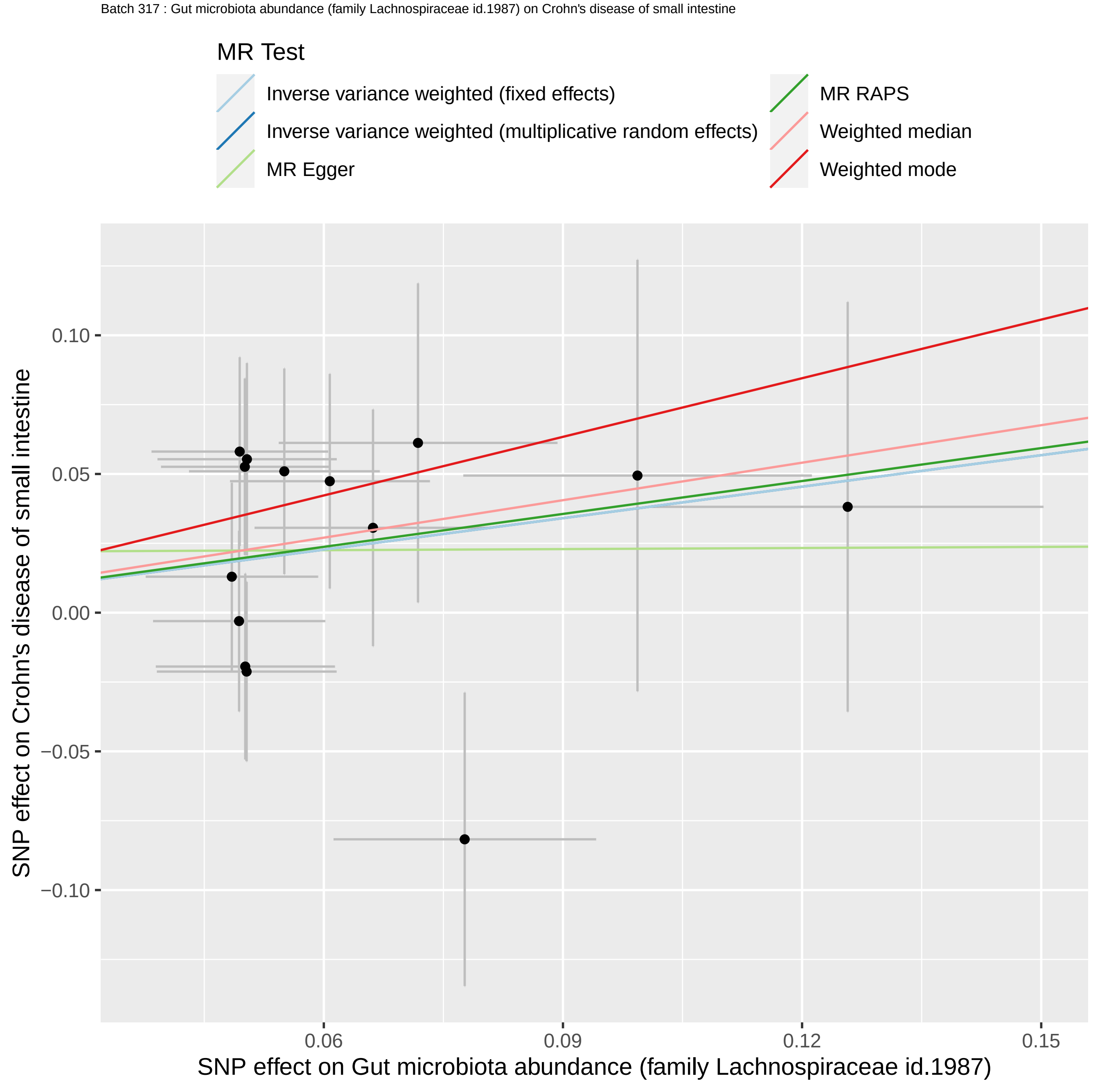

Batch 317 : Gut microbiota abundance (family Lachnospiraceae id.1987) on Crohn's disease of small intestine
MR Test
Inverse variance weighted (fixed effects)
Inverse variance weighted (multiplicative random effects)
MR Egger
MR RAPS
Weighted median
Weighted mode
0.10
0.05
0.00
−0.05
−0.10
0.06
0.09
0.12
0.15
SNP effect on Crohn's disease of small intestine
SNP effect on Gut microbiota abundance (family Lachnospiraceae id.1987)

Batch 318 : Gut microbiota abundance (family Lactobacillaceae id.1836) on Crohn's disease of small intestine

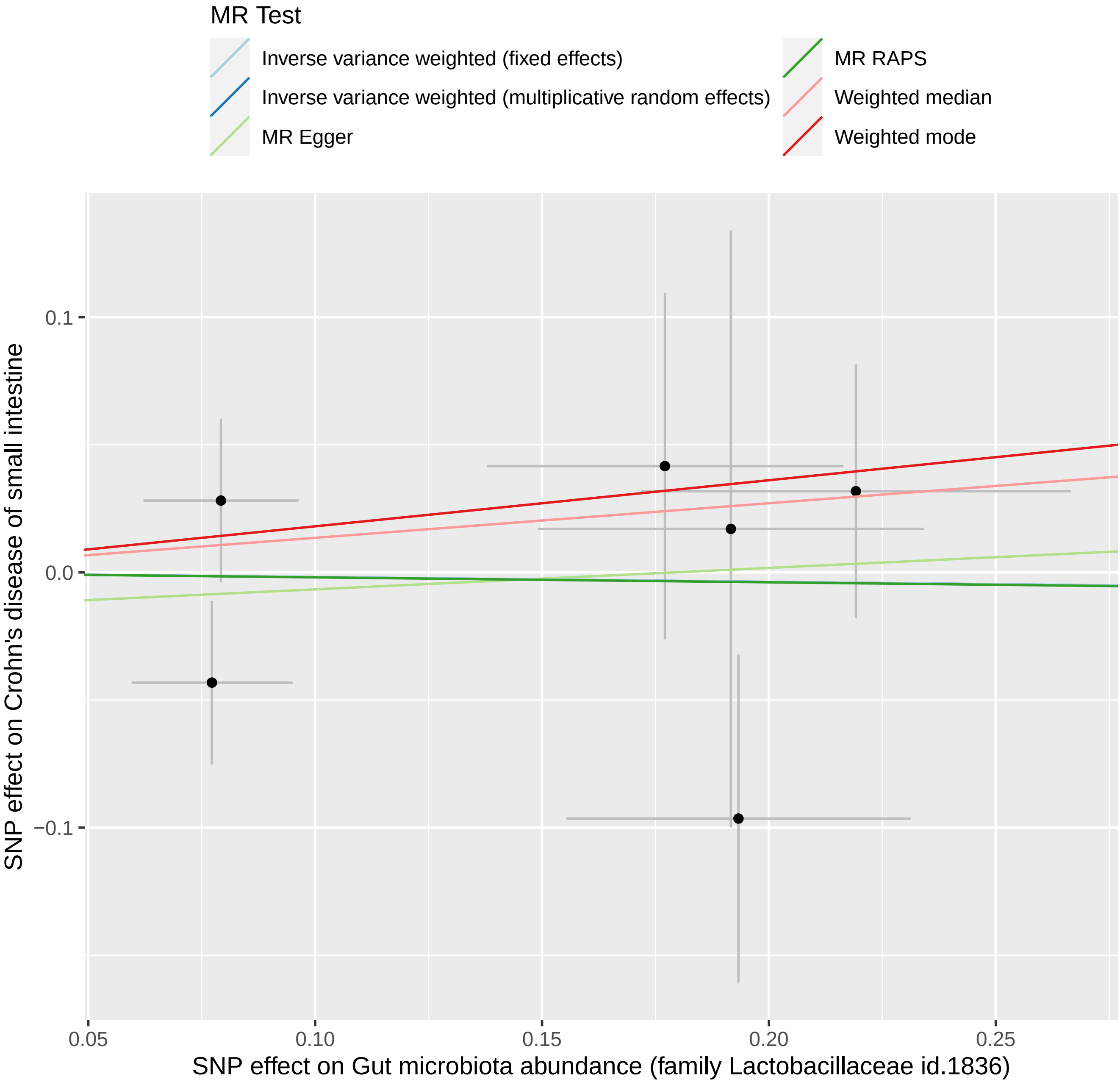

Batch 319 : Gut microbiota abundance (family Methanobacteriaceae id.121) on Crohn's disease of small intestine

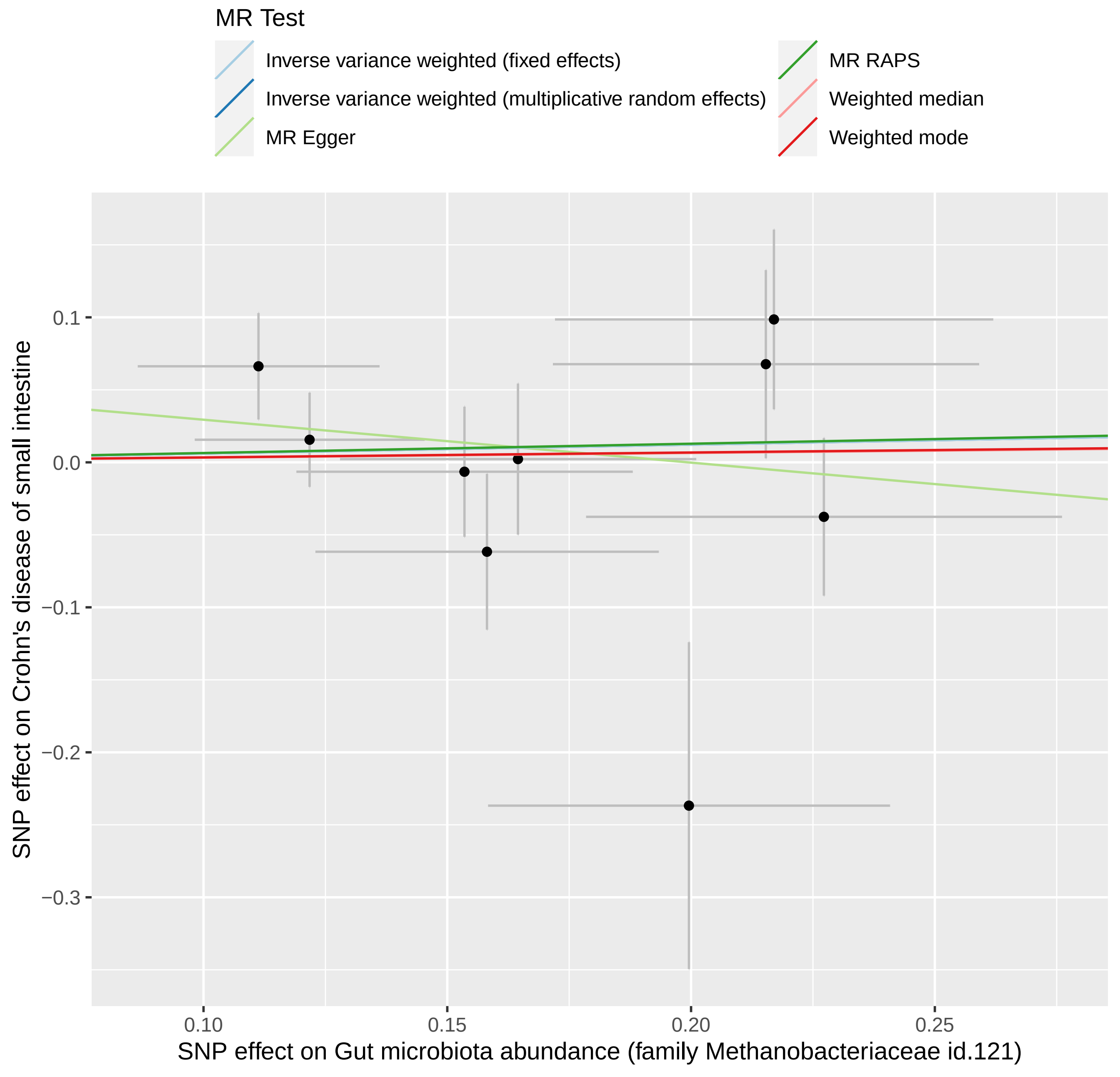

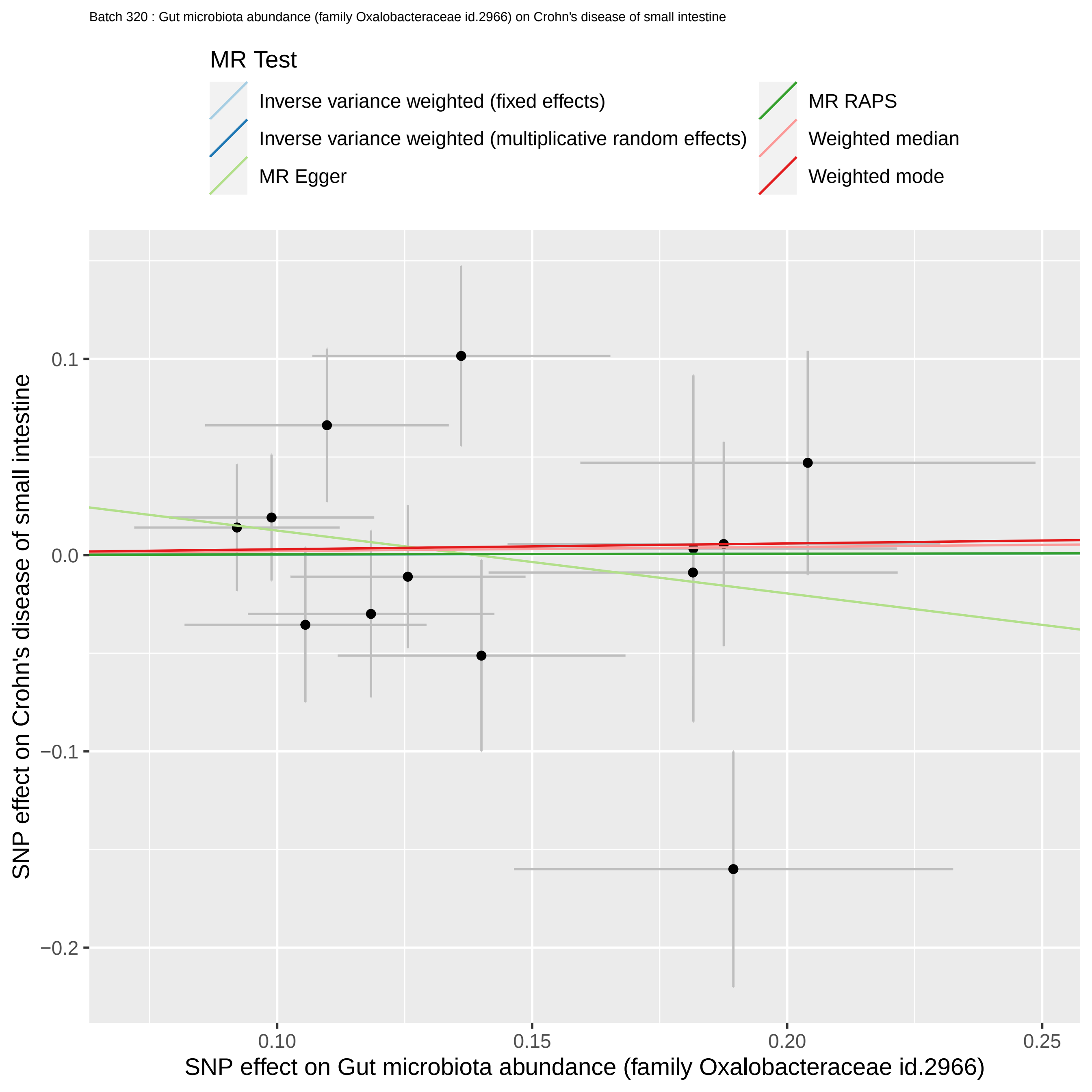

Batch 320 : Gut microbiota abundance (family Oxalobacteraceae id.2966) on Crohn's disease of small intestine
MR Test
Inverse variance weighted (fixed effects)
Inverse variance weighted (multiplicative random effects)
MR Egger
MR RAPS
Weighted median
Weighted mode
0.1
0.0
−0.1
−0.2
0.10
0.15
0.20
0.25
SNP effect on Crohn's disease of small intestine
SNP effect on Gut microbiota abundance (family Oxalobacteraceae id.2966)

Batch 321 : Gut microbiota abundance (family Pasteurellaceae id.3689) on Crohn's disease of small intestine

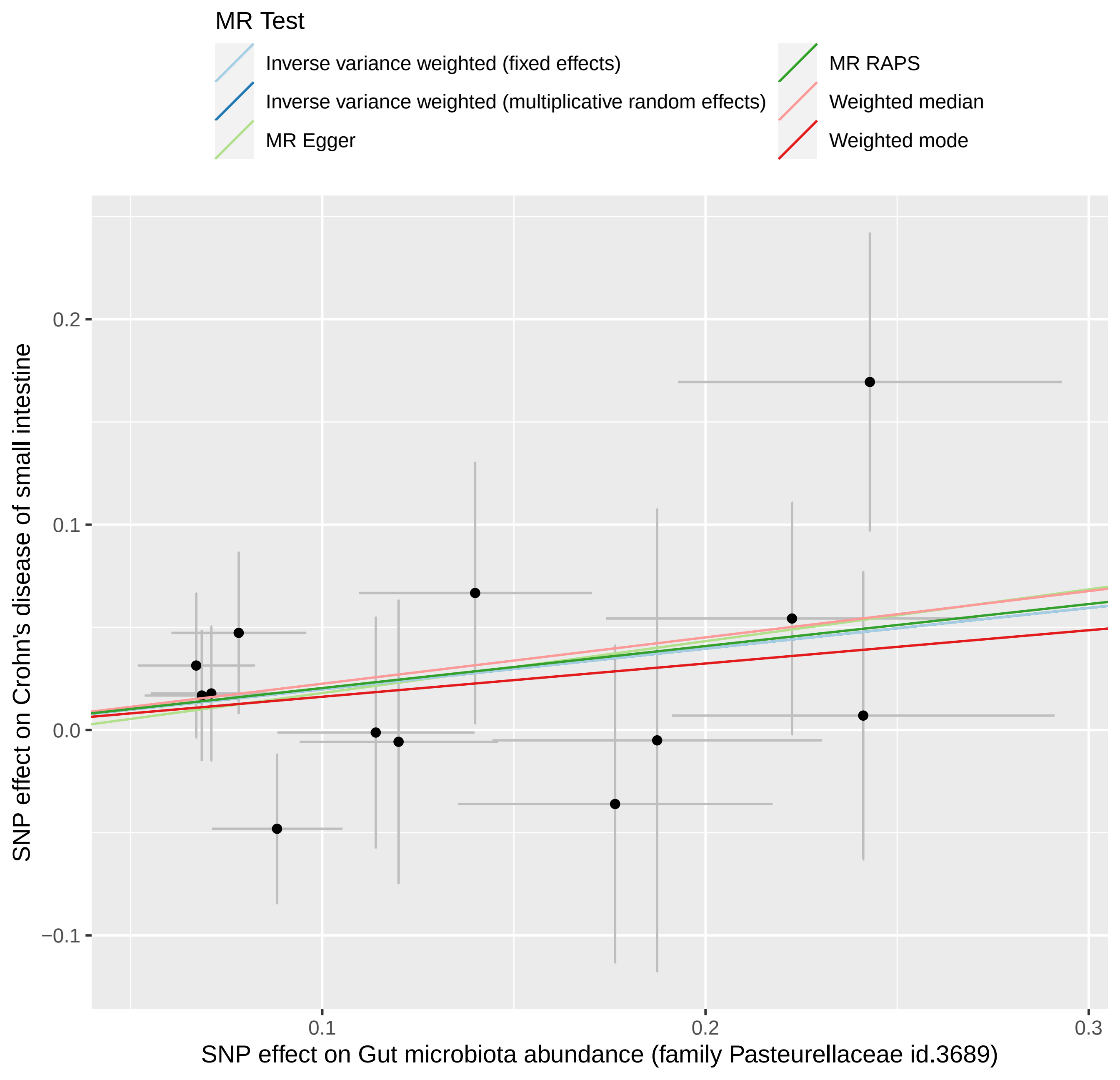

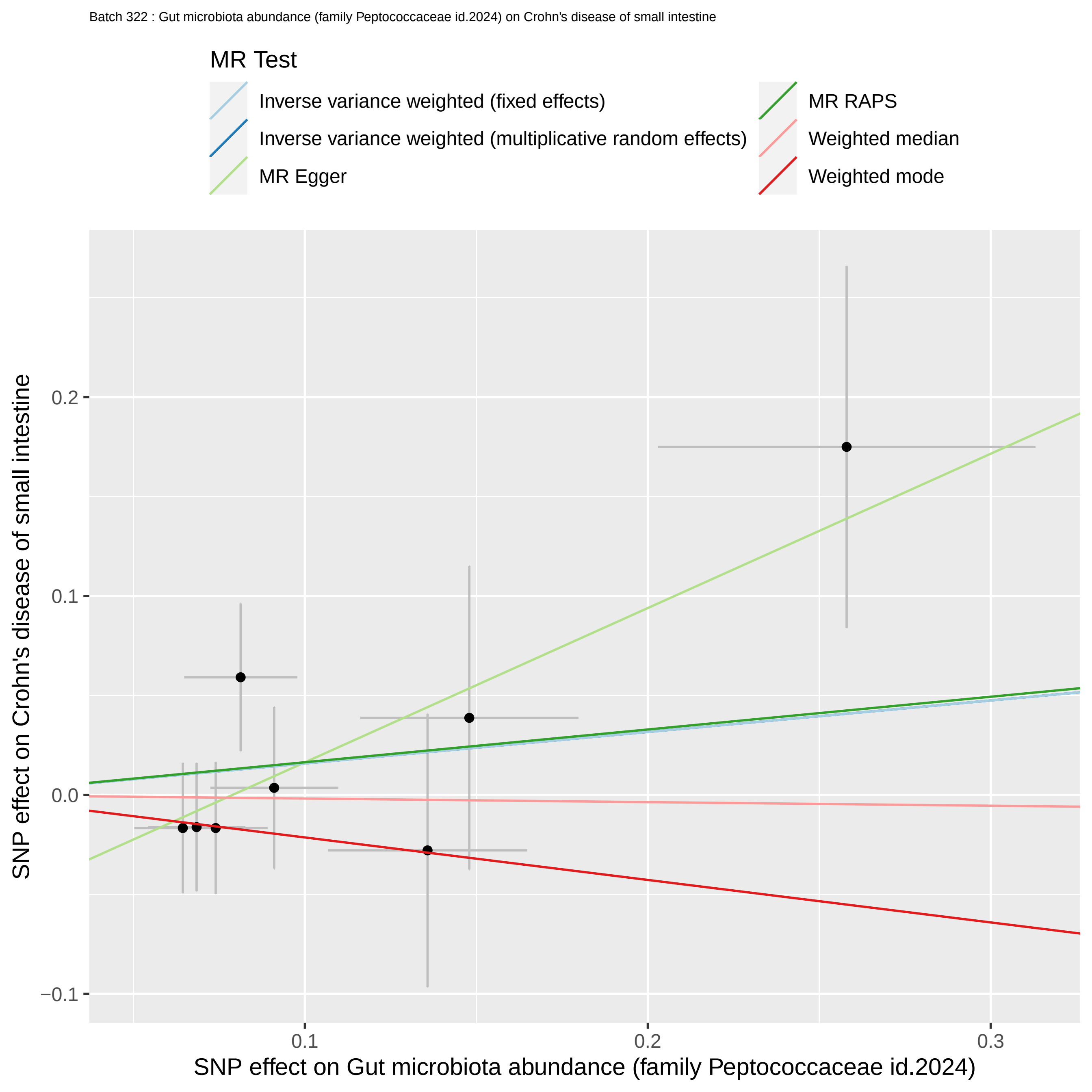
Batch 322 : Gut microbiota abundance (family Peptococcaceae id.2024) on Crohn's disease of small intestine
MR Test
Inverse variance weighted (fixed effects)
Inverse variance weighted (multiplicative random effects)
MR Egger
MR RAPS
Weighted median
Weighted mode
0.2
0.1
0.0
−0.1
0.1
0.2
0.3
SNP effect on Crohn's disease of small intestine
SNP effect on Gut microbiota abundance (family Peptococcaceae id.2024)

Batch 323 : Gut microbiota abundance (family Peptostreptococcaceae id.2042) on Crohn's disease of small intestine

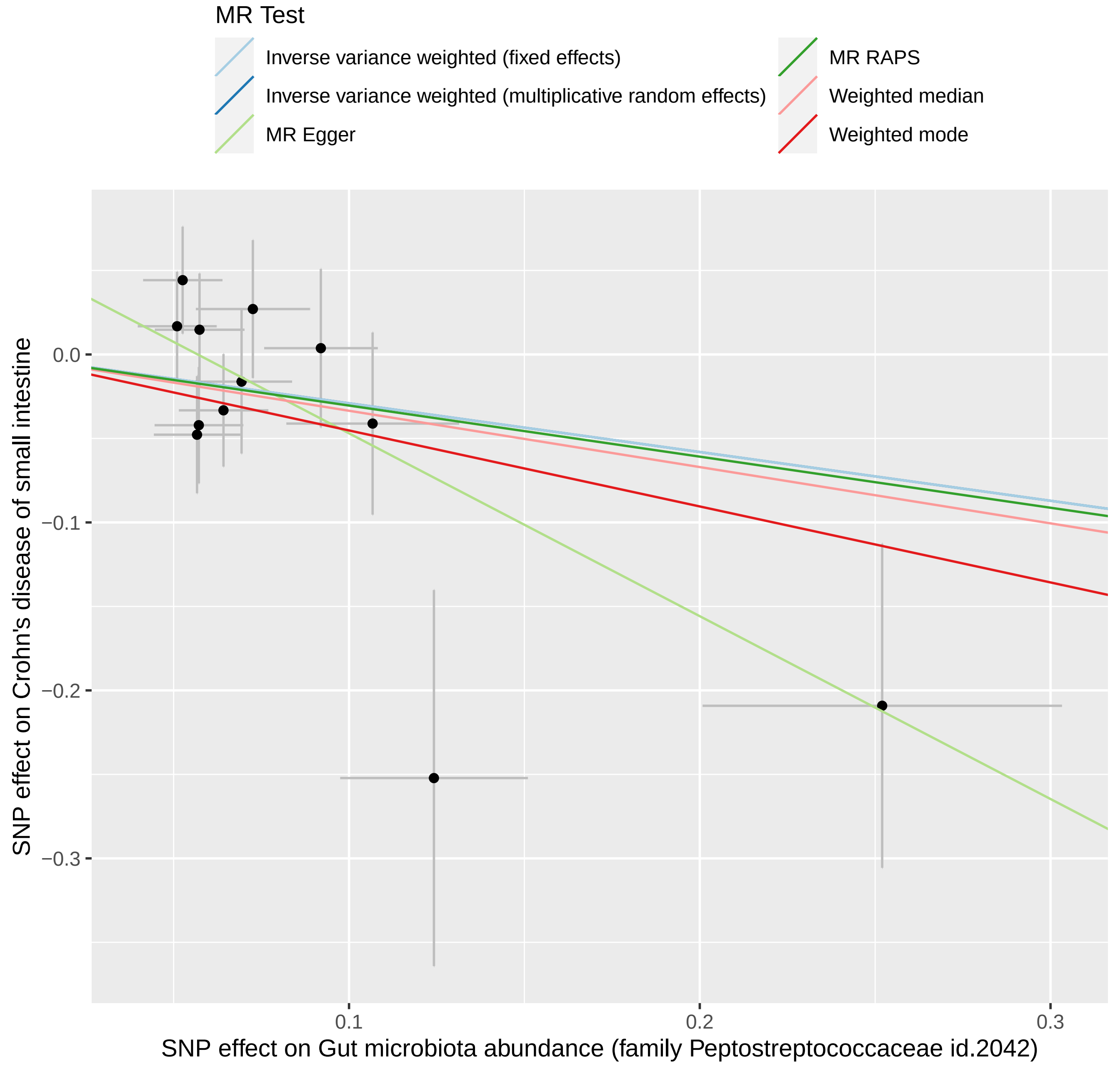

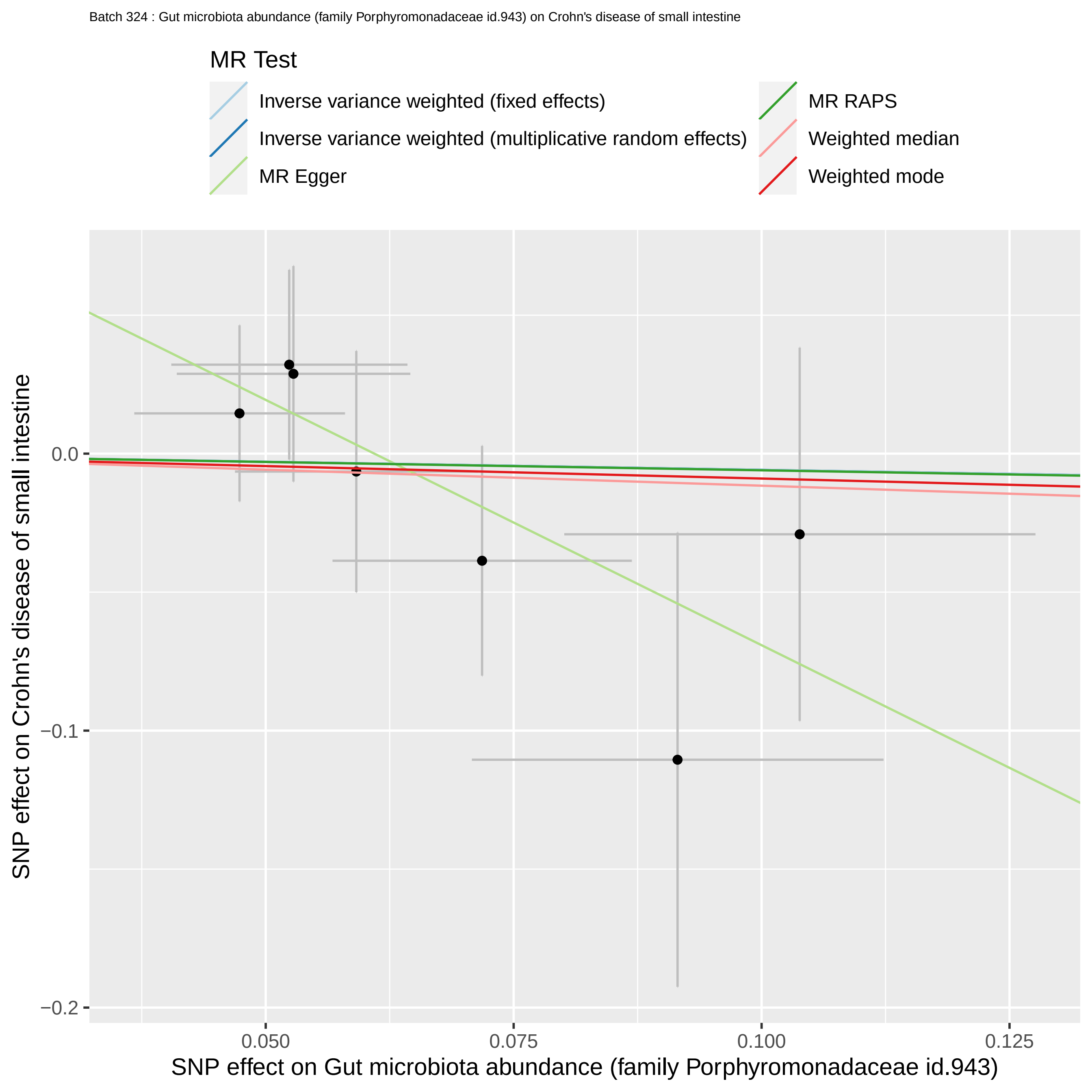

Batch 324 : Gut microbiota abundance (family Porphyromonadaceae id.943) on Crohn's disease of small intestine
MR Test
Inverse variance weighted (fixed effects)
Inverse variance weighted (multiplicative random effects)
MR Egger
MR RAPS
Weighted median
Weighted mode
0.0
−0.1
−0.2
0.050
0.075
0.100
0.125
SNP effect on Crohn's disease of small intestine
SNP effect on Gut microbiota abundance (family Porphyromonadaceae id.943)

Batch 325 : Gut microbiota abundance (family Prevotellaceae id.960) on Crohn's disease of small intestine

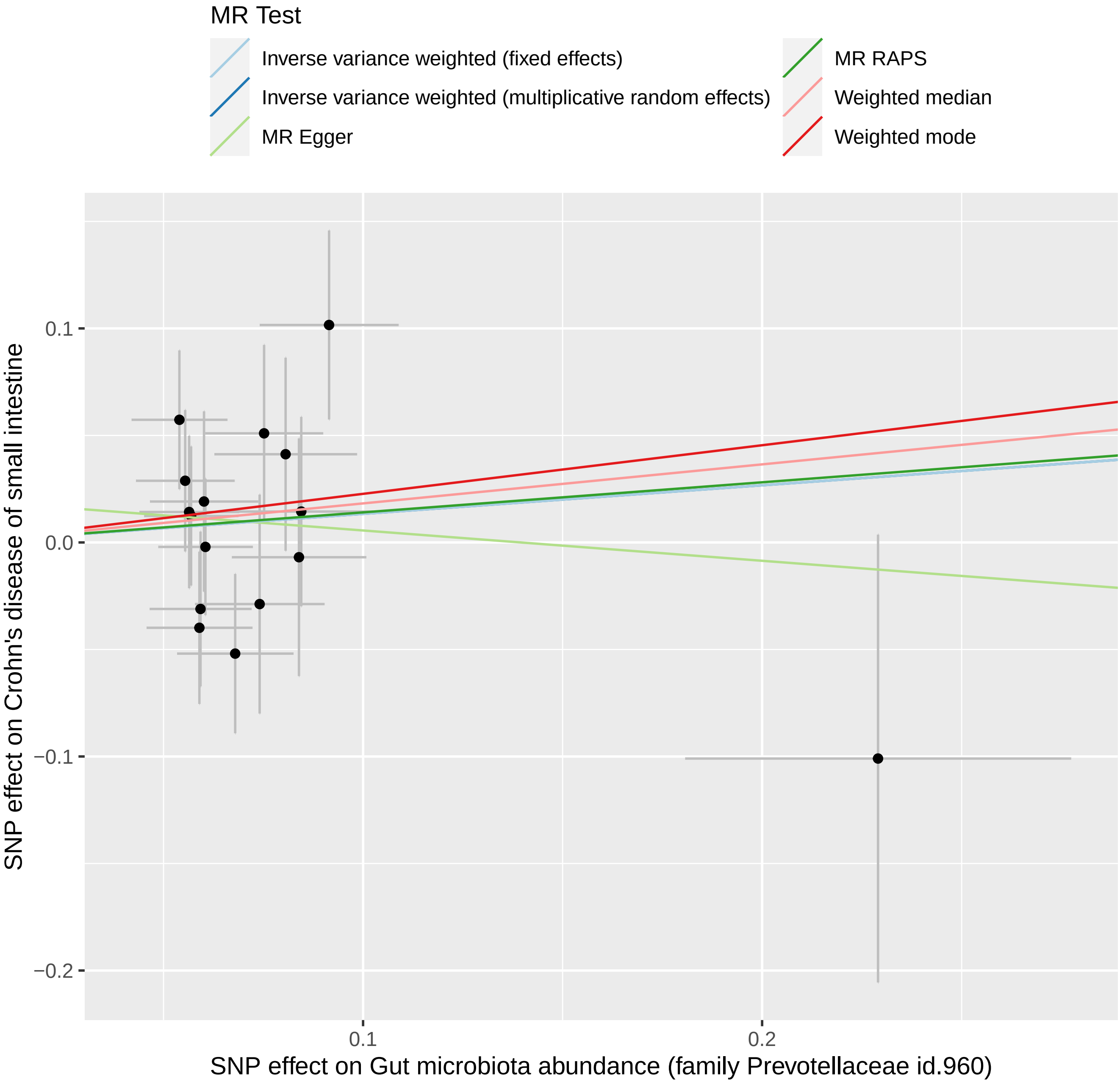

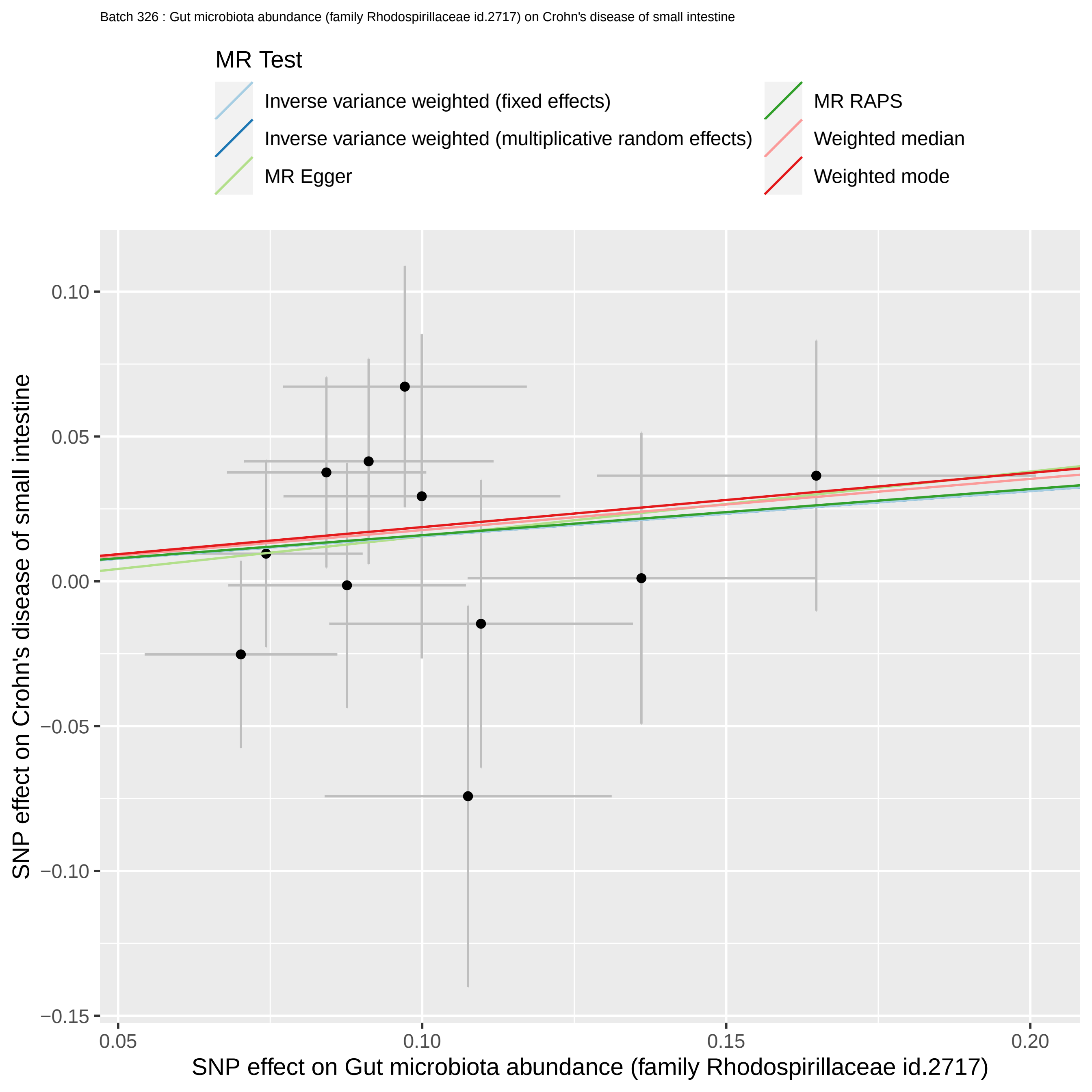

Batch 326 : Gut microbiota abundance (family Rhodospirillaceae id.2717) on Crohn's disease of small intestine
MR Test
Inverse variance weighted (fixed effects)
Inverse variance weighted (multiplicative random effects)
MR Egger
MR RAPS
Weighted median
Weighted mode
0.10
0.05
0.00
−0.05
−0.10
−0.15
0.05
0.10
0.15
0.20
SNP effect on Crohn's disease of small intestine
SNP effect on Gut microbiota abundance (family Rhodospirillaceae id.2717)

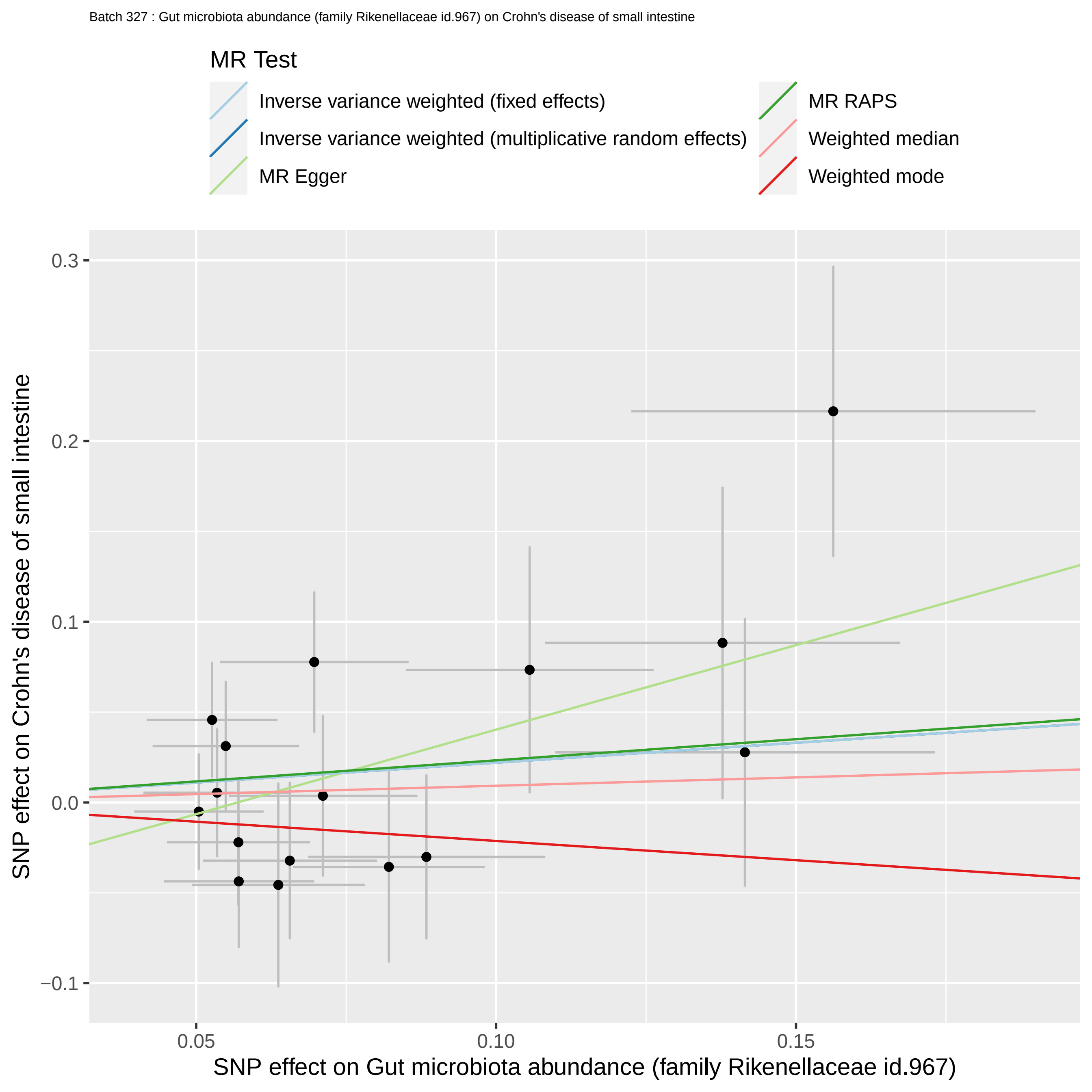

Batch 327 : Gut microbiota abundance (family Rikenellaceae id.967) on Crohn's disease of small intestine
MR Test
Inverse variance weighted (fixed effects)
Inverse variance weighted (multiplicative random effects)
MR Egger
MR RAPS
Weighted median
Weighted mode
SNP effect on Crohn's disease of small intestine
0.3
0.2
0.1
0.0
−0.1
0.05
0.10
0.15
SNP effect on Gut microbiota abundance (family Rikenellaceae id.967)

Batch 328 : Gut microbiota abundance (family Ruminococcaceae id.2050) on Crohn's disease of small intestine

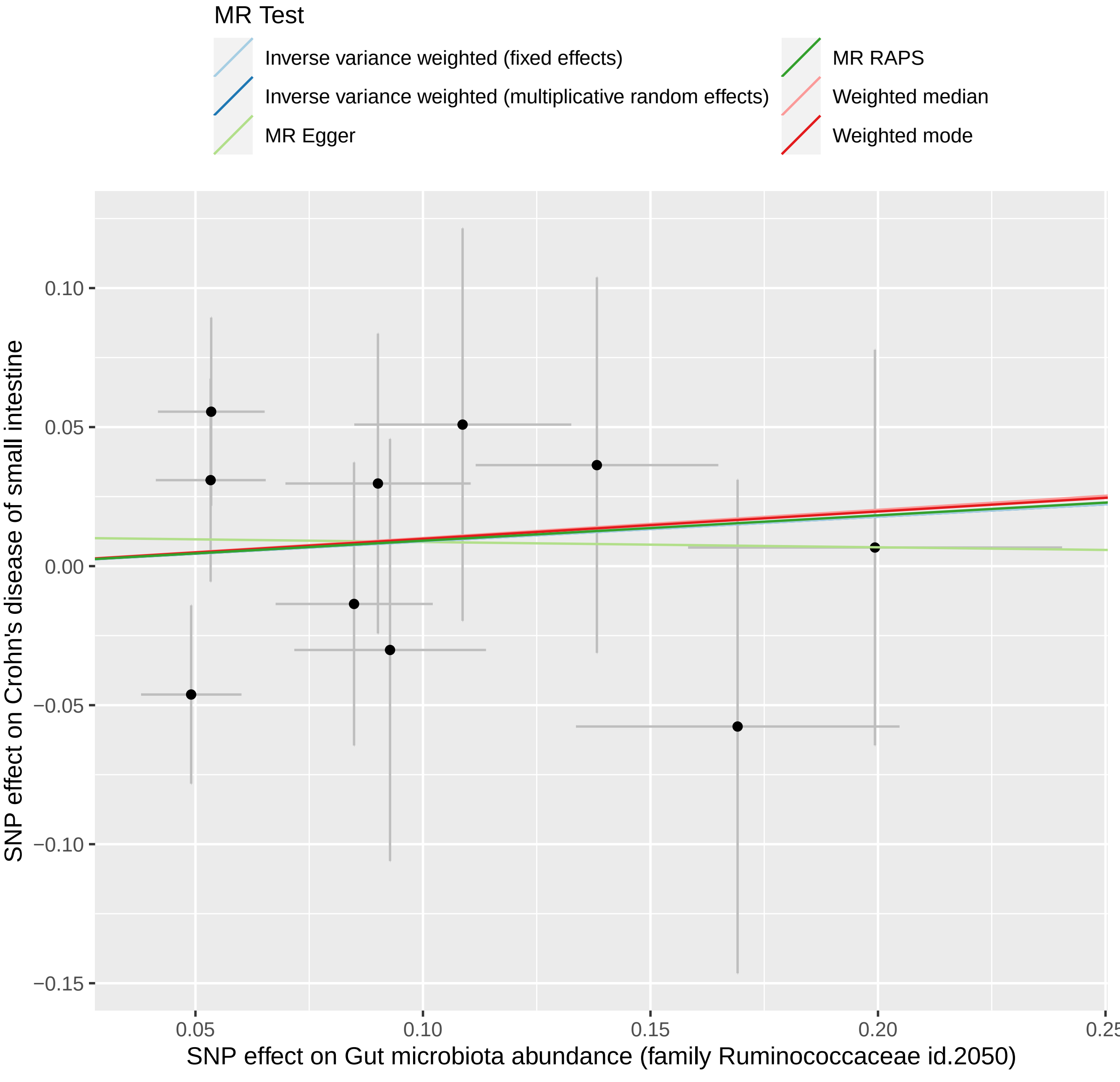

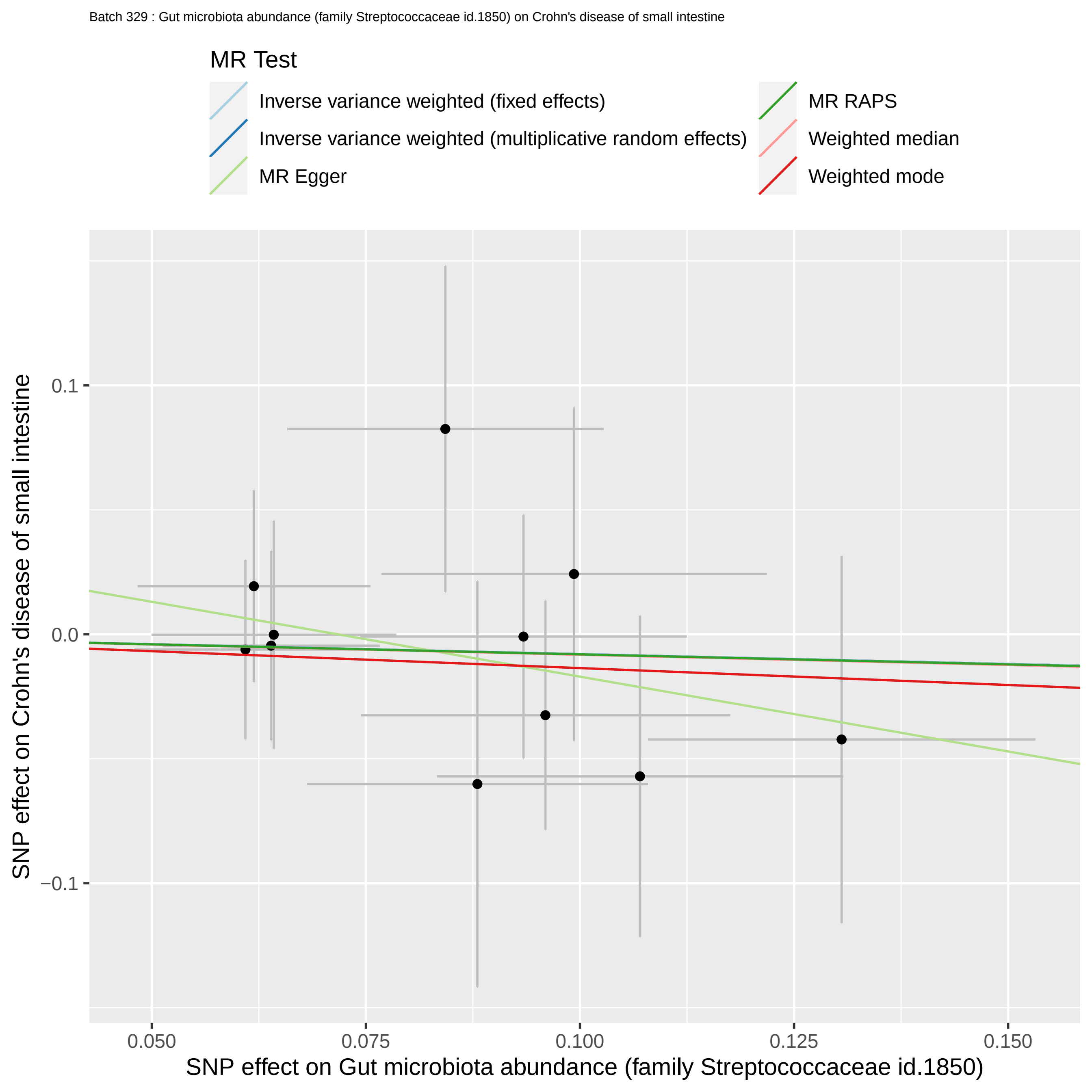

Batch 329 : Gut microbiota abundance (family Streptococcaceae id.1850) on Crohn's disease of small intestine
MR Test
Inverse variance weighted (fixed effects)
Inverse variance weighted (multiplicative random effects)
MR Egger
MR RAPS
Weighted median
Weighted mode
SNP effect on Crohn's disease of small intestine
0.1
0.0
−0.1
0.050
0.075
0.100
0.125
0.150
SNP effect on Gut microbiota abundance (family Streptococcaceae id.1850)

Batch 330 : Gut microbiota abundance (family Veillonellaceae id.2172) on Crohn's disease of small intestine

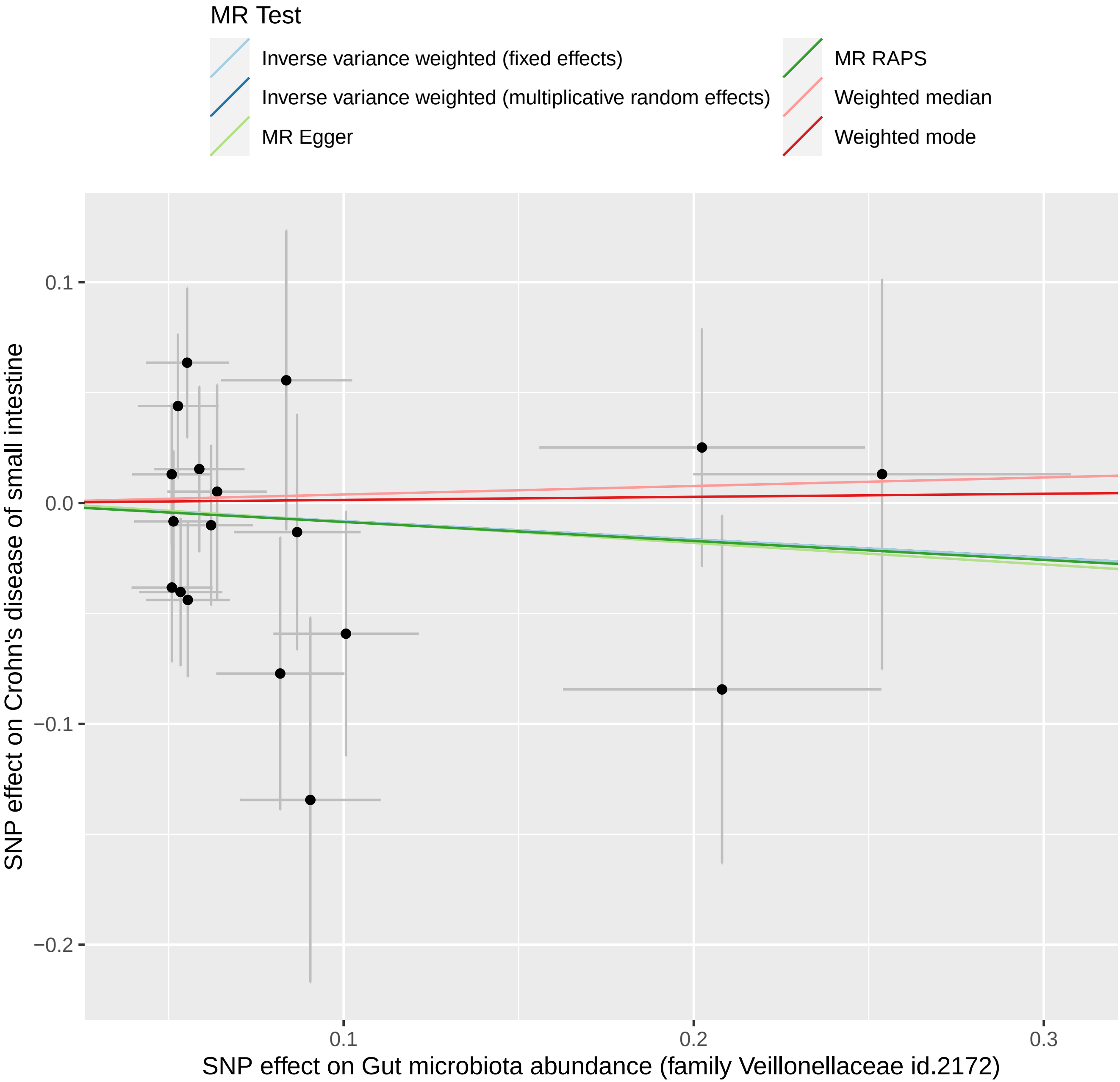

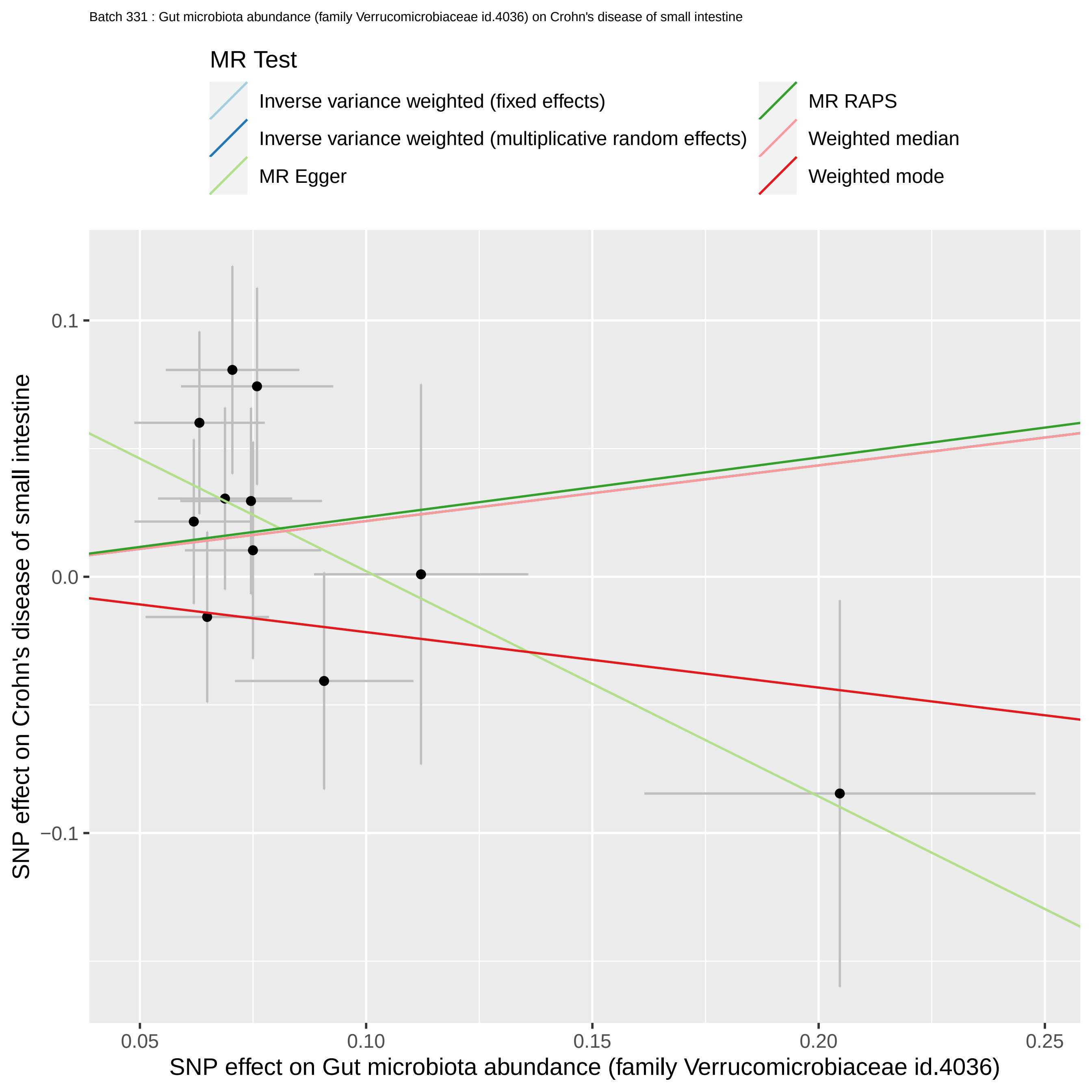

Batch 331 : Gut microbiota abundance (family Verrucomicrobiaceae id.4036) on Crohn's disease of small intestine
MR Test
Inverse variance weighted (fixed effects)
Inverse variance weighted (multiplicative random effects)
MR Egger
MR RAPS
Weighted median
Weighted mode
0.1
0.0
−0.1
0.05
0.10
0.15
0.20
0.25
SNP effect on Crohn's disease of small intestine
SNP effect on Gut microbiota abundance (family Verrucomicrobiaceae id.4036)

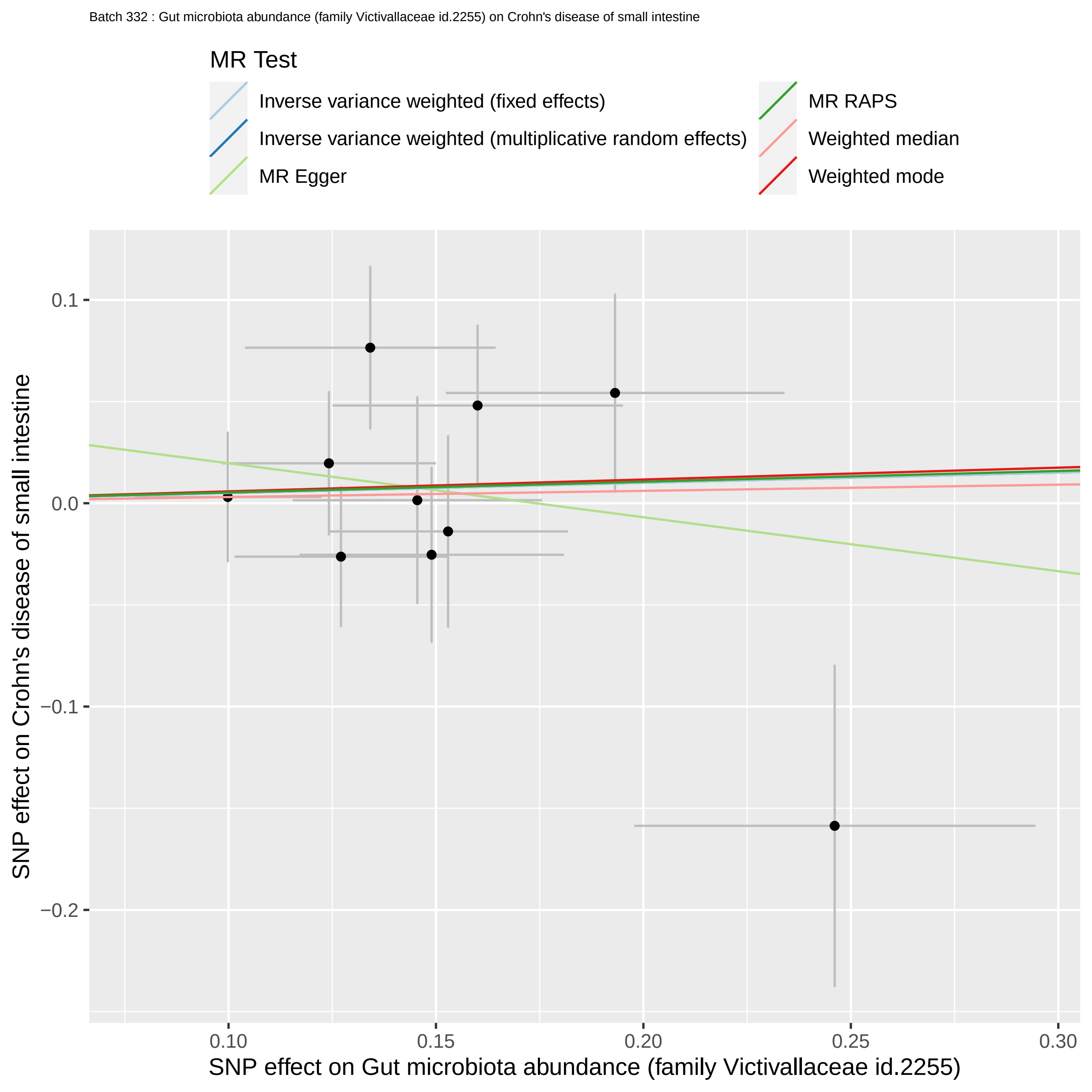
Batch 332 : Gut microbiota abundance (family Victivallaceae id.2255) on Crohn's disease of small intestine
MR Test
Inverse variance weighted (fixed effects)
Inverse variance weighted (multiplicative random effects)
MR Egger
MR RAPS
Weighted median
Weighted mode
0.1
0.0
−0.1
−0.2
0.10
0.15
0.20
0.25
0.30
SNP effect on Crohn's disease of small intestine
SNP effect on Gut microbiota abundance (family Victivallaceae id.2255)

Batch 333 : Gut microbiota abundance (genus Actinomyces id.423) on Crohn's disease of small intestine

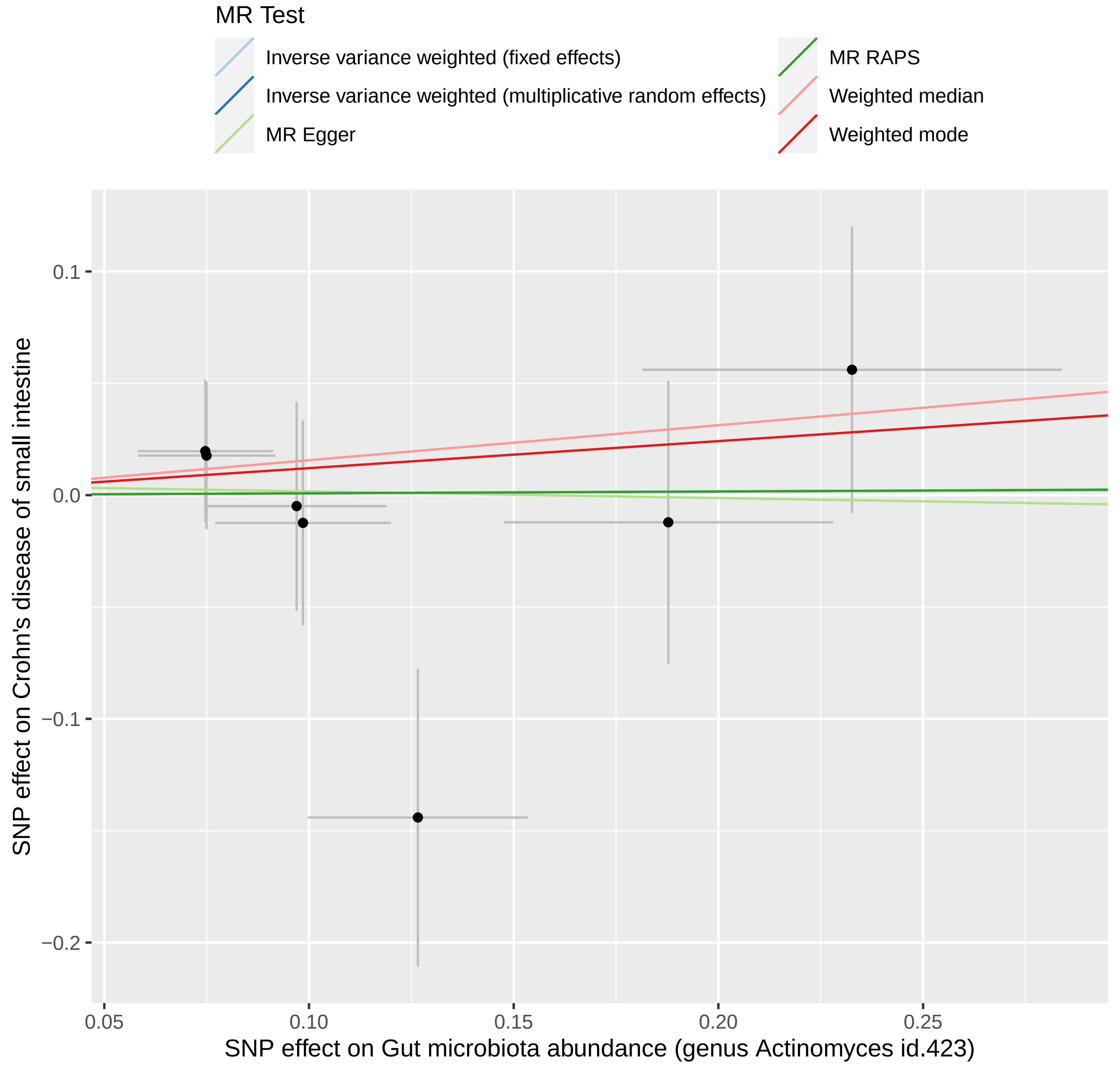

Batch 334 : Gut microbiota abundance (genus Adlercreutzia id.812) on Crohn's disease of small intestine

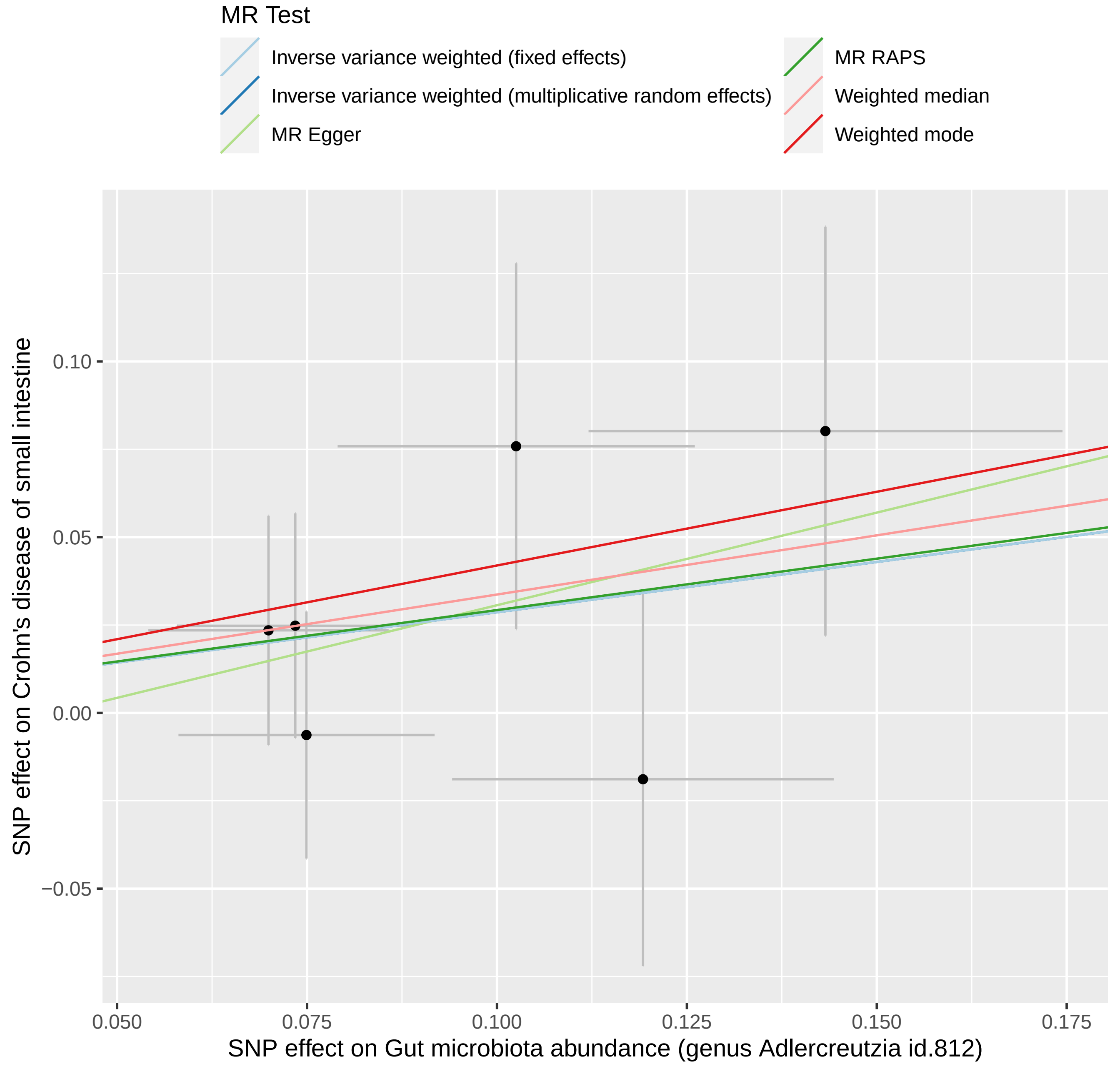

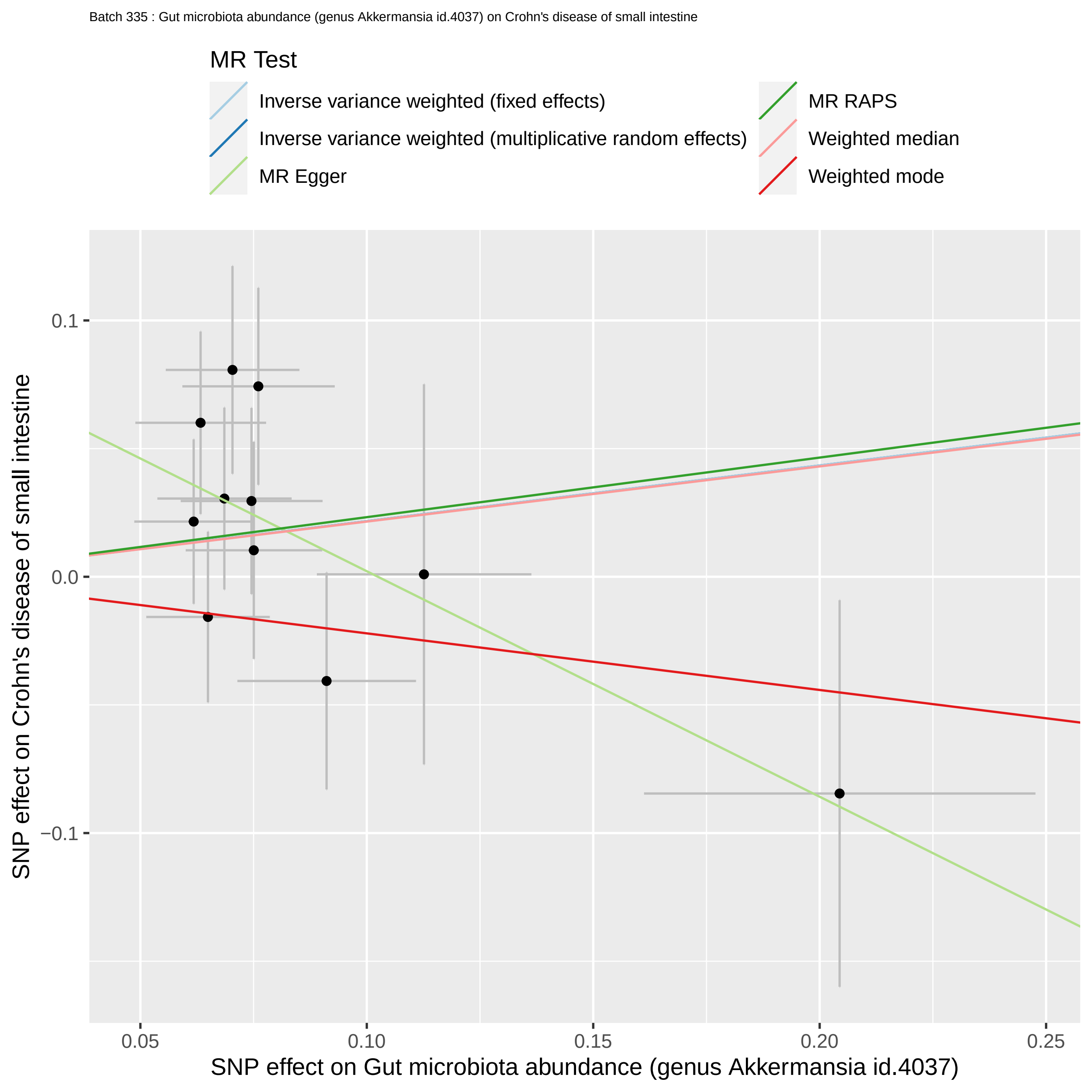

Batch 335 : Gut microbiota abundance (genus Akkermansia id.4037) on Crohn's disease of small intestine
MR Test
Inverse variance weighted (fixed effects)
Inverse variance weighted (multiplicative random effects)
MR Egger
MR RAPS
Weighted median
Weighted mode
0.1
0.0
−0.1
0.05
0.10
0.15
0.20
0.25
SNP effect on Crohn's disease of small intestine
SNP effect on Gut microbiota abundance (genus Akkermansia id.4037)

Batch 336 : Gut microbiota abundance (genus Alistipes id.968) on Crohn's disease of small intestine

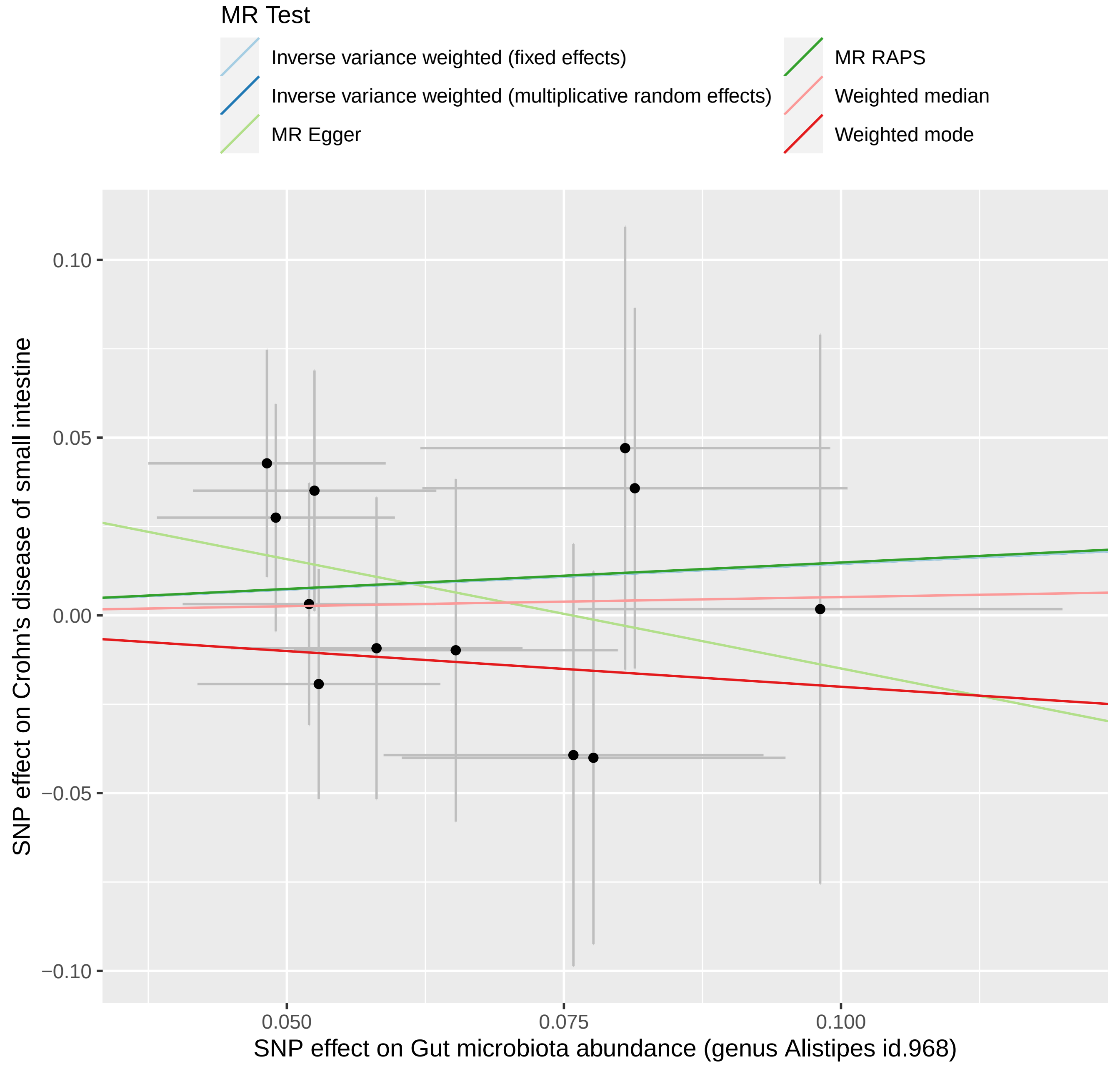

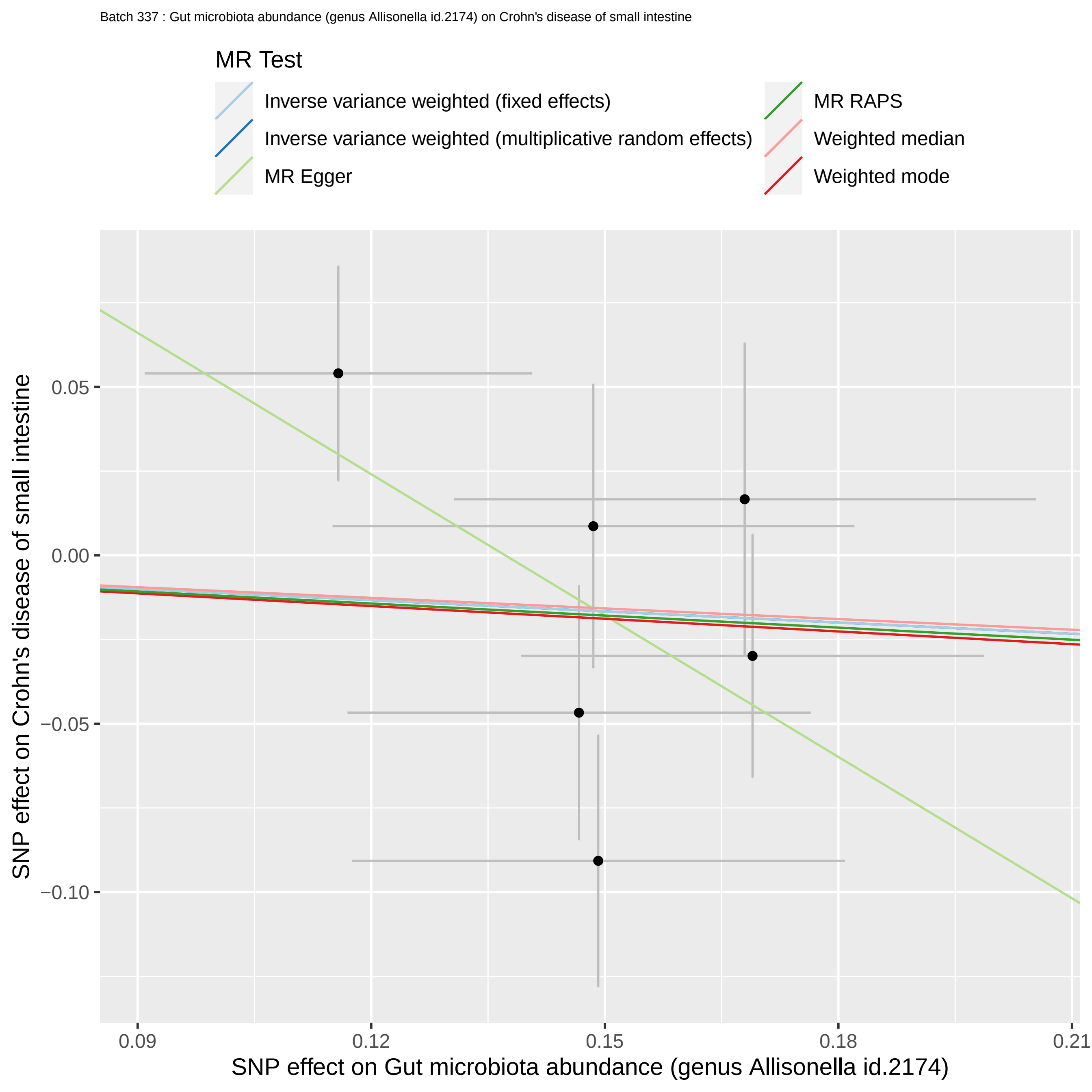

Batch 337 : Gut microbiota abundance (genus Allisonella id.2174) on Crohn's disease of small intestine
MR Test
Inverse variance weighted (fixed effects)
Inverse variance weighted (multiplicative random effects)
MR Egger
MR RAPS
Weighted median
Weighted mode
SNP effect on Crohn's disease of small intestine
0.05
0.00
−0.05
−0.10
0.09
0.12
0.15
0.18
0.21
SNP effect on Gut microbiota abundance (genus Allisonella id.2174)

Batch 338 : Gut microbiota abundance (genus Alloprevotella id.961) on Crohn's disease of small intestine

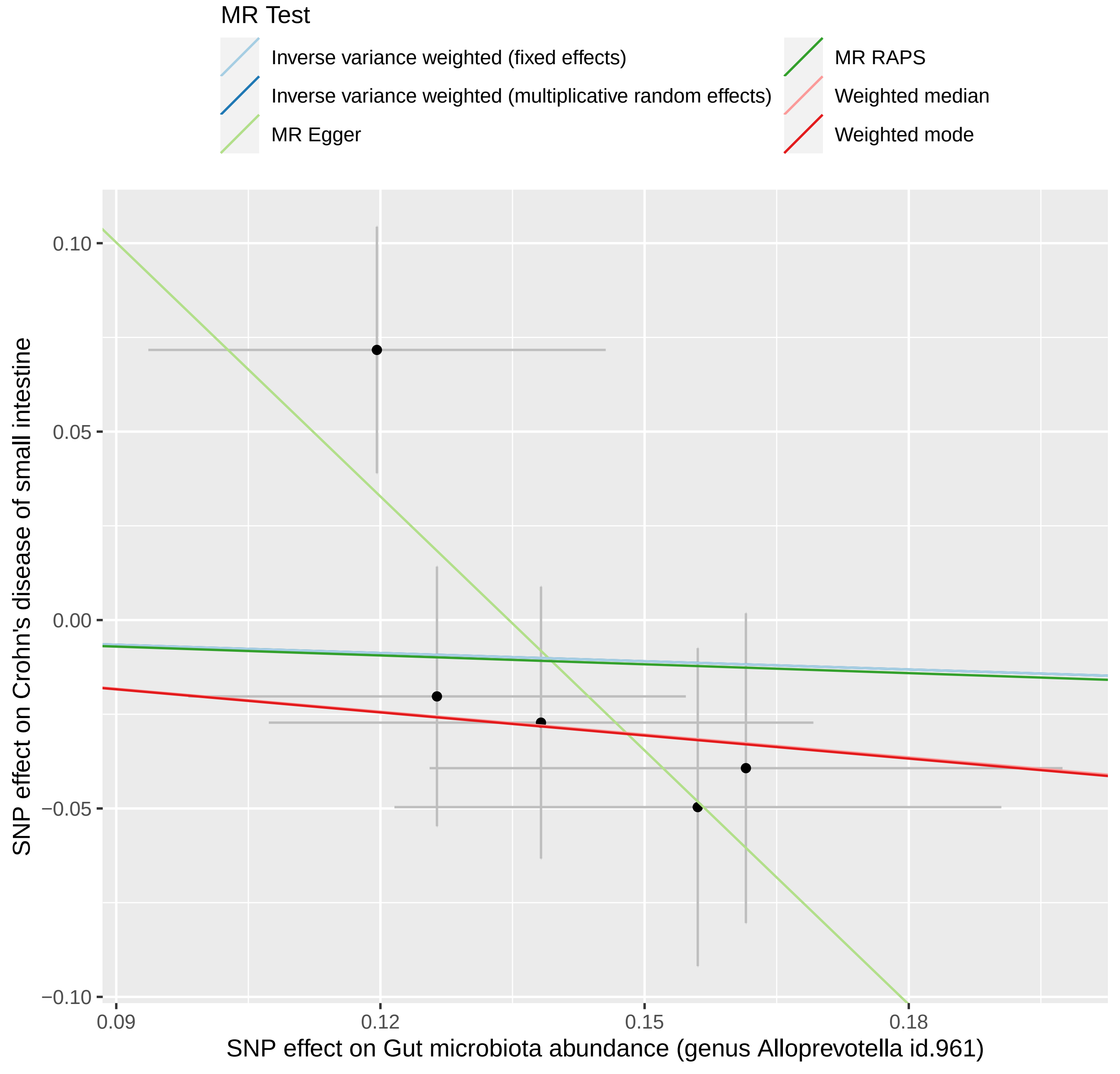

Batch 339 : Gut microbiota abundance (genus Anaerofilum id.2053) on Crohn's disease of small intestine

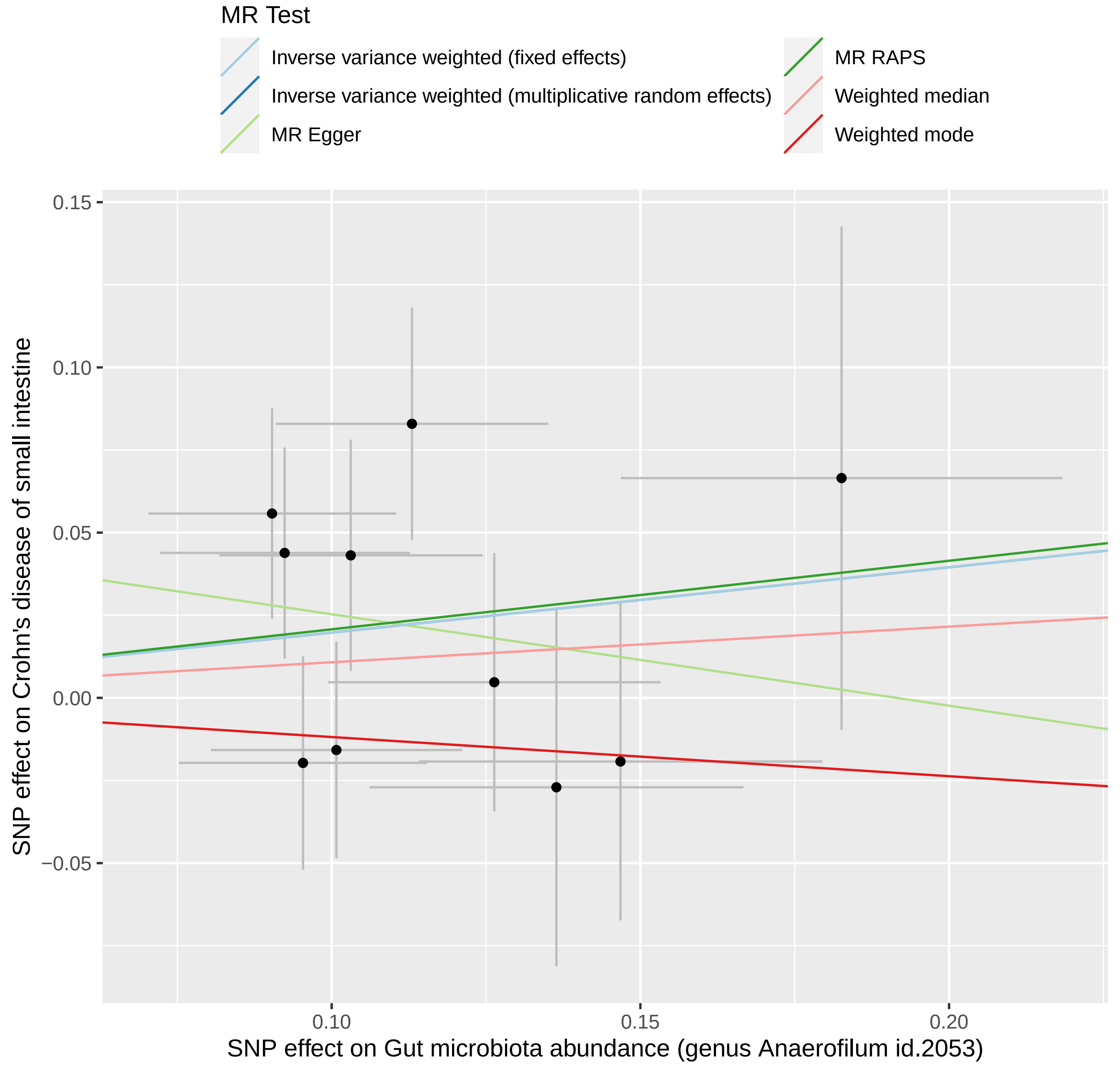

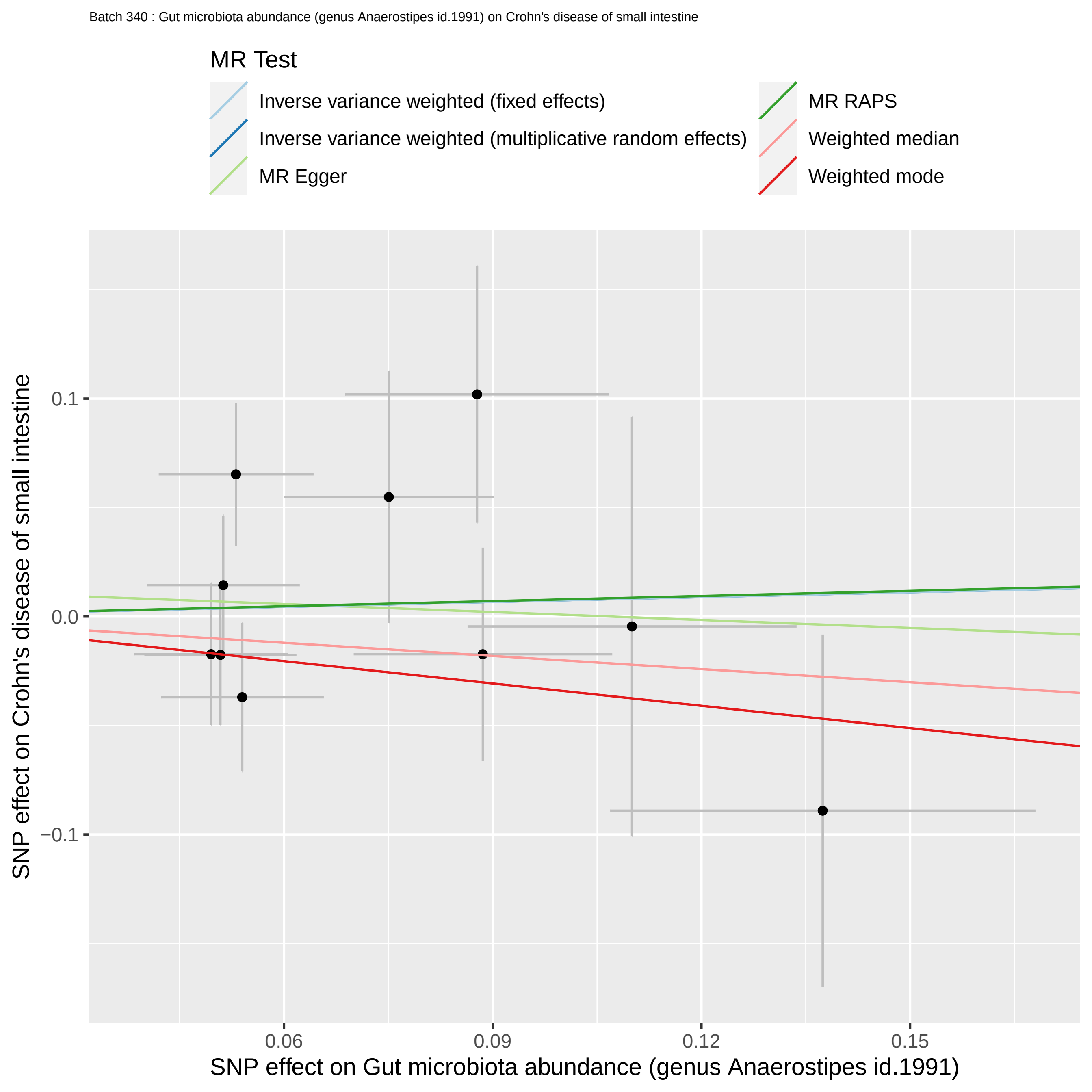

Batch 340 : Gut microbiota abundance (genus Anaerostipes id.1991) on Crohn's disease of small intestine
MR Test
Inverse variance weighted (fixed effects)
Inverse variance weighted (multiplicative random effects)
MR Egger
MR RAPS
Weighted median
Weighted mode
0.1
0.0
−0.1
0.06
0.09
0.12
0.15
SNP effect on Crohn's disease of small intestine
SNP effect on Gut microbiota abundance (genus Anaerostipes id.1991)

Batch 341 : Gut microbiota abundance (genus Anaerotruncus id.2054) on Crohn's disease of small intestine

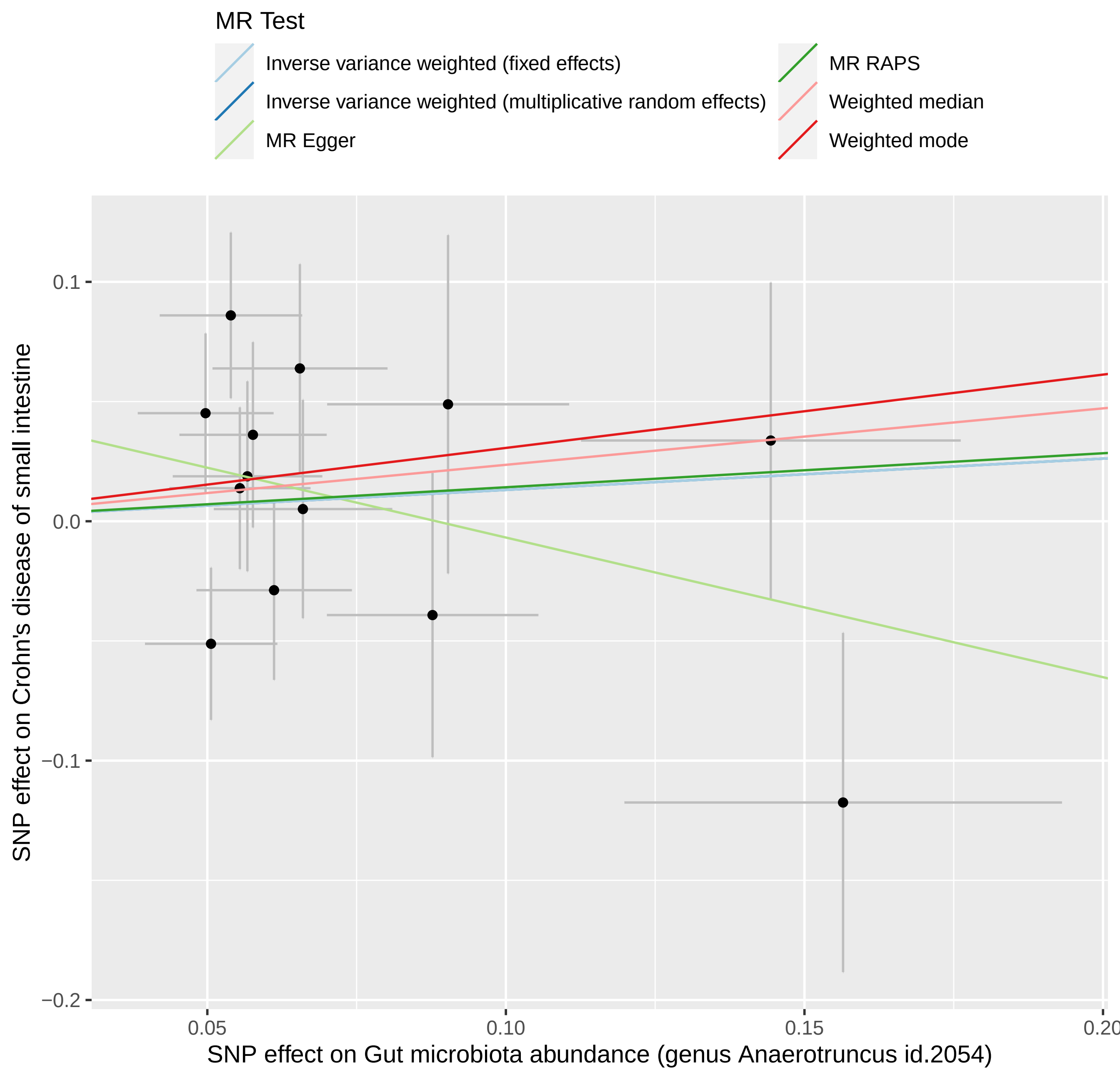

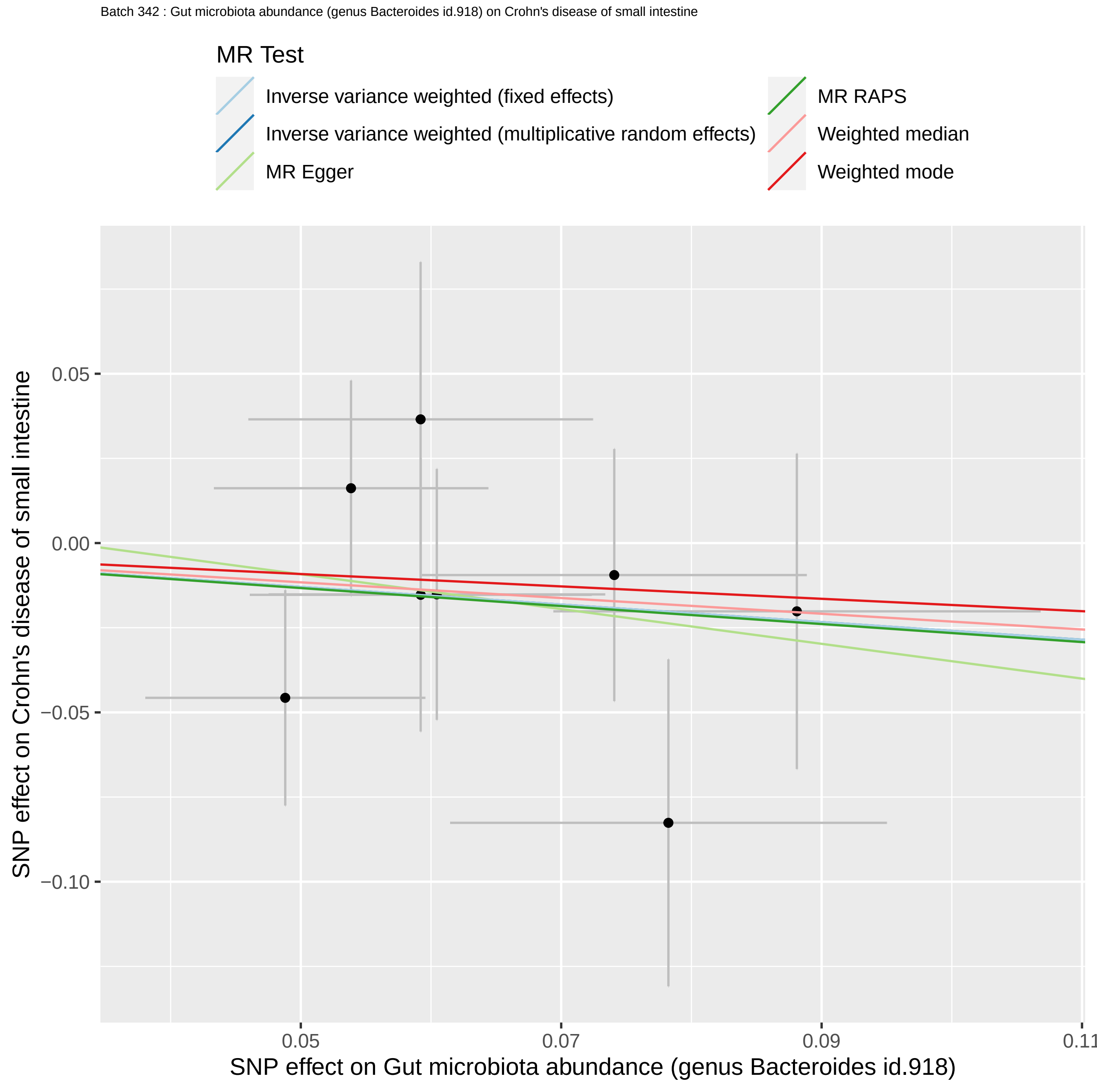

Batch 342 : Gut microbiota abundance (genus Bacteroides id.918) on Crohn's disease of small intestine
MR Test
Inverse variance weighted (fixed effects)
Inverse variance weighted (multiplicative random effects)
MR Egger
MR RAPS
Weighted median
Weighted mode
SNP effect on Crohn's disease of small intestine
0.05
0.00
−0.05
−0.10
0.05
0.07
0.09
0.11
SNP effect on Gut microbiota abundance (genus Bacteroides id.918)

Batch 343 : Gut microbiota abundance (genus Barnesiella id.944) on Crohn's disease of small intestine

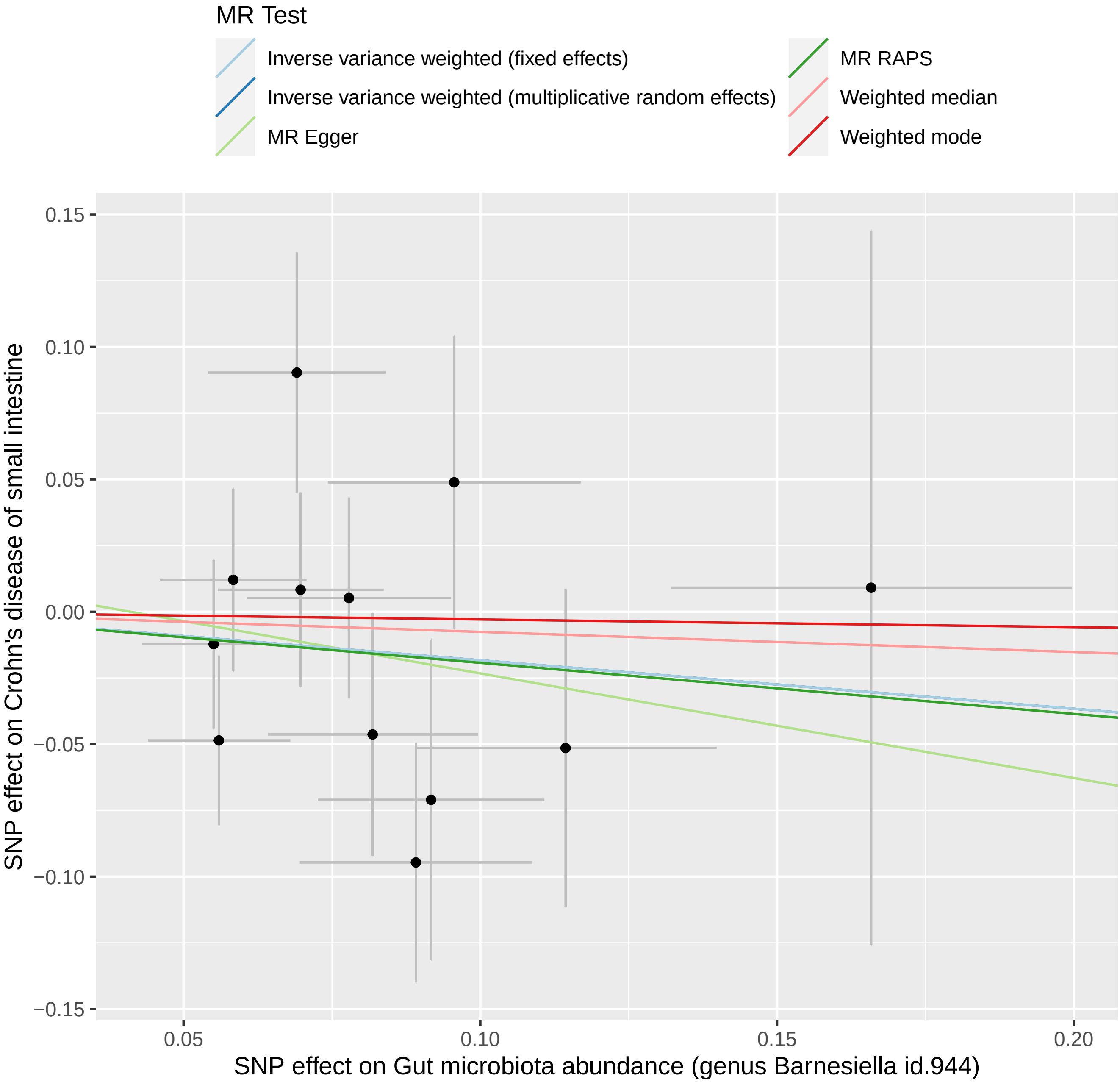

Batch 344 : Gut microbiota abundance (genus Bifidobacterium id.436) on Crohn's disease of small intestine

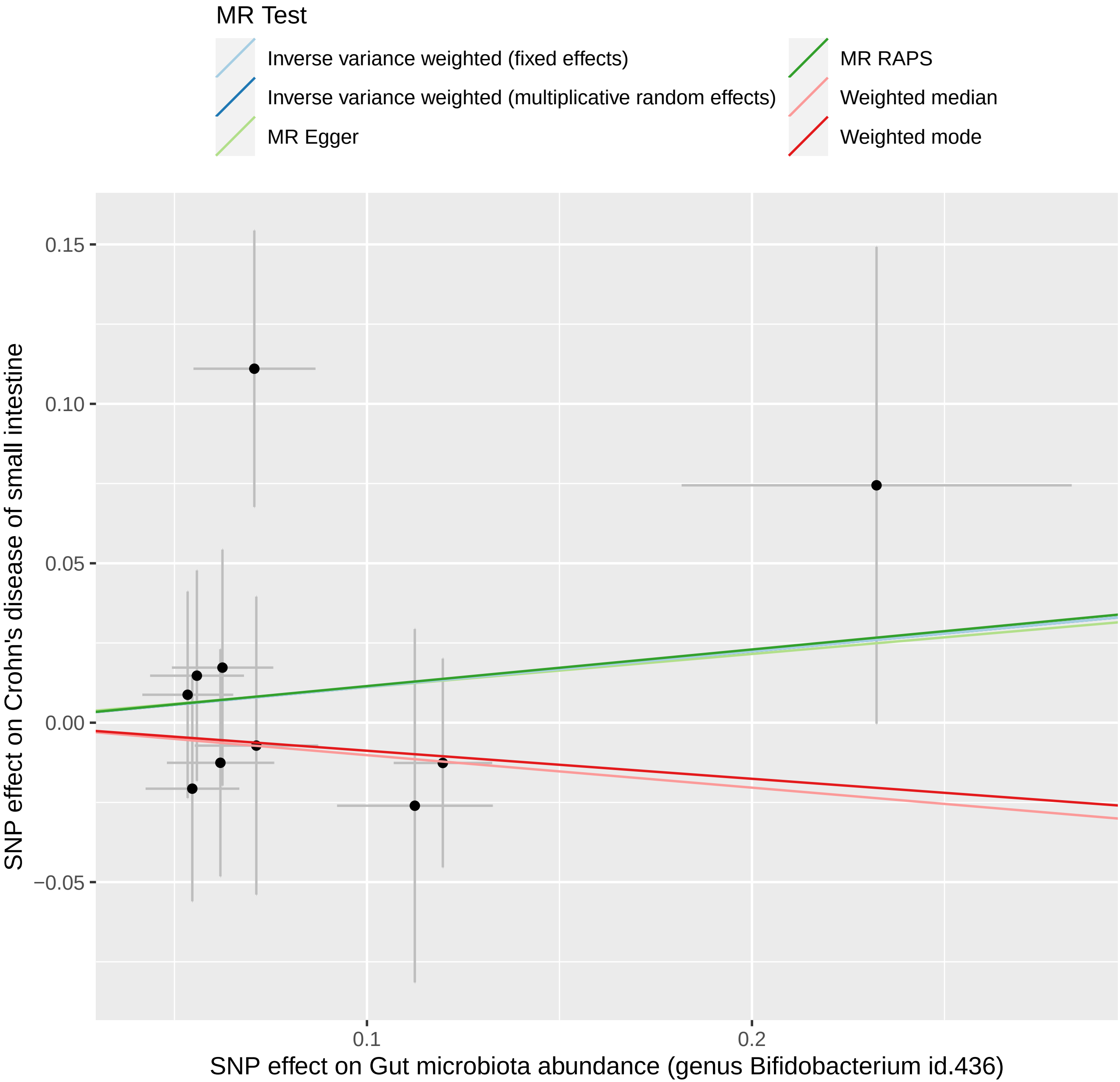

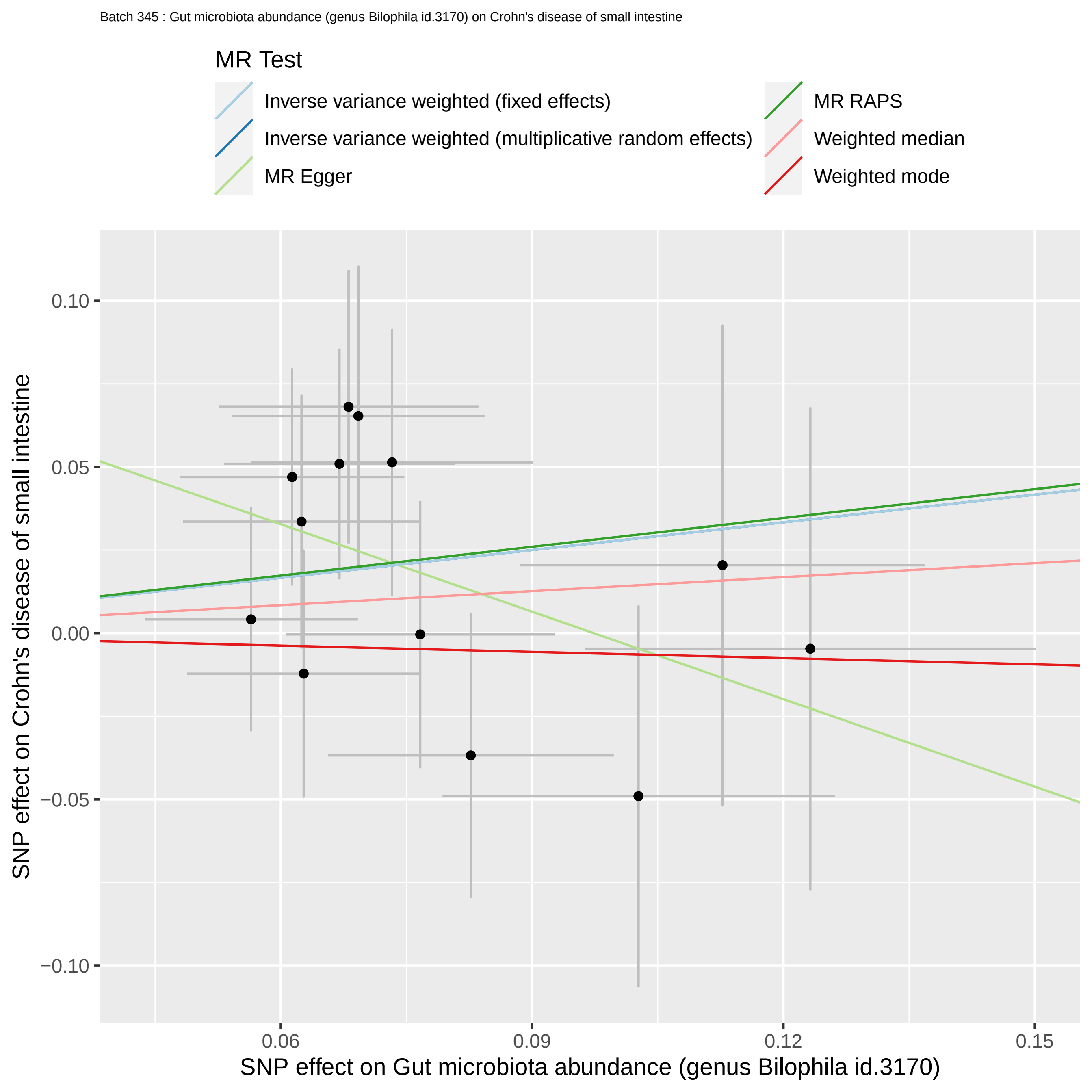

Batch 345 : Gut microbiota abundance (genus Bilophila id.3170) on Crohn's disease of small intestine
MR Test
Inverse variance weighted (fixed effects)
Inverse variance weighted (multiplicative random effects)
MR Egger
MR RAPS
Weighted median
Weighted mode
SNP effect on Crohn's disease of small intestine
0.10
0.05
0.00
−0.05
−0.10
0.06
0.09
0.12
0.15
SNP effect on Gut microbiota abundance (genus Bilophila id.3170)

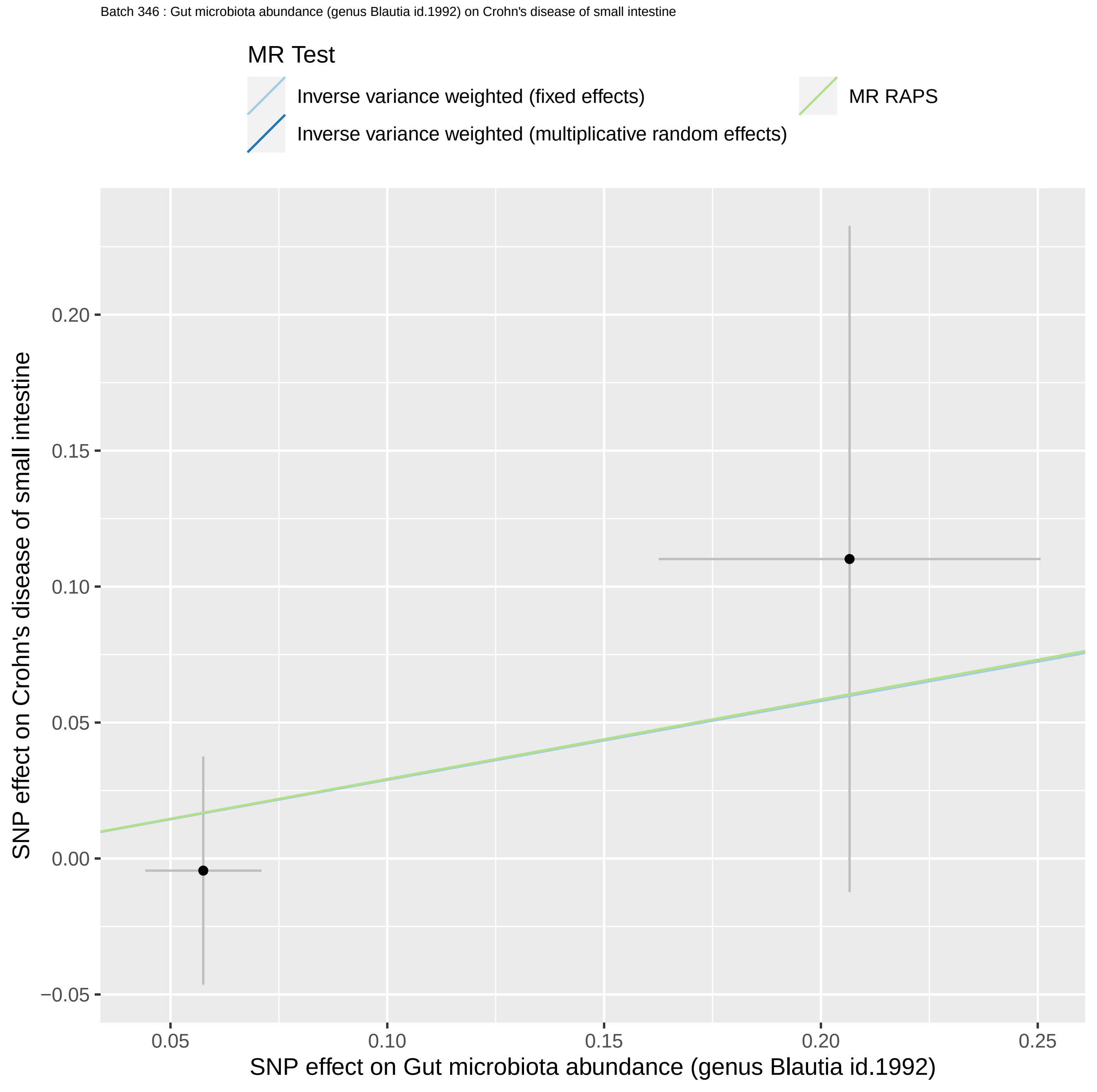

Batch 346 : Gut microbiota abundance (genus Blautia id.1992) on Crohn's disease of small intestine
MR Test
Inverse variance weighted (fixed effects)
Inverse variance weighted (multiplicative random effects)
MR RAPS
SNP effect on Crohn's disease of small intestine
0.20
0.15
0.10
0.05
0.00
−0.05
0.05
0.10
0.15
0.20
0.25
SNP effect on Gut microbiota abundance (genus Blautia id.1992)

Batch 347 : Gut microbiota abundance (genus Butyricicoccus id.2055) on Crohn's disease of small intestine

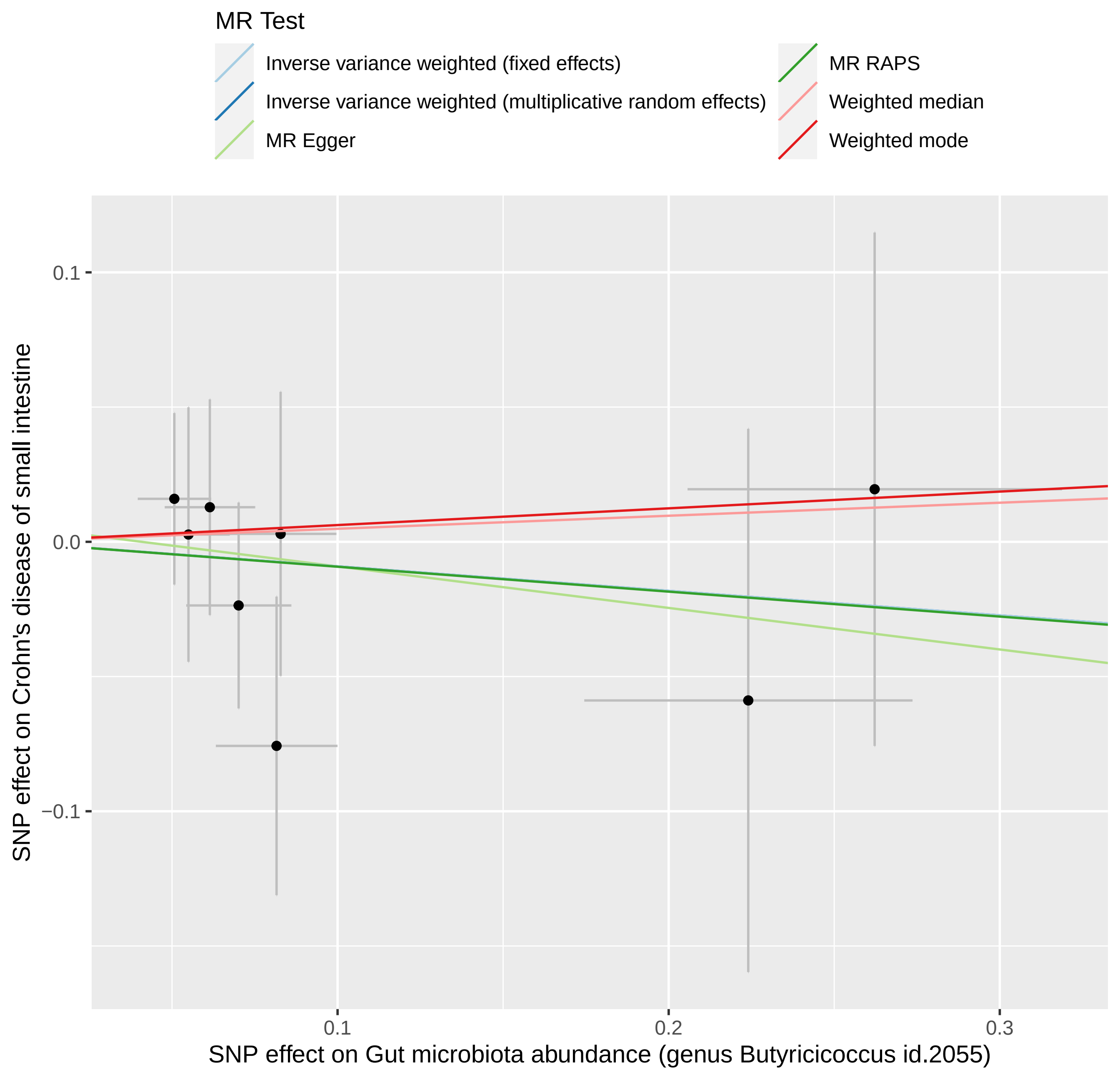

Batch 348 : Gut microbiota abundance (genus Butyricimonas id.945) on Crohn's disease of small intestine

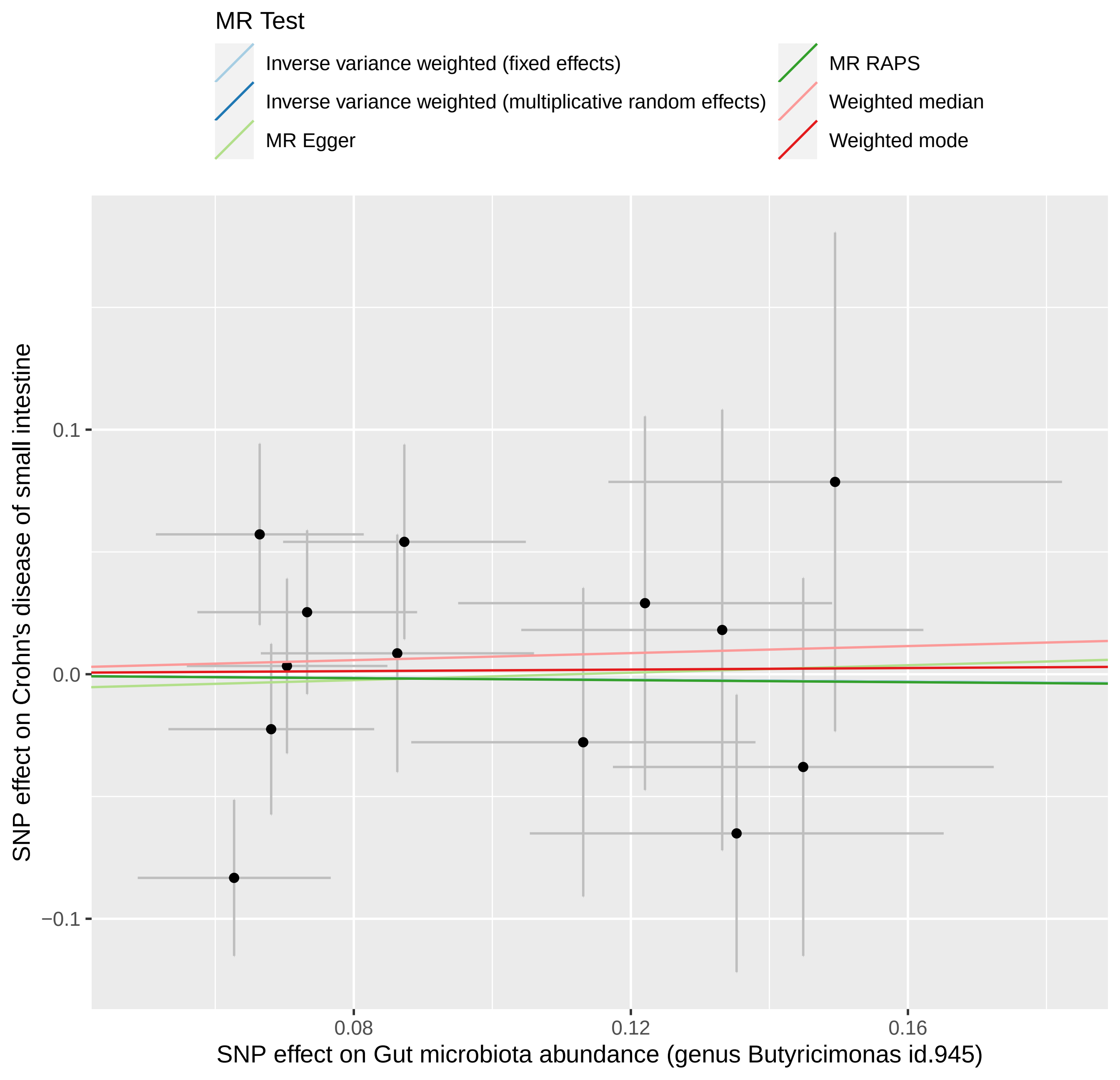

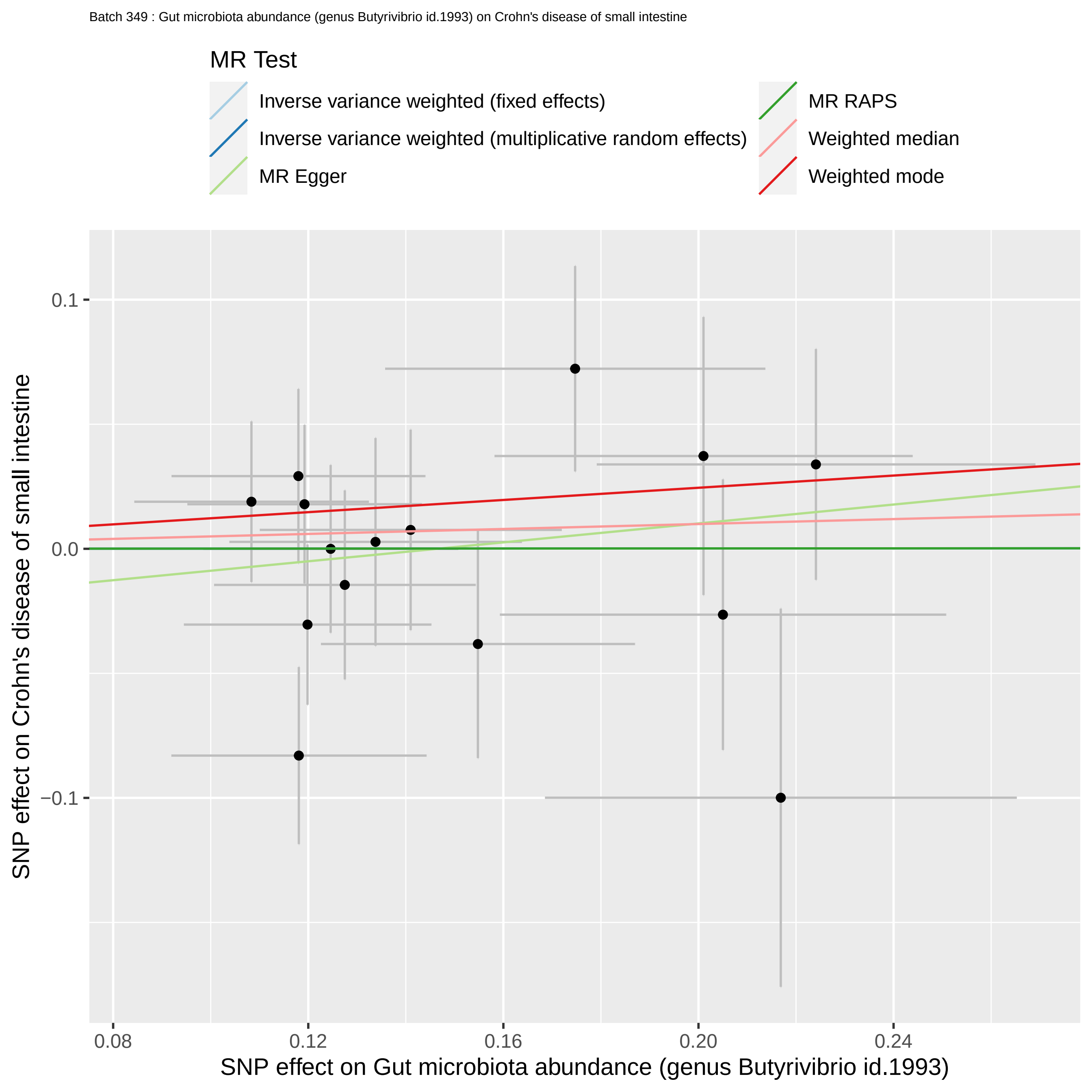

Batch 349 : Gut microbiota abundance (genus Butyrivibrio id.1993) on Crohn's disease of small intestine
MR Test
Inverse variance weighted (fixed effects)
Inverse variance weighted (multiplicative random effects)
MR Egger
MR RAPS
Weighted median
Weighted mode
0.1
0.0
−0.1
0.08
0.12
0.16
0.20
0.24
SNP effect on Crohn's disease of small intestine
SNP effect on Gut microbiota abundance (genus Butyrivibrio id.1993)

Batch 350 : Gut microbiota abundance (genus Candidatus Soleaferrea id.11350) on Crohn's disease of small intestine

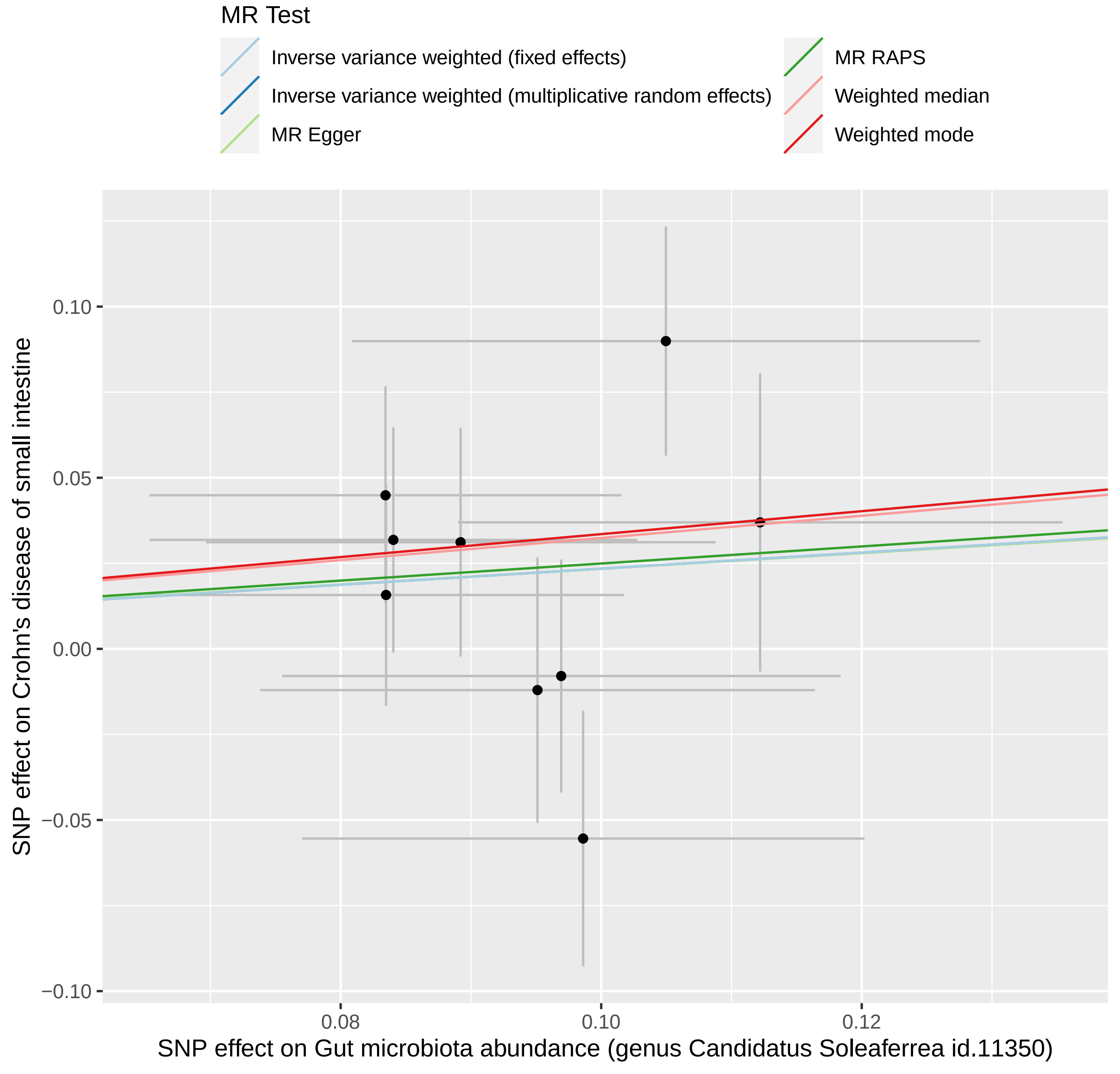

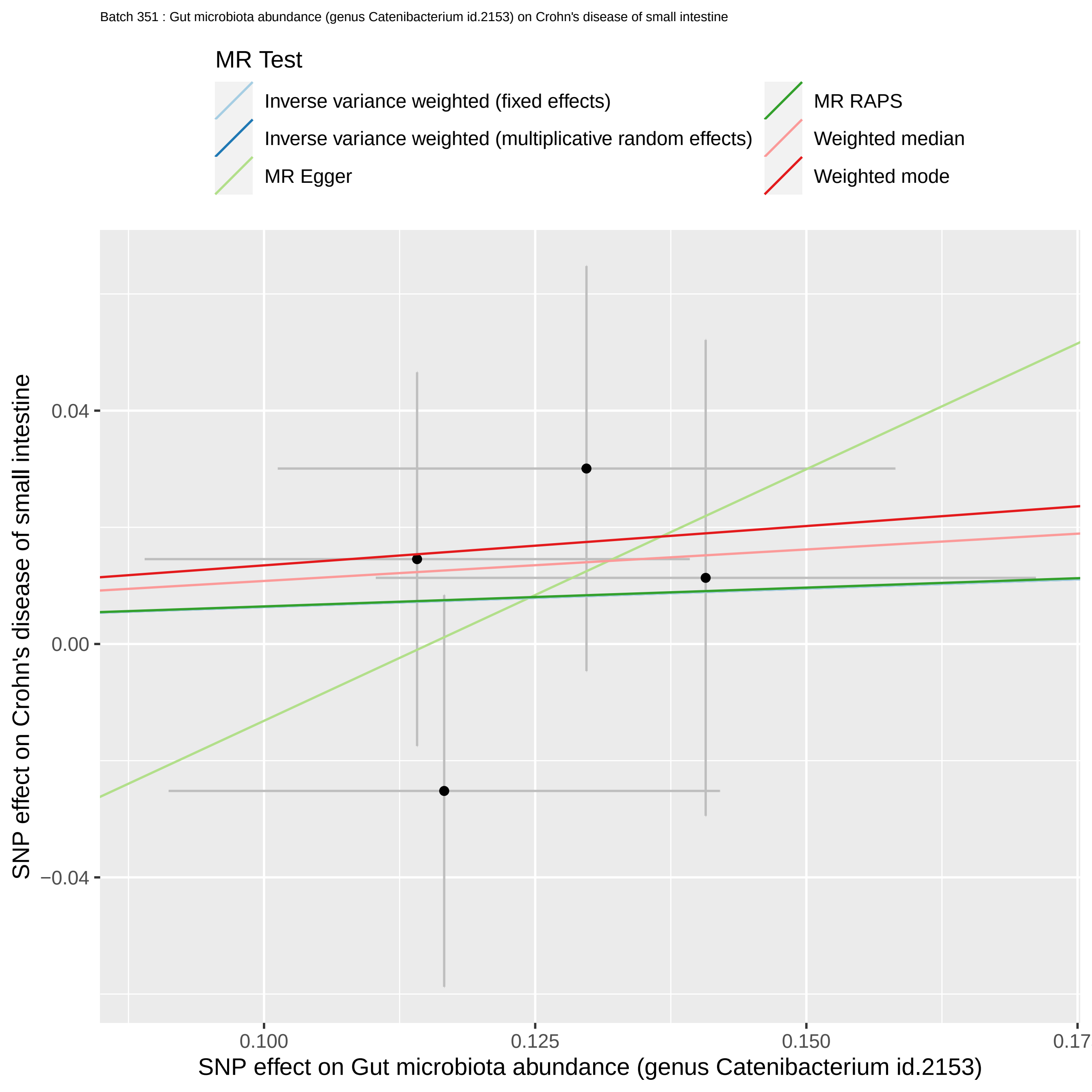

Batch 351 : Gut microbiota abundance (genus Catenibacterium id.2153) on Crohn's disease of small intestine
MR Test
Inverse variance weighted (fixed effects)
Inverse variance weighted (multiplicative random effects)
MR Egger
MR RAPS
Weighted median
Weighted mode
0.04
0.00
−0.04
0.100
0.125
0.150
0.17
SNP effect on Crohn's disease of small intestine
SNP effect on Gut microbiota abundance (genus Catenibacterium id.2153)

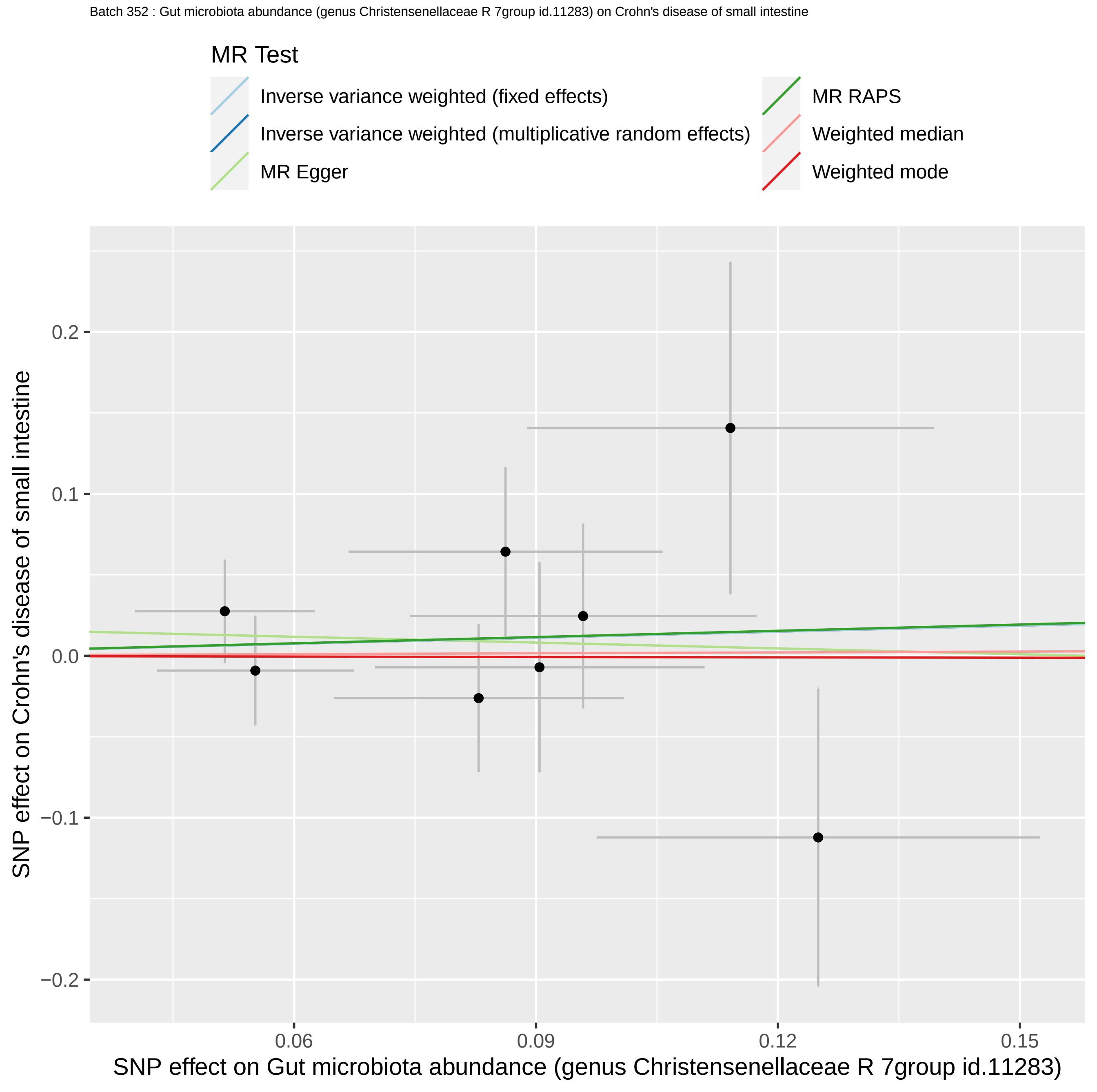

Batch 352 : Gut microbiota abundance (genus Christensenellaceae R 7group id.11283) on Crohn's disease of small intestine
MR Test
Inverse variance weighted (fixed effects)
Inverse variance weighted (multiplicative random effects)
MR Egger
MR RAPS
Weighted median
Weighted mode
SNP effect on Crohn's disease of small intestine
0.2
0.1
0.0
−0.1
−0.2
0.06
0.09
0.12
0.15
SNP effect on Gut microbiota abundance (genus Christensenellaceae R 7group id.11283)

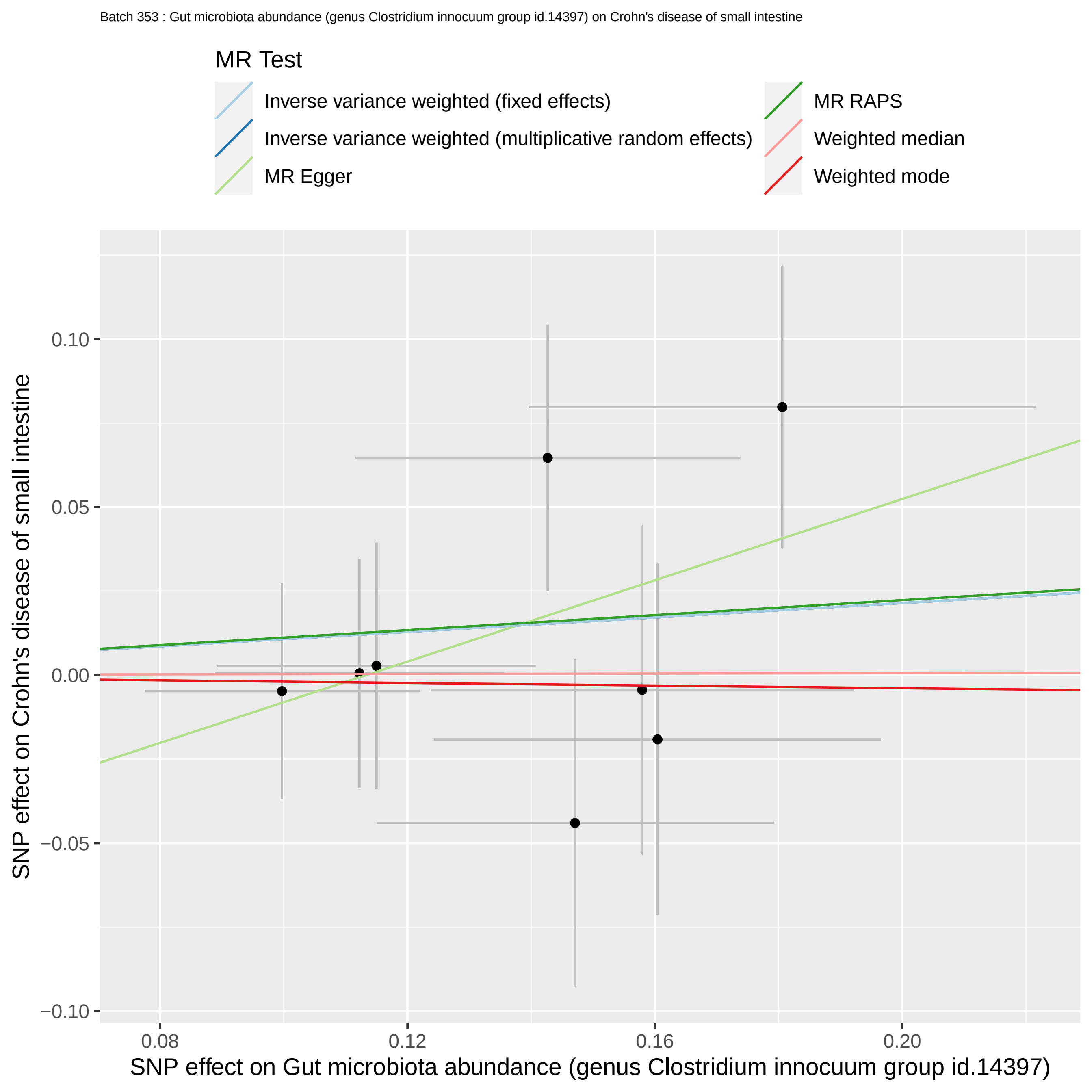

Batch 353 : Gut microbiota abundance (genus Clostridium innocuum group id.14397) on Crohn's disease of small intestine
MR Test
Inverse variance weighted (fixed effects)
Inverse variance weighted (multiplicative random effects)
MR Egger
MR RAPS
Weighted median
Weighted mode
SNP effect on Crohn's disease of small intestine
0.10
0.05
0.00
−0.05
−0.10
0.08
0.12
0.16
0.20
SNP effect on Gut microbiota abundance (genus Clostridium innocuum group id.14397)

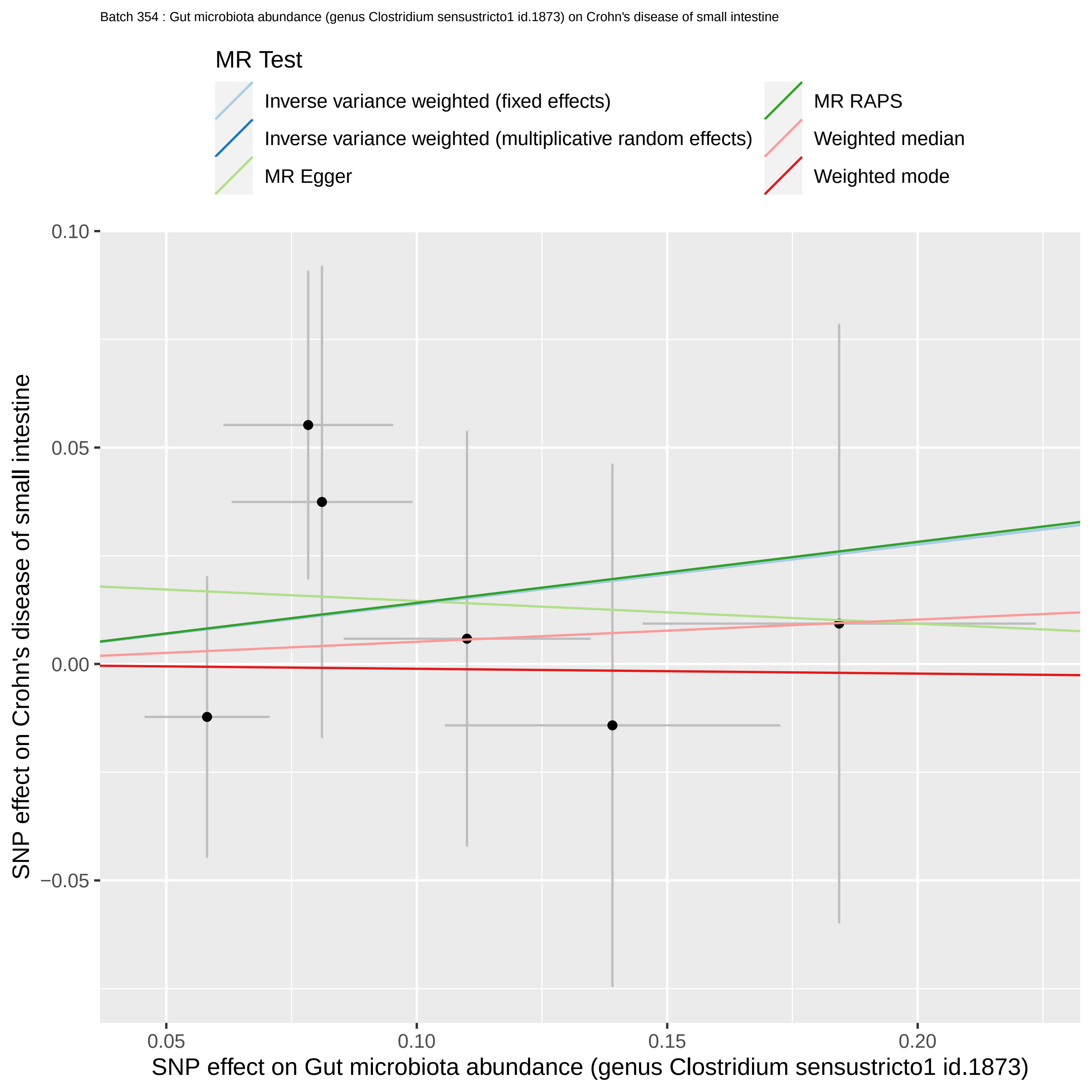

Batch 354 : Gut microbiota abundance (genus Clostridium sensustricto1 id.1873) on Crohn's disease of small intestine
MR Test
Inverse variance weighted (fixed effects)
Inverse variance weighted (multiplicative random effects)
MR Egger
MR RAPS
Weighted median
Weighted mode
0.10
0.05
0.00
−0.05
0.05
0.10
0.15
0.20
SNP effect on Crohn's disease of small intestine
SNP effect on Gut microbiota abundance (genus Clostridium sensustricto1 id.1873)

Batch 355 : Gut microbiota abundance (genus Collinsella id.815) on Crohn's disease of small intestine

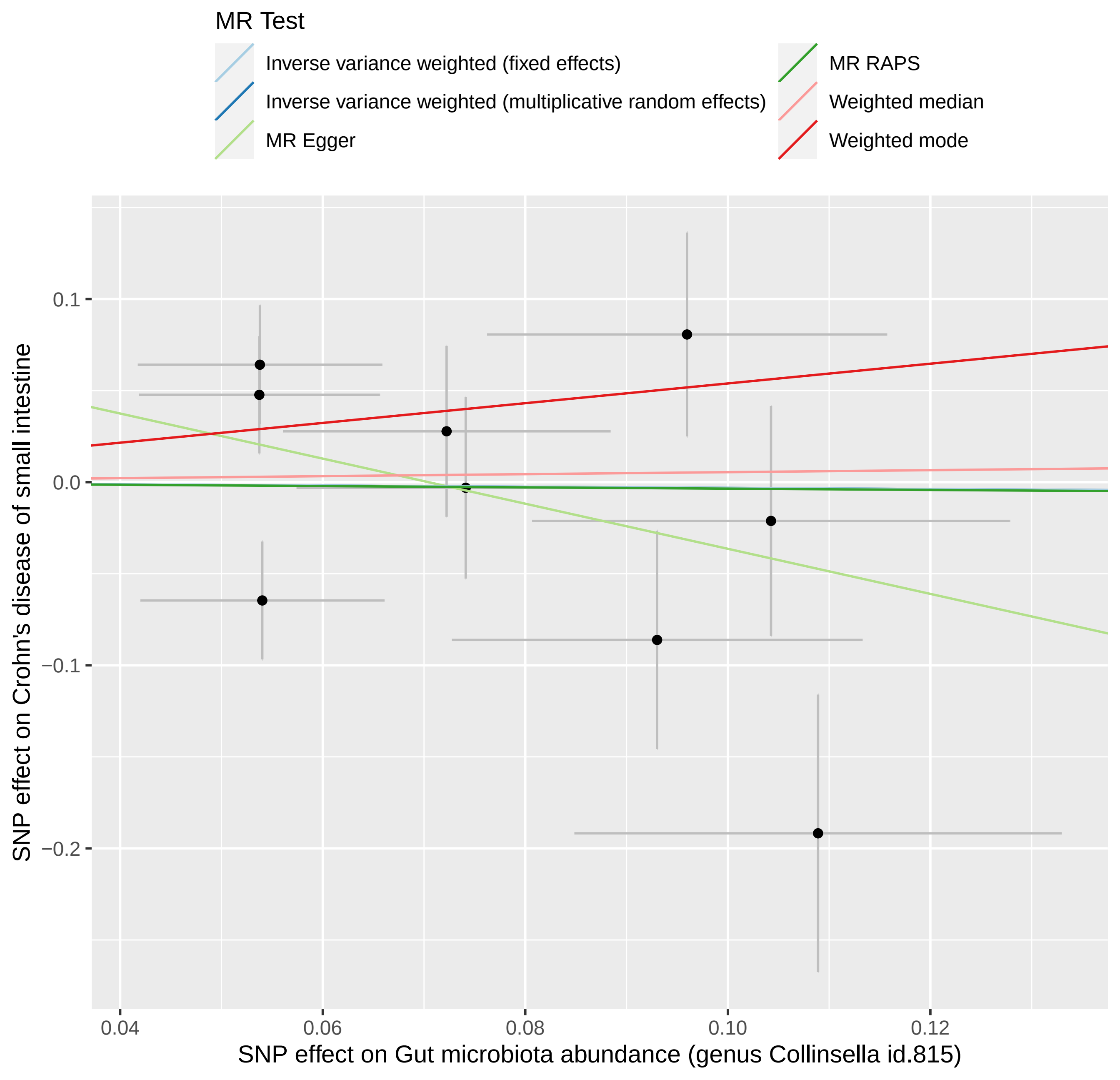

Batch 356 : Gut microbiota abundance (genus Coprobacter id.949) on Crohn's disease of small intestine

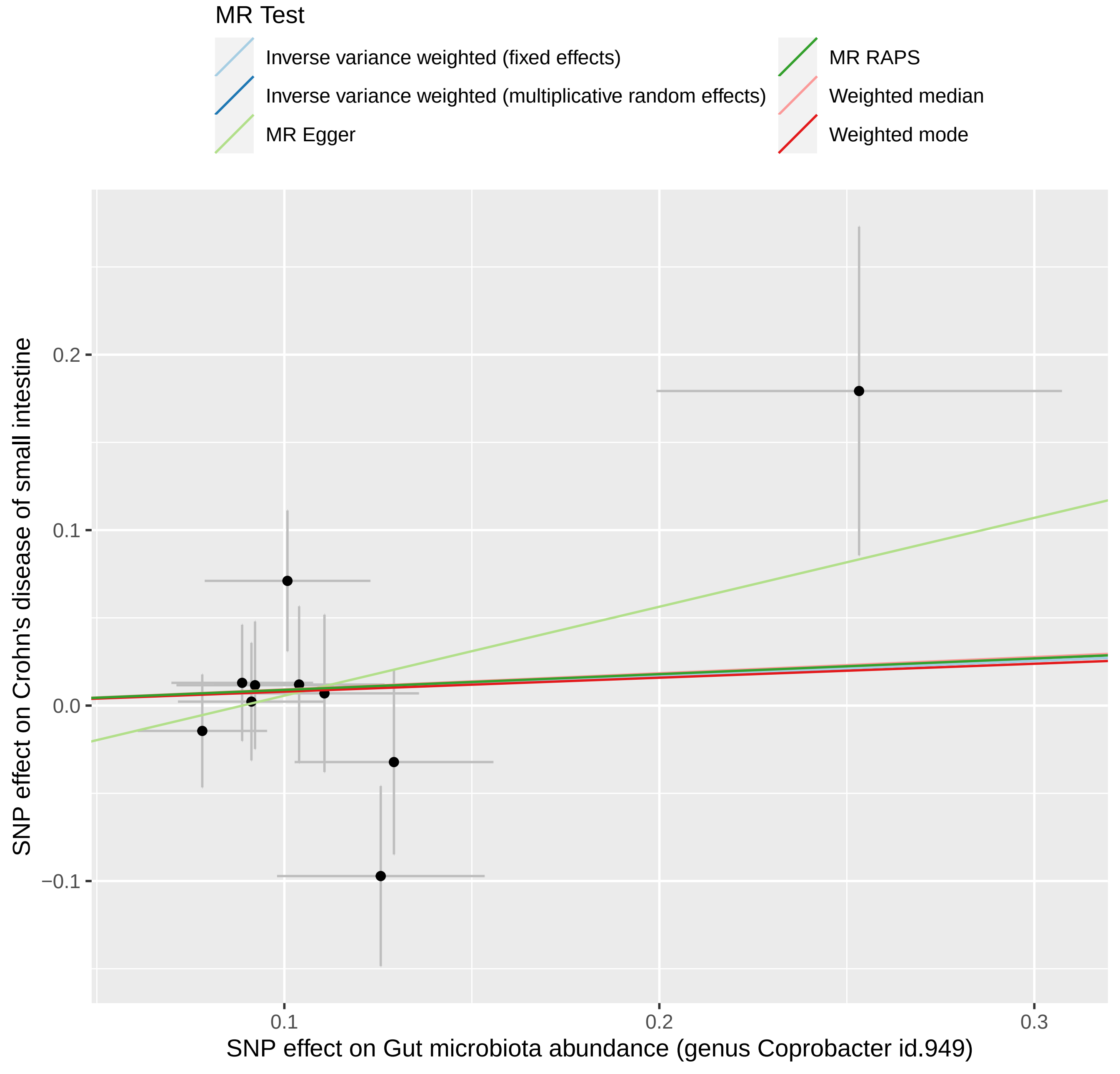

Batch 357 : Gut microbiota abundance (genus Coprococcus1 id.11301) on Crohn's disease of small intestine

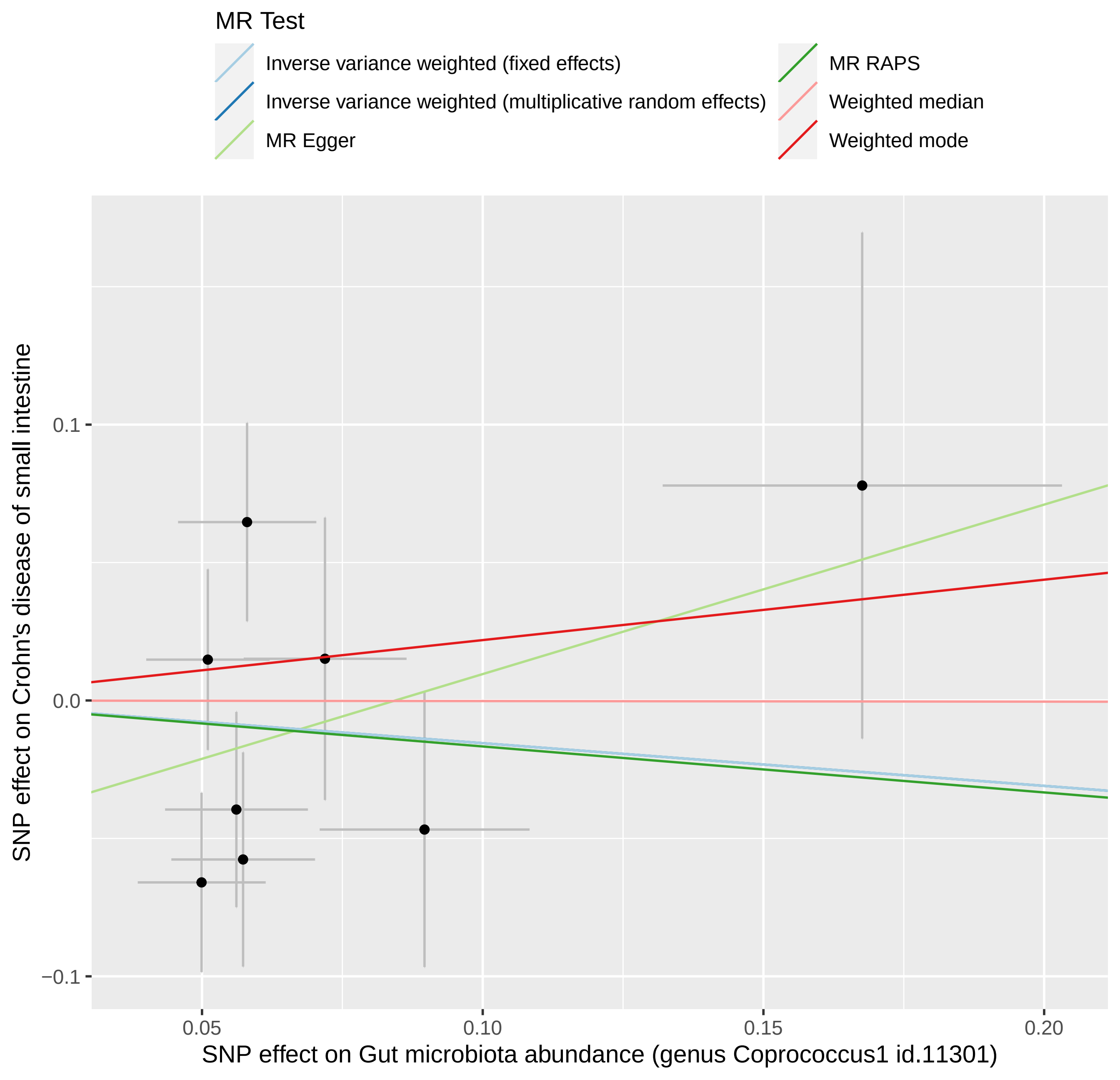

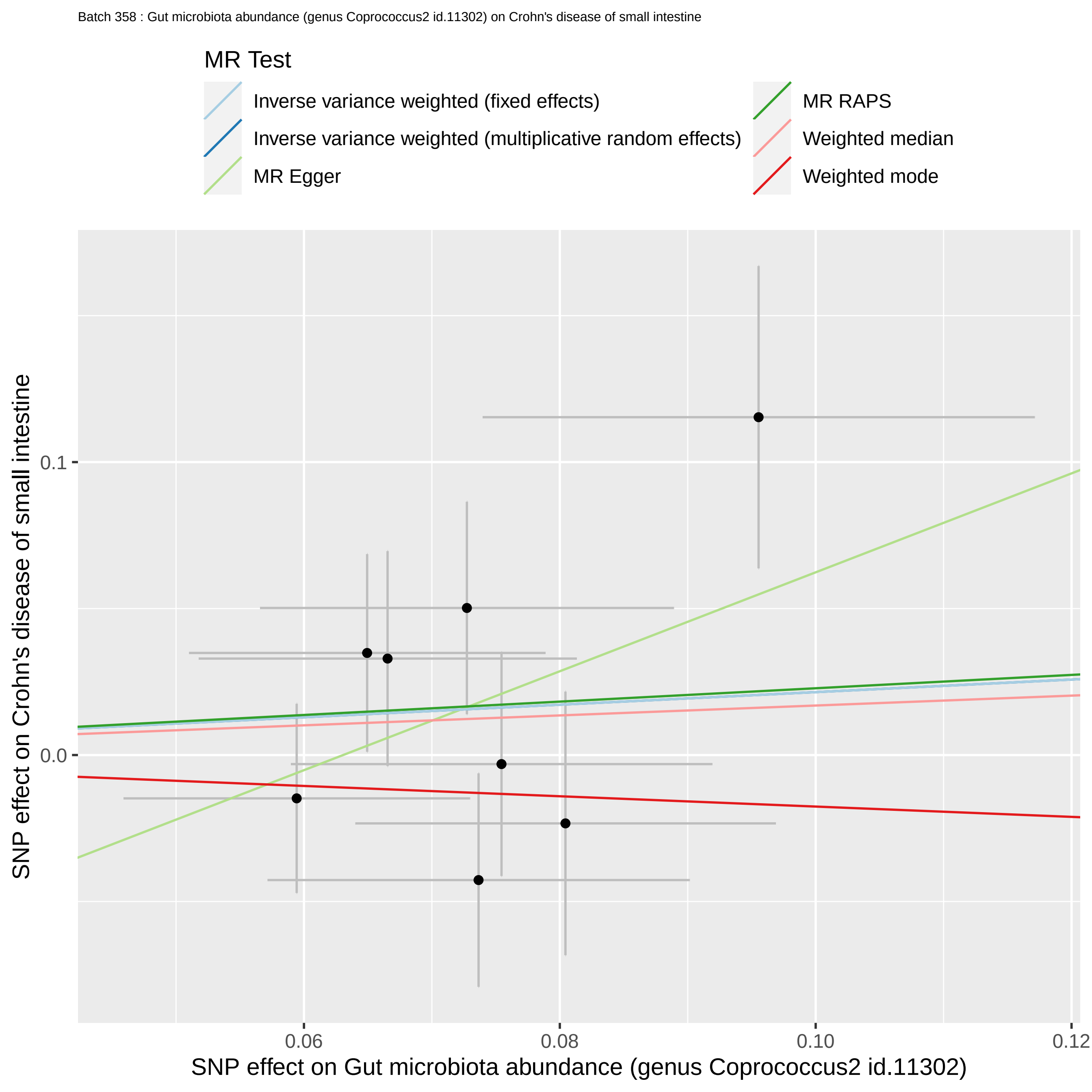
Batch 358 : Gut microbiota abundance (genus Coprococcus2 id.11302) on Crohn's disease of small intestine
MR Test
Inverse variance weighted (fixed effects)
Inverse variance weighted (multiplicative random effects)
MR Egger
MR RAPS
Weighted median
Weighted mode
SNP effect on Crohn's disease of small intestine
0.1
0.0
0.06
0.08
0.10
0.12
SNP effect on Gut microbiota abundance (genus Coprococcus2 id.11302)

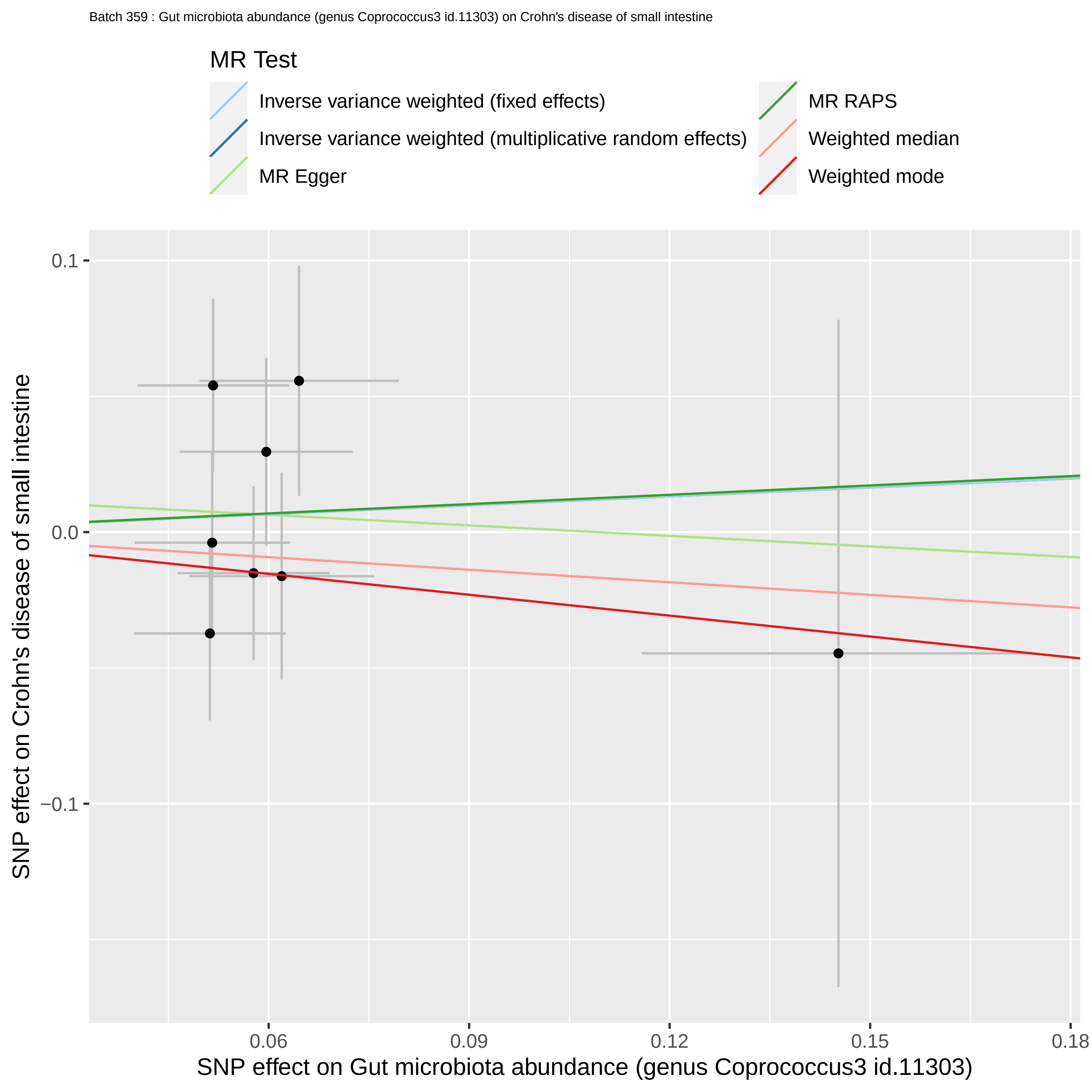
Batch 359 : Gut microbiota abundance (genus Coprococcus3 id.11303) on Crohn's disease of small intestine
MR Test
Inverse variance weighted (fixed effects)
Inverse variance weighted (multiplicative random effects)
MR Egger
MR RAPS
Weighted median
Weighted mode
0.1
0.0
−0.1
0.06
0.09
0.12
0.15
0.18
SNP effect on Crohn's disease of small intestine
SNP effect on Gut microbiota abundance (genus Coprococcus3 id.11303)

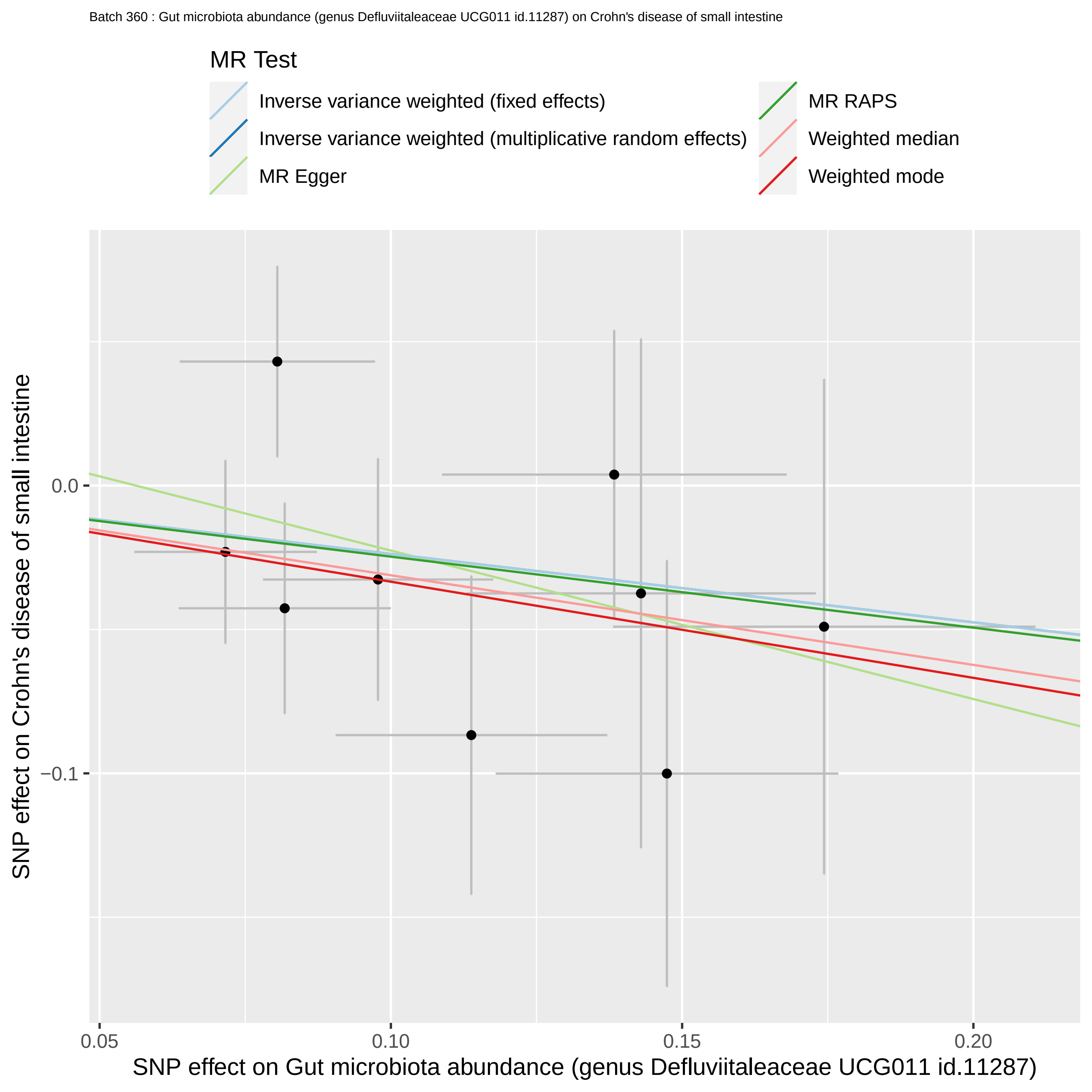

Batch 360 : Gut microbiota abundance (genus Defluviitaleaceae UCG011 id.11287) on Crohn's disease of small intestine
MR Test
Inverse variance weighted (fixed effects)
Inverse variance weighted (multiplicative random effects)
MR Egger
MR RAPS
Weighted median
Weighted mode
SNP effect on Crohn's disease of small intestine
0.0
−0.1
0.05
0.10
0.15
0.20
SNP effect on Gut microbiota abundance (genus Defluviitaleaceae UCG011 id.11287)

Batch 361 : Gut microbiota abundance (genus Desulfovibrio id.3173) on Crohn's disease of small intestine

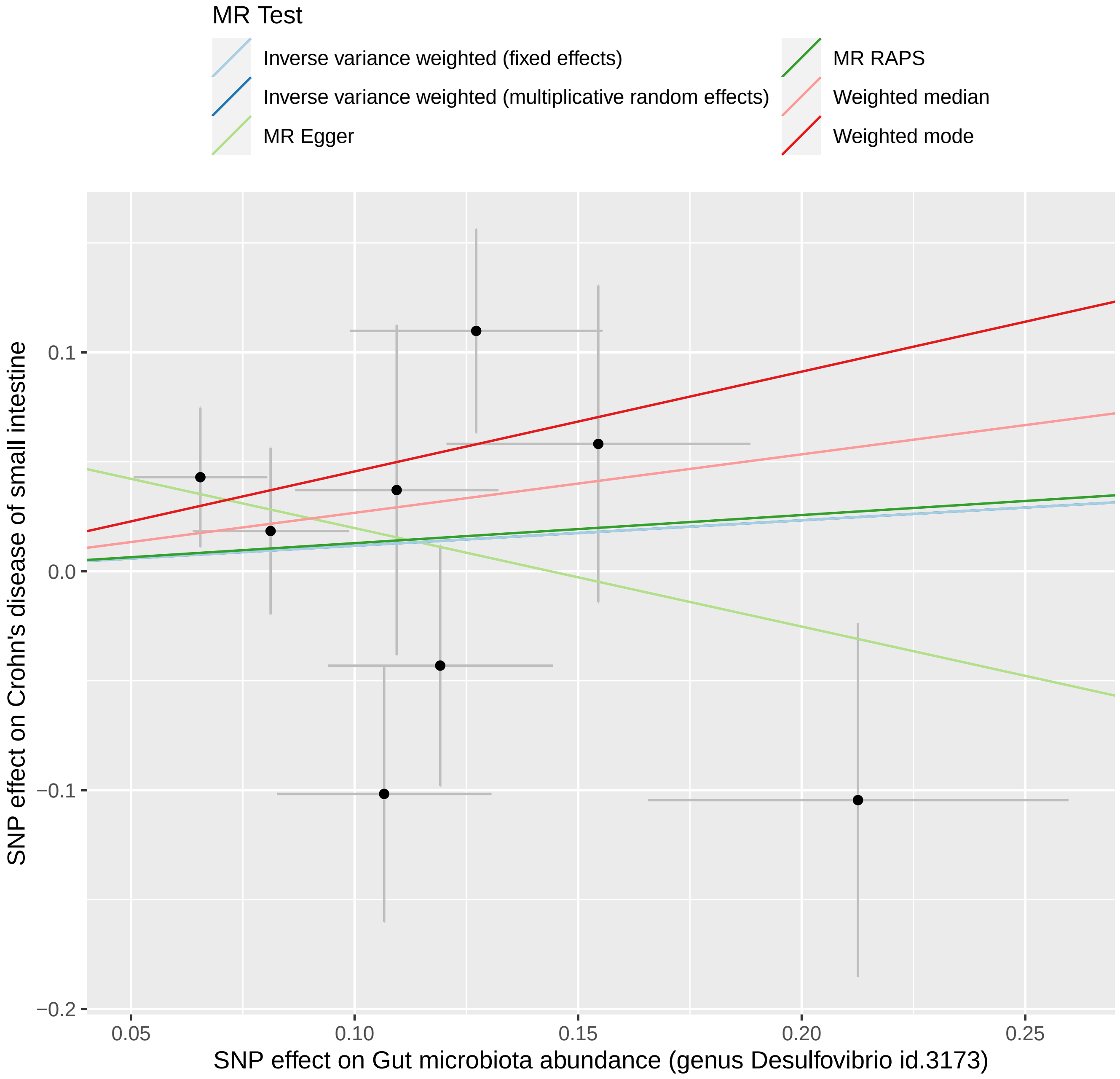

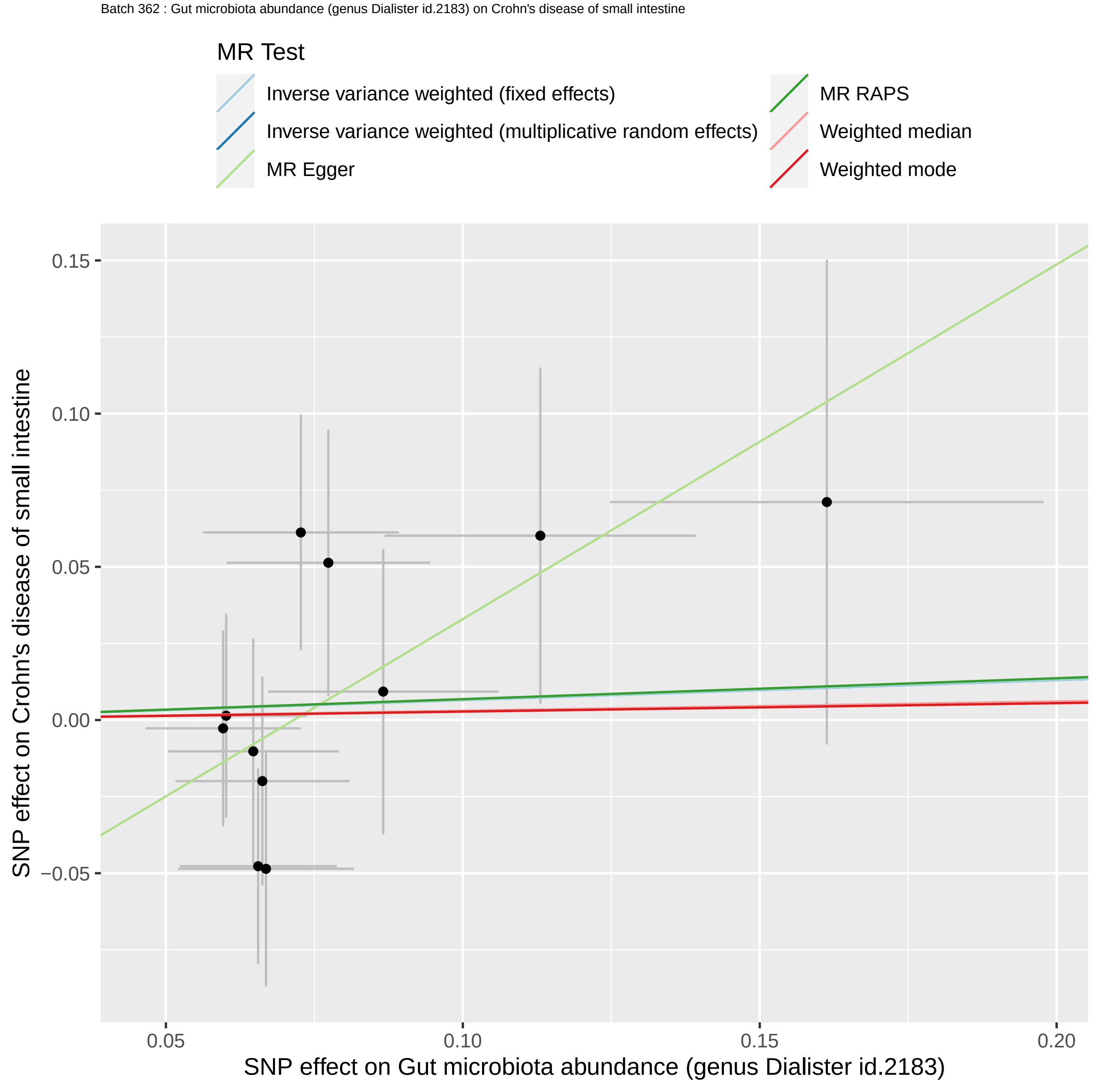
Batch 362 : Gut microbiota abundance (genus Dialister id.2183) on Crohn's disease of small intestine
MR Test
Inverse variance weighted (fixed effects)
Inverse variance weighted (multiplicative random effects)
MR Egger
MR RAPS
Weighted median
Weighted mode
0.15
0.10
0.05
0.00
−0.05
0.05
0.10
0.15
0.20
SNP effect on Crohn's disease of small intestine
SNP effect on Gut microbiota abundance (genus Dialister id.2183)

Batch 363 : Gut microbiota abundance (genus Dorea id.1997) on Crohn's disease of small intestine

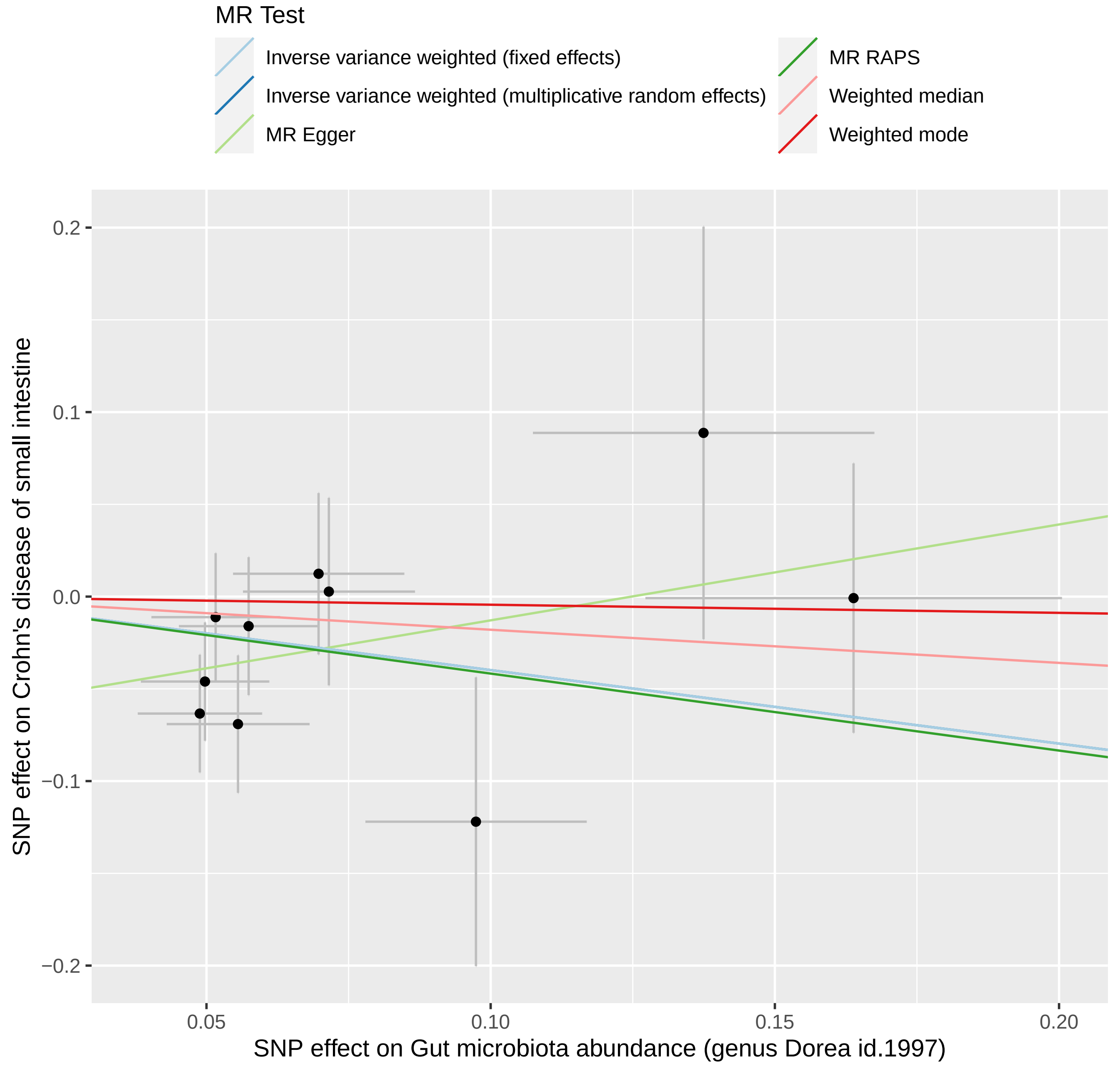

Batch 364 : Gut microbiota abundance (genus Eggerthella id.819) on Crohn's disease of small intestine

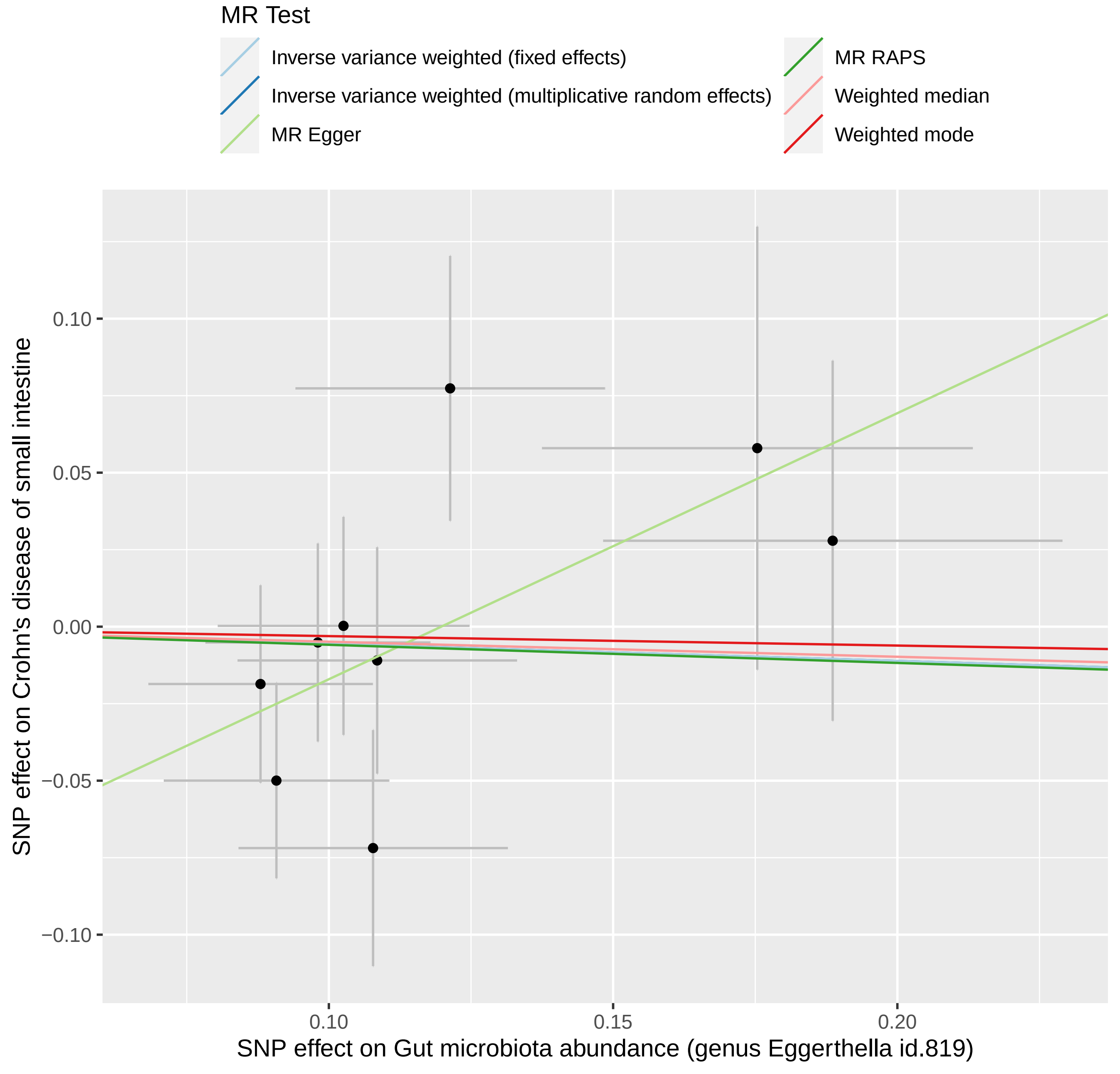

Batch 365 : Gut microbiota abundance (genus Eisenbergiella id.11304) on Crohn's disease of small intestine

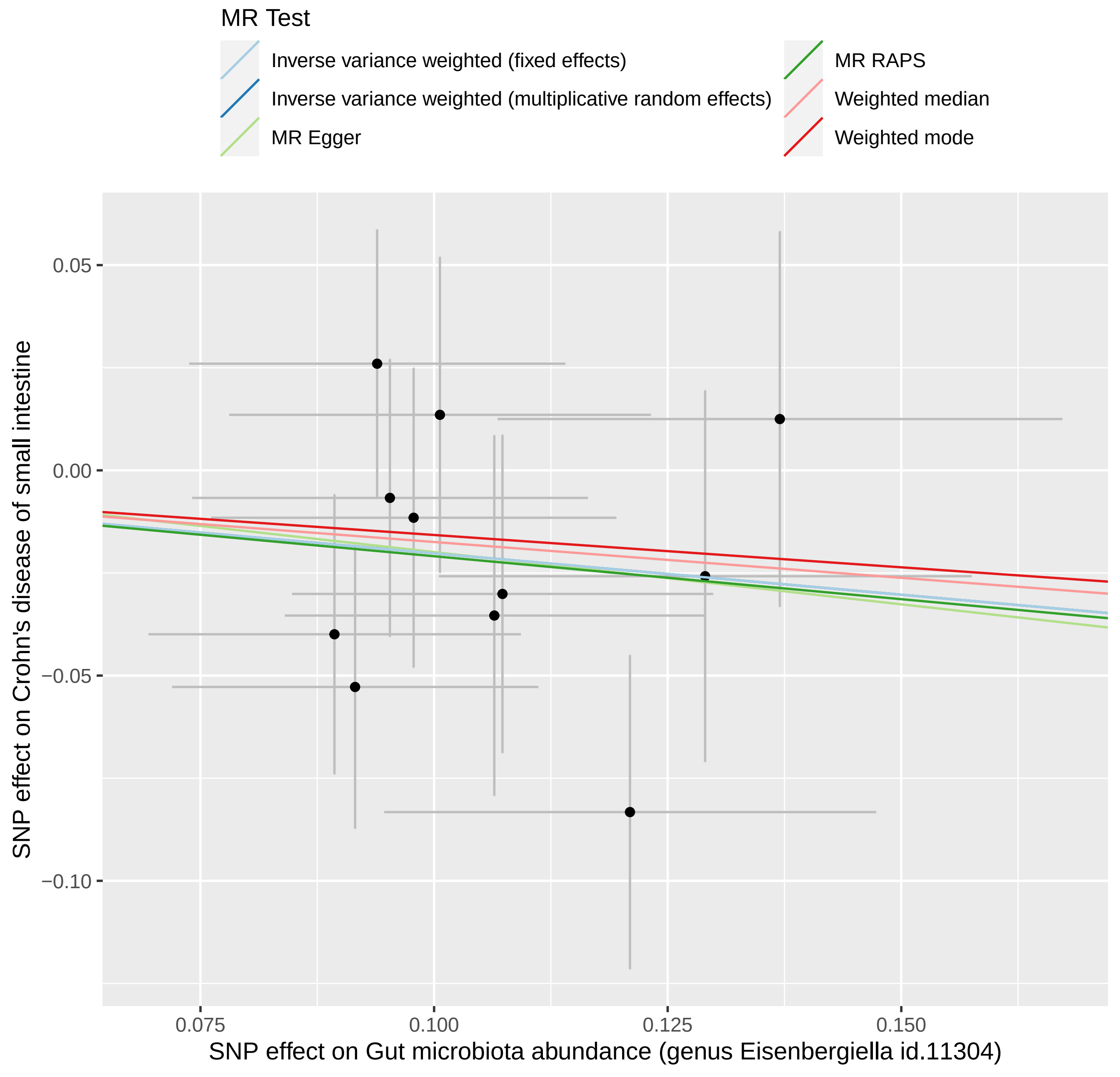

Batch 366 : Gut microbiota abundance (genus Enterorhabdus id.820) on Crohn's disease of small intestine

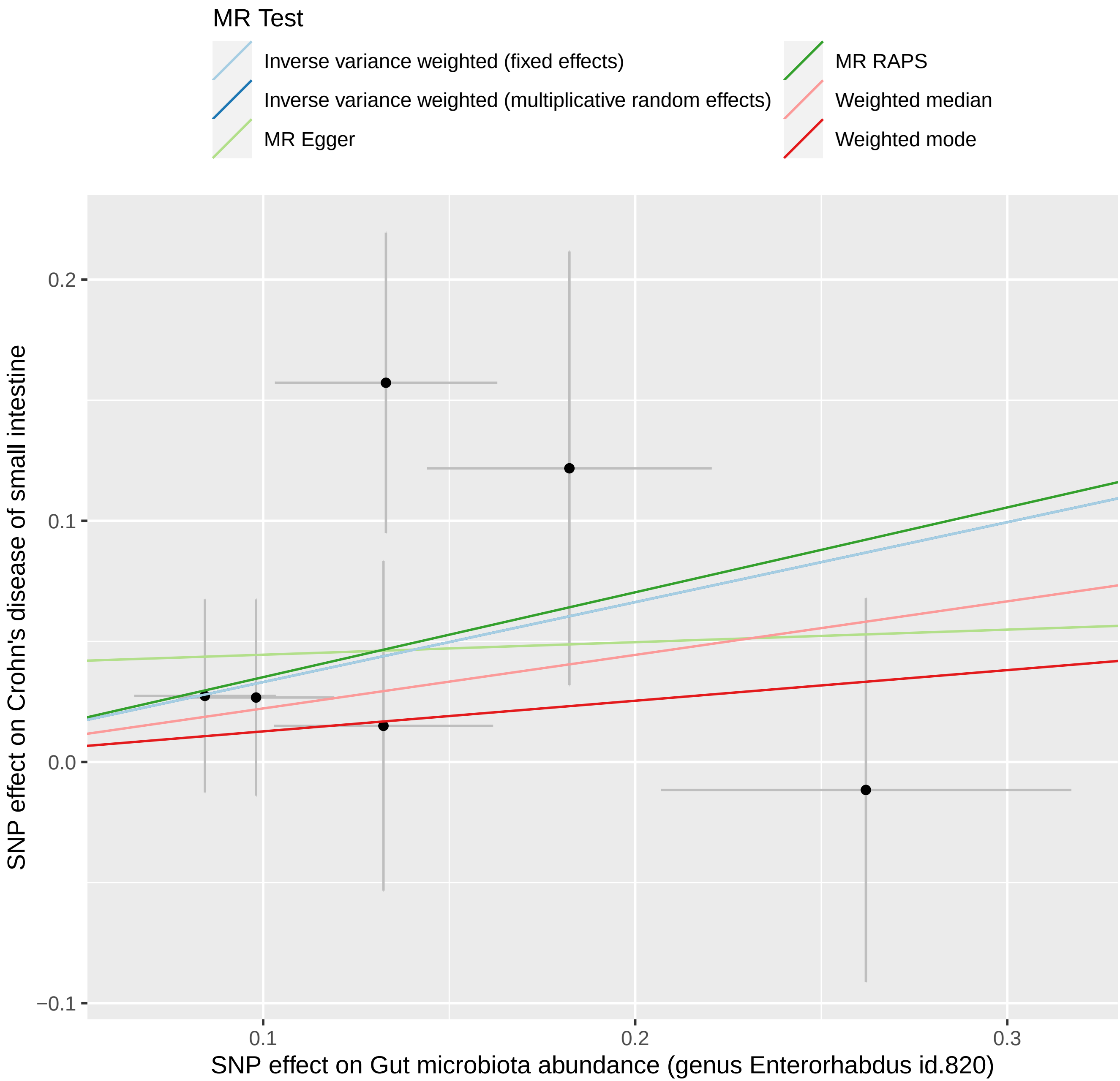

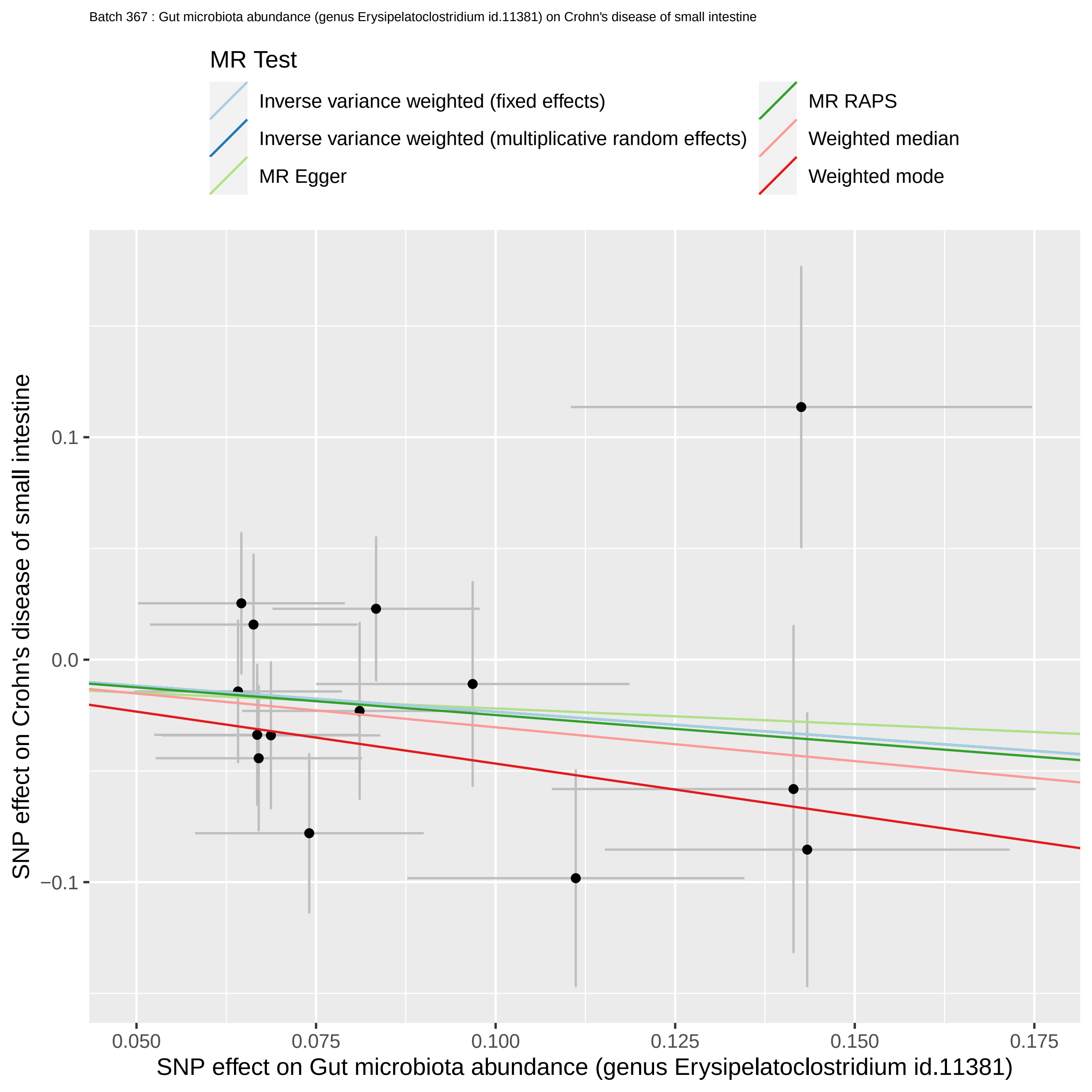
Batch 367 : Gut microbiota abundance (genus Erysipelatoclostridium id.11381) on Crohn's disease of small intestine
MR Test
Inverse variance weighted (fixed effects)
Inverse variance weighted (multiplicative random effects)
MR Egger
MR RAPS
Weighted median
Weighted mode
SNP effect on Crohn's disease of small intestine
0.1
0.0
−0.1
0.050
0.075
0.100
0.125
0.150
0.175
SNP effect on Gut microbiota abundance (genus Erysipelatoclostridium id.11381)

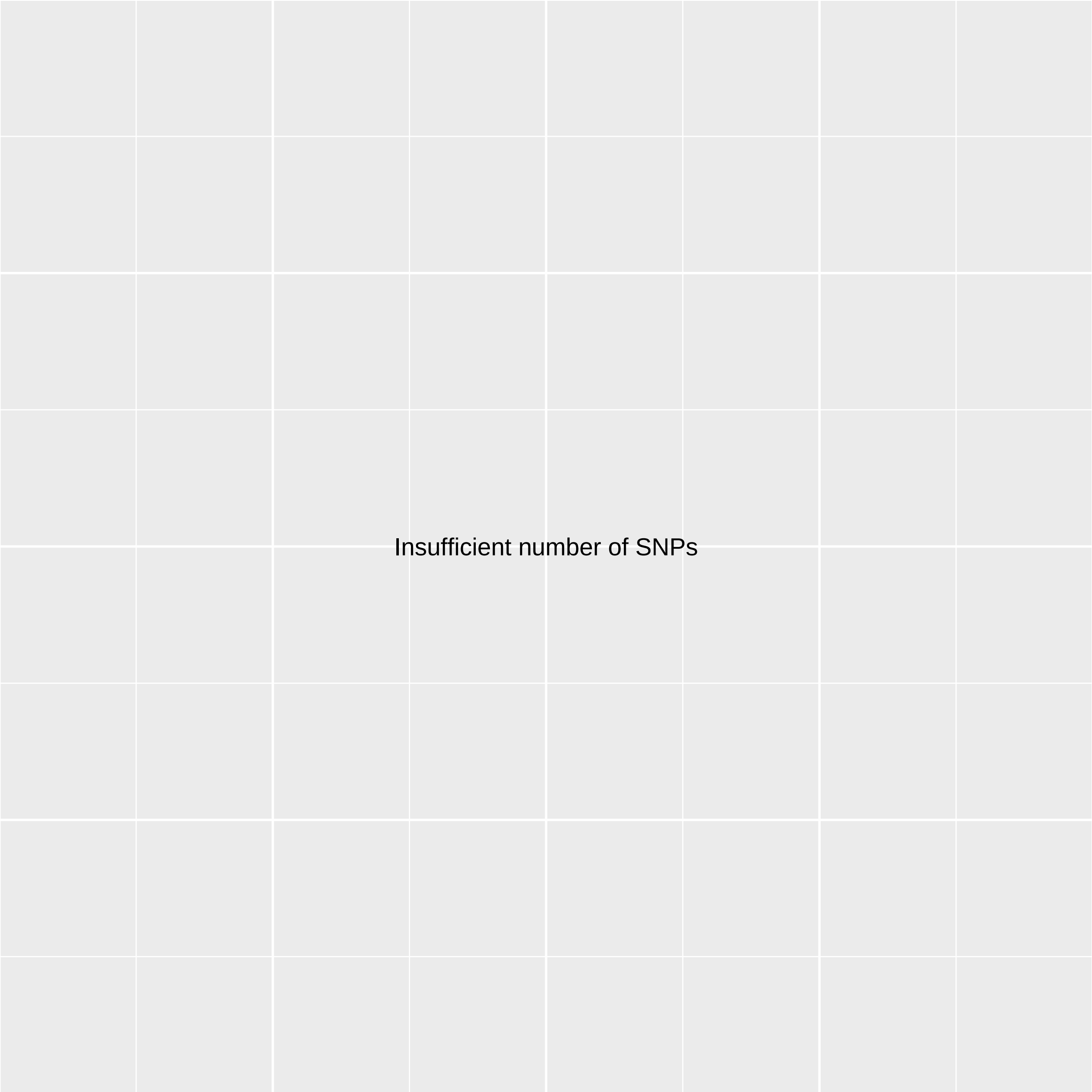
Insufficient number of SNPs

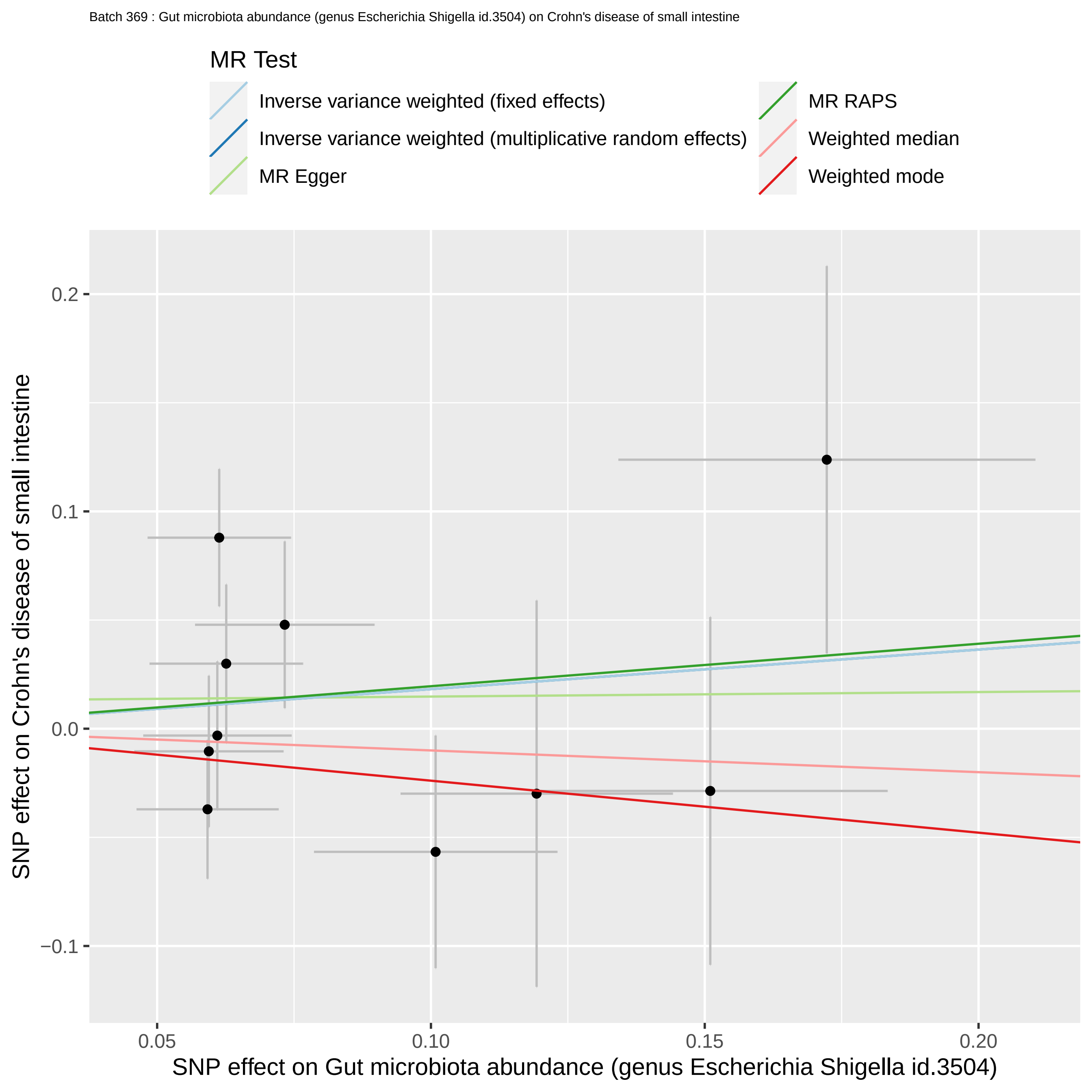
Batch 369 : Gut microbiota abundance (genus Escherichia Shigella id.3504) on Crohn's disease of small intestine
MR Test
Inverse variance weighted (fixed effects)
Inverse variance weighted (multiplicative random effects)
MR Egger
MR RAPS
Weighted median
Weighted mode
SNP effect on Crohn's disease of small intestine
0.2
0.1
0.0
−0.1
0.05
0.10
0.15
0.20
SNP effect on Gut microbiota abundance (genus Escherichia Shigella id.3504)

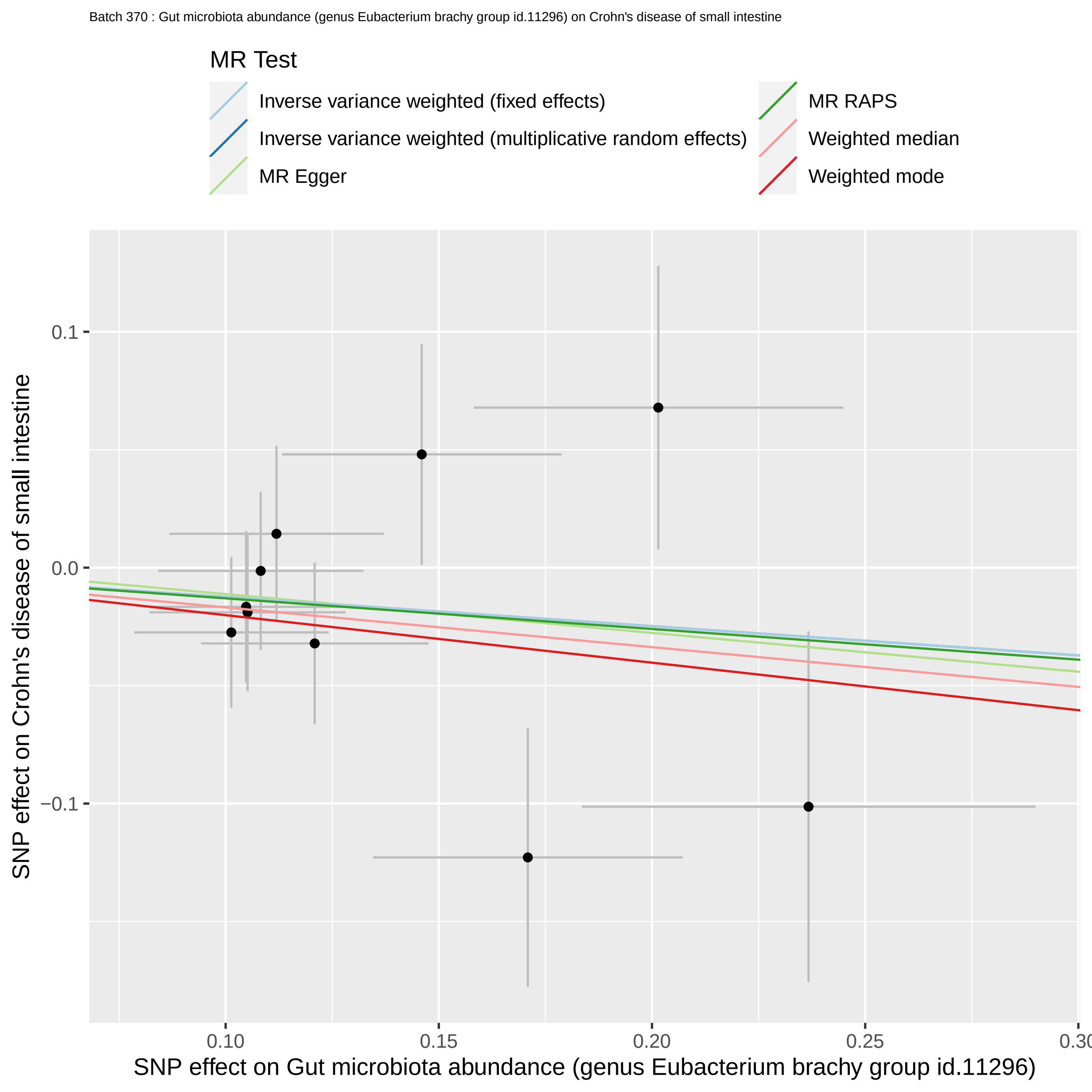
Batch 370 : Gut microbiota abundance (genus Eubacterium brachy group id.11296) on Crohn's disease of small intestine
MR Test
Inverse variance weighted (fixed effects)
Inverse variance weighted (multiplicative random effects)
MR Egger
MR RAPS
Weighted median
Weighted mode
0.1
0.0
−0.1
0.10
0.15
0.20
0.25
0.30
SNP effect on Crohn's disease of small intestine
SNP effect on Gut microbiota abundance (genus Eubacterium brachy group id.11296)

Batch 371 : Gut microbiota abundance (genus Eubacterium coprostanoligenes group id.11375) on Crohn's disease of small intestine

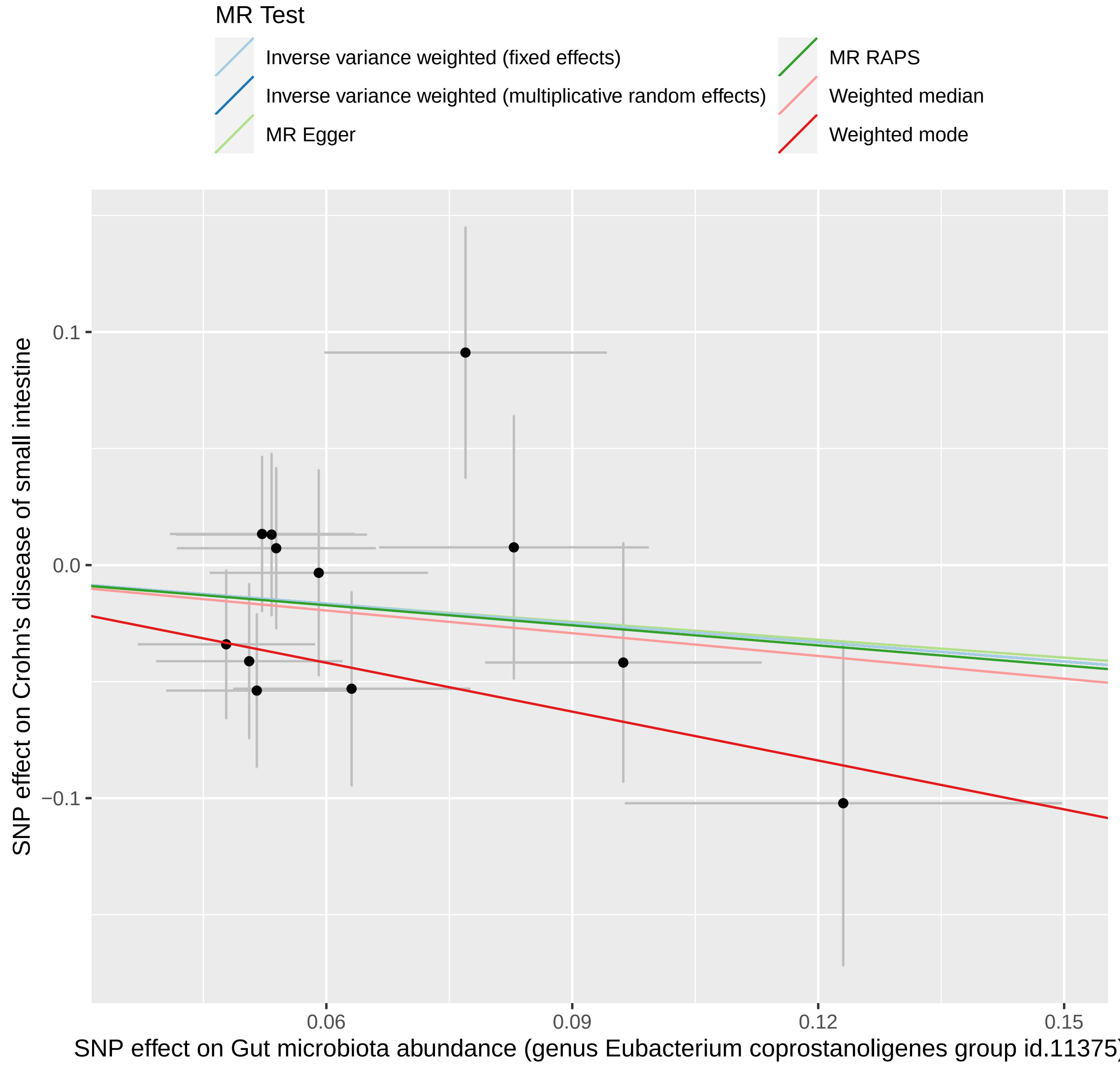

Batch 372 : Gut microbiota abundance (genus Eubacterium eligens group id.14372) on Crohn's disease of small intestine

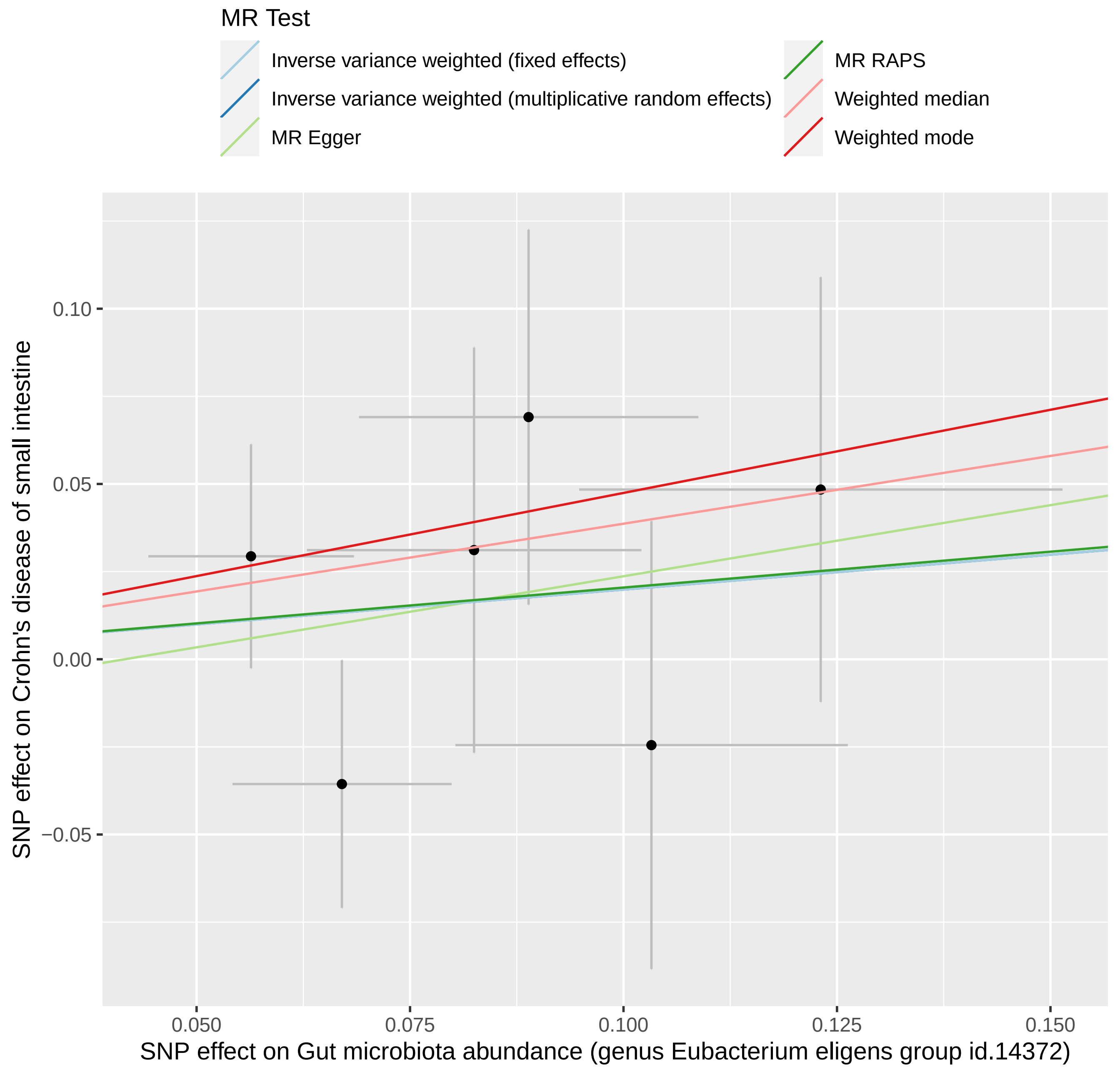

Batch 373 : Gut microbiota abundance (genus Eubacterium fissicatena group id.14373) on Crohn's disease of small intestine

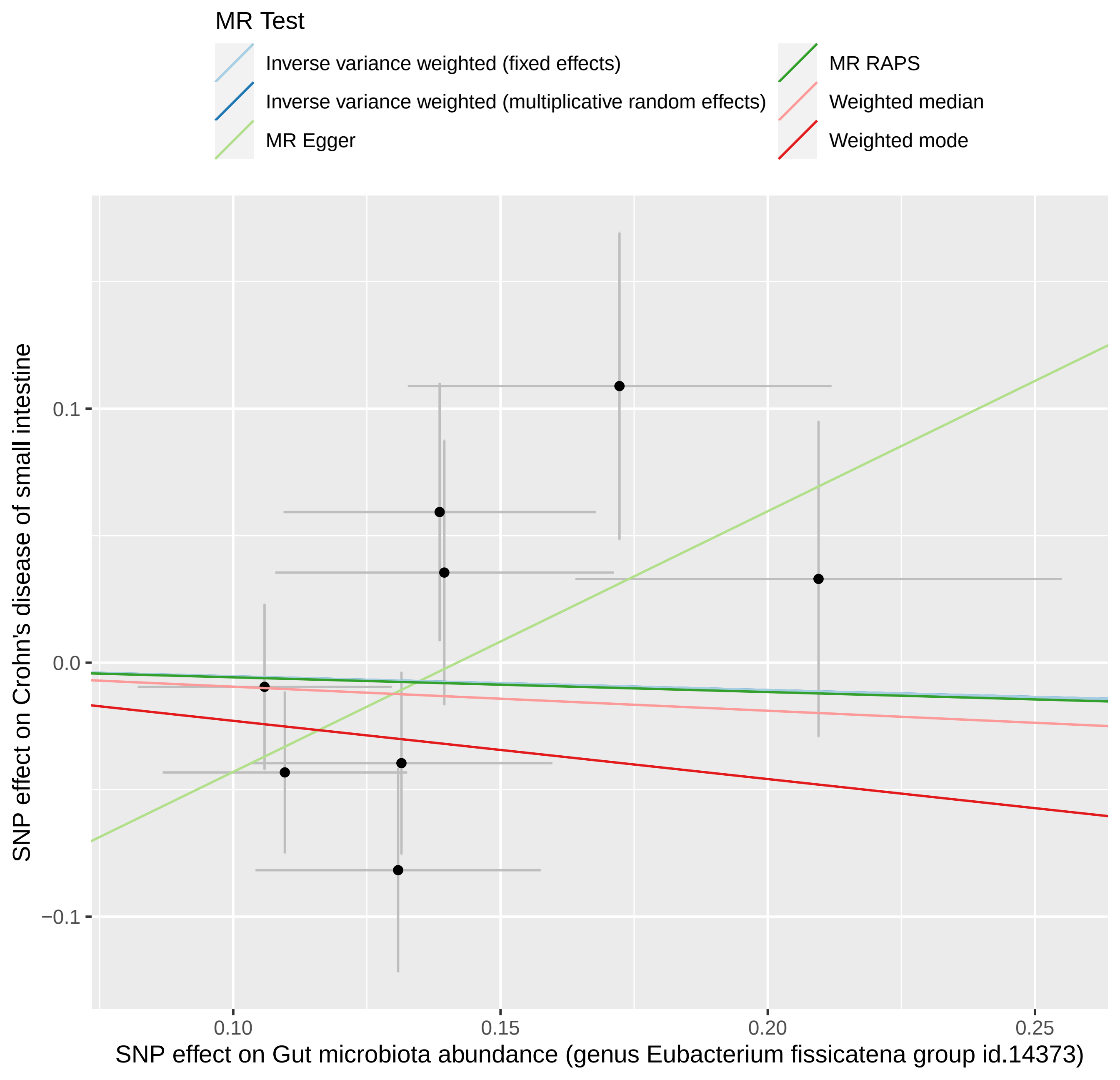

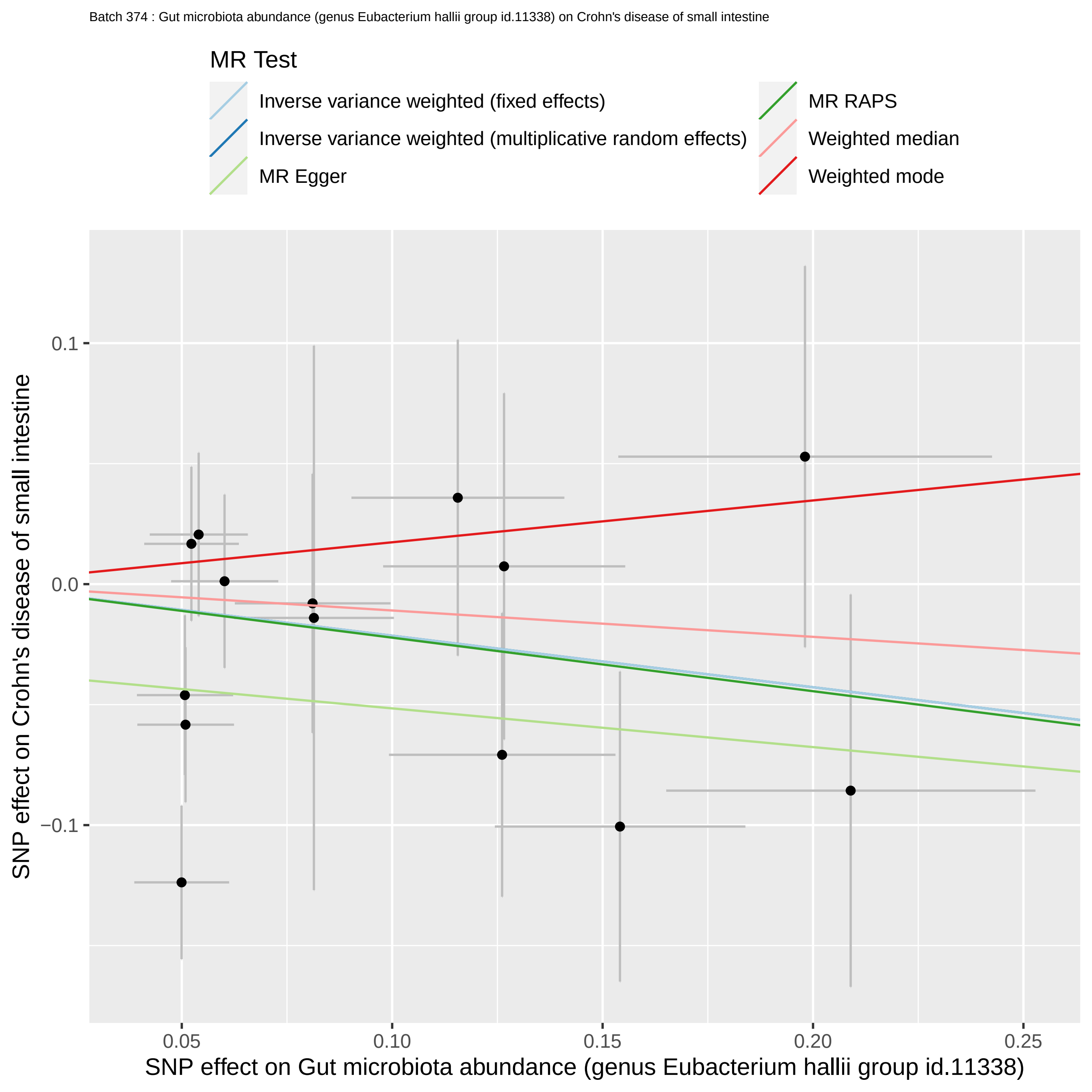
Batch 374 : Gut microbiota abundance (genus Eubacterium hallii group id.11338) on Crohn's disease of small intestine
MR Test
Inverse variance weighted (fixed effects)
Inverse variance weighted (multiplicative random effects)
MR Egger
MR RAPS
Weighted median
Weighted mode
0.1
0.0
−0.1
0.05
0.10
0.15
0.20
0.25
SNP effect on Crohn's disease of small intestine
SNP effect on Gut microbiota abundance (genus Eubacterium hallii group id.11338)

Batch 375 : Gut microbiota abundance (genus Eubacterium nodatum group id.11297) on Crohn's disease of small intestine

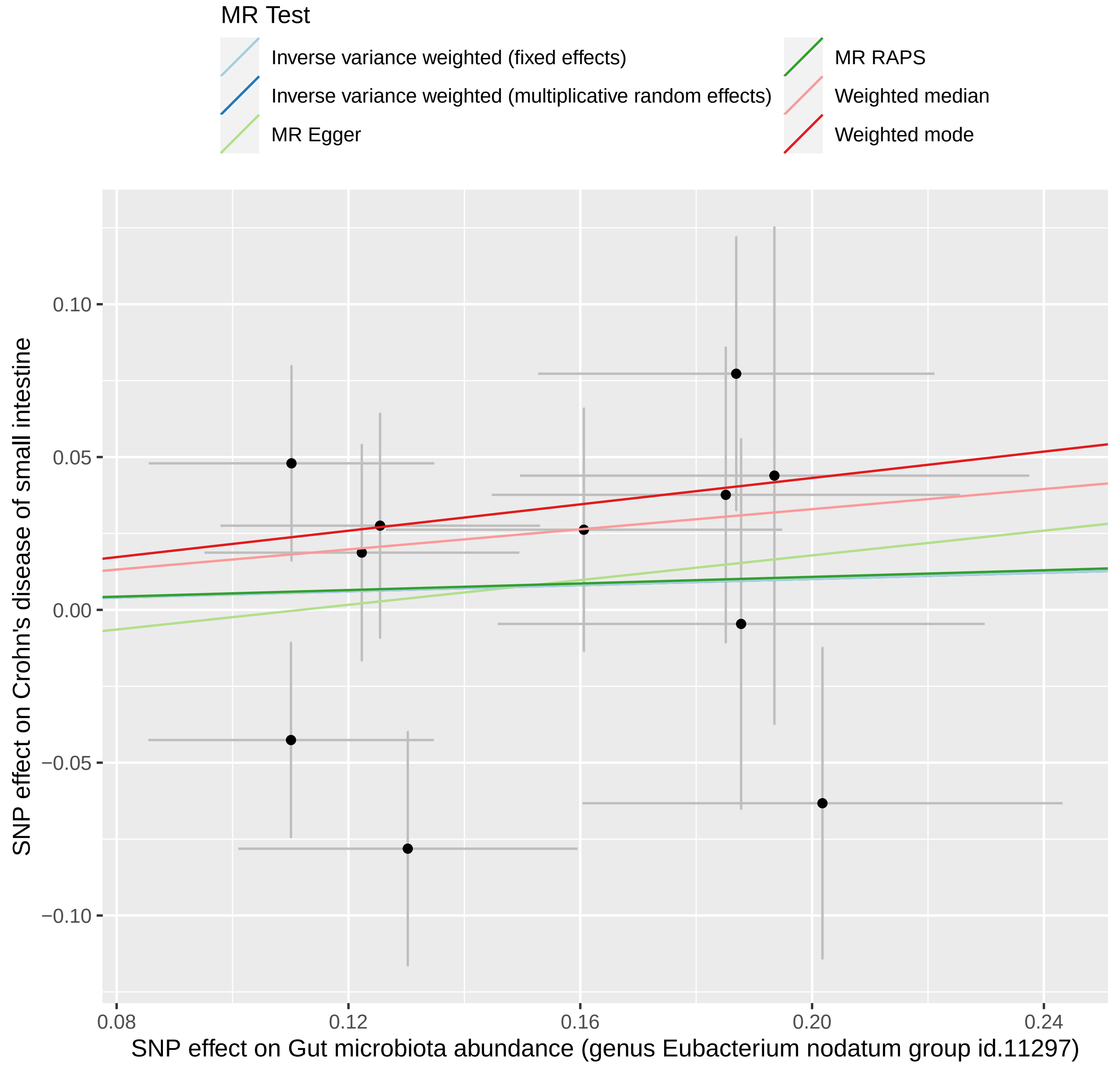

Batch 376 : Gut microbiota abundance (genus Eubacterium oxidoreducens group id.11339) on Crohn's disease of small intestine

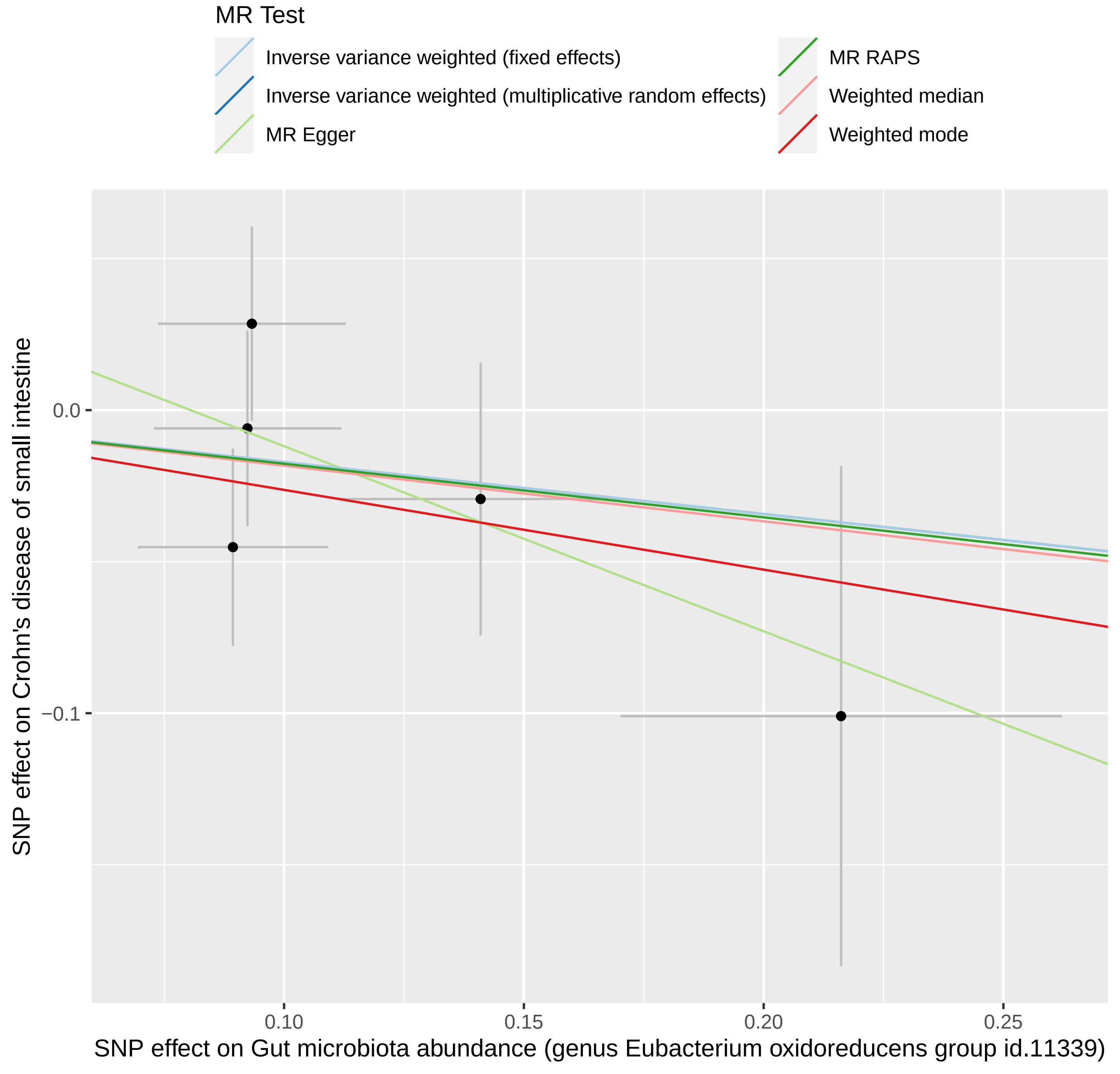

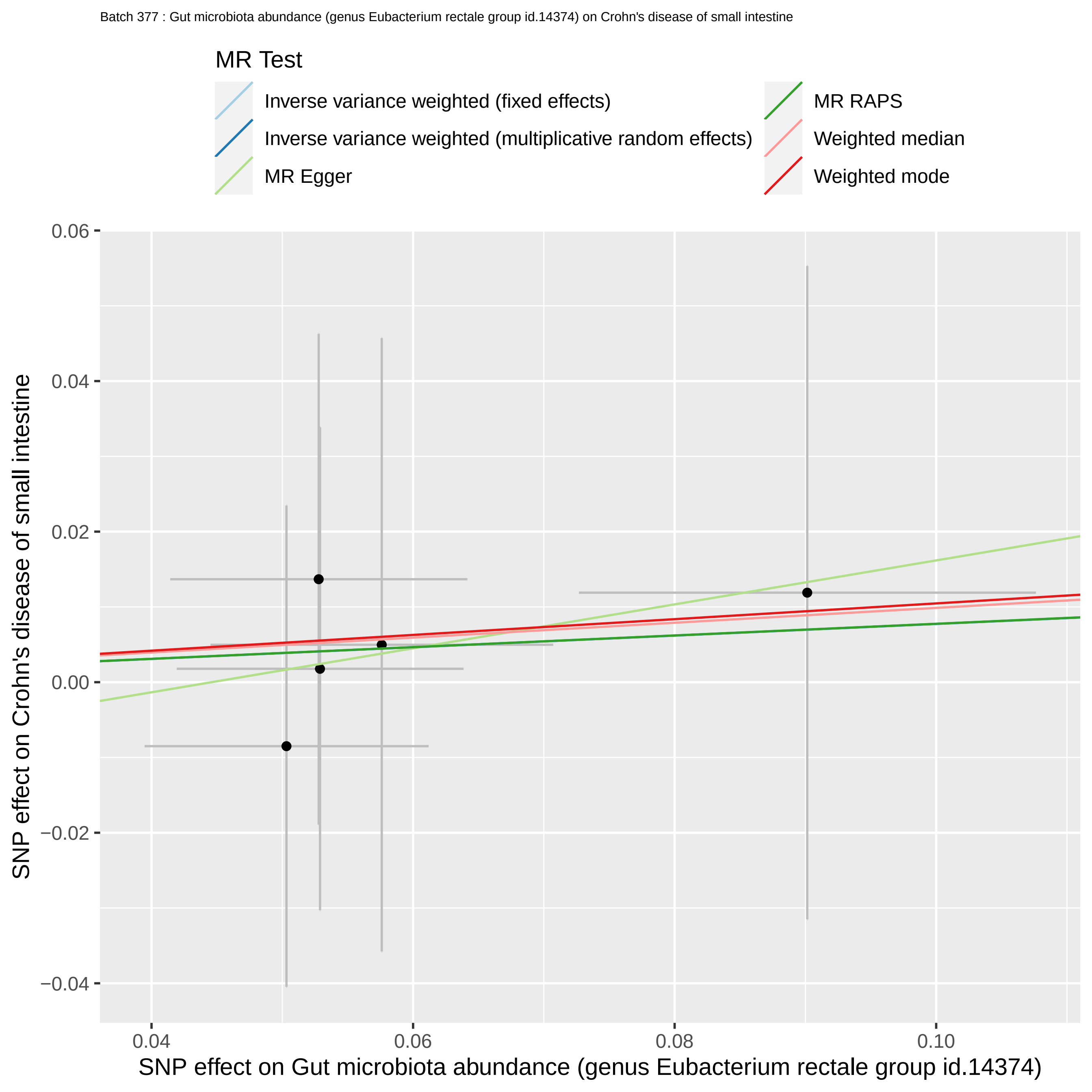
Batch 377 : Gut microbiota abundance (genus Eubacterium rectale group id.14374) on Crohn's disease of small intestine
MR Test
Inverse variance weighted (fixed effects)
Inverse variance weighted (multiplicative random effects)
MR Egger
MR RAPS
Weighted median
Weighted mode
0.06
0.04
0.02
0.00
−0.02
−0.04
0.04
0.06
0.08
0.10
SNP effect on Crohn's disease of small intestine
SNP effect on Gut microbiota abundance (genus Eubacterium rectale group id.14374)

Batch 378 : Gut microbiota abundance (genus Eubacterium ruminantium group id.11340) on Crohn's disease of small intestine

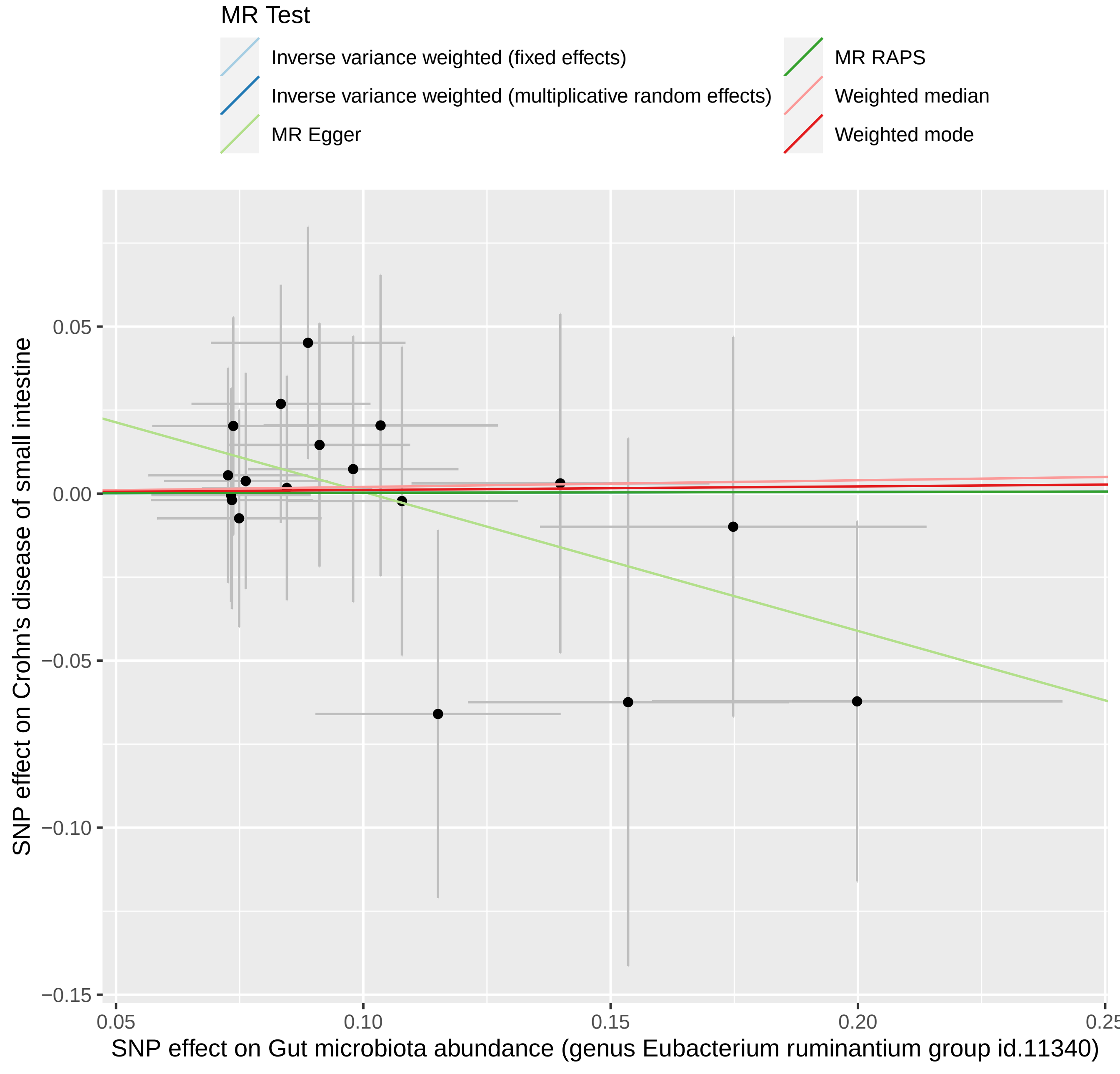

Batch 379 : Gut microbiota abundance (genus Eubacterium ventriosum group id.11341) on Crohn's disease of small intestine

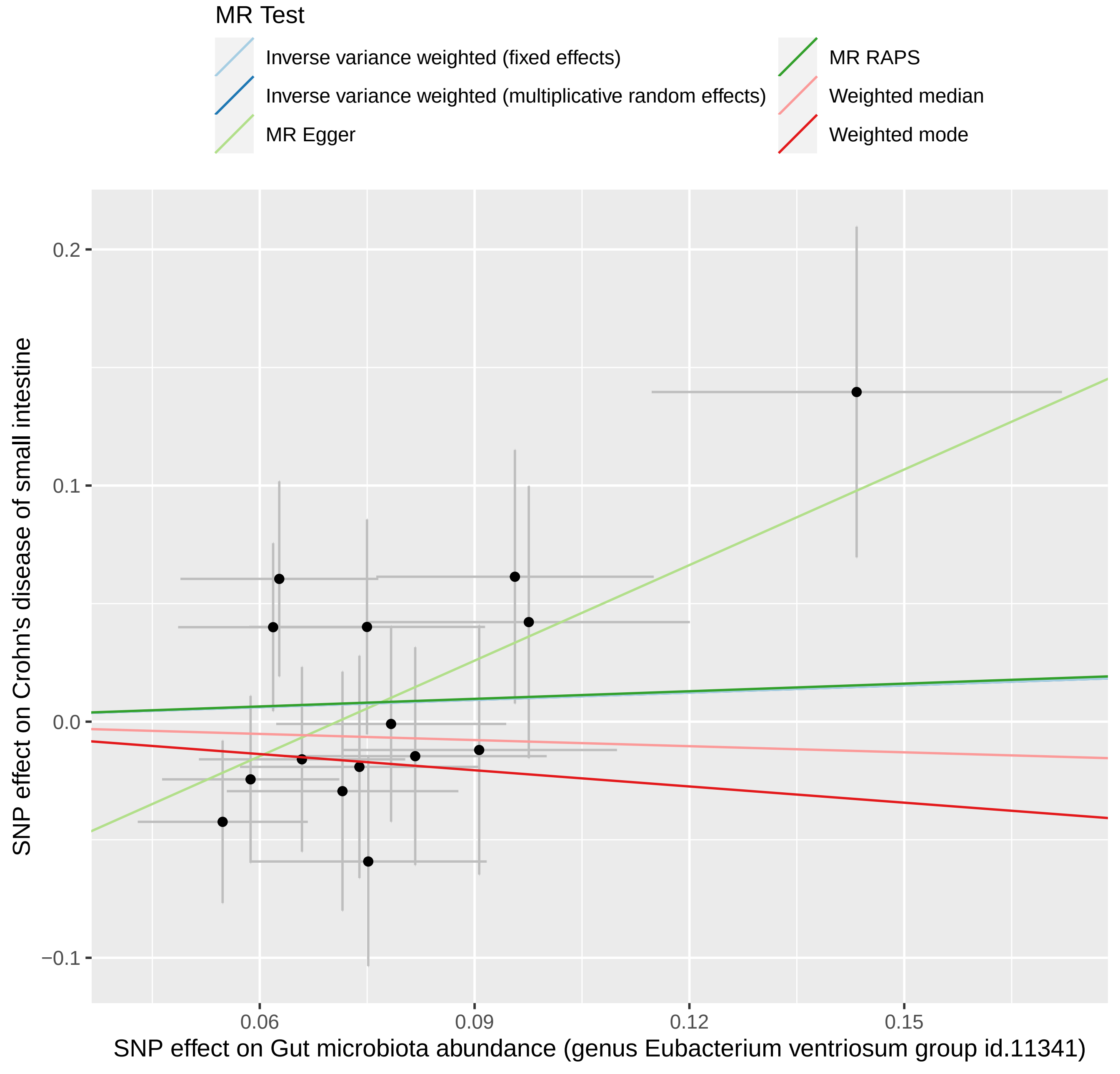

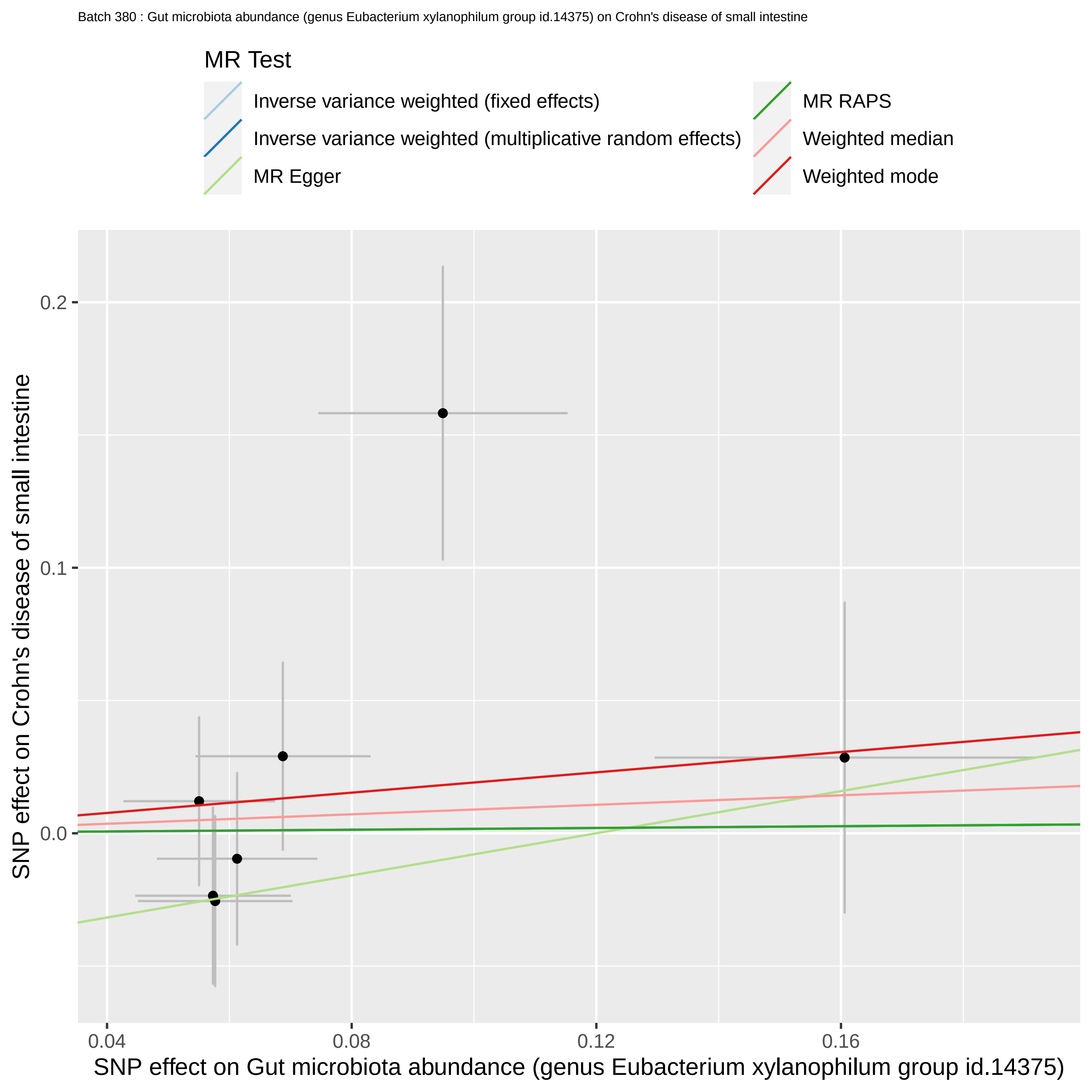

Batch 380 : Gut microbiota abundance (genus Eubacterium xylanophilum group id.14375) on Crohn's disease of small intestine
MR Test
Inverse variance weighted (fixed effects)
Inverse variance weighted (multiplicative random effects)
MR Egger
MR RAPS
Weighted median
Weighted mode
SNP effect on Crohn's disease of small intestine
0.2
0.1
0.0
0.04
0.08
0.12
0.16
SNP effect on Gut microbiota abundance (genus Eubacterium xylanophilum group id.14375)

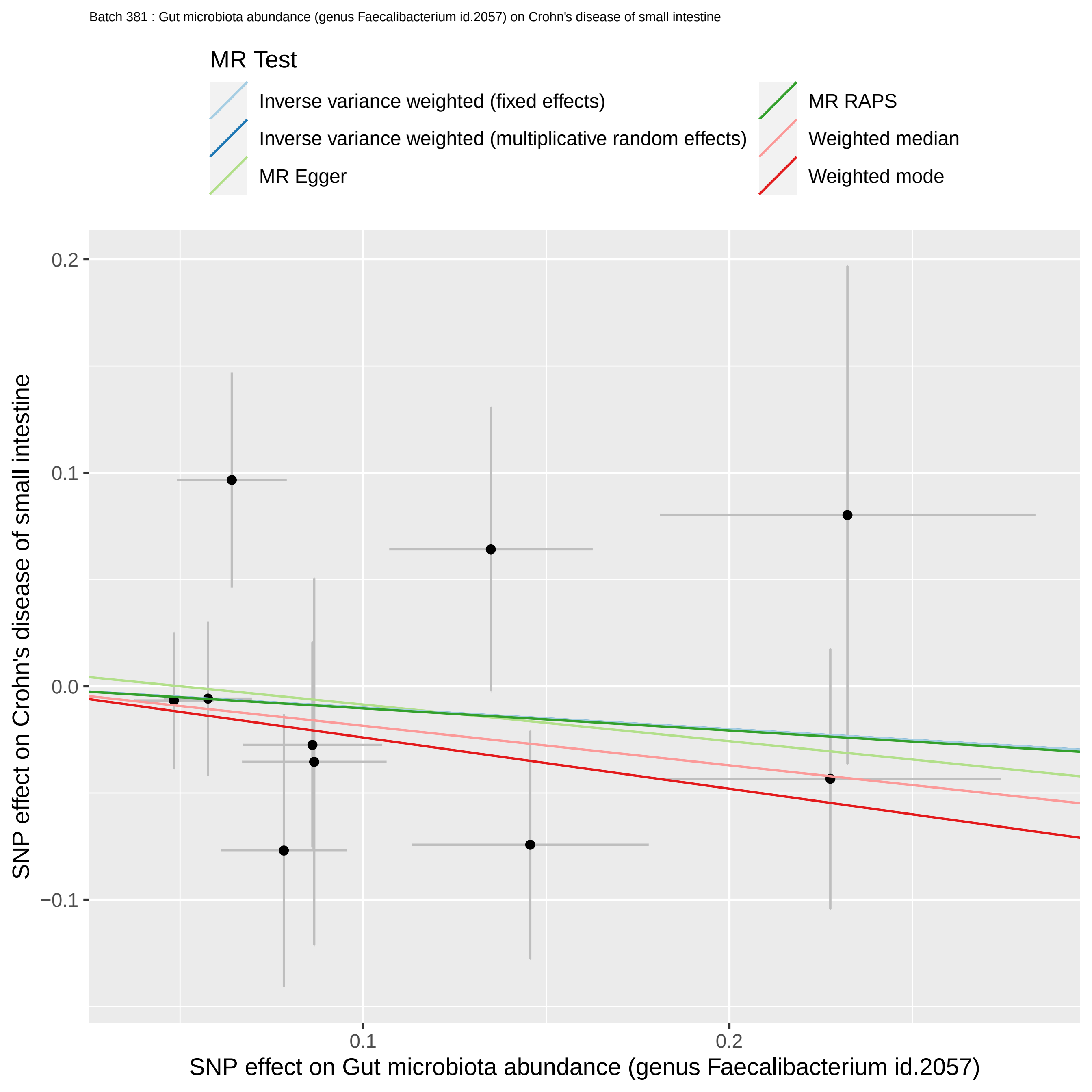

Batch 381 : Gut microbiota abundance (genus Faecalibacterium id.2057) on Crohn's disease of small intestine
MR Test
Inverse variance weighted (fixed effects)
Inverse variance weighted (multiplicative random effects)
MR Egger
MR RAPS
Weighted median
Weighted mode
0.2
0.1
0.0
−0.1
SNP effect on Crohn's disease of small intestine
0.1
0.2
SNP effect on Gut microbiota abundance (genus Faecalibacterium id.2057)

Batch 382 : Gut microbiota abundance (genus Family XIII AD3011 group id.11293) on Crohn's disease of small intestine

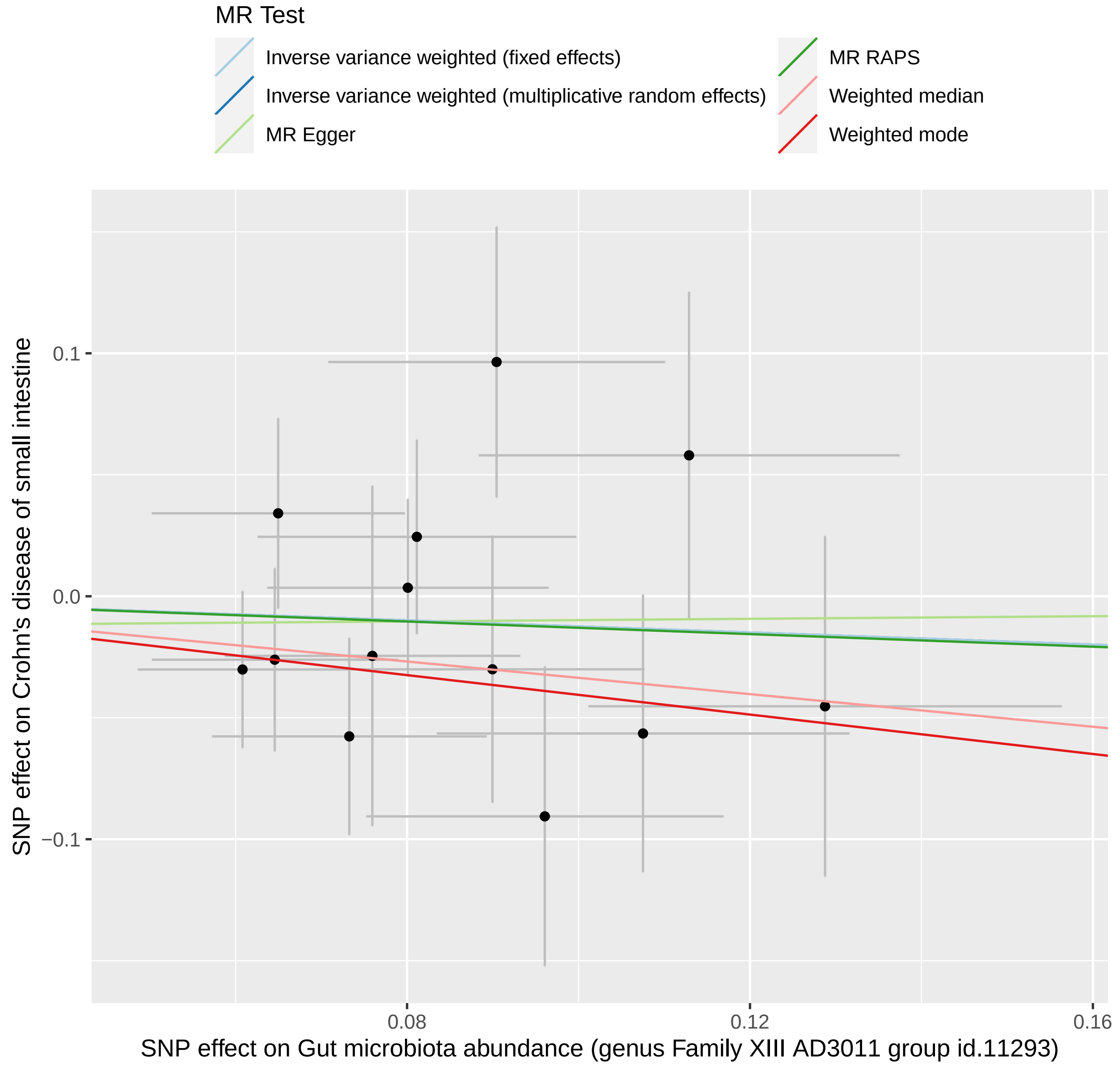

Batch 383 : Gut microbiota abundance (genus Family XIII UCG001 id.11294) on Crohn's disease of small intestine

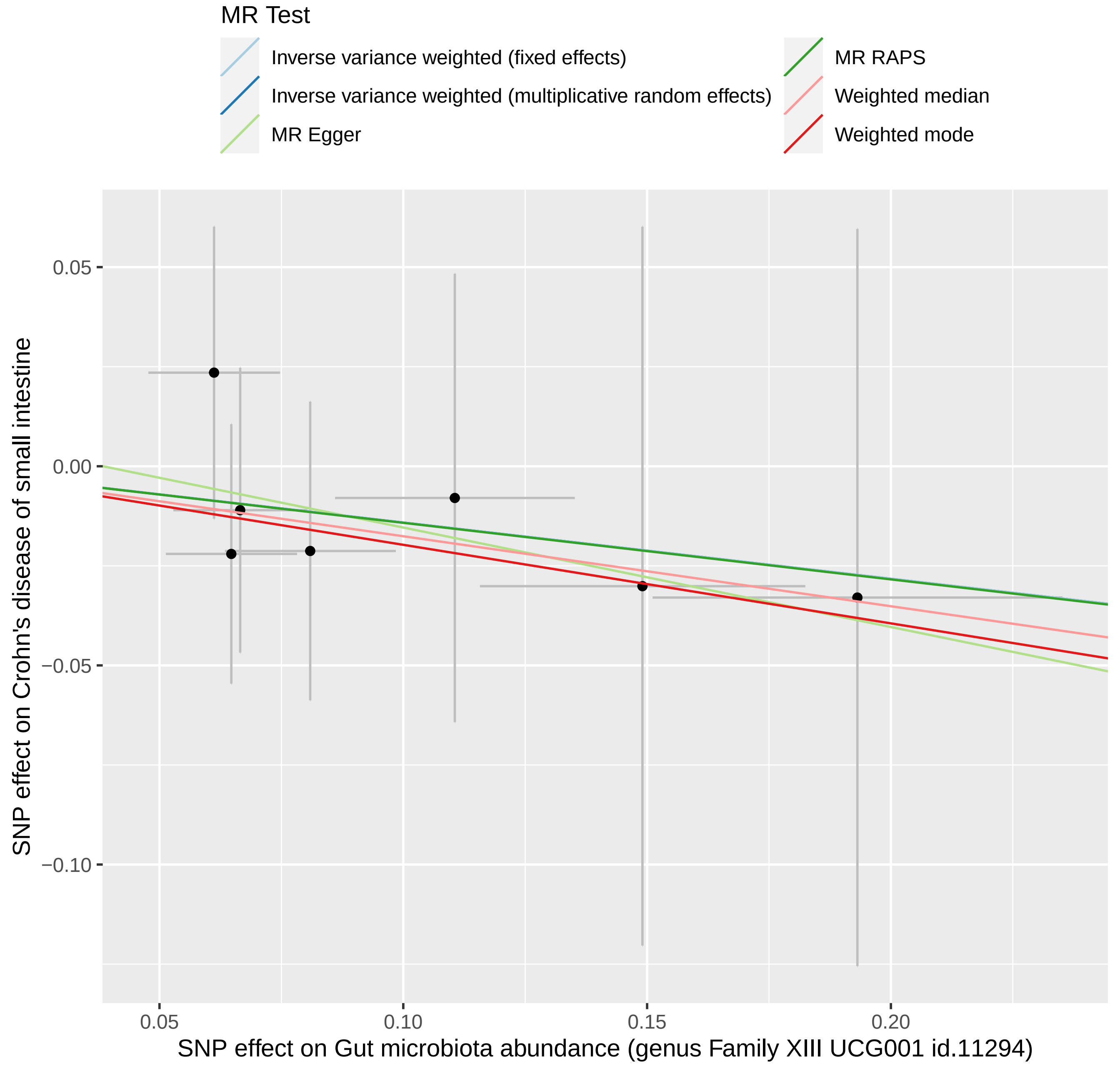

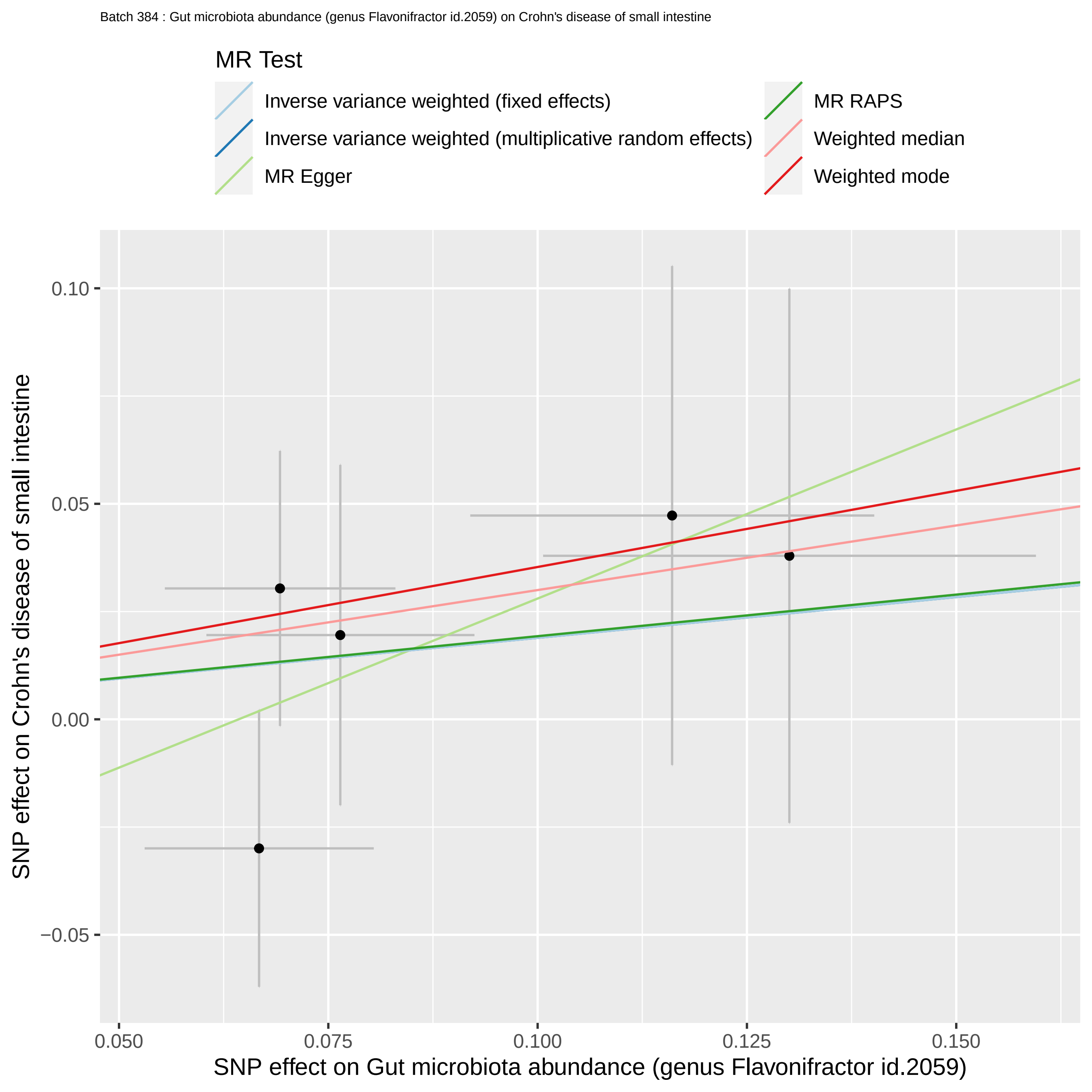
Batch 384 : Gut microbiota abundance (genus Flavonifractor id.2059) on Crohn's disease of small intestine
MR Test
Inverse variance weighted (fixed effects)
Inverse variance weighted (multiplicative random effects)
MR Egger
MR RAPS
Weighted median
Weighted mode
0.10
0.05
0.00
−0.05
0.050
0.075
0.100
0.125
0.150
SNP effect on Crohn's disease of small intestine
SNP effect on Gut microbiota abundance (genus Flavonifractor id.2059)

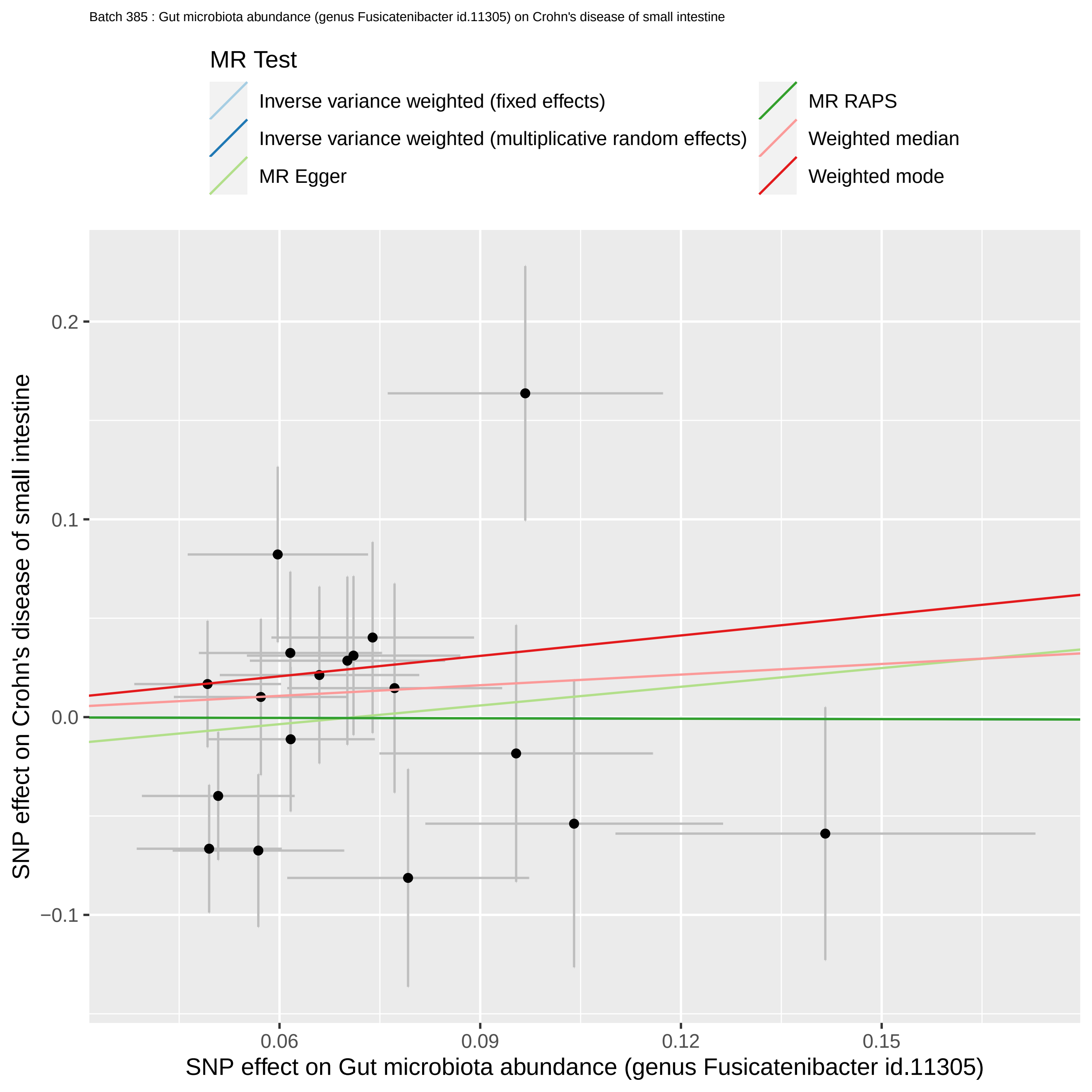
Batch 385 : Gut microbiota abundance (genus Fusicatenibacter id.11305) on Crohn's disease of small intestine
MR Test
Inverse variance weighted (fixed effects)
Inverse variance weighted (multiplicative random effects)
MR Egger
MR RAPS
Weighted median
Weighted mode
SNP effect on Crohn's disease of small intestine
0.2
0.1
0.0
−0.1
0.06
0.09
0.12
0.15
SNP effect on Gut microbiota abundance (genus Fusicatenibacter id.11305)

Batch 386 : Gut microbiota abundance (genus Gordonibacter id.821) on Crohn's disease of small intestine

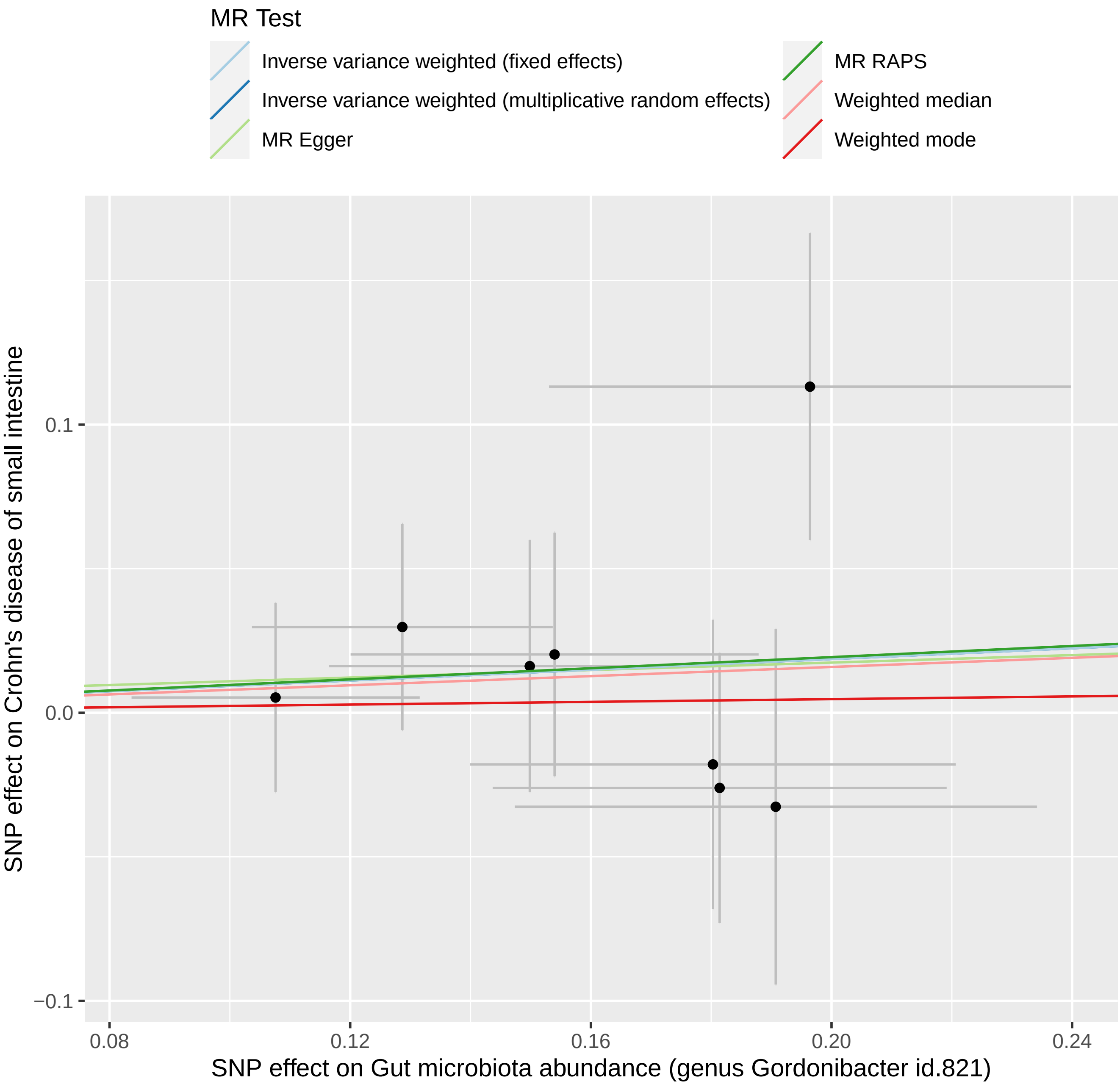

Batch 387 : Gut microbiota abundance (genus Haemophilus id.3698) on Crohn's disease of small intestine

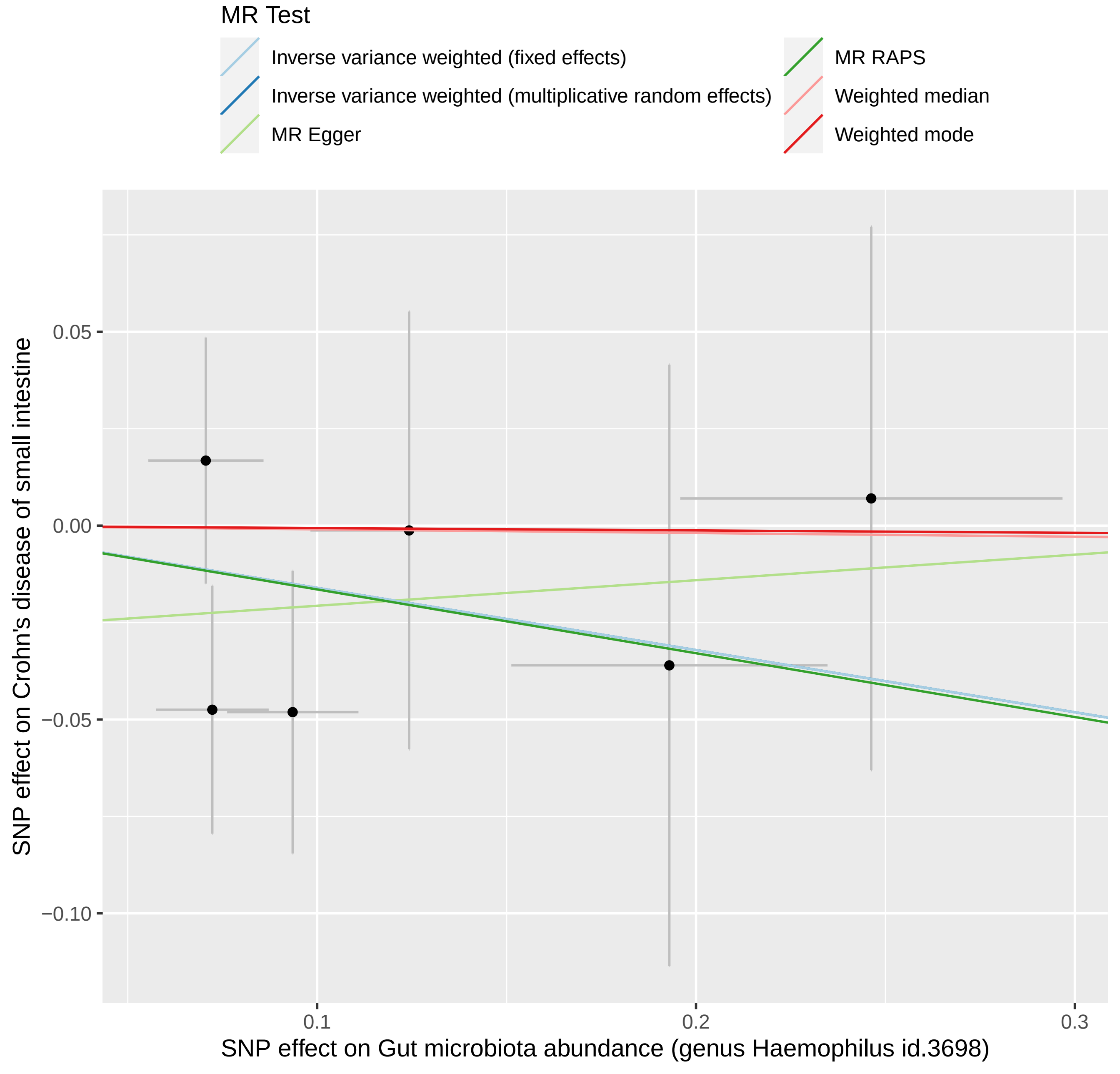

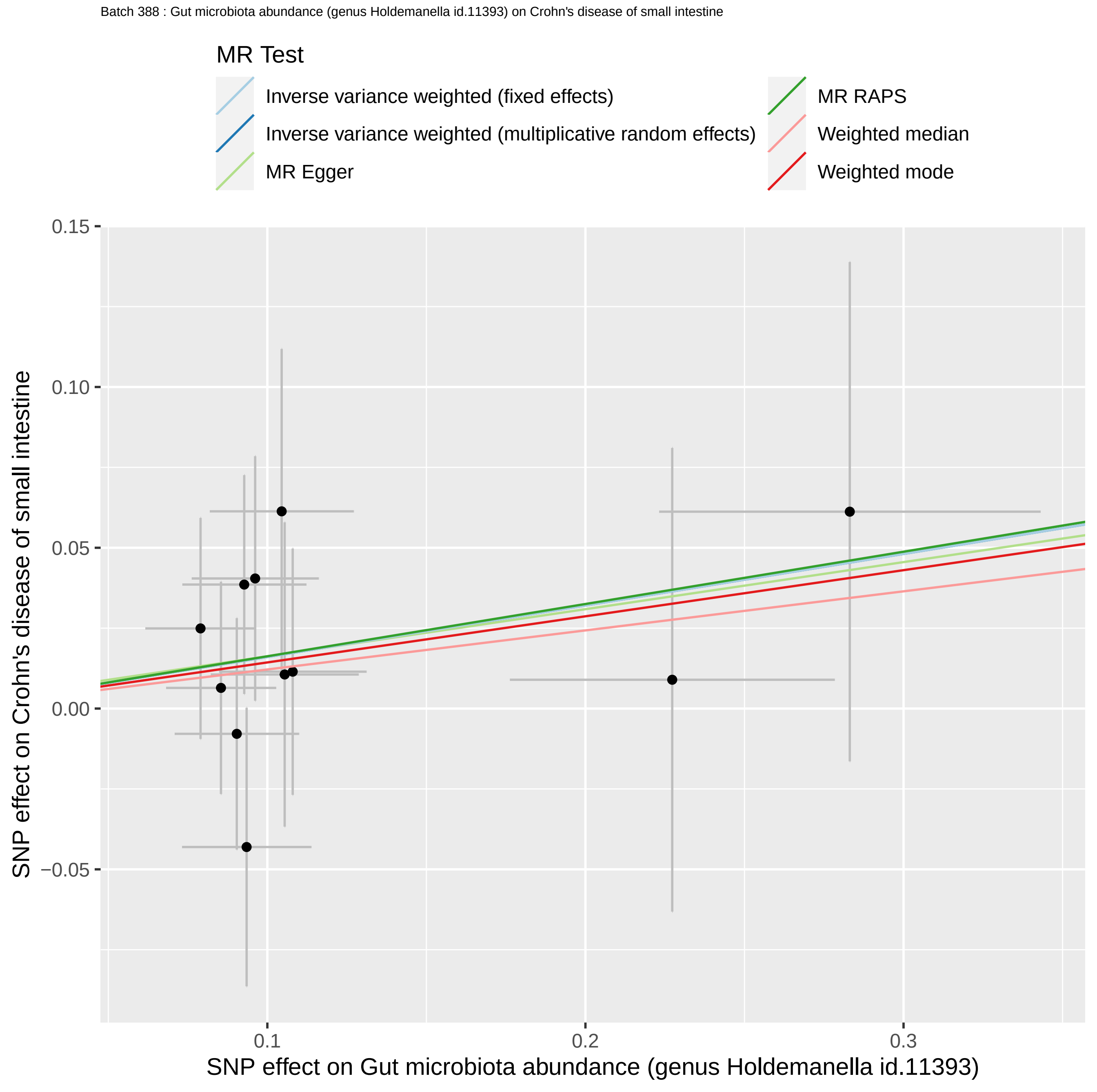

Batch 388 : Gut microbiota abundance (genus Holdemanella id.11393) on Crohn's disease of small intestine
MR Test
Inverse variance weighted (fixed effects)
Inverse variance weighted (multiplicative random effects)
MR Egger
MR RAPS
Weighted median
Weighted mode
0.15
0.10
0.05
0.00
−0.05
0.1
0.2
0.3
SNP effect on Crohn's disease of small intestine
SNP effect on Gut microbiota abundance (genus Holdemanella id.11393)

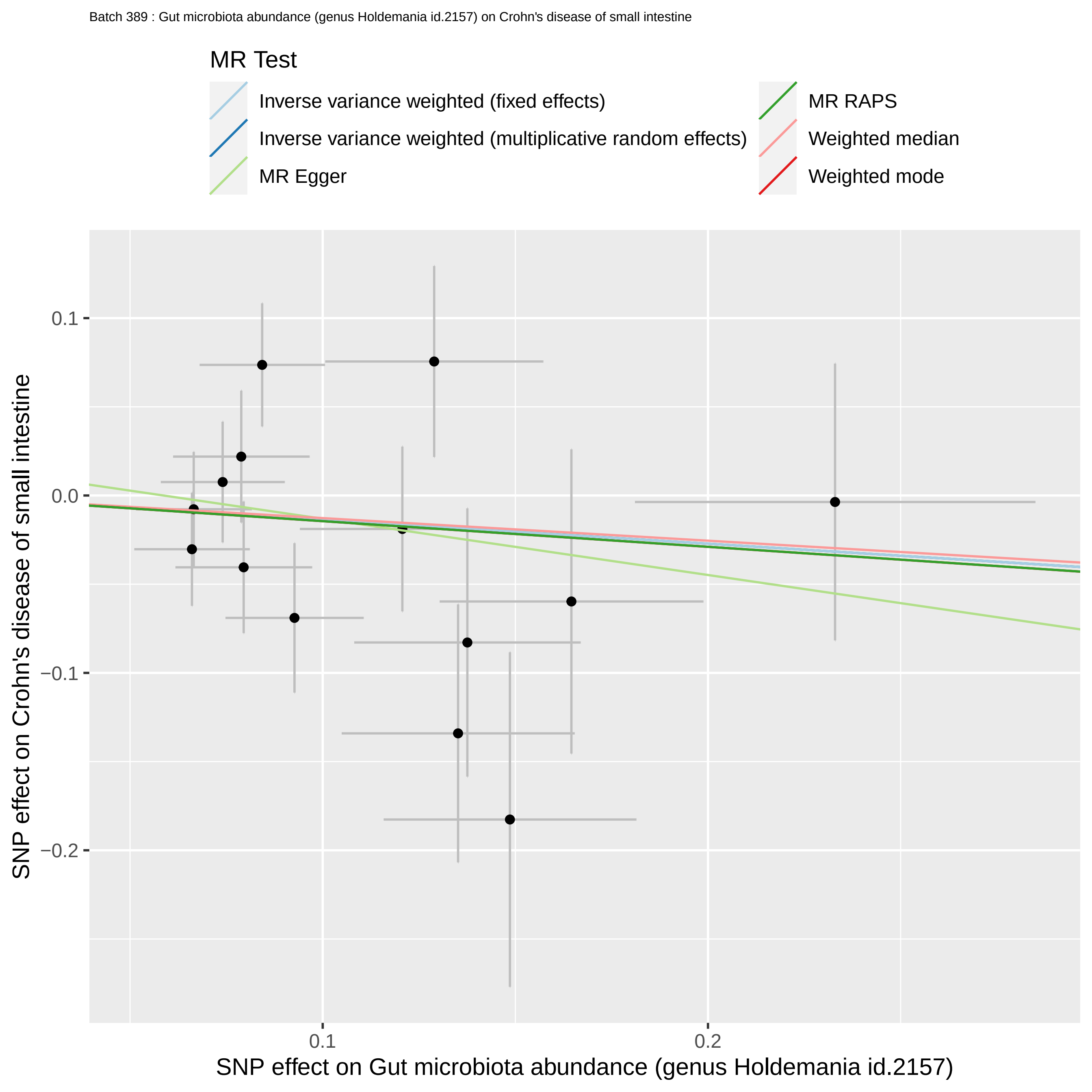

Batch 389 : Gut microbiota abundance (genus Holdemania id.2157) on Crohn's disease of small intestine
MR Test
Inverse variance weighted (fixed effects)
Inverse variance weighted (multiplicative random effects)
MR Egger
MR RAPS
Weighted median
Weighted mode
0.1
0.0
−0.1
−0.2
0.1
0.2
SNP effect on Crohn's disease of small intestine
SNP effect on Gut microbiota abundance (genus Holdemania id.2157)

Batch 390 : Gut microbiota abundance (genus Howardella id.2000) on Crohn's disease of small intestine

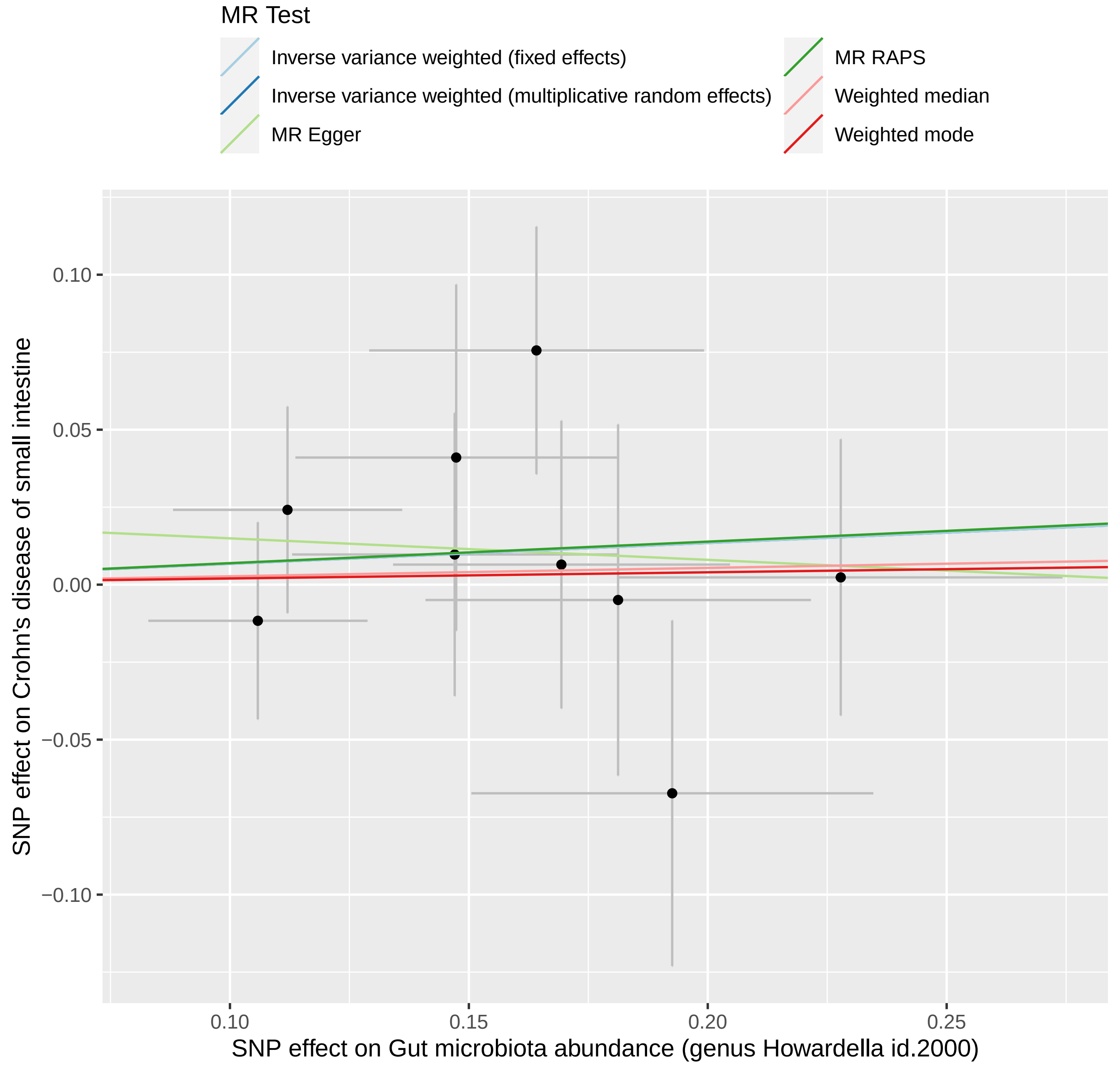

Batch 391 : Gut microbiota abundance (genus Hungatella id.11306) on Crohn's disease of small intestine

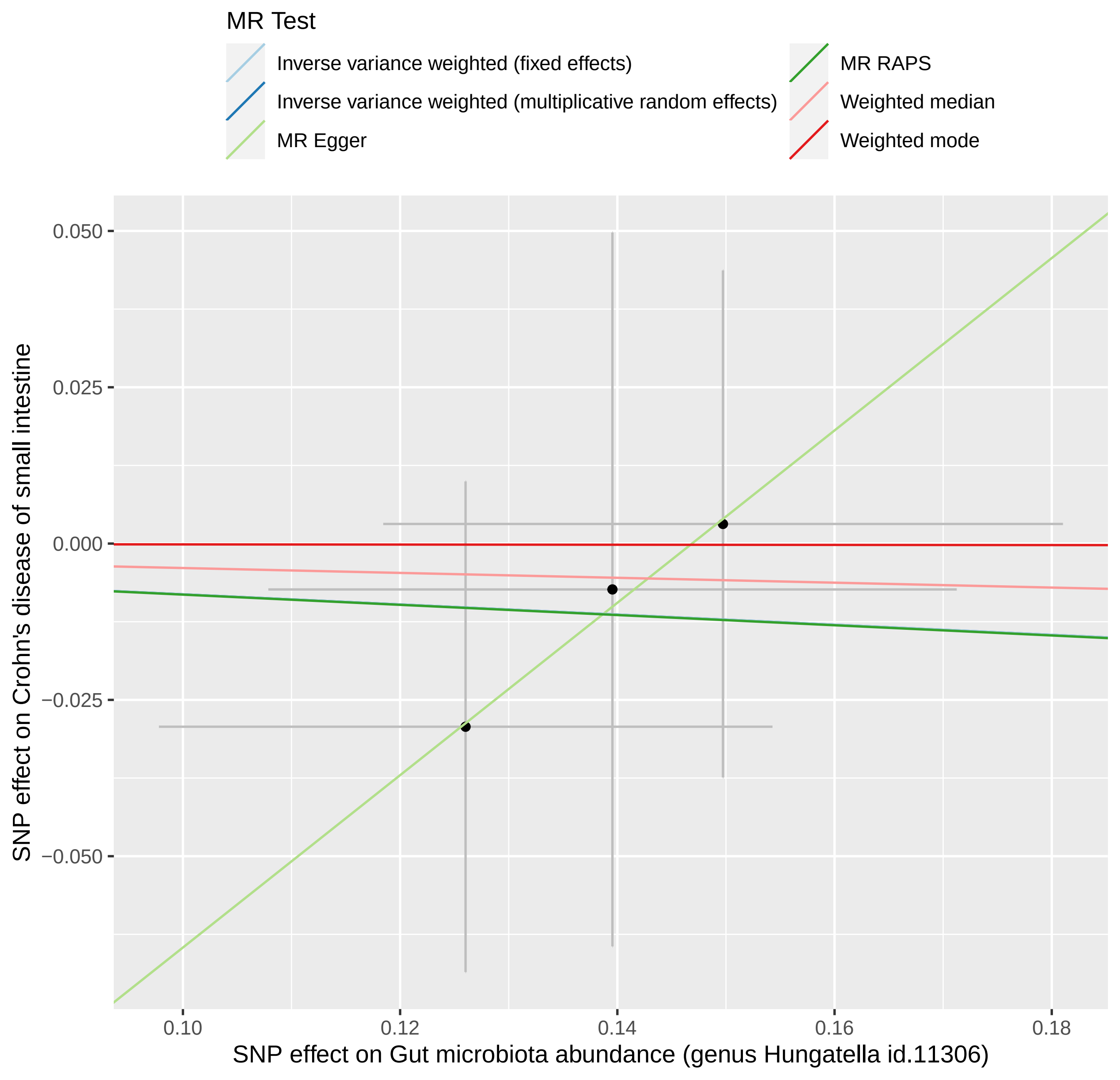

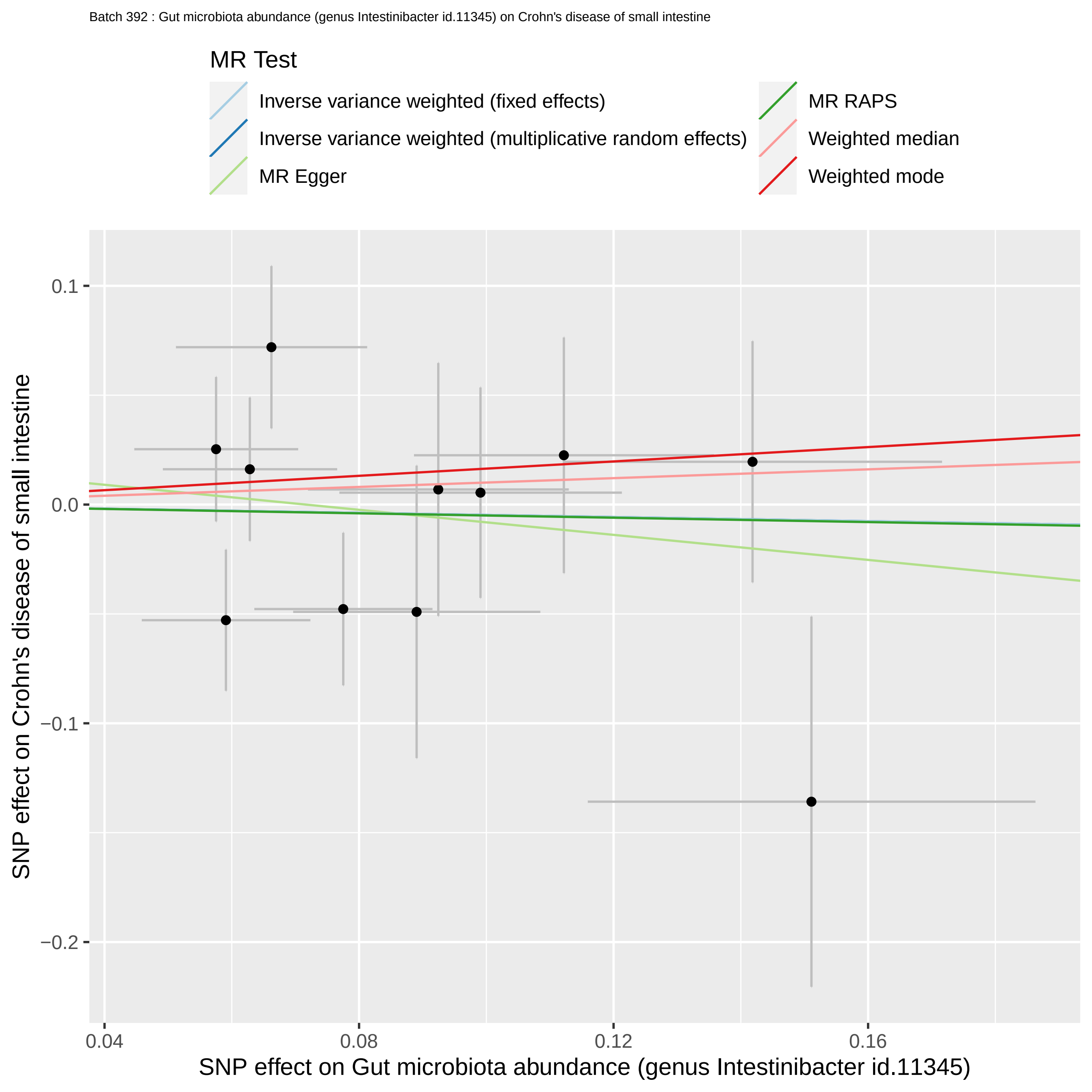

Batch 392 : Gut microbiota abundance (genus Intestinibacter id.11345) on Crohn's disease of small intestine
MR Test
Inverse variance weighted (fixed effects)
Inverse variance weighted (multiplicative random effects)
MR Egger
MR RAPS
Weighted median
Weighted mode
SNP effect on Crohn's disease of small intestine
0.1
0.0
−0.1
−0.2
0.04
0.08
0.12
0.16
SNP effect on Gut microbiota abundance (genus Intestinibacter id.11345)

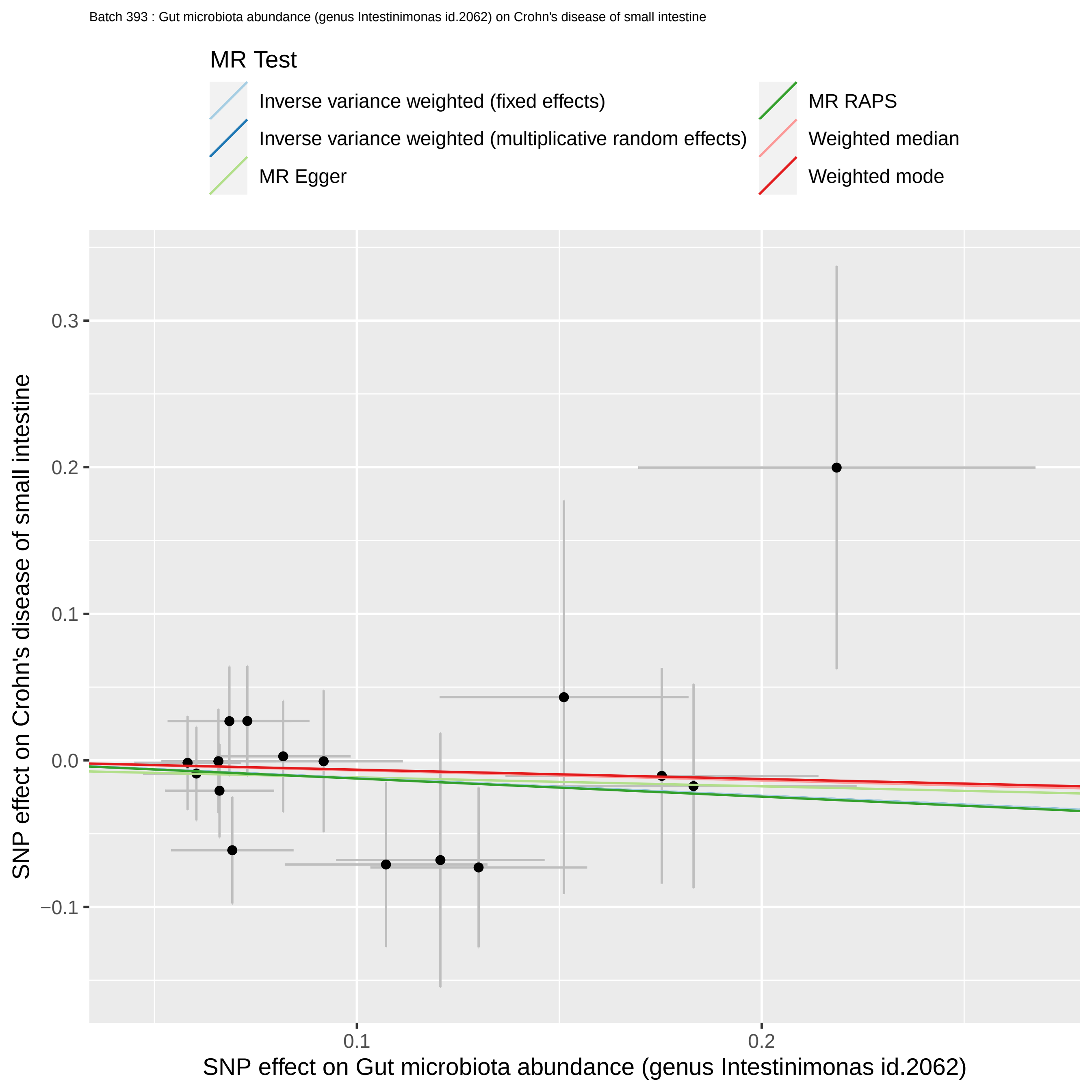
Batch 393 : Gut microbiota abundance (genus Intestinimonas id.2062) on Crohn's disease of small intestine
MR Test
Inverse variance weighted (fixed effects)
Inverse variance weighted (multiplicative random effects)
MR Egger
MR RAPS
Weighted median
Weighted mode
0.3
0.2
0.1
0.0
−0.1
0.1
0.2
SNP effect on Crohn's disease of small intestine
SNP effect on Gut microbiota abundance (genus Intestinimonas id.2062)

Batch 394 : Gut microbiota abundance (genus Lachnoclostridium id.11308) on Crohn's disease of small intestine

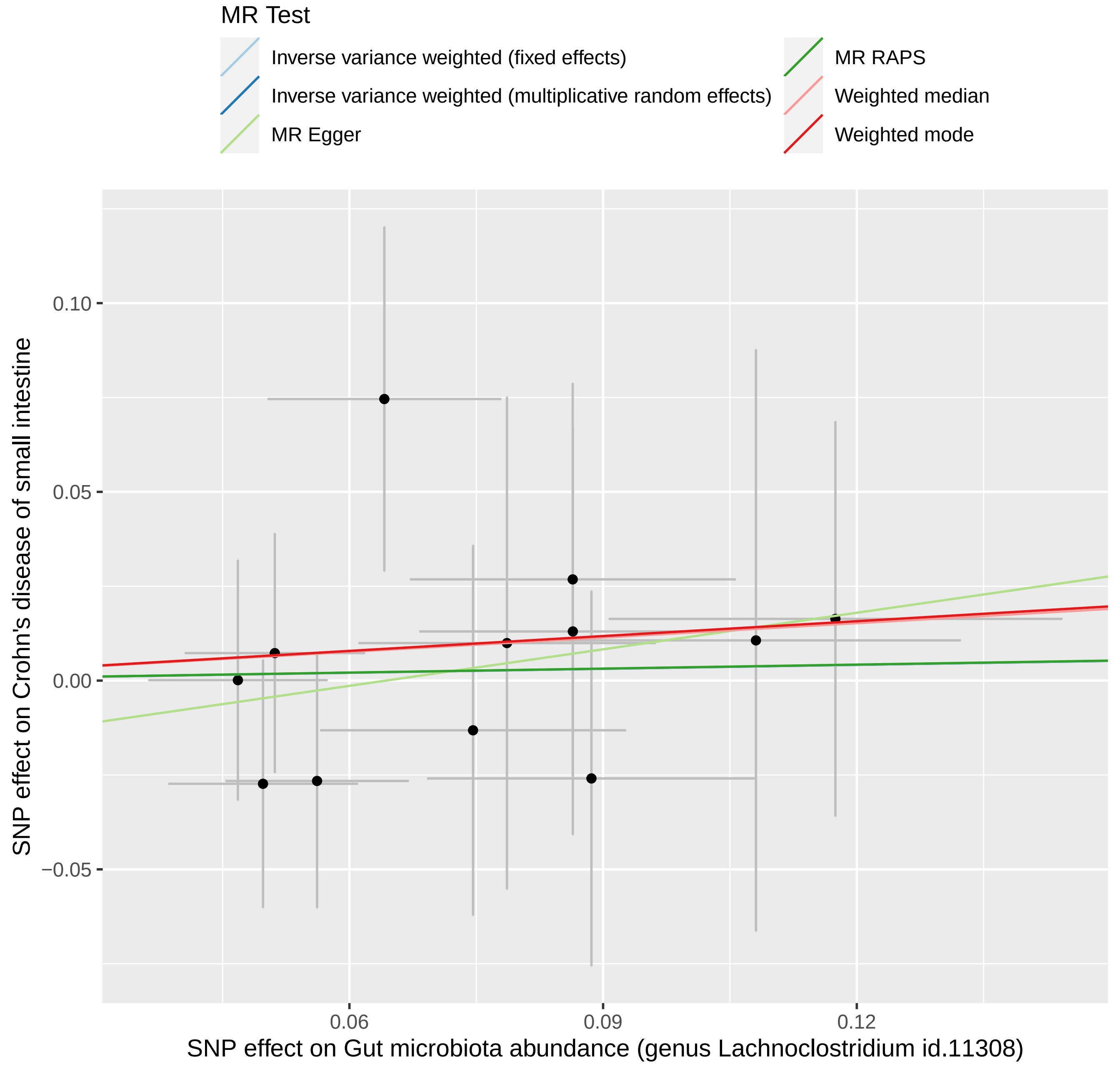

Batch 395 : Gut microbiota abundance (genus Lachnospiraceae FCS020 group id.11314) on Crohn's disease of small intestine

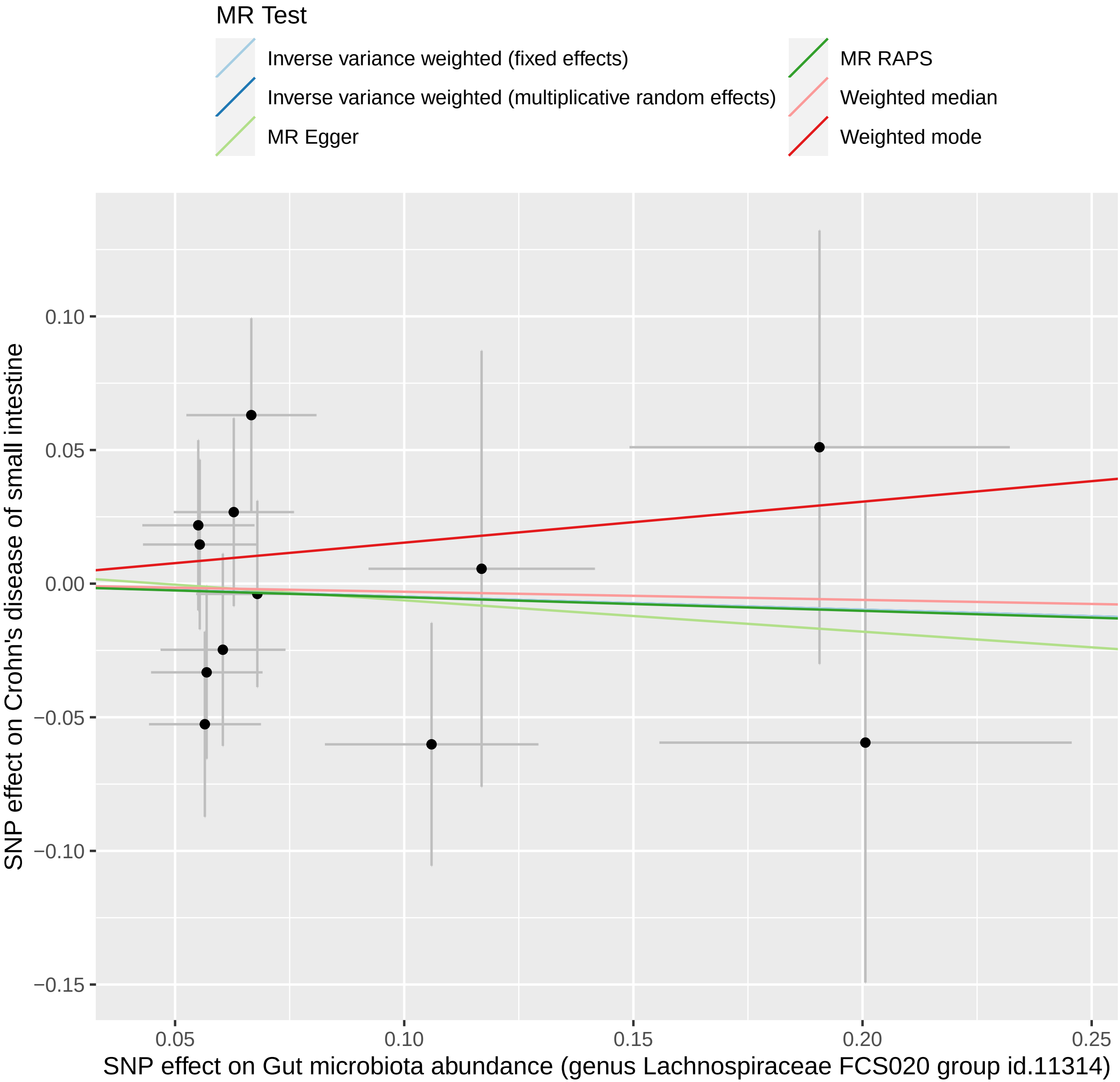

Batch 396 : Gut microbiota abundance (genus Lachnospiraceae NC2004 group id.11316) on Crohn's disease of small intestine

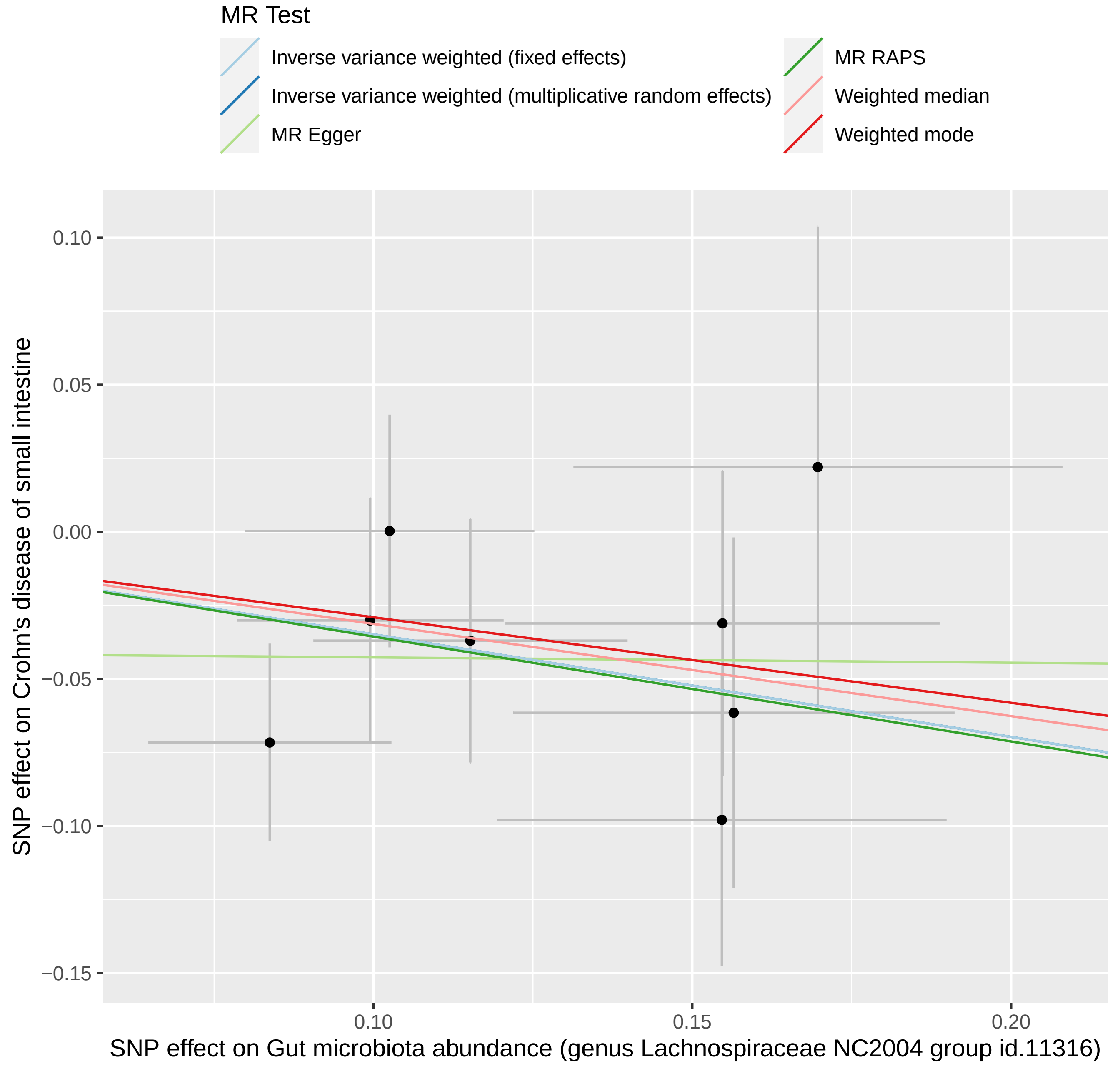

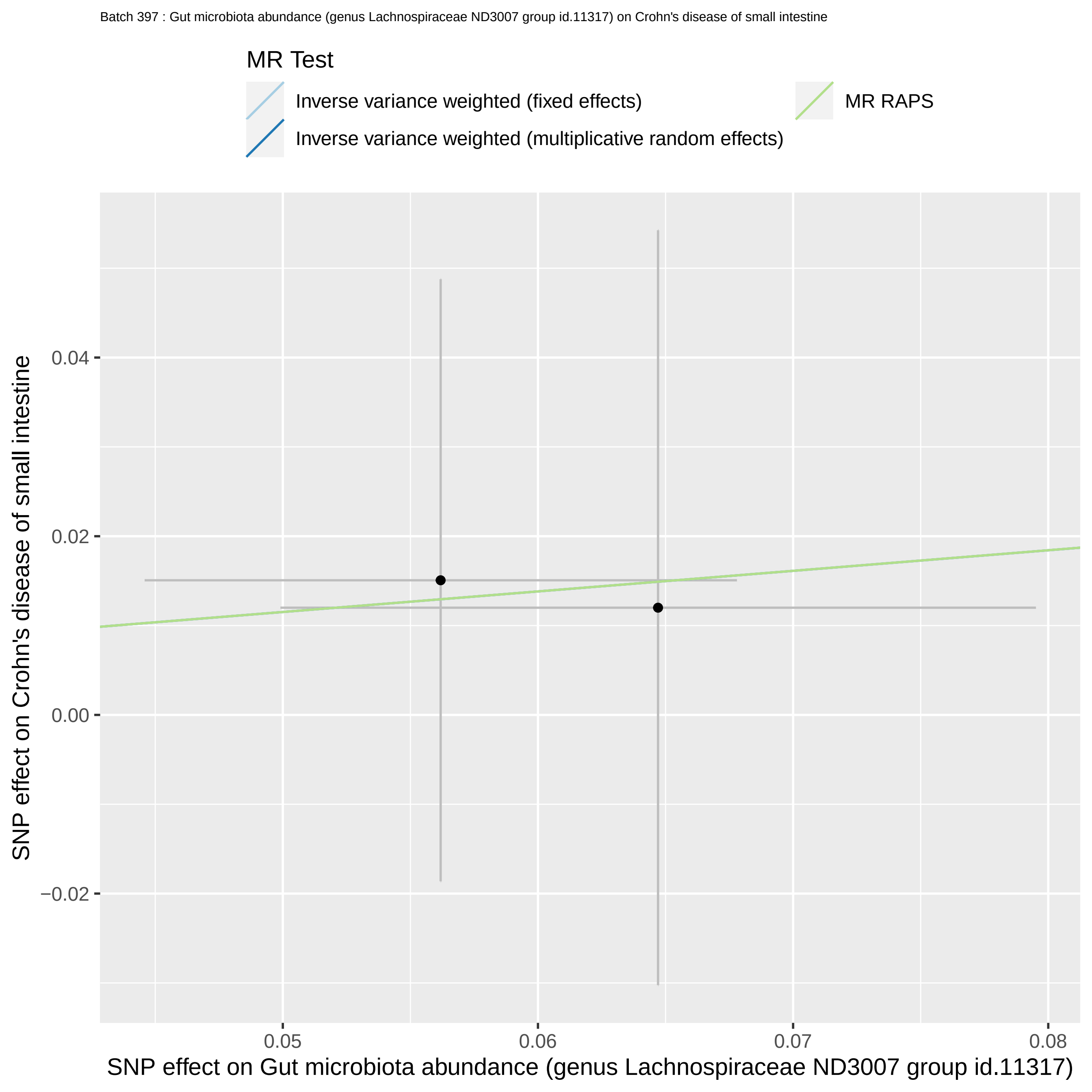

Batch 397 : Gut microbiota abundance (genus Lachnospiraceae ND3007 group id.11317) on Crohn's disease of small intestine
MR Test
Inverse variance weighted (fixed effects)
Inverse variance weighted (multiplicative random effects)
MR RAPS
0.04
0.02
0.00
−0.02
0.05
0.06
0.07
0.08
SNP effect on Crohn's disease of small intestine
SNP effect on Gut microbiota abundance (genus Lachnospiraceae ND3007 group id.11317)

Batch 398 : Gut microbiota abundance (genus Lachnospiraceae NK4A136 group id.11319) on Crohn's disease of small intestine

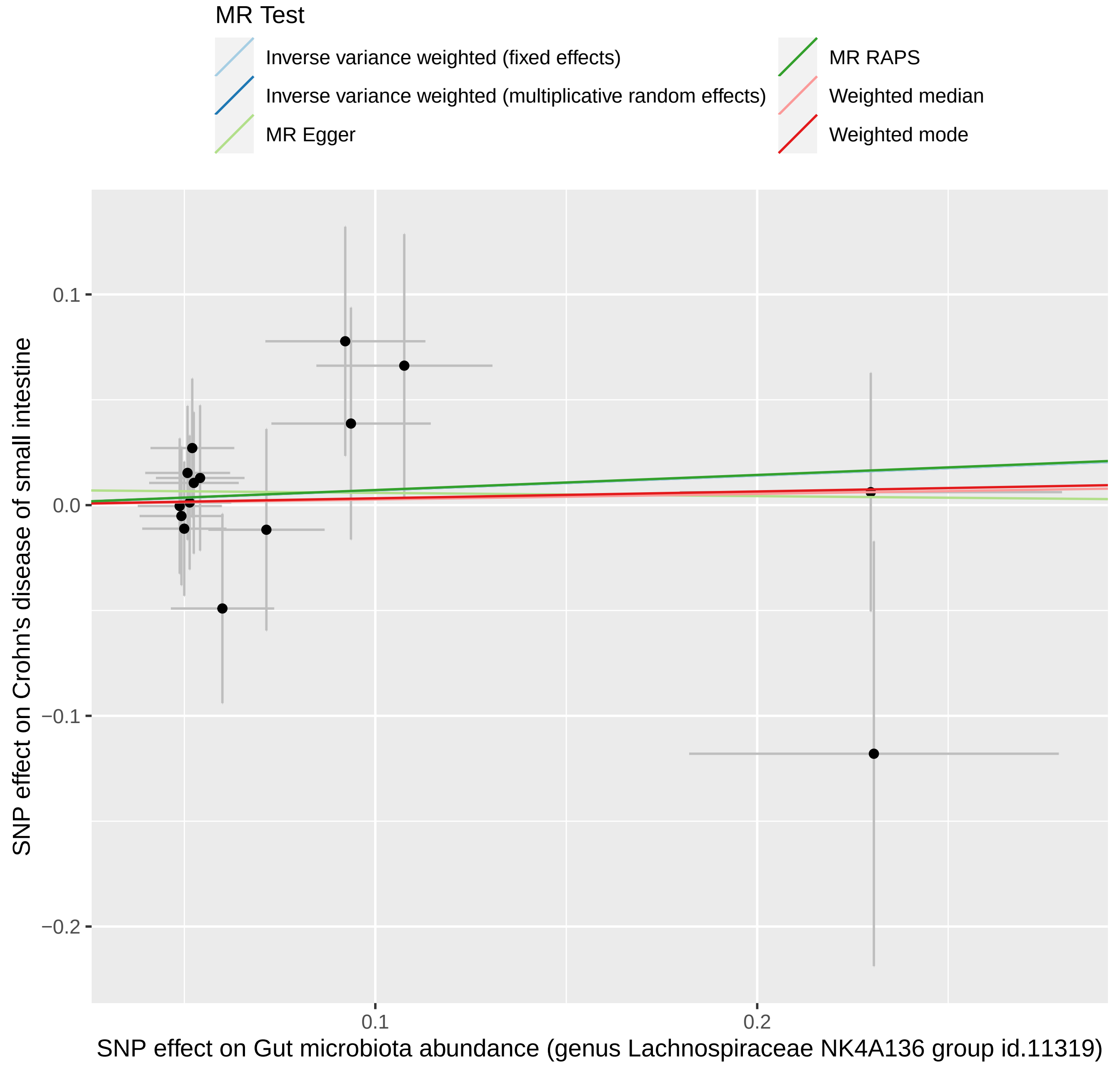

Batch 399 : Gut microbiota abundance (genus Lachnospiraceae UCG001 id.11321) on Crohn's disease of small intestine

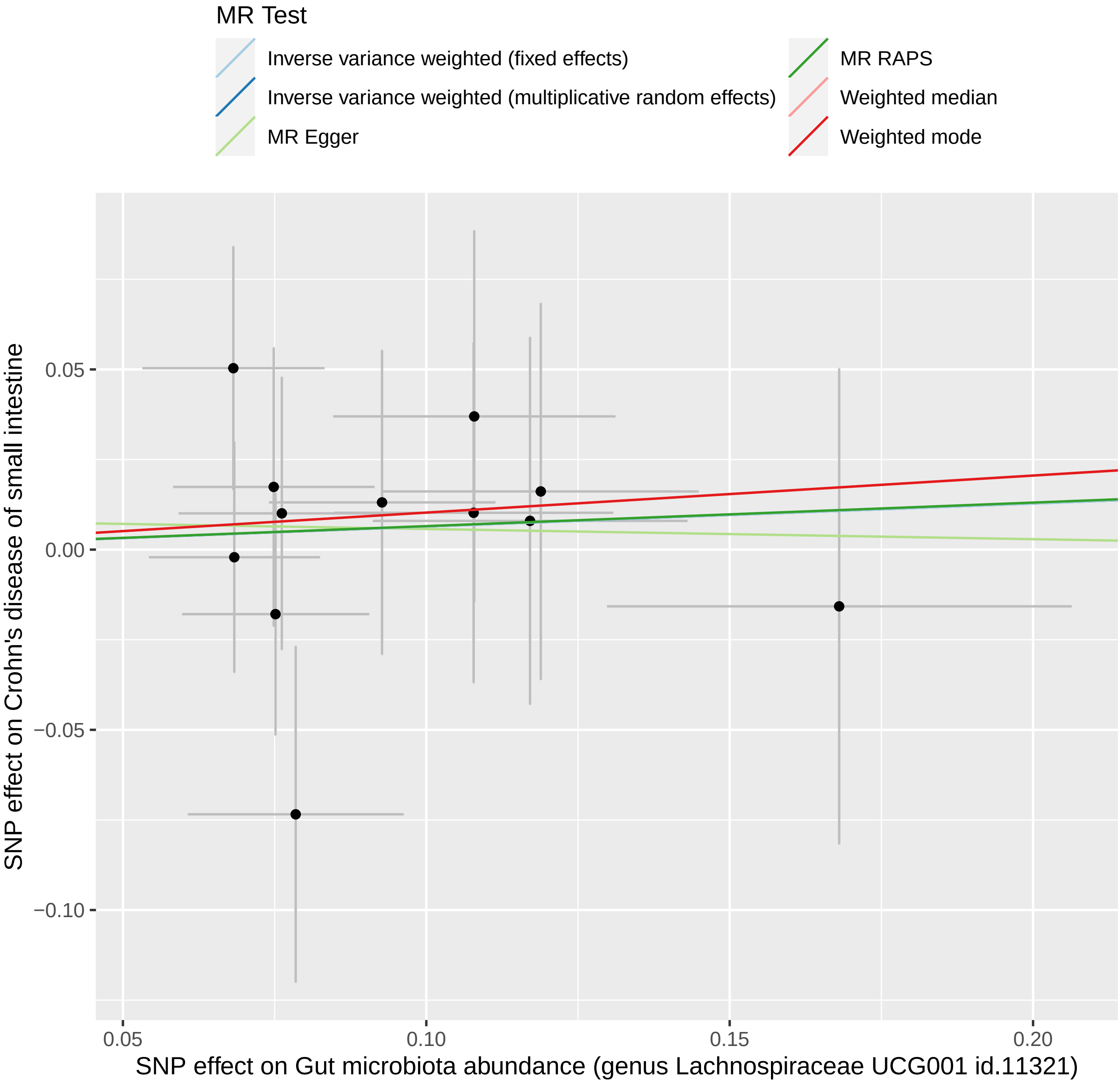

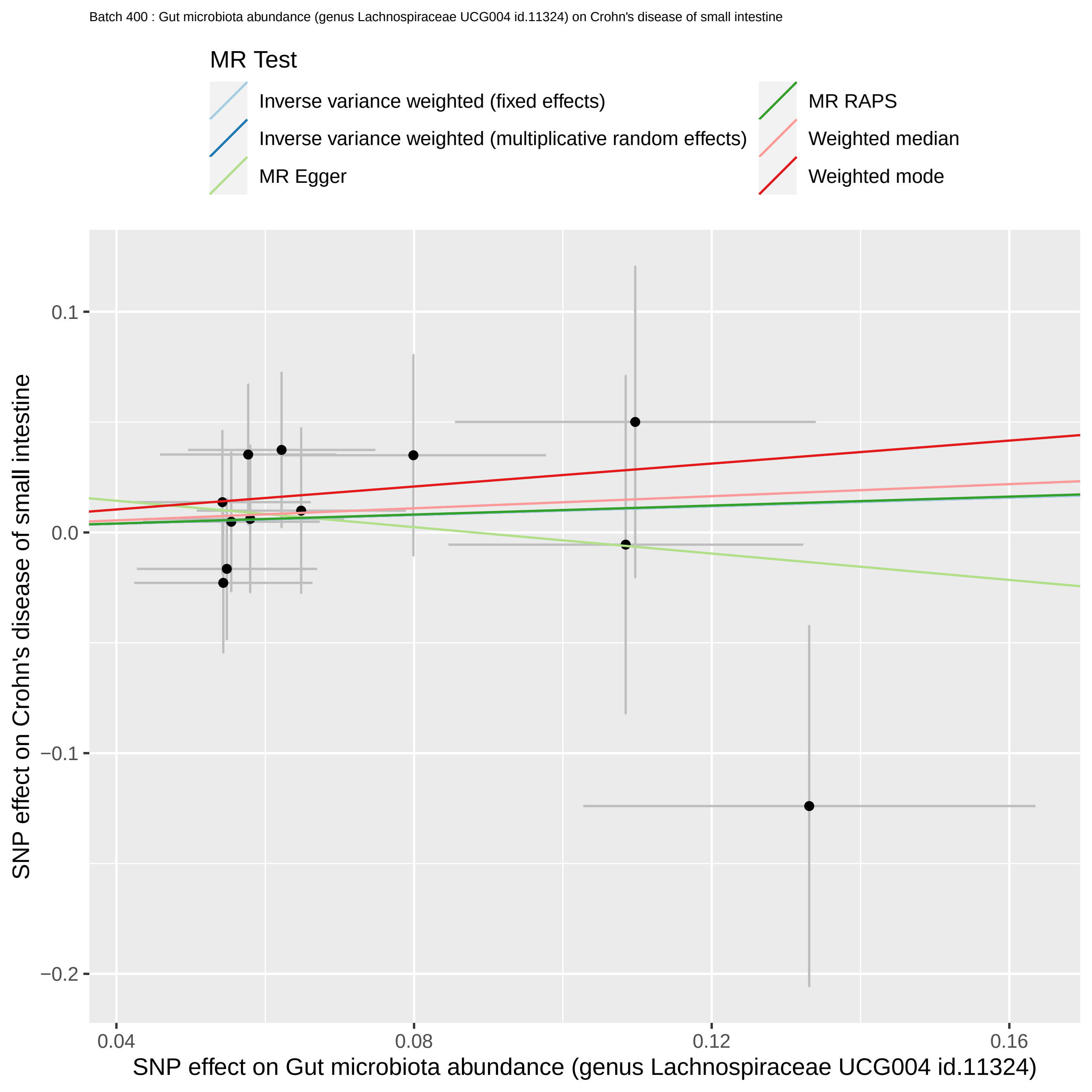
Batch 400 : Gut microbiota abundance (genus Lachnospiraceae UCG004 id.11324) on Crohn's disease of small intestine
MR Test
Inverse variance weighted (fixed effects)
Inverse variance weighted (multiplicative random effects)
MR Egger
MR RAPS
Weighted median
Weighted mode
0.1
0.0
−0.1
−0.2
0.04
0.08
0.12
0.16
SNP effect on Crohn's disease of small intestine
SNP effect on Gut microbiota abundance (genus Lachnospiraceae UCG004 id.11324)

Batch 401 : Gut microbiota abundance (genus Lachnospiraceae UCG008 id.11328) on Crohn's disease of small intestine

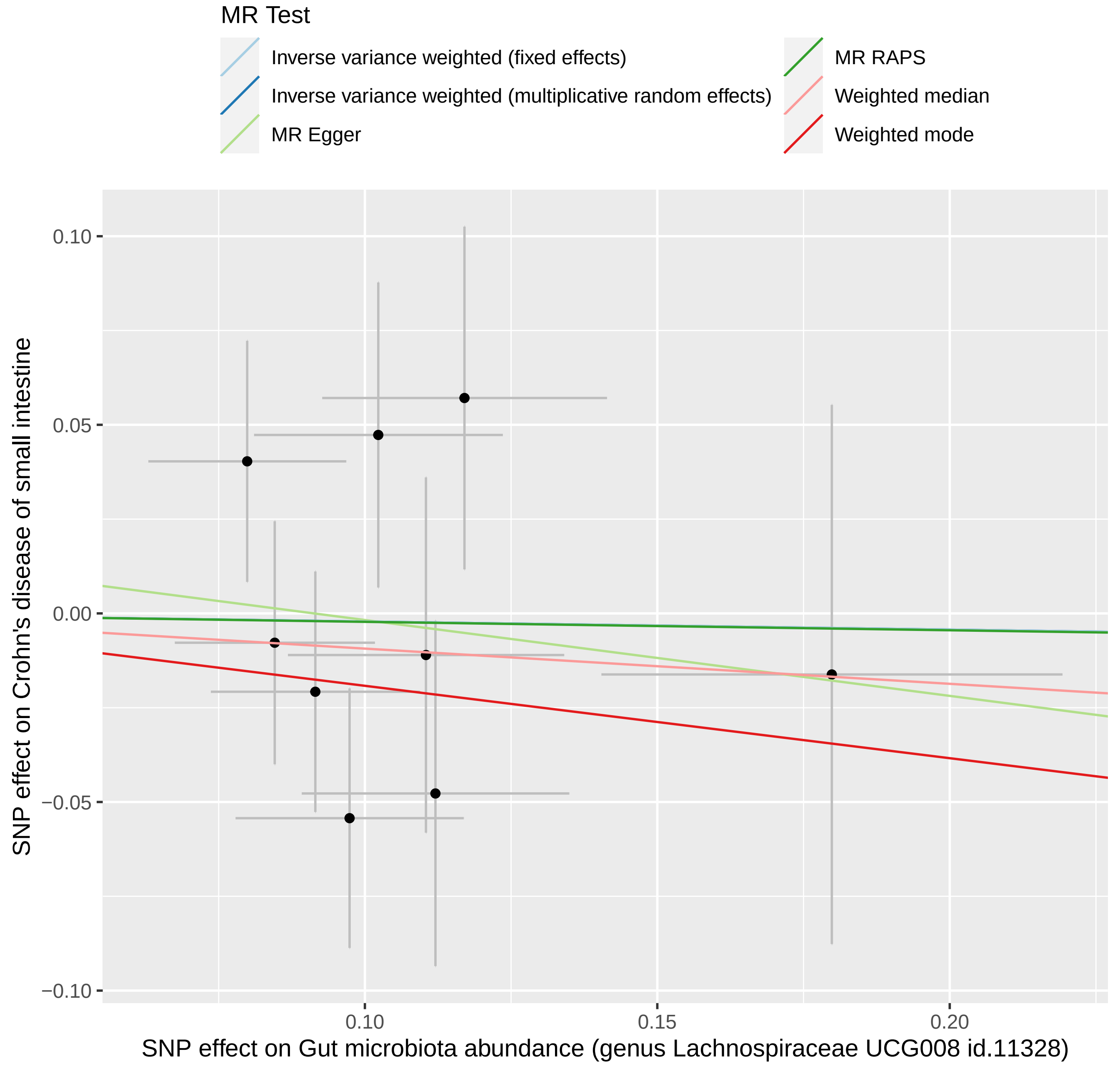

Batch 402 : Gut microbiota abundance (genus Lachnospiraceae UCG010 id.11330) on Crohn's disease of small intestine

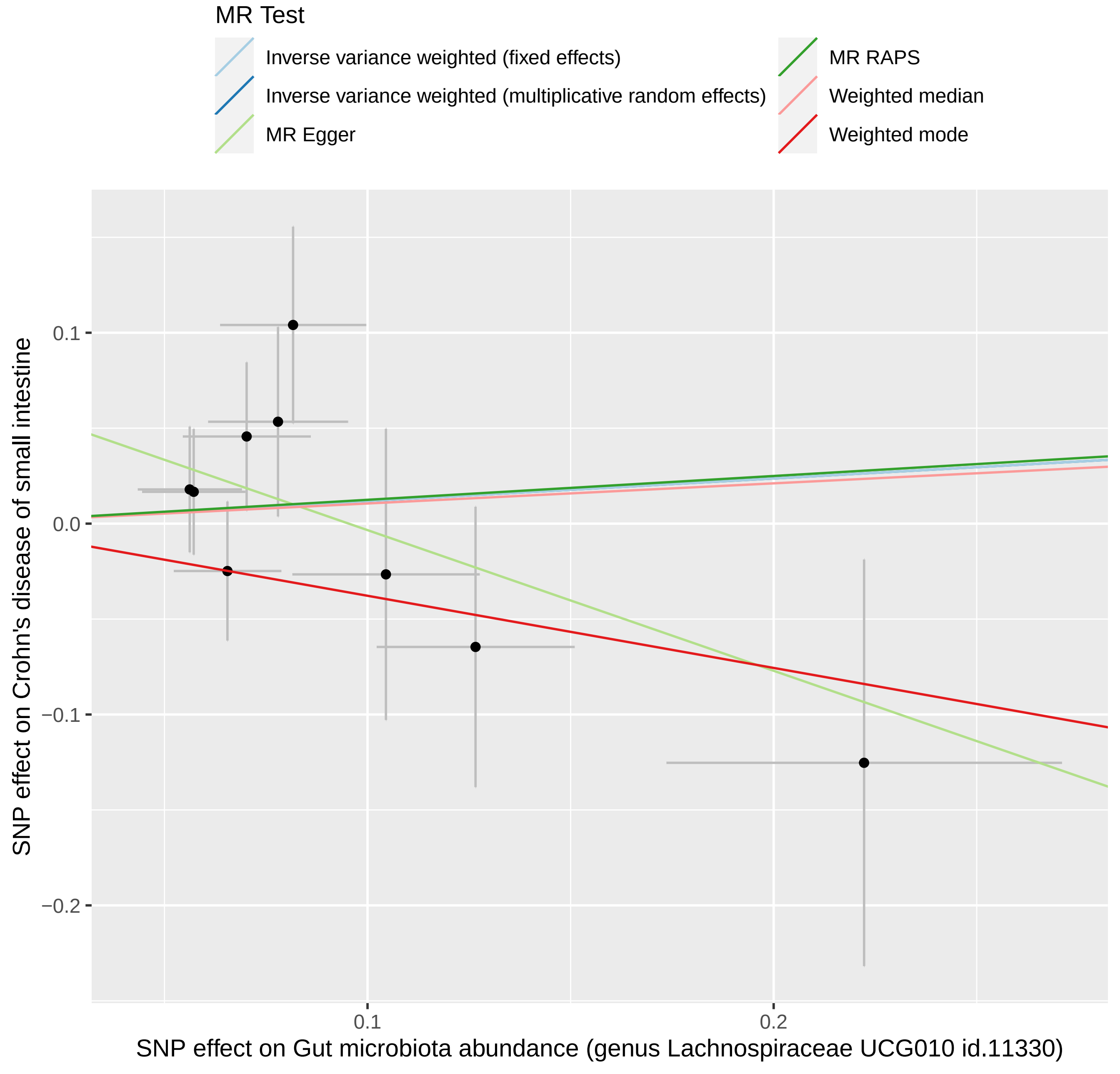

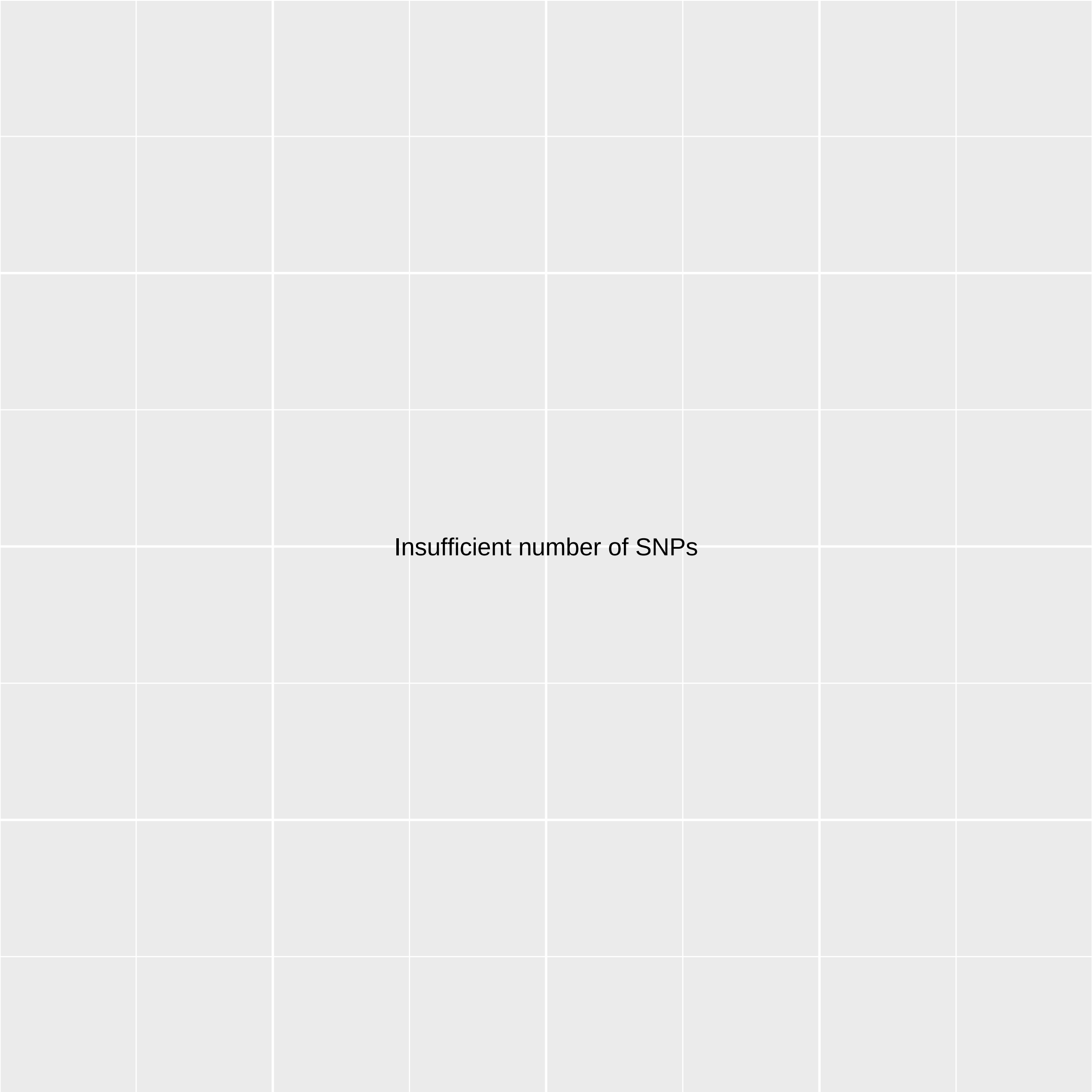
Insufficient number of SNPs

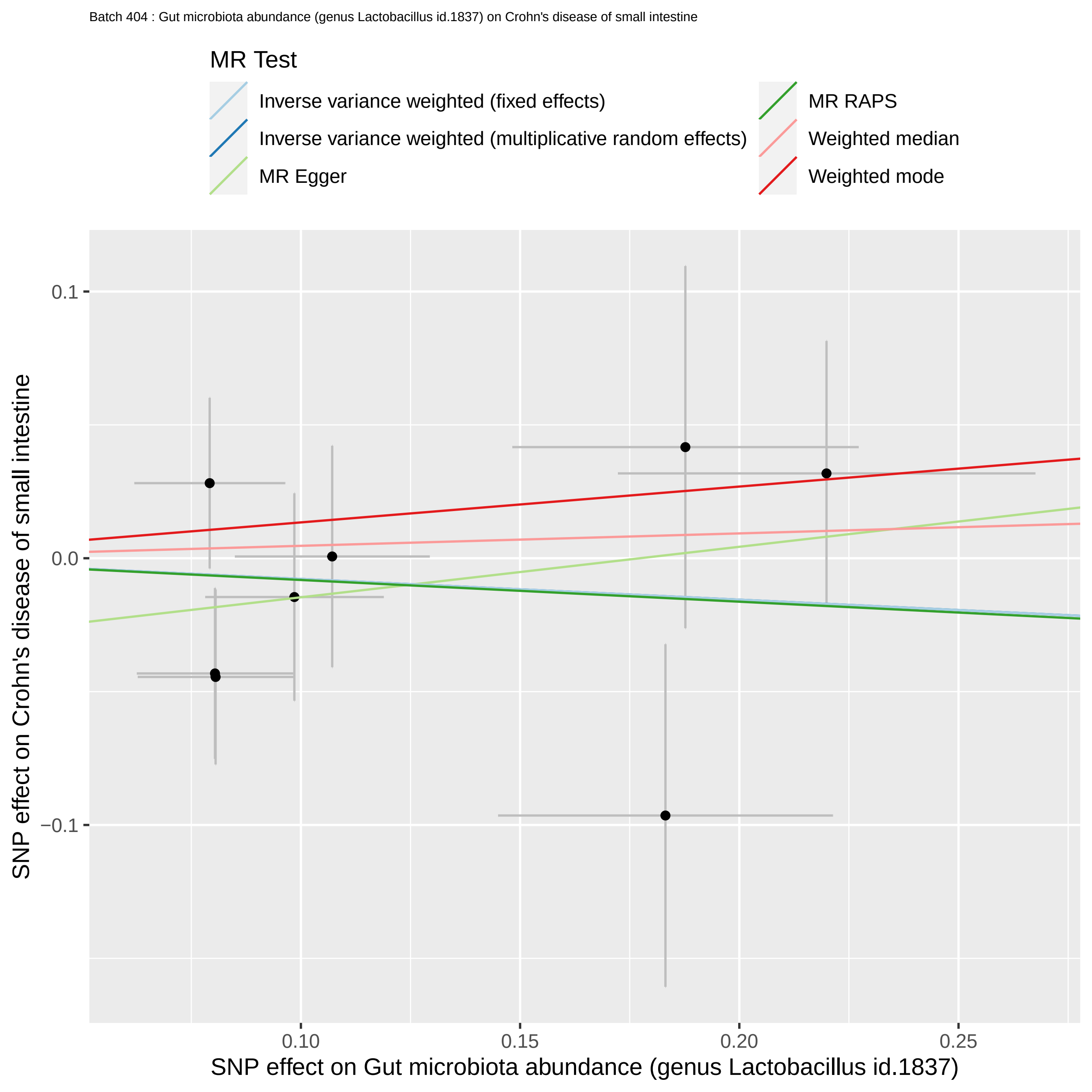

Batch 404 : Gut microbiota abundance (genus Lactobacillus id.1837) on Crohn's disease of small intestine
MR Test
Inverse variance weighted (fixed effects)
Inverse variance weighted (multiplicative random effects)
MR Egger
MR RAPS
Weighted median
Weighted mode
0.1
0.0
−0.1
0.10
0.15
0.20
0.25
SNP effect on Crohn's disease of small intestine
SNP effect on Gut microbiota abundance (genus Lactobacillus id.1837)

Batch 405 : Gut microbiota abundance (genus Lactococcus id.1851) on Crohn's disease of small intestine

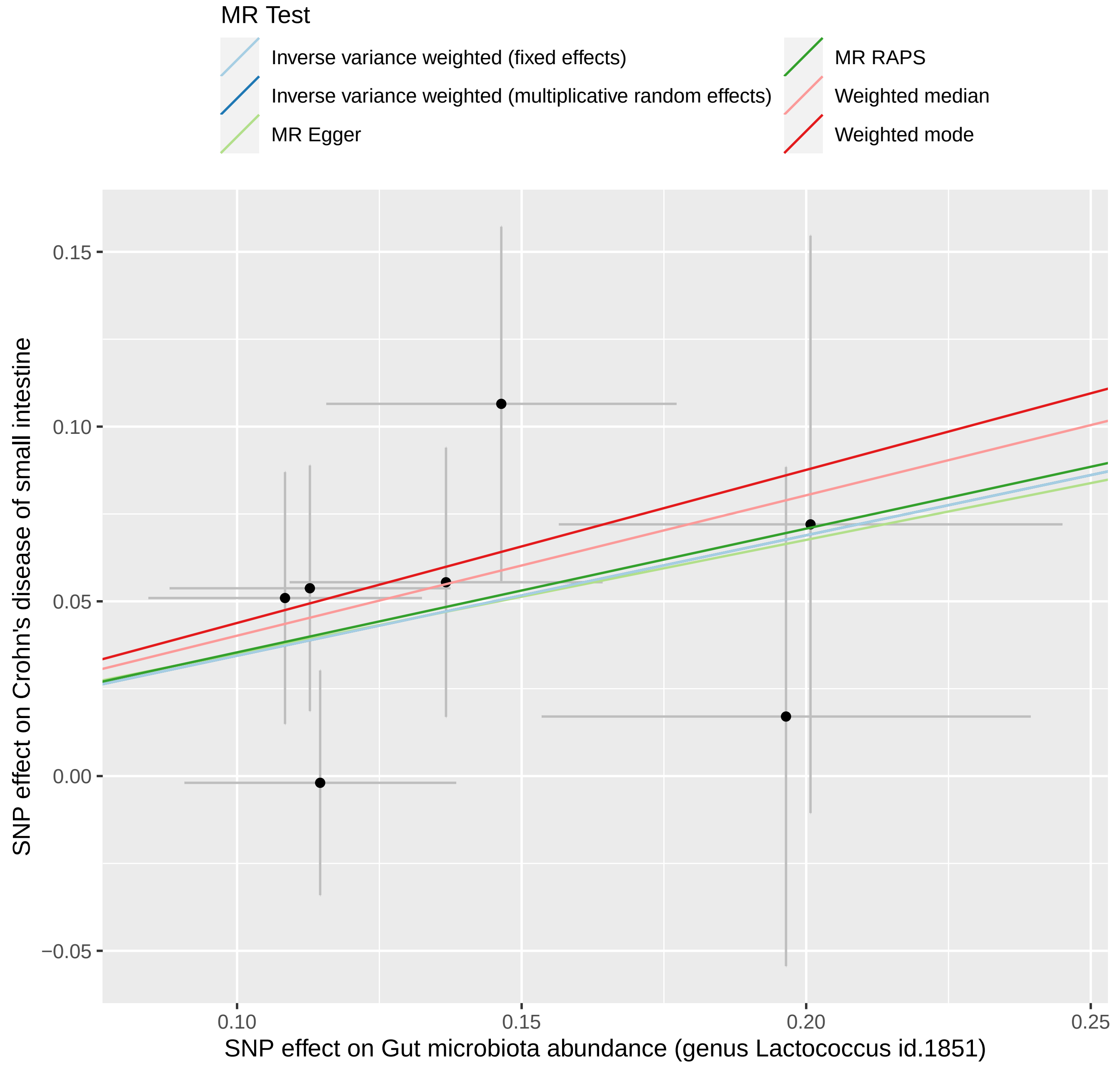

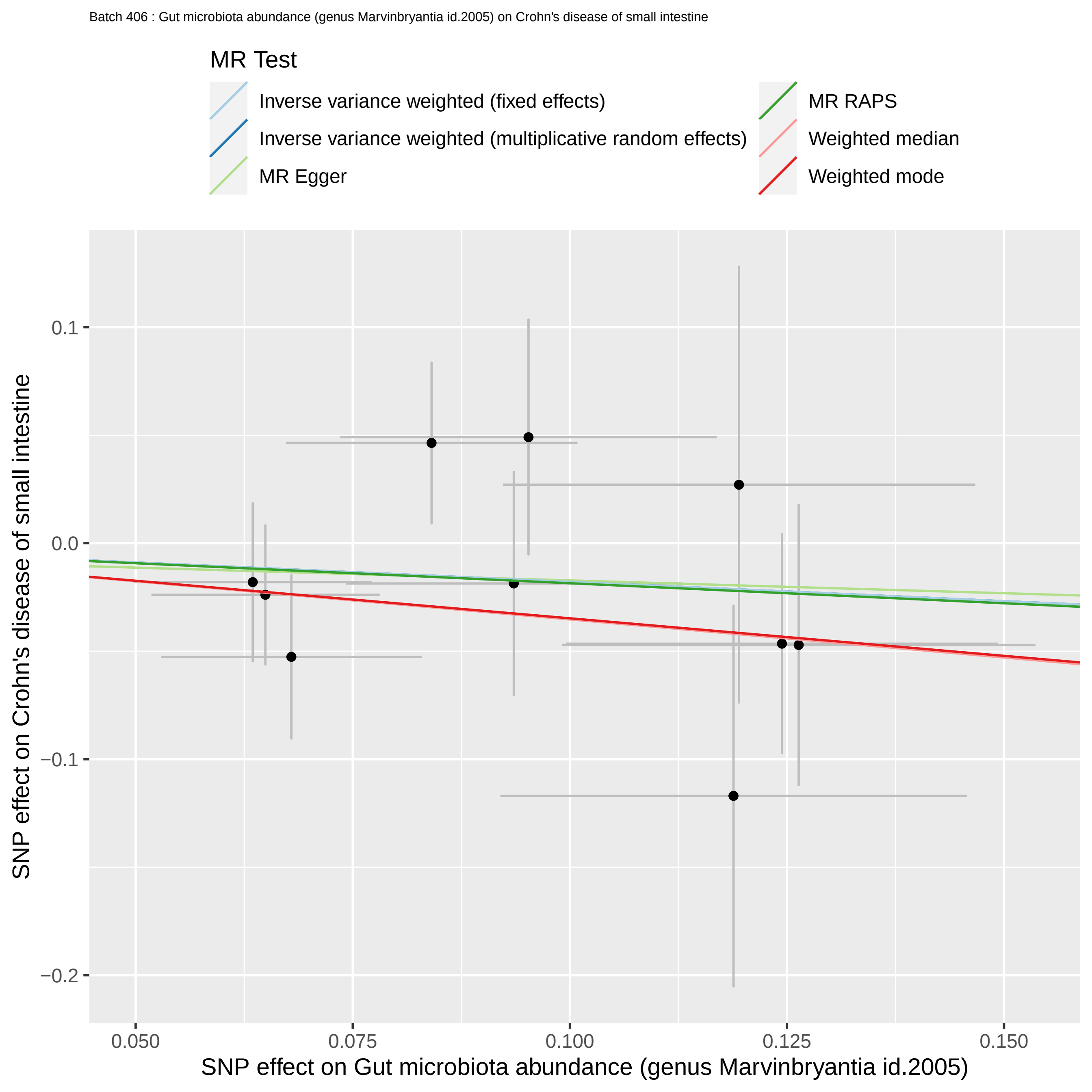
Batch 406 : Gut microbiota abundance (genus Marvinbryantia id.2005) on Crohn's disease of small intestine
MR Test
Inverse variance weighted (fixed effects)
Inverse variance weighted (multiplicative random effects)
MR Egger
MR RAPS
Weighted median
Weighted mode
0.1
0.0
−0.1
−0.2
0.050
0.075
0.100
0.125
0.150
SNP effect on Crohn's disease of small intestine
SNP effect on Gut microbiota abundance (genus Marvinbryantia id.2005)

Batch 407 : Gut microbiota abundance (genus Methanobrevibacter id.123) on Crohn's disease of small intestine

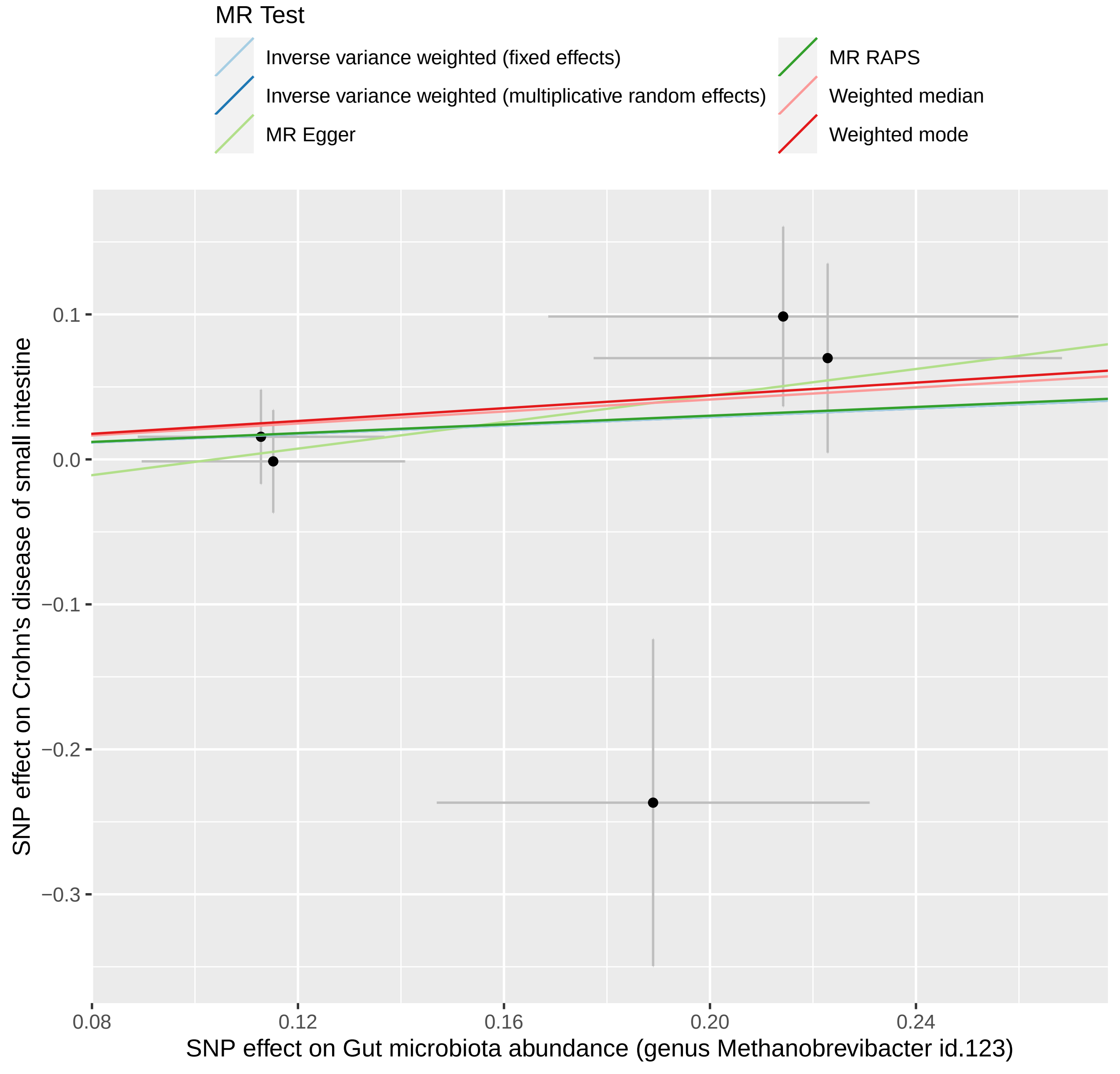

Batch 408 : Gut microbiota abundance (genus Odoribacter id.952) on Crohn's disease of small intestine

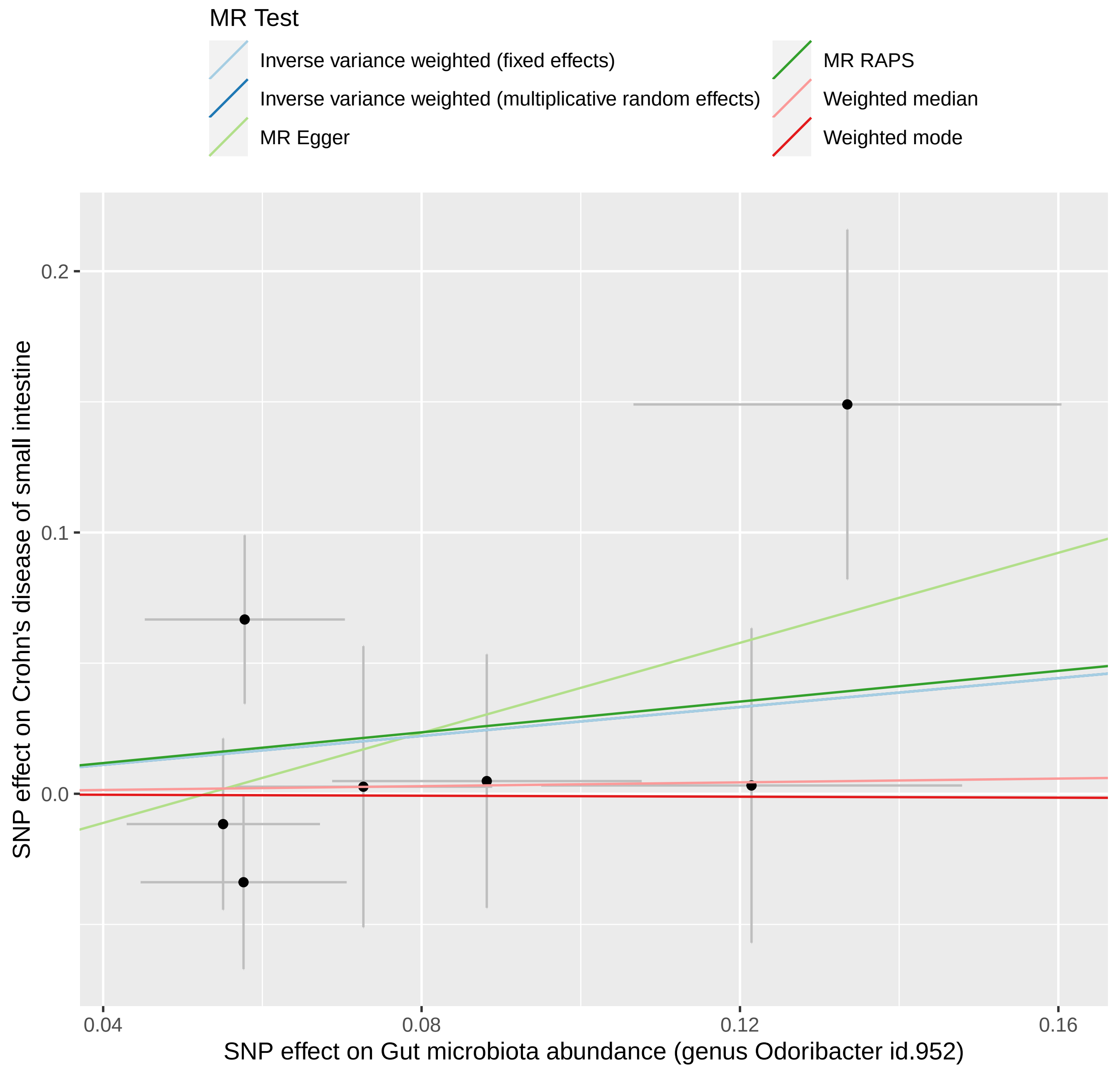

Batch 409 : Gut microbiota abundance (genus Olsenella id.822) on Crohn's disease of small intestine

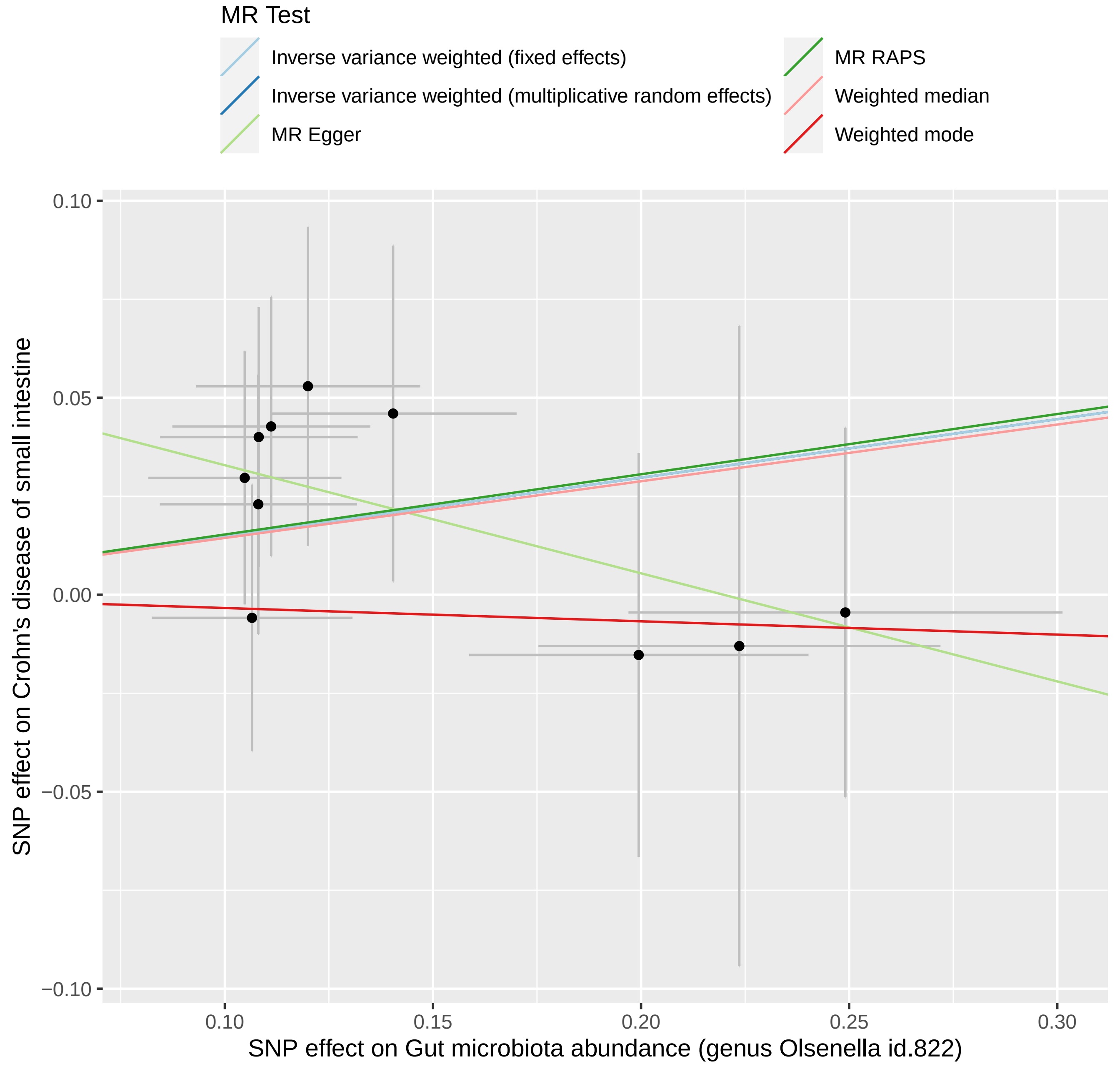

Batch 410 : Gut microbiota abundance (genus Oscillibacter id.2063) on Crohn's disease of small intestine

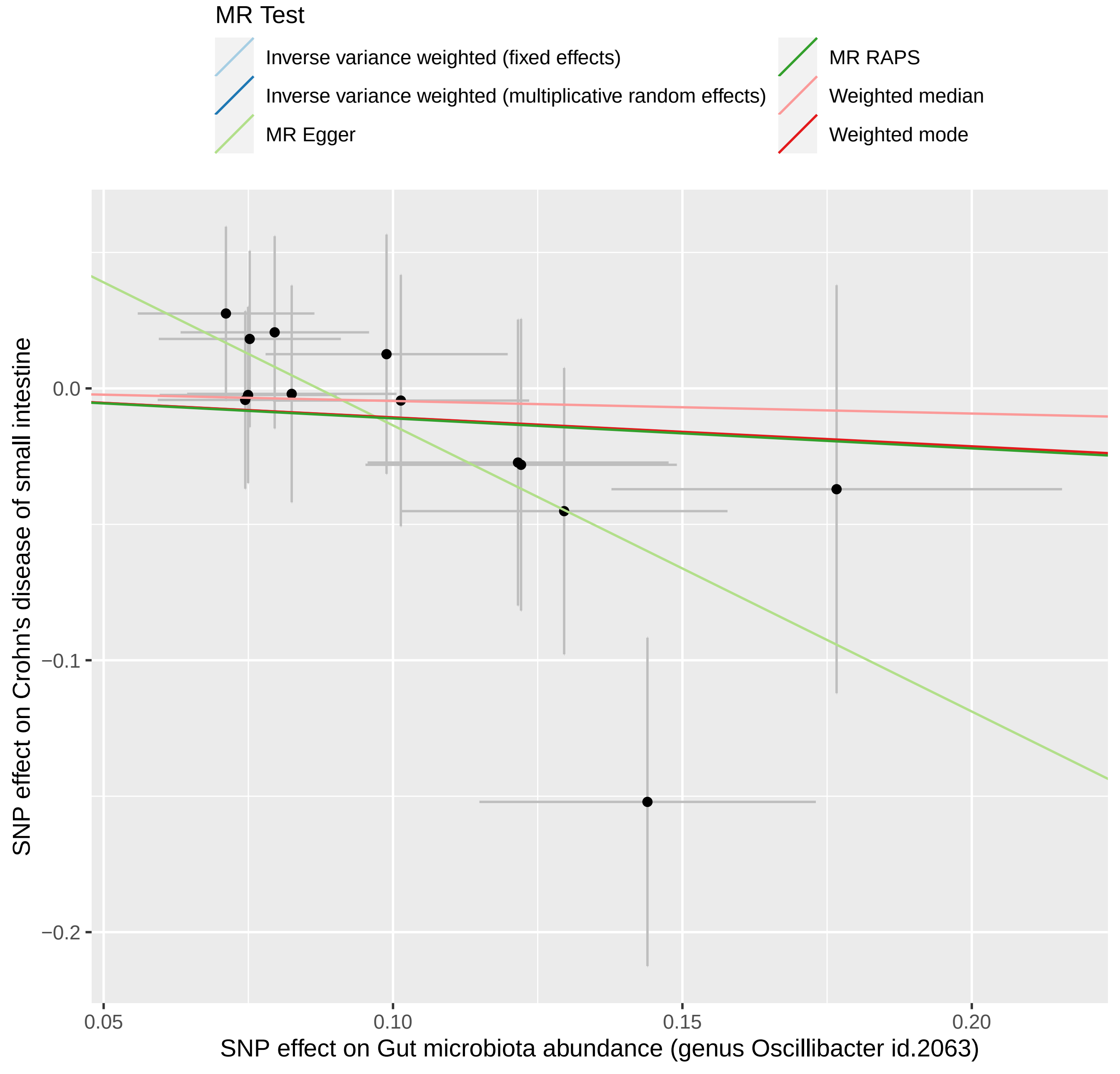

Batch 411 : Gut microbiota abundance (genus Oscillospira id.2064) on Crohn's disease of small intestine

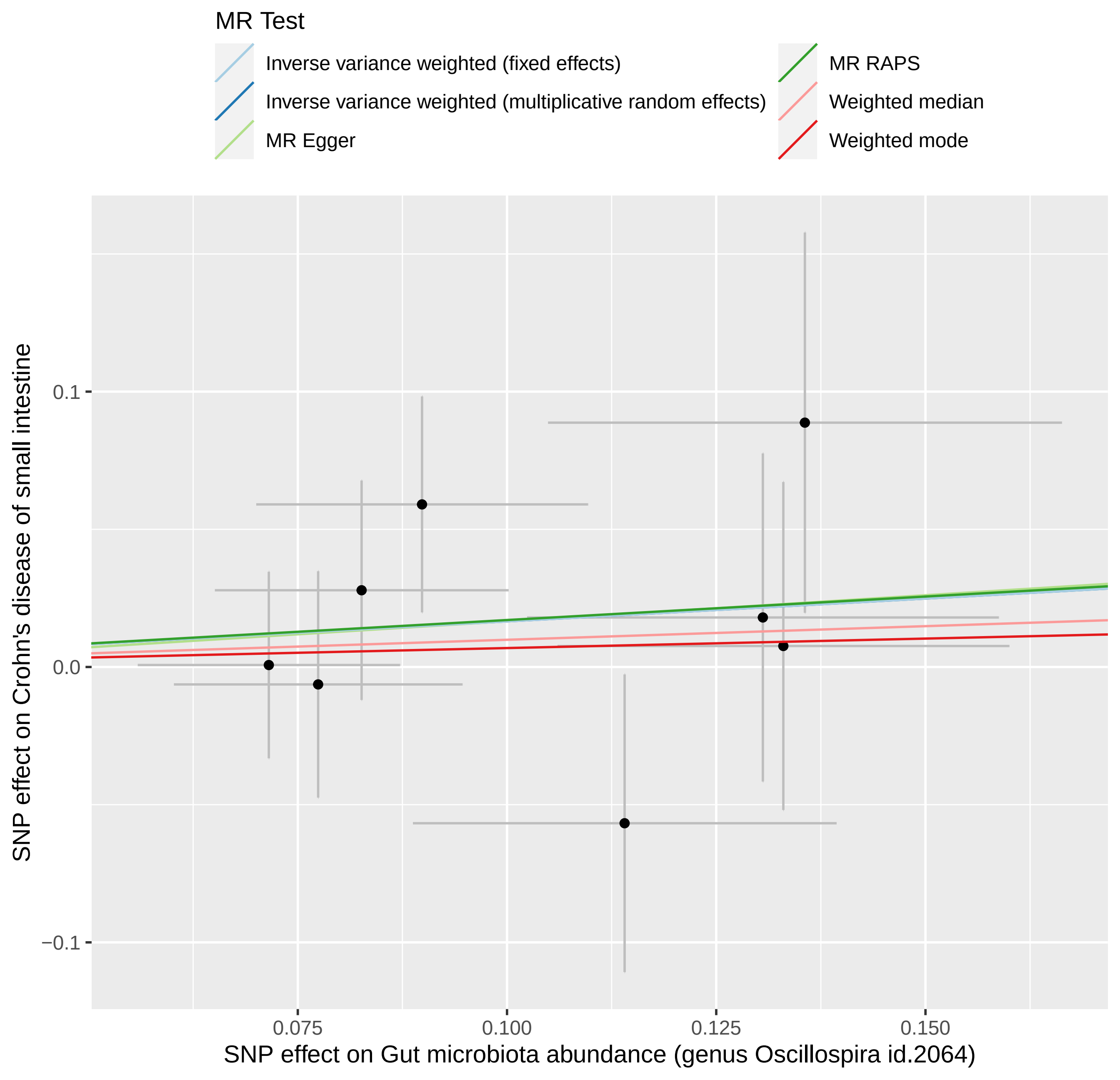

Batch 412 : Gut microbiota abundance (genus Oxalobacter id.2978) on Crohn's disease of small intestine

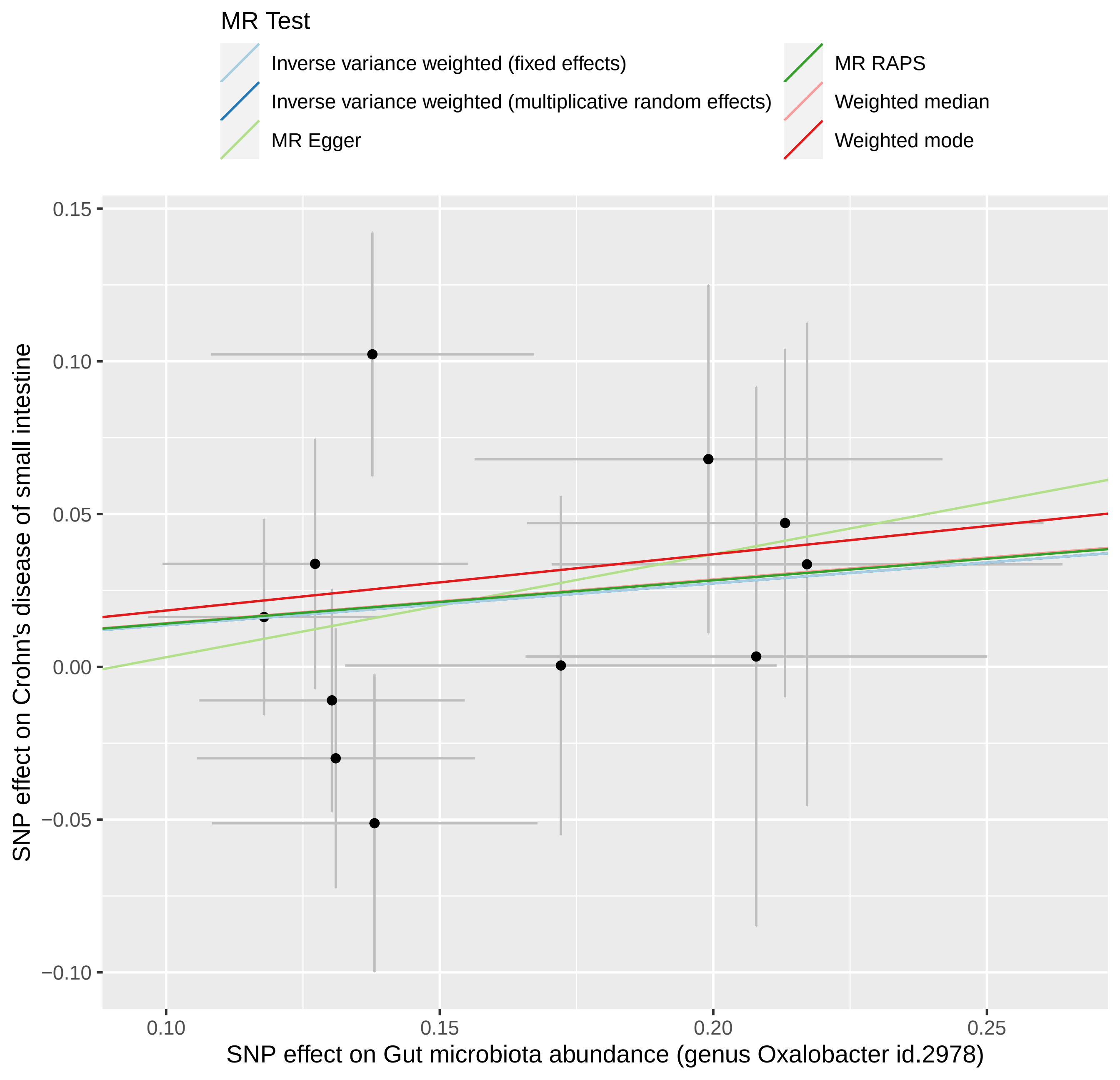

Batch 413 : Gut microbiota abundance (genus Parabacteroides id.954) on Crohn's disease of small intestine

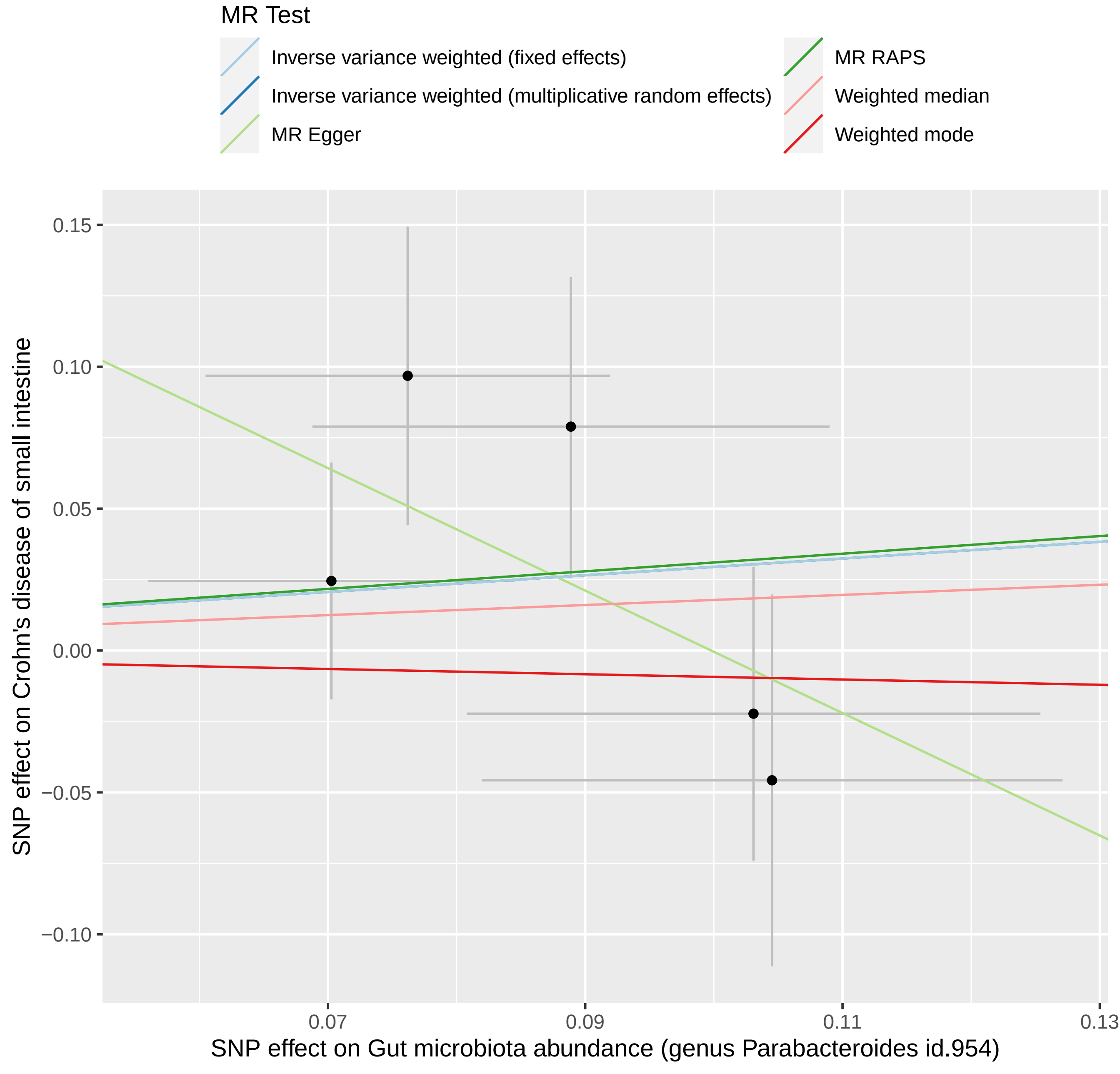

Batch 414 : Gut microbiota abundance (genus Paraprevotella id.962) on Crohn's disease of small intestine

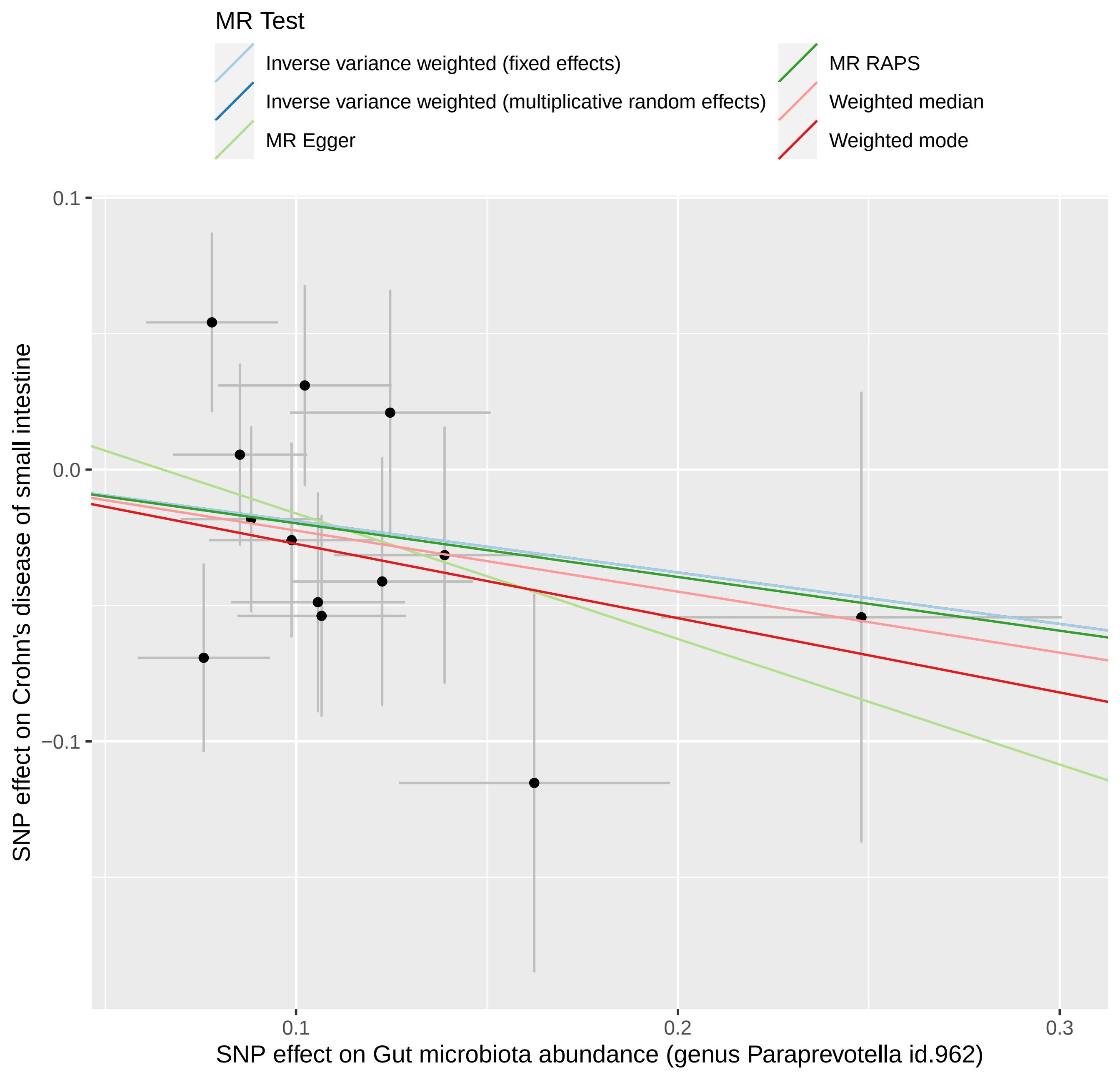

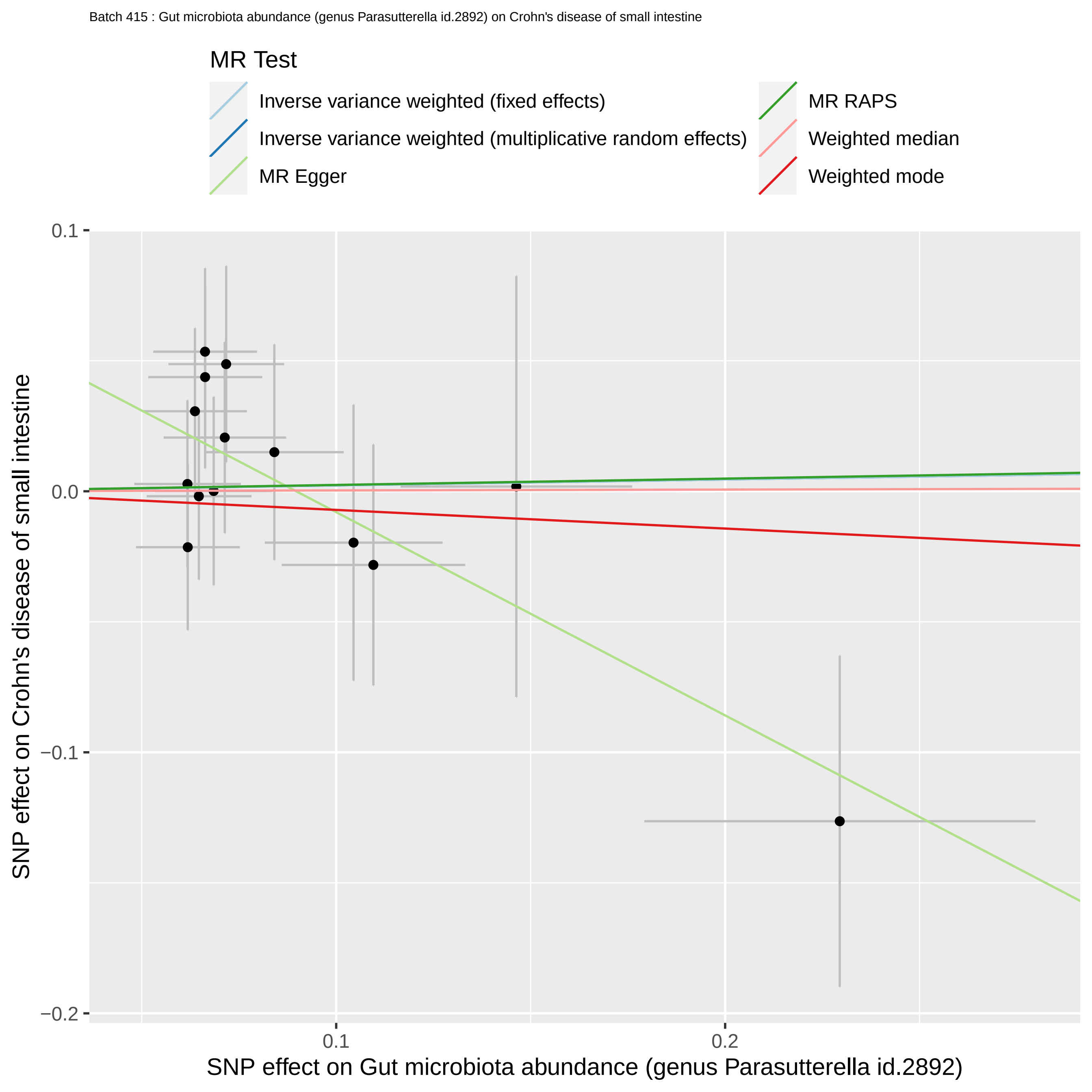

Batch 415 : Gut microbiota abundance (genus Parasutterella id.2892) on Crohn's disease of small intestine
MR Test
Inverse variance weighted (fixed effects)
Inverse variance weighted (multiplicative random effects)
MR Egger
MR RAPS
Weighted median
Weighted mode
0.1
0.0
−0.1
−0.2
0.1
0.2
SNP effect on Crohn's disease of small intestine
SNP effect on Gut microbiota abundance (genus Parasutterella id.2892)

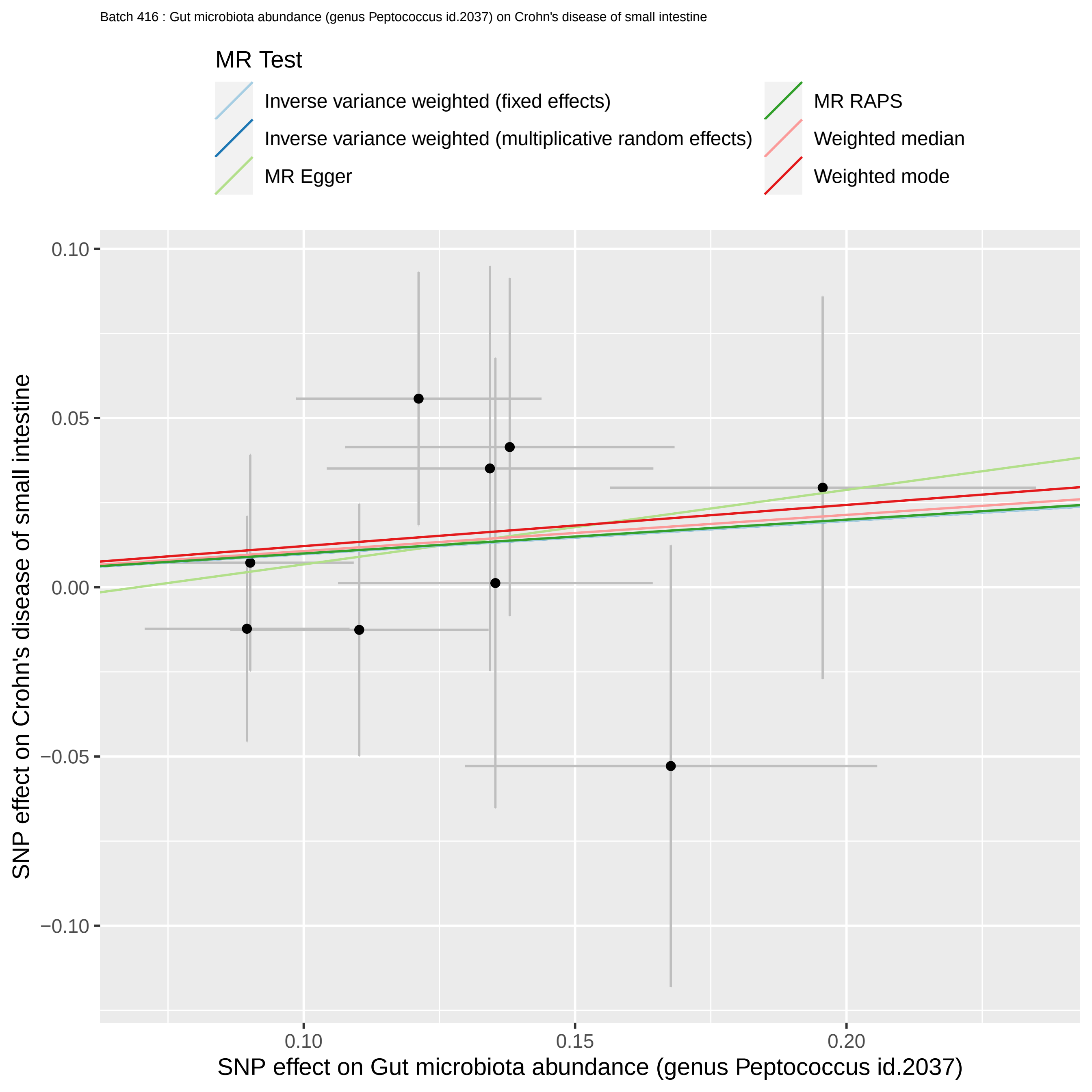
Batch 416 : Gut microbiota abundance (genus Peptococcus id.2037) on Crohn's disease of small intestine
MR Test
Inverse variance weighted (fixed effects)
Inverse variance weighted (multiplicative random effects)
MR Egger
MR RAPS
Weighted median
Weighted mode
0.10
0.05
0.00
−0.05
−0.10
0.10
0.15
0.20
SNP effect on Crohn's disease of small intestine
SNP effect on Gut microbiota abundance (genus Peptococcus id.2037)

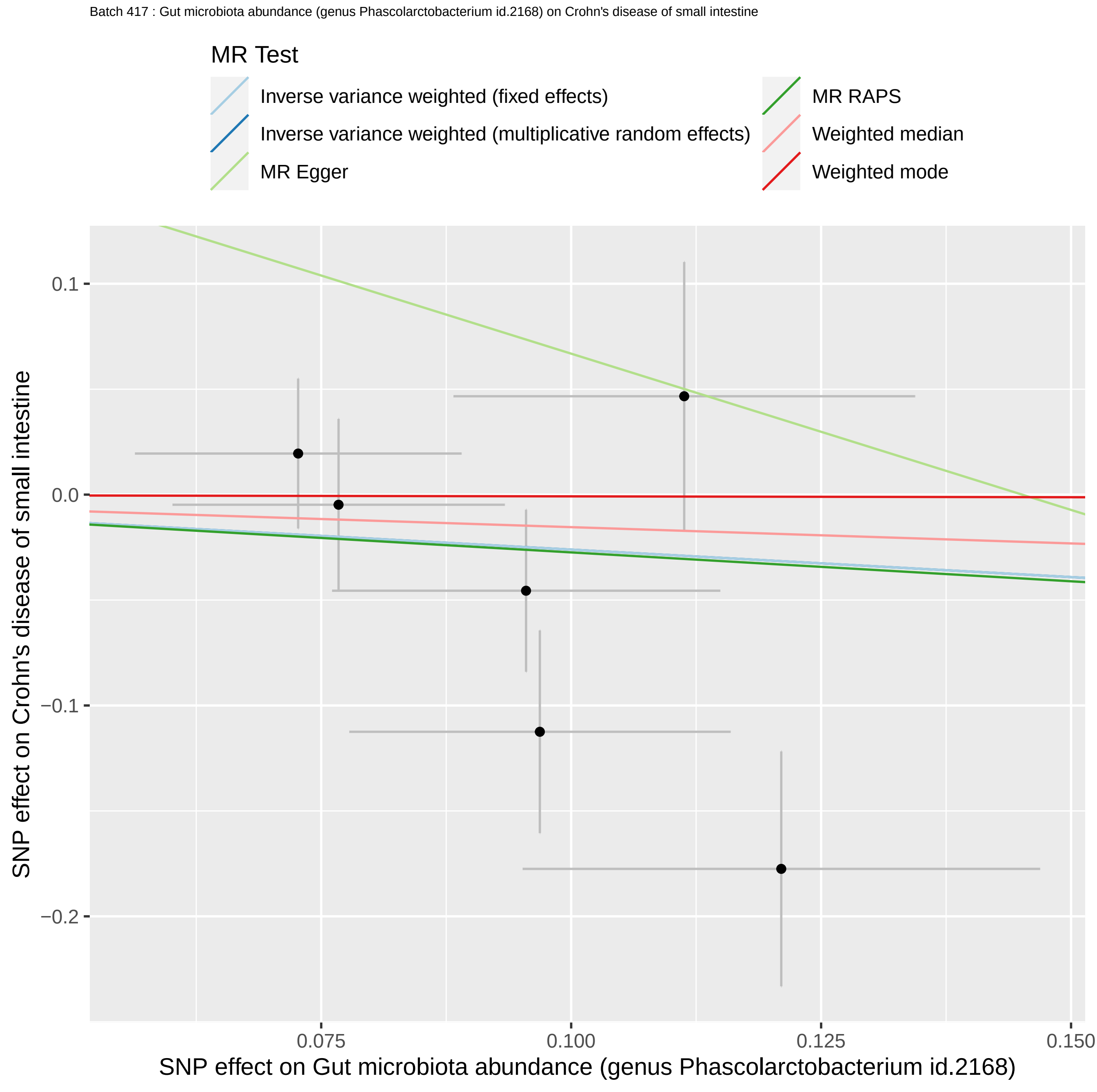

Batch 417 : Gut microbiota abundance (genus Phascolarctobacterium id.2168) on Crohn's disease of small intestine
MR Test
Inverse variance weighted (fixed effects)
Inverse variance weighted (multiplicative random effects)
MR Egger
MR RAPS
Weighted median
Weighted mode
SNP effect on Crohn's disease of small intestine
0.1
0.0
−0.1
−0.2
0.075
0.100
0.125
0.150
SNP effect on Gut microbiota abundance (genus Phascolarctobacterium id.2168)

Batch 418 : Gut microbiota abundance (genus Prevotella7 id.11182) on Crohn's disease of small intestine

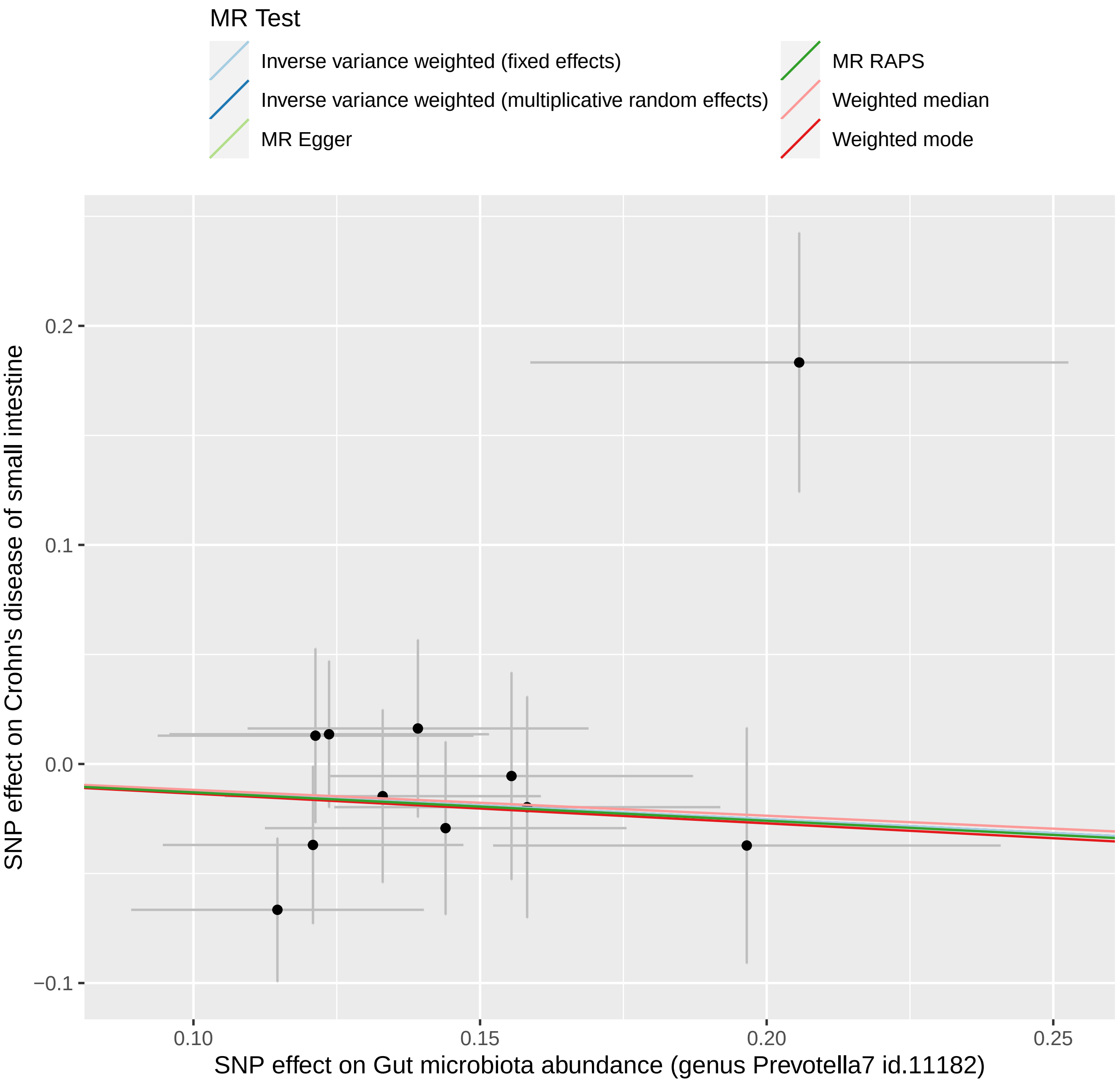

Batch 419 : Gut microbiota abundance (genus Prevotella9 id.11183) on Crohn's disease of small intestine

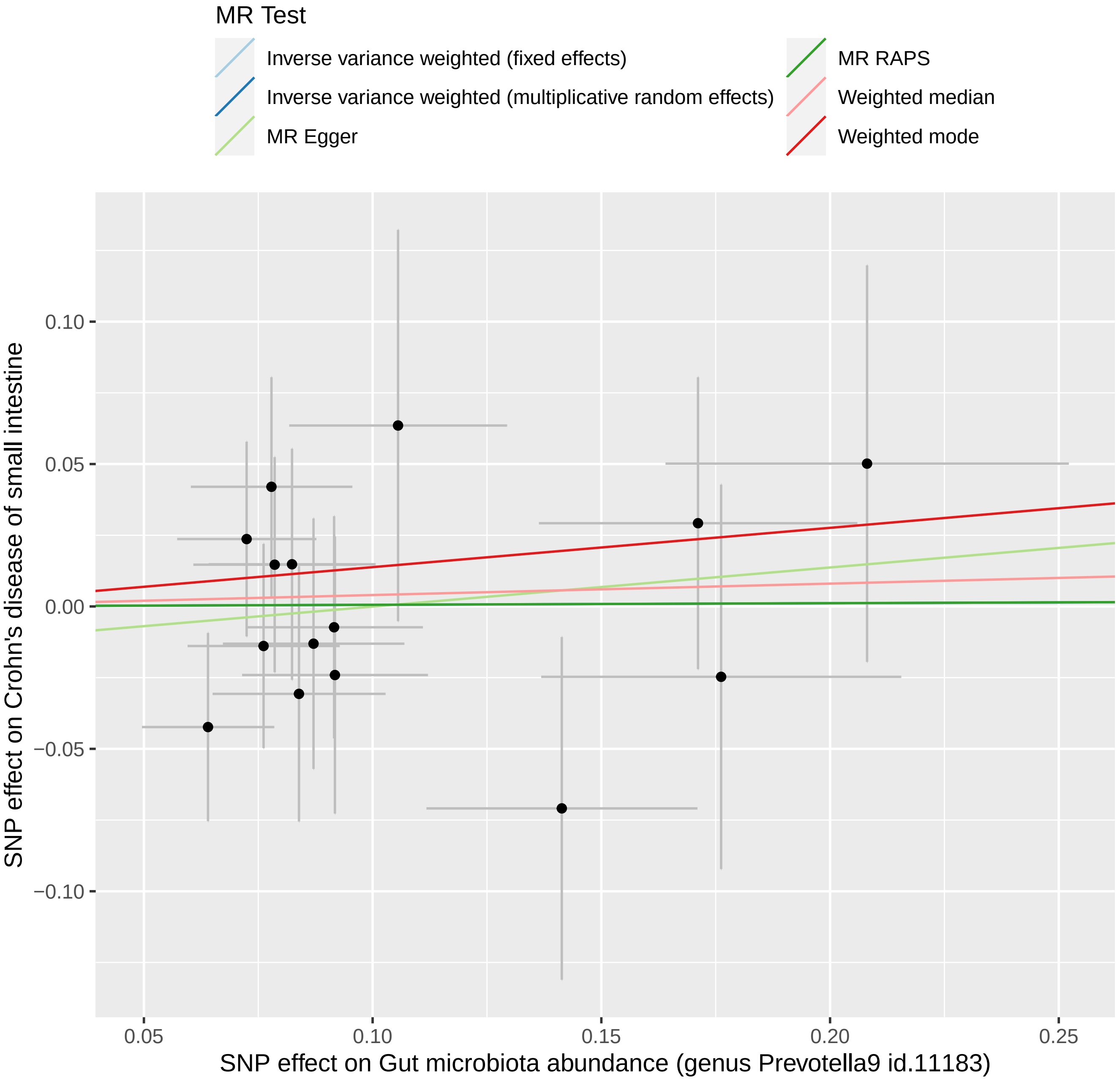

Batch 420 : Gut microbiota abundance (genus Rikenellaceae RC9 gut group id.11191) on Crohn's disease of small intestine

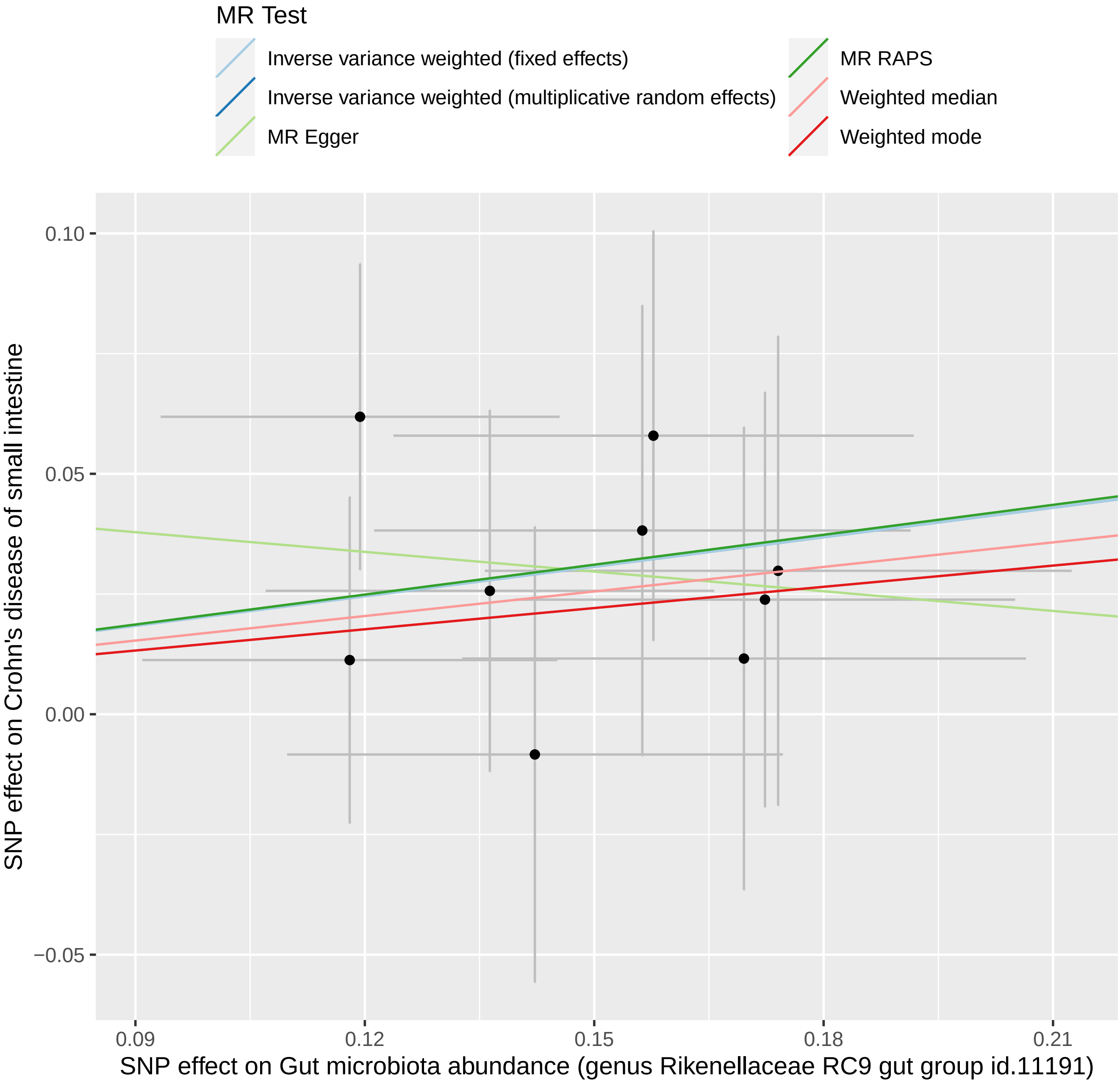

Batch 421 : Gut microbiota abundance (genus Romboutsia id.11347) on Crohn's disease of small intestine

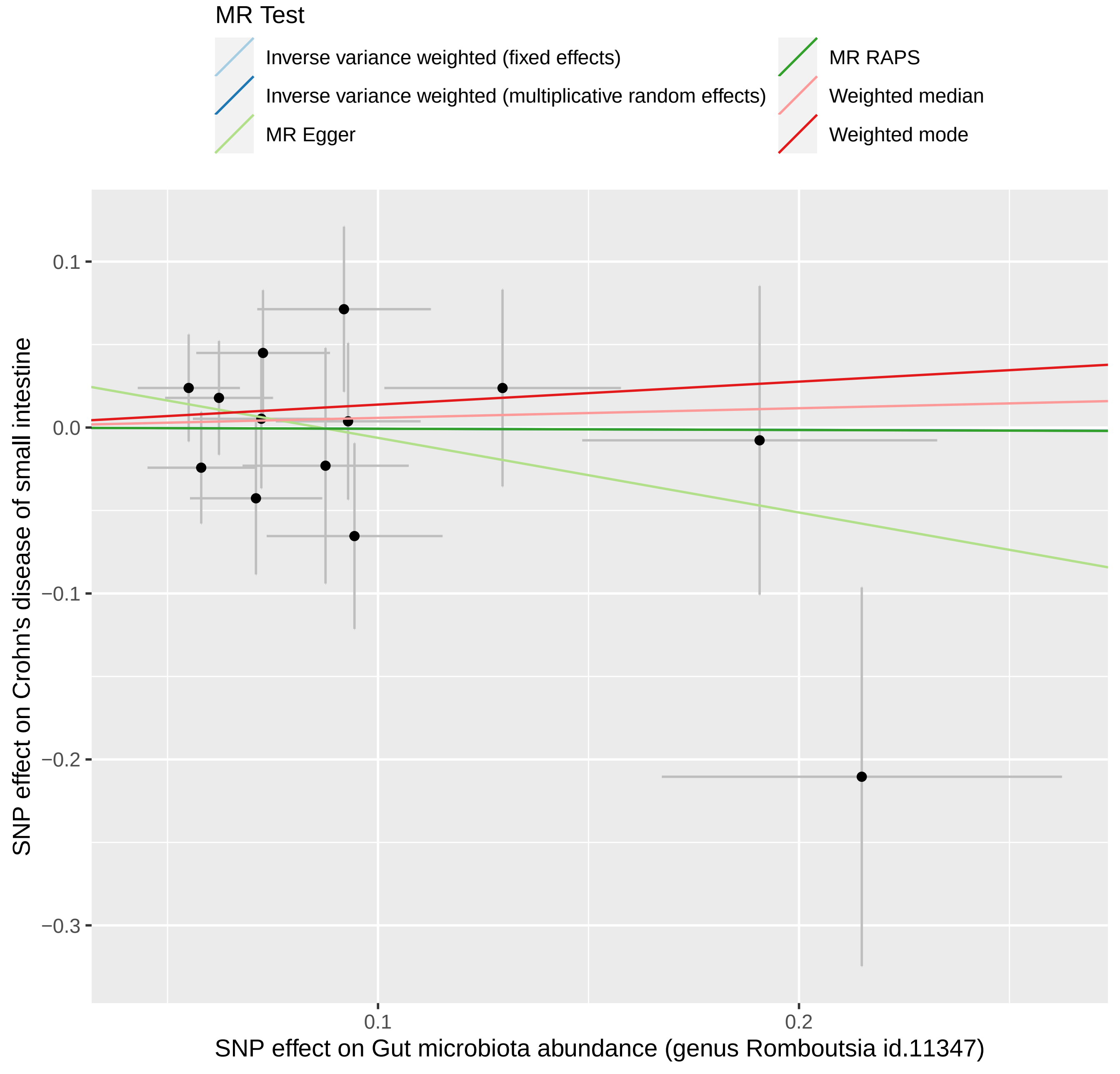

Batch 422 : Gut microbiota abundance (genus Roseburia id.2012) on Crohn's disease of small intestine

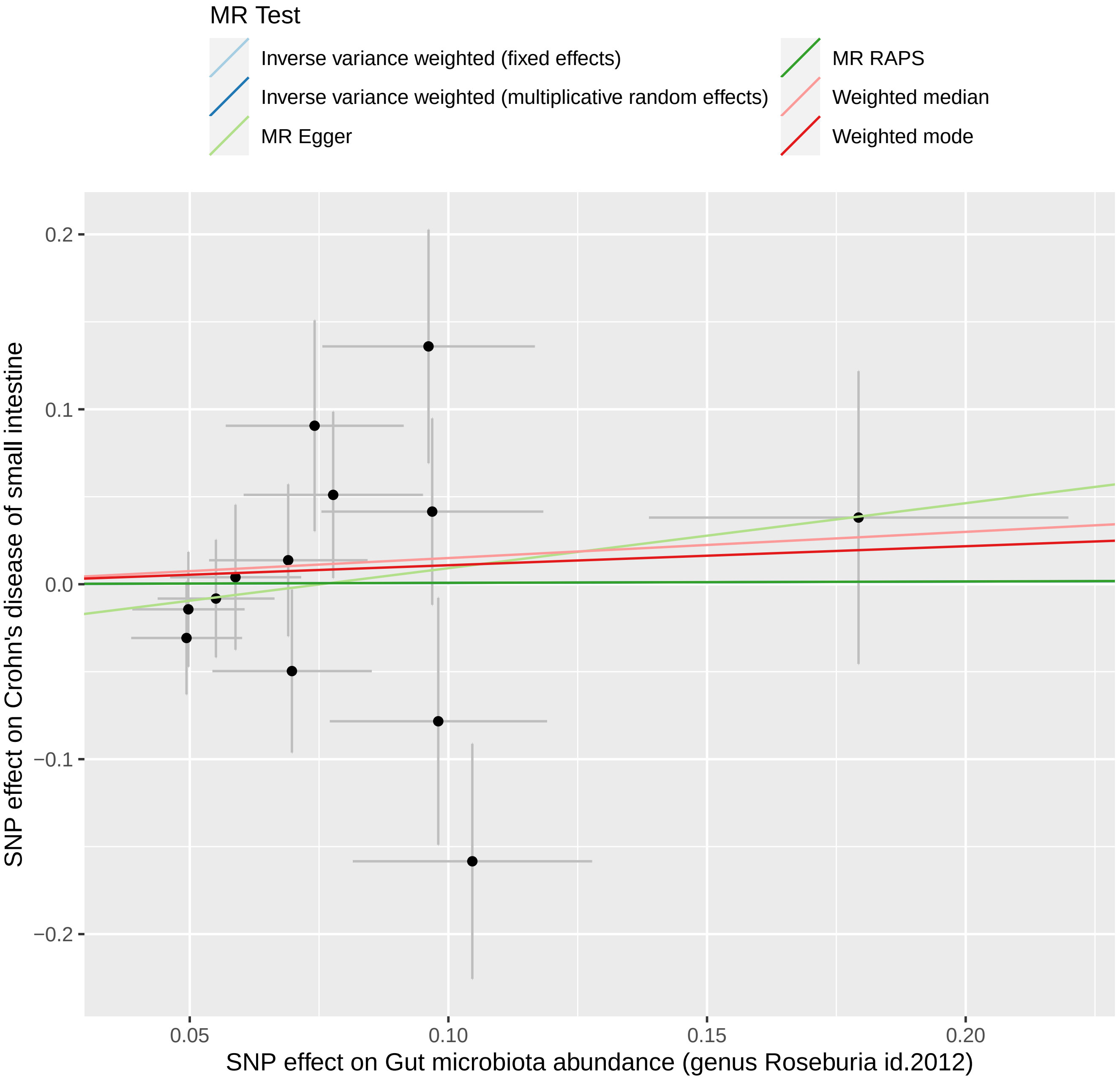

Batch 423 : Gut microbiota abundance (genus Ruminiclostridium5 id.11355) on Crohn's disease of small intestine

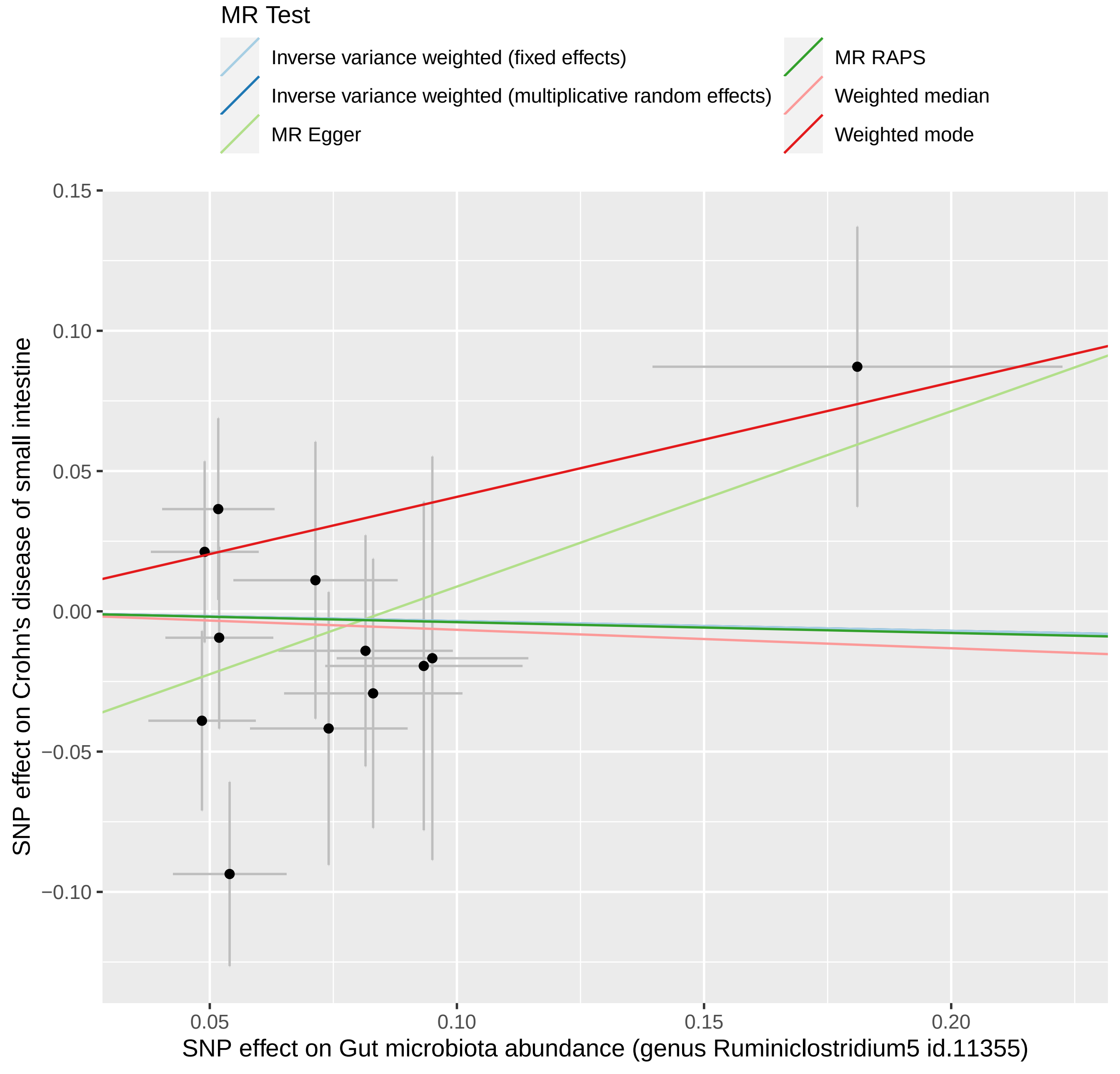

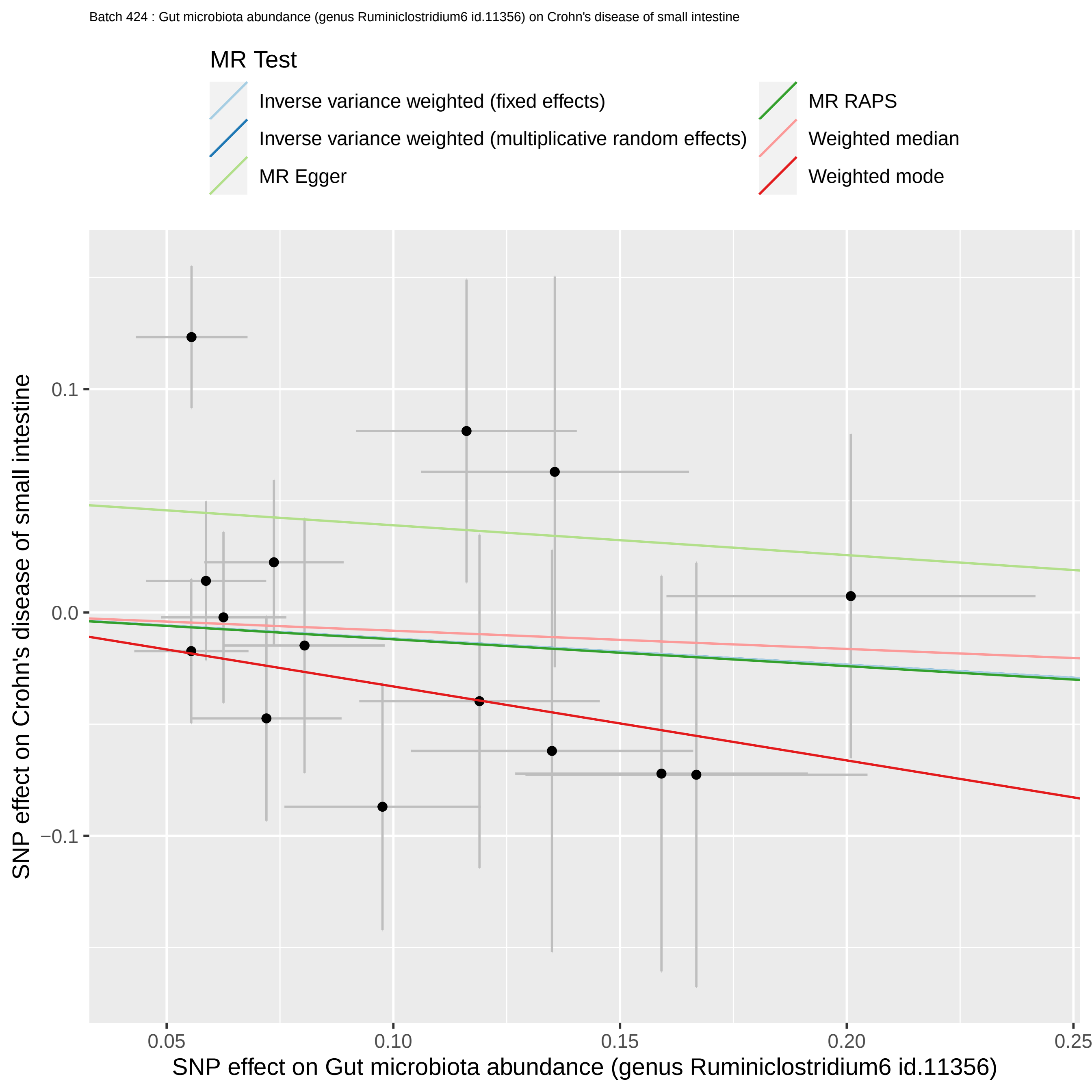

Batch 424 : Gut microbiota abundance (genus Ruminiclostridium6 id.11356) on Crohn's disease of small intestine
MR Test
Inverse variance weighted (fixed effects)
Inverse variance weighted (multiplicative random effects)
MR Egger
MR RAPS
Weighted median
Weighted mode
0.1
0.0
−0.1
0.05
0.10
0.15
0.20
0.25
SNP effect on Crohn's disease of small intestine
SNP effect on Gut microbiota abundance (genus Ruminiclostridium6 id.11356)

Batch 425 : Gut microbiota abundance (genus Ruminiclostridium9 id.11357) on Crohn's disease of small intestine

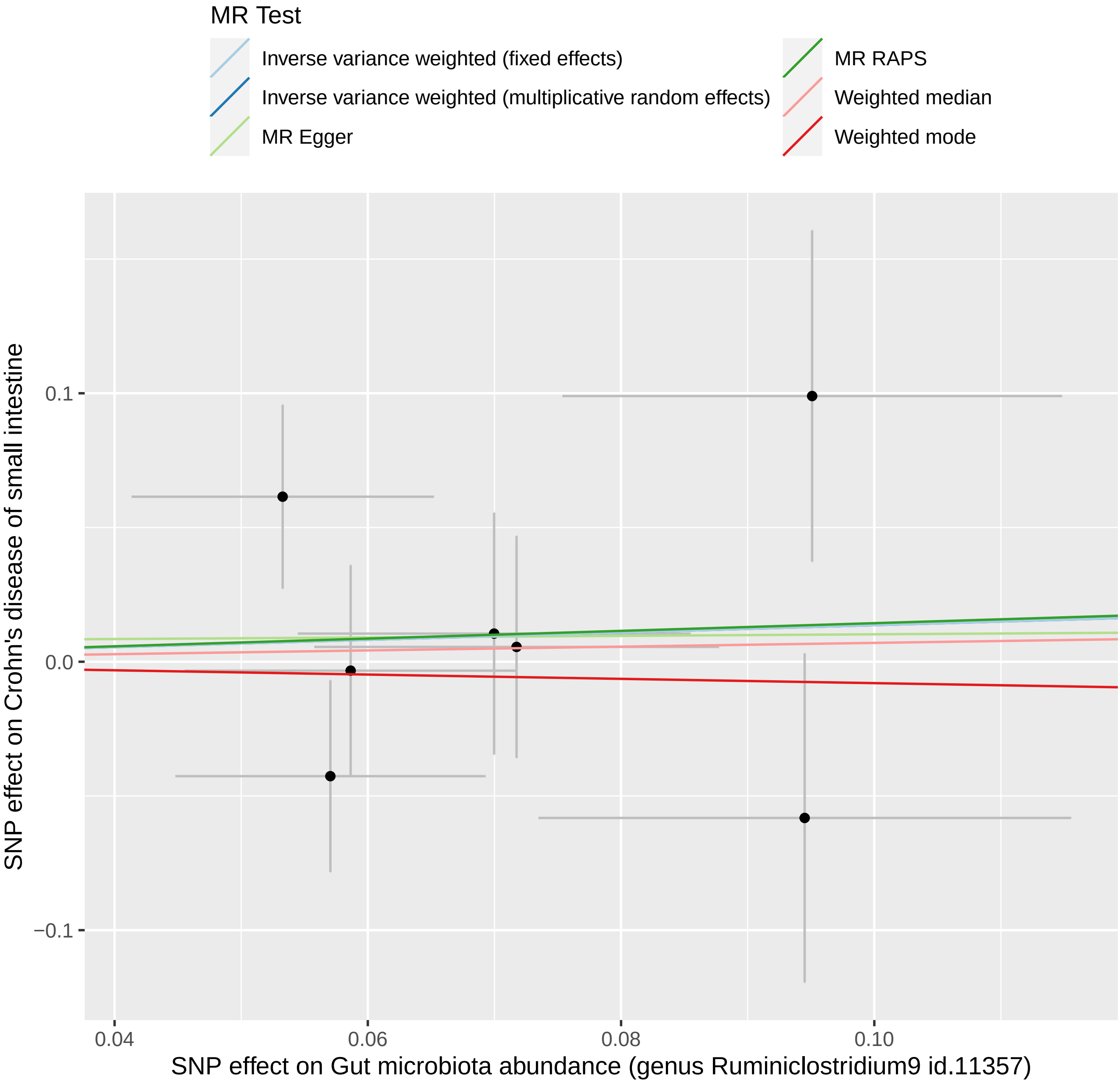

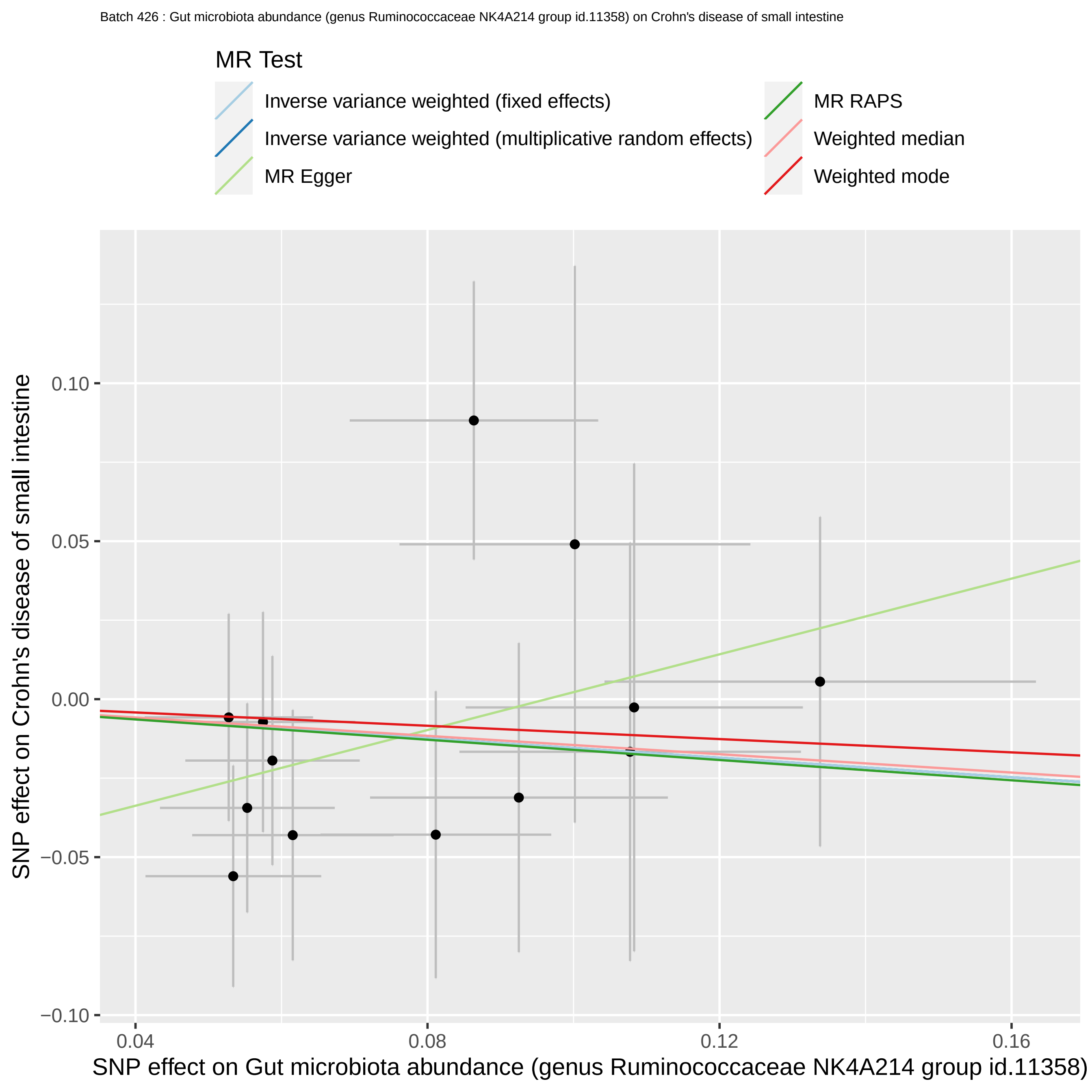
Batch 426 : Gut microbiota abundance (genus Ruminococcaceae NK4A214 group id.11358) on Crohn's disease of small intestine
MR Test
Inverse variance weighted (fixed effects)
Inverse variance weighted (multiplicative random effects)
MR Egger
MR RAPS
Weighted median
Weighted mode
SNP effect on Crohn's disease of small intestine
0.10
0.05
0.00
−0.05
−0.10
0.04
0.08
0.12
0.16
SNP effect on Gut microbiota abundance (genus Ruminococcaceae NK4A214 group id.11358)

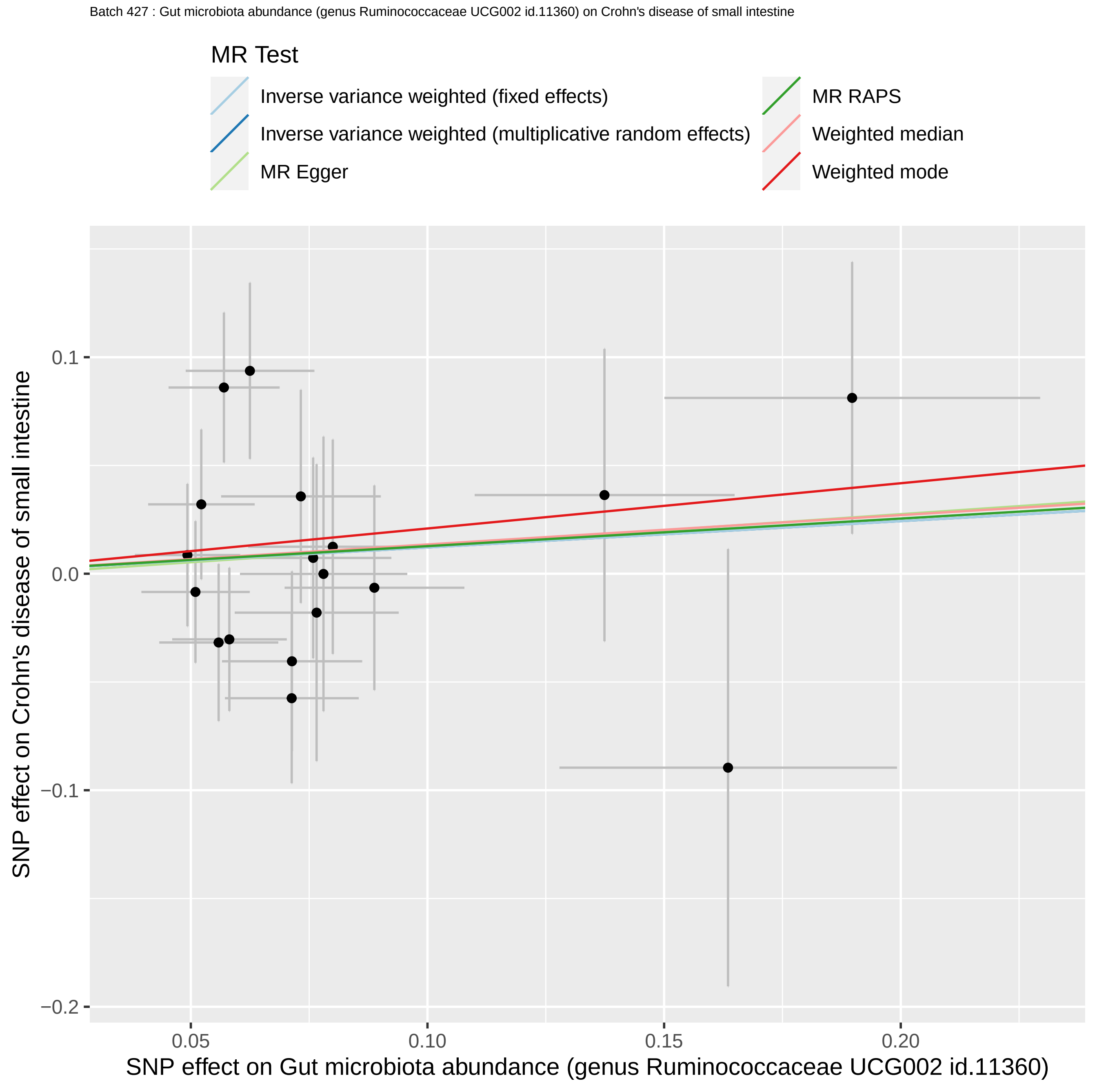

Batch 427 : Gut microbiota abundance (genus Ruminococcaceae UCG002 id.11360) on Crohn's disease of small intestine
MR Test
Inverse variance weighted (fixed effects)
Inverse variance weighted (multiplicative random effects)
MR Egger
MR RAPS
Weighted median
Weighted mode
SNP effect on Crohn's disease of small intestine
0.1
0.0
−0.1
−0.2
0.05
0.10
0.15
0.20
SNP effect on Gut microbiota abundance (genus Ruminococcaceae UCG002 id.11360)

Batch 428 : Gut microbiota abundance (genus Ruminococcaceae UCG003 id.11361) on Crohn's disease of small intestine

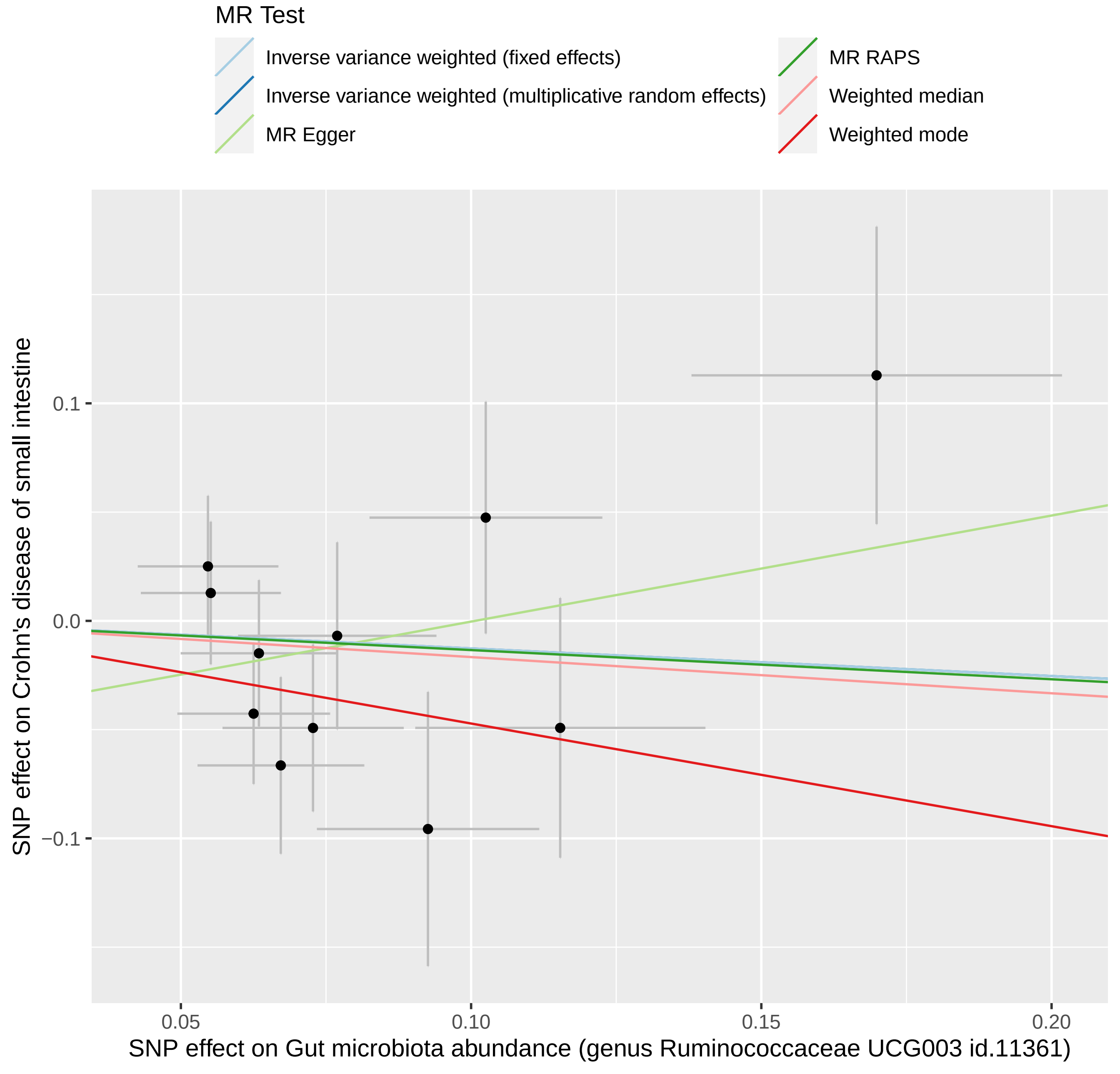

Batch 429 : Gut microbiota abundance (genus Ruminococcaceae UCG004 id.11362) on Crohn's disease of small intestine

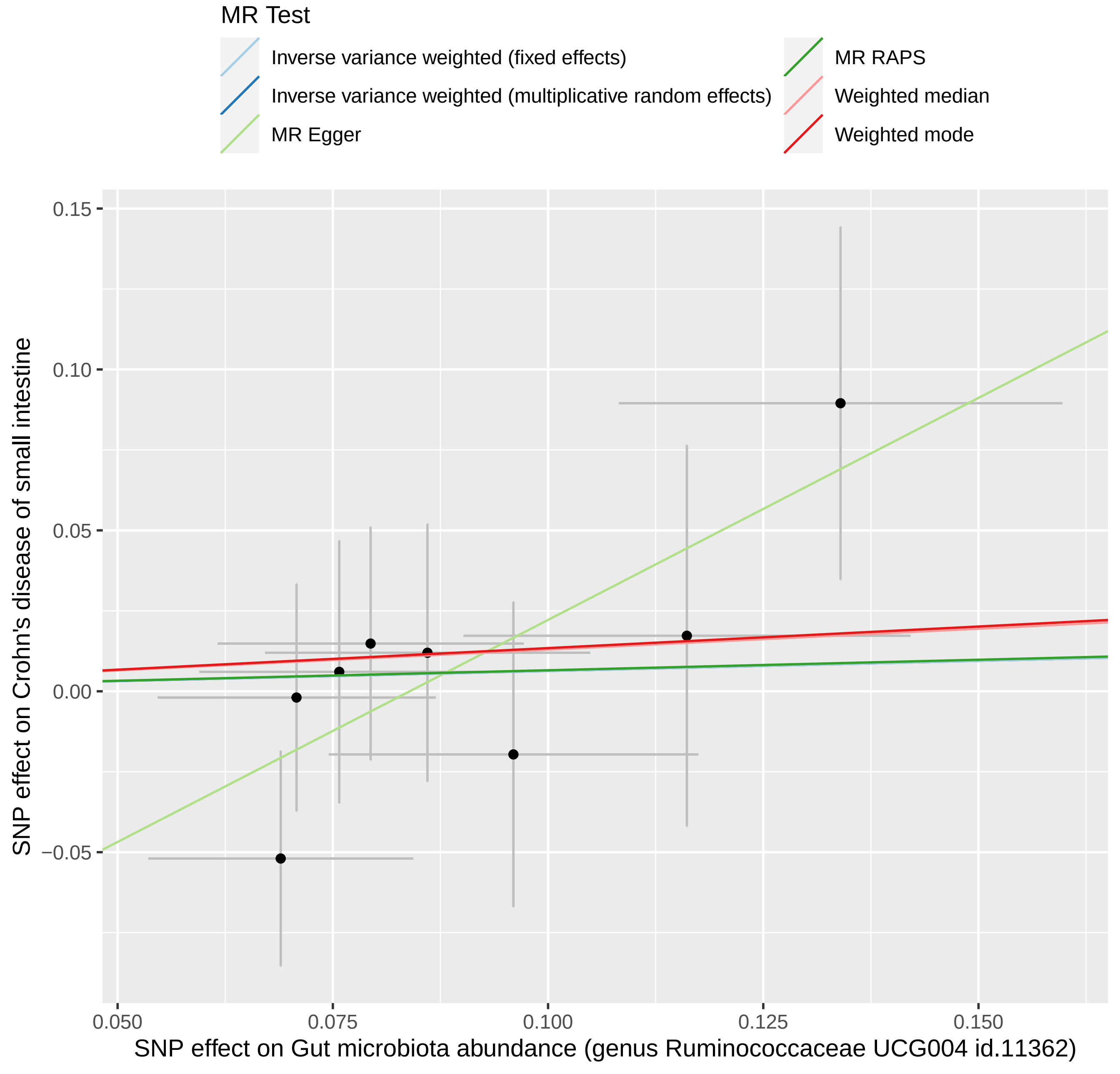

Batch 430 : Gut microbiota abundance (genus Ruminococcaceae UCG005 id.11363) on Crohn's disease of small intestine

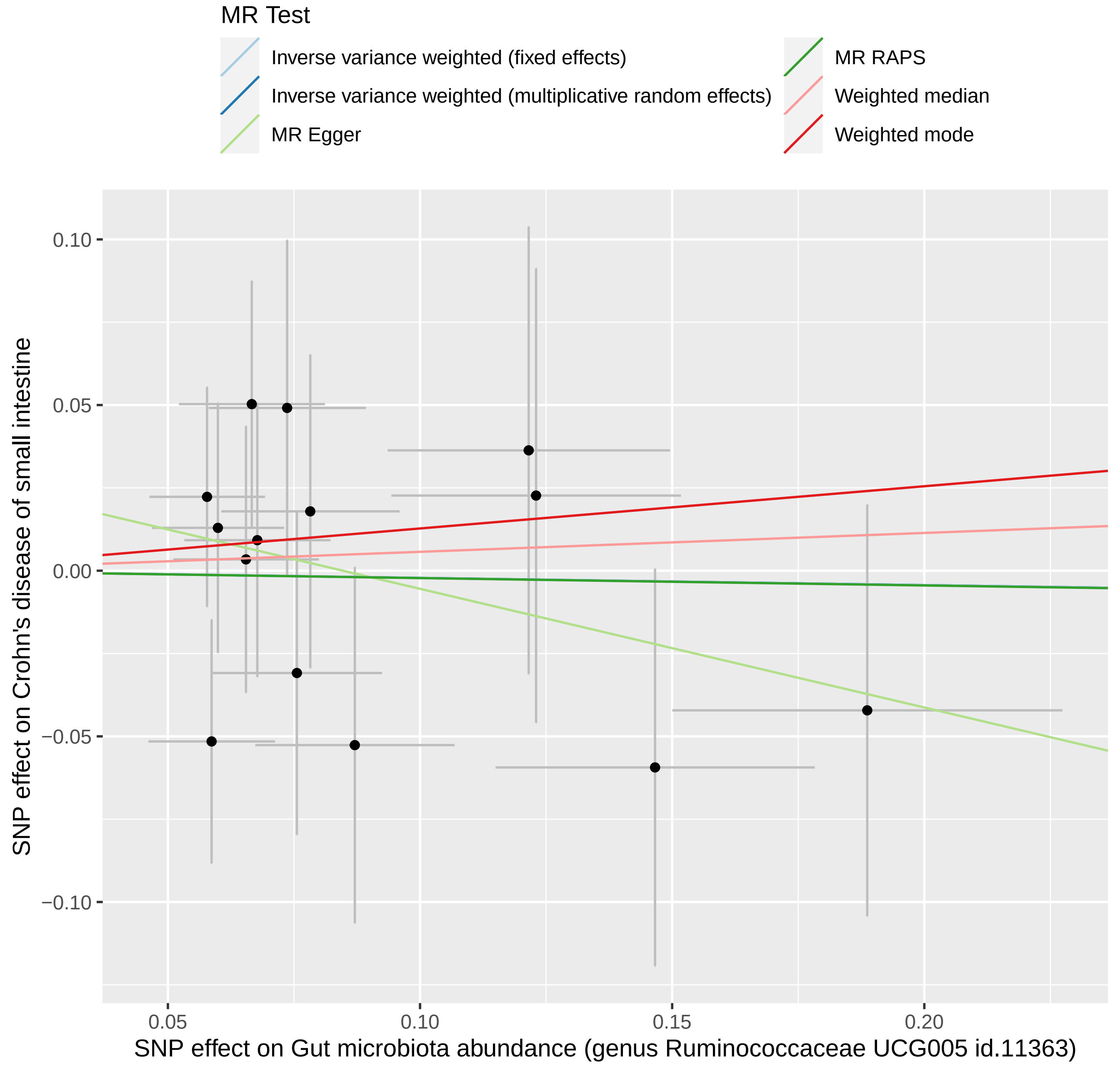

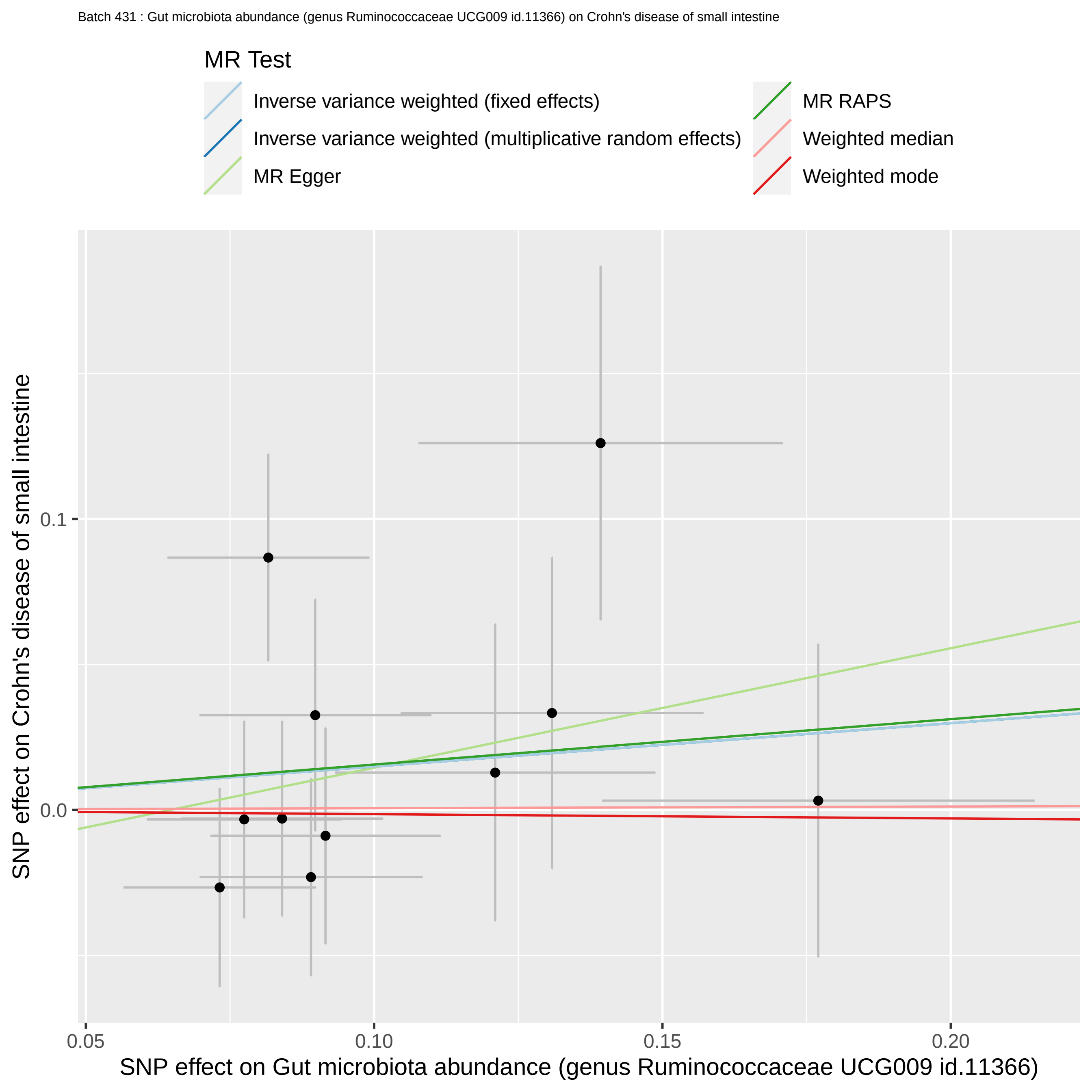
Batch 431 : Gut microbiota abundance (genus Ruminococcaceae UCG009 id.11366) on Crohn's disease of small intestine
MR Test
Inverse variance weighted (fixed effects)
Inverse variance weighted (multiplicative random effects)
MR Egger
MR RAPS
Weighted median
Weighted mode
SNP effect on Crohn's disease of small intestine
0.1
0.0
0.05
0.10
0.15
0.20
SNP effect on Gut microbiota abundance (genus Ruminococcaceae UCG009 id.11366)

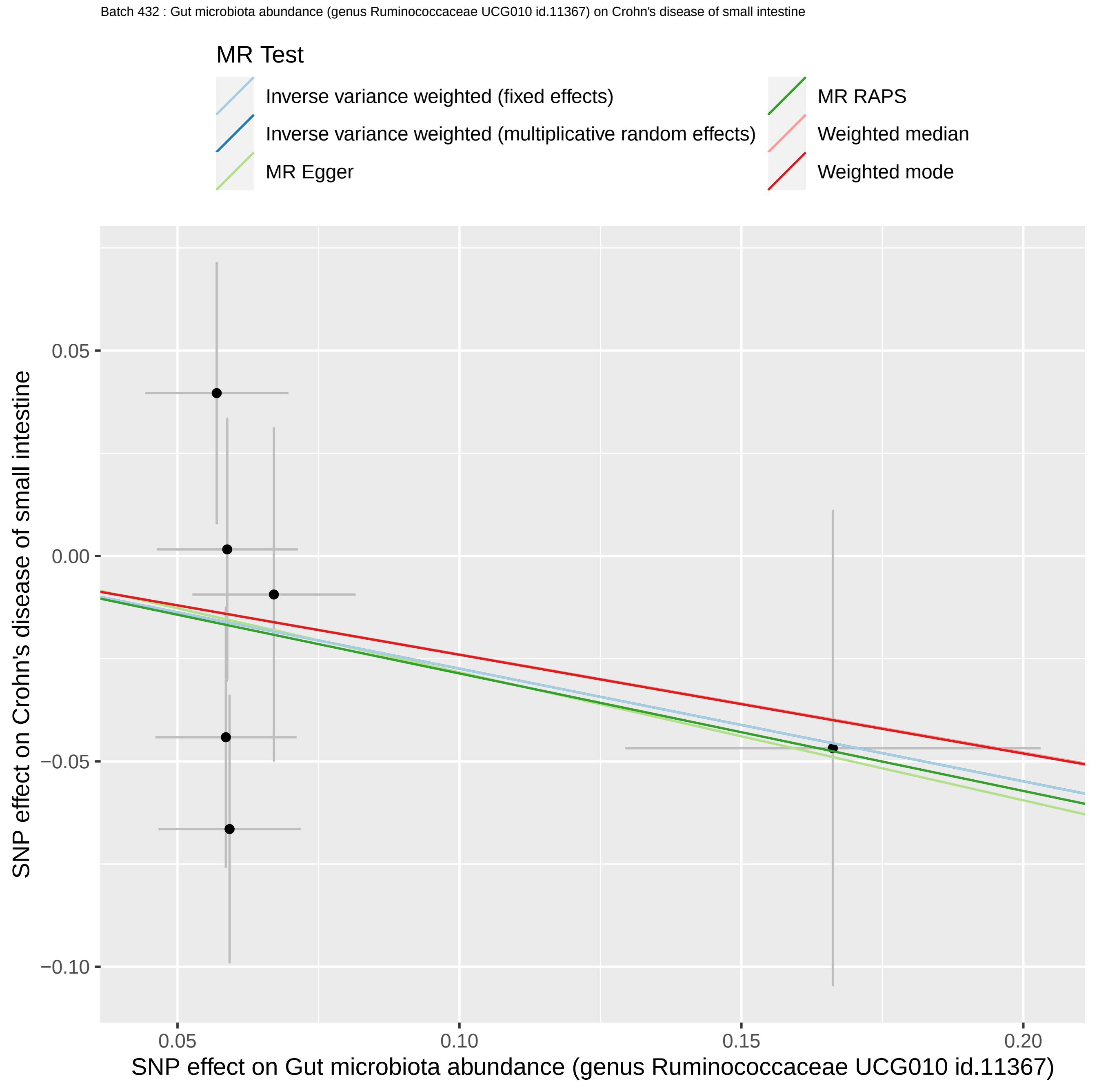
Batch 432 : Gut microbiota abundance (genus Ruminococcaceae UCG010 id.11367) on Crohn's disease of small intestine
MR Test
Inverse variance weighted (fixed effects)
Inverse variance weighted (multiplicative random effects)
MR Egger
MR RAPS
Weighted median
Weighted mode
0.05
0.00
−0.05
−0.10
0.05
0.10
0.15
0.20
SNP effect on Crohn's disease of small intestine
SNP effect on Gut microbiota abundance (genus Ruminococcaceae UCG010 id.11367)

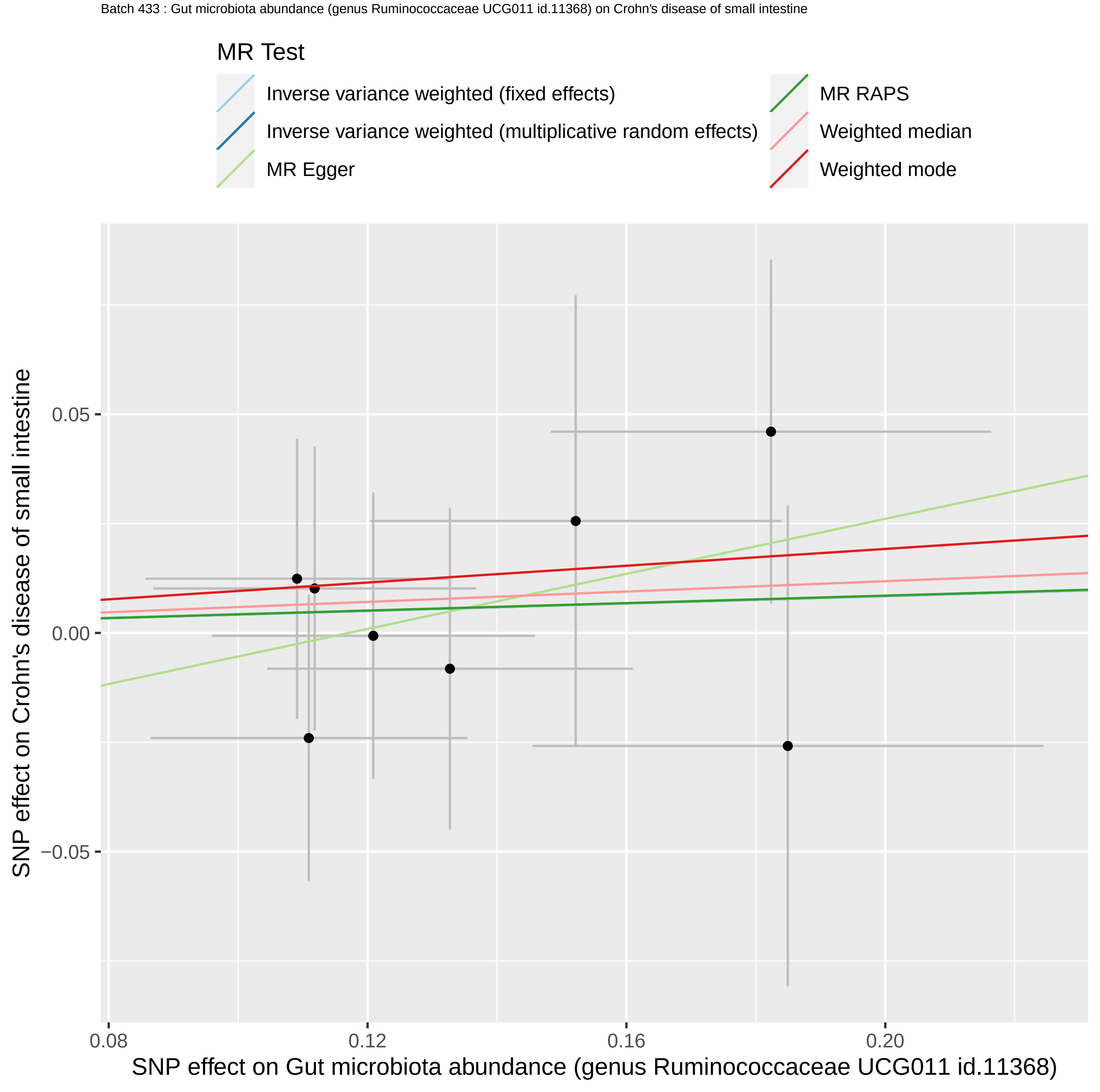
Batch 433 : Gut microbiota abundance (genus Ruminococcaceae UCG011 id.11368) on Crohn's disease of small intestine
MR Test
Inverse variance weighted (fixed effects)
Inverse variance weighted (multiplicative random effects)
MR Egger
MR RAPS
Weighted median
Weighted mode
SNP effect on Crohn's disease of small intestine
0.05
0.00
−0.05
0.08
0.12
0.16
0.20
SNP effect on Gut microbiota abundance (genus Ruminococcaceae UCG011 id.11368)

Batch 434 : Gut microbiota abundance (genus Ruminococcaceae UCG013 id.11370) on Crohn's disease of small intestine

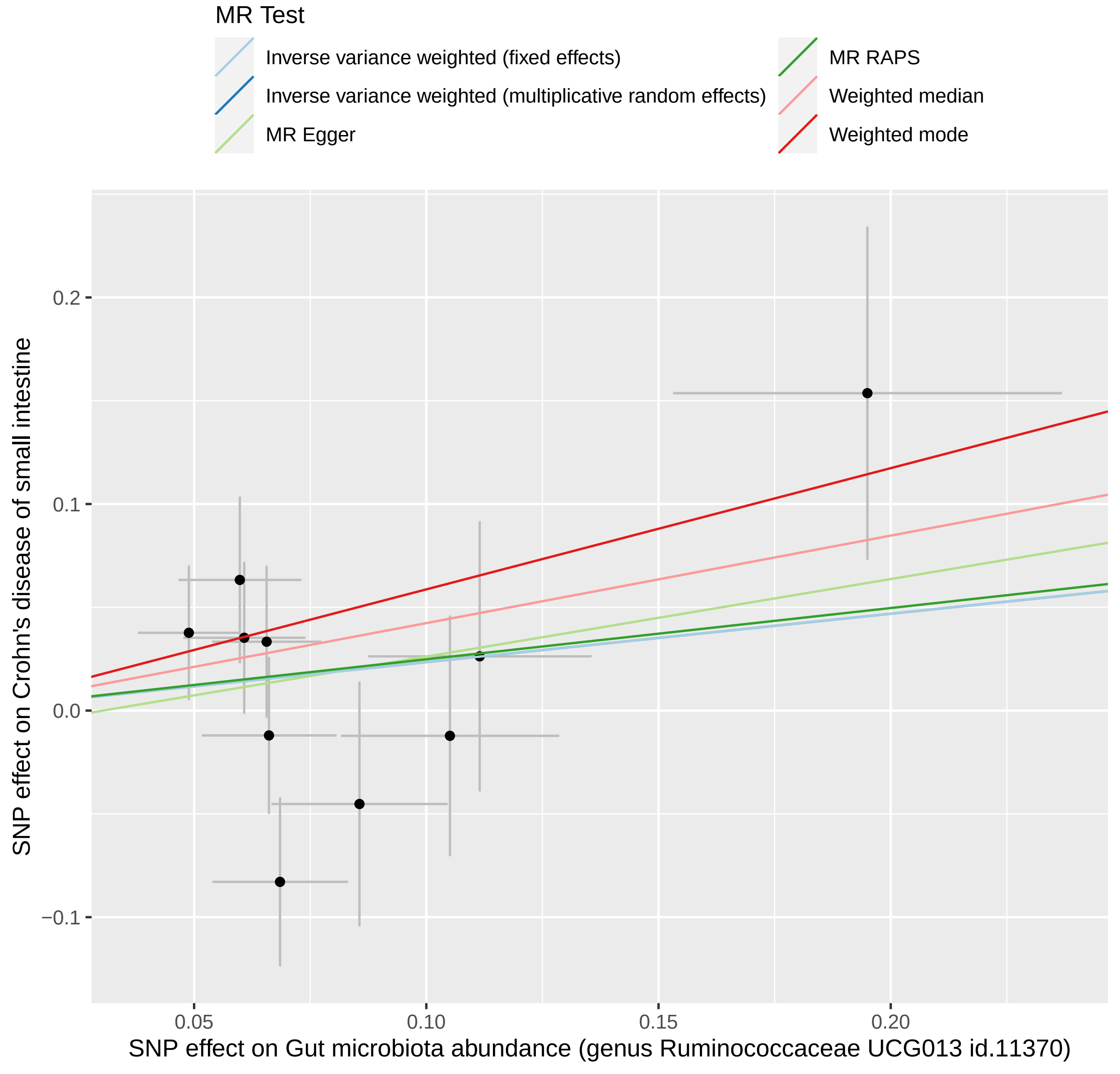

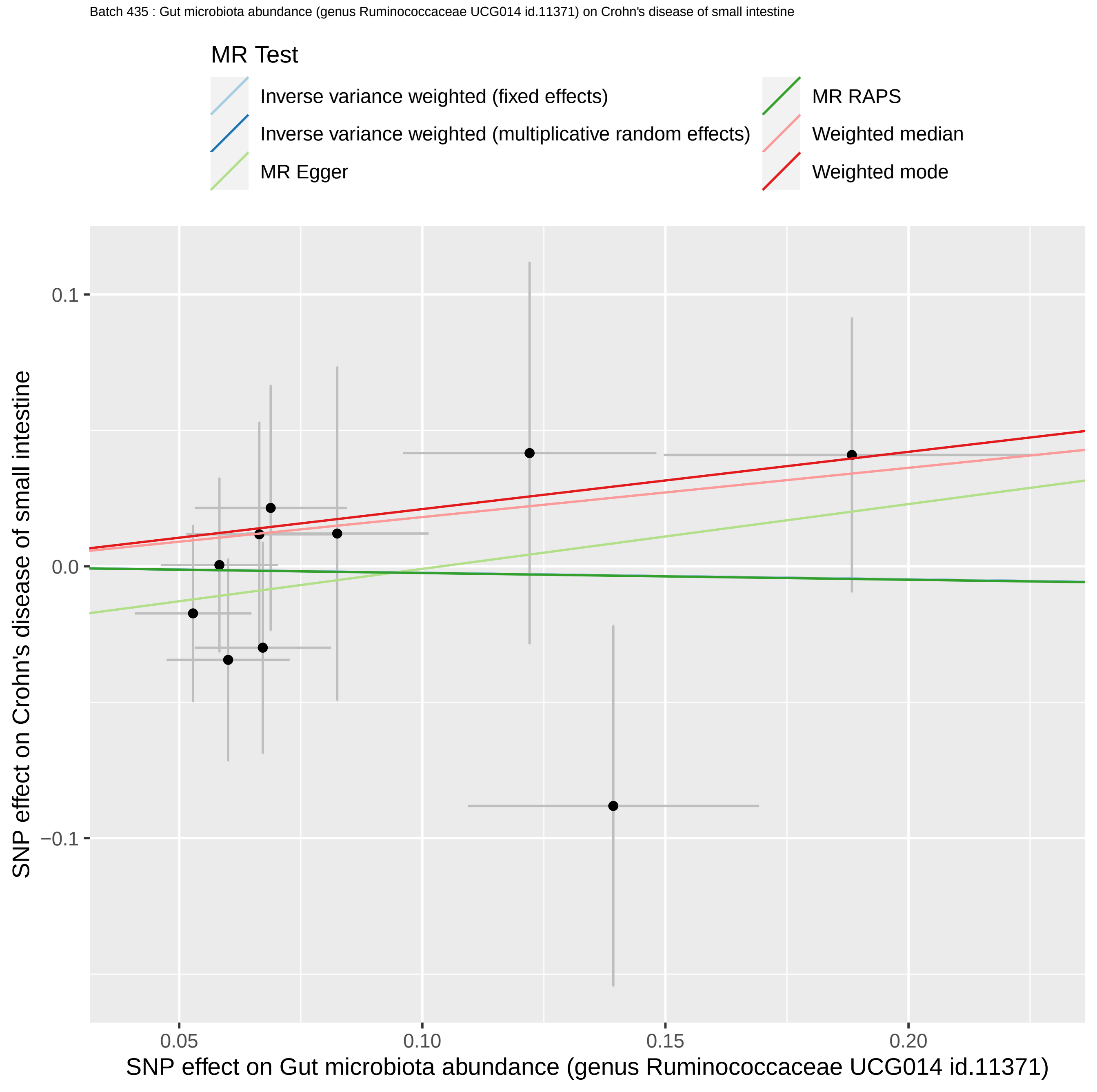

Batch 435 : Gut microbiota abundance (genus Ruminococcaceae UCG014 id.11371) on Crohn's disease of small intestine
MR Test
Inverse variance weighted (fixed effects)
Inverse variance weighted (multiplicative random effects)
MR Egger
MR RAPS
Weighted median
Weighted mode
0.1
0.0
−0.1
0.05
0.10
0.15
0.20
SNP effect on Crohn's disease of small intestine
SNP effect on Gut microbiota abundance (genus Ruminococcaceae UCG014 id.11371)

Batch 436 : Gut microbiota abundance (genus Ruminococcus1 id.11373) on Crohn's disease of small intestine

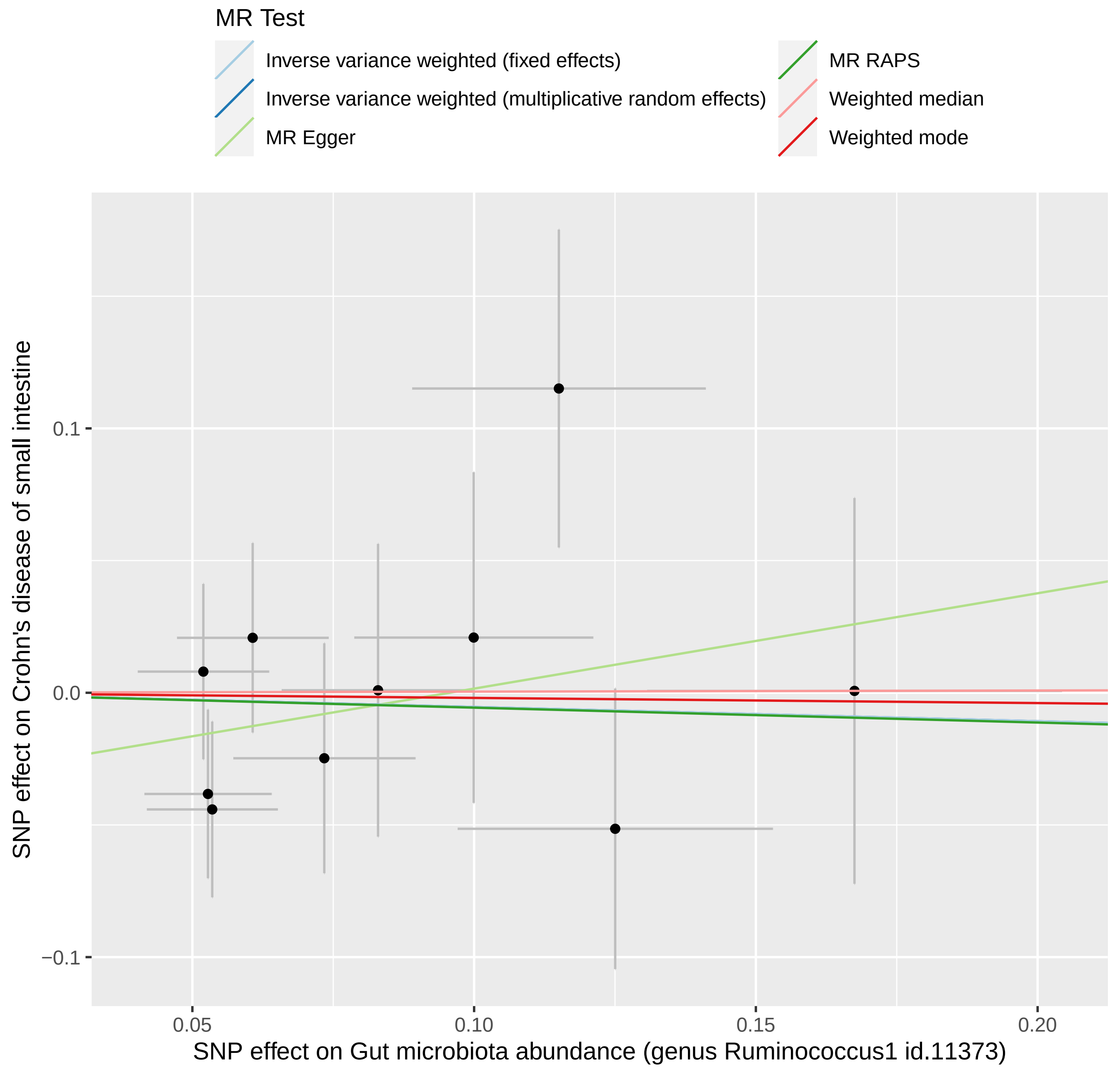

Batch 437 : Gut microbiota abundance (genus Ruminococcus2 id.11374) on Crohn's disease of small intestine

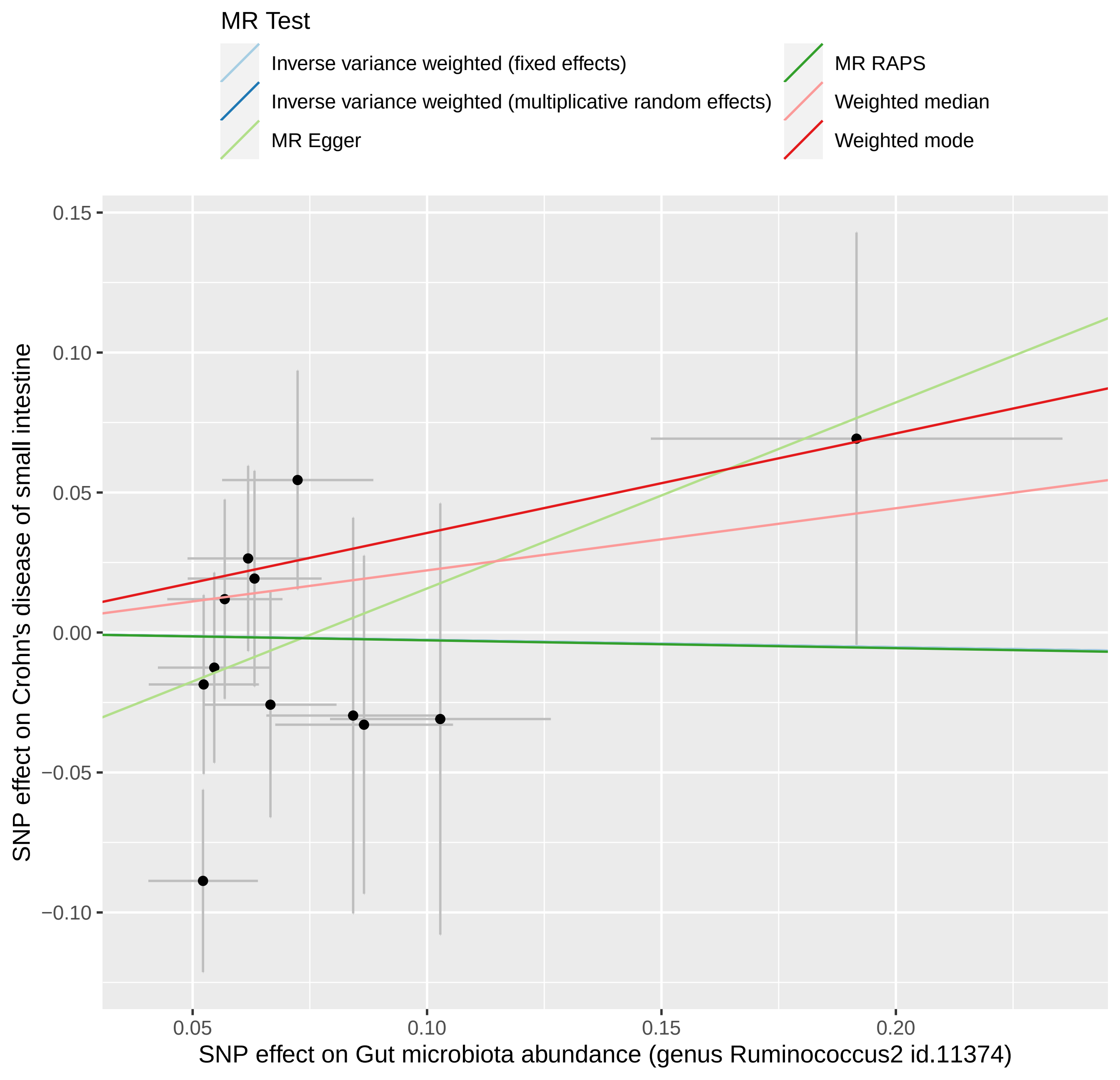

Batch 438 : Gut microbiota abundance (genus Ruminococcus gauvreauii group id.11342) on Crohn's disease of small intestine

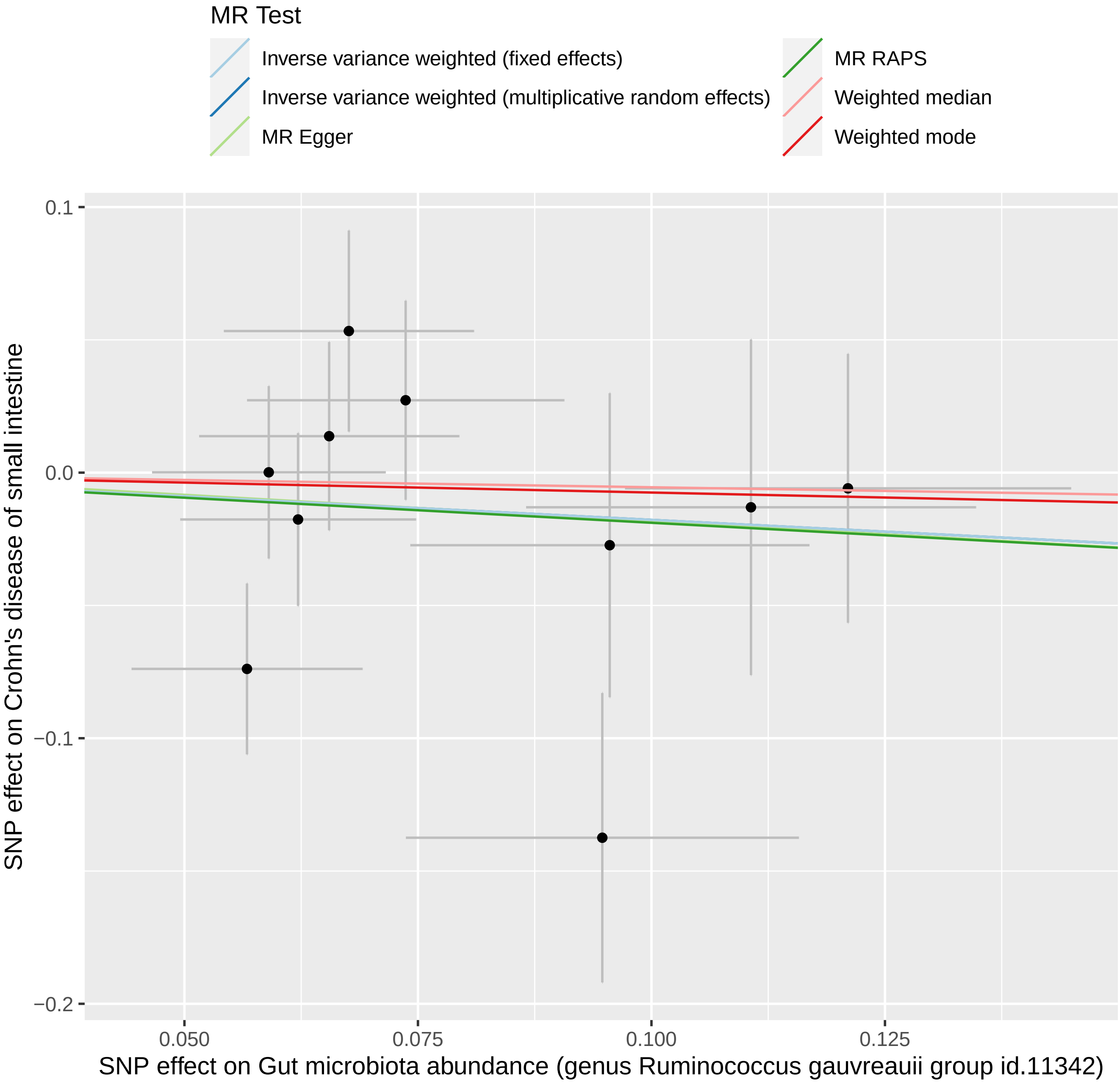

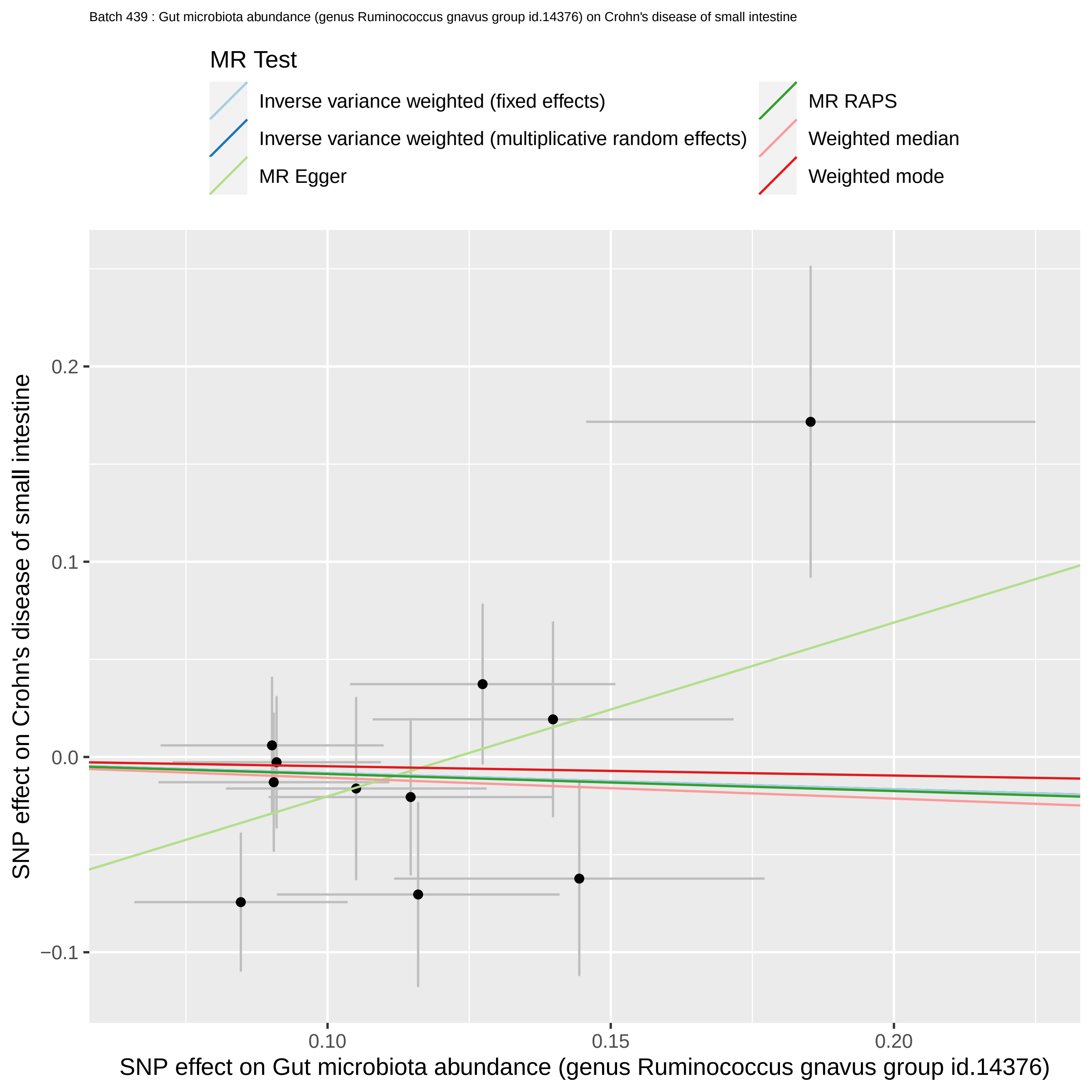
Batch 439 : Gut microbiota abundance (genus Ruminococcus gnavus group id.14376) on Crohn's disease of small intestine
MR Test
Inverse variance weighted (fixed effects)
Inverse variance weighted (multiplicative random effects)
MR Egger
MR RAPS
Weighted median
Weighted mode
SNP effect on Crohn's disease of small intestine
0.2
0.1
0.0
−0.1
0.10
0.15
0.20
SNP effect on Gut microbiota abundance (genus Ruminococcus gnavus group id.14376)

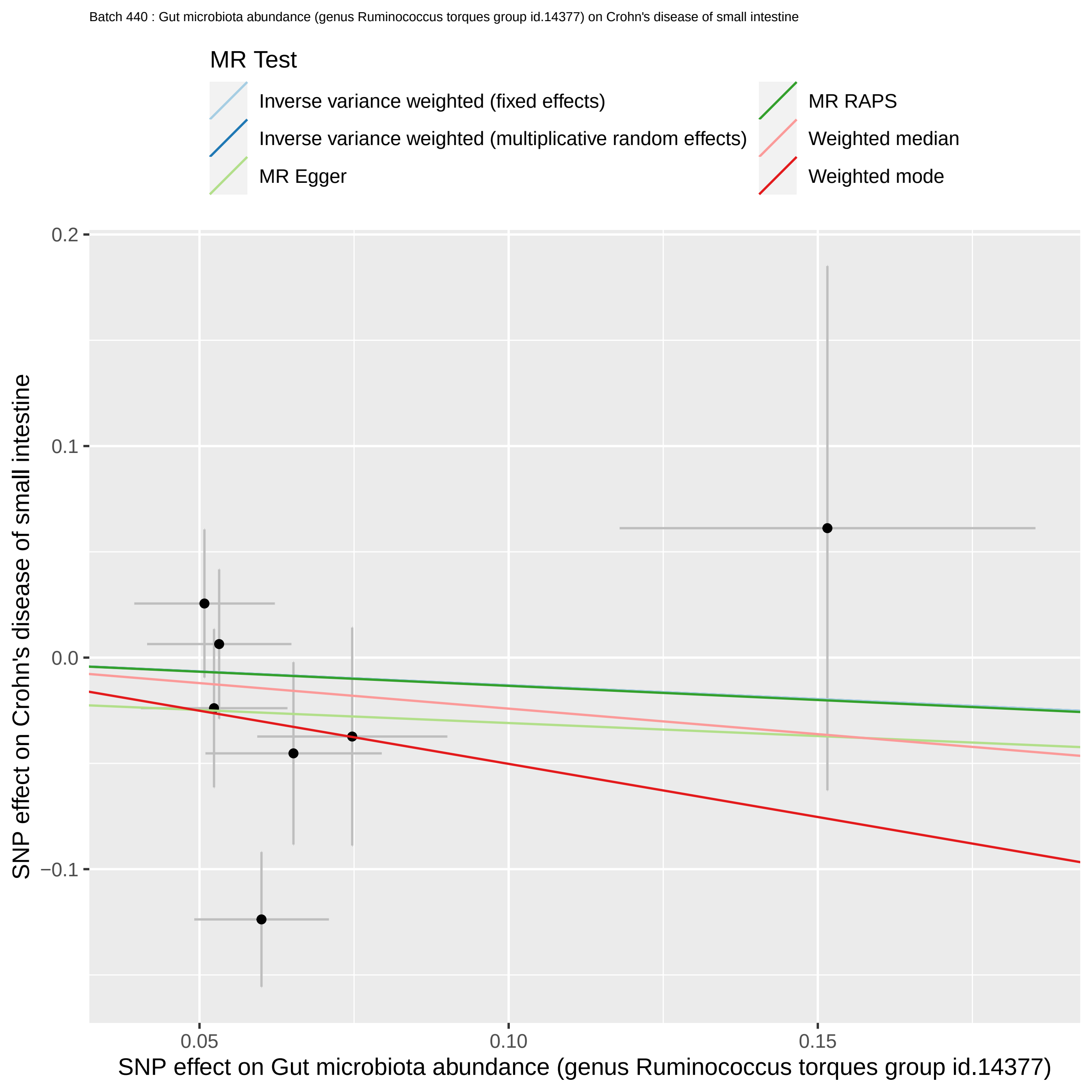

Batch 440 : Gut microbiota abundance (genus Ruminococcus torques group id.14377) on Crohn's disease of small intestine
MR Test
Inverse variance weighted (fixed effects)
Inverse variance weighted (multiplicative random effects)
MR Egger
MR RAPS
Weighted median
Weighted mode
0.2
0.1
0.0
−0.1
0.05
0.10
0.15
SNP effect on Crohn's disease of small intestine
SNP effect on Gut microbiota abundance (genus Ruminococcus torques group id.14377)

Batch 441 : Gut microbiota abundance (genus Sellimonas id.14369) on Crohn's disease of small intestine

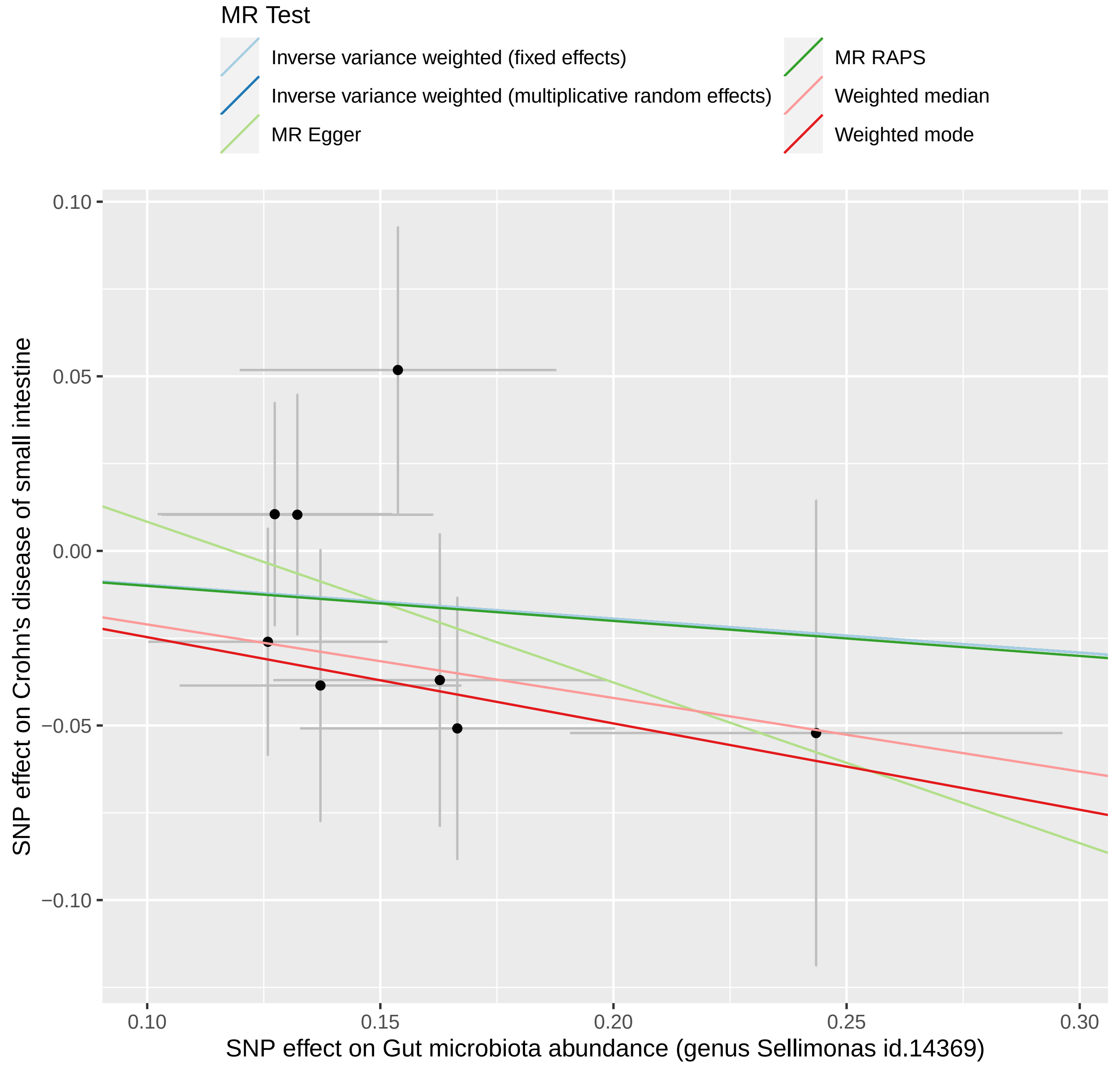

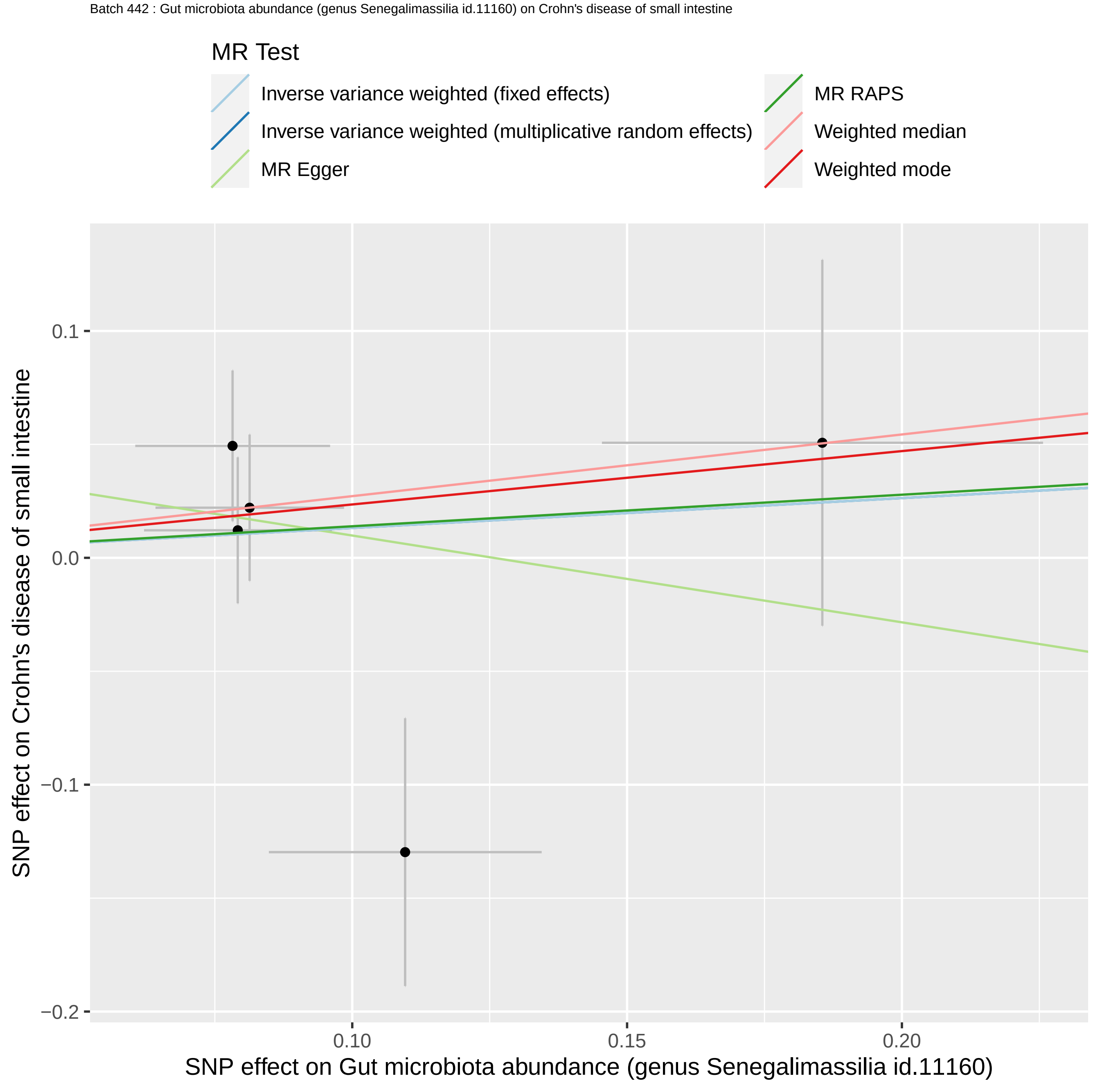
Batch 442 : Gut microbiota abundance (genus Senegalimassilia id.11160) on Crohn's disease of small intestine
MR Test
Inverse variance weighted (fixed effects)
Inverse variance weighted (multiplicative random effects)
MR Egger
MR RAPS
Weighted median
Weighted mode
SNP effect on Crohn's disease of small intestine
0.1
0.0
−0.1
−0.2
0.10
0.15
0.20
SNP effect on Gut microbiota abundance (genus Senegalimassilia id.11160)

Batch 443 : Gut microbiota abundance (genus Slackia id.825) on Crohn's disease of small intestine

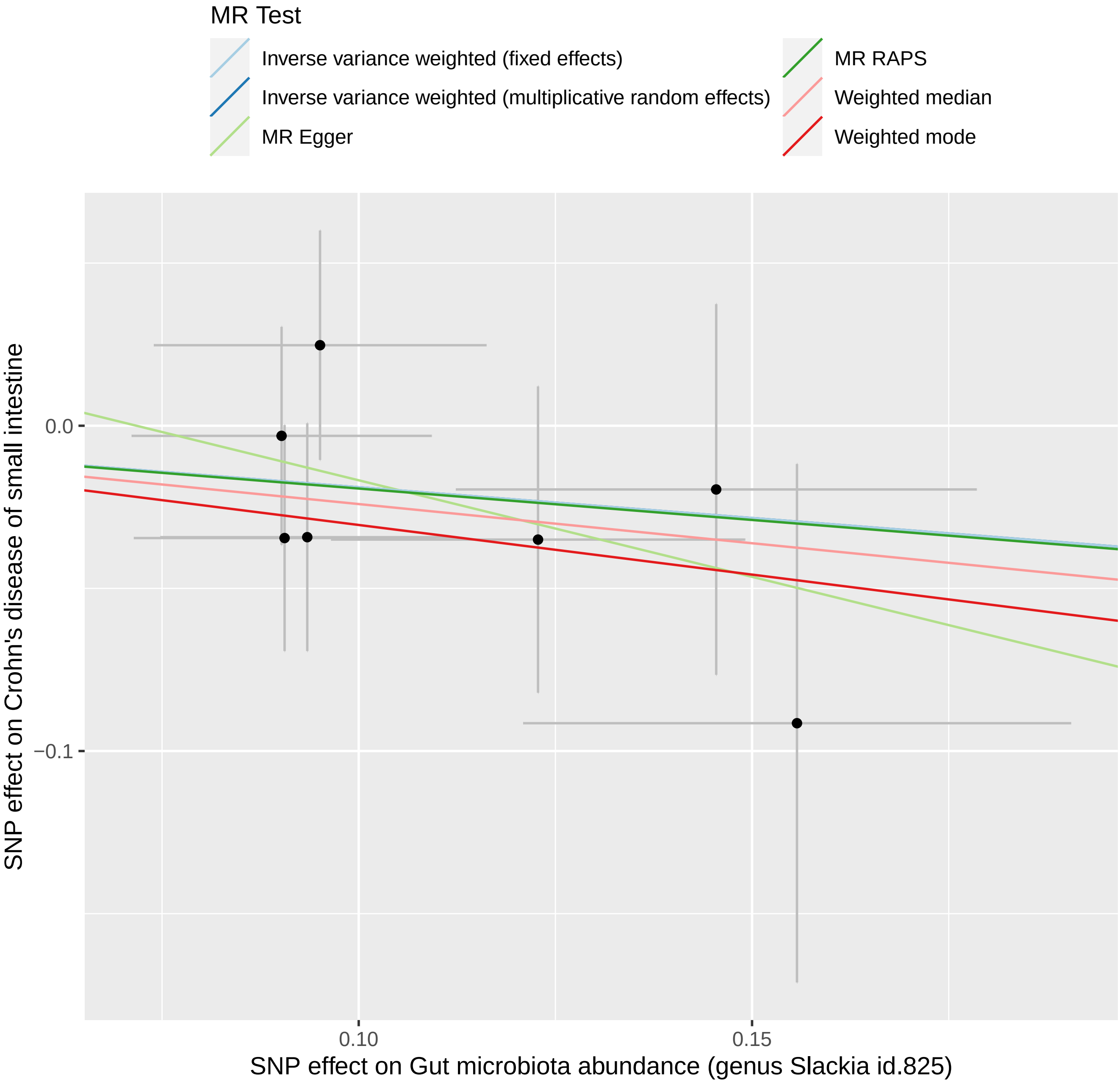

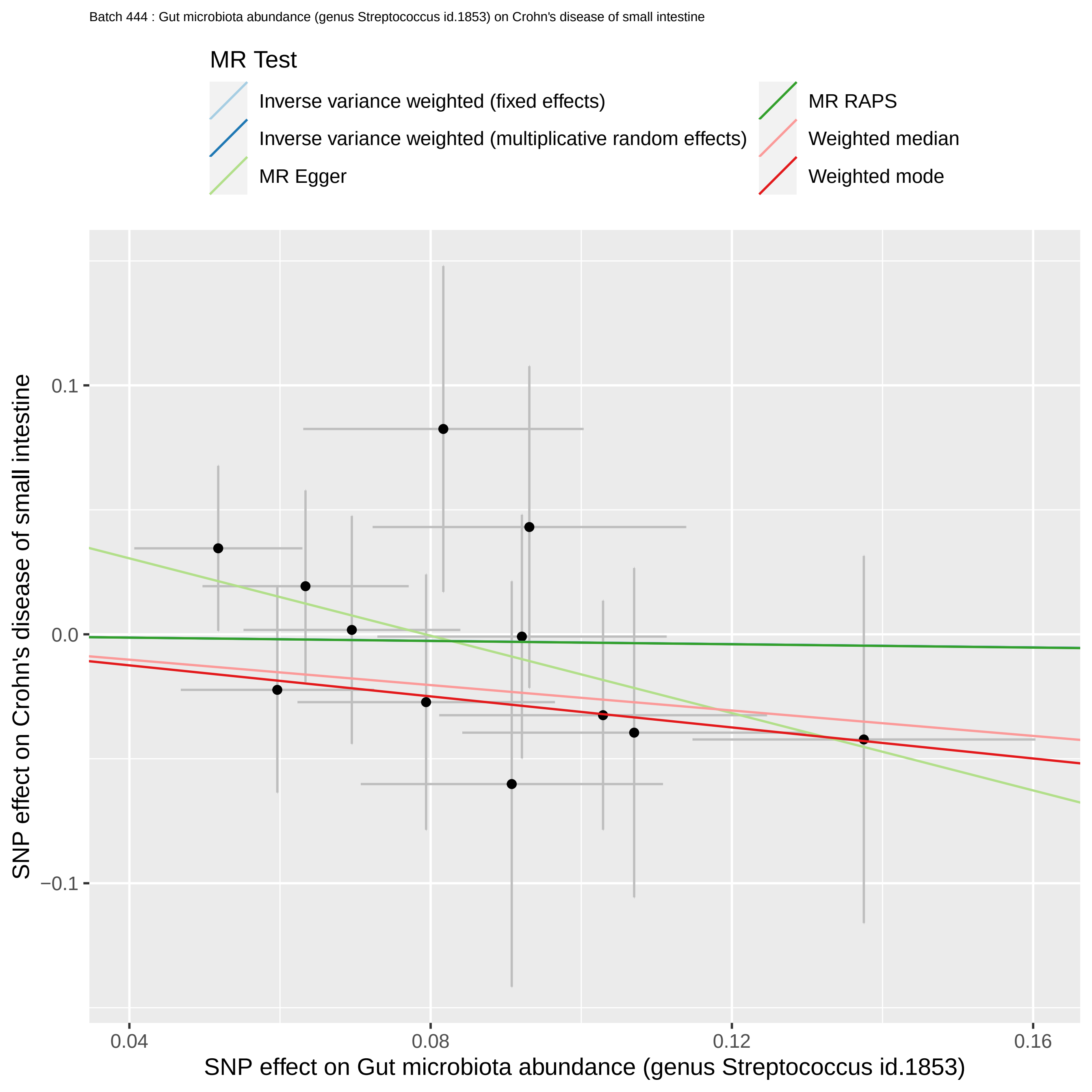
Batch 444 : Gut microbiota abundance (genus Streptococcus id.1853) on Crohn's disease of small intestine
MR Test
Inverse variance weighted (fixed effects)
Inverse variance weighted (multiplicative random effects)
MR Egger
MR RAPS
Weighted median
Weighted mode
0.1
0.0
−0.1
0.04
0.08
0.12
0.16
SNP effect on Crohn's disease of small intestine
SNP effect on Gut microbiota abundance (genus Streptococcus id.1853)

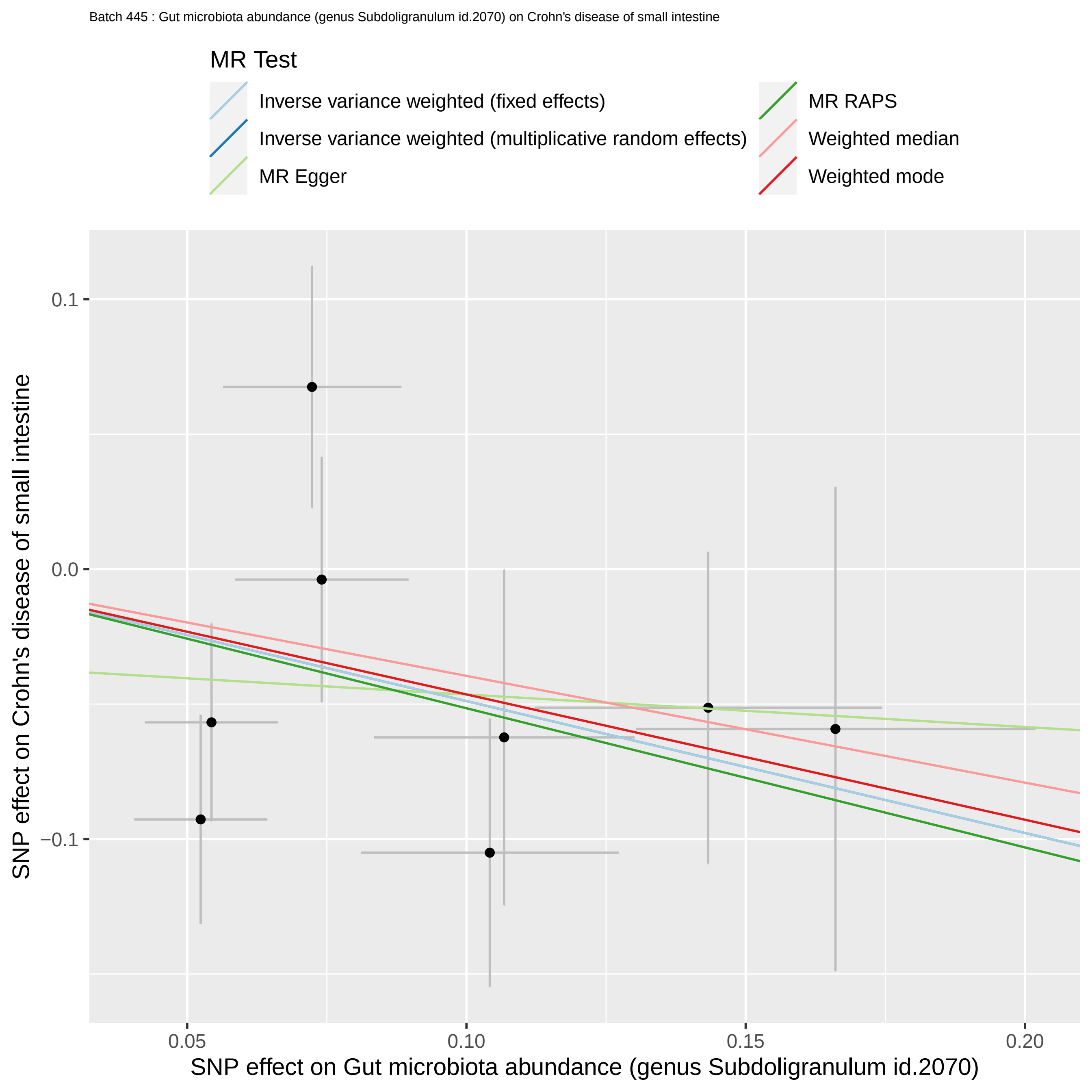
Batch 445 : Gut microbiota abundance (genus Subdoligranulum id.2070) on Crohn's disease of small intestine
MR Test
Inverse variance weighted (fixed effects)
Inverse variance weighted (multiplicative random effects)
MR Egger
MR RAPS
Weighted median
Weighted mode
0.1
0.0
−0.1
0.05
0.10
0.15
0.20
SNP effect on Crohn's disease of small intestine
SNP effect on Gut microbiota abundance (genus Subdoligranulum id.2070)

Batch 446 : Gut microbiota abundance (genus Sutterella id.2896) on Crohn's disease of small intestine

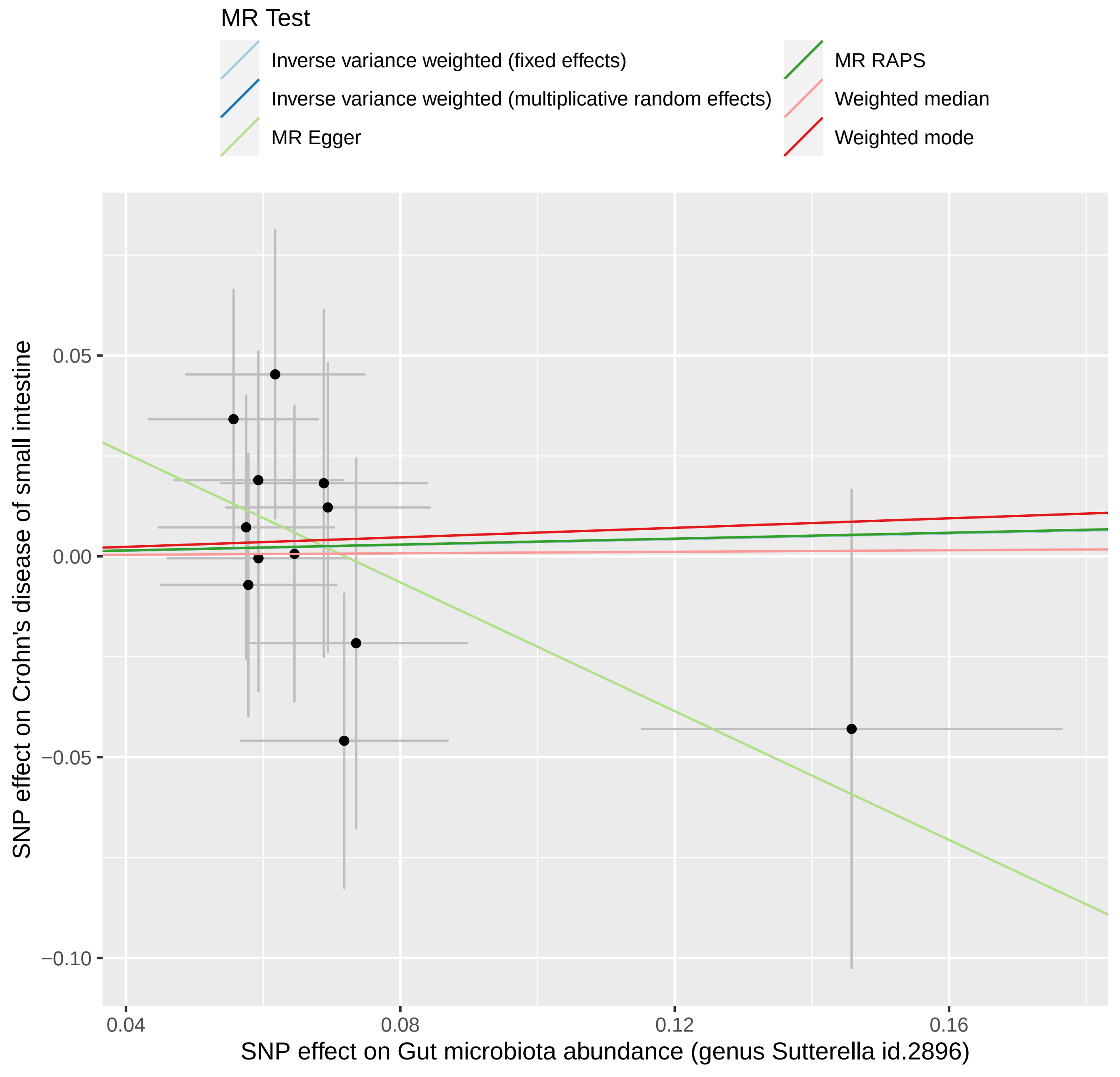

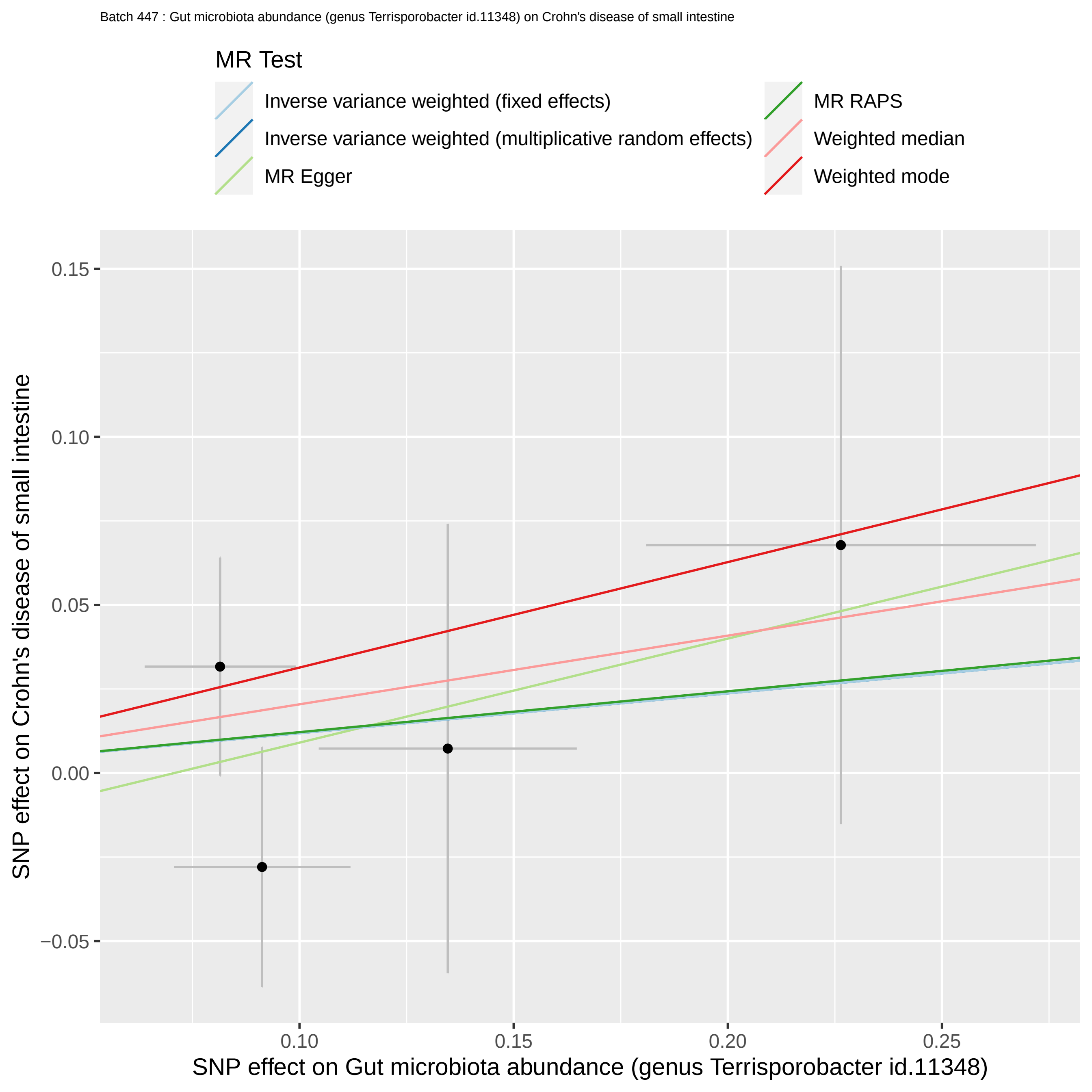

Batch 447 : Gut microbiota abundance (genus Terrisporobacter id.11348) on Crohn's disease of small intestine
MR Test
Inverse variance weighted (fixed effects)
Inverse variance weighted (multiplicative random effects)
MR Egger
MR RAPS
Weighted median
Weighted mode
0.15
0.10
0.05
0.00
−0.05
0.10
0.15
0.20
0.25
SNP effect on Crohn's disease of small intestine
SNP effect on Gut microbiota abundance (genus Terrisporobacter id.11348)

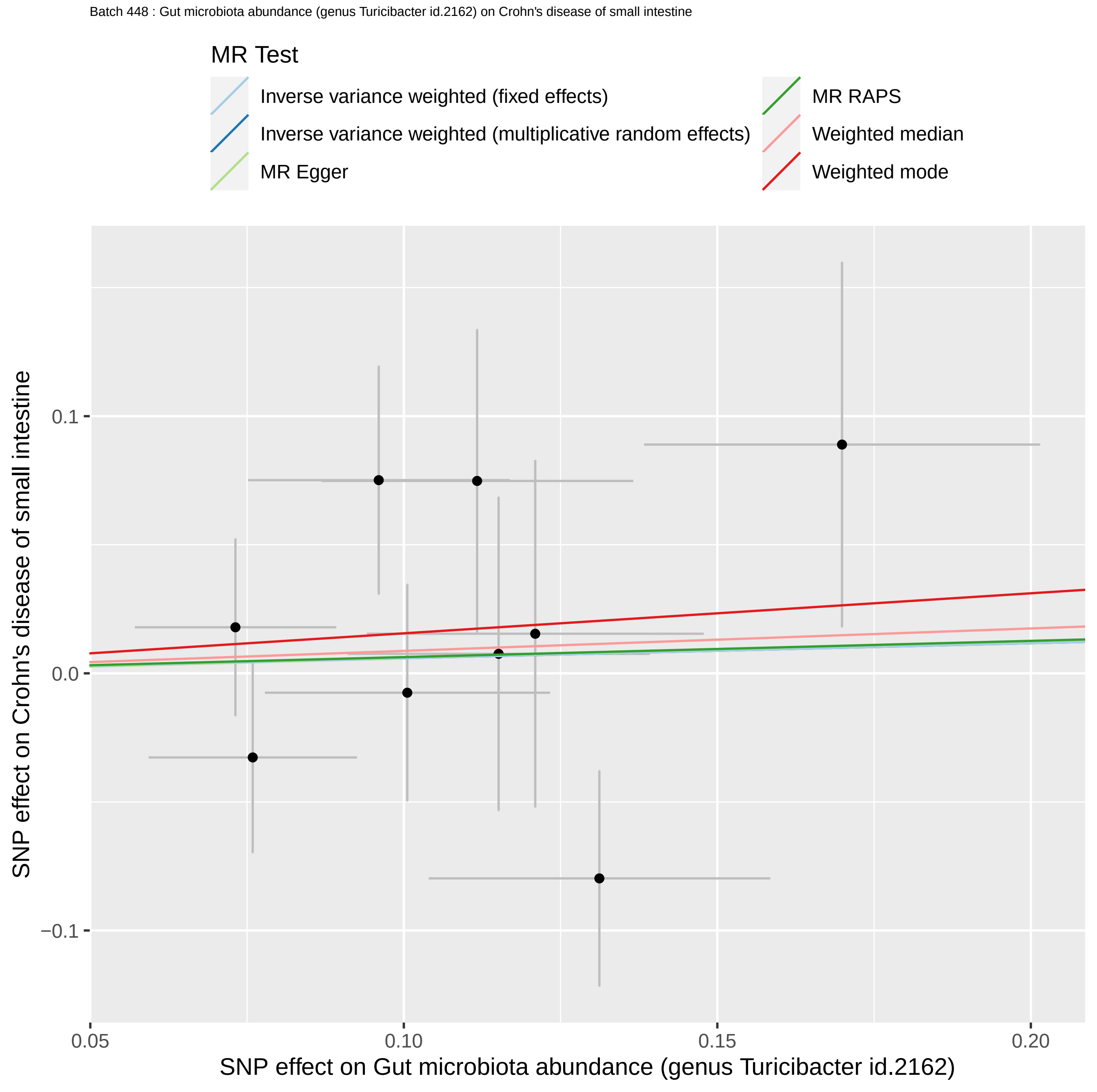
Batch 448 : Gut microbiota abundance (genus Turicibacter id.2162) on Crohn's disease of small intestine
MR Test
Inverse variance weighted (fixed effects)
Inverse variance weighted (multiplicative random effects)
MR Egger
MR RAPS
Weighted median
Weighted mode
SNP effect on Crohn's disease of small intestine
0.1
0.0
−0.1
0.05
0.10
0.15
0.20
SNP effect on Gut microbiota abundance (genus Turicibacter id.2162)

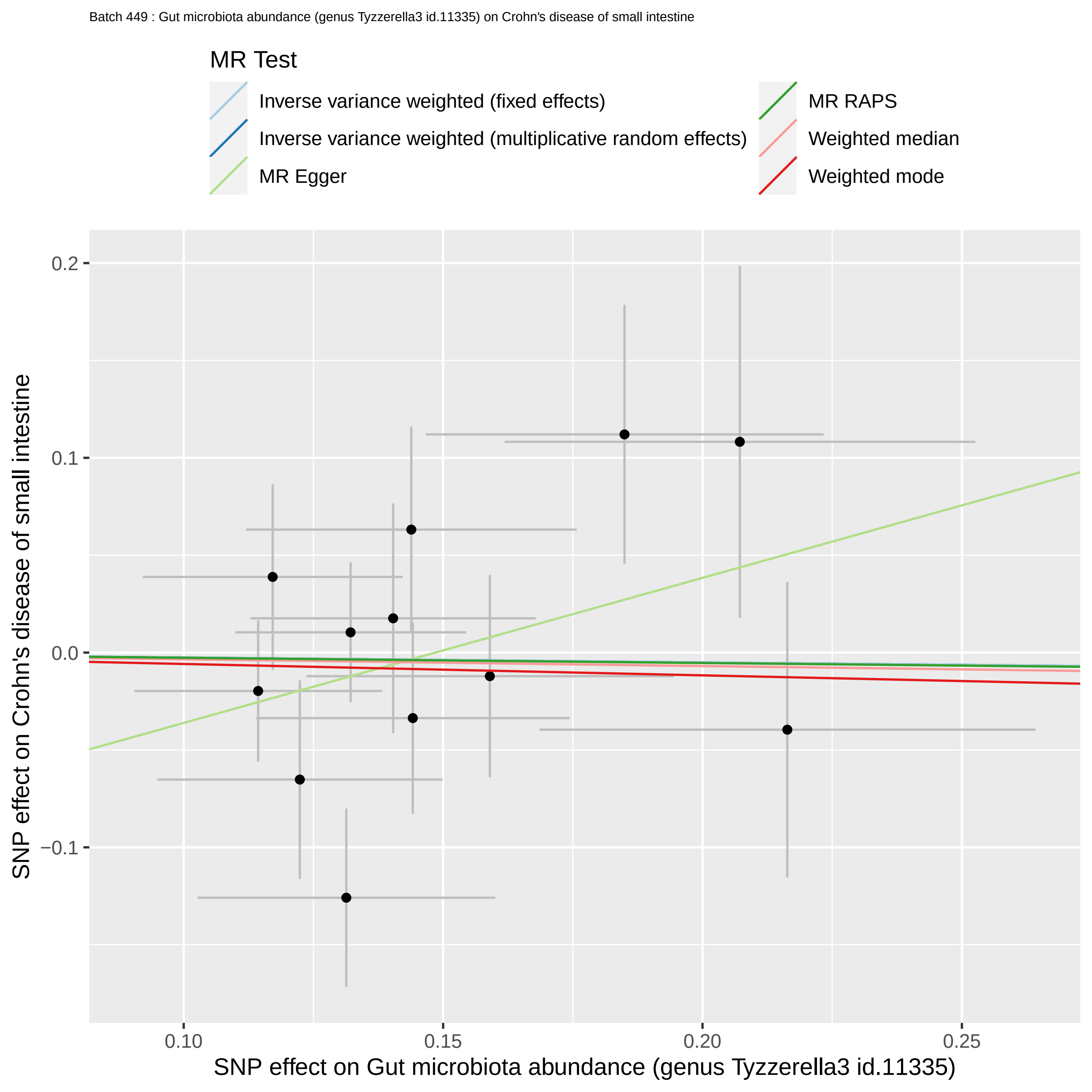

Batch 449 : Gut microbiota abundance (genus Tyzzerella3 id.11335) on Crohn's disease of small intestine
MR Test
Inverse variance weighted (fixed effects)
Inverse variance weighted (multiplicative random effects)
MR Egger
MR RAPS
Weighted median
Weighted mode
0.2
0.1
0.0
−0.1
0.10
0.15
0.20
0.25
SNP effect on Crohn's disease of small intestine
SNP effect on Gut microbiota abundance (genus Tyzzerella3 id.11335)

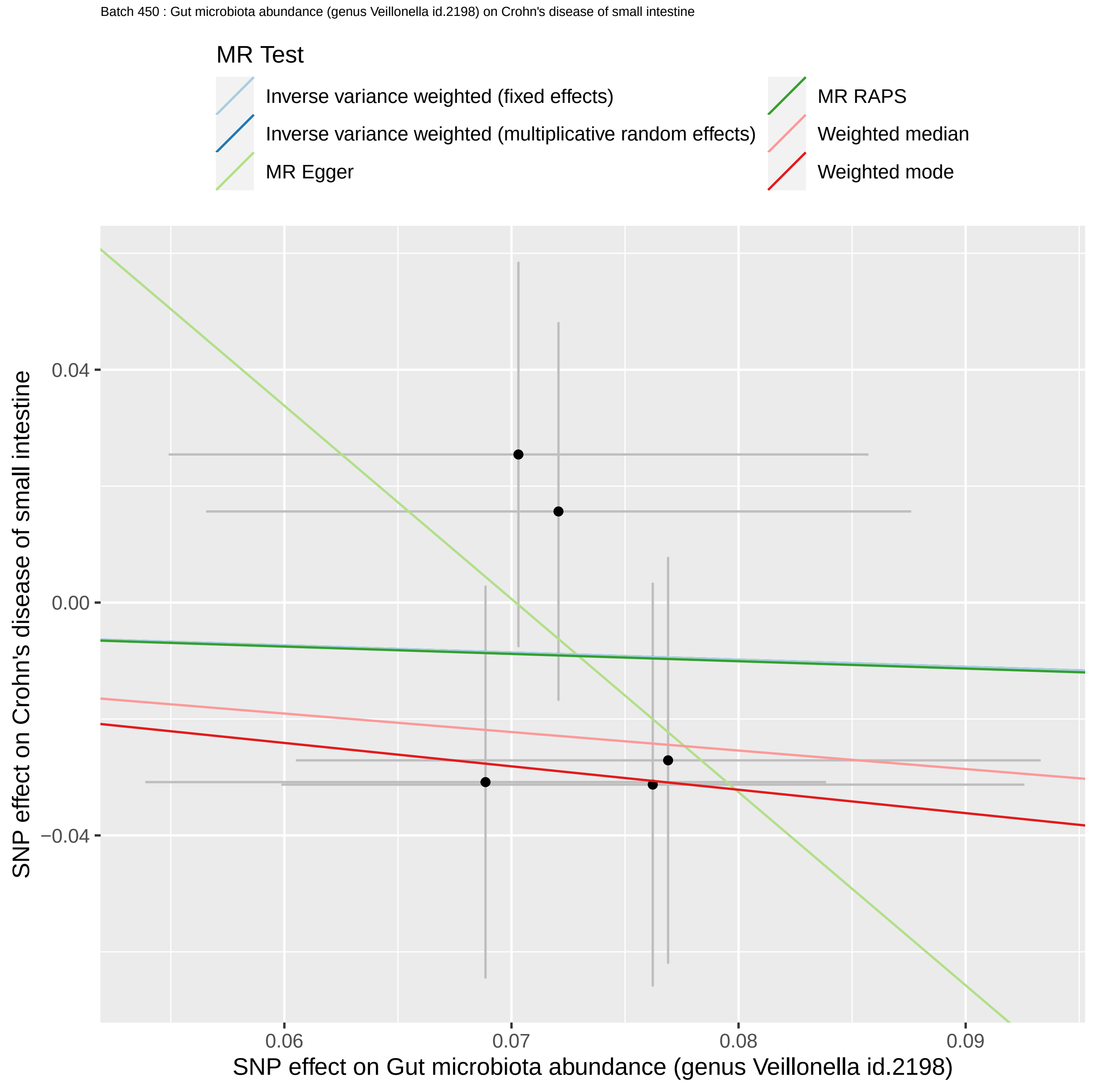

Batch 450 : Gut microbiota abundance (genus Veillonella id.2198) on Crohn's disease of small intestine
MR Test
Inverse variance weighted (fixed effects)
Inverse variance weighted (multiplicative random effects)
MR Egger
MR RAPS
Weighted median
Weighted mode
0.04
0.00
−0.04
0.06
0.07
0.08
0.09
SNP effect on Crohn's disease of small intestine
SNP effect on Gut microbiota abundance (genus Veillonella id.2198)

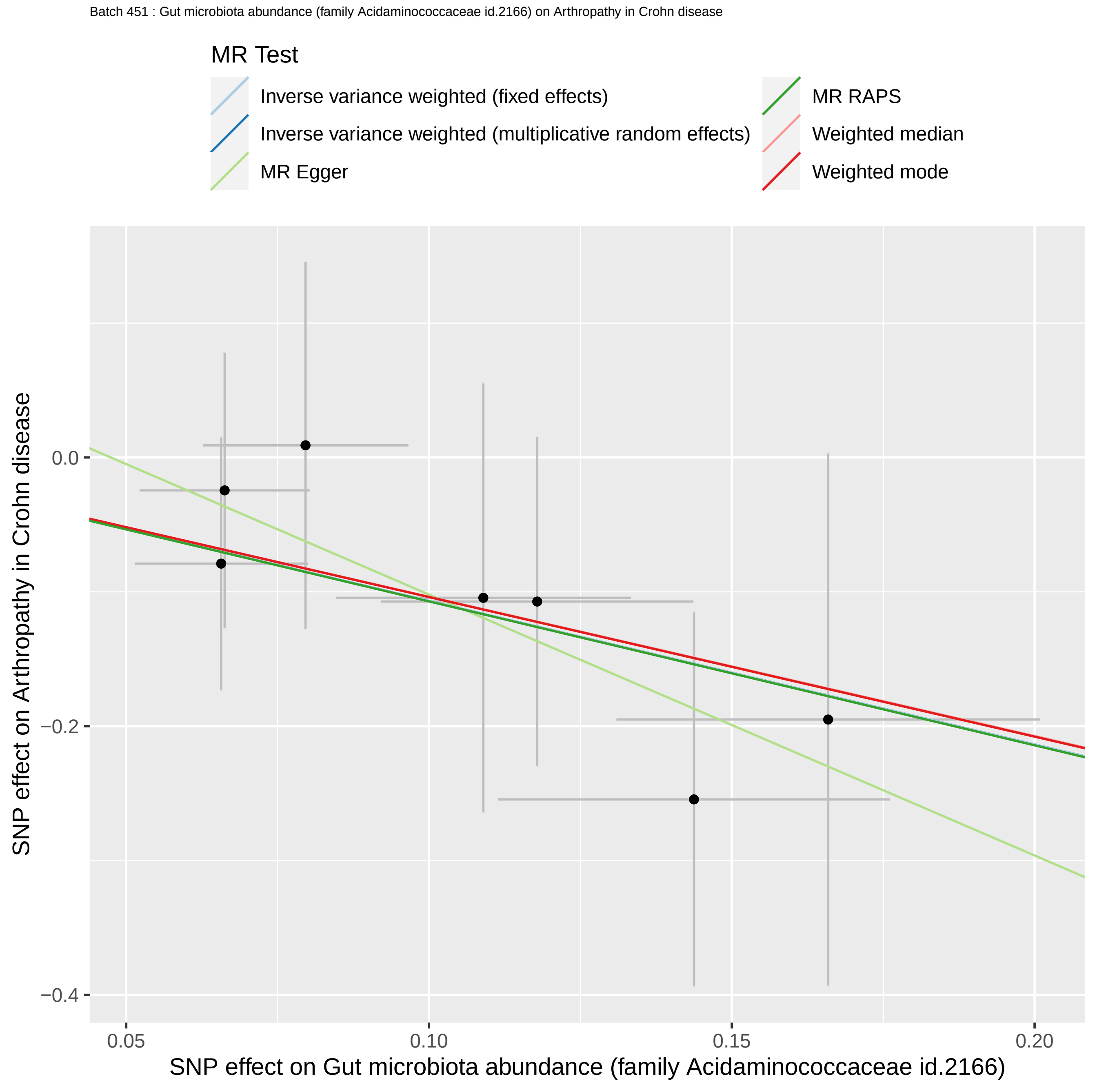

Batch 451 : Gut microbiota abundance (family Acidaminococcaceae id.2166) on Arthropathy in Crohn disease
MR Test
Inverse variance weighted (fixed effects)
Inverse variance weighted (multiplicative random effects)
MR Egger
MR RAPS
Weighted median
Weighted mode
0.0
−0.2
−0.4
0.05
0.10
0.15
0.20
SNP effect on Arthropathy in Crohn disease
SNP effect on Gut microbiota abundance (family Acidaminococcaceae id.2166)

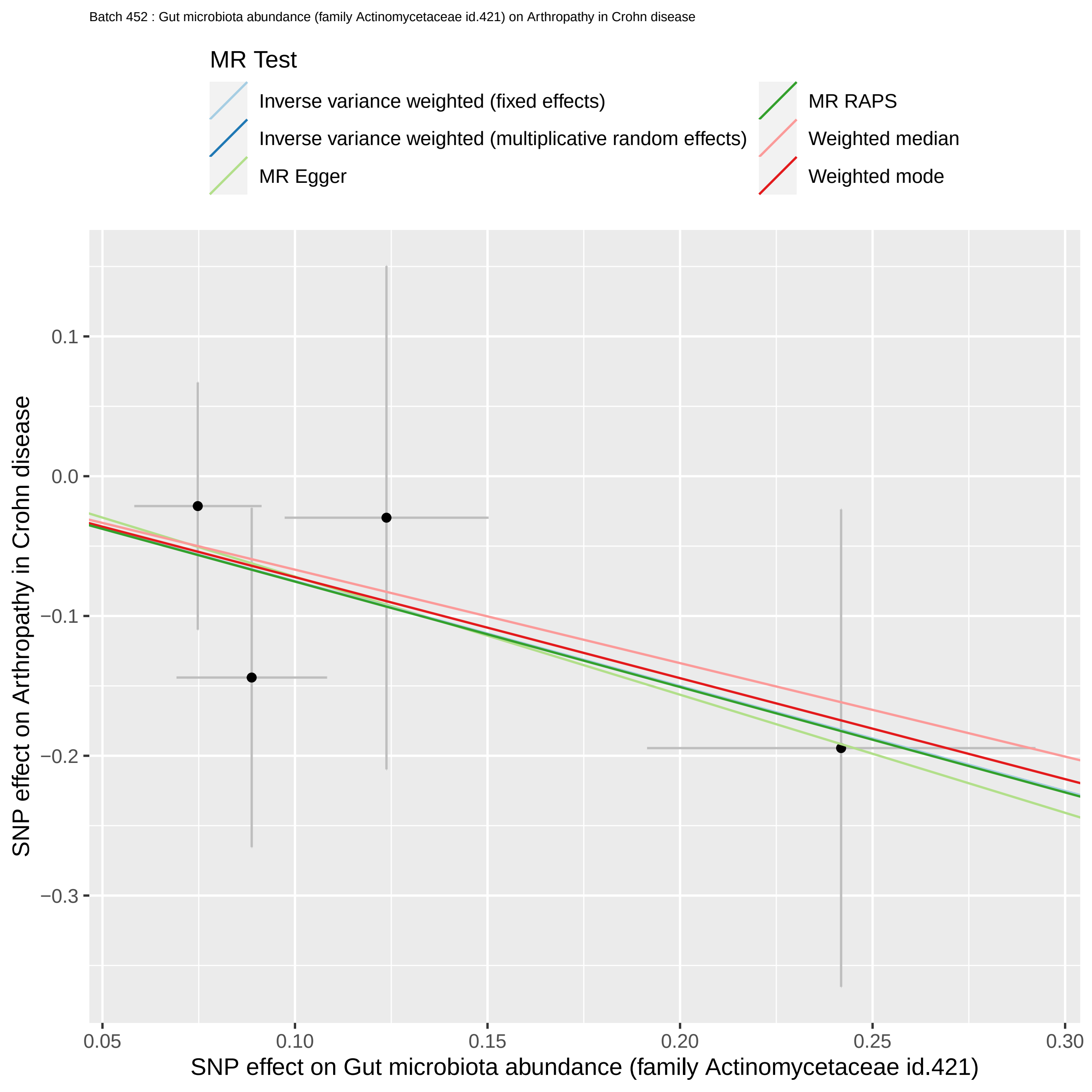

Batch 452 : Gut microbiota abundance (family Actinomycetaceae id.421) on Arthropathy in Crohn disease
MR Test
Inverse variance weighted (fixed effects)
Inverse variance weighted (multiplicative random effects)
MR Egger
MR RAPS
Weighted median
Weighted mode
SNP effect on Arthropathy in Crohn disease
0.1
0.0
−0.1
−0.2
−0.3
0.05
0.10
0.15
0.20
0.25
0.30
SNP effect on Gut microbiota abundance (family Actinomycetaceae id.421)

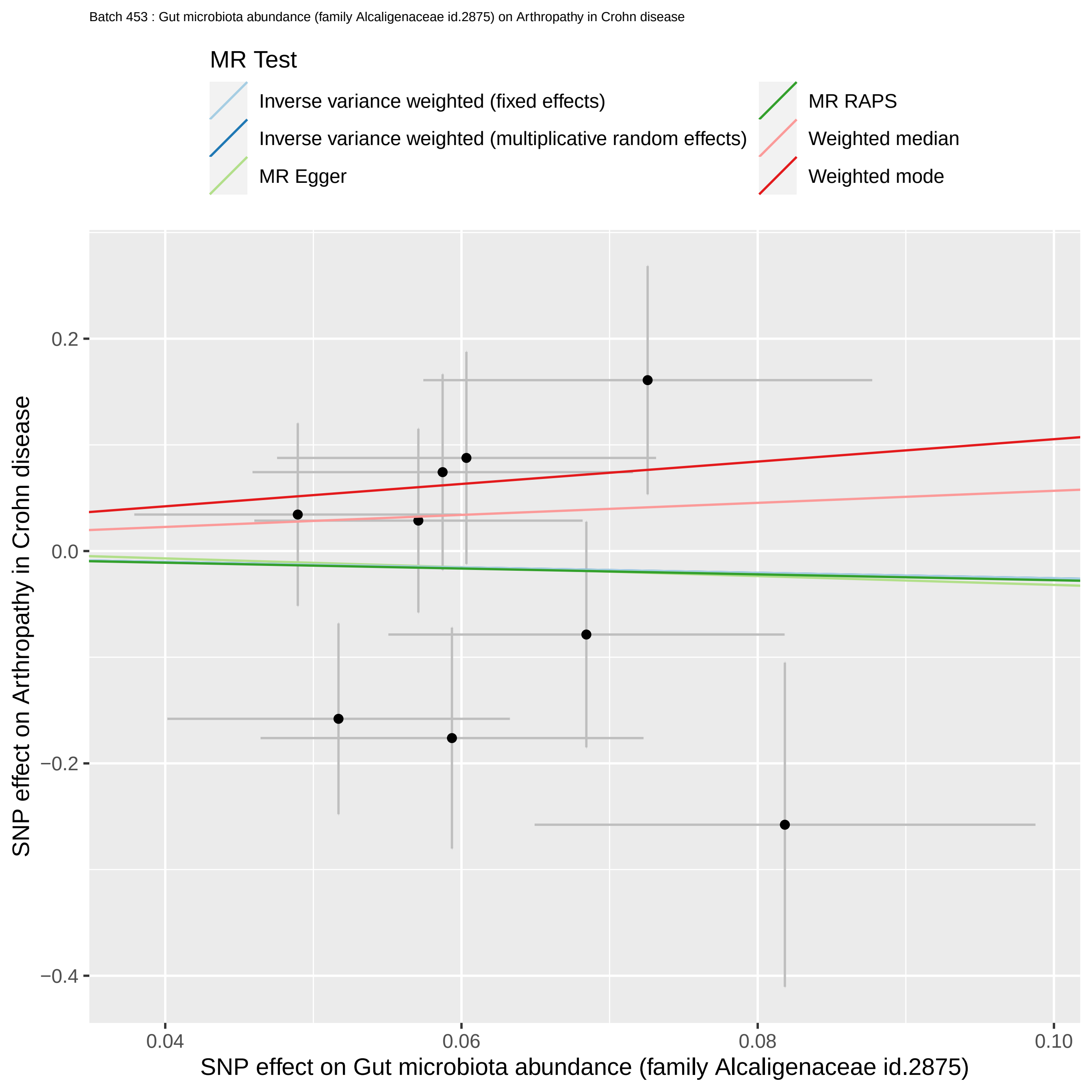

Batch 453 : Gut microbiota abundance (family Alcaligenaceae id.2875) on Arthropathy in Crohn disease
MR Test
Inverse variance weighted (fixed effects)
Inverse variance weighted (multiplicative random effects)
MR Egger
MR RAPS
Weighted median
Weighted mode
SNP effect on Arthropathy in Crohn disease
0.2
0.0
−0.2
−0.4
0.04
0.06
0.08
0.10
SNP effect on Gut microbiota abundance (family Alcaligenaceae id.2875)

Batch 454 : Gut microbiota abundance (family Bacteroidaceae id.917) on Arthropathy in Crohn disease

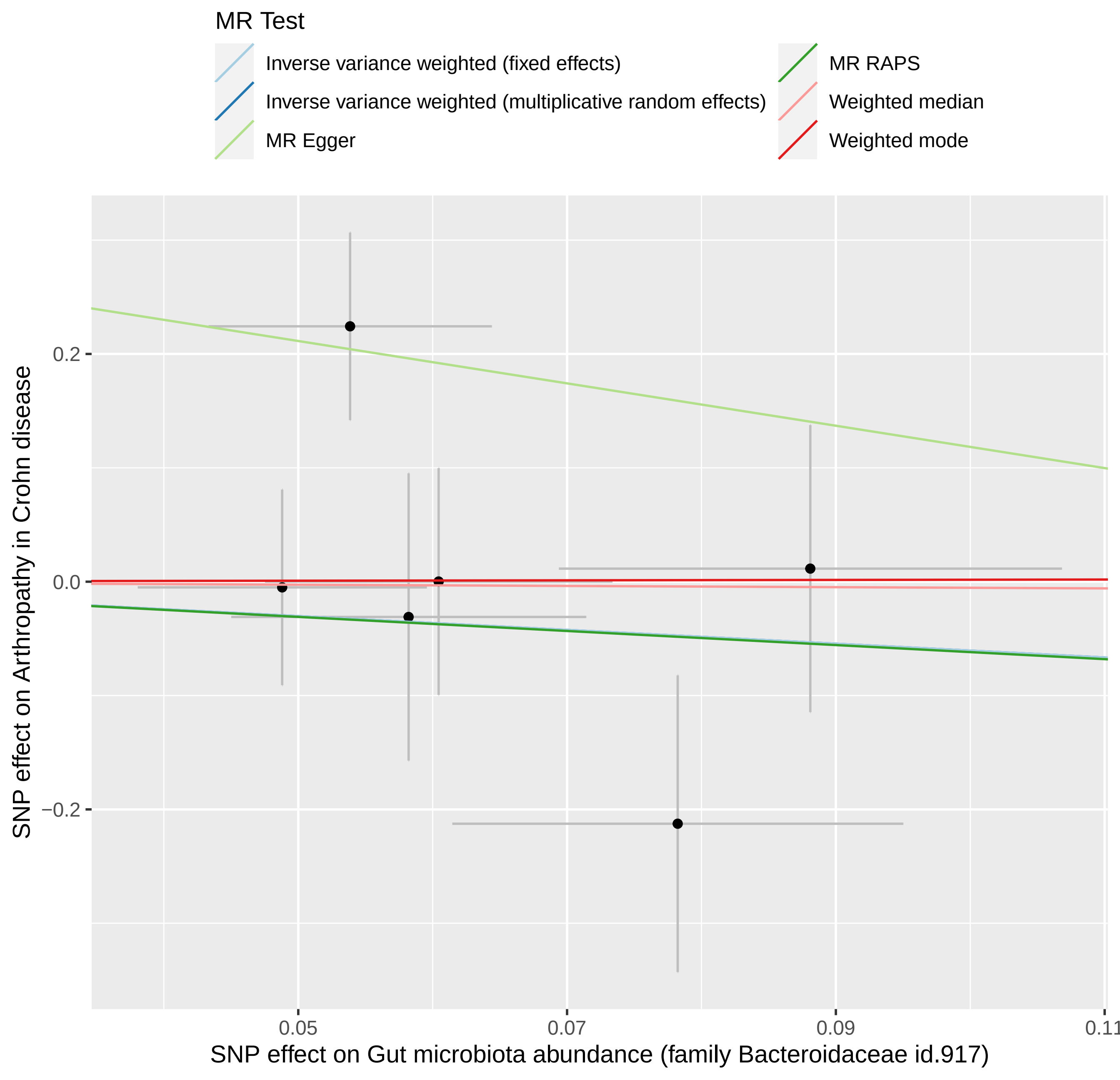

Batch 455 : Gut microbiota abundance (family Bacteroidales S24 7group id.11173) on Arthropathy in Crohn disease

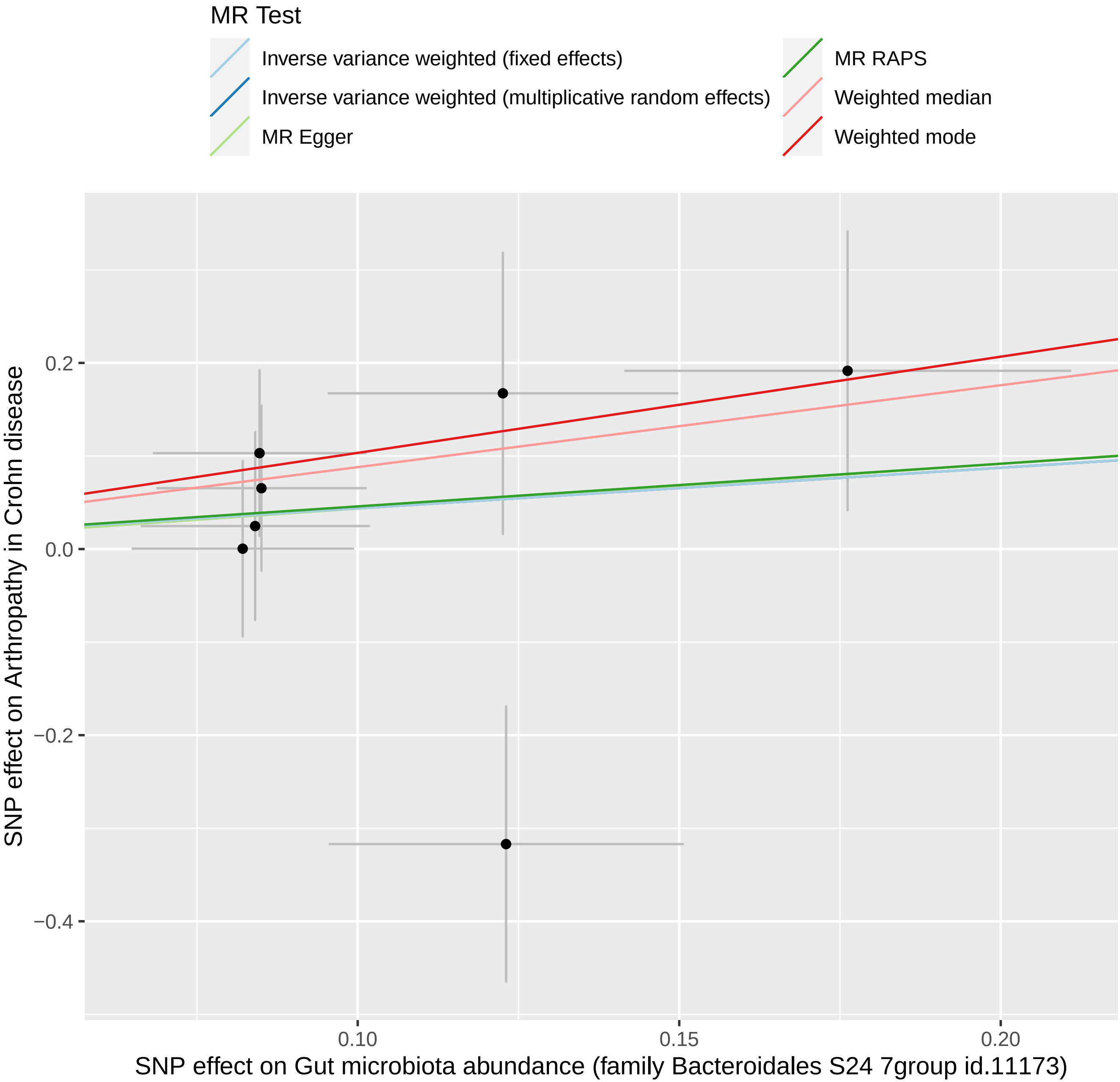

Batch 456 : Gut microbiota abundance (family Bifidobacteriaceae id.433) on Arthropathy in Crohn disease

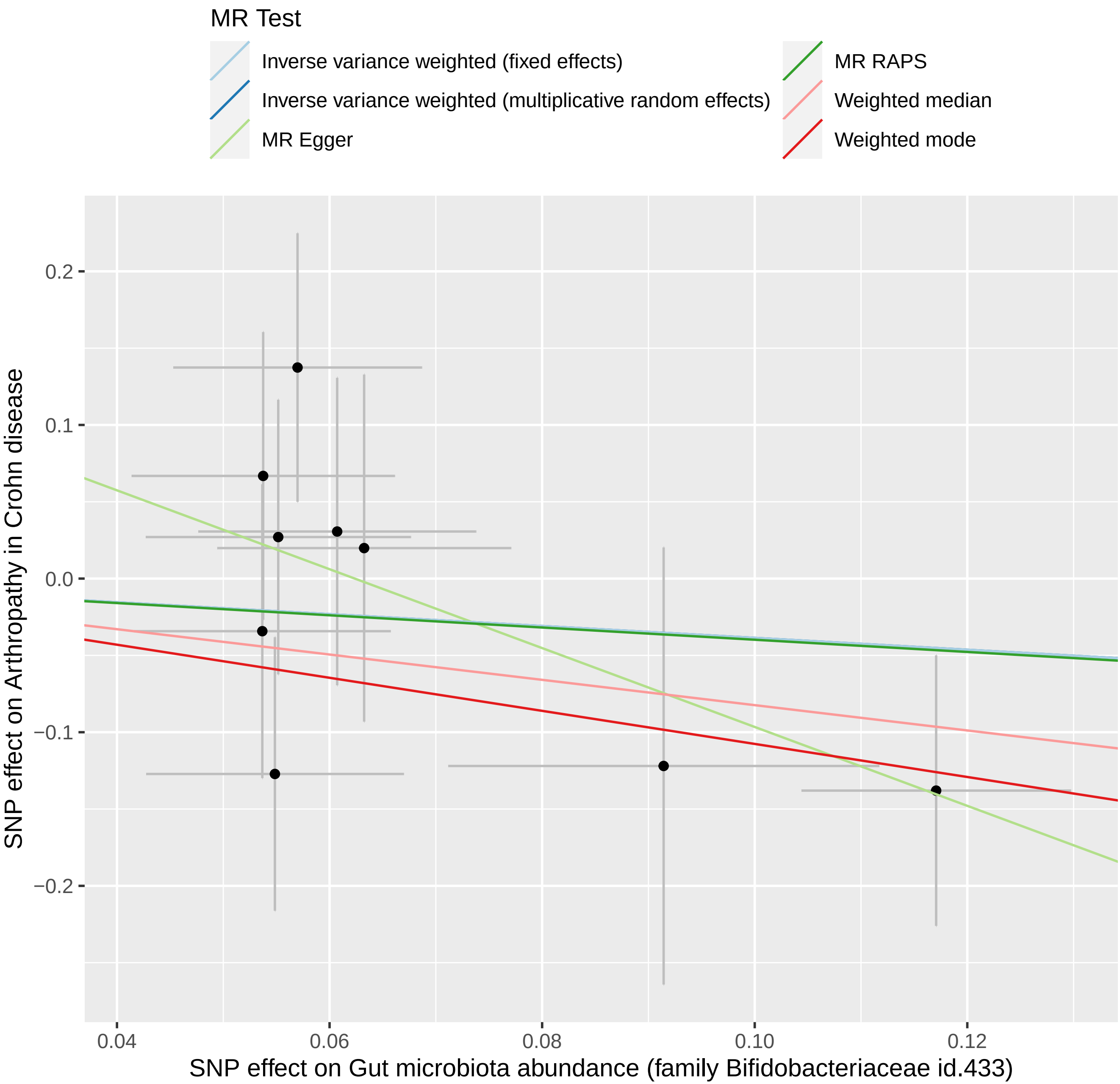

Batch 458 : Gut microbiota abundance (family Clostridiaceae1 id.1869) on Arthropathy in Crohn disease

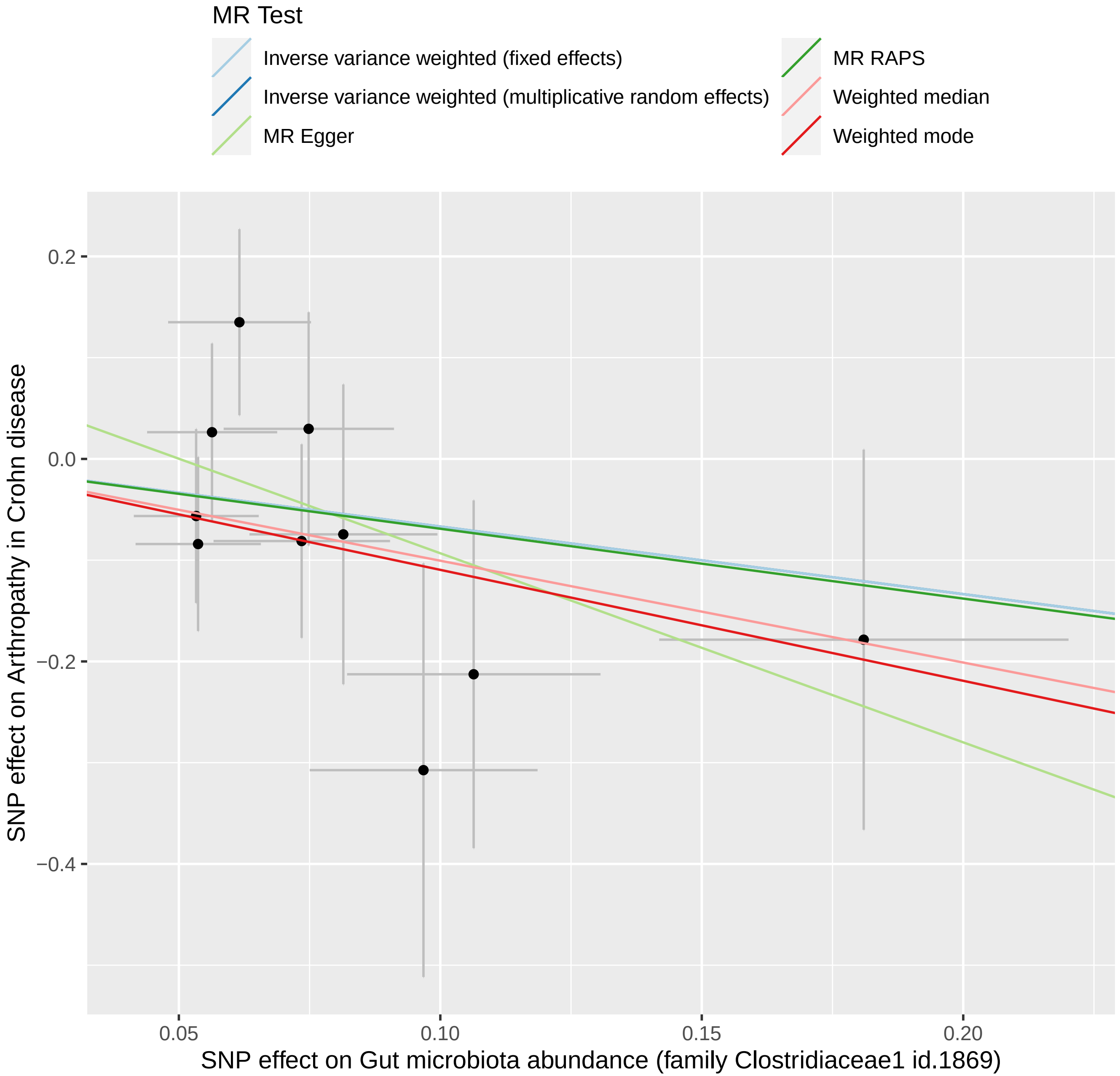

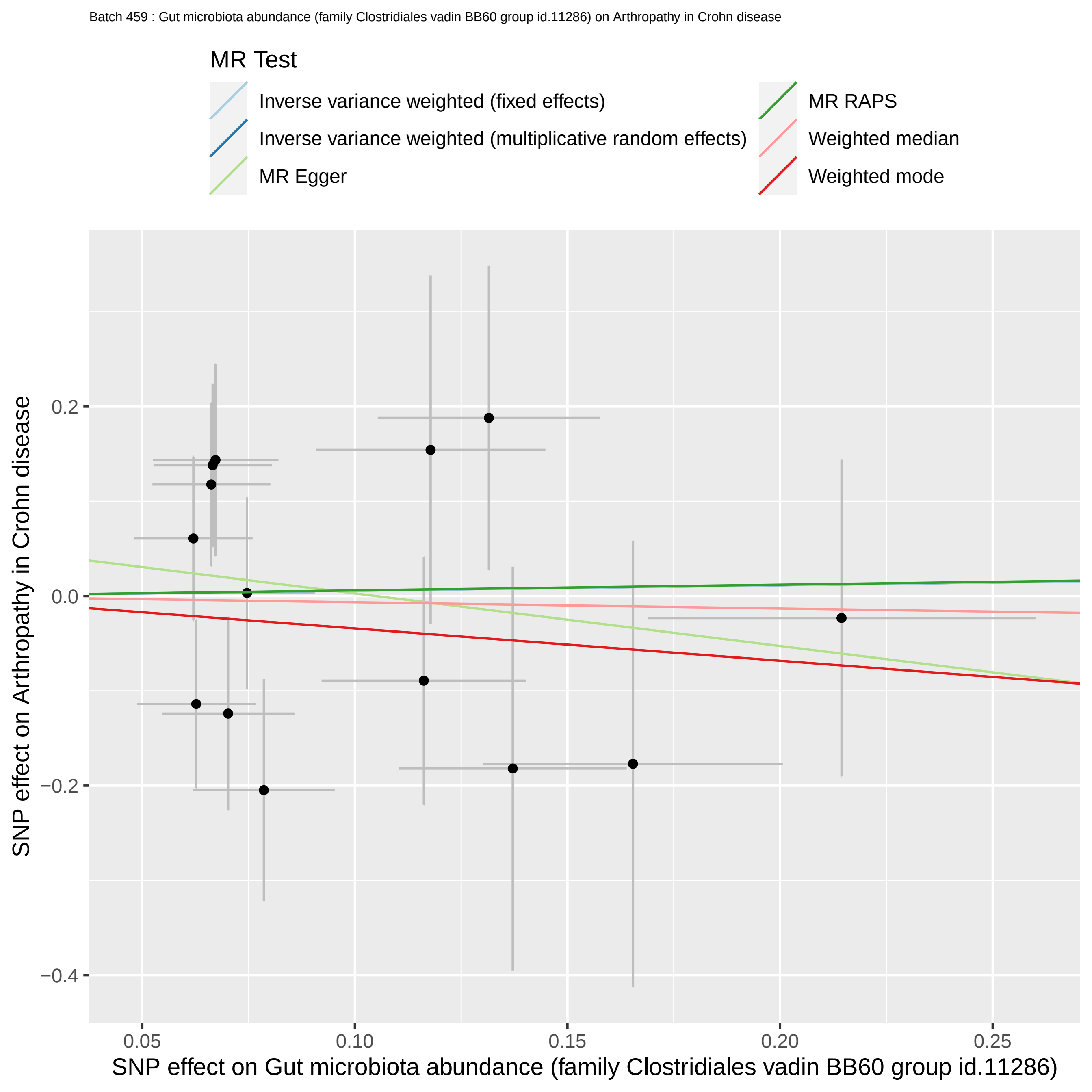

Batch 459 : Gut microbiota abundance (family Clostridiales vadin BB60 group id.11286) on Arthropathy in Crohn disease
MR Test
Inverse variance weighted (fixed effects)
Inverse variance weighted (multiplicative random effects)
MR Egger
MR RAPS
Weighted median
Weighted mode
SNP effect on Arthropathy in Crohn disease
0.2
0.0
−0.2
−0.4
0.05
0.10
0.15
0.20
0.25
SNP effect on Gut microbiota abundance (family Clostridiales vadin BB60 group id.11286)

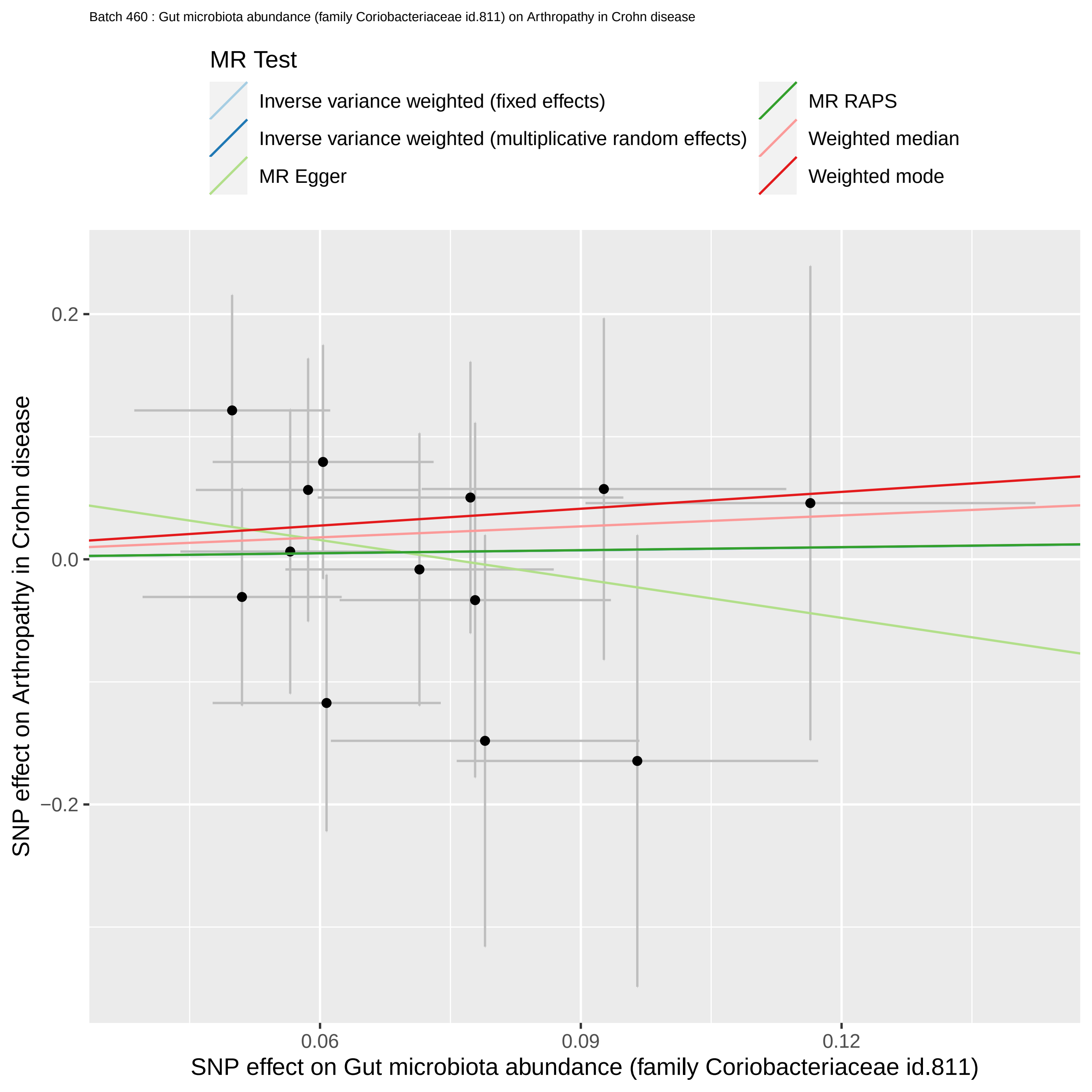

Batch 460 : Gut microbiota abundance (family Coriobacteriaceae id.811) on Arthropathy in Crohn disease
MR Test
Inverse variance weighted (fixed effects)
Inverse variance weighted (multiplicative random effects)
MR Egger
MR RAPS
Weighted median
Weighted mode
0.2
0.0
−0.2
0.06
0.09
0.12
SNP effect on Arthropathy in Crohn disease
SNP effect on Gut microbiota abundance (family Coriobacteriaceae id.811)

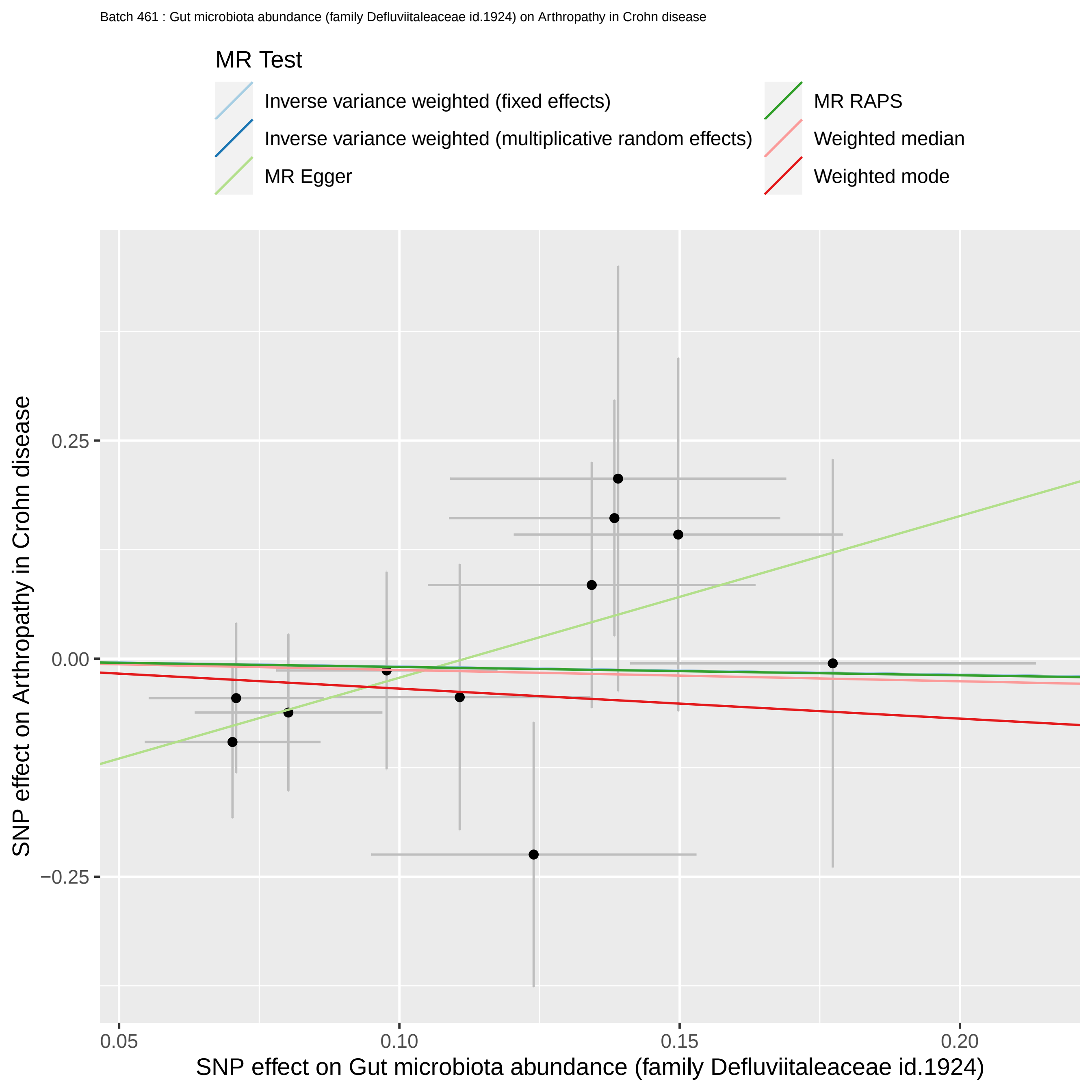

Batch 461 : Gut microbiota abundance (family Defluviitaleaceae id.1924) on Arthropathy in Crohn disease
MR Test
Inverse variance weighted (fixed effects)
Inverse variance weighted (multiplicative random effects)
MR Egger
MR RAPS
Weighted median
Weighted mode
0.25
0.00
−0.25
0.05
0.10
0.15
0.20
SNP effect on Arthropathy in Crohn disease
SNP effect on Gut microbiota abundance (family Defluviitaleaceae id.1924)

Batch 462 : Gut microbiota abundance (family Desulfovibrionaceae id.3169) on Arthropathy in Crohn disease

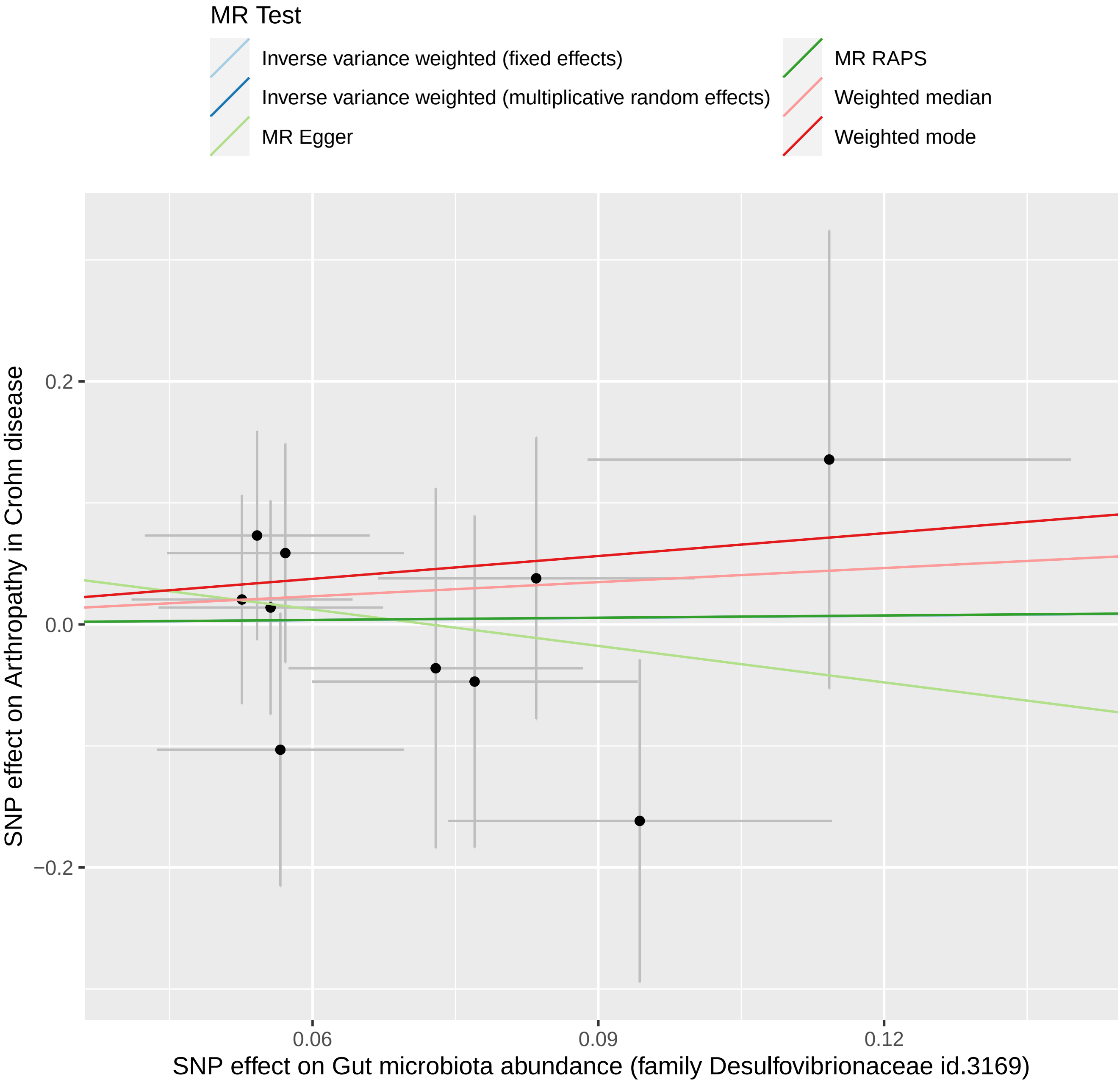

Batch 463 : Gut microbiota abundance (family Enterobacteriaceae id.3469) on Arthropathy in Crohn disease

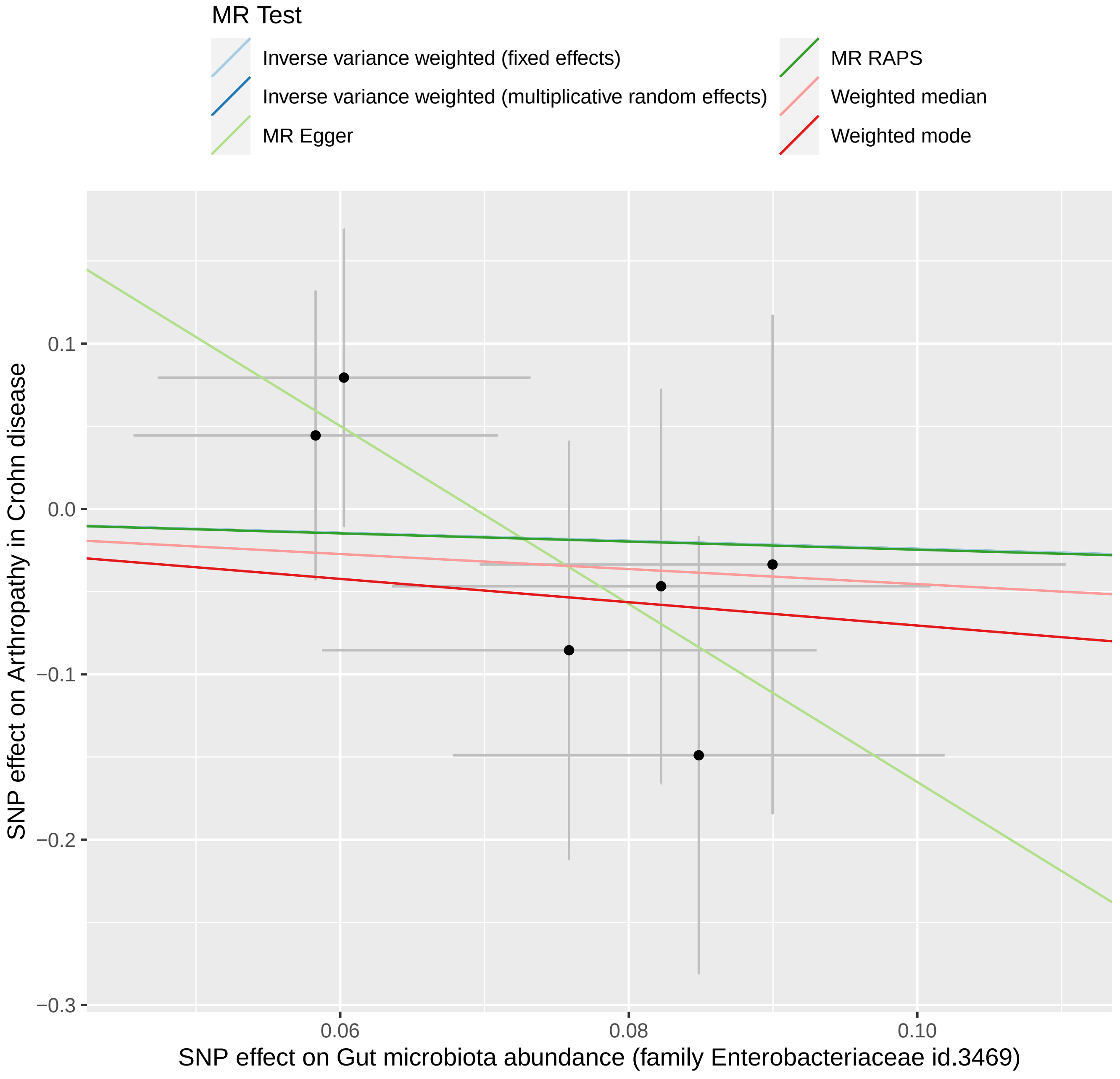

Batch 464 : Gut microbiota abundance (family Erysipelotrichaceae id.2149) on Arthropathy in Crohn disease

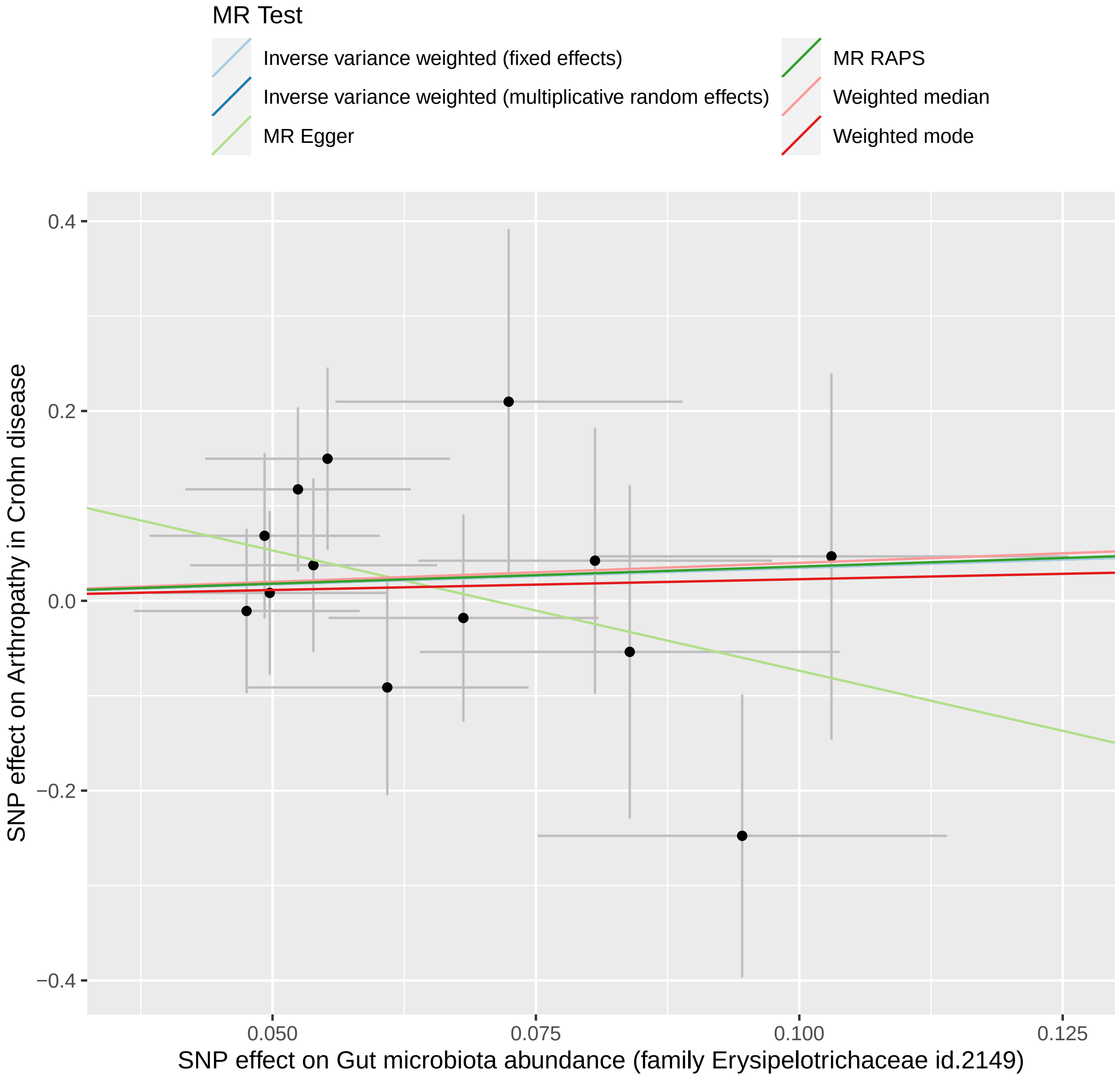

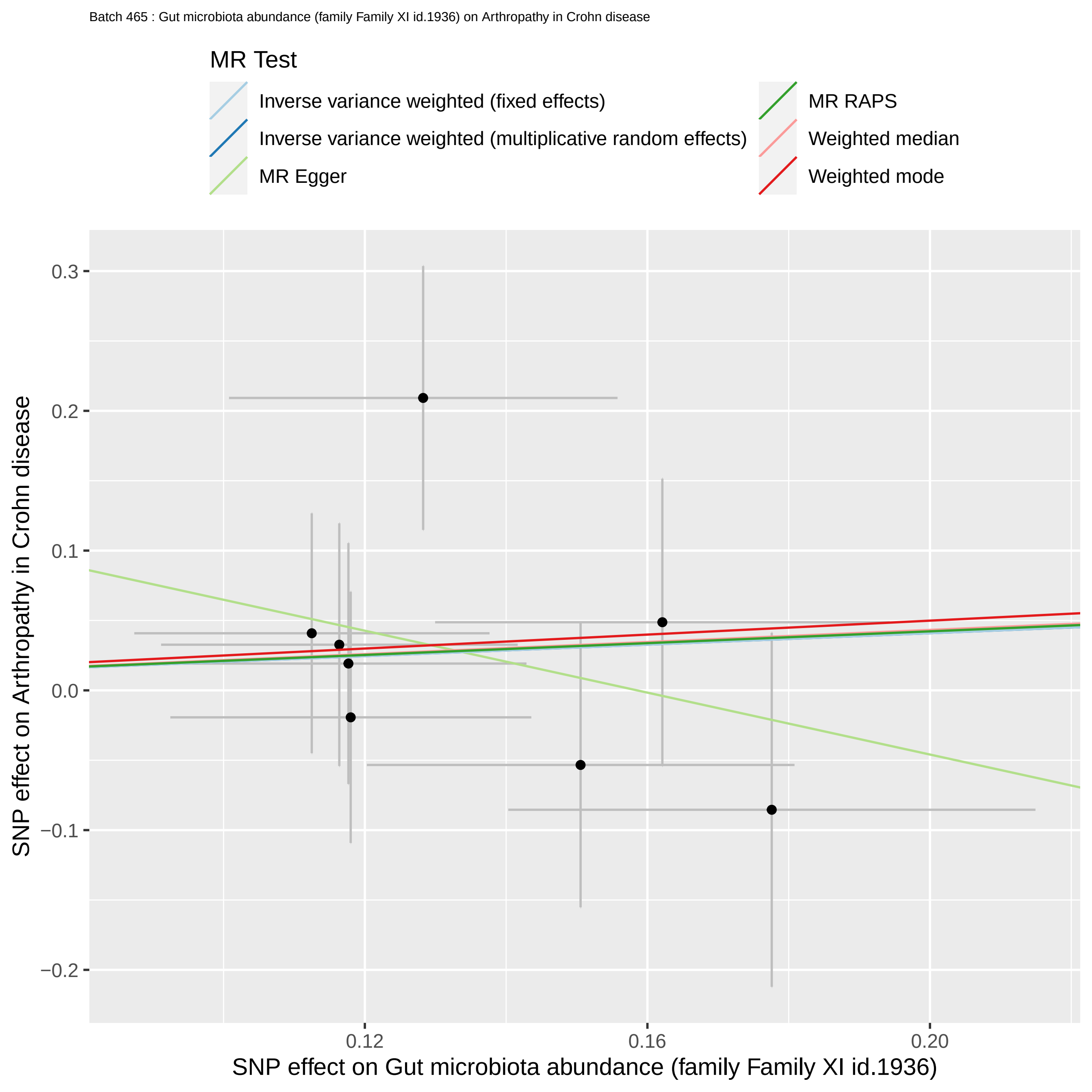
Batch 465 : Gut microbiota abundance (family Family XI id.1936) on Arthropathy in Crohn disease
MR Test
Inverse variance weighted (fixed effects)
Inverse variance weighted (multiplicative random effects)
MR Egger
MR RAPS
Weighted median
Weighted mode
0.3
0.2
0.1
0.0
−0.1
−0.2
0.12
0.16
0.20
SNP effect on Arthropathy in Crohn disease
SNP effect on Gut microbiota abundance (family Family XI id.1936)

Batch 466 : Gut microbiota abundance (family Family XIII id.1957) on Arthropathy in Crohn disease

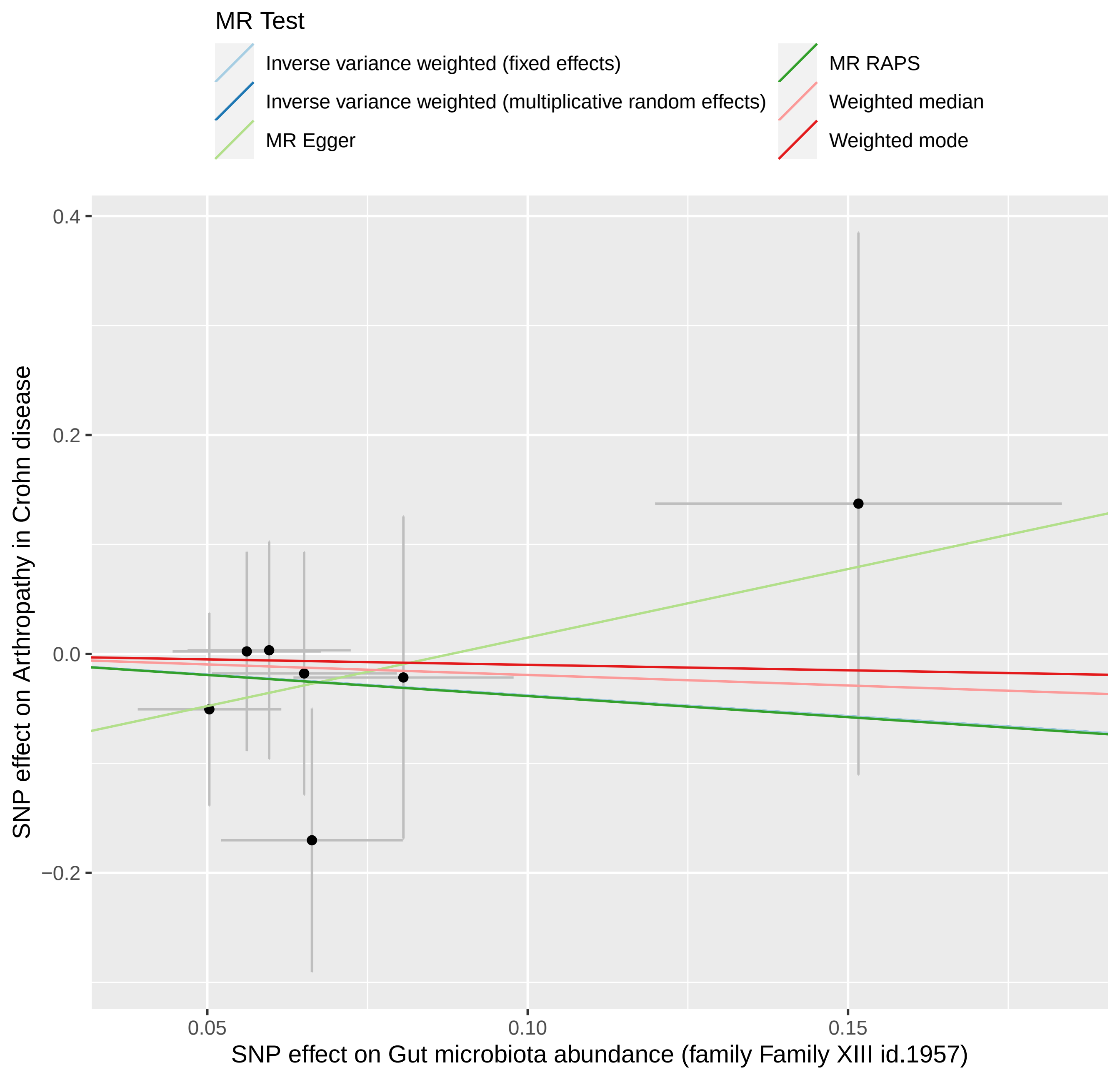

Batch 467 : Gut microbiota abundance (family Lachnospiraceae id.1987) on Arthropathy in Crohn disease

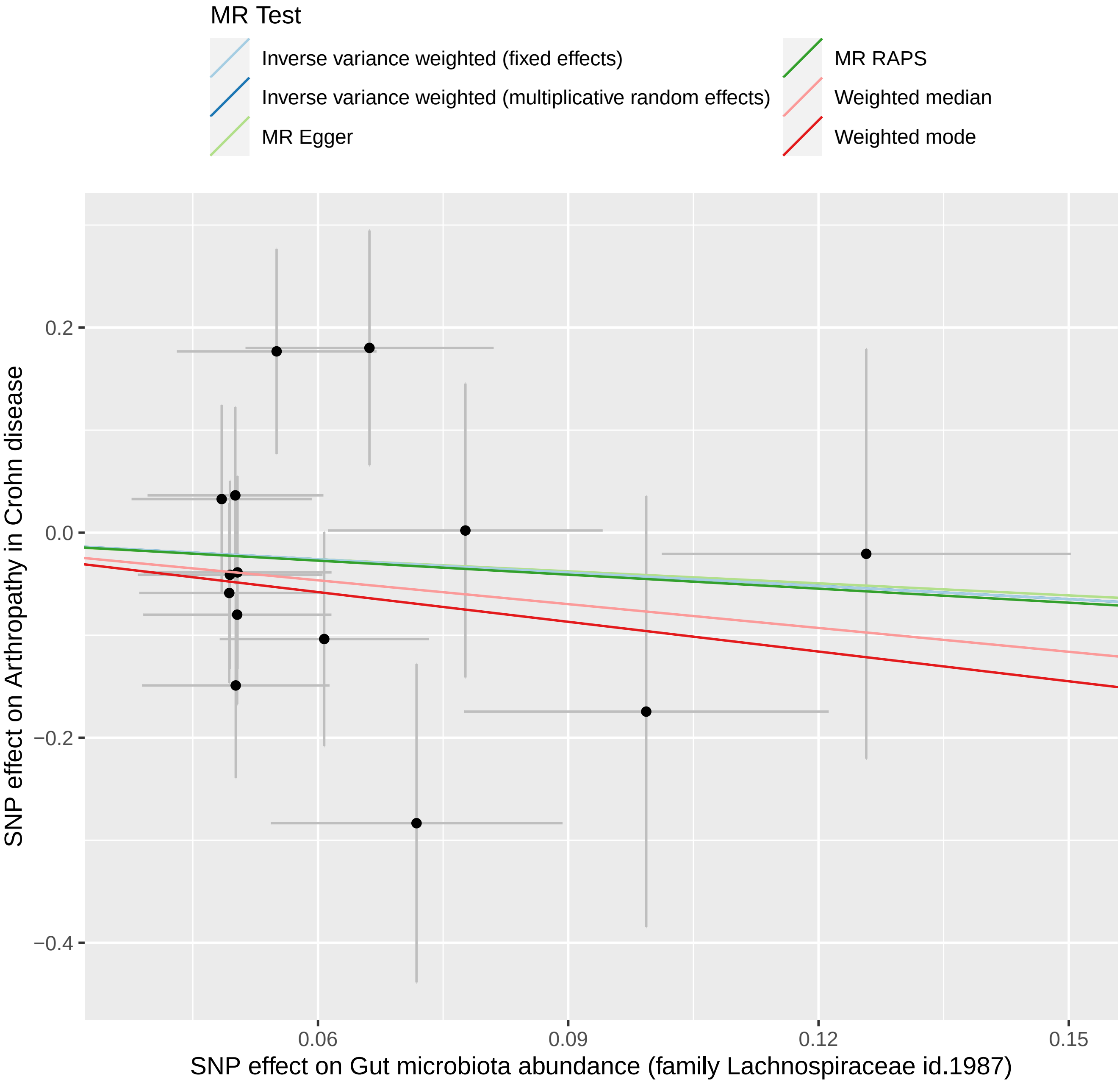

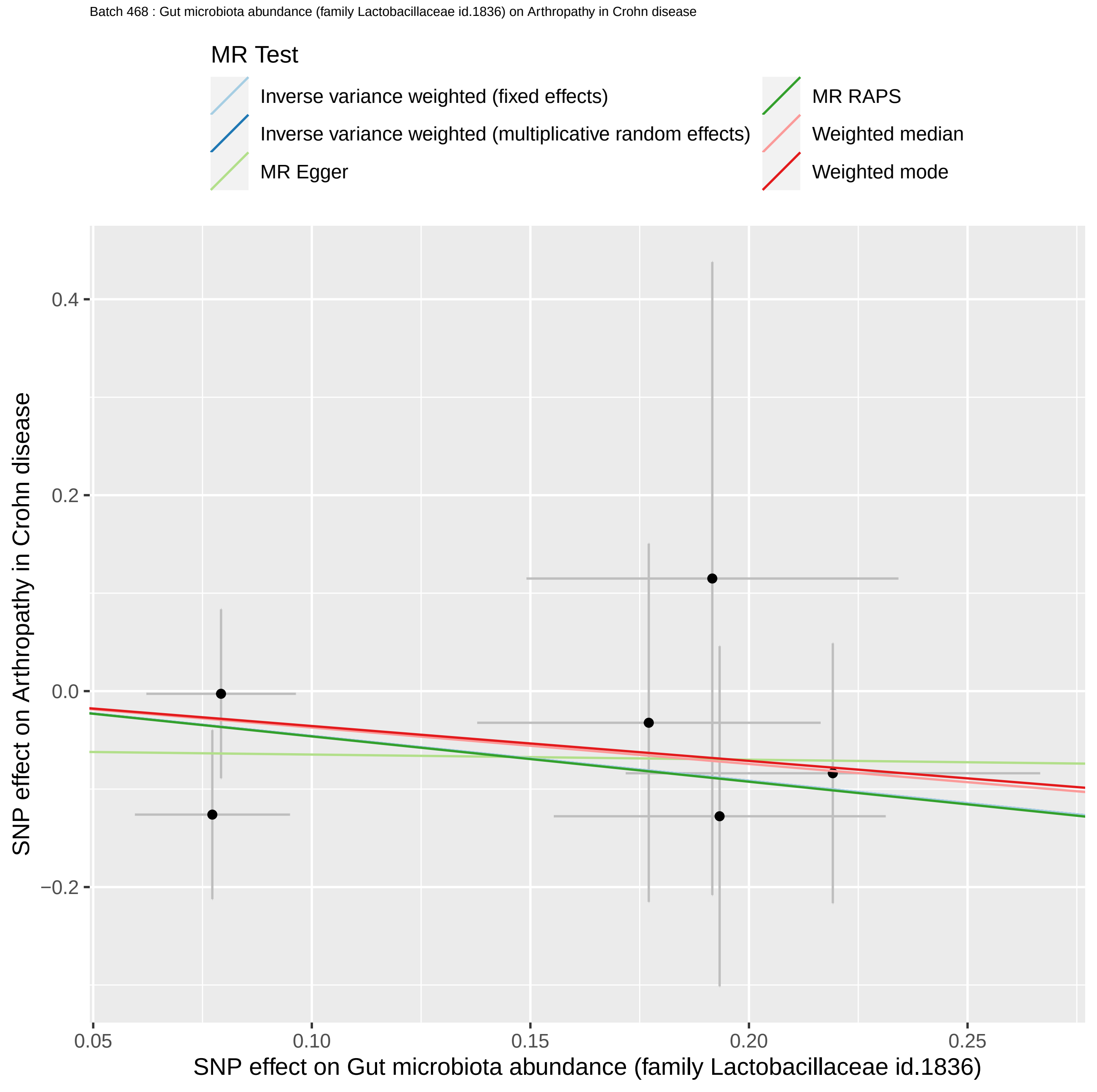

Batch 468 : Gut microbiota abundance (family Lactobacillaceae id.1836) on Arthropathy in Crohn disease
MR Test
Inverse variance weighted (fixed effects)
Inverse variance weighted (multiplicative random effects)
MR Egger
MR RAPS
Weighted median
Weighted mode
0.4
0.2
0.0
−0.2
0.05
0.10
0.15
0.20
0.25
SNP effect on Arthropathy in Crohn disease
SNP effect on Gut microbiota abundance (family Lactobacillaceae id.1836)

Batch 469 : Gut microbiota abundance (family Methanobacteriaceae id.121) on Arthropathy in Crohn disease

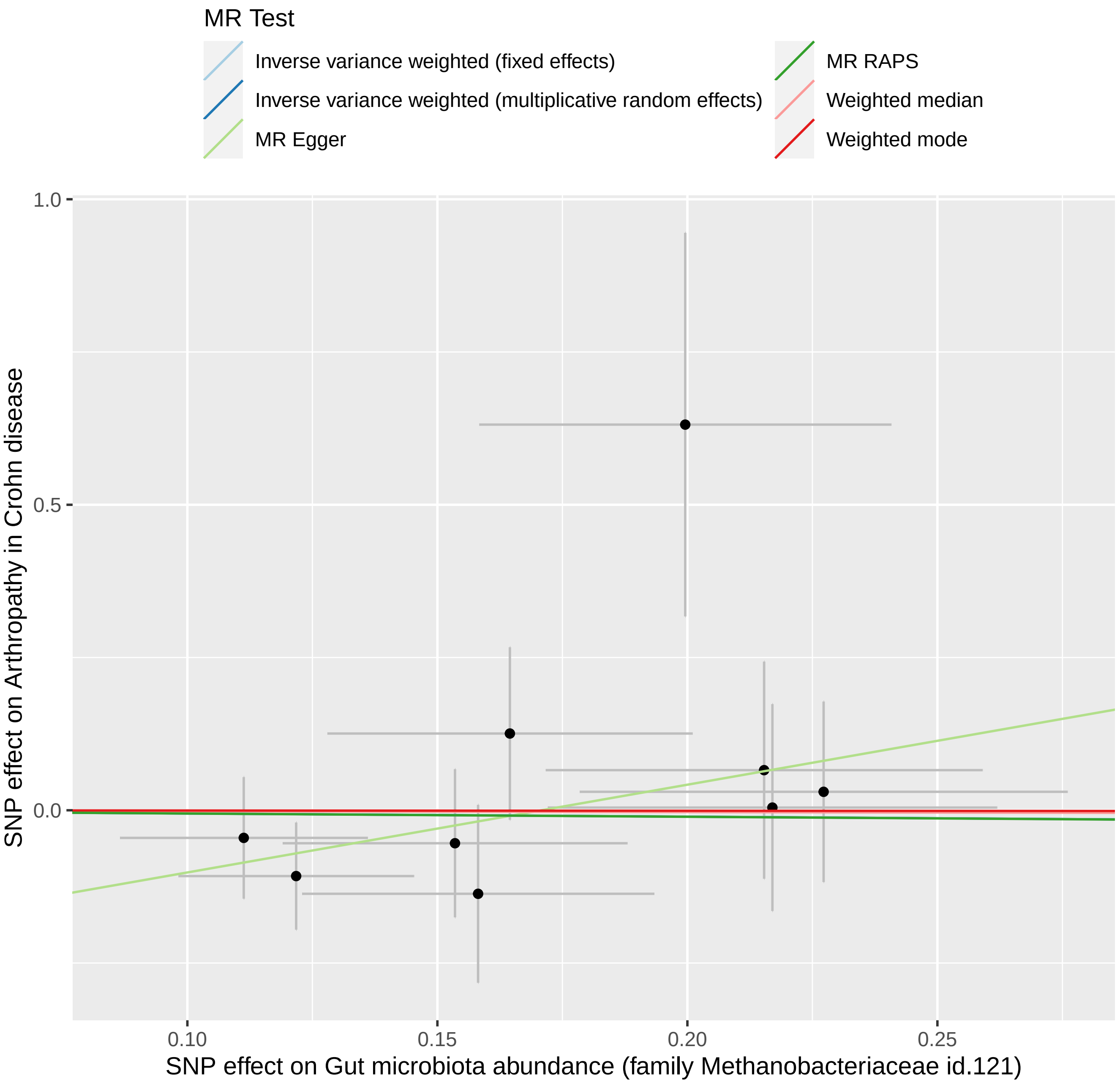

Batch 470 : Gut microbiota abundance (family Oxalobacteraceae id.2966) on Arthropathy in Crohn disease

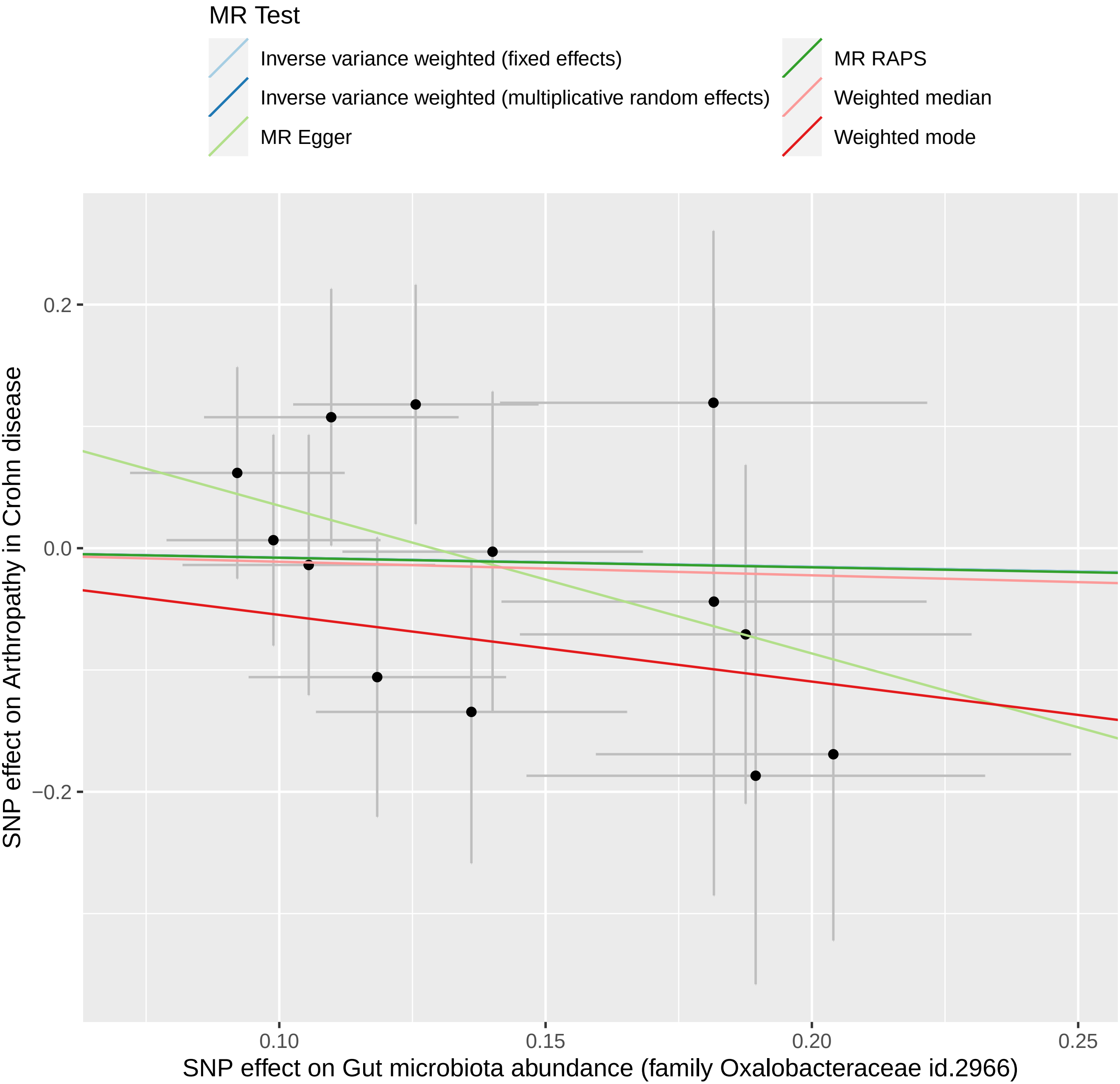

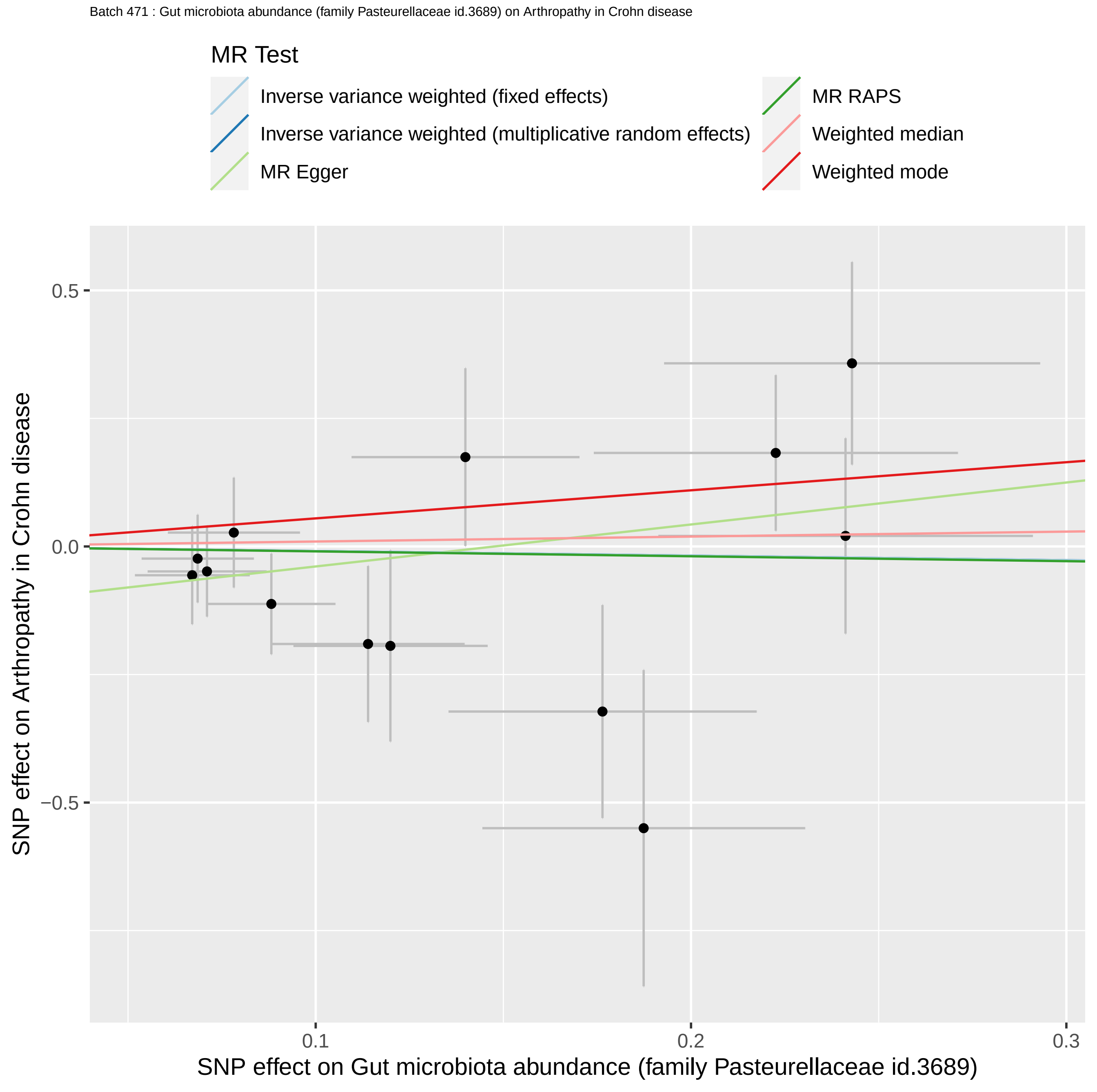

Batch 471 : Gut microbiota abundance (family Pasteurellaceae id.3689) on Arthropathy in Crohn disease
MR Test
Inverse variance weighted (fixed effects)
Inverse variance weighted (multiplicative random effects)
MR Egger
MR RAPS
Weighted median
Weighted mode
0.5
0.0
−0.5
0.1
0.2
0.3
SNP effect on Arthropathy in Crohn disease
SNP effect on Gut microbiota abundance (family Pasteurellaceae id.3689)

Batch 472 : Gut microbiota abundance (family Peptococcaceae id.2024) on Arthropathy in Crohn disease

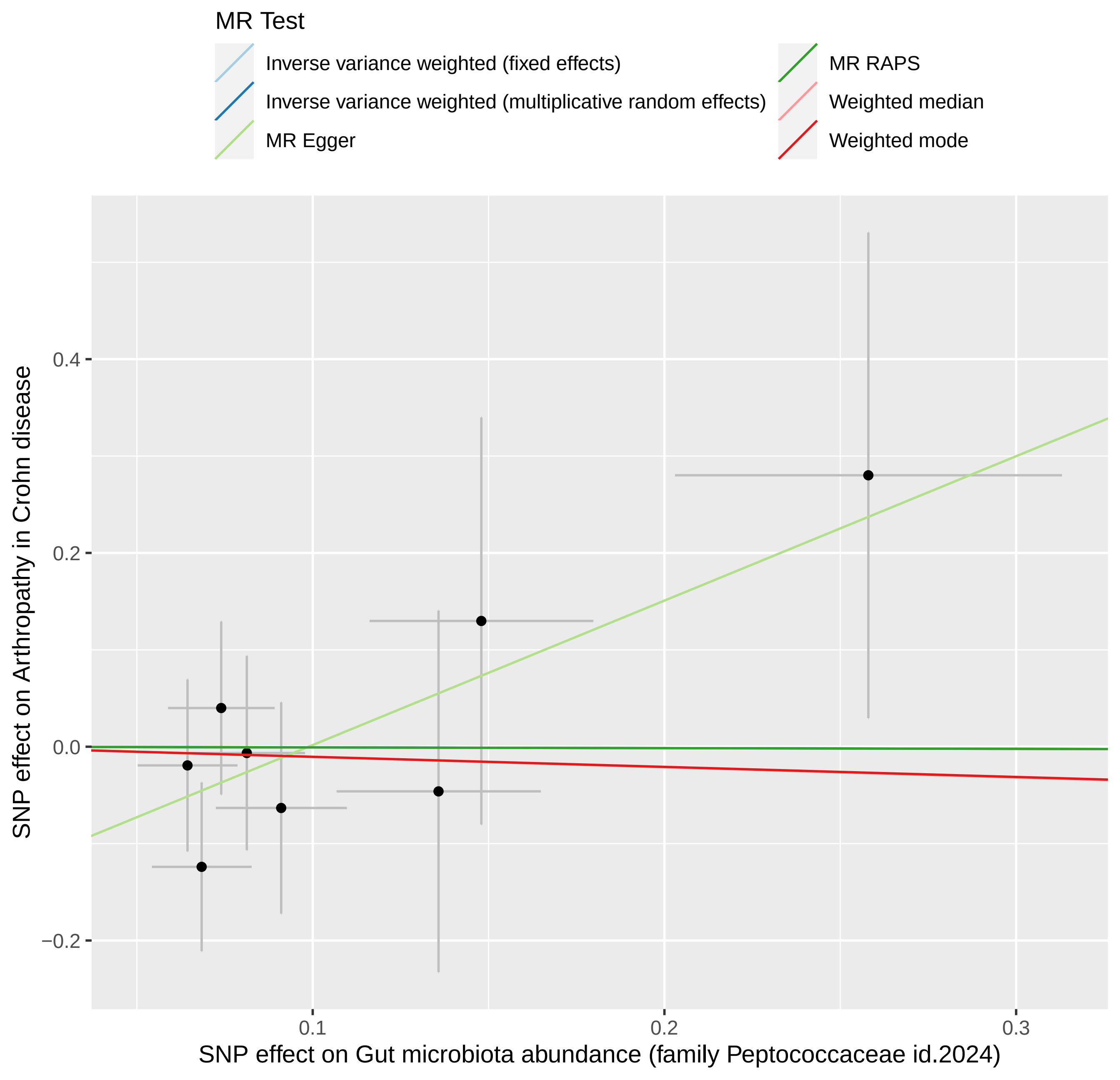

Batch 473 : Gut microbiota abundance (family Peptostreptococcaceae id.2042) on Arthropathy in Crohn disease

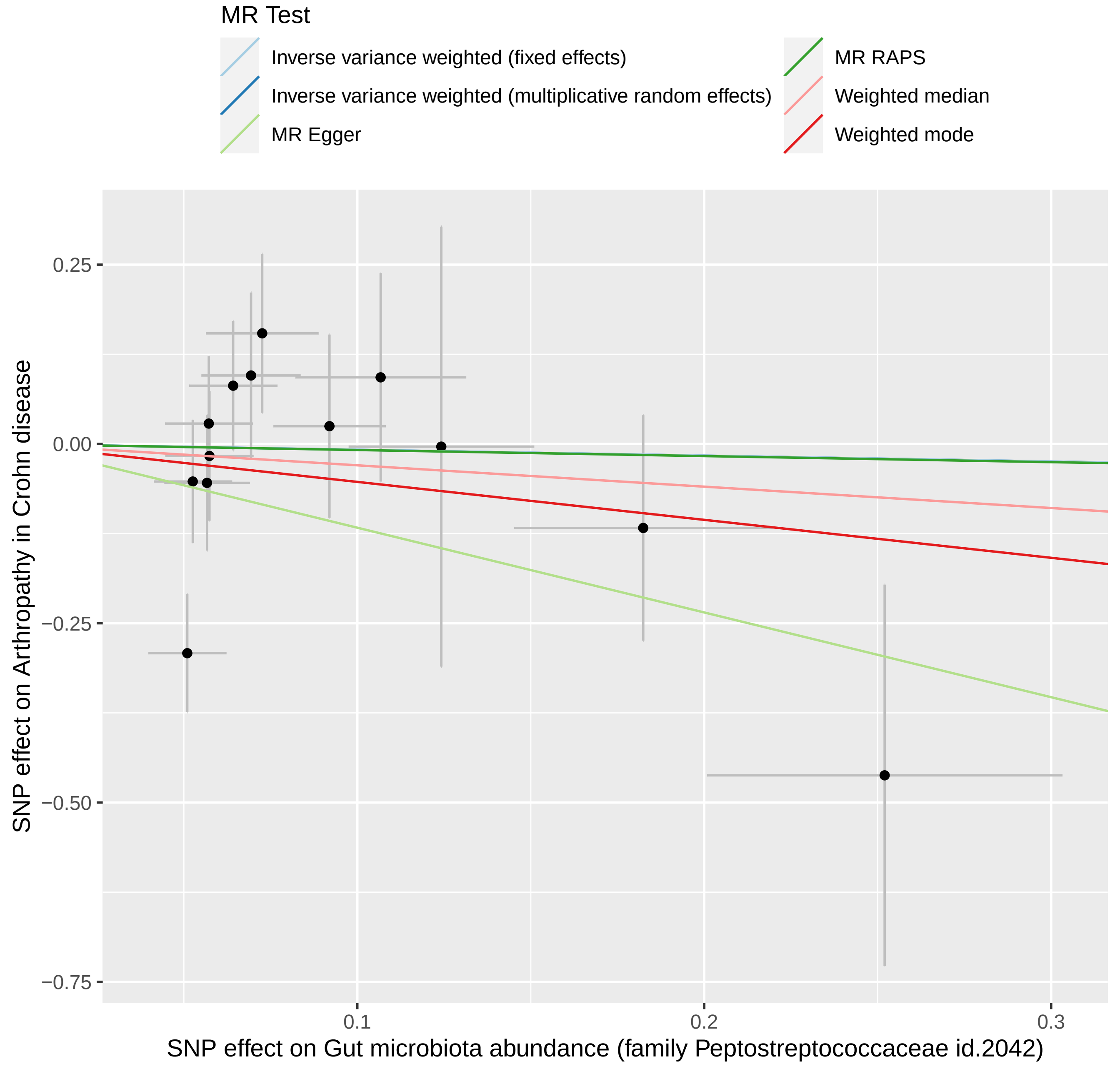

Batch 474 : Gut microbiota abundance (family Porphyromonadaceae id.943) on Arthropathy in Crohn disease

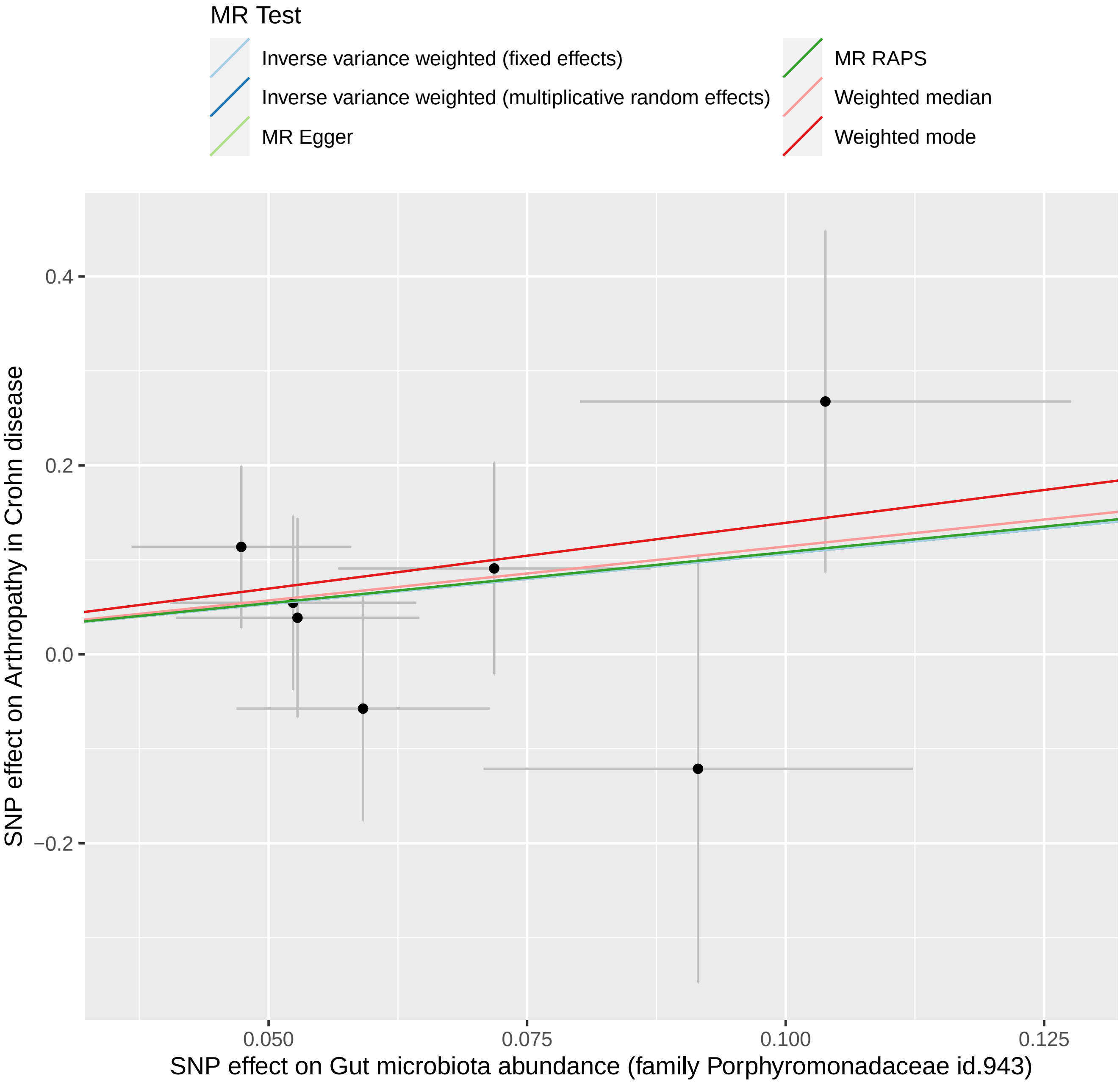

Batch 475 : Gut microbiota abundance (family Prevotellaceae id.960) on Arthropathy in Crohn disease

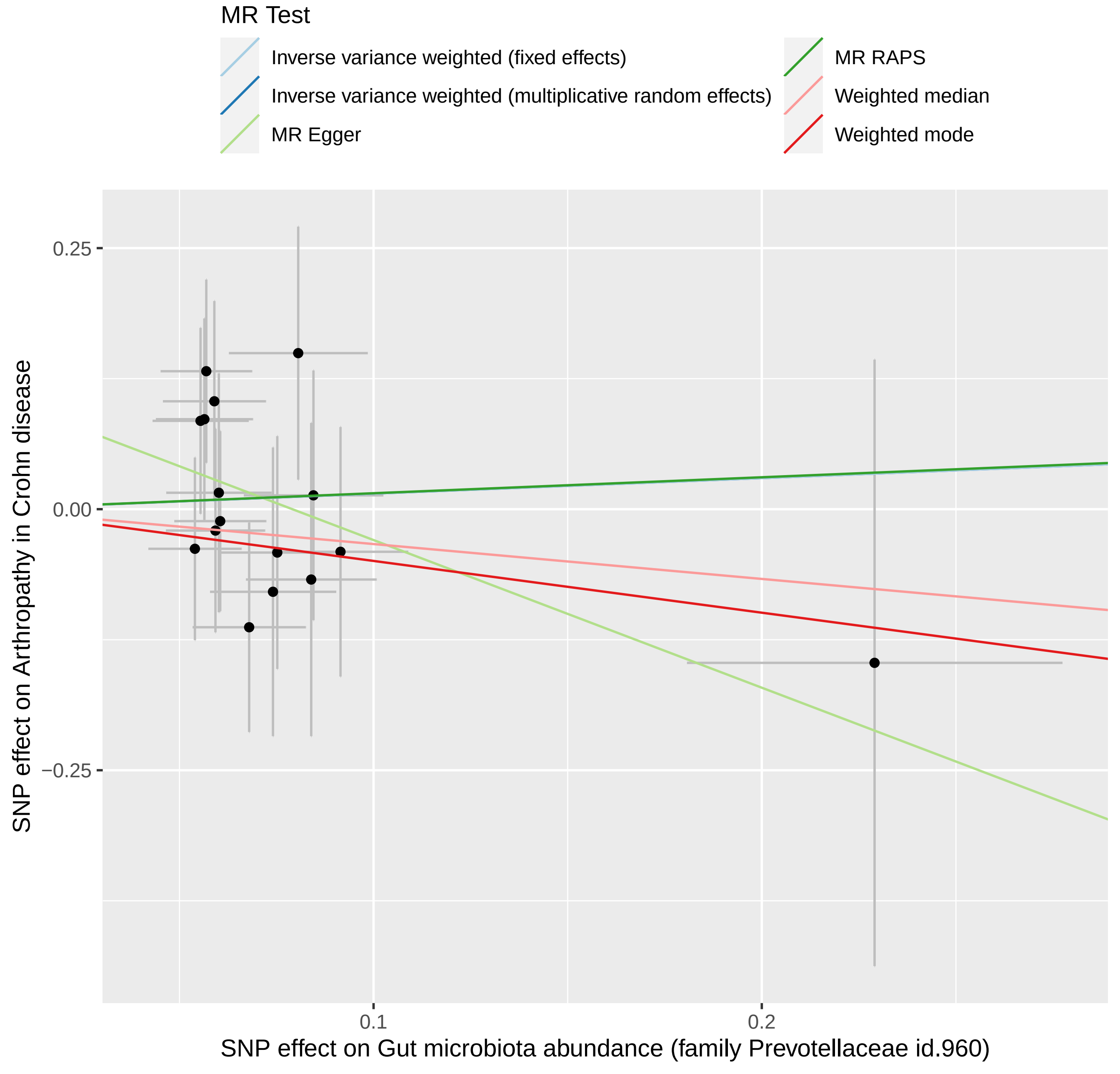

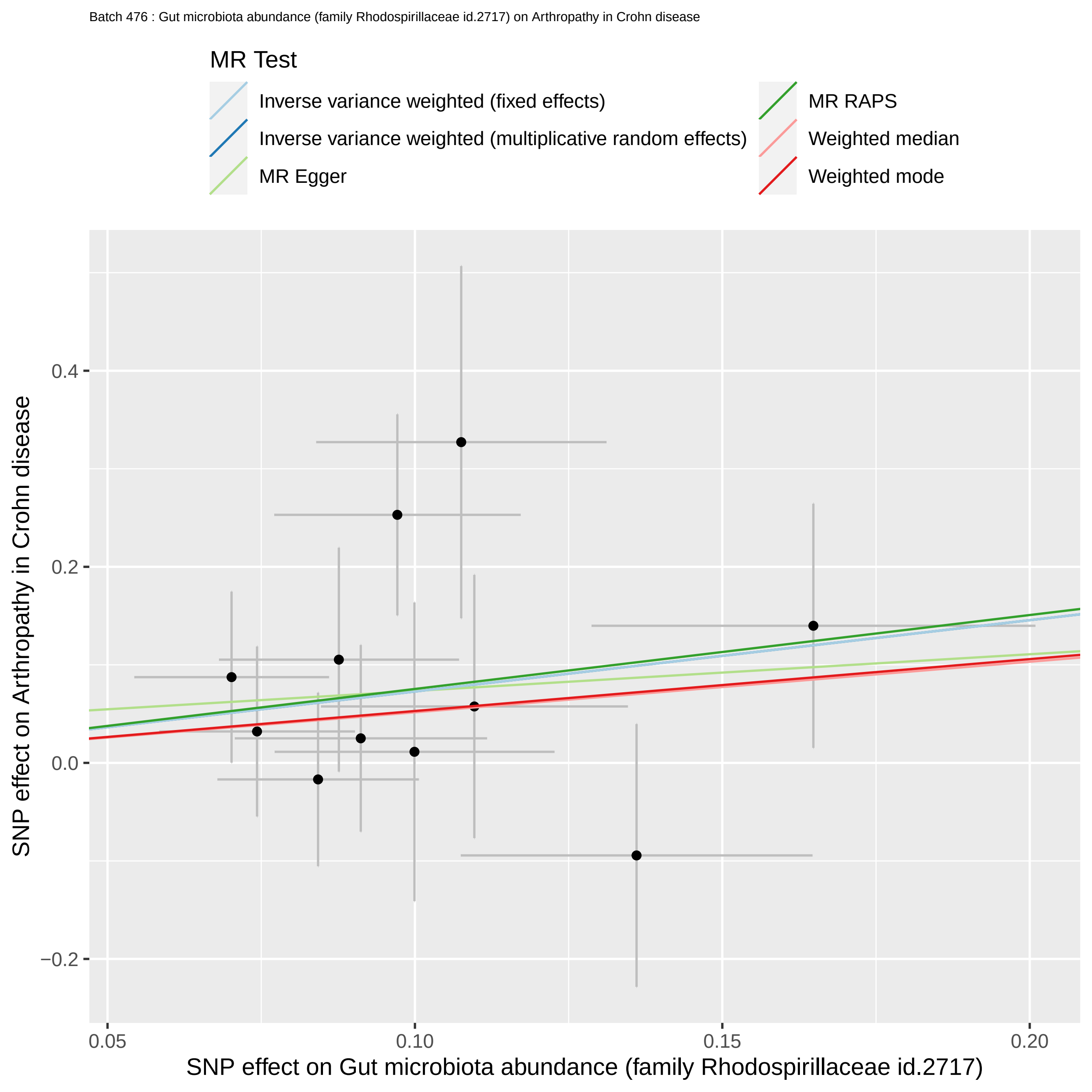
Batch 476 : Gut microbiota abundance (family Rhodospirillaceae id.2717) on Arthropathy in Crohn disease
MR Test
Inverse variance weighted (fixed effects)
Inverse variance weighted (multiplicative random effects)
MR Egger
MR RAPS
Weighted median
Weighted mode
0.4
0.2
0.0
−0.2
0.05
0.10
0.15
0.20
SNP effect on Arthropathy in Crohn disease
SNP effect on Gut microbiota abundance (family Rhodospirillaceae id.2717)

Batch 477 : Gut microbiota abundance (family Rikenellaceae id.967) on Arthropathy in Crohn disease

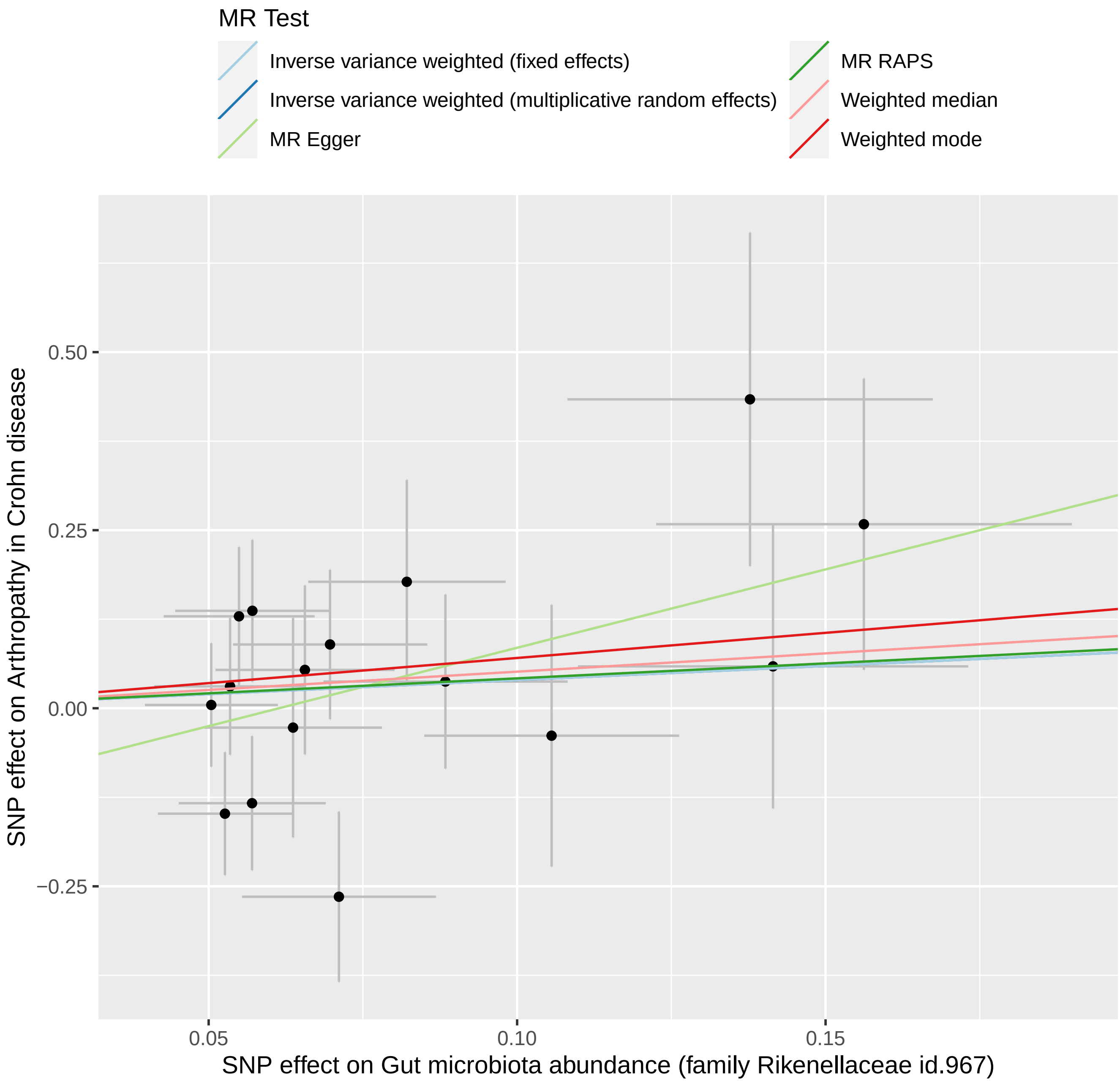

Batch 478 : Gut microbiota abundance (family Ruminococcaceae id.2050) on Arthropathy in Crohn disease

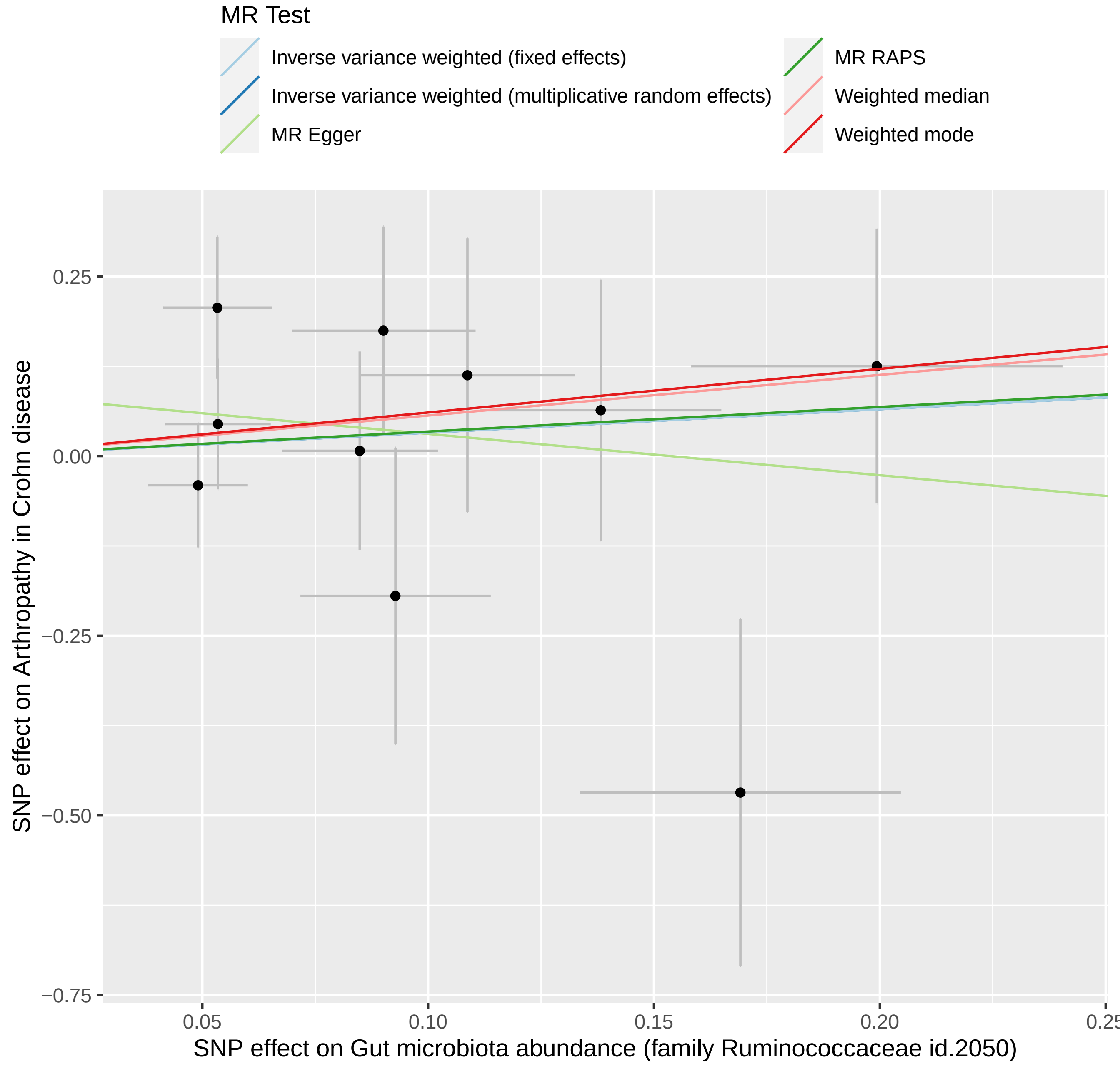

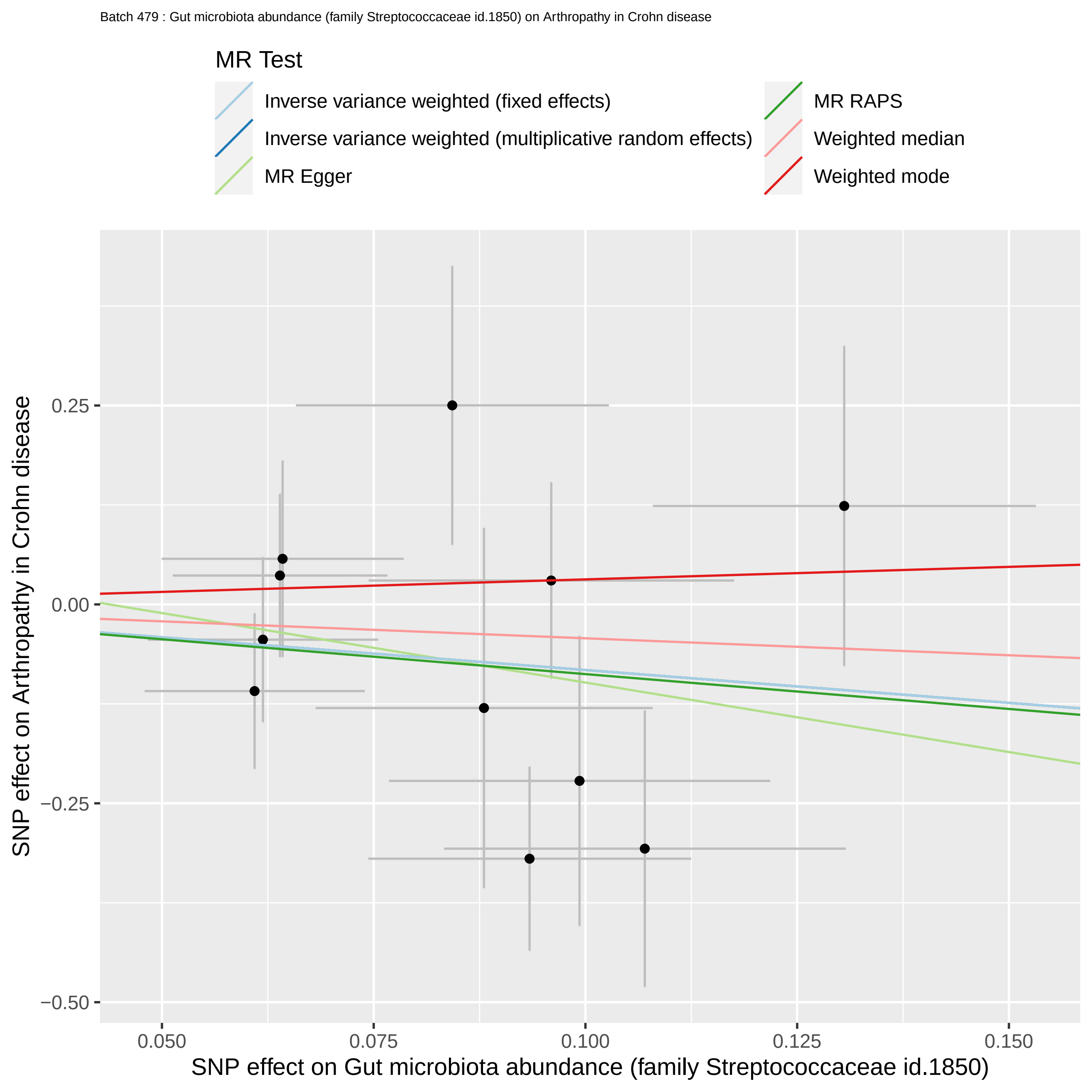

Batch 479 : Gut microbiota abundance (family Streptococcaceae id.1850) on Arthropathy in Crohn disease
MR Test
Inverse variance weighted (fixed effects)
Inverse variance weighted (multiplicative random effects)
MR Egger
MR RAPS
Weighted median
Weighted mode
0.25
0.00
−0.25
−0.50
0.050
0.075
0.100
0.125
0.150
SNP effect on Arthropathy in Crohn disease
SNP effect on Gut microbiota abundance (family Streptococcaceae id.1850)

Batch 480 : Gut microbiota abundance (family Veillonellaceae id.2172) on Arthropathy in Crohn disease

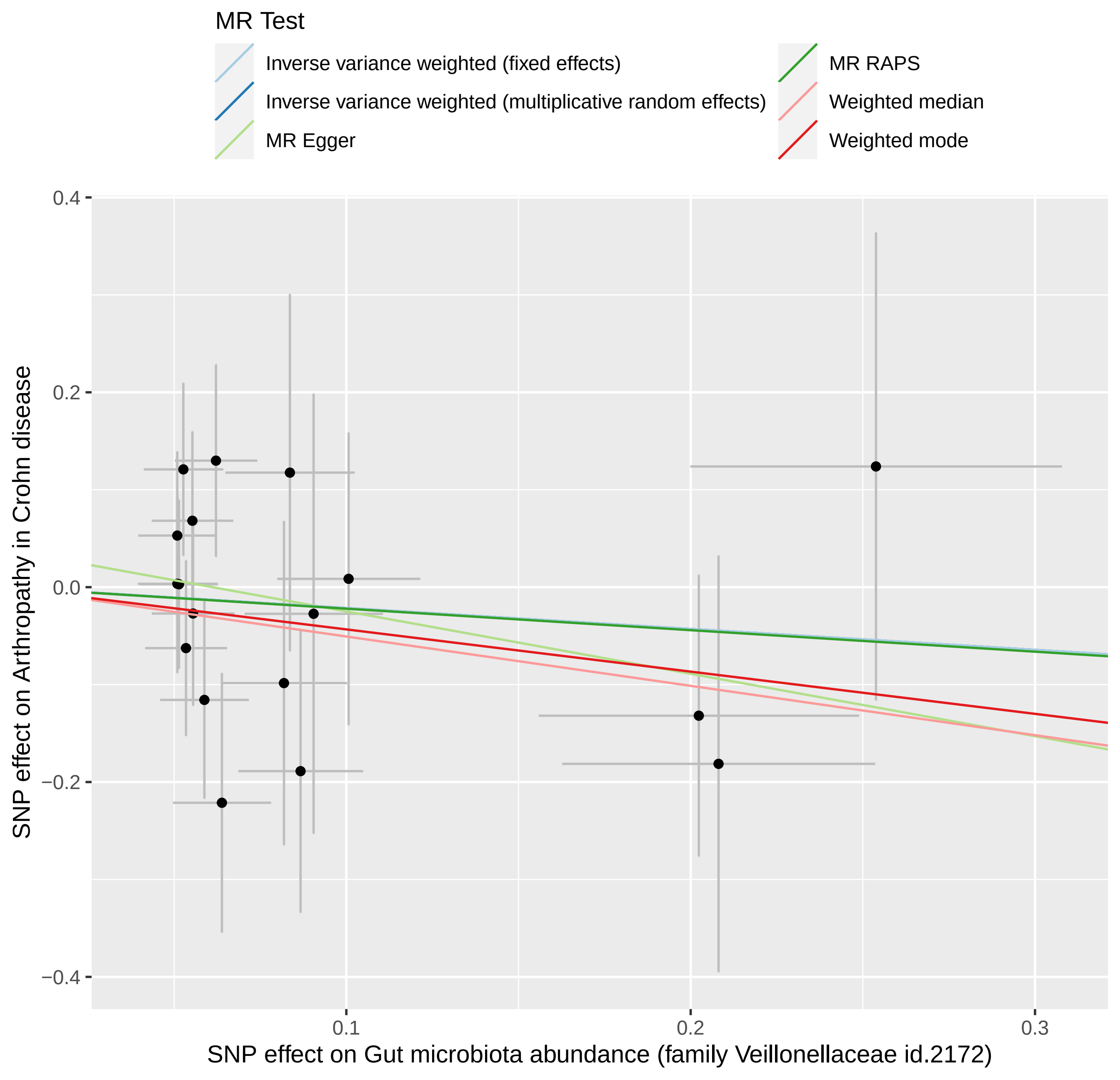

Batch 481 : Gut microbiota abundance (family Verrucomicrobiaceae id.4036) on Arthropathy in Crohn disease

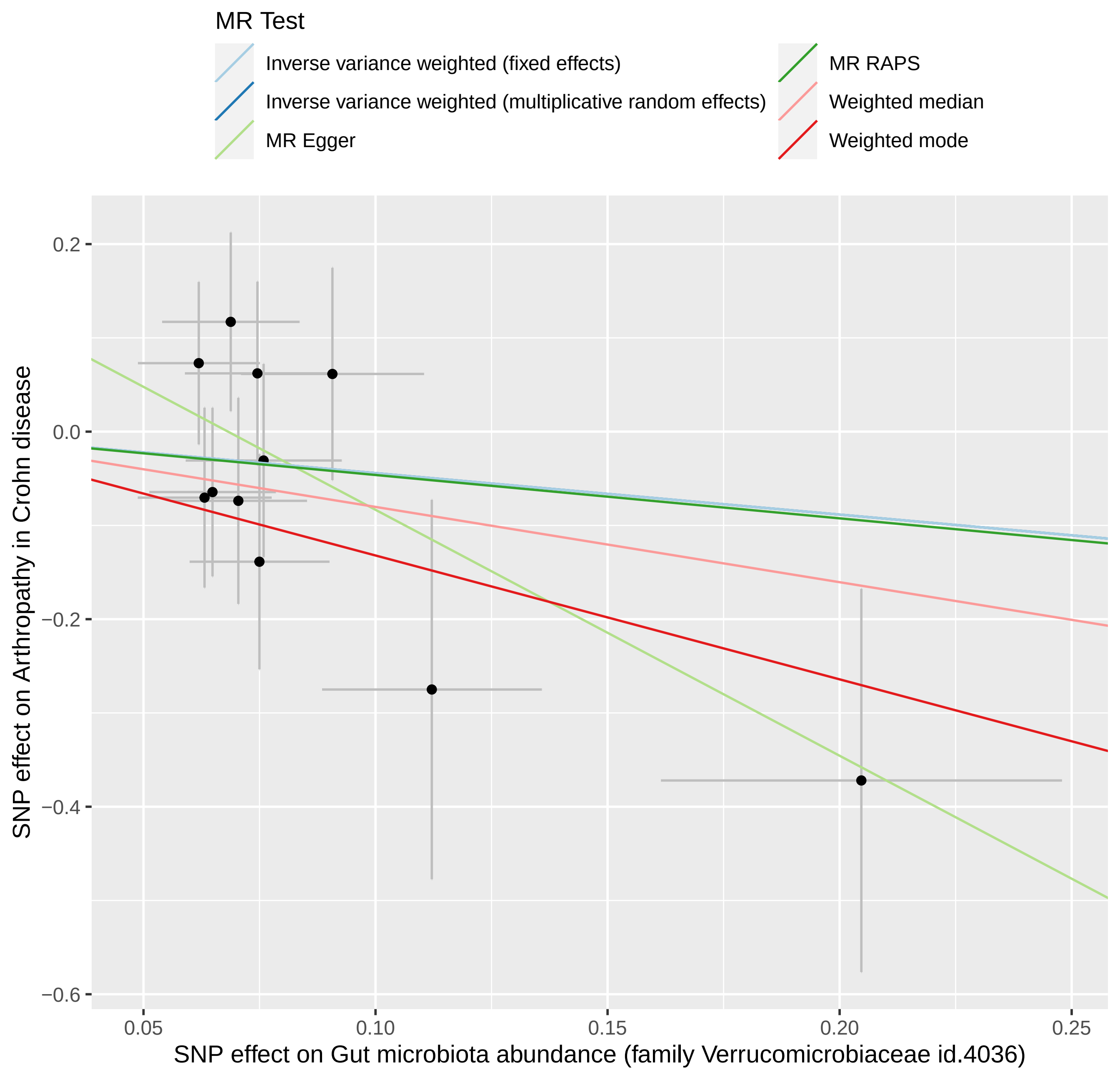

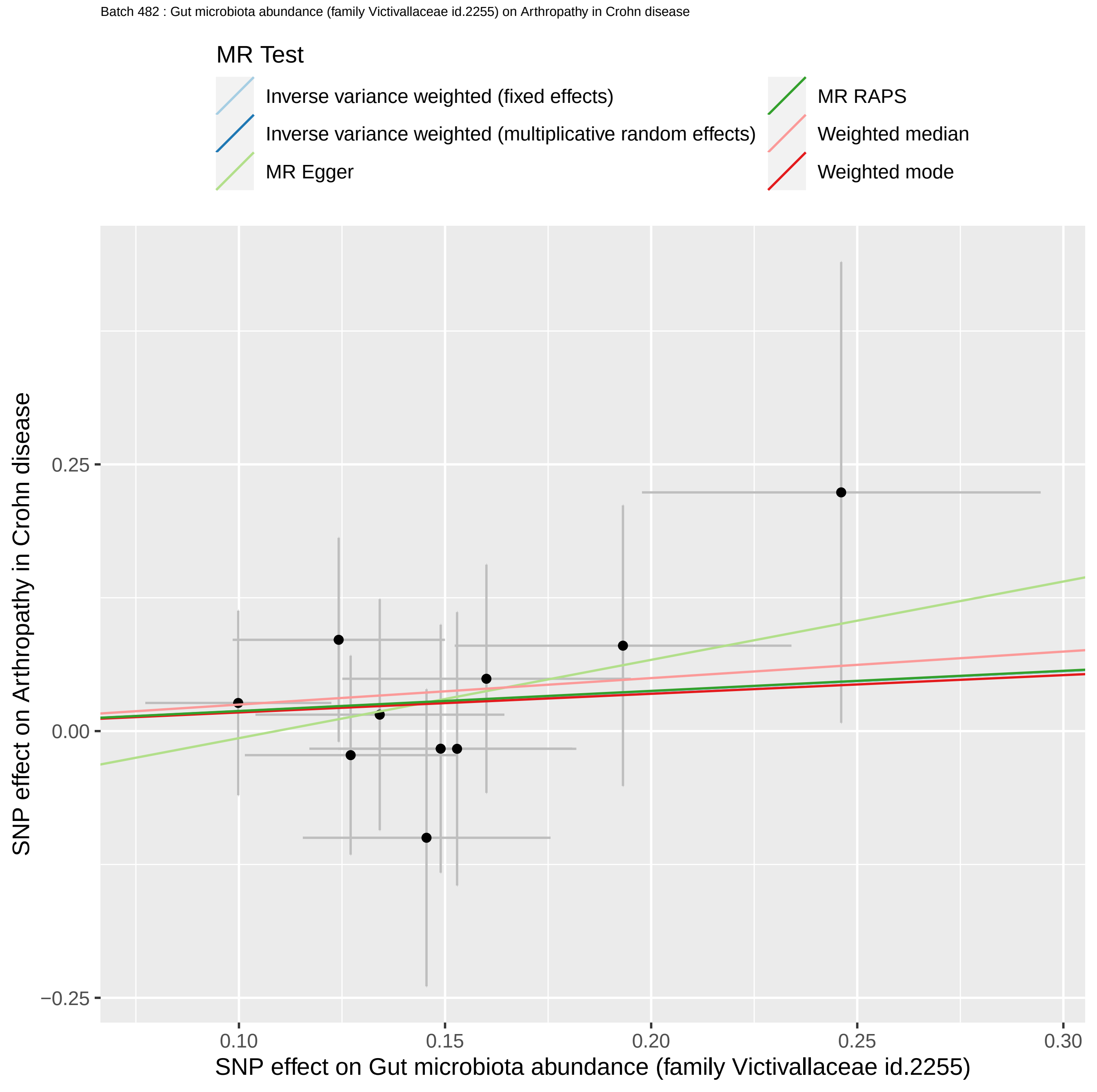

Batch 482 : Gut microbiota abundance (family Victivallaceae id.2255) on Arthropathy in Crohn disease
MR Test
Inverse variance weighted (fixed effects)
Inverse variance weighted (multiplicative random effects)
MR Egger
MR RAPS
Weighted median
Weighted mode
SNP effect on Arthropathy in Crohn disease
0.25
0.00
−0.25
0.10
0.15
0.20
0.25
0.30
SNP effect on Gut microbiota abundance (family Victivallaceae id.2255)

Batch 483 : Gut microbiota abundance (genus Actinomyces id.423) on Arthropathy in Crohn disease

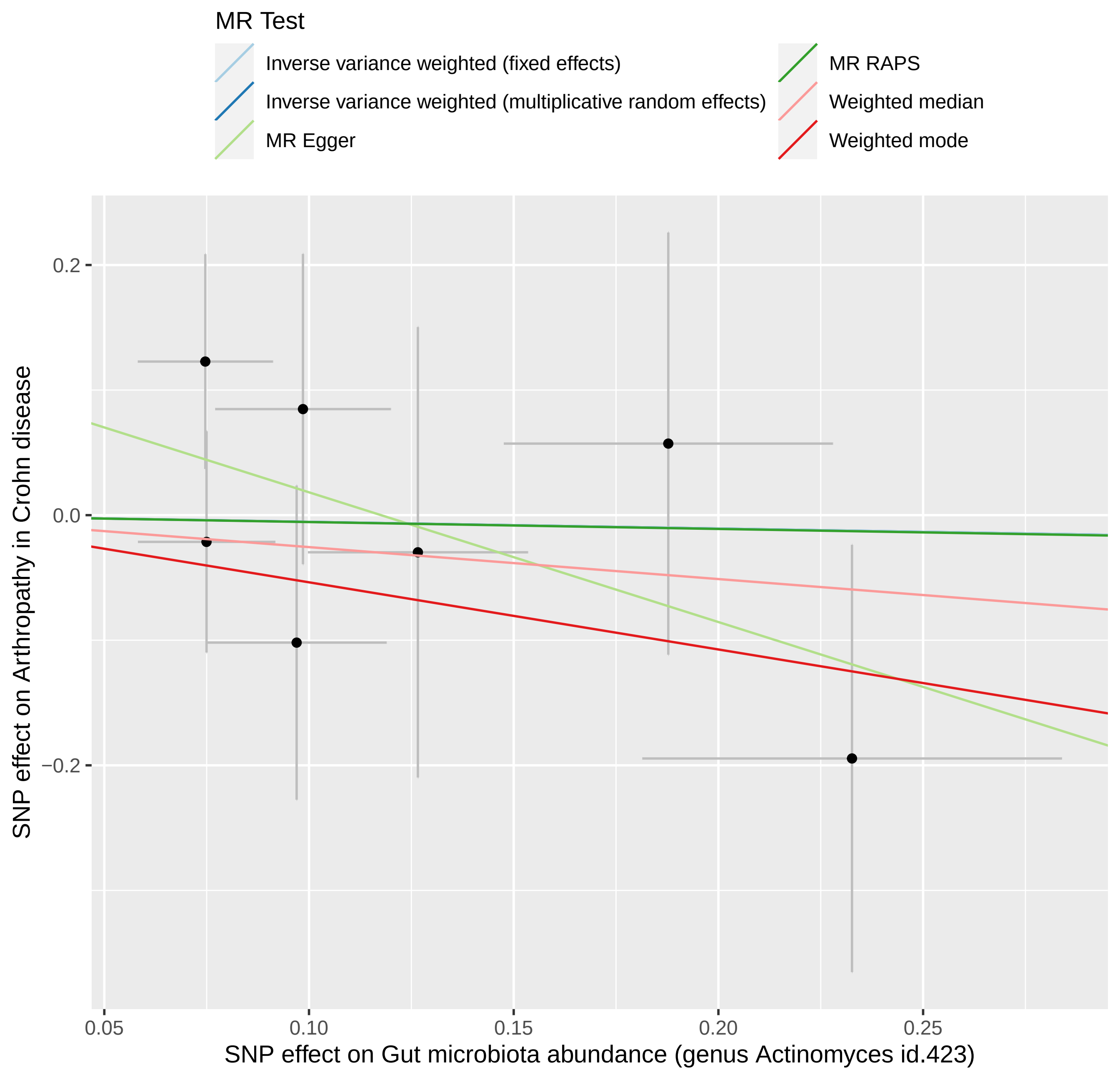

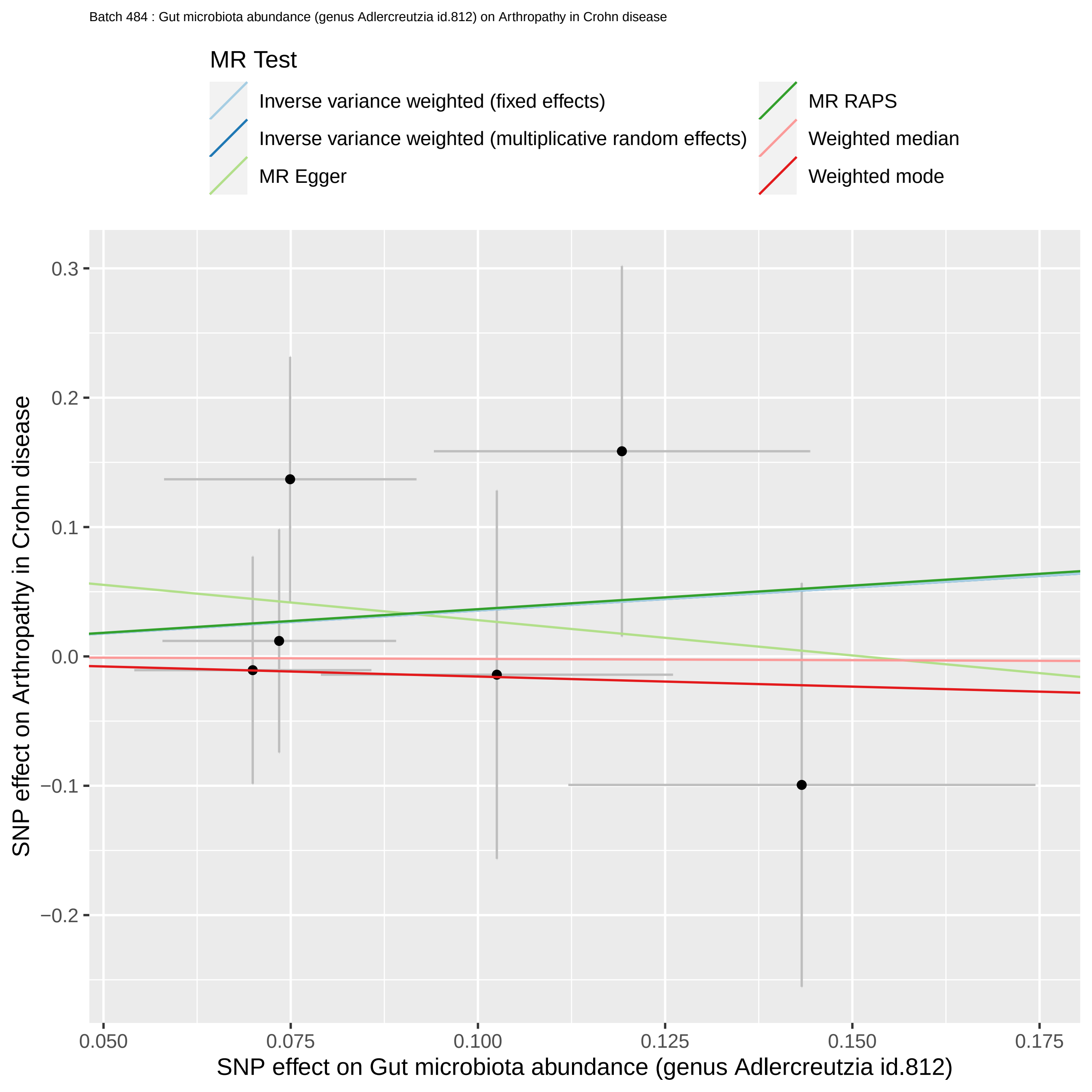

Batch 484 : Gut microbiota abundance (genus Adlercreutzia id.812) on Arthropathy in Crohn disease
MR Test
Inverse variance weighted (fixed effects)
Inverse variance weighted (multiplicative random effects)
MR Egger
MR RAPS
Weighted median
Weighted mode
0.3
0.2
0.1
0.0
−0.1
−0.2
0.050
0.075
0.100
0.125
0.150
0.175
SNP effect on Arthropathy in Crohn disease
SNP effect on Gut microbiota abundance (genus Adlercreutzia id.812)

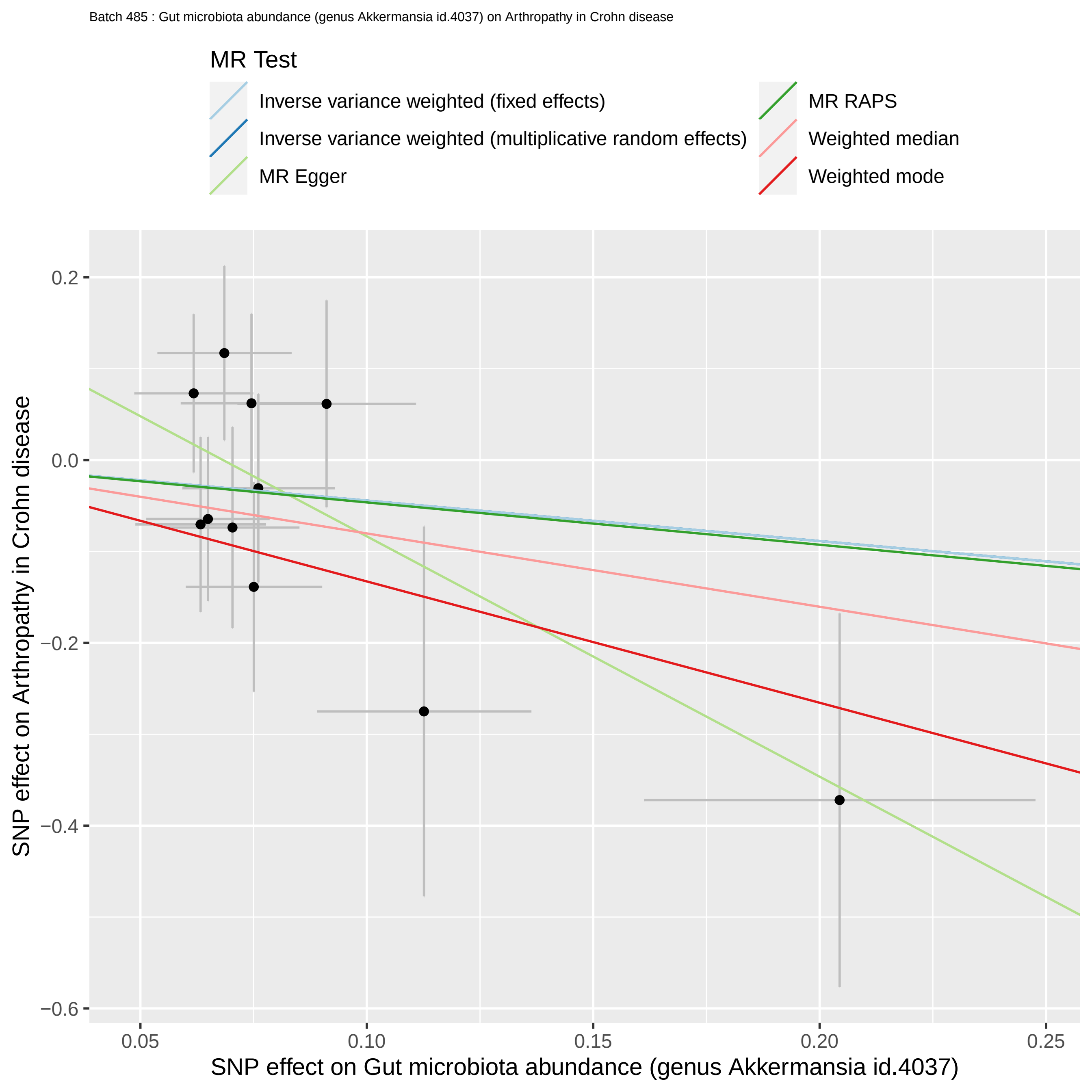

Batch 485 : Gut microbiota abundance (genus Akkermansia id.4037) on Arthropathy in Crohn disease
MR Test
Inverse variance weighted (fixed effects)
Inverse variance weighted (multiplicative random effects)
MR Egger
MR RAPS
Weighted median
Weighted mode
SNP effect on Arthropathy in Crohn disease
0.2
0.0
−0.2
−0.4
−0.6
0.05
0.10
0.15
0.20
0.25
SNP effect on Gut microbiota abundance (genus Akkermansia id.4037)

Batch 486 : Gut microbiota abundance (genus Alistipes id.968) on Arthropathy in Crohn disease

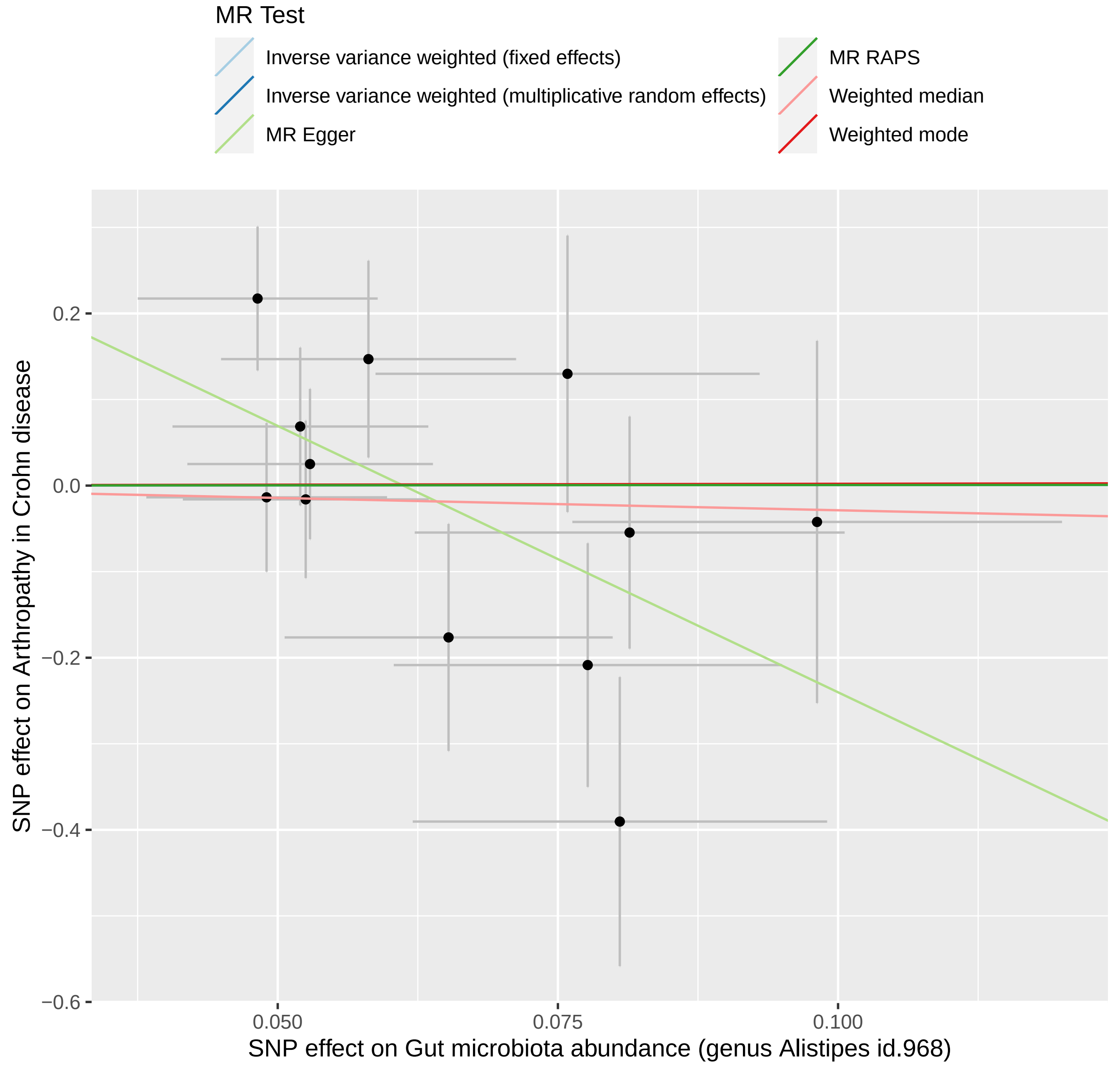

Batch 487 : Gut microbiota abundance (genus Allisonella id.2174) on Arthropathy in Crohn disease

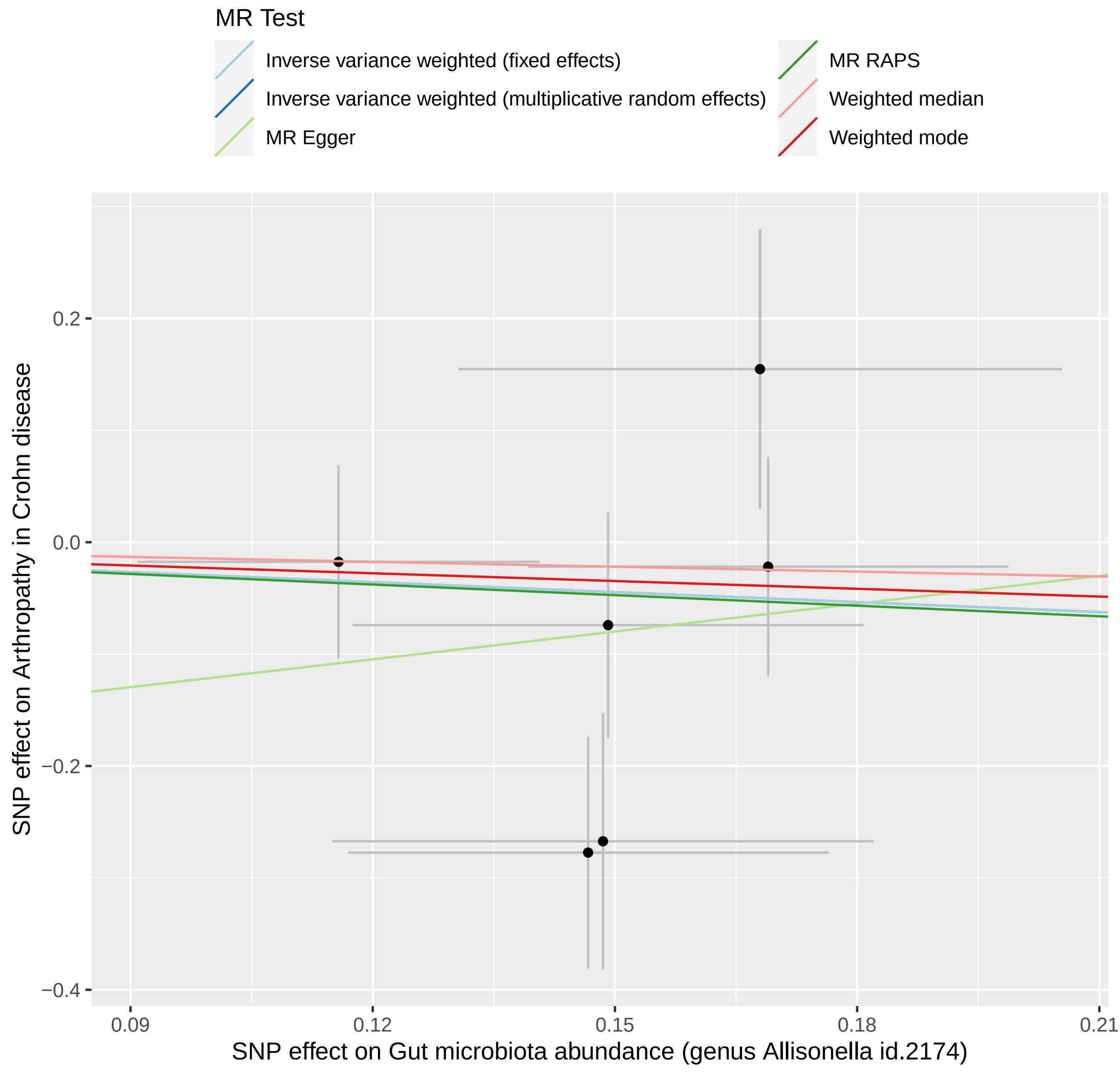

Batch 488 : Gut microbiota abundance (genus Alloprevotella id.961) on Arthropathy in Crohn disease

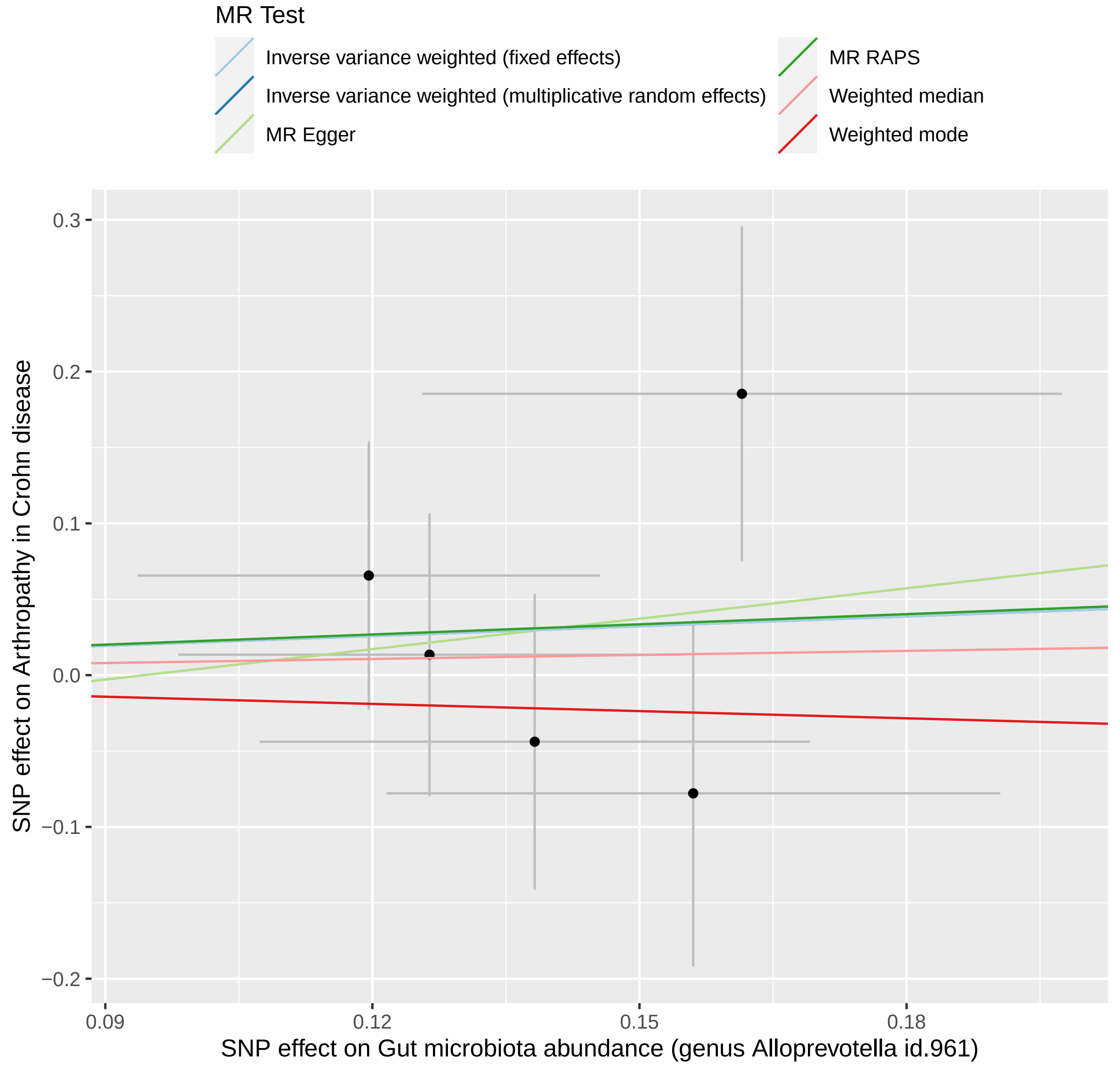

Batch 489 : Gut microbiota abundance (genus Anaerofilum id.2053) on Arthropathy in Crohn disease

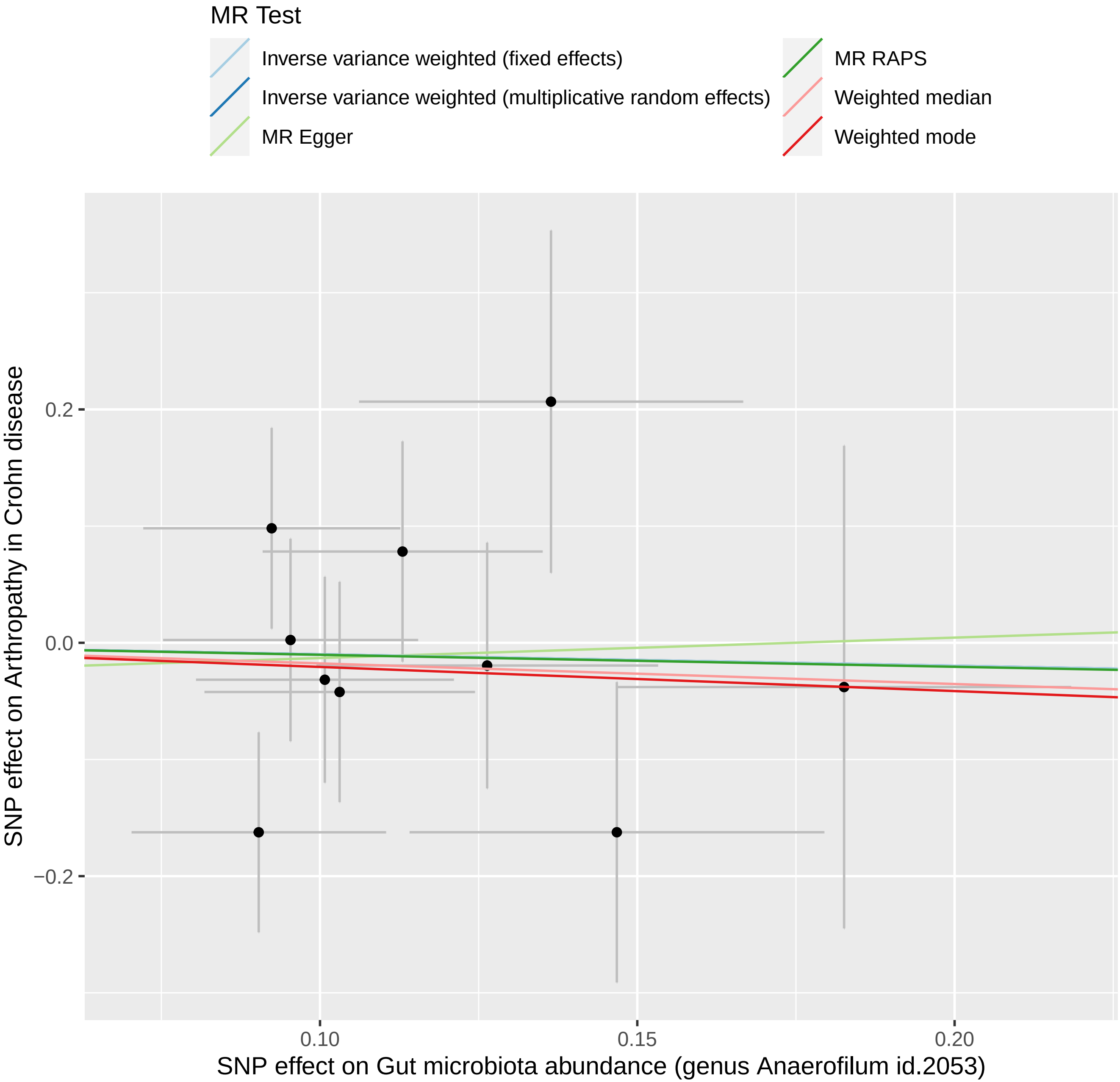

Batch 490 : Gut microbiota abundance (genus Anaerostipes id.1991) on Arthropathy in Crohn disease

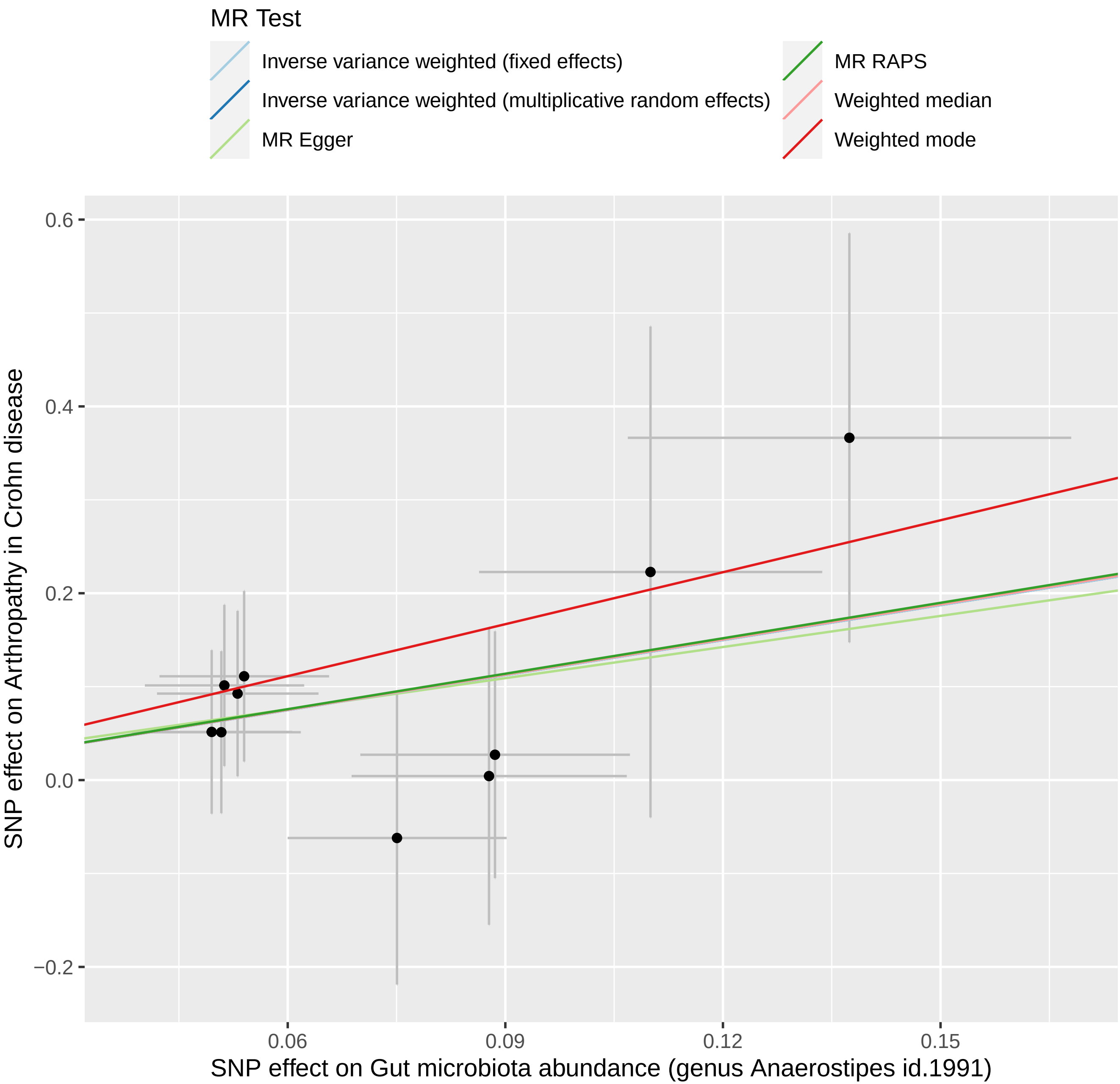

Batch 491 : Gut microbiota abundance (genus Anaerotruncus id.2054) on Arthropathy in Crohn disease

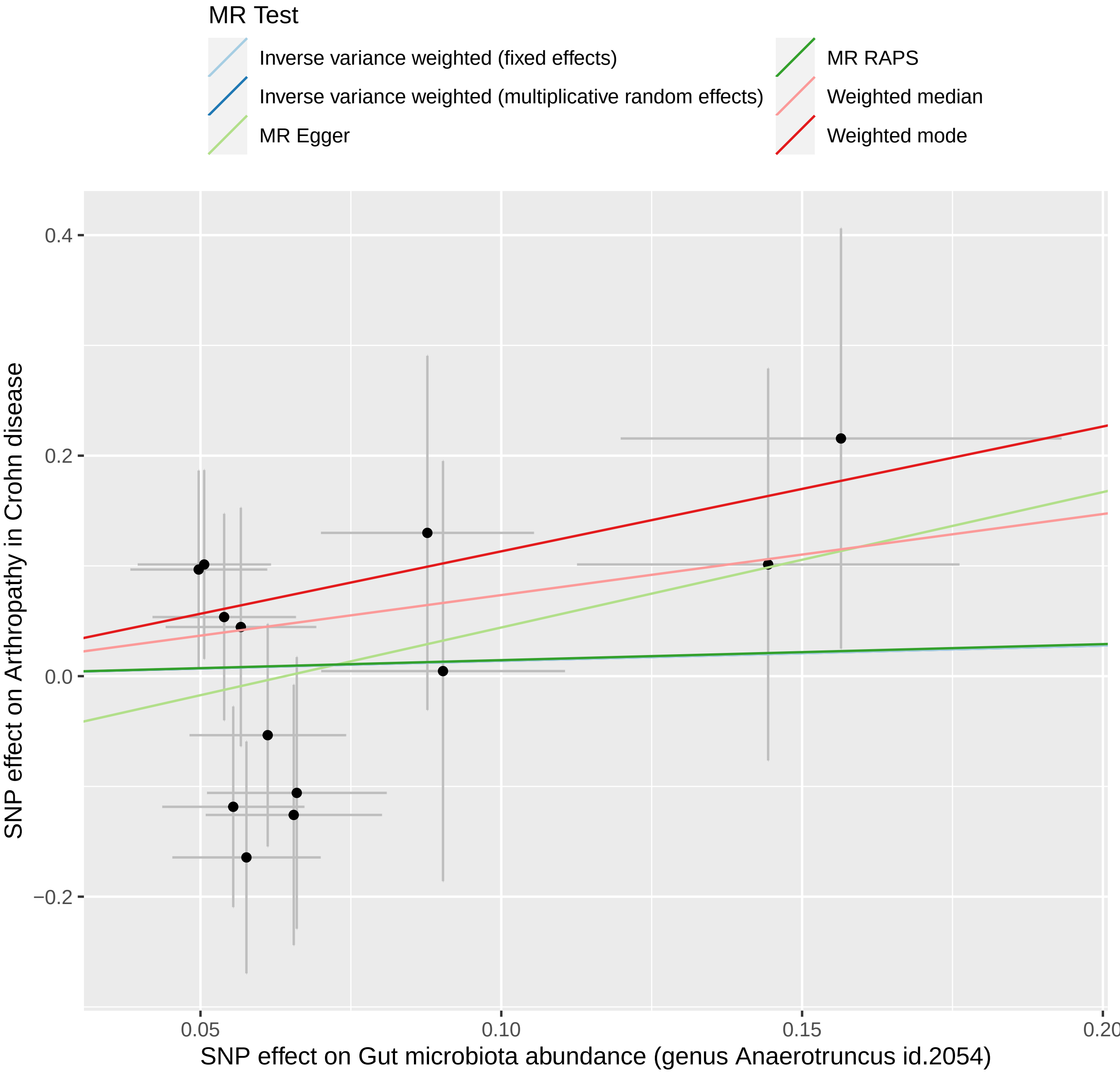

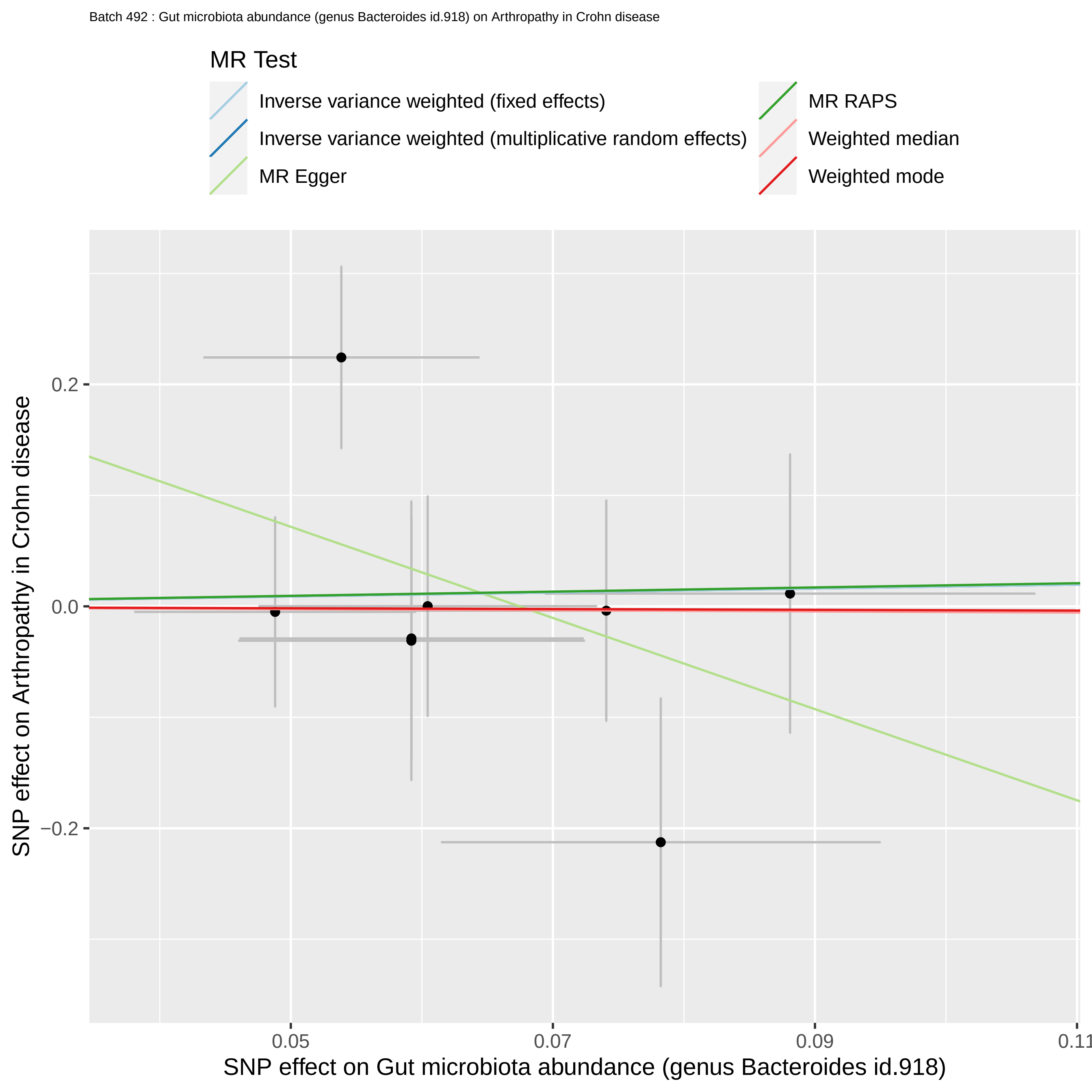
Batch 492 : Gut microbiota abundance (genus Bacteroides id.918) on Arthropathy in Crohn disease
MR Test
Inverse variance weighted (fixed effects)
Inverse variance weighted (multiplicative random effects)
MR Egger
MR RAPS
Weighted median
Weighted mode
0.2
0.0
−0.2
0.05
0.07
0.09
0.11
SNP effect on Arthropathy in Crohn disease
SNP effect on Gut microbiota abundance (genus Bacteroides id.918)

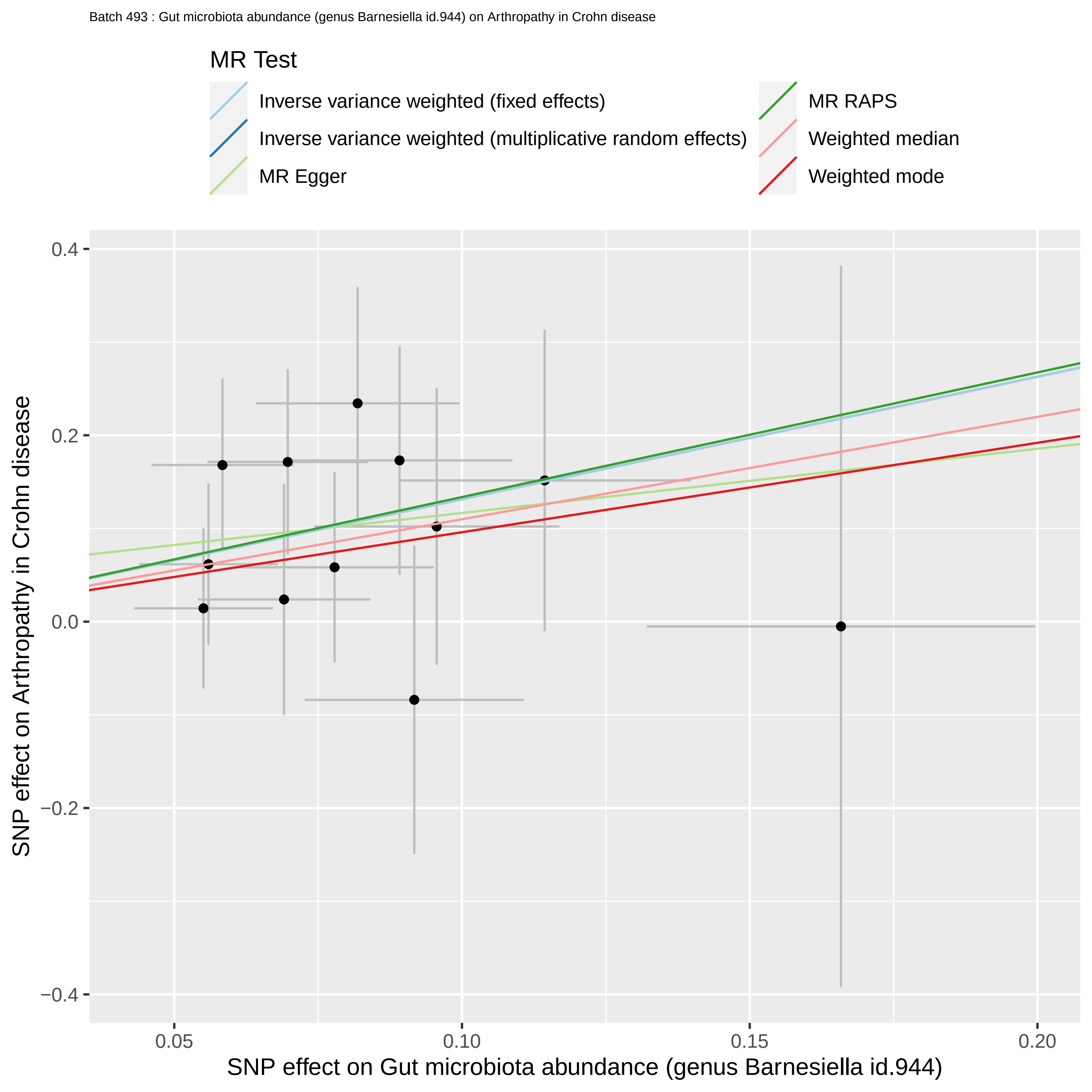

Batch 493 : Gut microbiota abundance (genus Barnesiella id.944) on Arthropathy in Crohn disease
MR Test
Inverse variance weighted (fixed effects)
Inverse variance weighted (multiplicative random effects)
MR Egger
MR RAPS
Weighted median
Weighted mode
SNP effect on Arthropathy in Crohn disease
0.4
0.2
0.0
−0.2
−0.4
0.05
0.10
0.15
0.20
SNP effect on Gut microbiota abundance (genus Barnesiella id.944)

Batch 494 : Gut microbiota abundance (genus Bifidobacterium id.436) on Arthropathy in Crohn disease

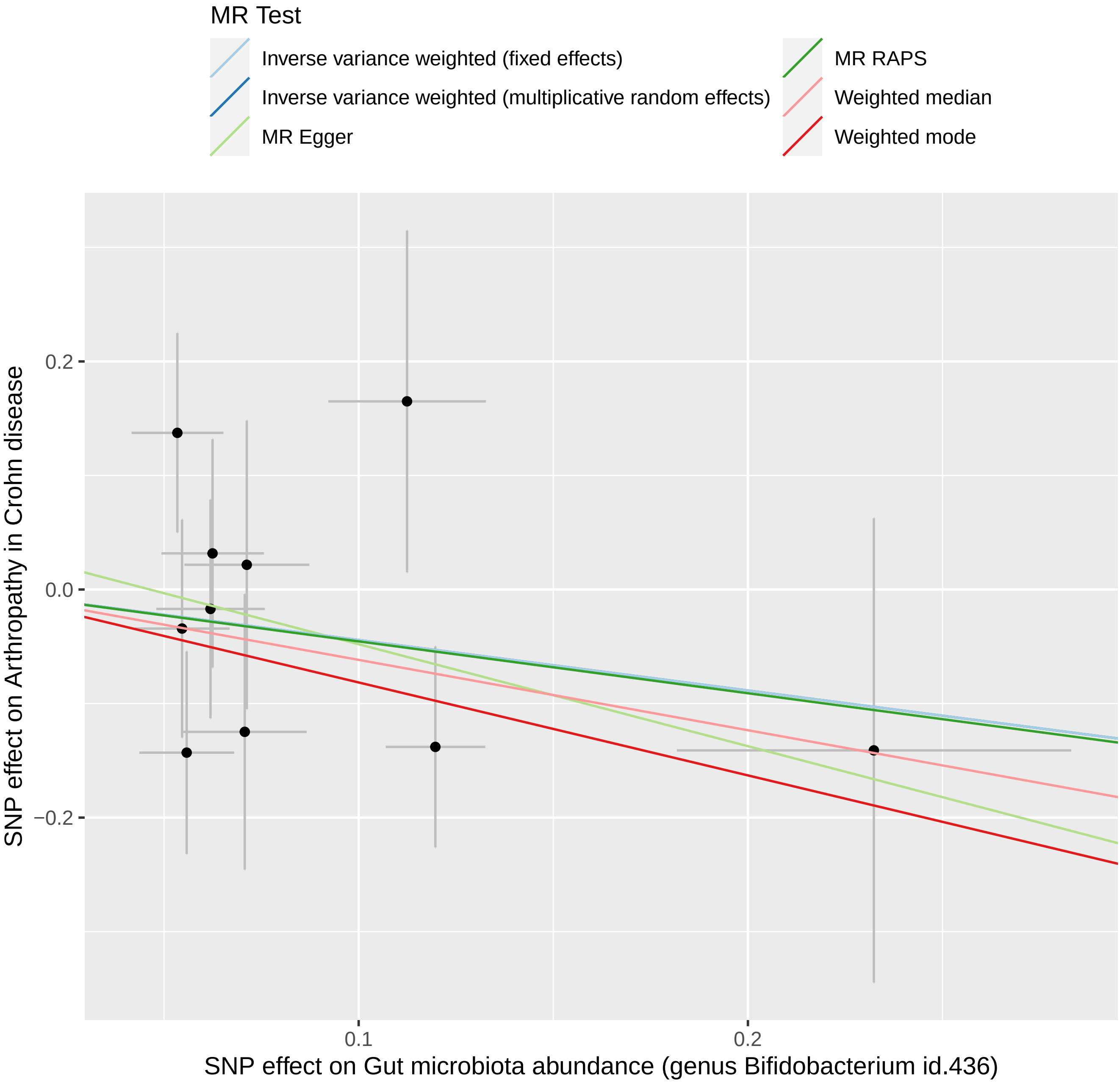

Batch 495 : Gut microbiota abundance (genus Bilophila id.3170) on Arthropathy in Crohn disease

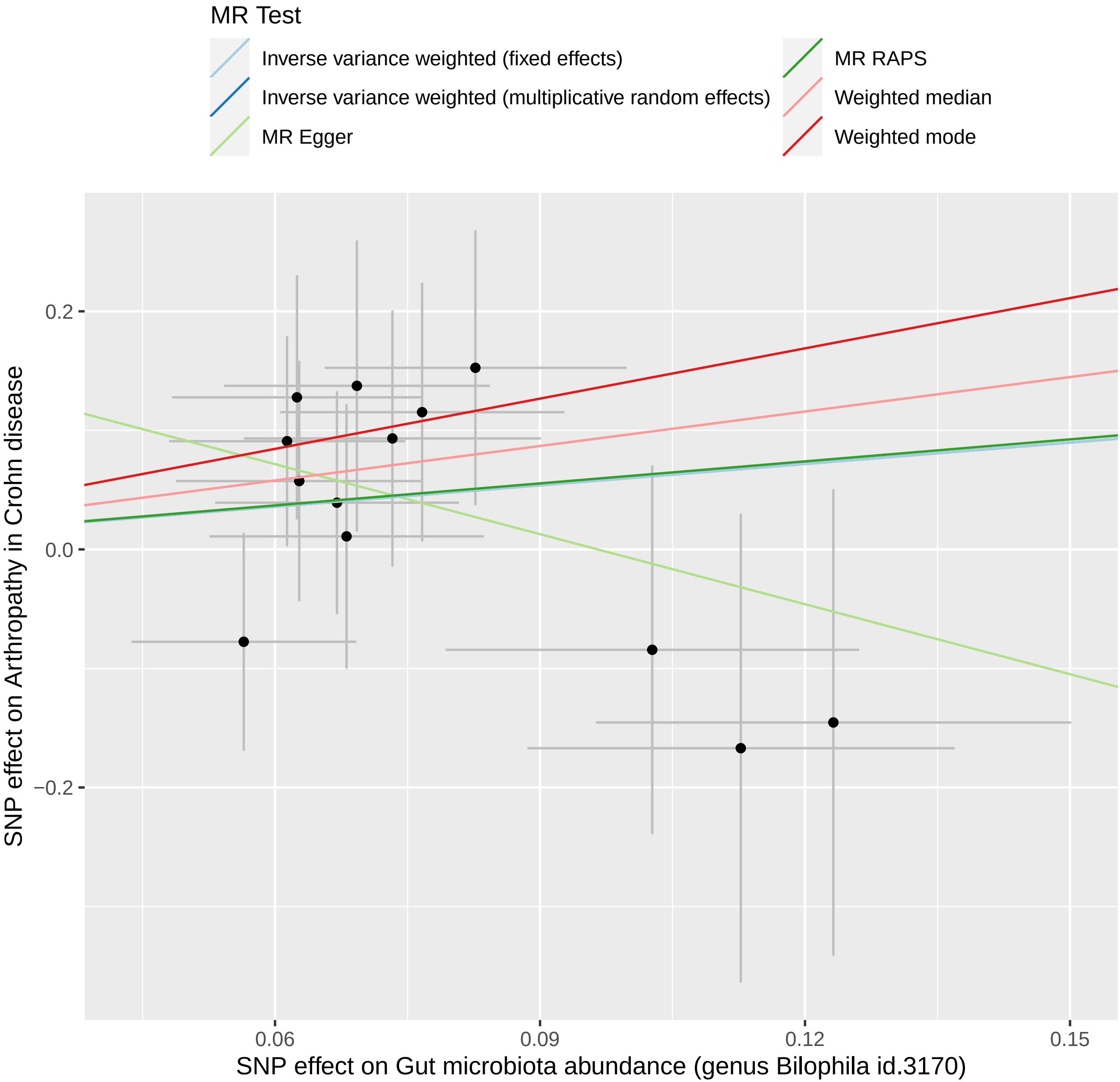

Batch 496 : Gut microbiota abundance (genus Blautia id.1992) on Arthropathy in Crohn disease

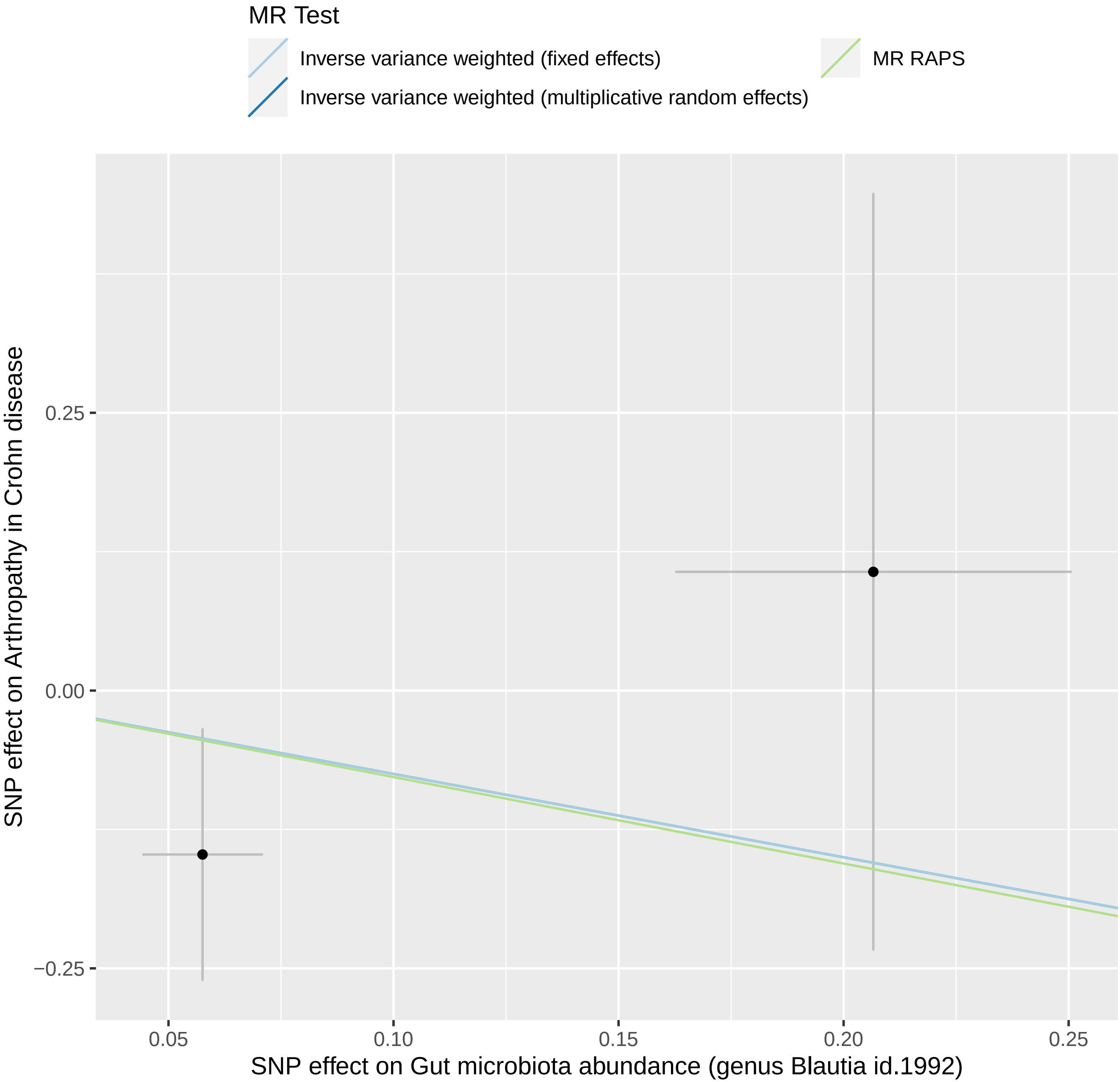

Batch 497 : Gut microbiota abundance (genus Butyricicoccus id.2055) on Arthropathy in Crohn disease

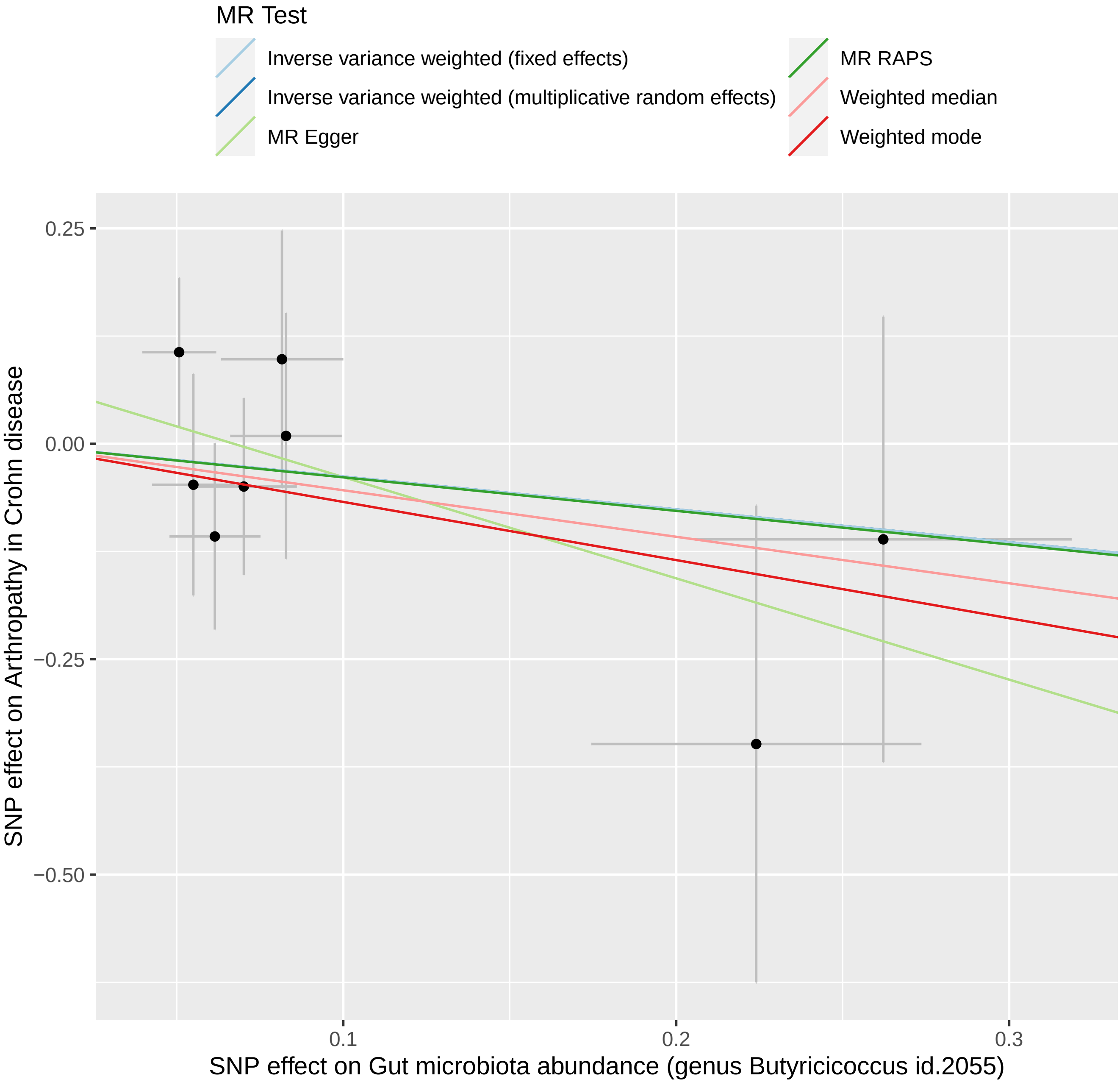

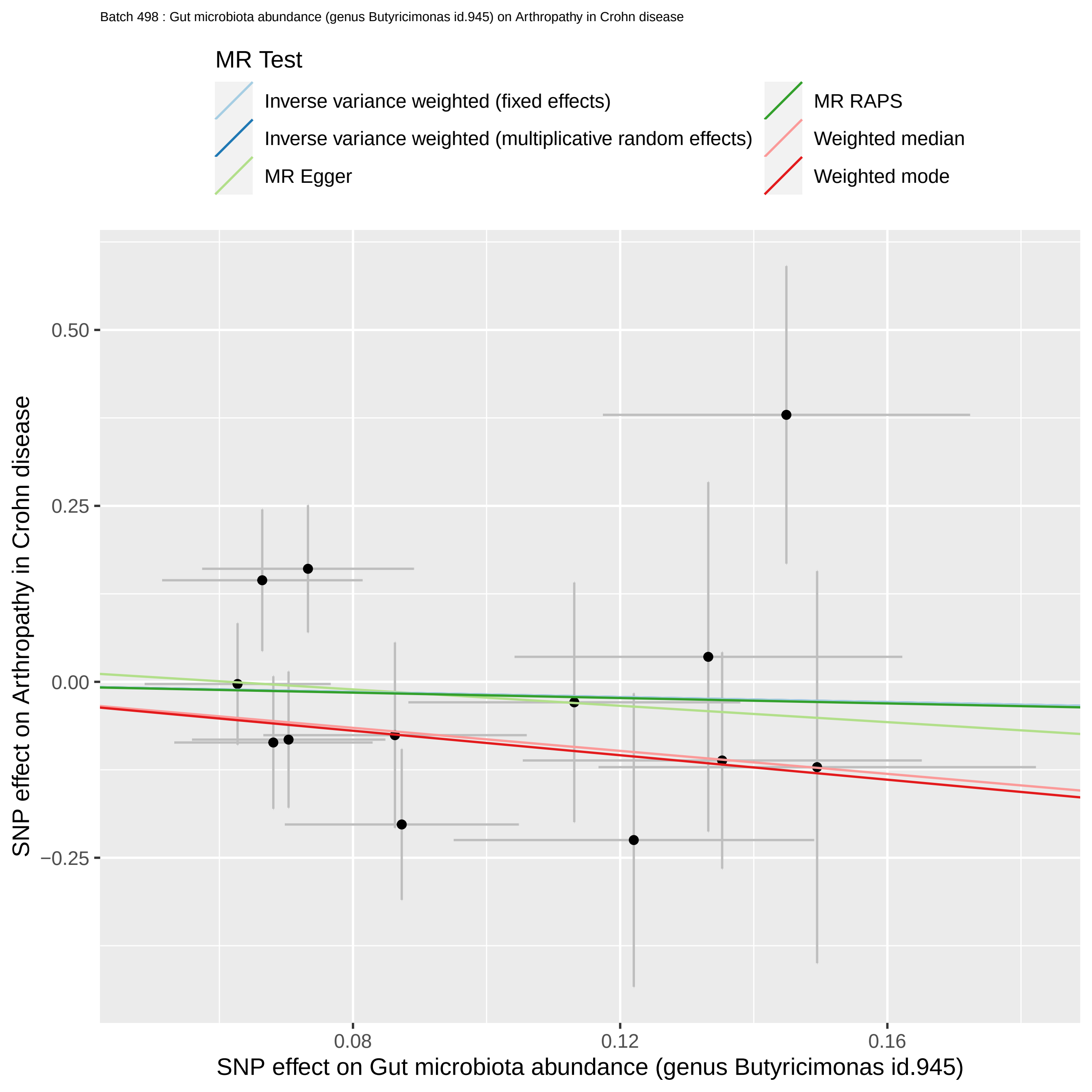

Batch 498 : Gut microbiota abundance (genus Butyricimonas id.945) on Arthropathy in Crohn disease
MR Test
Inverse variance weighted (fixed effects)
Inverse variance weighted (multiplicative random effects)
MR Egger
MR RAPS
Weighted median
Weighted mode
0.50
0.25
0.00
−0.25
SNP effect on Arthropathy in Crohn disease
0.08
0.12
0.16
SNP effect on Gut microbiota abundance (genus Butyricimonas id.945)

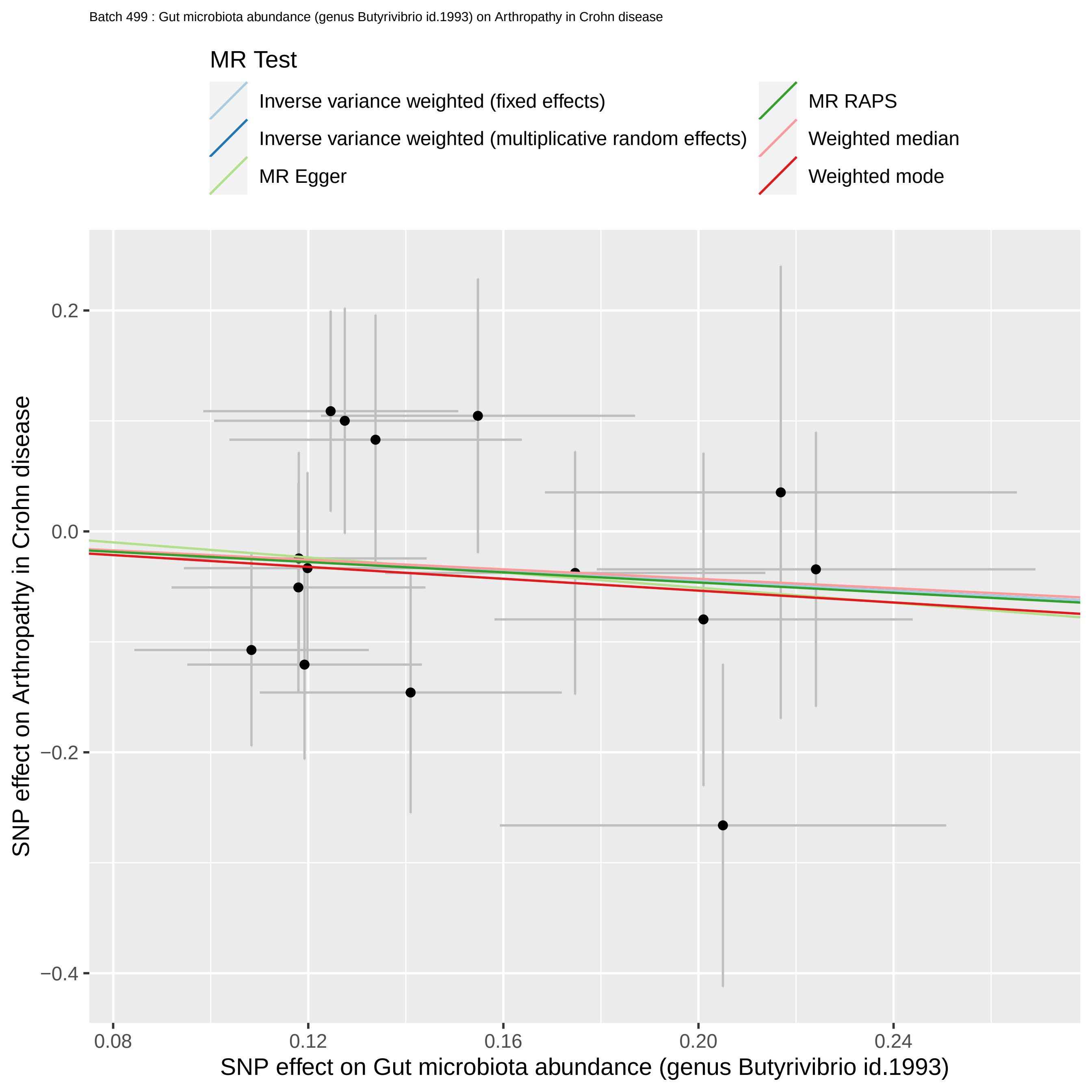

Batch 499 : Gut microbiota abundance (genus Butyrivibrio id.1993) on Arthropathy in Crohn disease
MR Test
Inverse variance weighted (fixed effects)
Inverse variance weighted (multiplicative random effects)
MR Egger
MR RAPS
Weighted median
Weighted mode
0.2
0.0
−0.2
−0.4
0.08
0.12
0.16
0.20
0.24
SNP effect on Arthropathy in Crohn disease
SNP effect on Gut microbiota abundance (genus Butyrivibrio id.1993)

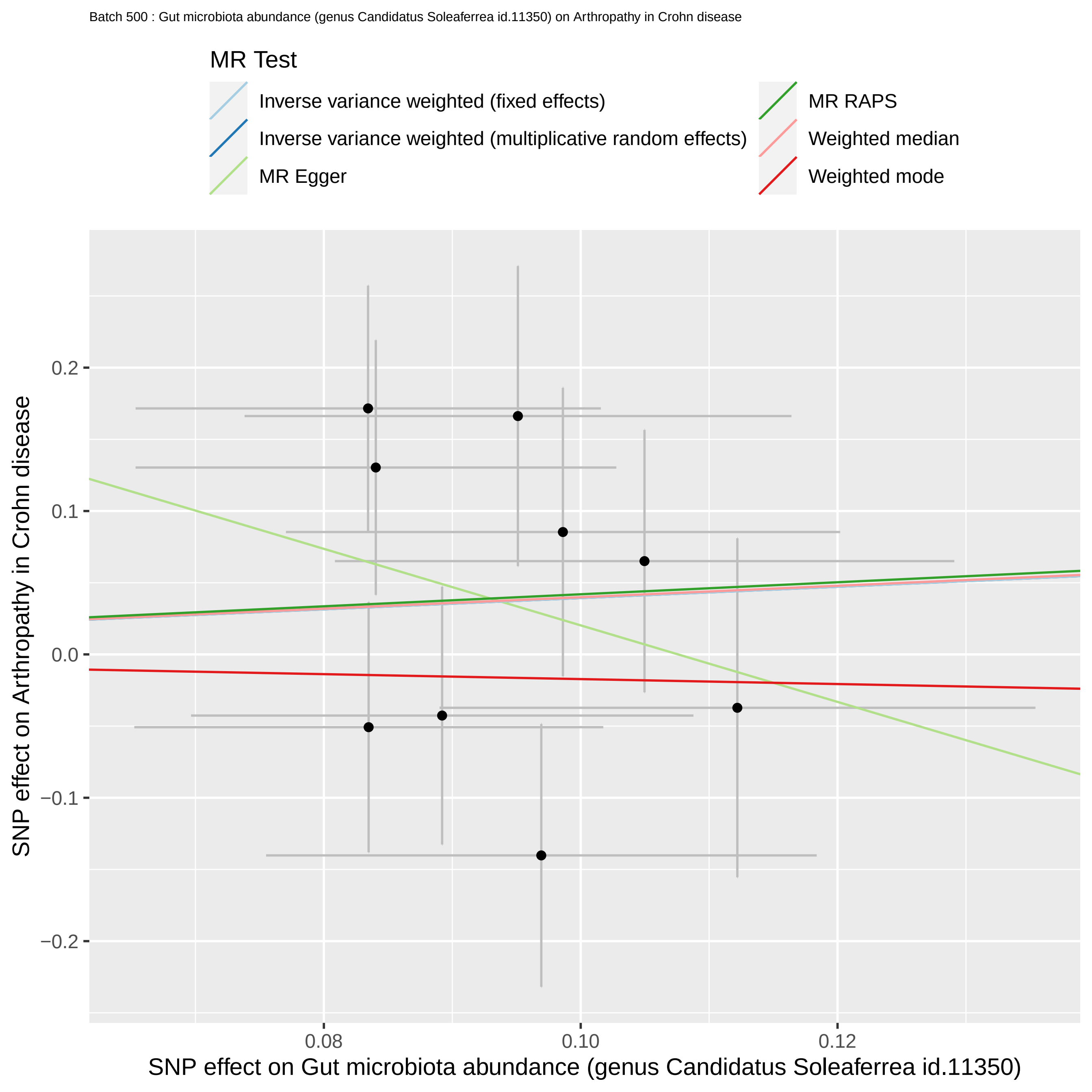

Batch 500 : Gut microbiota abundance (genus Candidatus Soleaferrea id.11350) on Arthropathy in Crohn disease
MR Test
Inverse variance weighted (fixed effects)
Inverse variance weighted (multiplicative random effects)
MR Egger
MR RAPS
Weighted median
Weighted mode
SNP effect on Arthropathy in Crohn disease
0.2
0.1
0.0
−0.1
−0.2
0.08
0.10
0.12
SNP effect on Gut microbiota abundance (genus Candidatus Soleaferrea id.11350)

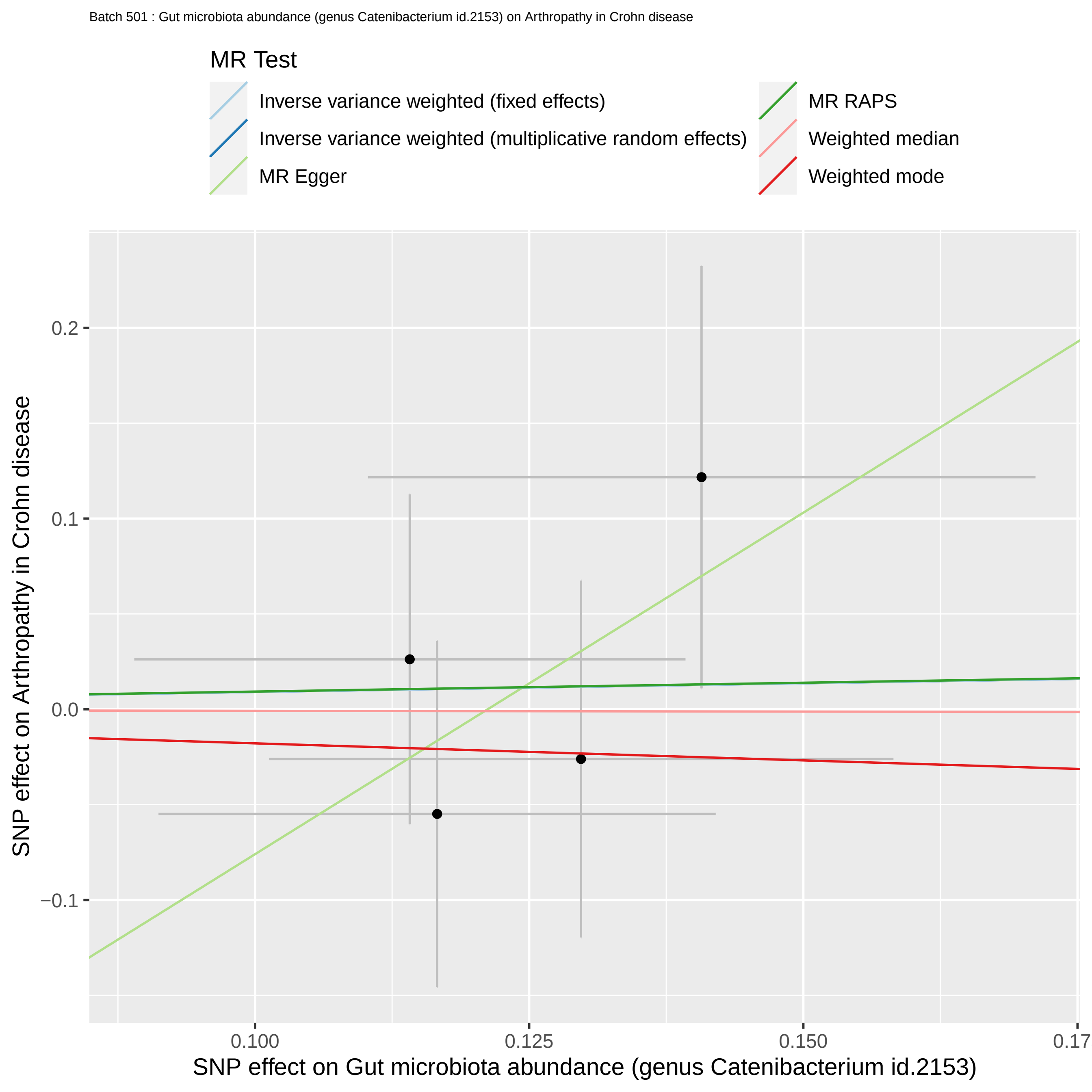
Batch 501 : Gut microbiota abundance (genus Catenibacterium id.2153) on Arthropathy in Crohn disease
MR Test
Inverse variance weighted (fixed effects)
Inverse variance weighted (multiplicative random effects)
MR Egger
MR RAPS
Weighted median
Weighted mode
SNP effect on Arthropathy in Crohn disease
0.2
0.1
0.0
−0.1
0.100
0.125
0.150
0.17
SNP effect on Gut microbiota abundance (genus Catenibacterium id.2153)

Batch 502 : Gut microbiota abundance (genus Christensenellaceae R 7group id.11283) on Arthropathy in Crohn disease

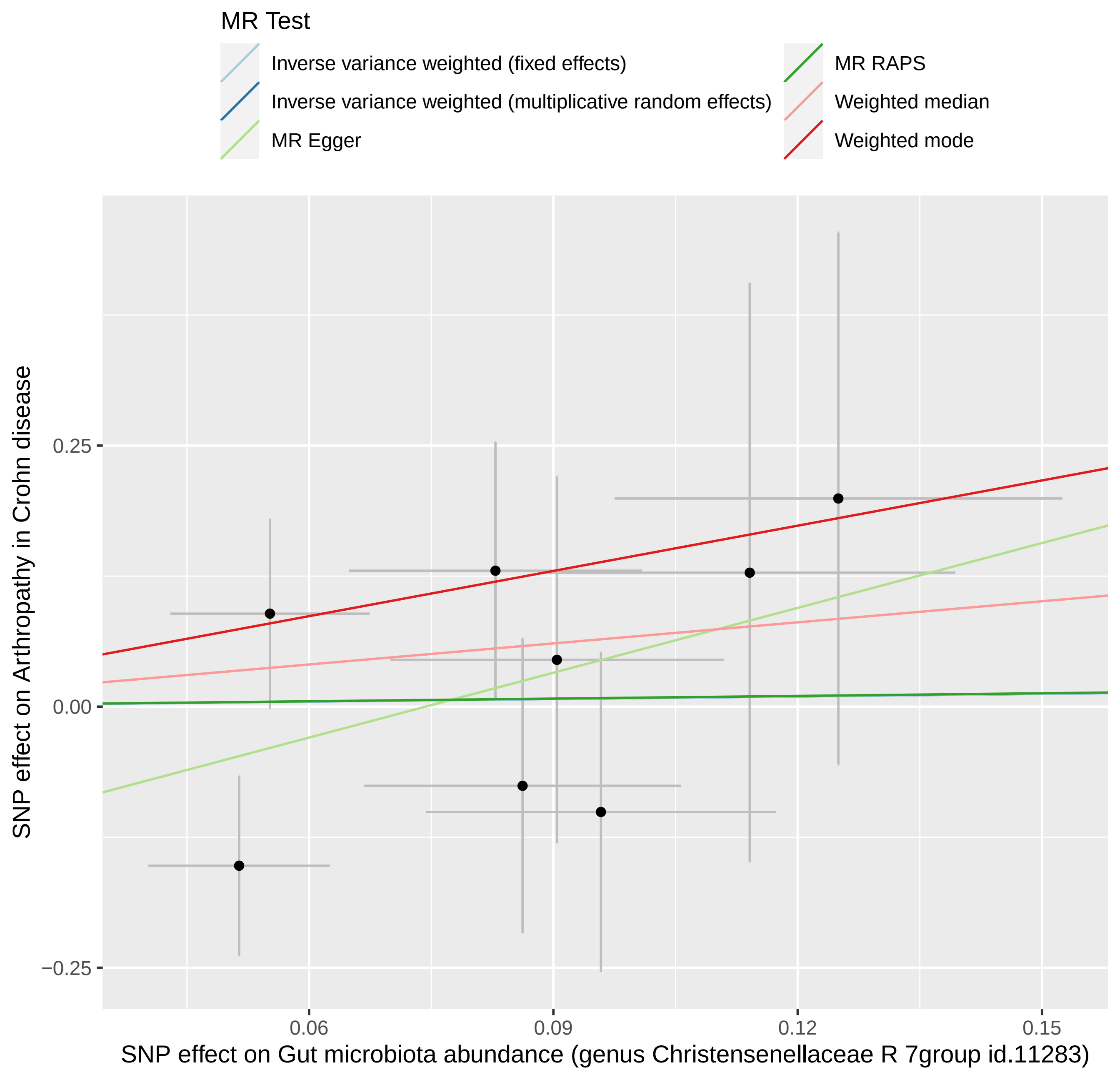

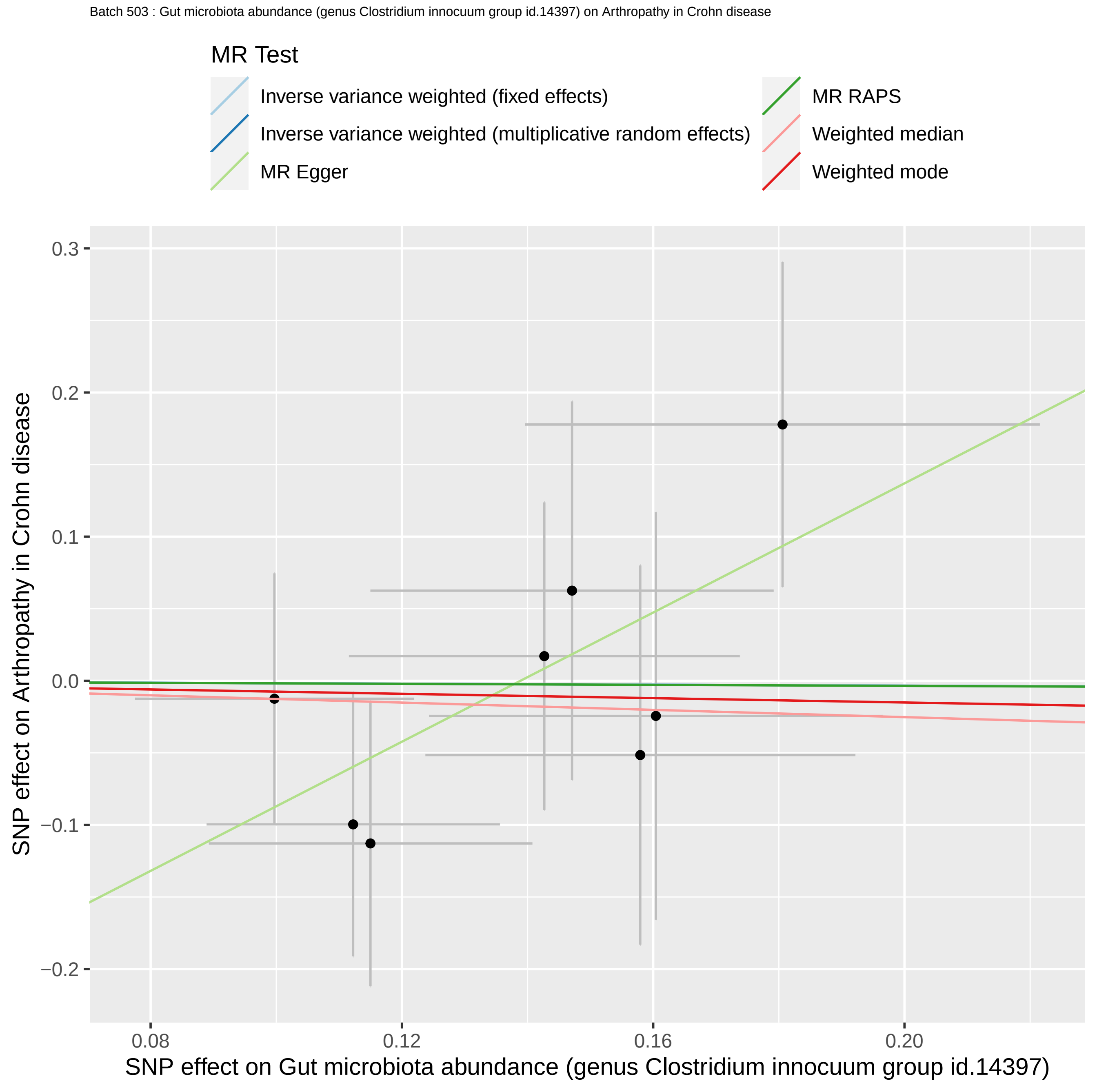
Batch 503 : Gut microbiota abundance (genus Clostridium innocuum group id.14397) on Arthropathy in Crohn disease
MR Test
Inverse variance weighted (fixed effects)
Inverse variance weighted (multiplicative random effects)
MR Egger
MR RAPS
Weighted median
Weighted mode
0.3
0.2
0.1
0.0
−0.1
−0.2
0.08
0.12
0.16
0.20
SNP effect on Arthropathy in Crohn disease
SNP effect on Gut microbiota abundance (genus Clostridium innocuum group id.14397)

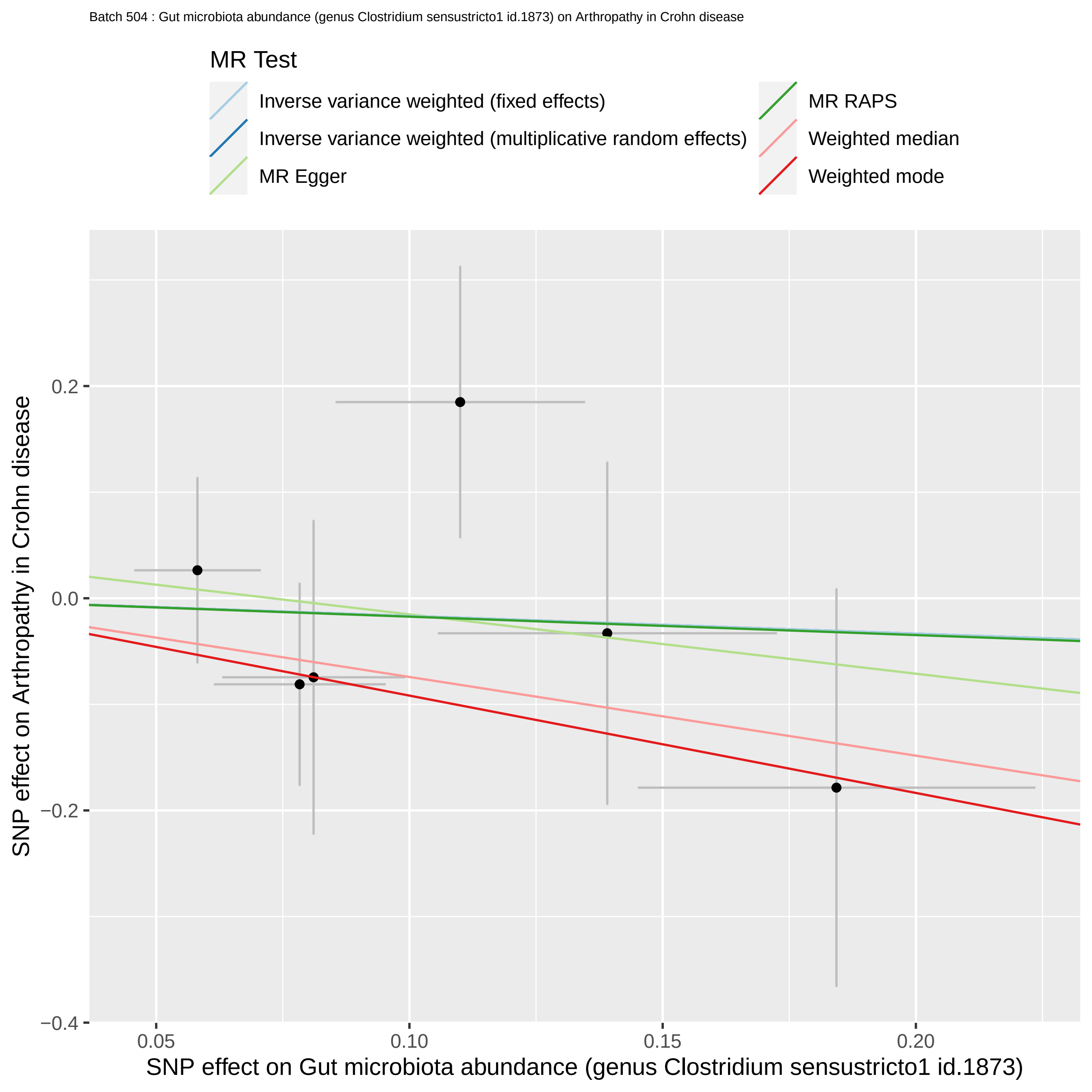

Batch 504 : Gut microbiota abundance (genus Clostridium sensustricto1 id.1873) on Arthropathy in Crohn disease
MR Test
Inverse variance weighted (fixed effects)
Inverse variance weighted (multiplicative random effects)
MR Egger
MR RAPS
Weighted median
Weighted mode
0.2
0.0
−0.2
−0.4
0.05
0.10
0.15
0.20
SNP effect on Arthropathy in Crohn disease
SNP effect on Gut microbiota abundance (genus Clostridium sensustricto1 id.1873)

Batch 505 : Gut microbiota abundance (genus Collinsella id.815) on Arthropathy in Crohn disease

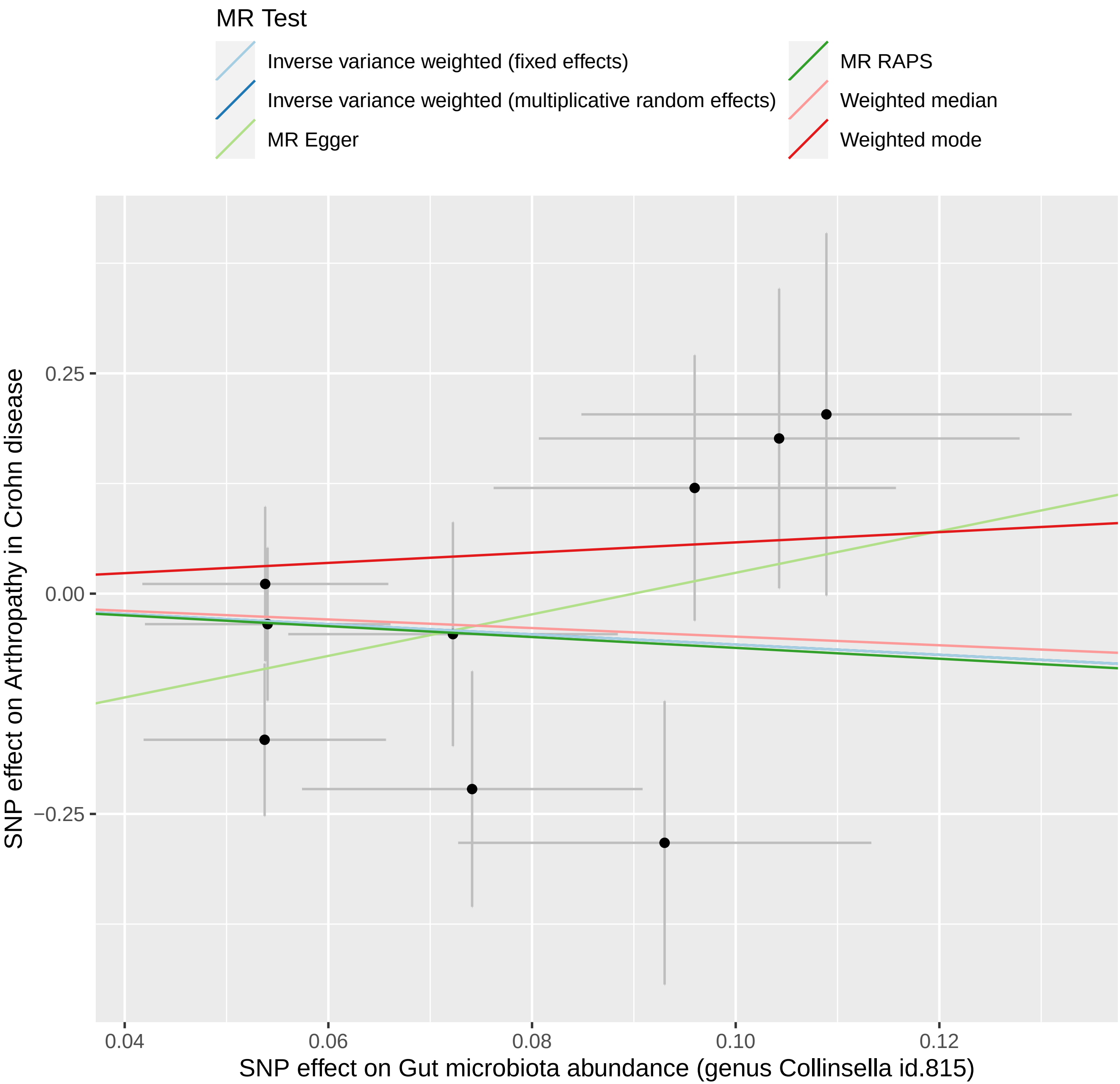

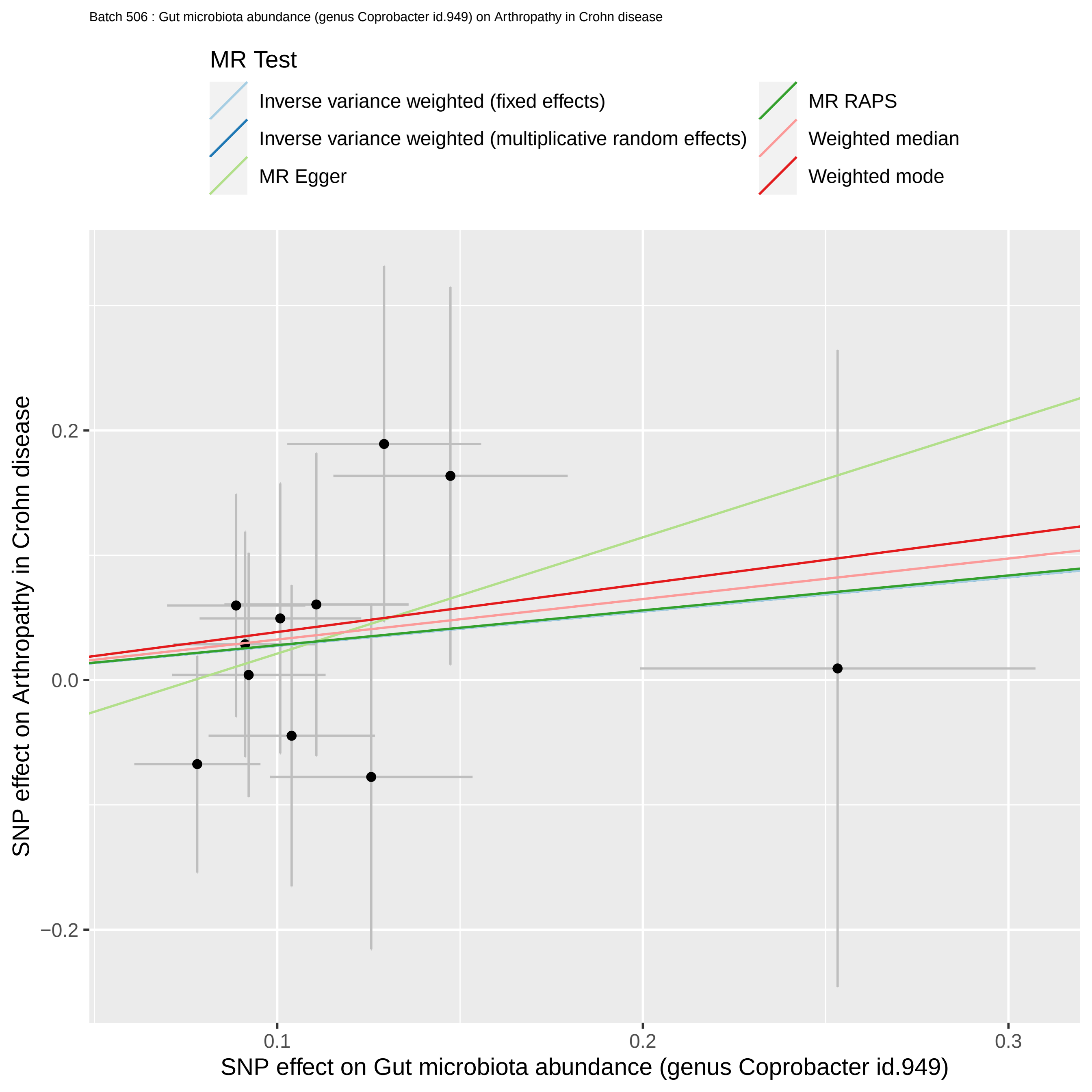

Batch 506 : Gut microbiota abundance (genus Coprobacter id.949) on Arthropathy in Crohn disease
MR Test
Inverse variance weighted (fixed effects)
Inverse variance weighted (multiplicative random effects)
MR Egger
MR RAPS
Weighted median
Weighted mode
SNP effect on Arthropathy in Crohn disease
0.2
0.0
−0.2
0.1
0.2
0.3
SNP effect on Gut microbiota abundance (genus Coprobacter id.949)

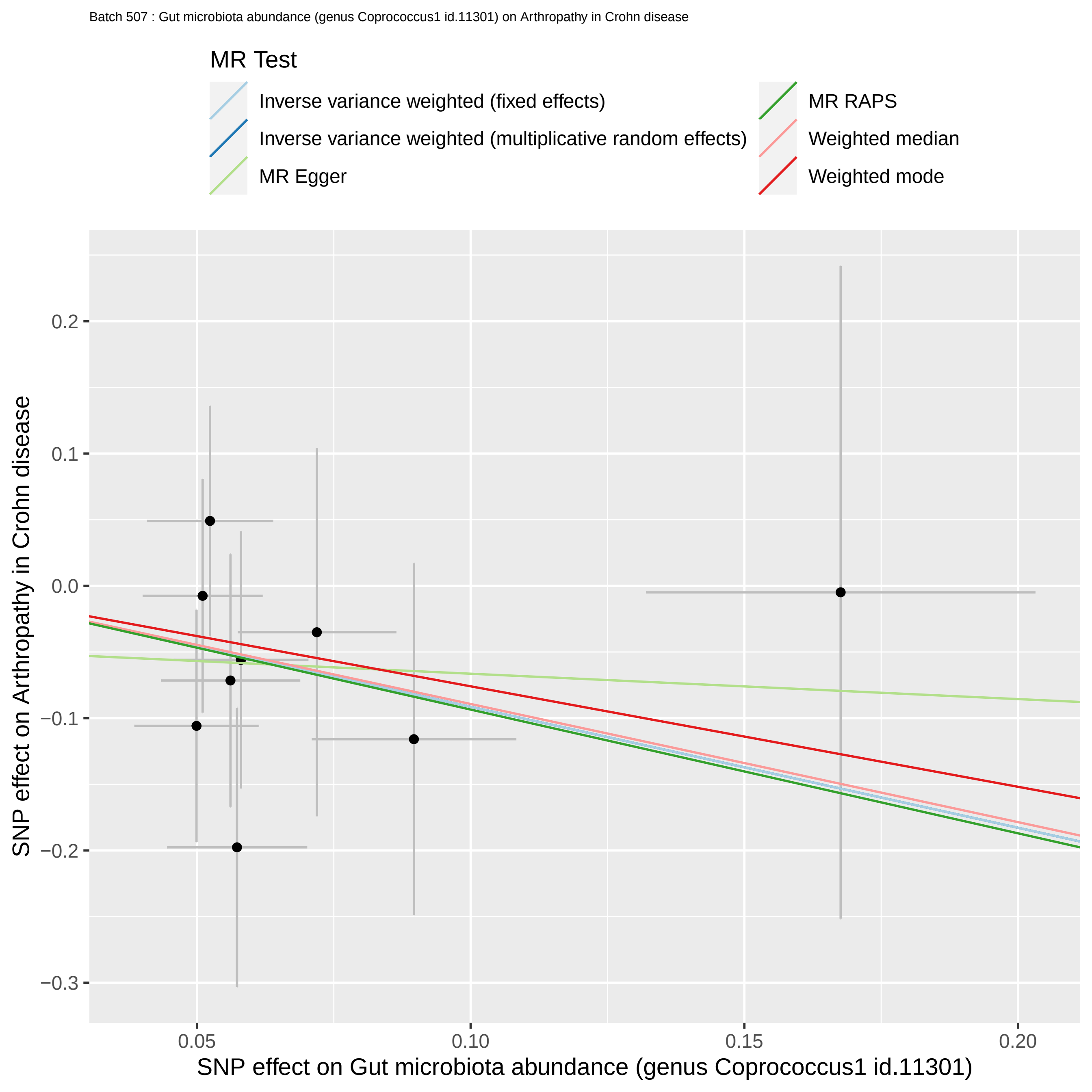
Batch 507 : Gut microbiota abundance (genus Coprococcus1 id.11301) on Arthropathy in Crohn disease
MR Test
Inverse variance weighted (fixed effects)
Inverse variance weighted (multiplicative random effects)
MR Egger
MR RAPS
Weighted median
Weighted mode
0.2
0.1
0.0
−0.1
−0.2
−0.3
0.05
0.10
0.15
0.20
SNP effect on Arthropathy in Crohn disease
SNP effect on Gut microbiota abundance (genus Coprococcus1 id.11301)

Batch 508 : Gut microbiota abundance (genus Coprococcus2 id.11302) on Arthropathy in Crohn disease

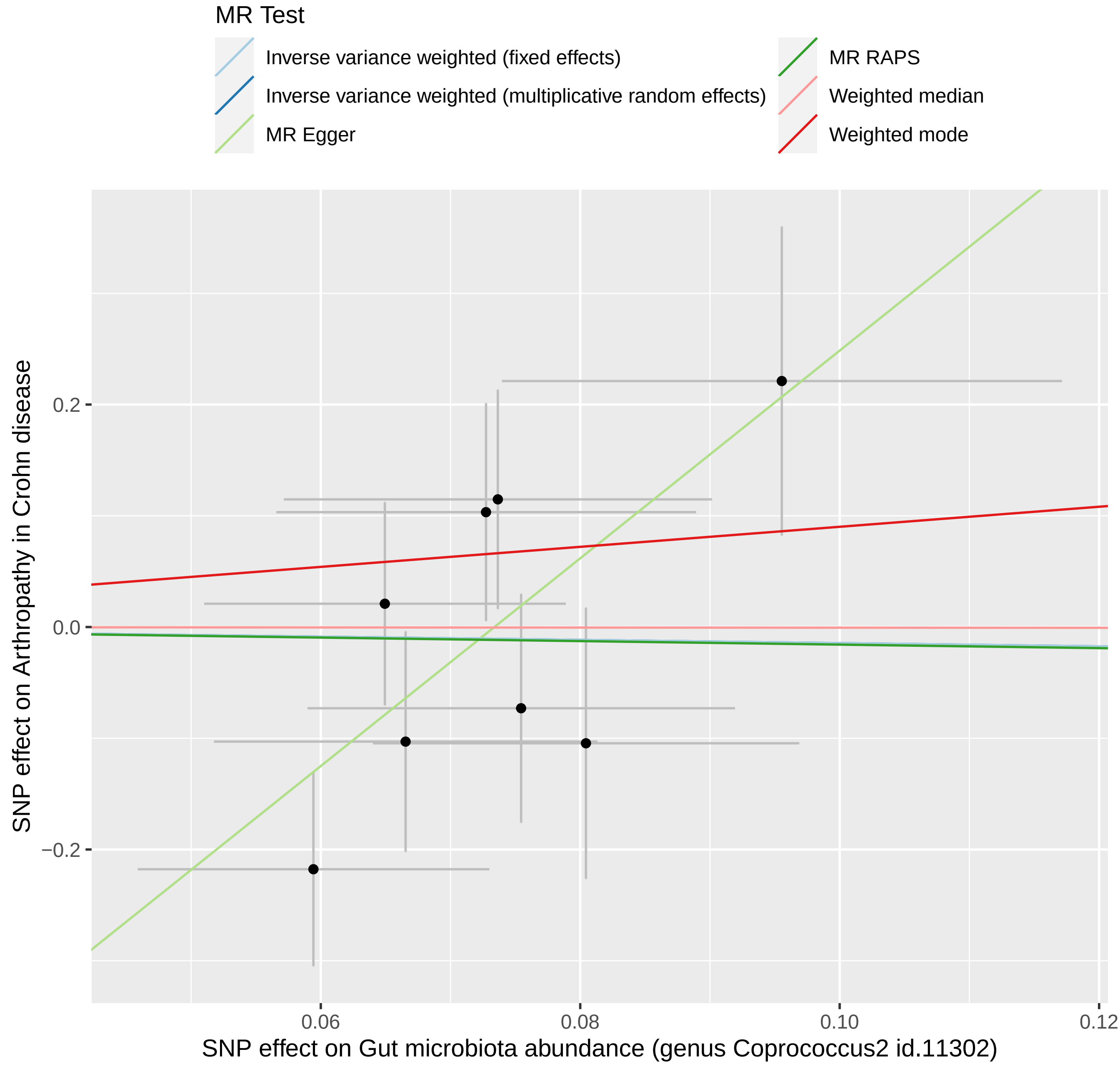

Batch 509 : Gut microbiota abundance (genus Coprococcus3 id.11303) on Arthropathy in Crohn disease

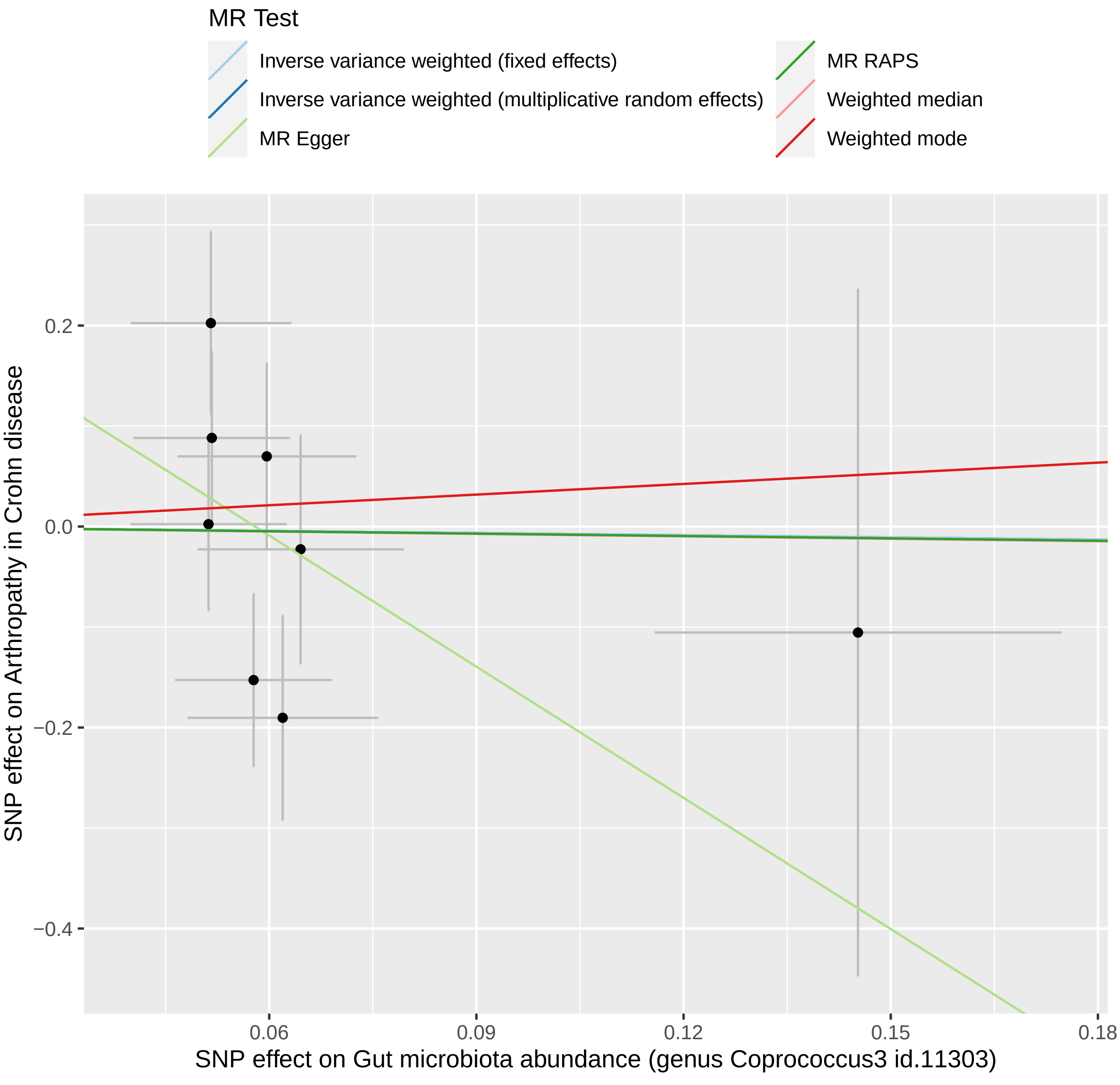

Batch 510 : Gut microbiota abundance (genus Defluviitaleaceae UCG011 id.11287) on Arthropathy in Crohn disease

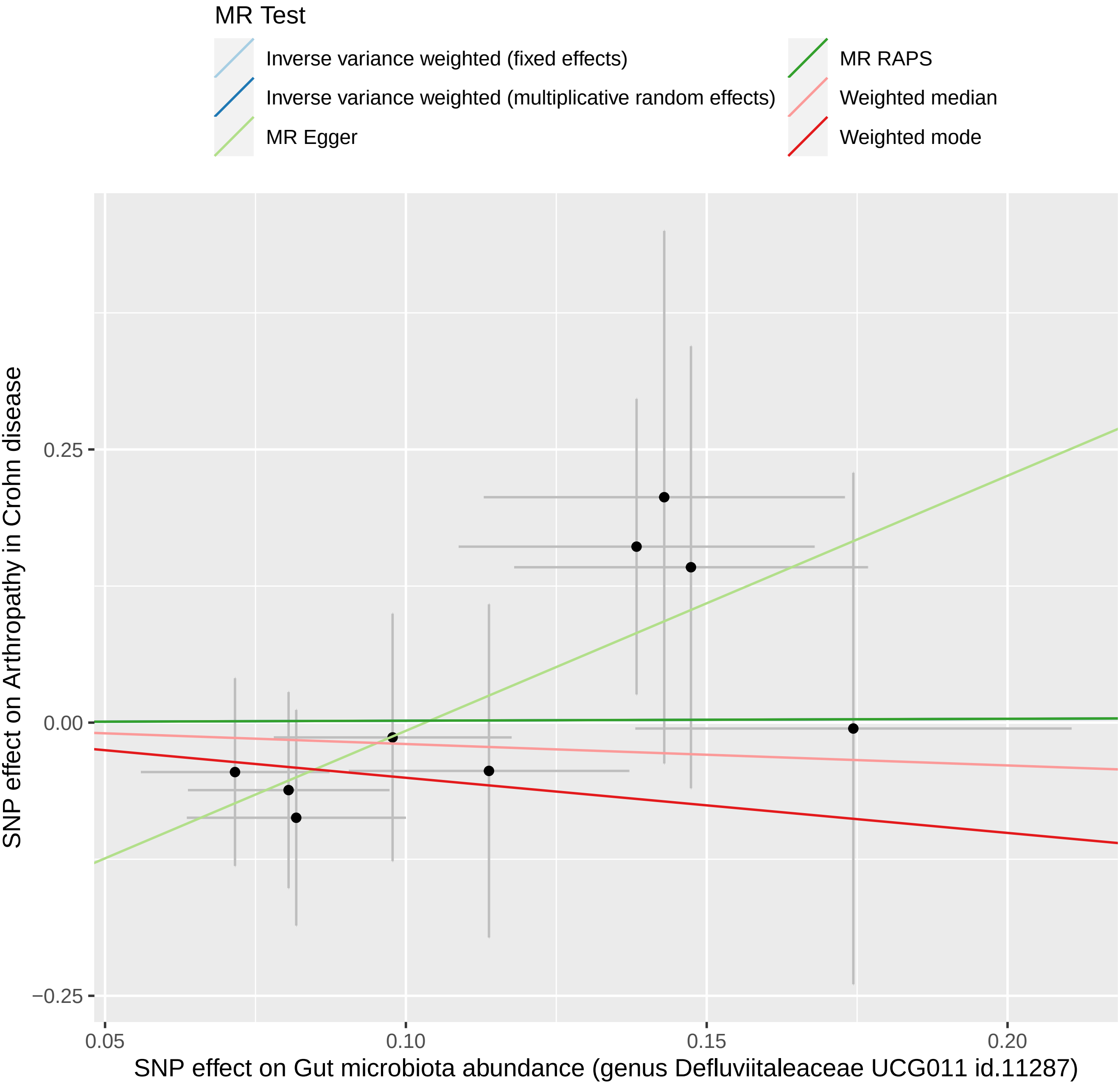

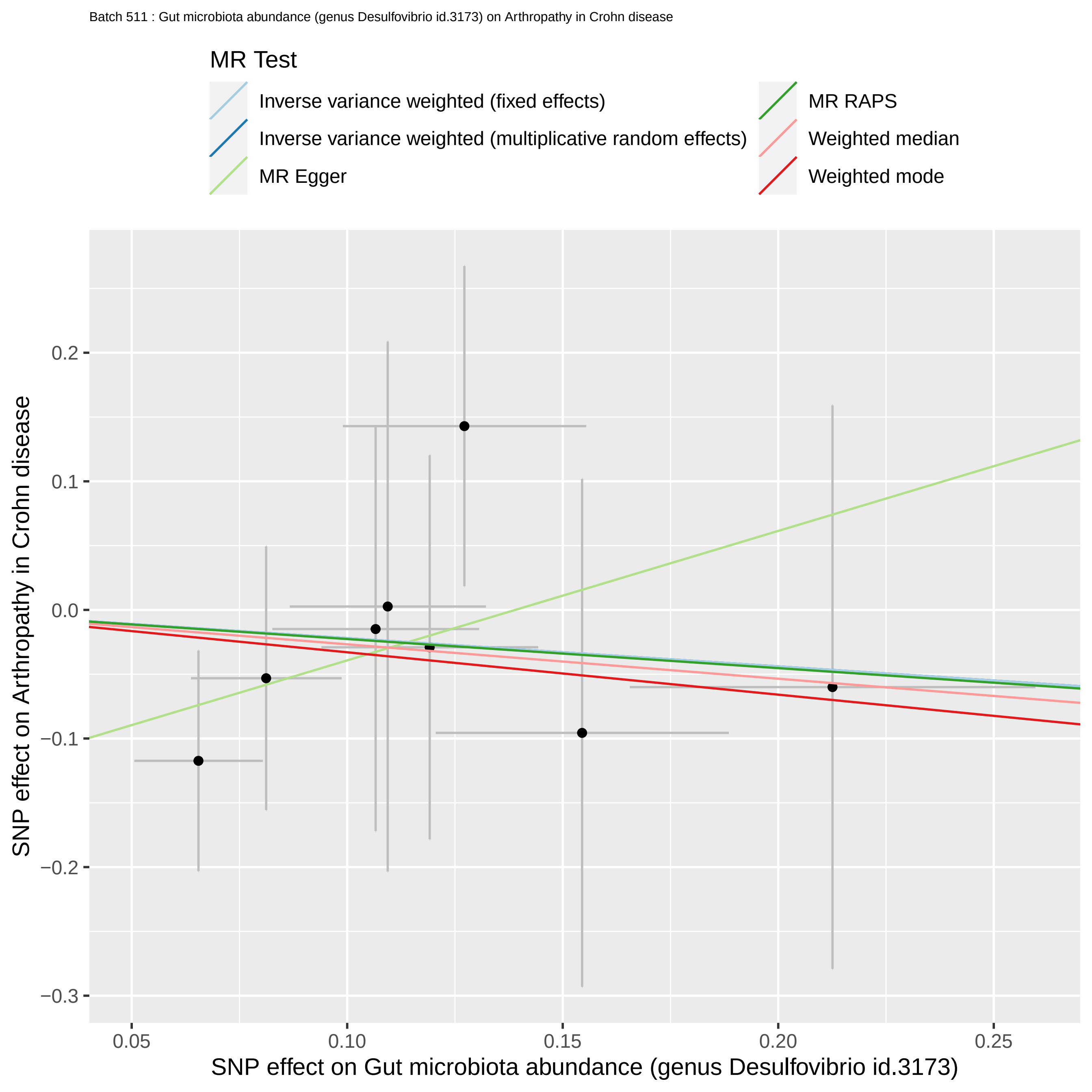
Batch 511 : Gut microbiota abundance (genus Desulfovibrio id.3173) on Arthropathy in Crohn disease
MR Test
Inverse variance weighted (fixed effects)
Inverse variance weighted (multiplicative random effects)
MR Egger
MR RAPS
Weighted median
Weighted mode
0.2
0.1
0.0
−0.1
−0.2
−0.3
0.05
0.10
0.15
0.20
0.25
SNP effect on Arthropathy in Crohn disease
SNP effect on Gut microbiota abundance (genus Desulfovibrio id.3173)

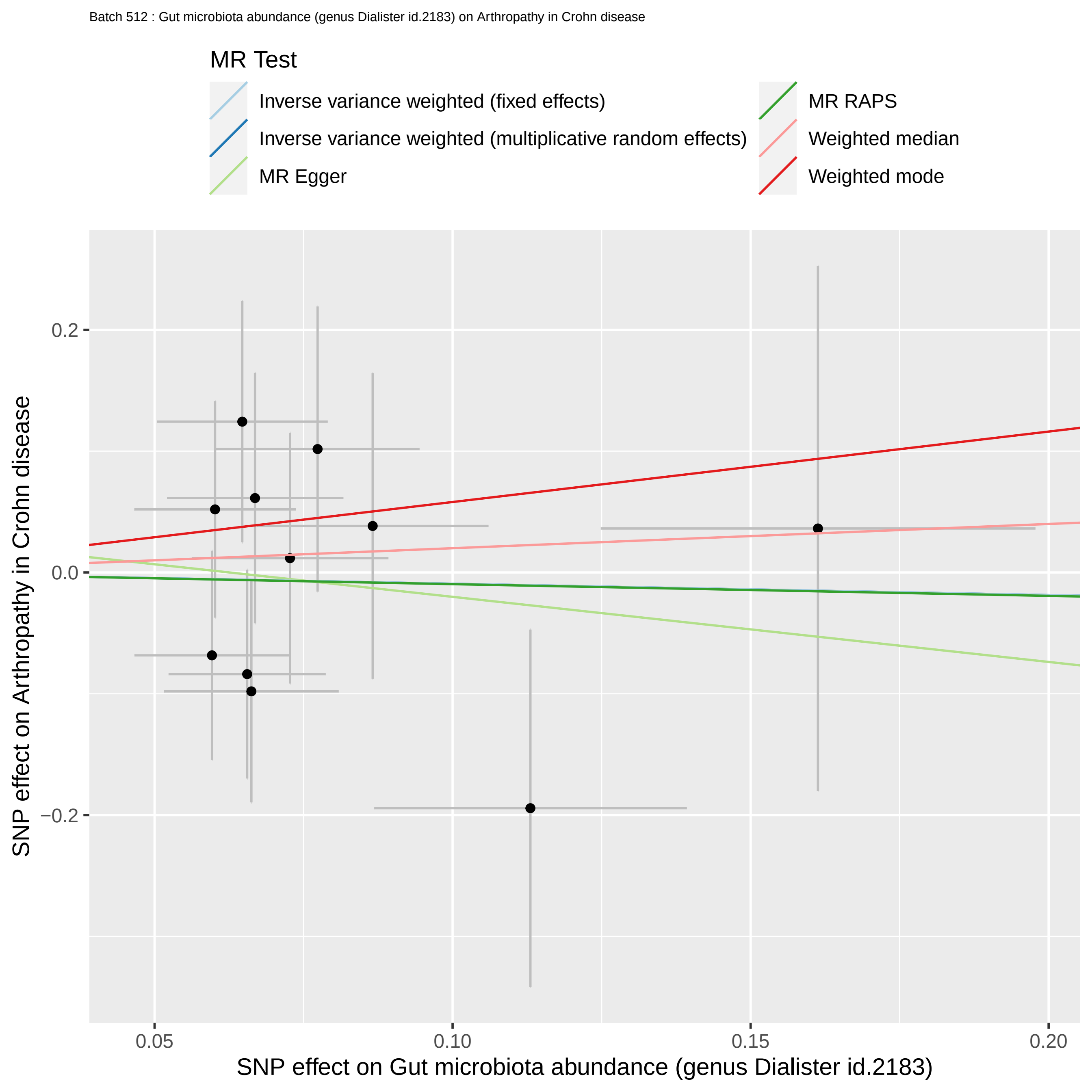

Batch 512 : Gut microbiota abundance (genus Dialister id.2183) on Arthropathy in Crohn disease
MR Test
Inverse variance weighted (fixed effects)
Inverse variance weighted (multiplicative random effects)
MR Egger
MR RAPS
Weighted median
Weighted mode
0.2
0.0
−0.2
0.05
0.10
0.15
0.20
SNP effect on Arthropathy in Crohn disease
SNP effect on Gut microbiota abundance (genus Dialister id.2183)

Batch 513 : Gut microbiota abundance (genus Dorea id.1997) on Arthropathy in Crohn disease

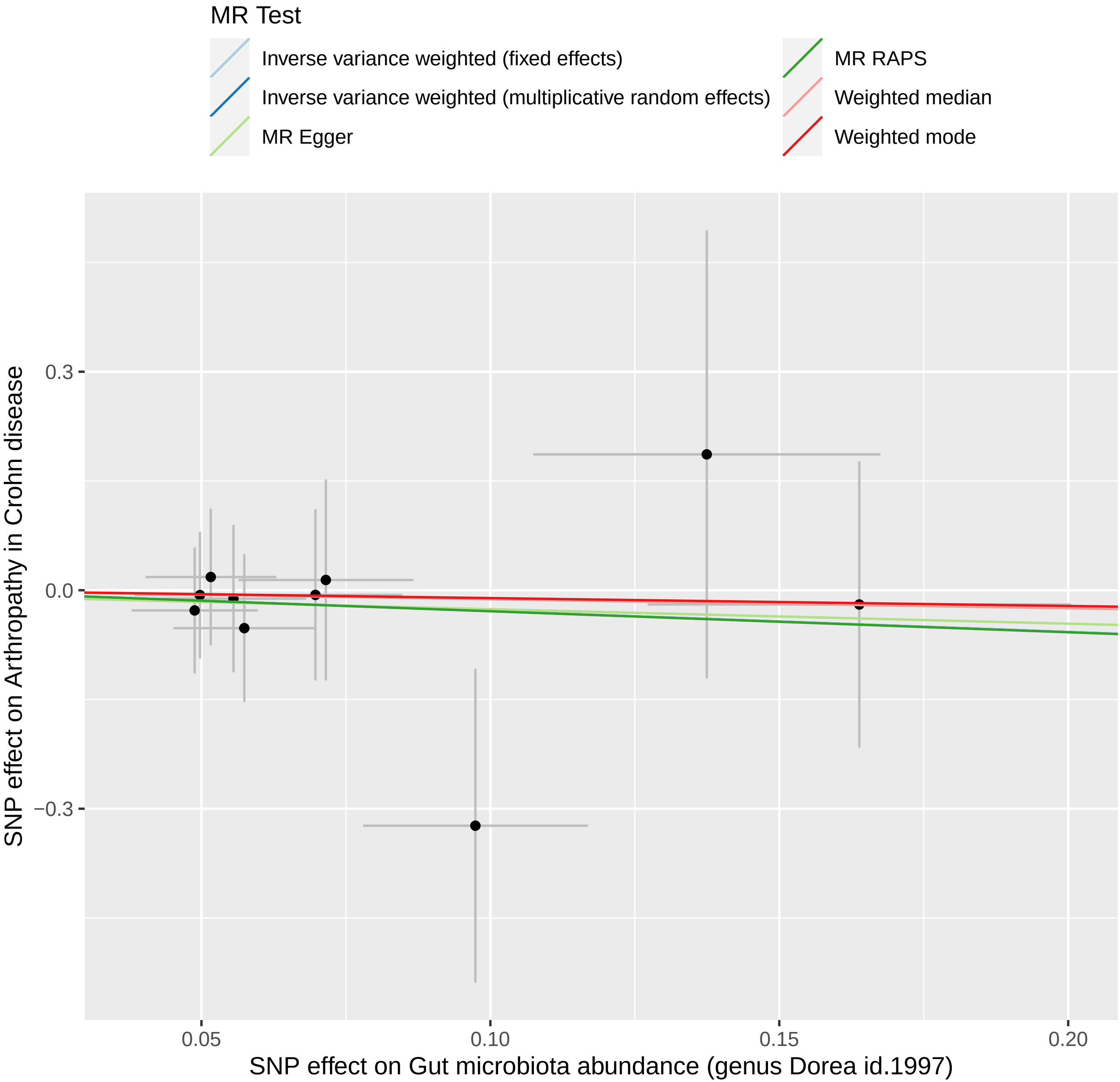

Batch 514 : Gut microbiota abundance (genus Eggerthella id.819) on Arthropathy in Crohn disease

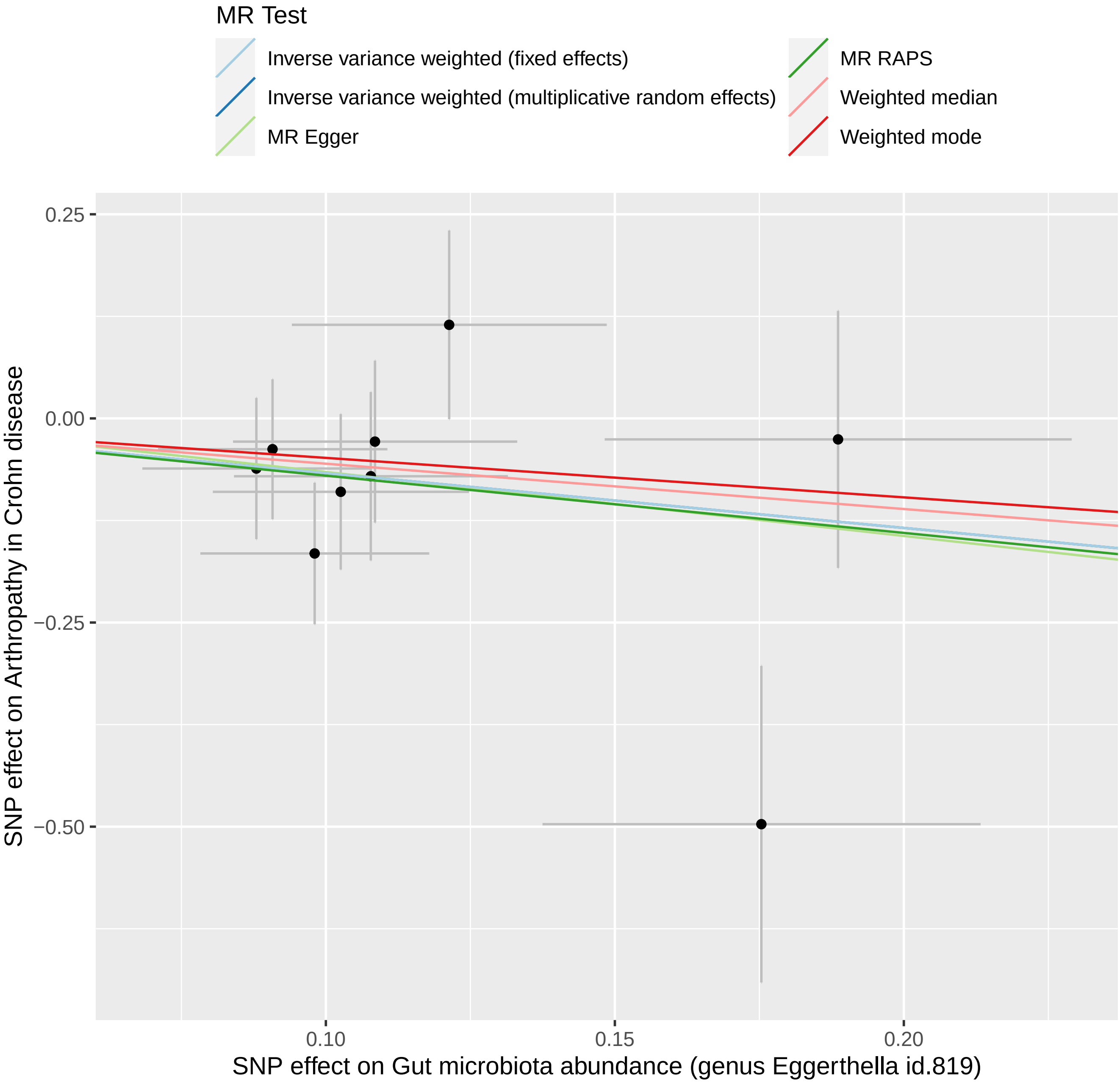

Batch 515 : Gut microbiota abundance (genus Eisenbergiella id.11304) on Arthropathy in Crohn disease

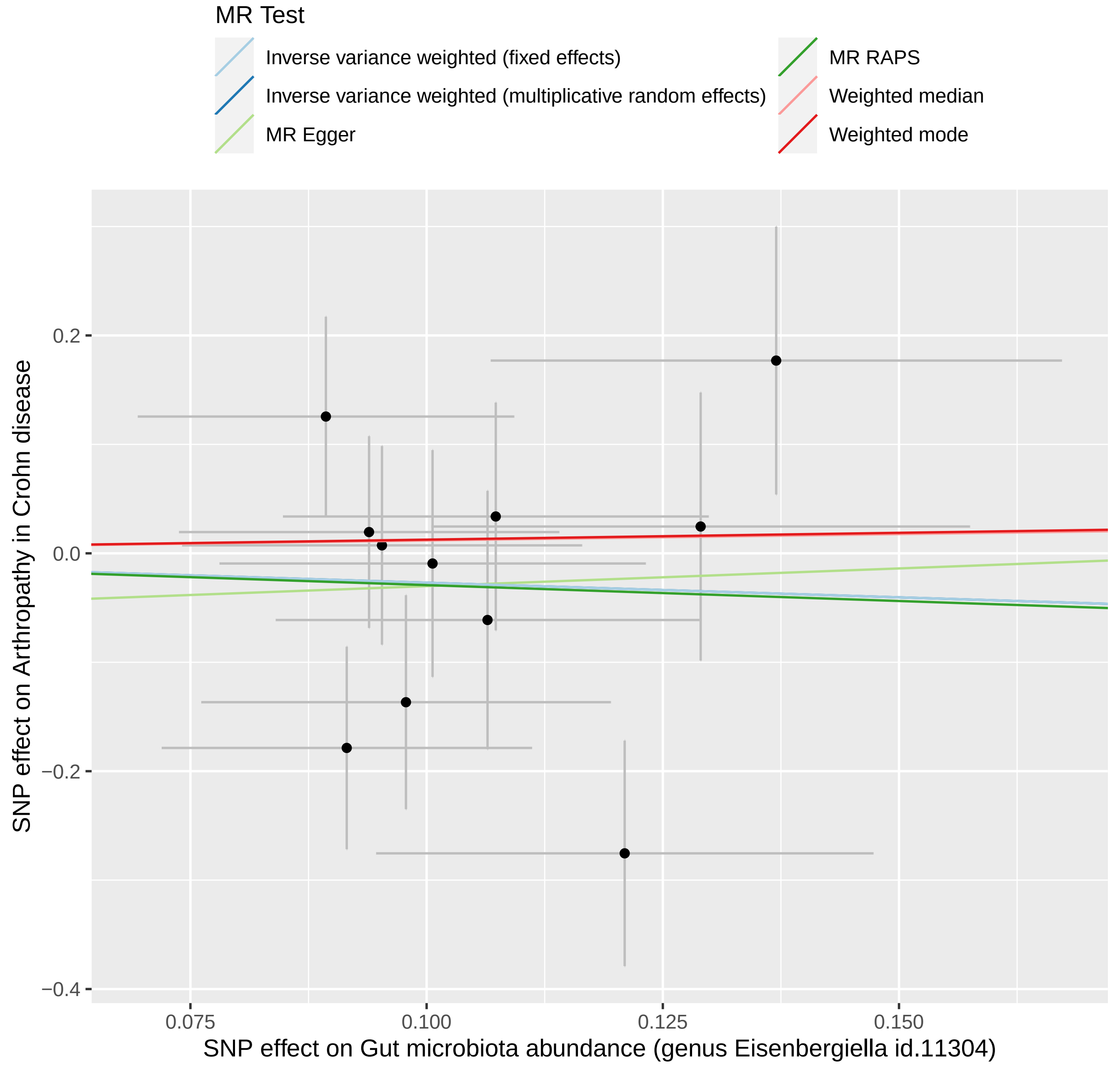

Batch 516 : Gut microbiota abundance (genus Enterorhabdus id.820) on Arthropathy in Crohn disease

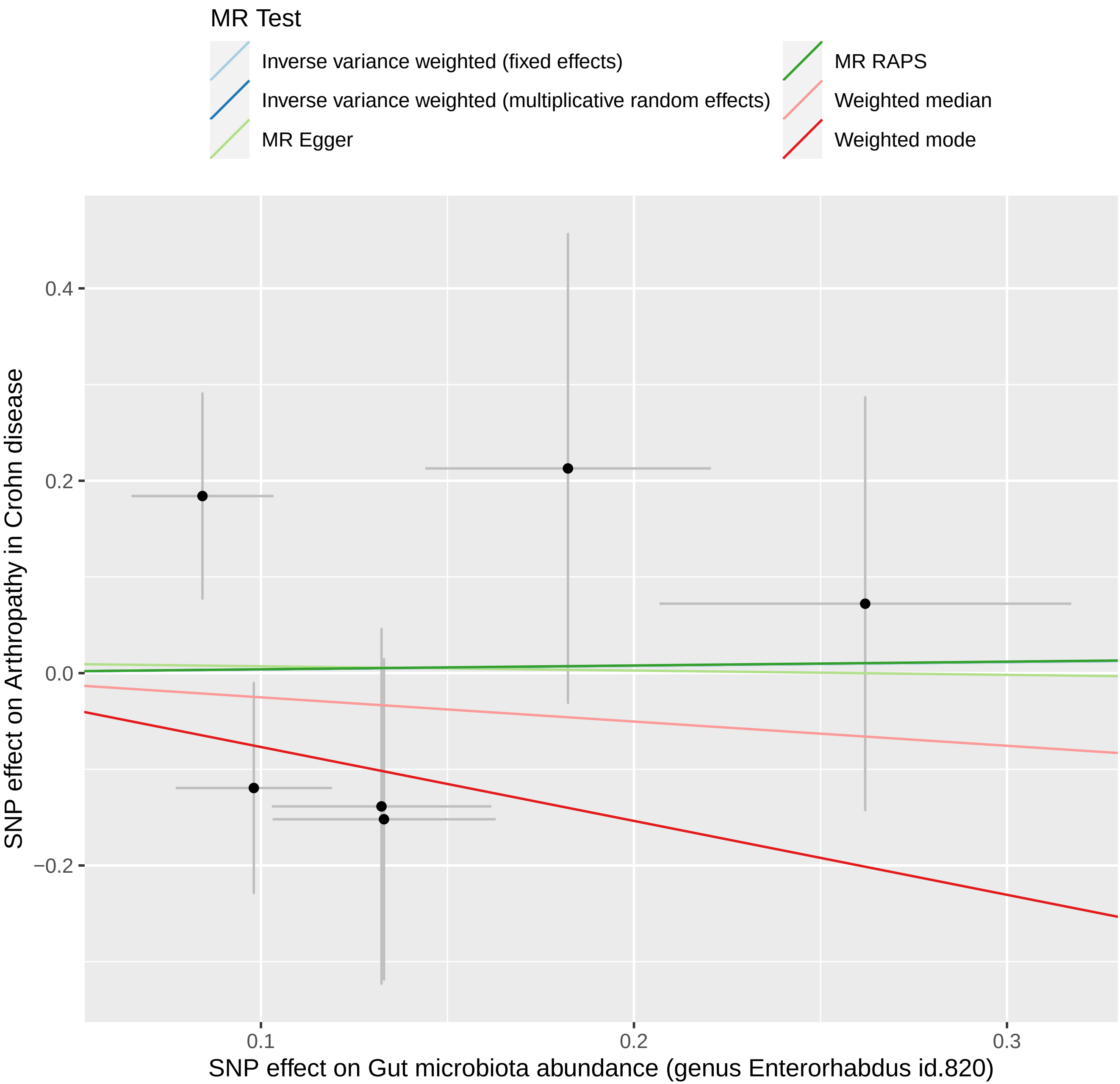

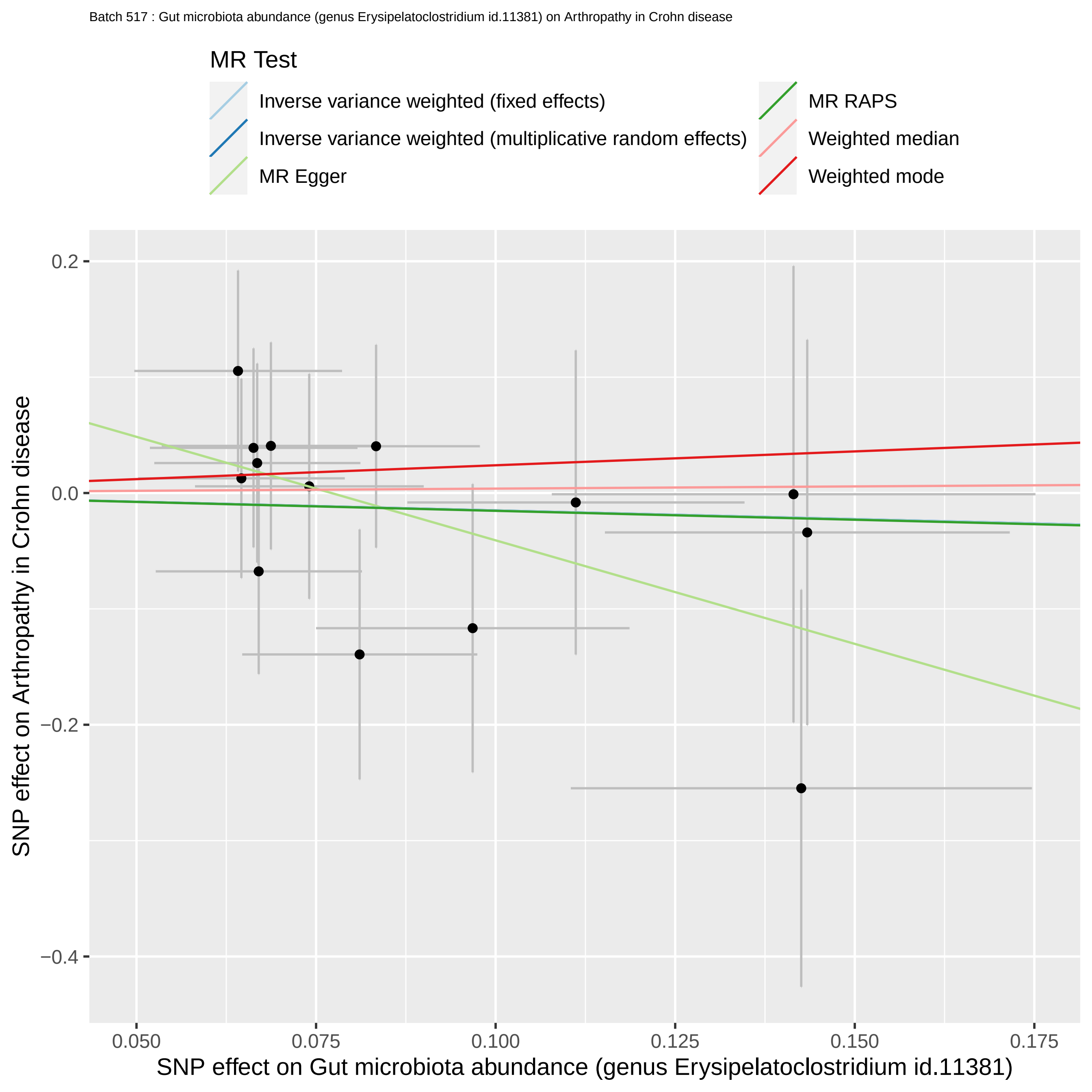

Batch 517 : Gut microbiota abundance (genus Erysipelatoclostridium id.11381) on Arthropathy in Crohn disease
MR Test
Inverse variance weighted (fixed effects)
Inverse variance weighted (multiplicative random effects)
MR Egger
MR RAPS
Weighted median
Weighted mode
0.2
0.0
−0.2
−0.4
0.050
0.075
0.100
0.125
0.150
0.175
SNP effect on Arthropathy in Crohn disease
SNP effect on Gut microbiota abundance (genus Erysipelatoclostridium id.11381)

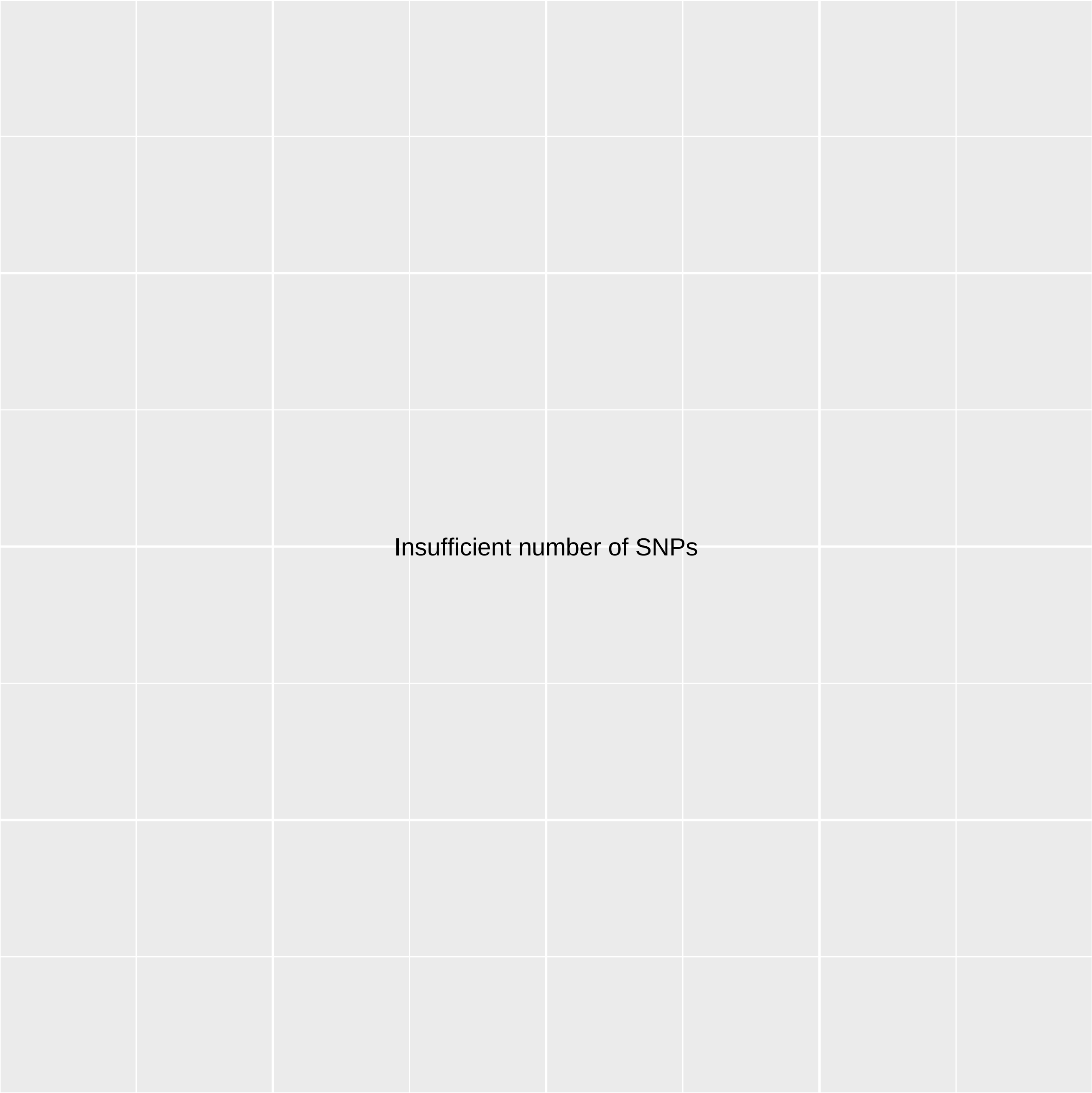
Insufficient number of SNPs

Batch 519 : Gut microbiota abundance (genus Escherichia Shigella id.3504) on Arthropathy in Crohn disease

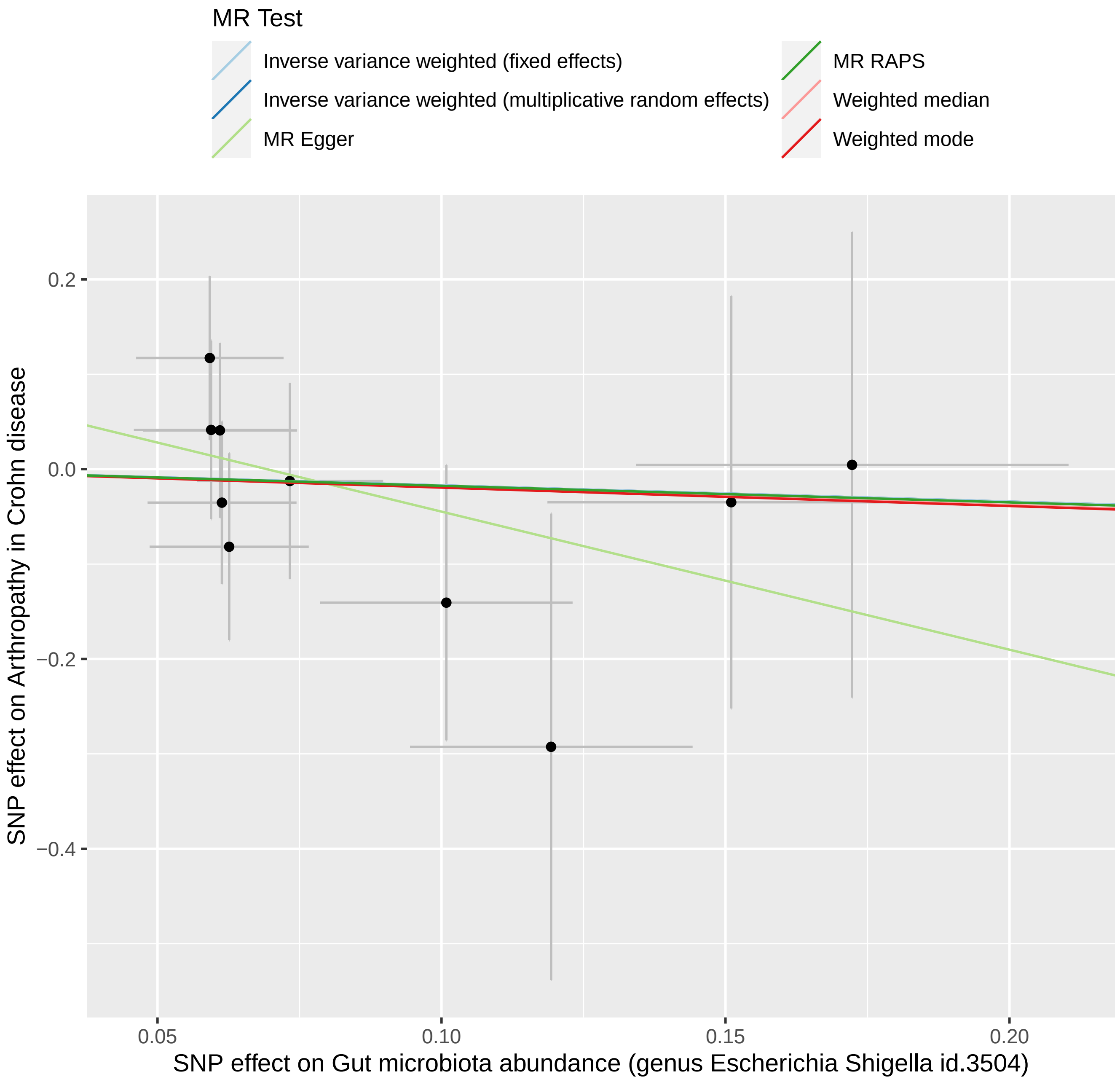

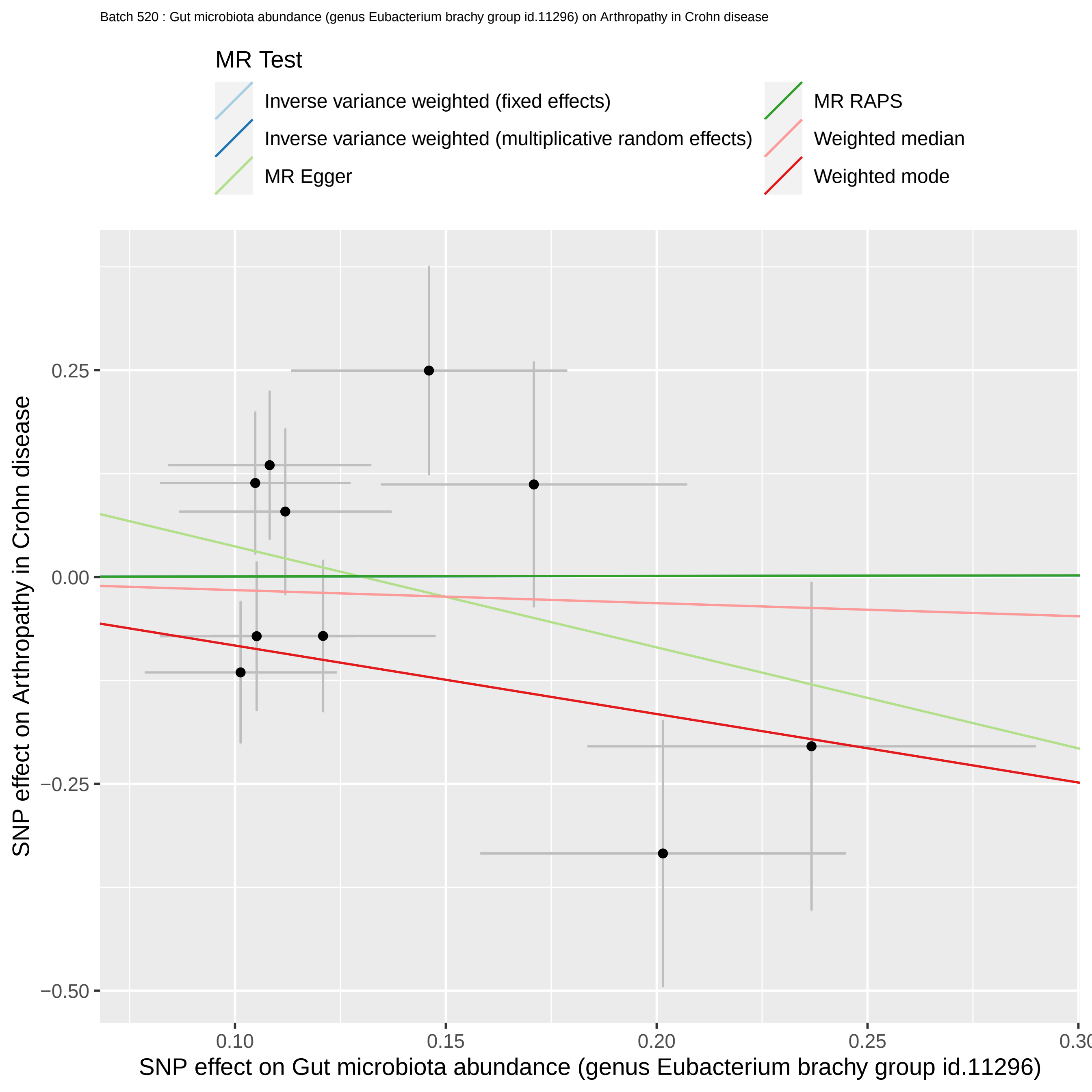

Batch 520 : Gut microbiota abundance (genus Eubacterium brachy group id.11296) on Arthropathy in Crohn disease
MR Test
Inverse variance weighted (fixed effects)
Inverse variance weighted (multiplicative random effects)
MR Egger
MR RAPS
Weighted median
Weighted mode
0.25
0.00
−0.25
−0.50
0.10
0.15
0.20
0.25
0.30
SNP effect on Arthropathy in Crohn disease
SNP effect on Gut microbiota abundance (genus Eubacterium brachy group id.11296)

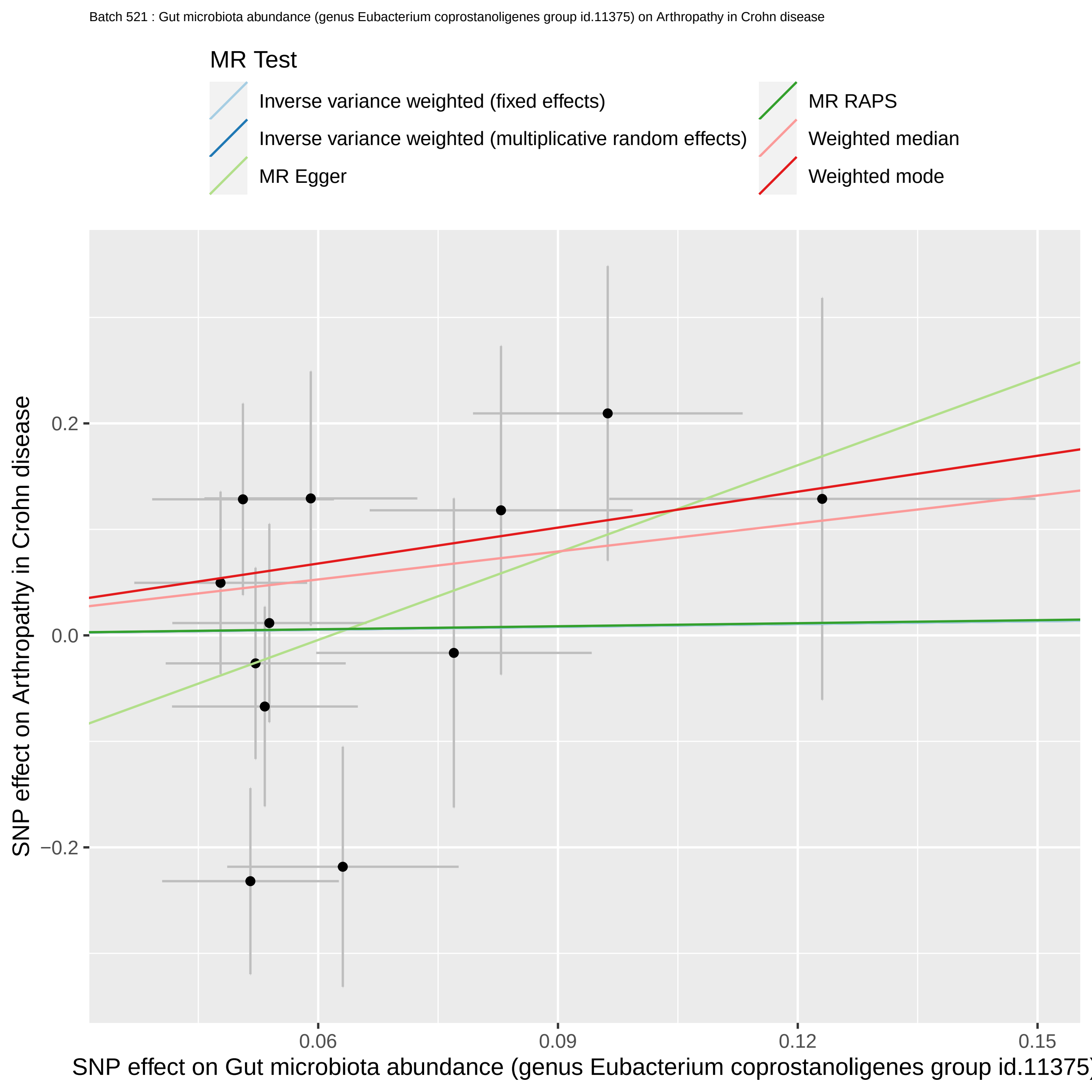

Batch 521 : Gut microbiota abundance (genus Eubacterium coprostanoligenes group id.11375) on Arthropathy in Crohn disease
MR Test
Inverse variance weighted (fixed effects)
Inverse variance weighted (multiplicative random effects)
MR Egger
MR RAPS
Weighted median
Weighted mode
SNP effect on Arthropathy in Crohn disease
0.2
0.0
−0.2
0.06
0.09
0.12
0.15
SNP effect on Gut microbiota abundance (genus Eubacterium coprostanoligenes group id.11375)

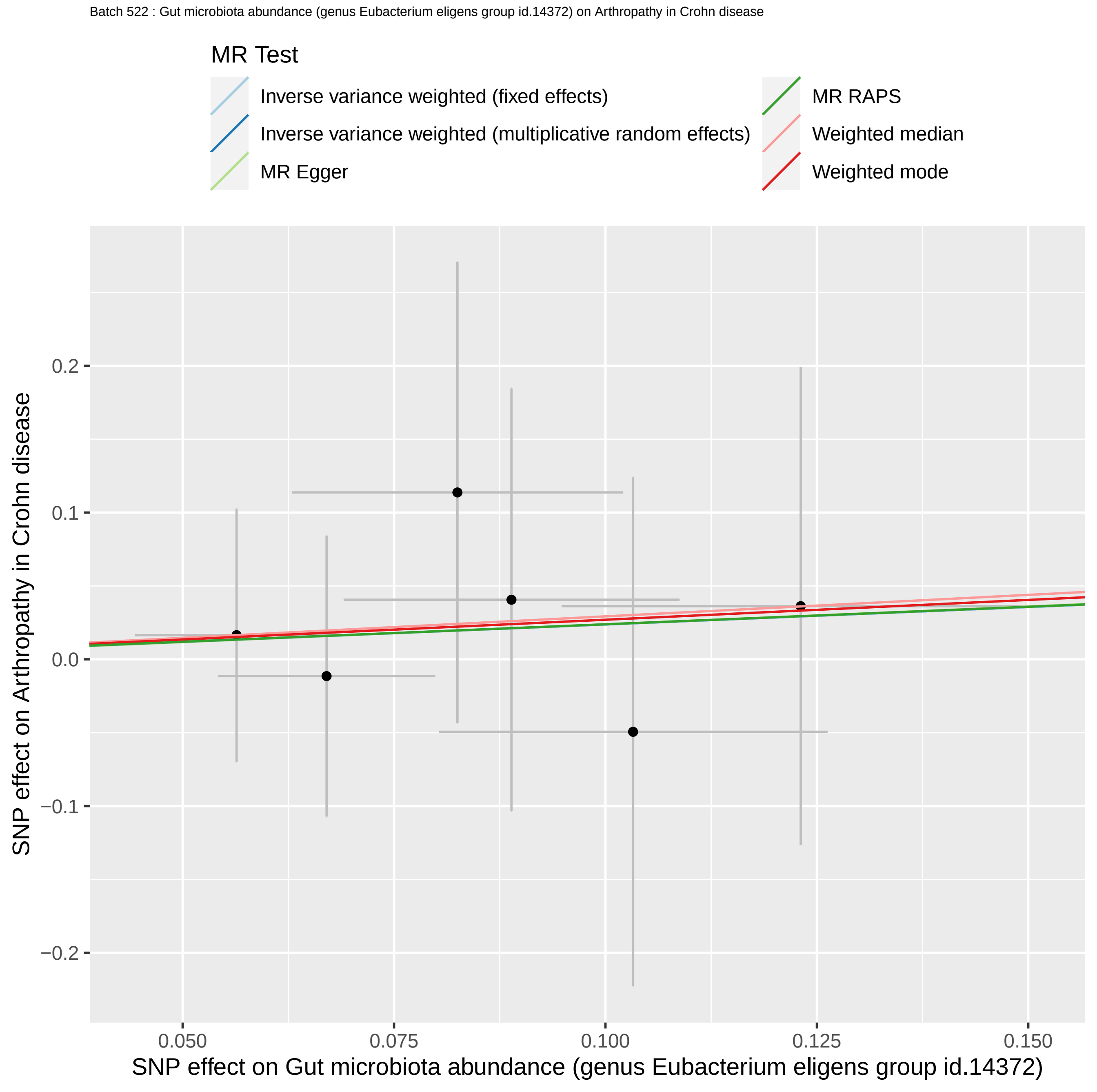
Batch 522 : Gut microbiota abundance (genus Eubacterium eligens group id.14372) on Arthropathy in Crohn disease
MR Test
Inverse variance weighted (fixed effects)
Inverse variance weighted (multiplicative random effects)
MR Egger
MR RAPS
Weighted median
Weighted mode
SNP effect on Arthropathy in Crohn disease
0.2
0.1
0.0
−0.1
−0.2
0.050
0.075
0.100
0.125
0.150
SNP effect on Gut microbiota abundance (genus Eubacterium eligens group id.14372)

Batch 523 : Gut microbiota abundance (genus Eubacterium fissicatena group id.14373) on Arthropathy in Crohn disease

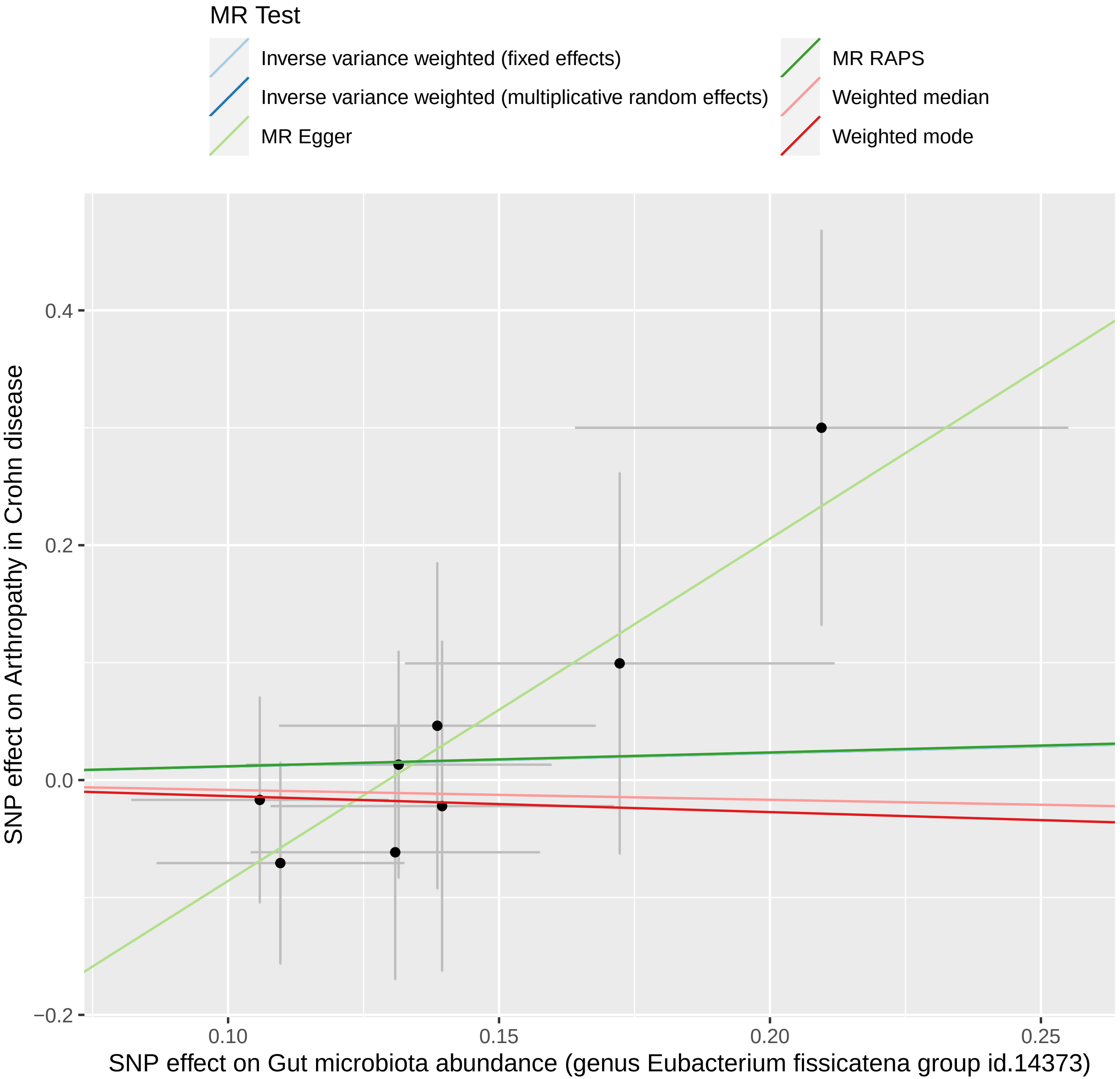

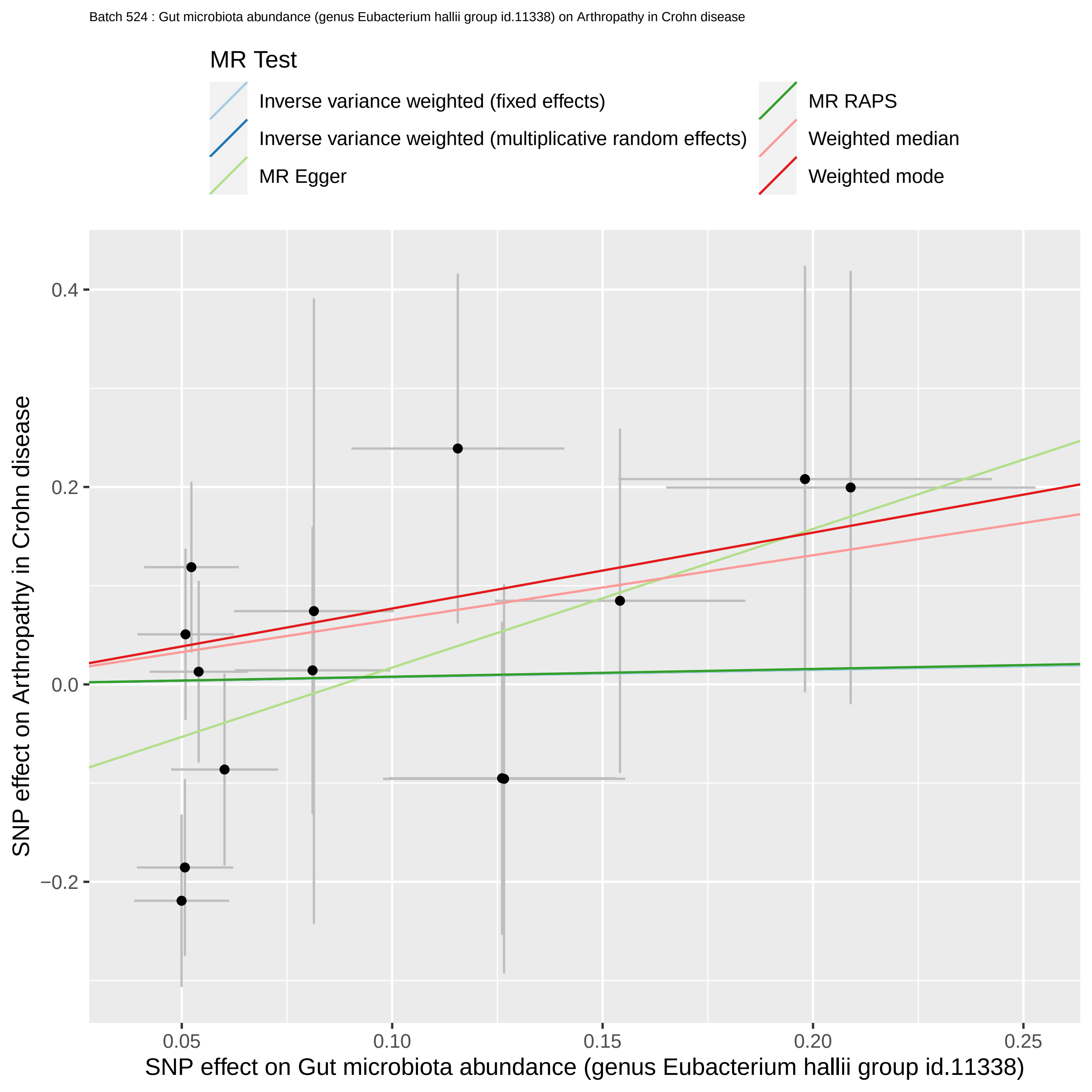

Batch 524 : Gut microbiota abundance (genus Eubacterium hallii group id.11338) on Arthropathy in Crohn disease
MR Test
Inverse variance weighted (fixed effects)
Inverse variance weighted (multiplicative random effects)
MR Egger
MR RAPS
Weighted median
Weighted mode
0.4
0.2
0.0
−0.2
0.05
0.10
0.15
0.20
0.25
SNP effect on Arthropathy in Crohn disease
SNP effect on Gut microbiota abundance (genus Eubacterium hallii group id.11338)

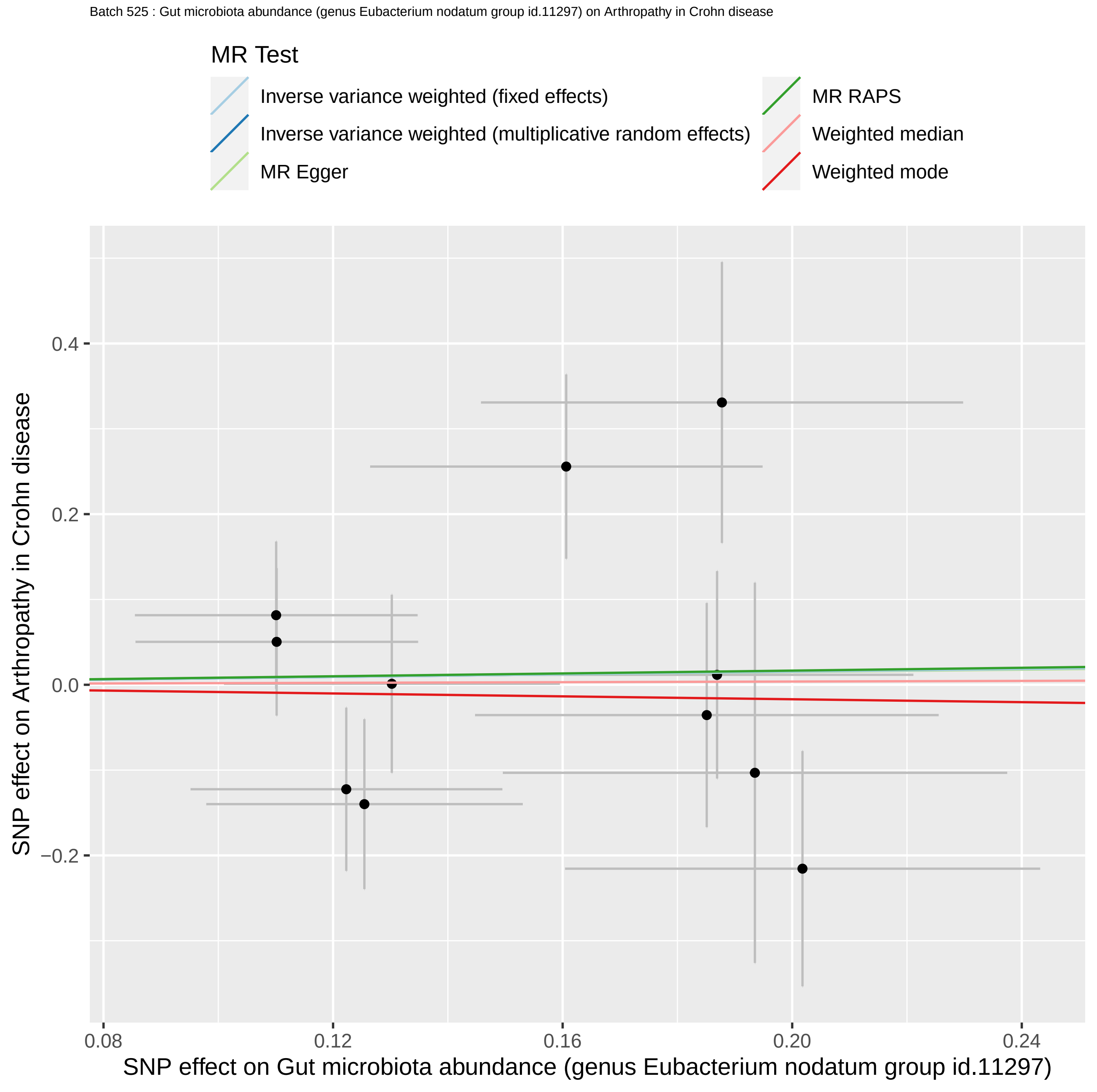

Batch 525 : Gut microbiota abundance (genus Eubacterium nodatum group id.11297) on Arthropathy in Crohn disease
MR Test
Inverse variance weighted (fixed effects)
Inverse variance weighted (multiplicative random effects)
MR Egger
MR RAPS
Weighted median
Weighted mode
SNP effect on Arthropathy in Crohn disease
0.4
0.2
0.0
−0.2
0.08
0.12
0.16
0.20
0.24
SNP effect on Gut microbiota abundance (genus Eubacterium nodatum group id.11297)

Batch 526 : Gut microbiota abundance (genus Eubacterium oxidoreducens group id.11339) on Arthropathy in Crohn disease

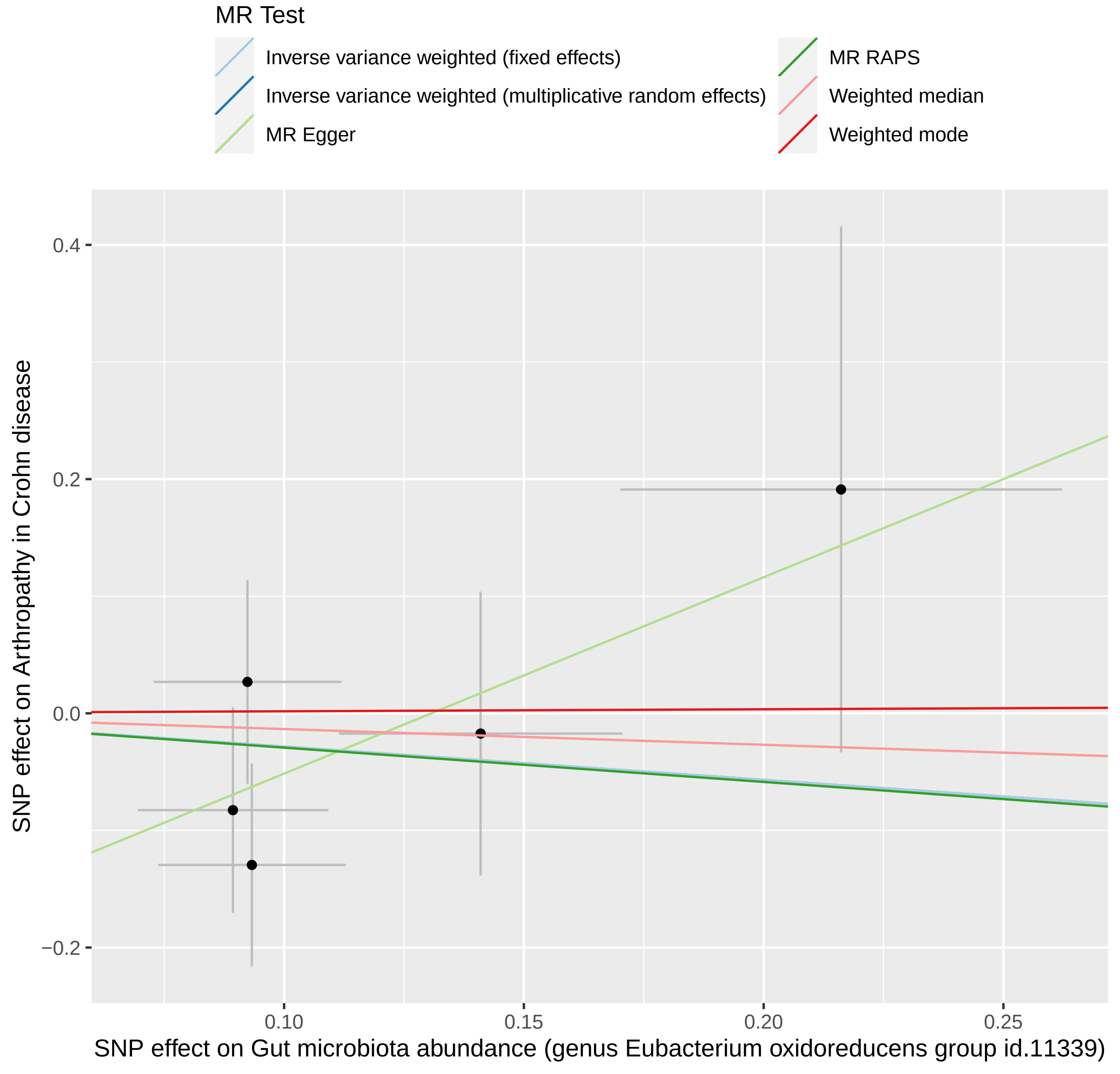

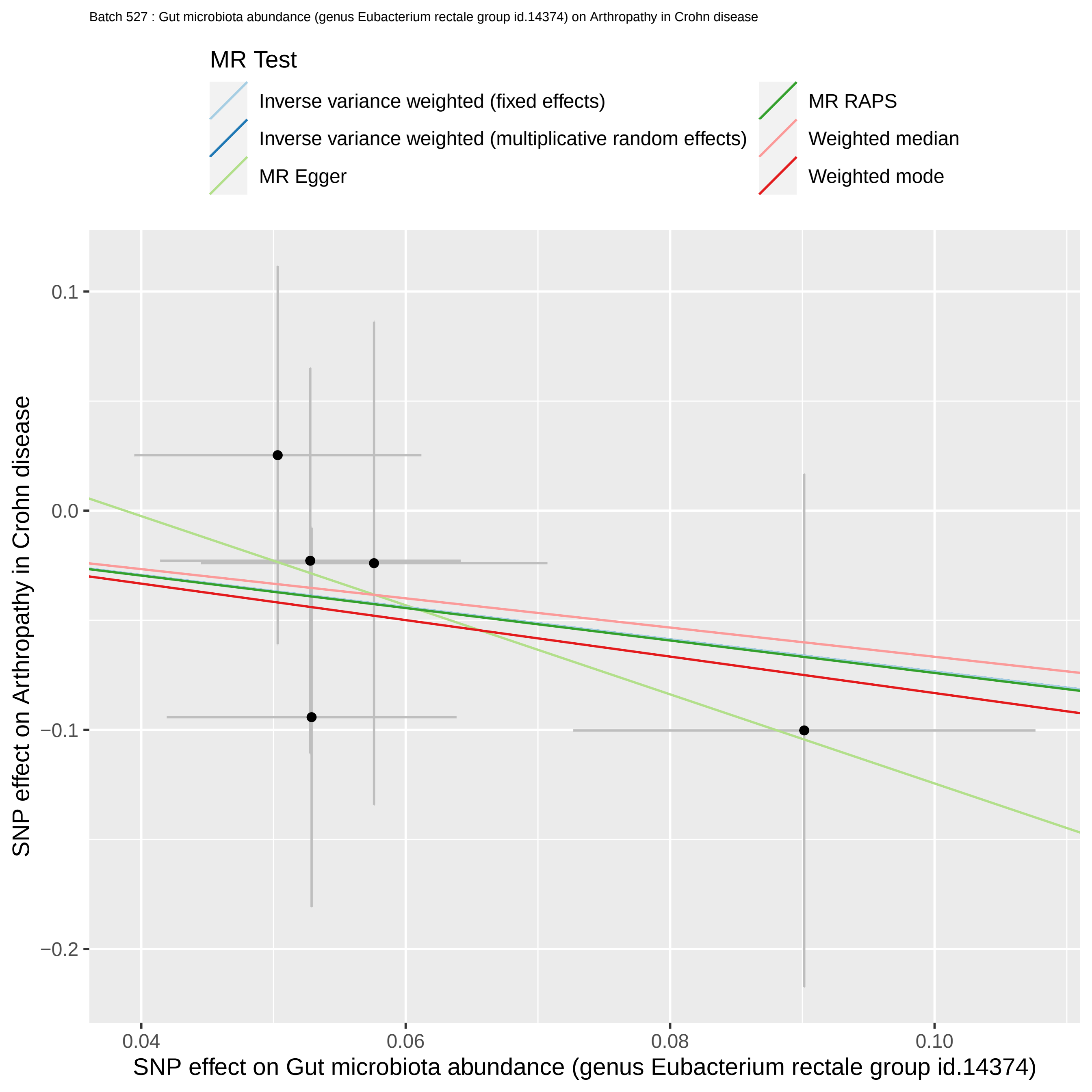
Batch 527 : Gut microbiota abundance (genus Eubacterium rectale group id.14374) on Arthropathy in Crohn disease
MR Test
Inverse variance weighted (fixed effects)
Inverse variance weighted (multiplicative random effects)
MR Egger
MR RAPS
Weighted median
Weighted mode
SNP effect on Arthropathy in Crohn disease
0.1
0.0
−0.1
−0.2
0.04
0.06
0.08
0.10
SNP effect on Gut microbiota abundance (genus Eubacterium rectale group id.14374)

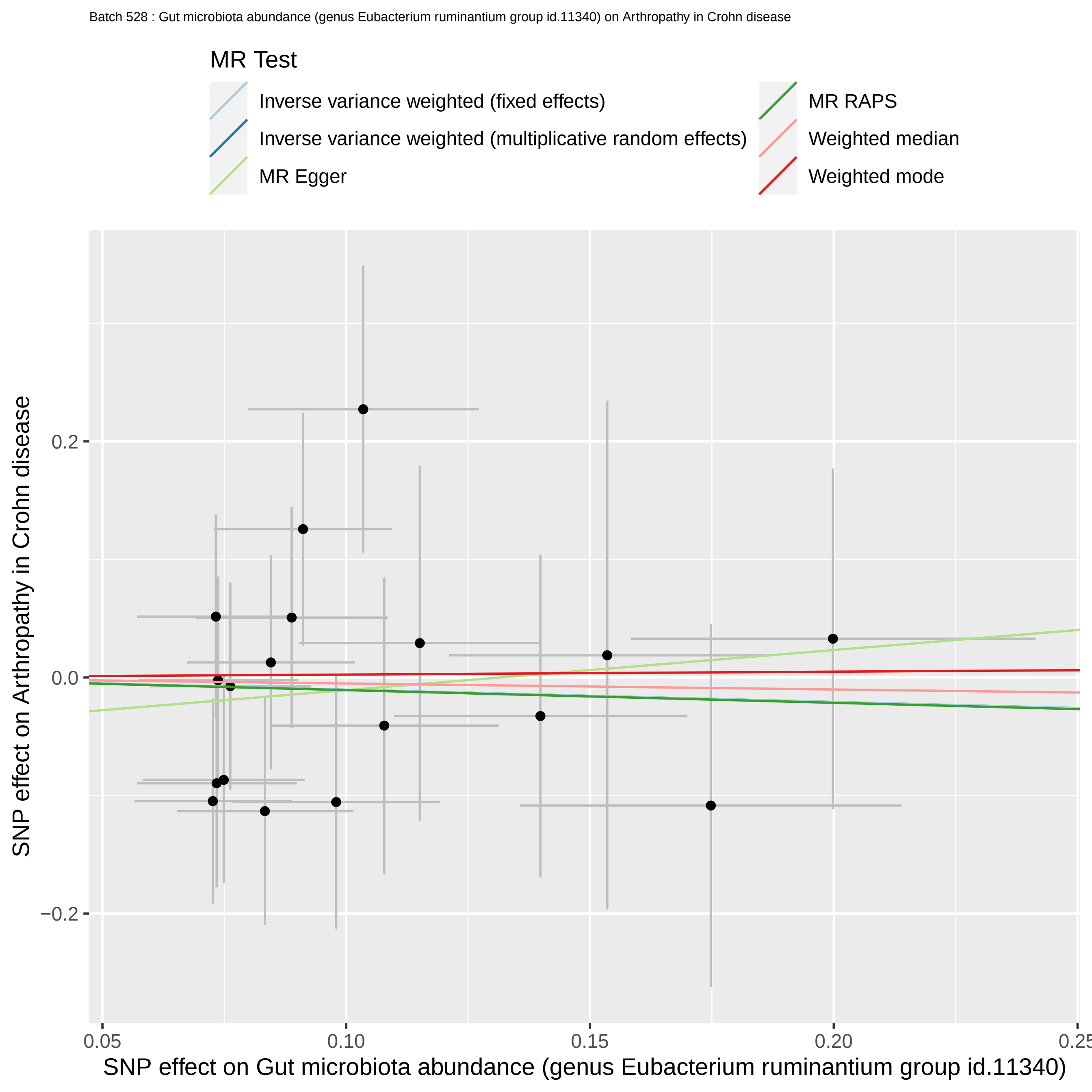

Batch 528 : Gut microbiota abundance (genus Eubacterium ruminantium group id.11340) on Arthropathy in Crohn disease
MR Test
Inverse variance weighted (fixed effects)
Inverse variance weighted (multiplicative random effects)
MR Egger
MR RAPS
Weighted median
Weighted mode
SNP effect on Arthropathy in Crohn disease
0.2
0.0
−0.2
0.05
0.10
0.15
0.20
SNP effect on Gut microbiota abundance (genus Eubacterium ruminantium group id.11340)

Batch 529 : Gut microbiota abundance (genus Eubacterium ventriosum group id.11341) on Arthropathy in Crohn disease

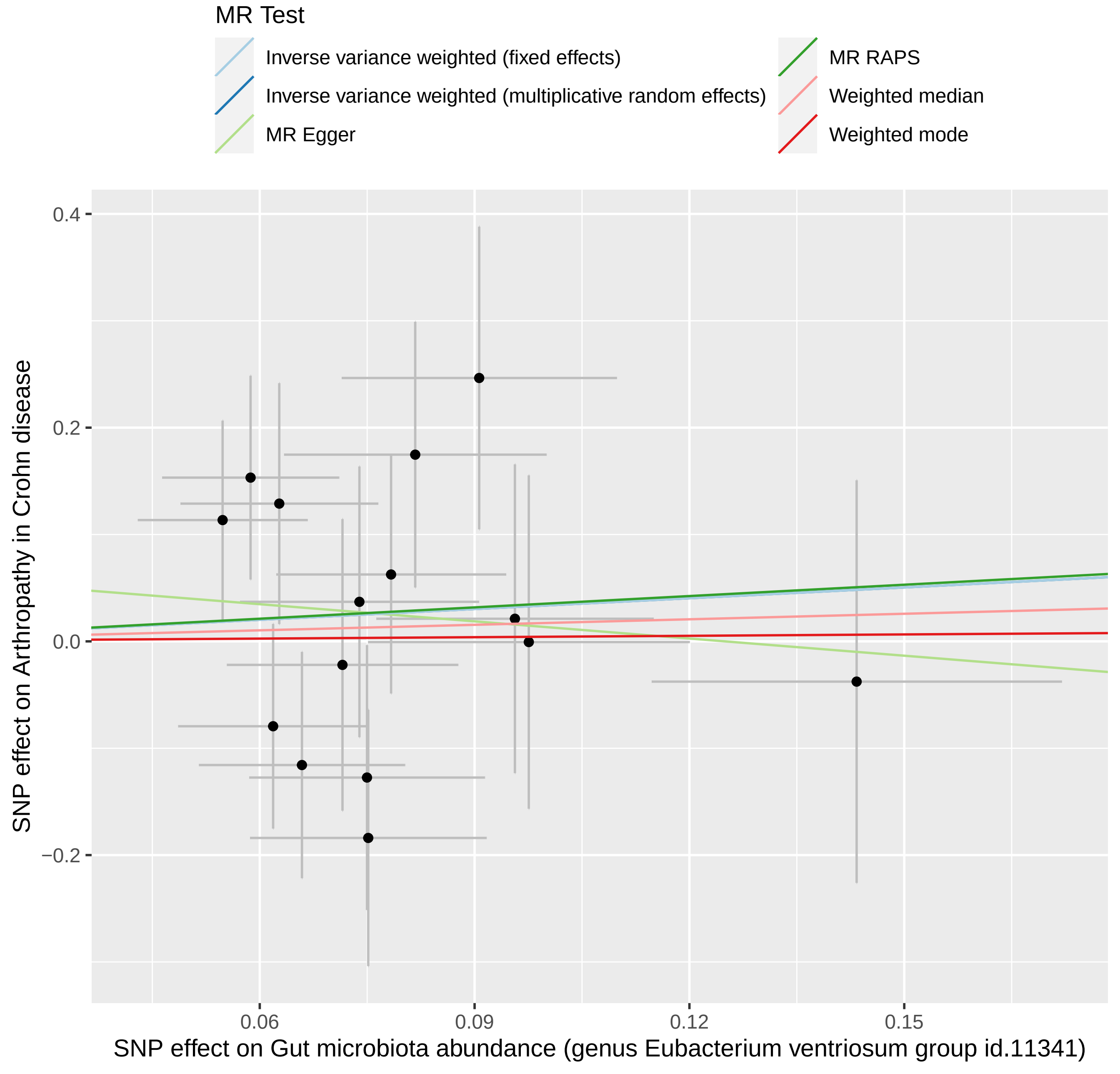

Batch 530 : Gut microbiota abundance (genus Eubacterium xylanophilum group id.14375) on Arthropathy in Crohn disease

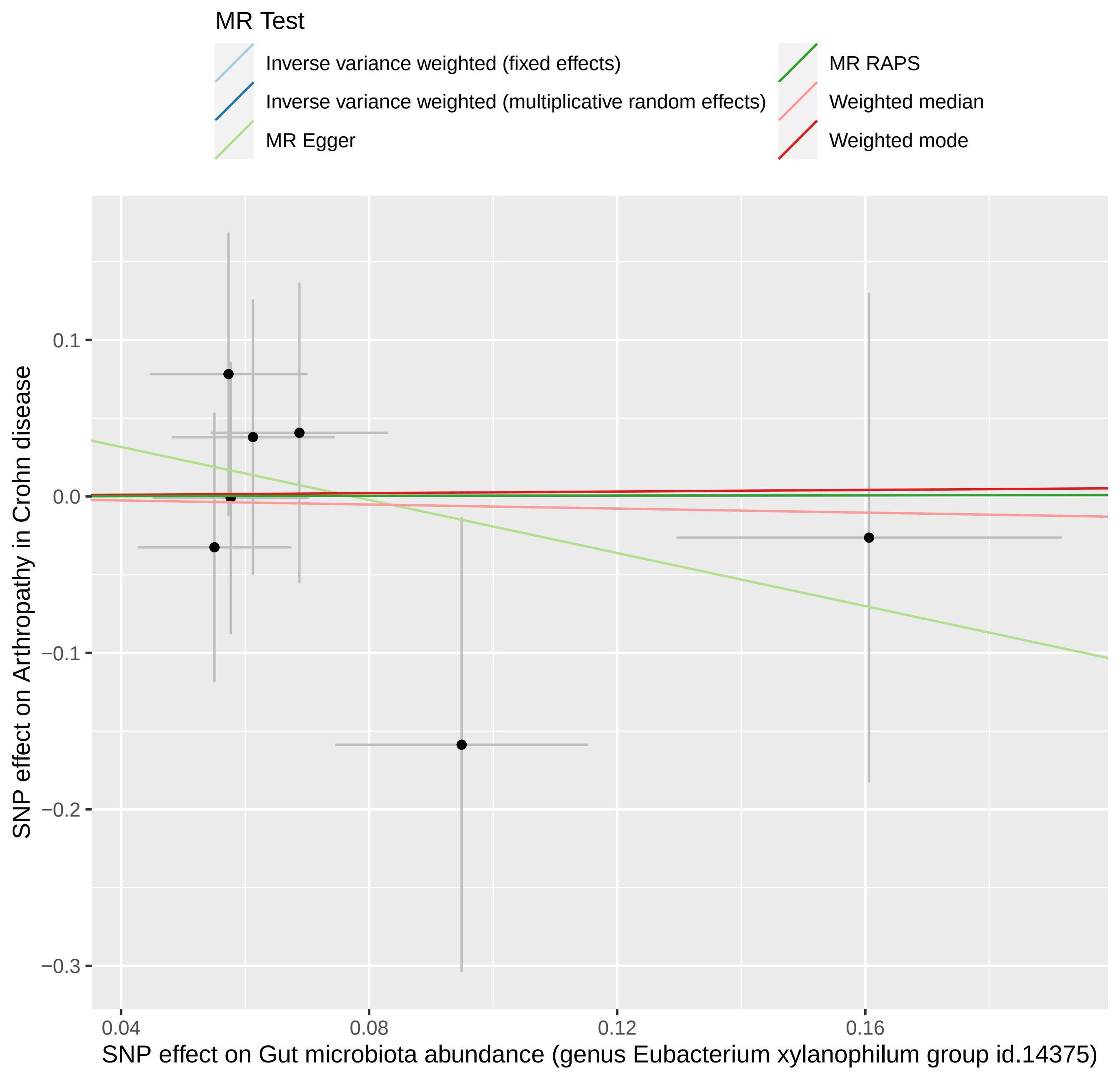

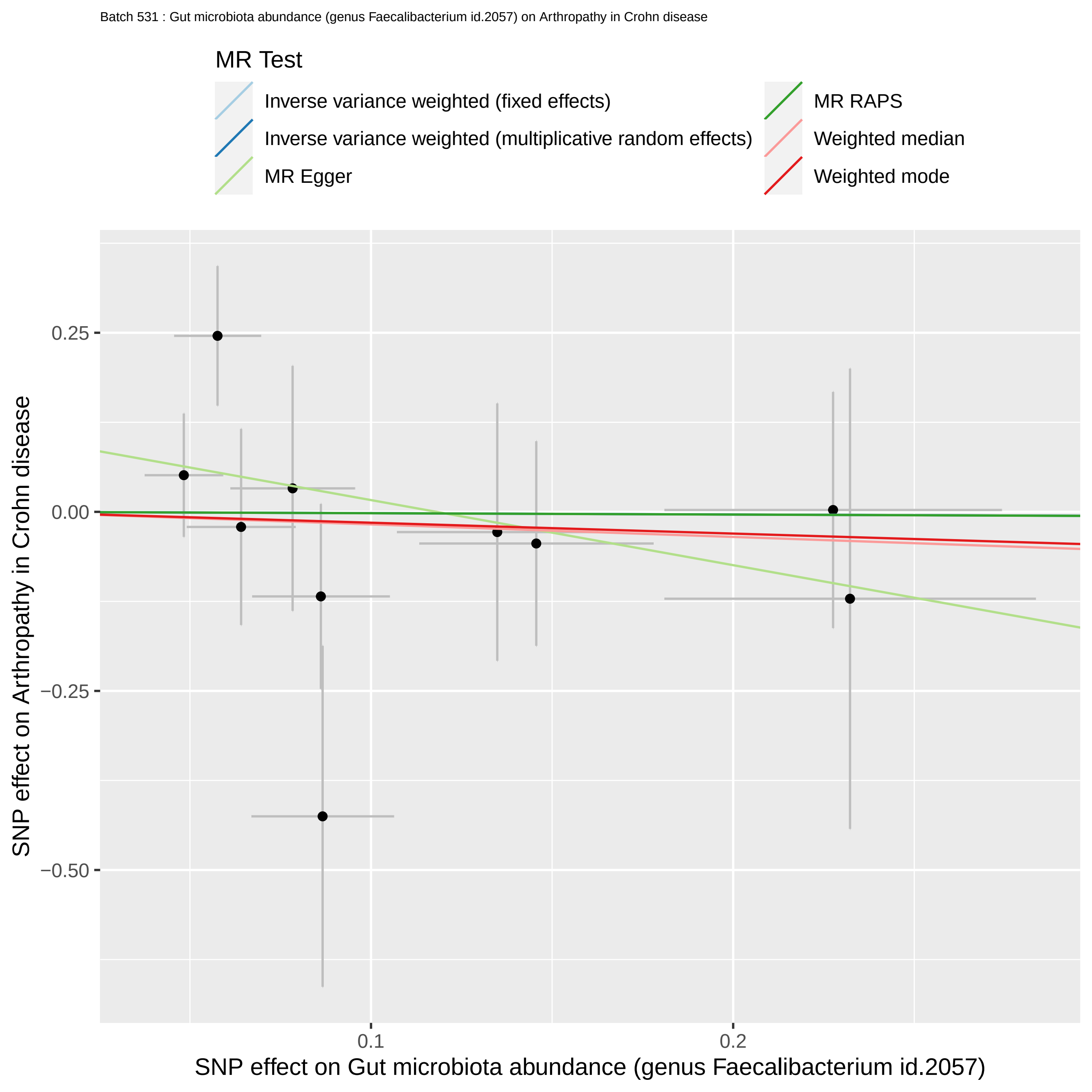
Batch 531 : Gut microbiota abundance (genus Faecalibacterium id.2057) on Arthropathy in Crohn disease
MR Test
Inverse variance weighted (fixed effects)
Inverse variance weighted (multiplicative random effects)
MR Egger
MR RAPS
Weighted median
Weighted mode
0.25
0.00
−0.25
−0.50
0.1
0.2
SNP effect on Arthropathy in Crohn disease
SNP effect on Gut microbiota abundance (genus Faecalibacterium id.2057)

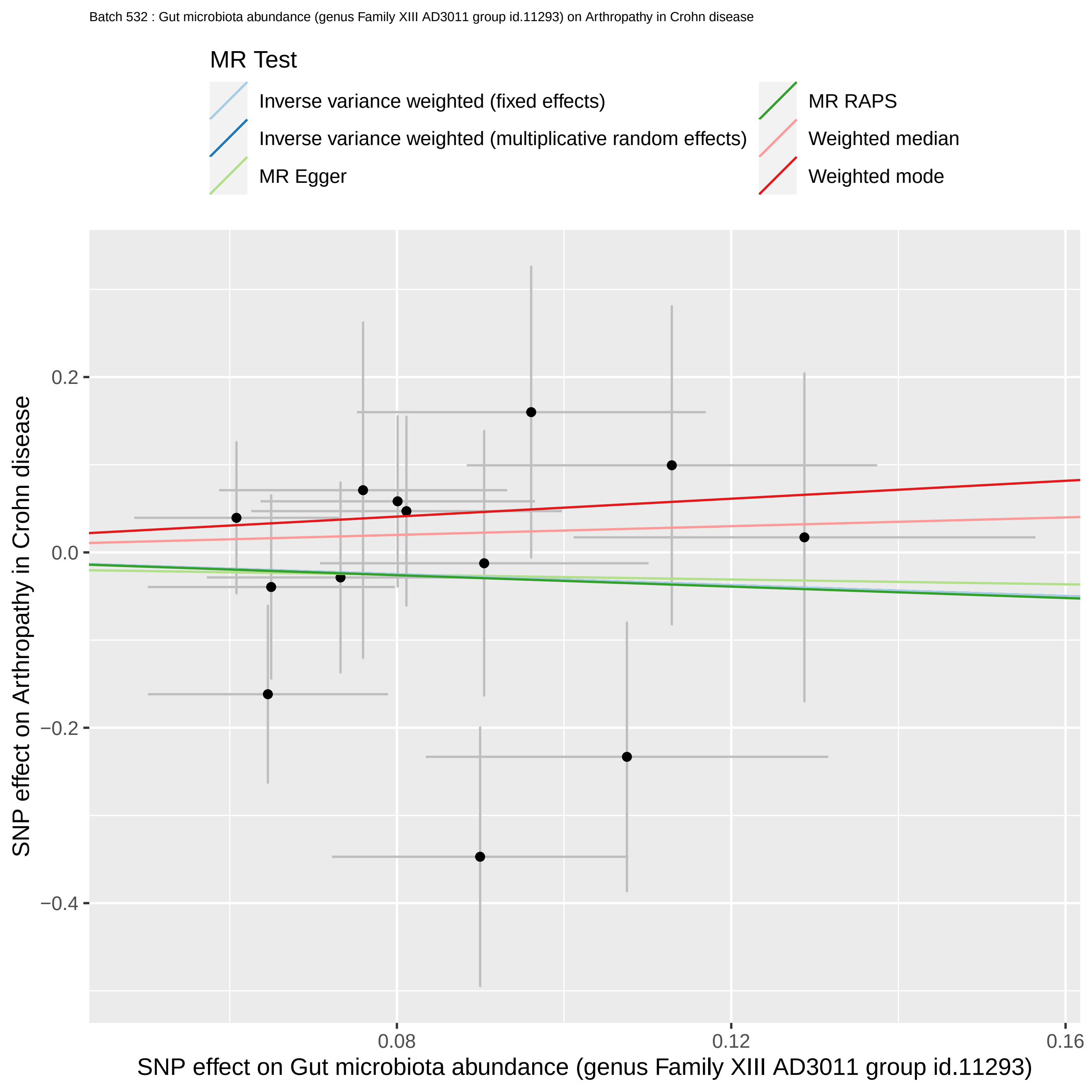
Batch 532 : Gut microbiota abundance (genus Family XIII AD3011 group id.11293) on Arthropathy in Crohn disease
MR Test
Inverse variance weighted (fixed effects)
Inverse variance weighted (multiplicative random effects)
MR Egger
MR RAPS
Weighted median
Weighted mode
0.2
0.0
−0.2
−0.4
0.08
0.12
0.16
SNP effect on Arthropathy in Crohn disease
SNP effect on Gut microbiota abundance (genus Family XIII AD3011 group id.11293)

Batch 533 : Gut microbiota abundance (genus Family XIII UCG001 id.11294) on Arthropathy in Crohn disease

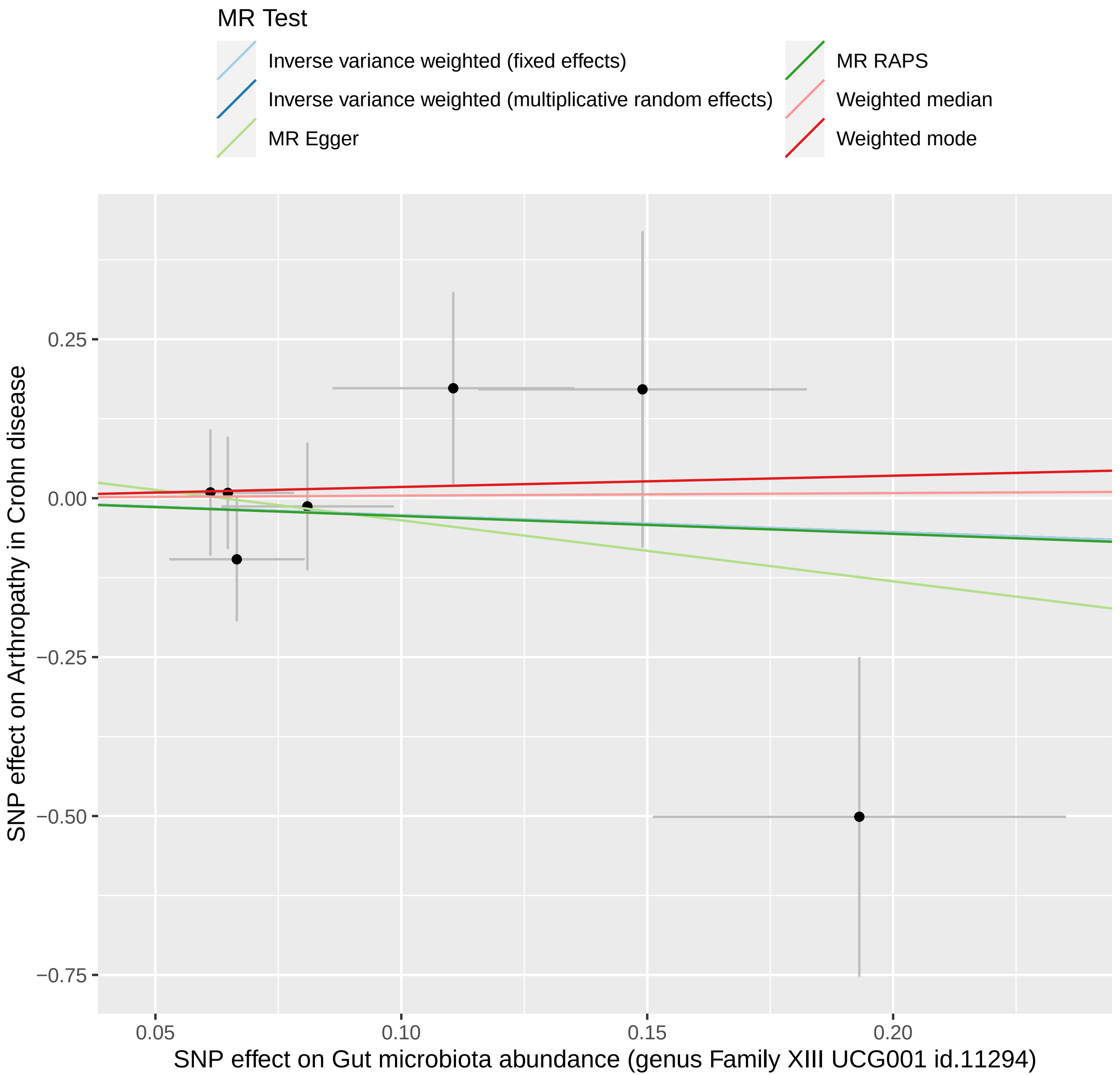

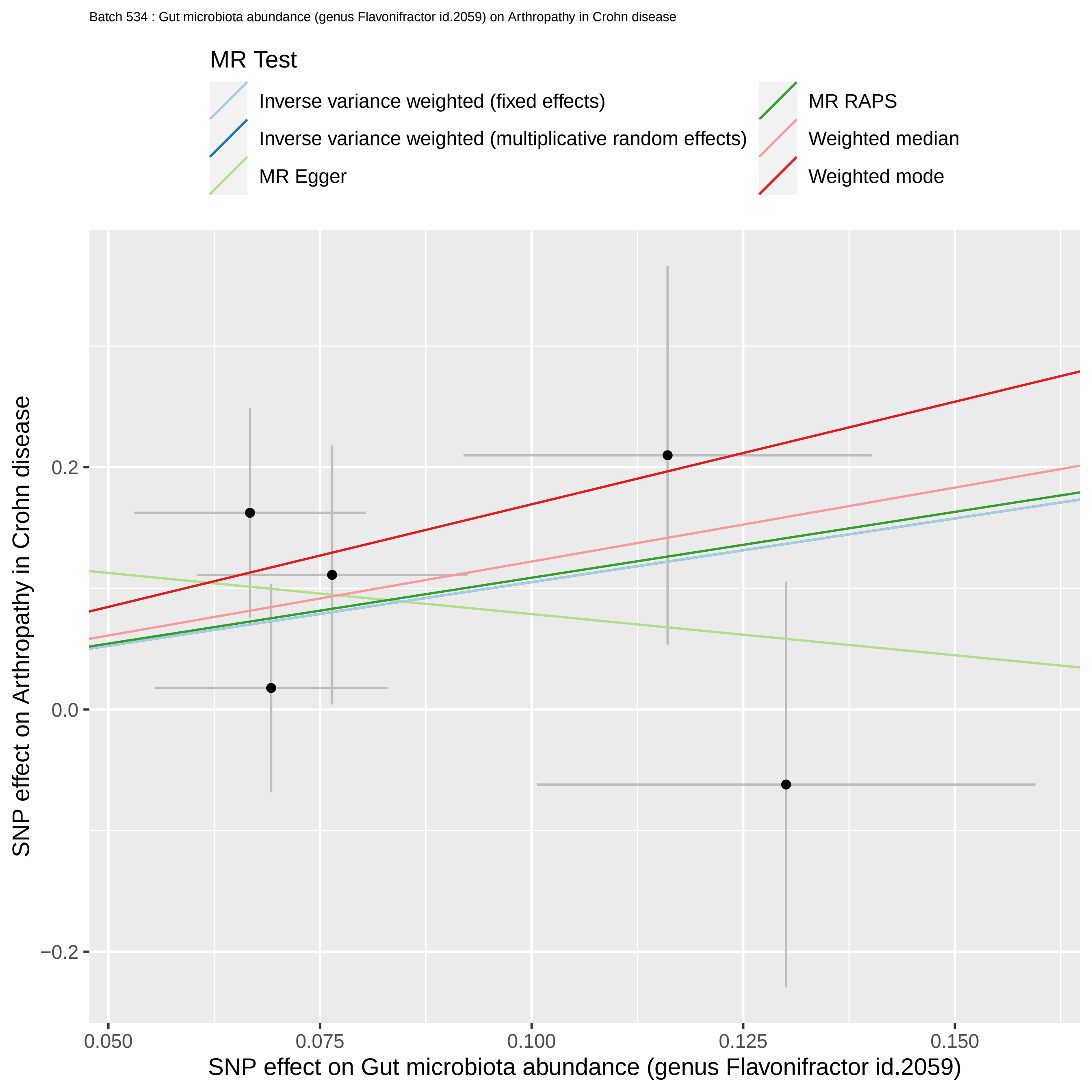
Batch 534 : Gut microbiota abundance (genus Flavonifractor id.2059) on Arthropathy in Crohn disease
MR Test
Inverse variance weighted (fixed effects)
Inverse variance weighted (multiplicative random effects)
MR Egger
MR RAPS
Weighted median
Weighted mode
SNP effect on Arthropathy in Crohn disease
0.2
0.0
−0.2
0.050
0.075
0.100
0.125
0.150
SNP effect on Gut microbiota abundance (genus Flavonifractor id.2059)

Batch 535 : Gut microbiota abundance (genus Fusicatenibacter id.11305) on Arthropathy in Crohn disease

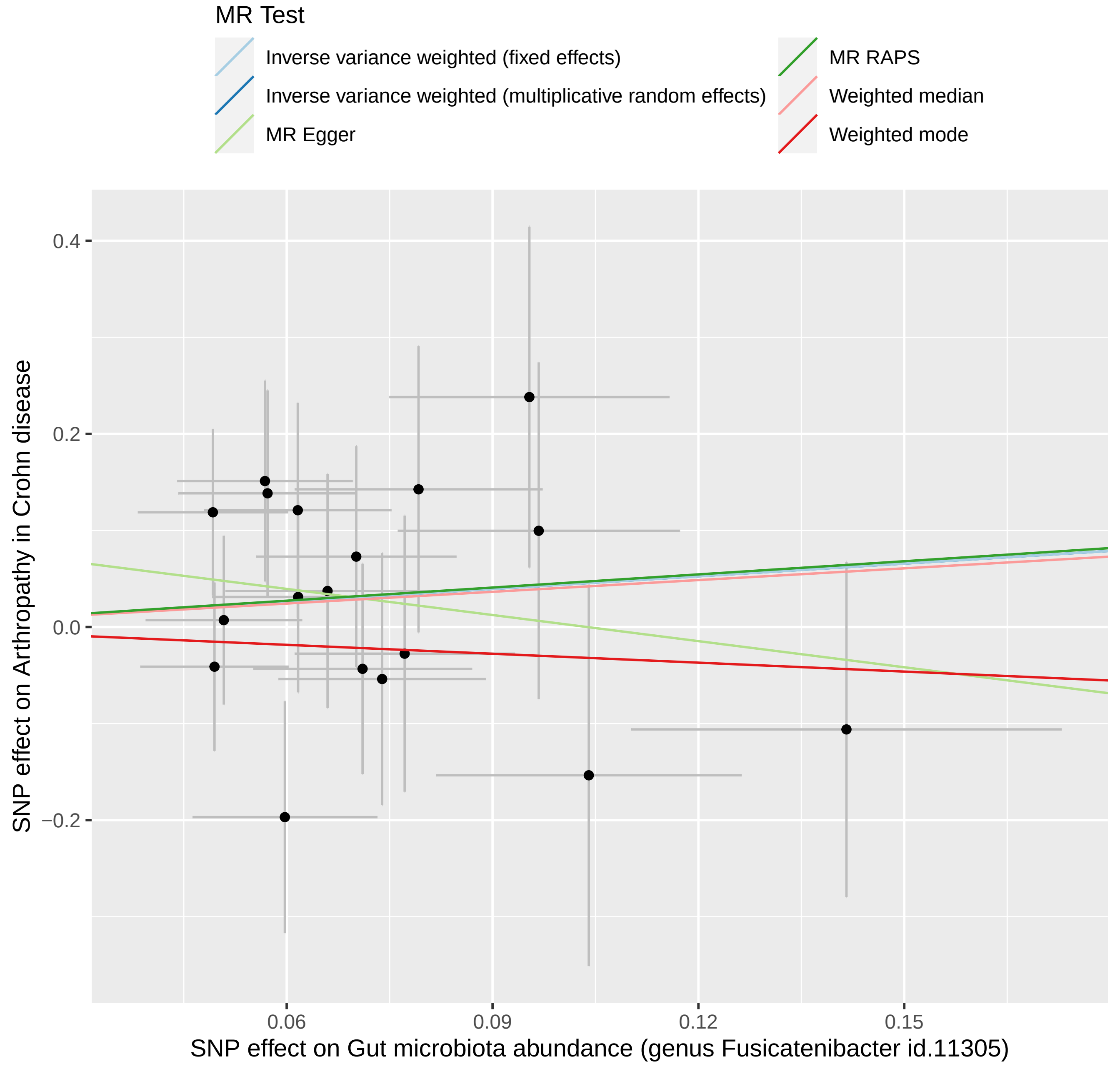

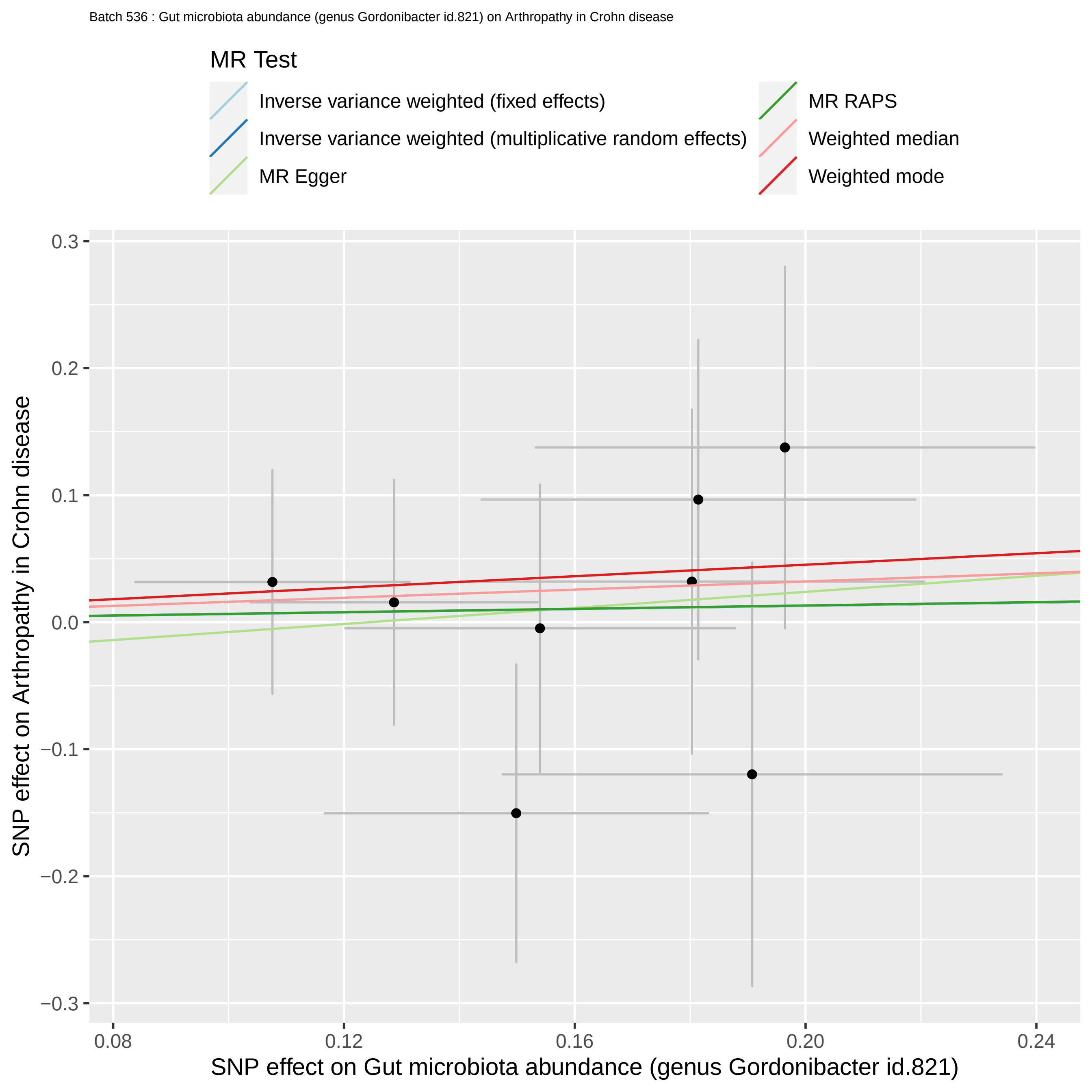

Batch 536 : Gut microbiota abundance (genus Gordonibacter id.821) on Arthropathy in Crohn disease
MR Test
Inverse variance weighted (fixed effects)
Inverse variance weighted (multiplicative random effects)
MR Egger
MR RAPS
Weighted median
Weighted mode
SNP effect on Arthropathy in Crohn disease
SNP effect on Gut microbiota abundance (genus Gordonibacter id.821)
0.3
0.2
0.1
0.0
−0.1
−0.2
−0.3
0.08
0.12
0.16
0.20
0.24

Batch 537 : Gut microbiota abundance (genus Haemophilus id.3698) on Arthropathy in Crohn disease

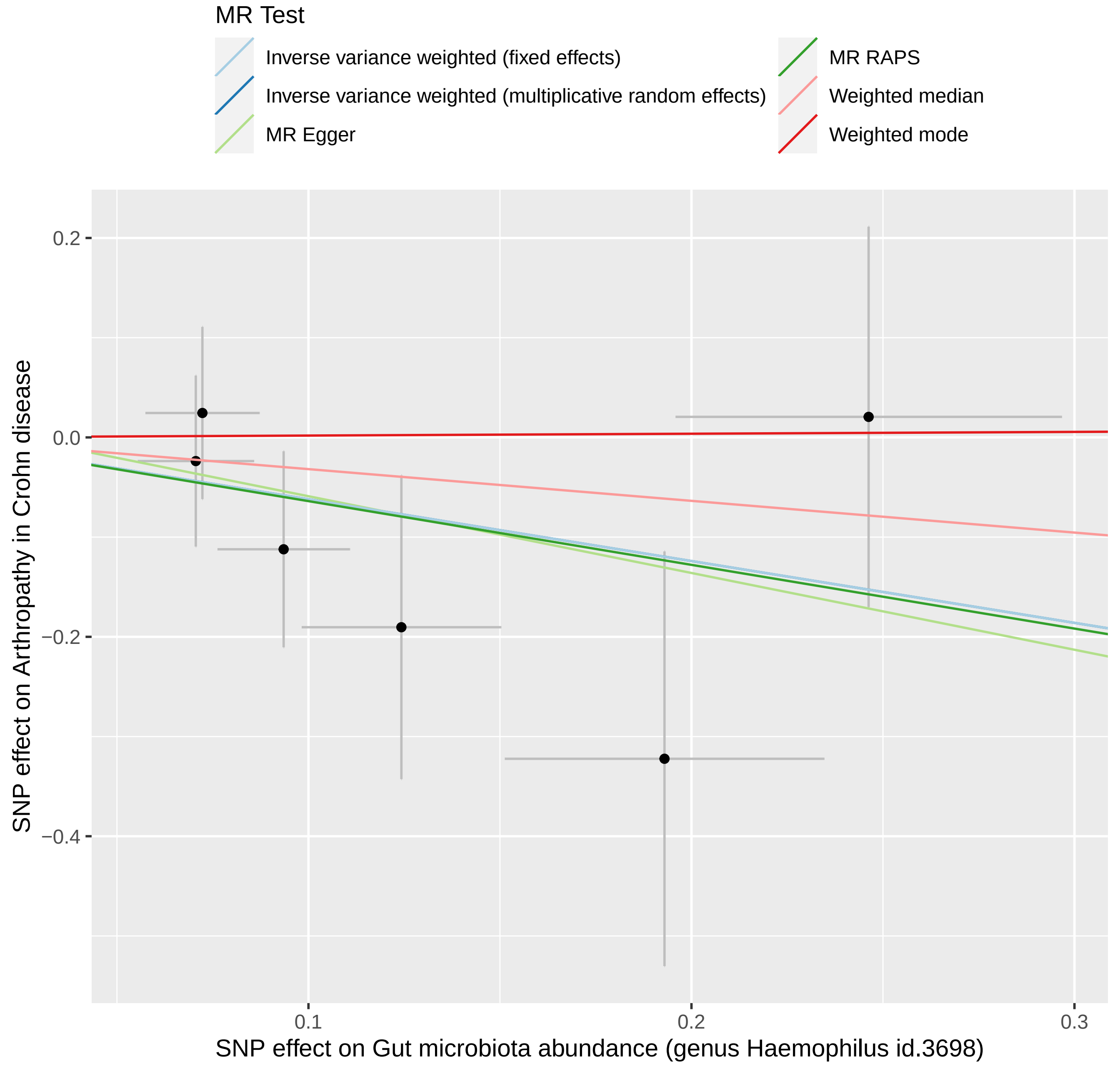

Batch 538 : Gut microbiota abundance (genus Holdemanella id.11393) on Arthropathy in Crohn disease

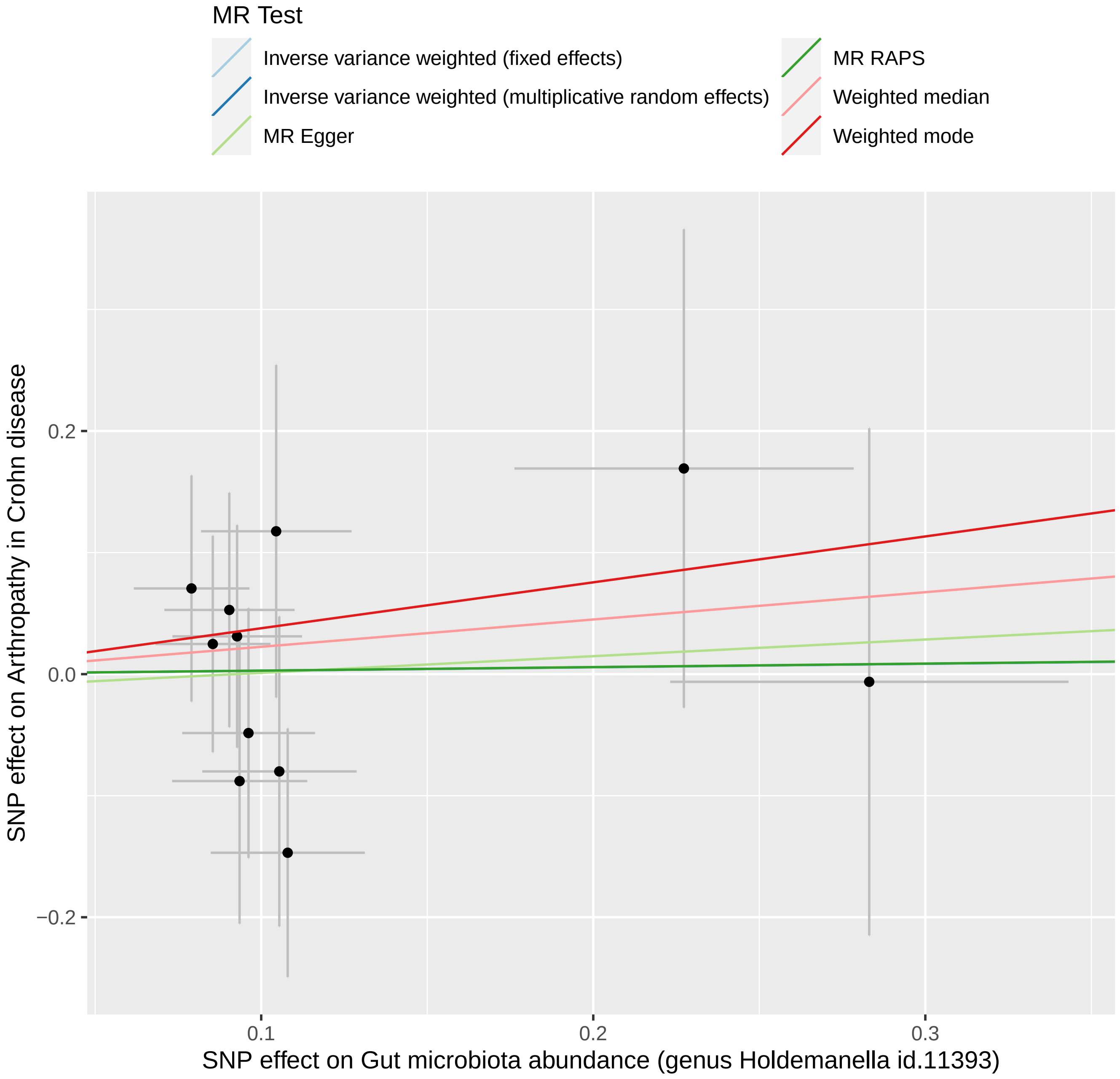

Batch 539 : Gut microbiota abundance (genus Holdemania id.2157) on Arthropathy in Crohn disease

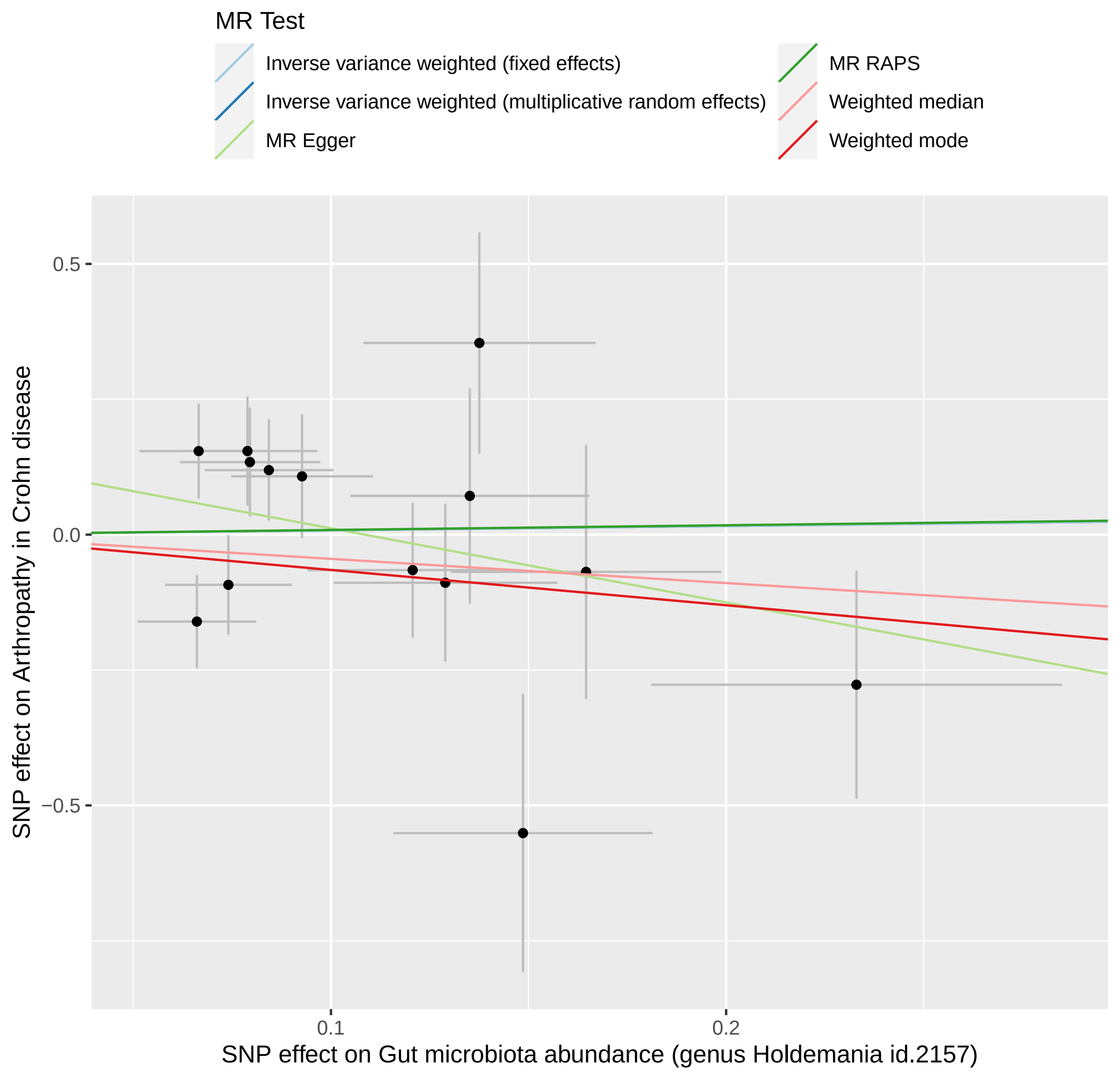

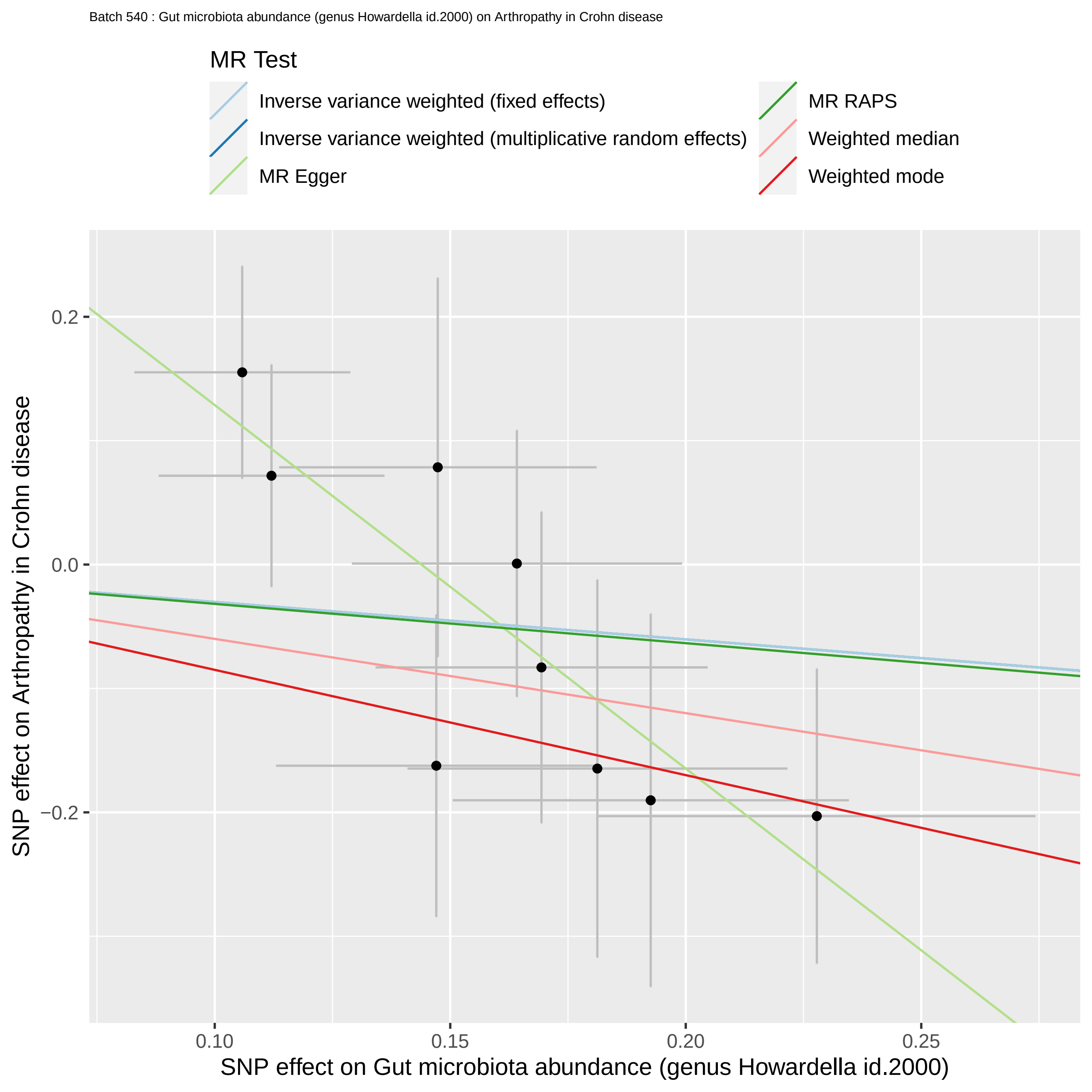

Batch 540 : Gut microbiota abundance (genus Howardella id.2000) on Arthropathy in Crohn disease
MR Test
Inverse variance weighted (fixed effects)
Inverse variance weighted (multiplicative random effects)
MR Egger
MR RAPS
Weighted median
Weighted mode
0.2
0.0
−0.2
0.10
0.15
0.20
0.25
SNP effect on Arthropathy in Crohn disease
SNP effect on Gut microbiota abundance (genus Howardella id.2000)

Batch 541 : Gut microbiota abundance (genus Hungatella id.11306) on Arthropathy in Crohn disease

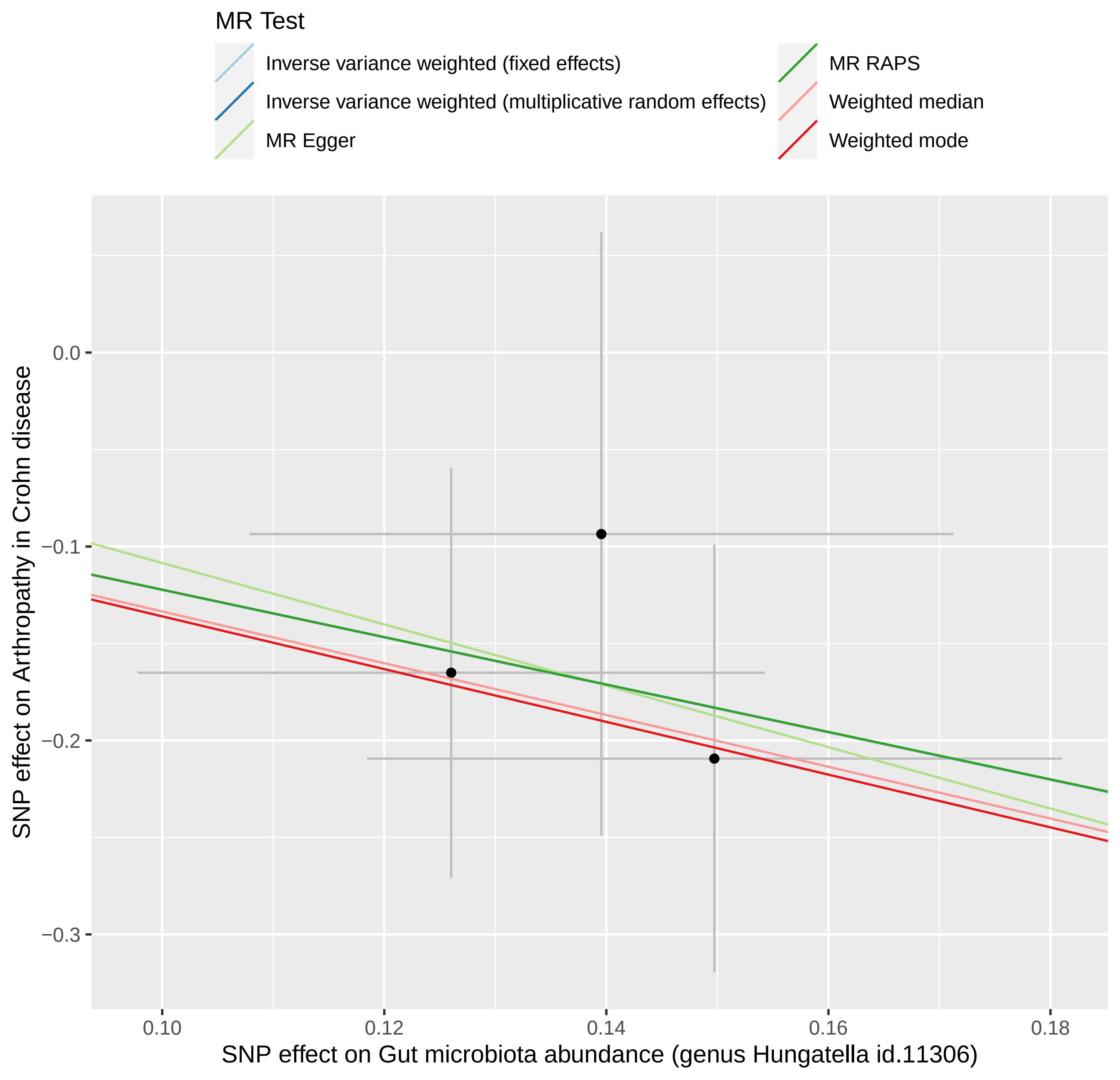

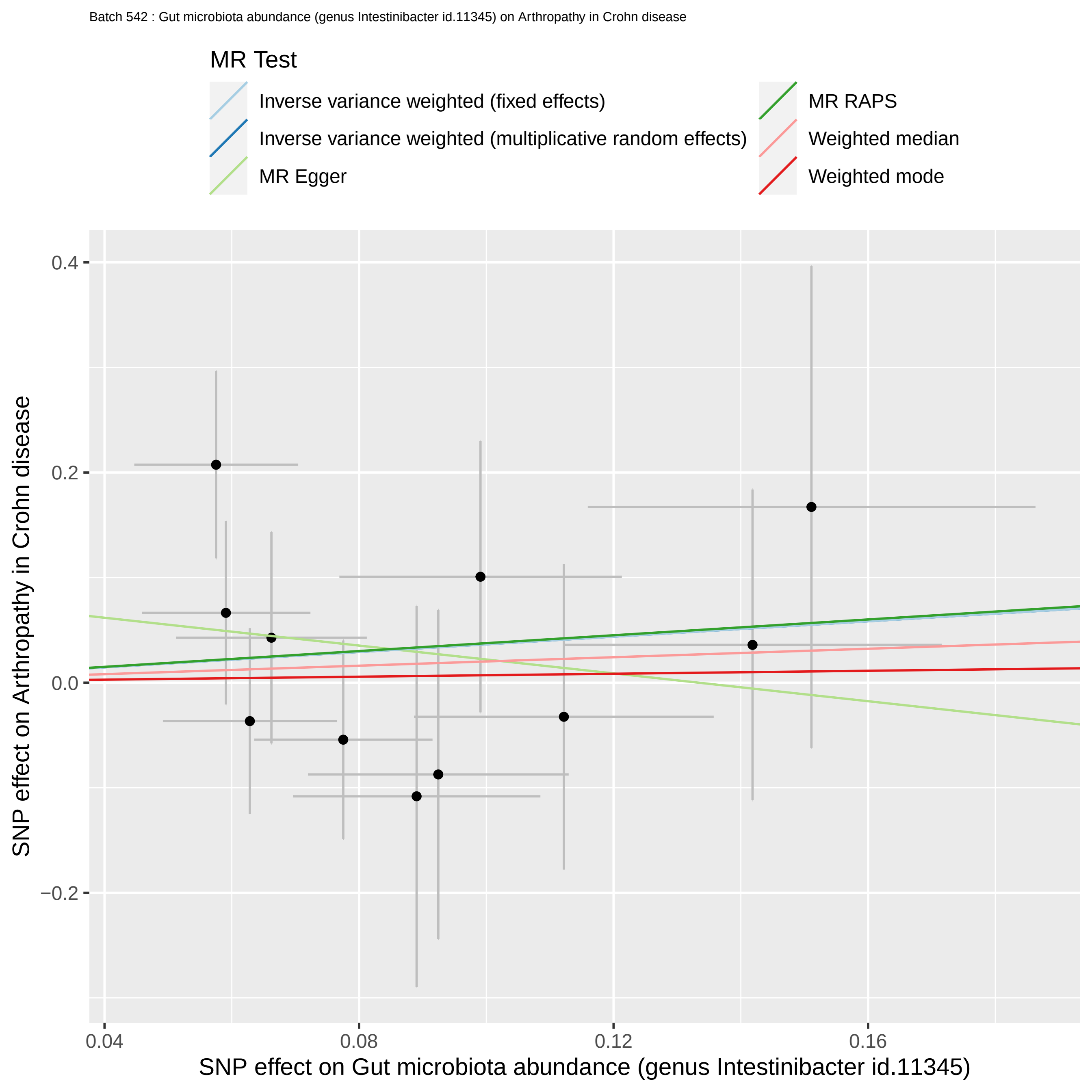

Batch 542 : Gut microbiota abundance (genus Intestinibacter id.11345) on Arthropathy in Crohn disease
MR Test
Inverse variance weighted (fixed effects)
Inverse variance weighted (multiplicative random effects)
MR Egger
MR RAPS
Weighted median
Weighted mode
0.4
0.2
0.0
−0.2
0.04
0.08
0.12
0.16
SNP effect on Arthropathy in Crohn disease
SNP effect on Gut microbiota abundance (genus Intestinibacter id.11345)

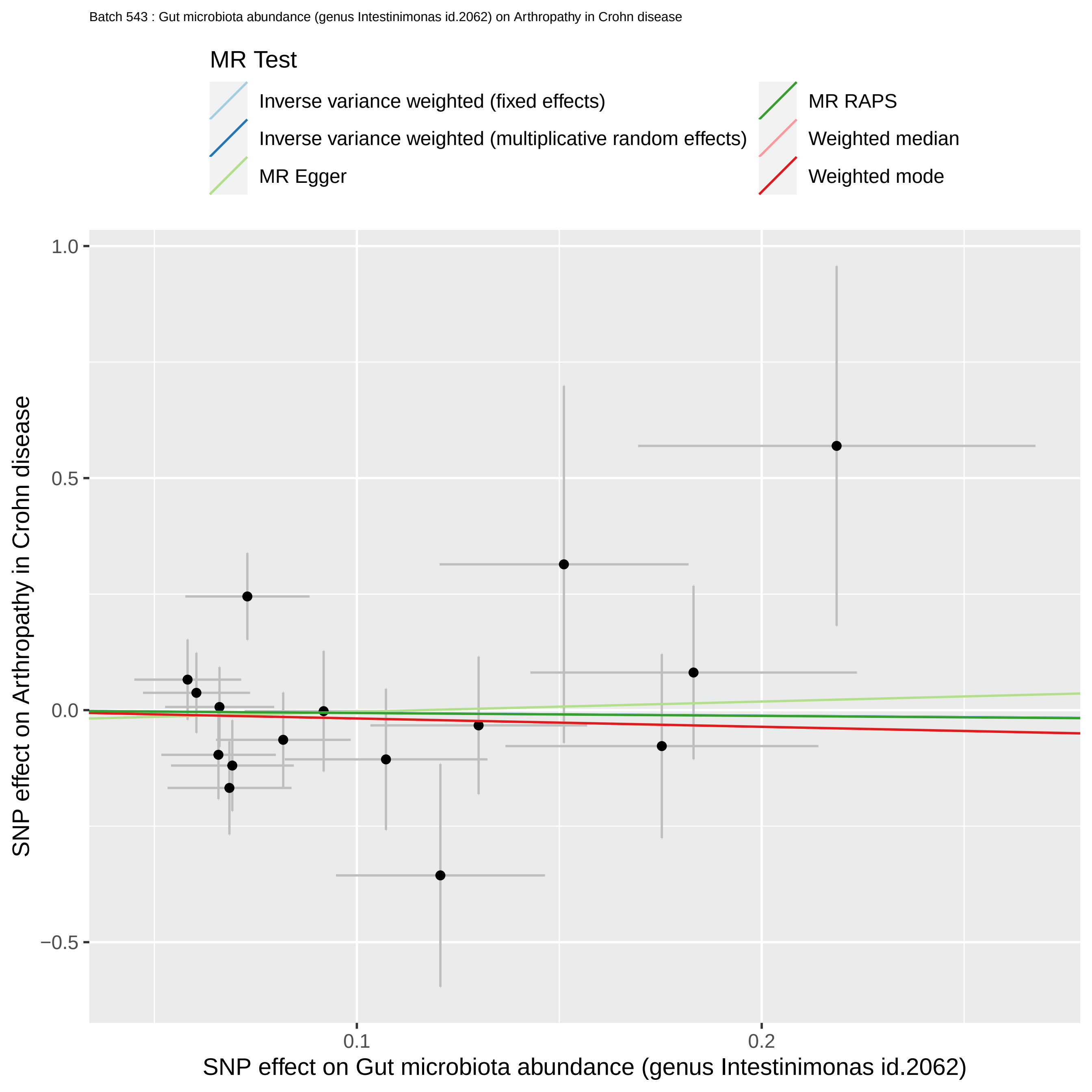

Batch 543 : Gut microbiota abundance (genus Intestinimonas id.2062) on Arthropathy in Crohn disease
MR Test
Inverse variance weighted (fixed effects)
Inverse variance weighted (multiplicative random effects)
MR Egger
MR RAPS
Weighted median
Weighted mode
1.0
0.5
0.0
−0.5
0.1
0.2
SNP effect on Arthropathy in Crohn disease
SNP effect on Gut microbiota abundance (genus Intestinimonas id.2062)

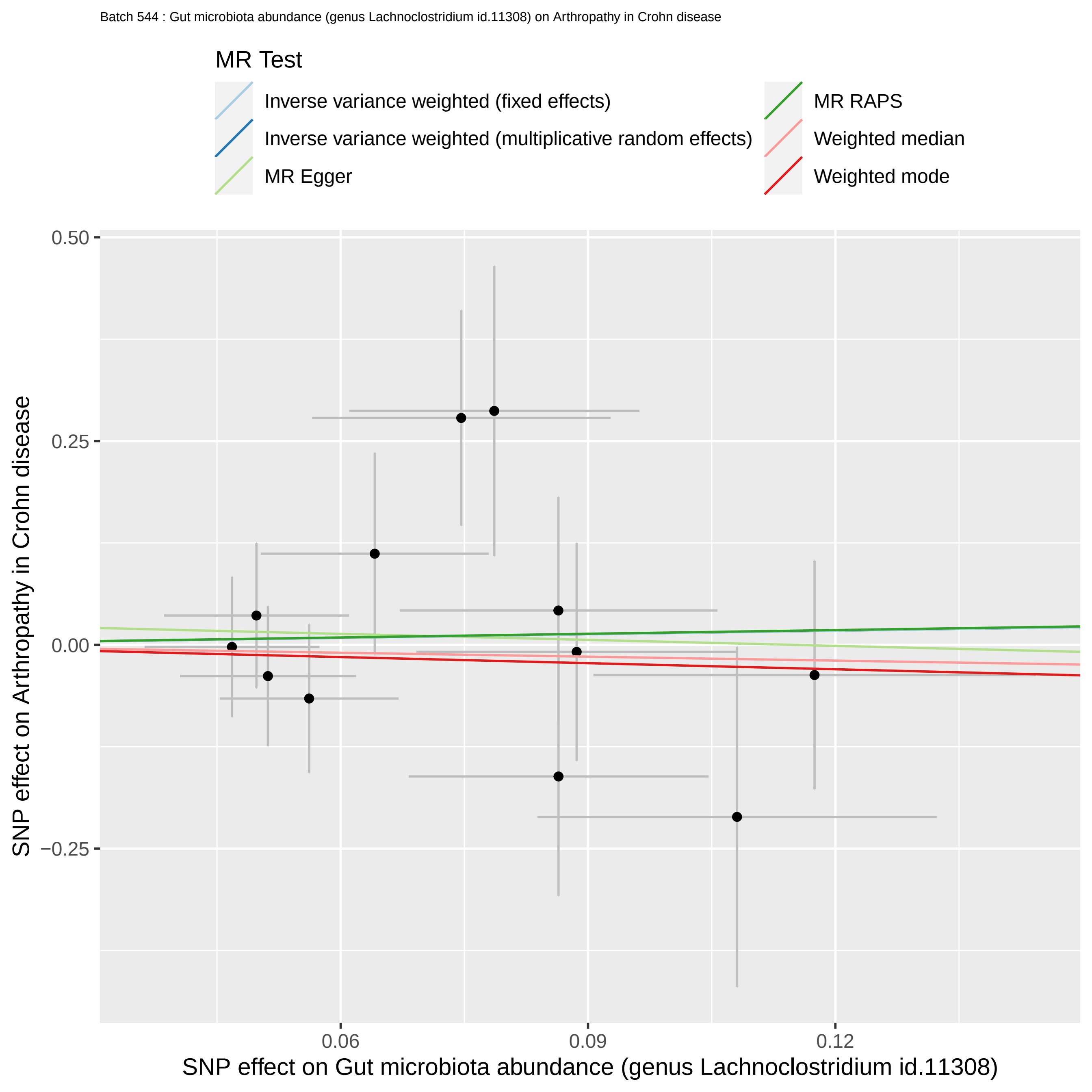
Batch 544 : Gut microbiota abundance (genus Lachnoclostridium id.11308) on Arthropathy in Crohn disease
MR Test
Inverse variance weighted (fixed effects)
Inverse variance weighted (multiplicative random effects)
MR Egger
MR RAPS
Weighted median
Weighted mode
0.50
0.25
0.00
−0.25
0.06
0.09
0.12
SNP effect on Arthropathy in Crohn disease
SNP effect on Gut microbiota abundance (genus Lachnoclostridium id.11308)

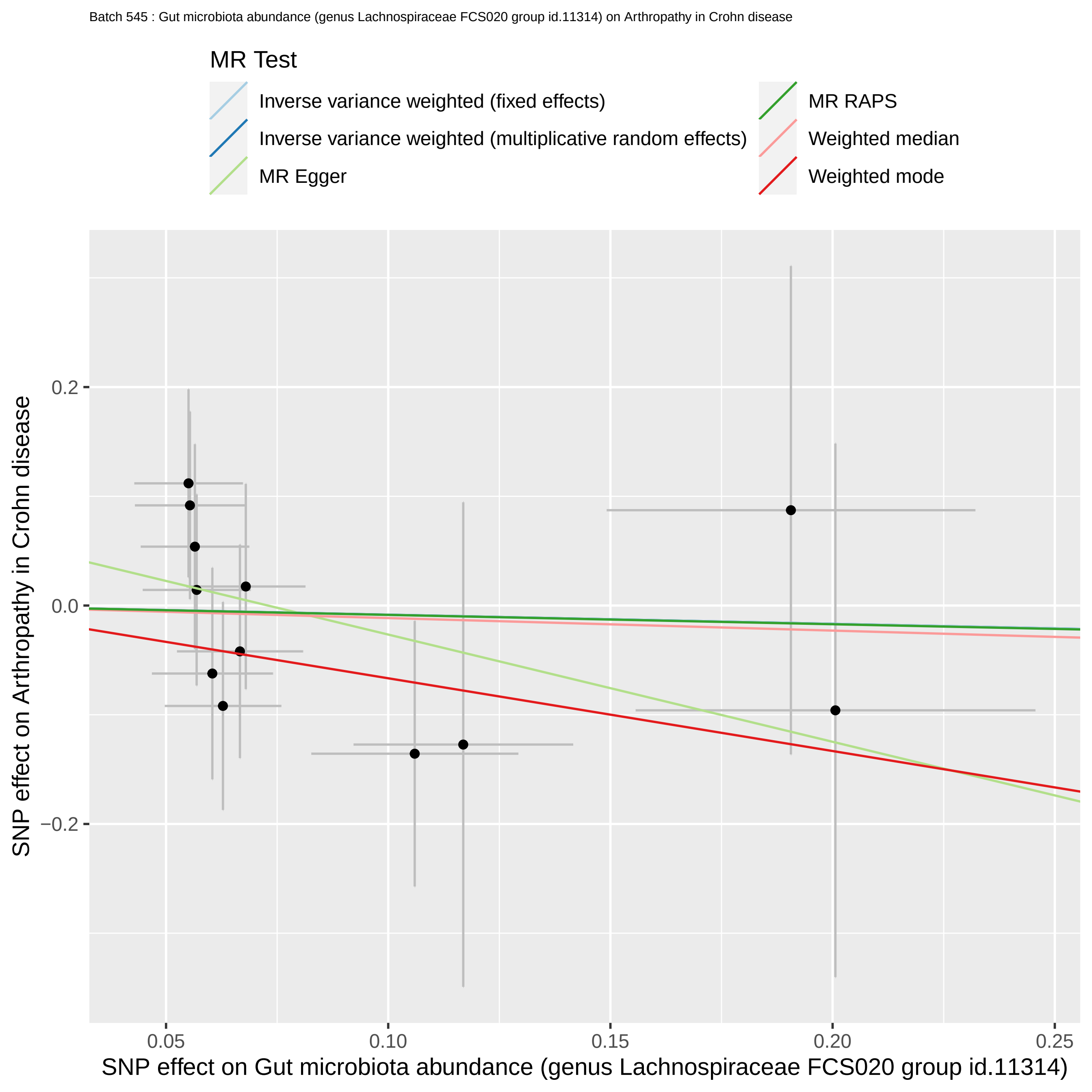

Batch 545 : Gut microbiota abundance (genus Lachnospiraceae FCS020 group id.11314) on Arthropathy in Crohn disease
MR Test
Inverse variance weighted (fixed effects)
Inverse variance weighted (multiplicative random effects)
MR Egger
MR RAPS
Weighted median
Weighted mode
0.2
0.0
−0.2
0.05
0.10
0.15
0.20
0.25
SNP effect on Arthropathy in Crohn disease
SNP effect on Gut microbiota abundance (genus Lachnospiraceae FCS020 group id.11314)

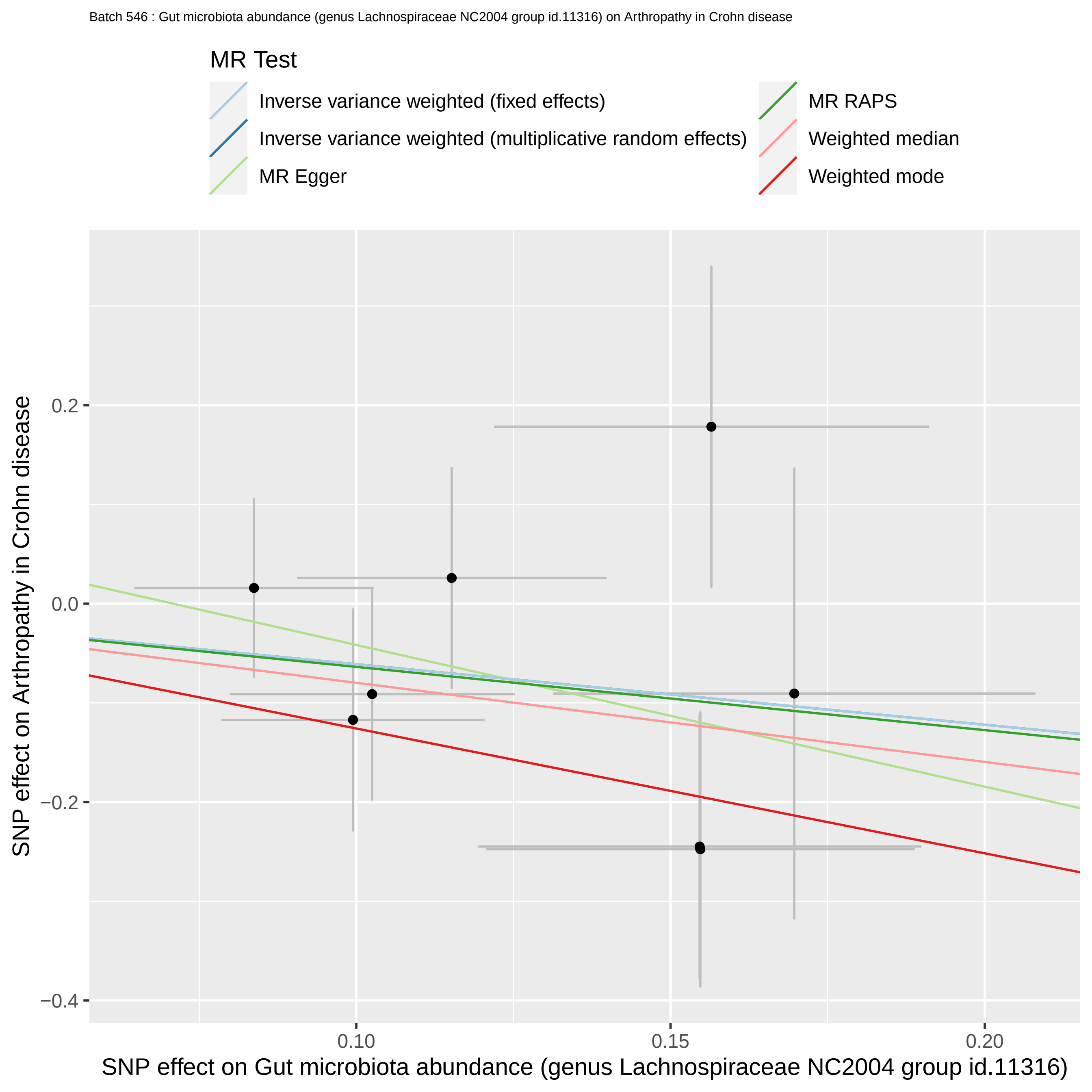
Batch 546 : Gut microbiota abundance (genus Lachnospiraceae NC2004 group id.11316) on Arthropathy in Crohn disease
MR Test
Inverse variance weighted (fixed effects)
Inverse variance weighted (multiplicative random effects)
MR Egger
MR RAPS
Weighted median
Weighted mode
0.2
0.0
−0.2
−0.4
0.10
0.15
0.20
SNP effect on Arthropathy in Crohn disease
SNP effect on Gut microbiota abundance (genus Lachnospiraceae NC2004 group id.11316)

Batch 547 : Gut microbiota abundance (genus Lachnospiraceae ND3007 group id.11317) on Arthropathy in Crohn disease

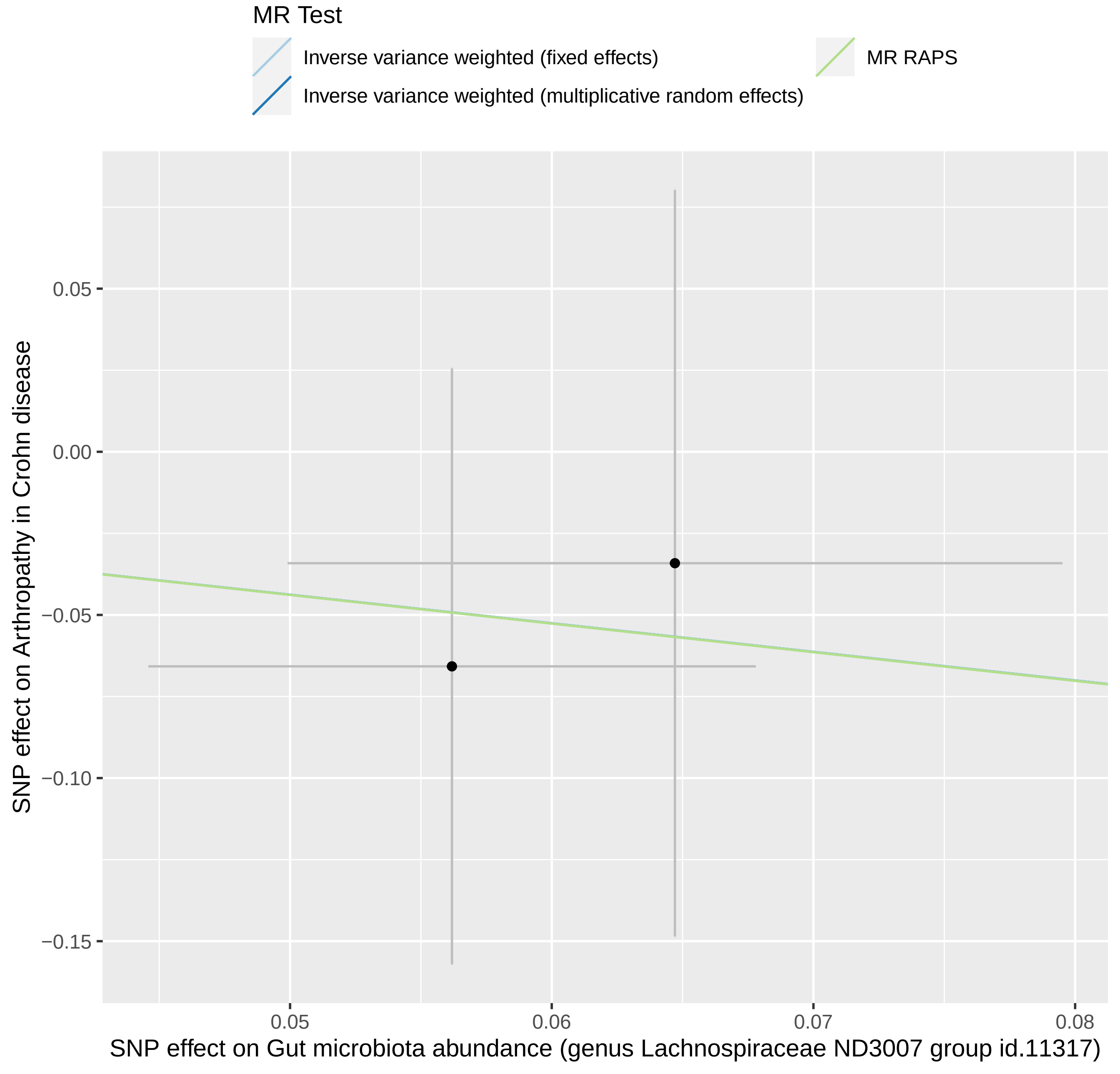

Batch 548 : Gut microbiota abundance (genus Lachnospiraceae NK4A136 group id.11319) on Arthropathy in Crohn disease

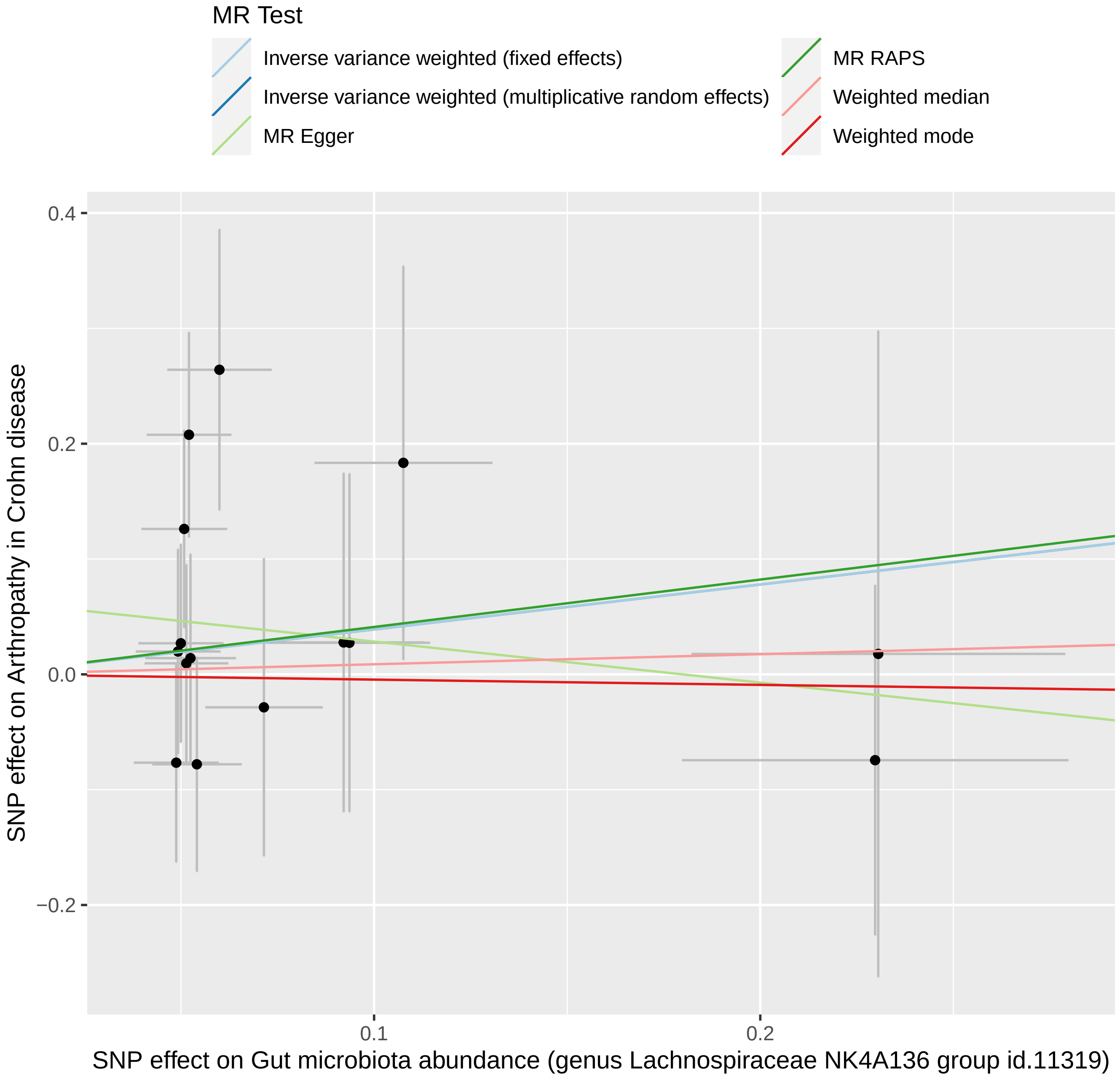

Batch 549 : Gut microbiota abundance (genus Lachnospiraceae UCG001 id.11321) on Arthropathy in Crohn disease

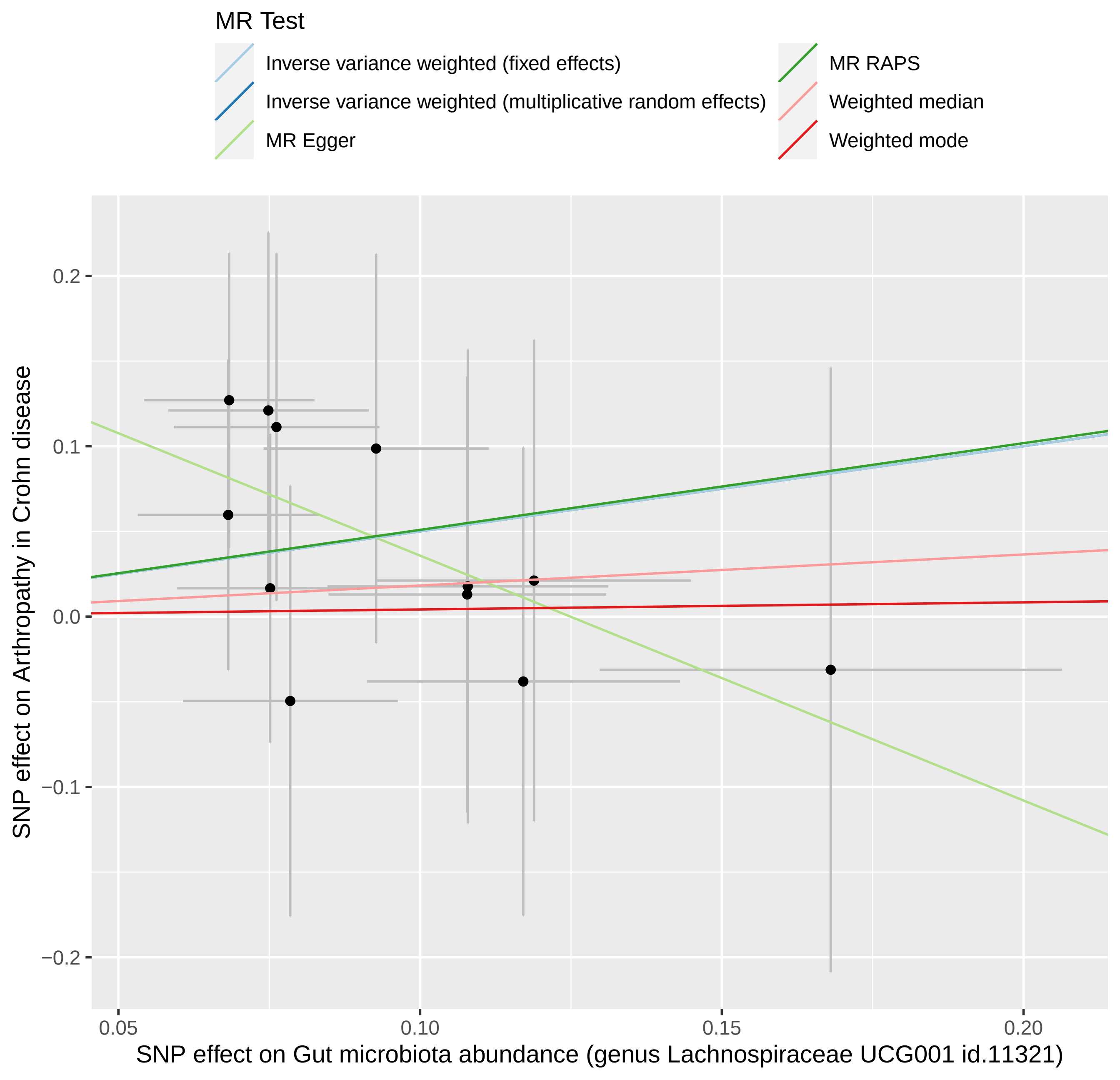

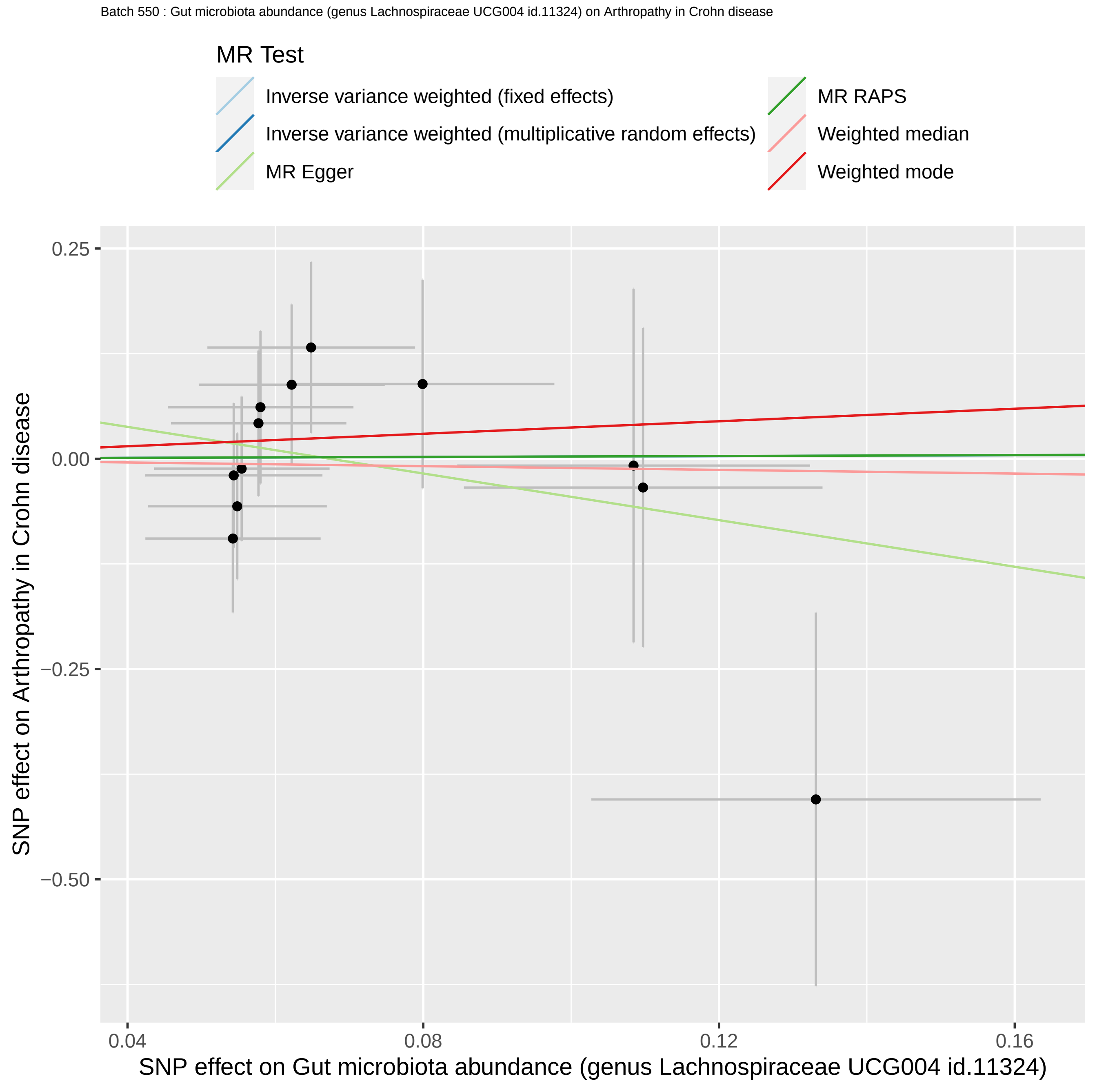
Batch 550 : Gut microbiota abundance (genus Lachnospiraceae UCG004 id.11324) on Arthropathy in Crohn disease
MR Test
Inverse variance weighted (fixed effects)
Inverse variance weighted (multiplicative random effects)
MR Egger
MR RAPS
Weighted median
Weighted mode
0.25
0.00
−0.25
−0.50
0.04
0.08
0.12
0.16
SNP effect on Arthropathy in Crohn disease
SNP effect on Gut microbiota abundance (genus Lachnospiraceae UCG004 id.11324)

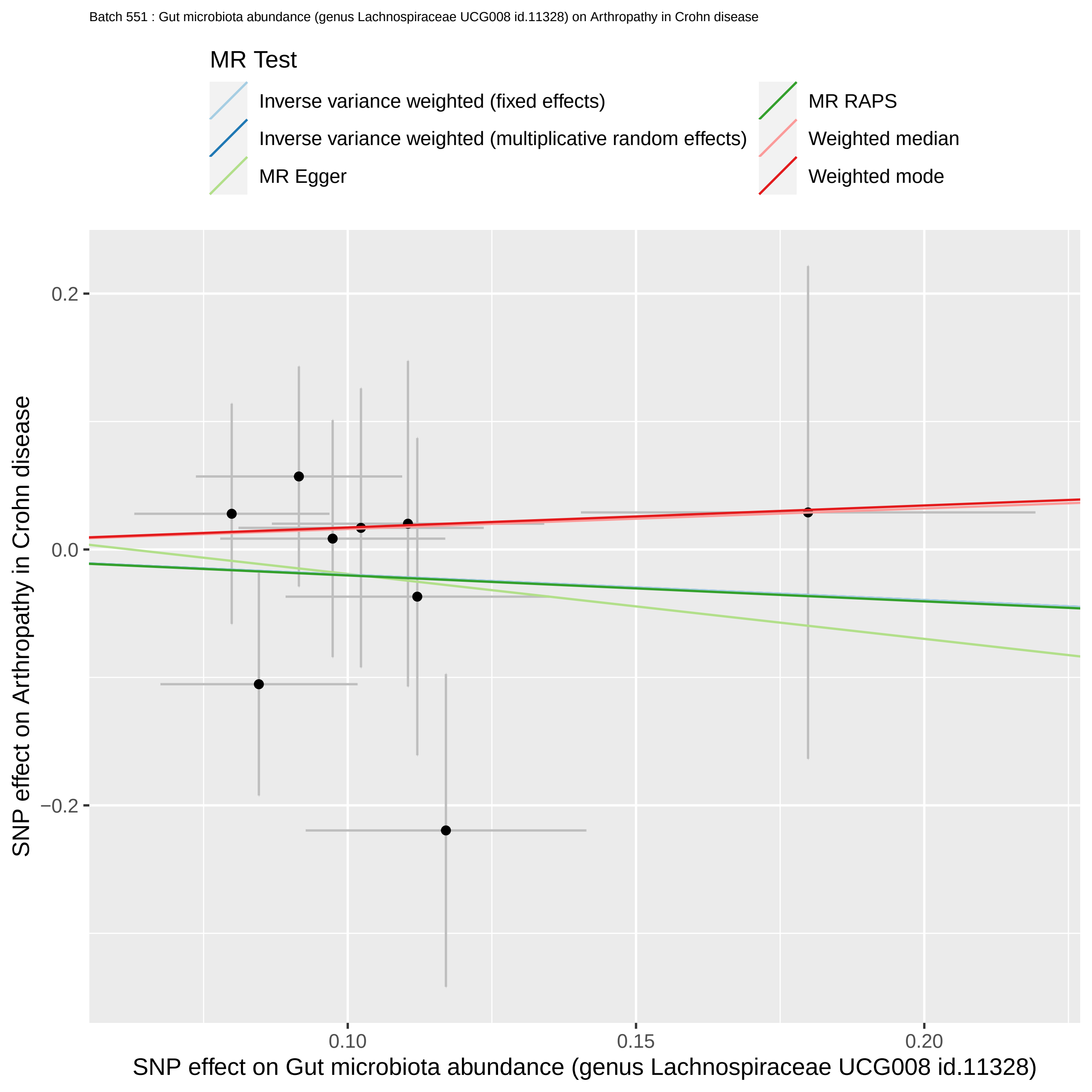

Batch 551 : Gut microbiota abundance (genus Lachnospiraceae UCG008 id.11328) on Arthropathy in Crohn disease
MR Test
Inverse variance weighted (fixed effects)
Inverse variance weighted (multiplicative random effects)
MR Egger
MR RAPS
Weighted median
Weighted mode
0.2
0.0
−0.2
0.10
0.15
0.20
SNP effect on Arthropathy in Crohn disease
SNP effect on Gut microbiota abundance (genus Lachnospiraceae UCG008 id.11328)

Batch 552 : Gut microbiota abundance (genus Lachnospiraceae UCG010 id.11330) on Arthropathy in Crohn disease

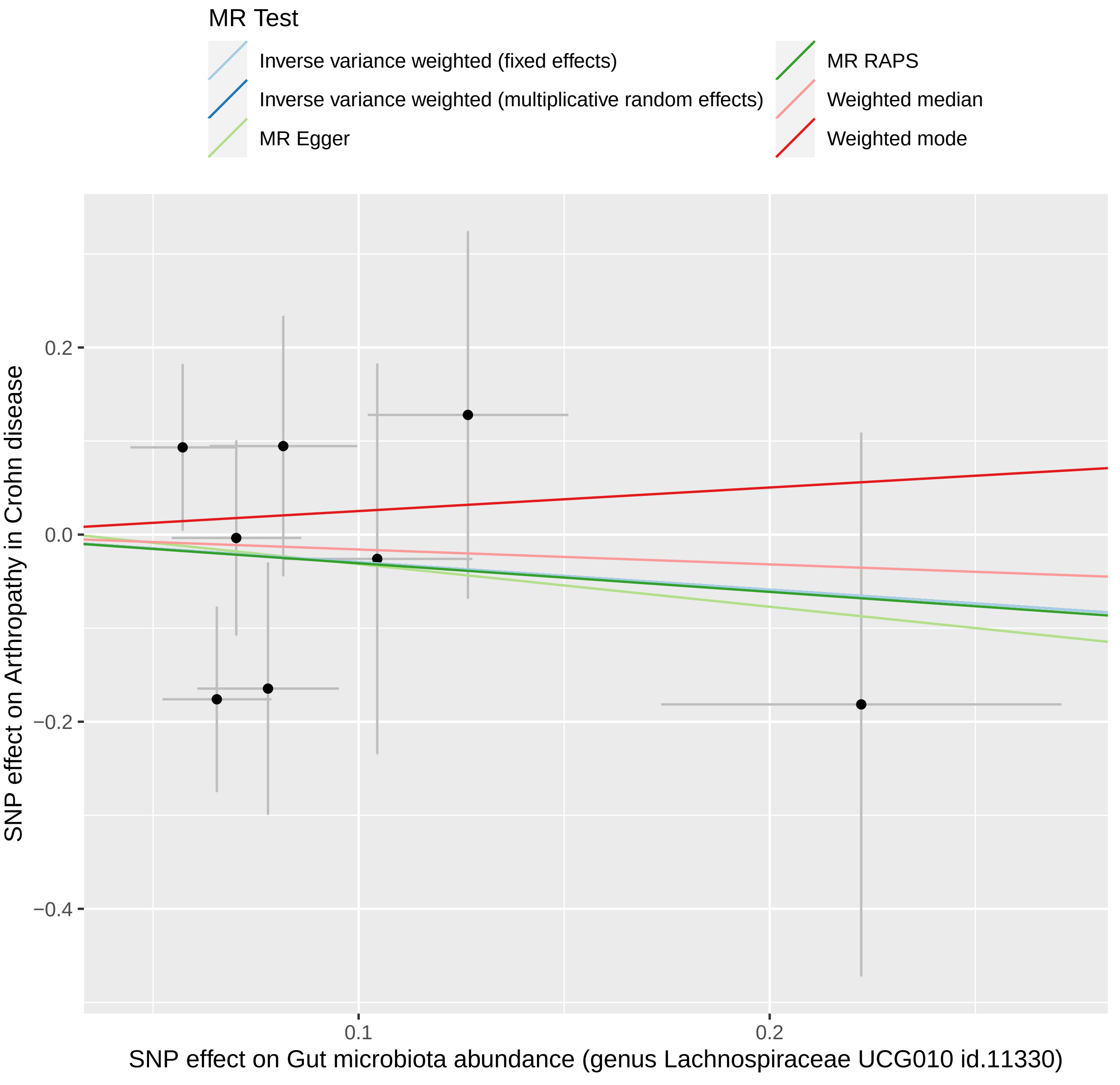

Insufficient number of SNPs

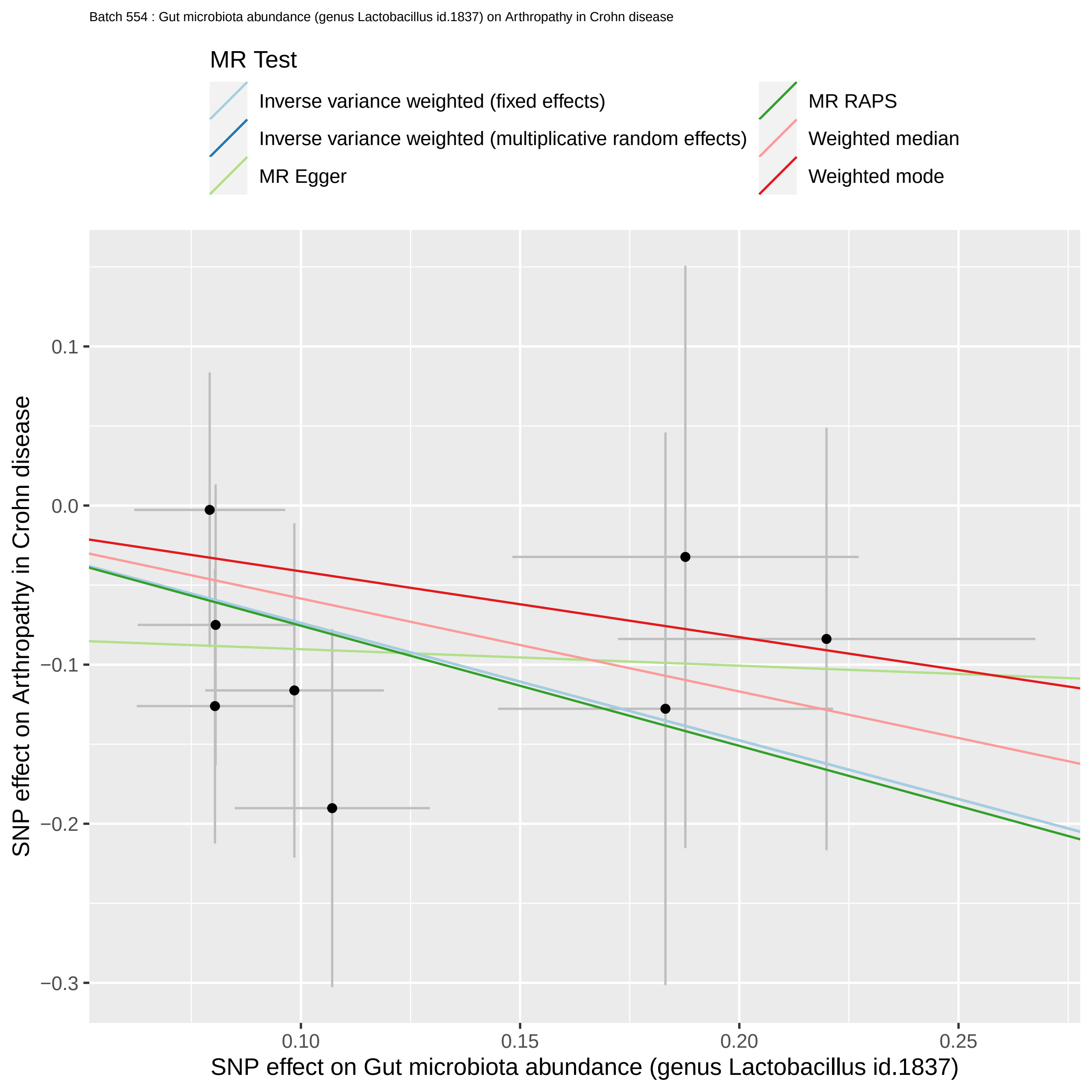
Batch 554 : Gut microbiota abundance (genus Lactobacillus id.1837) on Arthropathy in Crohn disease
MR Test
Inverse variance weighted (fixed effects)
Inverse variance weighted (multiplicative random effects)
MR Egger
MR RAPS
Weighted median
Weighted mode
0.1
0.0
−0.1
−0.2
−0.3
SNP effect on Arthropathy in Crohn disease
0.10
0.15
0.20
0.25
SNP effect on Gut microbiota abundance (genus Lactobacillus id.1837)

Batch 555 : Gut microbiota abundance (genus Lactococcus id.1851) on Arthropathy in Crohn disease

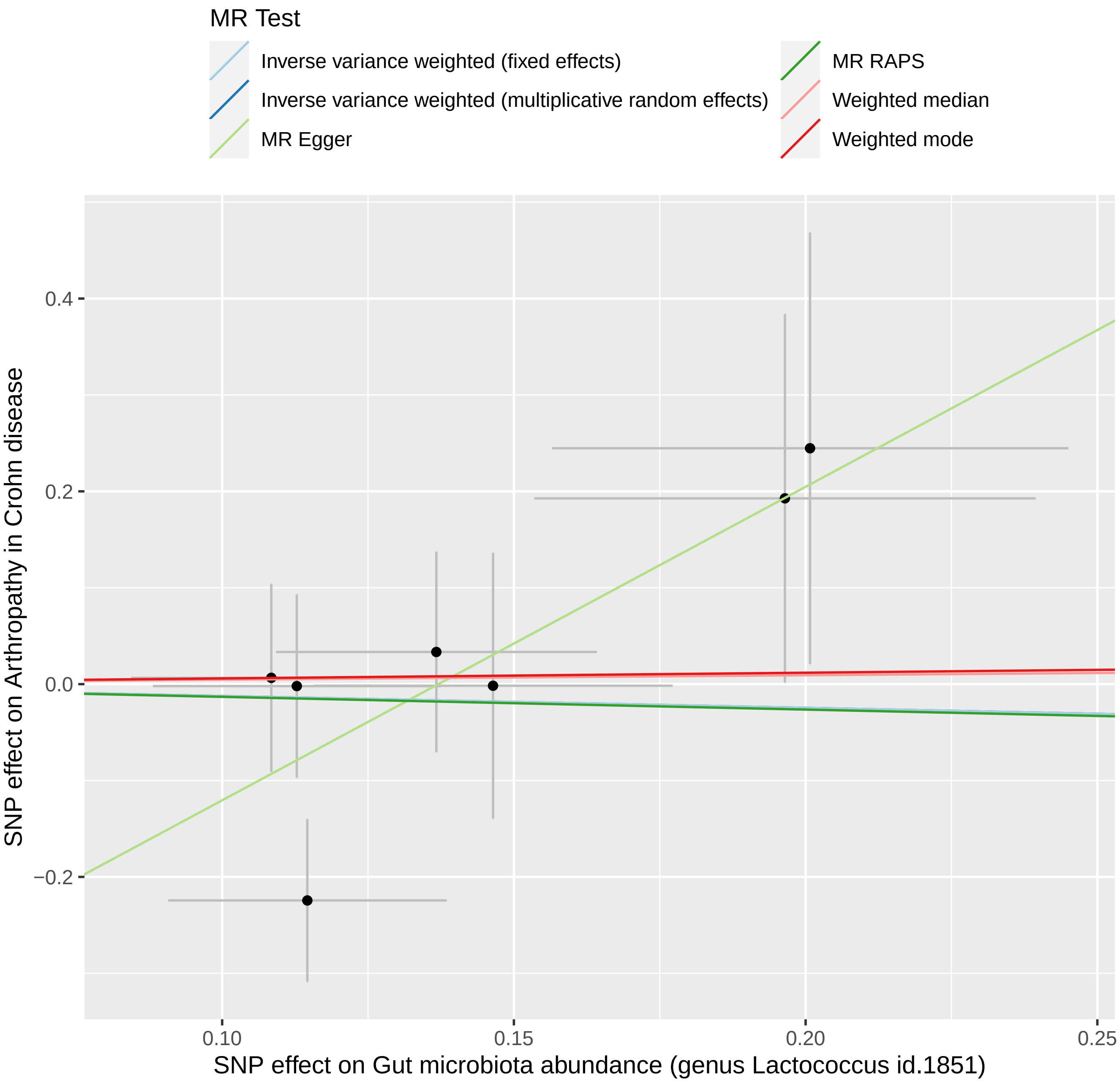

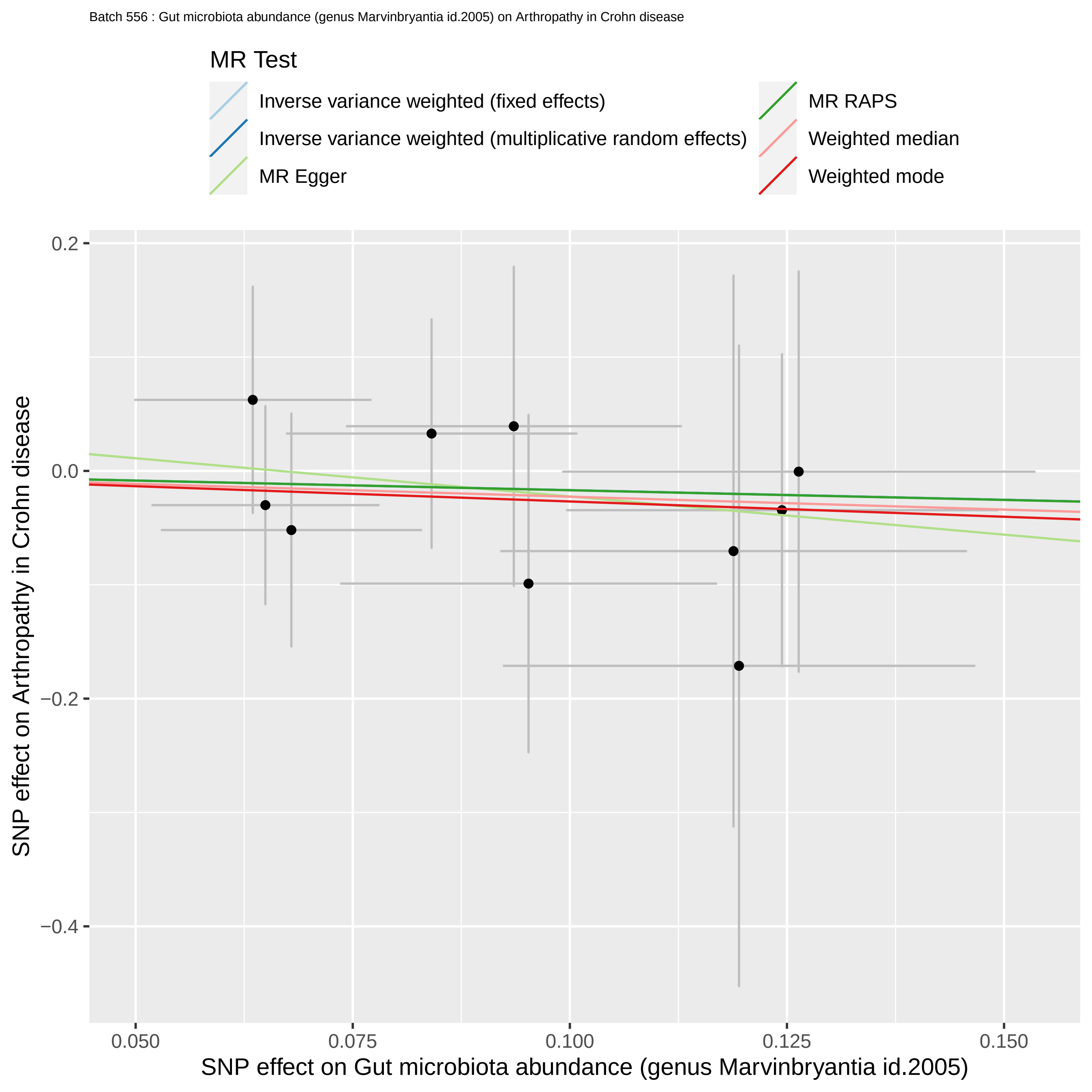

Batch 556 : Gut microbiota abundance (genus Marvinbryantia id.2005) on Arthropathy in Crohn disease
MR Test
Inverse variance weighted (fixed effects)
Inverse variance weighted (multiplicative random effects)
MR Egger
MR RAPS
Weighted median
Weighted mode
0.2
0.0
−0.2
−0.4
SNP effect on Arthropathy in Crohn disease
0.050
0.075
0.100
0.125
0.150
SNP effect on Gut microbiota abundance (genus Marvinbryantia id.2005)

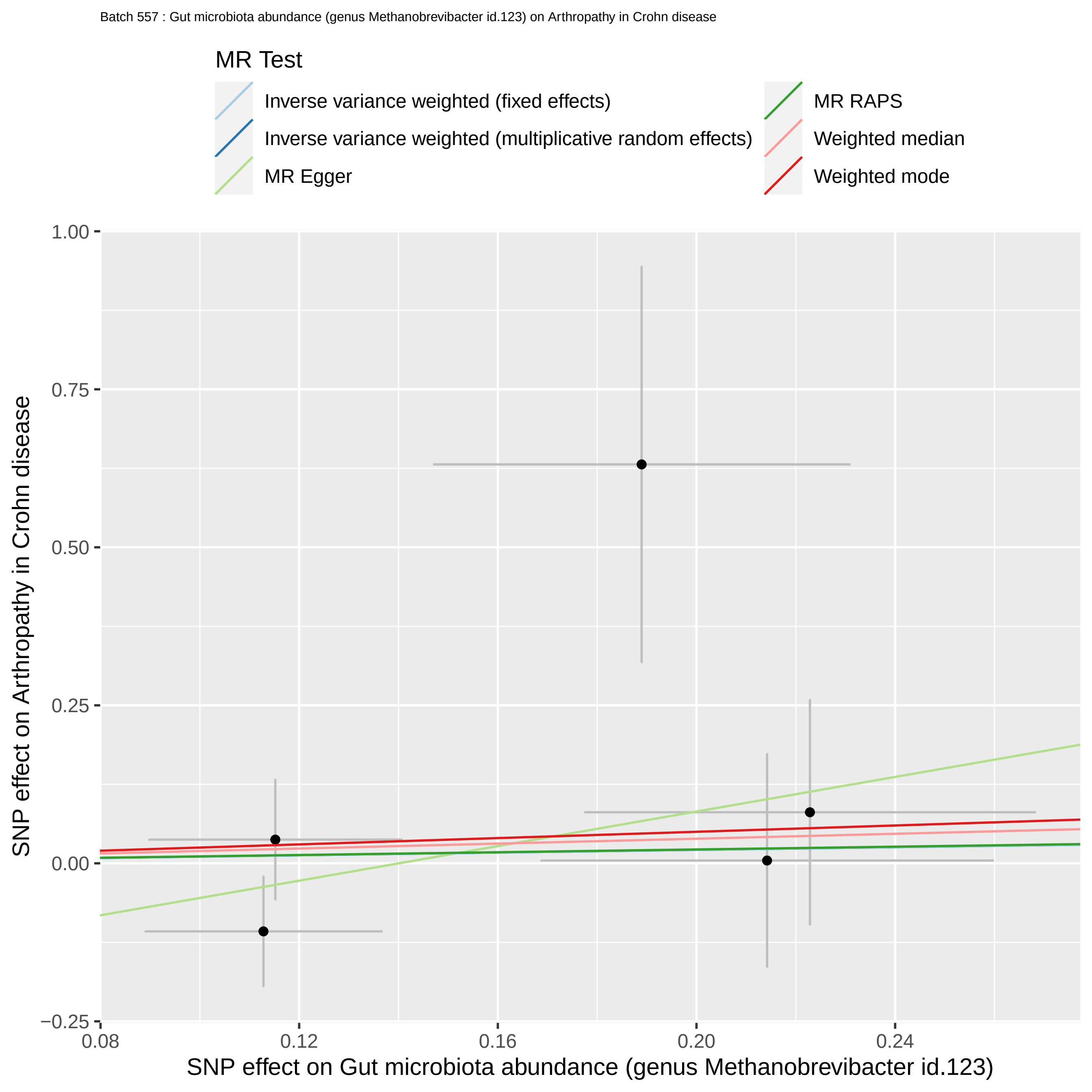
Batch 557 : Gut microbiota abundance (genus Methanobrevibacter id.123) on Arthropathy in Crohn disease
MR Test
Inverse variance weighted (fixed effects)
Inverse variance weighted (multiplicative random effects)
MR Egger
MR RAPS
Weighted median
Weighted mode
1.00
0.75
0.50
0.25
0.00
−0.25
0.08
0.12
0.16
0.20
0.24
SNP effect on Arthropathy in Crohn disease
SNP effect on Gut microbiota abundance (genus Methanobrevibacter id.123)

Batch 558 : Gut microbiota abundance (genus Odoribacter id.952) on Arthropathy in Crohn disease

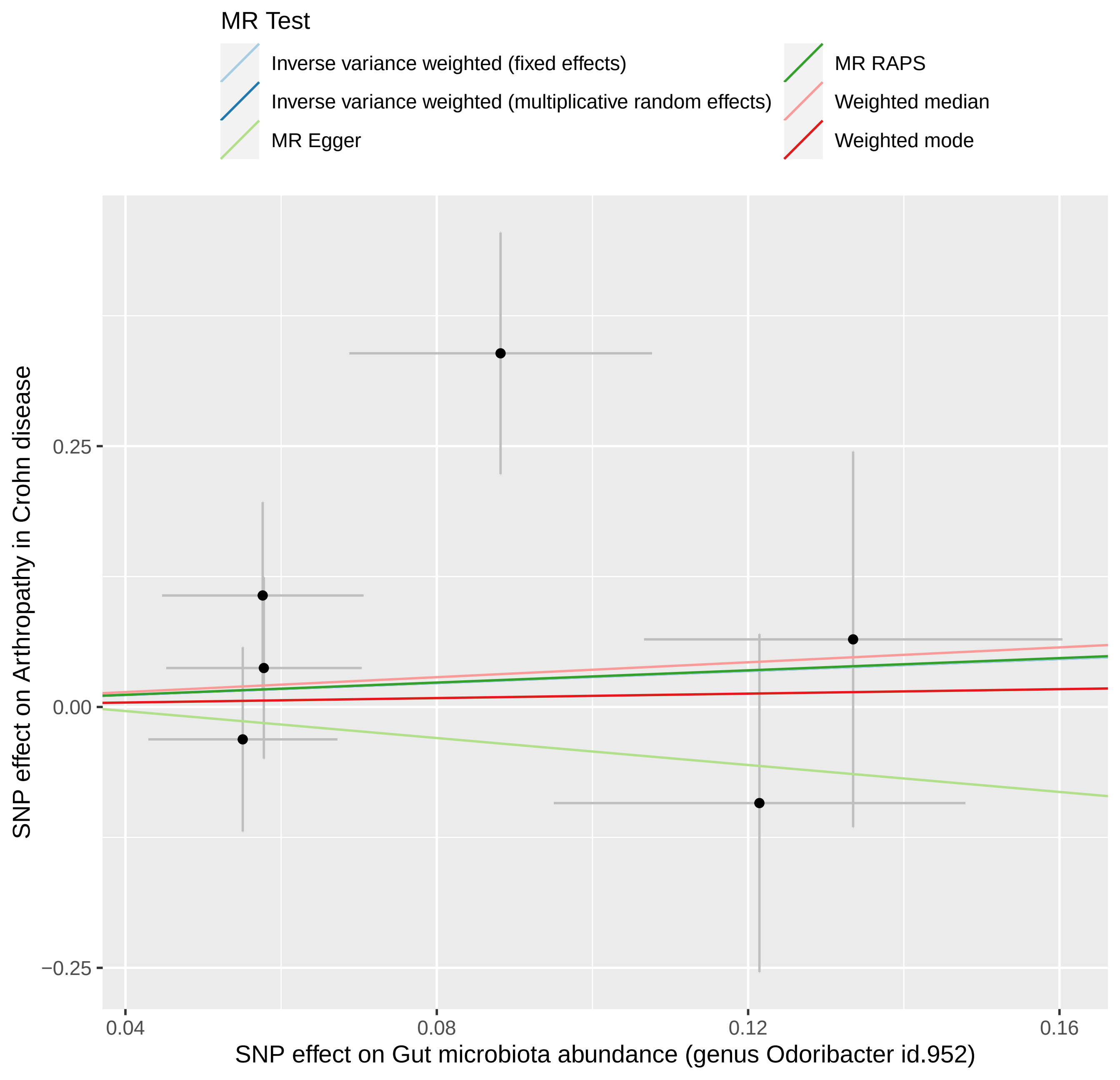

Batch 559 : Gut microbiota abundance (genus Olsenella id.822) on Arthropathy in Crohn disease

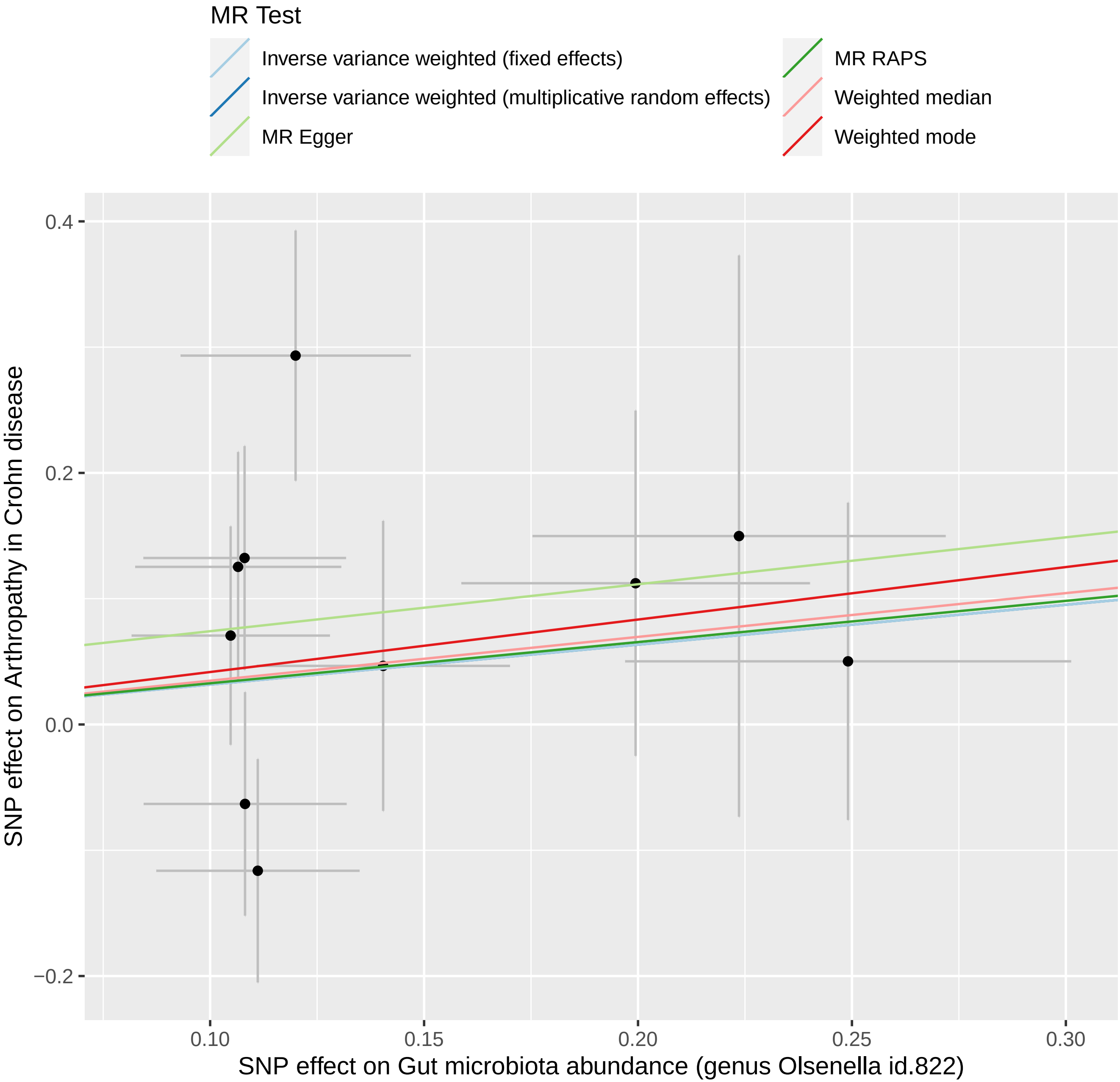

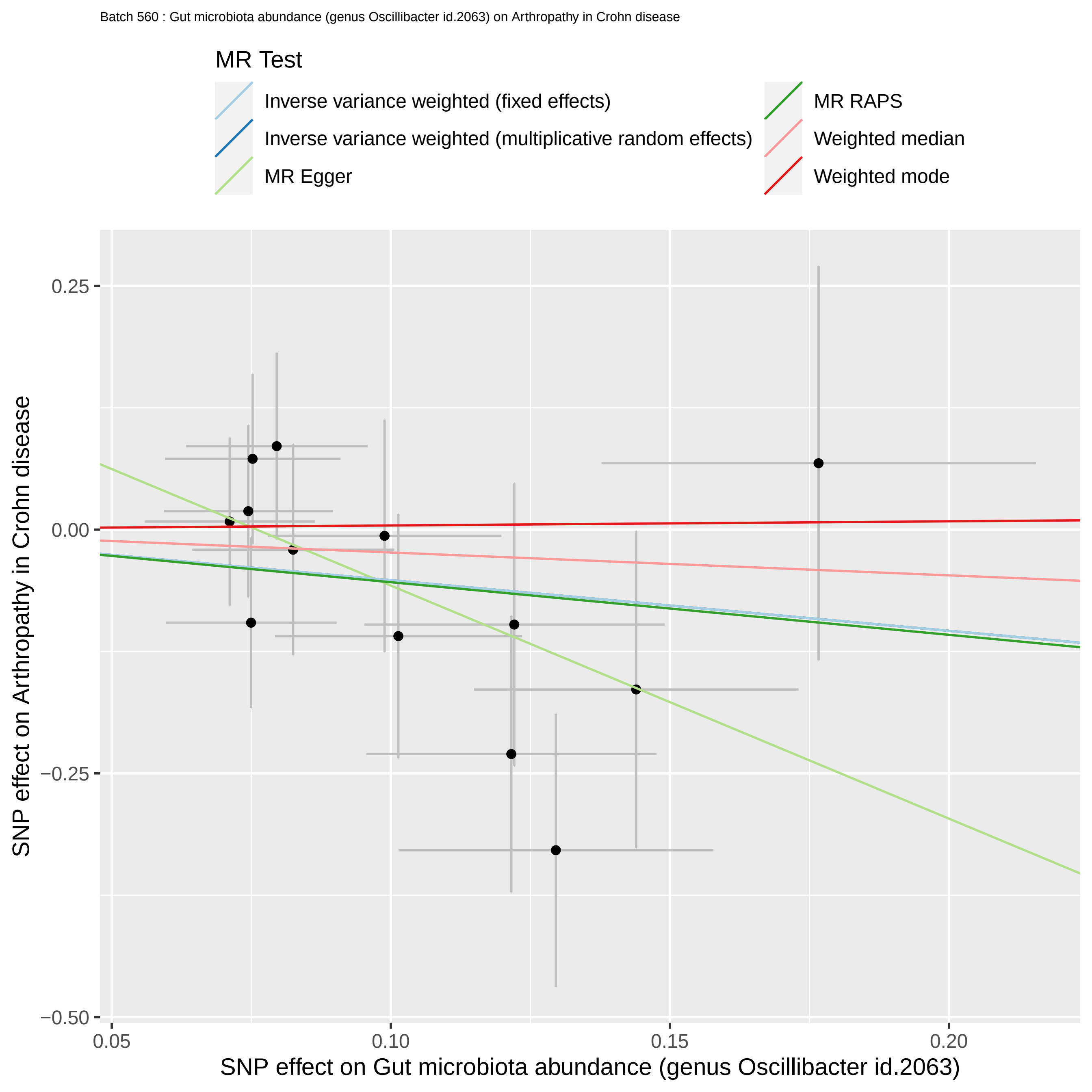
Batch 560 : Gut microbiota abundance (genus Oscillibacter id.2063) on Arthropathy in Crohn disease
MR Test
Inverse variance weighted (fixed effects)
Inverse variance weighted (multiplicative random effects)
MR Egger
MR RAPS
Weighted median
Weighted mode
0.25
0.00
−0.25
−0.50
0.05
0.10
0.15
0.20
SNP effect on Arthropathy in Crohn disease
SNP effect on Gut microbiota abundance (genus Oscillibacter id.2063)

Batch 561 : Gut microbiota abundance (genus Oscillospira id.2064) on Arthropathy in Crohn disease

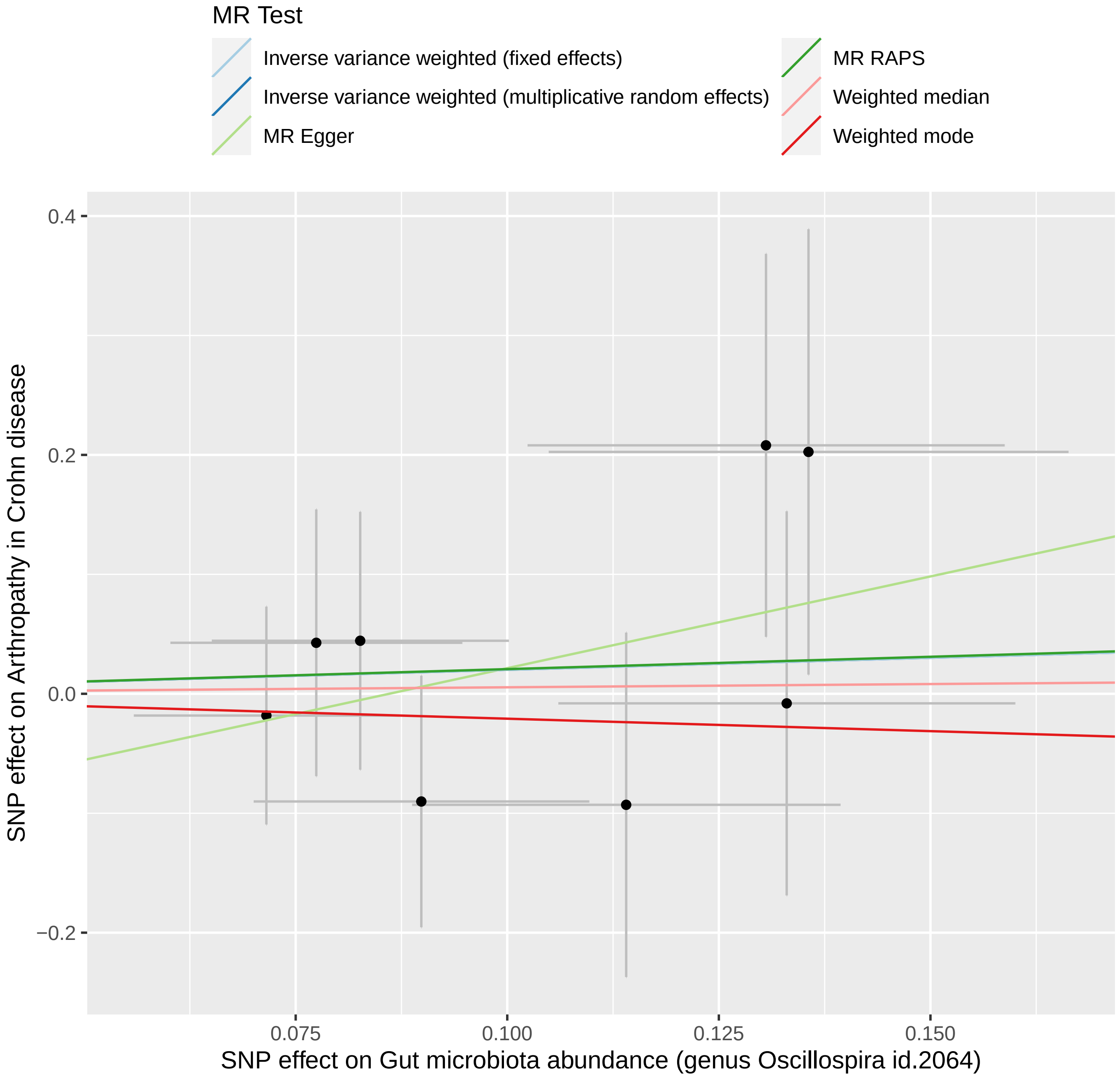

Batch 562 : Gut microbiota abundance (genus Oxalobacter id.2978) on Arthropathy in Crohn disease

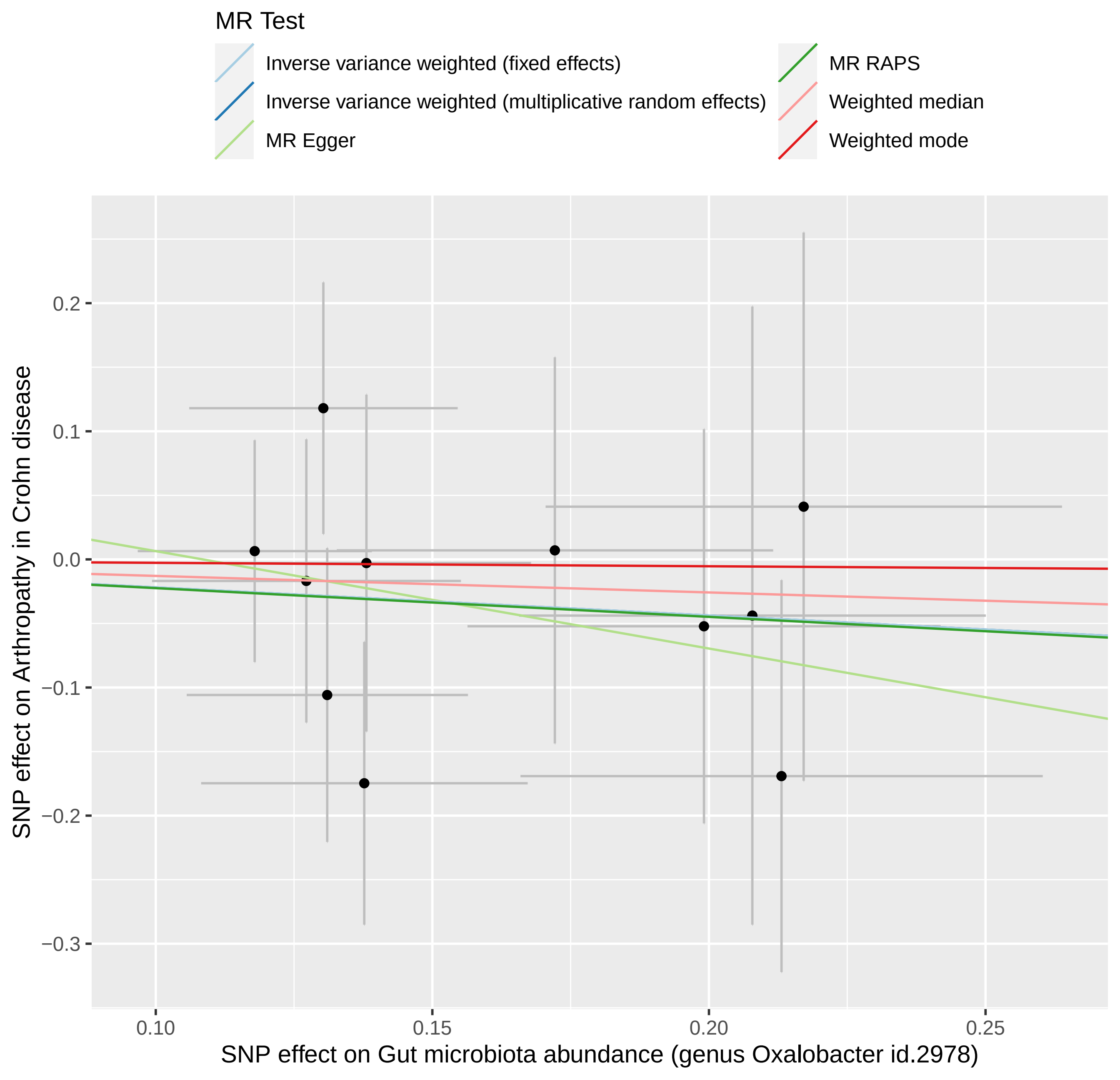

Batch 563 : Gut microbiota abundance (genus Parabacteroides id.954) on Arthropathy in Crohn disease

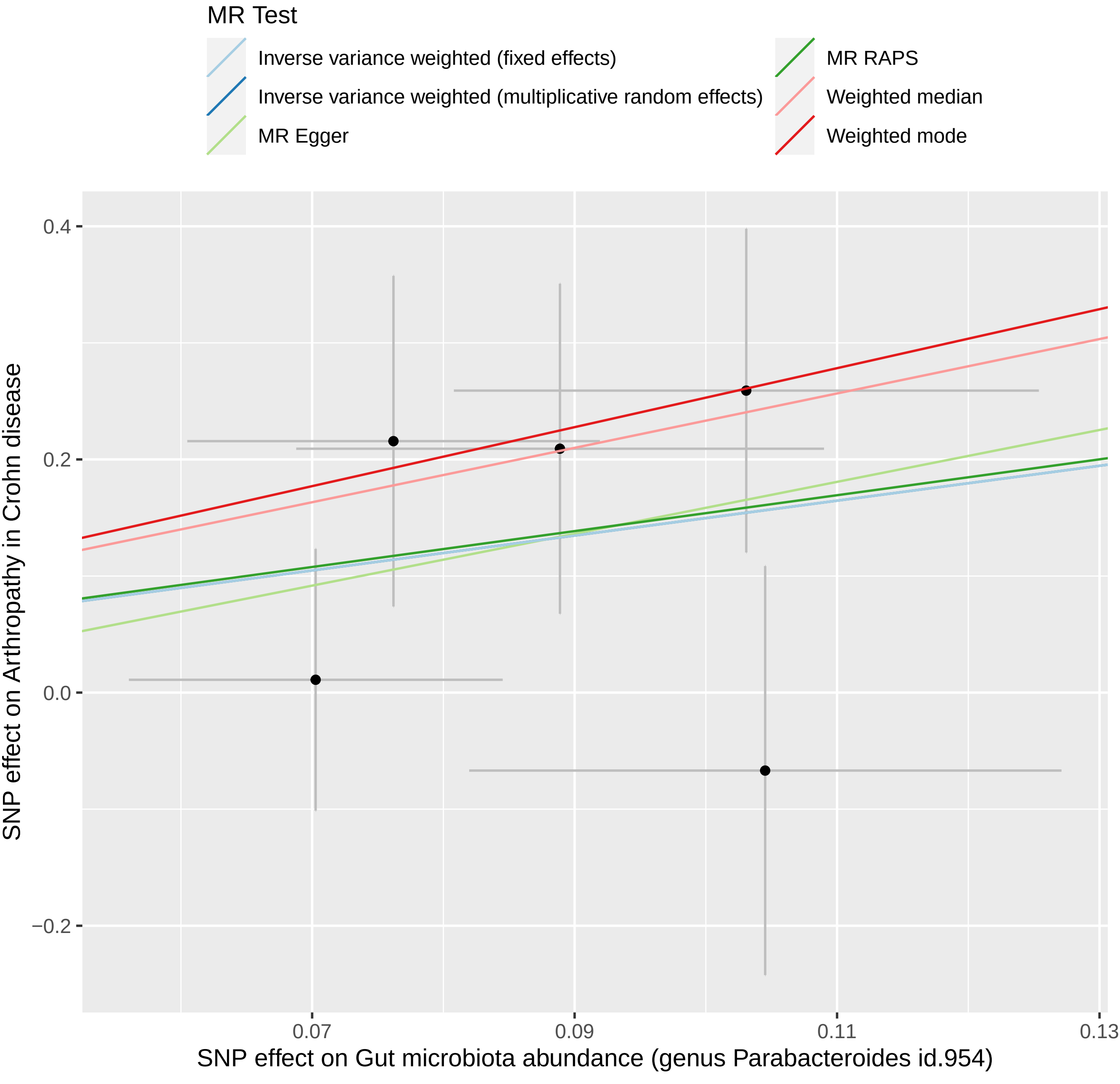

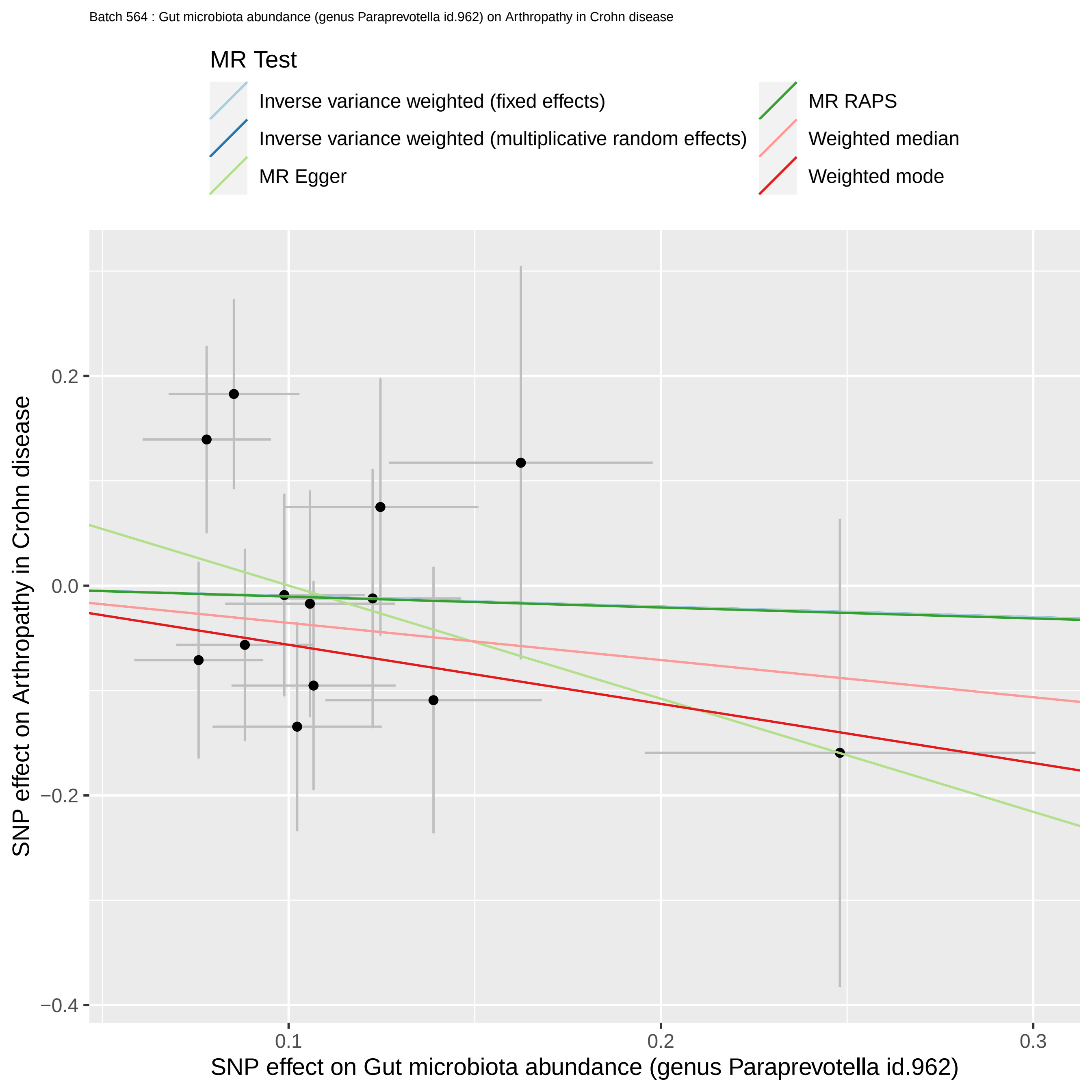
Batch 564 : Gut microbiota abundance (genus Paraprevotella id.962) on Arthropathy in Crohn disease
MR Test
Inverse variance weighted (fixed effects)
Inverse variance weighted (multiplicative random effects)
MR Egger
MR RAPS
Weighted median
Weighted mode
0.2
0.0
−0.2
−0.4
0.1
0.2
0.3
SNP effect on Arthropathy in Crohn disease
SNP effect on Gut microbiota abundance (genus Paraprevotella id.962)

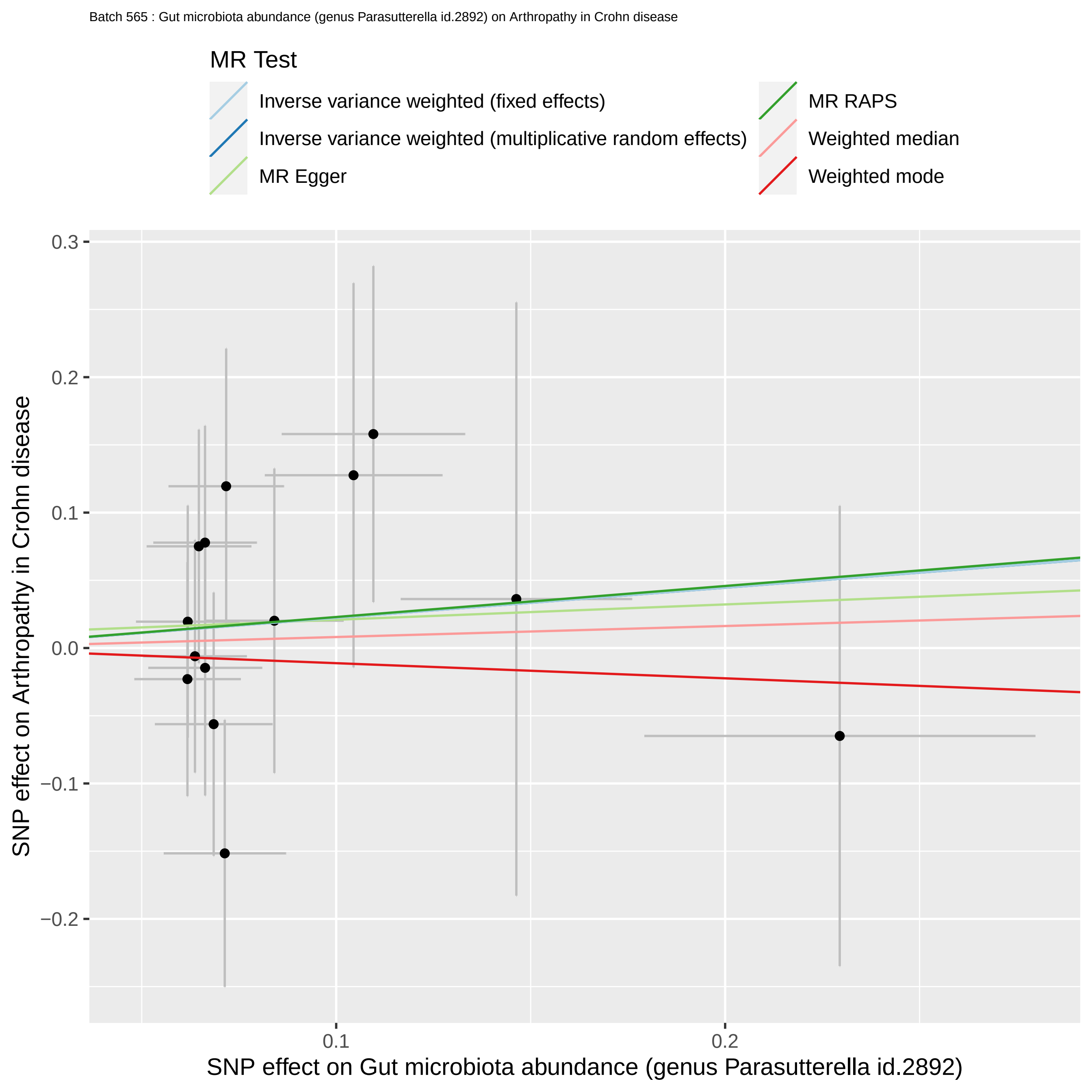

Batch 565 : Gut microbiota abundance (genus Parasutterella id.2892) on Arthropathy in Crohn disease
MR Test
Inverse variance weighted (fixed effects)
Inverse variance weighted (multiplicative random effects)
MR Egger
MR RAPS
Weighted median
Weighted mode
0.3
0.2
0.1
0.0
−0.1
−0.2
0.1
0.2
SNP effect on Arthropathy in Crohn disease
SNP effect on Gut microbiota abundance (genus Parasutterella id.2892)

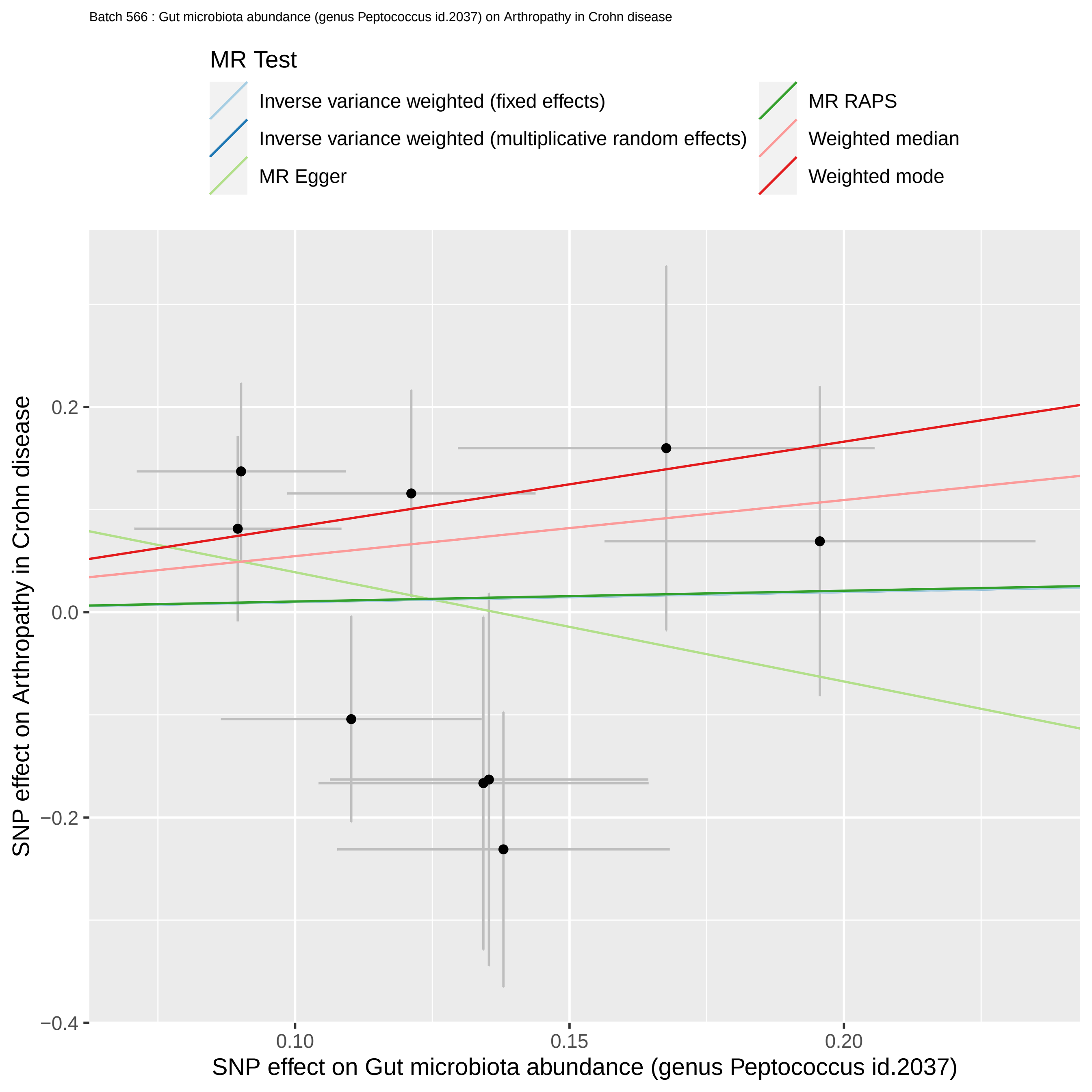

Batch 566 : Gut microbiota abundance (genus Peptococcus id.2037) on Arthropathy in Crohn disease
MR Test
Inverse variance weighted (fixed effects)
Inverse variance weighted (multiplicative random effects)
MR Egger
MR RAPS
Weighted median
Weighted mode
0.2
0.0
−0.2
−0.4
0.10
0.15
0.20
SNP effect on Arthropathy in Crohn disease
SNP effect on Gut microbiota abundance (genus Peptococcus id.2037)

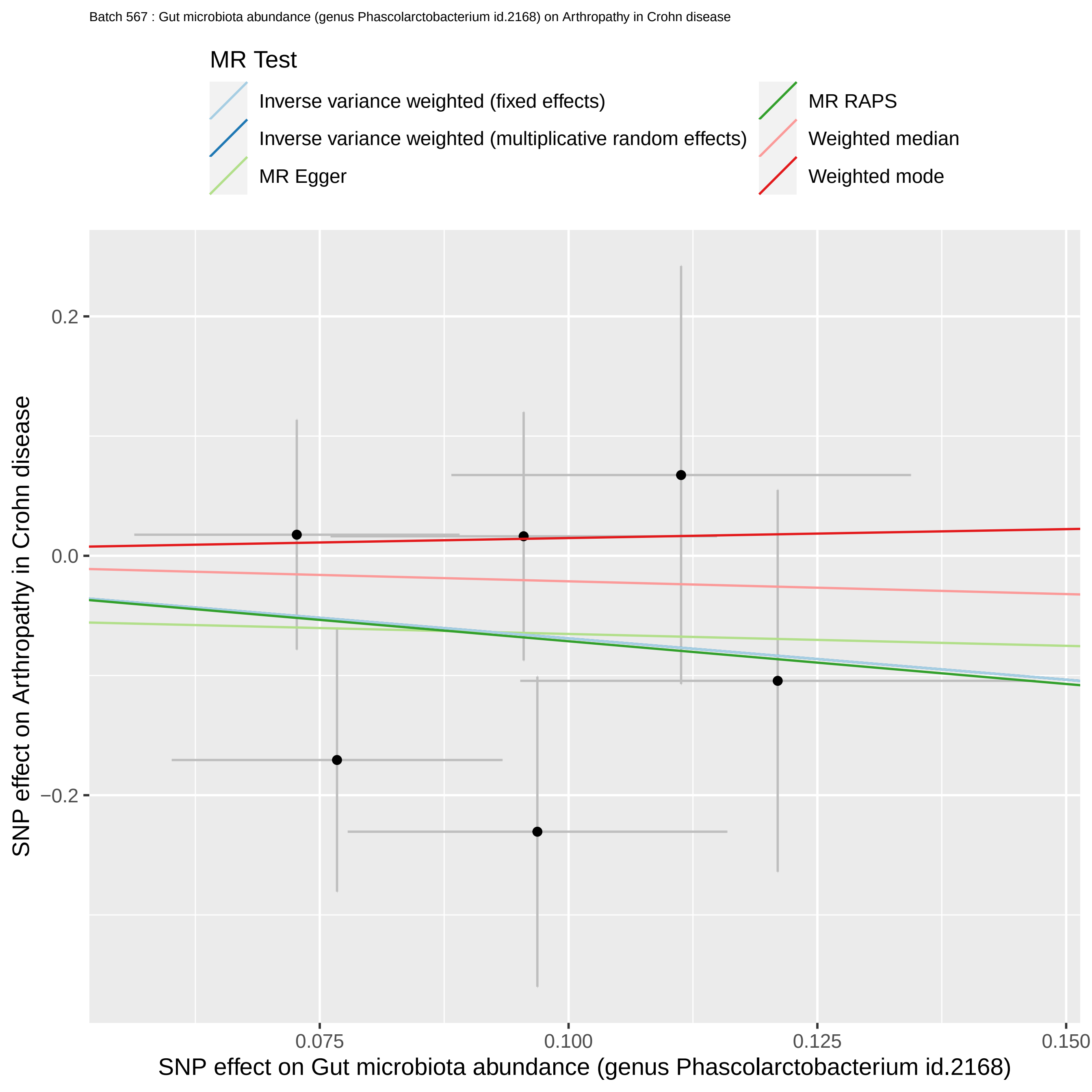
Batch 567 : Gut microbiota abundance (genus Phascolarctobacterium id.2168) on Arthropathy in Crohn disease
MR Test
Inverse variance weighted (fixed effects)
Inverse variance weighted (multiplicative random effects)
MR Egger
MR RAPS
Weighted median
Weighted mode
0.2
0.0
−0.2
0.075
0.100
0.125
0.150
SNP effect on Arthropathy in Crohn disease
SNP effect on Gut microbiota abundance (genus Phascolarctobacterium id.2168)

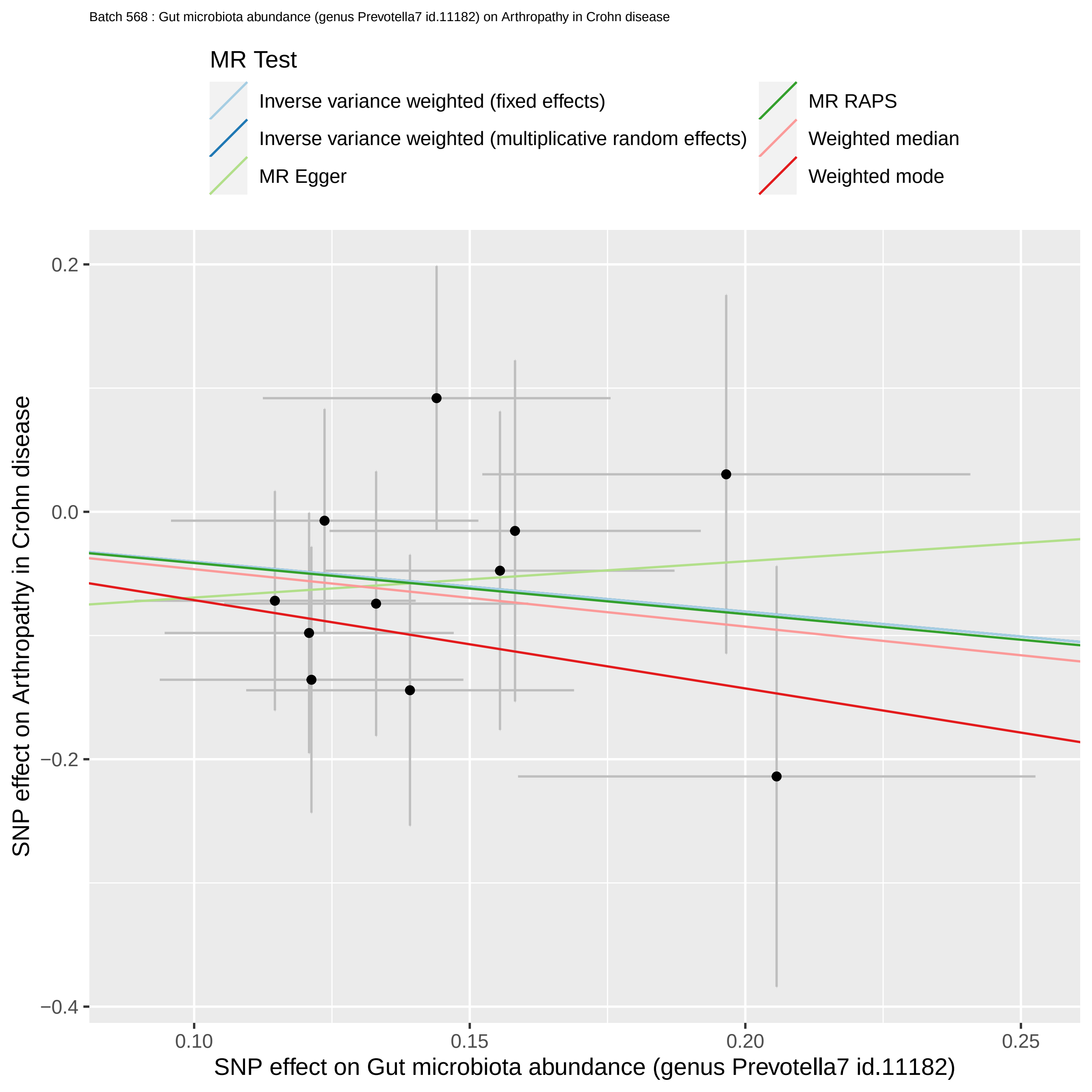

Batch 568 : Gut microbiota abundance (genus Prevotella7 id.11182) on Arthropathy in Crohn disease
MR Test
Inverse variance weighted (fixed effects)
Inverse variance weighted (multiplicative random effects)
MR Egger
MR RAPS
Weighted median
Weighted mode
0.2
0.0
−0.2
−0.4
0.10
0.15
0.20
0.25
SNP effect on Arthropathy in Crohn disease
SNP effect on Gut microbiota abundance (genus Prevotella7 id.11182)

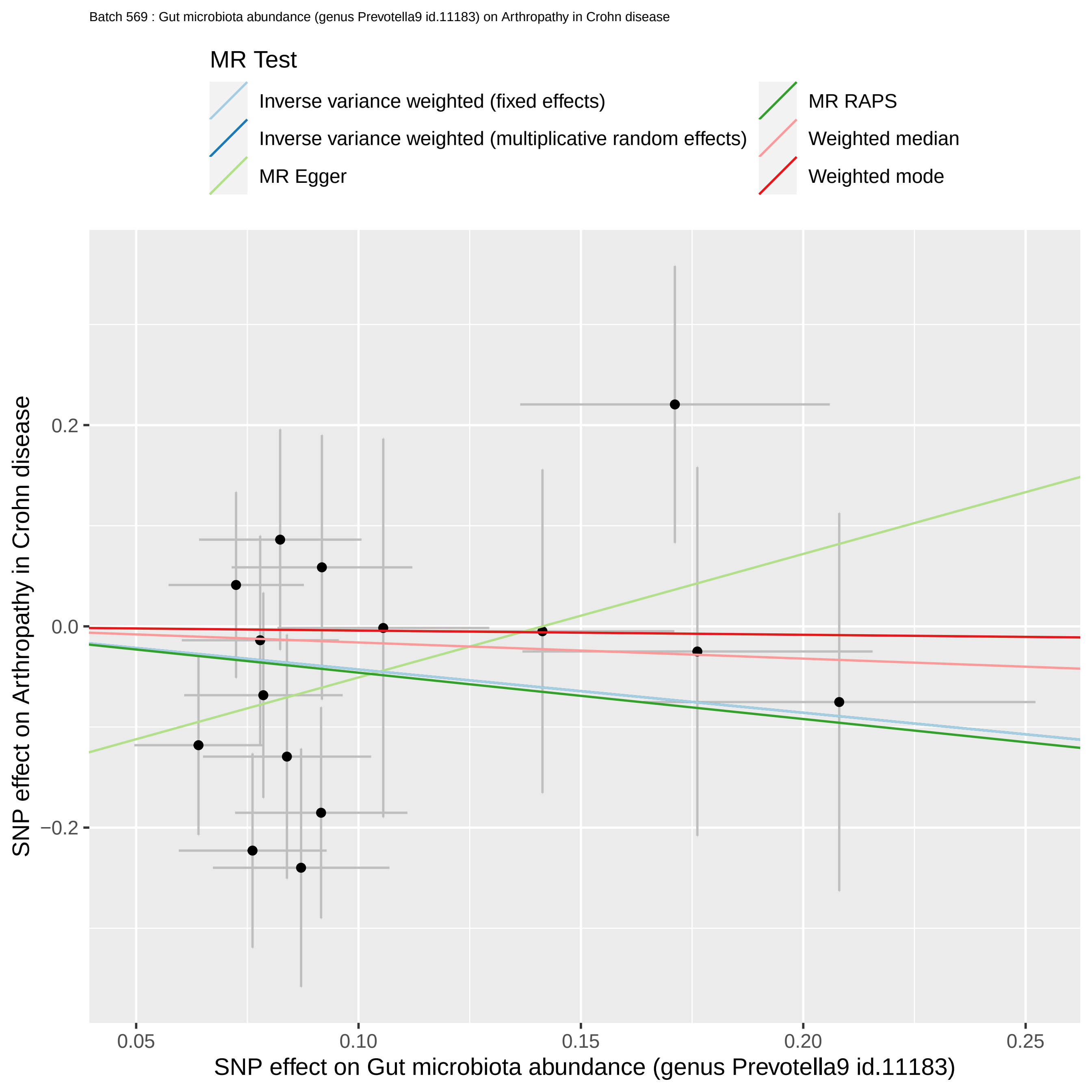
Batch 569 : Gut microbiota abundance (genus Prevotella9 id.11183) on Arthropathy in Crohn disease
MR Test
Inverse variance weighted (fixed effects)
Inverse variance weighted (multiplicative random effects)
MR Egger
MR RAPS
Weighted median
Weighted mode
0.2
0.0
−0.2
0.05
0.10
0.15
0.20
0.25
SNP effect on Arthropathy in Crohn disease
SNP effect on Gut microbiota abundance (genus Prevotella9 id.11183)

Batch 570 : Gut microbiota abundance (genus Rikenellaceae RC9 gut group id.11191) on Arthropathy in Crohn disease

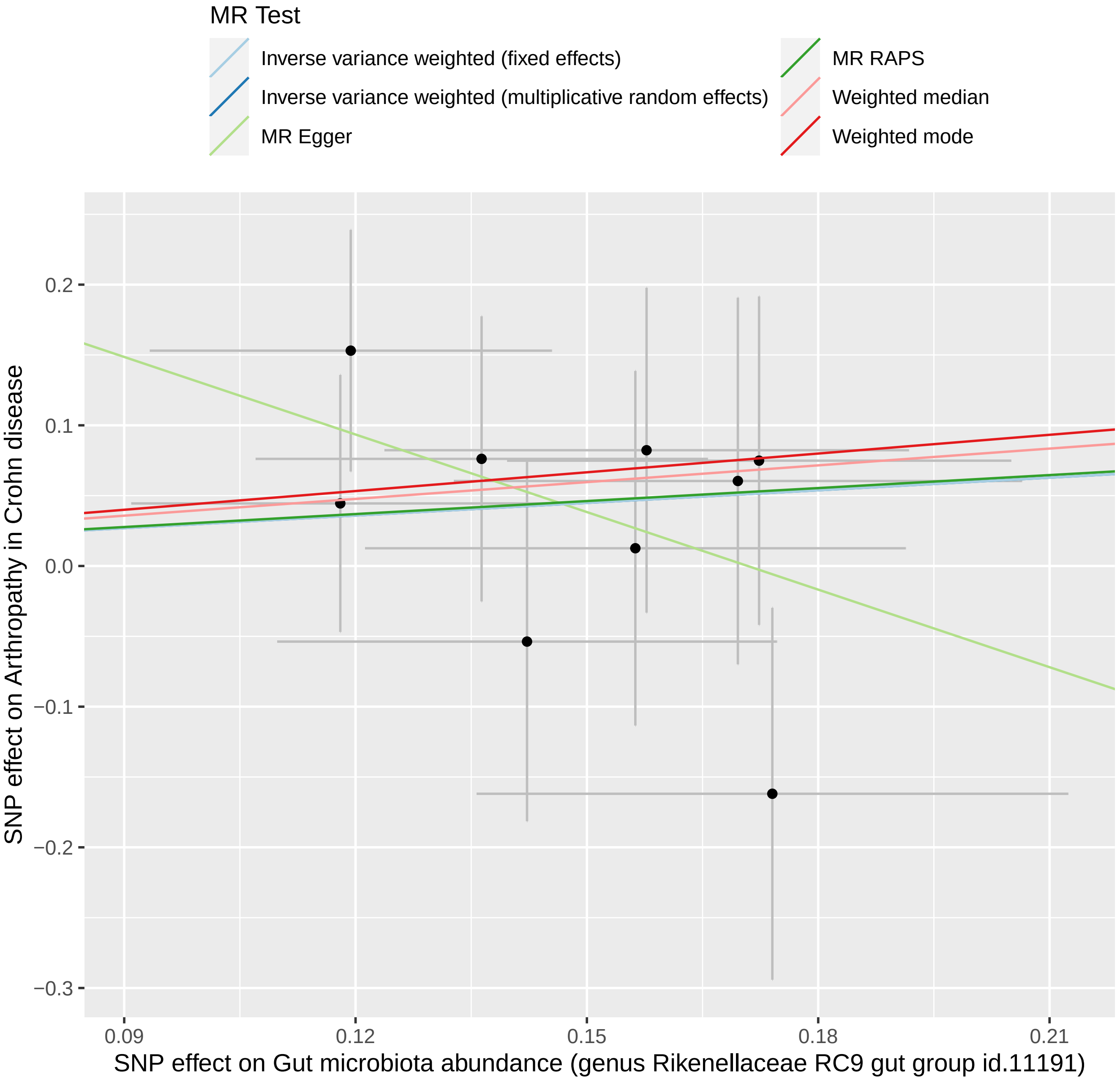

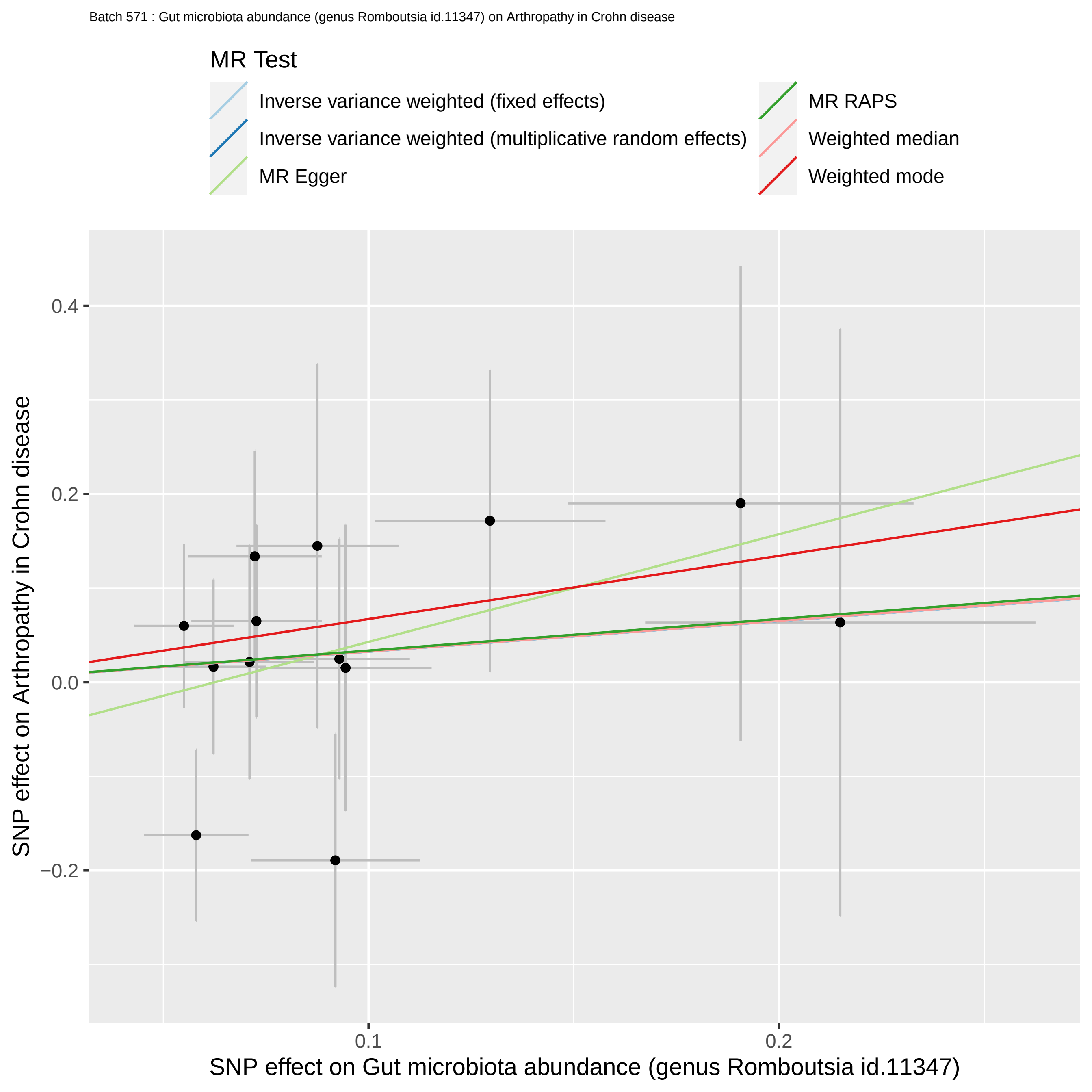
Batch 571 : Gut microbiota abundance (genus Romboutsia id.11347) on Arthropathy in Crohn disease
MR Test
Inverse variance weighted (fixed effects)
Inverse variance weighted (multiplicative random effects)
MR Egger
MR RAPS
Weighted median
Weighted mode
0.4
0.2
0.0
−0.2
0.1
0.2
SNP effect on Arthropathy in Crohn disease
SNP effect on Gut microbiota abundance (genus Romboutsia id.11347)

Batch 572 : Gut microbiota abundance (genus Roseburia id.2012) on Arthropathy in Crohn disease

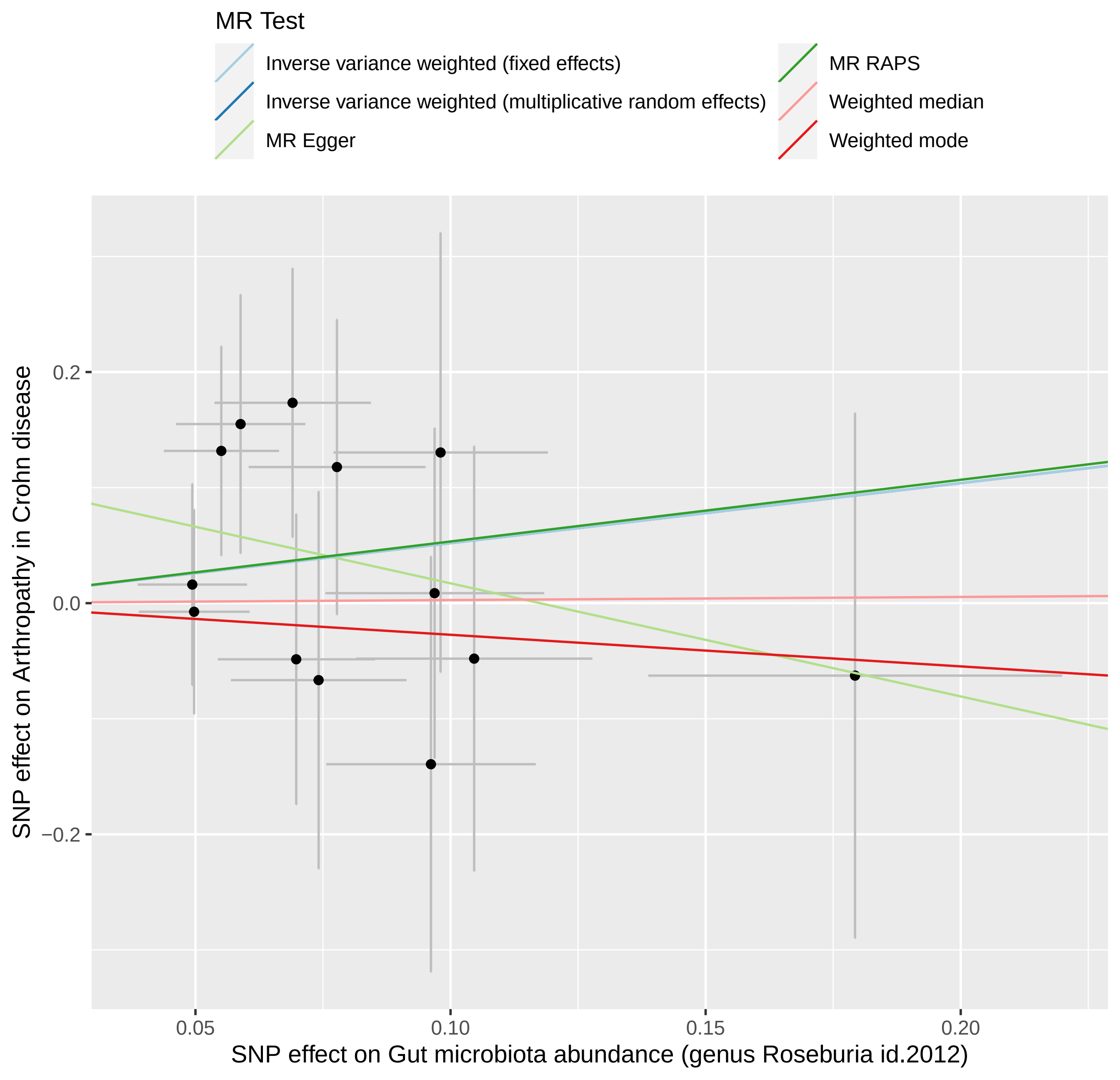

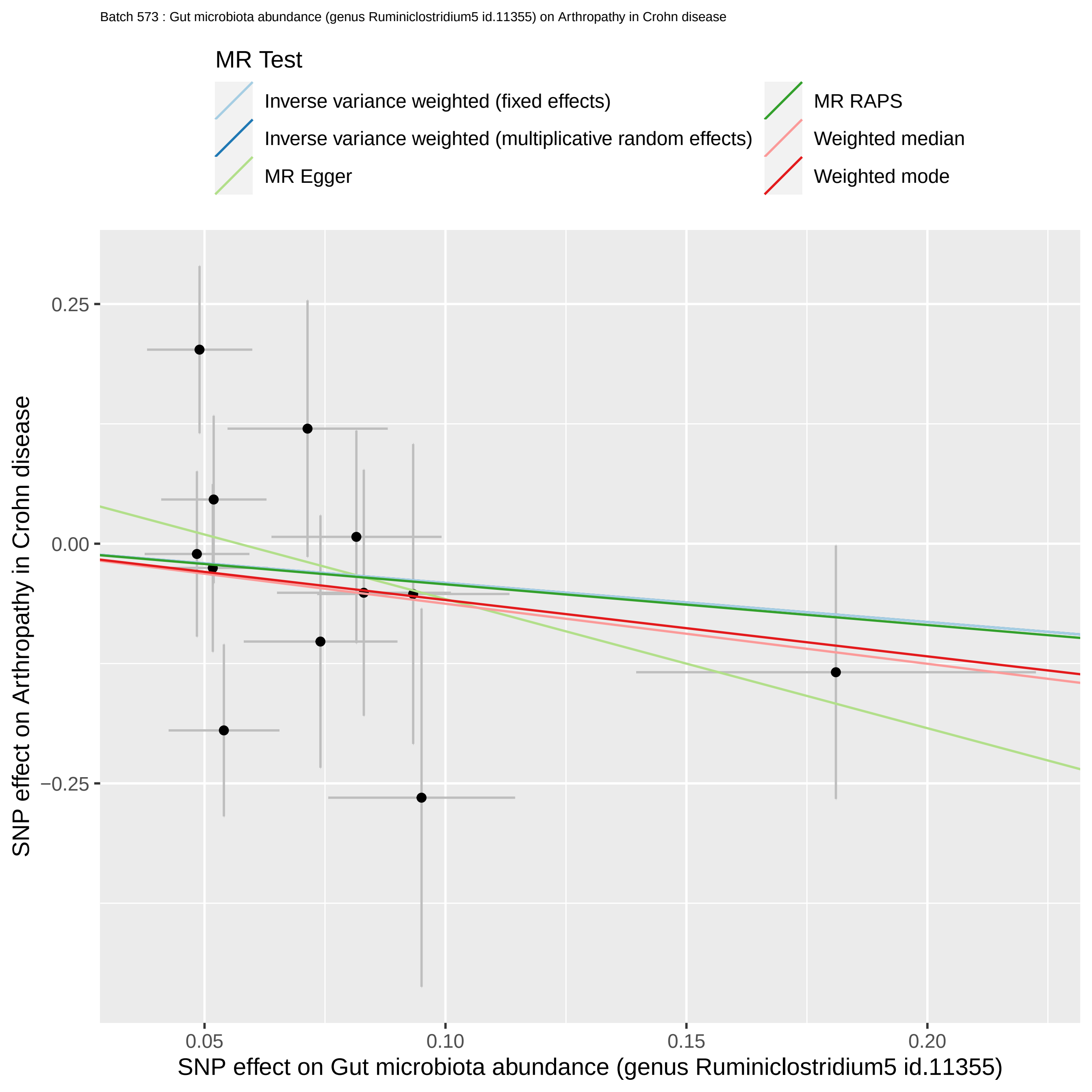

Batch 573 : Gut microbiota abundance (genus Ruminiclostridium5 id.11355) on Arthropathy in Crohn disease
MR Test
Inverse variance weighted (fixed effects)
Inverse variance weighted (multiplicative random effects)
MR Egger
MR RAPS
Weighted median
Weighted mode
0.25
0.00
−0.25
0.05
0.10
0.15
0.20
SNP effect on Arthropathy in Crohn disease
SNP effect on Gut microbiota abundance (genus Ruminiclostridium5 id.11355)

Batch 574 : Gut microbiota abundance (genus Ruminiclostridium6 id.11356) on Arthropathy in Crohn disease

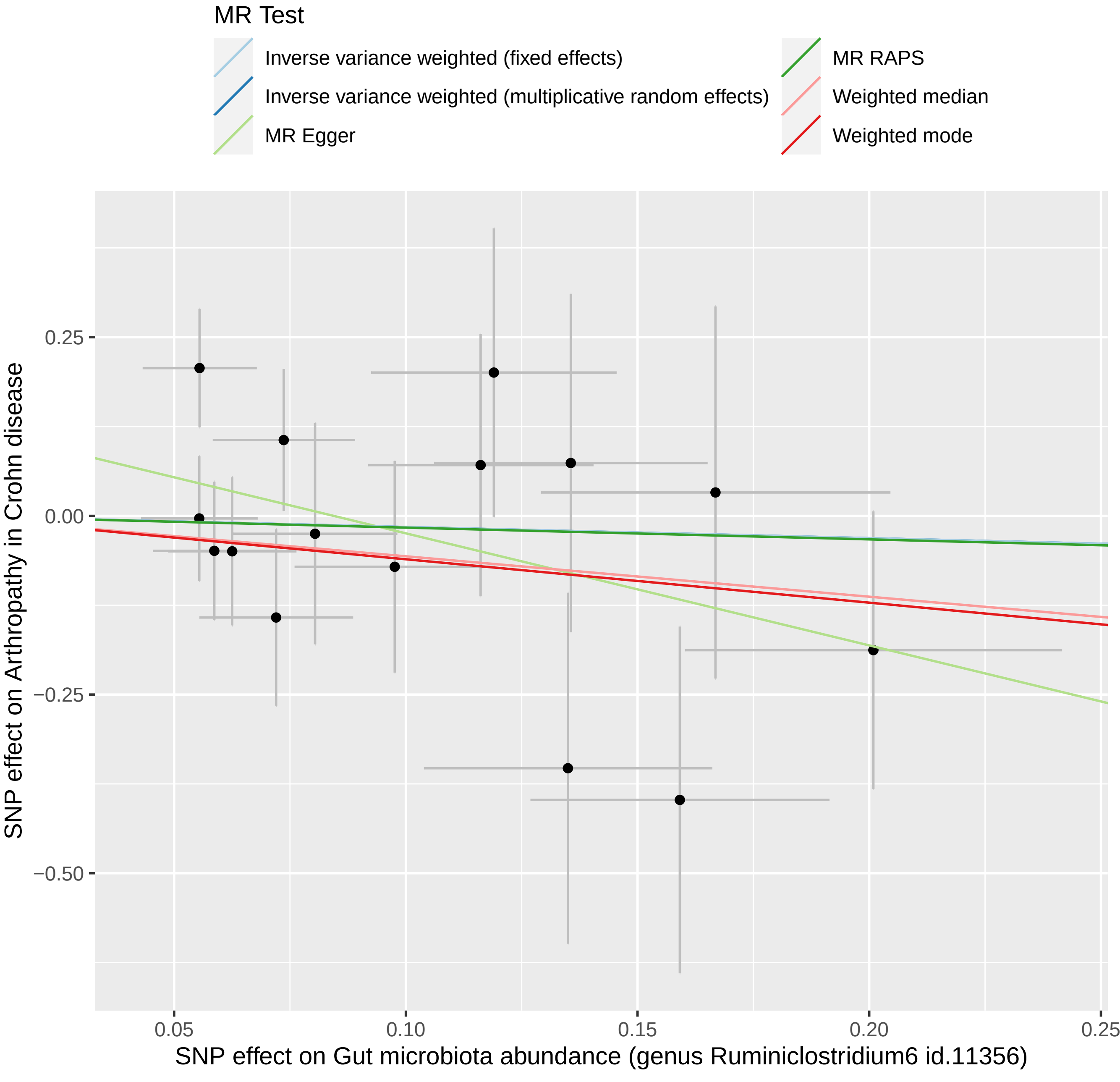

Batch 575 : Gut microbiota abundance (genus Ruminiclostridium9 id.11357) on Arthropathy in Crohn disease

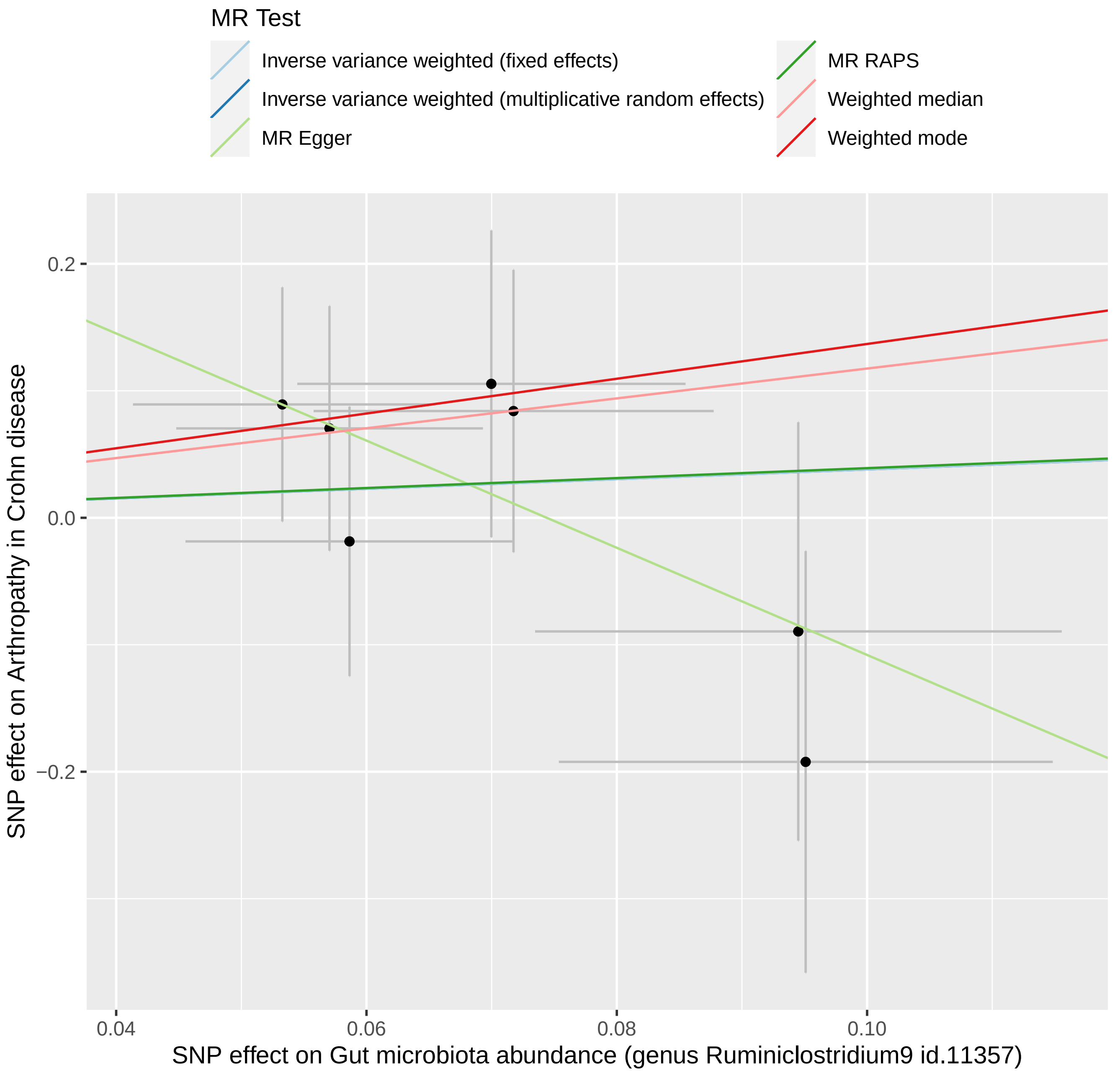

Batch 576 : Gut microbiota abundance (genus Ruminococcaceae NK4A214 group id.11358) on Arthropathy in Crohn disease

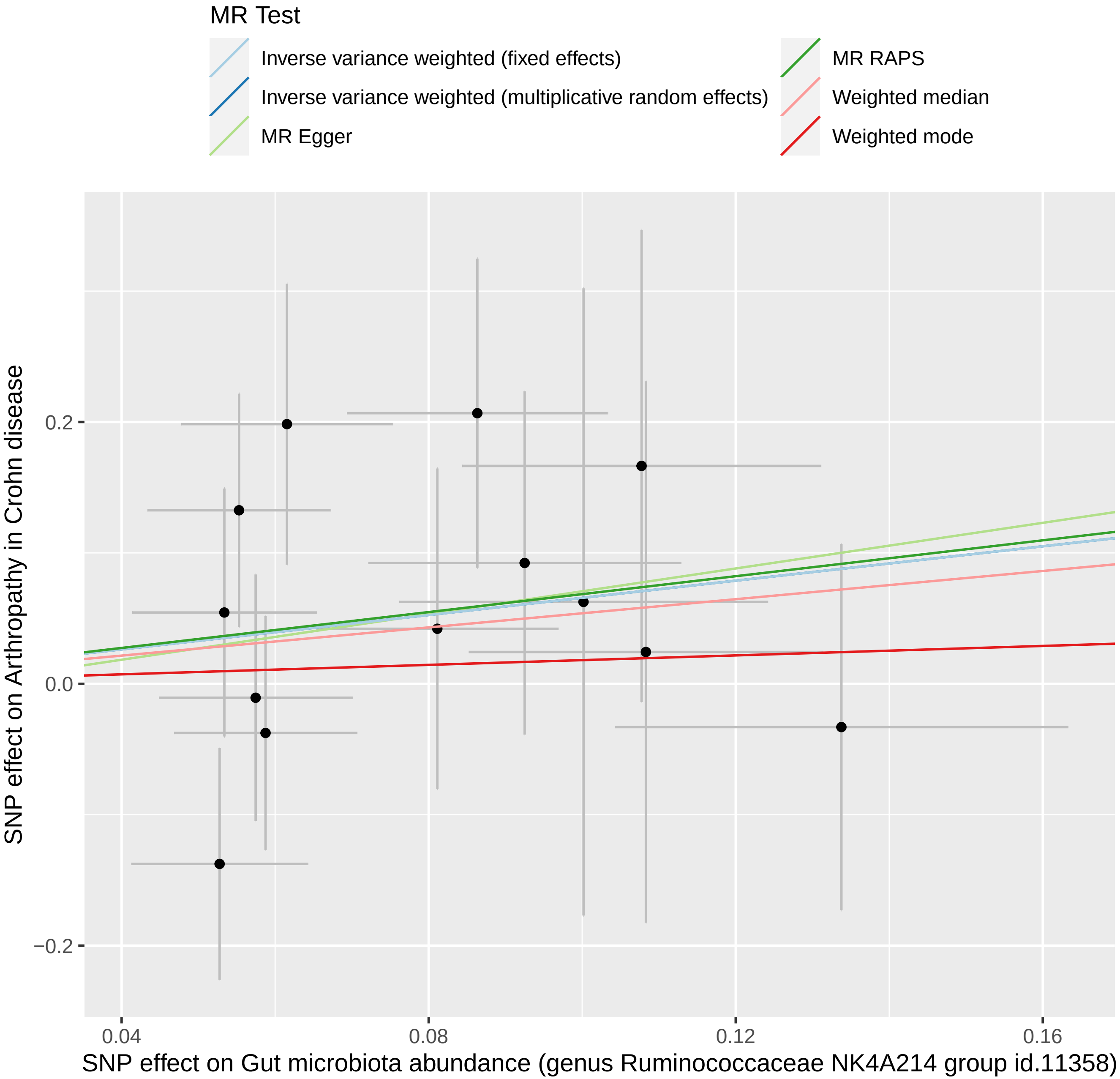

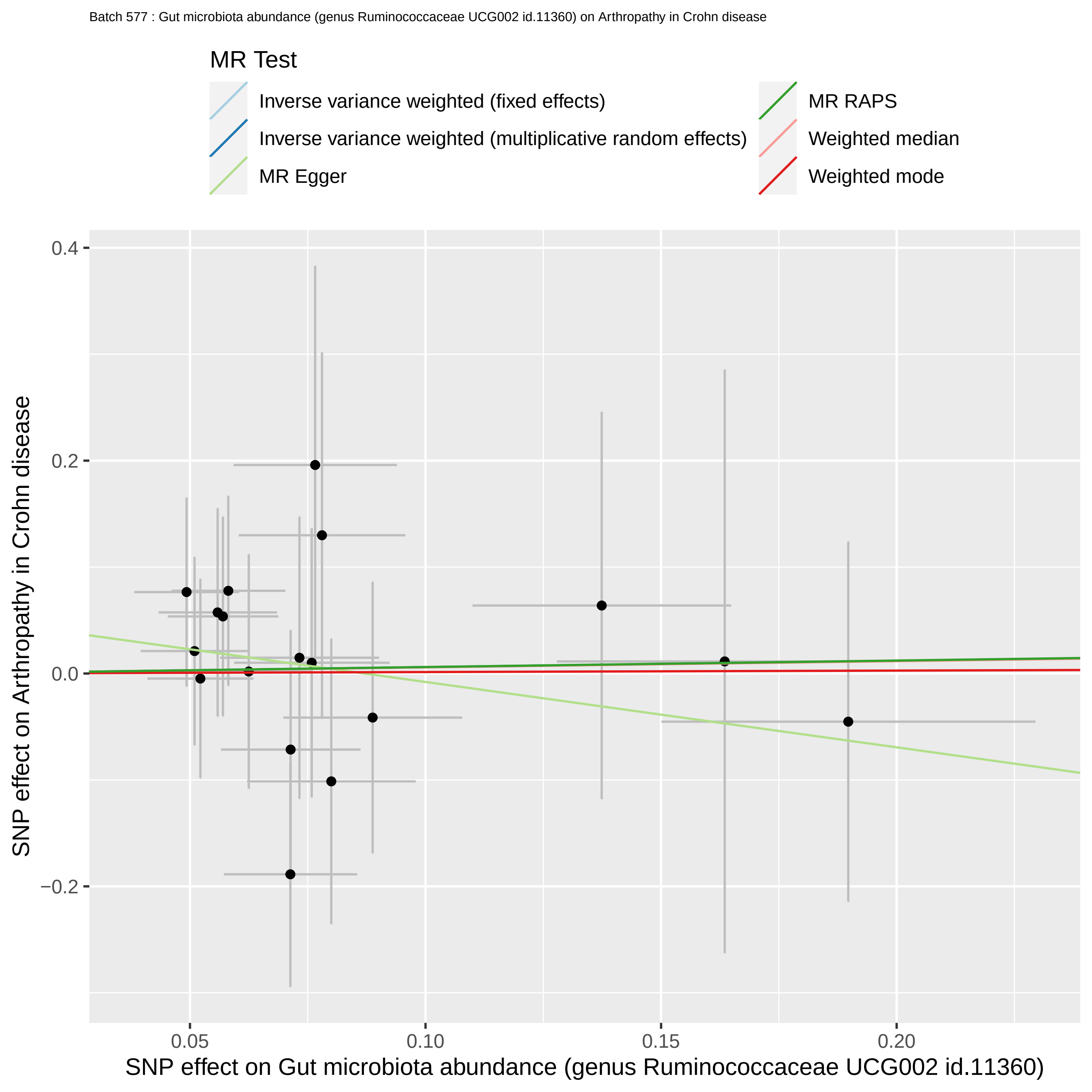

Batch 577 : Gut microbiota abundance (genus Ruminococcaceae UCG002 id.11360) on Arthropathy in Crohn disease
MR Test
Inverse variance weighted (fixed effects)
Inverse variance weighted (multiplicative random effects)
MR Egger
MR RAPS
Weighted median
Weighted mode
0.4
0.2
0.0
−0.2
0.05
0.10
0.15
0.20
SNP effect on Arthropathy in Crohn disease
SNP effect on Gut microbiota abundance (genus Ruminococcaceae UCG002 id.11360)

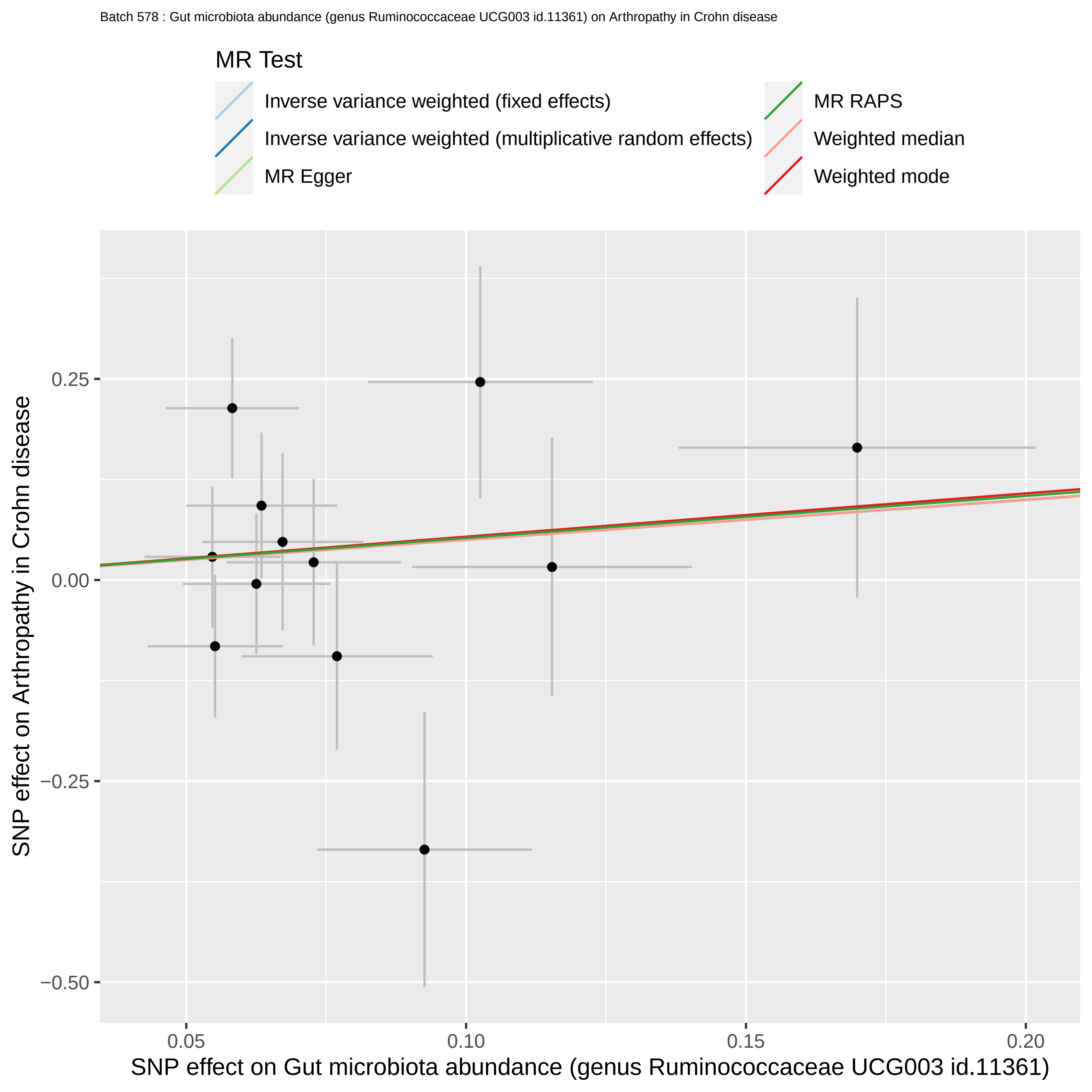

Batch 578 : Gut microbiota abundance (genus Ruminococcaceae UCG003 id.11361) on Arthropathy in Crohn disease
MR Test
Inverse variance weighted (fixed effects)
Inverse variance weighted (multiplicative random effects)
MR Egger
MR RAPS
Weighted median
Weighted mode
0.25
0.00
−0.25
−0.50
0.05
0.10
0.15
0.20
SNP effect on Arthropathy in Crohn disease
SNP effect on Gut microbiota abundance (genus Ruminococcaceae UCG003 id.11361)

Batch 579 : Gut microbiota abundance (genus Ruminococcaceae UCG004 id.11362) on Arthropathy in Crohn disease

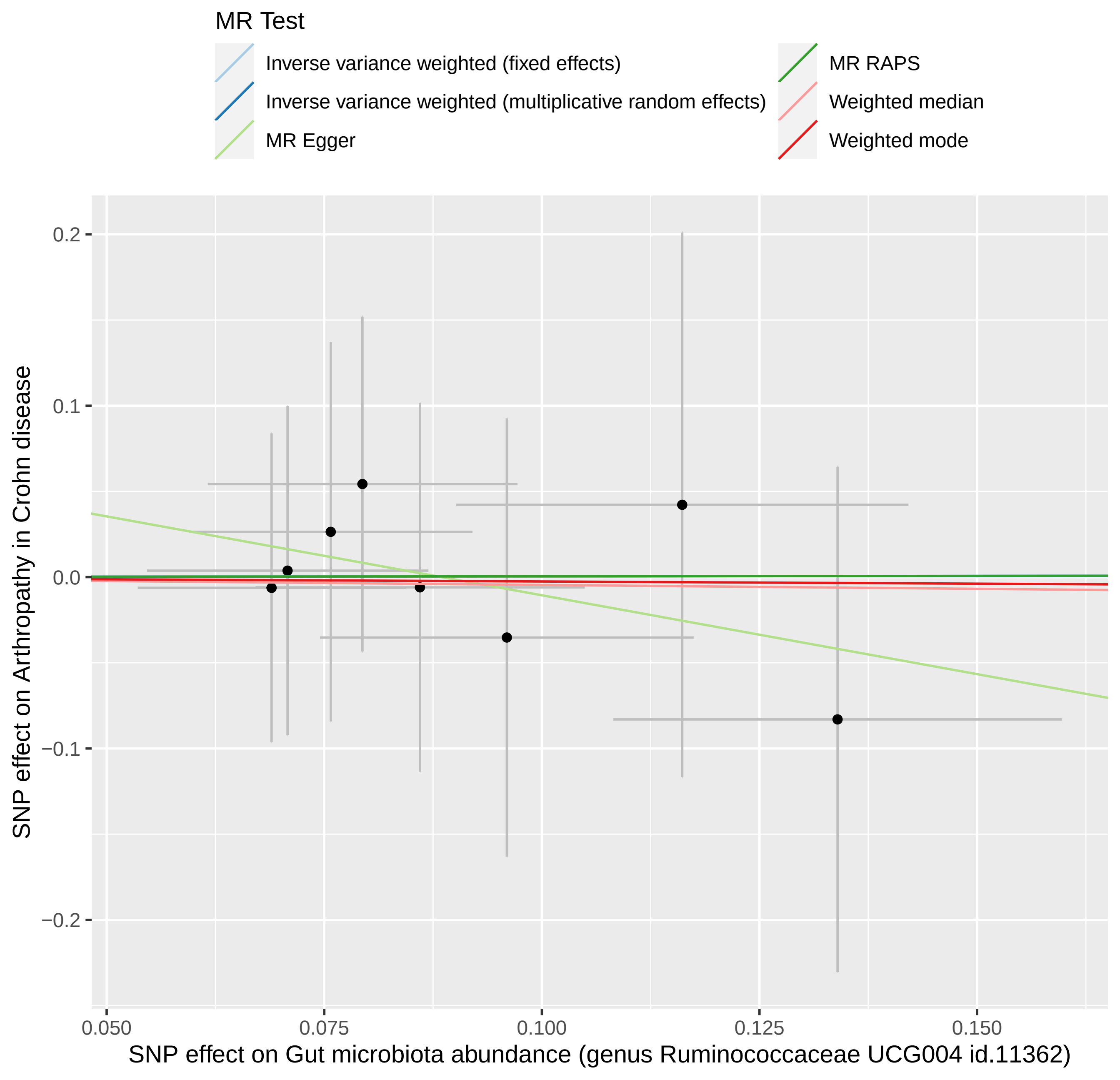

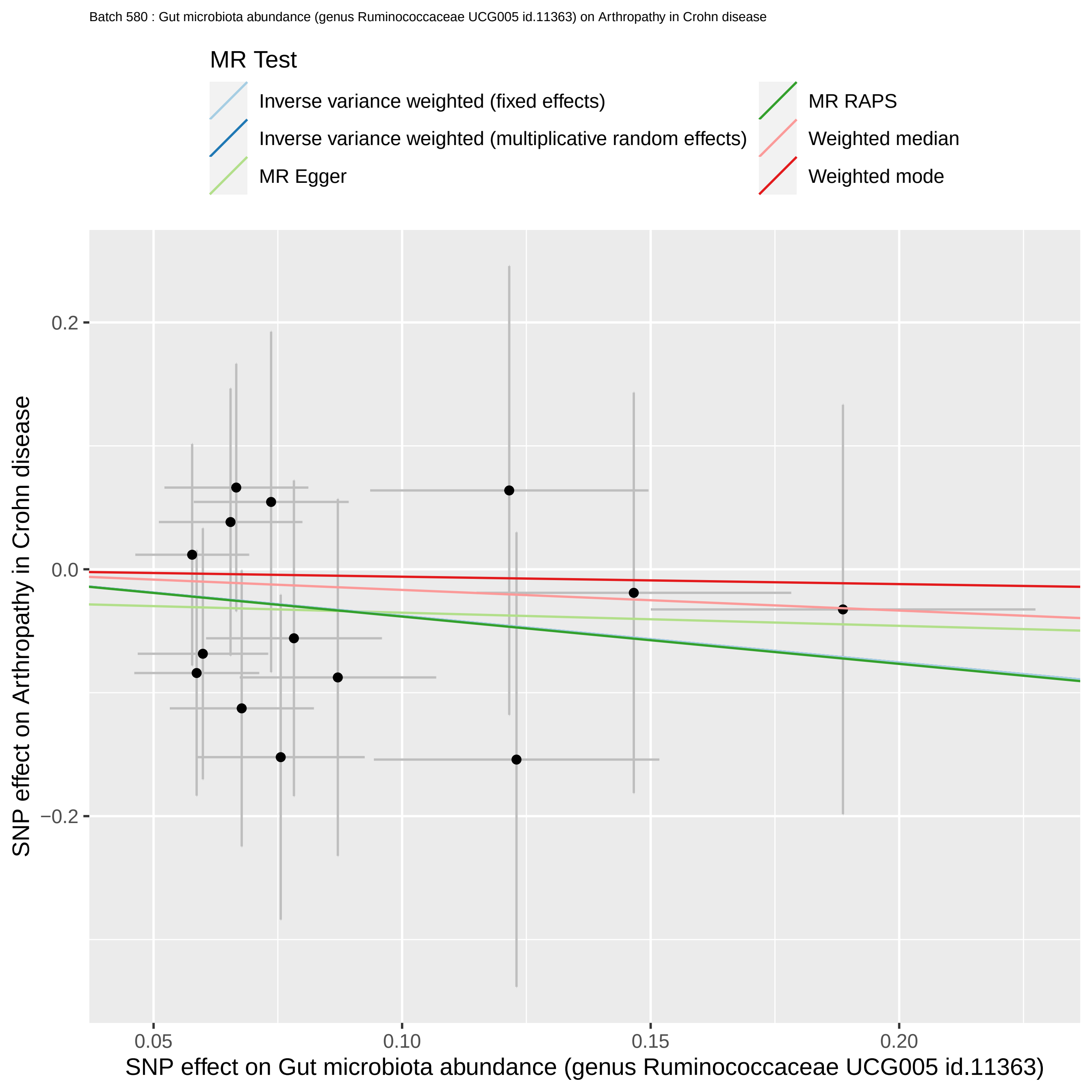
Batch 580 : Gut microbiota abundance (genus Ruminococcaceae UCG005 id.11363) on Arthropathy in Crohn disease
MR Test
Inverse variance weighted (fixed effects)
Inverse variance weighted (multiplicative random effects)
MR Egger
MR RAPS
Weighted median
Weighted mode
0.2
0.0
−0.2
0.05
0.10
0.15
0.20
SNP effect on Arthropathy in Crohn disease
SNP effect on Gut microbiota abundance (genus Ruminococcaceae UCG005 id.11363)

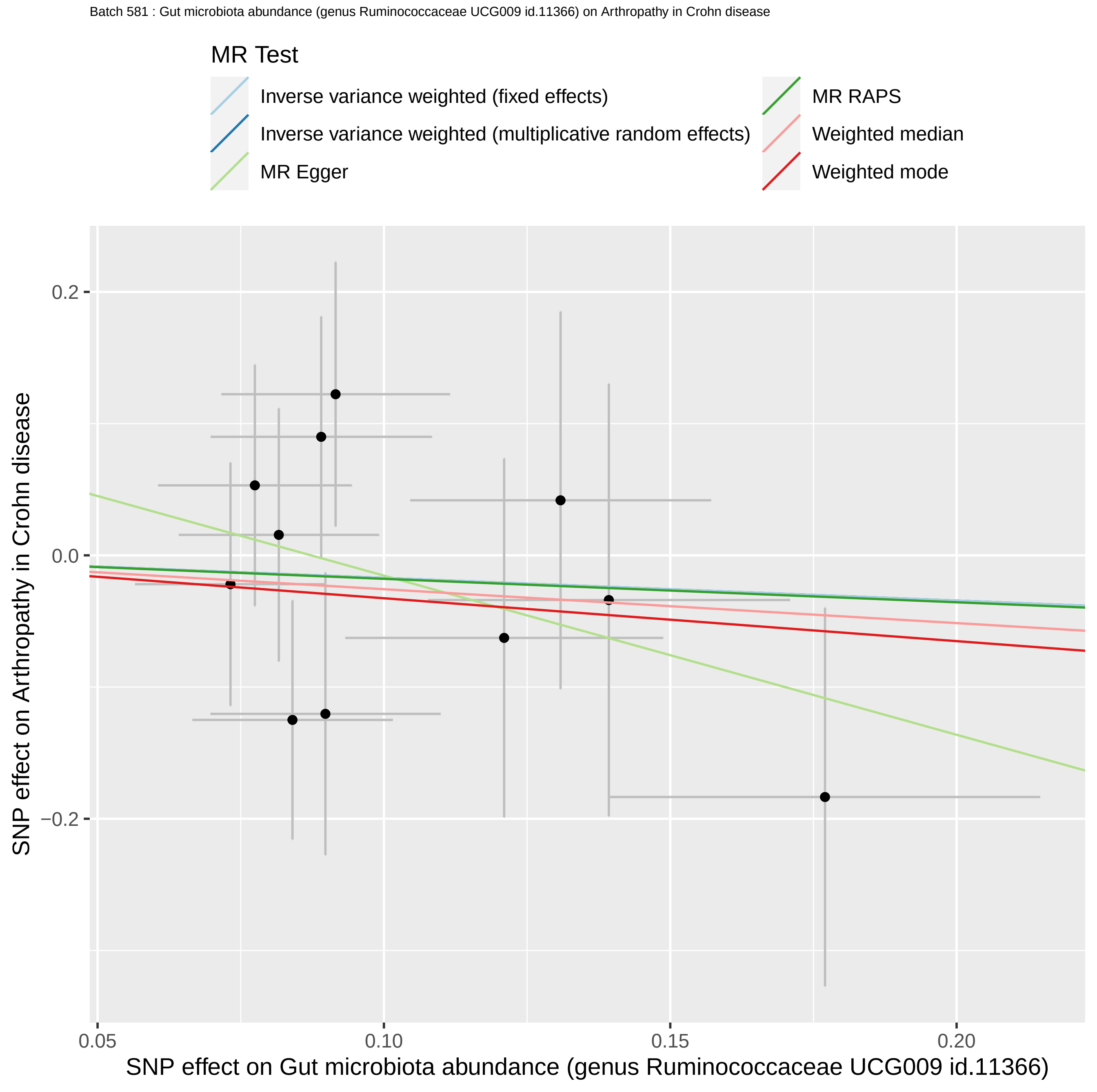

Batch 581 : Gut microbiota abundance (genus Ruminococcaceae UCG009 id.11366) on Arthropathy in Crohn disease
MR Test
Inverse variance weighted (fixed effects)
Inverse variance weighted (multiplicative random effects)
MR Egger
MR RAPS
Weighted median
Weighted mode
0.2
0.0
−0.2
0.05
0.10
0.15
0.20
SNP effect on Arthropathy in Crohn disease
SNP effect on Gut microbiota abundance (genus Ruminococcaceae UCG009 id.11366)

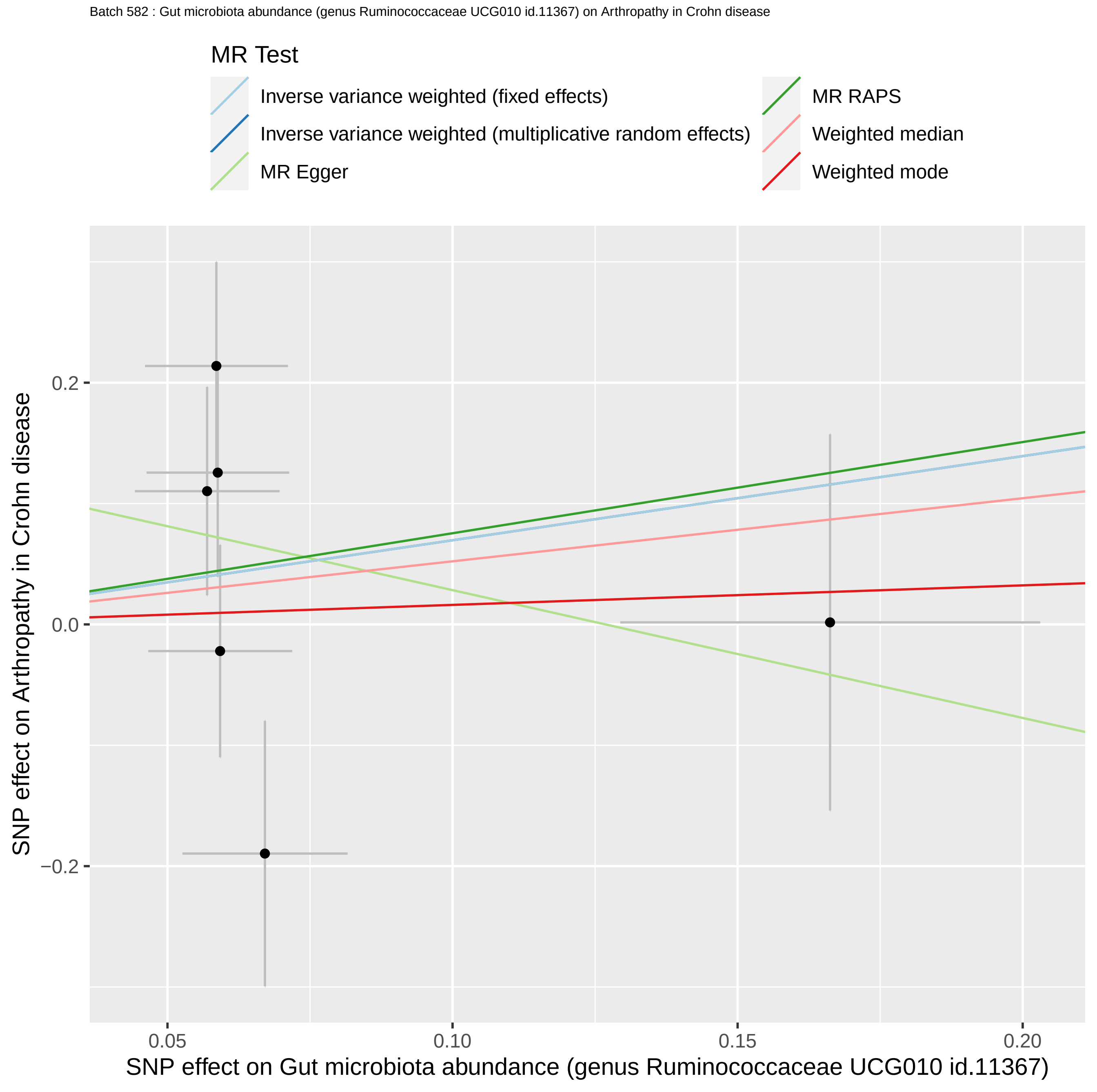

Batch 582 : Gut microbiota abundance (genus Ruminococcaceae UCG010 id.11367) on Arthropathy in Crohn disease
MR Test
Inverse variance weighted (fixed effects)
Inverse variance weighted (multiplicative random effects)
MR Egger
MR RAPS
Weighted median
Weighted mode
SNP effect on Arthropathy in Crohn disease
0.2
0.0
−0.2
0.05
0.10
0.15
0.20
SNP effect on Gut microbiota abundance (genus Ruminococcaceae UCG010 id.11367)

Batch 583 : Gut microbiota abundance (genus Ruminococcaceae UCG011 id.11368) on Arthropathy in Crohn disease

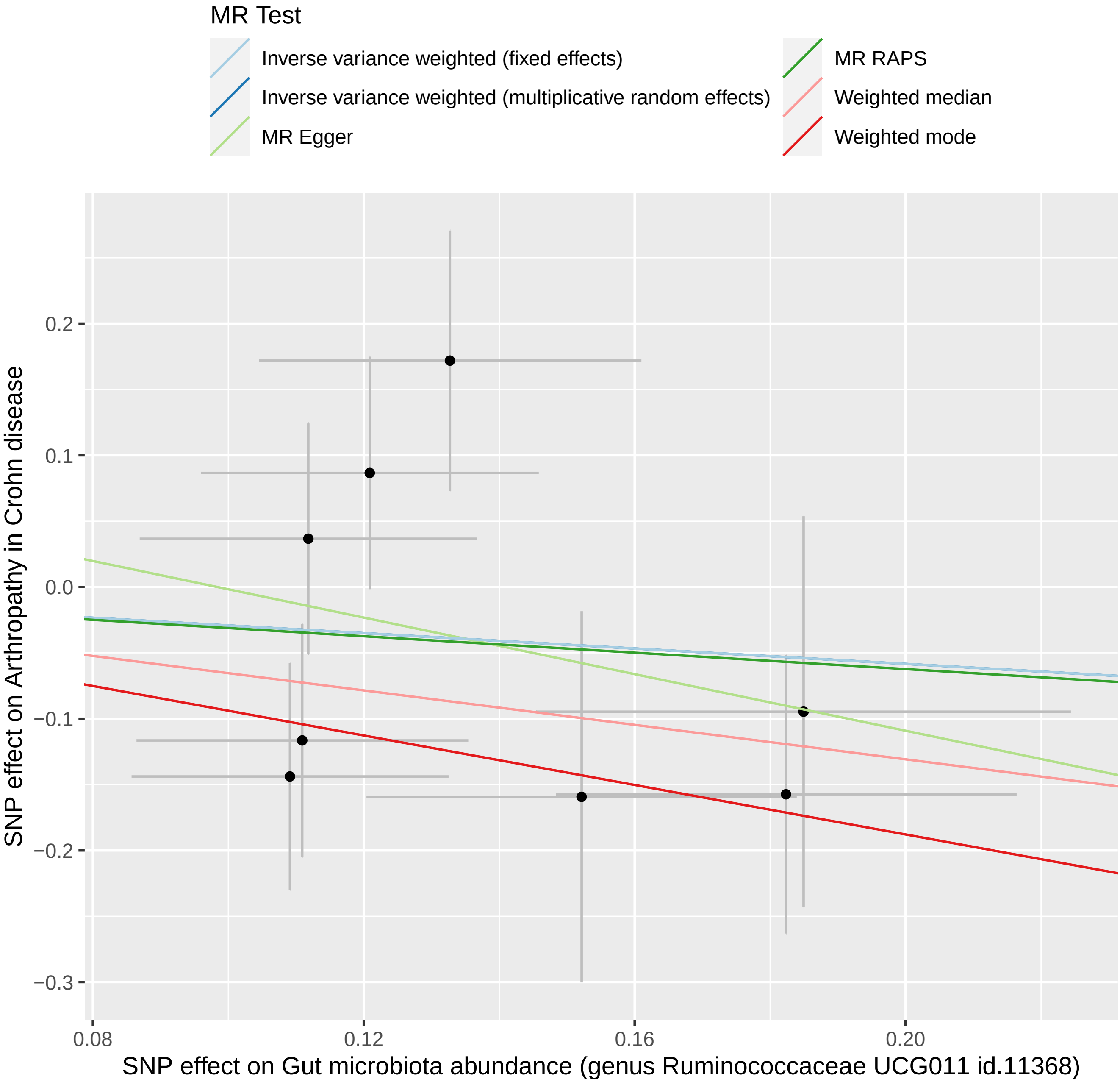

Batch 584 : Gut microbiota abundance (genus Ruminococcaceae UCG013 id.11370) on Arthropathy in Crohn disease

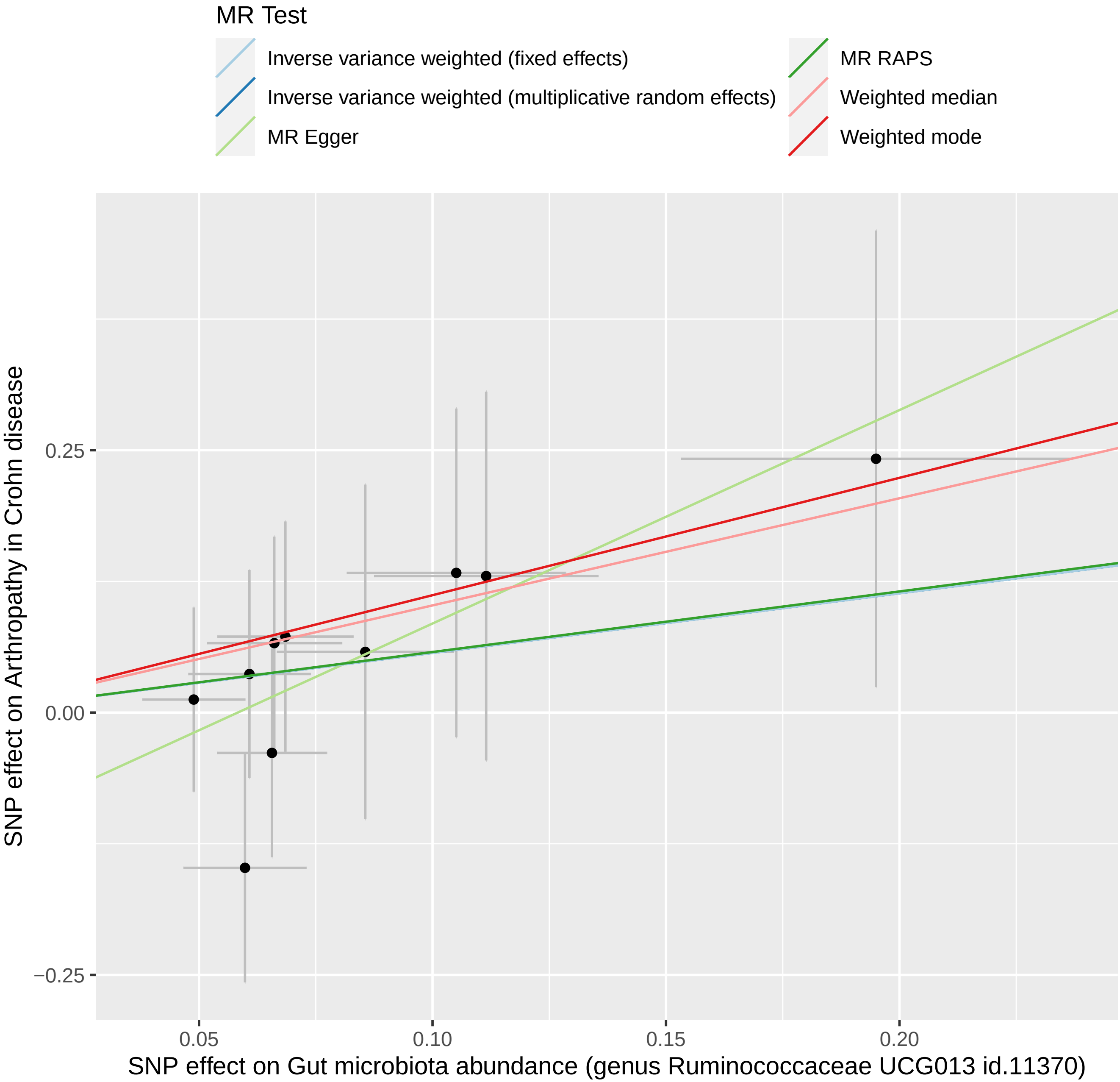

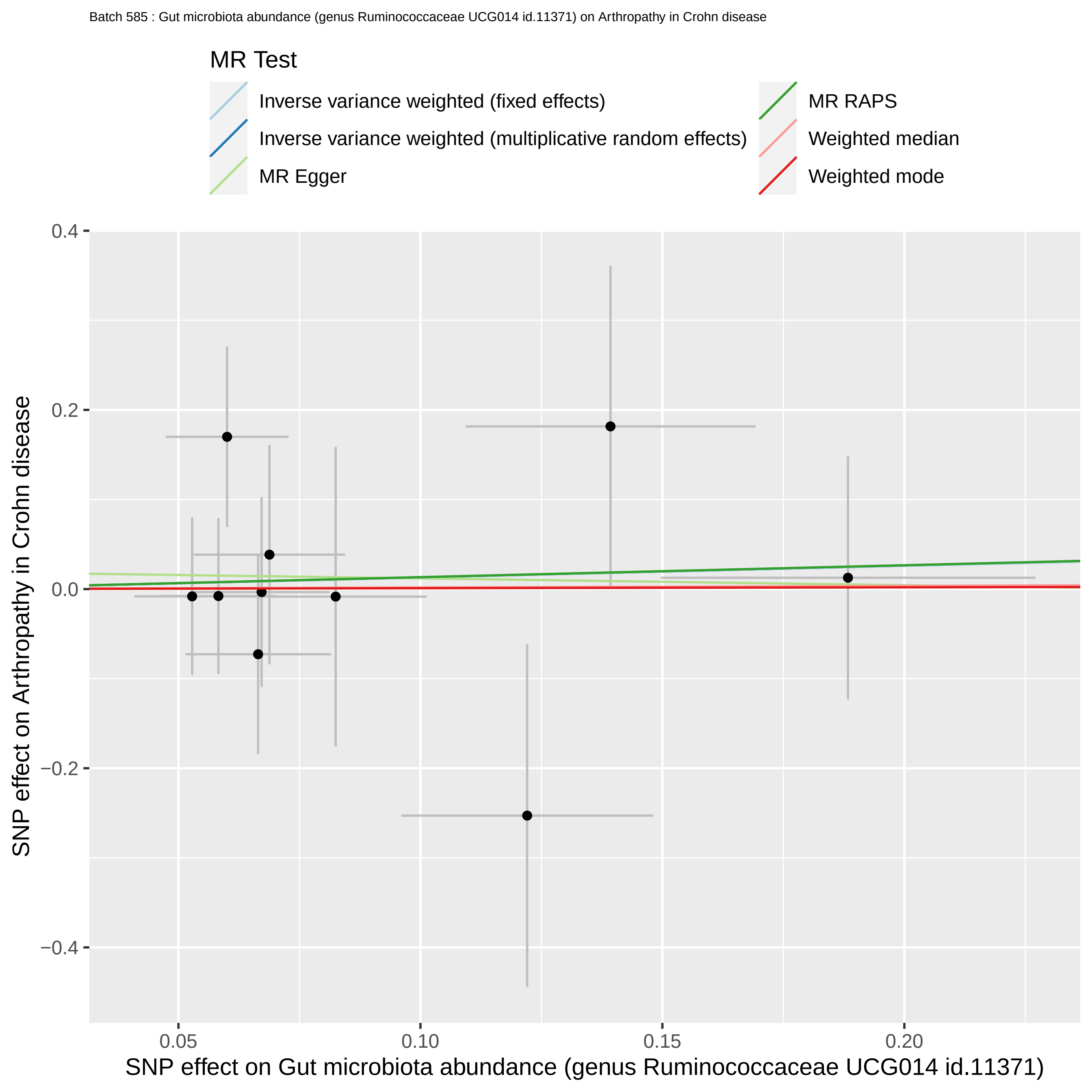
Batch 585 : Gut microbiota abundance (genus Ruminococcaceae UCG014 id.11371) on Arthropathy in Crohn disease
MR Test
Inverse variance weighted (fixed effects)
Inverse variance weighted (multiplicative random effects)
MR Egger
MR RAPS
Weighted median
Weighted mode
0.4
0.2
0.0
−0.2
−0.4
0.05
0.10
0.15
0.20
SNP effect on Arthropathy in Crohn disease
SNP effect on Gut microbiota abundance (genus Ruminococcaceae UCG014 id.11371)

Batch 586 : Gut microbiota abundance (genus Ruminococcus1 id.11373) on Arthropathy in Crohn disease

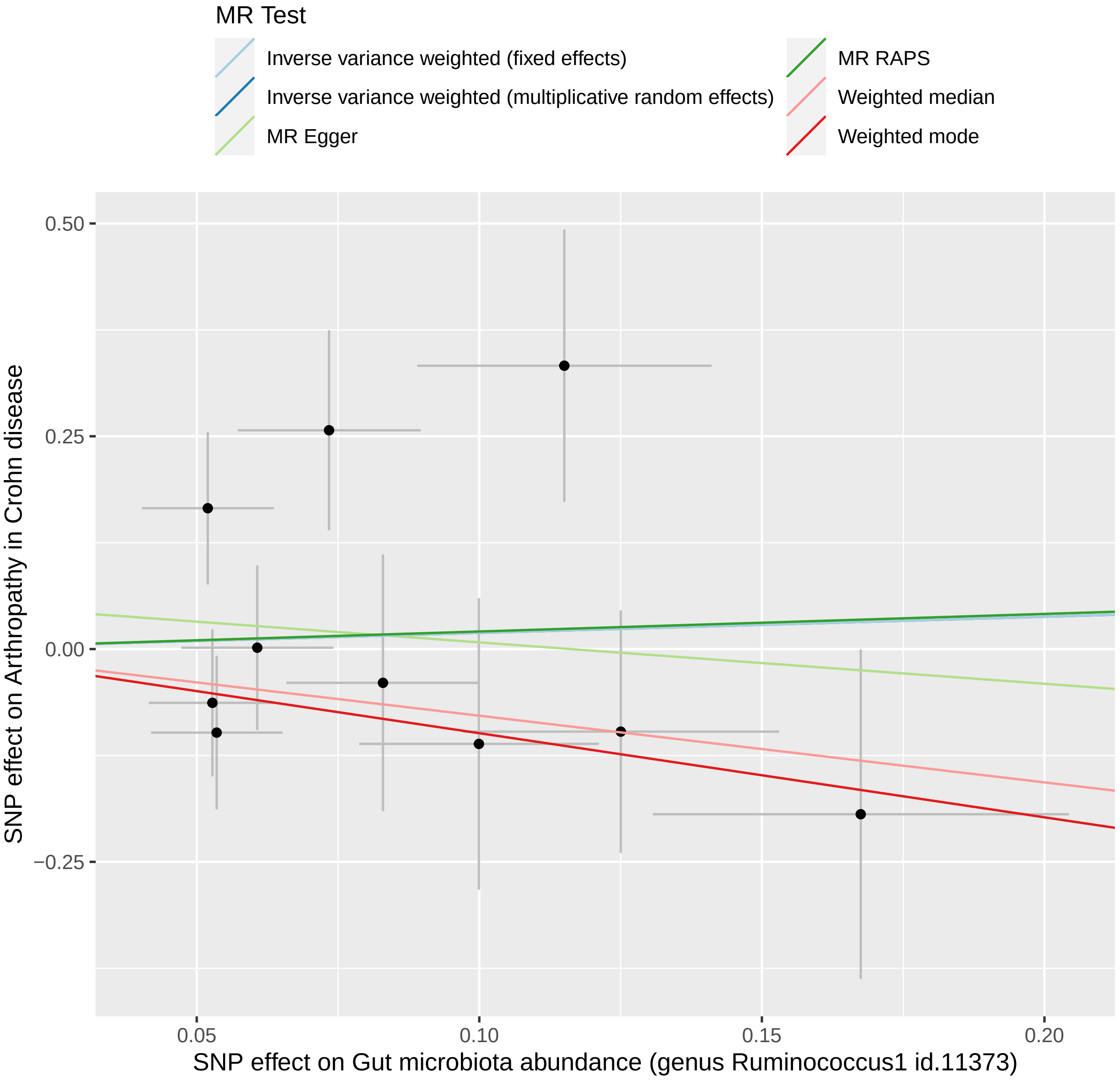

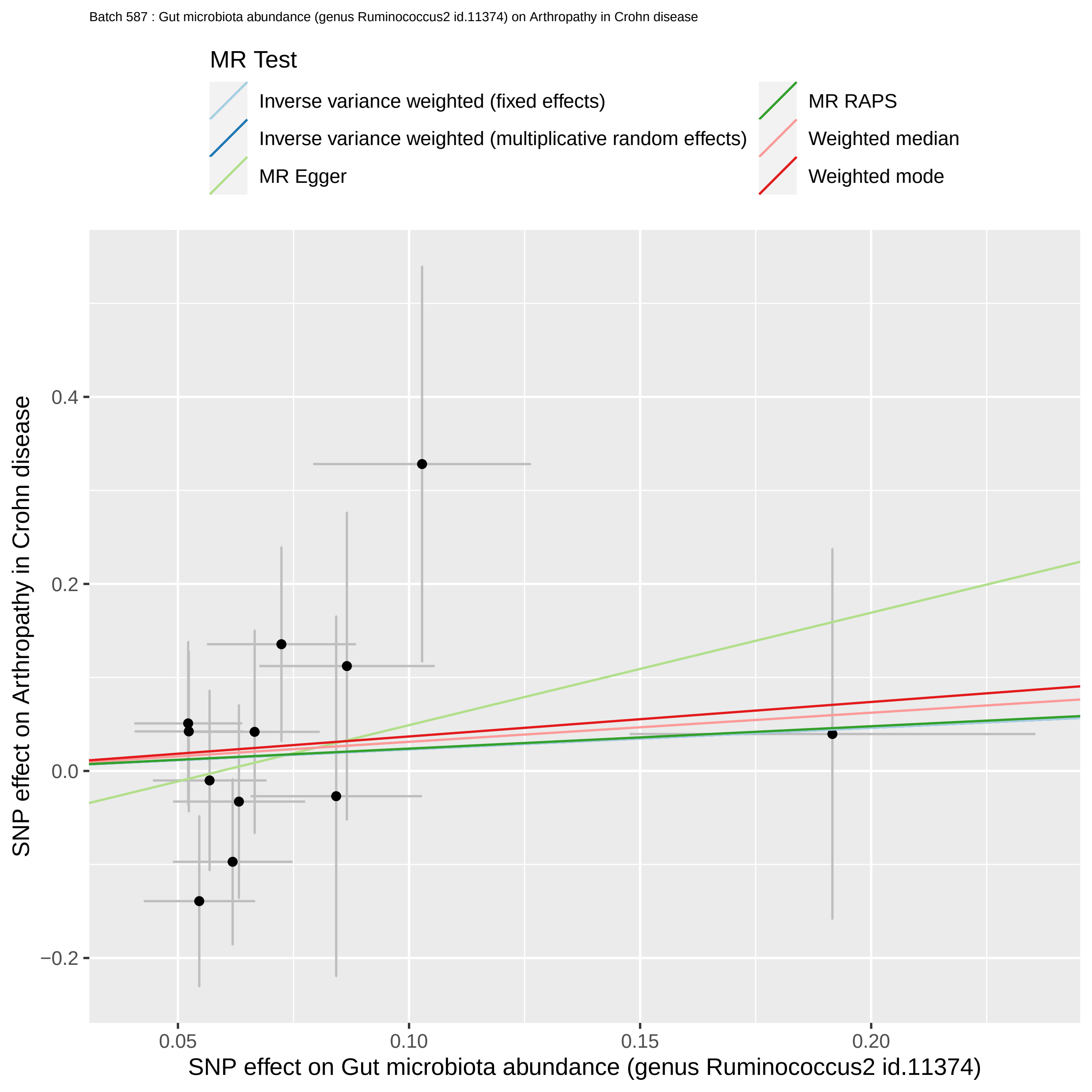
Batch 587 : Gut microbiota abundance (genus Ruminococcus2 id.11374) on Arthropathy in Crohn disease
MR Test
Inverse variance weighted (fixed effects)
Inverse variance weighted (multiplicative random effects)
MR Egger
MR RAPS
Weighted median
Weighted mode
SNP effect on Arthropathy in Crohn disease
0.4
0.2
0.0
−0.2
0.05
0.10
0.15
0.20
SNP effect on Gut microbiota abundance (genus Ruminococcus2 id.11374)

Batch 588 : Gut microbiota abundance (genus Ruminococcus gauvreauii group id.11342) on Arthropathy in Crohn disease

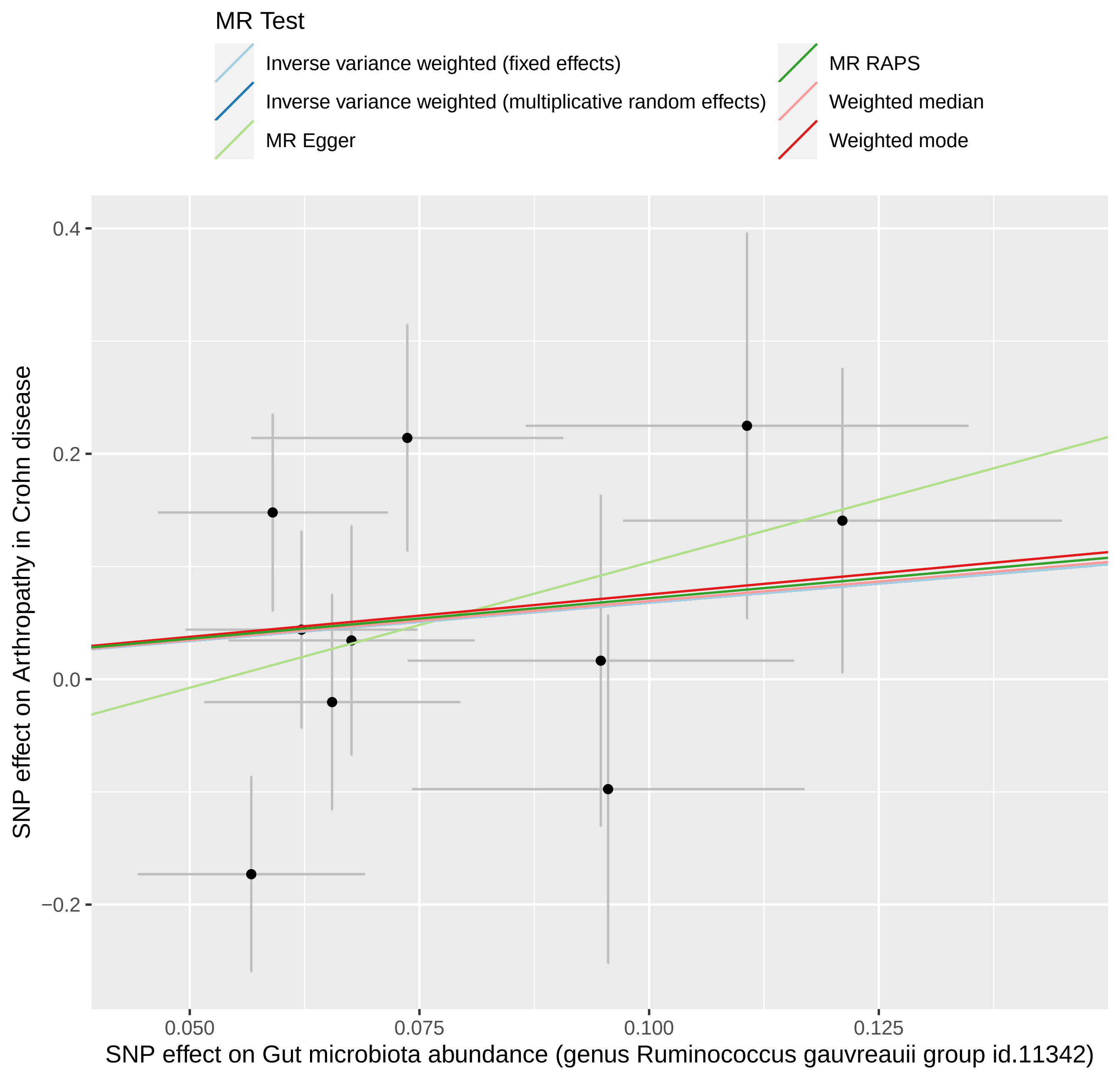

Batch 589 : Gut microbiota abundance (genus Ruminococcus gnavus group id.14376) on Arthropathy in Crohn disease

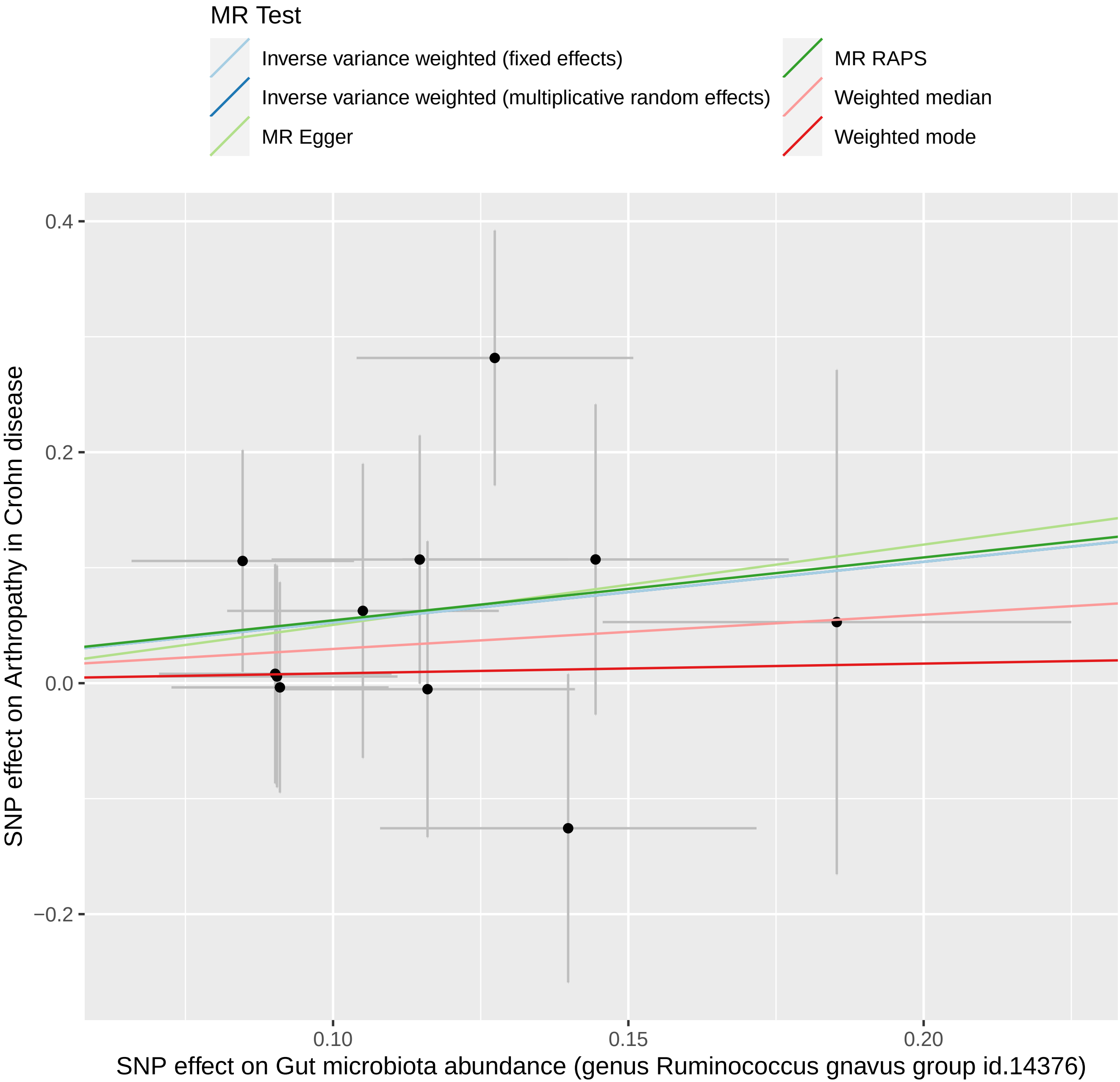

Batch 590 : Gut microbiota abundance (genus Ruminococcus torques group id.14377) on Arthropathy in Crohn disease

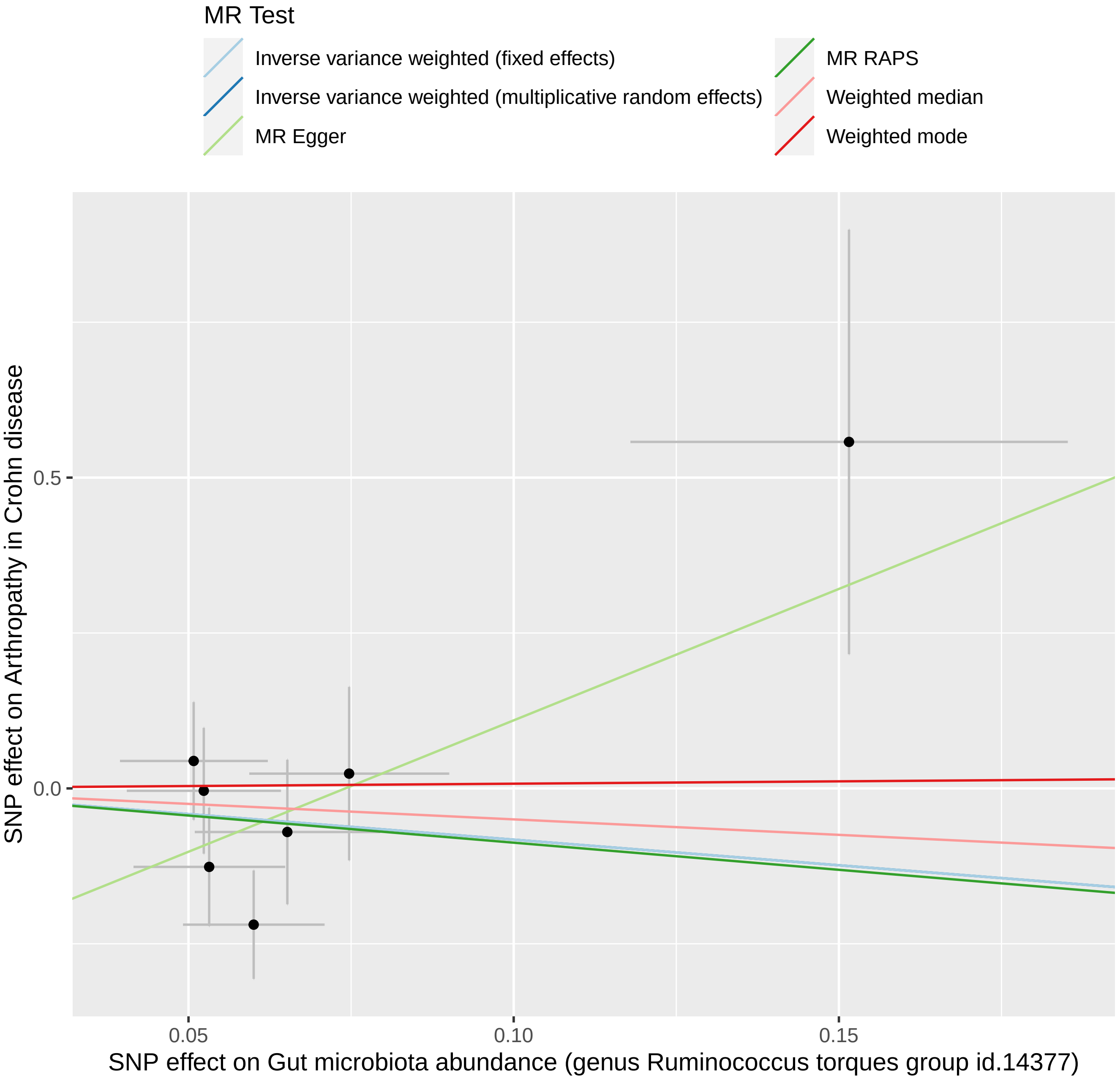

Batch 591 : Gut microbiota abundance (genus Sellimonas id.14369) on Arthropathy in Crohn disease

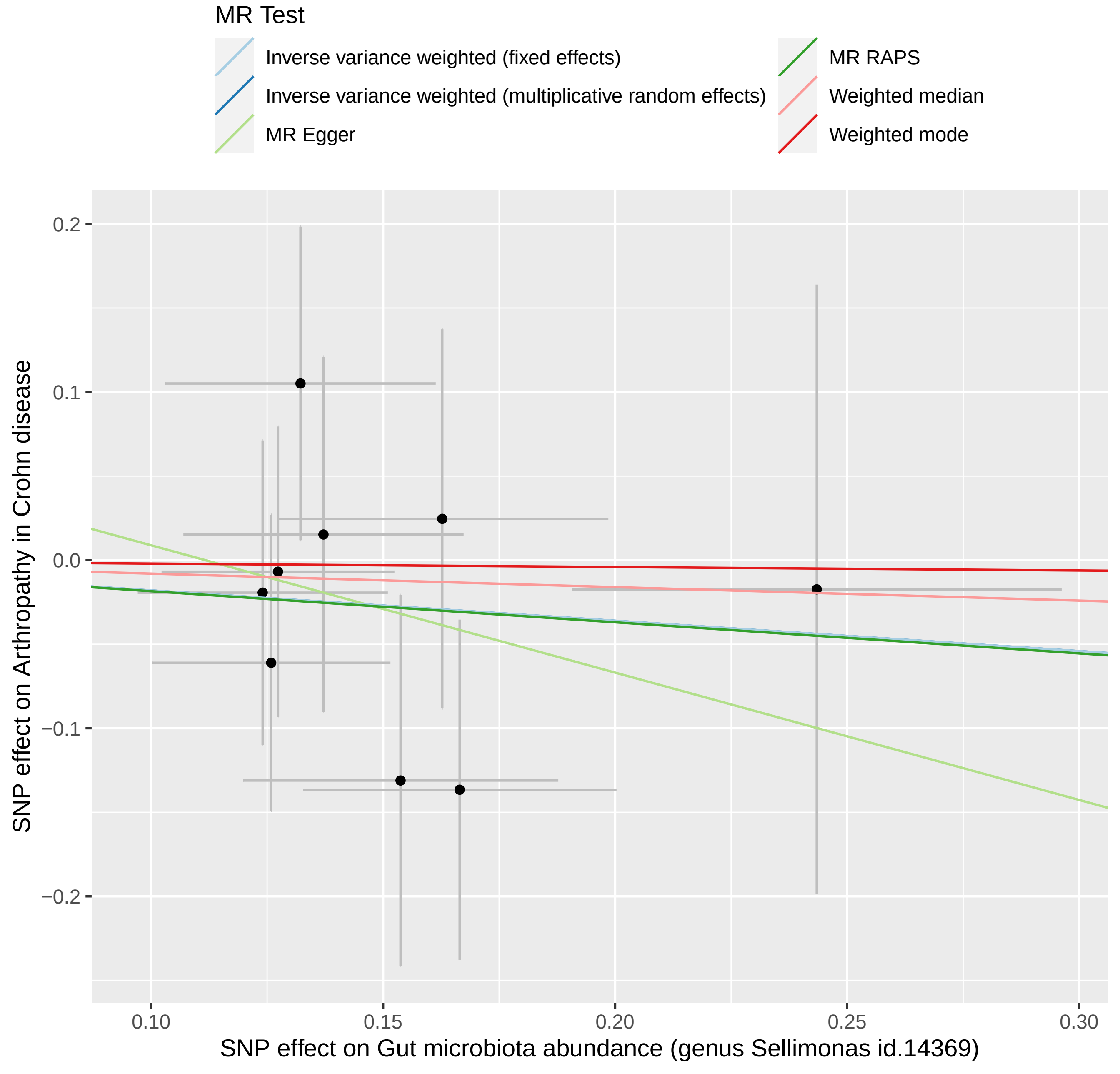

Batch 592 : Gut microbiota abundance (genus Senegalimassilia id.11160) on Arthropathy in Crohn disease

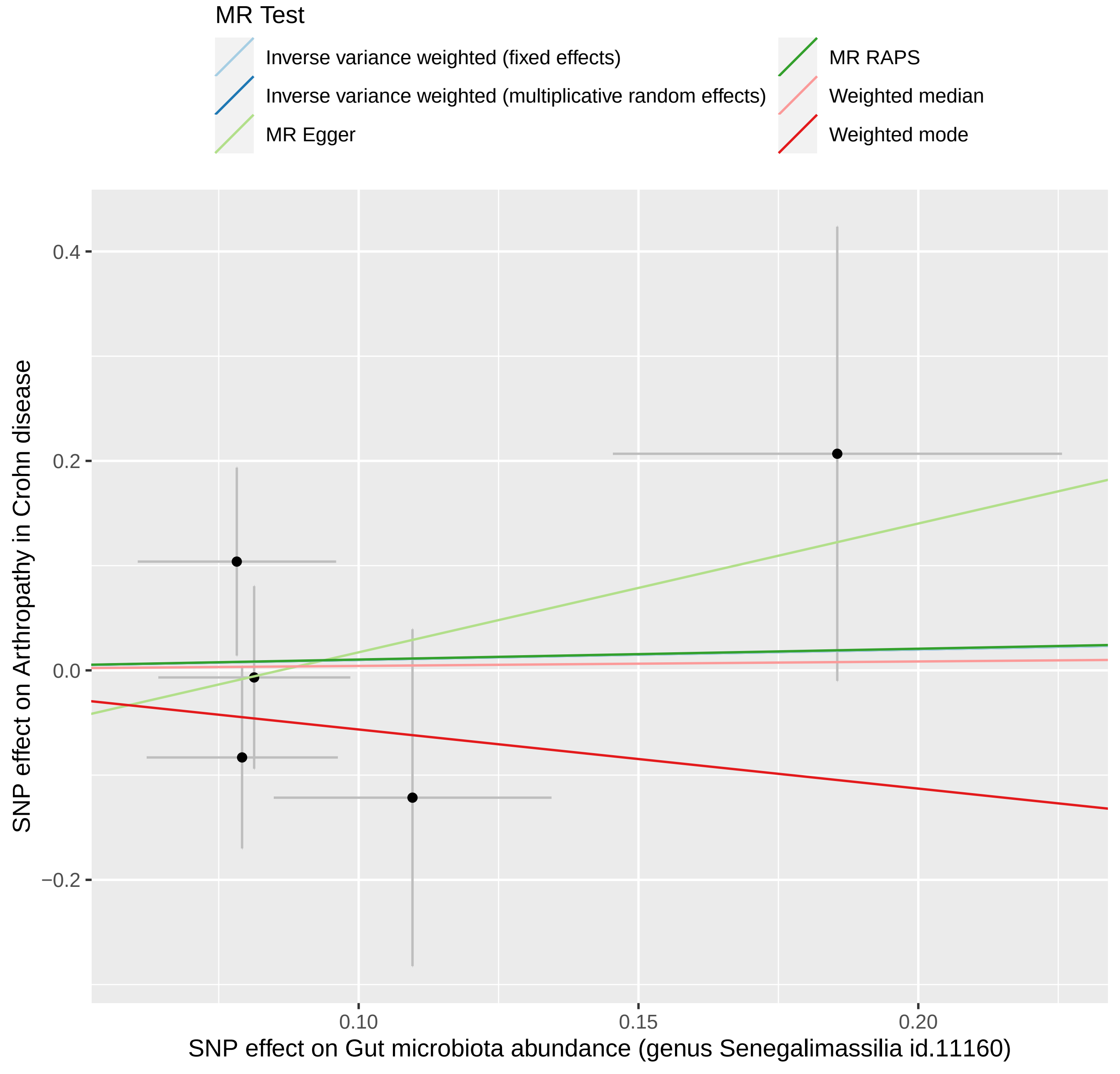

Batch 593 : Gut microbiota abundance (genus Slackia id.825) on Arthropathy in Crohn disease

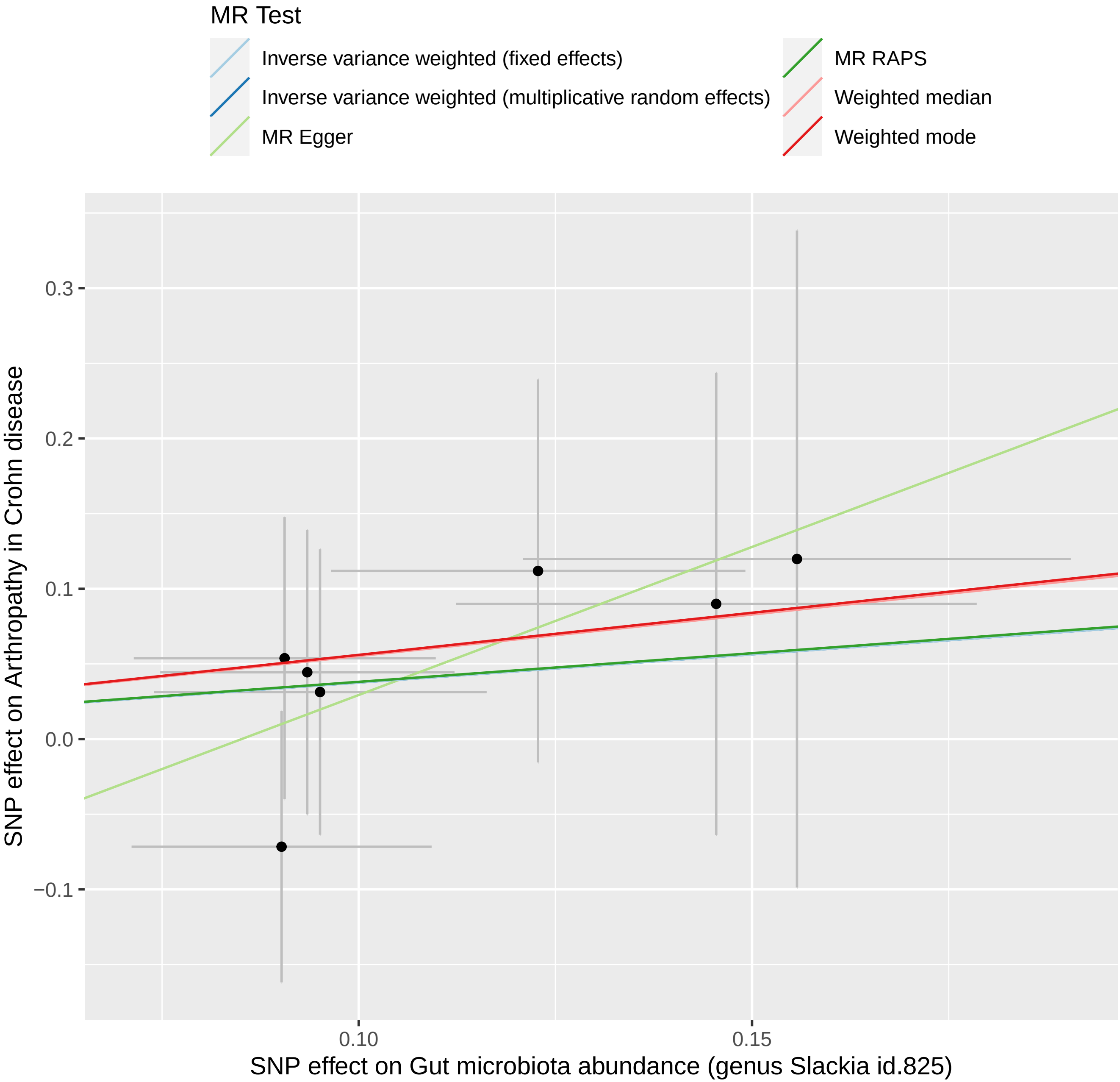

Batch 594 : Gut microbiota abundance (genus Streptococcus id.1853) on Arthropathy in Crohn disease

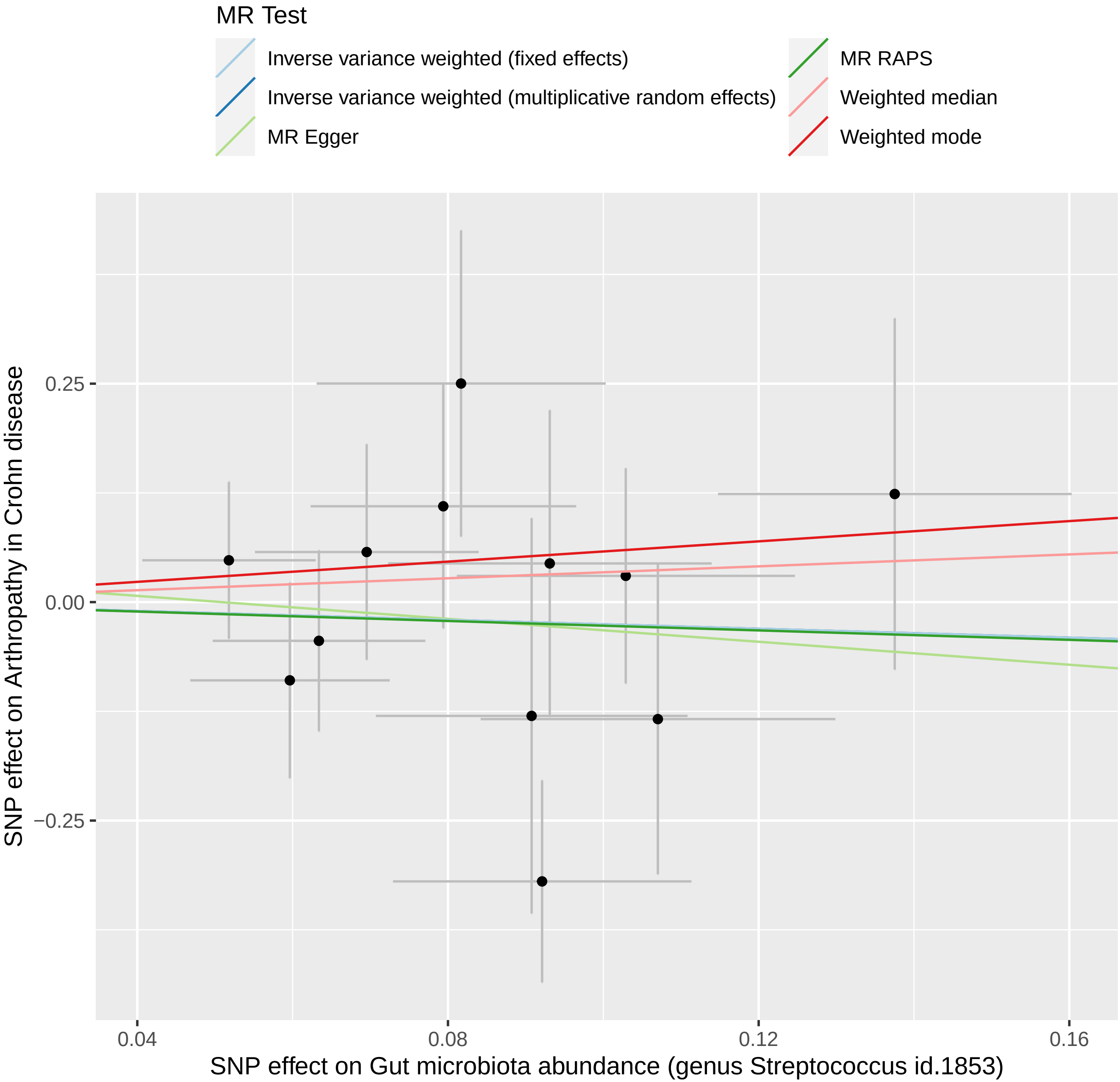

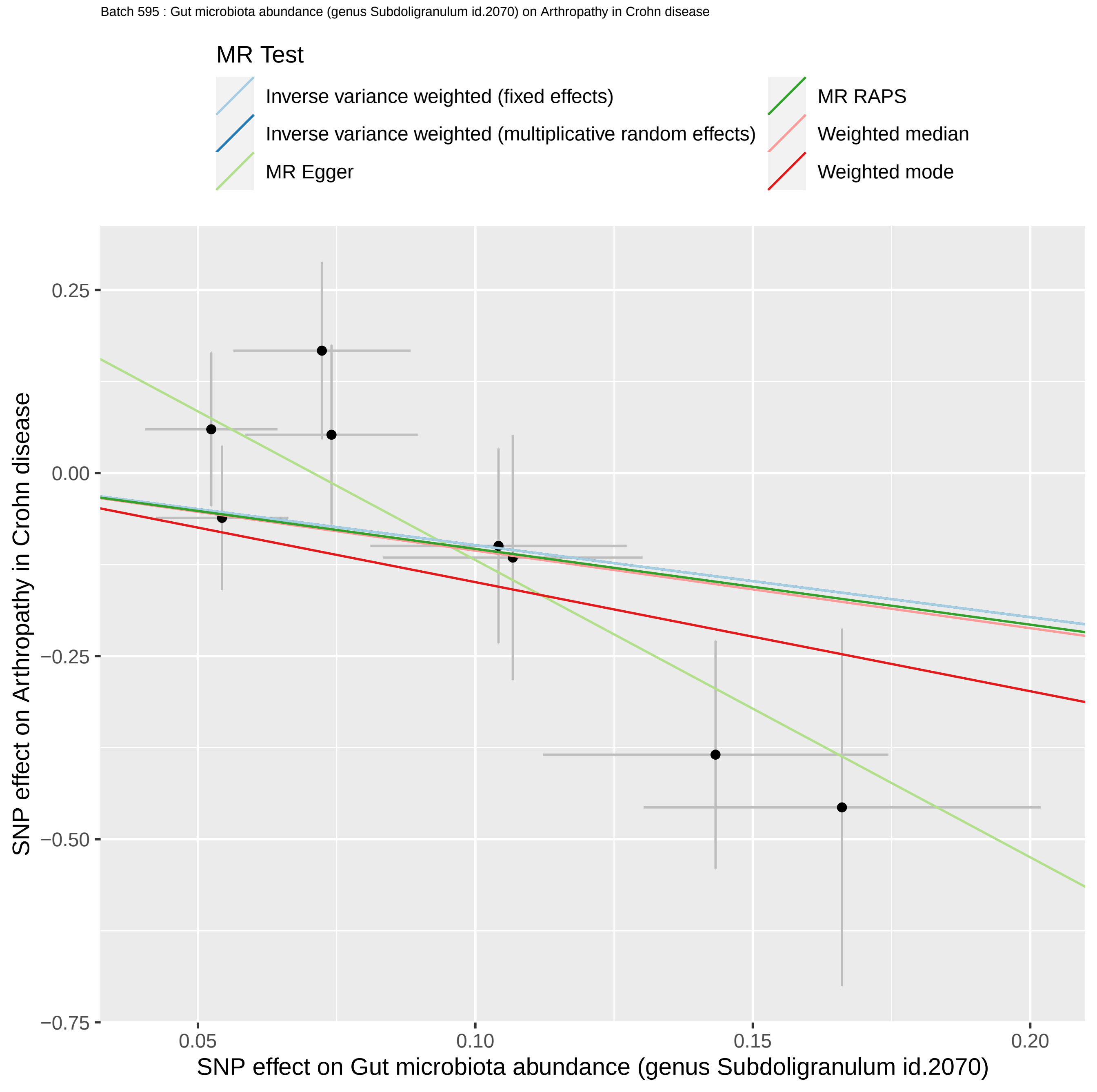

Batch 595 : Gut microbiota abundance (genus Subdoligranulum id.2070) on Arthropathy in Crohn disease
MR Test
Inverse variance weighted (fixed effects)
Inverse variance weighted (multiplicative random effects)
MR Egger
MR RAPS
Weighted median
Weighted mode
0.25
0.00
−0.25
−0.50
−0.75
0.05
0.10
0.15
0.20
SNP effect on Arthropathy in Crohn disease
SNP effect on Gut microbiota abundance (genus Subdoligranulum id.2070)

Batch 596 : Gut microbiota abundance (genus Sutterella id.2896) on Arthropathy in Crohn disease

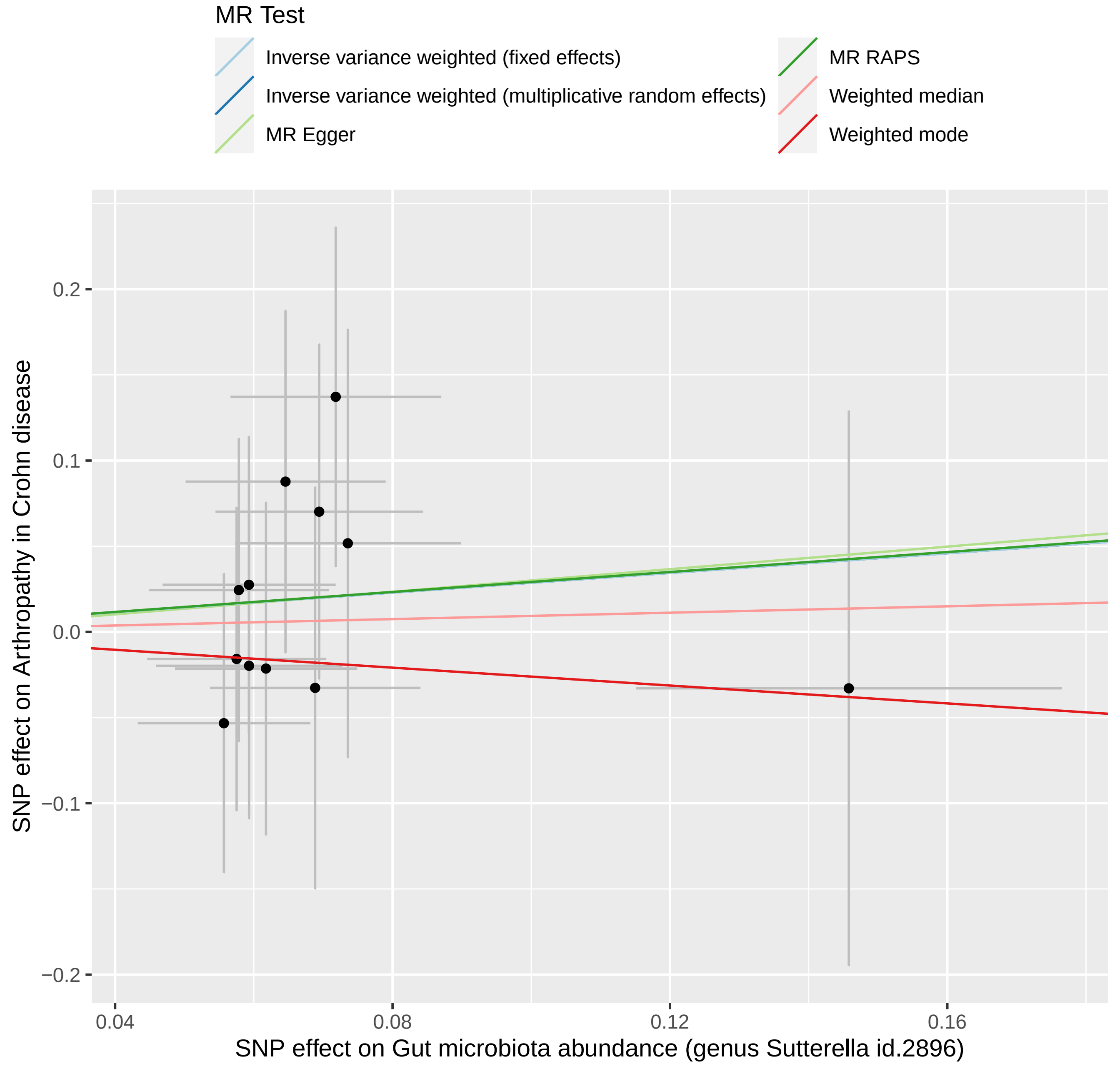

Batch 597 : Gut microbiota abundance (genus Terrisporobacter id.11348) on Arthropathy in Crohn disease

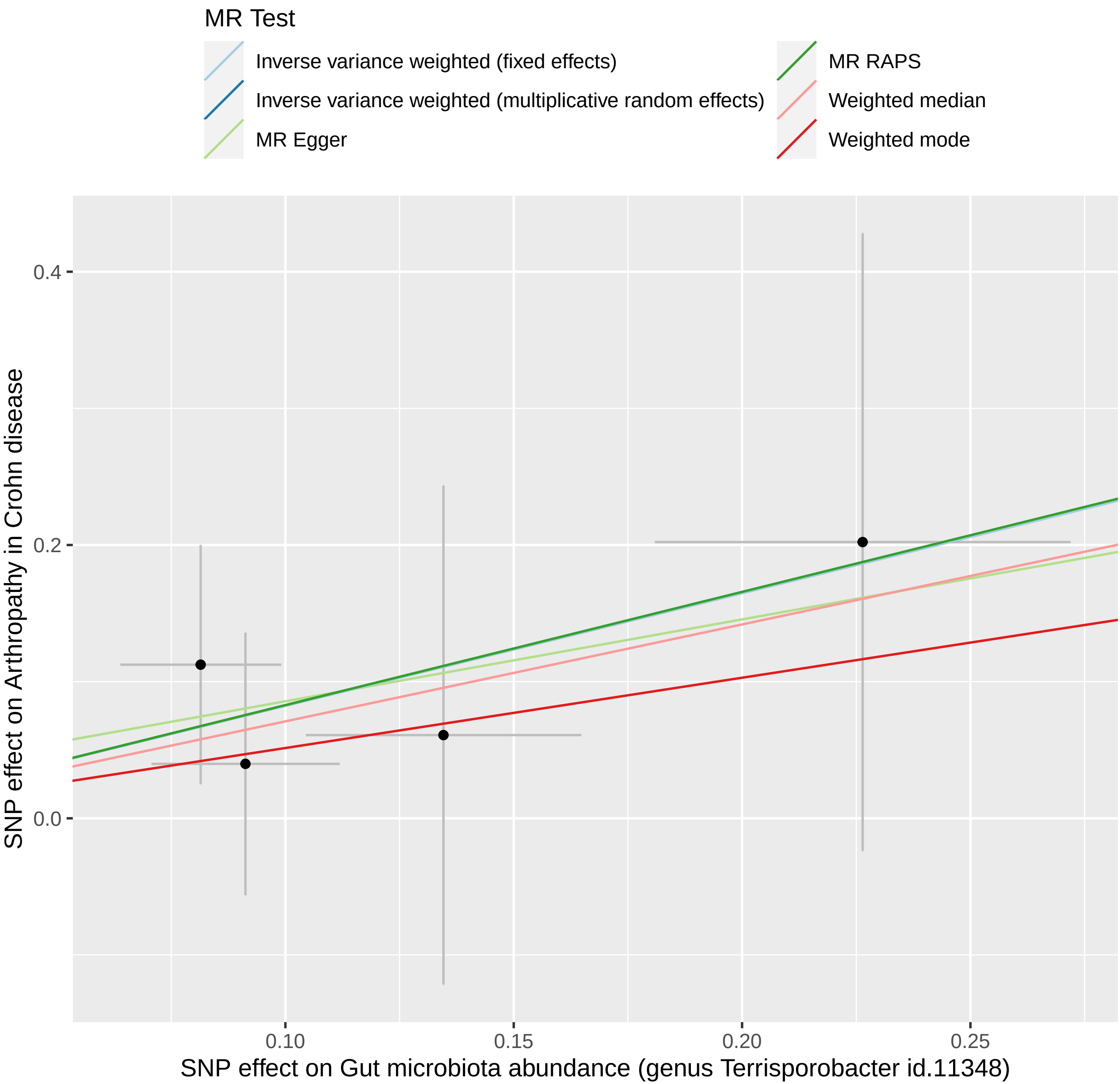

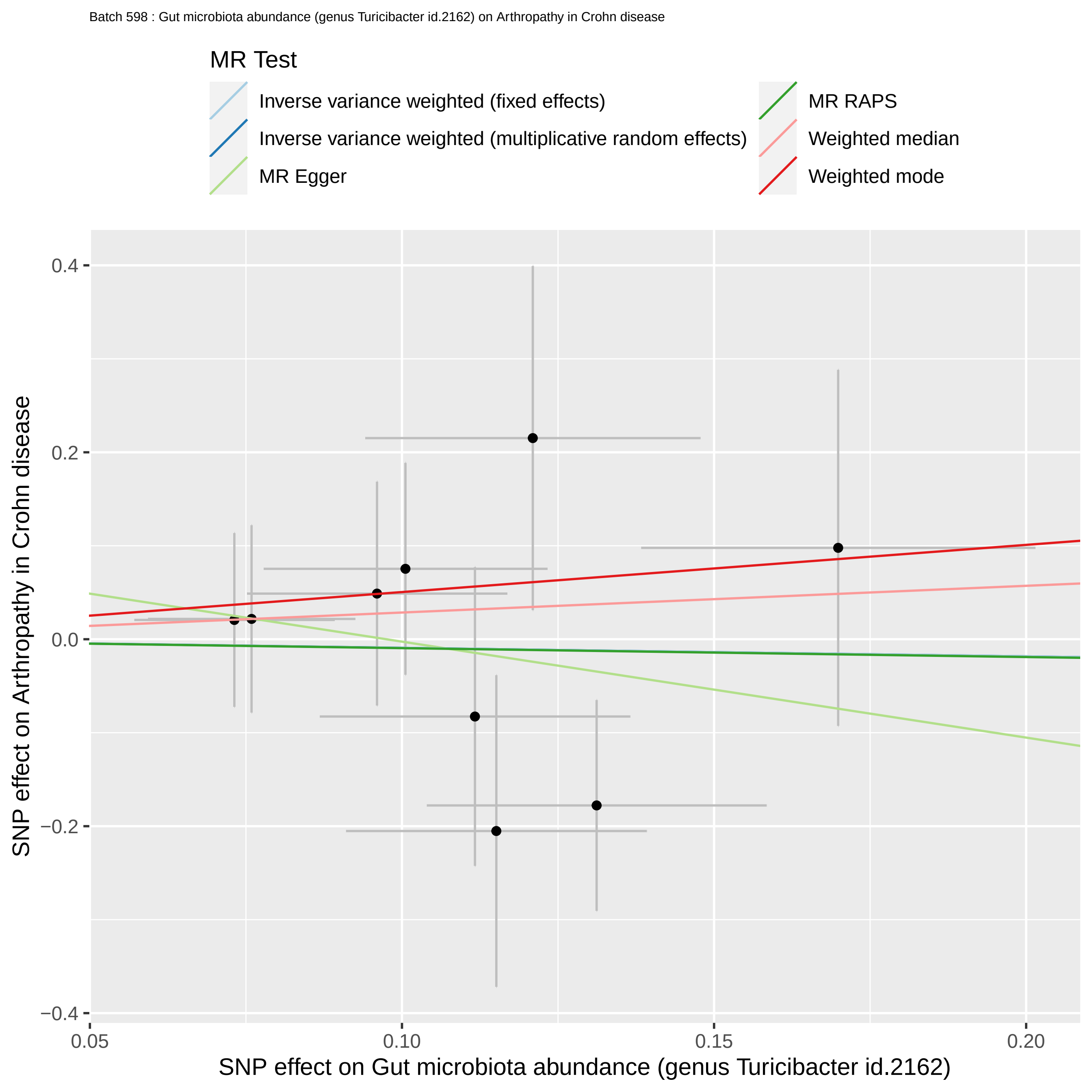

Batch 598 : Gut microbiota abundance (genus Turicibacter id.2162) on Arthropathy in Crohn disease
MR Test
Inverse variance weighted (fixed effects)
Inverse variance weighted (multiplicative random effects)
MR Egger
MR RAPS
Weighted median
Weighted mode
SNP effect on Arthropathy in Crohn disease
0.4
0.2
0.0
−0.2
−0.4
0.05
0.10
0.15
0.20
SNP effect on Gut microbiota abundance (genus Turicibacter id.2162)

Batch 599 : Gut microbiota abundance (genus Tyzzerella3 id.11335) on Arthropathy in Crohn disease

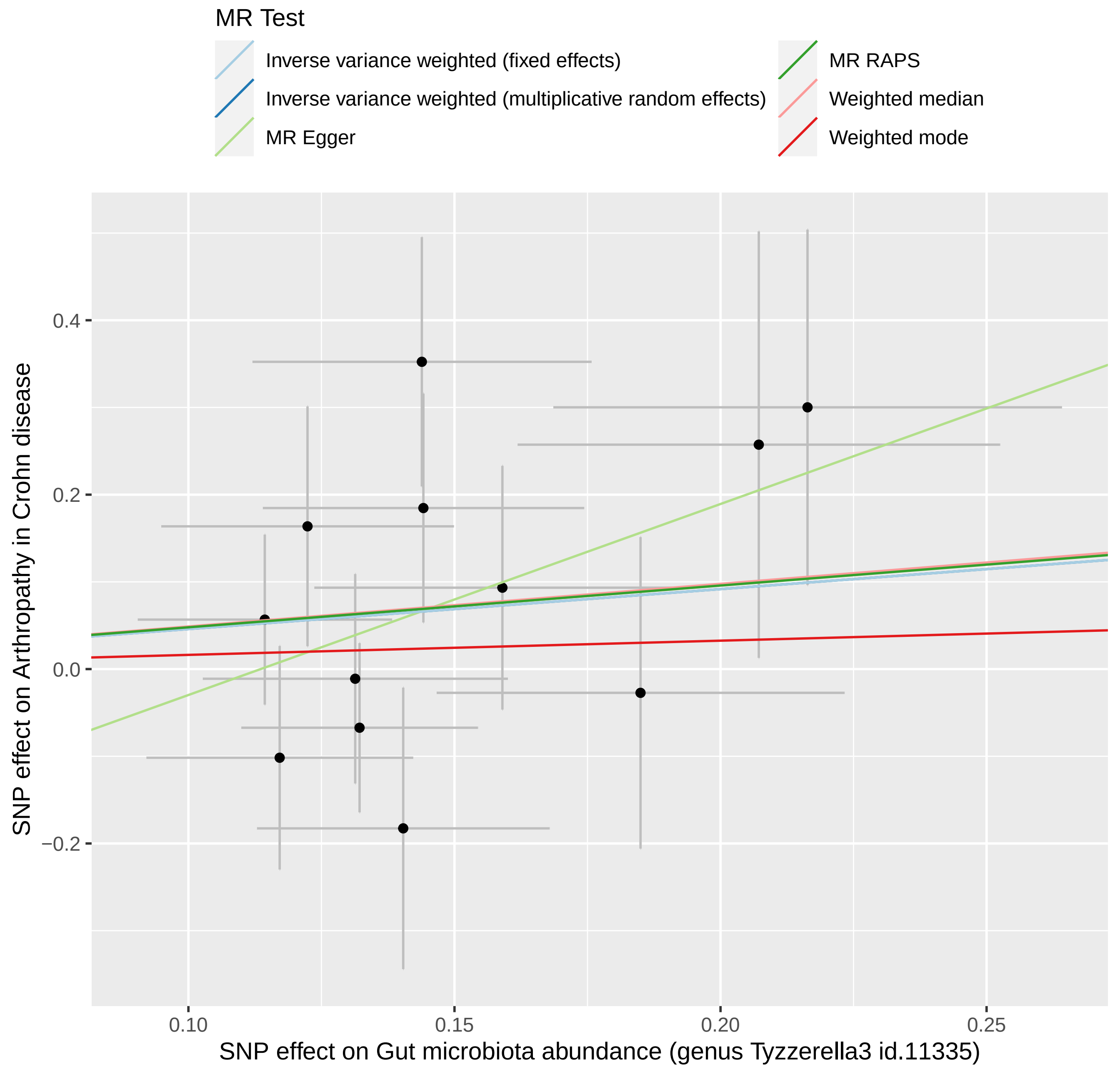

Batch 600 : Gut microbiota abundance (genus Veillonella id.2198) on Arthropathy in Crohn disease

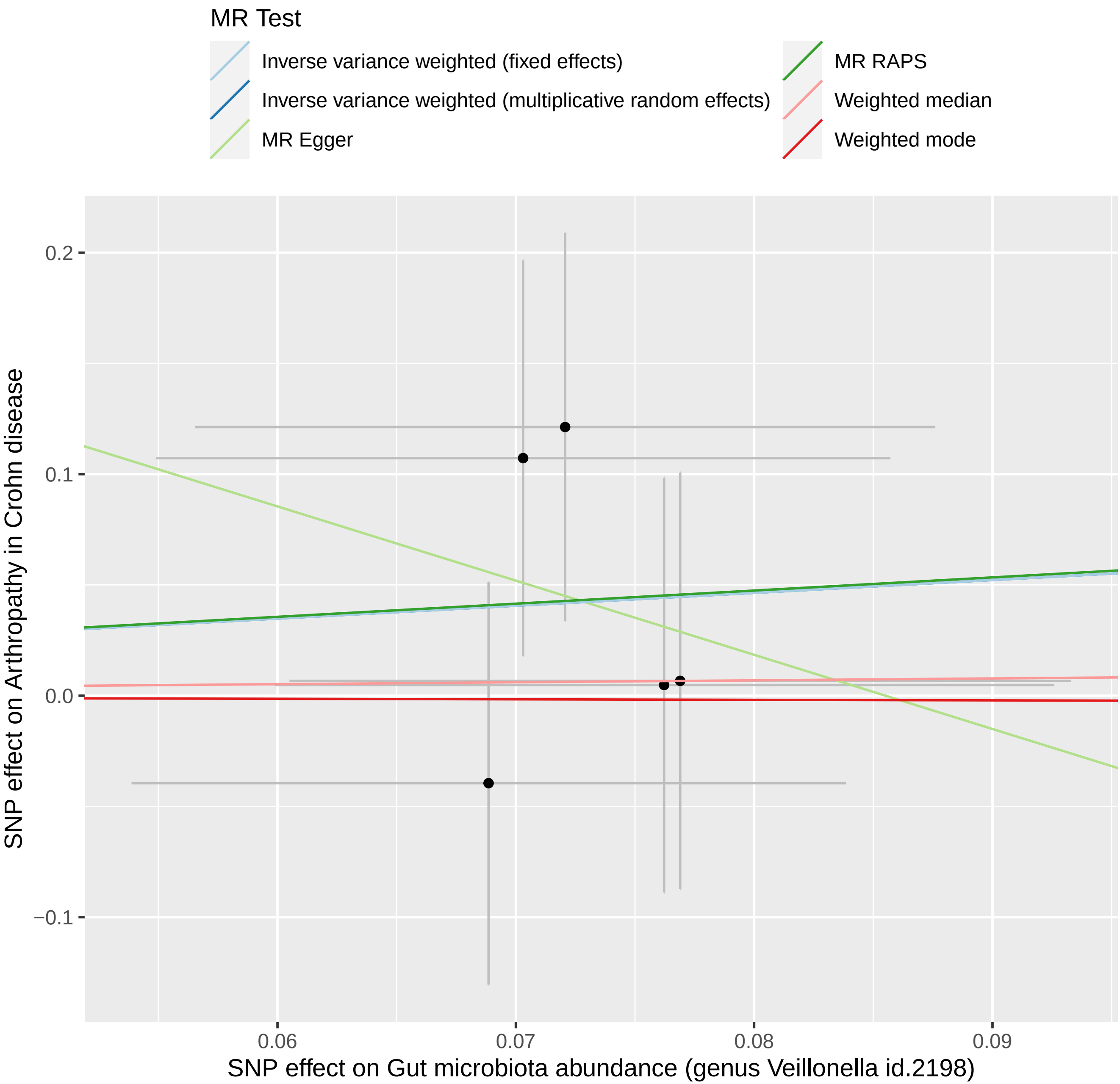

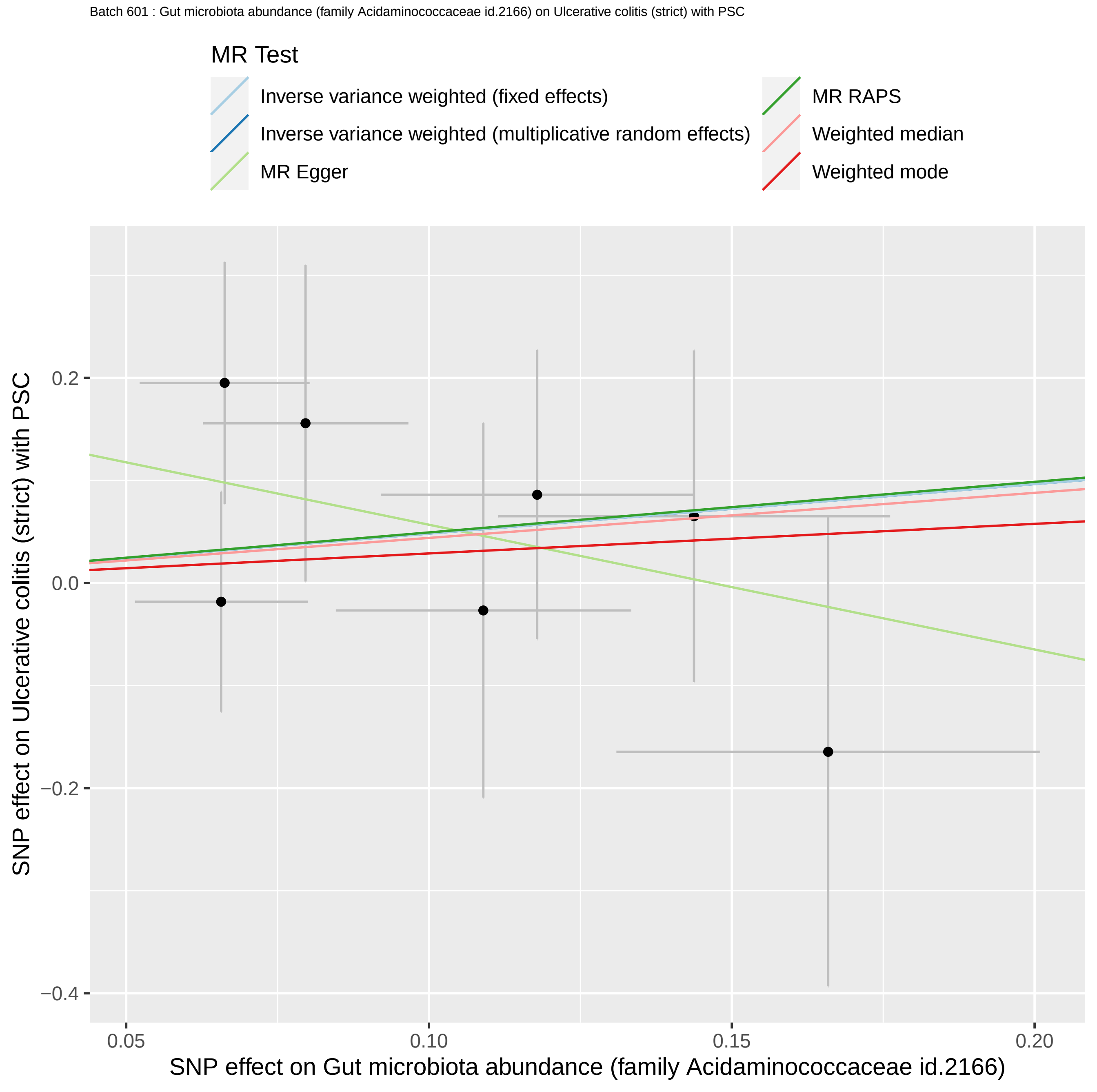

Batch 601 : Gut microbiota abundance (family Acidaminococcaceae id.2166) on Ulcerative colitis (strict) with PSC
MR Test
Inverse variance weighted (fixed effects)
Inverse variance weighted (multiplicative random effects)
MR Egger
MR RAPS
Weighted median
Weighted mode
0.2
0.0
−0.2
−0.4
0.05
0.10
0.15
0.20
SNP effect on Ulcerative colitis (strict) with PSC
SNP effect on Gut microbiota abundance (family Acidaminococcaceae id.2166)

Batch 602 : Gut microbiota abundance (family Actinomycetaceae id.421) on Ulcerative colitis (strict) with PSC

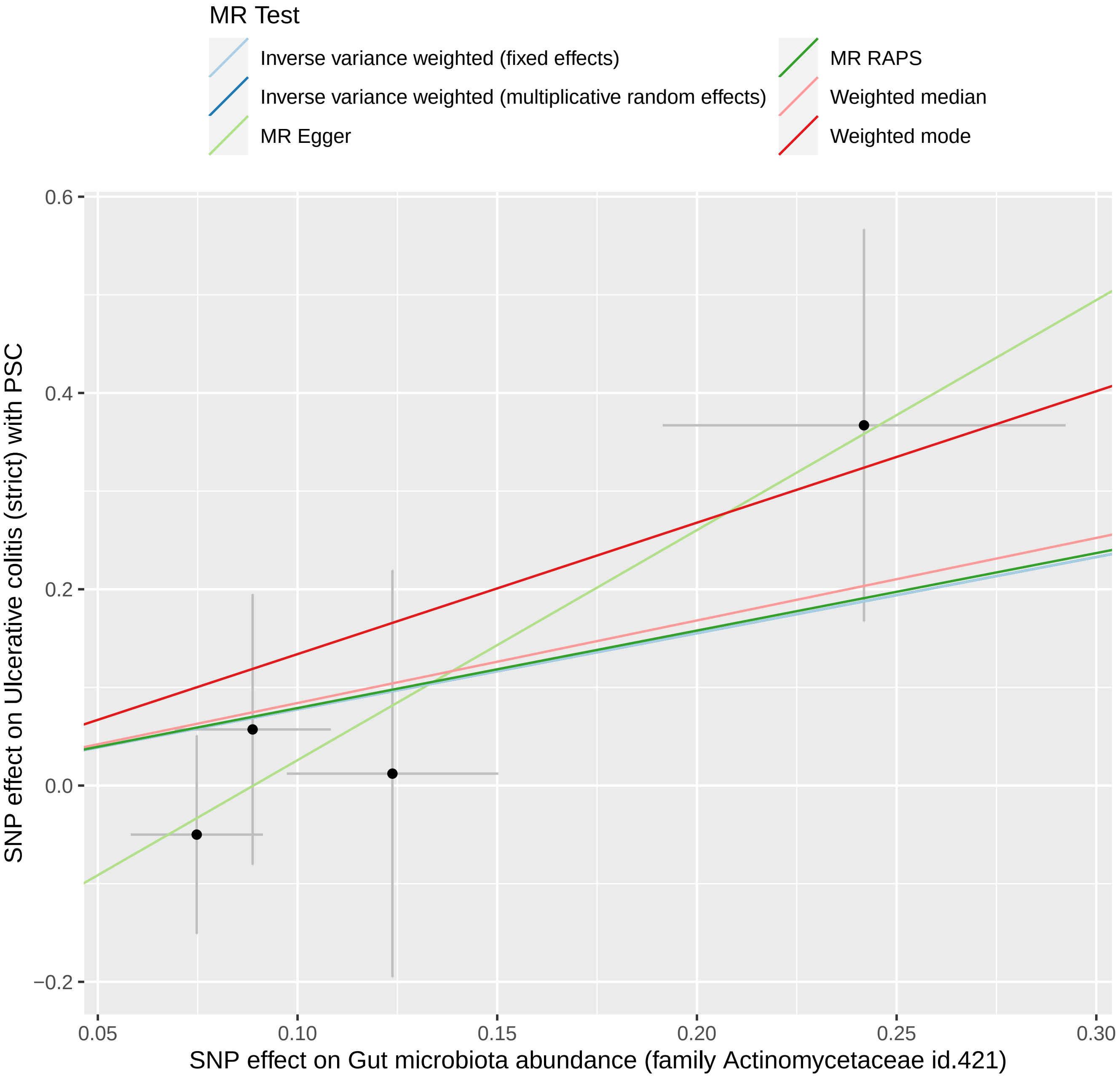

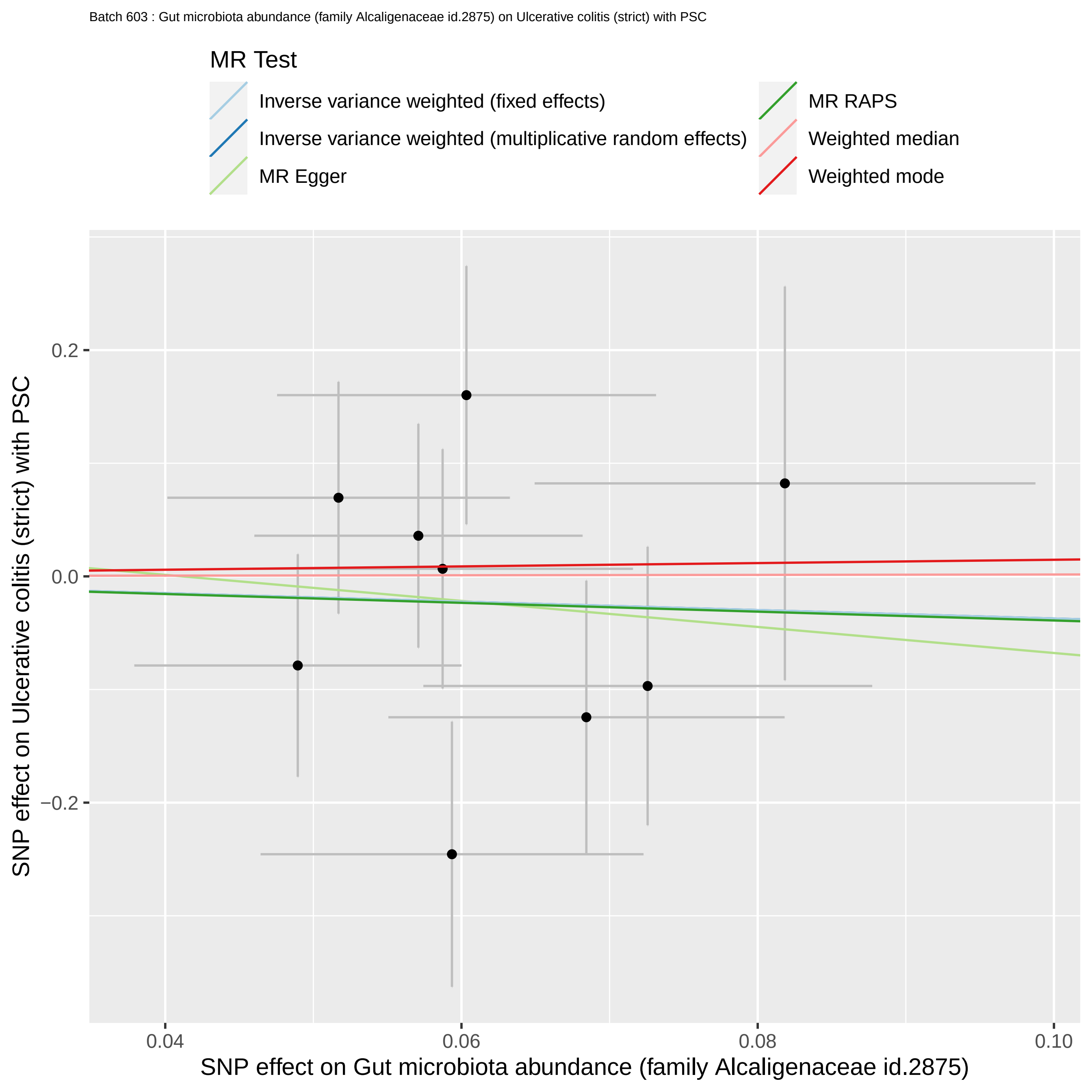

Batch 603 : Gut microbiota abundance (family Alcaligenaceae id.2875) on Ulcerative colitis (strict) with PSC
MR Test
Inverse variance weighted (fixed effects)
Inverse variance weighted (multiplicative random effects)
MR Egger
MR RAPS
Weighted median
Weighted mode
SNP effect on Ulcerative colitis (strict) with PSC
0.2
0.0
−0.2
0.04
0.06
0.08
0.10
SNP effect on Gut microbiota abundance (family Alcaligenaceae id.2875)

Batch 604 : Gut microbiota abundance (family Bacteroidaceae id.917) on Ulcerative colitis (strict) with PSC

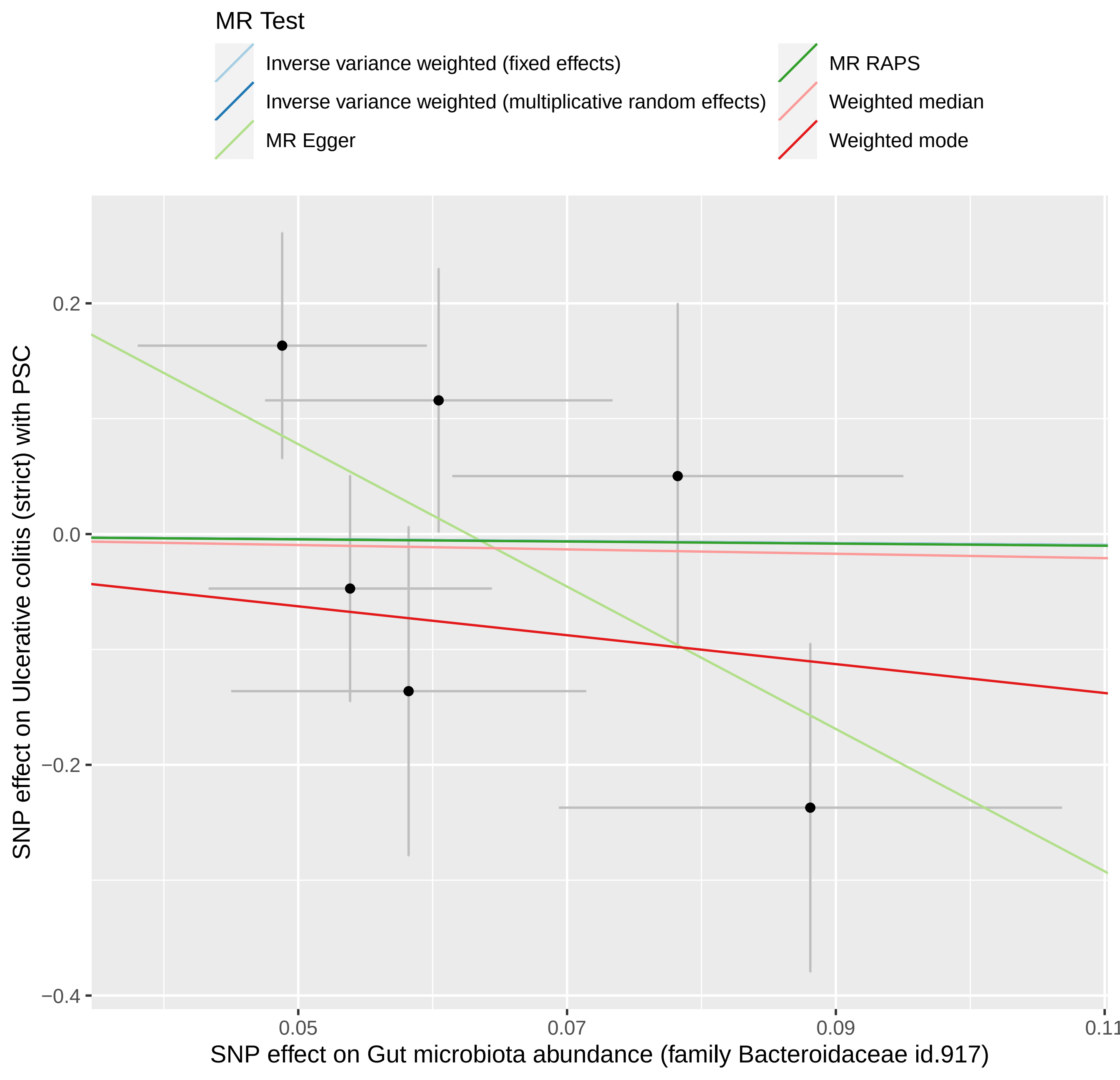

Batch 605 : Gut microbiota abundance (family Bacteroidales S24 7group id.11173) on Ulcerative colitis (strict) with PSC

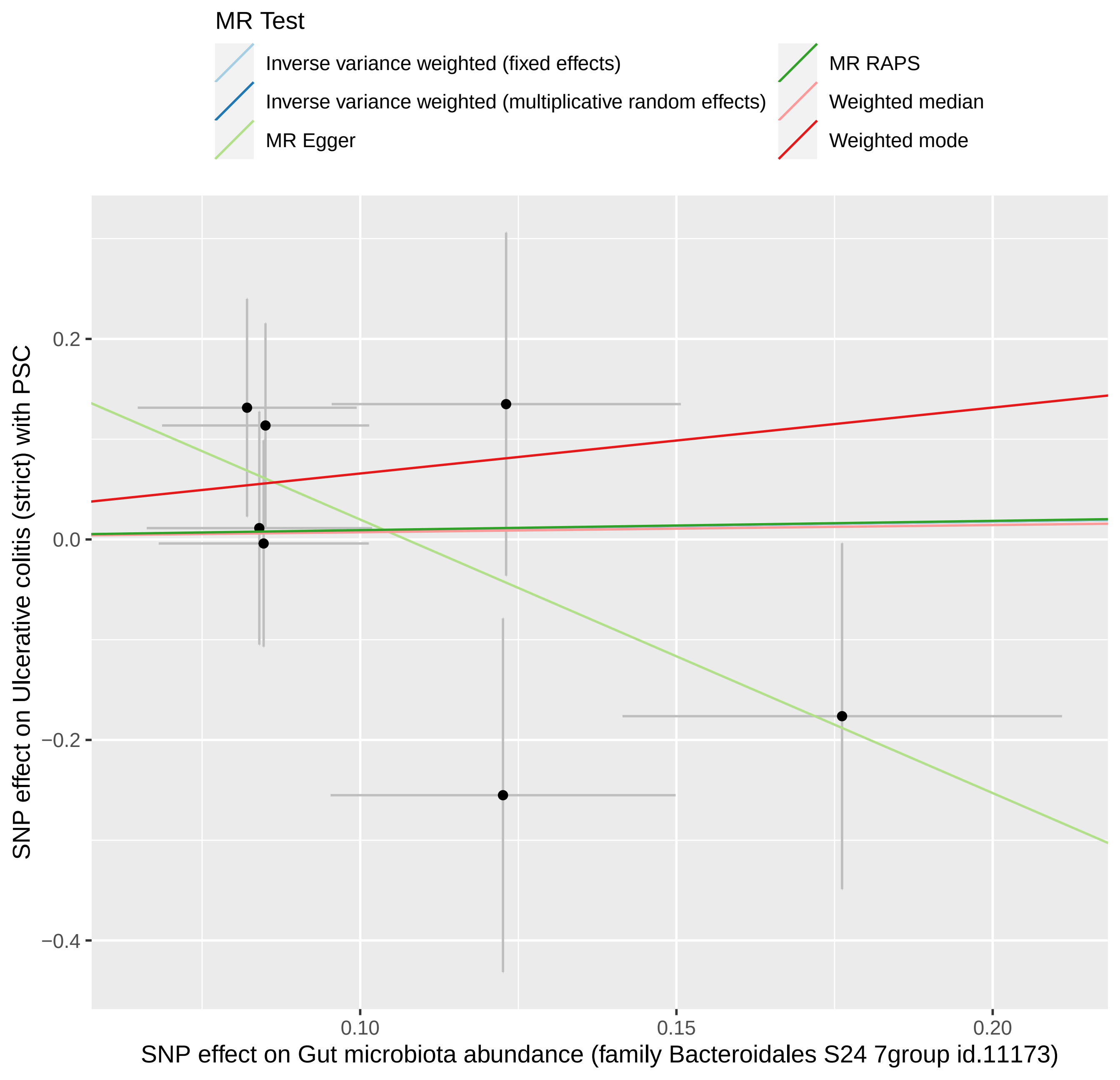

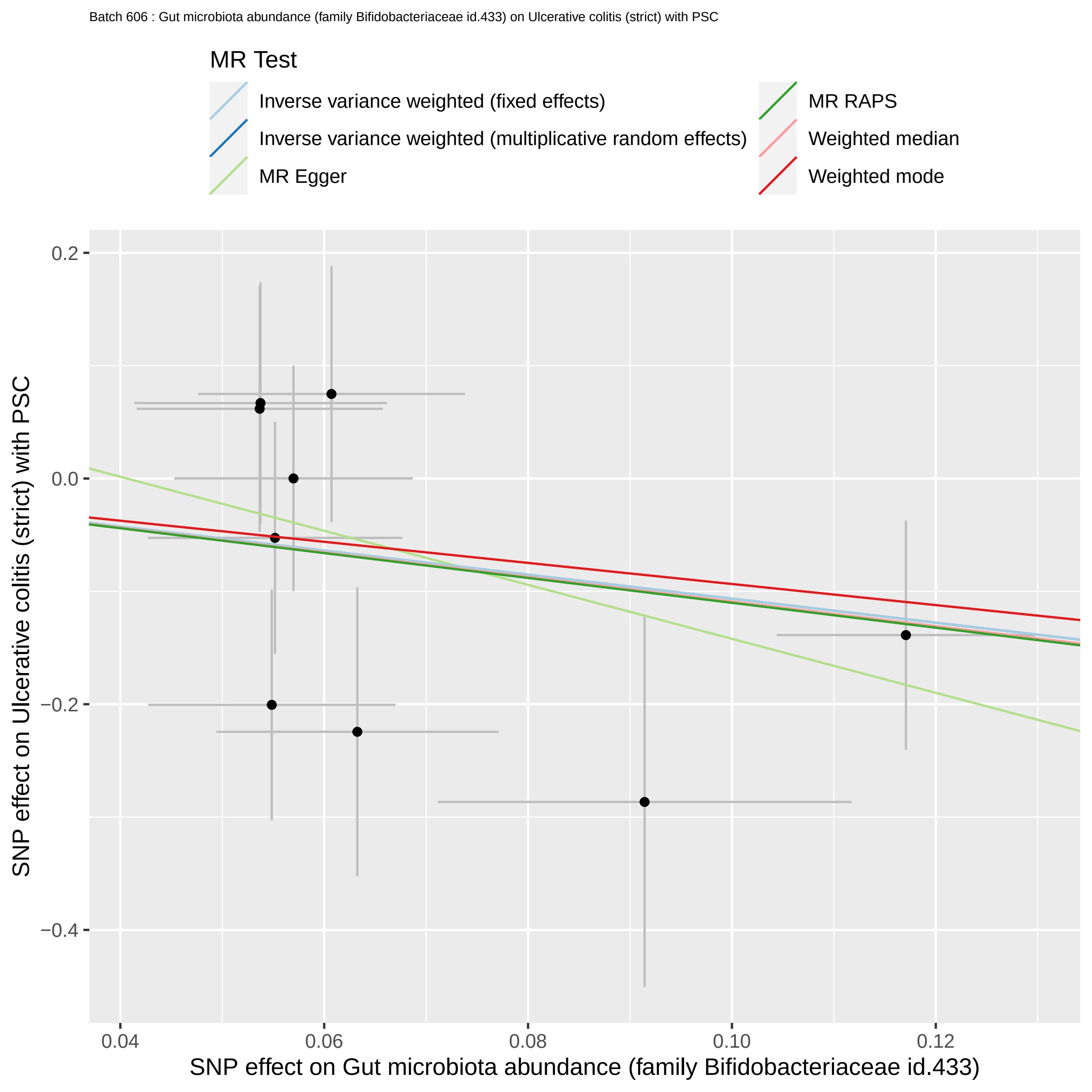
Batch 606 : Gut microbiota abundance (family Bifidobacteriaceae id.433) on Ulcerative colitis (strict) with PSC
MR Test
Inverse variance weighted (fixed effects)
Inverse variance weighted (multiplicative random effects)
MR Egger
MR RAPS
Weighted median
Weighted mode
0.2
0.0
−0.2
−0.4
0.04
0.06
0.08
0.10
0.12
SNP effect on Ulcerative colitis (strict) with PSC
SNP effect on Gut microbiota abundance (family Bifidobacteriaceae id.433)

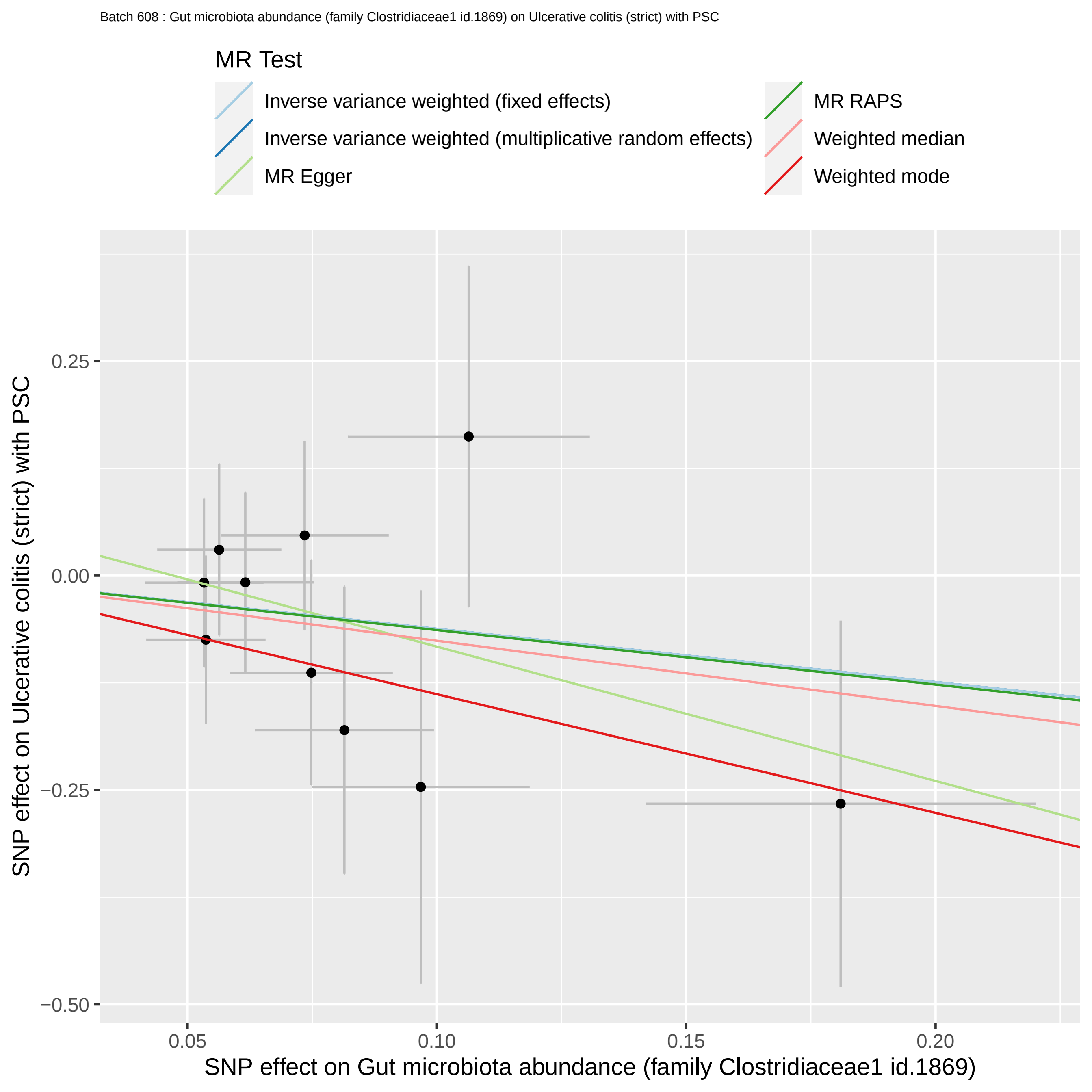
Batch 608 : Gut microbiota abundance (family Clostridiaceae1 id.1869) on Ulcerative colitis (strict) with PSC
MR Test
Inverse variance weighted (fixed effects)
Inverse variance weighted (multiplicative random effects)
MR Egger
MR RAPS
Weighted median
Weighted mode
SNP effect on Ulcerative colitis (strict) with PSC
0.25
0.00
−0.25
−0.50
0.05
0.10
0.15
0.20
SNP effect on Gut microbiota abundance (family Clostridiaceae1 id.1869)

Batch 609 : Gut microbiota abundance (family Clostridiales vadin BB60 group id.11286) on Ulcerative colitis (strict) with PSC

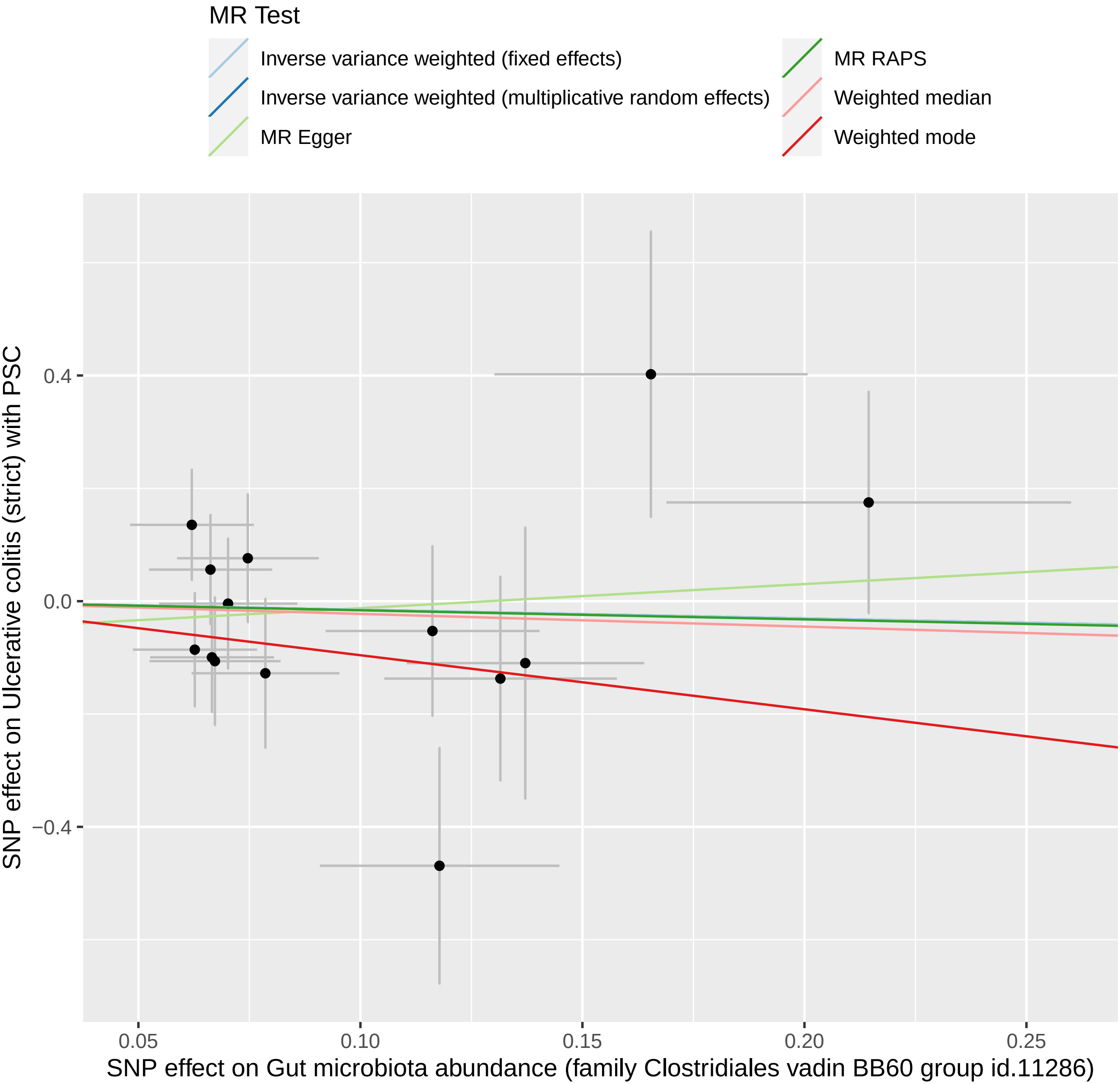

Batch 610 : Gut microbiota abundance (family Coriobacteriaceae id.811) on Ulcerative colitis (strict) with PSC

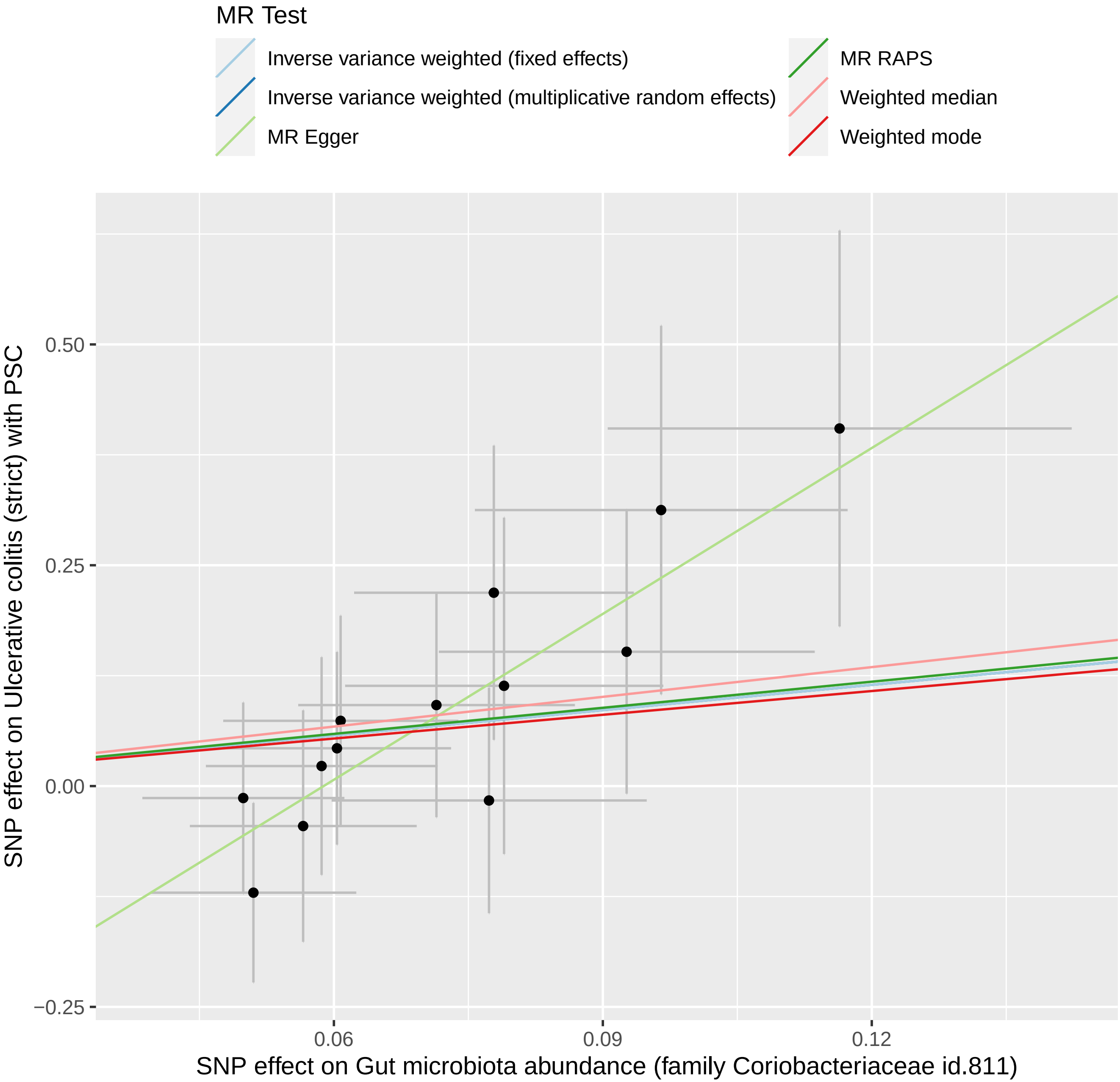

Batch 611 : Gut microbiota abundance (family Defluviitaleaceae id.1924) on Ulcerative colitis (strict) with PSC

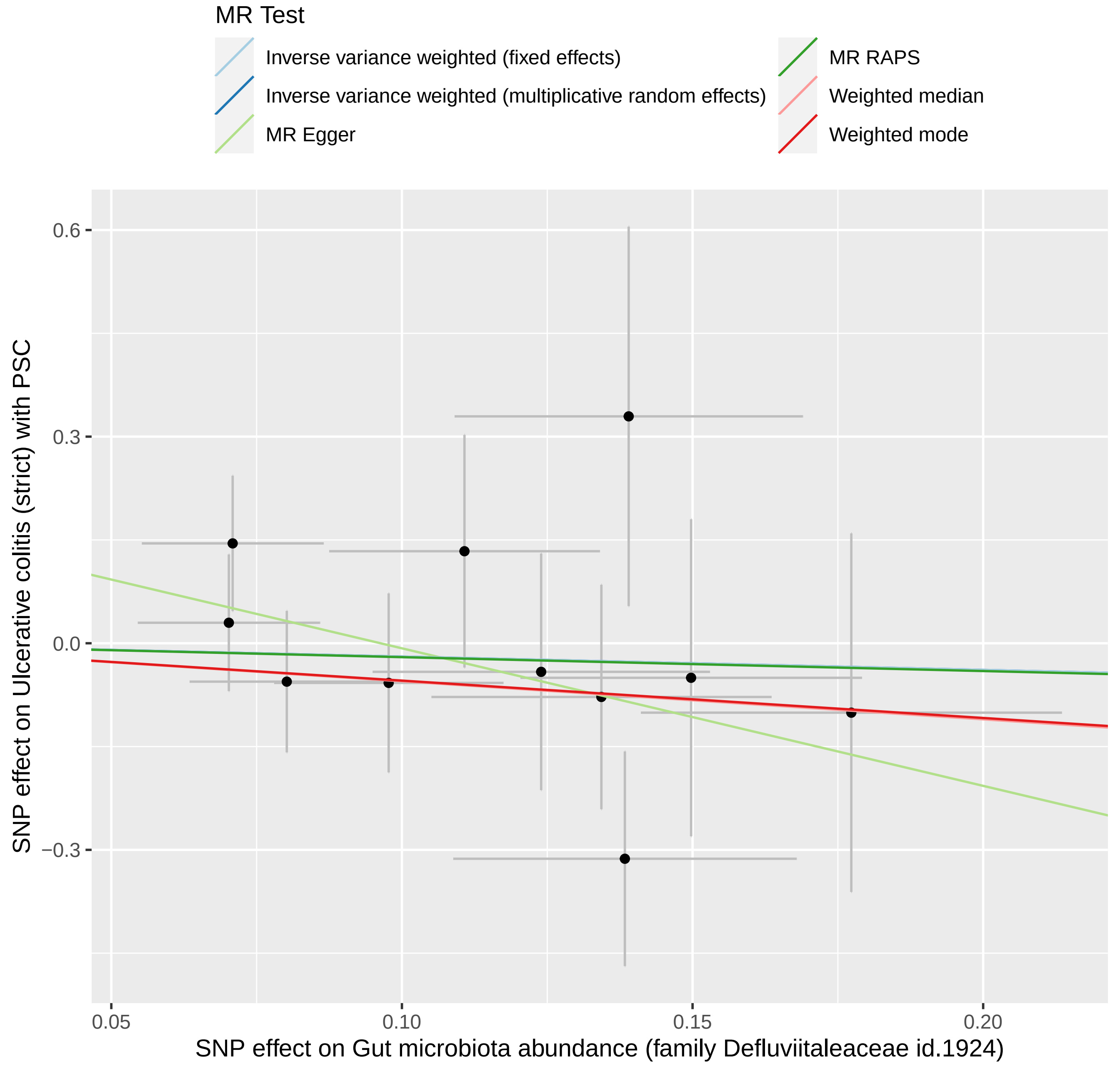

Batch 612 : Gut microbiota abundance (family Desulfovibrionaceae id.3169) on Ulcerative colitis (strict) with PSC

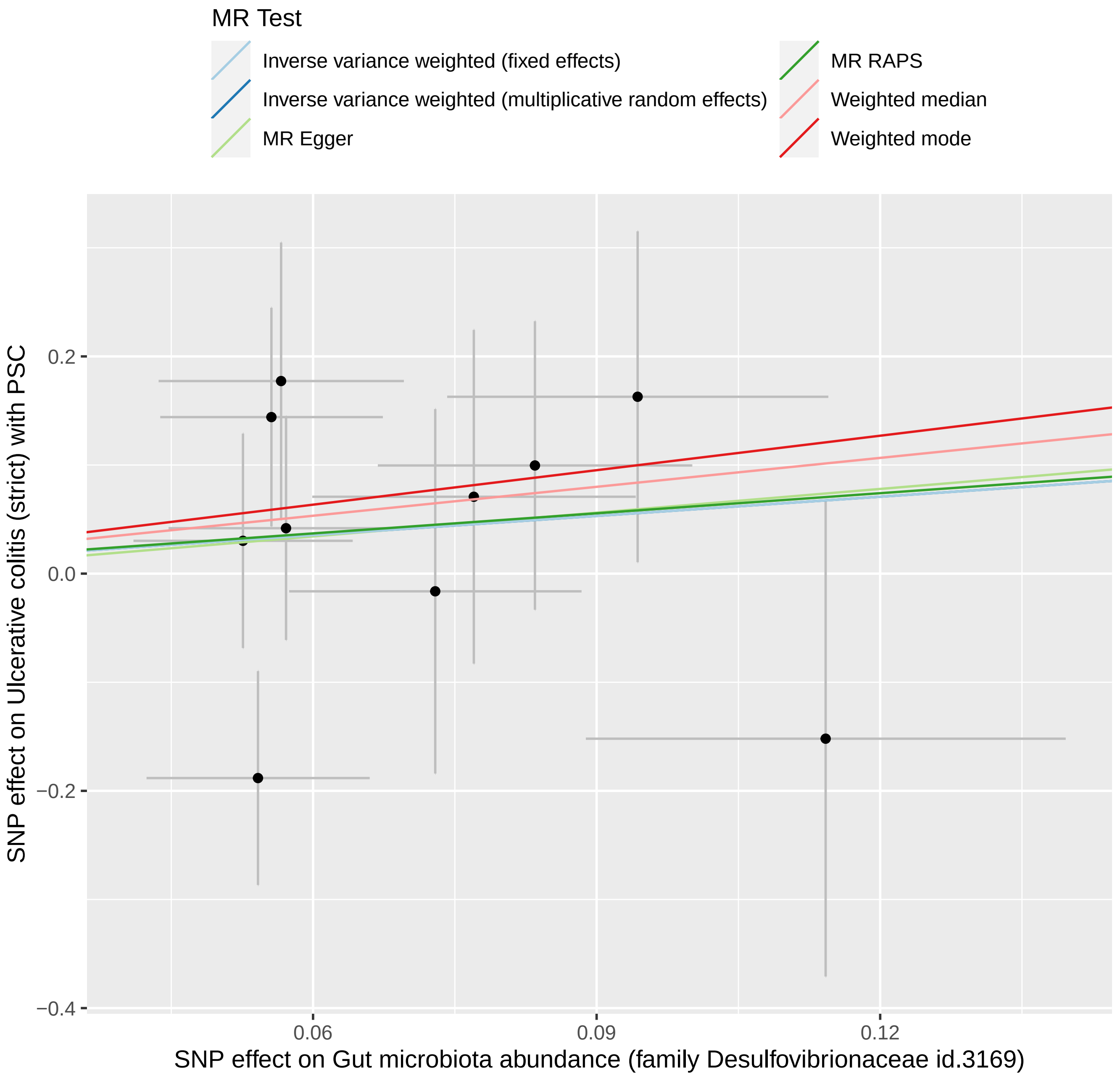

Batch 613 : Gut microbiota abundance (family Enterobacteriaceae id.3469) on Ulcerative colitis (strict) with PSC

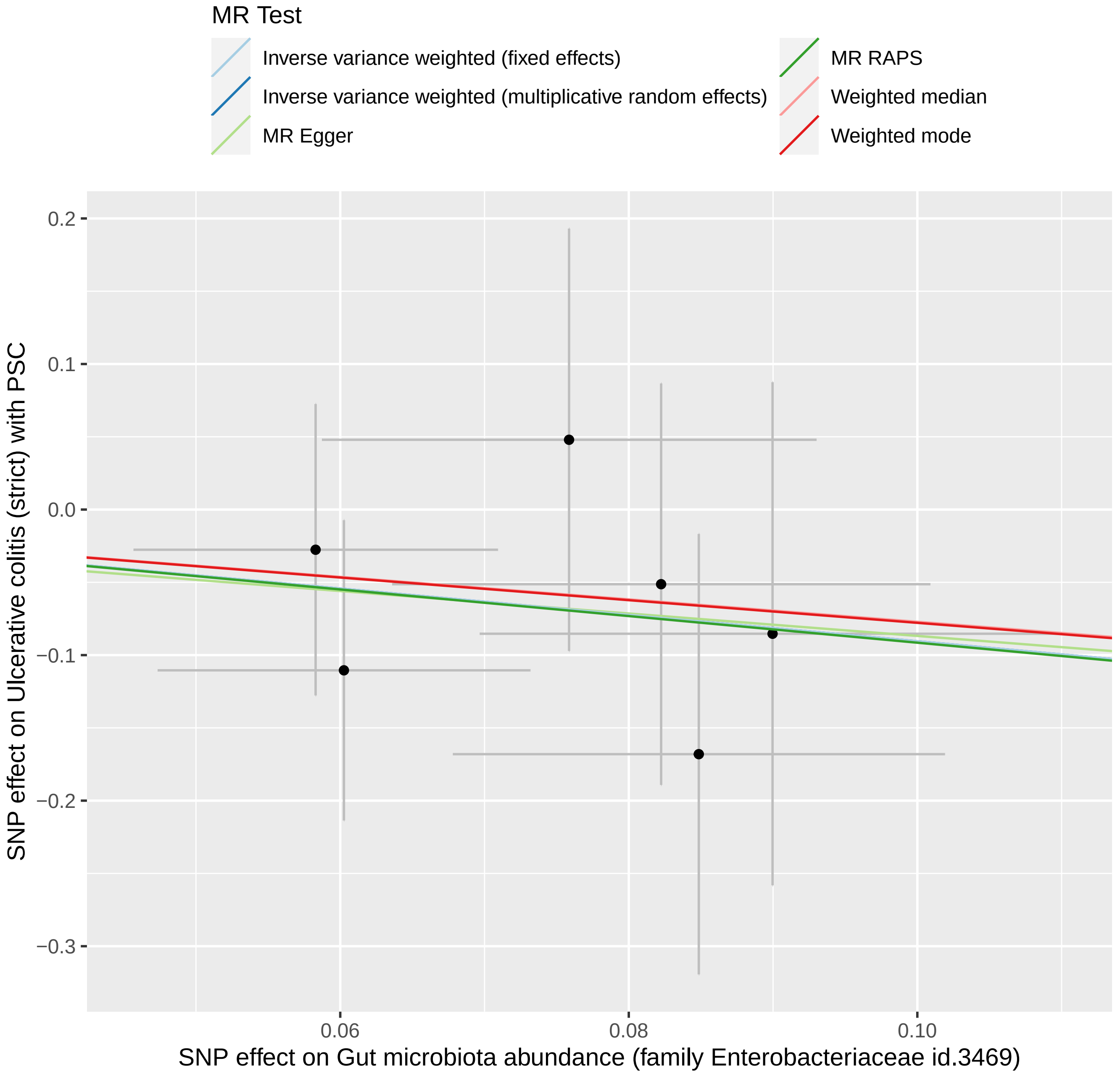

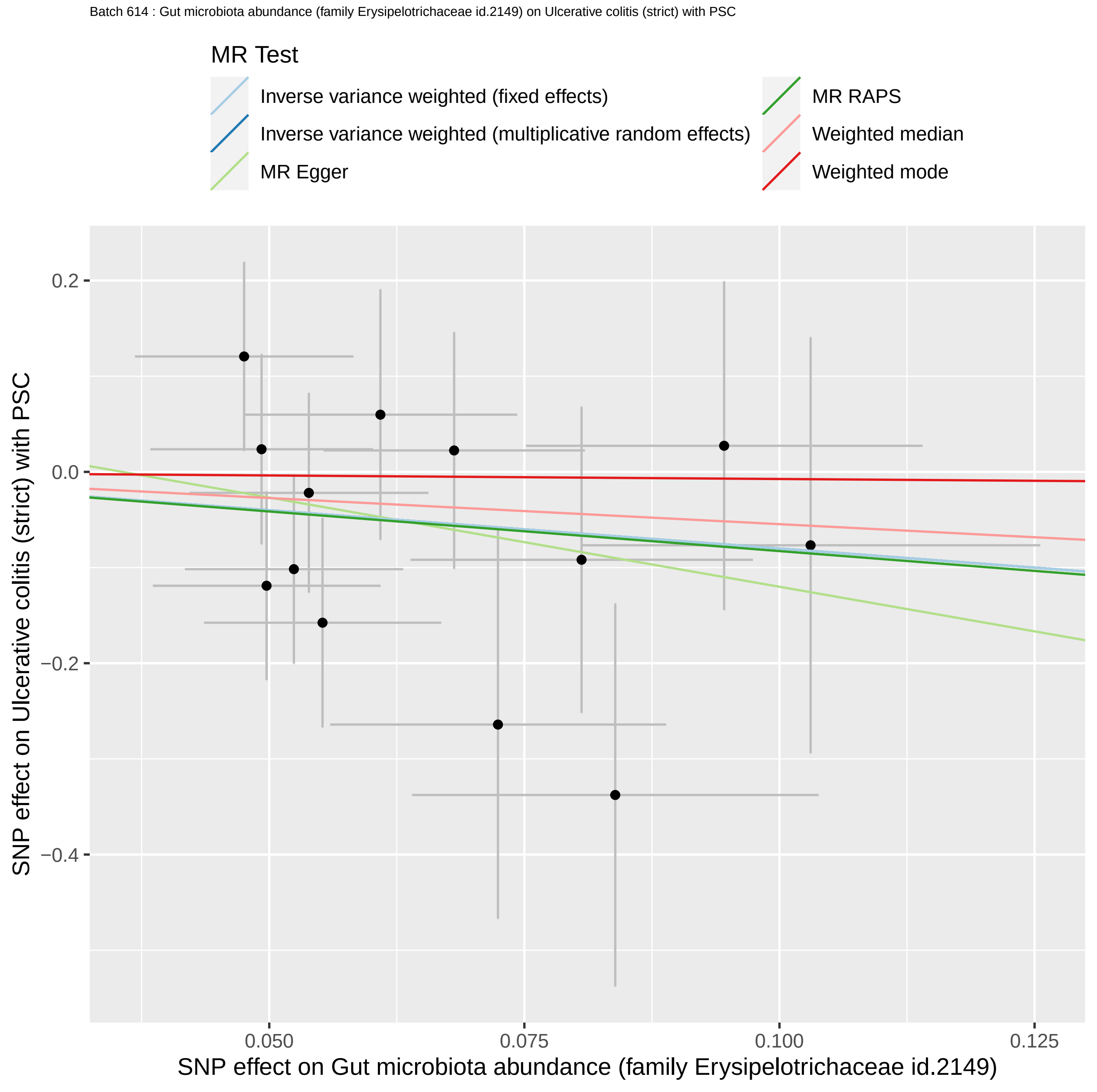
Batch 614 : Gut microbiota abundance (family Erysipelotrichaceae id.2149) on Ulcerative colitis (strict) with PSC
MR Test
Inverse variance weighted (fixed effects)
Inverse variance weighted (multiplicative random effects)
MR Egger
MR RAPS
Weighted median
Weighted mode
0.2
0.0
−0.2
−0.4
0.050
0.075
0.100
0.125
SNP effect on Ulcerative colitis (strict) with PSC
SNP effect on Gut microbiota abundance (family Erysipelotrichaceae id.2149)

Batch 615 : Gut microbiota abundance (family Family XI id.1936) on Ulcerative colitis (strict) with PSC

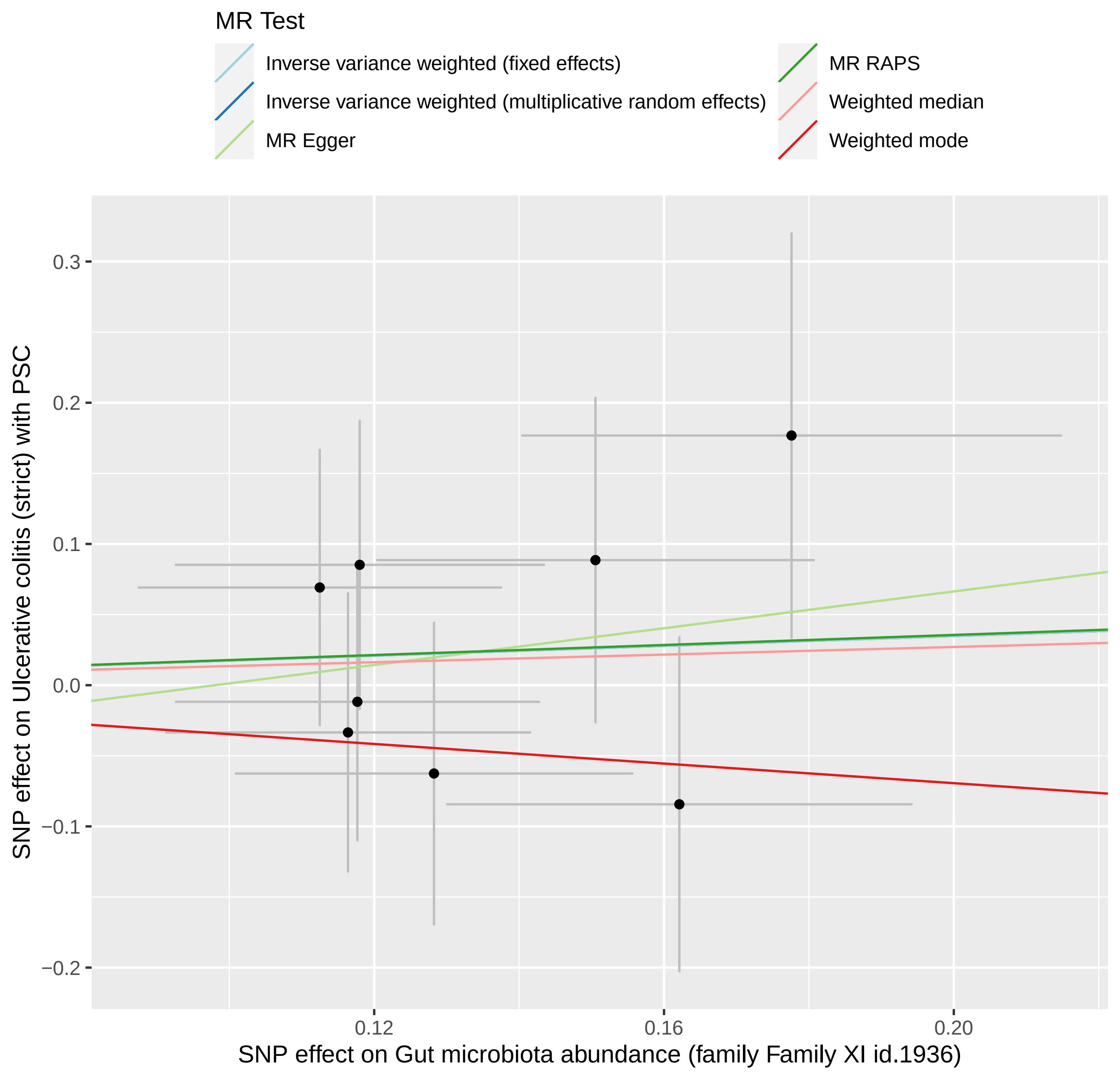

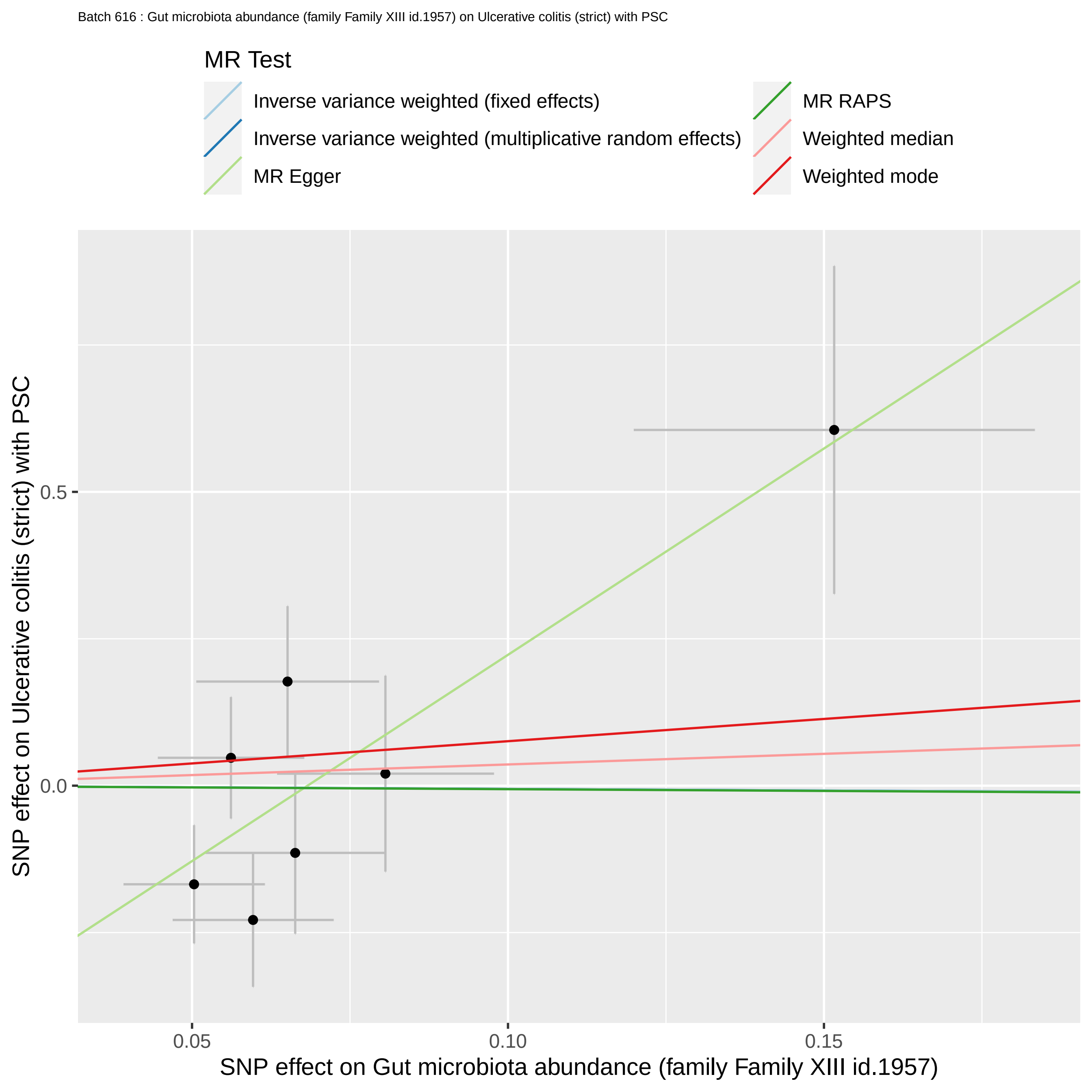

Batch 616 : Gut microbiota abundance (family Family XIII id.1957) on Ulcerative colitis (strict) with PSC
MR Test
Inverse variance weighted (fixed effects)
Inverse variance weighted (multiplicative random effects)
MR Egger
MR RAPS
Weighted median
Weighted mode
SNP effect on Ulcerative colitis (strict) with PSC
0.5
0.0
0.05
0.10
0.15
SNP effect on Gut microbiota abundance (family Family XIII id.1957)

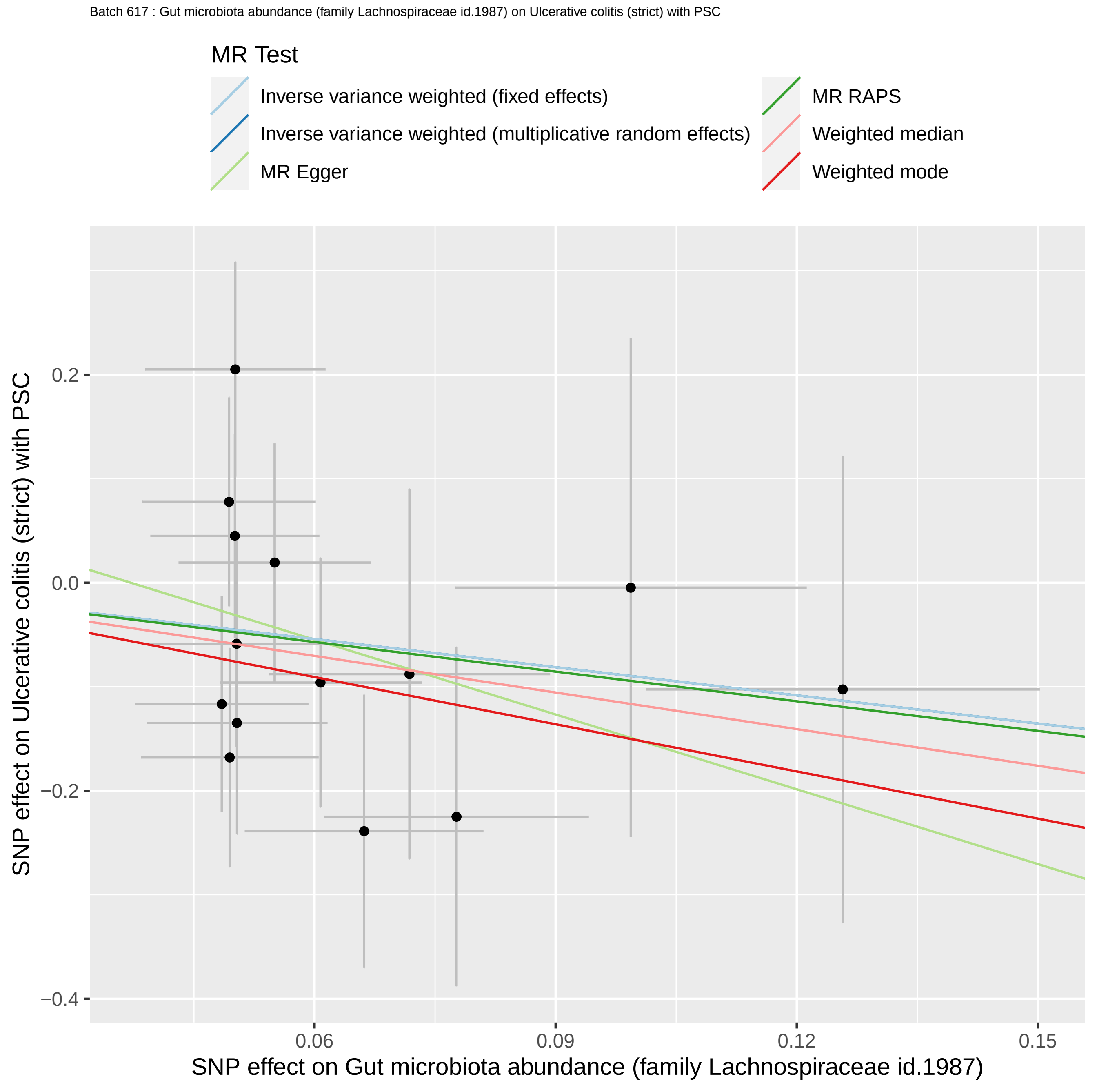

Batch 617 : Gut microbiota abundance (family Lachnospiraceae id.1987) on Ulcerative colitis (strict) with PSC
MR Test
Inverse variance weighted (fixed effects)
Inverse variance weighted (multiplicative random effects)
MR Egger
MR RAPS
Weighted median
Weighted mode
0.2
0.0
−0.2
−0.4
0.06
0.09
0.12
0.15
SNP effect on Ulcerative colitis (strict) with PSC
SNP effect on Gut microbiota abundance (family Lachnospiraceae id.1987)

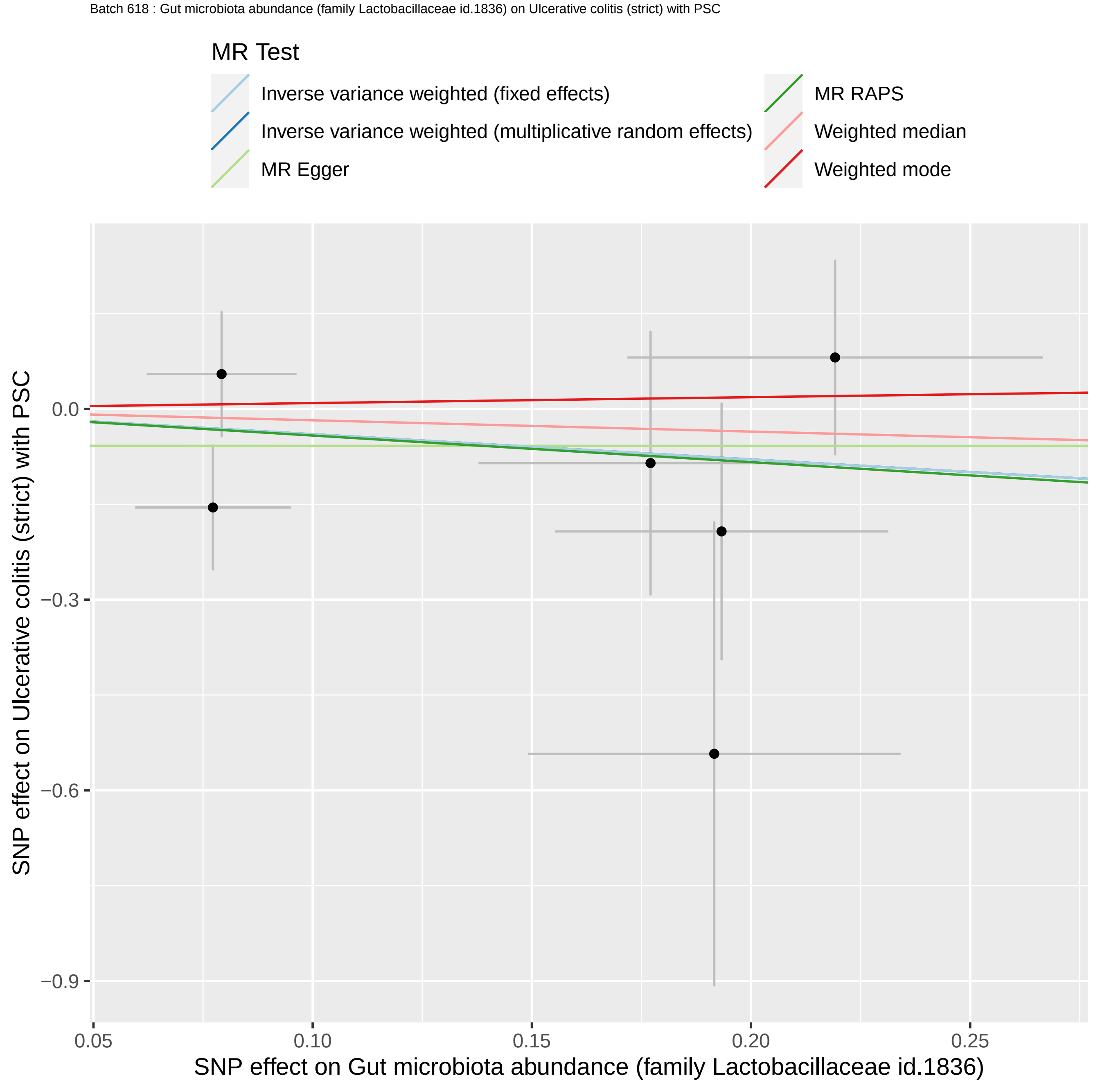
Batch 618 : Gut microbiota abundance (family Lactobacillaceae id.1836) on Ulcerative colitis (strict) with PSC
MR Test
Inverse variance weighted (fixed effects)
Inverse variance weighted (multiplicative random effects)
MR Egger
MR RAPS
Weighted median
Weighted mode
SNP effect on Ulcerative colitis (strict) with PSC
0.0
−0.3
−0.6
−0.9
0.05
0.10
0.15
0.20
0.25
SNP effect on Gut microbiota abundance (family Lactobacillaceae id.1836)

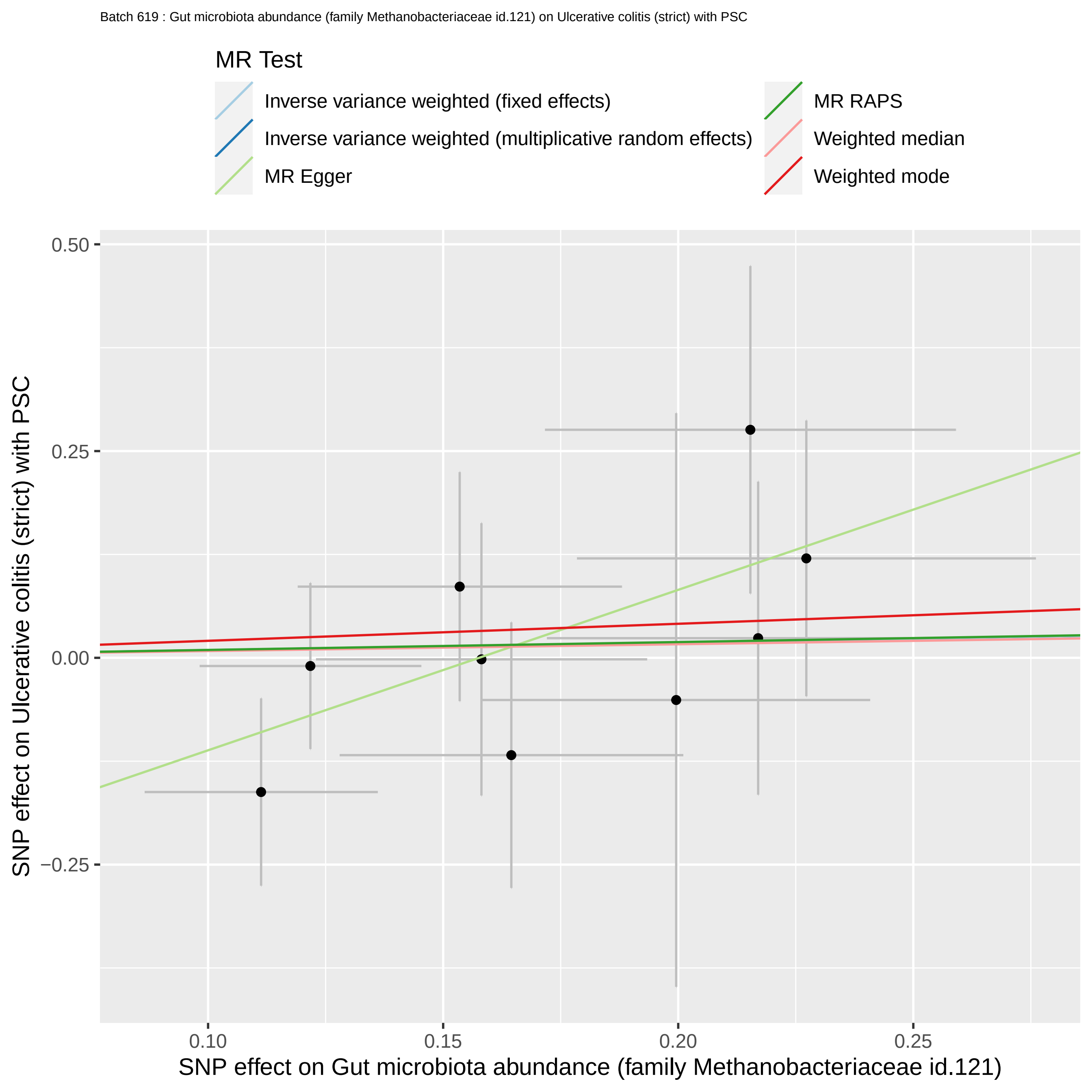

Batch 619 : Gut microbiota abundance (family Methanobacteriaceae id.121) on Ulcerative colitis (strict) with PSC
MR Test
Inverse variance weighted (fixed effects)
Inverse variance weighted (multiplicative random effects)
MR Egger
MR RAPS
Weighted median
Weighted mode
0.50
0.25
0.00
−0.25
0.10
0.15
0.20
0.25
SNP effect on Ulcerative colitis (strict) with PSC
SNP effect on Gut microbiota abundance (family Methanobacteriaceae id.121)

Batch 620 : Gut microbiota abundance (family Oxalobacteraceae id.2966) on Ulcerative colitis (strict) with PSC

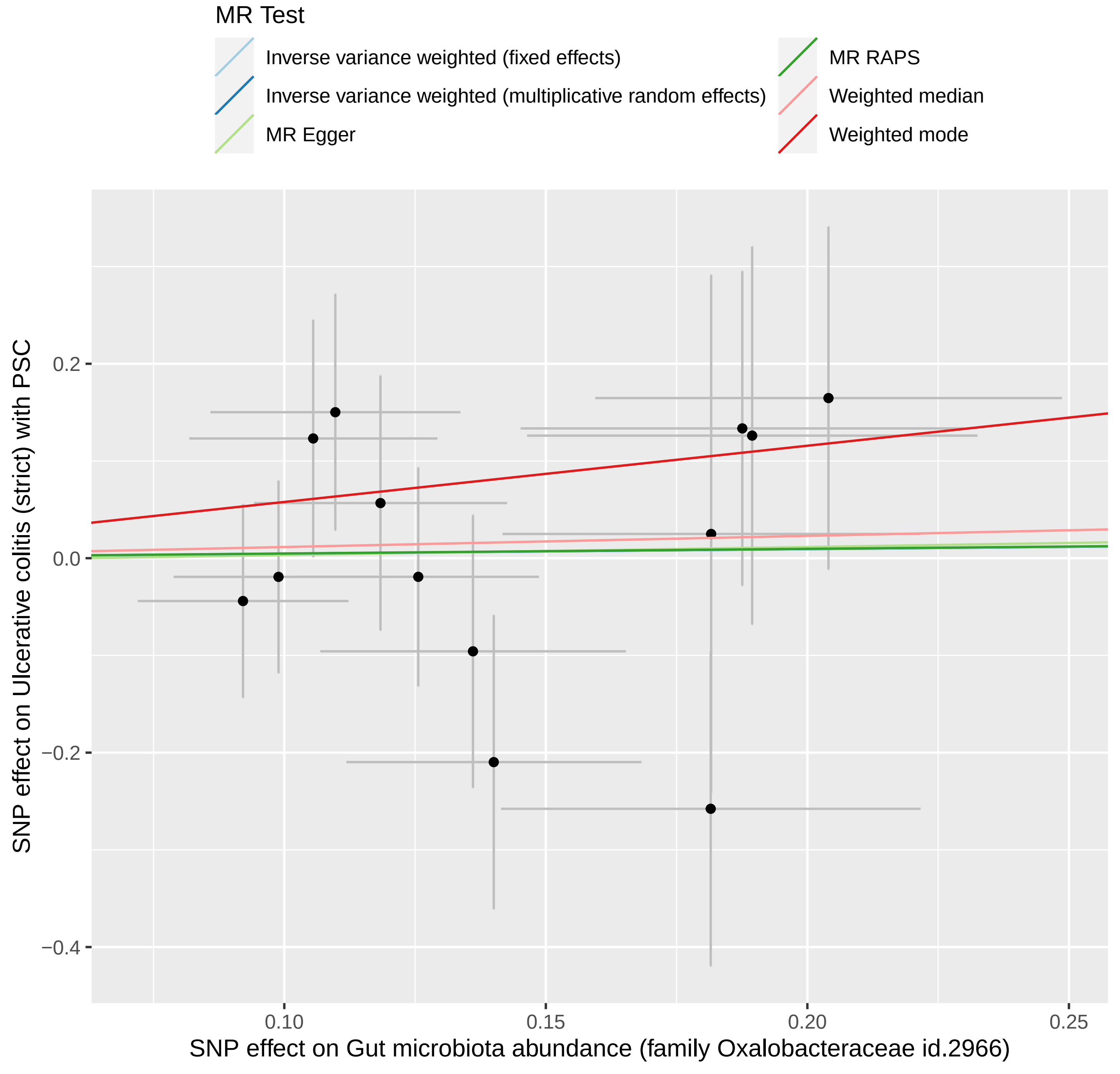

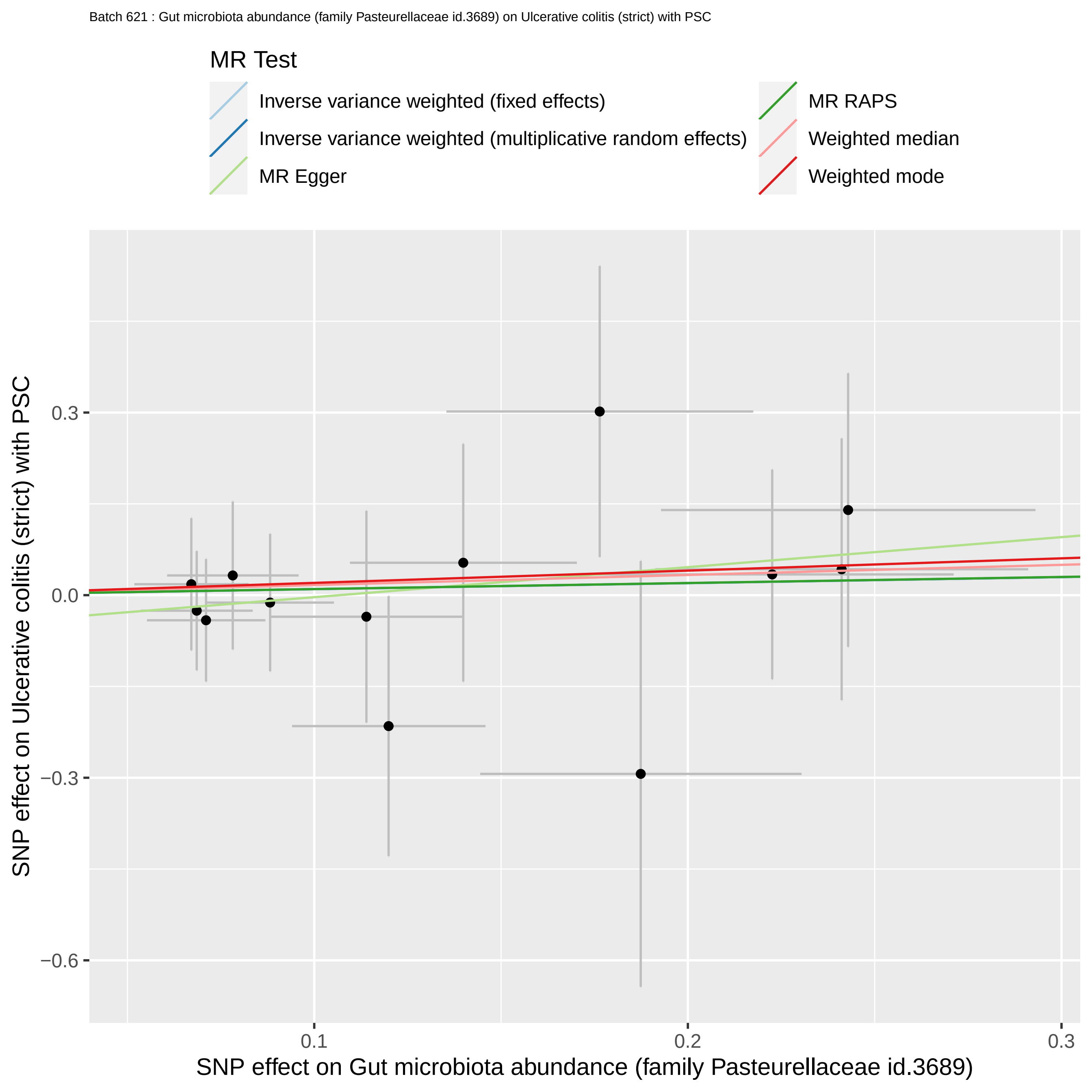

Batch 621 : Gut microbiota abundance (family Pasteurellaceae id.3689) on Ulcerative colitis (strict) with PSC
MR Test
Inverse variance weighted (fixed effects)
Inverse variance weighted (multiplicative random effects)
MR Egger
MR RAPS
Weighted median
Weighted mode
0.3
0.0
−0.3
−0.6
0.1
0.2
0.3
SNP effect on Ulcerative colitis (strict) with PSC
SNP effect on Gut microbiota abundance (family Pasteurellaceae id.3689)

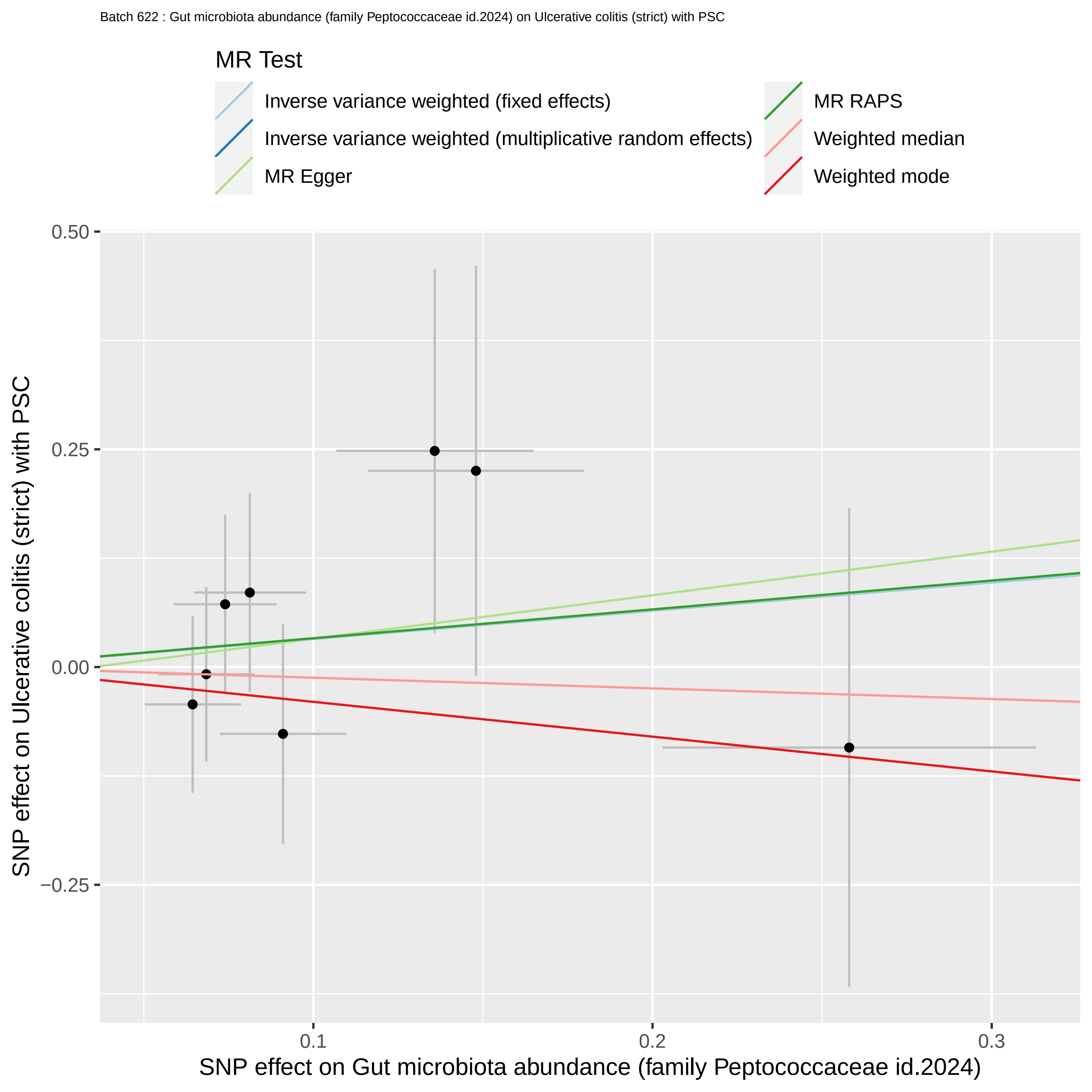

Batch 622 : Gut microbiota abundance (family Peptococcaceae id.2024) on Ulcerative colitis (strict) with PSC
MR Test
Inverse variance weighted (fixed effects)
Inverse variance weighted (multiplicative random effects)
MR Egger
MR RAPS
Weighted median
Weighted mode
0.50
0.25
0.00
−0.25
0.1
0.2
0.3
SNP effect on Ulcerative colitis (strict) with PSC
SNP effect on Gut microbiota abundance (family Peptococcaceae id.2024)

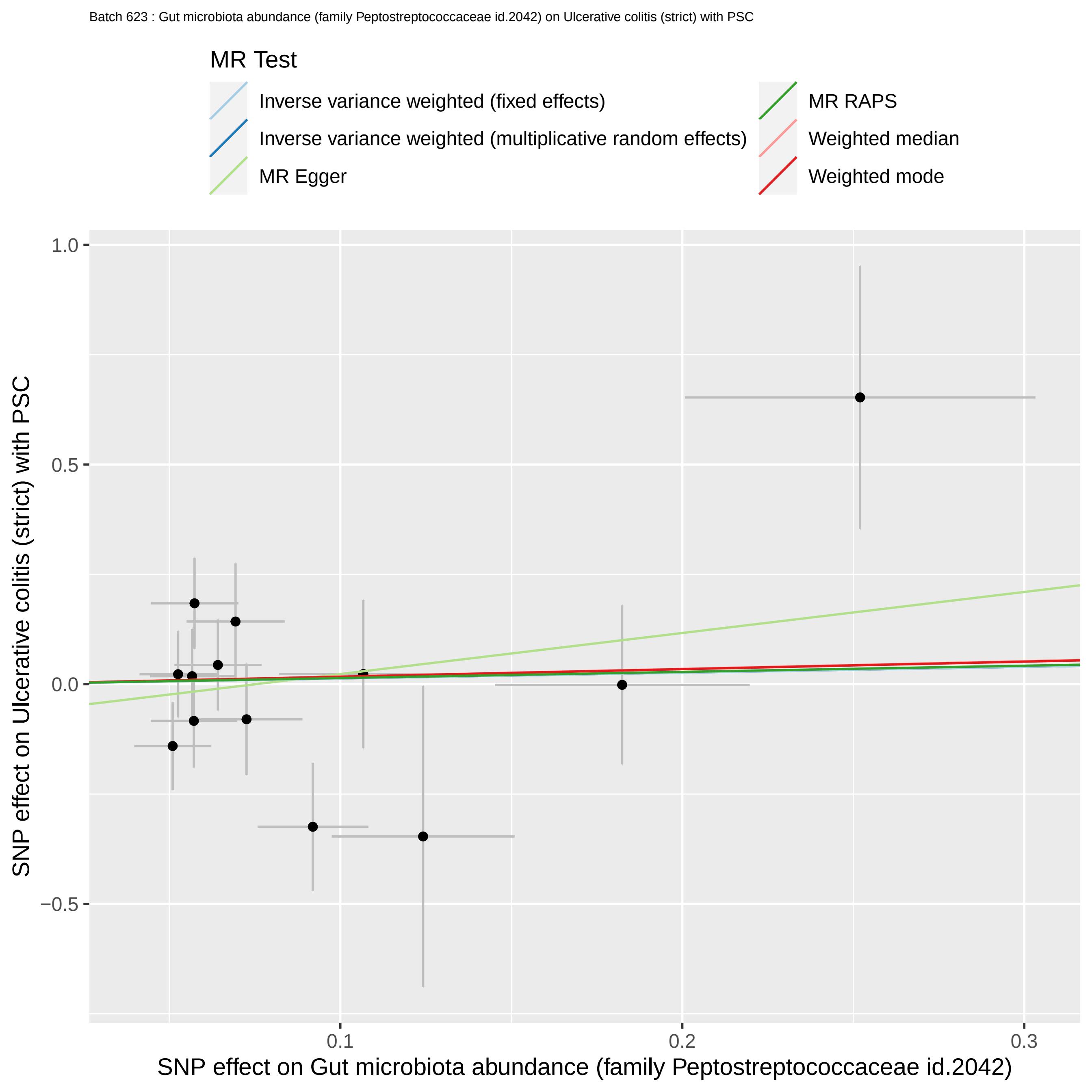

Batch 623 : Gut microbiota abundance (family Peptostreptococcaceae id.2042) on Ulcerative colitis (strict) with PSC
MR Test
Inverse variance weighted (fixed effects)
Inverse variance weighted (multiplicative random effects)
MR Egger
MR RAPS
Weighted median
Weighted mode
1.0
0.5
0.0
−0.5
0.1
0.2
0.3
SNP effect on Ulcerative colitis (strict) with PSC
SNP effect on Gut microbiota abundance (family Peptostreptococcaceae id.2042)

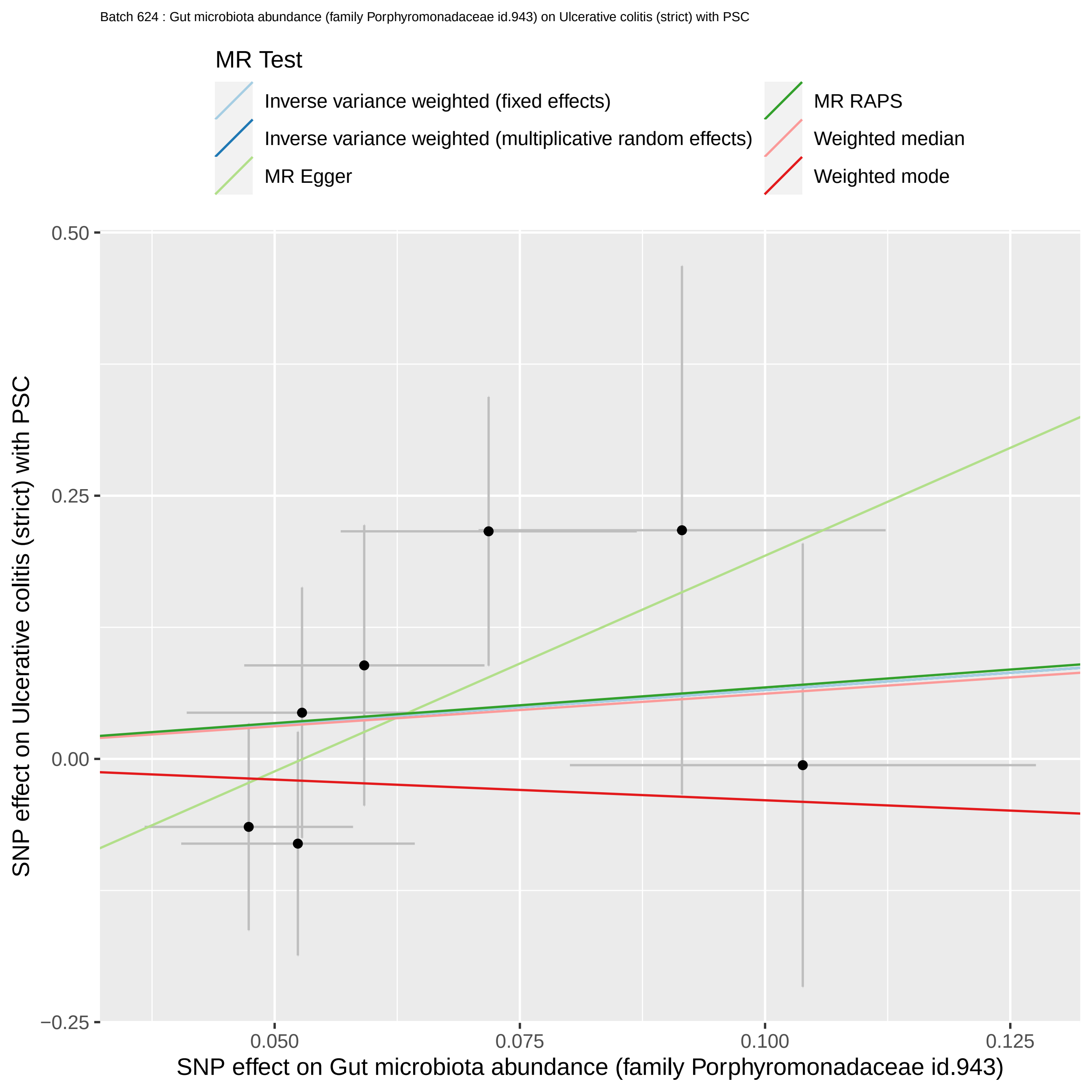
Batch 624 : Gut microbiota abundance (family Porphyromonadaceae id.943) on Ulcerative colitis (strict) with PSC
MR Test
Inverse variance weighted (fixed effects)
Inverse variance weighted (multiplicative random effects)
MR Egger
MR RAPS
Weighted median
Weighted mode
0.50
0.25
0.00
−0.25
0.050
0.075
0.100
0.125
SNP effect on Ulcerative colitis (strict) with PSC
SNP effect on Gut microbiota abundance (family Porphyromonadaceae id.943)

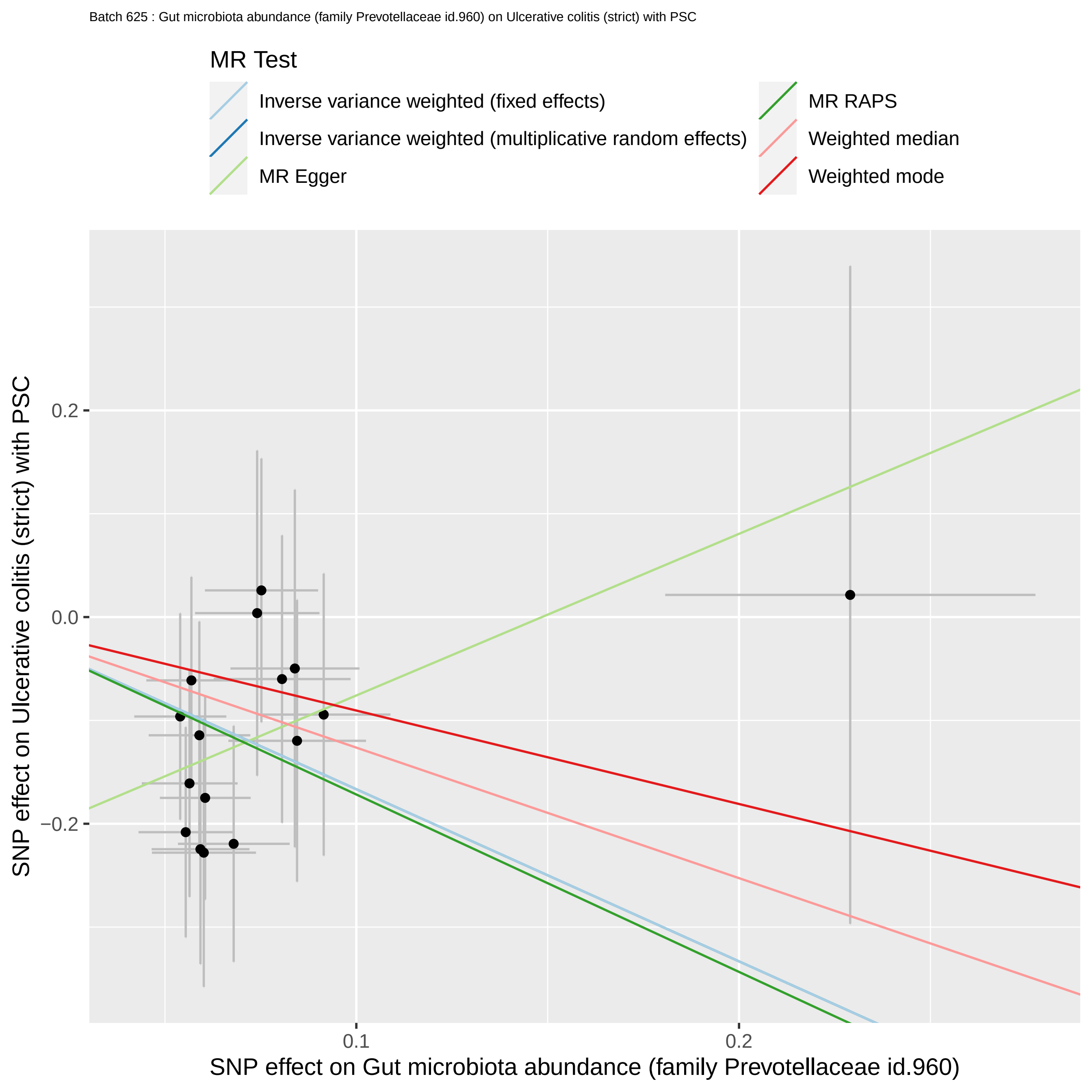

Batch 625 : Gut microbiota abundance (family Prevotellaceae id.960) on Ulcerative colitis (strict) with PSC
MR Test
Inverse variance weighted (fixed effects)
Inverse variance weighted (multiplicative random effects)
MR Egger
MR RAPS
Weighted median
Weighted mode
0.2
0.0
−0.2
0.1
0.2
SNP effect on Ulcerative colitis (strict) with PSC
SNP effect on Gut microbiota abundance (family Prevotellaceae id.960)

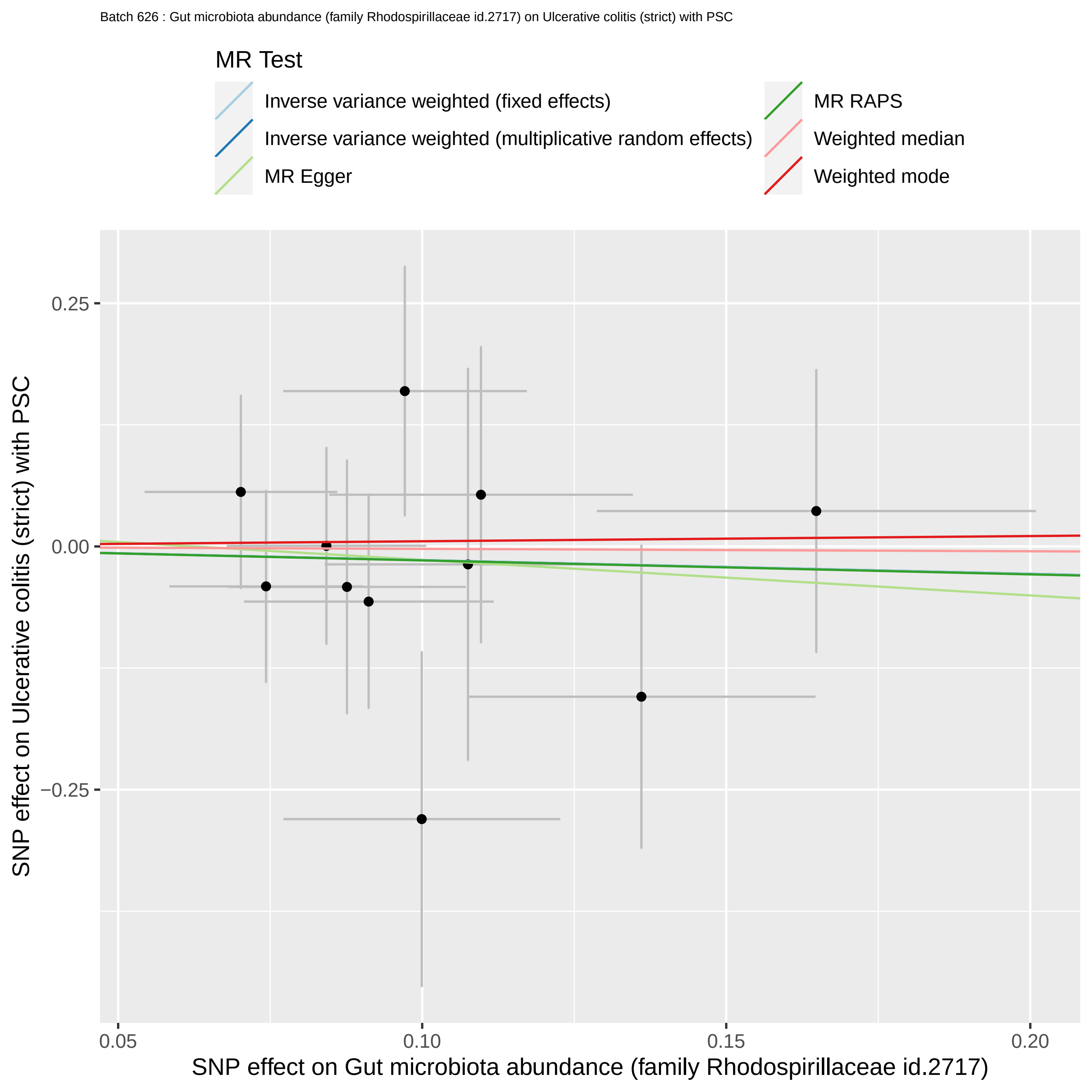
Batch 626 : Gut microbiota abundance (family Rhodospirillaceae id.2717) on Ulcerative colitis (strict) with PSC
MR Test
Inverse variance weighted (fixed effects)
Inverse variance weighted (multiplicative random effects)
MR Egger
MR RAPS
Weighted median
Weighted mode
0.25
0.00
−0.25
0.05
0.10
0.15
0.20
SNP effect on Ulcerative colitis (strict) with PSC
SNP effect on Gut microbiota abundance (family Rhodospirillaceae id.2717)

Batch 627 : Gut microbiota abundance (family Rikenellaceae id.967) on Ulcerative colitis (strict) with PSC

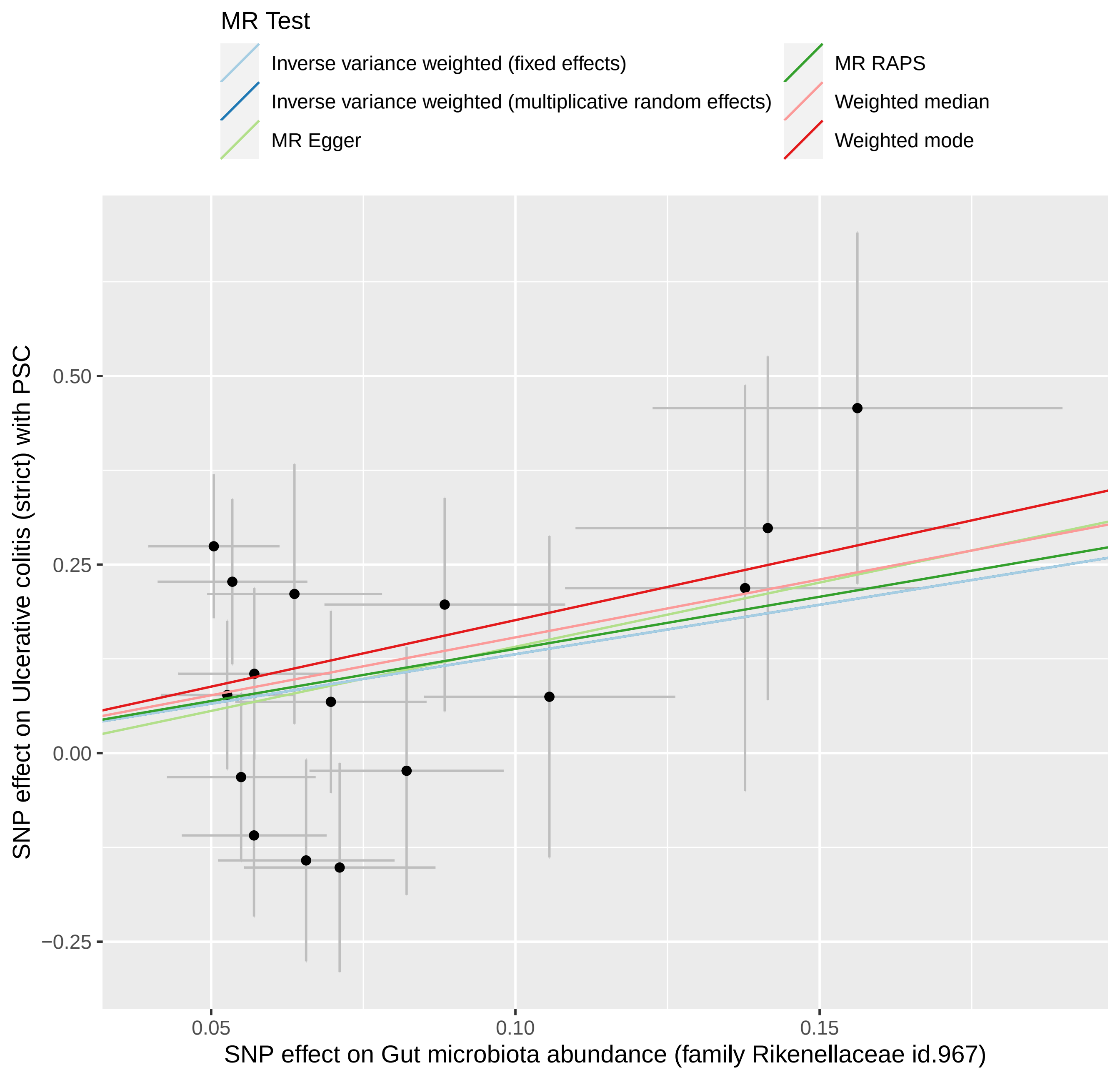

Batch 628 : Gut microbiota abundance (family Ruminococcaceae id.2050) on Ulcerative colitis (strict) with PSC

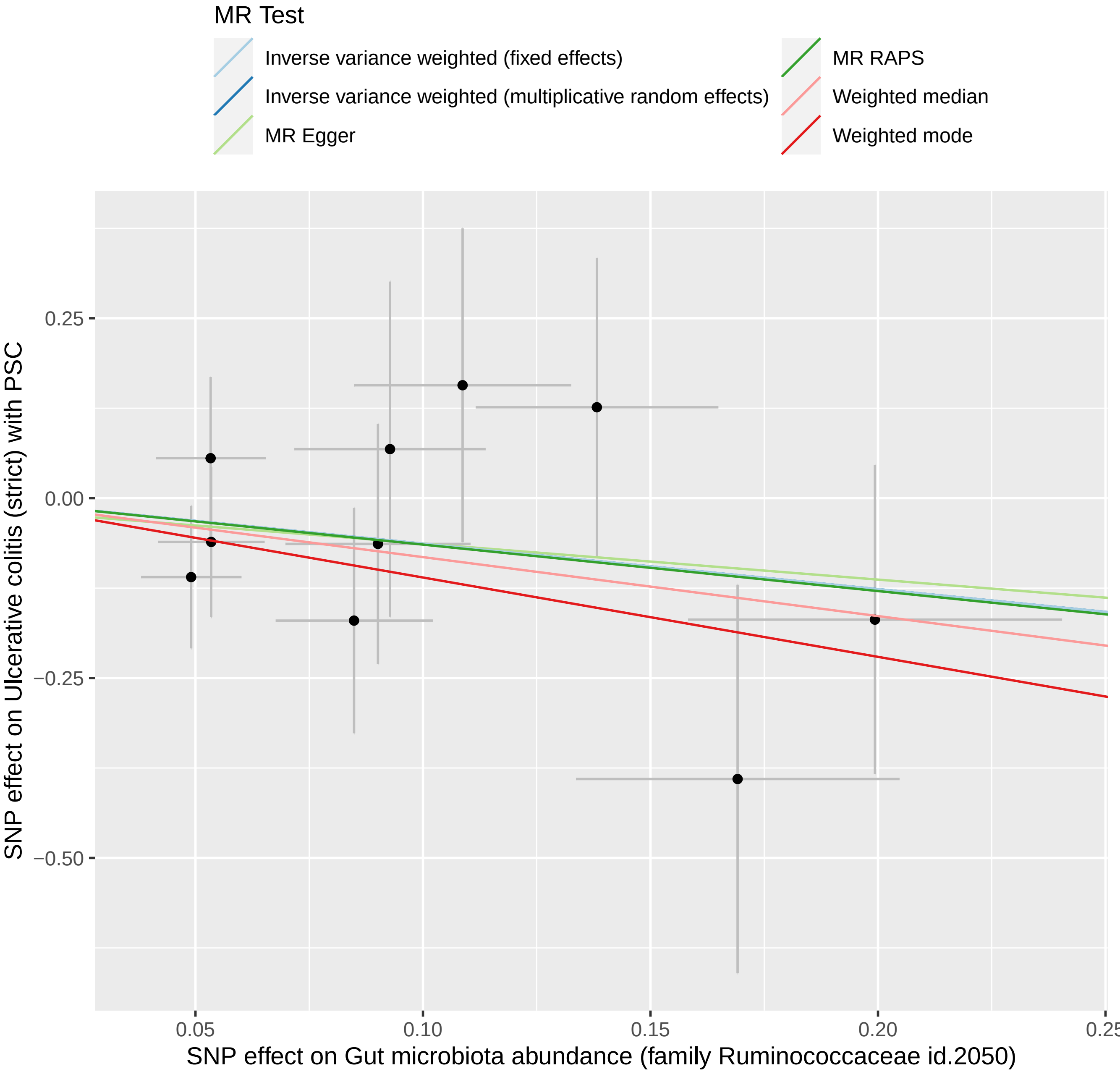

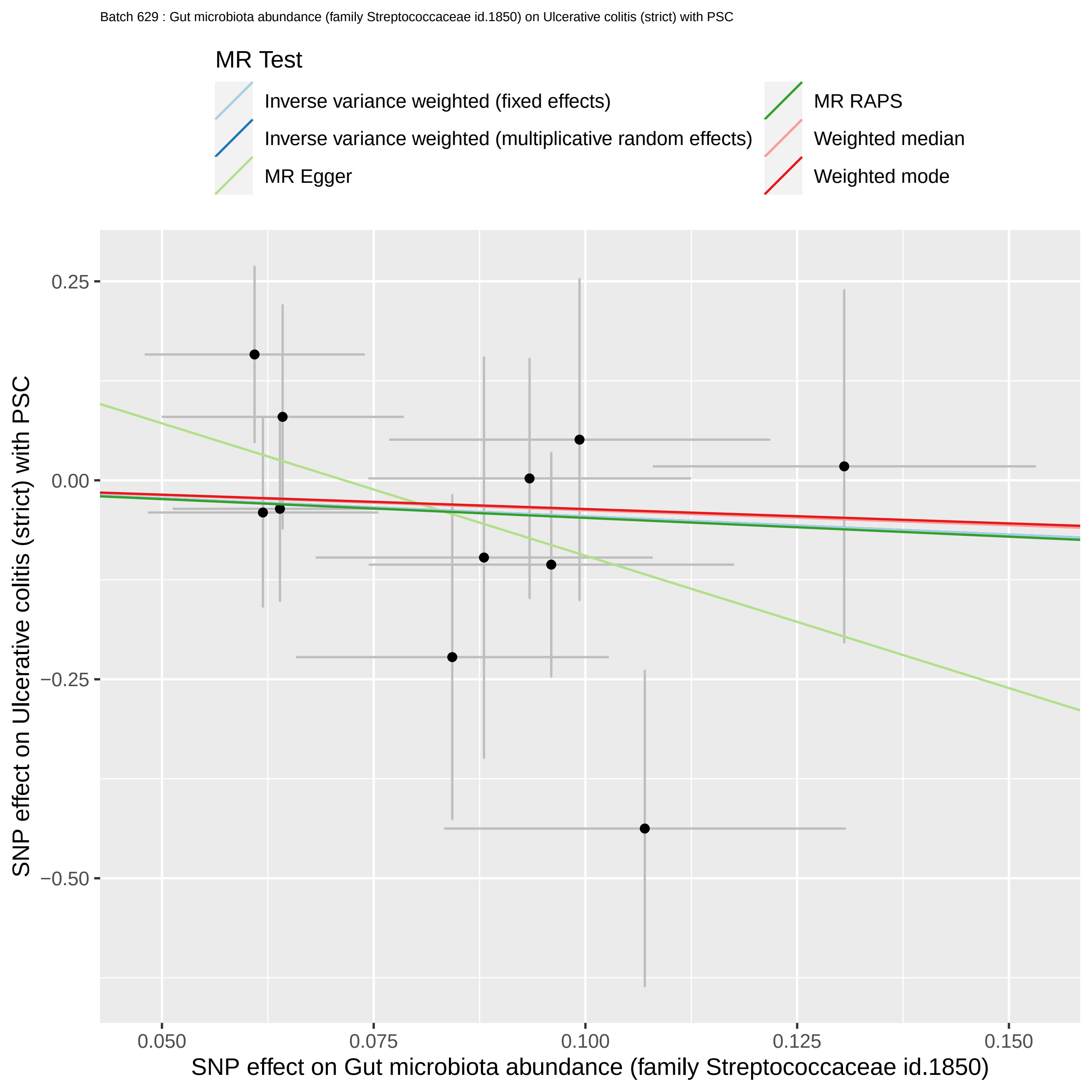

Batch 629 : Gut microbiota abundance (family Streptococcaceae id.1850) on Ulcerative colitis (strict) with PSC
MR Test
Inverse variance weighted (fixed effects)
Inverse variance weighted (multiplicative random effects)
MR Egger
MR RAPS
Weighted median
Weighted mode
0.25
0.00
−0.25
−0.50
0.050
0.075
0.100
0.125
0.150
SNP effect on Ulcerative colitis (strict) with PSC
SNP effect on Gut microbiota abundance (family Streptococcaceae id.1850)

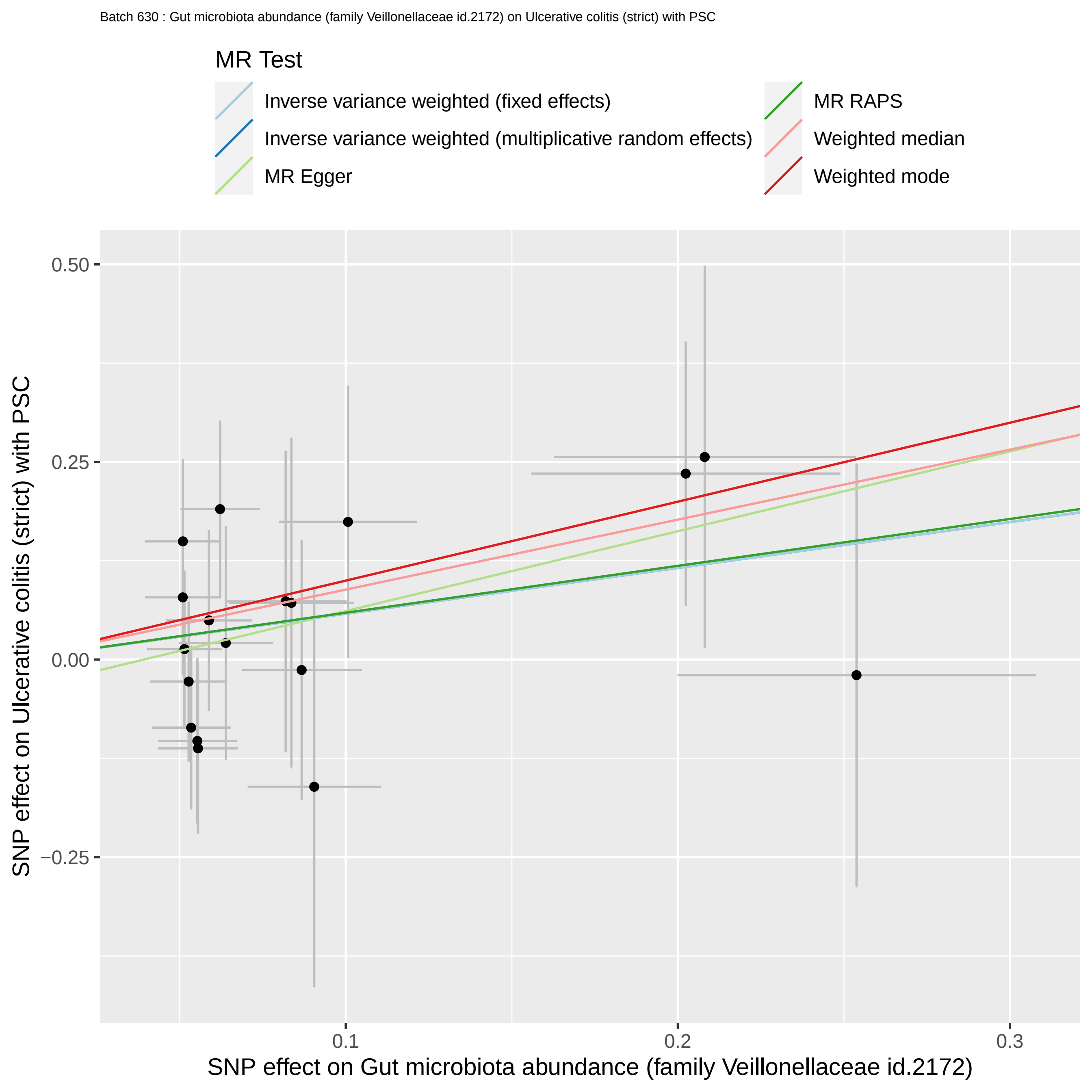

Batch 630 : Gut microbiota abundance (family Veillonellaceae id.2172) on Ulcerative colitis (strict) with PSC
MR Test
Inverse variance weighted (fixed effects)
Inverse variance weighted (multiplicative random effects)
MR Egger
MR RAPS
Weighted median
Weighted mode
0.50
0.25
0.00
−0.25
0.1
0.2
0.3
SNP effect on Ulcerative colitis (strict) with PSC
SNP effect on Gut microbiota abundance (family Veillonellaceae id.2172)

Batch 631 : Gut microbiota abundance (family Verrucomicrobiaceae id.4036) on Ulcerative colitis (strict) with PSC

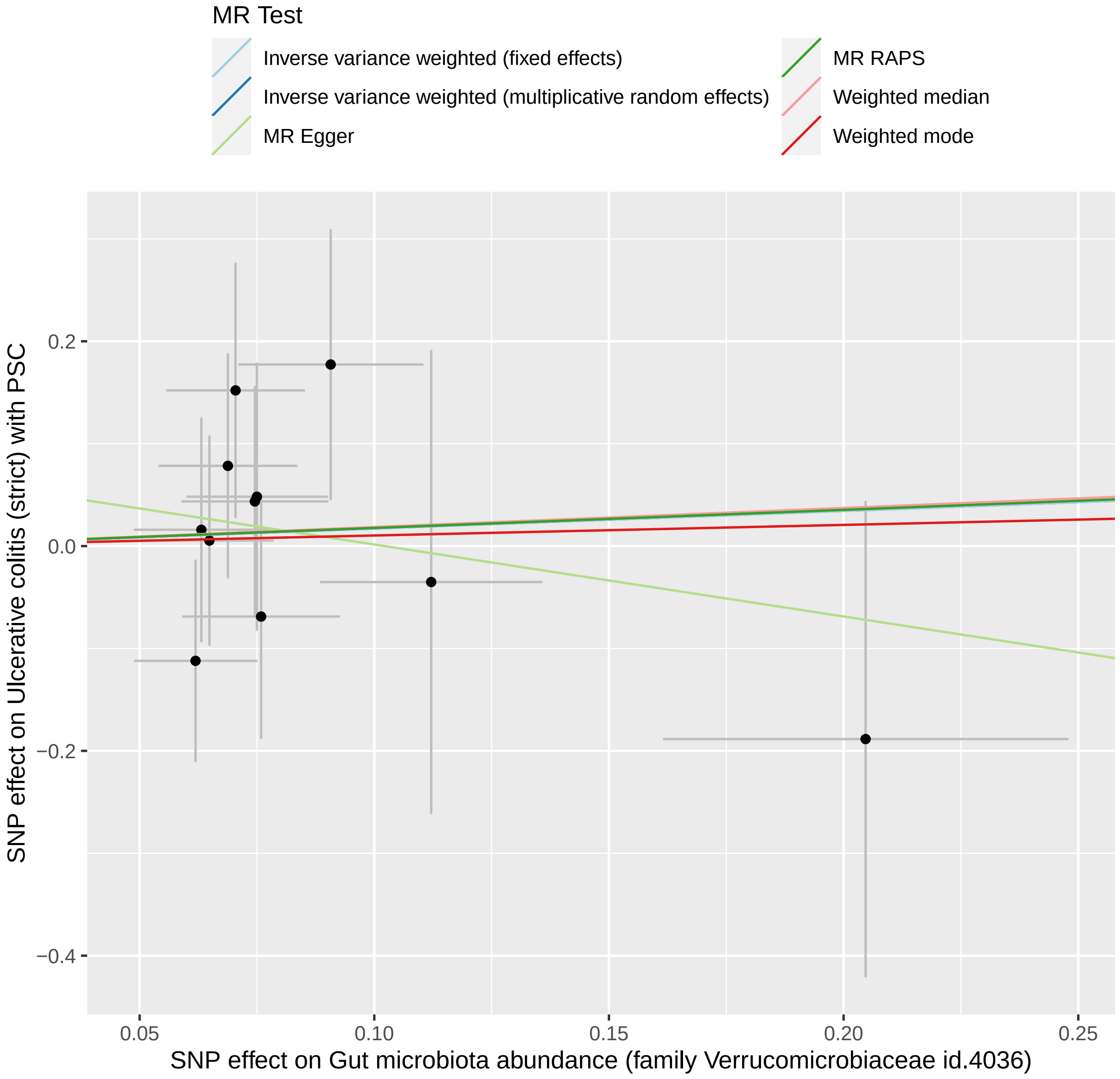

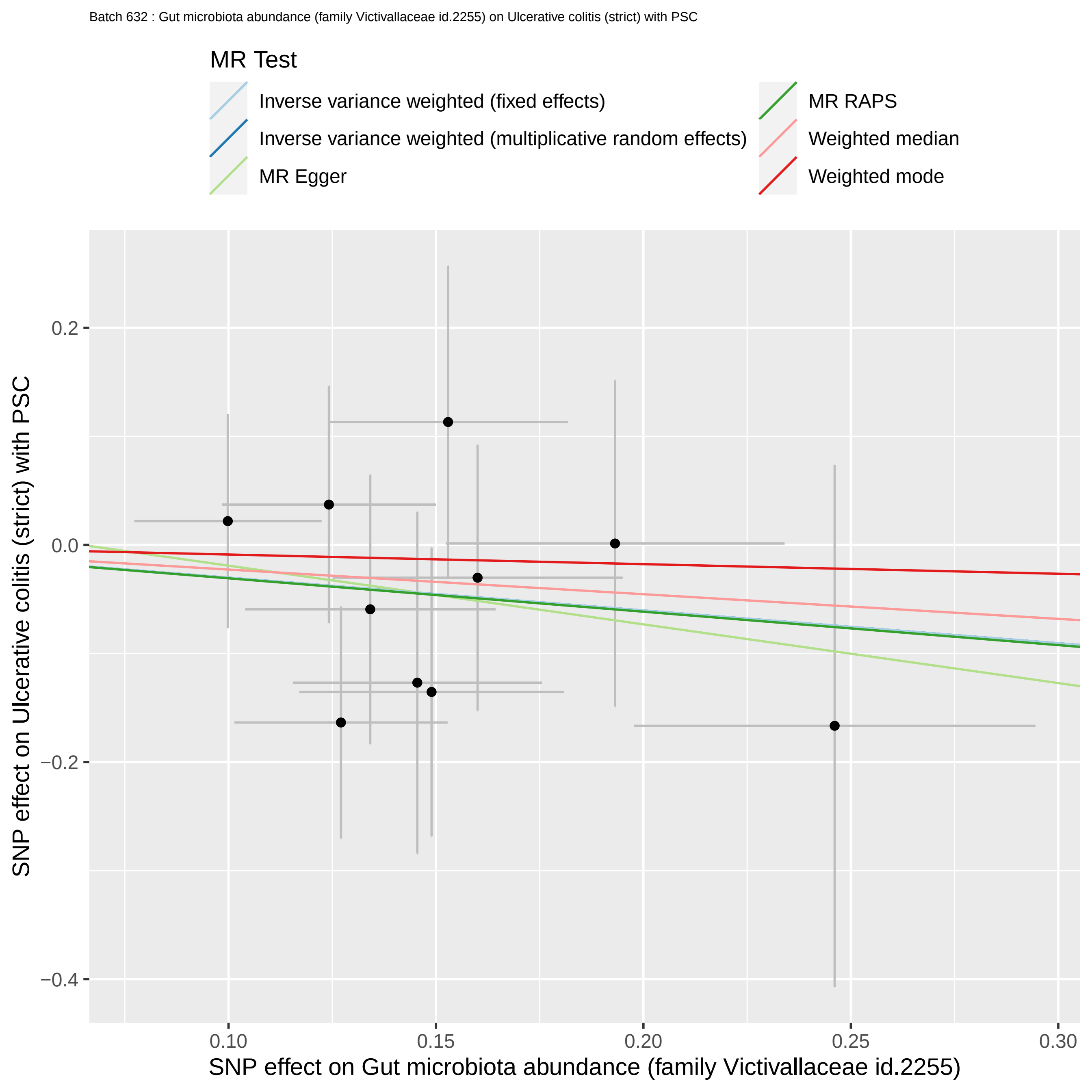
Batch 632 : Gut microbiota abundance (family Victivallaceae id.2255) on Ulcerative colitis (strict) with PSC
MR Test
Inverse variance weighted (fixed effects)
Inverse variance weighted (multiplicative random effects)
MR Egger
MR RAPS
Weighted median
Weighted mode
SNP effect on Ulcerative colitis (strict) with PSC
0.2
0.0
−0.2
−0.4
0.10
0.15
0.20
0.25
0.30
SNP effect on Gut microbiota abundance (family Victivallaceae id.2255)

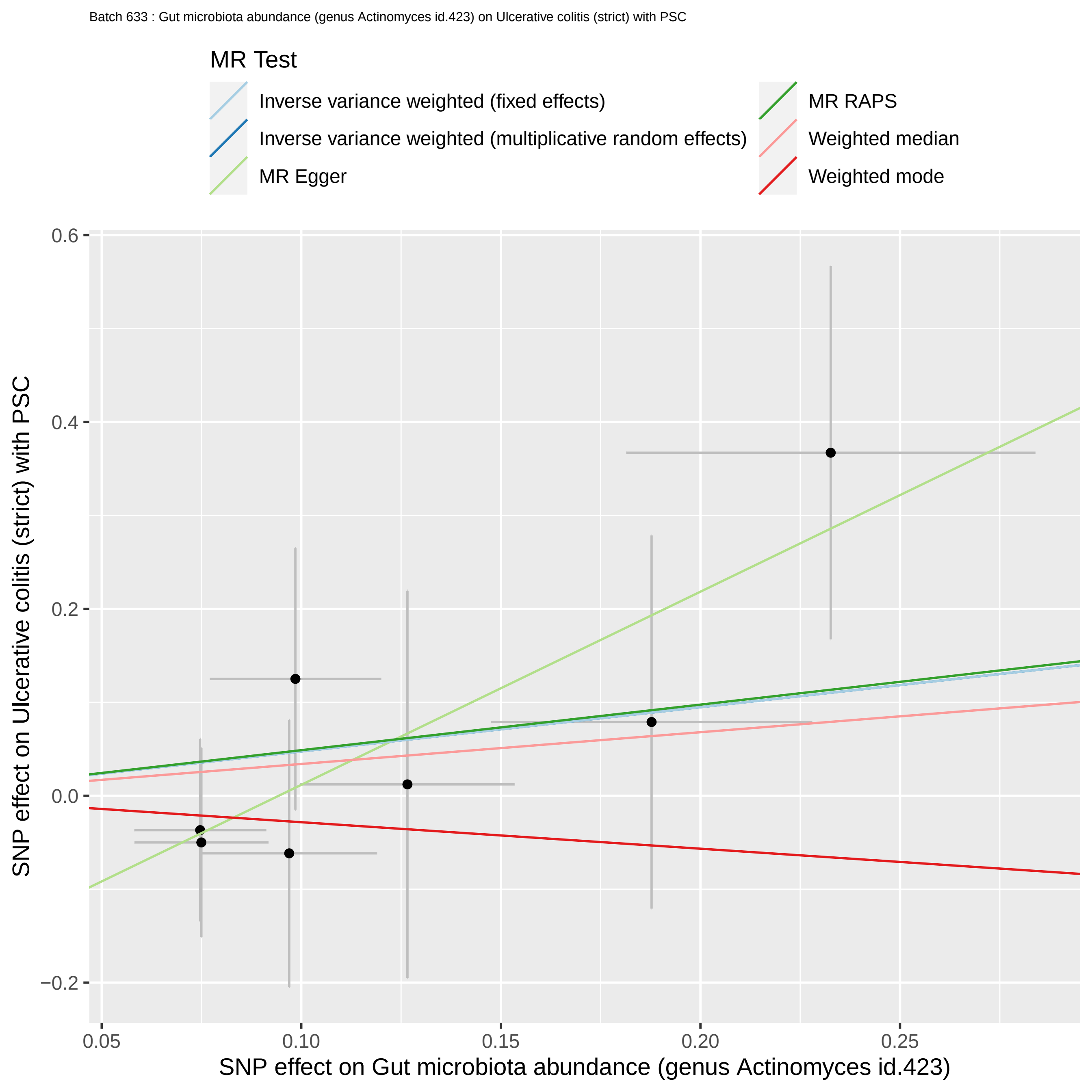

Batch 633 : Gut microbiota abundance (genus Actinomyces id.423) on Ulcerative colitis (strict) with PSC
MR Test
Inverse variance weighted (fixed effects)
Inverse variance weighted (multiplicative random effects)
MR Egger
MR RAPS
Weighted median
Weighted mode
0.6
0.4
0.2
0.0
−0.2
0.05
0.10
0.15
0.20
0.25
SNP effect on Ulcerative colitis (strict) with PSC
SNP effect on Gut microbiota abundance (genus Actinomyces id.423)

Batch 634 : Gut microbiota abundance (genus Adlercreutzia id.812) on Ulcerative colitis (strict) with PSC

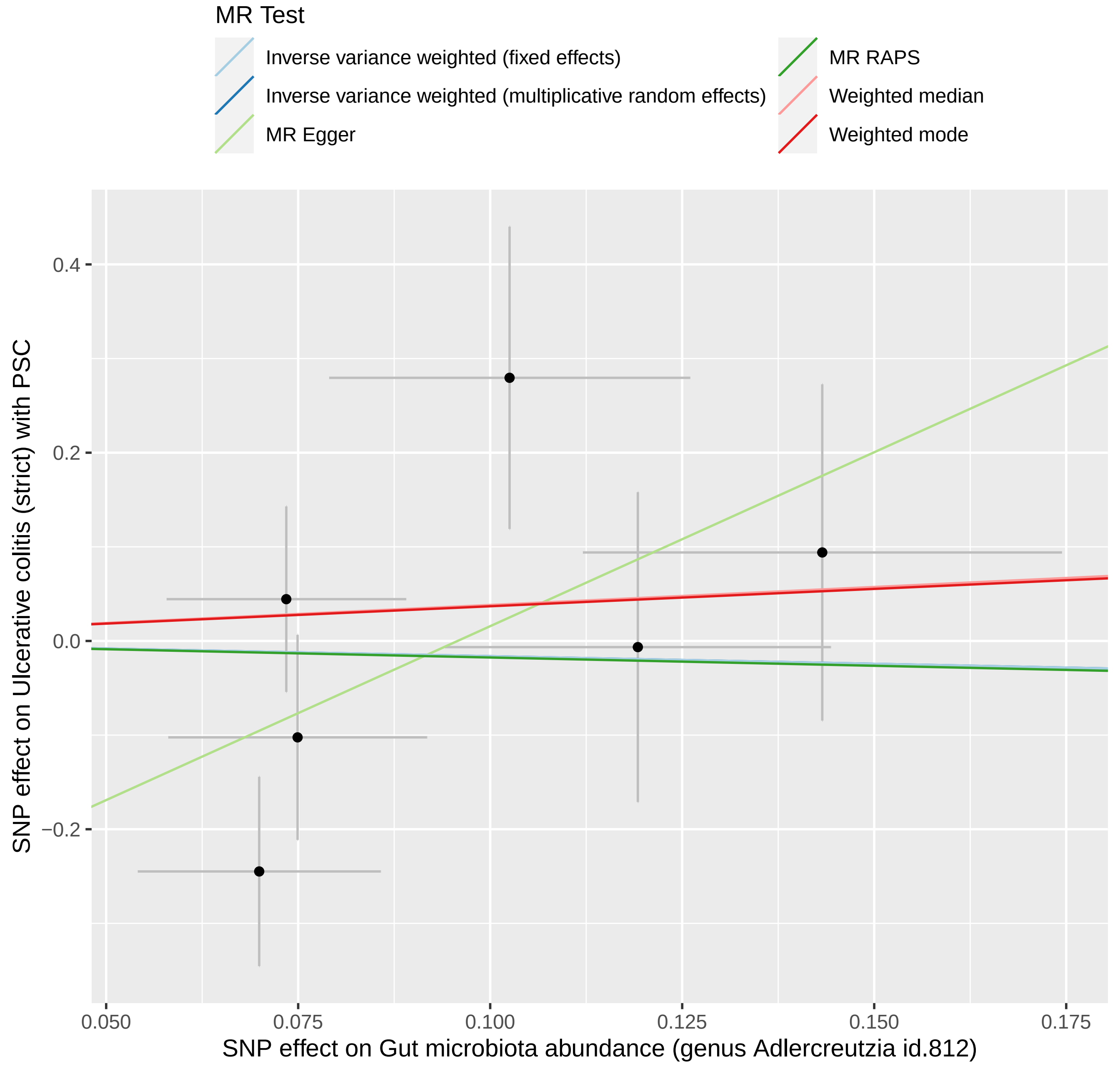

Batch 635 : Gut microbiota abundance (genus Akkermansia id.4037) on Ulcerative colitis (strict) with PSC

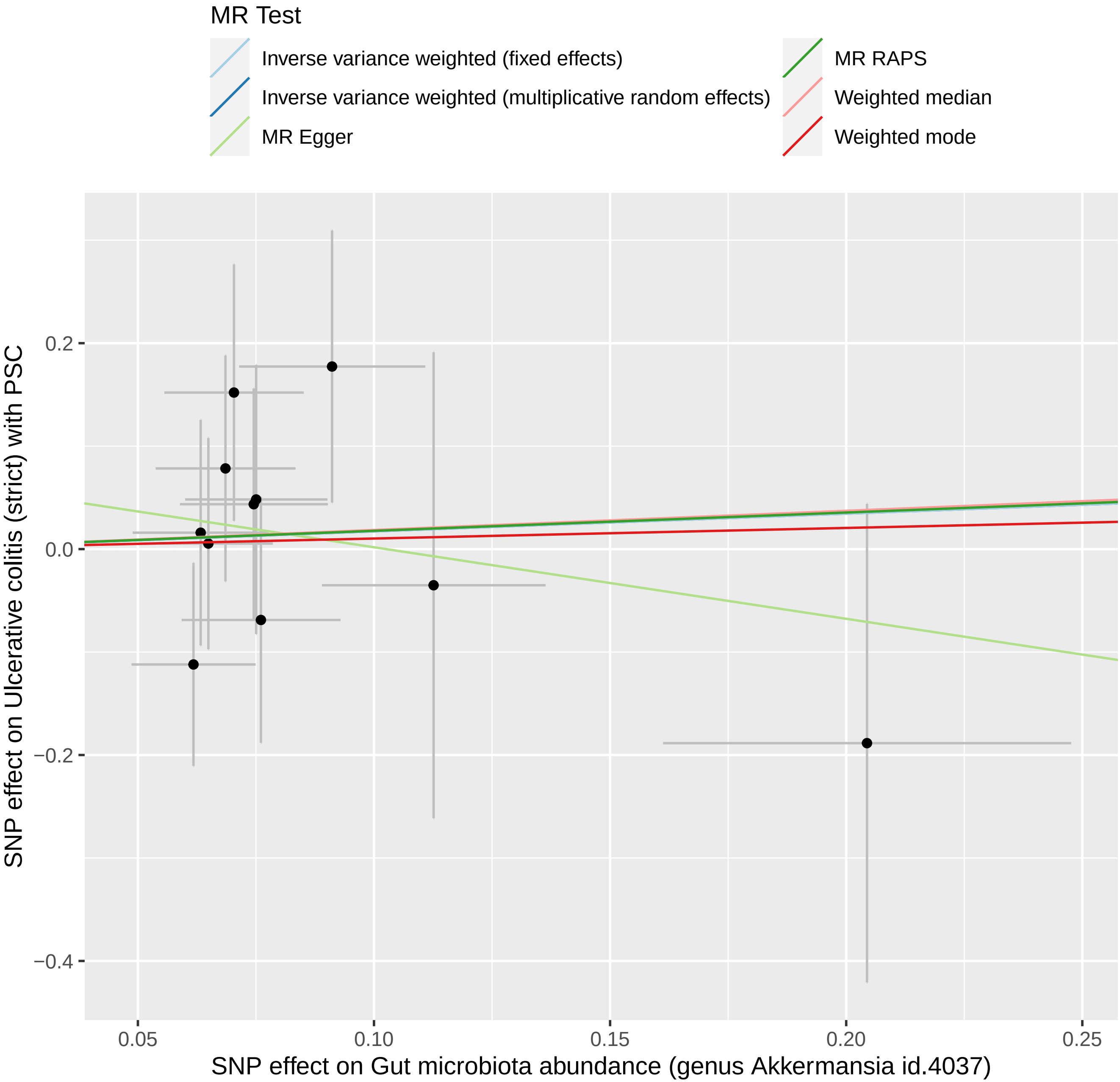

Batch 636 : Gut microbiota abundance (genus Alistipes id.968) on Ulcerative colitis (strict) with PSC

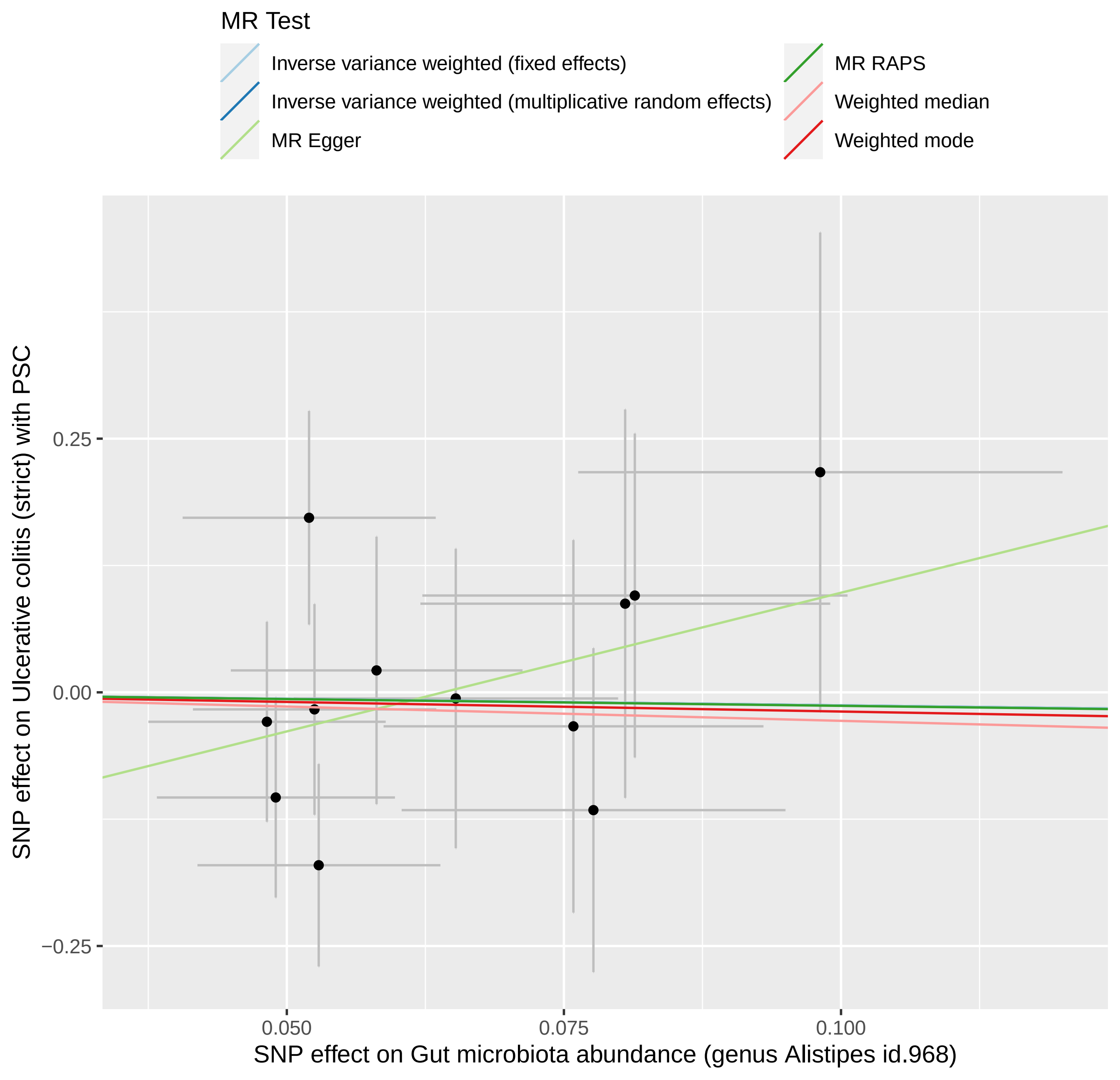

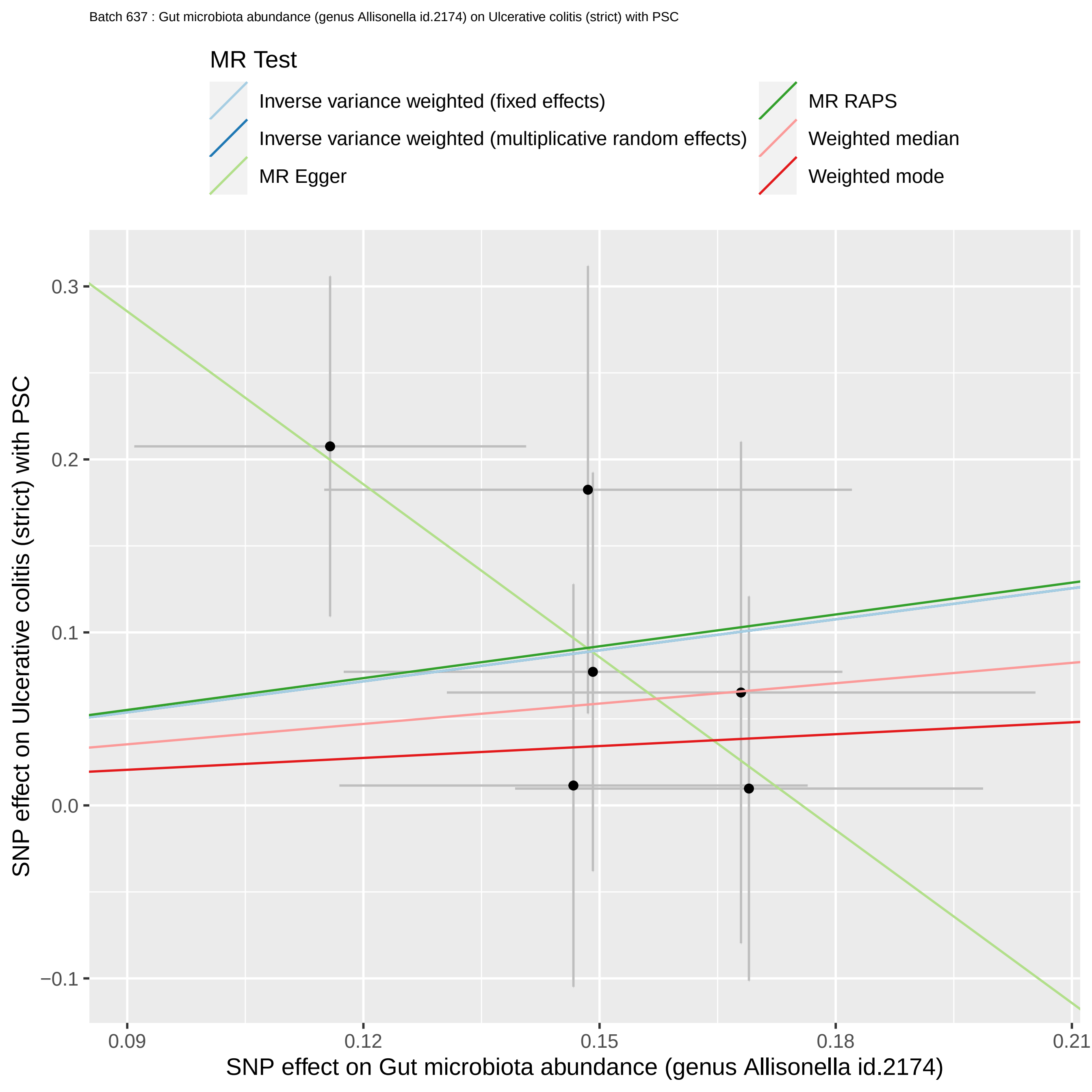

Batch 637 : Gut microbiota abundance (genus Allisonella id.2174) on Ulcerative colitis (strict) with PSC
MR Test
Inverse variance weighted (fixed effects)
Inverse variance weighted (multiplicative random effects)
MR Egger
MR RAPS
Weighted median
Weighted mode
0.3
0.2
0.1
0.0
−0.1
0.09
0.12
0.15
0.18
0.21
SNP effect on Ulcerative colitis (strict) with PSC
SNP effect on Gut microbiota abundance (genus Allisonella id.2174)

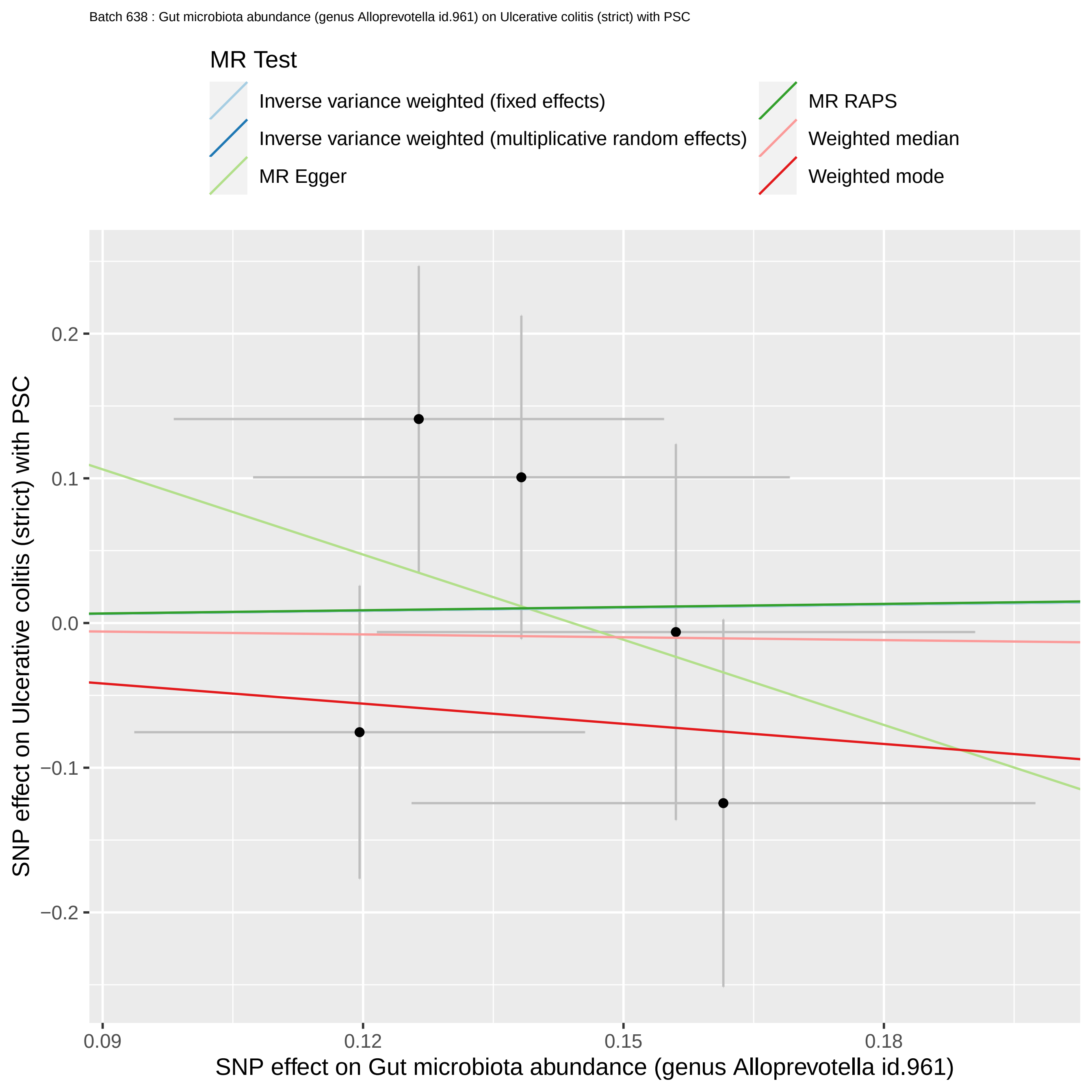

Batch 638 : Gut microbiota abundance (genus Alloprevotella id.961) on Ulcerative colitis (strict) with PSC
MR Test
Inverse variance weighted (fixed effects)
Inverse variance weighted (multiplicative random effects)
MR Egger
MR RAPS
Weighted median
Weighted mode
0.2
0.1
0.0
−0.1
−0.2
0.09
0.12
0.15
0.18
SNP effect on Ulcerative colitis (strict) with PSC
SNP effect on Gut microbiota abundance (genus Alloprevotella id.961)

Batch 639 : Gut microbiota abundance (genus Anaerofilum id.2053) on Ulcerative colitis (strict) with PSC

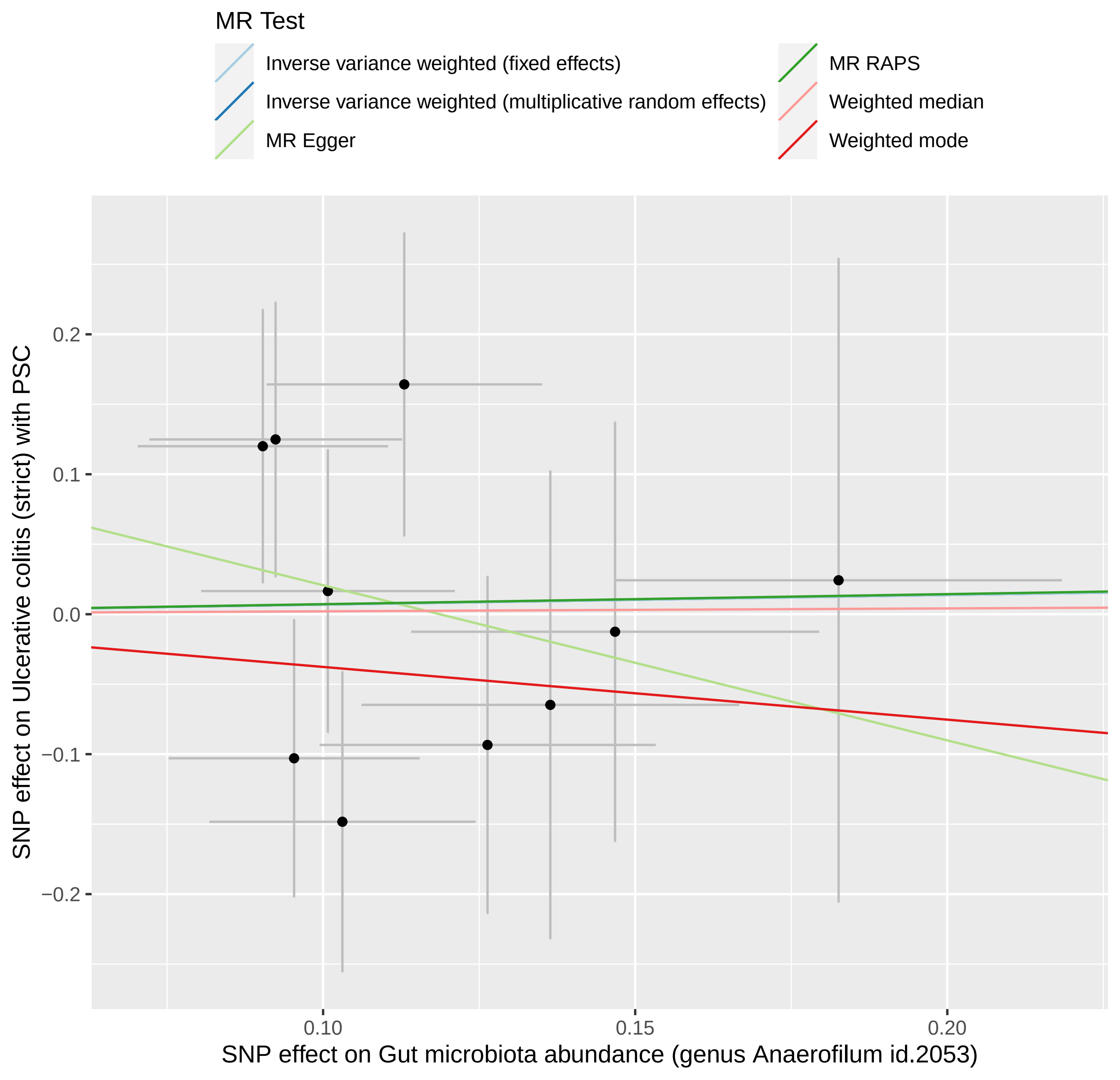

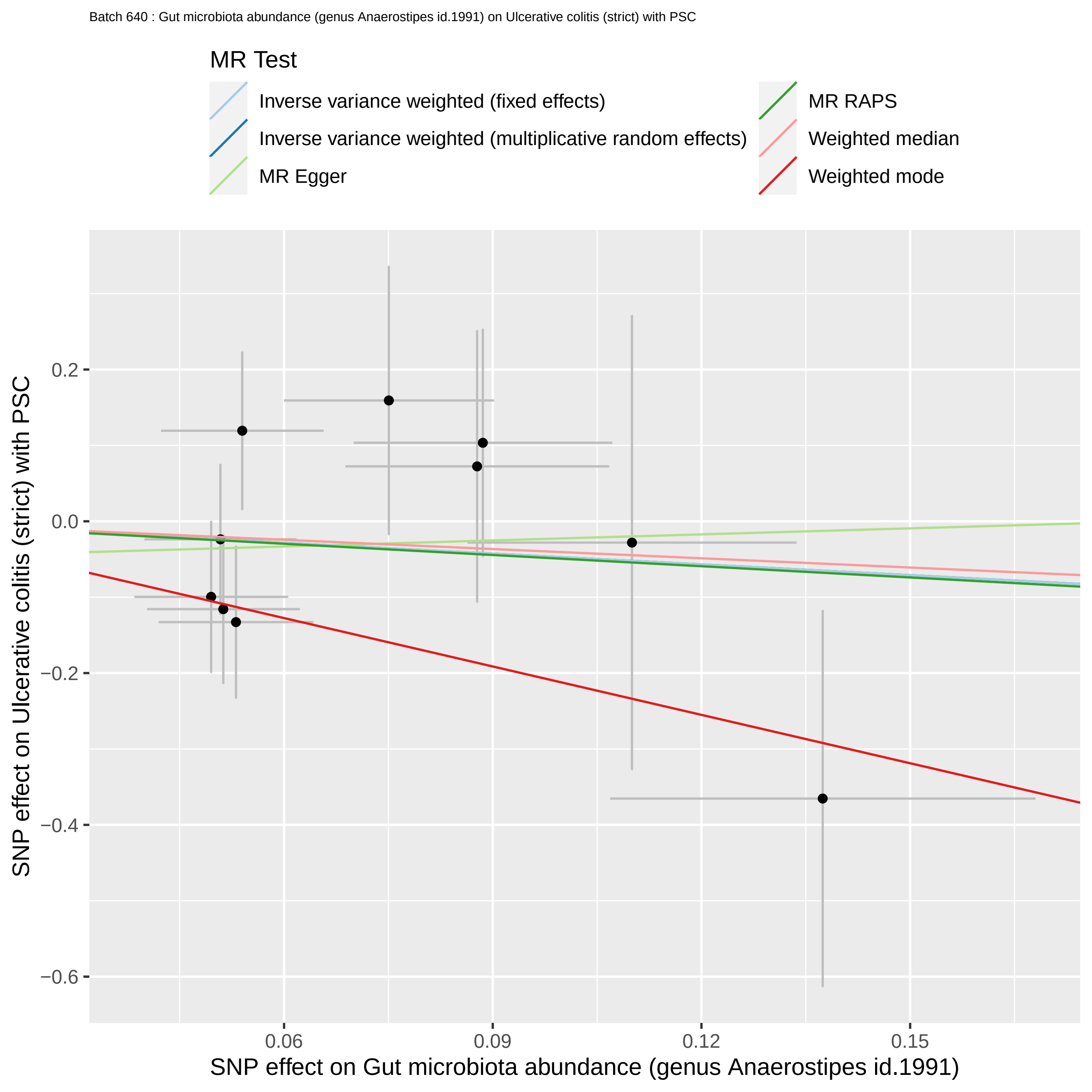
Batch 640 : Gut microbiota abundance (genus Anaerostipes id.1991) on Ulcerative colitis (strict) with PSC
MR Test
Inverse variance weighted (fixed effects)
Inverse variance weighted (multiplicative random effects)
MR Egger
MR RAPS
Weighted median
Weighted mode
SNP effect on Ulcerative colitis (strict) with PSC
0.2
0.0
−0.2
−0.4
−0.6
0.06
0.09
0.12
0.15
SNP effect on Gut microbiota abundance (genus Anaerostipes id.1991)

Batch 641 : Gut microbiota abundance (genus Anaerotruncus id.2054) on Ulcerative colitis (strict) with PSC

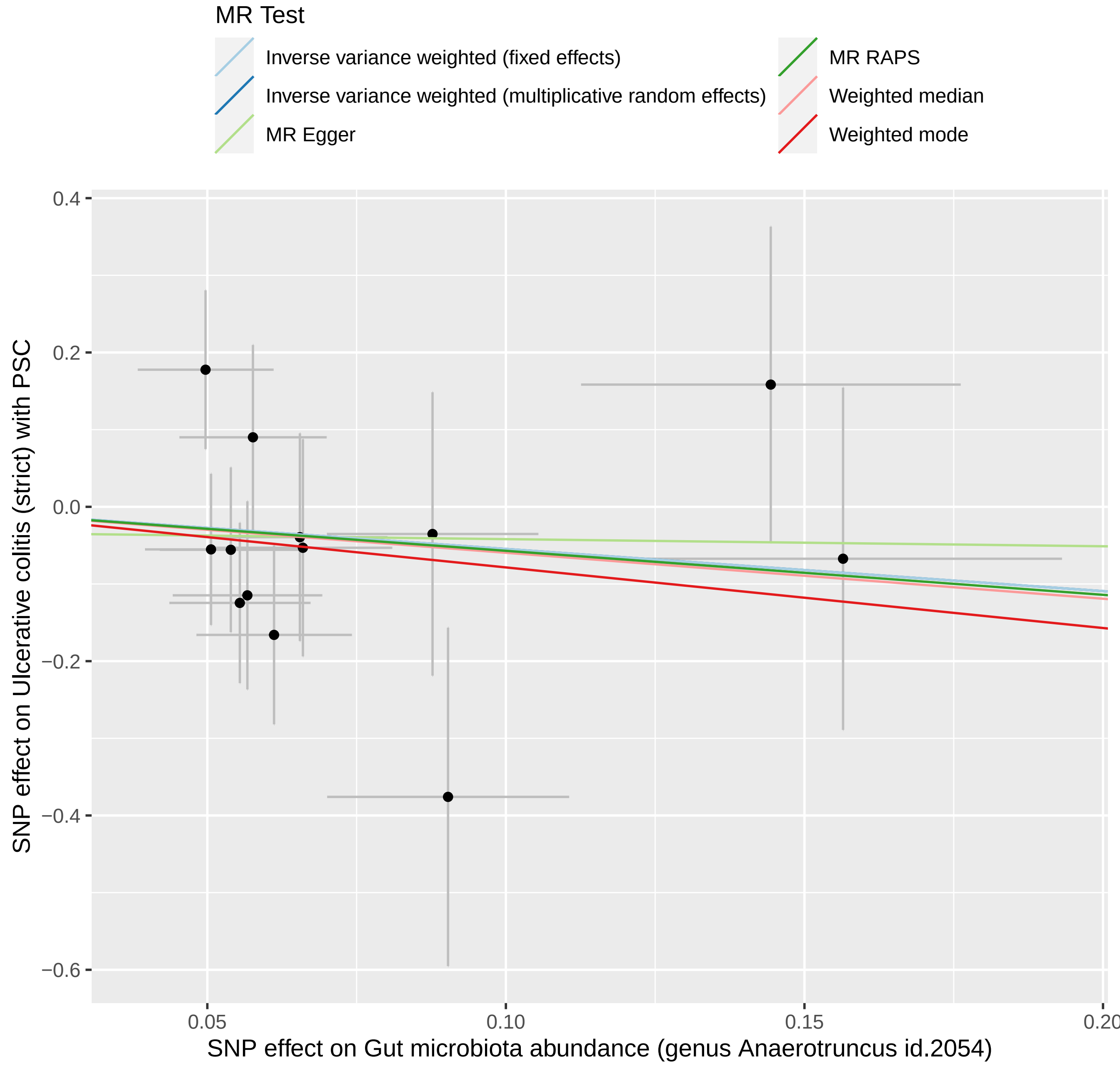

Batch 642 : Gut microbiota abundance (genus Bacteroides id.918) on Ulcerative colitis (strict) with PSC

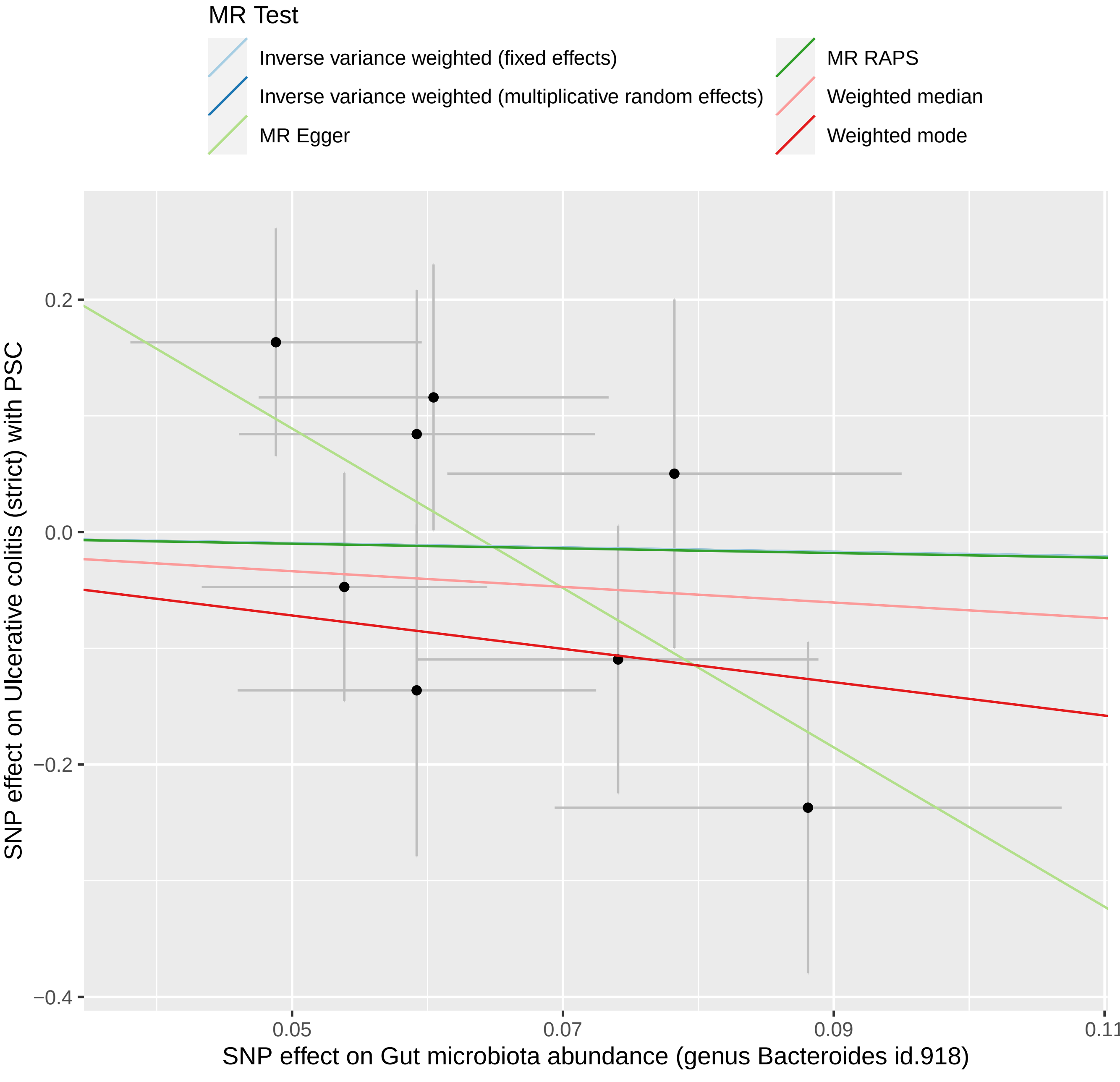

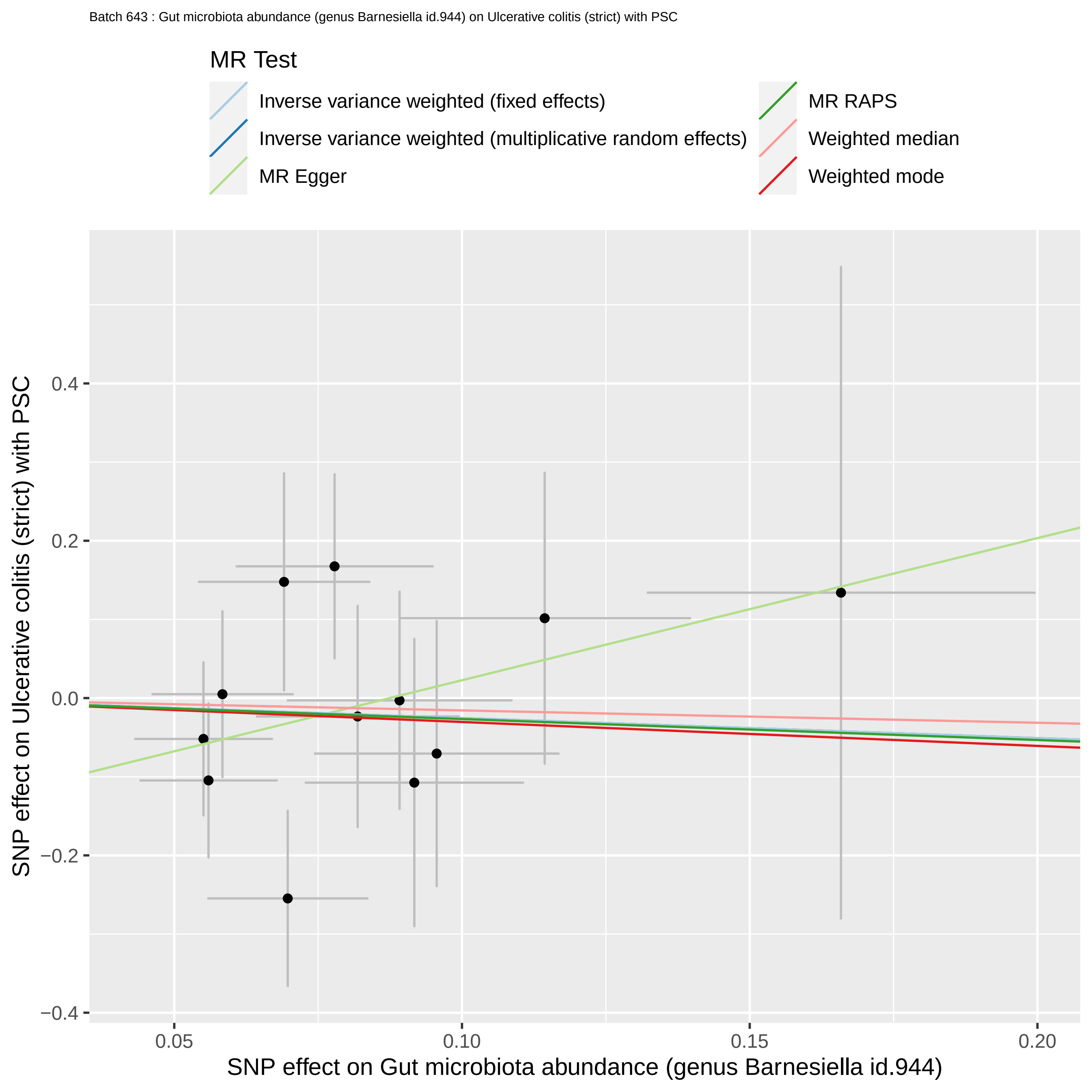

Batch 643 : Gut microbiota abundance (genus Barnesiella id.944) on Ulcerative colitis (strict) with PSC
MR Test
Inverse variance weighted (fixed effects)
Inverse variance weighted (multiplicative random effects)
MR Egger
MR RAPS
Weighted median
Weighted mode
SNP effect on Ulcerative colitis (strict) with PSC
0.4
0.2
0.0
−0.2
−0.4
0.05
0.10
0.15
0.20
SNP effect on Gut microbiota abundance (genus Barnesiella id.944)

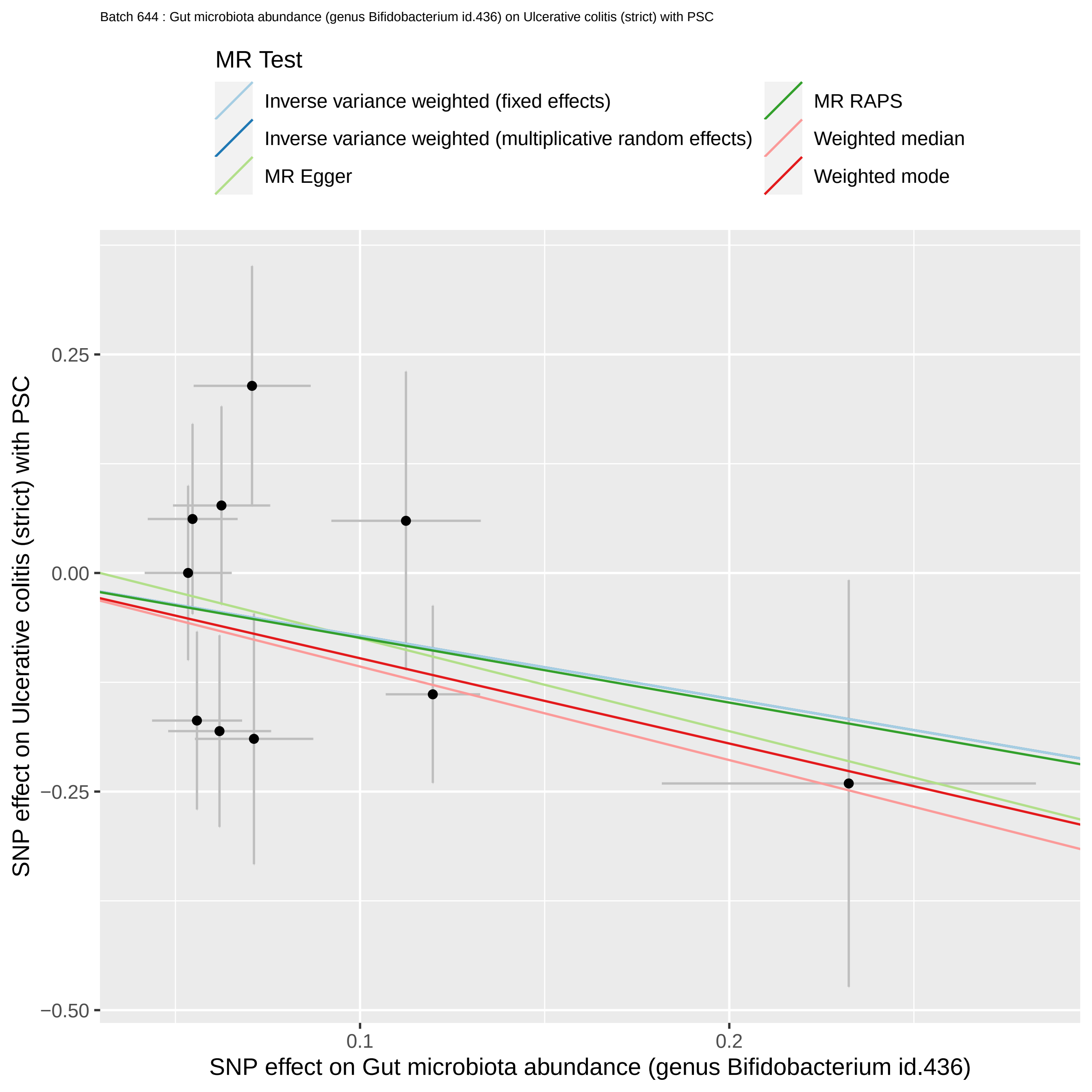

Batch 644 : Gut microbiota abundance (genus Bifidobacterium id.436) on Ulcerative colitis (strict) with PSC
MR Test
Inverse variance weighted (fixed effects)
Inverse variance weighted (multiplicative random effects)
MR Egger
MR RAPS
Weighted median
Weighted mode
0.25
0.00
−0.25
−0.50
0.1
0.2
SNP effect on Ulcerative colitis (strict) with PSC
SNP effect on Gut microbiota abundance (genus Bifidobacterium id.436)

Batch 645 : Gut microbiota abundance (genus Bilophila id.3170) on Ulcerative colitis (strict) with PSC

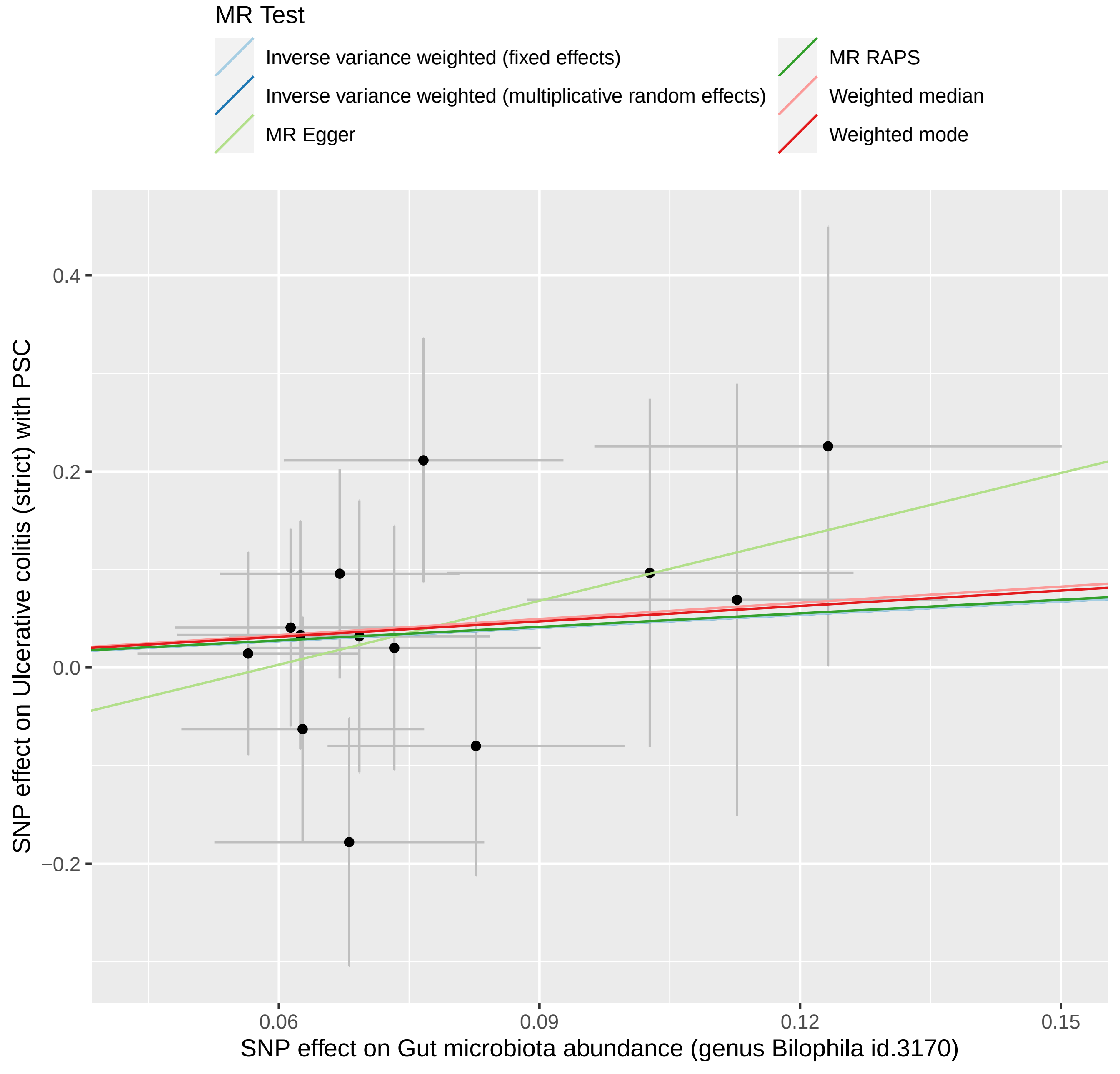

Batch 646 : Gut microbiota abundance (genus Blautia id.1992) on Ulcerative colitis (strict) with PSC

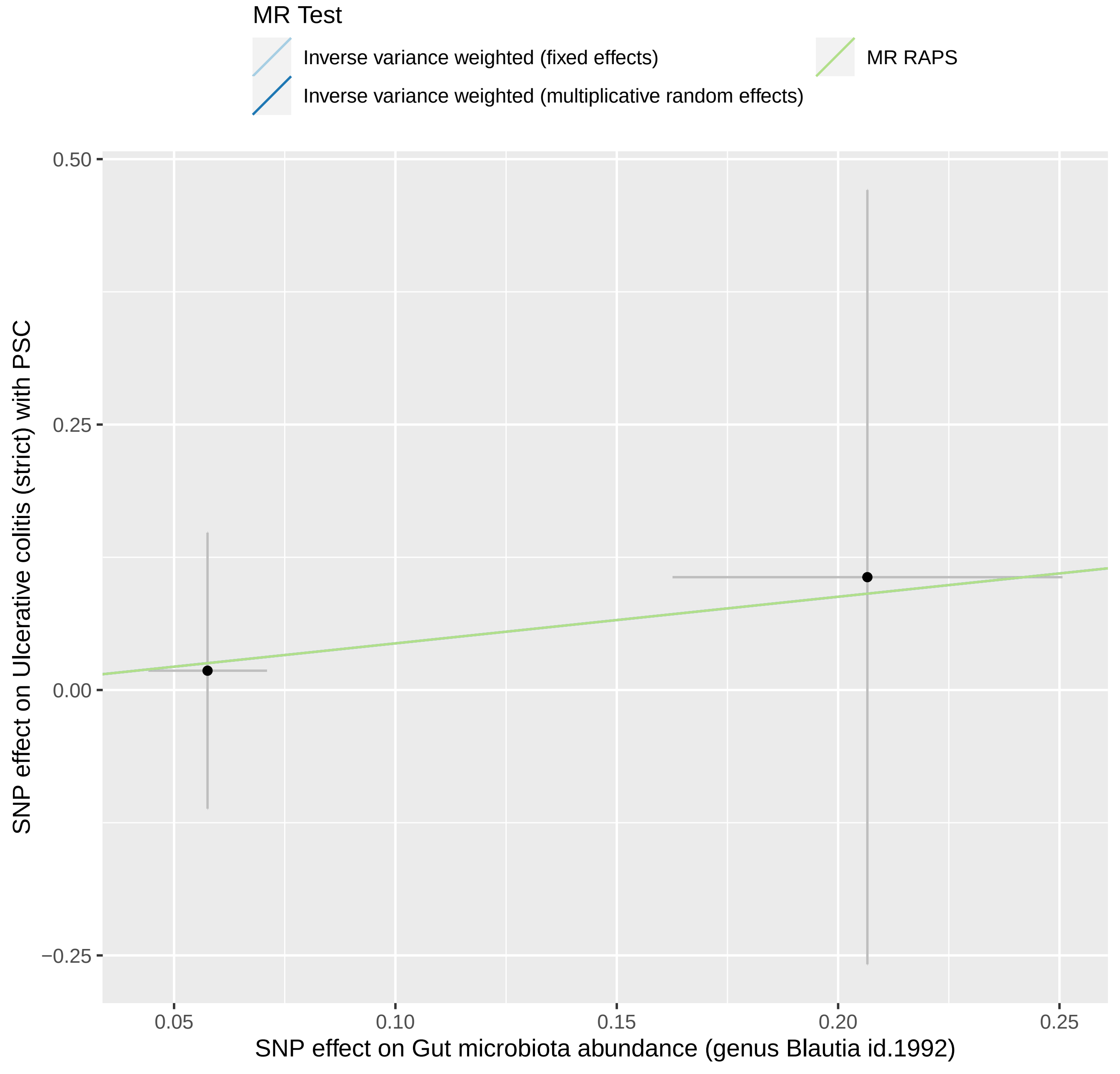

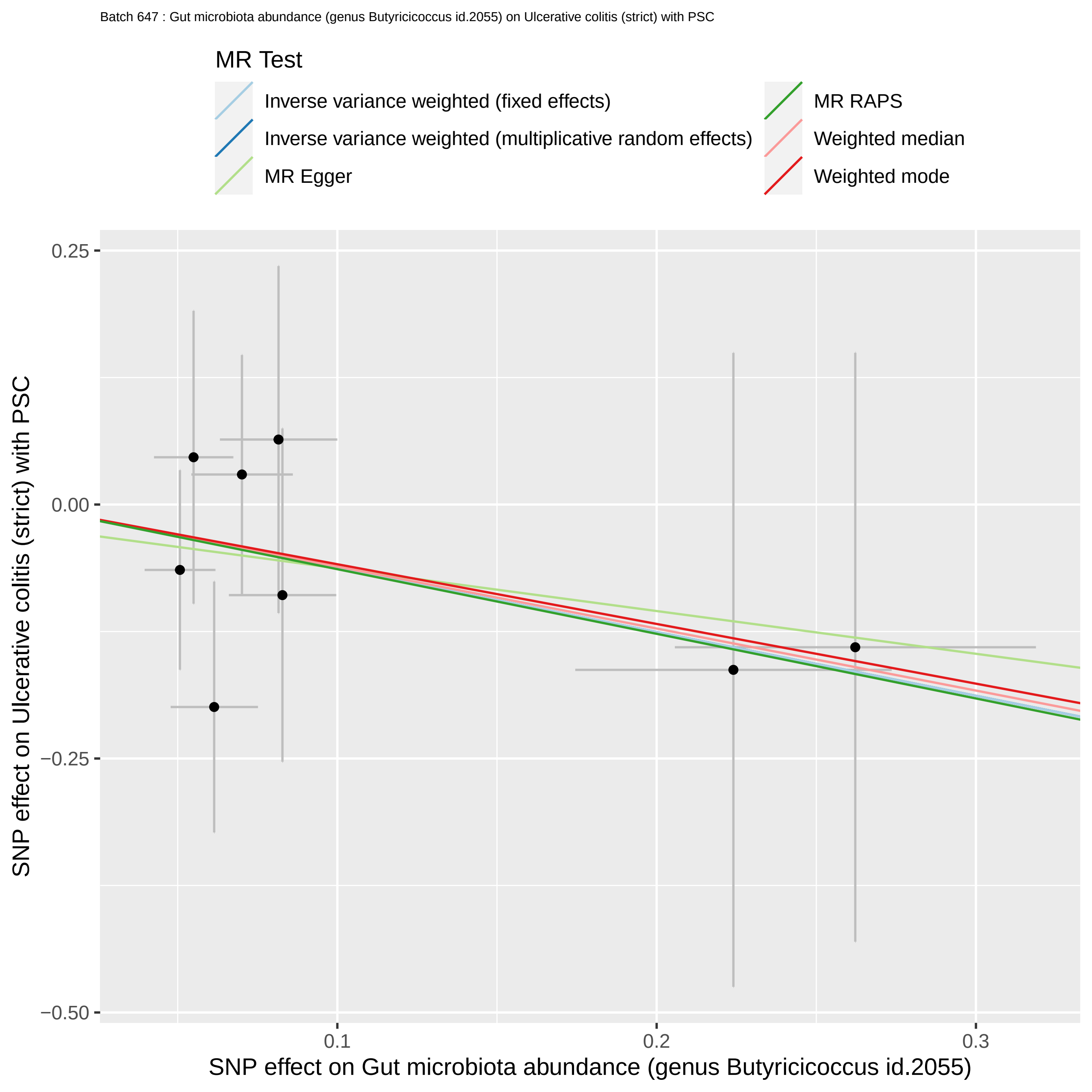
Batch 647 : Gut microbiota abundance (genus Butyricicoccus id.2055) on Ulcerative colitis (strict) with PSC
MR Test
Inverse variance weighted (fixed effects)
Inverse variance weighted (multiplicative random effects)
MR Egger
MR RAPS
Weighted median
Weighted mode
SNP effect on Ulcerative colitis (strict) with PSC
0.25
0.00
−0.25
−0.50
0.1
0.2
0.3
SNP effect on Gut microbiota abundance (genus Butyricicoccus id.2055)

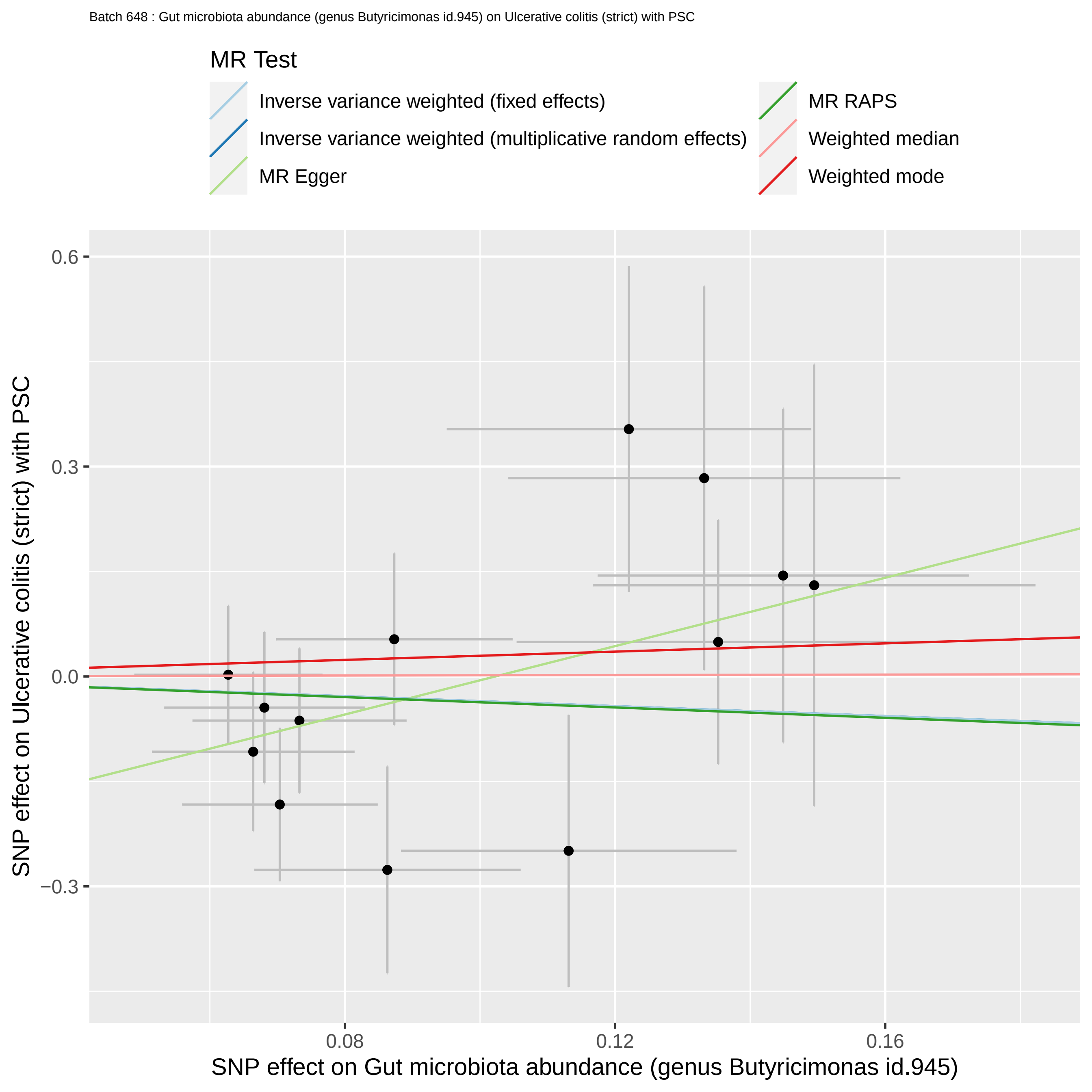
Batch 648 : Gut microbiota abundance (genus Butyricimonas id.945) on Ulcerative colitis (strict) with PSC
MR Test
Inverse variance weighted (fixed effects)
Inverse variance weighted (multiplicative random effects)
MR Egger
MR RAPS
Weighted median
Weighted mode
0.6
0.3
0.0
−0.3
0.08
0.12
0.16
SNP effect on Ulcerative colitis (strict) with PSC
SNP effect on Gut microbiota abundance (genus Butyricimonas id.945)

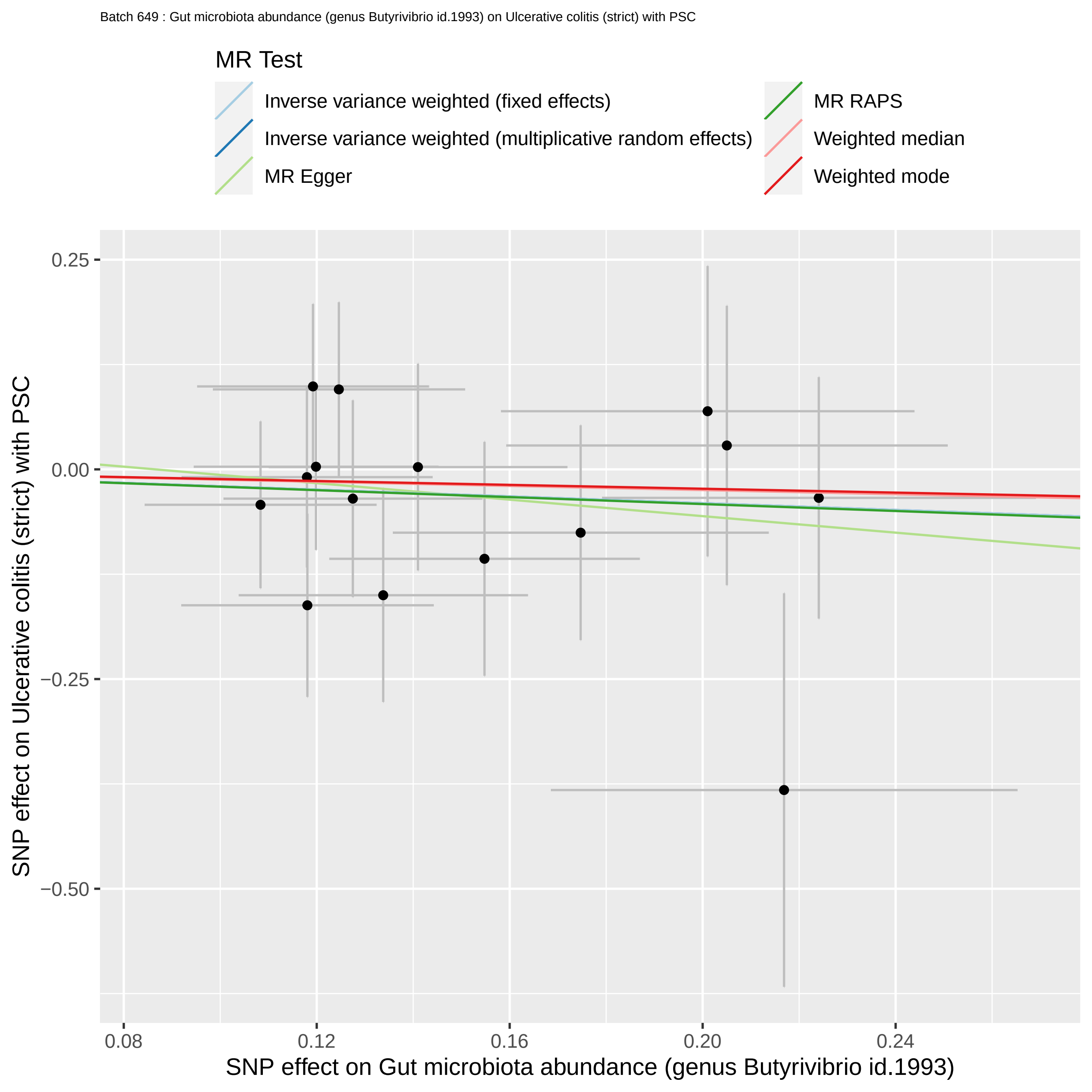

Batch 649 : Gut microbiota abundance (genus Butyrivibrio id.1993) on Ulcerative colitis (strict) with PSC
MR Test
Inverse variance weighted (fixed effects)
Inverse variance weighted (multiplicative random effects)
MR Egger
MR RAPS
Weighted median
Weighted mode
0.25
0.00
−0.25
−0.50
0.08
0.12
0.16
0.20
0.24
SNP effect on Ulcerative colitis (strict) with PSC
SNP effect on Gut microbiota abundance (genus Butyrivibrio id.1993)

Batch 650 : Gut microbiota abundance (genus Candidatus Soleaferrea id.11350) on Ulcerative colitis (strict) with PSC

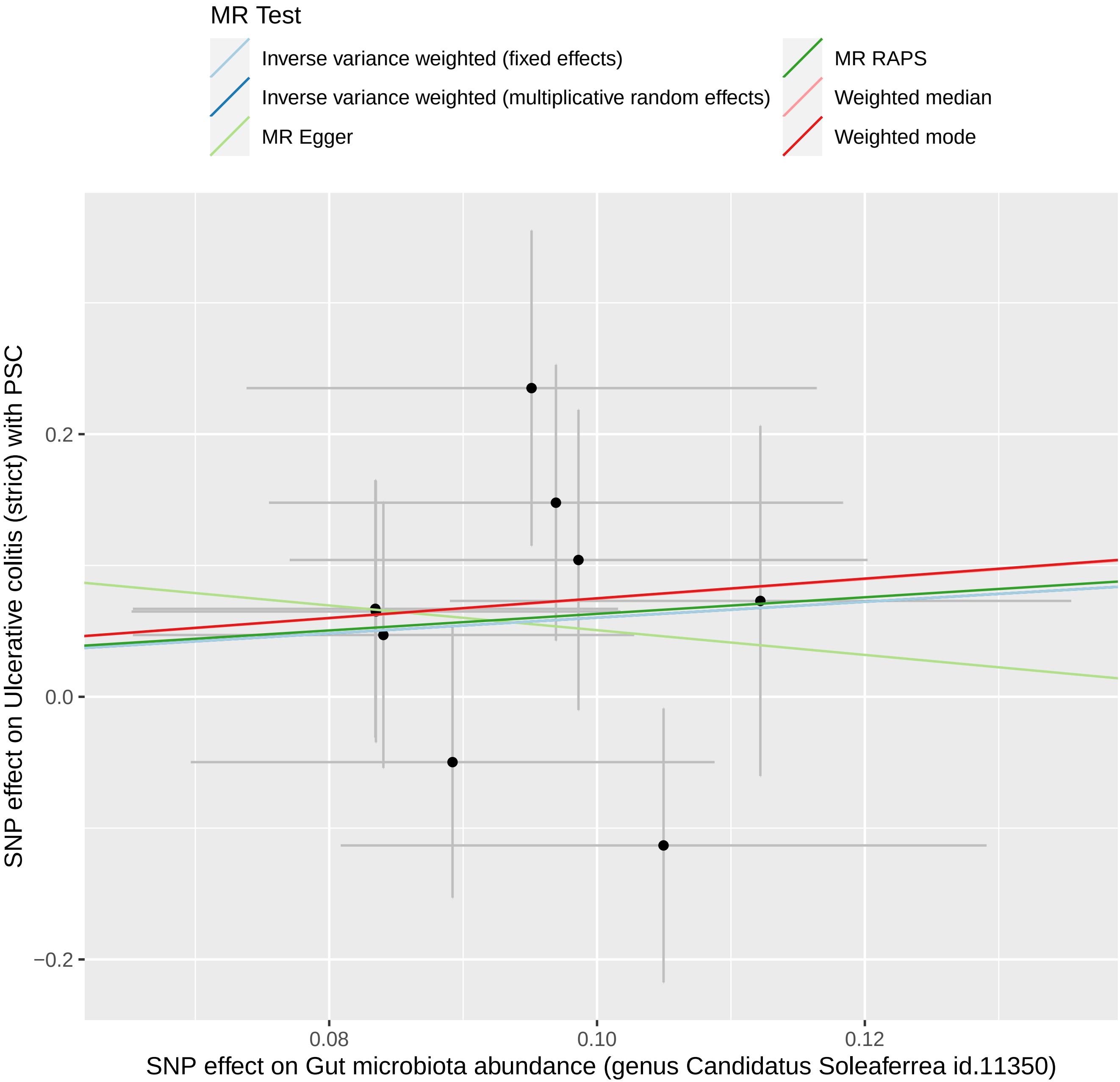

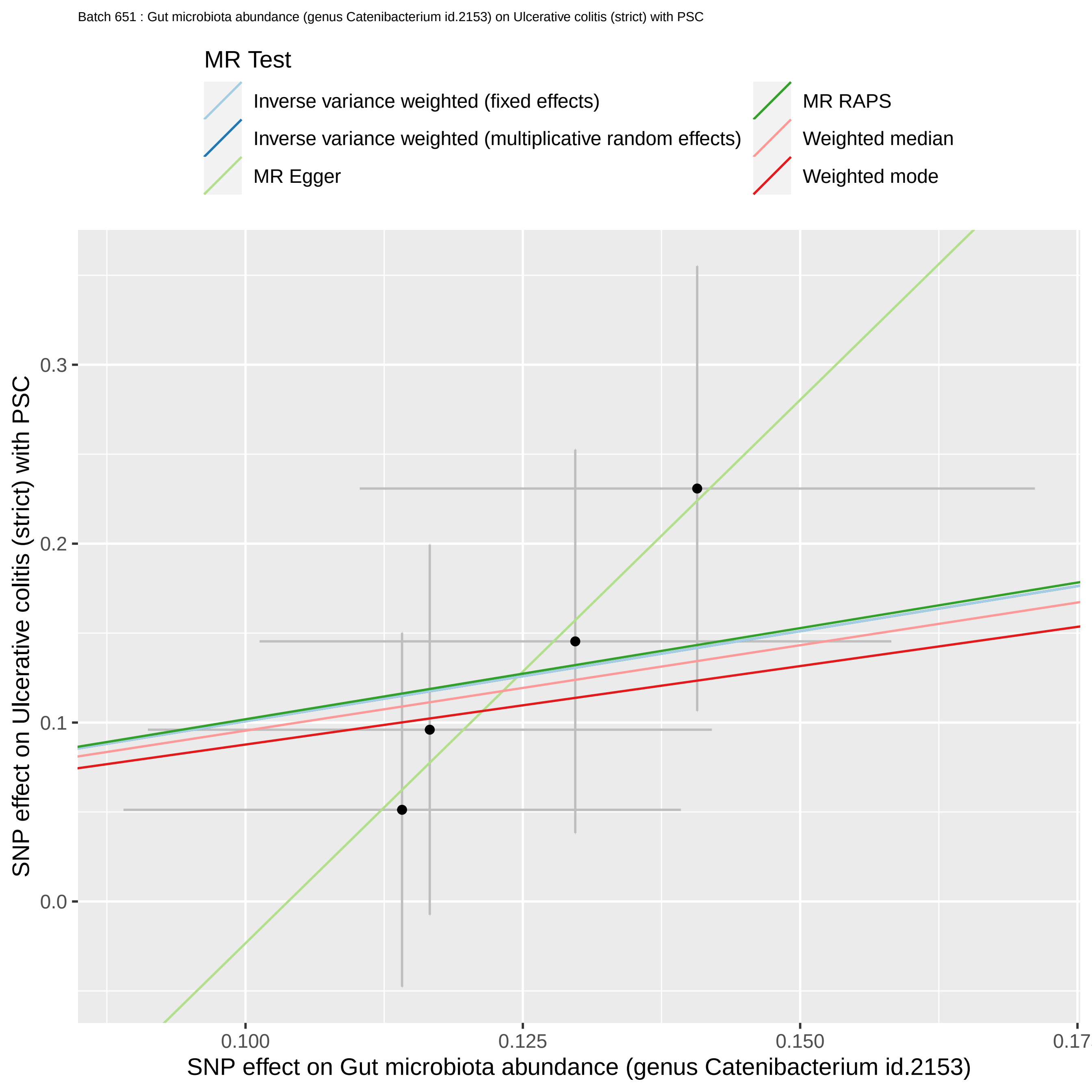

Batch 651 : Gut microbiota abundance (genus Catenibacterium id.2153) on Ulcerative colitis (strict) with PSC
MR Test
Inverse variance weighted (fixed effects)
Inverse variance weighted (multiplicative random effects)
MR Egger
MR RAPS
Weighted median
Weighted mode
SNP effect on Ulcerative colitis (strict) with PSC
0.0
0.1
0.2
0.3
0.100
0.125
0.150
0.17
SNP effect on Gut microbiota abundance (genus Catenibacterium id.2153)

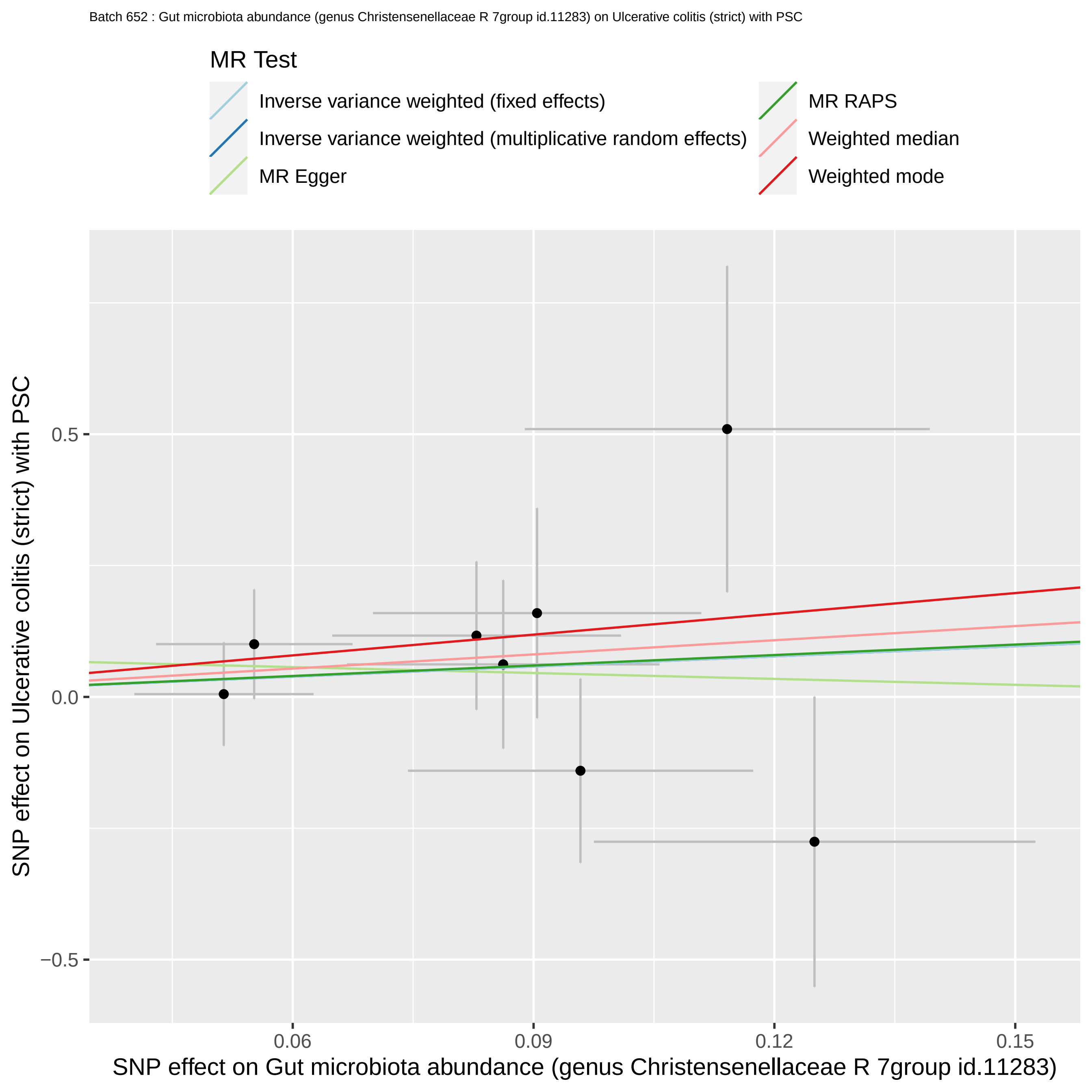

Batch 652 : Gut microbiota abundance (genus Christensenellaceae R 7group id.11283) on Ulcerative colitis (strict) with PSC
MR Test
Inverse variance weighted (fixed effects)
Inverse variance weighted (multiplicative random effects)
MR Egger
MR RAPS
Weighted median
Weighted mode
0.5
0.0
−0.5
0.06
0.09
0.12
0.15
SNP effect on Ulcerative colitis (strict) with PSC
SNP effect on Gut microbiota abundance (genus Christensenellaceae R 7group id.11283)

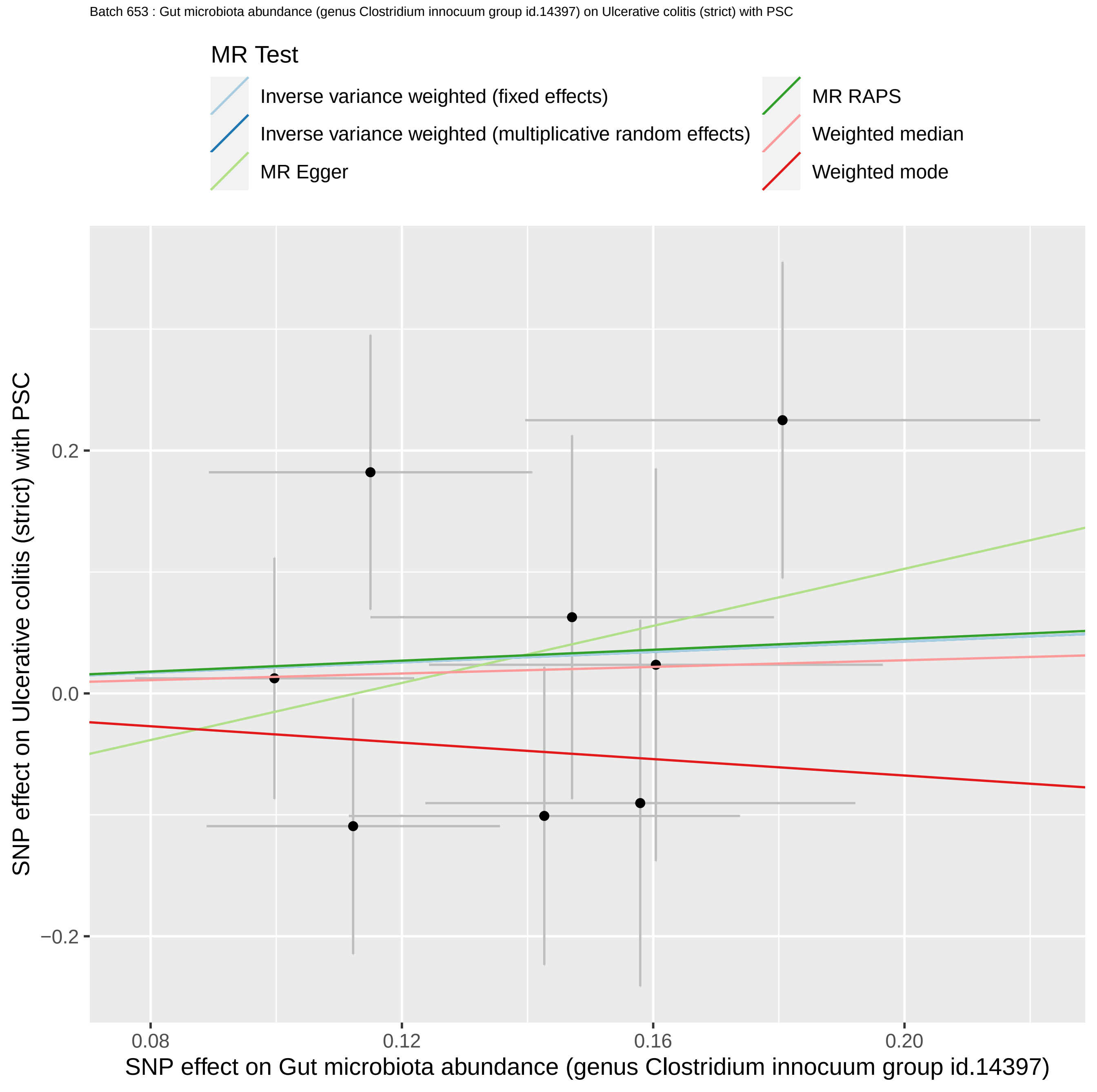
Batch 653 : Gut microbiota abundance (genus Clostridium innocuum group id.14397) on Ulcerative colitis (strict) with PSC
MR Test
Inverse variance weighted (fixed effects)
Inverse variance weighted (multiplicative random effects)
MR Egger
MR RAPS
Weighted median
Weighted mode
0.2
0.0
−0.2
0.08
0.12
0.16
0.20
SNP effect on Ulcerative colitis (strict) with PSC
SNP effect on Gut microbiota abundance (genus Clostridium innocuum group id.14397)

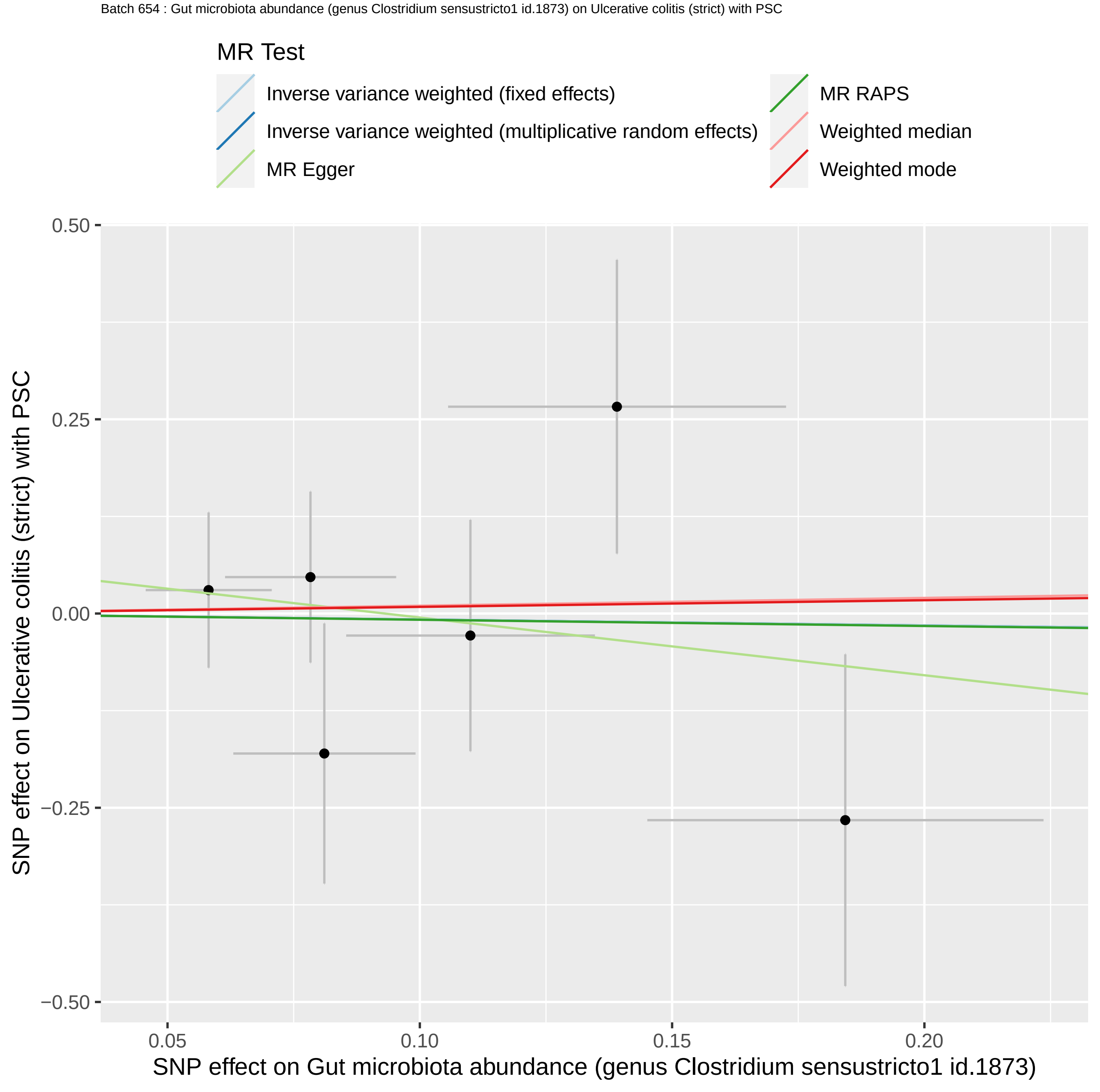

Batch 654 : Gut microbiota abundance (genus Clostridium sensustricto1 id.1873) on Ulcerative colitis (strict) with PSC
MR Test
Inverse variance weighted (fixed effects)
Inverse variance weighted (multiplicative random effects)
MR Egger
MR RAPS
Weighted median
Weighted mode
0.50
0.25
0.00
−0.25
−0.50
0.05
0.10
0.15
0.20
SNP effect on Ulcerative colitis (strict) with PSC
SNP effect on Gut microbiota abundance (genus Clostridium sensustricto1 id.1873)

Batch 655 : Gut microbiota abundance (genus Collinsella id.815) on Ulcerative colitis (strict) with PSC

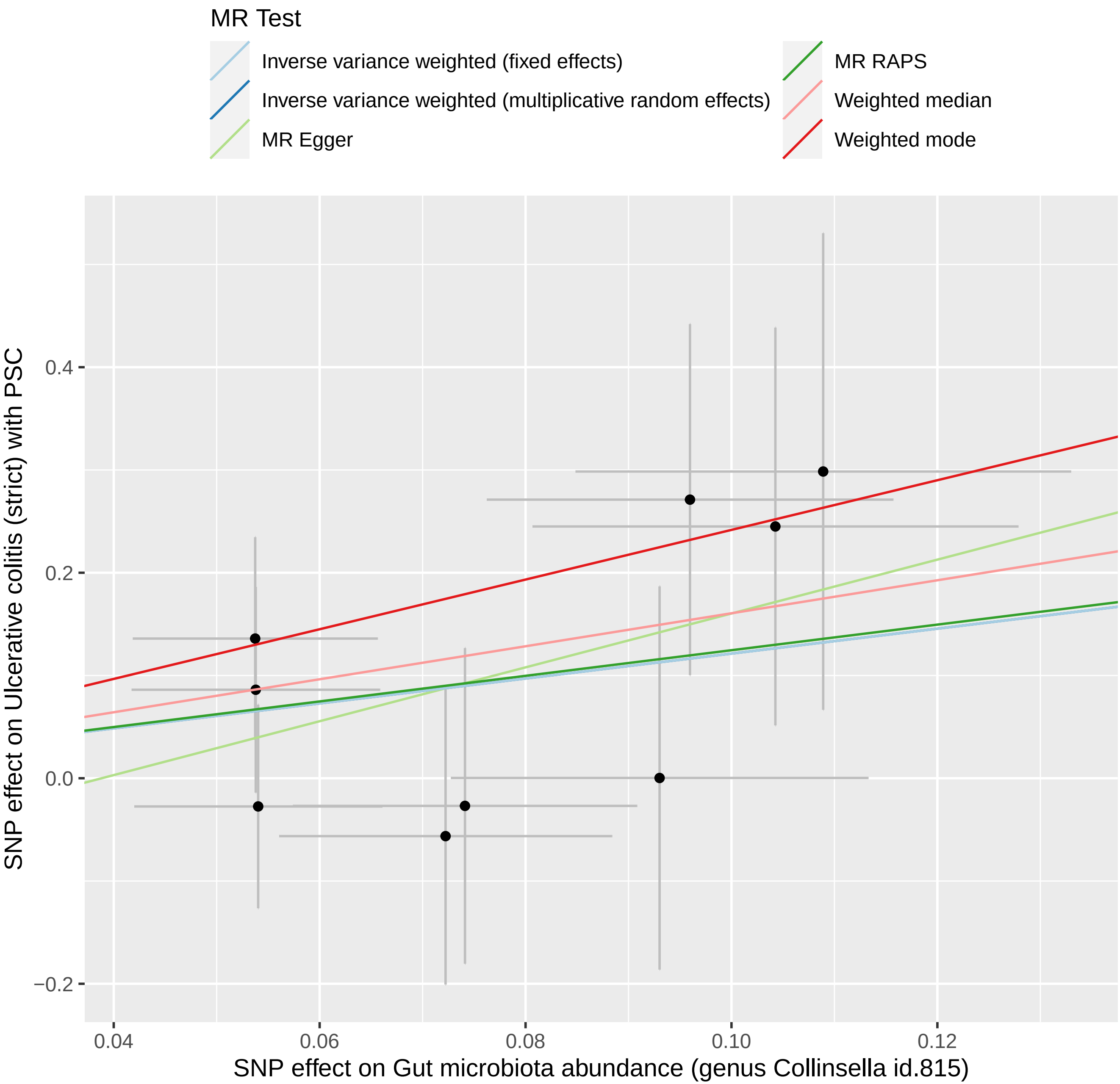

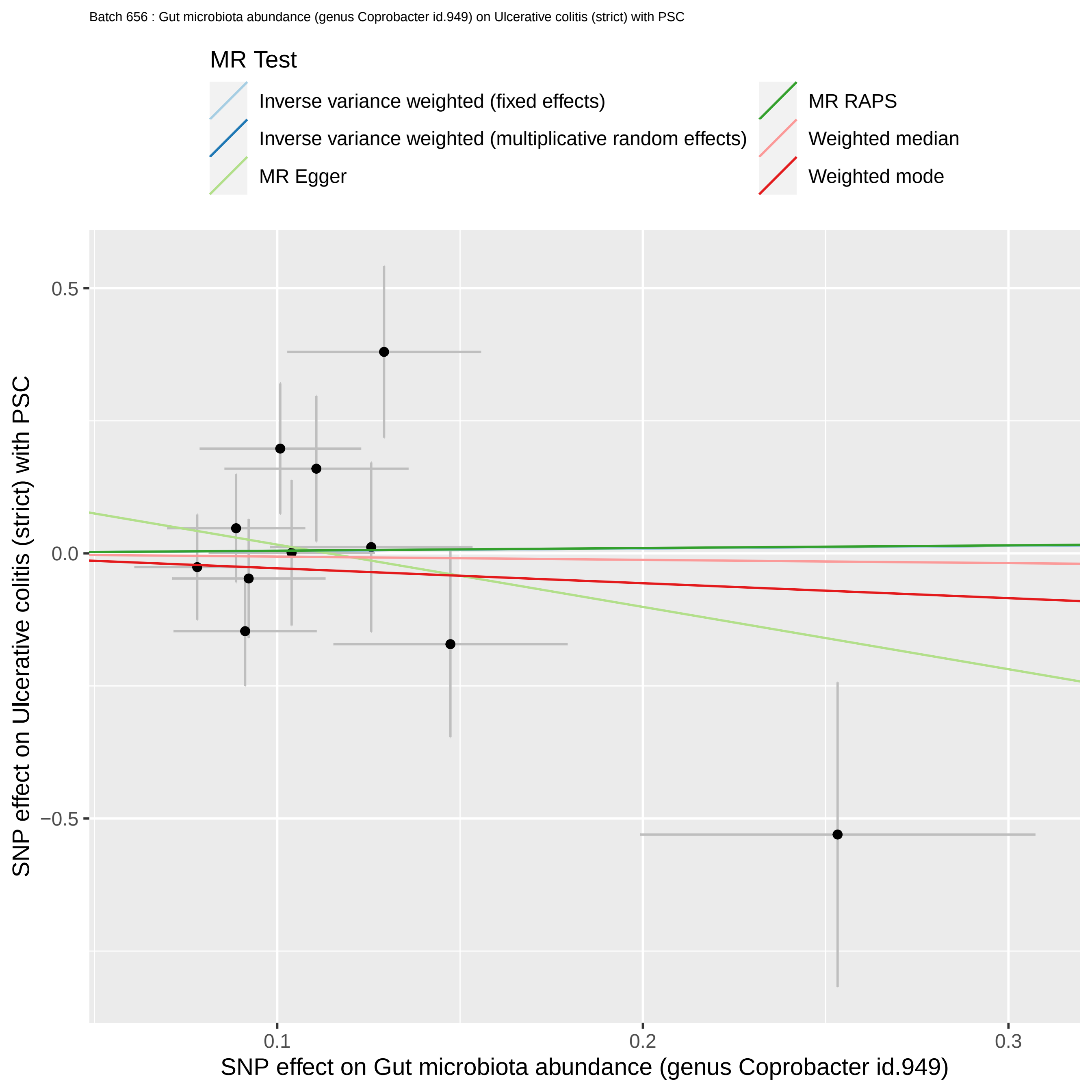

Batch 656 : Gut microbiota abundance (genus Coprobacter id.949) on Ulcerative colitis (strict) with PSC
MR Test
Inverse variance weighted (fixed effects)
Inverse variance weighted (multiplicative random effects)
MR Egger
MR RAPS
Weighted median
Weighted mode
0.5
0.0
−0.5
0.1
0.2
0.3
SNP effect on Ulcerative colitis (strict) with PSC
SNP effect on Gut microbiota abundance (genus Coprobacter id.949)

Batch 657 : Gut microbiota abundance (genus Coprococcus1 id.11301) on Ulcerative colitis (strict) with PSC

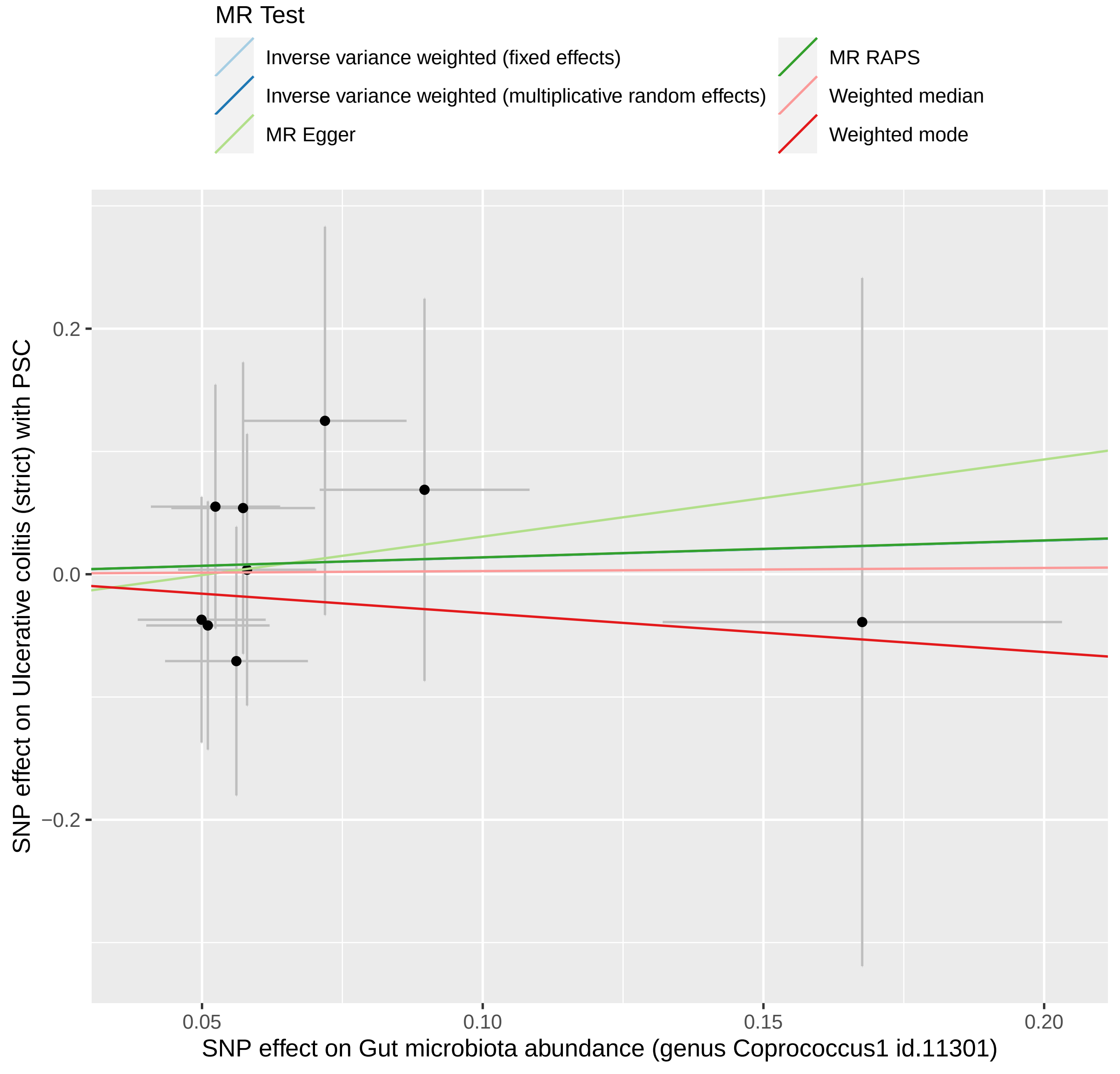

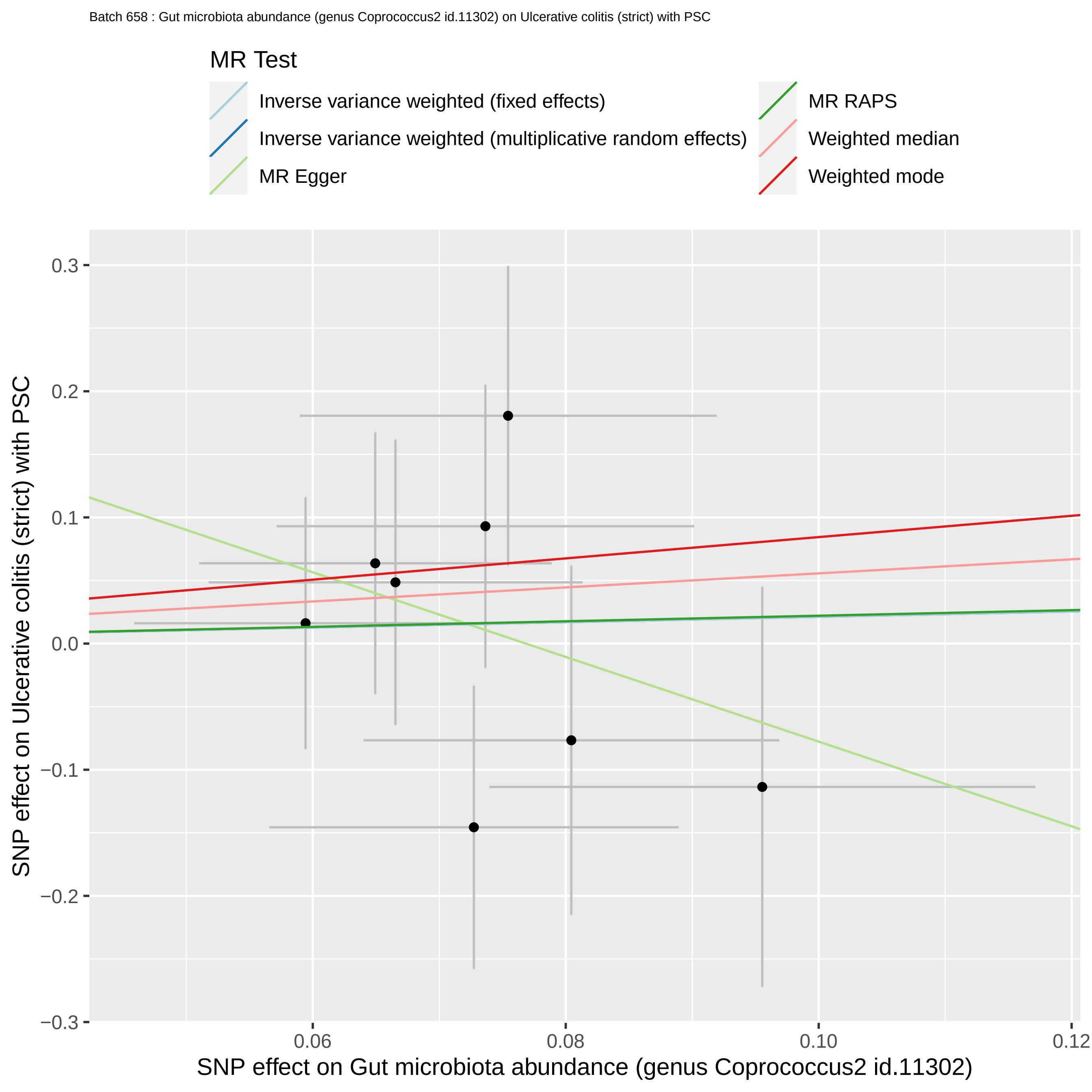

Batch 658 : Gut microbiota abundance (genus Coprococcus2 id.11302) on Ulcerative colitis (strict) with PSC
MR Test
Inverse variance weighted (fixed effects)
Inverse variance weighted (multiplicative random effects)
MR Egger
MR RAPS
Weighted median
Weighted mode
0.3
0.2
0.1
0.0
−0.1
−0.2
−0.3
0.06
0.08
0.10
0.12
SNP effect on Ulcerative colitis (strict) with PSC
SNP effect on Gut microbiota abundance (genus Coprococcus2 id.11302)

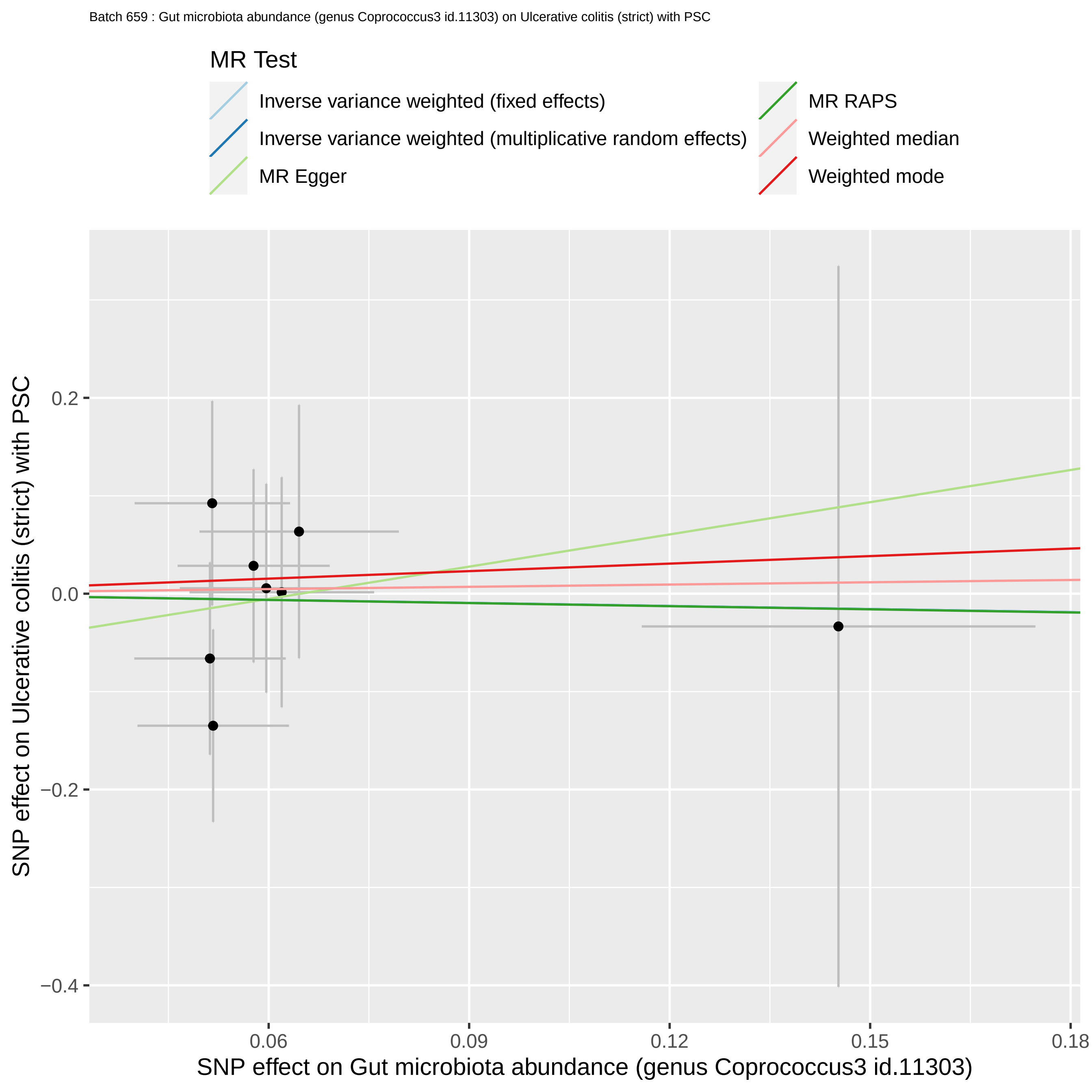
Batch 659 : Gut microbiota abundance (genus Coprococcus3 id.11303) on Ulcerative colitis (strict) with PSC
MR Test
Inverse variance weighted (fixed effects)
Inverse variance weighted (multiplicative random effects)
MR Egger
MR RAPS
Weighted median
Weighted mode
0.2
0.0
−0.2
−0.4
0.06
0.09
0.12
0.15
0.18
SNP effect on Ulcerative colitis (strict) with PSC
SNP effect on Gut microbiota abundance (genus Coprococcus3 id.11303)

Batch 660 : Gut microbiota abundance (genus Defluviitaleaceae UCG011 id.11287) on Ulcerative colitis (strict) with PSC

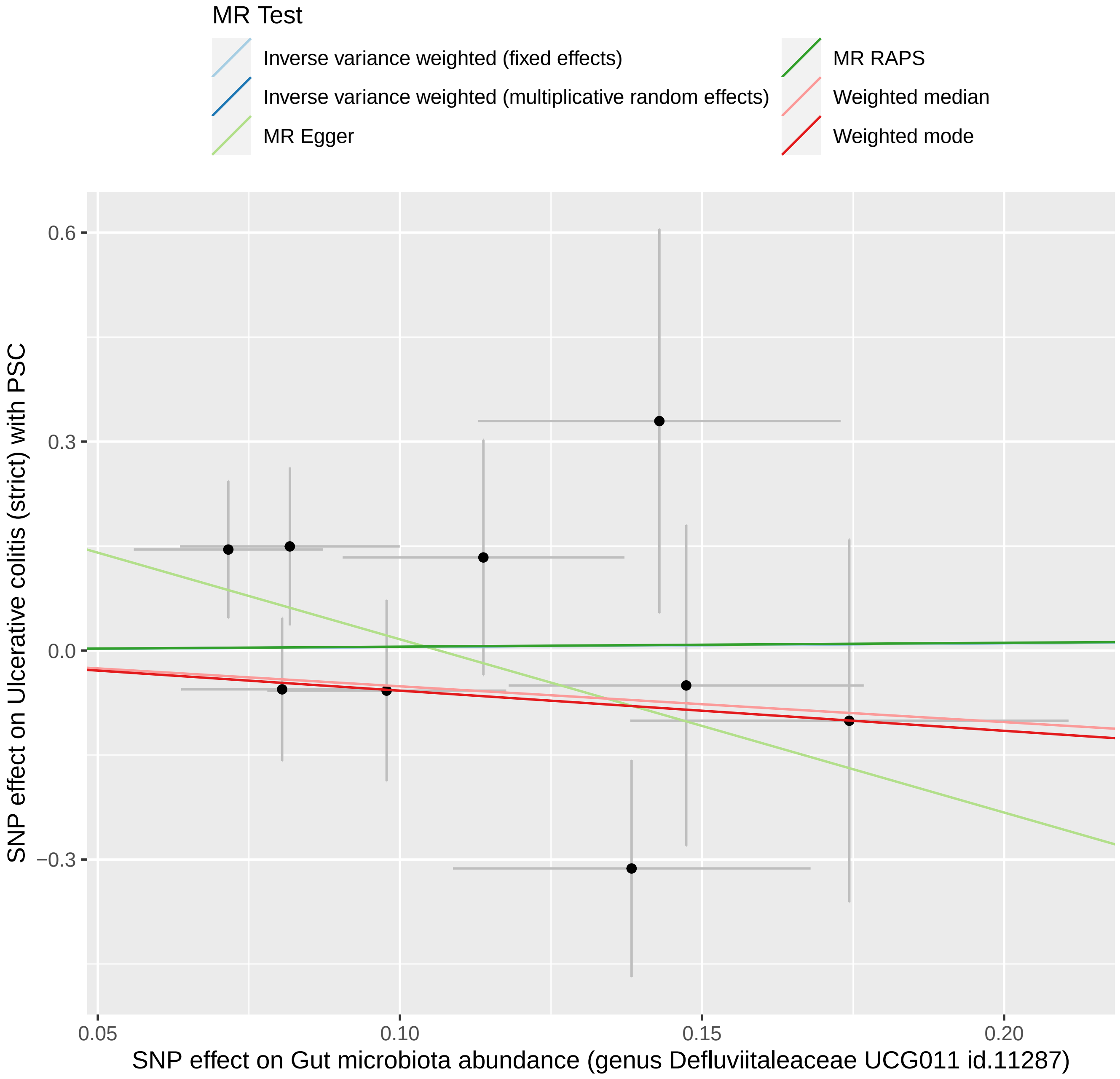

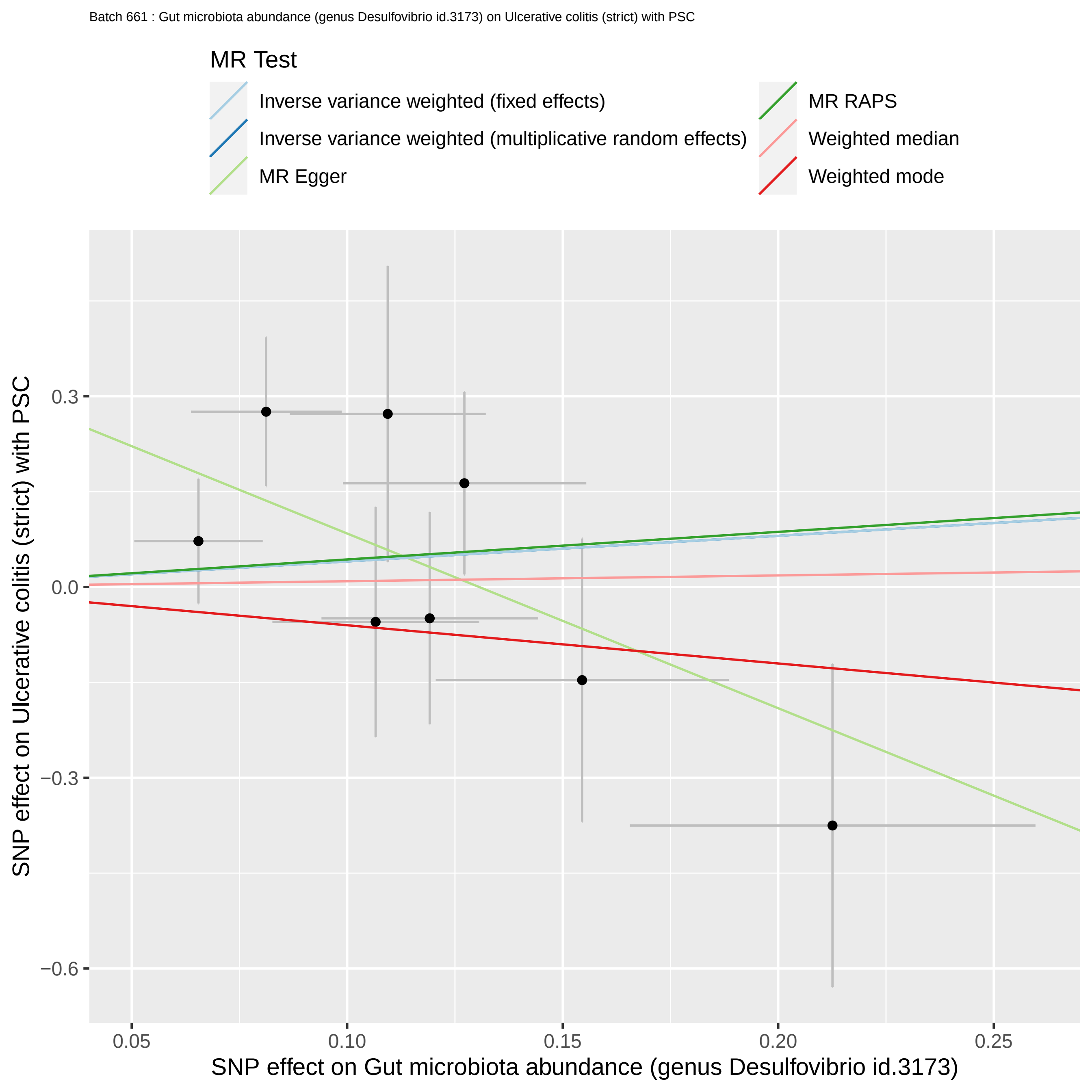

Batch 661 : Gut microbiota abundance (genus Desulfovibrio id.3173) on Ulcerative colitis (strict) with PSC
MR Test
Inverse variance weighted (fixed effects)
Inverse variance weighted (multiplicative random effects)
MR Egger
MR RAPS
Weighted median
Weighted mode
SNP effect on Ulcerative colitis (strict) with PSC
0.3
0.0
−0.3
−0.6
0.05
0.10
0.15
0.20
0.25
SNP effect on Gut microbiota abundance (genus Desulfovibrio id.3173)

Batch 662 : Gut microbiota abundance (genus Dialister id.2183) on Ulcerative colitis (strict) with PSC

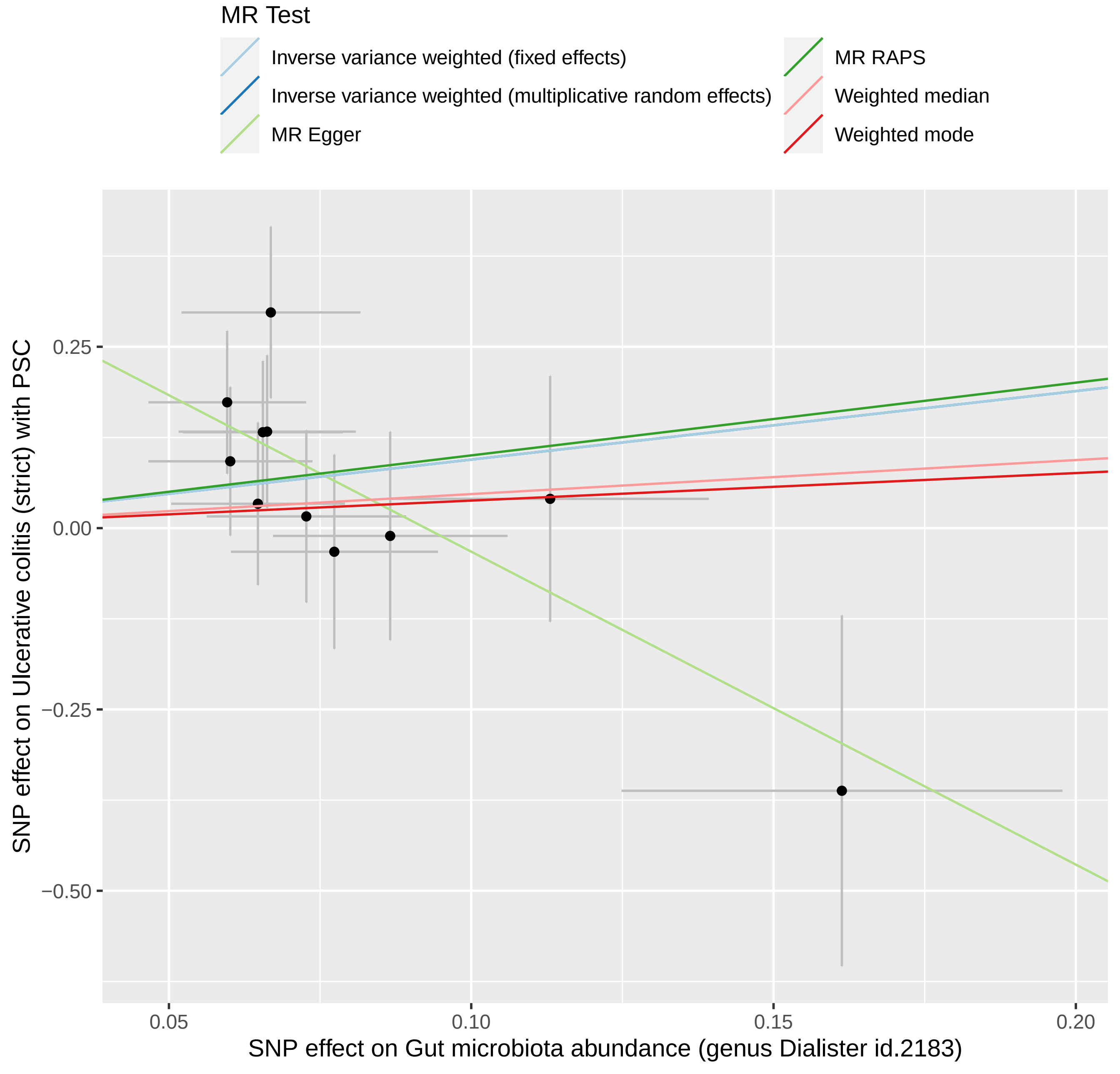

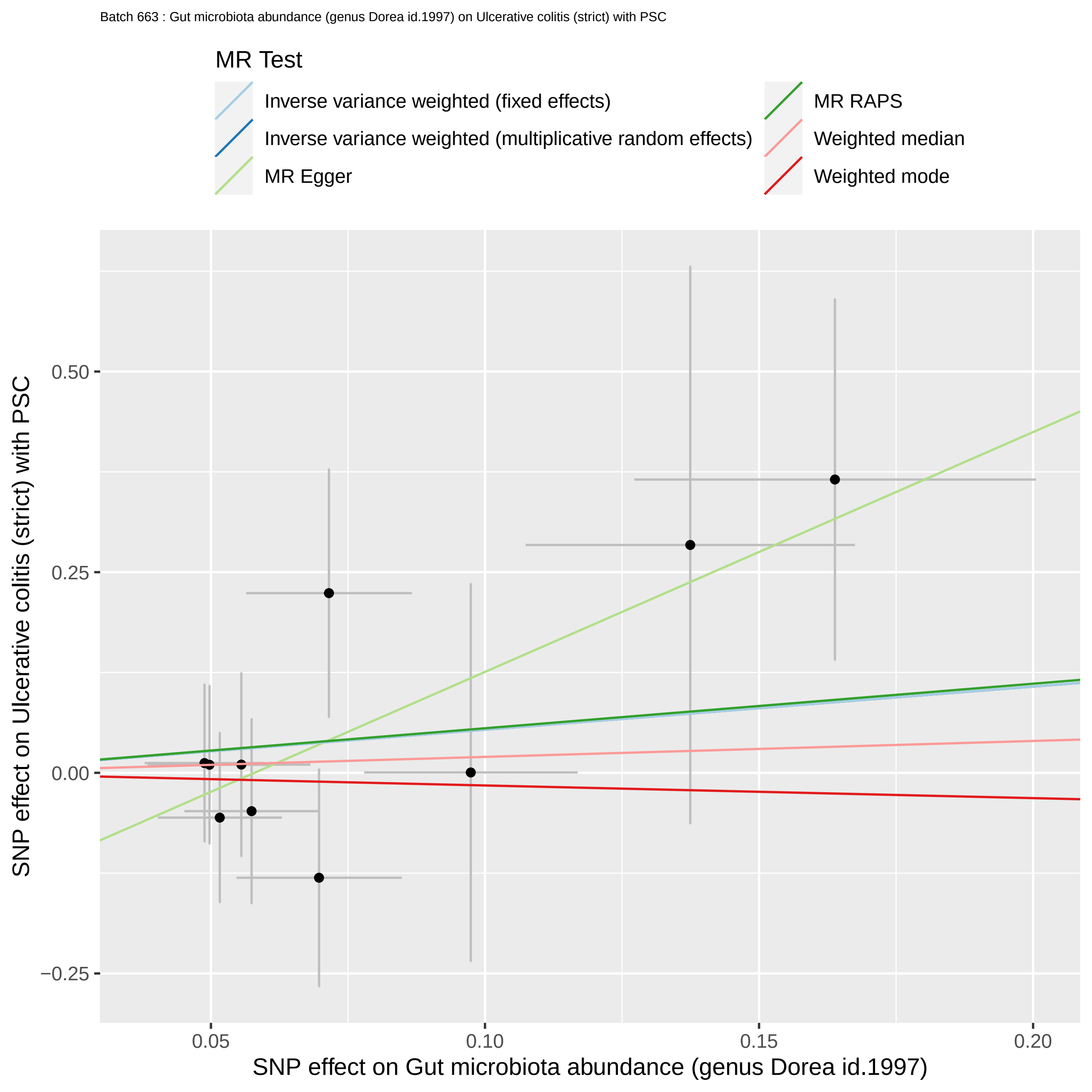

Batch 663 : Gut microbiota abundance (genus Dorea id.1997) on Ulcerative colitis (strict) with PSC
MR Test
Inverse variance weighted (fixed effects)
Inverse variance weighted (multiplicative random effects)
MR Egger
MR RAPS
Weighted median
Weighted mode
SNP effect on Ulcerative colitis (strict) with PSC
0.50
0.25
0.00
−0.25
0.05
0.10
0.15
0.20
SNP effect on Gut microbiota abundance (genus Dorea id.1997)

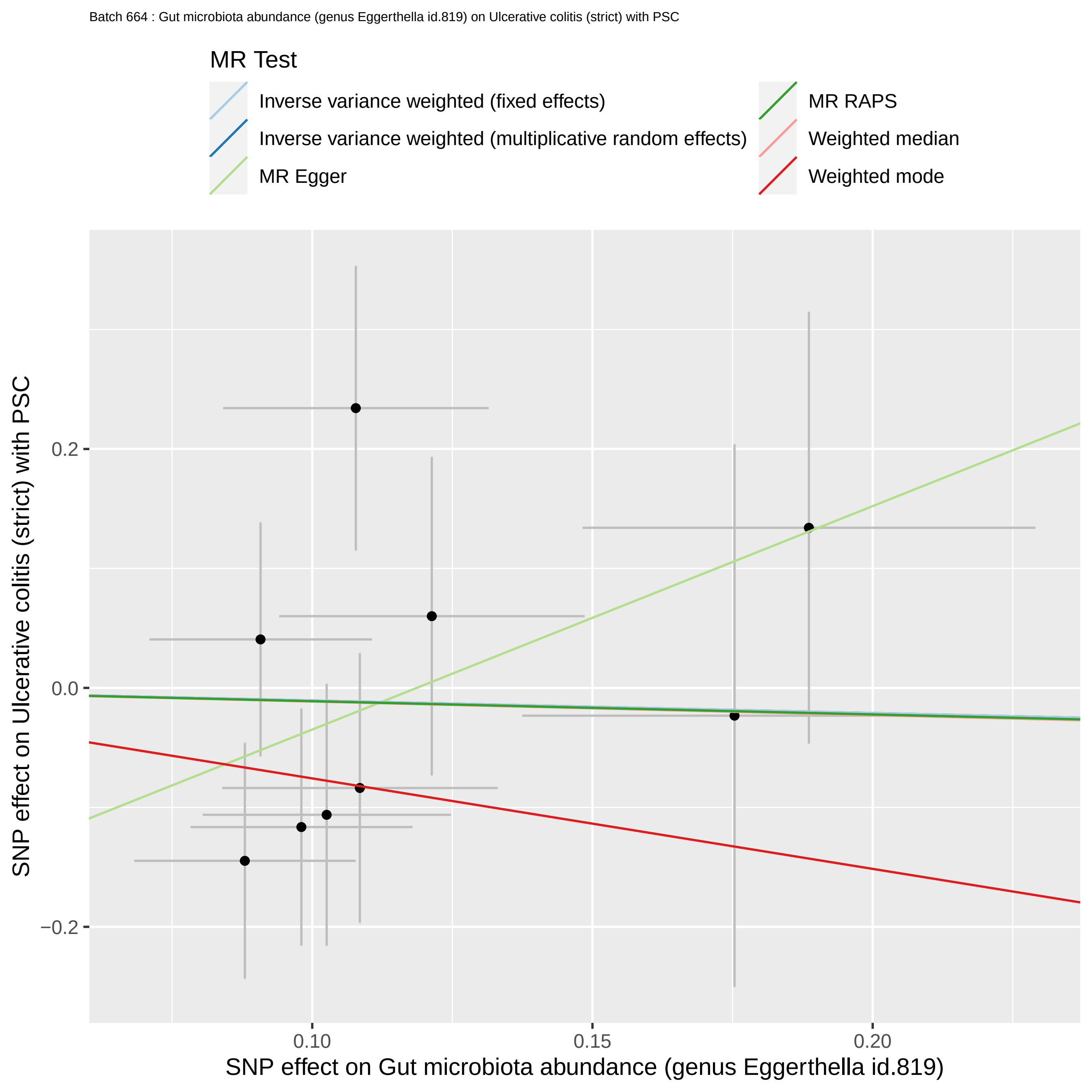

Batch 664 : Gut microbiota abundance (genus Eggerthella id.819) on Ulcerative colitis (strict) with PSC
MR Test
Inverse variance weighted (fixed effects)
Inverse variance weighted (multiplicative random effects)
MR Egger
MR RAPS
Weighted median
Weighted mode
SNP effect on Ulcerative colitis (strict) with PSC
0.2
0.0
−0.2
0.10
0.15
0.20
SNP effect on Gut microbiota abundance (genus Eggerthella id.819)

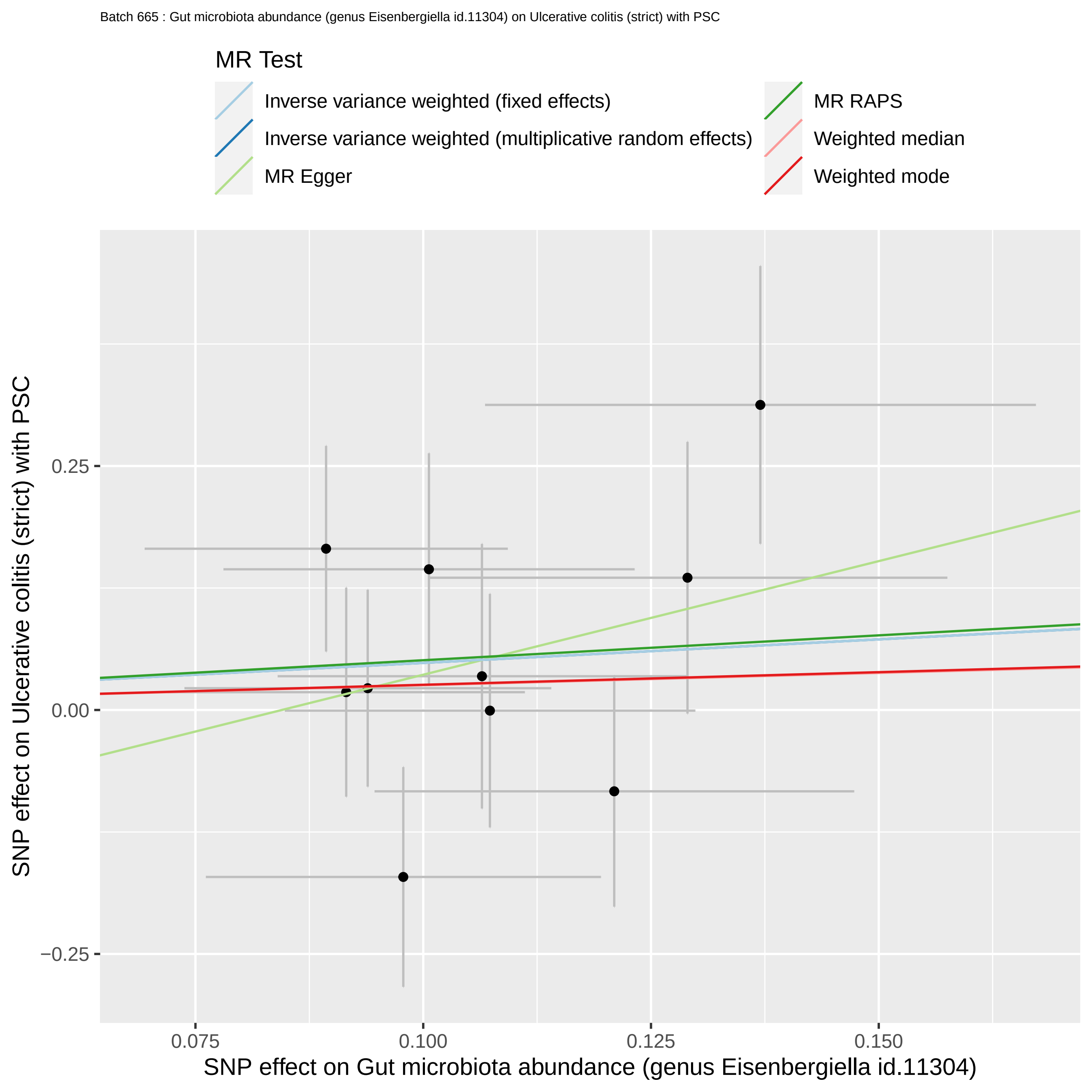
Batch 665 : Gut microbiota abundance (genus Eisenbergiella id.11304) on Ulcerative colitis (strict) with PSC
MR Test
Inverse variance weighted (fixed effects)
Inverse variance weighted (multiplicative random effects)
MR Egger
MR RAPS
Weighted median
Weighted mode
0.25
0.00
−0.25
0.075
0.100
0.125
0.150
SNP effect on Ulcerative colitis (strict) with PSC
SNP effect on Gut microbiota abundance (genus Eisenbergiella id.11304)

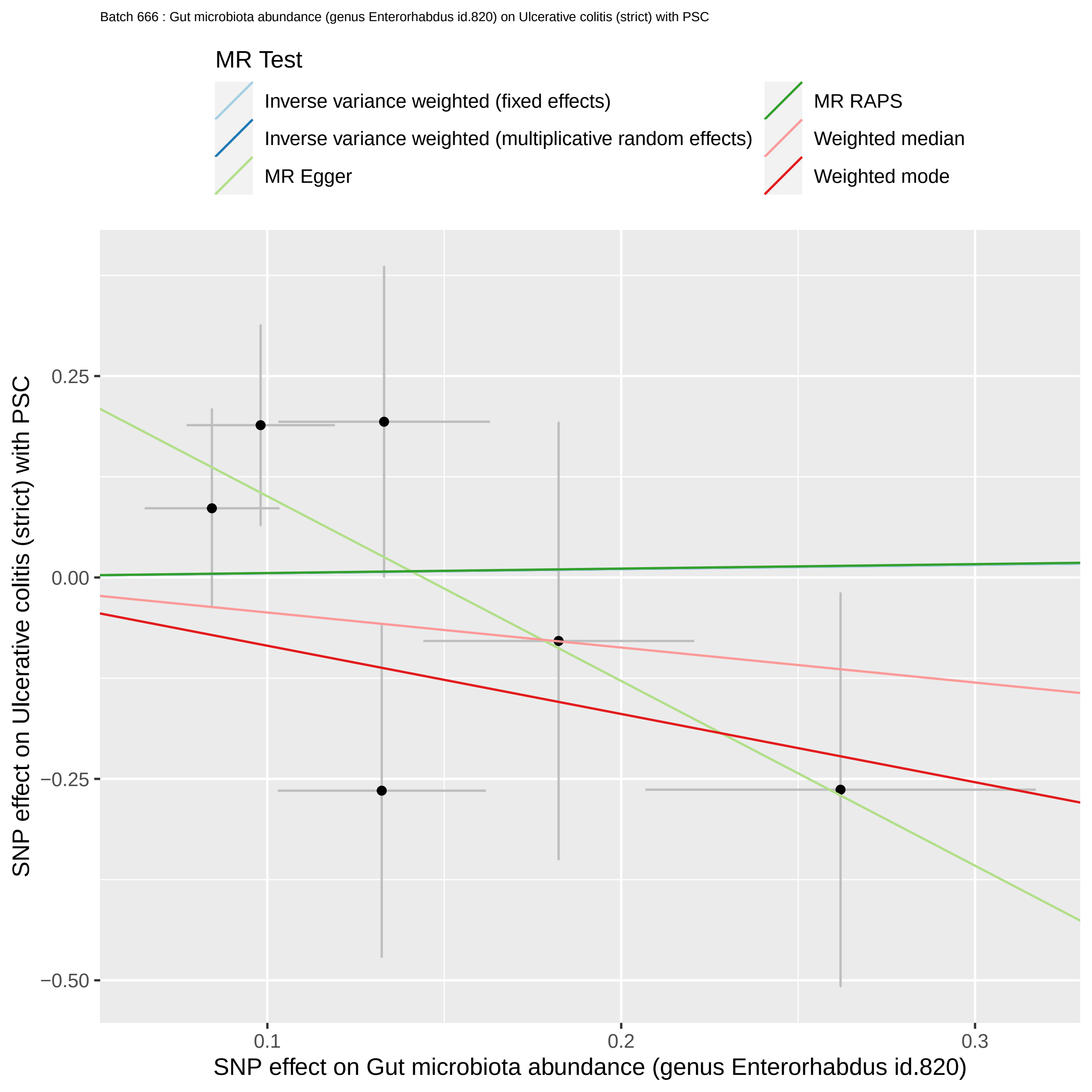

Batch 666 : Gut microbiota abundance (genus Enterorhabdus id.820) on Ulcerative colitis (strict) with PSC
MR Test
Inverse variance weighted (fixed effects)
Inverse variance weighted (multiplicative random effects)
MR Egger
MR RAPS
Weighted median
Weighted mode
0.25
0.00
−0.25
−0.50
0.1
0.2
0.3
SNP effect on Ulcerative colitis (strict) with PSC
SNP effect on Gut microbiota abundance (genus Enterorhabdus id.820)

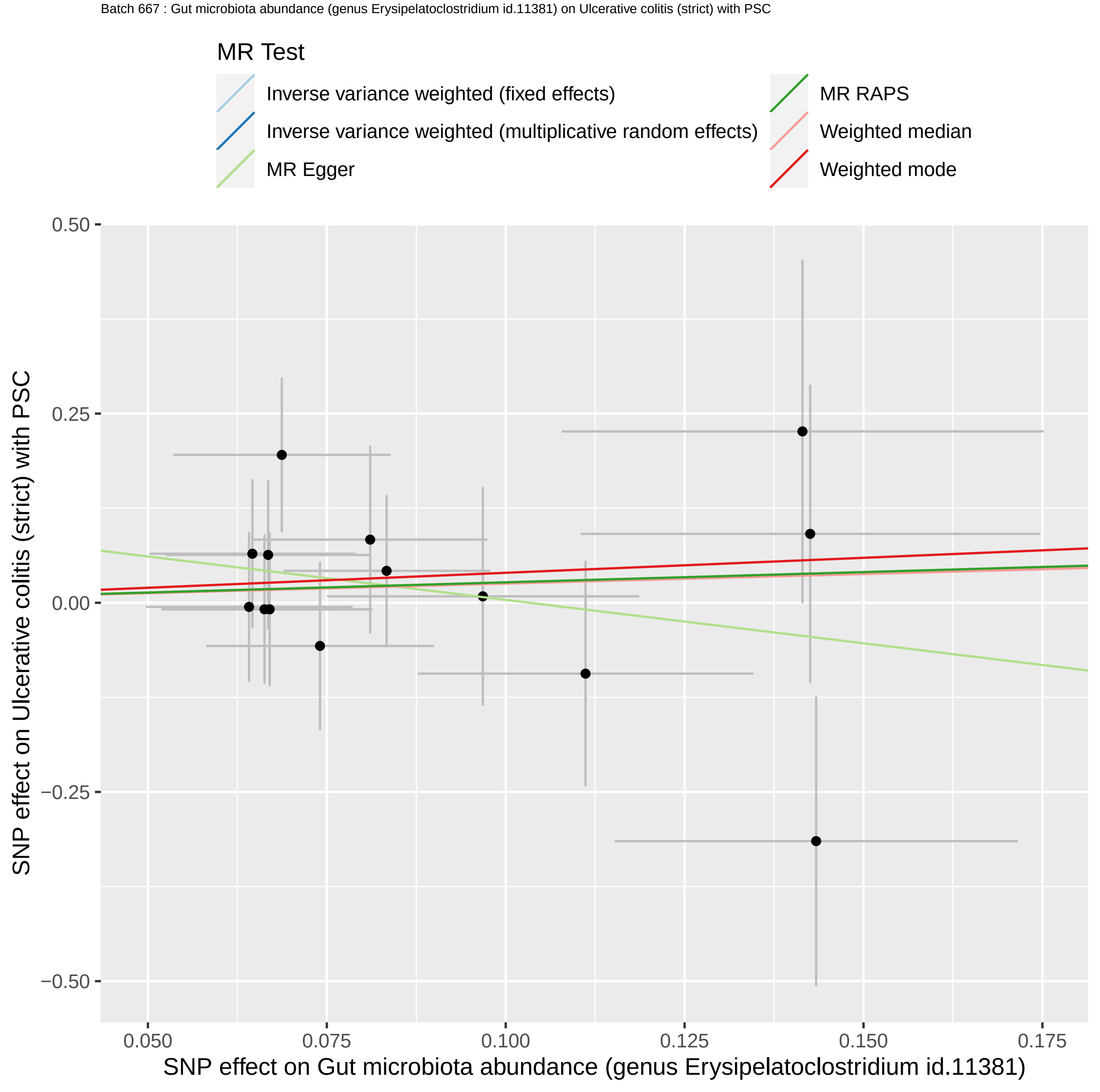

Batch 667 : Gut microbiota abundance (genus Erysipelatoclostridium id.11381) on Ulcerative colitis (strict) with PSC
MR Test
Inverse variance weighted (fixed effects)
Inverse variance weighted (multiplicative random effects)
MR Egger
MR RAPS
Weighted median
Weighted mode
0.50
0.25
0.00
−0.25
−0.50
SNP effect on Ulcerative colitis (strict) with PSC
0.050
0.075
0.100
0.125
0.150
0.175
SNP effect on Gut microbiota abundance (genus Erysipelatoclostridium id.11381)

Insufficient number of SNPs

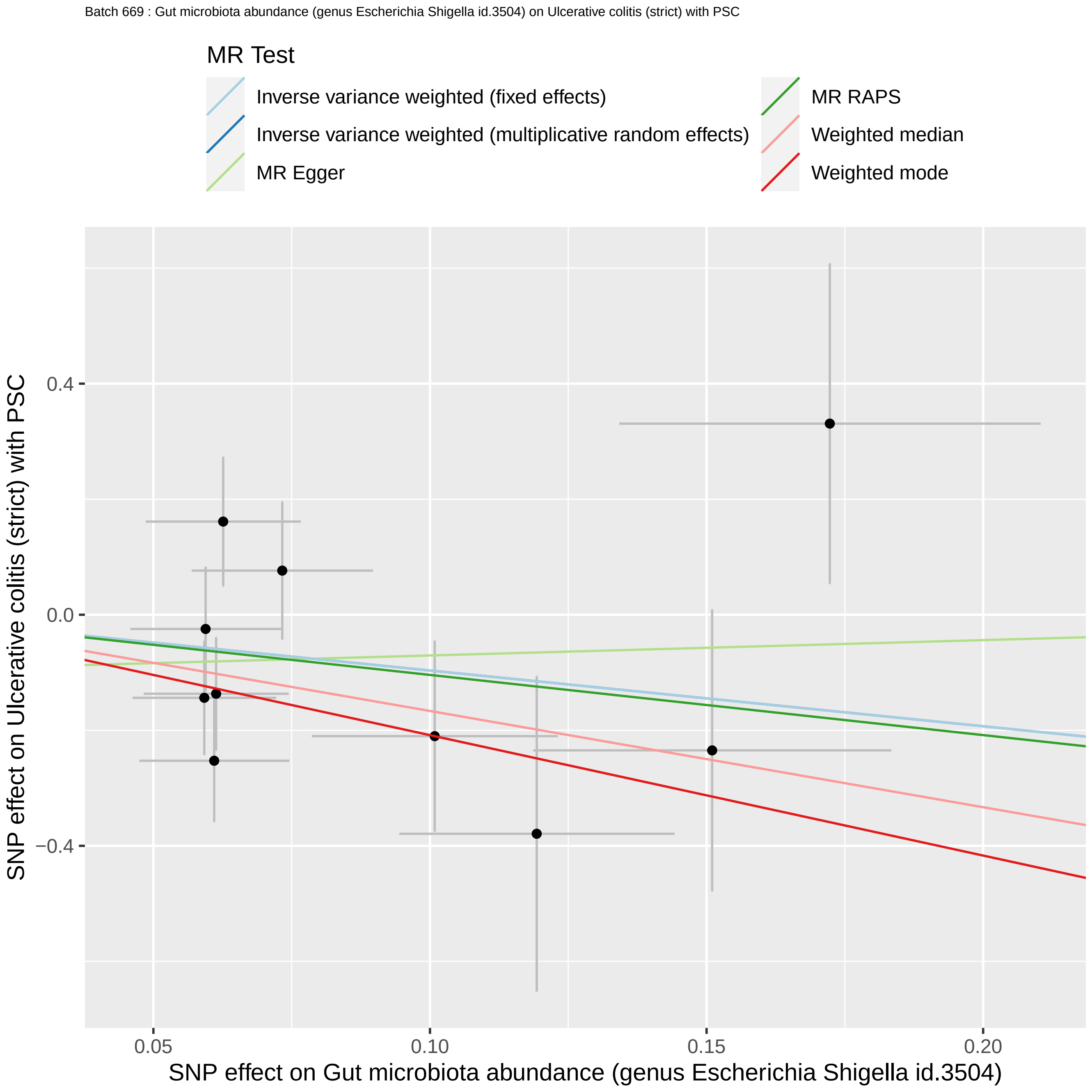
Batch 669 : Gut microbiota abundance (genus Escherichia Shigella id.3504) on Ulcerative colitis (strict) with PSC
MR Test
Inverse variance weighted (fixed effects)
Inverse variance weighted (multiplicative random effects)
MR Egger
MR RAPS
Weighted median
Weighted mode
SNP effect on Ulcerative colitis (strict) with PSC
0.4
0.0
−0.4
0.05
0.10
0.15
0.20
SNP effect on Gut microbiota abundance (genus Escherichia Shigella id.3504)

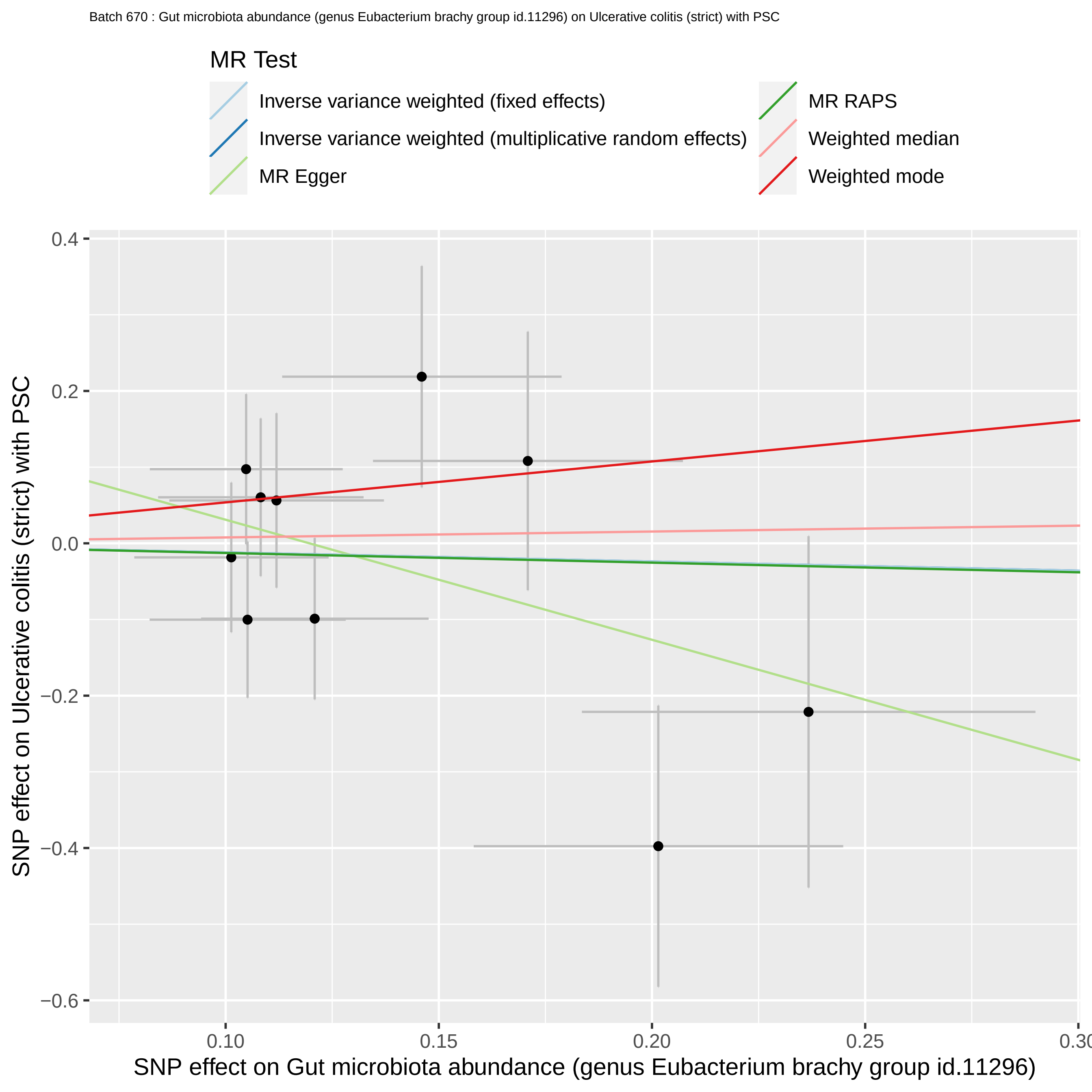
Batch 670 : Gut microbiota abundance (genus Eubacterium brachy group id.11296) on Ulcerative colitis (strict) with PSC
MR Test
Inverse variance weighted (fixed effects)
Inverse variance weighted (multiplicative random effects)
MR Egger
MR RAPS
Weighted median
Weighted mode
0.4
0.2
0.0
−0.2
−0.4
−0.6
0.10
0.15
0.20
0.25
0.30
SNP effect on Ulcerative colitis (strict) with PSC
SNP effect on Gut microbiota abundance (genus Eubacterium brachy group id.11296)

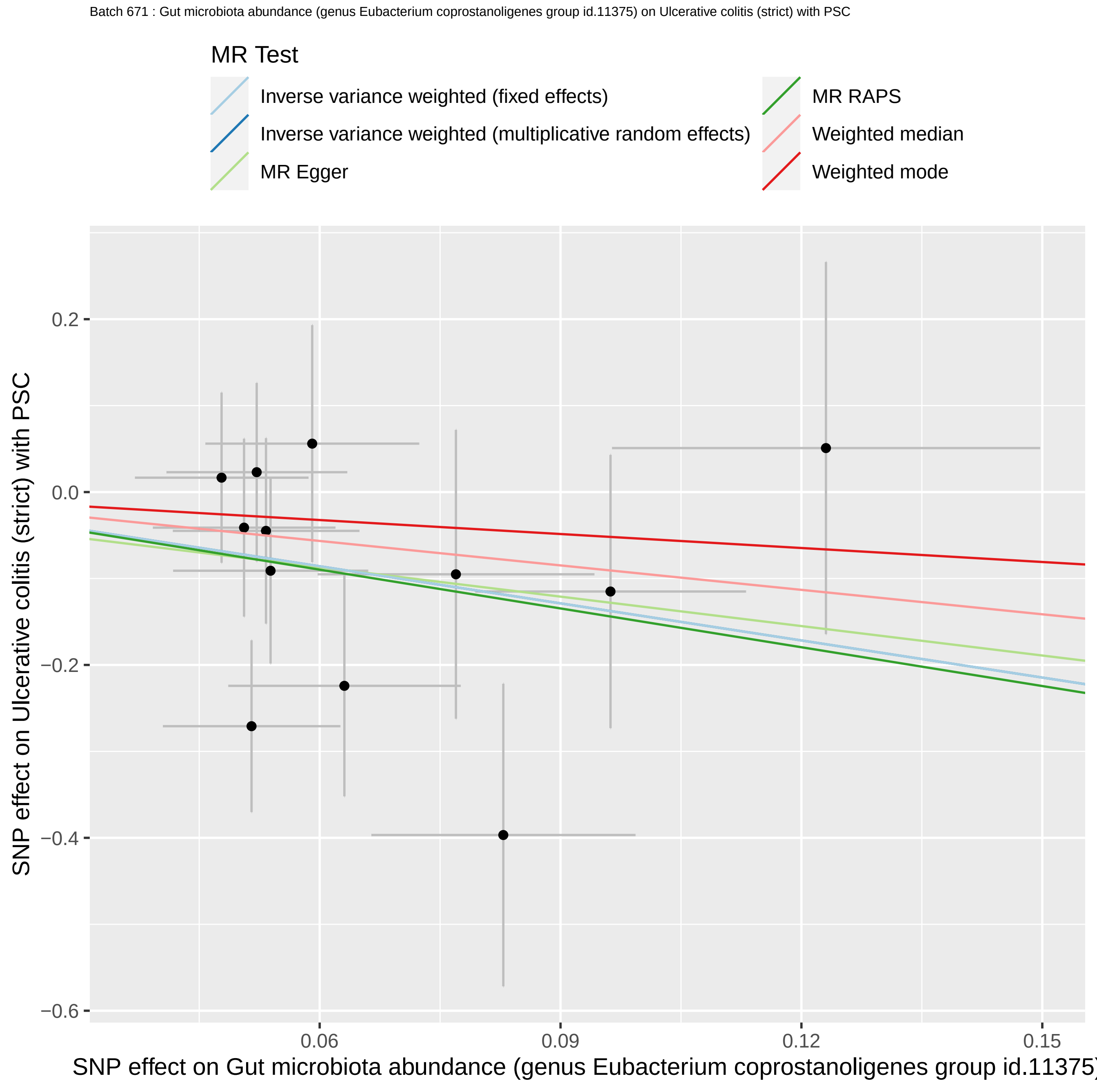

Batch 671 : Gut microbiota abundance (genus Eubacterium coprostanoligenes group id.11375) on Ulcerative colitis (strict) with PSC
MR Test
Inverse variance weighted (fixed effects)
Inverse variance weighted (multiplicative random effects)
MR Egger
MR RAPS
Weighted median
Weighted mode
0.2
0.0
−0.2
−0.4
−0.6
0.06
0.09
0.12
0.15
SNP effect on Ulcerative colitis (strict) with PSC
SNP effect on Gut microbiota abundance (genus Eubacterium coprostanoligenes group id.11375)

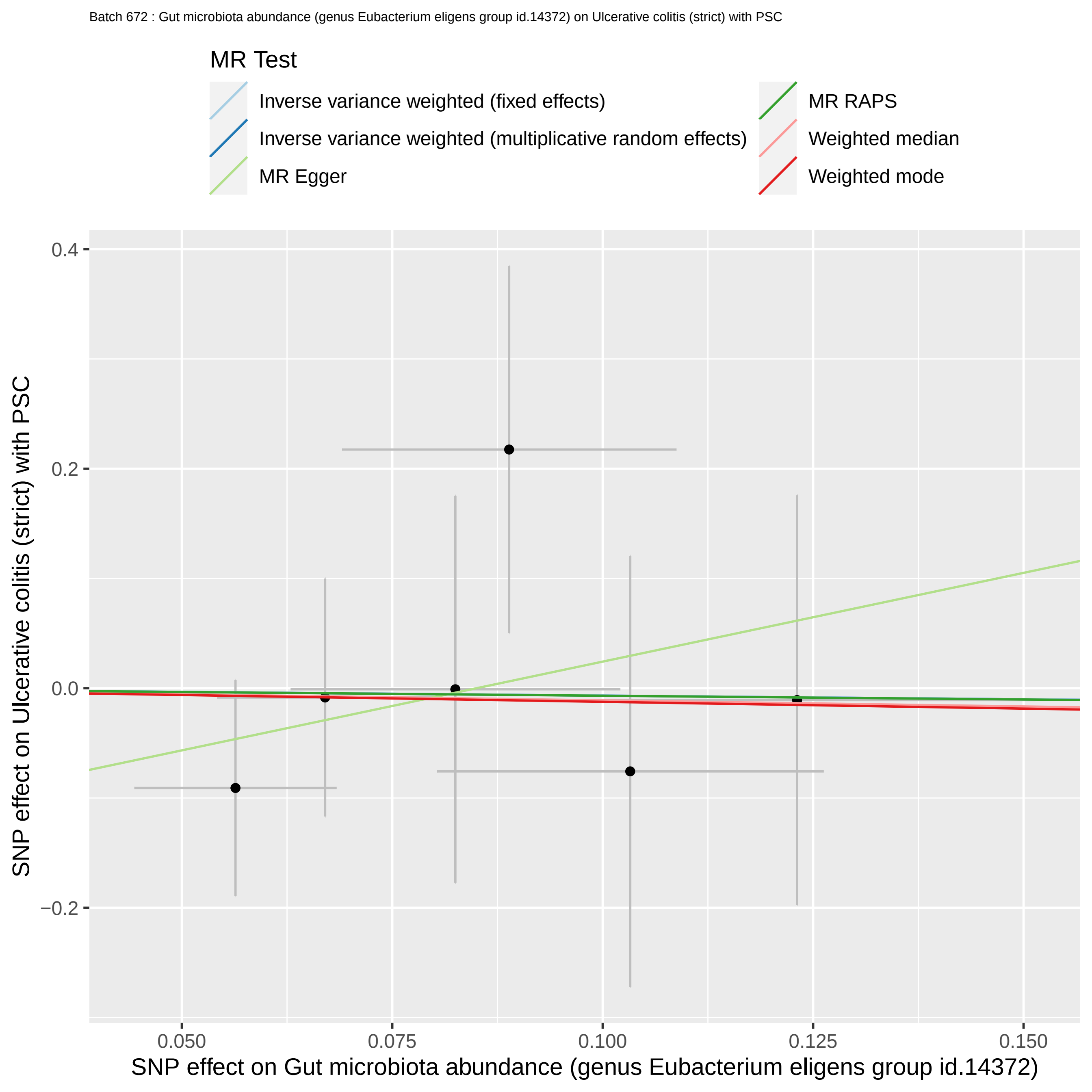

Batch 672 : Gut microbiota abundance (genus Eubacterium eligens group id.14372) on Ulcerative colitis (strict) with PSC
MR Test
Inverse variance weighted (fixed effects)
Inverse variance weighted (multiplicative random effects)
MR Egger
MR RAPS
Weighted median
Weighted mode
0.4
0.2
0.0
−0.2
0.050
0.075
0.100
0.125
0.150
SNP effect on Ulcerative colitis (strict) with PSC
SNP effect on Gut microbiota abundance (genus Eubacterium eligens group id.14372)

Batch 673 : Gut microbiota abundance (genus Eubacterium fissicatena group id.14373) on Ulcerative colitis (strict) with PSC

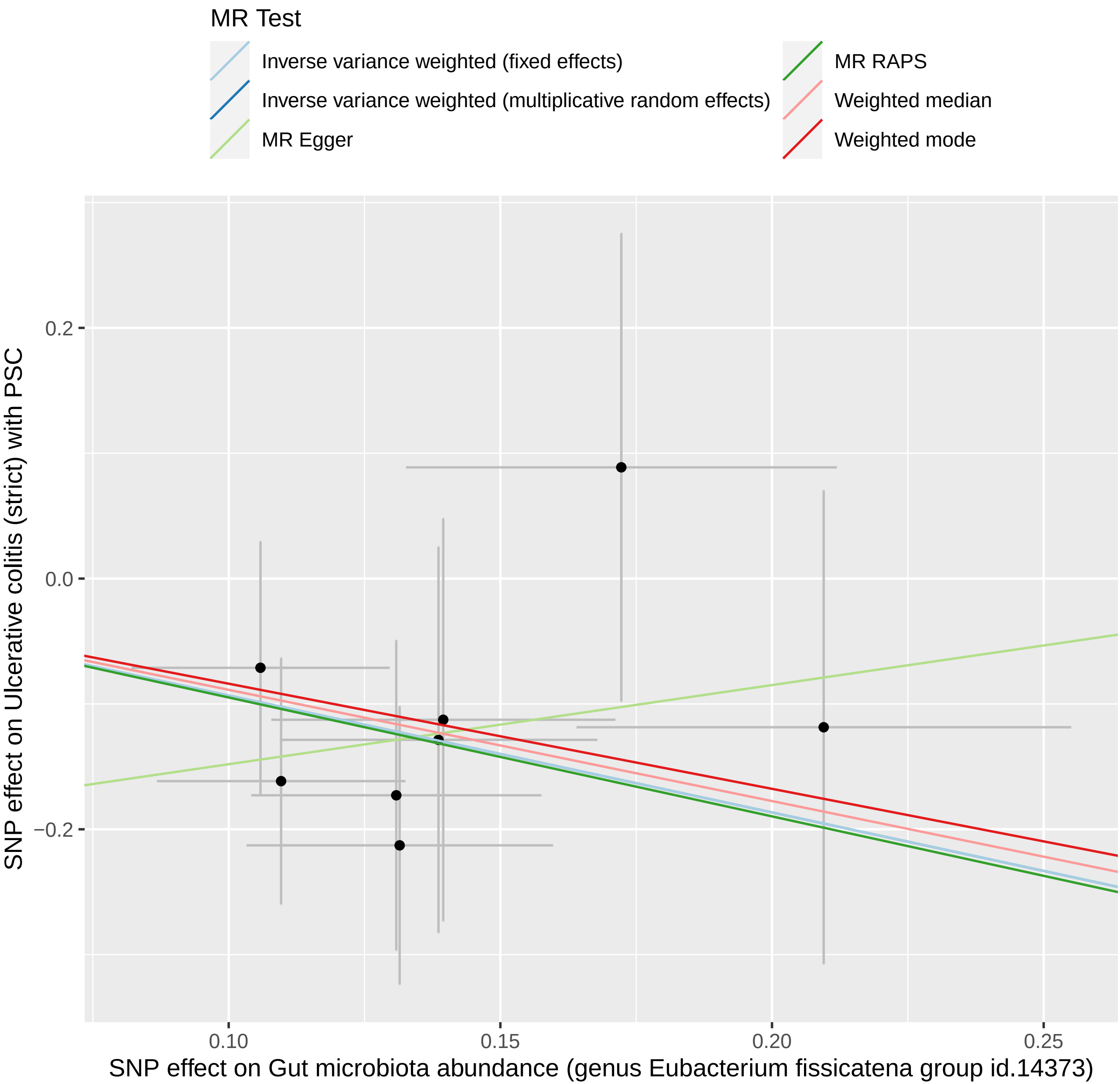

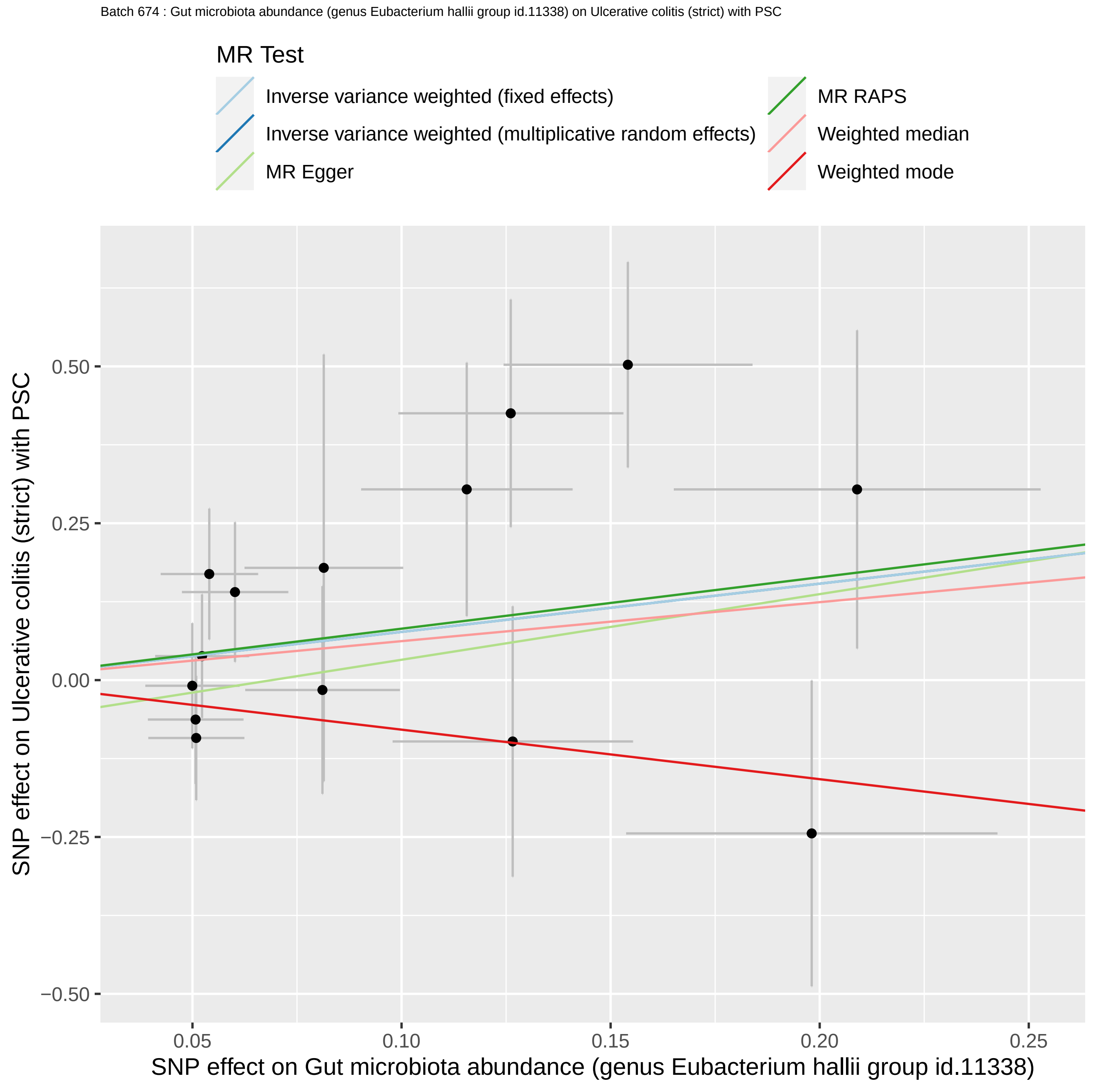

Batch 674 : Gut microbiota abundance (genus Eubacterium hallii group id.11338) on Ulcerative colitis (strict) with PSC
MR Test
Inverse variance weighted (fixed effects)
Inverse variance weighted (multiplicative random effects)
MR Egger
MR RAPS
Weighted median
Weighted mode
SNP effect on Ulcerative colitis (strict) with PSC
0.50
0.25
0.00
−0.25
−0.50
0.05
0.10
0.15
0.20
0.25
SNP effect on Gut microbiota abundance (genus Eubacterium hallii group id.11338)

Batch 675 : Gut microbiota abundance (genus Eubacterium nodatum group id.11297) on Ulcerative colitis (strict) with PSC

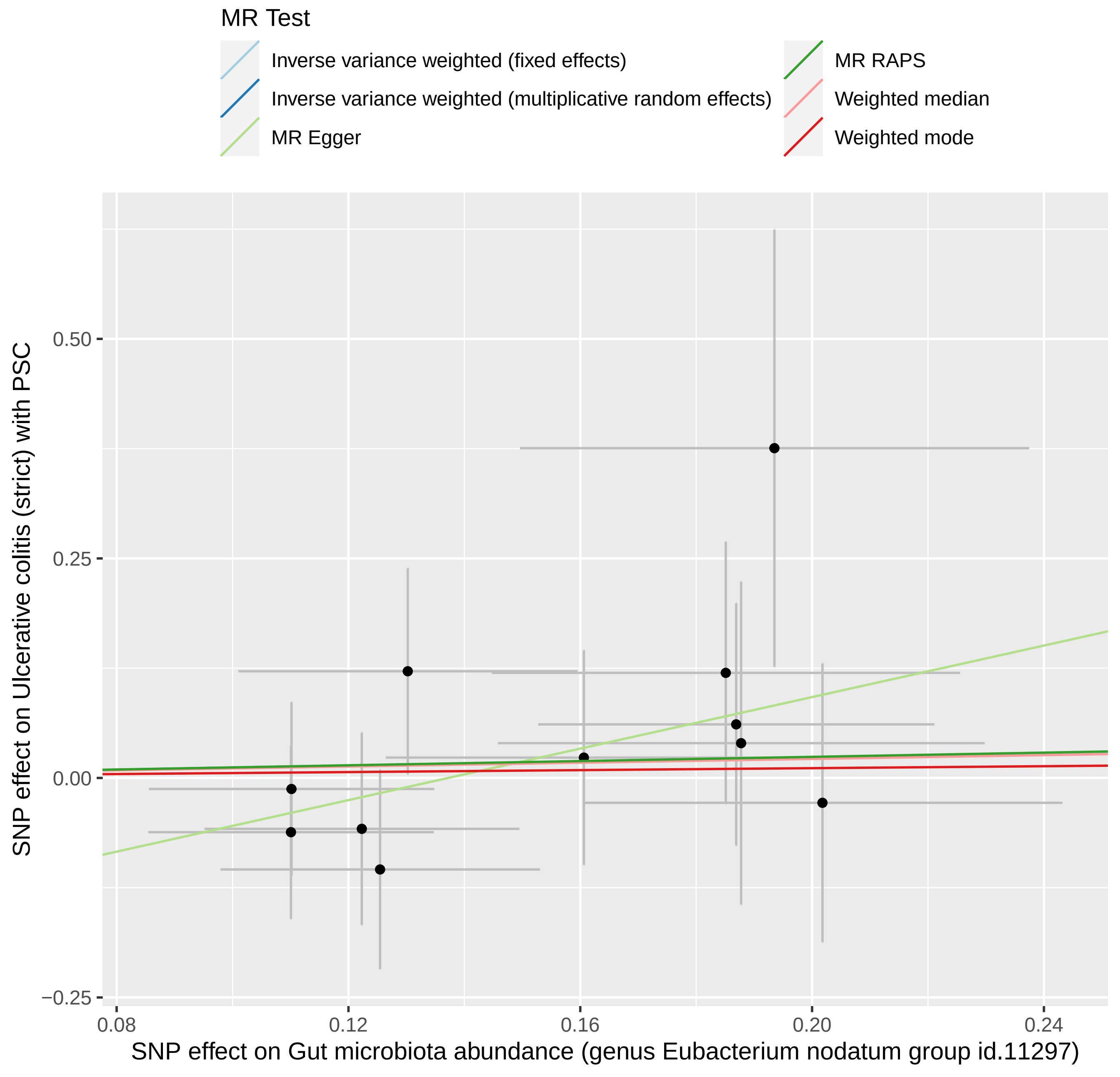

Batch 676 : Gut microbiota abundance (genus Eubacterium oxidoreducens group id.11339) on Ulcerative colitis (strict) with PSC

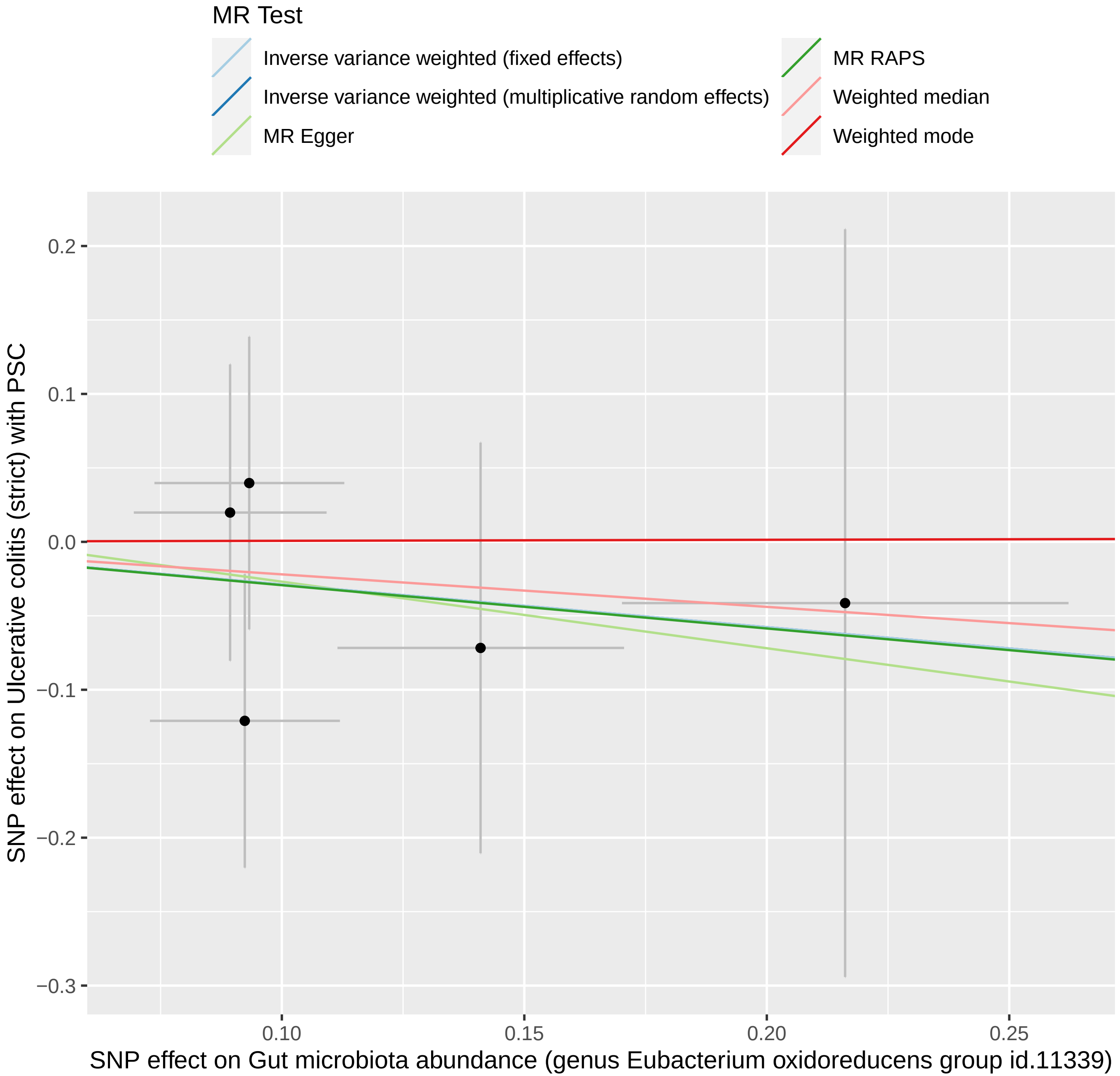

Batch 677 : Gut microbiota abundance (genus Eubacterium rectale group id.14374) on Ulcerative colitis (strict) with PSC

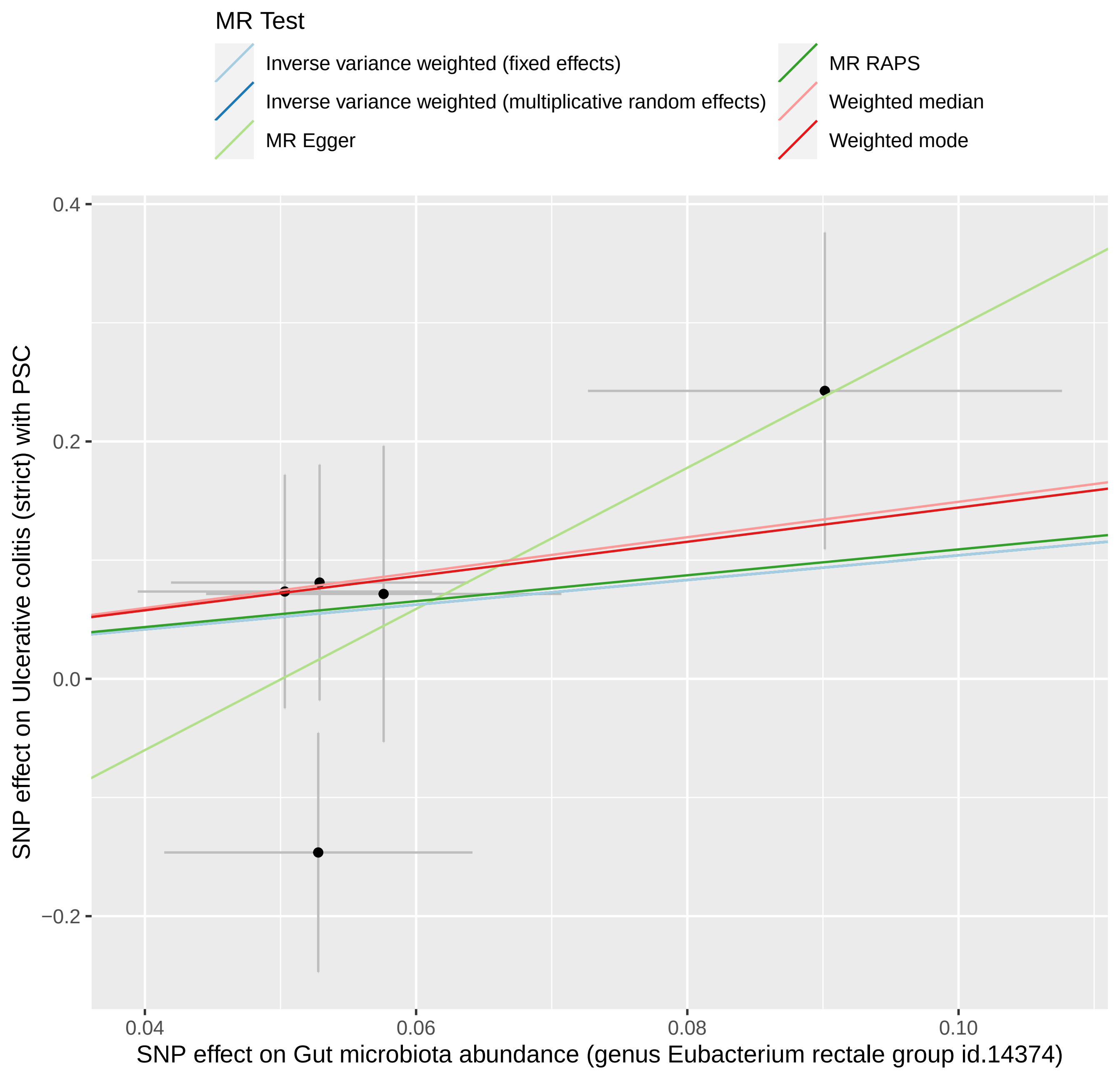

Batch 678 : Gut microbiota abundance (genus Eubacterium ruminantium group id.11340) on Ulcerative colitis (strict) with PSC

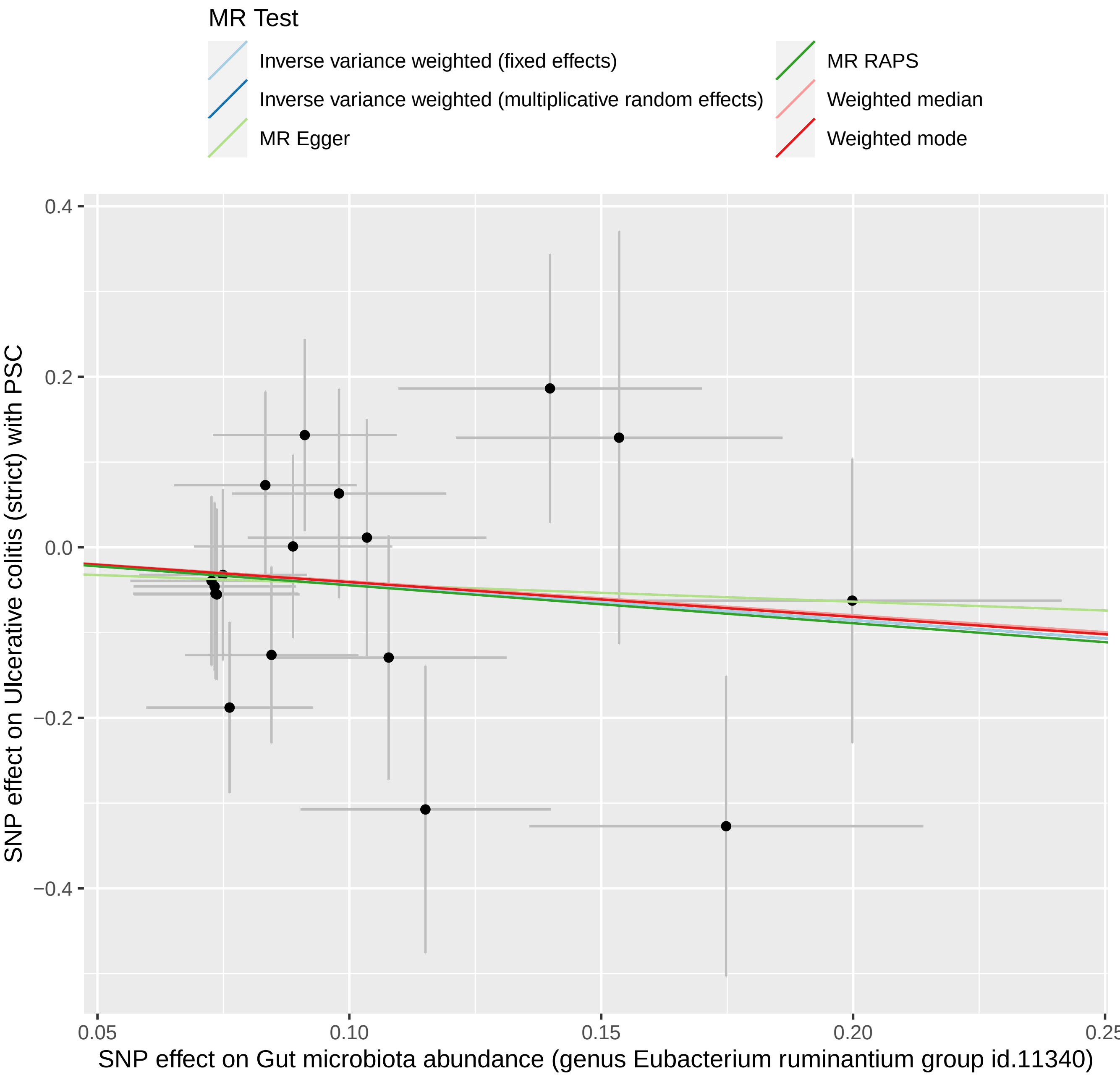

Batch 679 : Gut microbiota abundance (genus Eubacterium ventriosum group id.11341) on Ulcerative colitis (strict) with PSC

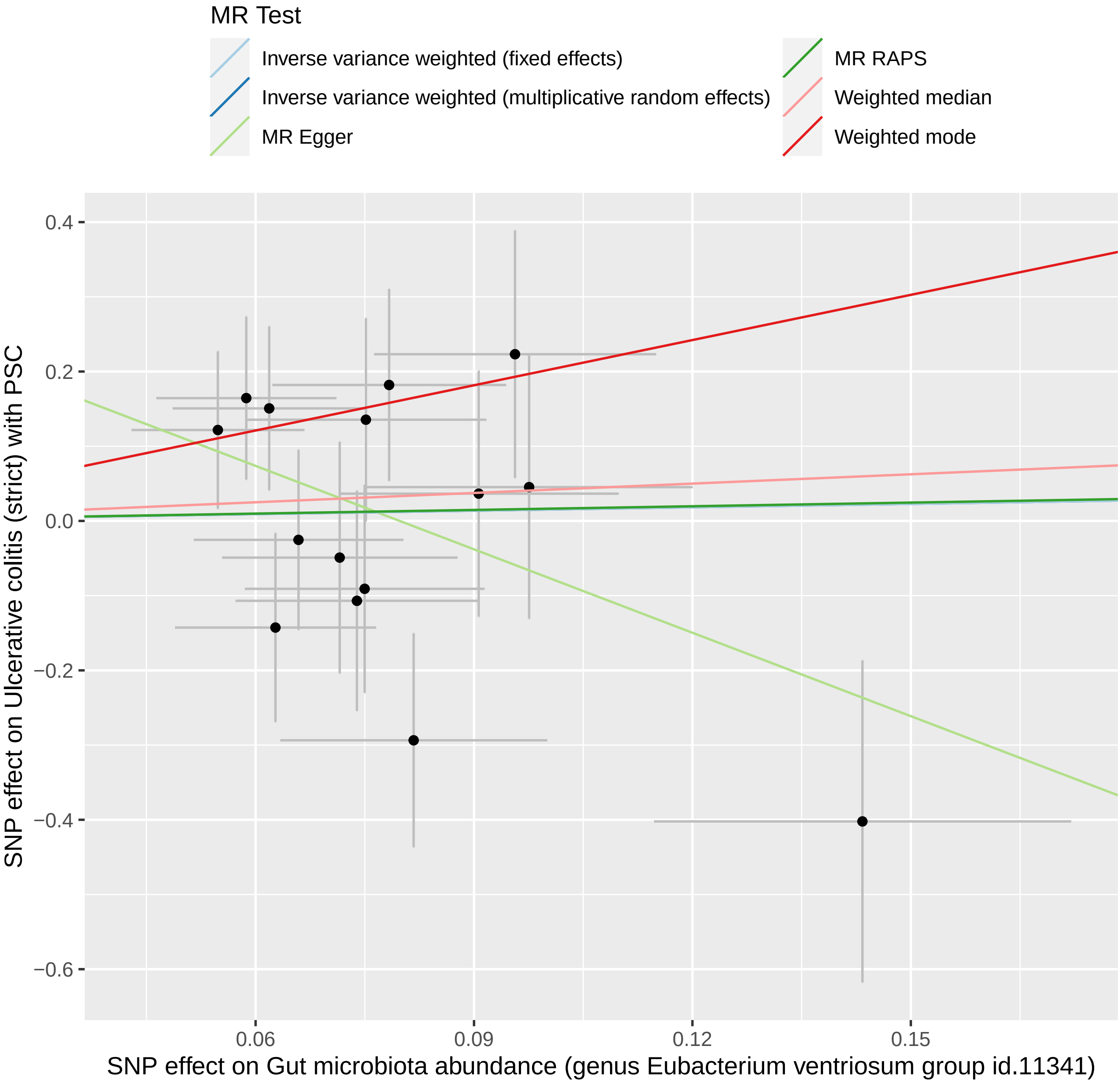

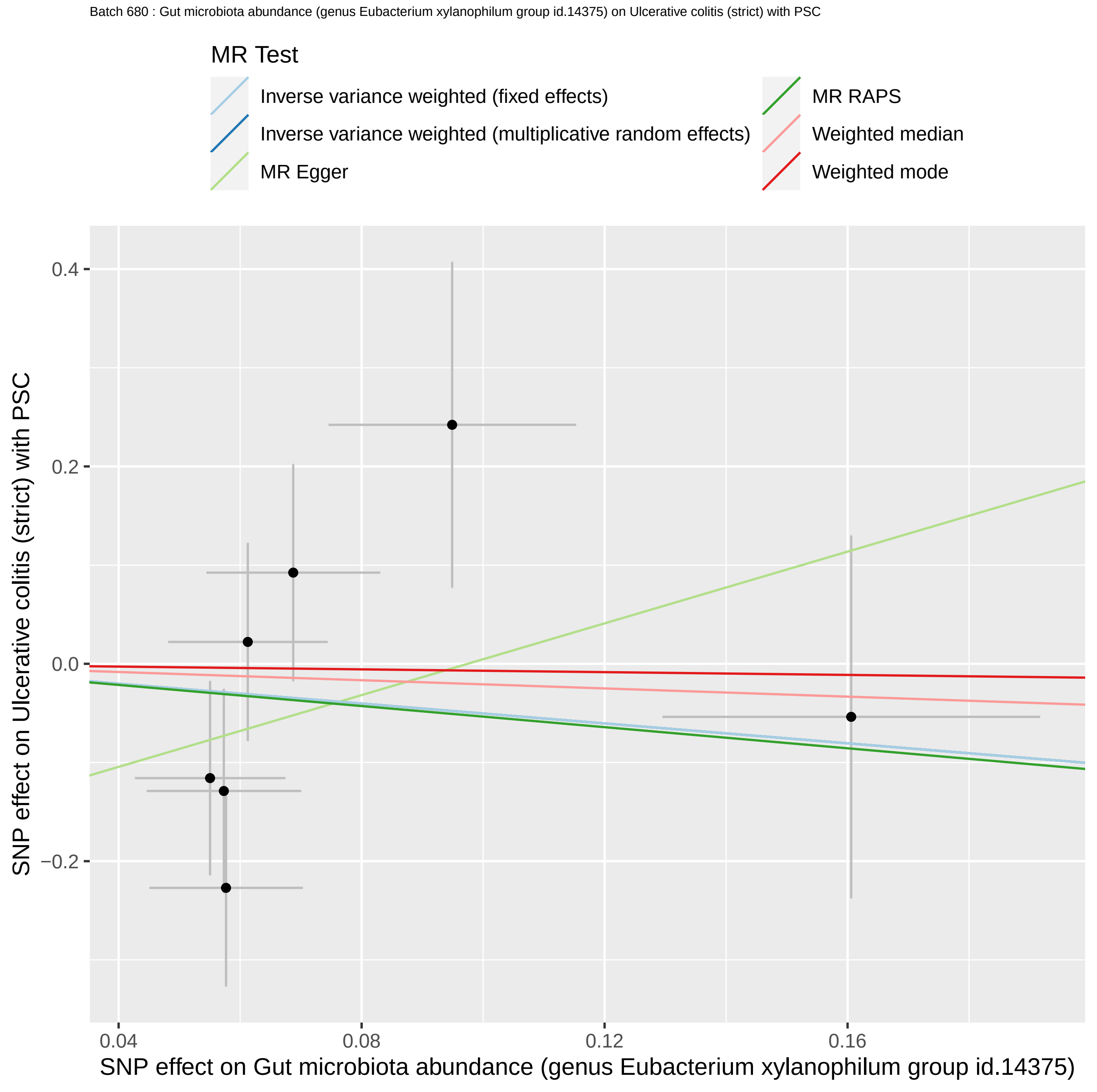

Batch 680 : Gut microbiota abundance (genus Eubacterium xylanophilum group id.14375) on Ulcerative colitis (strict) with PSC
MR Test
Inverse variance weighted (fixed effects)
Inverse variance weighted (multiplicative random effects)
MR Egger
MR RAPS
Weighted median
Weighted mode
SNP effect on Ulcerative colitis (strict) with PSC
0.4
0.2
0.0
−0.2
0.04
0.08
0.12
0.16
SNP effect on Gut microbiota abundance (genus Eubacterium xylanophilum group id.14375)

Batch 681 : Gut microbiota abundance (genus Faecalibacterium id.2057) on Ulcerative colitis (strict) with PSC

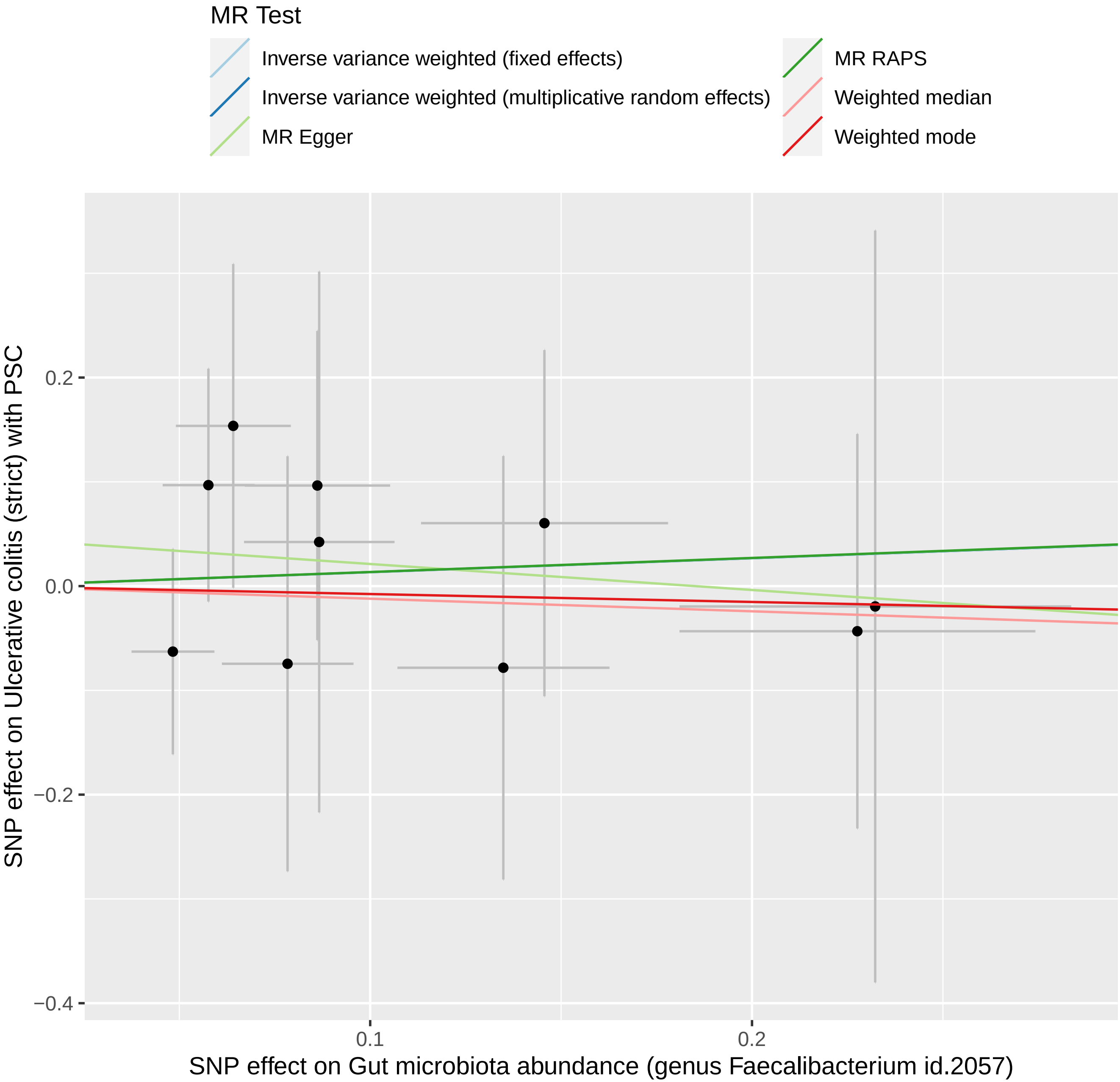

Batch 682 : Gut microbiota abundance (genus Family XIII AD3011 group id.11293) on Ulcerative colitis (strict) with PSC

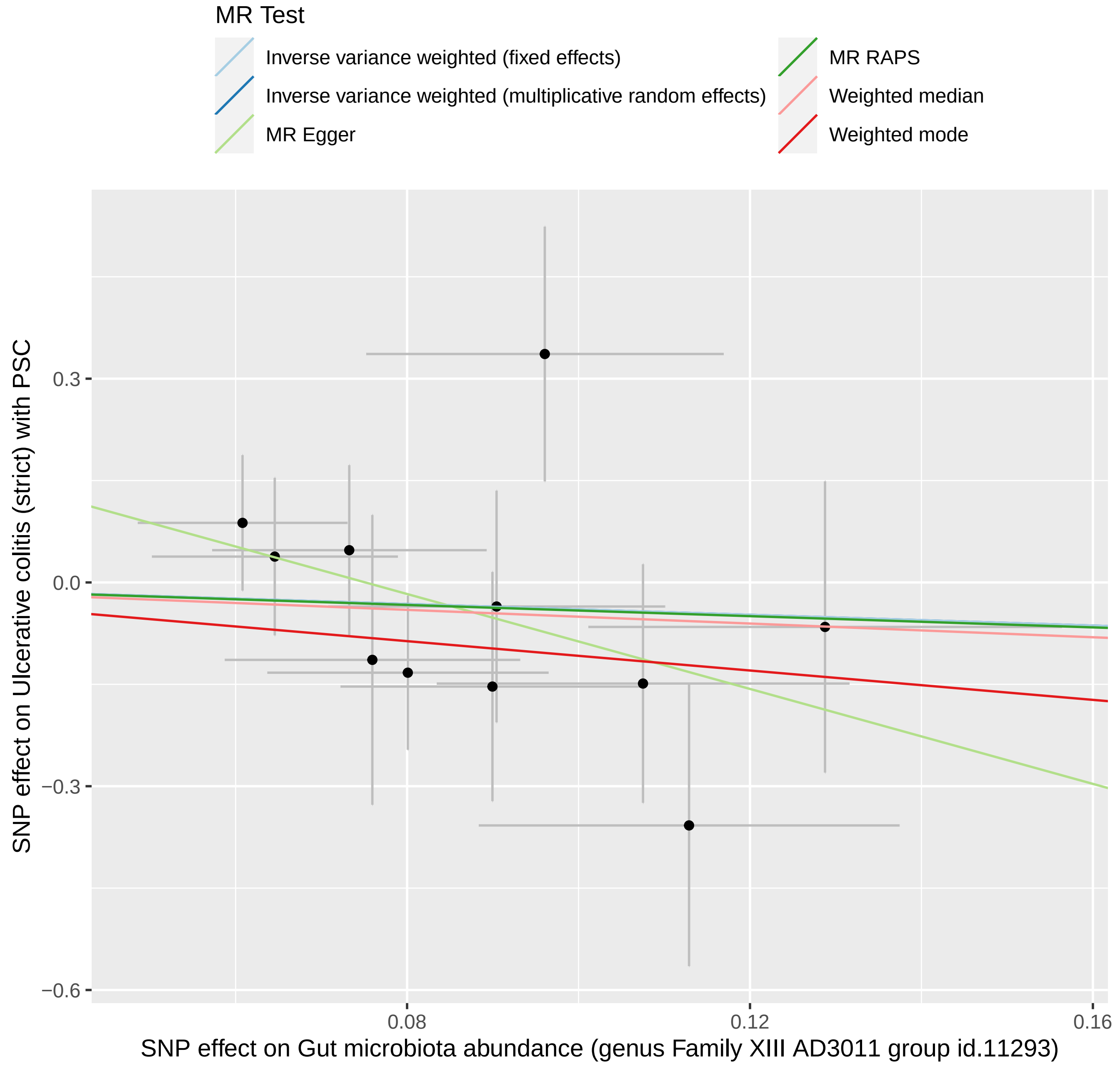

Batch 683 : Gut microbiota abundance (genus Family XIII UCG001 id.11294) on Ulcerative colitis (strict) with PSC

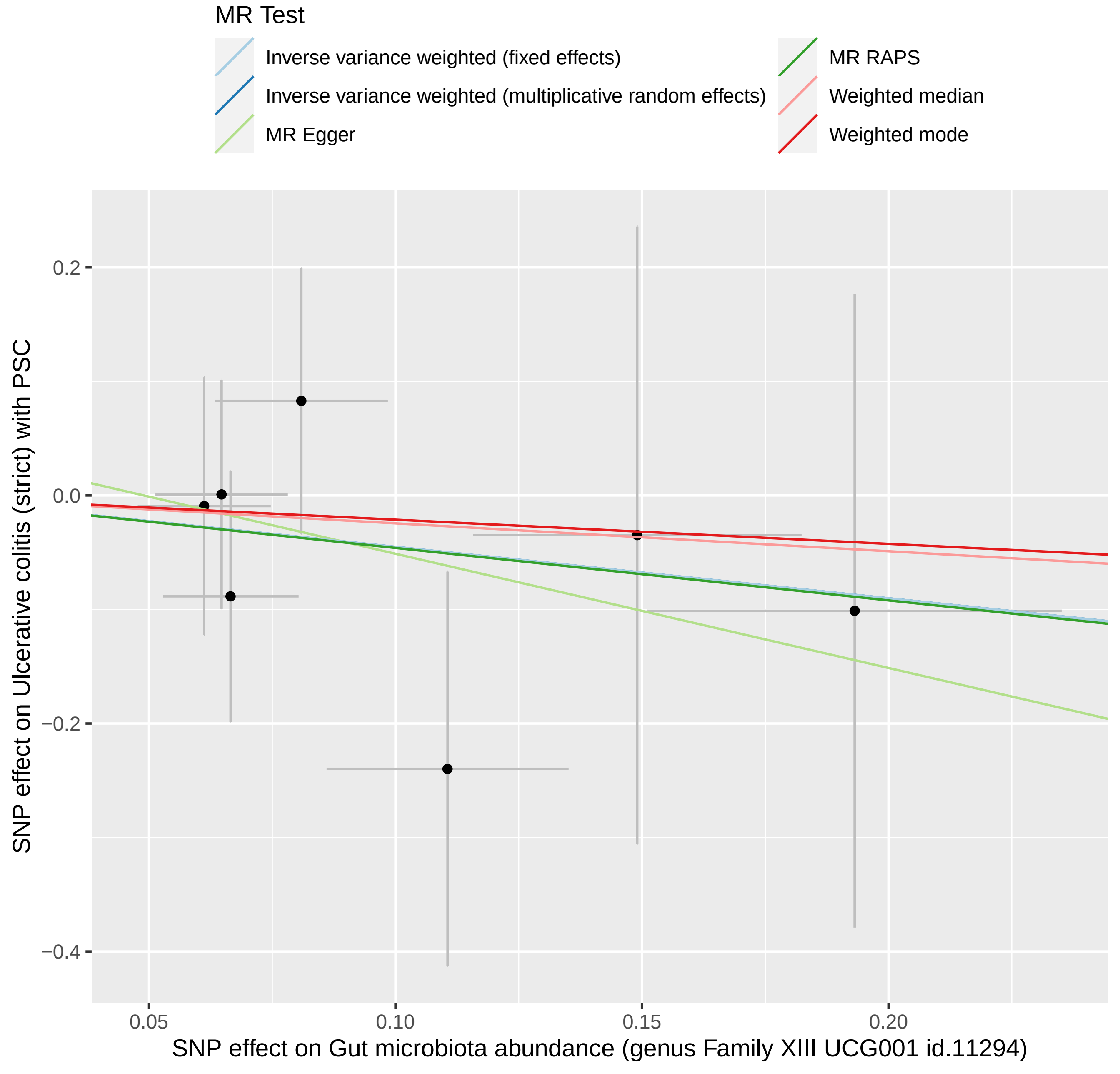

Batch 684 : Gut microbiota abundance (genus Flavonifractor id.2059) on Ulcerative colitis (strict) with PSC

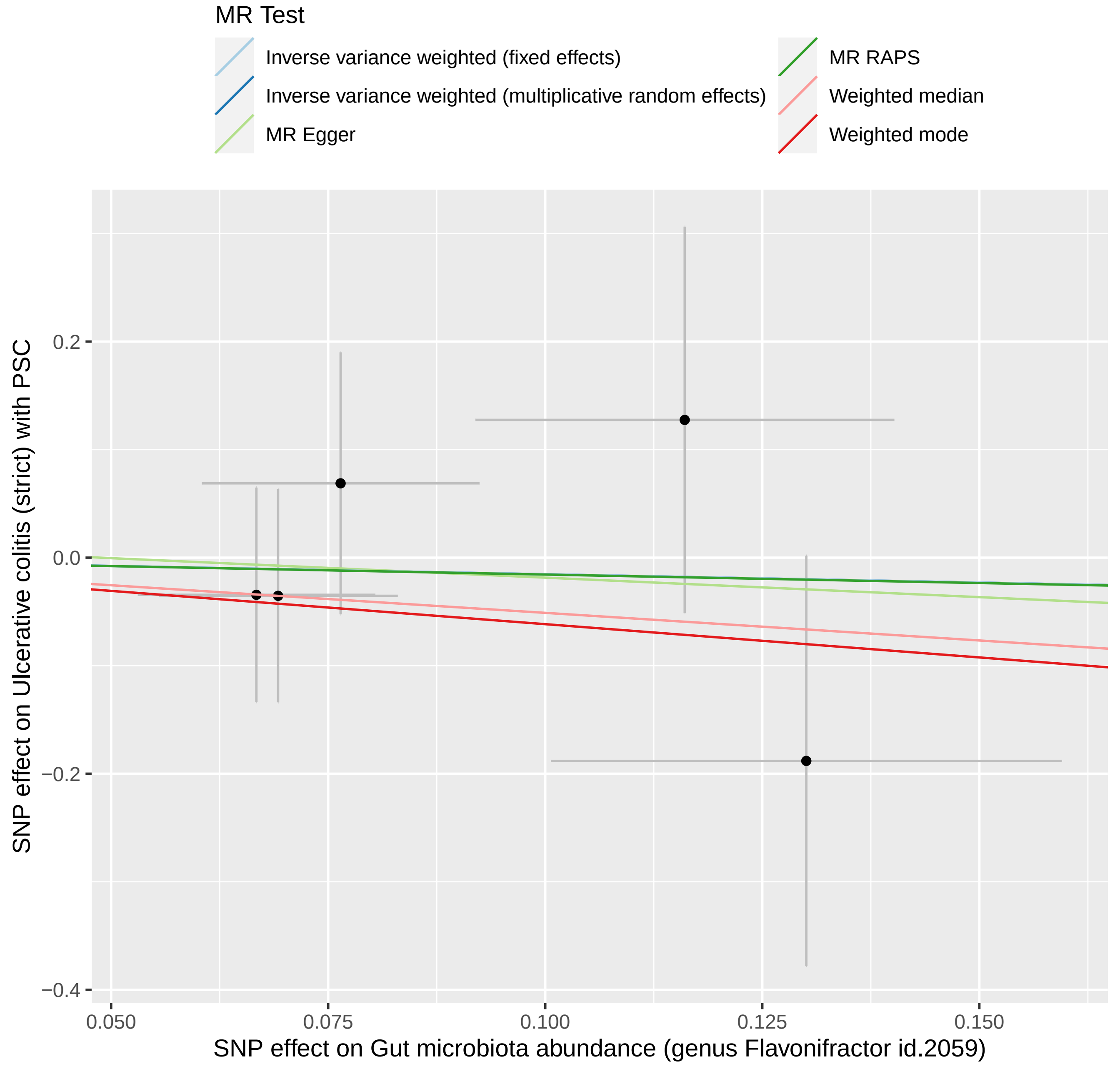

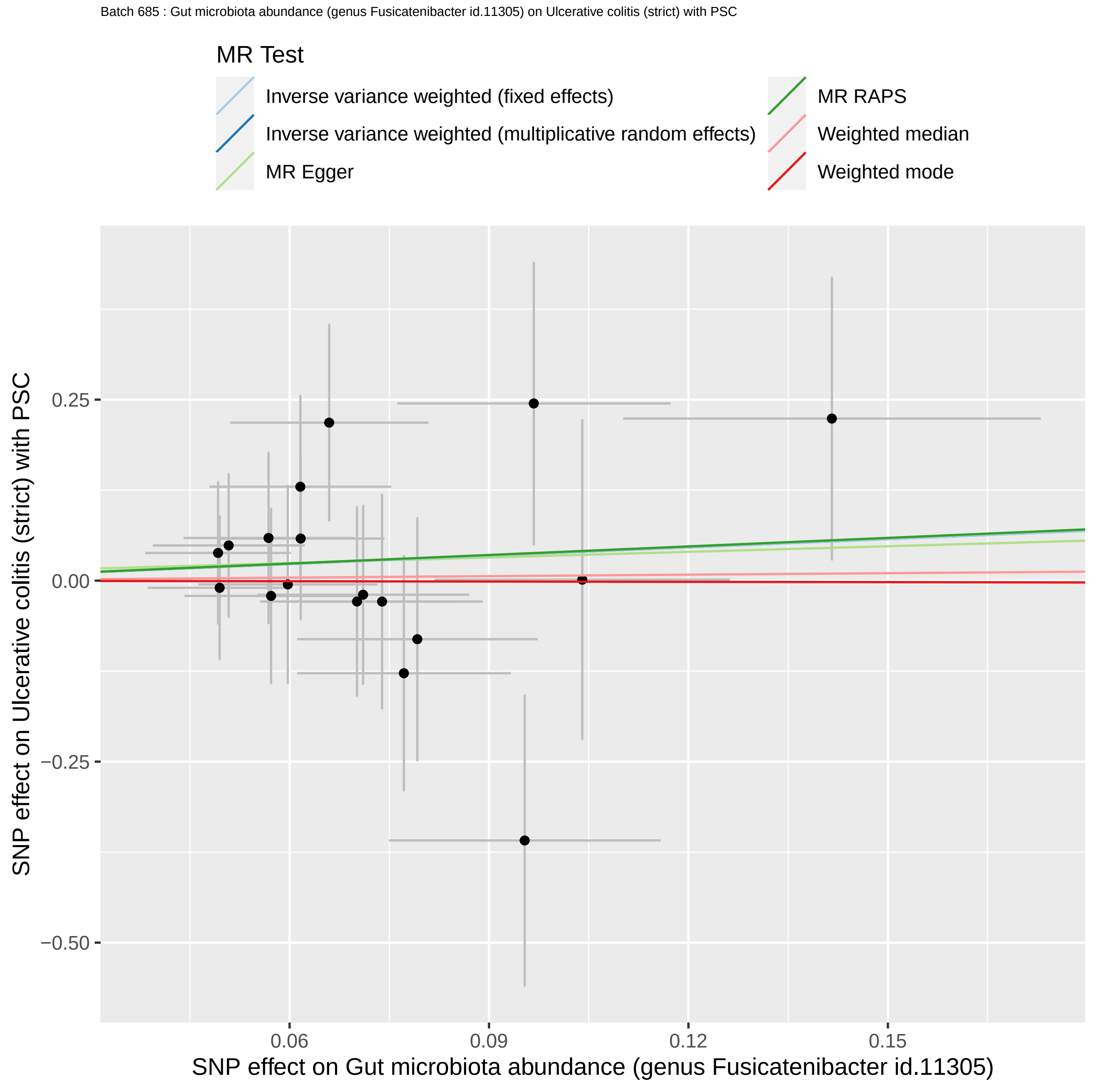

Batch 685 : Gut microbiota abundance (genus Fusicatenibacter id.11305) on Ulcerative colitis (strict) with PSC
MR Test
Inverse variance weighted (fixed effects)
Inverse variance weighted (multiplicative random effects)
MR Egger
MR RAPS
Weighted median
Weighted mode
SNP effect on Ulcerative colitis (strict) with PSC
0.25
0.00
−0.25
−0.50
0.06
0.09
0.12
0.15
SNP effect on Gut microbiota abundance (genus Fusicatenibacter id.11305)

Batch 686 : Gut microbiota abundance (genus Gordonibacter id.821) on Ulcerative colitis (strict) with PSC

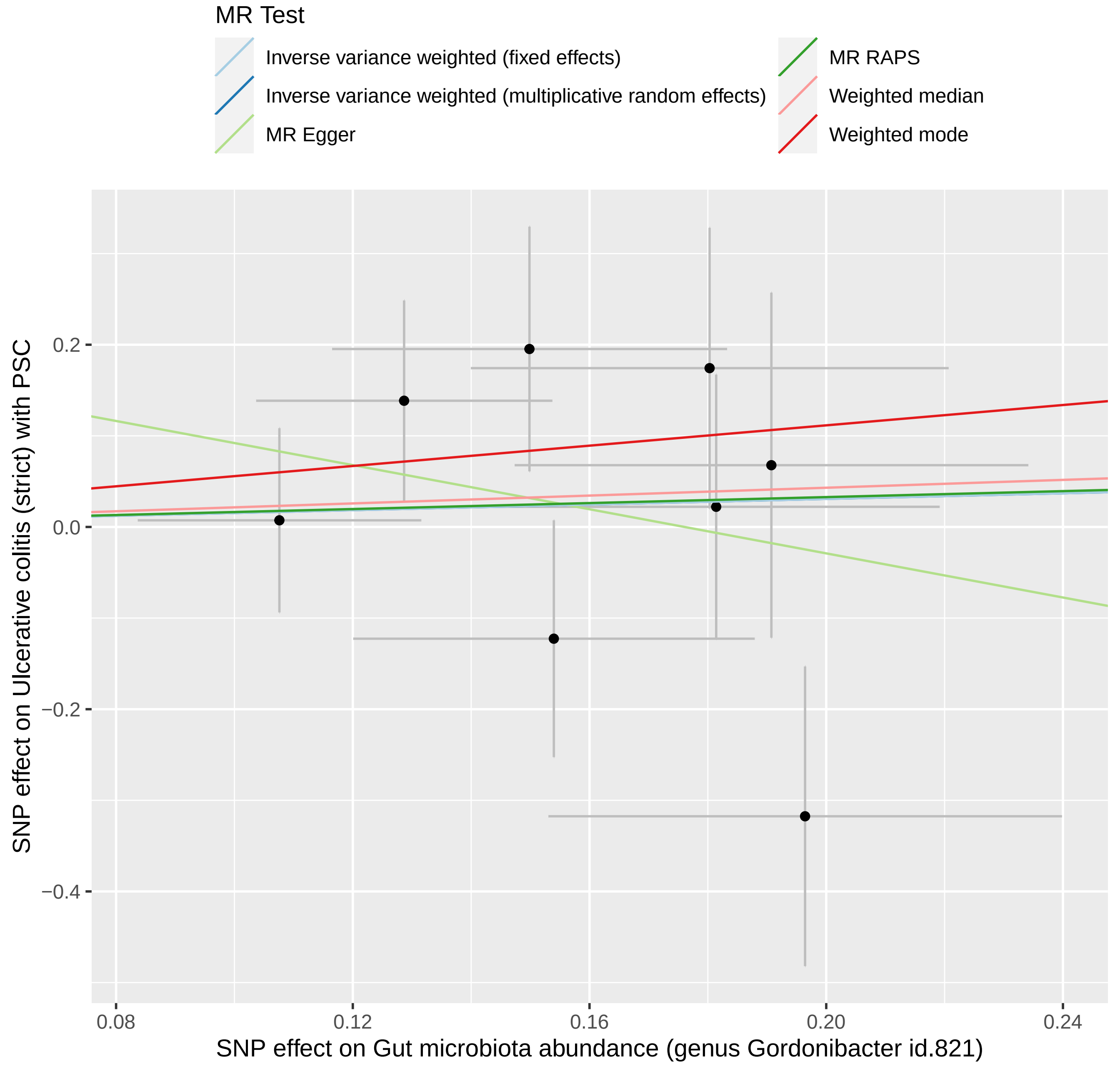

Batch 687 : Gut microbiota abundance (genus Haemophilus id.3698) on Ulcerative colitis (strict) with PSC

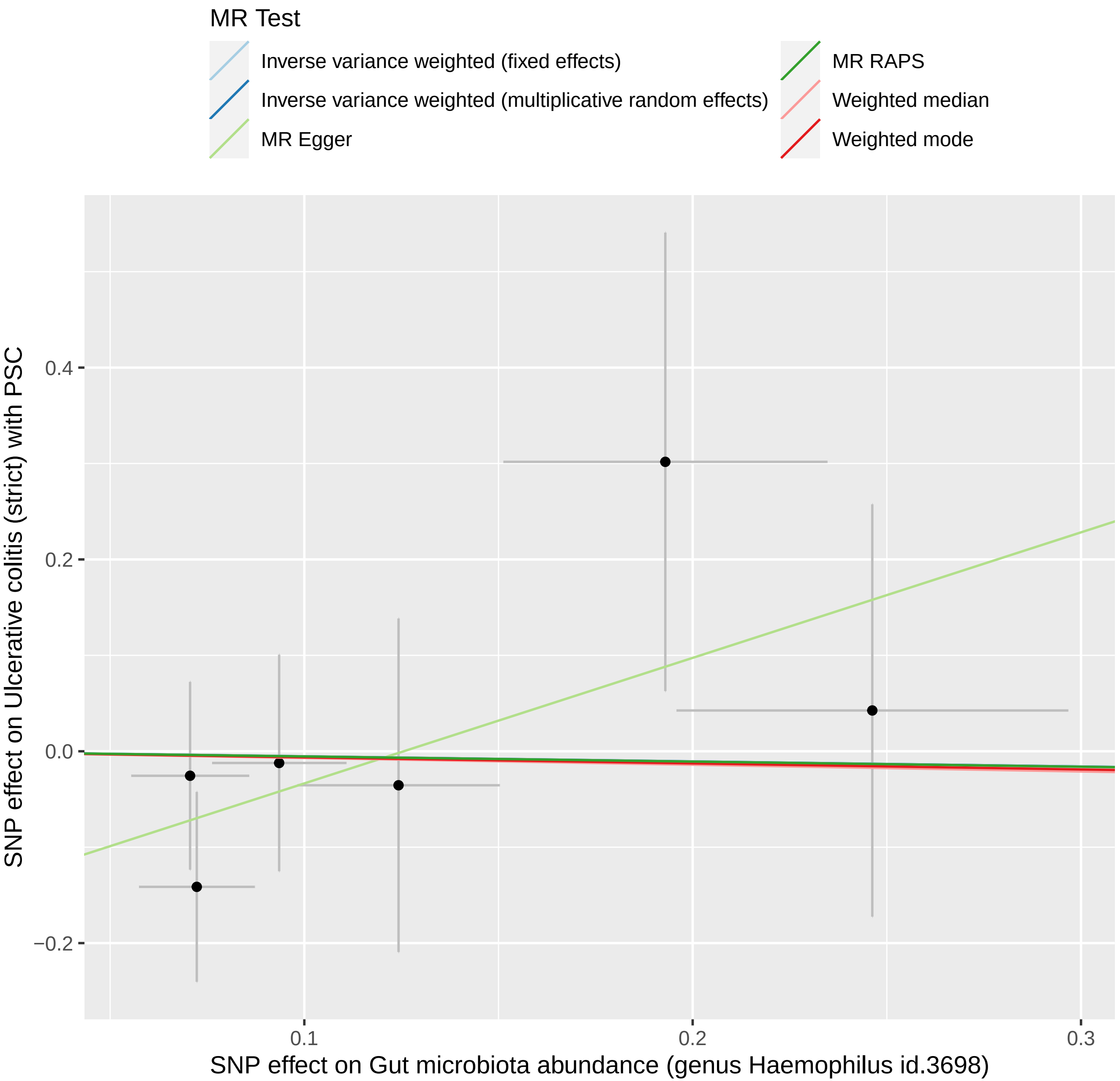

Batch 688 : Gut microbiota abundance (genus Holdemanella id.11393) on Ulcerative colitis (strict) with PSC

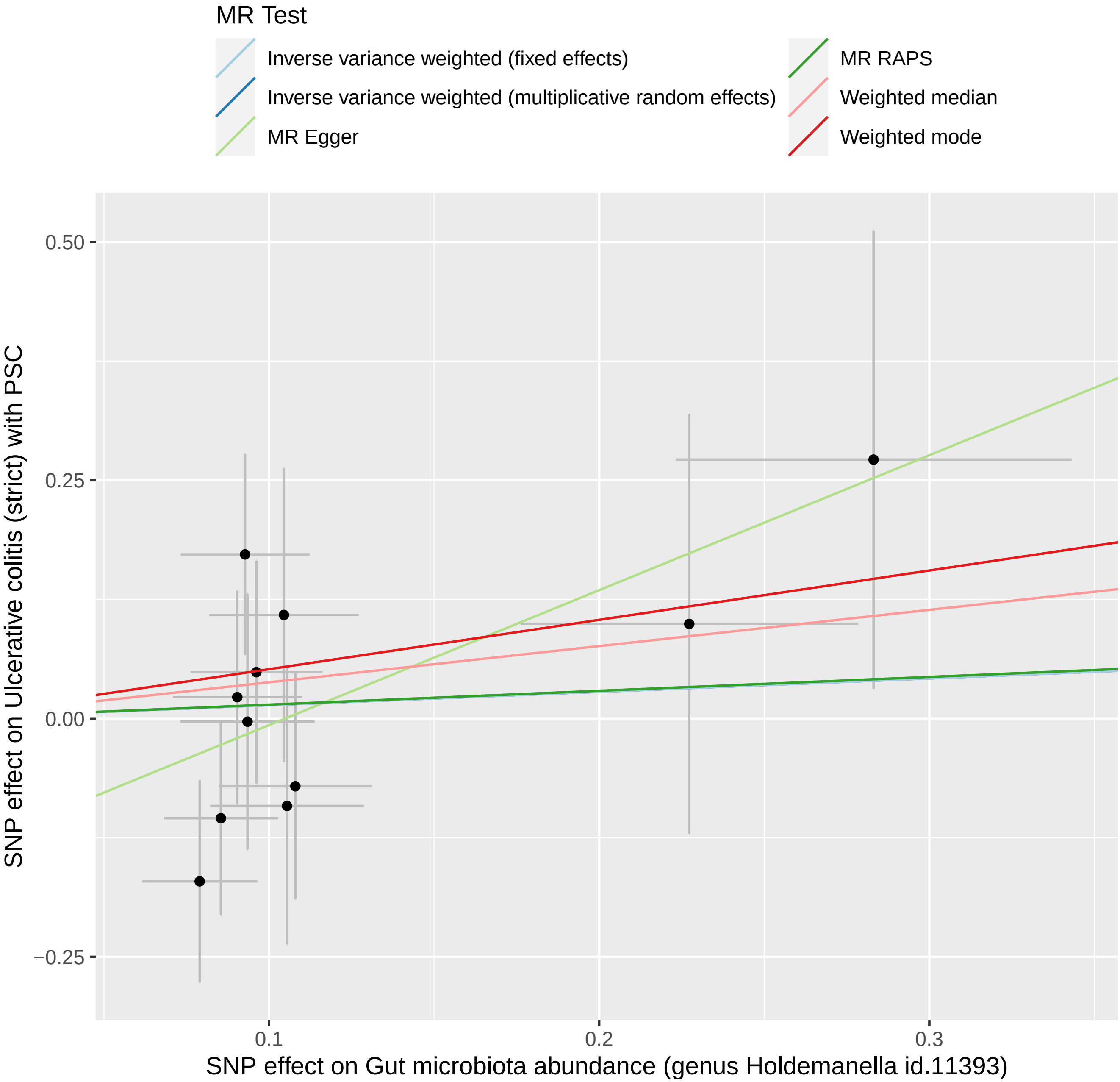

Batch 689 : Gut microbiota abundance (genus Holdemania id.2157) on Ulcerative colitis (strict) with PSC

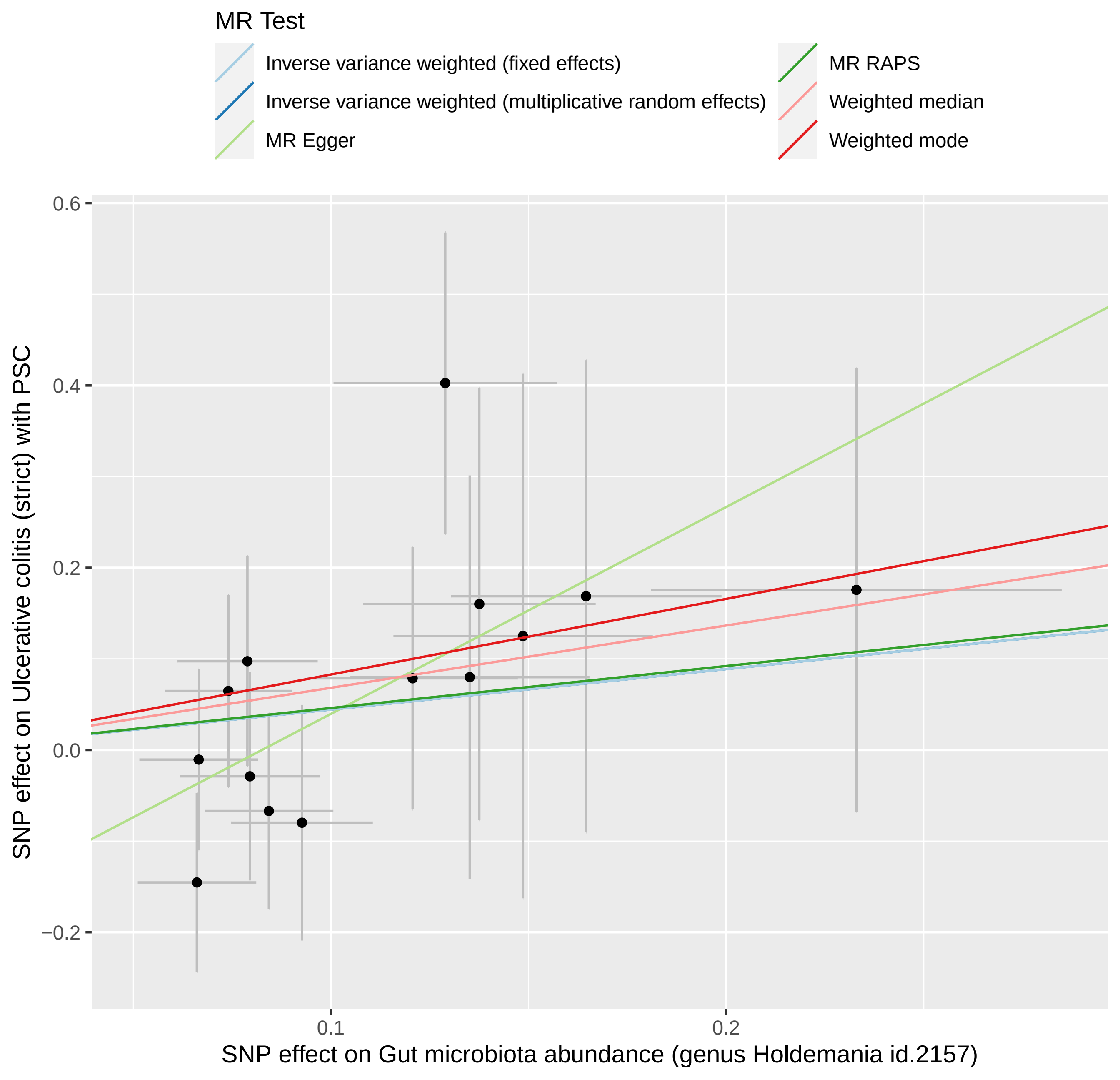

Batch 690 : Gut microbiota abundance (genus Howardella id.2000) on Ulcerative colitis (strict) with PSC

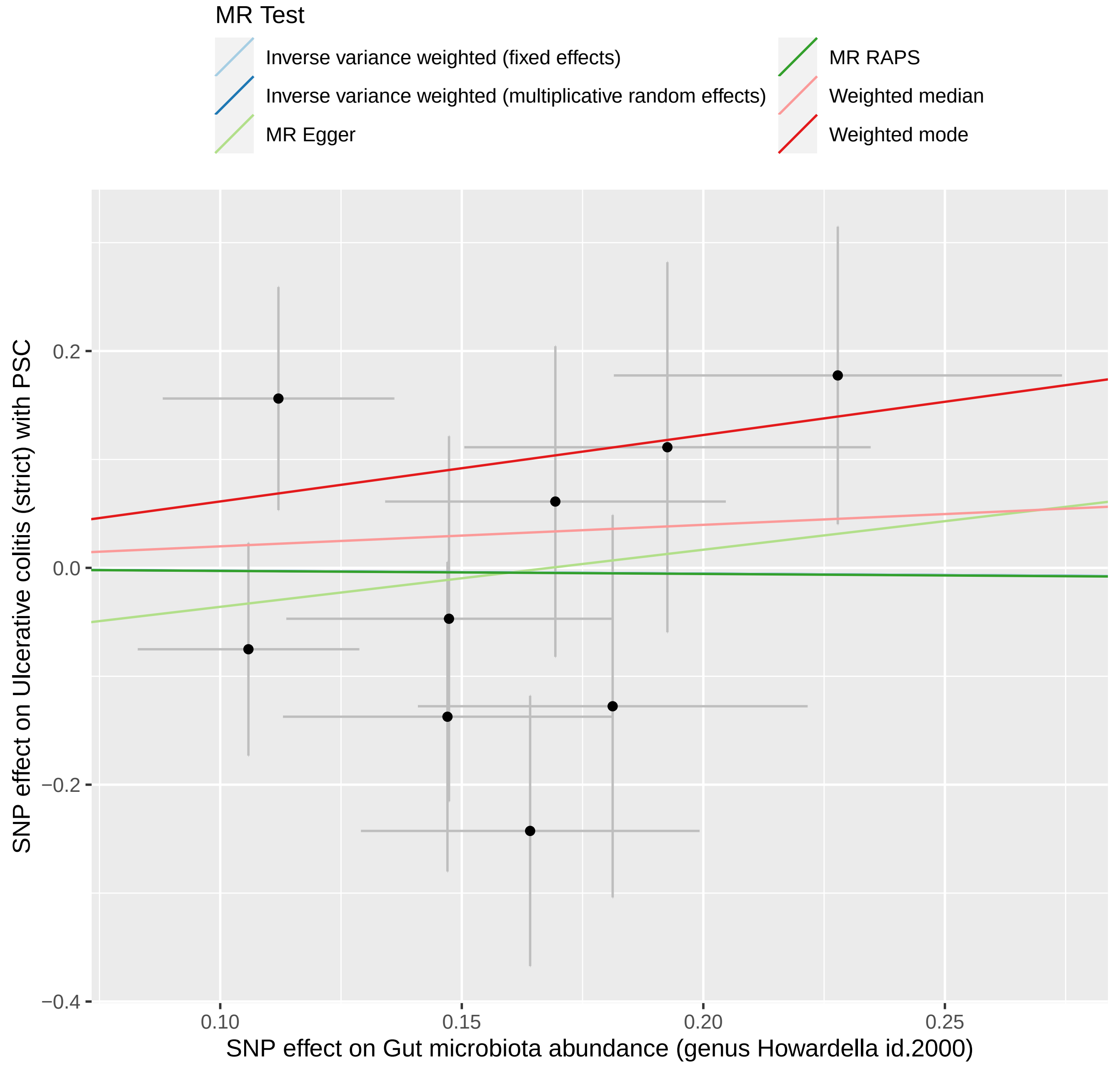

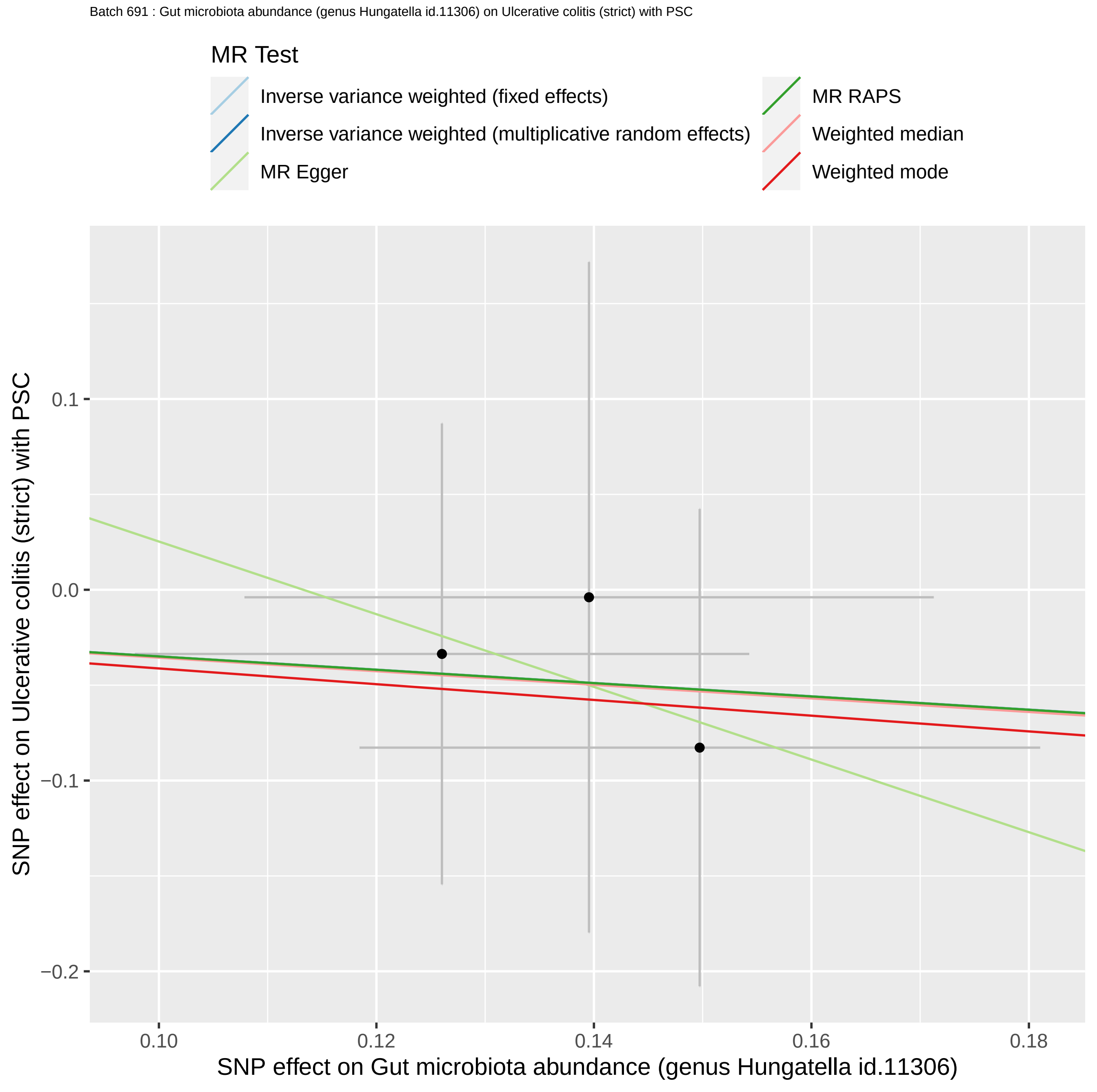
Batch 691 : Gut microbiota abundance (genus Hungatella id.11306) on Ulcerative colitis (strict) with PSC
MR Test
Inverse variance weighted (fixed effects)
Inverse variance weighted (multiplicative random effects)
MR Egger
MR RAPS
Weighted median
Weighted mode
SNP effect on Ulcerative colitis (strict) with PSC
0.1
0.0
−0.1
−0.2
0.10
0.12
0.14
0.16
0.18
SNP effect on Gut microbiota abundance (genus Hungatella id.11306)

Batch 692 : Gut microbiota abundance (genus Intestinibacter id.11345) on Ulcerative colitis (strict) with PSC

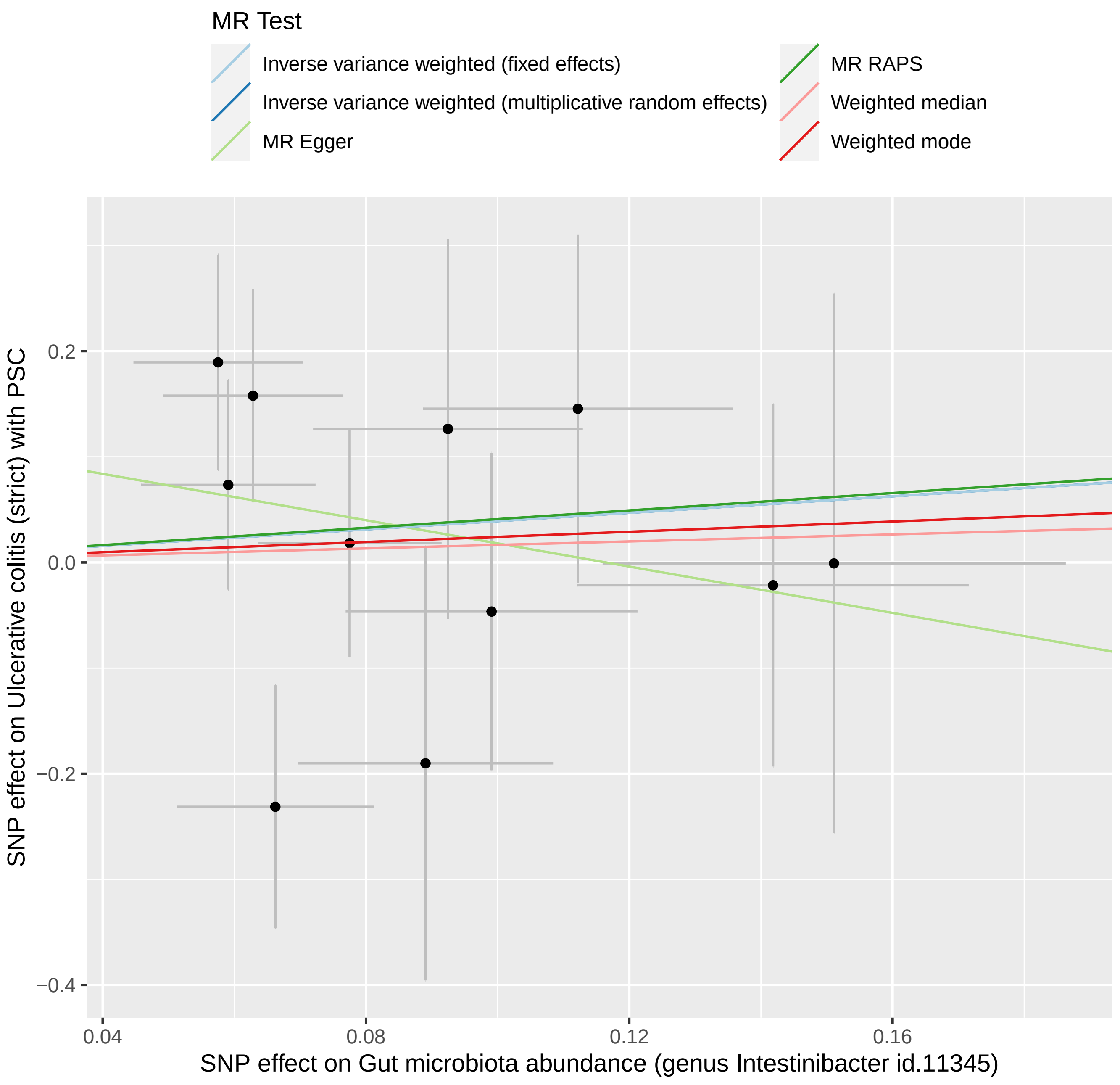

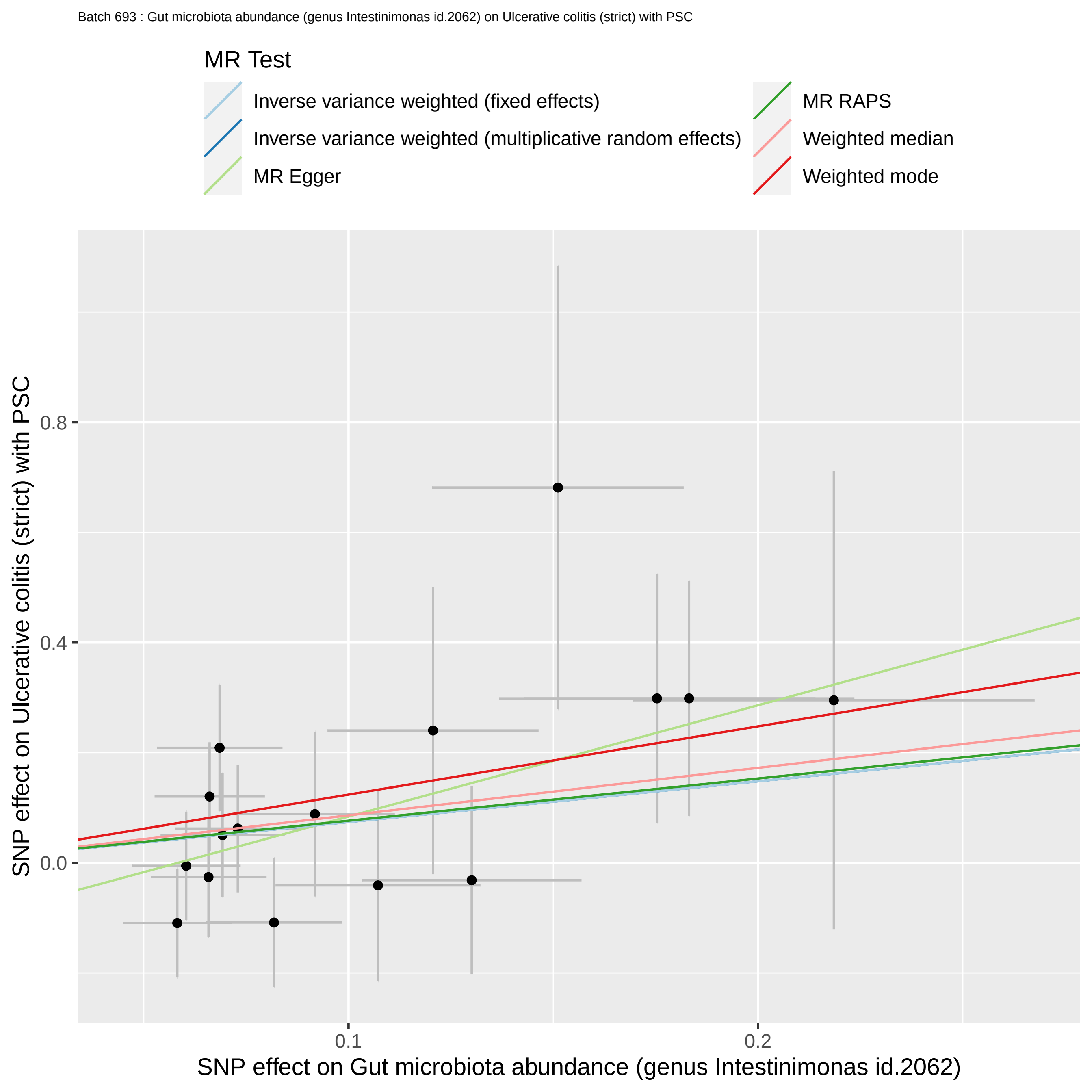
Batch 693 : Gut microbiota abundance (genus Intestinimonas id.2062) on Ulcerative colitis (strict) with PSC
MR Test
Inverse variance weighted (fixed effects)
Inverse variance weighted (multiplicative random effects)
MR Egger
MR RAPS
Weighted median
Weighted mode
SNP effect on Ulcerative colitis (strict) with PSC
0.8
0.4
0.0
0.1
0.2
SNP effect on Gut microbiota abundance (genus Intestinimonas id.2062)

Batch 694 : Gut microbiota abundance (genus Lachnoclostridium id.11308) on Ulcerative colitis (strict) with PSC

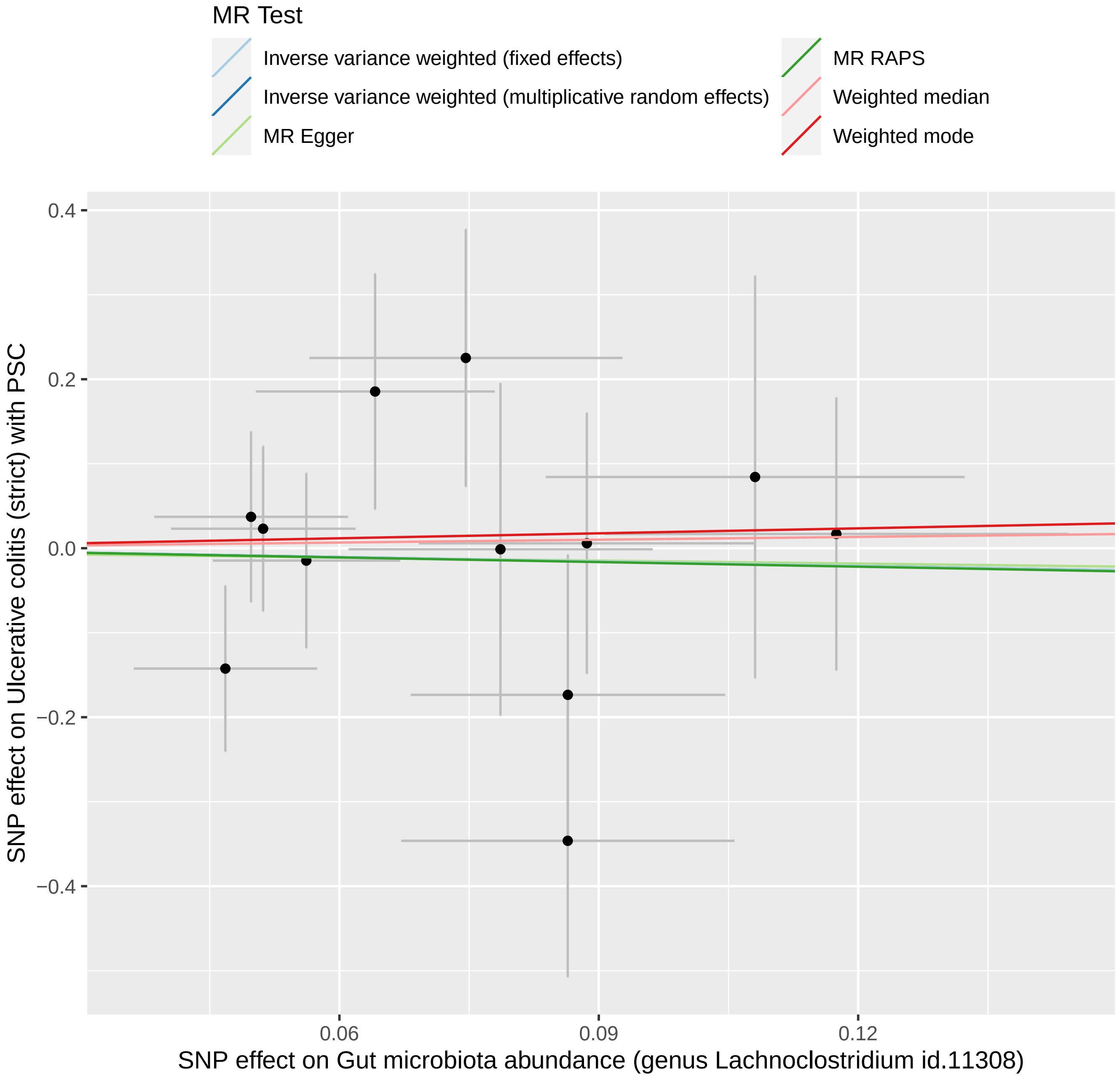

Batch 695 : Gut microbiota abundance (genus Lachnospiraceae FCS020 group id.11314) on Ulcerative colitis (strict) with PSC

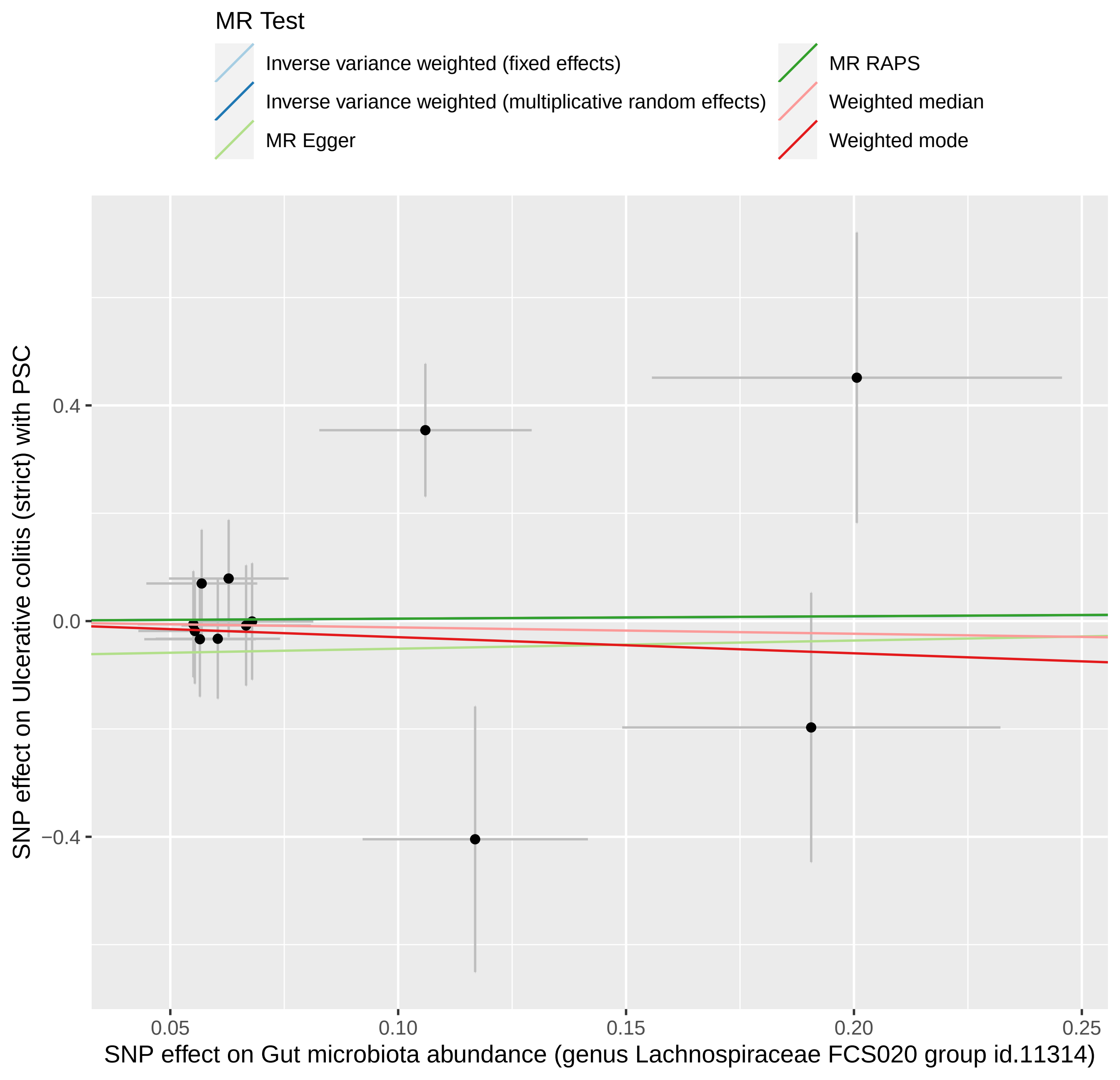

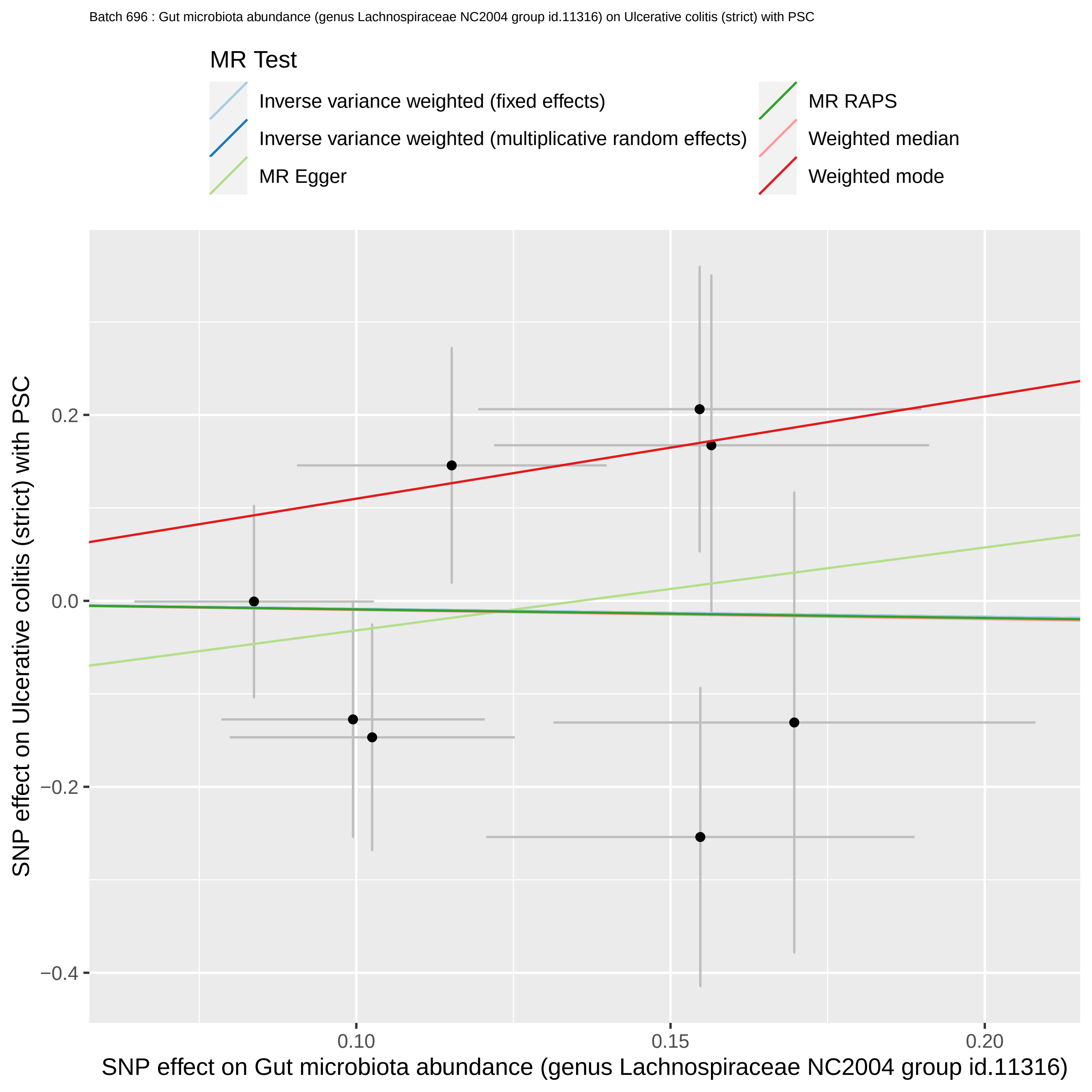
Batch 696 : Gut microbiota abundance (genus Lachnospiraceae NC2004 group id.11316) on Ulcerative colitis (strict) with PSC
MR Test
Inverse variance weighted (fixed effects)
Inverse variance weighted (multiplicative random effects)
MR Egger
MR RAPS
Weighted median
Weighted mode
SNP effect on Ulcerative colitis (strict) with PSC
0.2
0.0
−0.2
−0.4
0.10
0.15
0.20
SNP effect on Gut microbiota abundance (genus Lachnospiraceae NC2004 group id.11316)

Batch 697 : Gut microbiota abundance (genus Lachnospiraceae ND3007 group id.11317) on Ulcerative colitis (strict) with PSC

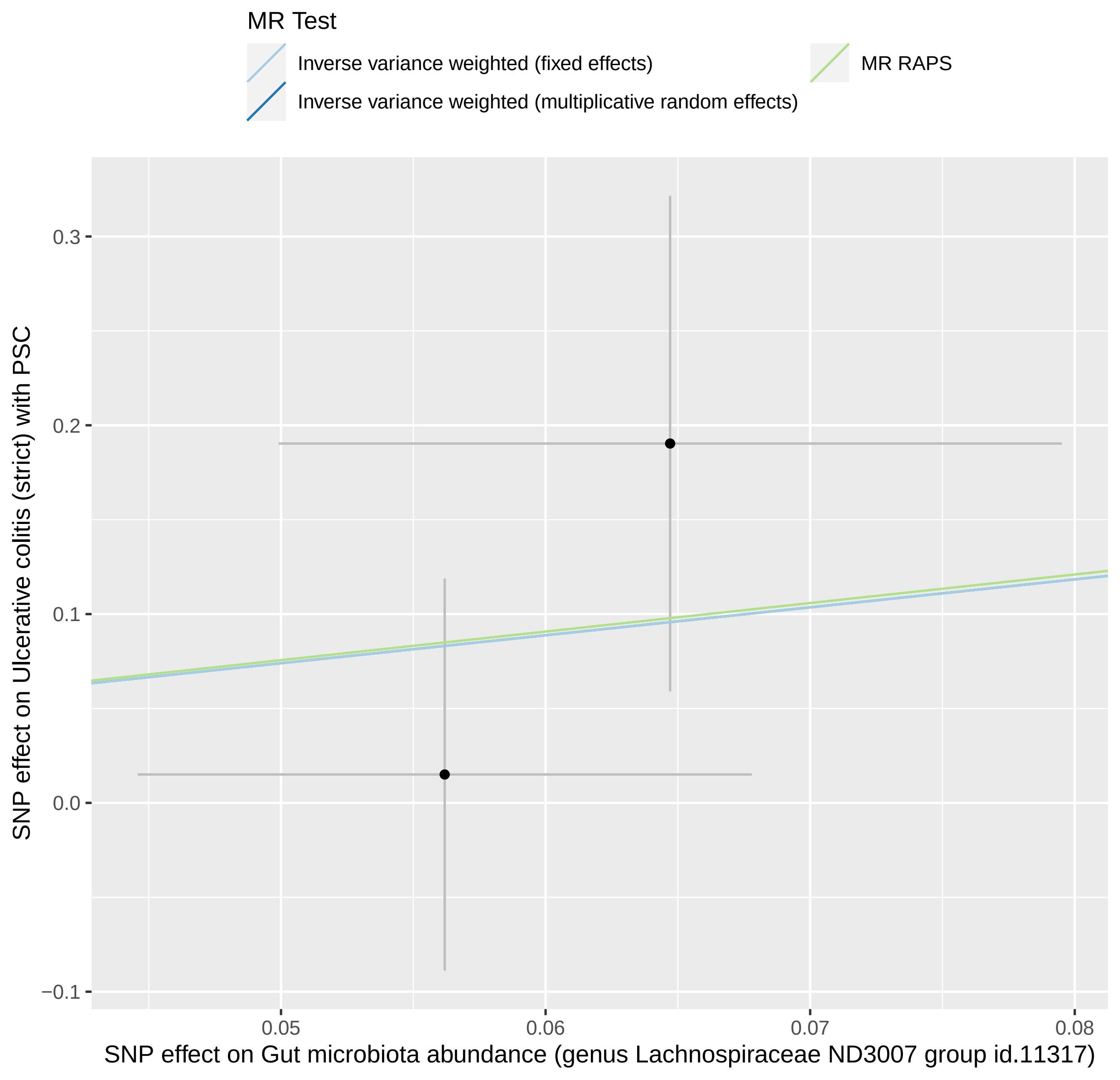

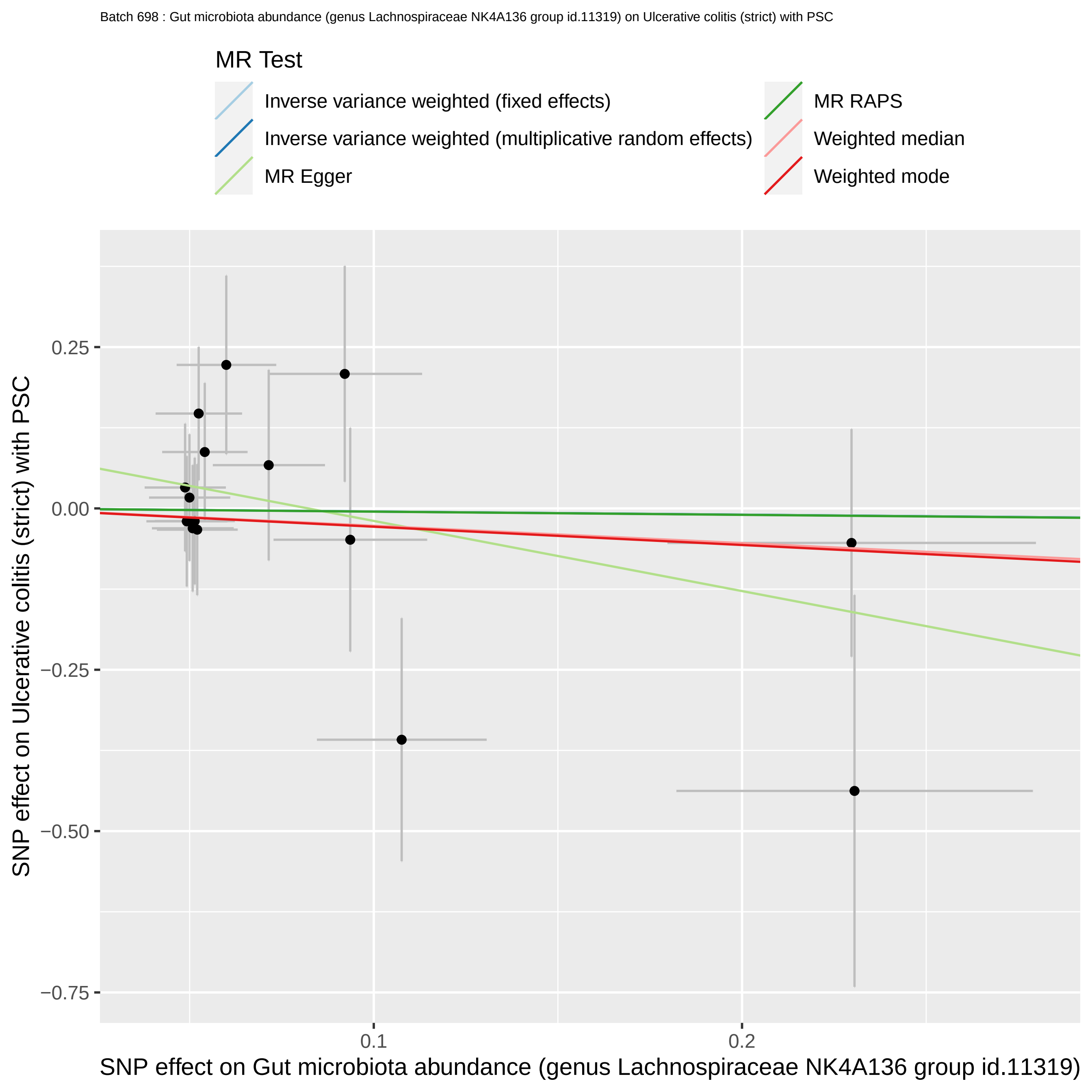

Batch 698 : Gut microbiota abundance (genus Lachnospiraceae NK4A136 group id.11319) on Ulcerative colitis (strict) with PSC
MR Test
Inverse variance weighted (fixed effects)
Inverse variance weighted (multiplicative random effects)
MR Egger
MR RAPS
Weighted median
Weighted mode
0.25
0.00
−0.25
−0.50
−0.75
0.1
0.2
SNP effect on Ulcerative colitis (strict) with PSC
SNP effect on Gut microbiota abundance (genus Lachnospiraceae NK4A136 group id.11319)

Batch 699 : Gut microbiota abundance (genus Lachnospiraceae UCG001 id.11321) on Ulcerative colitis (strict) with PSC

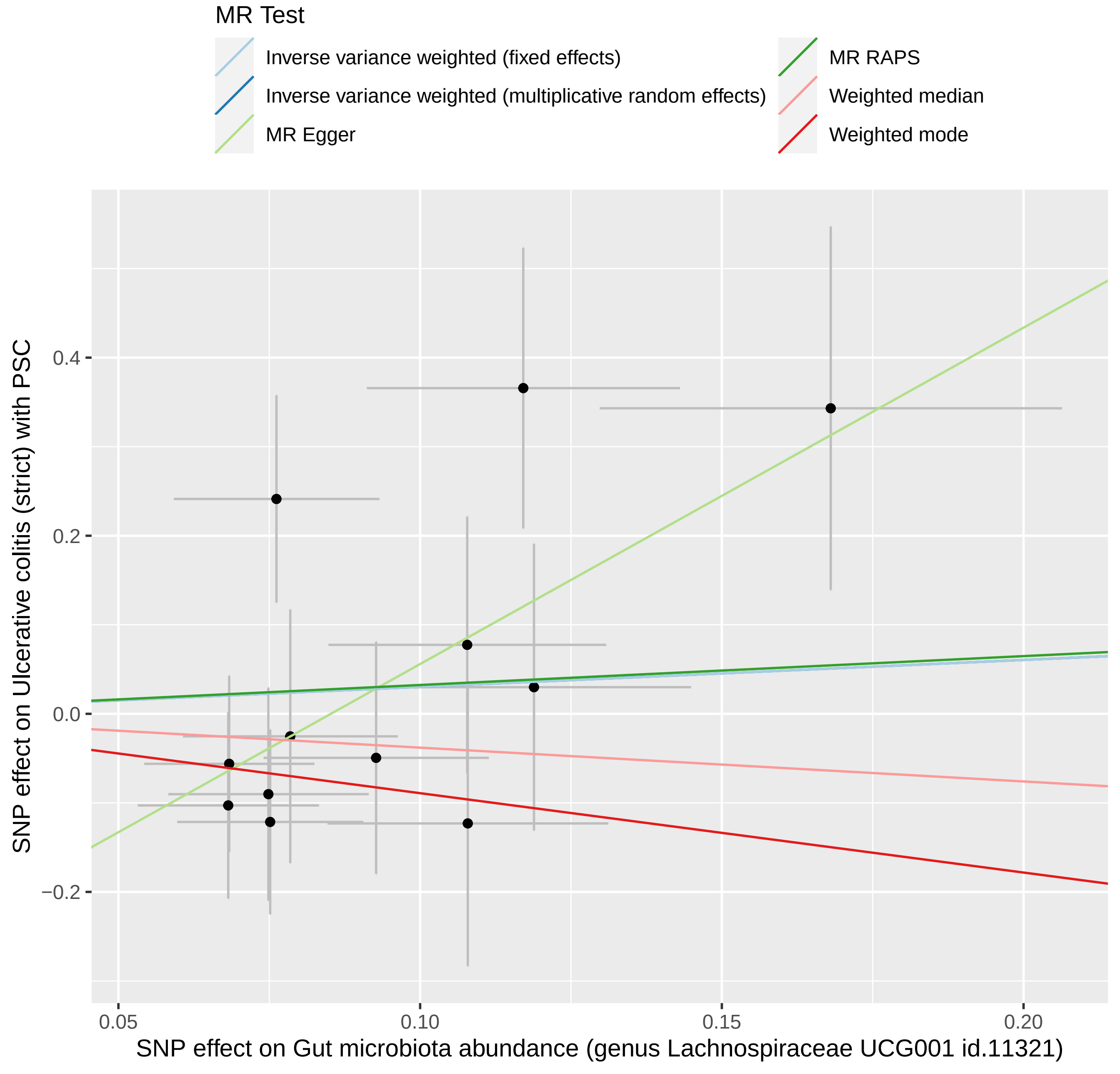

Batch 700 : Gut microbiota abundance (genus Lachnospiraceae UCG004 id.11324) on Ulcerative colitis (strict) with PSC

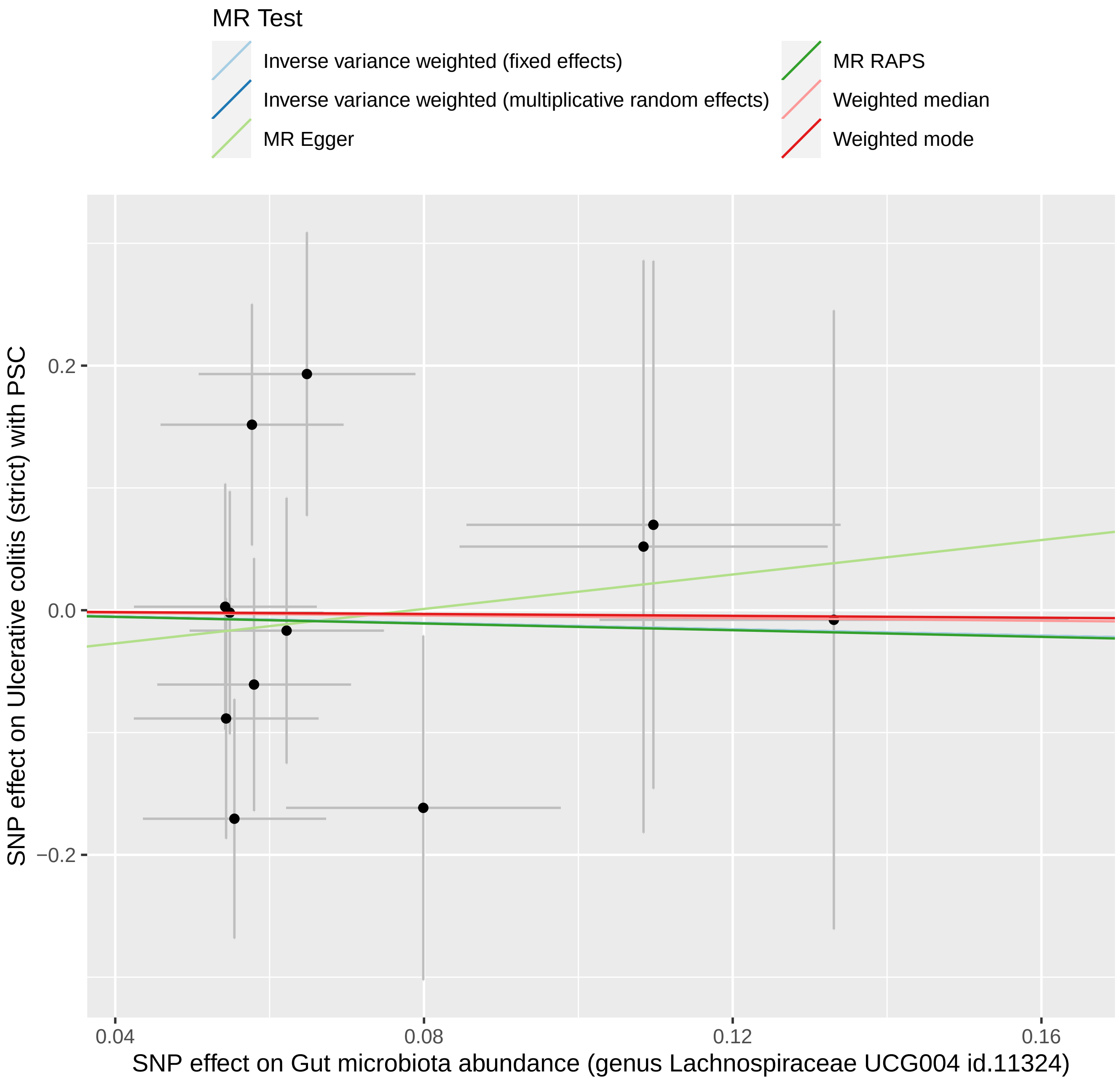

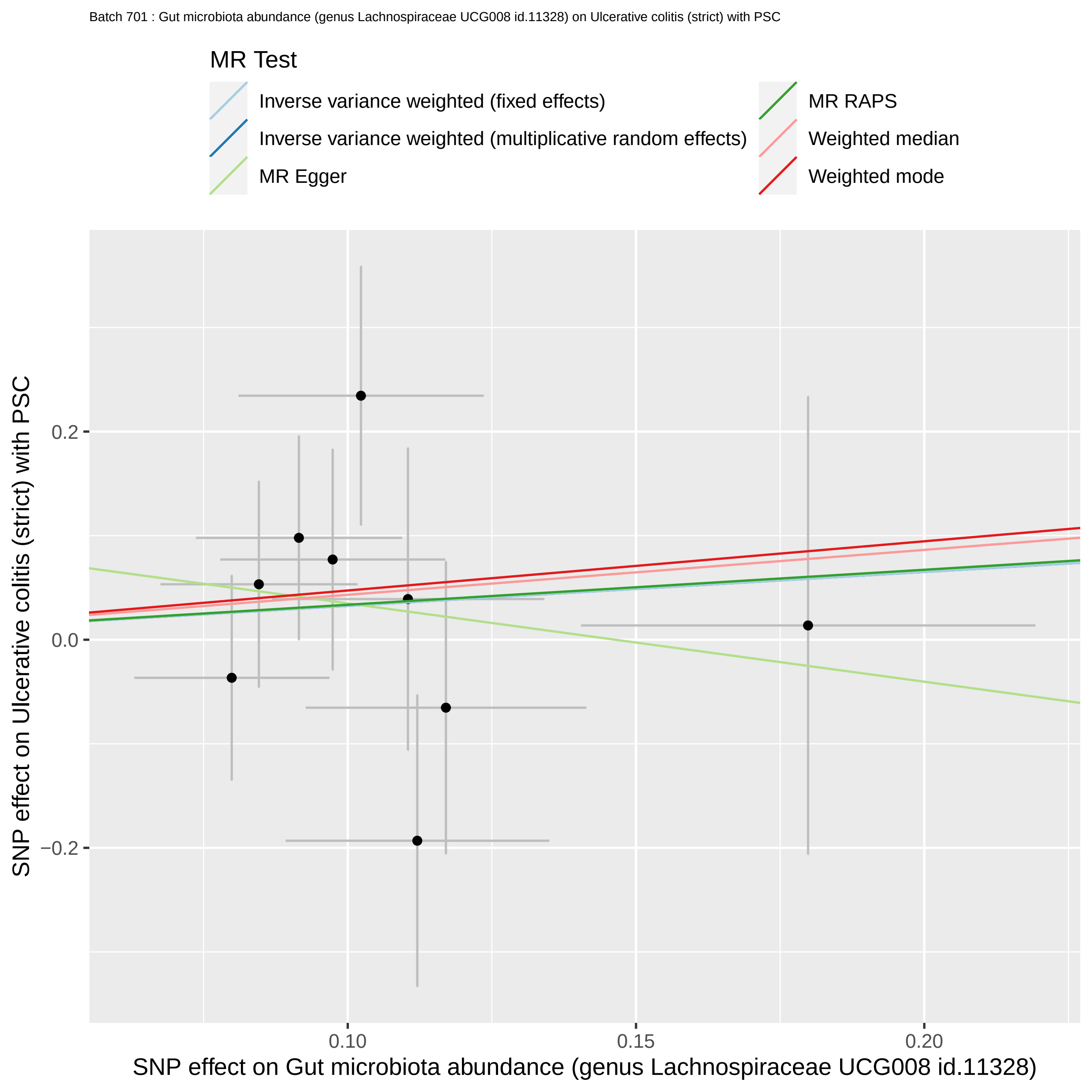
Batch 701 : Gut microbiota abundance (genus Lachnospiraceae UCG008 id.11328) on Ulcerative colitis (strict) with PSC
MR Test
Inverse variance weighted (fixed effects)
Inverse variance weighted (multiplicative random effects)
MR Egger
MR RAPS
Weighted median
Weighted mode
0.2
0.0
−0.2
0.10
0.15
0.20
SNP effect on Ulcerative colitis (strict) with PSC
SNP effect on Gut microbiota abundance (genus Lachnospiraceae UCG008 id.11328)

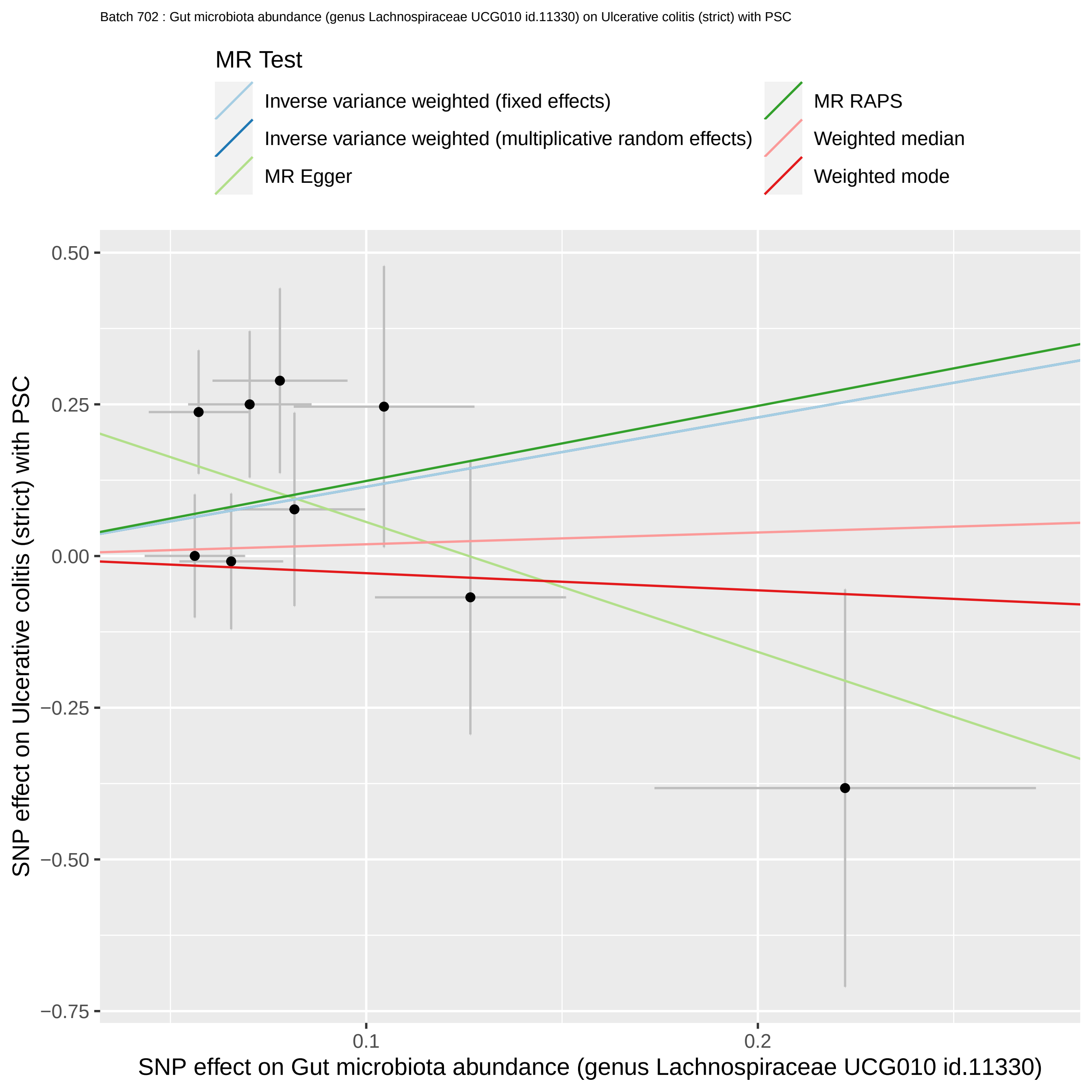

Batch 702 : Gut microbiota abundance (genus Lachnospiraceae UCG010 id.11330) on Ulcerative colitis (strict) with PSC
MR Test
Inverse variance weighted (fixed effects)
Inverse variance weighted (multiplicative random effects)
MR Egger
MR RAPS
Weighted median
Weighted mode
0.50
0.25
0.00
−0.25
−0.50
−0.75
0.1
0.2
SNP effect on Ulcerative colitis (strict) with PSC
SNP effect on Gut microbiota abundance (genus Lachnospiraceae UCG010 id.11330)

Insufficient number of SNPs

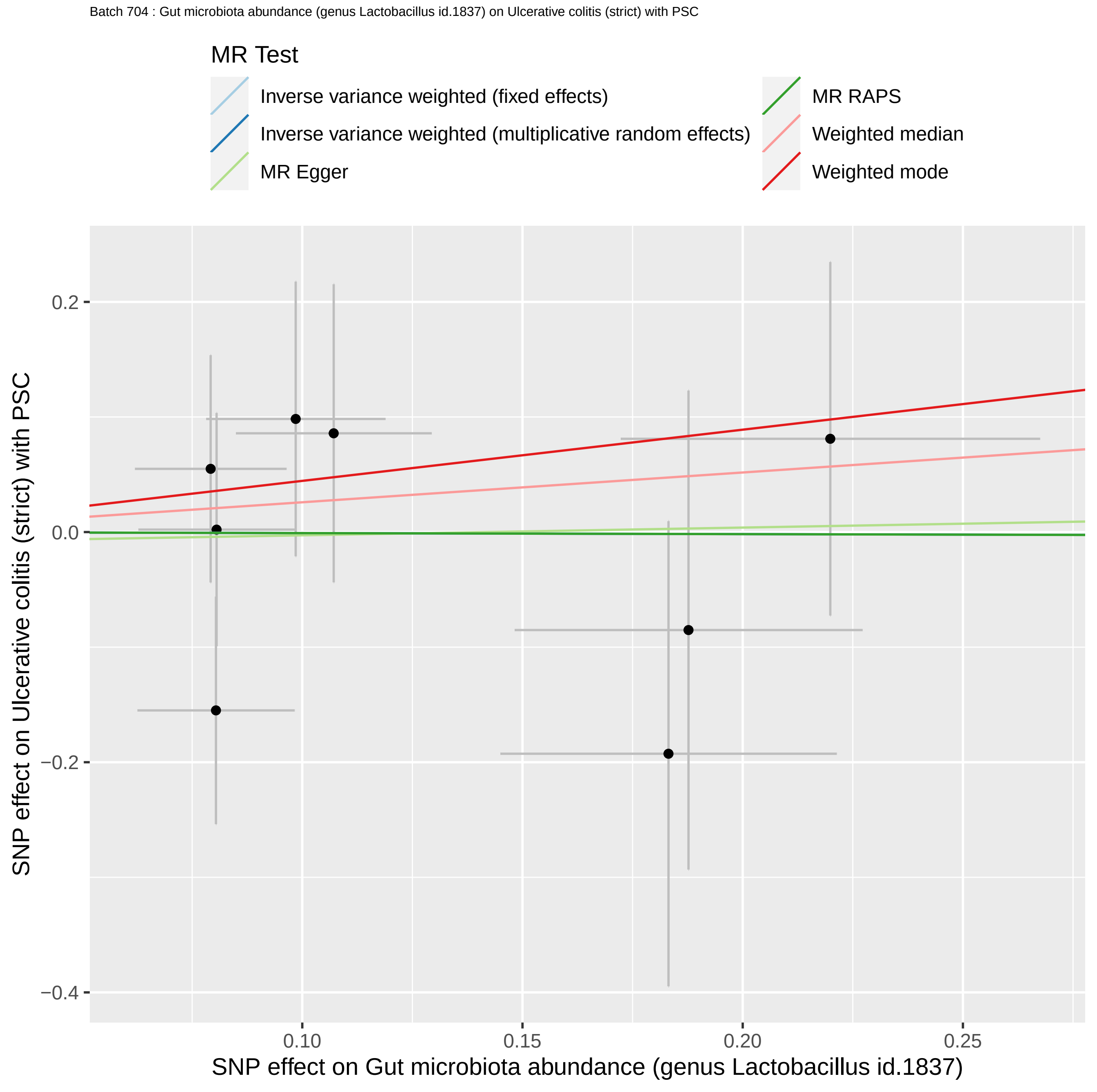

Batch 704 : Gut microbiota abundance (genus Lactobacillus id.1837) on Ulcerative colitis (strict) with PSC
MR Test
Inverse variance weighted (fixed effects)
Inverse variance weighted (multiplicative random effects)
MR Egger
MR RAPS
Weighted median
Weighted mode
0.2
0.0
−0.2
−0.4
0.10
0.15
0.20
0.25
SNP effect on Ulcerative colitis (strict) with PSC
SNP effect on Gut microbiota abundance (genus Lactobacillus id.1837)

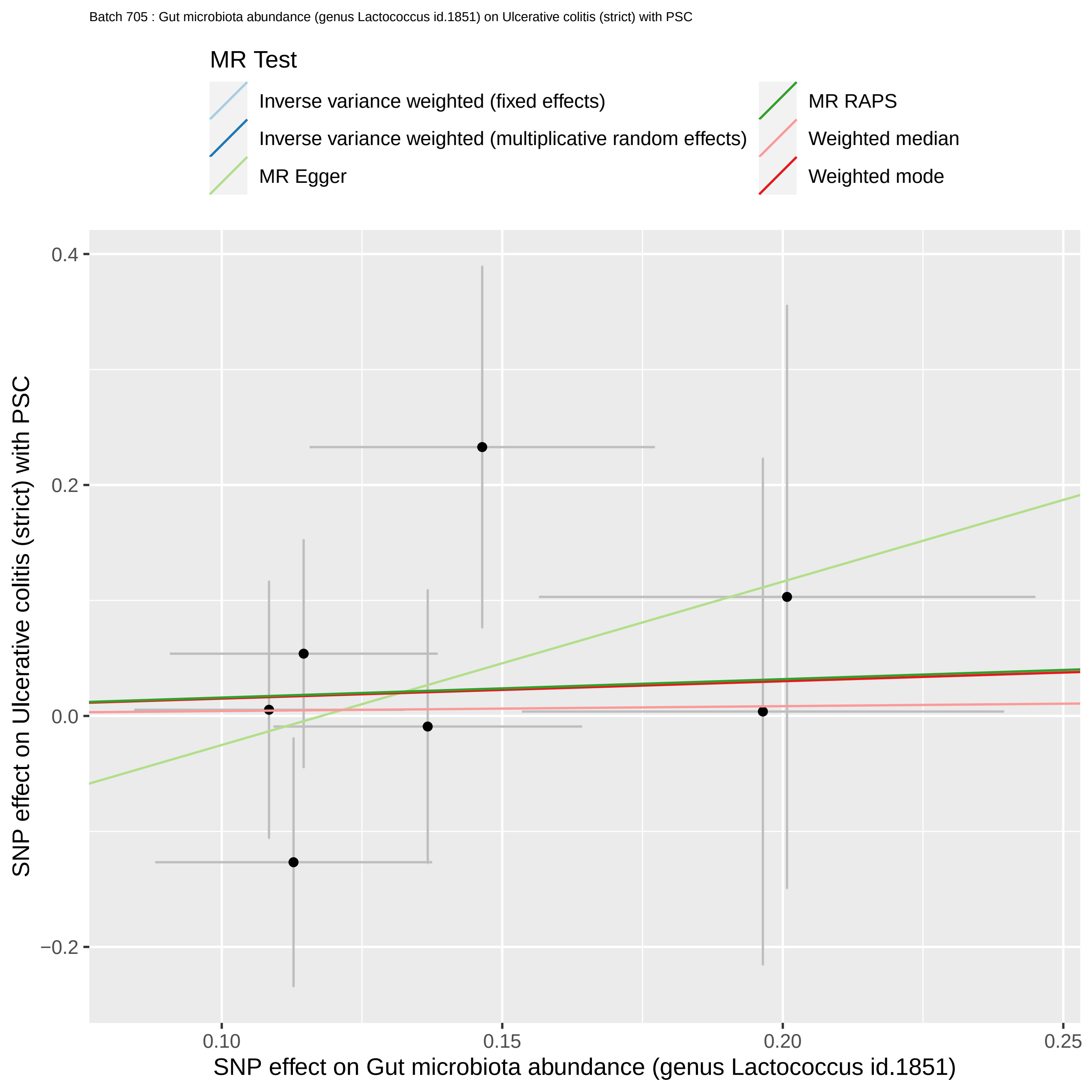
Batch 705 : Gut microbiota abundance (genus Lactococcus id.1851) on Ulcerative colitis (strict) with PSC
MR Test
Inverse variance weighted (fixed effects)
Inverse variance weighted (multiplicative random effects)
MR Egger
MR RAPS
Weighted median
Weighted mode
0.4
0.2
0.0
−0.2
0.10
0.15
0.20
0.25
SNP effect on Ulcerative colitis (strict) with PSC
SNP effect on Gut microbiota abundance (genus Lactococcus id.1851)

Batch 706 : Gut microbiota abundance (genus Marvinbryantia id.2005) on Ulcerative colitis (strict) with PSC

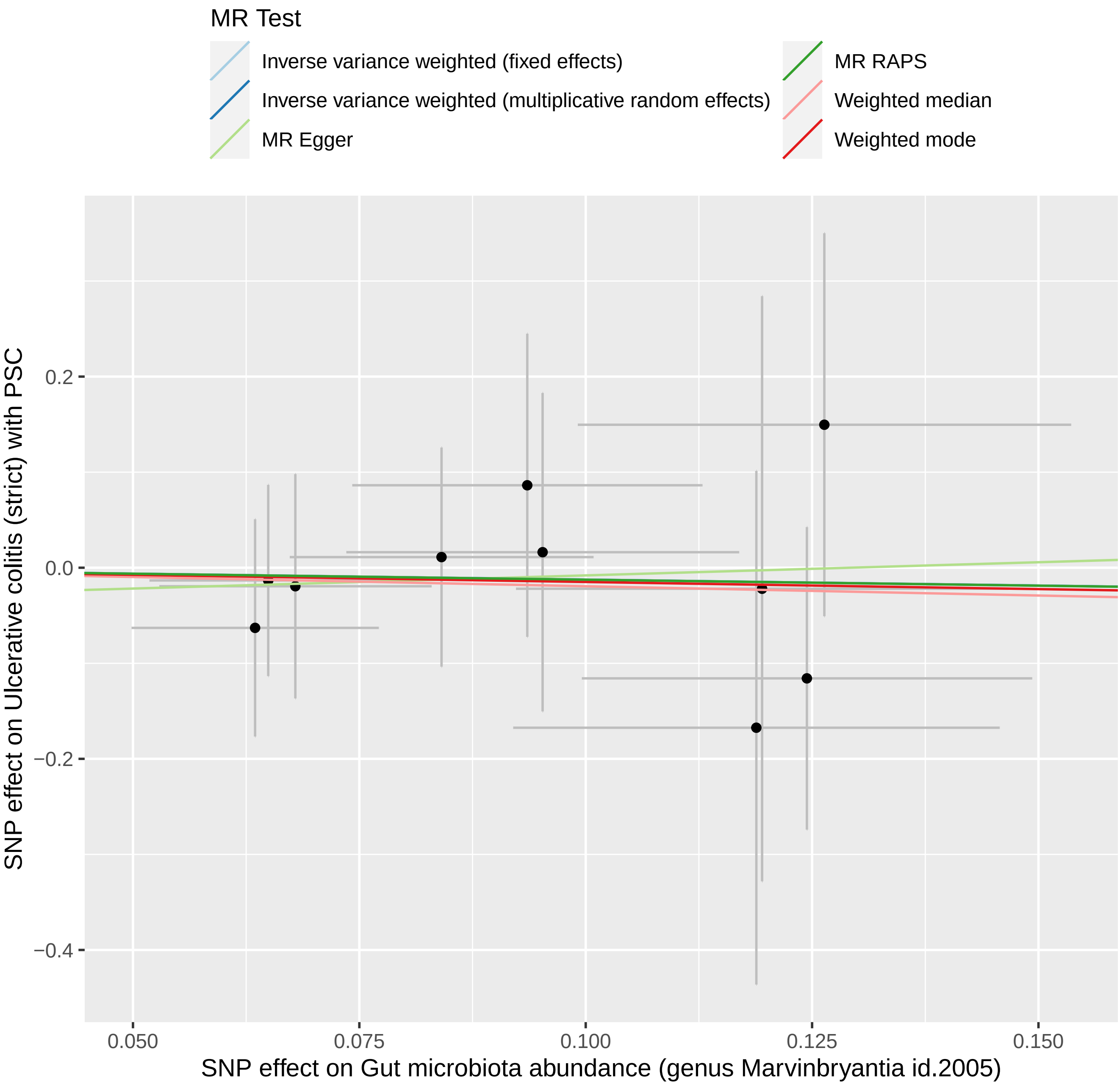

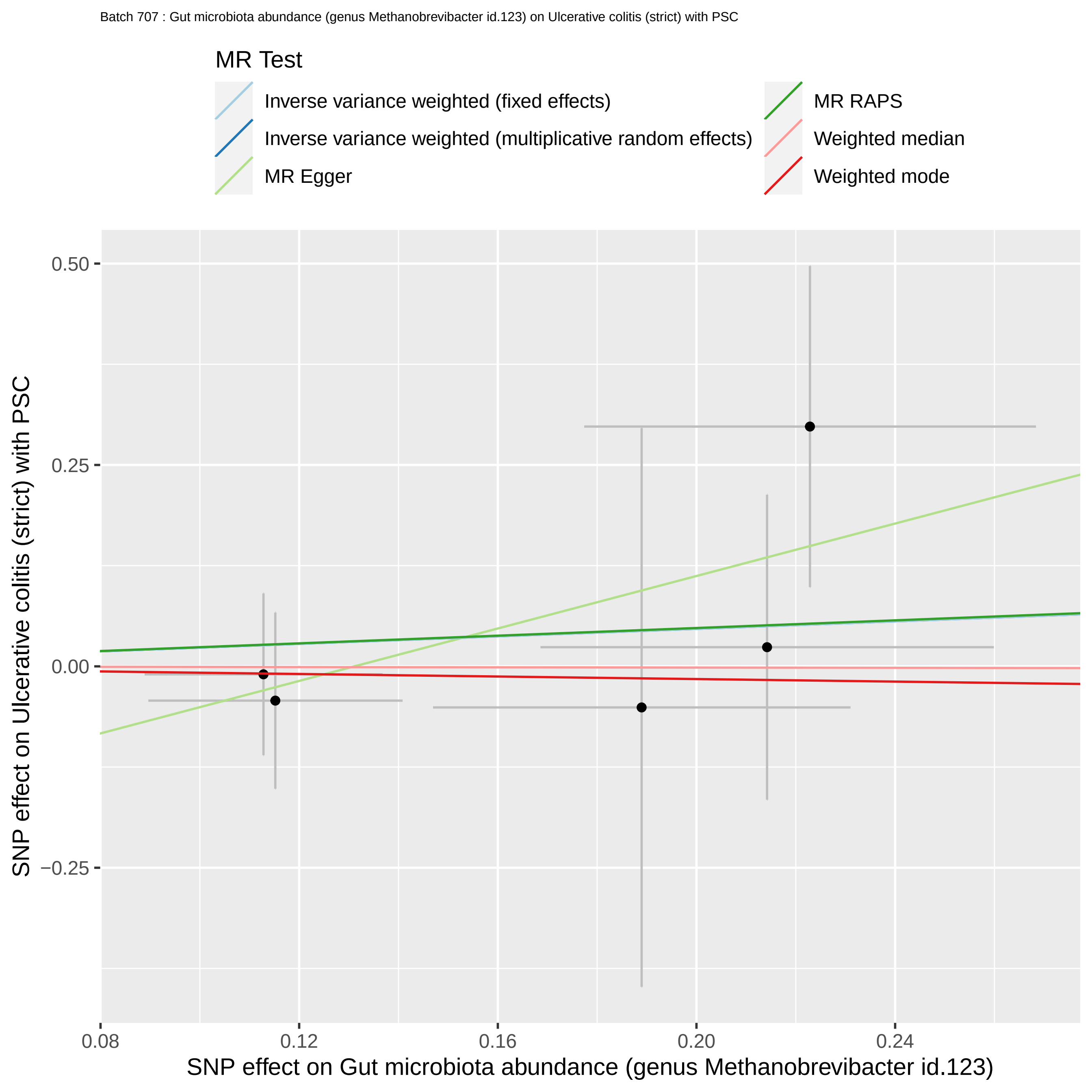

Batch 707 : Gut microbiota abundance (genus Methanobrevibacter id.123) on Ulcerative colitis (strict) with PSC
MR Test
Inverse variance weighted (fixed effects)
Inverse variance weighted (multiplicative random effects)
MR Egger
MR RAPS
Weighted median
Weighted mode
0.50
0.25
0.00
−0.25
0.08
0.12
0.16
0.20
0.24
SNP effect on Ulcerative colitis (strict) with PSC
SNP effect on Gut microbiota abundance (genus Methanobrevibacter id.123)

Batch 708 : Gut microbiota abundance (genus Odoribacter id.952) on Ulcerative colitis (strict) with PSC

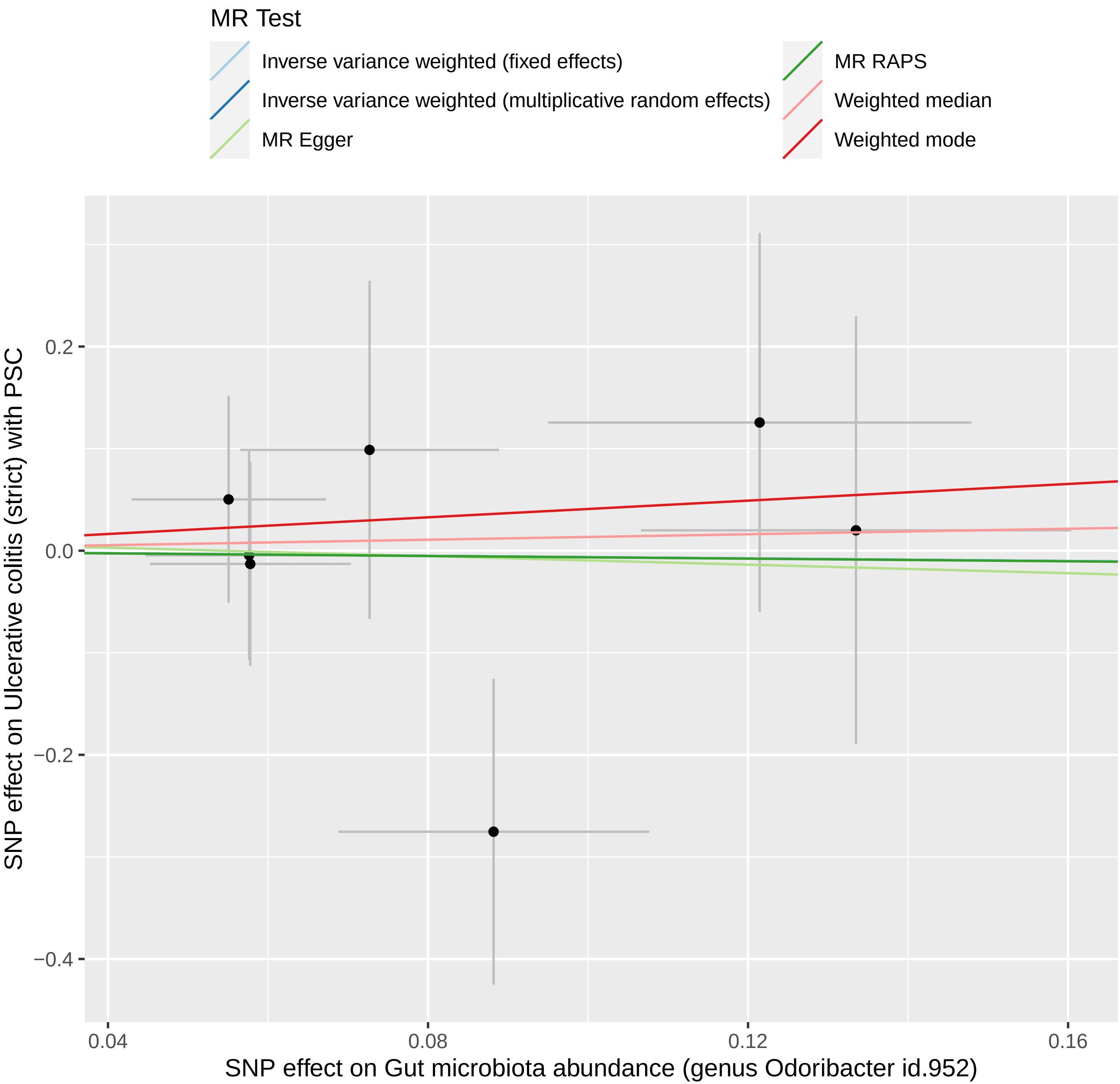

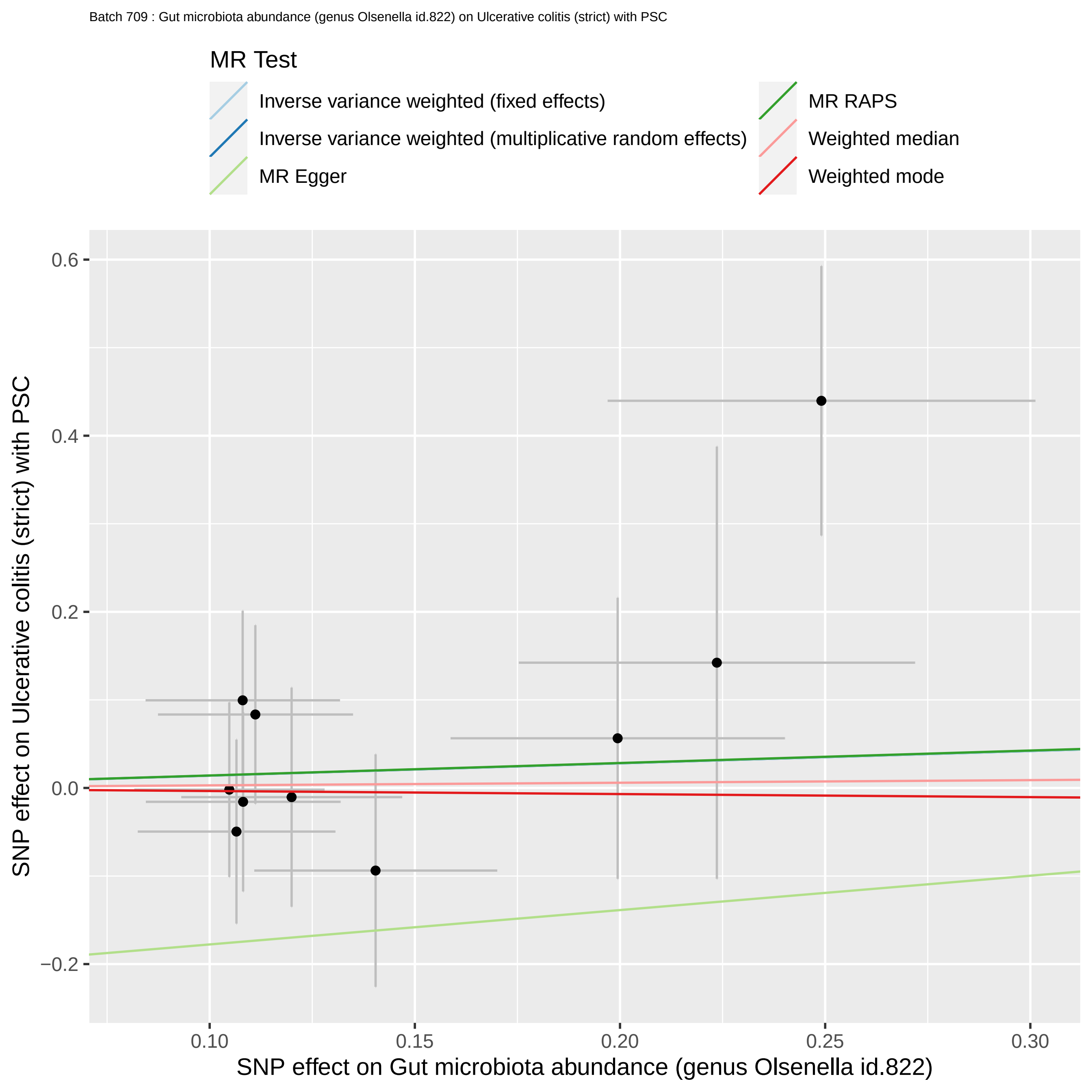

Batch 709 : Gut microbiota abundance (genus Olsenella id.822) on Ulcerative colitis (strict) with PSC
MR Test
Inverse variance weighted (fixed effects)
Inverse variance weighted (multiplicative random effects)
MR Egger
MR RAPS
Weighted median
Weighted mode
0.6
0.4
0.2
0.0
−0.2
0.10
0.15
0.20
0.25
0.30
SNP effect on Ulcerative colitis (strict) with PSC
SNP effect on Gut microbiota abundance (genus Olsenella id.822)

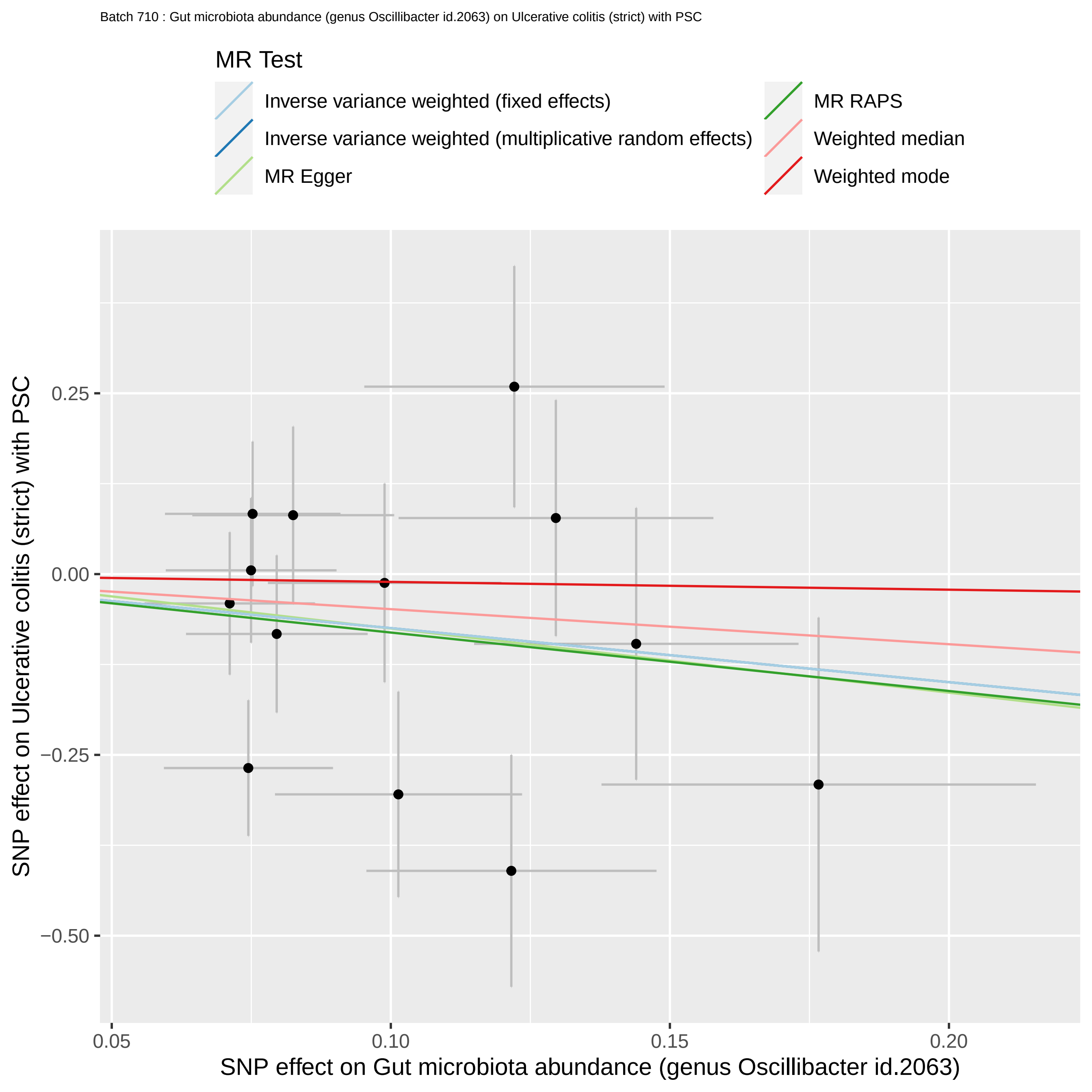

Batch 710 : Gut microbiota abundance (genus Oscillibacter id.2063) on Ulcerative colitis (strict) with PSC
MR Test
Inverse variance weighted (fixed effects)
Inverse variance weighted (multiplicative random effects)
MR Egger
MR RAPS
Weighted median
Weighted mode
0.25
0.00
−0.25
−0.50
0.05
0.10
0.15
0.20
SNP effect on Ulcerative colitis (strict) with PSC
SNP effect on Gut microbiota abundance (genus Oscillibacter id.2063)

Batch 711 : Gut microbiota abundance (genus Oscillospira id.2064) on Ulcerative colitis (strict) with PSC

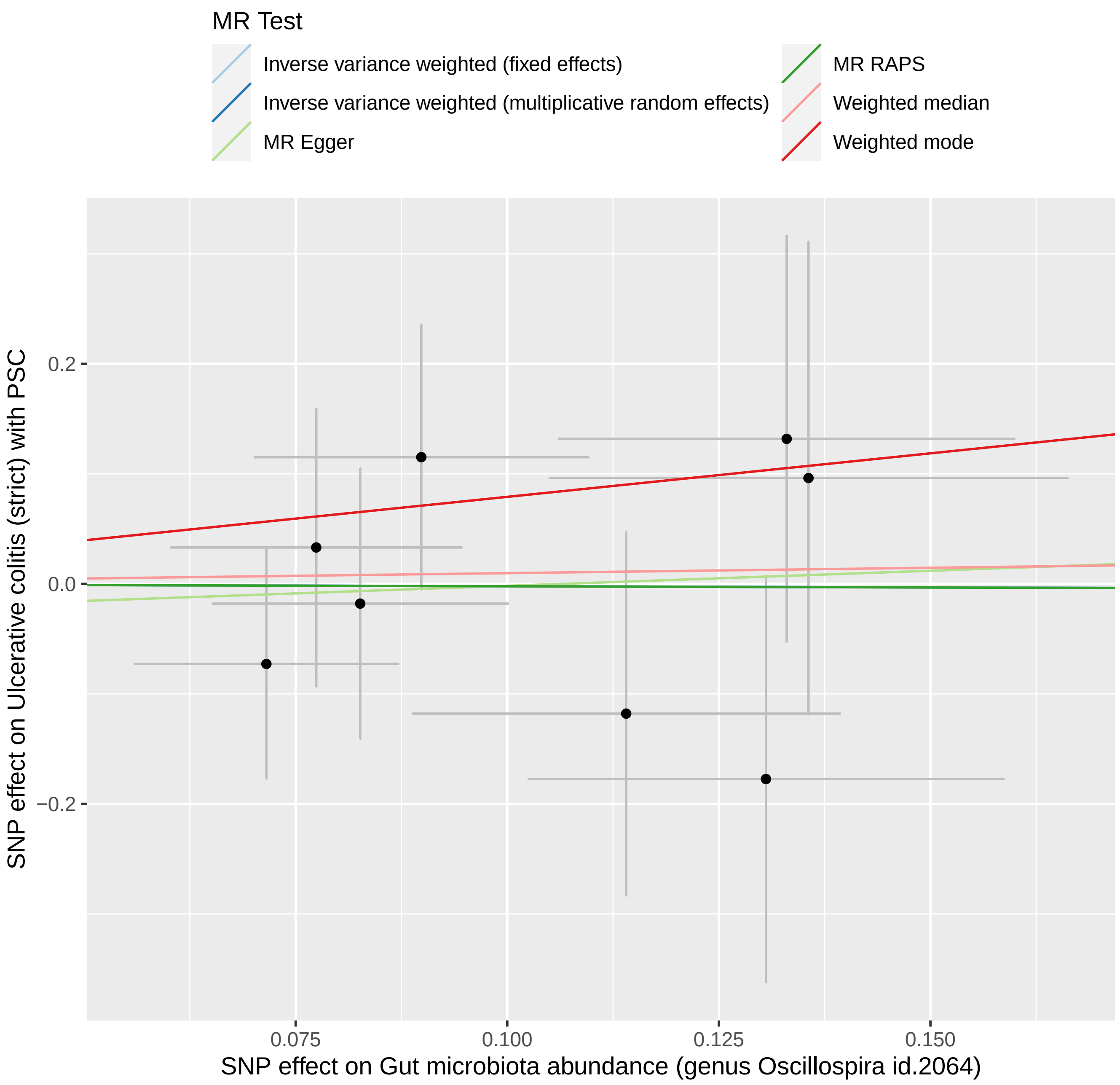

Batch 712 : Gut microbiota abundance (genus Oxalobacter id.2978) on Ulcerative colitis (strict) with PSC

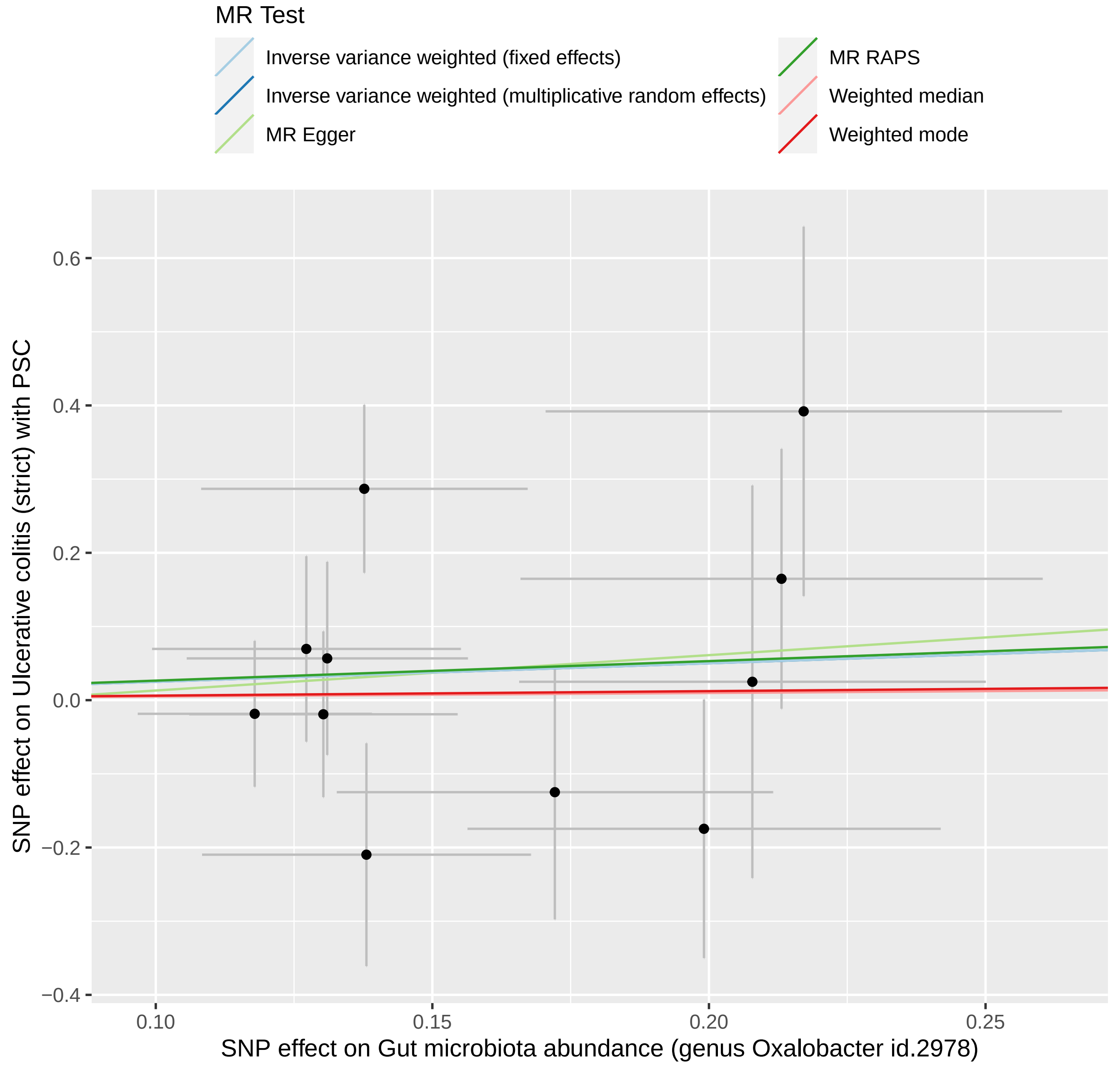

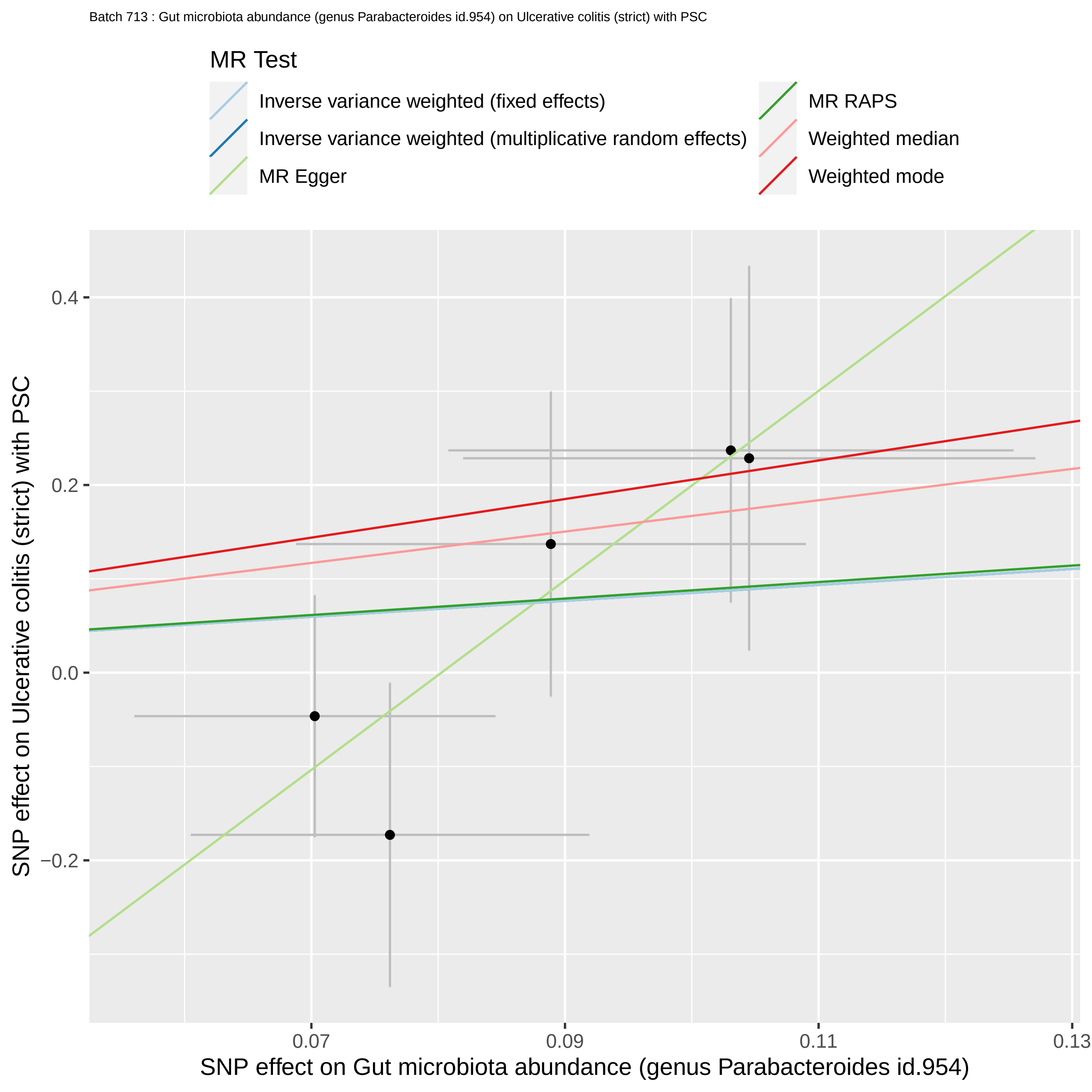

Batch 713 : Gut microbiota abundance (genus Parabacteroides id.954) on Ulcerative colitis (strict) with PSC
MR Test
Inverse variance weighted (fixed effects)
Inverse variance weighted (multiplicative random effects)
MR Egger
MR RAPS
Weighted median
Weighted mode
0.4
0.2
0.0
−0.2
0.07
0.09
0.11
0.13
SNP effect on Ulcerative colitis (strict) with PSC
SNP effect on Gut microbiota abundance (genus Parabacteroides id.954)

Batch 714 : Gut microbiota abundance (genus Paraprevotella id.962) on Ulcerative colitis (strict) with PSC

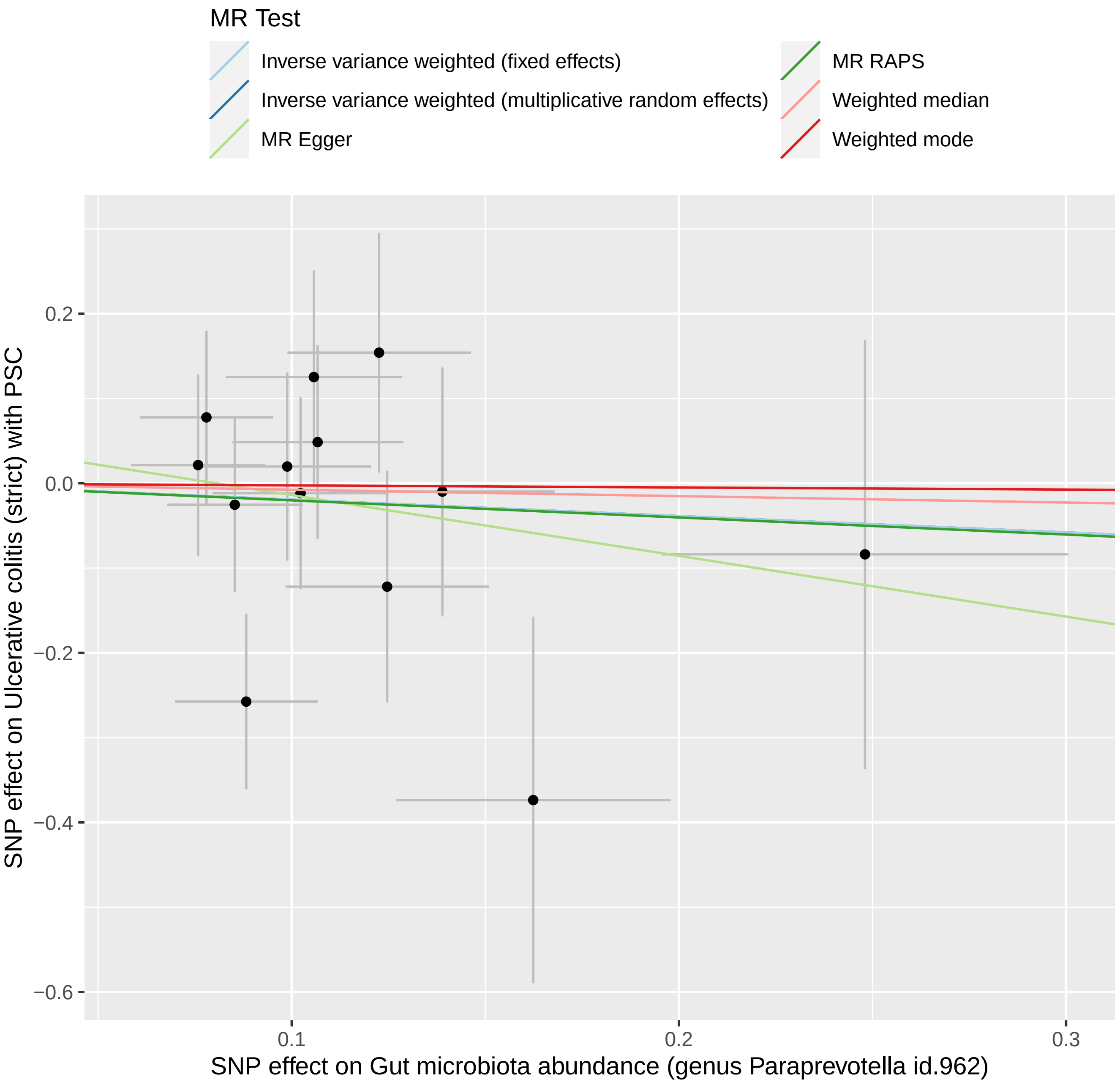

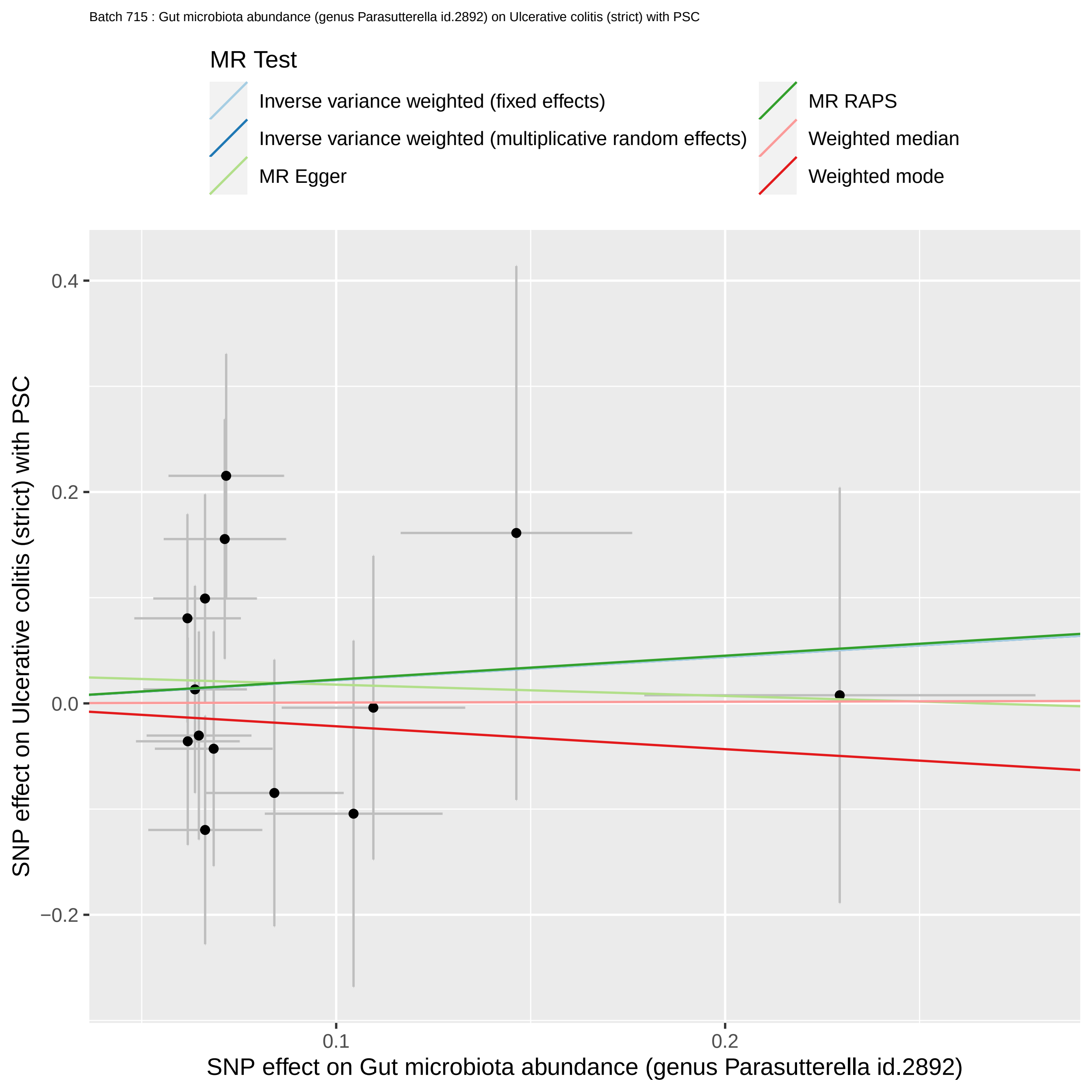
Batch 715 : Gut microbiota abundance (genus Parasutterella id.2892) on Ulcerative colitis (strict) with PSC
MR Test
Inverse variance weighted (fixed effects)
Inverse variance weighted (multiplicative random effects)
MR Egger
MR RAPS
Weighted median
Weighted mode
0.4
0.2
0.0
−0.2
0.1
0.2
SNP effect on Ulcerative colitis (strict) with PSC
SNP effect on Gut microbiota abundance (genus Parasutterella id.2892)

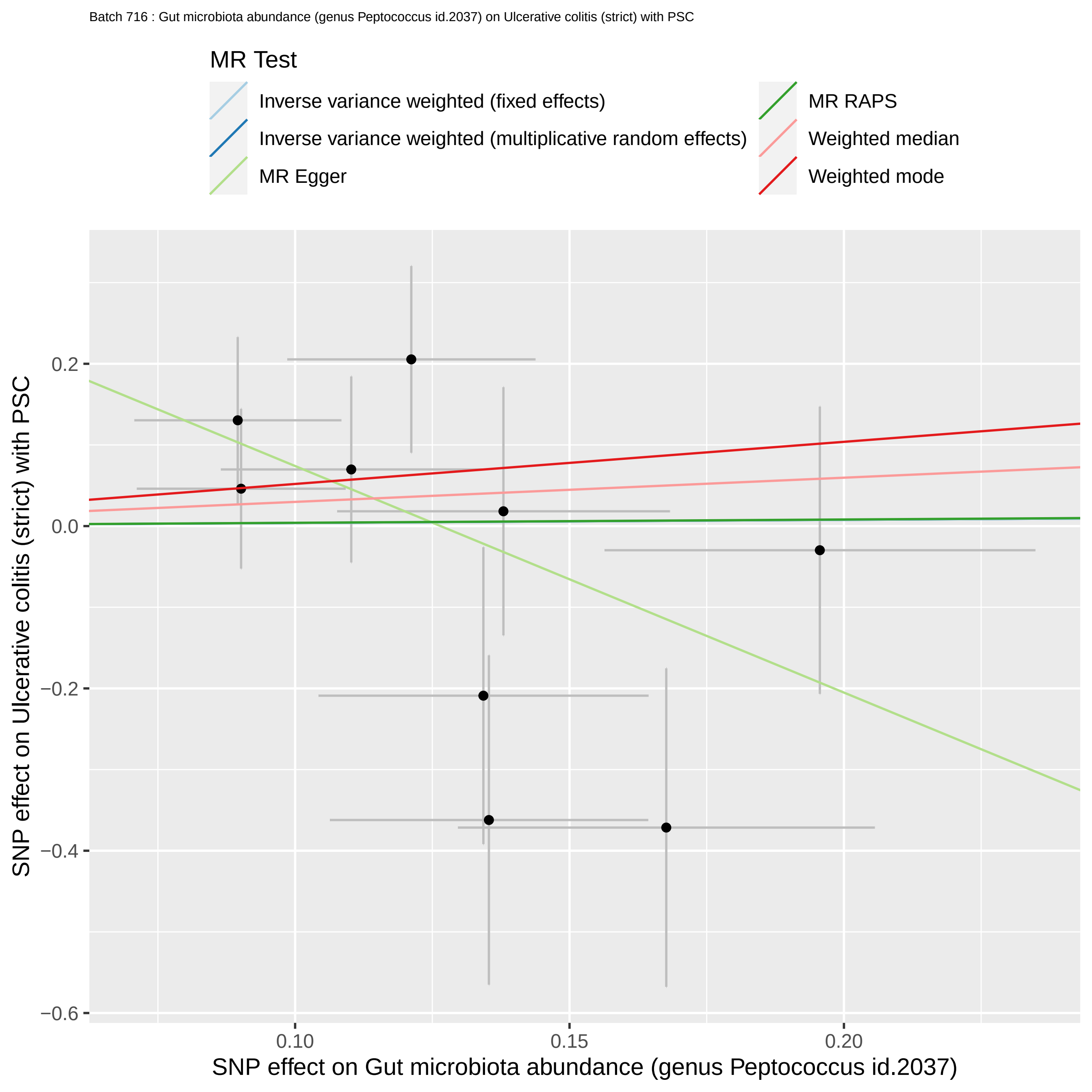

Batch 716 : Gut microbiota abundance (genus Peptococcus id.2037) on Ulcerative colitis (strict) with PSC
MR Test
Inverse variance weighted (fixed effects)
Inverse variance weighted (multiplicative random effects)
MR Egger
MR RAPS
Weighted median
Weighted mode
0.2
0.0
−0.2
−0.4
−0.6
0.10
0.15
0.20
SNP effect on Ulcerative colitis (strict) with PSC
SNP effect on Gut microbiota abundance (genus Peptococcus id.2037)

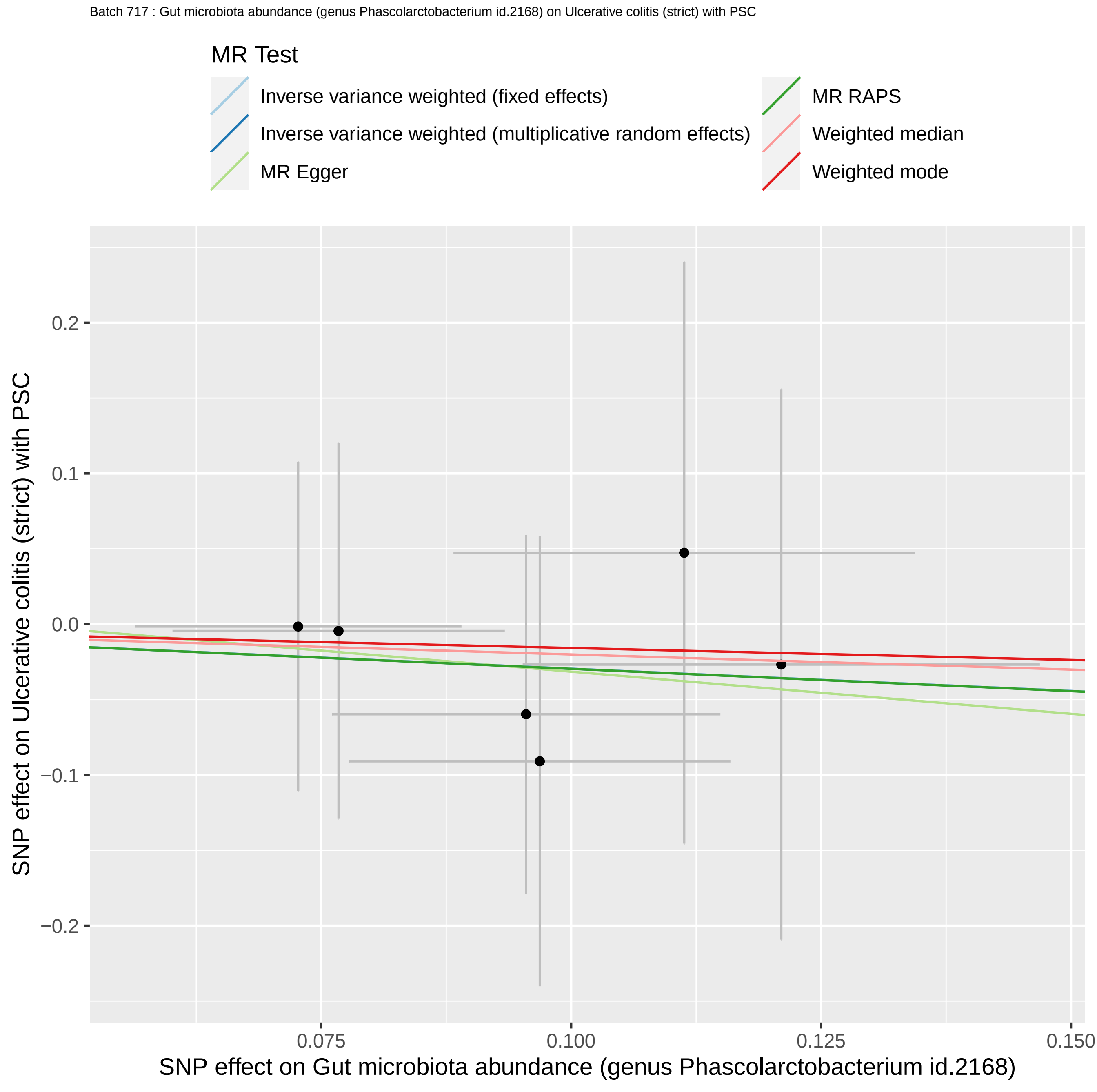

Batch 717 : Gut microbiota abundance (genus Phascolarctobacterium id.2168) on Ulcerative colitis (strict) with PSC
MR Test
Inverse variance weighted (fixed effects)
Inverse variance weighted (multiplicative random effects)
MR Egger
MR RAPS
Weighted median
Weighted mode
SNP effect on Ulcerative colitis (strict) with PSC
0.2
0.1
0.0
−0.1
−0.2
0.075
0.100
0.125
0.150
SNP effect on Gut microbiota abundance (genus Phascolarctobacterium id.2168)

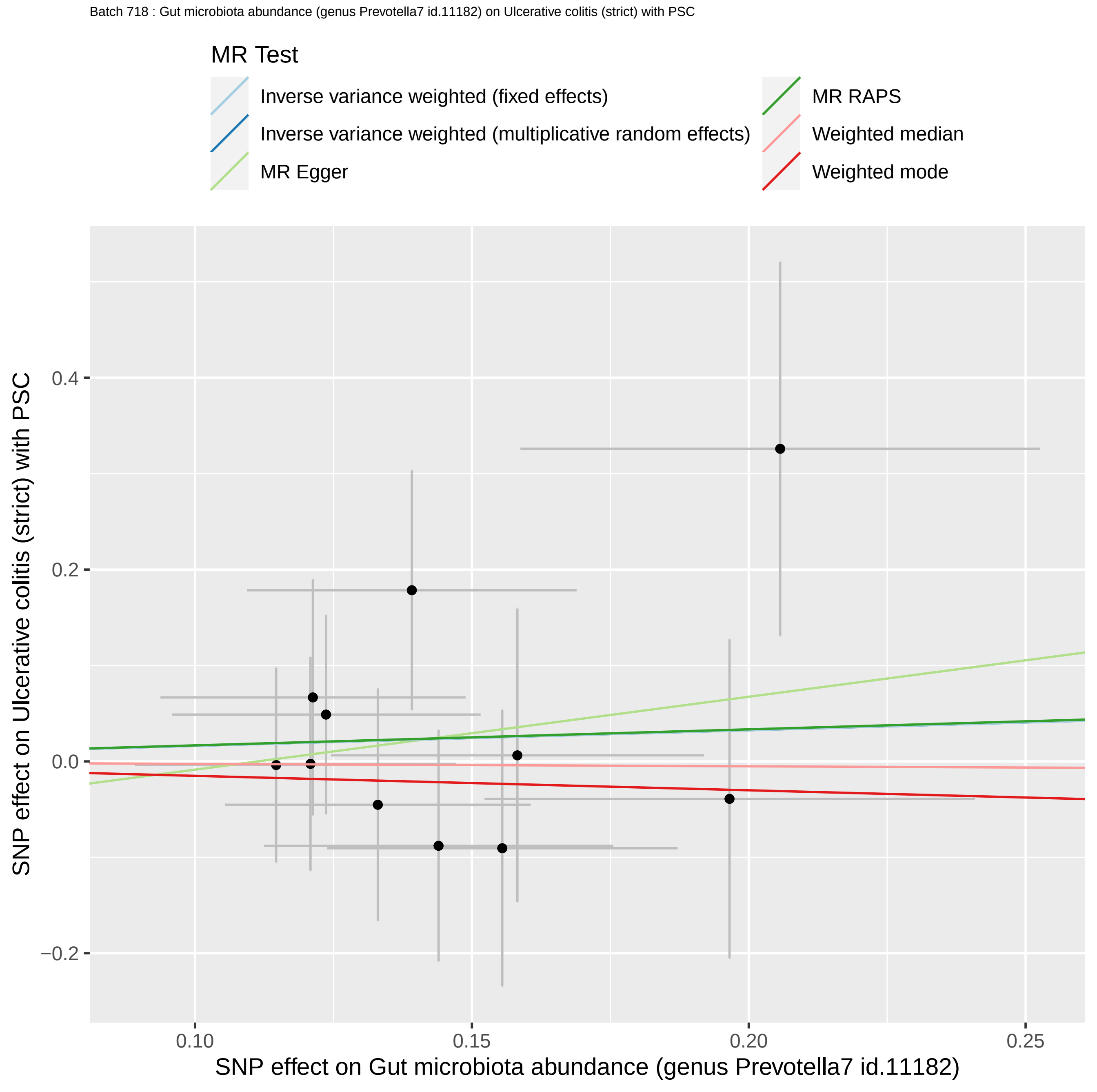
Batch 718 : Gut microbiota abundance (genus Prevotella7 id.11182) on Ulcerative colitis (strict) with PSC
MR Test
Inverse variance weighted (fixed effects)
Inverse variance weighted (multiplicative random effects)
MR Egger
MR RAPS
Weighted median
Weighted mode
SNP effect on Ulcerative colitis (strict) with PSC
0.4
0.2
0.0
−0.2
0.10
0.15
0.20
0.25
SNP effect on Gut microbiota abundance (genus Prevotella7 id.11182)

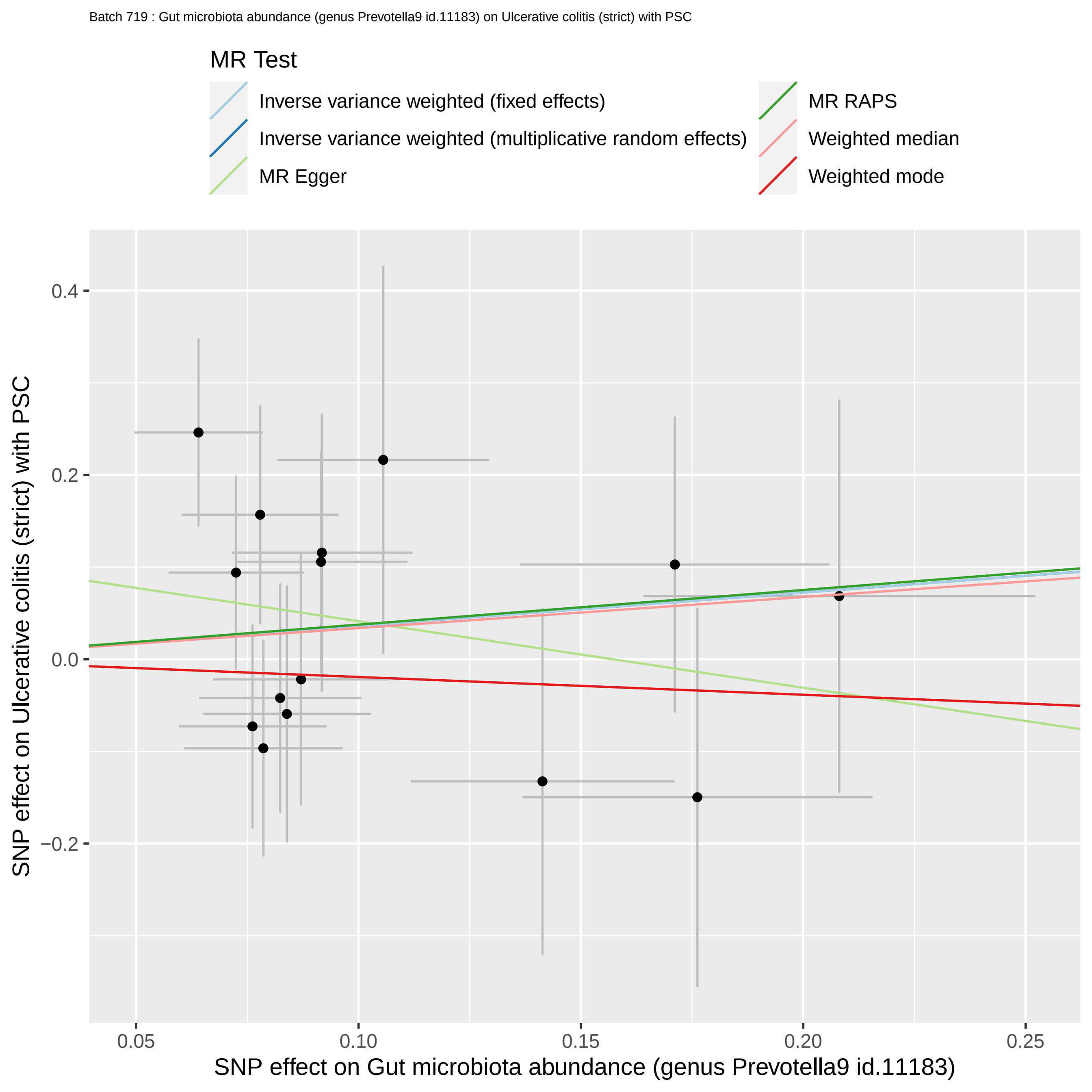

Batch 719 : Gut microbiota abundance (genus Prevotella9 id.11183) on Ulcerative colitis (strict) with PSC
MR Test
Inverse variance weighted (fixed effects)
Inverse variance weighted (multiplicative random effects)
MR Egger
MR RAPS
Weighted median
Weighted mode
0.4
0.2
0.0
−0.2
0.05
0.10
0.15
0.20
0.25
SNP effect on Ulcerative colitis (strict) with PSC
SNP effect on Gut microbiota abundance (genus Prevotella9 id.11183)

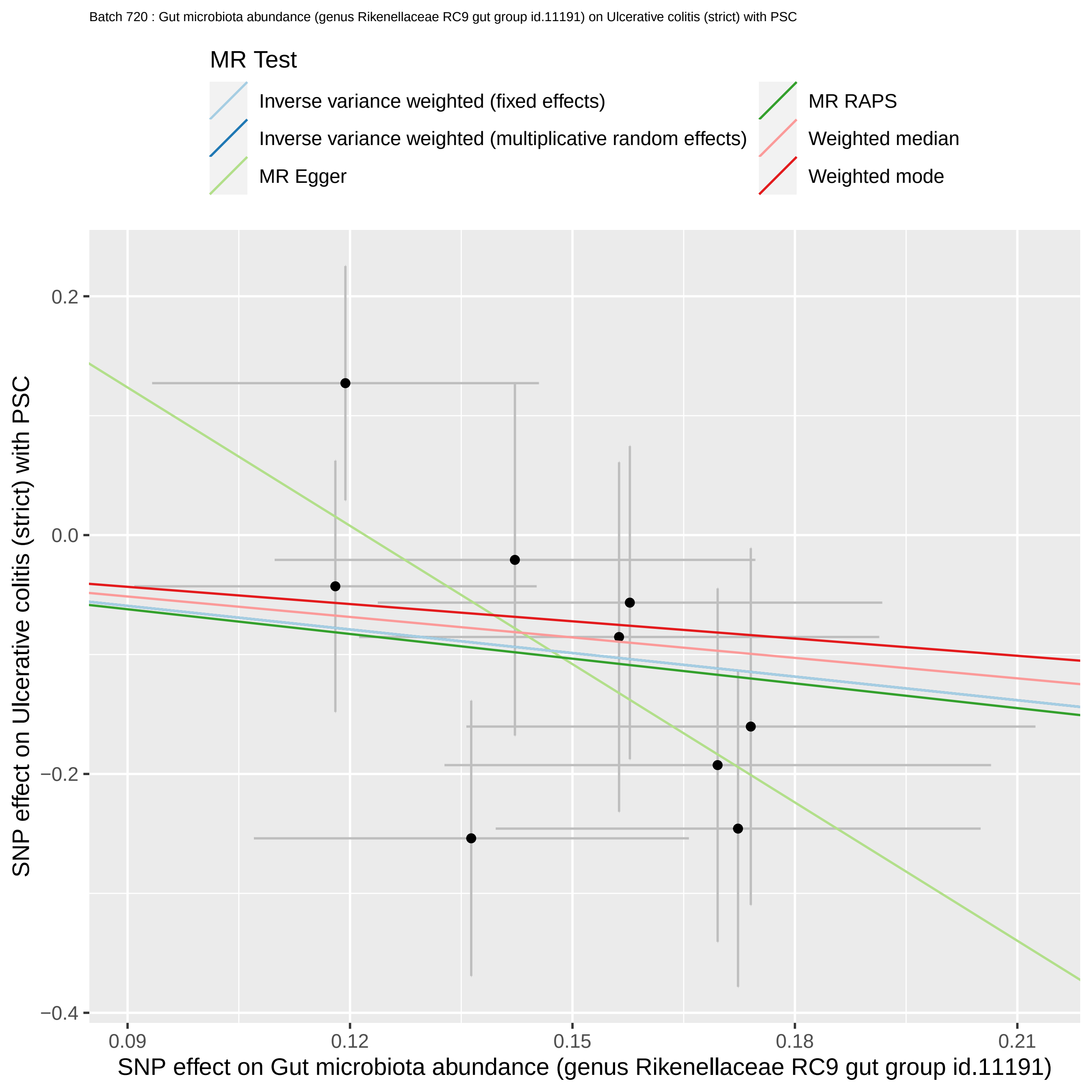
Batch 720 : Gut microbiota abundance (genus Rikenellaceae RC9 gut group id.11191) on Ulcerative colitis (strict) with PSC
MR Test
Inverse variance weighted (fixed effects)
Inverse variance weighted (multiplicative random effects)
MR Egger
MR RAPS
Weighted median
Weighted mode
0.2
0.0
−0.2
−0.4
0.09
0.12
0.15
0.18
0.21
SNP effect on Ulcerative colitis (strict) with PSC
SNP effect on Gut microbiota abundance (genus Rikenellaceae RC9 gut group id.11191)

Batch 721 : Gut microbiota abundance (genus Romboutsia id.11347) on Ulcerative colitis (strict) with PSC

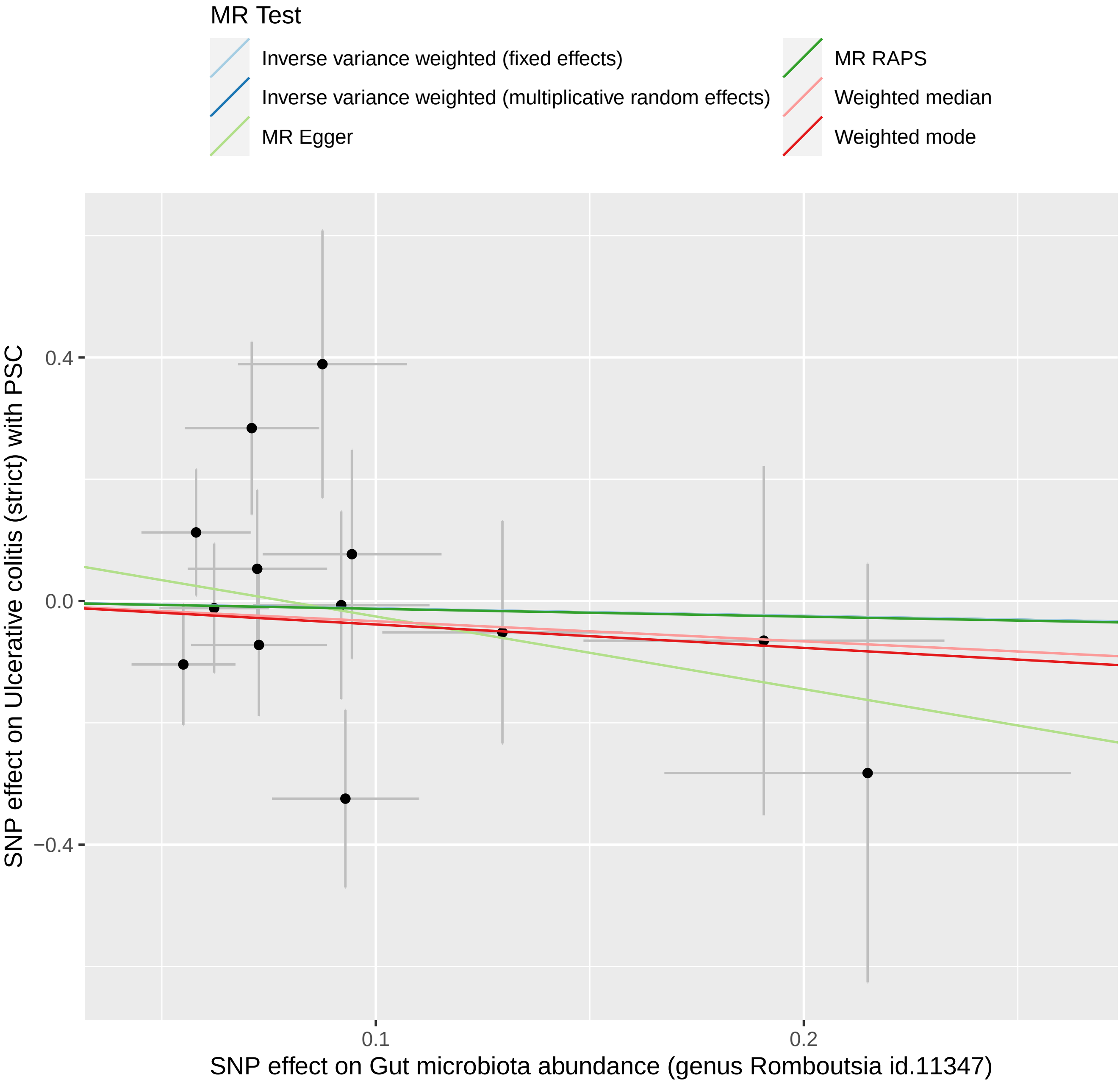

Batch 722 : Gut microbiota abundance (genus Roseburia id.2012) on Ulcerative colitis (strict) with PSC

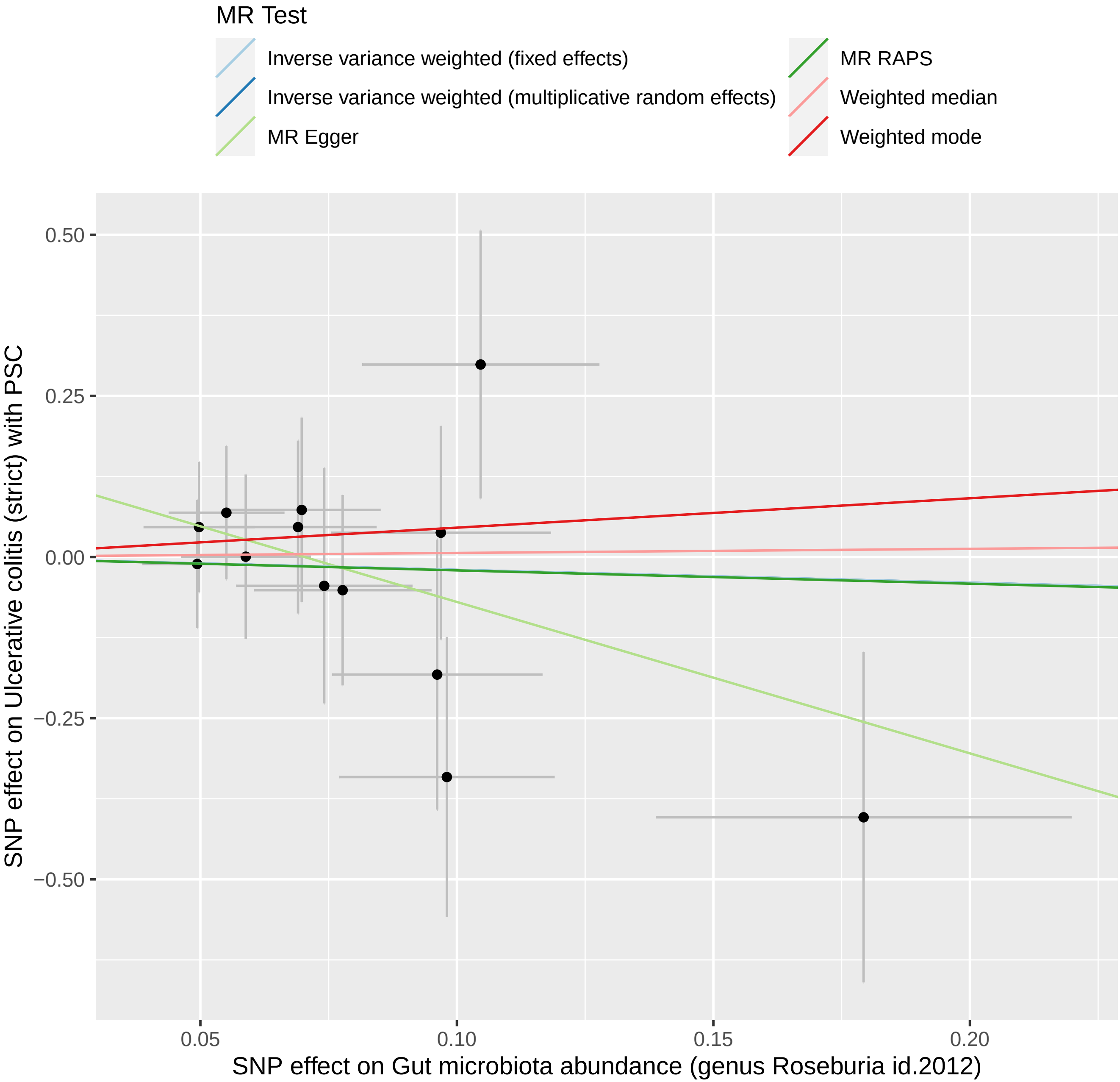

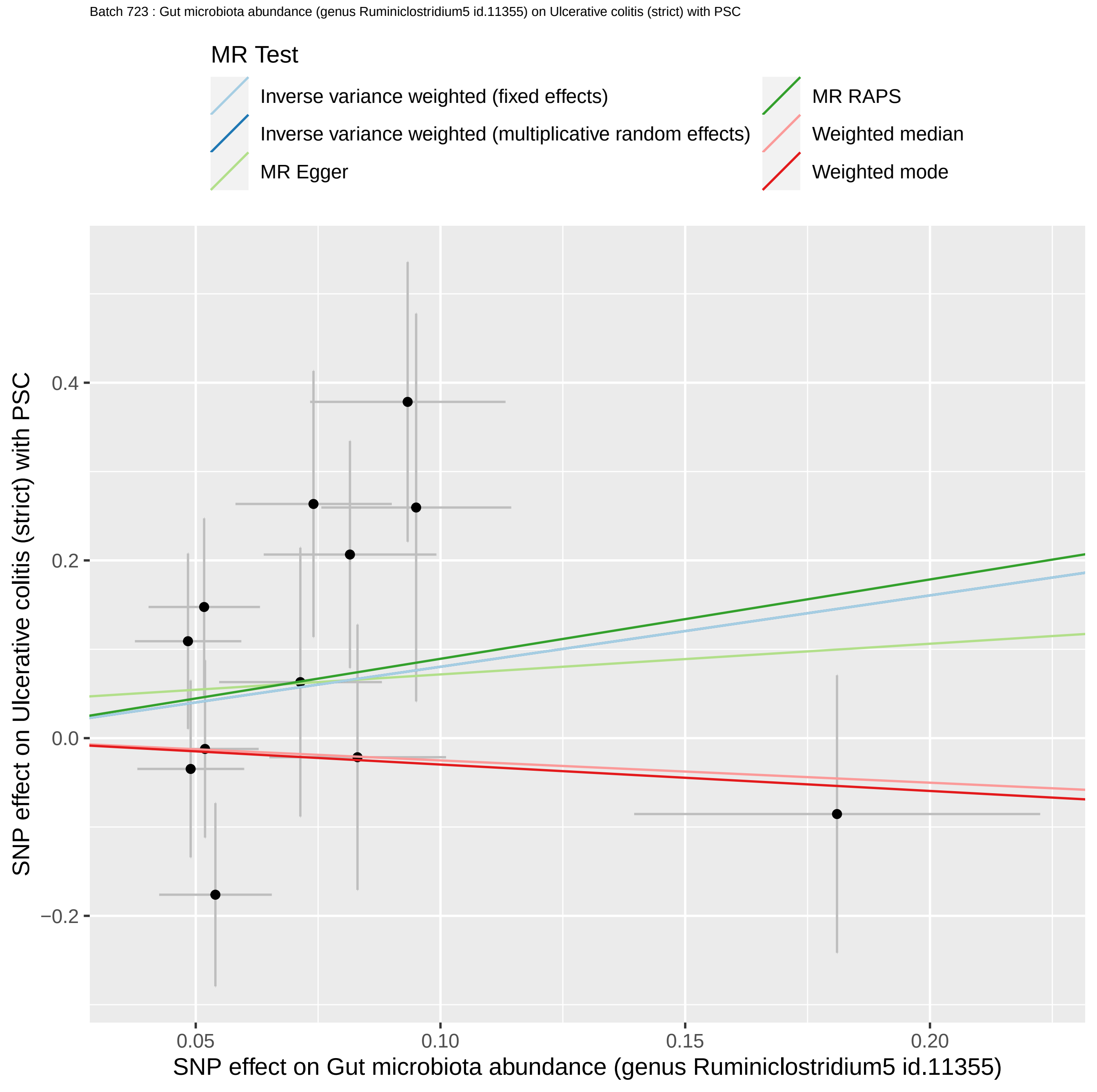

Batch 723 : Gut microbiota abundance (genus Ruminiclostridium5 id.11355) on Ulcerative colitis (strict) with PSC
MR Test
Inverse variance weighted (fixed effects)
Inverse variance weighted (multiplicative random effects)
MR Egger
MR RAPS
Weighted median
Weighted mode
SNP effect on Ulcerative colitis (strict) with PSC
0.4
0.2
0.0
−0.2
0.05
0.10
0.15
0.20
SNP effect on Gut microbiota abundance (genus Ruminiclostridium5 id.11355)

Batch 724 : Gut microbiota abundance (genus Ruminiclostridium6 id.11356) on Ulcerative colitis (strict) with PSC

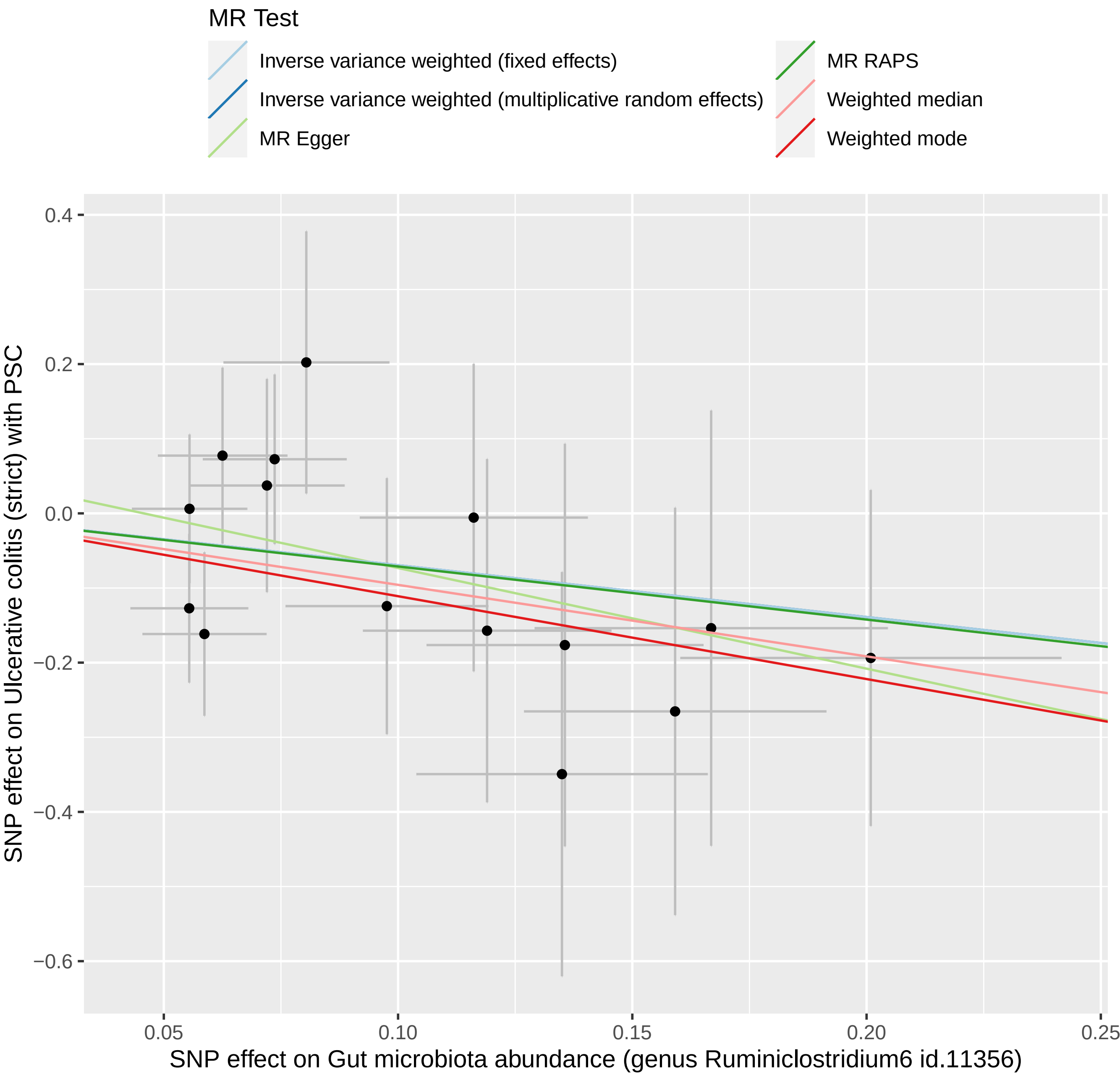

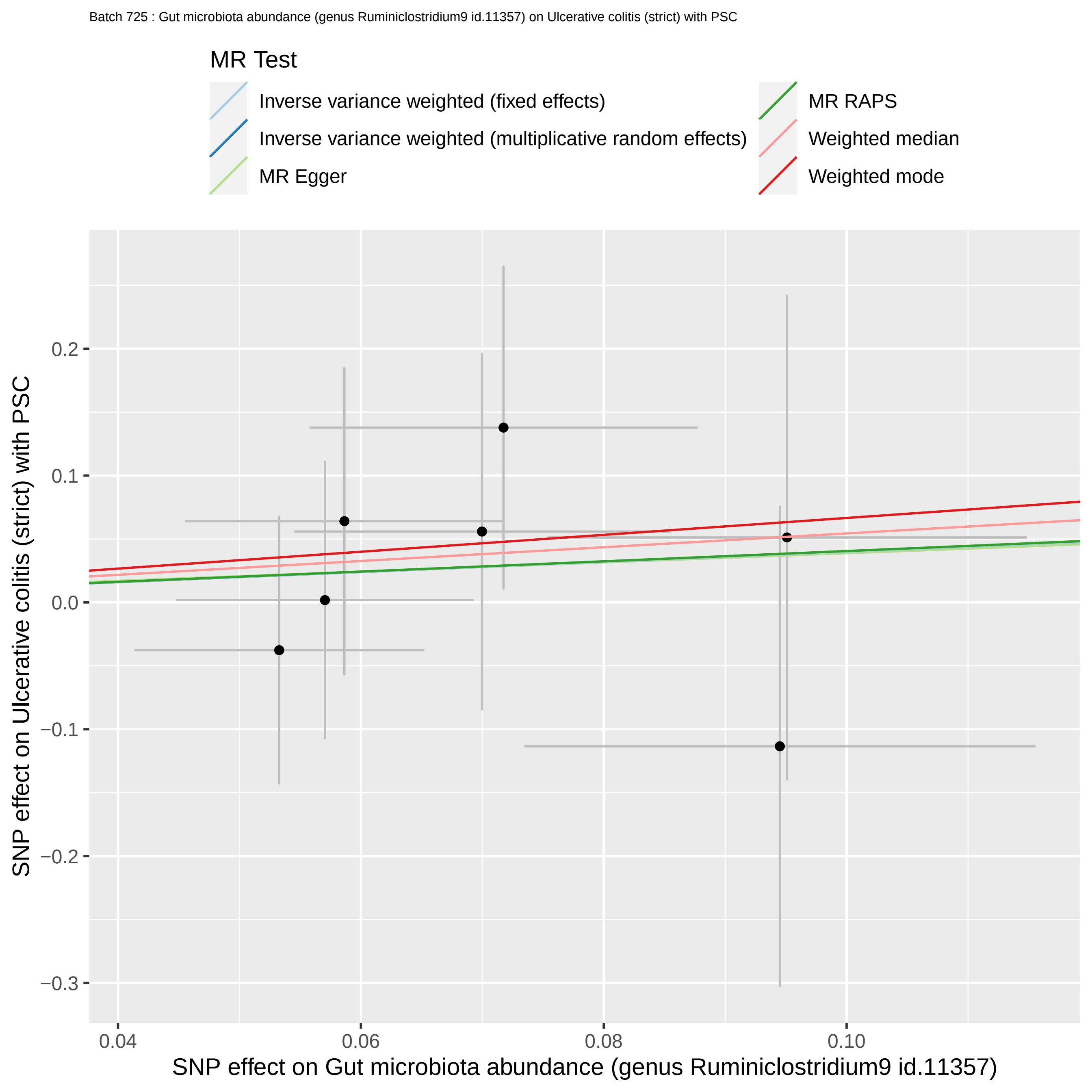

Batch 725 : Gut microbiota abundance (genus Ruminiclostridium9 id.11357) on Ulcerative colitis (strict) with PSC
MR Test
Inverse variance weighted (fixed effects)
Inverse variance weighted (multiplicative random effects)
MR Egger
MR RAPS
Weighted median
Weighted mode
SNP effect on Ulcerative colitis (strict) with PSC
0.2
0.1
0.0
−0.1
−0.2
−0.3
0.04
0.06
0.08
0.10
SNP effect on Gut microbiota abundance (genus Ruminiclostridium9 id.11357)

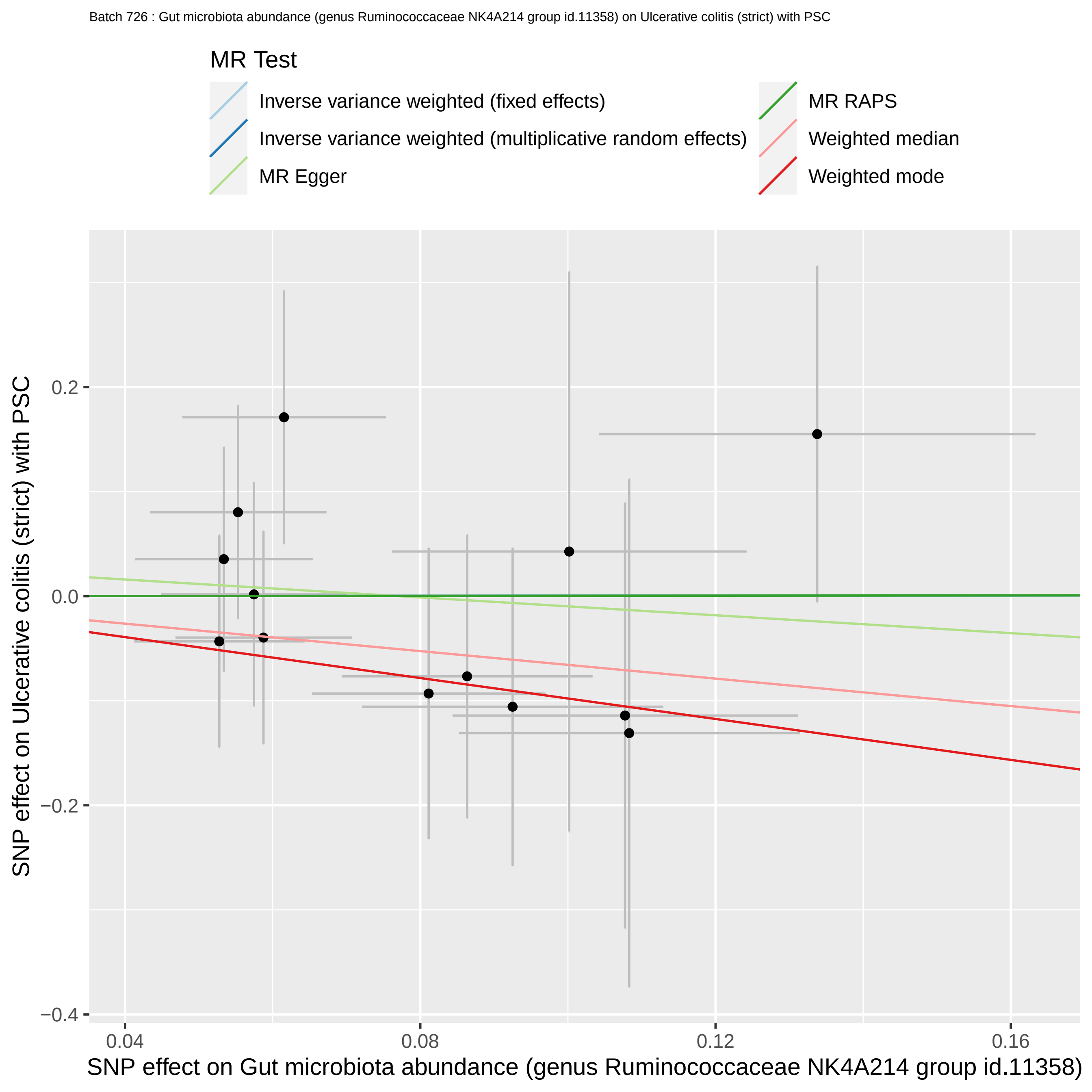

Batch 726 : Gut microbiota abundance (genus Ruminococcaceae NK4A214 group id.11358) on Ulcerative colitis (strict) with PSC
MR Test
Inverse variance weighted (fixed effects)
Inverse variance weighted (multiplicative random effects)
MR Egger
MR RAPS
Weighted median
Weighted mode
0.2
0.0
−0.2
−0.4
0.04
0.08
0.12
0.16
SNP effect on Ulcerative colitis (strict) with PSC
SNP effect on Gut microbiota abundance (genus Ruminococcaceae NK4A214 group id.11358)

Batch 727 : Gut microbiota abundance (genus Ruminococcaceae UCG002 id.11360) on Ulcerative colitis (strict) with PSC

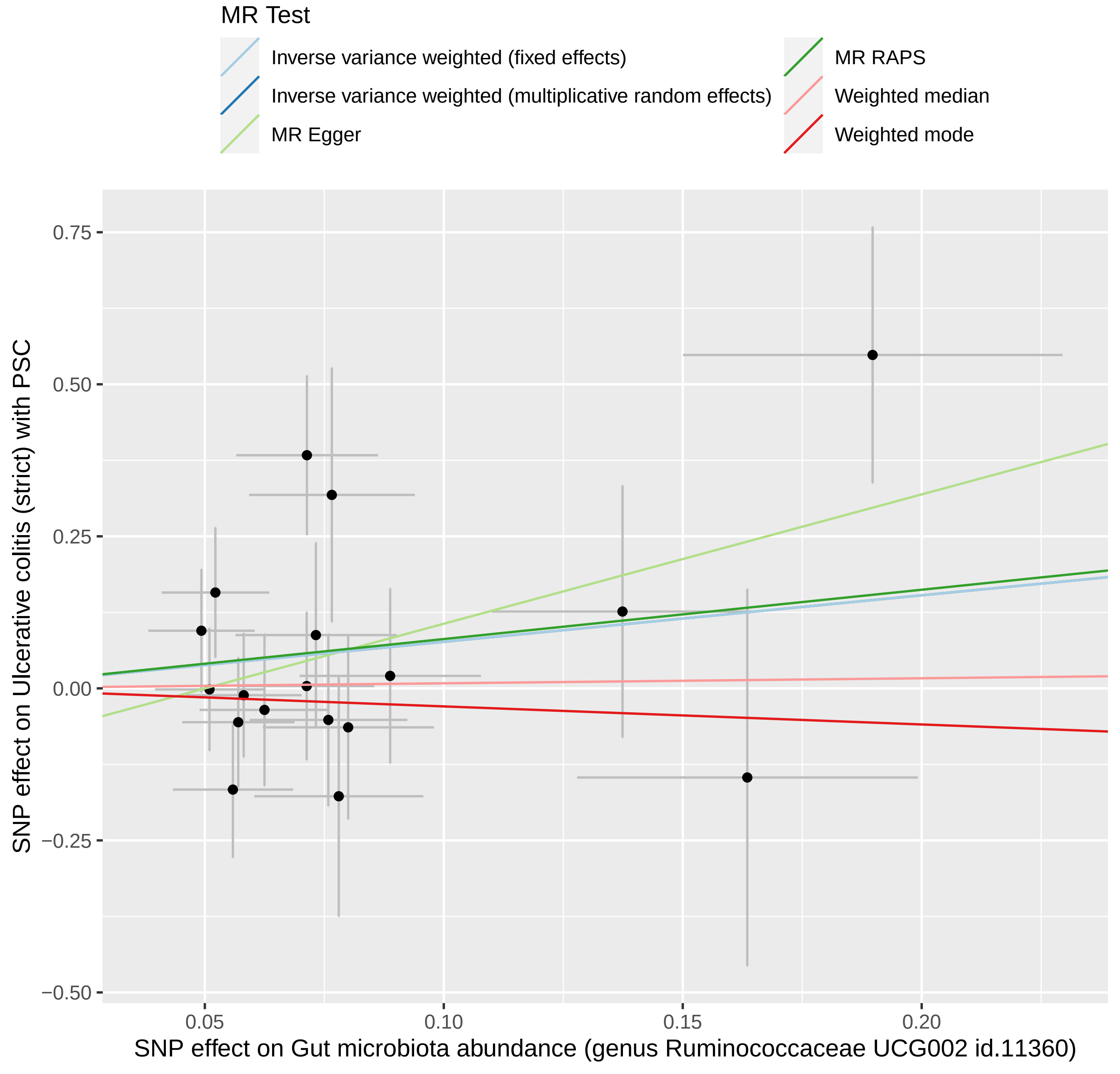

Batch 728 : Gut microbiota abundance (genus Ruminococcaceae UCG003 id.11361) on Ulcerative colitis (strict) with PSC

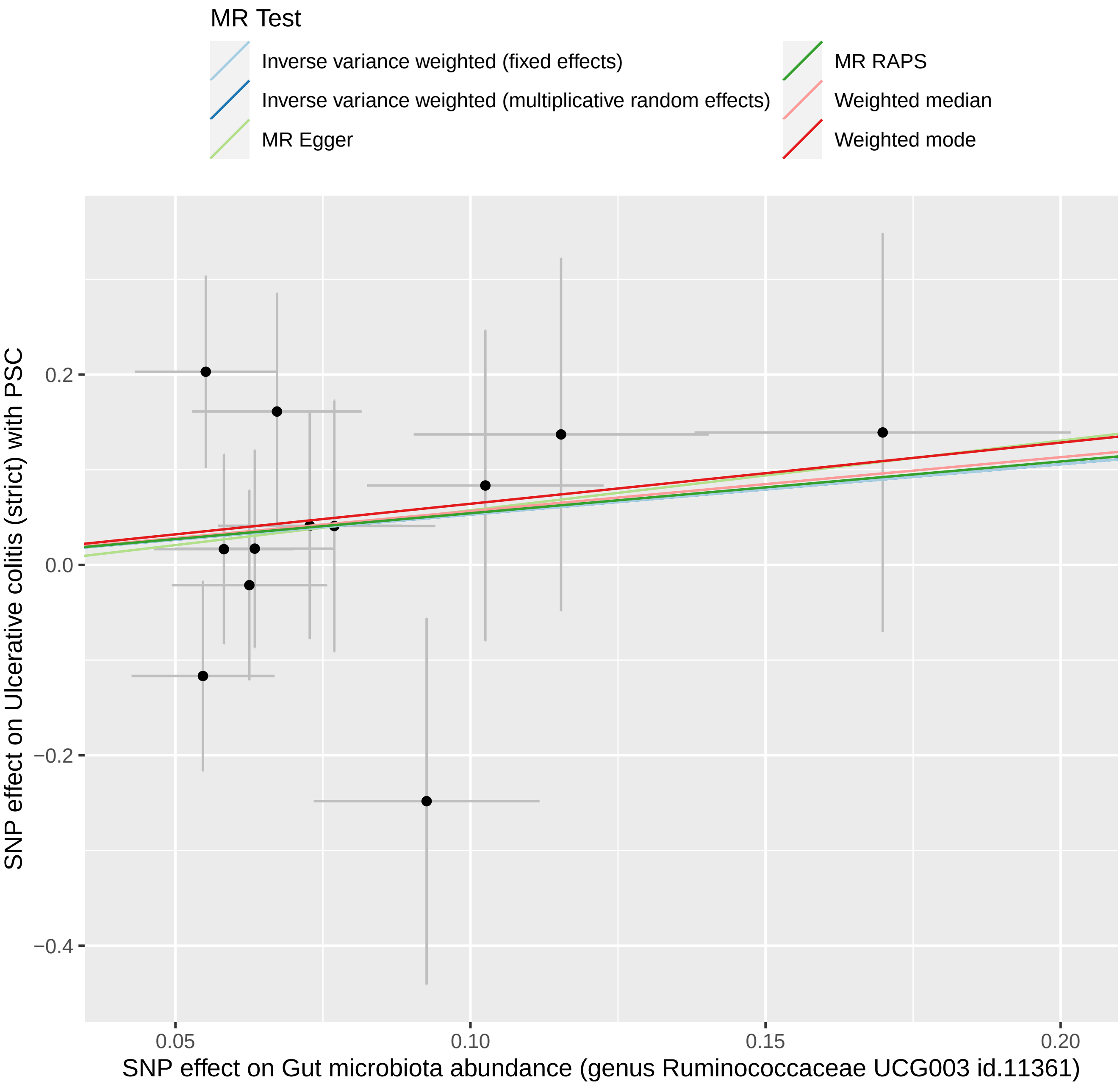

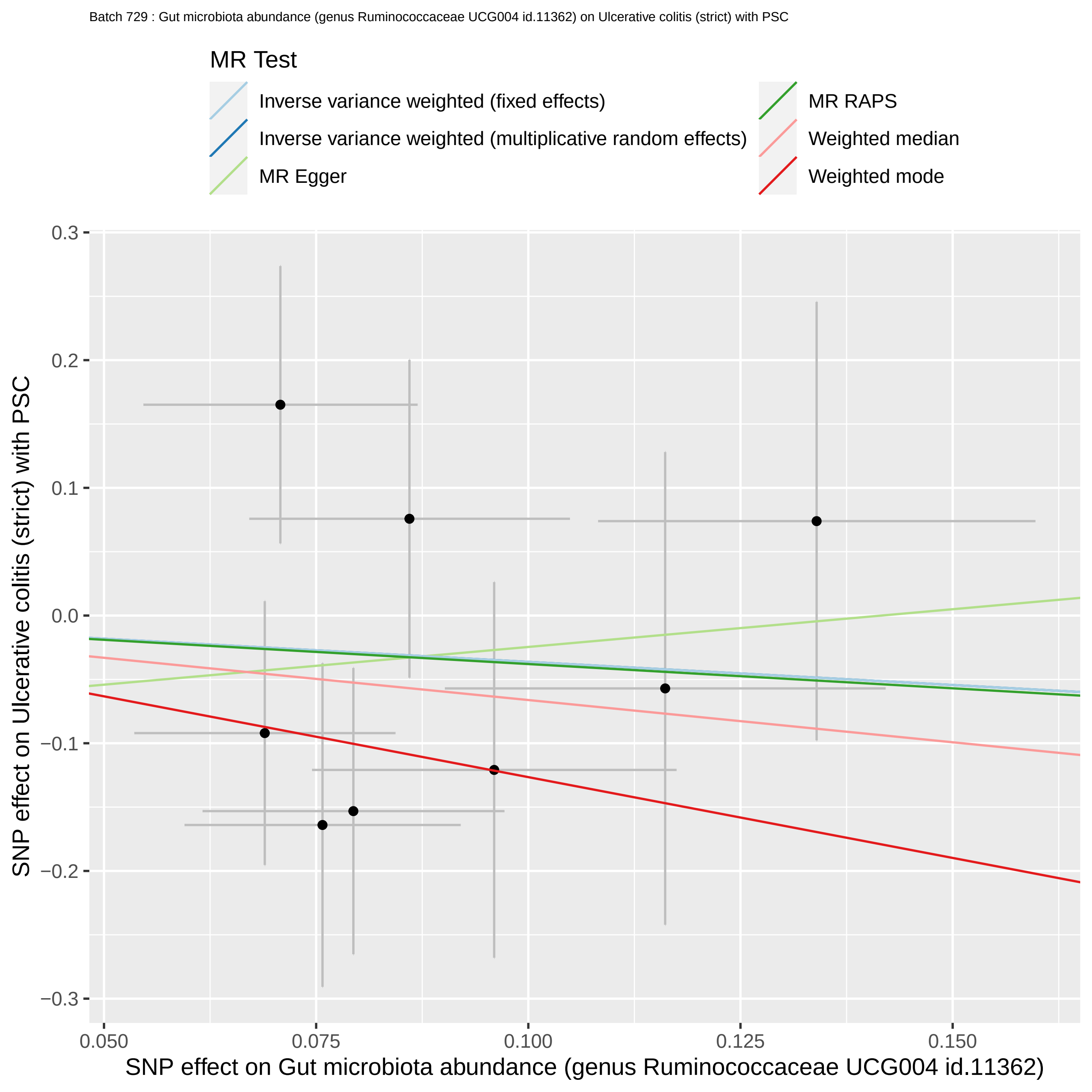
Batch 729 : Gut microbiota abundance (genus Ruminococcaceae UCG004 id.11362) on Ulcerative colitis (strict) with PSC
MR Test
Inverse variance weighted (fixed effects)
Inverse variance weighted (multiplicative random effects)
MR Egger
MR RAPS
Weighted median
Weighted mode
0.3
0.2
0.1
0.0
−0.1
−0.2
−0.3
0.050
0.075
0.100
0.125
0.150
SNP effect on Ulcerative colitis (strict) with PSC
SNP effect on Gut microbiota abundance (genus Ruminococcaceae UCG004 id.11362)

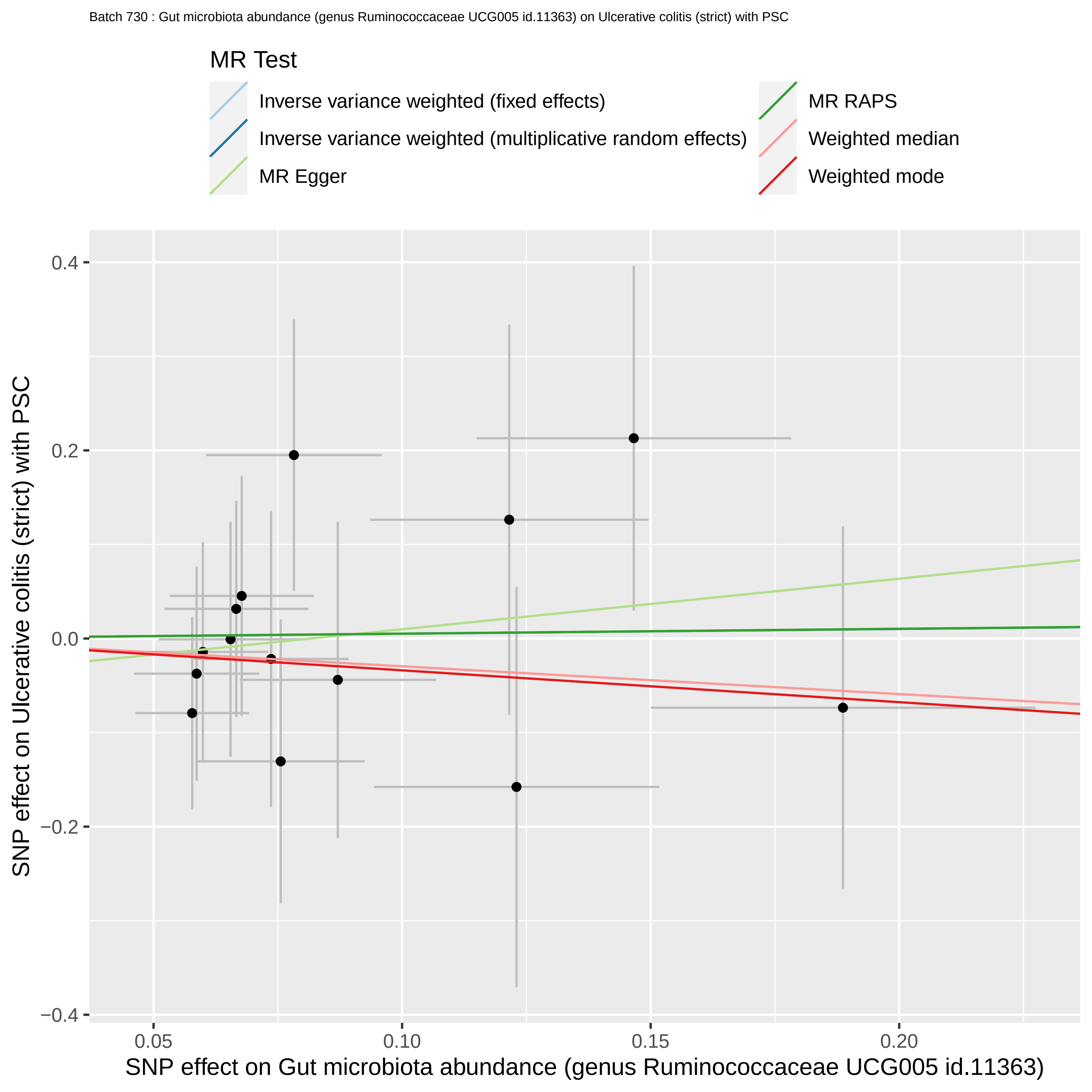
Batch 730 : Gut microbiota abundance (genus Ruminococcaceae UCG005 id.11363) on Ulcerative colitis (strict) with PSC
MR Test
Inverse variance weighted (fixed effects)
Inverse variance weighted (multiplicative random effects)
MR Egger
MR RAPS
Weighted median
Weighted mode
0.4
0.2
0.0
−0.2
−0.4
0.05
0.10
0.15
0.20
SNP effect on Ulcerative colitis (strict) with PSC
SNP effect on Gut microbiota abundance (genus Ruminococcaceae UCG005 id.11363)

Batch 731 : Gut microbiota abundance (genus Ruminococcaceae UCG009 id.11366) on Ulcerative colitis (strict) with PSC

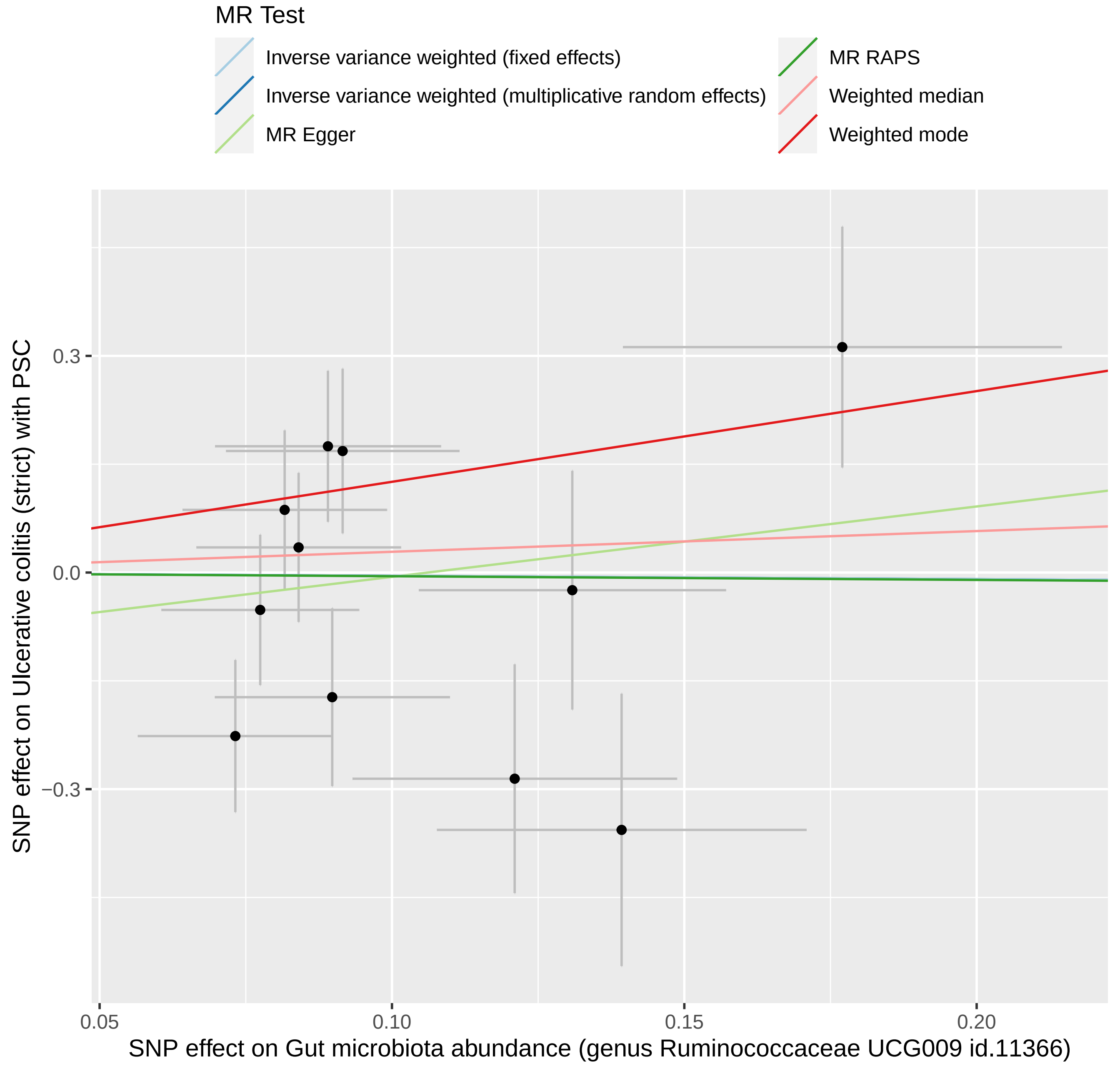

Batch 732 : Gut microbiota abundance (genus Ruminococcaceae UCG010 id.11367) on Ulcerative colitis (strict) with PSC

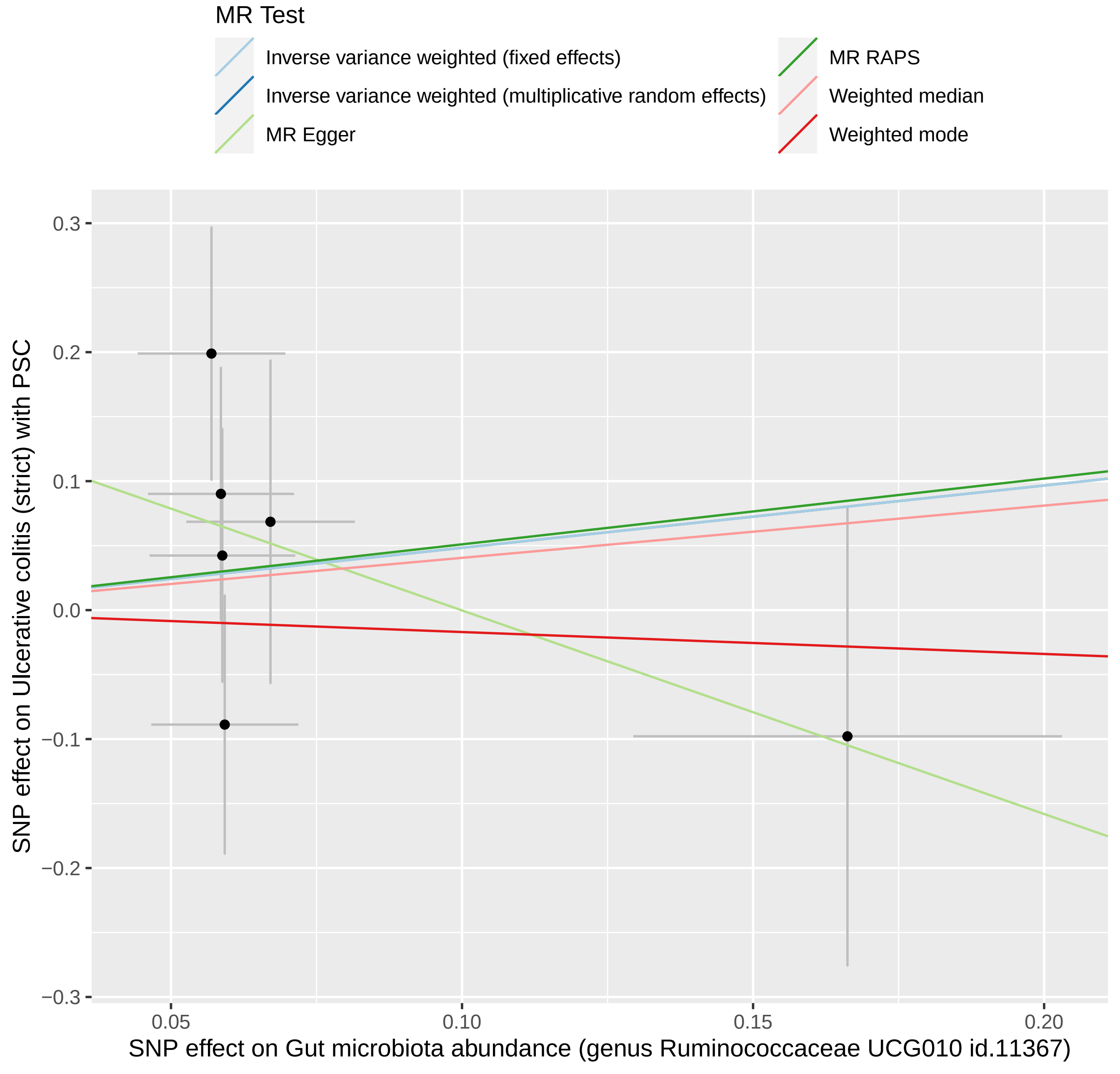

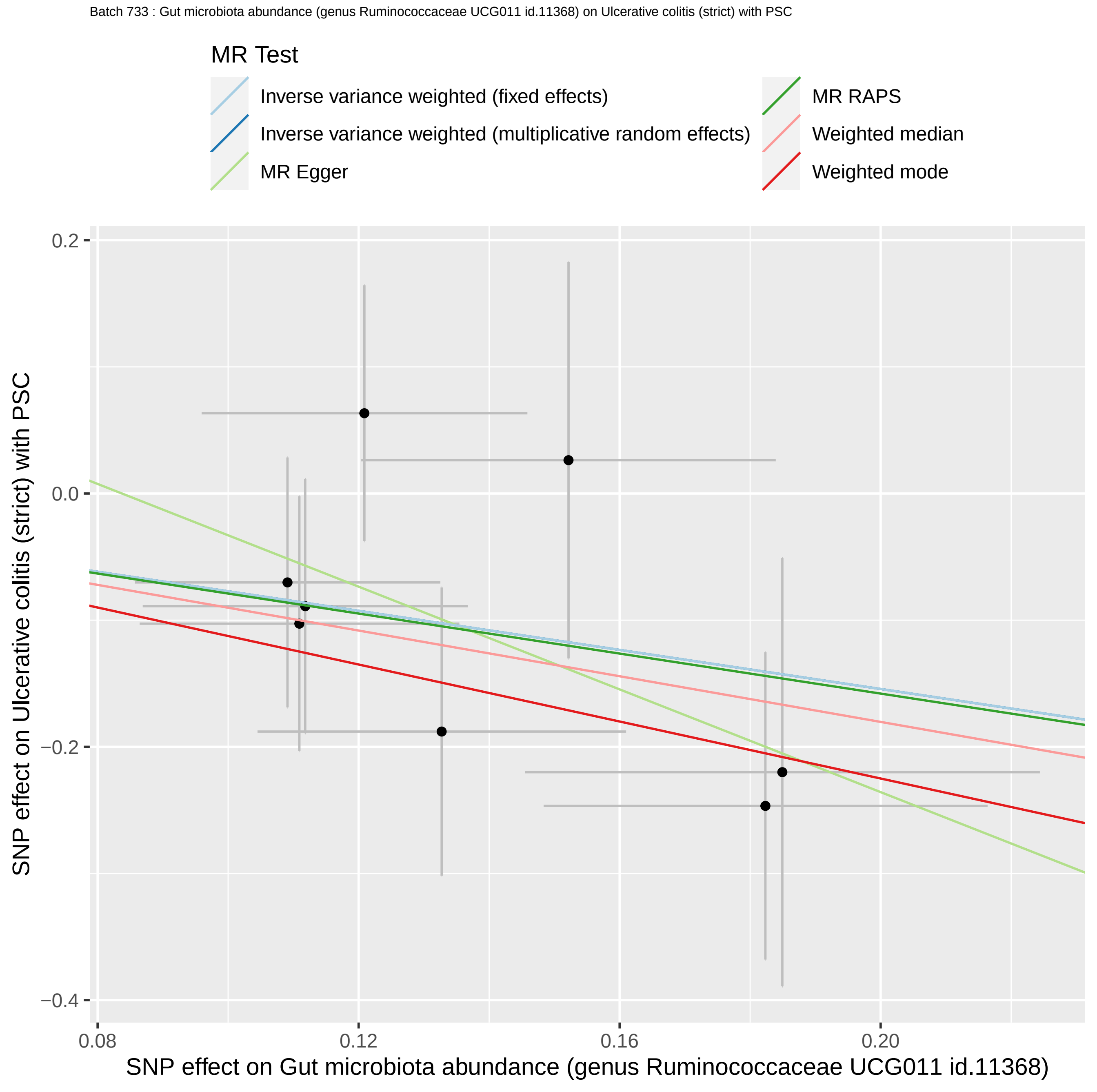

Batch 733 : Gut microbiota abundance (genus Ruminococcaceae UCG011 id.11368) on Ulcerative colitis (strict) with PSC
MR Test
Inverse variance weighted (fixed effects)
Inverse variance weighted (multiplicative random effects)
MR Egger
MR RAPS
Weighted median
Weighted mode
0.2
0.0
−0.2
−0.4
0.08
0.12
0.16
0.20
SNP effect on Ulcerative colitis (strict) with PSC
SNP effect on Gut microbiota abundance (genus Ruminococcaceae UCG011 id.11368)

Batch 734 : Gut microbiota abundance (genus Ruminococcaceae UCG013 id.11370) on Ulcerative colitis (strict) with PSC

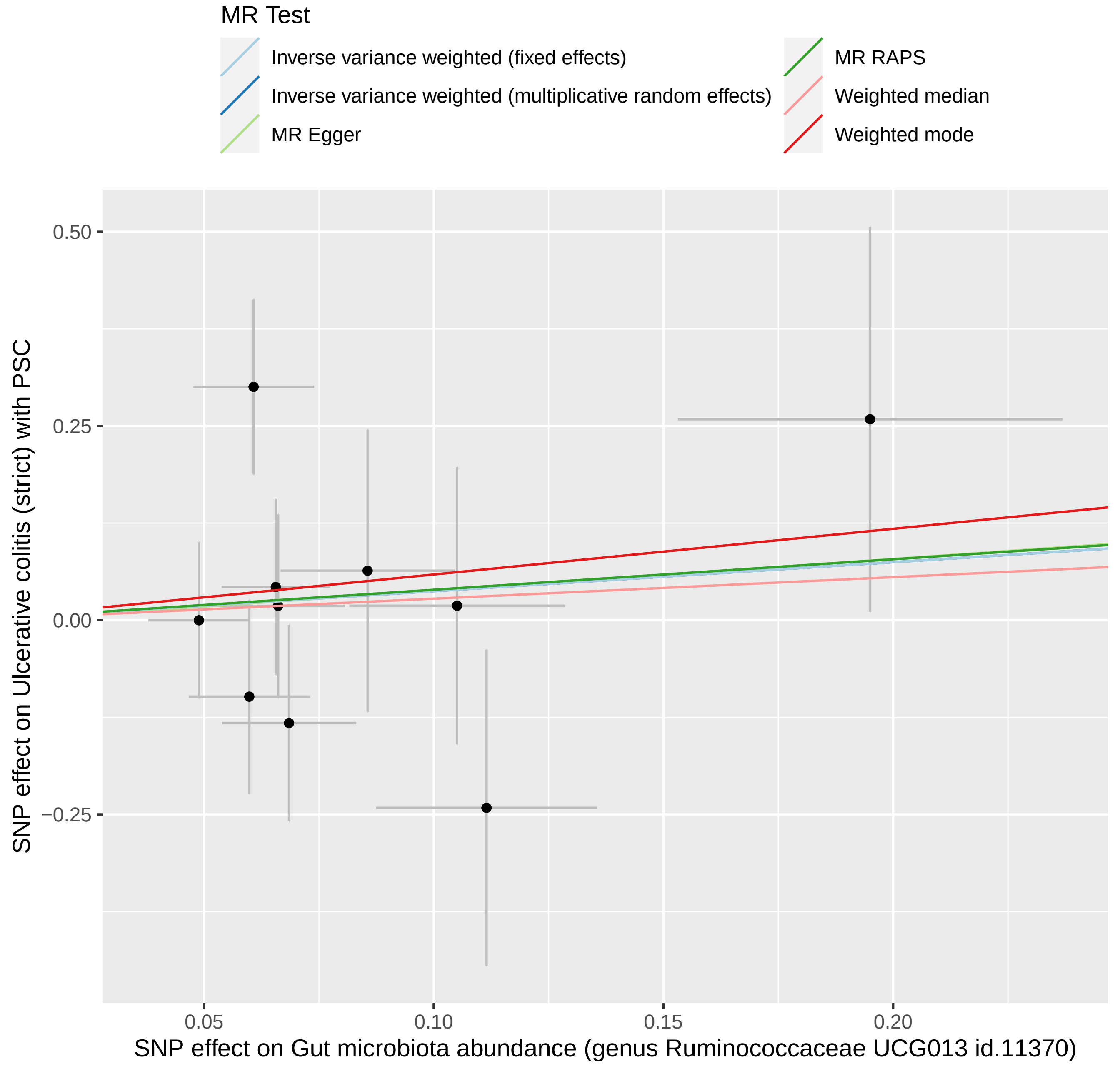

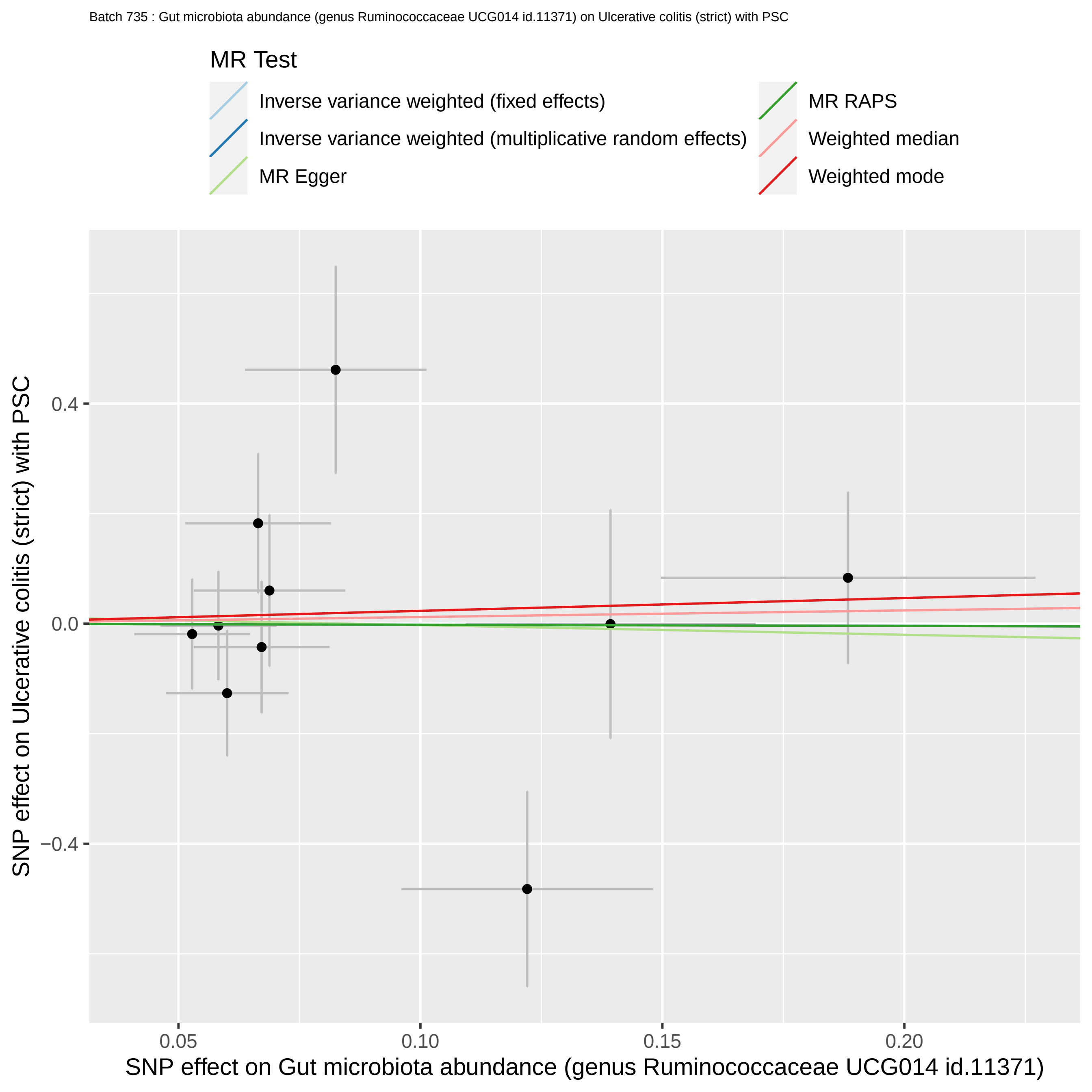
Batch 735 : Gut microbiota abundance (genus Ruminococcaceae UCG014 id.11371) on Ulcerative colitis (strict) with PSC
MR Test
Inverse variance weighted (fixed effects)
Inverse variance weighted (multiplicative random effects)
MR Egger
MR RAPS
Weighted median
Weighted mode
0.4
0.0
−0.4
0.05
0.10
0.15
0.20
SNP effect on Ulcerative colitis (strict) with PSC
SNP effect on Gut microbiota abundance (genus Ruminococcaceae UCG014 id.11371)

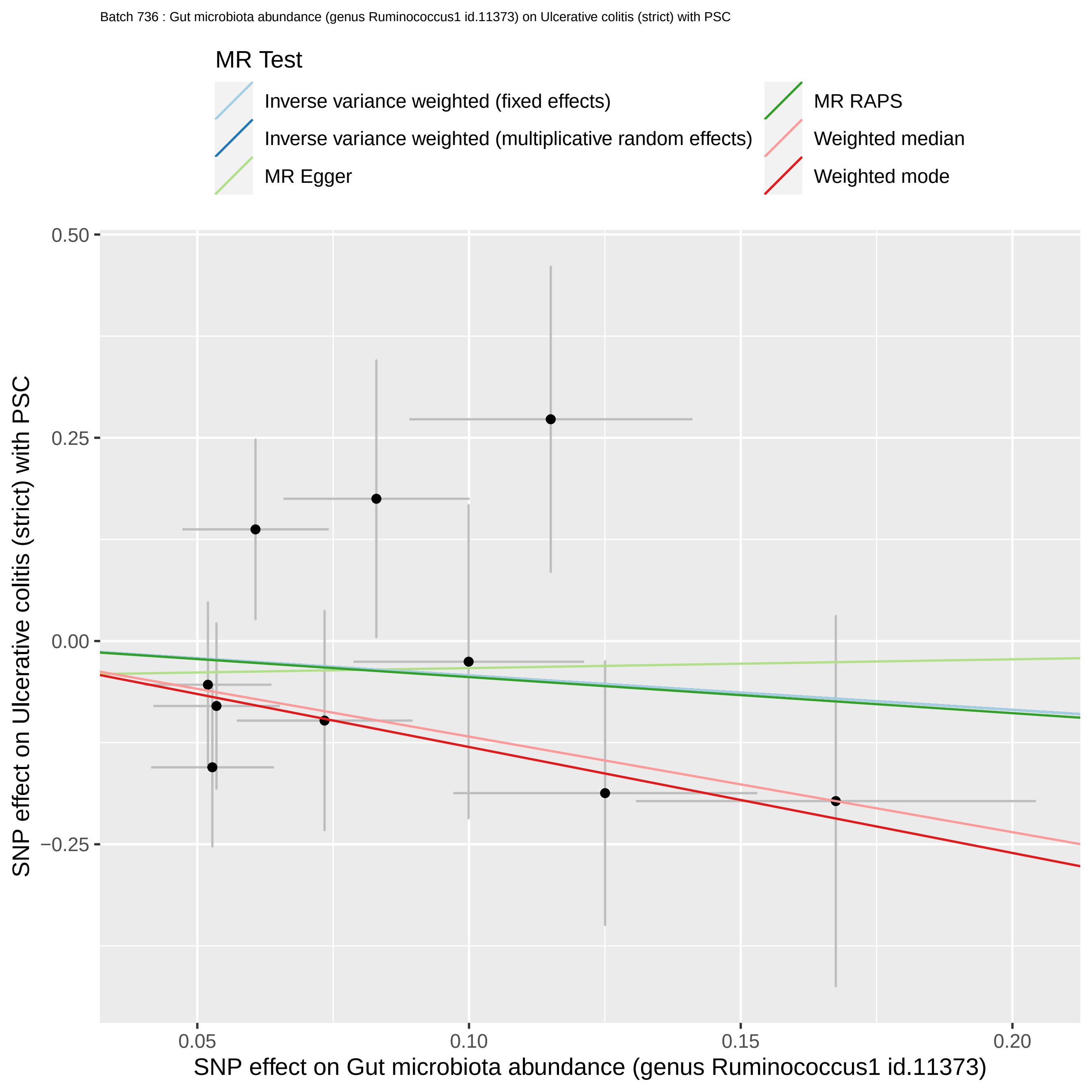

Batch 736 : Gut microbiota abundance (genus Ruminococcus1 id.11373) on Ulcerative colitis (strict) with PSC
MR Test
Inverse variance weighted (fixed effects)
Inverse variance weighted (multiplicative random effects)
MR Egger
MR RAPS
Weighted median
Weighted mode
0.50
0.25
0.00
−0.25
0.05
0.10
0.15
0.20
SNP effect on Ulcerative colitis (strict) with PSC
SNP effect on Gut microbiota abundance (genus Ruminococcus1 id.11373)

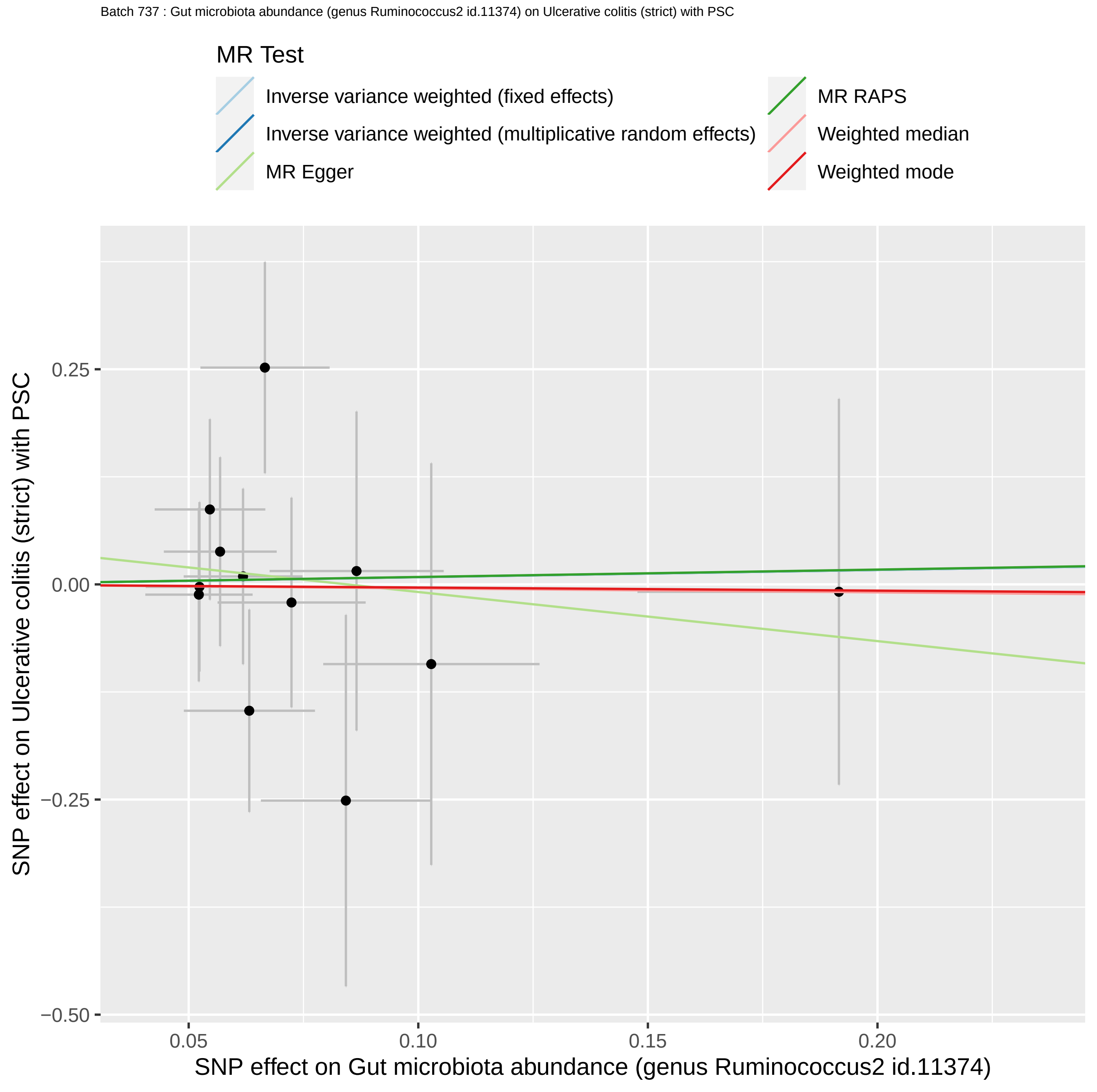

Batch 737 : Gut microbiota abundance (genus Ruminococcus2 id.11374) on Ulcerative colitis (strict) with PSC
MR Test
Inverse variance weighted (fixed effects)
Inverse variance weighted (multiplicative random effects)
MR Egger
MR RAPS
Weighted median
Weighted mode
0.25
0.00
−0.25
−0.50
0.05
0.10
0.15
0.20
SNP effect on Ulcerative colitis (strict) with PSC
SNP effect on Gut microbiota abundance (genus Ruminococcus2 id.11374)

Batch 738 : Gut microbiota abundance (genus Ruminococcus gauvreauii group id.11342) on Ulcerative colitis (strict) with PSC

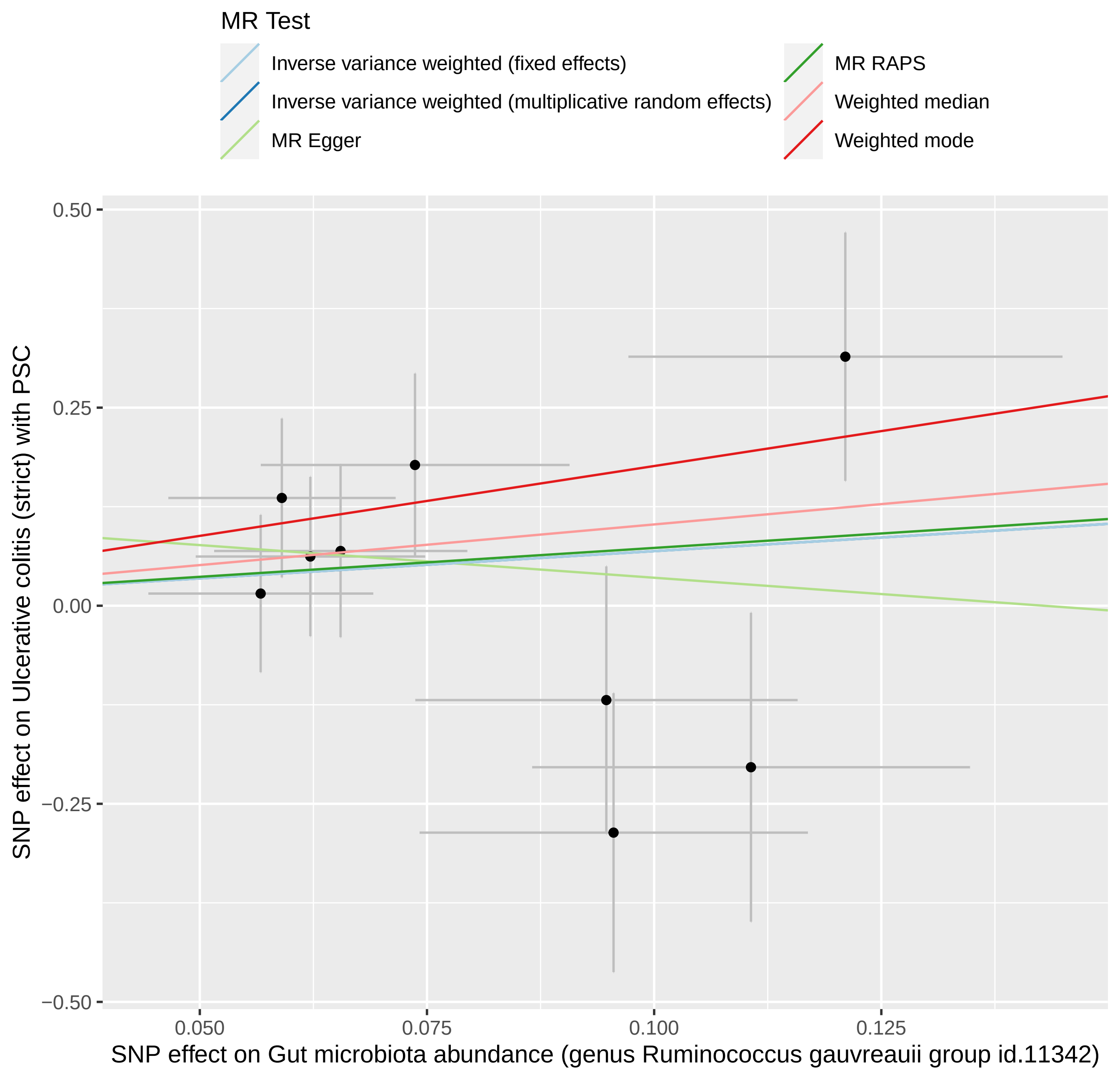

Batch 739 : Gut microbiota abundance (genus Ruminococcus gnavus group id.14376) on Ulcerative colitis (strict) with PSC

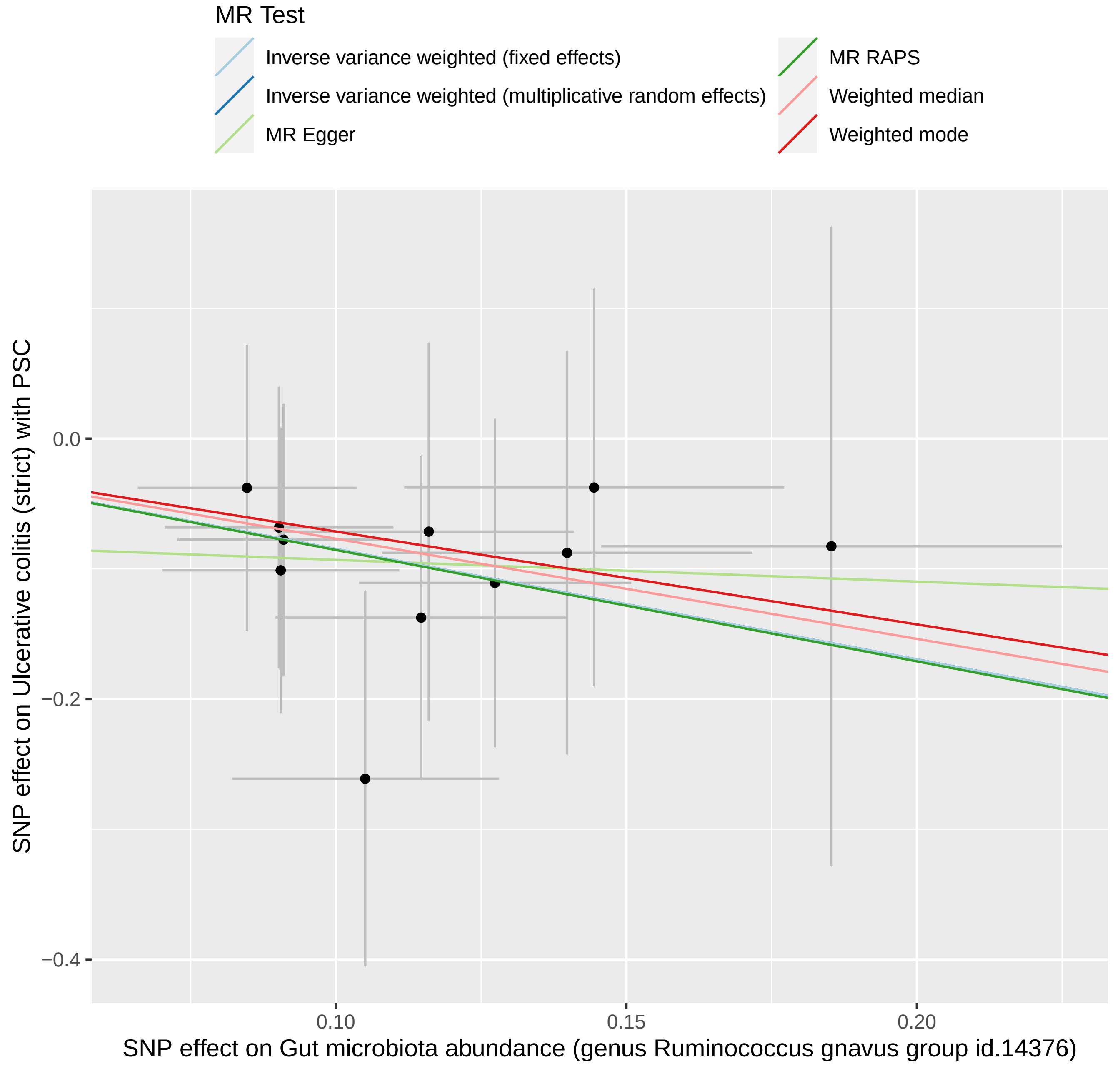

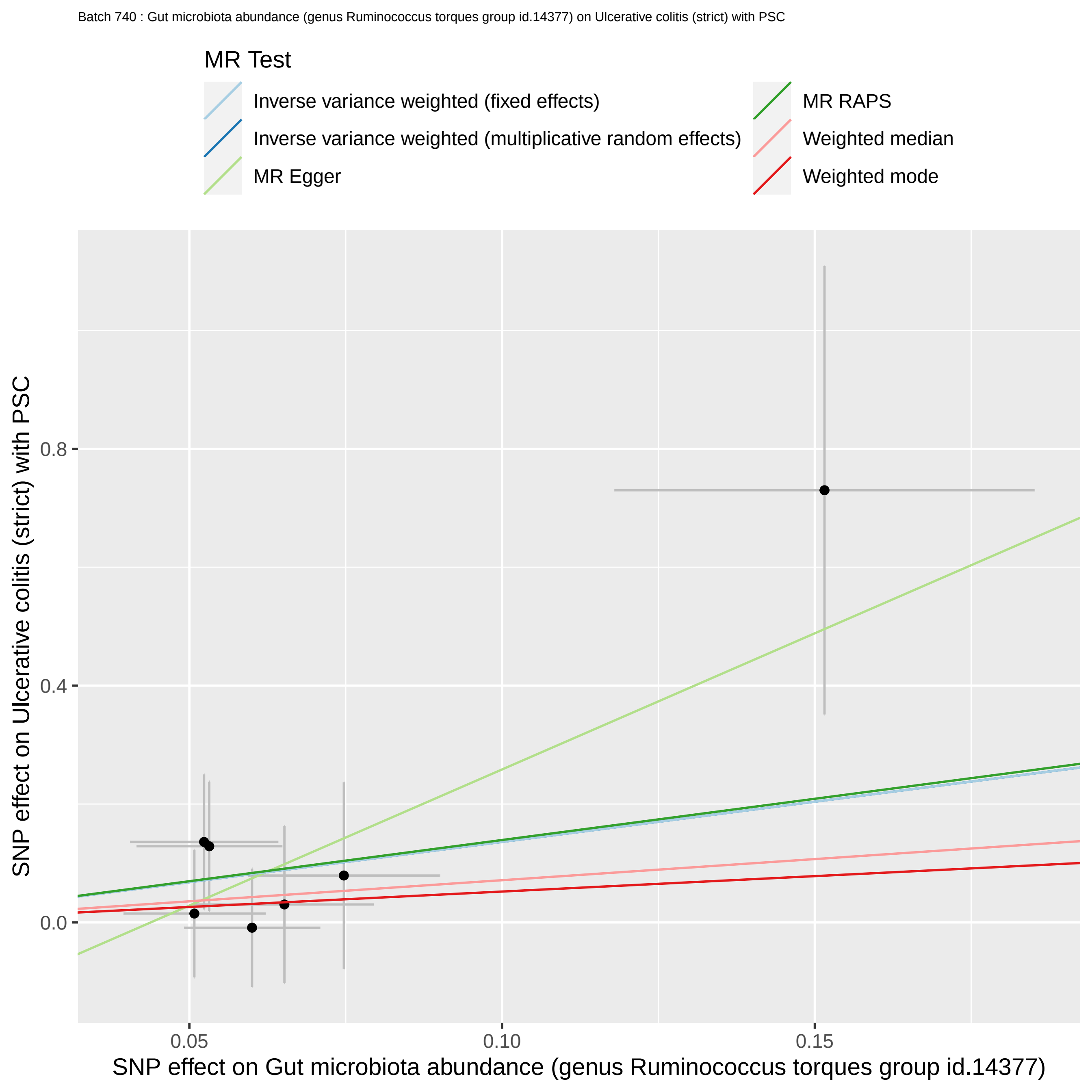

Batch 740 : Gut microbiota abundance (genus Ruminococcus torques group id.14377) on Ulcerative colitis (strict) with PSC
MR Test
Inverse variance weighted (fixed effects)
Inverse variance weighted (multiplicative random effects)
MR Egger
MR RAPS
Weighted median
Weighted mode
0.8
0.4
0.0
0.05
0.10
0.15
SNP effect on Ulcerative colitis (strict) with PSC
SNP effect on Gut microbiota abundance (genus Ruminococcus torques group id.14377)

Batch 741 : Gut microbiota abundance (genus Sellimonas id.14369) on Ulcerative colitis (strict) with PSC

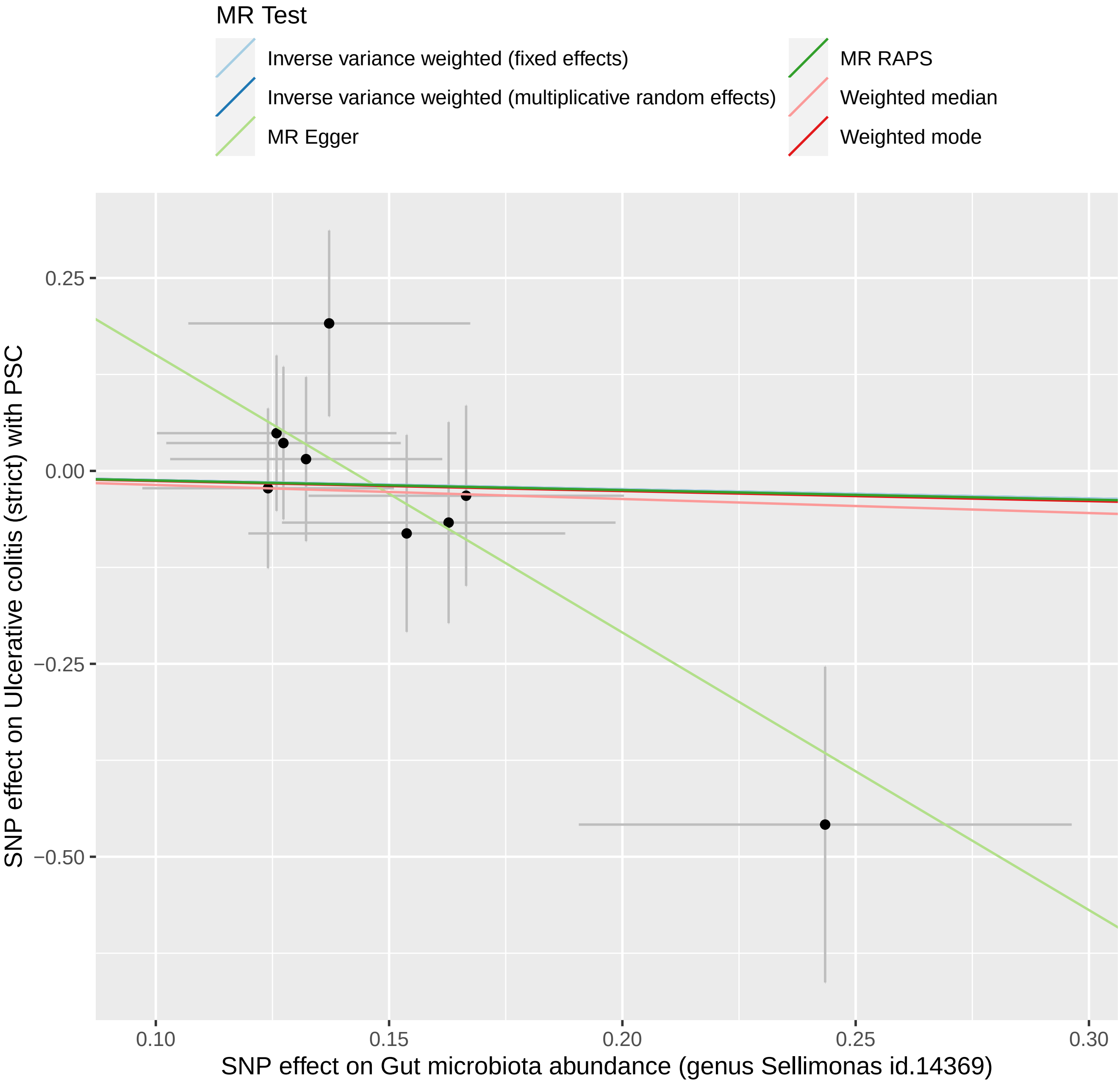

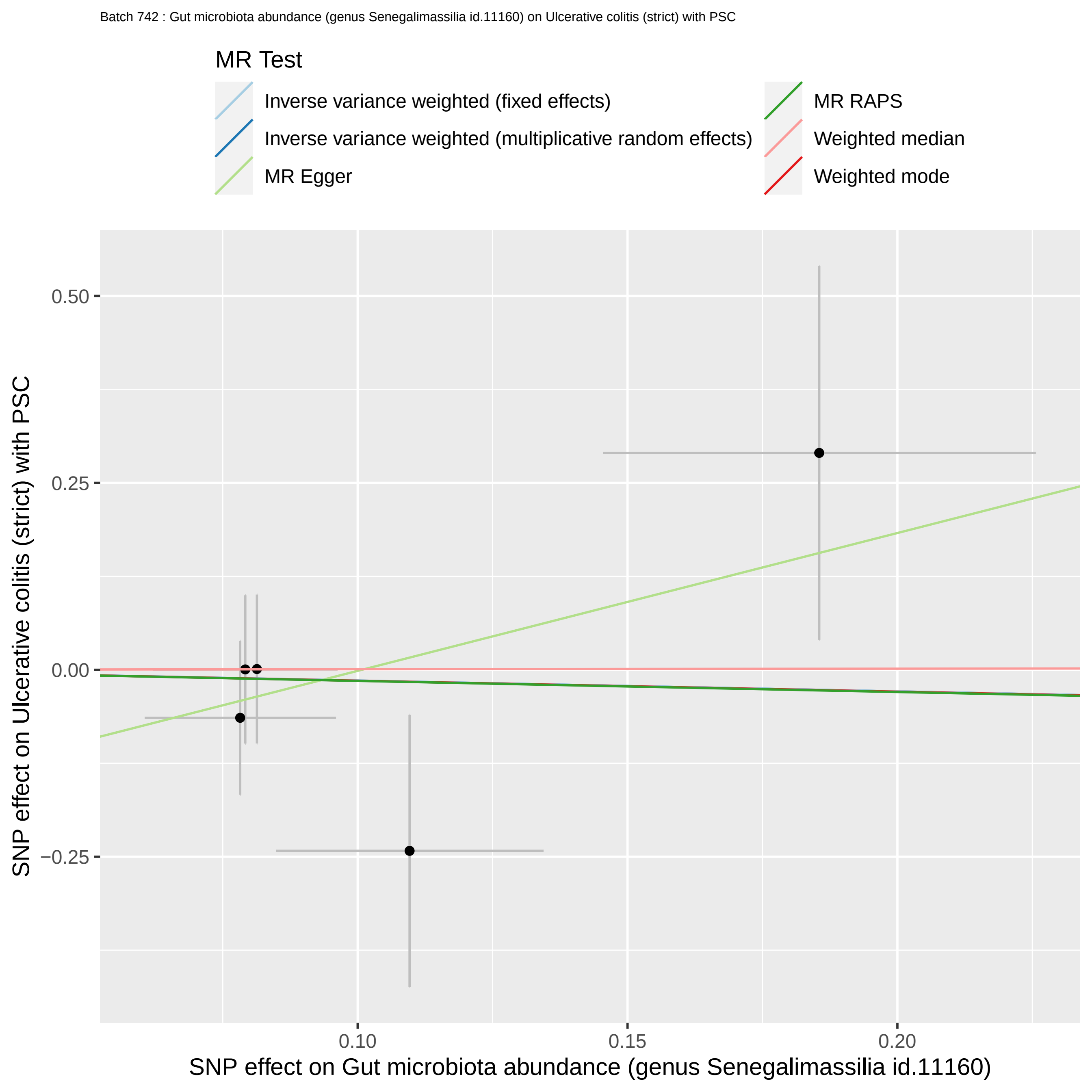
Batch 742 : Gut microbiota abundance (genus Senegalimassilia id.11160) on Ulcerative colitis (strict) with PSC
MR Test
Inverse variance weighted (fixed effects)
Inverse variance weighted (multiplicative random effects)
MR Egger
MR RAPS
Weighted median
Weighted mode
0.50
0.25
0.00
−0.25
0.10
0.15
0.20
SNP effect on Ulcerative colitis (strict) with PSC
SNP effect on Gut microbiota abundance (genus Senegalimassilia id.11160)

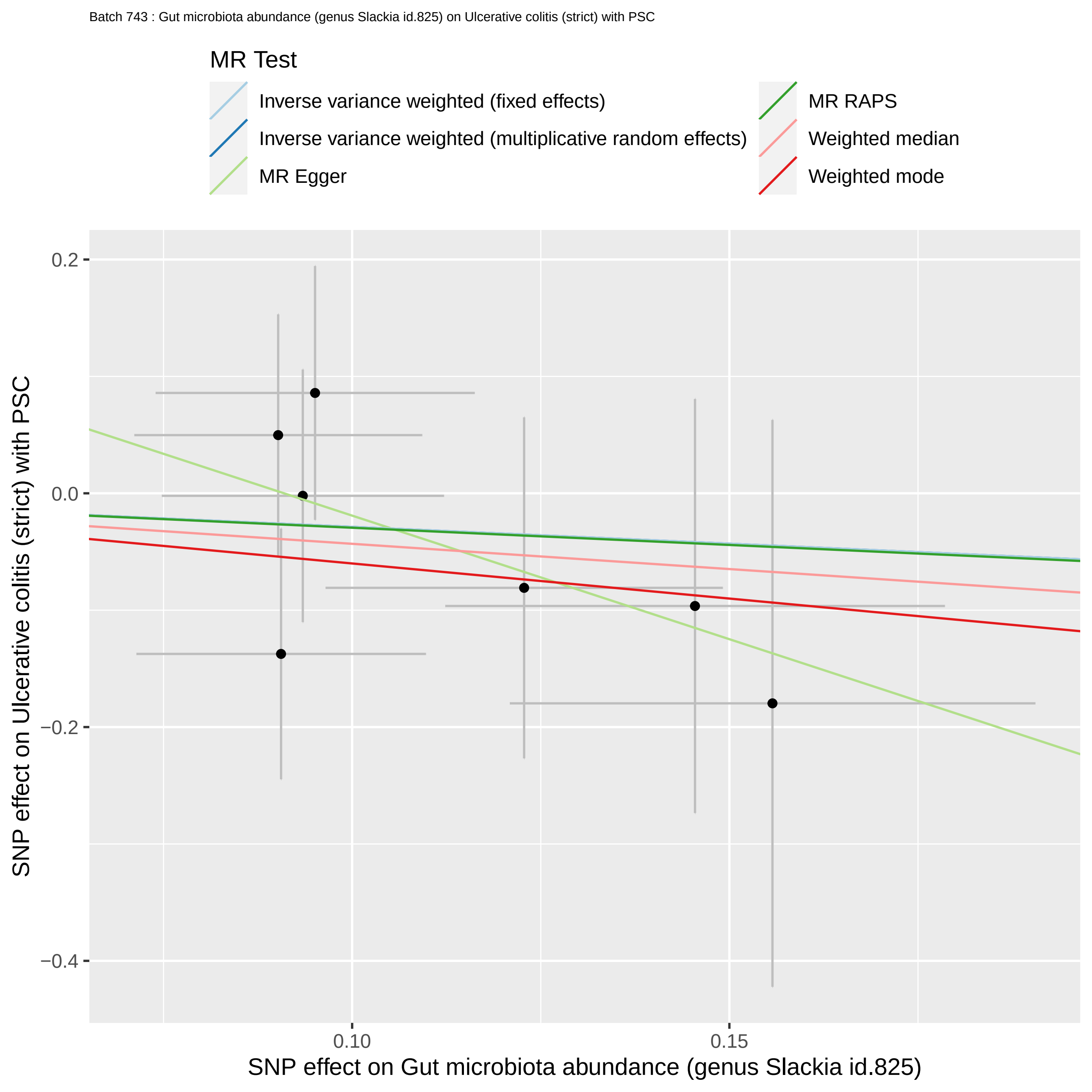
Batch 743 : Gut microbiota abundance (genus Slackia id.825) on Ulcerative colitis (strict) with PSC
MR Test
Inverse variance weighted (fixed effects)
Inverse variance weighted (multiplicative random effects)
MR Egger
MR RAPS
Weighted median
Weighted mode
0.2
0.0
−0.2
−0.4
0.10
0.15
SNP effect on Ulcerative colitis (strict) with PSC
SNP effect on Gut microbiota abundance (genus Slackia id.825)

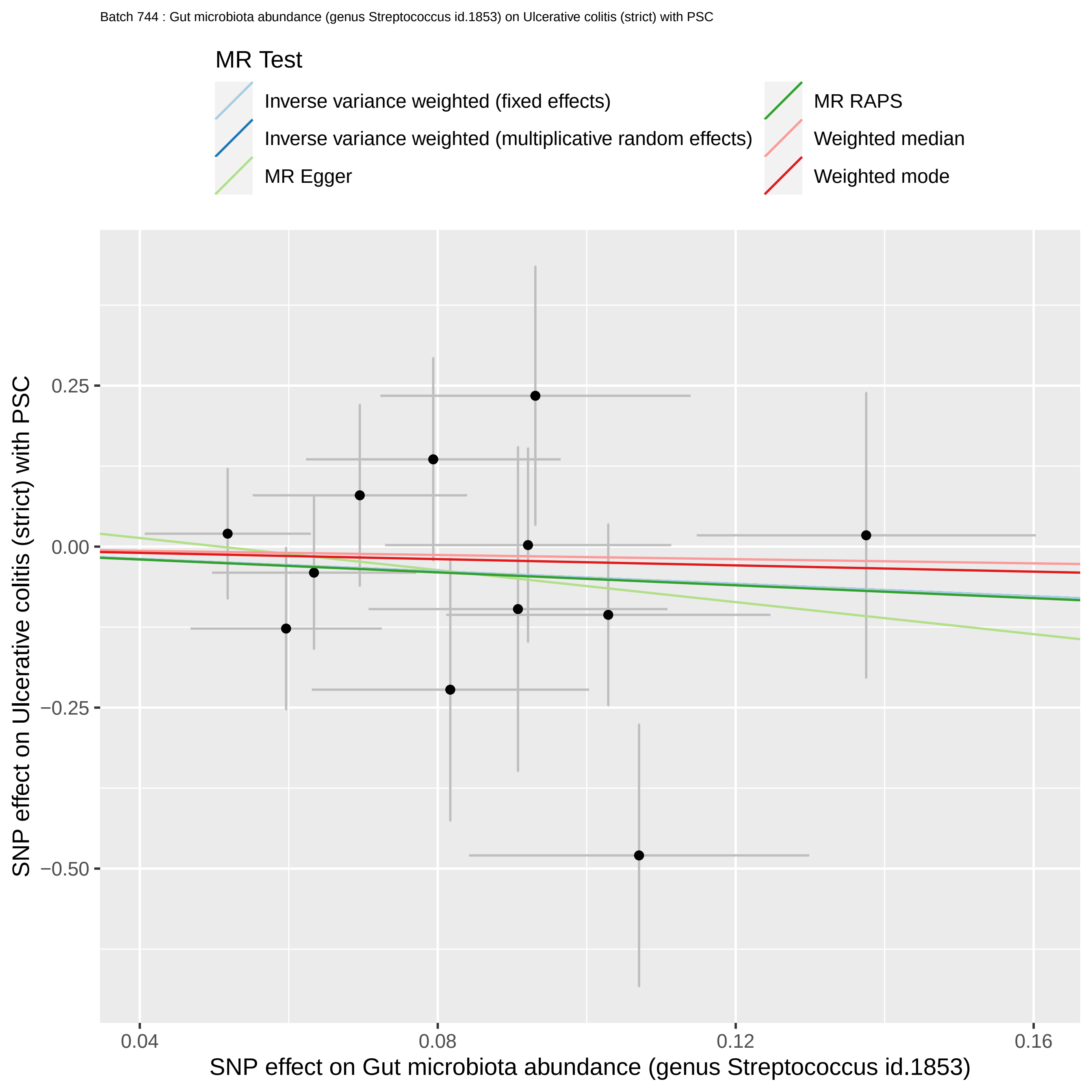

Batch 744 : Gut microbiota abundance (genus Streptococcus id.1853) on Ulcerative colitis (strict) with PSC
MR Test
Inverse variance weighted (fixed effects)
Inverse variance weighted (multiplicative random effects)
MR Egger
MR RAPS
Weighted median
Weighted mode
0.25
0.00
−0.25
−0.50
0.04
0.08
0.12
0.16
SNP effect on Ulcerative colitis (strict) with PSC
SNP effect on Gut microbiota abundance (genus Streptococcus id.1853)

Batch 745 : Gut microbiota abundance (genus Subdoligranulum id.2070) on Ulcerative colitis (strict) with PSC

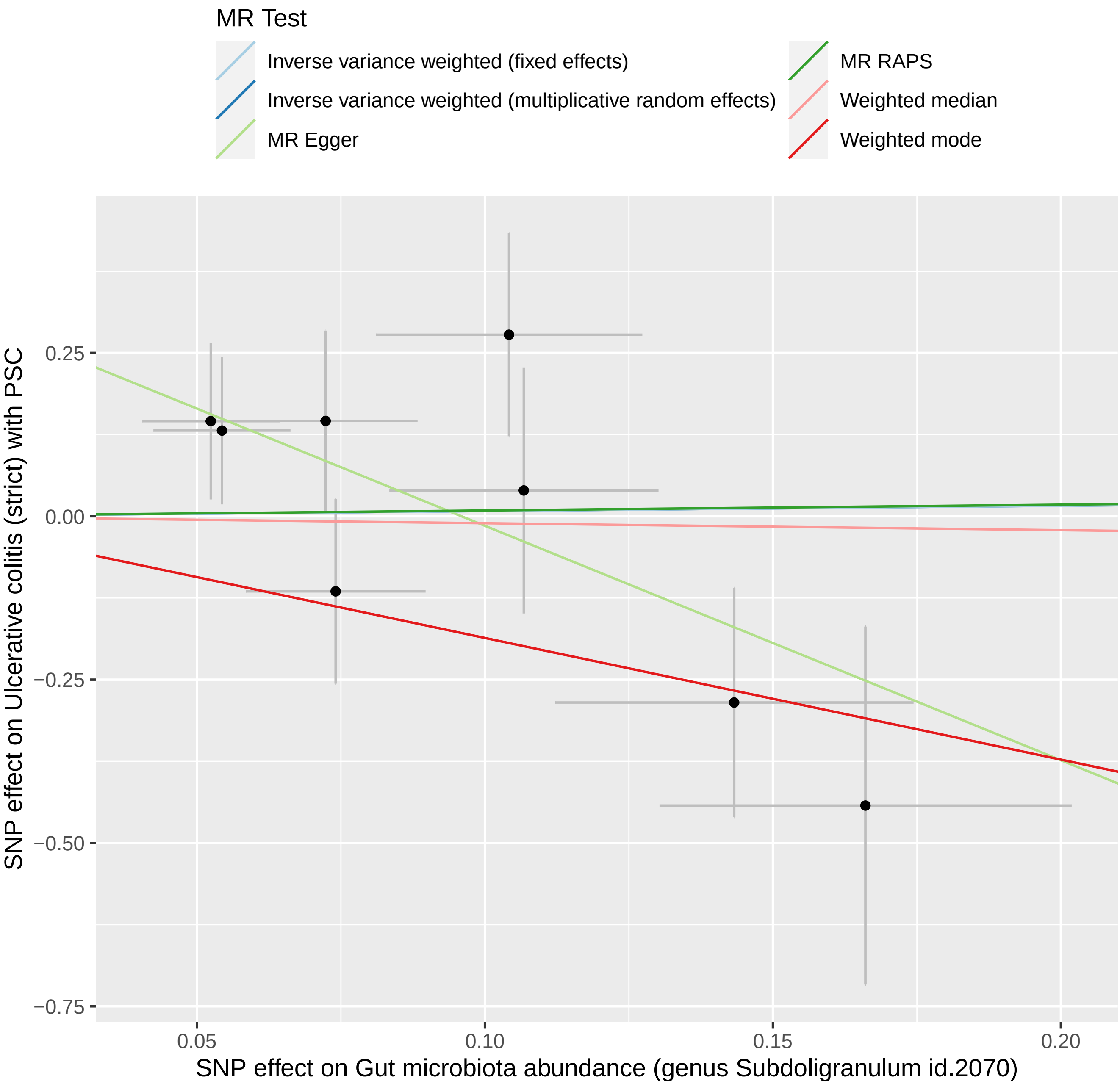

Batch 746 : Gut microbiota abundance (genus Sutterella id.2896) on Ulcerative colitis (strict) with PSC

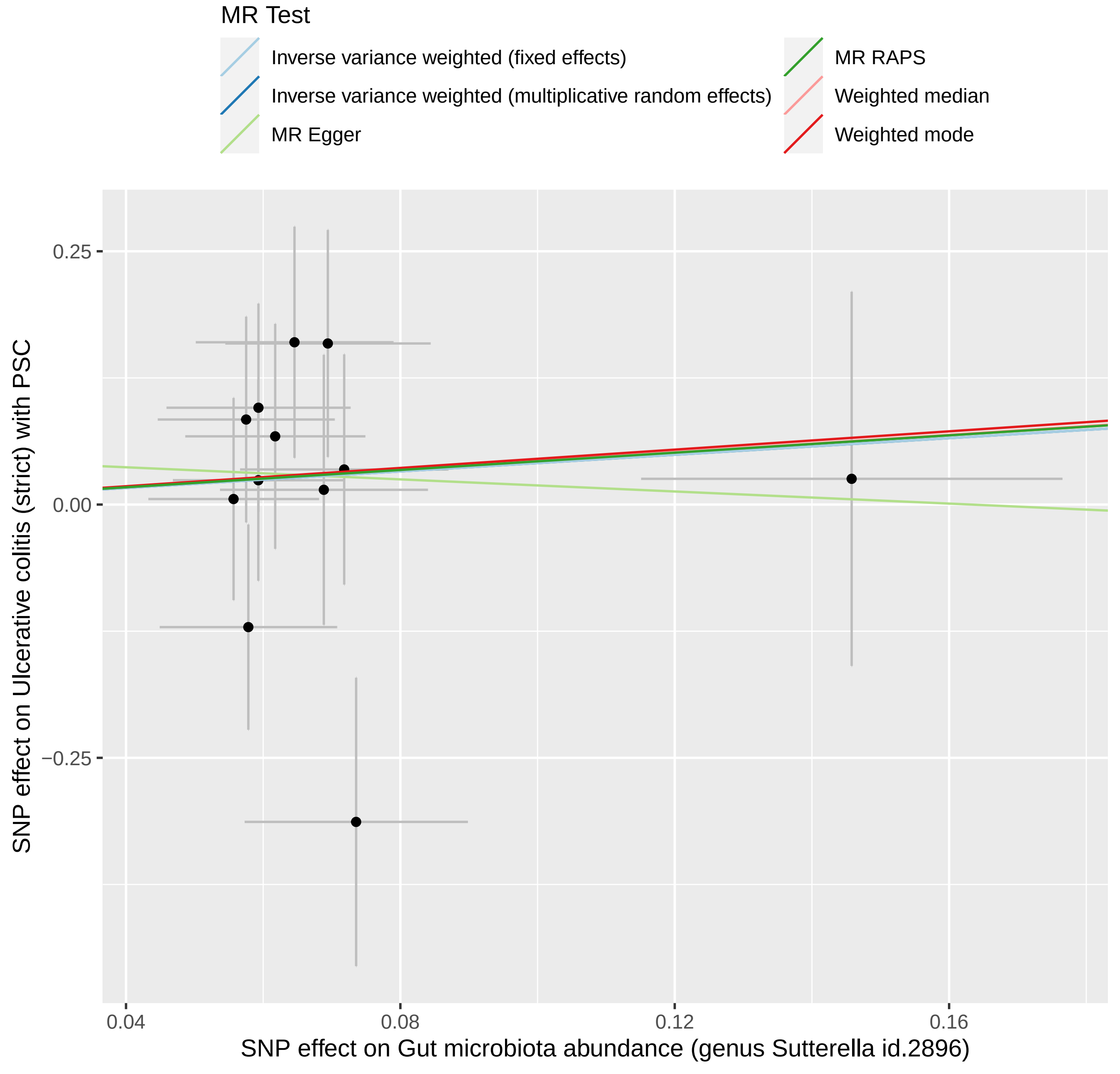

Batch 747 : Gut microbiota abundance (genus Terrisporobacter id.11348) on Ulcerative colitis (strict) with PSC

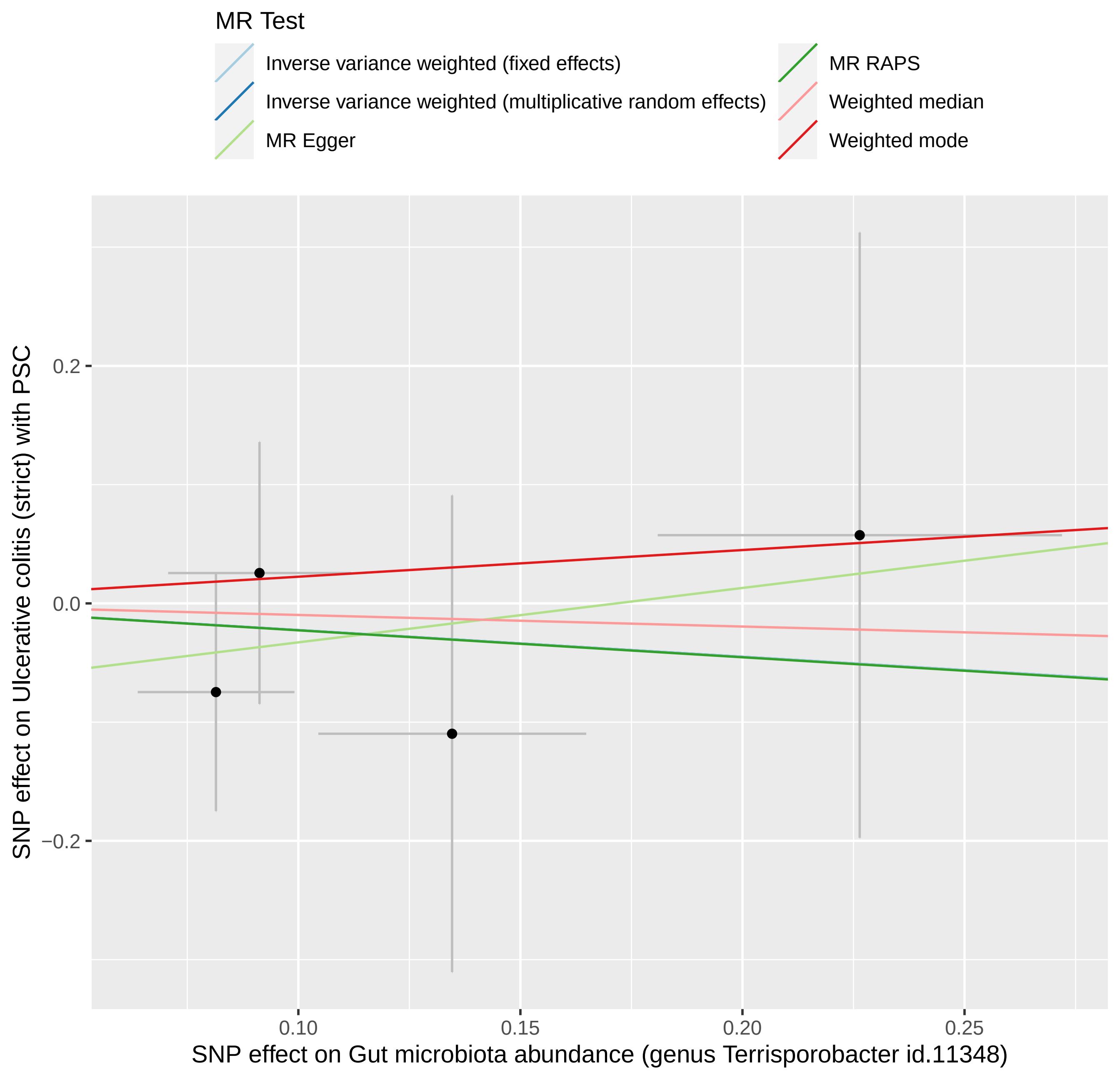

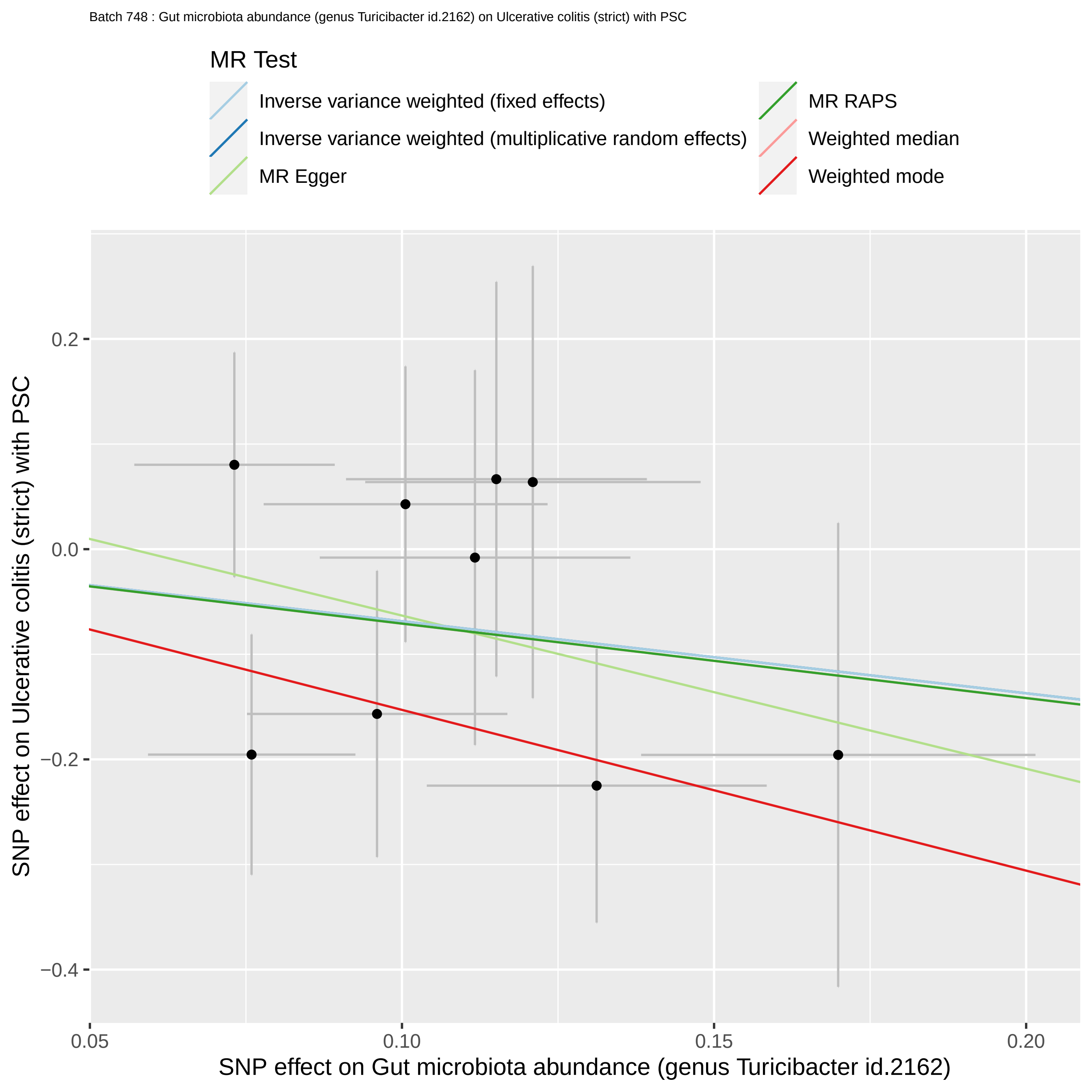
Batch 748 : Gut microbiota abundance (genus Turicibacter id.2162) on Ulcerative colitis (strict) with PSC
MR Test
Inverse variance weighted (fixed effects)
Inverse variance weighted (multiplicative random effects)
MR Egger
MR RAPS
Weighted median
Weighted mode
0.2
0.0
−0.2
−0.4
0.05
0.10
0.15
0.20
SNP effect on Ulcerative colitis (strict) with PSC
SNP effect on Gut microbiota abundance (genus Turicibacter id.2162)

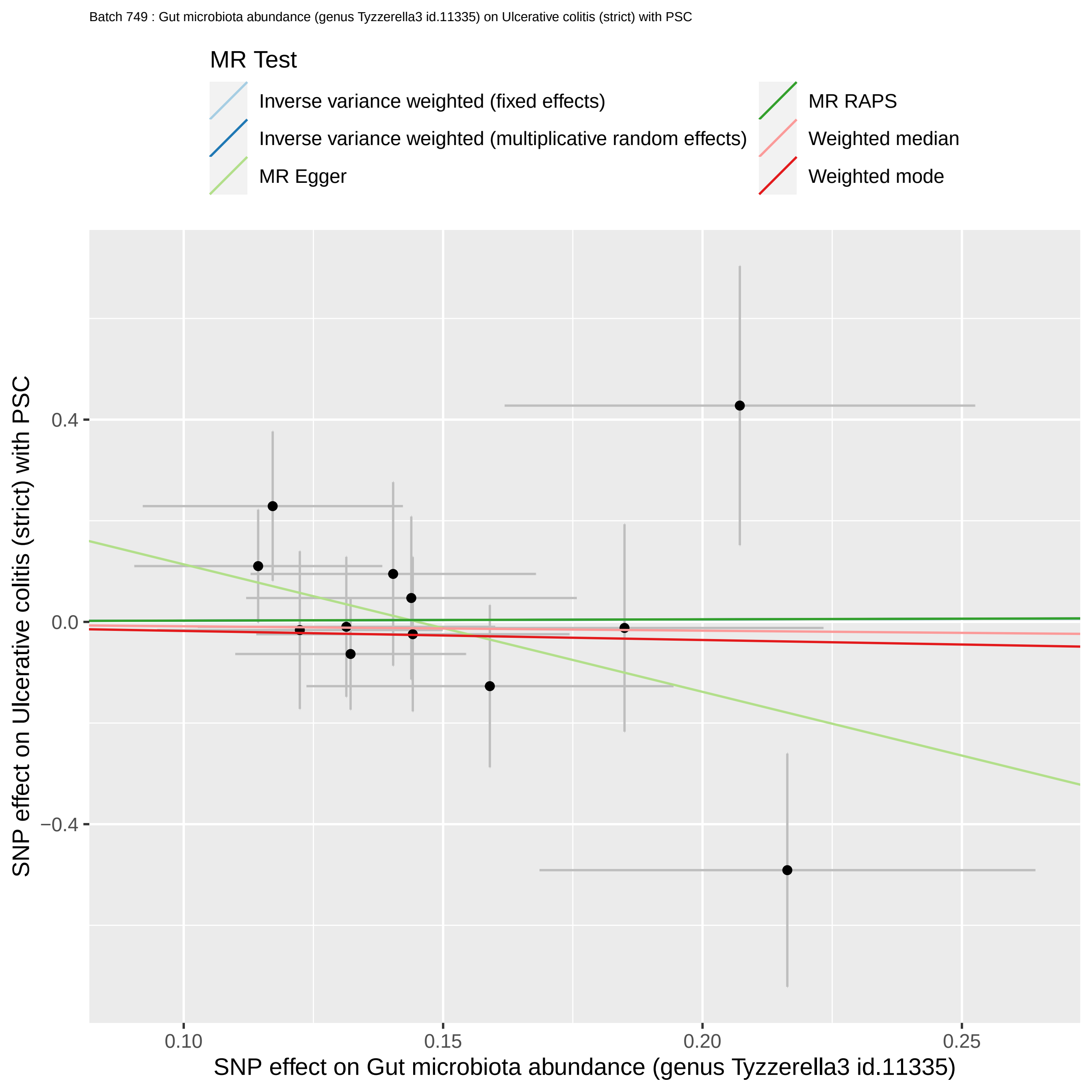

Batch 749 : Gut microbiota abundance (genus Tyzzerella3 id.11335) on Ulcerative colitis (strict) with PSC
MR Test
Inverse variance weighted (fixed effects)
Inverse variance weighted (multiplicative random effects)
MR Egger
MR RAPS
Weighted median
Weighted mode
SNP effect on Ulcerative colitis (strict) with PSC
0.4
0.0
−0.4
0.10
0.15
0.20
0.25
SNP effect on Gut microbiota abundance (genus Tyzzerella3 id.11335)

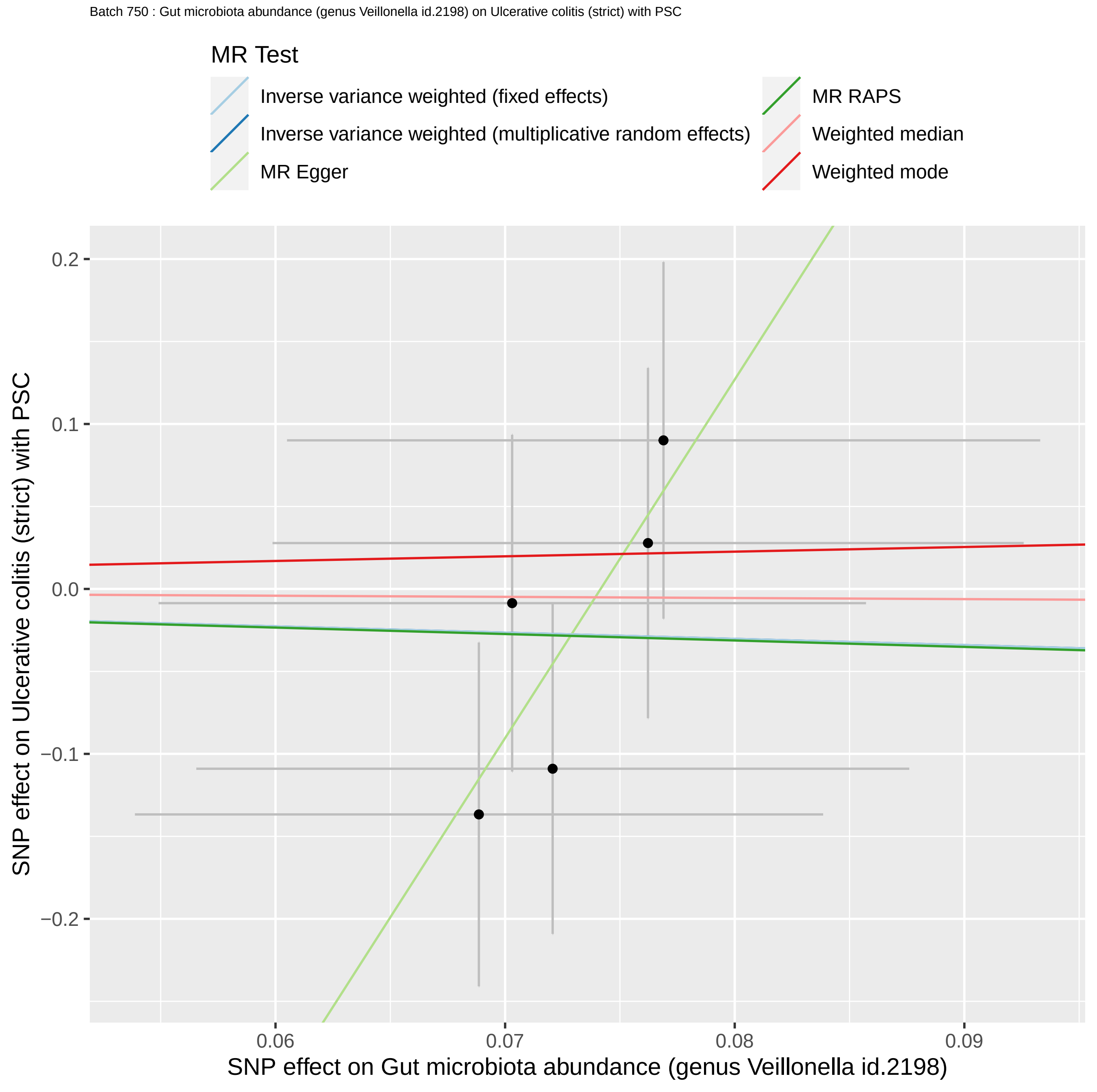

Batch 750 : Gut microbiota abundance (genus Veillonella id.2198) on Ulcerative colitis (strict) with PSC
MR Test
Inverse variance weighted (fixed effects)
Inverse variance weighted (multiplicative random effects)
MR Egger
MR RAPS
Weighted median
Weighted mode
0.2
0.1
0.0
−0.1
−0.2
0.06
0.07
0.08
0.09
SNP effect on Gut microbiota abundance (genus Veillonella id.2198)
SNP effect on Ulcerative colitis (strict) with PSC

Batch 751 : Gut microbiota abundance (family Acidaminococcaceae id.2166) on Arthropathy in ulcerative colitis

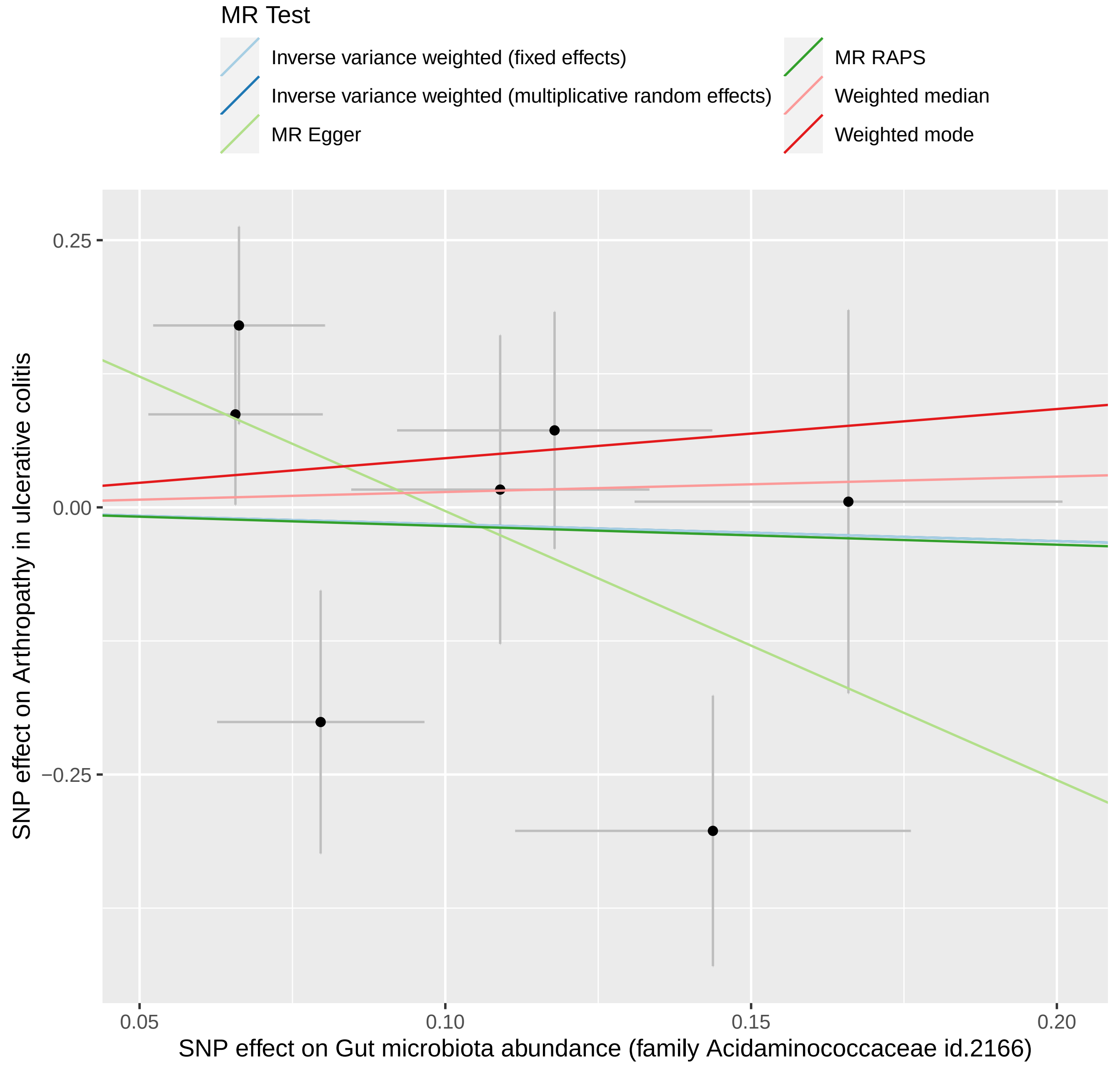

Batch 752 : Gut microbiota abundance (family Actinomycetaceae id.421) on Arthropathy in ulcerative colitis

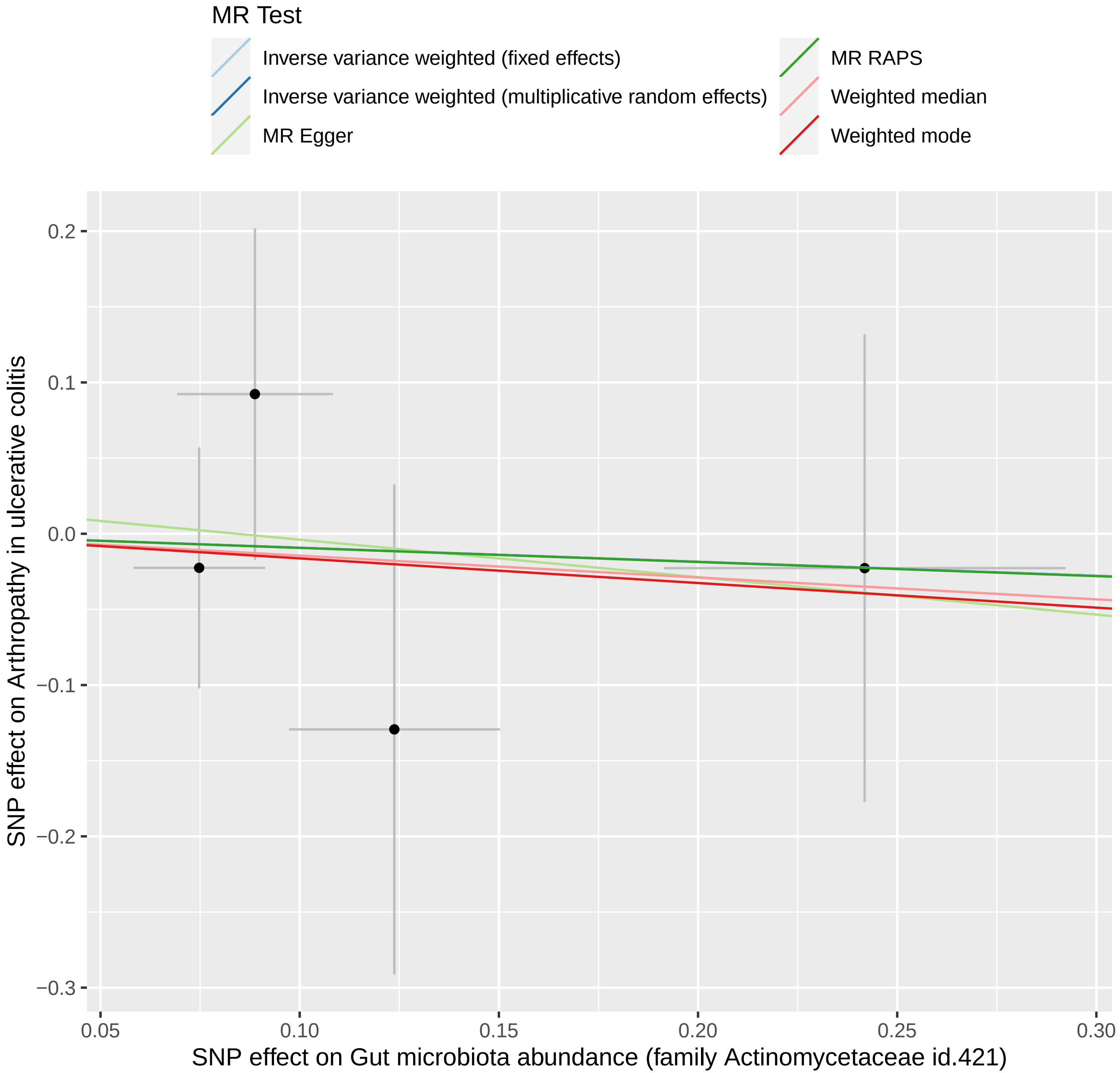

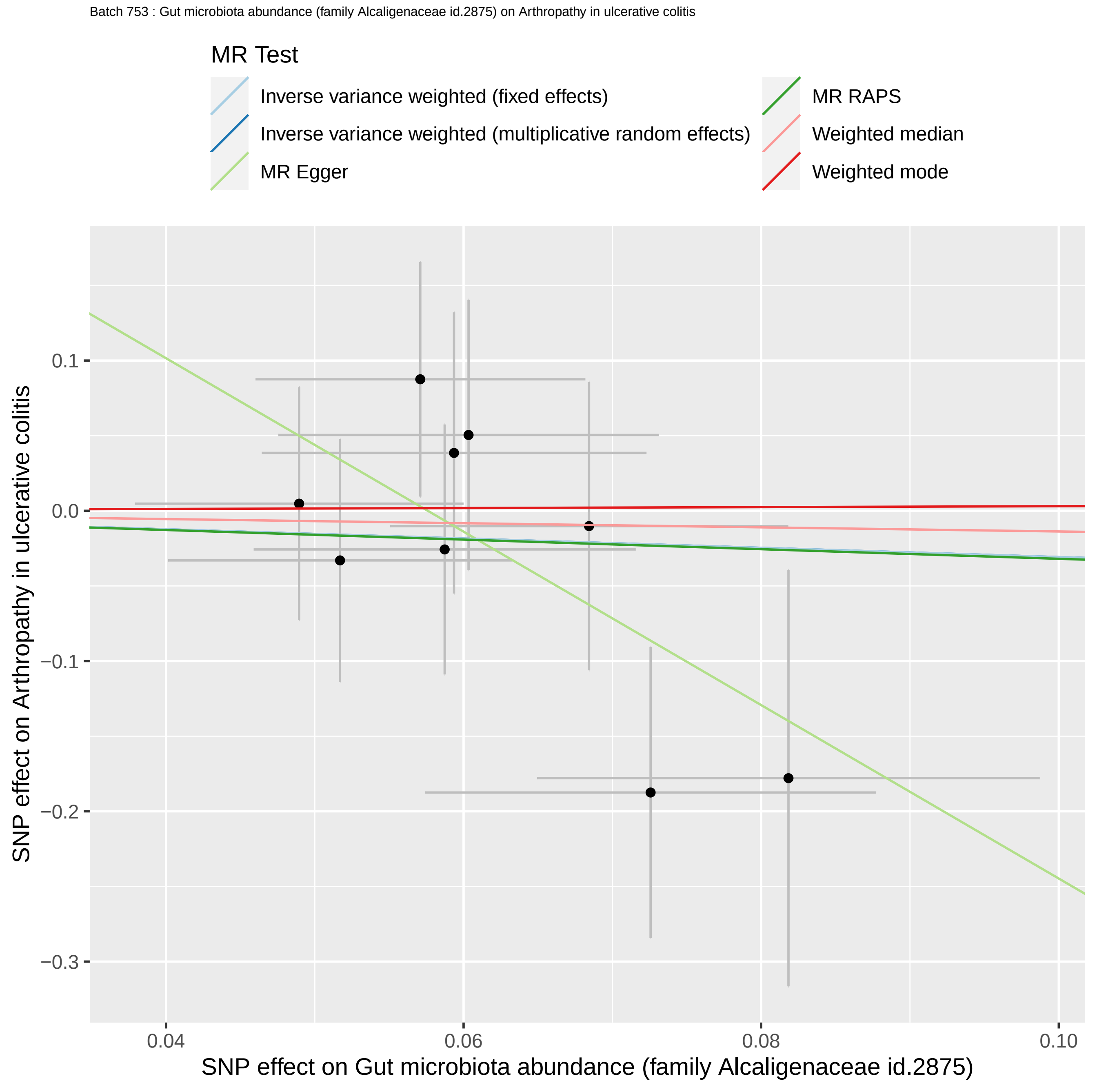

Batch 753 : Gut microbiota abundance (family Alcaligenaceae id.2875) on Arthropathy in ulcerative colitis
MR Test
Inverse variance weighted (fixed effects)
Inverse variance weighted (multiplicative random effects)
MR Egger
MR RAPS
Weighted median
Weighted mode
0.1
0.0
−0.1
−0.2
−0.3
0.04
0.06
0.08
0.10
SNP effect on Arthropathy in ulcerative colitis
SNP effect on Gut microbiota abundance (family Alcaligenaceae id.2875)

Batch 754 : Gut microbiota abundance (family Bacteroidaceae id.917) on Arthropathy in ulcerative colitis

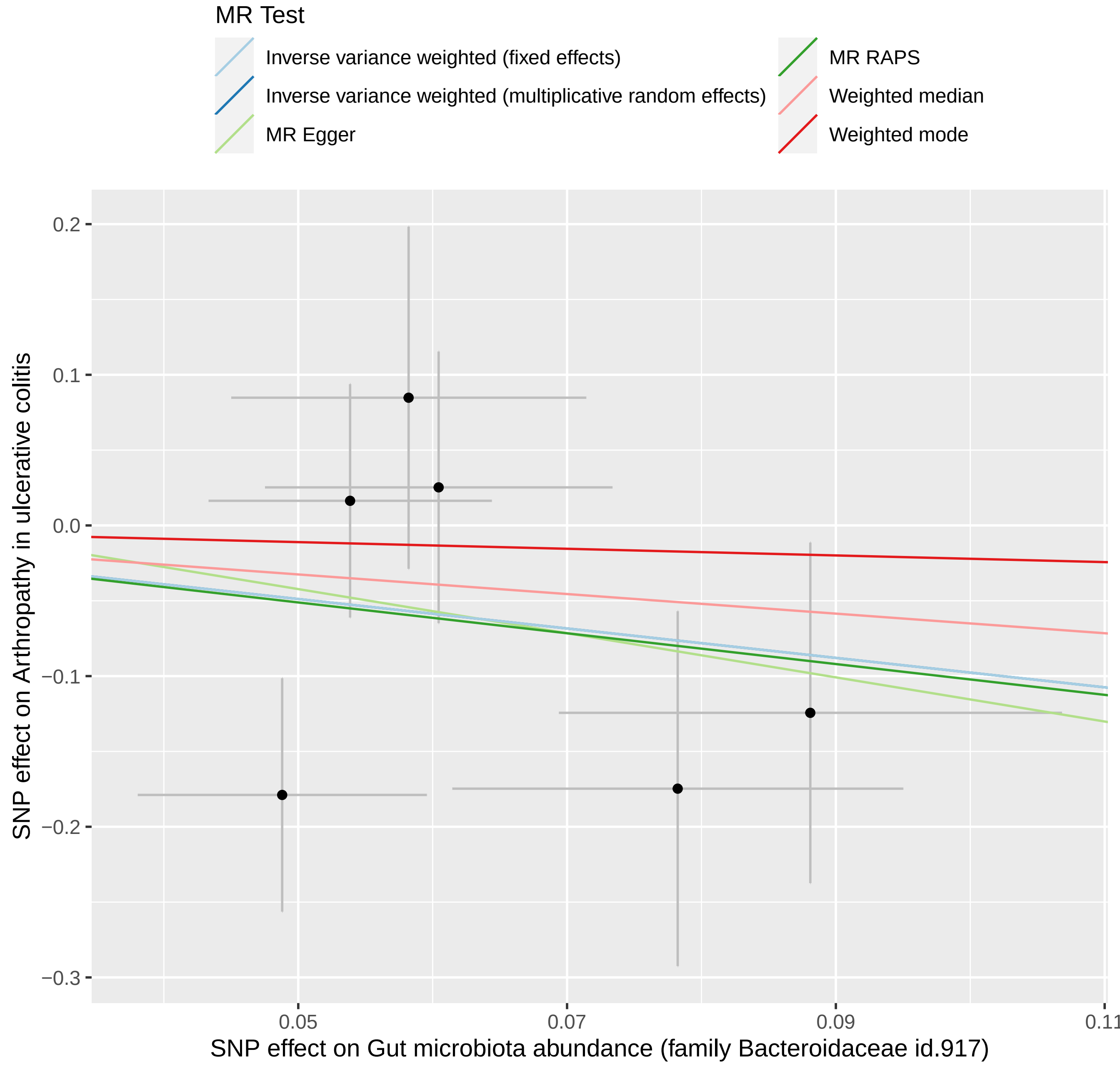

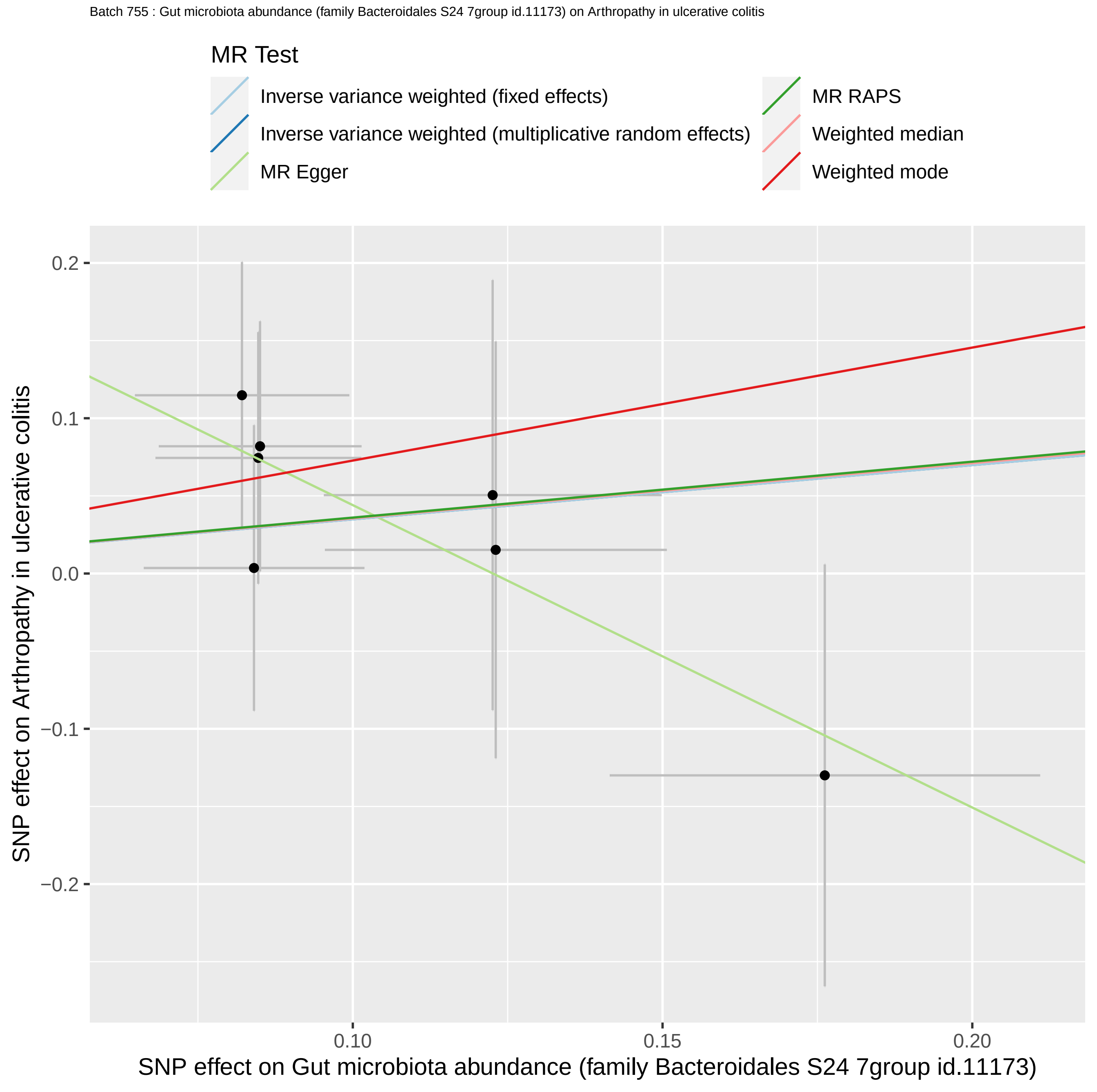

Batch 755 : Gut microbiota abundance (family Bacteroidales S24 7group id.11173) on Arthropathy in ulcerative colitis
MR Test
Inverse variance weighted (fixed effects)
Inverse variance weighted (multiplicative random effects)
MR Egger
MR RAPS
Weighted median
Weighted mode
0.2
0.1
0.0
−0.1
−0.2
0.10
0.15
0.20
SNP effect on Arthropathy in ulcerative colitis
SNP effect on Gut microbiota abundance (family Bacteroidales S24 7group id.11173)

Batch 756 : Gut microbiota abundance (family Bifidobacteriaceae id.433) on Arthropathy in ulcerative colitis

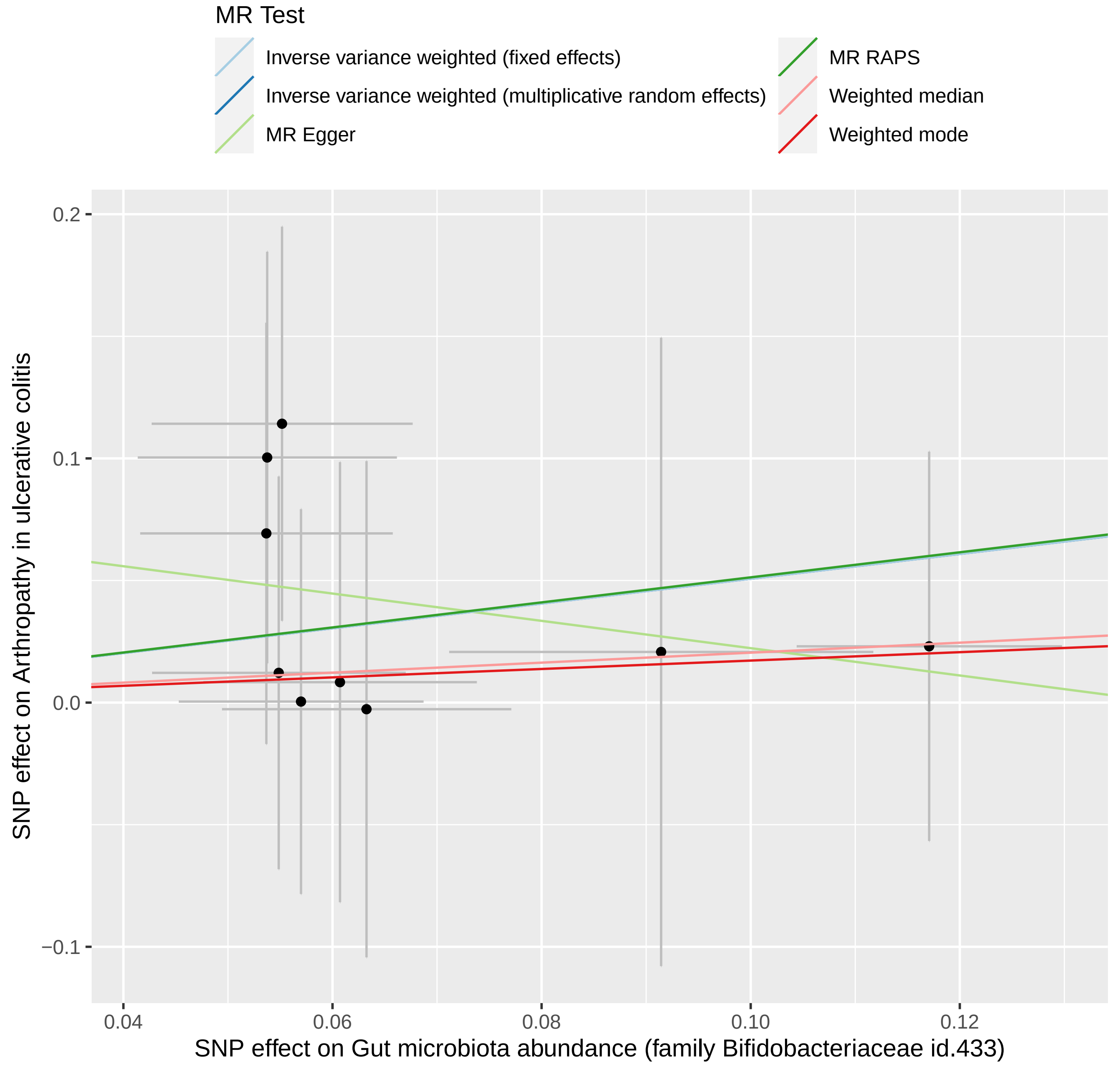

Batch 758 : Gut microbiota abundance (family Clostridiaceae1 id.1869) on Arthropathy in ulcerative colitis

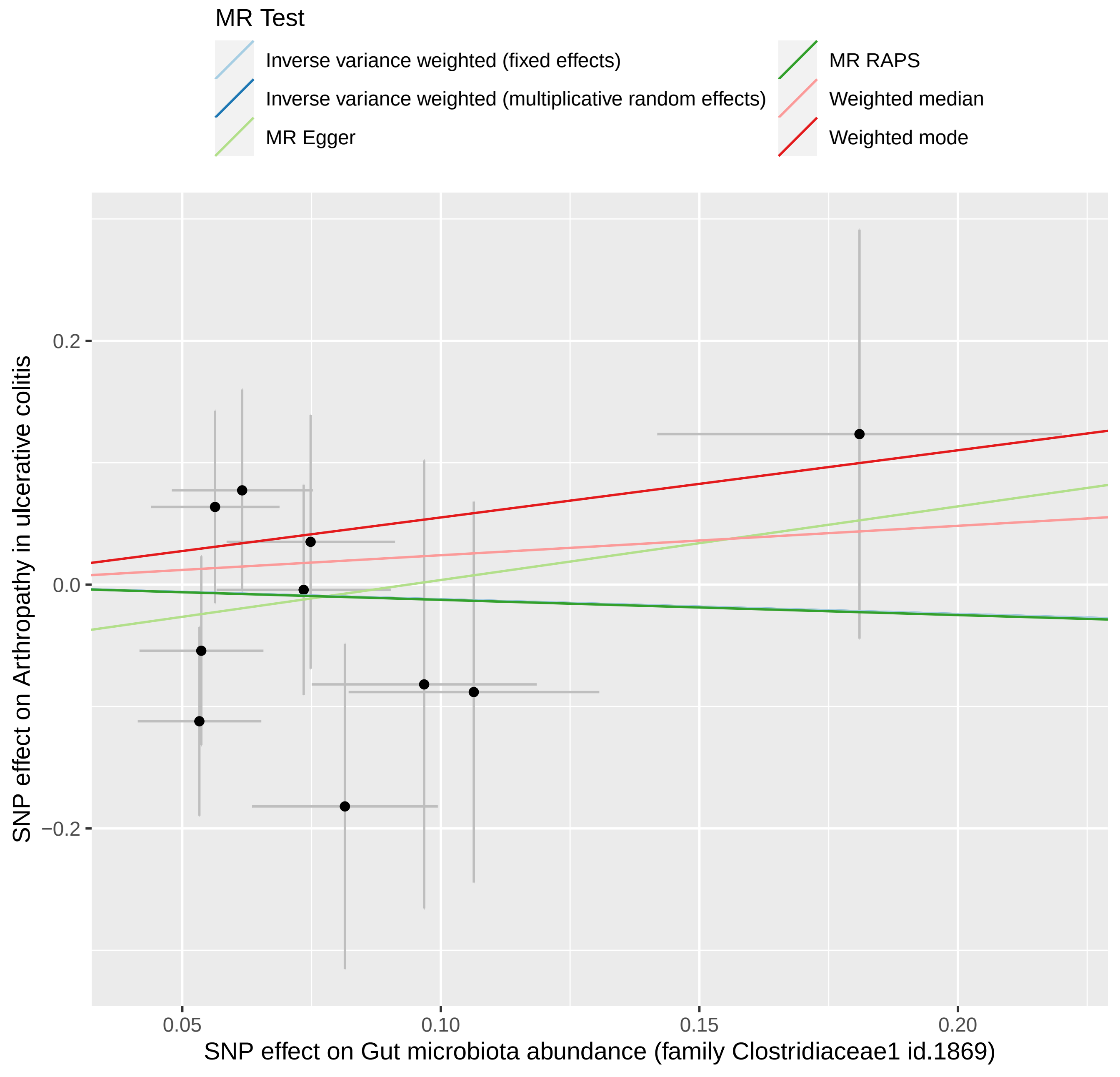

Batch 759 : Gut microbiota abundance (family Clostridiales vadin BB60 group id.11286) on Arthropathy in ulcerative colitis

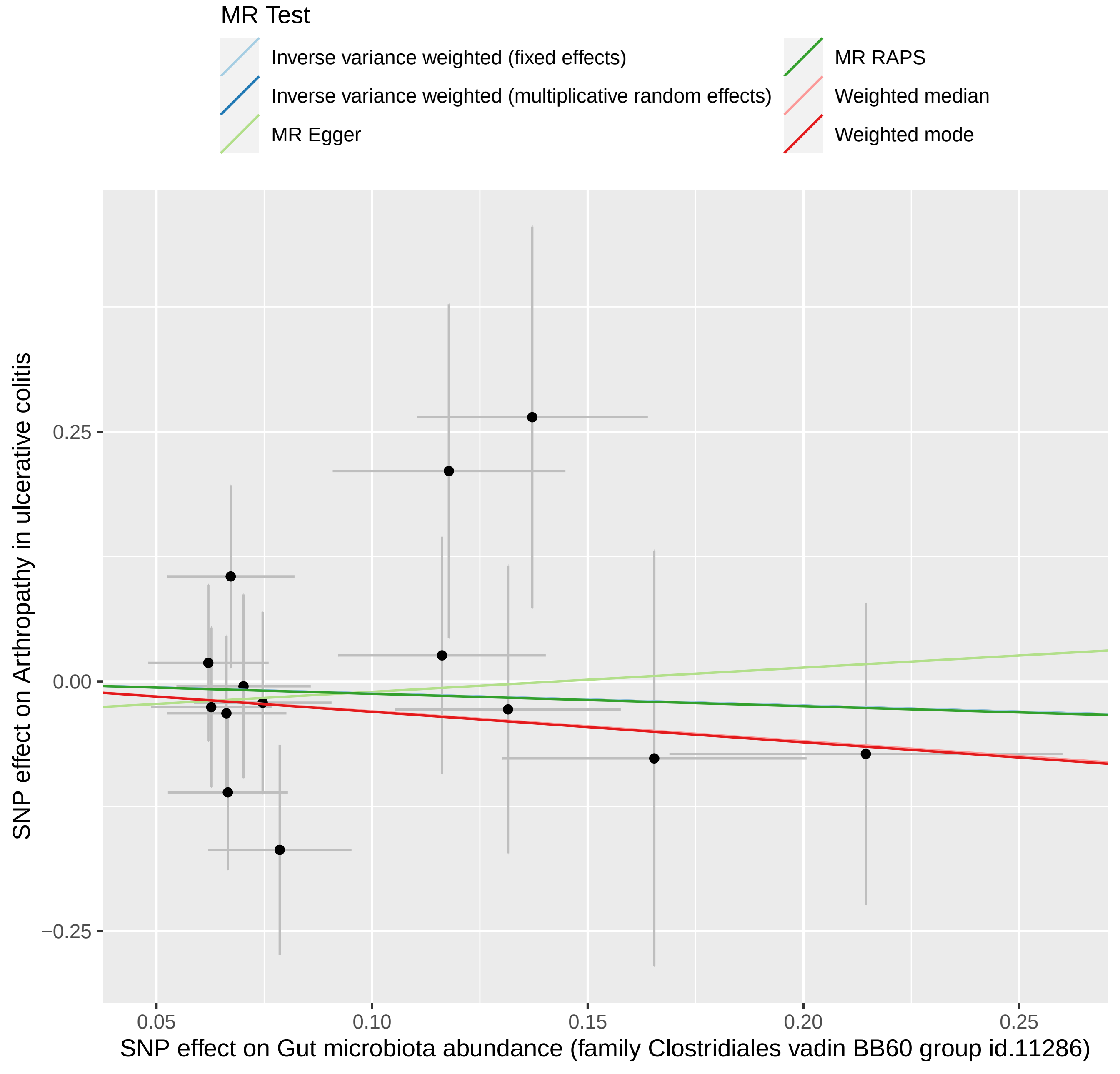

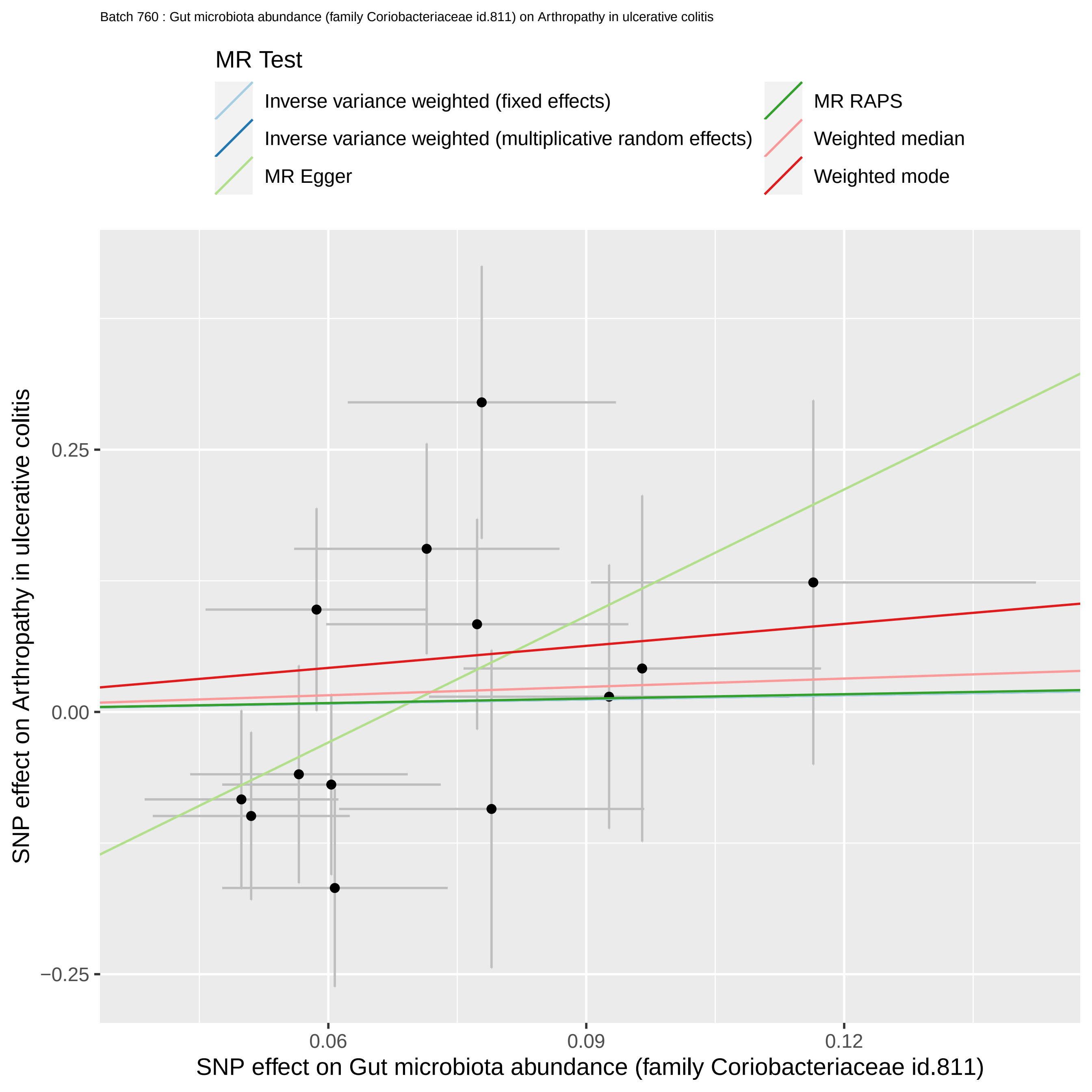
Batch 760 : Gut microbiota abundance (family Coriobacteriaceae id.811) on Arthropathy in ulcerative colitis
MR Test
Inverse variance weighted (fixed effects)
Inverse variance weighted (multiplicative random effects)
MR Egger
MR RAPS
Weighted median
Weighted mode
SNP effect on Arthropathy in ulcerative colitis
0.25
0.00
−0.25
0.06
0.09
0.12
SNP effect on Gut microbiota abundance (family Coriobacteriaceae id.811)

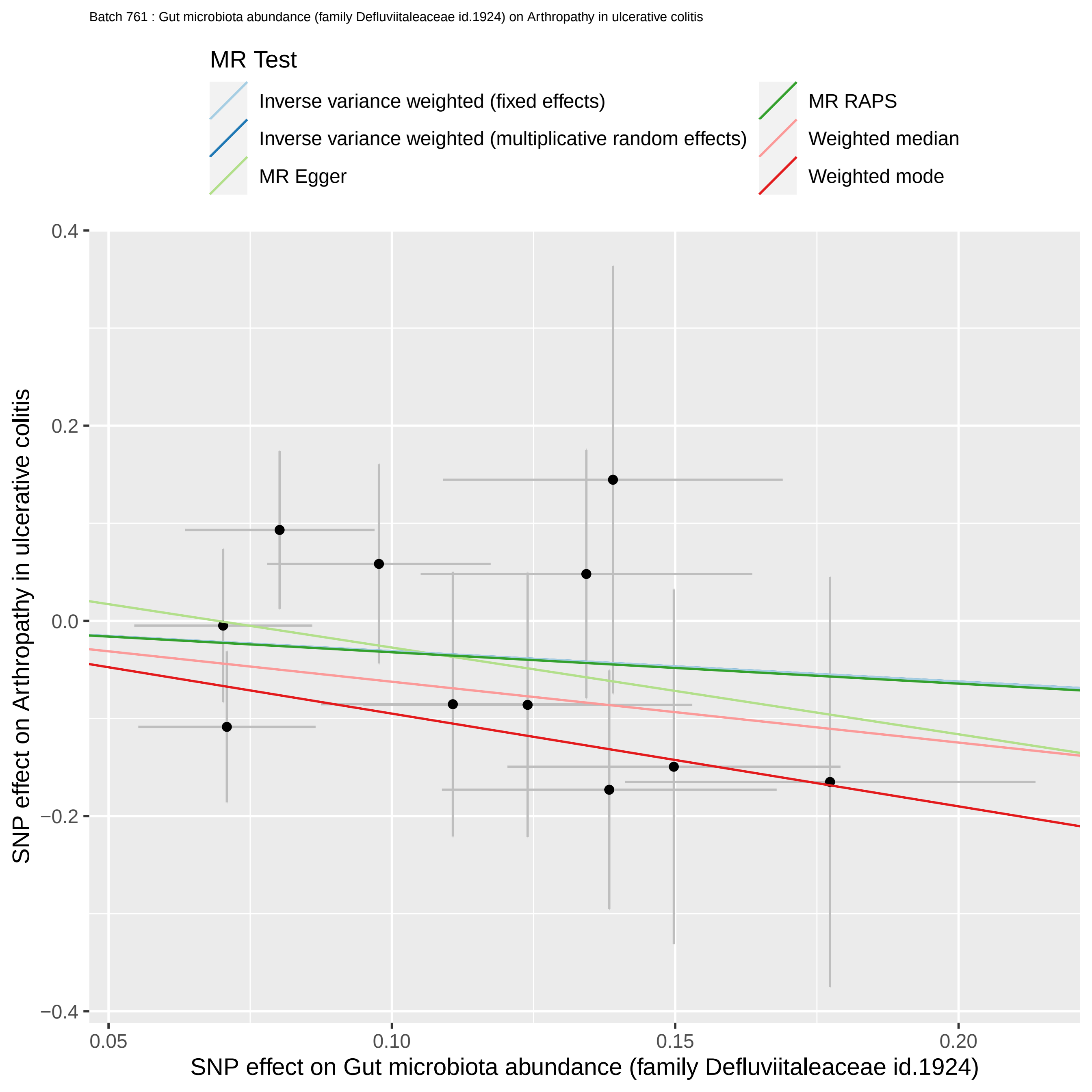

Batch 761 : Gut microbiota abundance (family Defluviitaleaceae id.1924) on Arthropathy in ulcerative colitis
MR Test
Inverse variance weighted (fixed effects)
Inverse variance weighted (multiplicative random effects)
MR Egger
MR RAPS
Weighted median
Weighted mode
0.4
0.2
0.0
−0.2
−0.4
0.05
0.10
0.15
0.20
SNP effect on Arthropathy in ulcerative colitis
SNP effect on Gut microbiota abundance (family Defluviitaleaceae id.1924)

Batch 762 : Gut microbiota abundance (family Desulfovibrionaceae id.3169) on Arthropathy in ulcerative colitis

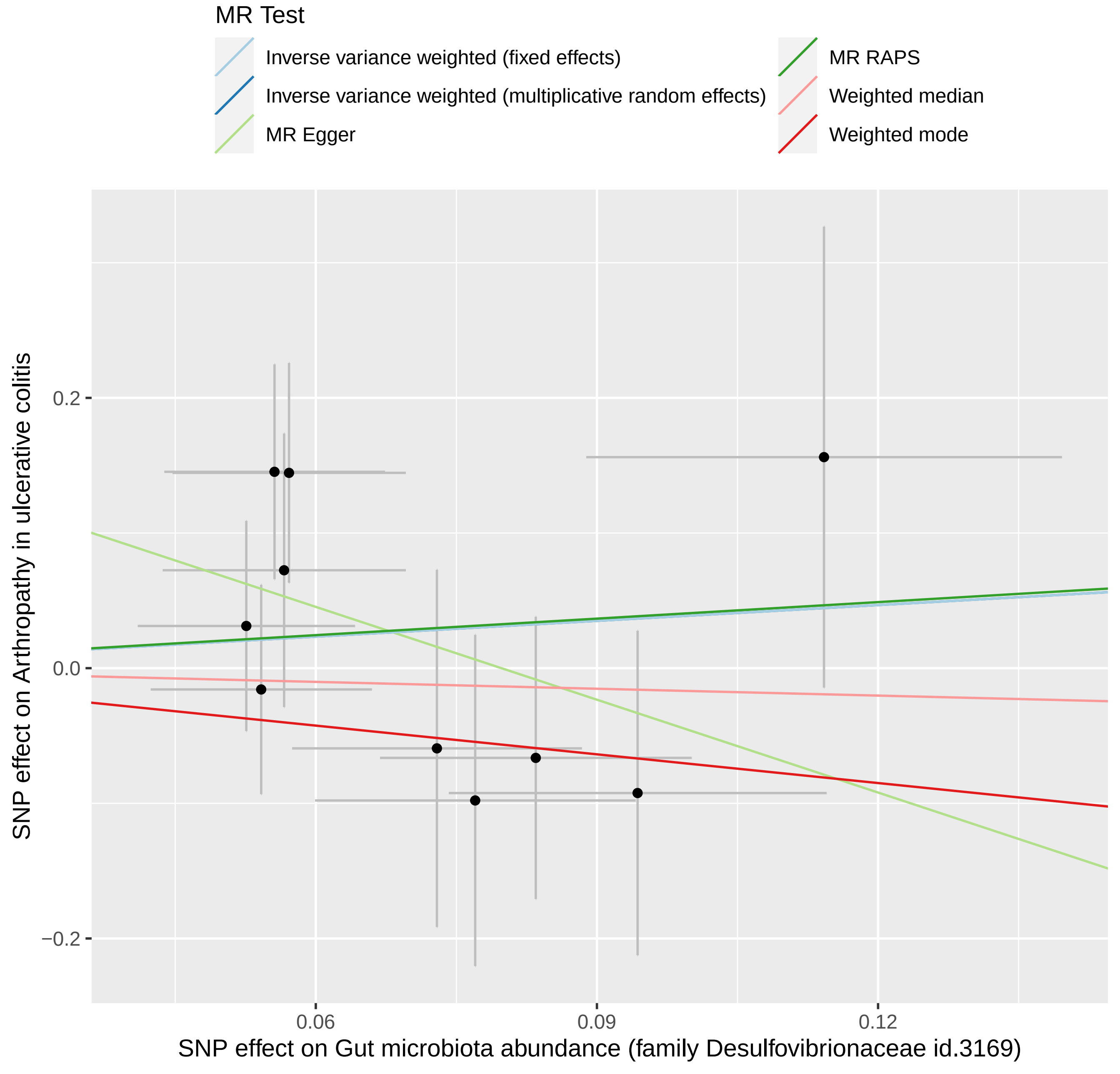

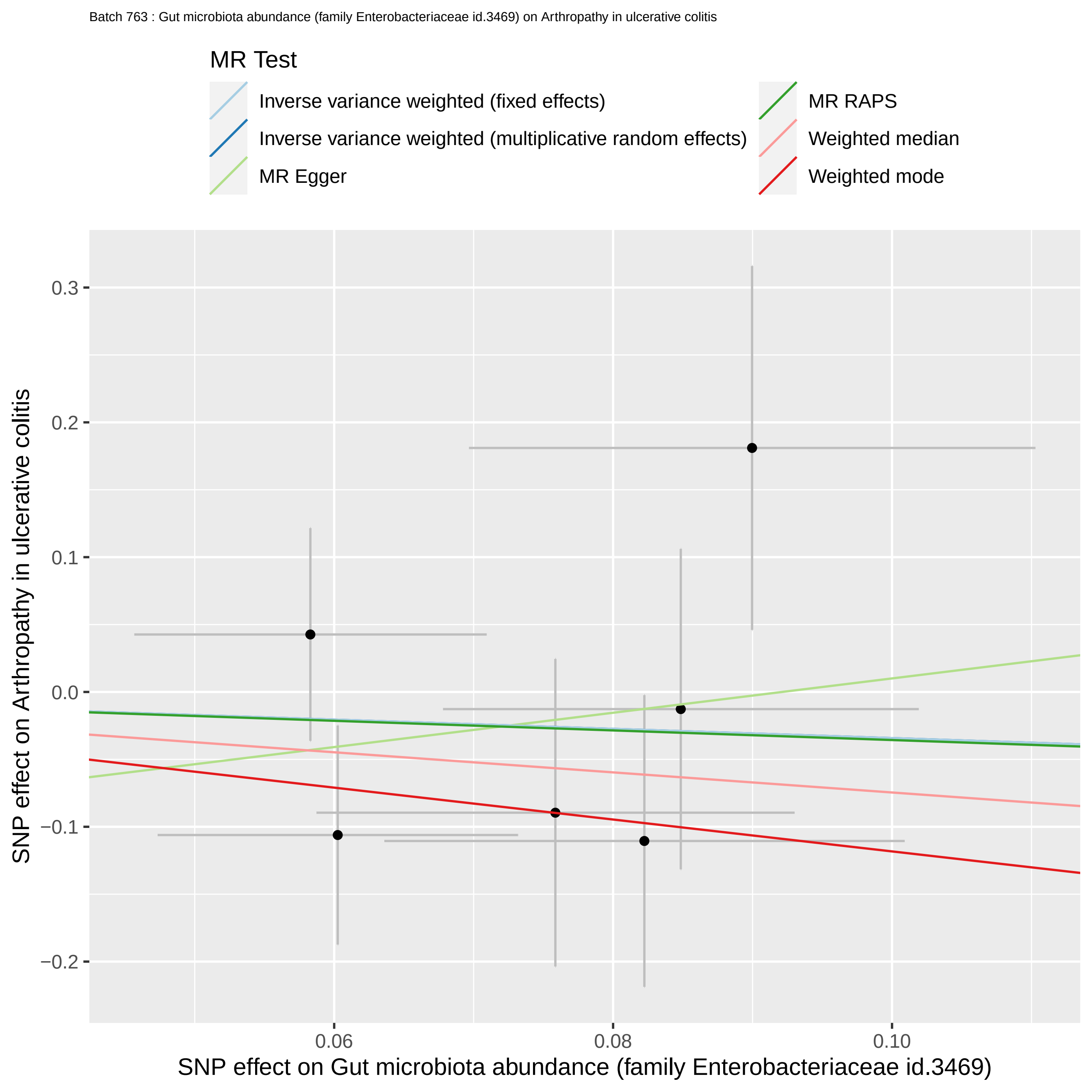

Batch 763 : Gut microbiota abundance (family Enterobacteriaceae id.3469) on Arthropathy in ulcerative colitis
MR Test
Inverse variance weighted (fixed effects)
Inverse variance weighted (multiplicative random effects)
MR Egger
MR RAPS
Weighted median
Weighted mode
0.3
0.2
0.1
0.0
−0.1
−0.2
0.06
0.08
0.10
SNP effect on Arthropathy in ulcerative colitis
SNP effect on Gut microbiota abundance (family Enterobacteriaceae id.3469)

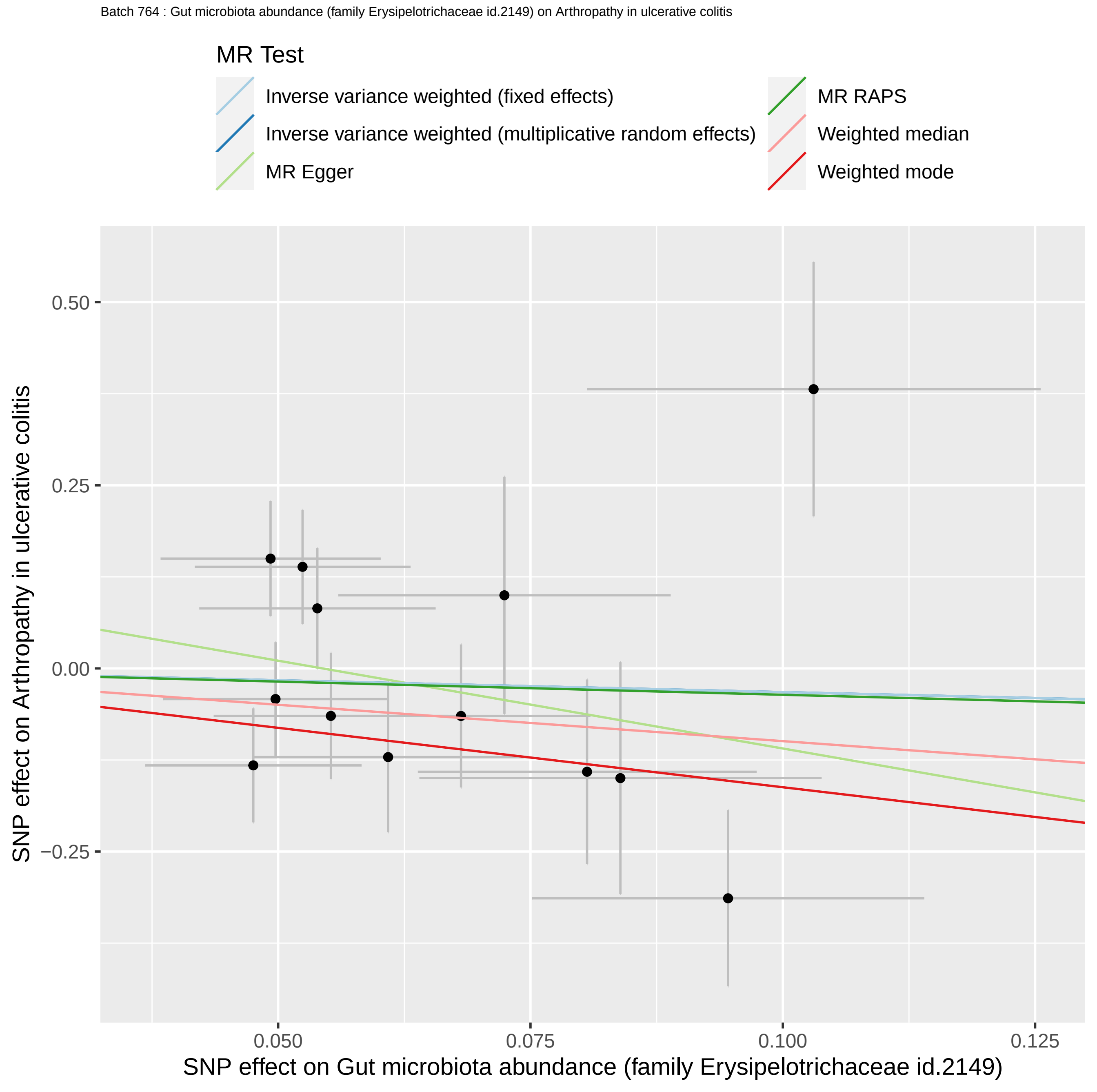

Batch 764 : Gut microbiota abundance (family Erysipelotrichaceae id.2149) on Arthropathy in ulcerative colitis
MR Test
Inverse variance weighted (fixed effects)
Inverse variance weighted (multiplicative random effects)
MR Egger
MR RAPS
Weighted median
Weighted mode
0.50
0.25
0.00
−0.25
0.050
0.075
0.100
0.125
SNP effect on Arthropathy in ulcerative colitis
SNP effect on Gut microbiota abundance (family Erysipelotrichaceae id.2149)

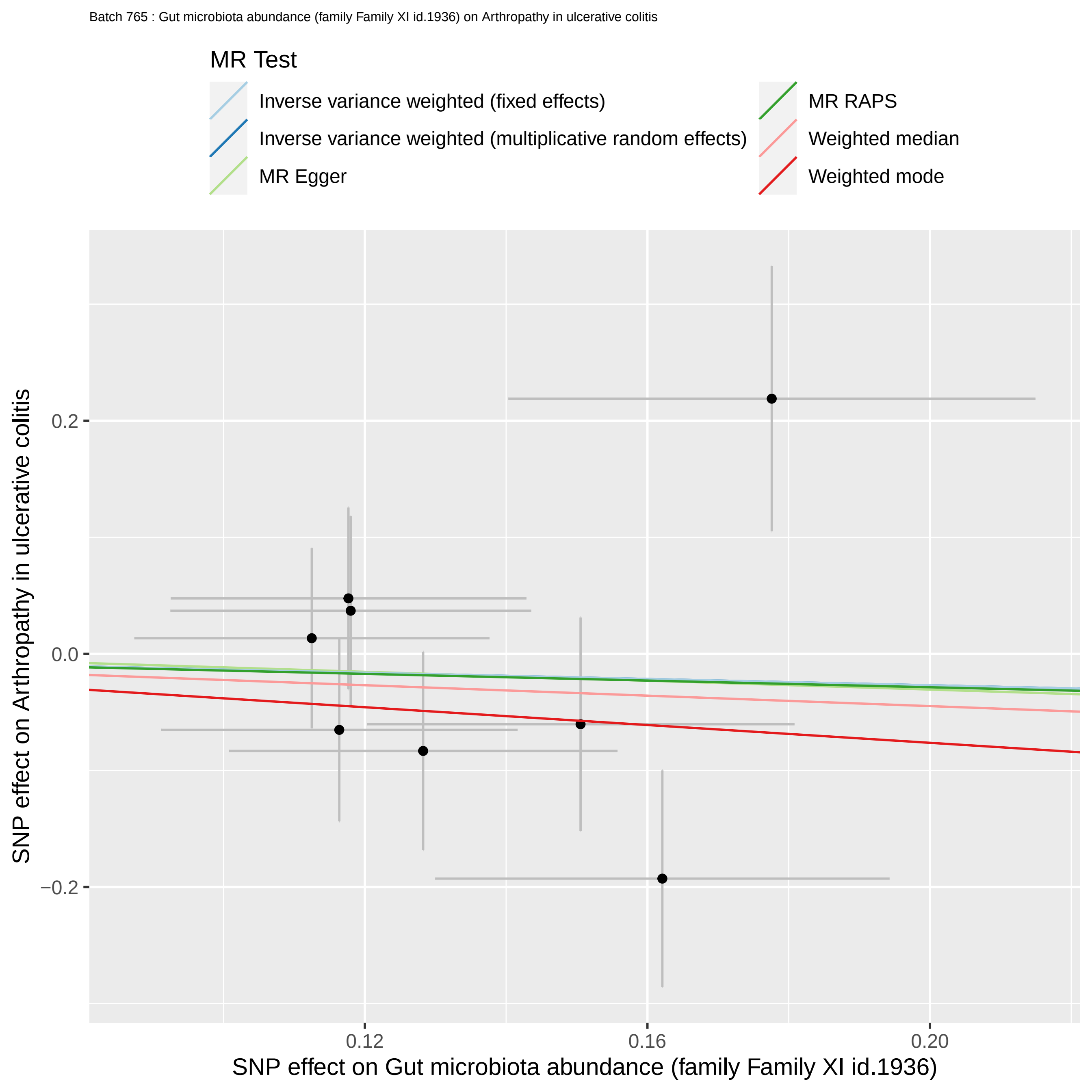

Batch 765 : Gut microbiota abundance (family Family XI id.1936) on Arthropathy in ulcerative colitis
MR Test
Inverse variance weighted (fixed effects)
Inverse variance weighted (multiplicative random effects)
MR Egger
MR RAPS
Weighted median
Weighted mode
0.2
0.0
−0.2
0.12
0.16
0.20
SNP effect on Arthropathy in ulcerative colitis
SNP effect on Gut microbiota abundance (family Family XI id.1936)

Batch 766 : Gut microbiota abundance (family Family XIII id.1957) on Arthropathy in ulcerative colitis

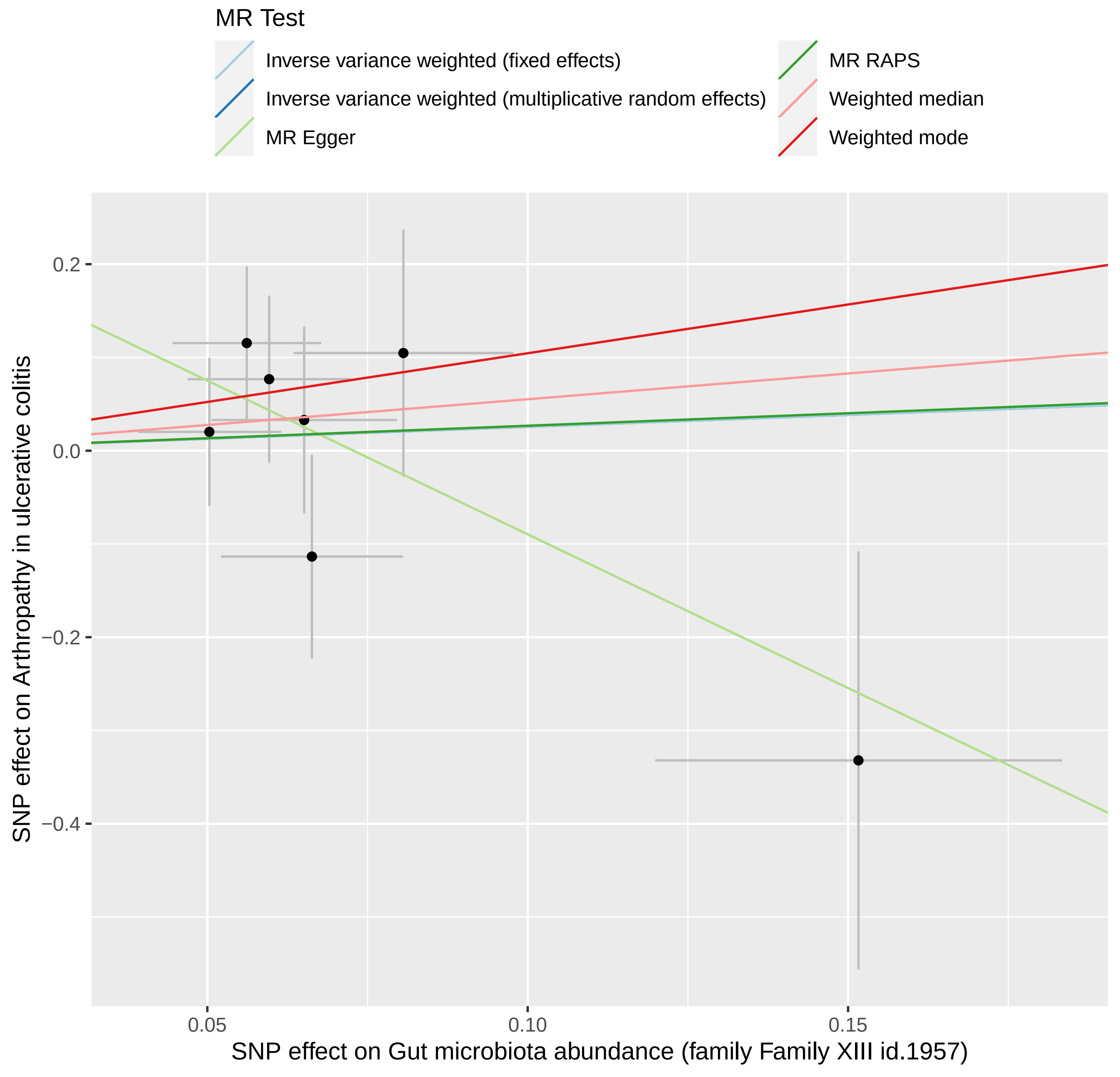

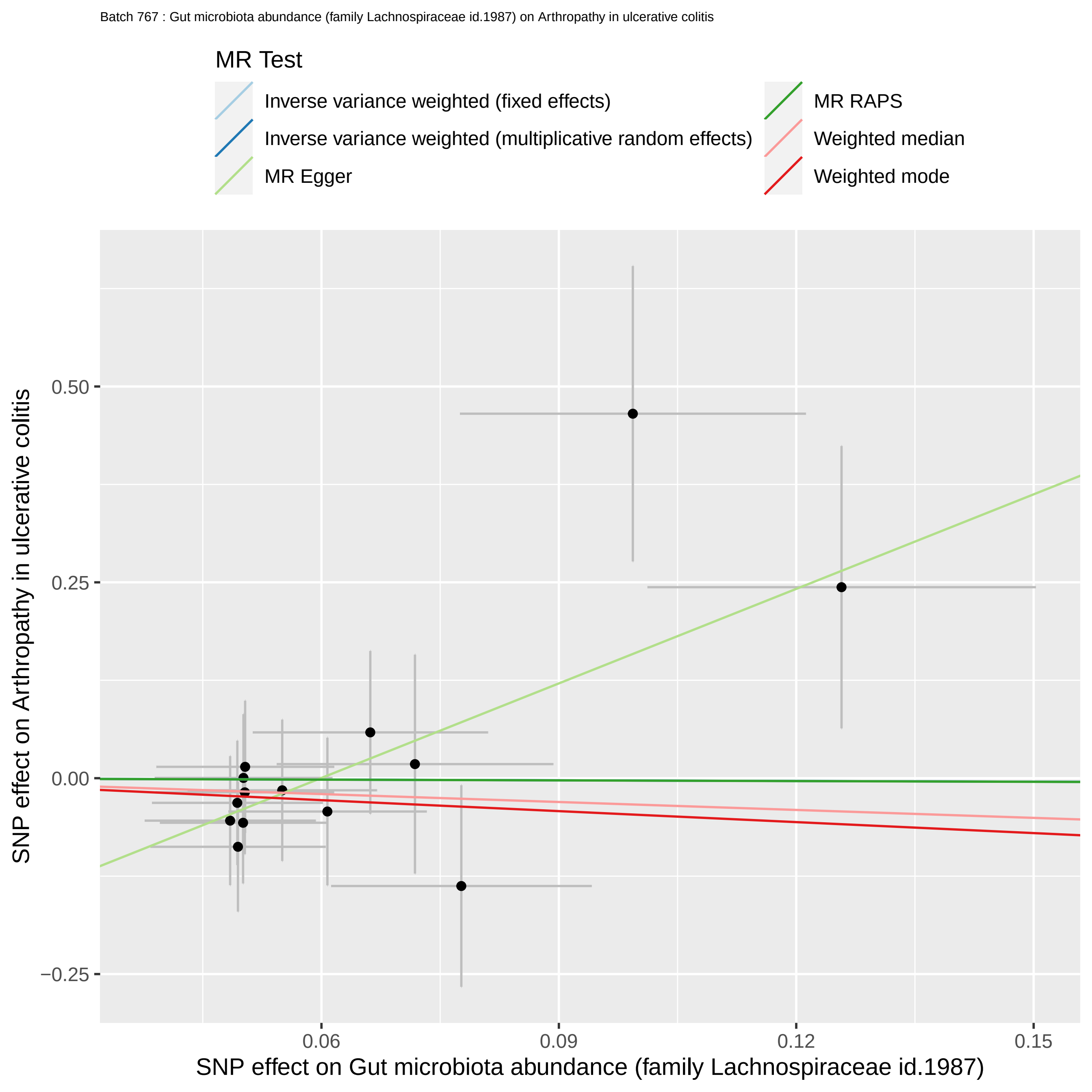
Batch 767 : Gut microbiota abundance (family Lachnospiraceae id.1987) on Arthropathy in ulcerative colitis
MR Test
Inverse variance weighted (fixed effects)
Inverse variance weighted (multiplicative random effects)
MR Egger
MR RAPS
Weighted median
Weighted mode
0.50
0.25
0.00
−0.25
0.06
0.09
0.12
0.15
SNP effect on Arthropathy in ulcerative colitis
SNP effect on Gut microbiota abundance (family Lachnospiraceae id.1987)

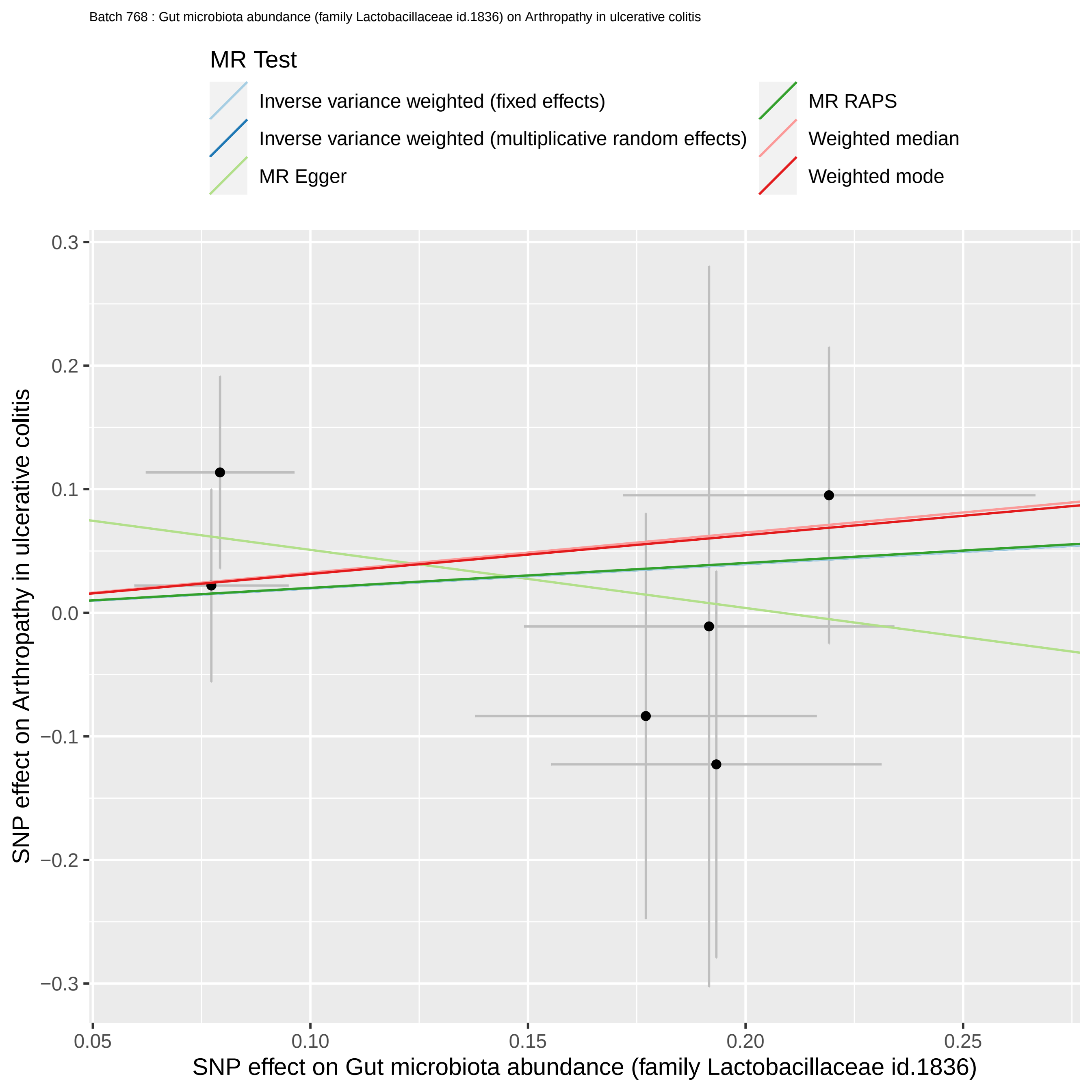

Batch 768 : Gut microbiota abundance (family Lactobacillaceae id.1836) on Arthropathy in ulcerative colitis
MR Test
Inverse variance weighted (fixed effects)
Inverse variance weighted (multiplicative random effects)
MR Egger
MR RAPS
Weighted median
Weighted mode
SNP effect on Arthropathy in ulcerative colitis
0.3
0.2
0.1
0.0
−0.1
−0.2
−0.3
0.05
0.10
0.15
0.20
0.25
SNP effect on Gut microbiota abundance (family Lactobacillaceae id.1836)

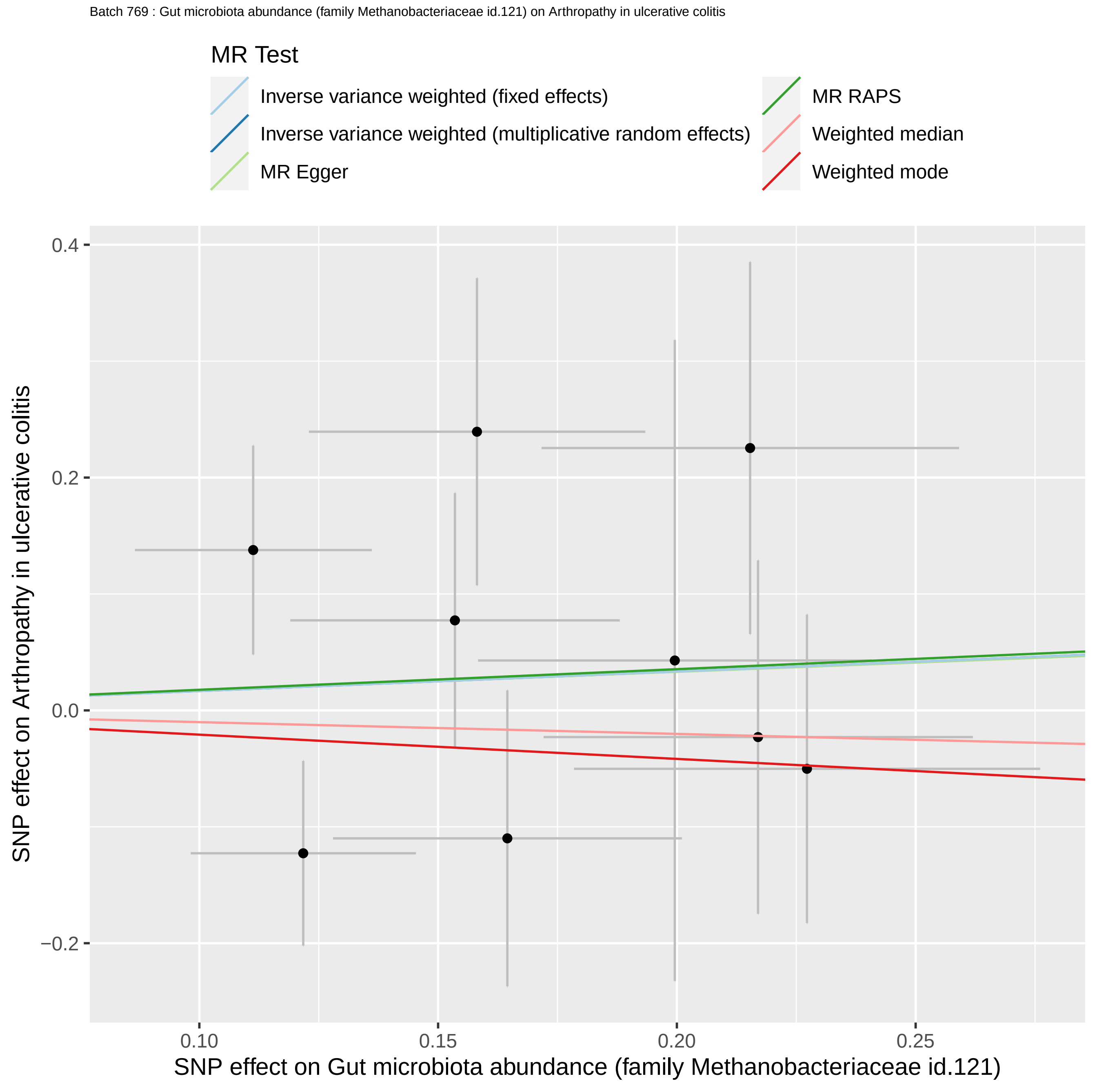

Batch 769 : Gut microbiota abundance (family Methanobacteriaceae id.121) on Arthropathy in ulcerative colitis
MR Test
Inverse variance weighted (fixed effects)
Inverse variance weighted (multiplicative random effects)
MR Egger
MR RAPS
Weighted median
Weighted mode
0.4
0.2
0.0
−0.2
0.10
0.15
0.20
0.25
SNP effect on Arthropathy in ulcerative colitis
SNP effect on Gut microbiota abundance (family Methanobacteriaceae id.121)

Batch 770 : Gut microbiota abundance (family Oxalobacteraceae id.2966) on Arthropathy in ulcerative colitis

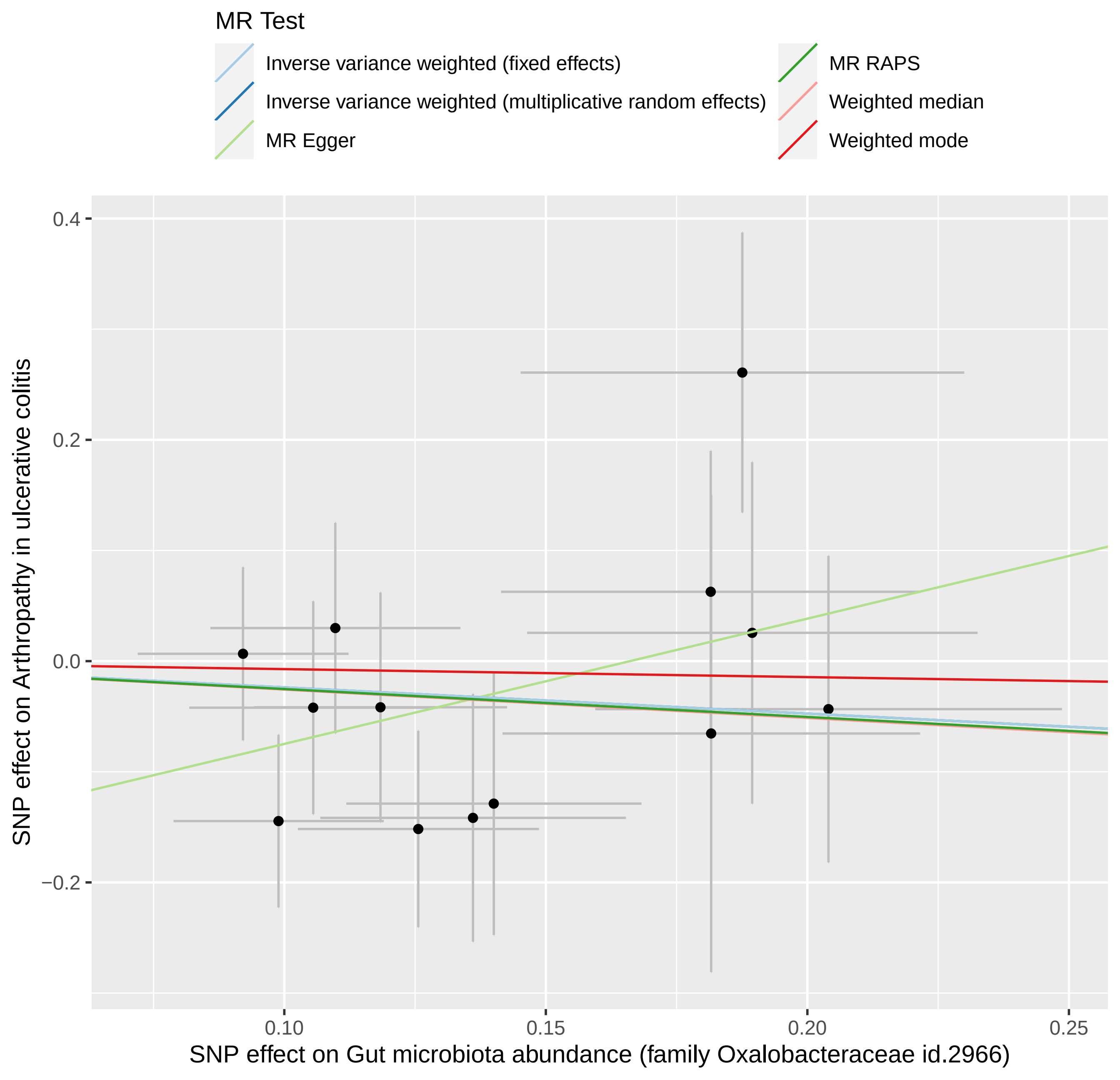

Batch 771 : Gut microbiota abundance (family Pasteurellaceae id.3689) on Arthropathy in ulcerative colitis

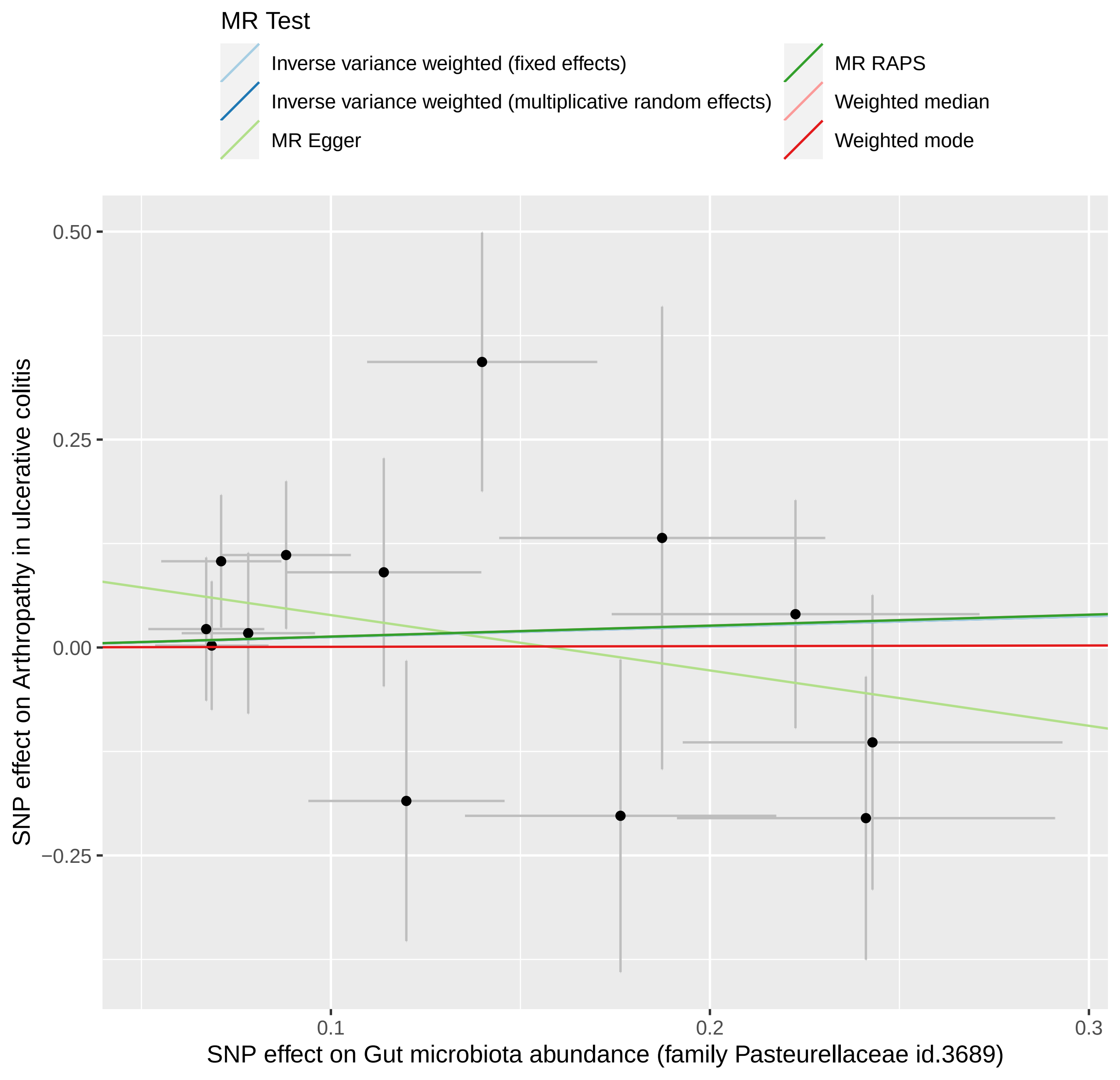

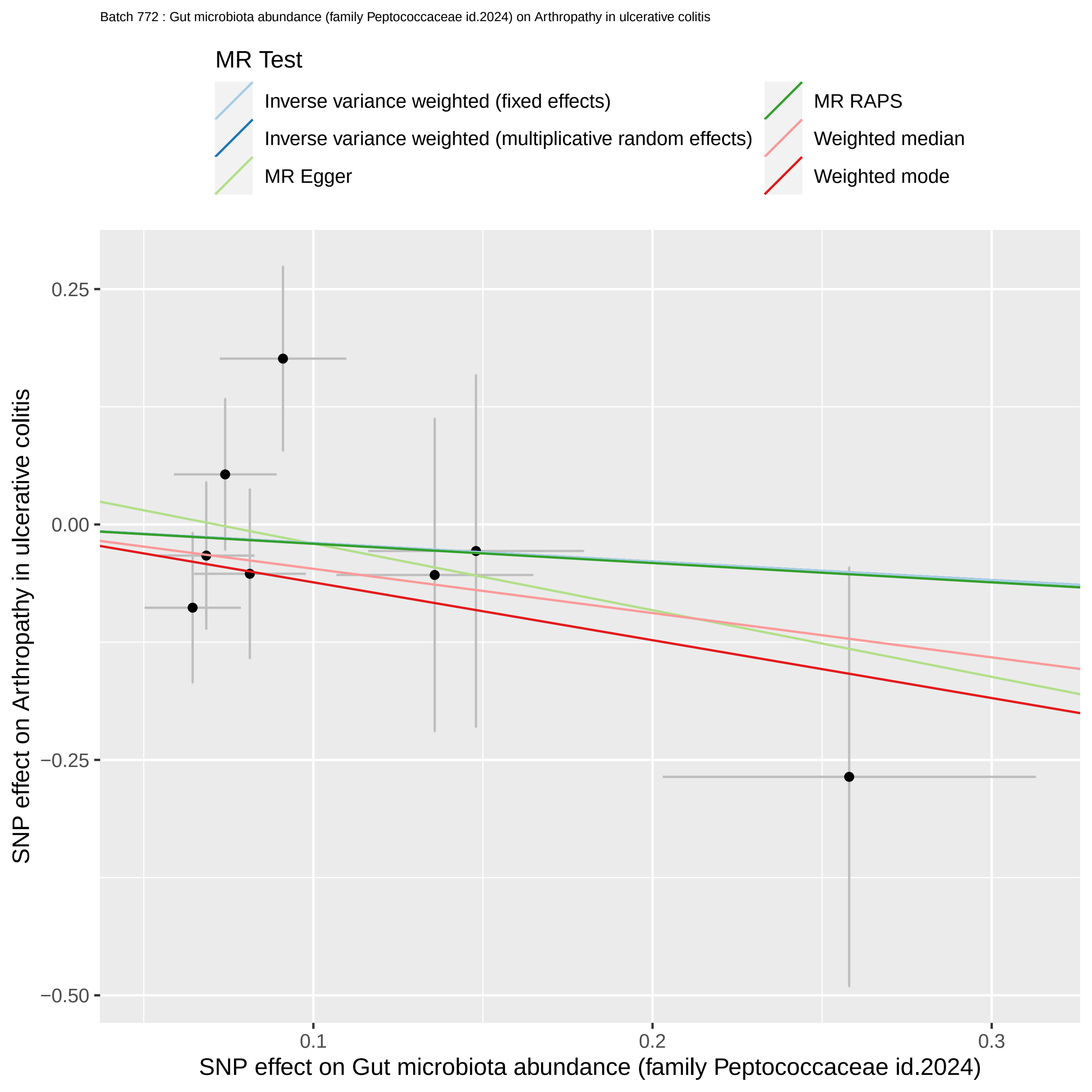
Batch 772 : Gut microbiota abundance (family Peptococcaceae id.2024) on Arthropathy in ulcerative colitis
MR Test
Inverse variance weighted (fixed effects)
Inverse variance weighted (multiplicative random effects)
MR Egger
MR RAPS
Weighted median
Weighted mode
0.25
0.00
−0.25
−0.50
0.1
0.2
0.3
SNP effect on Arthropathy in ulcerative colitis
SNP effect on Gut microbiota abundance (family Peptococcaceae id.2024)

Batch 773 : Gut microbiota abundance (family Peptostreptococcaceae id.2042) on Arthropathy in ulcerative colitis

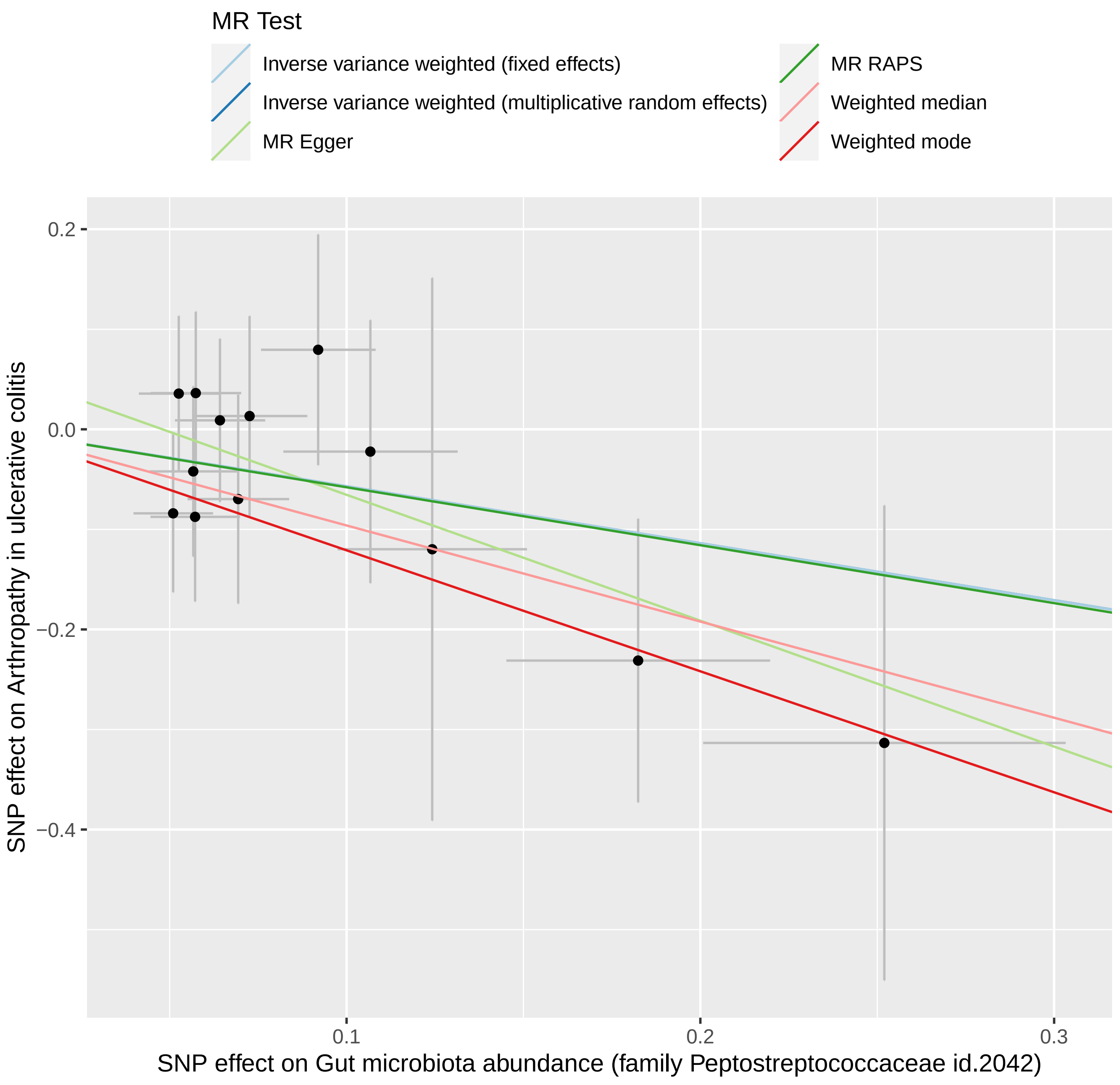

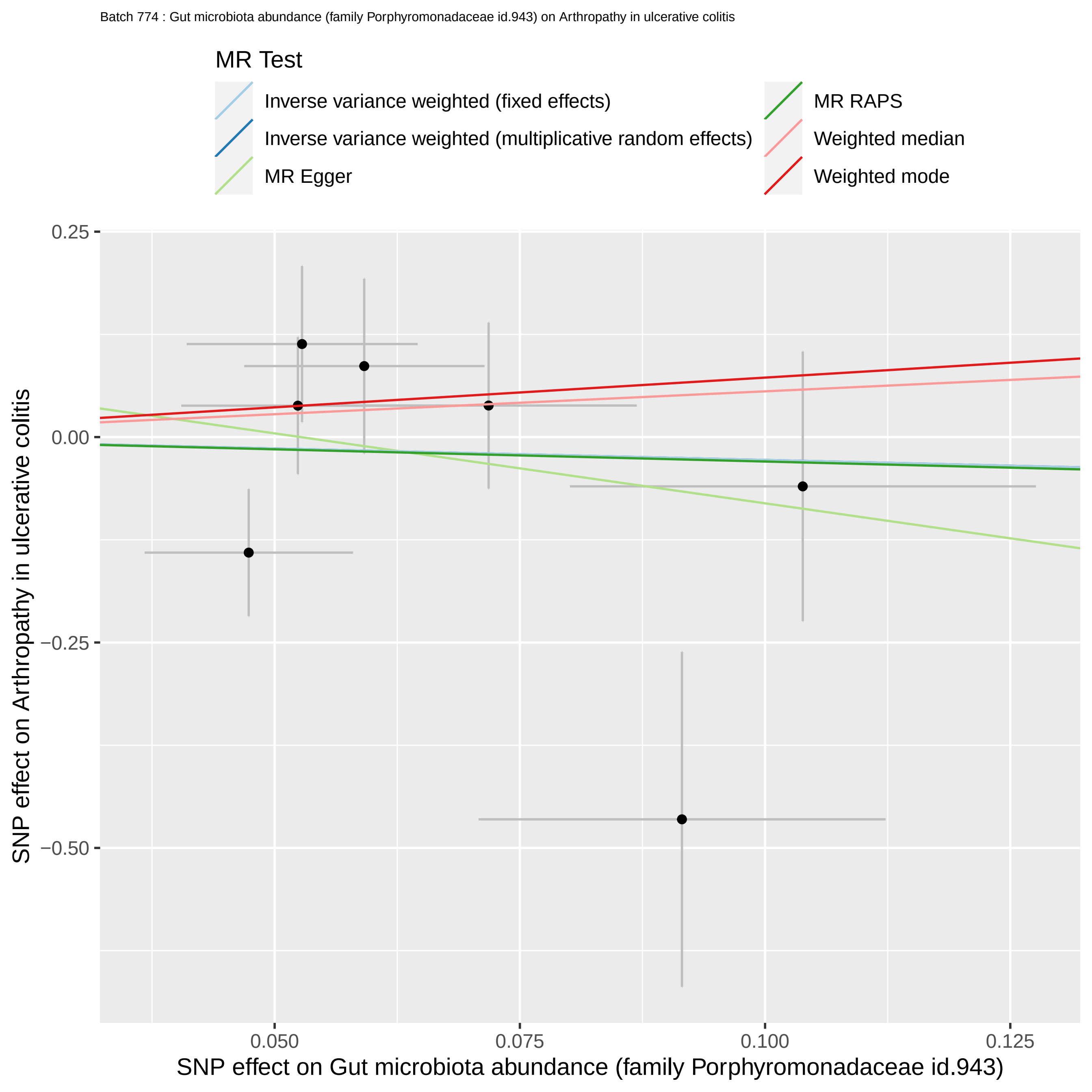

Batch 774 : Gut microbiota abundance (family Porphyromonadaceae id.943) on Arthropathy in ulcerative colitis
MR Test
Inverse variance weighted (fixed effects)
Inverse variance weighted (multiplicative random effects)
MR Egger
MR RAPS
Weighted median
Weighted mode
0.25
0.00
−0.25
−0.50
0.050
0.075
0.100
0.125
SNP effect on Arthropathy in ulcerative colitis
SNP effect on Gut microbiota abundance (family Porphyromonadaceae id.943)

Batch 775 : Gut microbiota abundance (family Prevotellaceae id.960) on Arthropathy in ulcerative colitis

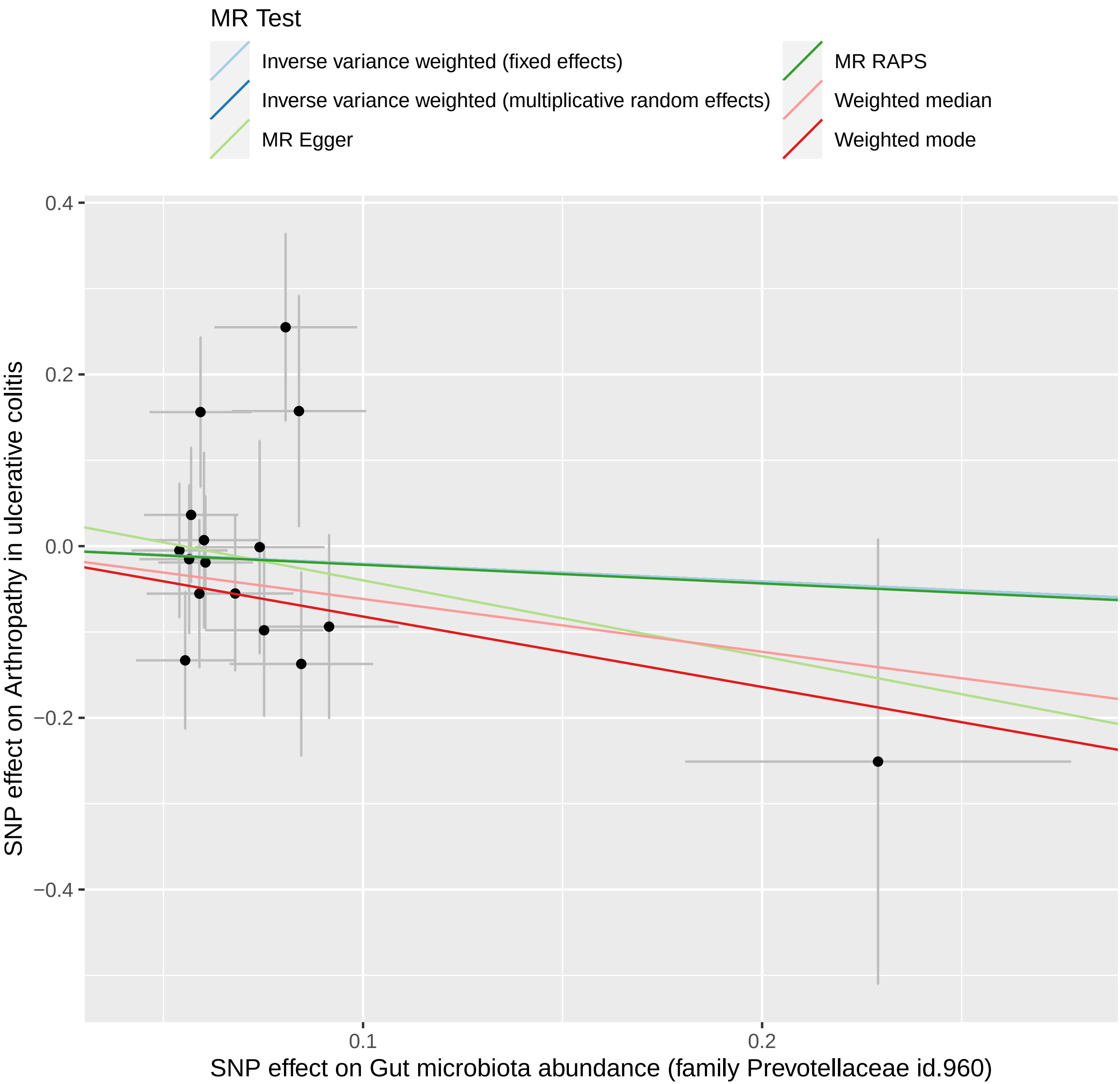

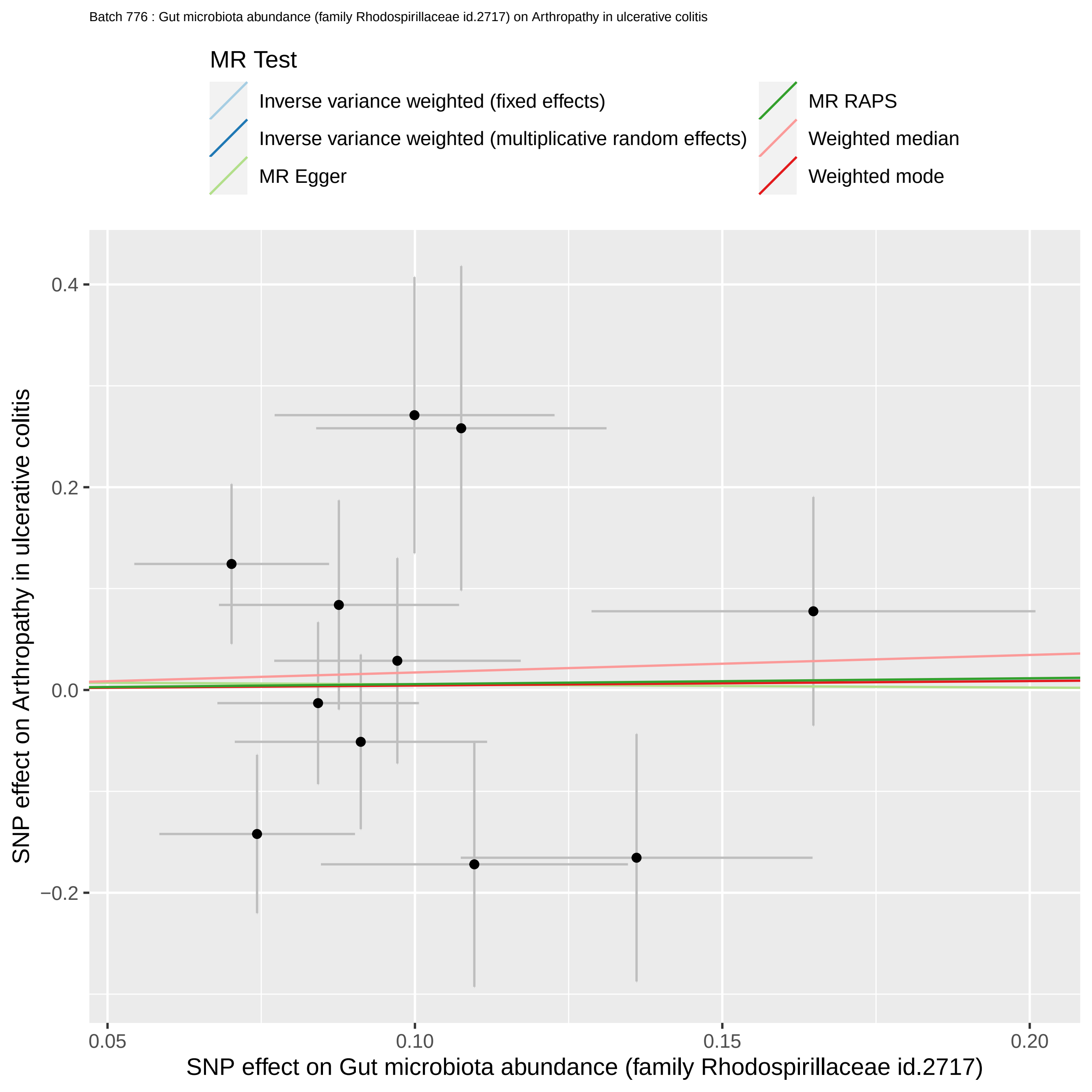
Batch 776 : Gut microbiota abundance (family Rhodospirillaceae id.2717) on Arthropathy in ulcerative colitis
MR Test
Inverse variance weighted (fixed effects)
Inverse variance weighted (multiplicative random effects)
MR Egger
MR RAPS
Weighted median
Weighted mode
0.4
0.2
0.0
−0.2
0.05
0.10
0.15
0.20
SNP effect on Arthropathy in ulcerative colitis
SNP effect on Gut microbiota abundance (family Rhodospirillaceae id.2717)

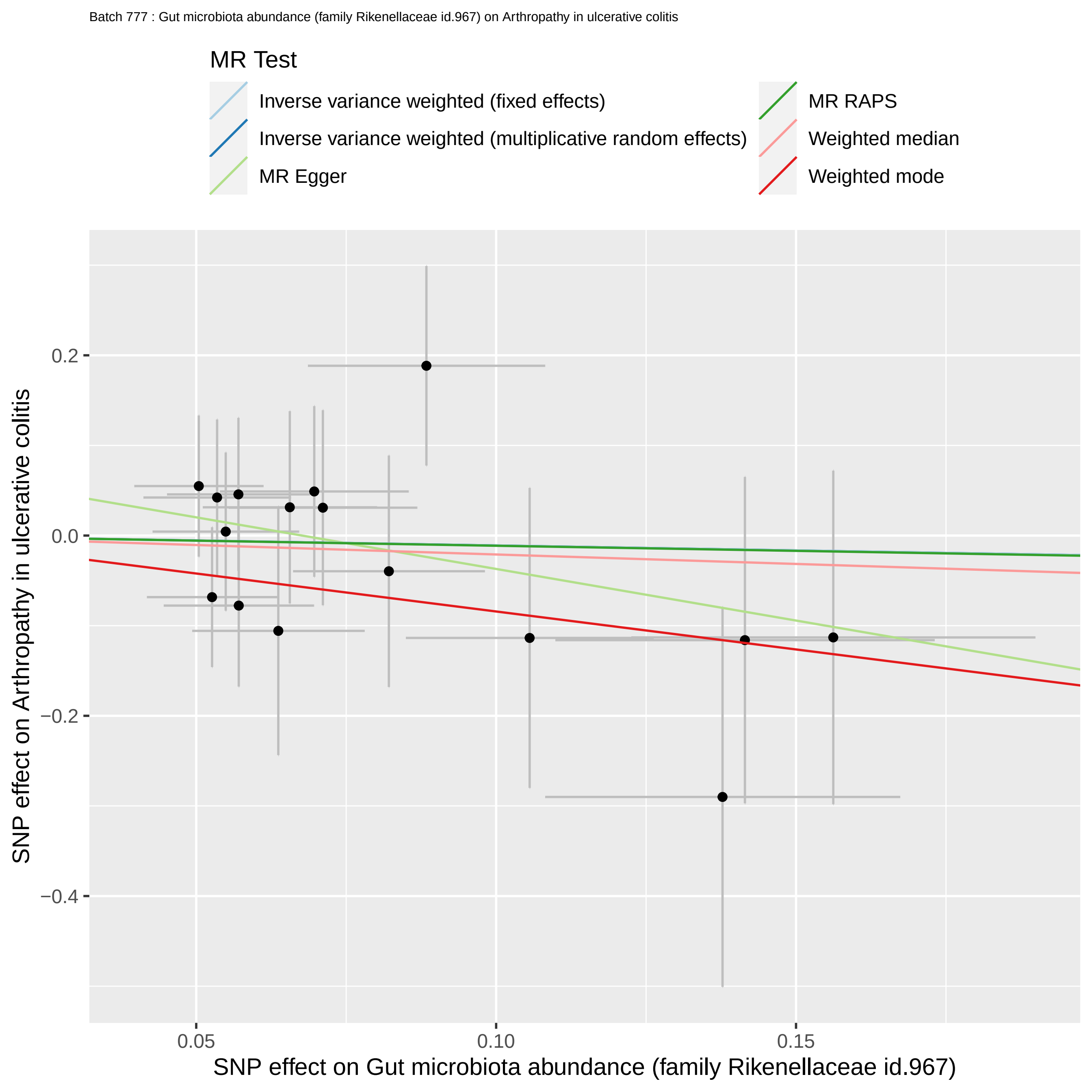
Batch 777 : Gut microbiota abundance (family Rikenellaceae id.967) on Arthropathy in ulcerative colitis
MR Test
Inverse variance weighted (fixed effects)
Inverse variance weighted (multiplicative random effects)
MR Egger
MR RAPS
Weighted median
Weighted mode
0.2
0.0
−0.2
−0.4
0.05
0.10
0.15
SNP effect on Arthropathy in ulcerative colitis
SNP effect on Gut microbiota abundance (family Rikenellaceae id.967)

Batch 778 : Gut microbiota abundance (family Ruminococcaceae id.2050) on Arthropathy in ulcerative colitis

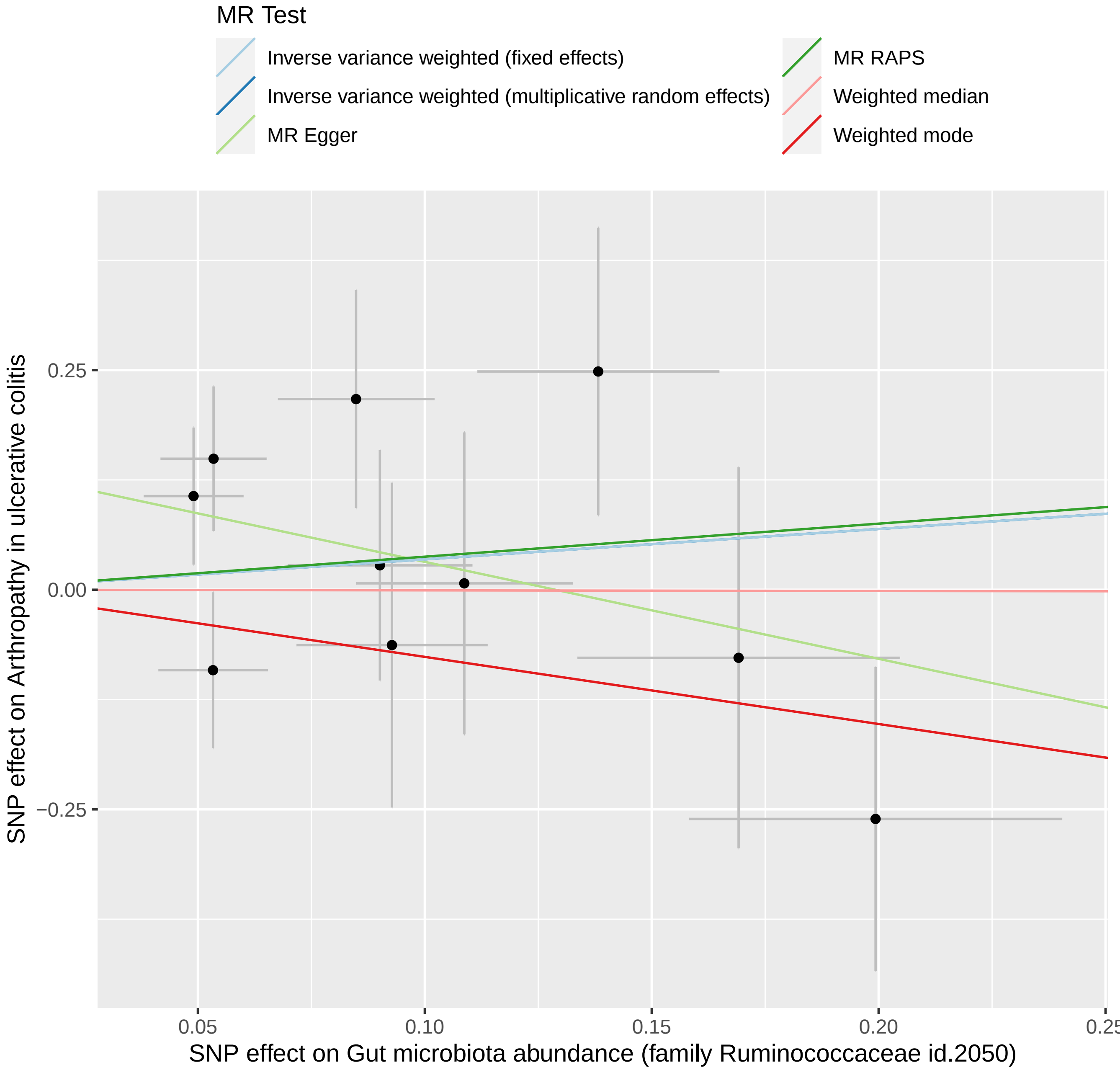

Batch 779 : Gut microbiota abundance (family Streptococcaceae id.1850) on Arthropathy in ulcerative colitis

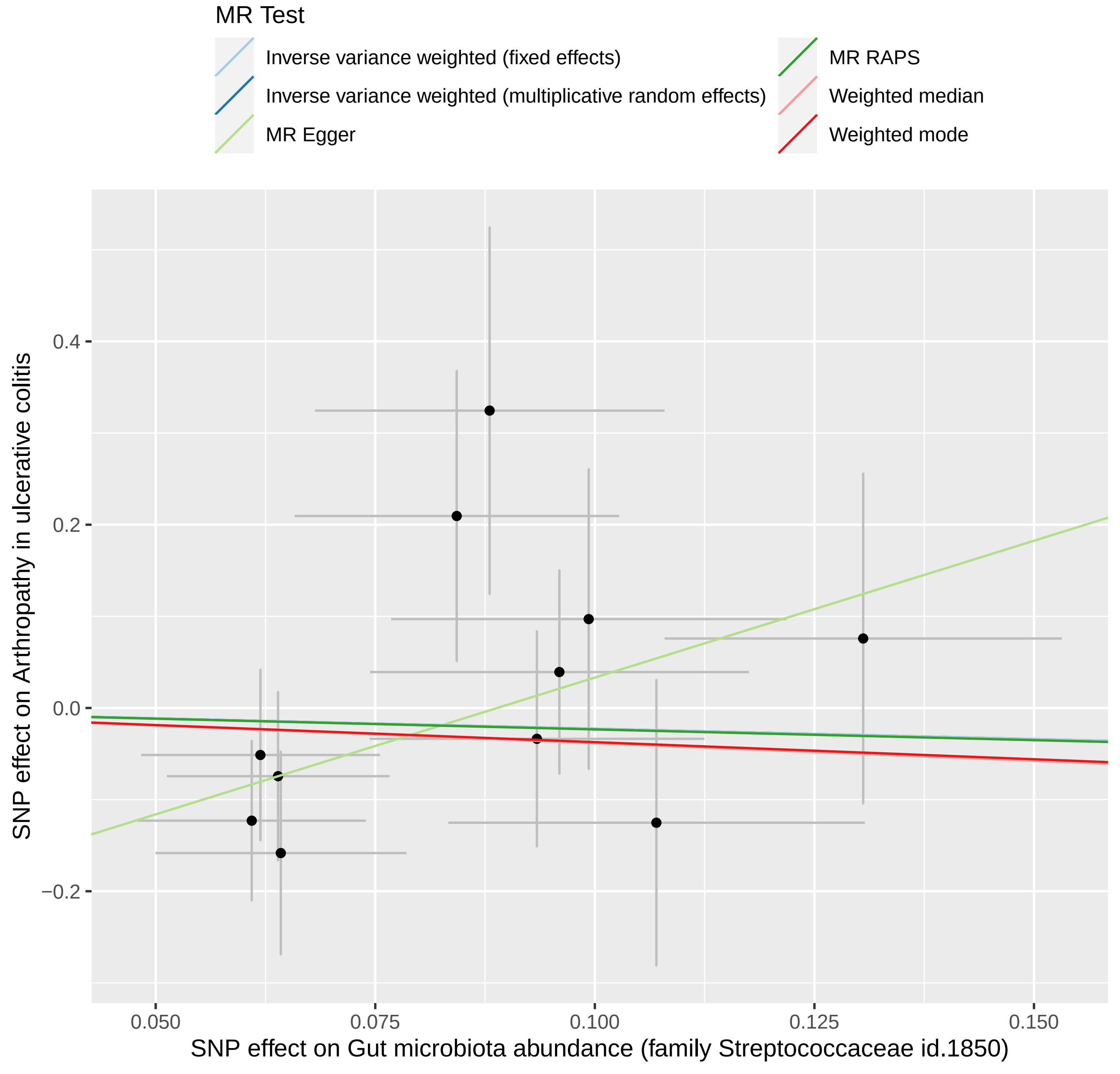

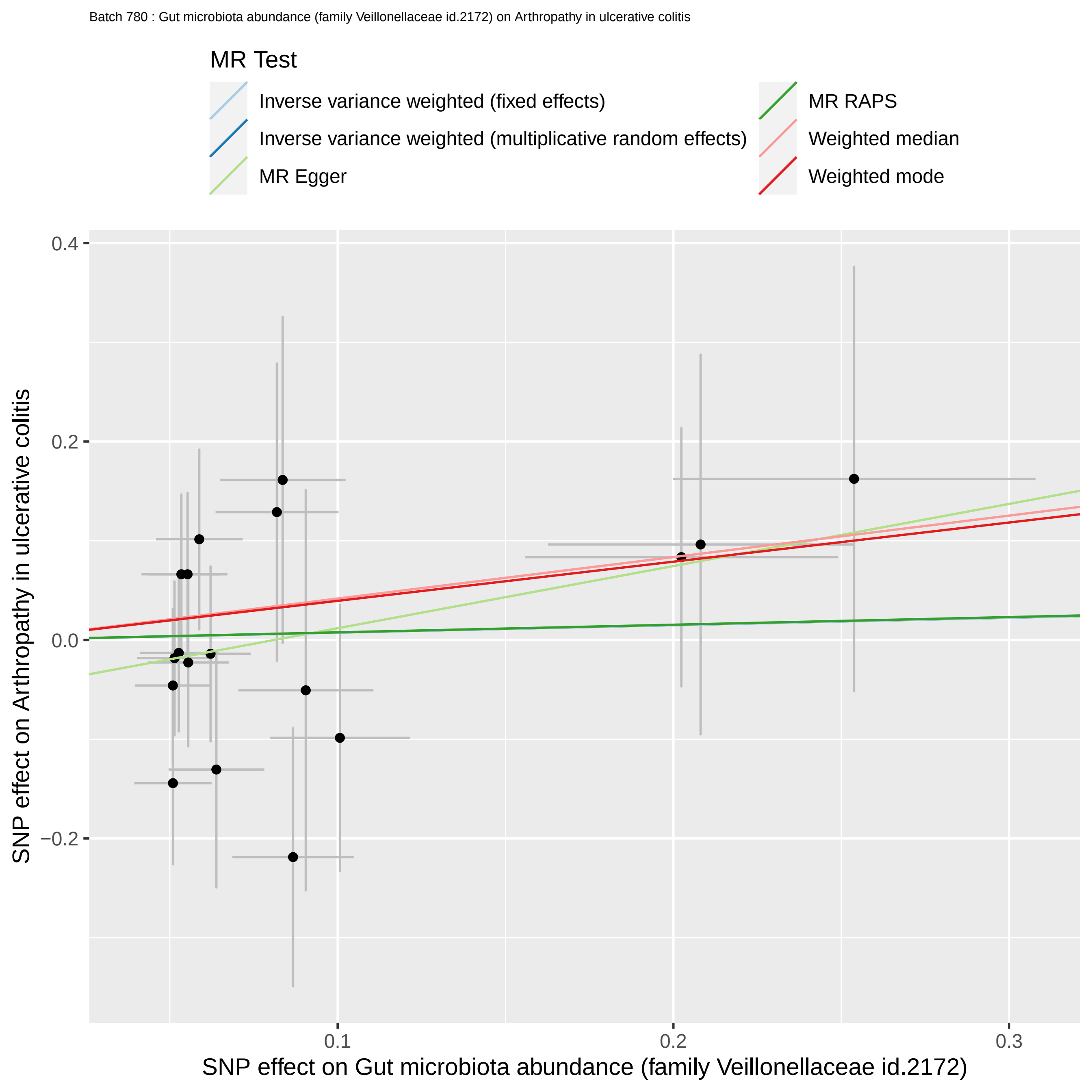

Batch 780 : Gut microbiota abundance (family Veillonellaceae id.2172) on Arthropathy in ulcerative colitis
MR Test
Inverse variance weighted (fixed effects)
Inverse variance weighted (multiplicative random effects)
MR Egger
MR RAPS
Weighted median
Weighted mode
0.4
0.2
0.0
−0.2
0.1
0.2
0.3
SNP effect on Arthropathy in ulcerative colitis
SNP effect on Gut microbiota abundance (family Veillonellaceae id.2172)

Batch 781 : Gut microbiota abundance (family Verrucomicrobiaceae id.4036) on Arthropathy in ulcerative colitis

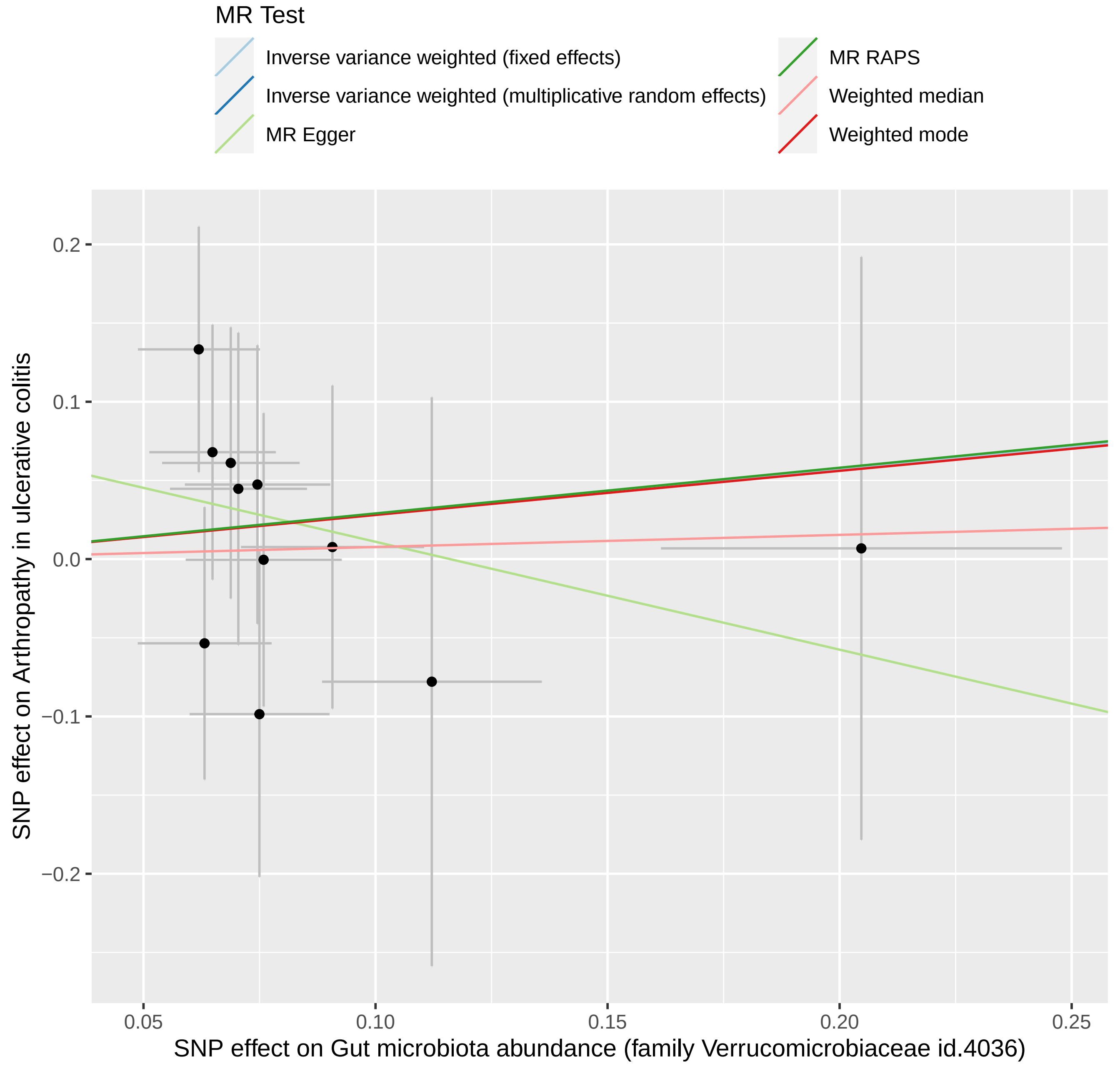

Batch 782 : Gut microbiota abundance (family Victivallaceae id.2255) on Arthropathy in ulcerative colitis

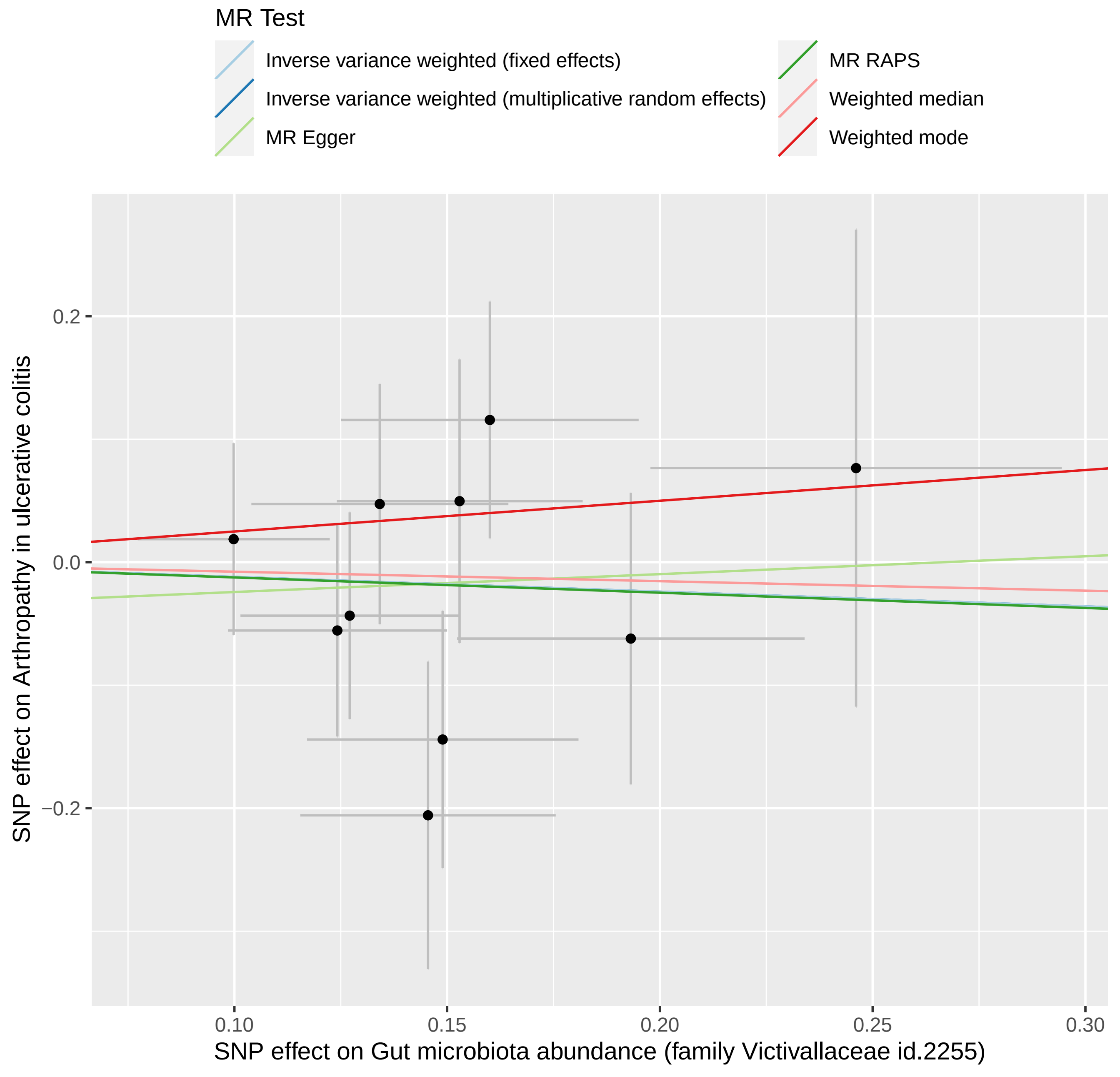

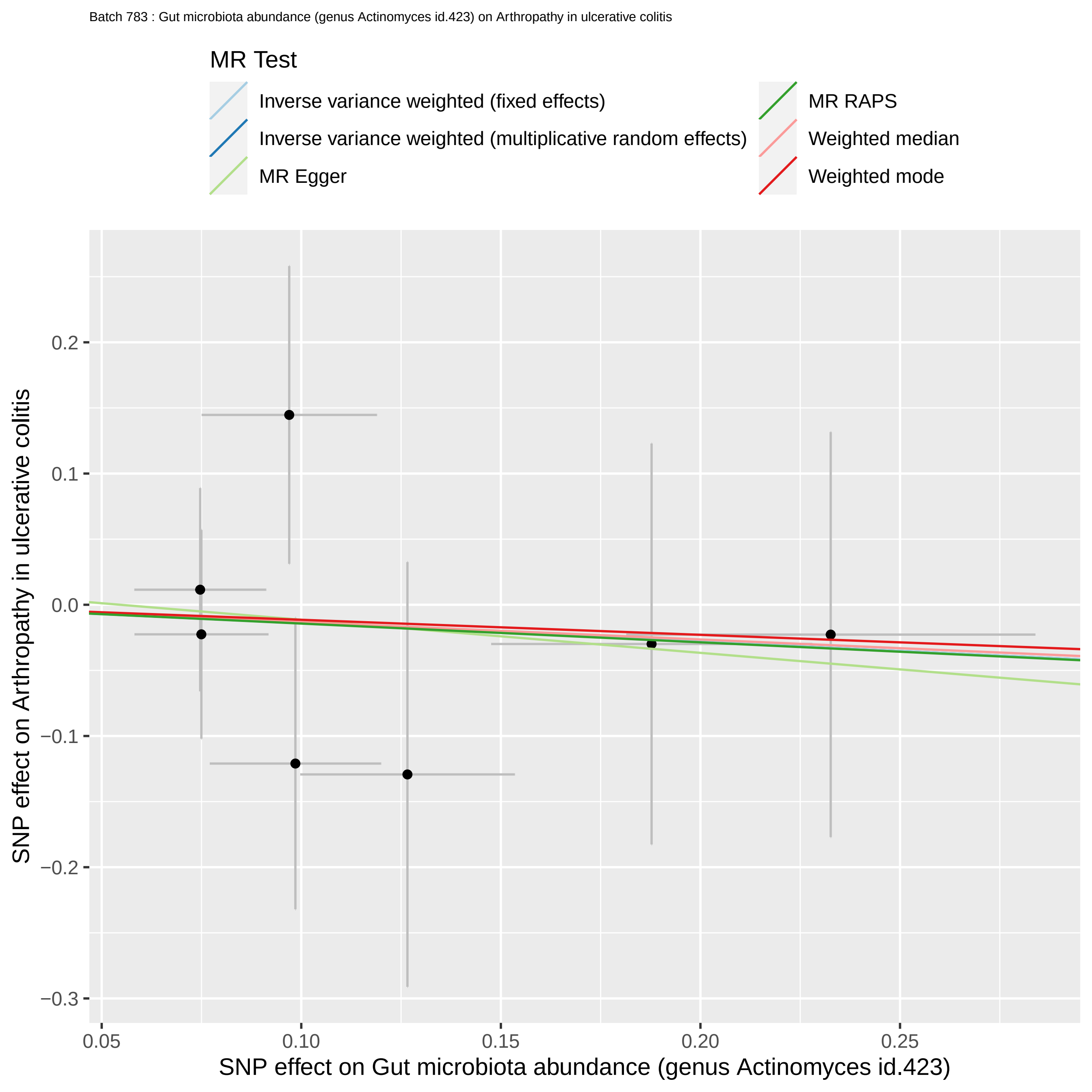

Batch 783 : Gut microbiota abundance (genus Actinomyces id.423) on Arthropathy in ulcerative colitis
MR Test
Inverse variance weighted (fixed effects)
Inverse variance weighted (multiplicative random effects)
MR Egger
MR RAPS
Weighted median
Weighted mode
SNP effect on Arthropathy in ulcerative colitis
0.2
0.1
0.0
−0.1
−0.2
−0.3
0.05
0.10
0.15
0.20
0.25
SNP effect on Gut microbiota abundance (genus Actinomyces id.423)

Batch 784 : Gut microbiota abundance (genus Adlercreutzia id.812) on Arthropathy in ulcerative colitis

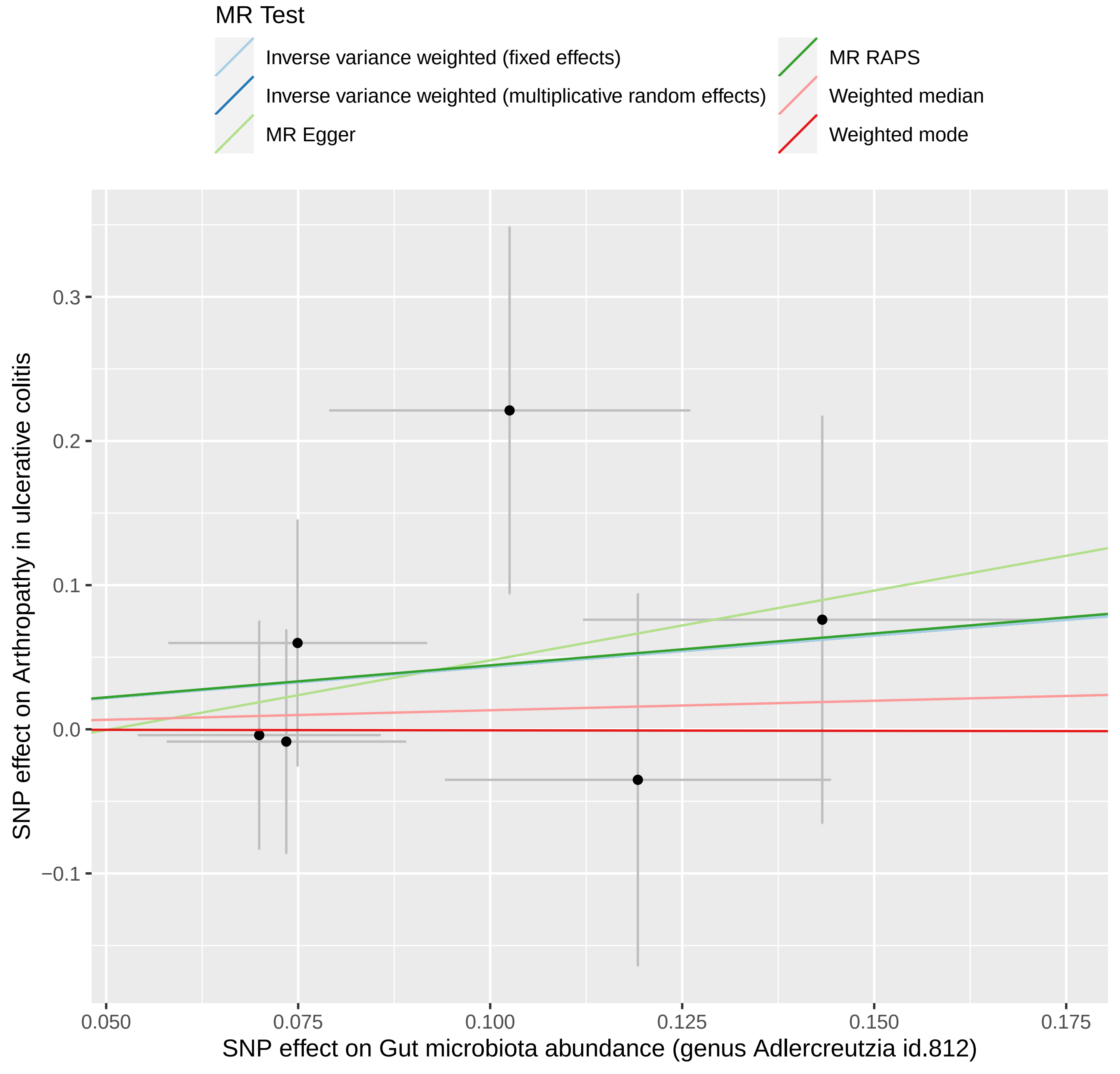

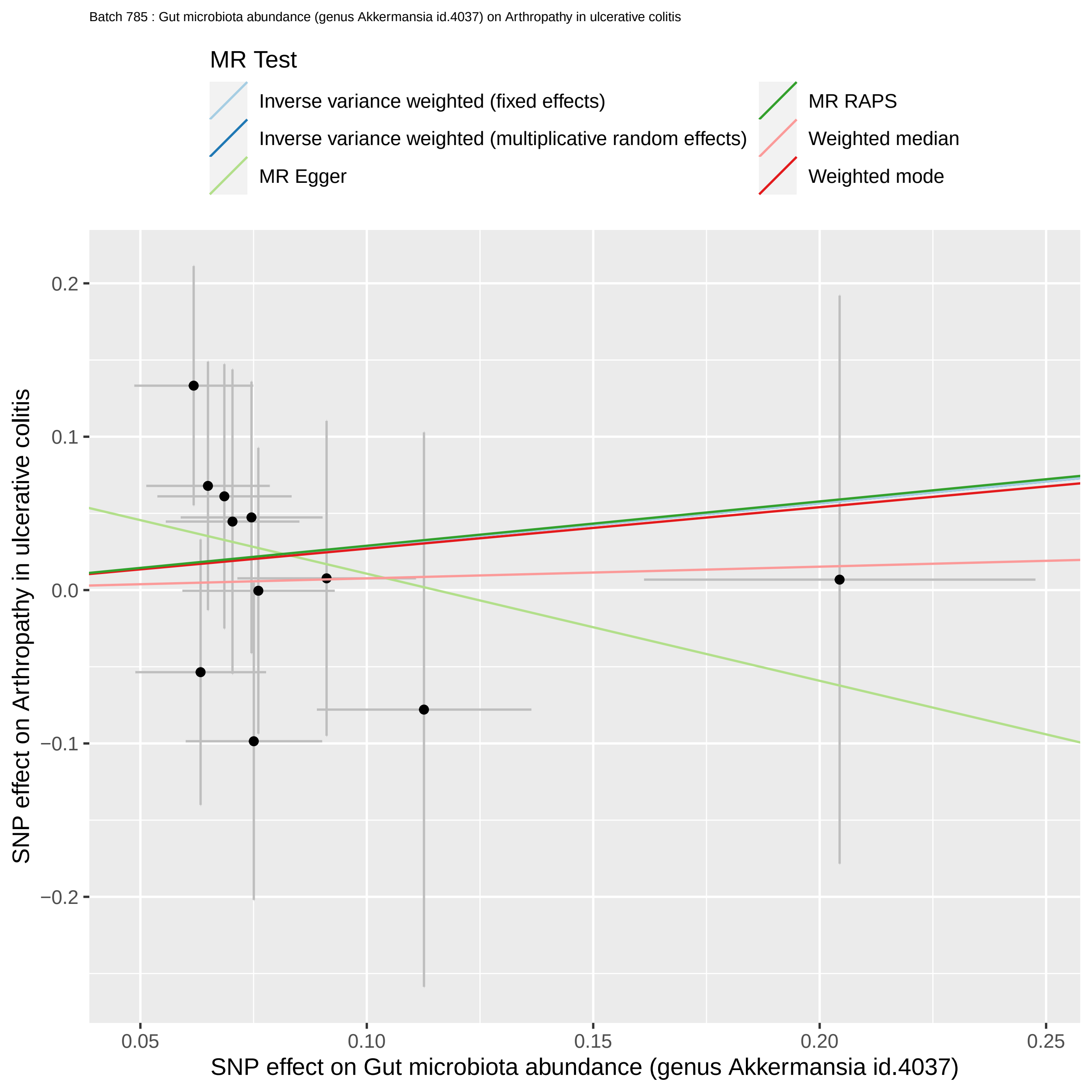
Batch 785 : Gut microbiota abundance (genus Akkermansia id.4037) on Arthropathy in ulcerative colitis
MR Test
Inverse variance weighted (fixed effects)
Inverse variance weighted (multiplicative random effects)
MR Egger
MR RAPS
Weighted median
Weighted mode
SNP effect on Arthropathy in ulcerative colitis
0.2
0.1
0.0
−0.1
−0.2
0.05
0.10
0.15
0.20
0.25
SNP effect on Gut microbiota abundance (genus Akkermansia id.4037)

Batch 786 : Gut microbiota abundance (genus Alistipes id.968) on Arthropathy in ulcerative colitis

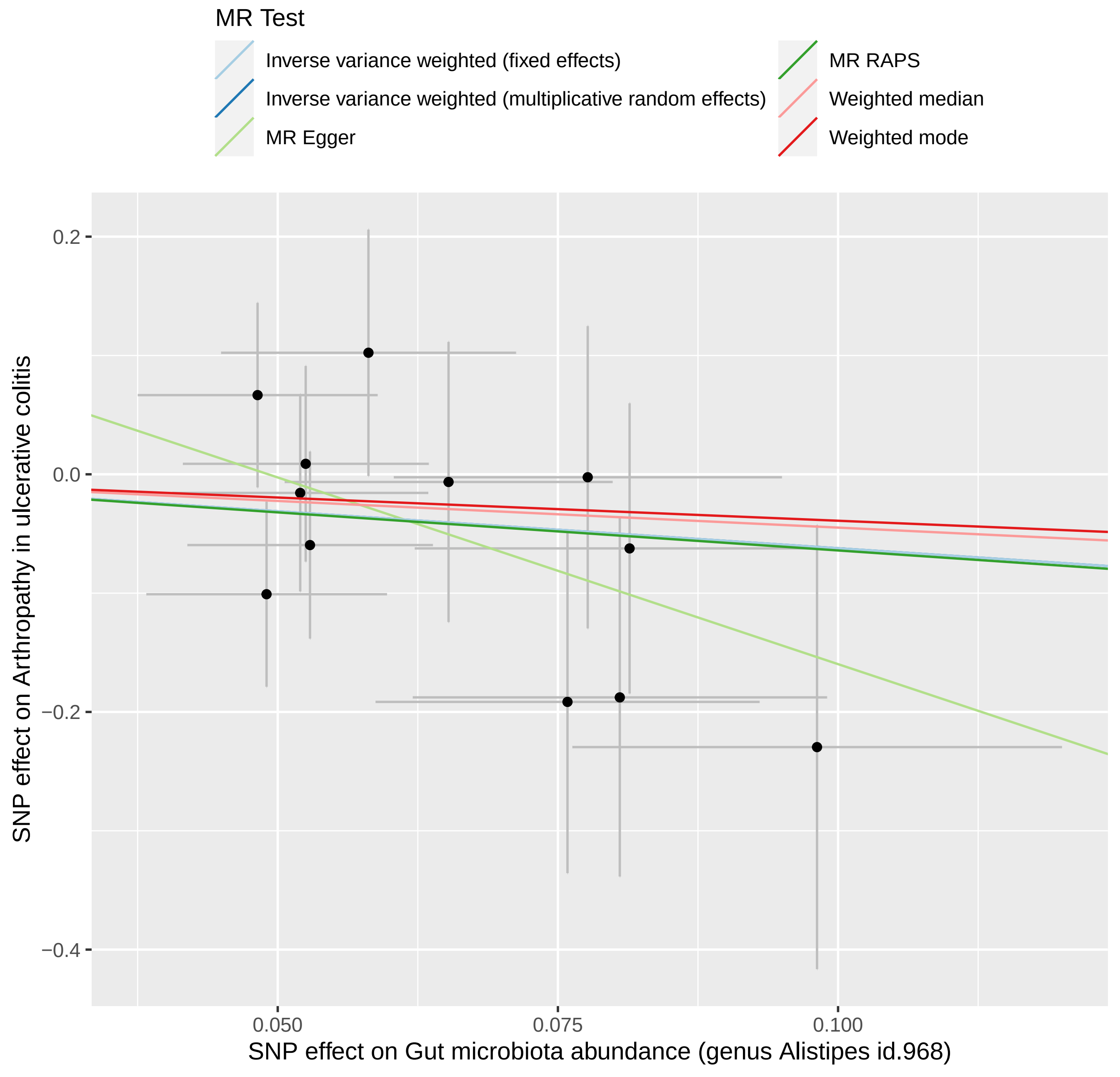

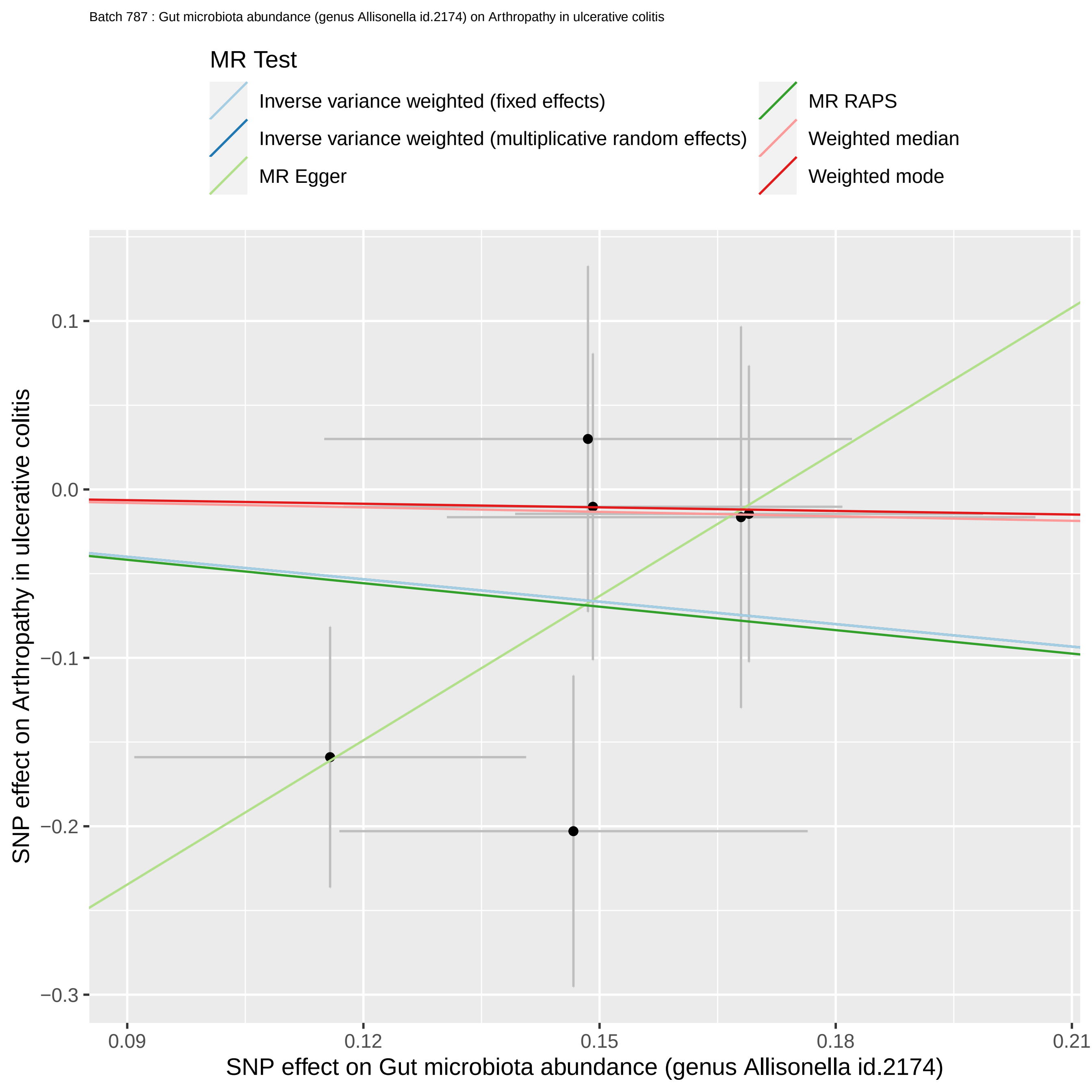
Batch 787 : Gut microbiota abundance (genus Allisonella id.2174) on Arthropathy in ulcerative colitis
MR Test
Inverse variance weighted (fixed effects)
Inverse variance weighted (multiplicative random effects)
MR Egger
MR RAPS
Weighted median
Weighted mode
0.1
0.0
−0.1
−0.2
−0.3
0.09
0.12
0.15
0.18
0.21
SNP effect on Arthropathy in ulcerative colitis
SNP effect on Gut microbiota abundance (genus Allisonella id.2174)

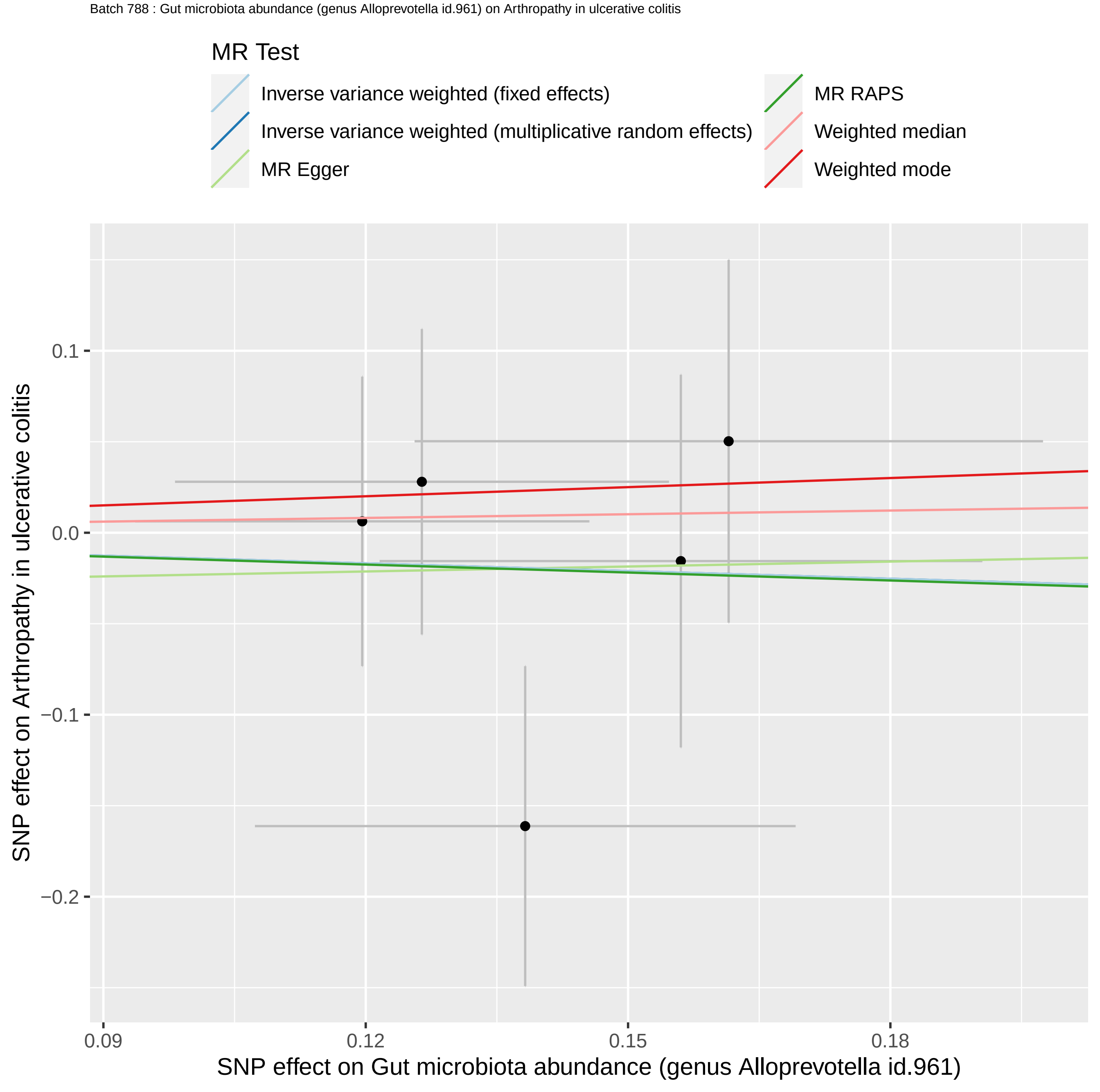

Batch 788 : Gut microbiota abundance (genus Alloprevotella id.961) on Arthropathy in ulcerative colitis
MR Test
Inverse variance weighted (fixed effects)
Inverse variance weighted (multiplicative random effects)
MR Egger
MR RAPS
Weighted median
Weighted mode
0.1
0.0
−0.1
−0.2
0.09
0.12
0.15
0.18
SNP effect on Arthropathy in ulcerative colitis
SNP effect on Gut microbiota abundance (genus Alloprevotella id.961)

Batch 789 : Gut microbiota abundance (genus Anaerofilum id.2053) on Arthropathy in ulcerative colitis

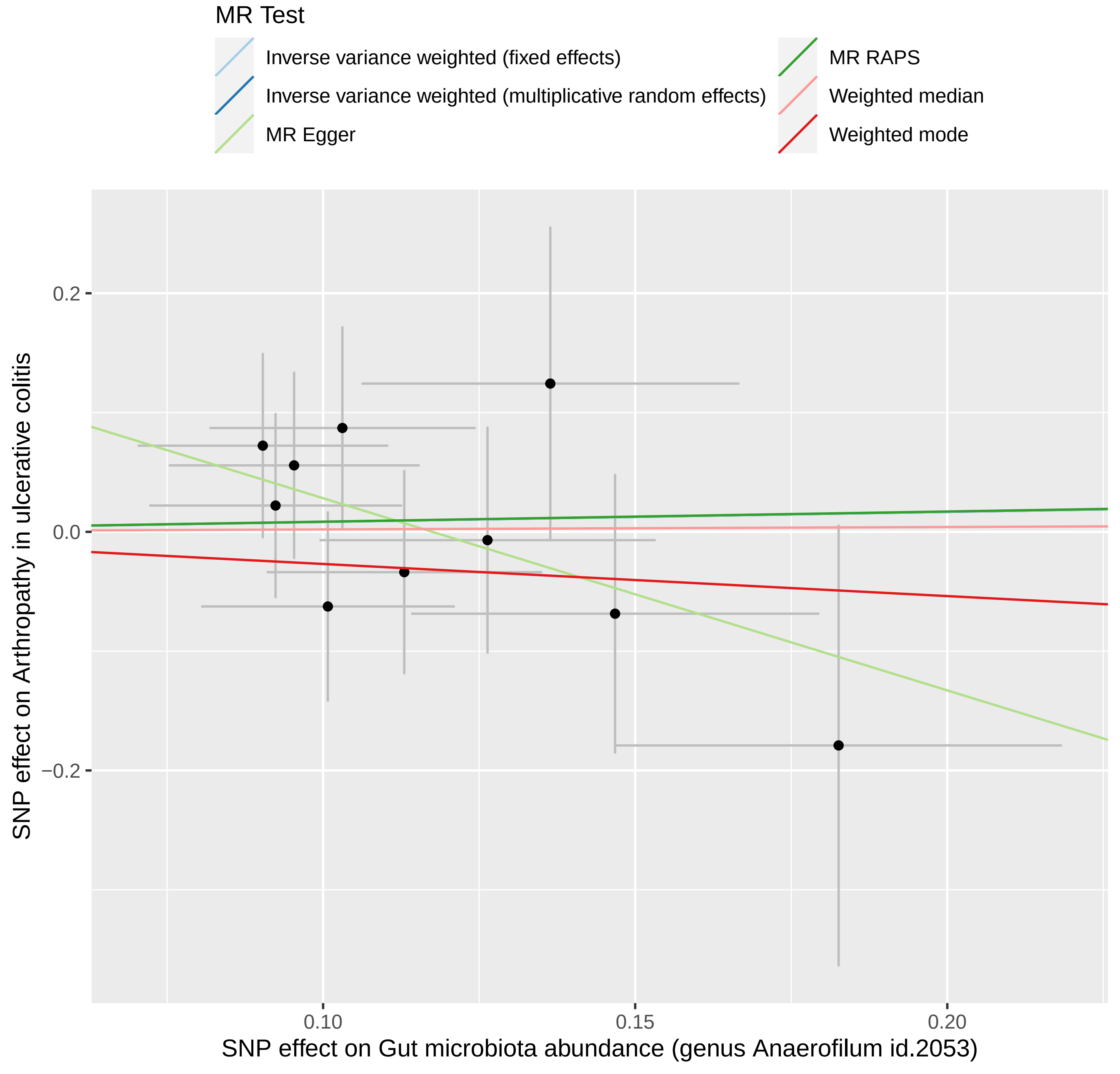

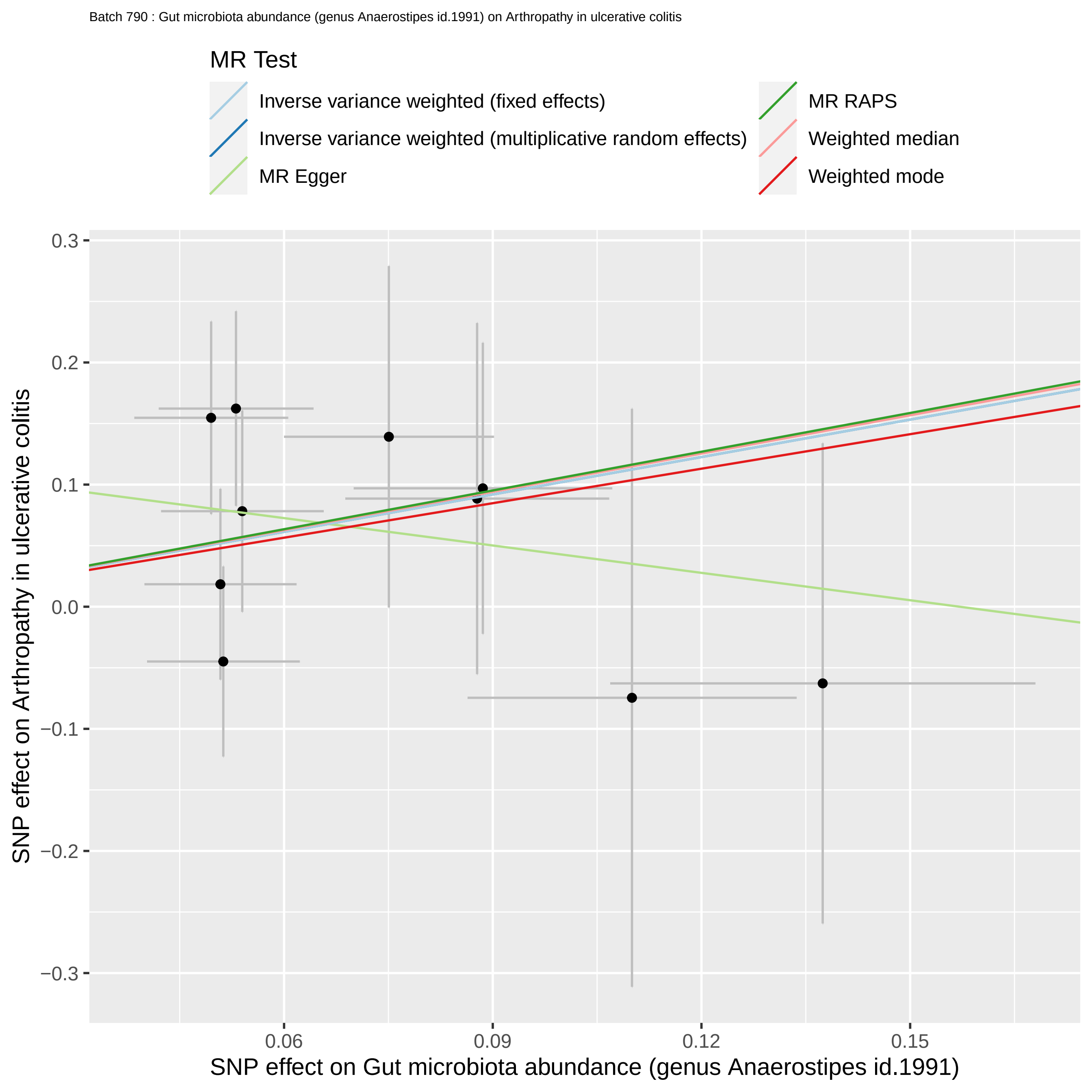

Batch 790 : Gut microbiota abundance (genus Anaerostipes id.1991) on Arthropathy in ulcerative colitis
MR Test
Inverse variance weighted (fixed effects)
Inverse variance weighted (multiplicative random effects)
MR Egger
MR RAPS
Weighted median
Weighted mode
0.3
0.2
0.1
0.0
−0.1
−0.2
−0.3
0.06
0.09
0.12
0.15
SNP effect on Arthropathy in ulcerative colitis
SNP effect on Gut microbiota abundance (genus Anaerostipes id.1991)

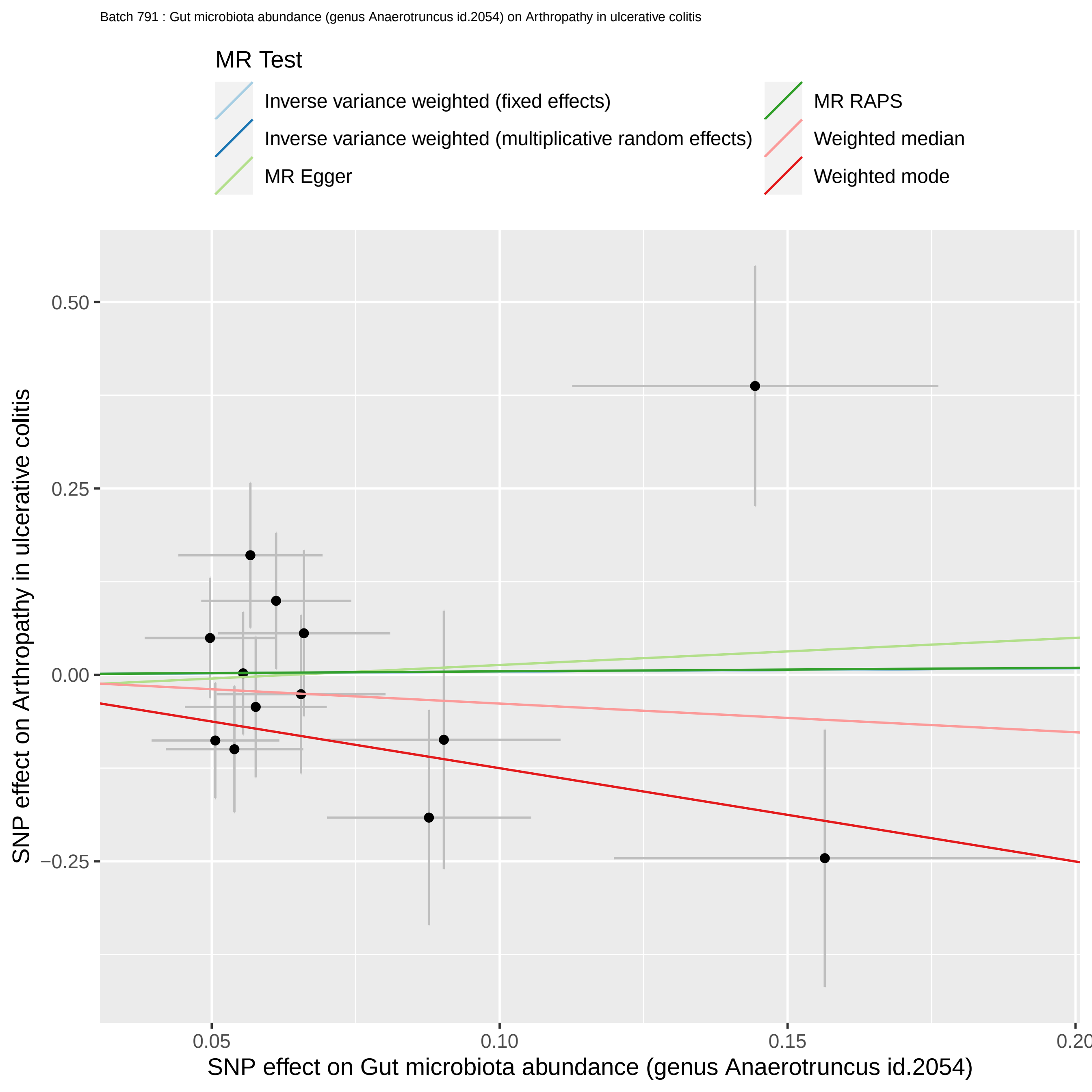
Batch 791 : Gut microbiota abundance (genus Anaerotruncus id.2054) on Arthropathy in ulcerative colitis
MR Test
Inverse variance weighted (fixed effects)
Inverse variance weighted (multiplicative random effects)
MR Egger
MR RAPS
Weighted median
Weighted mode
0.50
0.25
0.00
−0.25
0.05
0.10
0.15
0.20
SNP effect on Arthropathy in ulcerative colitis
SNP effect on Gut microbiota abundance (genus Anaerotruncus id.2054)

Batch 792 : Gut microbiota abundance (genus Bacteroides id.918) on Arthropathy in ulcerative colitis

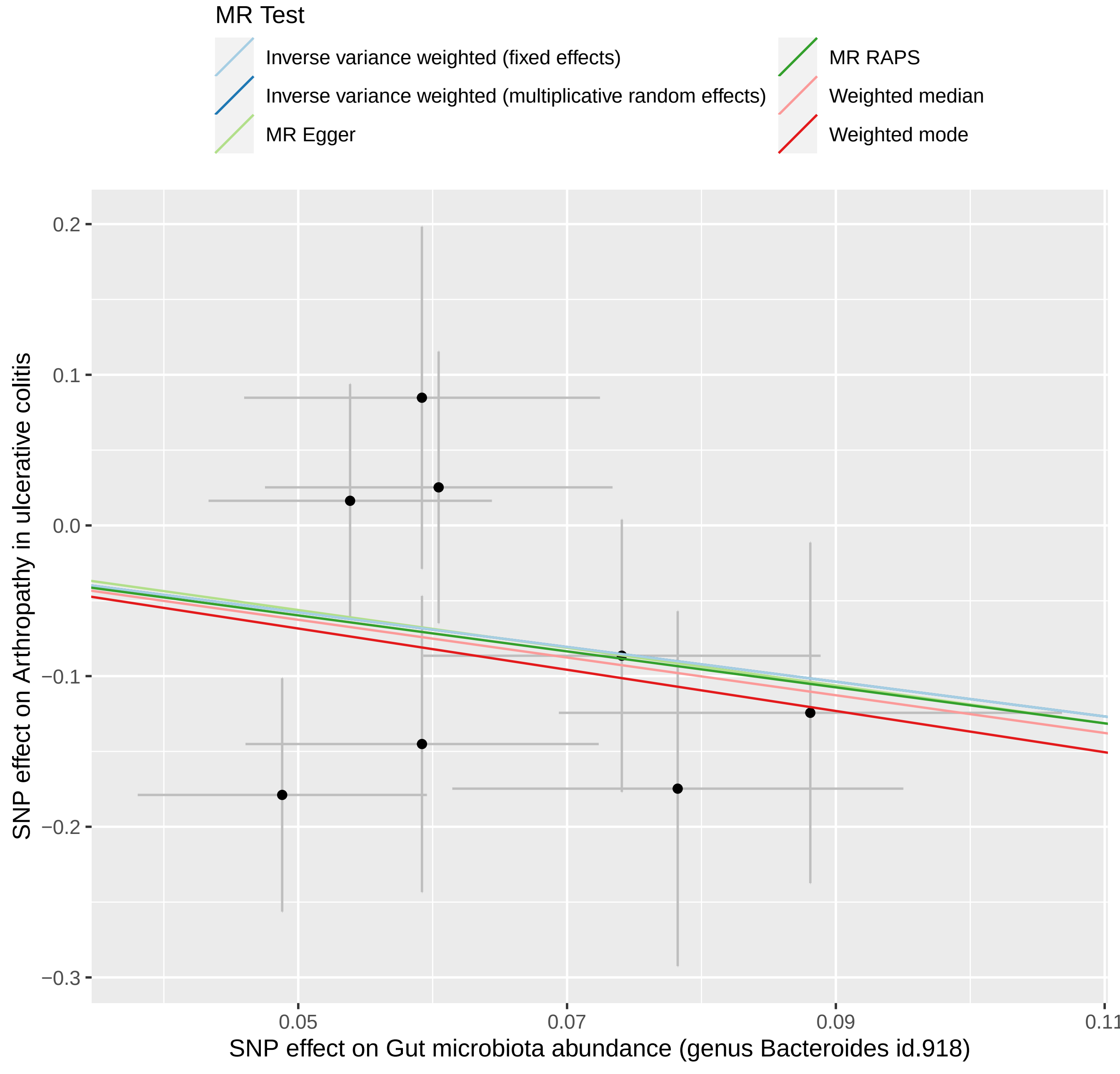

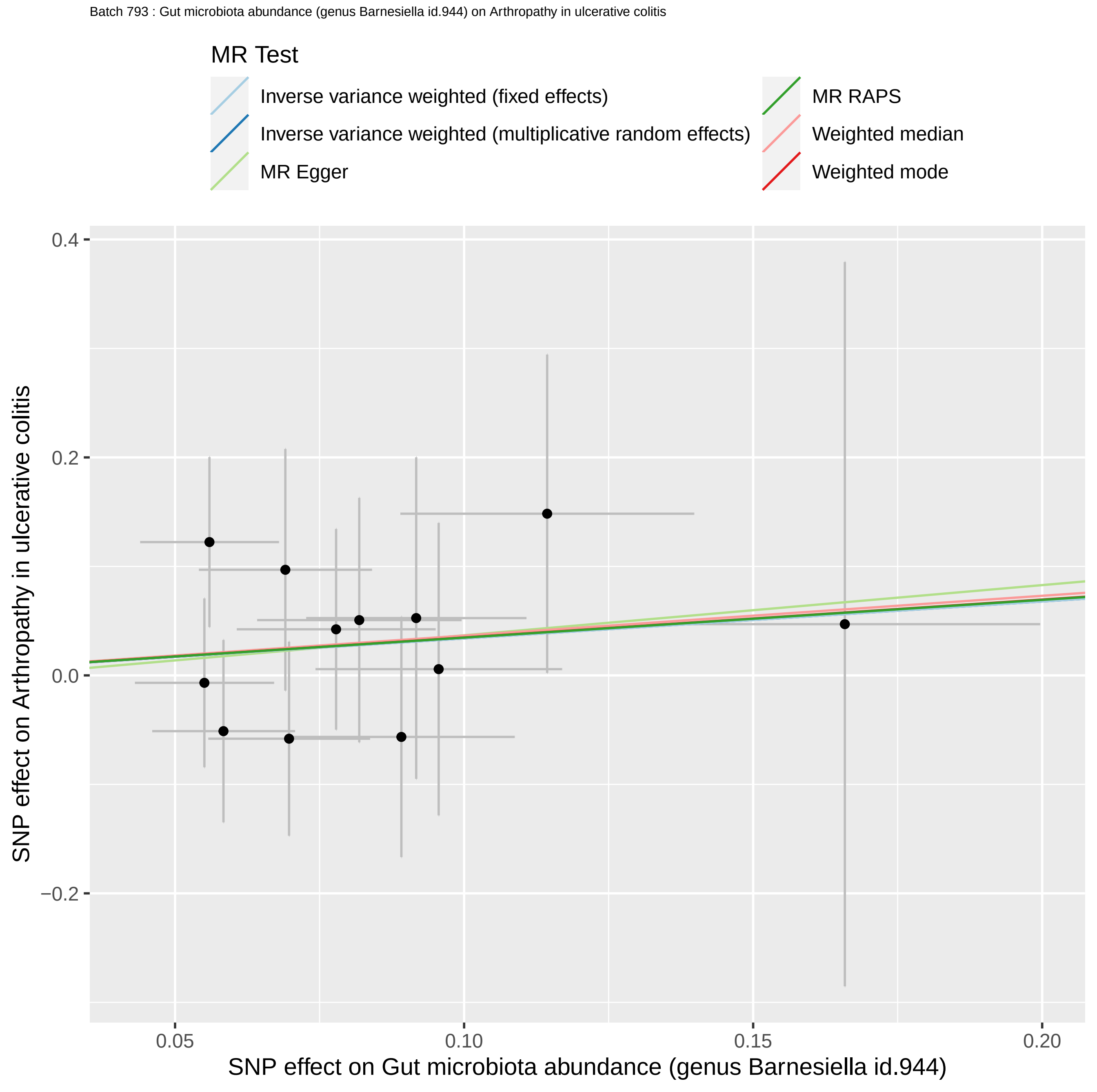

Batch 793 : Gut microbiota abundance (genus Barnesiella id.944) on Arthropathy in ulcerative colitis
MR Test
Inverse variance weighted (fixed effects)
Inverse variance weighted (multiplicative random effects)
MR Egger
MR RAPS
Weighted median
Weighted mode
0.4
0.2
0.0
−0.2
0.05
0.10
0.15
0.20
SNP effect on Arthropathy in ulcerative colitis
SNP effect on Gut microbiota abundance (genus Barnesiella id.944)

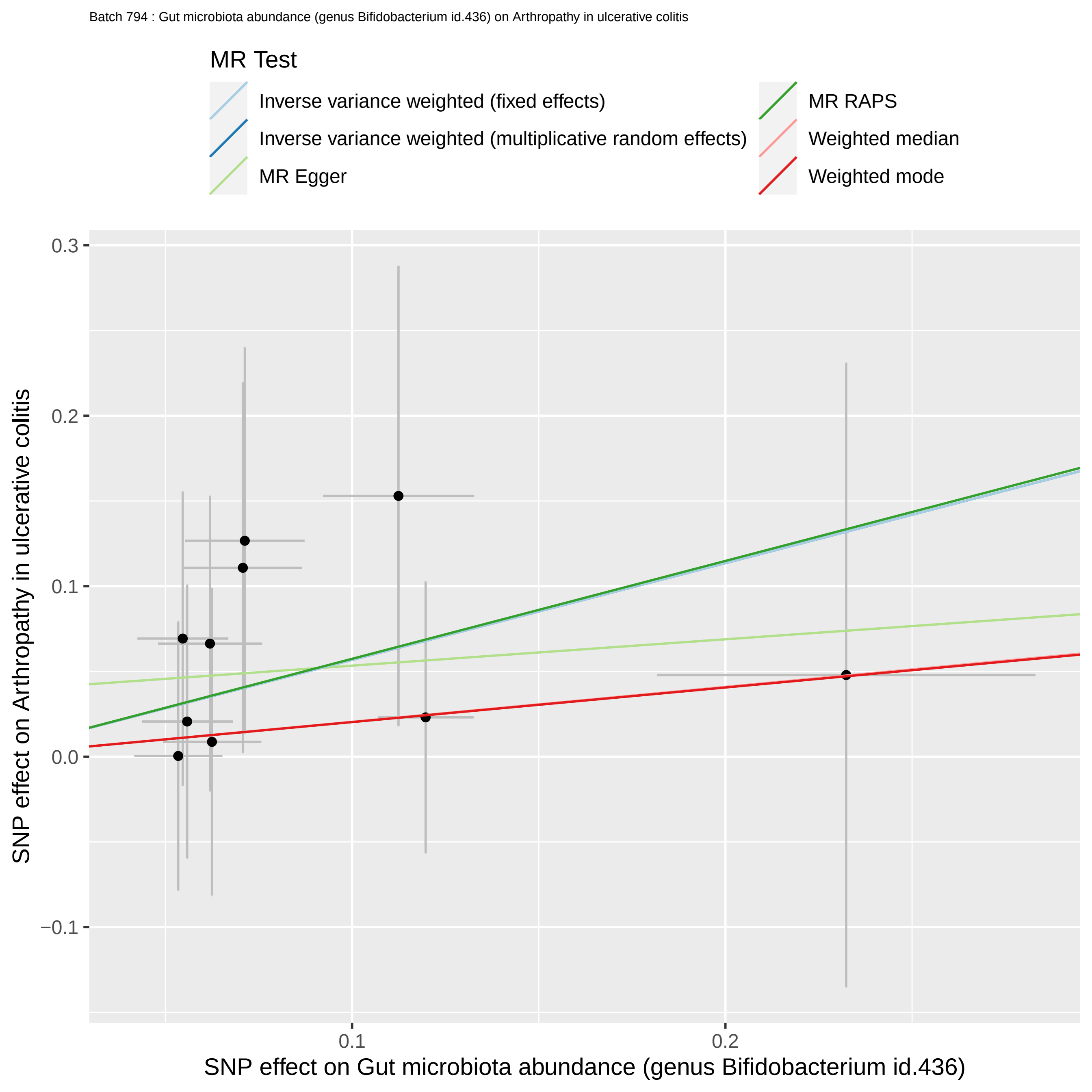

Batch 794 : Gut microbiota abundance (genus Bifidobacterium id.436) on Arthropathy in ulcerative colitis
MR Test
Inverse variance weighted (fixed effects)
Inverse variance weighted (multiplicative random effects)
MR Egger
MR RAPS
Weighted median
Weighted mode
0.3
0.2
0.1
0.0
−0.1
0.1
0.2
SNP effect on Arthropathy in ulcerative colitis
SNP effect on Gut microbiota abundance (genus Bifidobacterium id.436)

Batch 795 : Gut microbiota abundance (genus Bilophila id.3170) on Arthropathy in ulcerative colitis

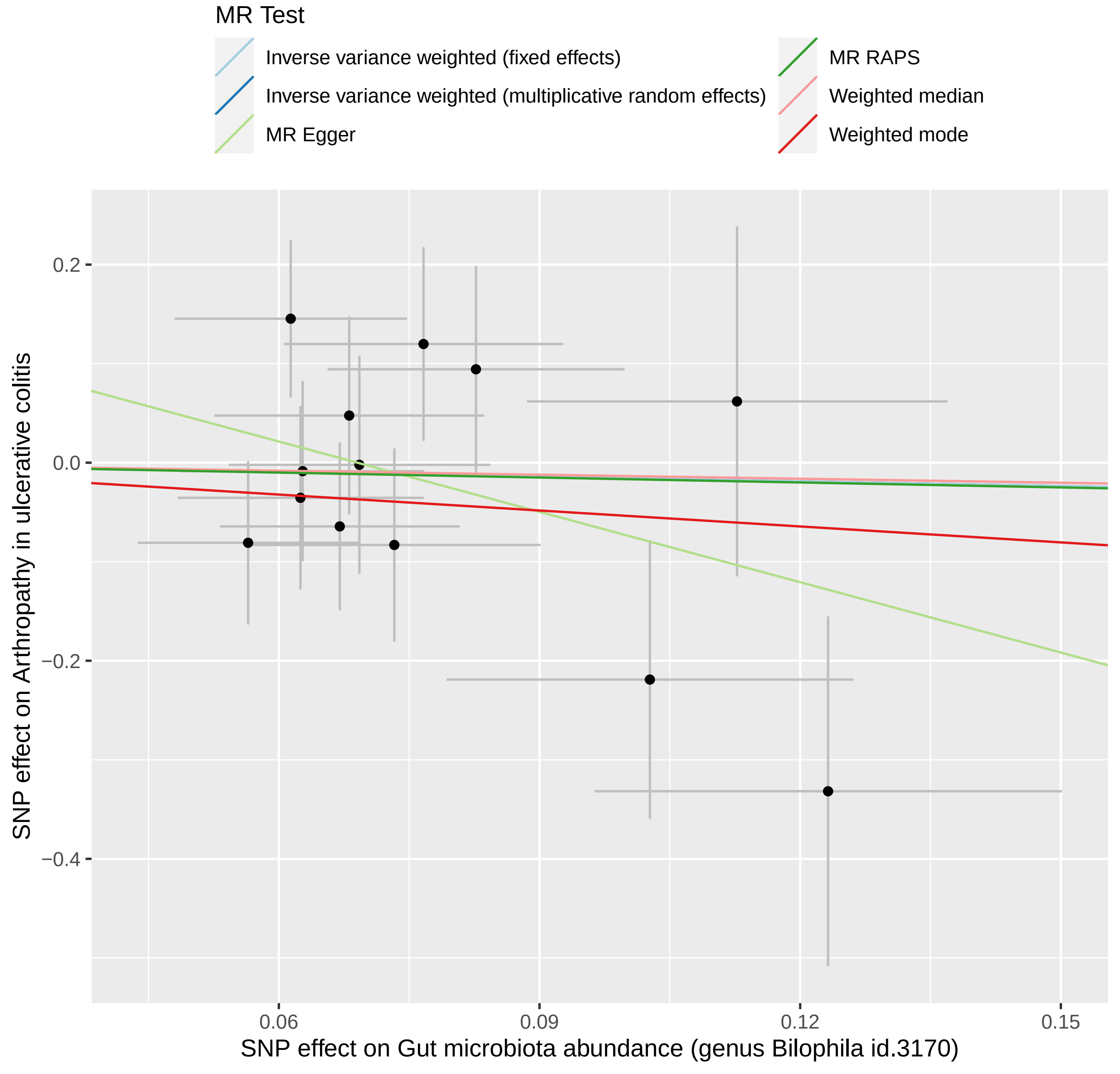

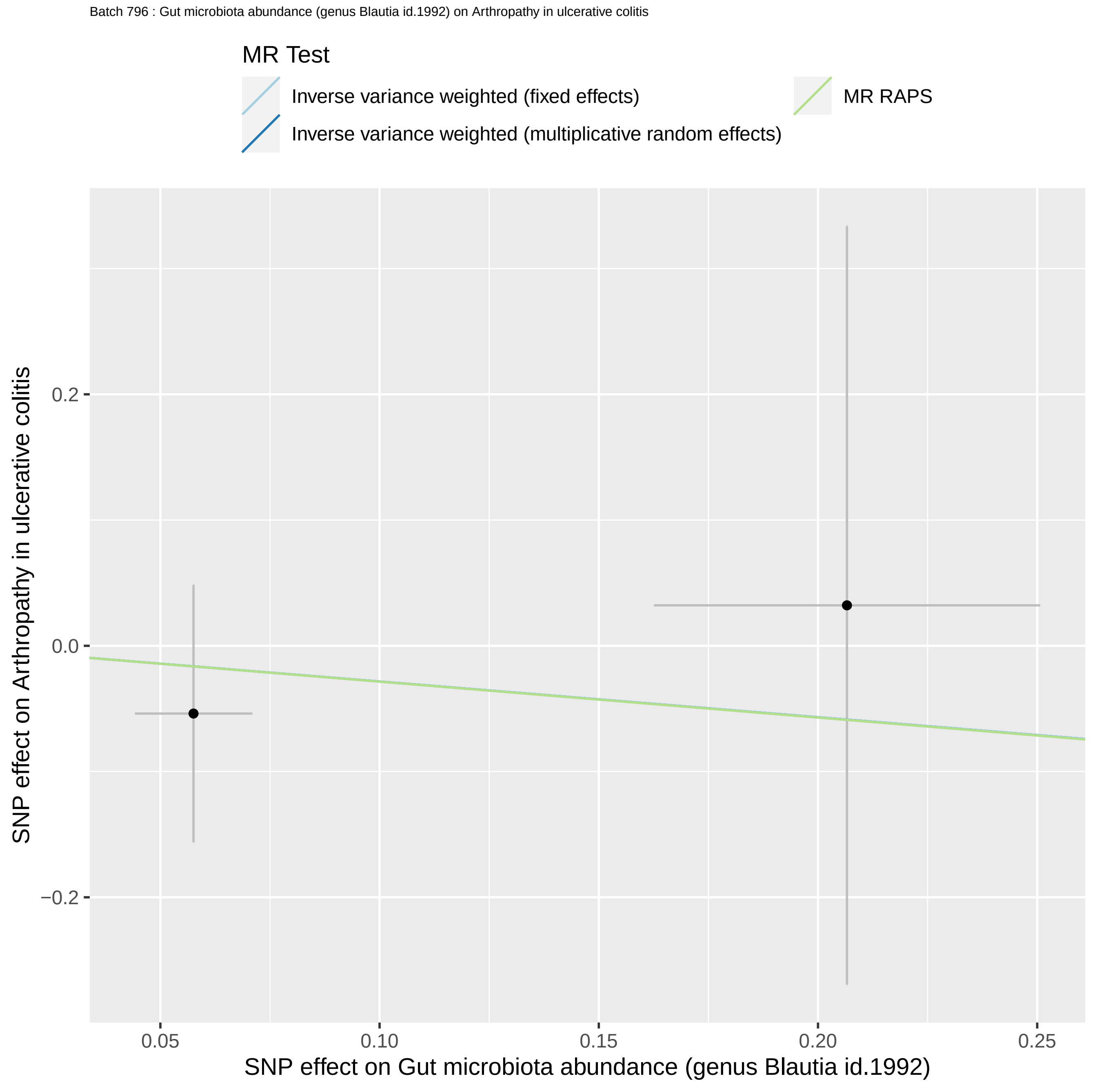

Batch 796 : Gut microbiota abundance (genus Blautia id.1992) on Arthropathy in ulcerative colitis
MR Test
Inverse variance weighted (fixed effects)
Inverse variance weighted (multiplicative random effects)
MR RAPS
0.2
0.0
−0.2
0.05
0.10
0.15
0.20
0.25
SNP effect on Arthropathy in ulcerative colitis
SNP effect on Gut microbiota abundance (genus Blautia id.1992)

Batch 797 : Gut microbiota abundance (genus Butyricicoccus id.2055) on Arthropathy in ulcerative colitis

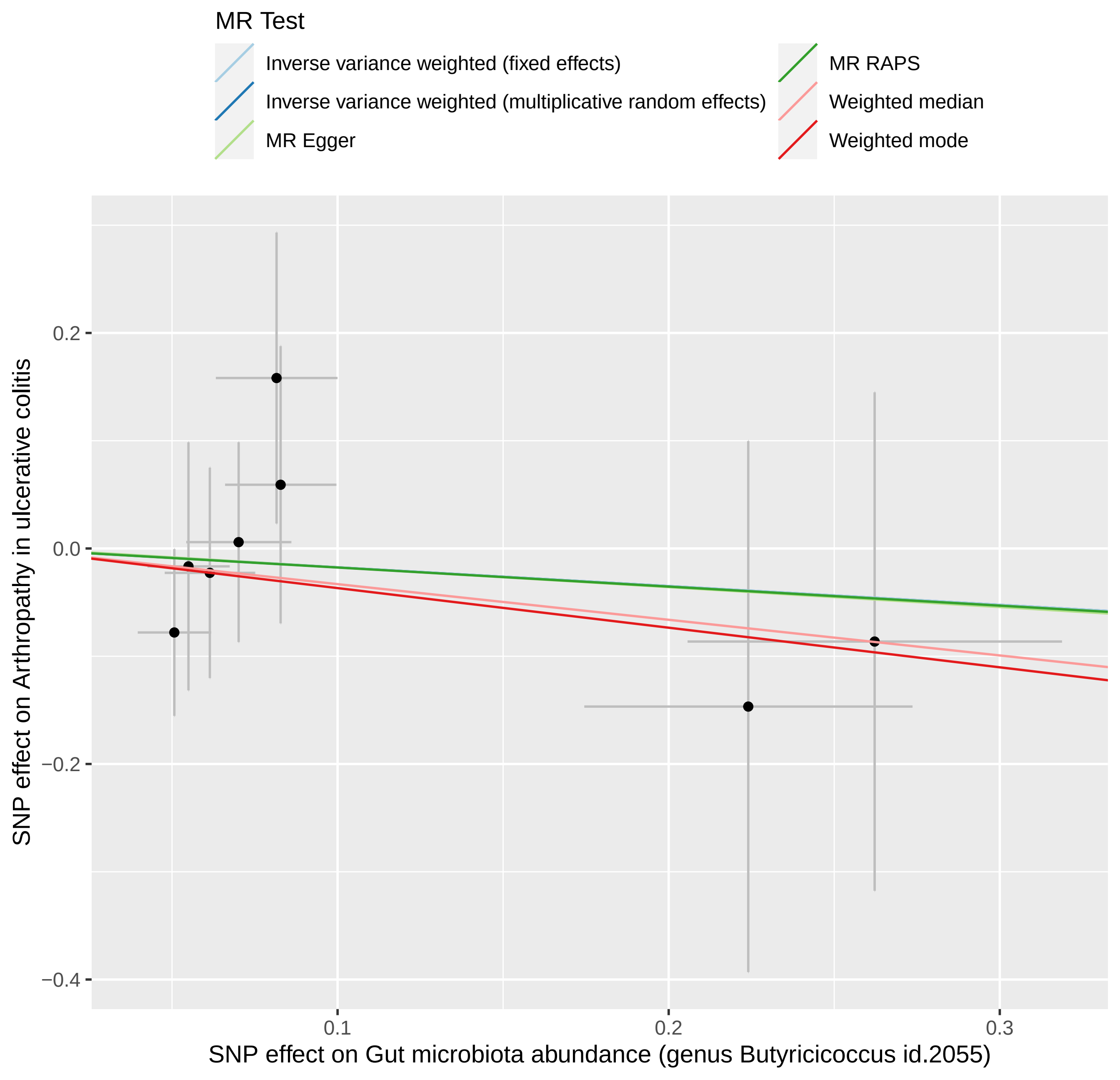

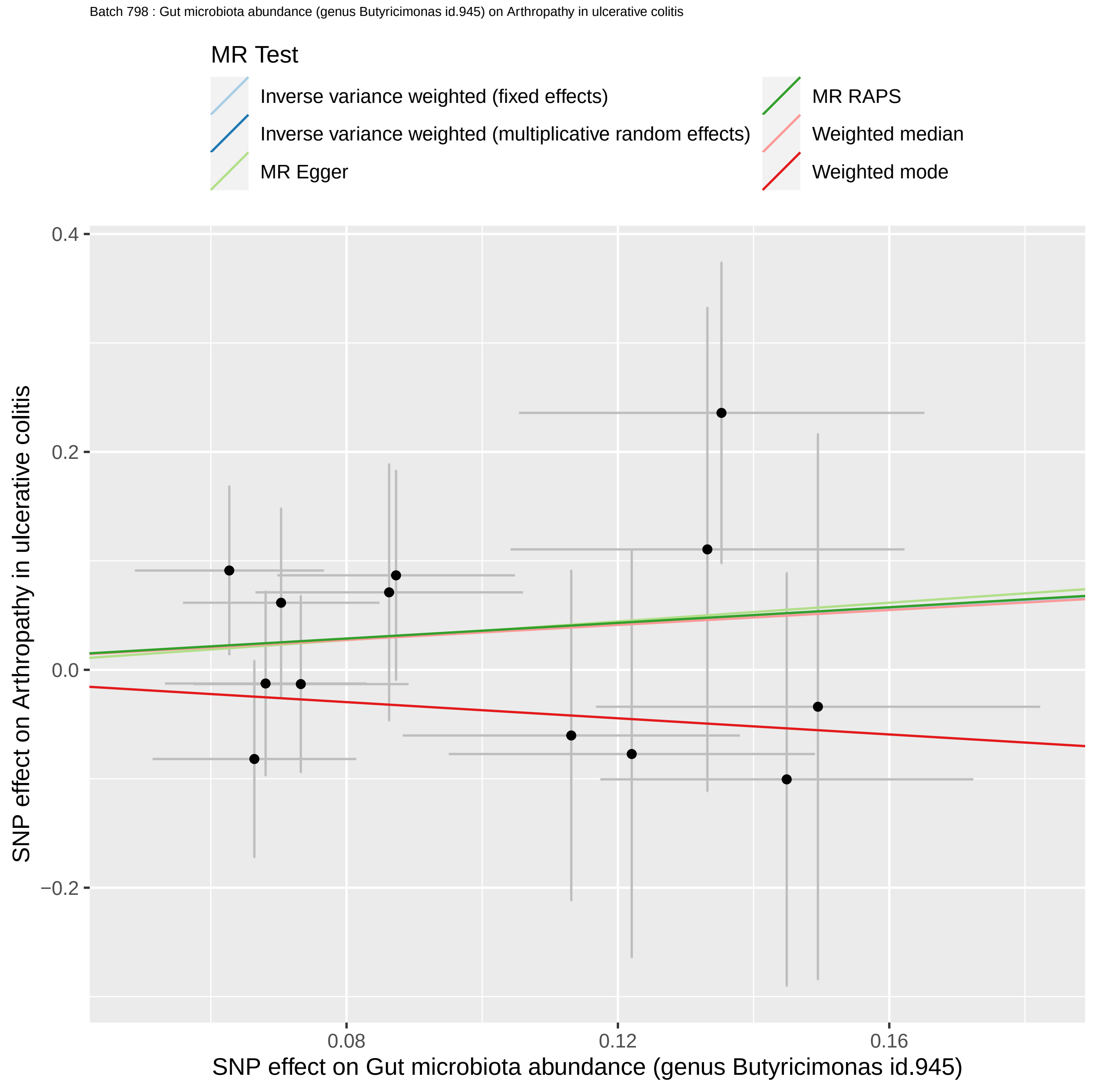

Batch 798 : Gut microbiota abundance (genus Butyricimonas id.945) on Arthropathy in ulcerative colitis
MR Test
Inverse variance weighted (fixed effects)
Inverse variance weighted (multiplicative random effects)
MR Egger
MR RAPS
Weighted median
Weighted mode
0.4
0.2
0.0
−0.2
0.08
0.12
0.16
SNP effect on Arthropathy in ulcerative colitis
SNP effect on Gut microbiota abundance (genus Butyricimonas id.945)

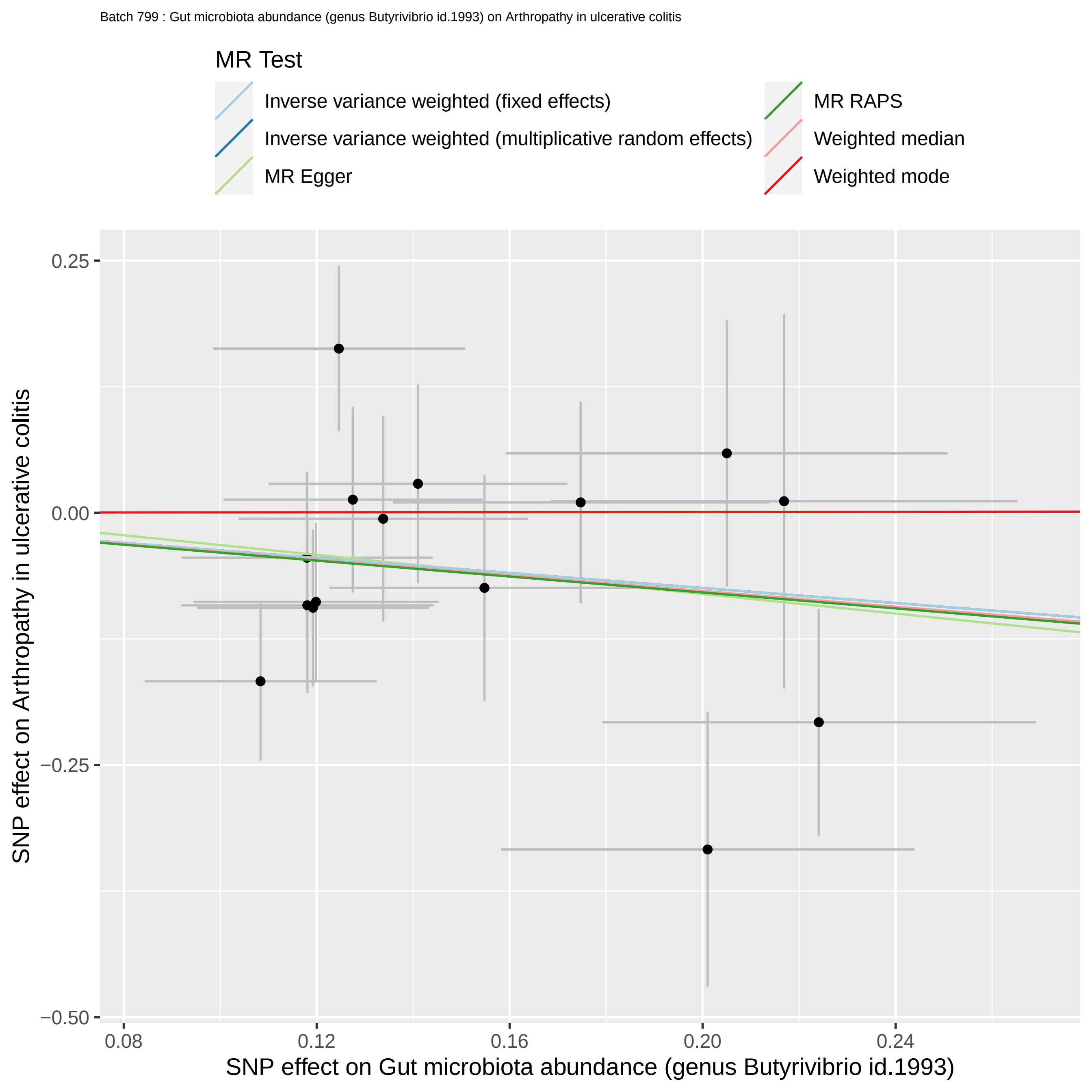

Batch 799 : Gut microbiota abundance (genus Butyrivibrio id.1993) on Arthropathy in ulcerative colitis
MR Test
Inverse variance weighted (fixed effects)
Inverse variance weighted (multiplicative random effects)
MR Egger
MR RAPS
Weighted median
Weighted mode
0.25
0.00
−0.25
−0.50
0.08
0.12
0.16
0.20
0.24
SNP effect on Arthropathy in ulcerative colitis
SNP effect on Gut microbiota abundance (genus Butyrivibrio id.1993)

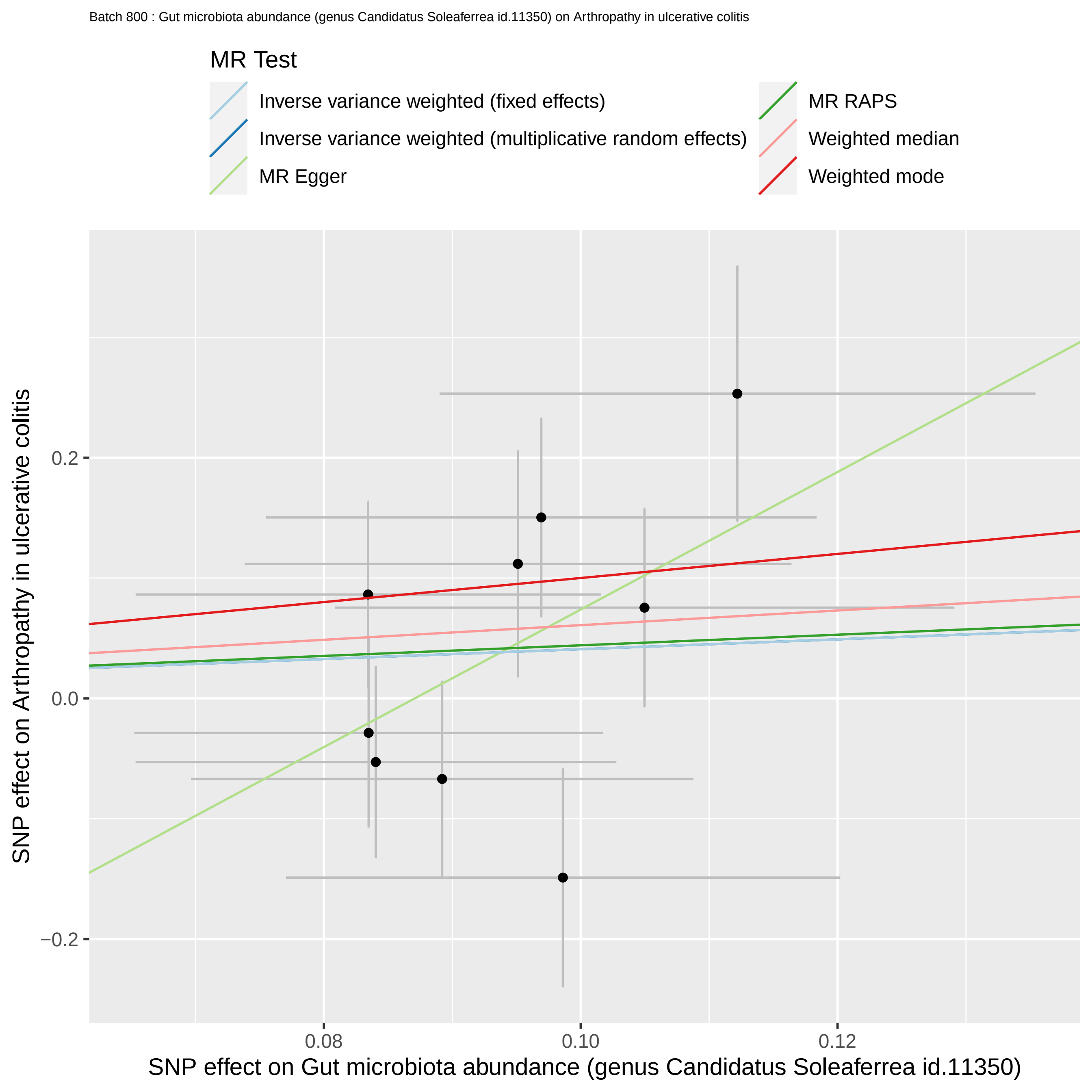
Batch 800 : Gut microbiota abundance (genus Candidatus Soleaferrea id.11350) on Arthropathy in ulcerative colitis
MR Test
Inverse variance weighted (fixed effects)
Inverse variance weighted (multiplicative random effects)
MR Egger
MR RAPS
Weighted median
Weighted mode
SNP effect on Arthropathy in ulcerative colitis
0.2
0.0
−0.2
0.08
0.10
0.12
SNP effect on Gut microbiota abundance (genus Candidatus Soleaferrea id.11350)

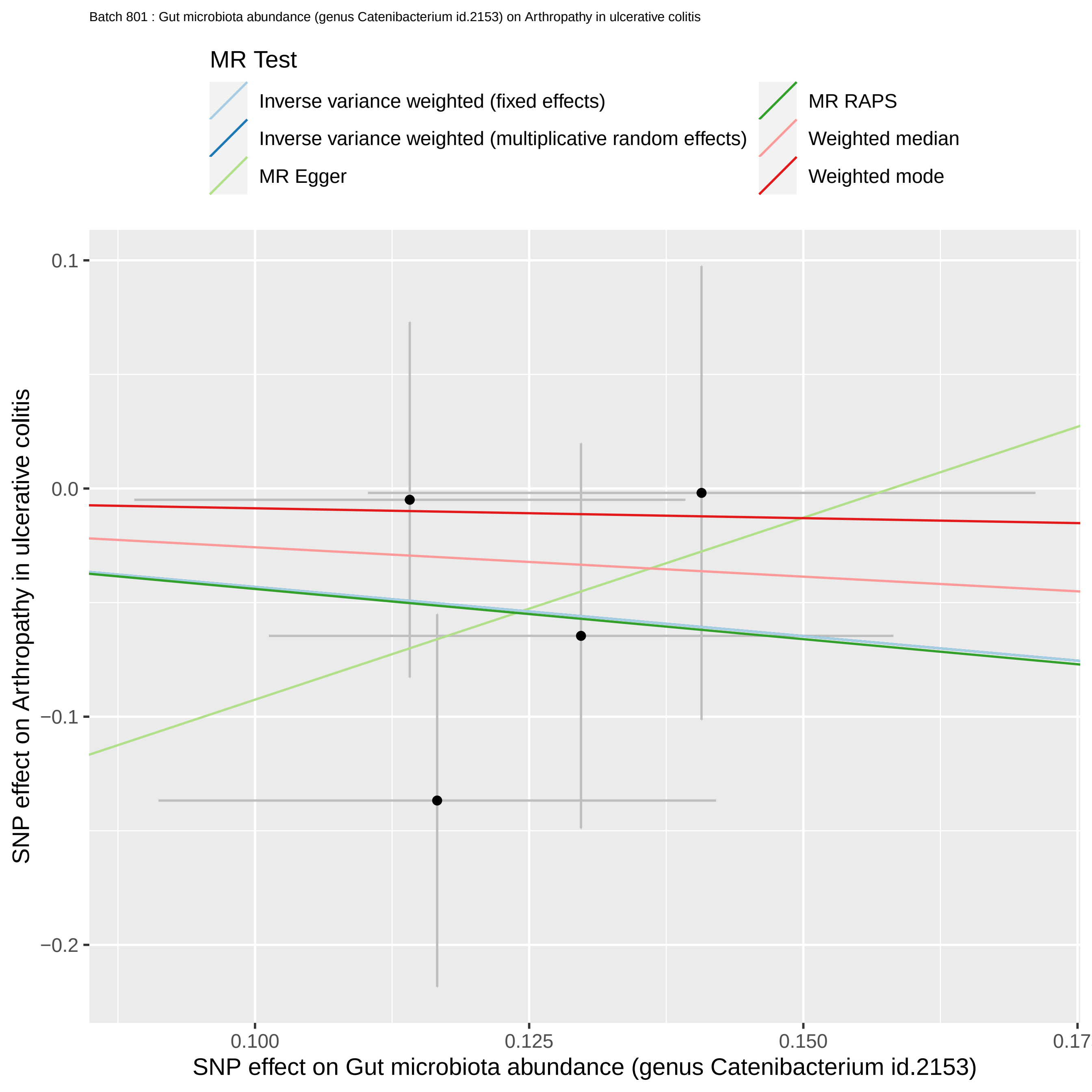

Batch 801 : Gut microbiota abundance (genus Catenibacterium id.2153) on Arthropathy in ulcerative colitis
MR Test
Inverse variance weighted (fixed effects)
Inverse variance weighted (multiplicative random effects)
MR Egger
MR RAPS
Weighted median
Weighted mode
0.1
0.0
−0.1
−0.2
0.100
0.125
0.150
0.17
SNP effect on Arthropathy in ulcerative colitis
SNP effect on Gut microbiota abundance (genus Catenibacterium id.2153)

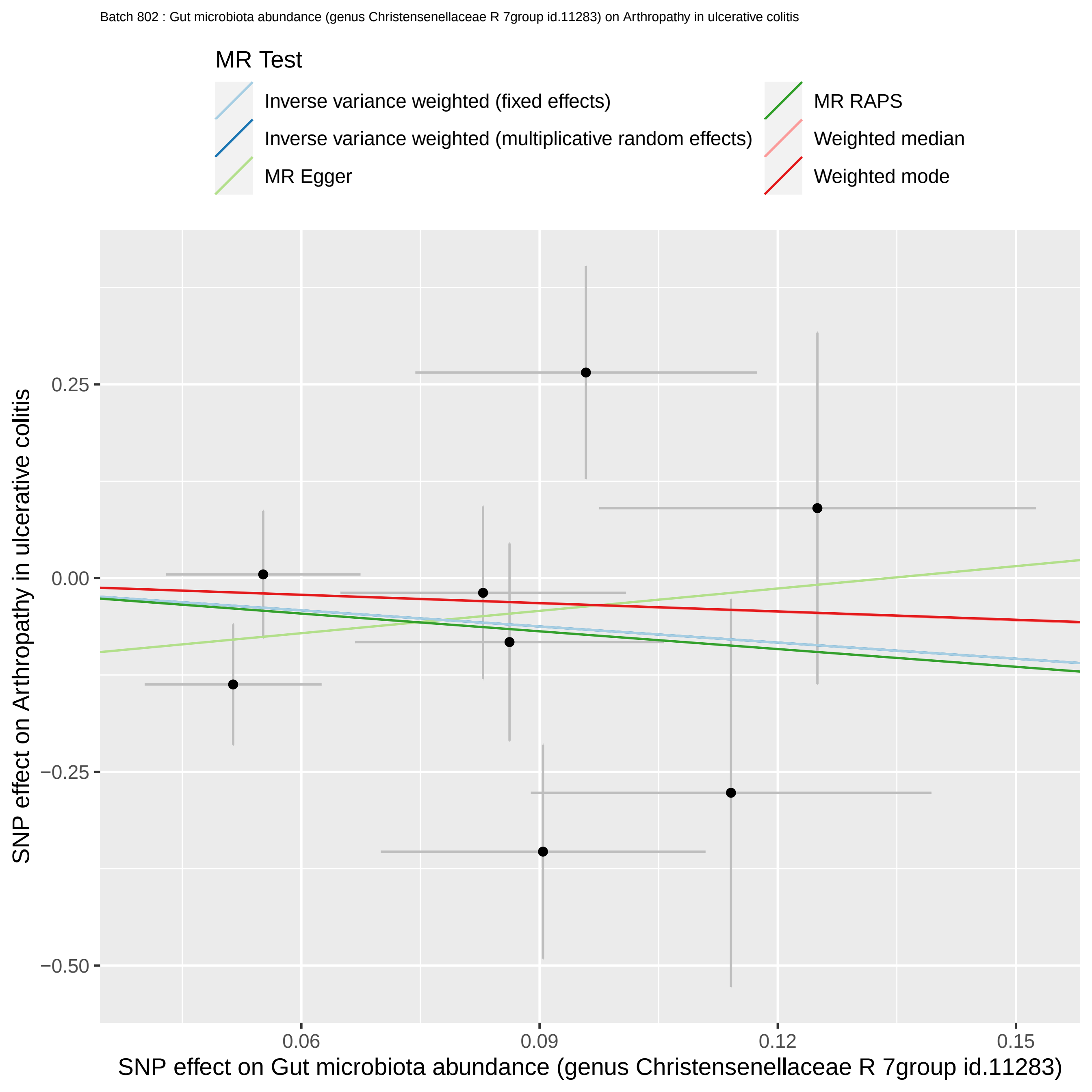

Batch 802 : Gut microbiota abundance (genus Christensenellaceae R 7group id.11283) on Arthropathy in ulcerative colitis
MR Test
Inverse variance weighted (fixed effects)
Inverse variance weighted (multiplicative random effects)
MR Egger
MR RAPS
Weighted median
Weighted mode
SNP effect on Arthropathy in ulcerative colitis
0.25
0.00
−0.25
−0.50
0.06
0.09
0.12
0.15
SNP effect on Gut microbiota abundance (genus Christensenellaceae R 7group id.11283)

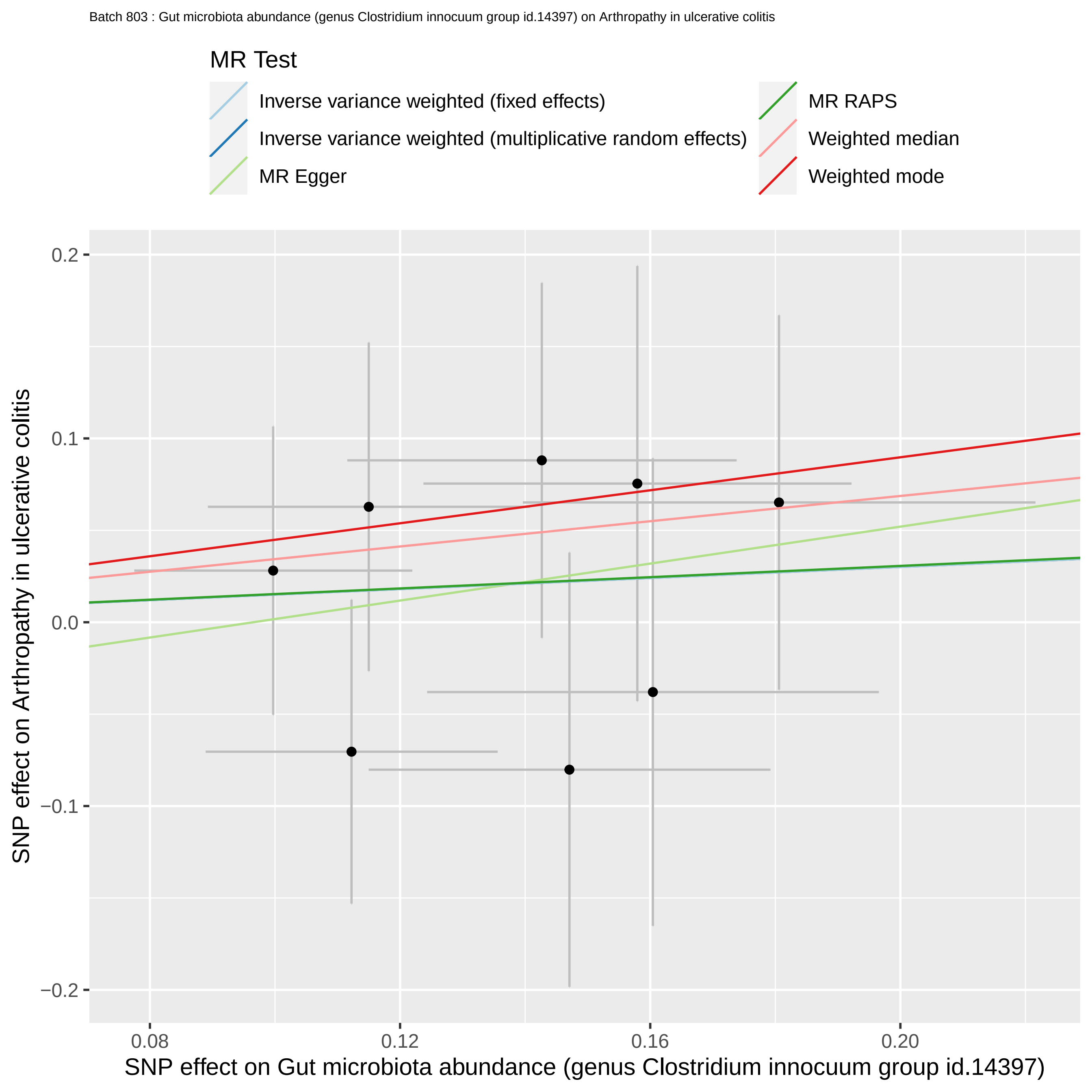

Batch 803 : Gut microbiota abundance (genus Clostridium innocuum group id.14397) on Arthropathy in ulcerative colitis
MR Test
Inverse variance weighted (fixed effects)
Inverse variance weighted (multiplicative random effects)
MR Egger
MR RAPS
Weighted median
Weighted mode
0.2
0.1
0.0
−0.1
−0.2
0.08
0.12
0.16
0.20
SNP effect on Arthropathy in ulcerative colitis
SNP effect on Gut microbiota abundance (genus Clostridium innocuum group id.14397)

Batch 804 : Gut microbiota abundance (genus Clostridium sensustricto1 id.1873) on Arthropathy in ulcerative colitis

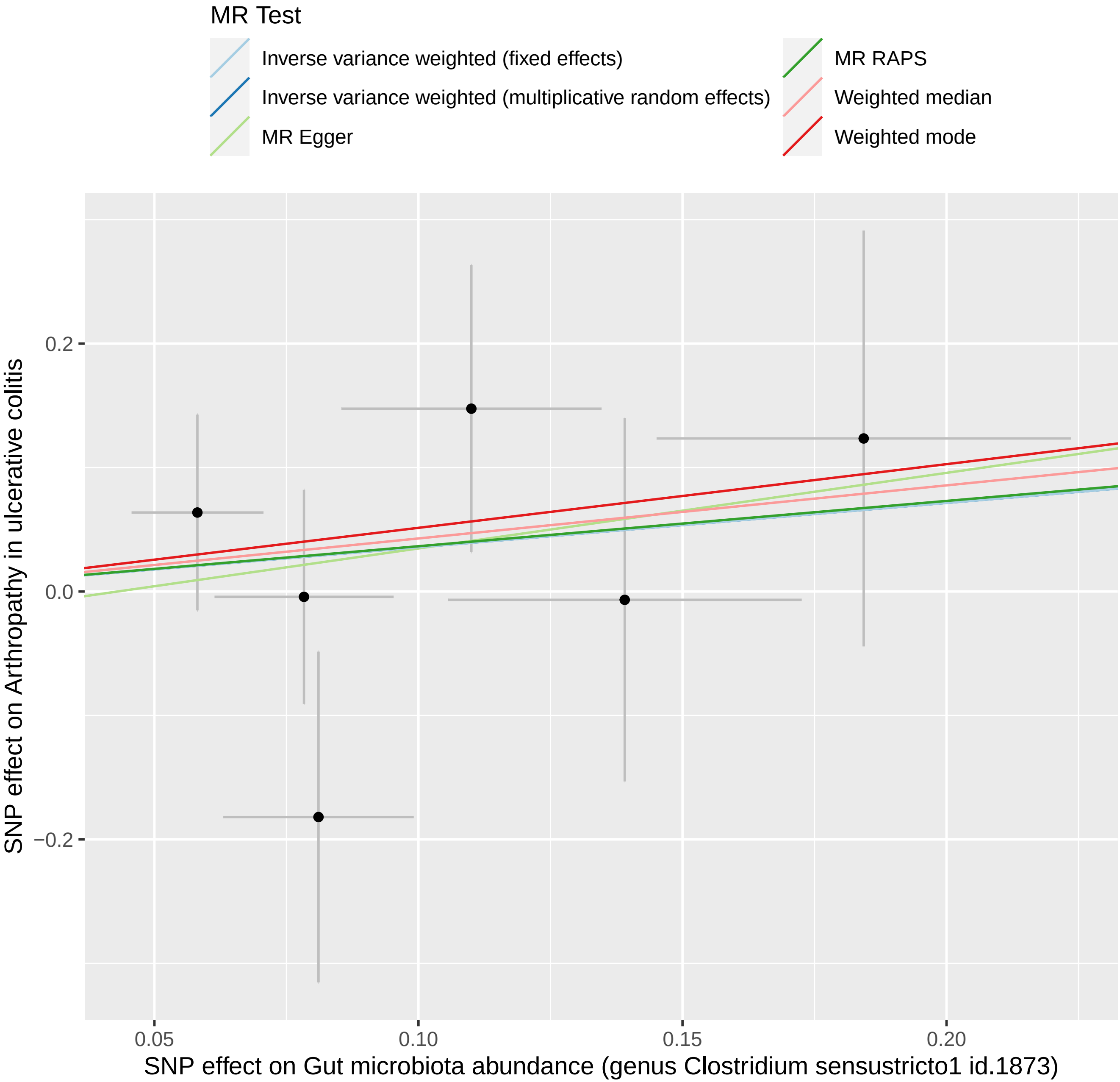

Batch 805 : Gut microbiota abundance (genus Collinsella id.815) on Arthropathy in ulcerative colitis

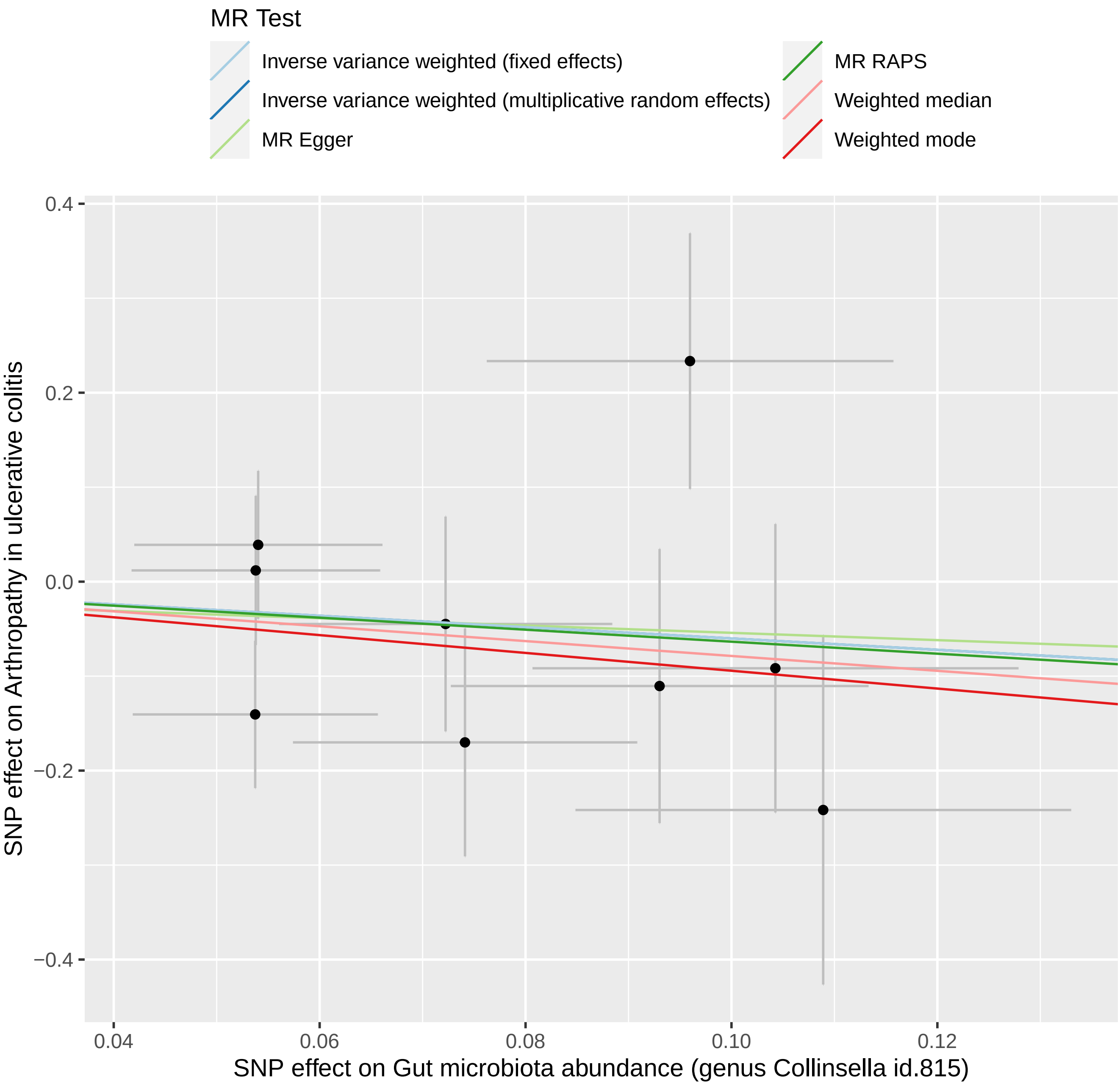

Batch 806 : Gut microbiota abundance (genus Coprobacter id.949) on Arthropathy in ulcerative colitis

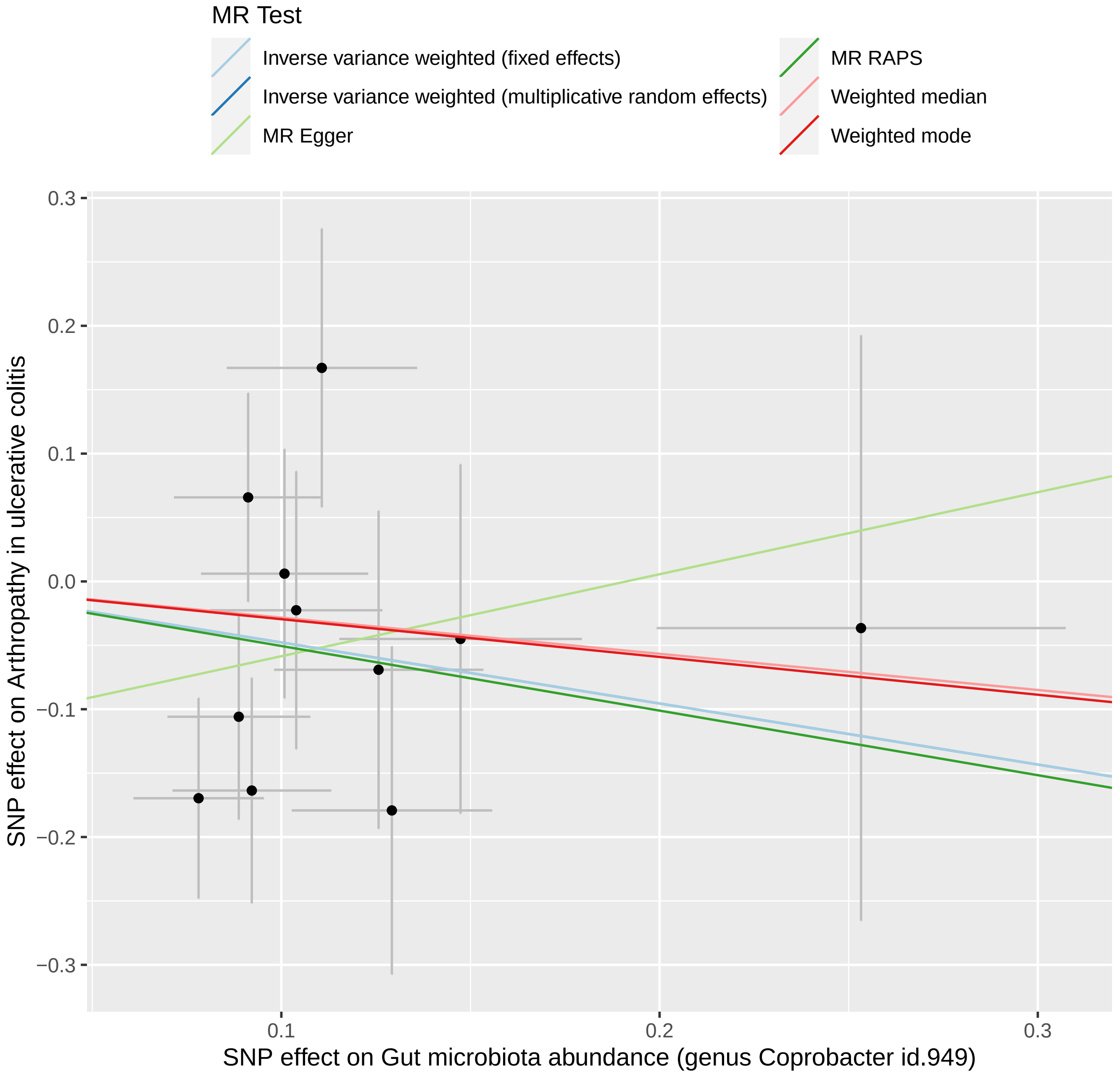

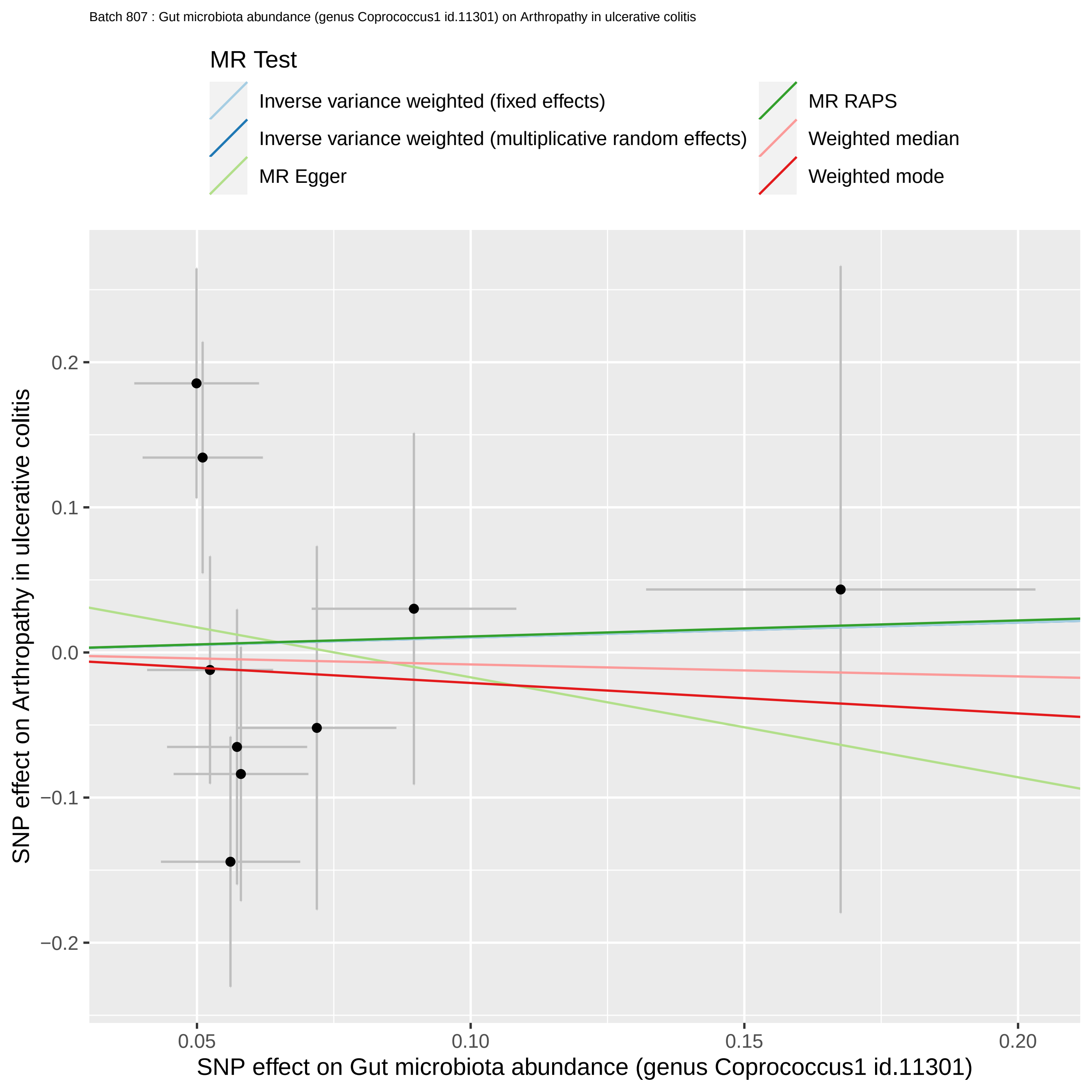
Batch 807 : Gut microbiota abundance (genus Coprococcus1 id.11301) on Arthropathy in ulcerative colitis
MR Test
Inverse variance weighted (fixed effects)
Inverse variance weighted (multiplicative random effects)
MR Egger
MR RAPS
Weighted median
Weighted mode
SNP effect on Arthropathy in ulcerative colitis
0.2
0.1
0.0
−0.1
−0.2
0.05
0.10
0.15
0.20
SNP effect on Gut microbiota abundance (genus Coprococcus1 id.11301)

Batch 808 : Gut microbiota abundance (genus Coprococcus2 id.11302) on Arthropathy in ulcerative colitis

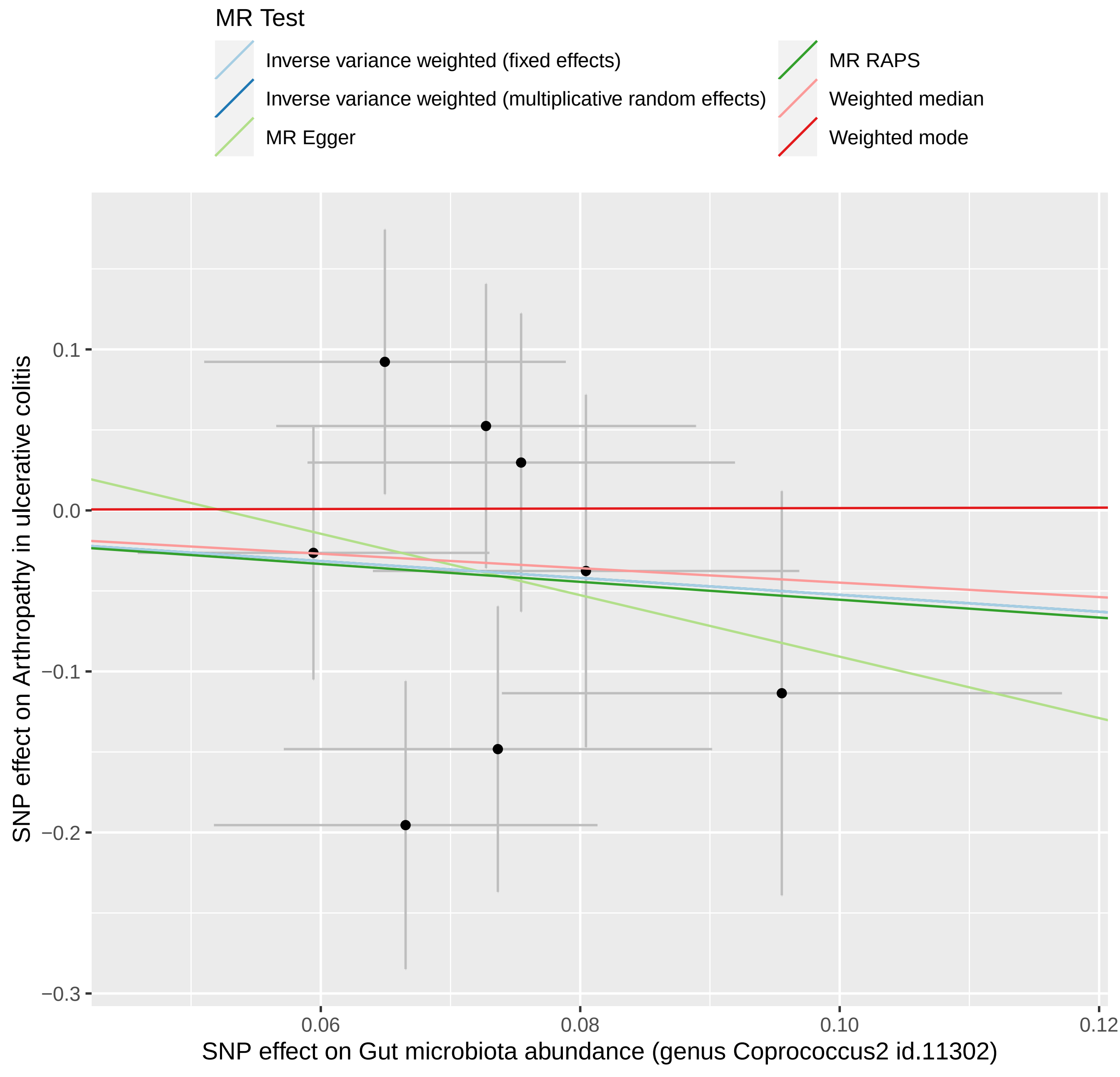

Batch 809 : Gut microbiota abundance (genus Coprococcus3 id.11303) on Arthropathy in ulcerative colitis

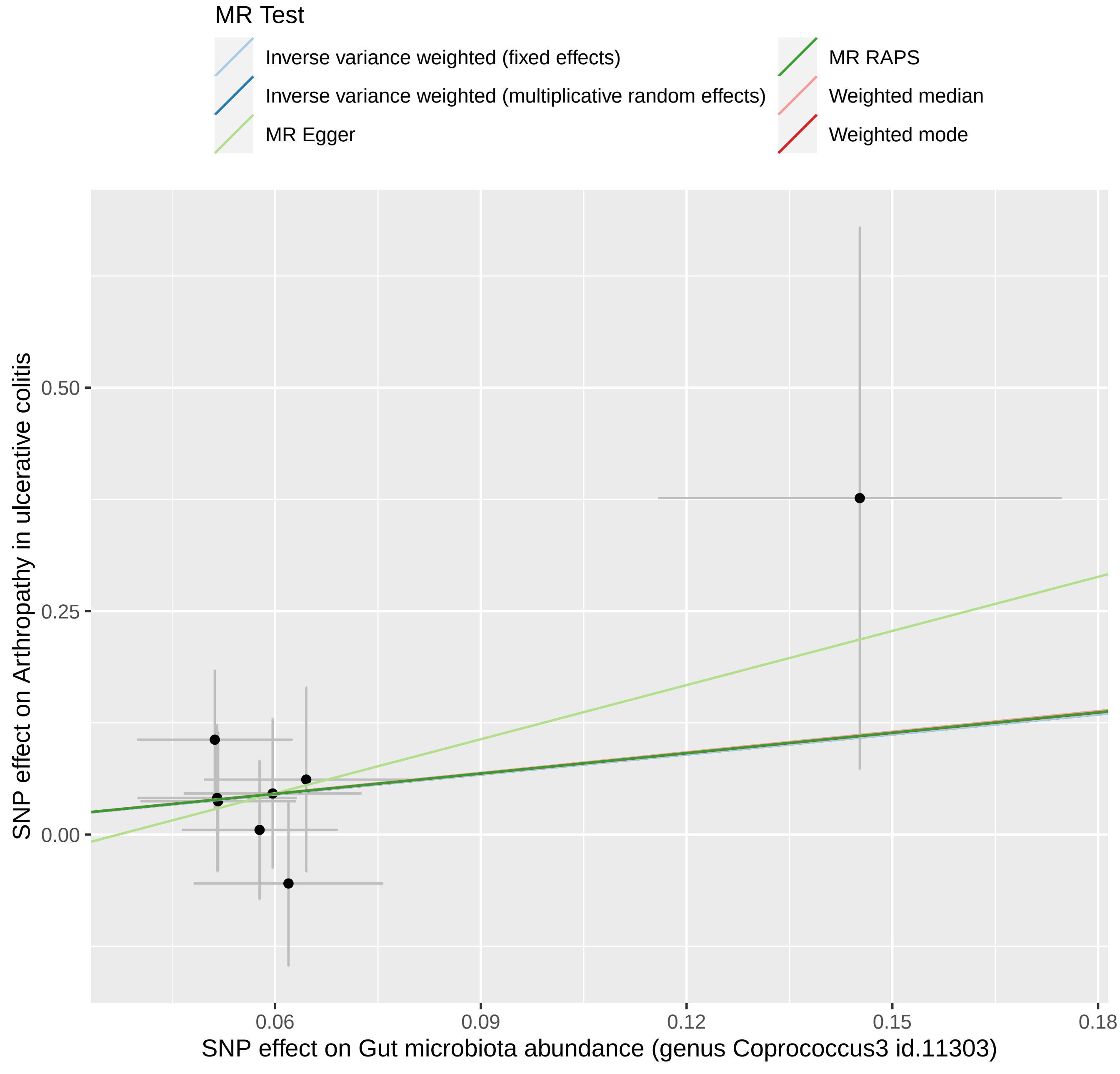

Batch 810 : Gut microbiota abundance (genus Defluviitaleaceae UCG011 id.11287) on Arthropathy in ulcerative colitis

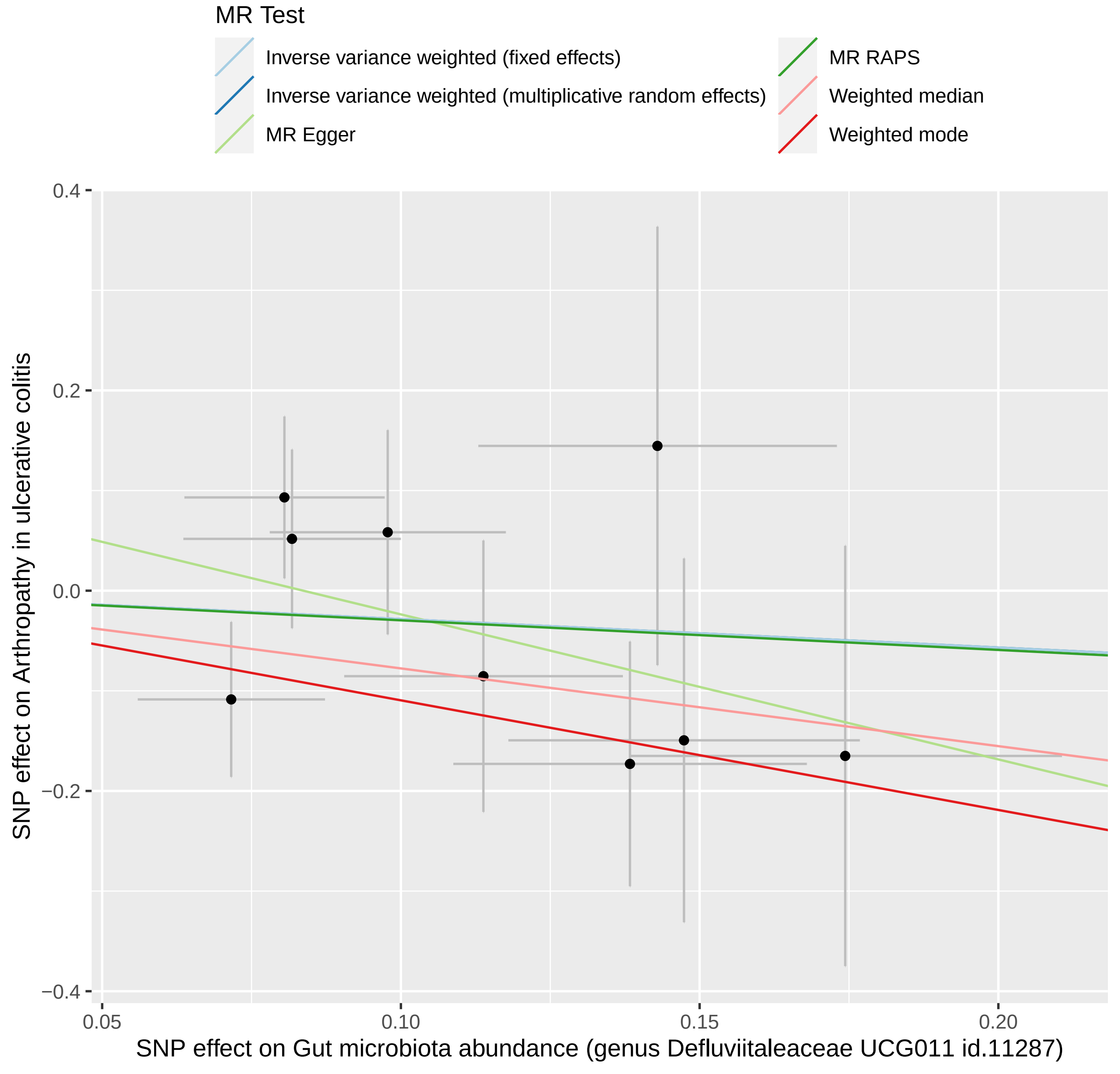

Batch 811 : Gut microbiota abundance (genus Desulfovibrio id.3173) on Arthropathy in ulcerative colitis

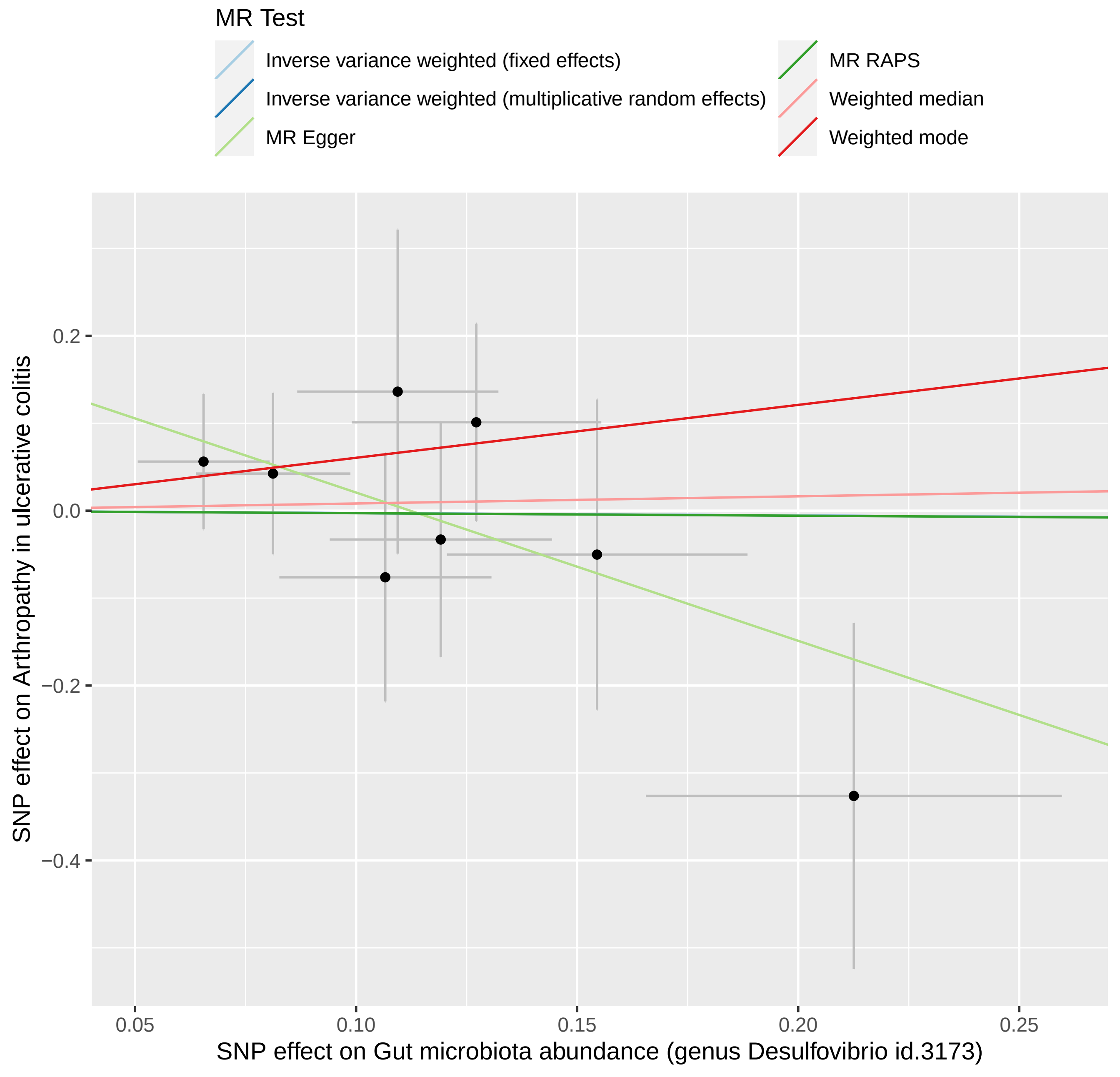

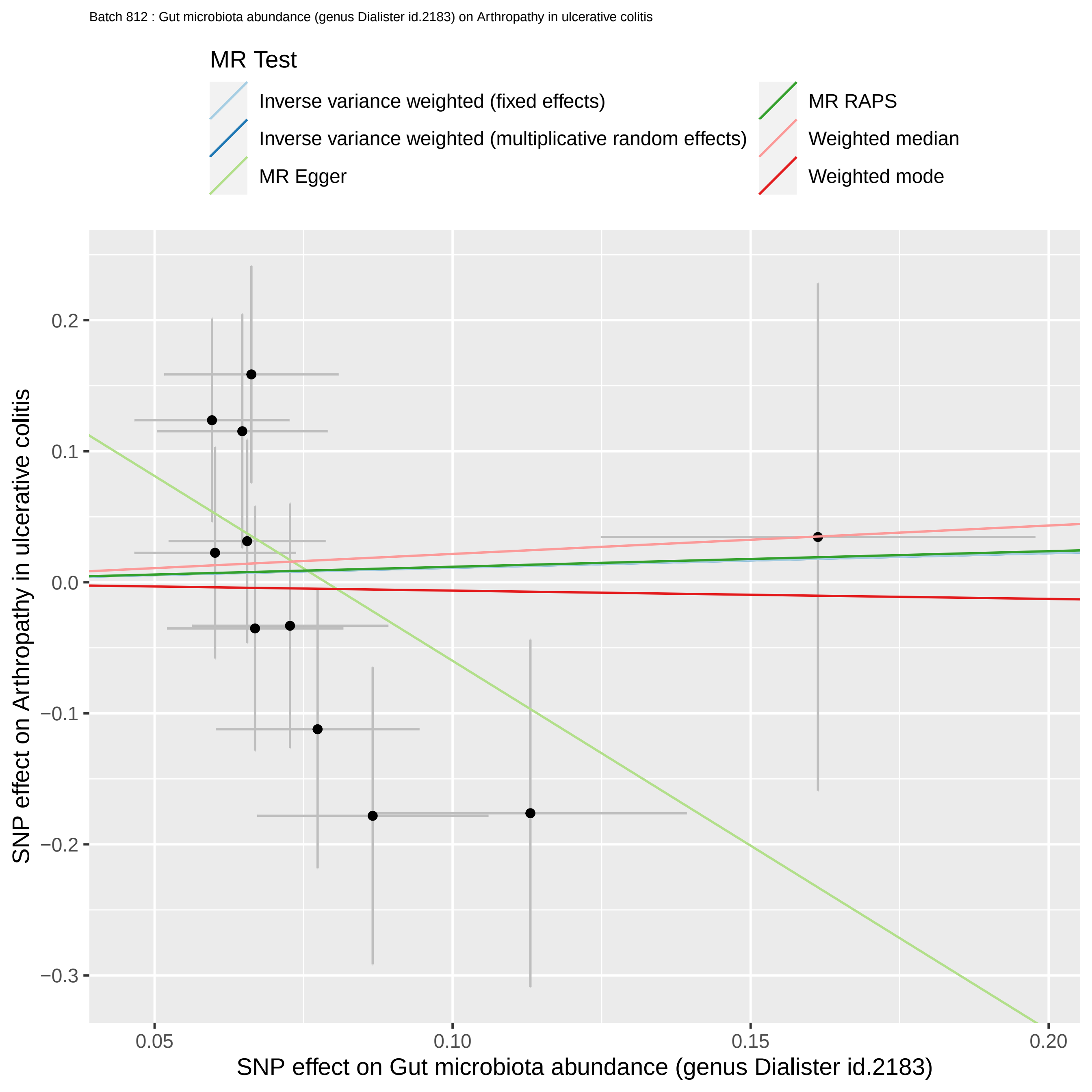

Batch 812 : Gut microbiota abundance (genus Dialister id.2183) on Arthropathy in ulcerative colitis
MR Test
Inverse variance weighted (fixed effects)
Inverse variance weighted (multiplicative random effects)
MR Egger
MR RAPS
Weighted median
Weighted mode
SNP effect on Arthropathy in ulcerative colitis
0.2
0.1
0.0
−0.1
−0.2
−0.3
0.05
0.10
0.15
0.20
SNP effect on Gut microbiota abundance (genus Dialister id.2183)

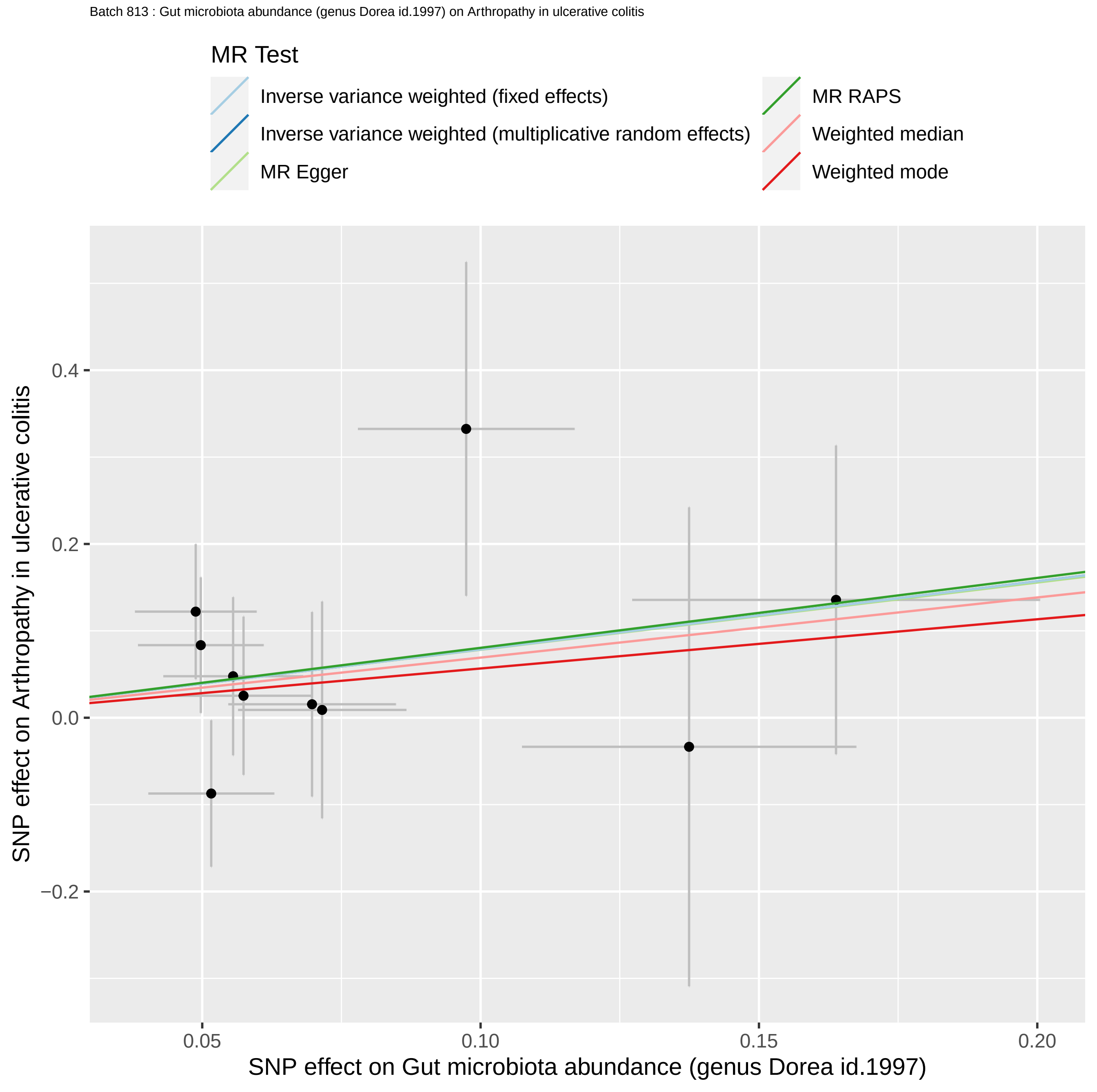
Batch 813 : Gut microbiota abundance (genus Dorea id.1997) on Arthropathy in ulcerative colitis
MR Test
Inverse variance weighted (fixed effects)
Inverse variance weighted (multiplicative random effects)
MR Egger
MR RAPS
Weighted median
Weighted mode
0.4
0.2
0.0
−0.2
0.05
0.10
0.15
0.20
SNP effect on Arthropathy in ulcerative colitis
SNP effect on Gut microbiota abundance (genus Dorea id.1997)

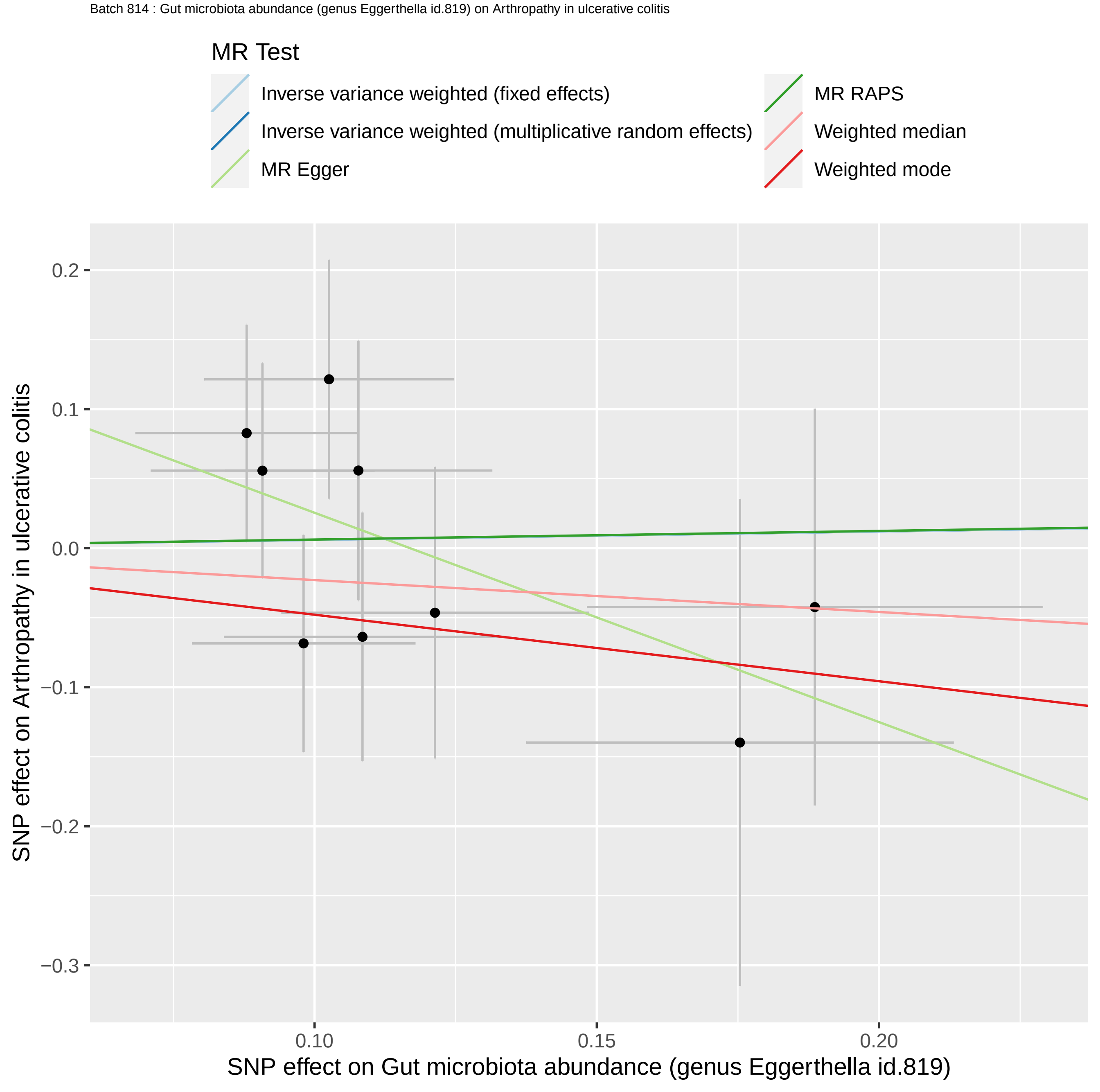
Batch 814 : Gut microbiota abundance (genus Eggerthella id.819) on Arthropathy in ulcerative colitis
MR Test
Inverse variance weighted (fixed effects)
Inverse variance weighted (multiplicative random effects)
MR Egger
MR RAPS
Weighted median
Weighted mode
SNP effect on Arthropathy in ulcerative colitis
0.2
0.1
0.0
−0.1
−0.2
−0.3
0.10
0.15
0.20
SNP effect on Gut microbiota abundance (genus Eggerthella id.819)

Batch 815 : Gut microbiota abundance (genus Eisenbergiella id.11304) on Arthropathy in ulcerative colitis

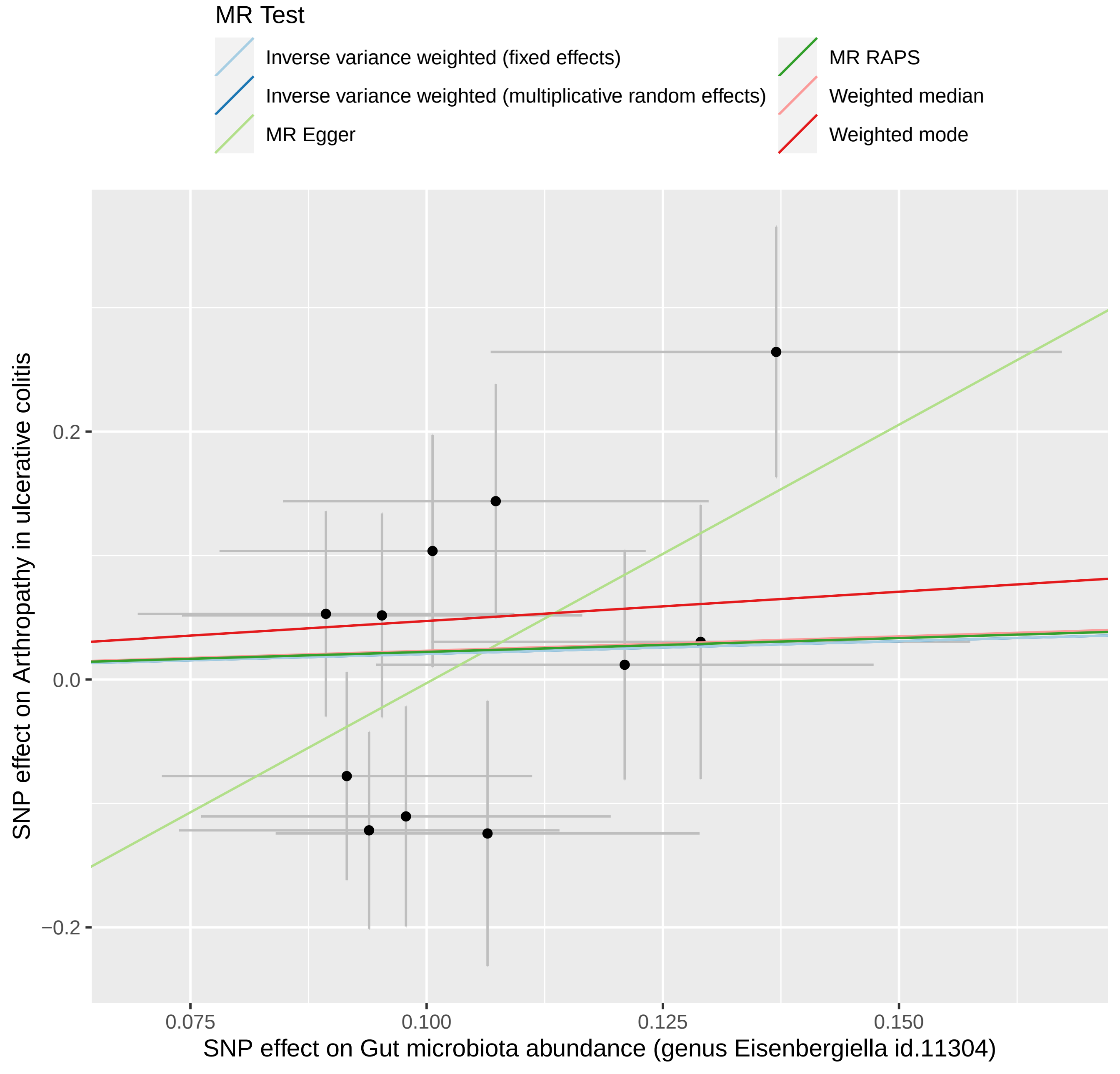

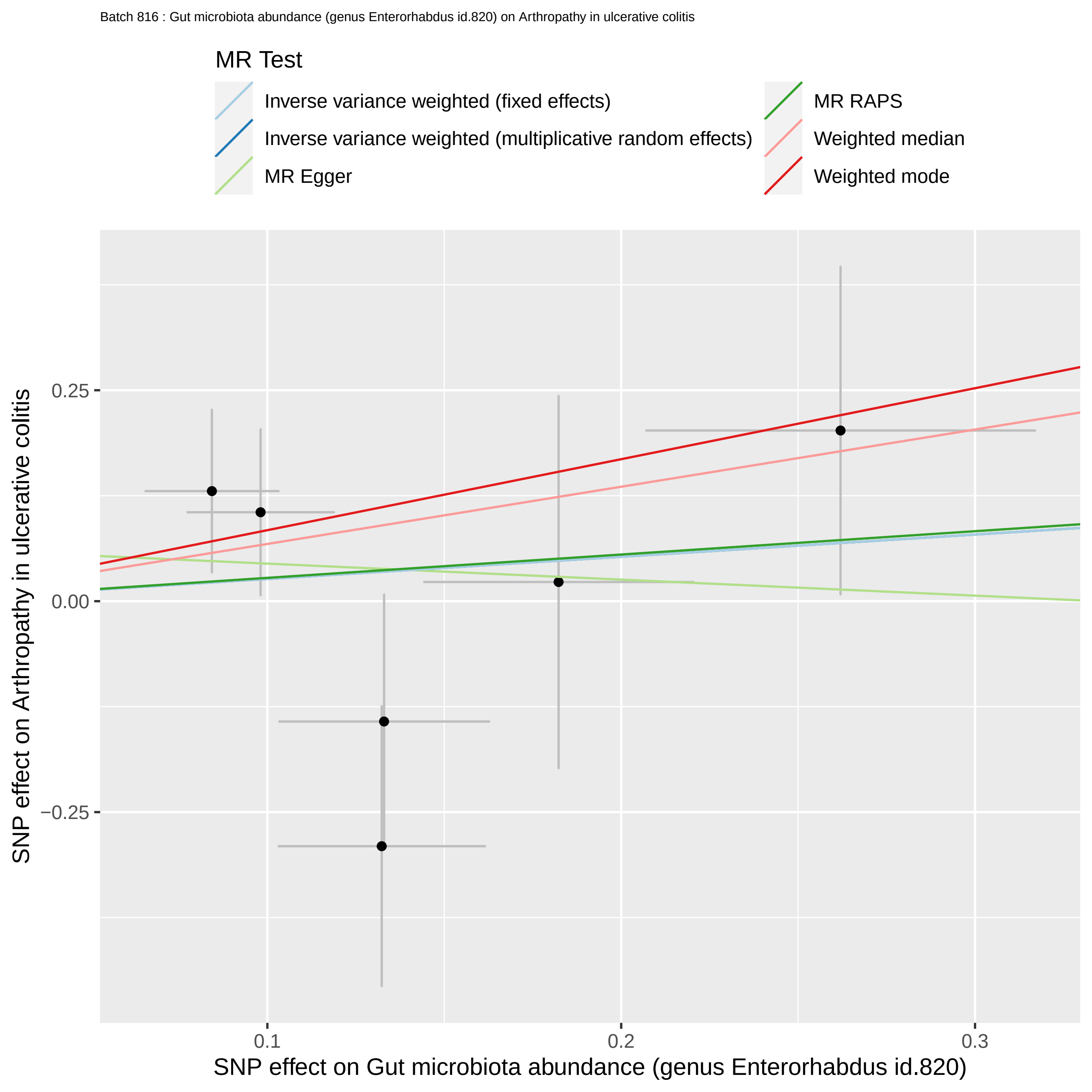

Batch 816 : Gut microbiota abundance (genus Enterorhabdus id.820) on Arthropathy in ulcerative colitis
MR Test
Inverse variance weighted (fixed effects)
Inverse variance weighted (multiplicative random effects)
MR Egger
MR RAPS
Weighted median
Weighted mode
0.25
0.00
−0.25
0.1
0.2
0.3
SNP effect on Arthropathy in ulcerative colitis
SNP effect on Gut microbiota abundance (genus Enterorhabdus id.820)

Batch 817 : Gut microbiota abundance (genus Erysipelatoclostridium id.11381) on Arthropathy in ulcerative colitis

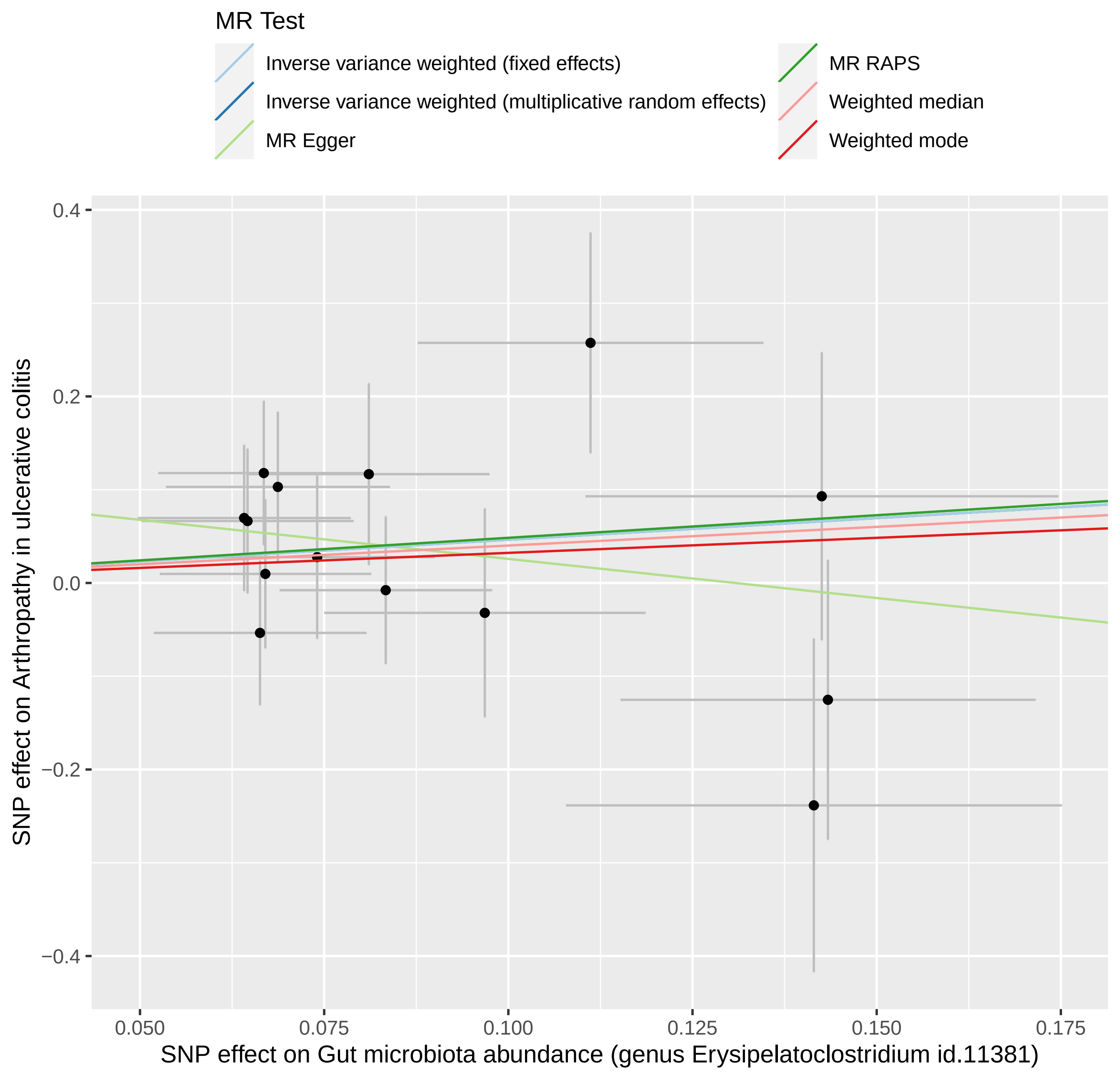

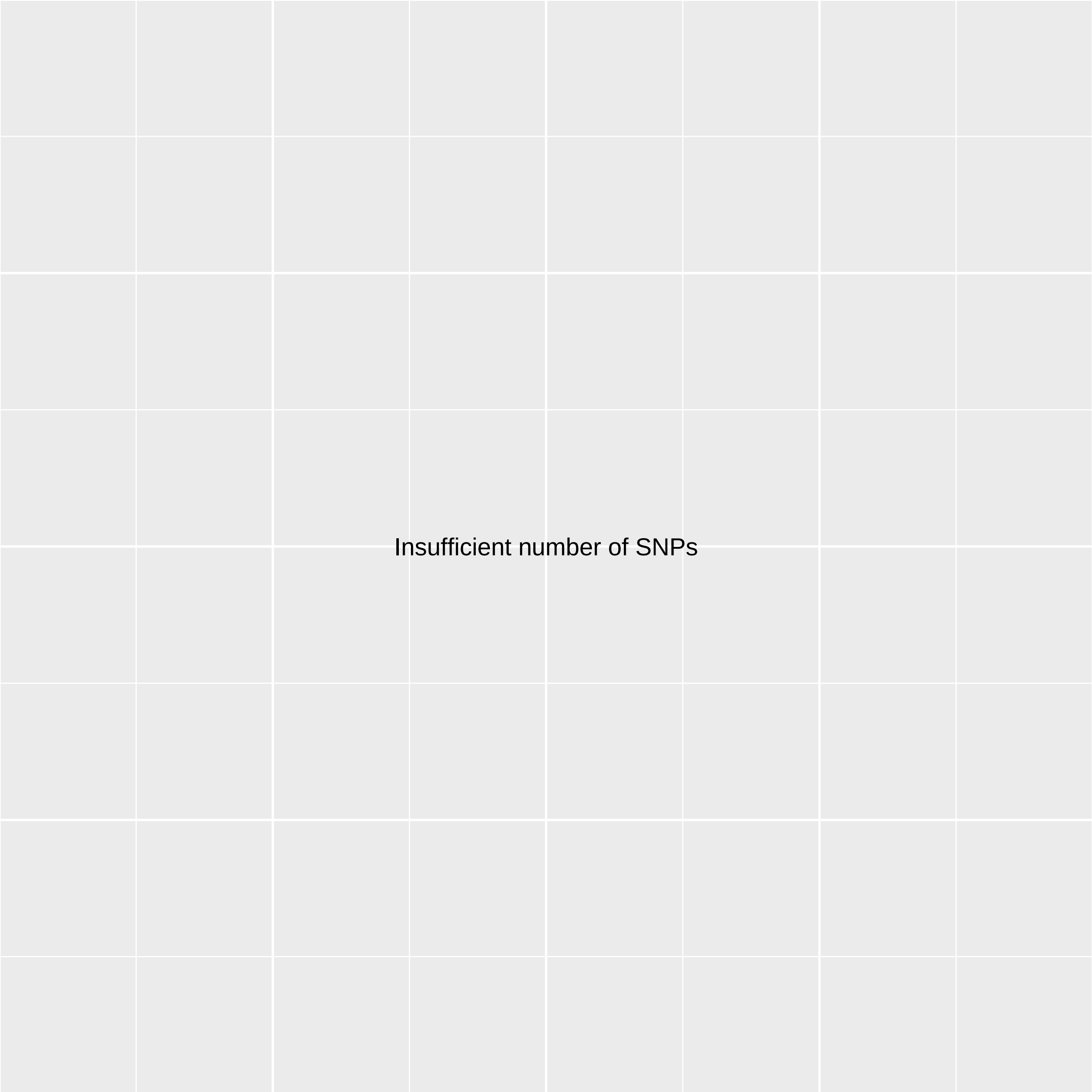
Insufficient number of SNPs

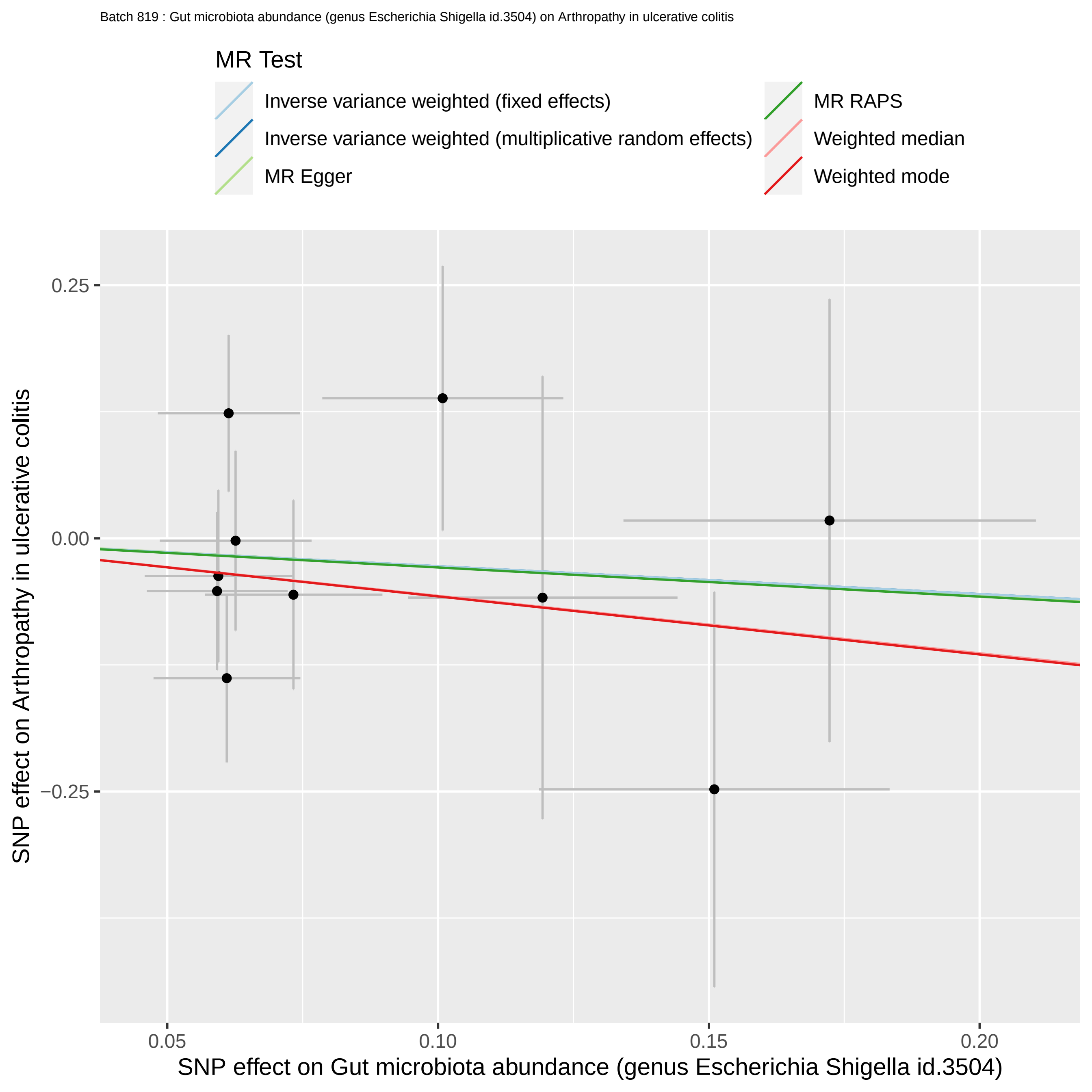

Batch 819 : Gut microbiota abundance (genus Escherichia Shigella id.3504) on Arthropathy in ulcerative colitis
MR Test
Inverse variance weighted (fixed effects)
Inverse variance weighted (multiplicative random effects)
MR Egger
MR RAPS
Weighted median
Weighted mode
0.25
0.00
−0.25
0.05
0.10
0.15
0.20
SNP effect on Arthropathy in ulcerative colitis
SNP effect on Gut microbiota abundance (genus Escherichia Shigella id.3504)

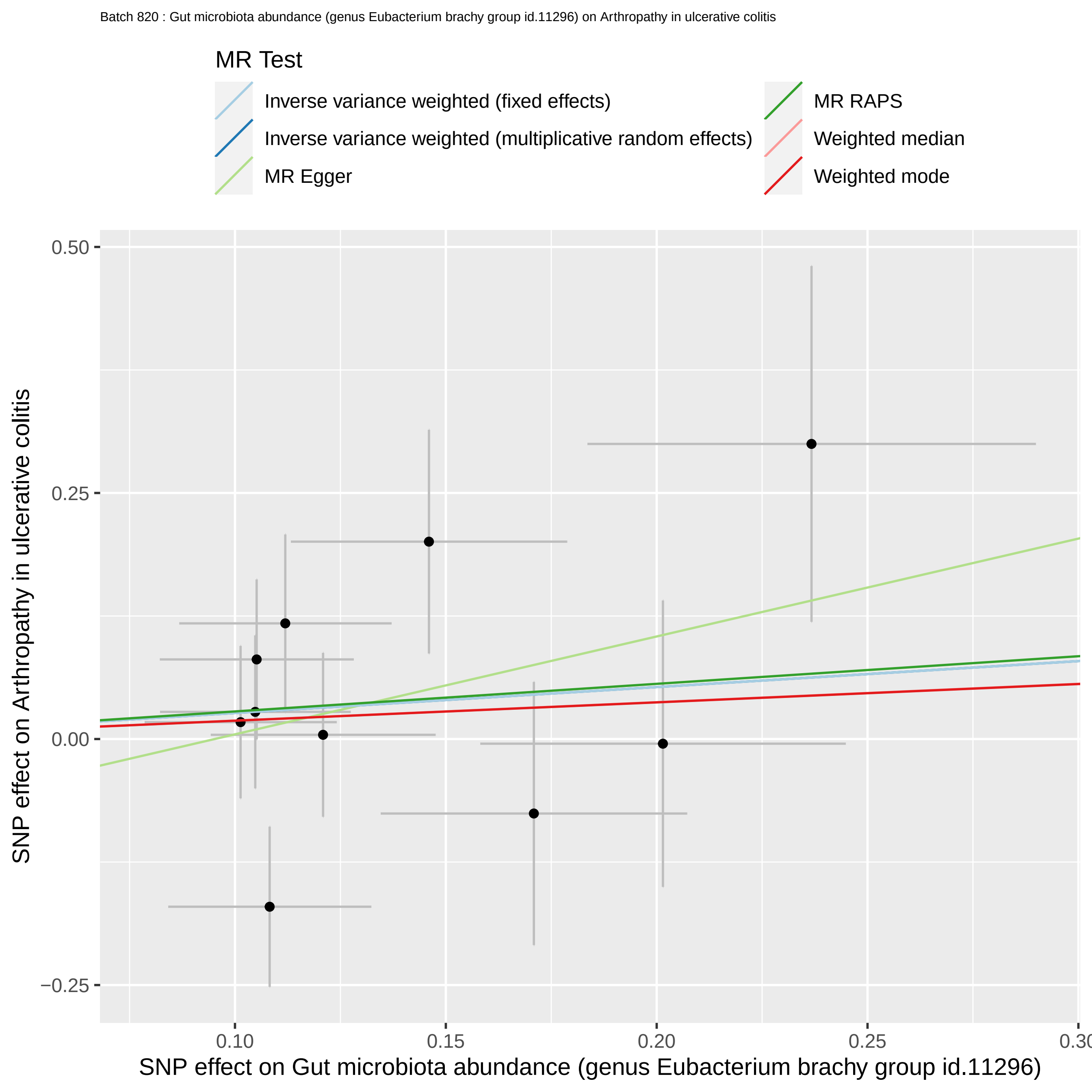
Batch 820 : Gut microbiota abundance (genus Eubacterium brachy group id.11296) on Arthropathy in ulcerative colitis
MR Test
Inverse variance weighted (fixed effects)
Inverse variance weighted (multiplicative random effects)
MR Egger
MR RAPS
Weighted median
Weighted mode
0.50
0.25
0.00
−0.25
0.10
0.15
0.20
0.25
SNP effect on Arthropathy in ulcerative colitis
SNP effect on Gut microbiota abundance (genus Eubacterium brachy group id.11296)

Batch 821 : Gut microbiota abundance (genus Eubacterium coprostanoligenes group id.11375) on Arthropathy in ulcerative colitis

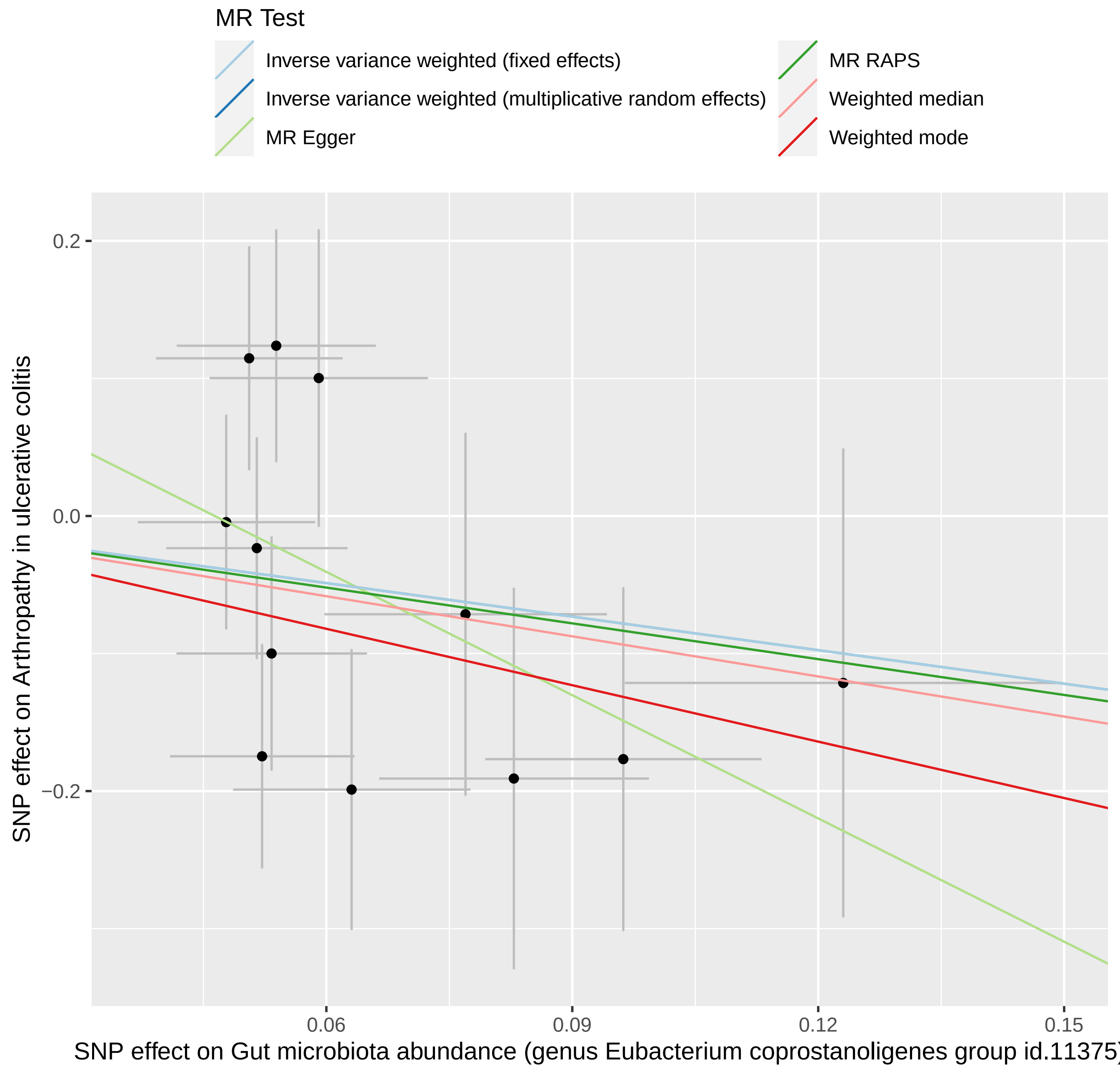

Batch 822 : Gut microbiota abundance (genus Eubacterium eligens group id.14372) on Arthropathy in ulcerative colitis

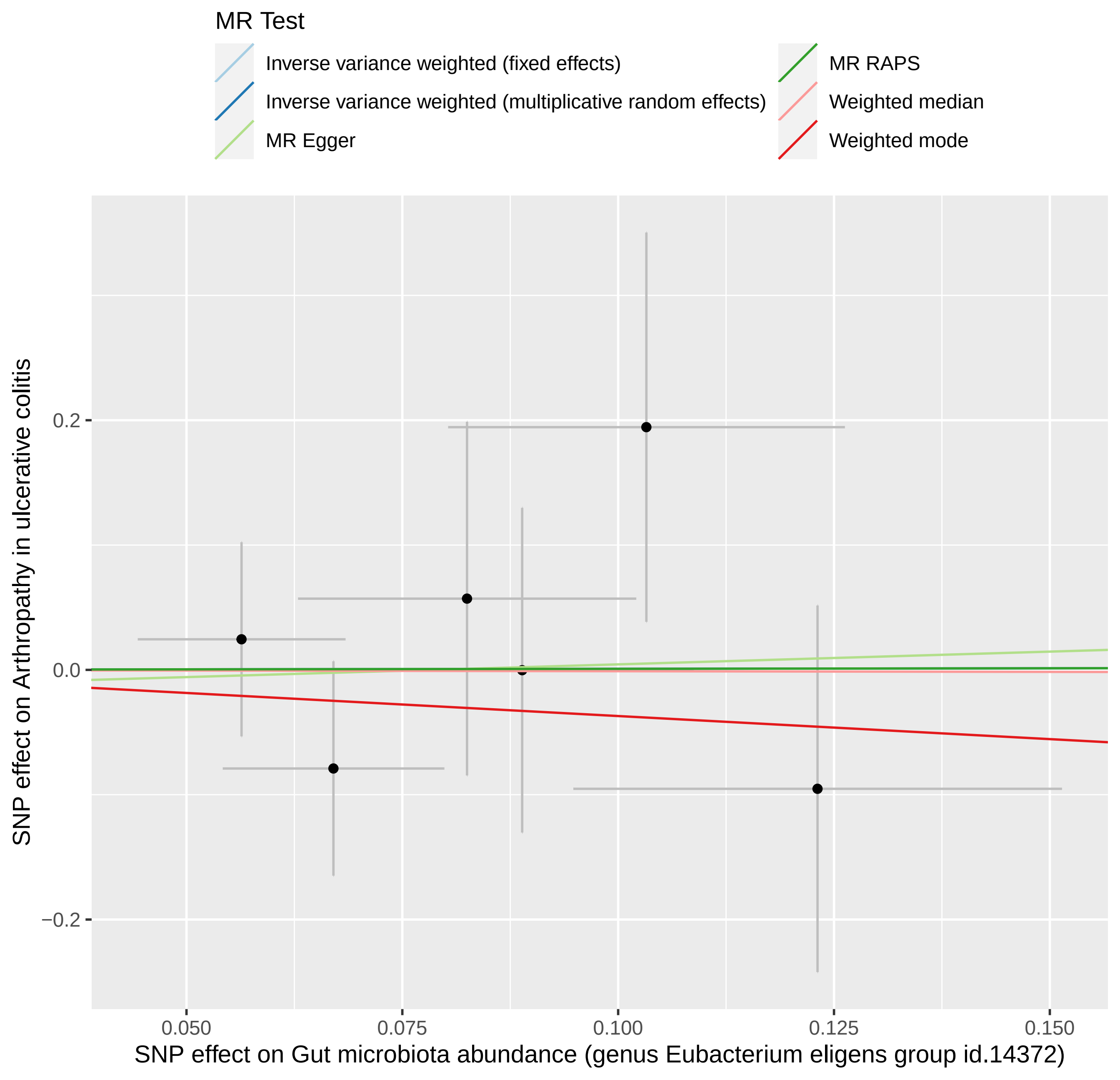

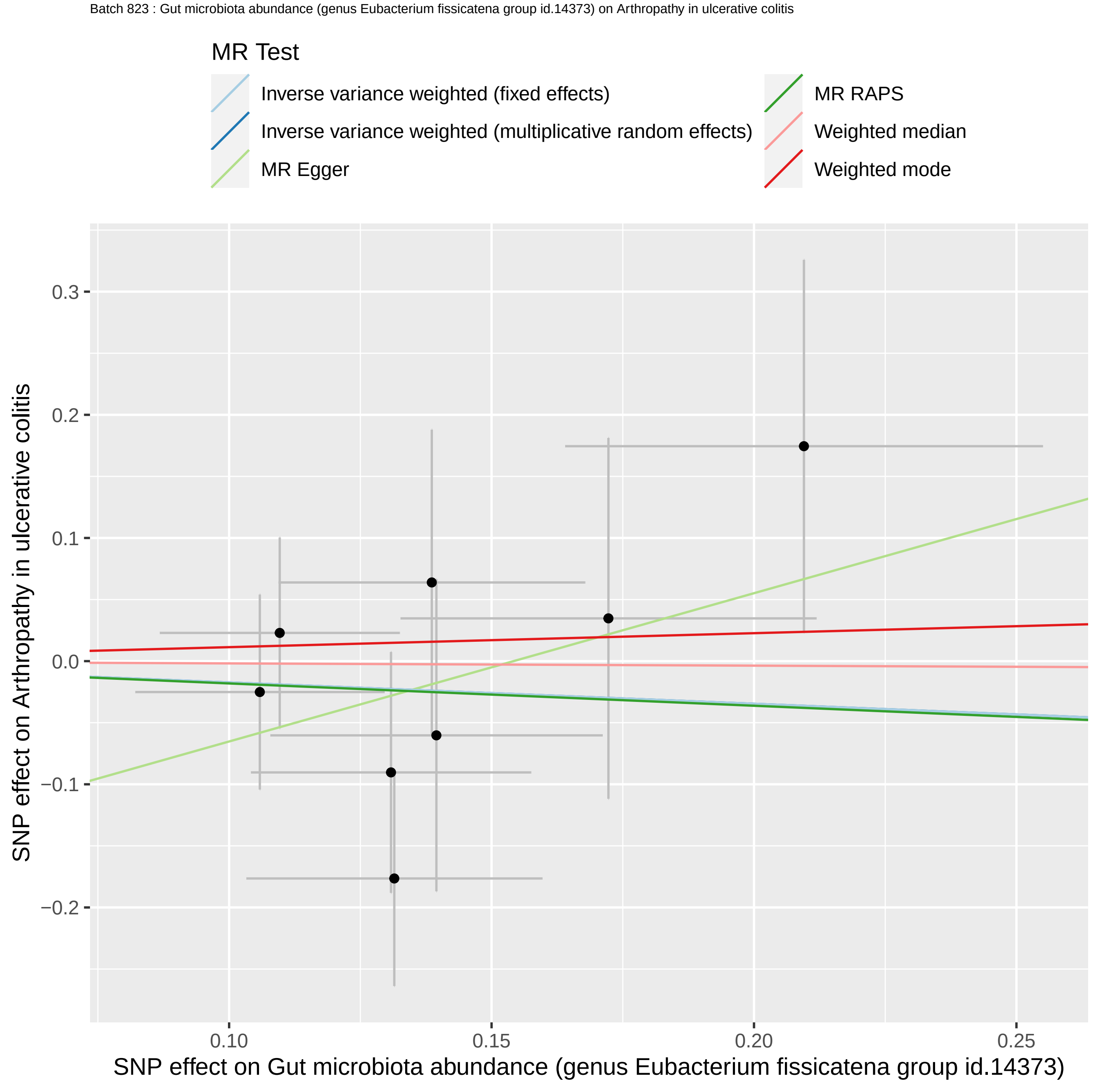

Batch 823 : Gut microbiota abundance (genus Eubacterium fissicatena group id.14373) on Arthropathy in ulcerative colitis
MR Test
Inverse variance weighted (fixed effects)
Inverse variance weighted (multiplicative random effects)
MR Egger
MR RAPS
Weighted median
Weighted mode
SNP effect on Arthropathy in ulcerative colitis
0.3
0.2
0.1
0.0
−0.1
−0.2
0.10
0.15
0.20
0.25
SNP effect on Gut microbiota abundance (genus Eubacterium fissicatena group id.14373)

Batch 824 : Gut microbiota abundance (genus Eubacterium hallii group id.11338) on Arthropathy in ulcerative colitis

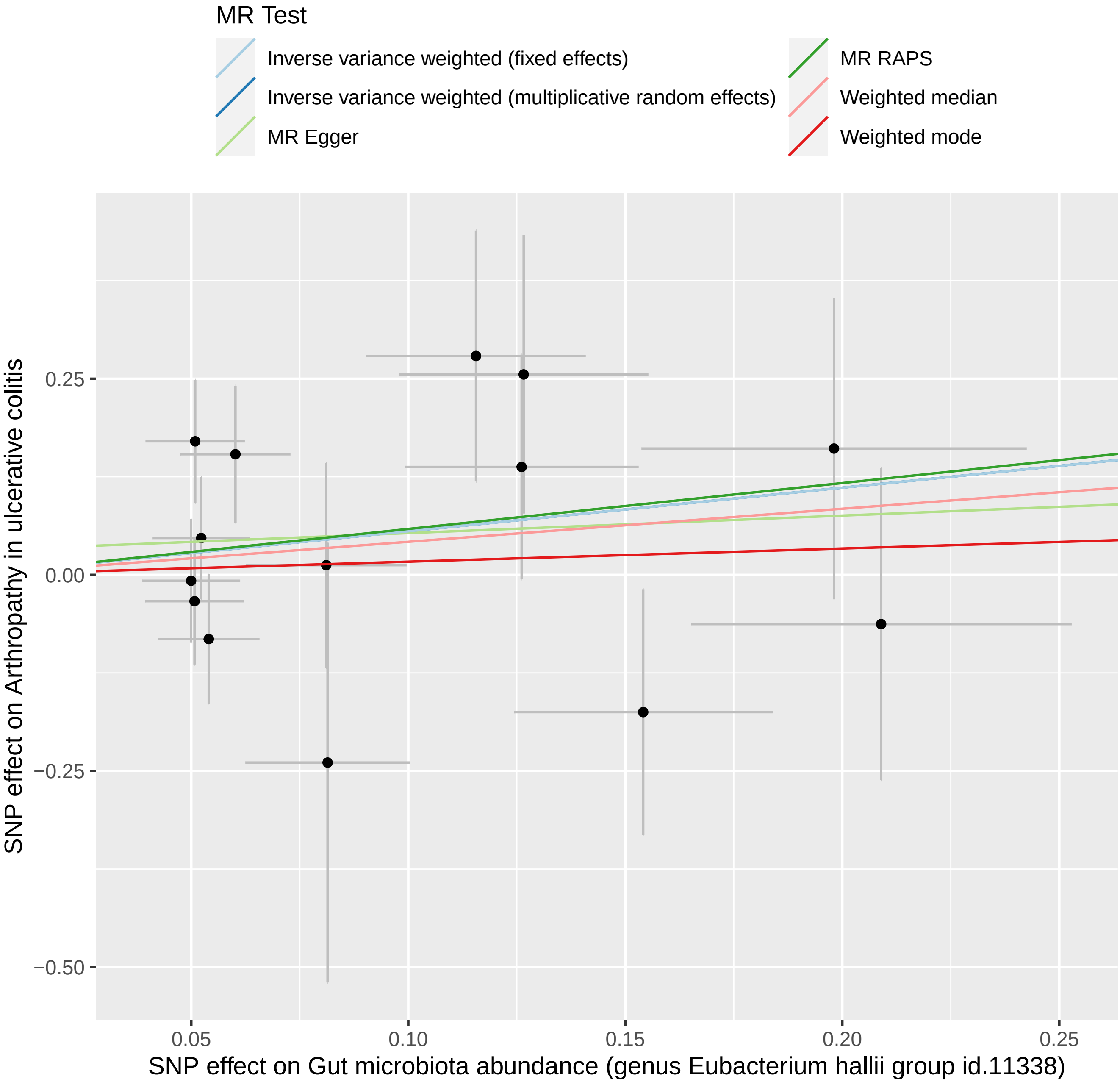

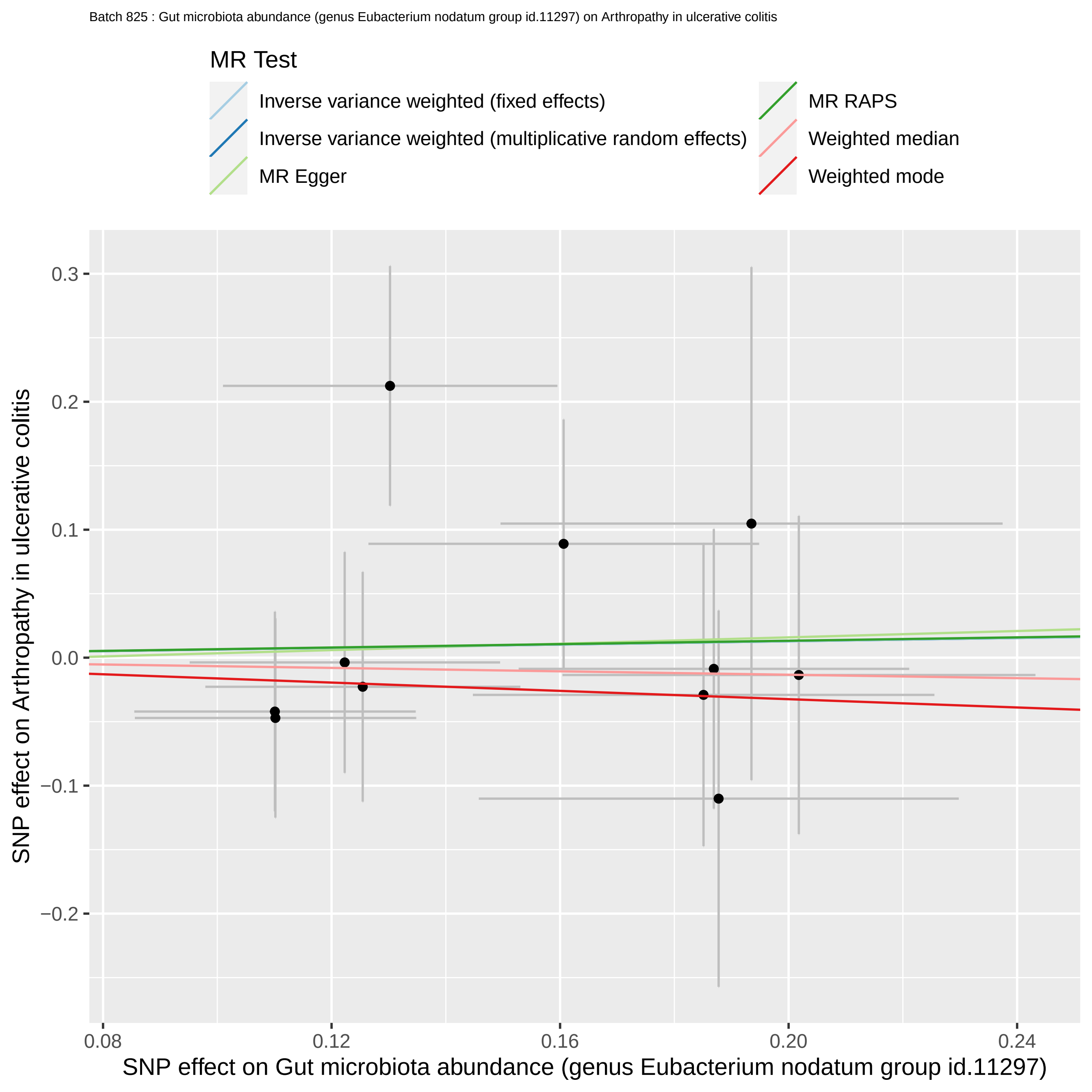
Batch 825 : Gut microbiota abundance (genus Eubacterium nodatum group id.11297) on Arthropathy in ulcerative colitis
MR Test
Inverse variance weighted (fixed effects)
Inverse variance weighted (multiplicative random effects)
MR Egger
MR RAPS
Weighted median
Weighted mode
SNP effect on Arthropathy in ulcerative colitis
0.3
0.2
0.1
0.0
−0.1
−0.2
0.08
0.12
0.16
0.20
0.24
SNP effect on Gut microbiota abundance (genus Eubacterium nodatum group id.11297)

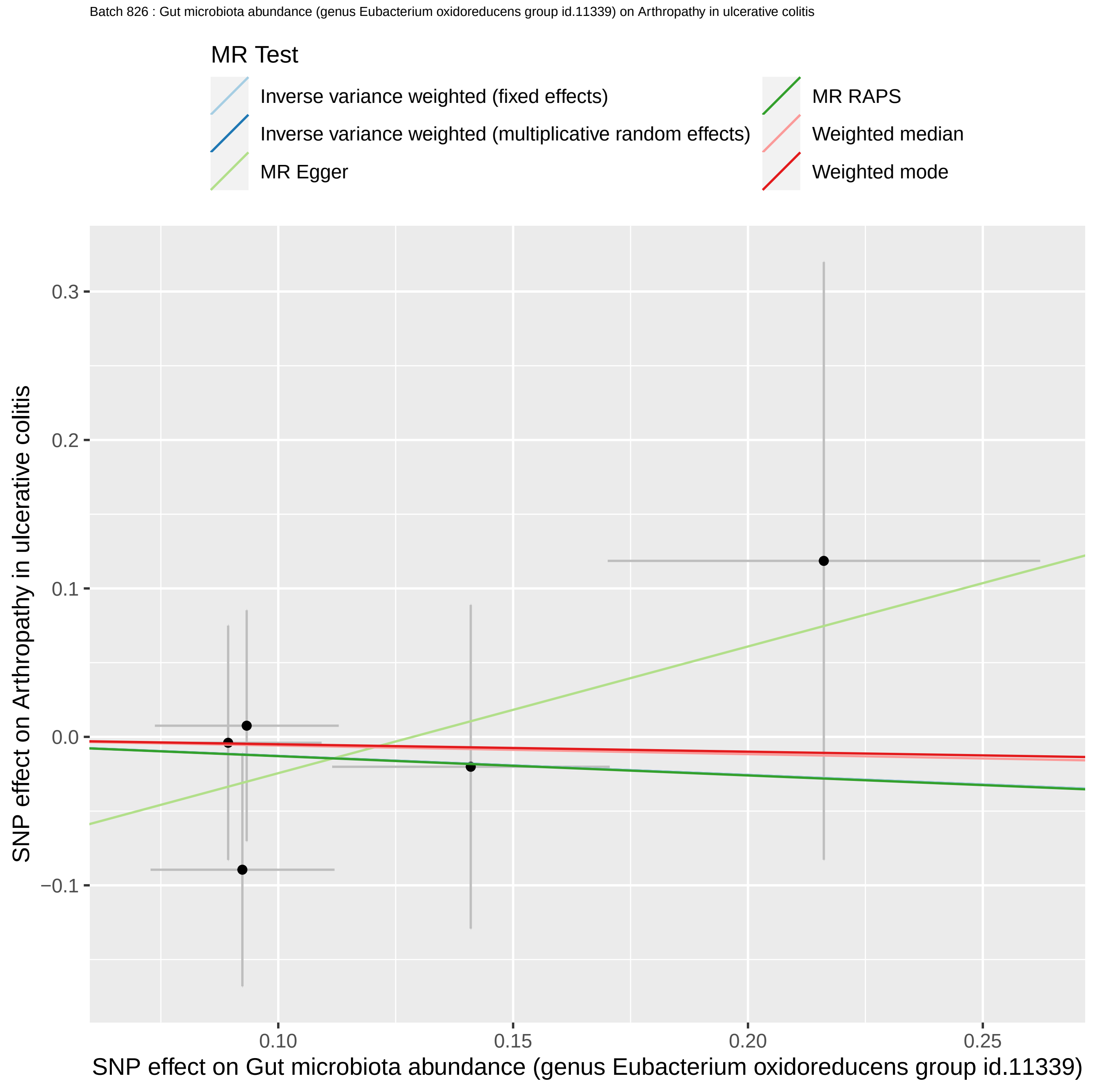

Batch 826 : Gut microbiota abundance (genus Eubacterium oxidoreducens group id.11339) on Arthropathy in ulcerative colitis
MR Test
Inverse variance weighted (fixed effects)
Inverse variance weighted (multiplicative random effects)
MR Egger
MR RAPS
Weighted median
Weighted mode
SNP effect on Arthropathy in ulcerative colitis
0.3
0.2
0.1
0.0
−0.1
0.10
0.15
0.20
0.25
SNP effect on Gut microbiota abundance (genus Eubacterium oxidoreducens group id.11339)

Batch 827 : Gut microbiota abundance (genus Eubacterium rectale group id.14374) on Arthropathy in ulcerative colitis

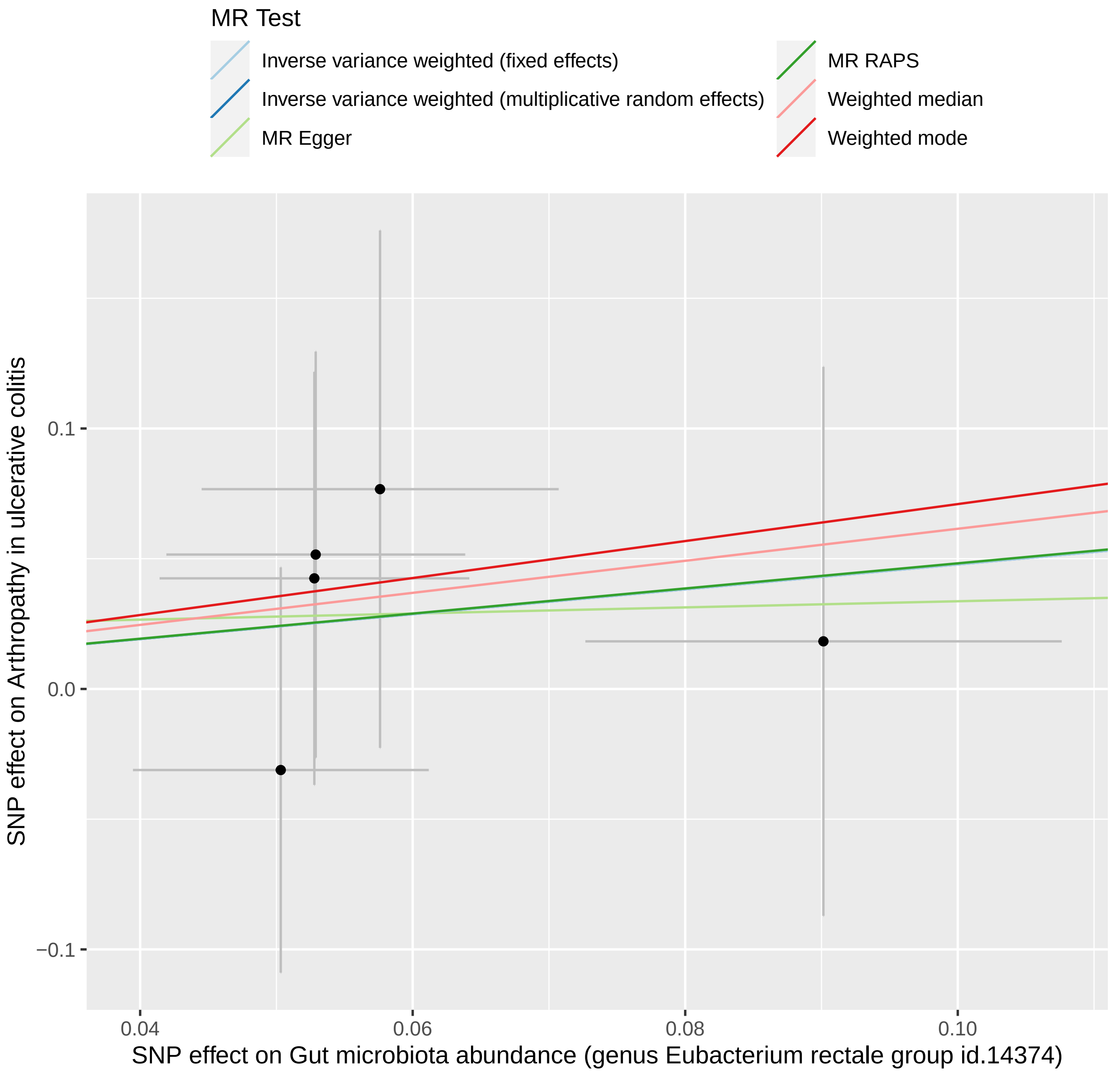

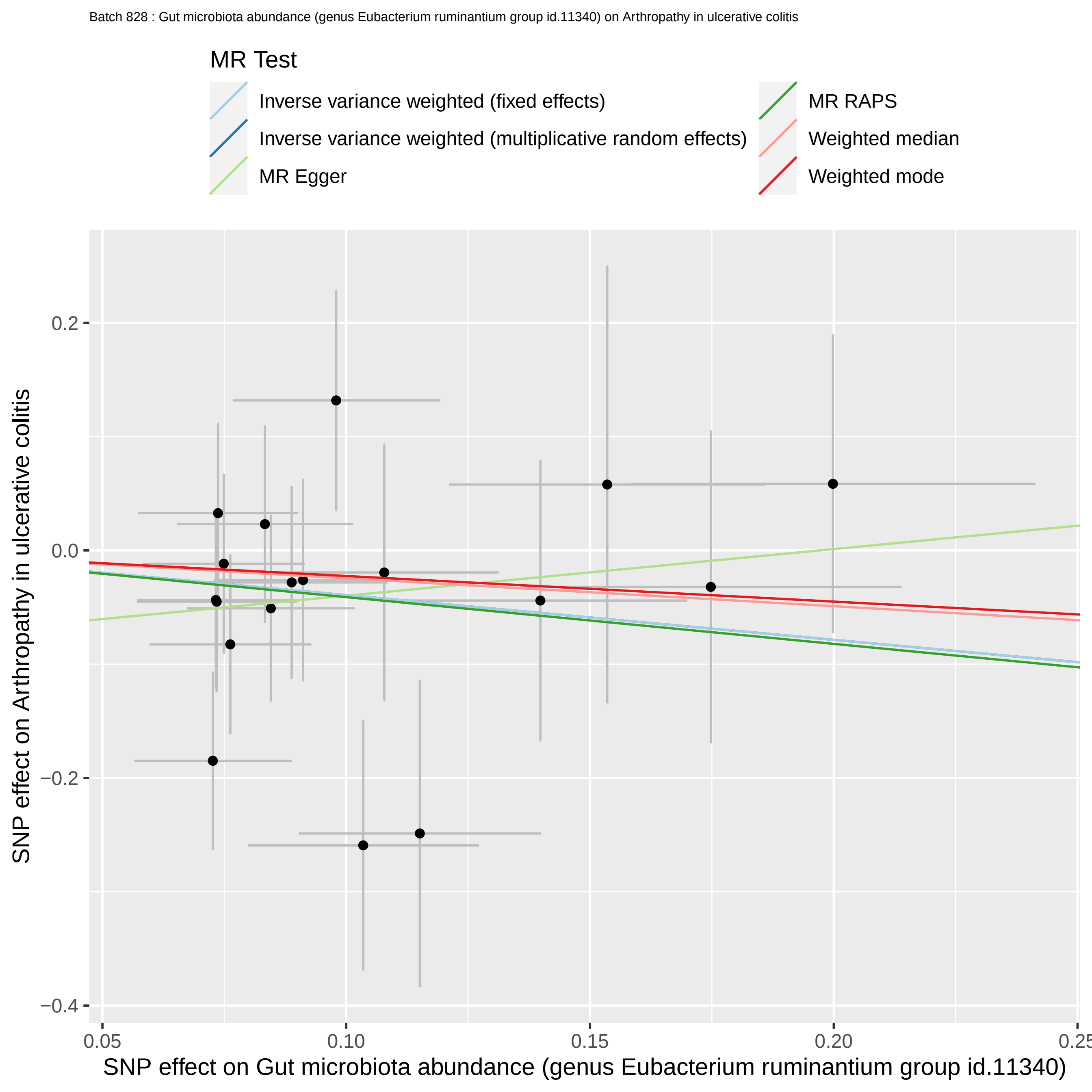

Batch 828 : Gut microbiota abundance (genus Eubacterium ruminantium group id.11340) on Arthropathy in ulcerative colitis
MR Test
Inverse variance weighted (fixed effects)
Inverse variance weighted (multiplicative random effects)
MR Egger
MR RAPS
Weighted median
Weighted mode
SNP effect on Arthropathy in ulcerative colitis
0.2
0.0
−0.2
−0.4
0.05
0.10
0.15
0.20
0.25
SNP effect on Gut microbiota abundance (genus Eubacterium ruminantium group id.11340)

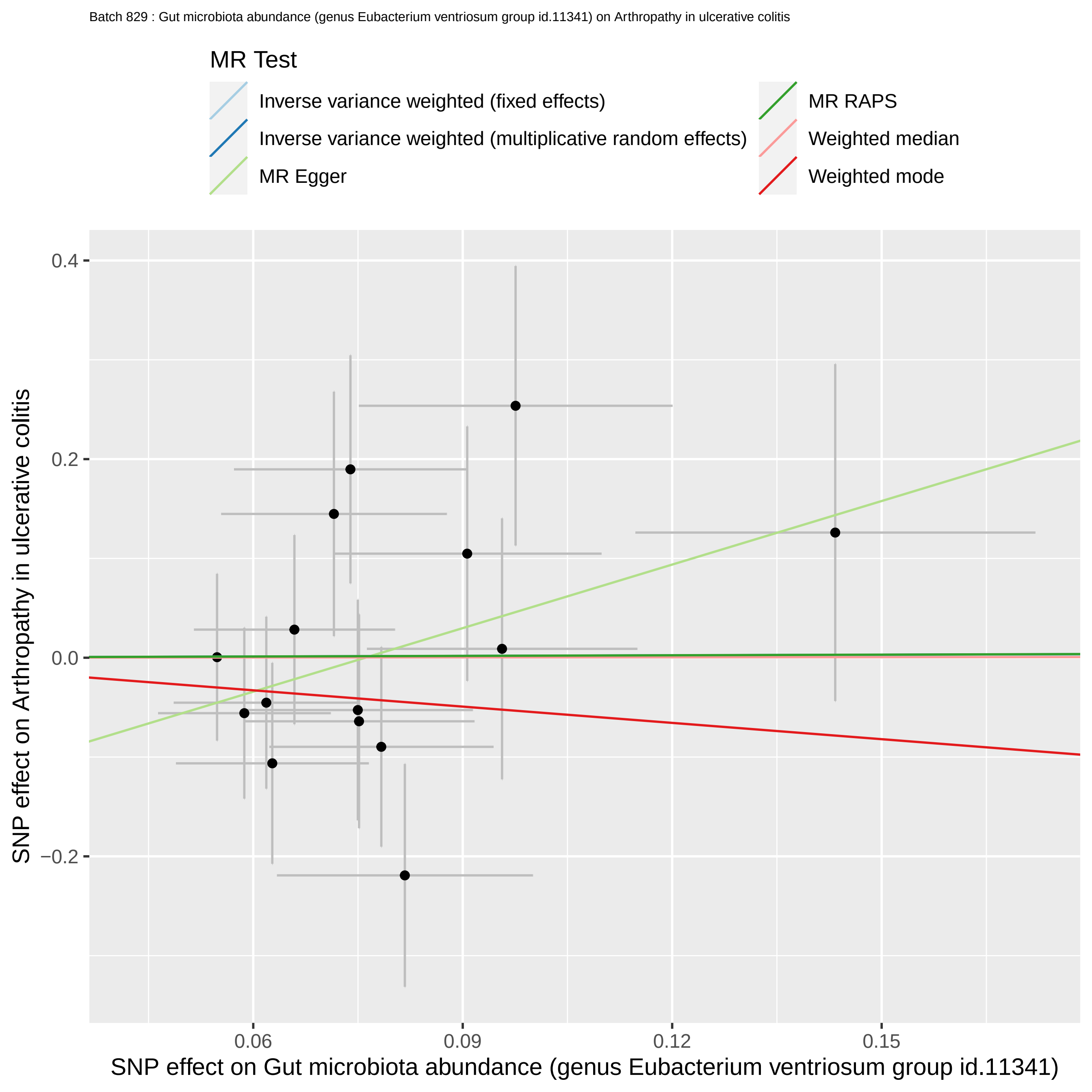

Batch 829 : Gut microbiota abundance (genus Eubacterium ventriosum group id.11341) on Arthropathy in ulcerative colitis
MR Test
Inverse variance weighted (fixed effects)
Inverse variance weighted (multiplicative random effects)
MR Egger
MR RAPS
Weighted median
Weighted mode
0.4
0.2
0.0
−0.2
0.06
0.09
0.12
0.15
SNP effect on Arthropathy in ulcerative colitis
SNP effect on Gut microbiota abundance (genus Eubacterium ventriosum group id.11341)

Batch 830 : Gut microbiota abundance (genus Eubacterium xylanophilum group id.14375) on Arthropathy in ulcerative colitis

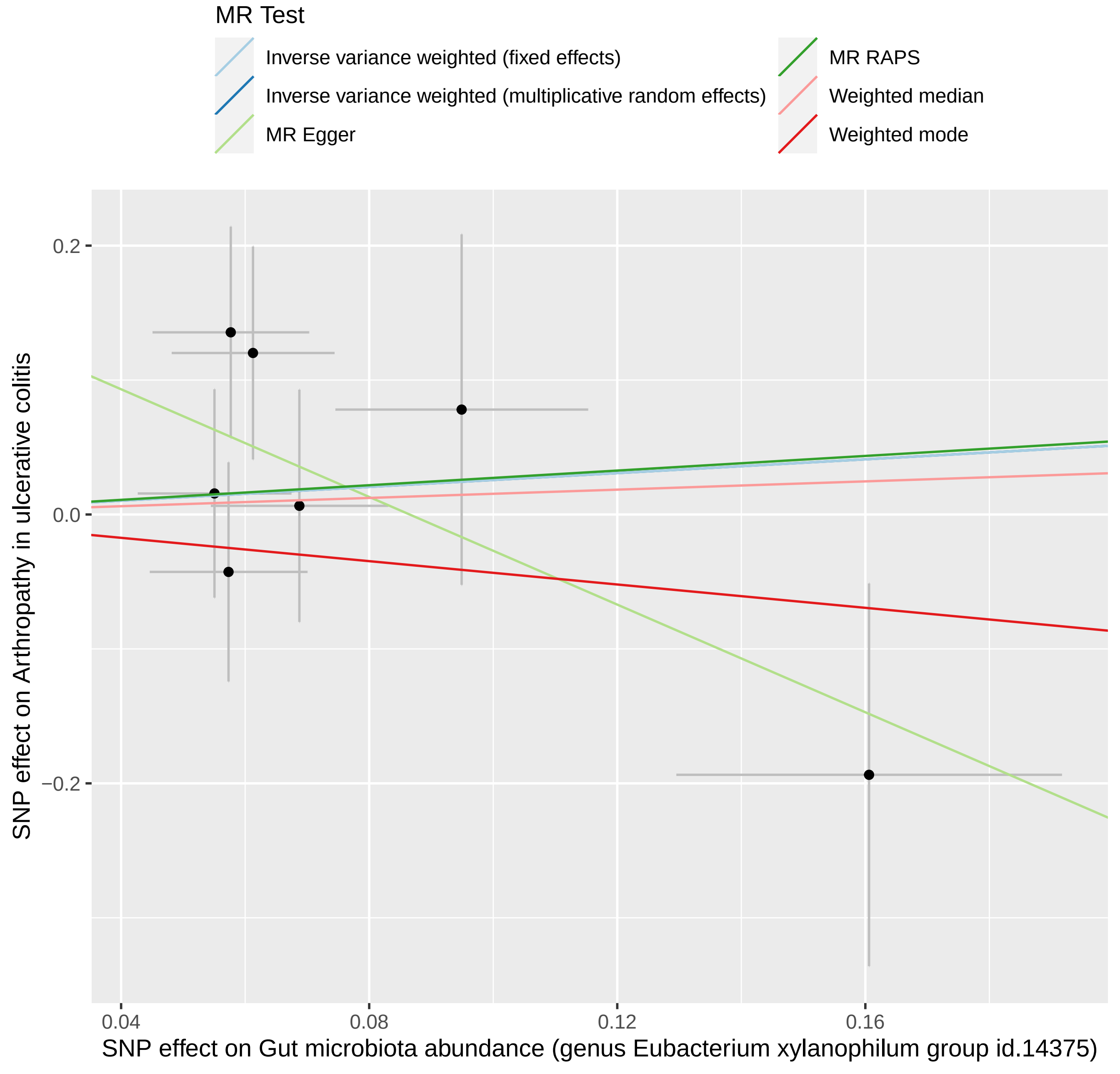

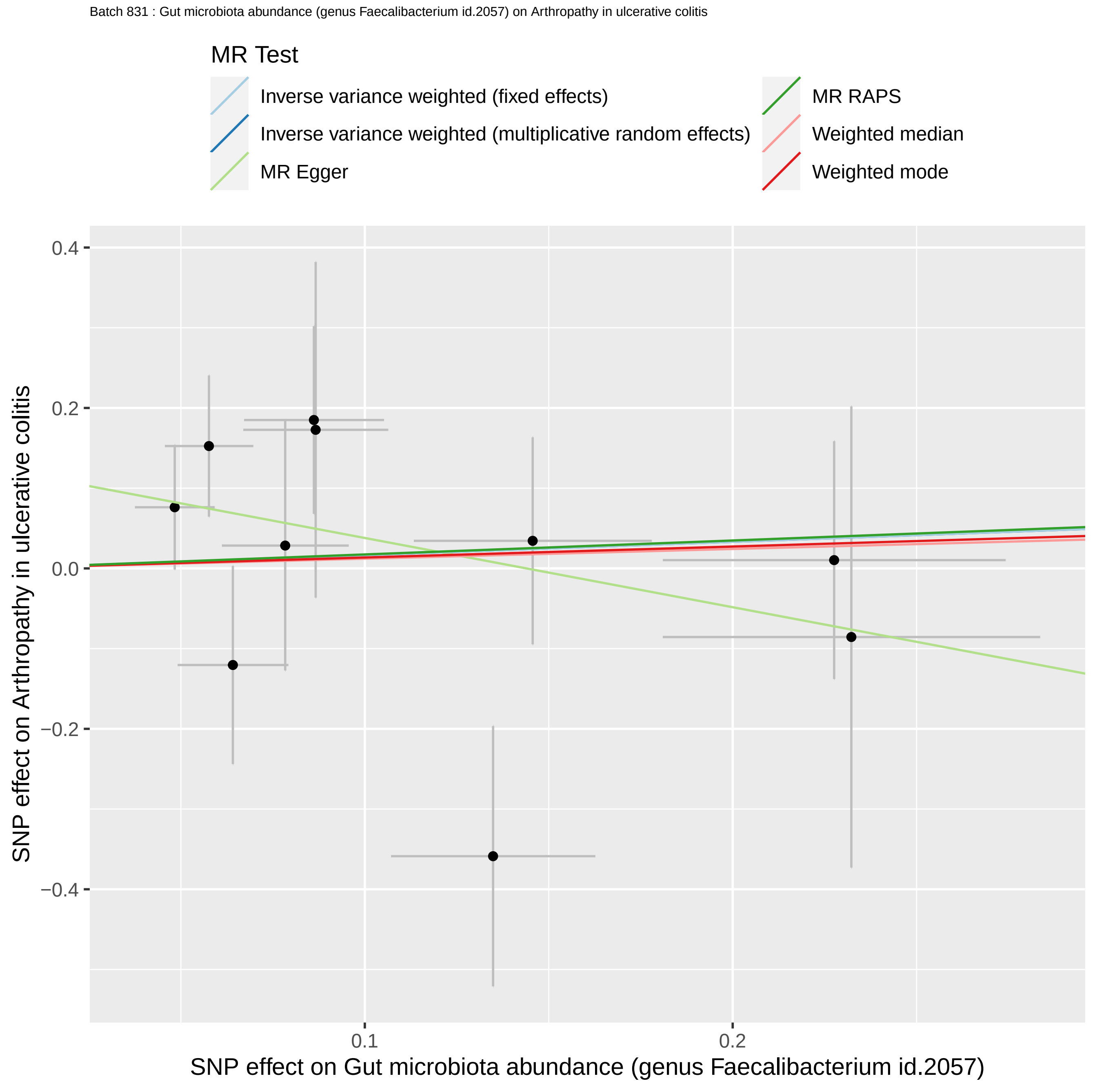

Batch 831 : Gut microbiota abundance (genus Faecalibacterium id.2057) on Arthropathy in ulcerative colitis
MR Test
Inverse variance weighted (fixed effects)
Inverse variance weighted (multiplicative random effects)
MR Egger
MR RAPS
Weighted median
Weighted mode
0.4
0.2
0.0
−0.2
−0.4
0.1
0.2
SNP effect on Arthropathy in ulcerative colitis
SNP effect on Gut microbiota abundance (genus Faecalibacterium id.2057)

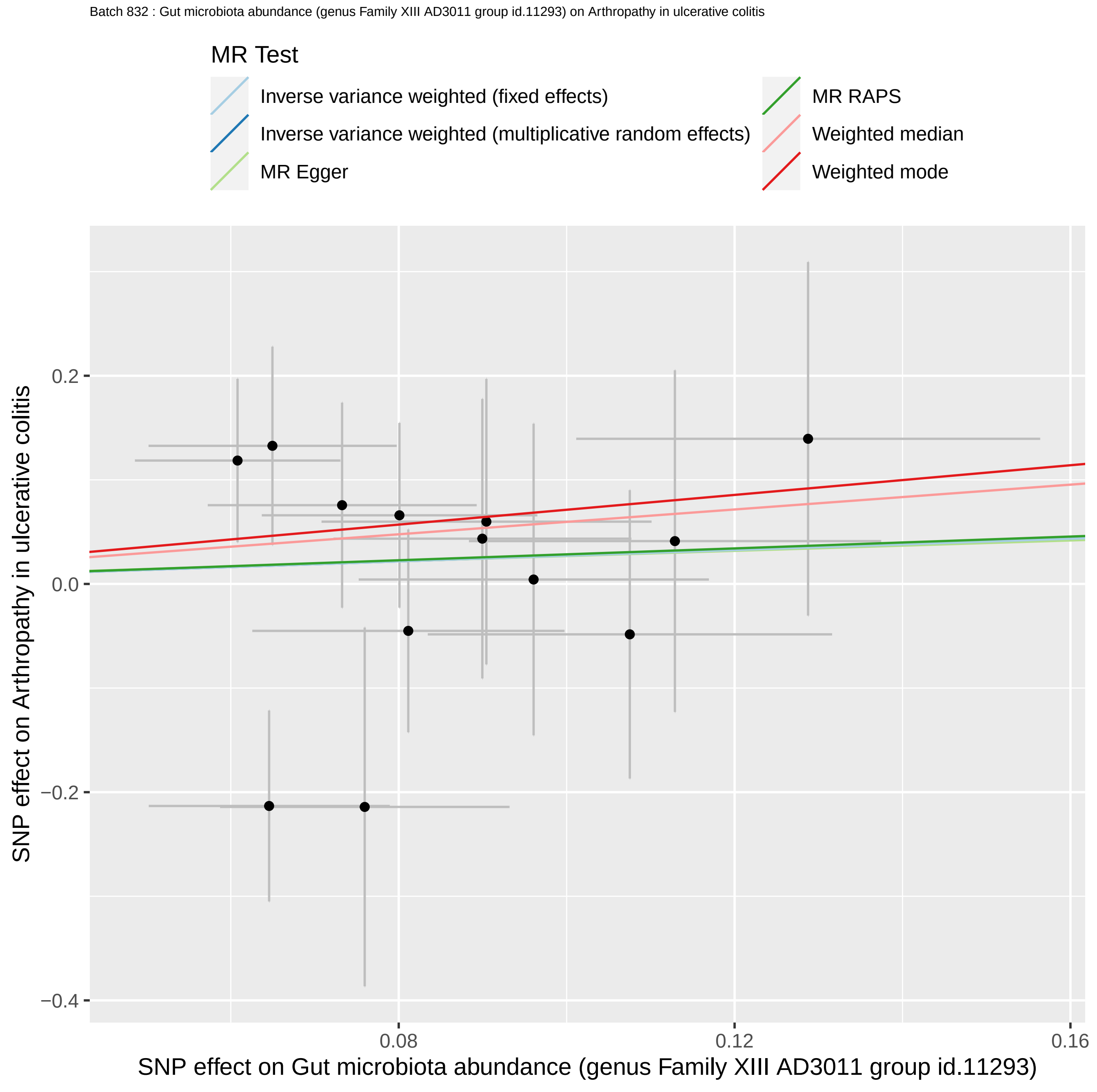

Batch 832 : Gut microbiota abundance (genus Family XIII AD3011 group id.11293) on Arthropathy in ulcerative colitis
MR Test
Inverse variance weighted (fixed effects)
Inverse variance weighted (multiplicative random effects)
MR Egger
MR RAPS
Weighted median
Weighted mode
SNP effect on Arthropathy in ulcerative colitis
0.2
0.0
−0.2
−0.4
0.08
0.12
0.16
SNP effect on Gut microbiota abundance (genus Family XIII AD3011 group id.11293)

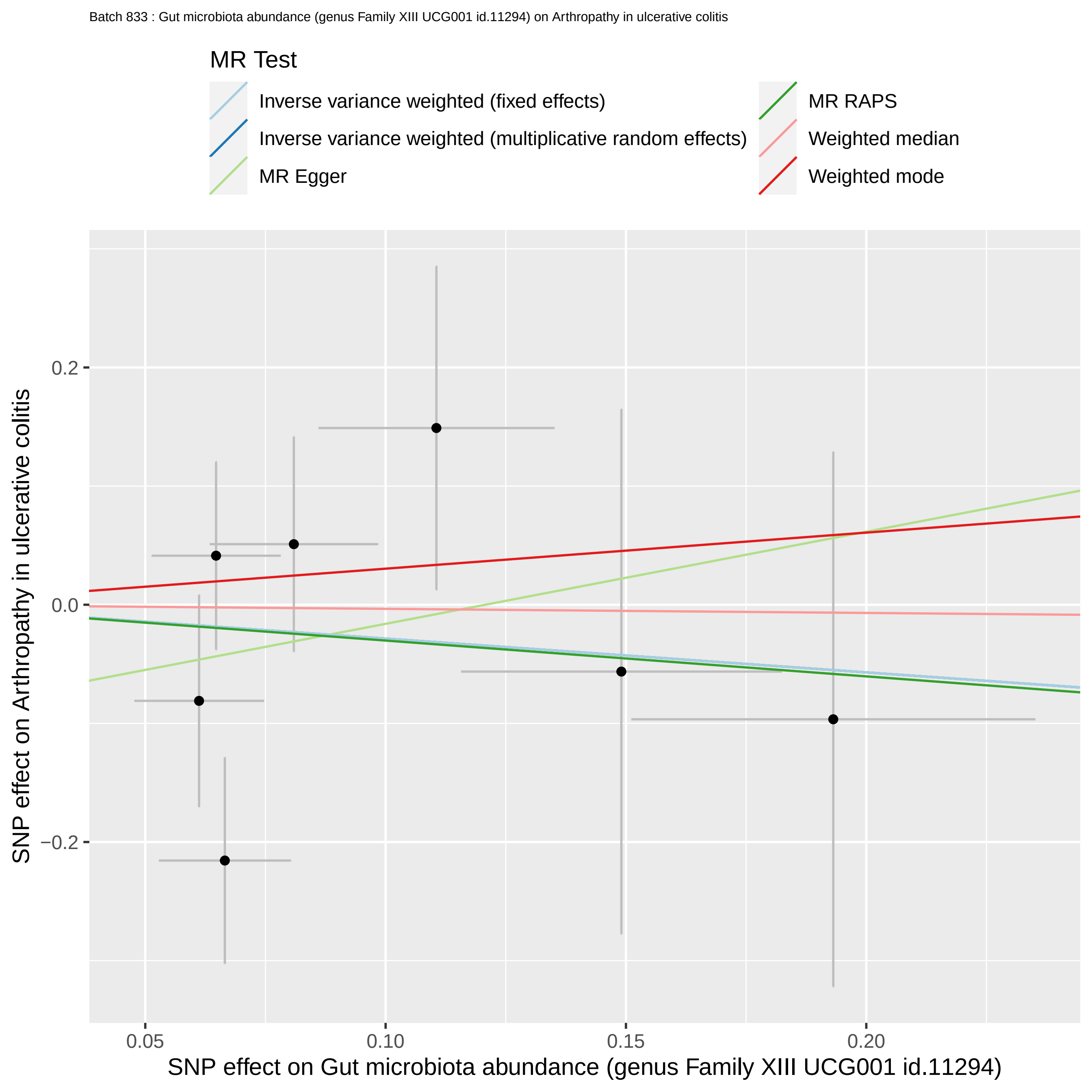

Batch 833 : Gut microbiota abundance (genus Family XIII UCG001 id.11294) on Arthropathy in ulcerative colitis
MR Test
Inverse variance weighted (fixed effects)
Inverse variance weighted (multiplicative random effects)
MR Egger
MR RAPS
Weighted median
Weighted mode
0.2
0.0
−0.2
0.05
0.10
0.15
0.20
SNP effect on Arthropathy in ulcerative colitis
SNP effect on Gut microbiota abundance (genus Family XIII UCG001 id.11294)

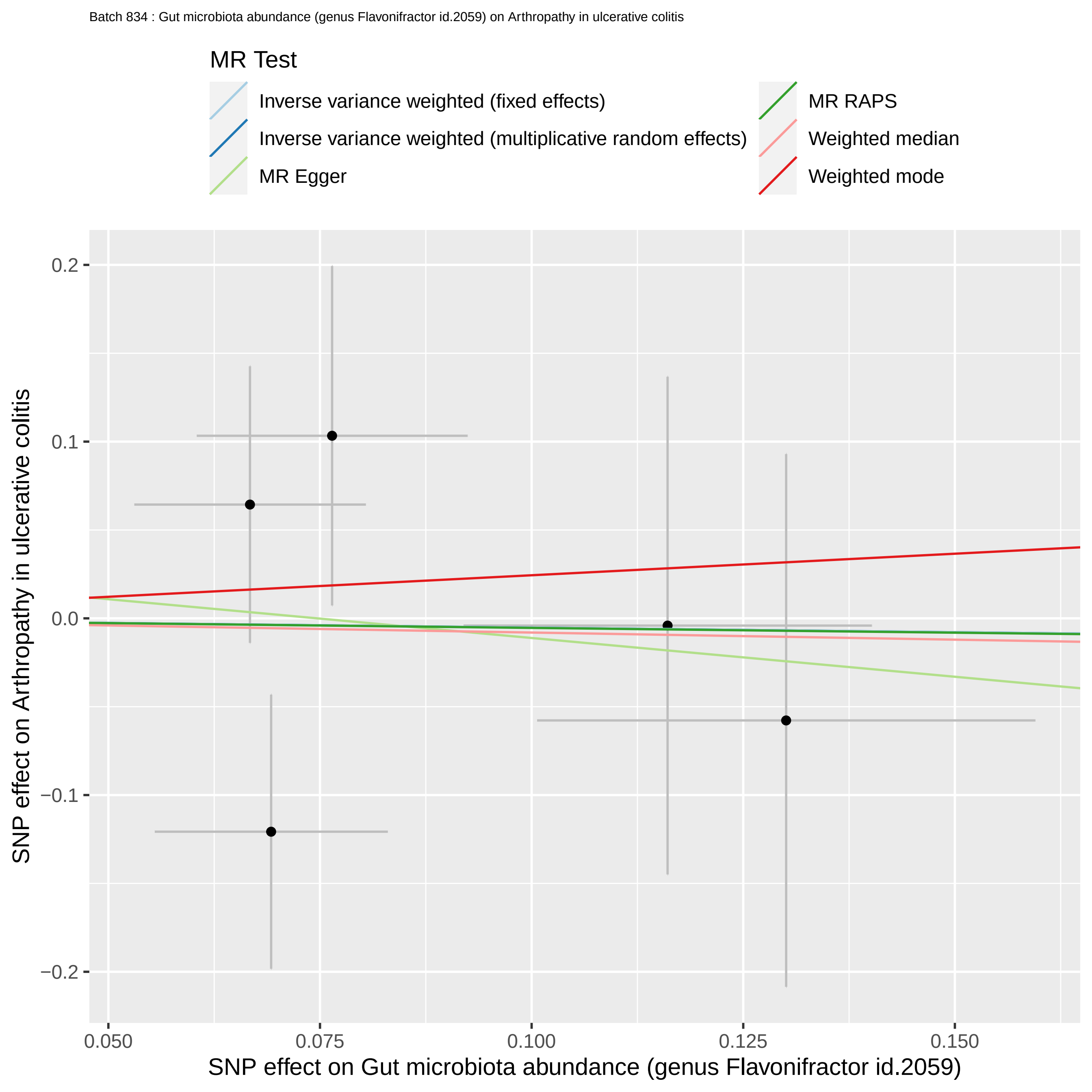

Batch 834 : Gut microbiota abundance (genus Flavonifractor id.2059) on Arthropathy in ulcerative colitis
MR Test
Inverse variance weighted (fixed effects)
Inverse variance weighted (multiplicative random effects)
MR Egger
MR RAPS
Weighted median
Weighted mode
SNP effect on Arthropathy in ulcerative colitis
0.2
0.1
0.0
−0.1
−0.2
0.050
0.075
0.100
0.125
0.150
SNP effect on Gut microbiota abundance (genus Flavonifractor id.2059)

Batch 835 : Gut microbiota abundance (genus Fusicatenibacter id.11305) on Arthropathy in ulcerative colitis

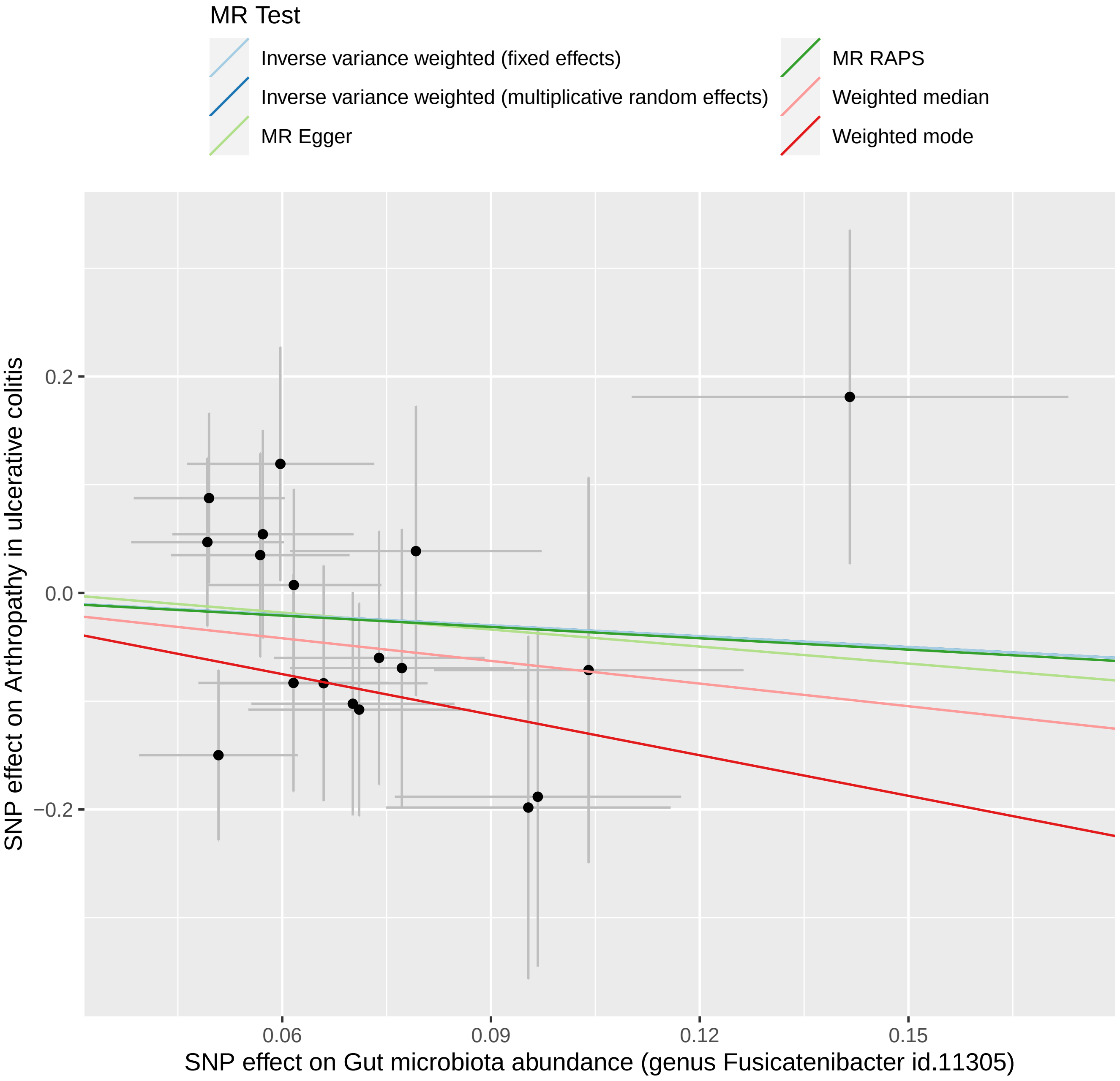

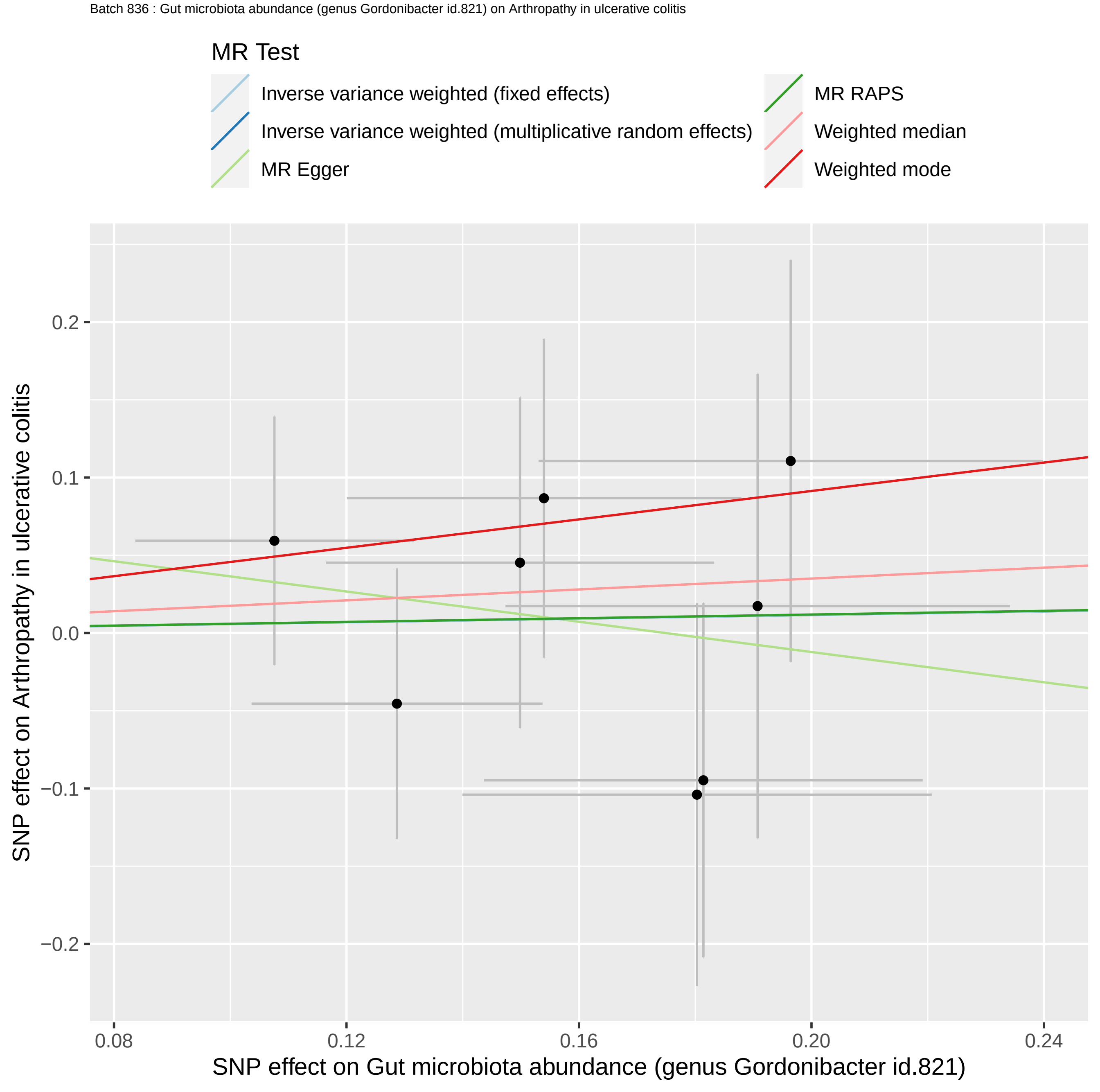
Batch 836 : Gut microbiota abundance (genus Gordonibacter id.821) on Arthropathy in ulcerative colitis
MR Test
Inverse variance weighted (fixed effects)
Inverse variance weighted (multiplicative random effects)
MR Egger
MR RAPS
Weighted median
Weighted mode
0.2
0.1
0.0
−0.1
−0.2
0.08
0.12
0.16
0.20
0.24
SNP effect on Arthropathy in ulcerative colitis
SNP effect on Gut microbiota abundance (genus Gordonibacter id.821)

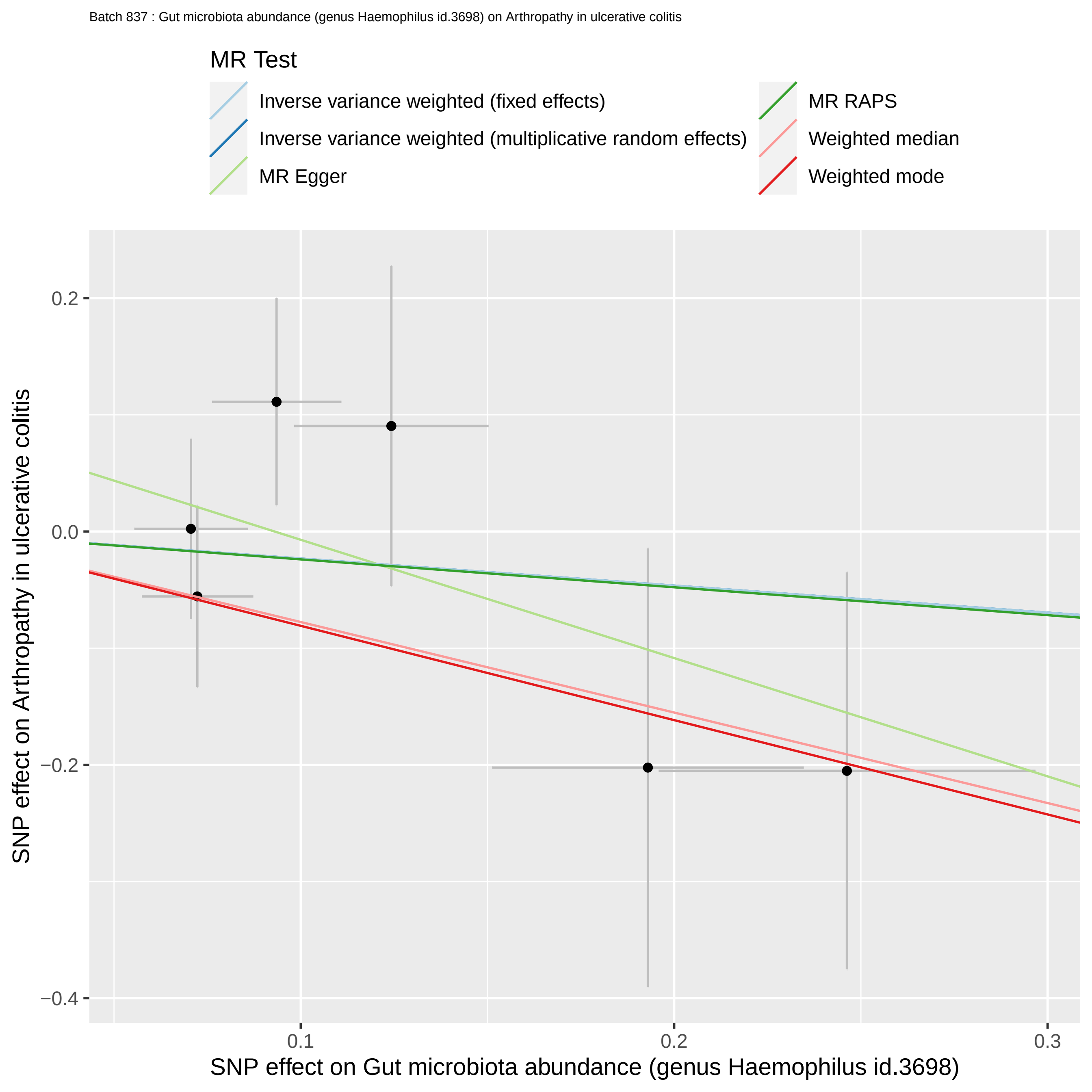

Batch 837 : Gut microbiota abundance (genus Haemophilus id.3698) on Arthropathy in ulcerative colitis
MR Test
Inverse variance weighted (fixed effects)
Inverse variance weighted (multiplicative random effects)
MR Egger
MR RAPS
Weighted median
Weighted mode
0.2
0.0
−0.2
−0.4
0.1
0.2
0.3
SNP effect on Arthropathy in ulcerative colitis
SNP effect on Gut microbiota abundance (genus Haemophilus id.3698)

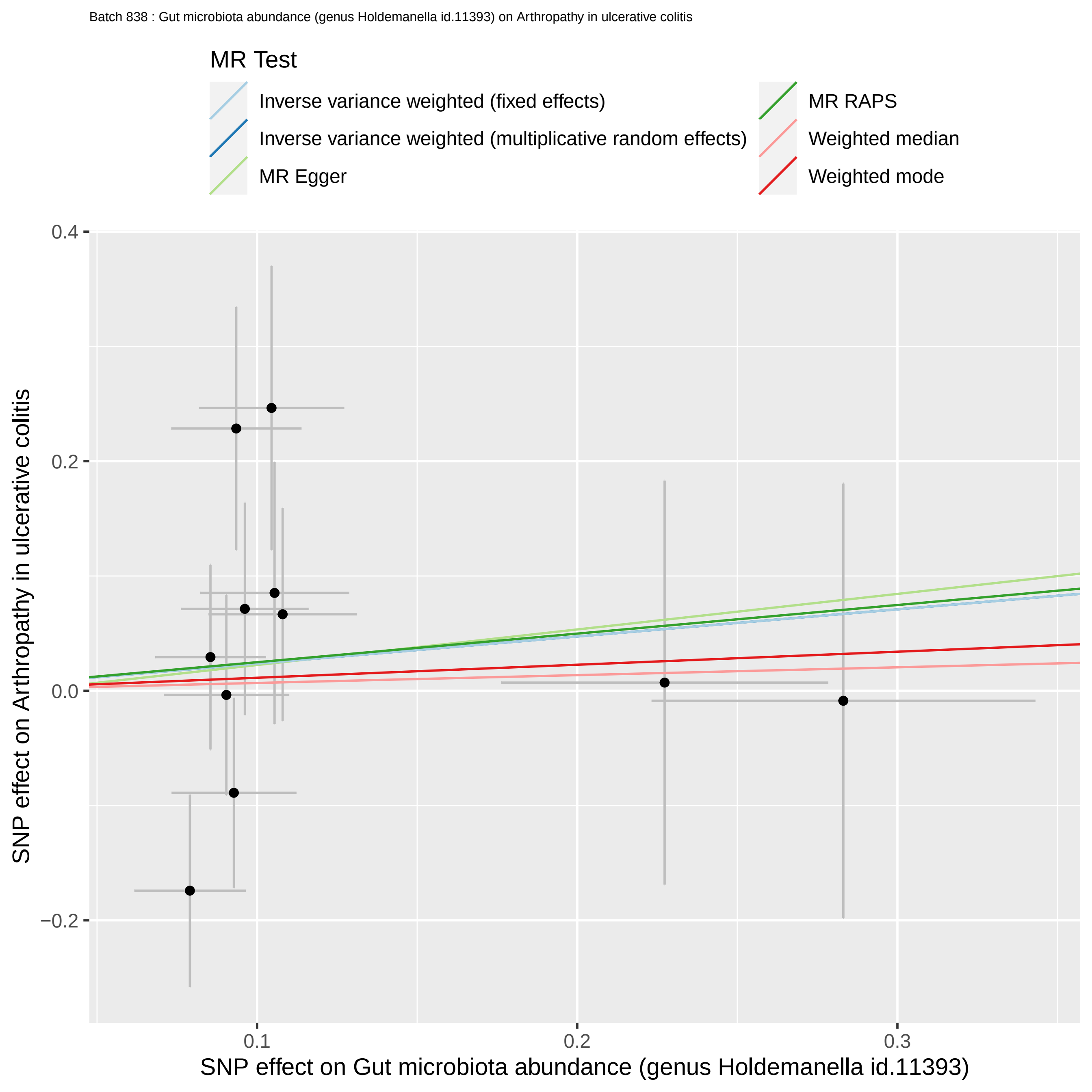

Batch 838 : Gut microbiota abundance (genus Holdemanella id.11393) on Arthropathy in ulcerative colitis
MR Test
Inverse variance weighted (fixed effects)
Inverse variance weighted (multiplicative random effects)
MR Egger
MR RAPS
Weighted median
Weighted mode
0.4
0.2
0.0
−0.2
0.1
0.2
0.3
SNP effect on Arthropathy in ulcerative colitis
SNP effect on Gut microbiota abundance (genus Holdemanella id.11393)

Batch 839 : Gut microbiota abundance (genus Holdemania id.2157) on Arthropathy in ulcerative colitis

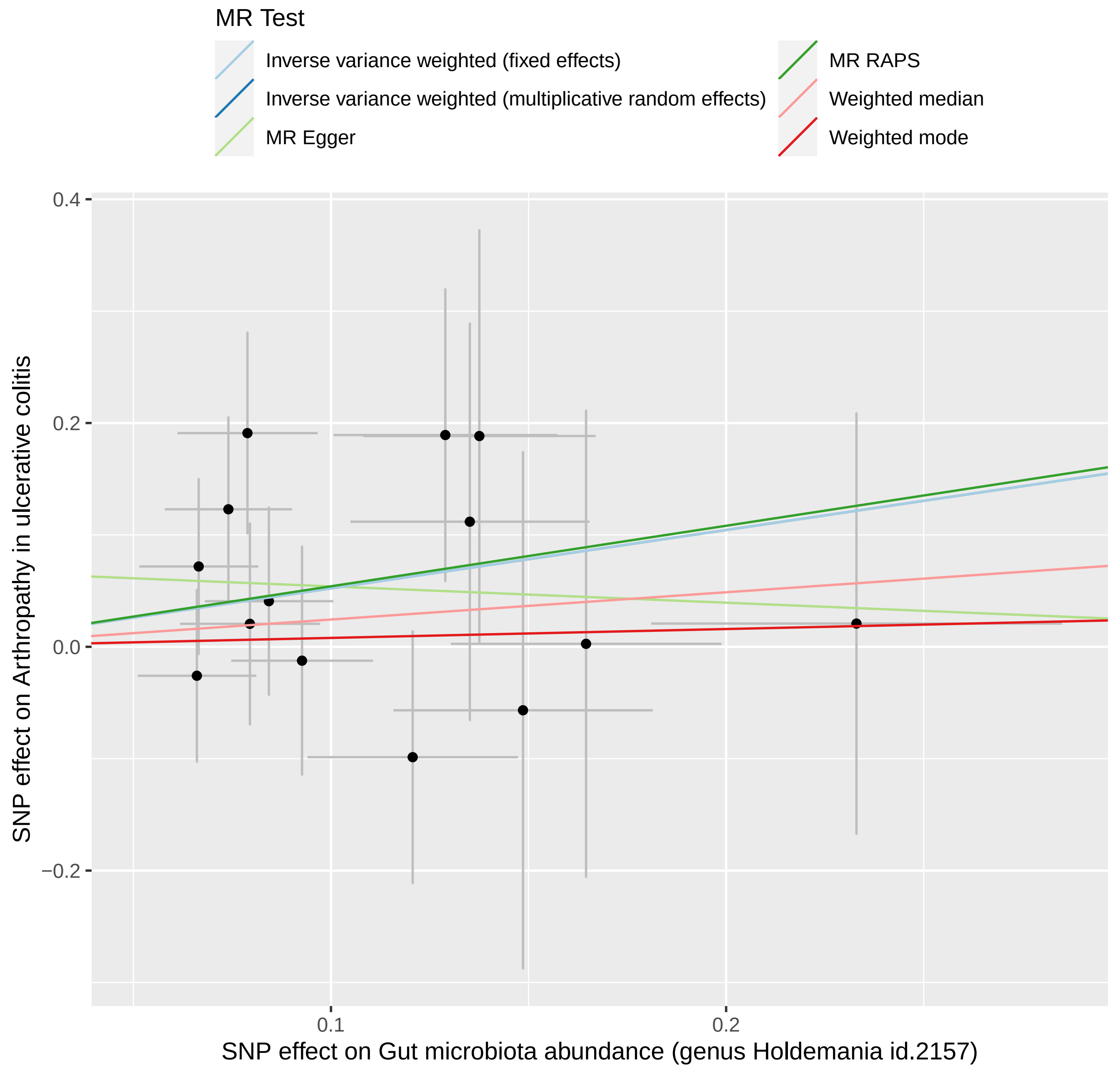

Batch 840 : Gut microbiota abundance (genus Howardella id.2000) on Arthropathy in ulcerative colitis

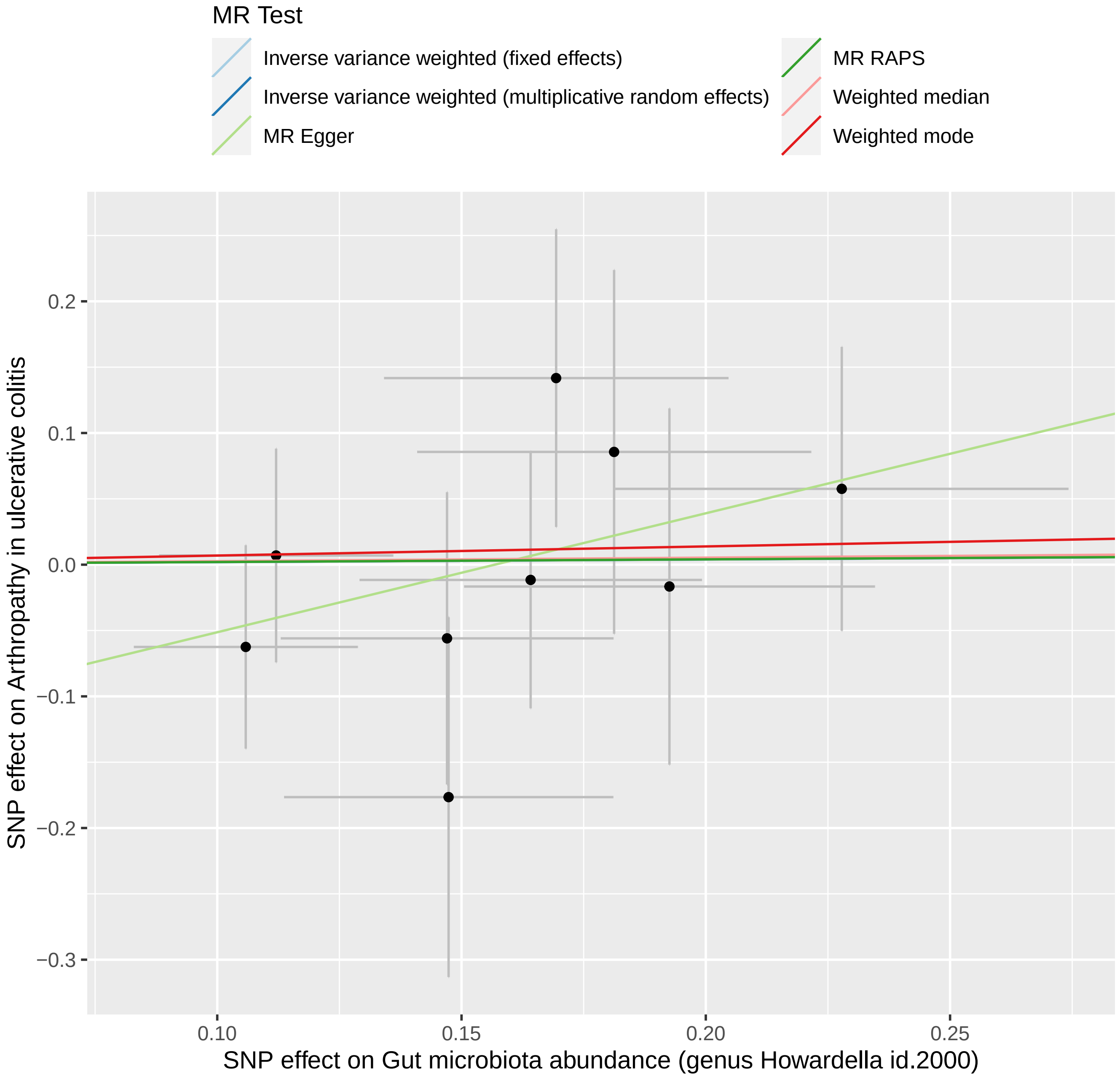

Batch 841 : Gut microbiota abundance (genus Hungatella id.11306) on Arthropathy in ulcerative colitis

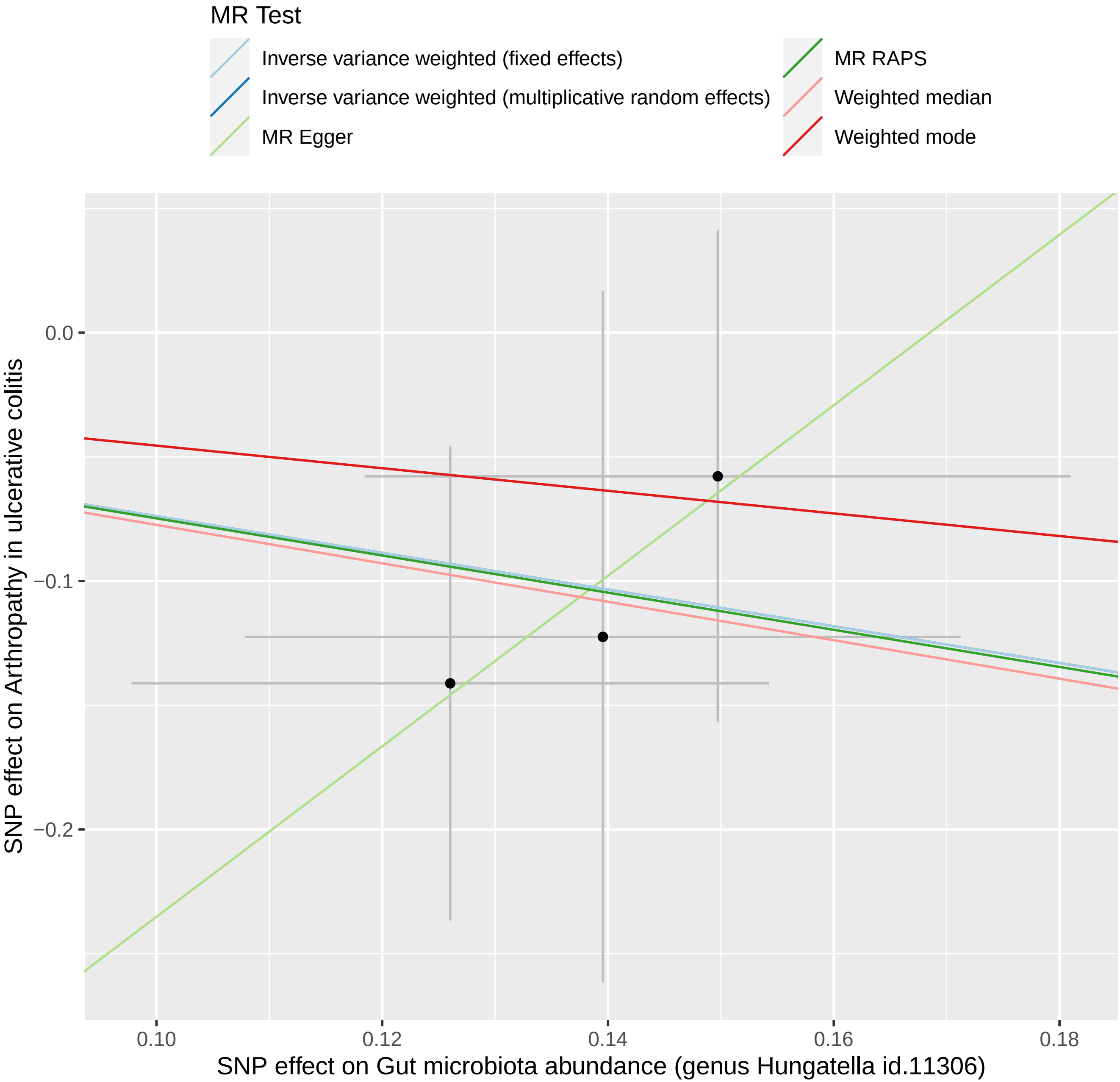

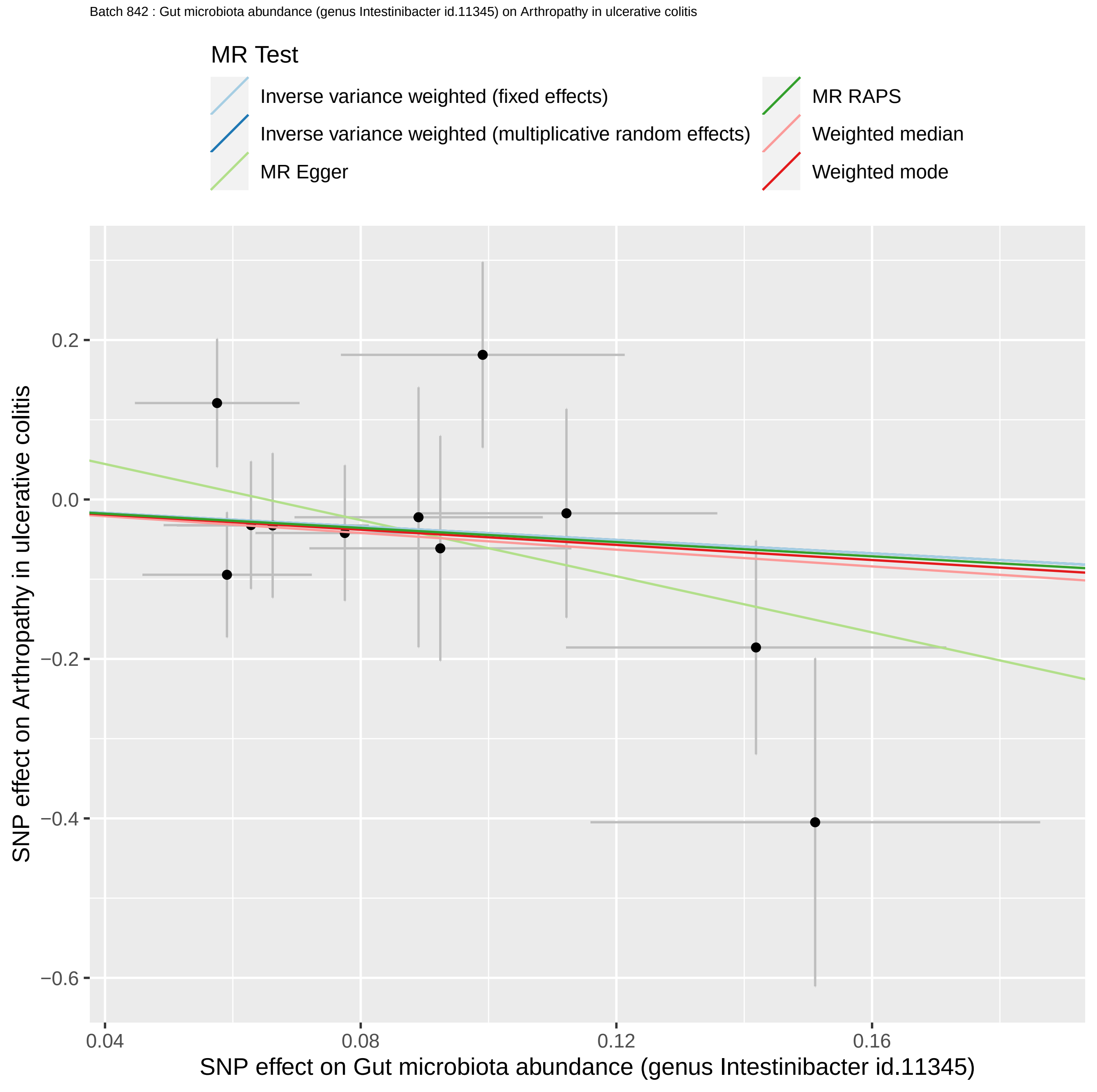

Batch 842 : Gut microbiota abundance (genus Intestinibacter id.11345) on Arthropathy in ulcerative colitis
MR Test
Inverse variance weighted (fixed effects)
Inverse variance weighted (multiplicative random effects)
MR Egger
MR RAPS
Weighted median
Weighted mode
0.2
0.0
−0.2
−0.4
−0.6
0.04
0.08
0.12
0.16
SNP effect on Arthropathy in ulcerative colitis
SNP effect on Gut microbiota abundance (genus Intestinibacter id.11345)

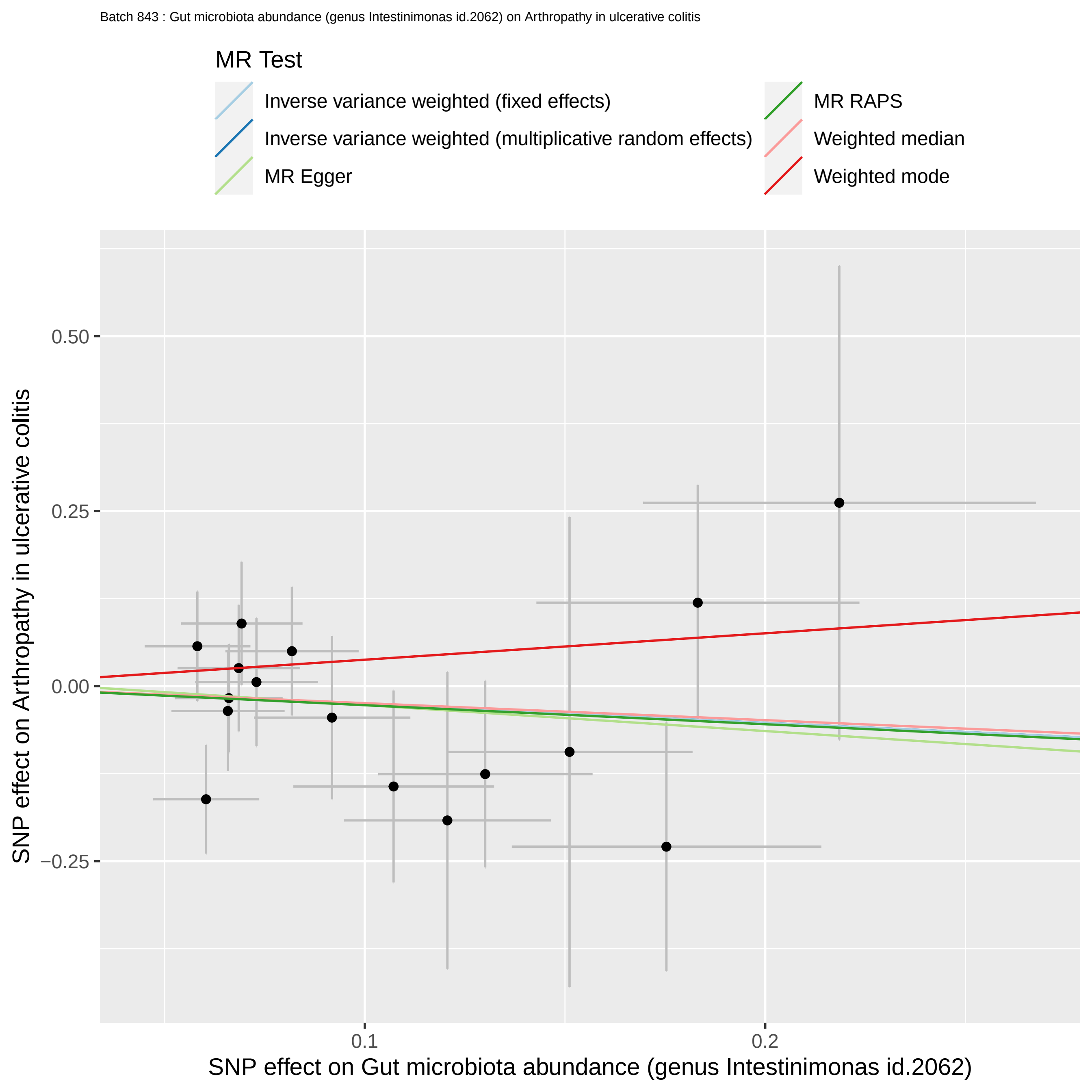

Batch 843 : Gut microbiota abundance (genus Intestinimonas id.2062) on Arthropathy in ulcerative colitis
MR Test
Inverse variance weighted (fixed effects)
Inverse variance weighted (multiplicative random effects)
MR Egger
MR RAPS
Weighted median
Weighted mode
0.50
0.25
0.00
−0.25
0.1
0.2
SNP effect on Arthropathy in ulcerative colitis
SNP effect on Gut microbiota abundance (genus Intestinimonas id.2062)

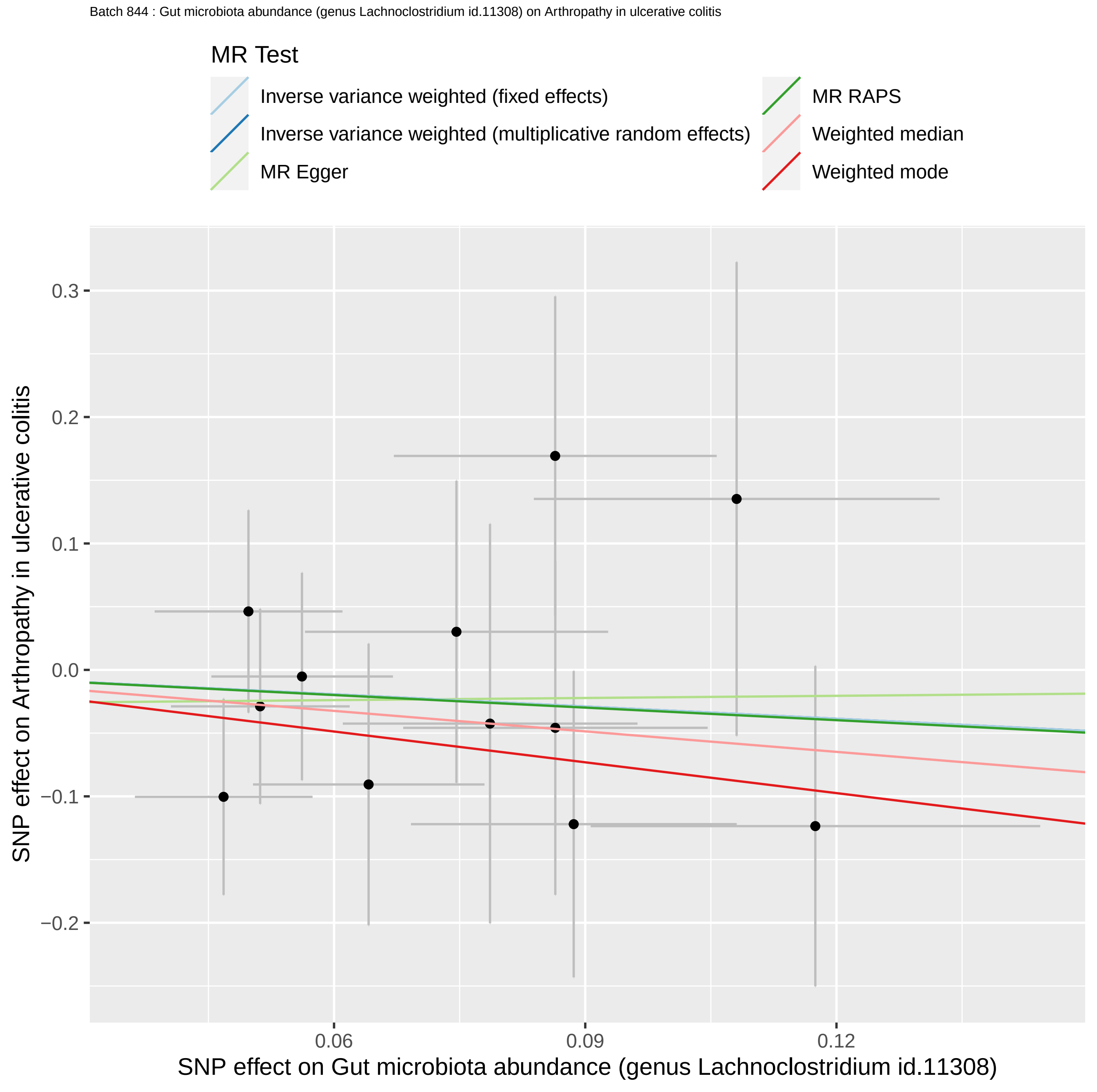

Batch 844 : Gut microbiota abundance (genus Lachnoclostridium id.11308) on Arthropathy in ulcerative colitis
MR Test
Inverse variance weighted (fixed effects)
Inverse variance weighted (multiplicative random effects)
MR Egger
MR RAPS
Weighted median
Weighted mode
SNP effect on Arthropathy in ulcerative colitis
0.3
0.2
0.1
0.0
−0.1
−0.2
0.06
0.09
0.12
SNP effect on Gut microbiota abundance (genus Lachnoclostridium id.11308)

Batch 845 : Gut microbiota abundance (genus Lachnospiraceae FCS020 group id.11314) on Arthropathy in ulcerative colitis

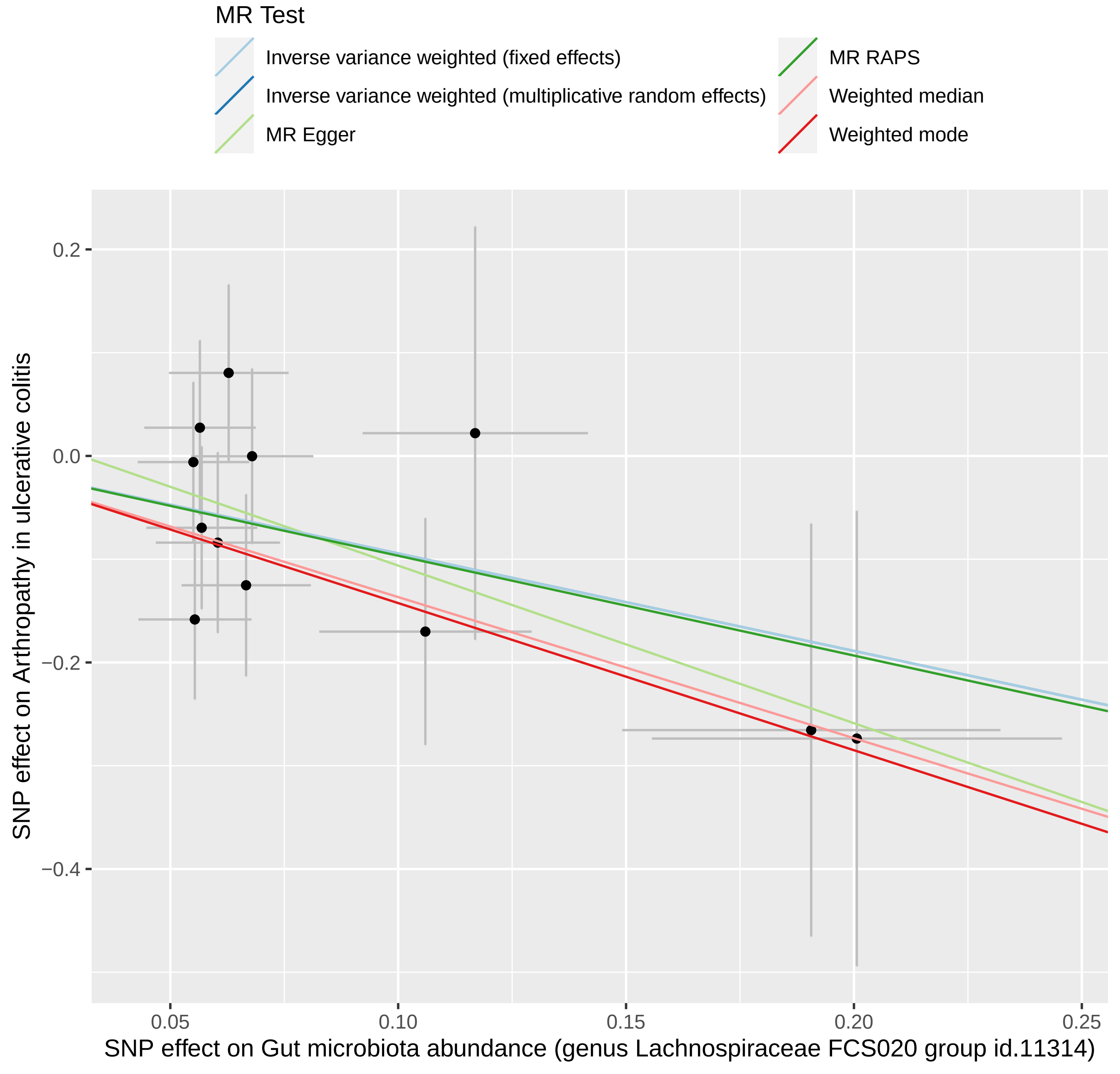

Batch 846 : Gut microbiota abundance (genus Lachnospiraceae NC2004 group id.11316) on Arthropathy in ulcerative colitis

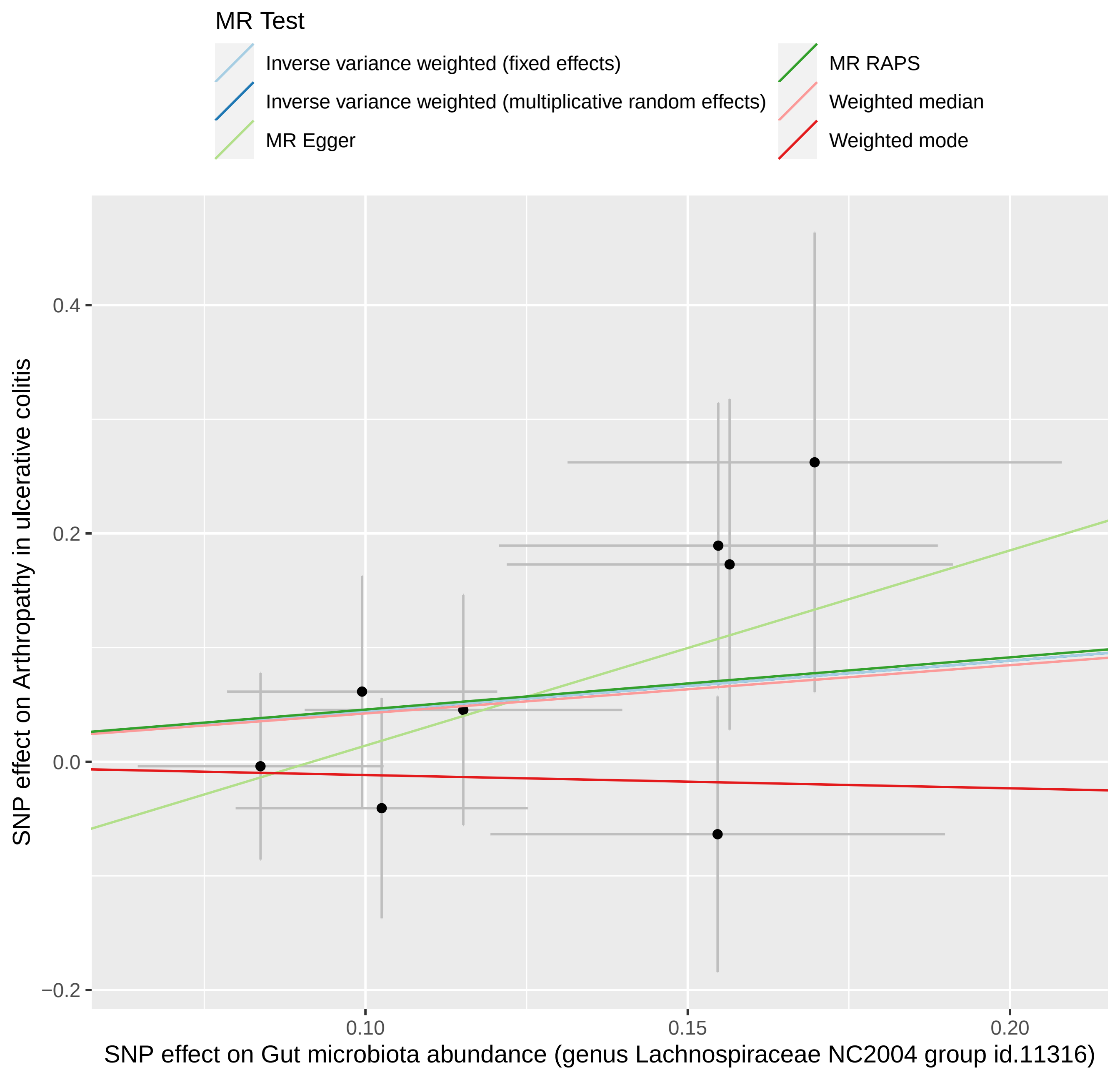

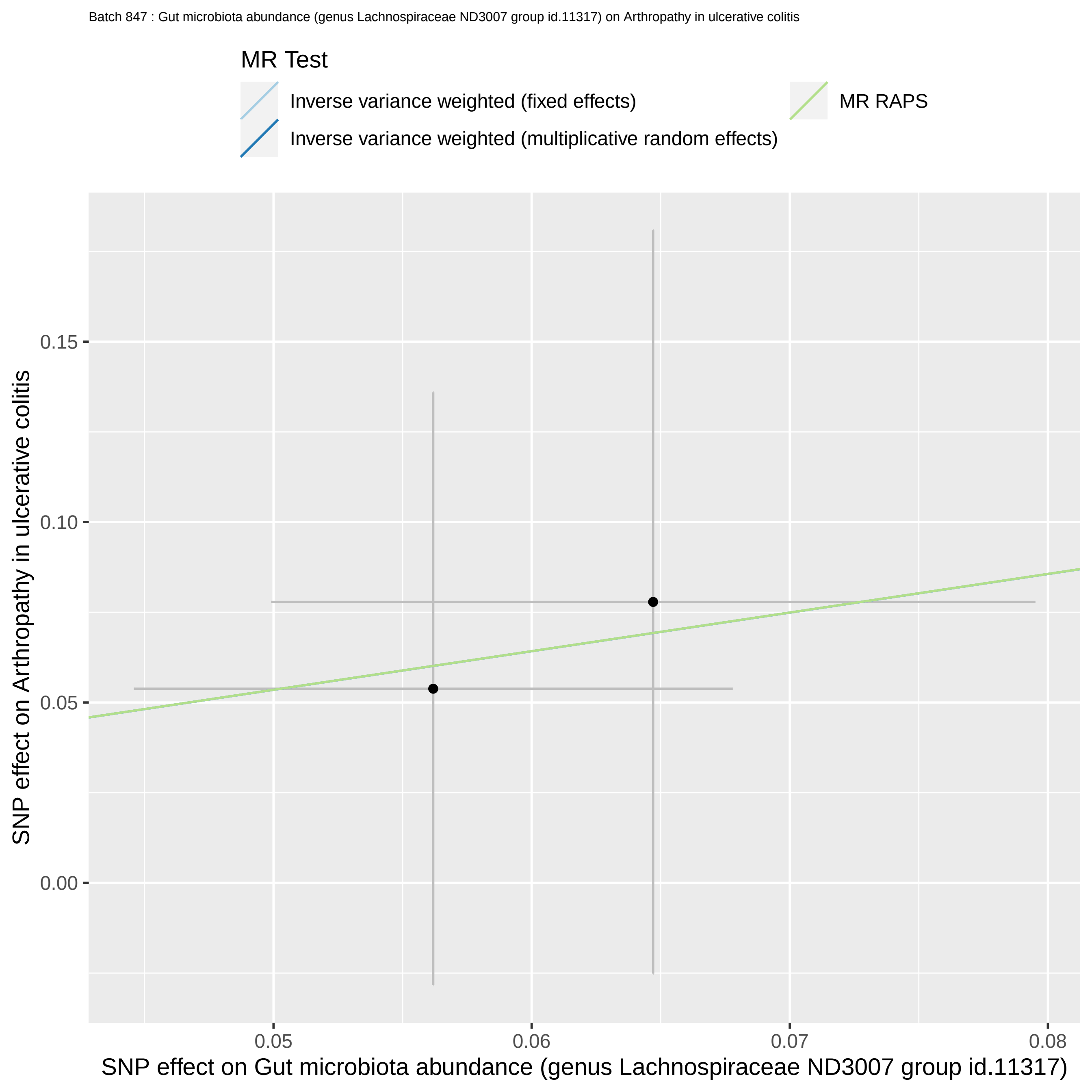

Batch 847 : Gut microbiota abundance (genus Lachnospiraceae ND3007 group id.11317) on Arthropathy in ulcerative colitis
MR Test
Inverse variance weighted (fixed effects)
Inverse variance weighted (multiplicative random effects)
MR RAPS
SNP effect on Arthropathy in ulcerative colitis
0.15
0.10
0.05
0.00
0.05
0.06
0.07
0.08
SNP effect on Gut microbiota abundance (genus Lachnospiraceae ND3007 group id.11317)

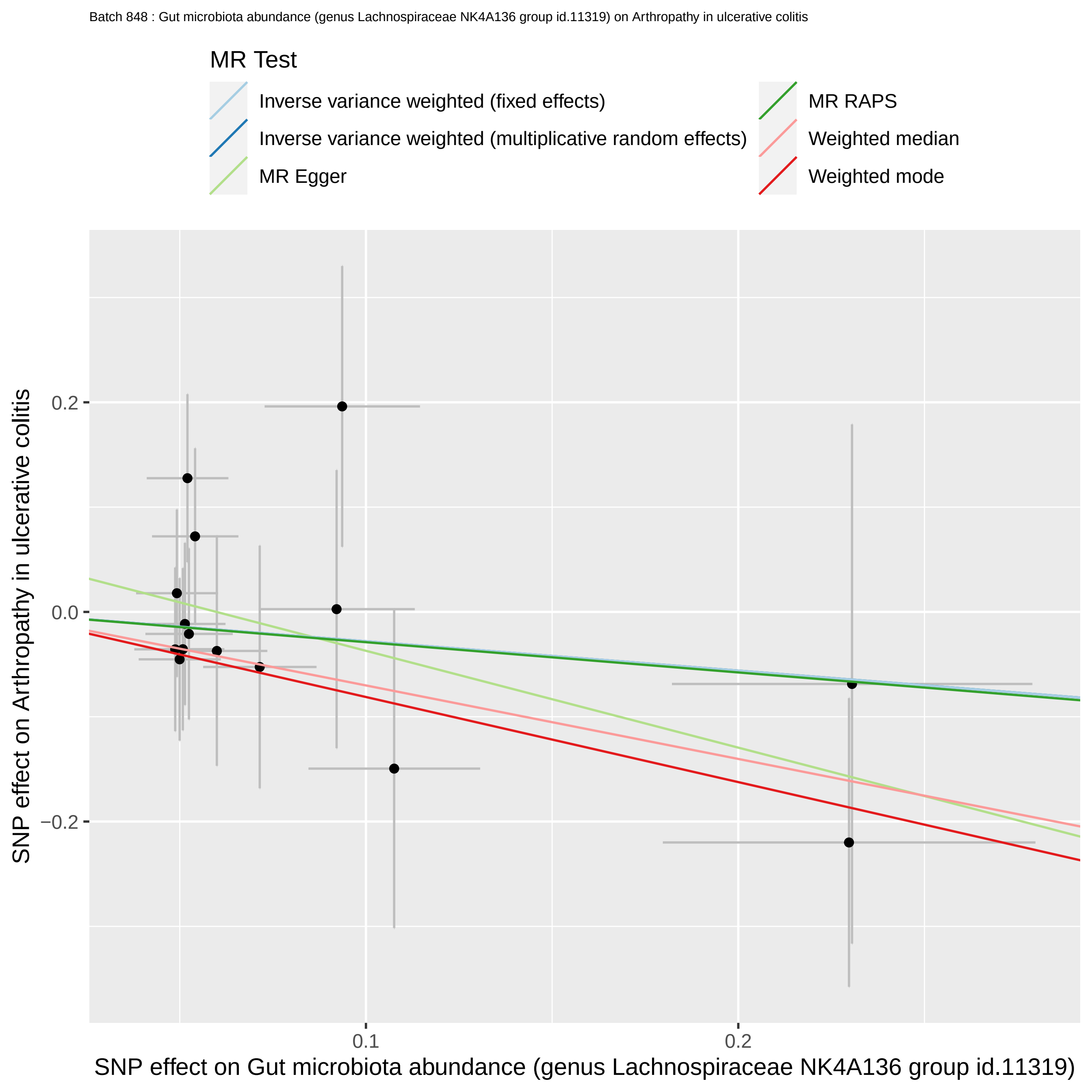
Batch 848 : Gut microbiota abundance (genus Lachnospiraceae NK4A136 group id.11319) on Arthropathy in ulcerative colitis
MR Test
Inverse variance weighted (fixed effects)
Inverse variance weighted (multiplicative random effects)
MR Egger
MR RAPS
Weighted median
Weighted mode
SNP effect on Arthropathy in ulcerative colitis
0.2
0.0
−0.2
0.1
0.2
SNP effect on Gut microbiota abundance (genus Lachnospiraceae NK4A136 group id.11319)

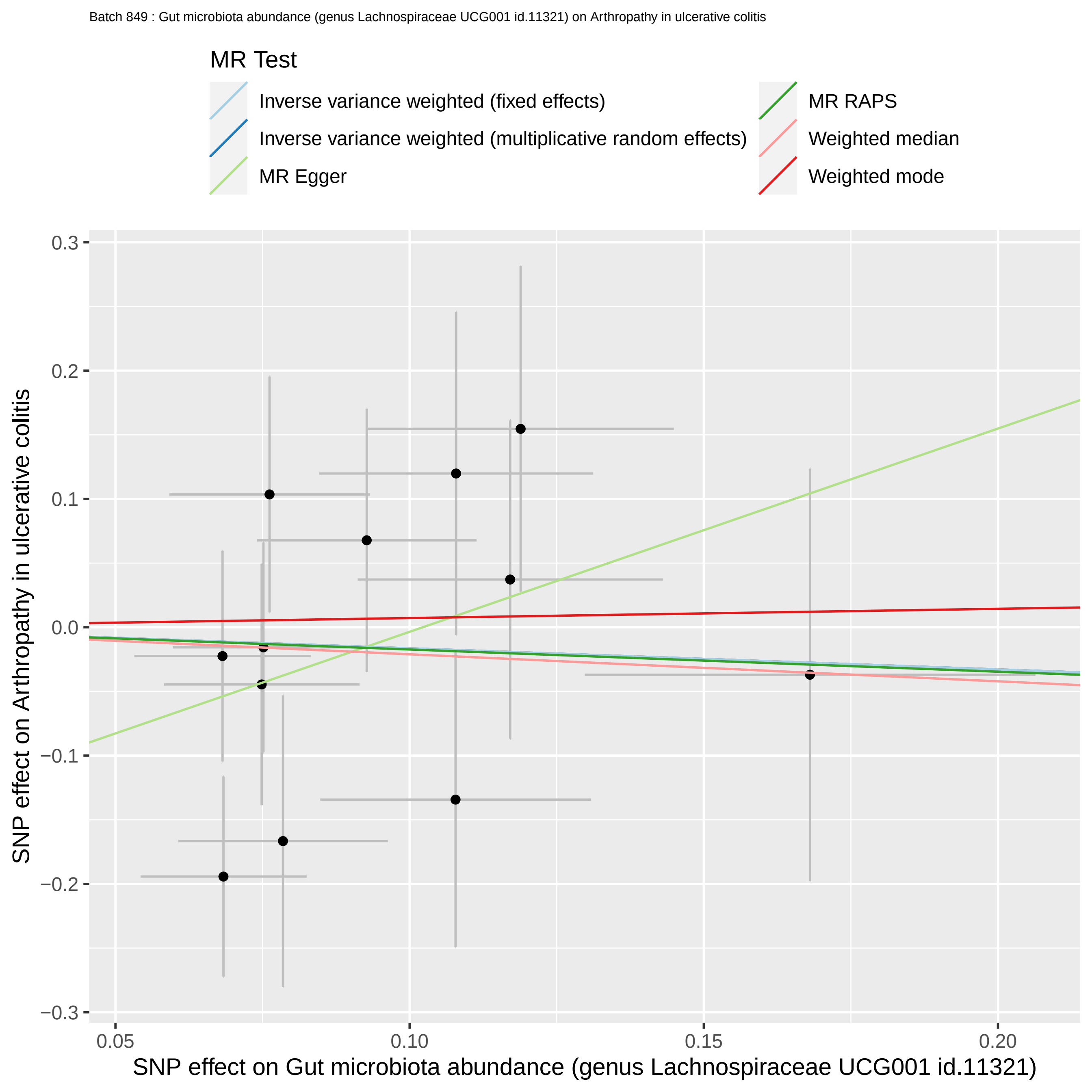

Batch 849 : Gut microbiota abundance (genus Lachnospiraceae UCG001 id.11321) on Arthropathy in ulcerative colitis
MR Test
Inverse variance weighted (fixed effects)
Inverse variance weighted (multiplicative random effects)
MR Egger
MR RAPS
Weighted median
Weighted mode
SNP effect on Arthropathy in ulcerative colitis
0.3
0.2
0.1
0.0
−0.1
−0.2
−0.3
0.05
0.10
0.15
0.20
SNP effect on Gut microbiota abundance (genus Lachnospiraceae UCG001 id.11321)

Batch 850 : Gut microbiota abundance (genus Lachnospiraceae UCG004 id.11324) on Arthropathy in ulcerative colitis

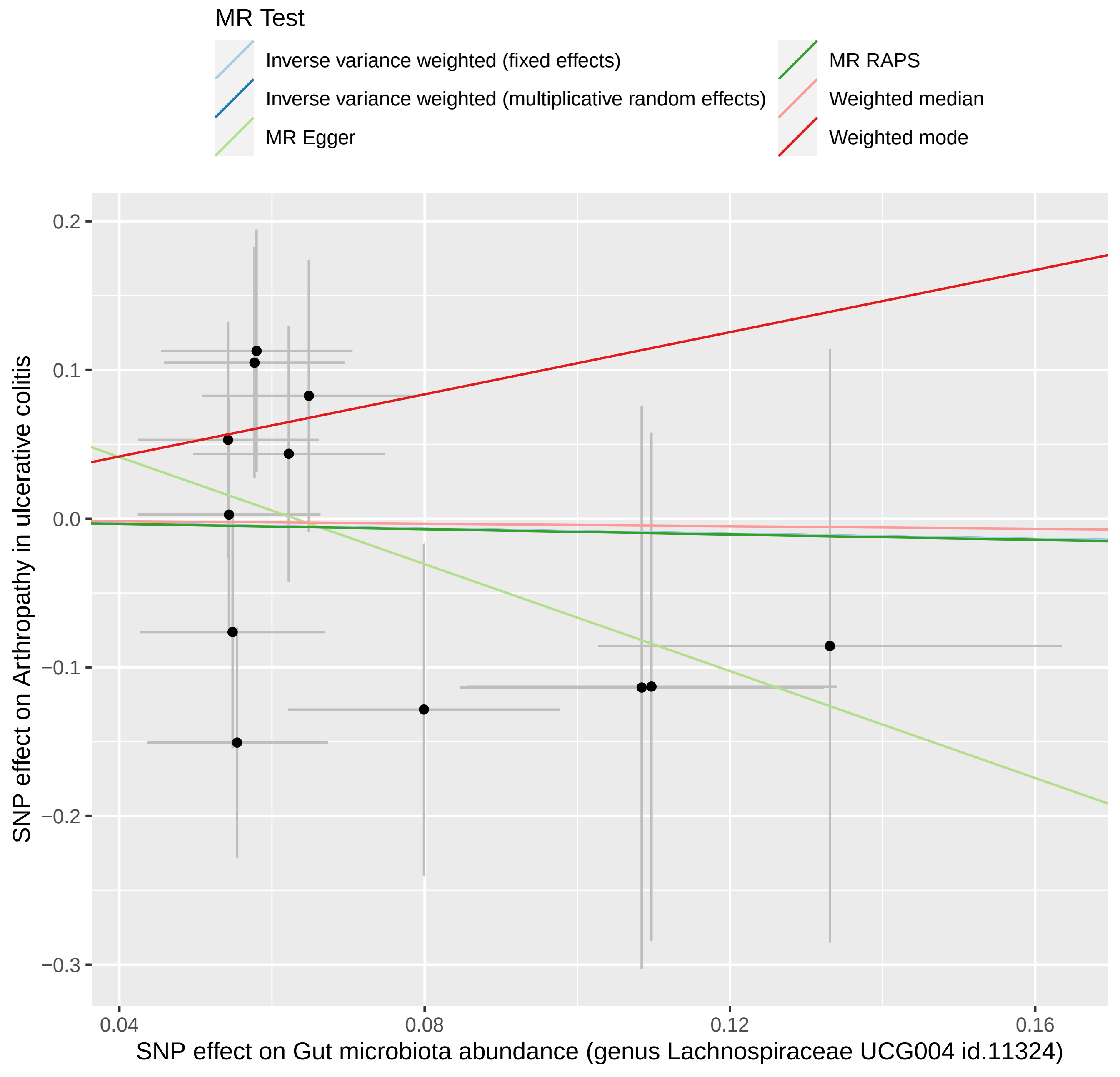

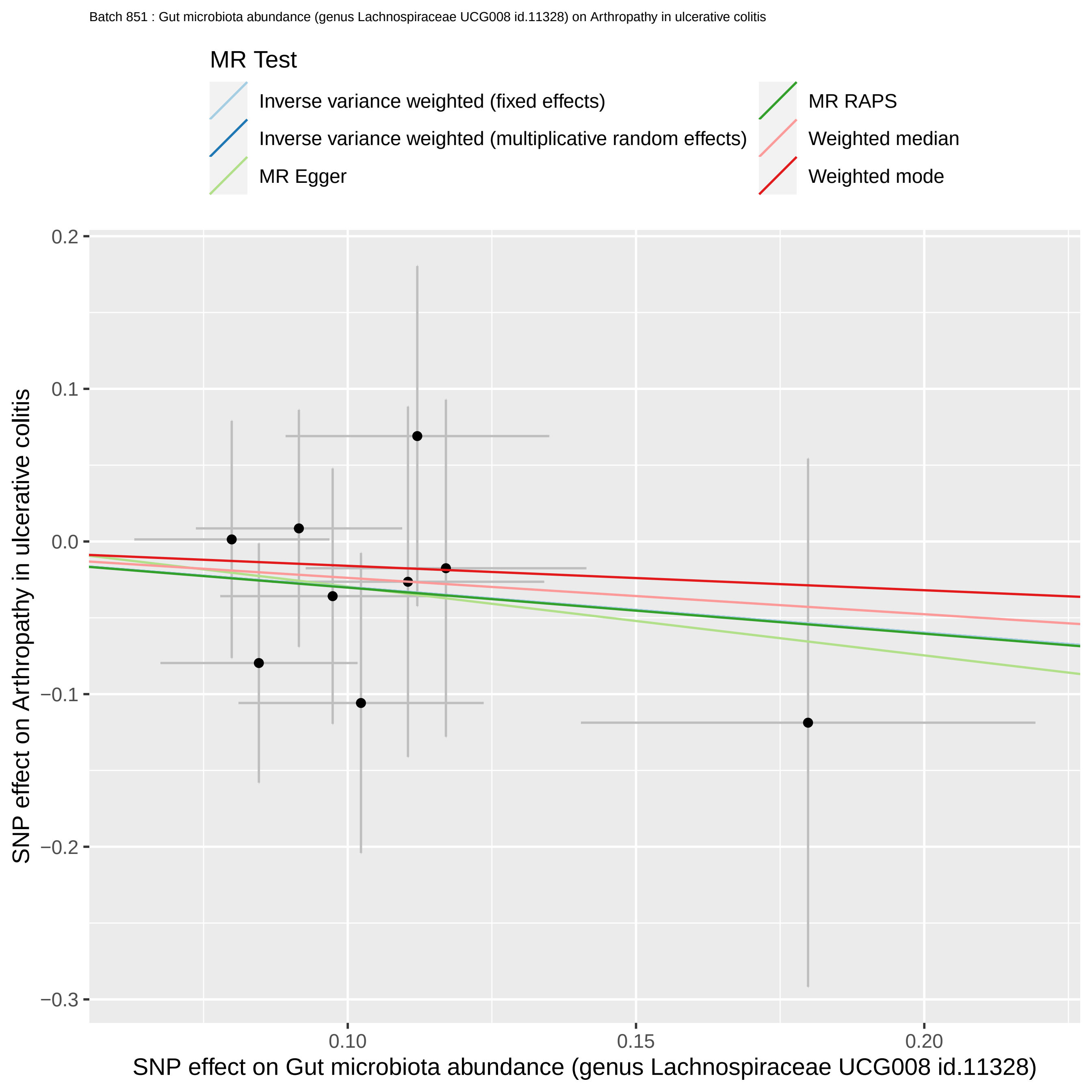
Batch 851 : Gut microbiota abundance (genus Lachnospiraceae UCG008 id.11328) on Arthropathy in ulcerative colitis
MR Test
Inverse variance weighted (fixed effects)
Inverse variance weighted (multiplicative random effects)
MR Egger
MR RAPS
Weighted median
Weighted mode
0.2
0.1
0.0
−0.1
−0.2
−0.3
0.10
0.15
0.20
SNP effect on Arthropathy in ulcerative colitis
SNP effect on Gut microbiota abundance (genus Lachnospiraceae UCG008 id.11328)

Batch 852 : Gut microbiota abundance (genus Lachnospiraceae UCG010 id.11330) on Arthropathy in ulcerative colitis

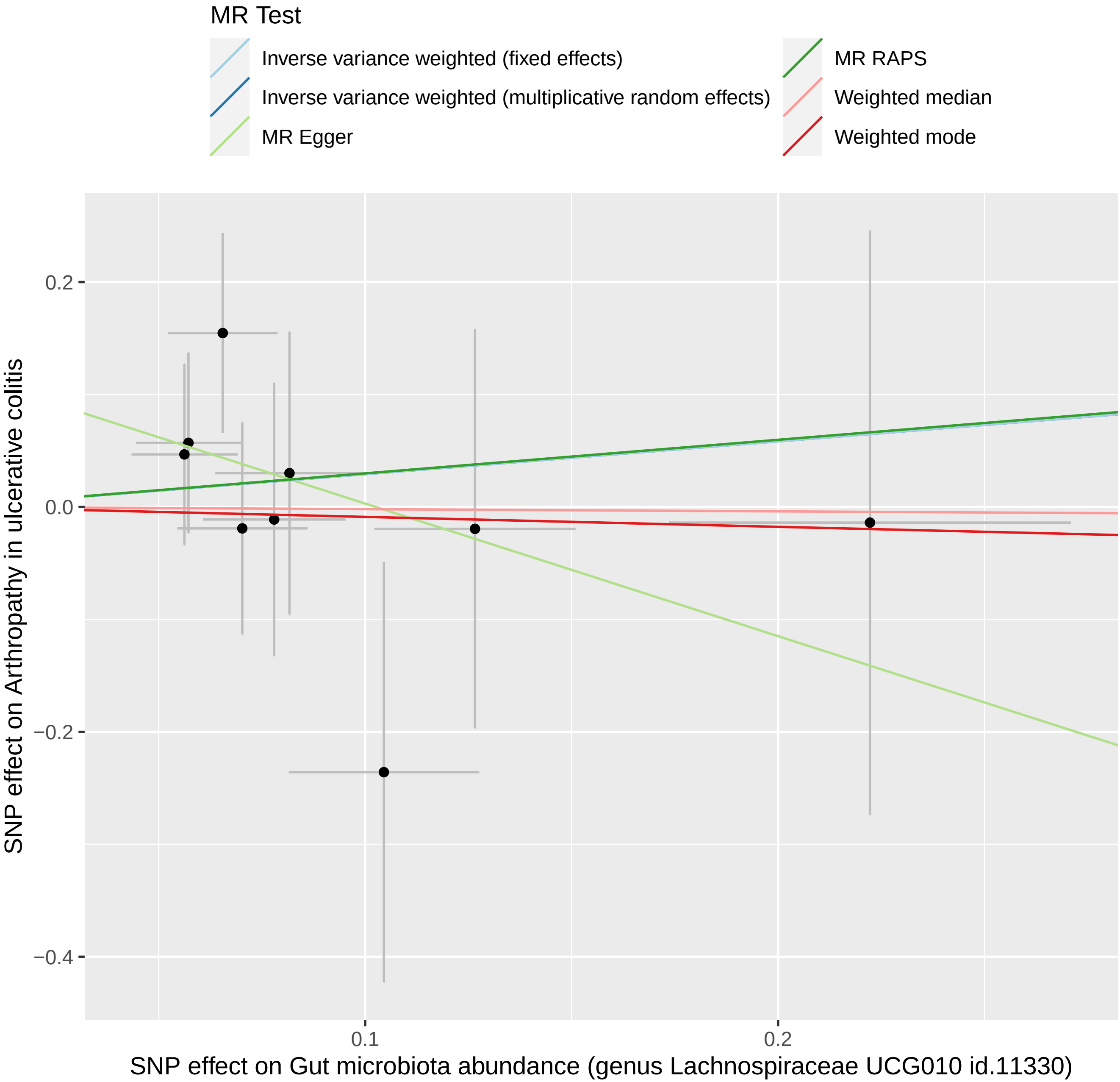

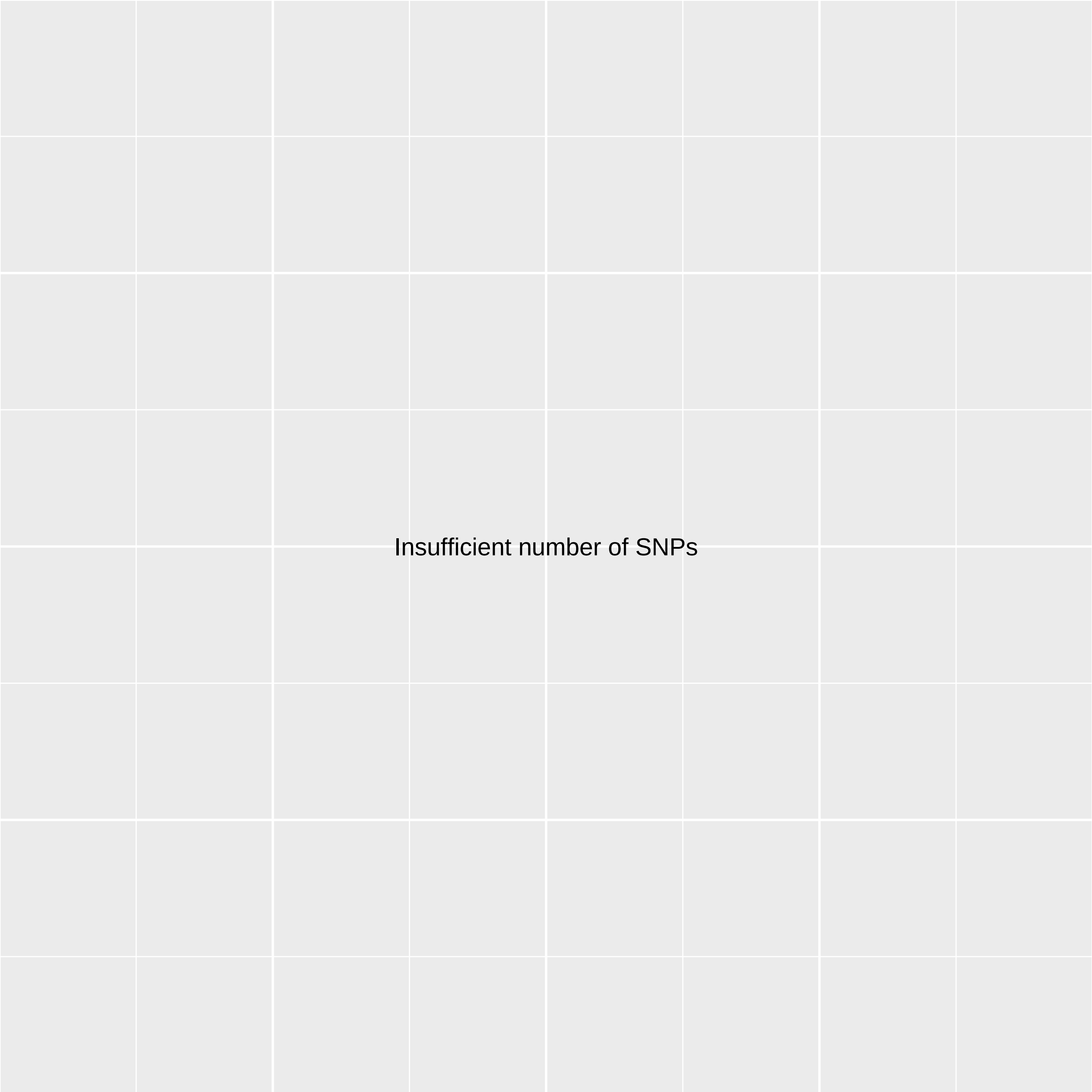
Insufficient number of SNPs

Batch 854 : Gut microbiota abundance (genus Lactobacillus id.1837) on Arthropathy in ulcerative colitis

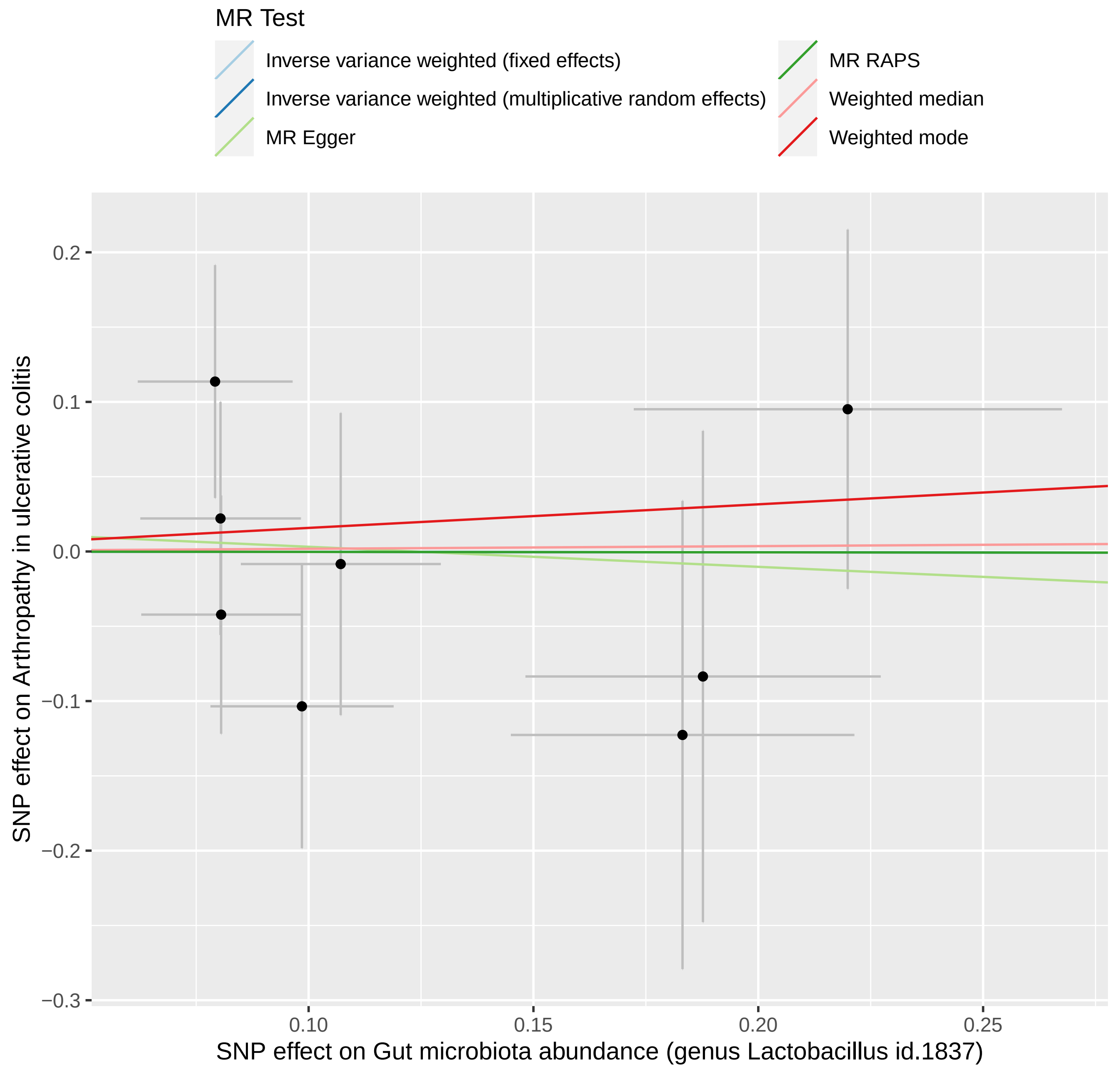

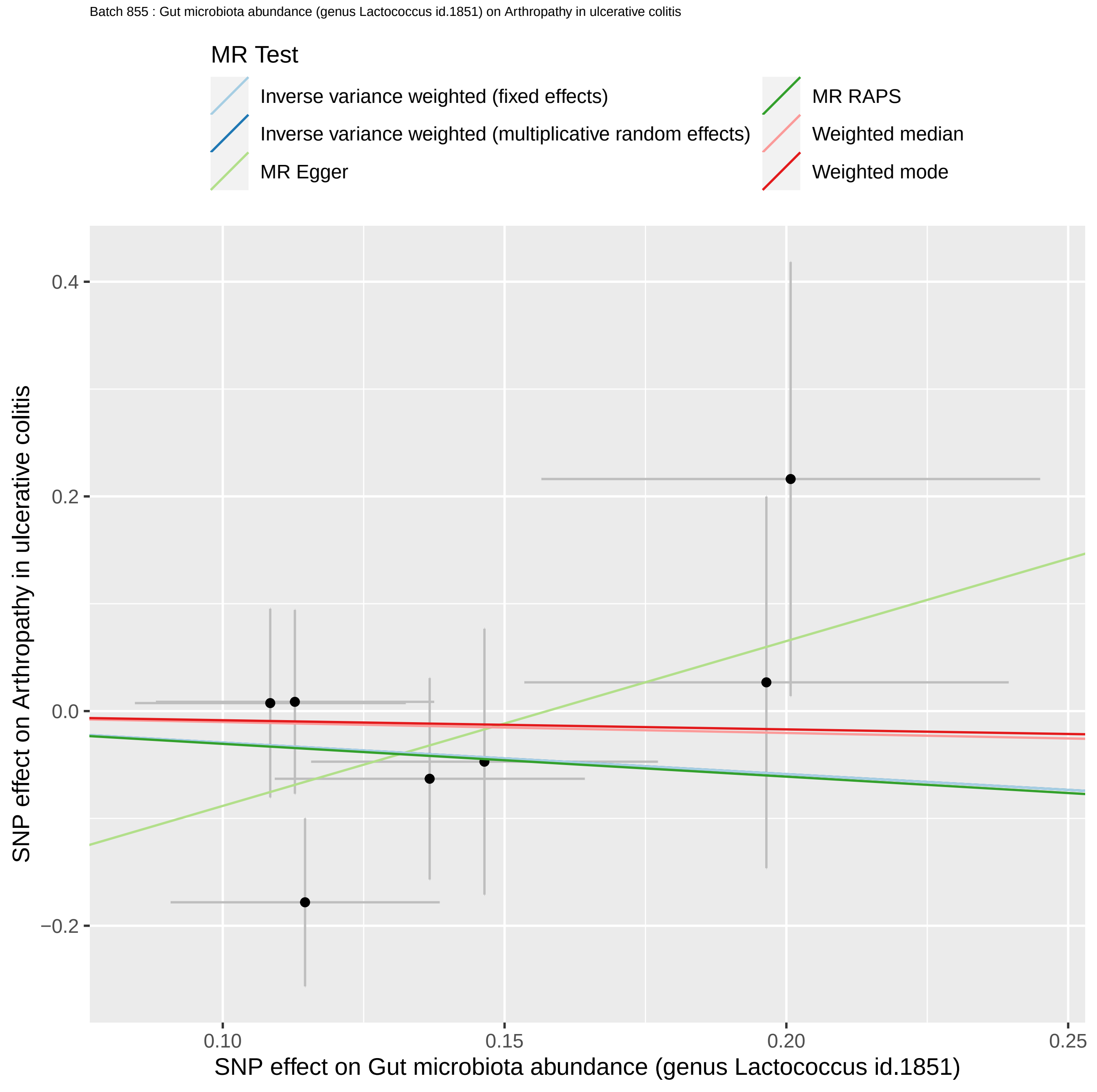

Batch 855 : Gut microbiota abundance (genus Lactococcus id.1851) on Arthropathy in ulcerative colitis
MR Test
Inverse variance weighted (fixed effects)
Inverse variance weighted (multiplicative random effects)
MR Egger
MR RAPS
Weighted median
Weighted mode
0.4
0.2
0.0
−0.2
0.10
0.15
0.20
0.25
SNP effect on Arthropathy in ulcerative colitis
SNP effect on Gut microbiota abundance (genus Lactococcus id.1851)

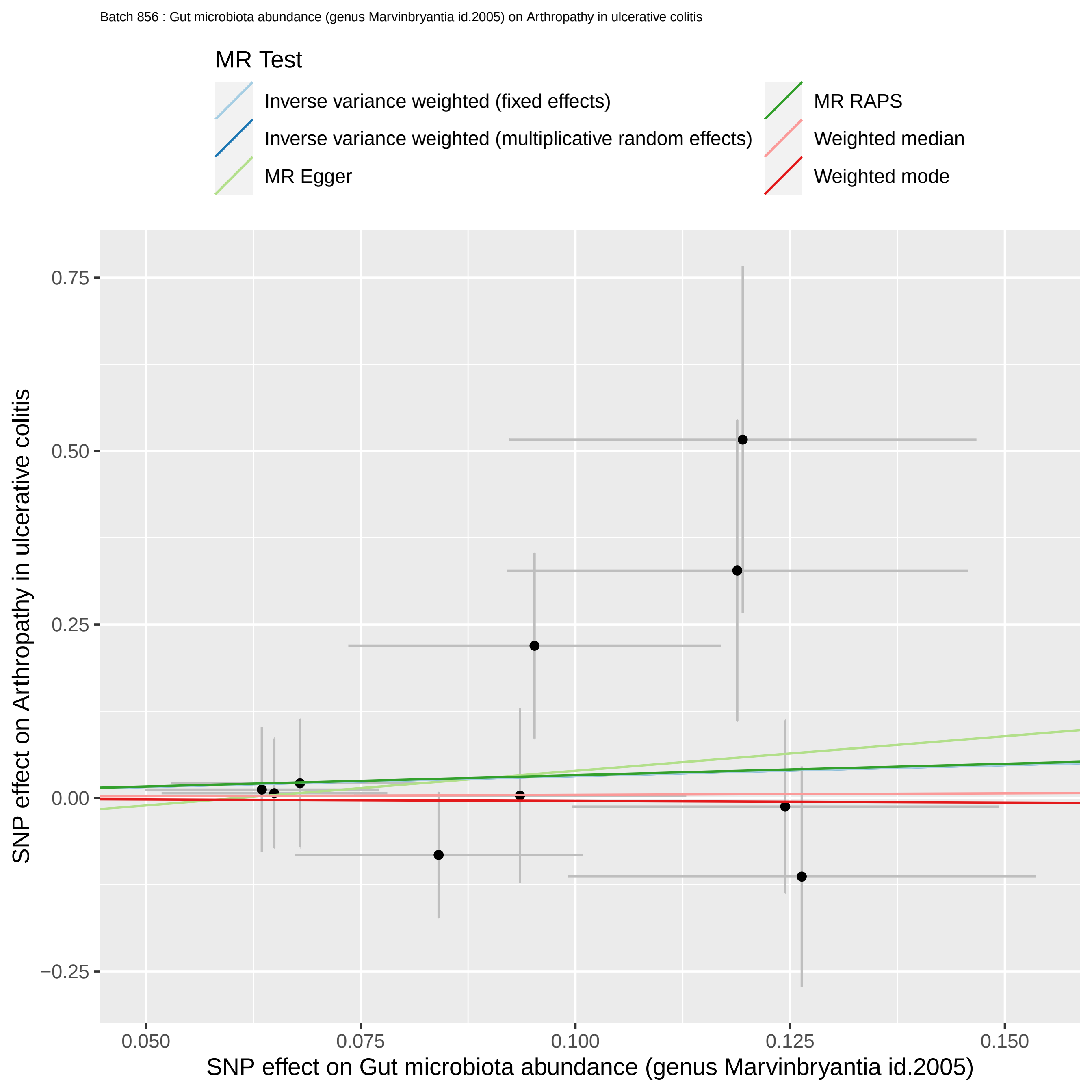
Batch 856 : Gut microbiota abundance (genus Marvinbryantia id.2005) on Arthropathy in ulcerative colitis
MR Test
Inverse variance weighted (fixed effects)
Inverse variance weighted (multiplicative random effects)
MR Egger
MR RAPS
Weighted median
Weighted mode
0.75
0.50
0.25
0.00
−0.25
0.050
0.075
0.100
0.125
0.150
SNP effect on Arthropathy in ulcerative colitis
SNP effect on Gut microbiota abundance (genus Marvinbryantia id.2005)

Batch 857 : Gut microbiota abundance (genus Methanobrevibacter id.123) on Arthropathy in ulcerative colitis

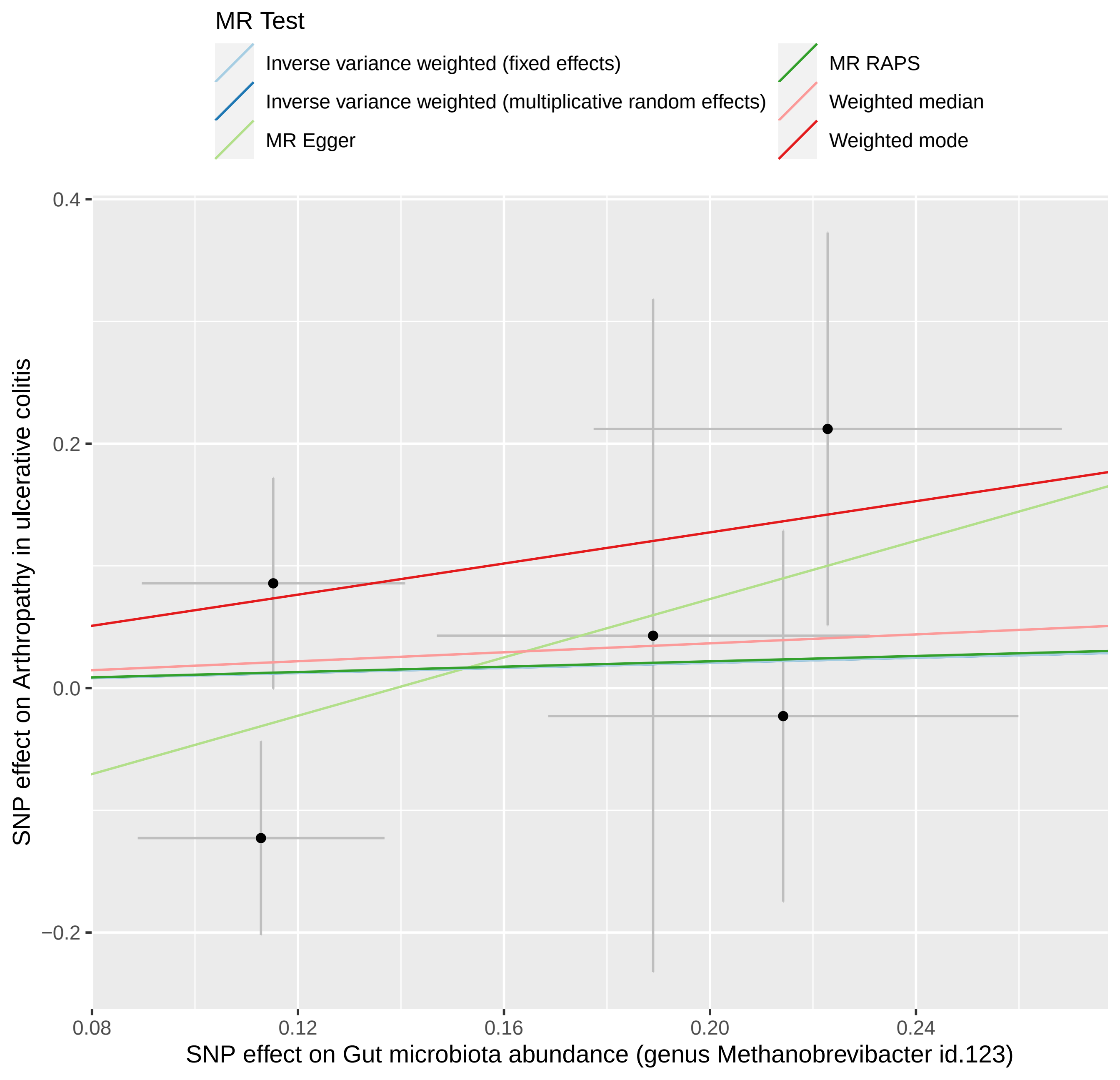

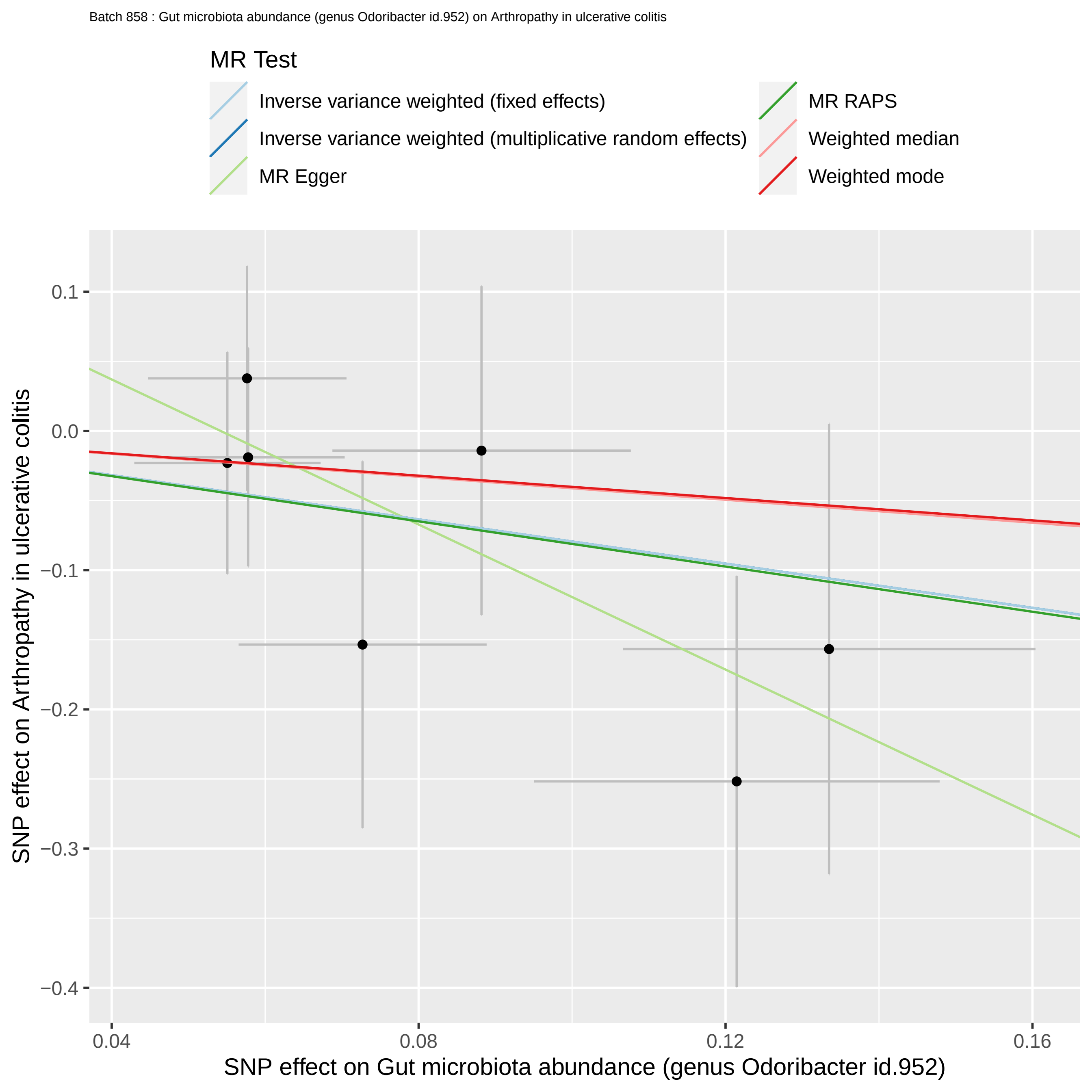

Batch 858 : Gut microbiota abundance (genus Odoribacter id.952) on Arthropathy in ulcerative colitis
MR Test
Inverse variance weighted (fixed effects)
Inverse variance weighted (multiplicative random effects)
MR Egger
MR RAPS
Weighted median
Weighted mode
0.1
0.0
−0.1
−0.2
−0.3
−0.4
0.04
0.08
0.12
0.16
SNP effect on Arthropathy in ulcerative colitis
SNP effect on Gut microbiota abundance (genus Odoribacter id.952)

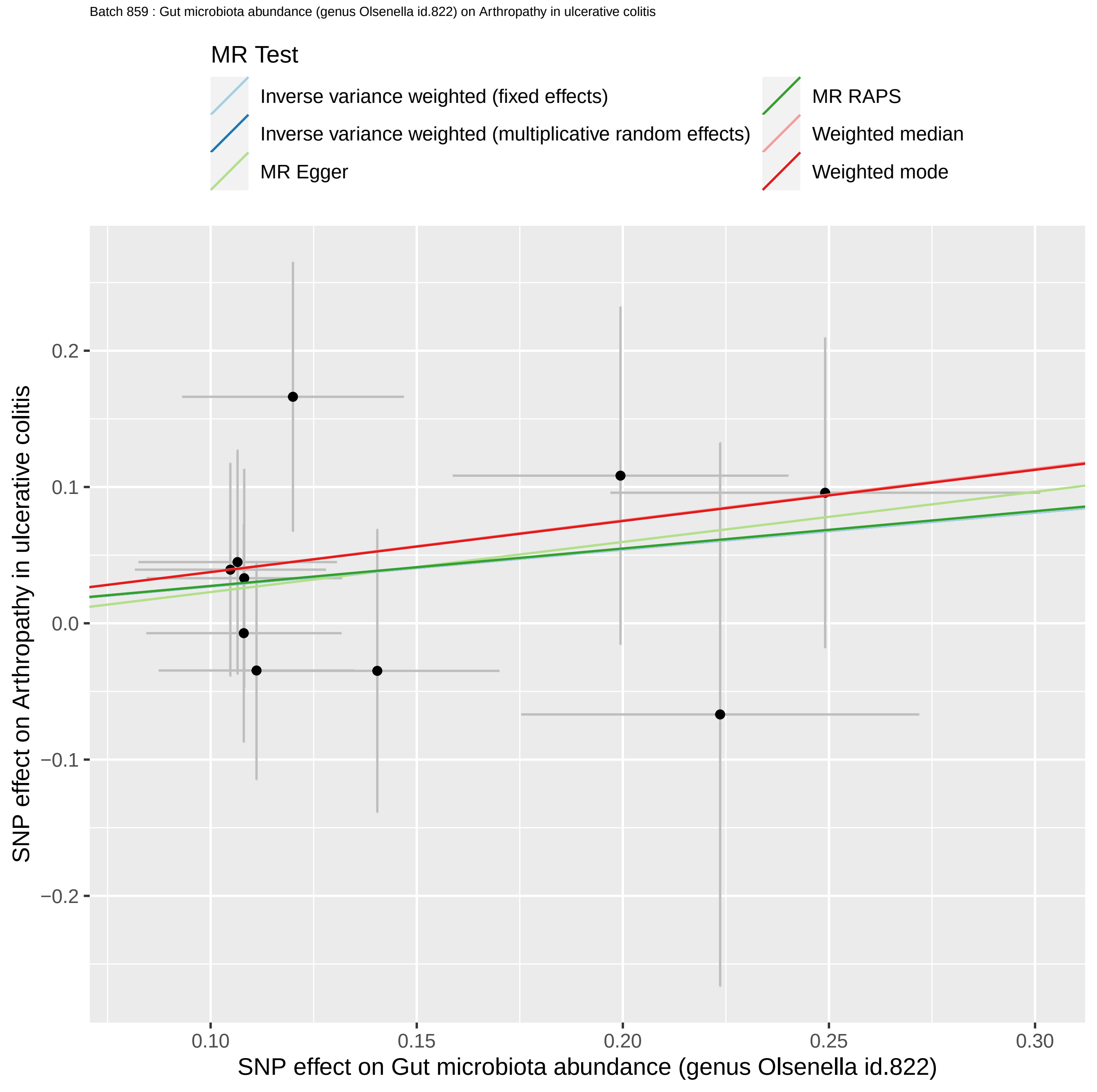
Batch 859 : Gut microbiota abundance (genus Olsenella id.822) on Arthropathy in ulcerative colitis
MR Test
Inverse variance weighted (fixed effects)
Inverse variance weighted (multiplicative random effects)
MR Egger
MR RAPS
Weighted median
Weighted mode
0.2
0.1
0.0
−0.1
−0.2
0.10
0.15
0.20
0.25
0.30
SNP effect on Arthropathy in ulcerative colitis
SNP effect on Gut microbiota abundance (genus Olsenella id.822)

Batch 860 : Gut microbiota abundance (genus Oscillibacter id.2063) on Arthropathy in ulcerative colitis

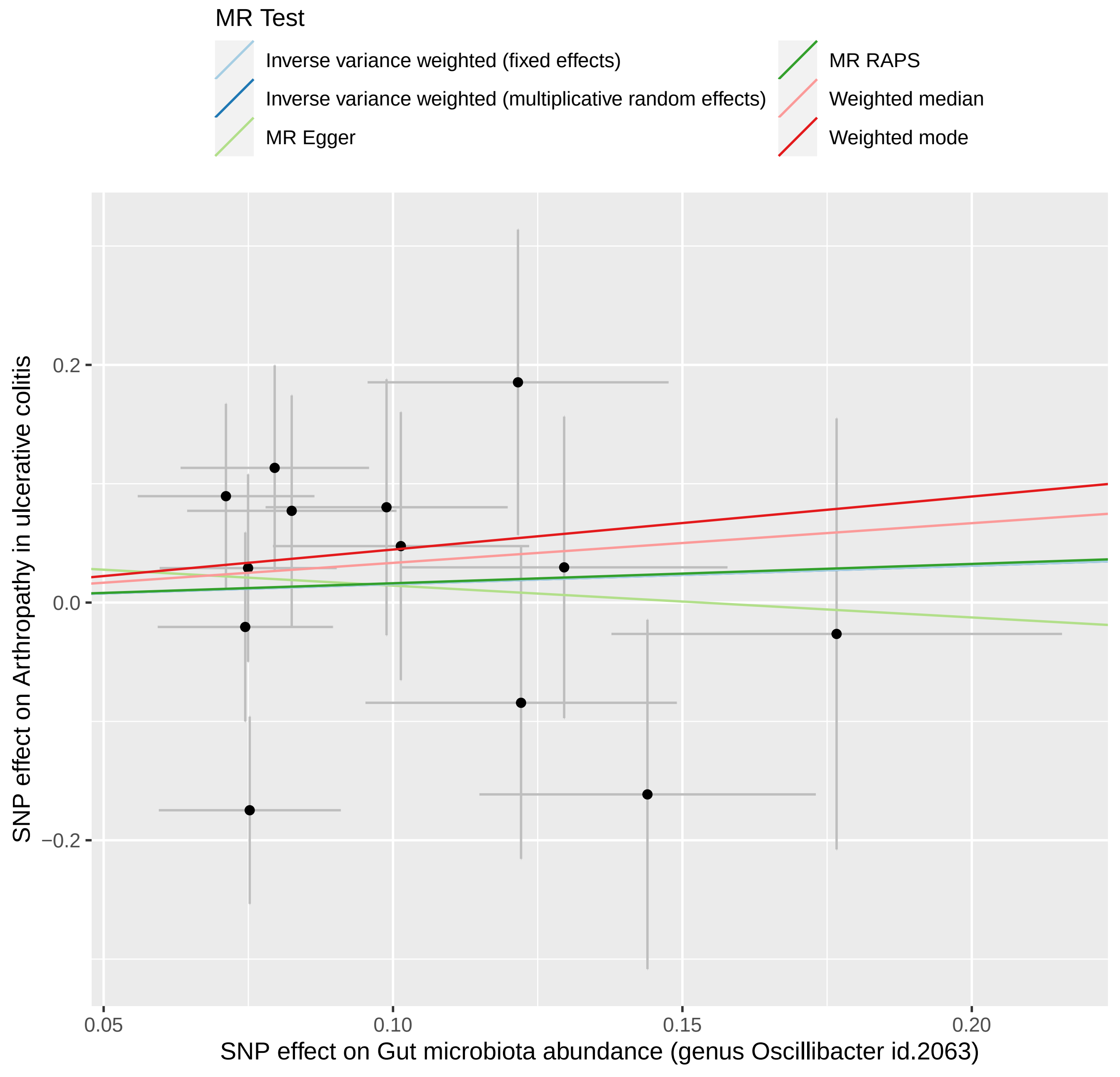

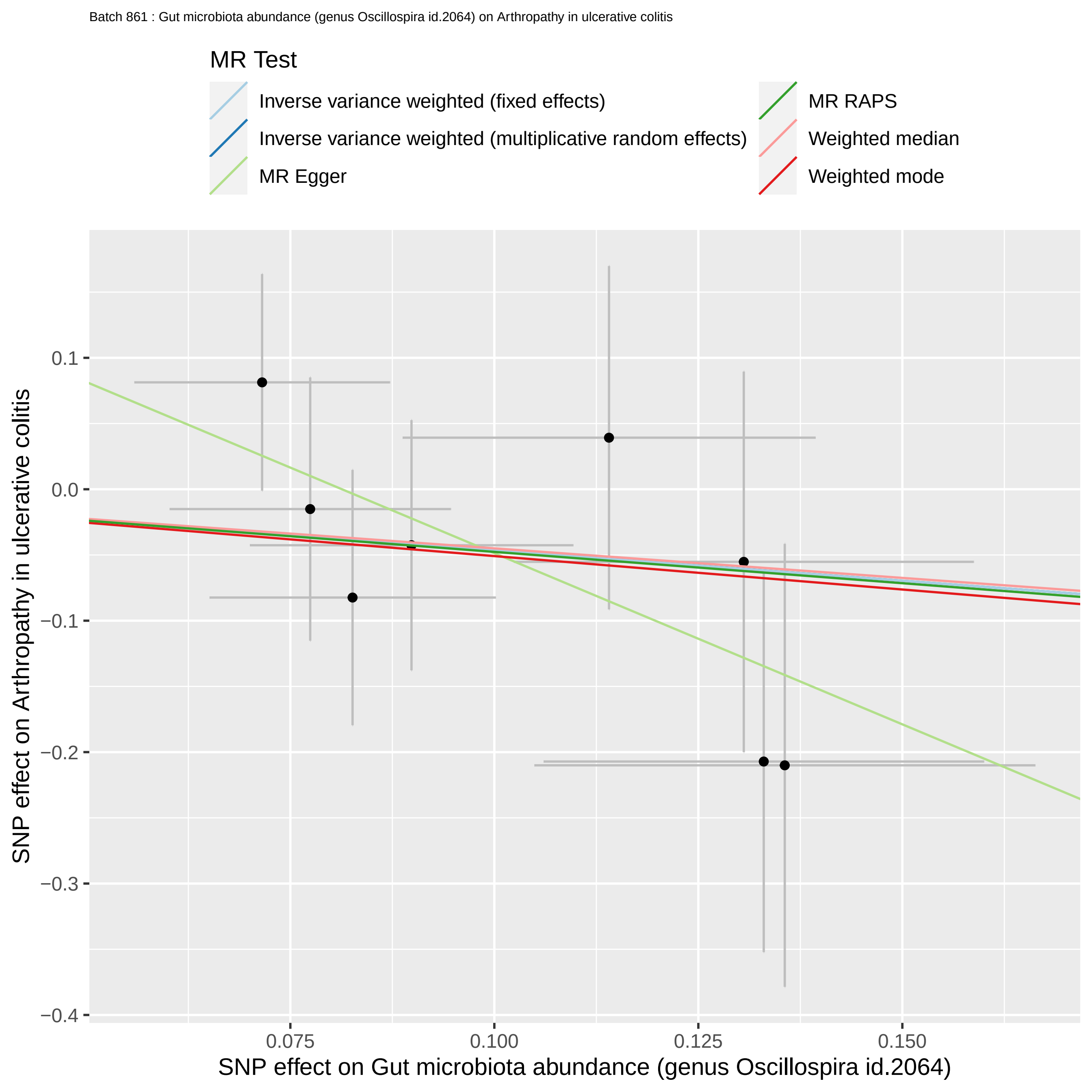

Batch 861 : Gut microbiota abundance (genus Oscillospira id.2064) on Arthropathy in ulcerative colitis
MR Test
Inverse variance weighted (fixed effects)
Inverse variance weighted (multiplicative random effects)
MR Egger
MR RAPS
Weighted median
Weighted mode
SNP effect on Arthropathy in ulcerative colitis
0.1
0.0
−0.1
−0.2
−0.3
−0.4
0.075
0.100
0.125
0.150
SNP effect on Gut microbiota abundance (genus Oscillospira id.2064)

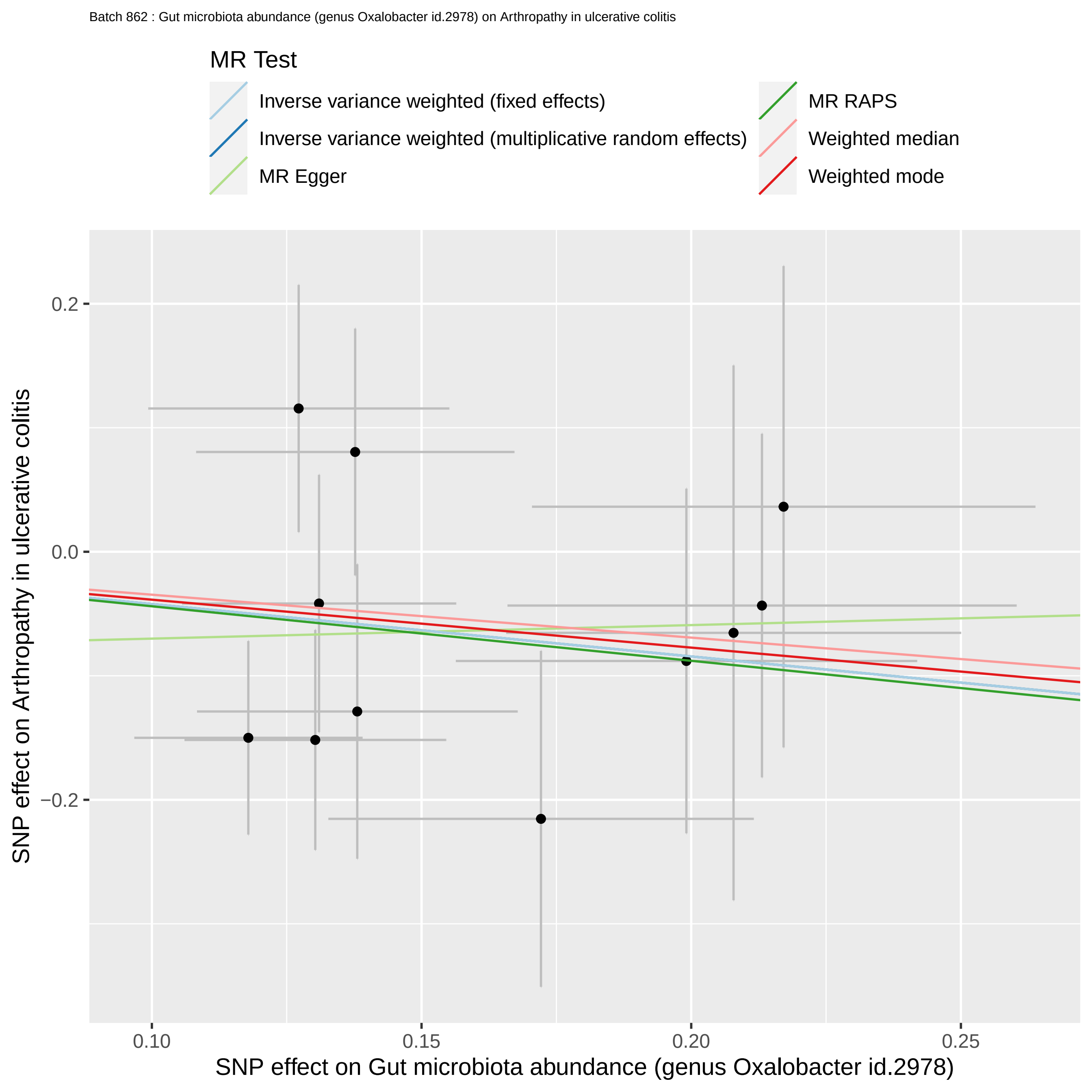

Batch 862 : Gut microbiota abundance (genus Oxalobacter id.2978) on Arthropathy in ulcerative colitis
MR Test
Inverse variance weighted (fixed effects)
Inverse variance weighted (multiplicative random effects)
MR Egger
MR RAPS
Weighted median
Weighted mode
0.2
0.0
−0.2
0.10
0.15
0.20
0.25
SNP effect on Arthropathy in ulcerative colitis
SNP effect on Gut microbiota abundance (genus Oxalobacter id.2978)

Batch 863 : Gut microbiota abundance (genus Parabacteroides id.954) on Arthropathy in ulcerative colitis

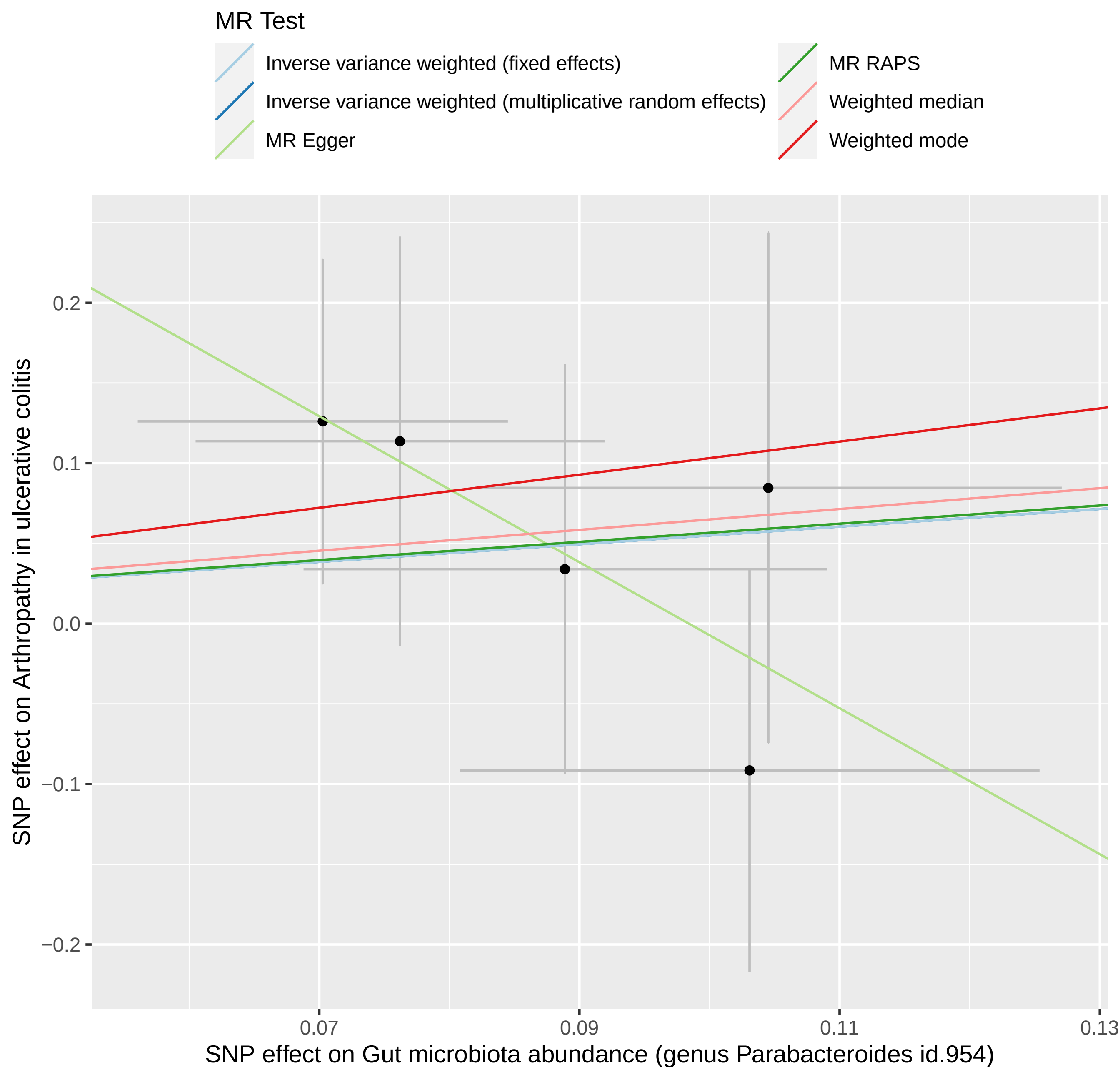

Batch 864 : Gut microbiota abundance (genus Paraprevotella id.962) on Arthropathy in ulcerative colitis

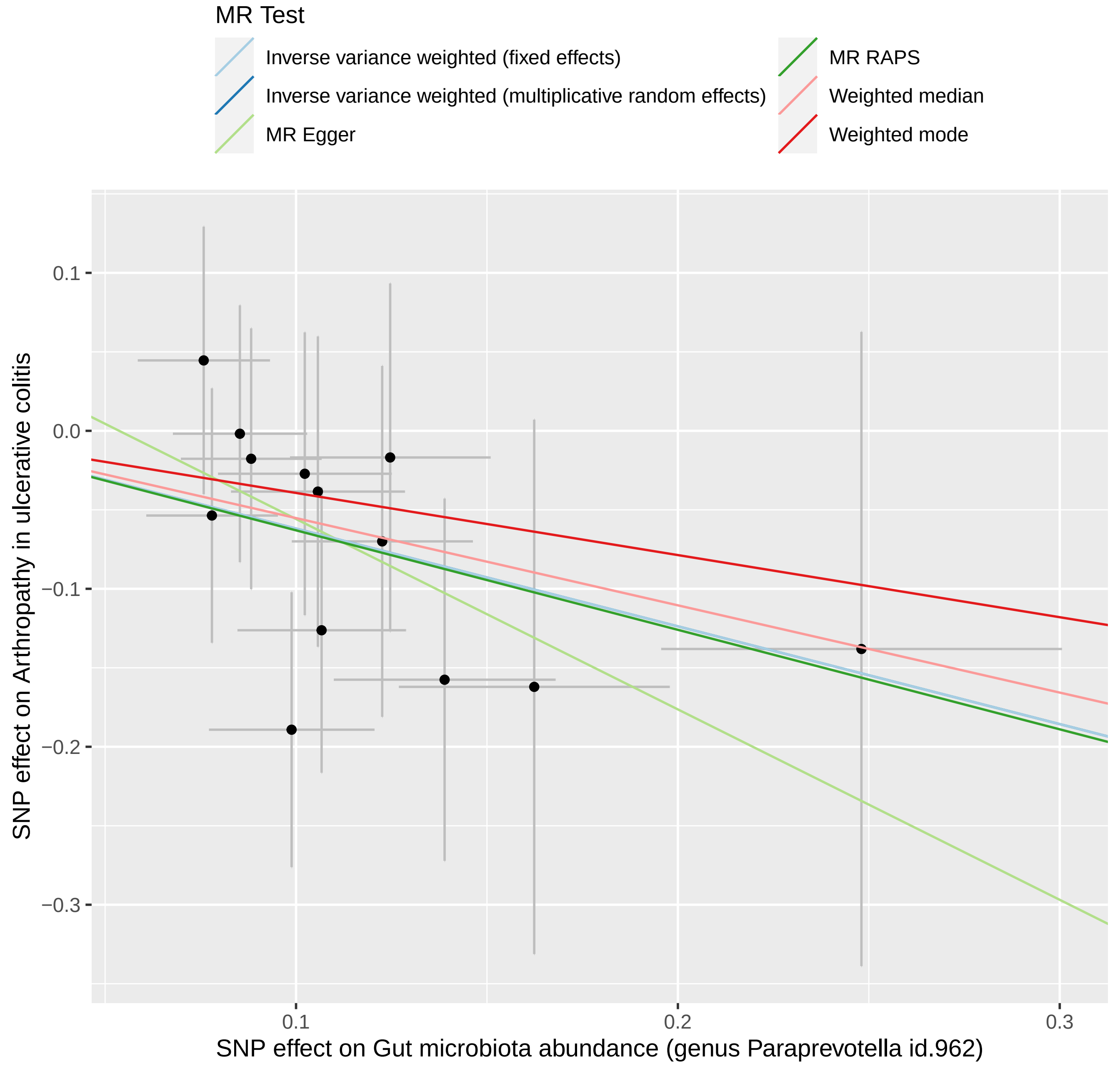

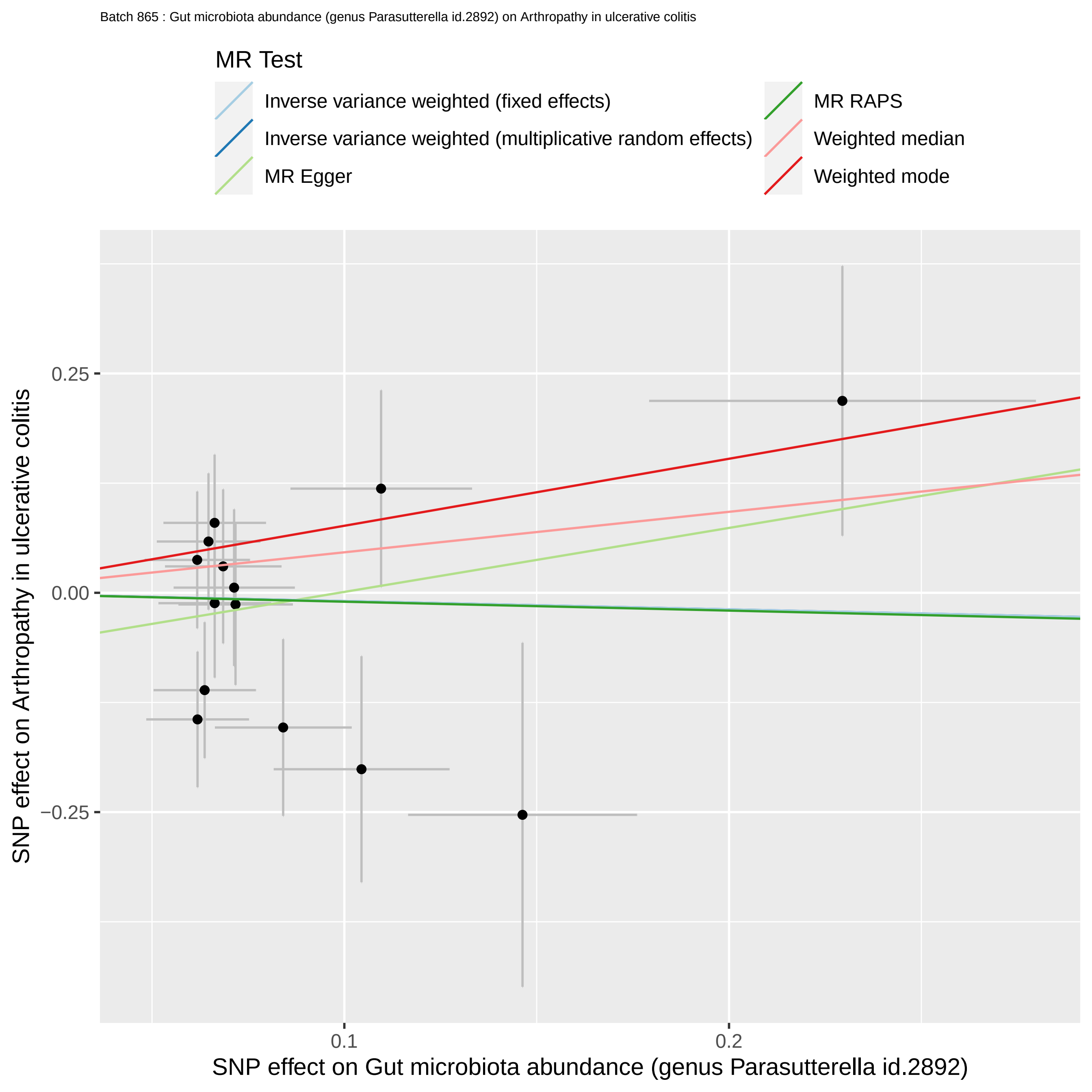

Batch 865 : Gut microbiota abundance (genus Parasutterella id.2892) on Arthropathy in ulcerative colitis
MR Test
Inverse variance weighted (fixed effects)
Inverse variance weighted (multiplicative random effects)
MR Egger
MR RAPS
Weighted median
Weighted mode
0.25
0.00
−0.25
0.1
0.2
SNP effect on Arthropathy in ulcerative colitis
SNP effect on Gut microbiota abundance (genus Parasutterella id.2892)

Batch 866 : Gut microbiota abundance (genus Peptococcus id.2037) on Arthropathy in ulcerative colitis

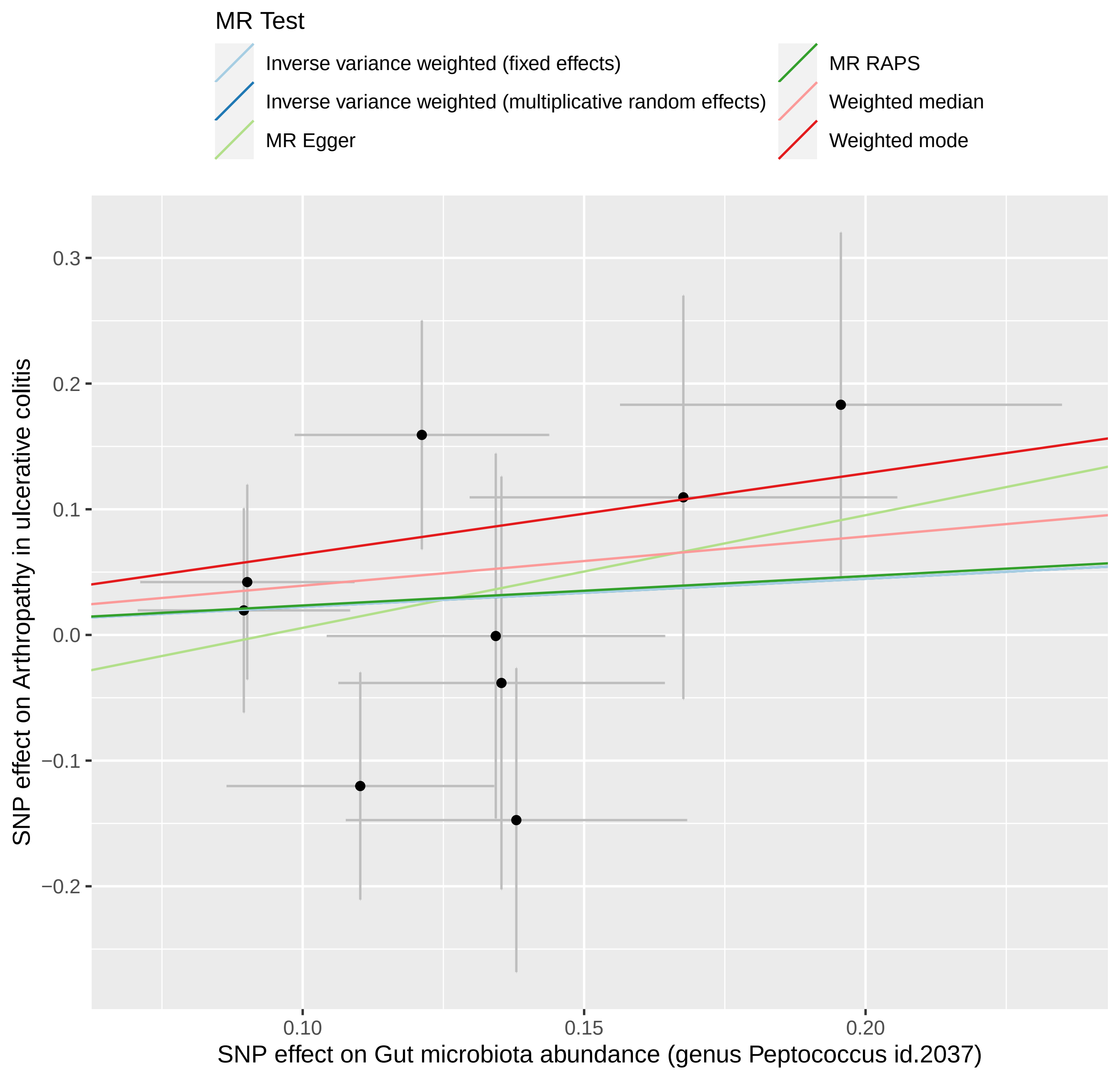

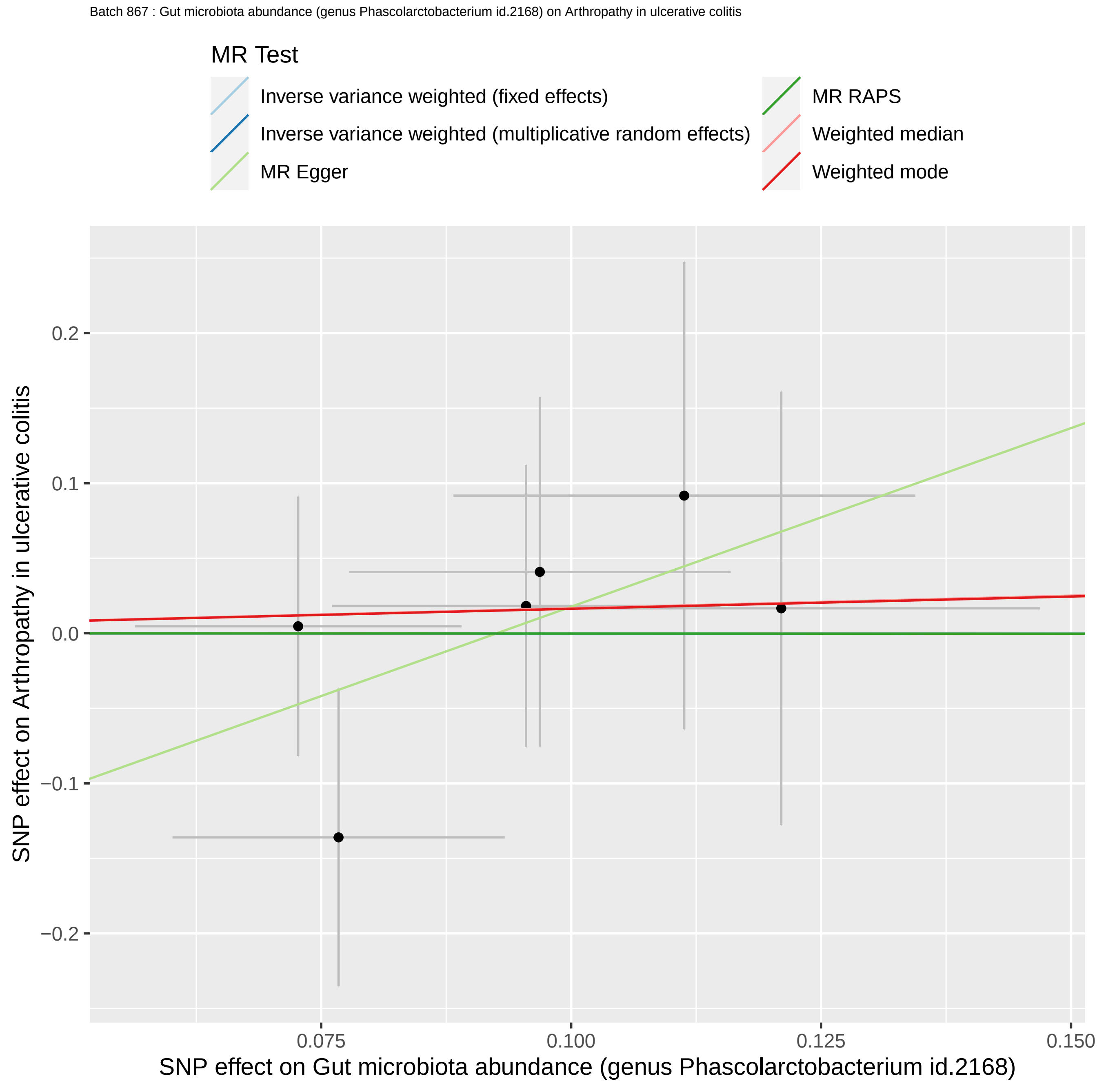
Batch 867 : Gut microbiota abundance (genus Phascolarctobacterium id.2168) on Arthropathy in ulcerative colitis
MR Test
Inverse variance weighted (fixed effects)
Inverse variance weighted (multiplicative random effects)
MR Egger
MR RAPS
Weighted median
Weighted mode
0.2
0.1
0.0
−0.1
−0.2
0.075
0.100
0.125
0.150
SNP effect on Arthropathy in ulcerative colitis
SNP effect on Gut microbiota abundance (genus Phascolarctobacterium id.2168)

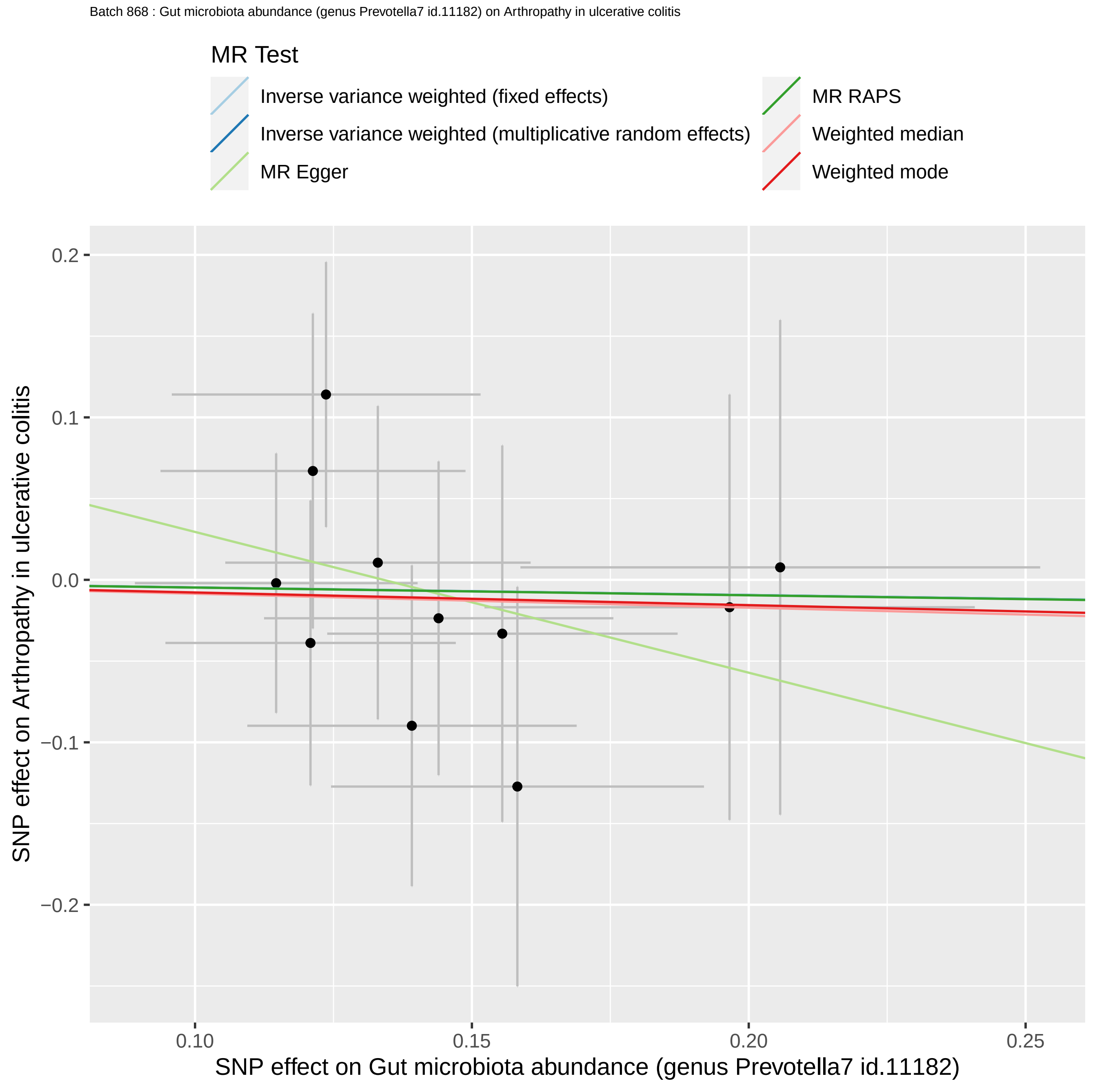
Batch 868 : Gut microbiota abundance (genus Prevotella7 id.11182) on Arthropathy in ulcerative colitis
MR Test
Inverse variance weighted (fixed effects)
Inverse variance weighted (multiplicative random effects)
MR Egger
MR RAPS
Weighted median
Weighted mode
SNP effect on Arthropathy in ulcerative colitis
0.2
0.1
0.0
−0.1
−0.2
0.10
0.15
0.20
0.25
SNP effect on Gut microbiota abundance (genus Prevotella7 id.11182)

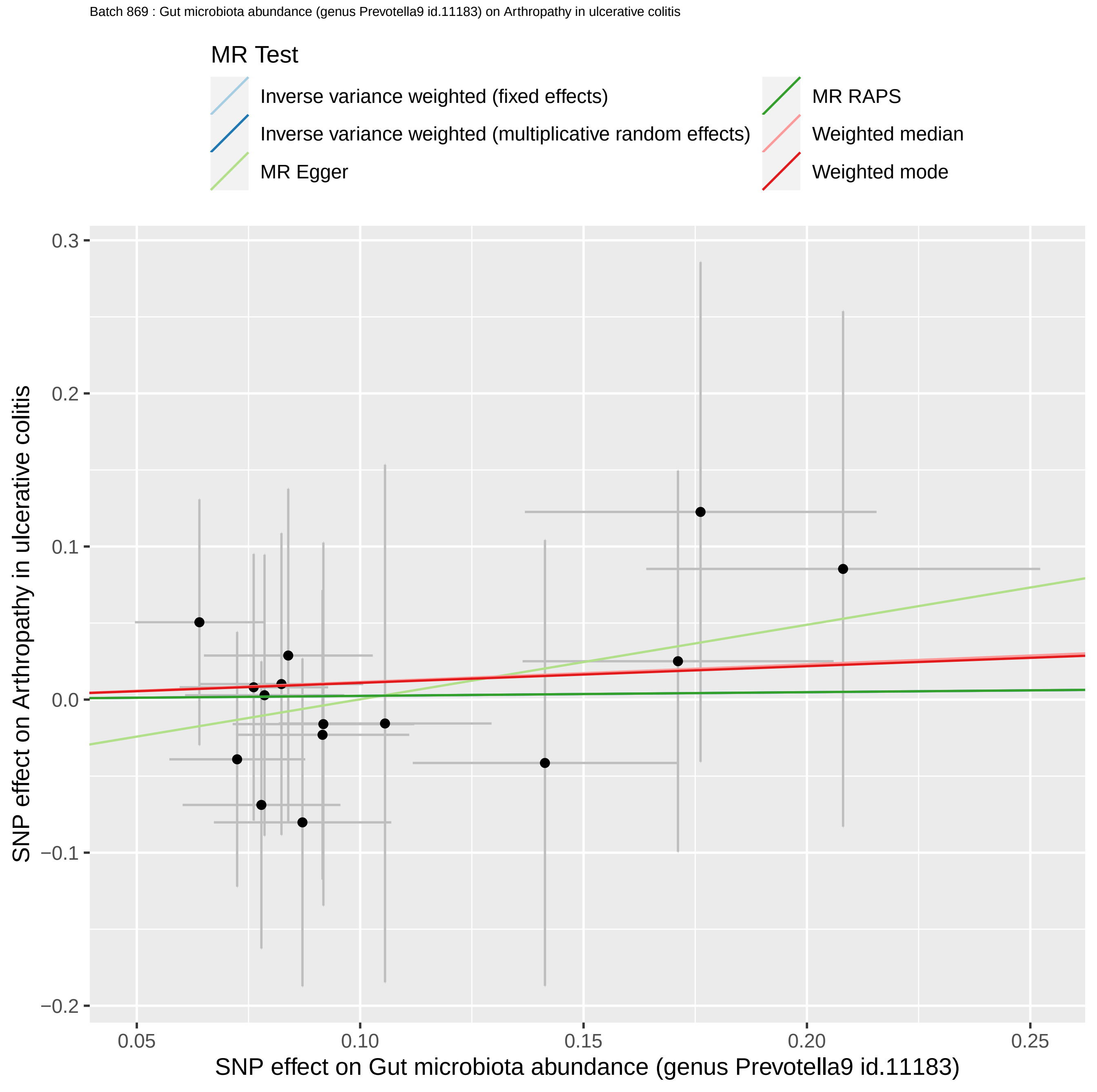
Batch 869 : Gut microbiota abundance (genus Prevotella9 id.11183) on Arthropathy in ulcerative colitis
MR Test
Inverse variance weighted (fixed effects)
Inverse variance weighted (multiplicative random effects)
MR Egger
MR RAPS
Weighted median
Weighted mode
SNP effect on Arthropathy in ulcerative colitis
0.3
0.2
0.1
0.0
−0.1
−0.2
0.05
0.10
0.15
0.20
0.25
SNP effect on Gut microbiota abundance (genus Prevotella9 id.11183)

Batch 870 : Gut microbiota abundance (genus Rikenellaceae RC9 gut group id.11191) on Arthropathy in ulcerative colitis

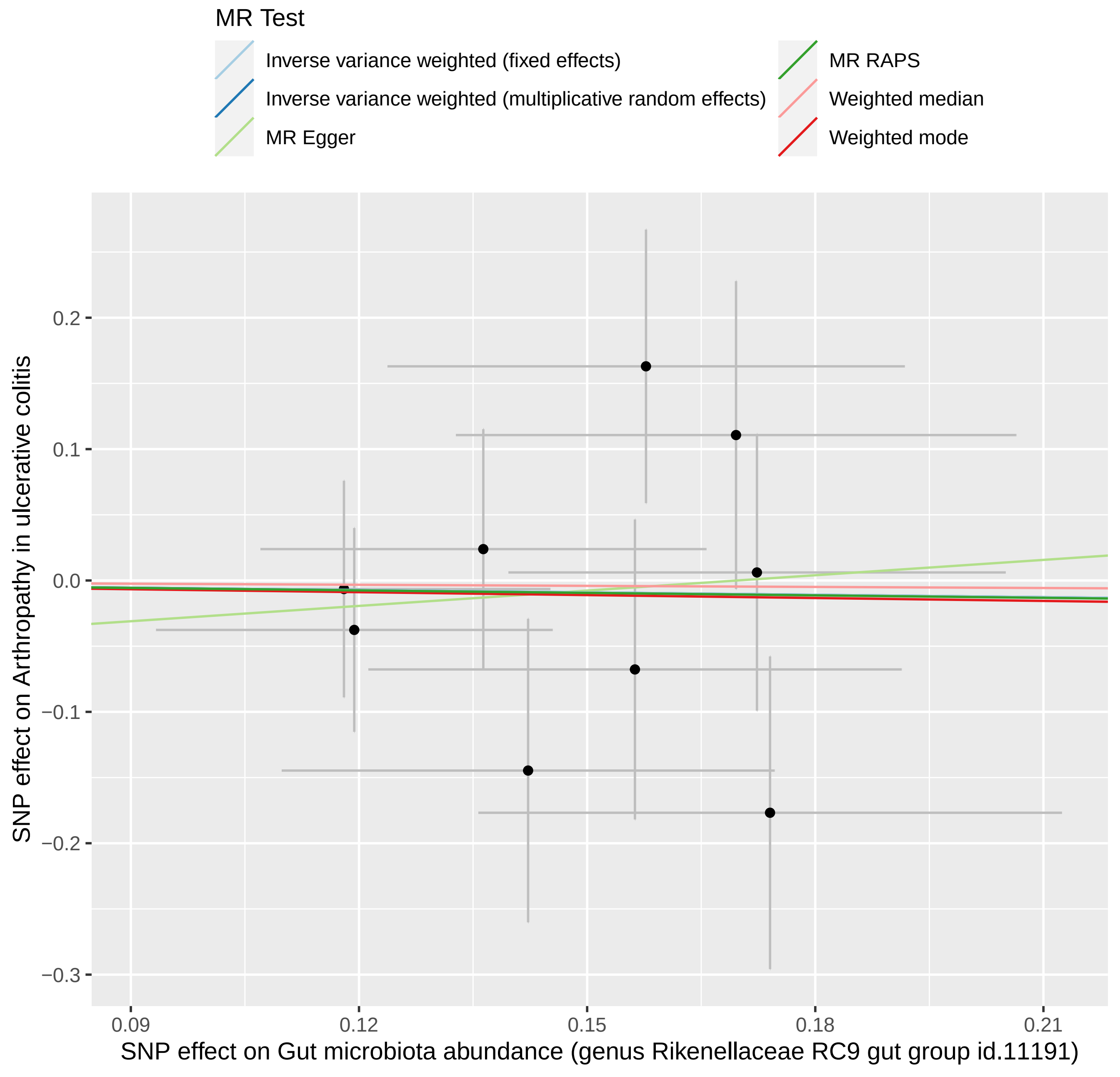

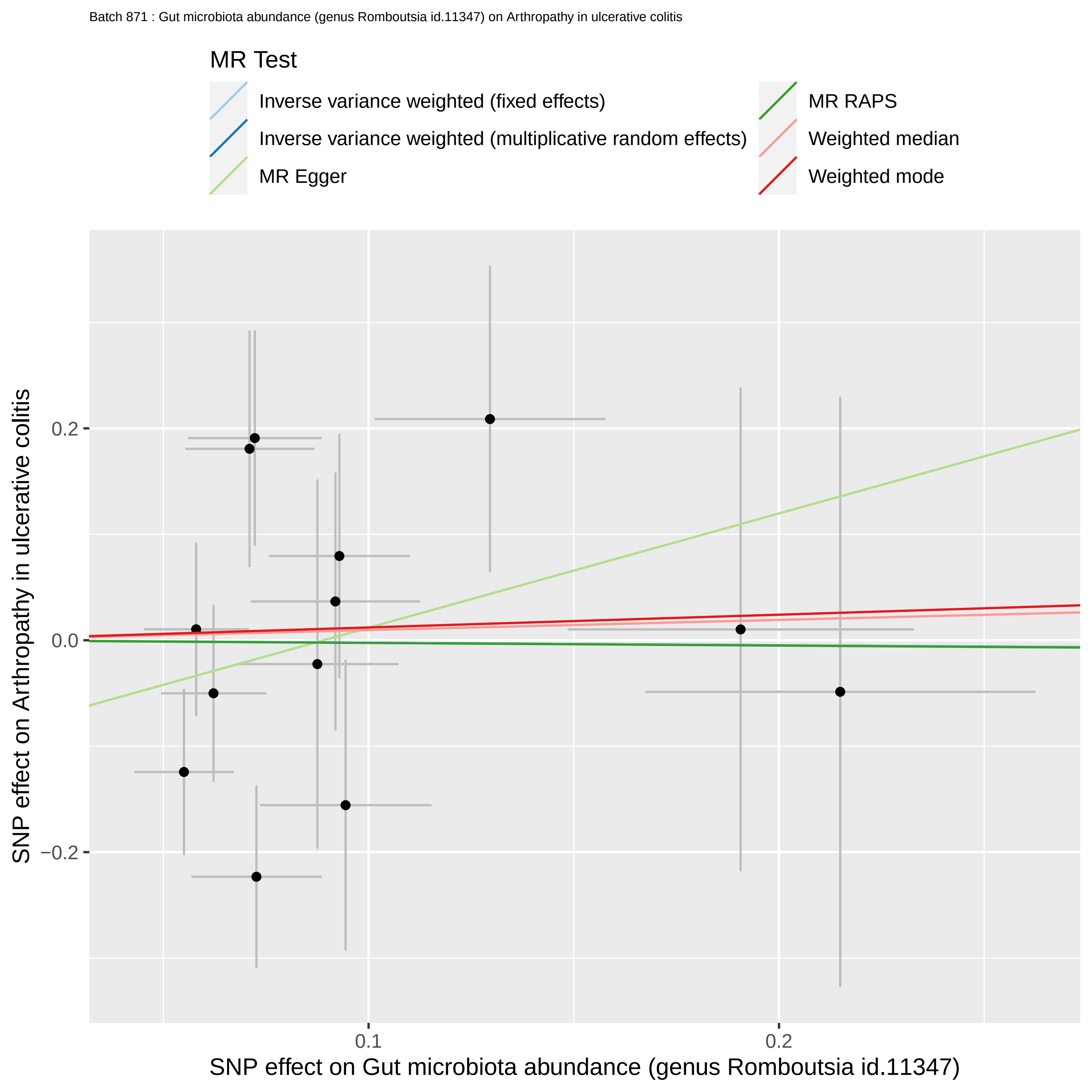
Batch 871 : Gut microbiota abundance (genus Romboutsia id.11347) on Arthropathy in ulcerative colitis
MR Test
Inverse variance weighted (fixed effects)
Inverse variance weighted (multiplicative random effects)
MR Egger
MR RAPS
Weighted median
Weighted mode
SNP effect on Arthropathy in ulcerative colitis
0.2
0.0
−0.2
0.1
0.2
SNP effect on Gut microbiota abundance (genus Romboutsia id.11347)

Batch 872 : Gut microbiota abundance (genus Roseburia id.2012) on Arthropathy in ulcerative colitis

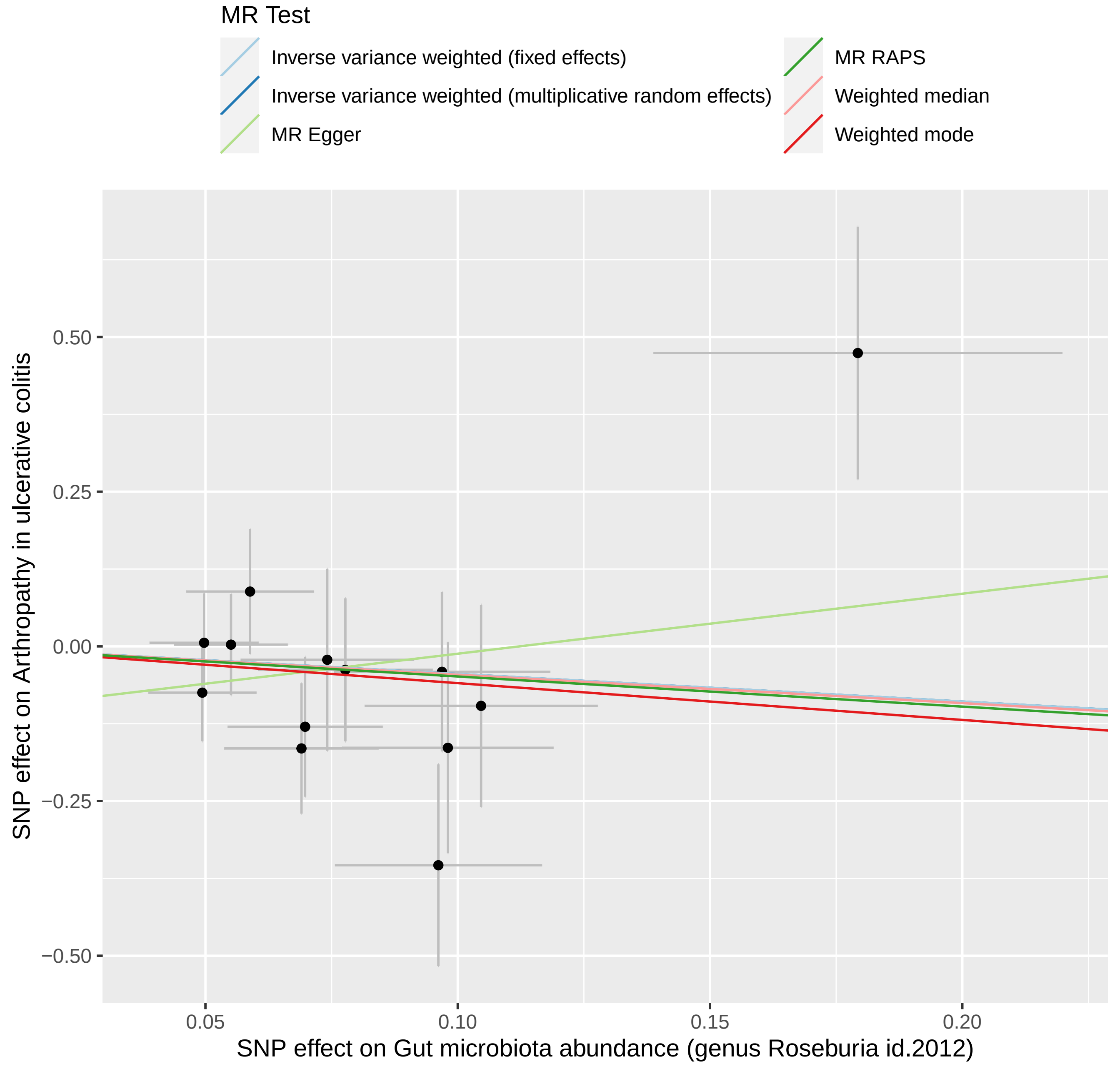

Batch 873 : Gut microbiota abundance (genus Ruminiclostridium5 id.11355) on Arthropathy in ulcerative colitis

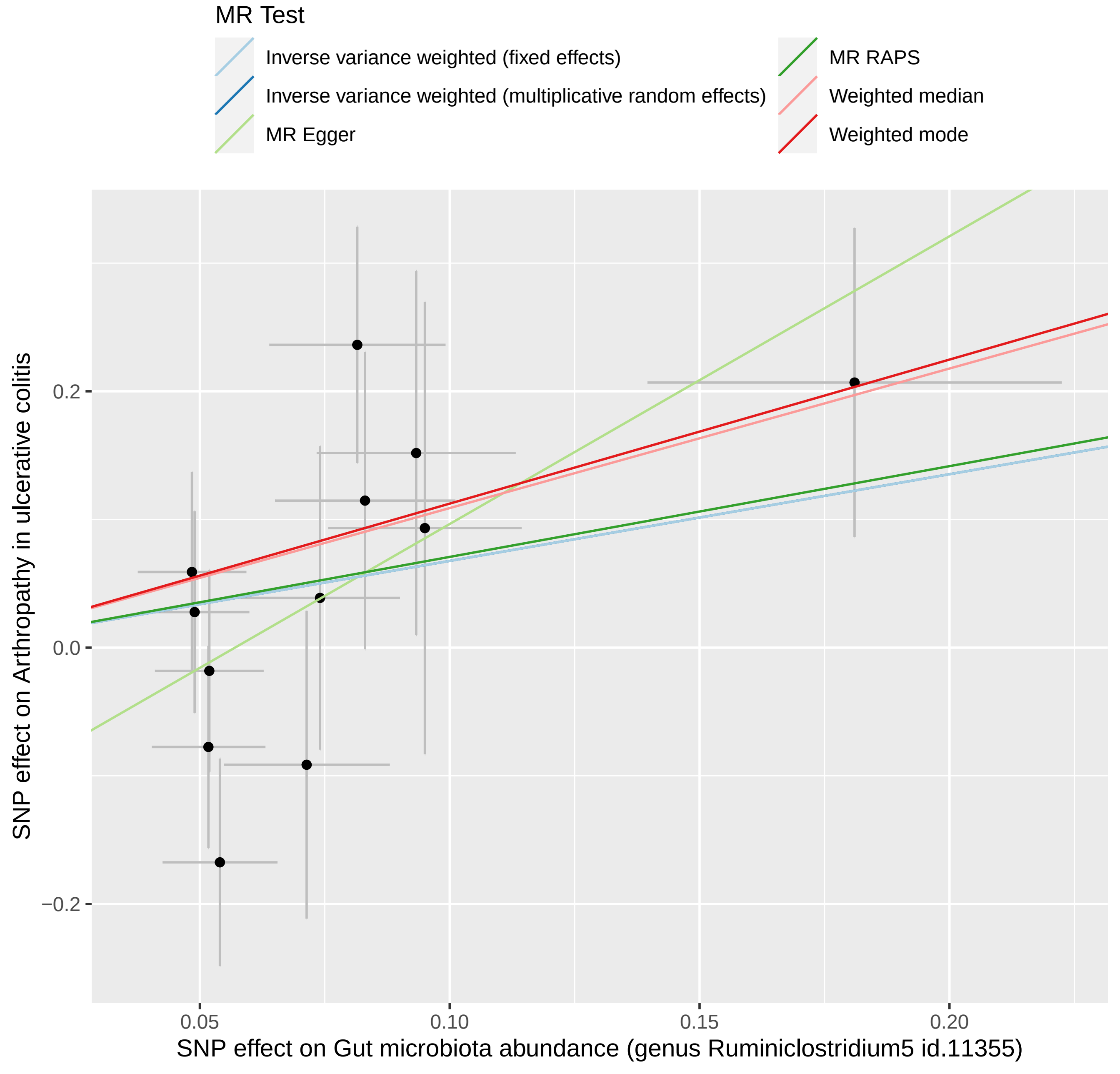

Batch 874 : Gut microbiota abundance (genus Ruminiclostridium6 id.11356) on Arthropathy in ulcerative colitis

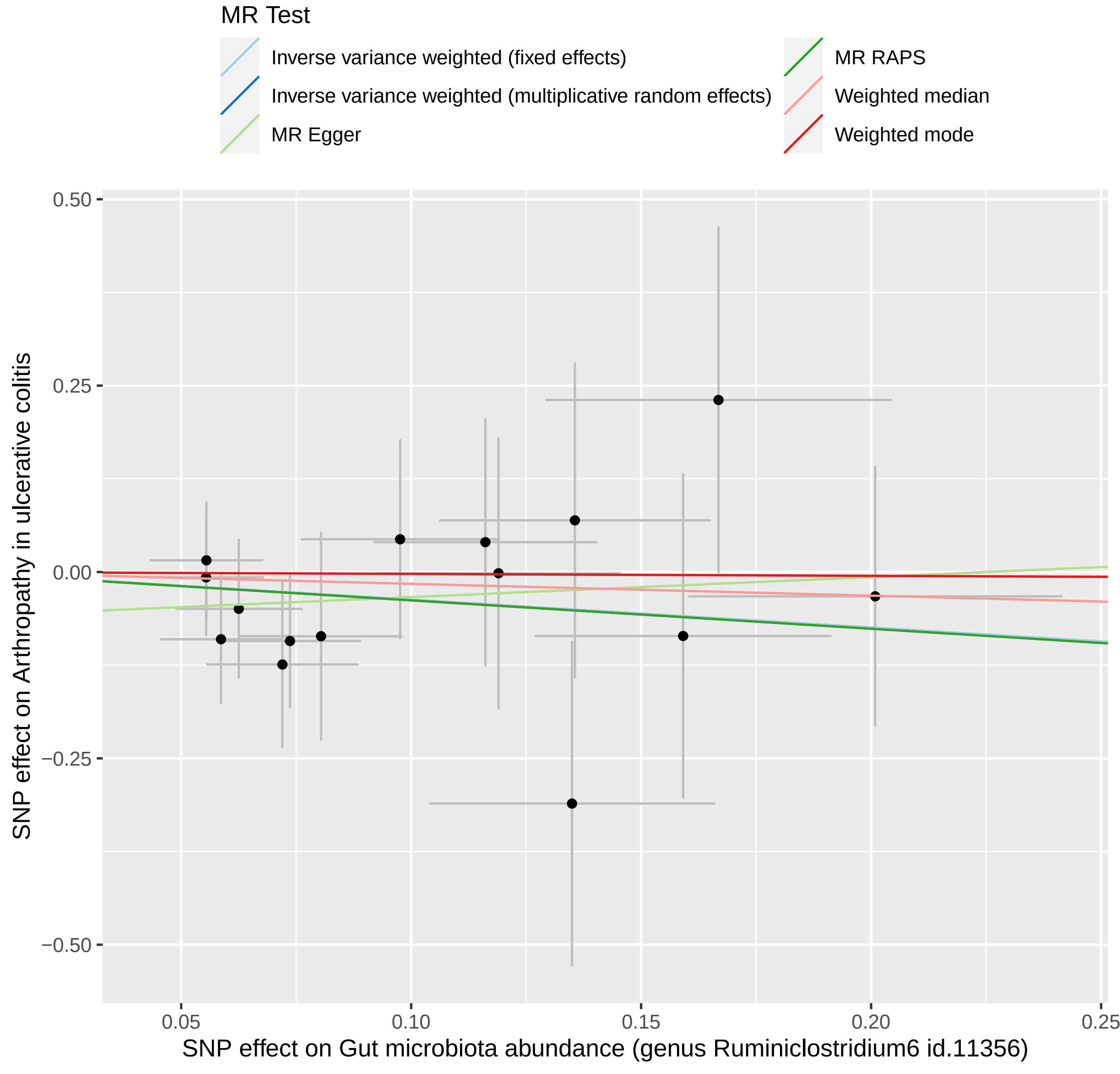

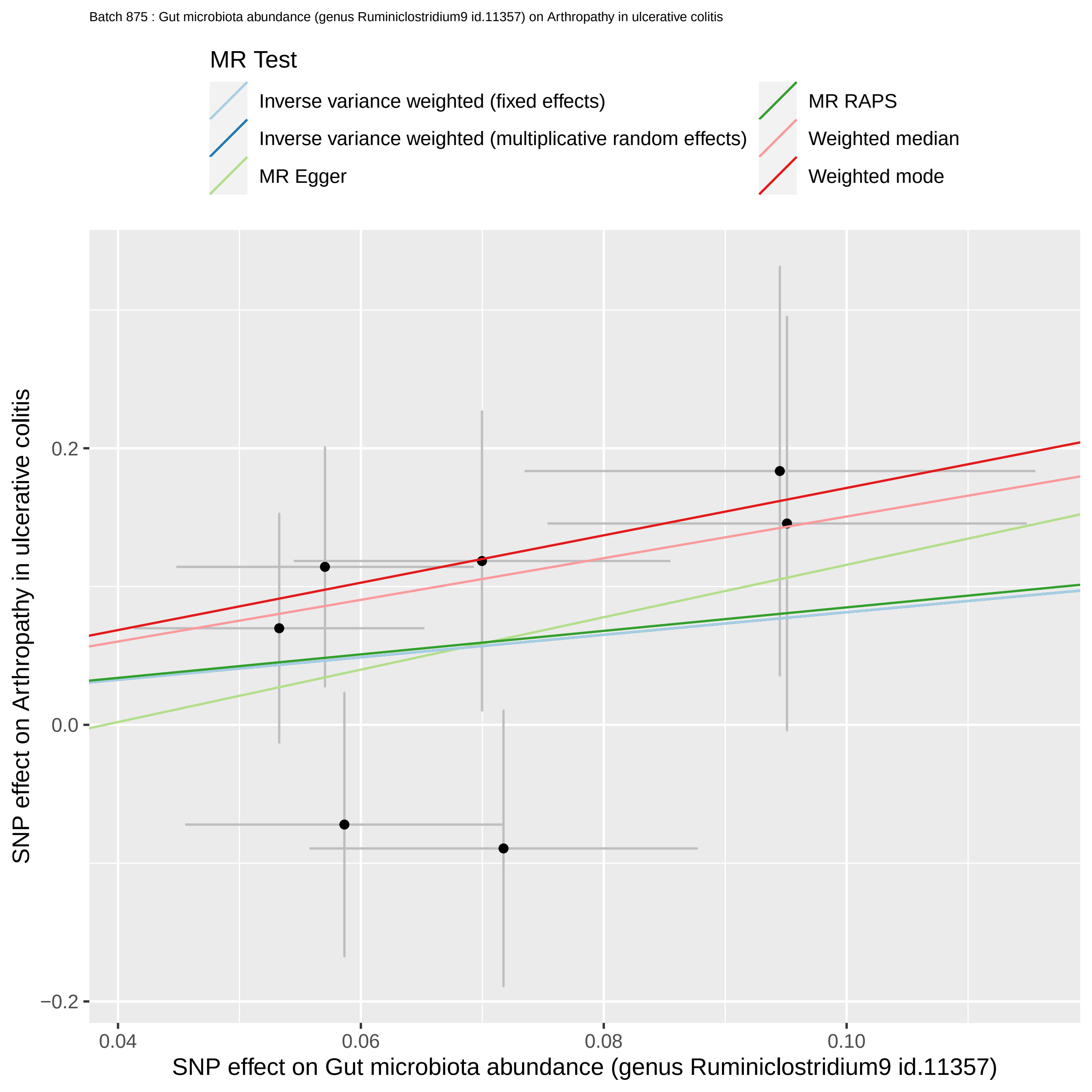
Batch 875 : Gut microbiota abundance (genus Ruminiclostridium9 id.11357) on Arthropathy in ulcerative colitis
MR Test
Inverse variance weighted (fixed effects)
Inverse variance weighted (multiplicative random effects)
MR Egger
MR RAPS
Weighted median
Weighted mode
0.2
0.0
−0.2
0.04
0.06
0.08
0.10
SNP effect on Arthropathy in ulcerative colitis
SNP effect on Gut microbiota abundance (genus Ruminiclostridium9 id.11357)

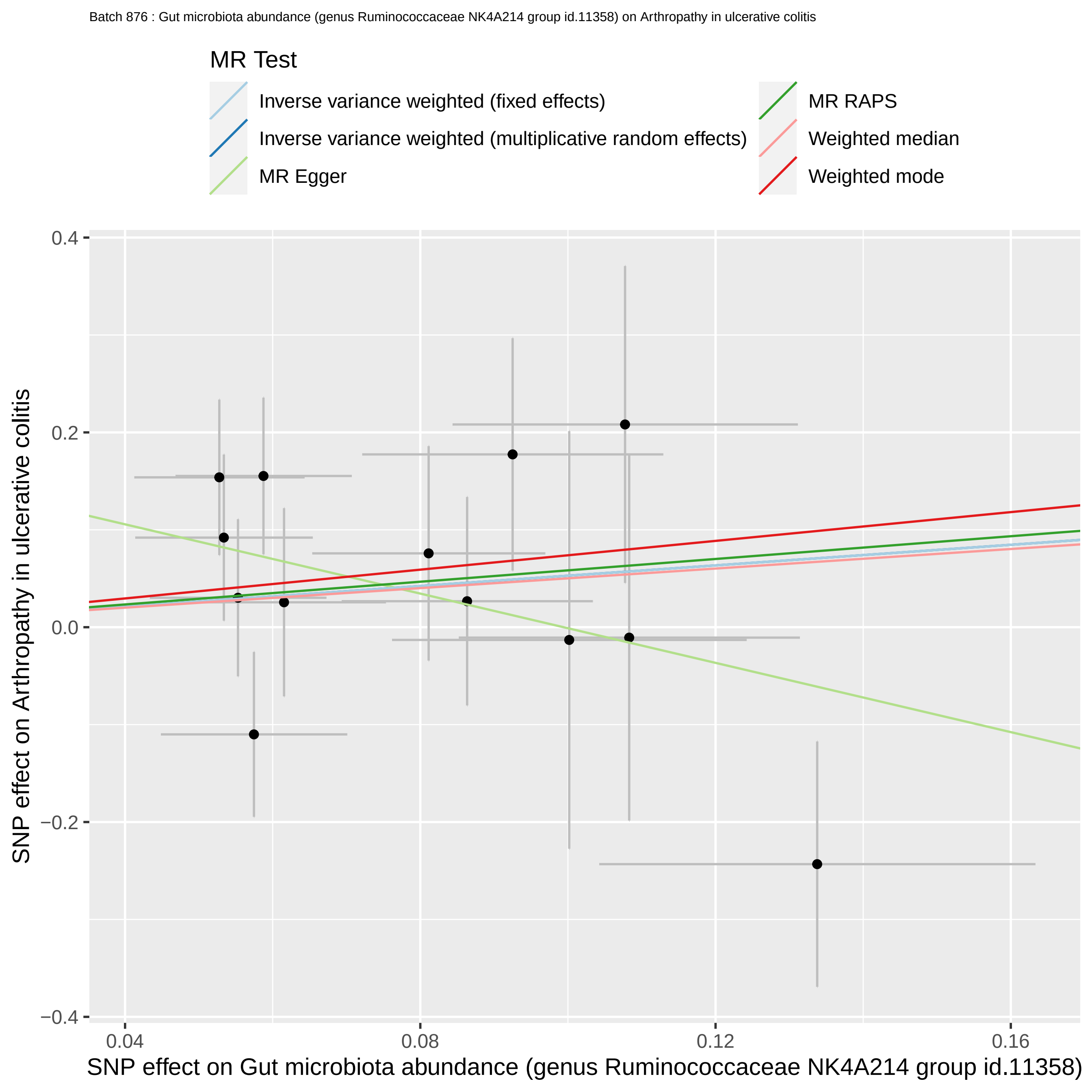
Batch 876 : Gut microbiota abundance (genus Ruminococcaceae NK4A214 group id.11358) on Arthropathy in ulcerative colitis
MR Test
Inverse variance weighted (fixed effects)
Inverse variance weighted (multiplicative random effects)
MR Egger
MR RAPS
Weighted median
Weighted mode
0.4
0.2
0.0
−0.2
−0.4
0.04
0.08
0.12
0.16
SNP effect on Arthropathy in ulcerative colitis
SNP effect on Gut microbiota abundance (genus Ruminococcaceae NK4A214 group id.11358)

Batch 877 : Gut microbiota abundance (genus Ruminococcaceae UCG002 id.11360) on Arthropathy in ulcerative colitis

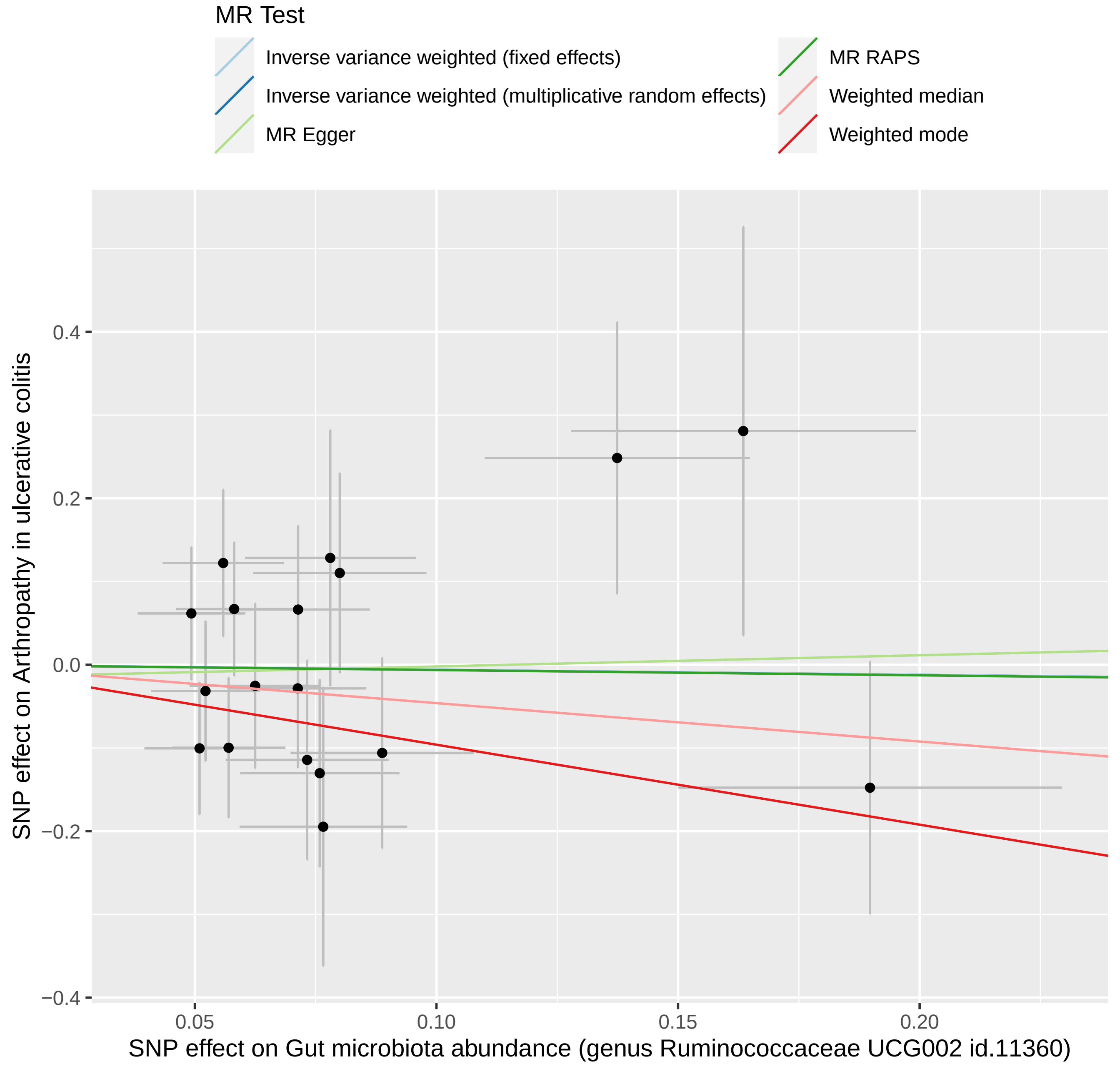

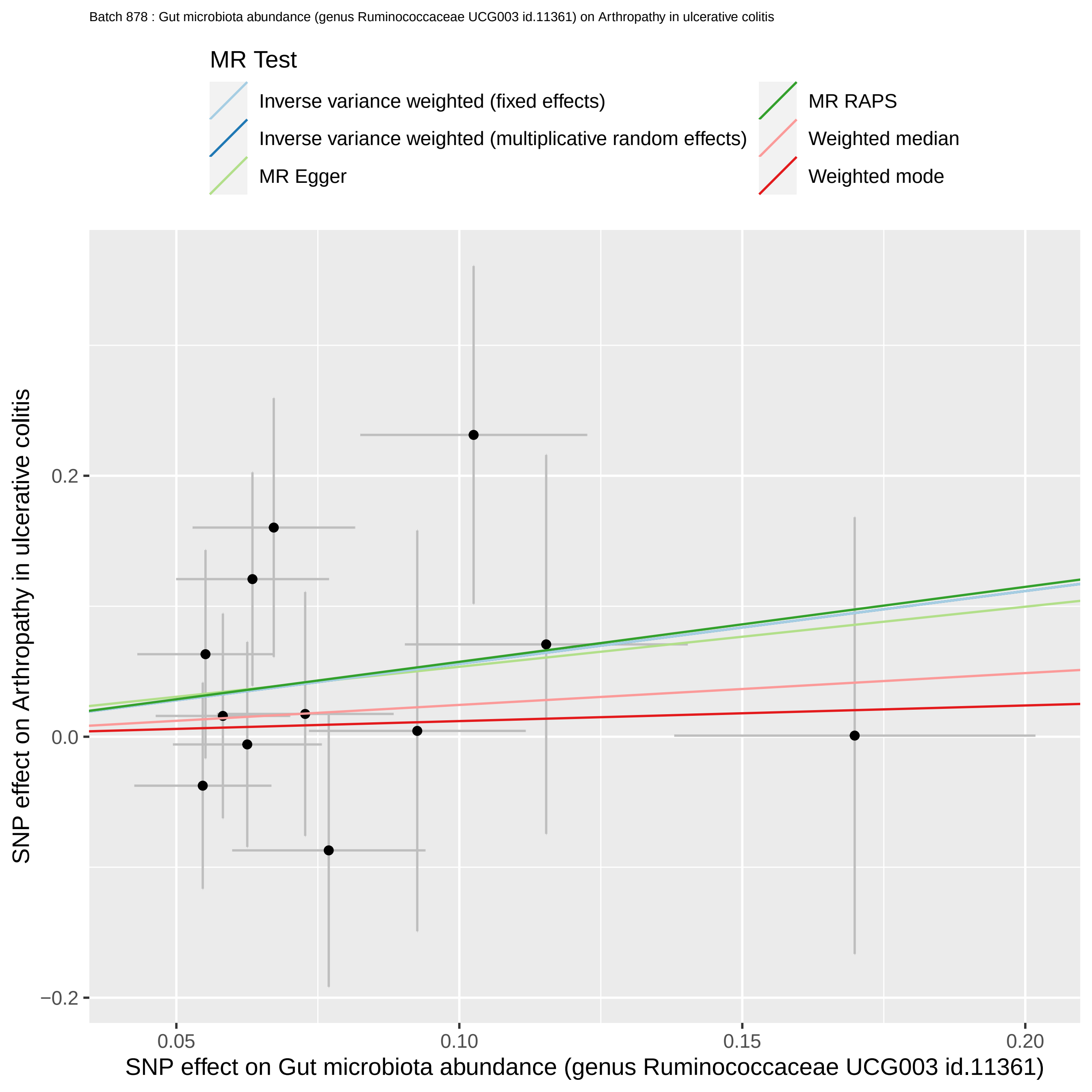
Batch 878 : Gut microbiota abundance (genus Ruminococcaceae UCG003 id.11361) on Arthropathy in ulcerative colitis
MR Test
Inverse variance weighted (fixed effects)
Inverse variance weighted (multiplicative random effects)
MR Egger
MR RAPS
Weighted median
Weighted mode
SNP effect on Arthropathy in ulcerative colitis
0.2
0.0
−0.2
0.05
0.10
0.15
0.20
SNP effect on Gut microbiota abundance (genus Ruminococcaceae UCG003 id.11361)

Batch 879 : Gut microbiota abundance (genus Ruminococcaceae UCG004 id.11362) on Arthropathy in ulcerative colitis

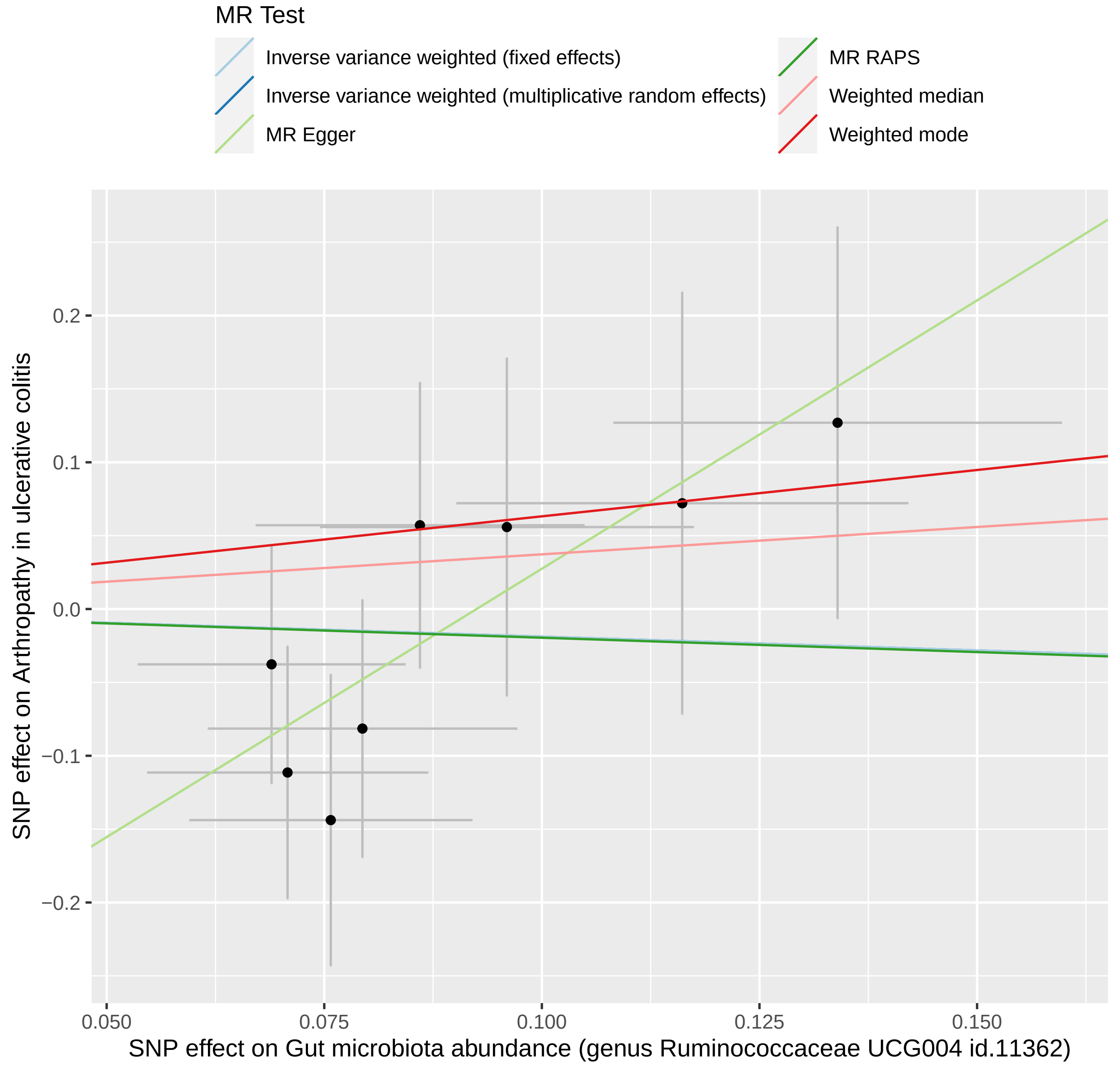

Batch 880 : Gut microbiota abundance (genus Ruminococcaceae UCG005 id.11363) on Arthropathy in ulcerative colitis

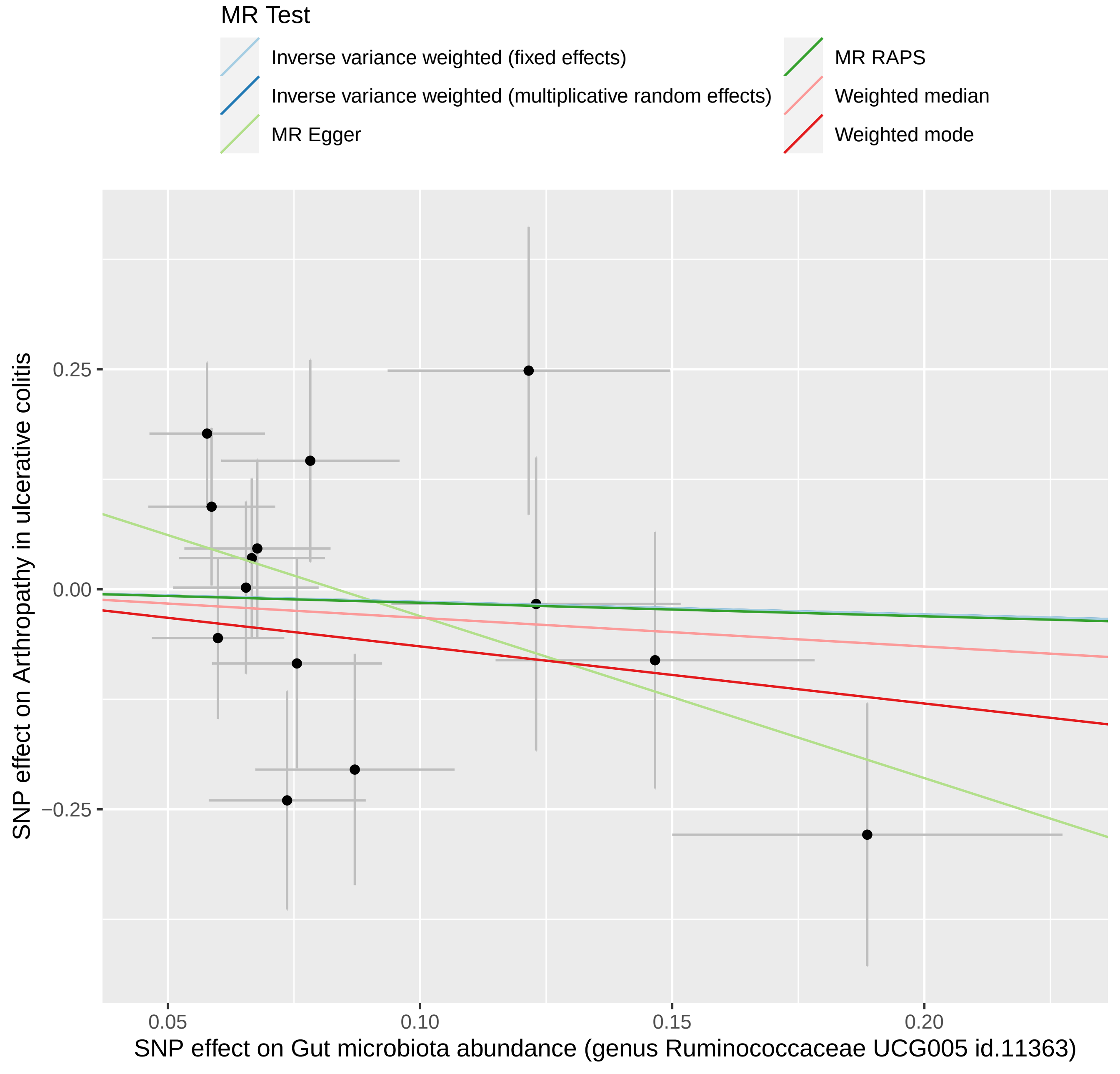

Batch 881 : Gut microbiota abundance (genus Ruminococcaceae UCG009 id.11366) on Arthropathy in ulcerative colitis

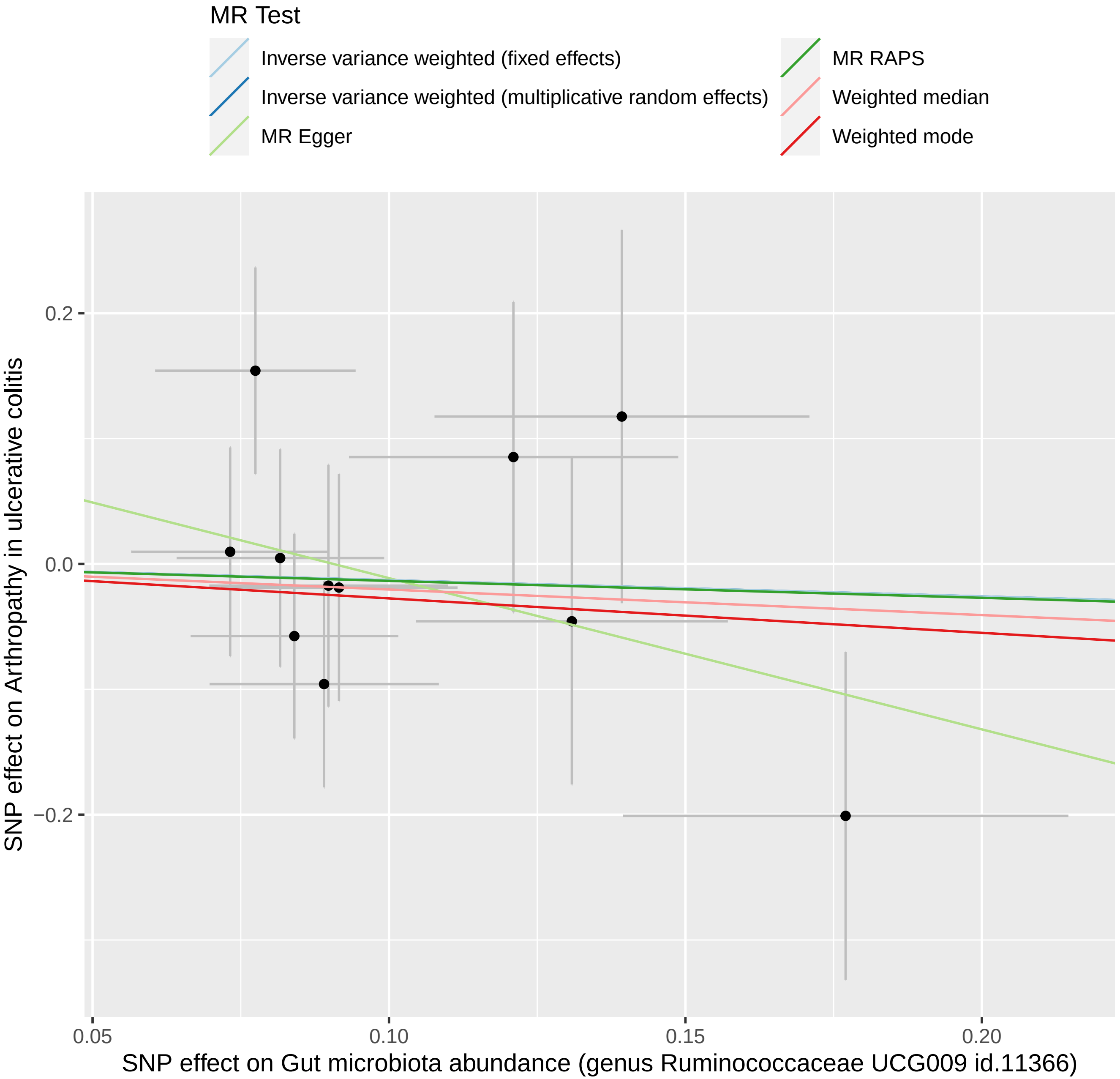

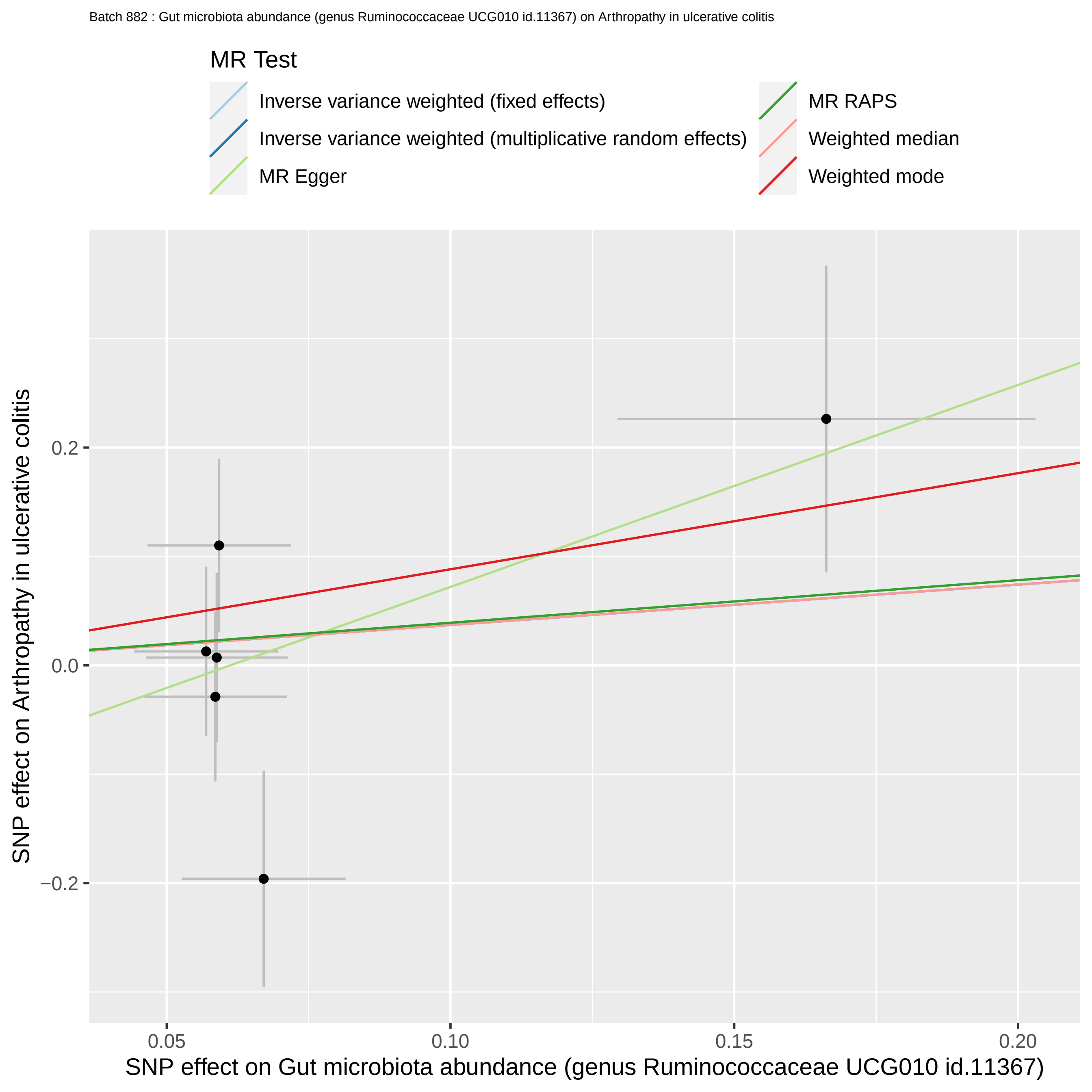
Batch 882 : Gut microbiota abundance (genus Ruminococcaceae UCG010 id.11367) on Arthropathy in ulcerative colitis
MR Test
Inverse variance weighted (fixed effects)
Inverse variance weighted (multiplicative random effects)
MR Egger
MR RAPS
Weighted median
Weighted mode
0.2
0.0
−0.2
0.05
0.10
0.15
0.20
SNP effect on Arthropathy in ulcerative colitis
SNP effect on Gut microbiota abundance (genus Ruminococcaceae UCG010 id.11367)

Batch 883 : Gut microbiota abundance (genus Ruminococcaceae UCG011 id.11368) on Arthropathy in ulcerative colitis

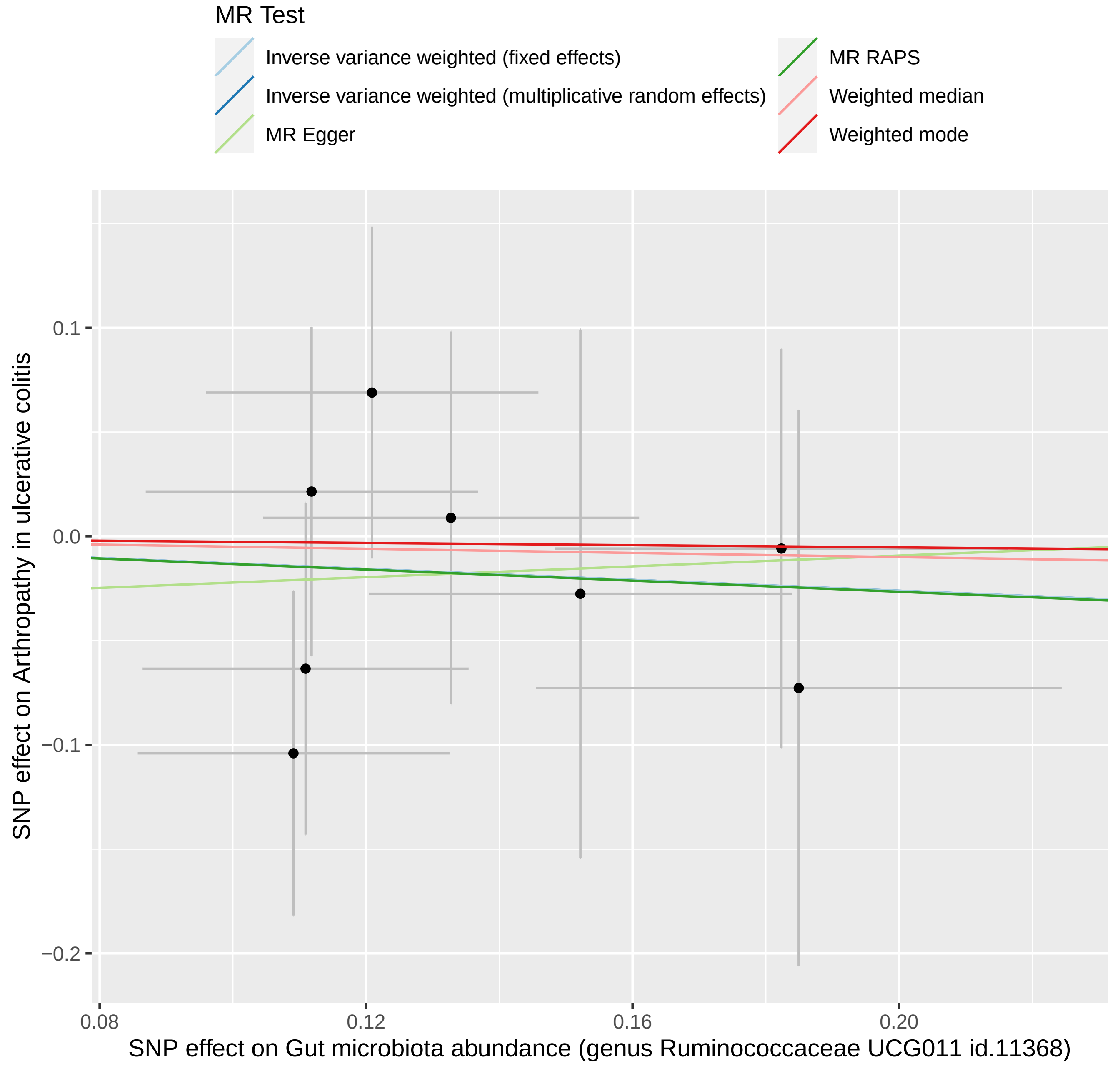

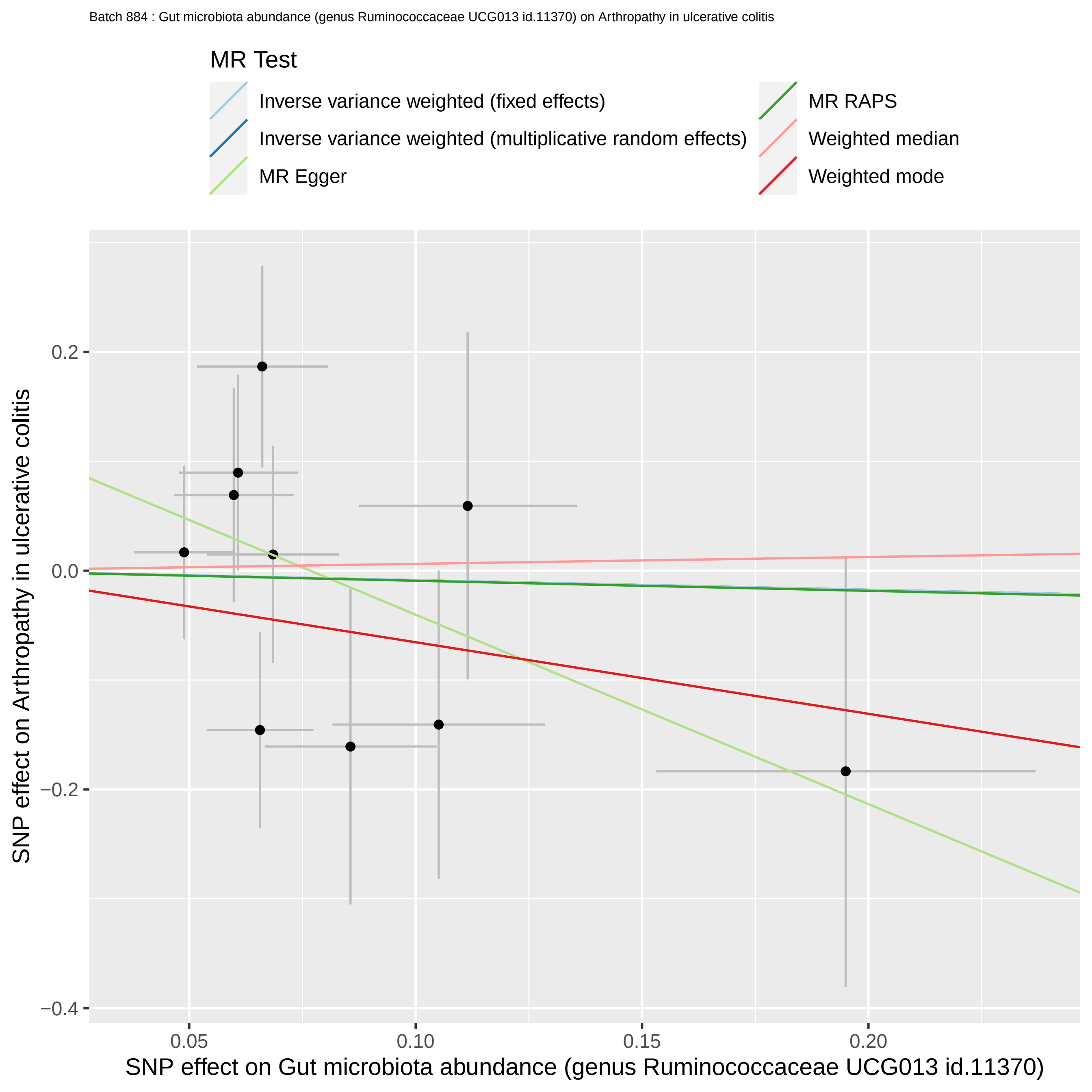
Batch 884 : Gut microbiota abundance (genus Ruminococcaceae UCG013 id.11370) on Arthropathy in ulcerative colitis
MR Test
Inverse variance weighted (fixed effects)
Inverse variance weighted (multiplicative random effects)
MR Egger
MR RAPS
Weighted median
Weighted mode
SNP effect on Arthropathy in ulcerative colitis
0.2
0.0
−0.2
−0.4
0.05
0.10
0.15
0.20
SNP effect on Gut microbiota abundance (genus Ruminococcaceae UCG013 id.11370)

Batch 885 : Gut microbiota abundance (genus Ruminococcaceae UCG014 id.11371) on Arthropathy in ulcerative colitis

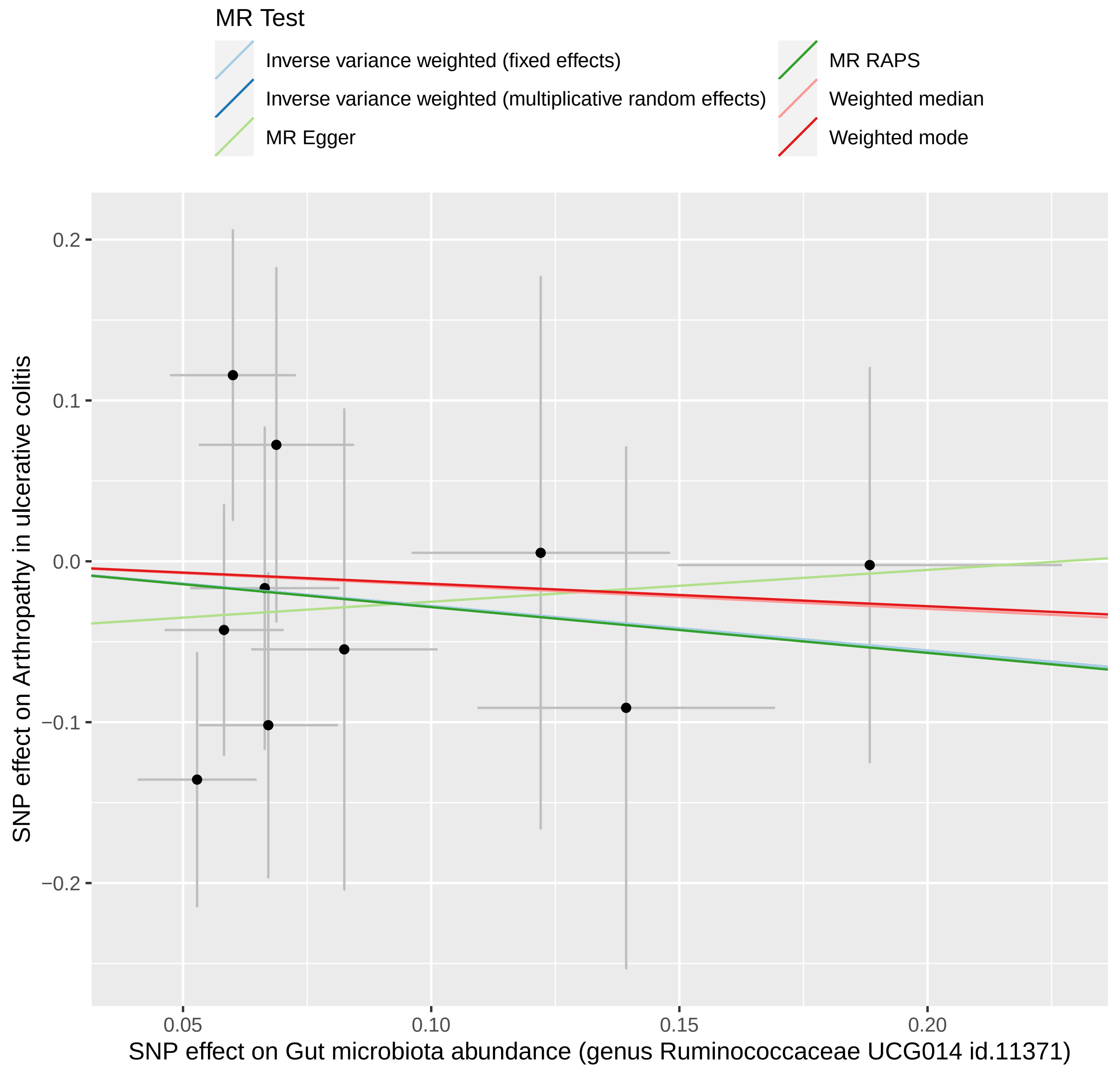

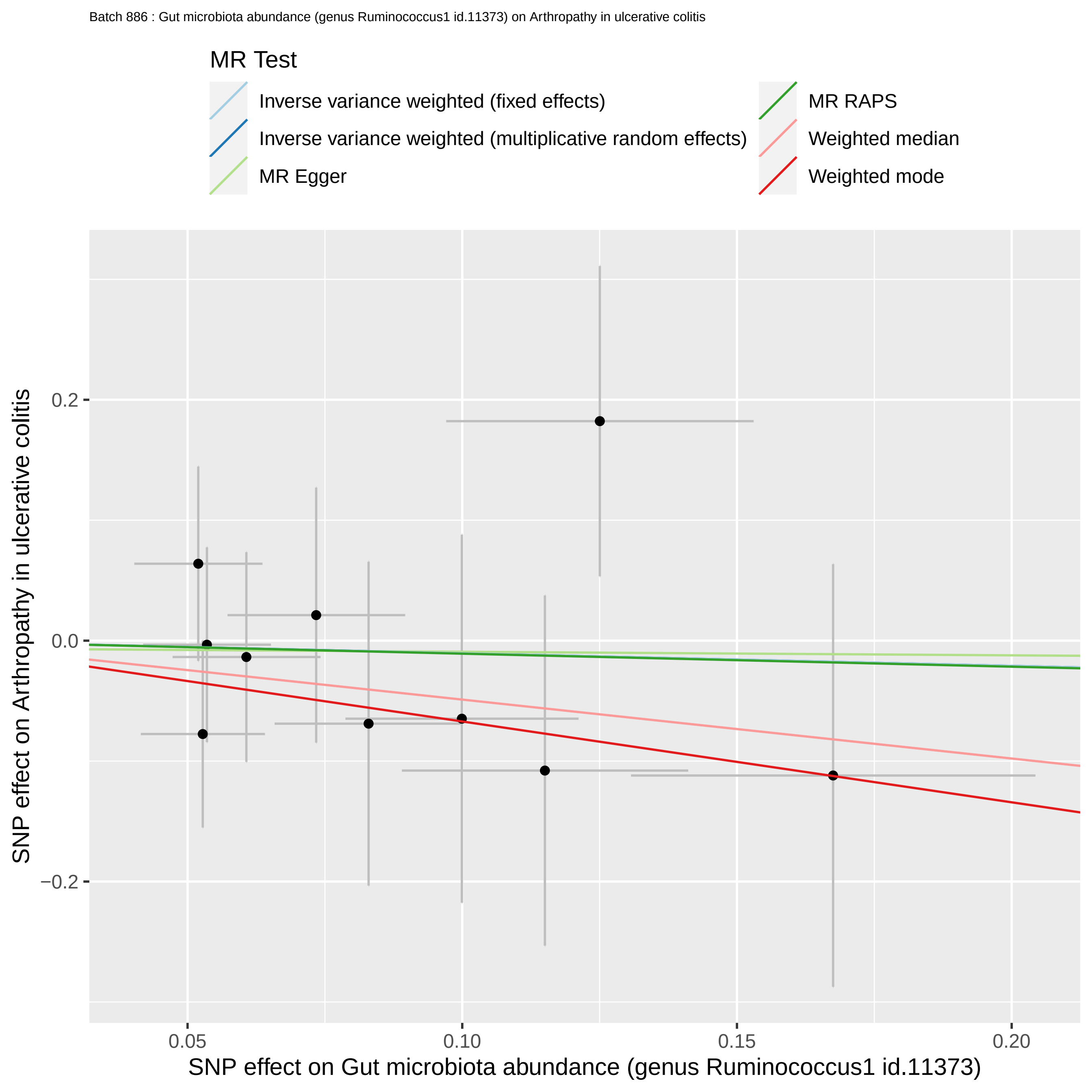

Batch 886 : Gut microbiota abundance (genus Ruminococcus1 id.11373) on Arthropathy in ulcerative colitis
MR Test
Inverse variance weighted (fixed effects)
Inverse variance weighted (multiplicative random effects)
MR Egger
MR RAPS
Weighted median
Weighted mode
0.2
0.0
−0.2
0.05
0.10
0.15
0.20
SNP effect on Arthropathy in ulcerative colitis
SNP effect on Gut microbiota abundance (genus Ruminococcus1 id.11373)

Batch 887 : Gut microbiota abundance (genus Ruminococcus2 id.11374) on Arthropathy in ulcerative colitis

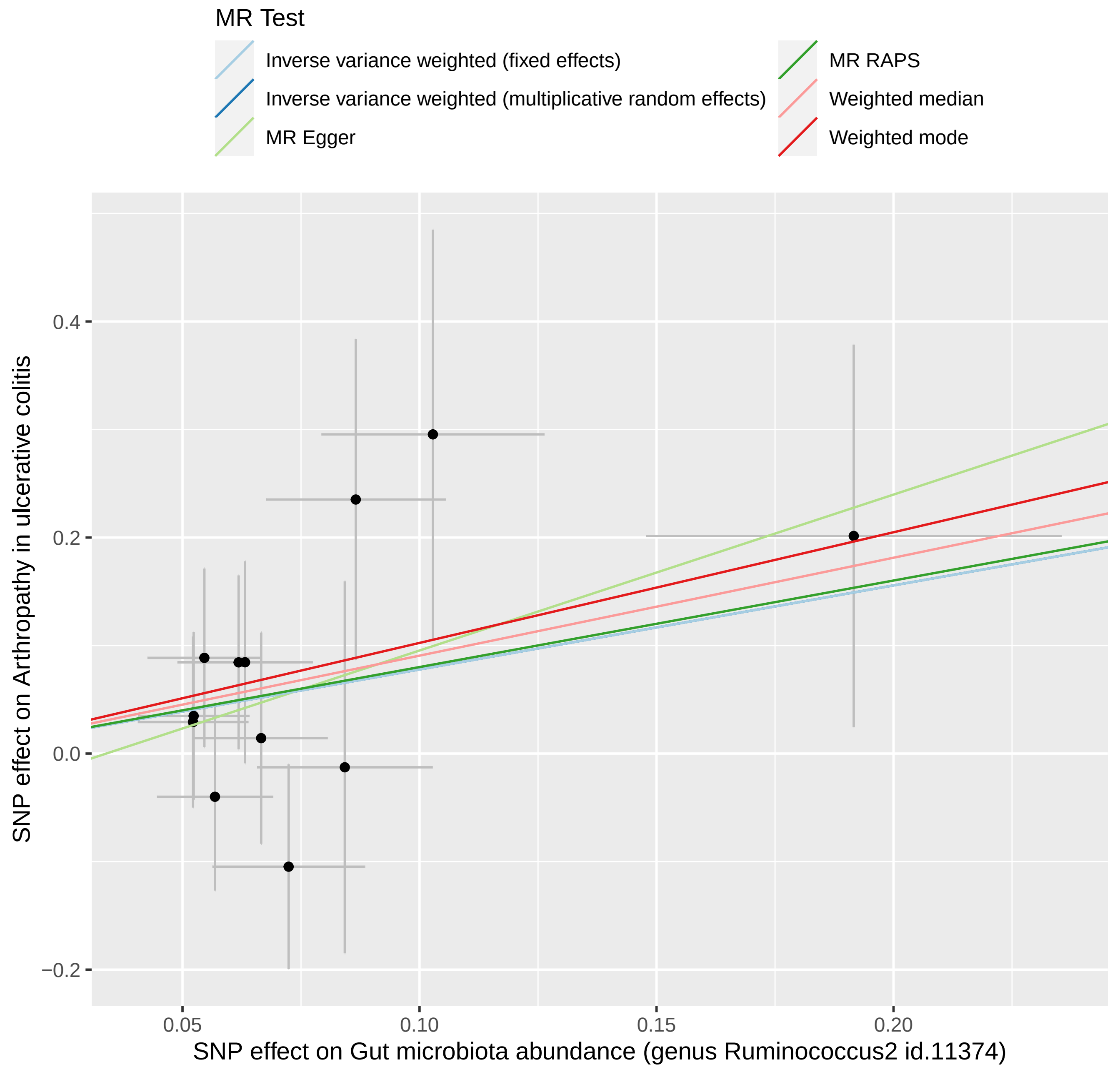

Batch 888 : Gut microbiota abundance (genus Ruminococcus gauvreauii group id.11342) on Arthropathy in ulcerative colitis

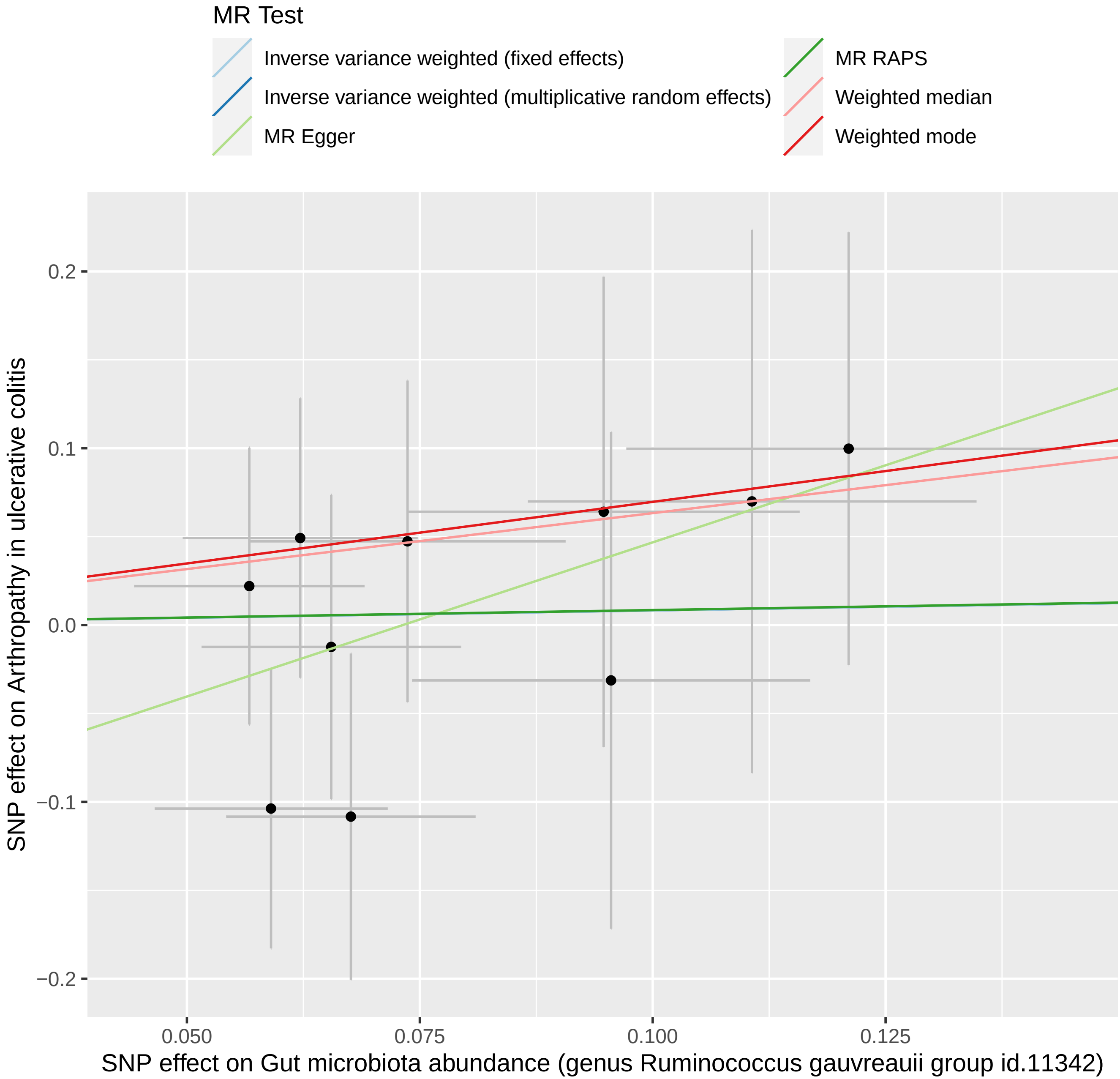

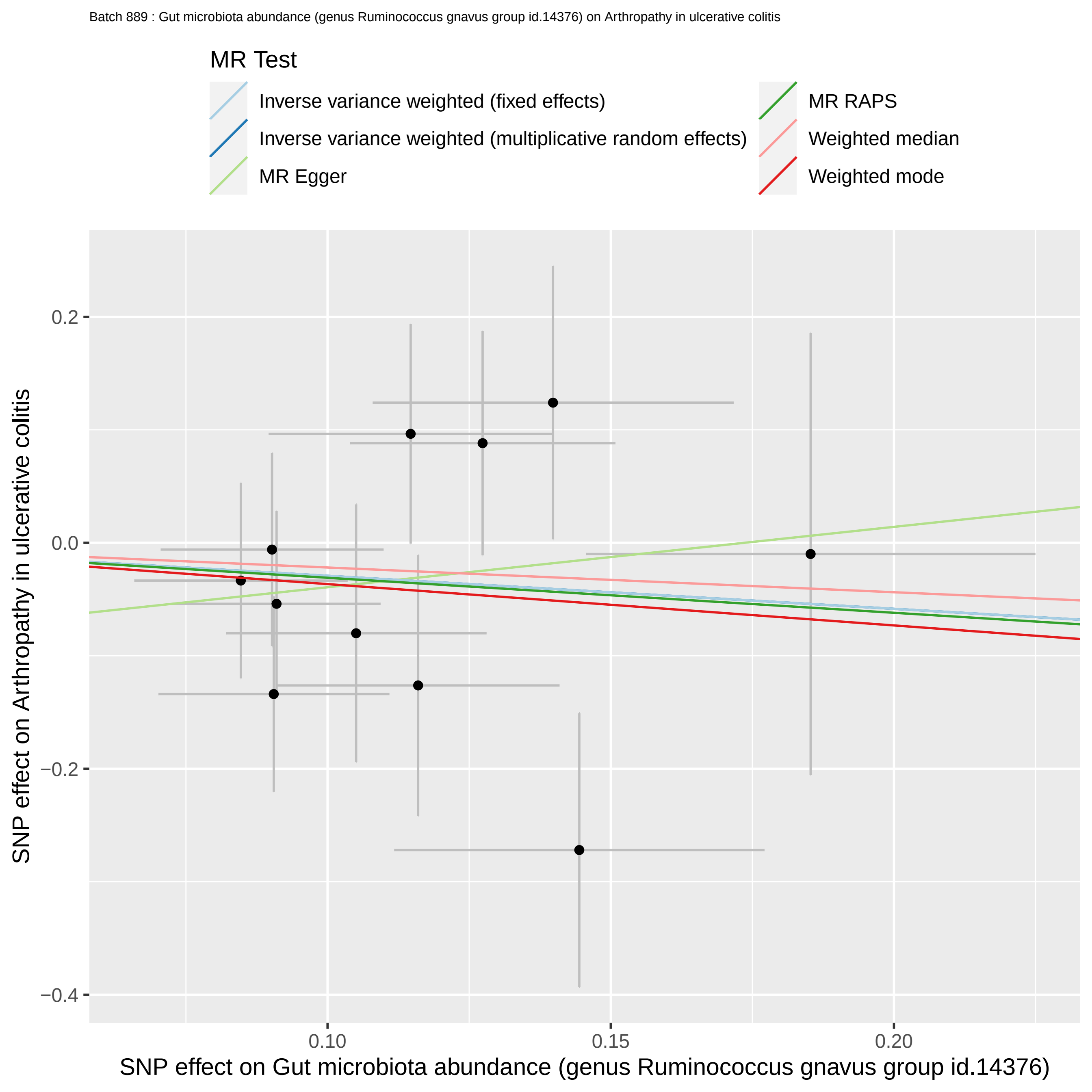
Batch 889 : Gut microbiota abundance (genus Ruminococcus gnavus group id.14376) on Arthropathy in ulcerative colitis
MR Test
Inverse variance weighted (fixed effects)
Inverse variance weighted (multiplicative random effects)
MR Egger
MR RAPS
Weighted median
Weighted mode
0.2
0.0
−0.2
−0.4
0.10
0.15
0.20
SNP effect on Arthropathy in ulcerative colitis
SNP effect on Gut microbiota abundance (genus Ruminococcus gnavus group id.14376)

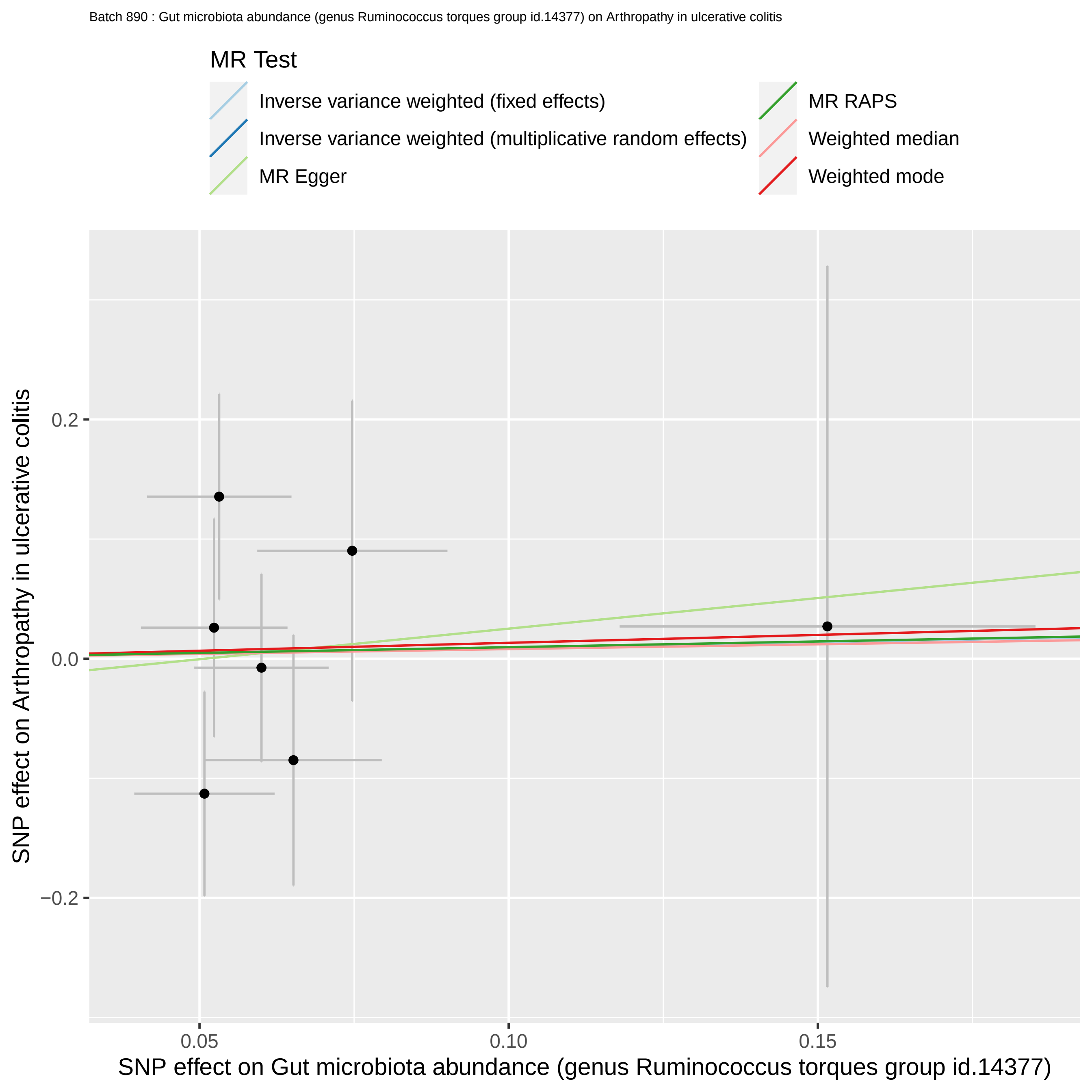

Batch 890 : Gut microbiota abundance (genus Ruminococcus torques group id.14377) on Arthropathy in ulcerative colitis
MR Test
Inverse variance weighted (fixed effects)
Inverse variance weighted (multiplicative random effects)
MR Egger
MR RAPS
Weighted median
Weighted mode
SNP effect on Arthropathy in ulcerative colitis
0.2
0.0
−0.2
0.05
0.10
0.15
SNP effect on Gut microbiota abundance (genus Ruminococcus torques group id.14377)

Batch 891 : Gut microbiota abundance (genus Sellimonas id.14369) on Arthropathy in ulcerative colitis

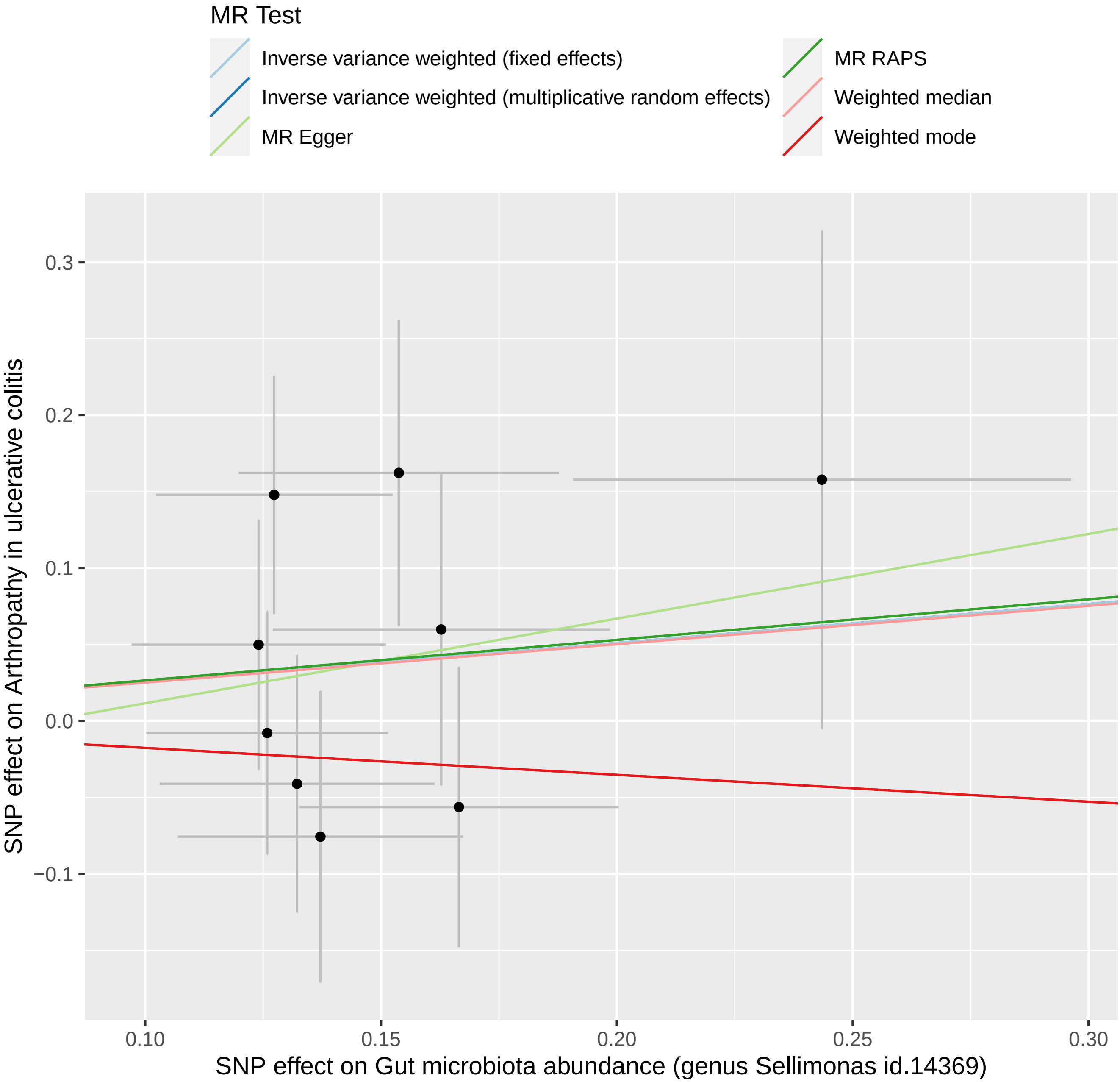

Batch 892 : Gut microbiota abundance (genus Senegalimassilia id.11160) on Arthropathy in ulcerative colitis

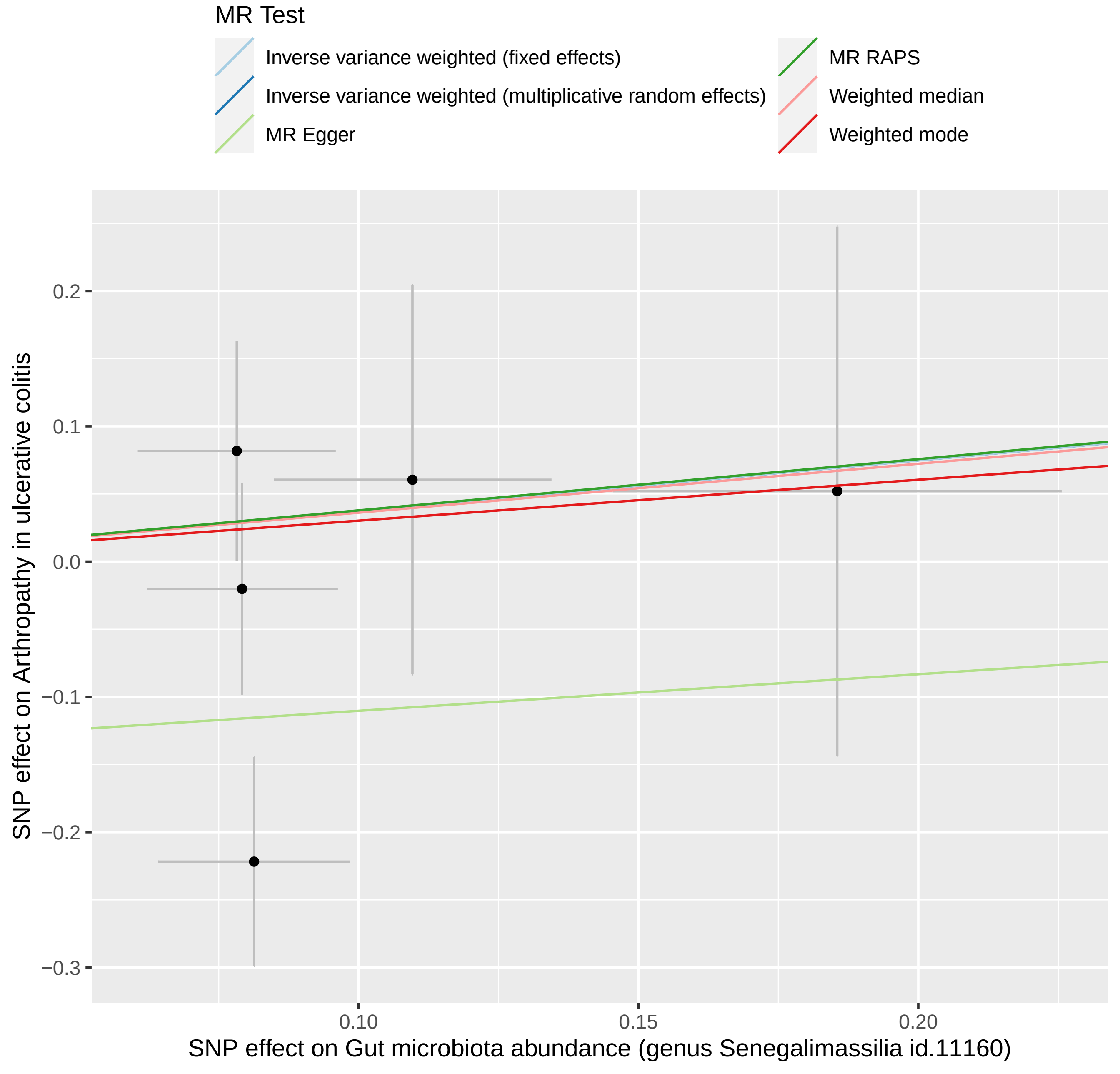

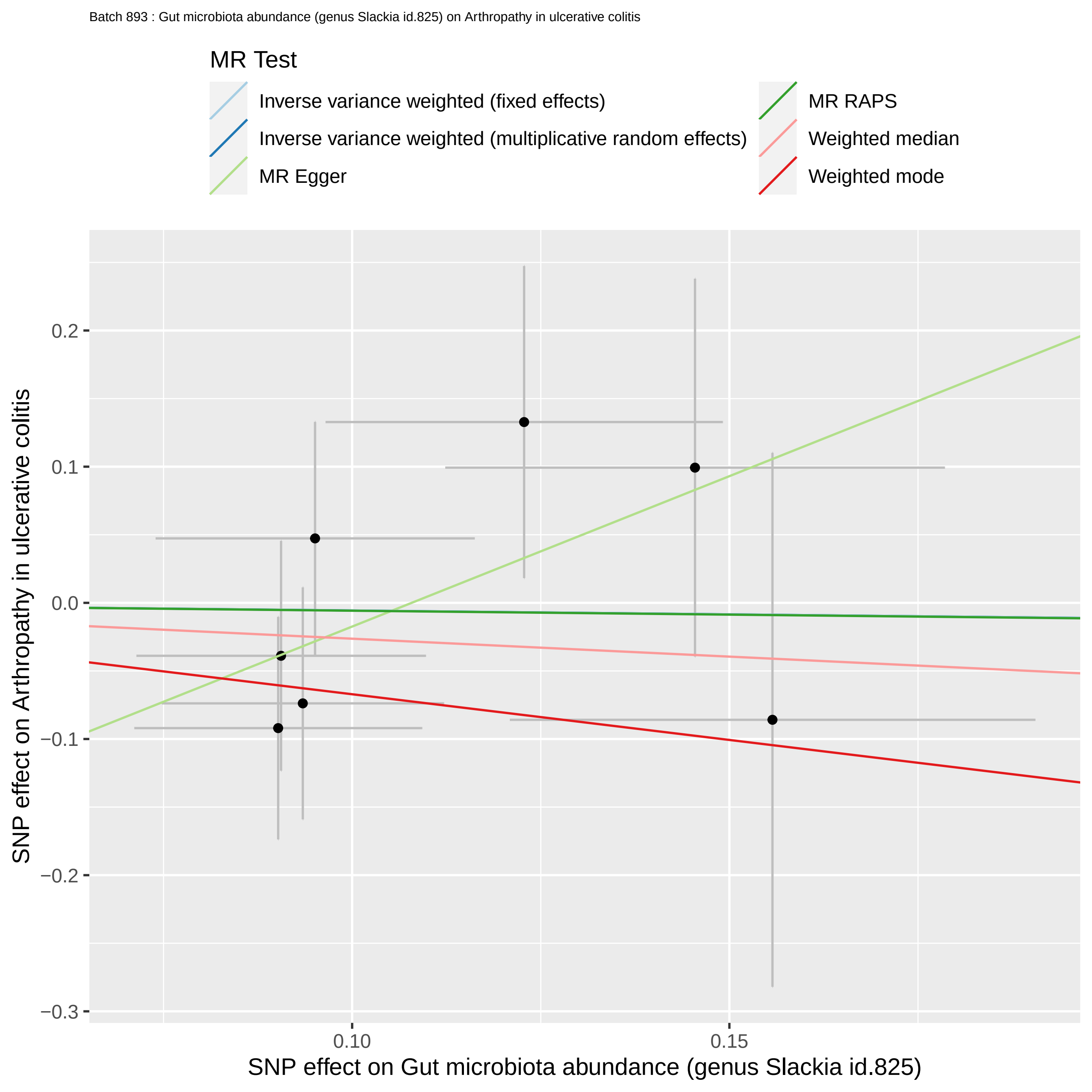
Batch 893 : Gut microbiota abundance (genus Slackia id.825) on Arthropathy in ulcerative colitis
MR Test
Inverse variance weighted (fixed effects)
Inverse variance weighted (multiplicative random effects)
MR Egger
MR RAPS
Weighted median
Weighted mode
SNP effect on Arthropathy in ulcerative colitis
0.2
0.1
0.0
−0.1
−0.2
−0.3
0.10
0.15
SNP effect on Gut microbiota abundance (genus Slackia id.825)

Batch 894 : Gut microbiota abundance (genus Streptococcus id.1853) on Arthropathy in ulcerative colitis

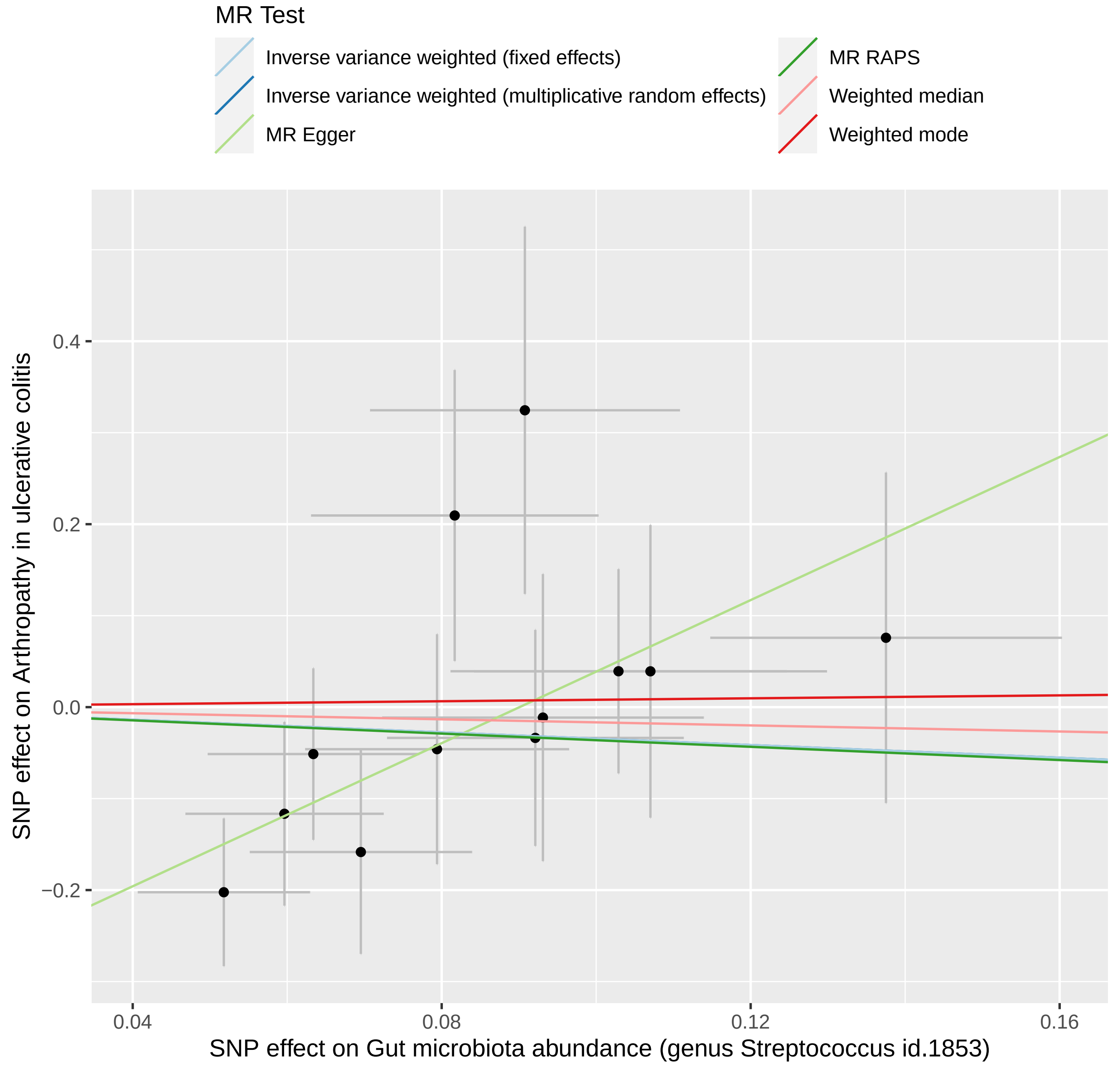

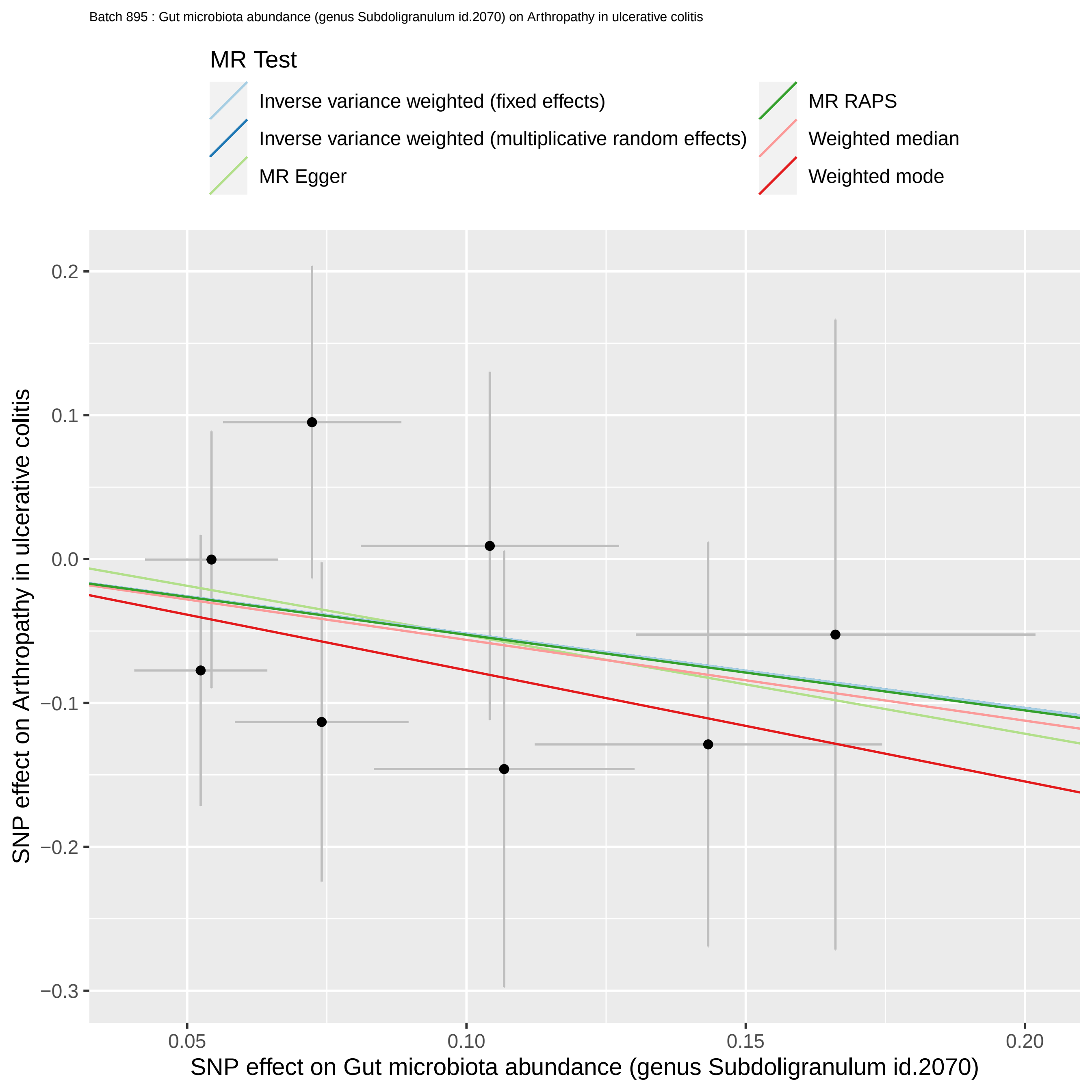

Batch 895 : Gut microbiota abundance (genus Subdoligranulum id.2070) on Arthropathy in ulcerative colitis
MR Test
Inverse variance weighted (fixed effects)
Inverse variance weighted (multiplicative random effects)
MR Egger
MR RAPS
Weighted median
Weighted mode
0.2
0.1
0.0
−0.1
−0.2
−0.3
0.05
0.10
0.15
0.20
SNP effect on Arthropathy in ulcerative colitis
SNP effect on Gut microbiota abundance (genus Subdoligranulum id.2070)

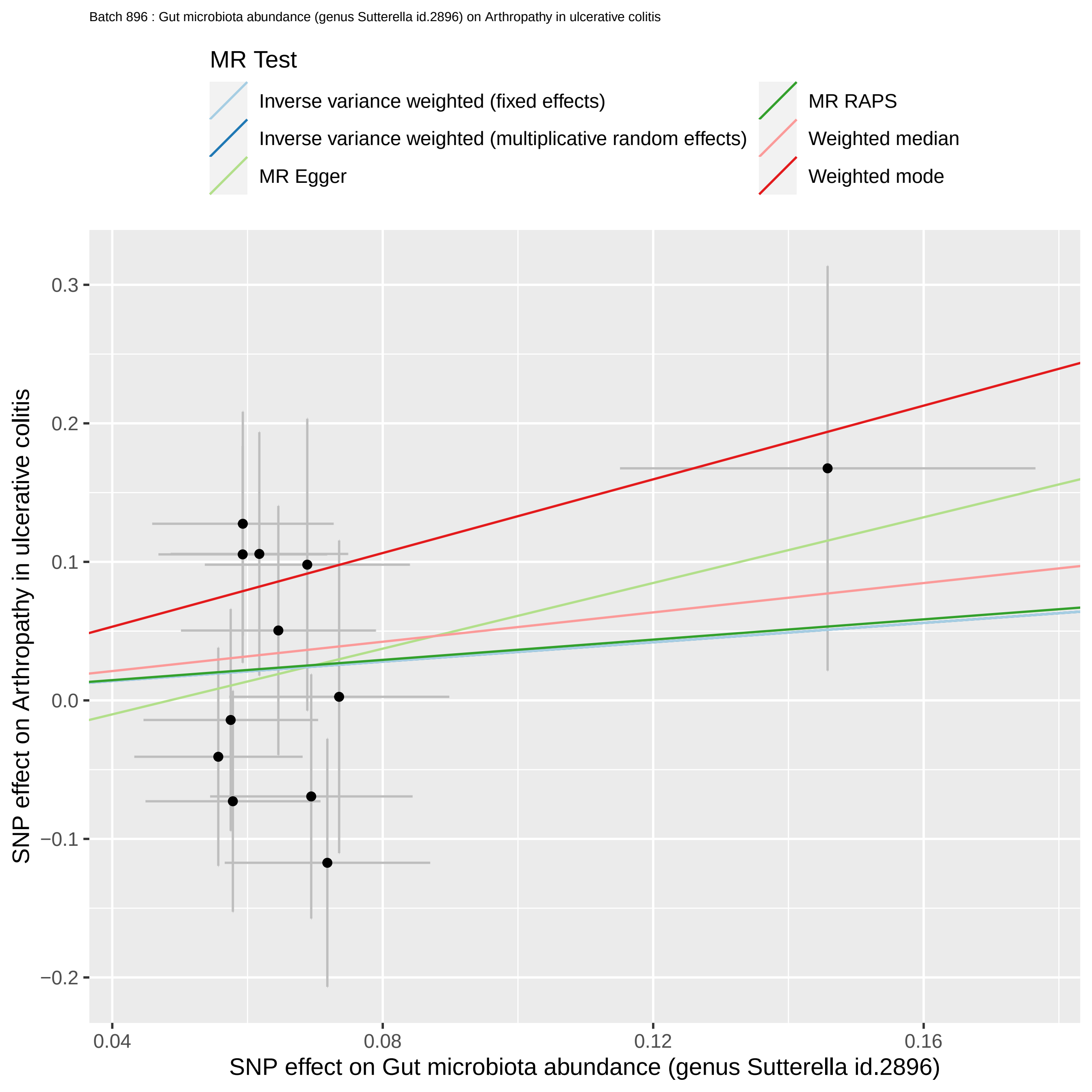
Batch 896 : Gut microbiota abundance (genus Sutterella id.2896) on Arthropathy in ulcerative colitis
MR Test
Inverse variance weighted (fixed effects)
Inverse variance weighted (multiplicative random effects)
MR Egger
MR RAPS
Weighted median
Weighted mode
0.3
0.2
0.1
0.0
−0.1
−0.2
0.04
0.08
0.12
0.16
SNP effect on Arthropathy in ulcerative colitis
SNP effect on Gut microbiota abundance (genus Sutterella id.2896)

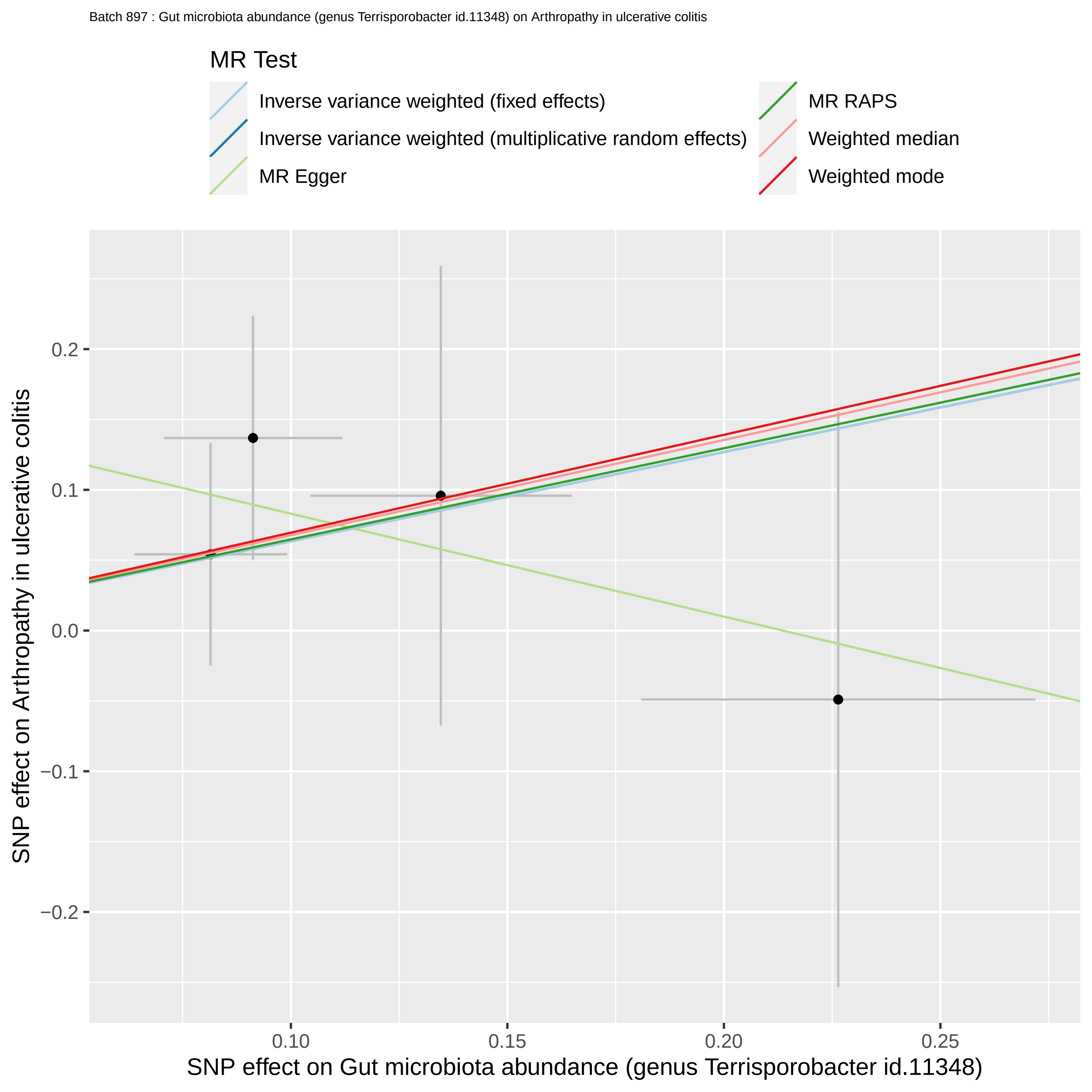
Batch 897 : Gut microbiota abundance (genus Terrisporobacter id.11348) on Arthropathy in ulcerative colitis
MR Test
Inverse variance weighted (fixed effects)
Inverse variance weighted (multiplicative random effects)
MR Egger
MR RAPS
Weighted median
Weighted mode
SNP effect on Arthropathy in ulcerative colitis
0.2
0.1
0.0
−0.1
−0.2
0.10
0.15
0.20
0.25
SNP effect on Gut microbiota abundance (genus Terrisporobacter id.11348)

Batch 898 : Gut microbiota abundance (genus Turicibacter id.2162) on Arthropathy in ulcerative colitis

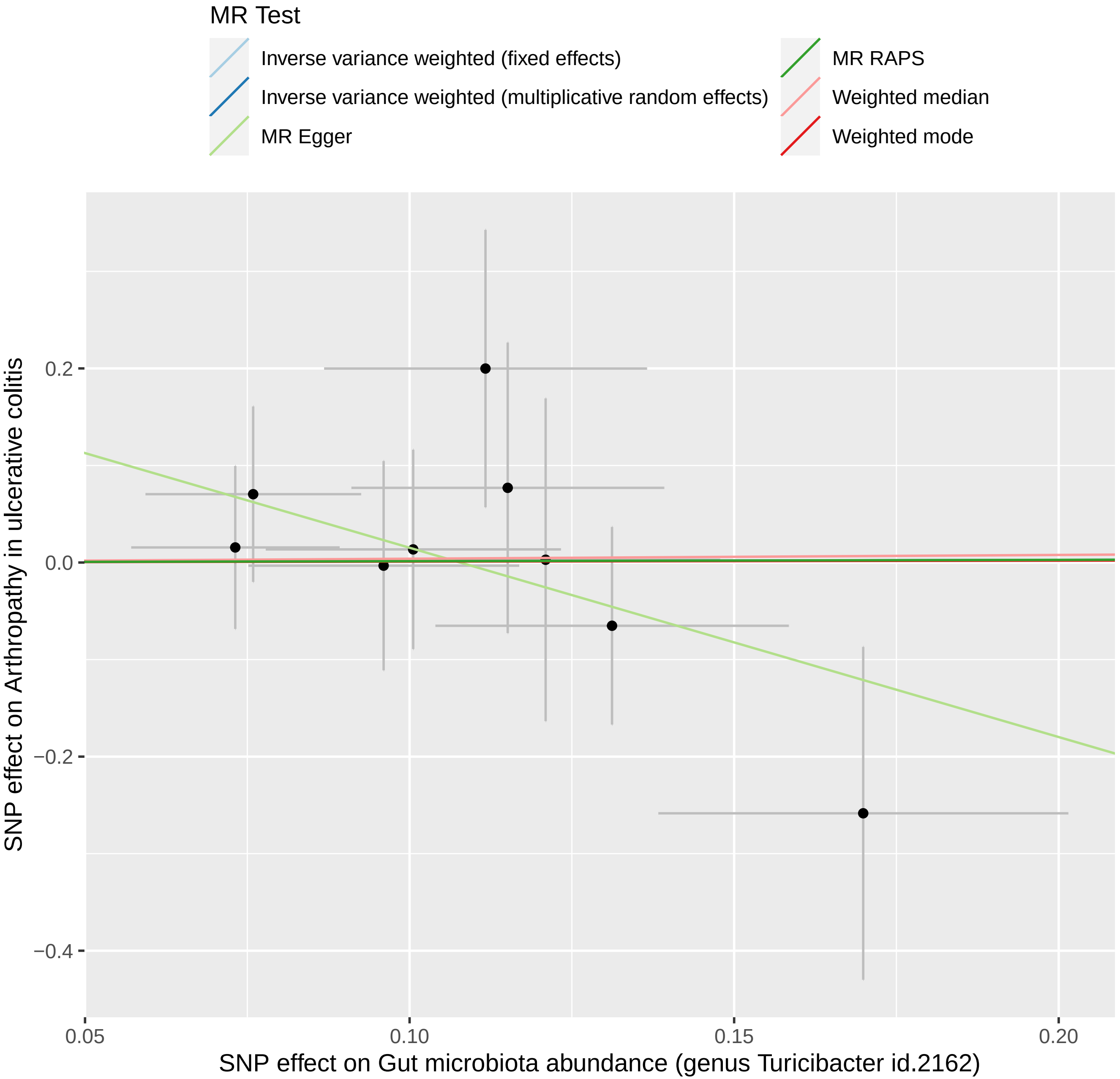

Batch 899 : Gut microbiota abundance (genus Tyzzerella3 id.11335) on Arthropathy in ulcerative colitis

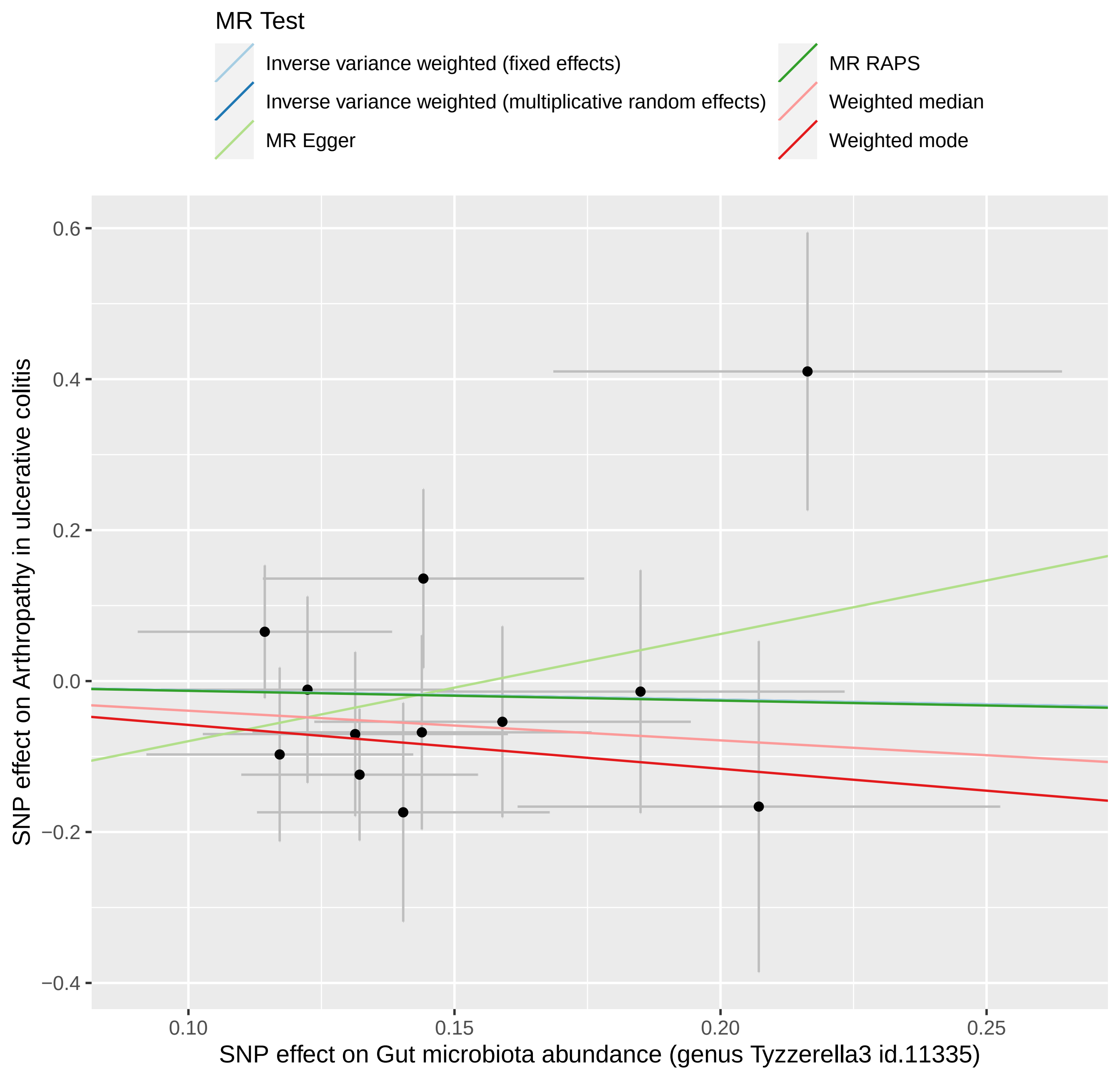

Batch 900 : Gut microbiota abundance (genus Veillonella id.2198) on Arthropathy in ulcerative colitis

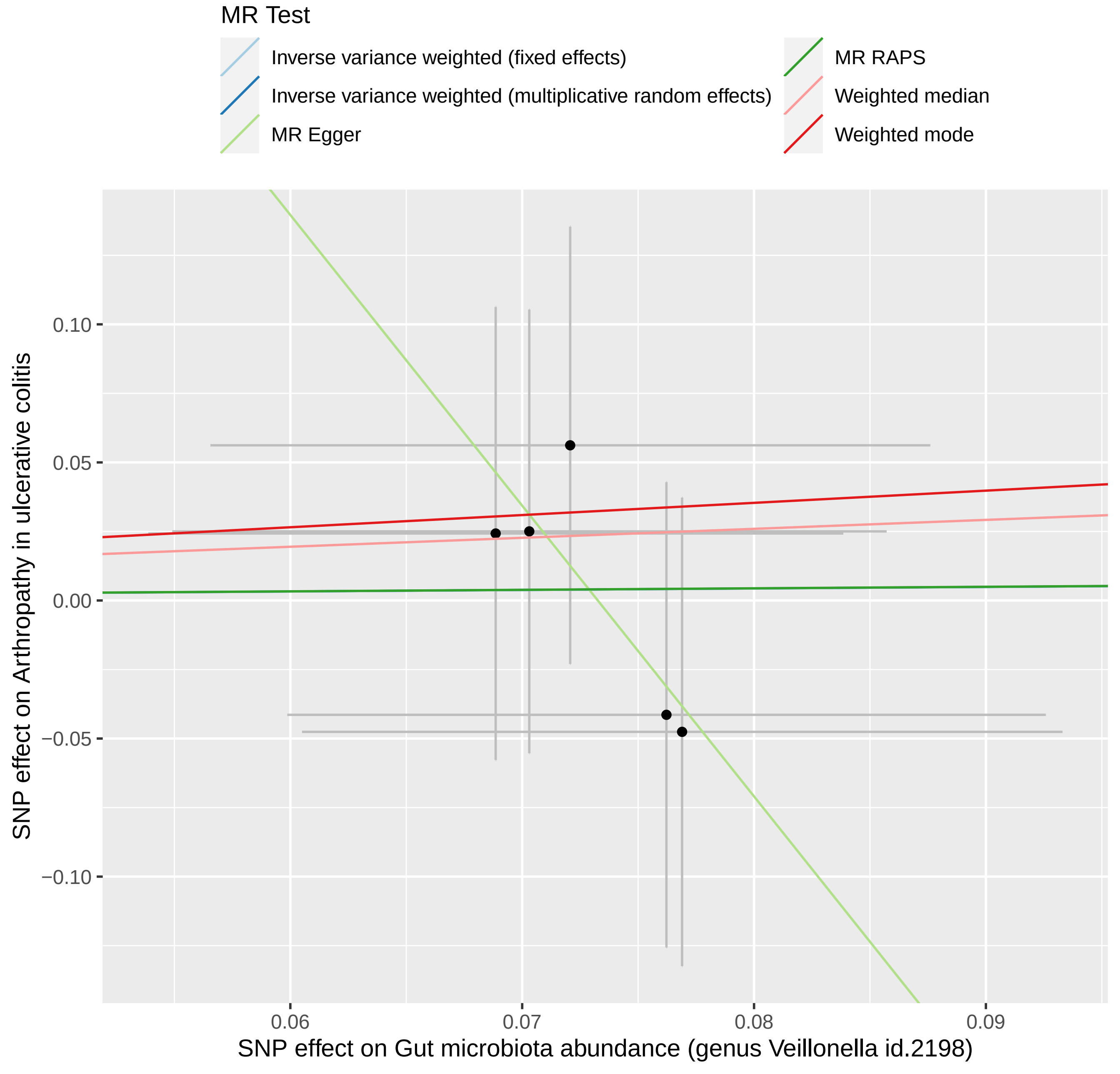

Batch 901 : Gut microbiota abundance (family Acidaminococcaceae id.2166) on Ulcerative enterocolitis

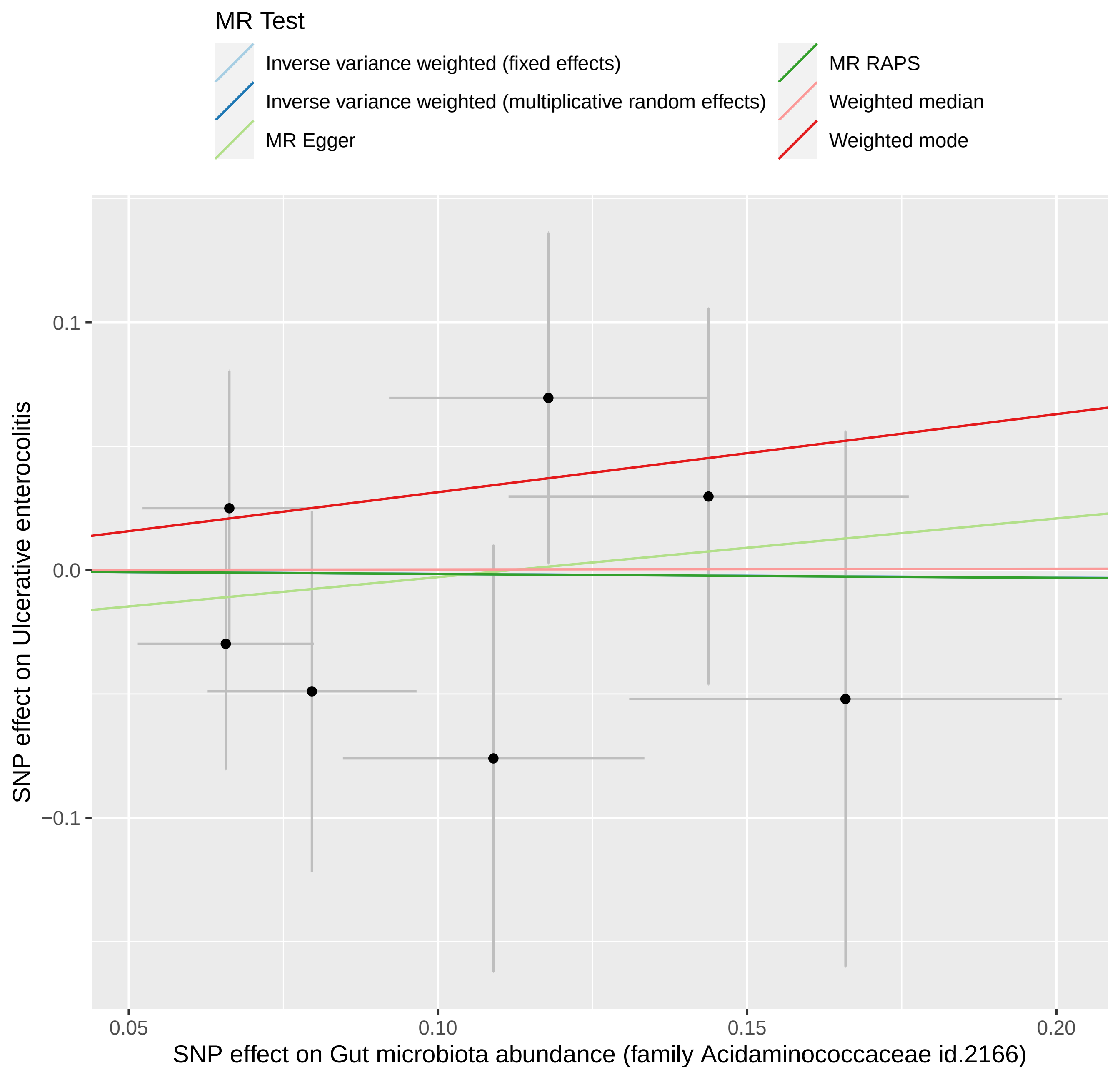

Batch 902 : Gut microbiota abundance (family Actinomycetaceae id.421) on Ulcerative enterocolitis

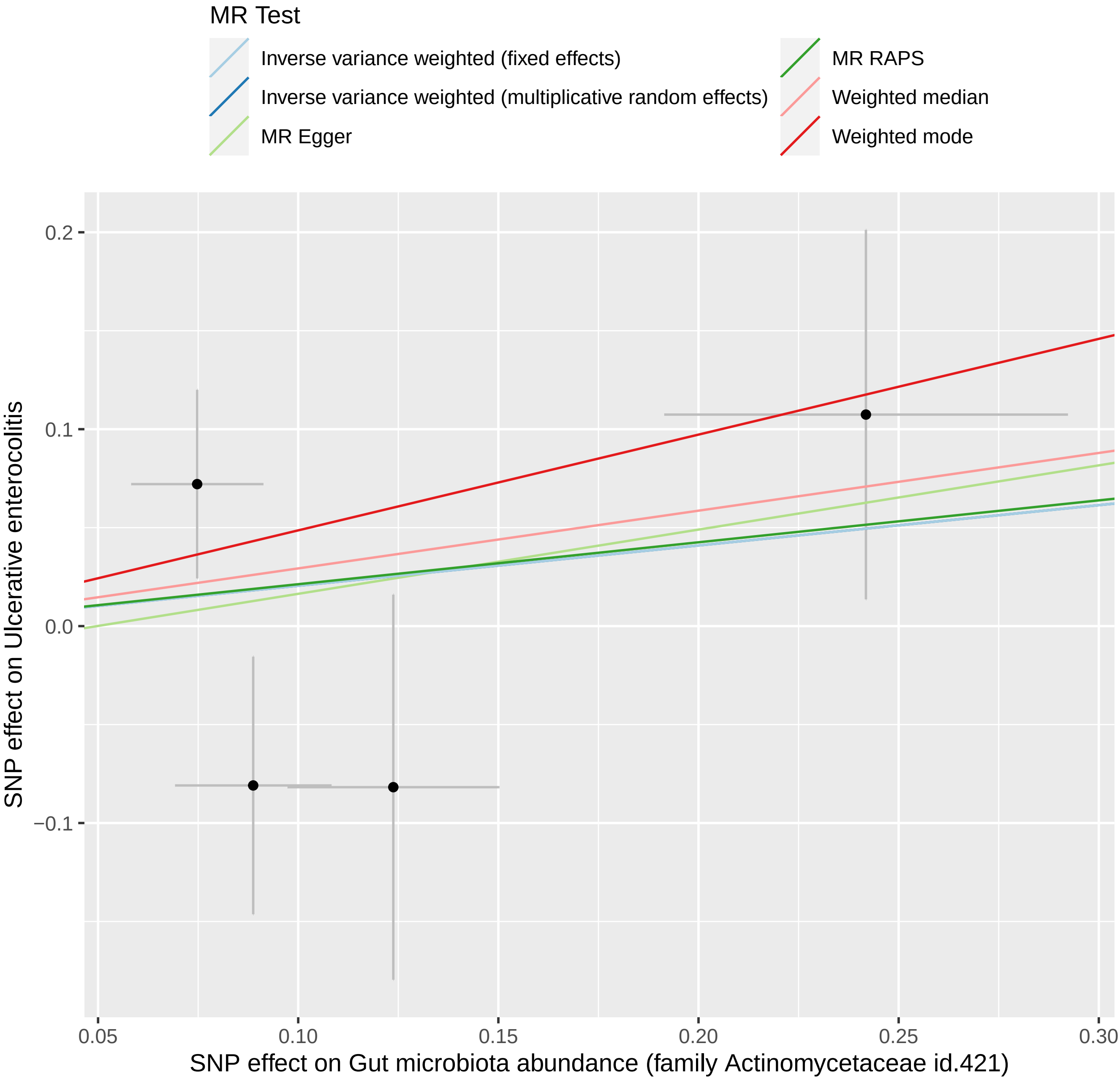

Batch 903 : Gut microbiota abundance (family Alcaligenaceae id.2875) on Ulcerative enterocolitis

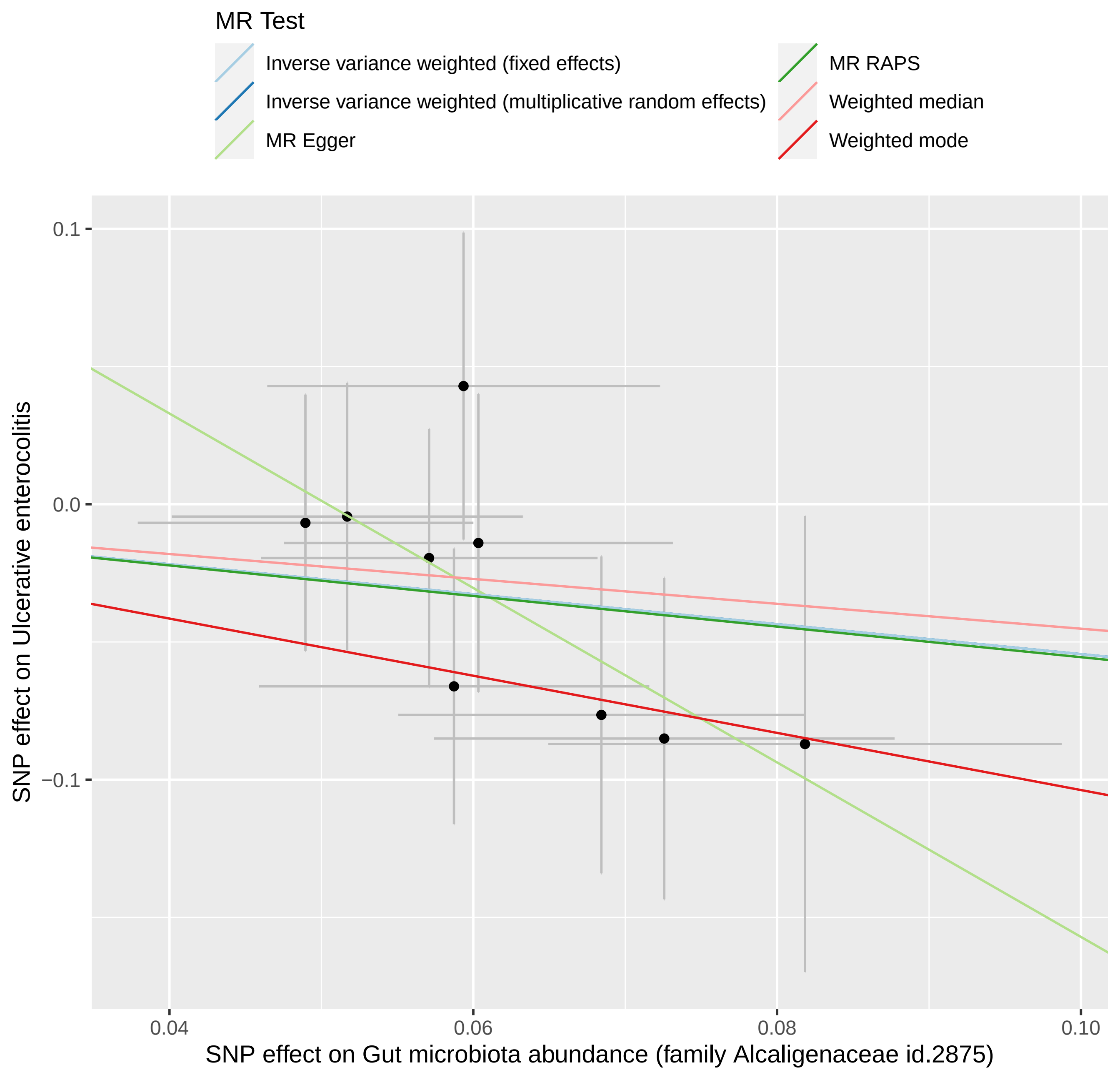

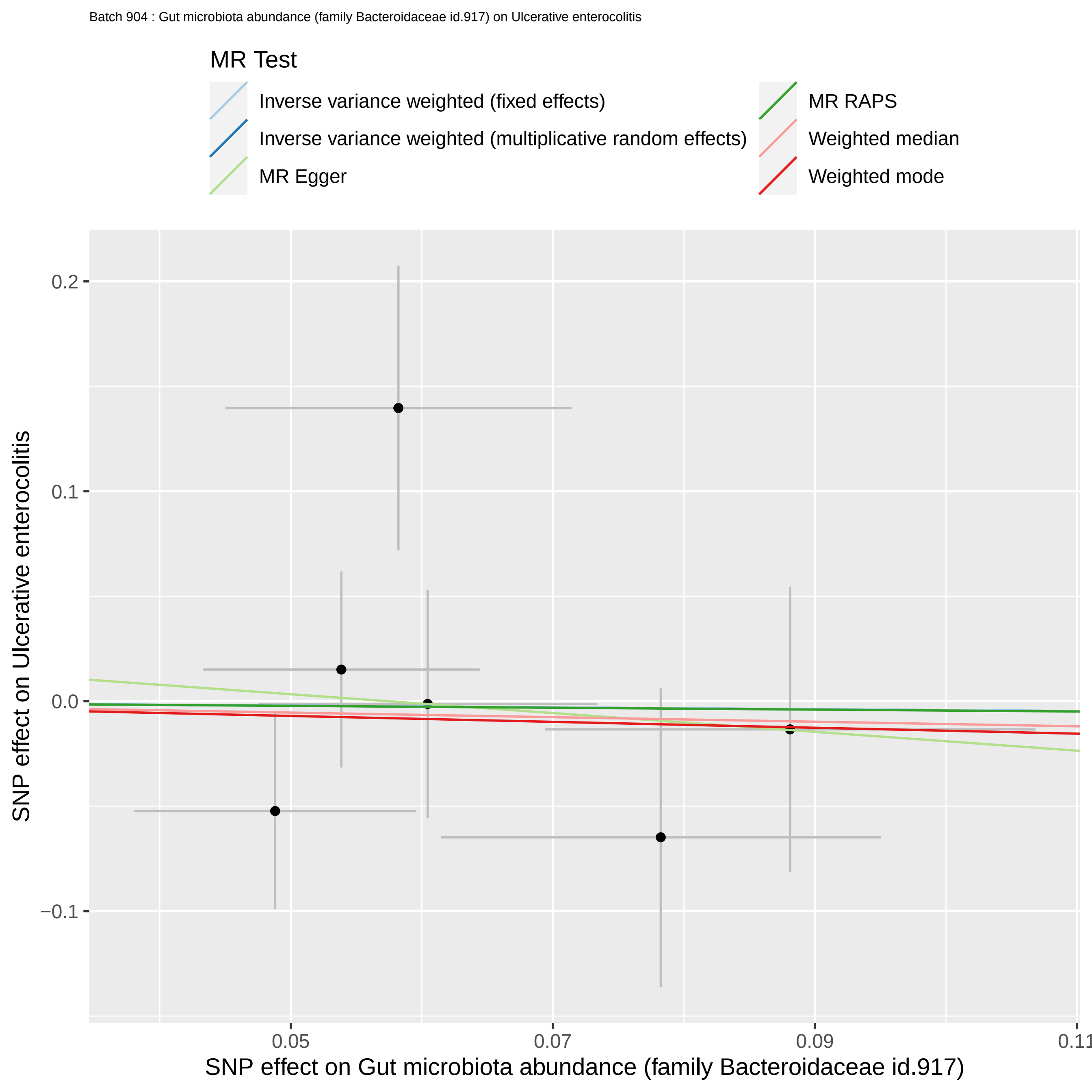
Batch 904 : Gut microbiota abundance (family Bacteroidaceae id.917) on Ulcerative enterocolitis
MR Test
Inverse variance weighted (fixed effects)
Inverse variance weighted (multiplicative random effects)
MR Egger
MR RAPS
Weighted median
Weighted mode
0.2
0.1
0.0
−0.1
0.05
0.07
0.09
0.11
SNP effect on Ulcerative enterocolitis
SNP effect on Gut microbiota abundance (family Bacteroidaceae id.917)

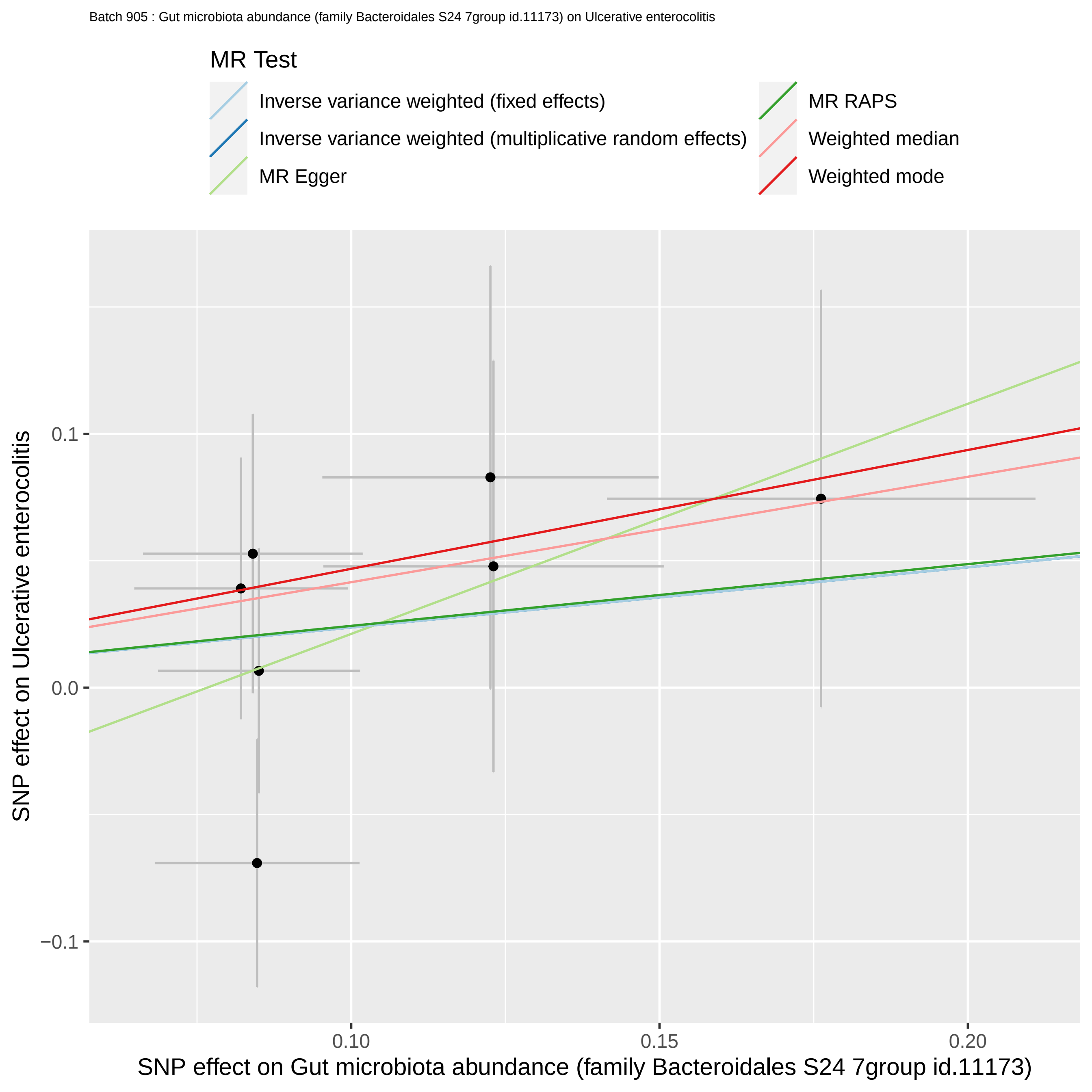

Batch 905 : Gut microbiota abundance (family Bacteroidales S24 7group id.11173) on Ulcerative enterocolitis
MR Test
Inverse variance weighted (fixed effects)
Inverse variance weighted (multiplicative random effects)
MR Egger
MR RAPS
Weighted median
Weighted mode
0.1
0.0
−0.1
0.10
0.15
0.20
SNP effect on Ulcerative enterocolitis
SNP effect on Gut microbiota abundance (family Bacteroidales S24 7group id.11173)

Batch 906 : Gut microbiota abundance (family Bifidobacteriaceae id.433) on Ulcerative enterocolitis

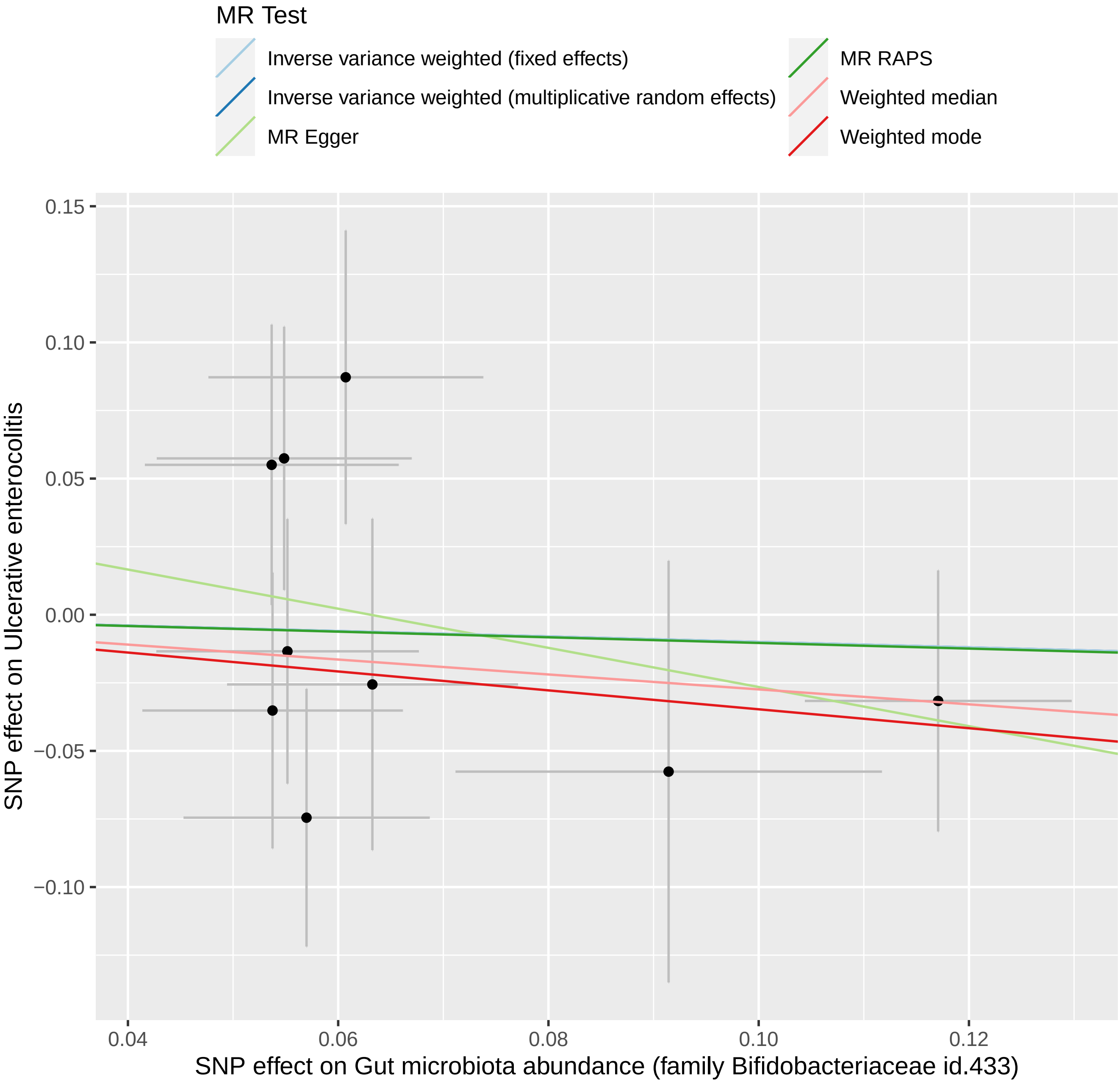

Batch 908 : Gut microbiota abundance (family Clostridiaceae1 id.1869) on Ulcerative enterocolitis

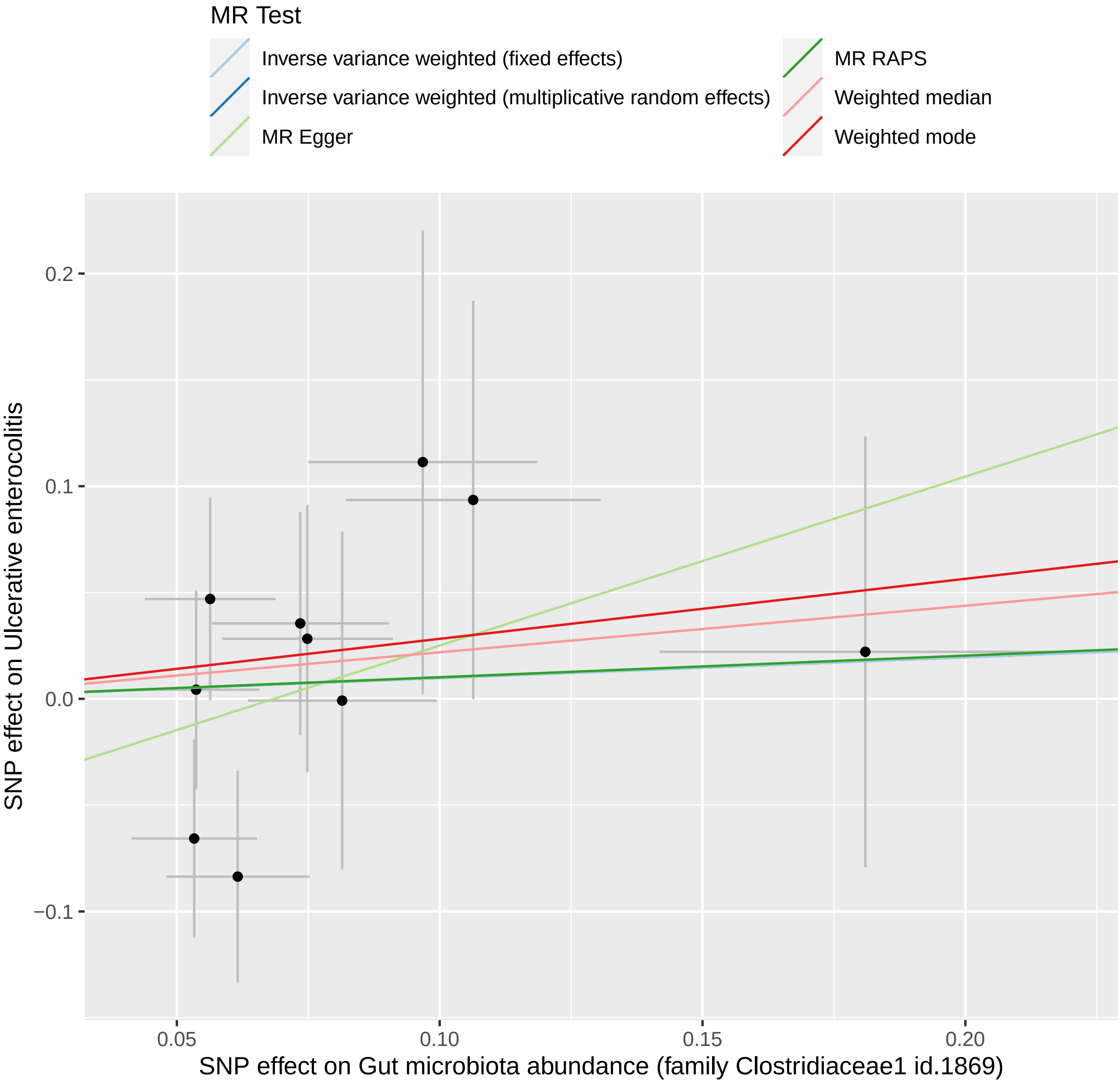

Batch 909 : Gut microbiota abundance (family Clostridiales vadin BB60 group id.11286) on Ulcerative enterocolitis

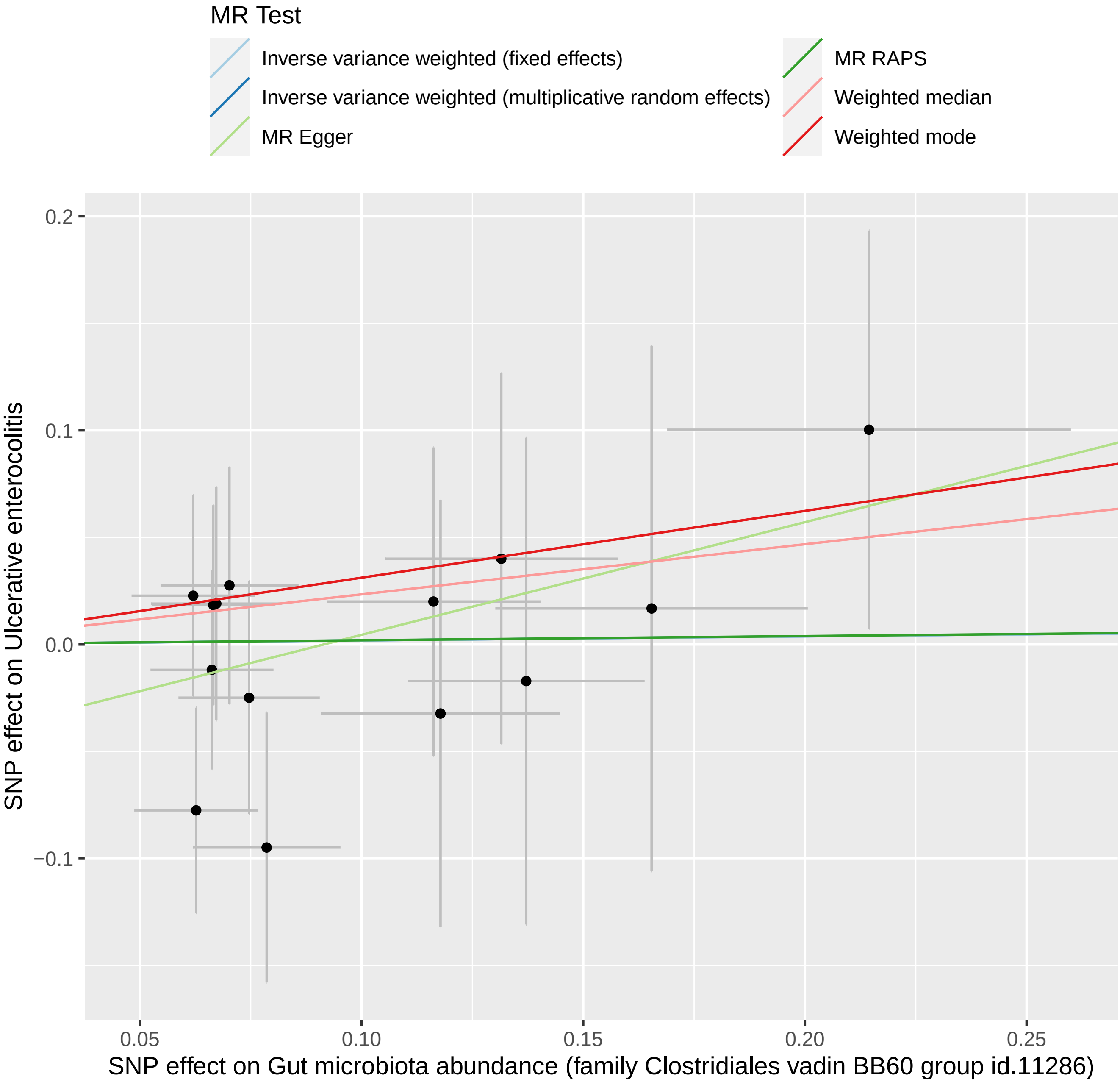

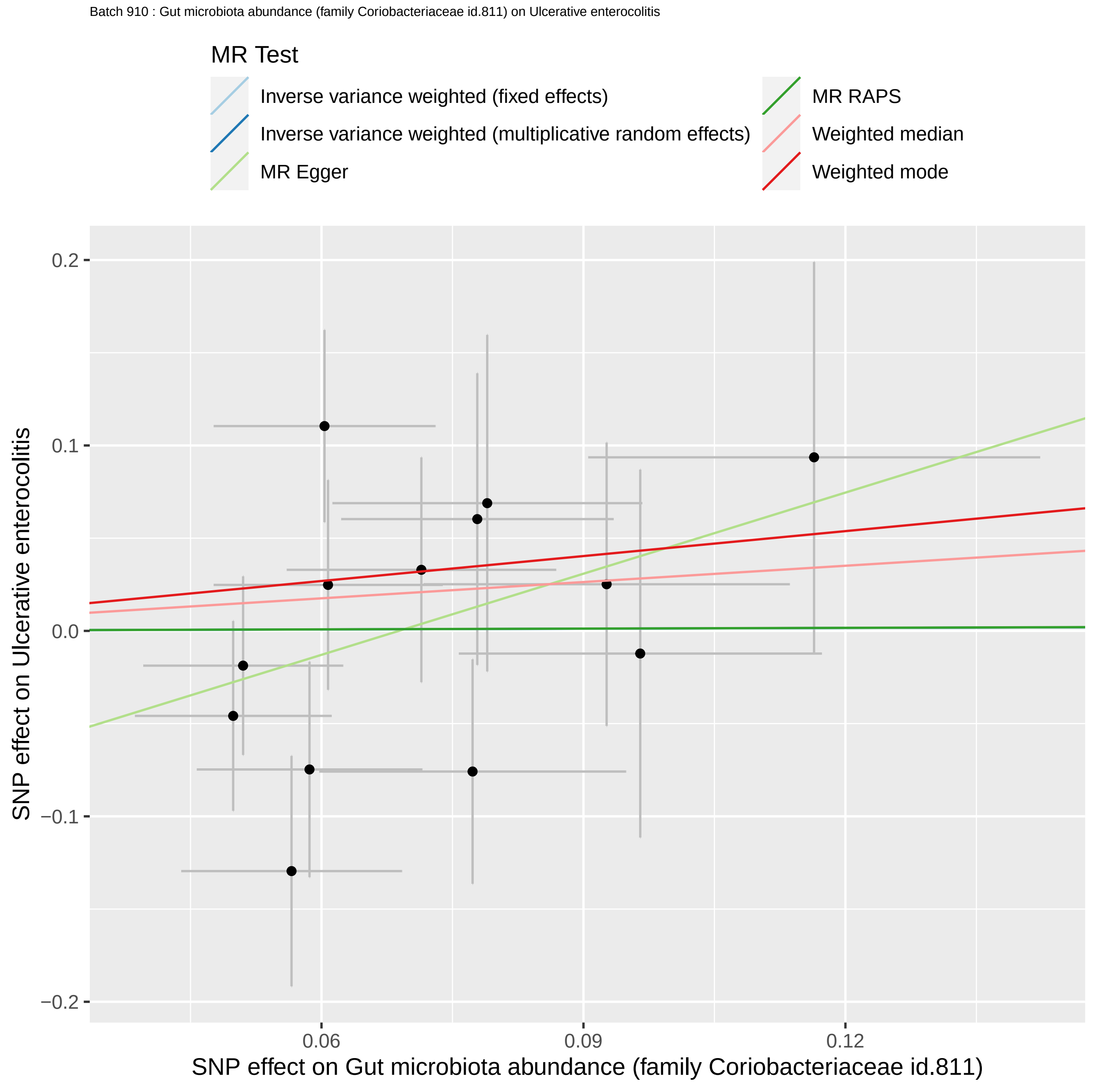
Batch 910 : Gut microbiota abundance (family Coriobacteriaceae id.811) on Ulcerative enterocolitis
MR Test
Inverse variance weighted (fixed effects)
Inverse variance weighted (multiplicative random effects)
MR Egger
MR RAPS
Weighted median
Weighted mode
0.2
0.1
0.0
−0.1
−0.2
0.06
0.09
0.12
SNP effect on Ulcerative enterocolitis
SNP effect on Gut microbiota abundance (family Coriobacteriaceae id.811)

Batch 911 : Gut microbiota abundance (family Defluviitaleaceae id.1924) on Ulcerative enterocolitis

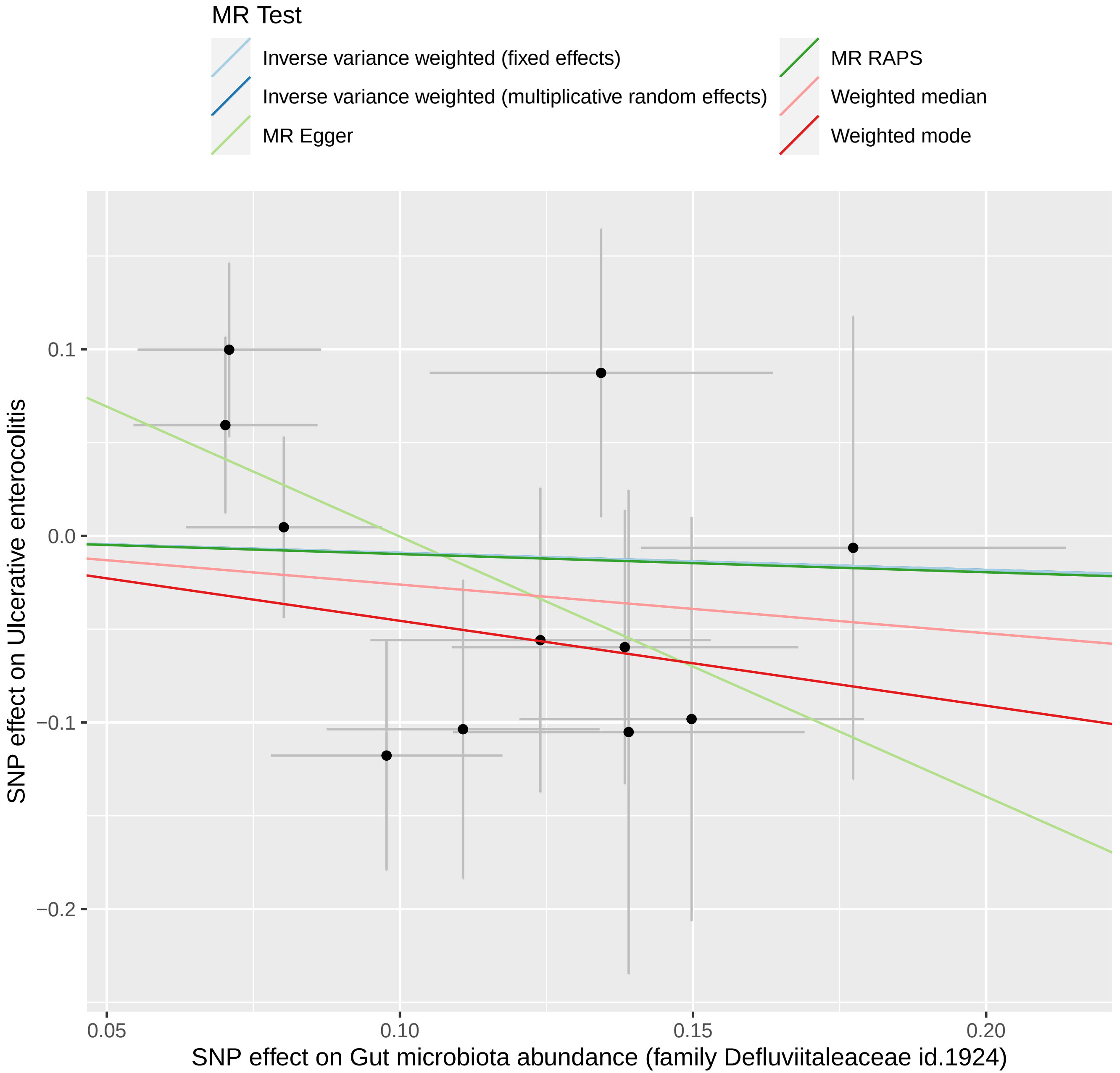

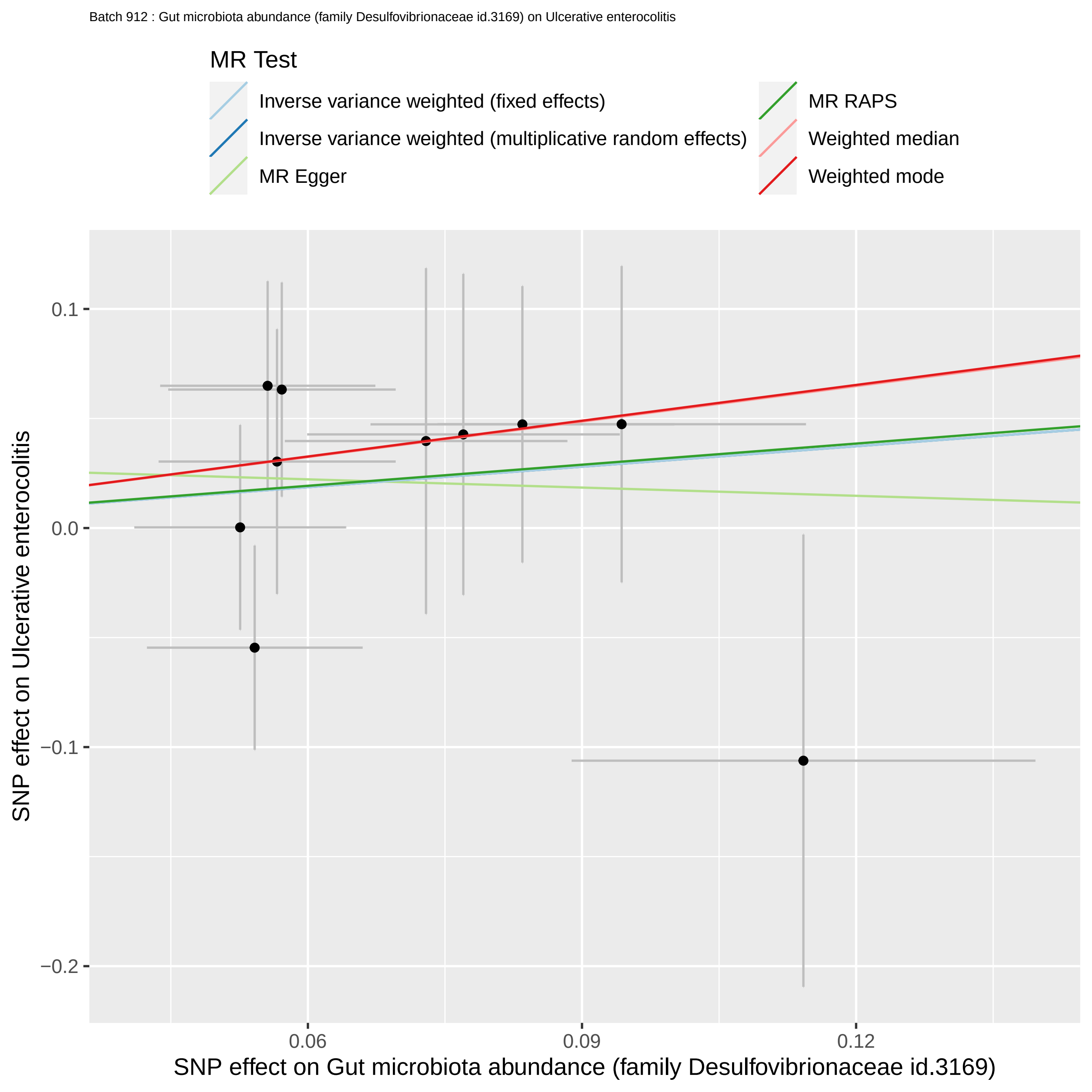
Batch 912 : Gut microbiota abundance (family Desulfovibrionaceae id.3169) on Ulcerative enterocolitis
MR Test
Inverse variance weighted (fixed effects)
Inverse variance weighted (multiplicative random effects)
MR Egger
MR RAPS
Weighted median
Weighted mode
SNP effect on Ulcerative enterocolitis
0.1
0.0
−0.1
−0.2
0.06
0.09
0.12
SNP effect on Gut microbiota abundance (family Desulfovibrionaceae id.3169)

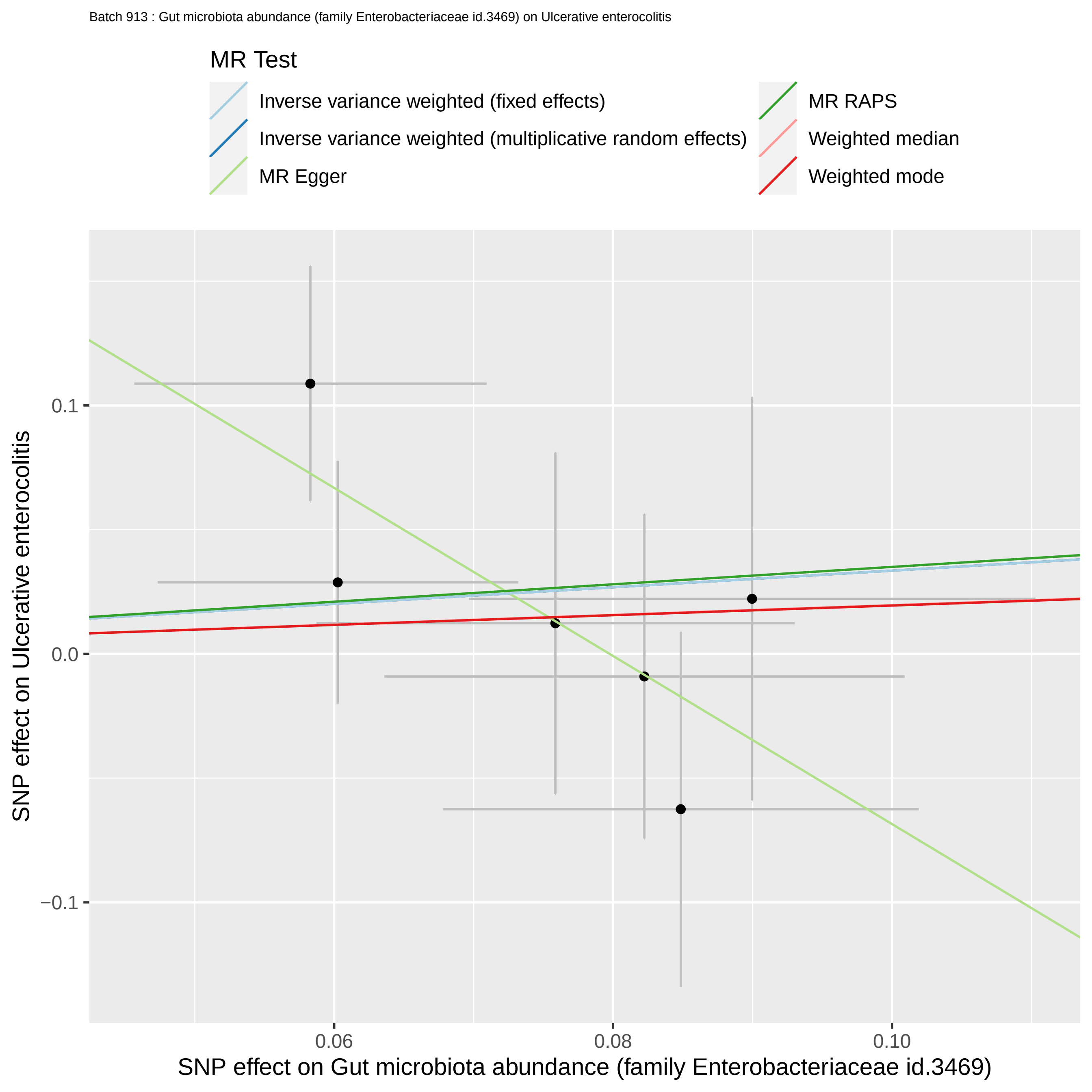
Batch 913 : Gut microbiota abundance (family Enterobacteriaceae id.3469) on Ulcerative enterocolitis
MR Test
Inverse variance weighted (fixed effects)
Inverse variance weighted (multiplicative random effects)
MR Egger
MR RAPS
Weighted median
Weighted mode
0.1
0.0
−0.1
0.06
0.08
0.10
SNP effect on Ulcerative enterocolitis
SNP effect on Gut microbiota abundance (family Enterobacteriaceae id.3469)

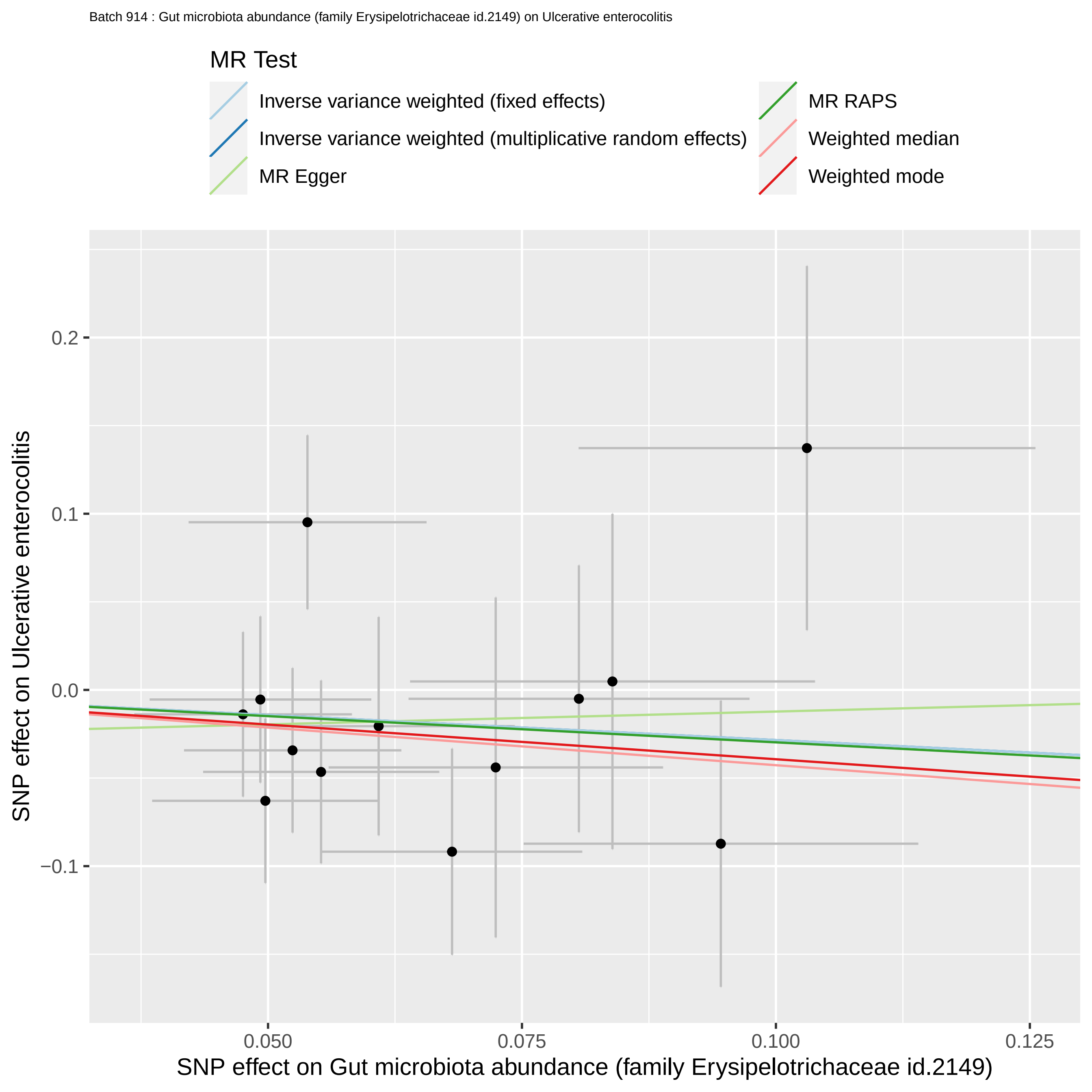

Batch 914 : Gut microbiota abundance (family Erysipelotrichaceae id.2149) on Ulcerative enterocolitis
MR Test
Inverse variance weighted (fixed effects)
Inverse variance weighted (multiplicative random effects)
MR Egger
MR RAPS
Weighted median
Weighted mode
0.2
0.1
0.0
−0.1
0.050
0.075
0.100
0.125
SNP effect on Ulcerative enterocolitis
SNP effect on Gut microbiota abundance (family Erysipelotrichaceae id.2149)

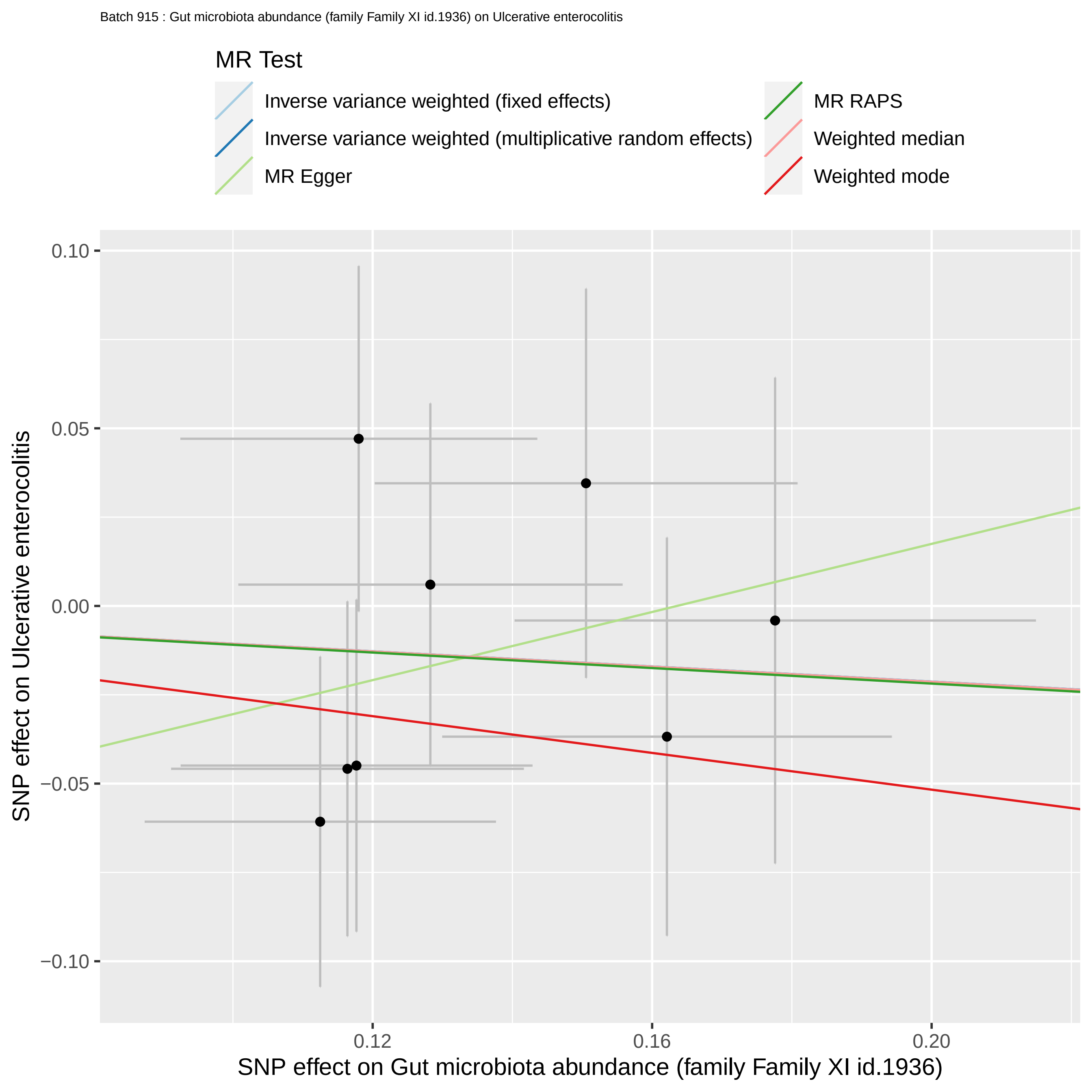

Batch 915 : Gut microbiota abundance (family Family XI id.1936) on Ulcerative enterocolitis
MR Test
Inverse variance weighted (fixed effects)
Inverse variance weighted (multiplicative random effects)
MR Egger
MR RAPS
Weighted median
Weighted mode
0.10
0.05
0.00
−0.05
−0.10
0.12
0.16
0.20
SNP effect on Ulcerative enterocolitis
SNP effect on Gut microbiota abundance (family Family XI id.1936)

Batch 916 : Gut microbiota abundance (family Family XIII id.1957) on Ulcerative enterocolitis

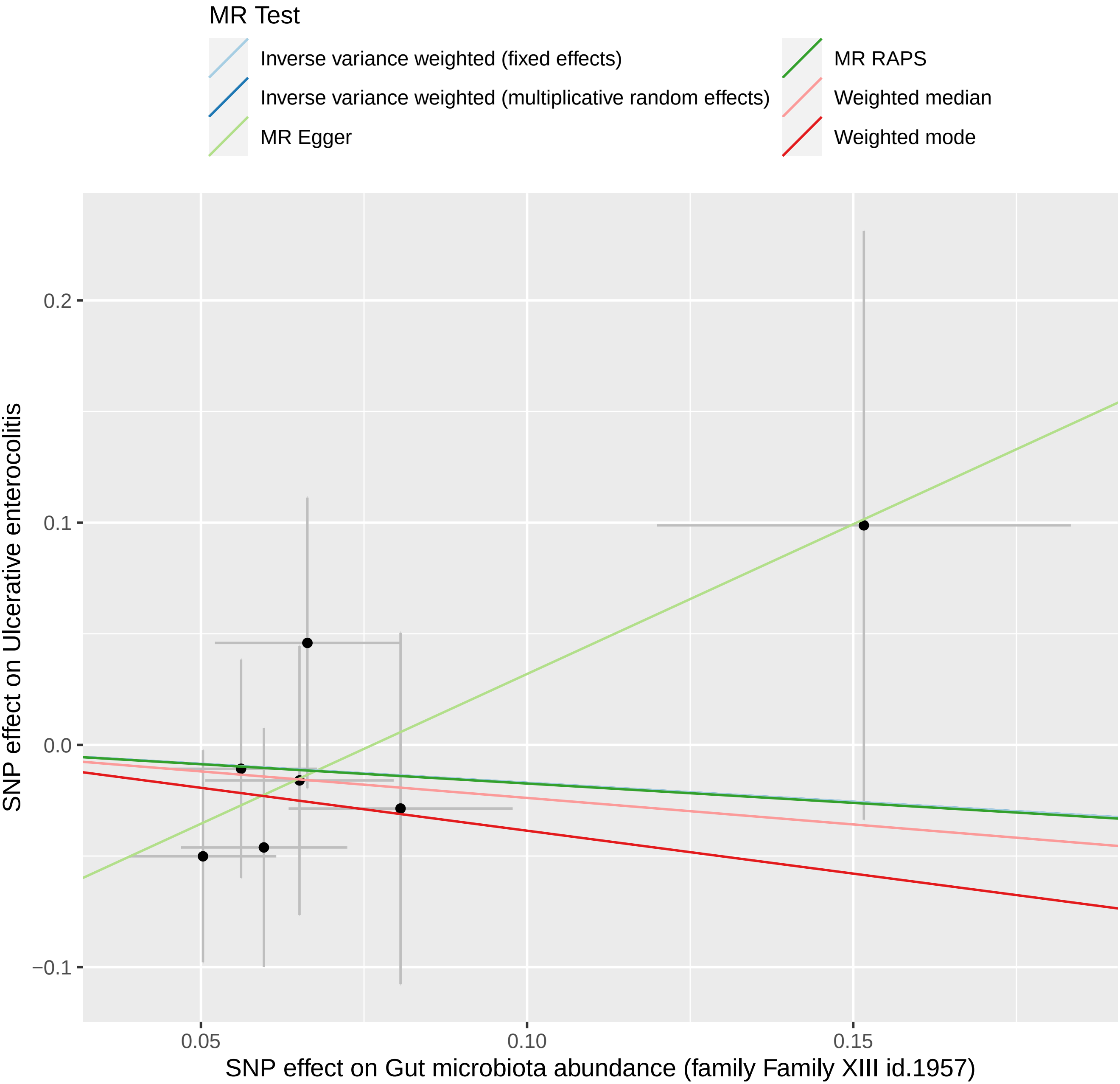

Batch 917 : Gut microbiota abundance (family Lachnospiraceae id.1987) on Ulcerative enterocolitis

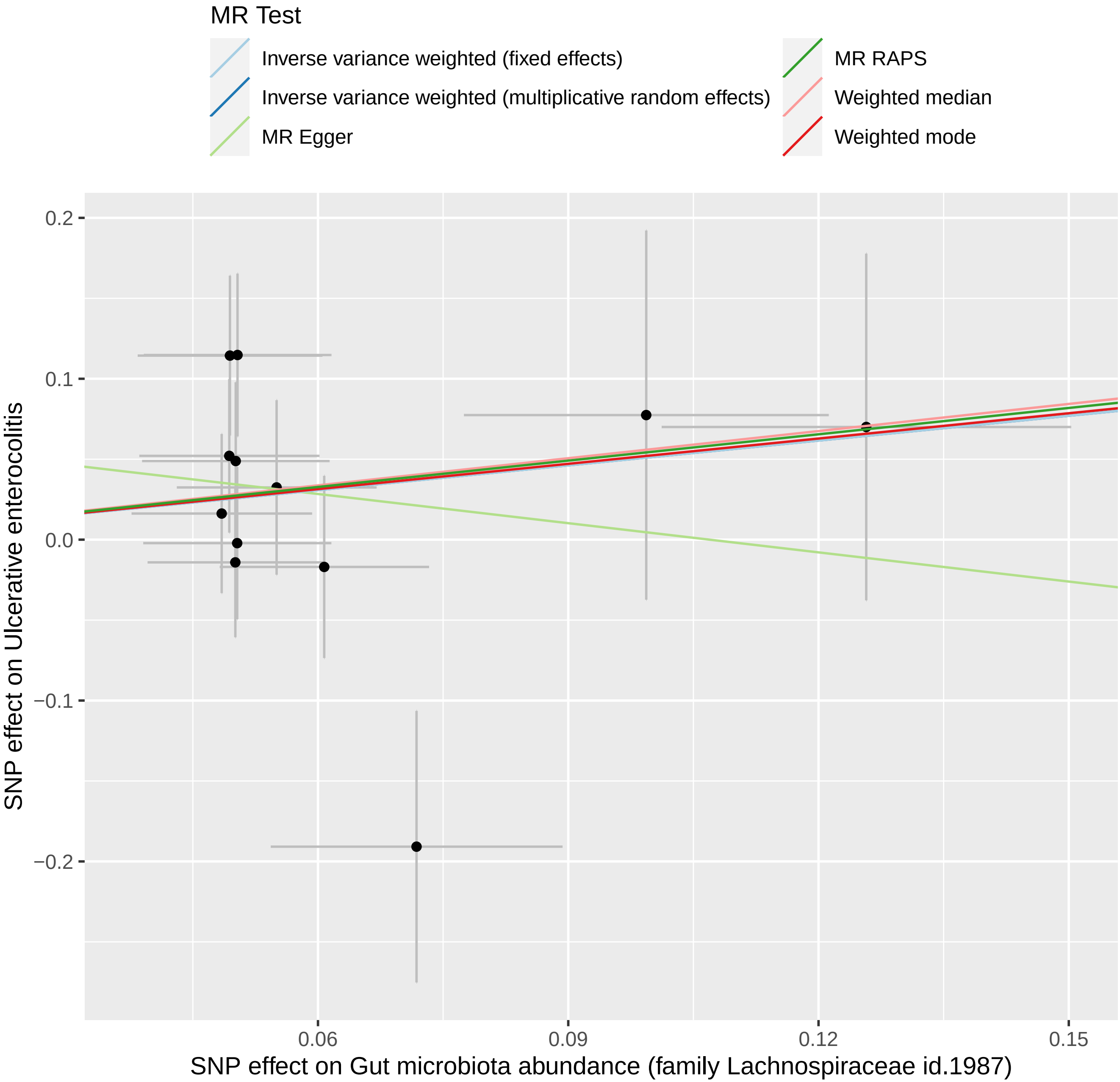

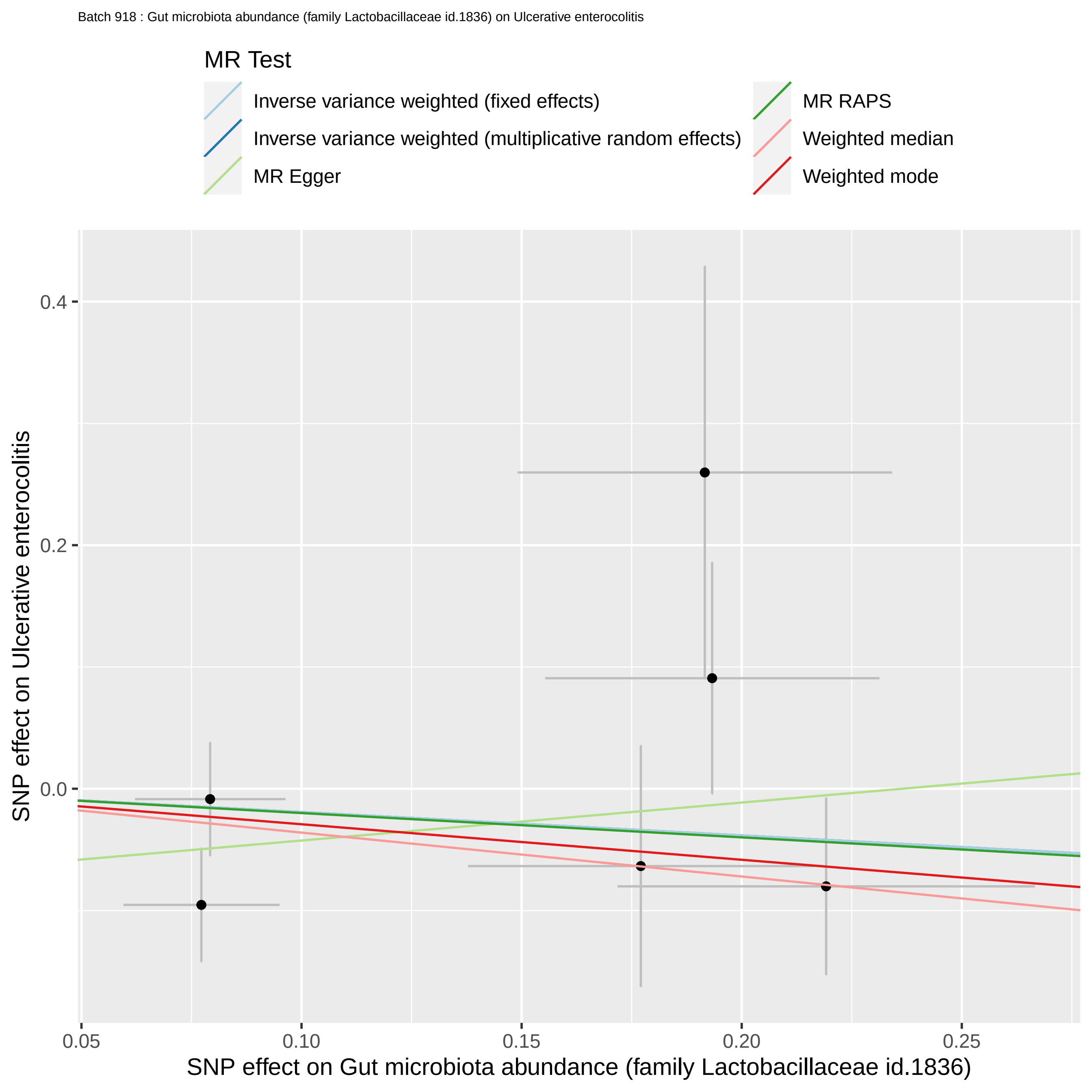

Batch 918 : Gut microbiota abundance (family Lactobacillaceae id.1836) on Ulcerative enterocolitis
MR Test
Inverse variance weighted (fixed effects)
Inverse variance weighted (multiplicative random effects)
MR Egger
MR RAPS
Weighted median
Weighted mode
SNP effect on Ulcerative enterocolitis
0.4
0.2
0.0
0.05
0.10
0.15
0.20
0.25
SNP effect on Gut microbiota abundance (family Lactobacillaceae id.1836)

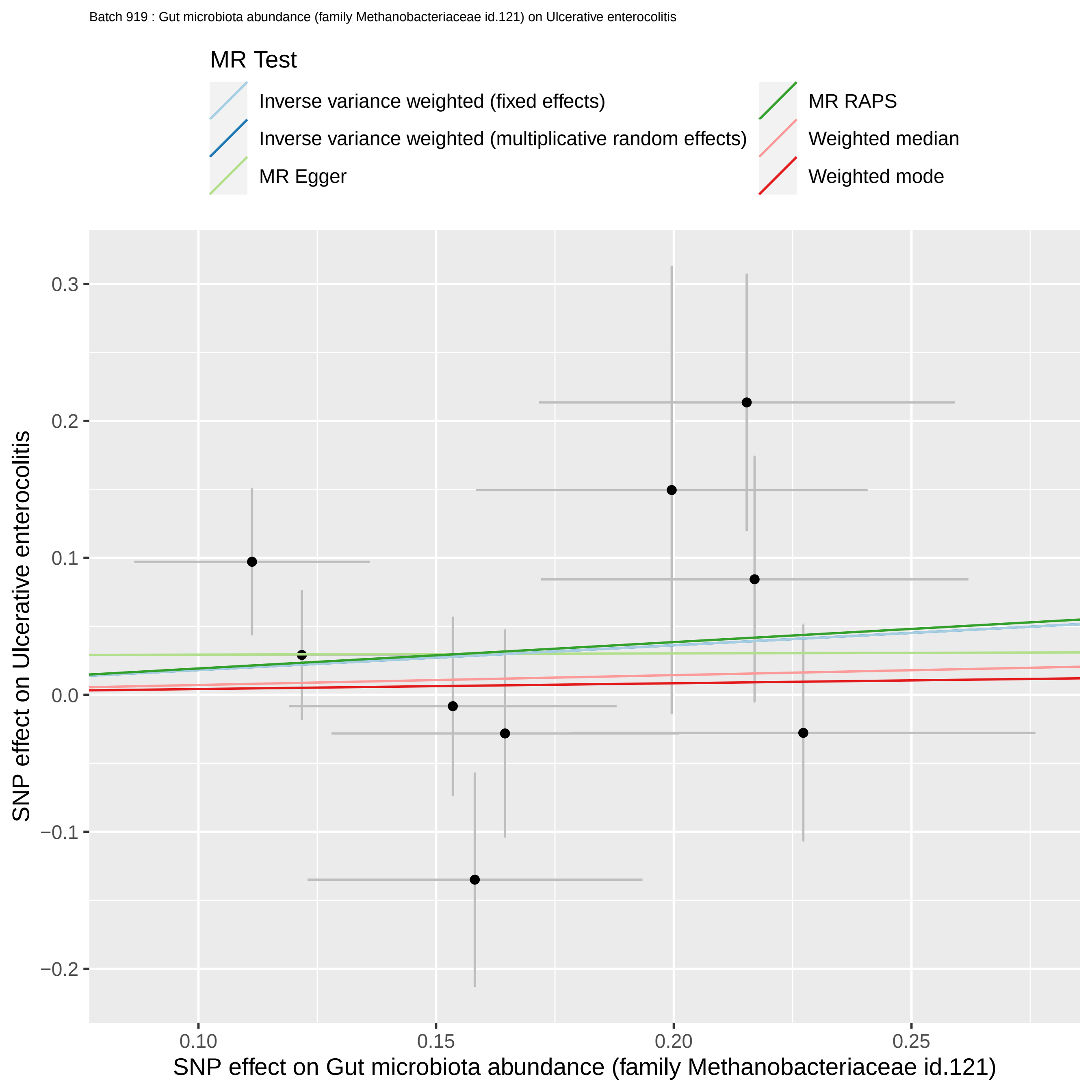
Batch 919 : Gut microbiota abundance (family Methanobacteriaceae id.121) on Ulcerative enterocolitis
MR Test
Inverse variance weighted (fixed effects)
Inverse variance weighted (multiplicative random effects)
MR Egger
MR RAPS
Weighted median
Weighted mode
SNP effect on Ulcerative enterocolitis
SNP effect on Gut microbiota abundance (family Methanobacteriaceae id.121)
0.3
0.2
0.1
0.0
−0.1
−0.2
0.10
0.15
0.20
0.25

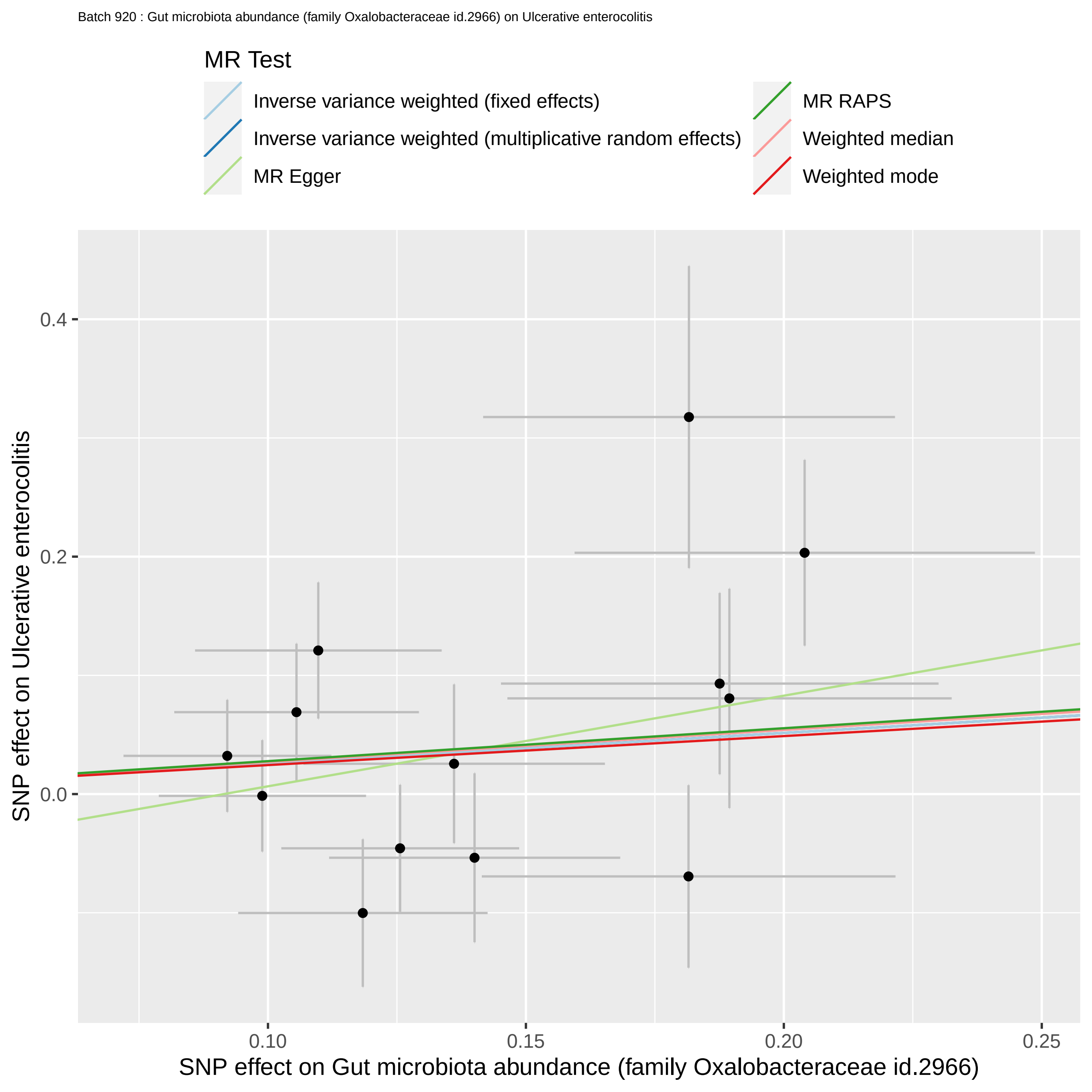
Batch 920 : Gut microbiota abundance (family Oxalobacteraceae id.2966) on Ulcerative enterocolitis
MR Test
Inverse variance weighted (fixed effects)
Inverse variance weighted (multiplicative random effects)
MR Egger
MR RAPS
Weighted median
Weighted mode
0.4
0.2
0.0
0.10
0.15
0.20
0.25
SNP effect on Ulcerative enterocolitis
SNP effect on Gut microbiota abundance (family Oxalobacteraceae id.2966)

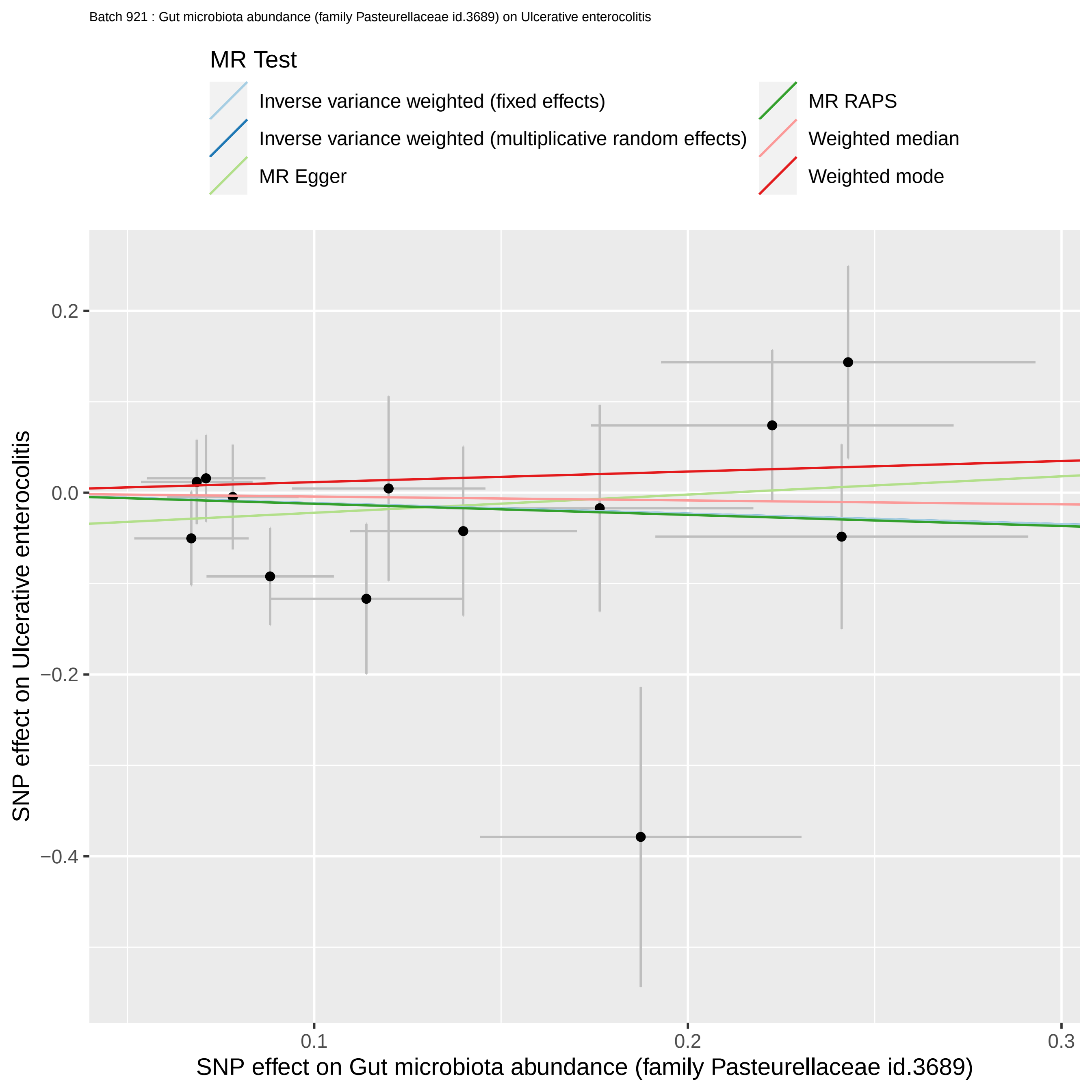

Batch 921 : Gut microbiota abundance (family Pasteurellaceae id.3689) on Ulcerative enterocolitis
MR Test
Inverse variance weighted (fixed effects)
Inverse variance weighted (multiplicative random effects)
MR Egger
MR RAPS
Weighted median
Weighted mode
0.2
0.0
−0.2
−0.4
0.1
0.2
0.3
SNP effect on Ulcerative enterocolitis
SNP effect on Gut microbiota abundance (family Pasteurellaceae id.3689)

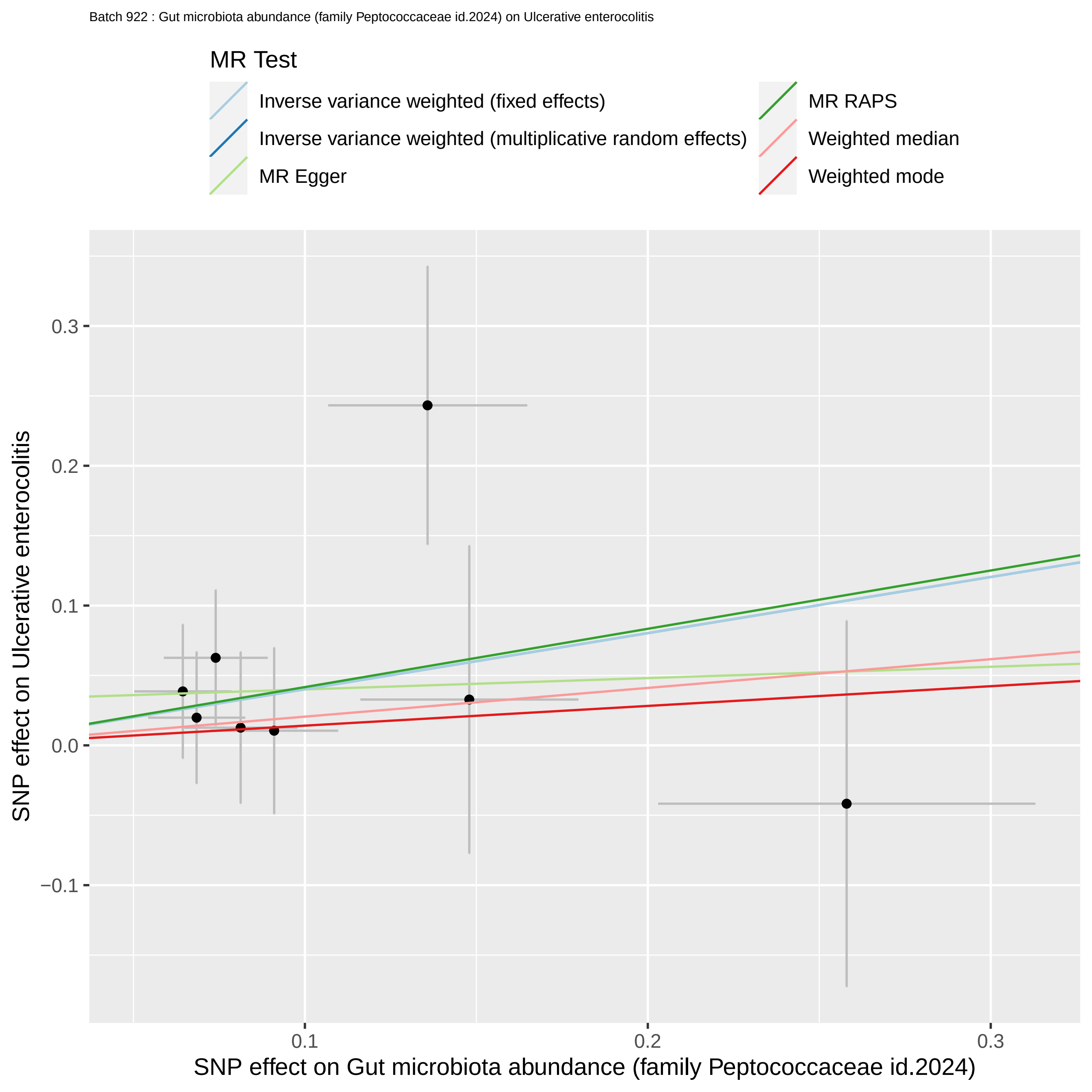
Batch 922 : Gut microbiota abundance (family Peptococcaceae id.2024) on Ulcerative enterocolitis
MR Test
Inverse variance weighted (fixed effects)
Inverse variance weighted (multiplicative random effects)
MR Egger
MR RAPS
Weighted median
Weighted mode
0.3
0.2
0.1
0.0
−0.1
0.1
0.2
0.3
SNP effect on Ulcerative enterocolitis
SNP effect on Gut microbiota abundance (family Peptococcaceae id.2024)

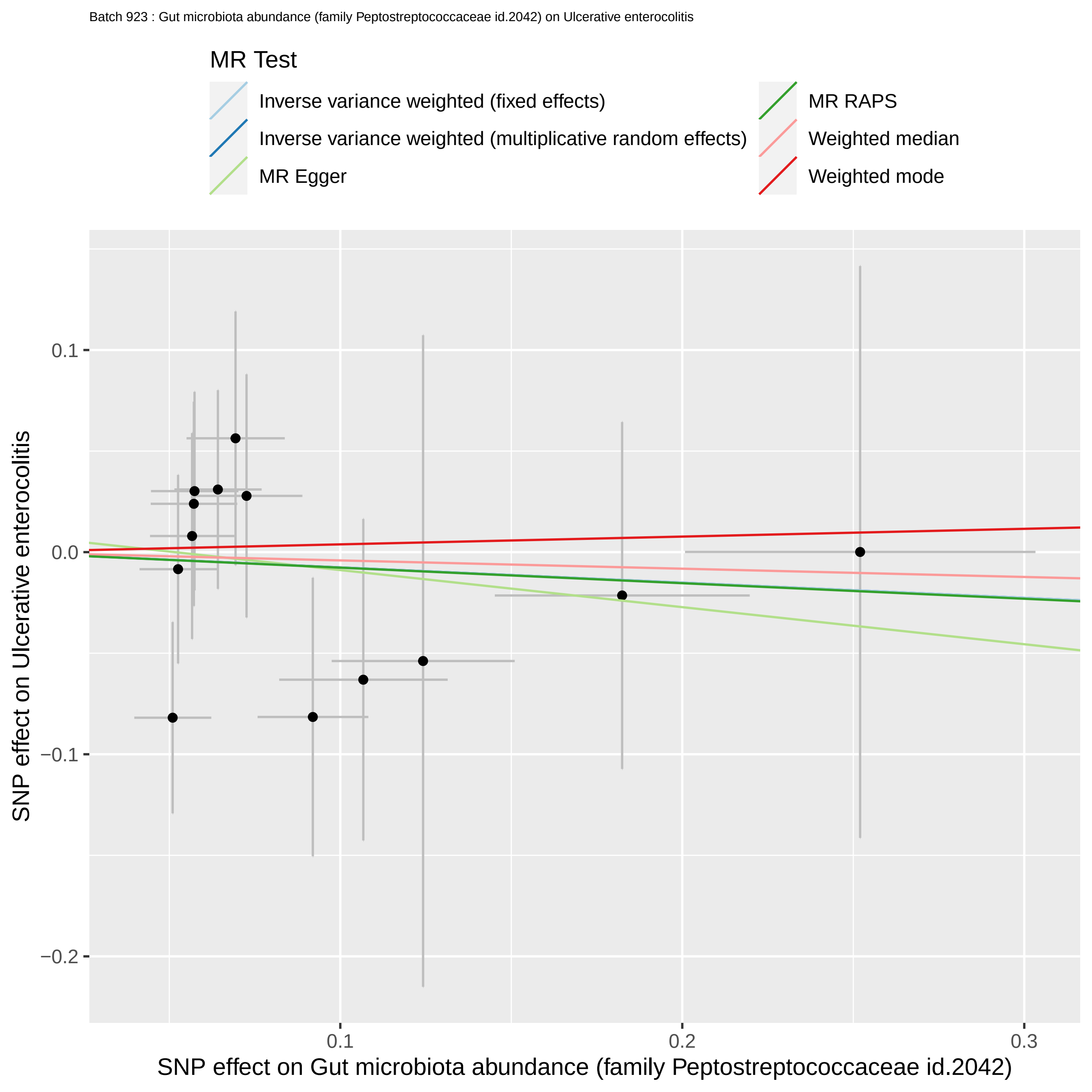

Batch 923 : Gut microbiota abundance (family Peptostreptococcaceae id.2042) on Ulcerative enterocolitis
MR Test
Inverse variance weighted (fixed effects)
Inverse variance weighted (multiplicative random effects)
MR Egger
MR RAPS
Weighted median
Weighted mode
0.1
0.0
−0.1
−0.2
0.1
0.2
0.3
SNP effect on Ulcerative enterocolitis
SNP effect on Gut microbiota abundance (family Peptostreptococcaceae id.2042)

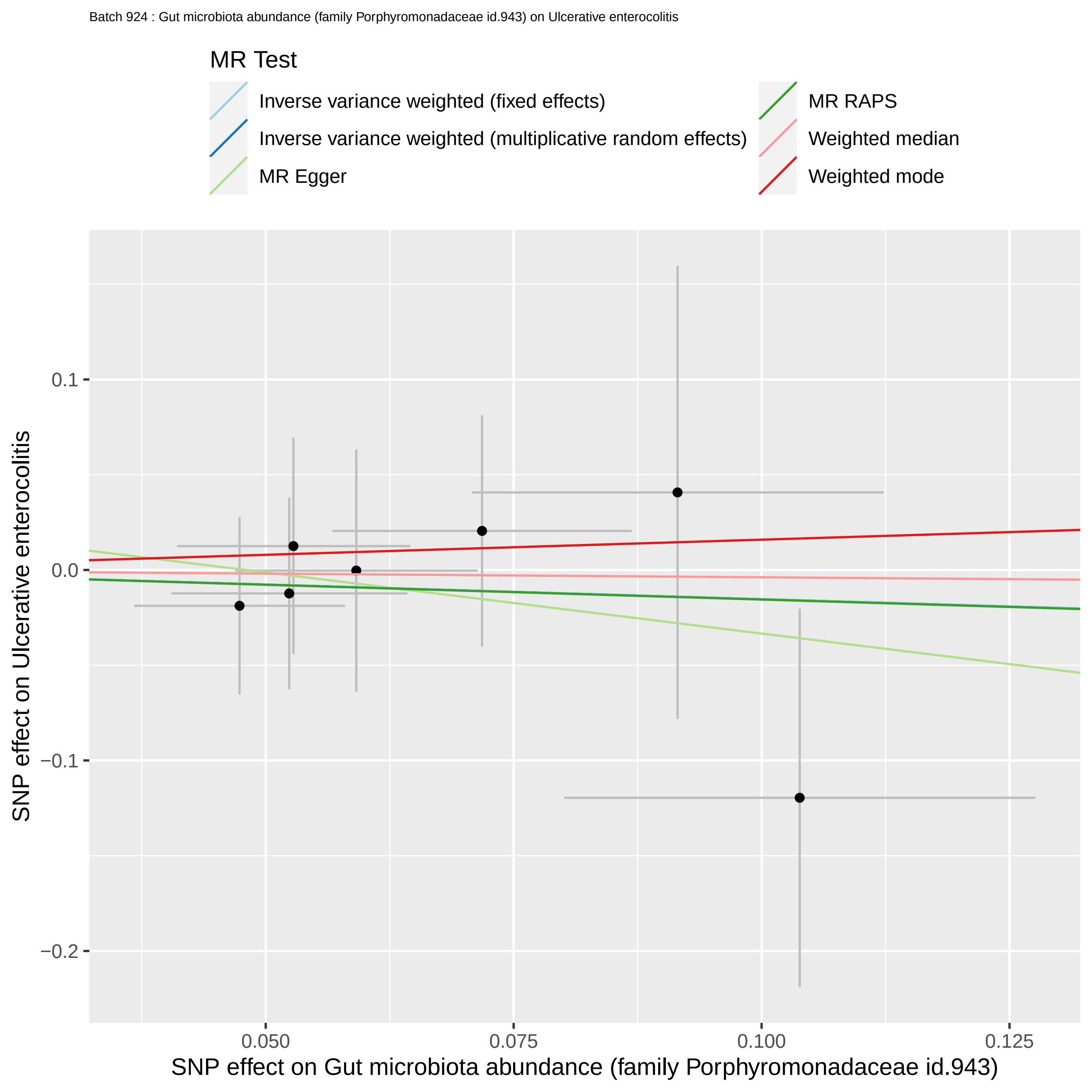

Batch 924 : Gut microbiota abundance (family Porphyromonadaceae id.943) on Ulcerative enterocolitis
MR Test
Inverse variance weighted (fixed effects)
Inverse variance weighted (multiplicative random effects)
MR Egger
MR RAPS
Weighted median
Weighted mode
0.1
0.0
−0.1
−0.2
0.050
0.075
0.100
0.125
SNP effect on Ulcerative enterocolitis
SNP effect on Gut microbiota abundance (family Porphyromonadaceae id.943)

Batch 925 : Gut microbiota abundance (family Prevotellaceae id.960) on Ulcerative enterocolitis

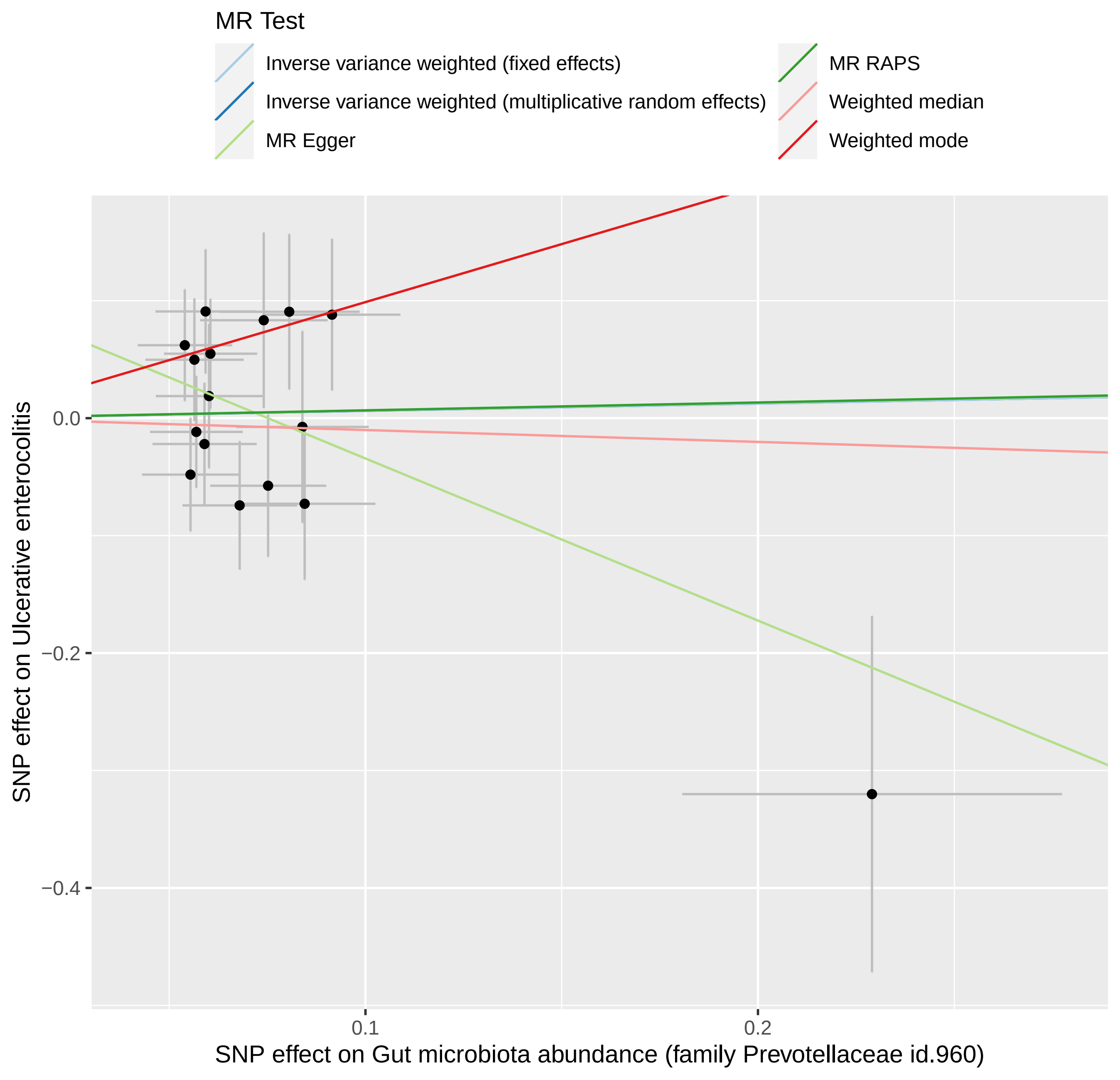

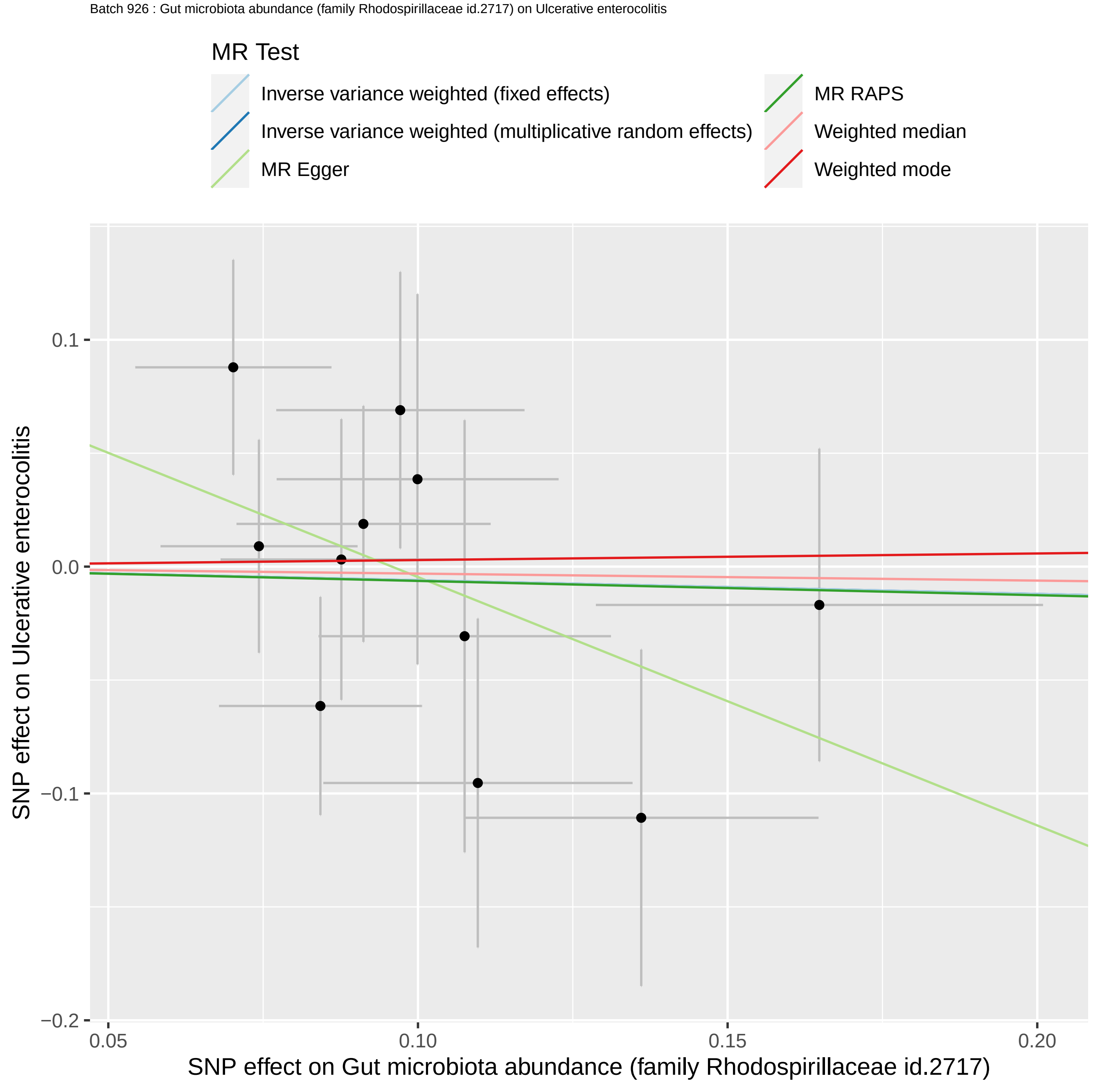

Batch 926 : Gut microbiota abundance (family Rhodospirillaceae id.2717) on Ulcerative enterocolitis
MR Test
Inverse variance weighted (fixed effects)
Inverse variance weighted (multiplicative random effects)
MR Egger
MR RAPS
Weighted median
Weighted mode
0.1
0.0
−0.1
−0.2
0.05
0.10
0.15
0.20
SNP effect on Ulcerative enterocolitis
SNP effect on Gut microbiota abundance (family Rhodospirillaceae id.2717)

Batch 927 : Gut microbiota abundance (family Rikenellaceae id.967) on Ulcerative enterocolitis

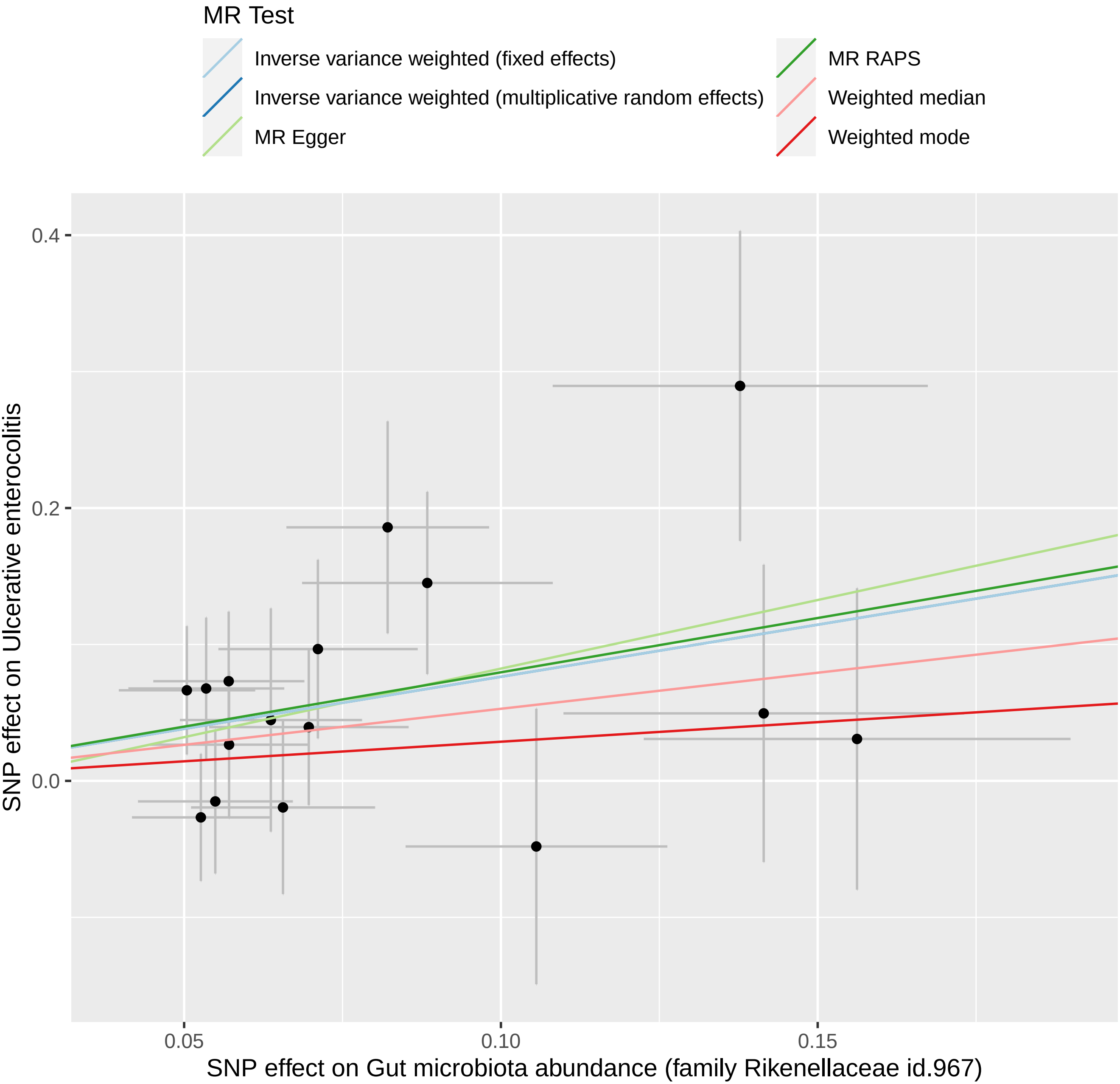

Batch 928 : Gut microbiota abundance (family Ruminococcaceae id.2050) on Ulcerative enterocolitis

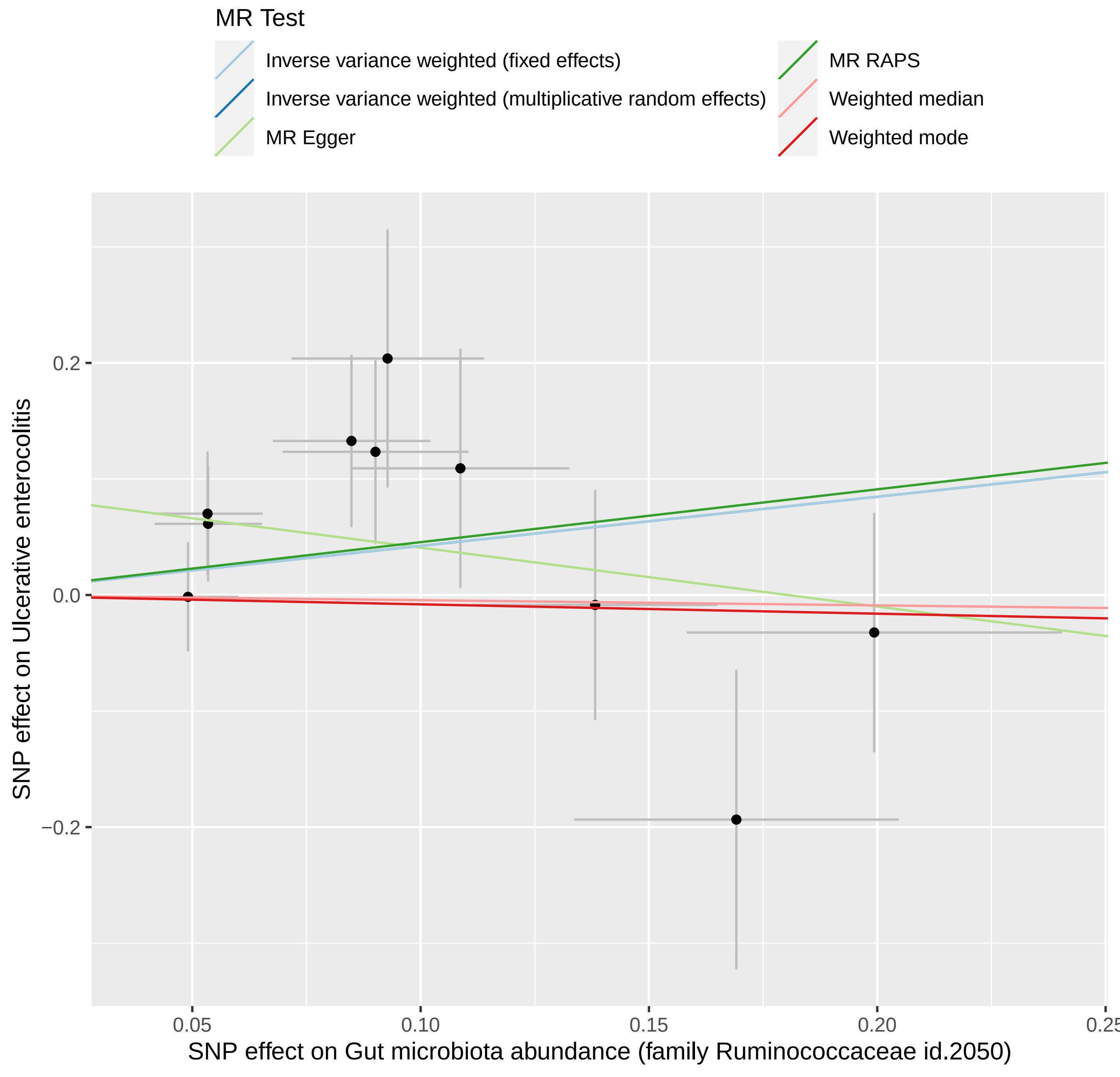

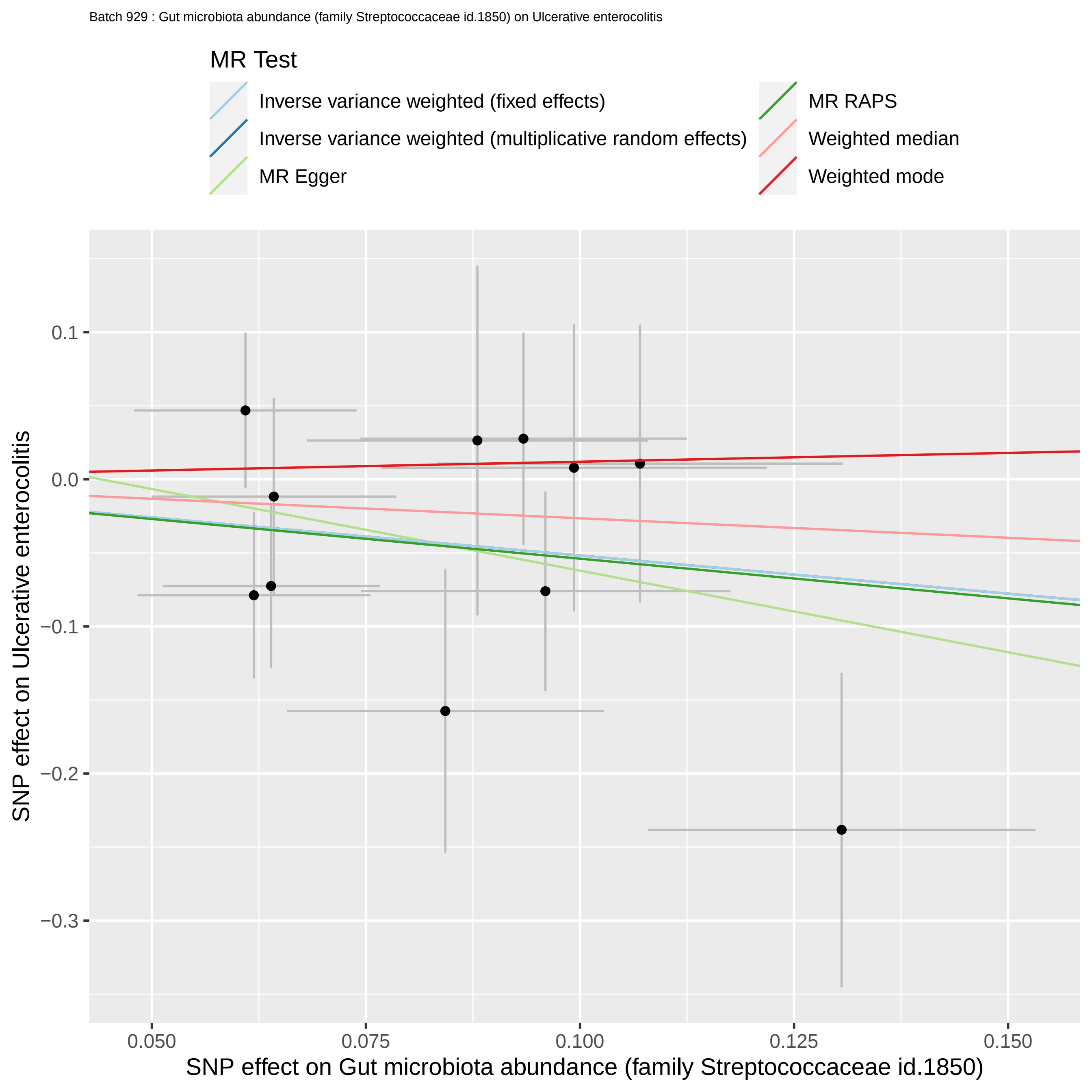
Batch 929 : Gut microbiota abundance (family Streptococcaceae id.1850) on Ulcerative enterocolitis
MR Test
Inverse variance weighted (fixed effects)
Inverse variance weighted (multiplicative random effects)
MR Egger
MR RAPS
Weighted median
Weighted mode
0.1
0.0
−0.1
−0.2
−0.3
0.050
0.075
0.100
0.125
0.150
SNP effect on Ulcerative enterocolitis
SNP effect on Gut microbiota abundance (family Streptococcaceae id.1850)

Batch 930 : Gut microbiota abundance (family Veillonellaceae id.2172) on Ulcerative enterocolitis

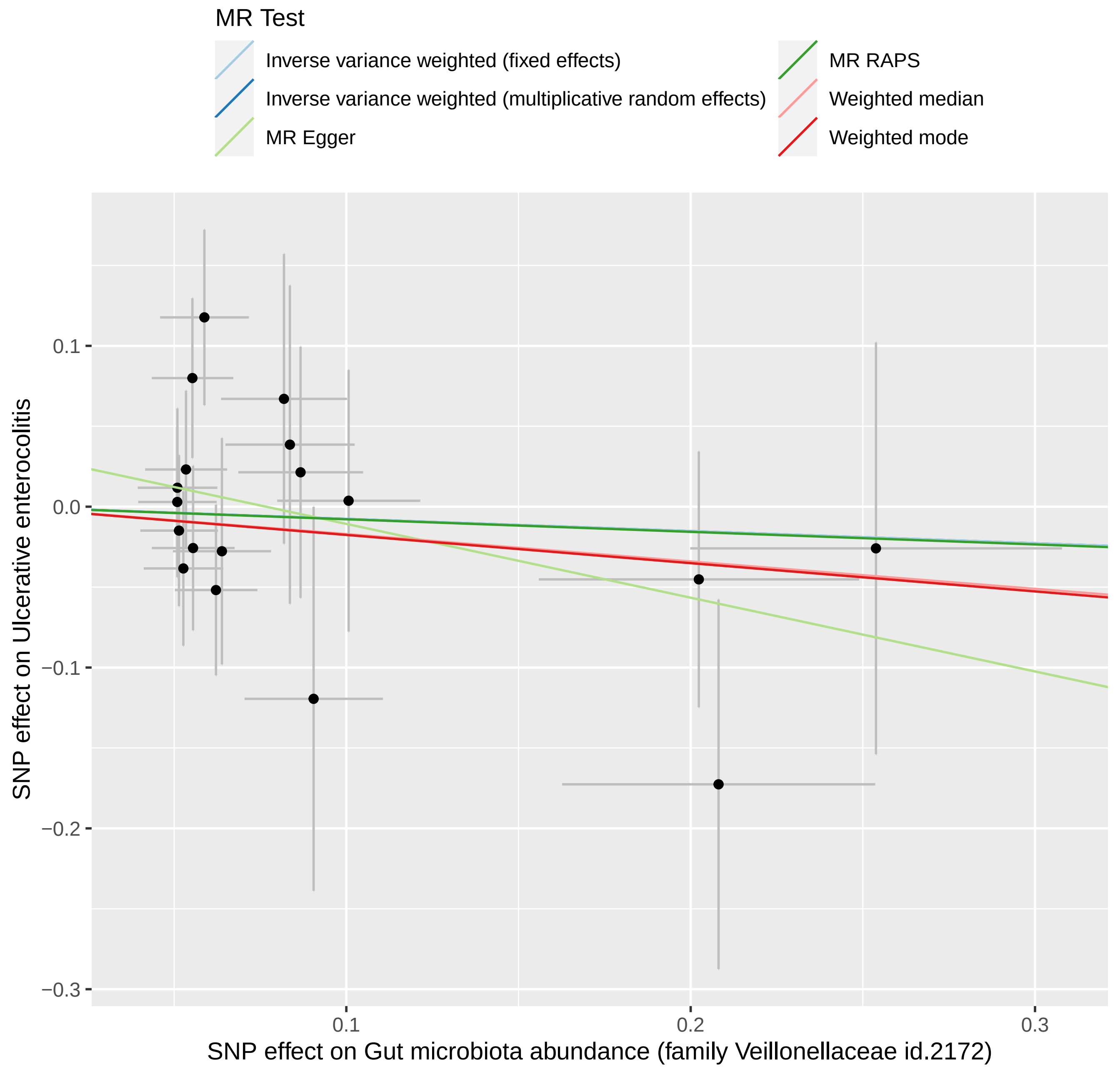

Batch 931 : Gut microbiota abundance (family Verrucomicrobiaceae id.4036) on Ulcerative enterocolitis

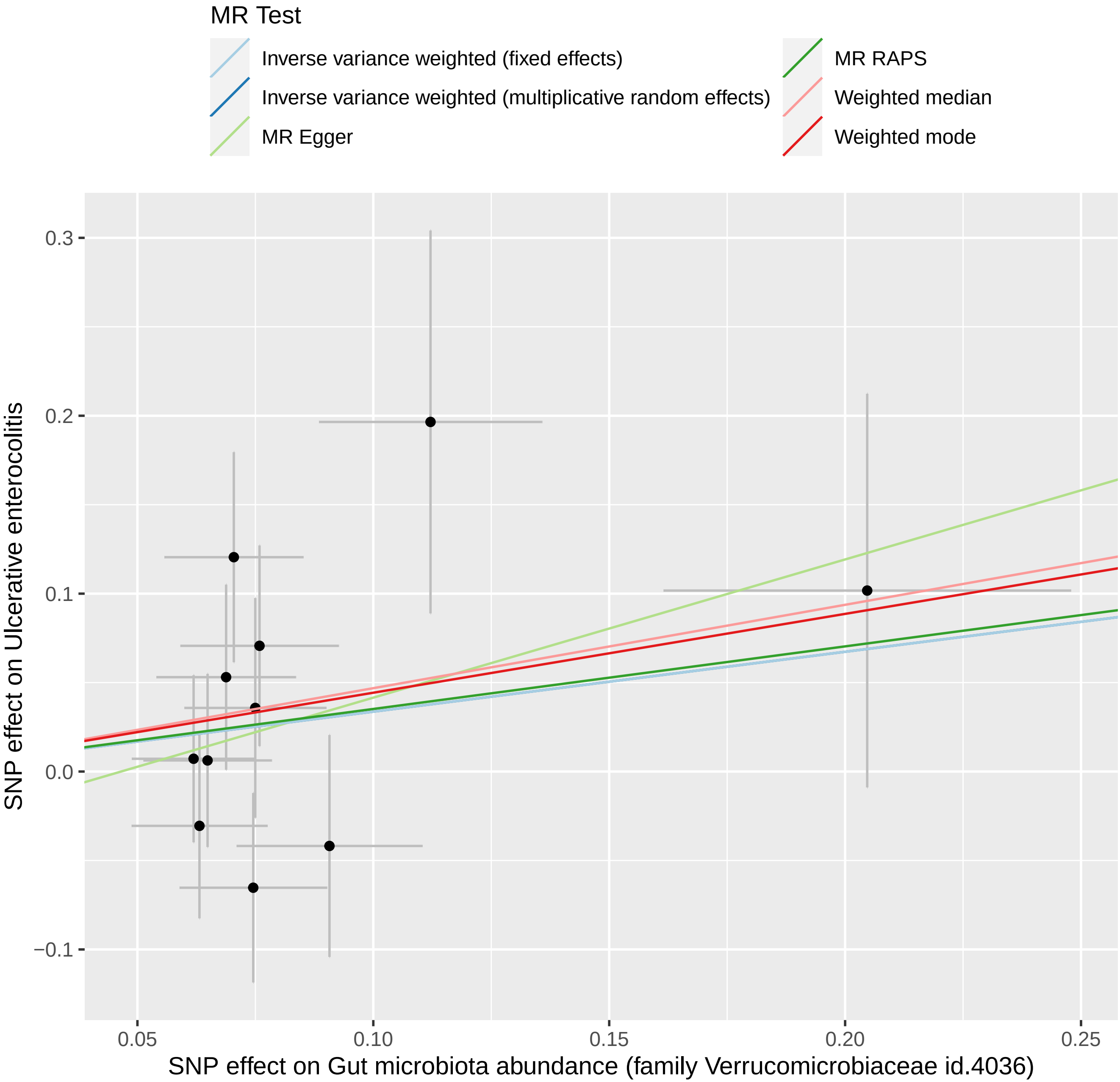

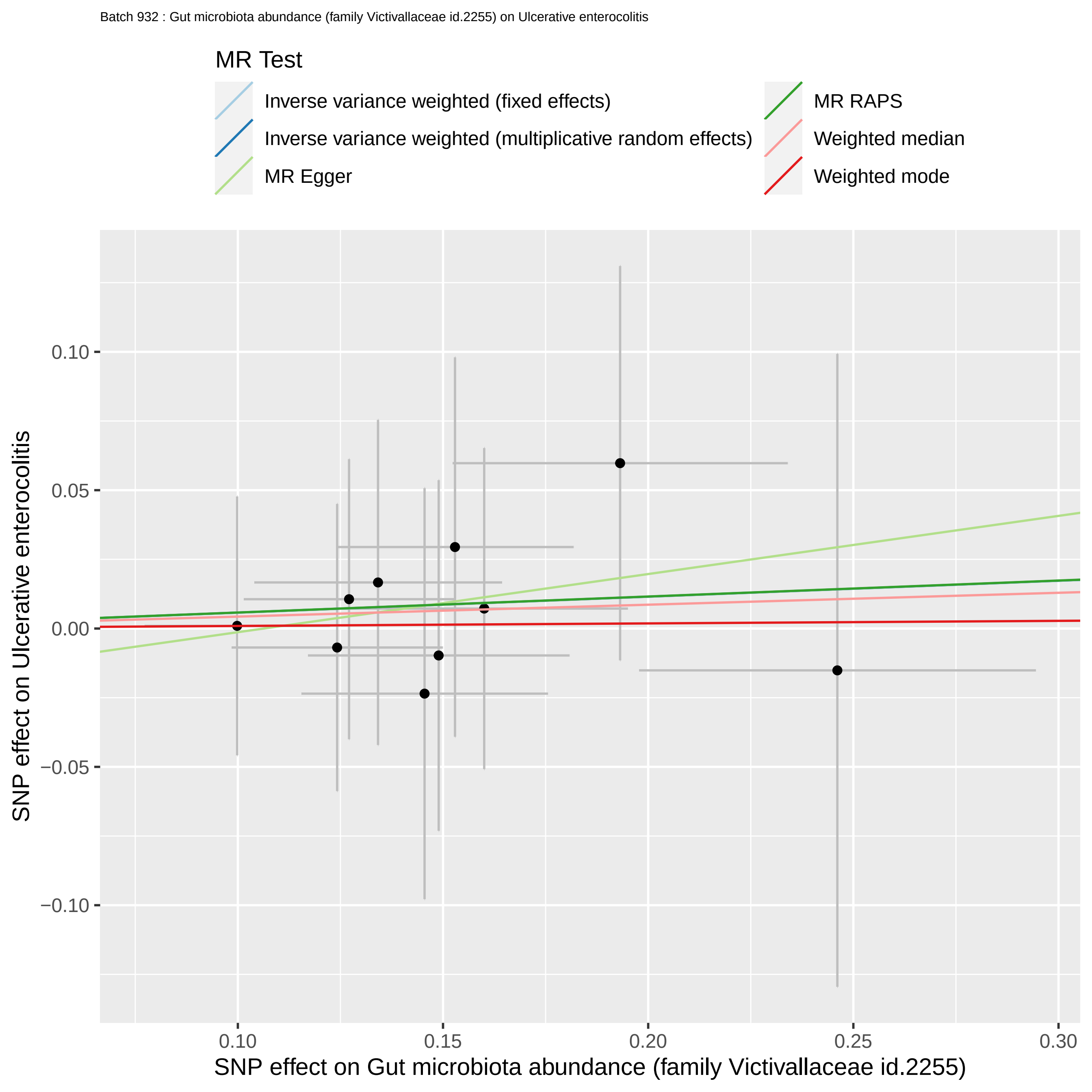

Batch 932 : Gut microbiota abundance (family Victivallaceae id.2255) on Ulcerative enterocolitis
MR Test
Inverse variance weighted (fixed effects)
Inverse variance weighted (multiplicative random effects)
MR Egger
MR RAPS
Weighted median
Weighted mode
SNP effect on Ulcerative enterocolitis
0.10
0.05
0.00
−0.05
−0.10
0.10
0.15
0.20
0.25
0.30
SNP effect on Gut microbiota abundance (family Victivallaceae id.2255)

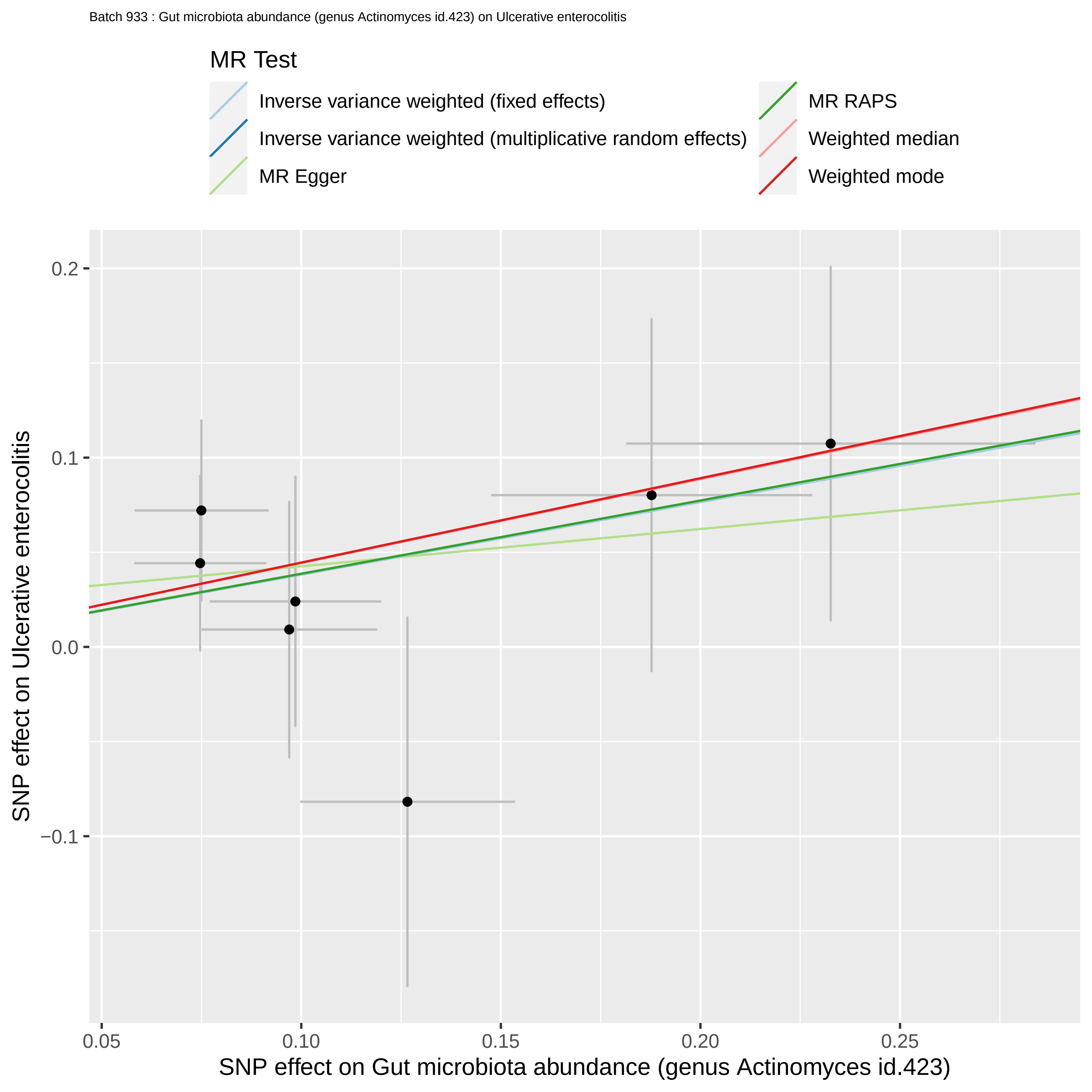

Batch 933 : Gut microbiota abundance (genus Actinomyces id.423) on Ulcerative enterocolitis
MR Test
Inverse variance weighted (fixed effects)
Inverse variance weighted (multiplicative random effects)
MR Egger
MR RAPS
Weighted median
Weighted mode
SNP effect on Ulcerative enterocolitis
0.2
0.1
0.0
−0.1
0.05
0.10
0.15
0.20
0.25
SNP effect on Gut microbiota abundance (genus Actinomyces id.423)

Batch 934 : Gut microbiota abundance (genus Adlercreutzia id.812) on Ulcerative enterocolitis

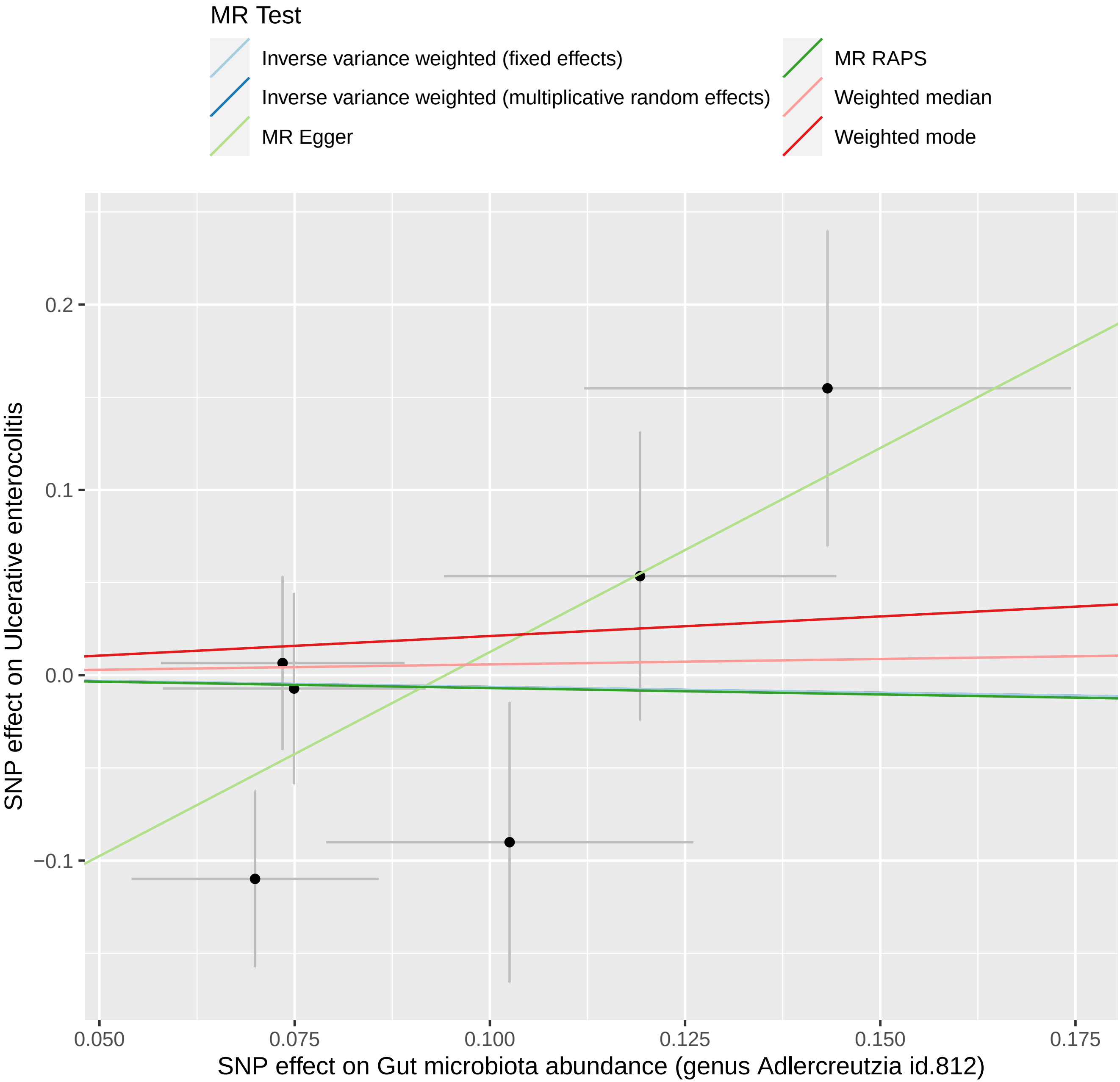

Batch 935 : Gut microbiota abundance (genus Akkermansia id.4037) on Ulcerative enterocolitis

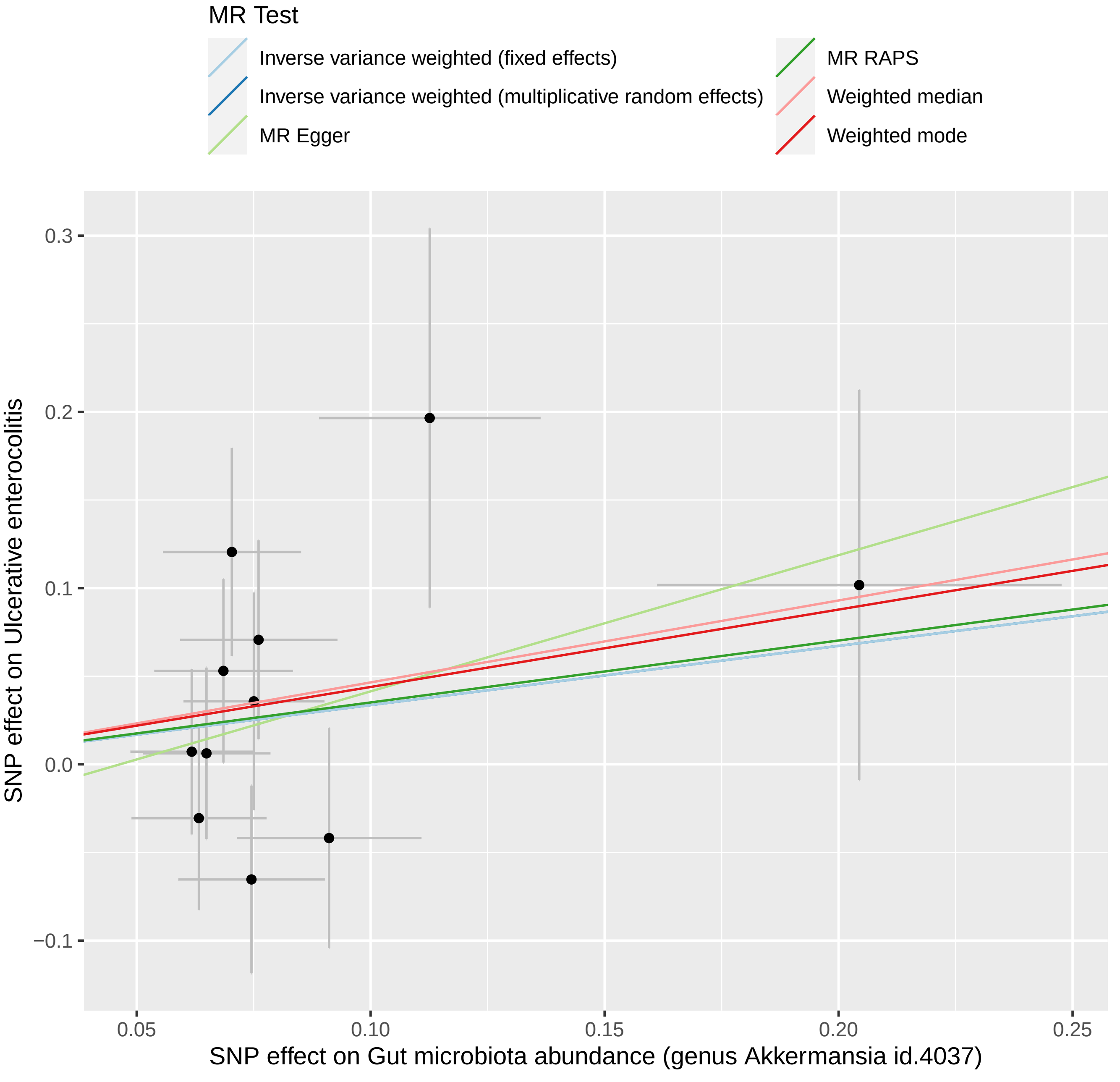

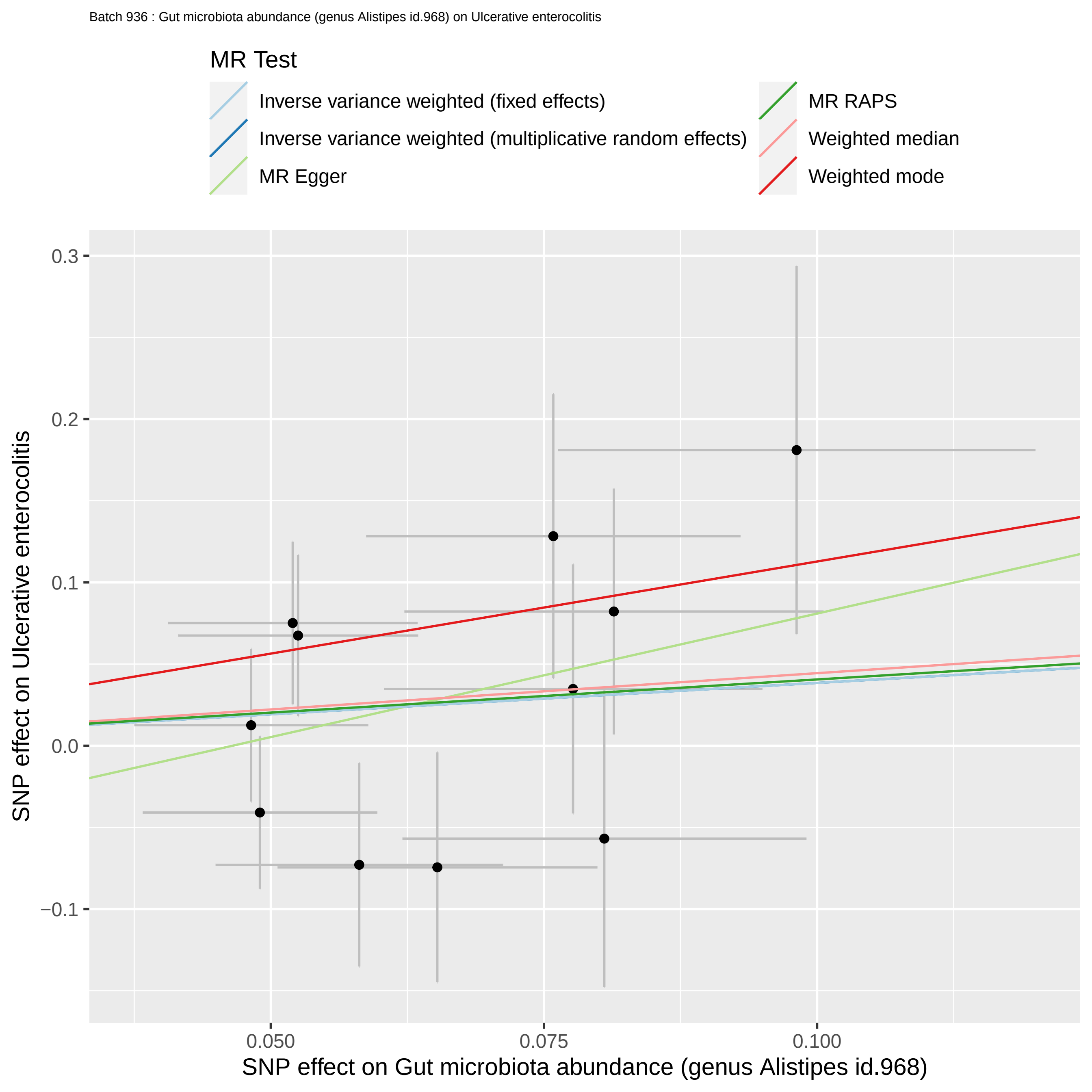

Batch 936 : Gut microbiota abundance (genus Alistipes id.968) on Ulcerative enterocolitis
MR Test
Inverse variance weighted (fixed effects)
Inverse variance weighted (multiplicative random effects)
MR Egger
MR RAPS
Weighted median
Weighted mode
0.3
0.2
0.1
0.0
−0.1
0.050
0.075
0.100
SNP effect on Ulcerative enterocolitis
SNP effect on Gut microbiota abundance (genus Alistipes id.968)

Batch 937 : Gut microbiota abundance (genus Allisonella id.2174) on Ulcerative enterocolitis

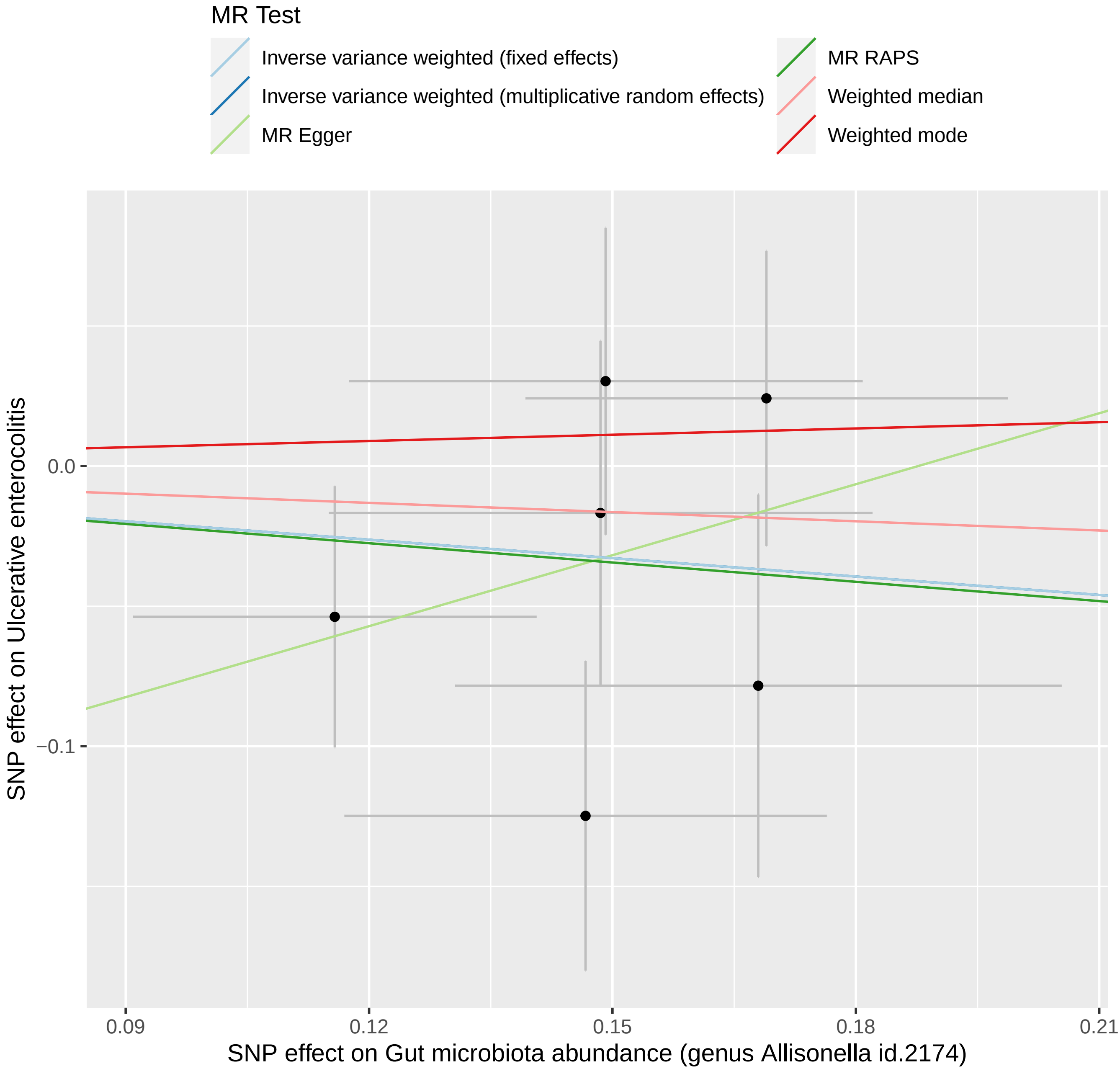

Batch 938 : Gut microbiota abundance (genus Alloprevotella id.961) on Ulcerative enterocolitis

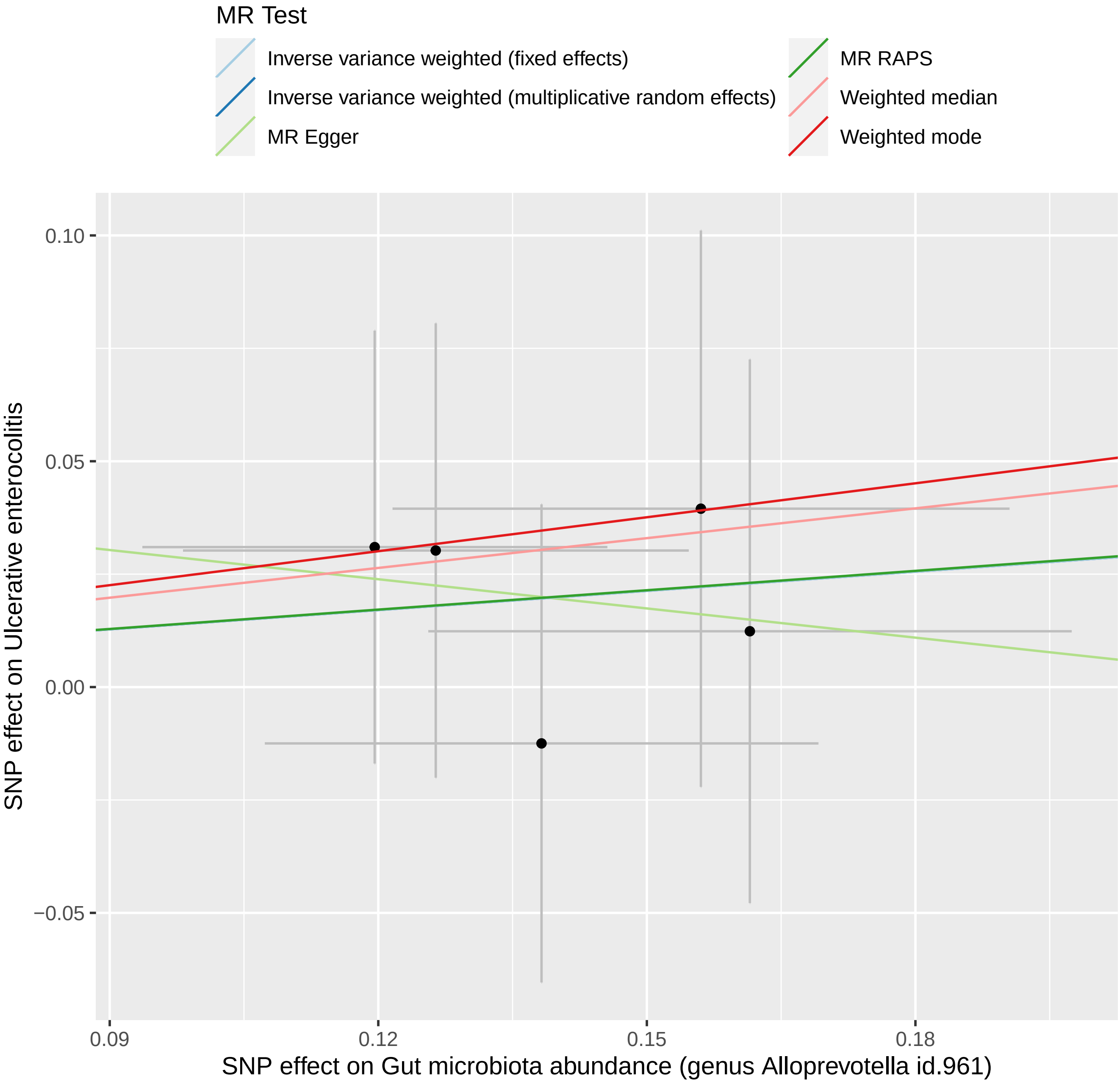

Batch 939 : Gut microbiota abundance (genus Anaerofilum id.2053) on Ulcerative enterocolitis

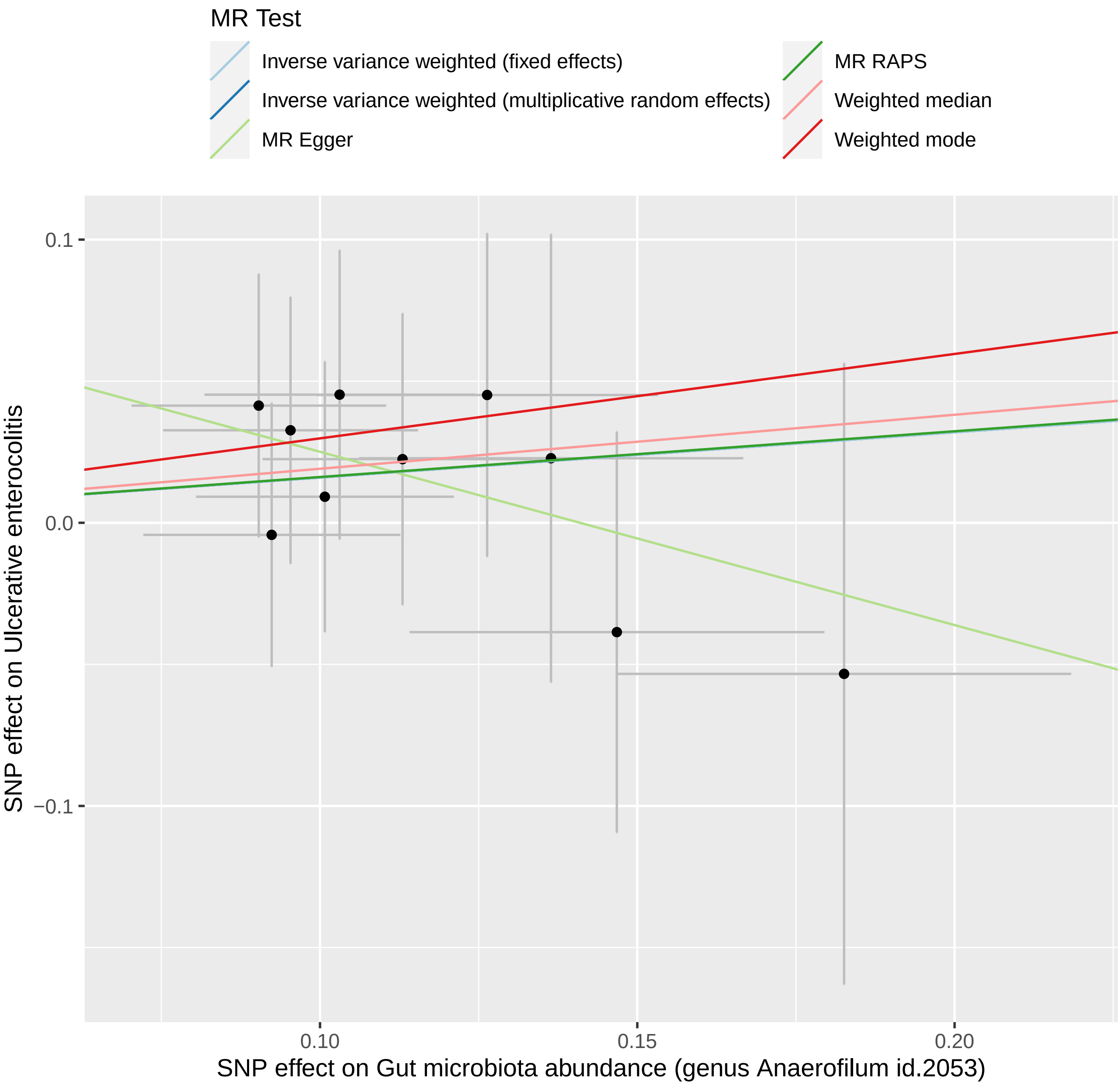

Batch 940 : Gut microbiota abundance (genus Anaerostipes id.1991) on Ulcerative enterocolitis

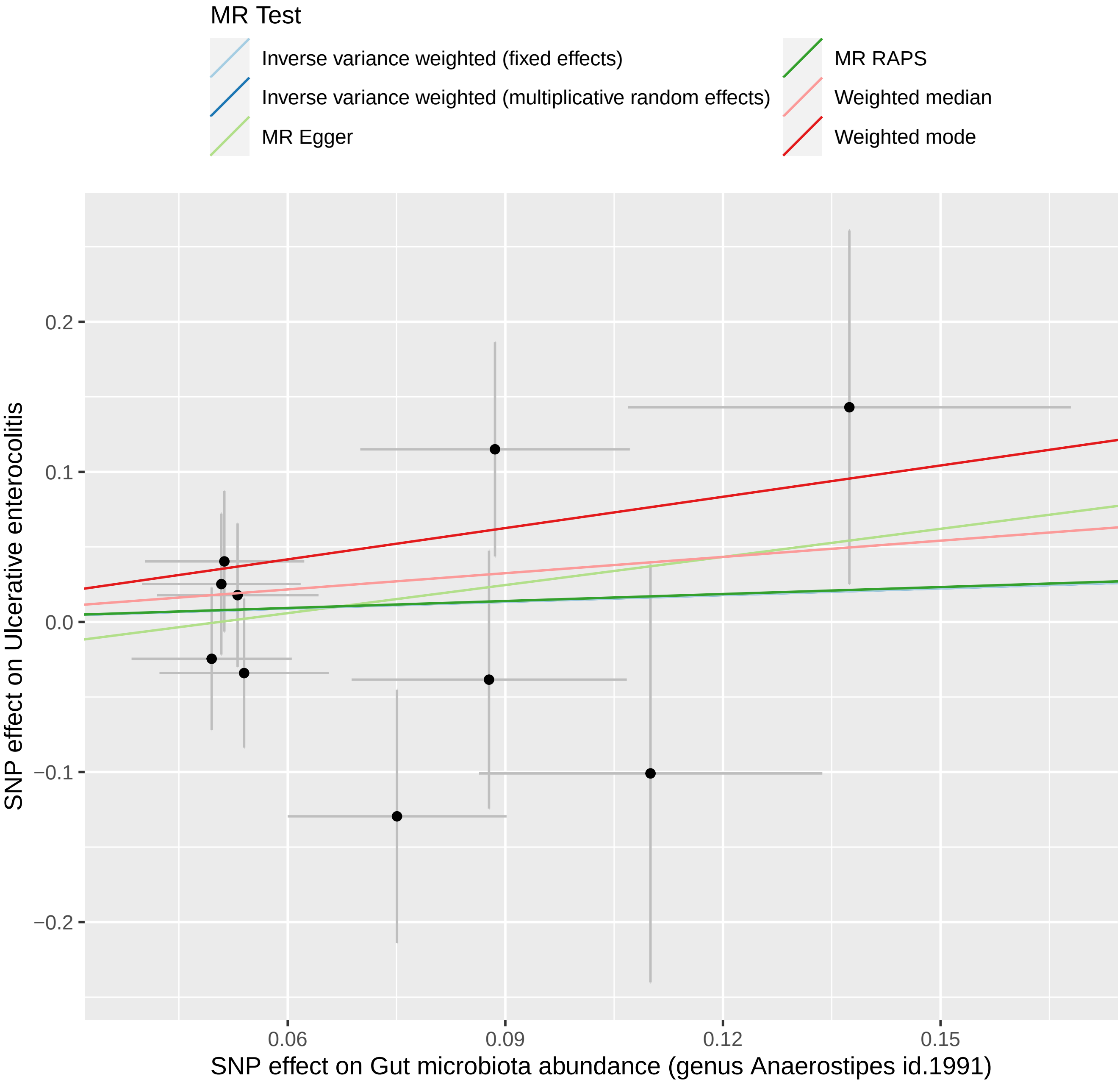

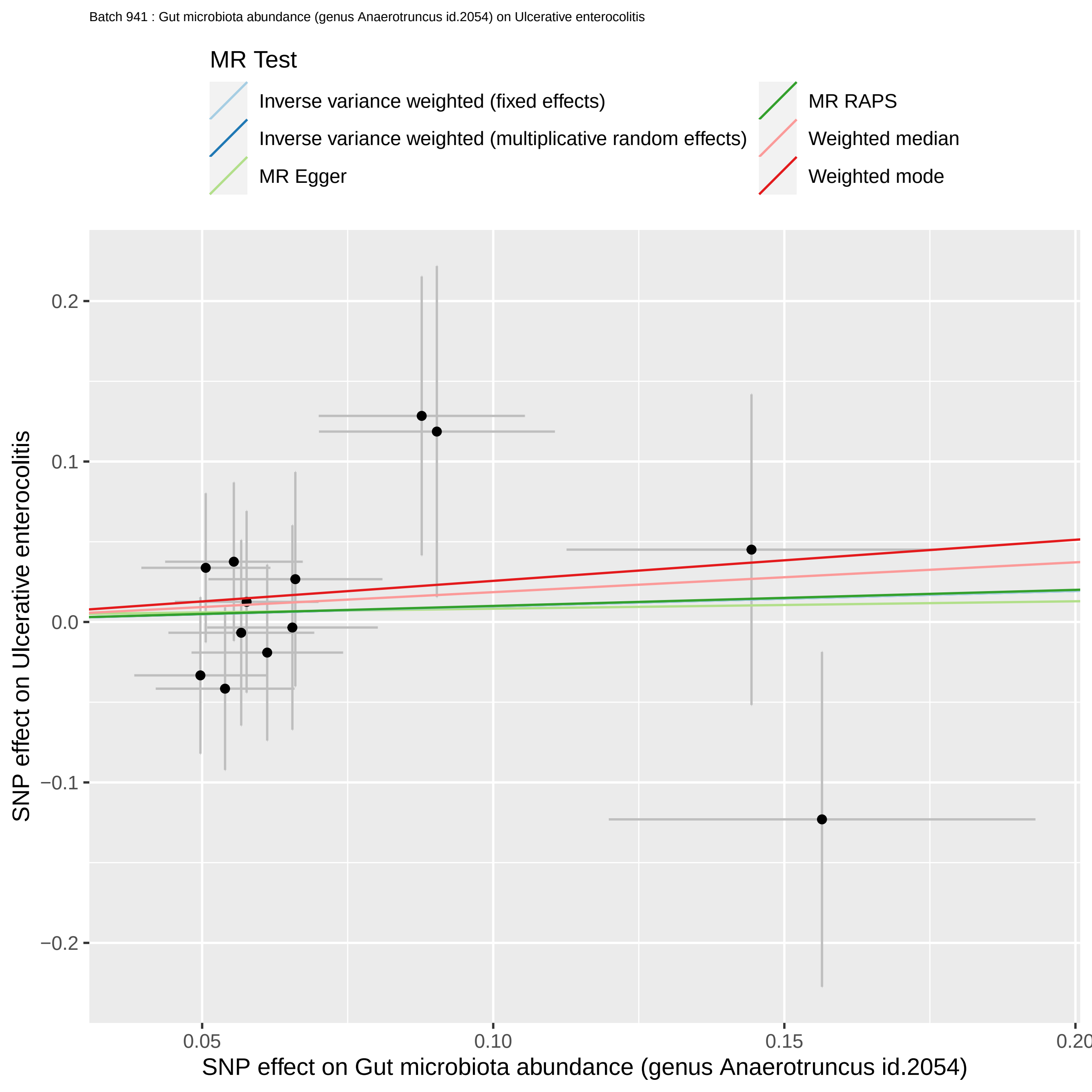
Batch 941 : Gut microbiota abundance (genus Anaerotruncus id.2054) on Ulcerative enterocolitis
MR Test
Inverse variance weighted (fixed effects)
Inverse variance weighted (multiplicative random effects)
MR Egger
MR RAPS
Weighted median
Weighted mode
SNP effect on Ulcerative enterocolitis
0.2
0.1
0.0
−0.1
−0.2
0.05
0.10
0.15
0.20
SNP effect on Gut microbiota abundance (genus Anaerotruncus id.2054)

Batch 942 : Gut microbiota abundance (genus Bacteroides id.918) on Ulcerative enterocolitis

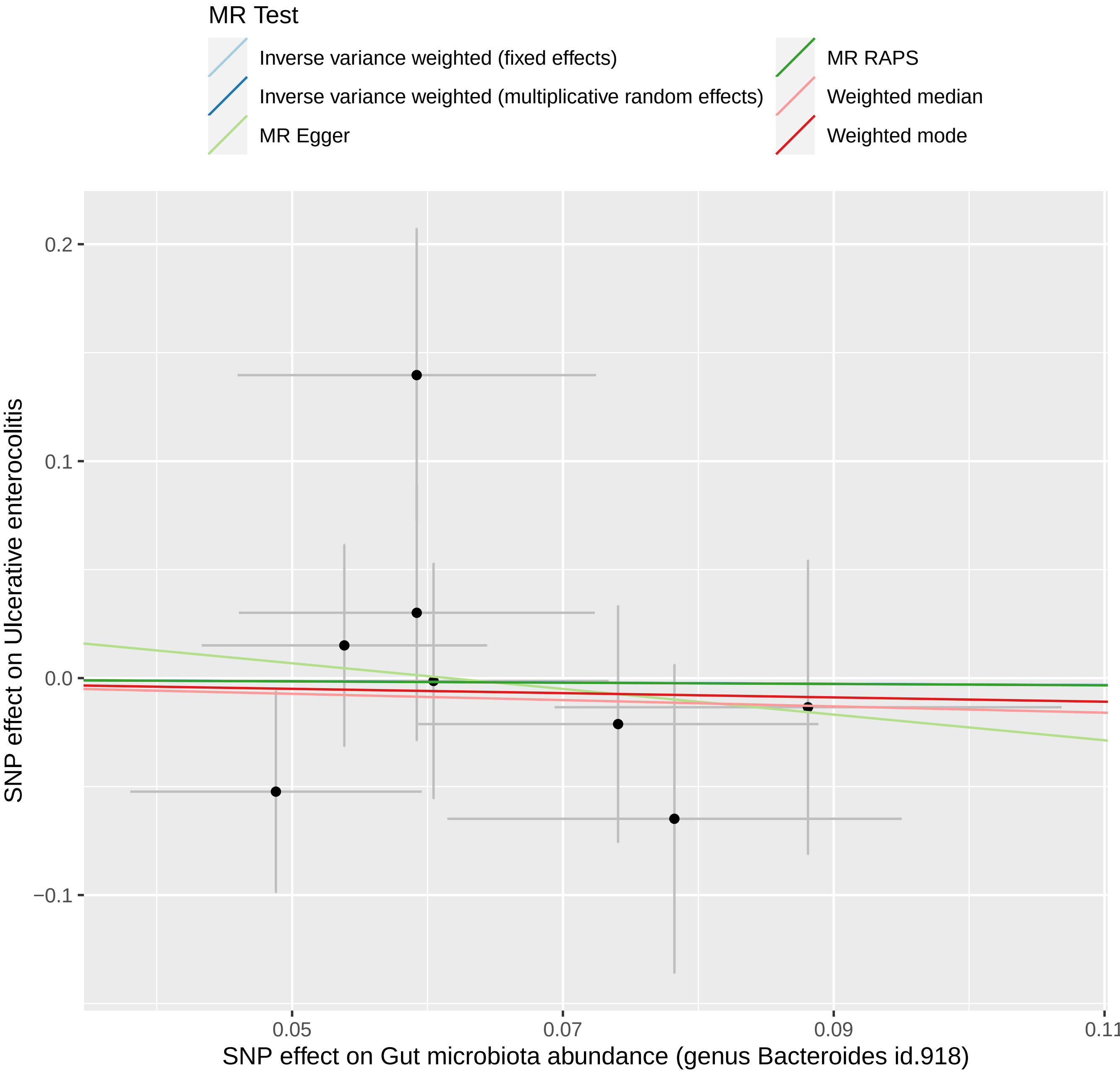

Batch 943 : Gut microbiota abundance (genus Barnesiella id.944) on Ulcerative enterocolitis

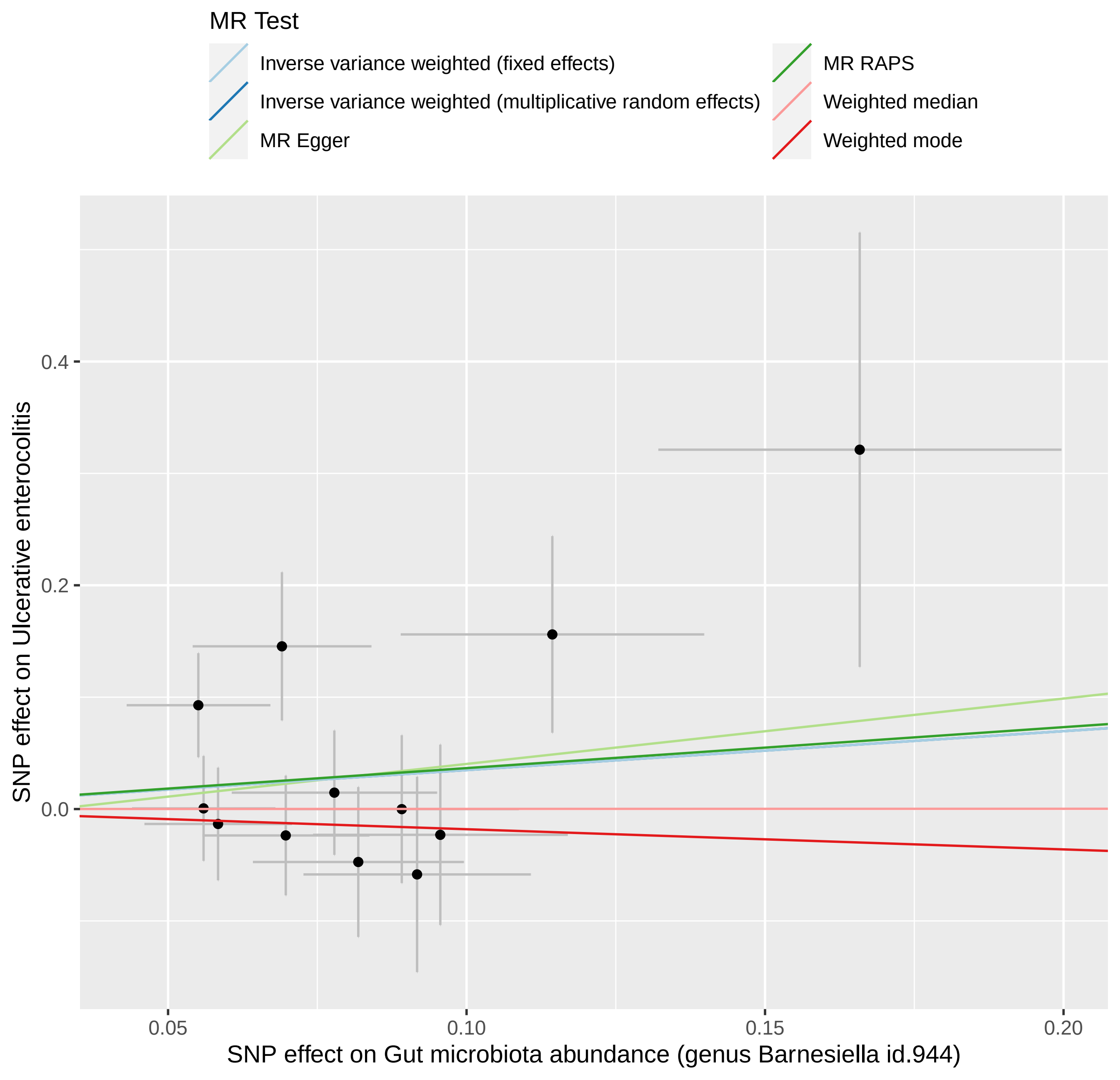

Batch 944 : Gut microbiota abundance (genus Bifidobacterium id.436) on Ulcerative enterocolitis

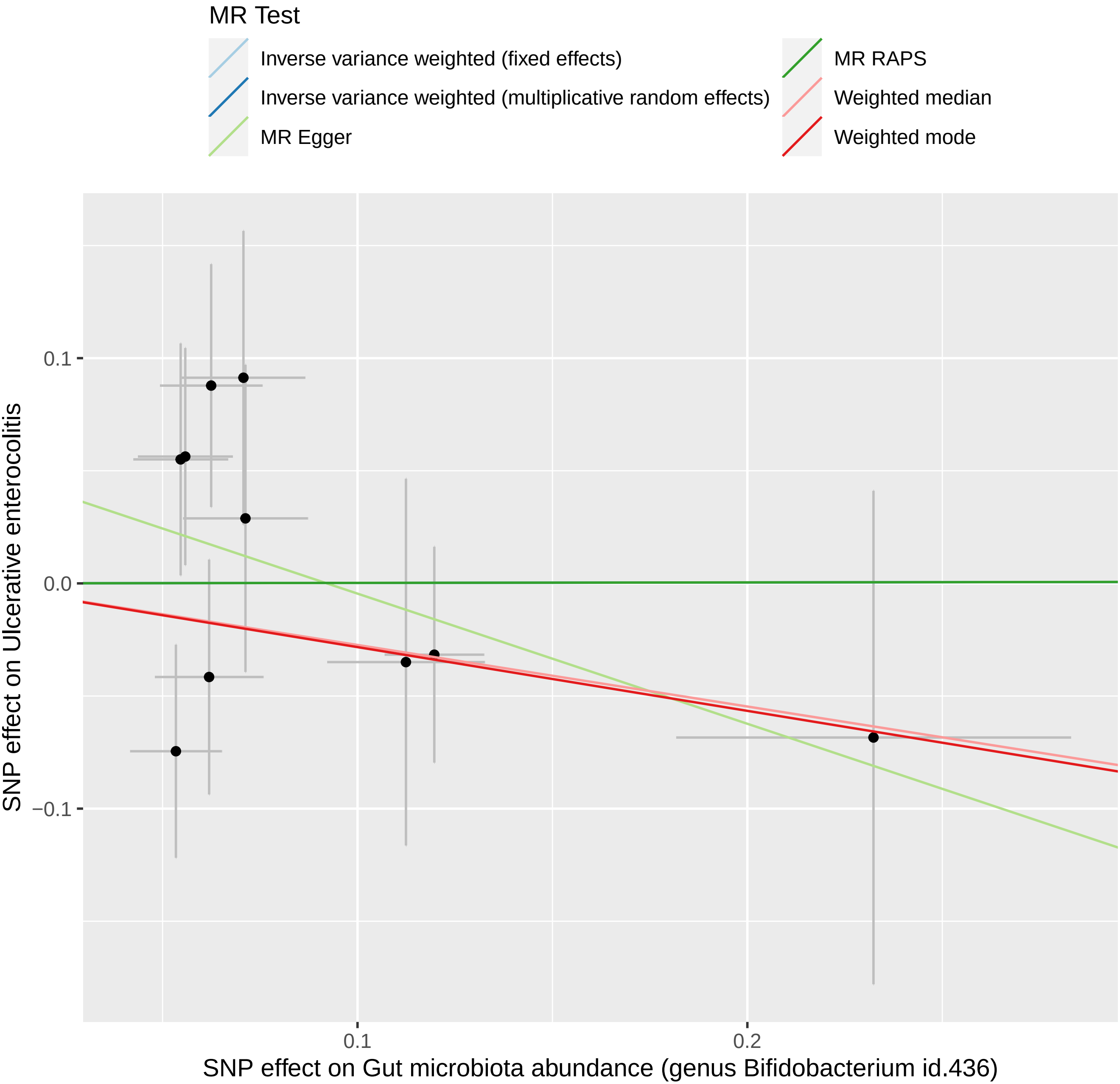

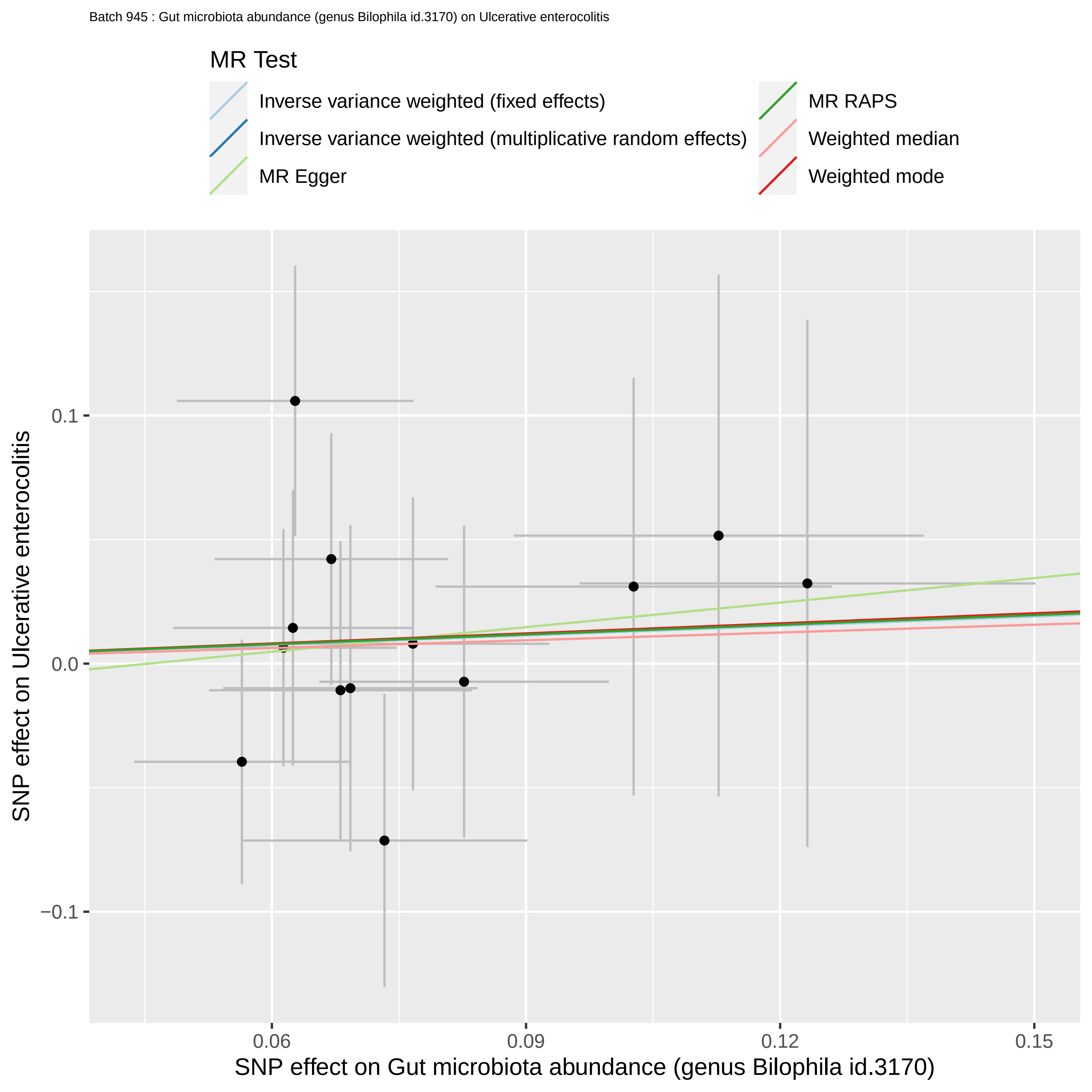
Batch 945 : Gut microbiota abundance (genus Bilophila id.3170) on Ulcerative enterocolitis
MR Test
Inverse variance weighted (fixed effects)
Inverse variance weighted (multiplicative random effects)
MR Egger
MR RAPS
Weighted median
Weighted mode
SNP effect on Ulcerative enterocolitis
0.1
0.0
−0.1
0.06
0.09
0.12
0.15
SNP effect on Gut microbiota abundance (genus Bilophila id.3170)

Batch 946 : Gut microbiota abundance (genus Blautia id.1992) on Ulcerative enterocolitis

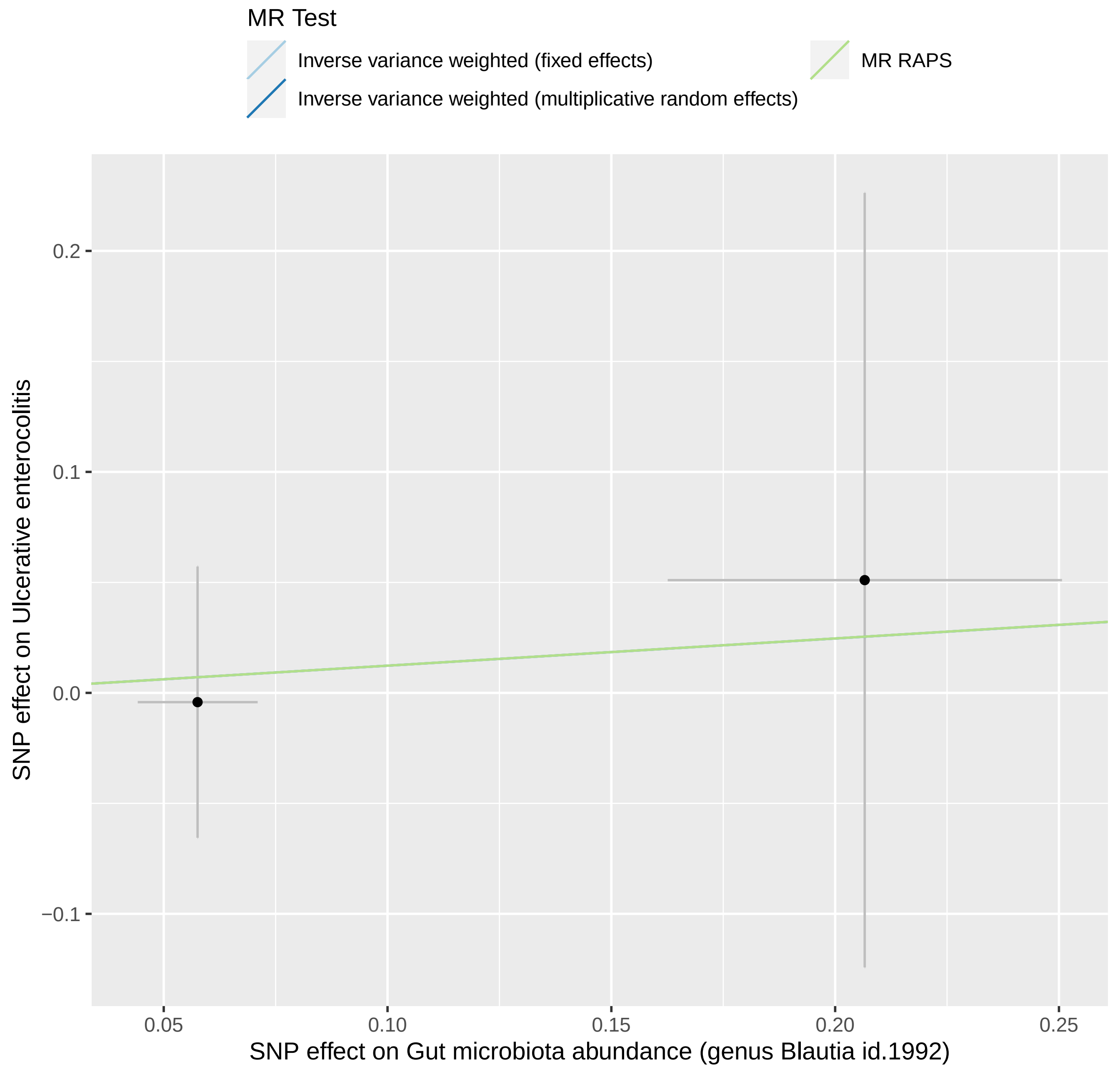

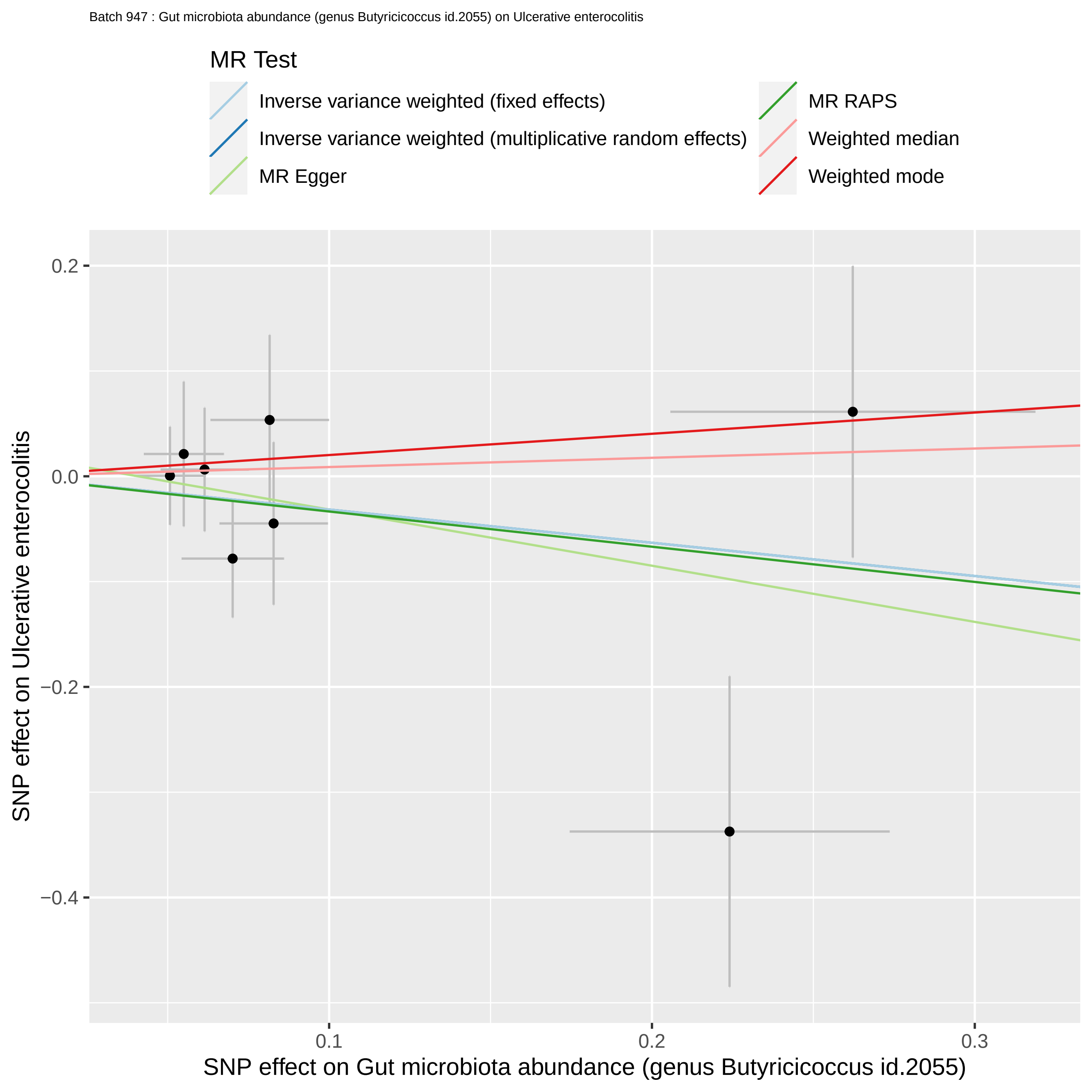

Batch 947 : Gut microbiota abundance (genus Butyricicoccus id.2055) on Ulcerative enterocolitis
MR Test
Inverse variance weighted (fixed effects)
Inverse variance weighted (multiplicative random effects)
MR Egger
MR RAPS
Weighted median
Weighted mode
0.2
0.0
−0.2
−0.4
0.1
0.2
0.3
SNP effect on Ulcerative enterocolitis
SNP effect on Gut microbiota abundance (genus Butyricicoccus id.2055)

Batch 948 : Gut microbiota abundance (genus Butyricimonas id.945) on Ulcerative enterocolitis

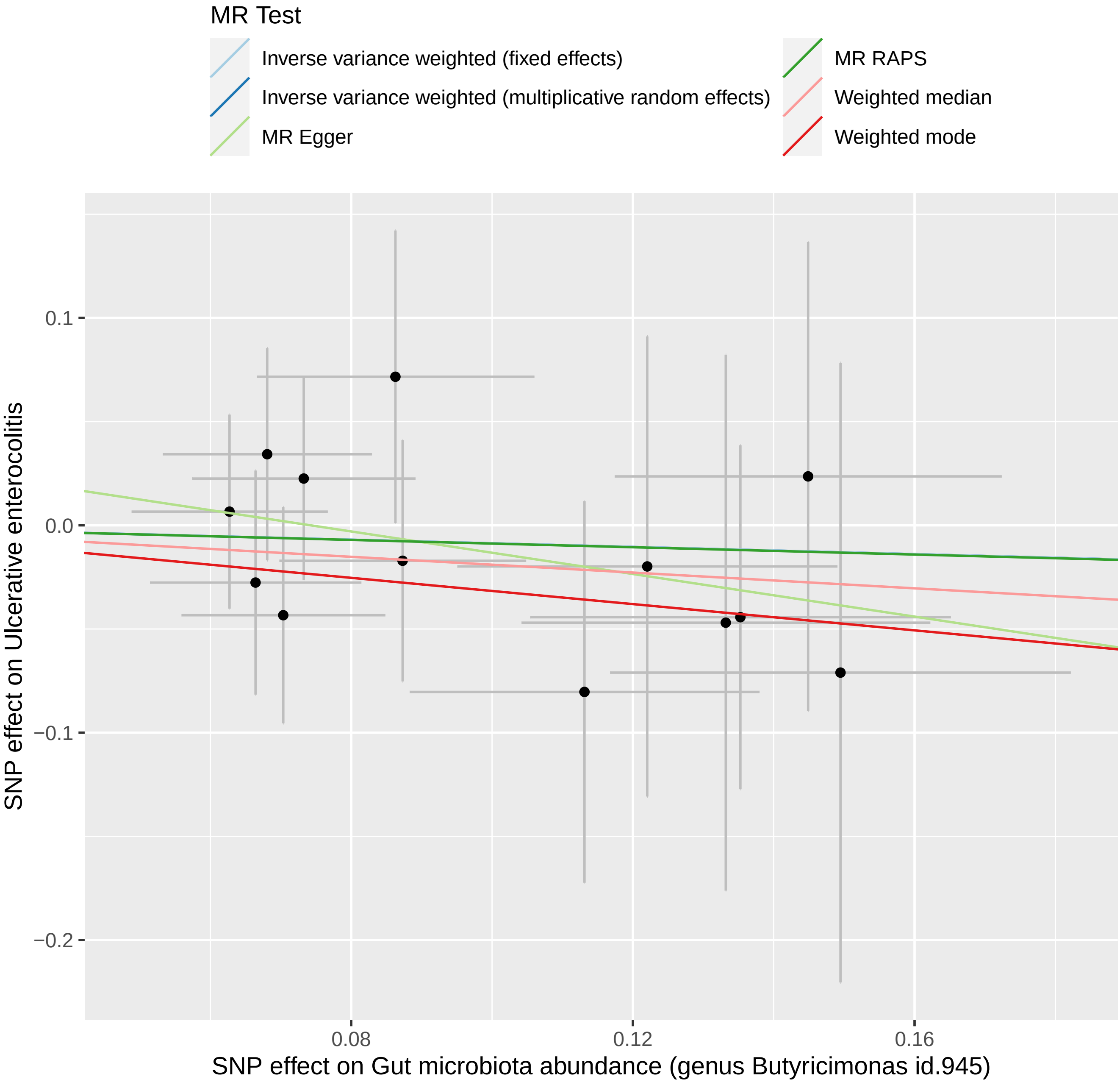

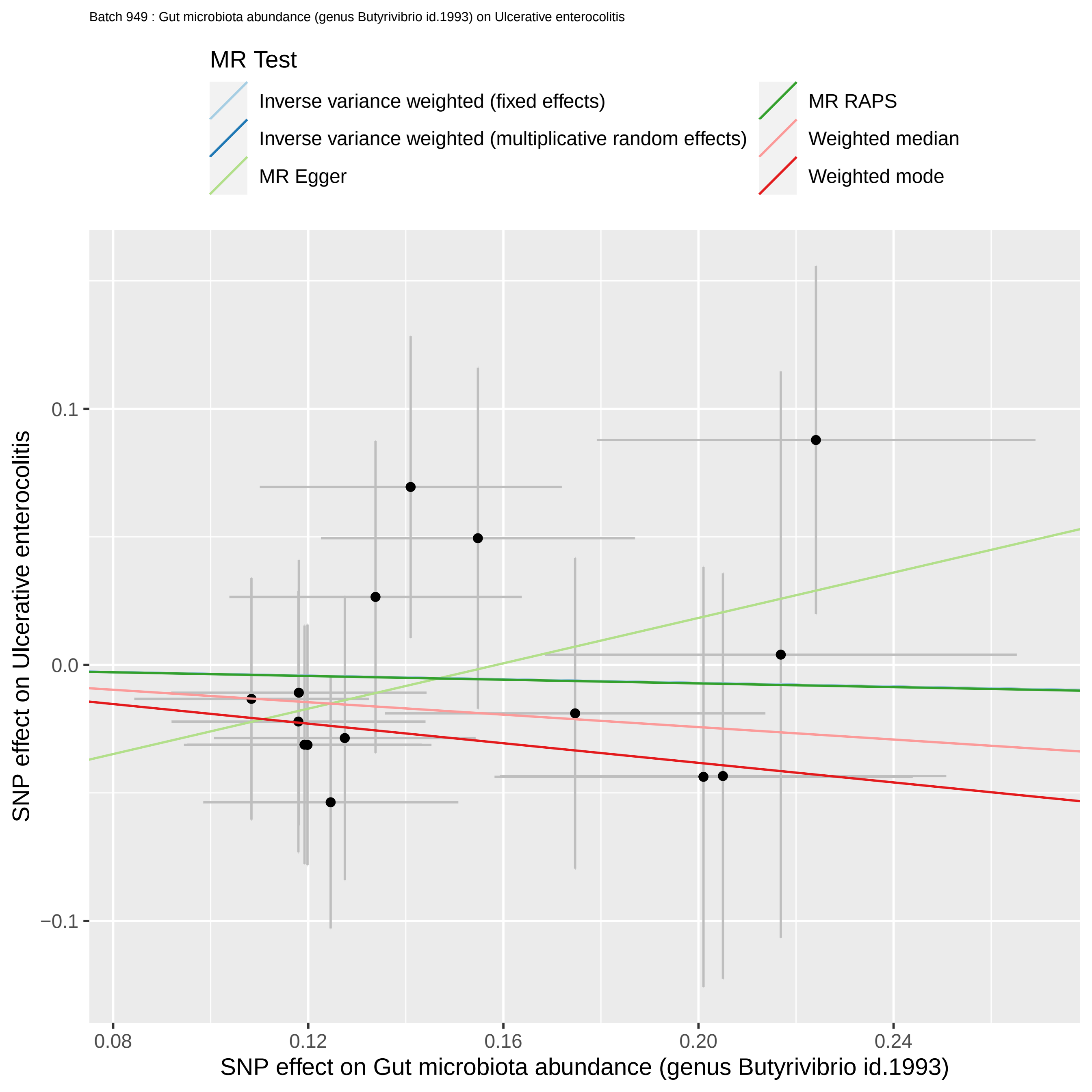

Batch 949 : Gut microbiota abundance (genus Butyrivibrio id.1993) on Ulcerative enterocolitis
MR Test
Inverse variance weighted (fixed effects)
Inverse variance weighted (multiplicative random effects)
MR Egger
MR RAPS
Weighted median
Weighted mode
0.1
0.0
−0.1
0.08
0.12
0.16
0.20
0.24
SNP effect on Ulcerative enterocolitis
SNP effect on Gut microbiota abundance (genus Butyrivibrio id.1993)

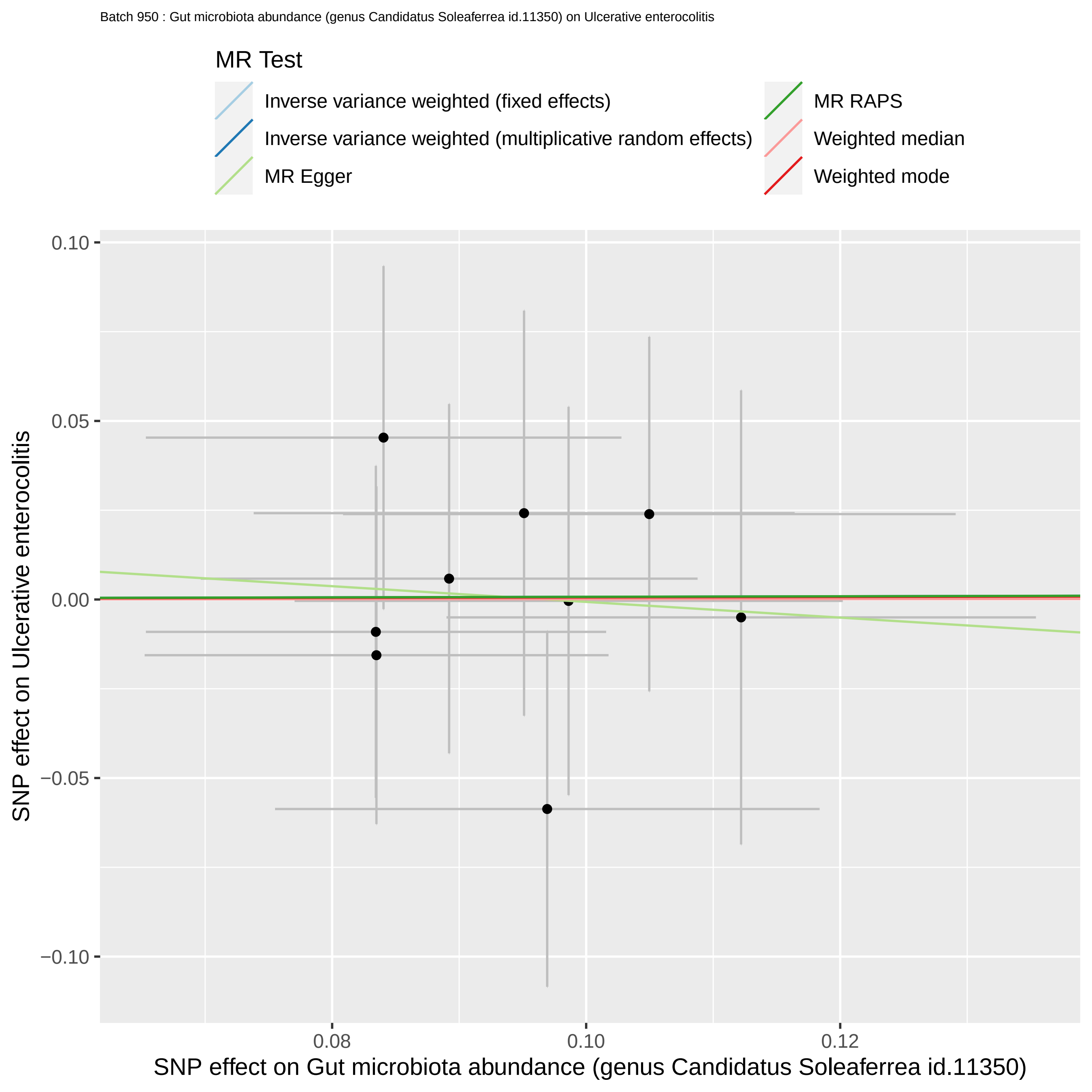
Batch 950 : Gut microbiota abundance (genus Candidatus Soleaferrea id.11350) on Ulcerative enterocolitis
MR Test
Inverse variance weighted (fixed effects)
Inverse variance weighted (multiplicative random effects)
MR Egger
MR RAPS
Weighted median
Weighted mode
0.10
0.05
0.00
−0.05
−0.10
0.08
0.10
0.12
SNP effect on Ulcerative enterocolitis
SNP effect on Gut microbiota abundance (genus Candidatus Soleaferrea id.11350)

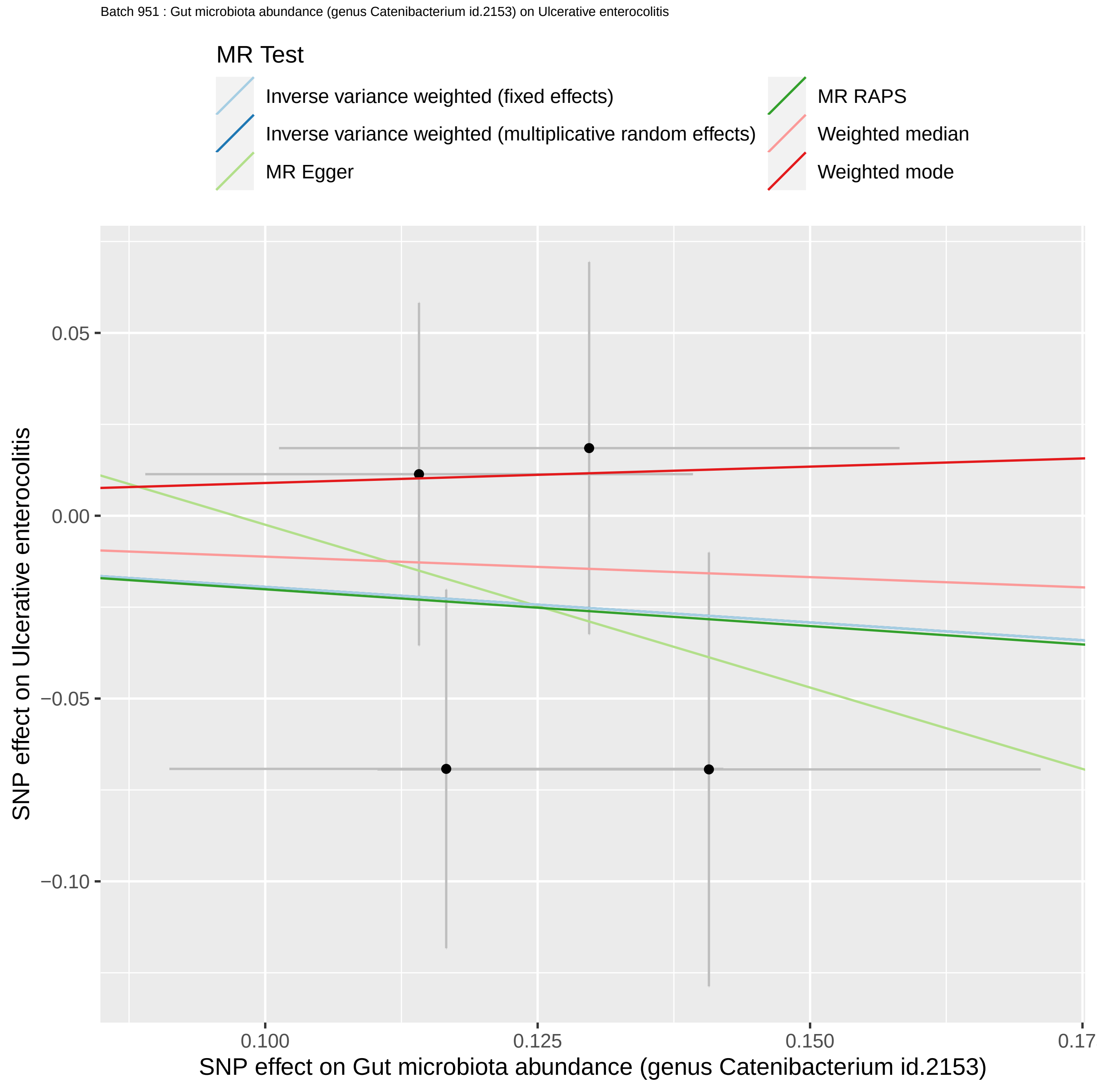

Batch 951 : Gut microbiota abundance (genus Catenibacterium id.2153) on Ulcerative enterocolitis
MR Test
Inverse variance weighted (fixed effects)
Inverse variance weighted (multiplicative random effects)
MR Egger
MR RAPS
Weighted median
Weighted mode
0.05
0.00
−0.05
−0.10
0.100
0.125
0.150
0.17
SNP effect on Ulcerative enterocolitis
SNP effect on Gut microbiota abundance (genus Catenibacterium id.2153)

Batch 952 : Gut microbiota abundance (genus Christensenellaceae R 7group id.11283) on Ulcerative enterocolitis

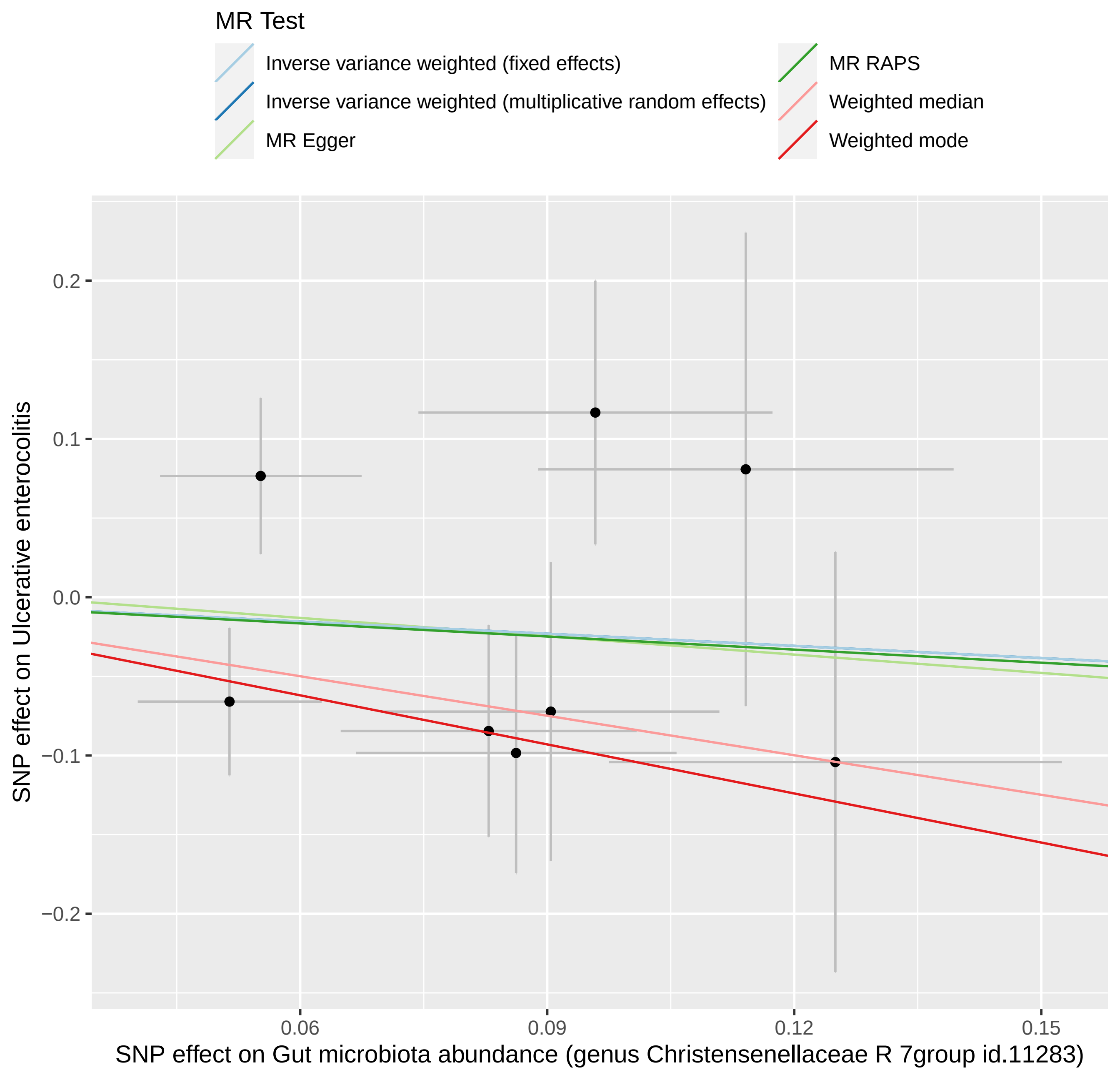

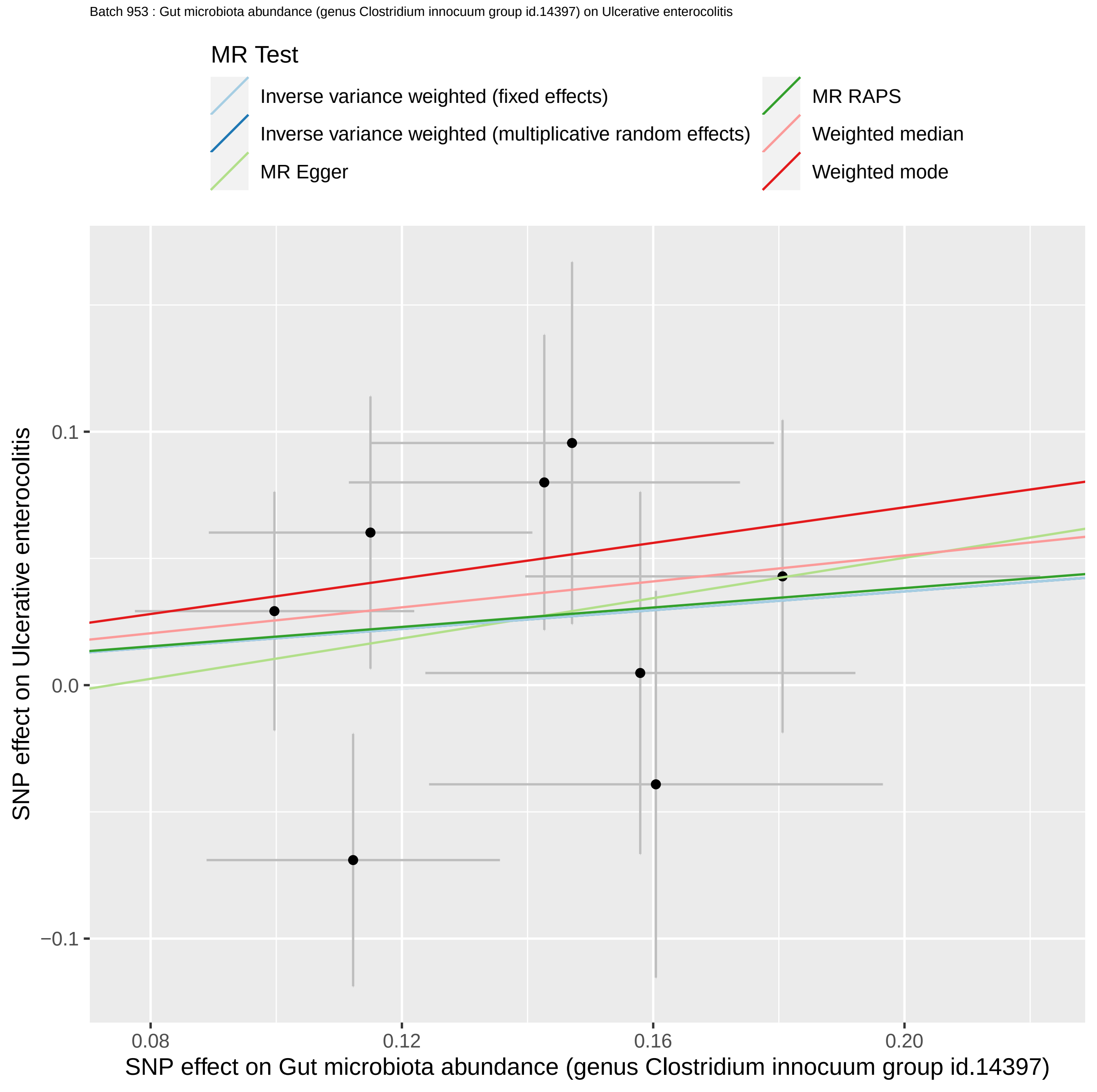

Batch 953 : Gut microbiota abundance (genus Clostridium innocuum group id.14397) on Ulcerative enterocolitis
MR Test
Inverse variance weighted (fixed effects)
Inverse variance weighted (multiplicative random effects)
MR Egger
MR RAPS
Weighted median
Weighted mode
0.1
0.0
−0.1
0.08
0.12
0.16
0.20
SNP effect on Ulcerative enterocolitis
SNP effect on Gut microbiota abundance (genus Clostridium innocuum group id.14397)

Batch 954 : Gut microbiota abundance (genus Clostridium sensustricto1 id.1873) on Ulcerative enterocolitis

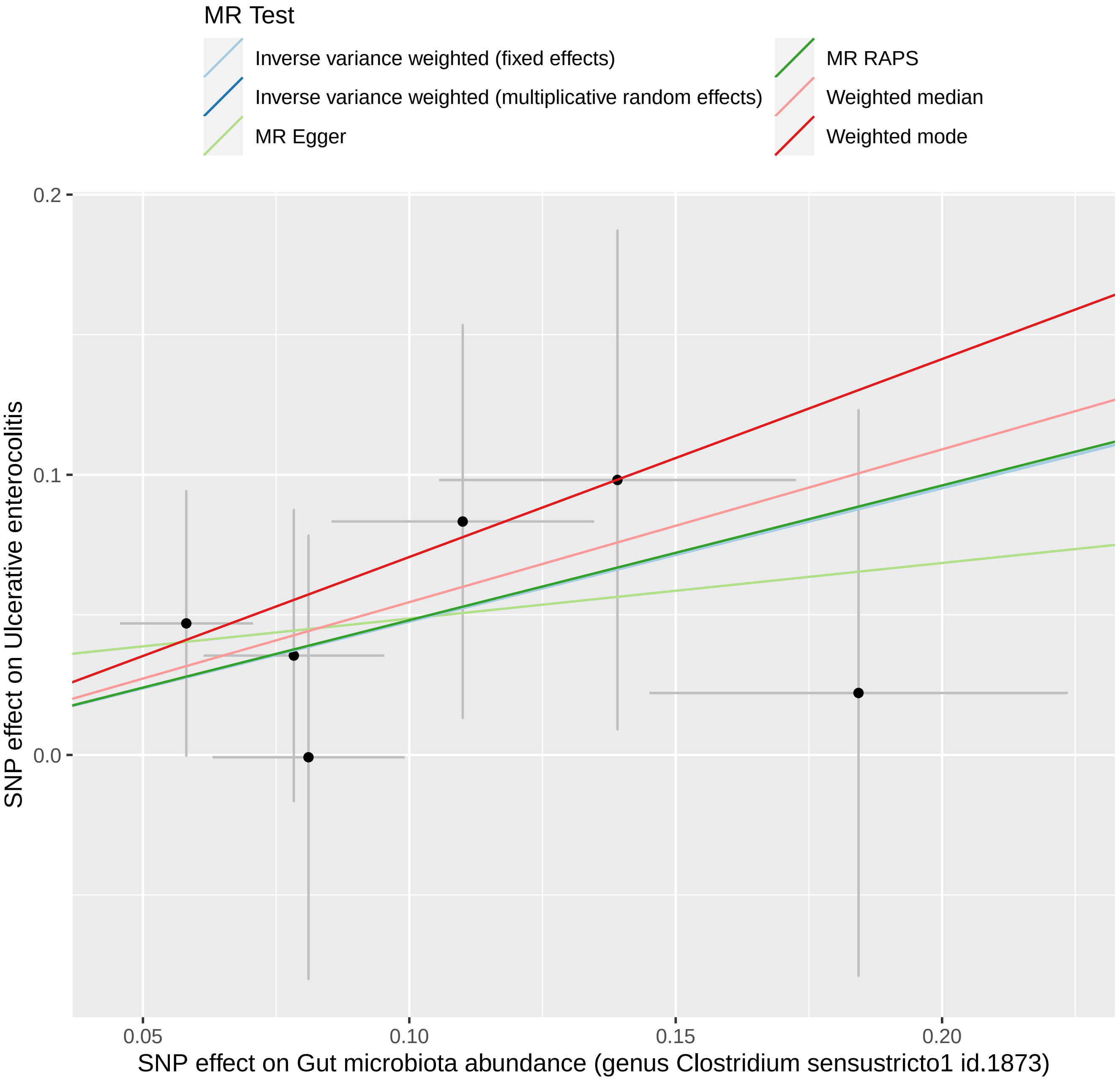

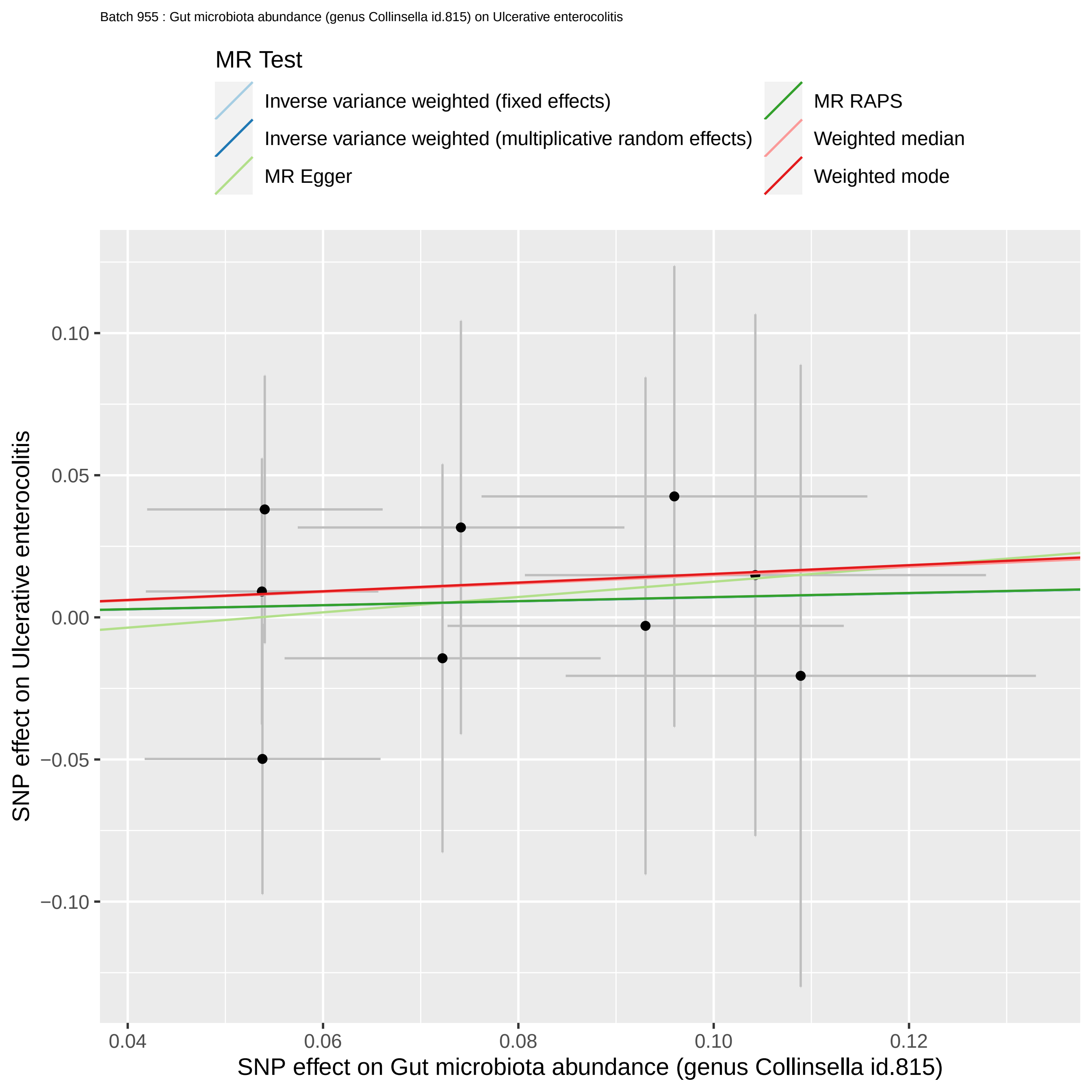
Batch 955 : Gut microbiota abundance (genus Collinsella id.815) on Ulcerative enterocolitis
MR Test
Inverse variance weighted (fixed effects)
Inverse variance weighted (multiplicative random effects)
MR Egger
MR RAPS
Weighted median
Weighted mode
SNP effect on Ulcerative enterocolitis
0.10
0.05
0.00
−0.05
−0.10
0.04
0.06
0.08
0.10
0.12
SNP effect on Gut microbiota abundance (genus Collinsella id.815)

Batch 956 : Gut microbiota abundance (genus Coprobacter id.949) on Ulcerative enterocolitis

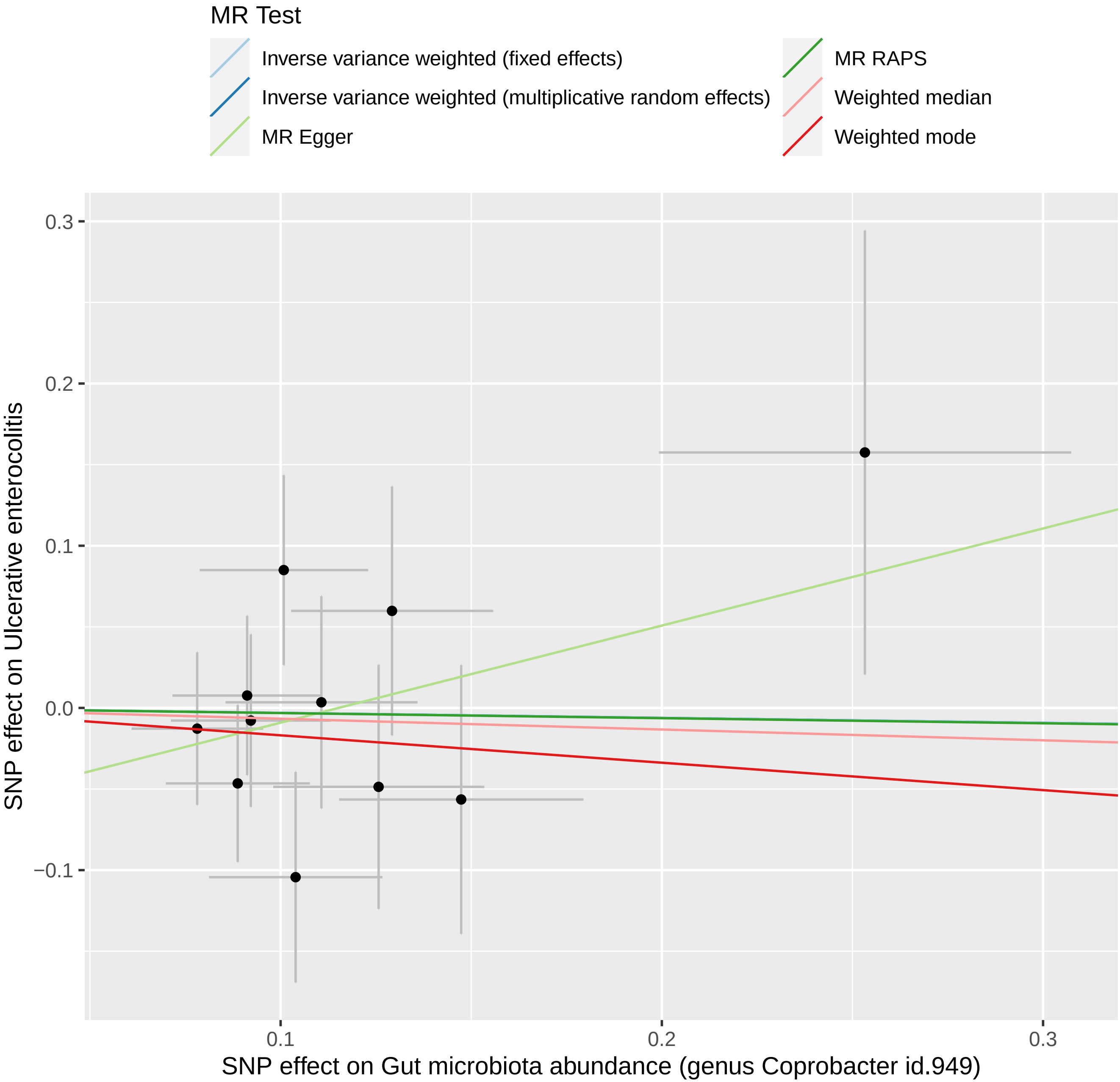

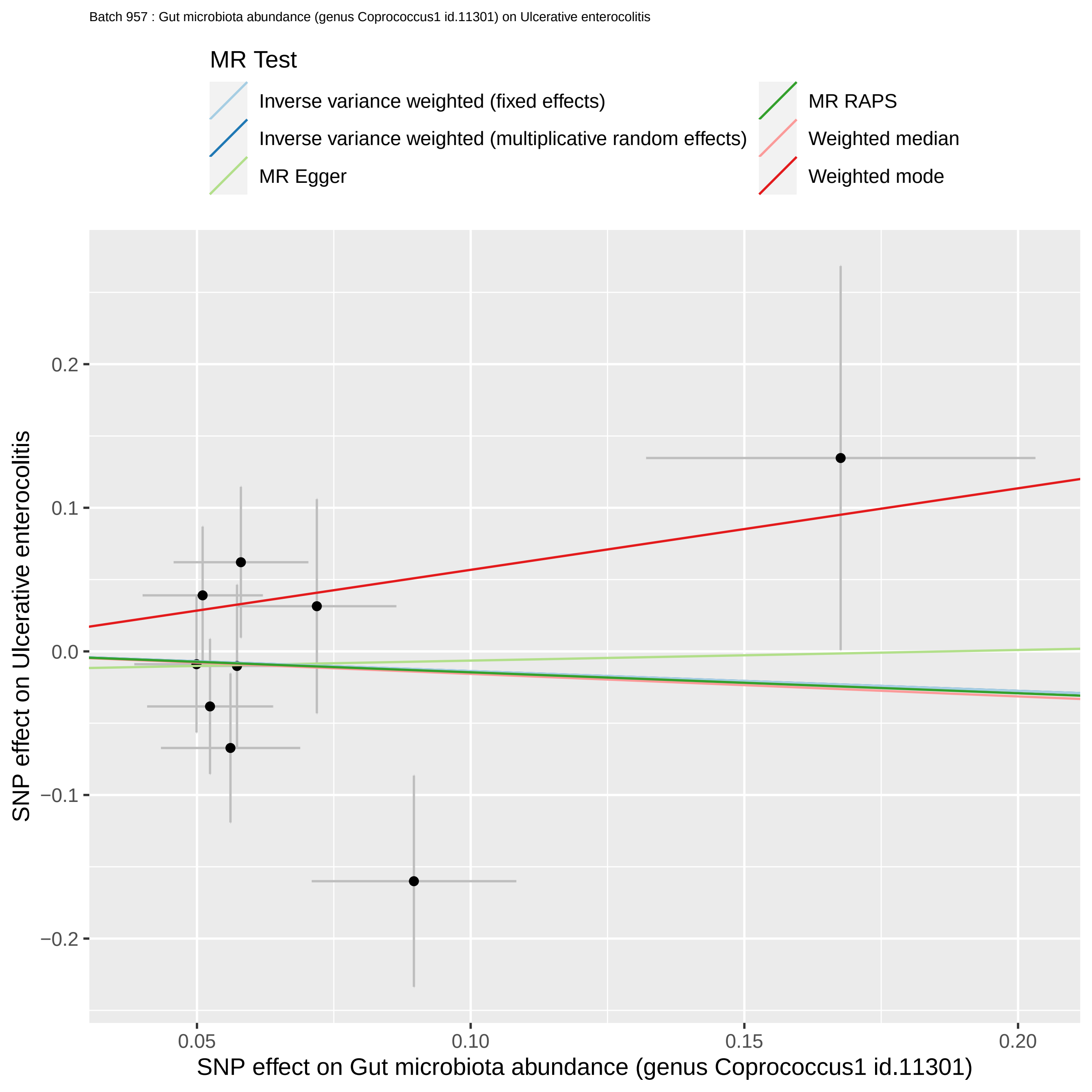

Batch 957 : Gut microbiota abundance (genus Coprococcus1 id.11301) on Ulcerative enterocolitis
MR Test
Inverse variance weighted (fixed effects)
Inverse variance weighted (multiplicative random effects)
MR Egger
MR RAPS
Weighted median
Weighted mode
0.2
0.1
0.0
−0.1
−0.2
0.05
0.10
0.15
0.20
SNP effect on Ulcerative enterocolitis
SNP effect on Gut microbiota abundance (genus Coprococcus1 id.11301)

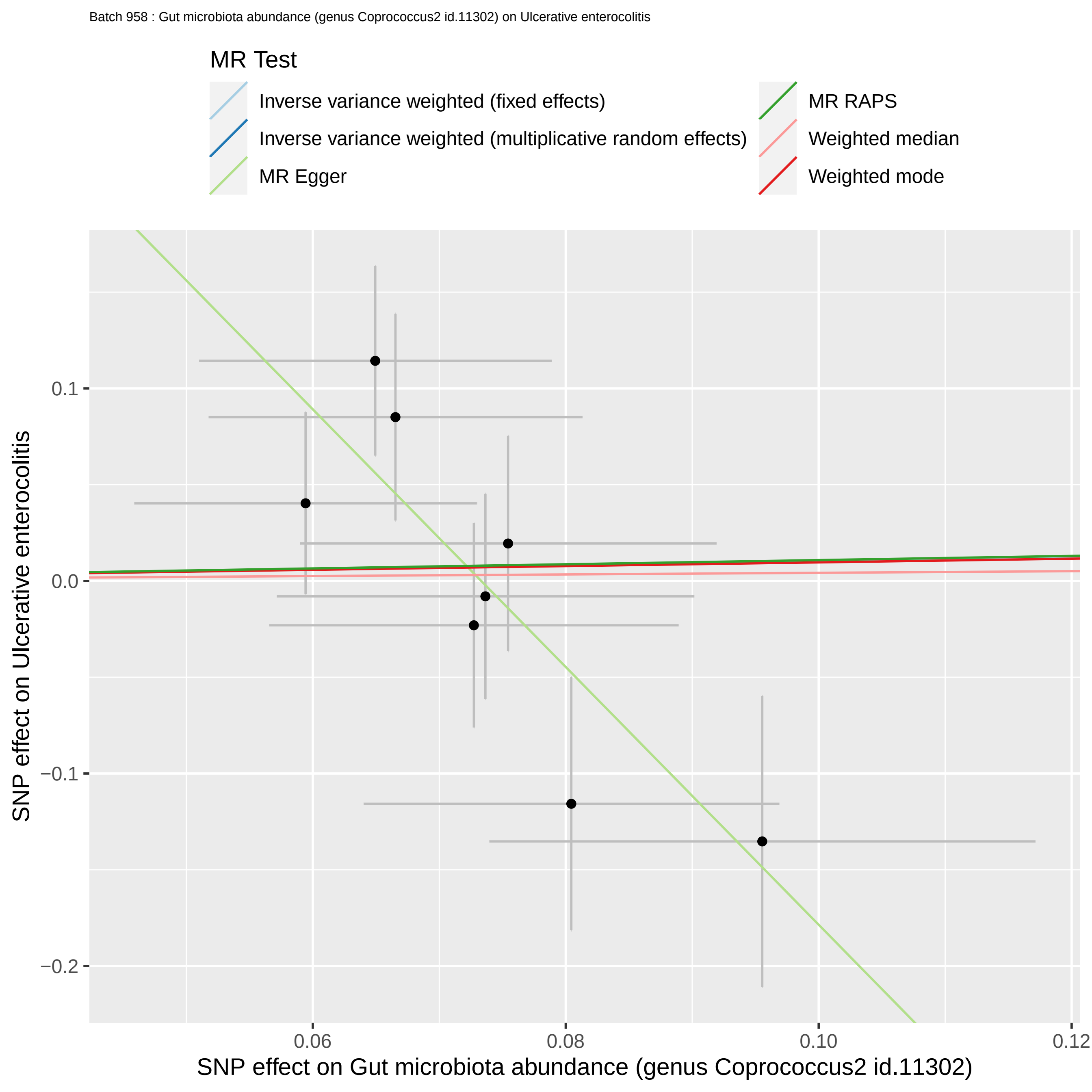

Batch 958 : Gut microbiota abundance (genus Coprococcus2 id.11302) on Ulcerative enterocolitis
MR Test
Inverse variance weighted (fixed effects)
Inverse variance weighted (multiplicative random effects)
MR Egger
MR RAPS
Weighted median
Weighted mode
0.1
0.0
−0.1
−0.2
0.06
0.08
0.10
0.12
SNP effect on Ulcerative enterocolitis
SNP effect on Gut microbiota abundance (genus Coprococcus2 id.11302)

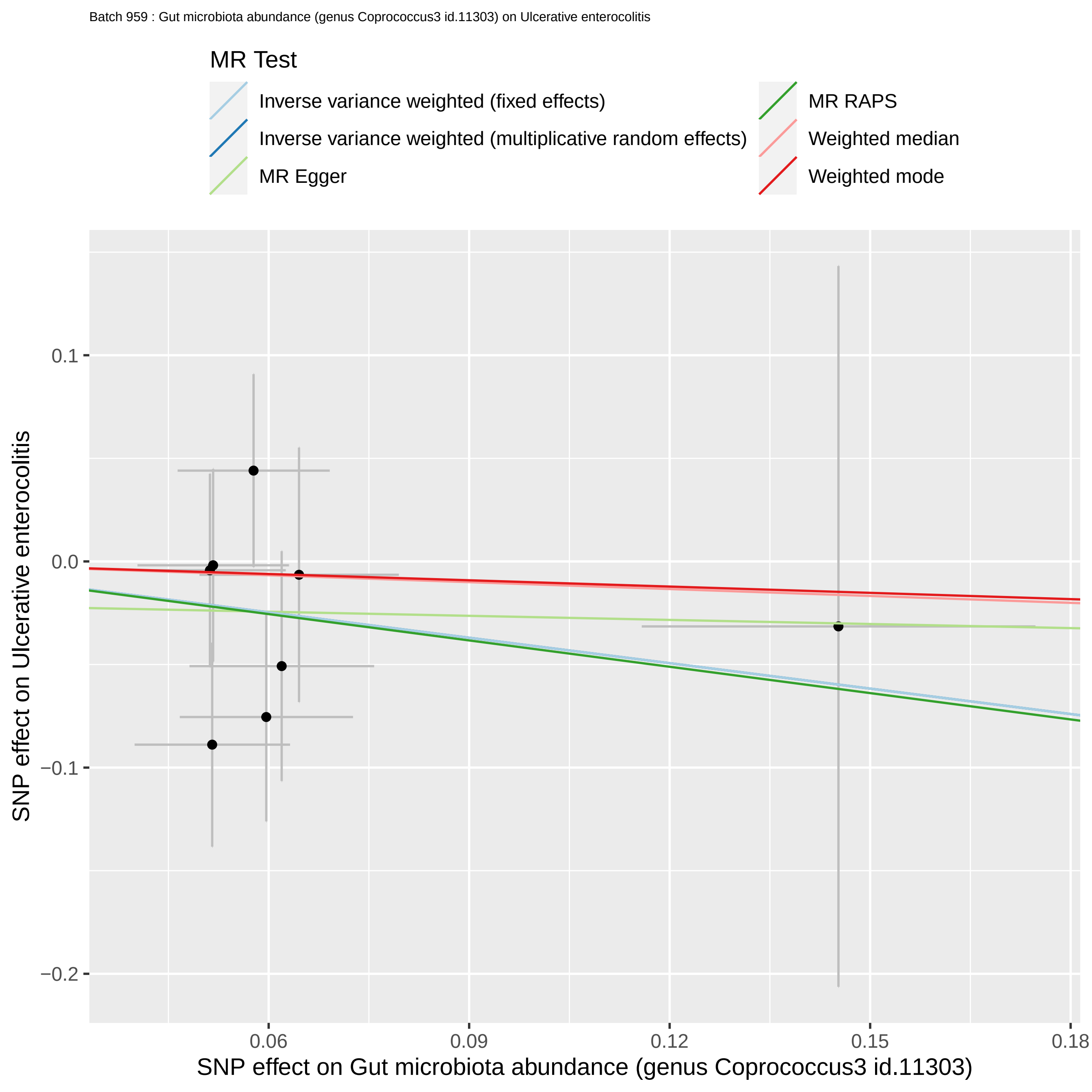

Batch 959 : Gut microbiota abundance (genus Coprococcus3 id.11303) on Ulcerative enterocolitis
MR Test
Inverse variance weighted (fixed effects)
Inverse variance weighted (multiplicative random effects)
MR Egger
MR RAPS
Weighted median
Weighted mode
0.1
0.0
−0.1
−0.2
0.06
0.09
0.12
0.15
0.18
SNP effect on Ulcerative enterocolitis
SNP effect on Gut microbiota abundance (genus Coprococcus3 id.11303)

Batch 960 : Gut microbiota abundance (genus Defluviitaleaceae UCG011 id.11287) on Ulcerative enterocolitis

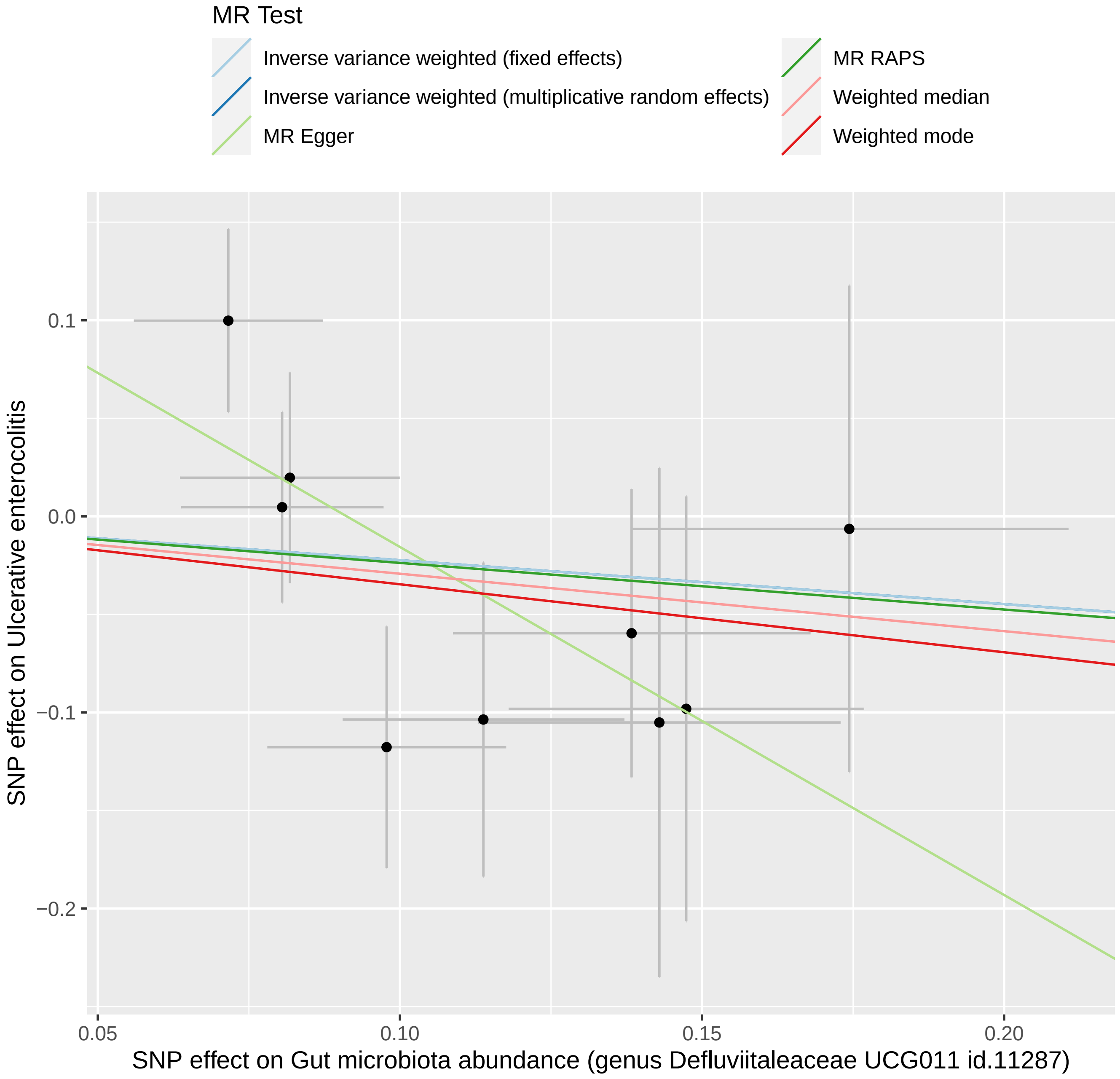

Batch 961 : Gut microbiota abundance (genus Desulfovibrio id.3173) on Ulcerative enterocolitis

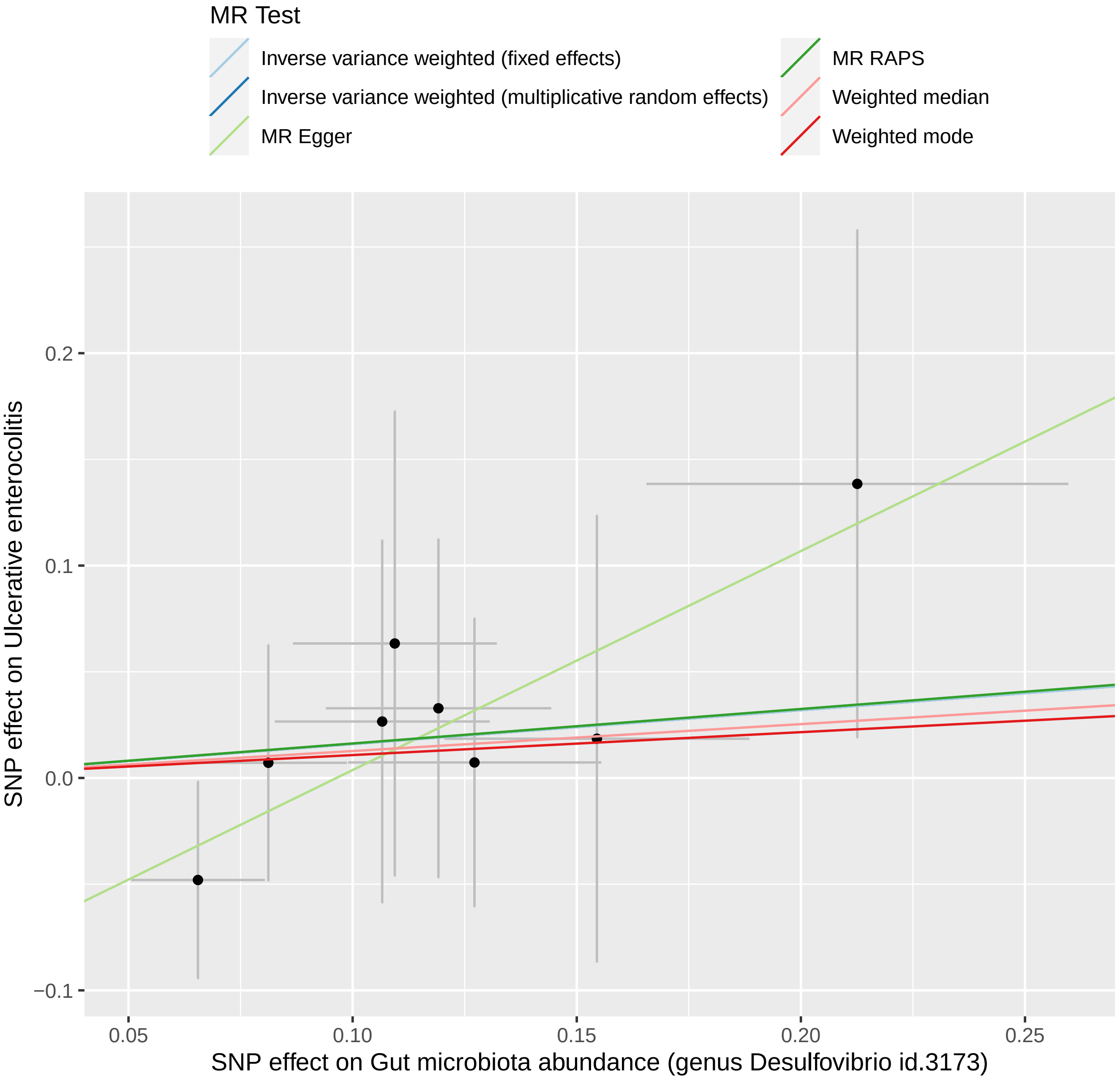

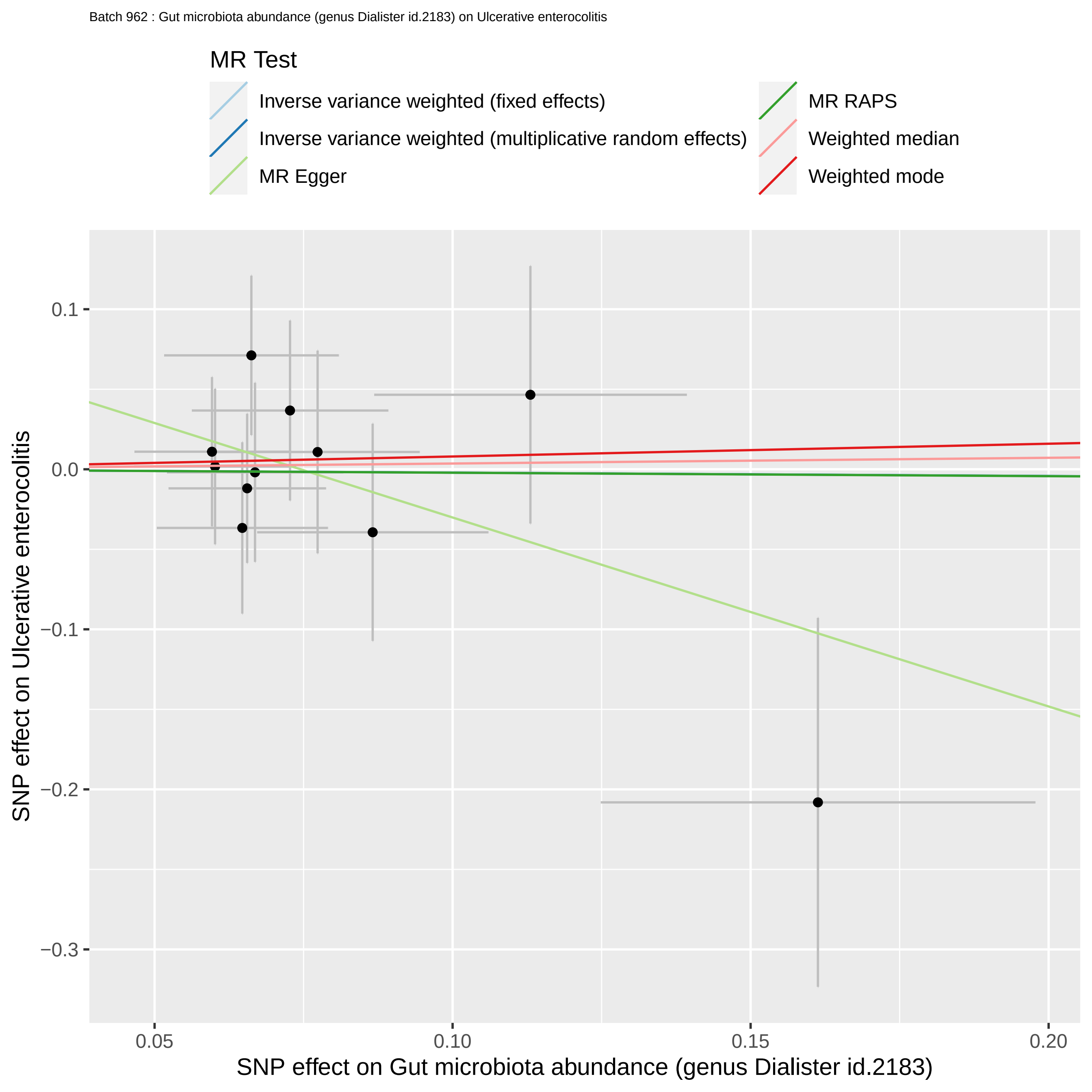

Batch 962 : Gut microbiota abundance (genus Dialister id.2183) on Ulcerative enterocolitis
MR Test
Inverse variance weighted (fixed effects)
Inverse variance weighted (multiplicative random effects)
MR Egger
MR RAPS
Weighted median
Weighted mode
SNP effect on Ulcerative enterocolitis
0.1
0.0
−0.1
−0.2
−0.3
0.05
0.10
0.15
0.20
SNP effect on Gut microbiota abundance (genus Dialister id.2183)

Batch 963 : Gut microbiota abundance (genus Dorea id.1997) on Ulcerative enterocolitis

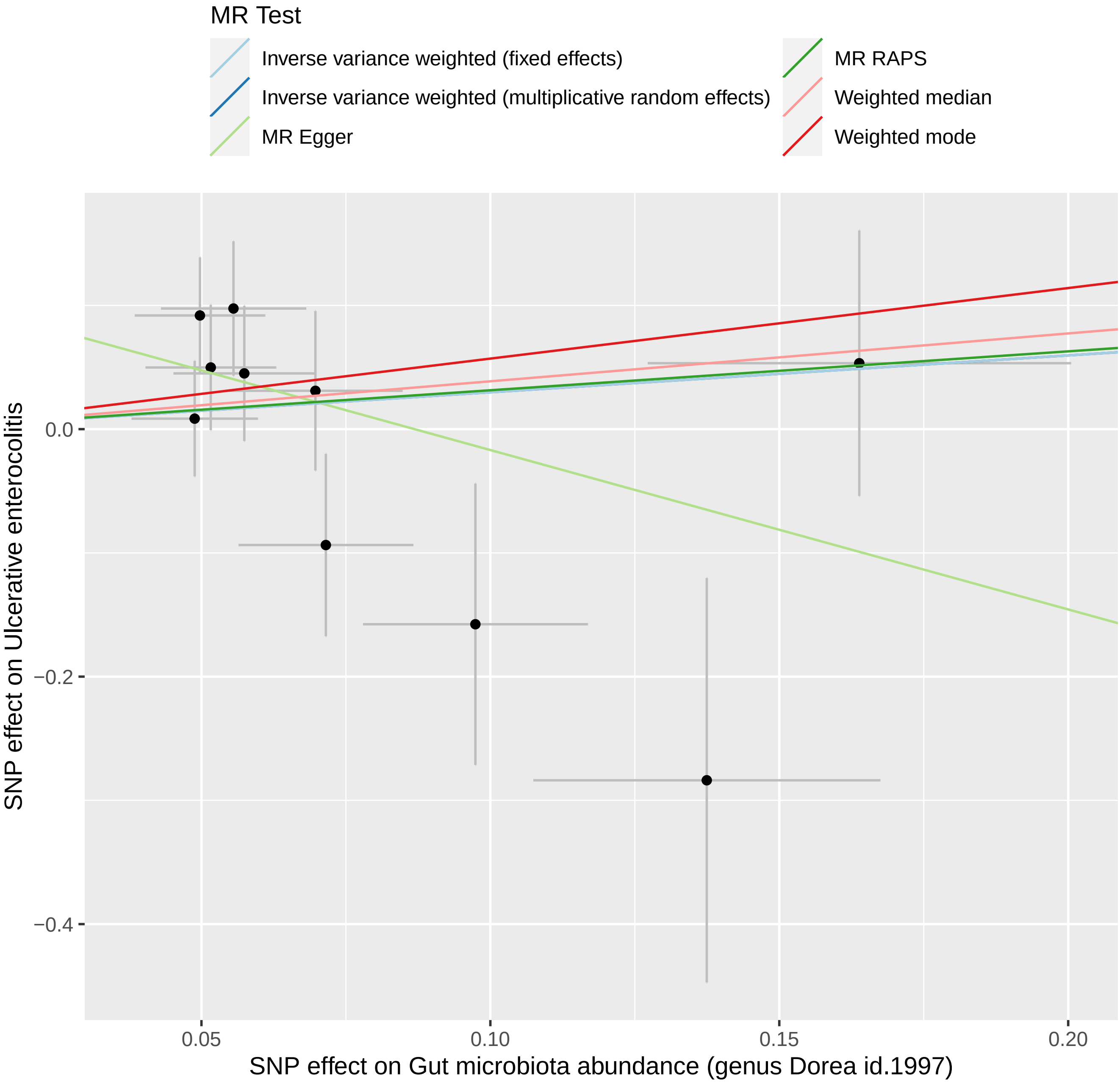

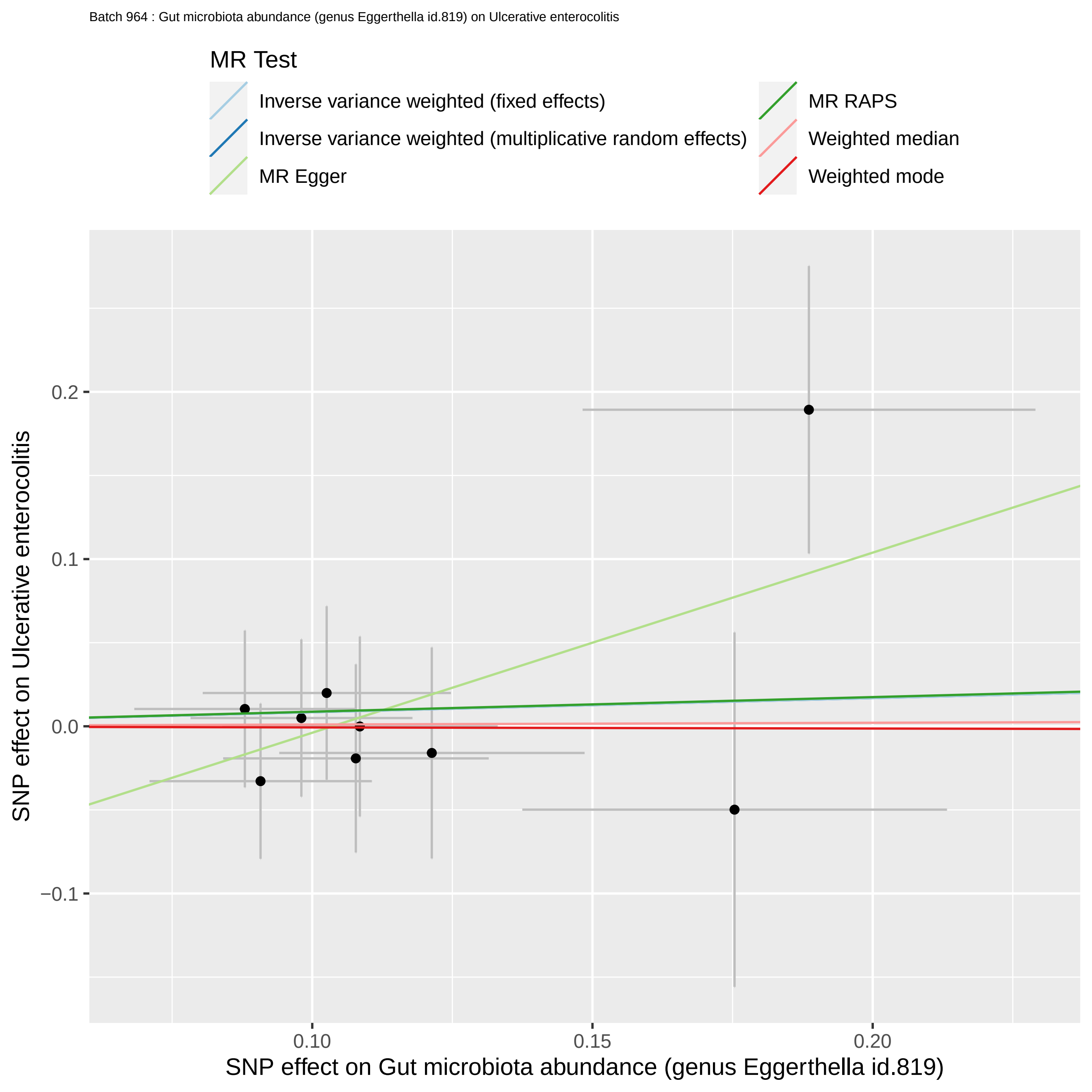
Batch 964 : Gut microbiota abundance (genus Eggerthella id.819) on Ulcerative enterocolitis
MR Test
Inverse variance weighted (fixed effects)
Inverse variance weighted (multiplicative random effects)
MR Egger
MR RAPS
Weighted median
Weighted mode
0.2
0.1
0.0
−0.1
0.10
0.15
0.20
SNP effect on Ulcerative enterocolitis
SNP effect on Gut microbiota abundance (genus Eggerthella id.819)

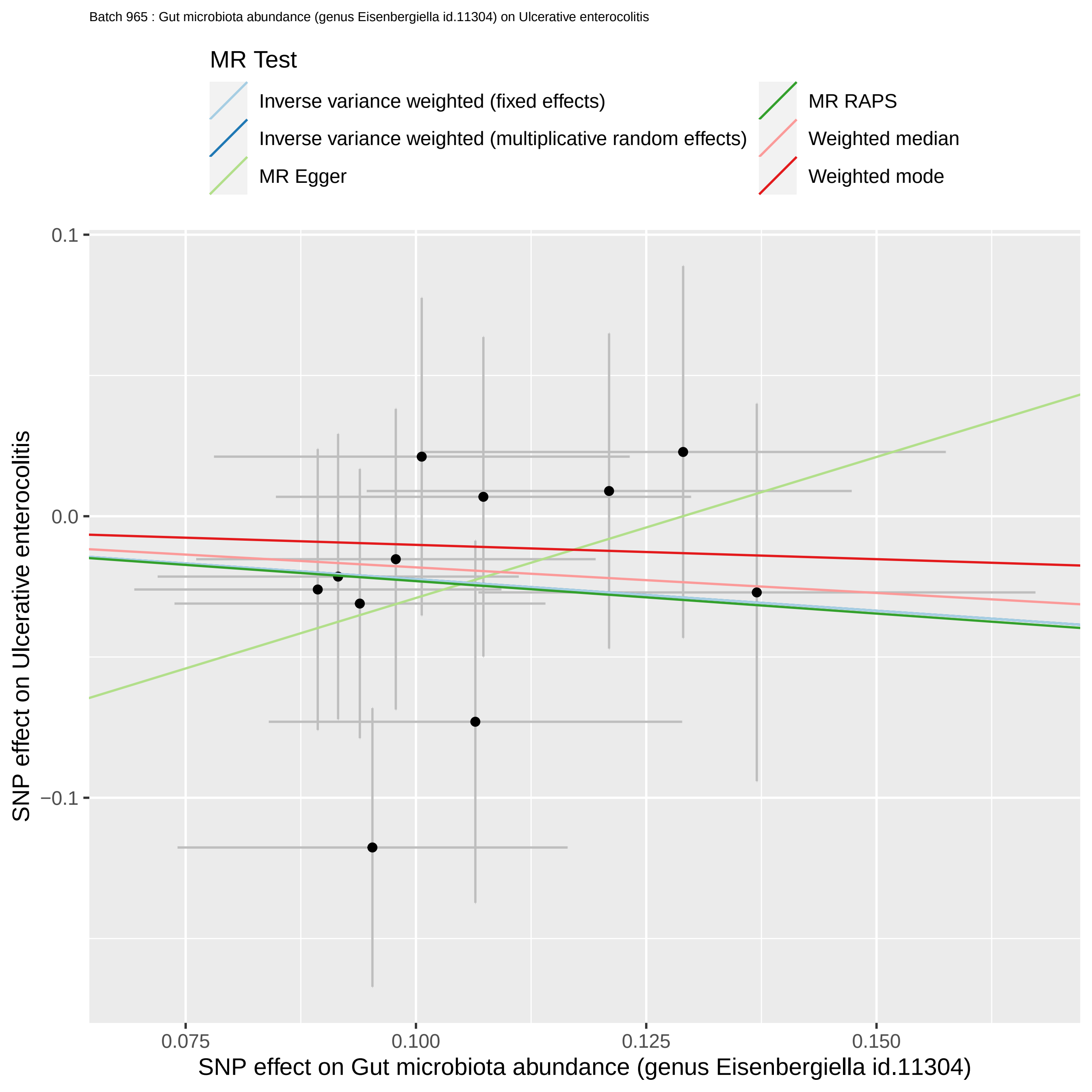
Batch 965 : Gut microbiota abundance (genus Eisenbergiella id.11304) on Ulcerative enterocolitis
MR Test
Inverse variance weighted (fixed effects)
Inverse variance weighted (multiplicative random effects)
MR Egger
MR RAPS
Weighted median
Weighted mode
0.1
0.0
−0.1
0.075
0.100
0.125
0.150
SNP effect on Ulcerative enterocolitis
SNP effect on Gut microbiota abundance (genus Eisenbergiella id.11304)

Batch 966 : Gut microbiota abundance (genus Enterorhabdus id.820) on Ulcerative enterocolitis

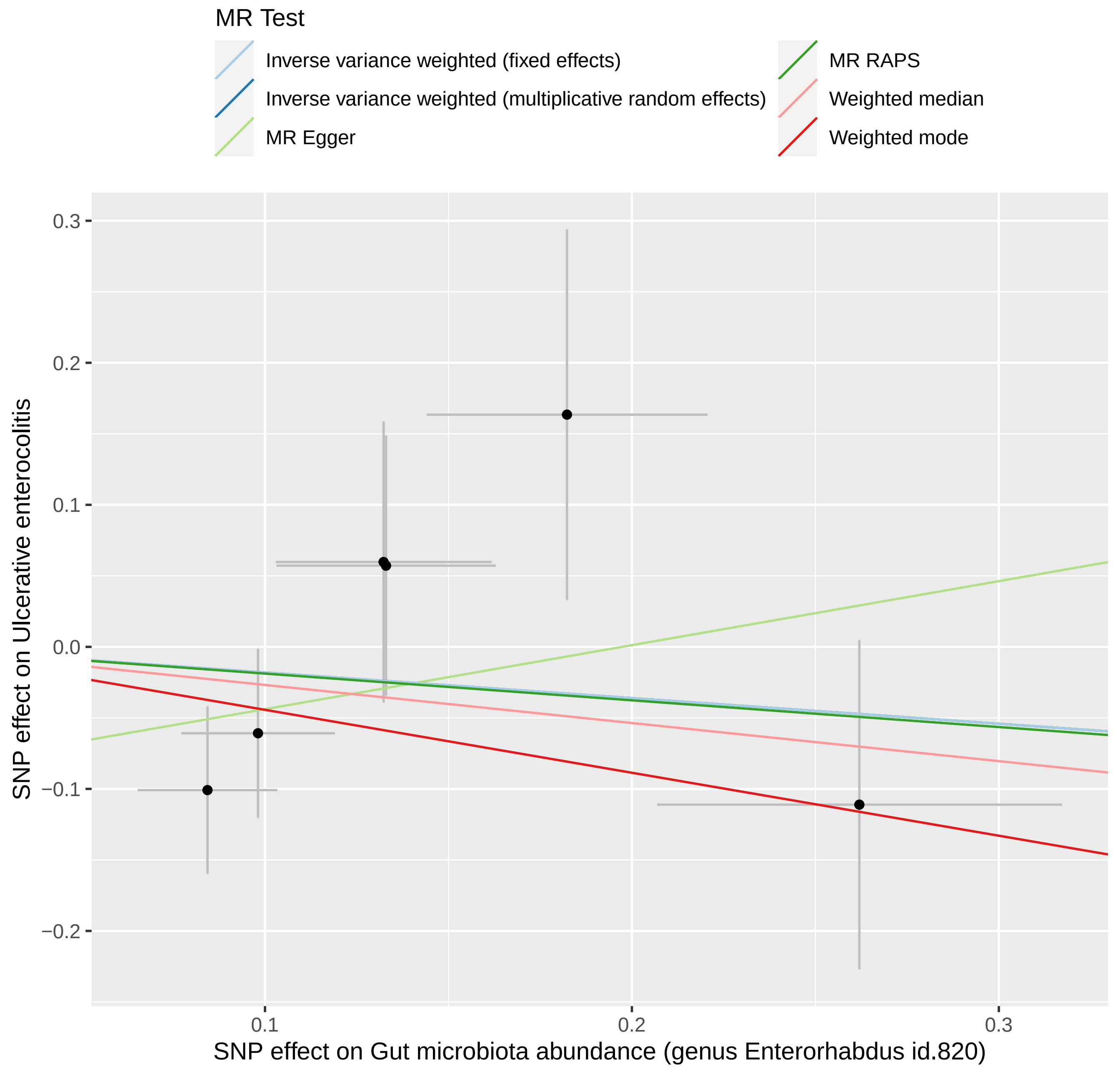

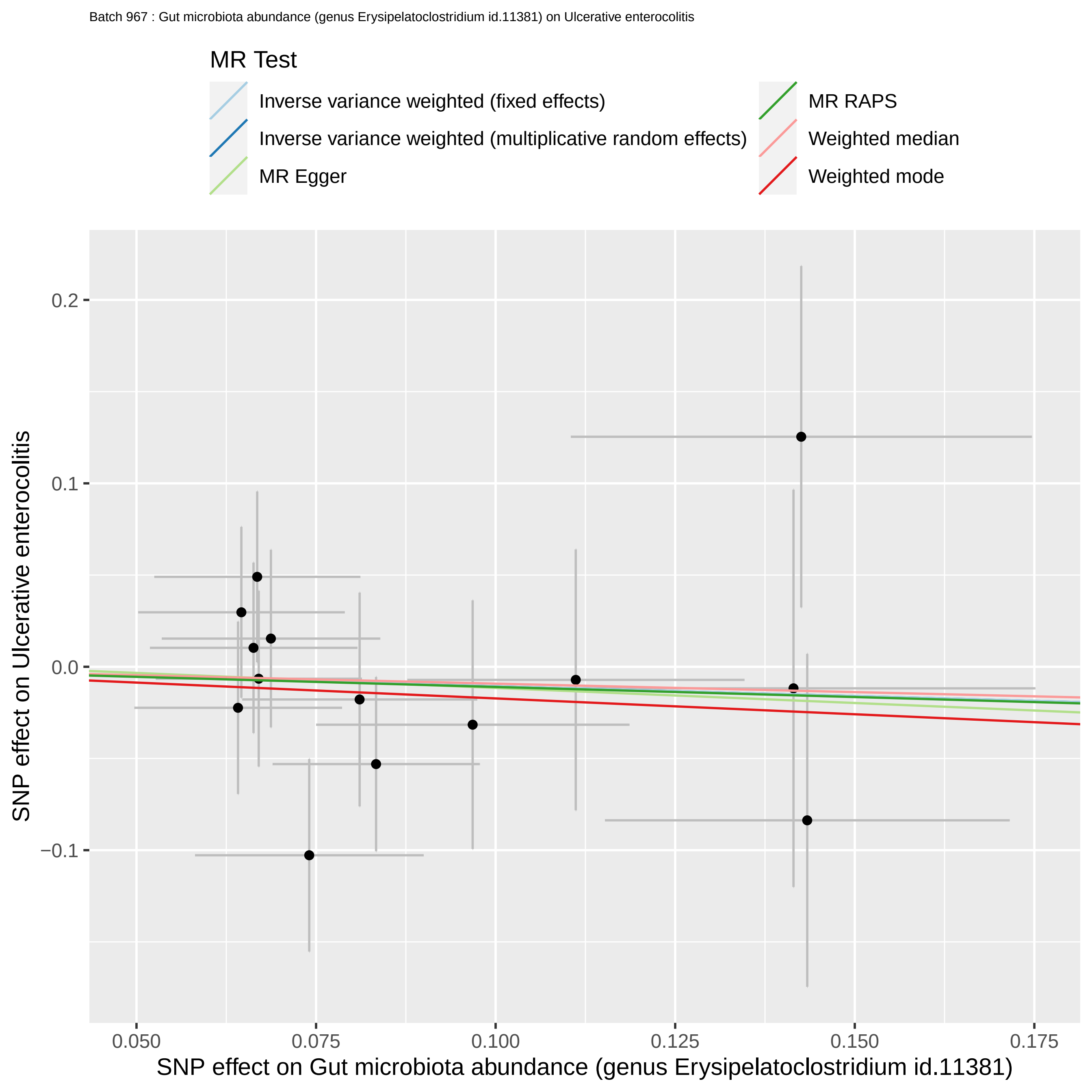

Batch 967 : Gut microbiota abundance (genus Erysipelatoclostridium id.11381) on Ulcerative enterocolitis
MR Test
Inverse variance weighted (fixed effects)
Inverse variance weighted (multiplicative random effects)
MR Egger
MR RAPS
Weighted median
Weighted mode
SNP effect on Ulcerative enterocolitis
0.2
0.1
0.0
−0.1
0.050
0.075
0.100
0.125
0.150
0.175
SNP effect on Gut microbiota abundance (genus Erysipelatoclostridium id.11381)

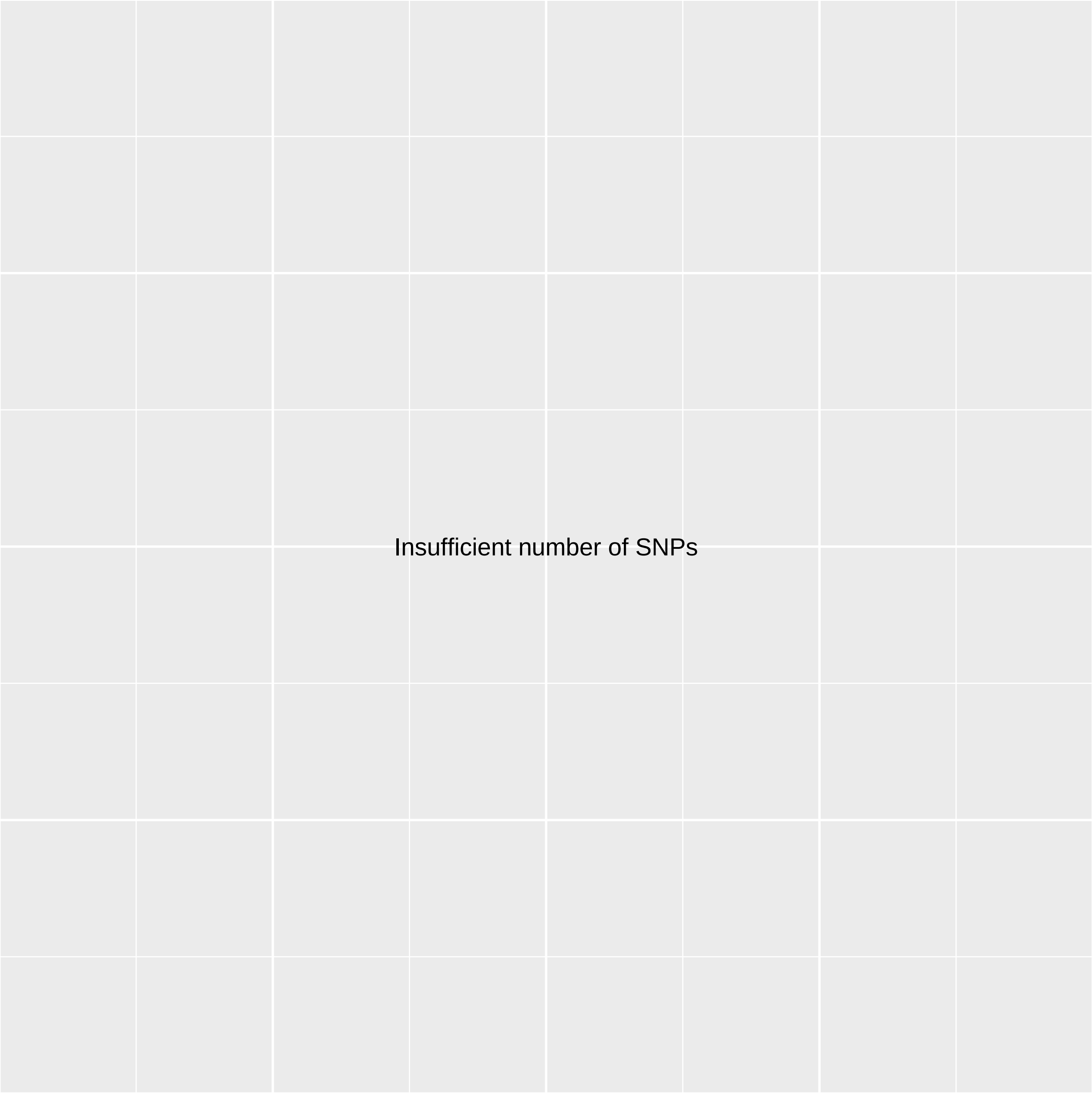
Insufficient number of SNPs

Batch 969 : Gut microbiota abundance (genus Escherichia Shigella id.3504) on Ulcerative enterocolitis

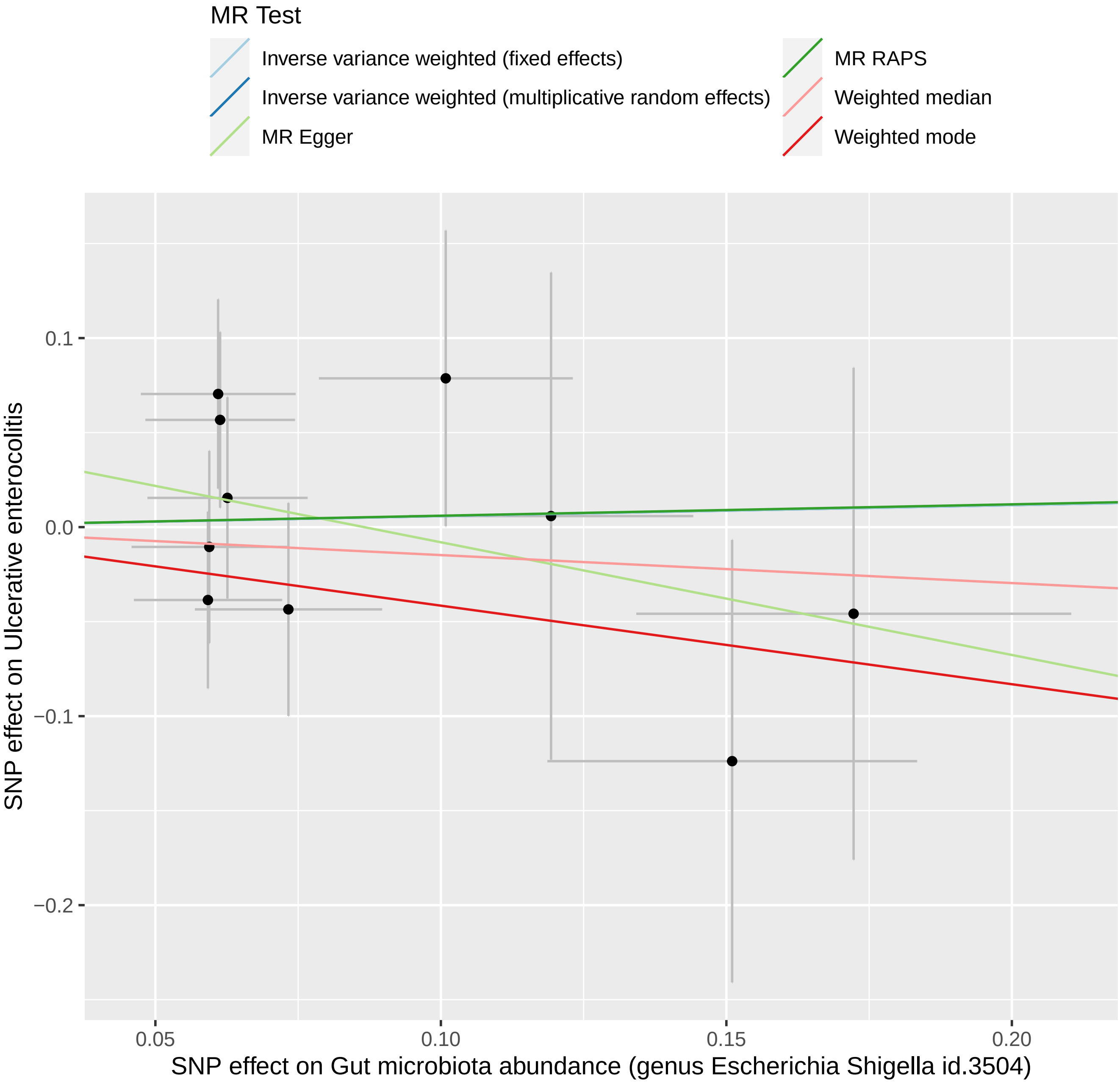

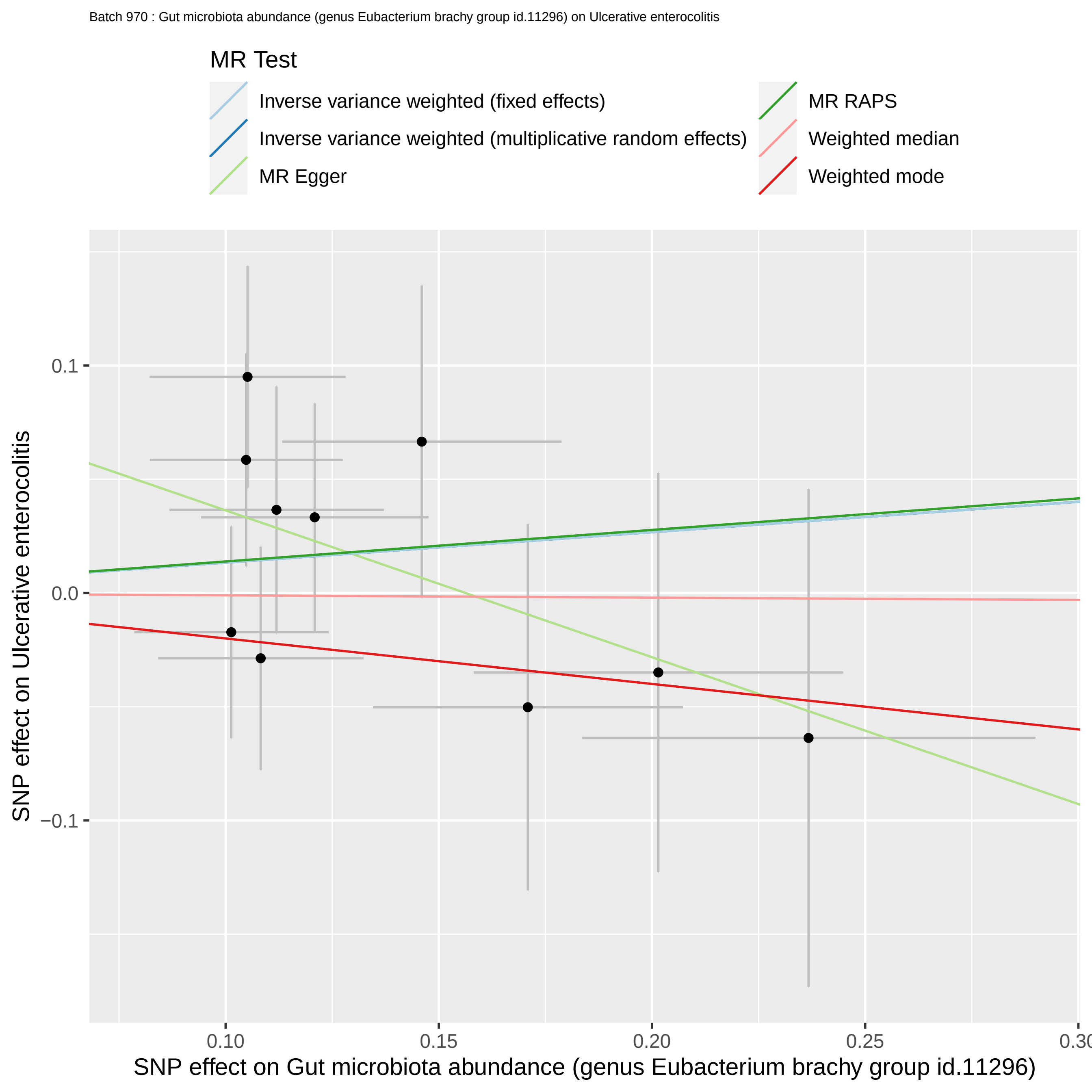

Batch 970 : Gut microbiota abundance (genus Eubacterium brachy group id.11296) on Ulcerative enterocolitis
MR Test
Inverse variance weighted (fixed effects)
Inverse variance weighted (multiplicative random effects)
MR Egger
MR RAPS
Weighted median
Weighted mode
0.1
0.0
−0.1
0.10
0.15
0.20
0.25
0.30
SNP effect on Ulcerative enterocolitis
SNP effect on Gut microbiota abundance (genus Eubacterium brachy group id.11296)

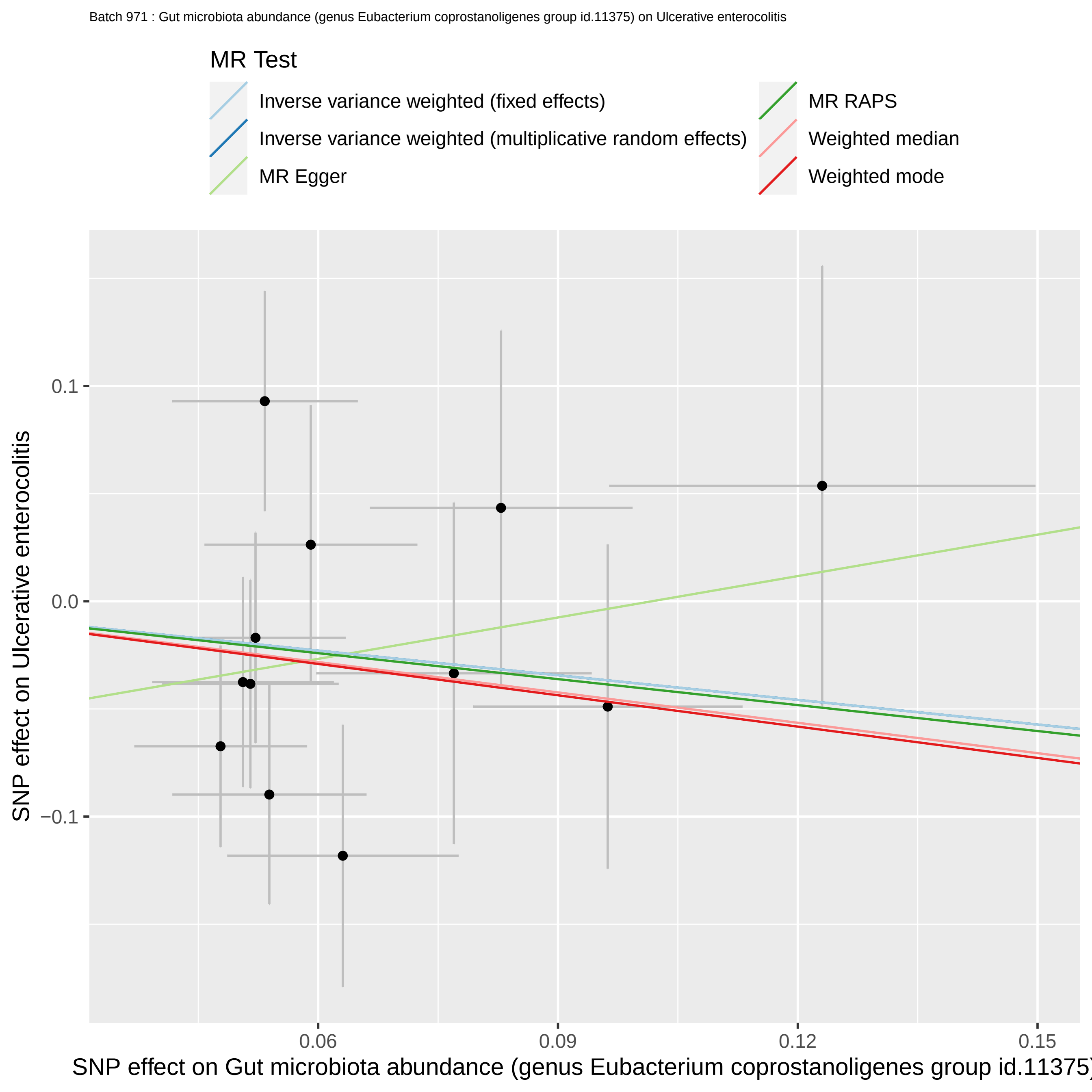
Batch 971 : Gut microbiota abundance (genus Eubacterium coprostanoligenes group id.11375) on Ulcerative enterocolitis
MR Test
Inverse variance weighted (fixed effects)
Inverse variance weighted (multiplicative random effects)
MR Egger
MR RAPS
Weighted median
Weighted mode
SNP effect on Ulcerative enterocolitis
0.1
0.0
−0.1
0.06
0.09
0.12
0.15
SNP effect on Gut microbiota abundance (genus Eubacterium coprostanoligenes group id.11375)

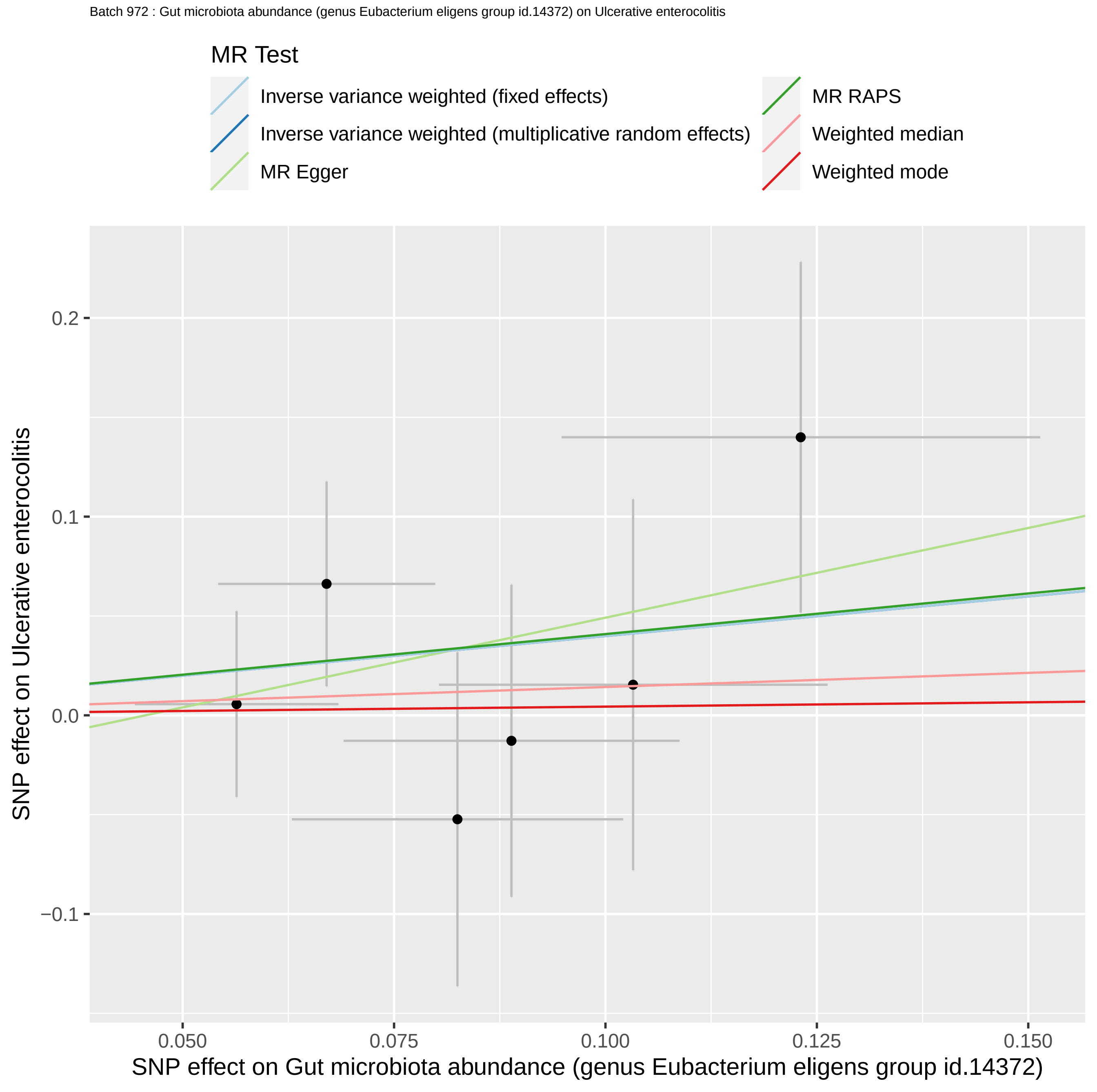

Batch 972 : Gut microbiota abundance (genus Eubacterium eligens group id.14372) on Ulcerative enterocolitis
MR Test
Inverse variance weighted (fixed effects)
Inverse variance weighted (multiplicative random effects)
MR Egger
MR RAPS
Weighted median
Weighted mode
0.2
0.1
0.0
−0.1
0.050
0.075
0.100
0.125
0.150
SNP effect on Ulcerative enterocolitis
SNP effect on Gut microbiota abundance (genus Eubacterium eligens group id.14372)

Batch 973 : Gut microbiota abundance (genus Eubacterium fissicatena group id.14373) on Ulcerative enterocolitis

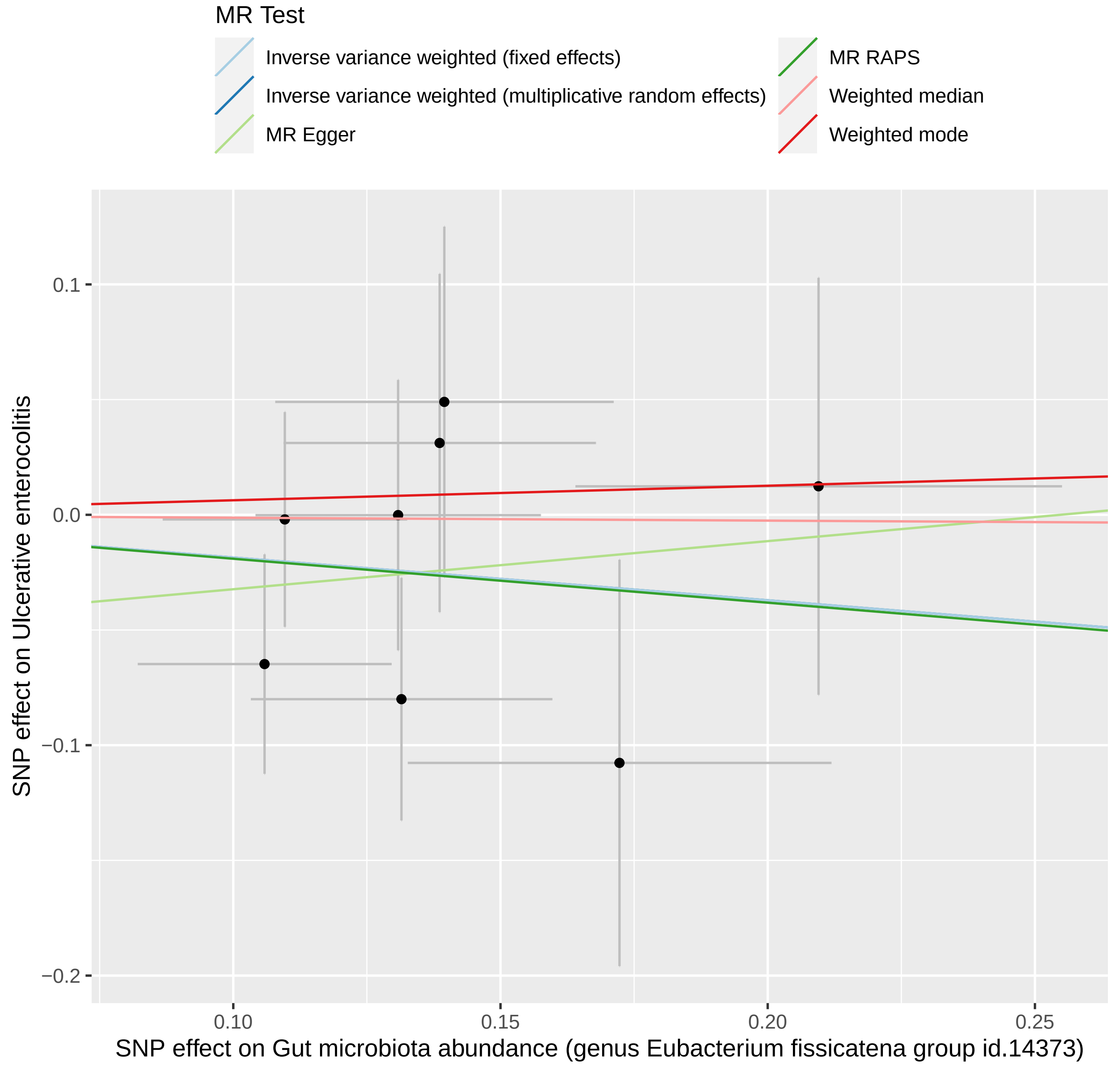

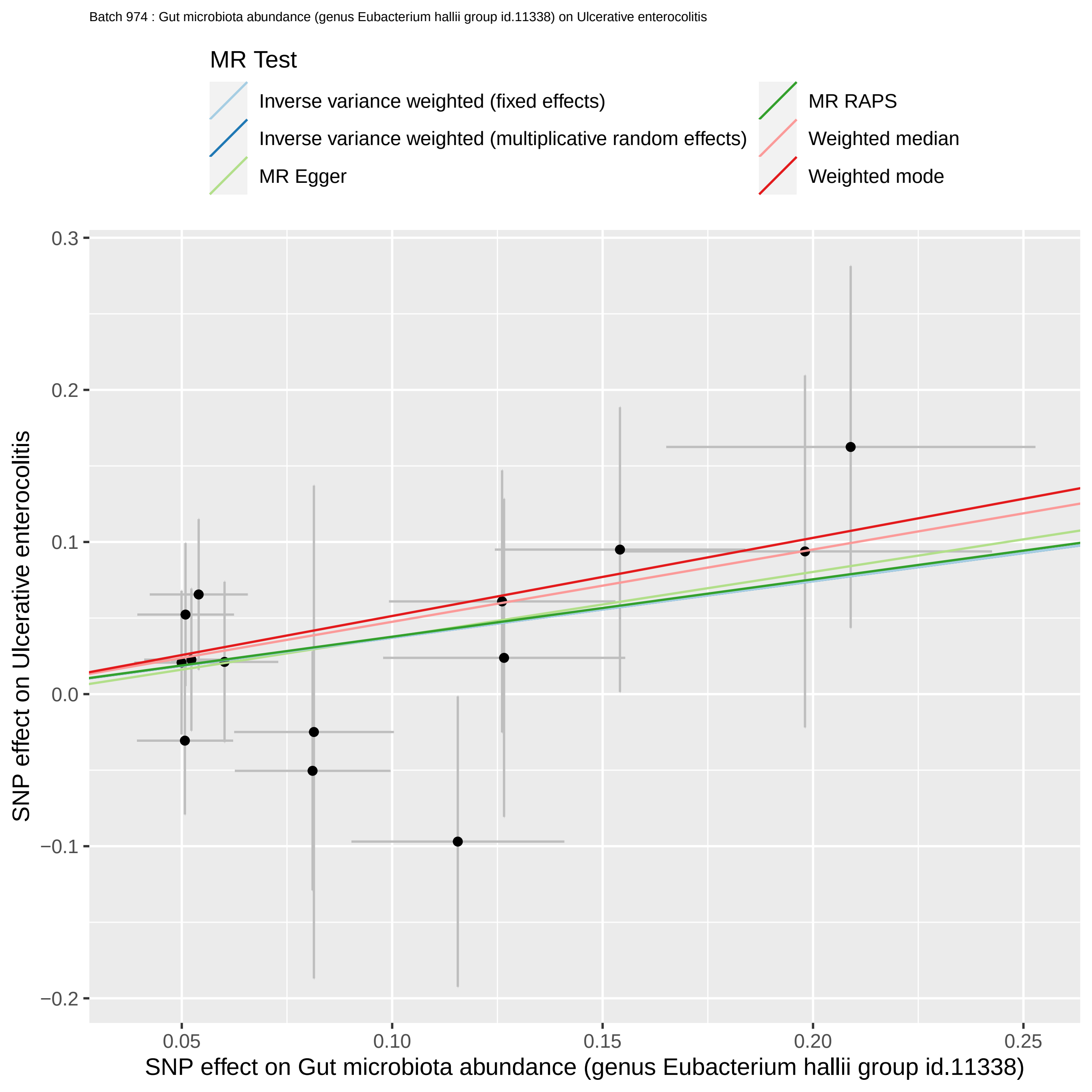

Batch 974 : Gut microbiota abundance (genus Eubacterium hallii group id.11338) on Ulcerative enterocolitis
MR Test
Inverse variance weighted (fixed effects)
Inverse variance weighted (multiplicative random effects)
MR Egger
MR RAPS
Weighted median
Weighted mode
0.3
0.2
0.1
0.0
−0.1
−0.2
0.05
0.10
0.15
0.20
0.25
SNP effect on Ulcerative enterocolitis
SNP effect on Gut microbiota abundance (genus Eubacterium hallii group id.11338)

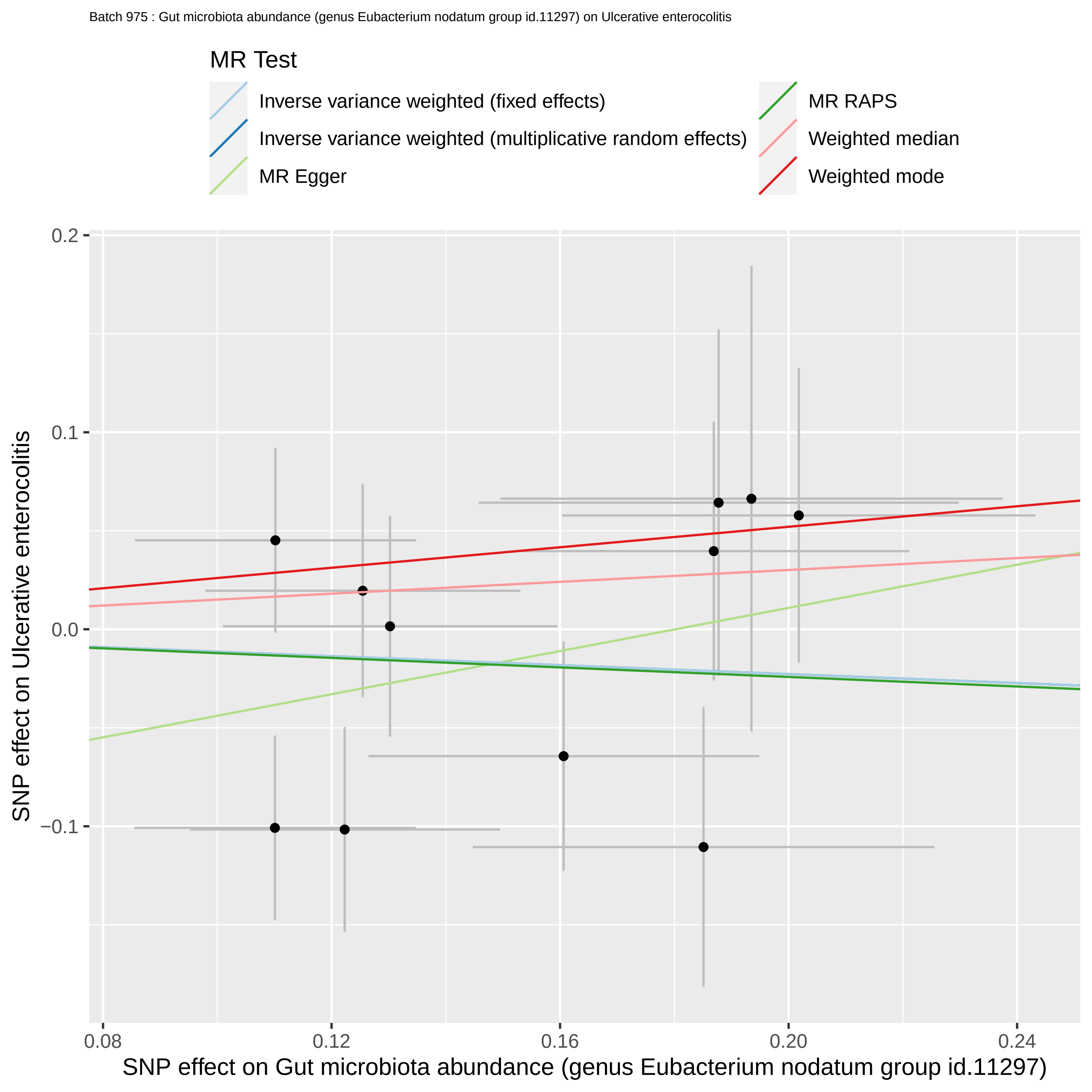

Batch 975 : Gut microbiota abundance (genus Eubacterium nodatum group id.11297) on Ulcerative enterocolitis
MR Test
Inverse variance weighted (fixed effects)
Inverse variance weighted (multiplicative random effects)
MR Egger
MR RAPS
Weighted median
Weighted mode
0.2
0.1
0.0
−0.1
0.08
0.12
0.16
0.20
0.24
SNP effect on Ulcerative enterocolitis
SNP effect on Gut microbiota abundance (genus Eubacterium nodatum group id.11297)

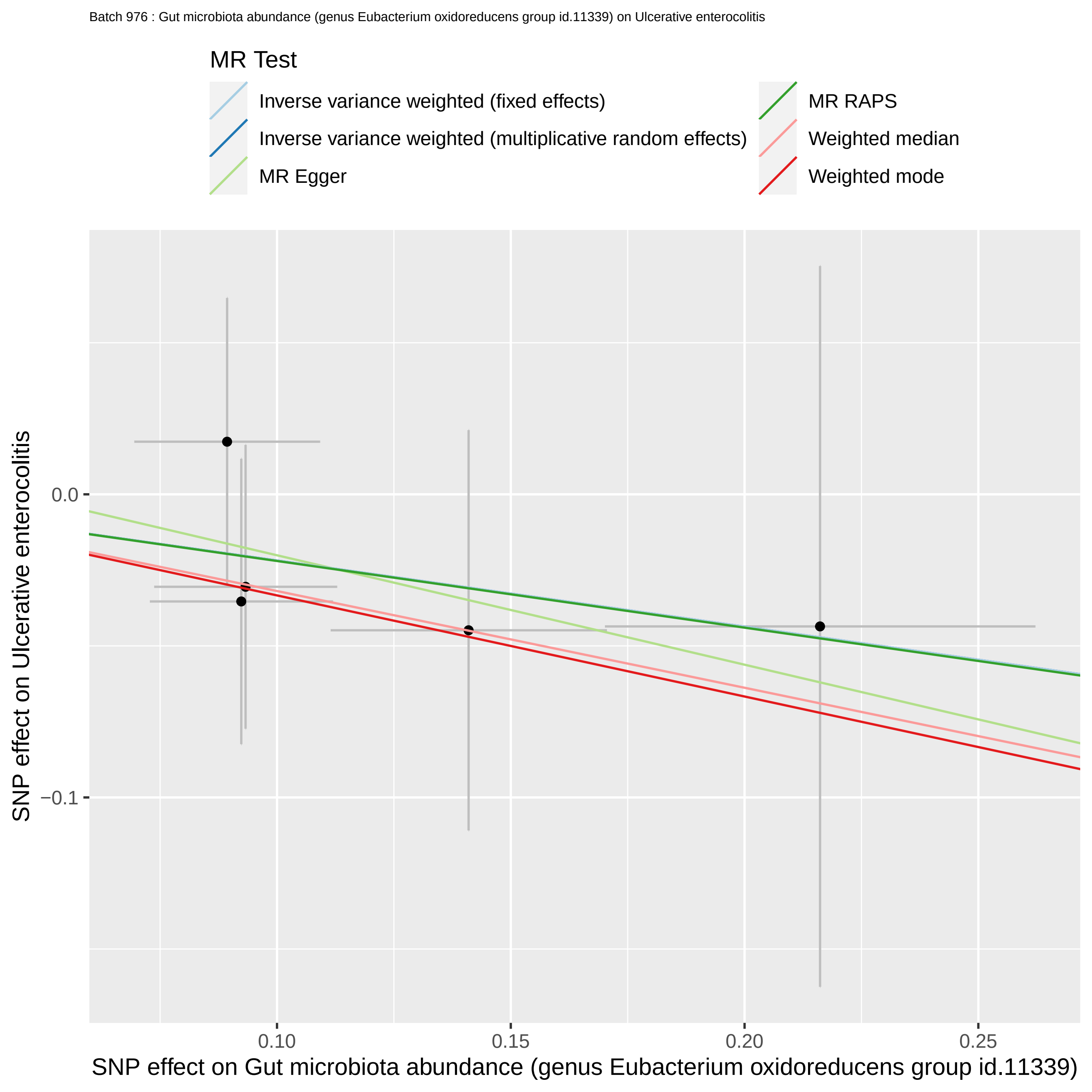

Batch 976 : Gut microbiota abundance (genus Eubacterium oxidoreducens group id.11339) on Ulcerative enterocolitis
MR Test
Inverse variance weighted (fixed effects)
Inverse variance weighted (multiplicative random effects)
MR Egger
MR RAPS
Weighted median
Weighted mode
0.0
−0.1
0.10
0.15
0.20
0.25
SNP effect on Ulcerative enterocolitis
SNP effect on Gut microbiota abundance (genus Eubacterium oxidoreducens group id.11339)

Batch 977 : Gut microbiota abundance (genus Eubacterium rectale group id.14374) on Ulcerative enterocolitis

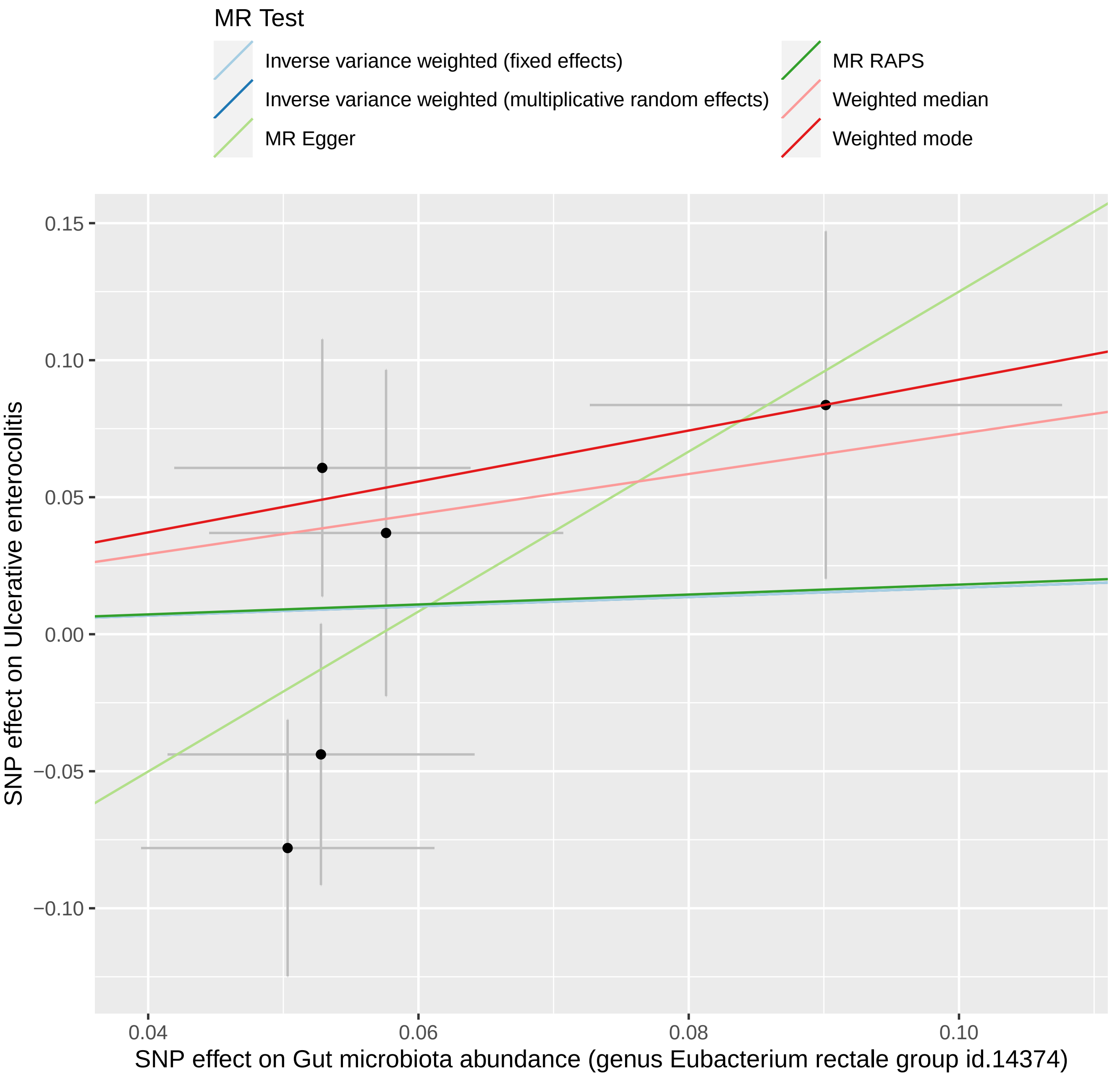

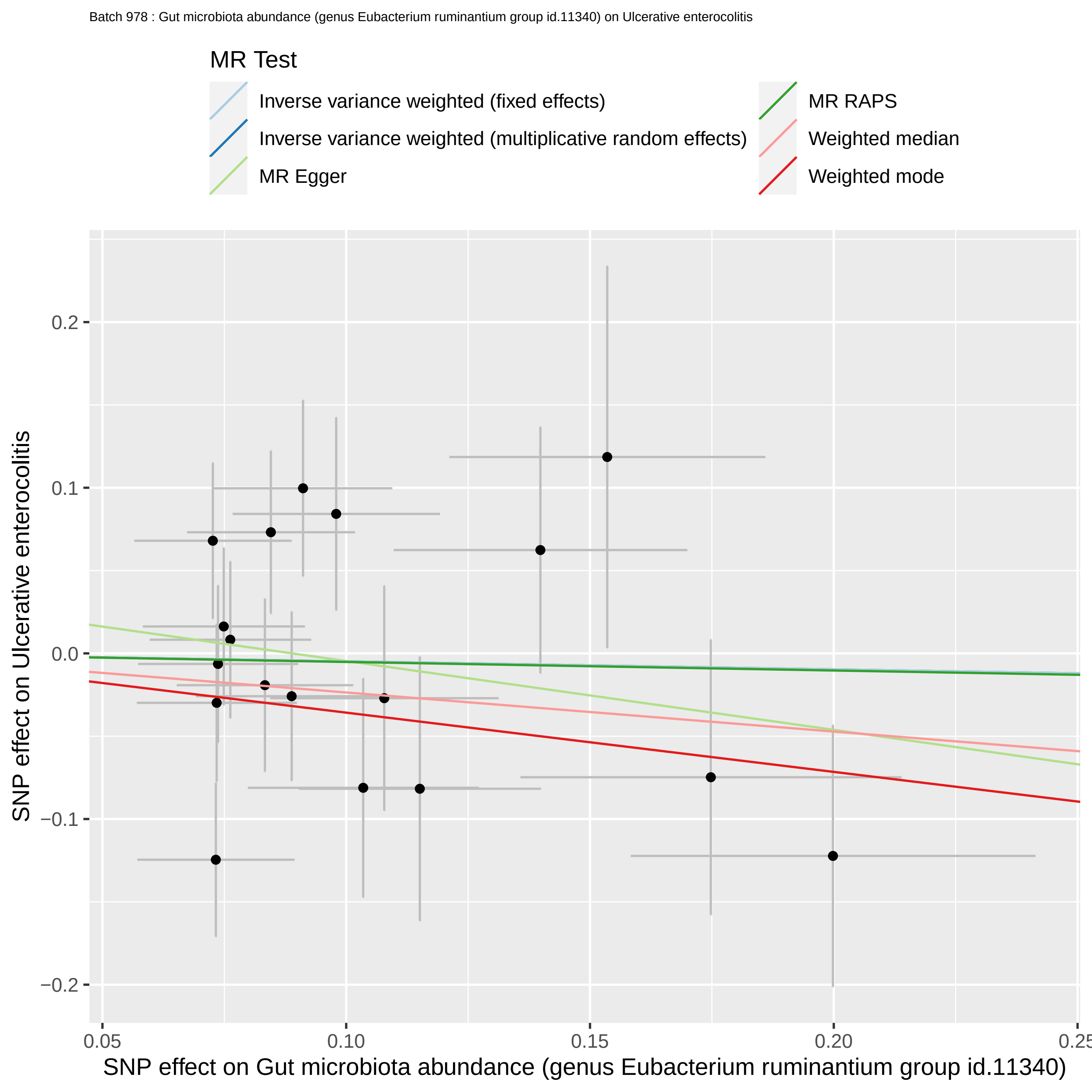
Batch 978 : Gut microbiota abundance (genus Eubacterium ruminantium group id.11340) on Ulcerative enterocolitis
MR Test
Inverse variance weighted (fixed effects)
Inverse variance weighted (multiplicative random effects)
MR Egger
MR RAPS
Weighted median
Weighted mode
0.2
0.1
0.0
−0.1
−0.2
0.05
0.10
0.15
0.20
0.25
SNP effect on Ulcerative enterocolitis
SNP effect on Gut microbiota abundance (genus Eubacterium ruminantium group id.11340)

Batch 979 : Gut microbiota abundance (genus Eubacterium ventriosum group id.11341) on Ulcerative enterocolitis

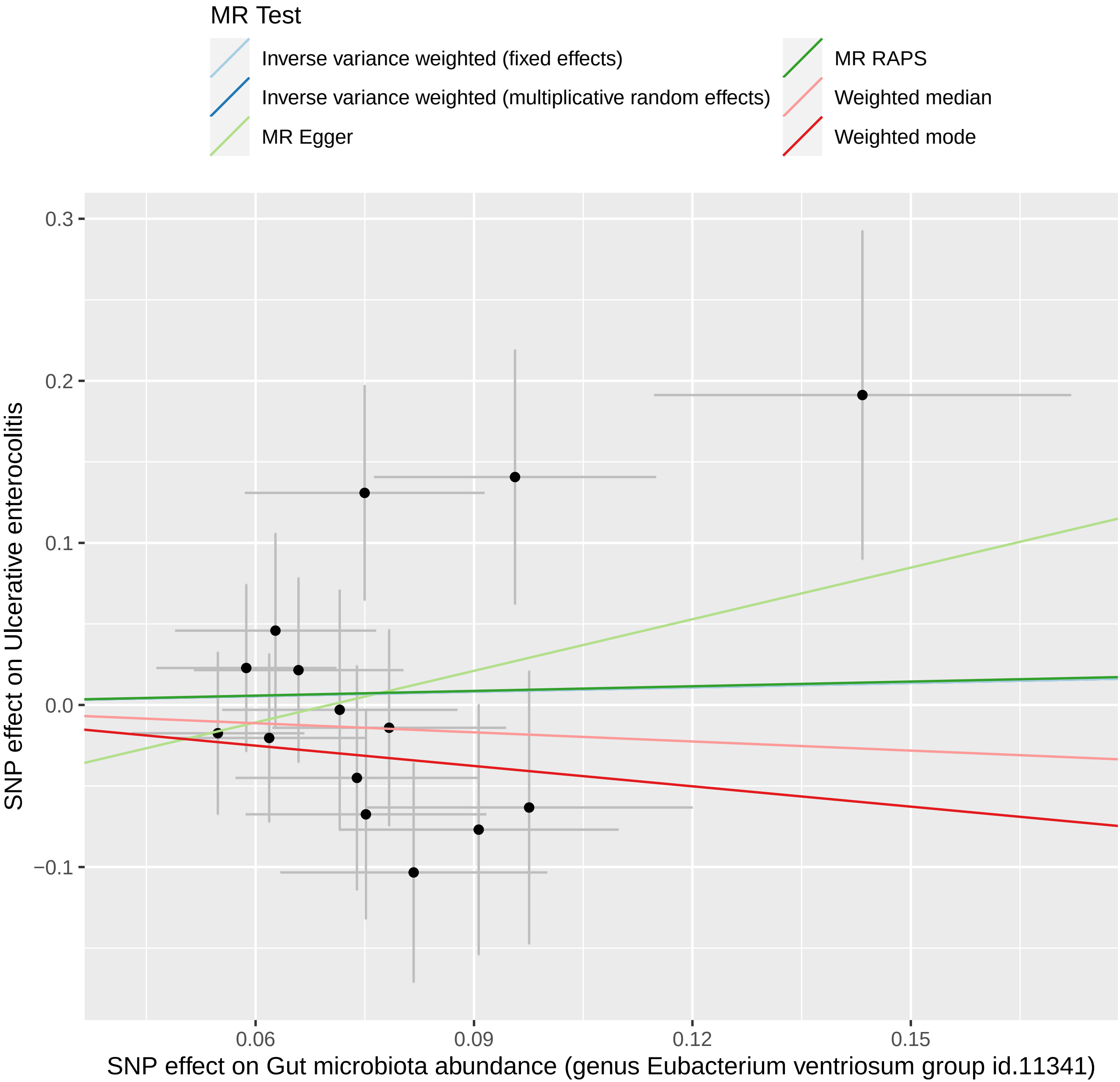

Batch 980 : Gut microbiota abundance (genus Eubacterium xylanophilum group id.14375) on Ulcerative enterocolitis

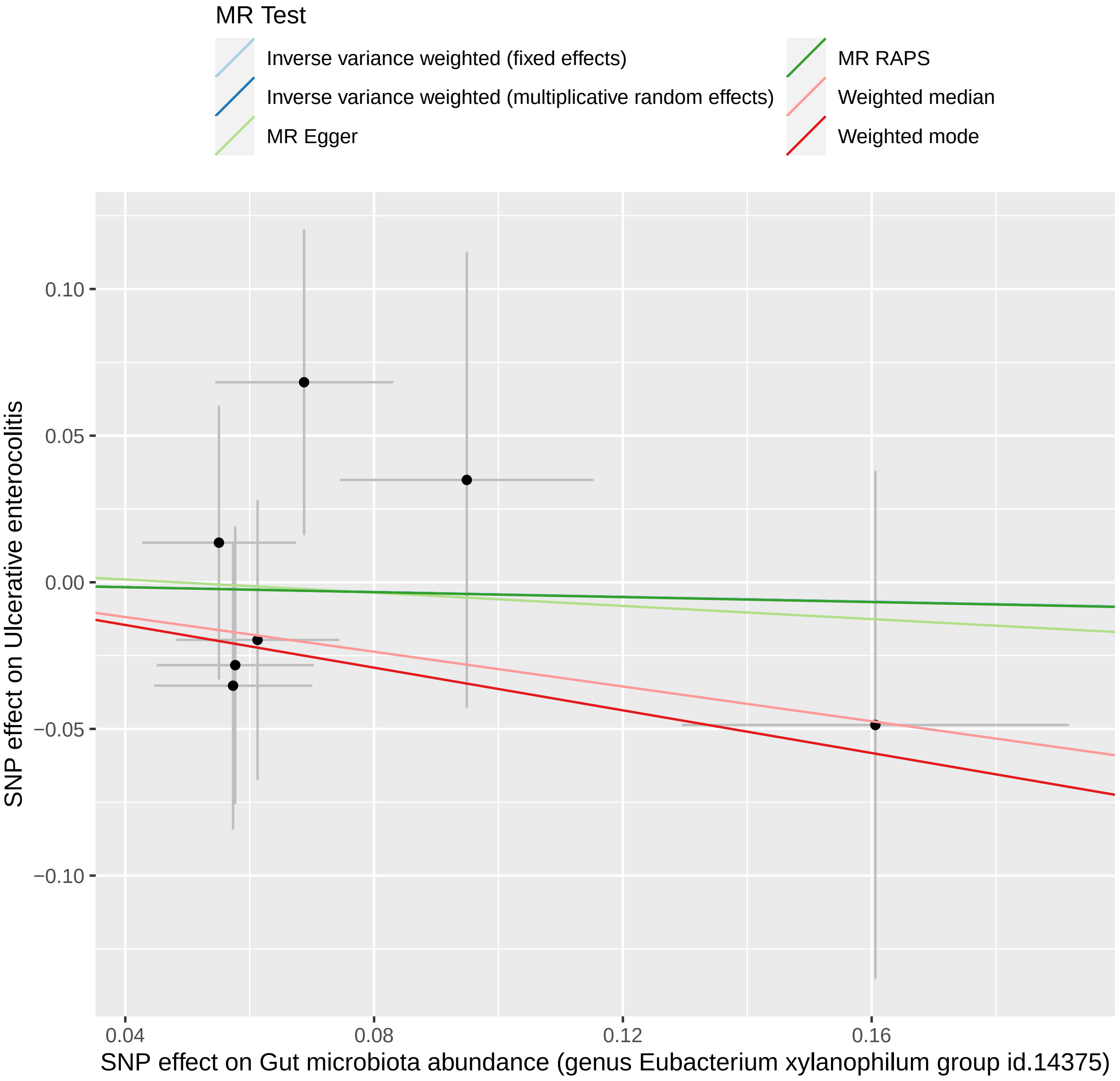

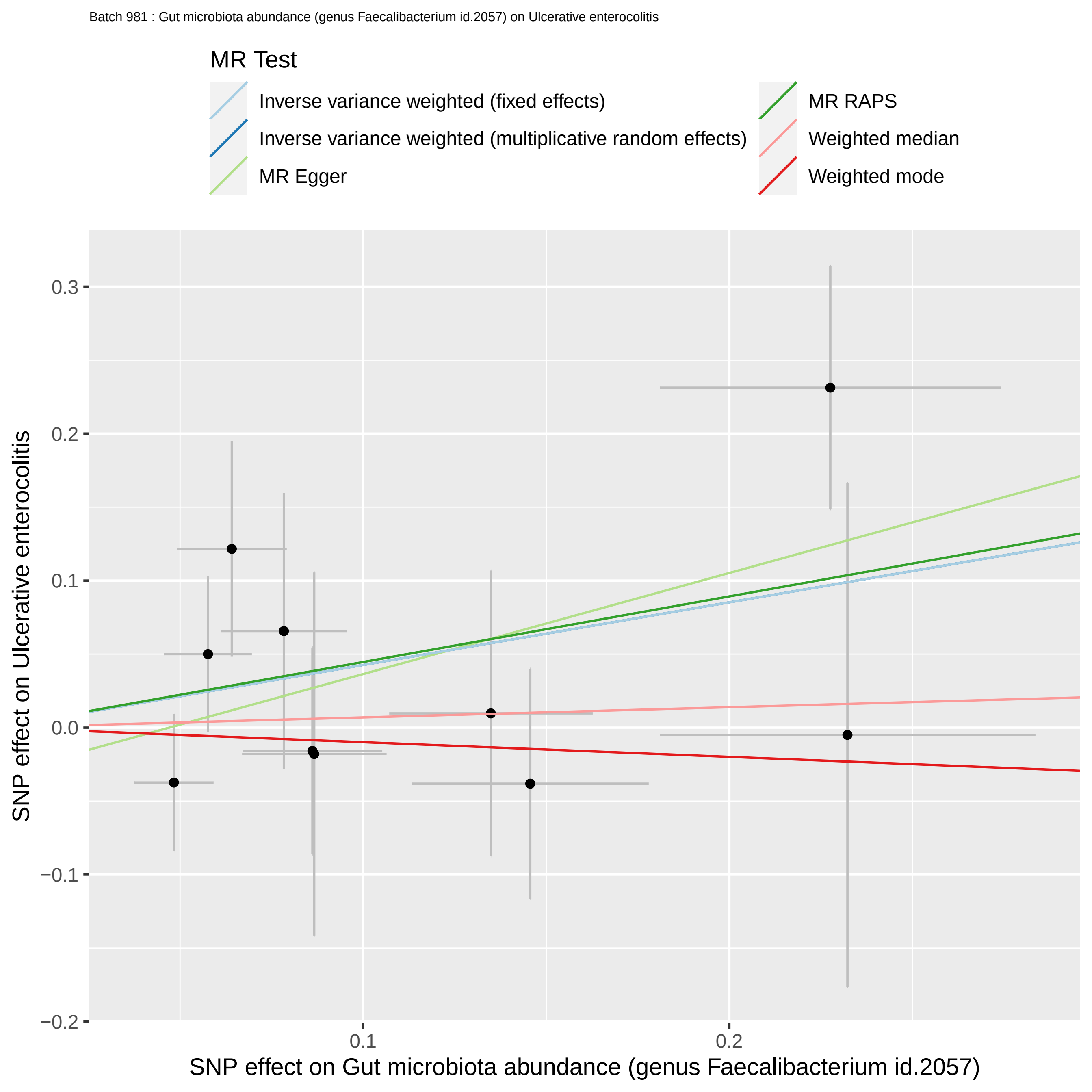
Batch 981 : Gut microbiota abundance (genus Faecalibacterium id.2057) on Ulcerative enterocolitis
MR Test
Inverse variance weighted (fixed effects)
Inverse variance weighted (multiplicative random effects)
MR Egger
MR RAPS
Weighted median
Weighted mode
0.3
0.2
0.1
0.0
−0.1
−0.2
0.1
0.2
SNP effect on Ulcerative enterocolitis
SNP effect on Gut microbiota abundance (genus Faecalibacterium id.2057)

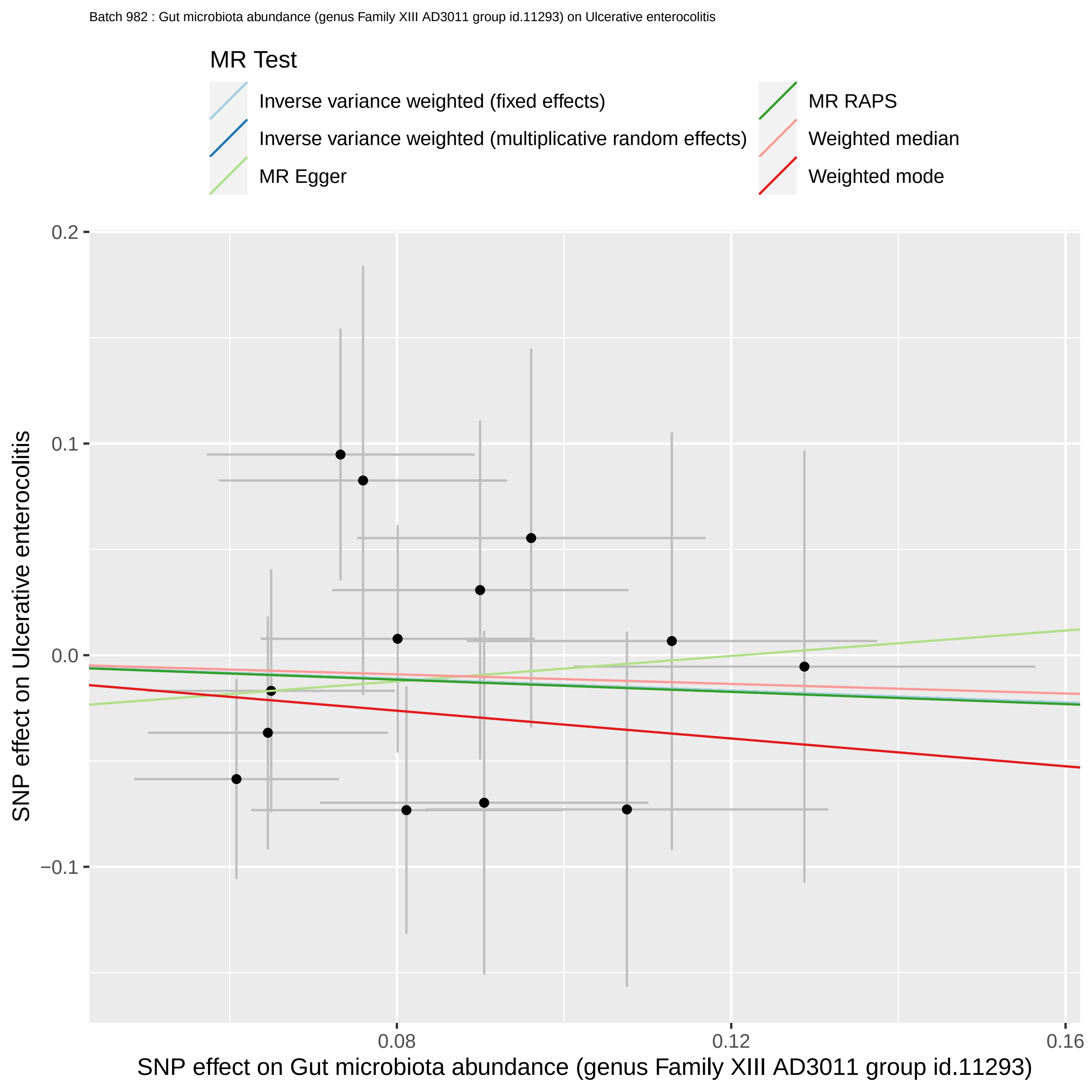

Batch 982 : Gut microbiota abundance (genus Family XIII AD3011 group id.11293) on Ulcerative enterocolitis
MR Test
Inverse variance weighted (fixed effects)
Inverse variance weighted (multiplicative random effects)
MR Egger
MR RAPS
Weighted median
Weighted mode
0.2
0.1
0.0
−0.1
0.08
0.12
0.16
SNP effect on Ulcerative enterocolitis
SNP effect on Gut microbiota abundance (genus Family XIII AD3011 group id.11293)

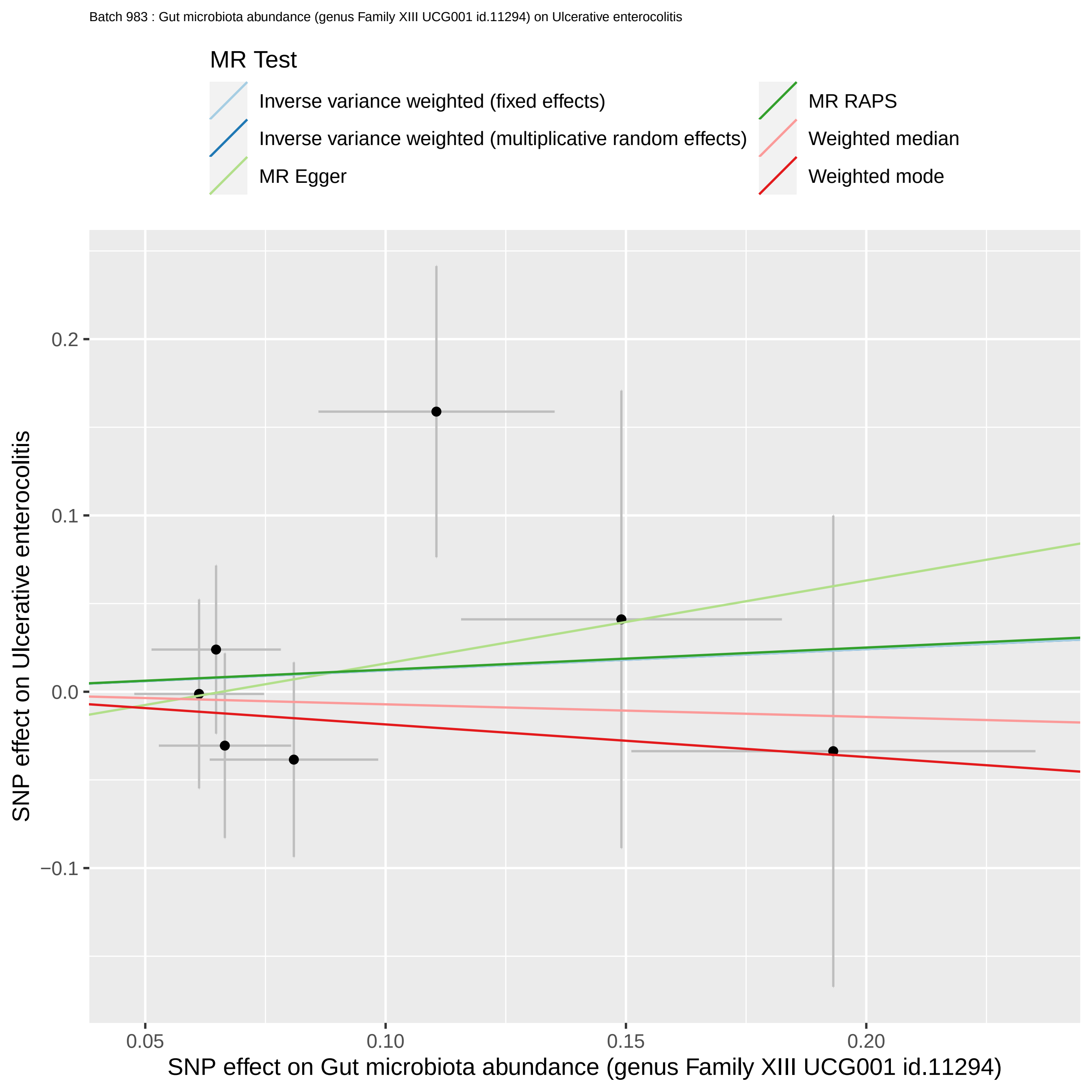
Batch 983 : Gut microbiota abundance (genus Family XIII UCG001 id.11294) on Ulcerative enterocolitis
MR Test
Inverse variance weighted (fixed effects)
Inverse variance weighted (multiplicative random effects)
MR Egger
MR RAPS
Weighted median
Weighted mode
0.2
0.1
0.0
−0.1
0.05
0.10
0.15
0.20
SNP effect on Ulcerative enterocolitis
SNP effect on Gut microbiota abundance (genus Family XIII UCG001 id.11294)

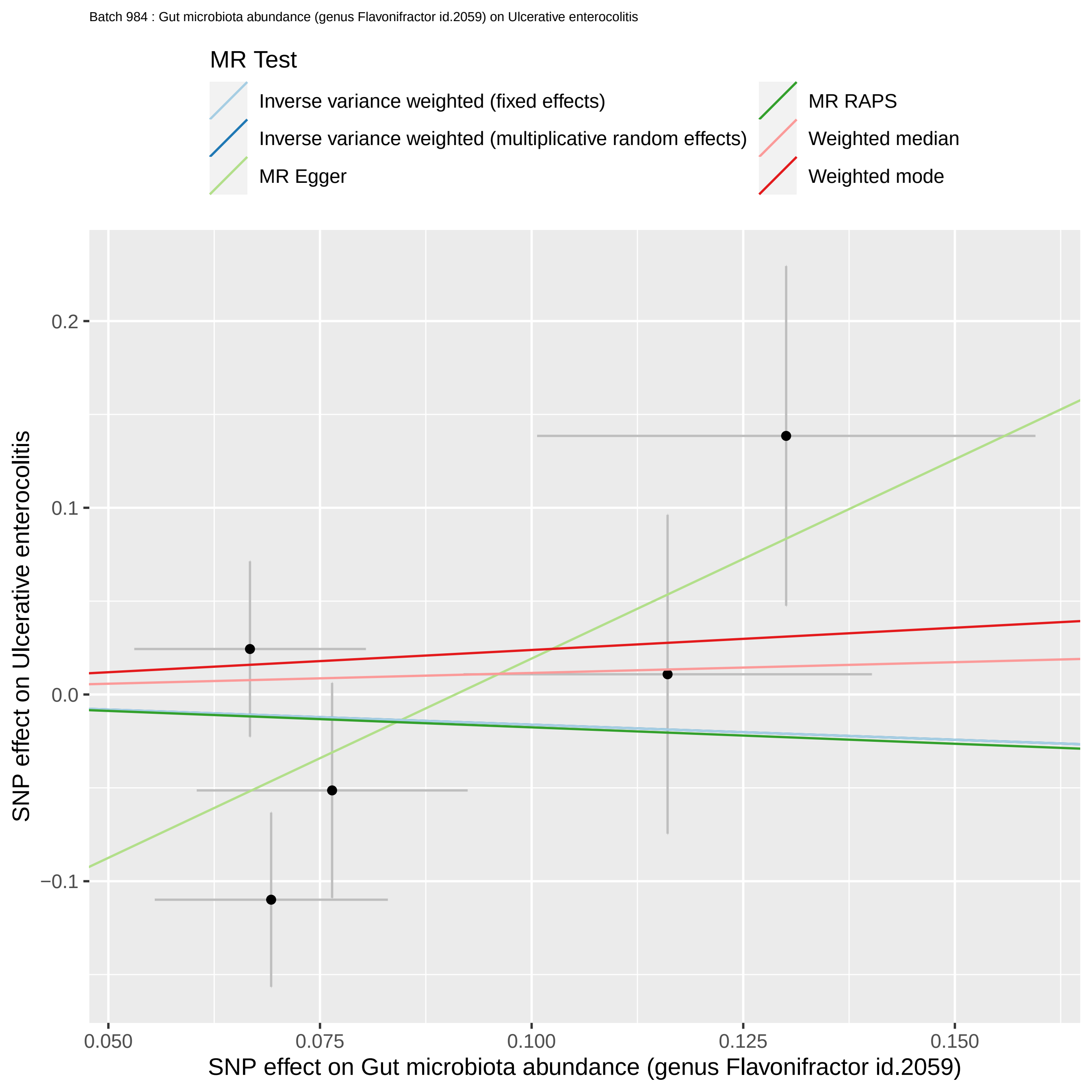
Batch 984 : Gut microbiota abundance (genus Flavonifractor id.2059) on Ulcerative enterocolitis
MR Test
Inverse variance weighted (fixed effects)
Inverse variance weighted (multiplicative random effects)
MR Egger
MR RAPS
Weighted median
Weighted mode
0.2
0.1
0.0
−0.1
0.050
0.075
0.100
0.125
0.150
SNP effect on Ulcerative enterocolitis
SNP effect on Gut microbiota abundance (genus Flavonifractor id.2059)

Batch 985 : Gut microbiota abundance (genus Fusicatenibacter id.11305) on Ulcerative enterocolitis

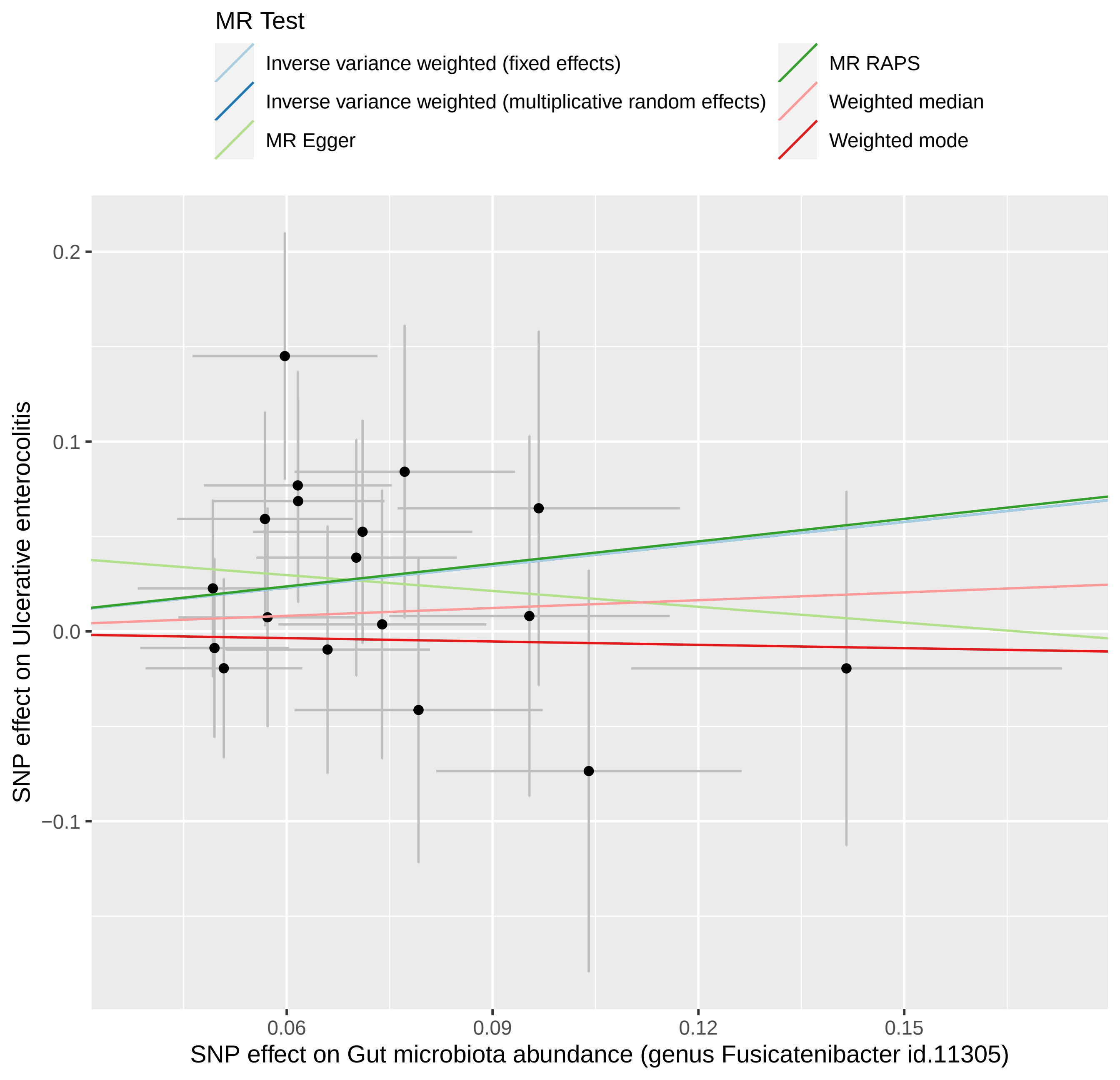

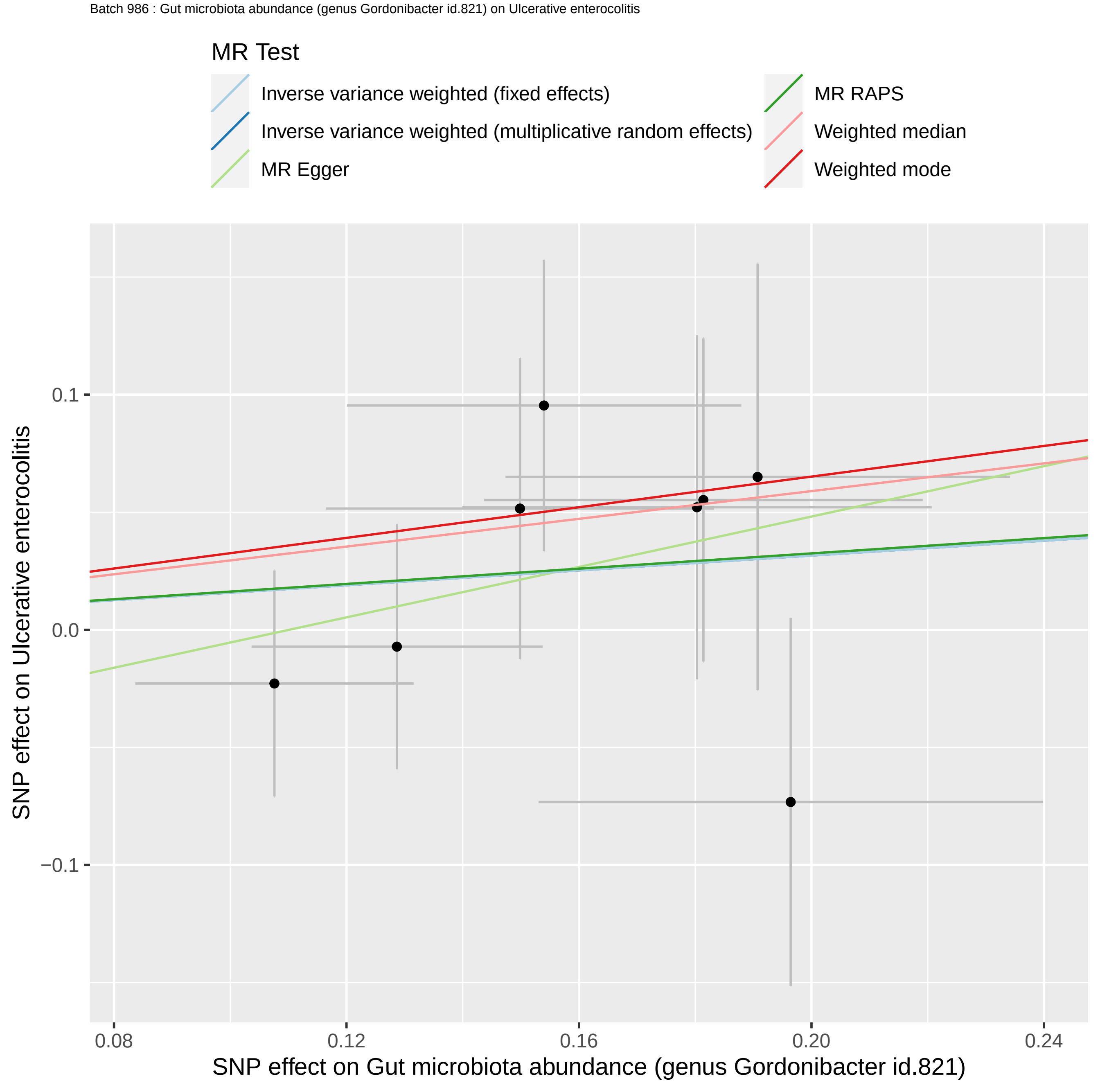
Batch 986 : Gut microbiota abundance (genus Gordonibacter id.821) on Ulcerative enterocolitis
MR Test
Inverse variance weighted (fixed effects)
Inverse variance weighted (multiplicative random effects)
MR Egger
MR RAPS
Weighted median
Weighted mode
0.1
0.0
−0.1
0.08
0.12
0.16
0.20
0.24
SNP effect on Ulcerative enterocolitis
SNP effect on Gut microbiota abundance (genus Gordonibacter id.821)

Batch 987 : Gut microbiota abundance (genus Haemophilus id.3698) on Ulcerative enterocolitis

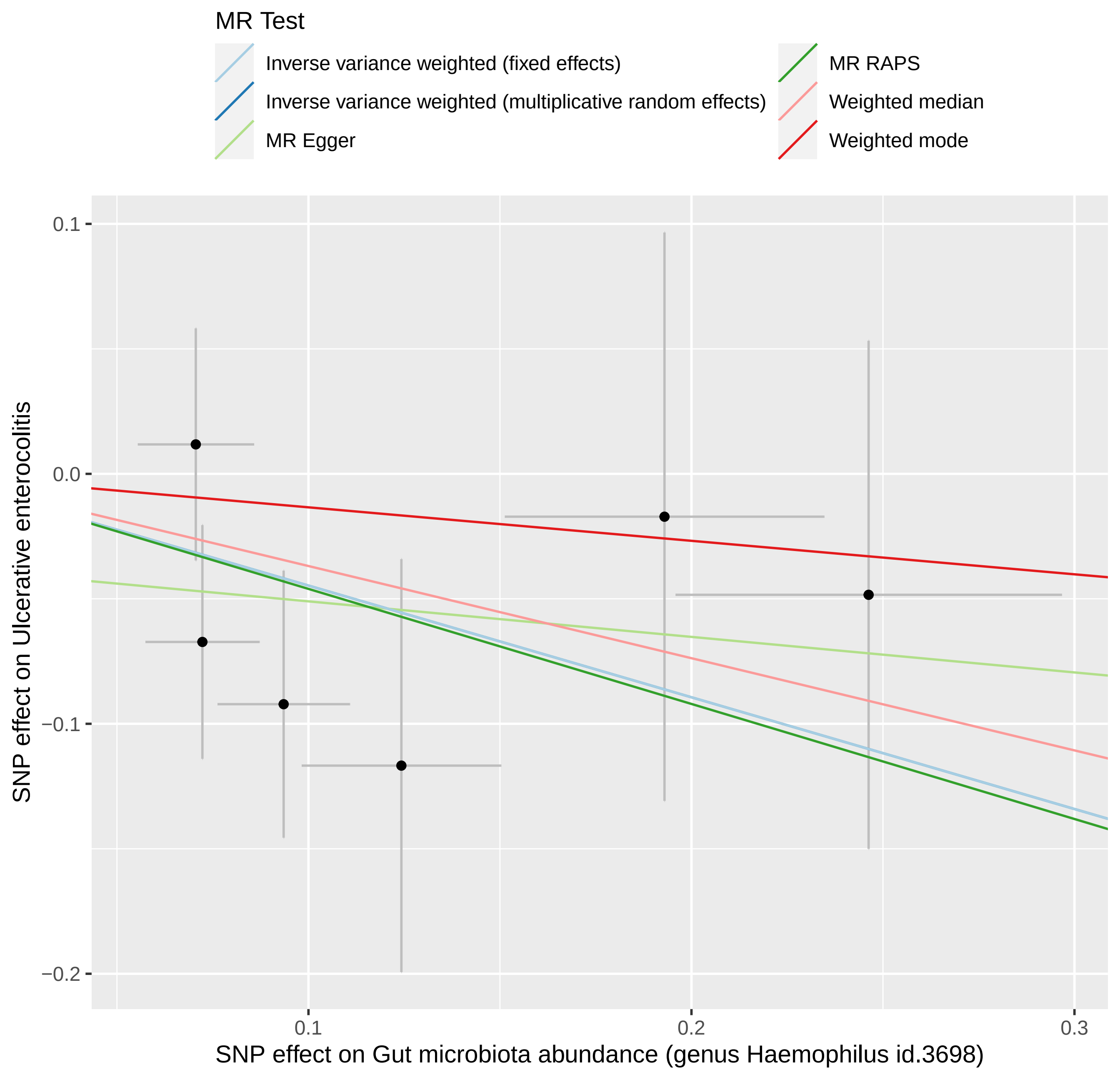

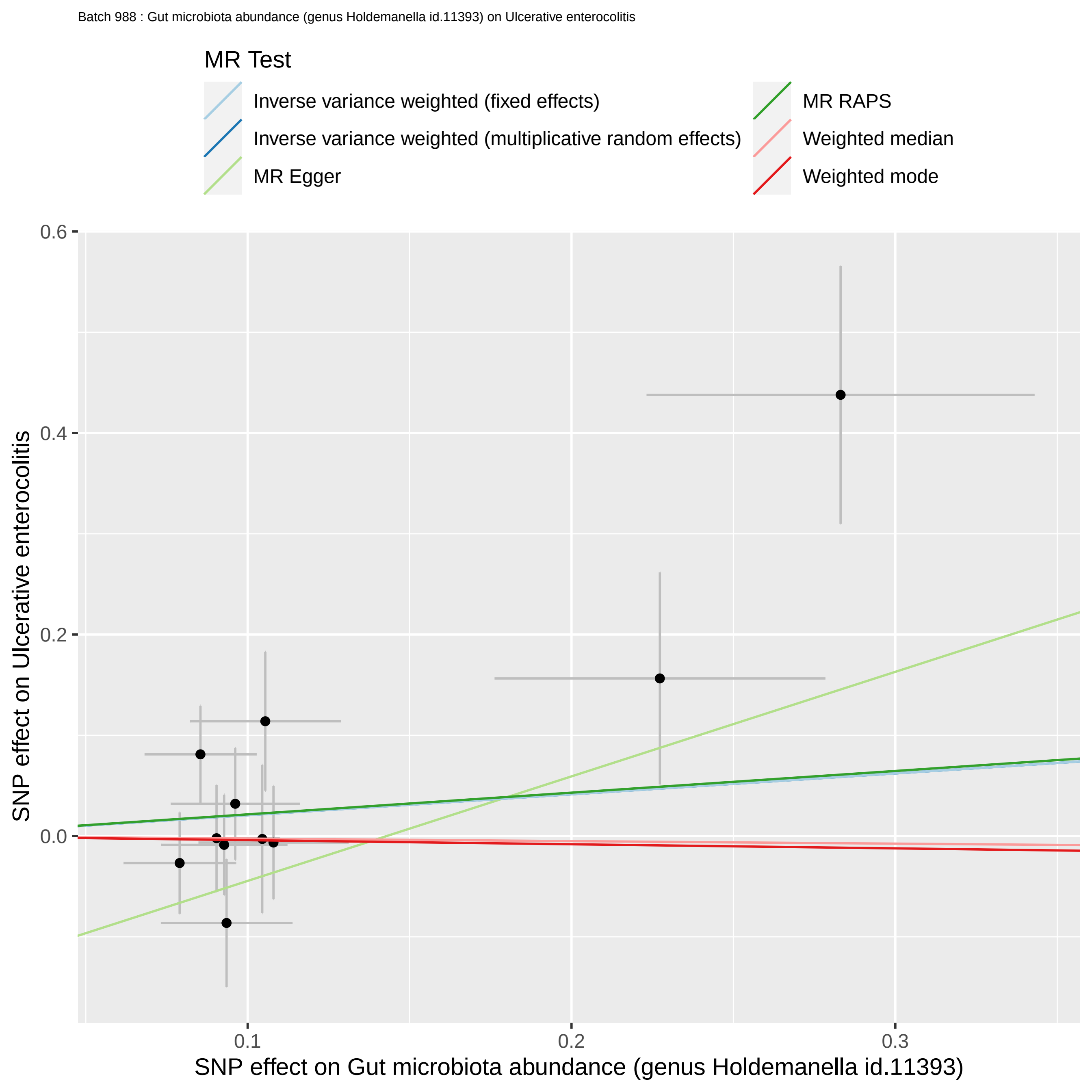

Batch 988 : Gut microbiota abundance (genus Holdemanella id.11393) on Ulcerative enterocolitis
MR Test
Inverse variance weighted (fixed effects)
Inverse variance weighted (multiplicative random effects)
MR Egger
MR RAPS
Weighted median
Weighted mode
0.6
0.4
0.2
0.0
0.1
0.2
0.3
SNP effect on Ulcerative enterocolitis
SNP effect on Gut microbiota abundance (genus Holdemanella id.11393)

Batch 989 : Gut microbiota abundance (genus Holdemania id.2157) on Ulcerative enterocolitis

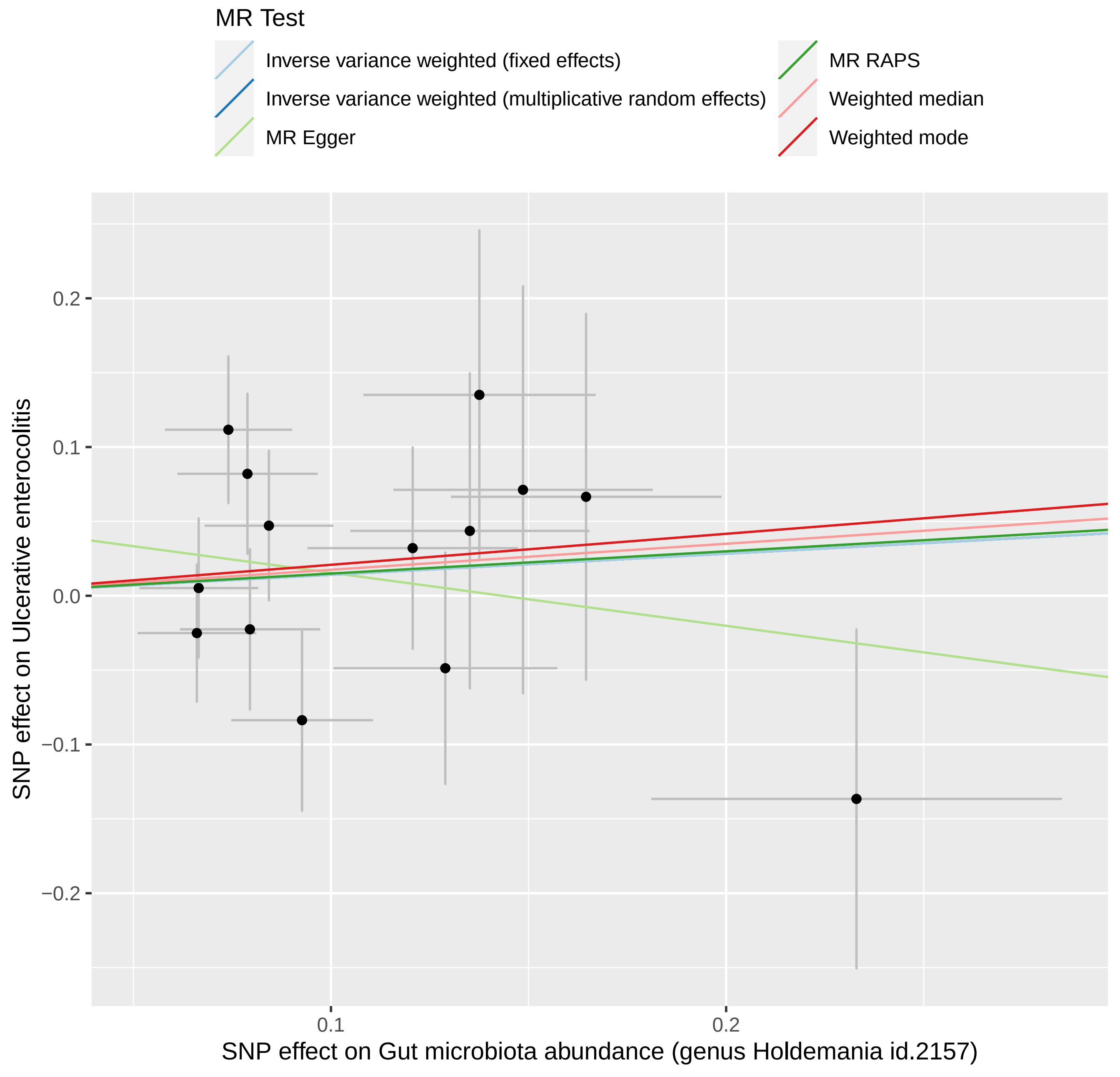

Batch 990 : Gut microbiota abundance (genus Howardella id.2000) on Ulcerative enterocolitis

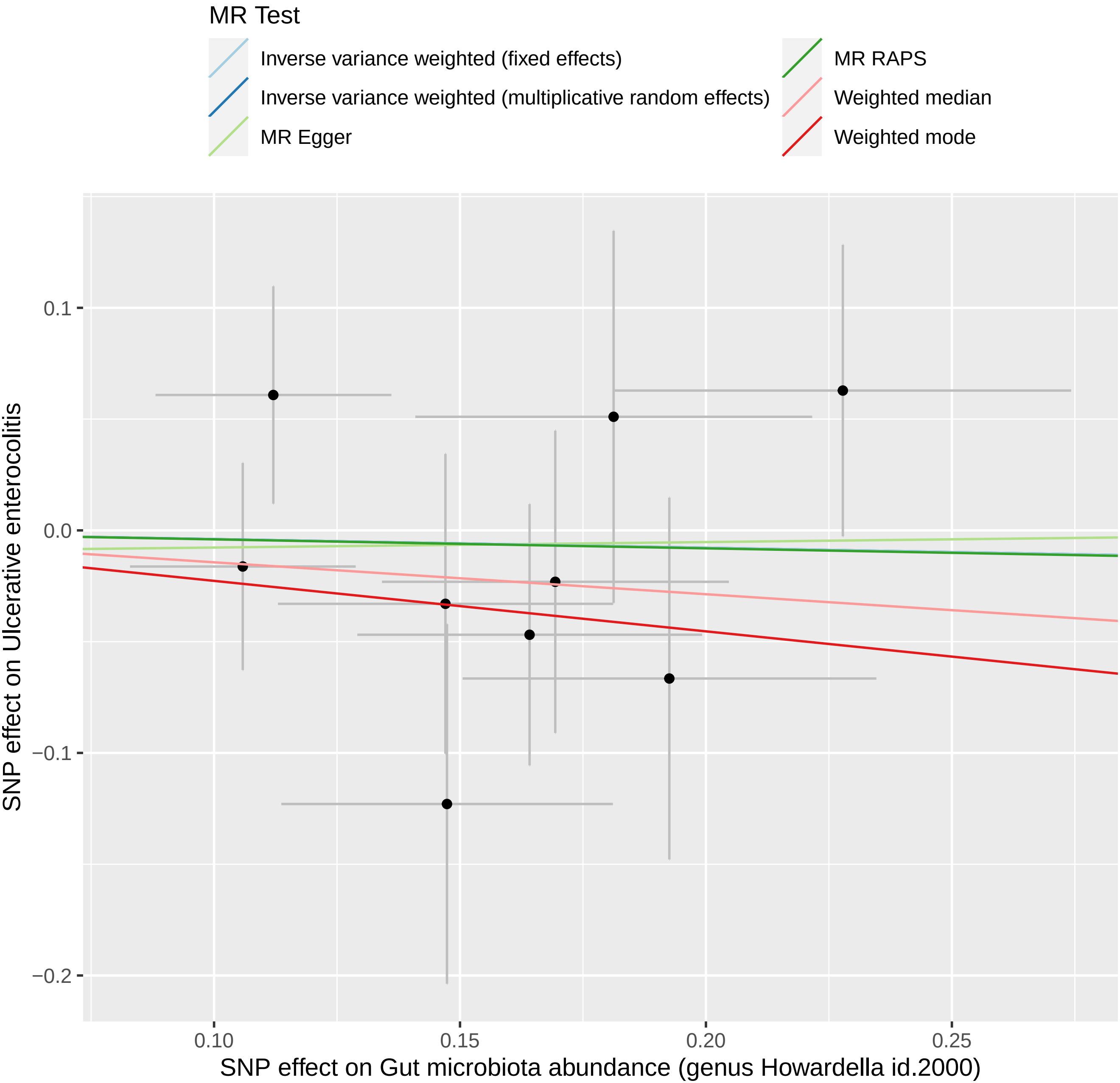

Batch 991 : Gut microbiota abundance (genus Hungatella id.11306) on Ulcerative enterocolitis

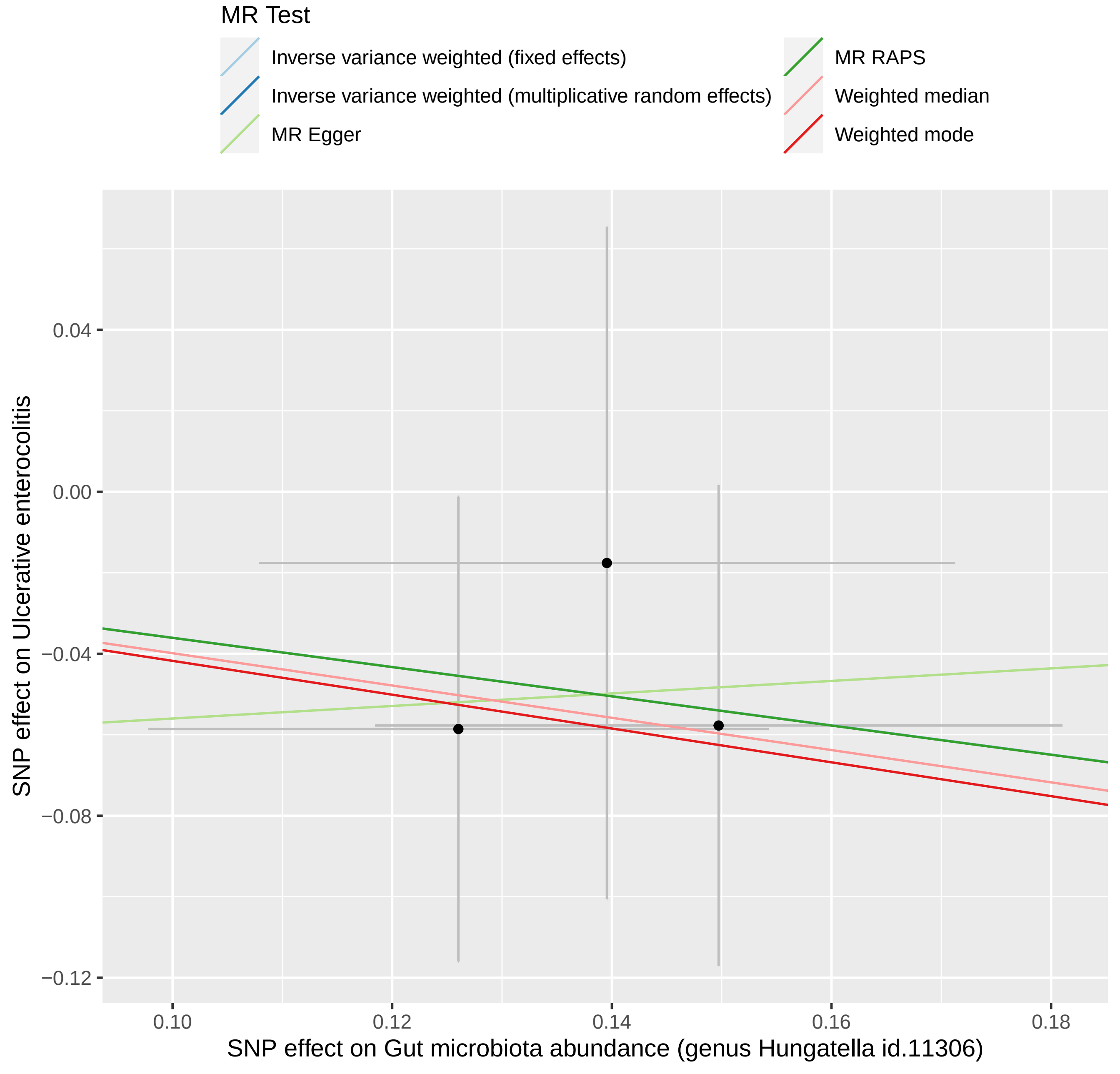

Batch 992 : Gut microbiota abundance (genus Intestinibacter id.11345) on Ulcerative enterocolitis

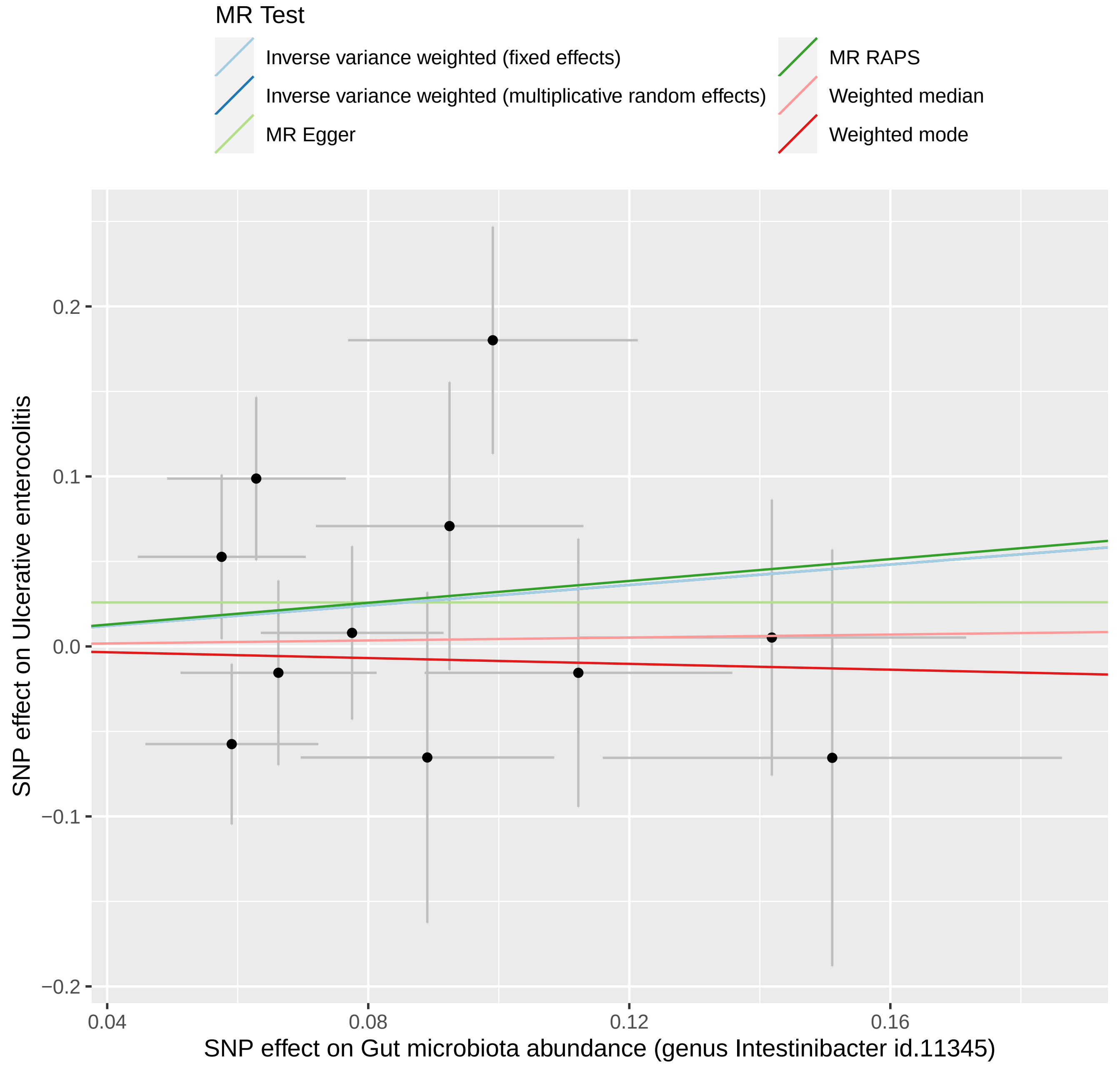

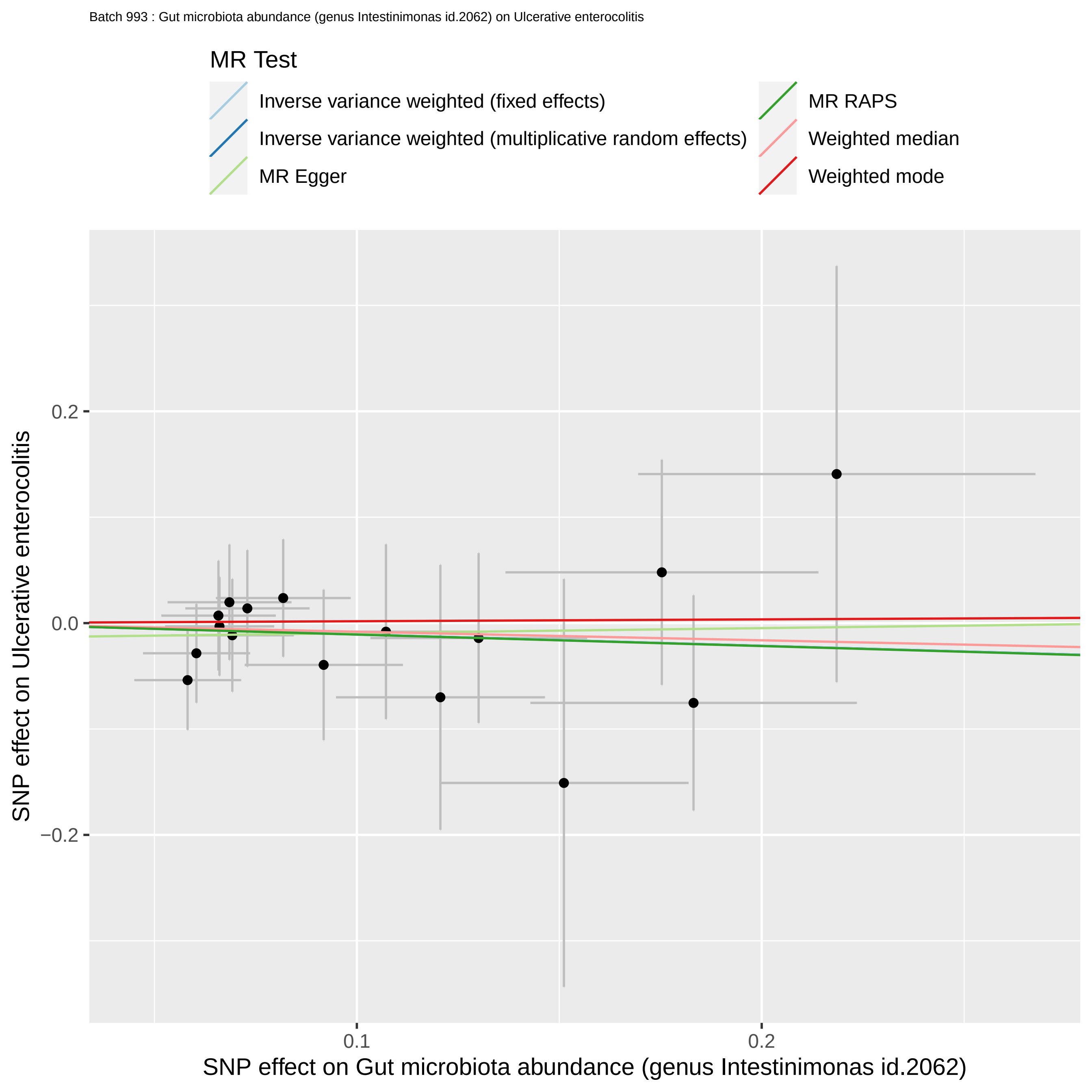
Batch 993 : Gut microbiota abundance (genus Intestinimonas id.2062) on Ulcerative enterocolitis
MR Test
Inverse variance weighted (fixed effects)
Inverse variance weighted (multiplicative random effects)
MR Egger
MR RAPS
Weighted median
Weighted mode
0.2
0.0
−0.2
0.1
0.2
SNP effect on Ulcerative enterocolitis
SNP effect on Gut microbiota abundance (genus Intestinimonas id.2062)

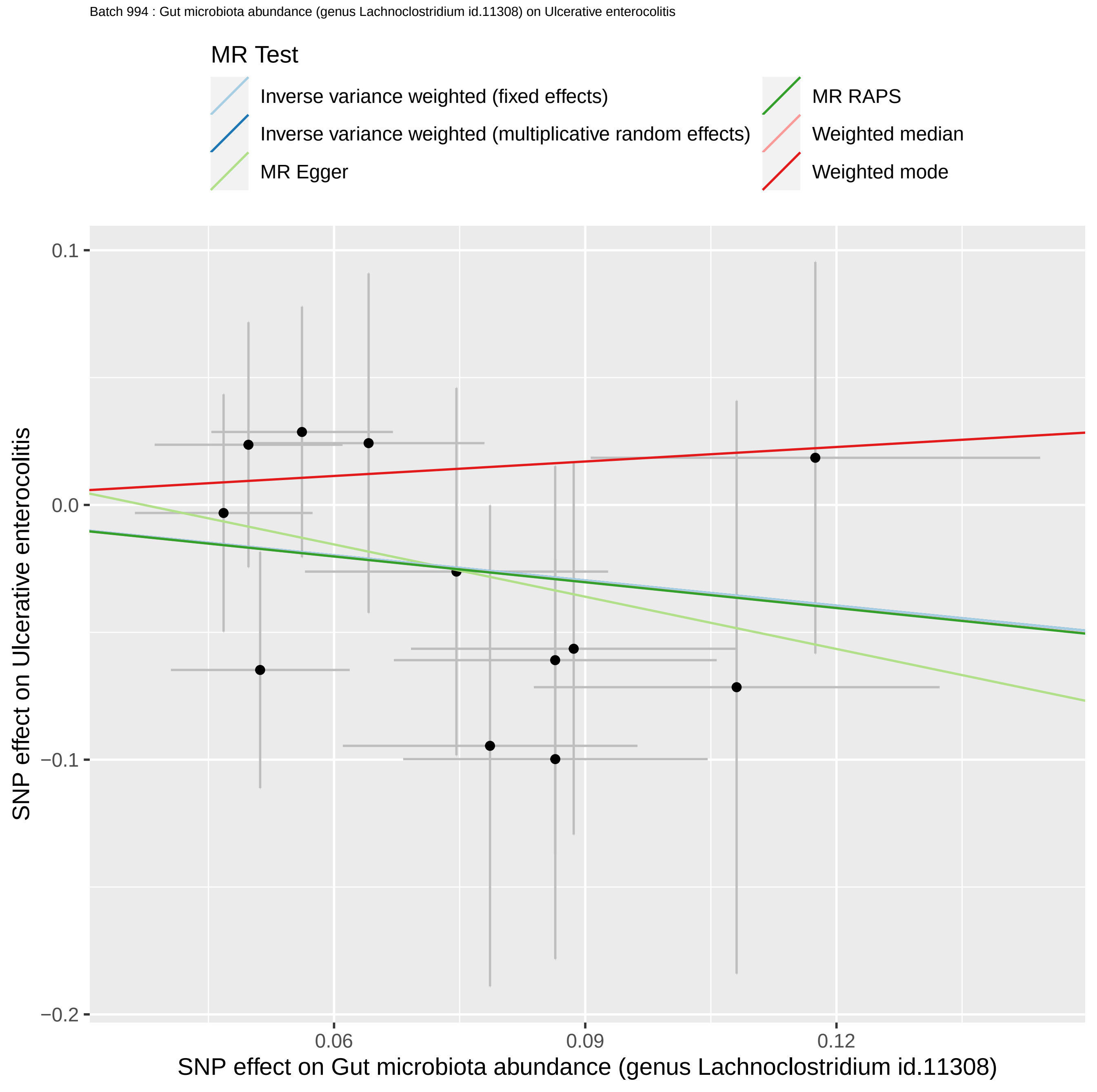

Batch 994 : Gut microbiota abundance (genus Lachnoclostridium id.11308) on Ulcerative enterocolitis
MR Test
Inverse variance weighted (fixed effects)
Inverse variance weighted (multiplicative random effects)
MR Egger
MR RAPS
Weighted median
Weighted mode
0.1
0.0
−0.1
−0.2
0.06
0.09
0.12
SNP effect on Ulcerative enterocolitis
SNP effect on Gut microbiota abundance (genus Lachnoclostridium id.11308)

Batch 995 : Gut microbiota abundance (genus Lachnospiraceae FCS020 group id.11314) on Ulcerative enterocolitis

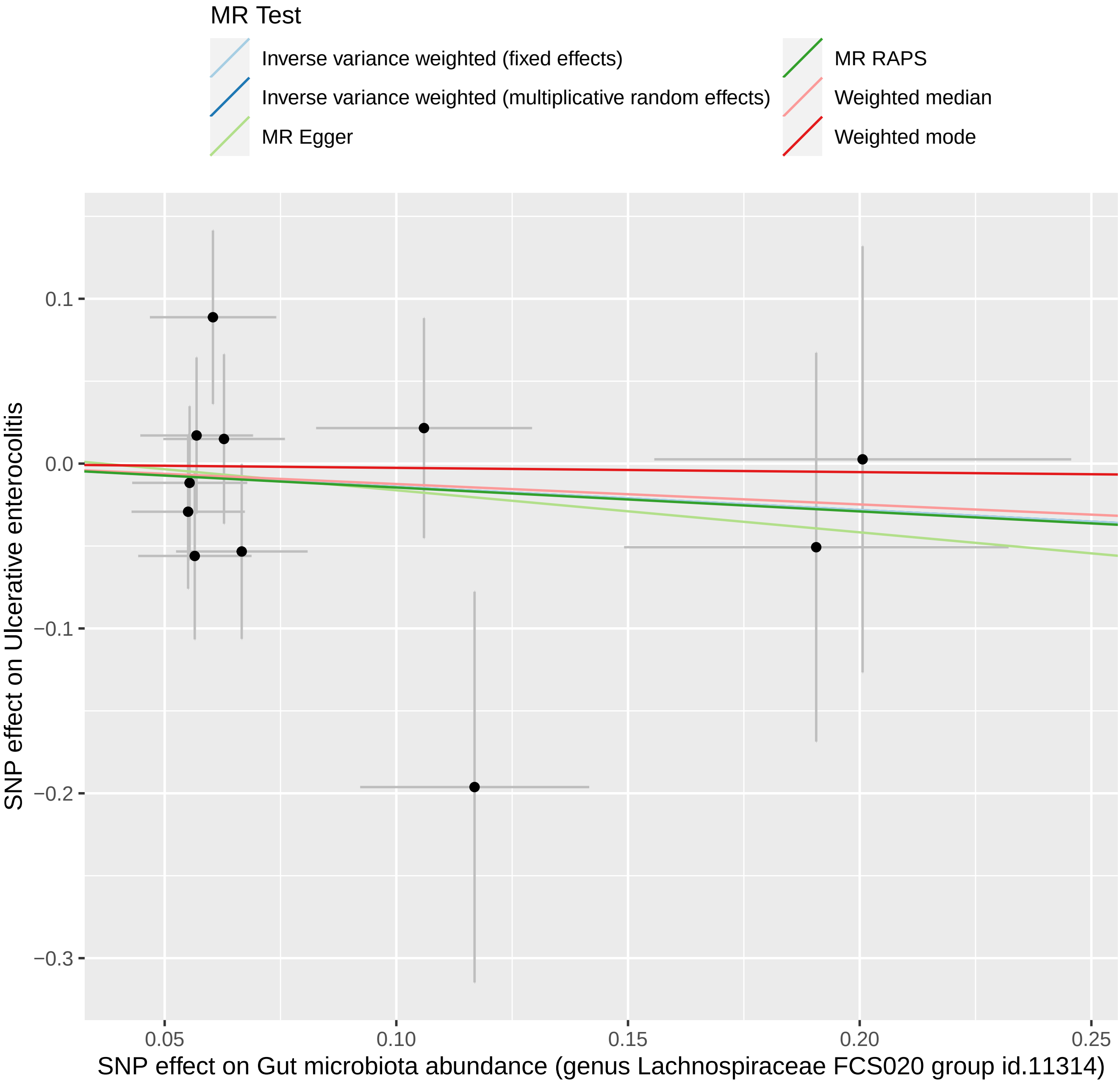

Batch 996 : Gut microbiota abundance (genus Lachnospiraceae NC2004 group id.11316) on Ulcerative enterocolitis

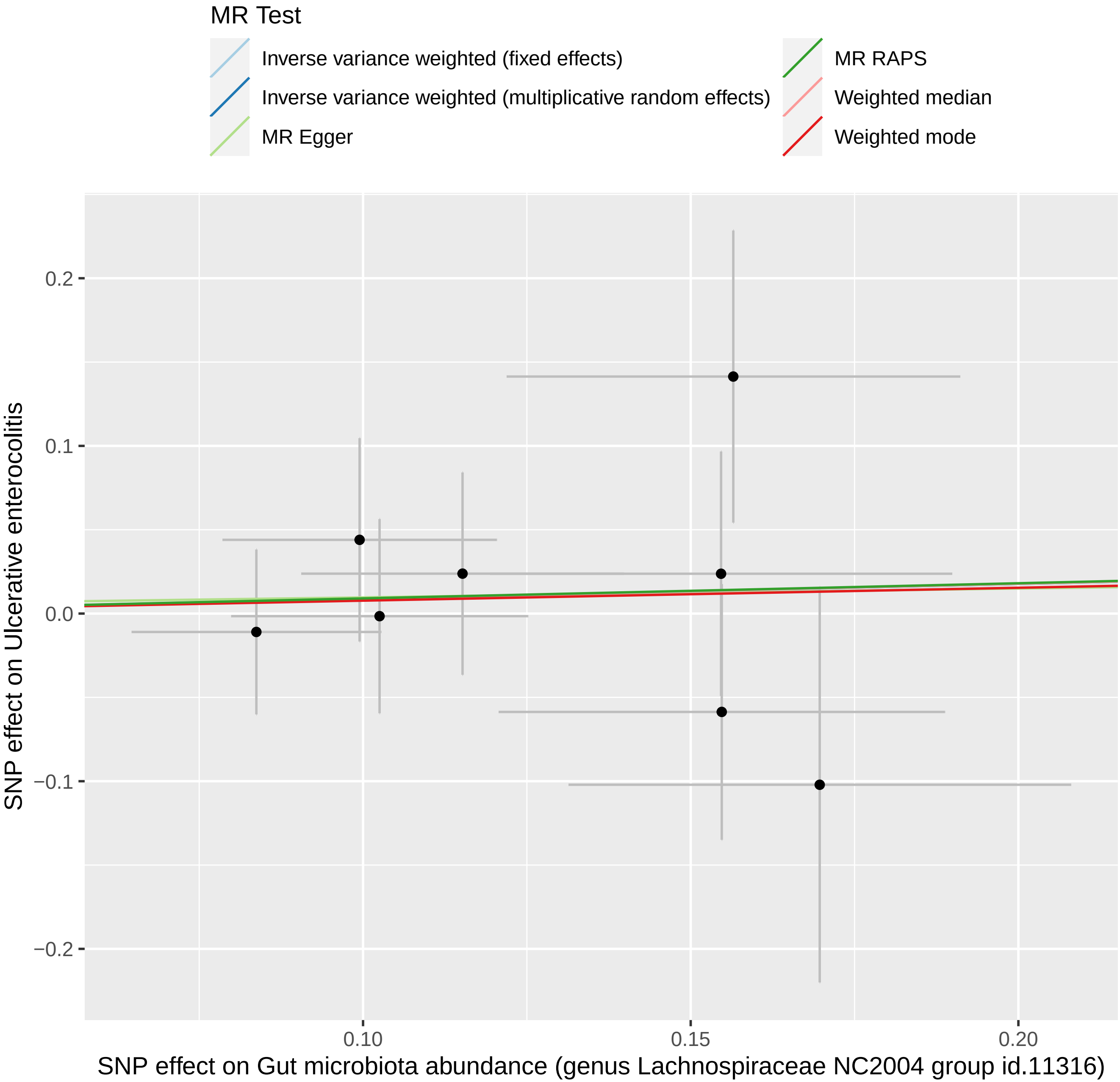

Batch 997 : Gut microbiota abundance (genus Lachnospiraceae ND3007 group id.11317) on Ulcerative enterocolitis

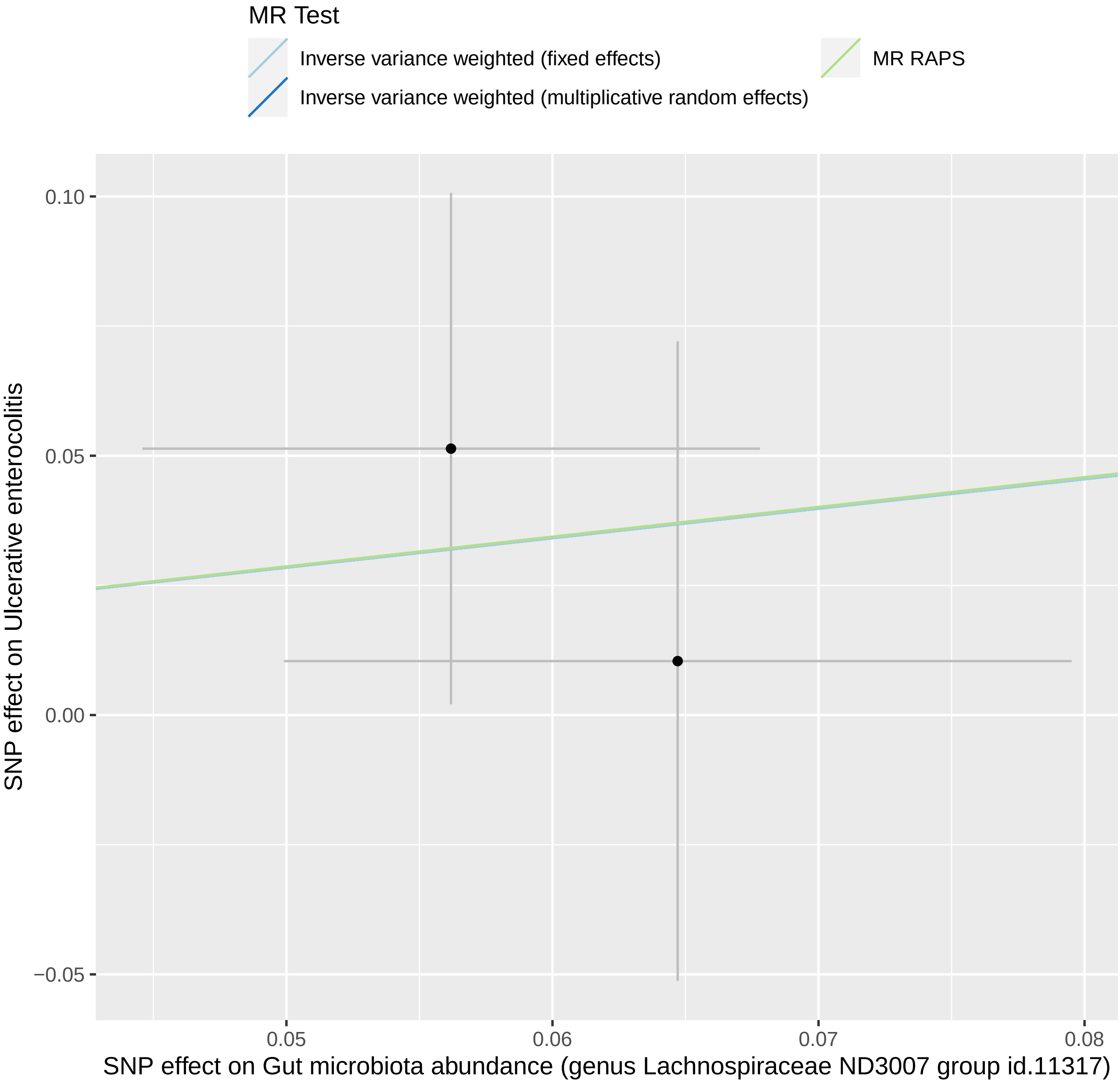

Batch 998 : Gut microbiota abundance (genus Lachnospiraceae NK4A136 group id.11319) on Ulcerative enterocolitis

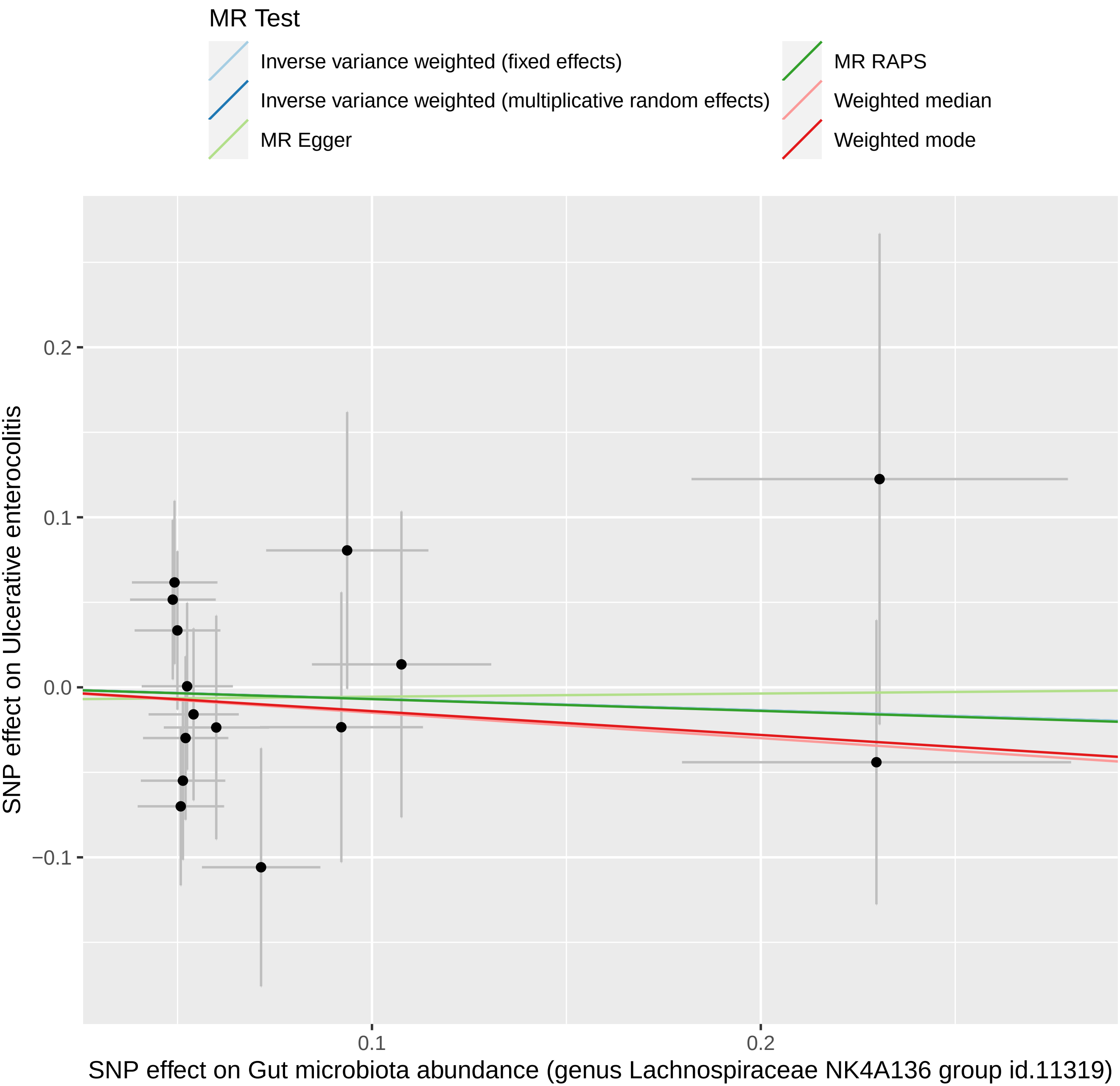

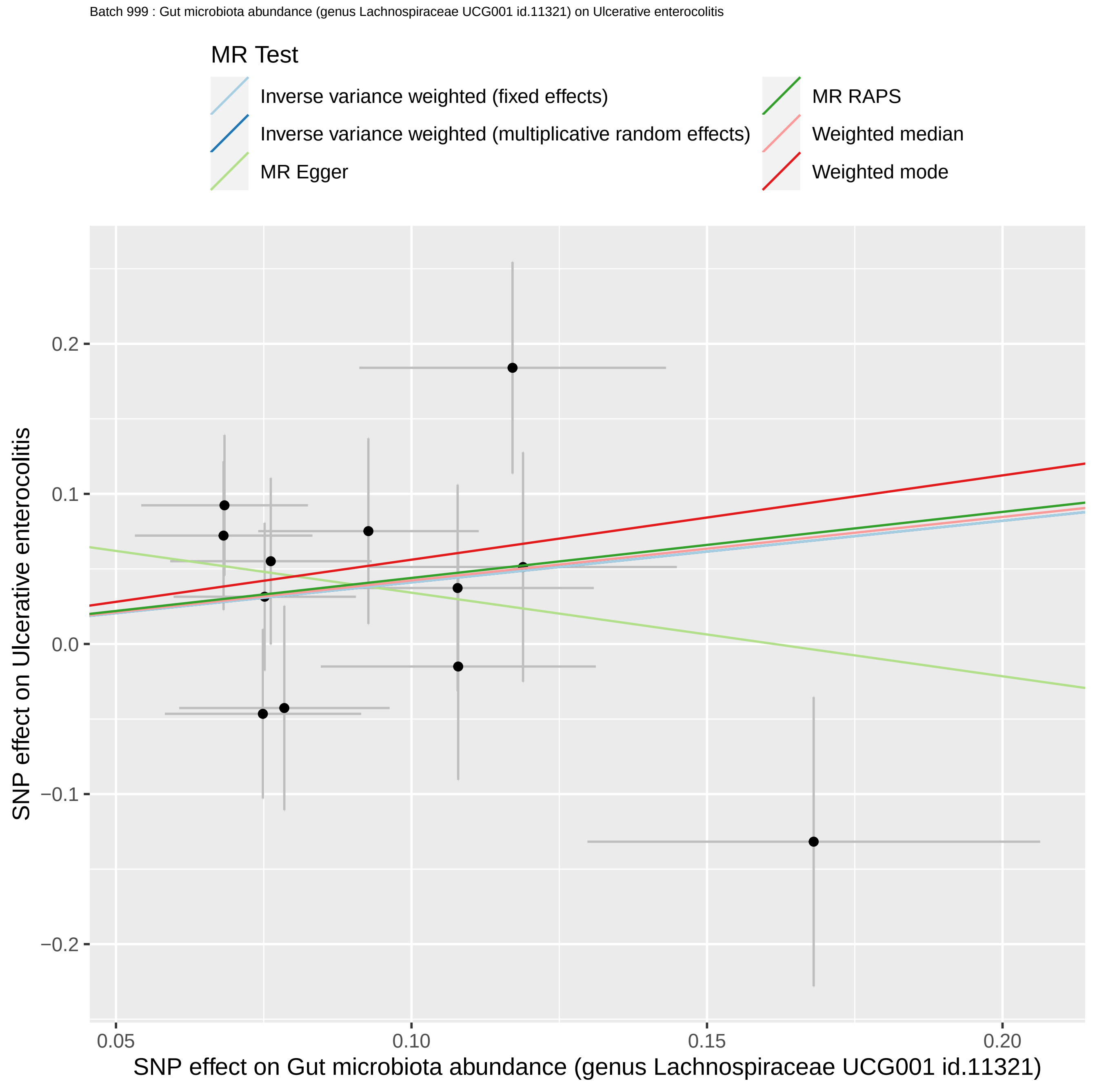

Batch 999 : Gut microbiota abundance (genus Lachnospiraceae UCG001 id.11321) on Ulcerative enterocolitis
MR Test
Inverse variance weighted (fixed effects)
Inverse variance weighted (multiplicative random effects)
MR Egger
MR RAPS
Weighted median
Weighted mode
SNP effect on Ulcerative enterocolitis
0.2
0.1
0.0
−0.1
−0.2
0.05
0.10
0.15
0.20
SNP effect on Gut microbiota abundance (genus Lachnospiraceae UCG001 id.11321)

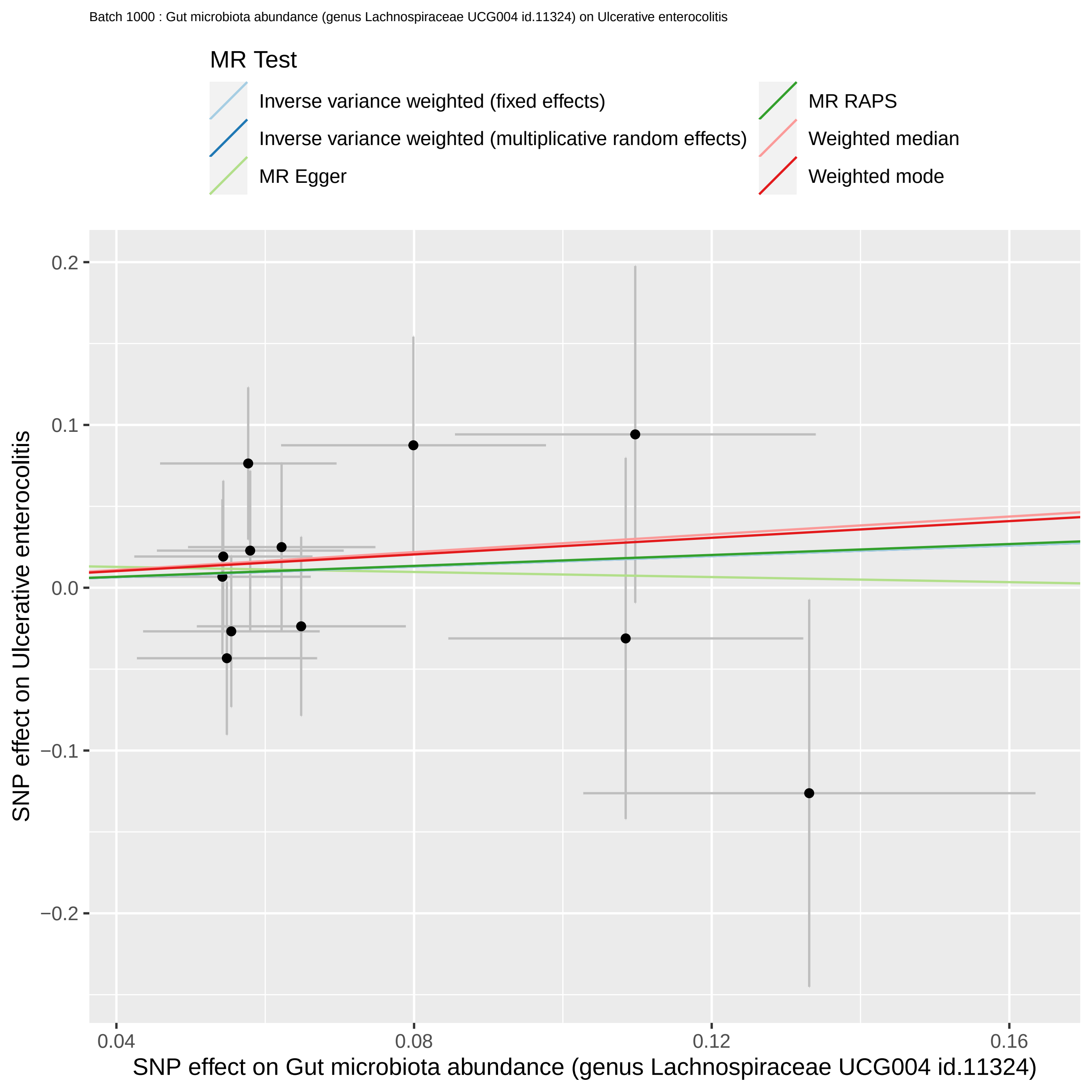

Batch 1000 : Gut microbiota abundance (genus Lachnospiraceae UCG004 id.11324) on Ulcerative enterocolitis
MR Test
Inverse variance weighted (fixed effects)
Inverse variance weighted (multiplicative random effects)
MR Egger
MR RAPS
Weighted median
Weighted mode
0.2
0.1
0.0
−0.1
−0.2
0.04
0.08
0.12
0.16
SNP effect on Ulcerative enterocolitis
SNP effect on Gut microbiota abundance (genus Lachnospiraceae UCG004 id.11324)

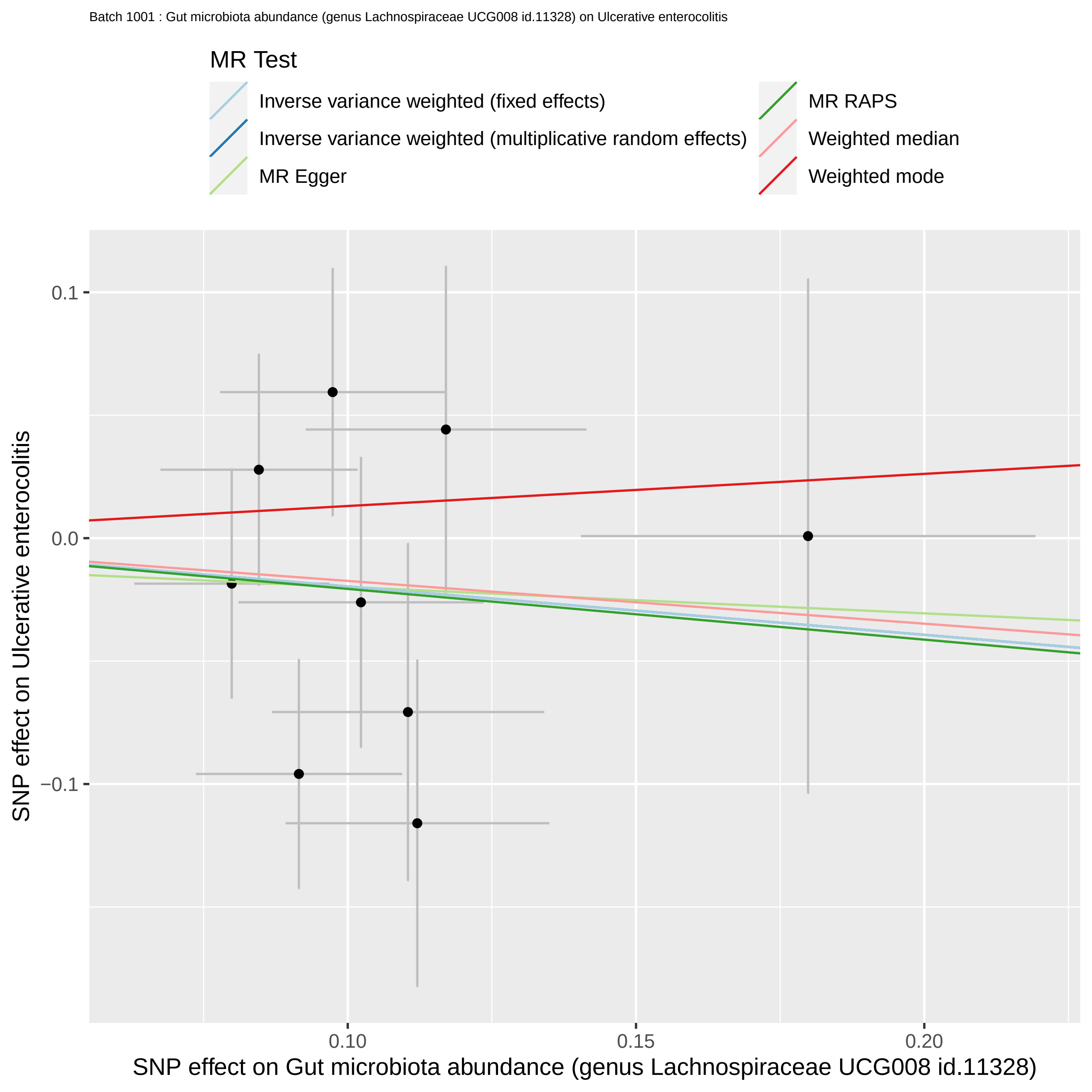

Batch 1001 : Gut microbiota abundance (genus Lachnospiraceae UCG008 id.11328) on Ulcerative enterocolitis
MR Test
Inverse variance weighted (fixed effects)
Inverse variance weighted (multiplicative random effects)
MR Egger
MR RAPS
Weighted median
Weighted mode
SNP effect on Ulcerative enterocolitis
0.1
0.0
−0.1
0.10
0.15
0.20
SNP effect on Gut microbiota abundance (genus Lachnospiraceae UCG008 id.11328)

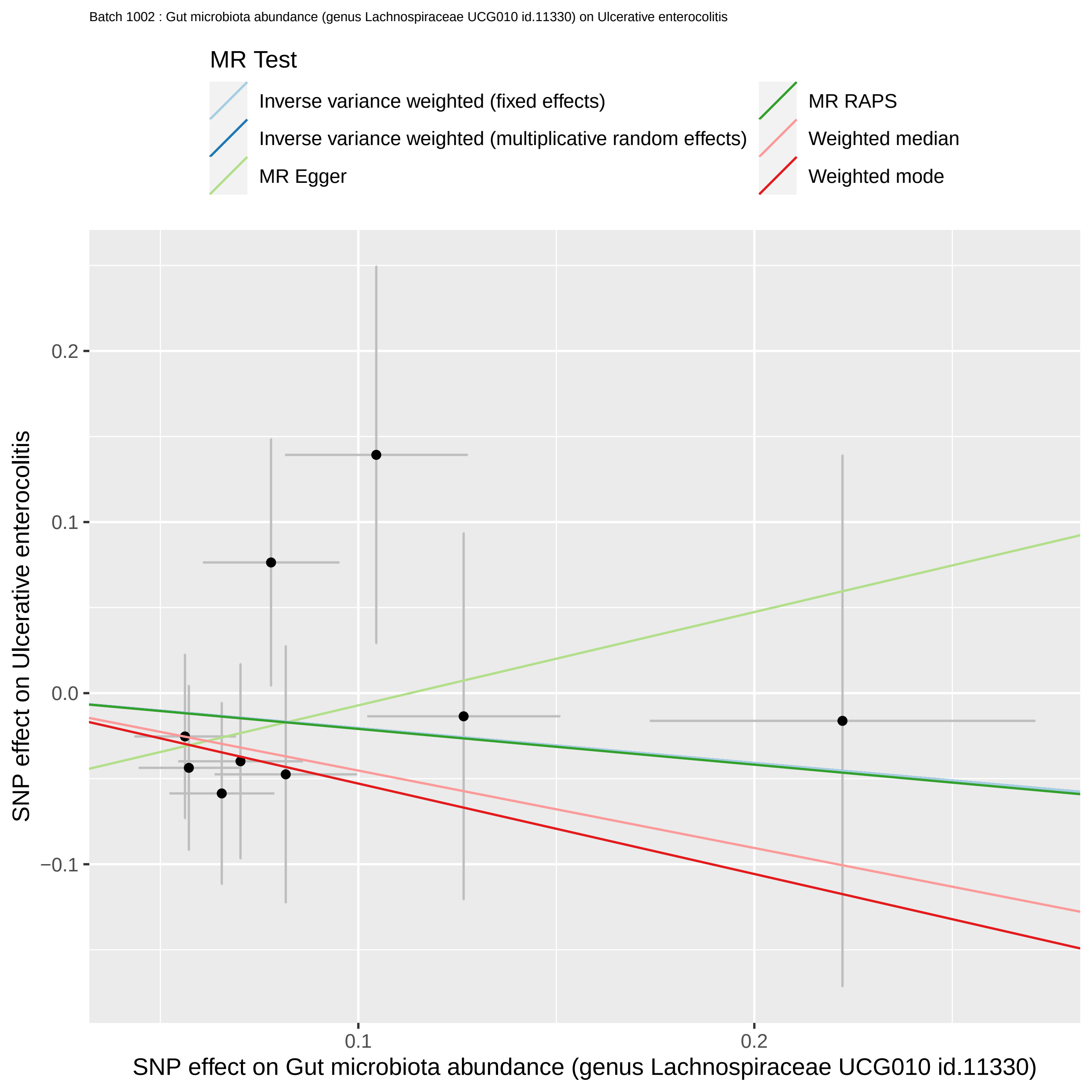

Batch 1002 : Gut microbiota abundance (genus Lachnospiraceae UCG010 id.11330) on Ulcerative enterocolitis
MR Test
Inverse variance weighted (fixed effects)
Inverse variance weighted (multiplicative random effects)
MR Egger
MR RAPS
Weighted median
Weighted mode
0.2
0.1
0.0
−0.1
0.1
0.2
SNP effect on Ulcerative enterocolitis
SNP effect on Gut microbiota abundance (genus Lachnospiraceae UCG010 id.11330)

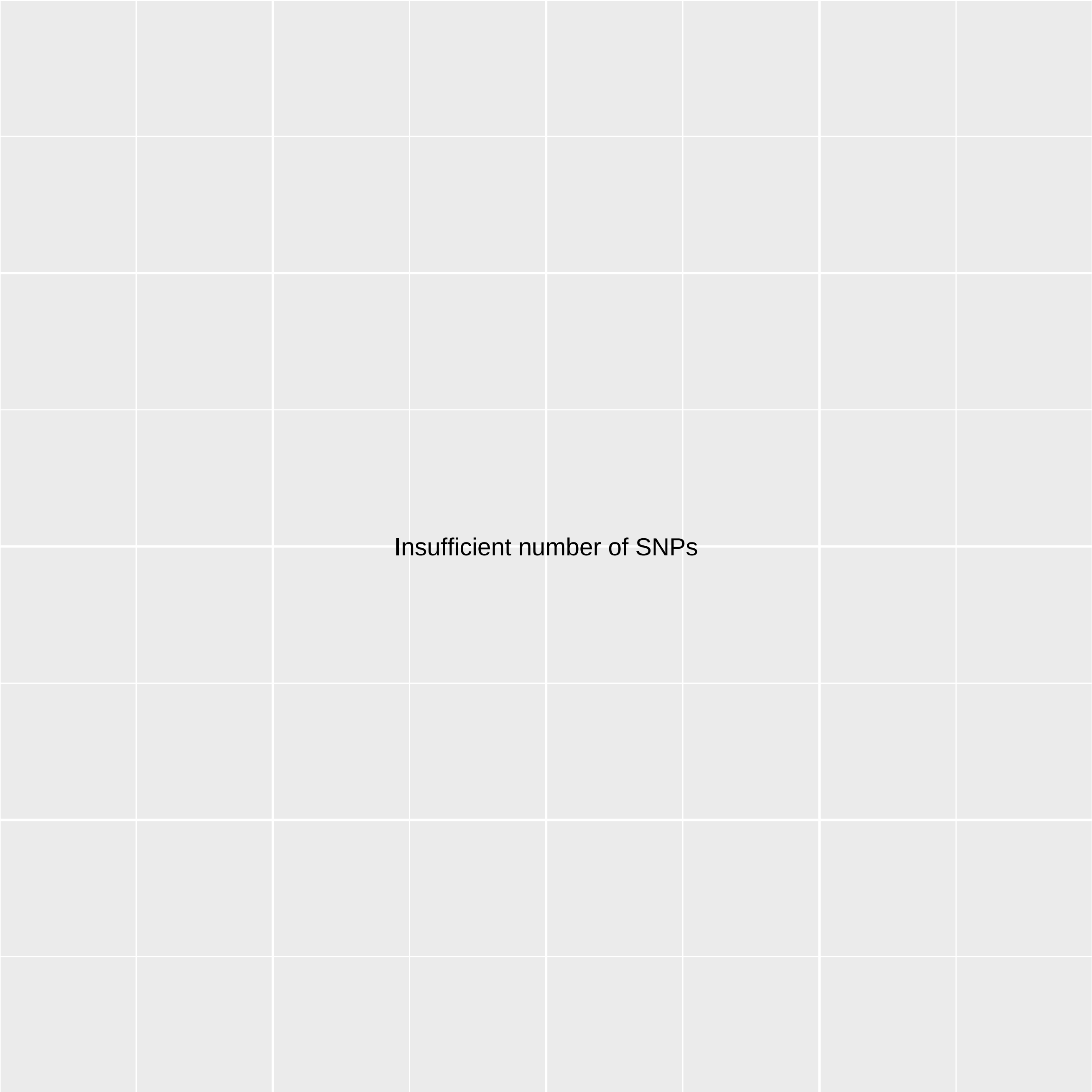
Insufficient number of SNPs

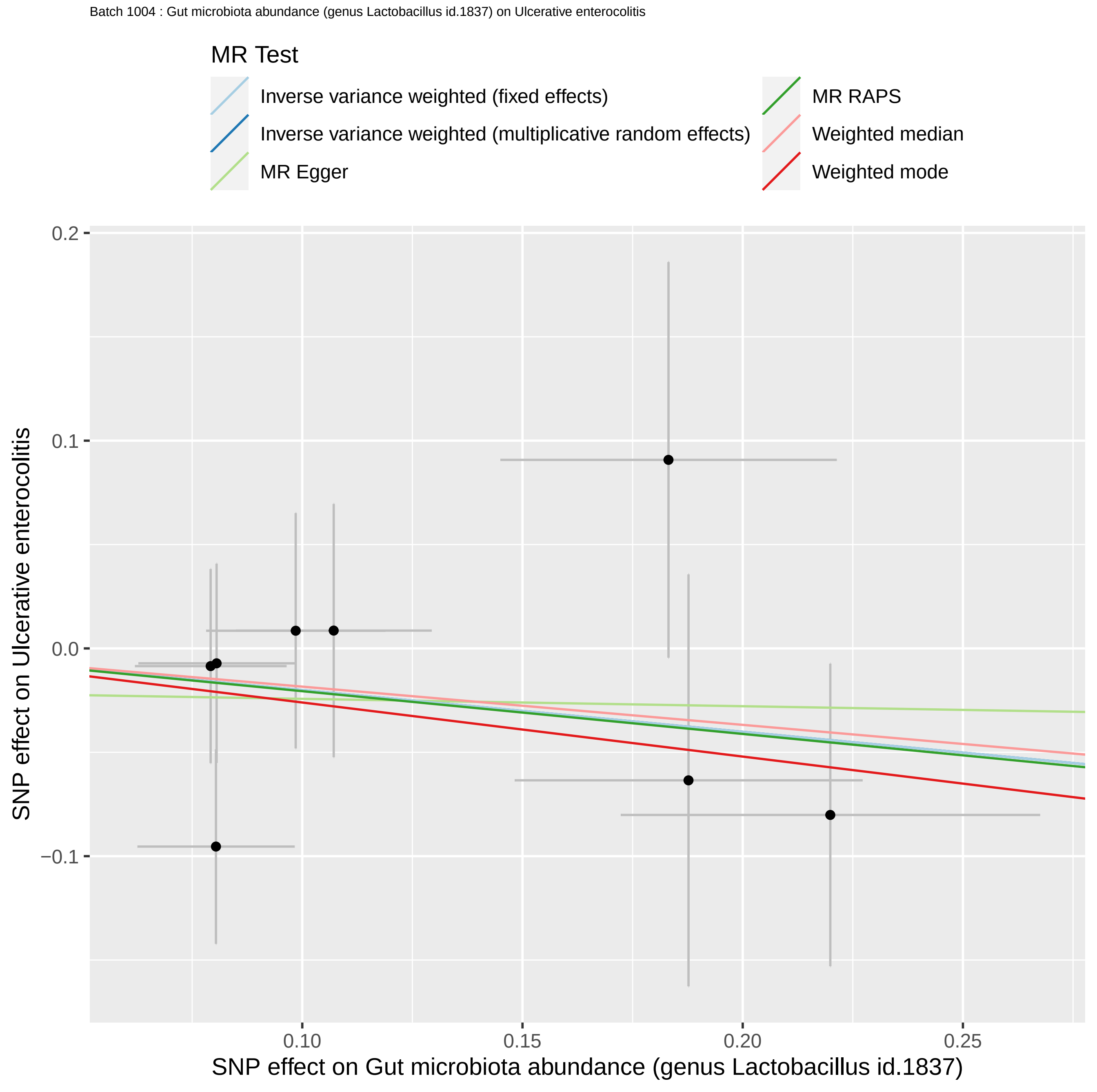

Batch 1004 : Gut microbiota abundance (genus Lactobacillus id.1837) on Ulcerative enterocolitis
MR Test
Inverse variance weighted (fixed effects)
Inverse variance weighted (multiplicative random effects)
MR Egger
MR RAPS
Weighted median
Weighted mode
0.2
0.1
0.0
−0.1
0.10
0.15
0.20
0.25
SNP effect on Ulcerative enterocolitis
SNP effect on Gut microbiota abundance (genus Lactobacillus id.1837)

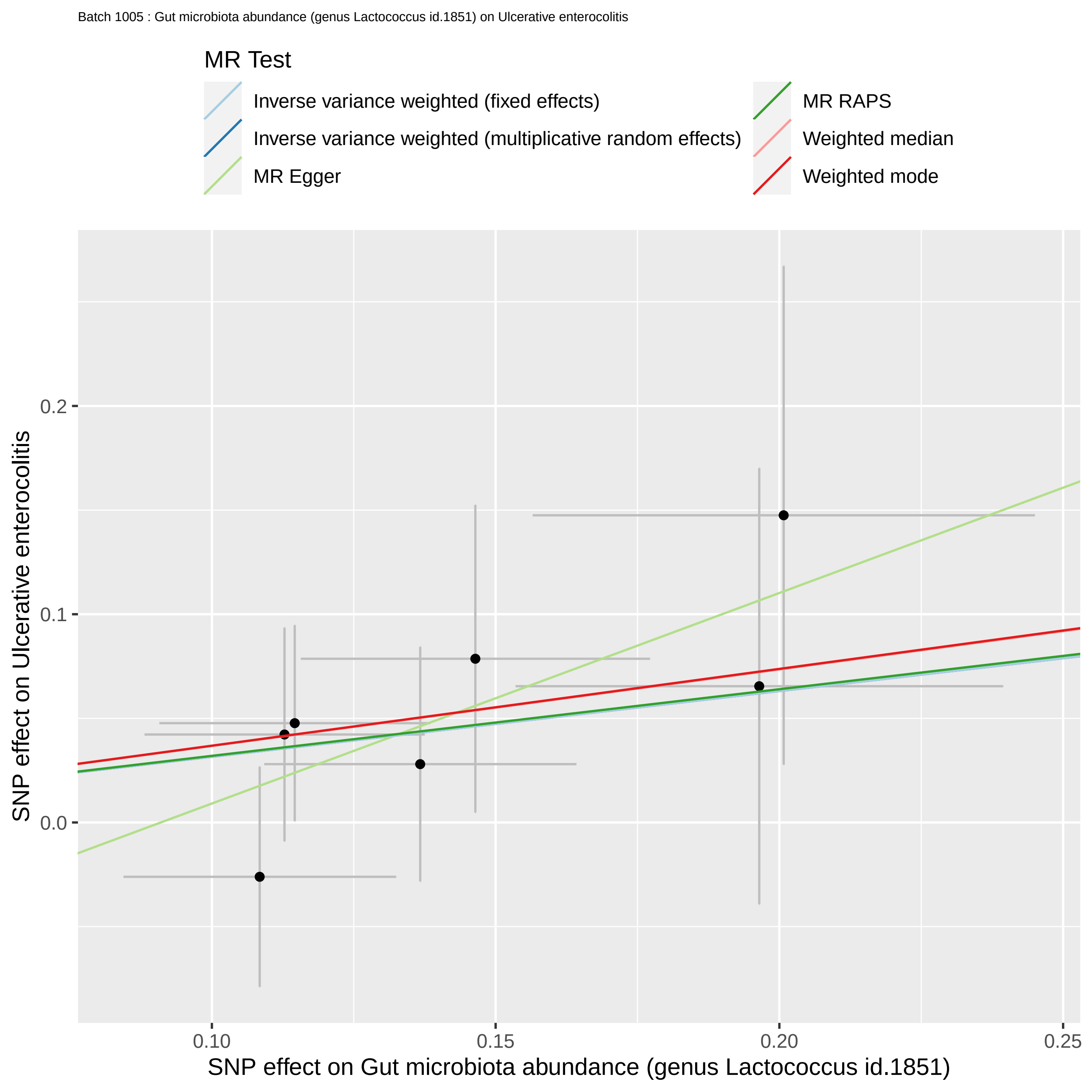
Batch 1005 : Gut microbiota abundance (genus Lactococcus id.1851) on Ulcerative enterocolitis
MR Test
Inverse variance weighted (fixed effects)
Inverse variance weighted (multiplicative random effects)
MR Egger
MR RAPS
Weighted median
Weighted mode
0.2
0.1
0.0
0.10
0.15
0.20
0.25
SNP effect on Ulcerative enterocolitis
SNP effect on Gut microbiota abundance (genus Lactococcus id.1851)

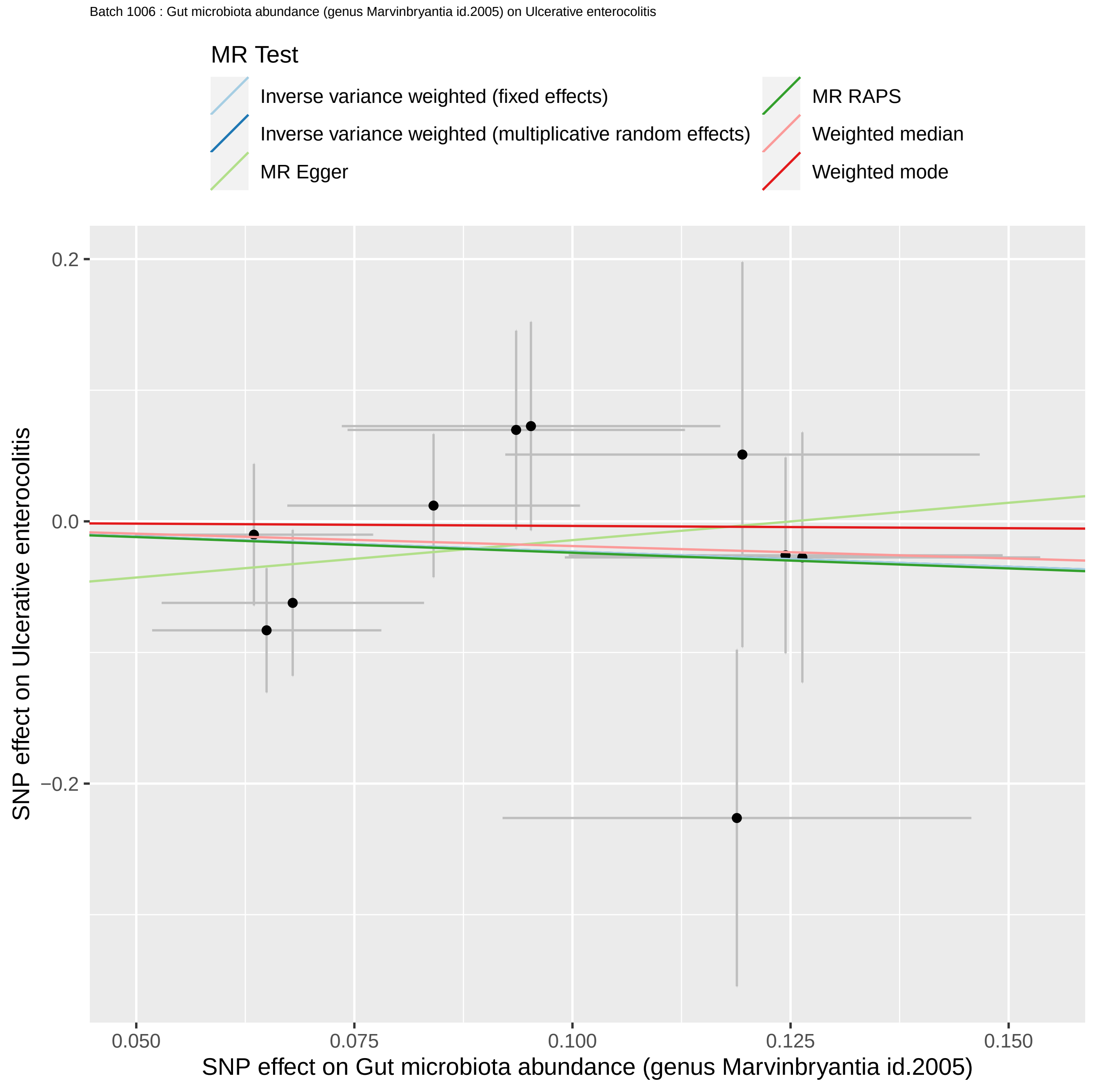

Batch 1006 : Gut microbiota abundance (genus Marvinbryantia id.2005) on Ulcerative enterocolitis
MR Test
Inverse variance weighted (fixed effects)
Inverse variance weighted (multiplicative random effects)
MR Egger
MR RAPS
Weighted median
Weighted mode
0.2
0.0
−0.2
0.050
0.075
0.100
0.125
0.150
SNP effect on Ulcerative enterocolitis
SNP effect on Gut microbiota abundance (genus Marvinbryantia id.2005)

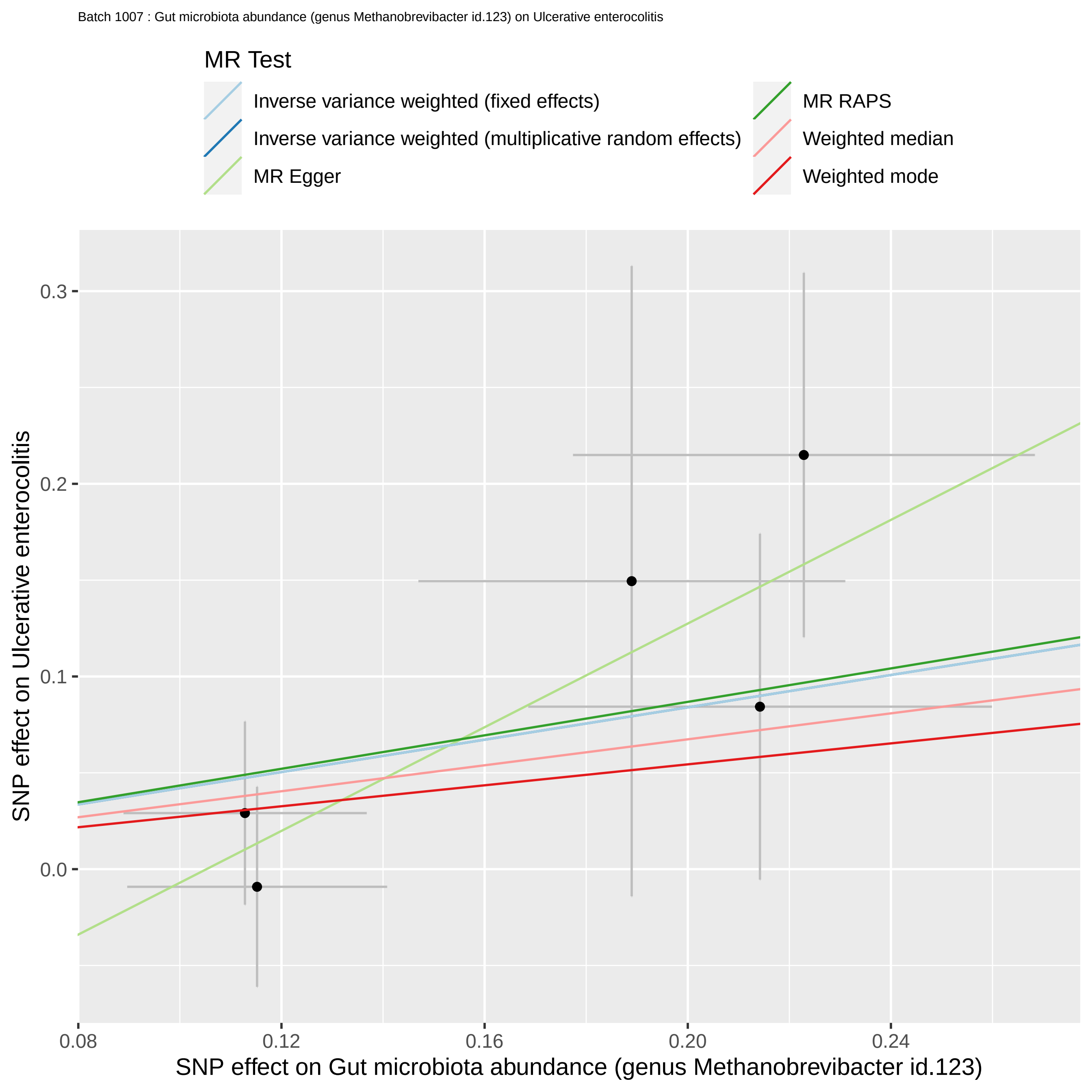
Batch 1007 : Gut microbiota abundance (genus Methanobrevibacter id.123) on Ulcerative enterocolitis
MR Test
Inverse variance weighted (fixed effects)
Inverse variance weighted (multiplicative random effects)
MR Egger
MR RAPS
Weighted median
Weighted mode
SNP effect on Ulcerative enterocolitis
0.3
0.2
0.1
0.0
0.08
0.12
0.16
0.20
0.24
SNP effect on Gut microbiota abundance (genus Methanobrevibacter id.123)

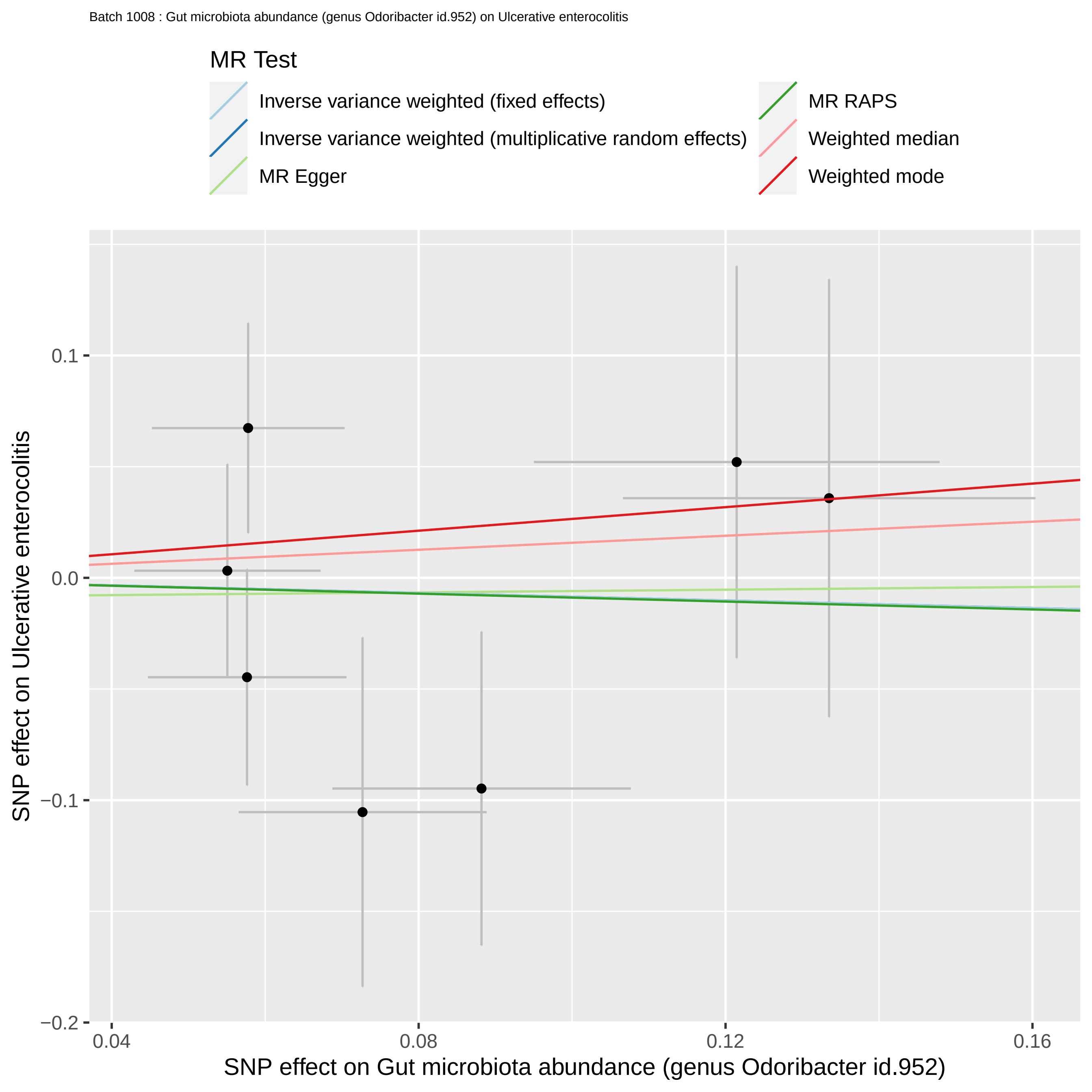
Batch 1008 : Gut microbiota abundance (genus Odoribacter id.952) on Ulcerative enterocolitis
MR Test
Inverse variance weighted (fixed effects)
Inverse variance weighted (multiplicative random effects)
MR Egger
MR RAPS
Weighted median
Weighted mode
SNP effect on Ulcerative enterocolitis
0.1
0.0
−0.1
−0.2
0.04
0.08
0.12
0.16
SNP effect on Gut microbiota abundance (genus Odoribacter id.952)

Batch 1009 : Gut microbiota abundance (genus Olsenella id.822) on Ulcerative enterocolitis

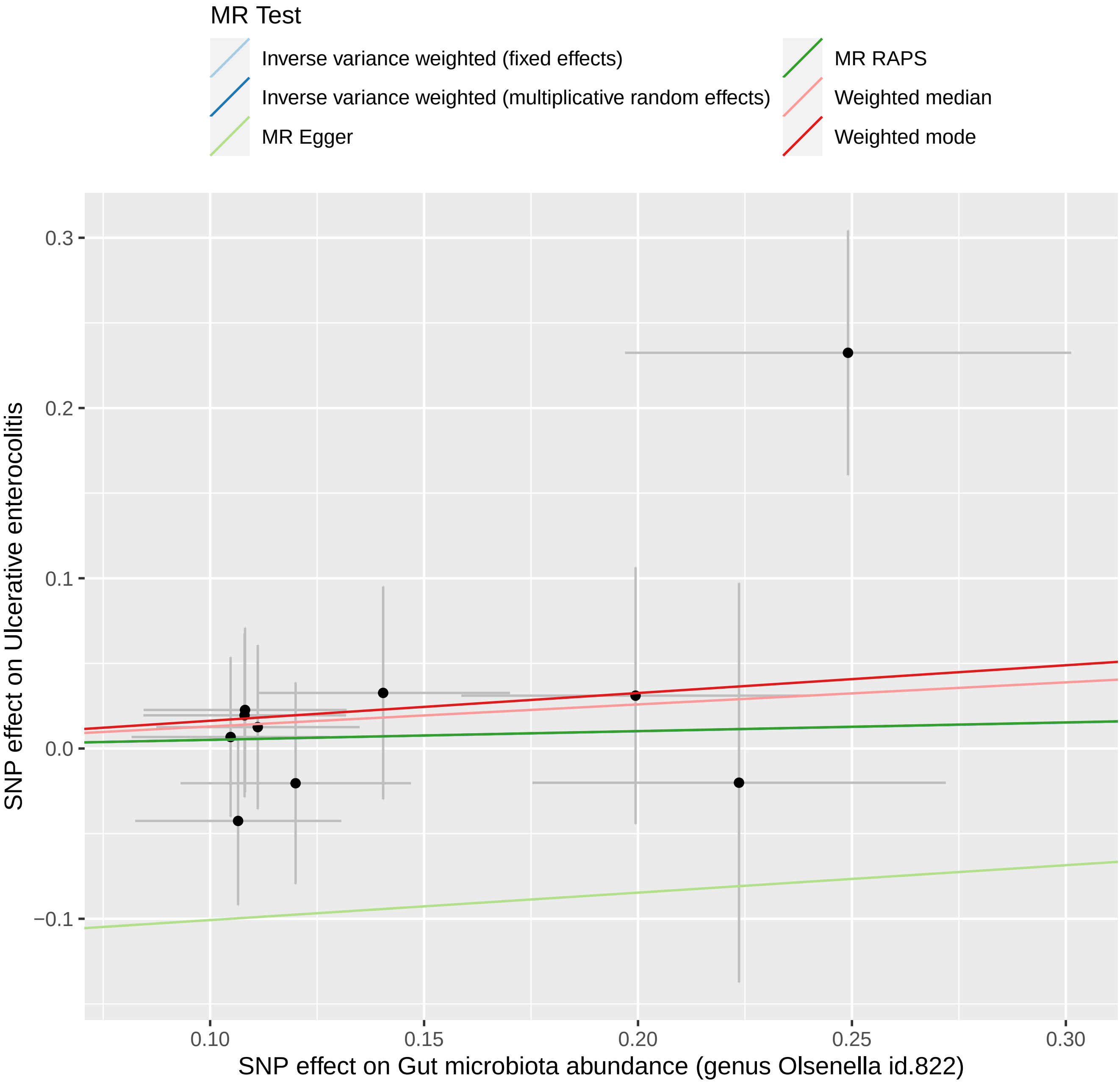

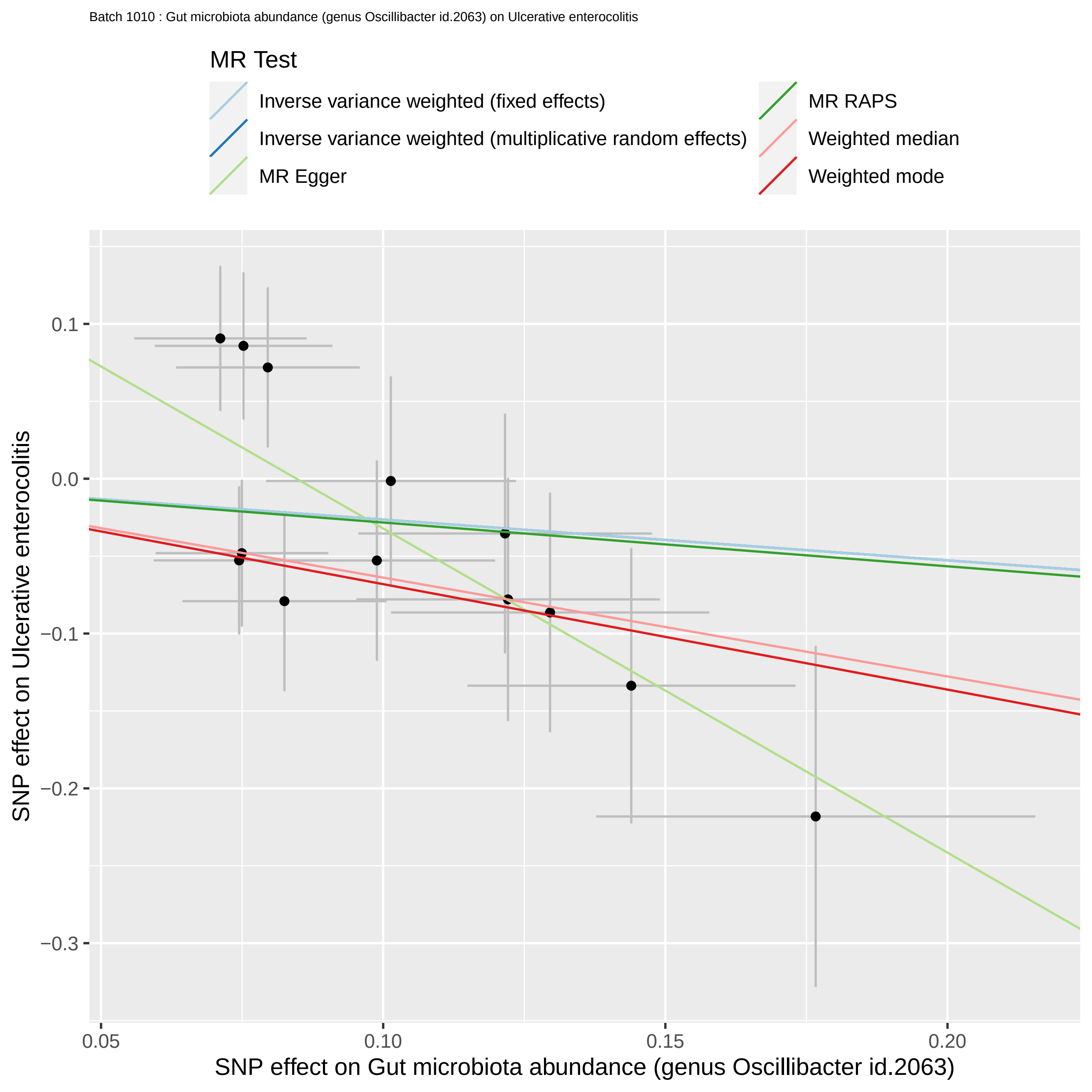

Batch 1010 : Gut microbiota abundance (genus Oscillibacter id.2063) on Ulcerative enterocolitis
MR Test
Inverse variance weighted (fixed effects)
Inverse variance weighted (multiplicative random effects)
MR Egger
MR RAPS
Weighted median
Weighted mode
0.1
0.0
−0.1
−0.2
−0.3
0.05
0.10
0.15
0.20
SNP effect on Ulcerative enterocolitis
SNP effect on Gut microbiota abundance (genus Oscillibacter id.2063)

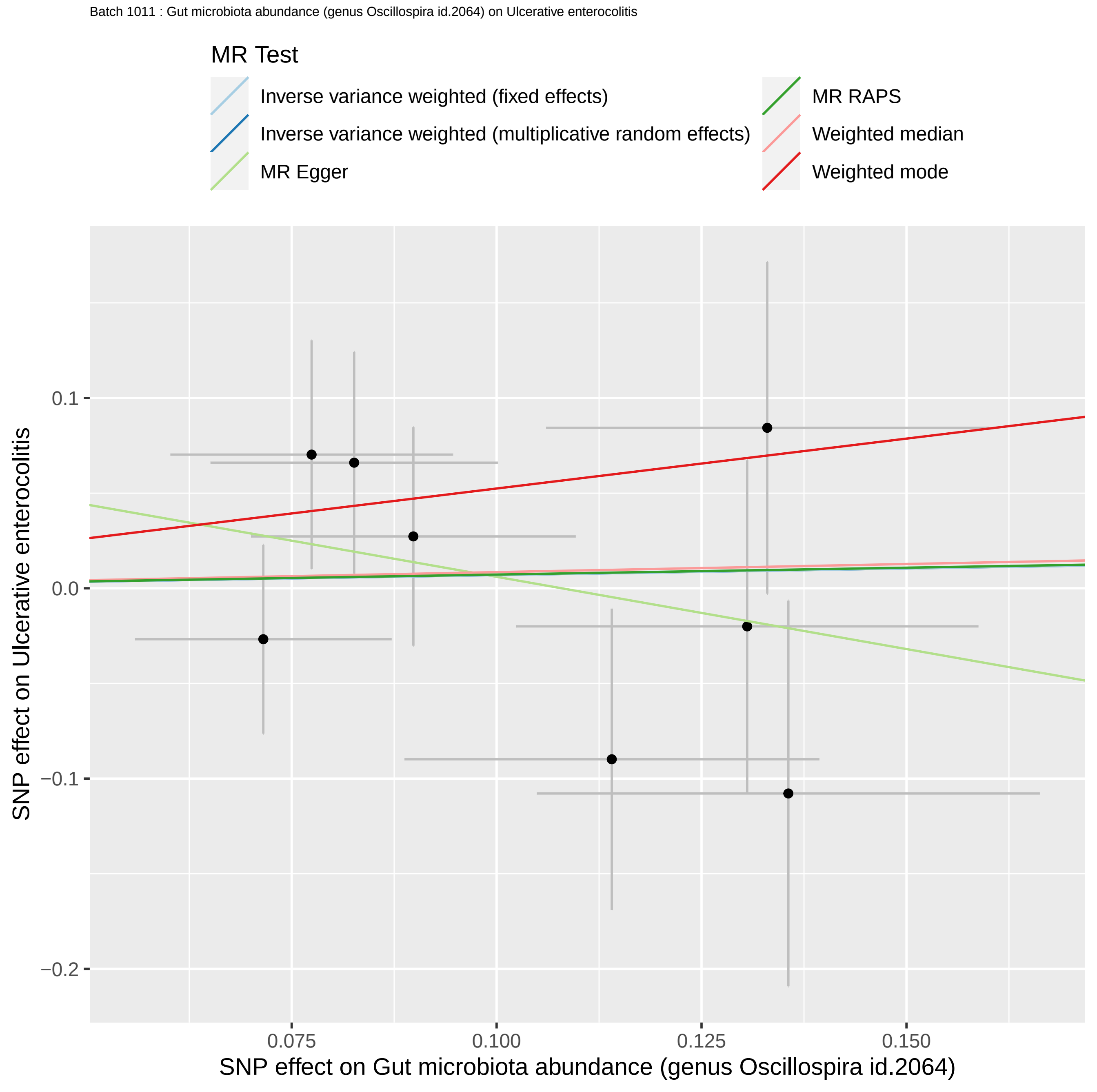

Batch 1011 : Gut microbiota abundance (genus Oscillospira id.2064) on Ulcerative enterocolitis
MR Test
Inverse variance weighted (fixed effects)
Inverse variance weighted (multiplicative random effects)
MR Egger
MR RAPS
Weighted median
Weighted mode
SNP effect on Ulcerative enterocolitis
0.1
0.0
−0.1
−0.2
0.075
0.100
0.125
0.150
SNP effect on Gut microbiota abundance (genus Oscillospira id.2064)

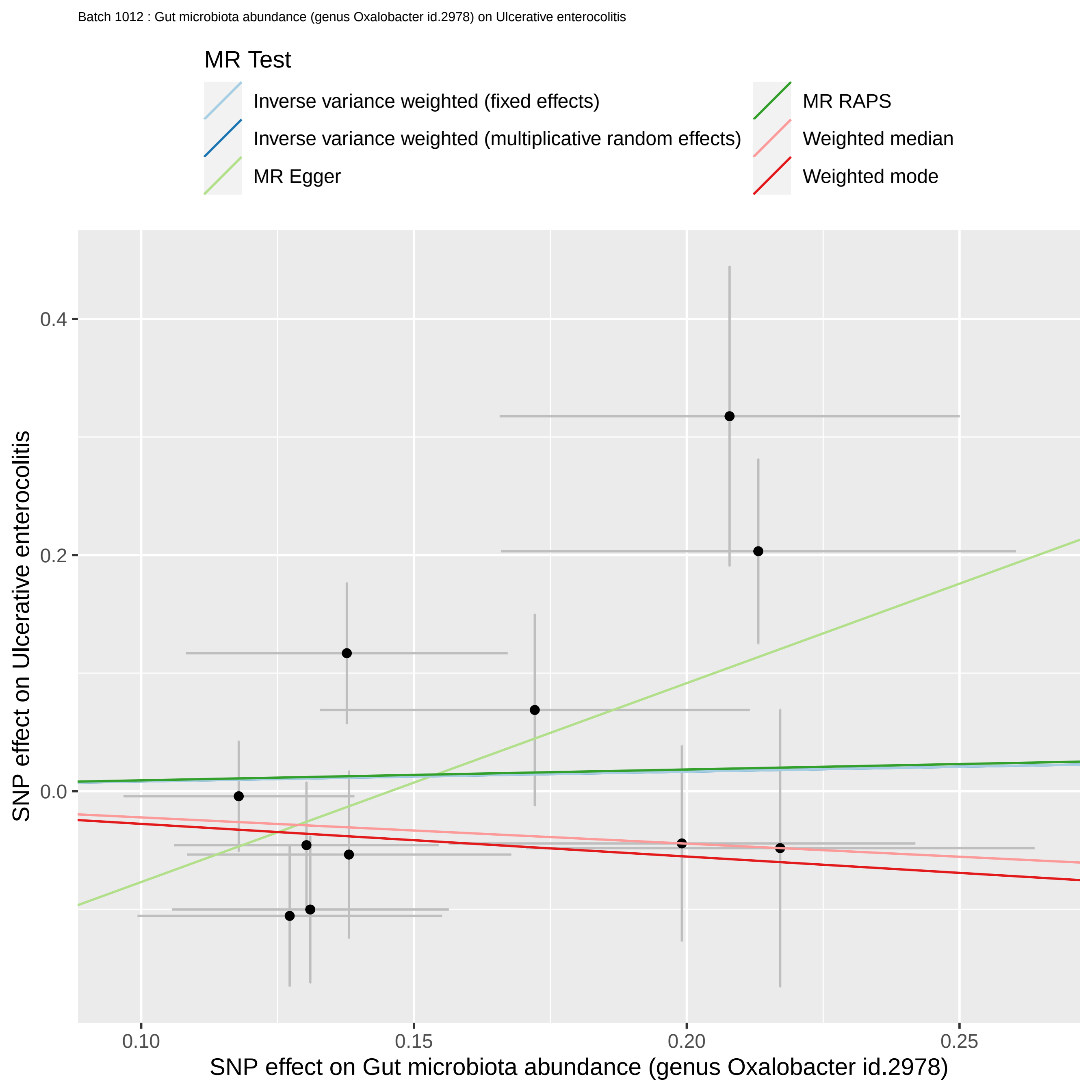

Batch 1012 : Gut microbiota abundance (genus Oxalobacter id.2978) on Ulcerative enterocolitis
MR Test
Inverse variance weighted (fixed effects)
Inverse variance weighted (multiplicative random effects)
MR Egger
MR RAPS
Weighted median
Weighted mode
SNP effect on Ulcerative enterocolitis
0.4
0.2
0.0
0.10
0.15
0.20
0.25
SNP effect on Gut microbiota abundance (genus Oxalobacter id.2978)

Batch 1013 : Gut microbiota abundance (genus Parabacteroides id.954) on Ulcerative enterocolitis

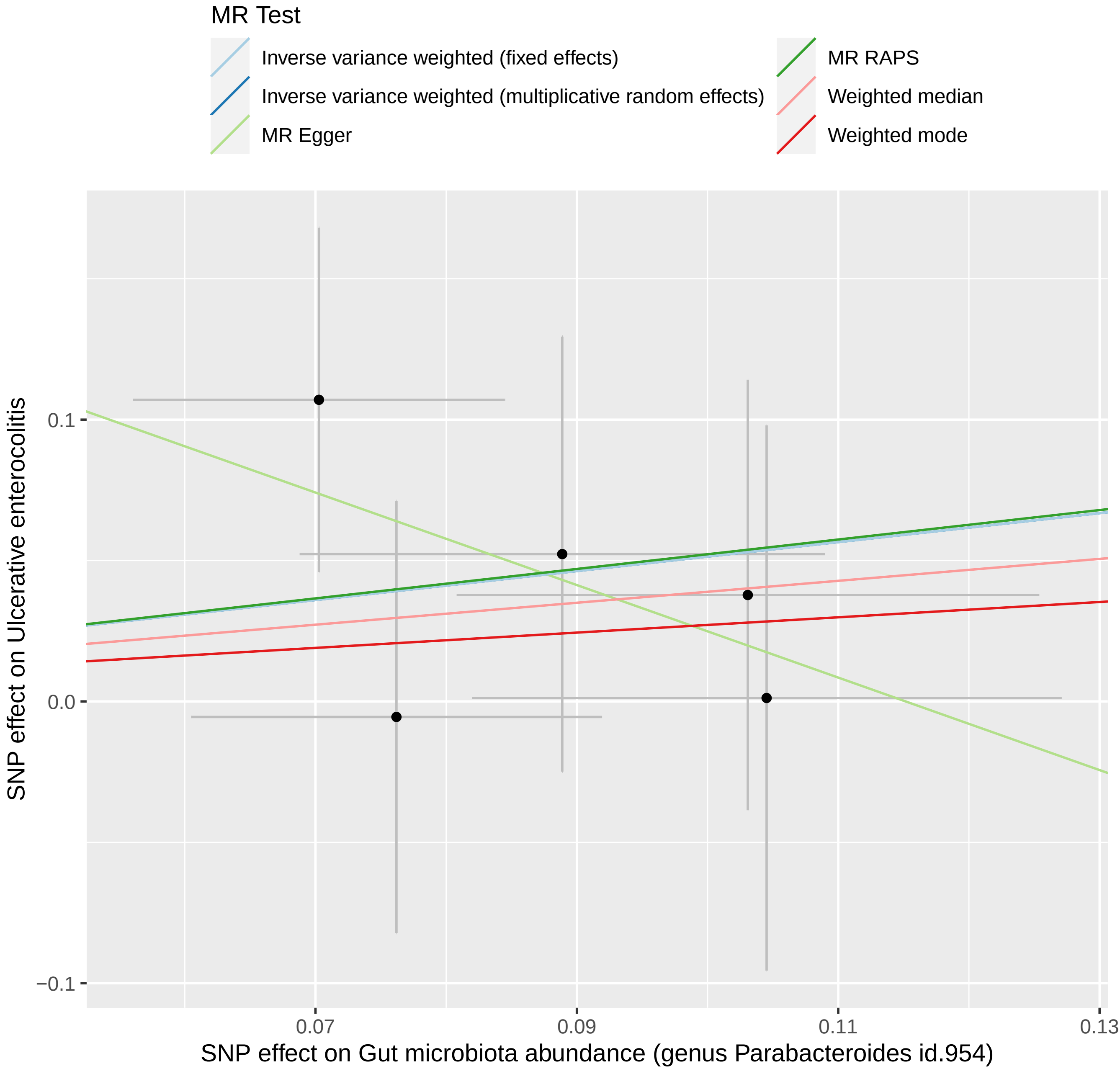

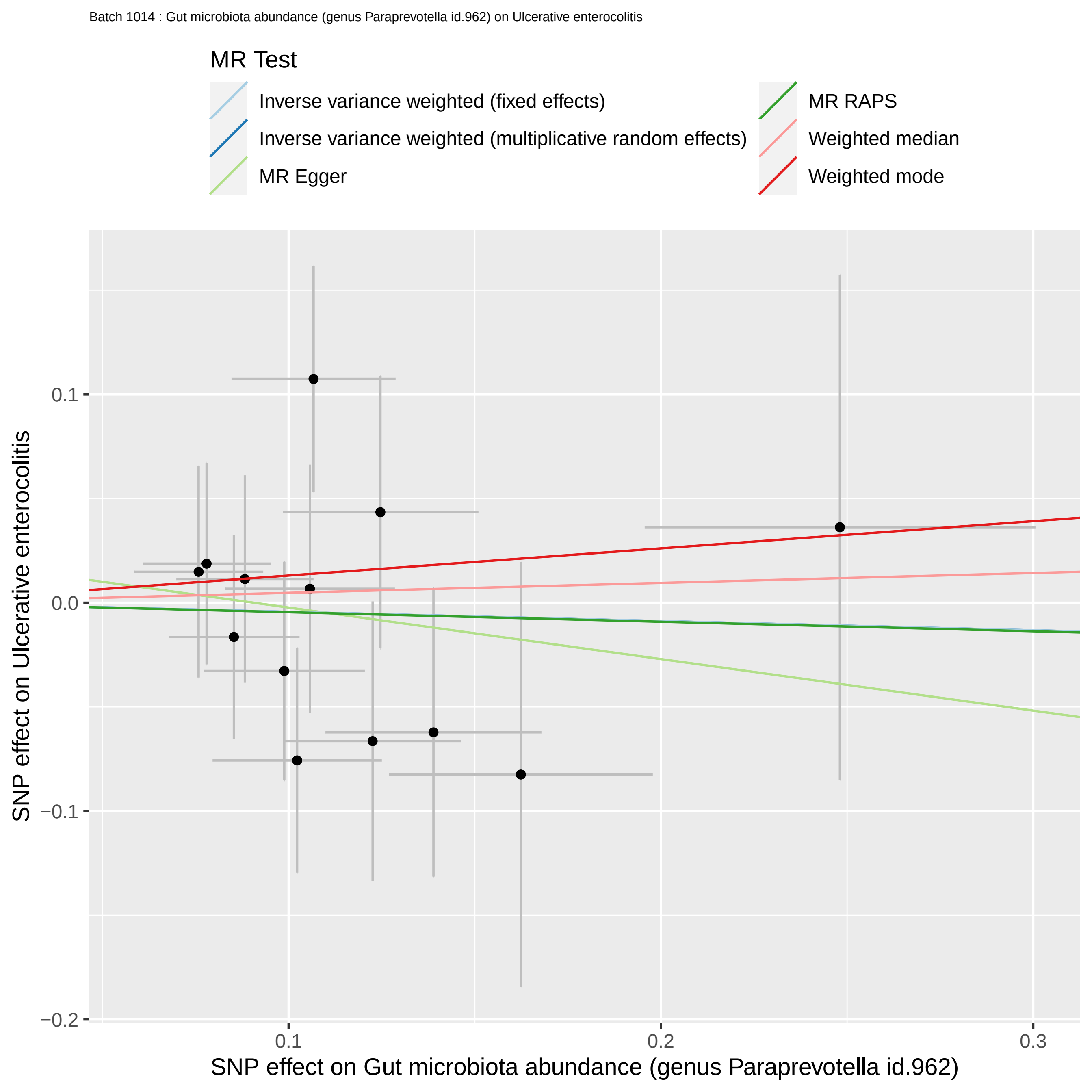
Batch 1014 : Gut microbiota abundance (genus Paraprevotella id.962) on Ulcerative enterocolitis
MR Test
Inverse variance weighted (fixed effects)
Inverse variance weighted (multiplicative random effects)
MR Egger
MR RAPS
Weighted median
Weighted mode
0.1
0.0
−0.1
−0.2
0.1
0.2
0.3
SNP effect on Ulcerative enterocolitis
SNP effect on Gut microbiota abundance (genus Paraprevotella id.962)

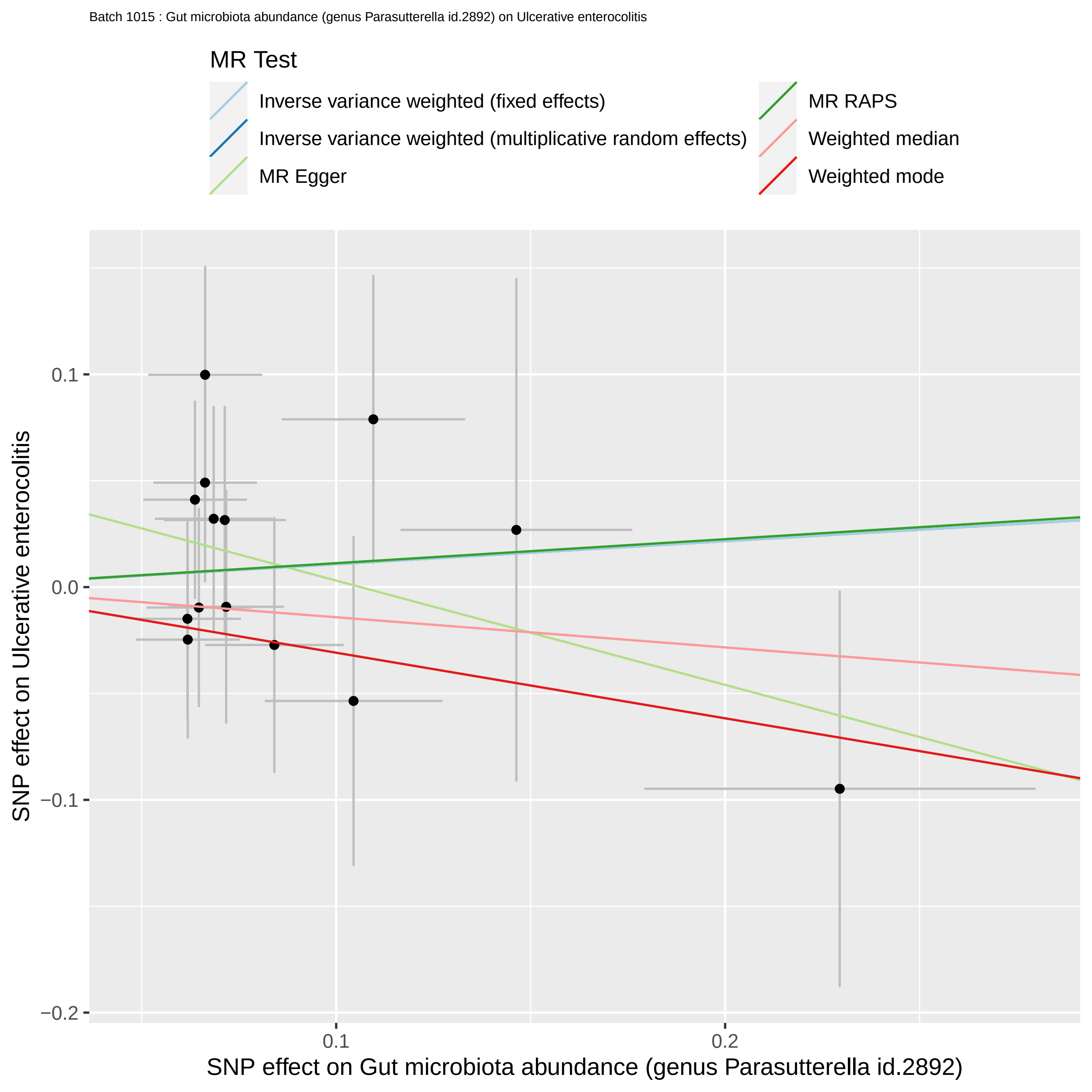

Batch 1015 : Gut microbiota abundance (genus Parasutterella id.2892) on Ulcerative enterocolitis
MR Test
Inverse variance weighted (fixed effects)
Inverse variance weighted (multiplicative random effects)
MR Egger
MR RAPS
Weighted median
Weighted mode
0.1
0.0
−0.1
−0.2
0.1
0.2
SNP effect on Ulcerative enterocolitis
SNP effect on Gut microbiota abundance (genus Parasutterella id.2892)

Batch 1016 : Gut microbiota abundance (genus Peptococcus id.2037) on Ulcerative enterocolitis

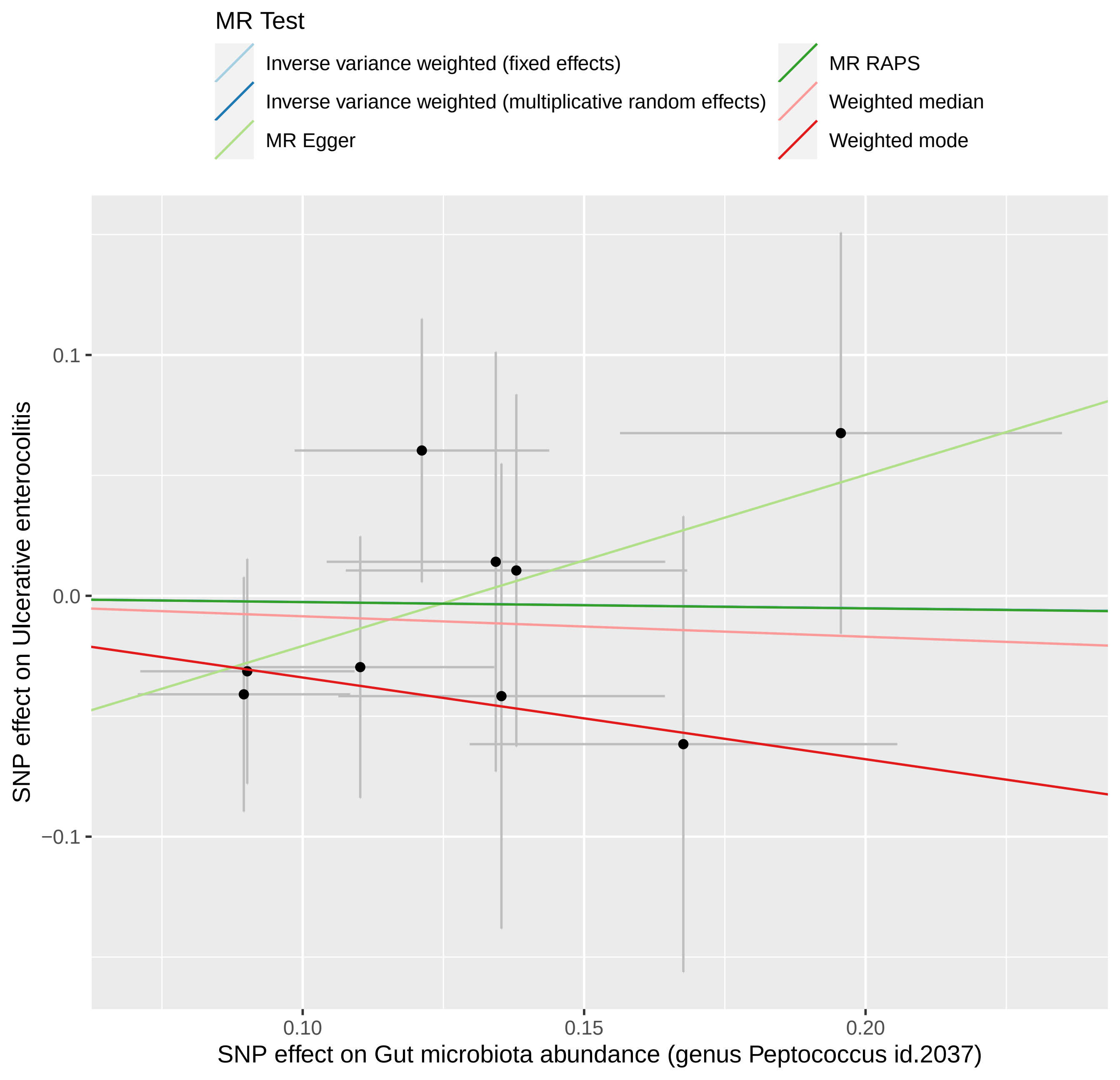

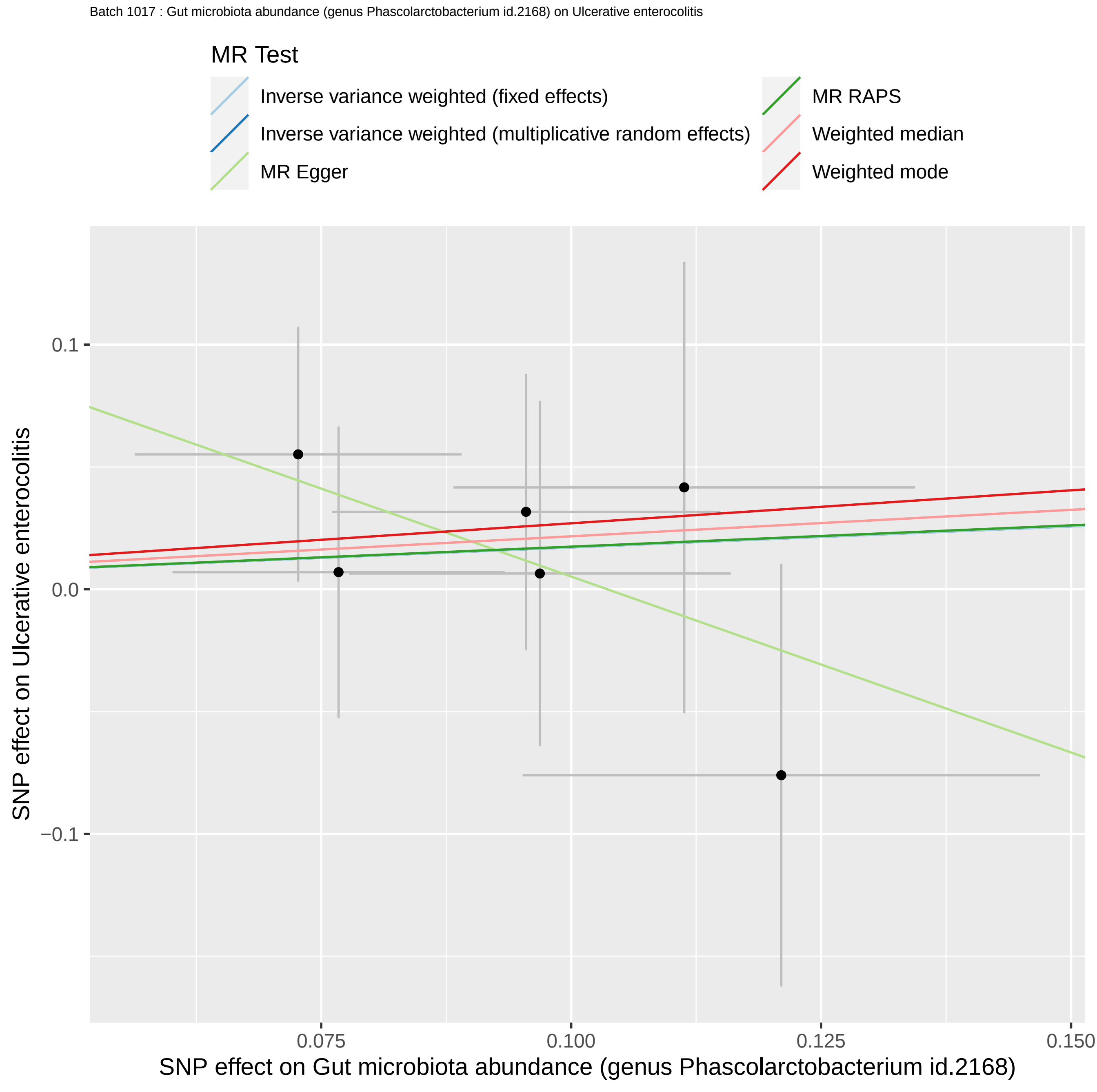
Batch 1017 : Gut microbiota abundance (genus Phascolarctobacterium id.2168) on Ulcerative enterocolitis
MR Test
Inverse variance weighted (fixed effects)
Inverse variance weighted (multiplicative random effects)
MR Egger
MR RAPS
Weighted median
Weighted mode
0.1
0.0
−0.1
0.075
0.100
0.125
0.150
SNP effect on Ulcerative enterocolitis
SNP effect on Gut microbiota abundance (genus Phascolarctobacterium id.2168)

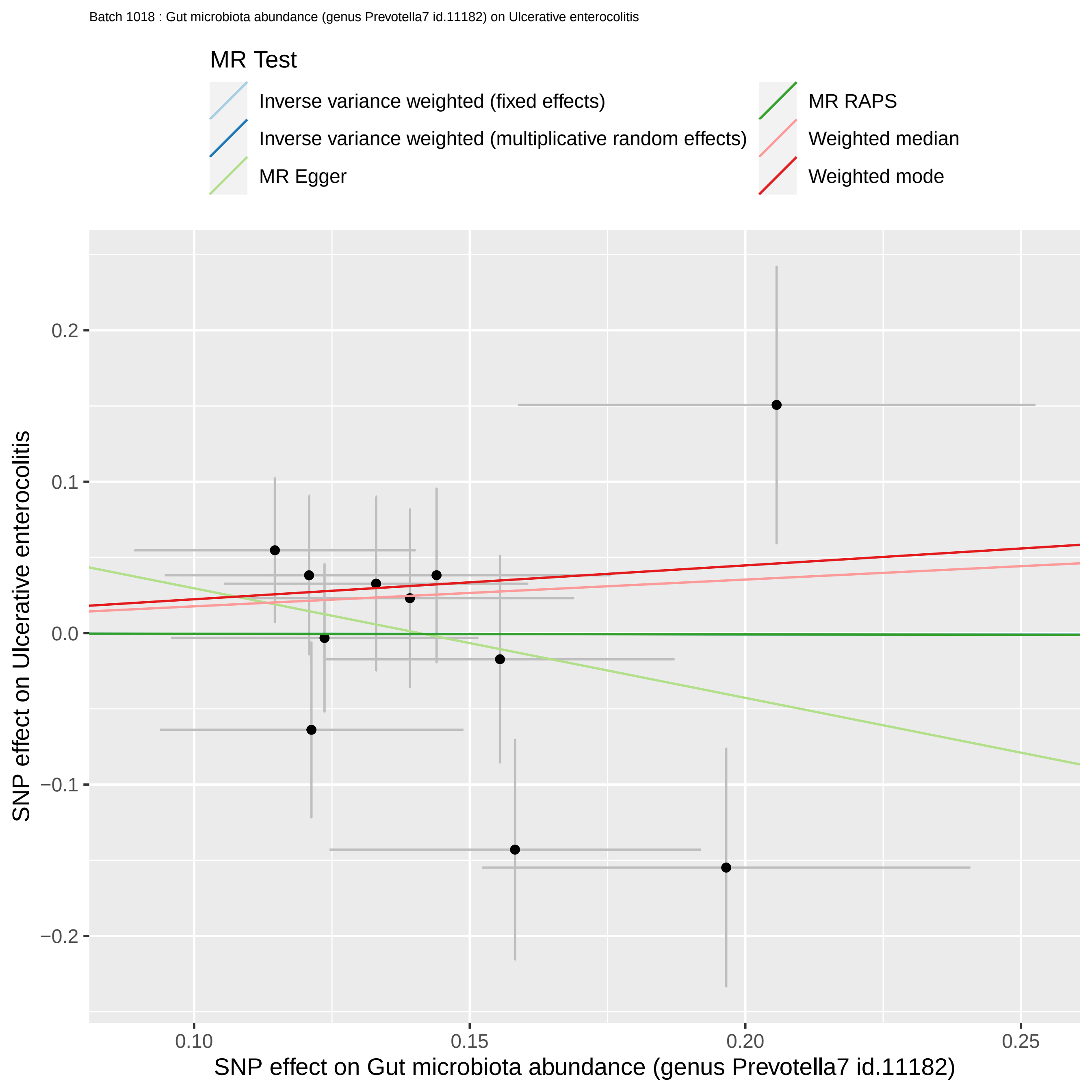

Batch 1018 : Gut microbiota abundance (genus Prevotella7 id.11182) on Ulcerative enterocolitis
MR Test
Inverse variance weighted (fixed effects)
Inverse variance weighted (multiplicative random effects)
MR Egger
MR RAPS
Weighted median
Weighted mode
SNP effect on Ulcerative enterocolitis
0.2
0.1
0.0
−0.1
−0.2
0.10
0.15
0.20
0.25
SNP effect on Gut microbiota abundance (genus Prevotella7 id.11182)

Batch 1019 : Gut microbiota abundance (genus Prevotella9 id.11183) on Ulcerative enterocolitis

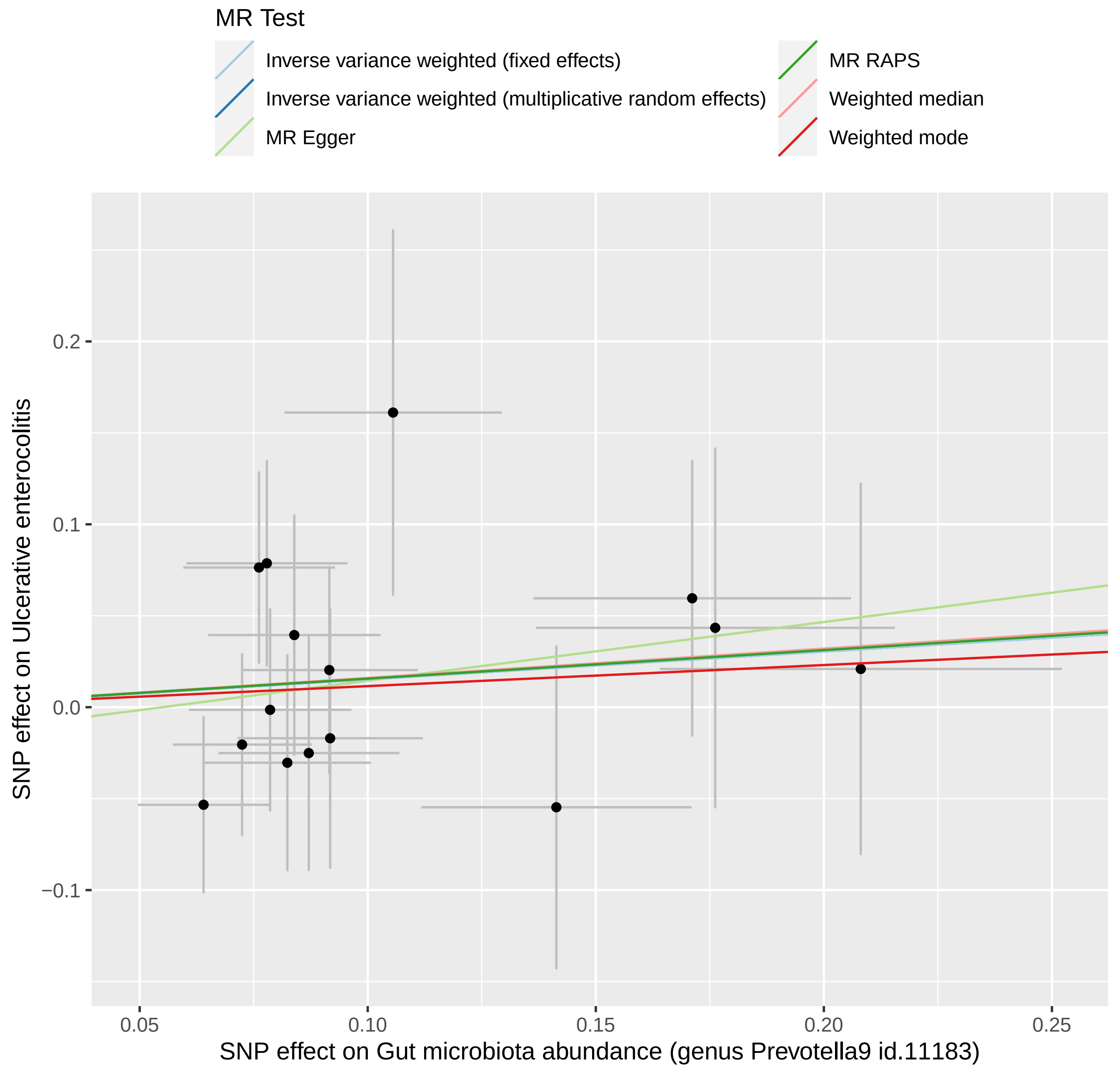

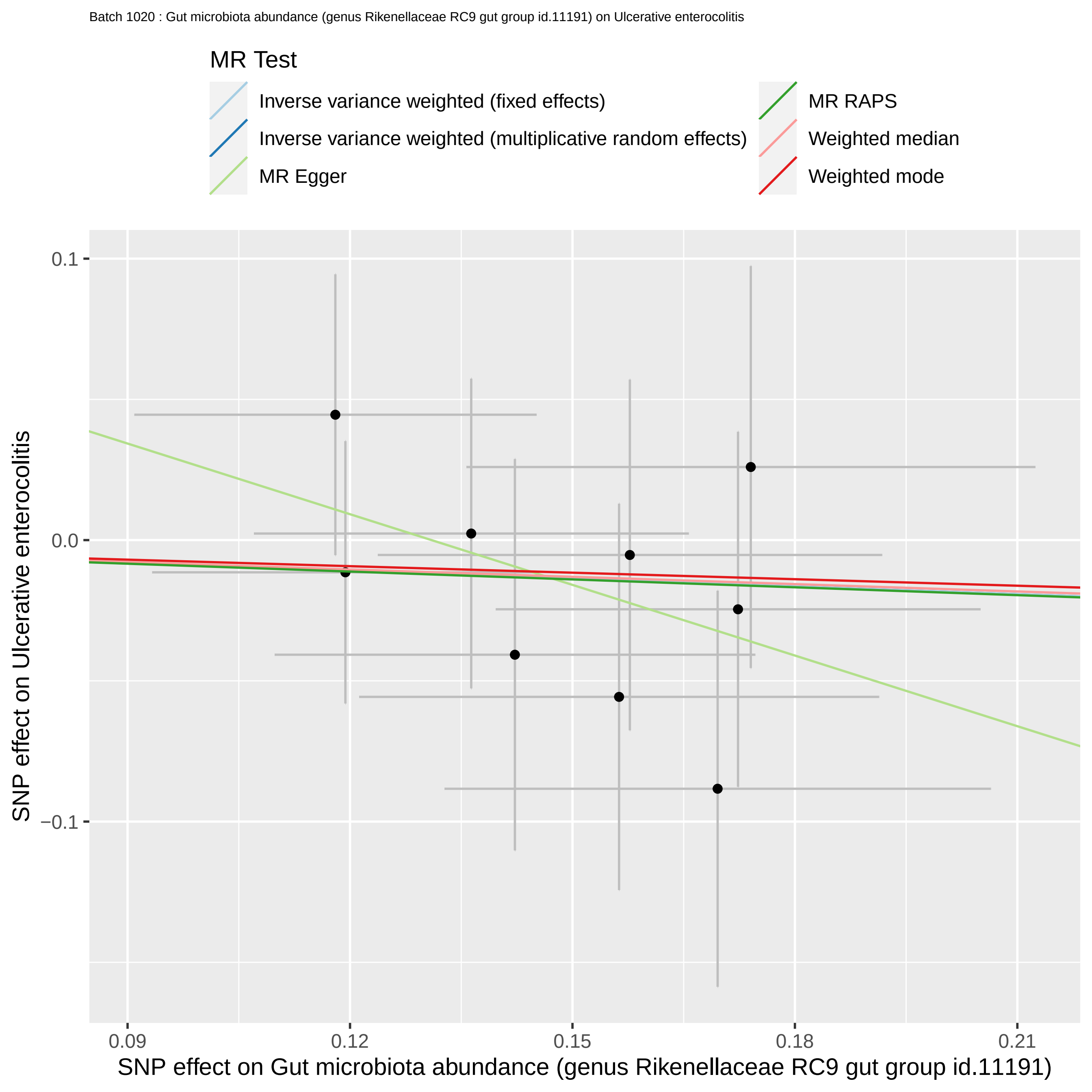

Batch 1020 : Gut microbiota abundance (genus Rikenellaceae RC9 gut group id.11191) on Ulcerative enterocolitis
MR Test
Inverse variance weighted (fixed effects)
Inverse variance weighted (multiplicative random effects)
MR Egger
MR RAPS
Weighted median
Weighted mode
0.1
0.0
−0.1
0.09
0.12
0.15
0.18
0.21
SNP effect on Ulcerative enterocolitis
SNP effect on Gut microbiota abundance (genus Rikenellaceae RC9 gut group id.11191)

Batch 1021 : Gut microbiota abundance (genus Romboutsia id.11347) on Ulcerative enterocolitis

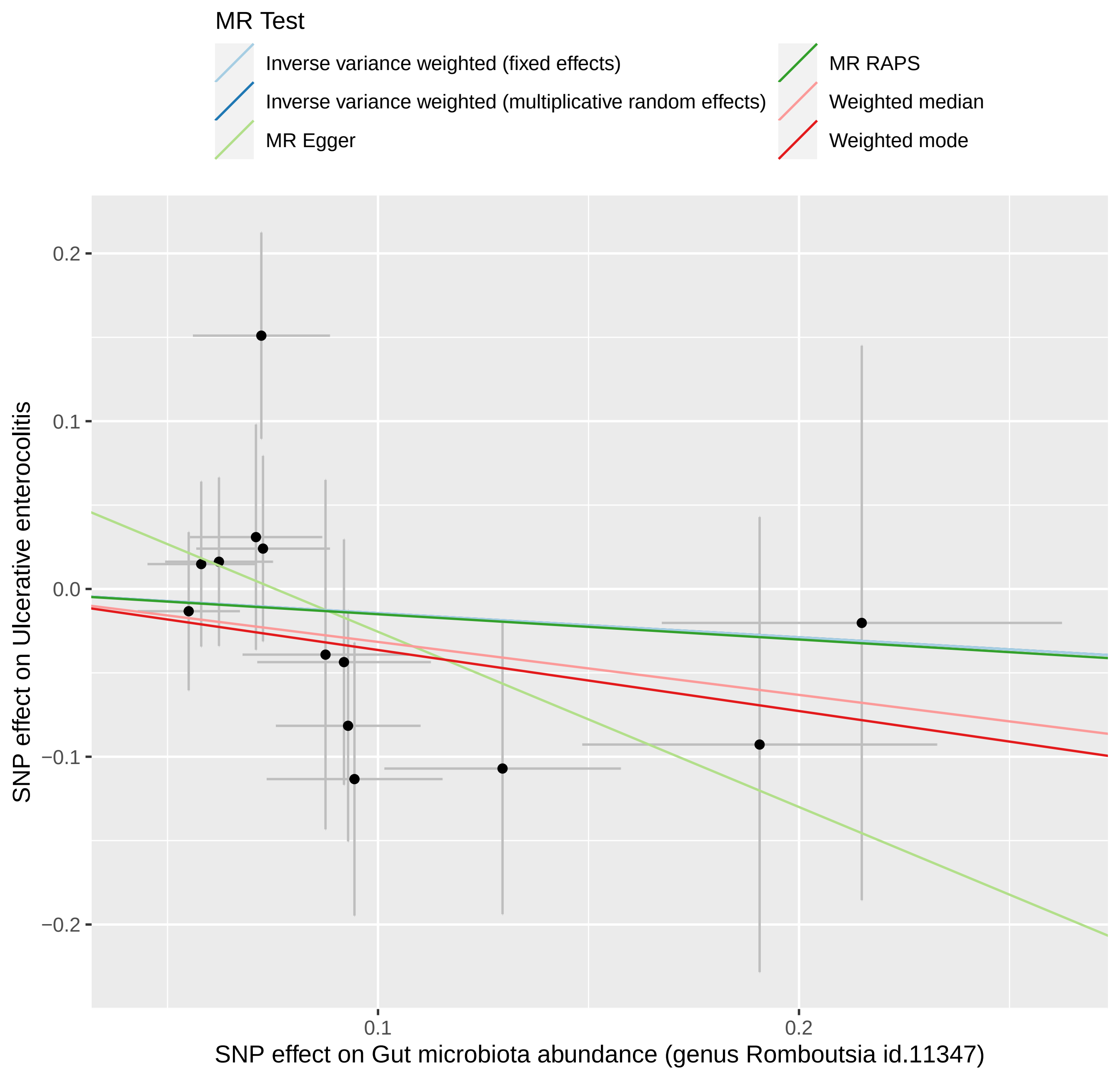

Batch 1022 : Gut microbiota abundance (genus Roseburia id.2012) on Ulcerative enterocolitis

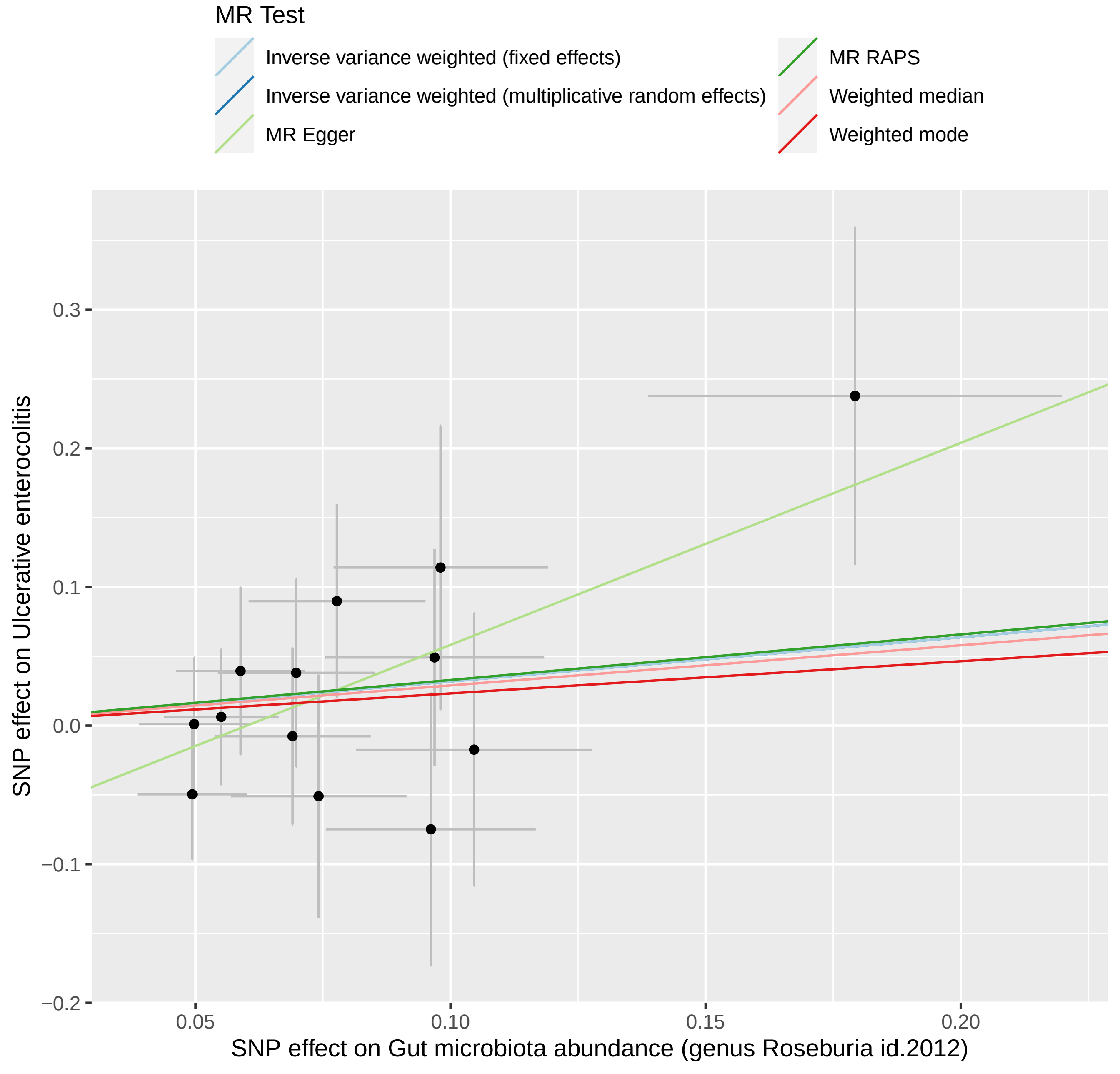

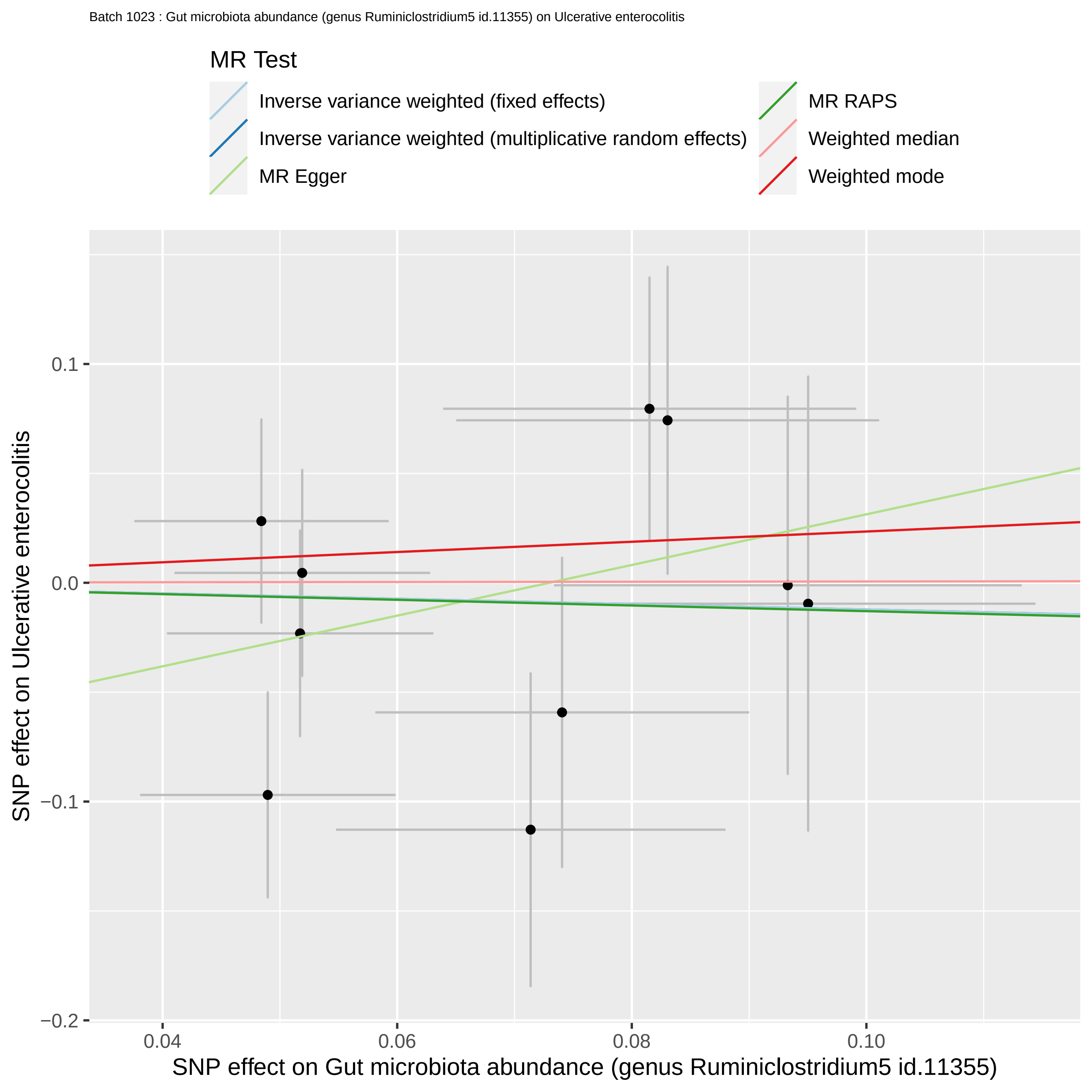
Batch 1023 : Gut microbiota abundance (genus Ruminiclostridium5 id.11355) on Ulcerative enterocolitis
MR Test
Inverse variance weighted (fixed effects)
Inverse variance weighted (multiplicative random effects)
MR Egger
MR RAPS
Weighted median
Weighted mode
0.1
0.0
−0.1
−0.2
0.04
0.06
0.08
0.10
SNP effect on Ulcerative enterocolitis
SNP effect on Gut microbiota abundance (genus Ruminiclostridium5 id.11355)

Batch 1024 : Gut microbiota abundance (genus Ruminiclostridium6 id.11356) on Ulcerative enterocolitis

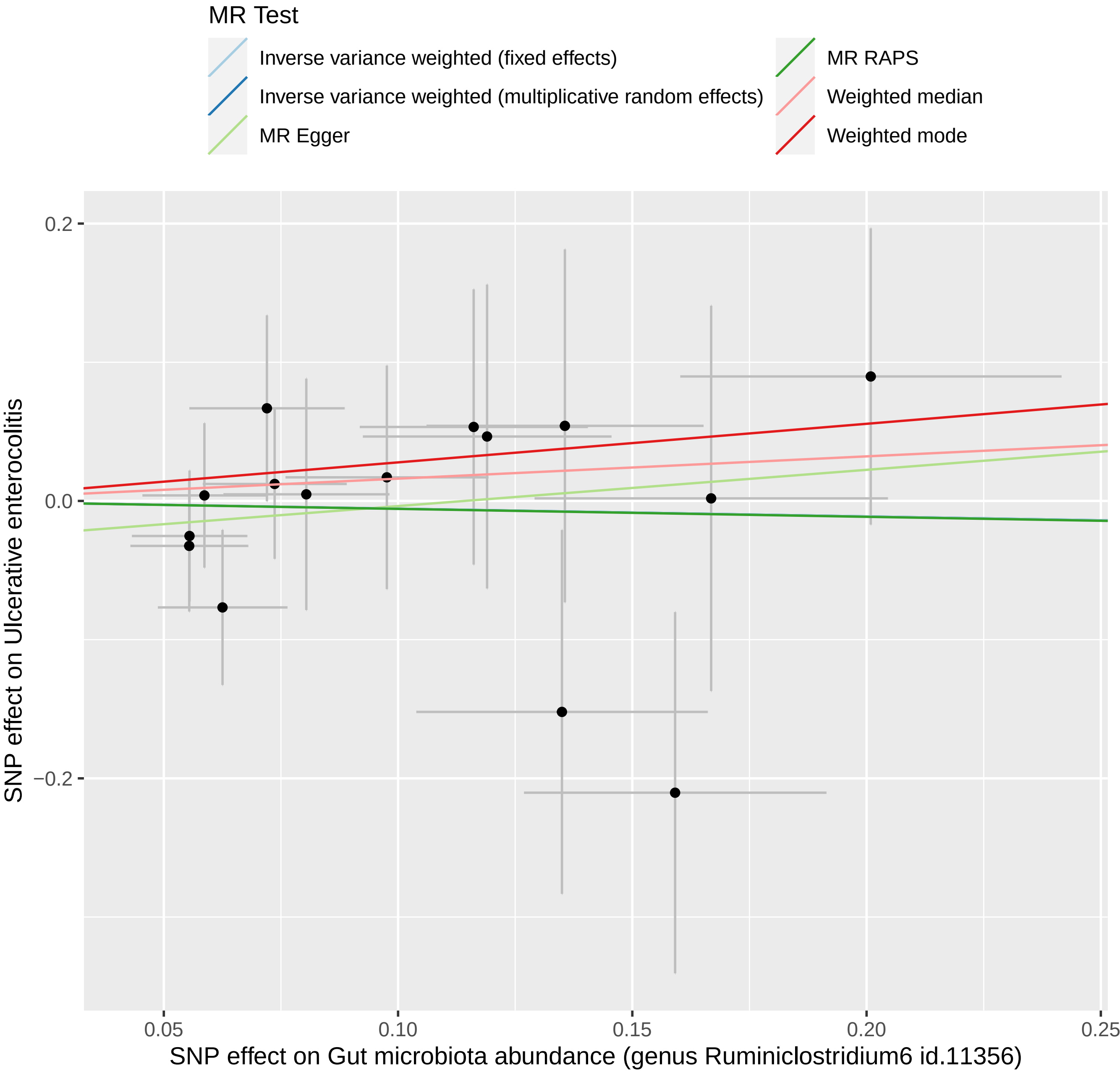

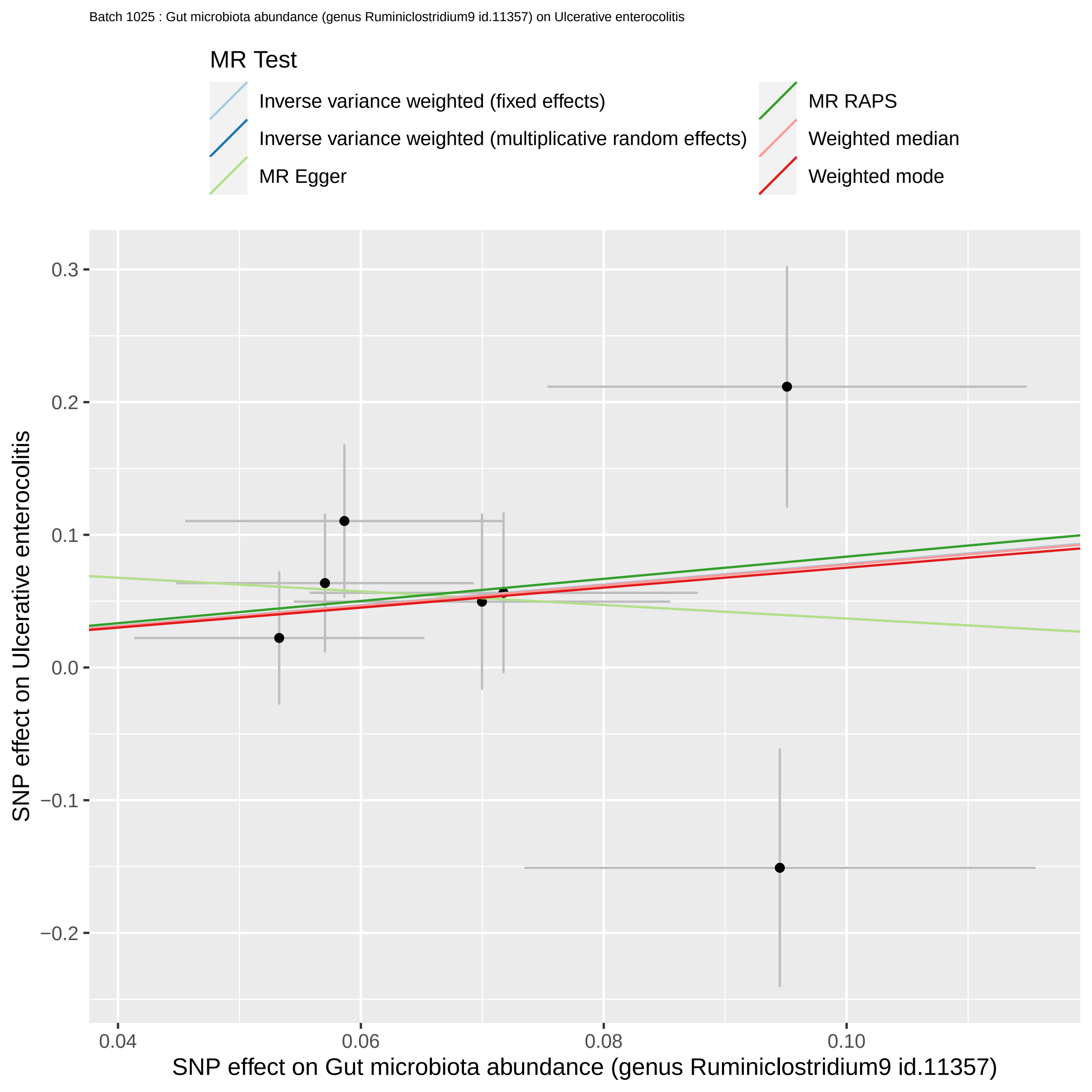

Batch 1025 : Gut microbiota abundance (genus Ruminiclostridium9 id.11357) on Ulcerative enterocolitis
MR Test
Inverse variance weighted (fixed effects)
Inverse variance weighted (multiplicative random effects)
MR Egger
MR RAPS
Weighted median
Weighted mode
0.3
0.2
0.1
0.0
−0.1
−0.2
0.04
0.06
0.08
0.10
SNP effect on Ulcerative enterocolitis
SNP effect on Gut microbiota abundance (genus Ruminiclostridium9 id.11357)

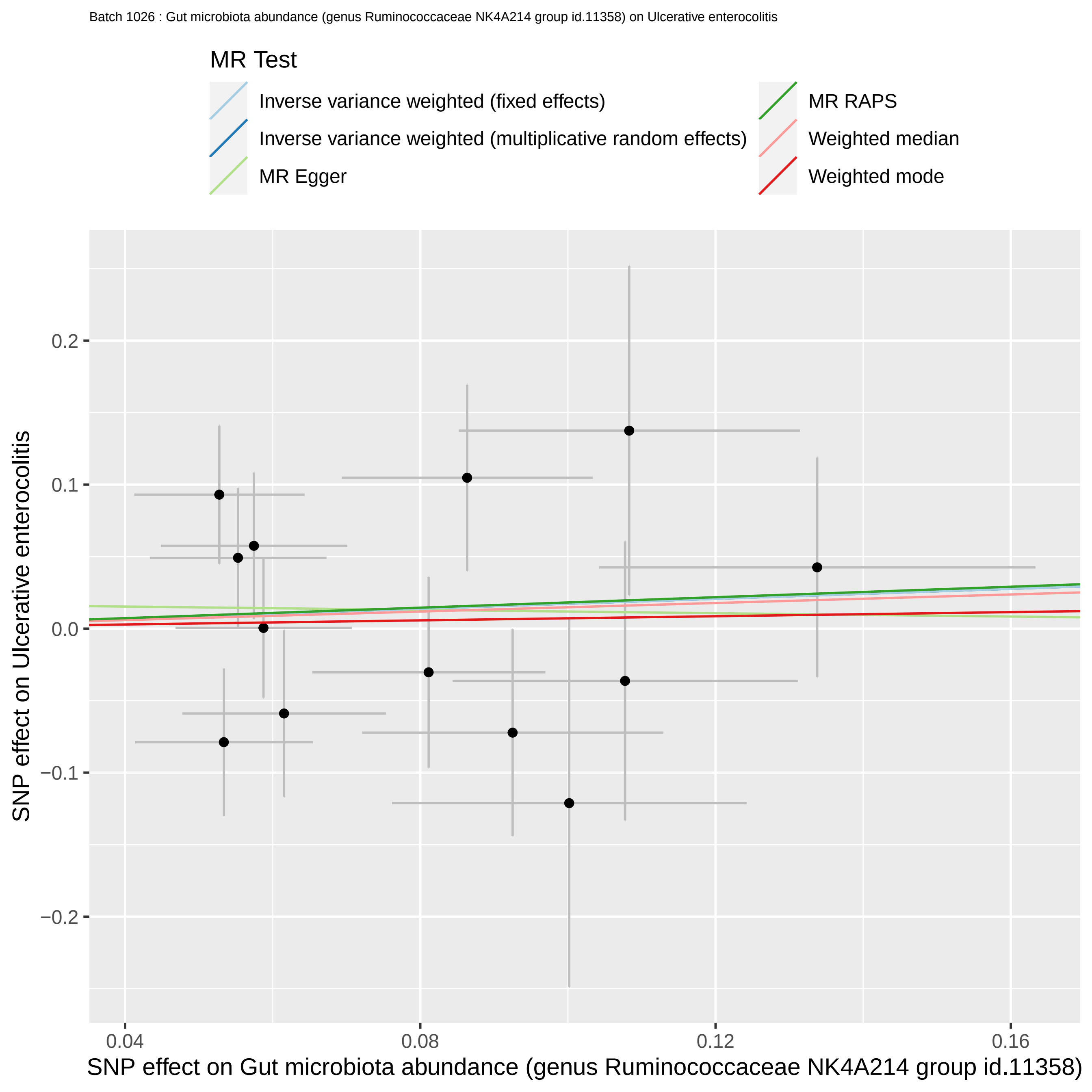
Batch 1026 : Gut microbiota abundance (genus Ruminococcaceae NK4A214 group id.11358) on Ulcerative enterocolitis
MR Test
Inverse variance weighted (fixed effects)
Inverse variance weighted (multiplicative random effects)
MR Egger
MR RAPS
Weighted median
Weighted mode
0.2
0.1
0.0
−0.1
−0.2
0.04
0.08
0.12
0.16
SNP effect on Ulcerative enterocolitis
SNP effect on Gut microbiota abundance (genus Ruminococcaceae NK4A214 group id.11358)

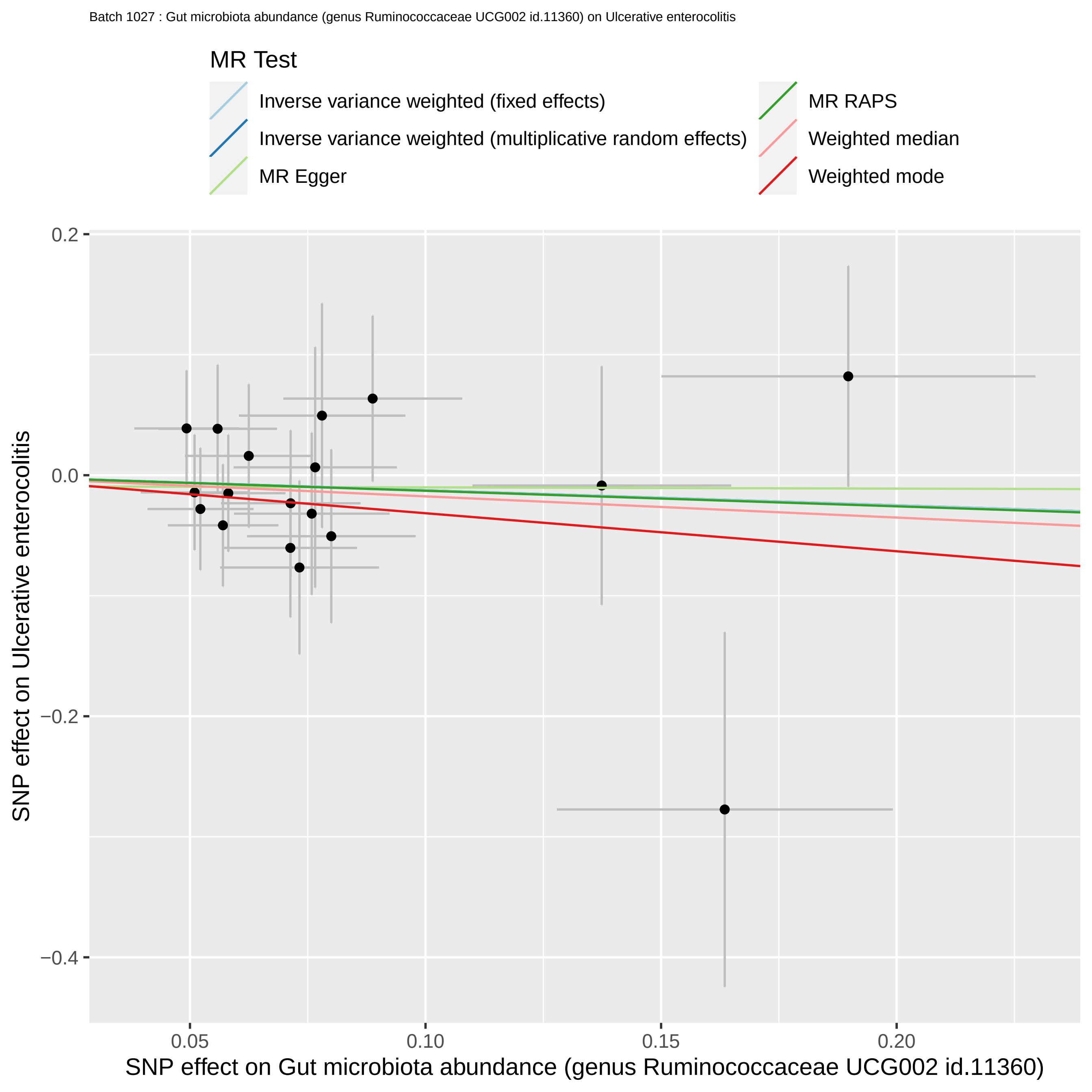
Batch 1027 : Gut microbiota abundance (genus Ruminococcaceae UCG002 id.11360) on Ulcerative enterocolitis
MR Test
Inverse variance weighted (fixed effects)
Inverse variance weighted (multiplicative random effects)
MR Egger
MR RAPS
Weighted median
Weighted mode
0.2
0.0
−0.2
−0.4
0.05
0.10
0.15
0.20
SNP effect on Ulcerative enterocolitis
SNP effect on Gut microbiota abundance (genus Ruminococcaceae UCG002 id.11360)

Batch 1028 : Gut microbiota abundance (genus Ruminococcaceae UCG003 id.11361) on Ulcerative enterocolitis

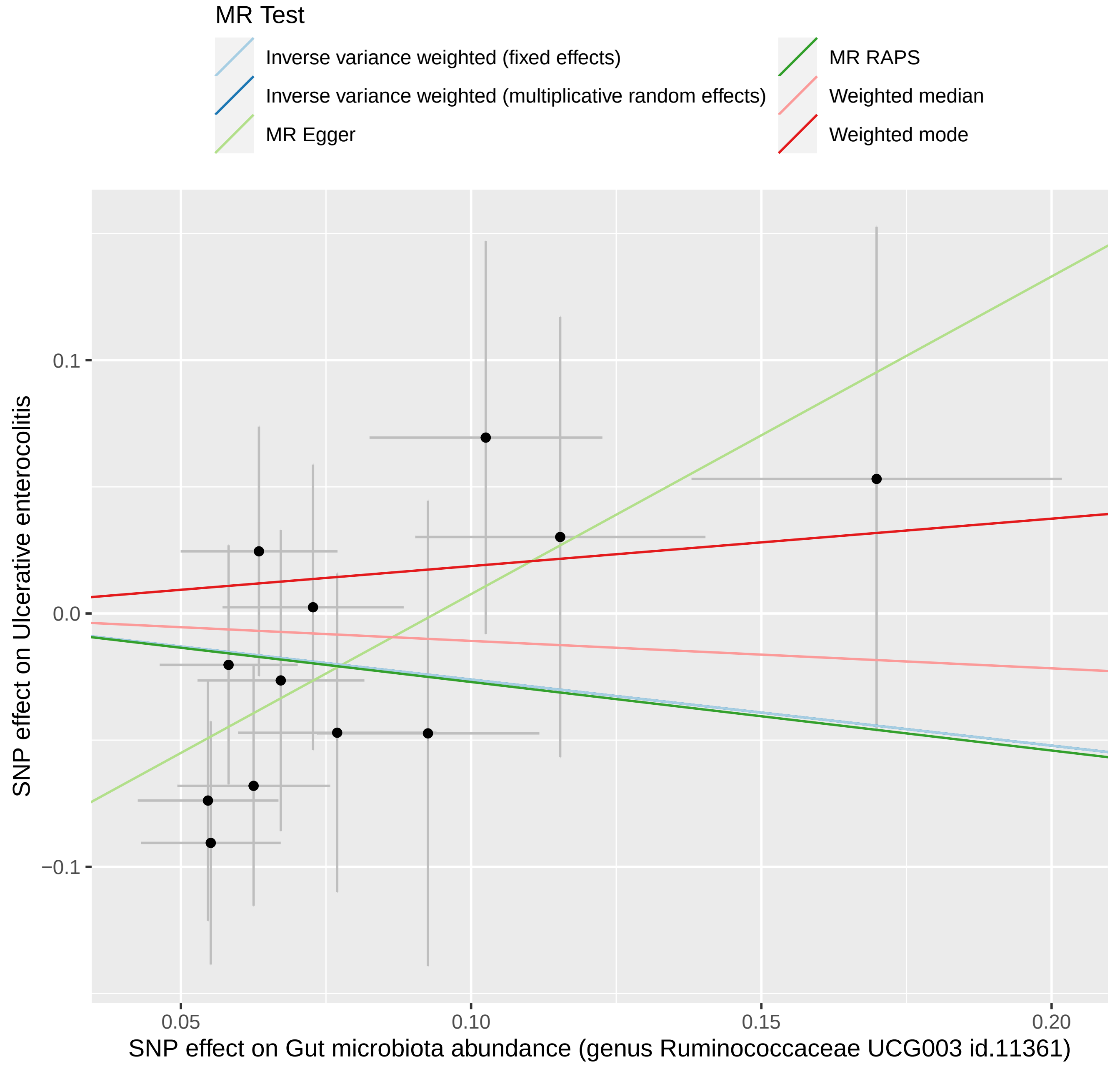

Batch 1029 : Gut microbiota abundance (genus Ruminococcaceae UCG004 id.11362) on Ulcerative enterocolitis

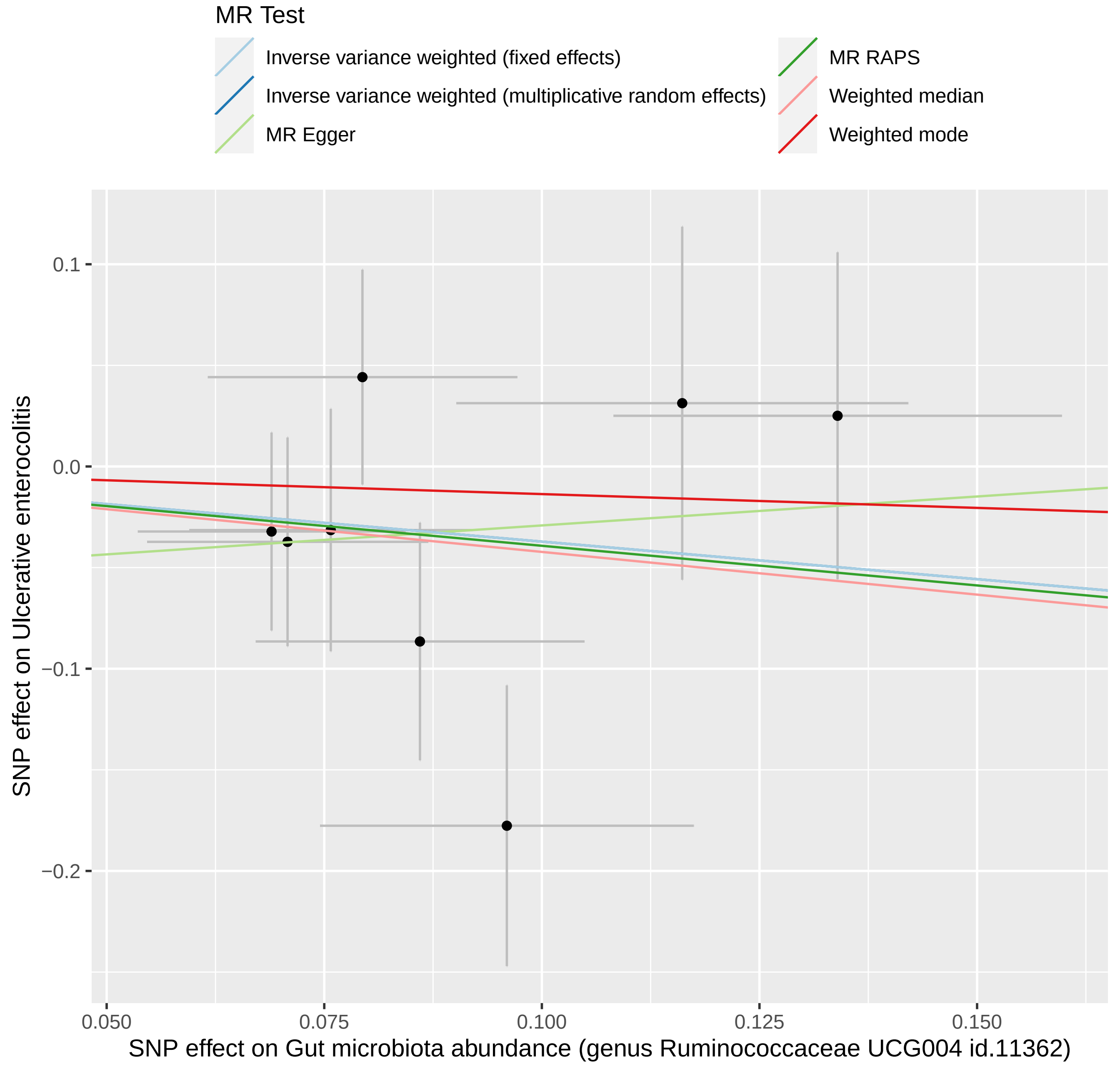

Batch 1030 : Gut microbiota abundance (genus Ruminococcaceae UCG005 id.11363) on Ulcerative enterocolitis

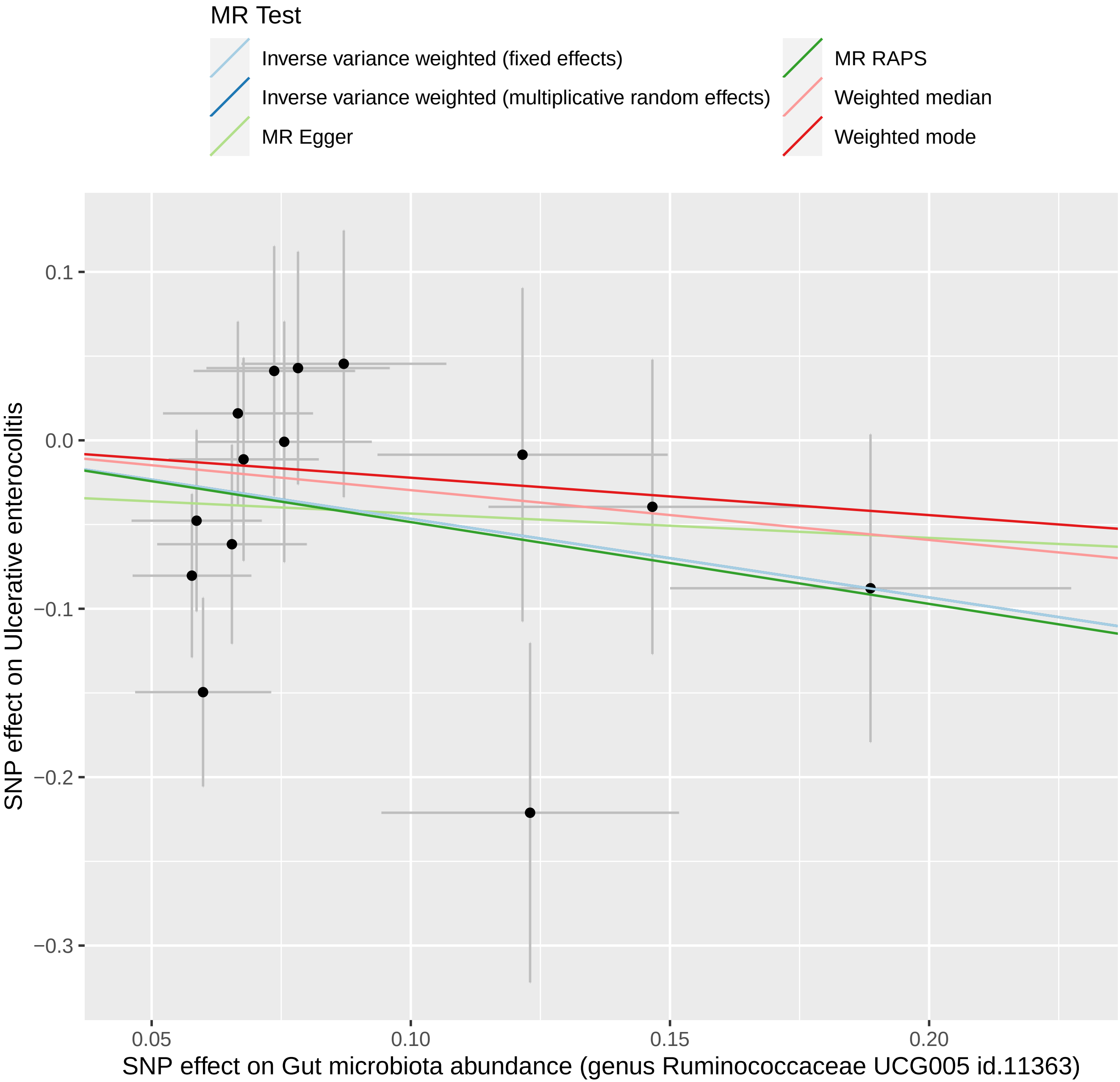

Batch 1031 : Gut microbiota abundance (genus Ruminococcaceae UCG009 id.11366) on Ulcerative enterocolitis

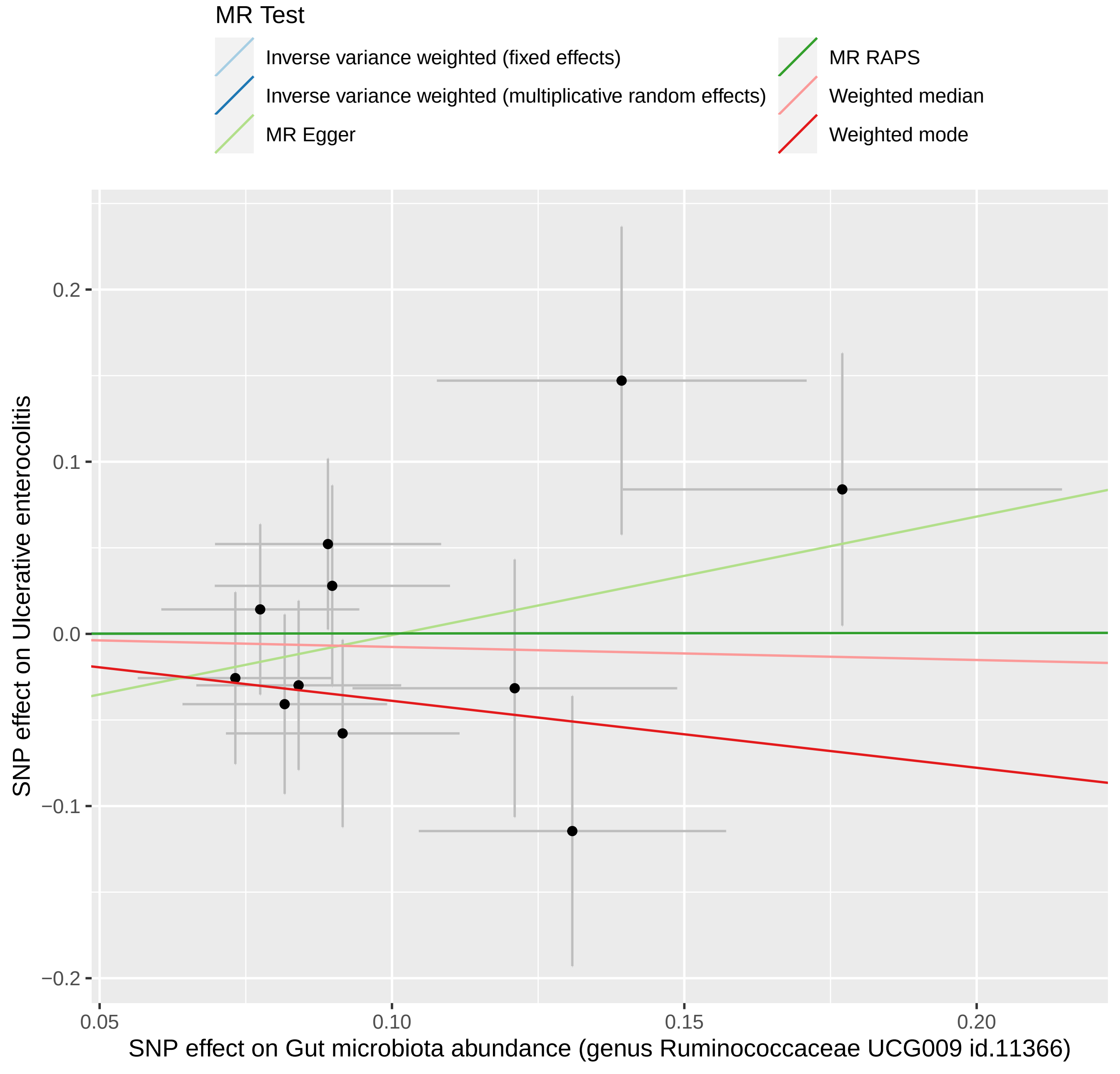

Batch 1032 : Gut microbiota abundance (genus Ruminococcaceae UCG010 id.11367) on Ulcerative enterocolitis

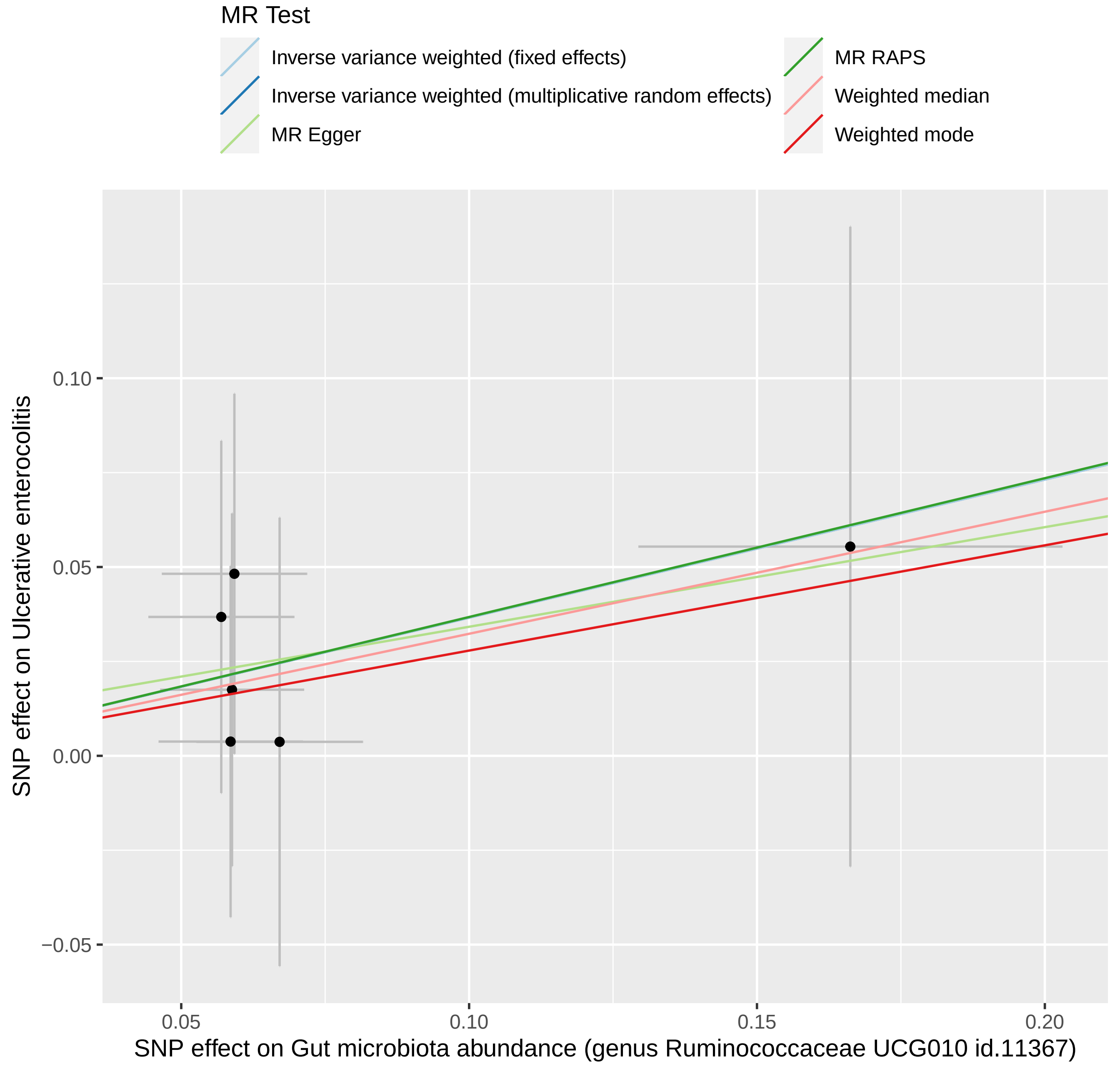

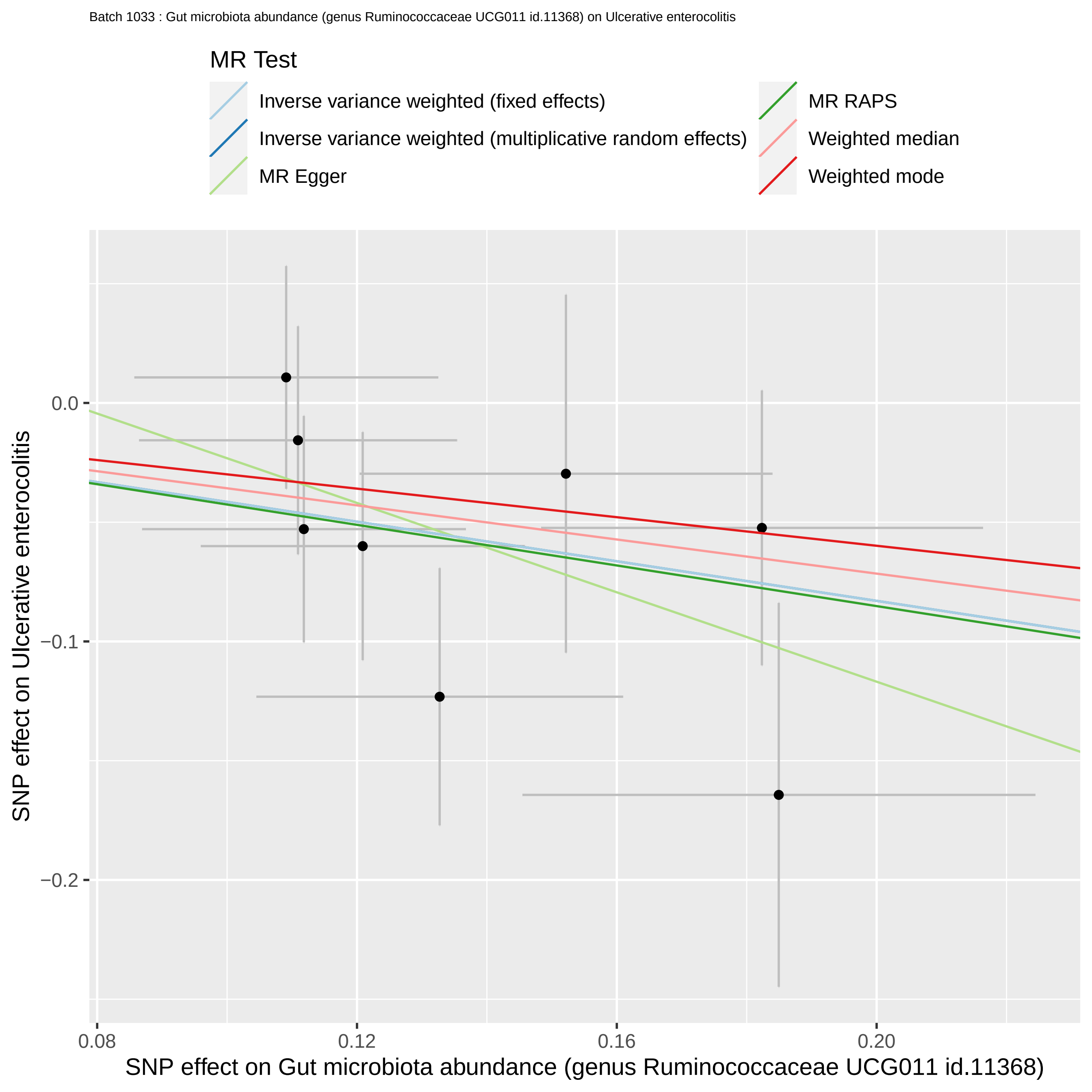
Batch 1033 : Gut microbiota abundance (genus Ruminococcaceae UCG011 id.11368) on Ulcerative enterocolitis
MR Test
Inverse variance weighted (fixed effects)
Inverse variance weighted (multiplicative random effects)
MR Egger
MR RAPS
Weighted median
Weighted mode
SNP effect on Ulcerative enterocolitis
0.0
−0.1
−0.2
0.08
0.12
0.16
0.20
SNP effect on Gut microbiota abundance (genus Ruminococcaceae UCG011 id.11368)

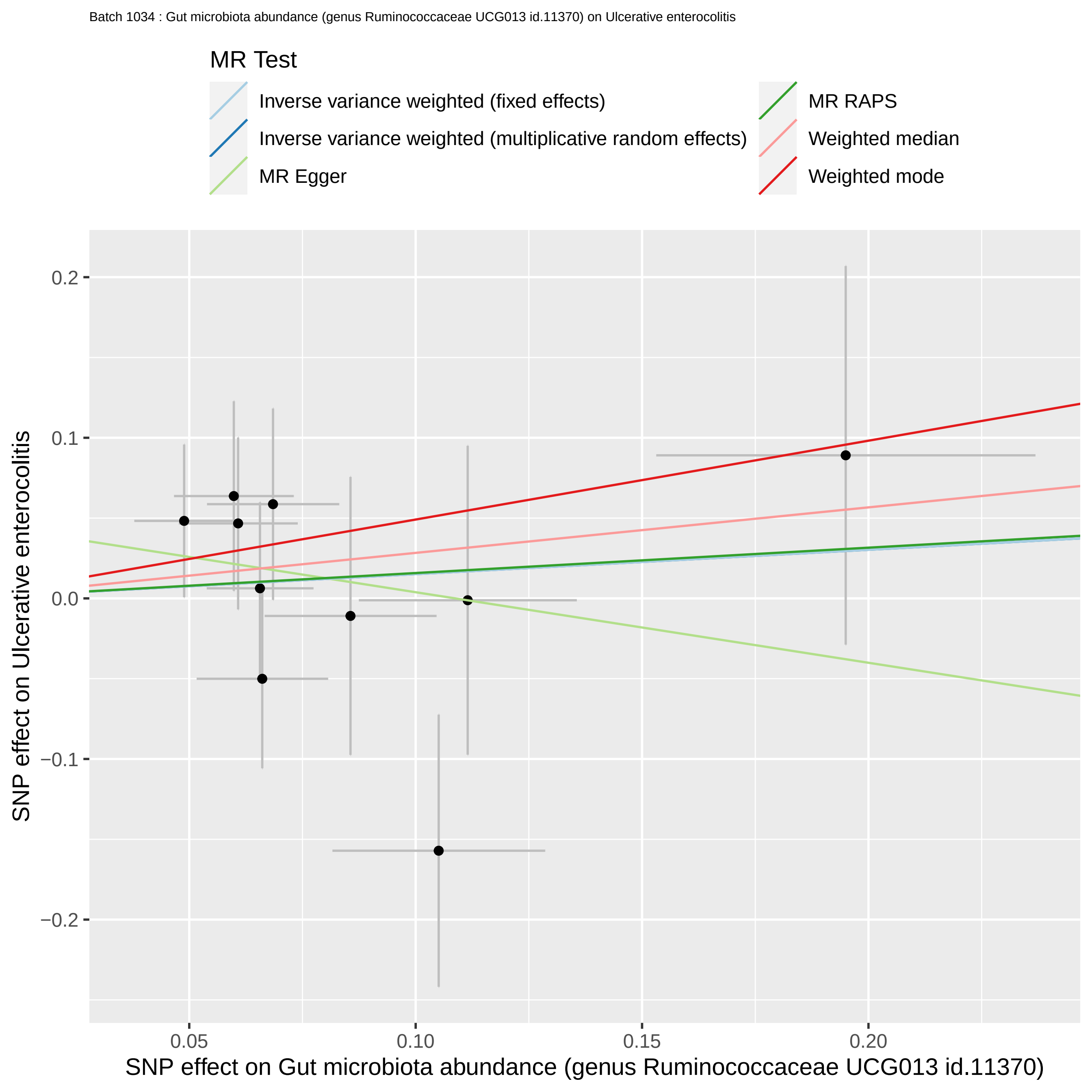

Batch 1034 : Gut microbiota abundance (genus Ruminococcaceae UCG013 id.11370) on Ulcerative enterocolitis
MR Test
Inverse variance weighted (fixed effects)
Inverse variance weighted (multiplicative random effects)
MR Egger
MR RAPS
Weighted median
Weighted mode
SNP effect on Ulcerative enterocolitis
0.2
0.1
0.0
−0.1
−0.2
0.05
0.10
0.15
0.20
SNP effect on Gut microbiota abundance (genus Ruminococcaceae UCG013 id.11370)

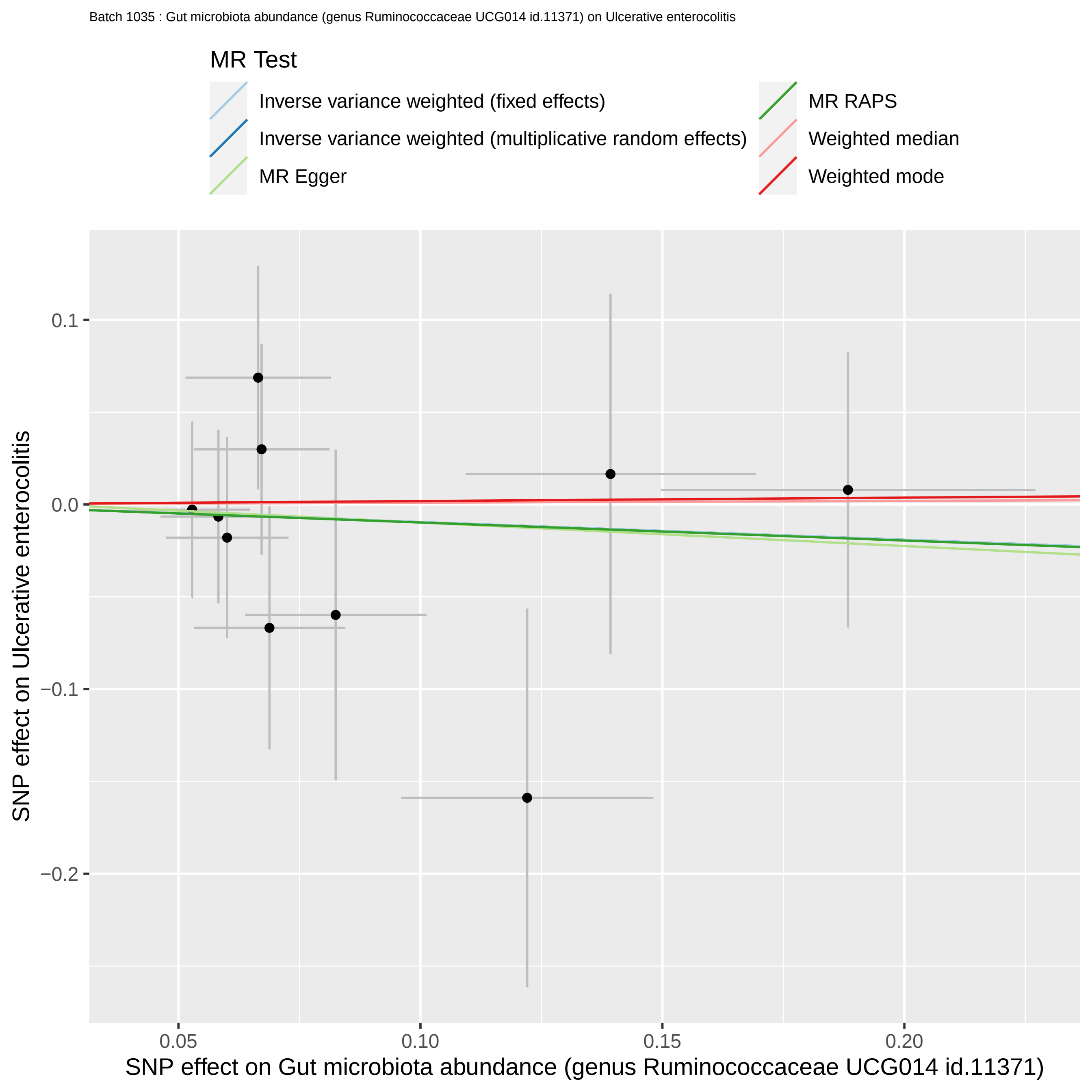

Batch 1035 : Gut microbiota abundance (genus Ruminococcaceae UCG014 id.11371) on Ulcerative enterocolitis
MR Test
Inverse variance weighted (fixed effects)
Inverse variance weighted (multiplicative random effects)
MR Egger
MR RAPS
Weighted median
Weighted mode
0.1
0.0
−0.1
−0.2
0.05
0.10
0.15
0.20
SNP effect on Ulcerative enterocolitis
SNP effect on Gut microbiota abundance (genus Ruminococcaceae UCG014 id.11371)

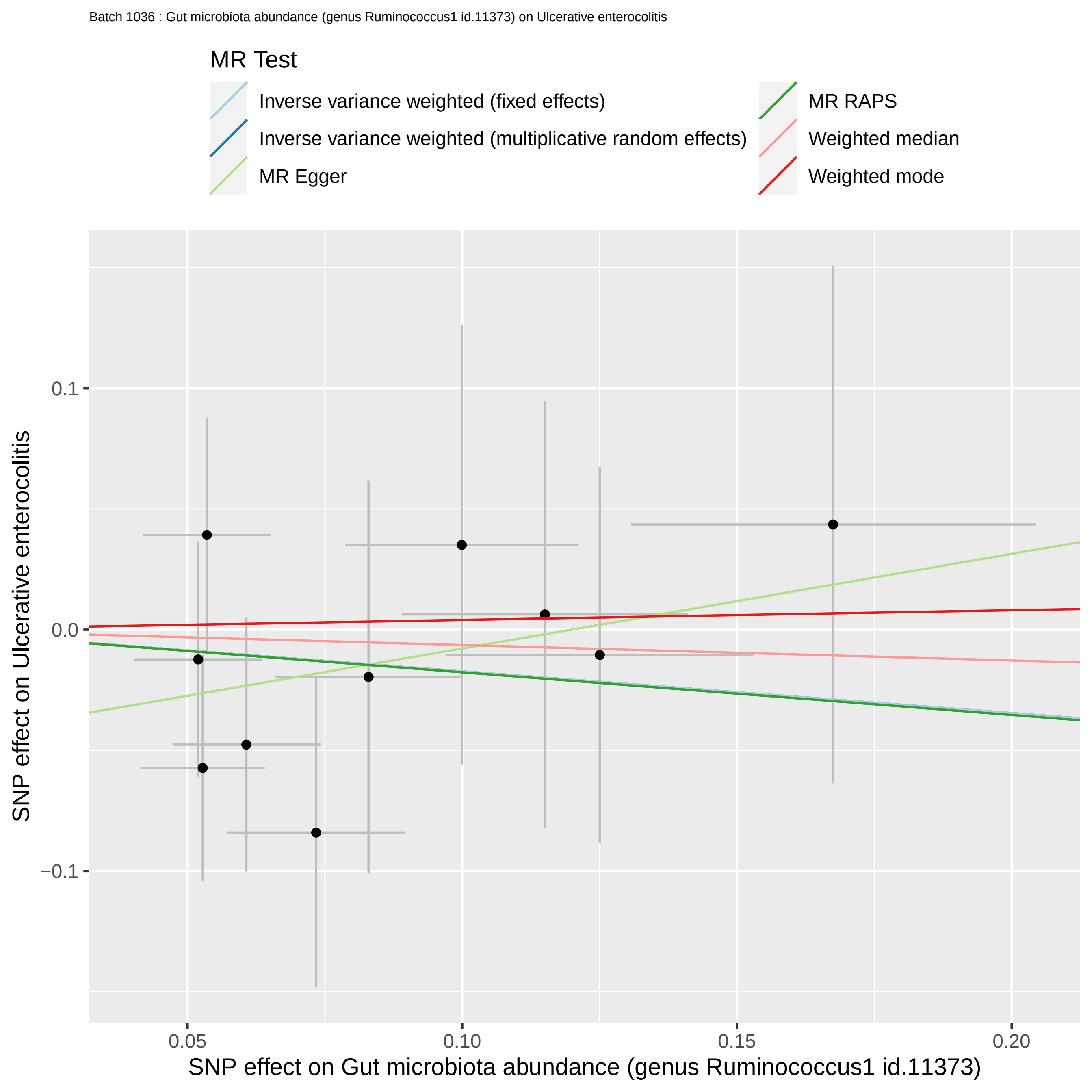

Batch 1036 : Gut microbiota abundance (genus Ruminococcus1 id.11373) on Ulcerative enterocolitis
MR Test
Inverse variance weighted (fixed effects)
Inverse variance weighted (multiplicative random effects)
MR Egger
MR RAPS
Weighted median
Weighted mode
0.1
0.0
−0.1
0.05
0.10
0.15
0.20
SNP effect on Ulcerative enterocolitis
SNP effect on Gut microbiota abundance (genus Ruminococcus1 id.11373)

Batch 1037 : Gut microbiota abundance (genus Ruminococcus2 id.11374) on Ulcerative enterocolitis

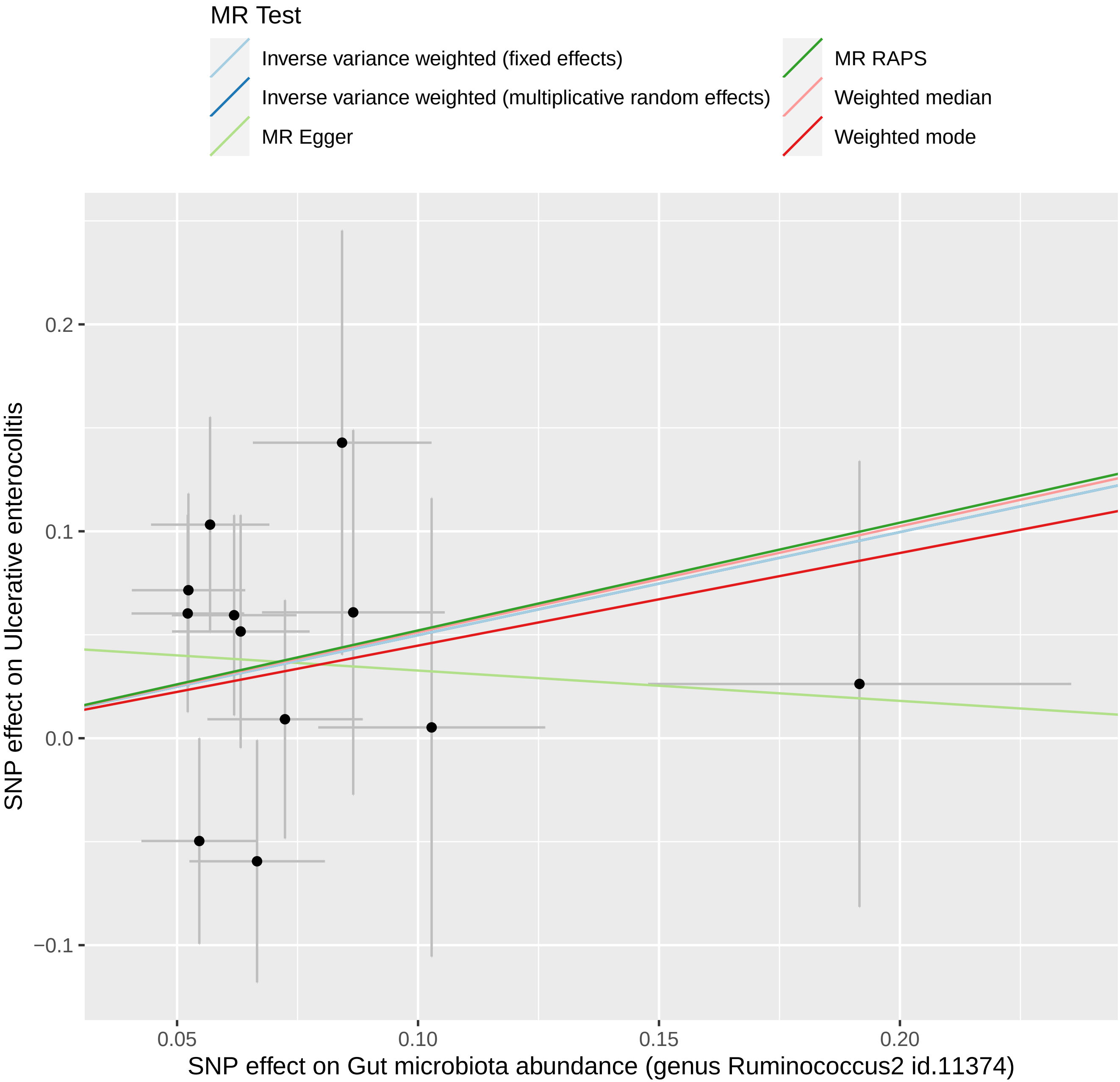

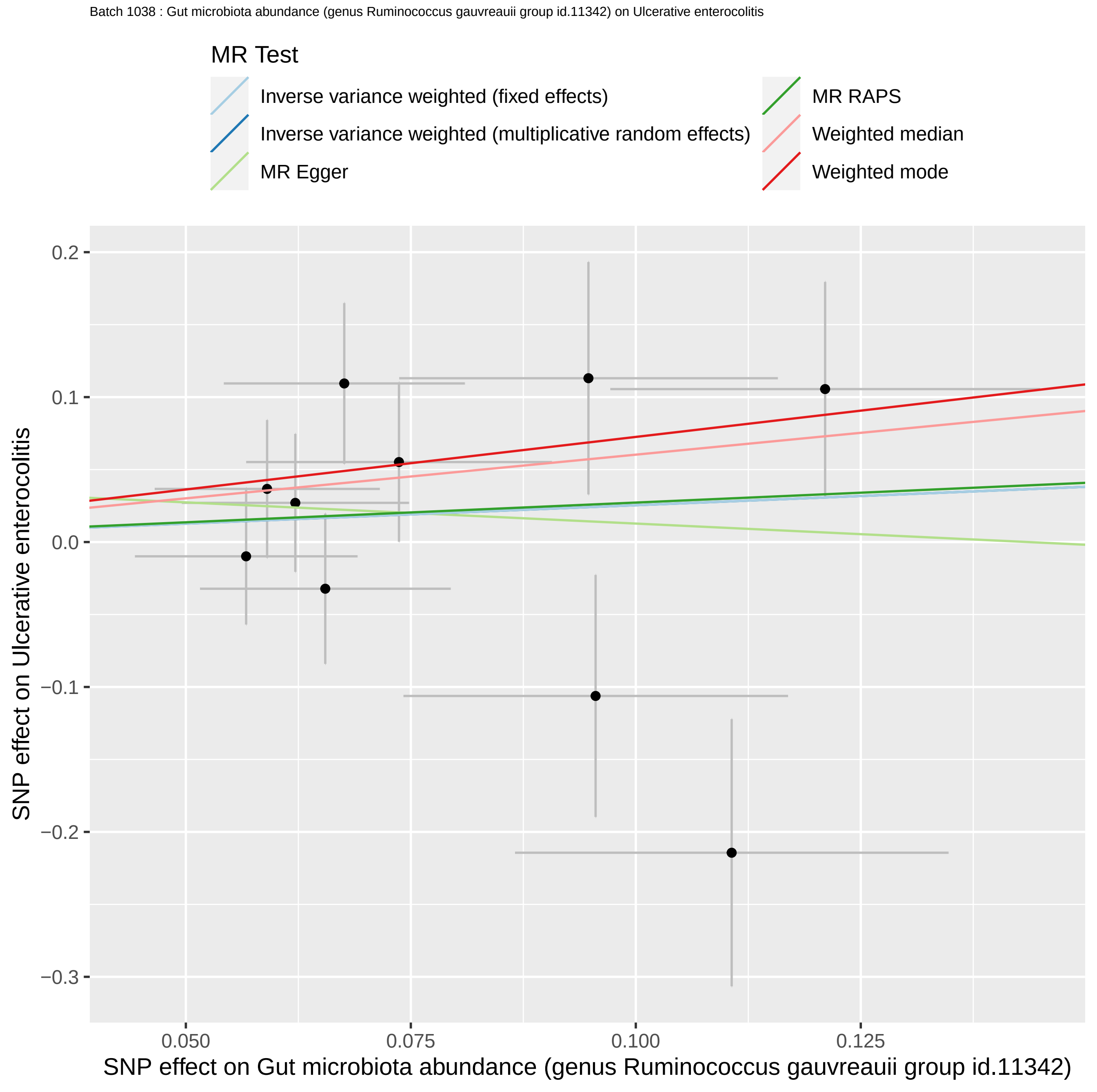

Batch 1038 : Gut microbiota abundance (genus Ruminococcus gauvreauii group id.11342) on Ulcerative enterocolitis
MR Test
Inverse variance weighted (fixed effects)
Inverse variance weighted (multiplicative random effects)
MR Egger
MR RAPS
Weighted median
Weighted mode
0.2
0.1
0.0
−0.1
−0.2
−0.3
0.050
0.075
0.100
0.125
SNP effect on Ulcerative enterocolitis
SNP effect on Gut microbiota abundance (genus Ruminococcus gauvreauii group id.11342)

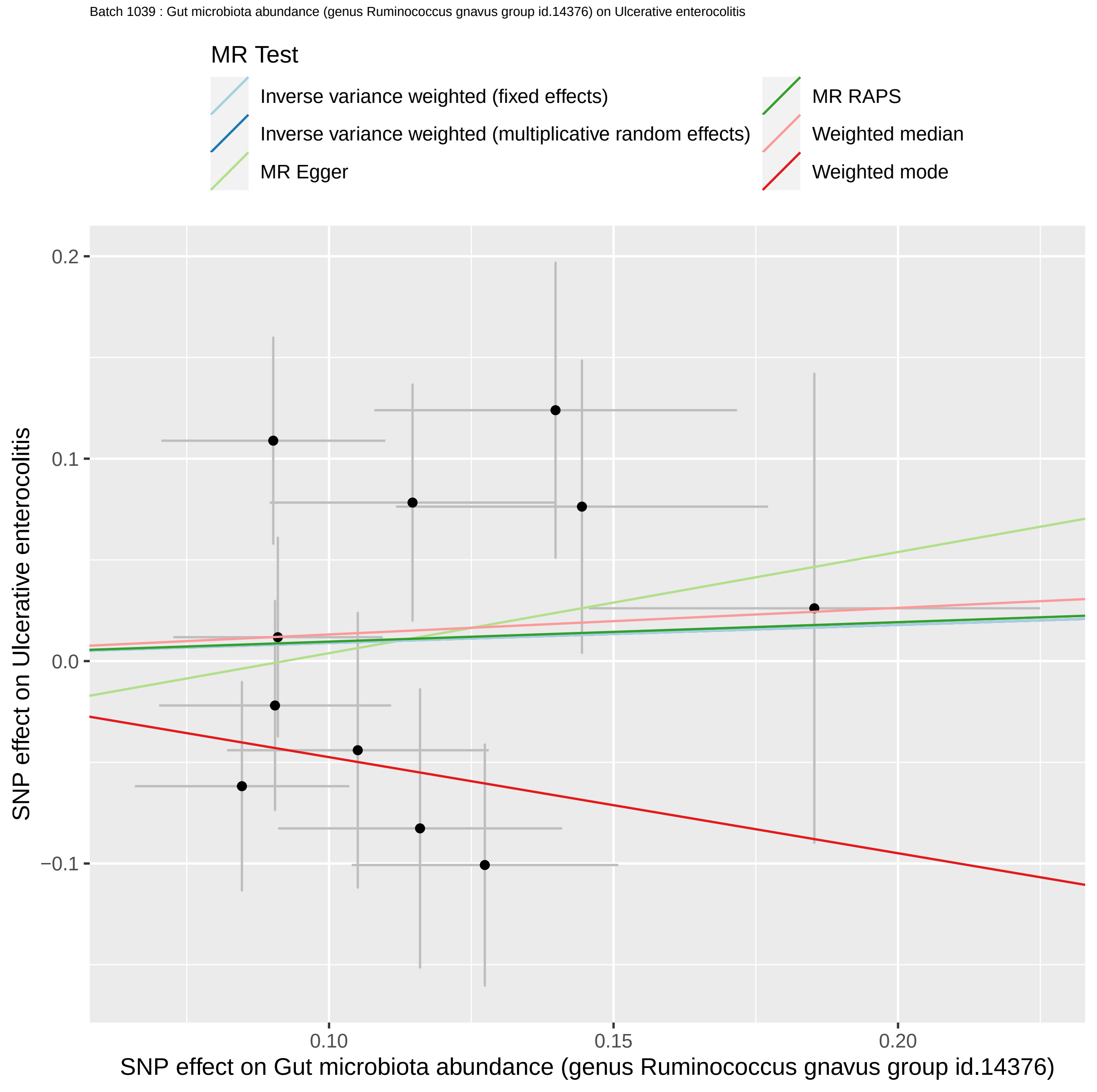

Batch 1039 : Gut microbiota abundance (genus Ruminococcus gnavus group id.14376) on Ulcerative enterocolitis
MR Test
Inverse variance weighted (fixed effects)
Inverse variance weighted (multiplicative random effects)
MR Egger
MR RAPS
Weighted median
Weighted mode
0.2
0.1
0.0
−0.1
0.10
0.15
0.20
SNP effect on Ulcerative enterocolitis
SNP effect on Gut microbiota abundance (genus Ruminococcus gnavus group id.14376)

Batch 1040 : Gut microbiota abundance (genus Ruminococcus torques group id.14377) on Ulcerative enterocolitis

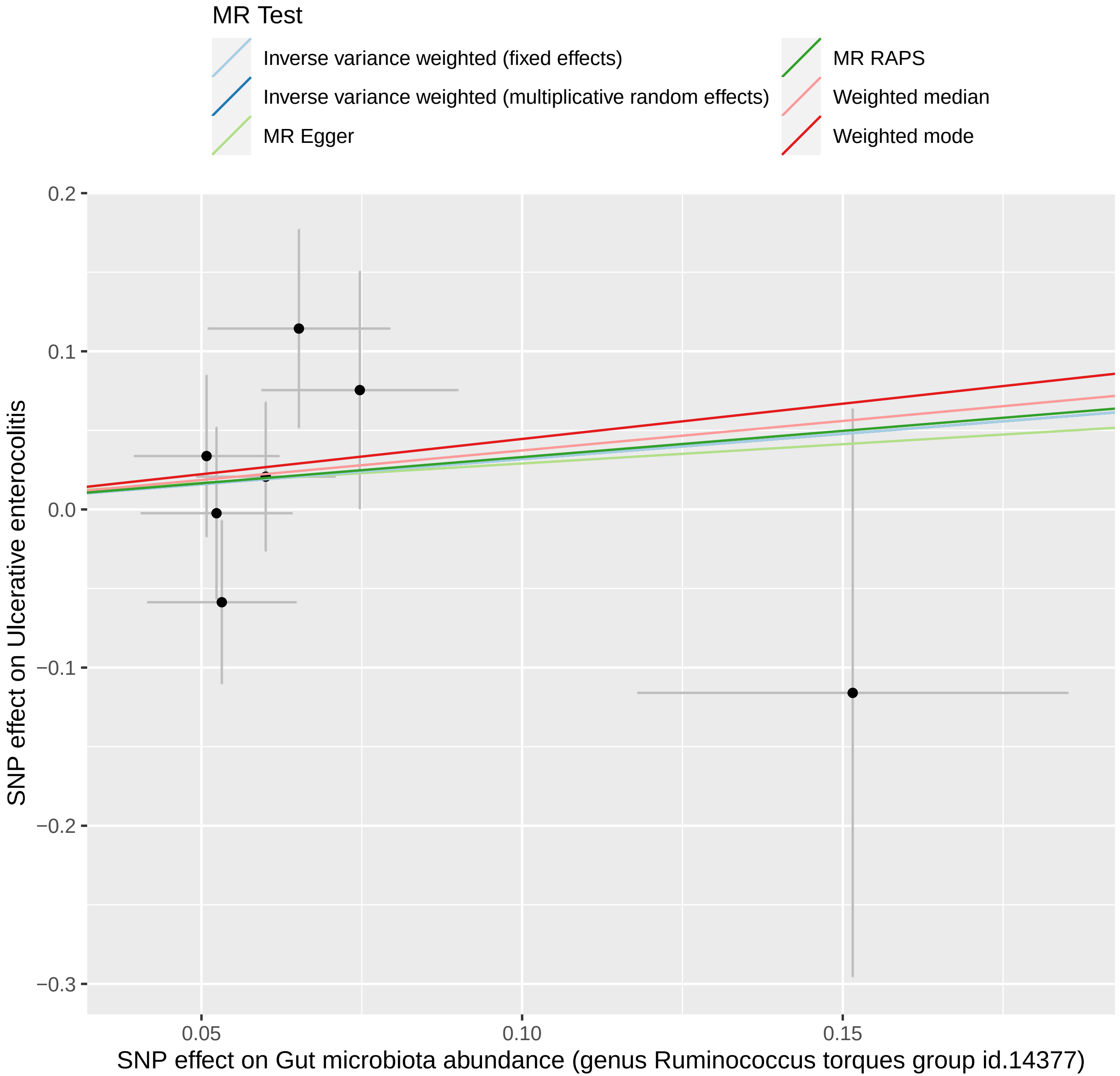

Batch 1041 : Gut microbiota abundance (genus Sellimonas id.14369) on Ulcerative enterocolitis

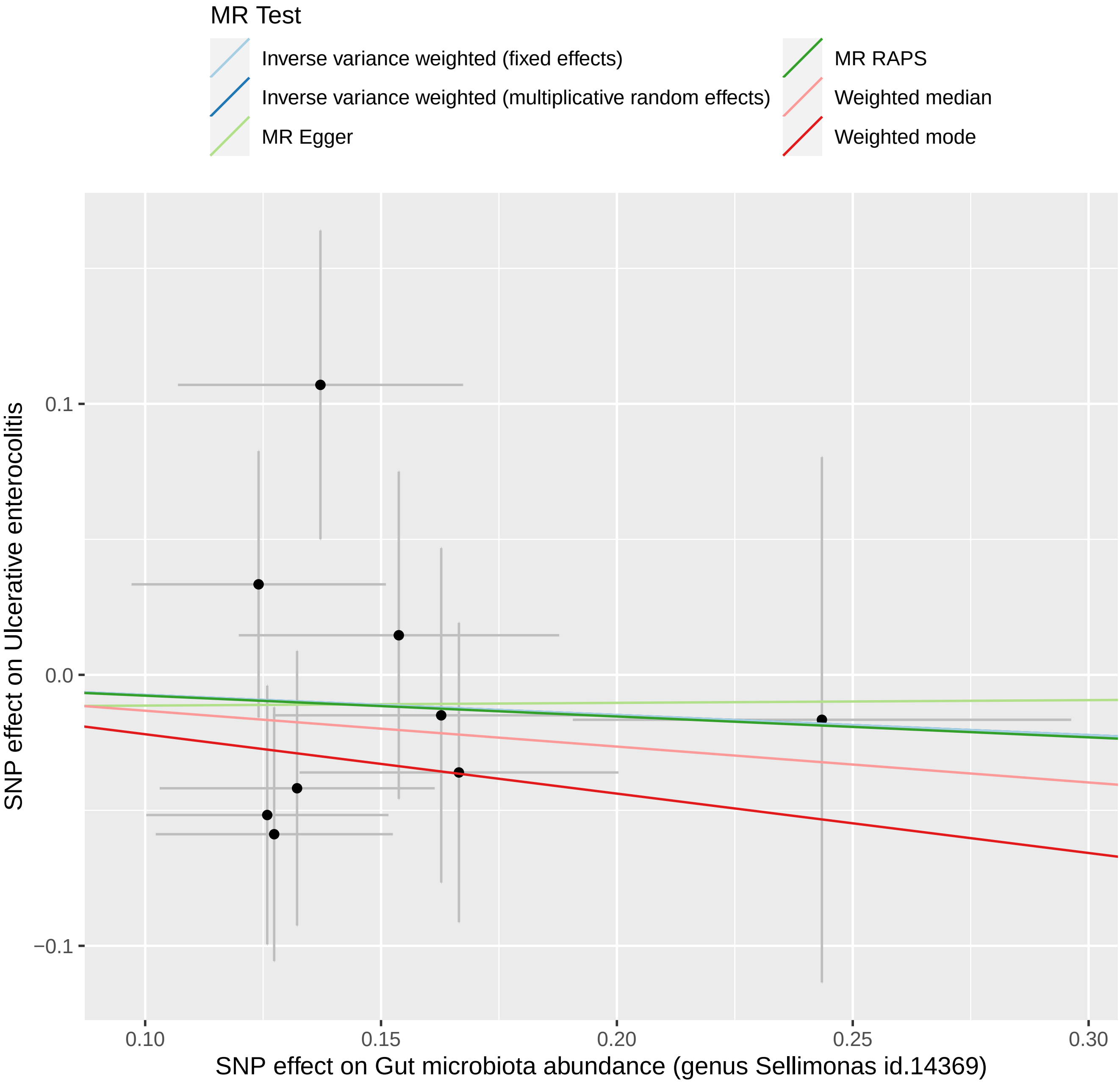

Batch 1042 : Gut microbiota abundance (genus Senegalimassilia id.11160) on Ulcerative enterocolitis

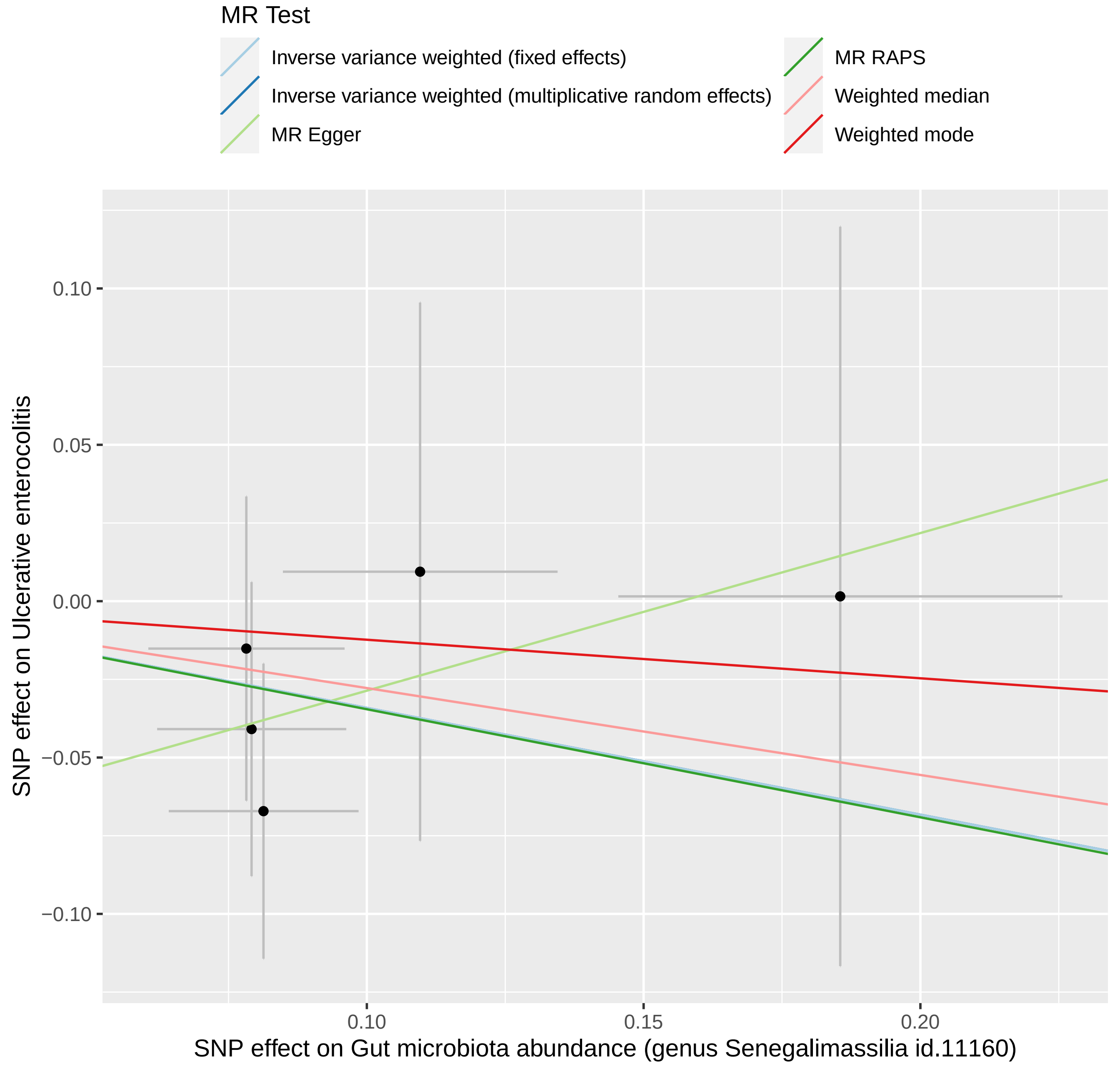

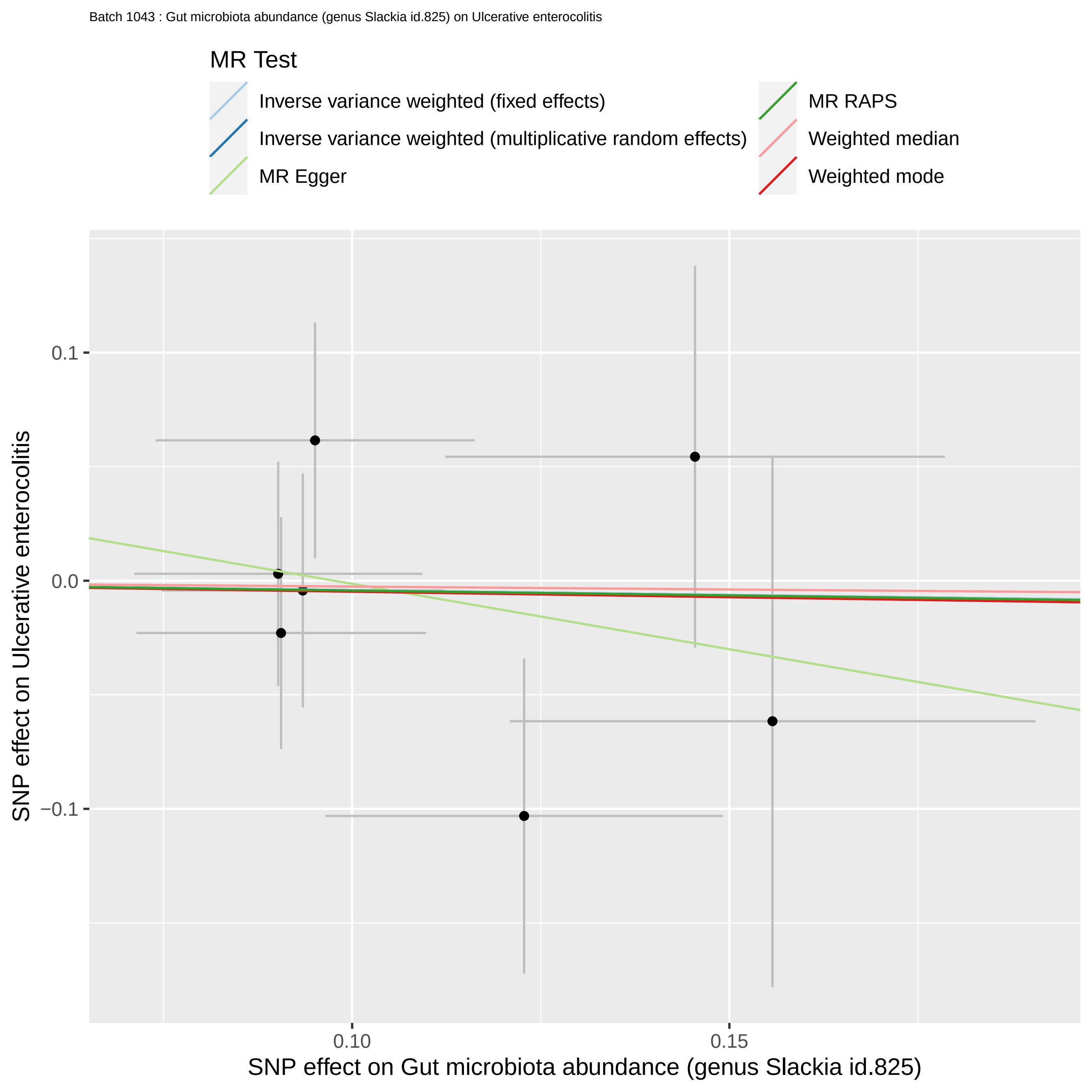

Batch 1043 : Gut microbiota abundance (genus Slackia id.825) on Ulcerative enterocolitis
MR Test
Inverse variance weighted (fixed effects)
Inverse variance weighted (multiplicative random effects)
MR Egger
MR RAPS
Weighted median
Weighted mode
0.1
0.0
−0.1
0.10
0.15
SNP effect on Ulcerative enterocolitis
SNP effect on Gut microbiota abundance (genus Slackia id.825)

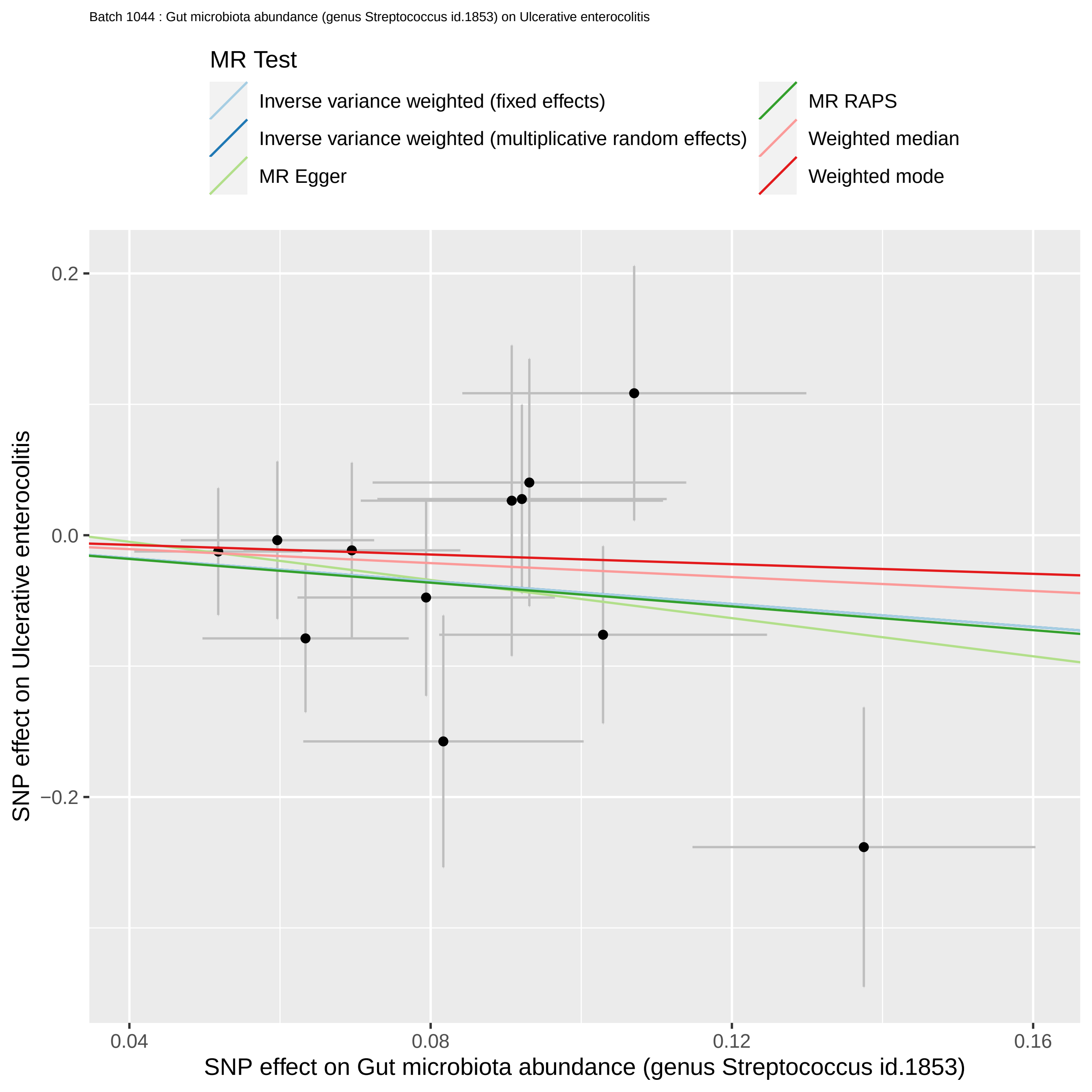
Batch 1044 : Gut microbiota abundance (genus Streptococcus id.1853) on Ulcerative enterocolitis
MR Test
Inverse variance weighted (fixed effects)
Inverse variance weighted (multiplicative random effects)
MR Egger
MR RAPS
Weighted median
Weighted mode
0.2
0.0
−0.2
SNP effect on Ulcerative enterocolitis
0.04
0.08
0.12
0.16
SNP effect on Gut microbiota abundance (genus Streptococcus id.1853)

Batch 1045 : Gut microbiota abundance (genus Subdoligranulum id.2070) on Ulcerative enterocolitis

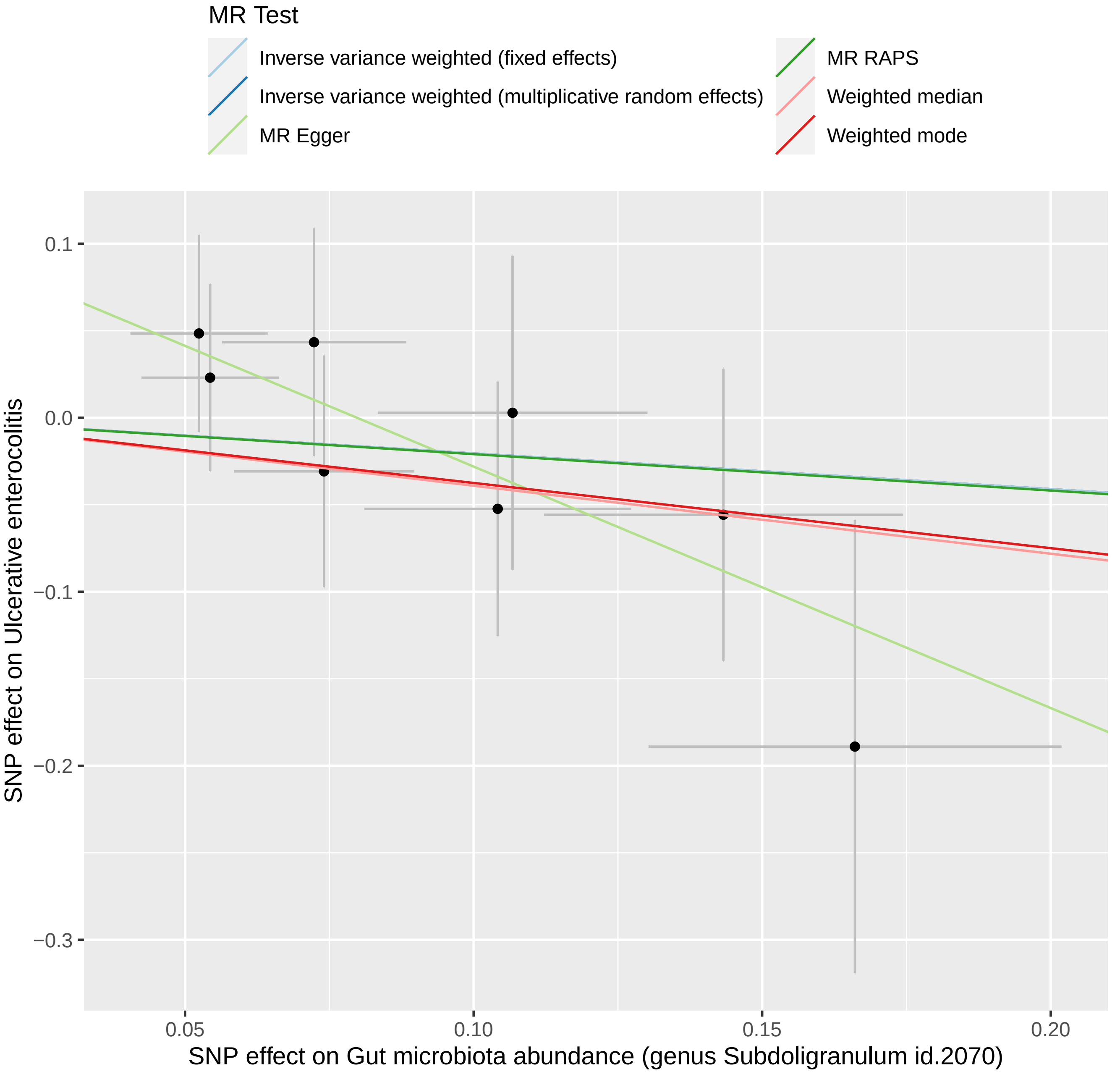

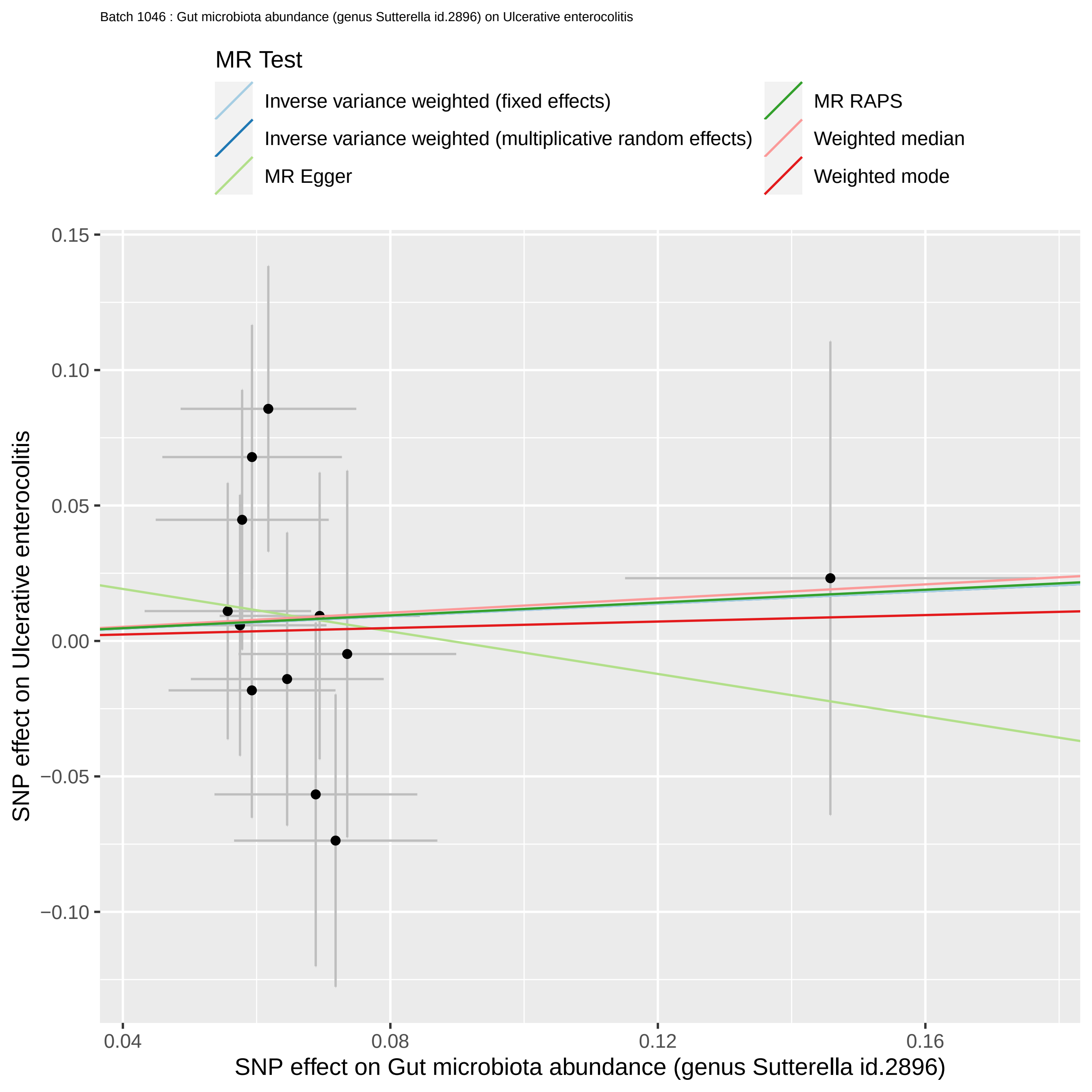

Batch 1046 : Gut microbiota abundance (genus Sutterella id.2896) on Ulcerative enterocolitis
MR Test
Inverse variance weighted (fixed effects)
Inverse variance weighted (multiplicative random effects)
MR Egger
MR RAPS
Weighted median
Weighted mode
0.15
0.10
0.05
0.00
−0.05
−0.10
0.04
0.08
0.12
0.16
SNP effect on Ulcerative enterocolitis
SNP effect on Gut microbiota abundance (genus Sutterella id.2896)

Batch 1047 : Gut microbiota abundance (genus Terrisporobacter id.11348) on Ulcerative enterocolitis

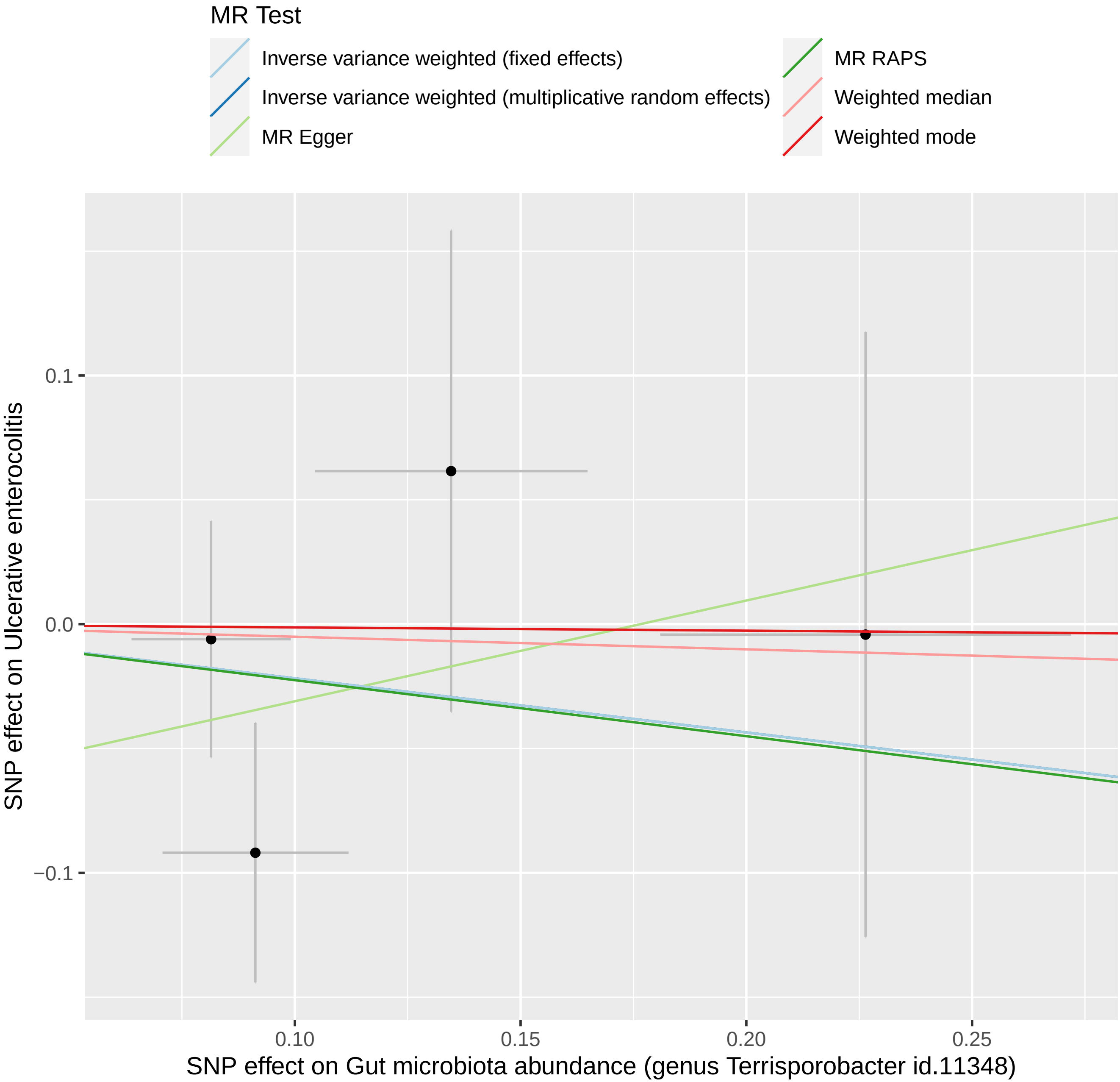

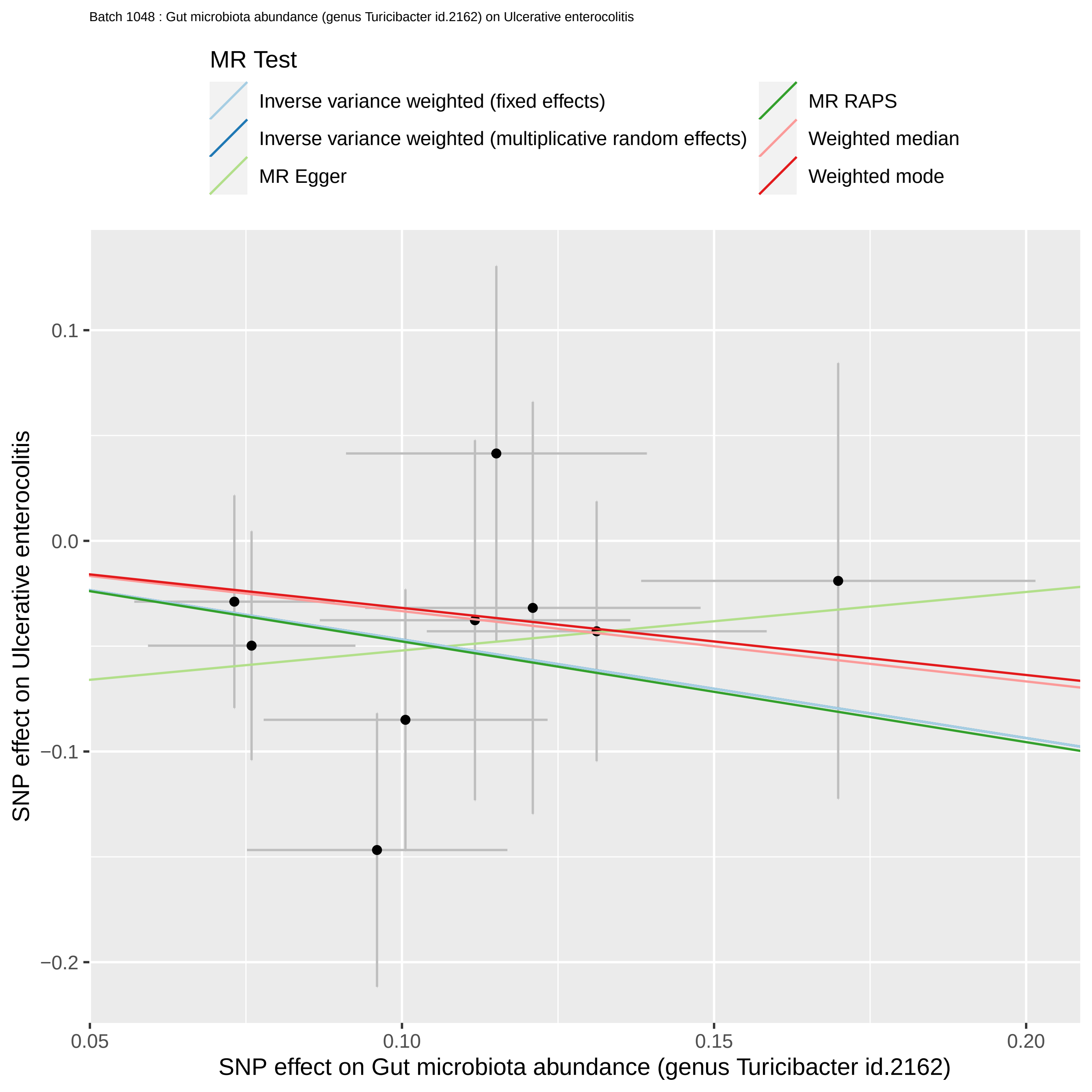

Batch 1048 : Gut microbiota abundance (genus Turicibacter id.2162) on Ulcerative enterocolitis
MR Test
Inverse variance weighted (fixed effects)
Inverse variance weighted (multiplicative random effects)
MR Egger
MR RAPS
Weighted median
Weighted mode
0.1
0.0
−0.1
−0.2
0.05
0.10
0.15
0.20
SNP effect on Ulcerative enterocolitis
SNP effect on Gut microbiota abundance (genus Turicibacter id.2162)

Batch 1049 : Gut microbiota abundance (genus Tyzzerella3 id.11335) on Ulcerative enterocolitis

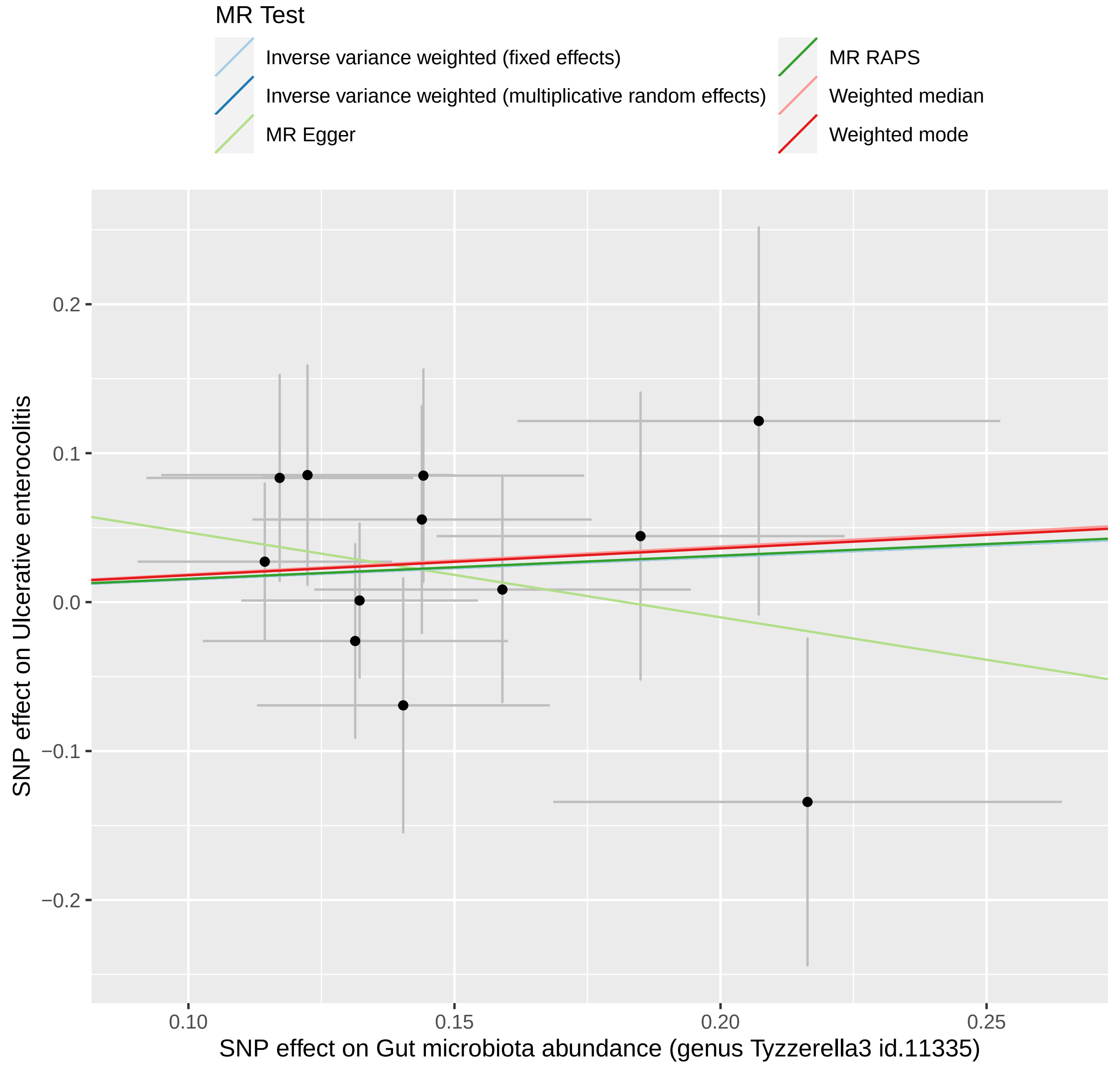

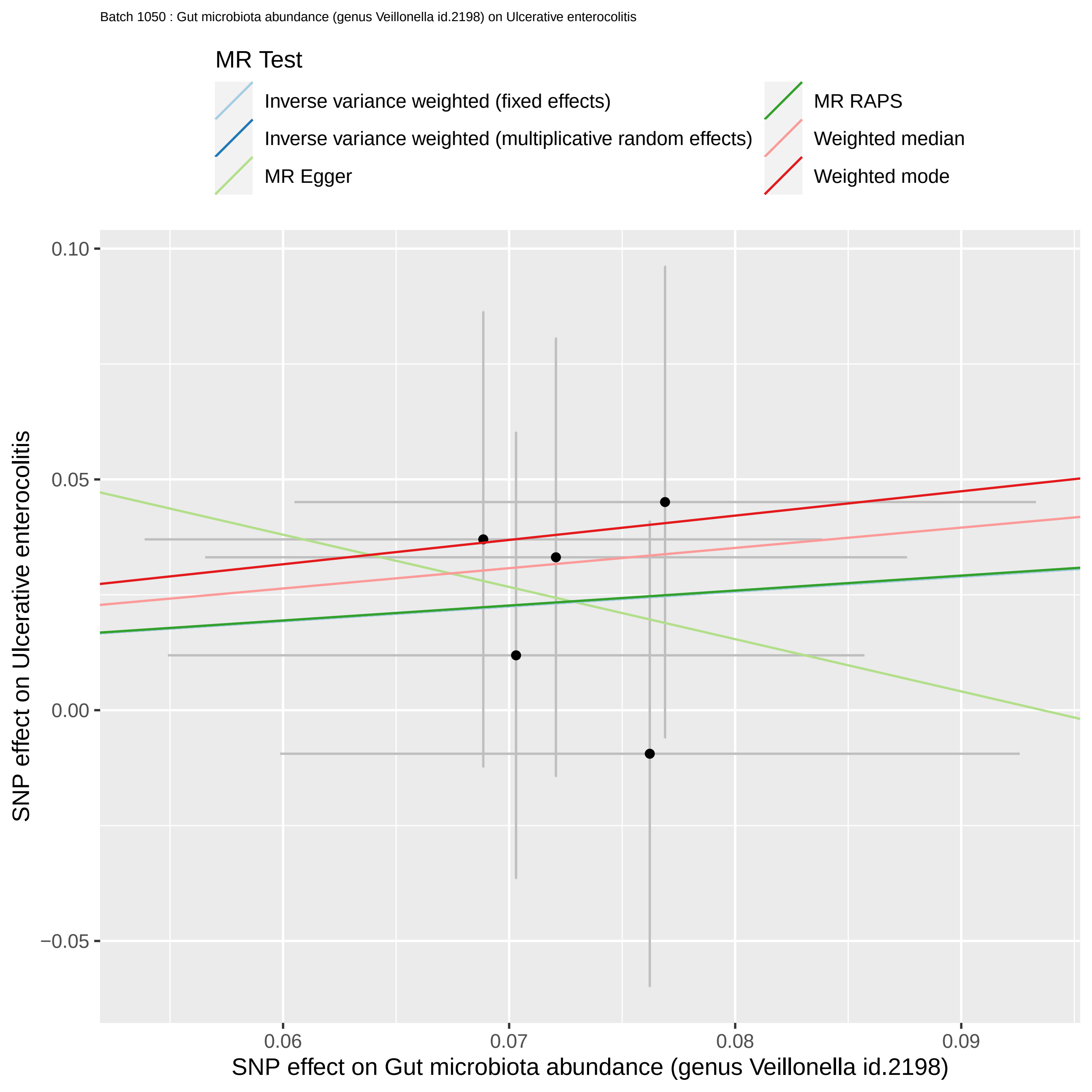
Batch 1050 : Gut microbiota abundance (genus Veillonella id.2198) on Ulcerative enterocolitis
MR Test
Inverse variance weighted (fixed effects)
Inverse variance weighted (multiplicative random effects)
MR Egger
MR RAPS
Weighted median
Weighted mode
0.10
0.05
0.00
−0.05
0.06
0.07
0.08
0.09
SNP effect on Ulcerative enterocolitis
SNP effect on Gut microbiota abundance (genus Veillonella id.2198)

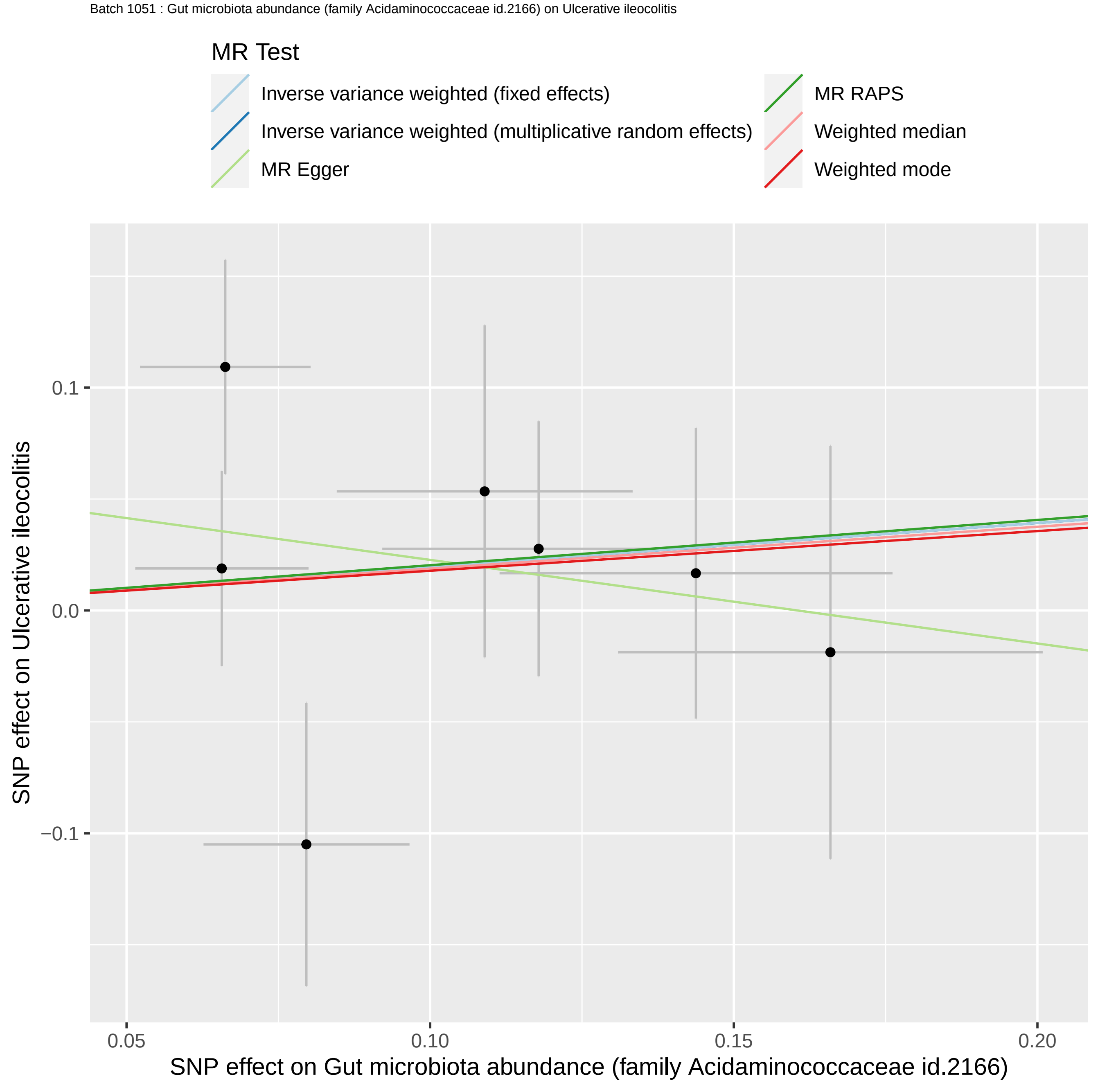

Batch 1051 : Gut microbiota abundance (family Acidaminococcaceae id.2166) on Ulcerative ileocolitis
MR Test
Inverse variance weighted (fixed effects)
Inverse variance weighted (multiplicative random effects)
MR Egger
MR RAPS
Weighted median
Weighted mode
SNP effect on Ulcerative ileocolitis
0.1
0.0
−0.1
0.05
0.10
0.15
0.20
SNP effect on Gut microbiota abundance (family Acidaminococcaceae id.2166)

Batch 1052 : Gut microbiota abundance (family Actinomycetaceae id.421) on Ulcerative ileocolitis

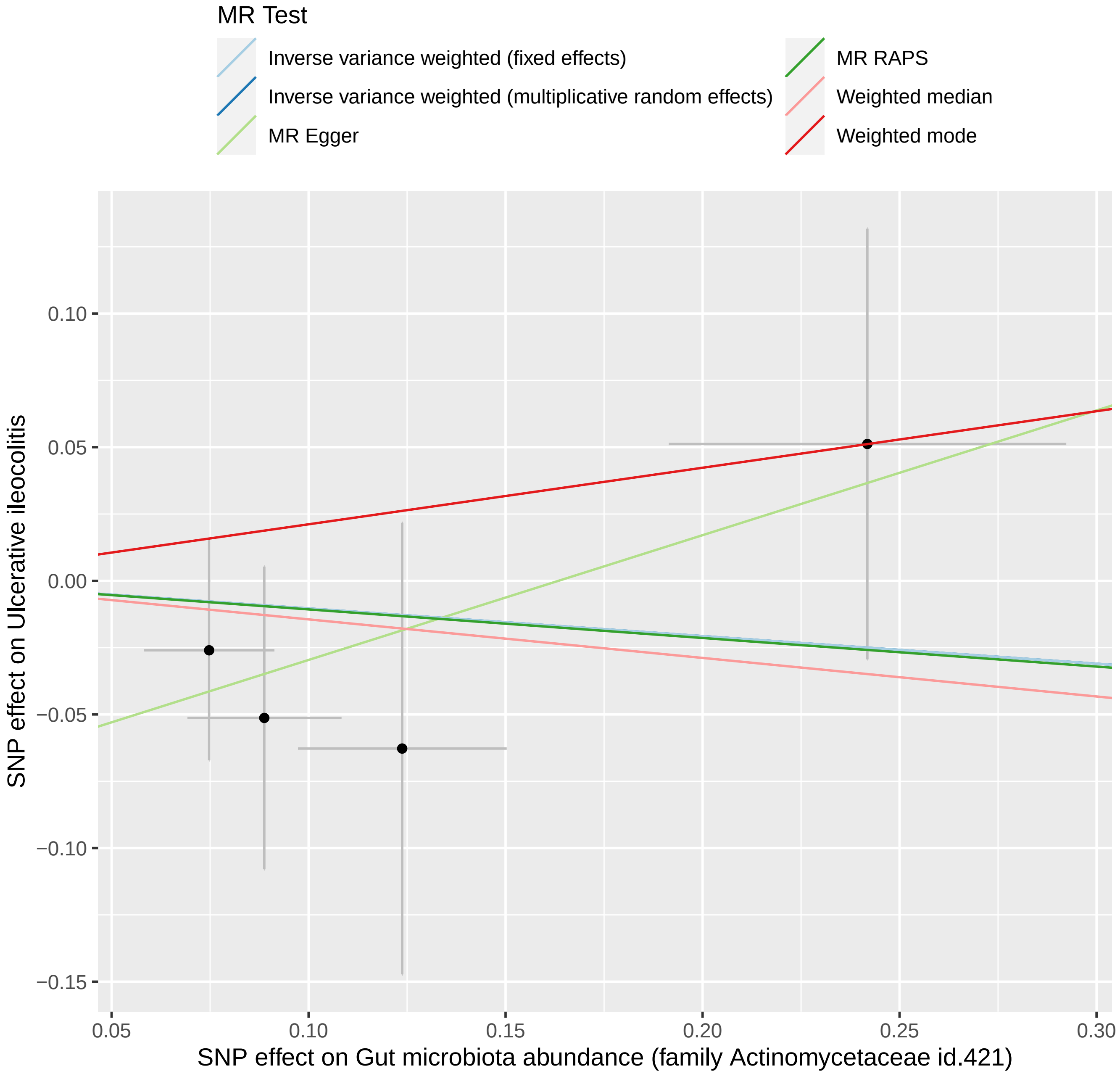

Batch 1053 : Gut microbiota abundance (family Alcaligenaceae id.2875) on Ulcerative ileocolitis

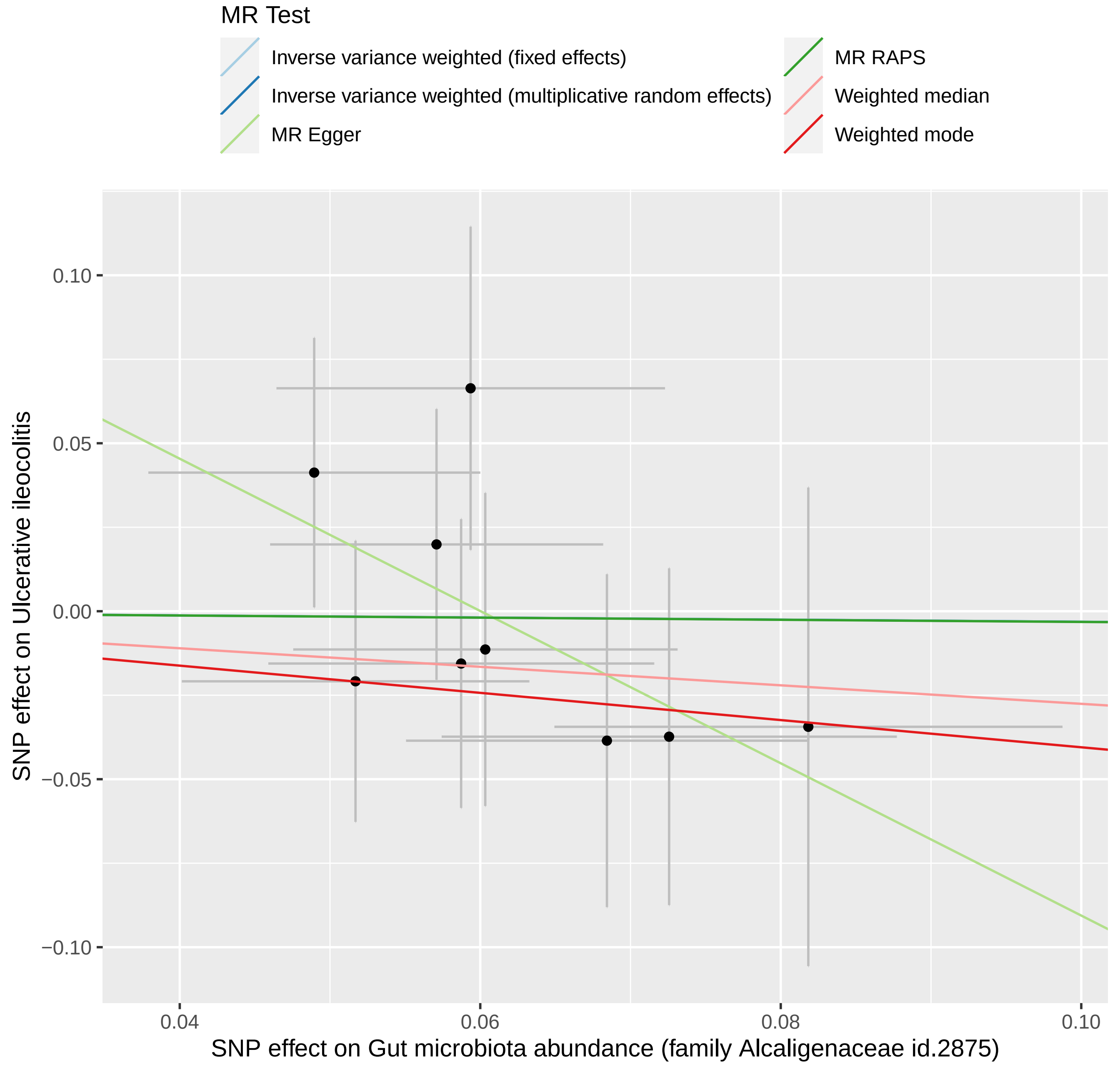

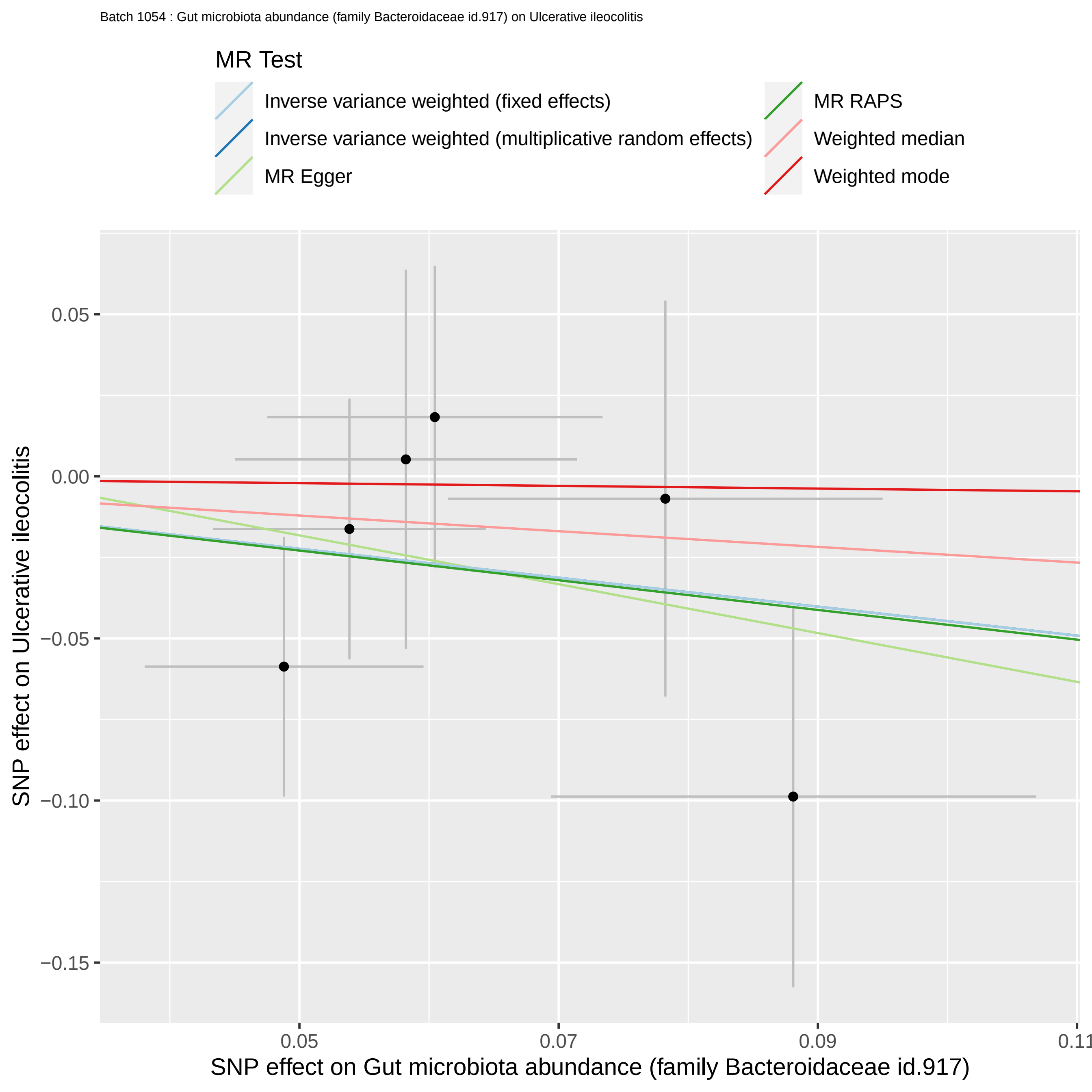
Batch 1054 : Gut microbiota abundance (family Bacteroidaceae id.917) on Ulcerative ileocolitis
MR Test
Inverse variance weighted (fixed effects)
Inverse variance weighted (multiplicative random effects)
MR Egger
MR RAPS
Weighted median
Weighted mode
0.05
0.00
−0.05
−0.10
−0.15
0.05
0.07
0.09
0.11
SNP effect on Ulcerative ileocolitis
SNP effect on Gut microbiota abundance (family Bacteroidaceae id.917)

Batch 1055 : Gut microbiota abundance (family Bacteroidales S24 7group id.11173) on Ulcerative ileocolitis

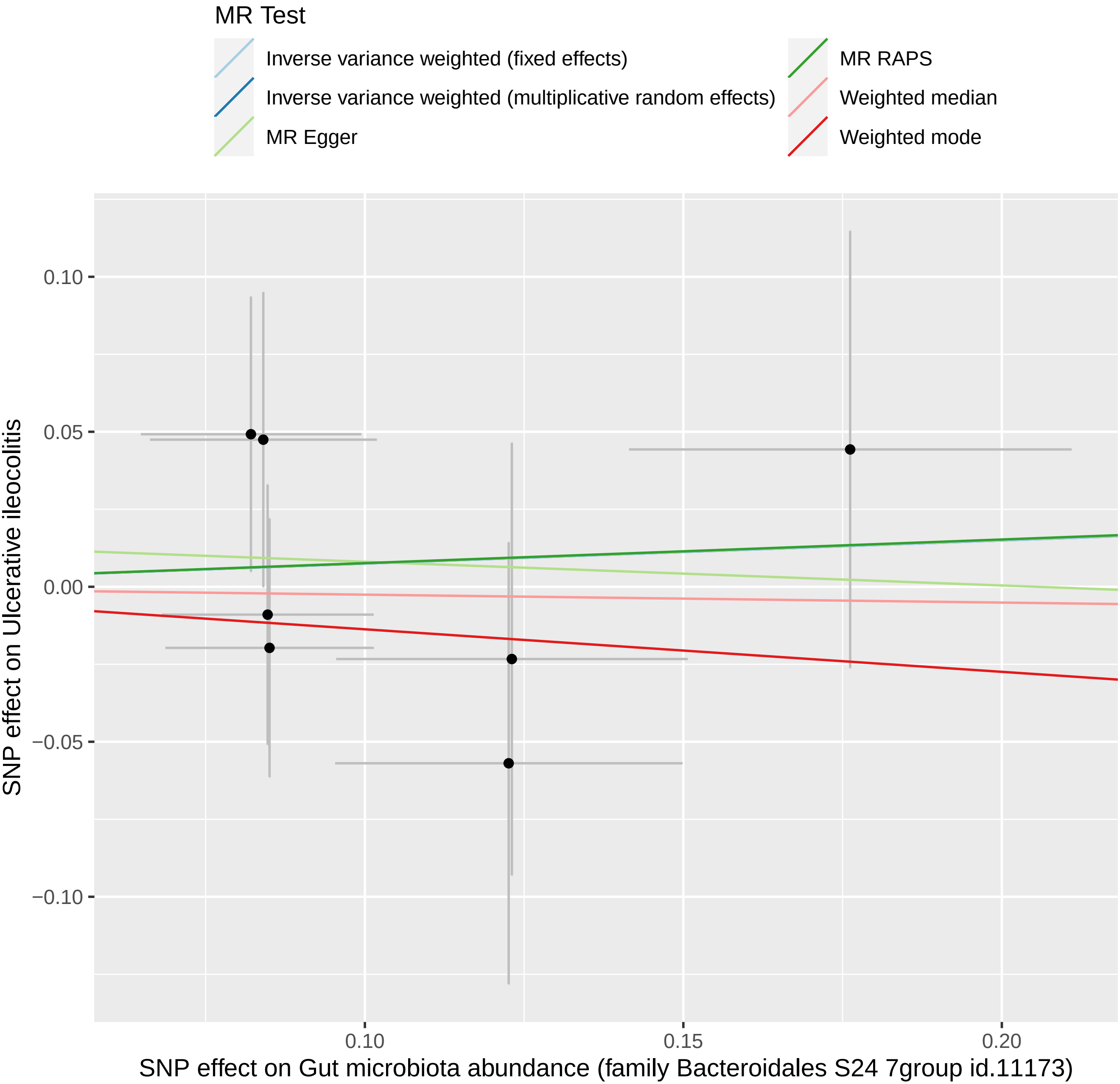

Batch 1056 : Gut microbiota abundance (family Bifidobacteriaceae id.433) on Ulcerative ileocolitis

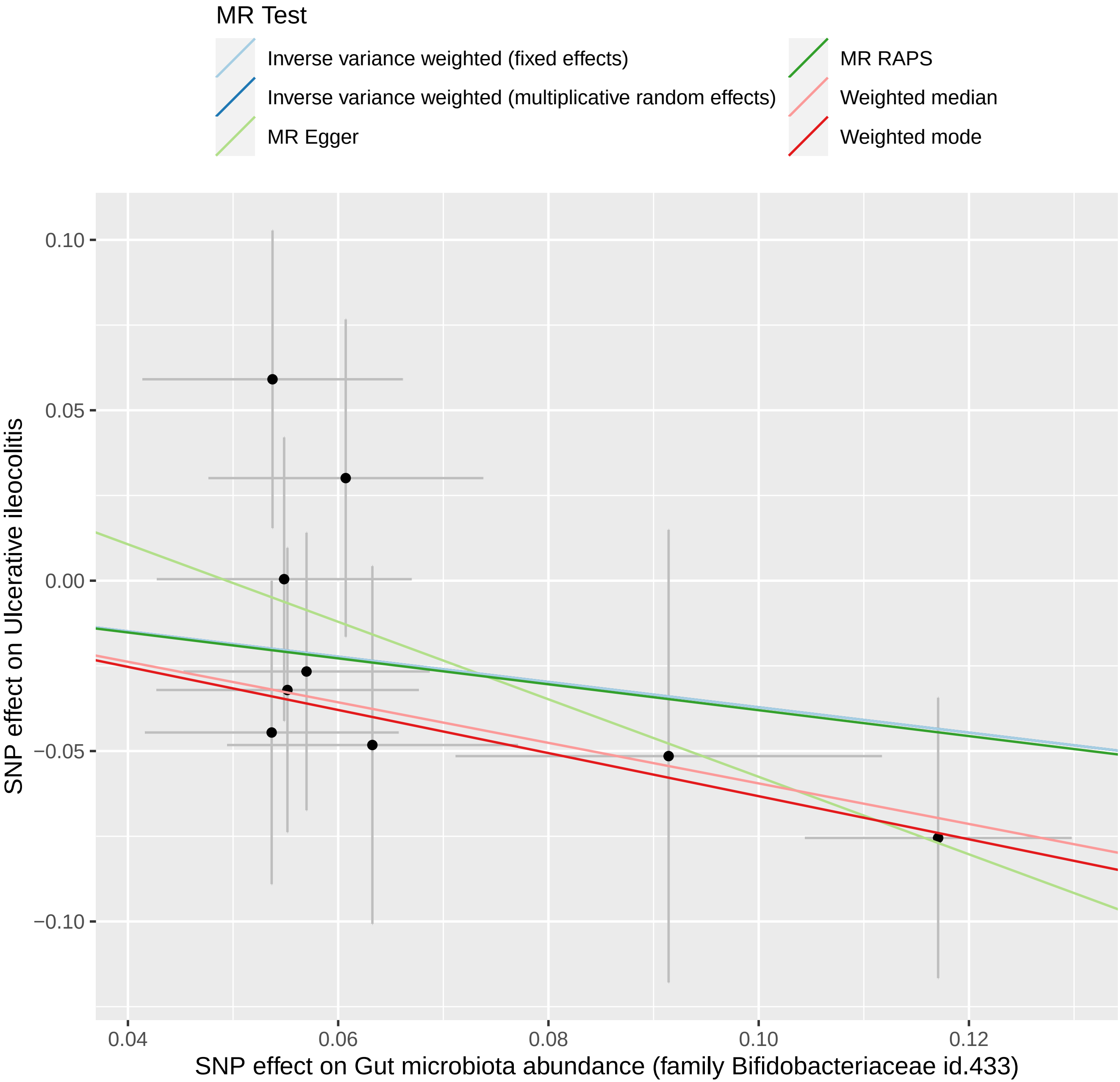

Batch 1058 : Gut microbiota abundance (family Clostridiaceae1 id.1869) on Ulcerative ileocolitis

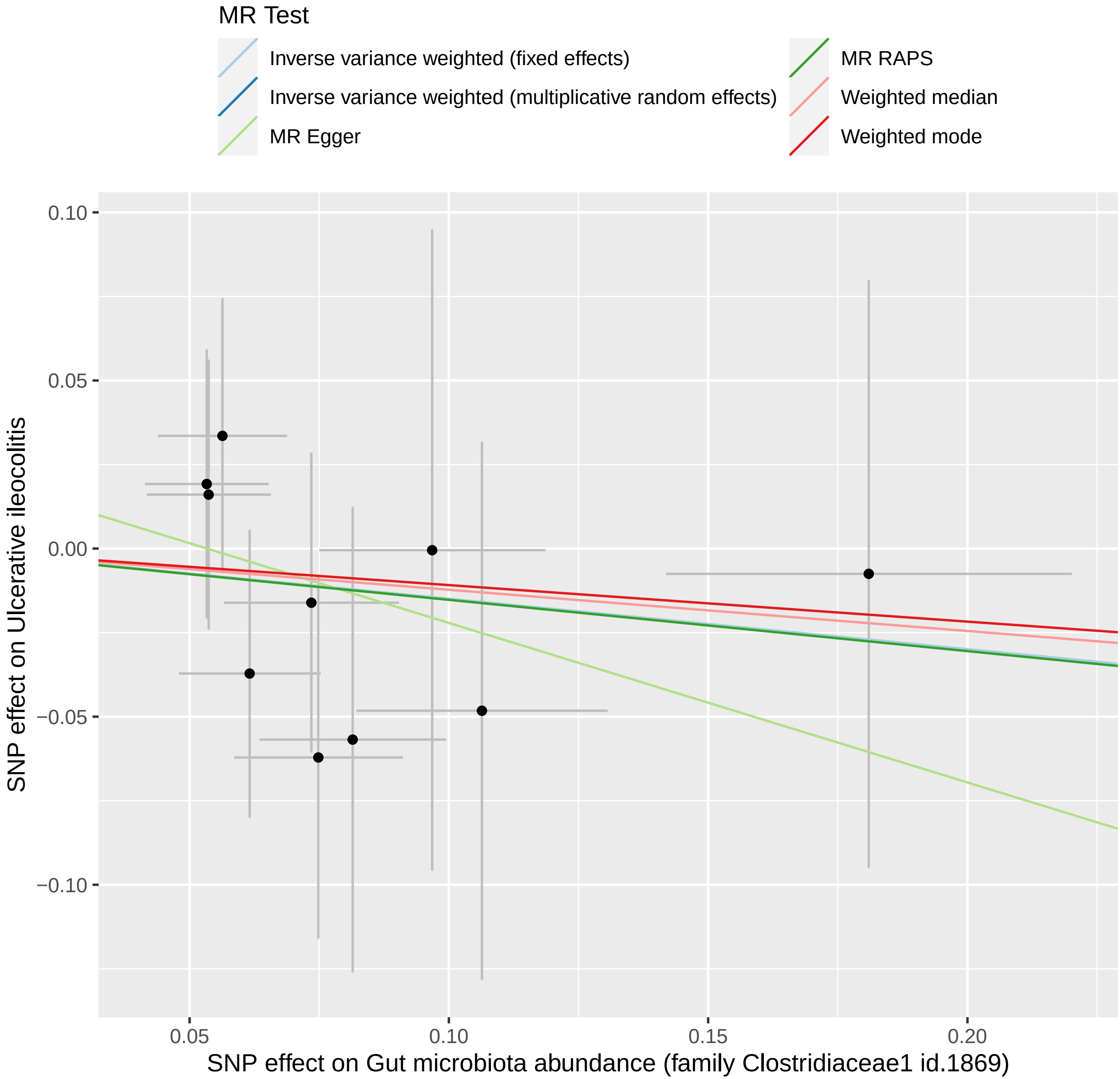

Batch 1059 : Gut microbiota abundance (family Clostridiales vadin BB60 group id.11286) on Ulcerative ileocolitis

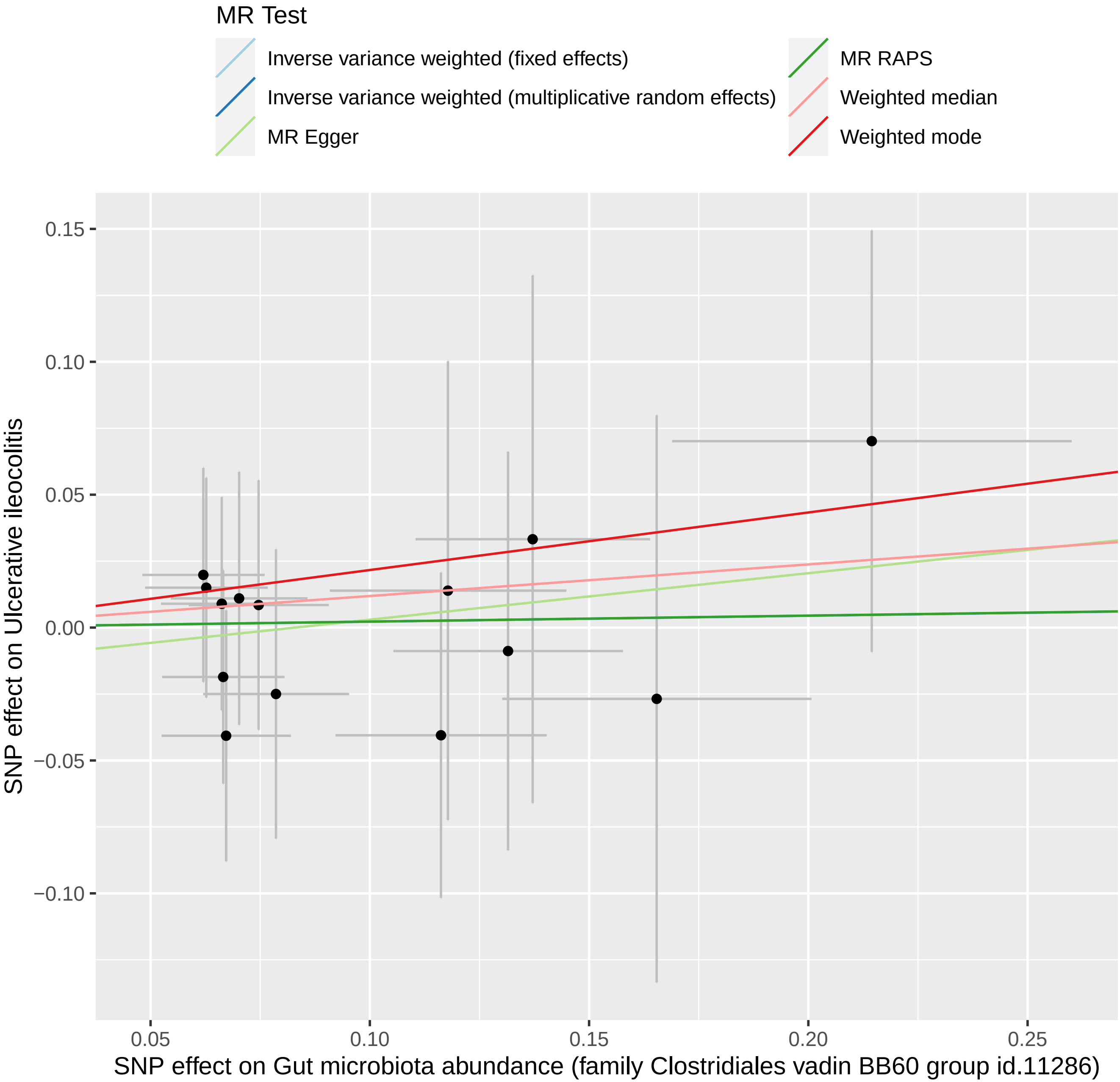

Batch 1060 : Gut microbiota abundance (family Coriobacteriaceae id.811) on Ulcerative ileocolitis

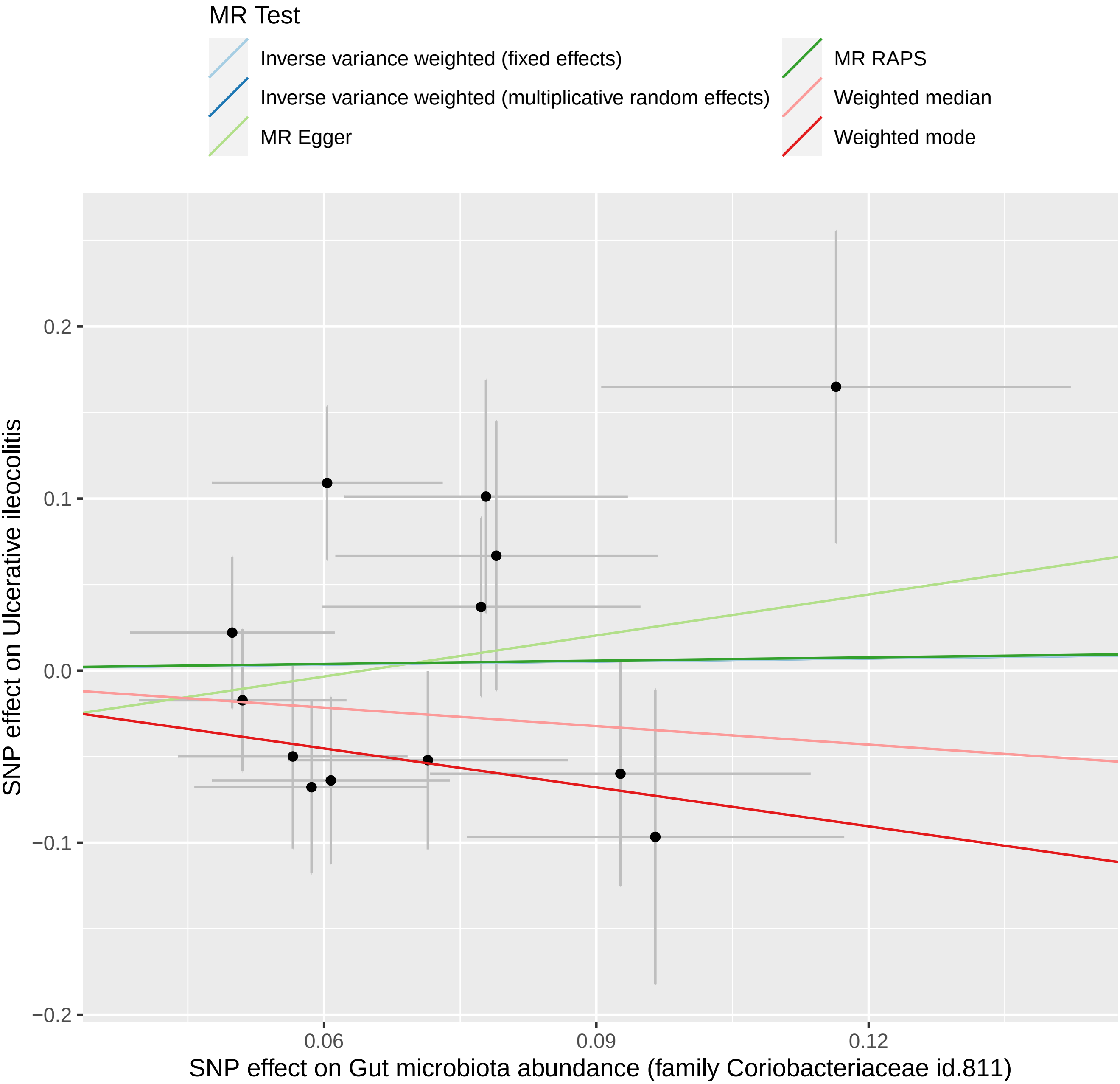

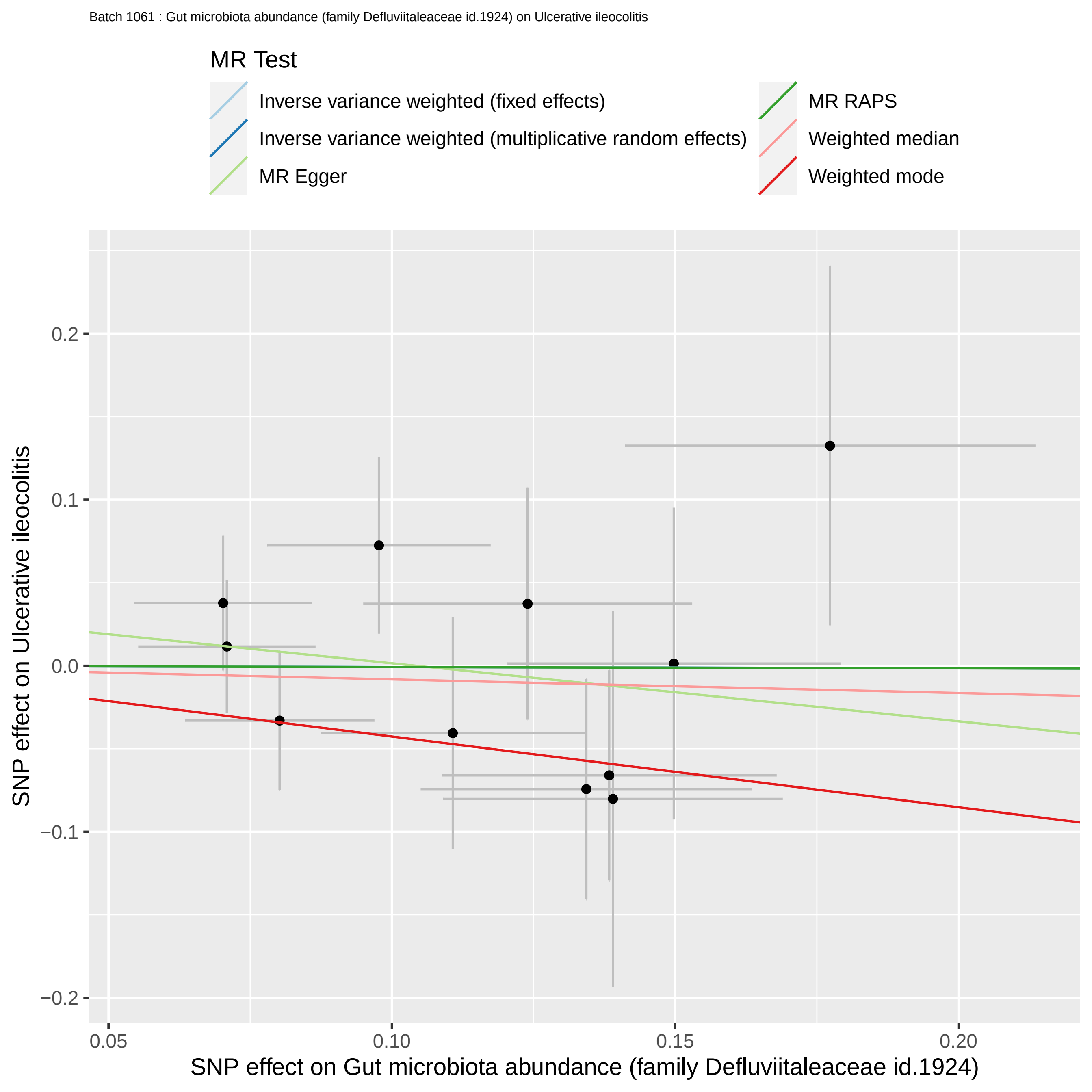

Batch 1061 : Gut microbiota abundance (family Defluviitaleaceae id.1924) on Ulcerative ileocolitis
MR Test
Inverse variance weighted (fixed effects)
Inverse variance weighted (multiplicative random effects)
MR Egger
MR RAPS
Weighted median
Weighted mode
SNP effect on Ulcerative ileocolitis
0.2
0.1
0.0
−0.1
−0.2
0.05
0.10
0.15
0.20
SNP effect on Gut microbiota abundance (family Defluviitaleaceae id.1924)

Batch 1062 : Gut microbiota abundance (family Desulfovibrionaceae id.3169) on Ulcerative ileocolitis

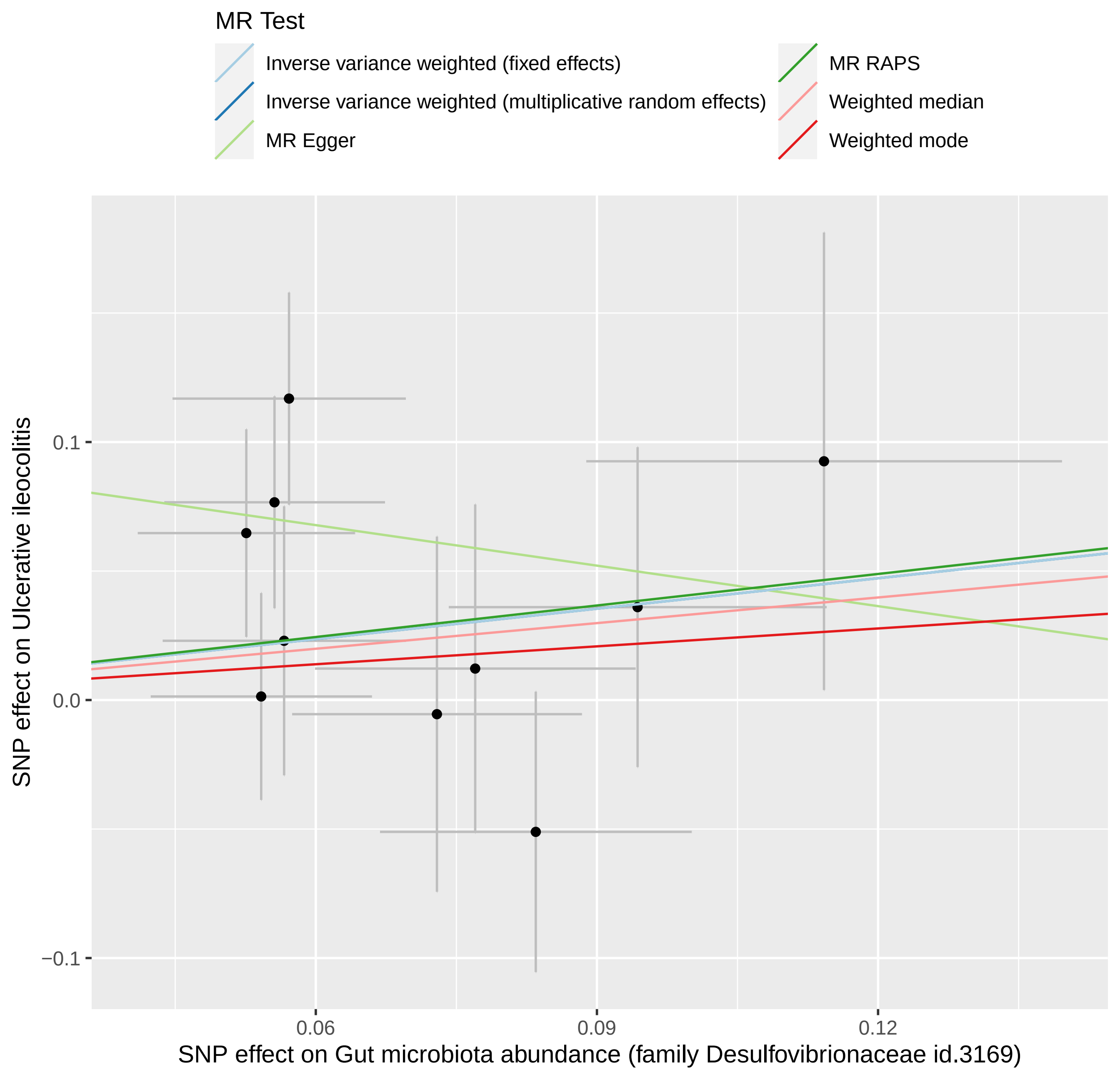

Batch 1063 : Gut microbiota abundance (family Enterobacteriaceae id.3469) on Ulcerative ileocolitis

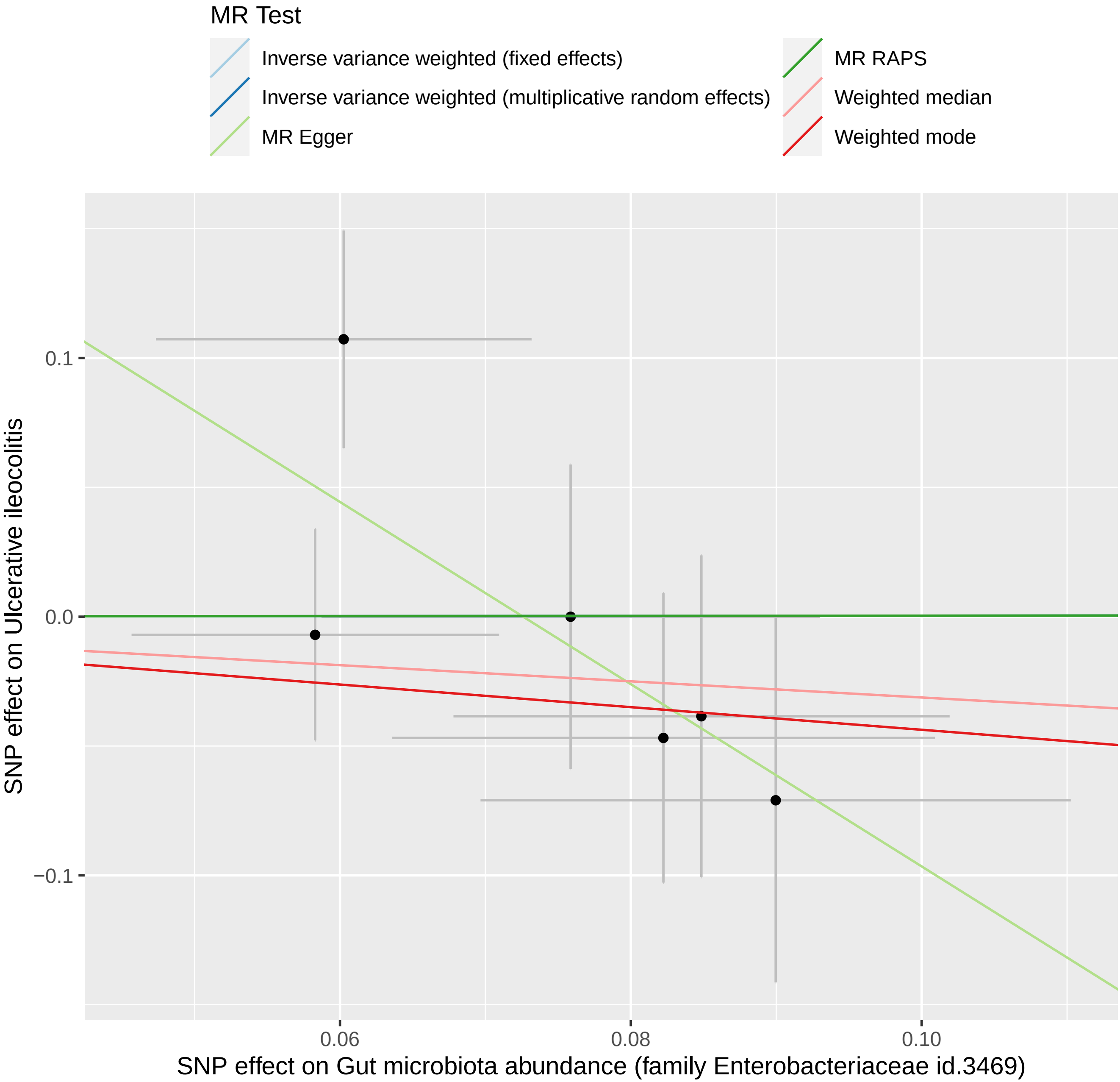

Batch 1064 : Gut microbiota abundance (family Erysipelotrichaceae id.2149) on Ulcerative ileocolitis

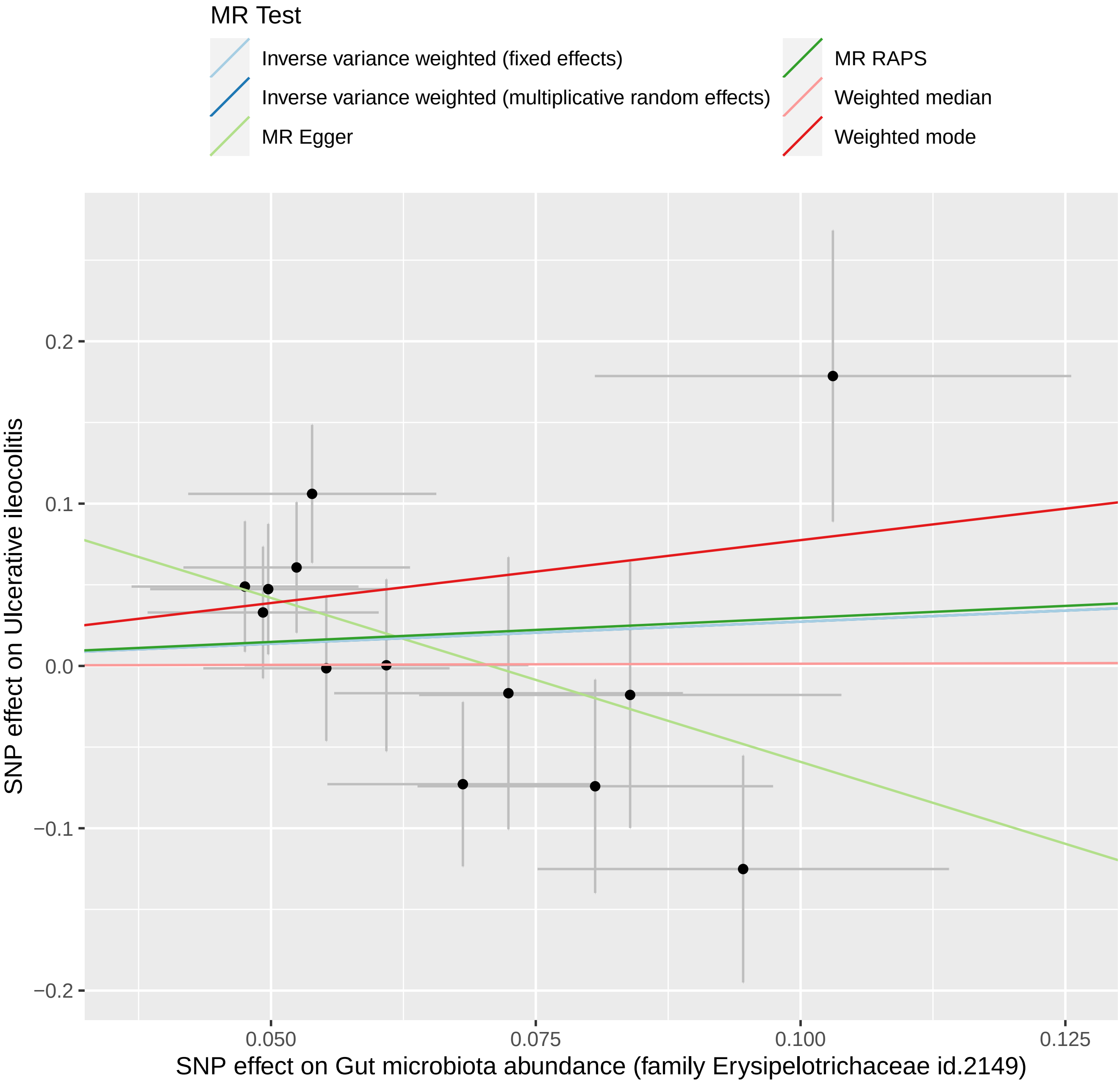

Batch 1065 : Gut microbiota abundance (family Family XI id.1936) on Ulcerative ileocolitis

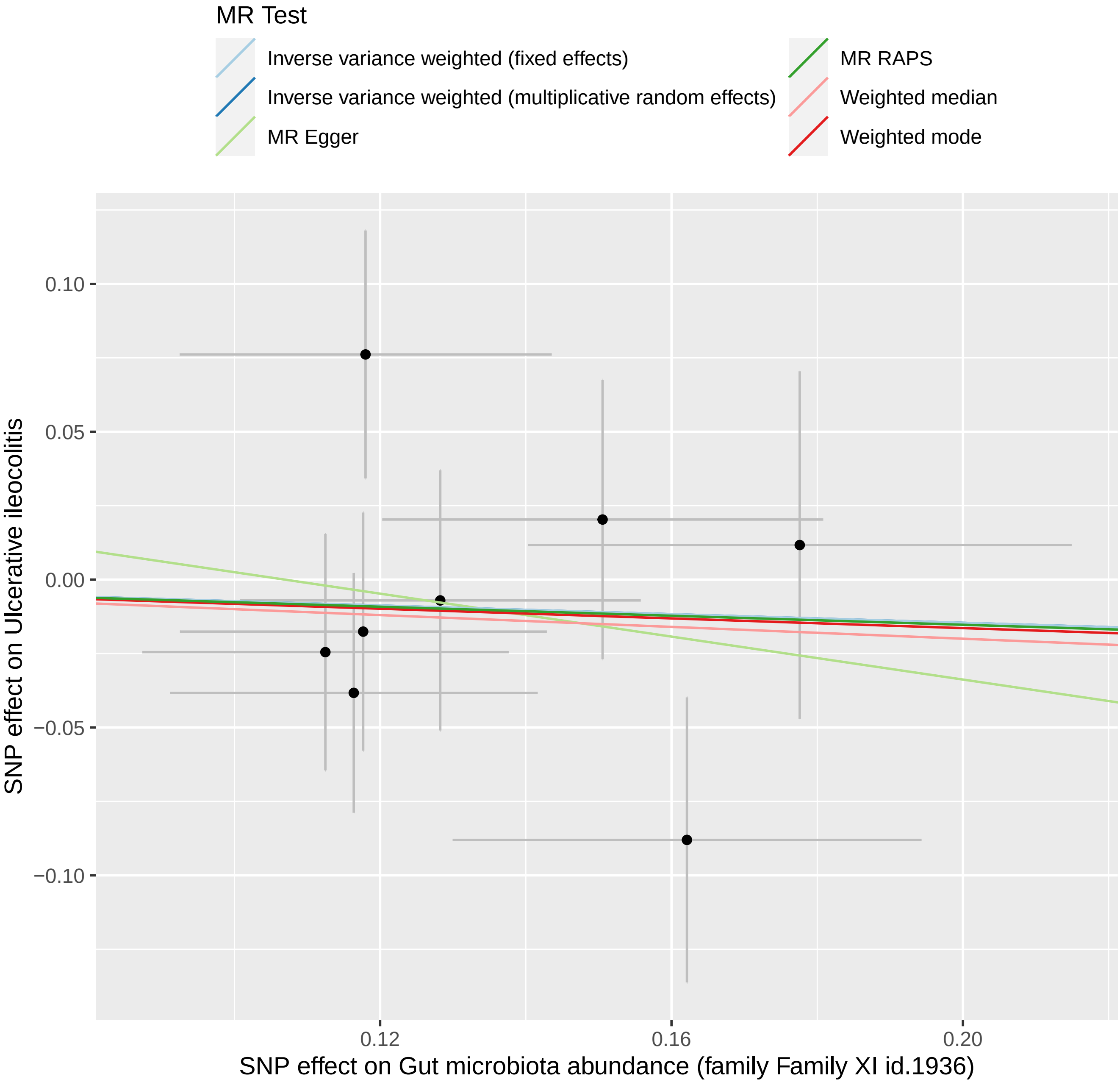

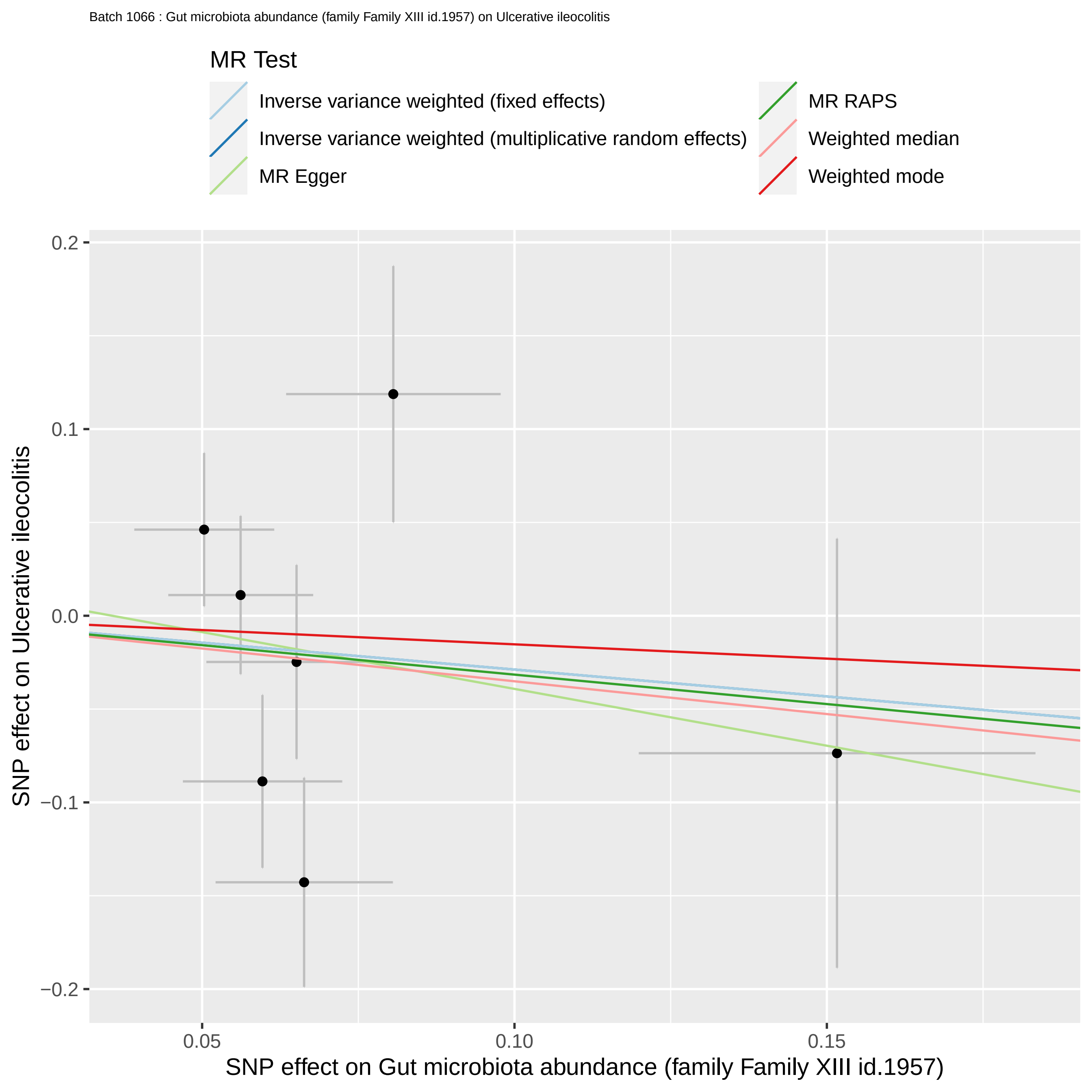
Batch 1066 : Gut microbiota abundance (family Family XIII id.1957) on Ulcerative ileocolitis
MR Test
Inverse variance weighted (fixed effects)
Inverse variance weighted (multiplicative random effects)
MR Egger
MR RAPS
Weighted median
Weighted mode
0.2
0.1
0.0
−0.1
−0.2
0.05
0.10
0.15
SNP effect on Ulcerative ileocolitis
SNP effect on Gut microbiota abundance (family Family XIII id.1957)

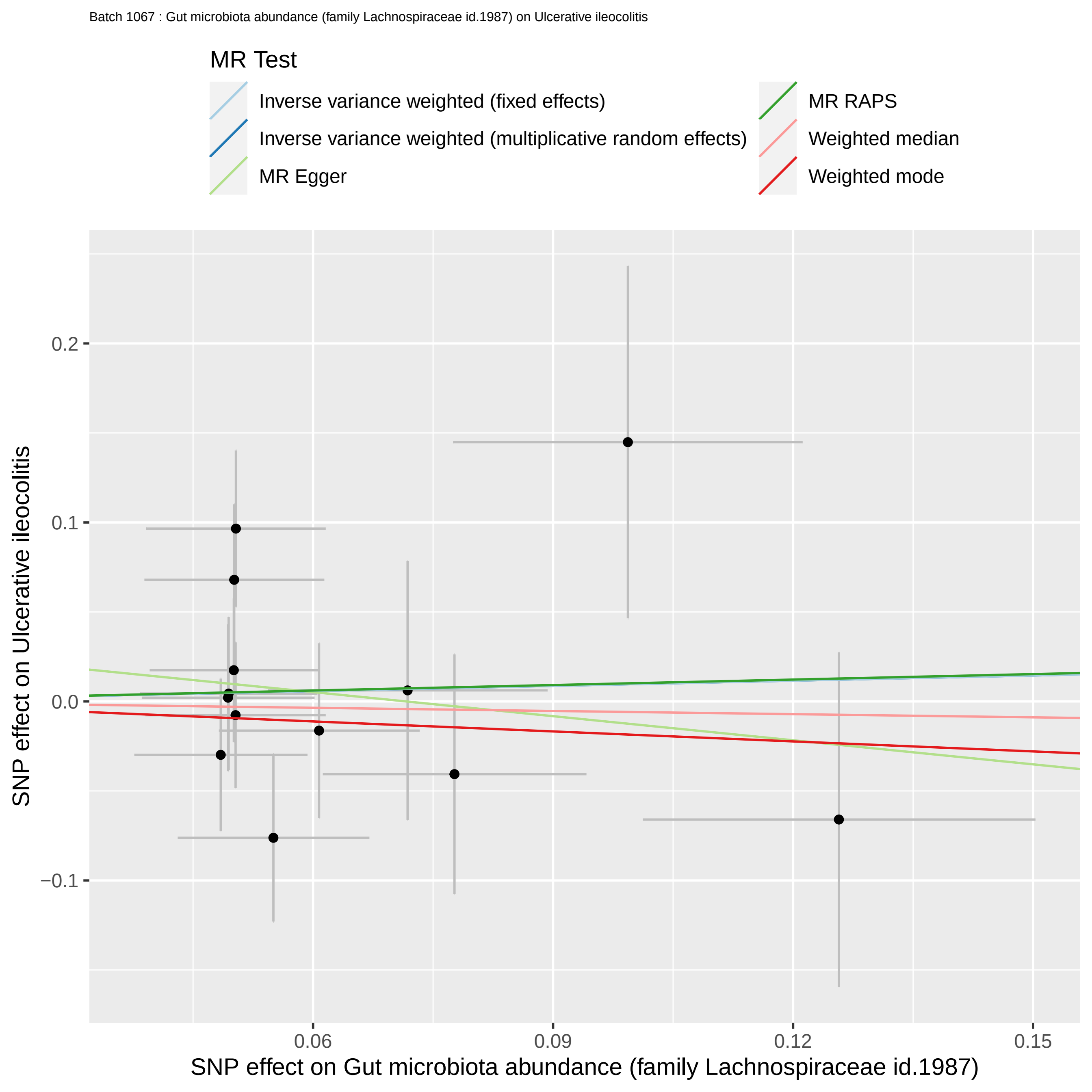

Batch 1067 : Gut microbiota abundance (family Lachnospiraceae id.1987) on Ulcerative ileocolitis
MR Test
Inverse variance weighted (fixed effects)
Inverse variance weighted (multiplicative random effects)
MR Egger
MR RAPS
Weighted median
Weighted mode
0.2
0.1
0.0
−0.1
0.06
0.09
0.12
0.15
SNP effect on Ulcerative ileocolitis
SNP effect on Gut microbiota abundance (family Lachnospiraceae id.1987)

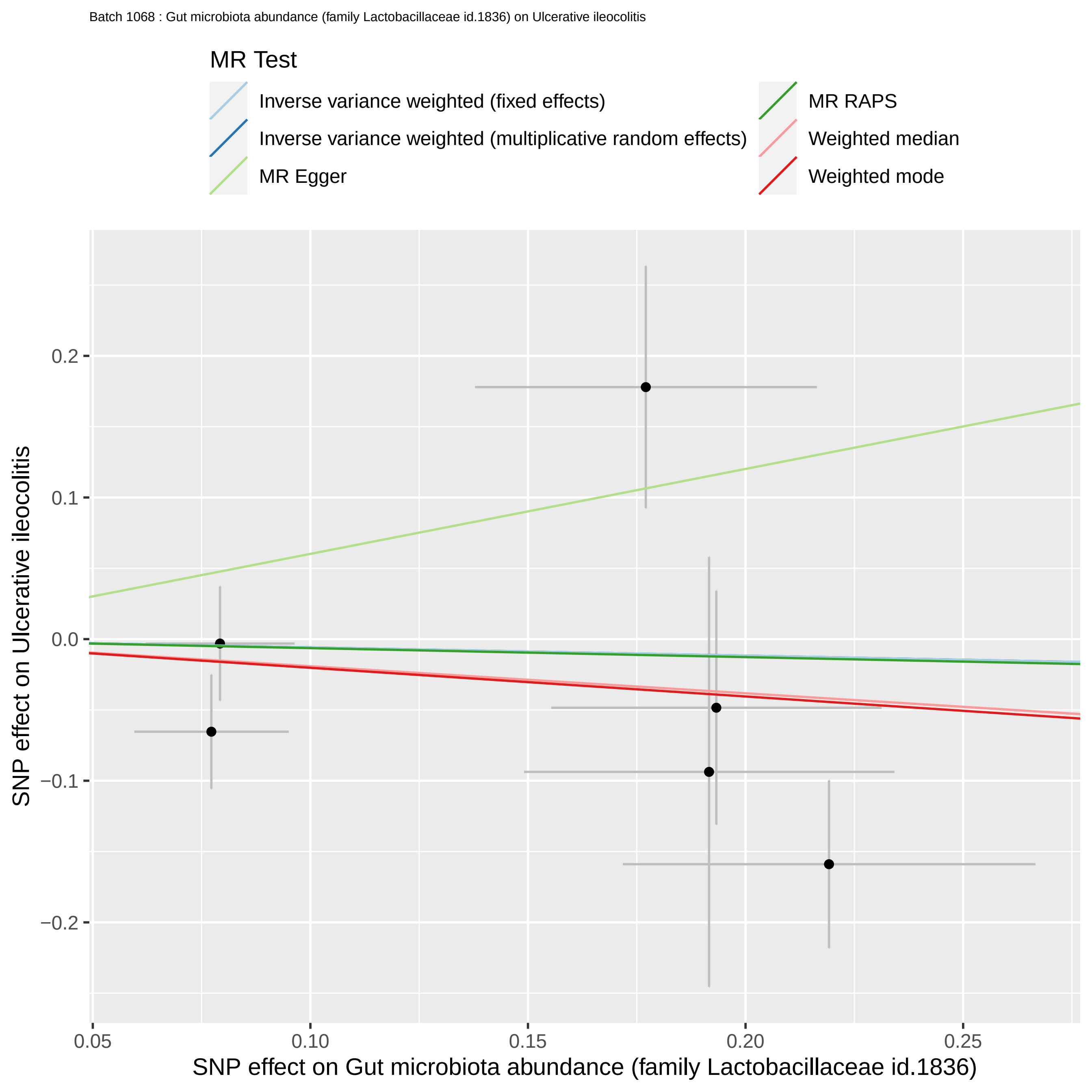

Batch 1068 : Gut microbiota abundance (family Lactobacillaceae id.1836) on Ulcerative ileocolitis
MR Test
Inverse variance weighted (fixed effects)
Inverse variance weighted (multiplicative random effects)
MR Egger
MR RAPS
Weighted median
Weighted mode
0.2
0.1
0.0
−0.1
−0.2
0.05
0.10
0.15
0.20
0.25
SNP effect on Ulcerative ileocolitis
SNP effect on Gut microbiota abundance (family Lactobacillaceae id.1836)

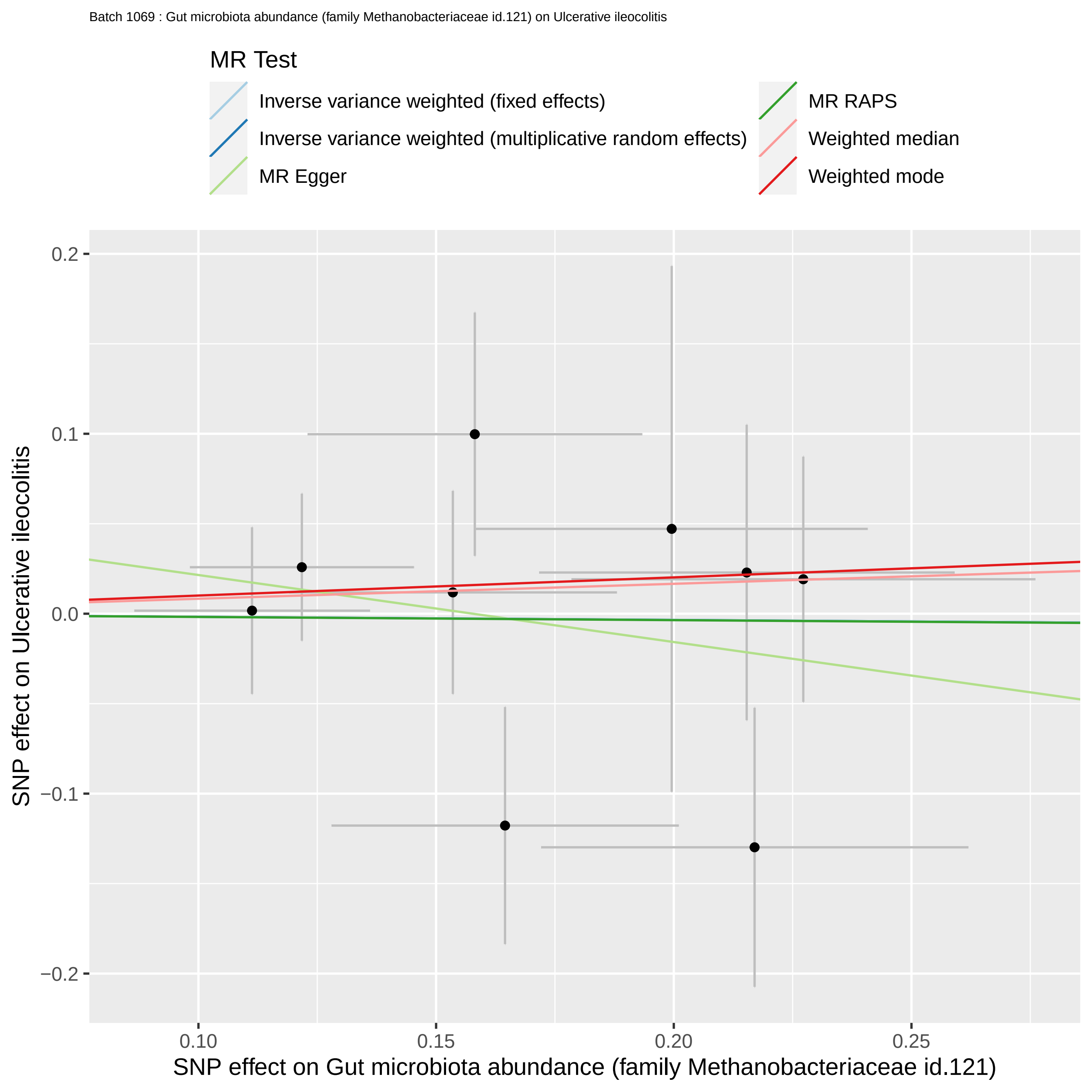

Batch 1069 : Gut microbiota abundance (family Methanobacteriaceae id.121) on Ulcerative ileocolitis
MR Test
Inverse variance weighted (fixed effects)
Inverse variance weighted (multiplicative random effects)
MR Egger
MR RAPS
Weighted median
Weighted mode
0.2
0.1
0.0
−0.1
−0.2
0.10
0.15
0.20
0.25
SNP effect on Ulcerative ileocolitis
SNP effect on Gut microbiota abundance (family Methanobacteriaceae id.121)

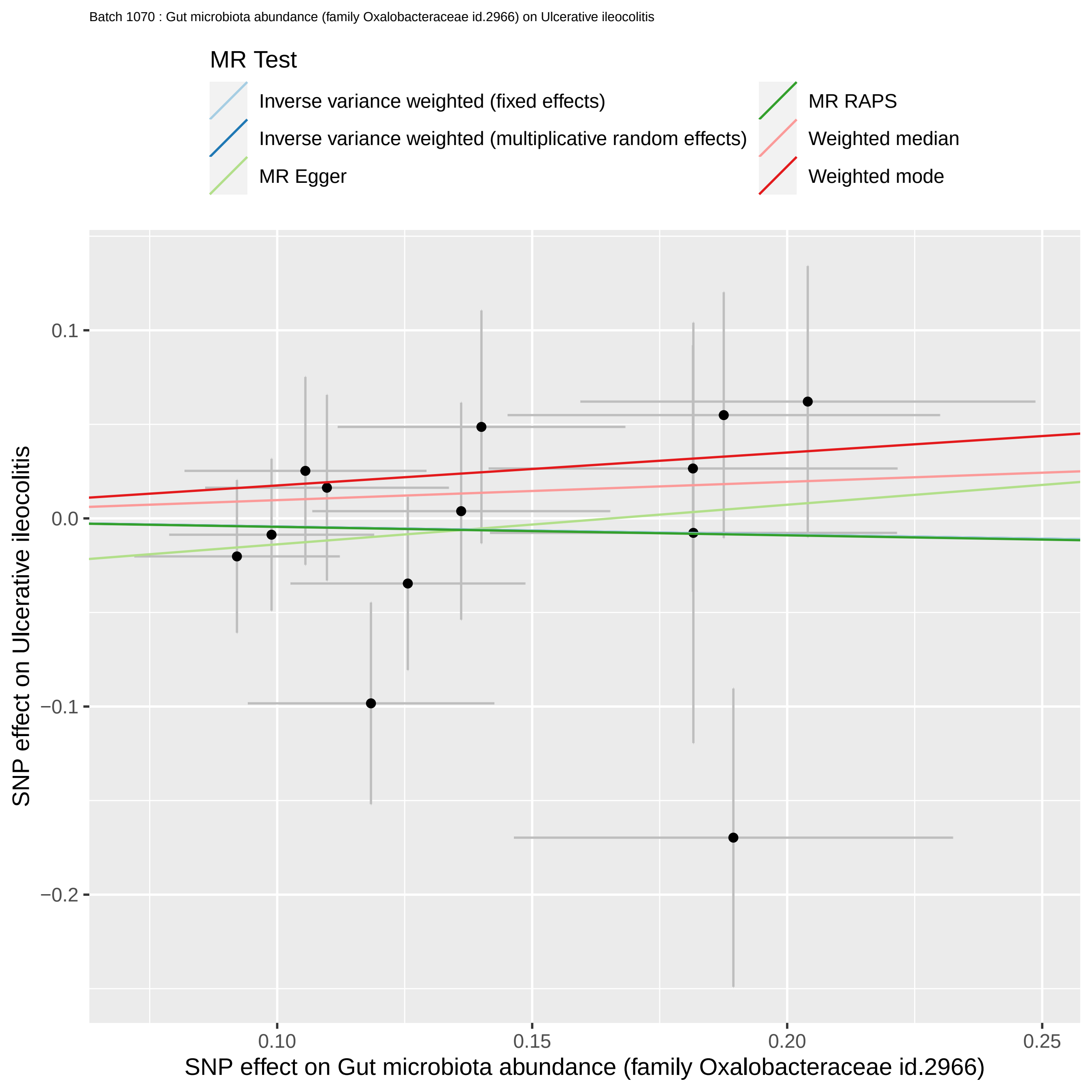

Batch 1070 : Gut microbiota abundance (family Oxalobacteraceae id.2966) on Ulcerative ileocolitis
MR Test
Inverse variance weighted (fixed effects)
Inverse variance weighted (multiplicative random effects)
MR Egger
MR RAPS
Weighted median
Weighted mode
0.1
0.0
−0.1
−0.2
0.10
0.15
0.20
0.25
SNP effect on Ulcerative ileocolitis
SNP effect on Gut microbiota abundance (family Oxalobacteraceae id.2966)

Batch 1071 : Gut microbiota abundance (family Pasteurellaceae id.3689) on Ulcerative ileocolitis

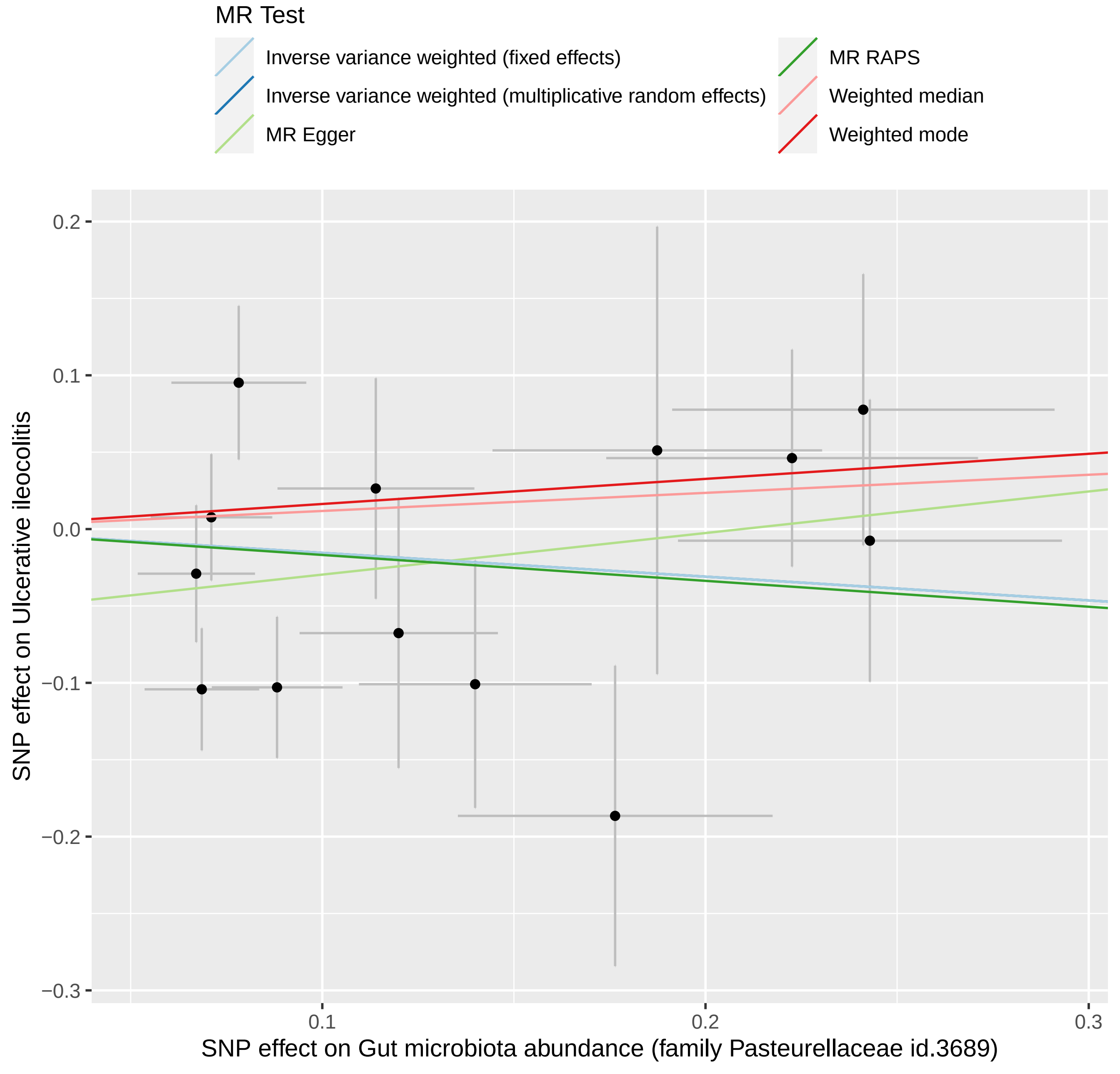

Batch 1072 : Gut microbiota abundance (family Peptococcaceae id.2024) on Ulcerative ileocolitis

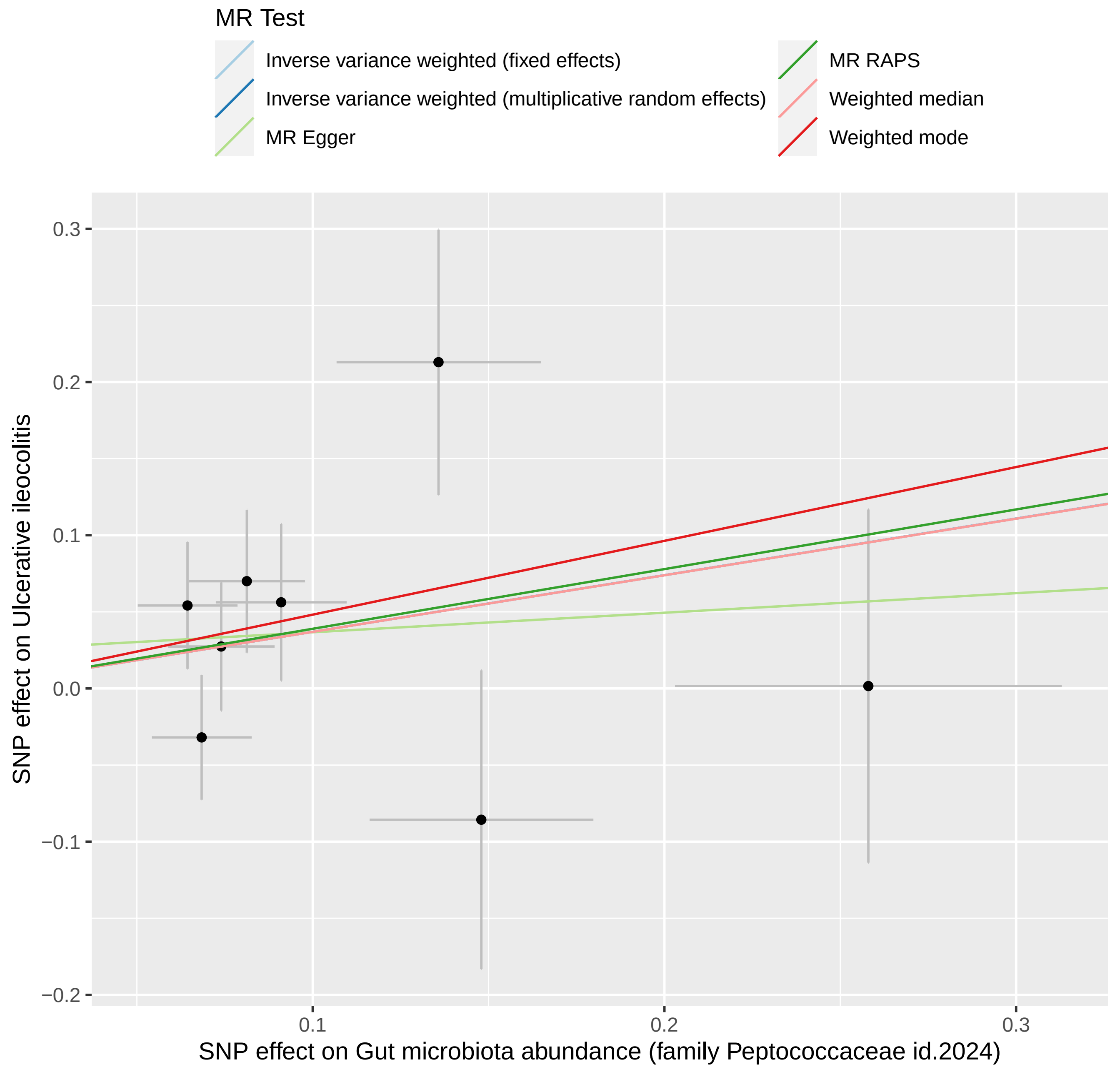

Batch 1073 : Gut microbiota abundance (family Peptostreptococcaceae id.2042) on Ulcerative ileocolitis

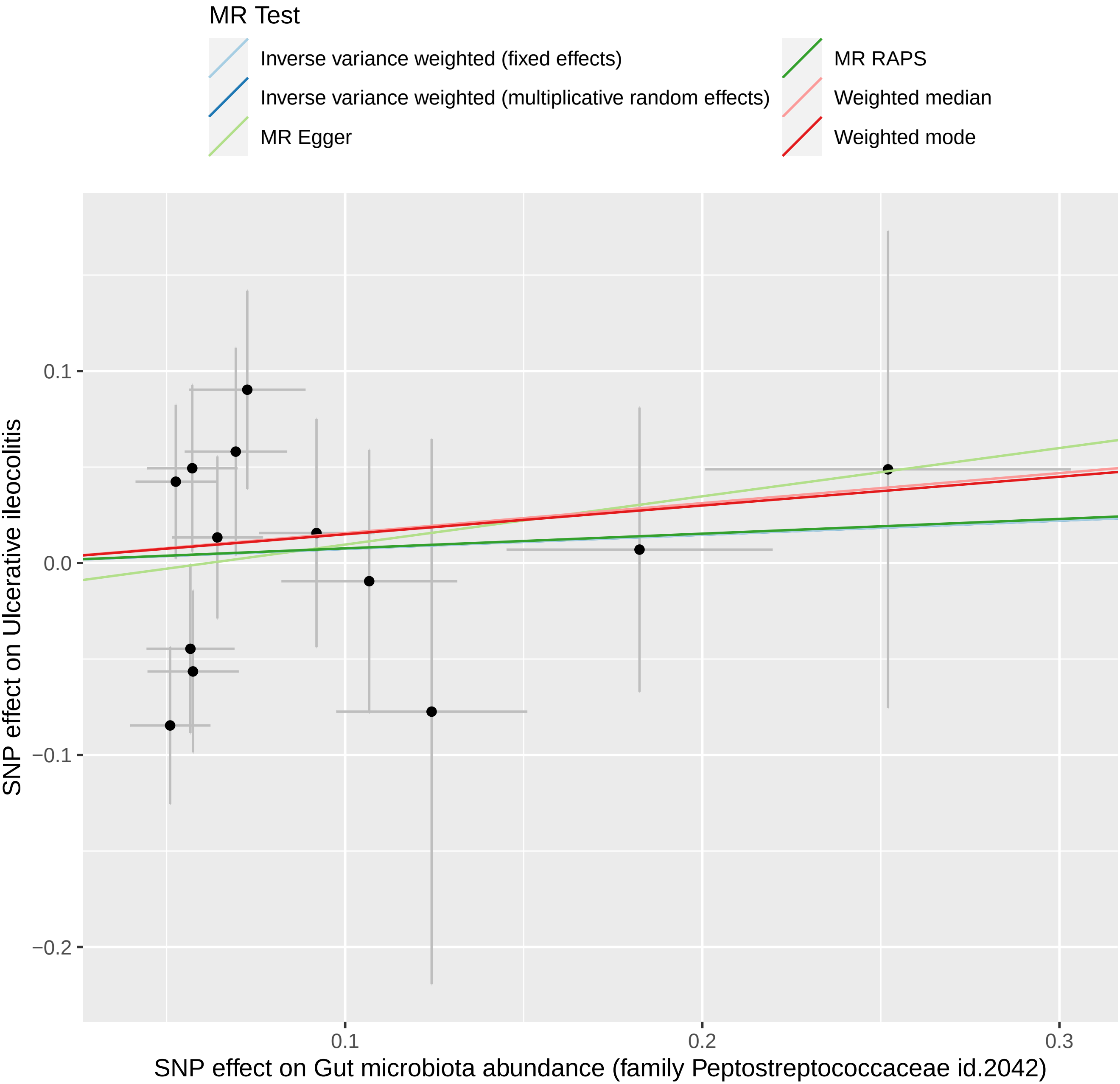

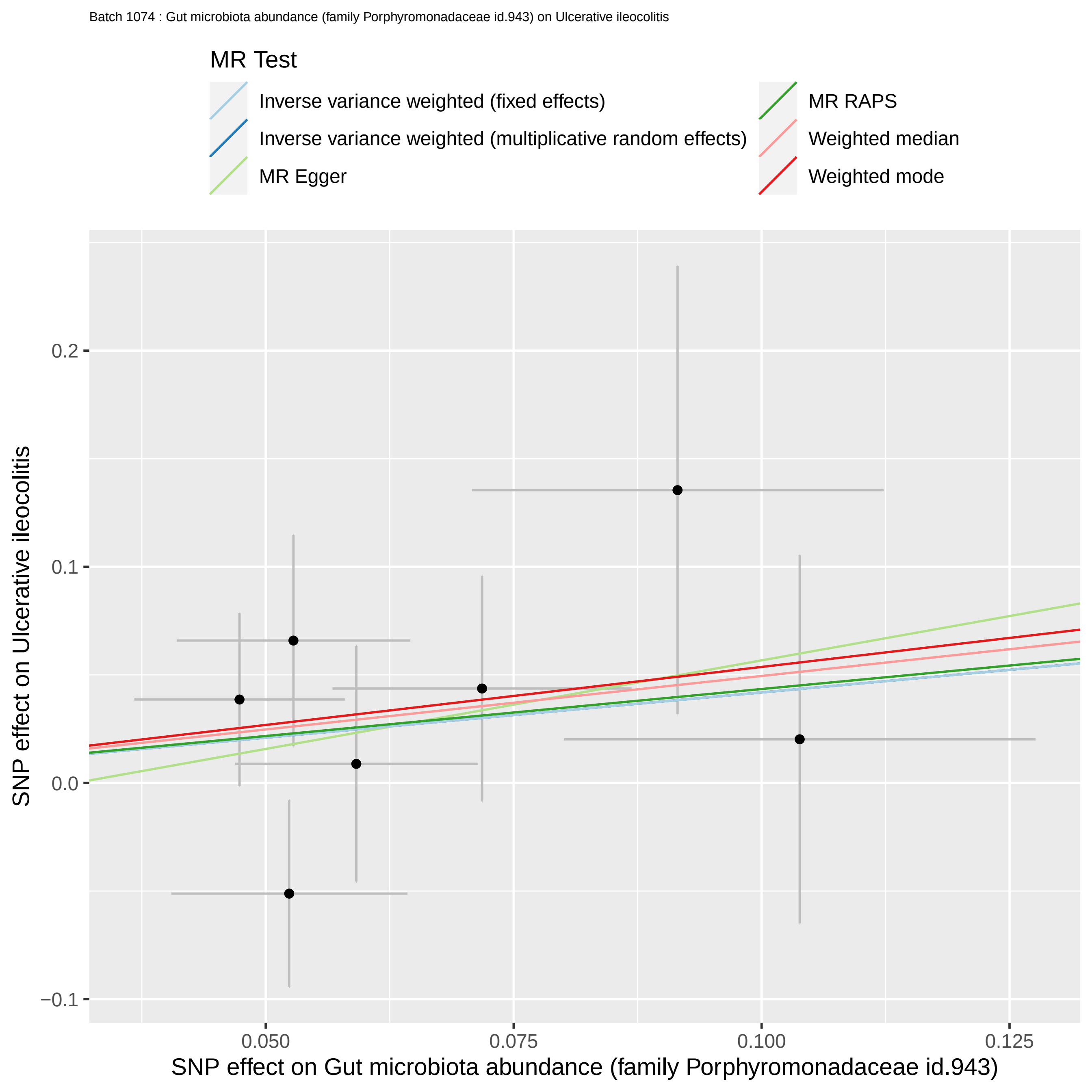
Batch 1074 : Gut microbiota abundance (family Porphyromonadaceae id.943) on Ulcerative ileocolitis
MR Test
Inverse variance weighted (fixed effects)
Inverse variance weighted (multiplicative random effects)
MR Egger
MR RAPS
Weighted median
Weighted mode
SNP effect on Ulcerative ileocolitis
0.2
0.1
0.0
−0.1
0.050
0.075
0.100
0.125
SNP effect on Gut microbiota abundance (family Porphyromonadaceae id.943)

Batch 1075 : Gut microbiota abundance (family Prevotellaceae id.960) on Ulcerative ileocolitis

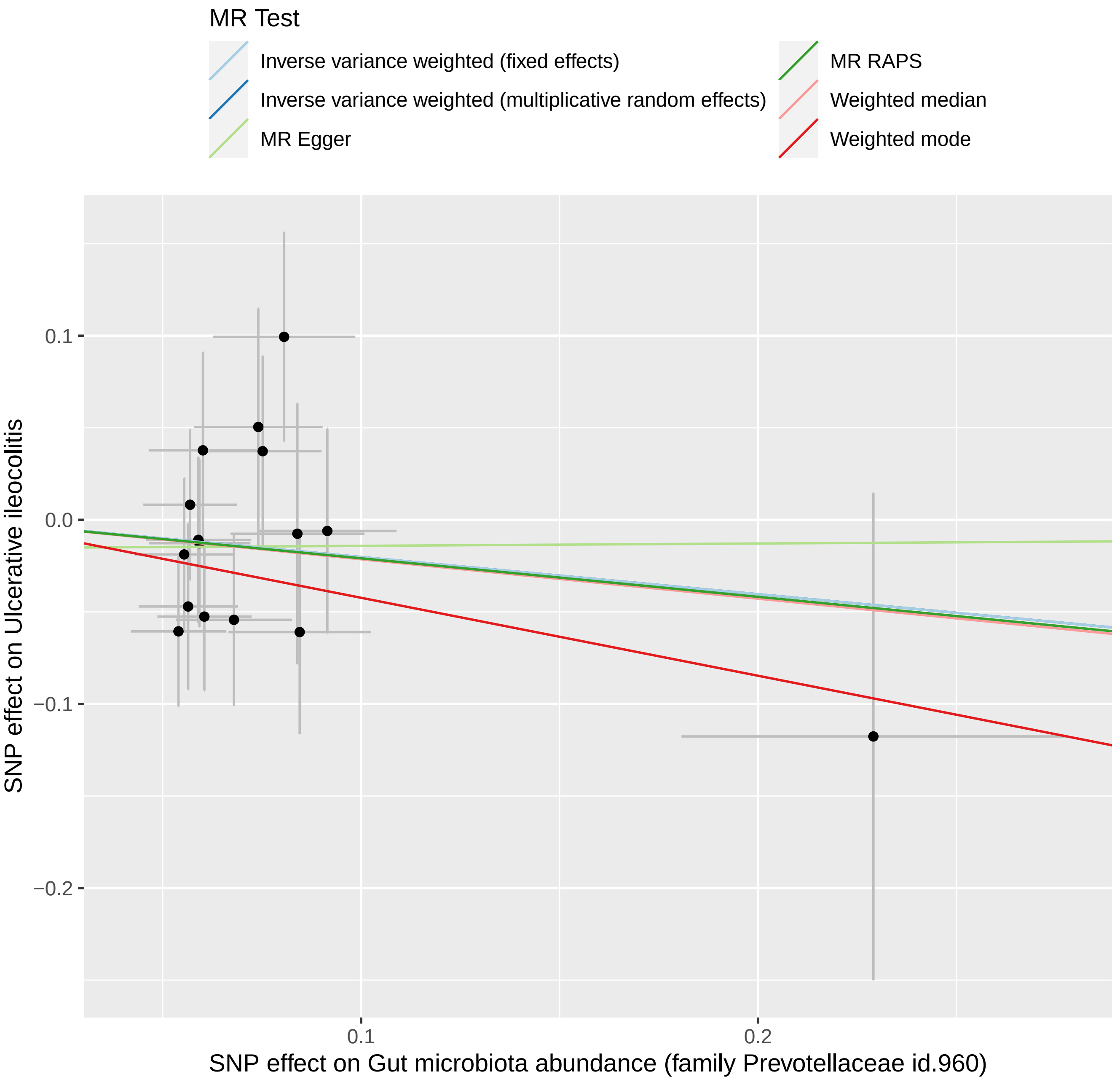

Batch 1076 : Gut microbiota abundance (family Rhodospirillaceae id.2717) on Ulcerative ileocolitis

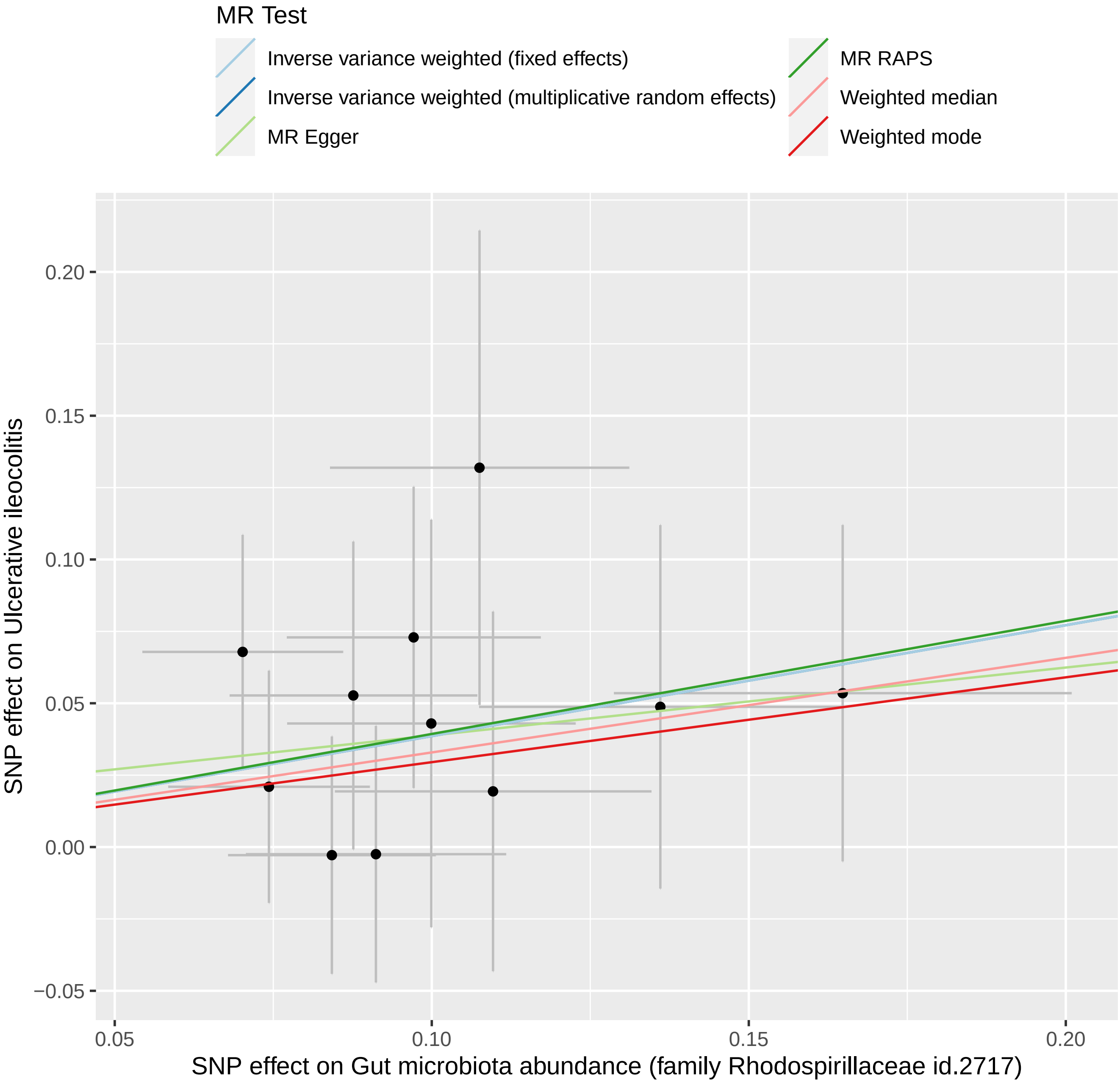

Batch 1077 : Gut microbiota abundance (family Rikenellaceae id.967) on Ulcerative ileocolitis

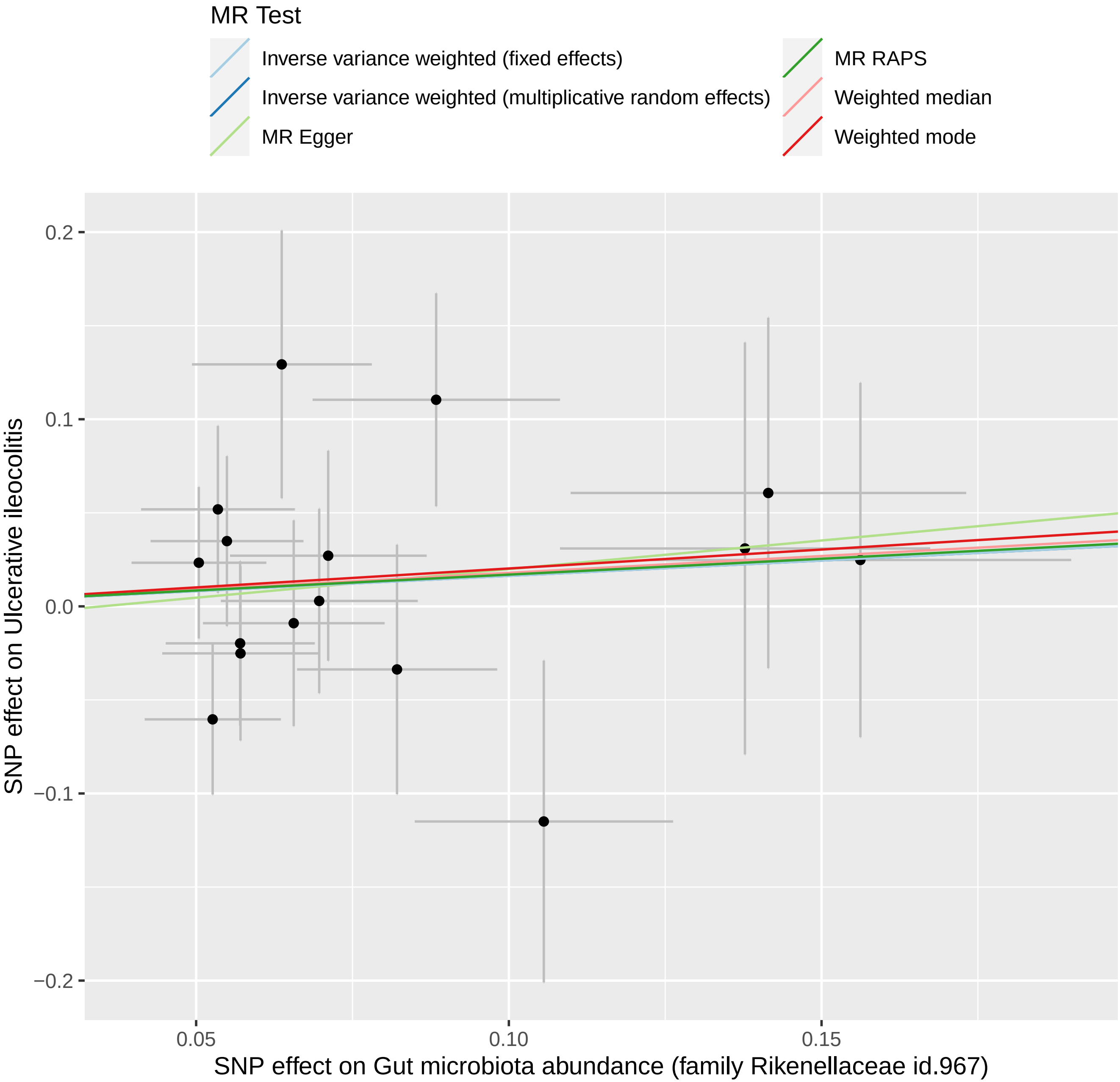

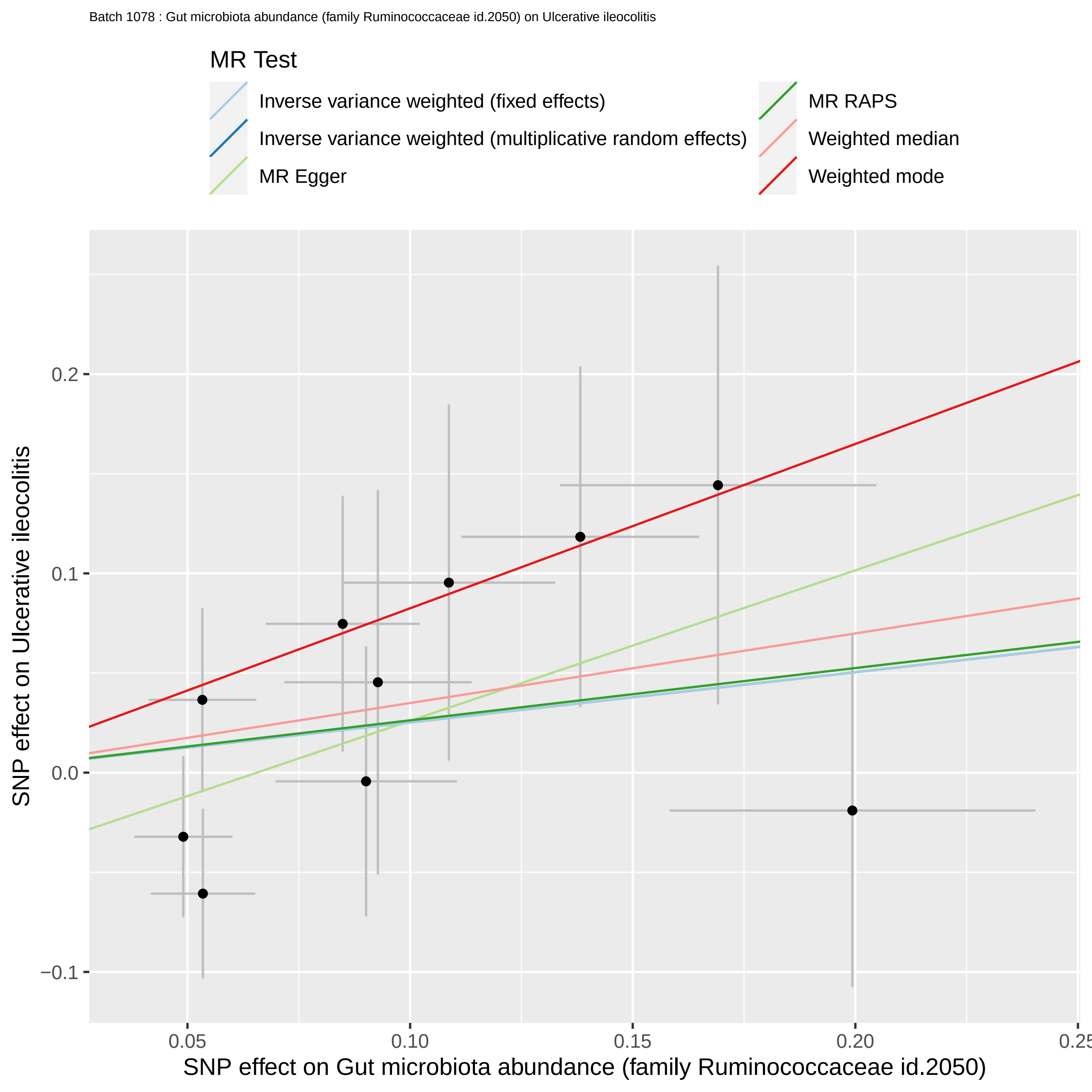
Batch 1078 : Gut microbiota abundance (family Ruminococcaceae id.2050) on Ulcerative ileocolitis
MR Test
Inverse variance weighted (fixed effects)
Inverse variance weighted (multiplicative random effects)
MR Egger
MR RAPS
Weighted median
Weighted mode
0.2
0.1
0.0
−0.1
0.05
0.10
0.15
0.20
0.25
SNP effect on Ulcerative ileocolitis
SNP effect on Gut microbiota abundance (family Ruminococcaceae id.2050)

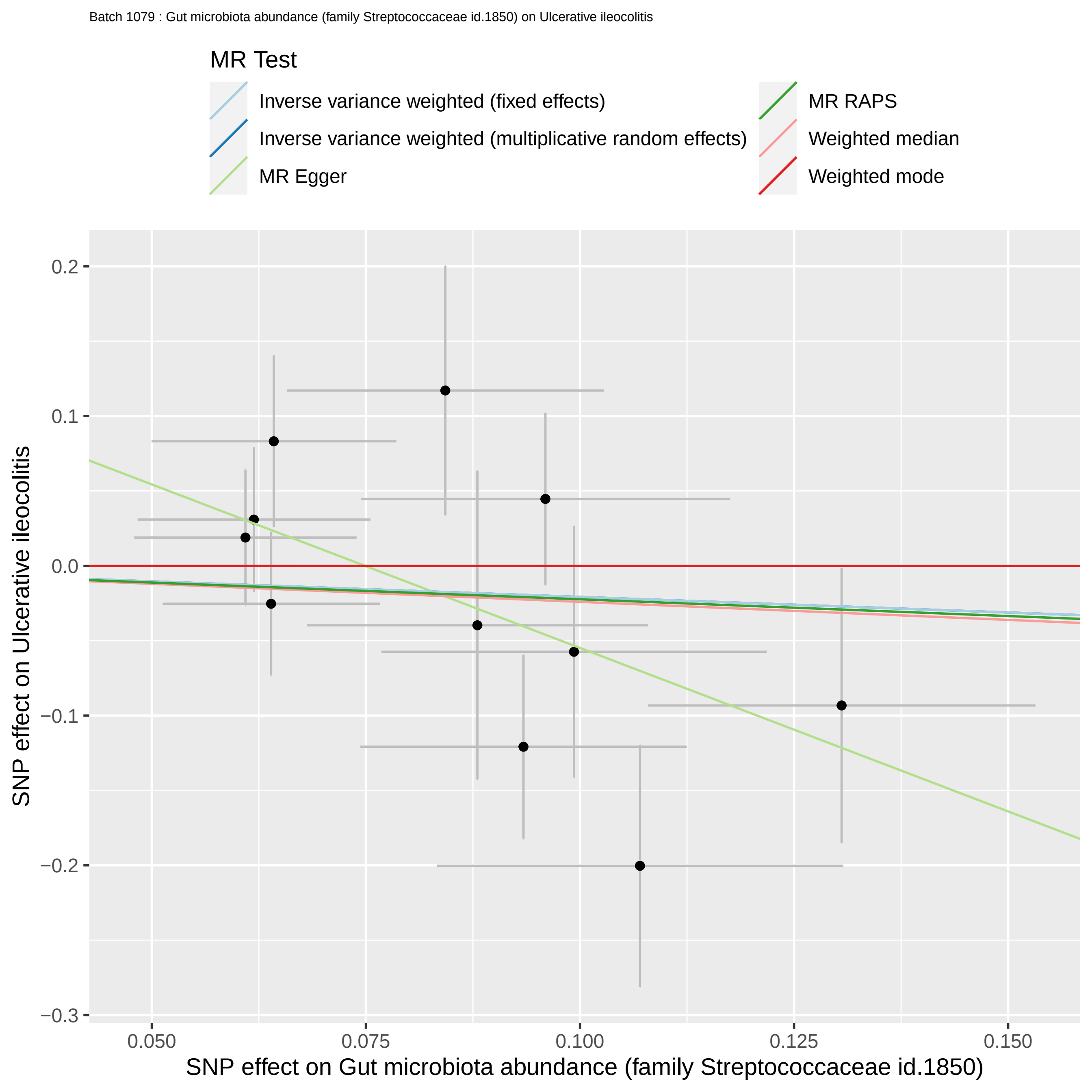

Batch 1079 : Gut microbiota abundance (family Streptococcaceae id.1850) on Ulcerative ileocolitis
MR Test
Inverse variance weighted (fixed effects)
Inverse variance weighted (multiplicative random effects)
MR Egger
MR RAPS
Weighted median
Weighted mode
SNP effect on Ulcerative ileocolitis
0.2
0.1
0.0
−0.1
−0.2
−0.3
0.050
0.075
0.100
0.125
0.150
SNP effect on Gut microbiota abundance (family Streptococcaceae id.1850)

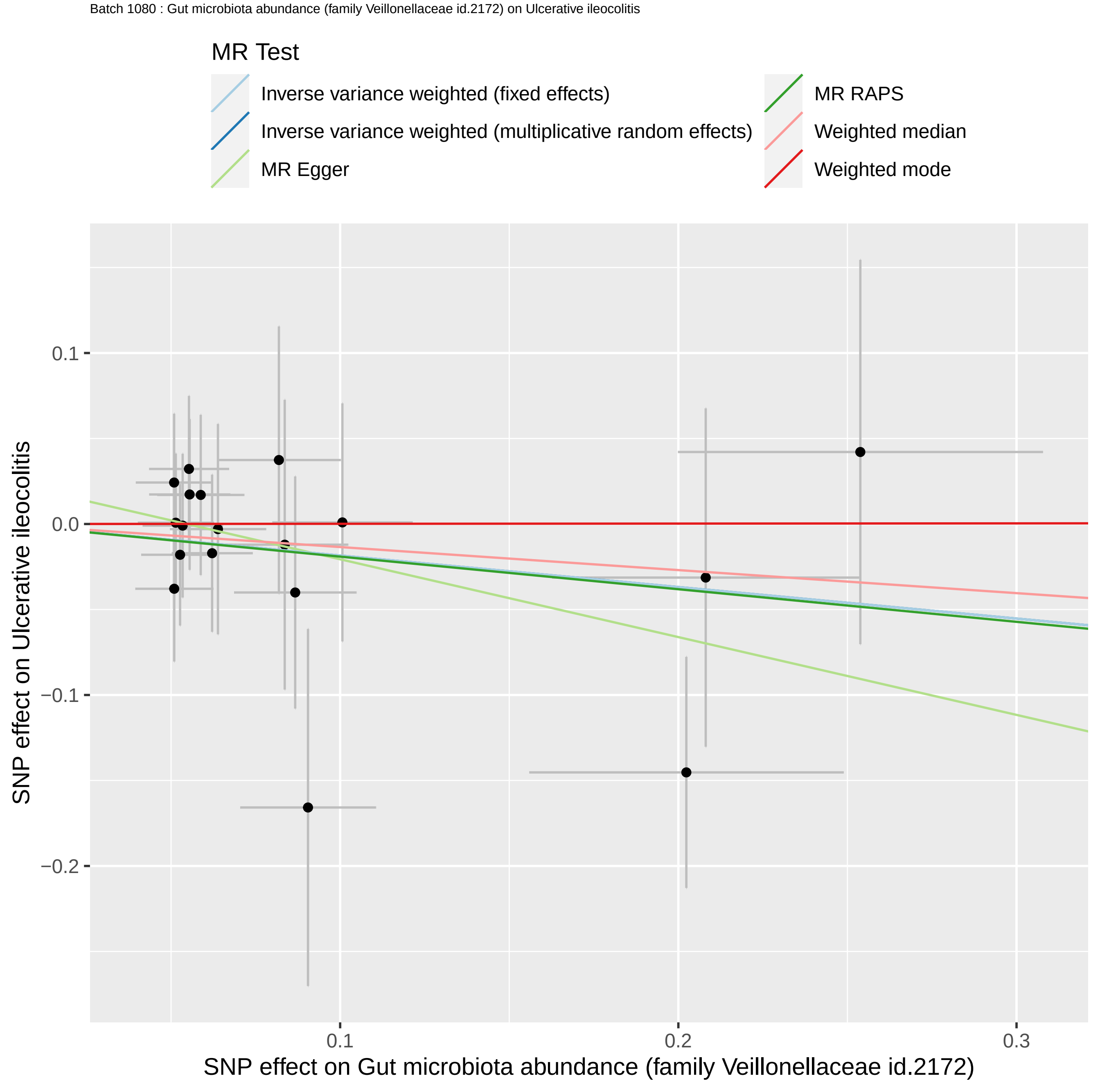
Batch 1080 : Gut microbiota abundance (family Veillonellaceae id.2172) on Ulcerative ileocolitis
MR Test
Inverse variance weighted (fixed effects)
Inverse variance weighted (multiplicative random effects)
MR Egger
MR RAPS
Weighted median
Weighted mode
SNP effect on Ulcerative ileocolitis
0.1
0.0
−0.1
−0.2
0.1
0.2
0.3
SNP effect on Gut microbiota abundance (family Veillonellaceae id.2172)

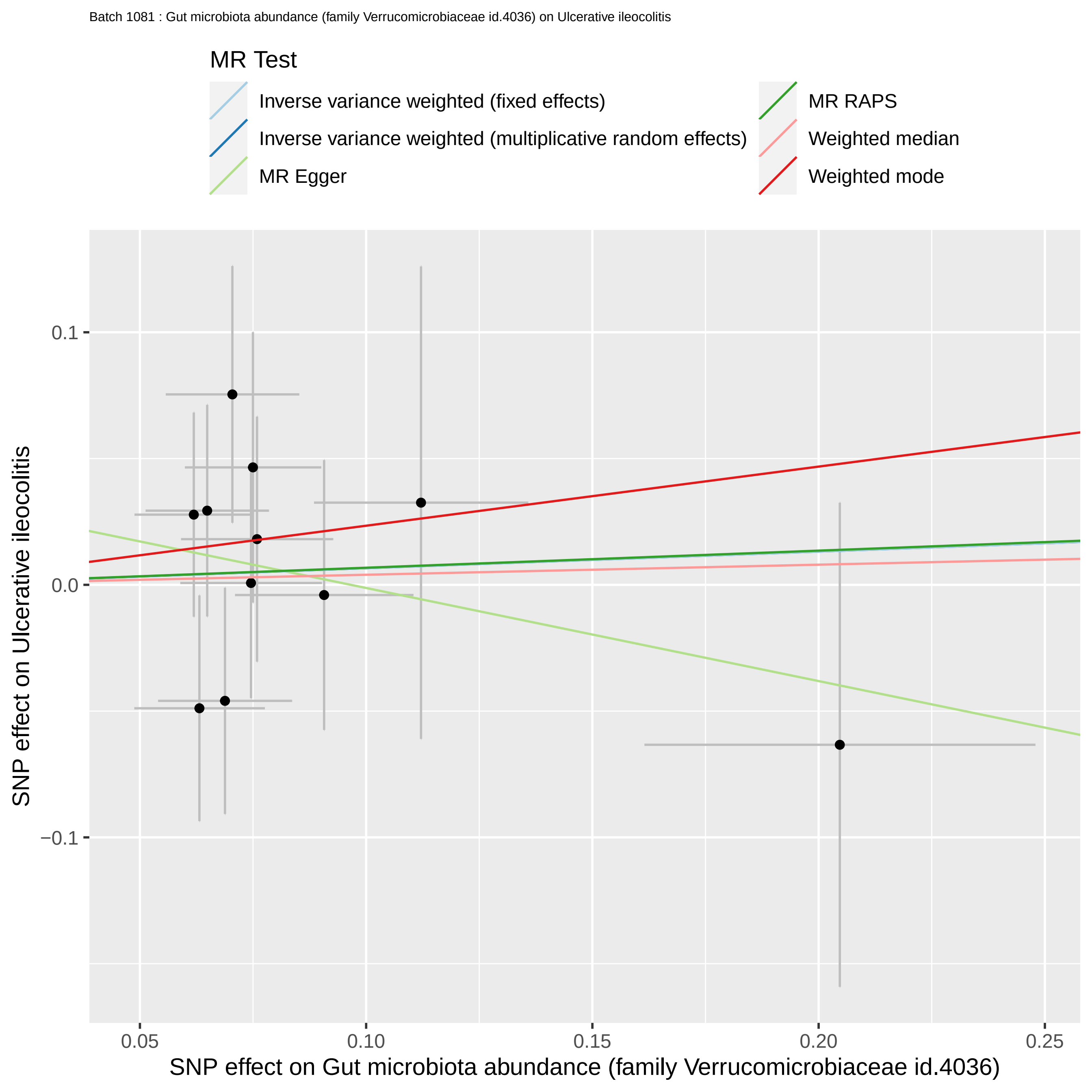
Batch 1081 : Gut microbiota abundance (family Verrucomicrobiaceae id.4036) on Ulcerative ileocolitis
MR Test
Inverse variance weighted (fixed effects)
Inverse variance weighted (multiplicative random effects)
MR Egger
MR RAPS
Weighted median
Weighted mode
0.1
0.0
−0.1
0.05
0.10
0.15
0.20
0.25
SNP effect on Ulcerative ileocolitis
SNP effect on Gut microbiota abundance (family Verrucomicrobiaceae id.4036)

Batch 1082 : Gut microbiota abundance (family Victivallaceae id.2255) on Ulcerative ileocolitis

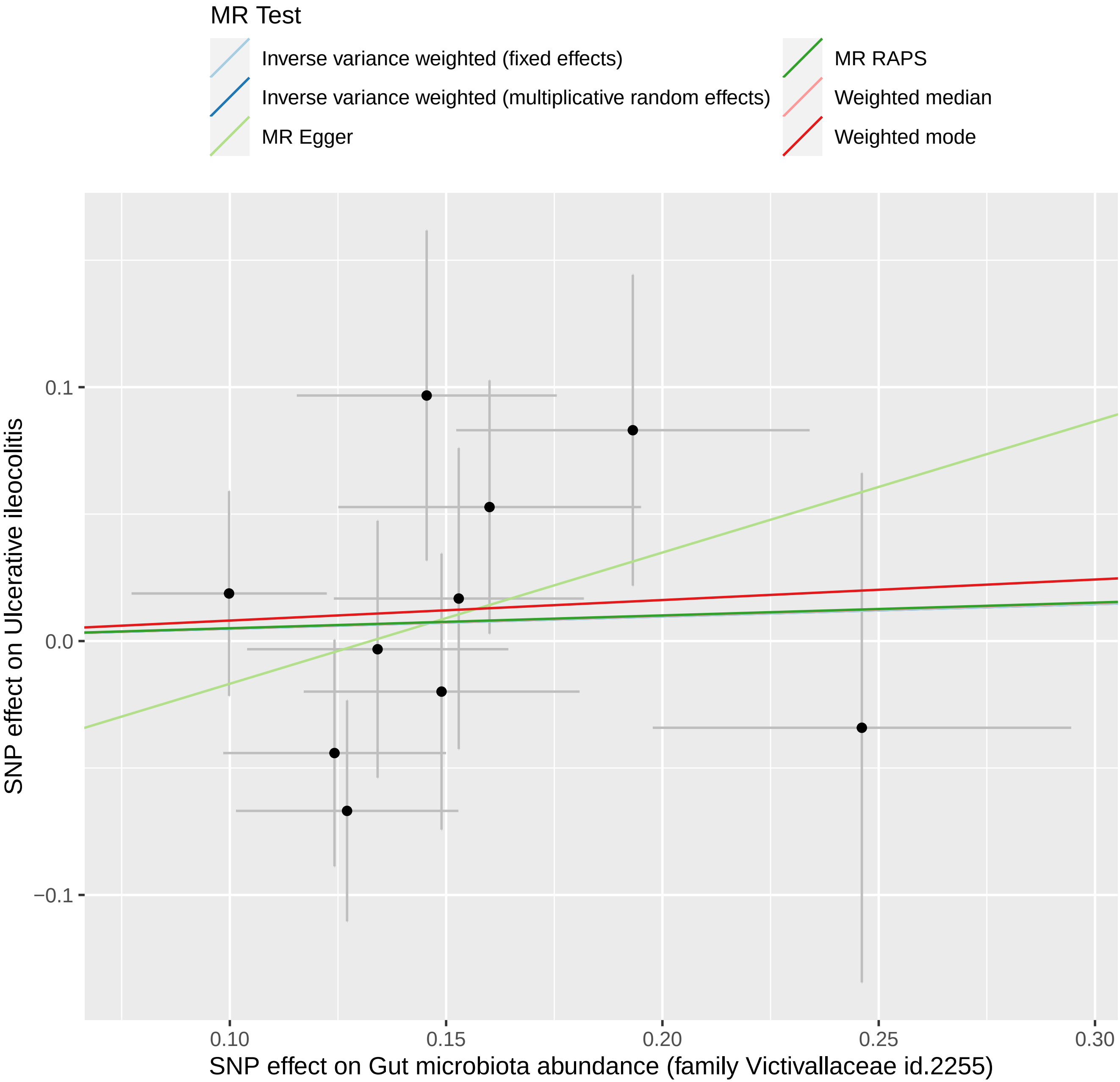

Batch 1083 : Gut microbiota abundance (genus Actinomyces id.423) on Ulcerative ileocolitis

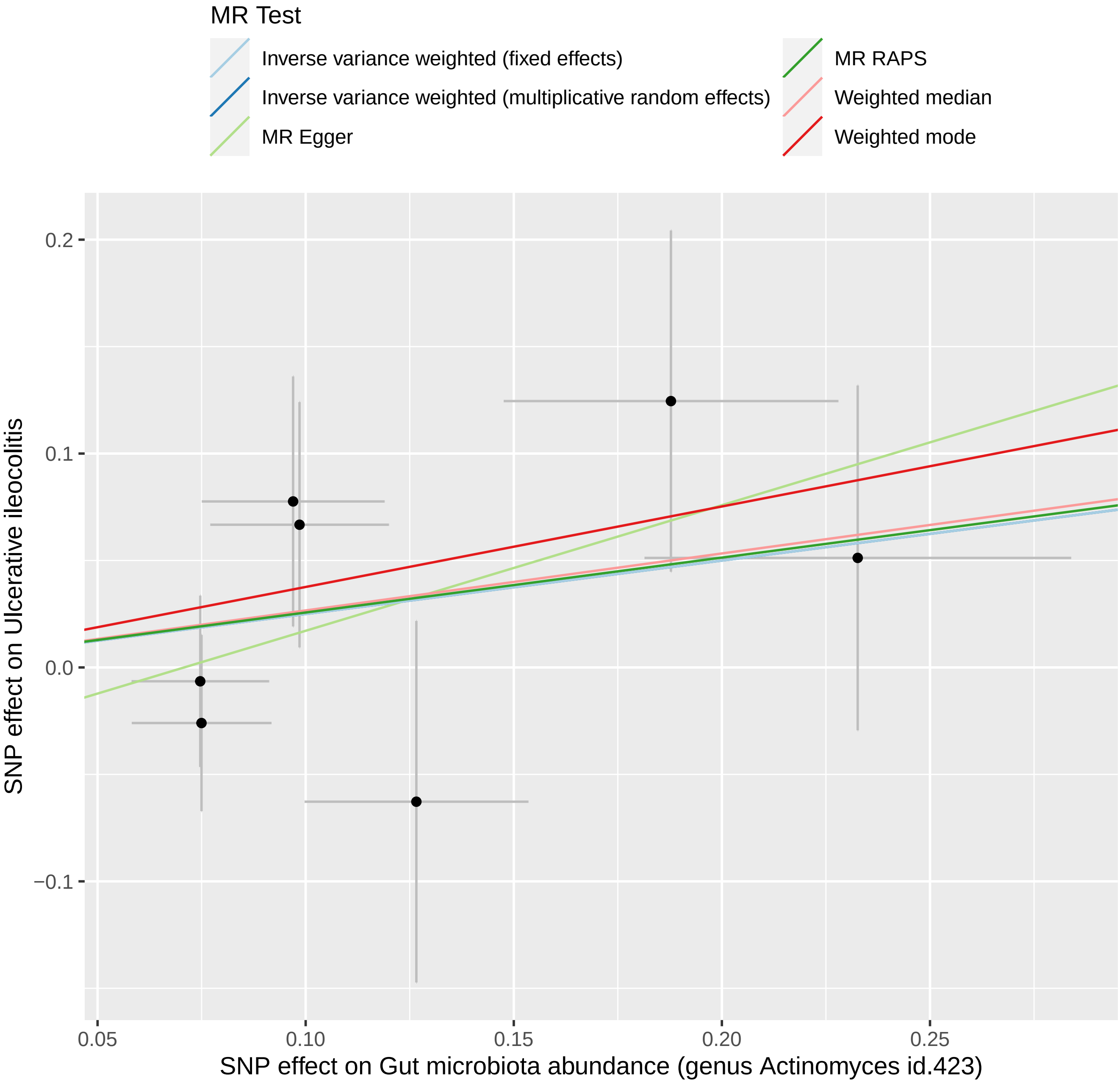

Batch 1084 : Gut microbiota abundance (genus Adlercreutzia id.812) on Ulcerative ileocolitis

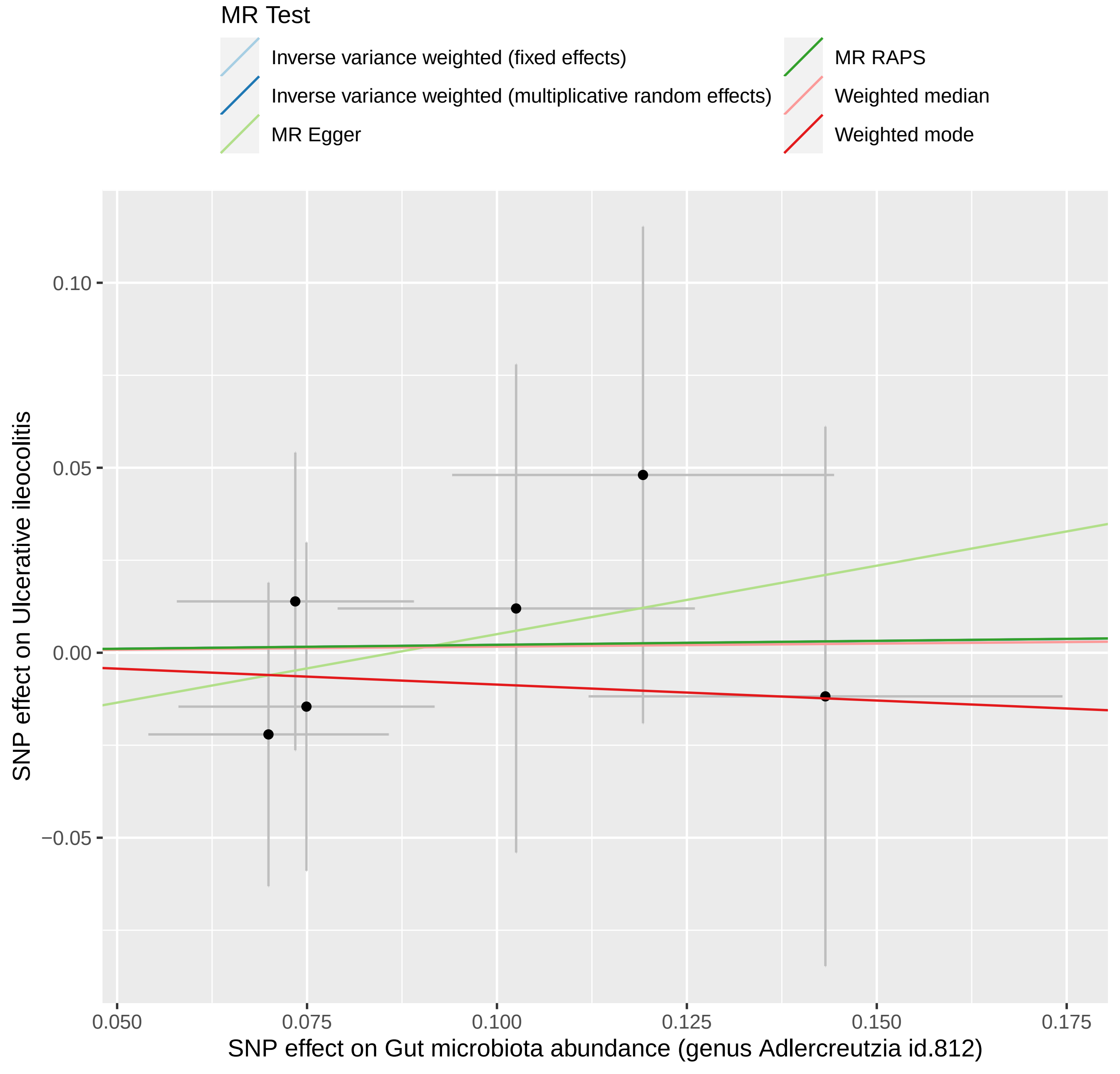

Batch 1085 : Gut microbiota abundance (genus Akkermansia id.4037) on Ulcerative ileocolitis

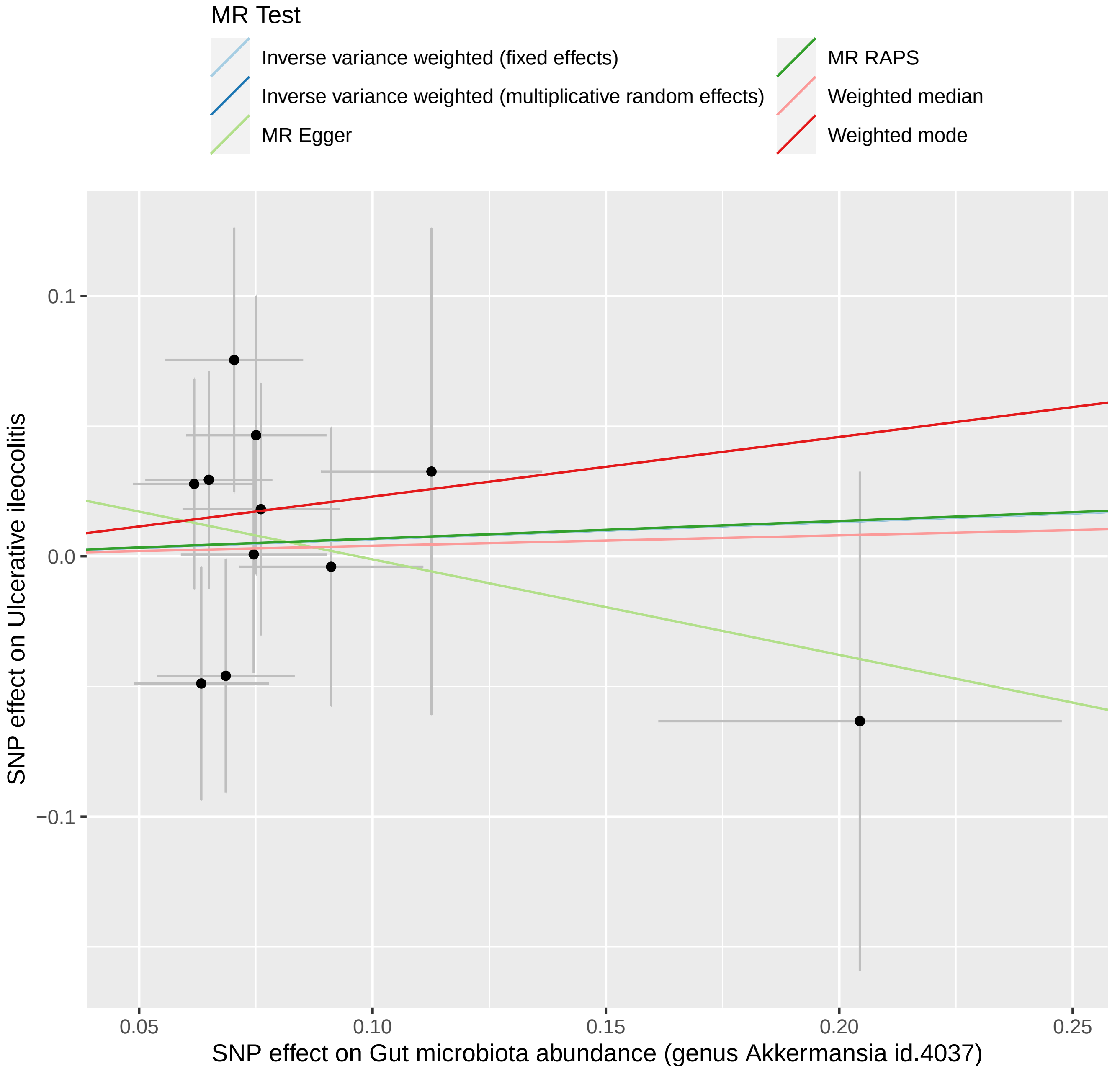

Batch 1086 : Gut microbiota abundance (genus Alistipes id.968) on Ulcerative ileocolitis

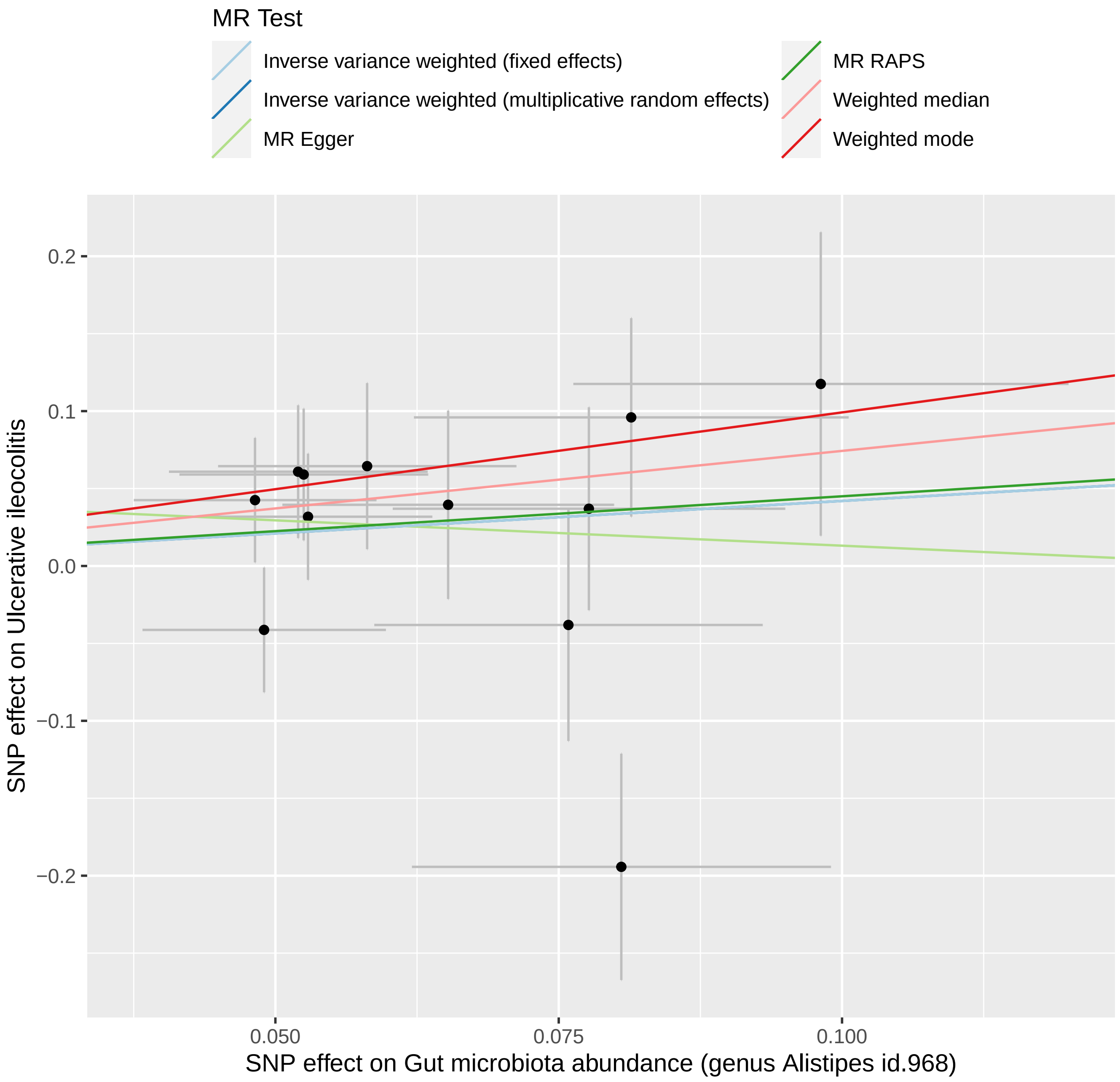

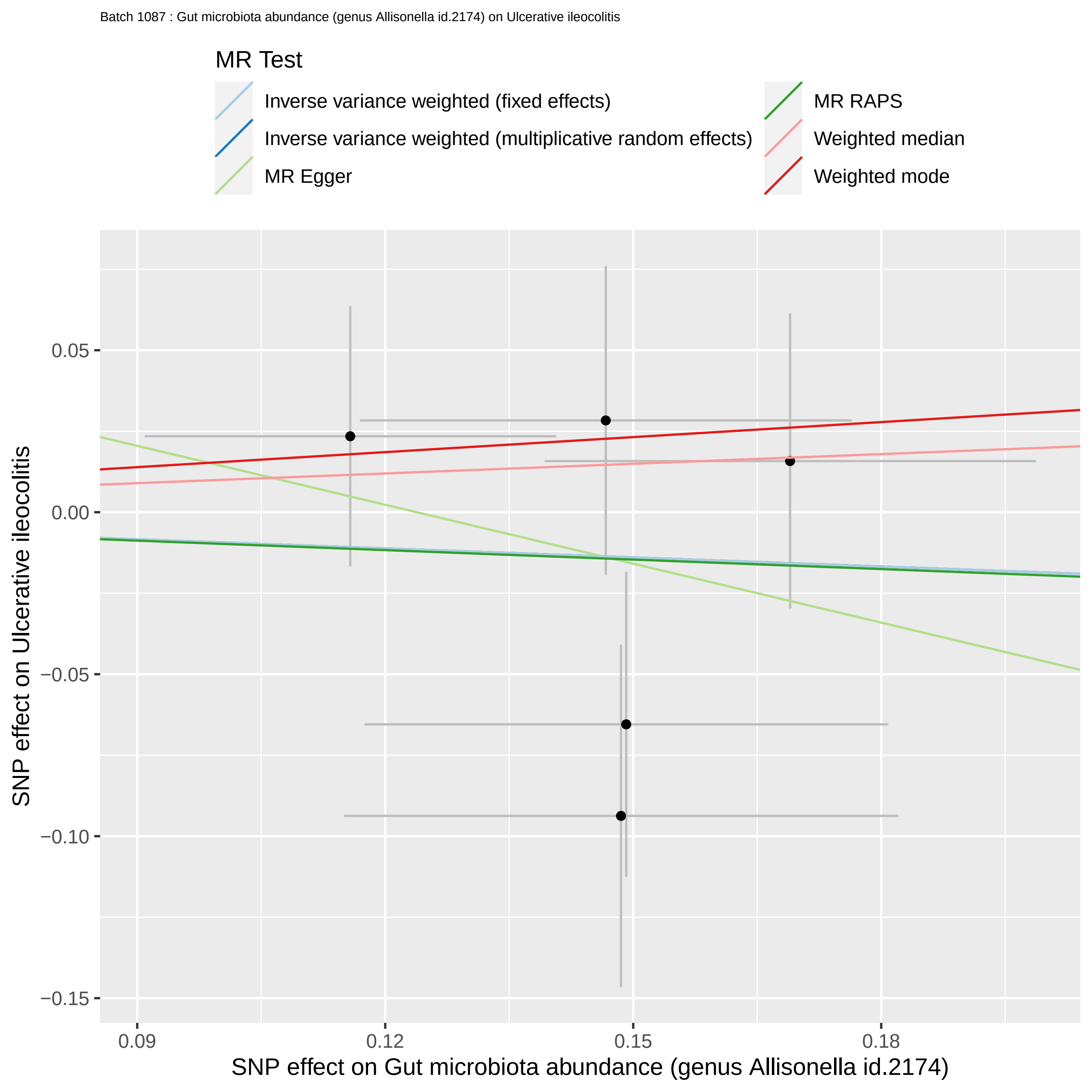
Batch 1087 : Gut microbiota abundance (genus Allisonella id.2174) on Ulcerative ileocolitis
MR Test
Inverse variance weighted (fixed effects)
Inverse variance weighted (multiplicative random effects)
MR Egger
MR RAPS
Weighted median
Weighted mode
0.05
0.00
−0.05
−0.10
−0.15
0.09
0.12
0.15
0.18
SNP effect on Ulcerative ileocolitis
SNP effect on Gut microbiota abundance (genus Allisonella id.2174)

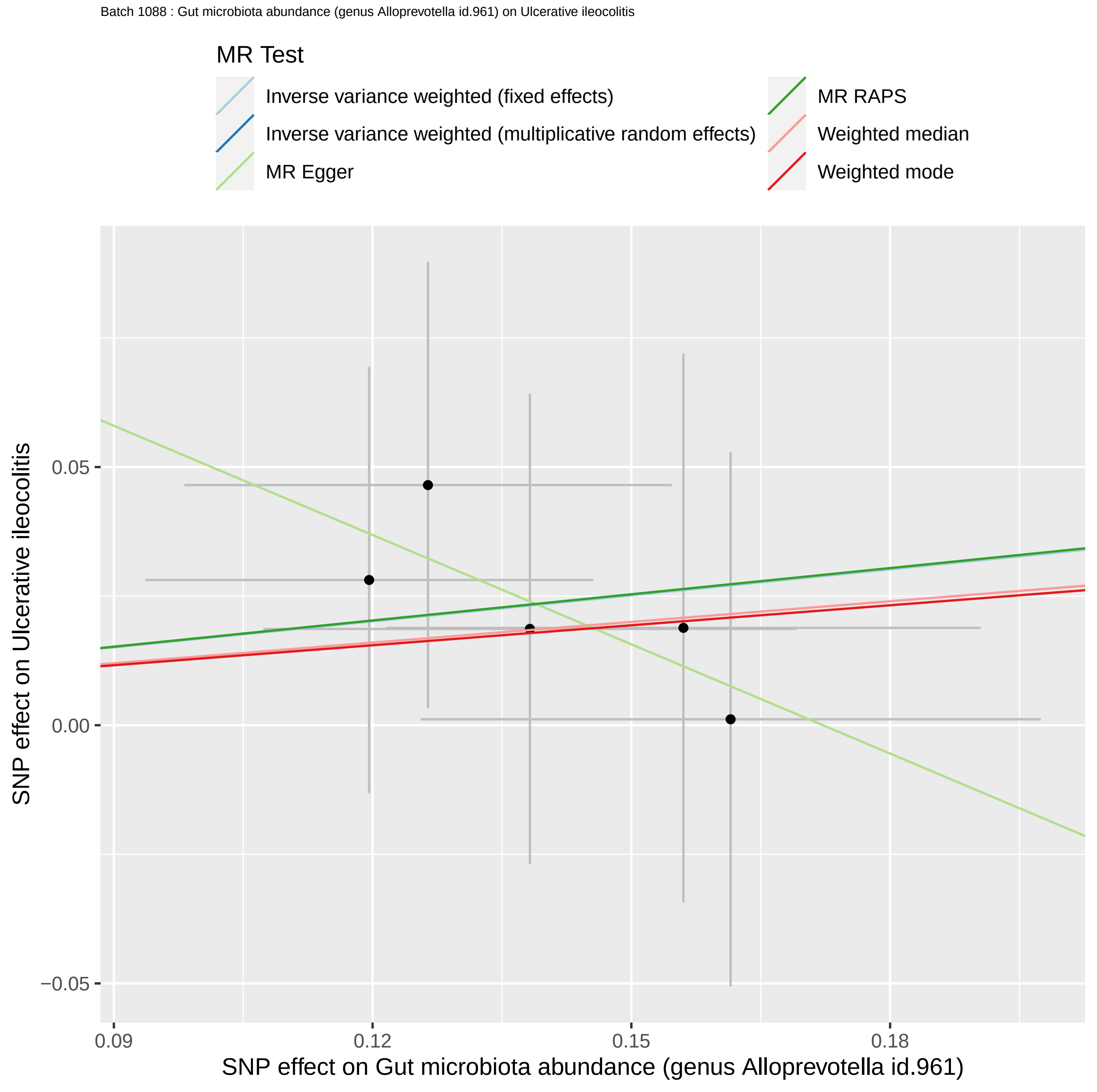

Batch 1088 : Gut microbiota abundance (genus Alloprevotella id.961) on Ulcerative ileocolitis
MR Test
Inverse variance weighted (fixed effects)
Inverse variance weighted (multiplicative random effects)
MR Egger
MR RAPS
Weighted median
Weighted mode
SNP effect on Ulcerative ileocolitis
0.05
0.00
−0.05
0.09
0.12
0.15
0.18
SNP effect on Gut microbiota abundance (genus Alloprevotella id.961)

Batch 1089 : Gut microbiota abundance (genus Anaerofilum id.2053) on Ulcerative ileocolitis

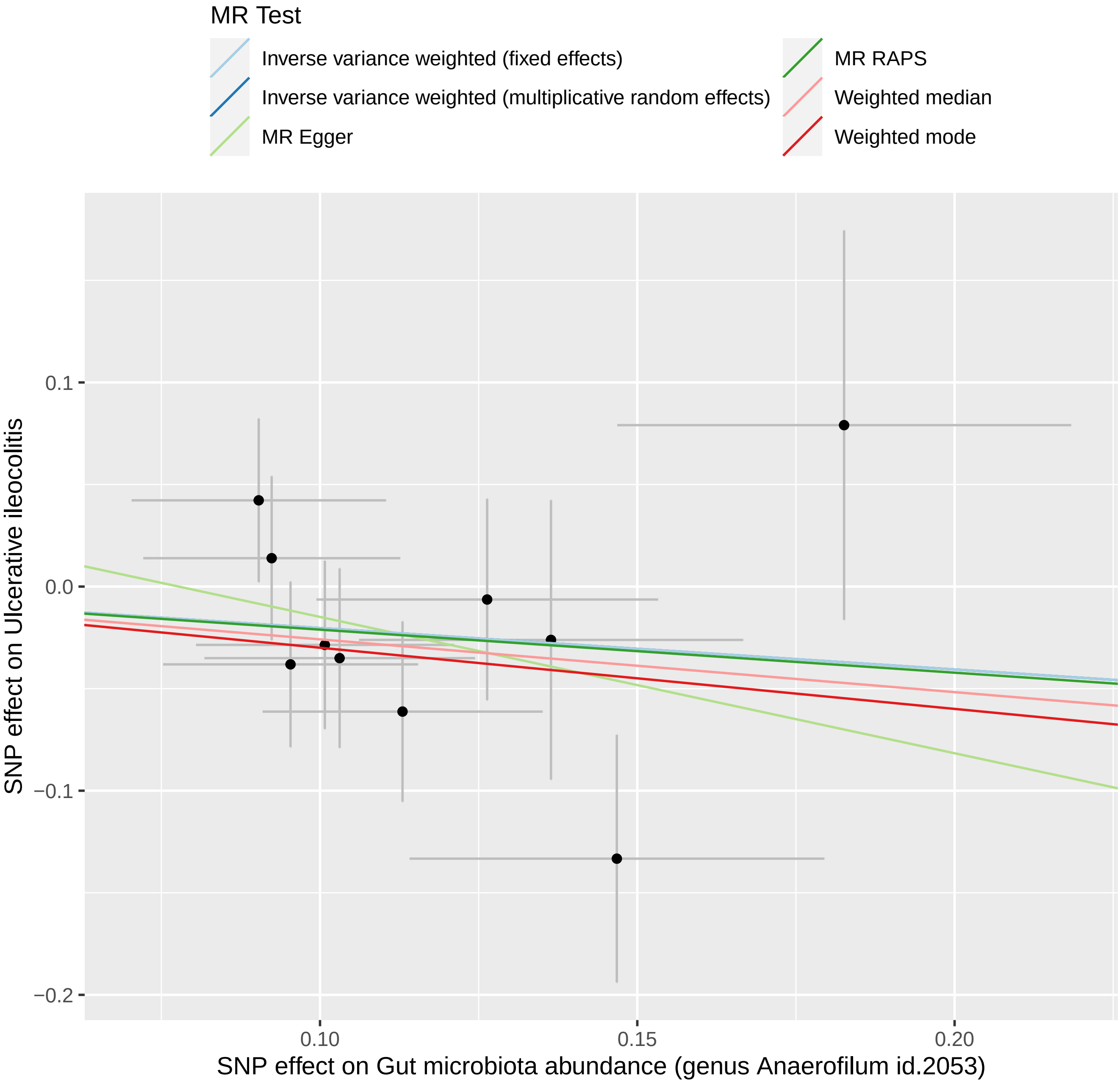

Batch 1090 : Gut microbiota abundance (genus Anaerostipes id.1991) on Ulcerative ileocolitis

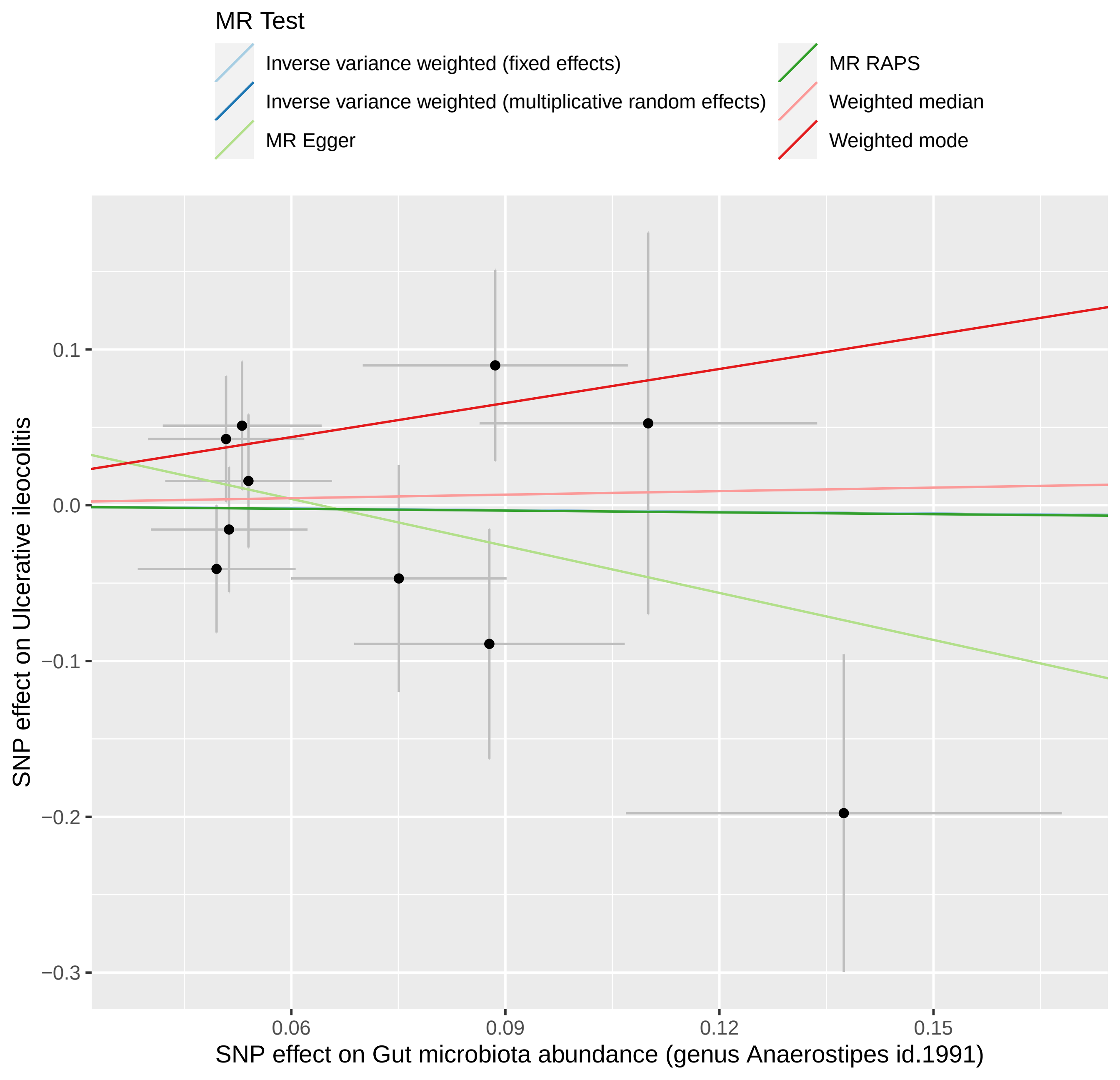

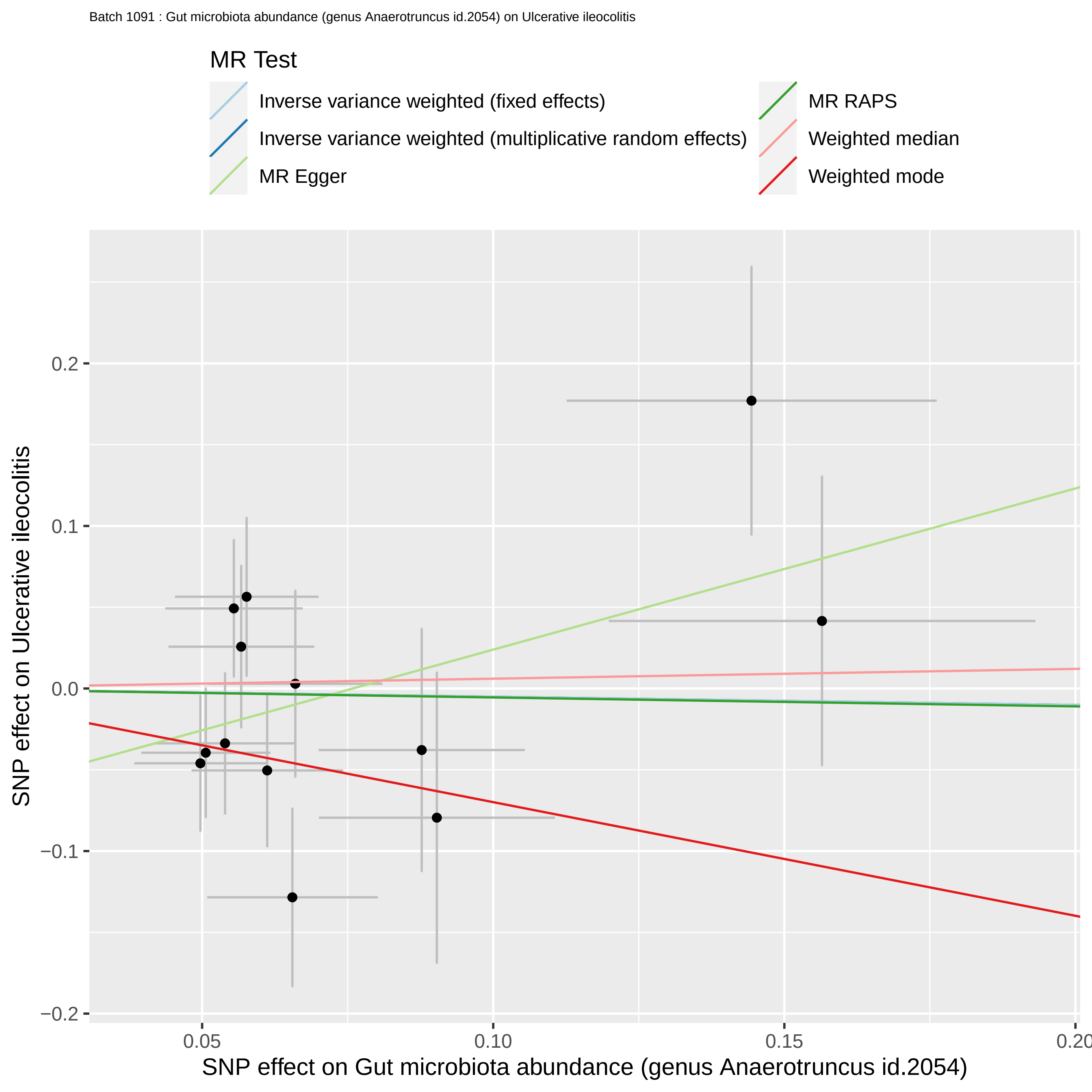

Batch 1091 : Gut microbiota abundance (genus Anaerotruncus id.2054) on Ulcerative ileocolitis
MR Test
Inverse variance weighted (fixed effects)
Inverse variance weighted (multiplicative random effects)
MR Egger
MR RAPS
Weighted median
Weighted mode
0.2
0.1
0.0
−0.1
−0.2
0.05
0.10
0.15
0.20
SNP effect on Ulcerative ileocolitis
SNP effect on Gut microbiota abundance (genus Anaerotruncus id.2054)

Batch 1092 : Gut microbiota abundance (genus Bacteroides id.918) on Ulcerative ileocolitis

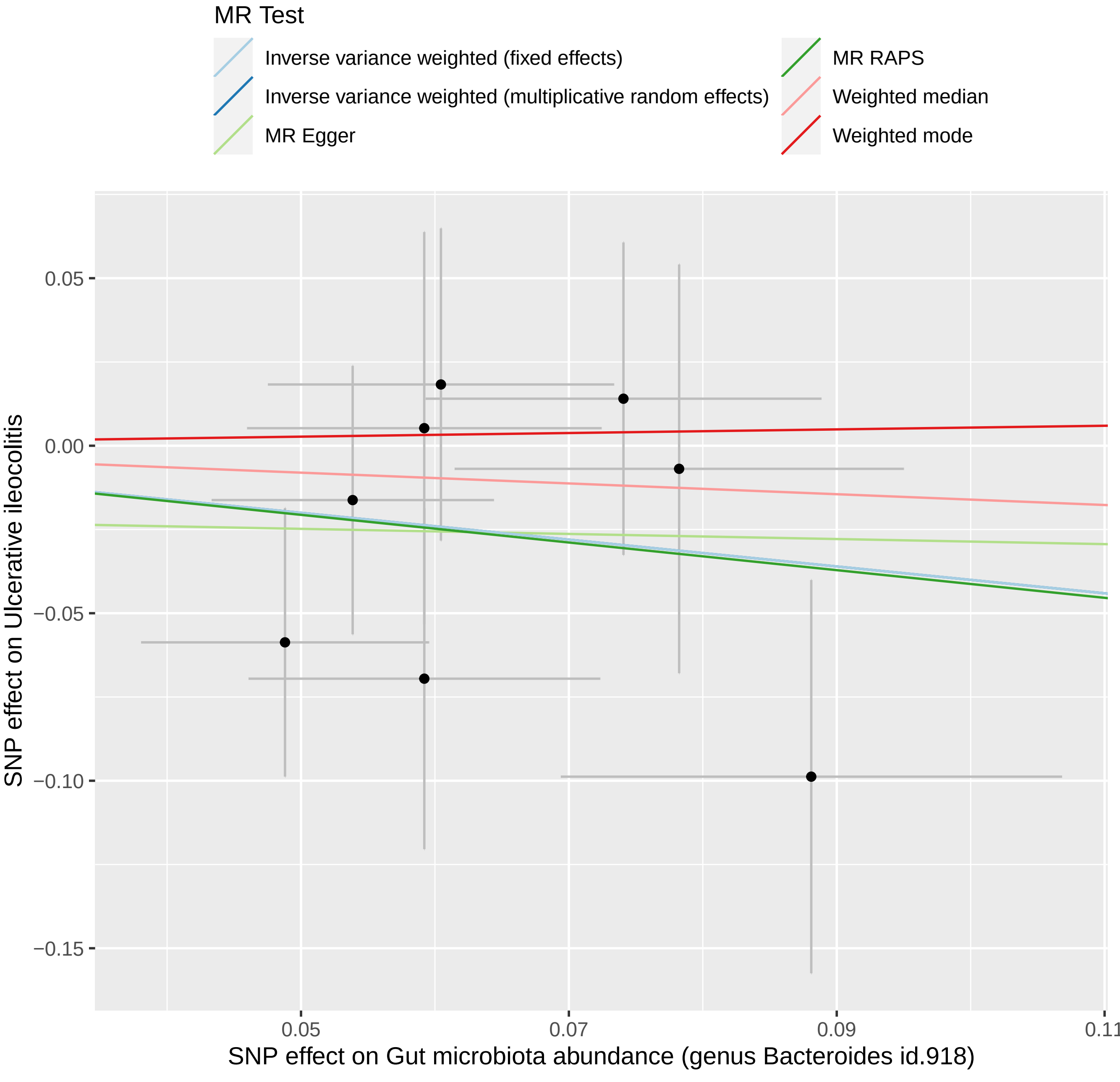

Batch 1093 : Gut microbiota abundance (genus Barnesiella id.944) on Ulcerative ileocolitis

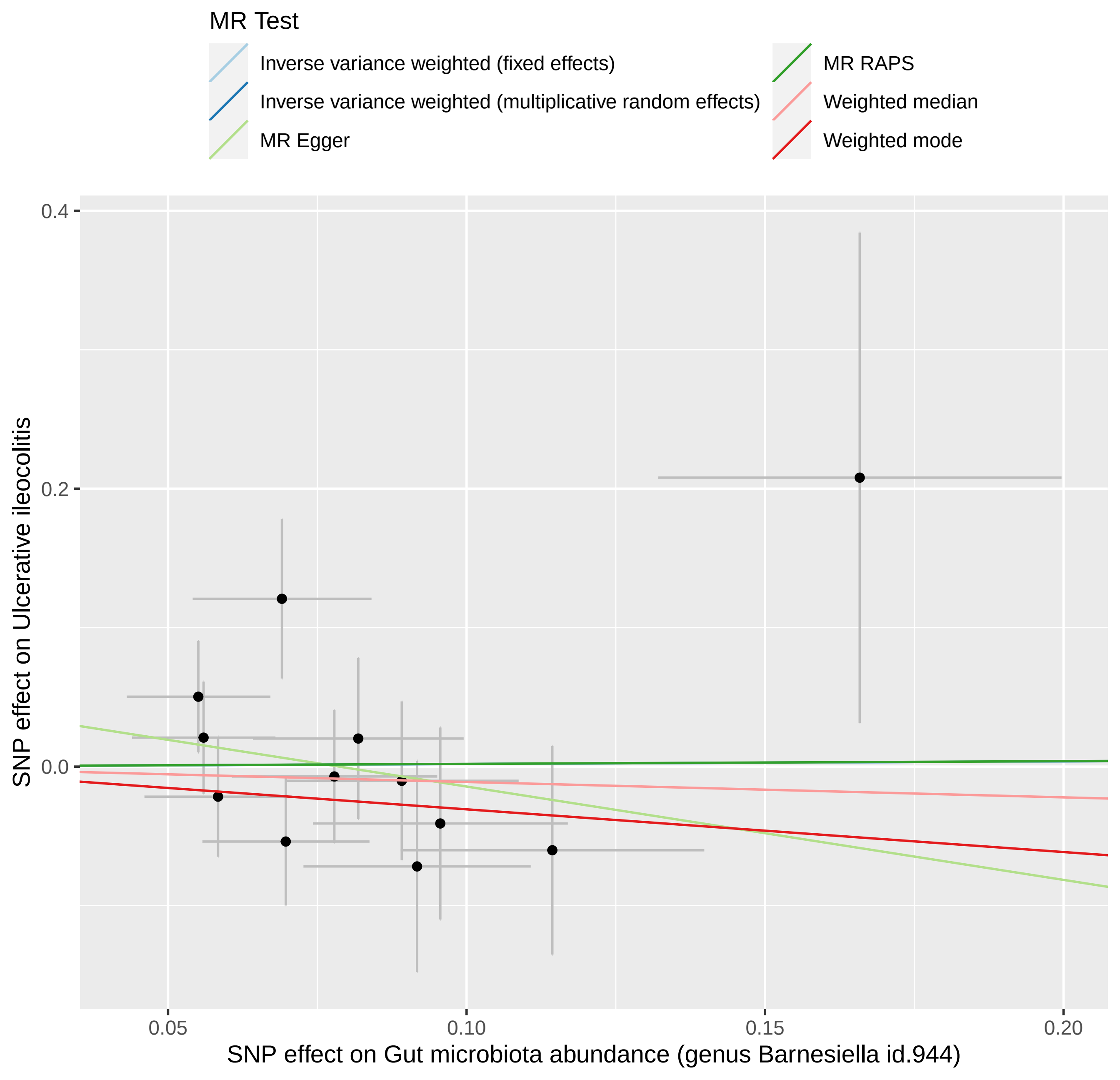

Batch 1094 : Gut microbiota abundance (genus Bifidobacterium id.436) on Ulcerative ileocolitis

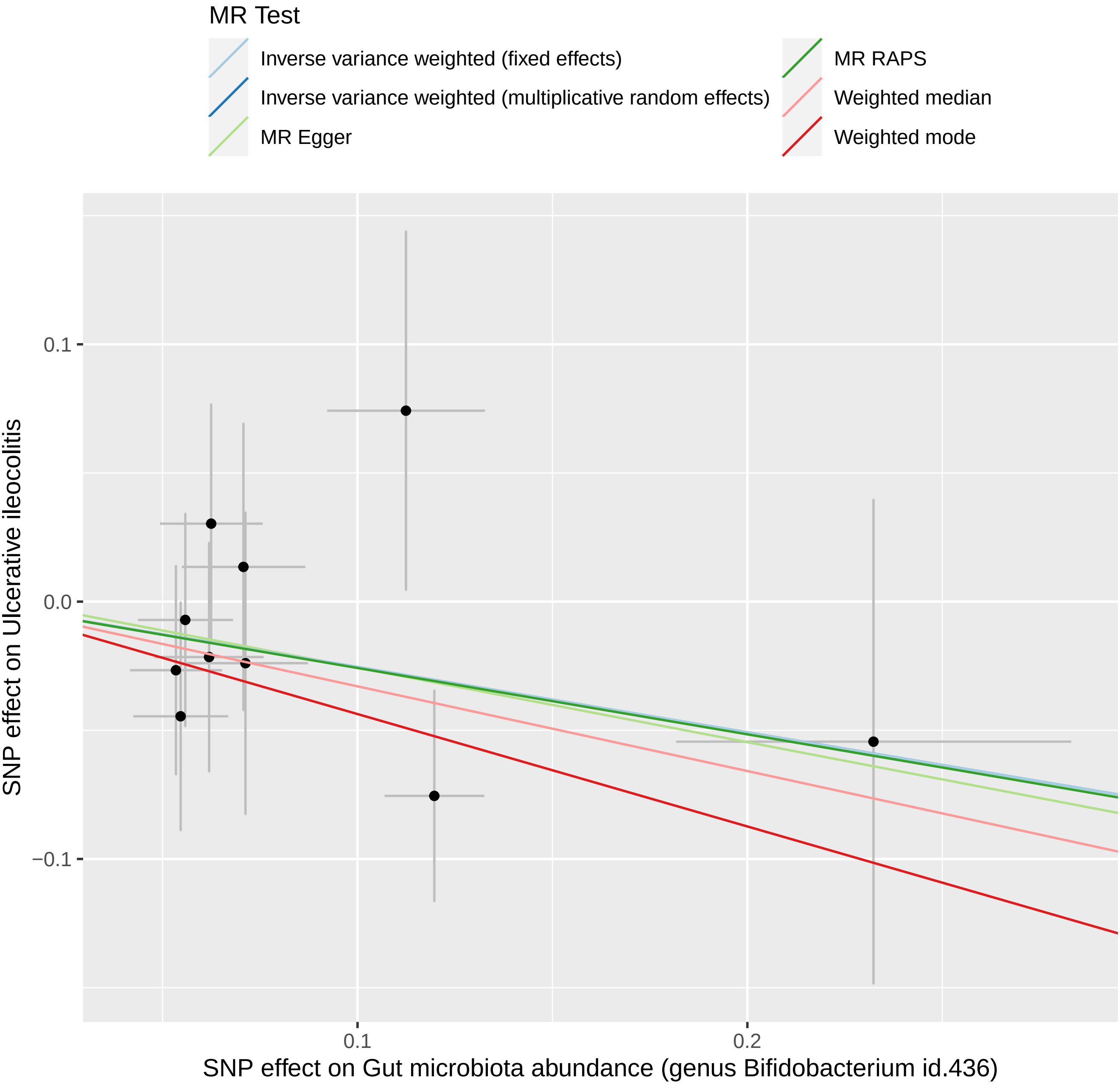

Batch 1095 : Gut microbiota abundance (genus Bilophila id.3170) on Ulcerative ileocolitis

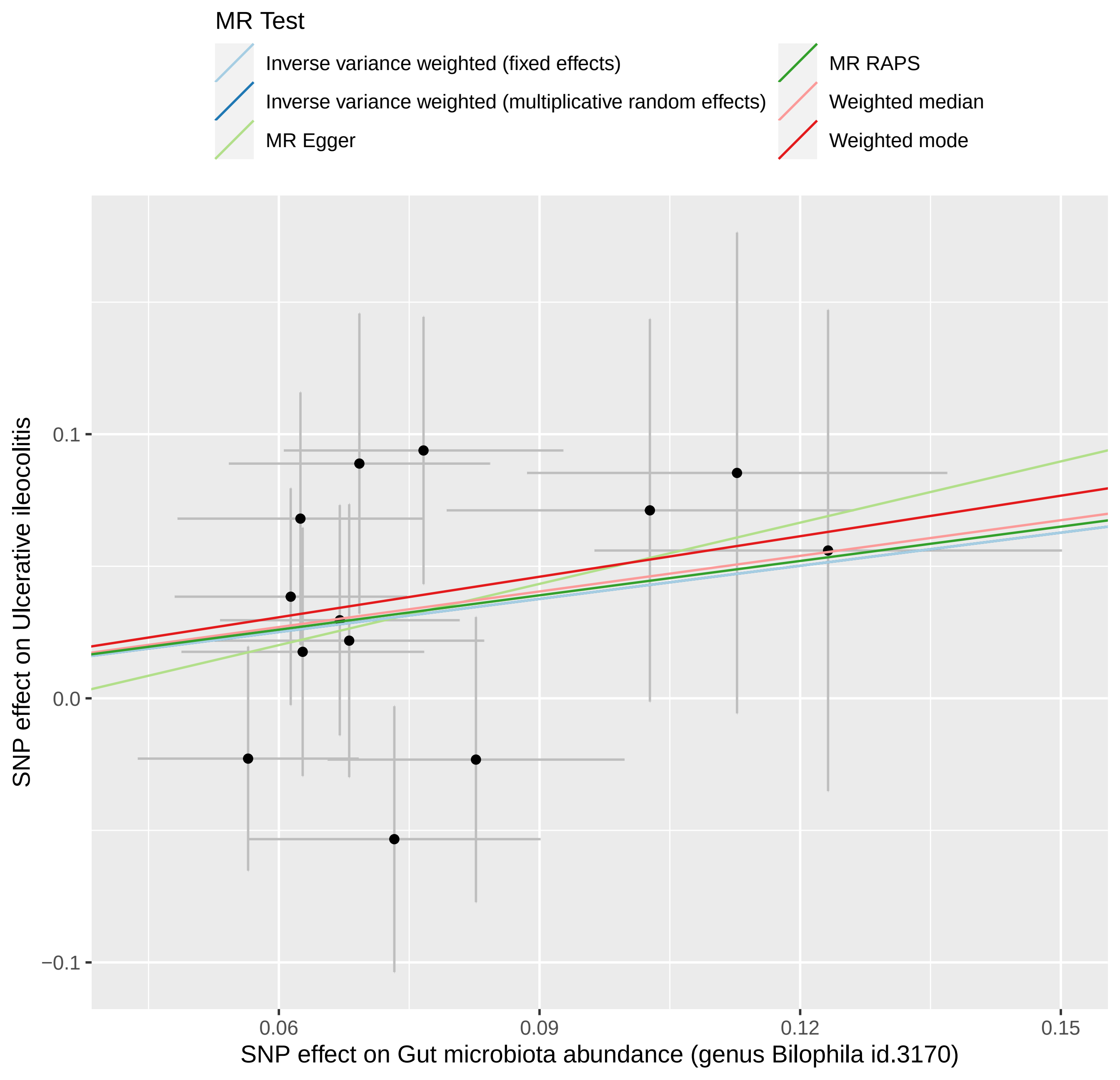

Batch 1096 : Gut microbiota abundance (genus Blautia id.1992) on Ulcerative ileocolitis

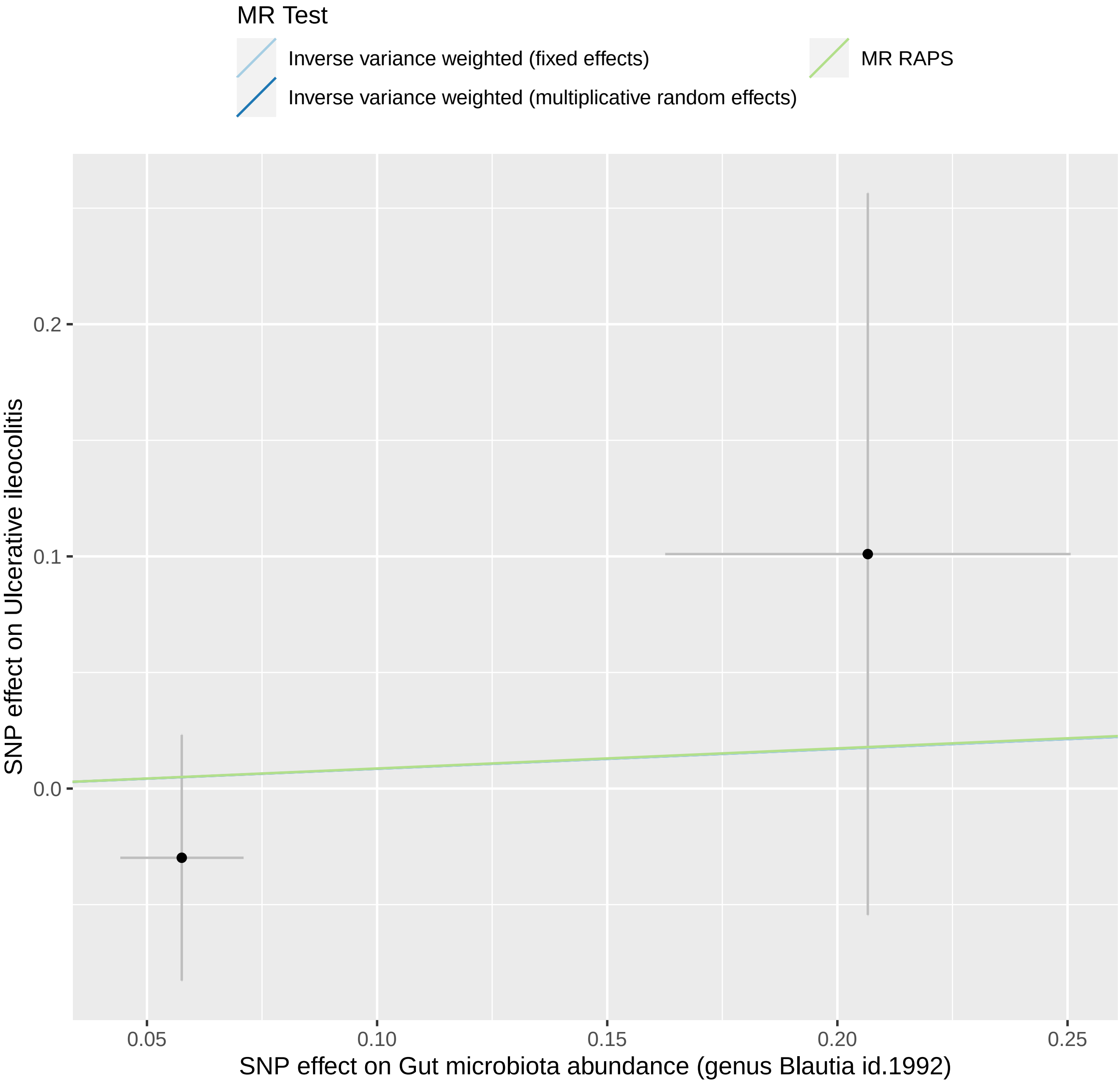

Batch 1097 : Gut microbiota abundance (genus Butyricicoccus id.2055) on Ulcerative ileocolitis

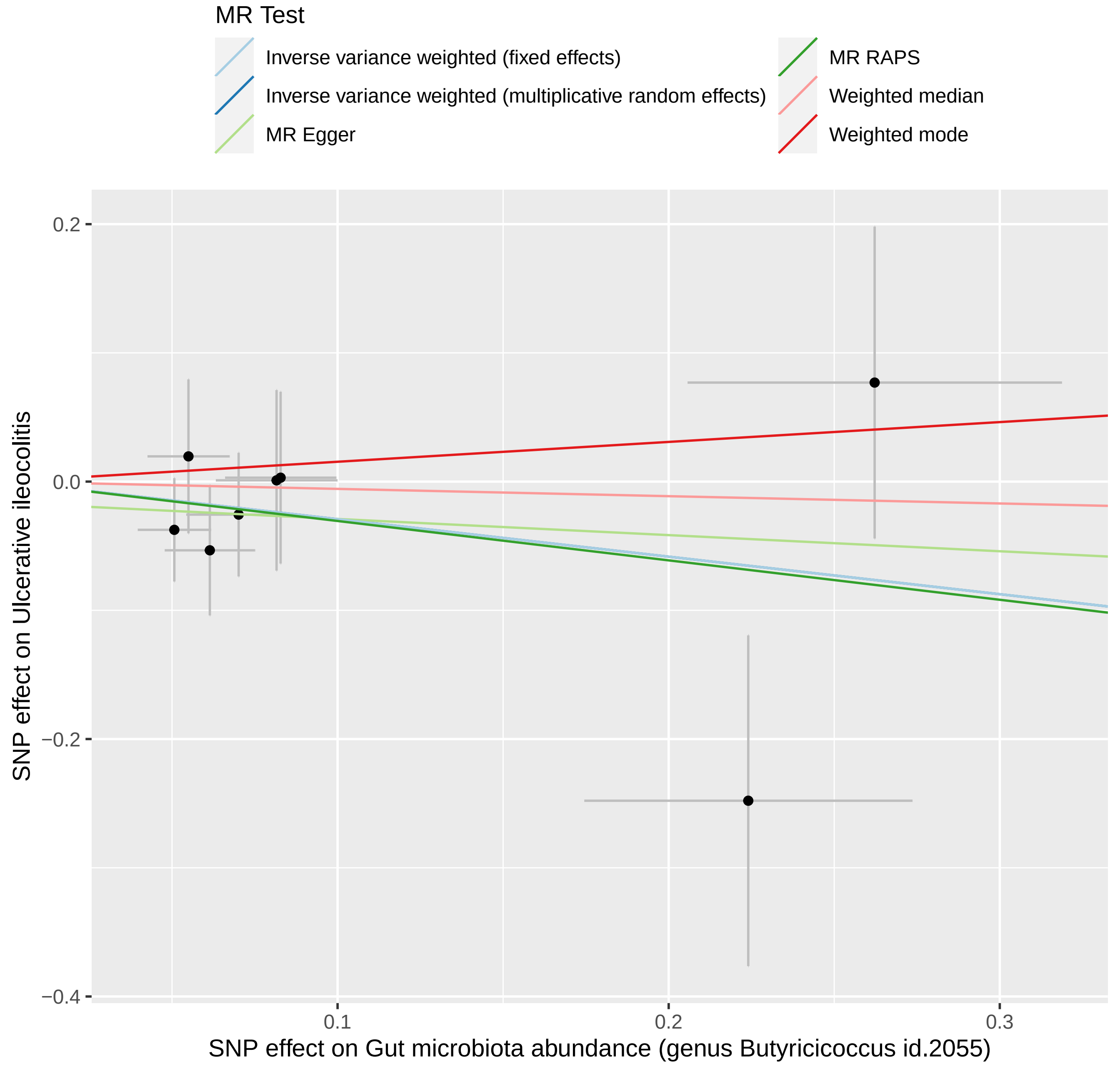

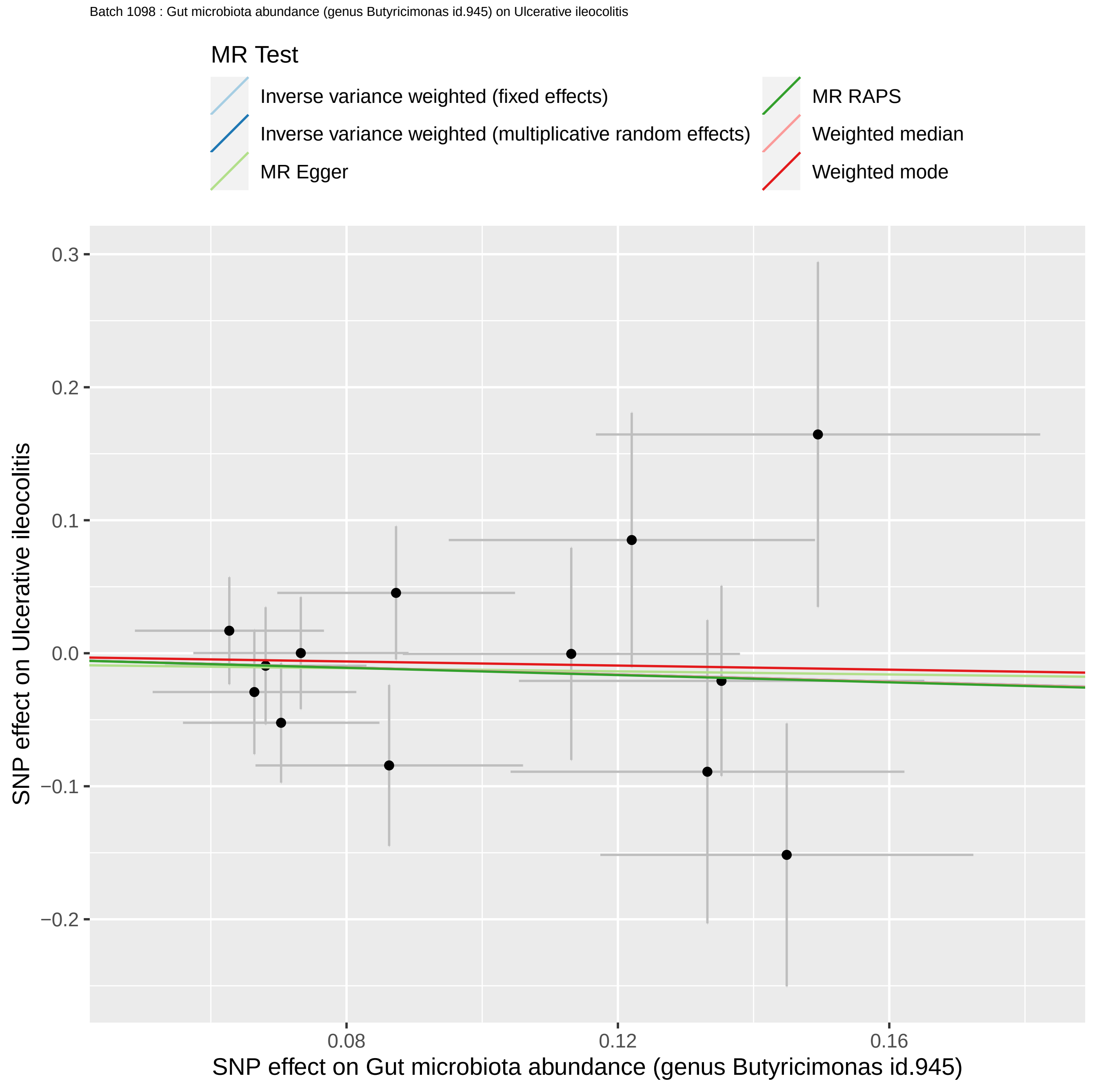

Batch 1098 : Gut microbiota abundance (genus Butyricimonas id.945) on Ulcerative ileocolitis
MR Test
Inverse variance weighted (fixed effects)
Inverse variance weighted (multiplicative random effects)
MR Egger
MR RAPS
Weighted median
Weighted mode
0.3
0.2
0.1
0.0
−0.1
−0.2
0.08
0.12
0.16
SNP effect on Ulcerative ileocolitis
SNP effect on Gut microbiota abundance (genus Butyricimonas id.945)

Batch 1099 : Gut microbiota abundance (genus Butyrivibrio id.1993) on Ulcerative ileocolitis

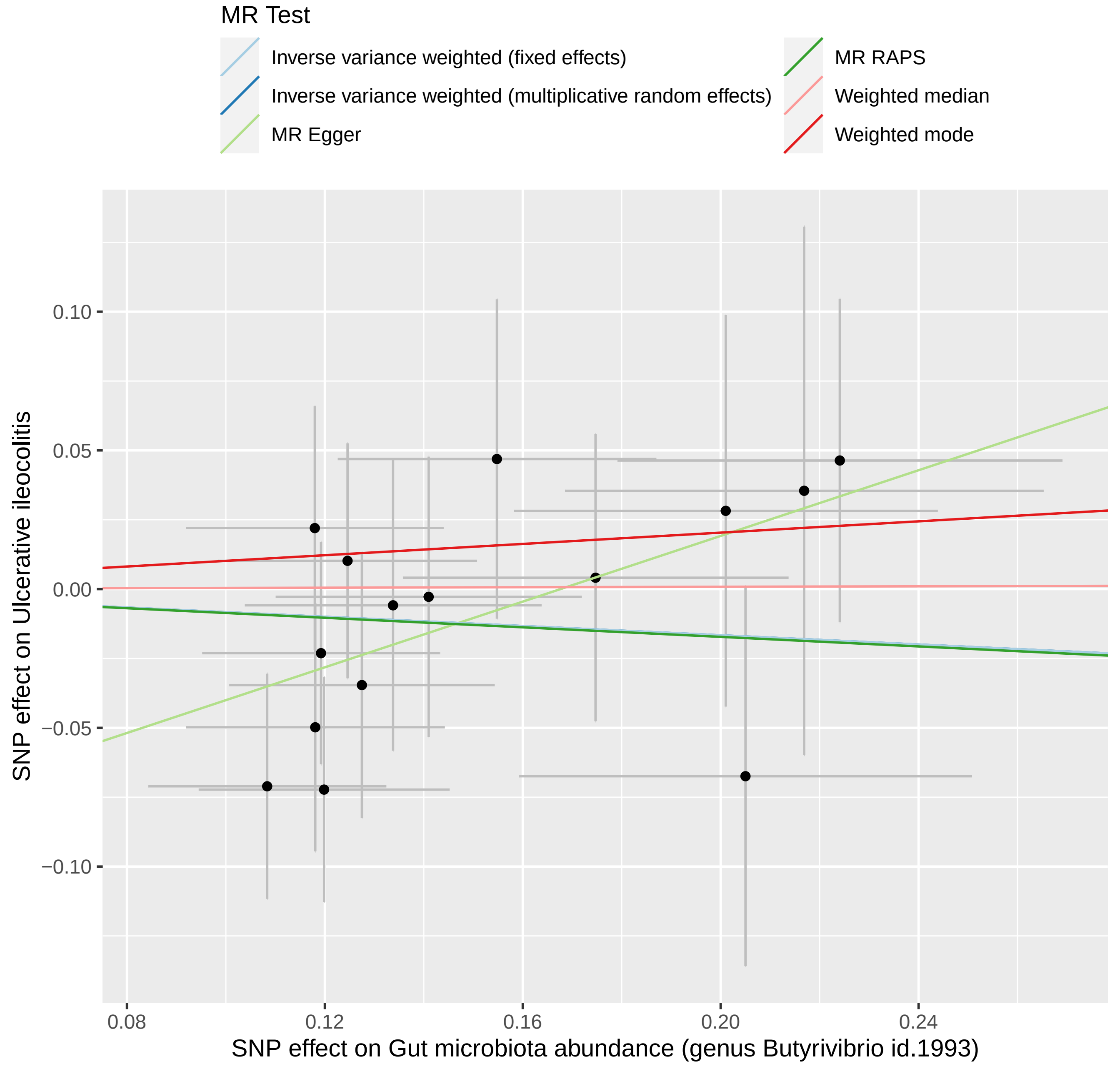

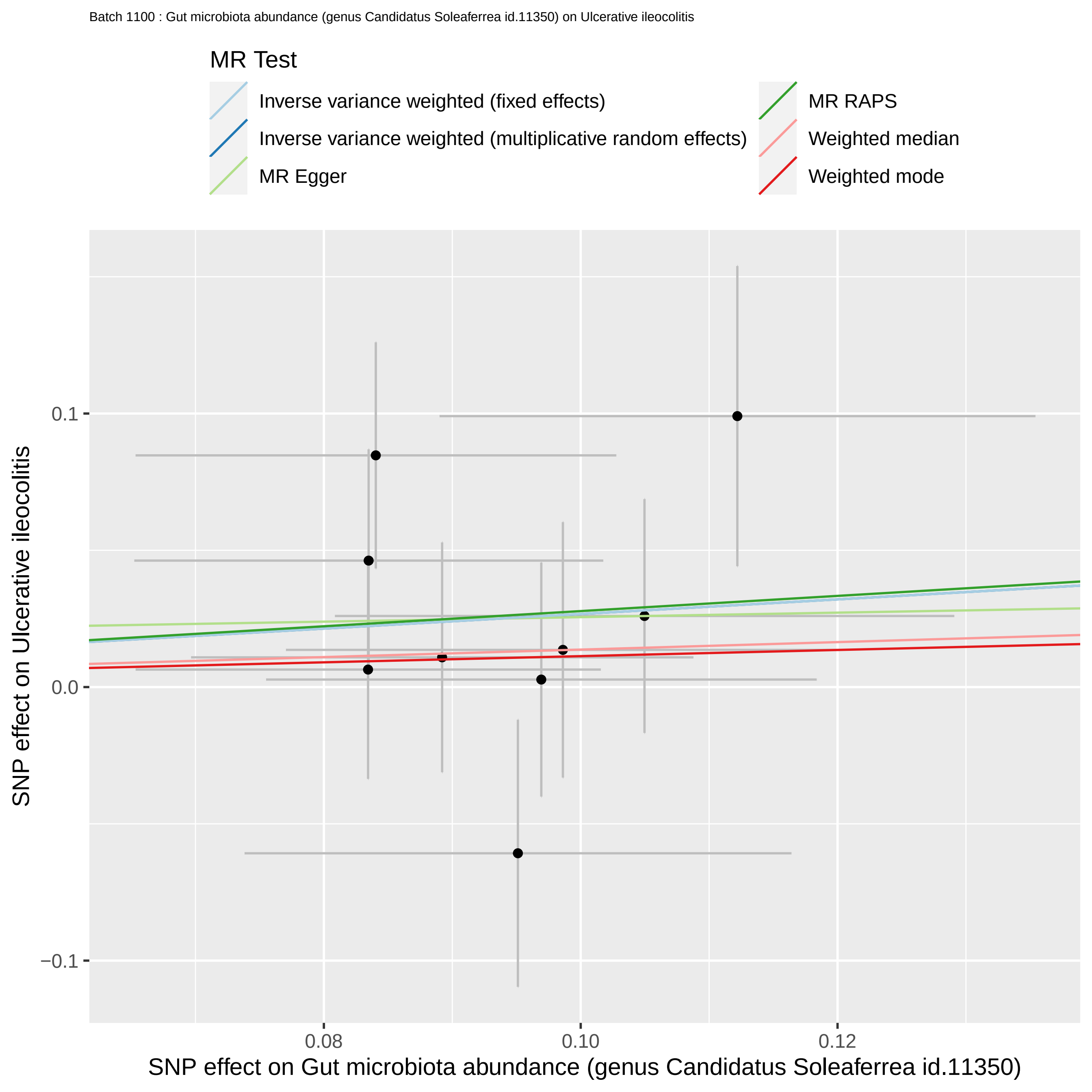
Batch 1100 : Gut microbiota abundance (genus Candidatus Soleaferrea id.11350) on Ulcerative ileocolitis
MR Test
Inverse variance weighted (fixed effects)
Inverse variance weighted (multiplicative random effects)
MR Egger
MR RAPS
Weighted median
Weighted mode
0.1
0.0
−0.1
0.08
0.10
0.12
SNP effect on Ulcerative ileocolitis
SNP effect on Gut microbiota abundance (genus Candidatus Soleaferrea id.11350)

Batch 1101 : Gut microbiota abundance (genus Catenibacterium id.2153) on Ulcerative ileocolitis

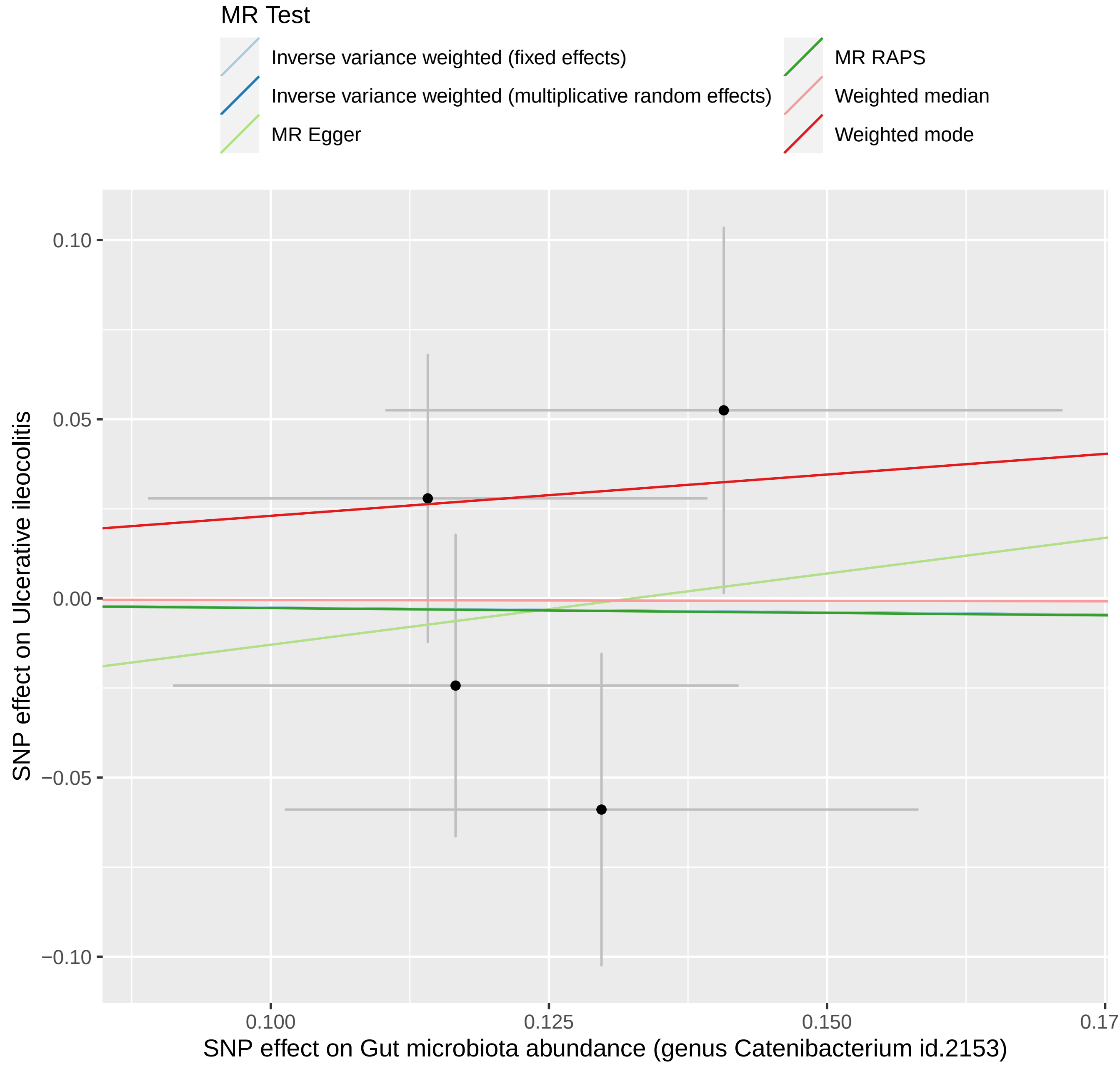

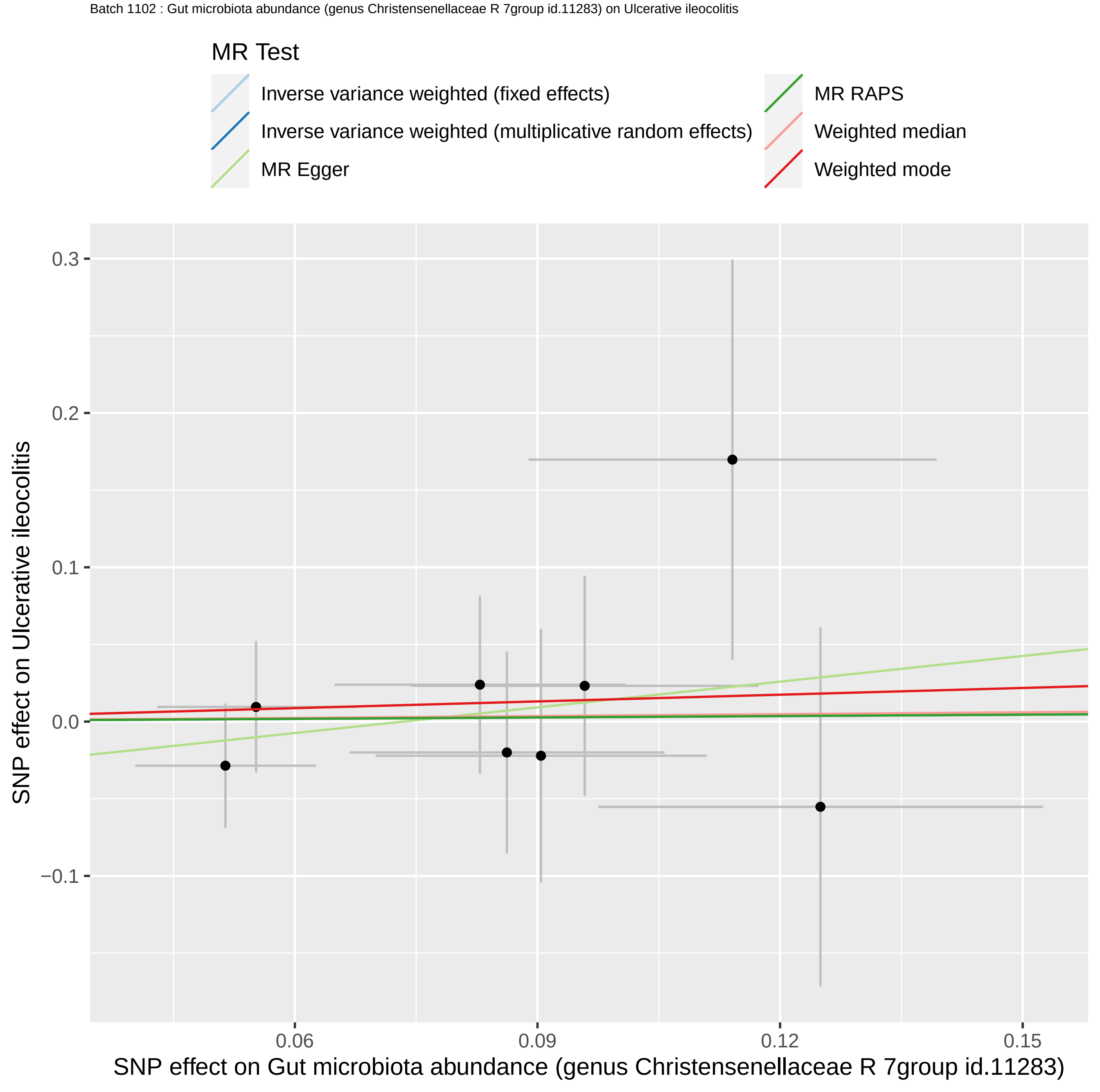

Batch 1102 : Gut microbiota abundance (genus Christensenellaceae R 7group id.11283) on Ulcerative ileocolitis
MR Test
Inverse variance weighted (fixed effects)
Inverse variance weighted (multiplicative random effects)
MR Egger
MR RAPS
Weighted median
Weighted mode
SNP effect on Ulcerative ileocolitis
0.3
0.2
0.1
0.0
−0.1
0.06
0.09
0.12
0.15
SNP effect on Gut microbiota abundance (genus Christensenellaceae R 7group id.11283)

Batch 1103 : Gut microbiota abundance (genus Clostridium innocuum group id.14397) on Ulcerative ileocolitis

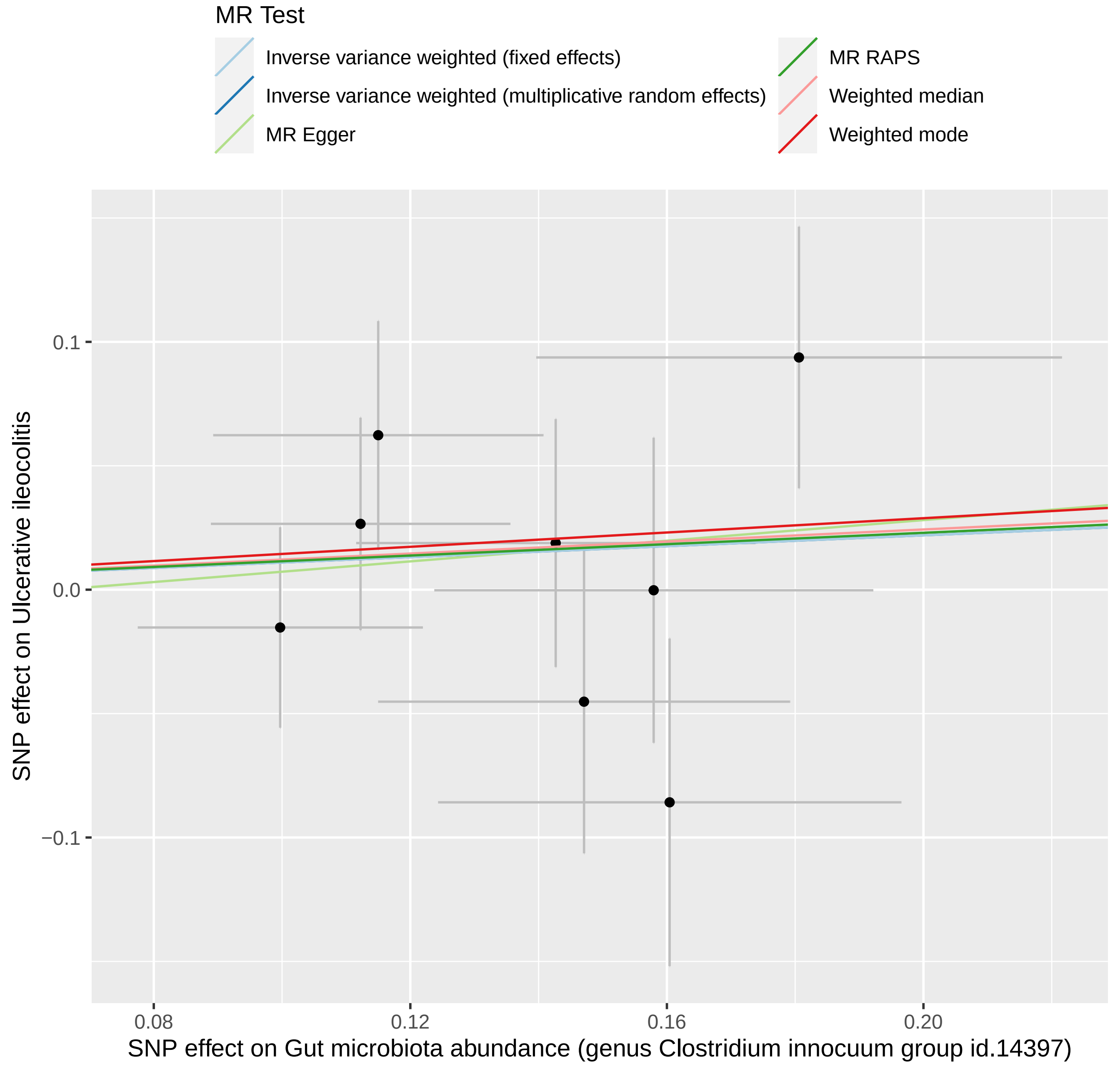

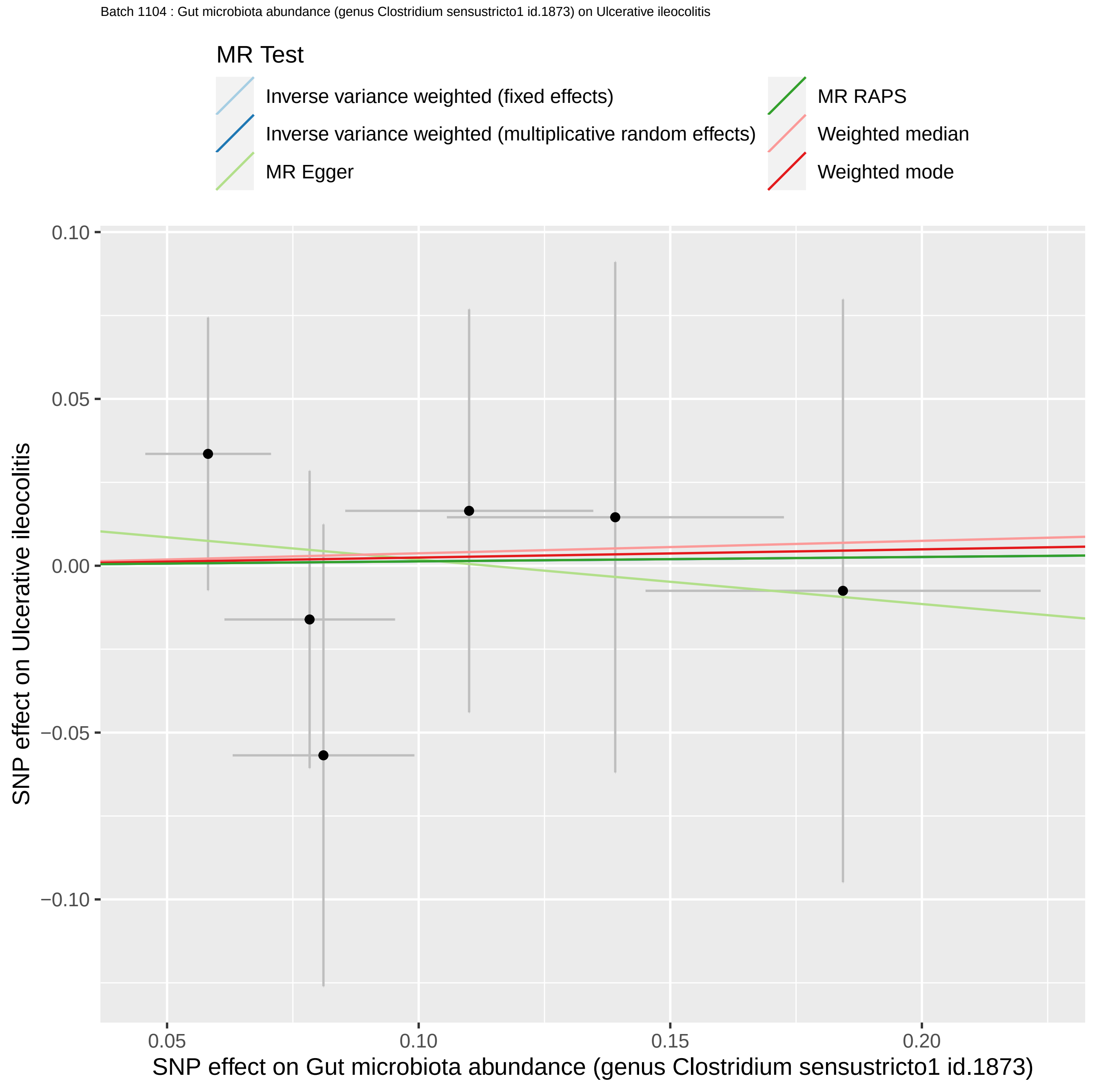

Batch 1104 : Gut microbiota abundance (genus Clostridium sensustricto1 id.1873) on Ulcerative ileocolitis
MR Test
Inverse variance weighted (fixed effects)
Inverse variance weighted (multiplicative random effects)
MR Egger
MR RAPS
Weighted median
Weighted mode
0.10
0.05
0.00
−0.05
−0.10
SNP effect on Ulcerative ileocolitis
0.05
0.10
0.15
0.20
SNP effect on Gut microbiota abundance (genus Clostridium sensustricto1 id.1873)

Batch 1105 : Gut microbiota abundance (genus Collinsella id.815) on Ulcerative ileocolitis

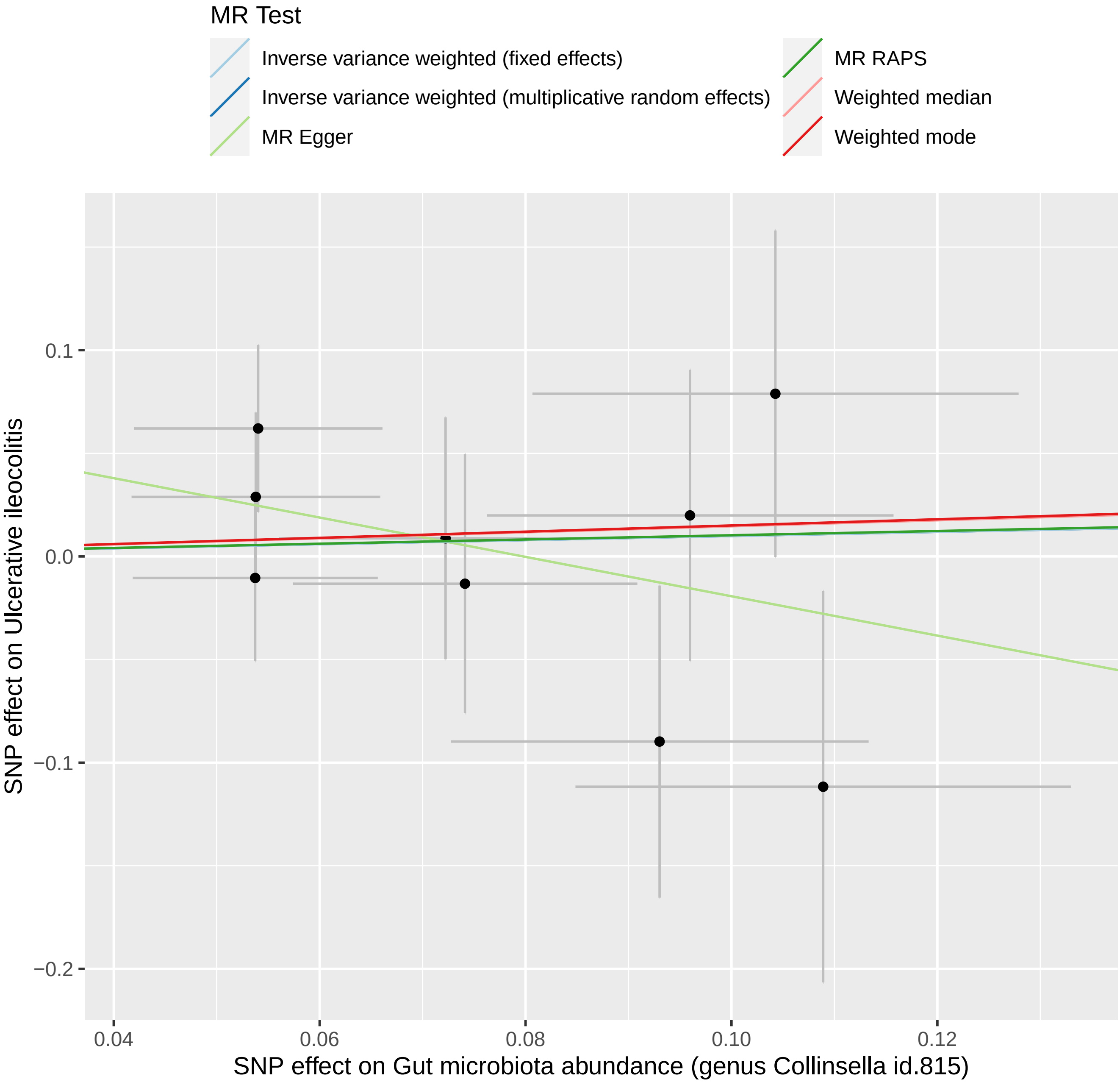

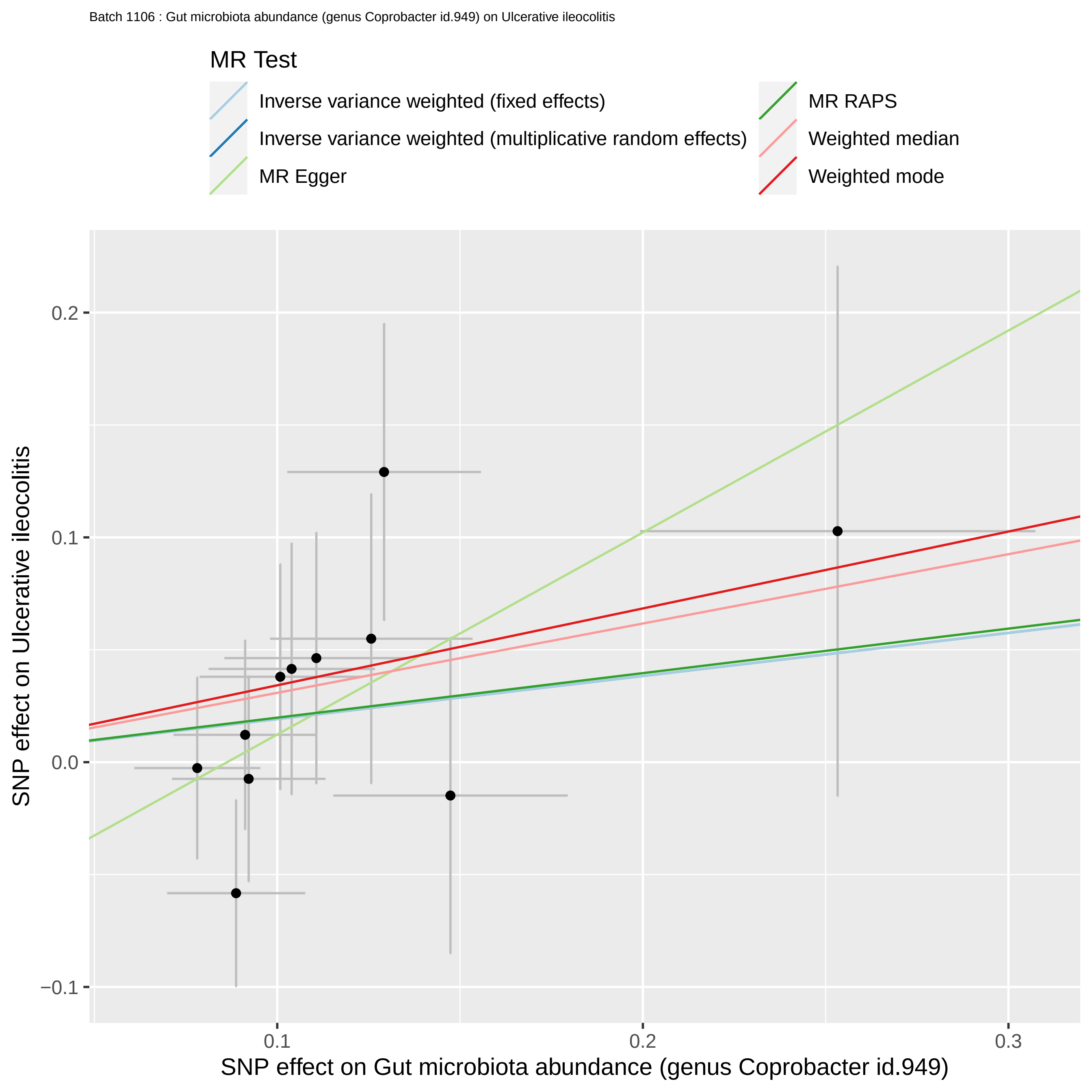

Batch 1106 : Gut microbiota abundance (genus Coprobacter id.949) on Ulcerative ileocolitis
MR Test
Inverse variance weighted (fixed effects)
Inverse variance weighted (multiplicative random effects)
MR Egger
MR RAPS
Weighted median
Weighted mode
0.2
0.1
0.0
−0.1
0.1
0.2
0.3
SNP effect on Ulcerative ileocolitis
SNP effect on Gut microbiota abundance (genus Coprobacter id.949)

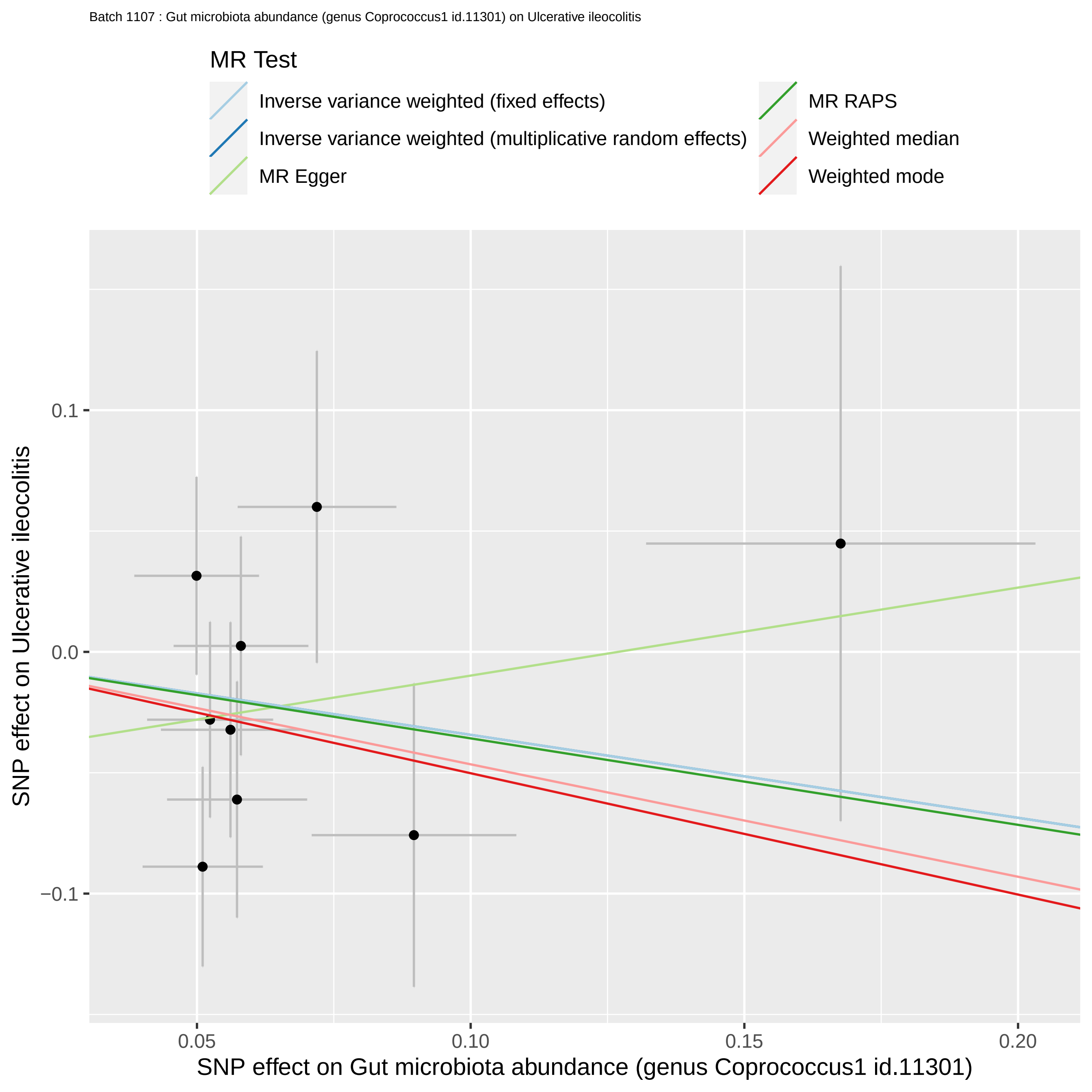

Batch 1107 : Gut microbiota abundance (genus Coprococcus1 id.11301) on Ulcerative ileocolitis
MR Test
Inverse variance weighted (fixed effects)
Inverse variance weighted (multiplicative random effects)
MR Egger
MR RAPS
Weighted median
Weighted mode
SNP effect on Ulcerative ileocolitis
0.1
0.0
−0.1
0.05
0.10
0.15
0.20
SNP effect on Gut microbiota abundance (genus Coprococcus1 id.11301)

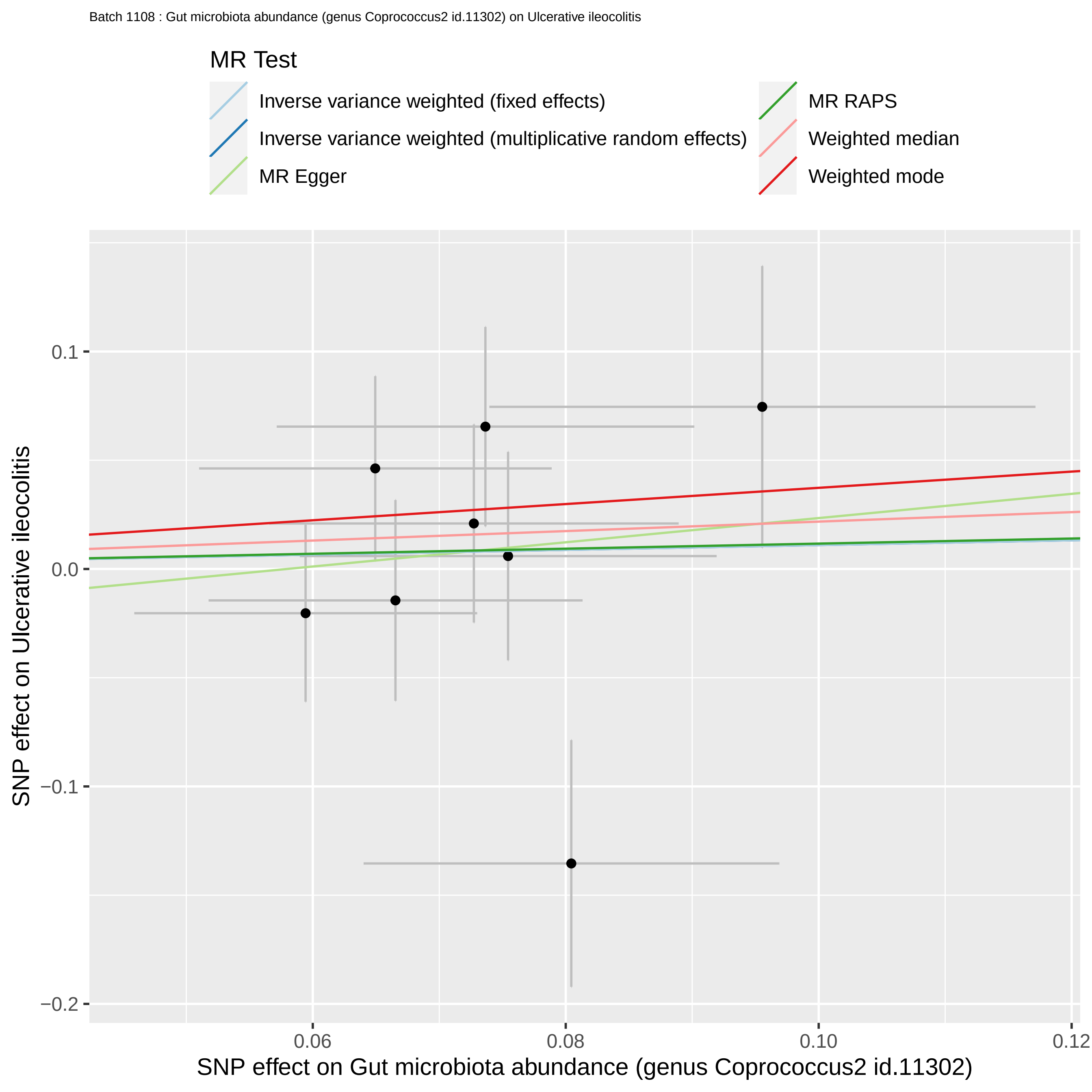

Batch 1108 : Gut microbiota abundance (genus Coprococcus2 id.11302) on Ulcerative ileocolitis
MR Test
Inverse variance weighted (fixed effects)
Inverse variance weighted (multiplicative random effects)
MR Egger
MR RAPS
Weighted median
Weighted mode
SNP effect on Ulcerative ileocolitis
0.1
0.0
−0.1
−0.2
0.06
0.08
0.10
0.12
SNP effect on Gut microbiota abundance (genus Coprococcus2 id.11302)

Batch 1109 : Gut microbiota abundance (genus Coprococcus3 id.11303) on Ulcerative ileocolitis

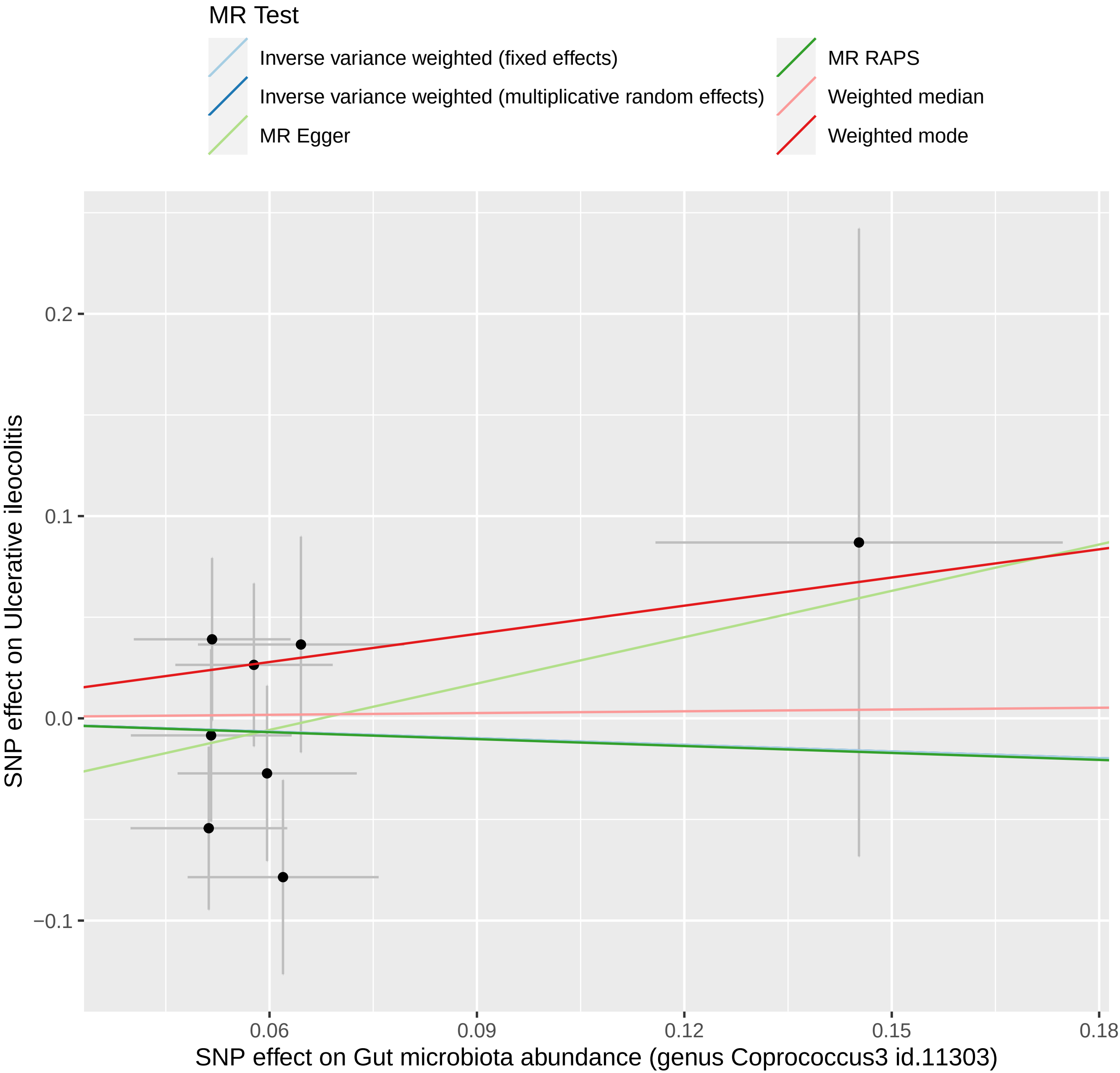

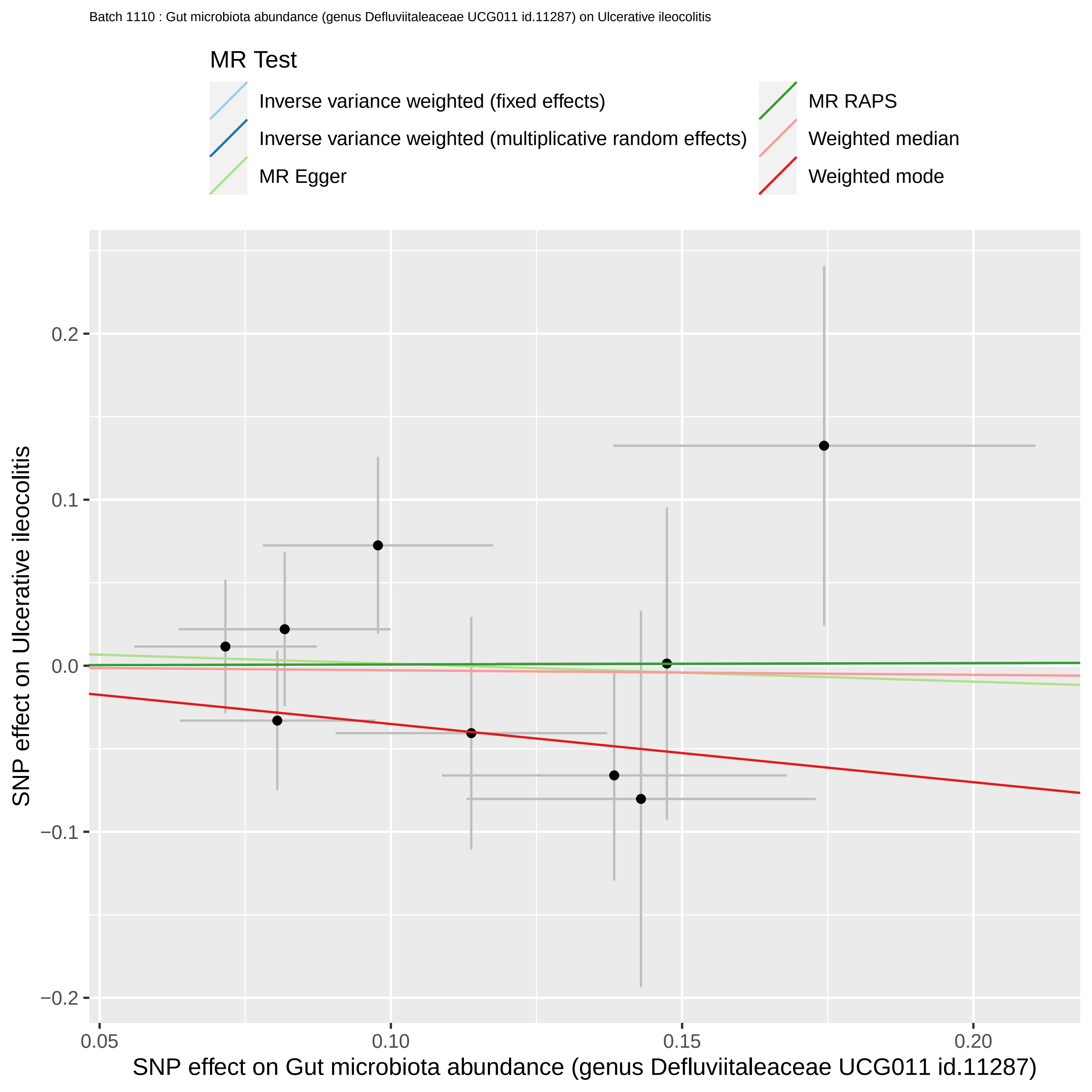

Batch 1110 : Gut microbiota abundance (genus Defluviitaleaceae UCG011 id.11287) on Ulcerative ileocolitis
MR Test
Inverse variance weighted (fixed effects)
Inverse variance weighted (multiplicative random effects)
MR Egger
MR RAPS
Weighted median
Weighted mode
0.2
0.1
0.0
−0.1
−0.2
0.05
0.10
0.15
0.20
SNP effect on Ulcerative ileocolitis
SNP effect on Gut microbiota abundance (genus Defluviitaleaceae UCG011 id.11287)

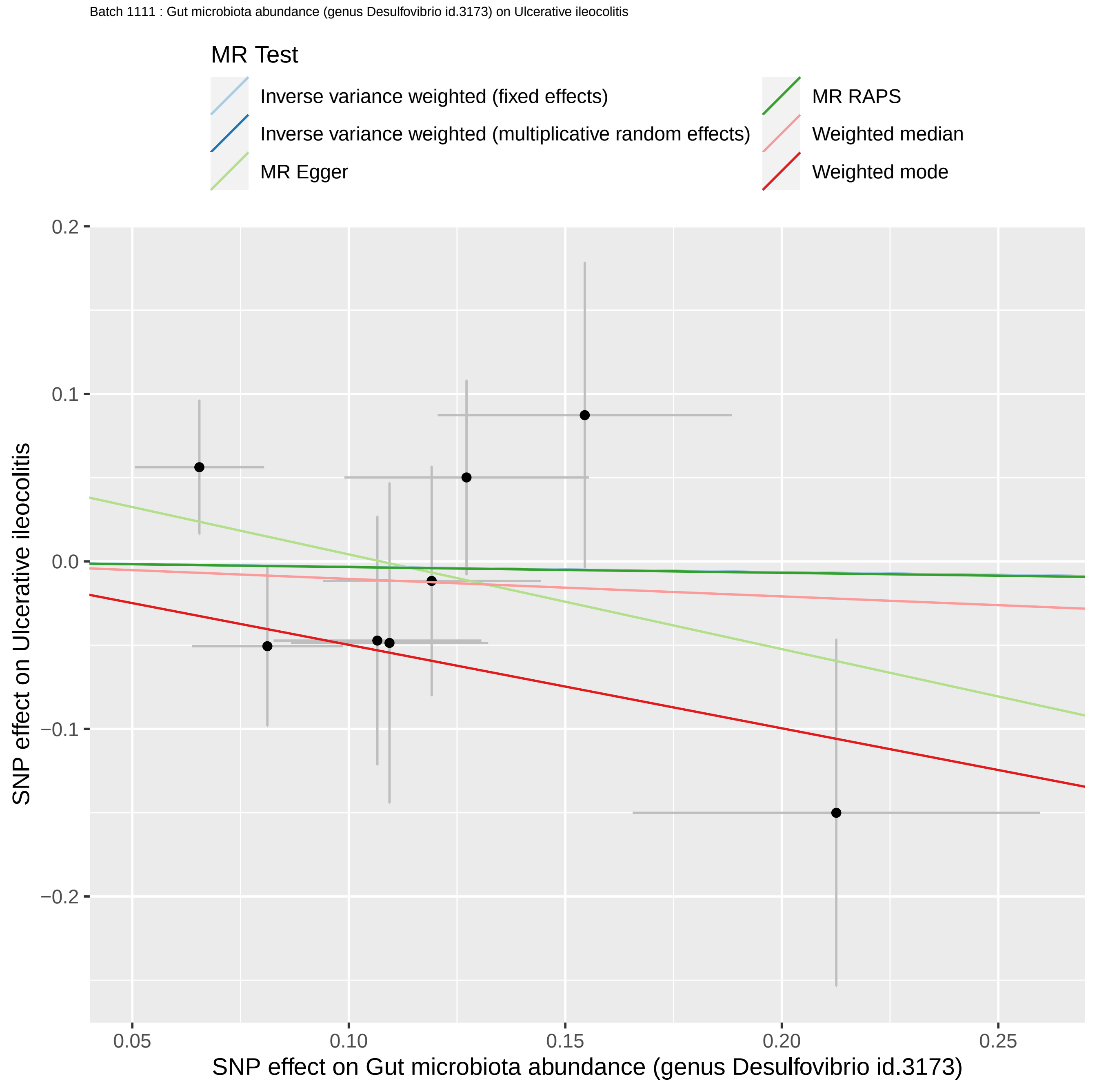
Batch 1111 : Gut microbiota abundance (genus Desulfovibrio id.3173) on Ulcerative ileocolitis
MR Test
Inverse variance weighted (fixed effects)
Inverse variance weighted (multiplicative random effects)
MR Egger
MR RAPS
Weighted median
Weighted mode
0.2
0.1
0.0
−0.1
−0.2
0.05
0.10
0.15
0.20
0.25
SNP effect on Ulcerative ileocolitis
SNP effect on Gut microbiota abundance (genus Desulfovibrio id.3173)

Batch 1112 : Gut microbiota abundance (genus Dialister id.2183) on Ulcerative ileocolitis

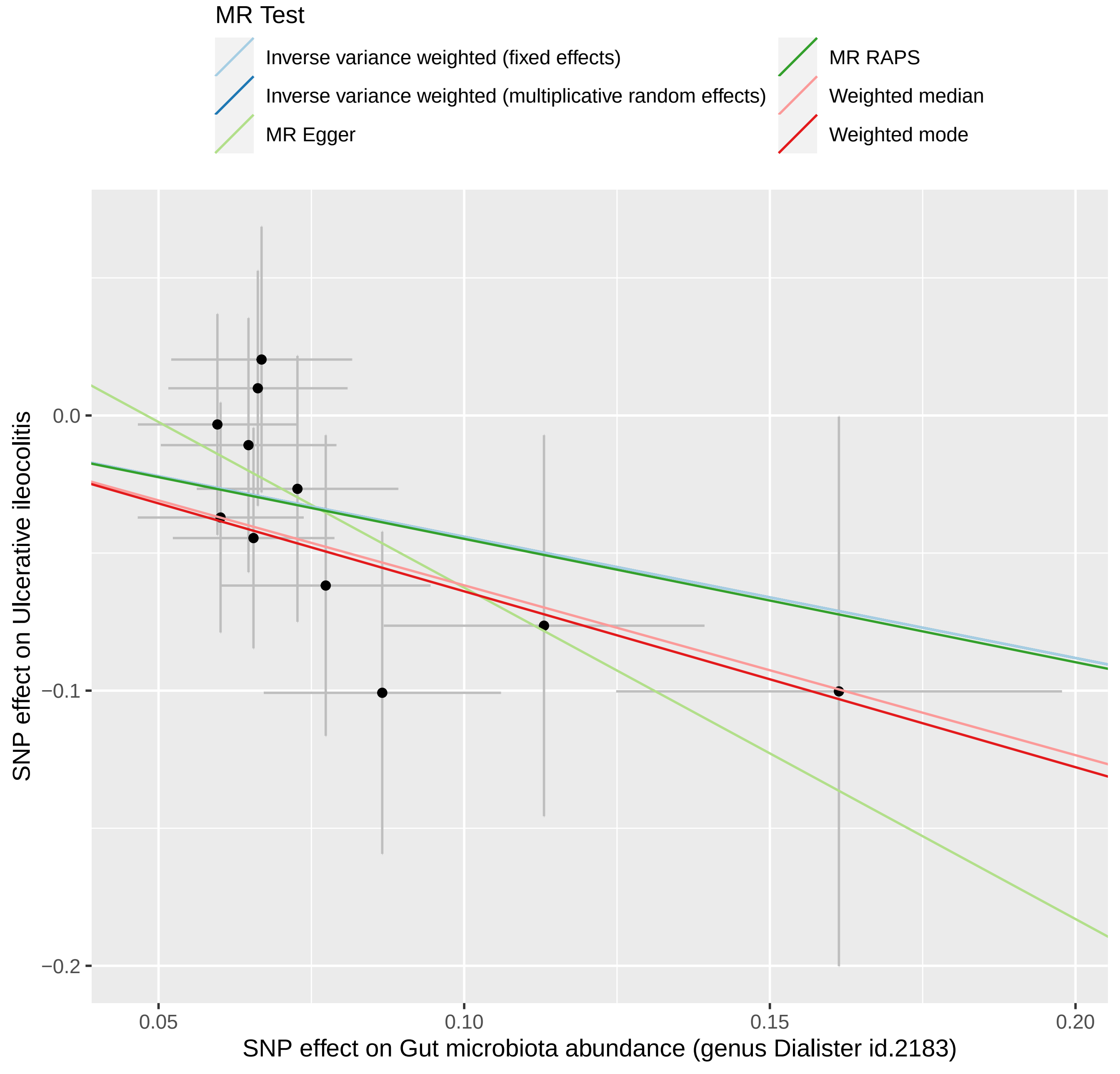

Batch 1113 : Gut microbiota abundance (genus Dorea id.1997) on Ulcerative ileocolitis

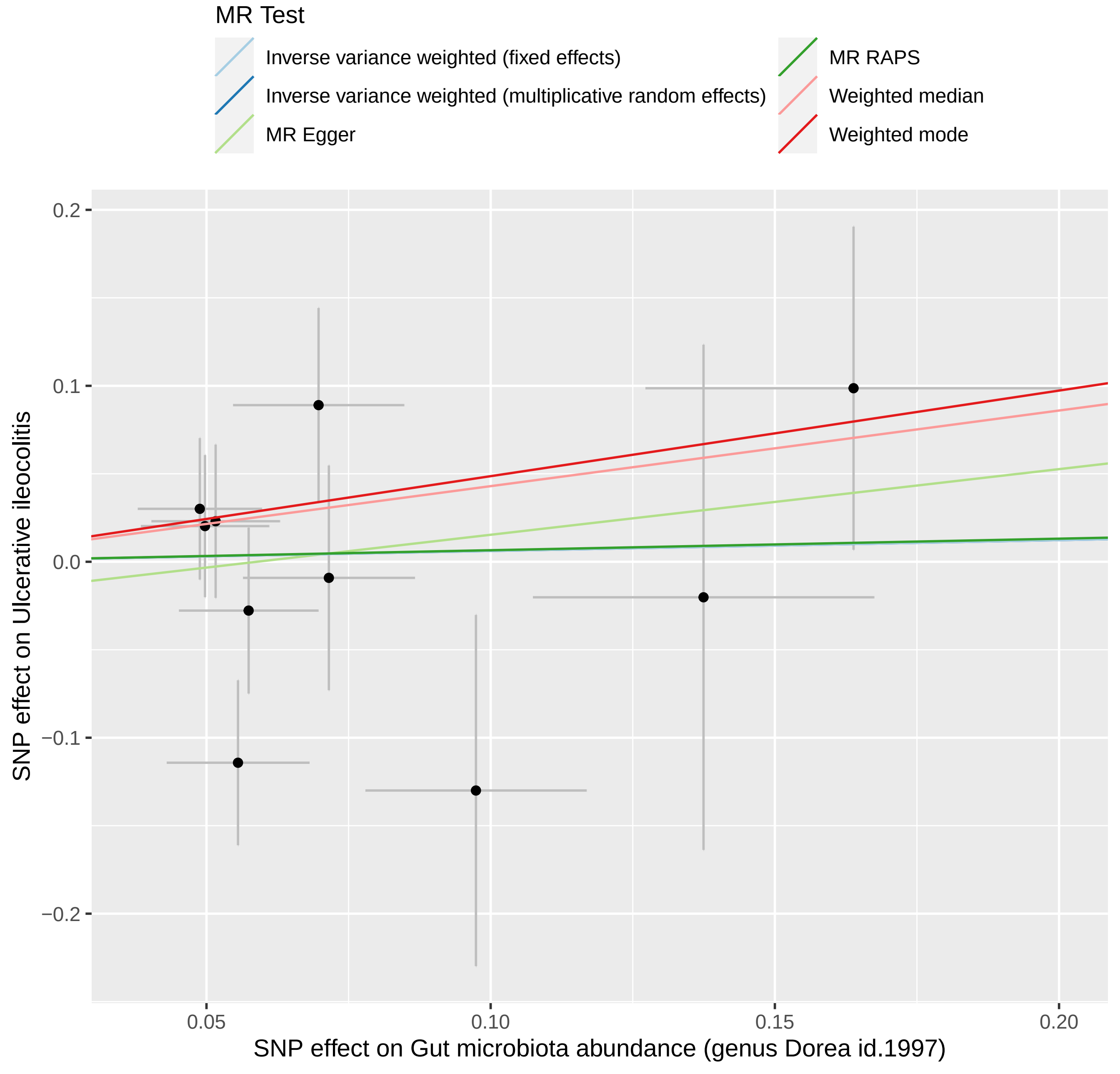

Batch 1114 : Gut microbiota abundance (genus Eggerthella id.819) on Ulcerative ileocolitis

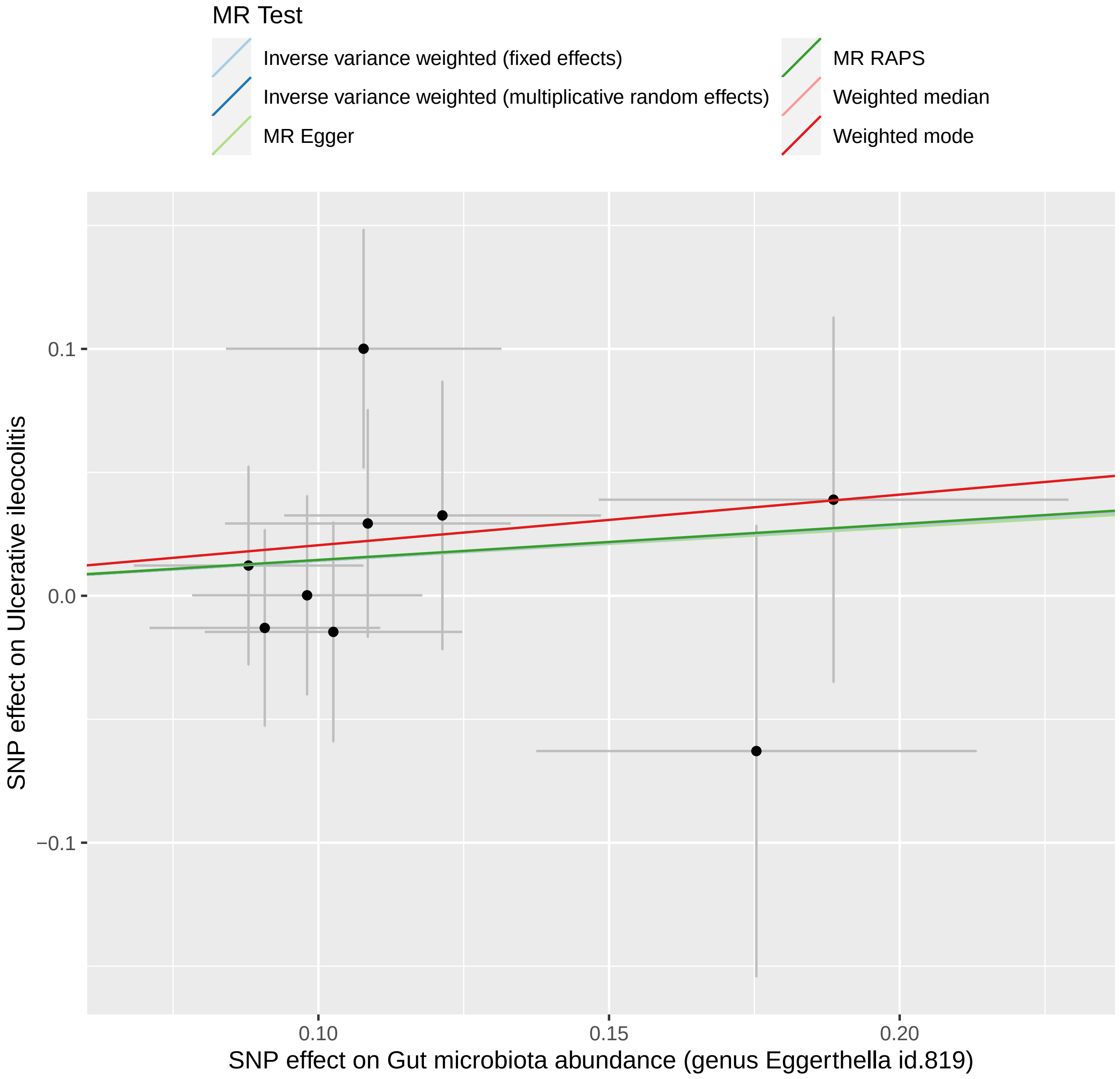

Batch 1115 : Gut microbiota abundance (genus Eisenbergiella id.11304) on Ulcerative ileocolitis

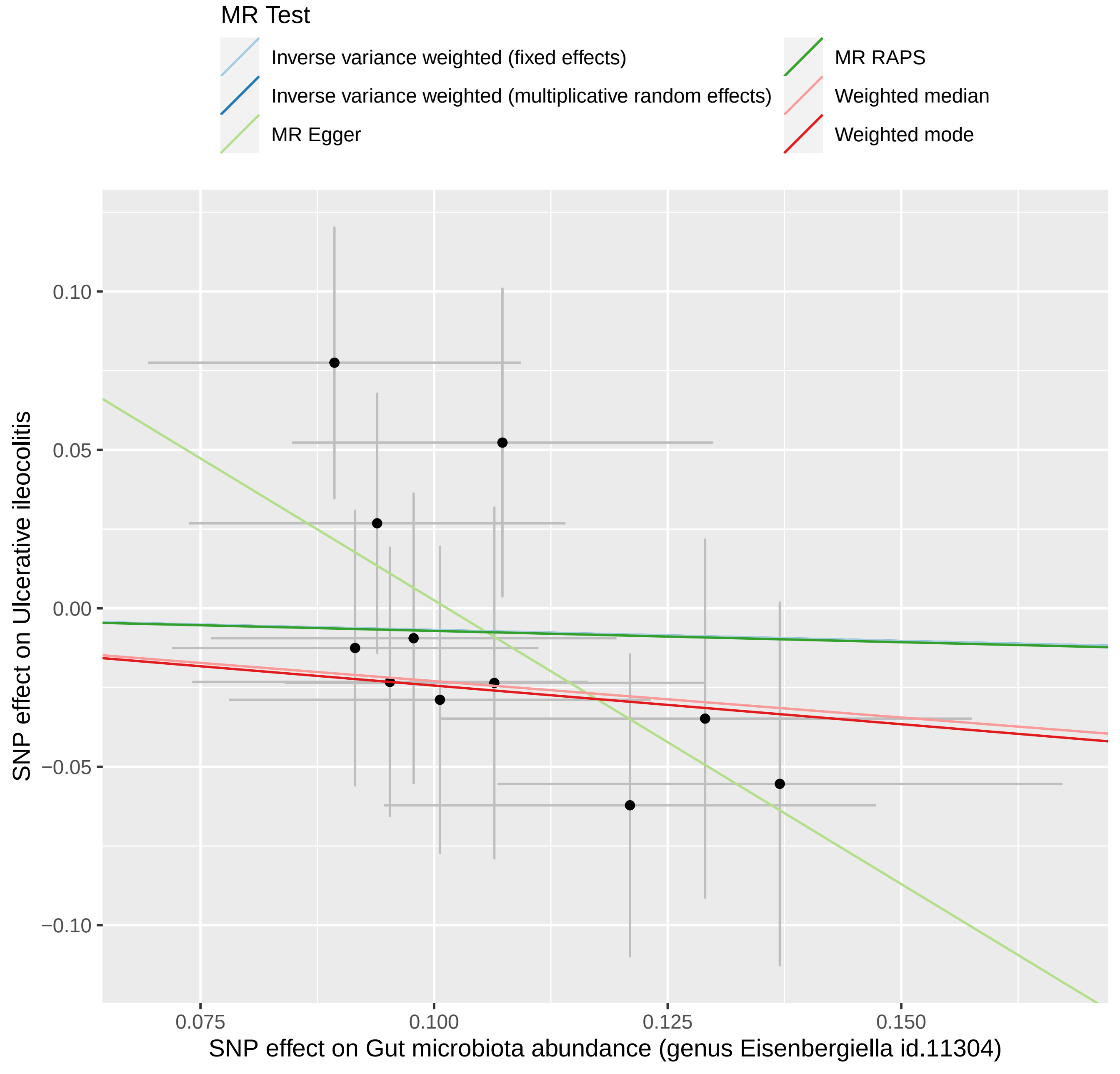

Batch 1116 : Gut microbiota abundance (genus Enterorhabdus id.820) on Ulcerative ileocolitis

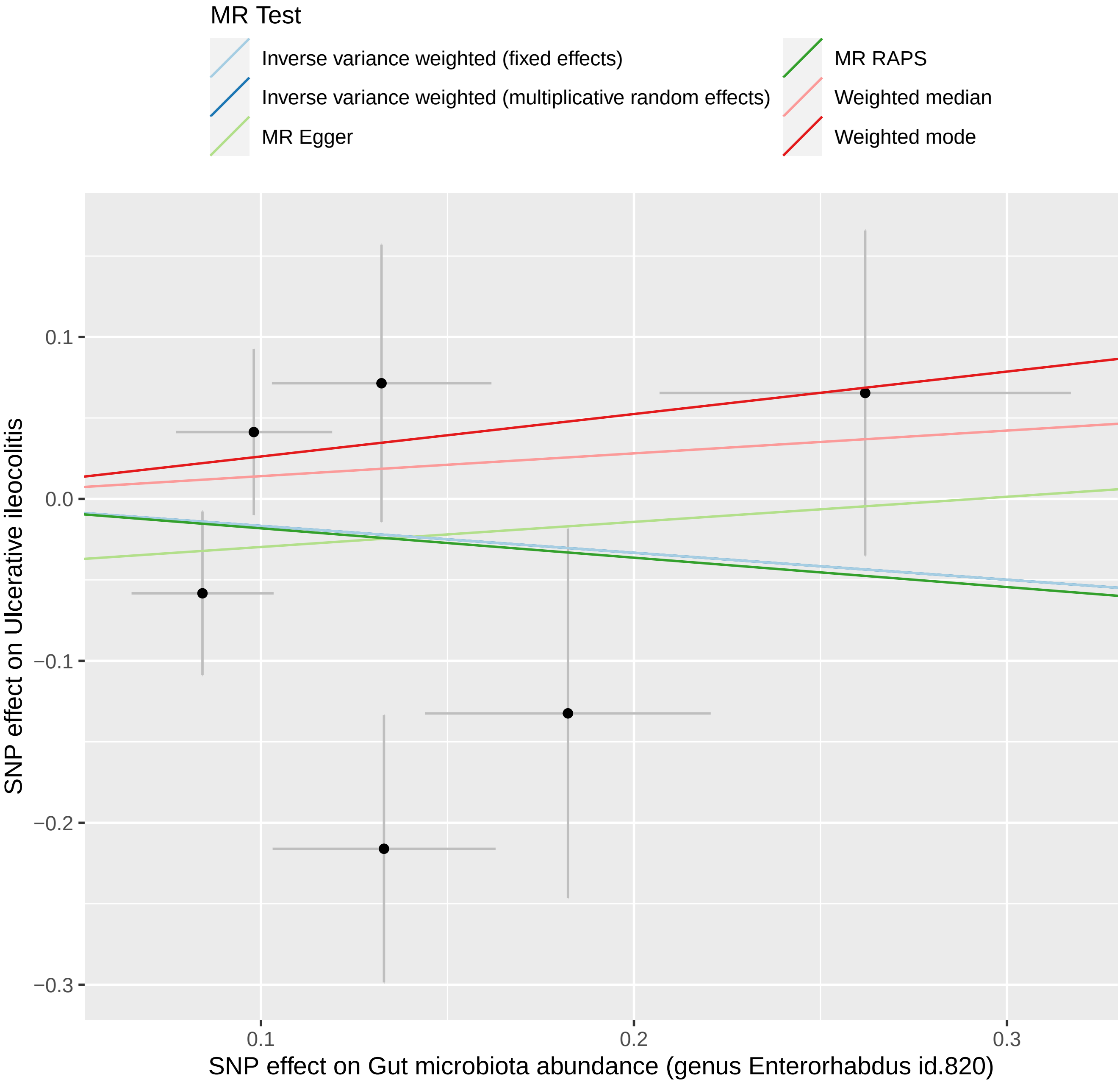

Batch 1117 : Gut microbiota abundance (genus Erysipelatoclostridium id.11381) on Ulcerative ileocolitis

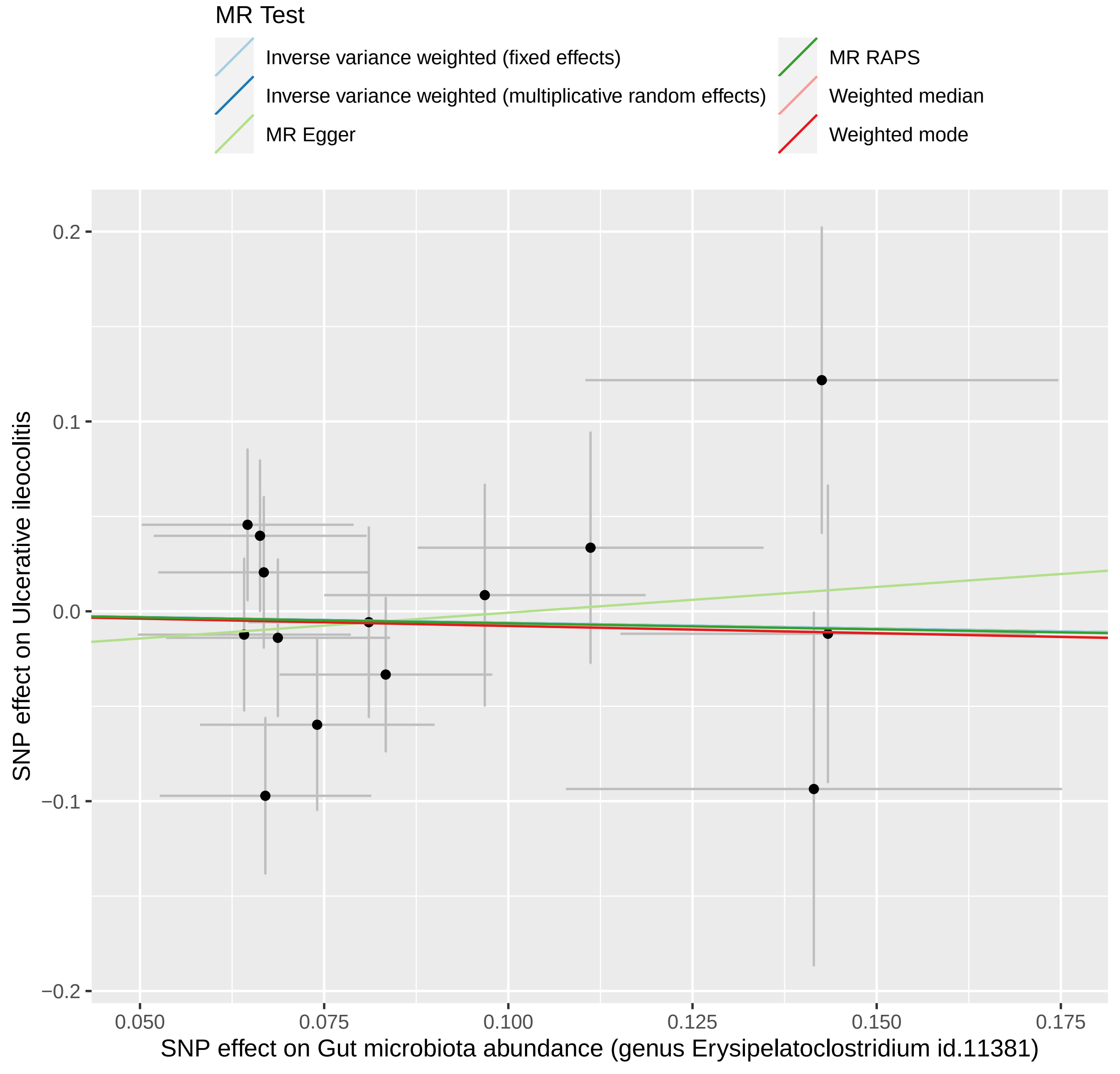

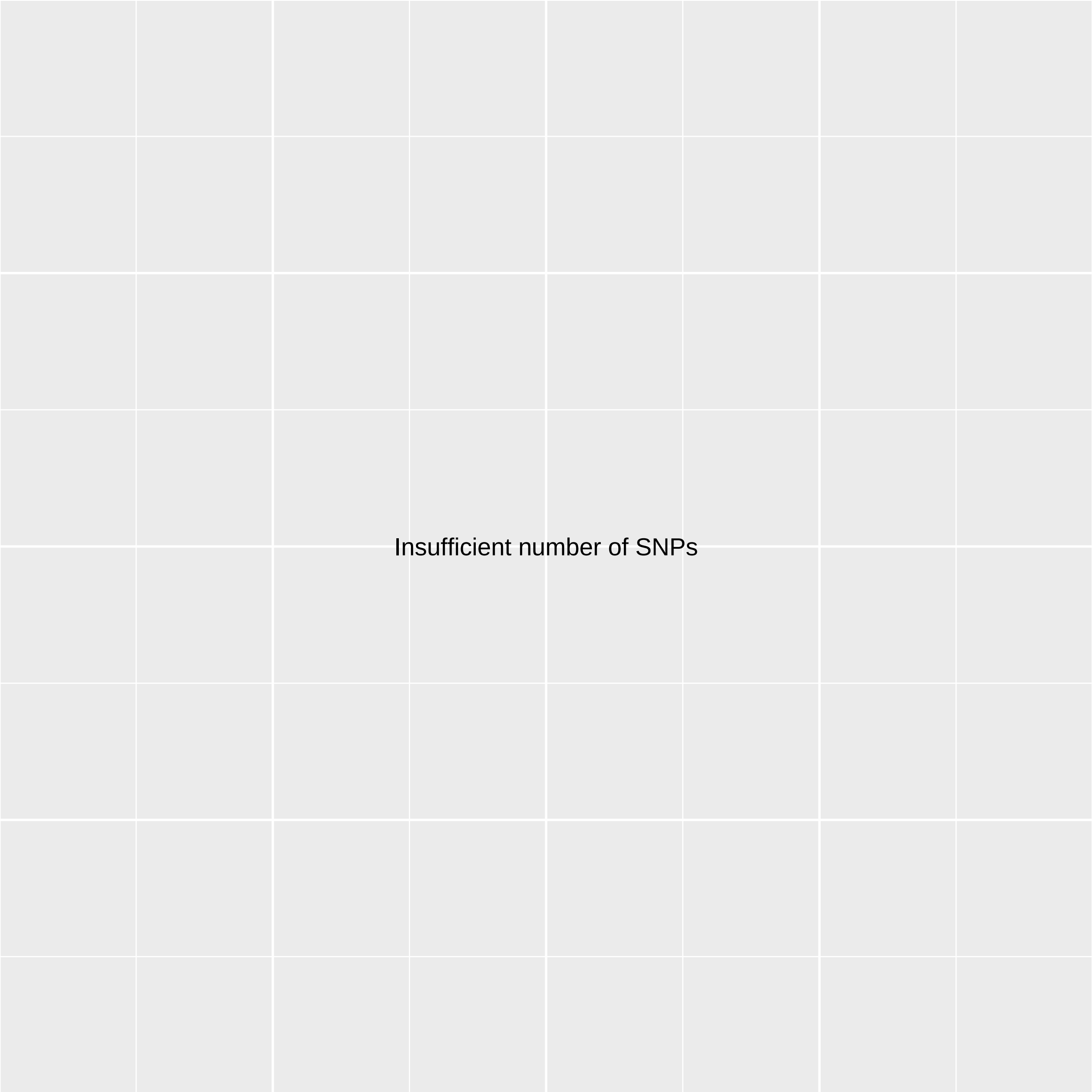
Insufficient number of SNPs

Batch 1119 : Gut microbiota abundance (genus Escherichia Shigella id.3504) on Ulcerative ileocolitis

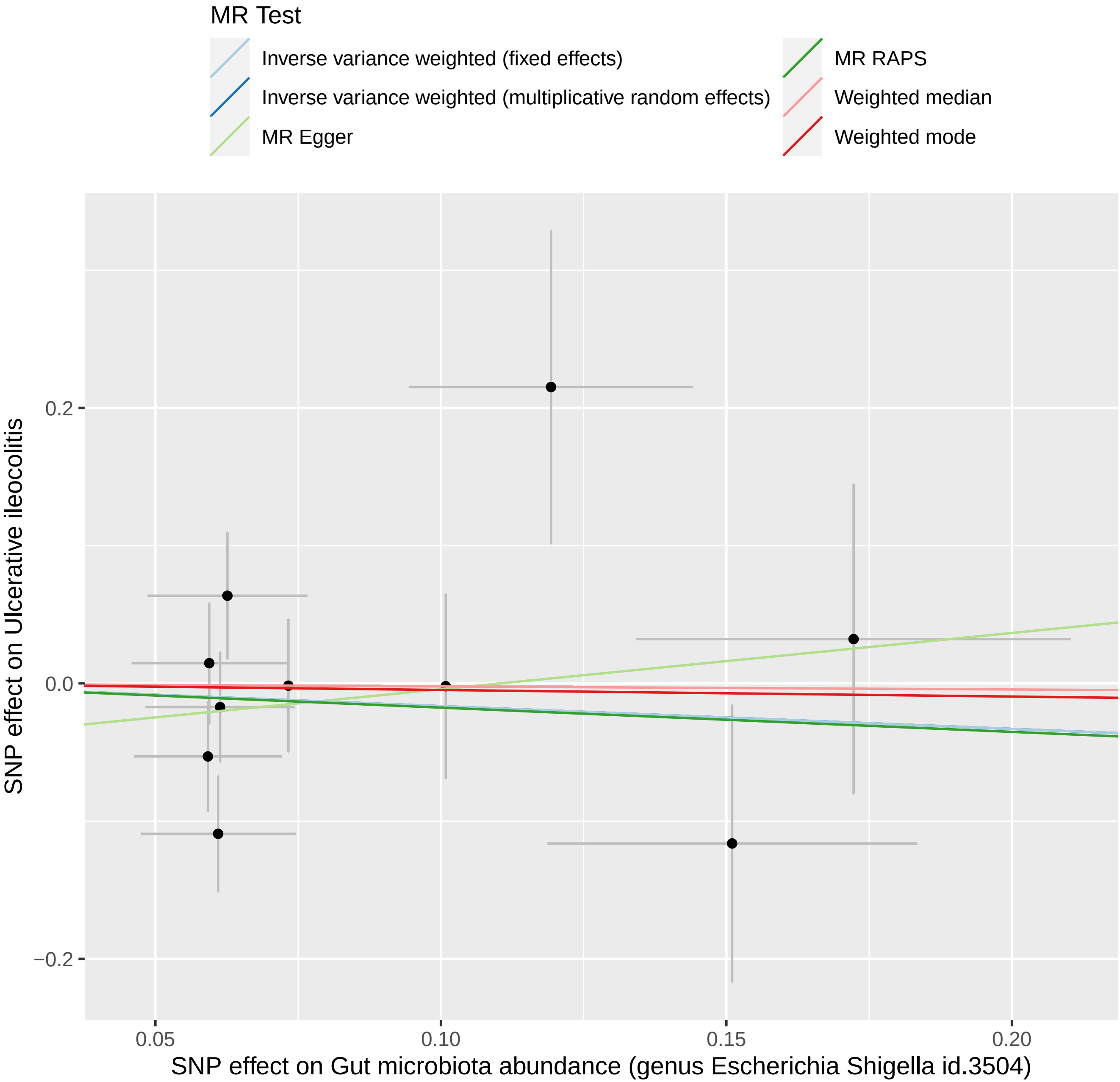

Batch 1120 : Gut microbiota abundance (genus Eubacterium brachy group id.11296) on Ulcerative ileocolitis

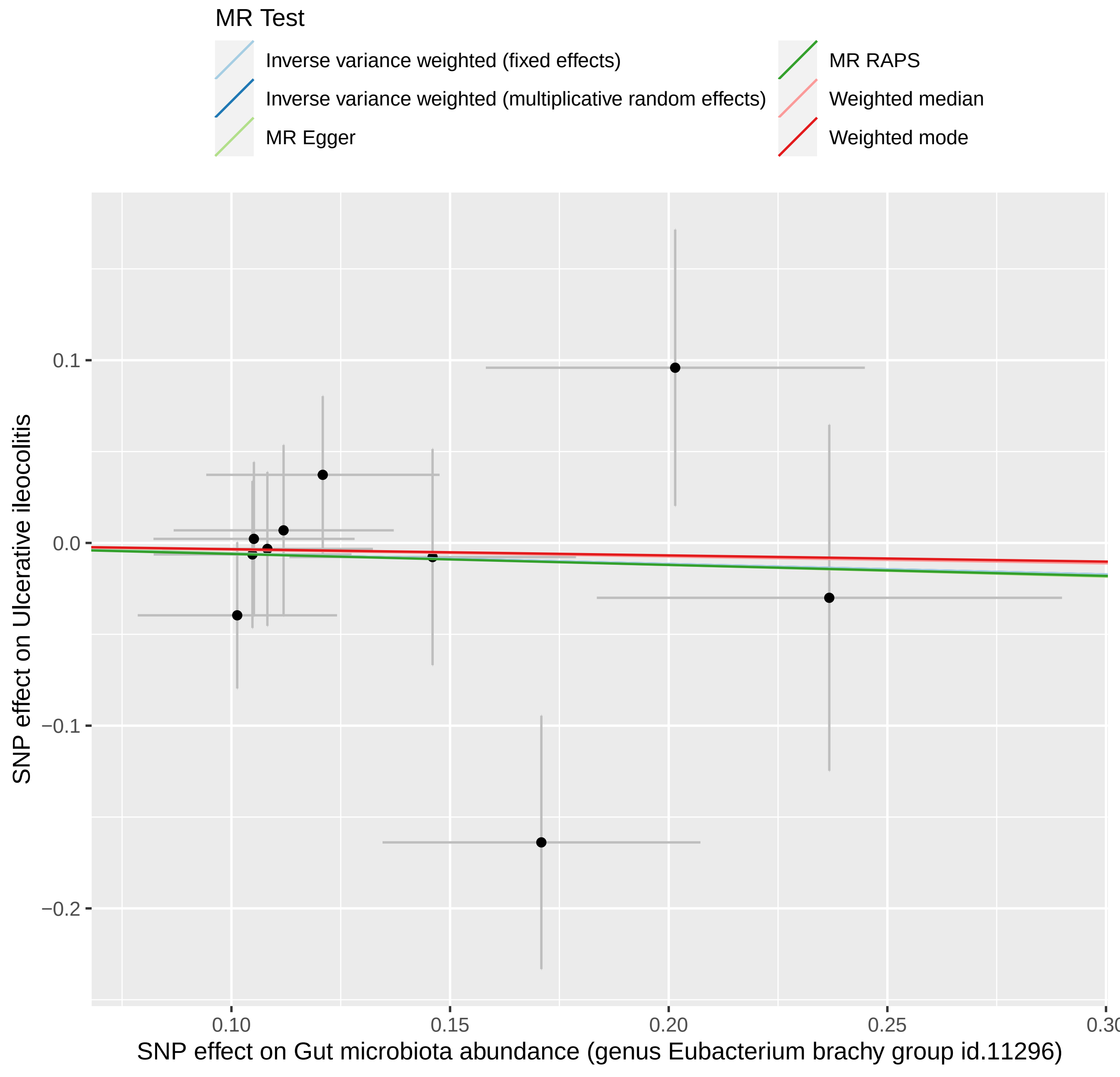

Batch 1121 : Gut microbiota abundance (genus Eubacterium coprostanoligenes group id.11375) on Ulcerative ileocolitis

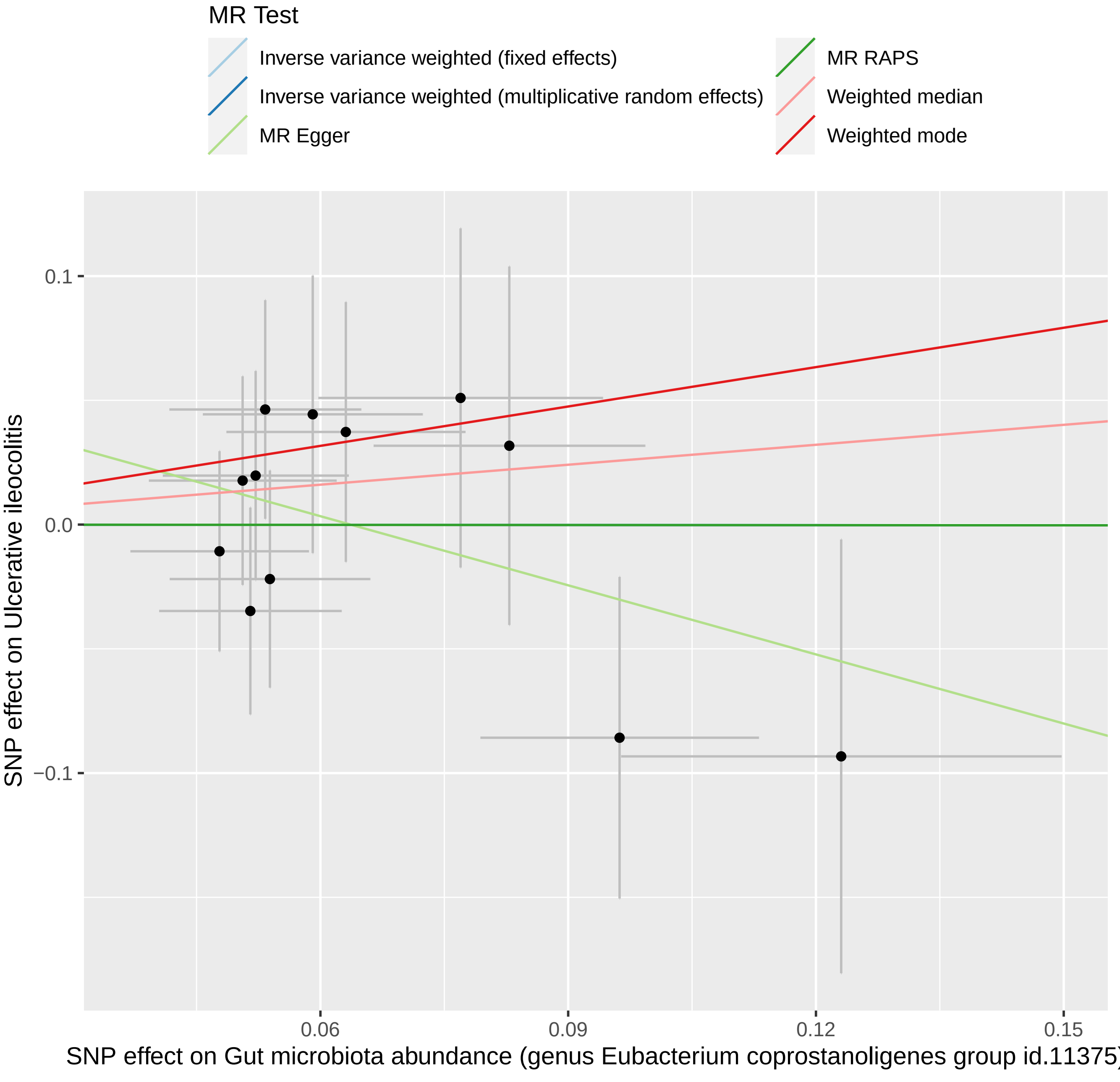

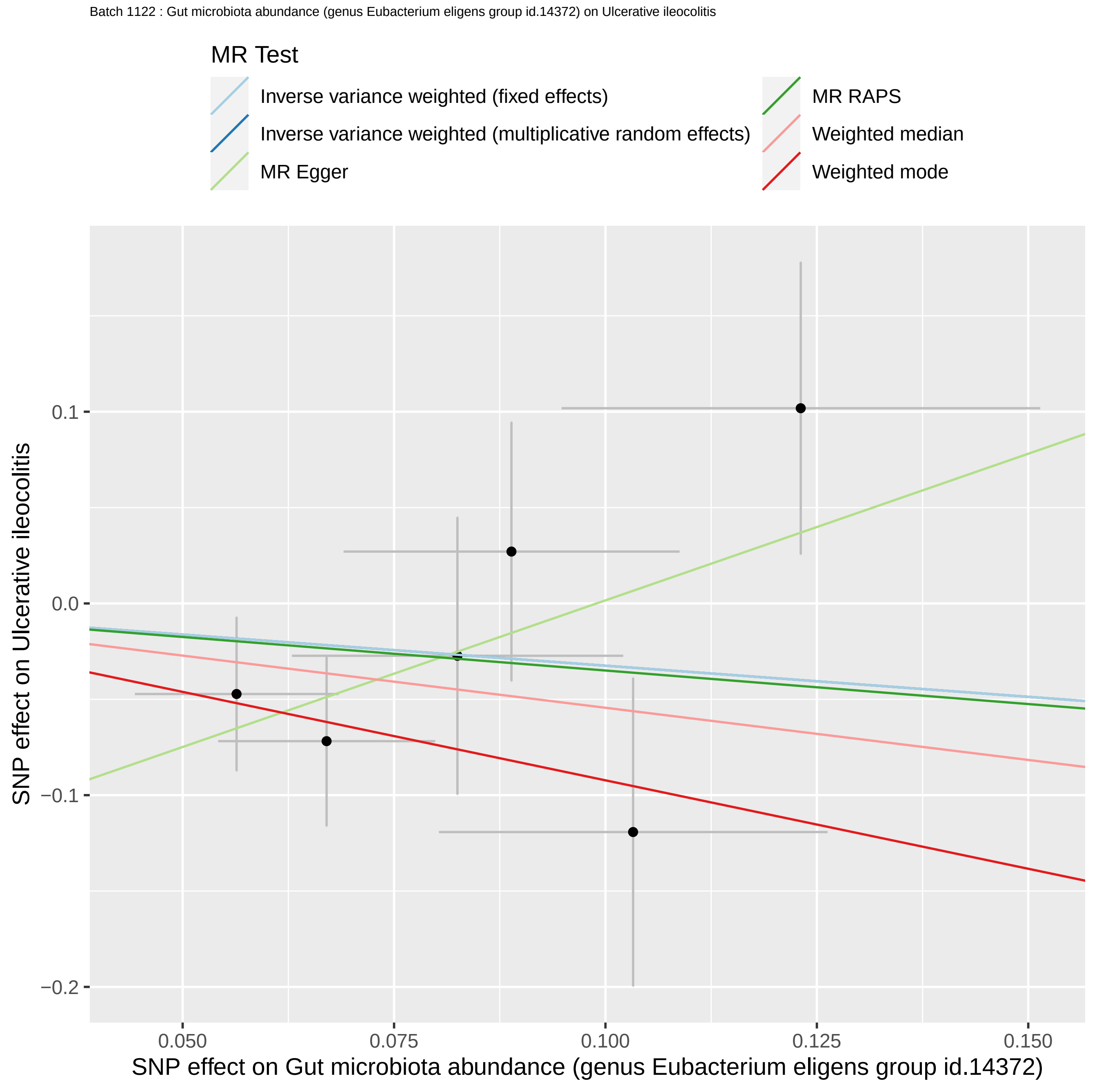
Batch 1122 : Gut microbiota abundance (genus Eubacterium eligens group id.14372) on Ulcerative ileocolitis
MR Test
Inverse variance weighted (fixed effects)
Inverse variance weighted (multiplicative random effects)
MR Egger
MR RAPS
Weighted median
Weighted mode
0.1
0.0
−0.1
−0.2
0.050
0.075
0.100
0.125
0.150
SNP effect on Ulcerative ileocolitis
SNP effect on Gut microbiota abundance (genus Eubacterium eligens group id.14372)

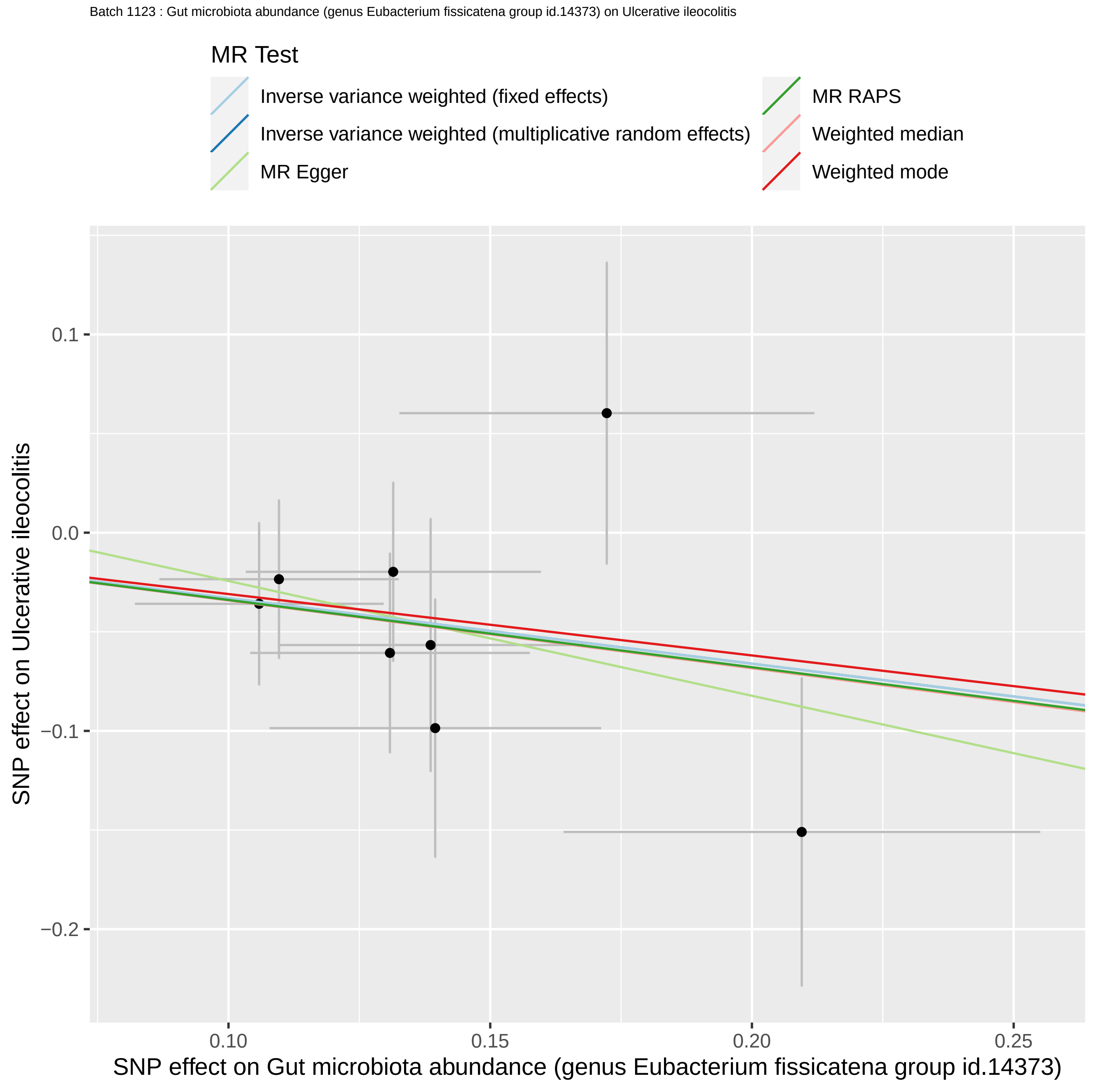
Batch 1123 : Gut microbiota abundance (genus Eubacterium fissicatena group id.14373) on Ulcerative ileocolitis
MR Test
Inverse variance weighted (fixed effects)
Inverse variance weighted (multiplicative random effects)
MR Egger
MR RAPS
Weighted median
Weighted mode
0.1
0.0
−0.1
−0.2
0.10
0.15
0.20
0.25
SNP effect on Ulcerative ileocolitis
SNP effect on Gut microbiota abundance (genus Eubacterium fissicatena group id.14373)

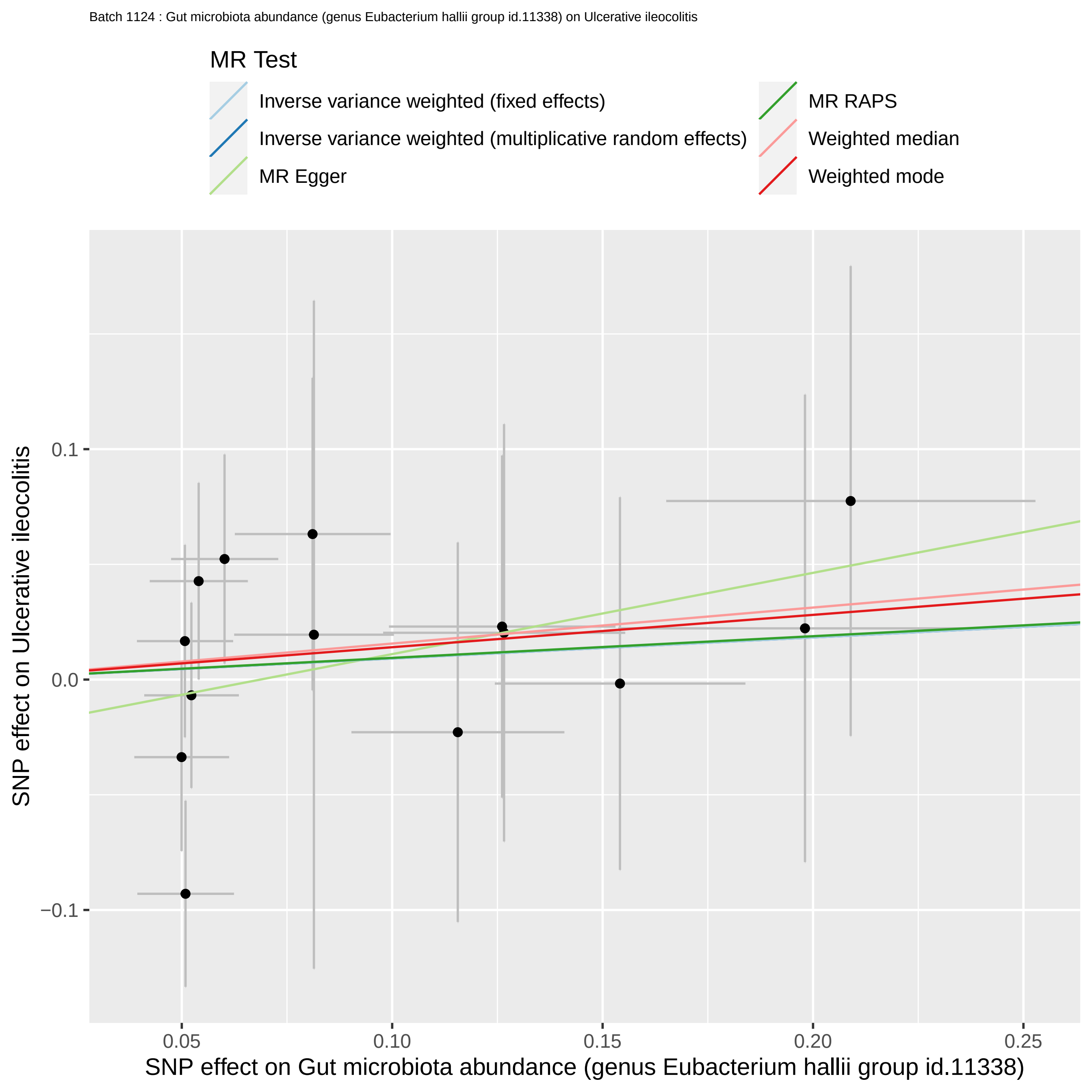
Batch 1124 : Gut microbiota abundance (genus Eubacterium hallii group id.11338) on Ulcerative ileocolitis
MR Test
Inverse variance weighted (fixed effects)
Inverse variance weighted (multiplicative random effects)
MR Egger
MR RAPS
Weighted median
Weighted mode
0.1
0.0
−0.1
0.05
0.10
0.15
0.20
0.25
SNP effect on Ulcerative ileocolitis
SNP effect on Gut microbiota abundance (genus Eubacterium hallii group id.11338)

Batch 1125 : Gut microbiota abundance (genus Eubacterium nodatum group id.11297) on Ulcerative ileocolitis

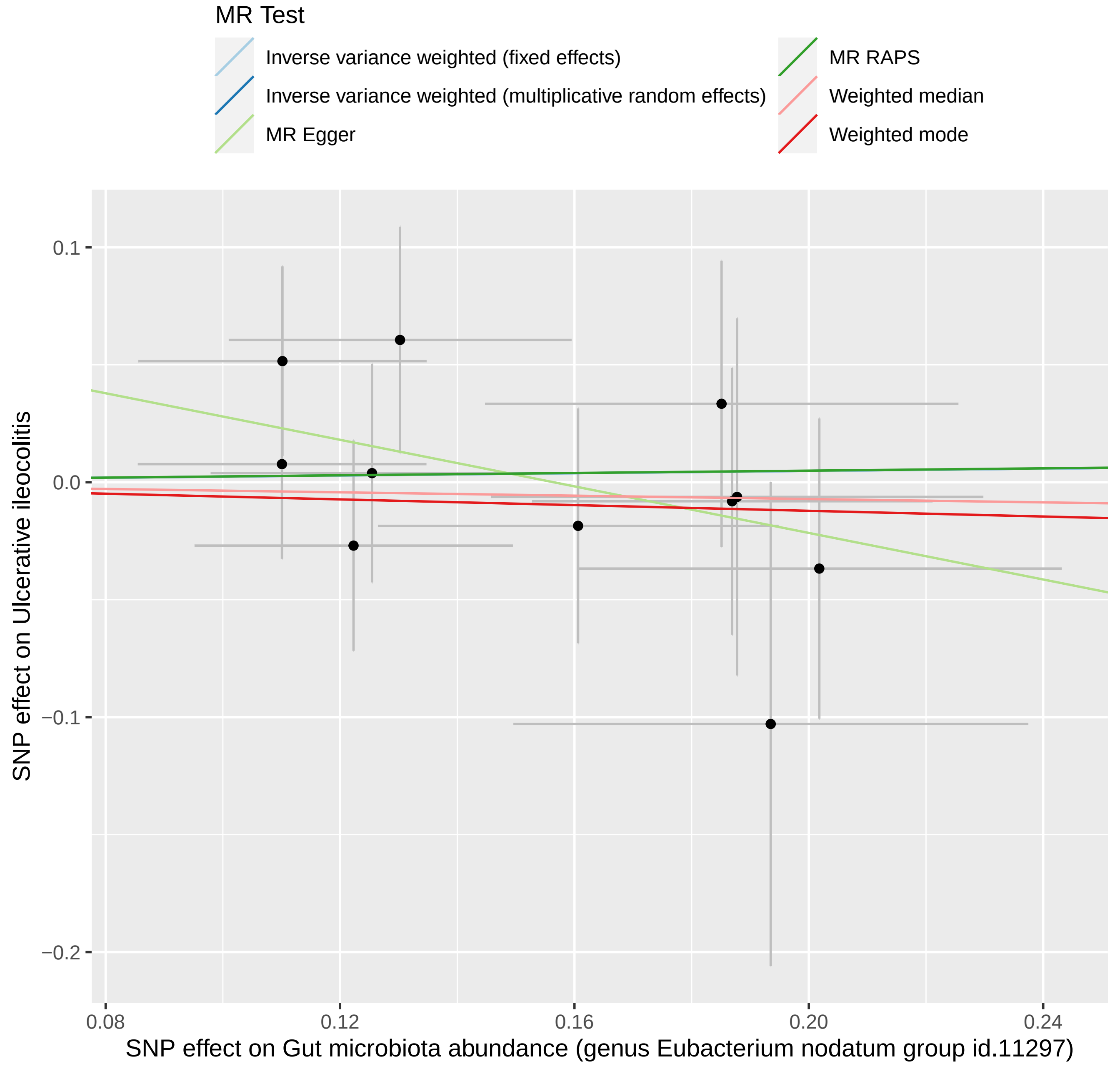

Batch 1126 : Gut microbiota abundance (genus Eubacterium oxidoreducens group id.11339) on Ulcerative ileocolitis

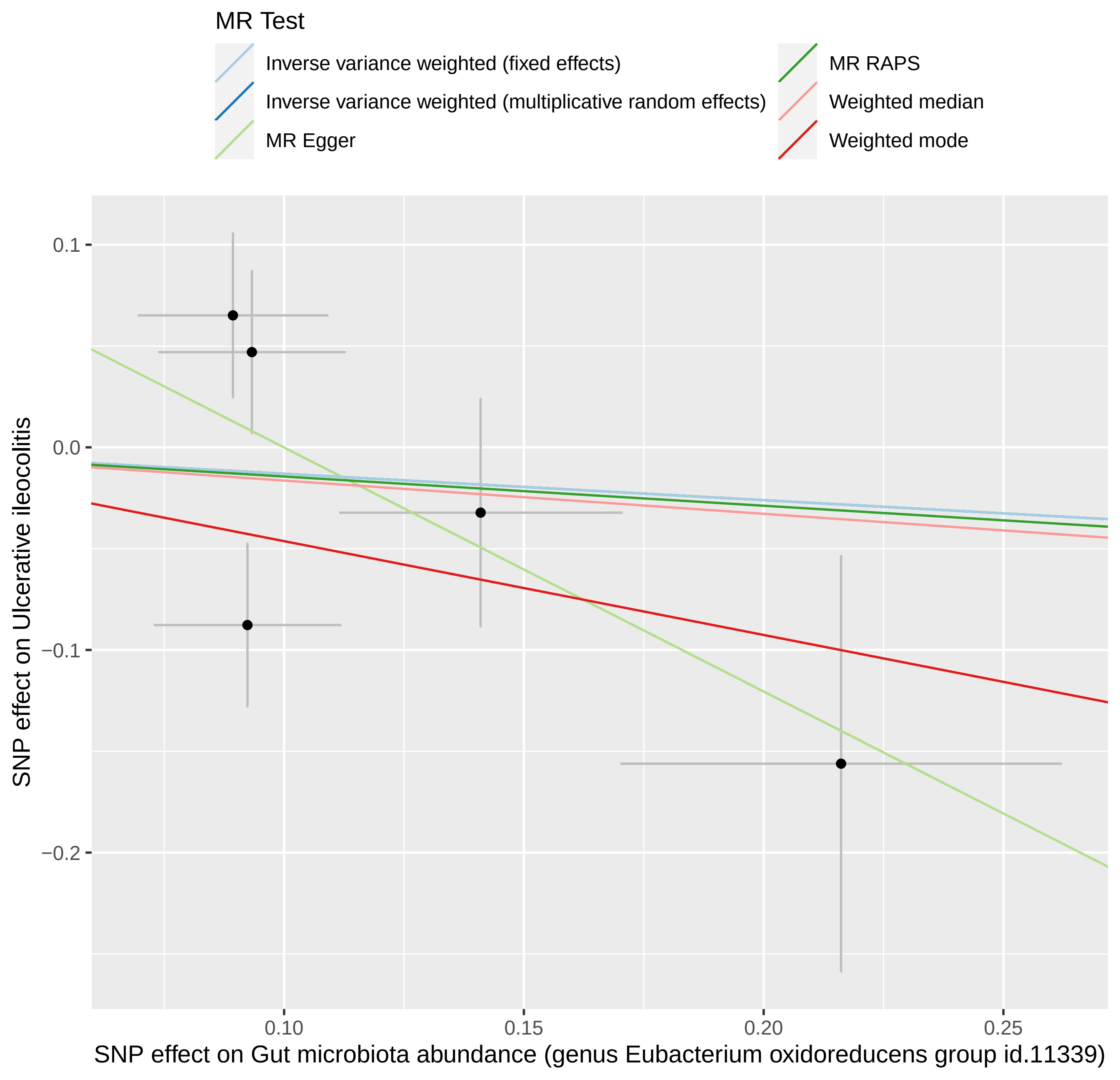

Batch 1127 : Gut microbiota abundance (genus Eubacterium rectale group id.14374) on Ulcerative ileocolitis

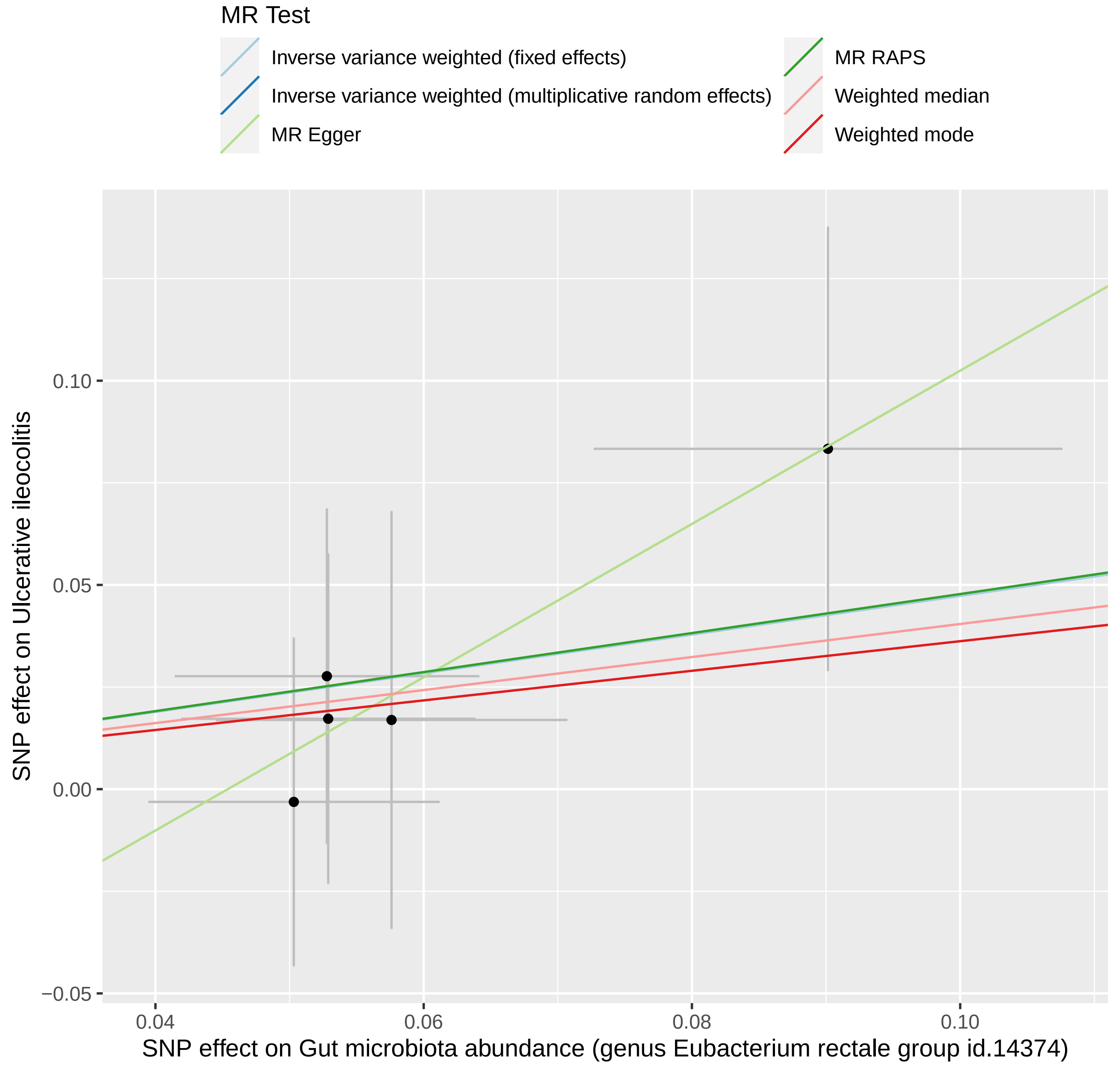

Batch 1128 : Gut microbiota abundance (genus Eubacterium ruminantium group id.11340) on Ulcerative ileocolitis

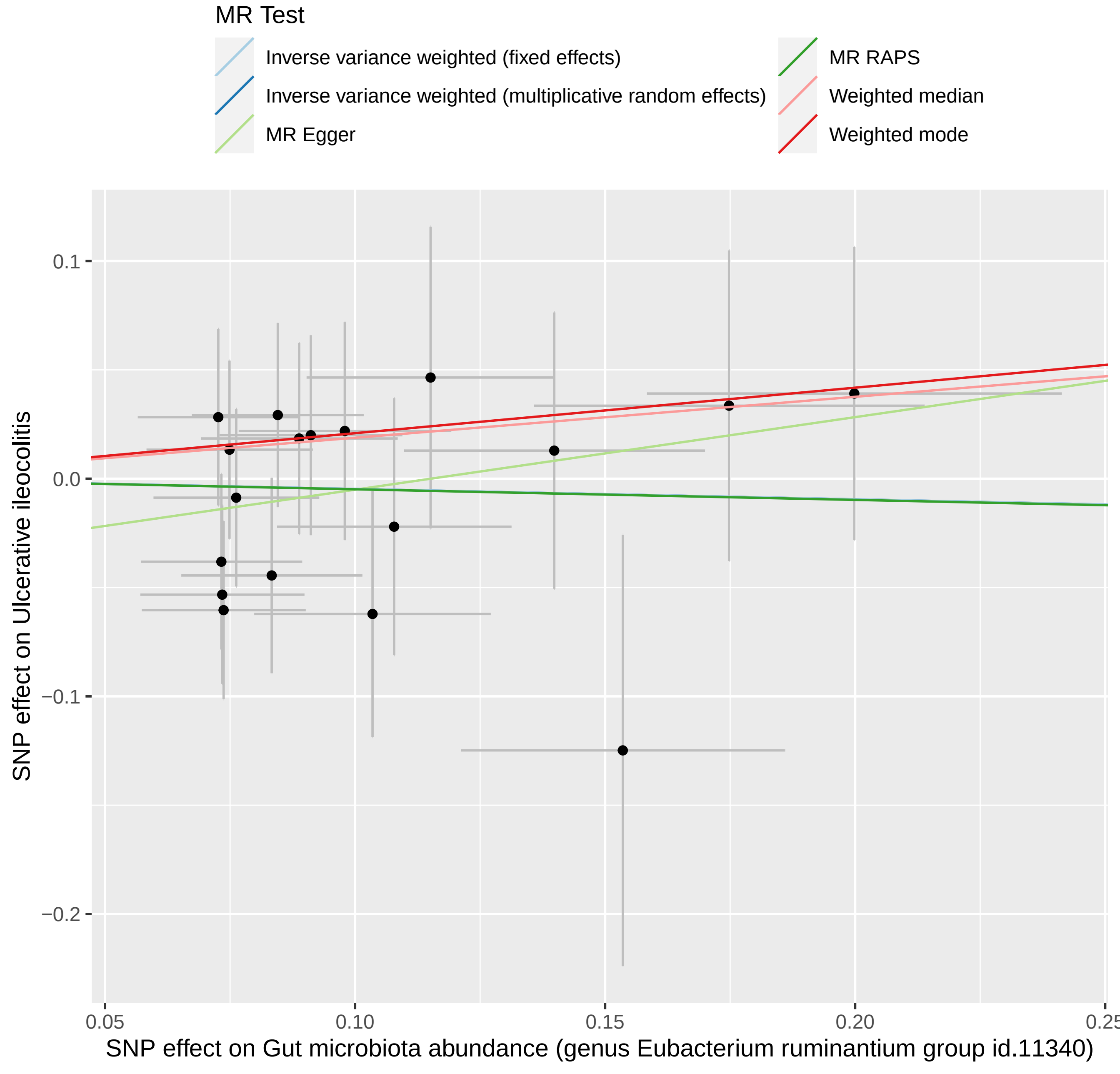

Batch 1129 : Gut microbiota abundance (genus Eubacterium ventriosum group id.11341) on Ulcerative ileocolitis

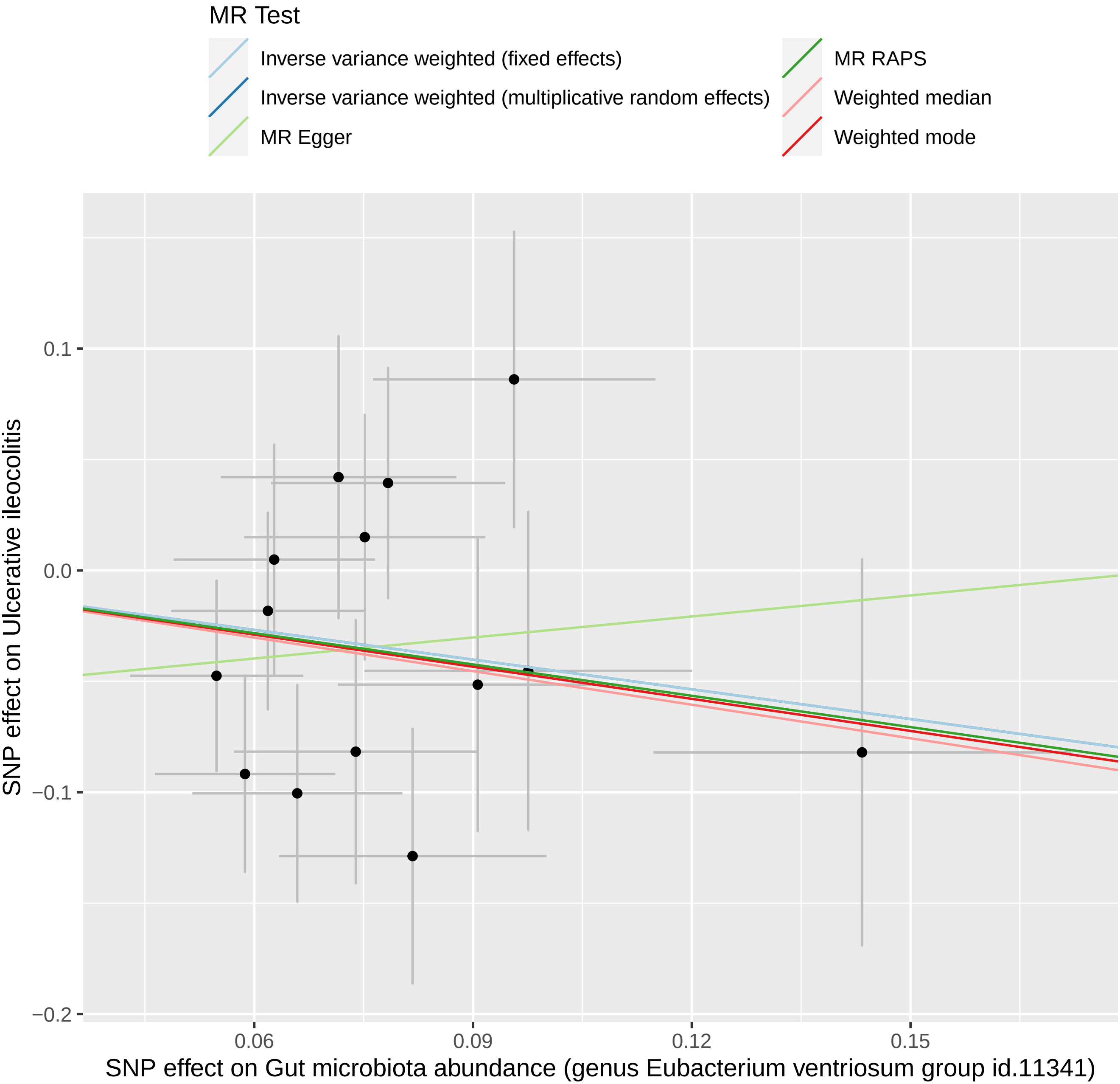

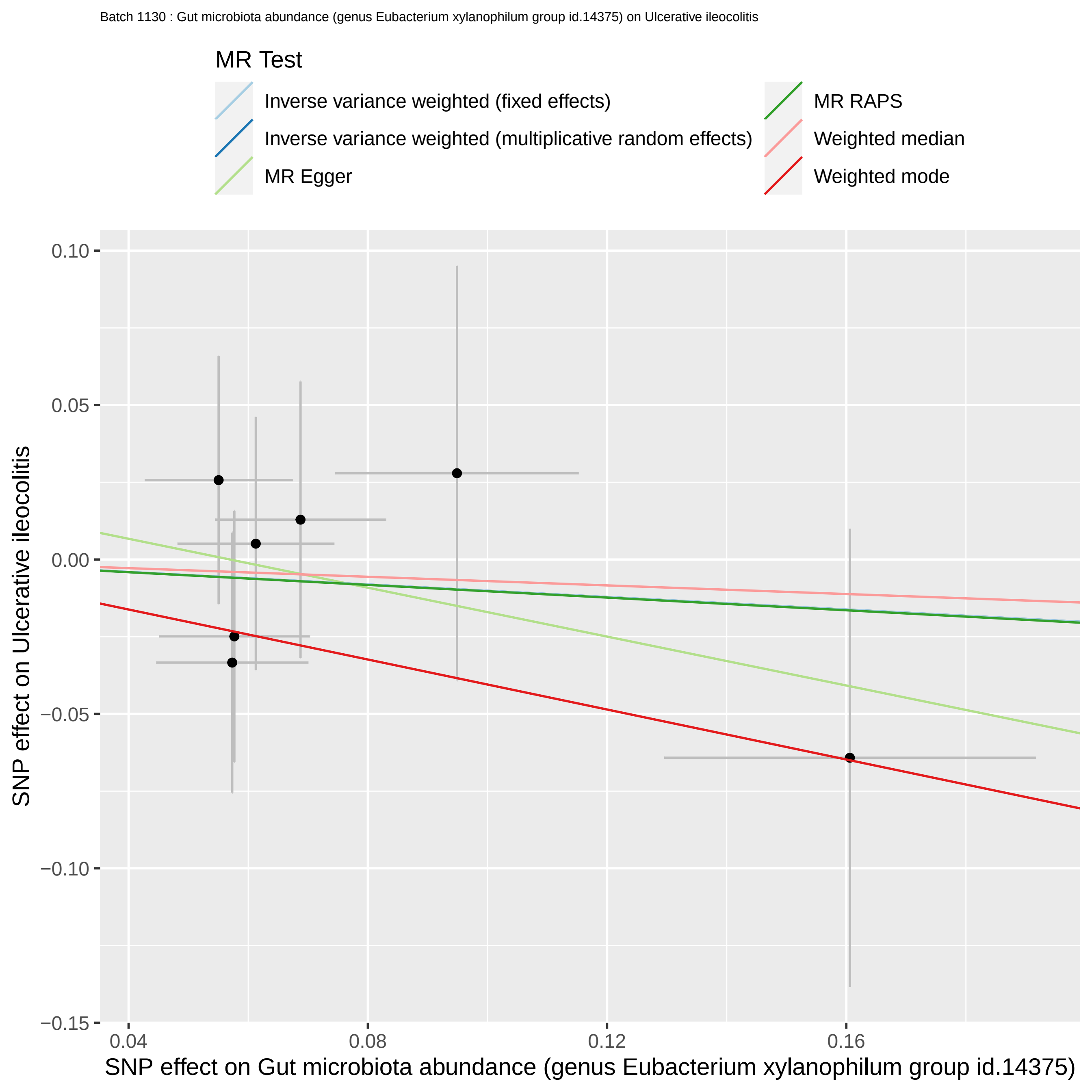

Batch 1130 : Gut microbiota abundance (genus Eubacterium xylanophilum group id.14375) on Ulcerative ileocolitis
MR Test
Inverse variance weighted (fixed effects)
Inverse variance weighted (multiplicative random effects)
MR Egger
MR RAPS
Weighted median
Weighted mode
0.10
0.05
0.00
−0.05
−0.10
−0.15
0.04
0.08
0.12
0.16
SNP effect on Ulcerative ileocolitis
SNP effect on Gut microbiota abundance (genus Eubacterium xylanophilum group id.14375)

Batch 1131 : Gut microbiota abundance (genus Faecalibacterium id.2057) on Ulcerative ileocolitis

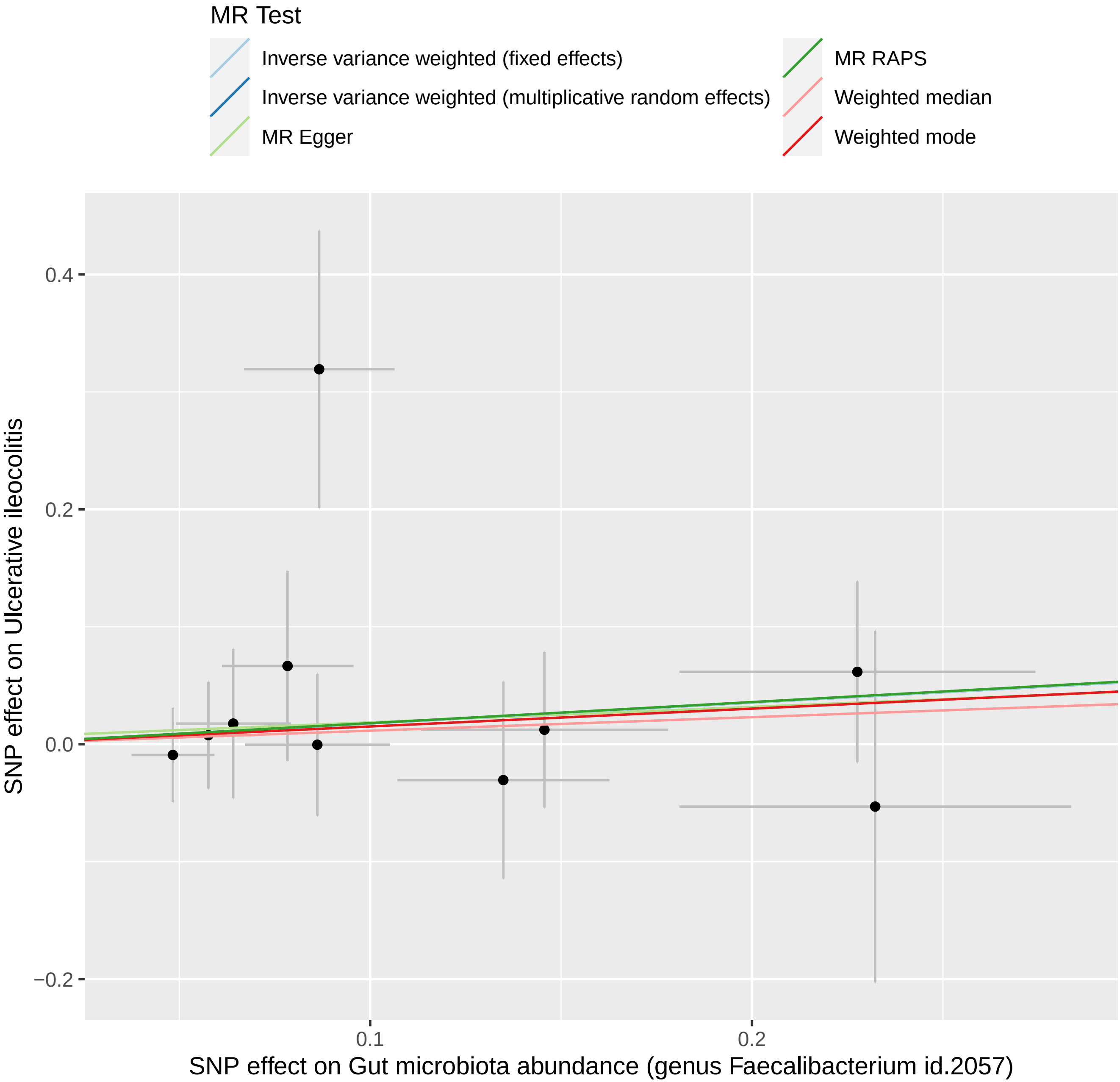

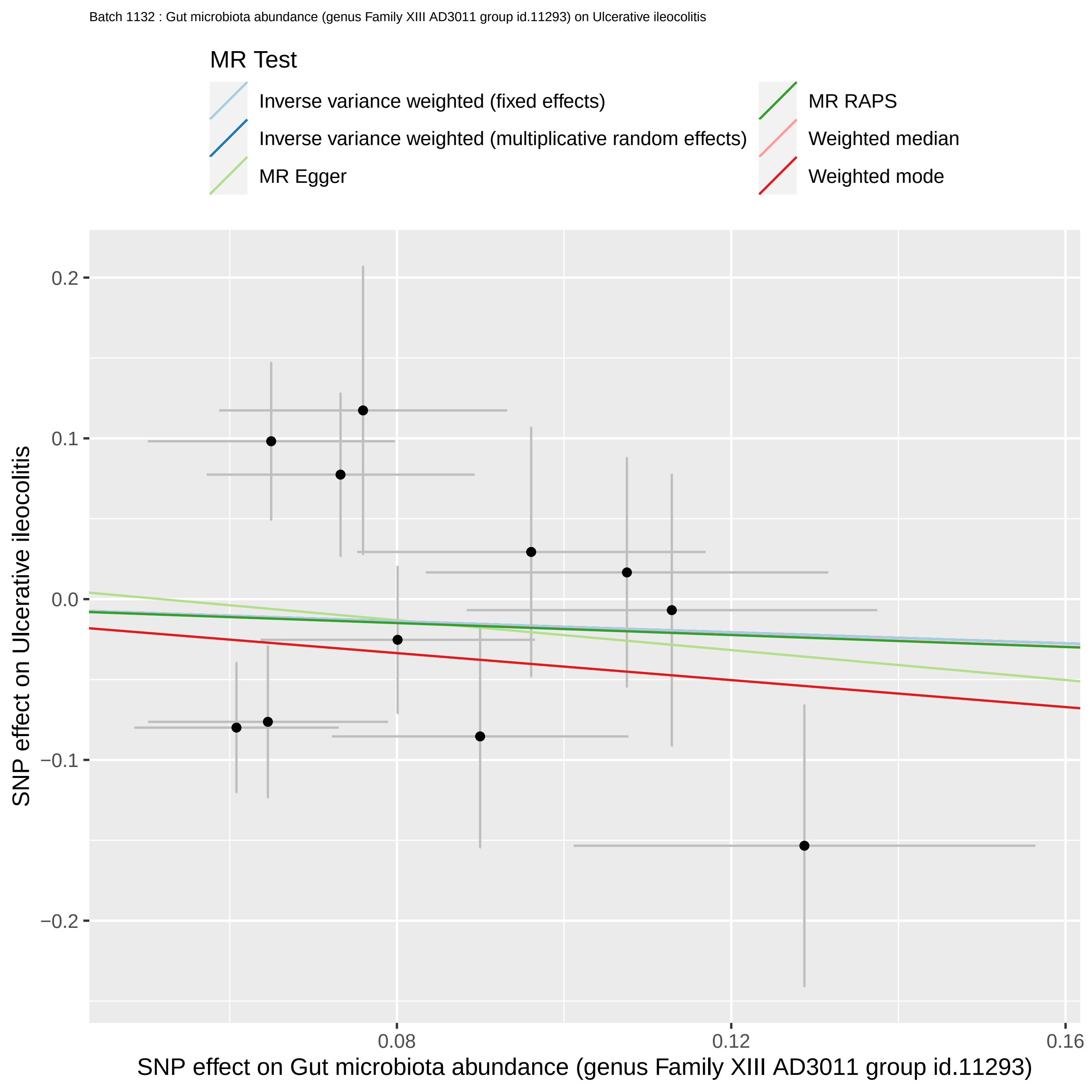

Batch 1132 : Gut microbiota abundance (genus Family XIII AD3011 group id.11293) on Ulcerative ileocolitis
MR Test
Inverse variance weighted (fixed effects)
Inverse variance weighted (multiplicative random effects)
MR Egger
MR RAPS
Weighted median
Weighted mode
0.2
0.1
0.0
−0.1
−0.2
0.08
0.12
0.16
SNP effect on Ulcerative ileocolitis
SNP effect on Gut microbiota abundance (genus Family XIII AD3011 group id.11293)

Batch 1133 : Gut microbiota abundance (genus Family XIII UCG001 id.11294) on Ulcerative ileocolitis

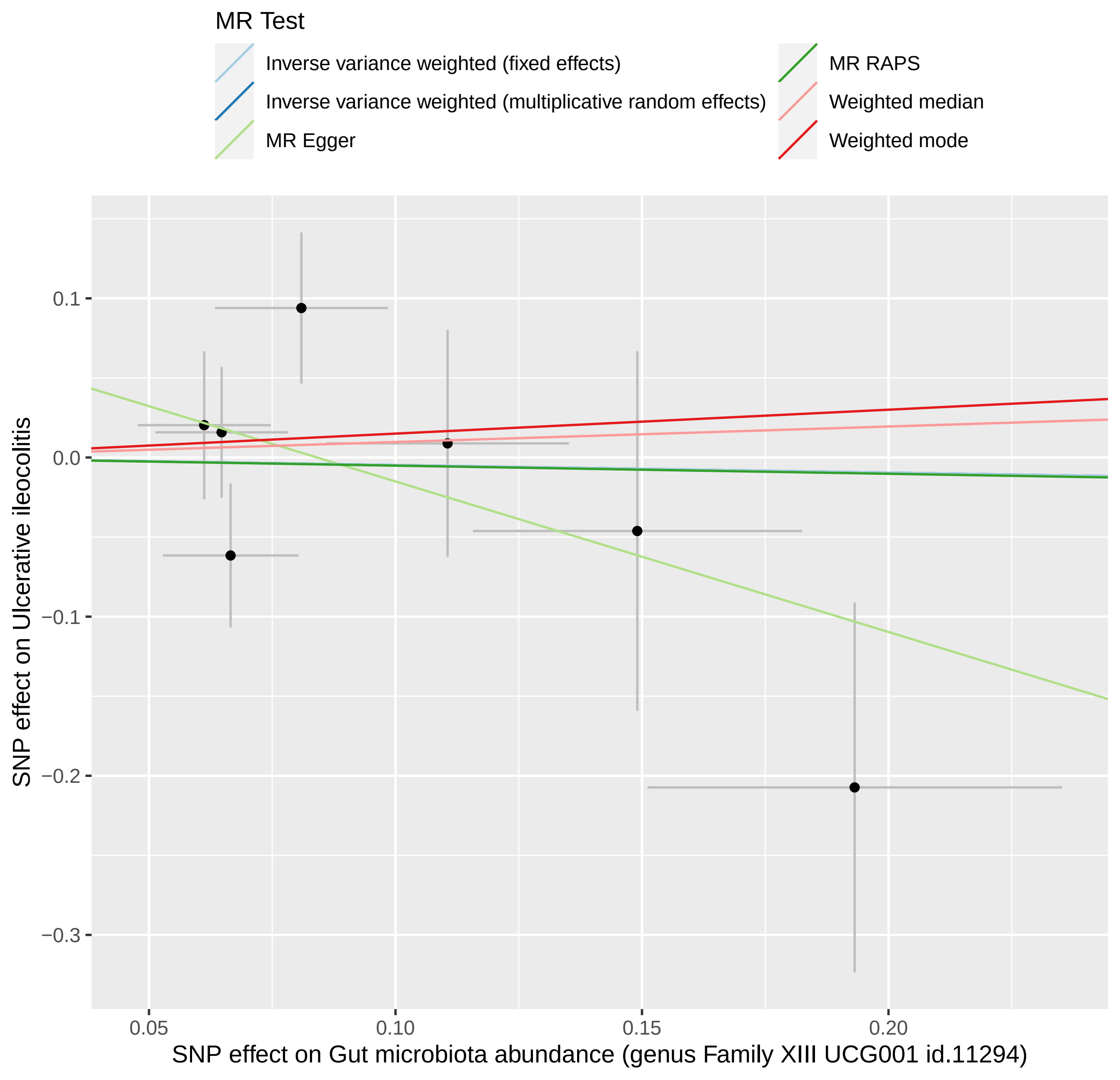

Batch 1134 : Gut microbiota abundance (genus Flavonifractor id.2059) on Ulcerative ileocolitis

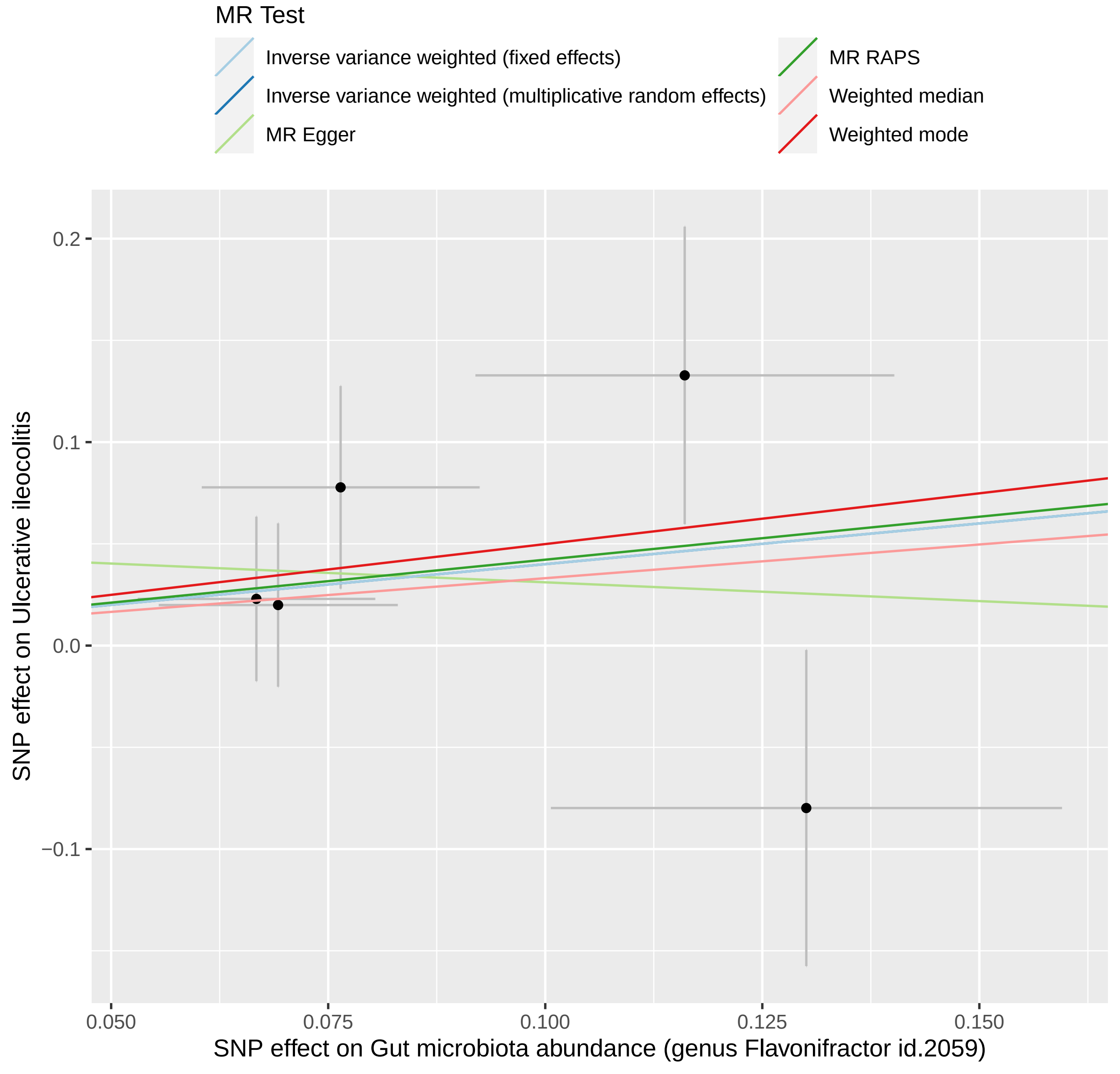

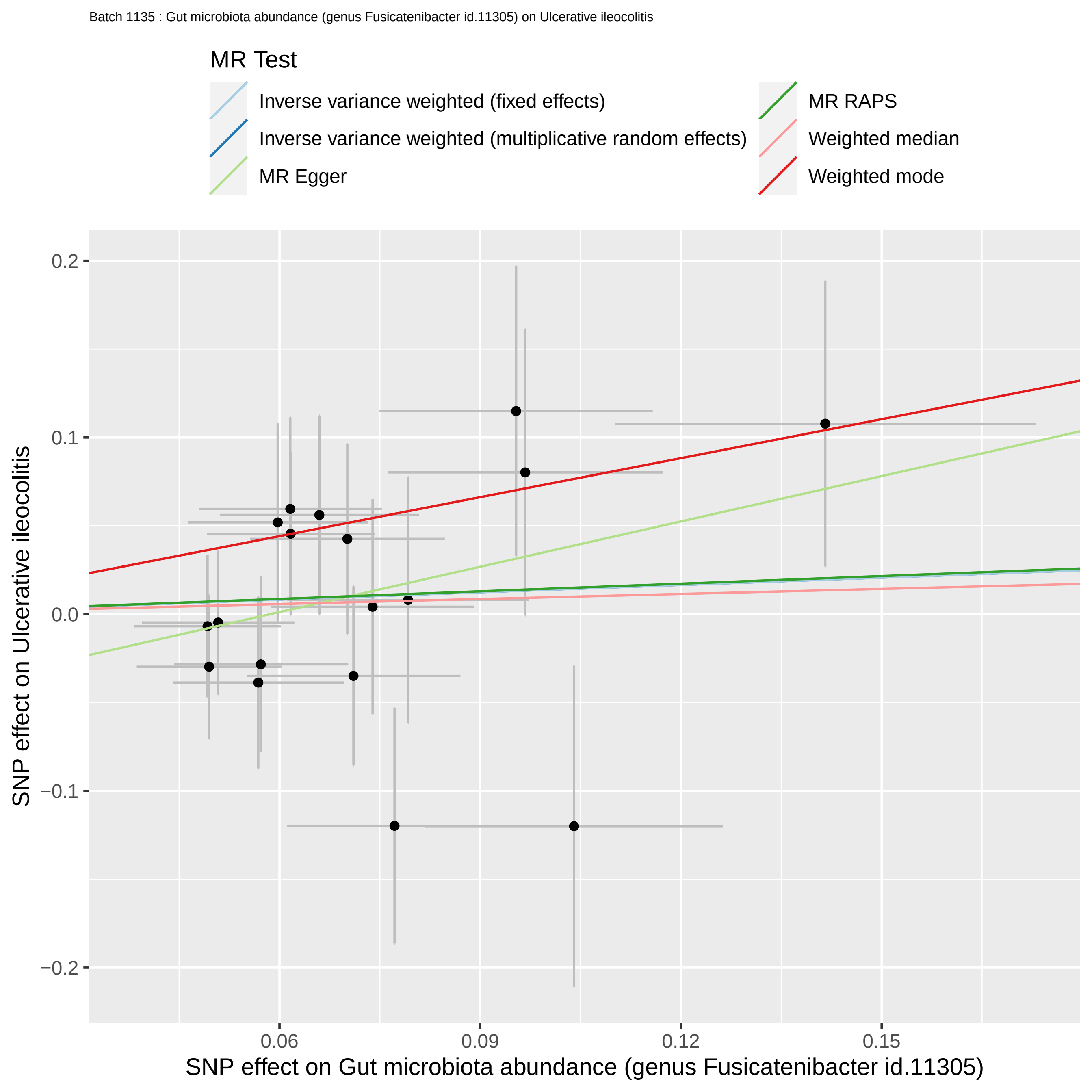

Batch 1135 : Gut microbiota abundance (genus Fusicatenibacter id.11305) on Ulcerative ileocolitis
MR Test
Inverse variance weighted (fixed effects)
Inverse variance weighted (multiplicative random effects)
MR Egger
MR RAPS
Weighted median
Weighted mode
SNP effect on Ulcerative ileocolitis
0.2
0.1
0.0
−0.1
−0.2
0.06
0.09
0.12
0.15
SNP effect on Gut microbiota abundance (genus Fusicatenibacter id.11305)

Batch 1136 : Gut microbiota abundance (genus Gordonibacter id.821) on Ulcerative ileocolitis

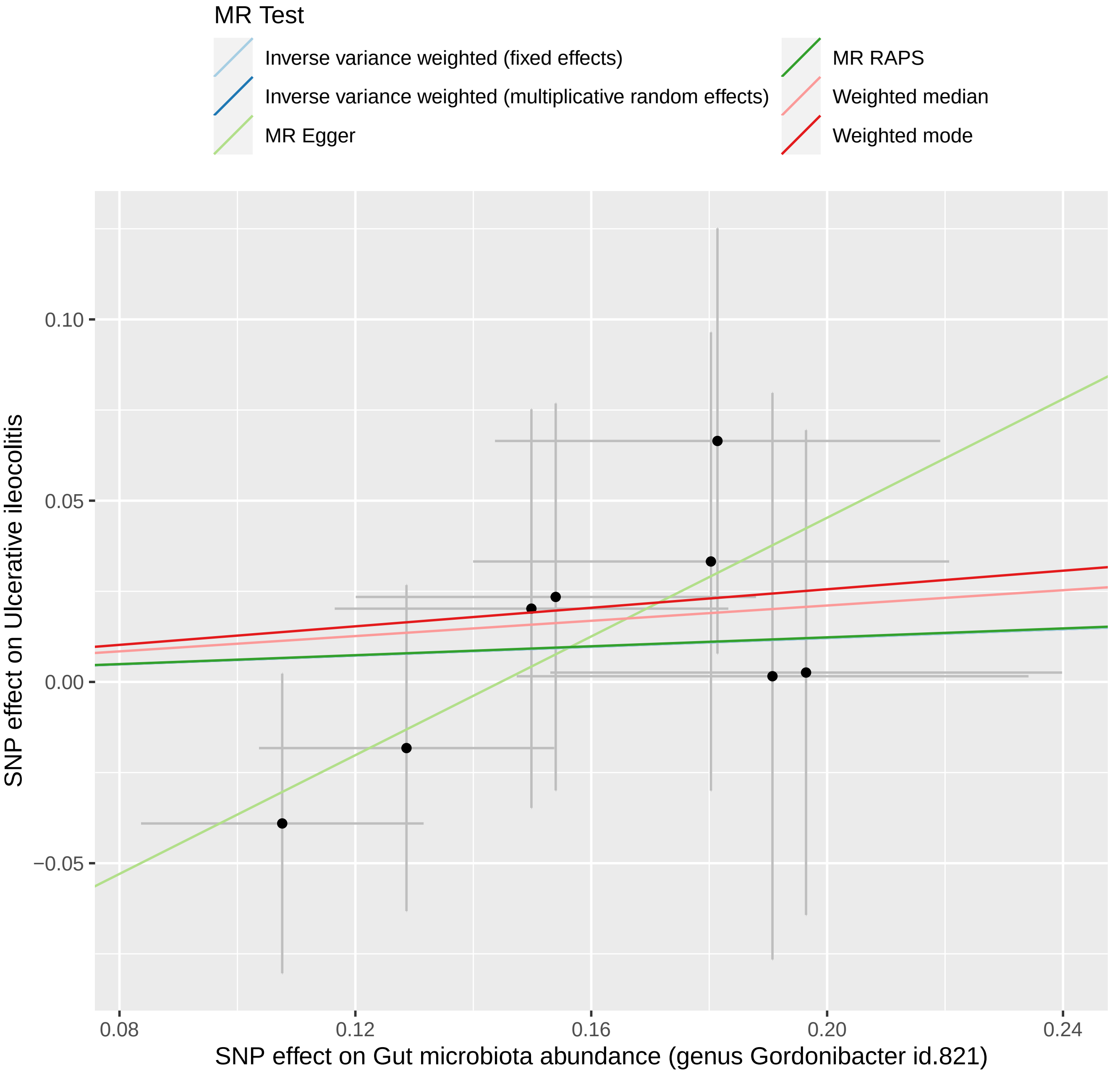

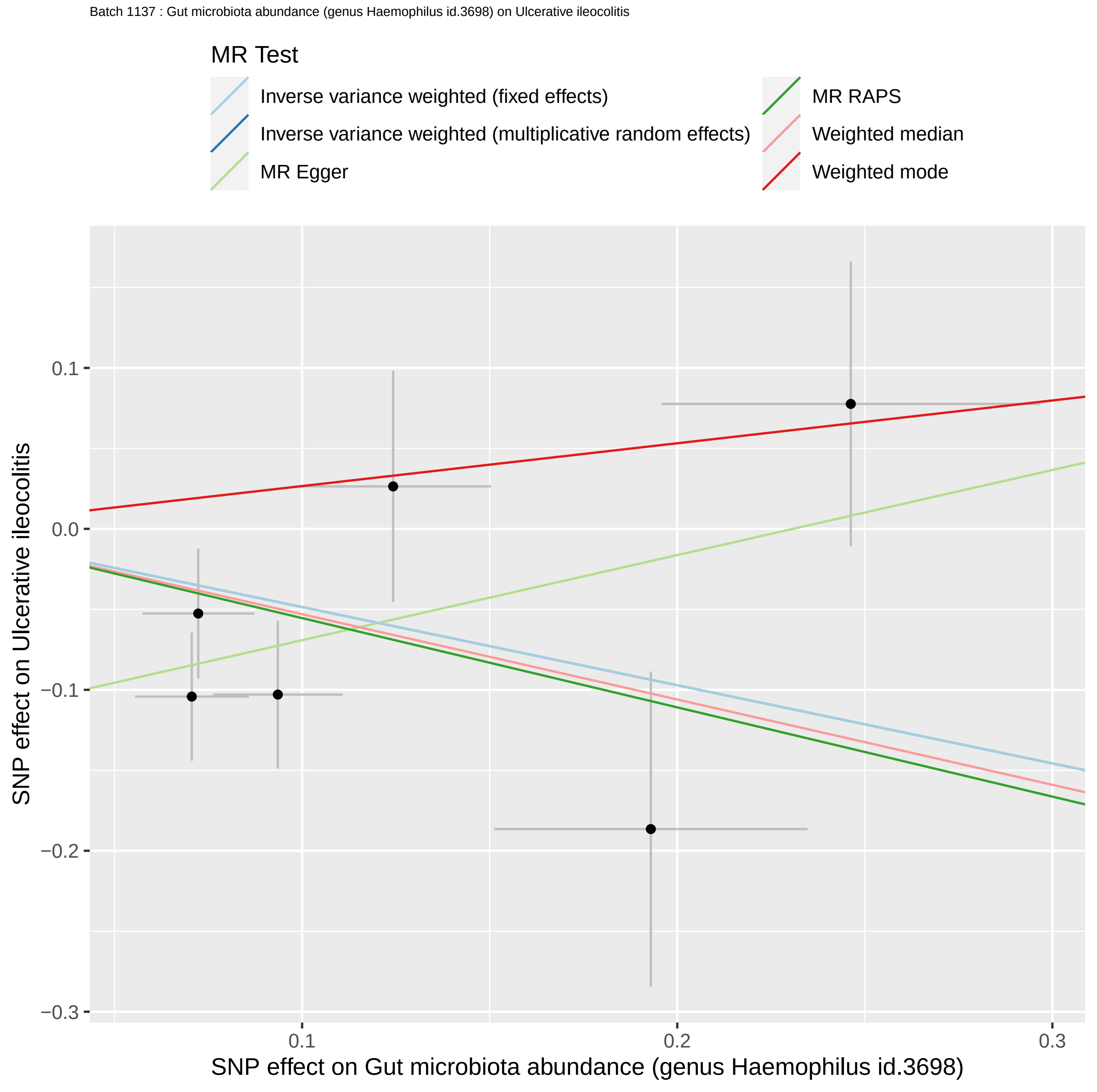

Batch 1137 : Gut microbiota abundance (genus Haemophilus id.3698) on Ulcerative ileocolitis
MR Test
Inverse variance weighted (fixed effects)
Inverse variance weighted (multiplicative random effects)
MR Egger
MR RAPS
Weighted median
Weighted mode
SNP effect on Ulcerative ileocolitis
0.1
0.0
−0.1
−0.2
−0.3
0.1
0.2
0.3
SNP effect on Gut microbiota abundance (genus Haemophilus id.3698)

Batch 1138 : Gut microbiota abundance (genus Holdemanella id.11393) on Ulcerative ileocolitis

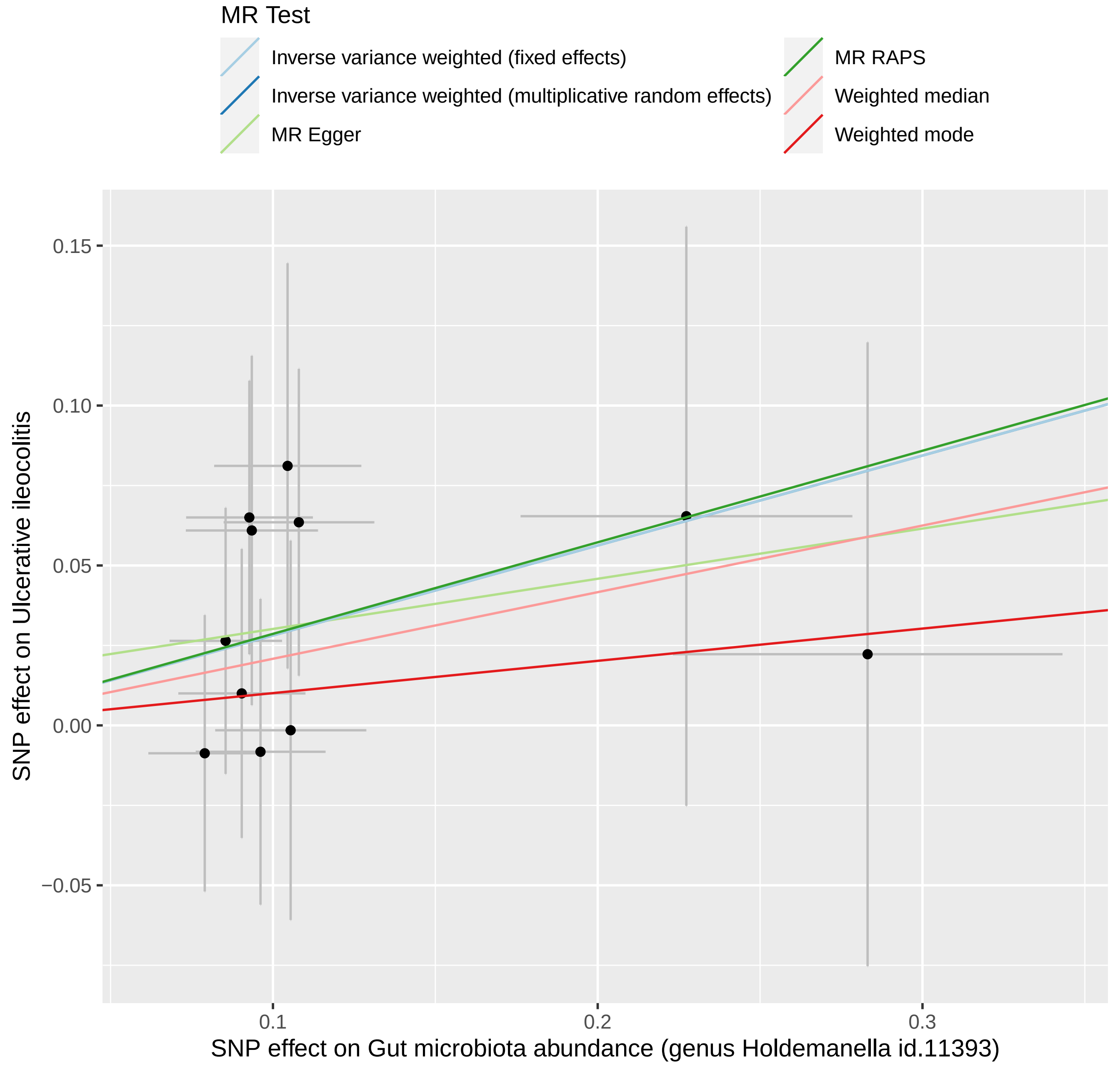

Batch 1139 : Gut microbiota abundance (genus Holdemania id.2157) on Ulcerative ileocolitis

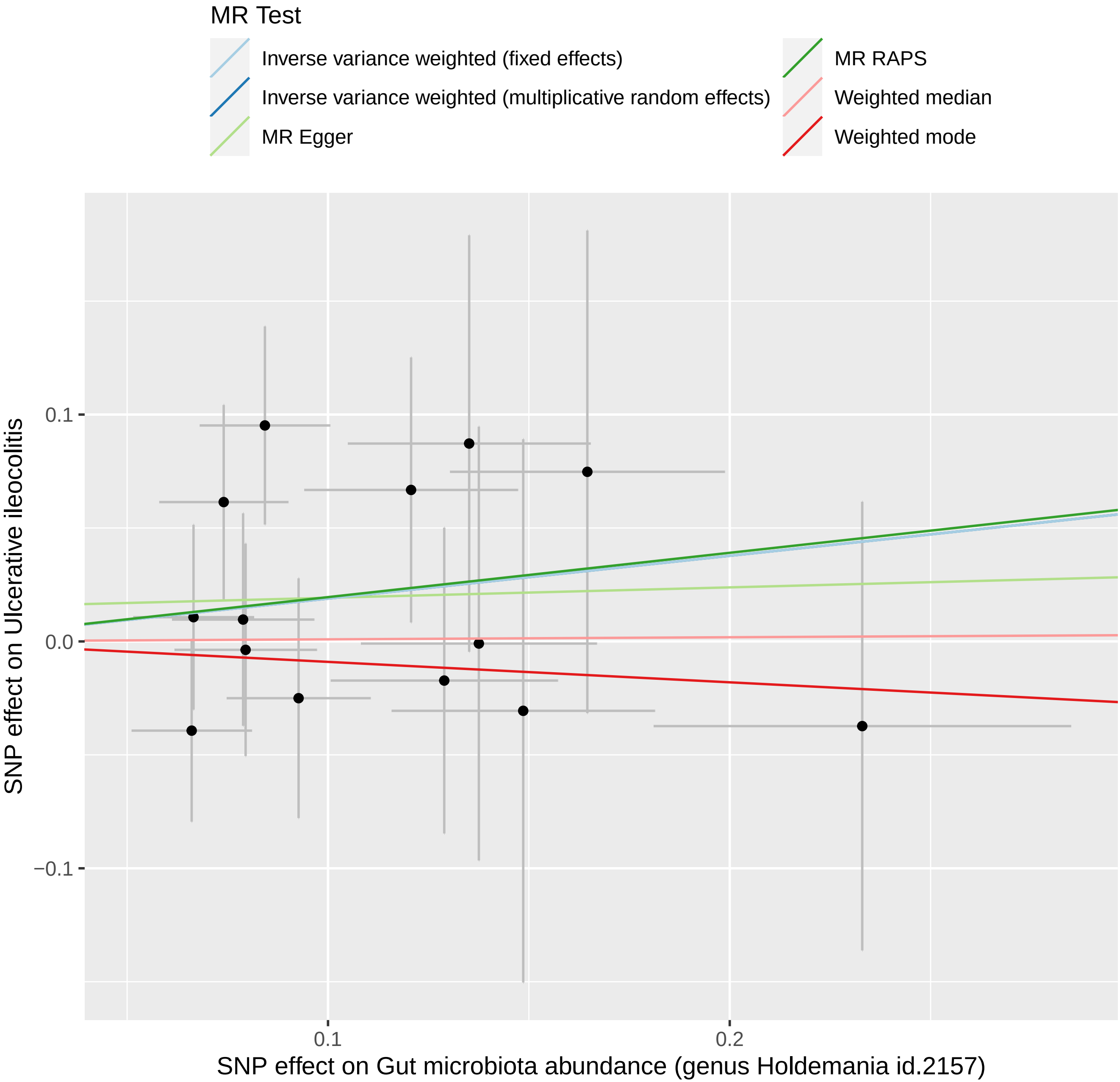

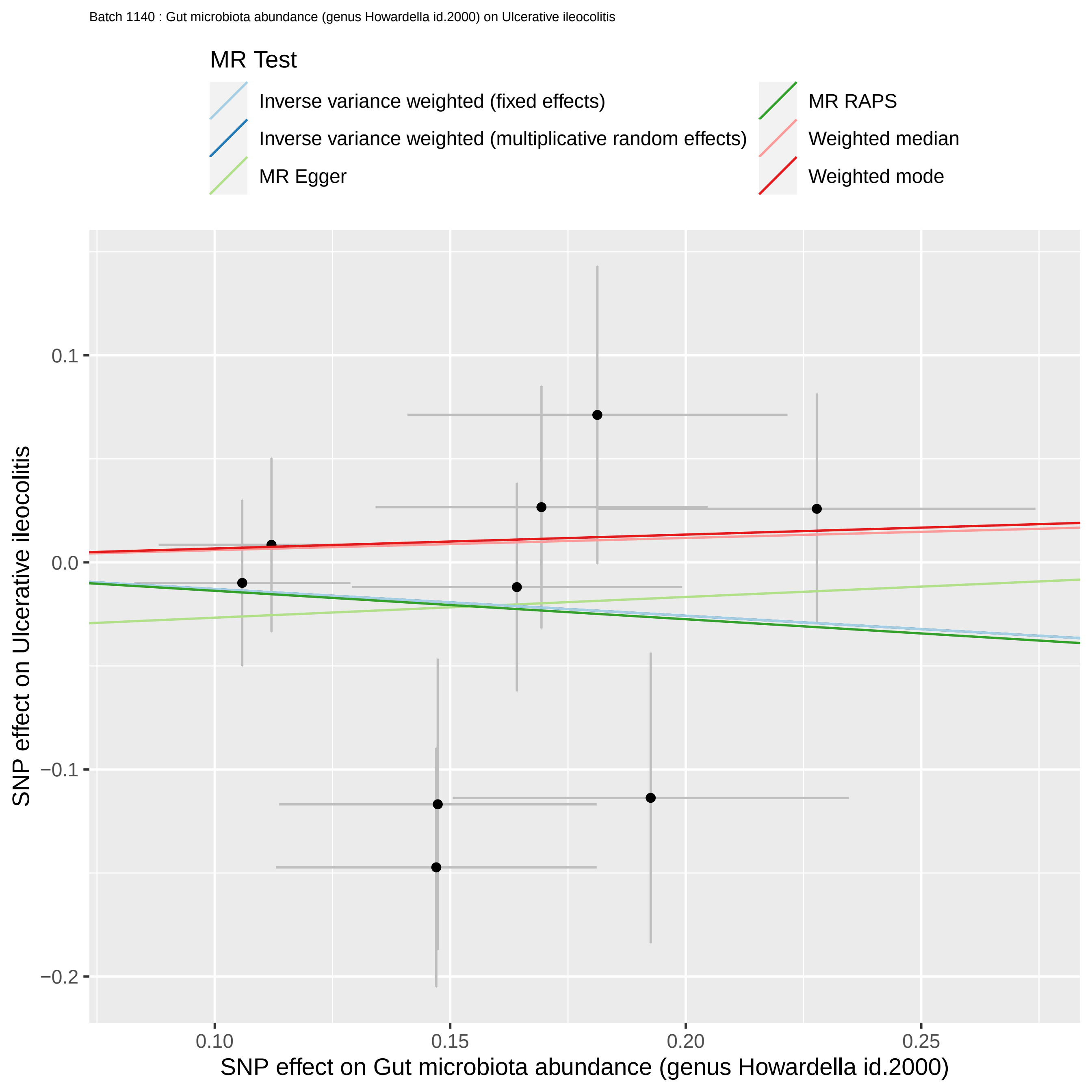

Batch 1140 : Gut microbiota abundance (genus Howardella id.2000) on Ulcerative ileocolitis
MR Test
Inverse variance weighted (fixed effects)
Inverse variance weighted (multiplicative random effects)
MR Egger
MR RAPS
Weighted median
Weighted mode
0.1
0.0
−0.1
−0.2
0.10
0.15
0.20
0.25
SNP effect on Ulcerative ileocolitis
SNP effect on Gut microbiota abundance (genus Howardella id.2000)

Batch 1141 : Gut microbiota abundance (genus Hungatella id.11306) on Ulcerative ileocolitis

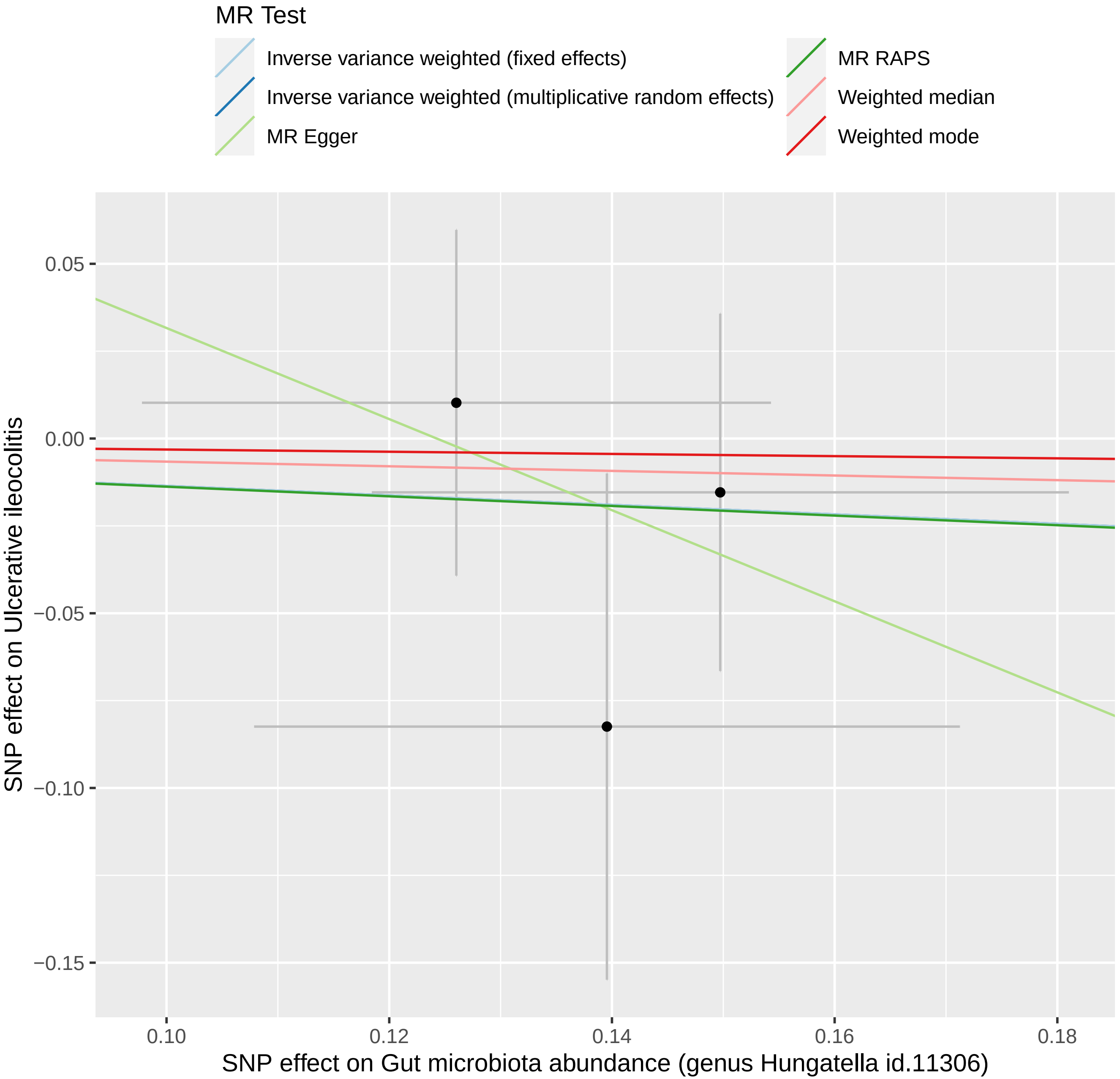

Batch 1142 : Gut microbiota abundance (genus Intestinibacter id.11345) on Ulcerative ileocolitis

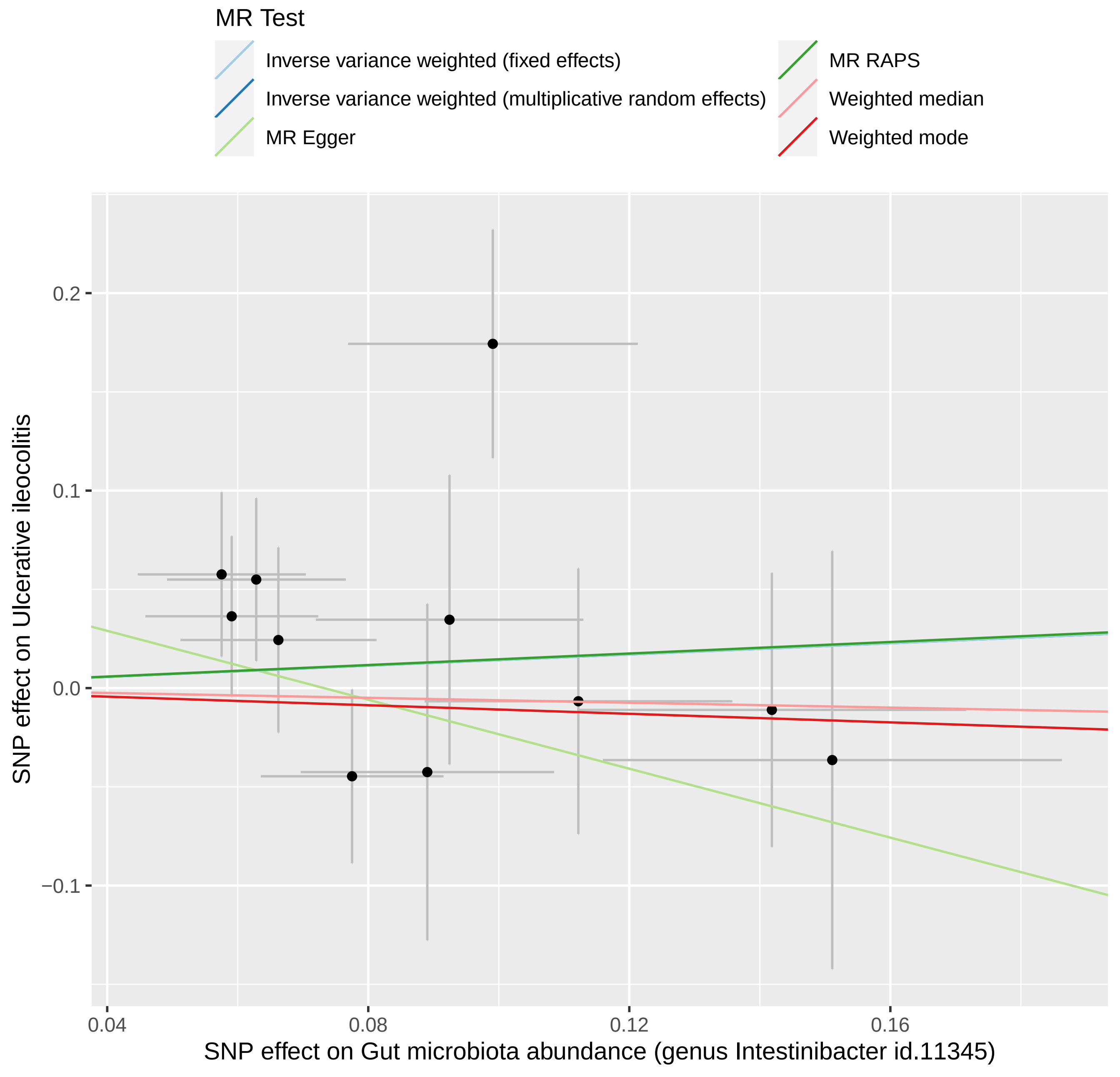

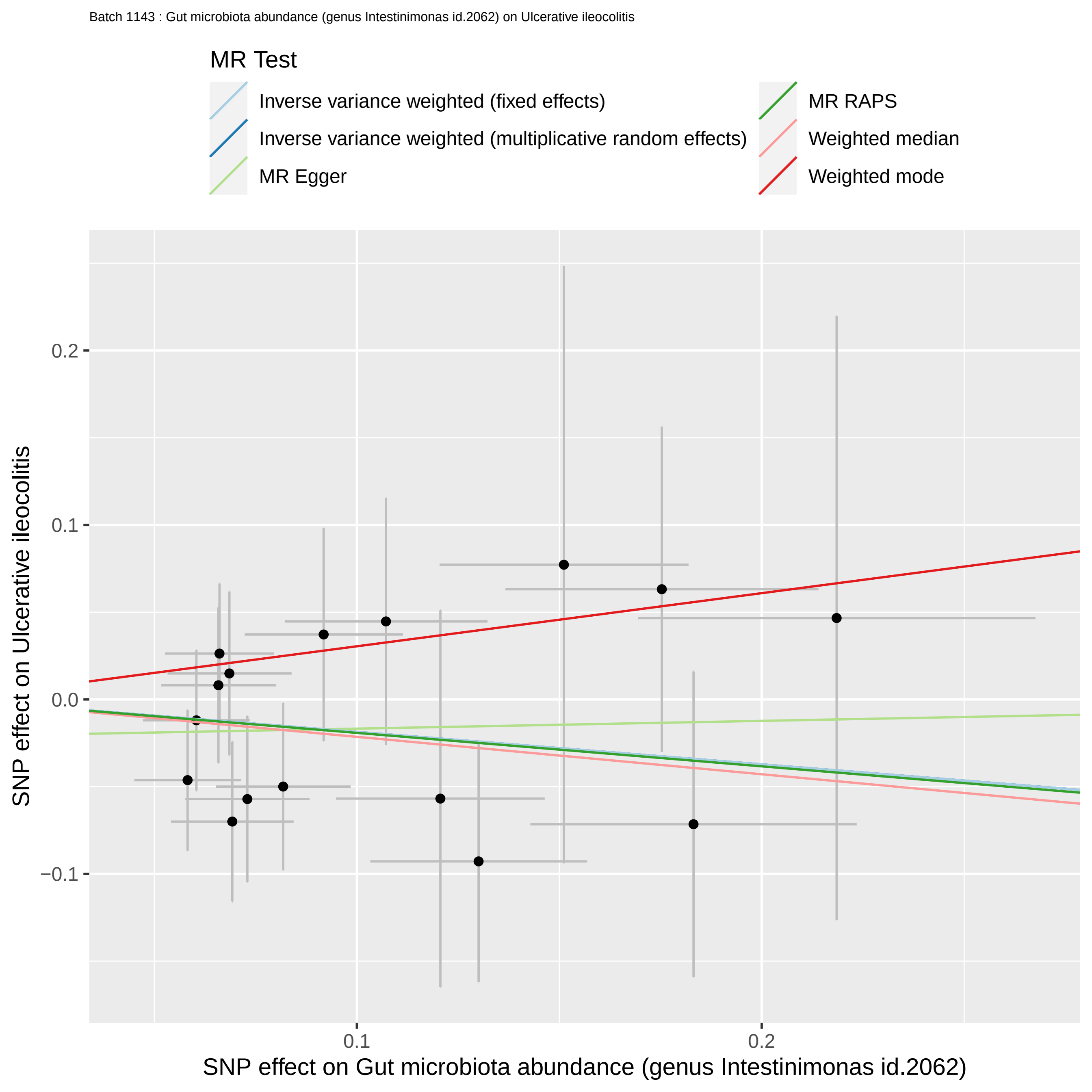

Batch 1143 : Gut microbiota abundance (genus Intestinimonas id.2062) on Ulcerative ileocolitis
MR Test
Inverse variance weighted (fixed effects)
Inverse variance weighted (multiplicative random effects)
MR Egger
MR RAPS
Weighted median
Weighted mode
0.2
0.1
0.0
−0.1
0.1
0.2
SNP effect on Ulcerative ileocolitis
SNP effect on Gut microbiota abundance (genus Intestinimonas id.2062)

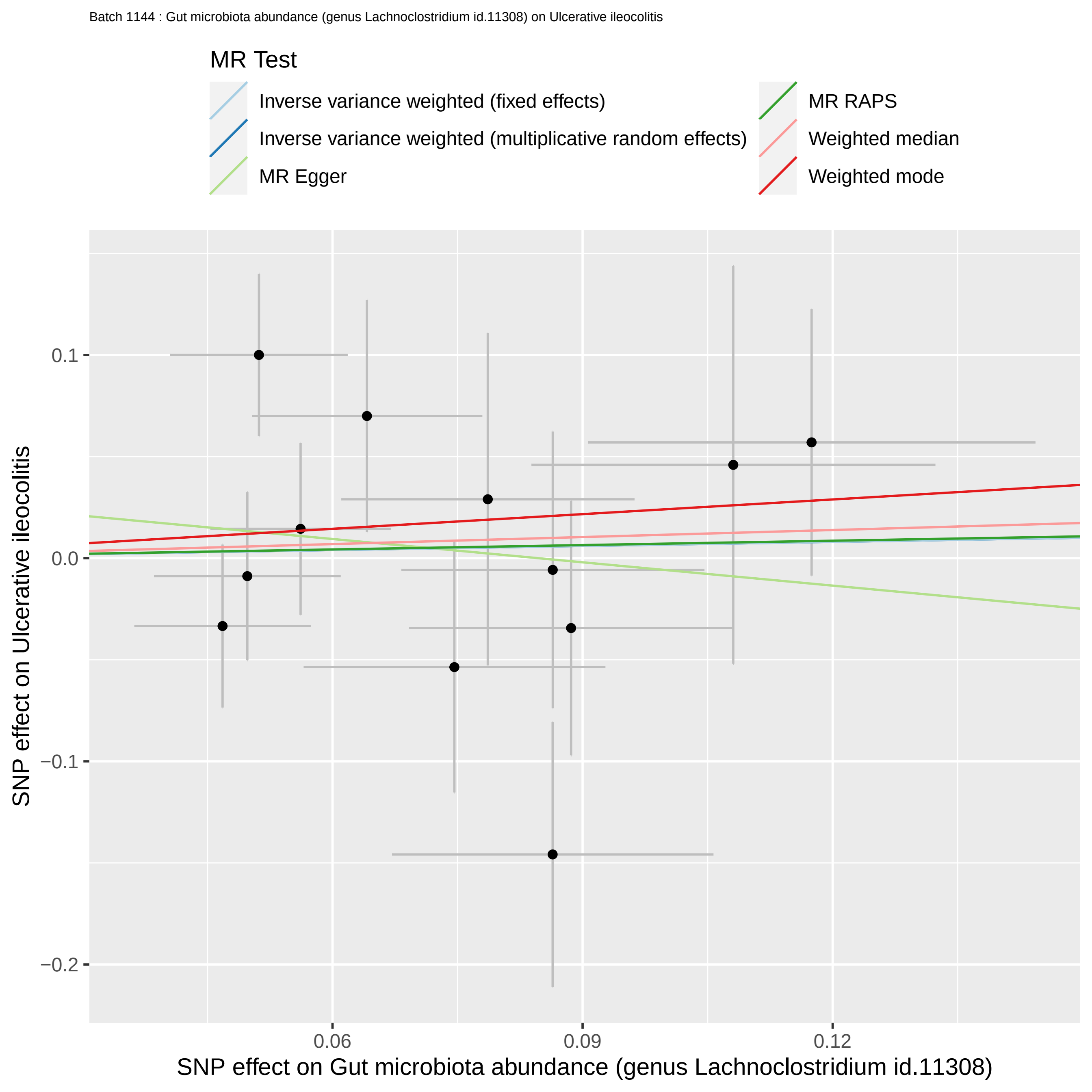

Batch 1144 : Gut microbiota abundance (genus Lachnoclostridium id.11308) on Ulcerative ileocolitis
MR Test
Inverse variance weighted (fixed effects)
Inverse variance weighted (multiplicative random effects)
MR Egger
MR RAPS
Weighted median
Weighted mode
0.1
0.0
−0.1
−0.2
0.06
0.09
0.12
SNP effect on Ulcerative ileocolitis
SNP effect on Gut microbiota abundance (genus Lachnoclostridium id.11308)

Batch 1145 : Gut microbiota abundance (genus Lachnospiraceae FCS020 group id.11314) on Ulcerative ileocolitis

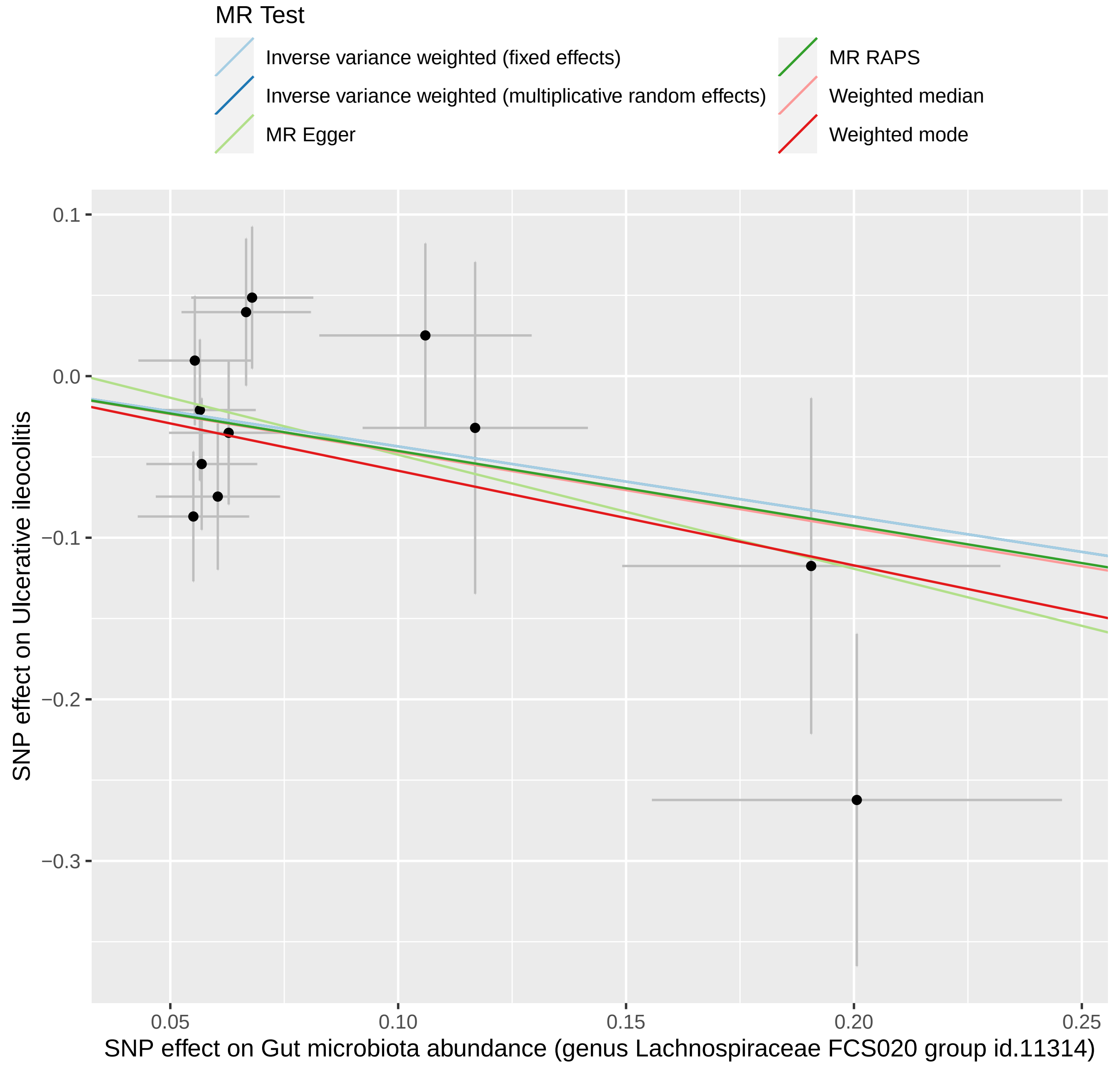

Batch 1146 : Gut microbiota abundance (genus Lachnospiraceae NC2004 group id.11316) on Ulcerative ileocolitis

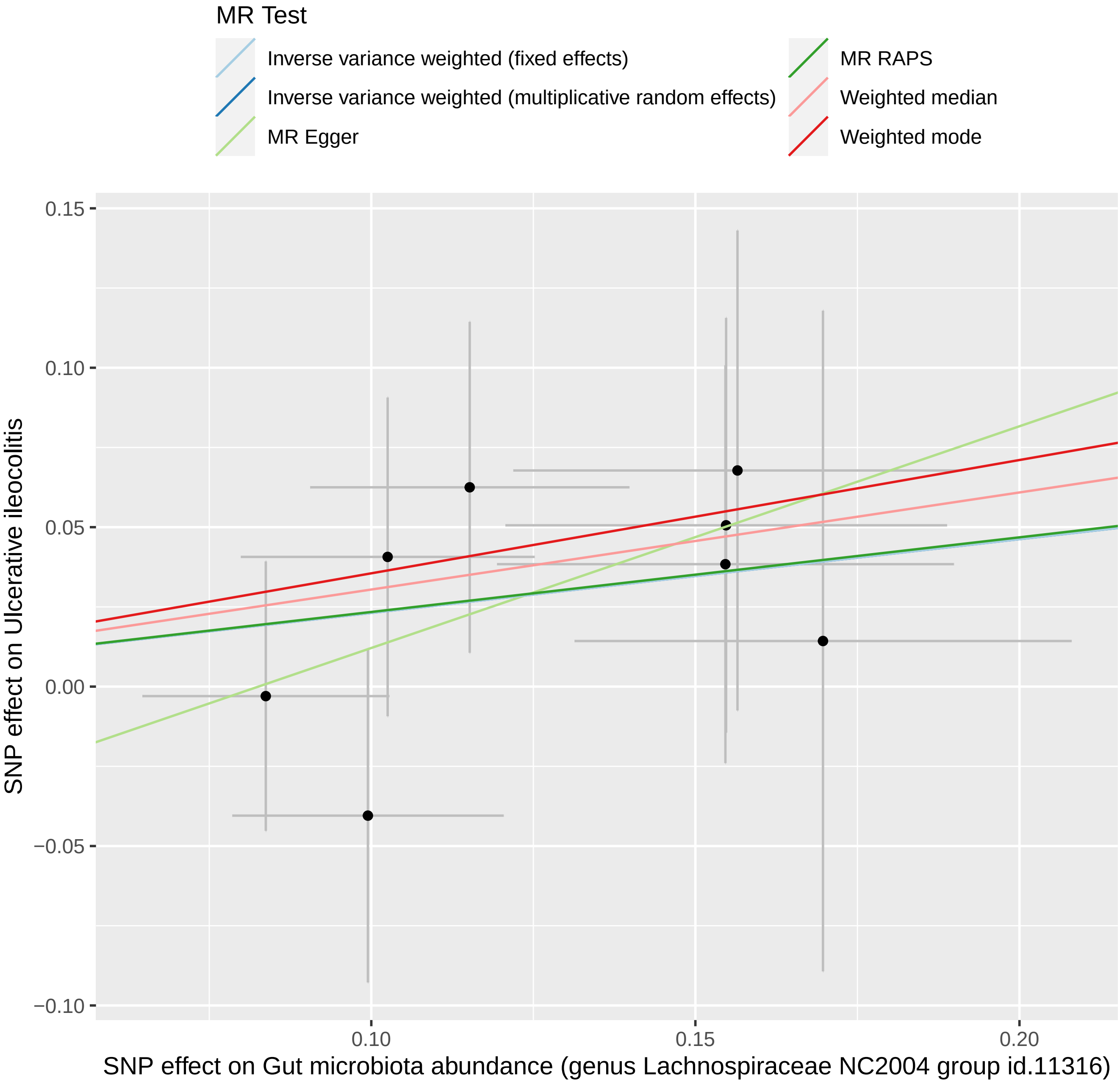

Batch 1147 : Gut microbiota abundance (genus Lachnospiraceae ND3007 group id.11317) on Ulcerative ileocolitis

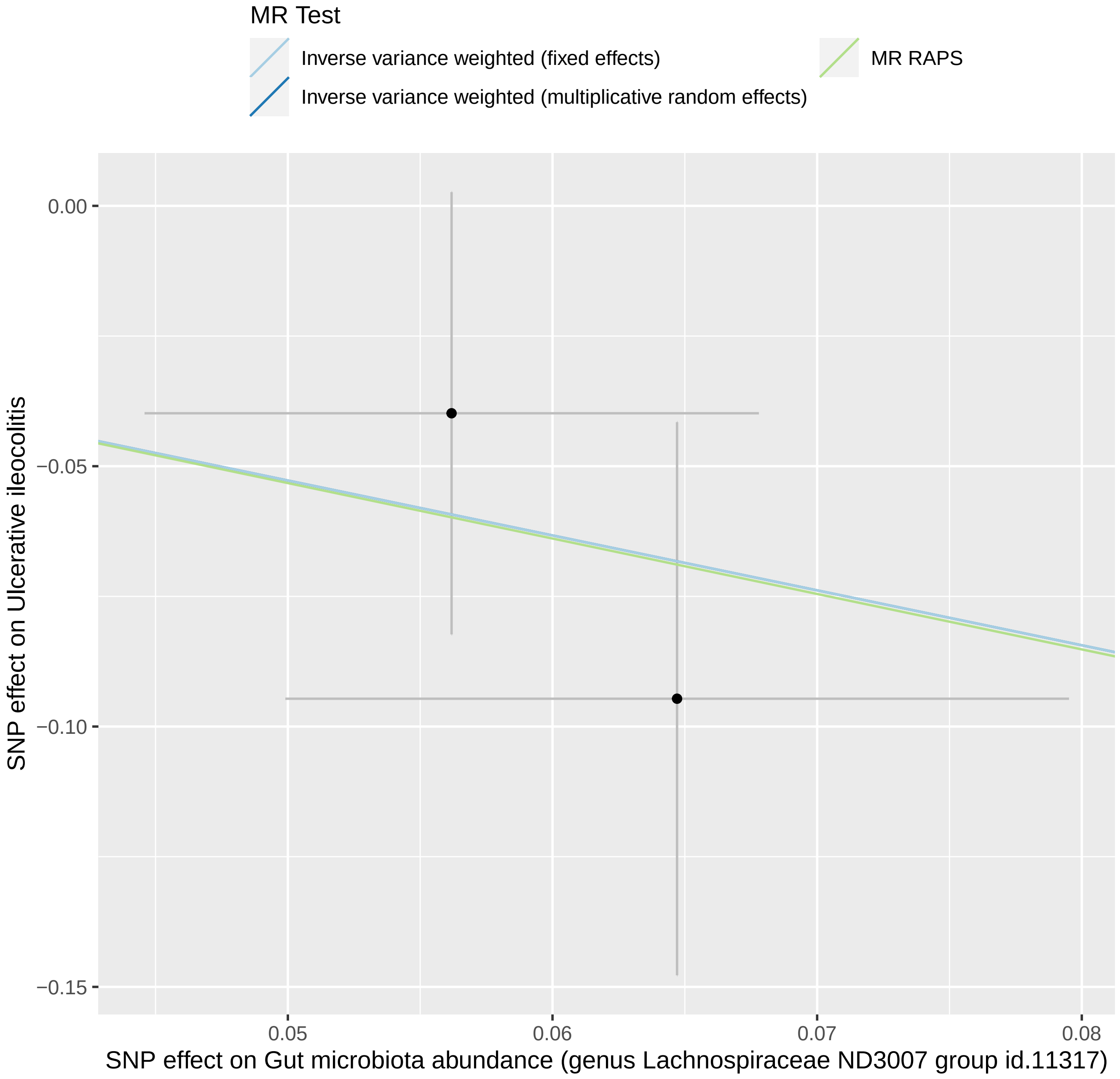

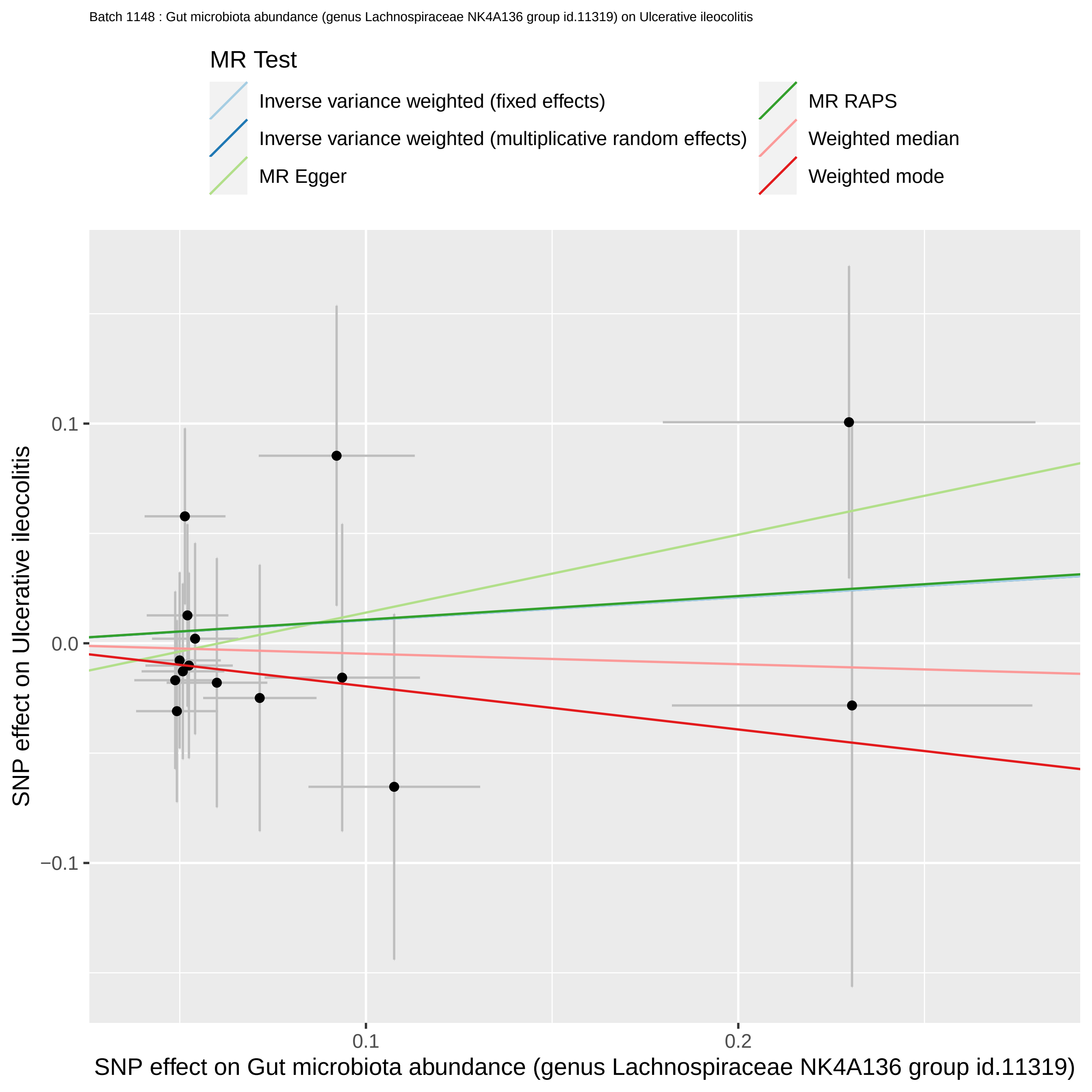

Batch 1148 : Gut microbiota abundance (genus Lachnospiraceae NK4A136 group id.11319) on Ulcerative ileocolitis
MR Test
Inverse variance weighted (fixed effects)
Inverse variance weighted (multiplicative random effects)
MR Egger
MR RAPS
Weighted median
Weighted mode
0.1
0.0
−0.1
0.1
0.2
SNP effect on Ulcerative ileocolitis
SNP effect on Gut microbiota abundance (genus Lachnospiraceae NK4A136 group id.11319)

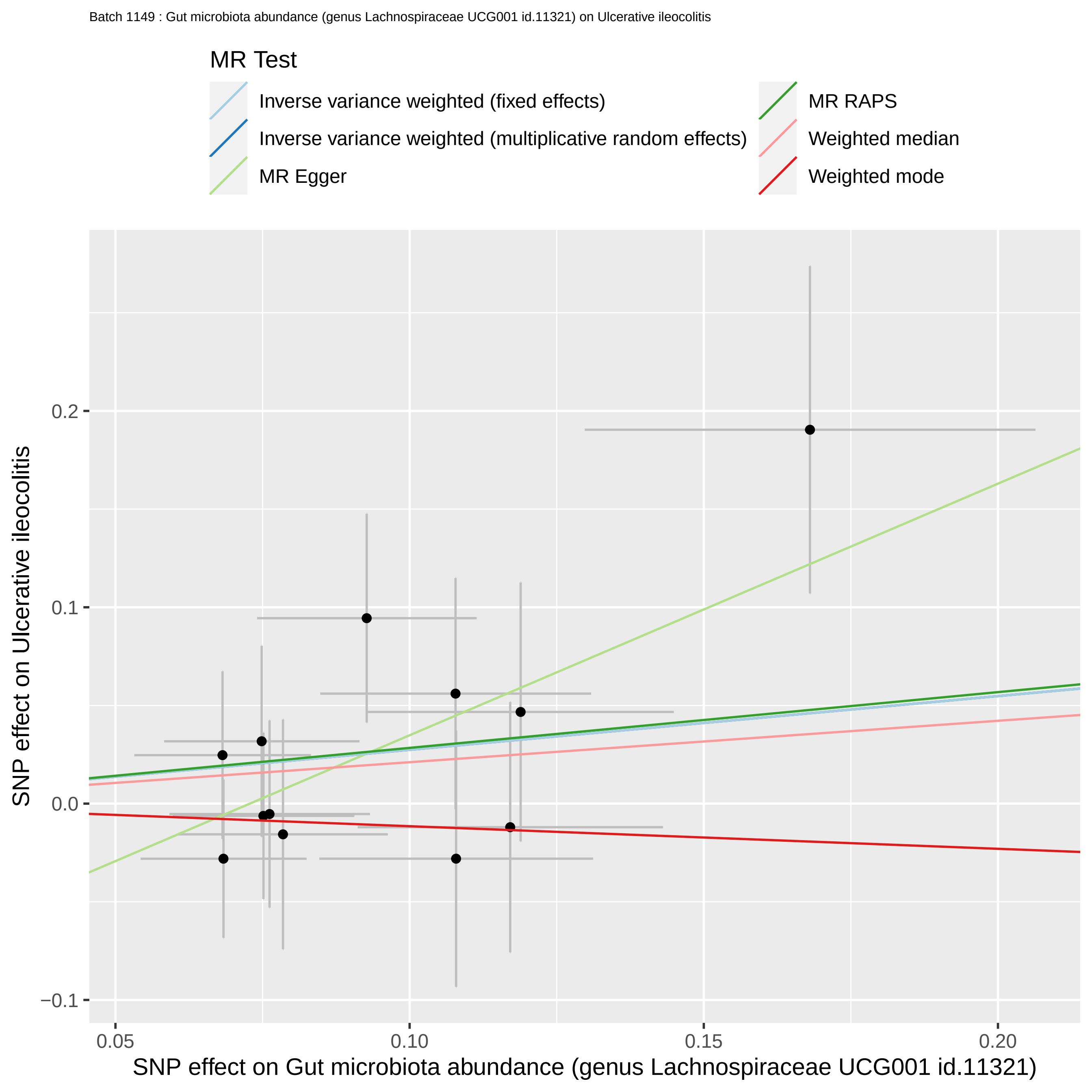
Batch 1149 : Gut microbiota abundance (genus Lachnospiraceae UCG001 id.11321) on Ulcerative ileocolitis
MR Test
Inverse variance weighted (fixed effects)
Inverse variance weighted (multiplicative random effects)
MR Egger
MR RAPS
Weighted median
Weighted mode
SNP effect on Ulcerative ileocolitis
0.2
0.1
0.0
−0.1
0.05
0.10
0.15
0.20
SNP effect on Gut microbiota abundance (genus Lachnospiraceae UCG001 id.11321)

Batch 1150 : Gut microbiota abundance (genus Lachnospiraceae UCG004 id.11324) on Ulcerative ileocolitis

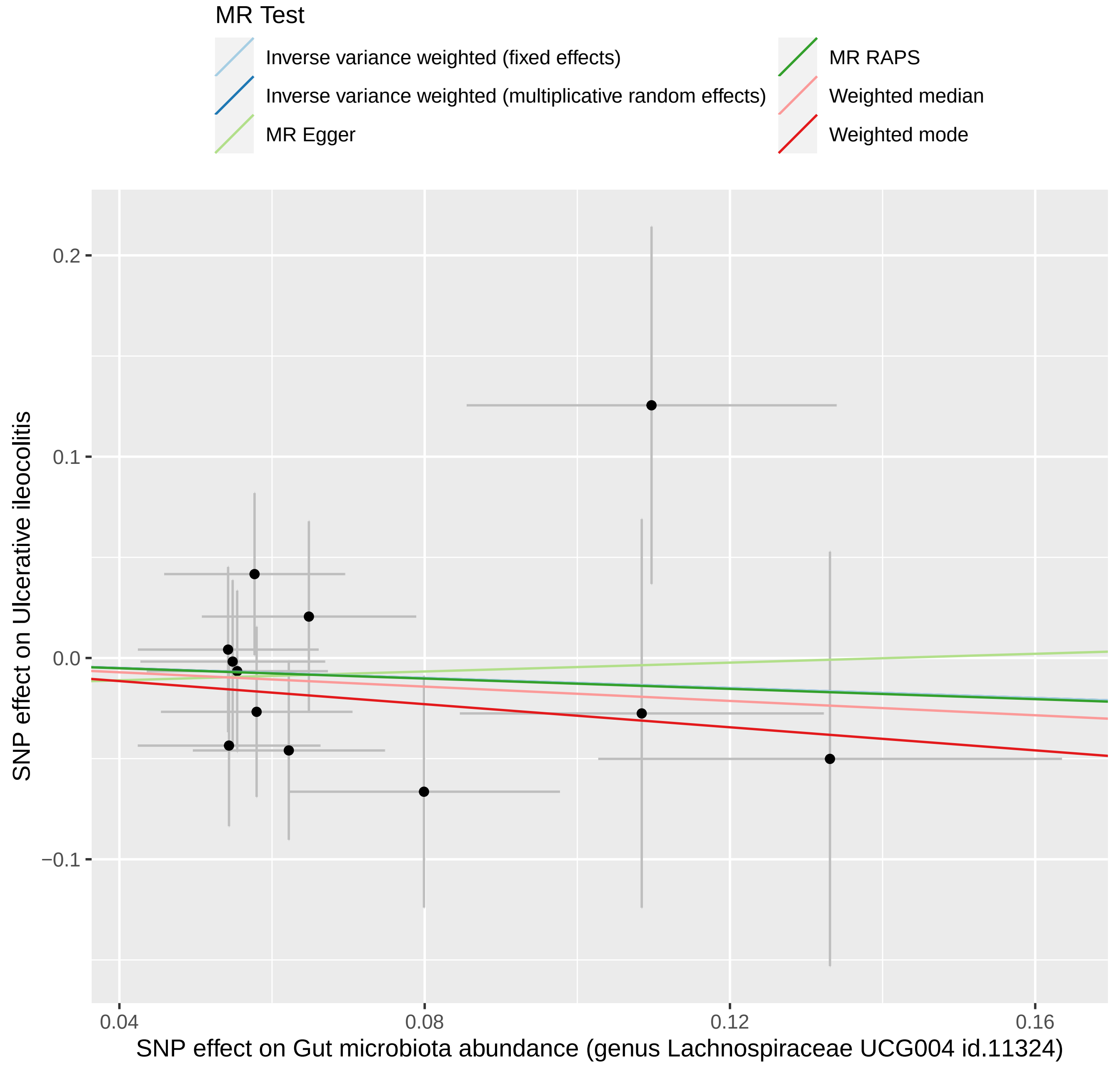

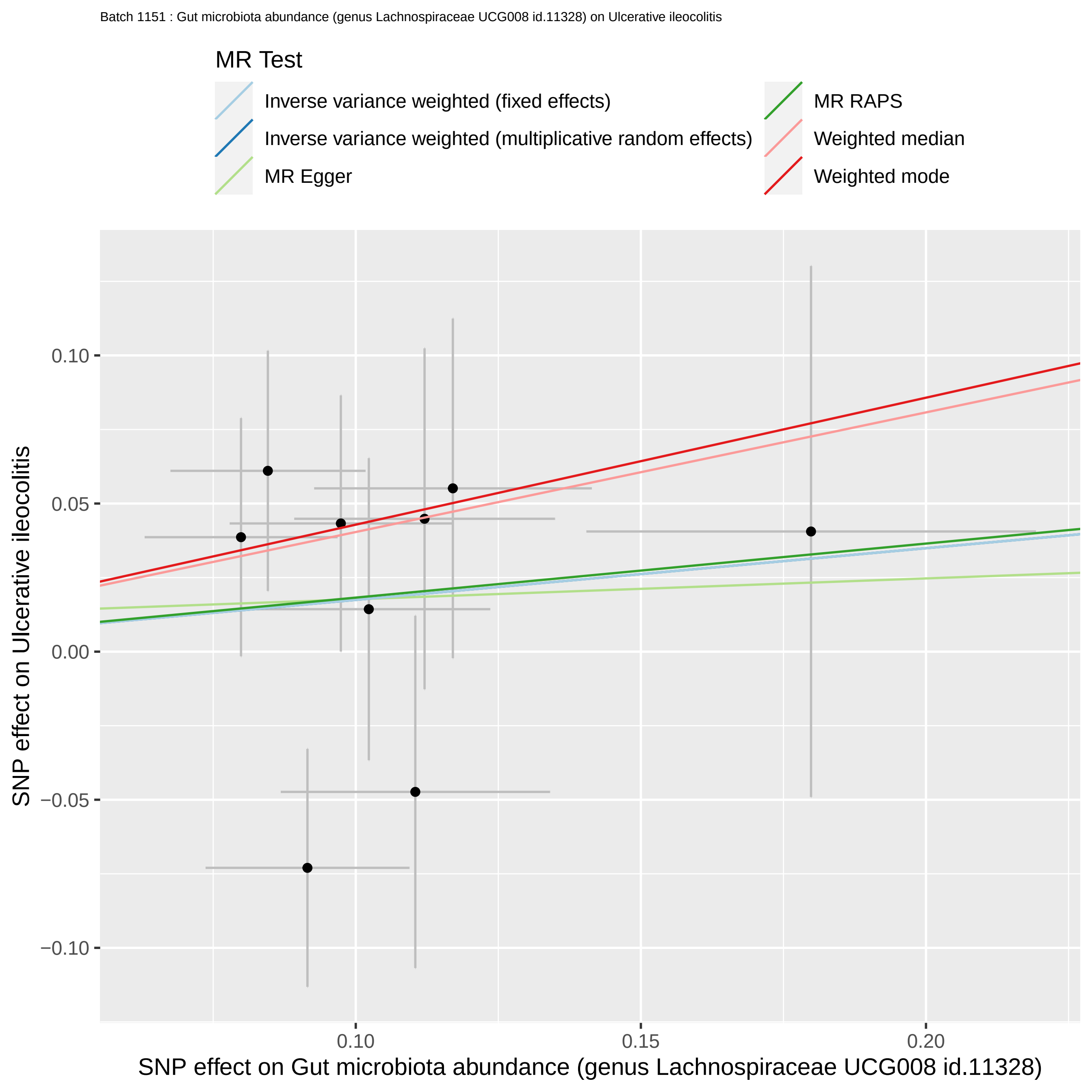

Batch 1151 : Gut microbiota abundance (genus Lachnospiraceae UCG008 id.11328) on Ulcerative ileocolitis
MR Test
Inverse variance weighted (fixed effects)
Inverse variance weighted (multiplicative random effects)
MR Egger
MR RAPS
Weighted median
Weighted mode
SNP effect on Ulcerative ileocolitis
0.10
0.05
0.00
−0.05
−0.10
0.10
0.15
0.20
SNP effect on Gut microbiota abundance (genus Lachnospiraceae UCG008 id.11328)

Batch 1152 : Gut microbiota abundance (genus Lachnospiraceae UCG010 id.11330) on Ulcerative ileocolitis

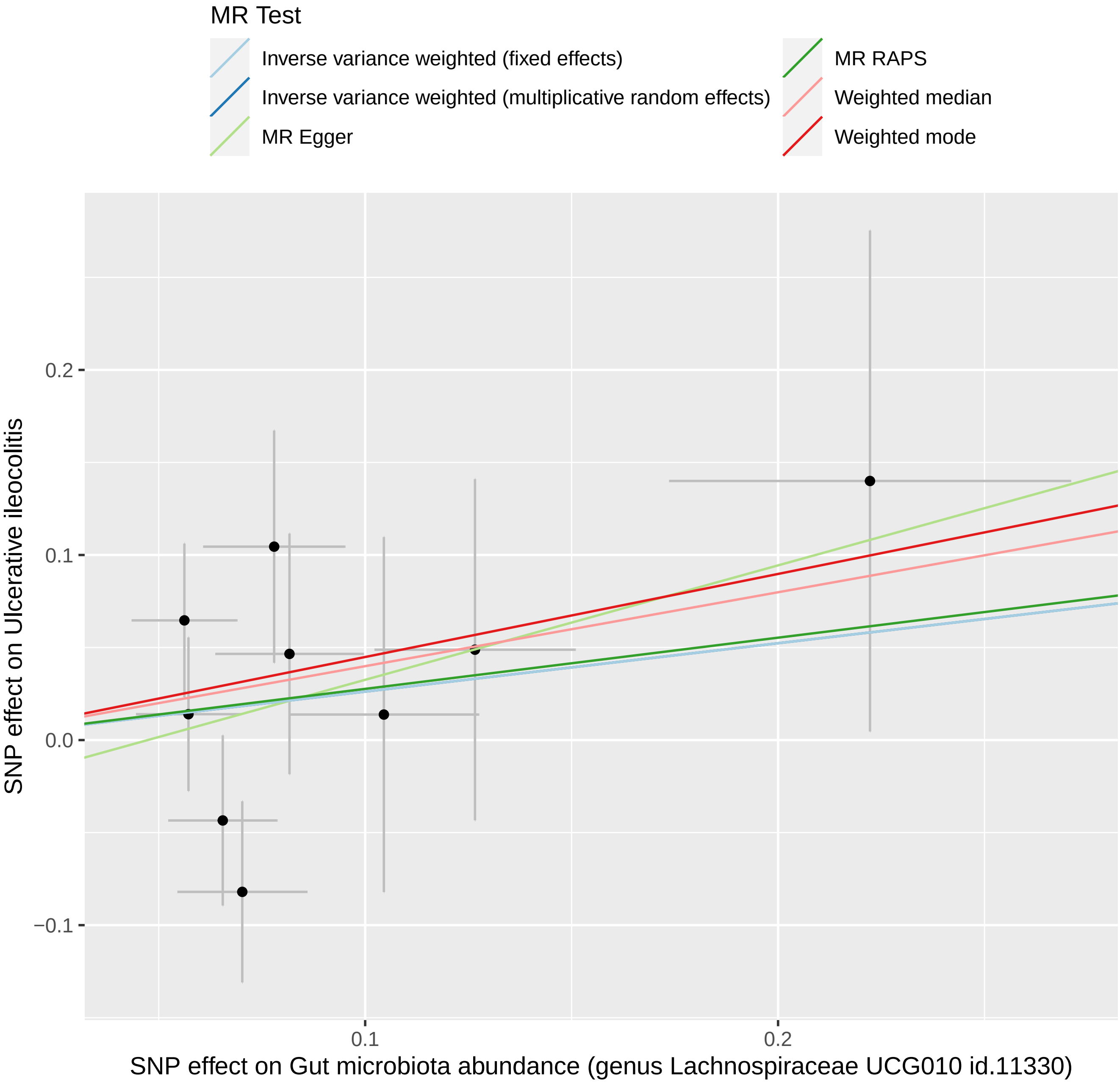

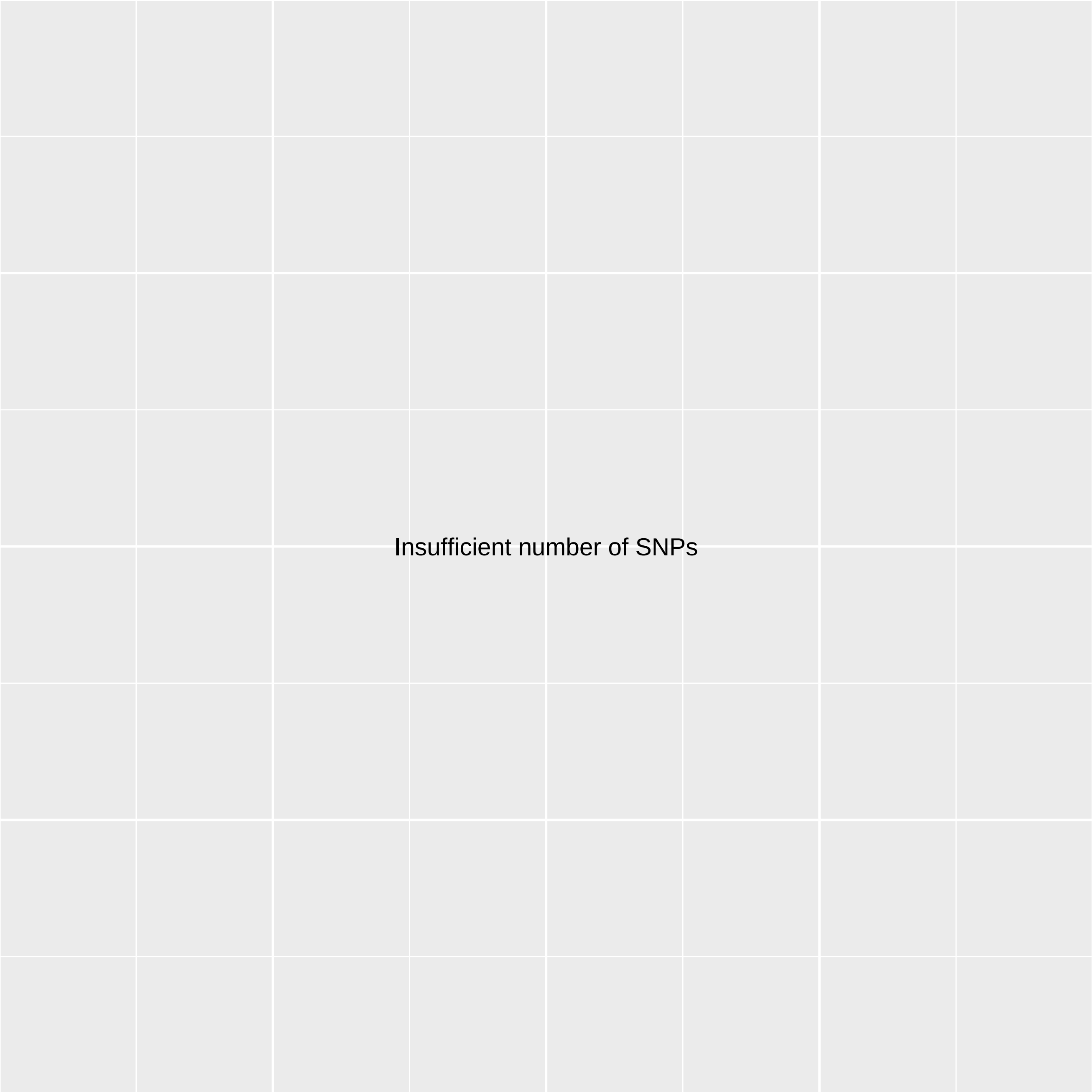
Insufficient number of SNPs

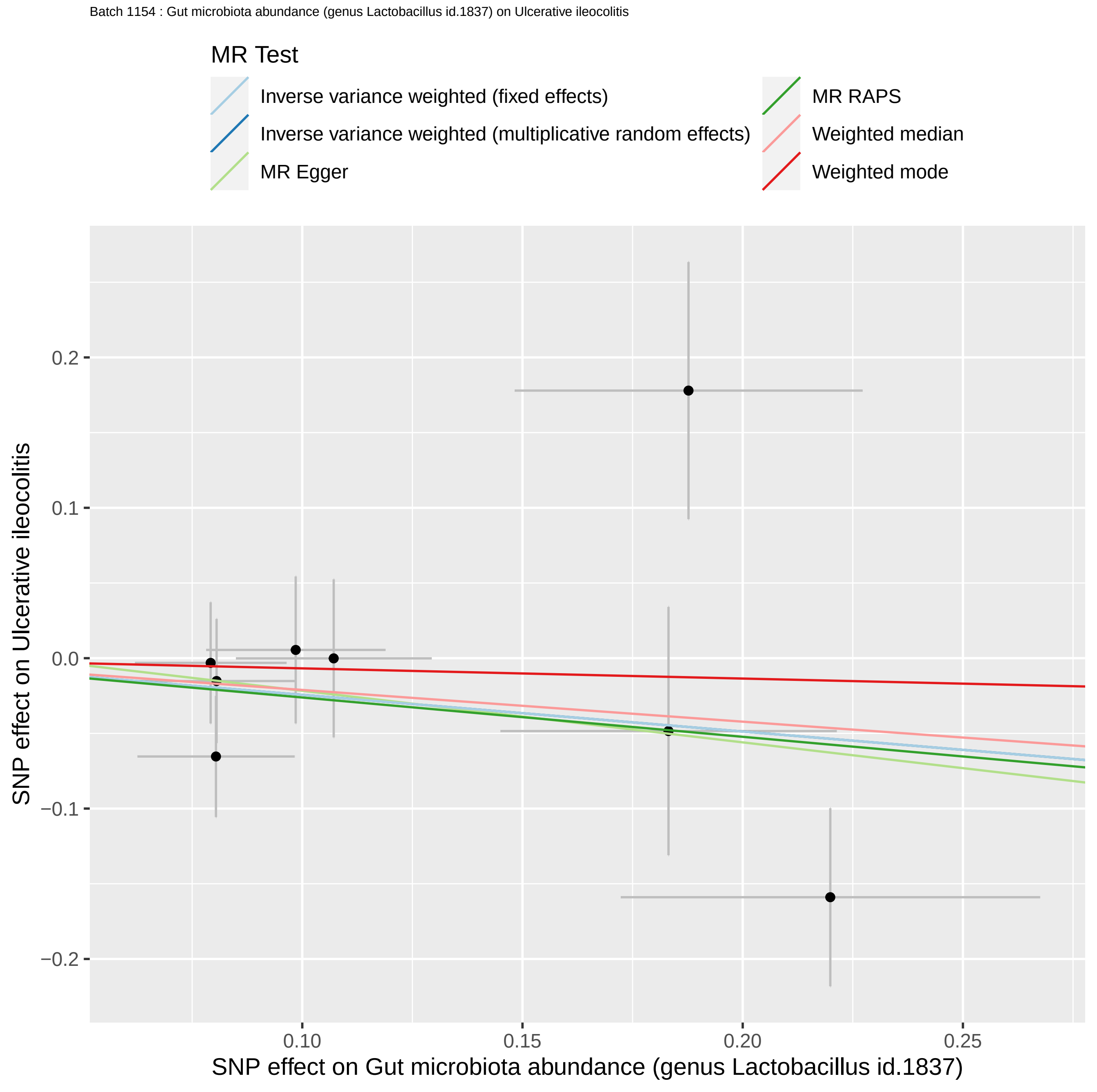
Batch 1154 : Gut microbiota abundance (genus Lactobacillus id.1837) on Ulcerative ileocolitis
MR Test
Inverse variance weighted (fixed effects)
Inverse variance weighted (multiplicative random effects)
MR Egger
MR RAPS
Weighted median
Weighted mode
SNP effect on Ulcerative ileocolitis
0.2
0.1
0.0
−0.1
−0.2
0.10
0.15
0.20
0.25
SNP effect on Gut microbiota abundance (genus Lactobacillus id.1837)

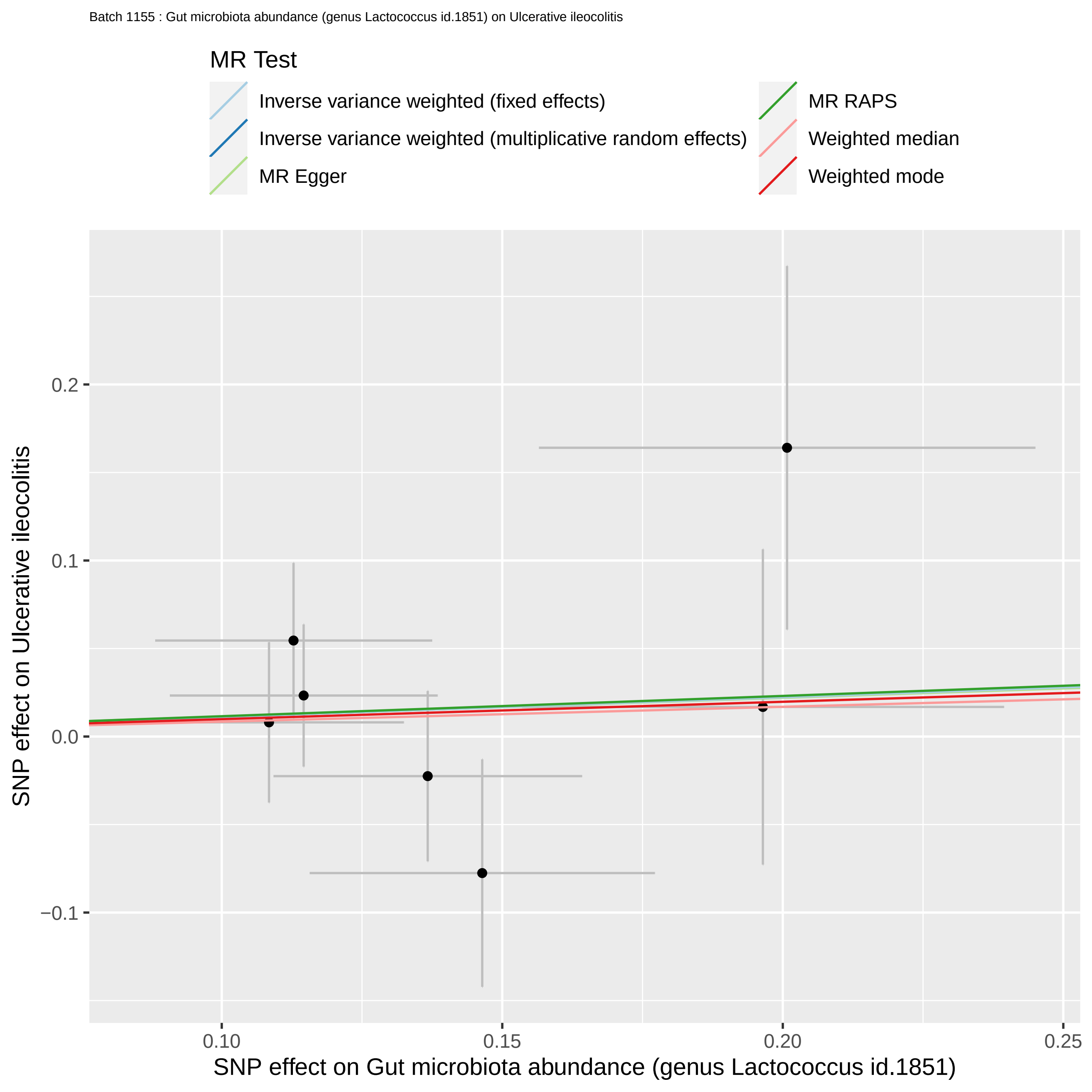

Batch 1155 : Gut microbiota abundance (genus Lactococcus id.1851) on Ulcerative ileocolitis
MR Test
Inverse variance weighted (fixed effects)
Inverse variance weighted (multiplicative random effects)
MR Egger
MR RAPS
Weighted median
Weighted mode
SNP effect on Ulcerative ileocolitis
0.2
0.1
0.0
−0.1
0.10
0.15
0.20
0.25
SNP effect on Gut microbiota abundance (genus Lactococcus id.1851)

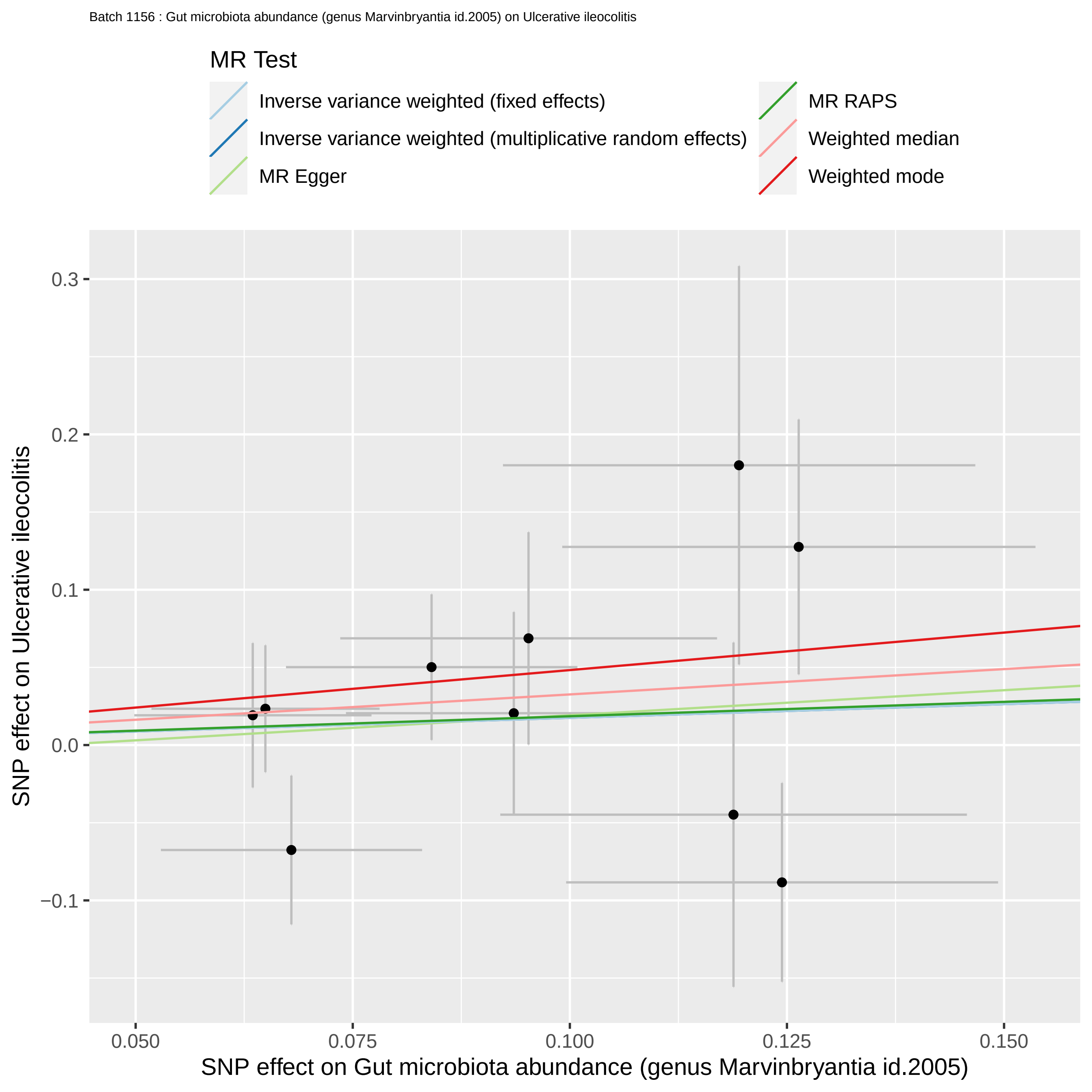
Batch 1156 : Gut microbiota abundance (genus Marvinbryantia id.2005) on Ulcerative ileocolitis
MR Test
Inverse variance weighted (fixed effects)
Inverse variance weighted (multiplicative random effects)
MR Egger
MR RAPS
Weighted median
Weighted mode
SNP effect on Ulcerative ileocolitis
0.3
0.2
0.1
0.0
−0.1
0.050
0.075
0.100
0.125
0.150
SNP effect on Gut microbiota abundance (genus Marvinbryantia id.2005)

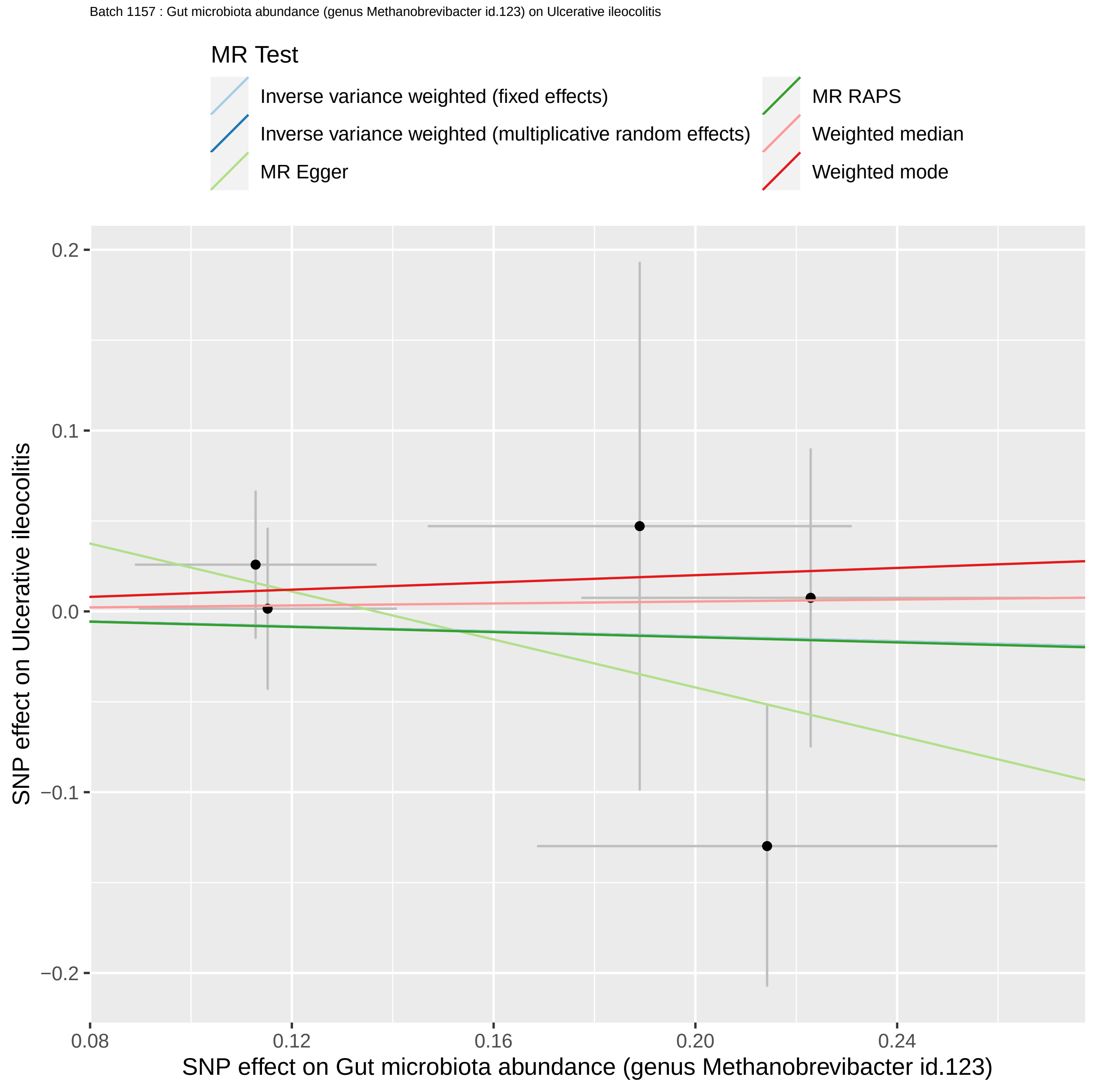
Batch 1157 : Gut microbiota abundance (genus Methanobrevibacter id.123) on Ulcerative ileocolitis
MR Test
Inverse variance weighted (fixed effects)
Inverse variance weighted (multiplicative random effects)
MR Egger
MR RAPS
Weighted median
Weighted mode
SNP effect on Ulcerative ileocolitis
0.2
0.1
0.0
−0.1
−0.2
0.08
0.12
0.16
0.20
0.24
SNP effect on Gut microbiota abundance (genus Methanobrevibacter id.123)

Batch 1158 : Gut microbiota abundance (genus Odoribacter id.952) on Ulcerative ileocolitis

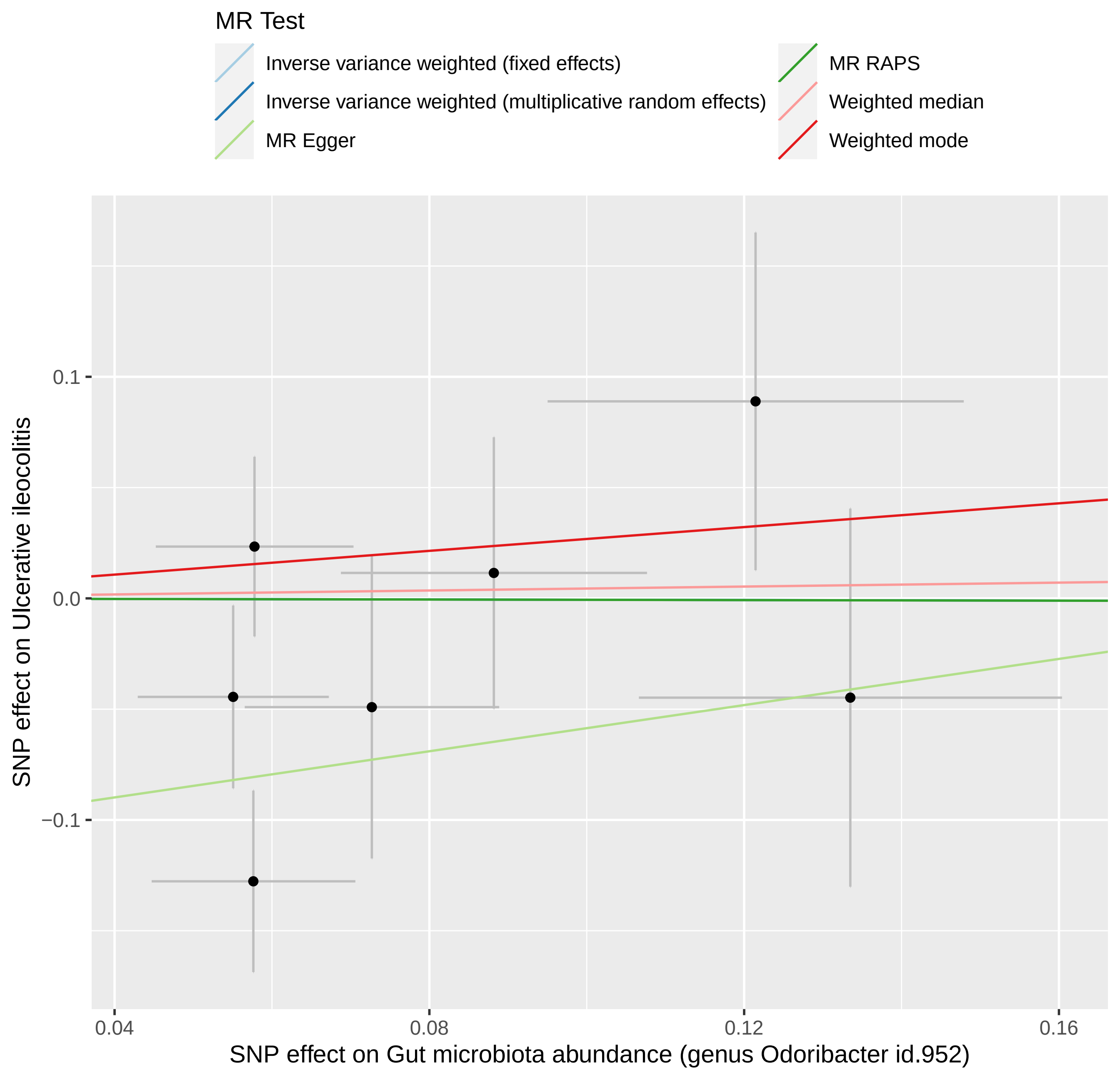

Batch 1159 : Gut microbiota abundance (genus Olsenella id.822) on Ulcerative ileocolitis

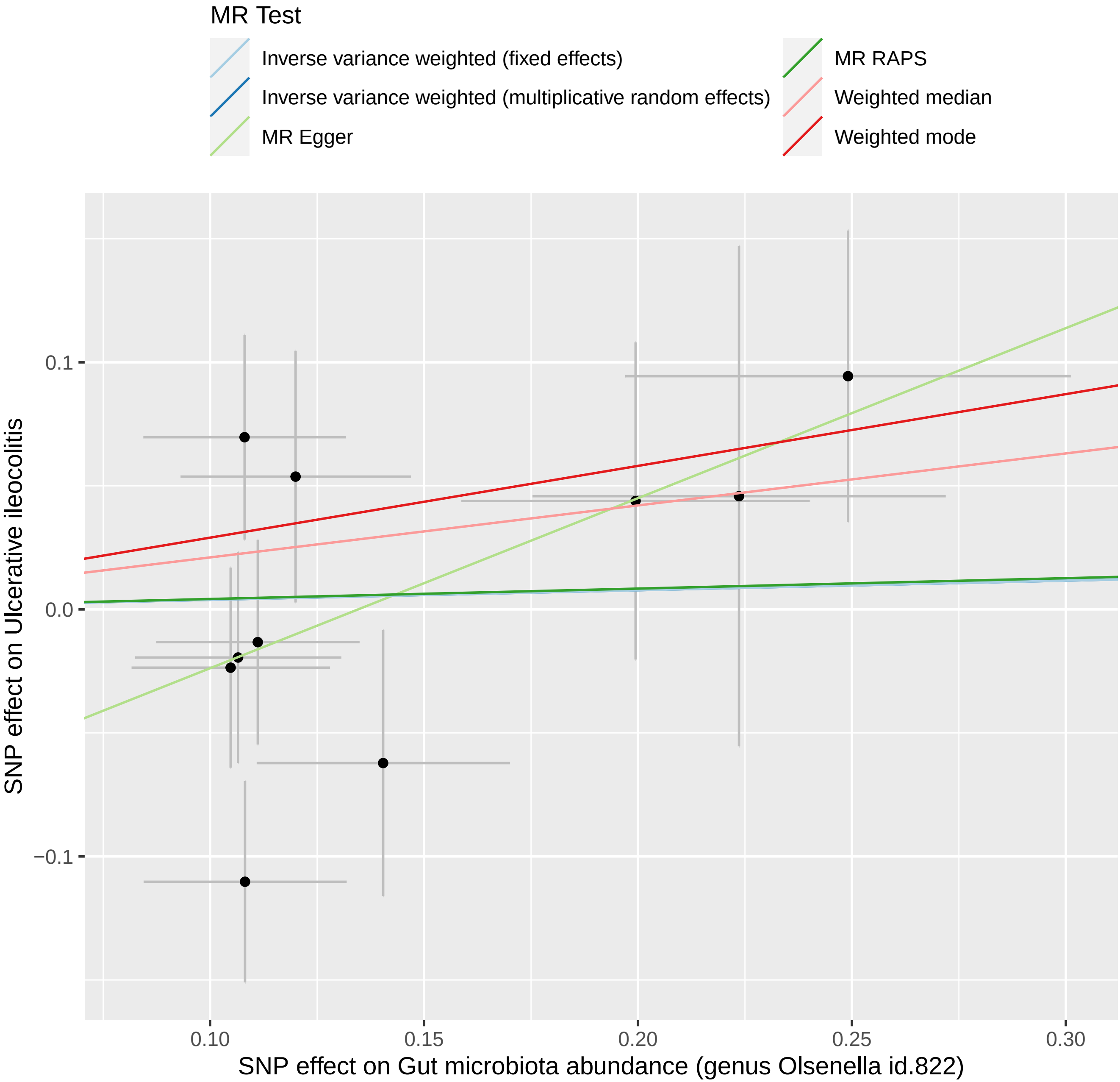

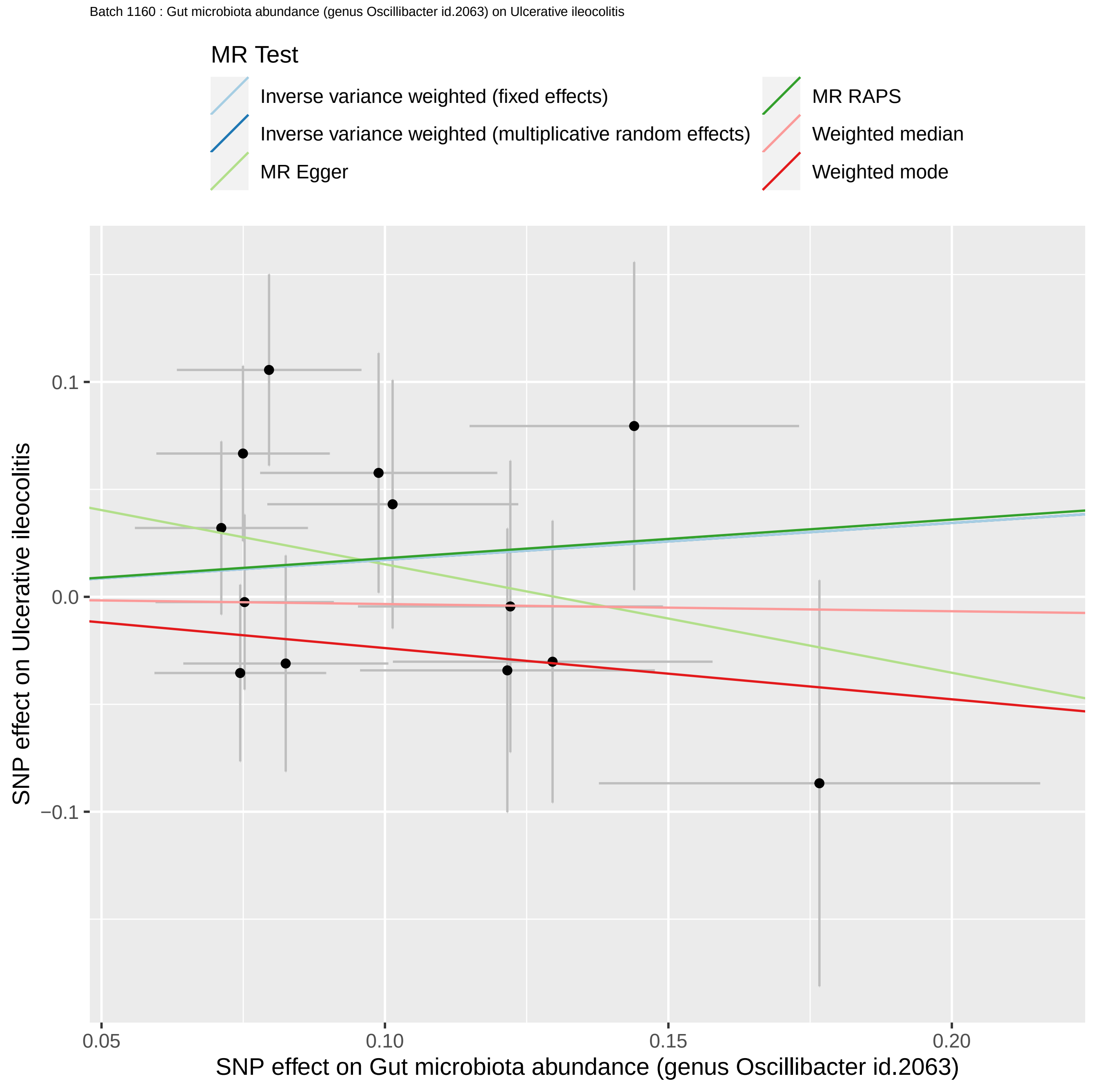
Batch 1160 : Gut microbiota abundance (genus Oscillibacter id.2063) on Ulcerative ileocolitis
MR Test
Inverse variance weighted (fixed effects)
Inverse variance weighted (multiplicative random effects)
MR Egger
MR RAPS
Weighted median
Weighted mode
0.1
0.0
−0.1
0.05
0.10
0.15
0.20
SNP effect on Ulcerative ileocolitis
SNP effect on Gut microbiota abundance (genus Oscillibacter id.2063)

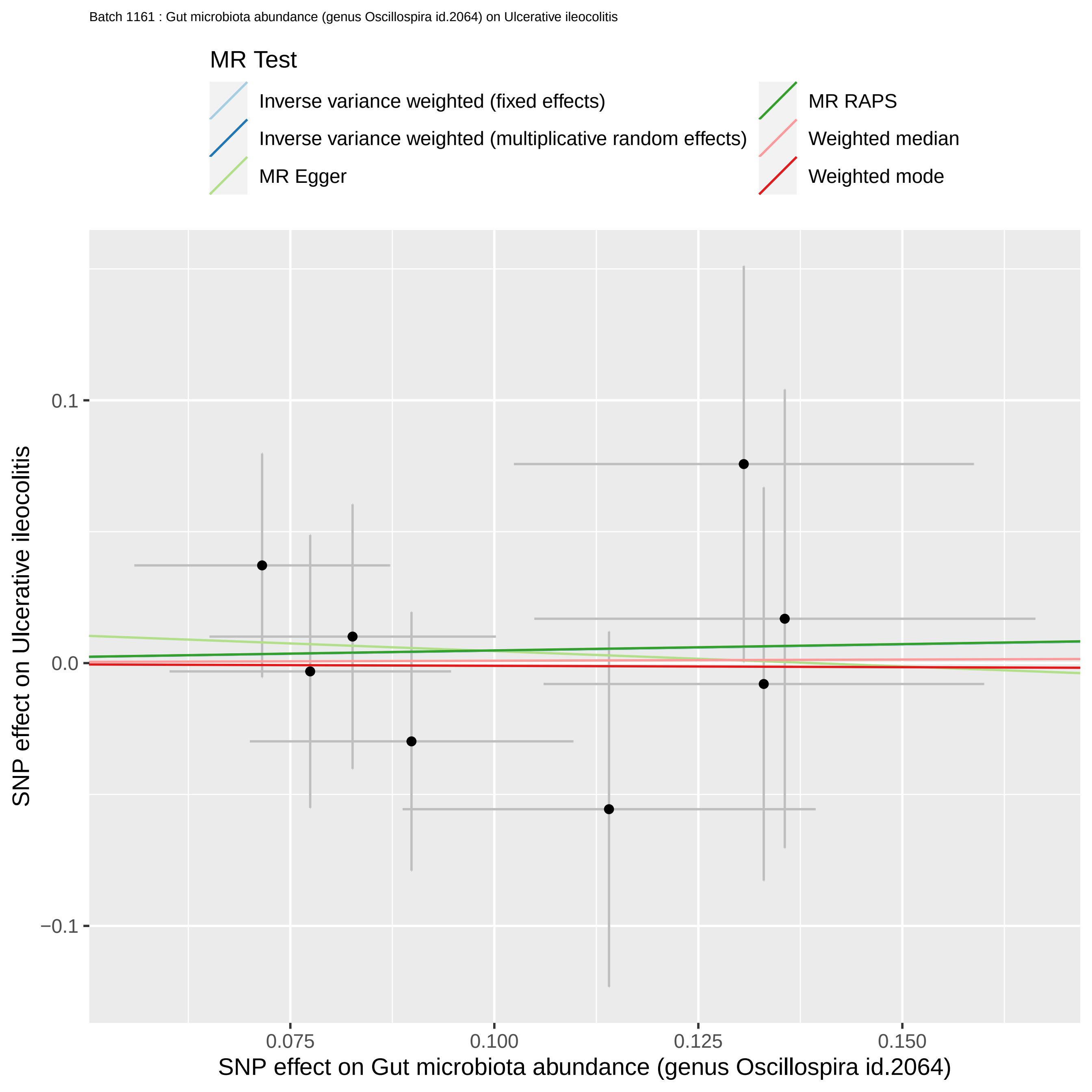

Batch 1161 : Gut microbiota abundance (genus Oscillospira id.2064) on Ulcerative ileocolitis
MR Test
Inverse variance weighted (fixed effects)
Inverse variance weighted (multiplicative random effects)
MR Egger
MR RAPS
Weighted median
Weighted mode
0.1
0.0
−0.1
0.075
0.100
0.125
0.150
SNP effect on Ulcerative ileocolitis
SNP effect on Gut microbiota abundance (genus Oscillospira id.2064)

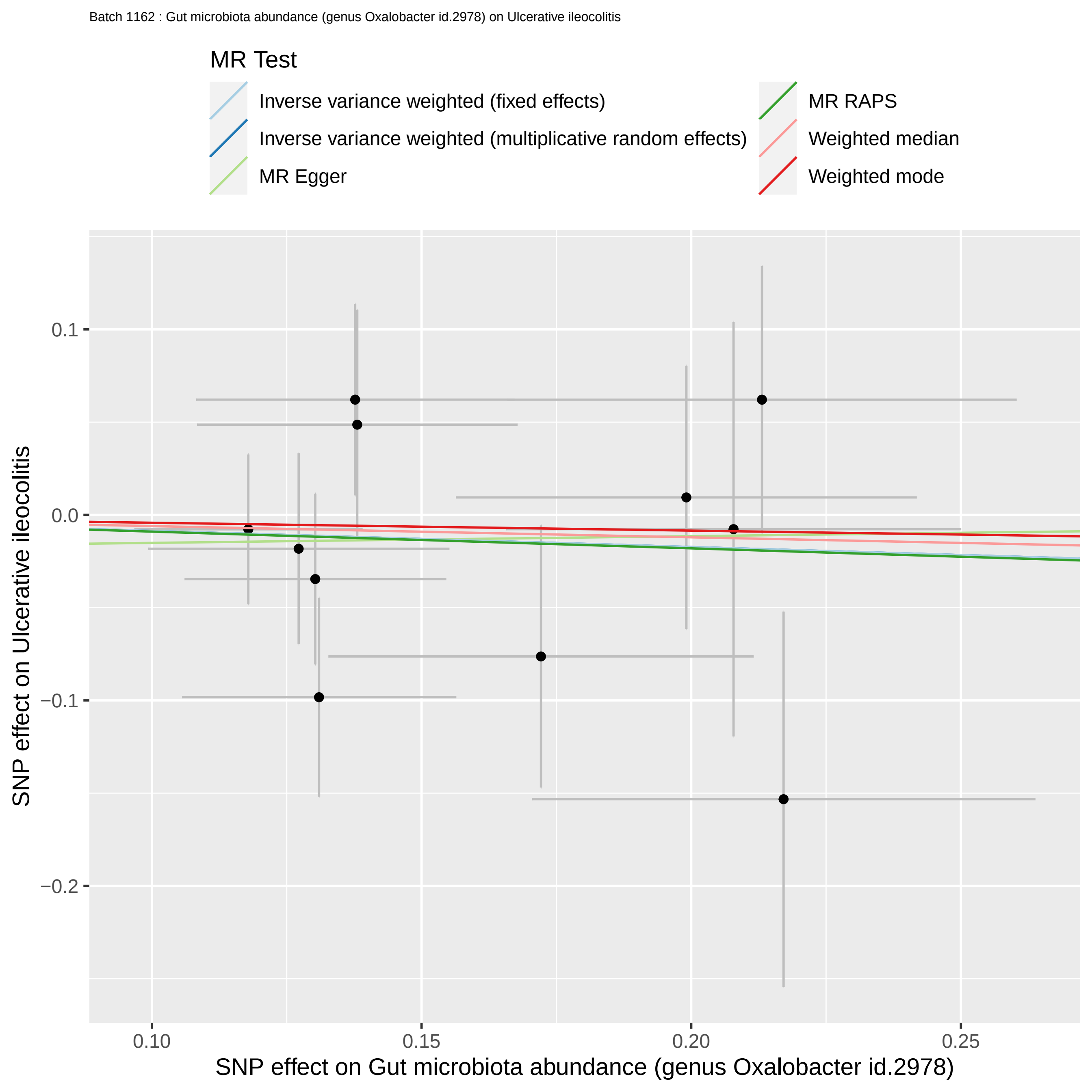
Batch 1162 : Gut microbiota abundance (genus Oxalobacter id.2978) on Ulcerative ileocolitis
MR Test
Inverse variance weighted (fixed effects)
Inverse variance weighted (multiplicative random effects)
MR Egger
MR RAPS
Weighted median
Weighted mode
0.1
0.0
−0.1
−0.2
0.10
0.15
0.20
0.25
SNP effect on Ulcerative ileocolitis
SNP effect on Gut microbiota abundance (genus Oxalobacter id.2978)

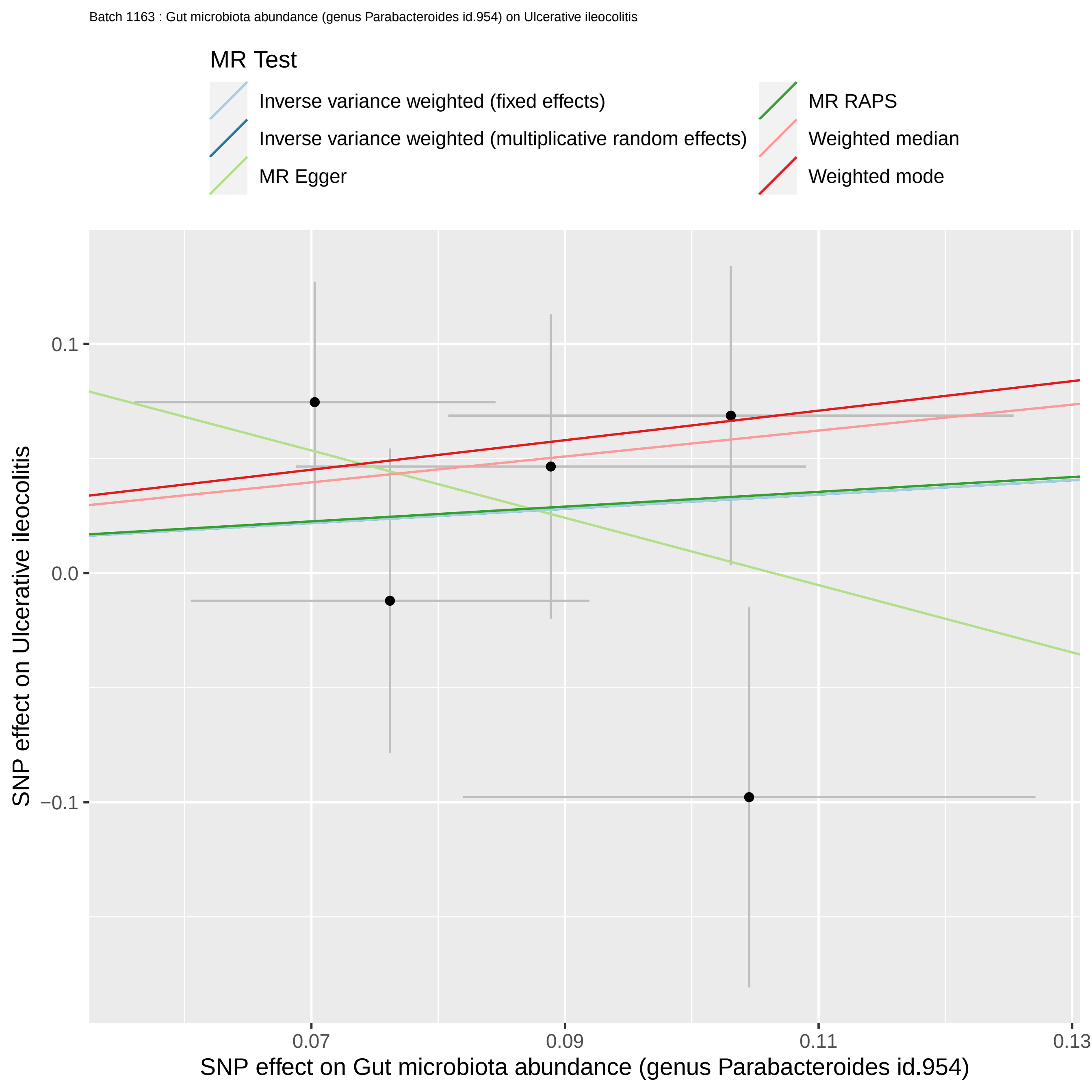

Batch 1163 : Gut microbiota abundance (genus Parabacteroides id.954) on Ulcerative ileocolitis
MR Test
Inverse variance weighted (fixed effects)
Inverse variance weighted (multiplicative random effects)
MR Egger
MR RAPS
Weighted median
Weighted mode
0.1
0.0
−0.1
0.07
0.09
0.11
0.13
SNP effect on Ulcerative ileocolitis
SNP effect on Gut microbiota abundance (genus Parabacteroides id.954)

Batch 1164 : Gut microbiota abundance (genus Paraprevotella id.962) on Ulcerative ileocolitis

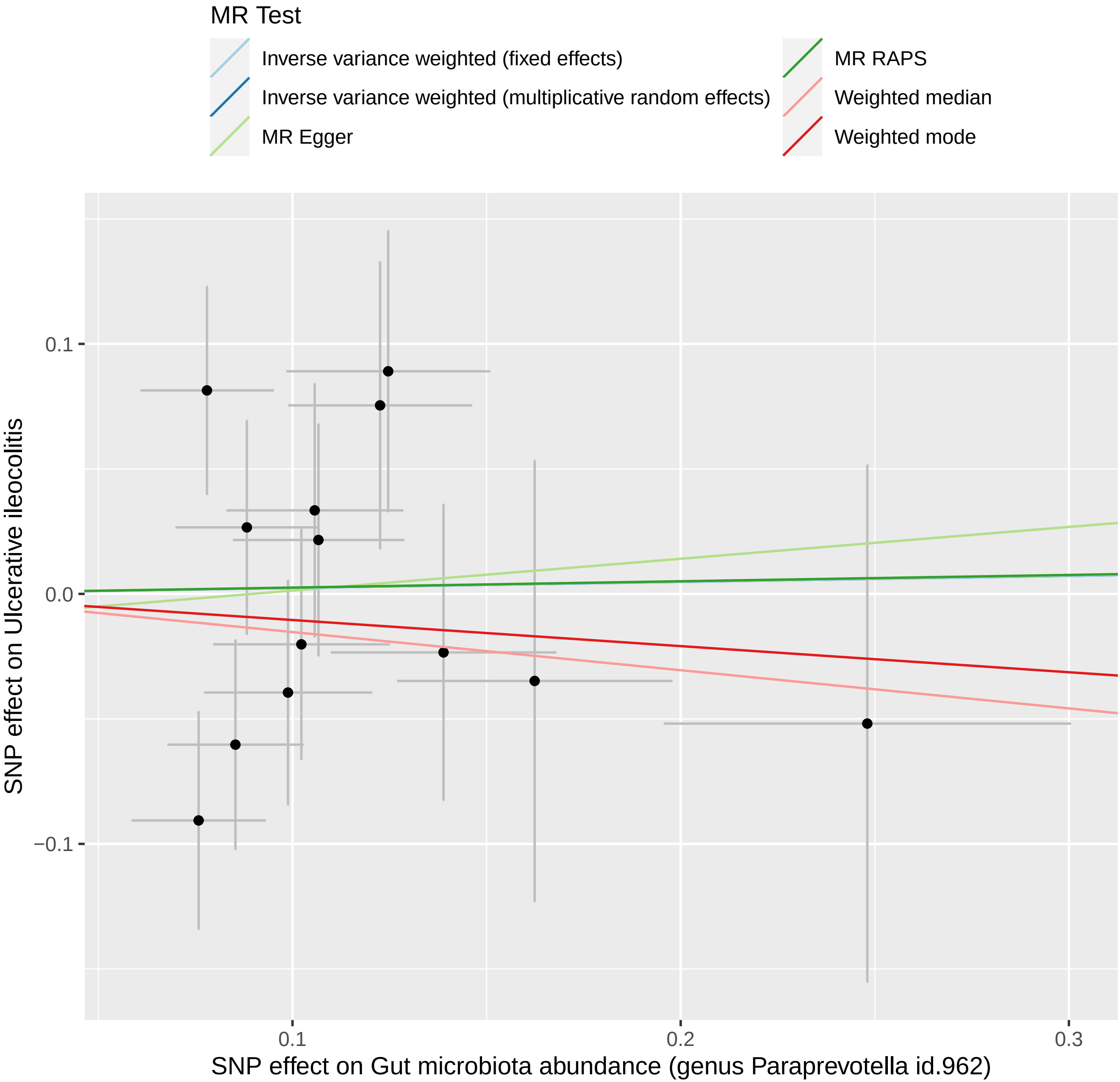

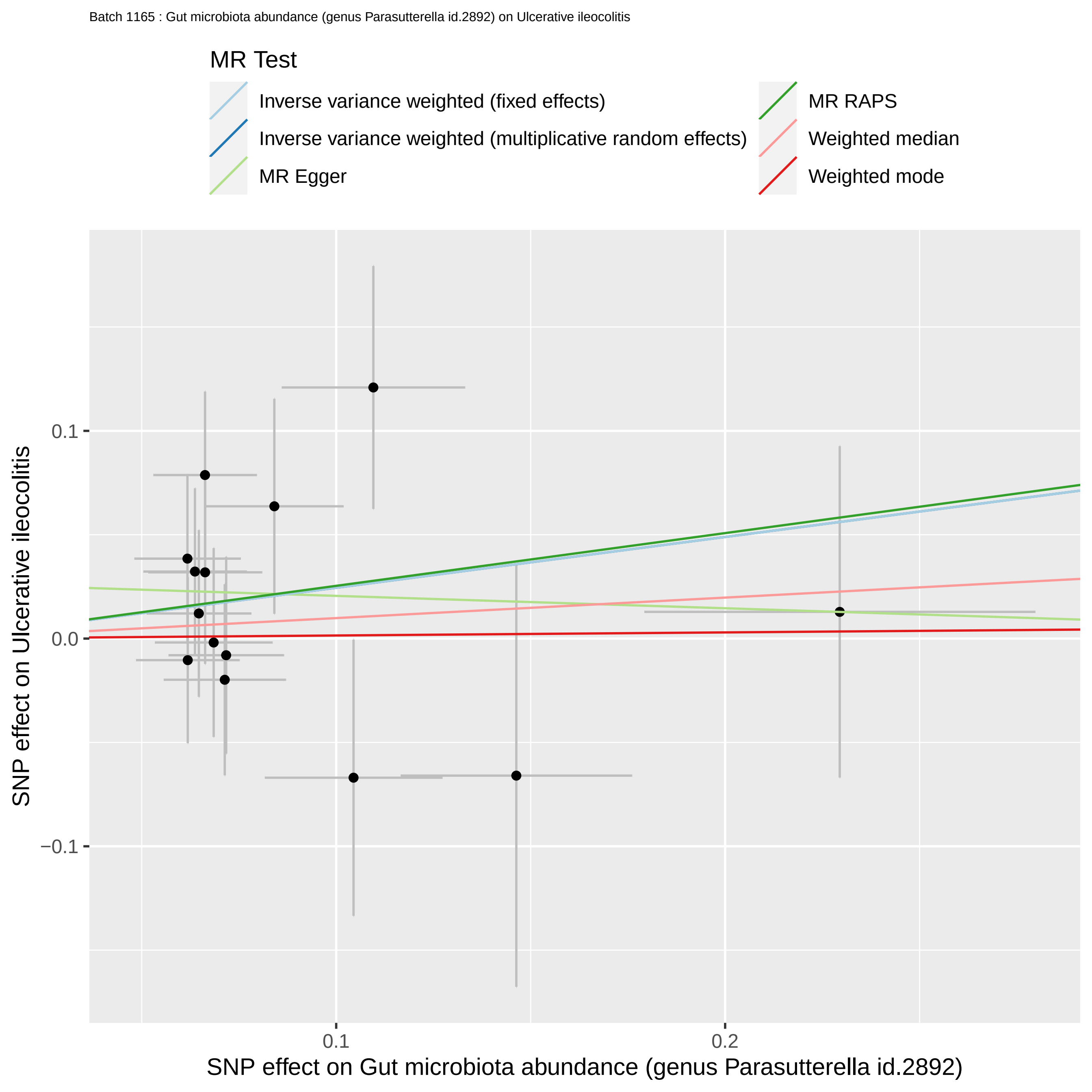

Batch 1165 : Gut microbiota abundance (genus Parasutterella id.2892) on Ulcerative ileocolitis
MR Test
Inverse variance weighted (fixed effects)
Inverse variance weighted (multiplicative random effects)
MR Egger
MR RAPS
Weighted median
Weighted mode
SNP effect on Ulcerative ileocolitis
0.1
0.0
−0.1
0.1
0.2
SNP effect on Gut microbiota abundance (genus Parasutterella id.2892)

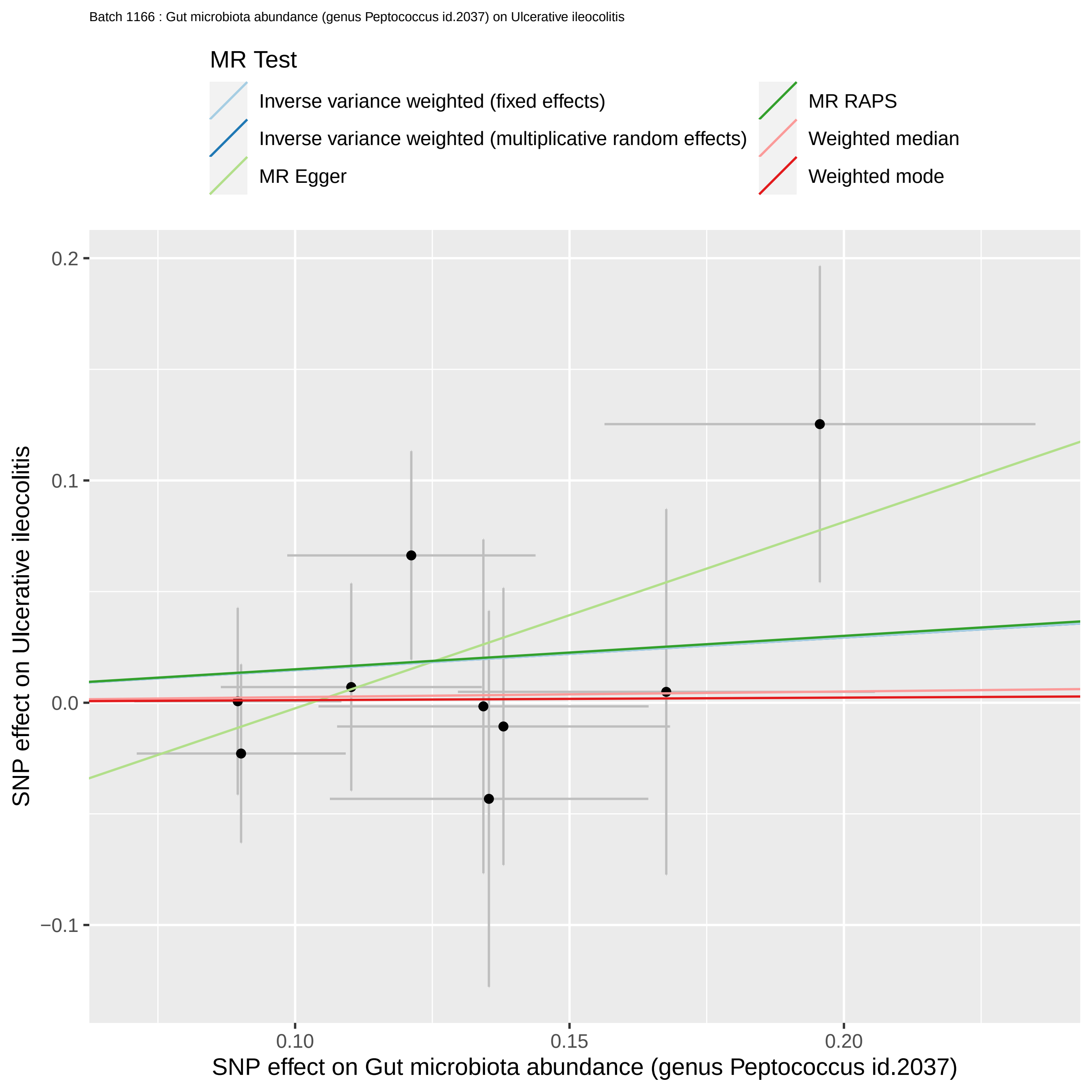

Batch 1166 : Gut microbiota abundance (genus Peptococcus id.2037) on Ulcerative ileocolitis
MR Test
Inverse variance weighted (fixed effects)
Inverse variance weighted (multiplicative random effects)
MR Egger
MR RAPS
Weighted median
Weighted mode
0.2
0.1
0.0
−0.1
0.10
0.15
0.20
SNP effect on Ulcerative ileocolitis
SNP effect on Gut microbiota abundance (genus Peptococcus id.2037)

Batch 1167 : Gut microbiota abundance (genus Phascolarctobacterium id.2168) on Ulcerative ileocolitis

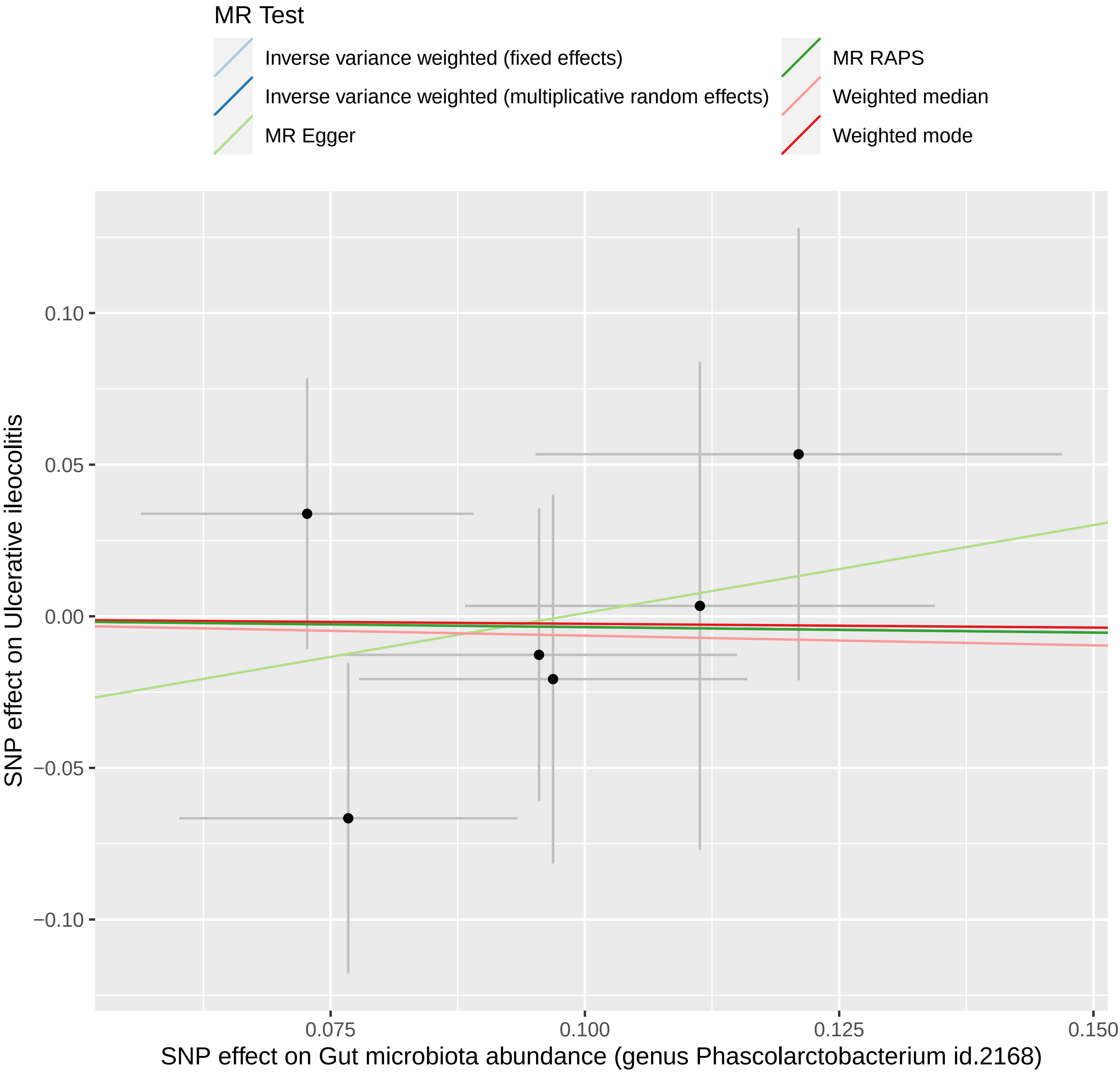

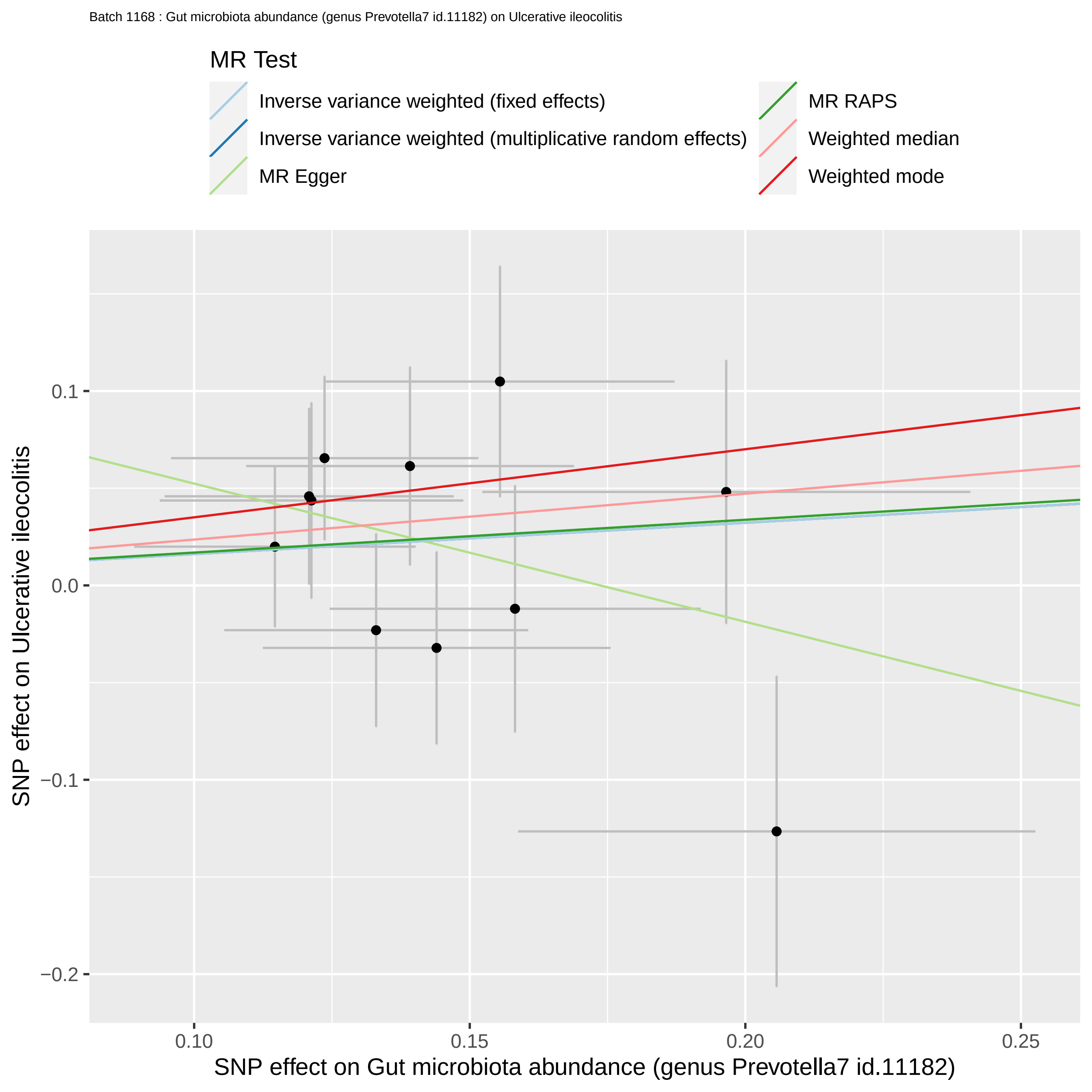
Batch 1168 : Gut microbiota abundance (genus Prevotella7 id.11182) on Ulcerative ileocolitis
MR Test
Inverse variance weighted (fixed effects)
Inverse variance weighted (multiplicative random effects)
MR Egger
MR RAPS
Weighted median
Weighted mode
0.1
0.0
−0.1
−0.2
0.10
0.15
0.20
0.25
SNP effect on Ulcerative ileocolitis
SNP effect on Gut microbiota abundance (genus Prevotella7 id.11182)

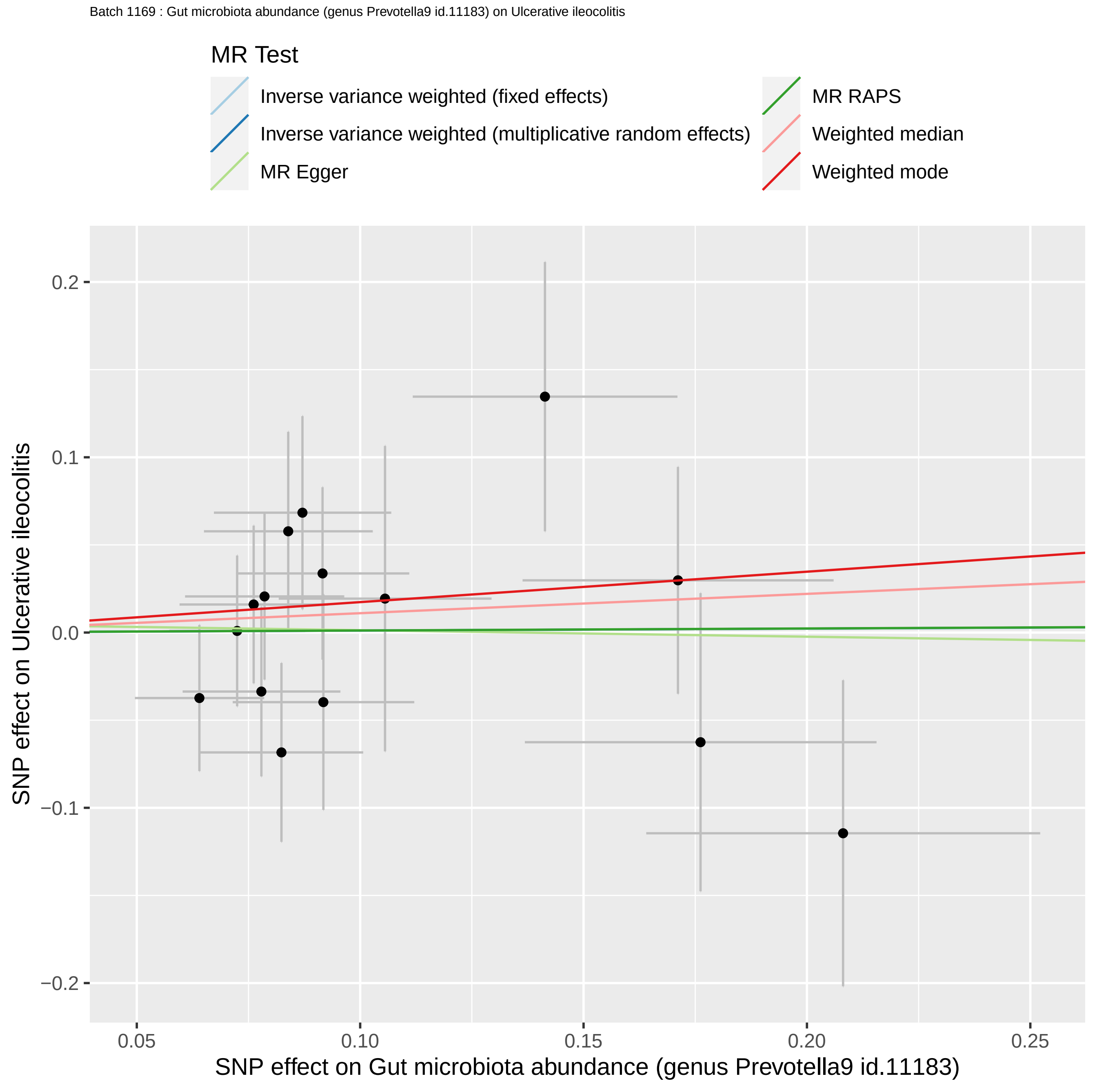

Batch 1169 : Gut microbiota abundance (genus Prevotella9 id.11183) on Ulcerative ileocolitis
MR Test
Inverse variance weighted (fixed effects)
Inverse variance weighted (multiplicative random effects)
MR Egger
MR RAPS
Weighted median
Weighted mode
0.2
0.1
0.0
−0.1
−0.2
SNP effect on Ulcerative ileocolitis
0.05
0.10
0.15
0.20
0.25
SNP effect on Gut microbiota abundance (genus Prevotella9 id.11183)

Batch 1170 : Gut microbiota abundance (genus Rikenellaceae RC9 gut group id.11191) on Ulcerative ileocolitis

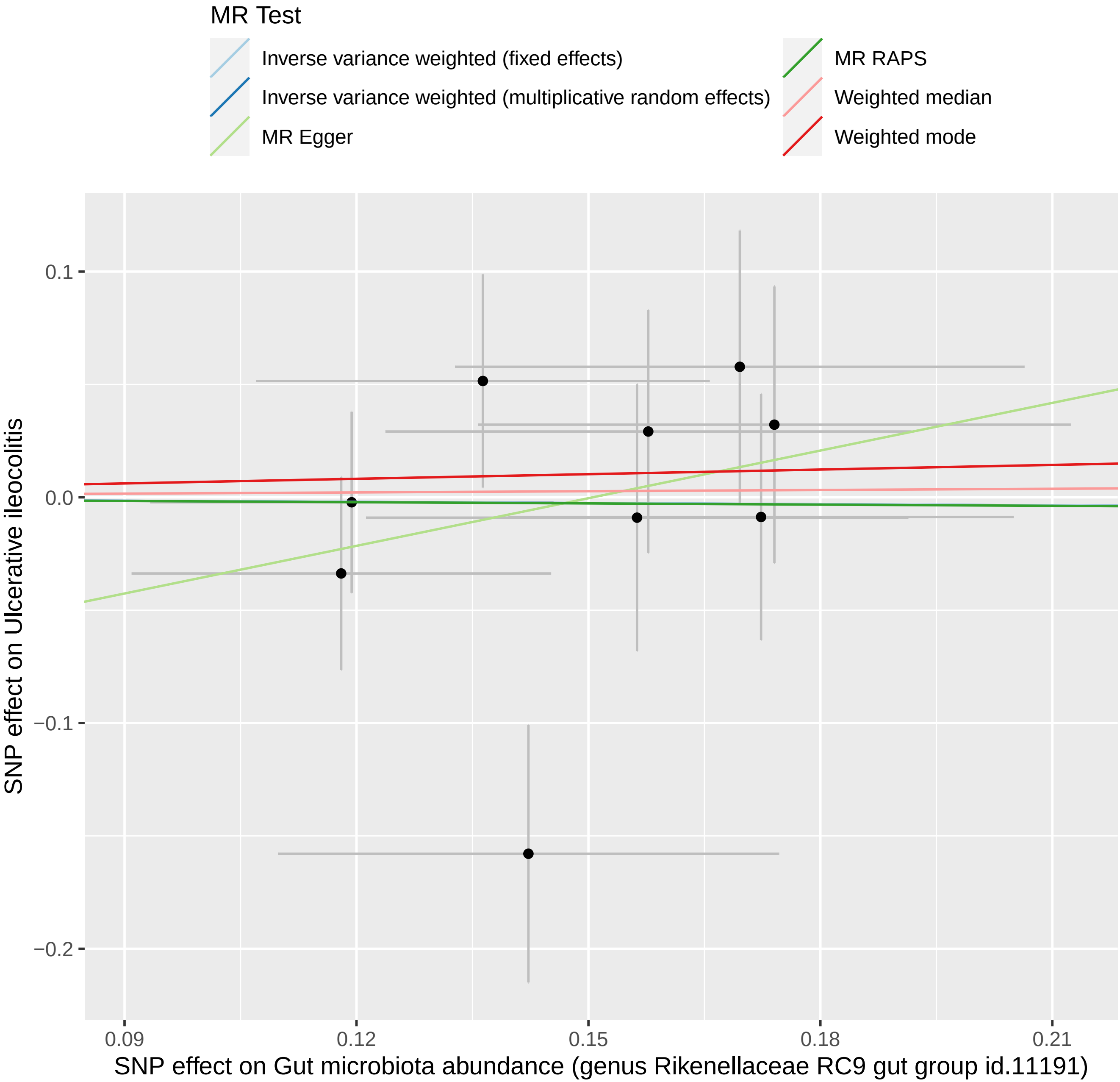

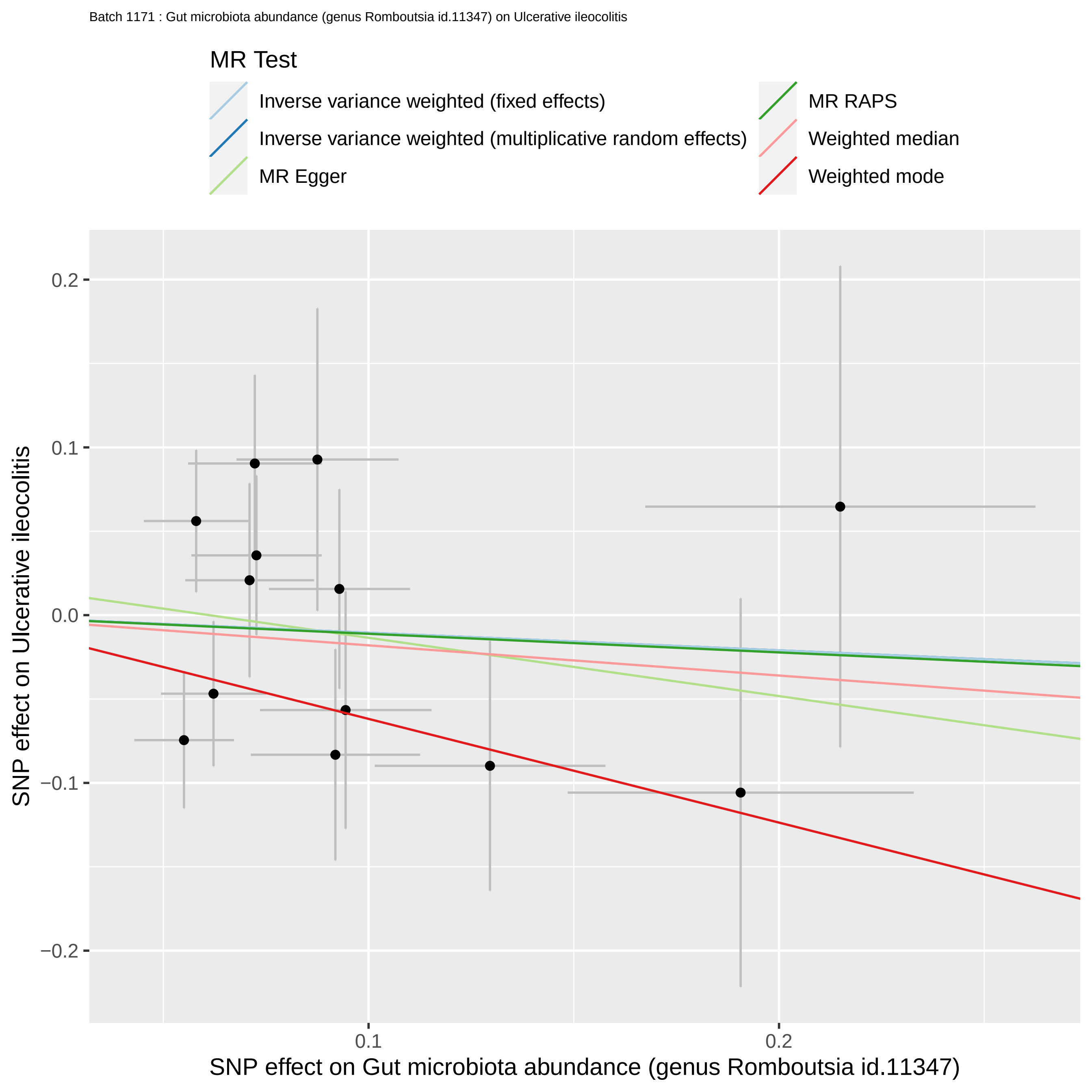

Batch 1171 : Gut microbiota abundance (genus Romboutsia id.11347) on Ulcerative ileocolitis
MR Test
Inverse variance weighted (fixed effects)
Inverse variance weighted (multiplicative random effects)
MR Egger
MR RAPS
Weighted median
Weighted mode
0.2
0.1
0.0
−0.1
−0.2
0.1
0.2
SNP effect on Ulcerative ileocolitis
SNP effect on Gut microbiota abundance (genus Romboutsia id.11347)

Batch 1172 : Gut microbiota abundance (genus Roseburia id.2012) on Ulcerative ileocolitis

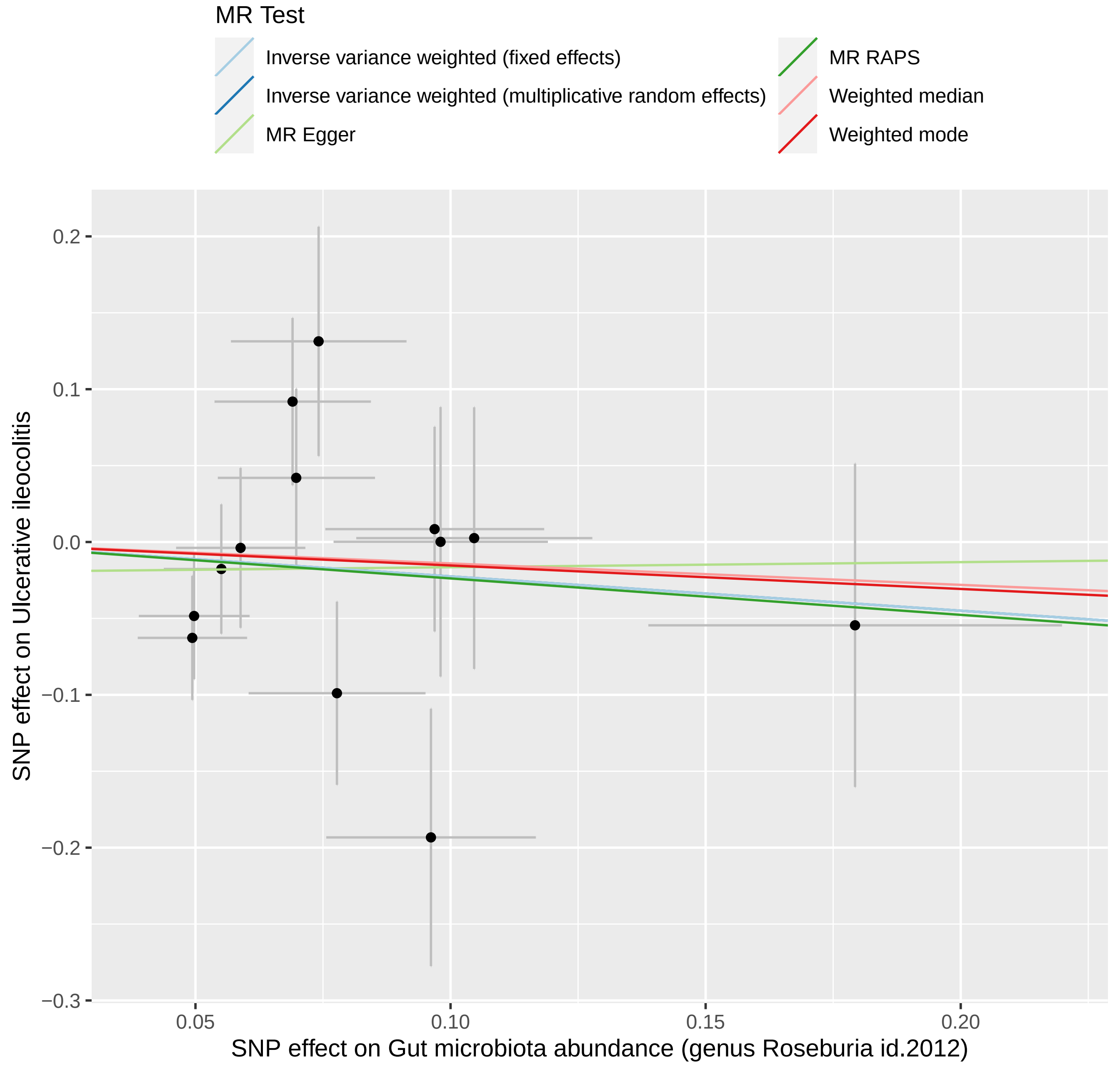

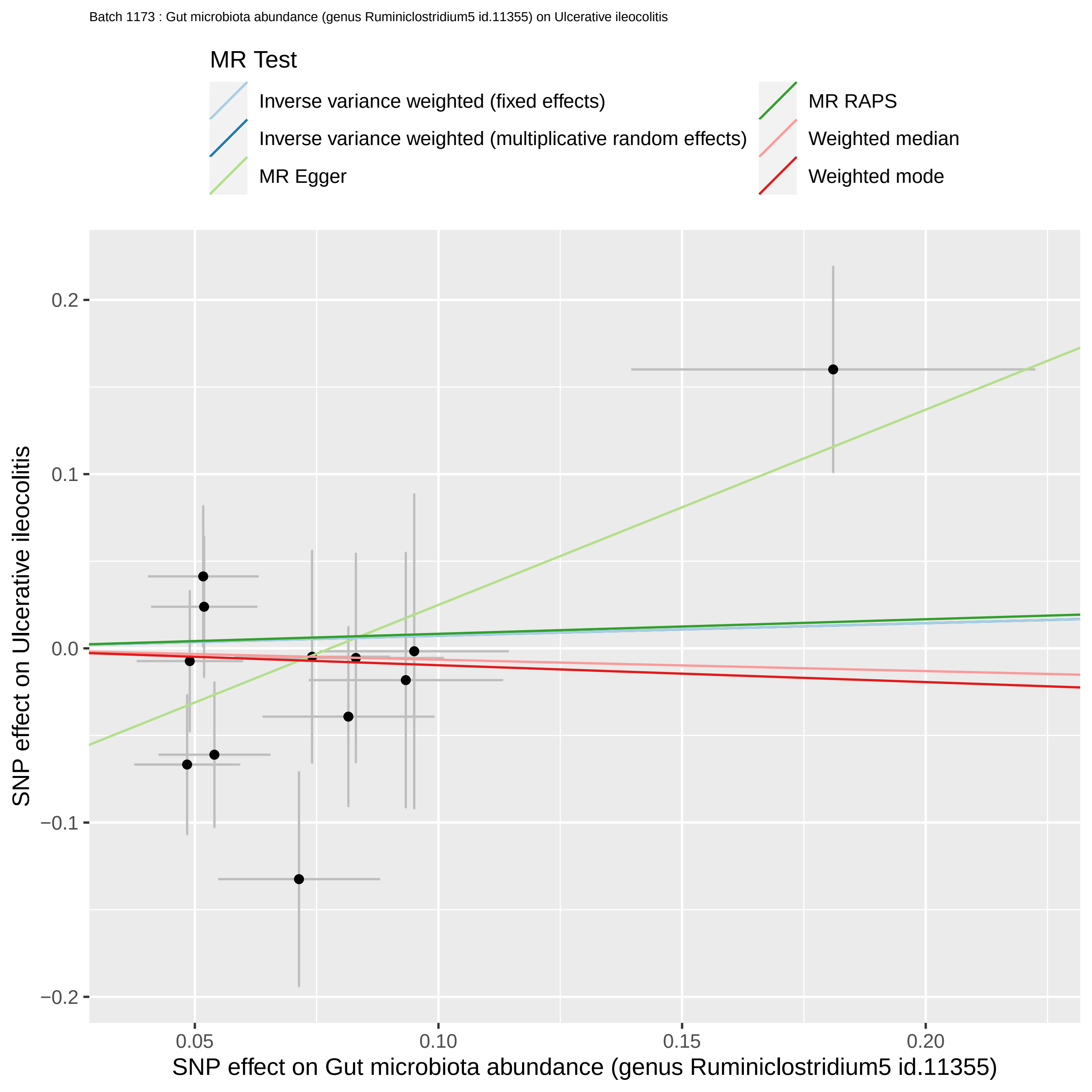

Batch 1173 : Gut microbiota abundance (genus Ruminiclostridium5 id.11355) on Ulcerative ileocolitis
MR Test
Inverse variance weighted (fixed effects)
Inverse variance weighted (multiplicative random effects)
MR Egger
MR RAPS
Weighted median
Weighted mode
SNP effect on Ulcerative ileocolitis
0.2
0.1
0.0
−0.1
−0.2
0.05
0.10
0.15
0.20
SNP effect on Gut microbiota abundance (genus Ruminiclostridium5 id.11355)

Batch 1174 : Gut microbiota abundance (genus Ruminiclostridium6 id.11356) on Ulcerative ileocolitis

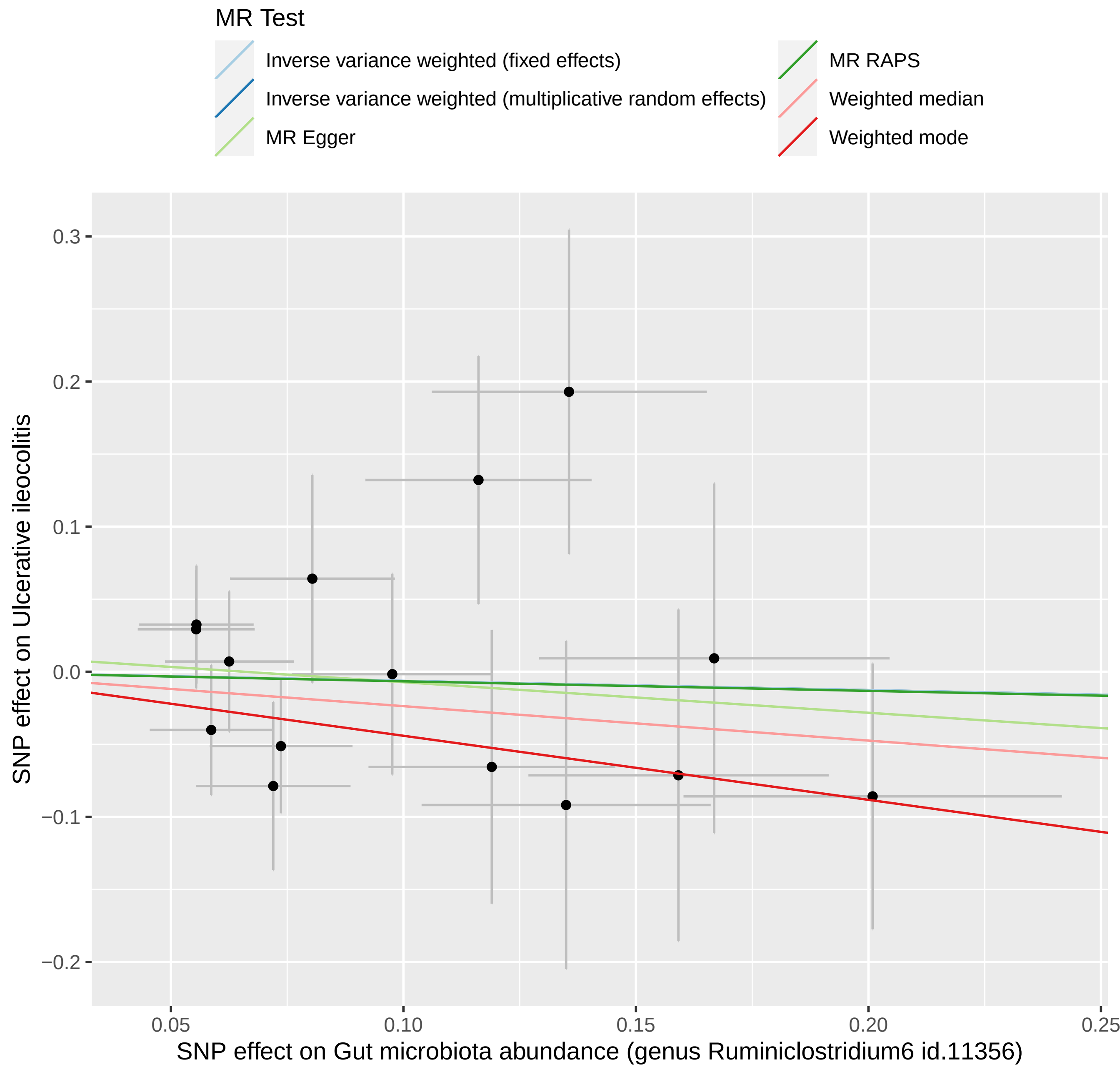

Batch 1175 : Gut microbiota abundance (genus Ruminiclostridium9 id.11357) on Ulcerative ileocolitis

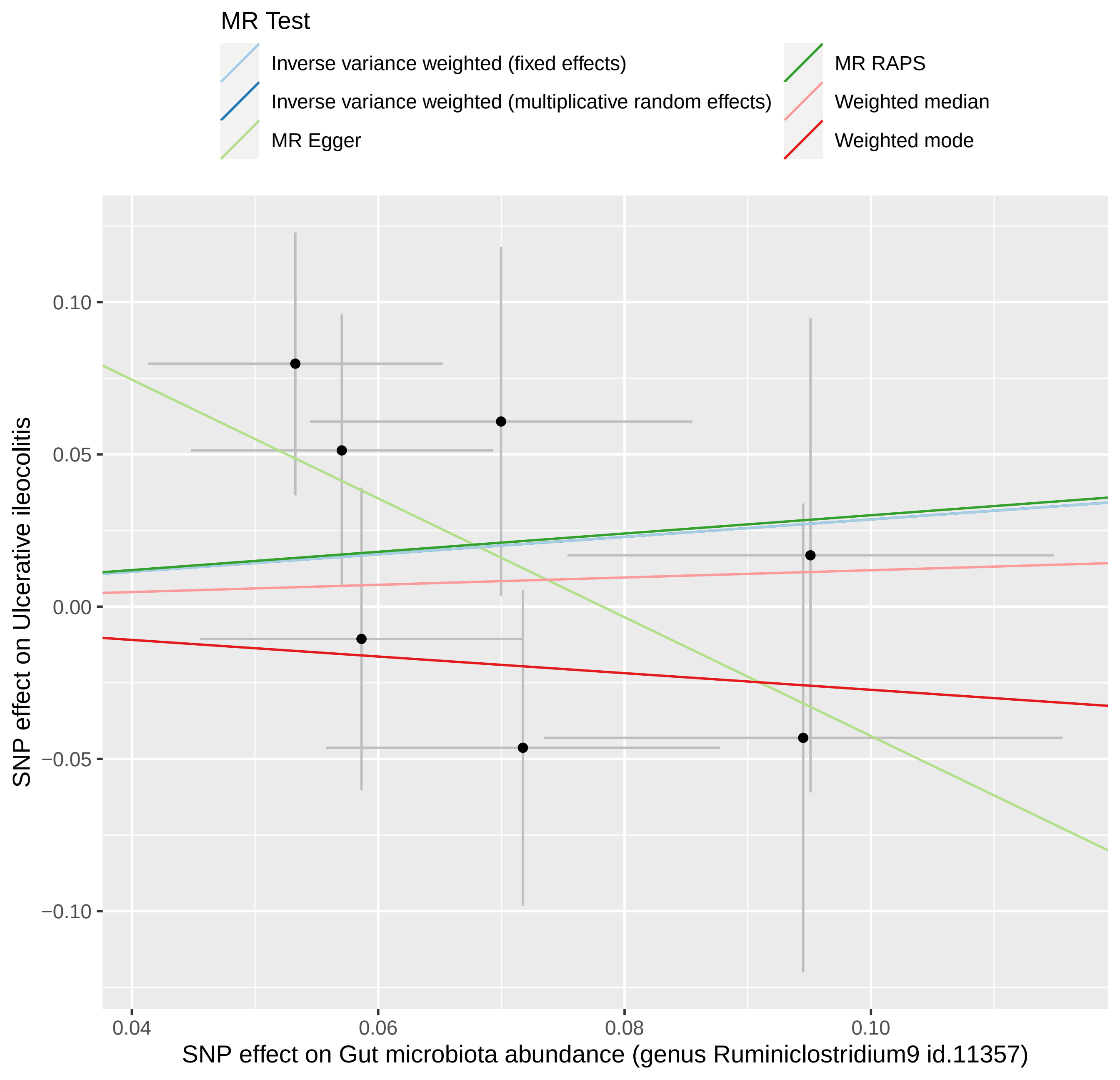

Batch 1176 : Gut microbiota abundance (genus Ruminococcaceae NK4A214 group id.11358) on Ulcerative ileocolitis

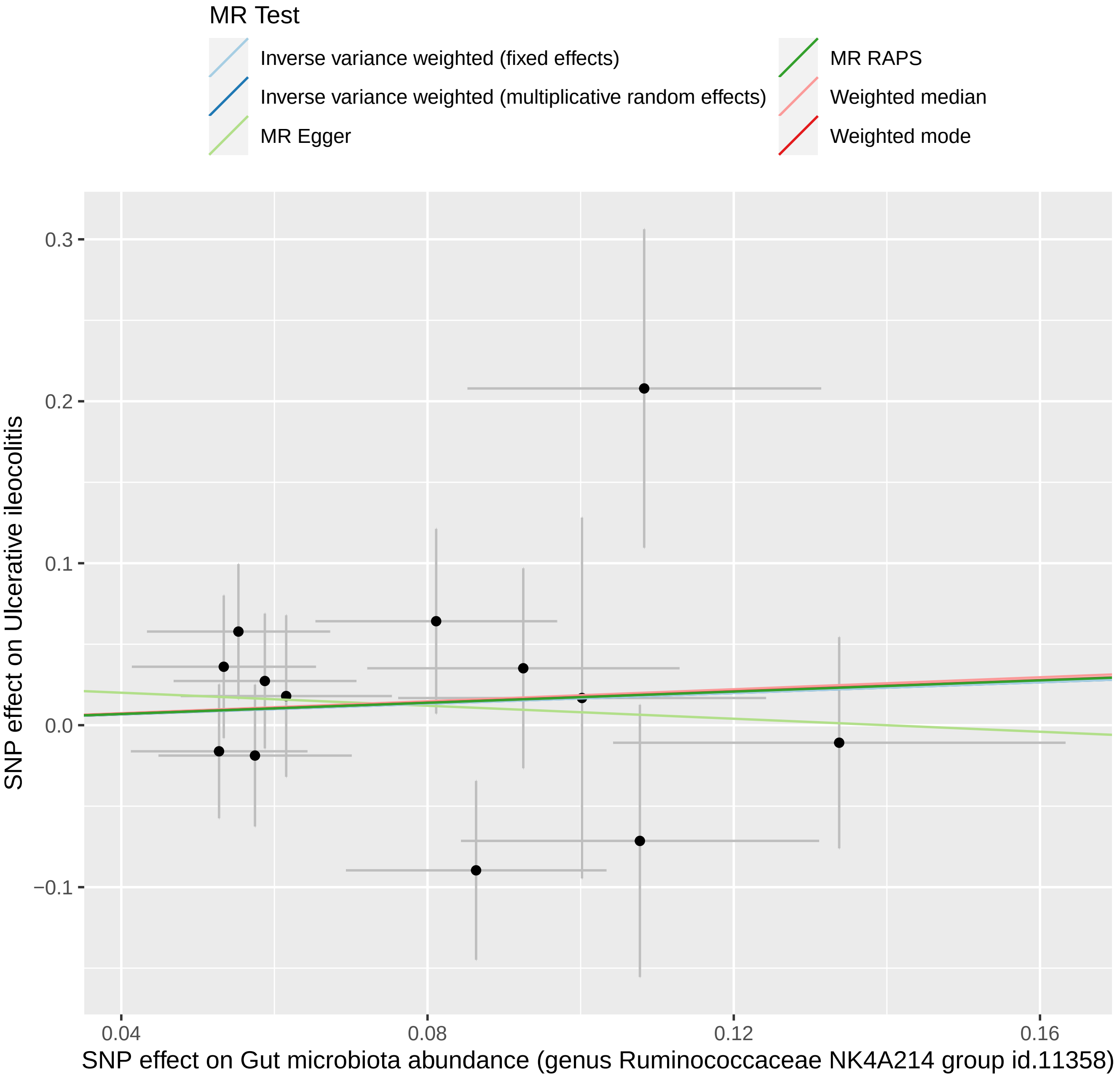

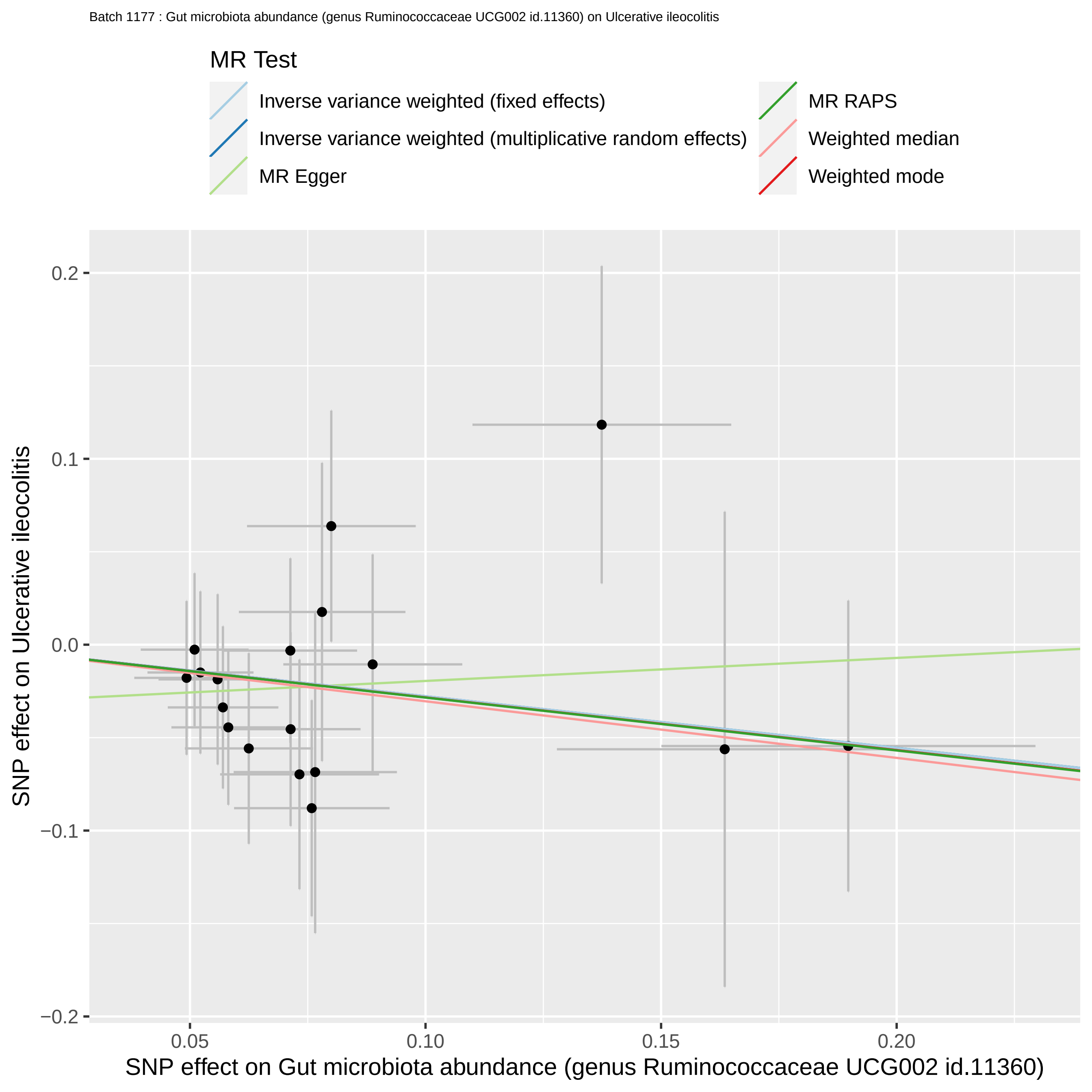

Batch 1177 : Gut microbiota abundance (genus Ruminococcaceae UCG002 id.11360) on Ulcerative ileocolitis
MR Test
Inverse variance weighted (fixed effects)
Inverse variance weighted (multiplicative random effects)
MR Egger
MR RAPS
Weighted median
Weighted mode
0.2
0.1
0.0
−0.1
−0.2
0.05
0.10
0.15
0.20
SNP effect on Ulcerative ileocolitis
SNP effect on Gut microbiota abundance (genus Ruminococcaceae UCG002 id.11360)

Batch 1178 : Gut microbiota abundance (genus Ruminococcaceae UCG003 id.11361) on Ulcerative ileocolitis

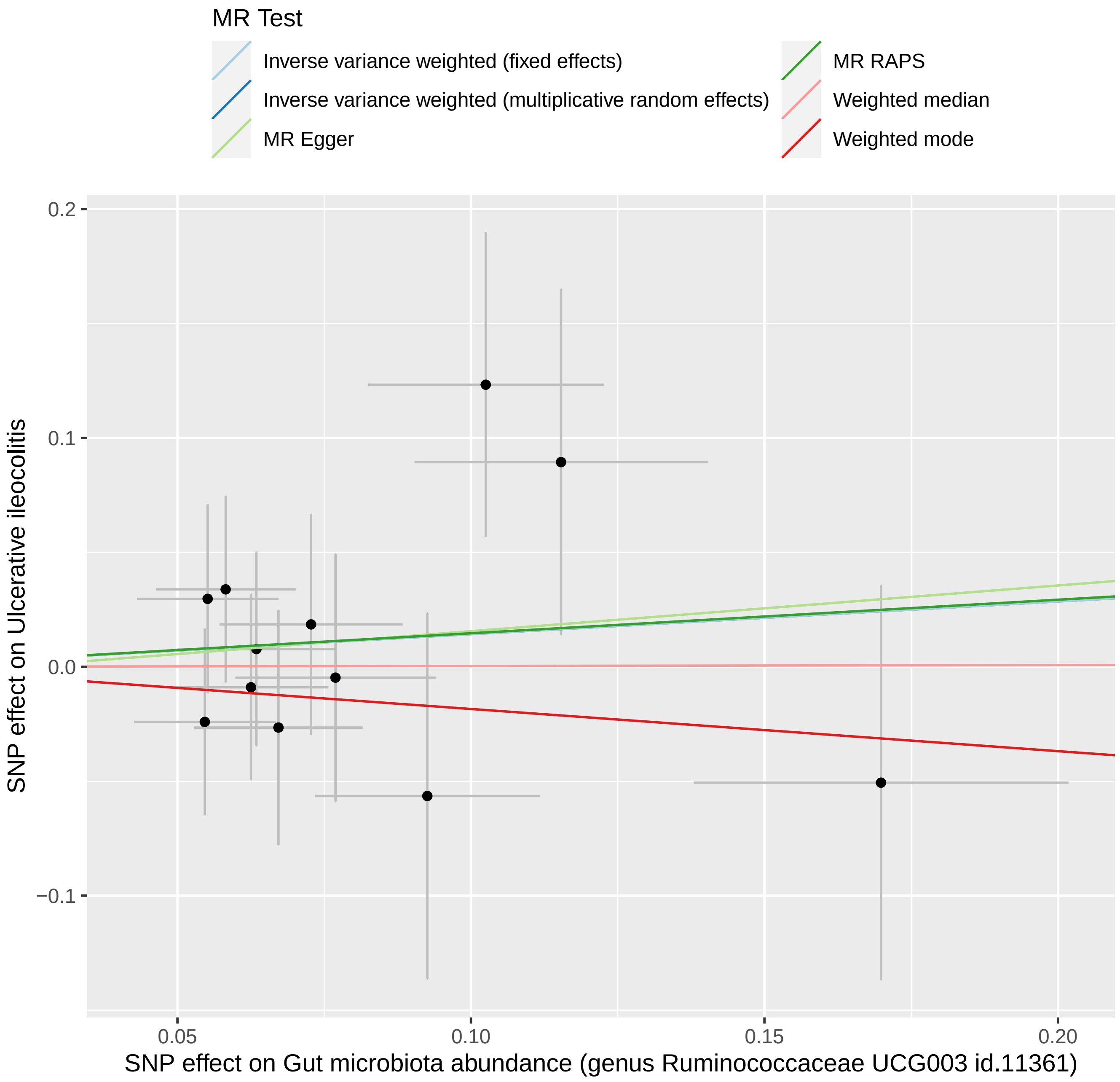

Batch 1179 : Gut microbiota abundance (genus Ruminococcaceae UCG004 id.11362) on Ulcerative ileocolitis

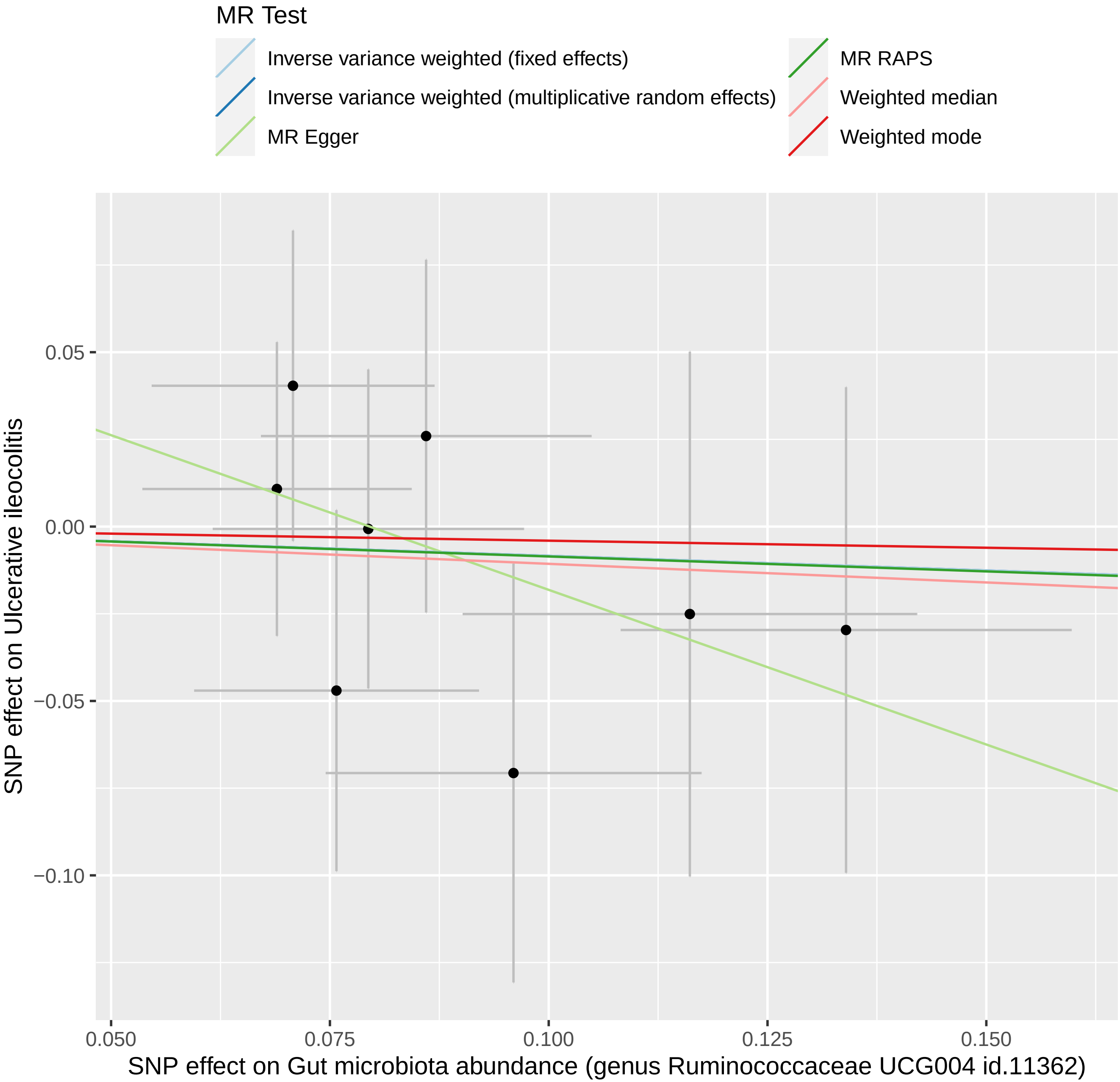

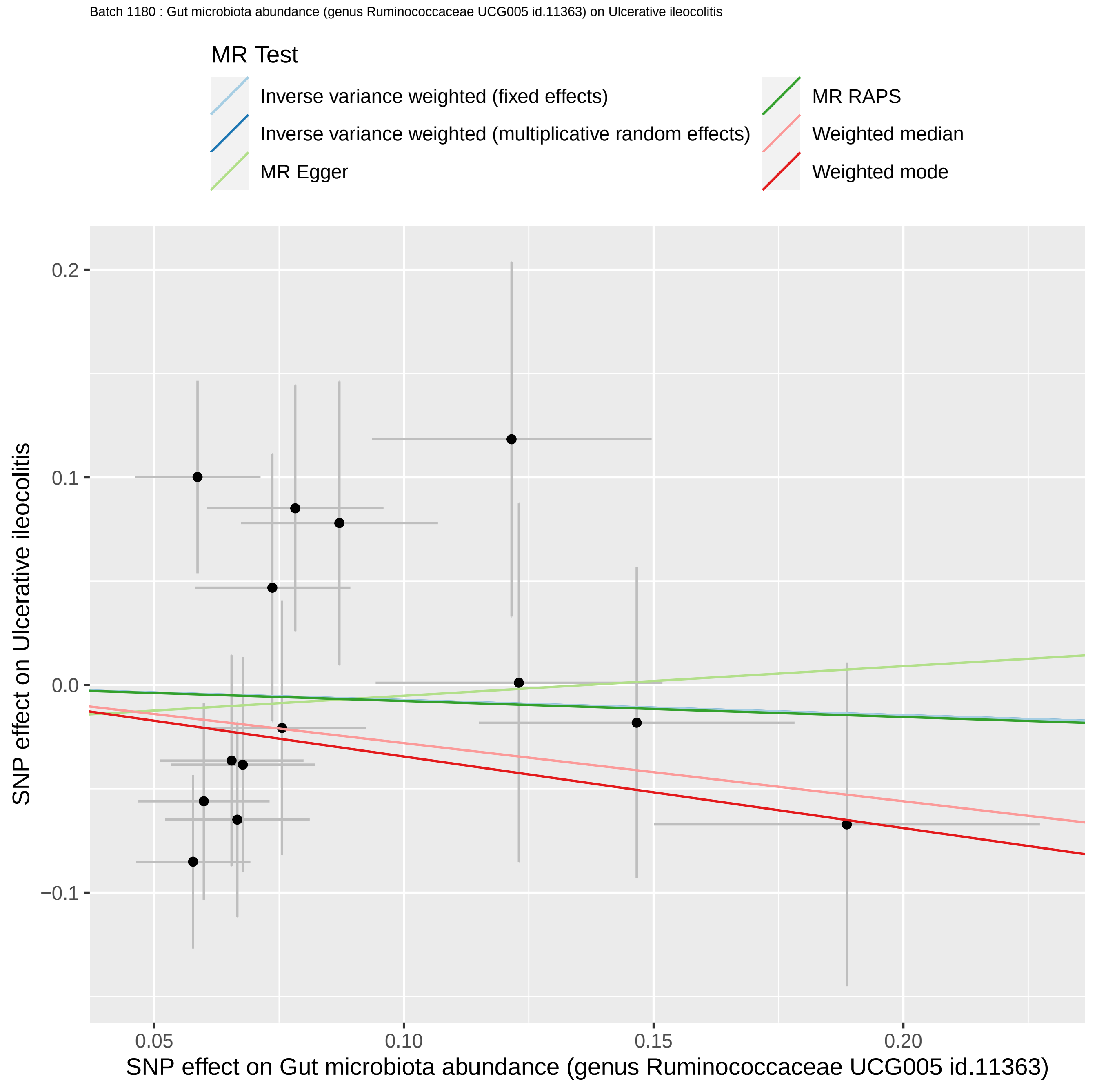
Batch 1180 : Gut microbiota abundance (genus Ruminococcaceae UCG005 id.11363) on Ulcerative ileocolitis
MR Test
Inverse variance weighted (fixed effects)
Inverse variance weighted (multiplicative random effects)
MR Egger
MR RAPS
Weighted median
Weighted mode
0.2
0.1
0.0
−0.1
0.05
0.10
0.15
0.20
SNP effect on Ulcerative ileocolitis
SNP effect on Gut microbiota abundance (genus Ruminococcaceae UCG005 id.11363)

Batch 1181 : Gut microbiota abundance (genus Ruminococcaceae UCG009 id.11366) on Ulcerative ileocolitis

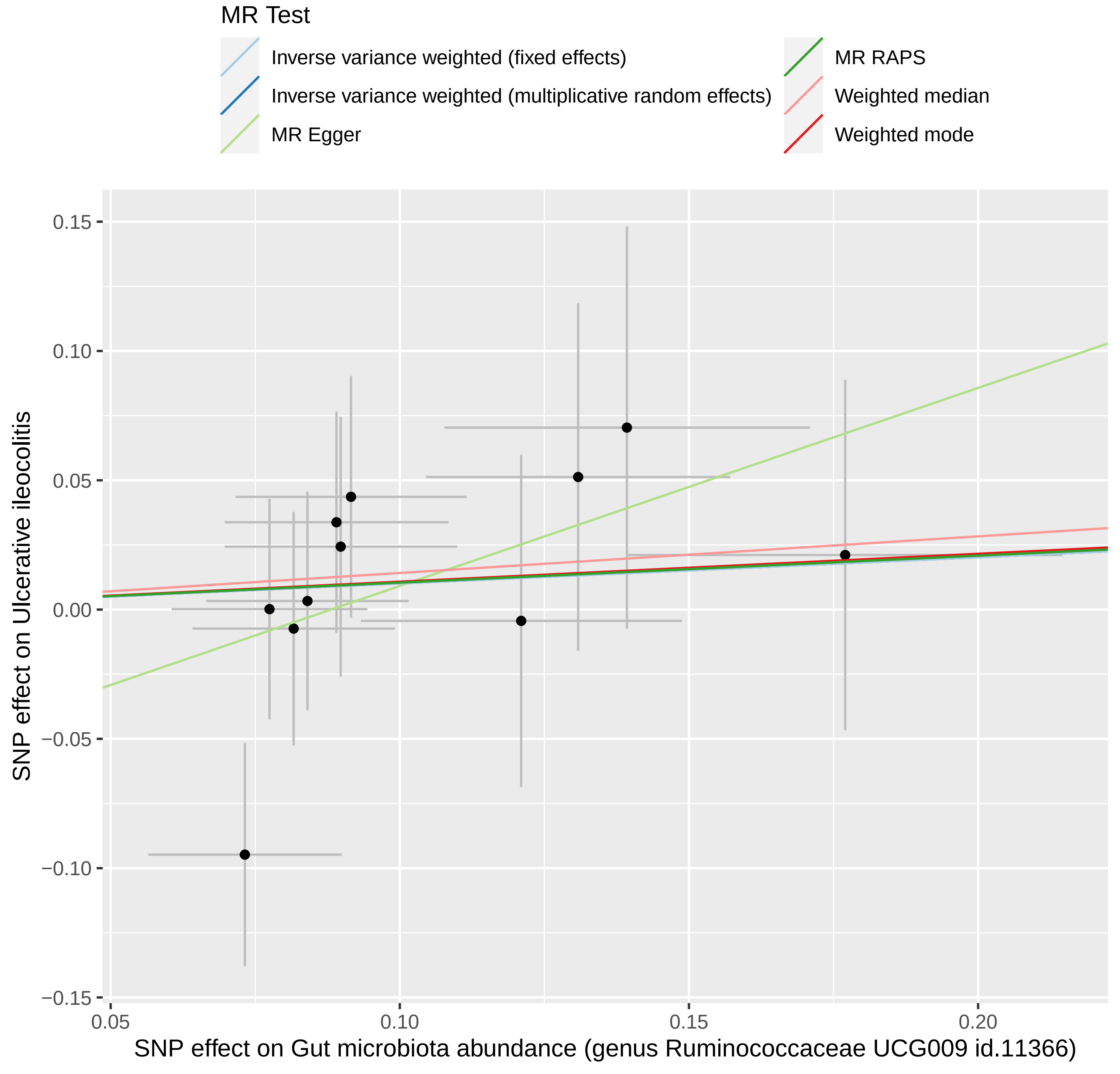

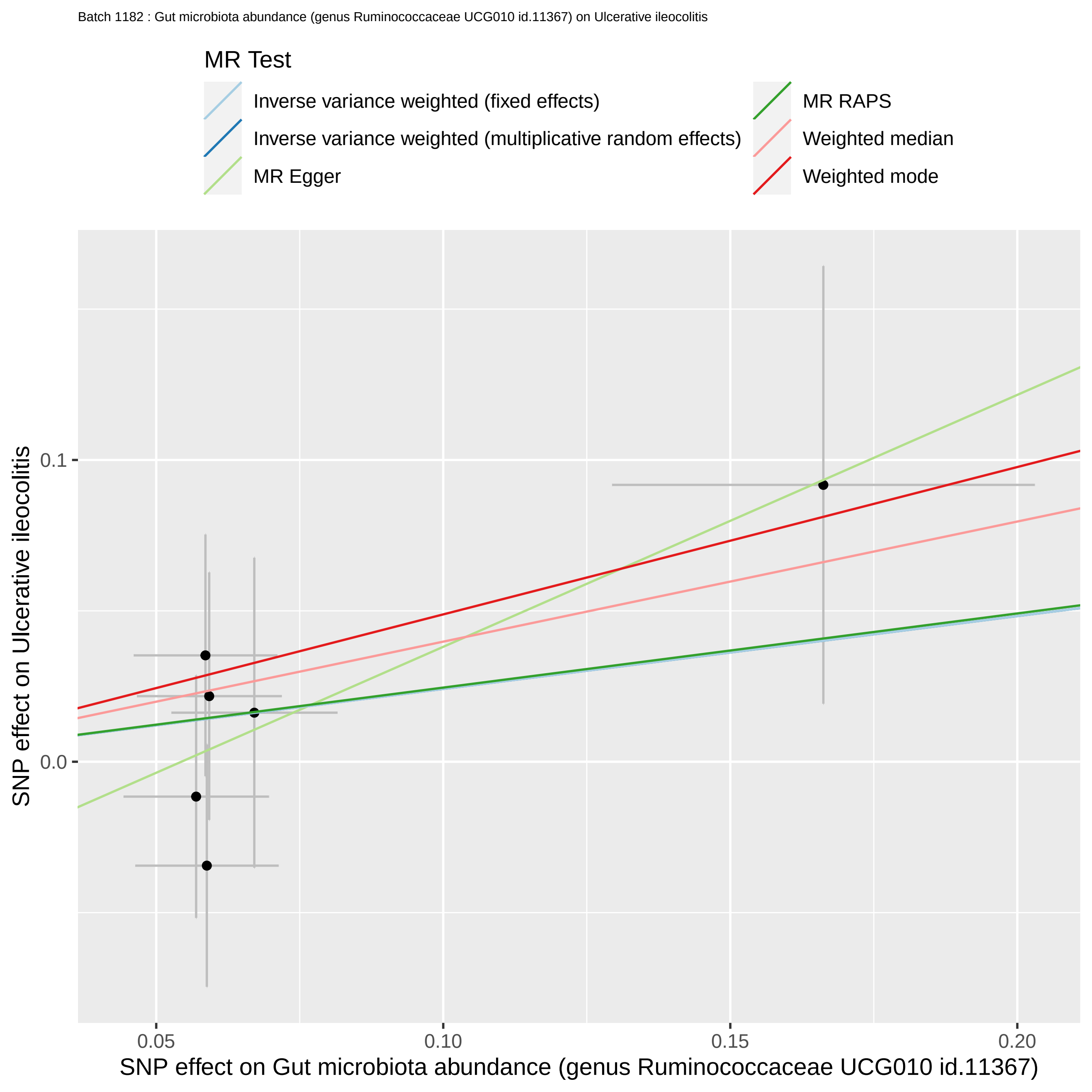
Batch 1182 : Gut microbiota abundance (genus Ruminococcaceae UCG010 id.11367) on Ulcerative ileocolitis
MR Test
Inverse variance weighted (fixed effects)
Inverse variance weighted (multiplicative random effects)
MR Egger
MR RAPS
Weighted median
Weighted mode
0.1
0.0
0.05
0.10
0.15
0.20
SNP effect on Ulcerative ileocolitis
SNP effect on Gut microbiota abundance (genus Ruminococcaceae UCG010 id.11367)

Batch 1183 : Gut microbiota abundance (genus Ruminococcaceae UCG011 id.11368) on Ulcerative ileocolitis

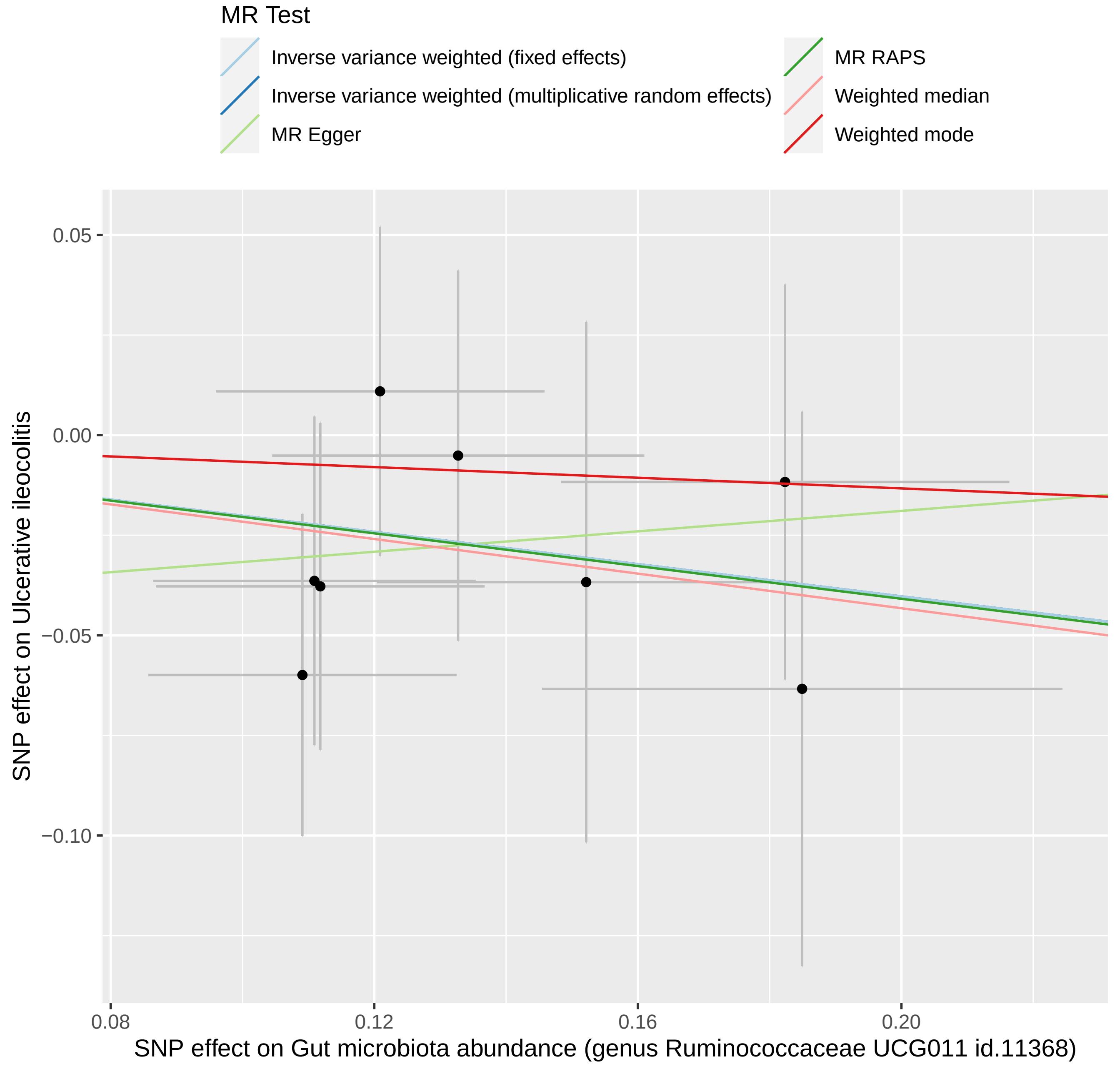

Batch 1184 : Gut microbiota abundance (genus Ruminococcaceae UCG013 id.11370) on Ulcerative ileocolitis

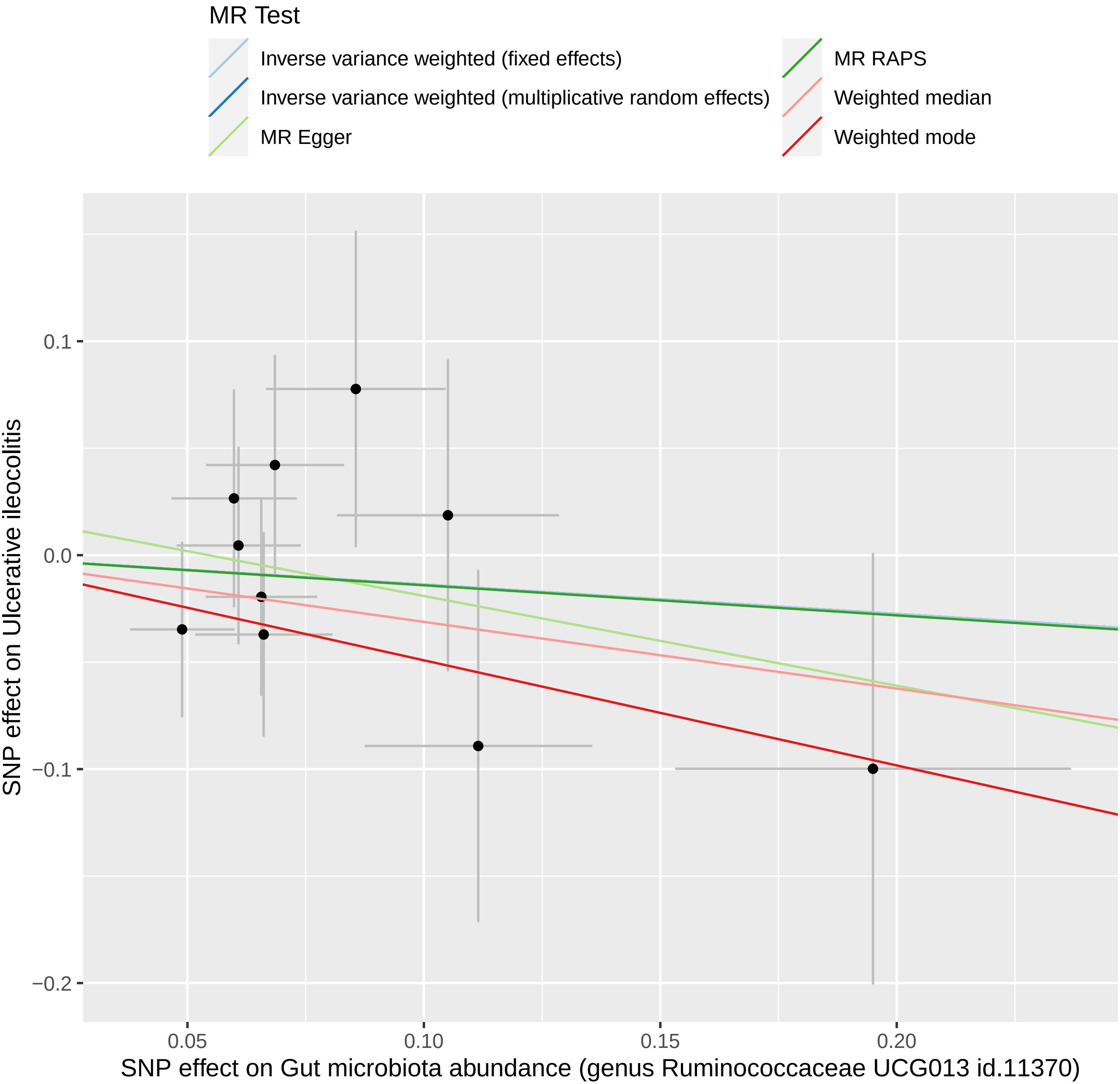

Batch 1185 : Gut microbiota abundance (genus Ruminococcaceae UCG014 id.11371) on Ulcerative ileocolitis

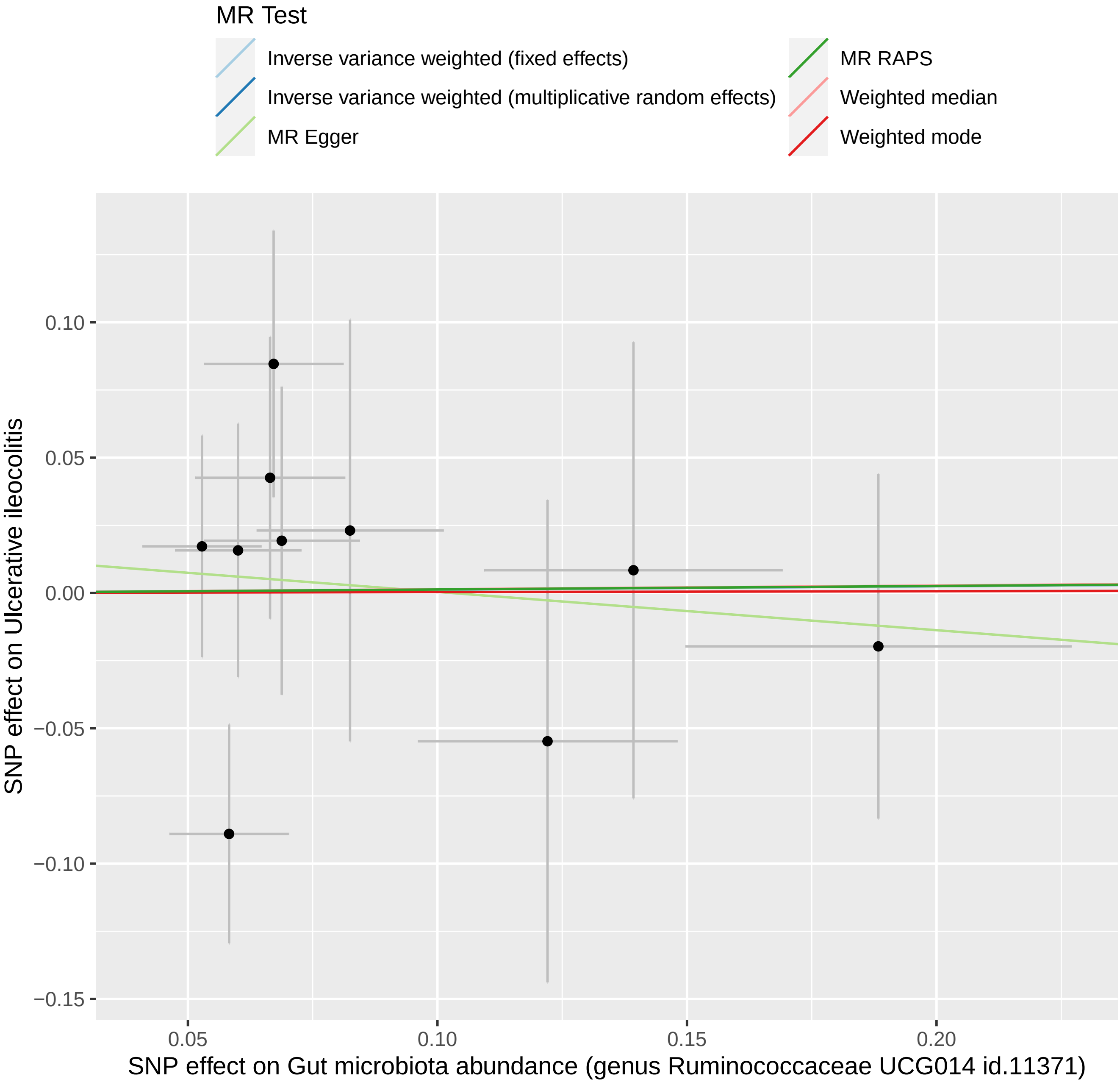

Batch 1186 : Gut microbiota abundance (genus Ruminococcus1 id.11373) on Ulcerative ileocolitis

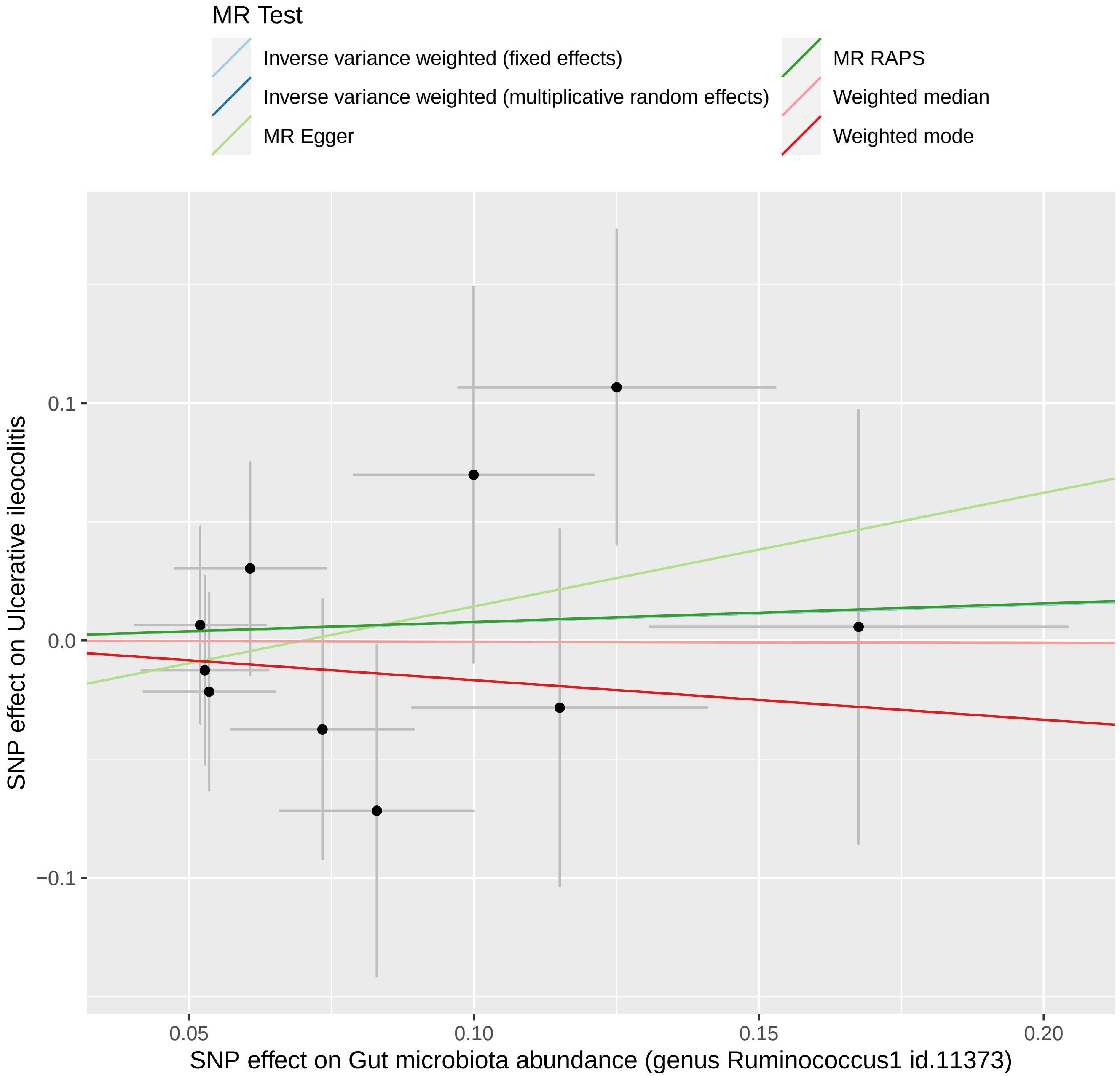

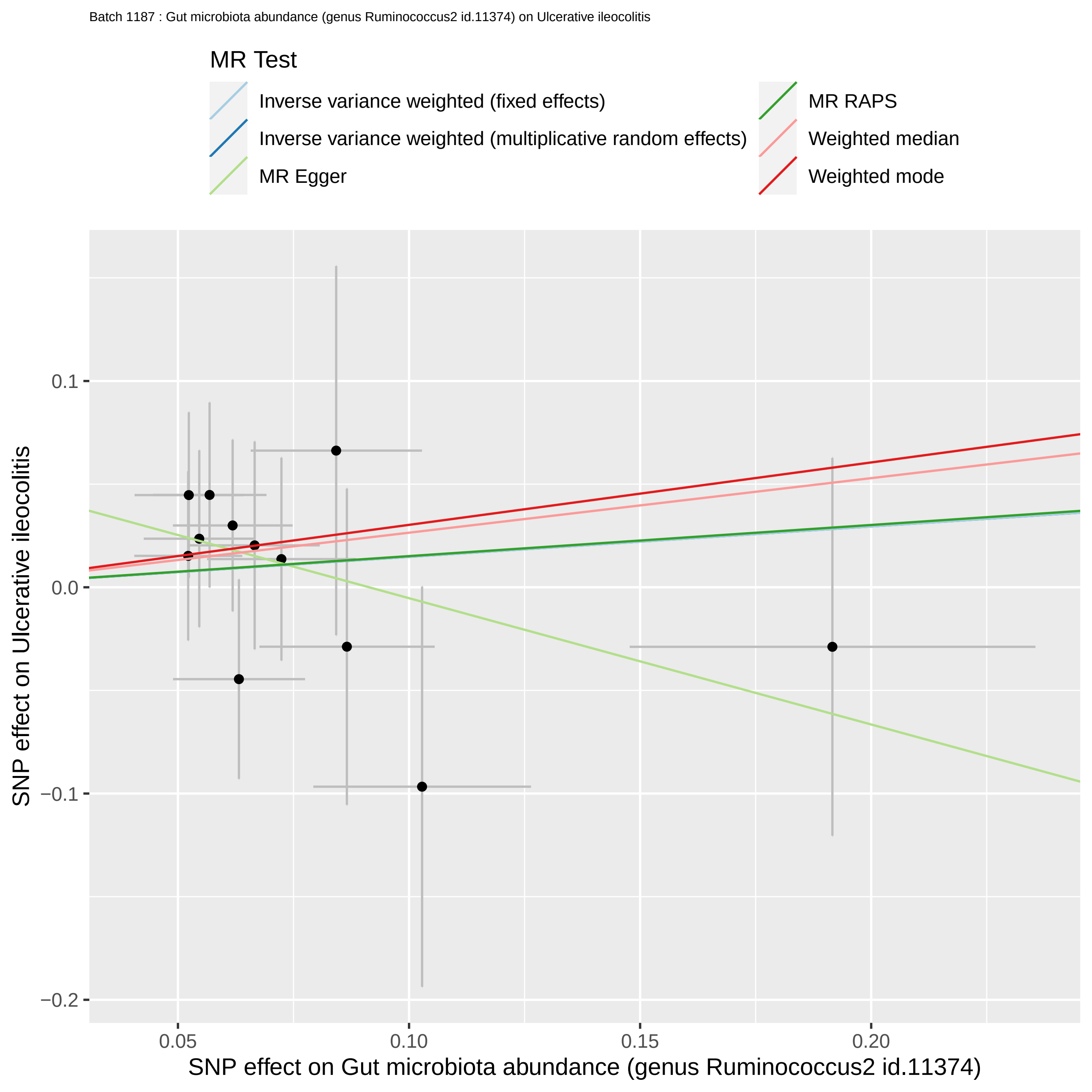
Batch 1187 : Gut microbiota abundance (genus Ruminococcus2 id.11374) on Ulcerative ileocolitis
MR Test
Inverse variance weighted (fixed effects)
Inverse variance weighted (multiplicative random effects)
MR Egger
MR RAPS
Weighted median
Weighted mode
0.1
0.0
−0.1
−0.2
0.05
0.10
0.15
0.20
SNP effect on Ulcerative ileocolitis
SNP effect on Gut microbiota abundance (genus Ruminococcus2 id.11374)

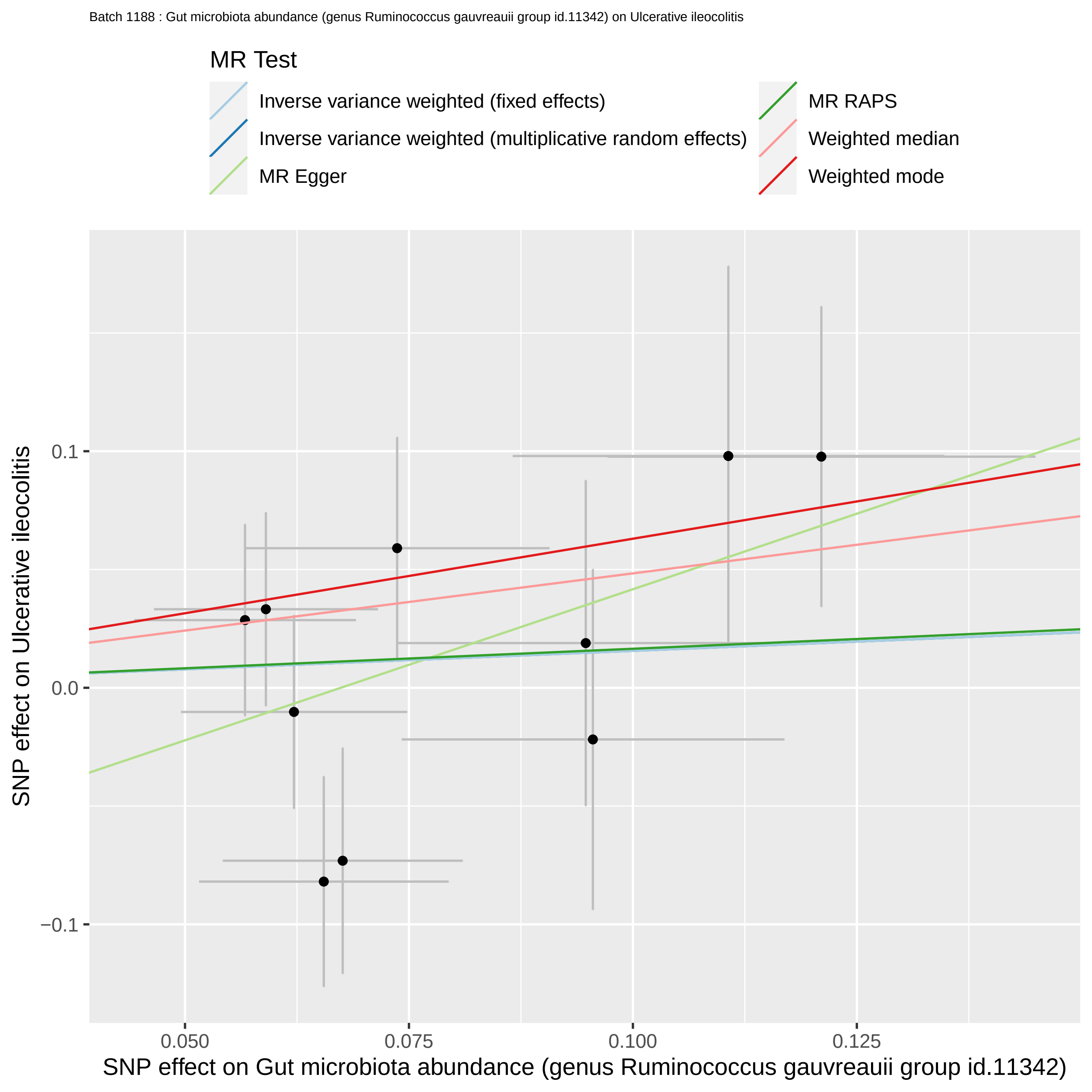
Batch 1188 : Gut microbiota abundance (genus Ruminococcus gauvreauii group id.11342) on Ulcerative ileocolitis
MR Test
Inverse variance weighted (fixed effects)
Inverse variance weighted (multiplicative random effects)
MR Egger
MR RAPS
Weighted median
Weighted mode
0.1
0.0
−0.1
0.050
0.075
0.100
0.125
SNP effect on Ulcerative ileocolitis
SNP effect on Gut microbiota abundance (genus Ruminococcus gauvreauii group id.11342)

Batch 1189 : Gut microbiota abundance (genus Ruminococcus gnavus group id.14376) on Ulcerative ileocolitis

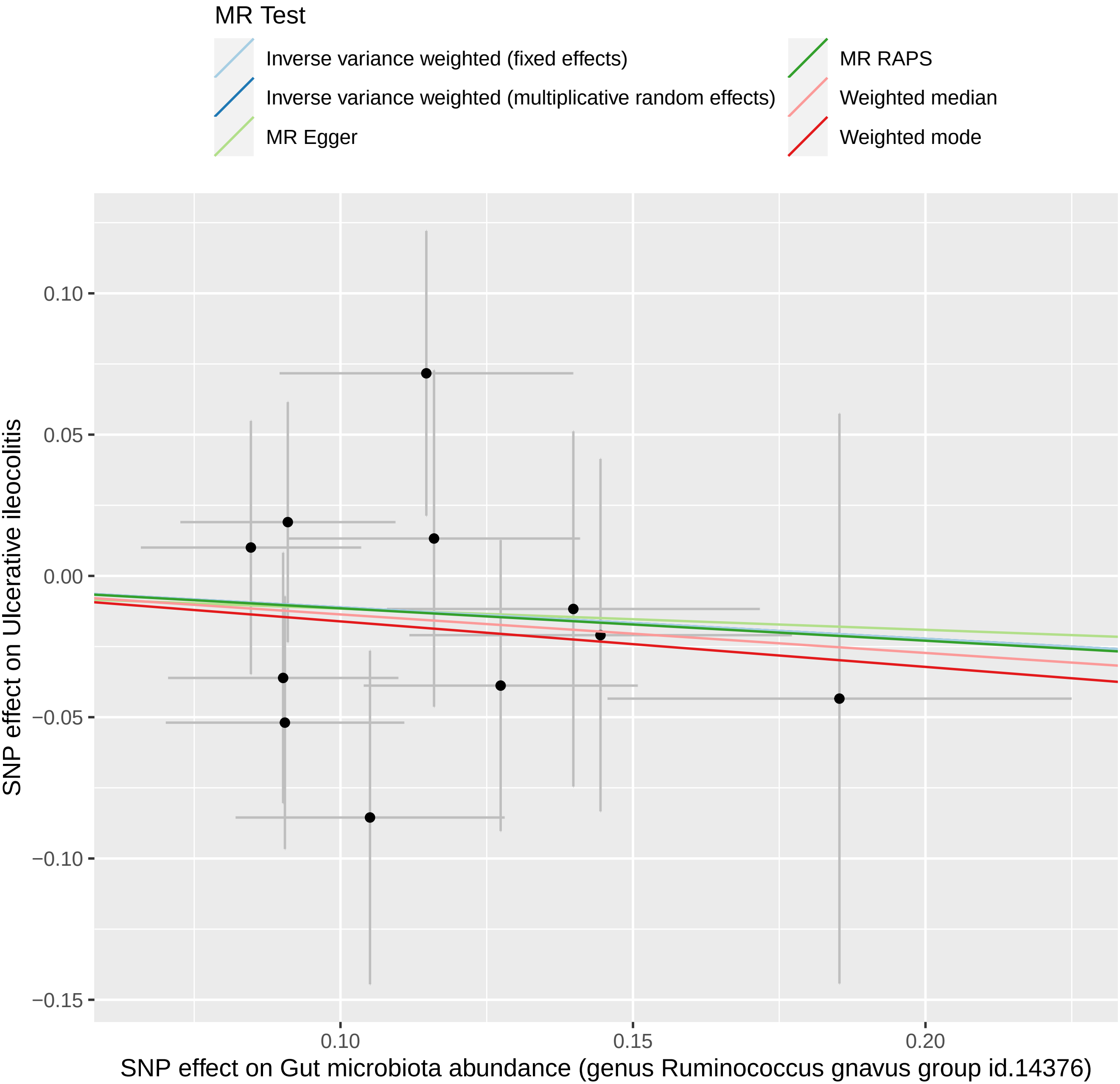

Batch 1190 : Gut microbiota abundance (genus Ruminococcus torques group id.14377) on Ulcerative ileocolitis

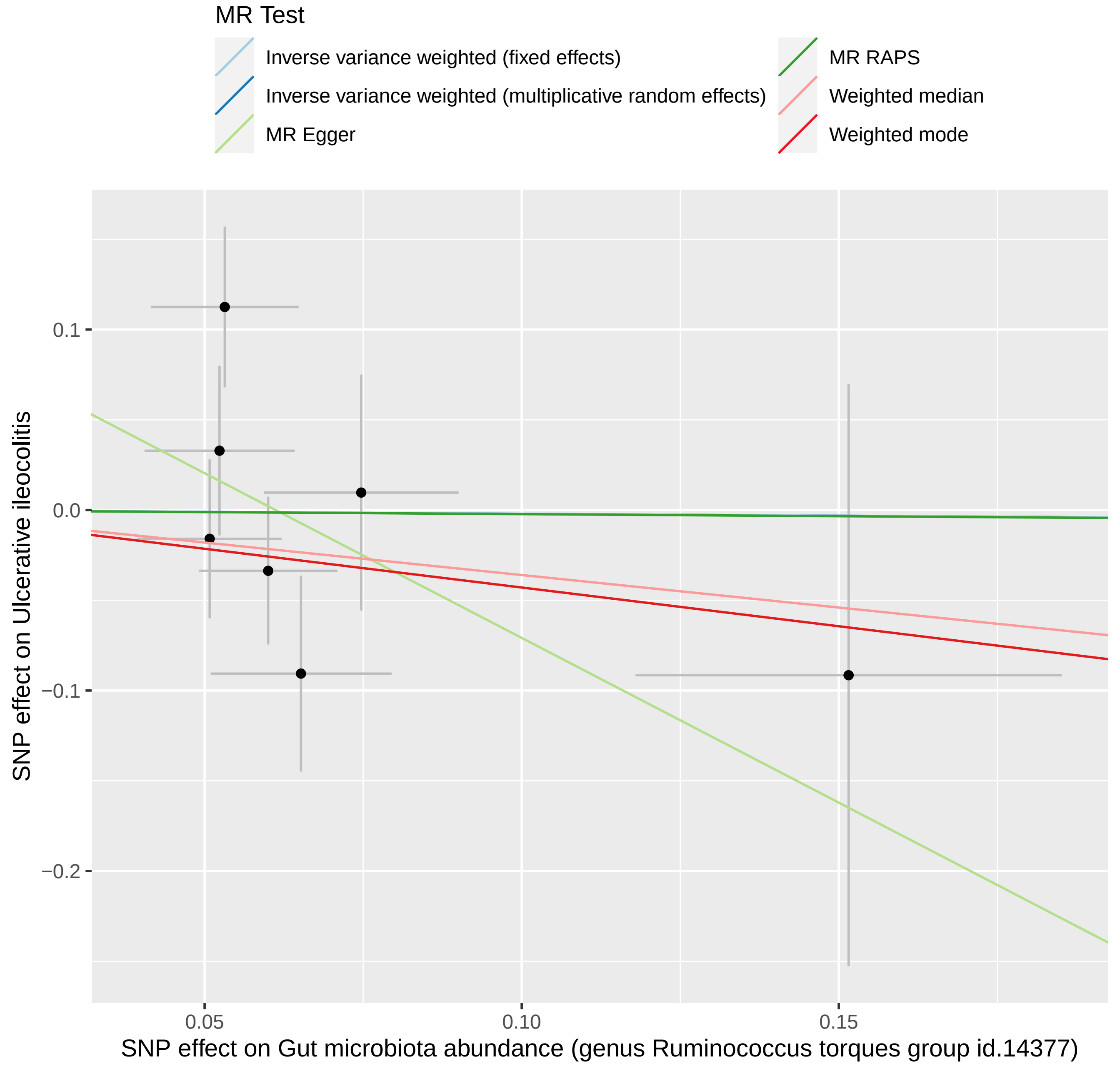

Batch 1191 : Gut microbiota abundance (genus Sellimonas id.14369) on Ulcerative ileocolitis

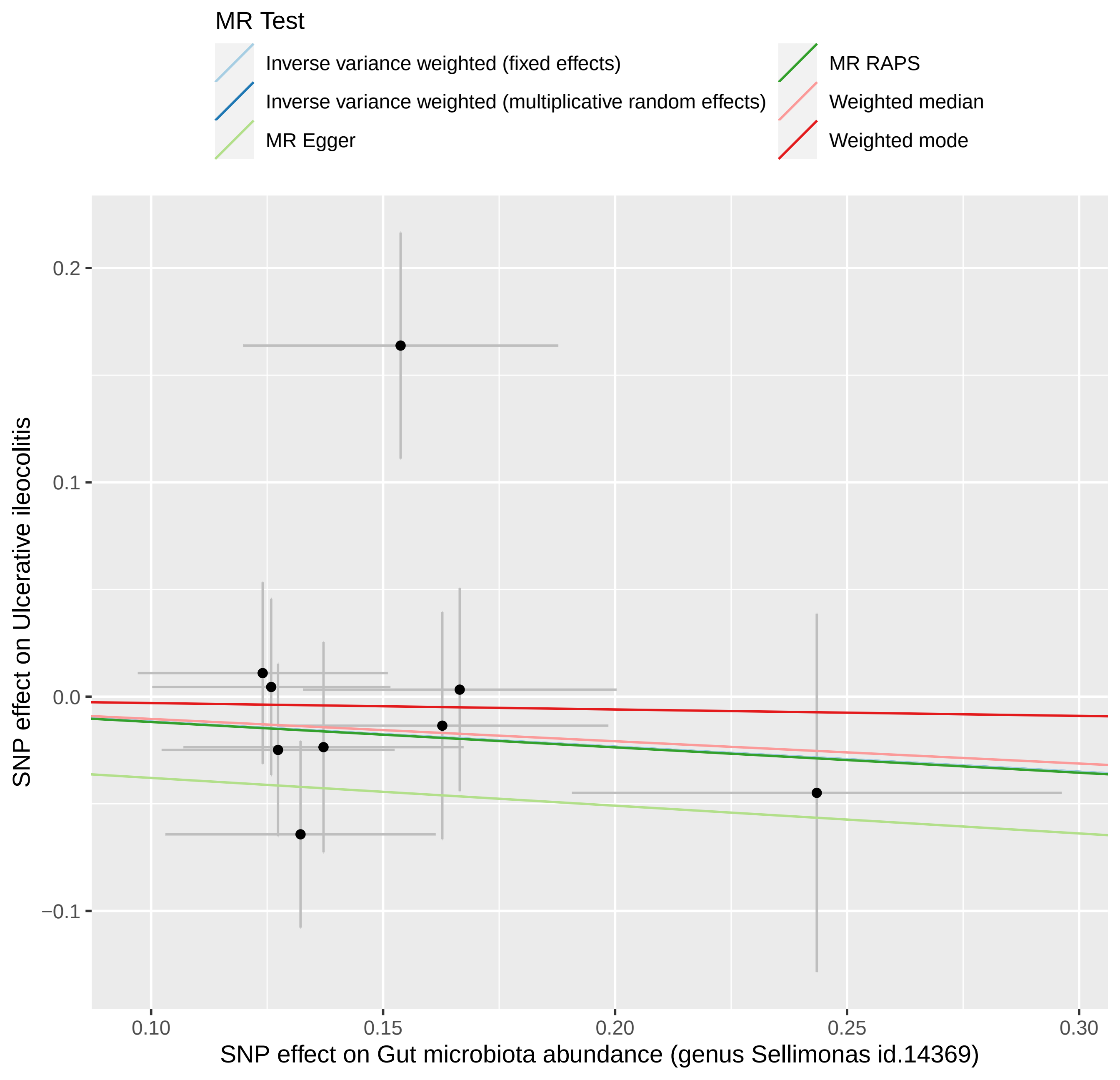

Batch 1192 : Gut microbiota abundance (genus Senegalimassilia id.11160) on Ulcerative ileocolitis

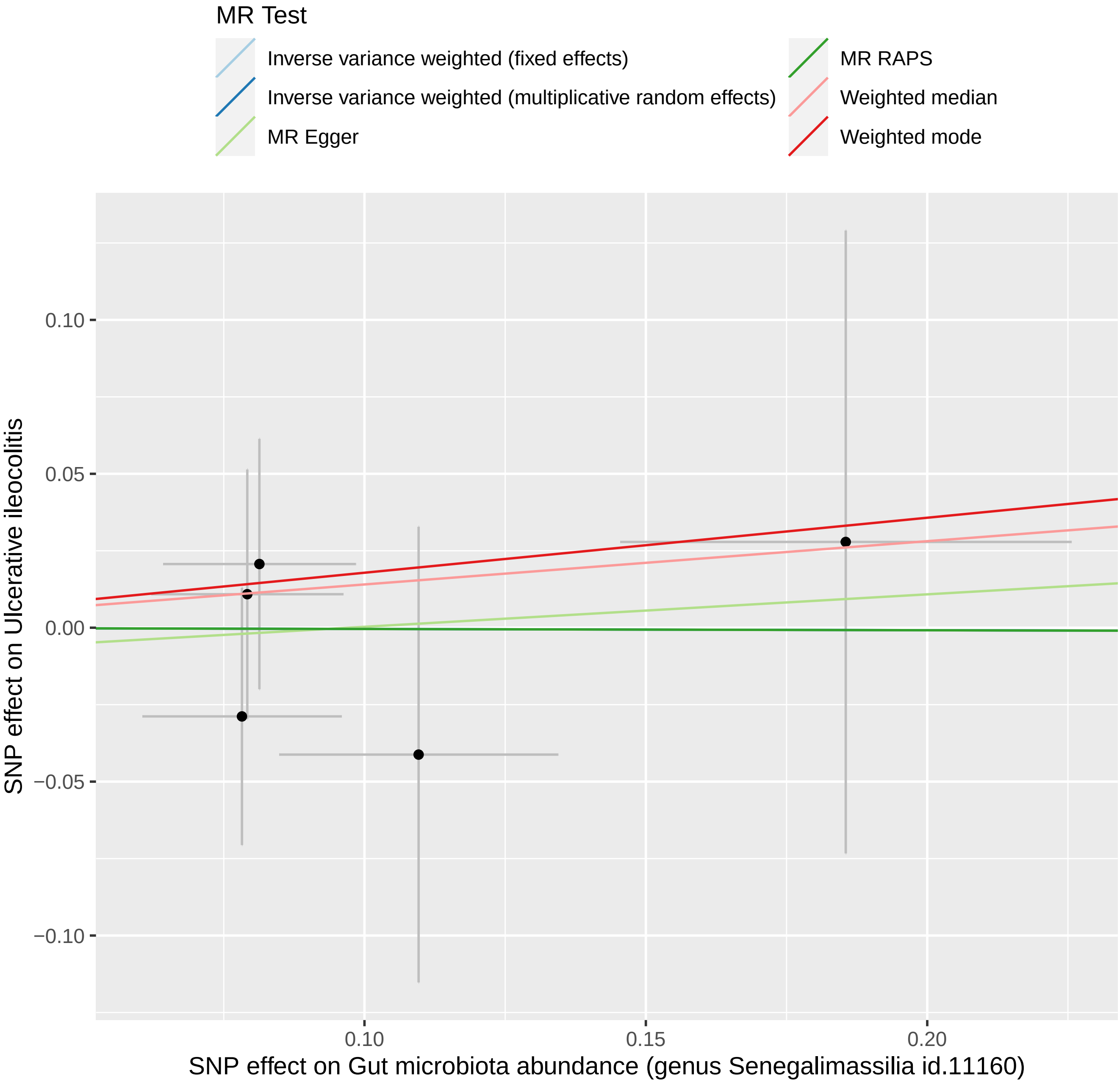

Batch 1193 : Gut microbiota abundance (genus Slackia id.825) on Ulcerative ileocolitis

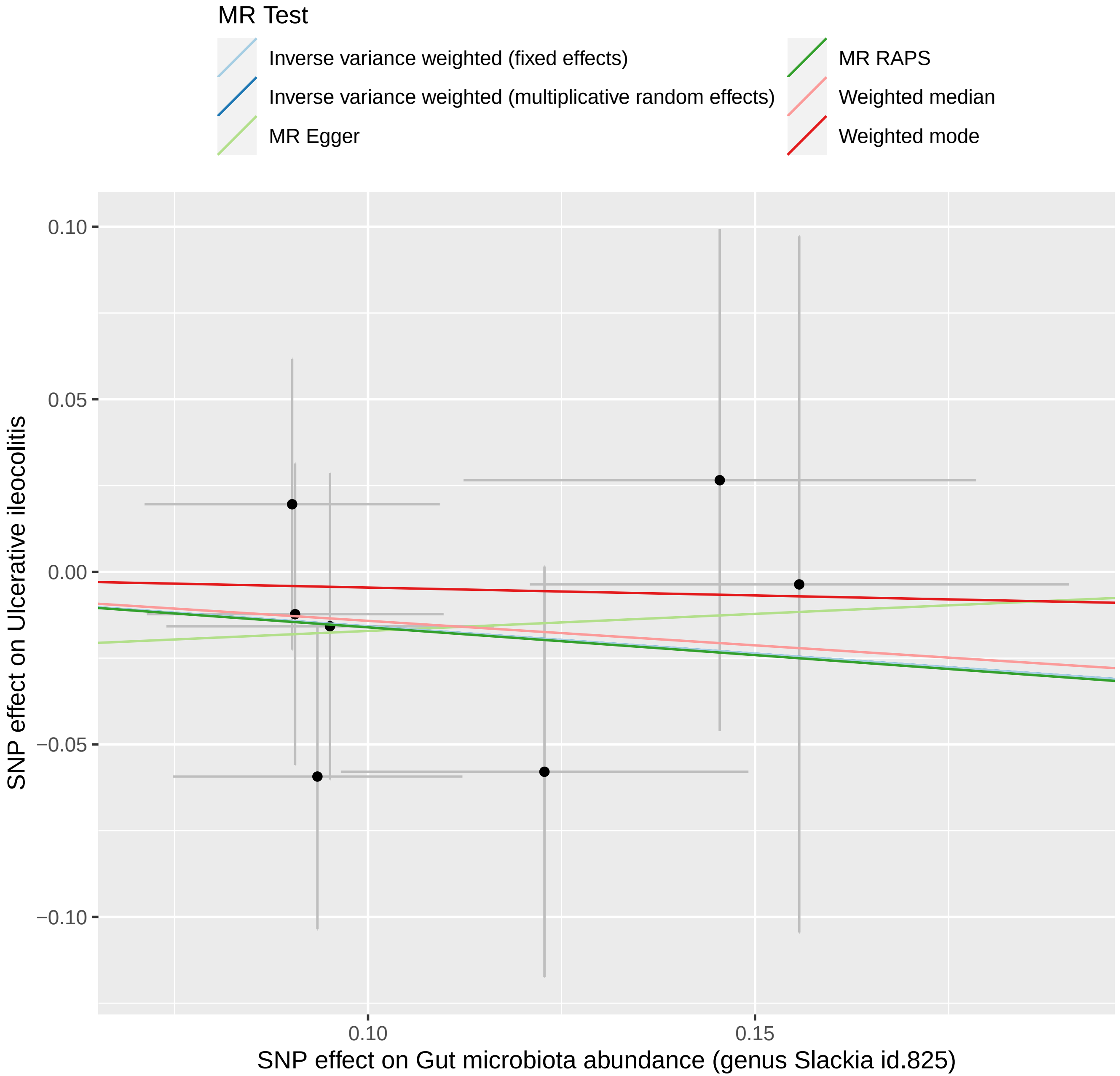

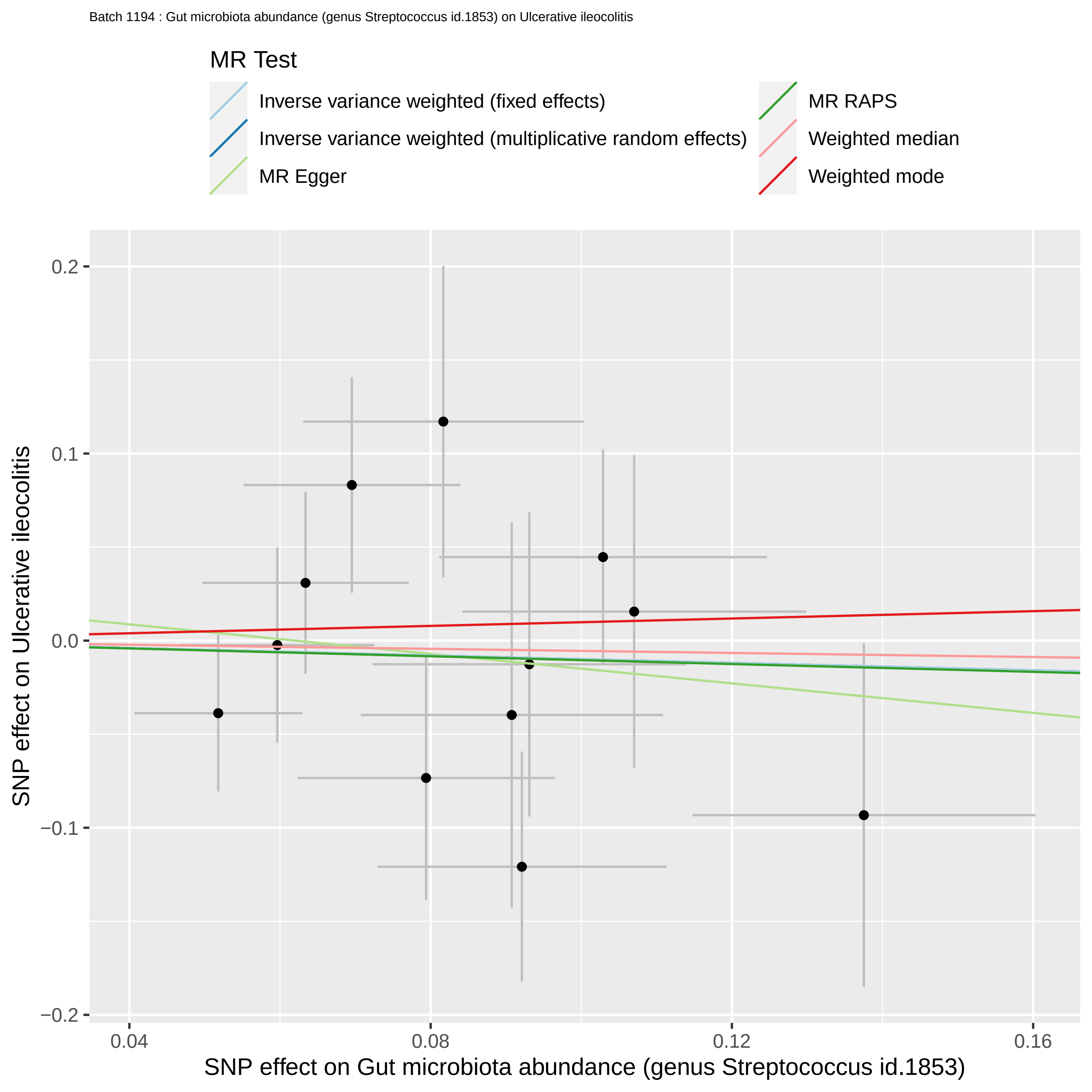

Batch 1194 : Gut microbiota abundance (genus Streptococcus id.1853) on Ulcerative ileocolitis
MR Test
Inverse variance weighted (fixed effects)
Inverse variance weighted (multiplicative random effects)
MR Egger
MR RAPS
Weighted median
Weighted mode
0.2
0.1
0.0
−0.1
−0.2
0.04
0.08
0.12
0.16
SNP effect on Ulcerative ileocolitis
SNP effect on Gut microbiota abundance (genus Streptococcus id.1853)

Batch 1195 : Gut microbiota abundance (genus Subdoligranulum id.2070) on Ulcerative ileocolitis

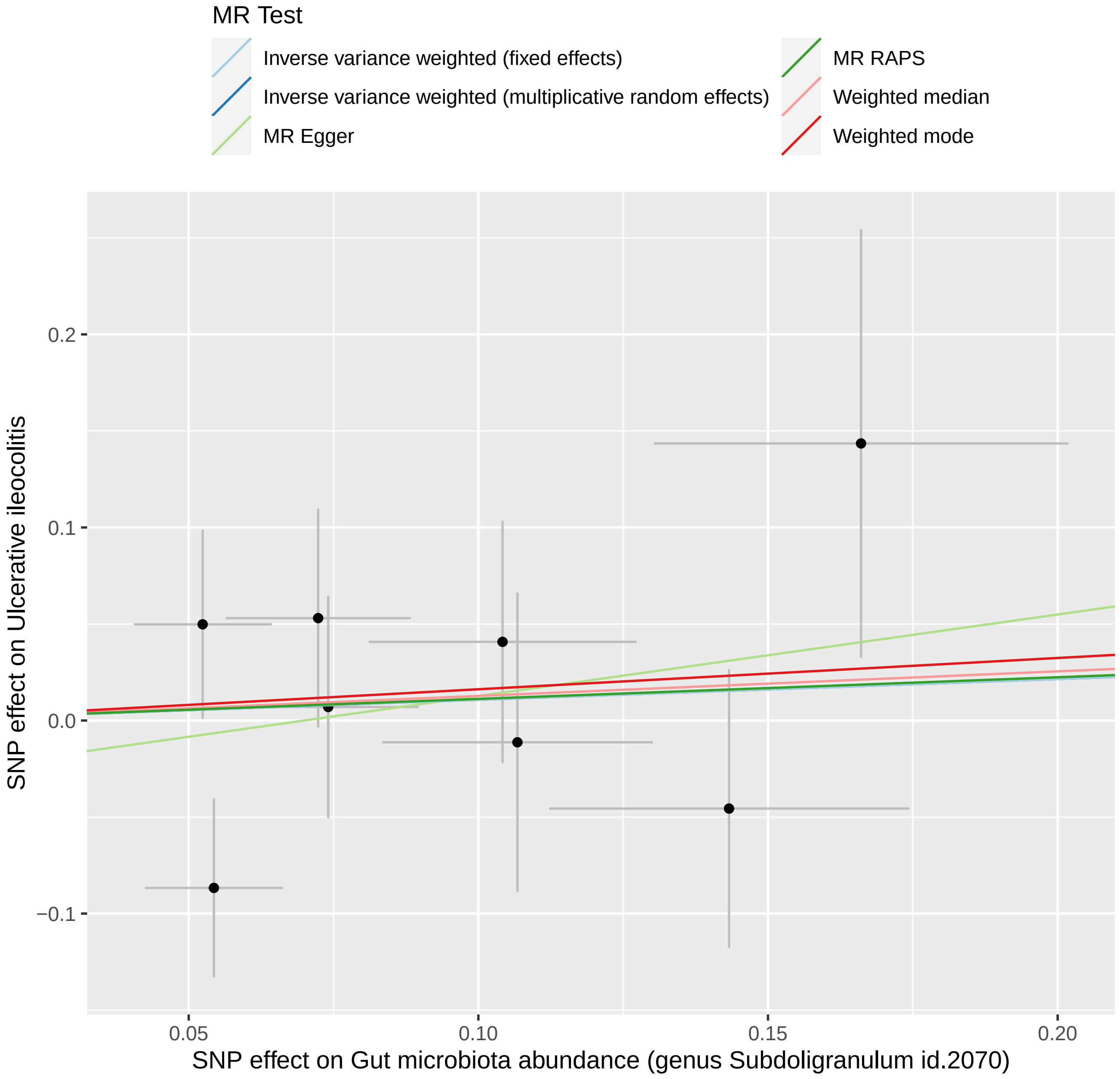

Batch 1196 : Gut microbiota abundance (genus Sutterella id.2896) on Ulcerative ileocolitis

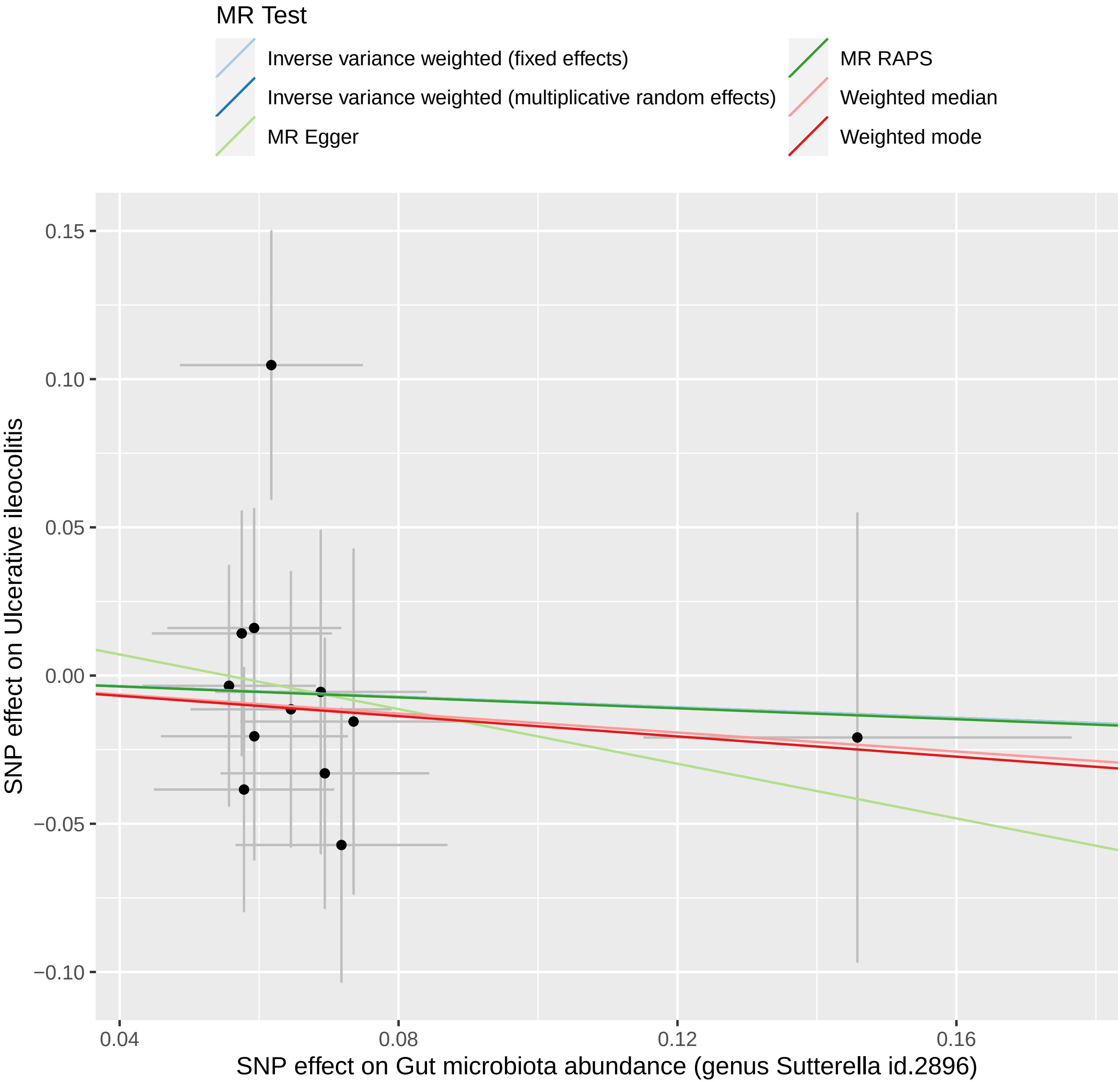

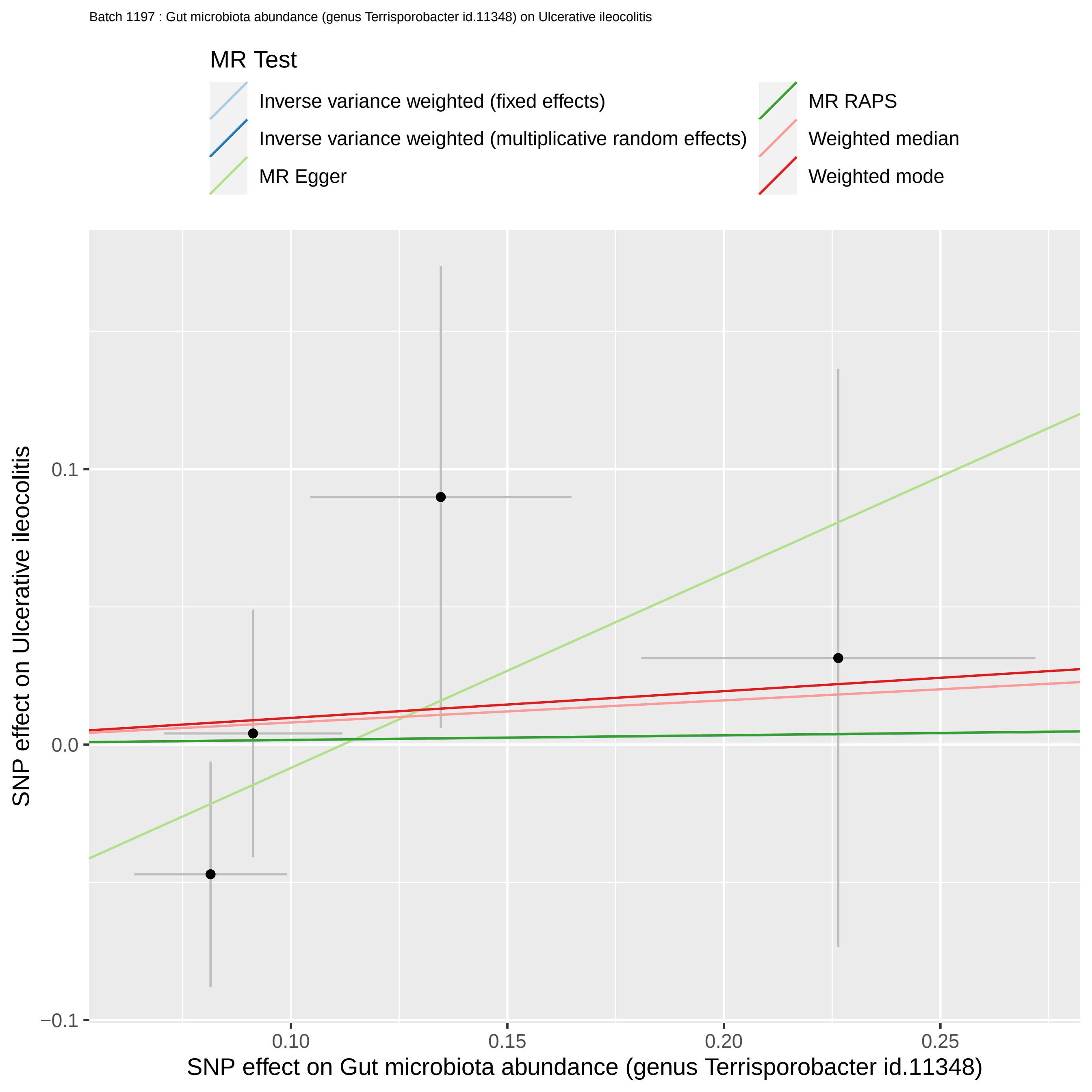

Batch 1197 : Gut microbiota abundance (genus Terrisporobacter id.11348) on Ulcerative ileocolitis
MR Test
Inverse variance weighted (fixed effects)
Inverse variance weighted (multiplicative random effects)
MR Egger
MR RAPS
Weighted median
Weighted mode
0.1
0.0
−0.1
0.10
0.15
0.20
0.25
SNP effect on Ulcerative ileocolitis
SNP effect on Gut microbiota abundance (genus Terrisporobacter id.11348)

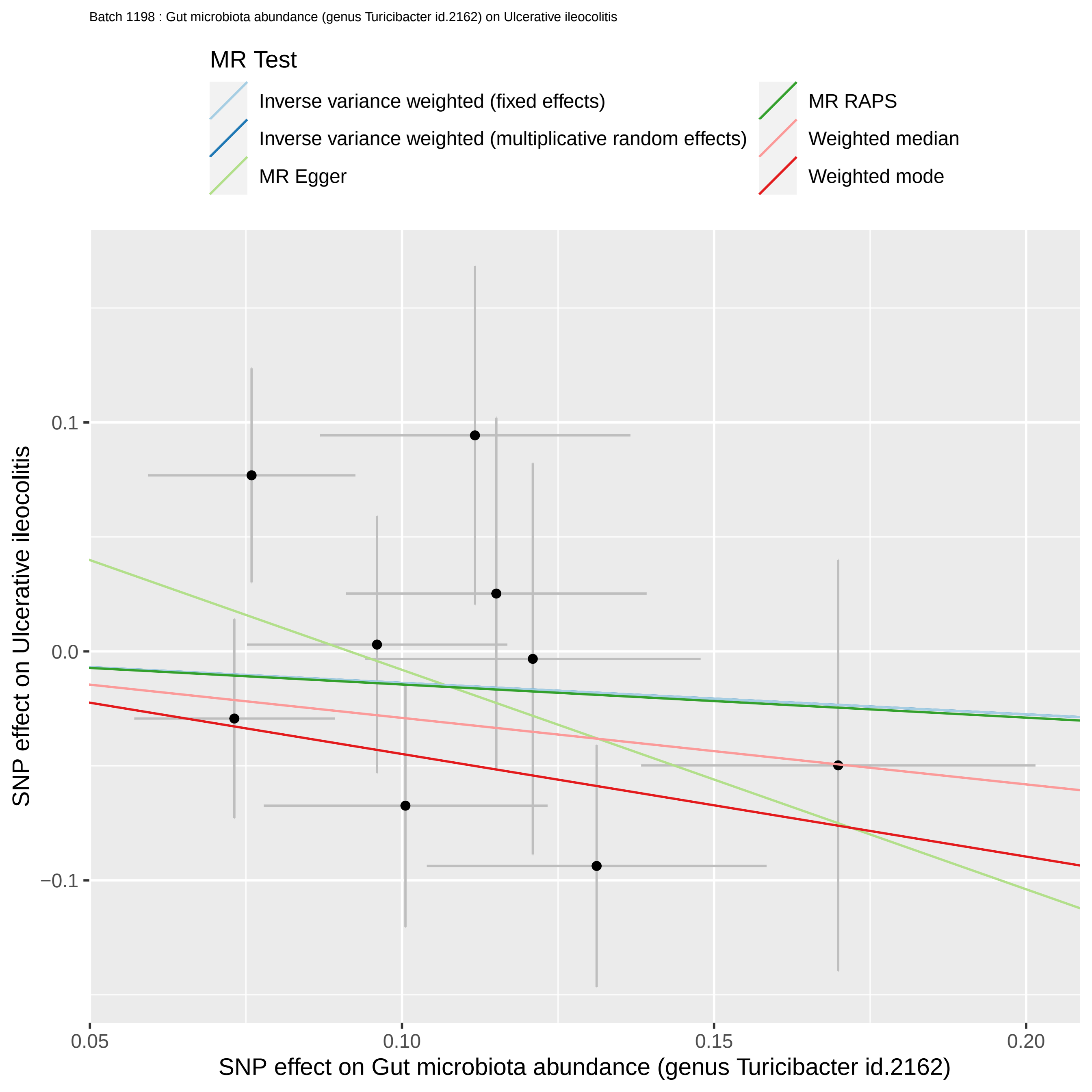

Batch 1198 : Gut microbiota abundance (genus Turicibacter id.2162) on Ulcerative ileocolitis
MR Test
Inverse variance weighted (fixed effects)
Inverse variance weighted (multiplicative random effects)
MR Egger
MR RAPS
Weighted median
Weighted mode
0.1
0.0
−0.1
0.05
0.10
0.15
0.20
SNP effect on Ulcerative ileocolitis
SNP effect on Gut microbiota abundance (genus Turicibacter id.2162)

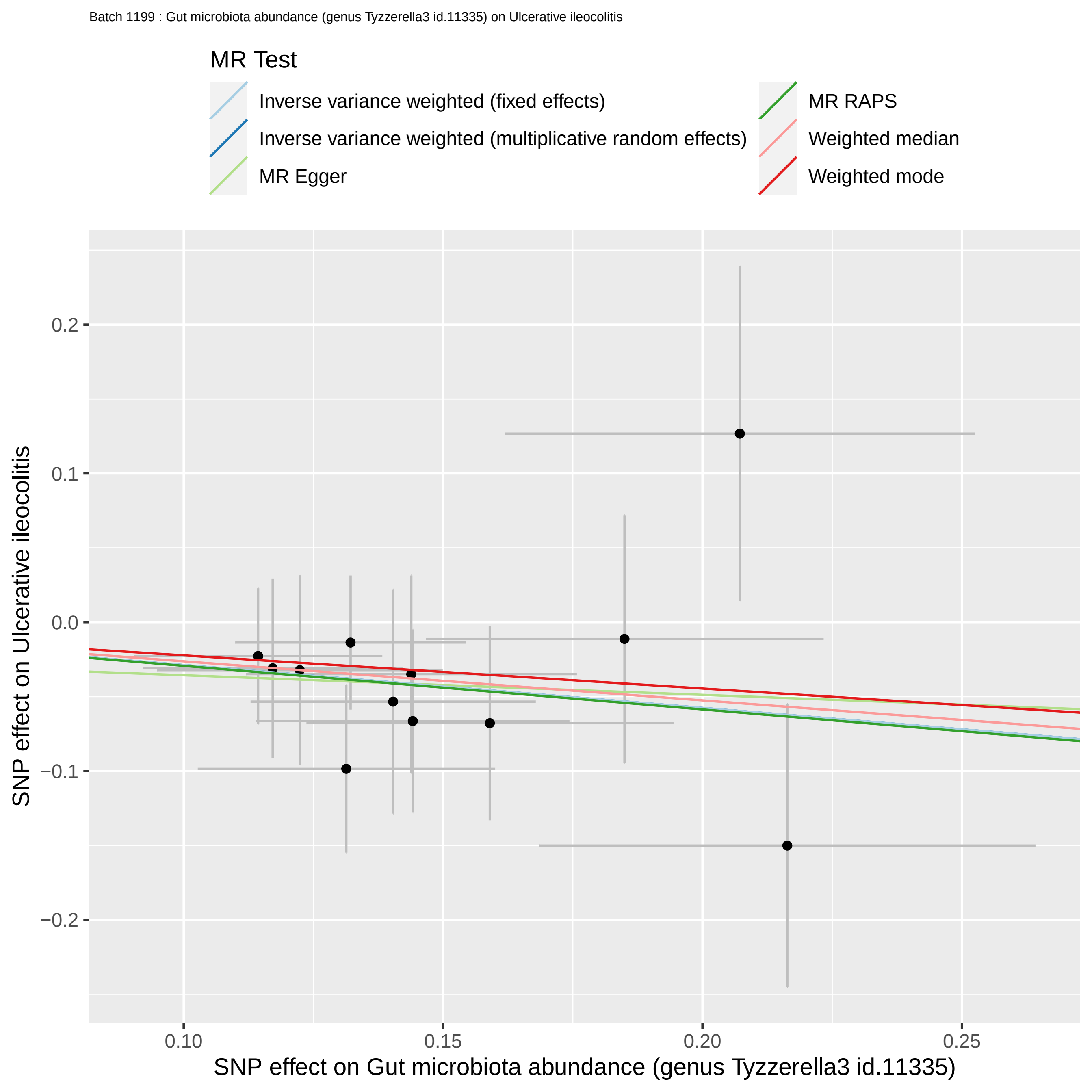
Batch 1199 : Gut microbiota abundance (genus Tyzzerella3 id.11335) on Ulcerative ileocolitis
MR Test
Inverse variance weighted (fixed effects)
Inverse variance weighted (multiplicative random effects)
MR Egger
MR RAPS
Weighted median
Weighted mode
SNP effect on Ulcerative ileocolitis
SNP effect on Gut microbiota abundance (genus Tyzzerella3 id.11335)
0.2
0.1
0.0
−0.1
−0.2
0.10
0.15
0.20
0.25

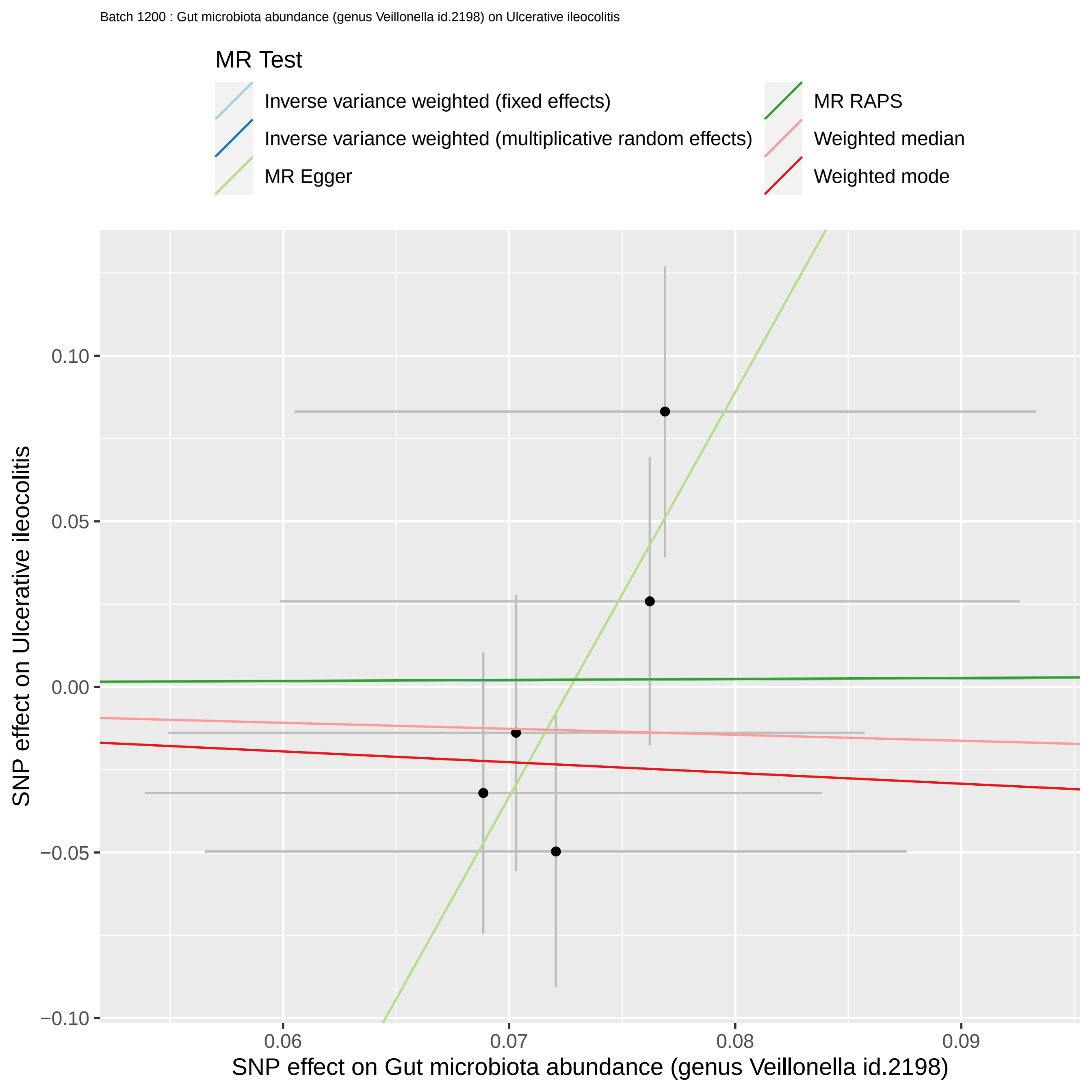

Batch 1200 : Gut microbiota abundance (genus Veillonella id.2198) on Ulcerative ileocolitis
MR Test
Inverse variance weighted (fixed effects)
Inverse variance weighted (multiplicative random effects)
MR Egger
MR RAPS
Weighted median
Weighted mode
0.10
0.05
0.00
−0.05
−0.10
SNP effect on Ulcerative ileocolitis
0.06
0.07
0.08
0.09
SNP effect on Gut microbiota abundance (genus Veillonella id.2198)

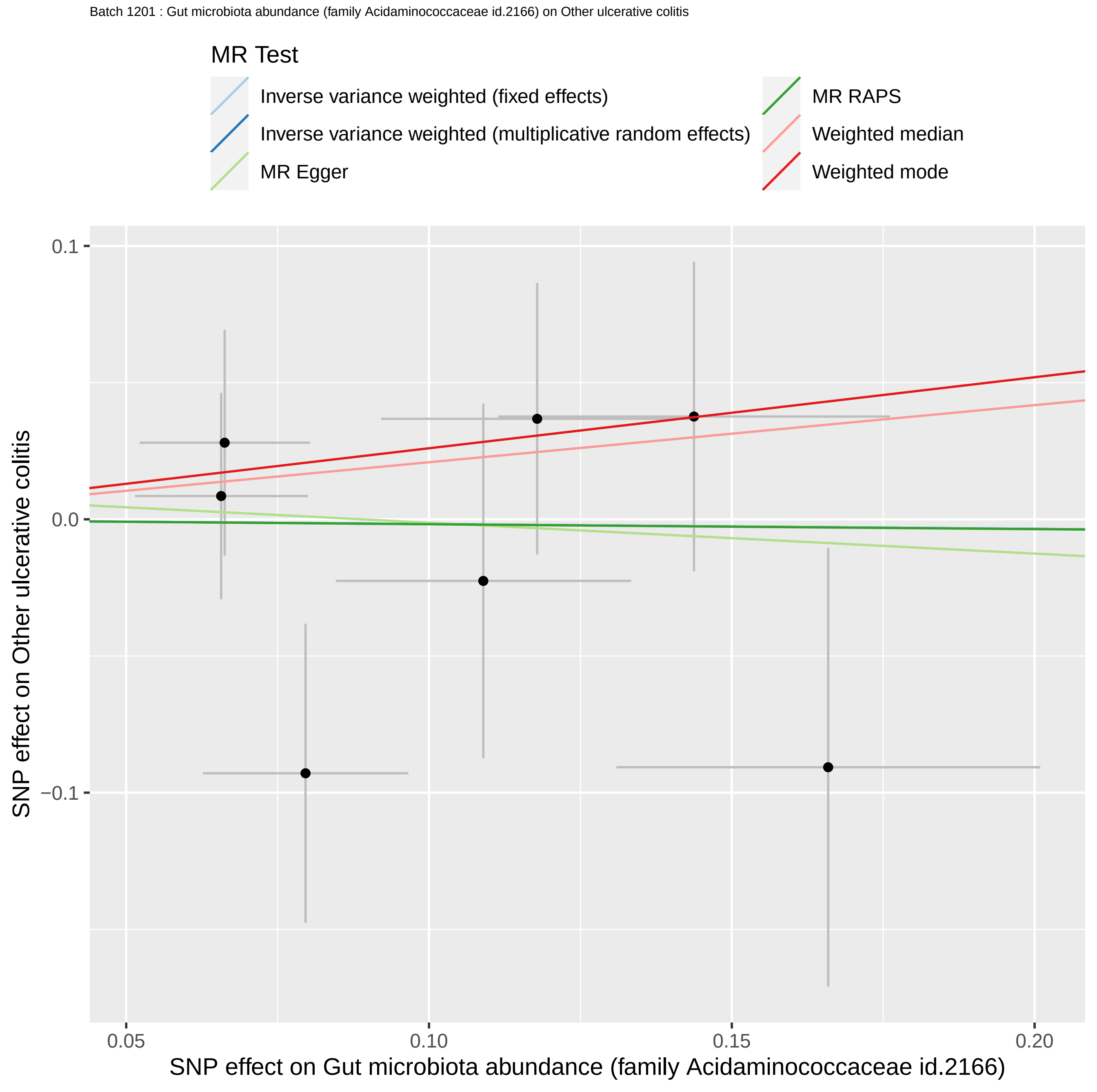
Batch 1201 : Gut microbiota abundance (family Acidaminococcaceae id.2166) on Other ulcerative colitis
MR Test
Inverse variance weighted (fixed effects)
Inverse variance weighted (multiplicative random effects)
MR Egger
MR RAPS
Weighted median
Weighted mode
0.1
0.0
−0.1
0.05
0.10
0.15
0.20
SNP effect on Other ulcerative colitis
SNP effect on Gut microbiota abundance (family Acidaminococcaceae id.2166)

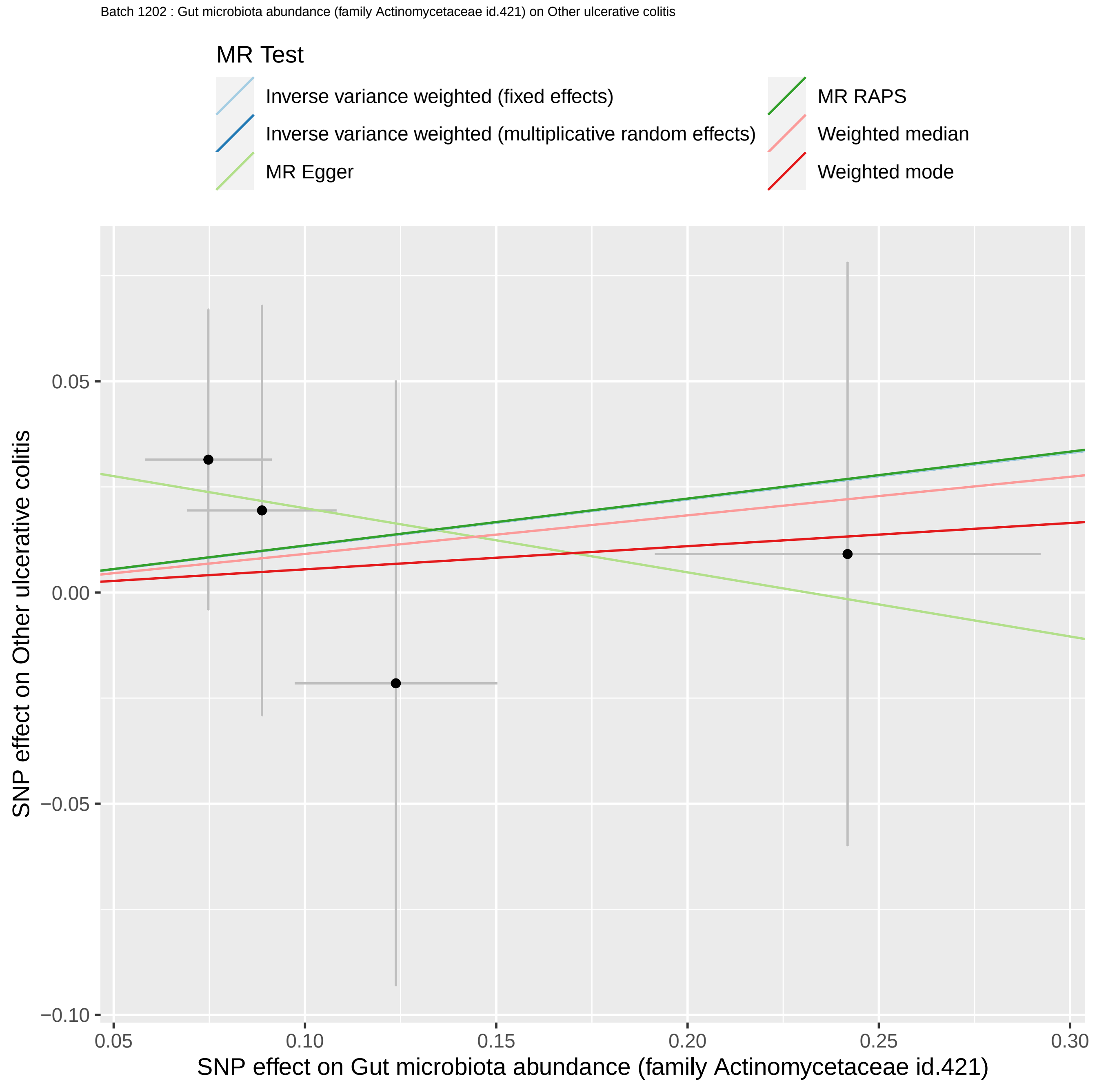

Batch 1202 : Gut microbiota abundance (family Actinomycetaceae id.421) on Other ulcerative colitis
MR Test
Inverse variance weighted (fixed effects)
Inverse variance weighted (multiplicative random effects)
MR Egger
MR RAPS
Weighted median
Weighted mode
0.05
0.00
−0.05
−0.10
0.05
0.10
0.15
0.20
0.25
0.30
SNP effect on Other ulcerative colitis
SNP effect on Gut microbiota abundance (family Actinomycetaceae id.421)

Batch 1203 : Gut microbiota abundance (family Alcaligenaceae id.2875) on Other ulcerative colitis

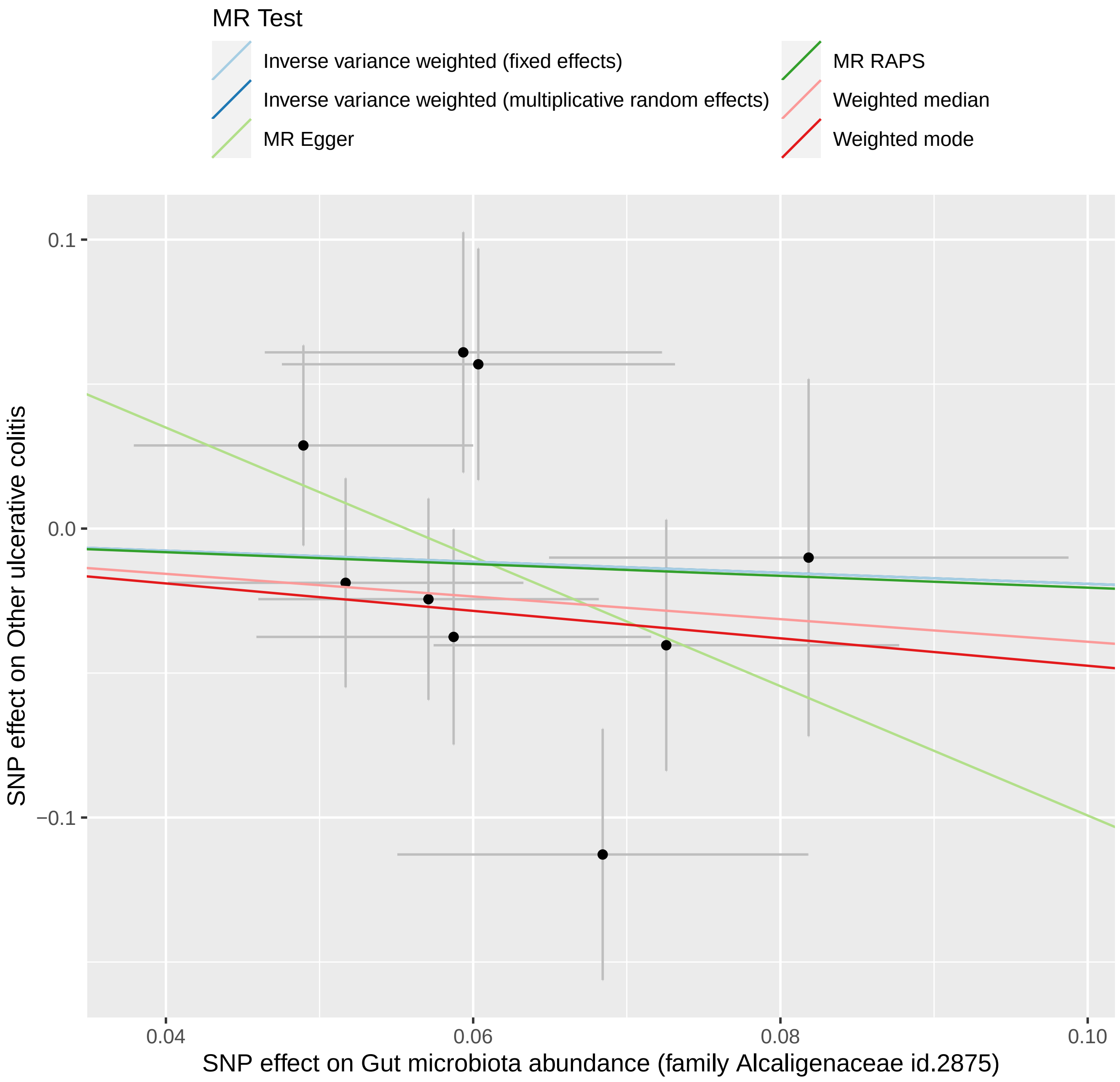

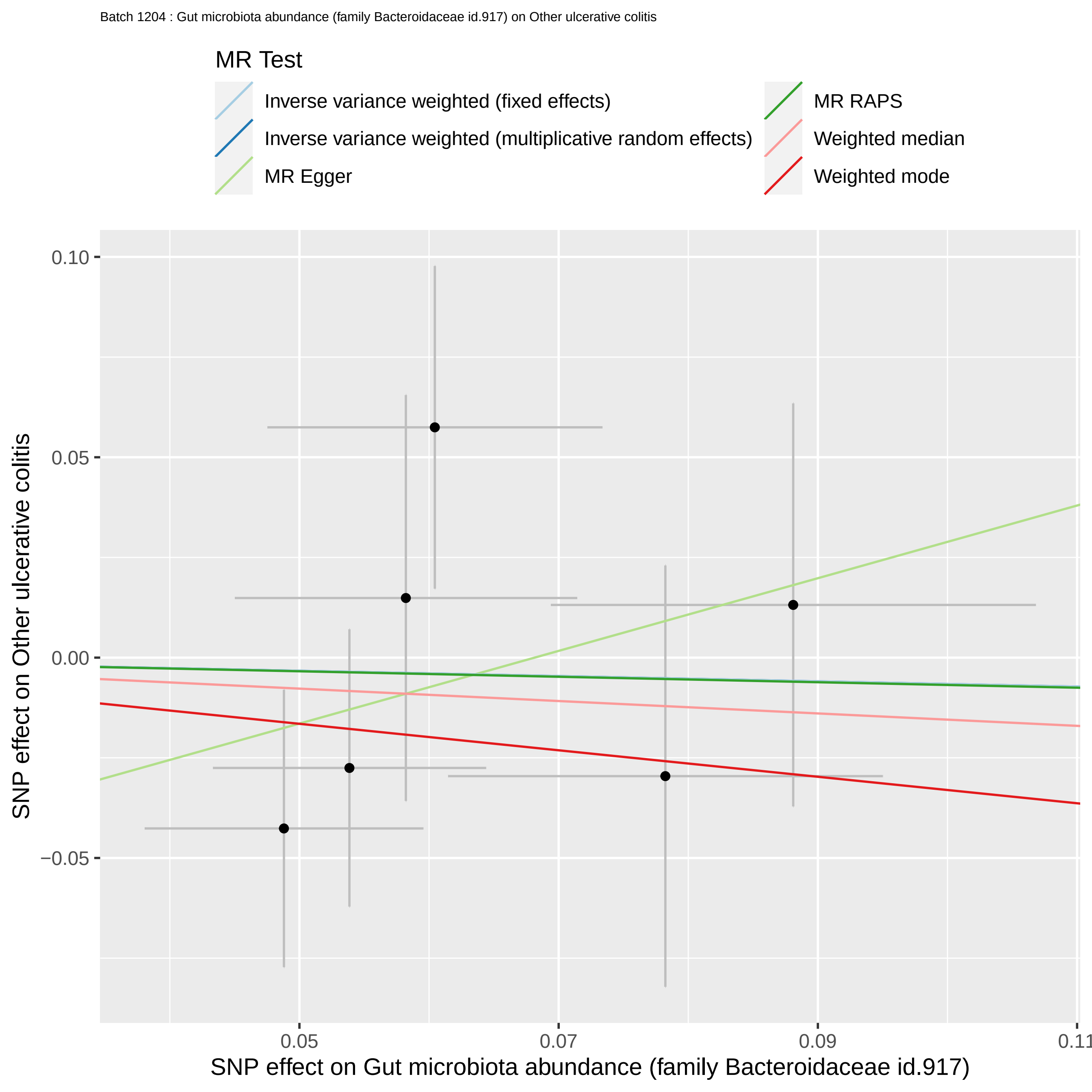

Batch 1204 : Gut microbiota abundance (family Bacteroidaceae id.917) on Other ulcerative colitis
MR Test
Inverse variance weighted (fixed effects)
Inverse variance weighted (multiplicative random effects)
MR Egger
MR RAPS
Weighted median
Weighted mode
0.10
0.05
0.00
−0.05
0.05
0.07
0.09
0.11
SNP effect on Other ulcerative colitis
SNP effect on Gut microbiota abundance (family Bacteroidaceae id.917)

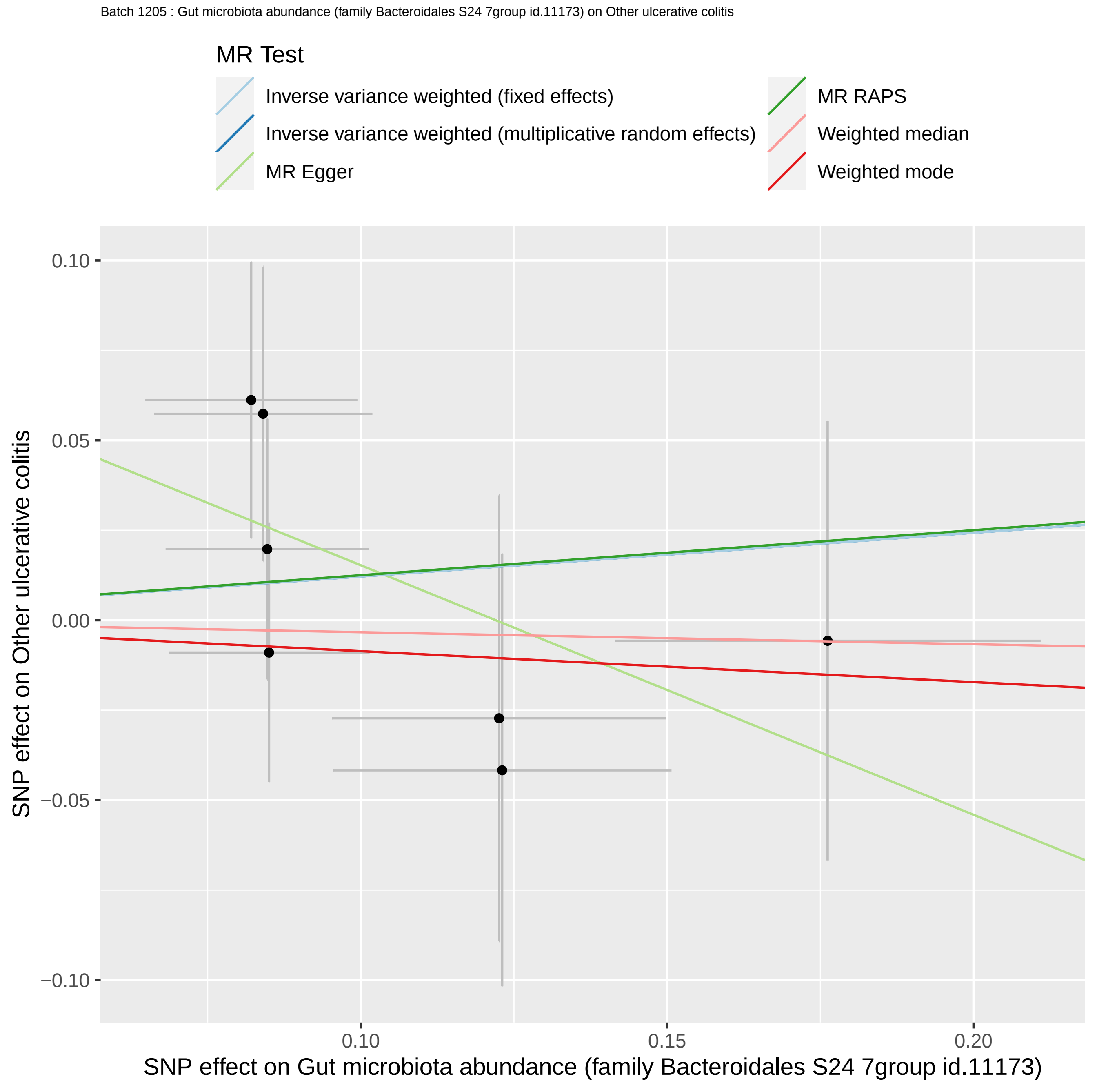

Batch 1205 : Gut microbiota abundance (family Bacteroidales S24 7group id.11173) on Other ulcerative colitis
MR Test
Inverse variance weighted (fixed effects)
Inverse variance weighted (multiplicative random effects)
MR Egger
MR RAPS
Weighted median
Weighted mode
SNP effect on Other ulcerative colitis
0.10
0.05
0.00
−0.05
−0.10
0.10
0.15
0.20
SNP effect on Gut microbiota abundance (family Bacteroidales S24 7group id.11173)

Batch 1206 : Gut microbiota abundance (family Bifidobacteriaceae id.433) on Other ulcerative colitis

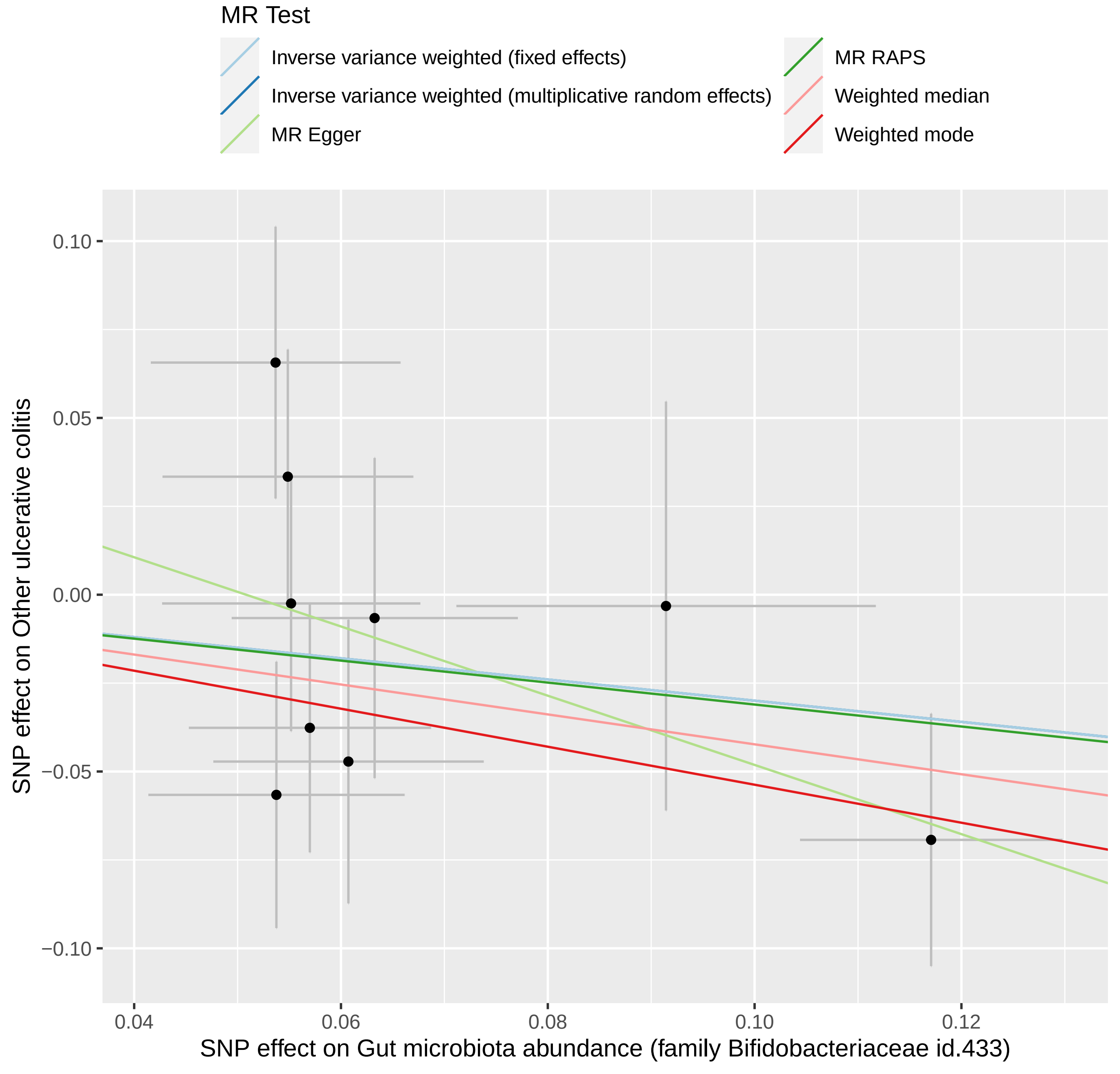

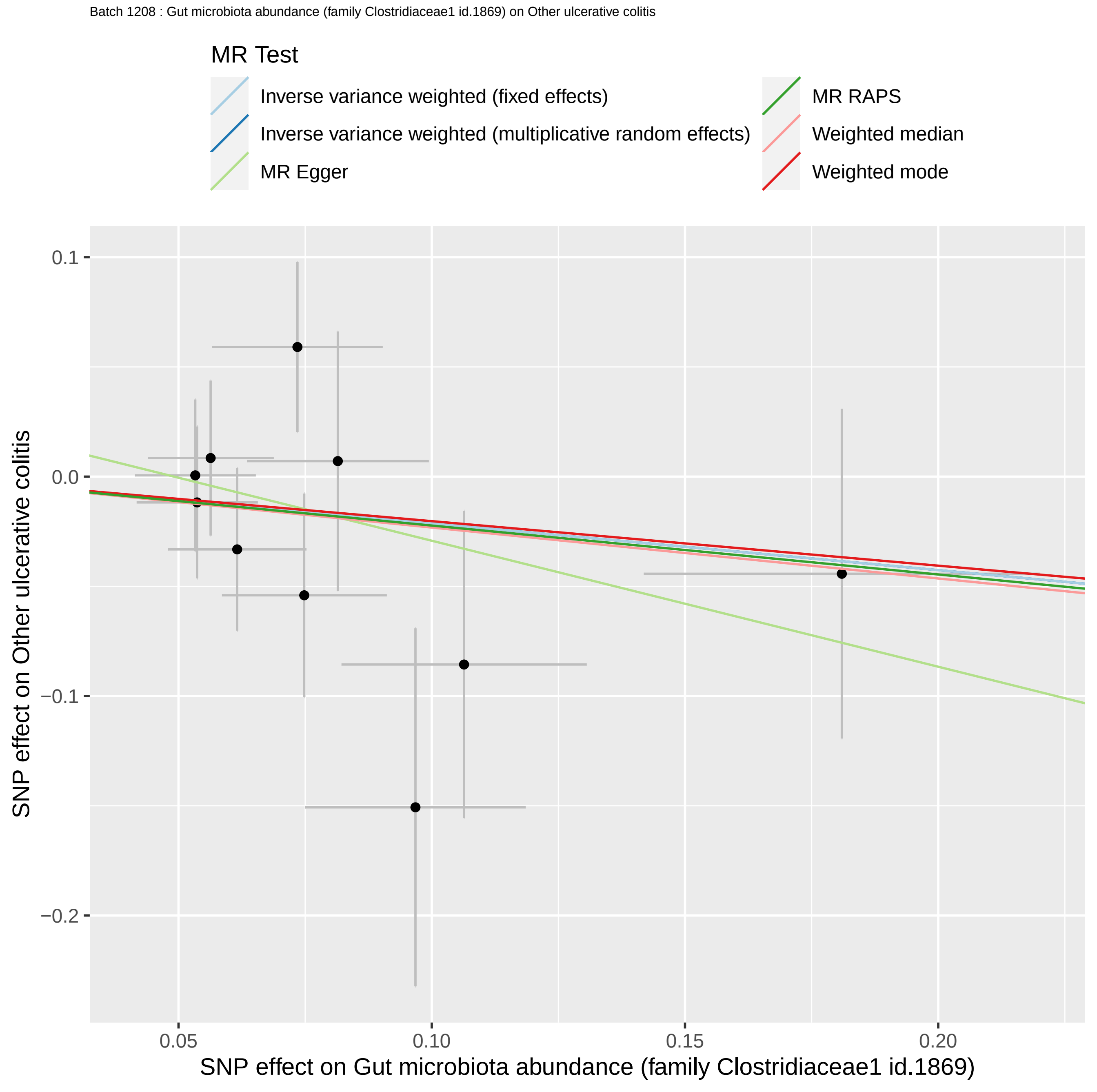

Batch 1208 : Gut microbiota abundance (family Clostridiaceae1 id.1869) on Other ulcerative colitis
MR Test
Inverse variance weighted (fixed effects)
Inverse variance weighted (multiplicative random effects)
MR Egger
MR RAPS
Weighted median
Weighted mode
SNP effect on Other ulcerative colitis
0.1
0.0
−0.1
−0.2
0.05
0.10
0.15
0.20
SNP effect on Gut microbiota abundance (family Clostridiaceae1 id.1869)

Batch 1209 : Gut microbiota abundance (family Clostridiales vadin BB60 group id.11286) on Other ulcerative colitis

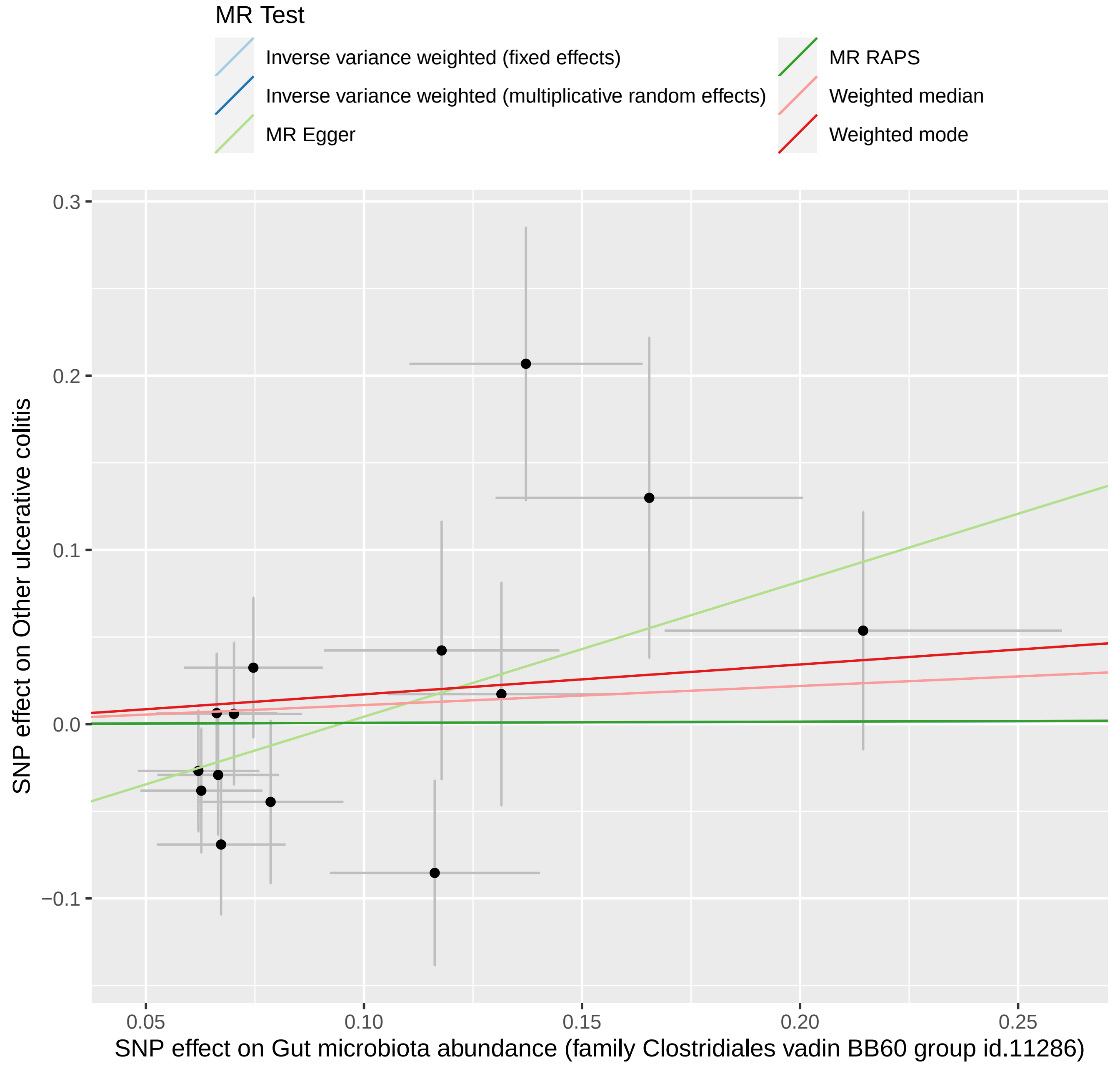

Batch 1210 : Gut microbiota abundance (family Coriobacteriaceae id.811) on Other ulcerative colitis

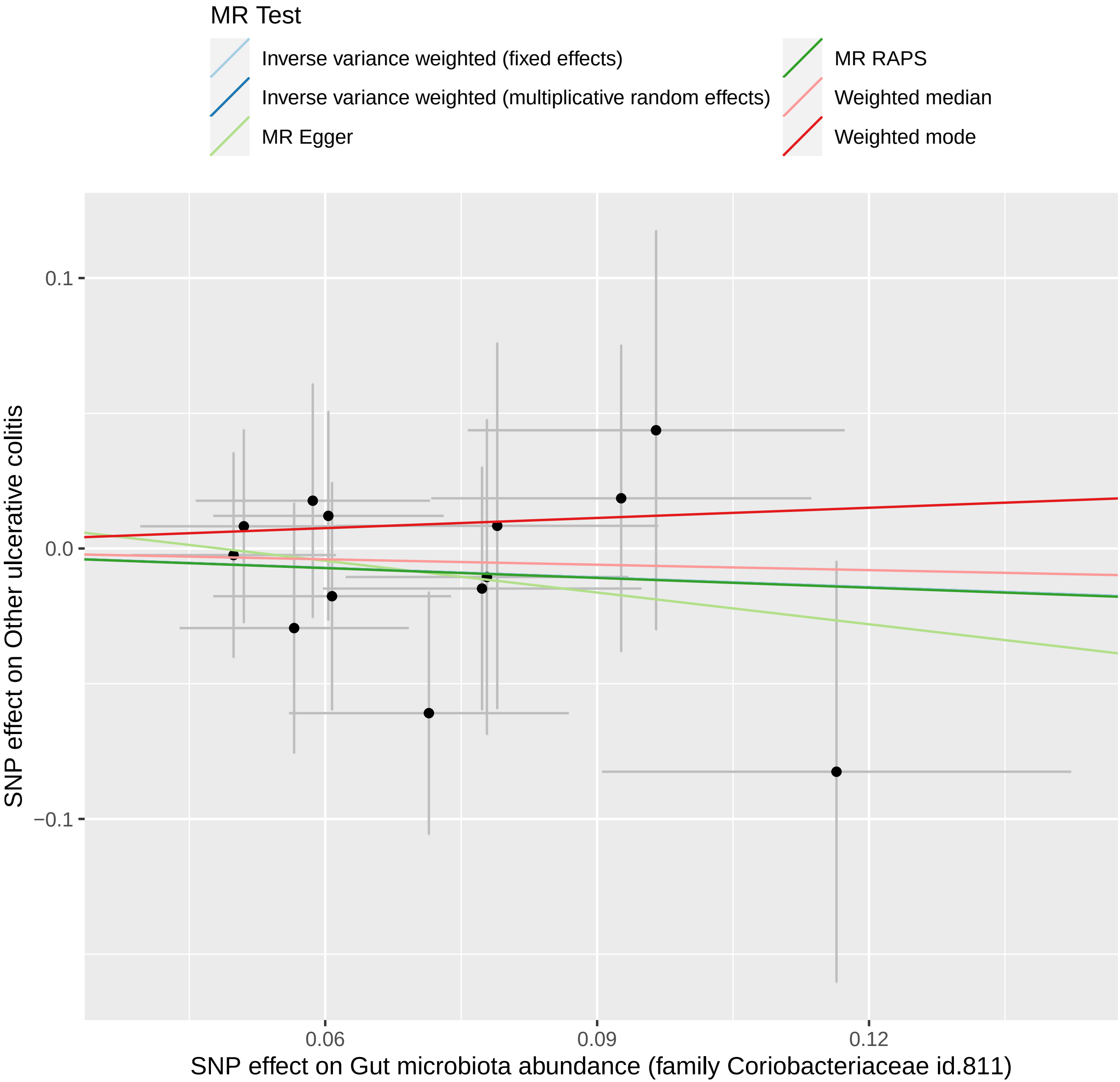

Batch 1211 : Gut microbiota abundance (family Defluviitaleaceae id.1924) on Other ulcerative colitis

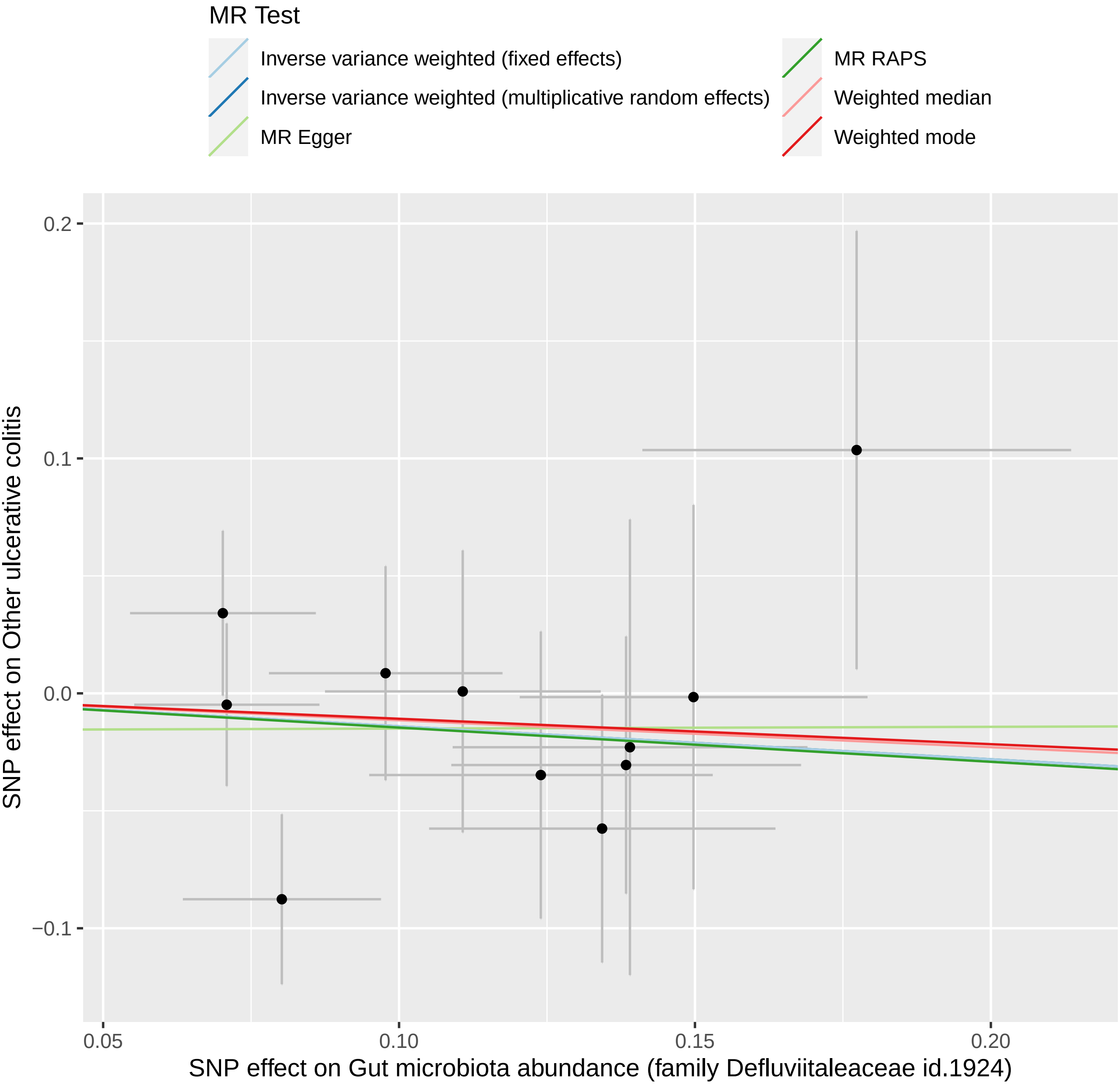

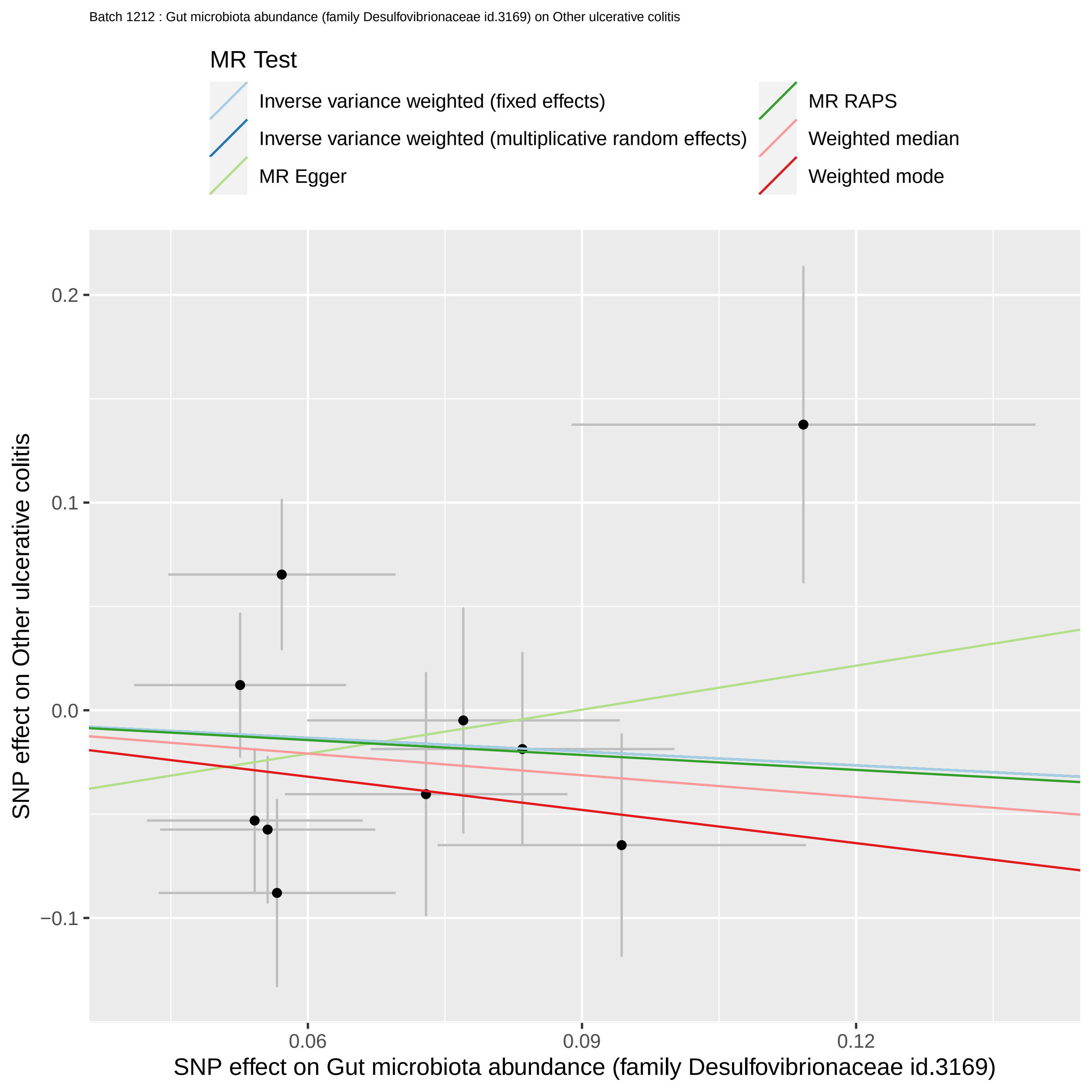

Batch 1212 : Gut microbiota abundance (family Desulfovibrionaceae id.3169) on Other ulcerative colitis
MR Test
Inverse variance weighted (fixed effects)
Inverse variance weighted (multiplicative random effects)
MR Egger
MR RAPS
Weighted median
Weighted mode
0.2
0.1
0.0
−0.1
0.06
0.09
0.12
SNP effect on Other ulcerative colitis
SNP effect on Gut microbiota abundance (family Desulfovibrionaceae id.3169)

Batch 1213 : Gut microbiota abundance (family Enterobacteriaceae id.3469) on Other ulcerative colitis

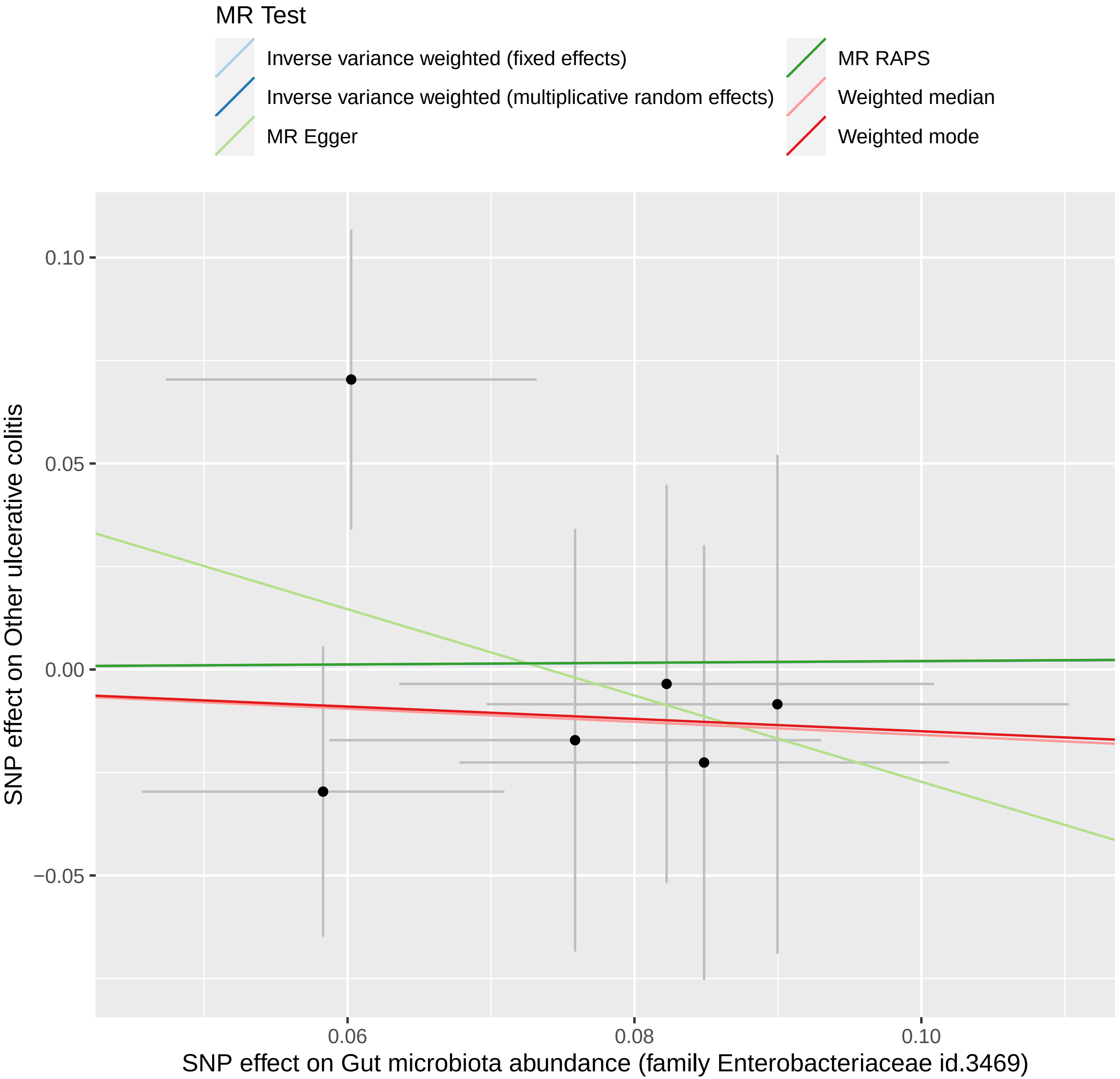

Batch 1214 : Gut microbiota abundance (family Erysipelotrichaceae id.2149) on Other ulcerative colitis

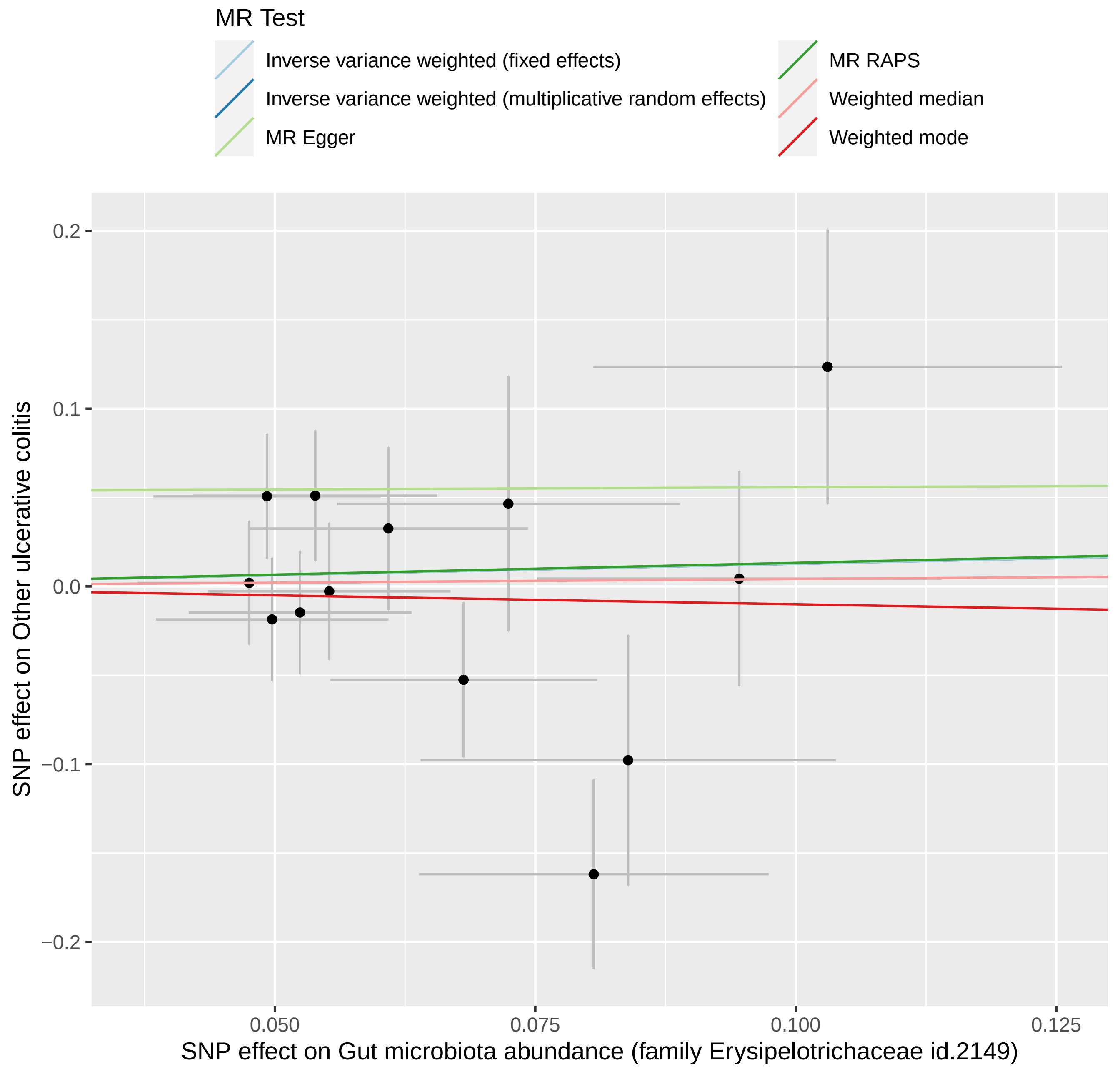

Batch 1215 : Gut microbiota abundance (family Family XI id.1936) on Other ulcerative colitis

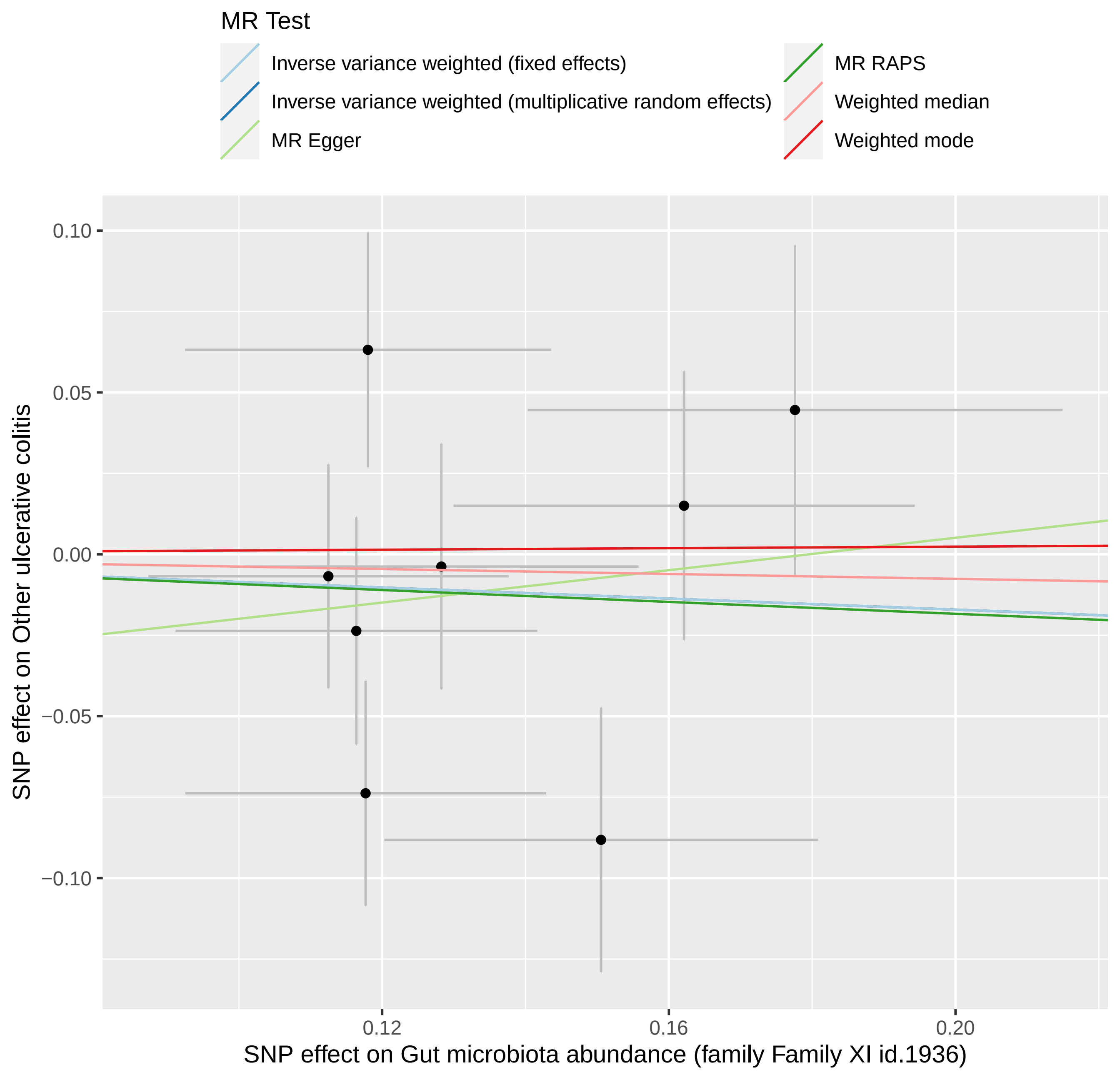

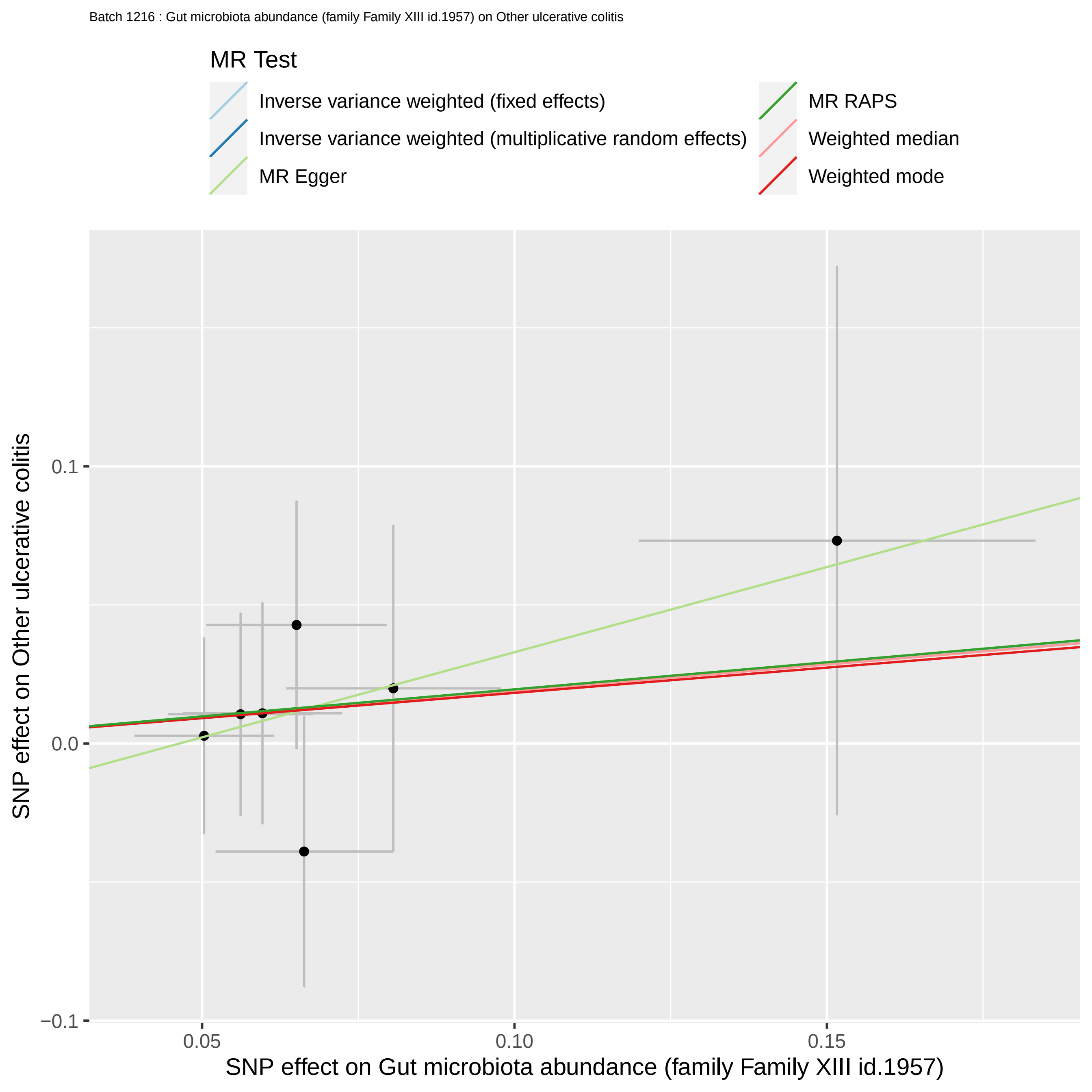

Batch 1216 : Gut microbiota abundance (family Family XIII id.1957) on Other ulcerative colitis
MR Test
Inverse variance weighted (fixed effects)
Inverse variance weighted (multiplicative random effects)
MR Egger
MR RAPS
Weighted median
Weighted mode
SNP effect on Other ulcerative colitis
0.1
0.0
−0.1
0.05
0.10
0.15
SNP effect on Gut microbiota abundance (family Family XIII id.1957)

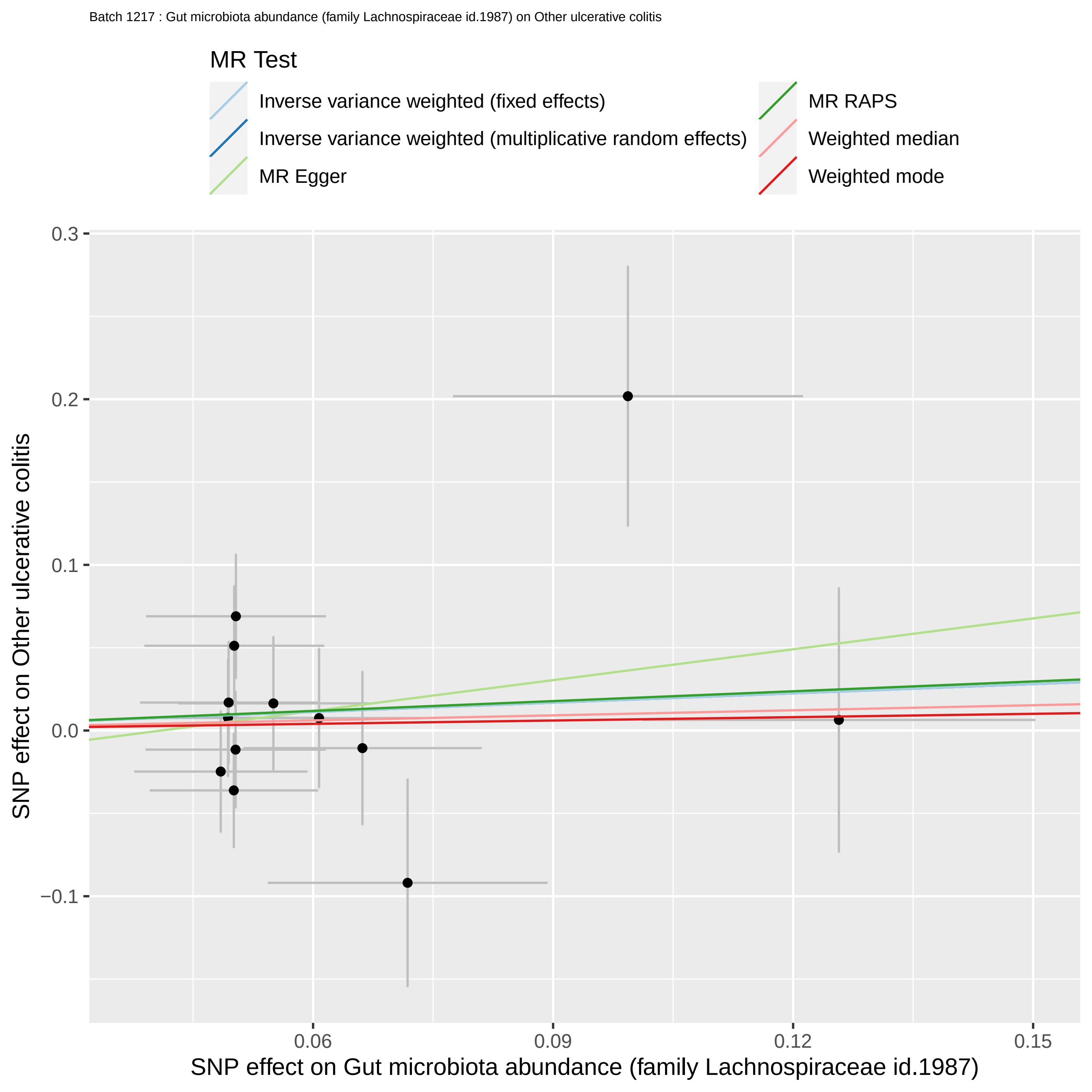
Batch 1217 : Gut microbiota abundance (family Lachnospiraceae id.1987) on Other ulcerative colitis
MR Test
Inverse variance weighted (fixed effects)
Inverse variance weighted (multiplicative random effects)
MR Egger
MR RAPS
Weighted median
Weighted mode
0.3
0.2
0.1
0.0
−0.1
0.06
0.09
0.12
0.15
SNP effect on Other ulcerative colitis
SNP effect on Gut microbiota abundance (family Lachnospiraceae id.1987)

Batch 1218 : Gut microbiota abundance (family Lactobacillaceae id.1836) on Other ulcerative colitis

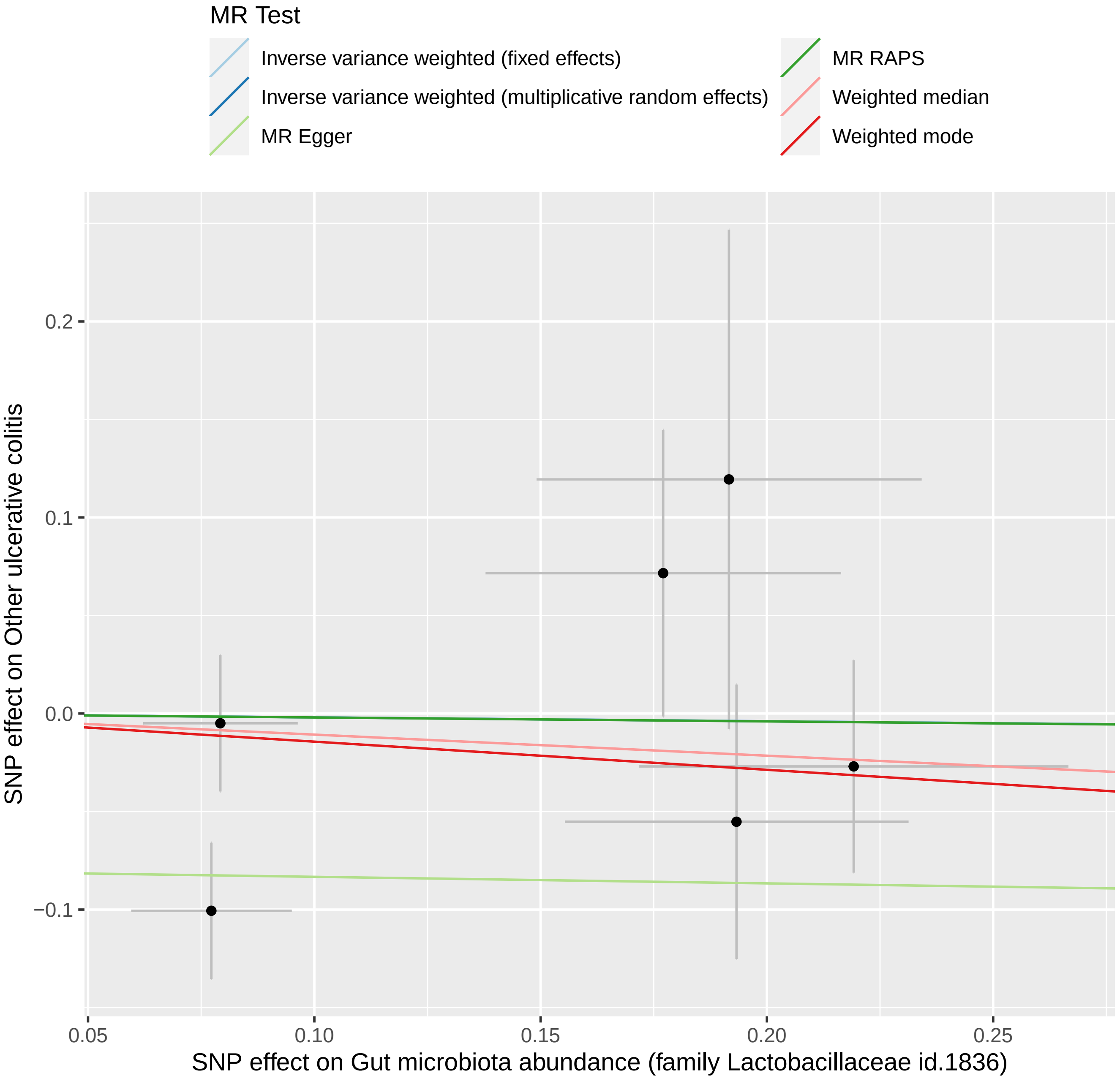

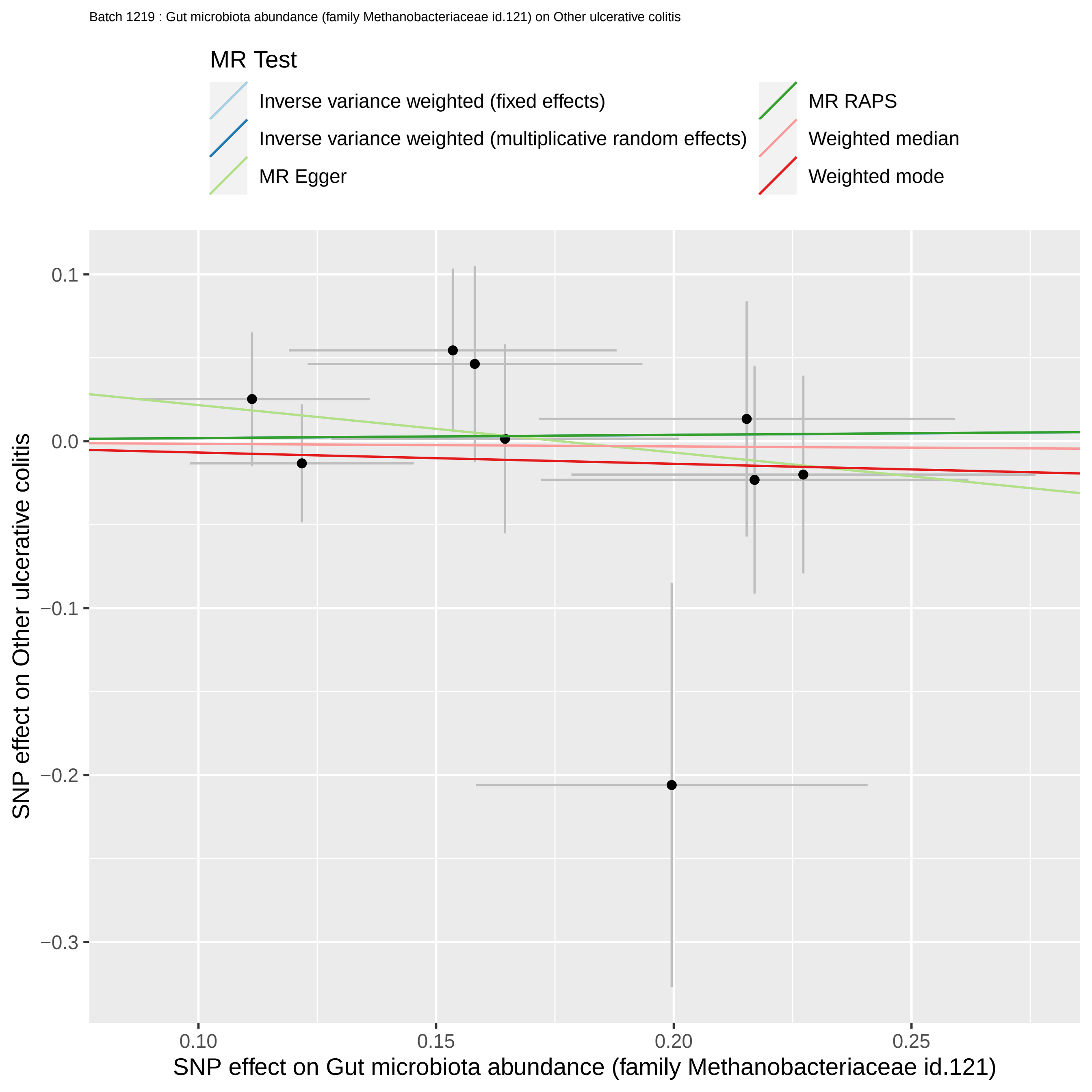
Batch 1219 : Gut microbiota abundance (family Methanobacteriaceae id.121) on Other ulcerative colitis
MR Test
Inverse variance weighted (fixed effects)
Inverse variance weighted (multiplicative random effects)
MR Egger
MR RAPS
Weighted median
Weighted mode
0.1
0.0
−0.1
−0.2
−0.3
0.10
0.15
0.20
0.25
SNP effect on Other ulcerative colitis
SNP effect on Gut microbiota abundance (family Methanobacteriaceae id.121)

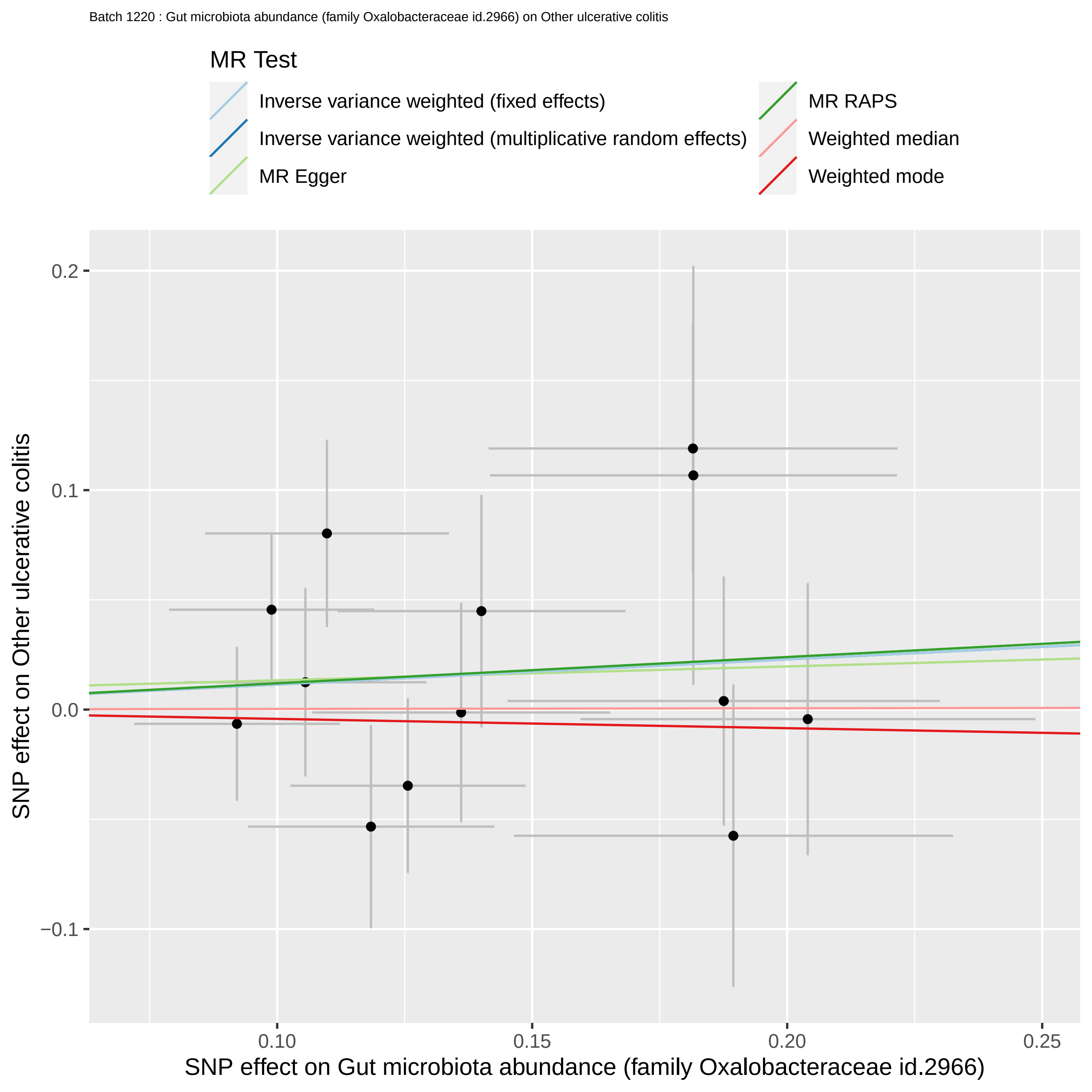
Batch 1220 : Gut microbiota abundance (family Oxalobacteraceae id.2966) on Other ulcerative colitis
MR Test
Inverse variance weighted (fixed effects)
Inverse variance weighted (multiplicative random effects)
MR Egger
MR RAPS
Weighted median
Weighted mode
SNP effect on Other ulcerative colitis
0.2
0.1
0.0
−0.1
0.10
0.15
0.20
0.25
SNP effect on Gut microbiota abundance (family Oxalobacteraceae id.2966)

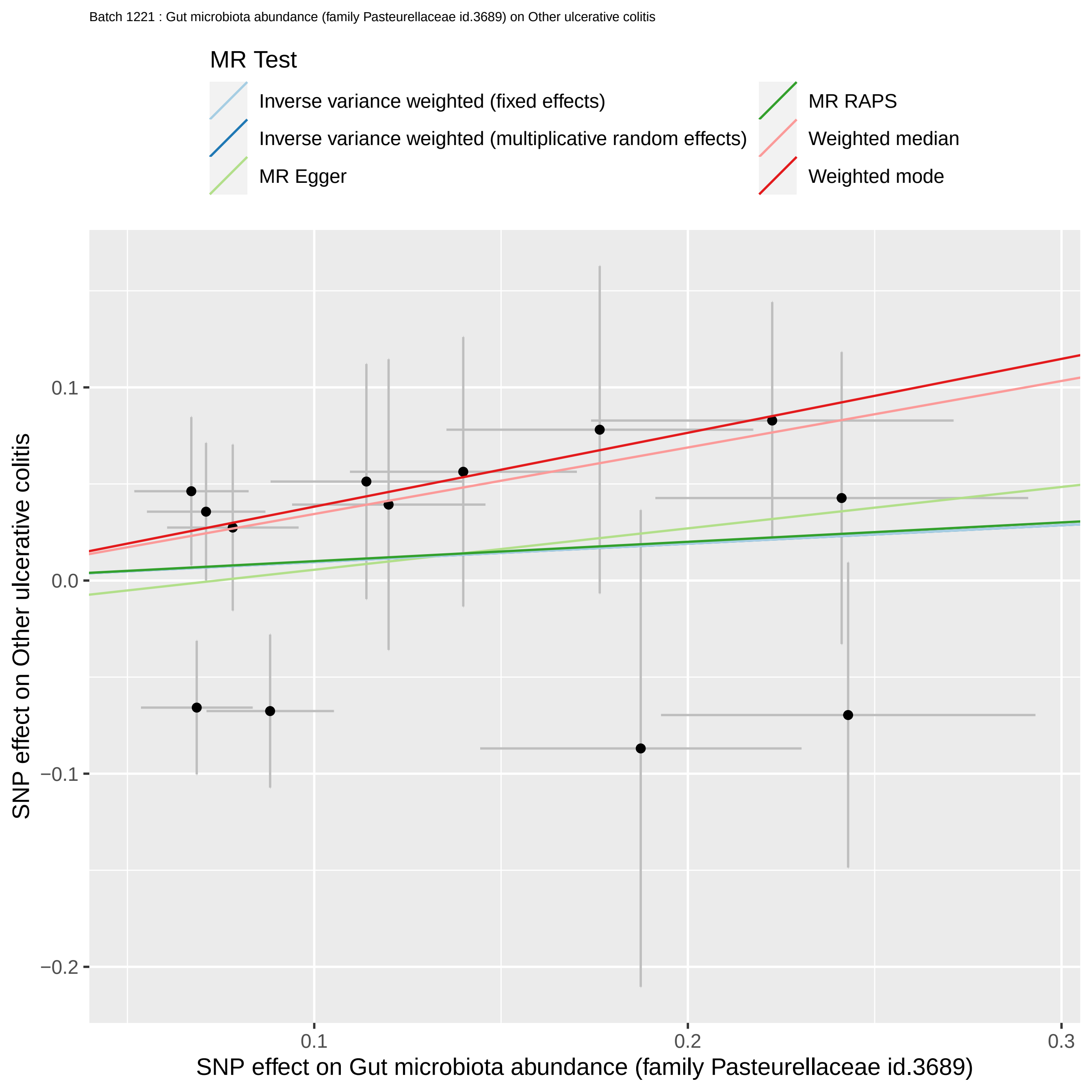

Batch 1221 : Gut microbiota abundance (family Pasteurellaceae id.3689) on Other ulcerative colitis
MR Test
Inverse variance weighted (fixed effects)
Inverse variance weighted (multiplicative random effects)
MR Egger
MR RAPS
Weighted median
Weighted mode
SNP effect on Other ulcerative colitis
0.1
0.0
−0.1
−0.2
0.1
0.2
0.3
SNP effect on Gut microbiota abundance (family Pasteurellaceae id.3689)

Batch 1222 : Gut microbiota abundance (family Peptococcaceae id.2024) on Other ulcerative colitis

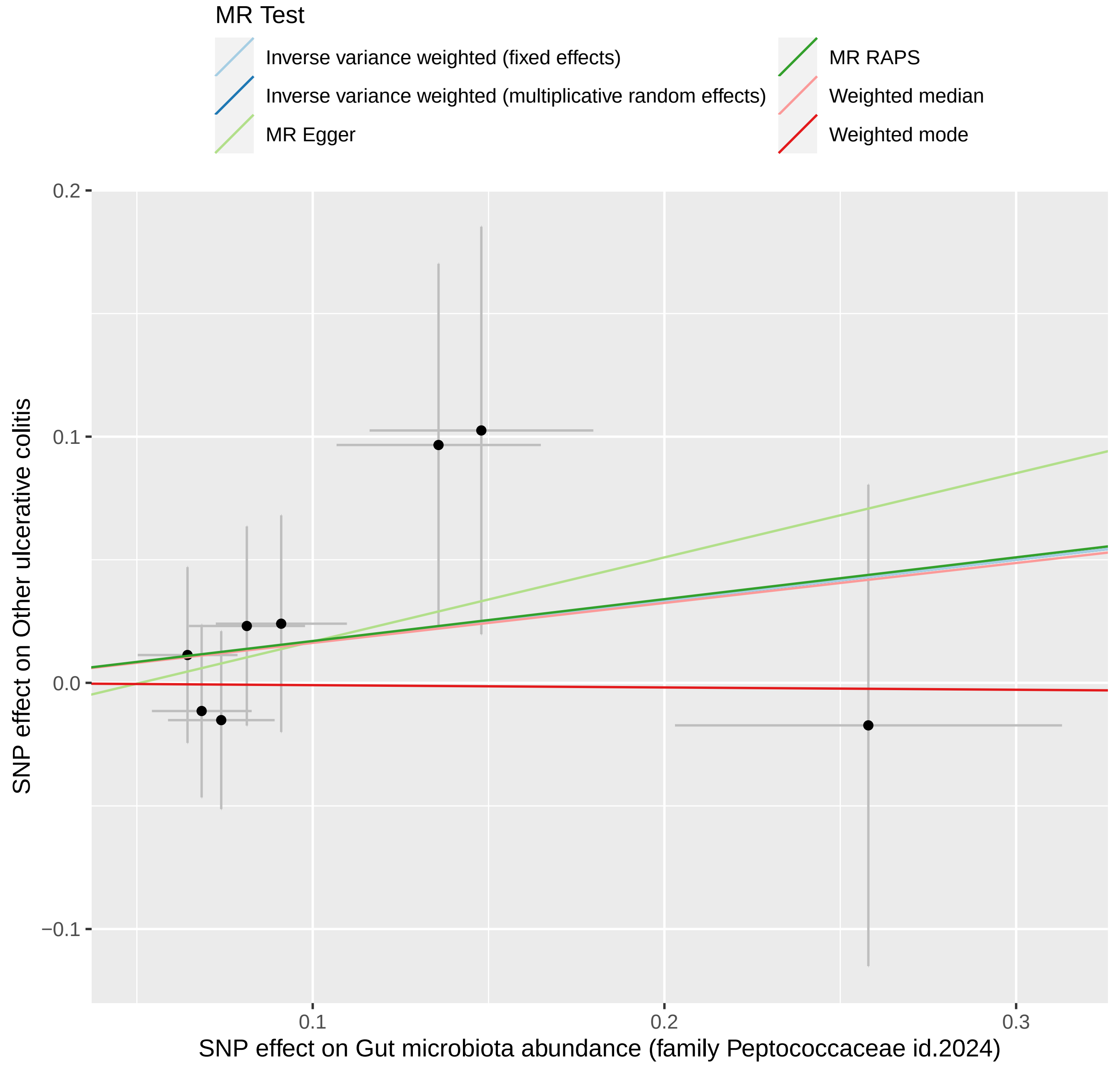

Batch 1223 : Gut microbiota abundance (family Peptostreptococcaceae id.2042) on Other ulcerative colitis

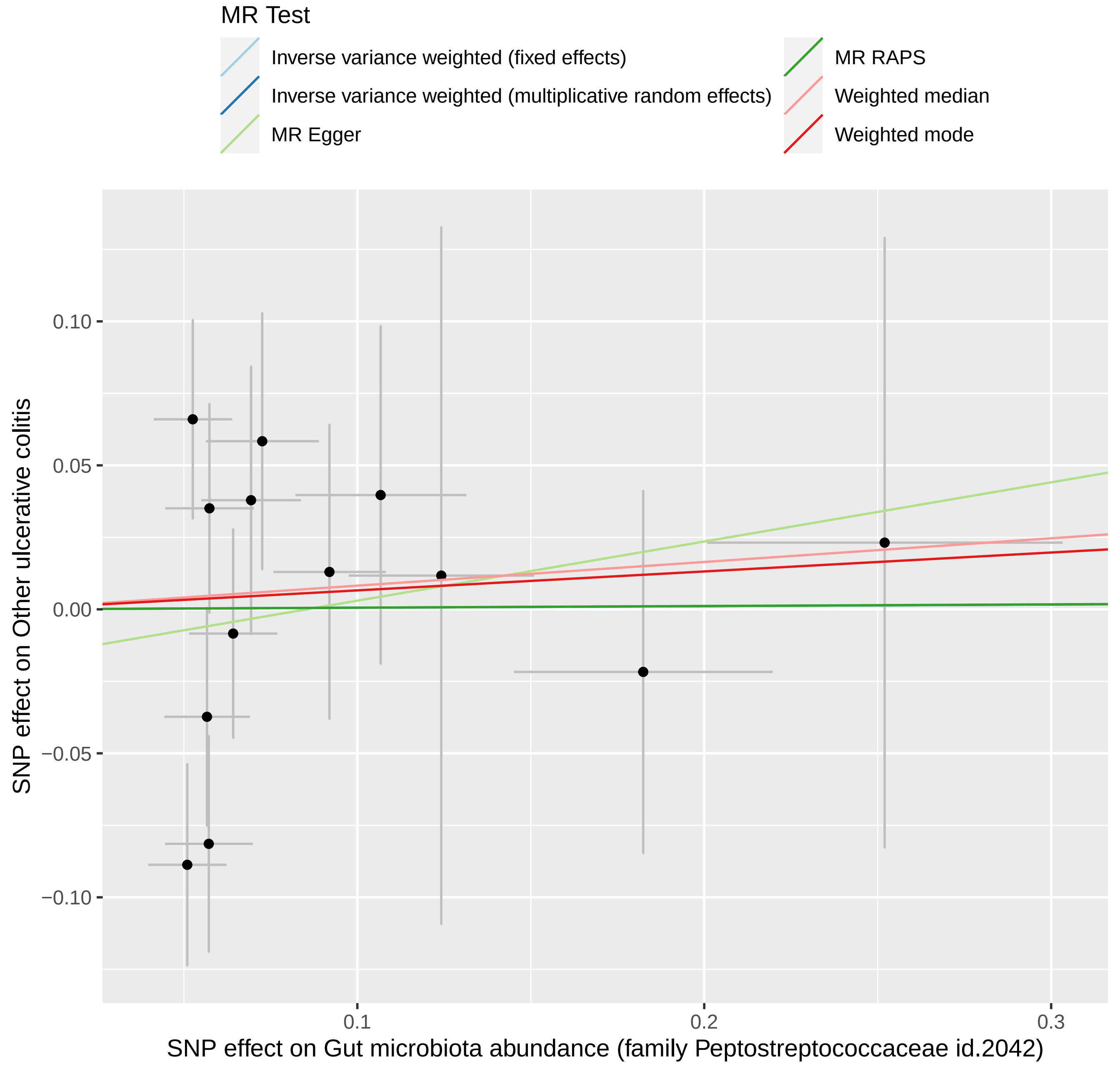

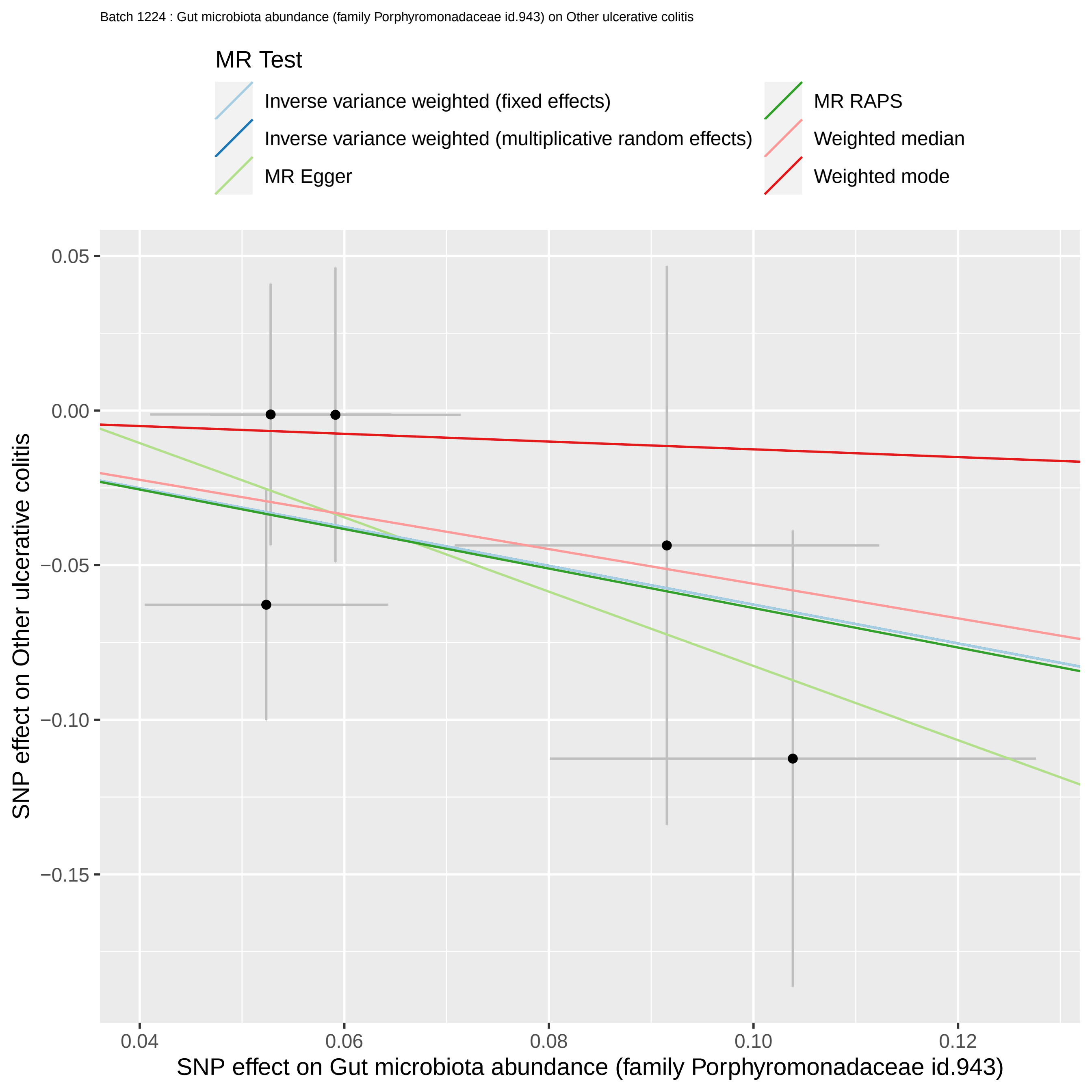

Batch 1224 : Gut microbiota abundance (family Porphyromonadaceae id.943) on Other ulcerative colitis
MR Test
Inverse variance weighted (fixed effects)
Inverse variance weighted (multiplicative random effects)
MR Egger
MR RAPS
Weighted median
Weighted mode
SNP effect on Other ulcerative colitis
0.05
0.00
−0.05
−0.10
−0.15
0.04
0.06
0.08
0.10
0.12
SNP effect on Gut microbiota abundance (family Porphyromonadaceae id.943)

Batch 1225 : Gut microbiota abundance (family Prevotellaceae id.960) on Other ulcerative colitis

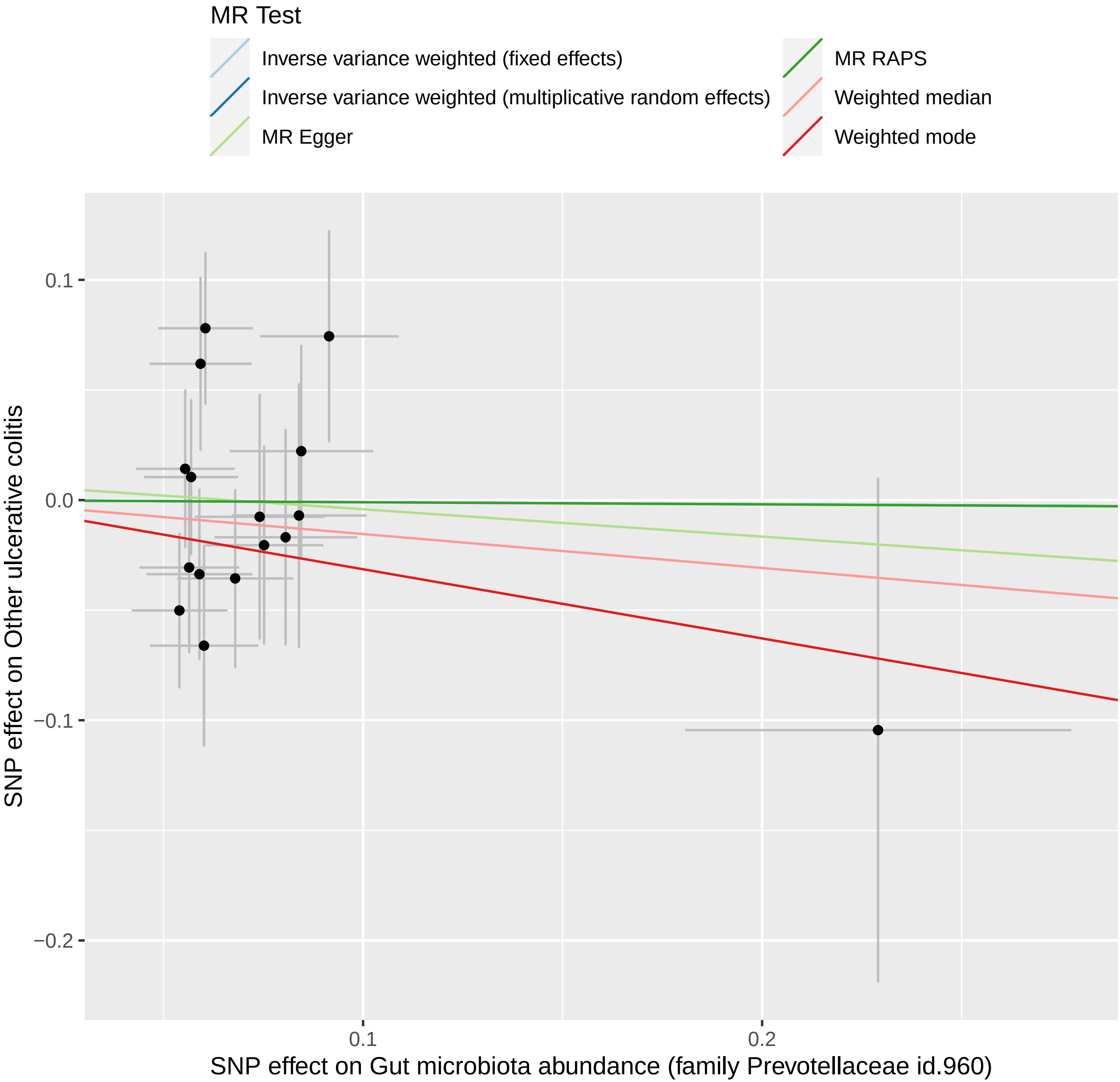

Batch 1226 : Gut microbiota abundance (family Rhodospirillaceae id.2717) on Other ulcerative colitis

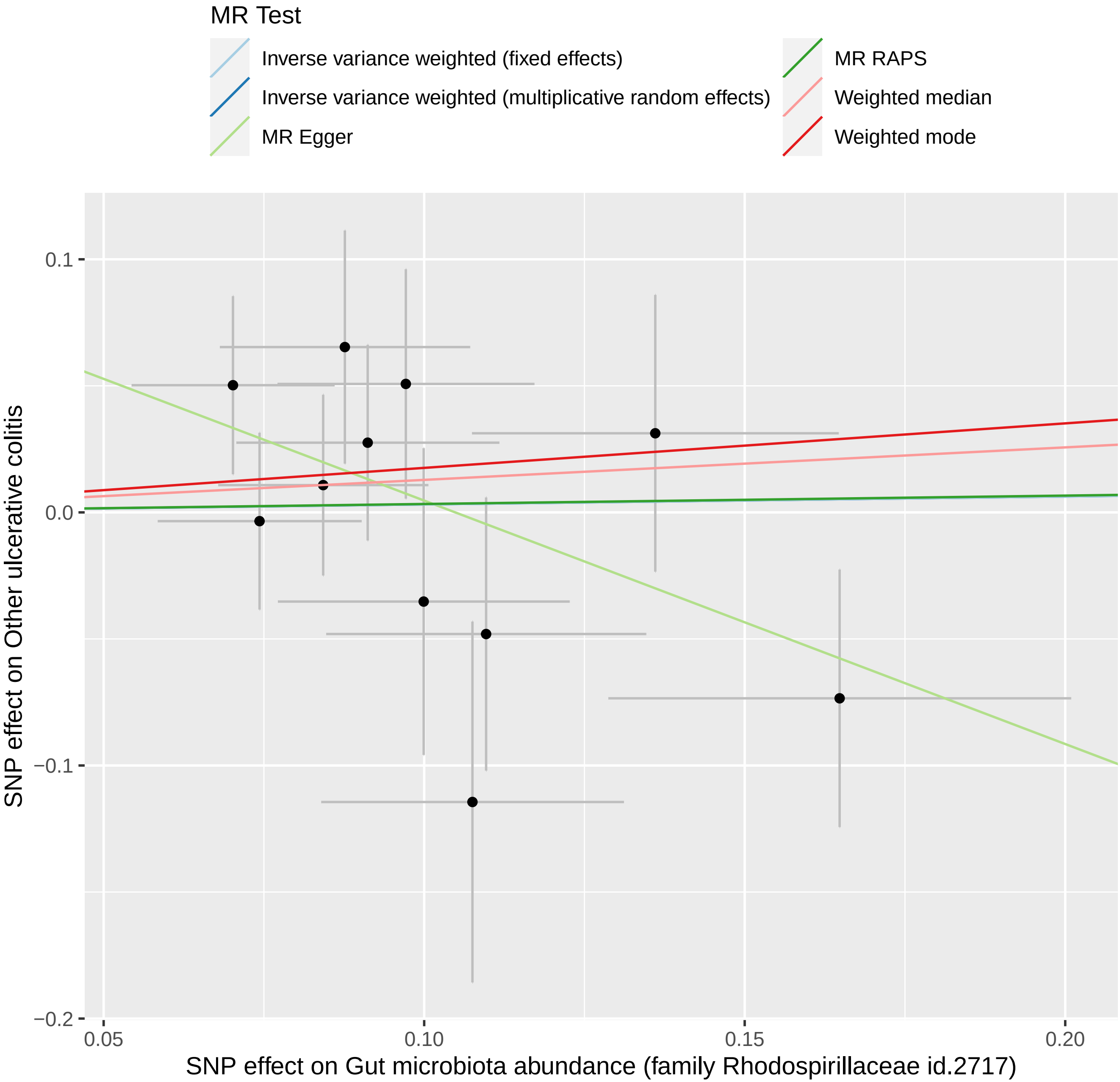

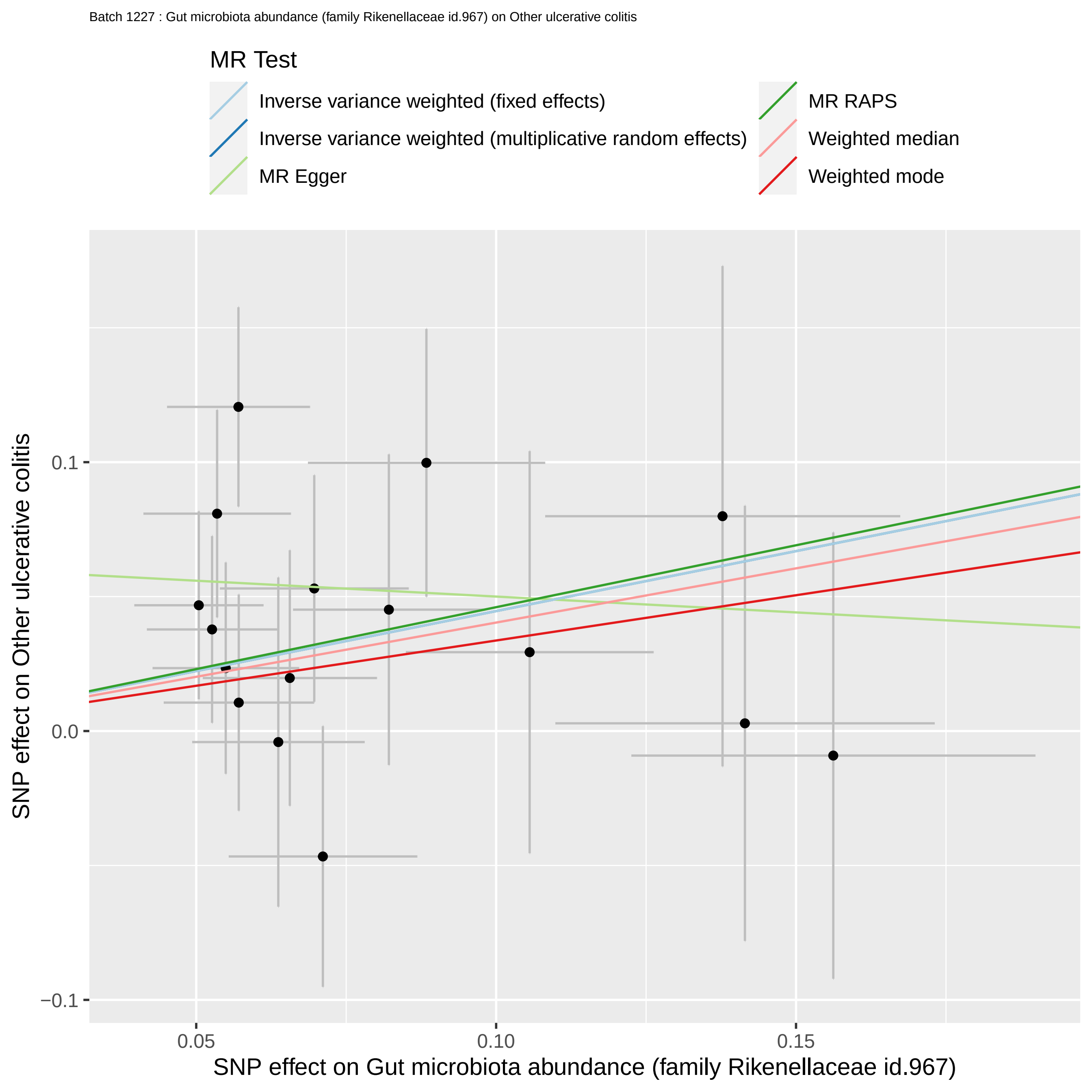
Batch 1227 : Gut microbiota abundance (family Rikenellaceae id.967) on Other ulcerative colitis
MR Test
Inverse variance weighted (fixed effects)
Inverse variance weighted (multiplicative random effects)
MR Egger
MR RAPS
Weighted median
Weighted mode
SNP effect on Other ulcerative colitis
0.1
0.0
−0.1
0.05
0.10
0.15
SNP effect on Gut microbiota abundance (family Rikenellaceae id.967)

Batch 1228 : Gut microbiota abundance (family Ruminococcaceae id.2050) on Other ulcerative colitis

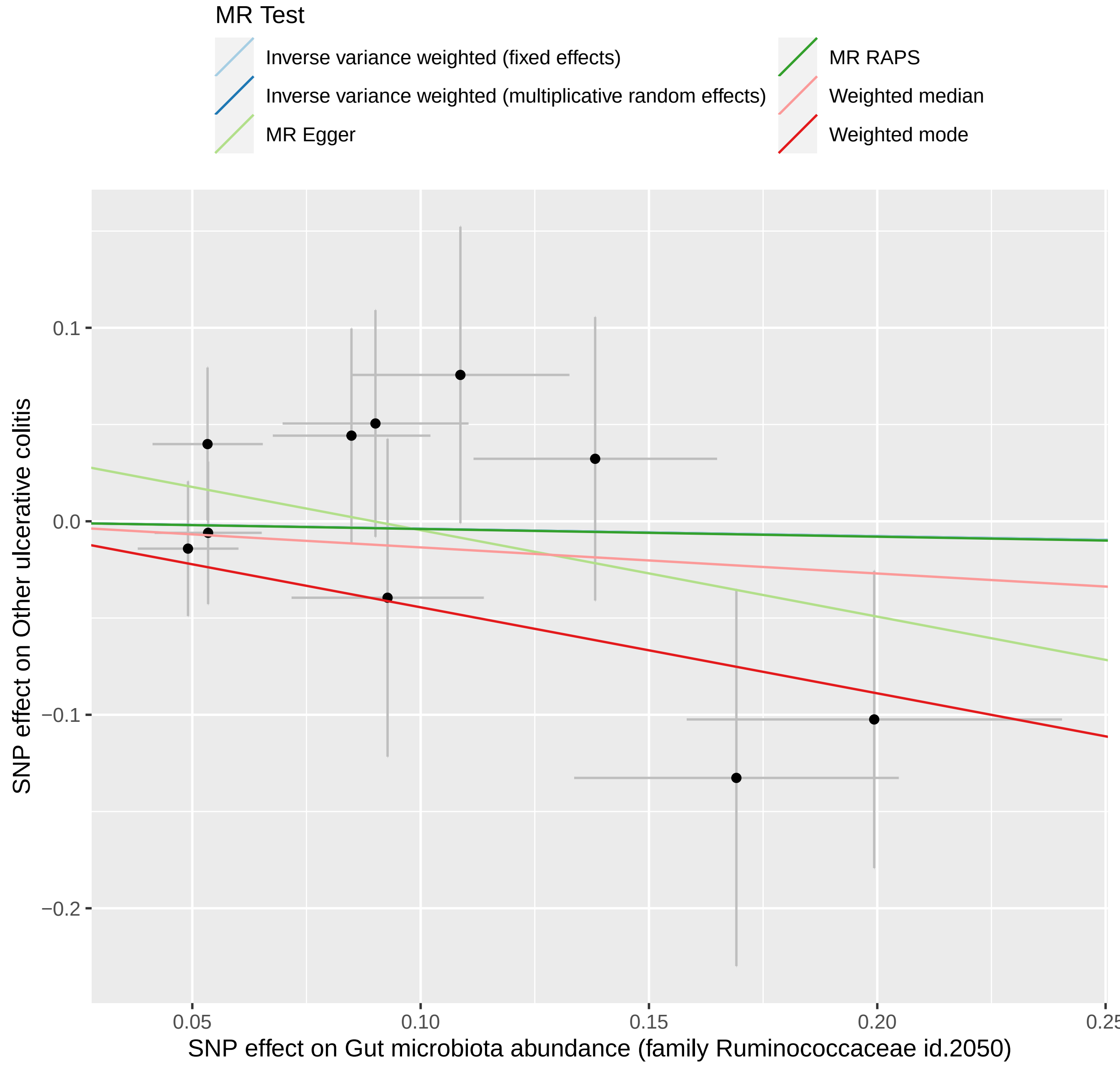

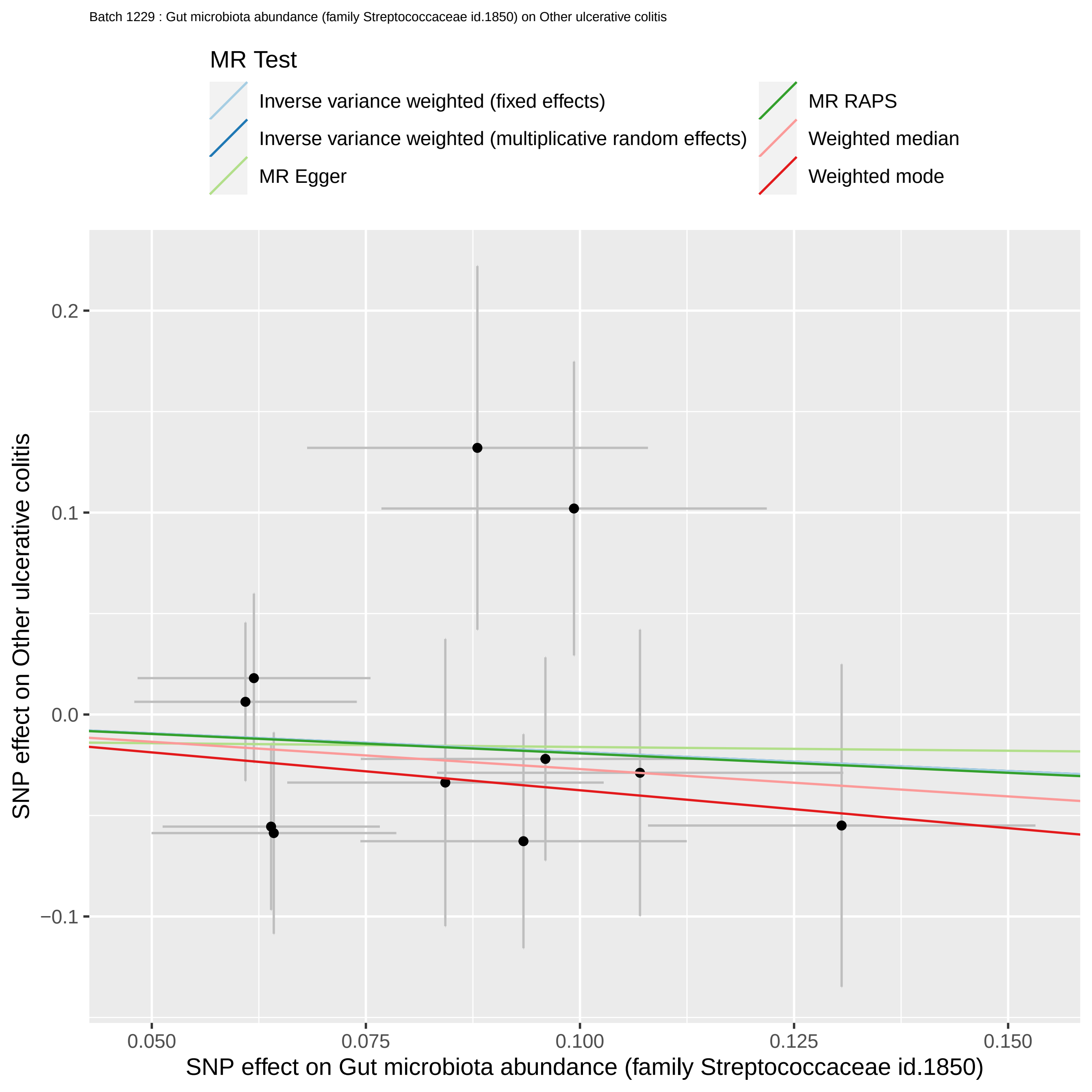

Batch 1229 : Gut microbiota abundance (family Streptococcaceae id.1850) on Other ulcerative colitis
MR Test
Inverse variance weighted (fixed effects)
Inverse variance weighted (multiplicative random effects)
MR Egger
MR RAPS
Weighted median
Weighted mode
0.2
0.1
0.0
−0.1
0.050
0.075
0.100
0.125
0.150
SNP effect on Other ulcerative colitis
SNP effect on Gut microbiota abundance (family Streptococcaceae id.1850)

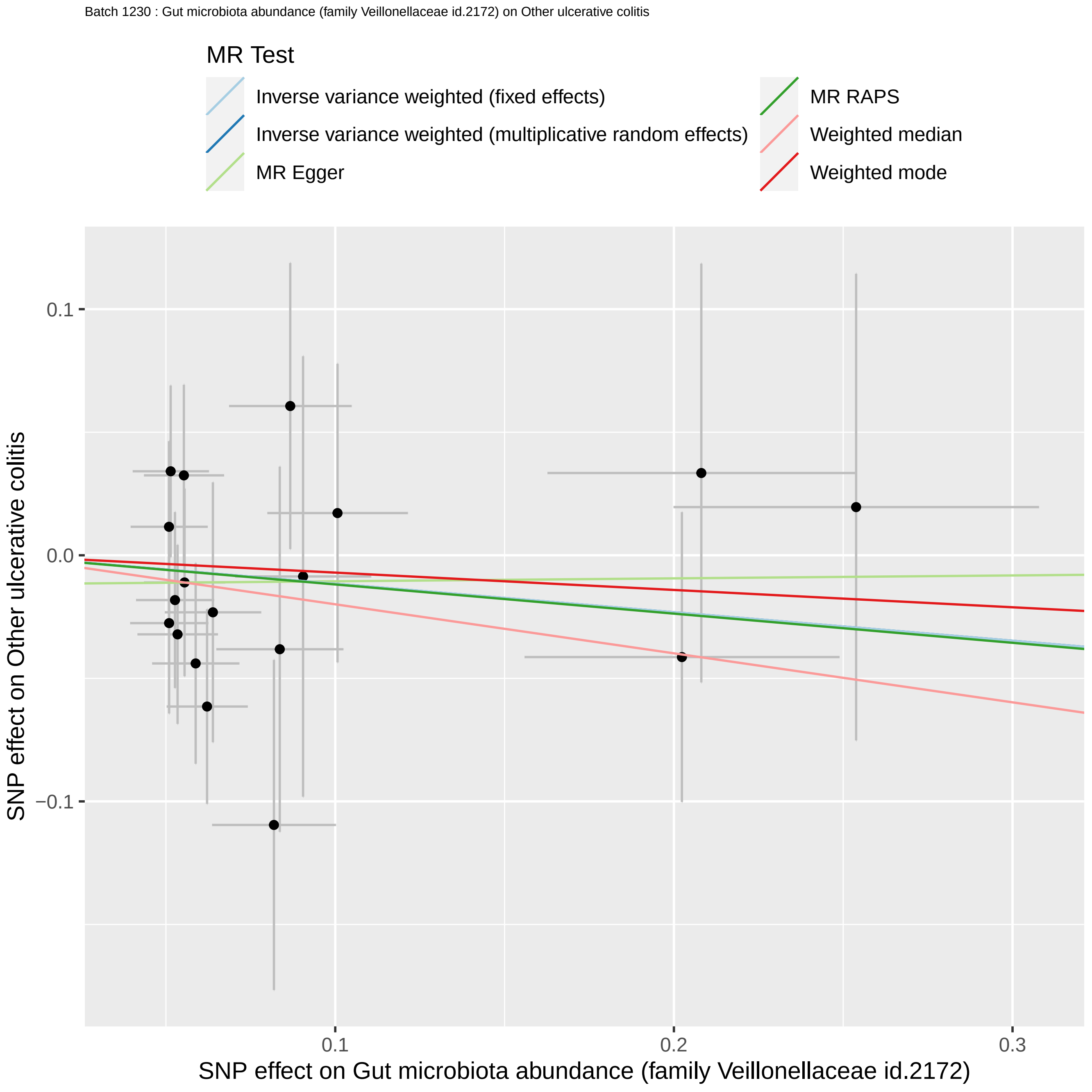

Batch 1230 : Gut microbiota abundance (family Veillonellaceae id.2172) on Other ulcerative colitis
MR Test
Inverse variance weighted (fixed effects)
Inverse variance weighted (multiplicative random effects)
MR Egger
MR RAPS
Weighted median
Weighted mode
0.1
0.0
−0.1
0.1
0.2
0.3
SNP effect on Other ulcerative colitis
SNP effect on Gut microbiota abundance (family Veillonellaceae id.2172)

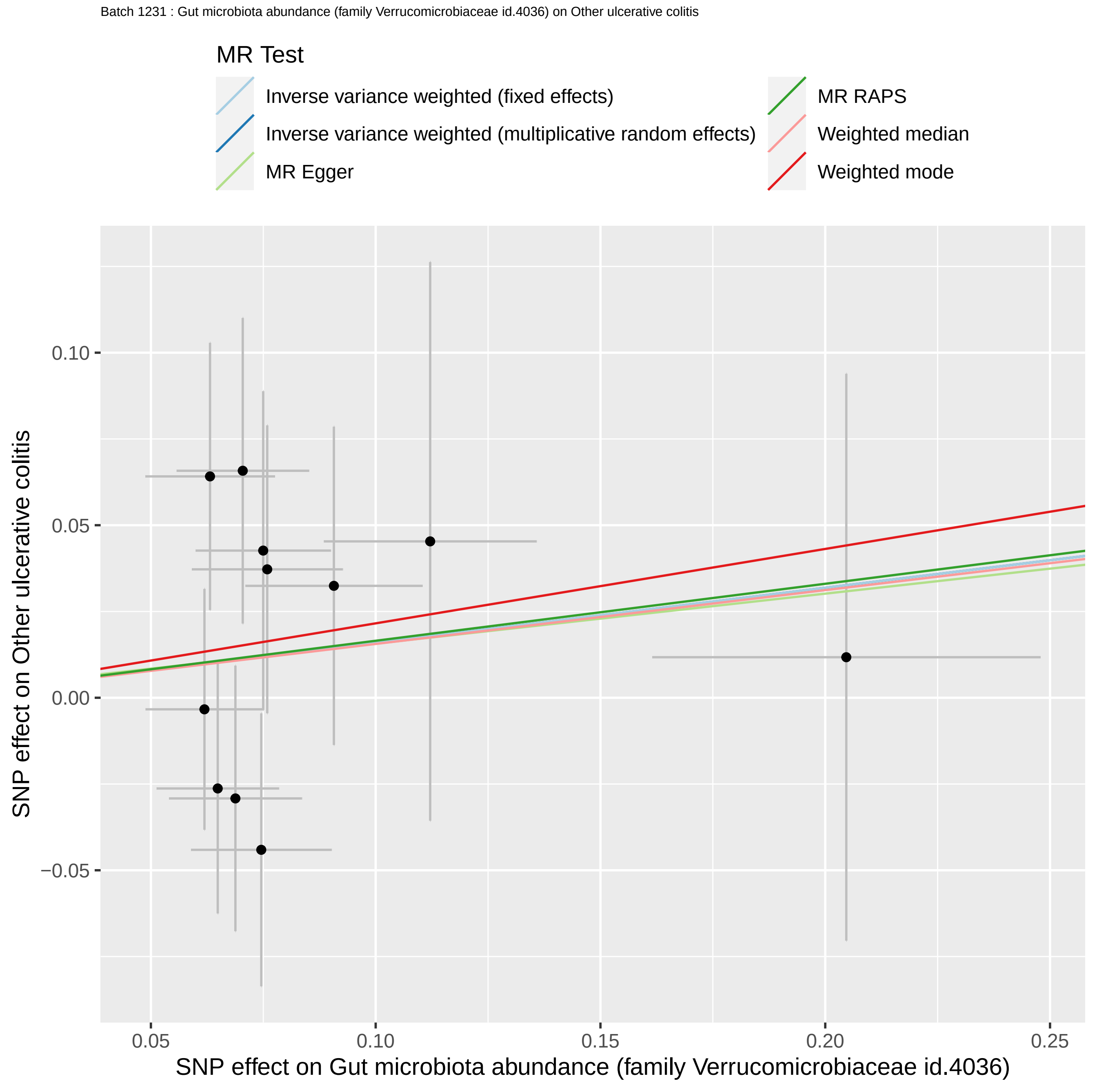

Batch 1231 : Gut microbiota abundance (family Verrucomicrobiaceae id.4036) on Other ulcerative colitis
MR Test
Inverse variance weighted (fixed effects)
Inverse variance weighted (multiplicative random effects)
MR Egger
MR RAPS
Weighted median
Weighted mode
0.10
0.05
0.00
−0.05
0.05
0.10
0.15
0.20
0.25
SNP effect on Gut microbiota abundance (family Verrucomicrobiaceae id.4036)
SNP effect on Other ulcerative colitis

Batch 1232 : Gut microbiota abundance (family Victivallaceae id.2255) on Other ulcerative colitis

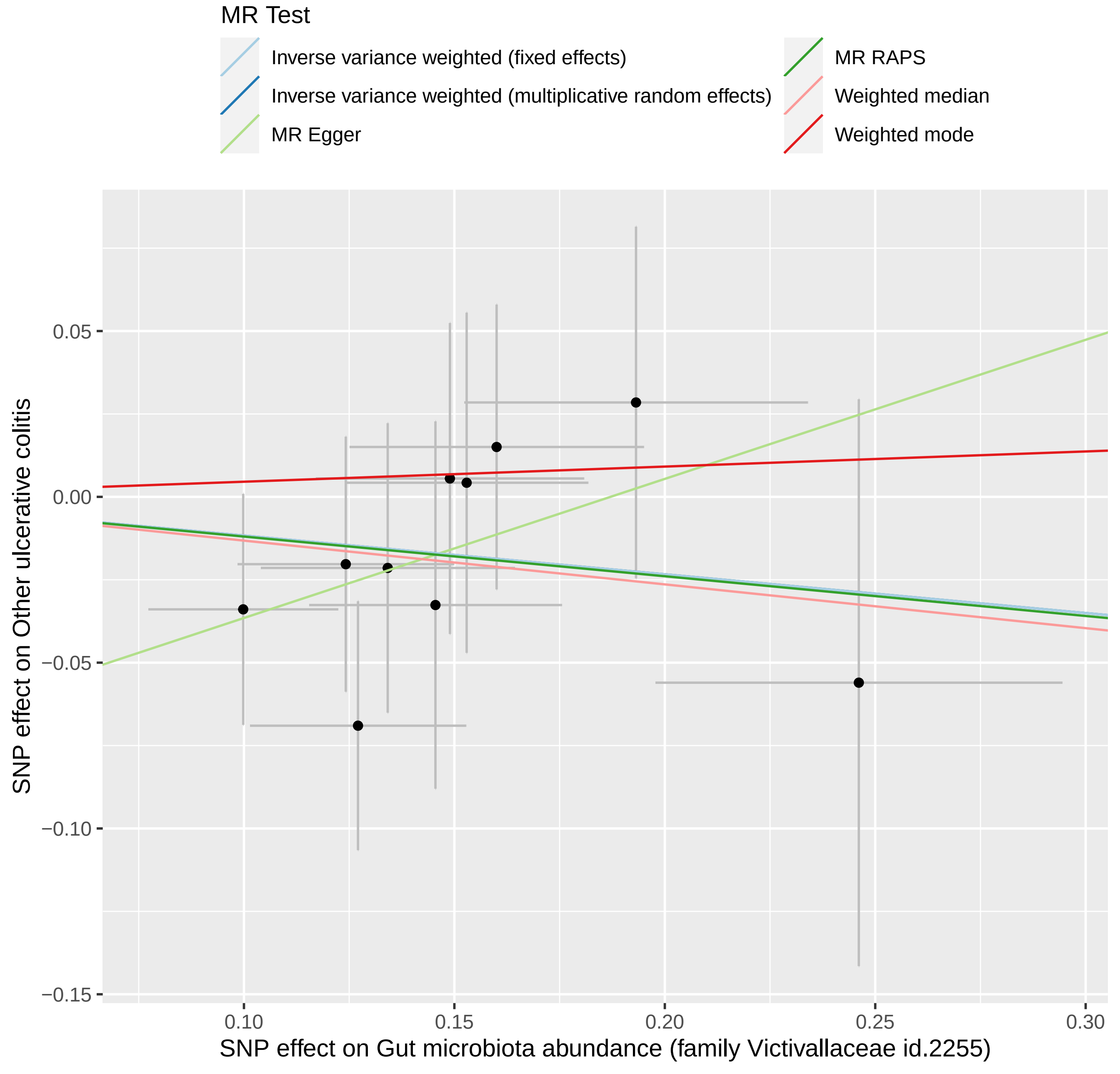

Batch 1233 : Gut microbiota abundance (genus Actinomyces id.423) on Other ulcerative colitis

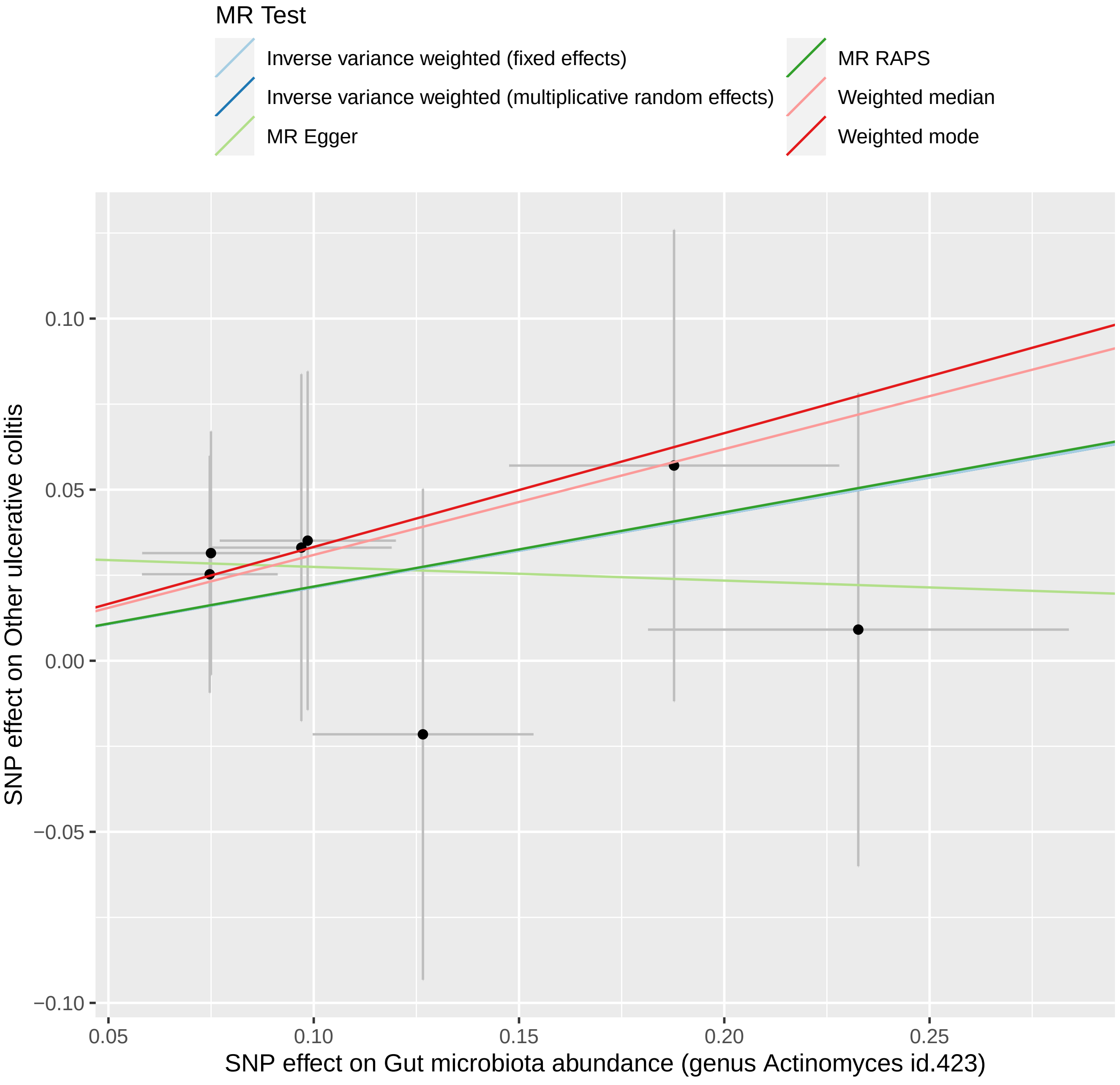

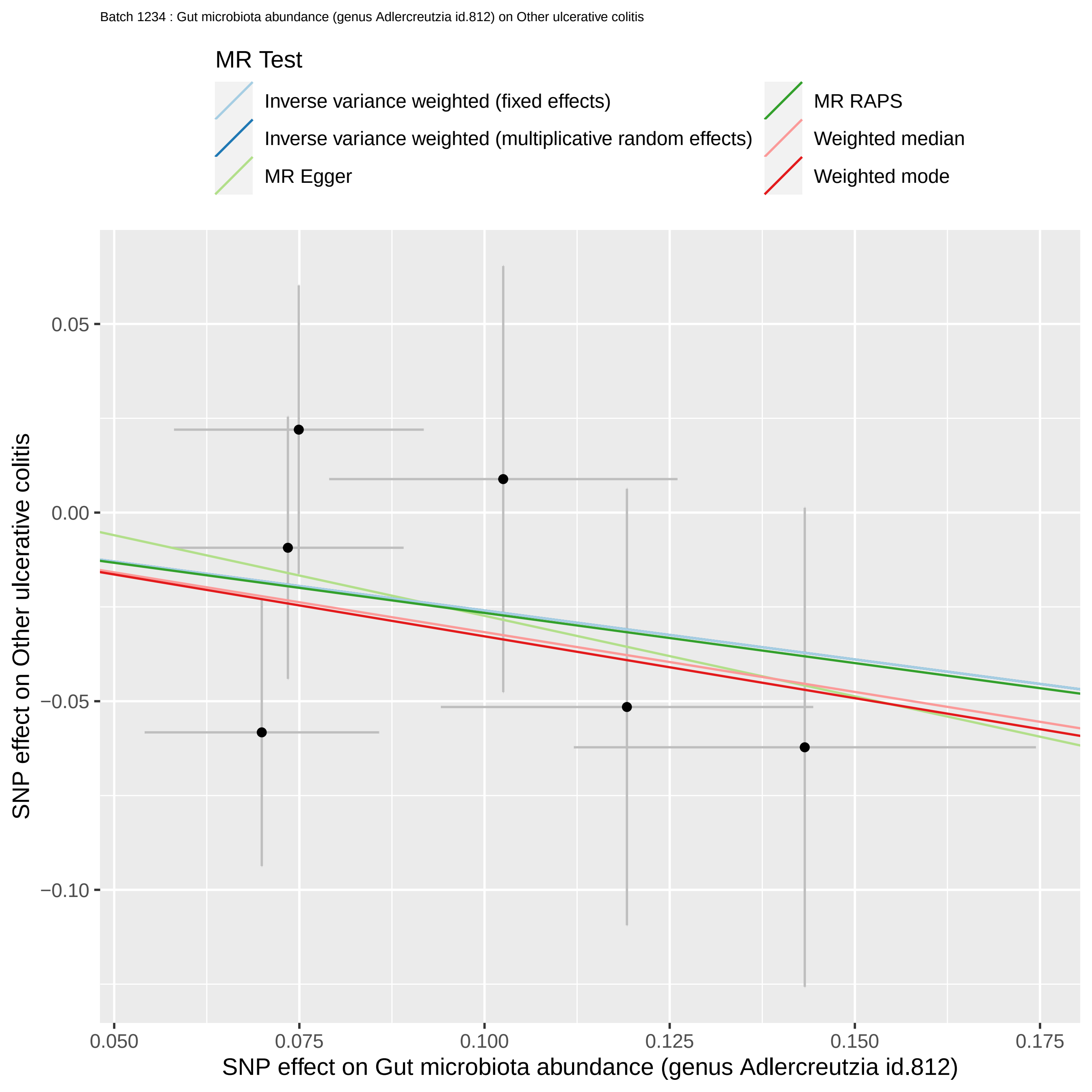

Batch 1234 : Gut microbiota abundance (genus Adlercreutzia id.812) on Other ulcerative colitis
MR Test
Inverse variance weighted (fixed effects)
Inverse variance weighted (multiplicative random effects)
MR Egger
MR RAPS
Weighted median
Weighted mode
0.05
0.00
−0.05
−0.10
0.050
0.075
0.100
0.125
0.150
0.175
SNP effect on Other ulcerative colitis
SNP effect on Gut microbiota abundance (genus Adlercreutzia id.812)

Batch 1235 : Gut microbiota abundance (genus Akkermansia id.4037) on Other ulcerative colitis

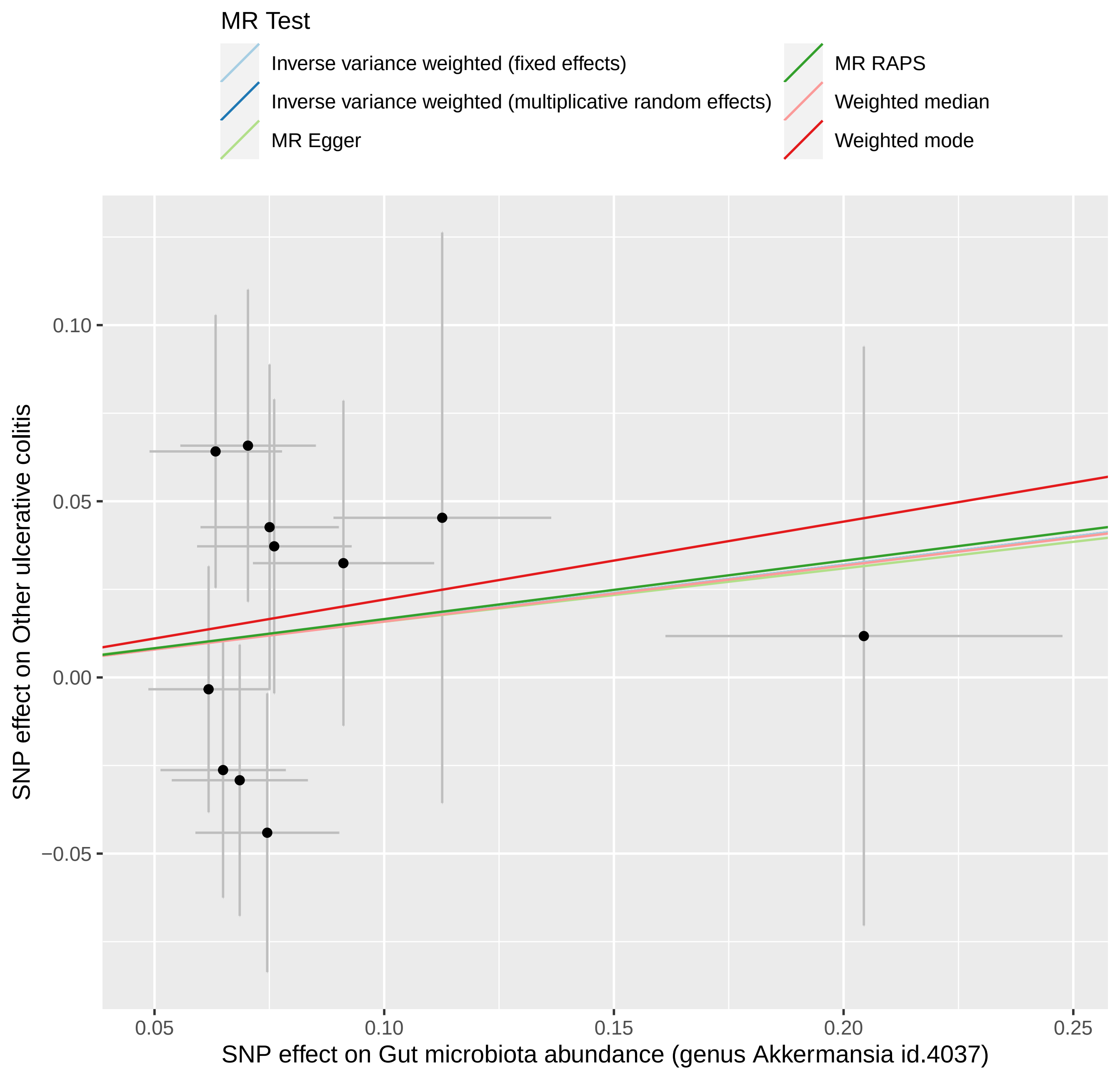

Batch 1236 : Gut microbiota abundance (genus Alistipes id.968) on Other ulcerative colitis

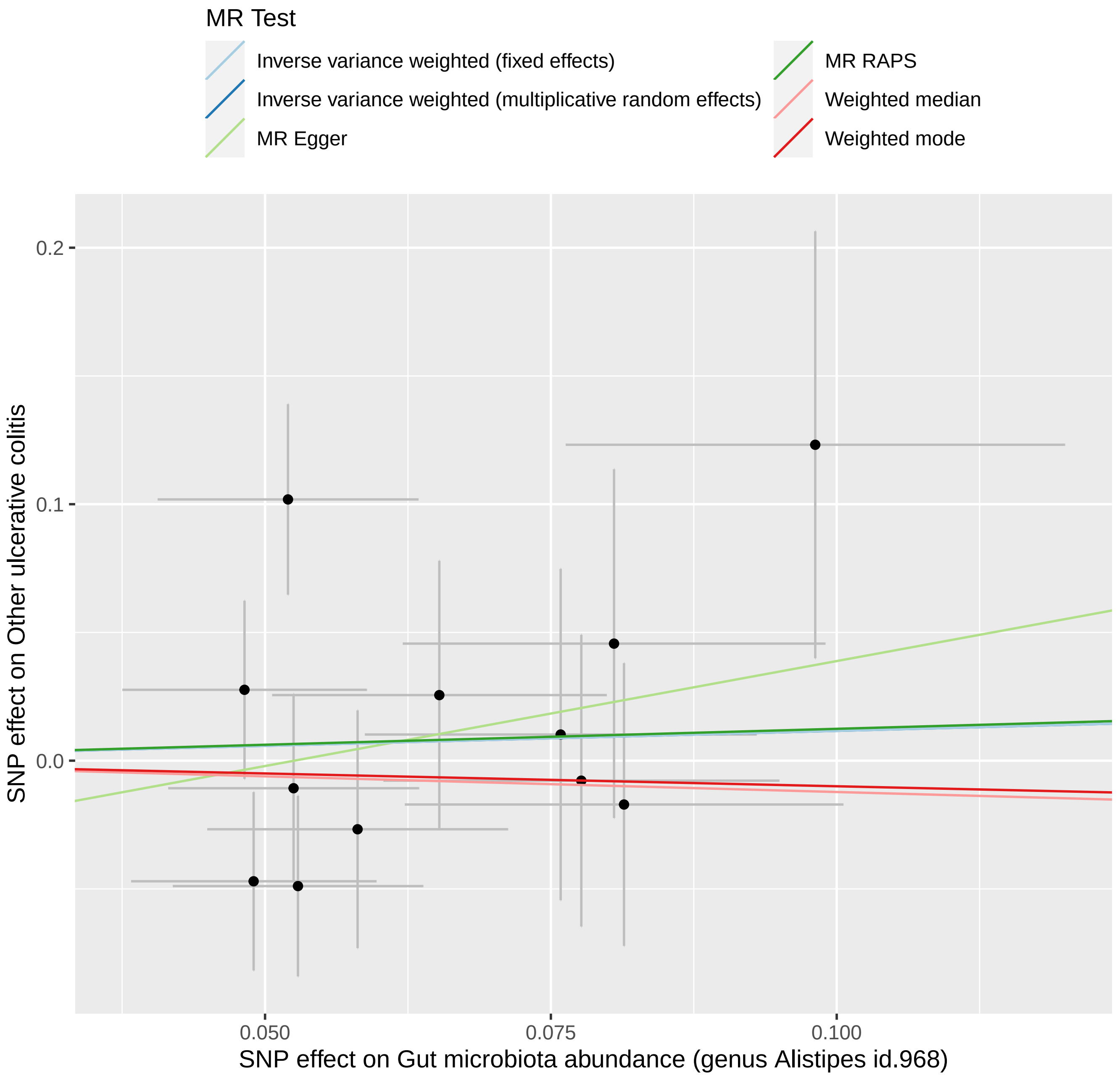

Batch 1237 : Gut microbiota abundance (genus Allisonella id.2174) on Other ulcerative colitis

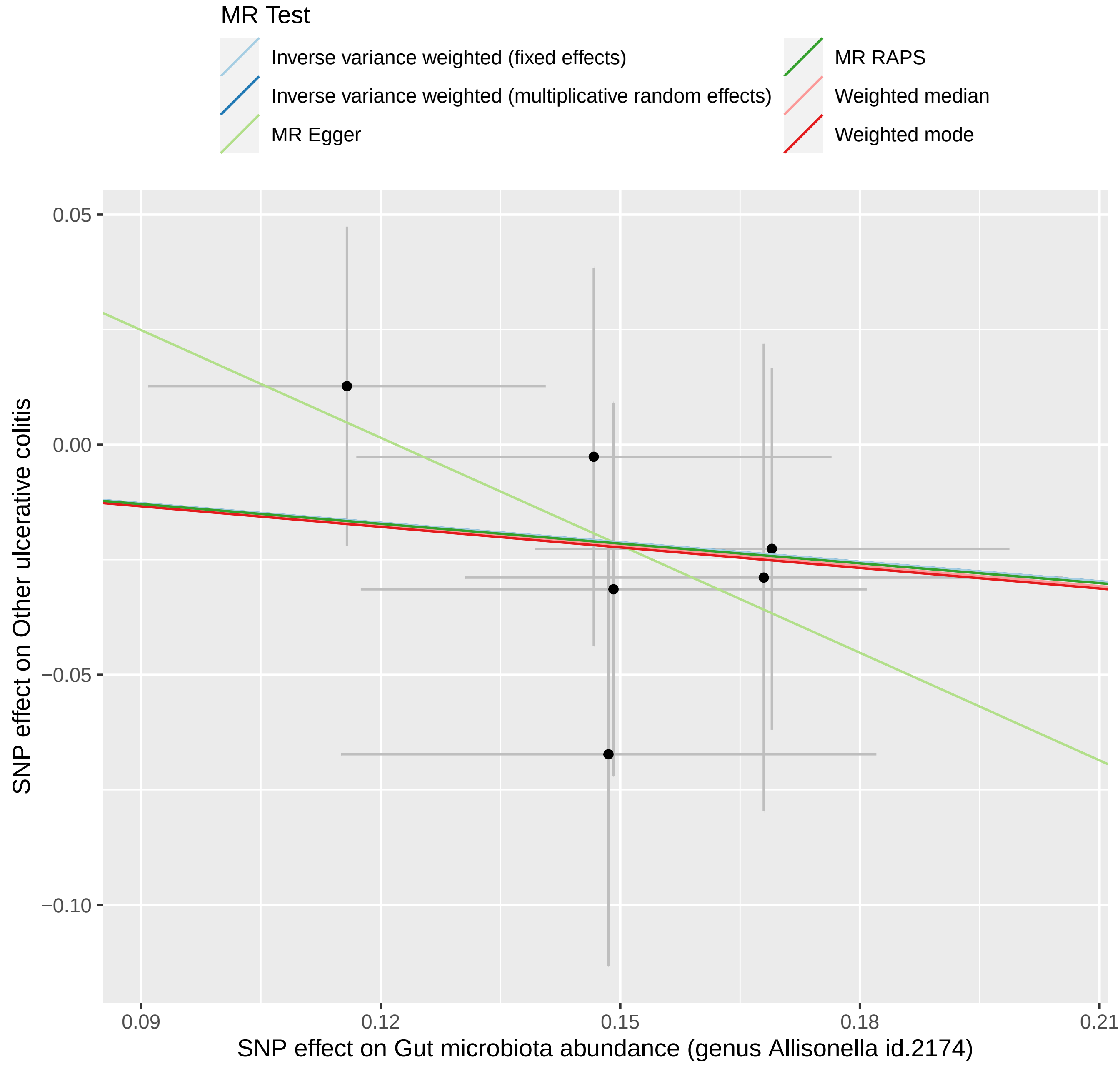

Batch 1238 : Gut microbiota abundance (genus Alloprevotella id.961) on Other ulcerative colitis

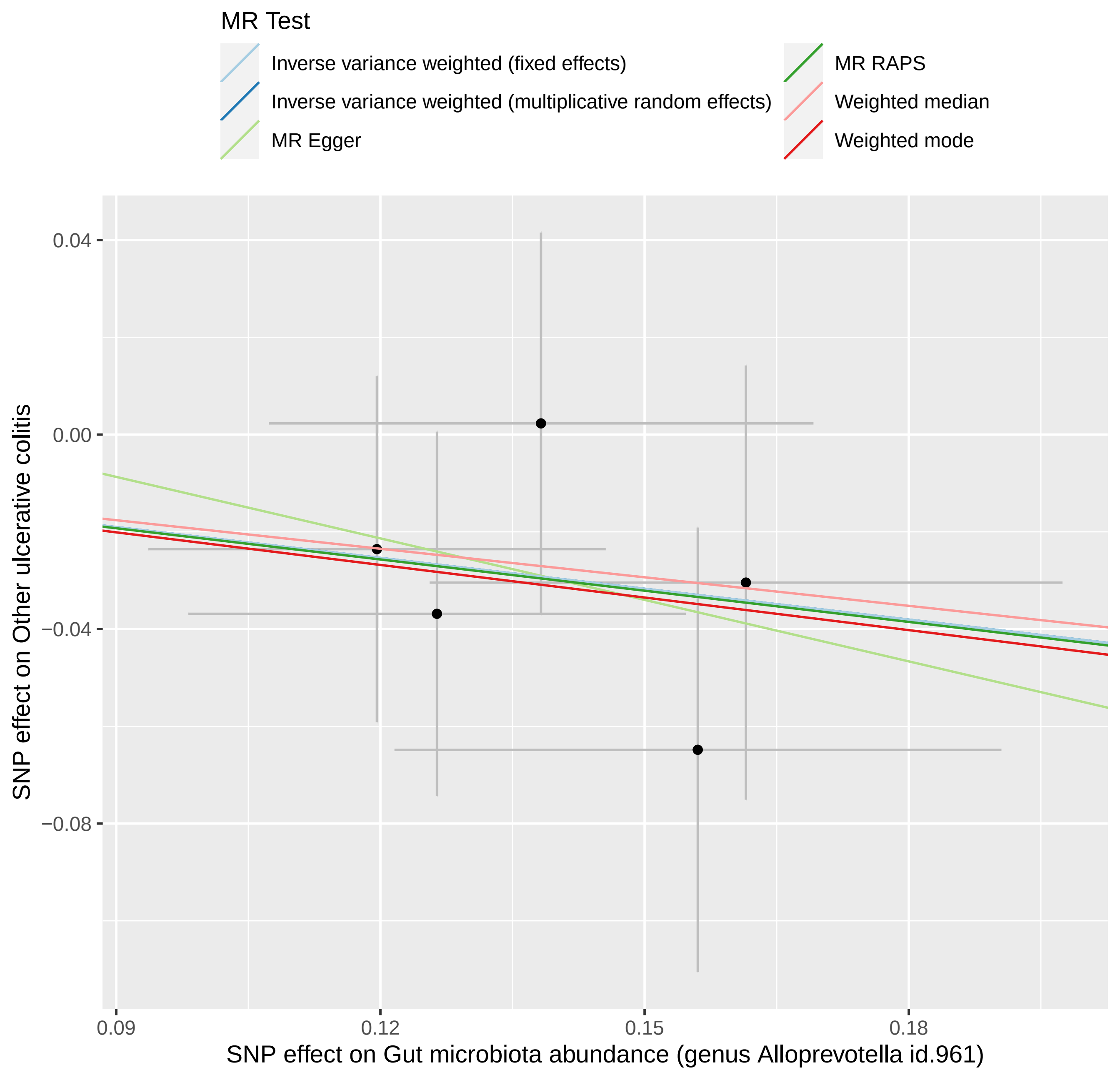

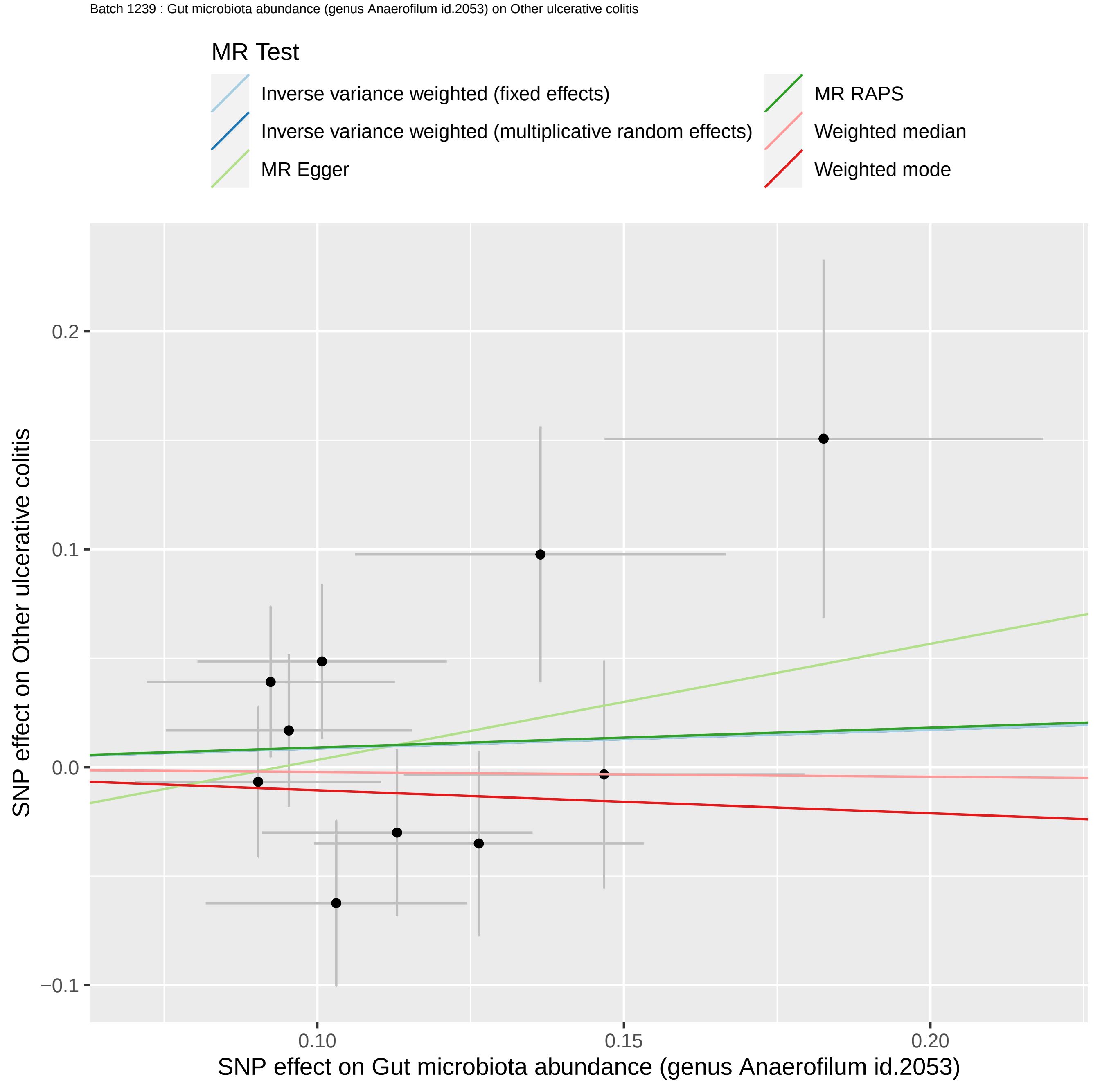
Batch 1239 : Gut microbiota abundance (genus Anaerofilum id.2053) on Other ulcerative colitis
MR Test
Inverse variance weighted (fixed effects)
Inverse variance weighted (multiplicative random effects)
MR Egger
MR RAPS
Weighted median
Weighted mode
0.2
0.1
0.0
−0.1
0.10
0.15
0.20
SNP effect on Other ulcerative colitis
SNP effect on Gut microbiota abundance (genus Anaerofilum id.2053)

Batch 1240 : Gut microbiota abundance (genus Anaerostipes id.1991) on Other ulcerative colitis

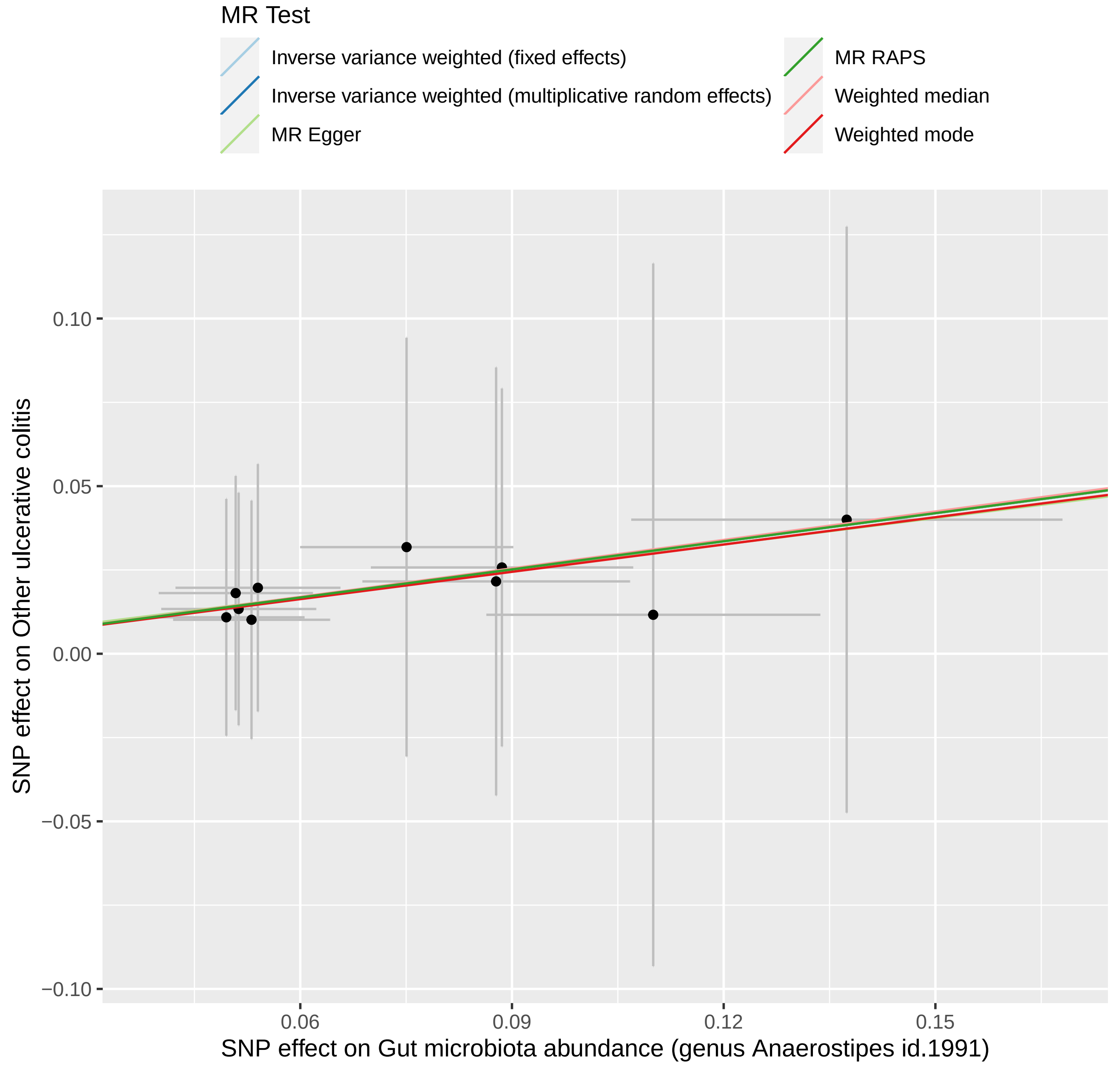

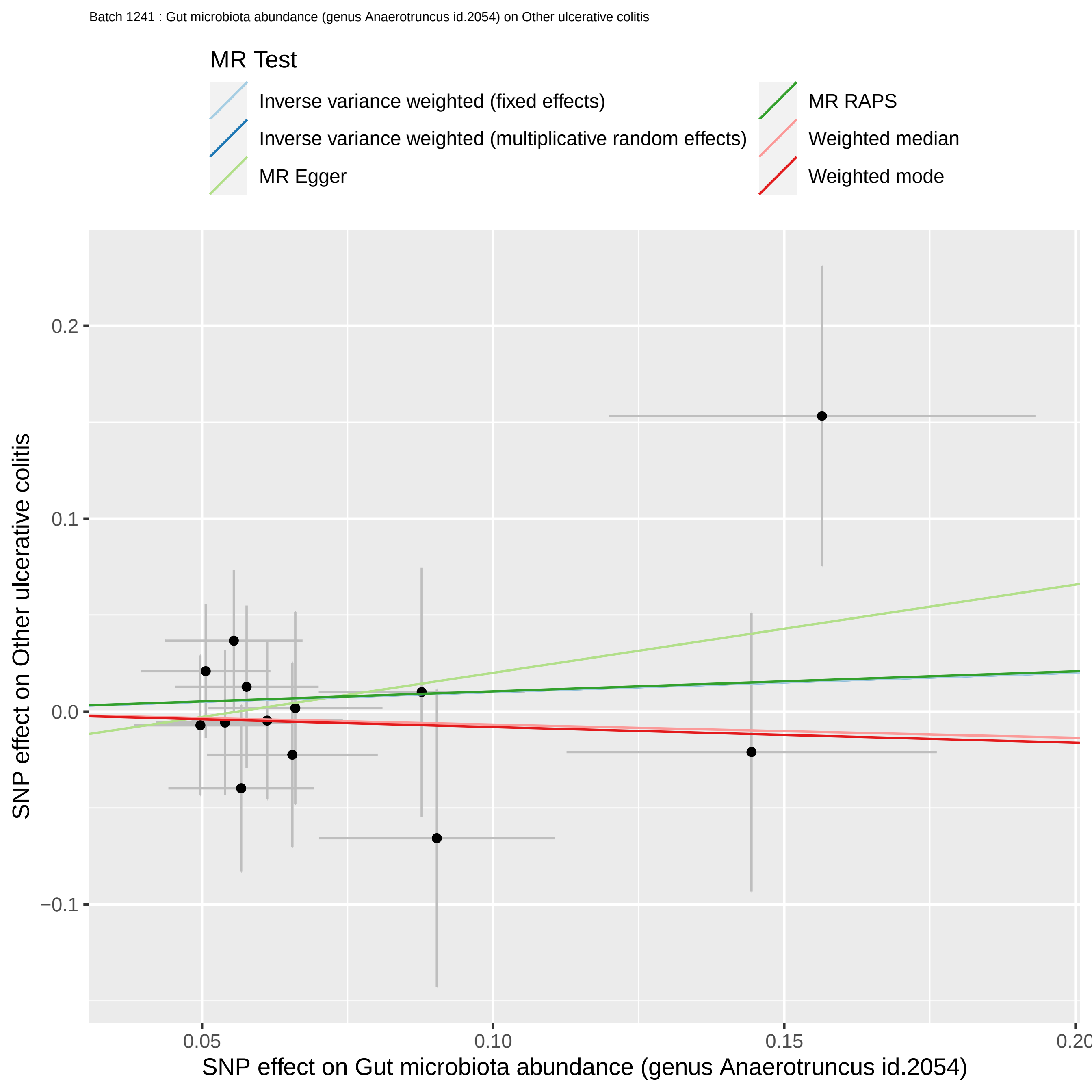
Batch 1241 : Gut microbiota abundance (genus Anaerotruncus id.2054) on Other ulcerative colitis
MR Test
Inverse variance weighted (fixed effects)
Inverse variance weighted (multiplicative random effects)
MR Egger
MR RAPS
Weighted median
Weighted mode
0.2
0.1
0.0
−0.1
0.05
0.10
0.15
0.20
SNP effect on Other ulcerative colitis
SNP effect on Gut microbiota abundance (genus Anaerotruncus id.2054)

Batch 1242 : Gut microbiota abundance (genus Bacteroides id.918) on Other ulcerative colitis

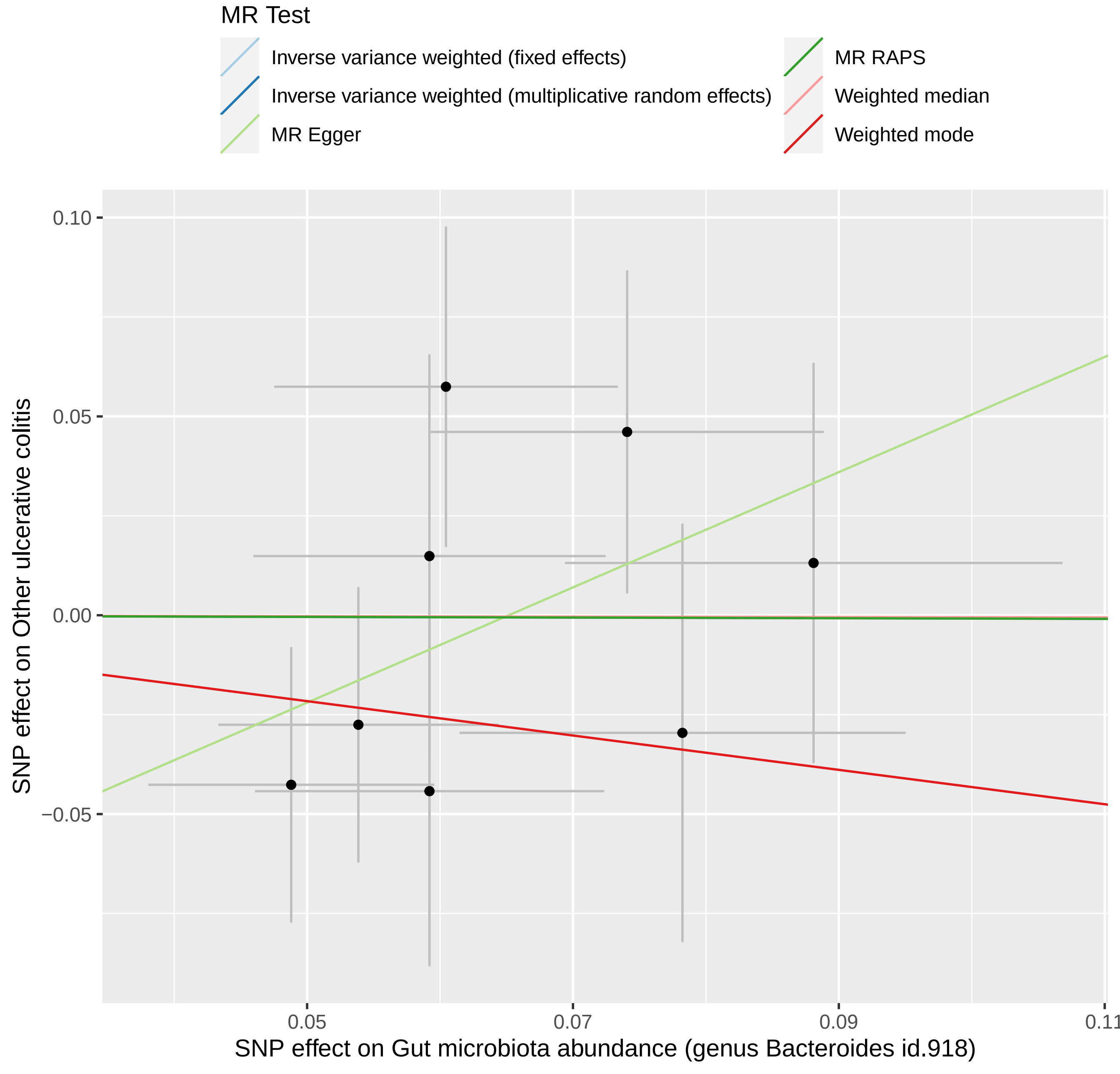

Batch 1243 : Gut microbiota abundance (genus Barnesiella id.944) on Other ulcerative colitis

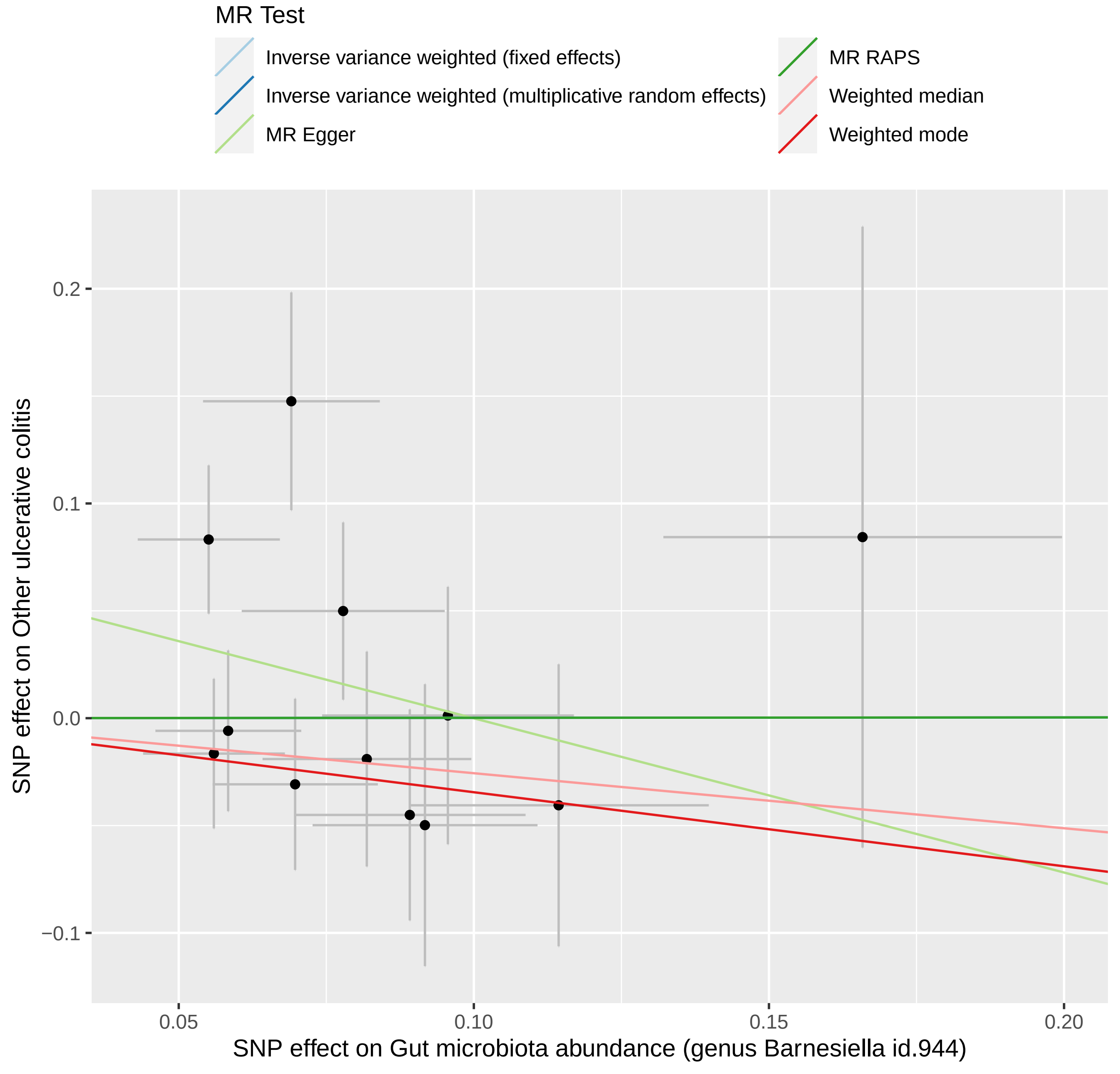

Batch 1244 : Gut microbiota abundance (genus Bifidobacterium id.436) on Other ulcerative colitis

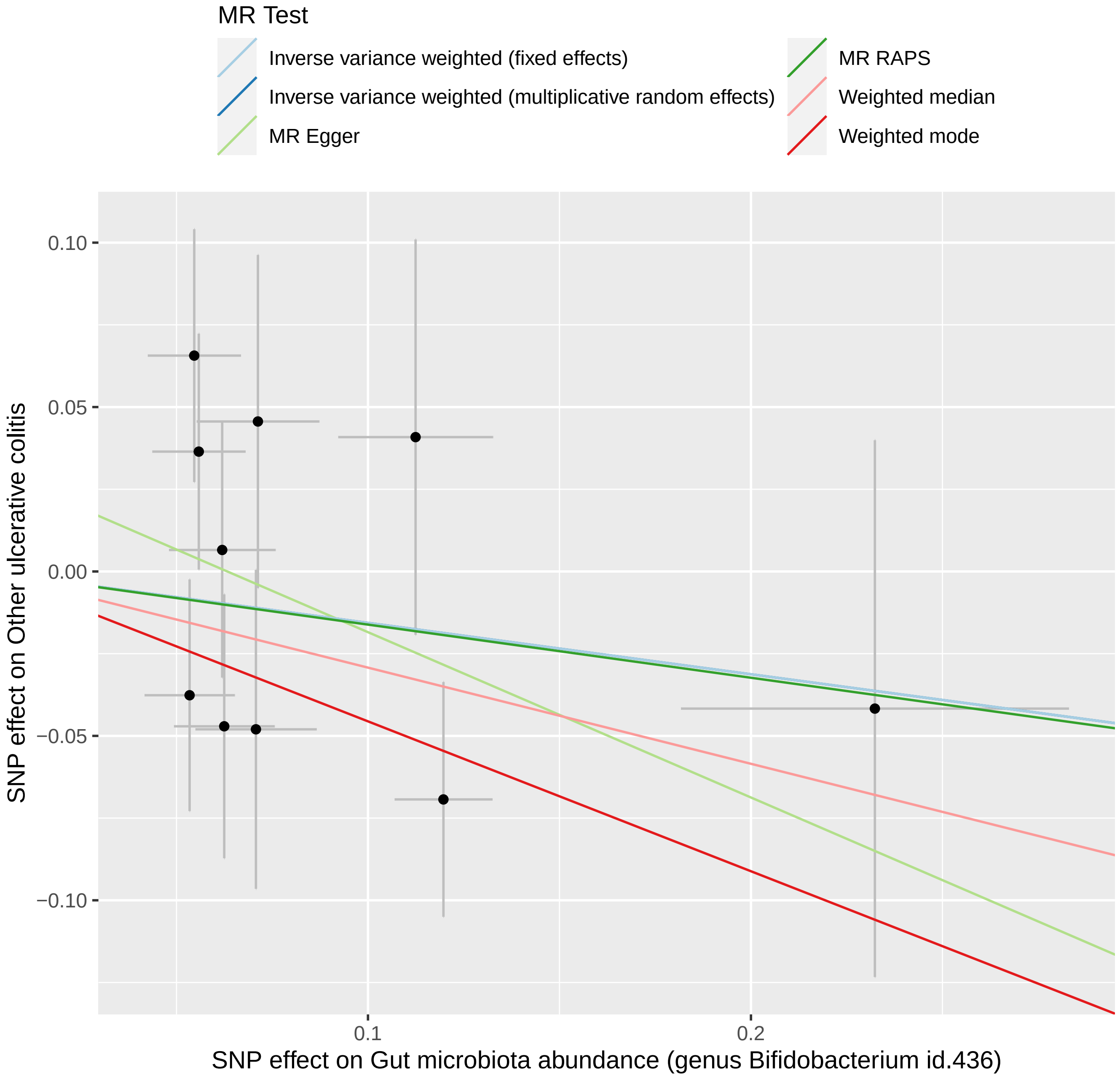

Batch 1245 : Gut microbiota abundance (genus Bilophila id.3170) on Other ulcerative colitis

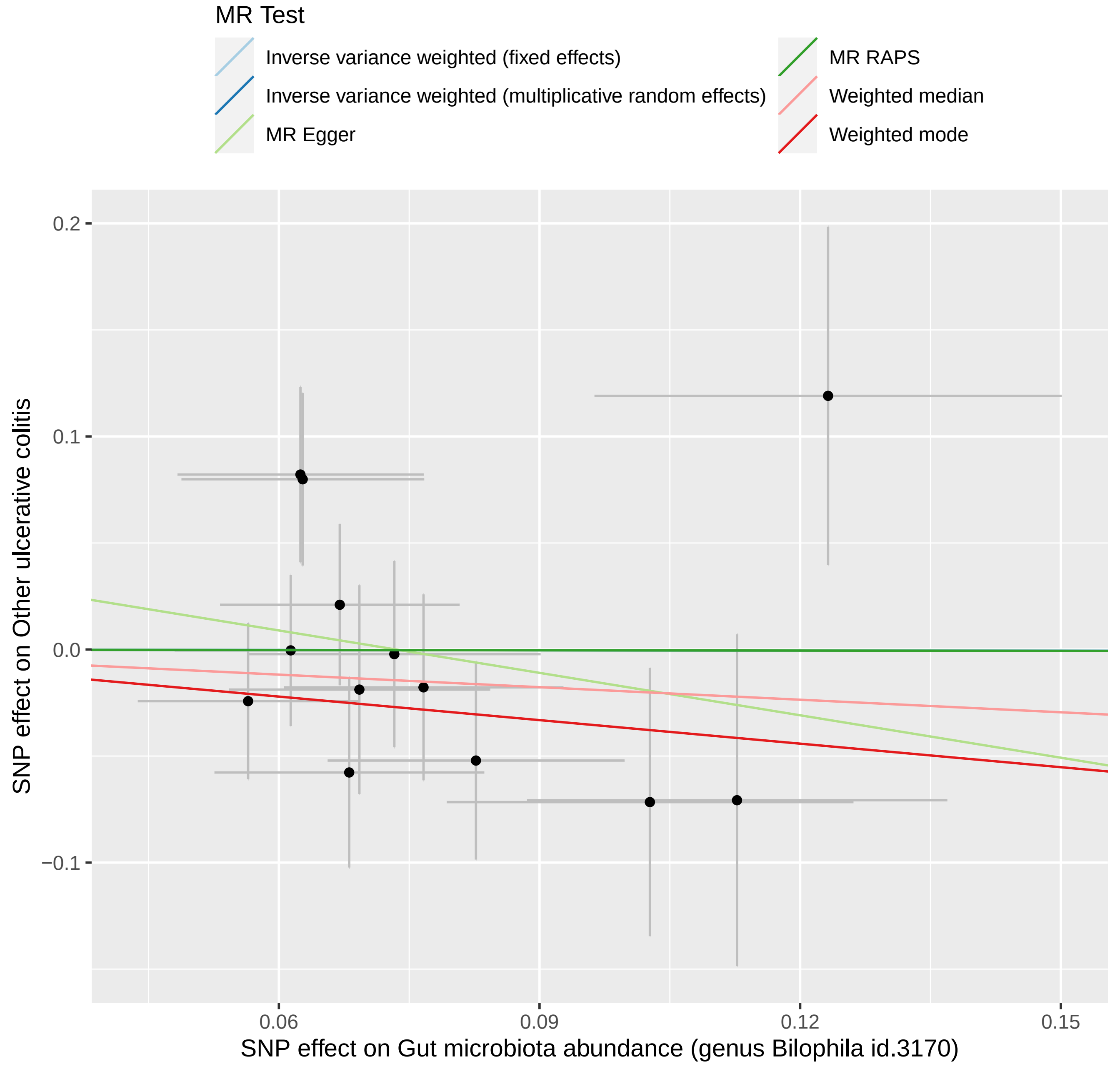

Batch 1246 : Gut microbiota abundance (genus Blautia id.1992) on Other ulcerative colitis

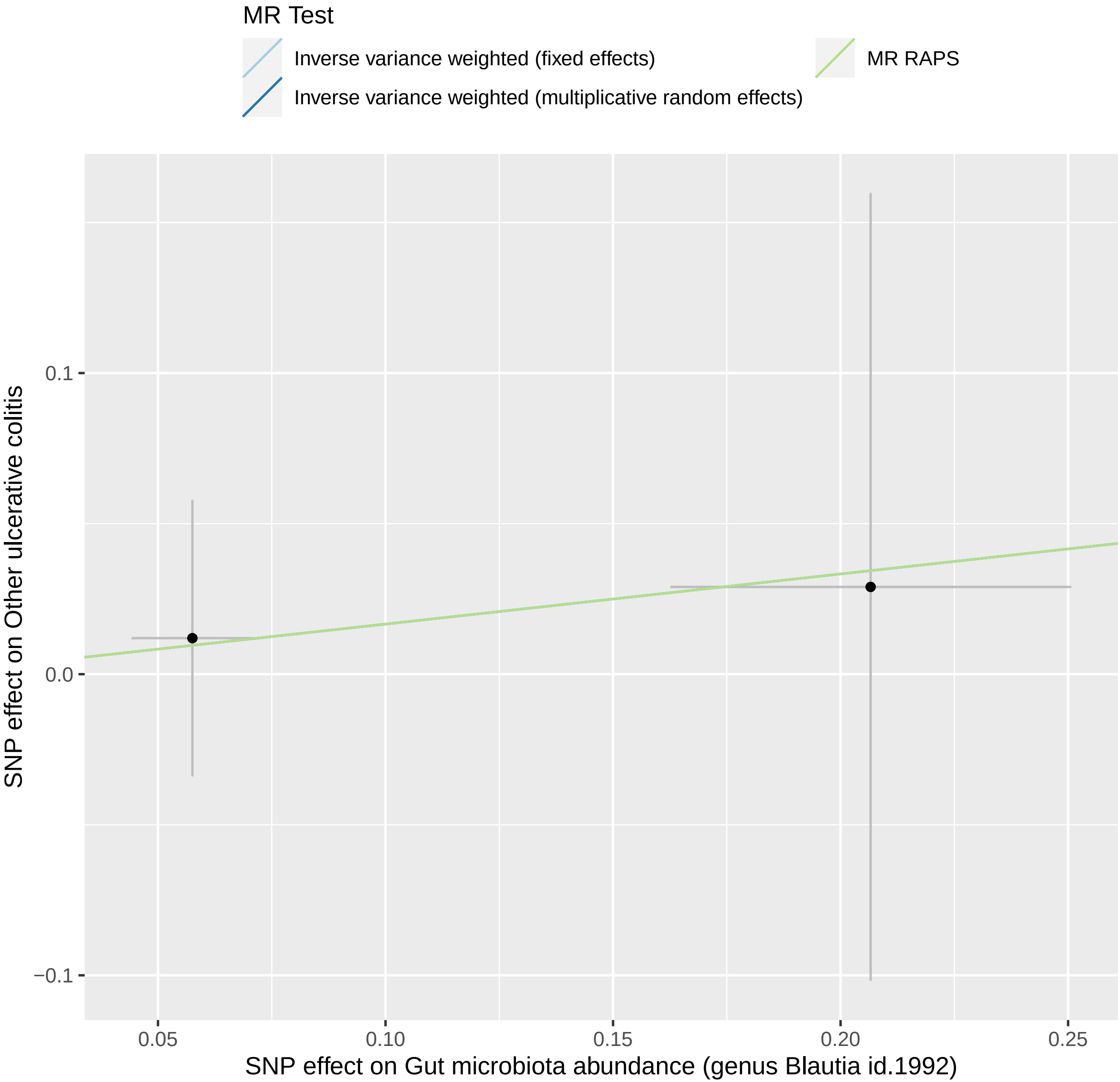

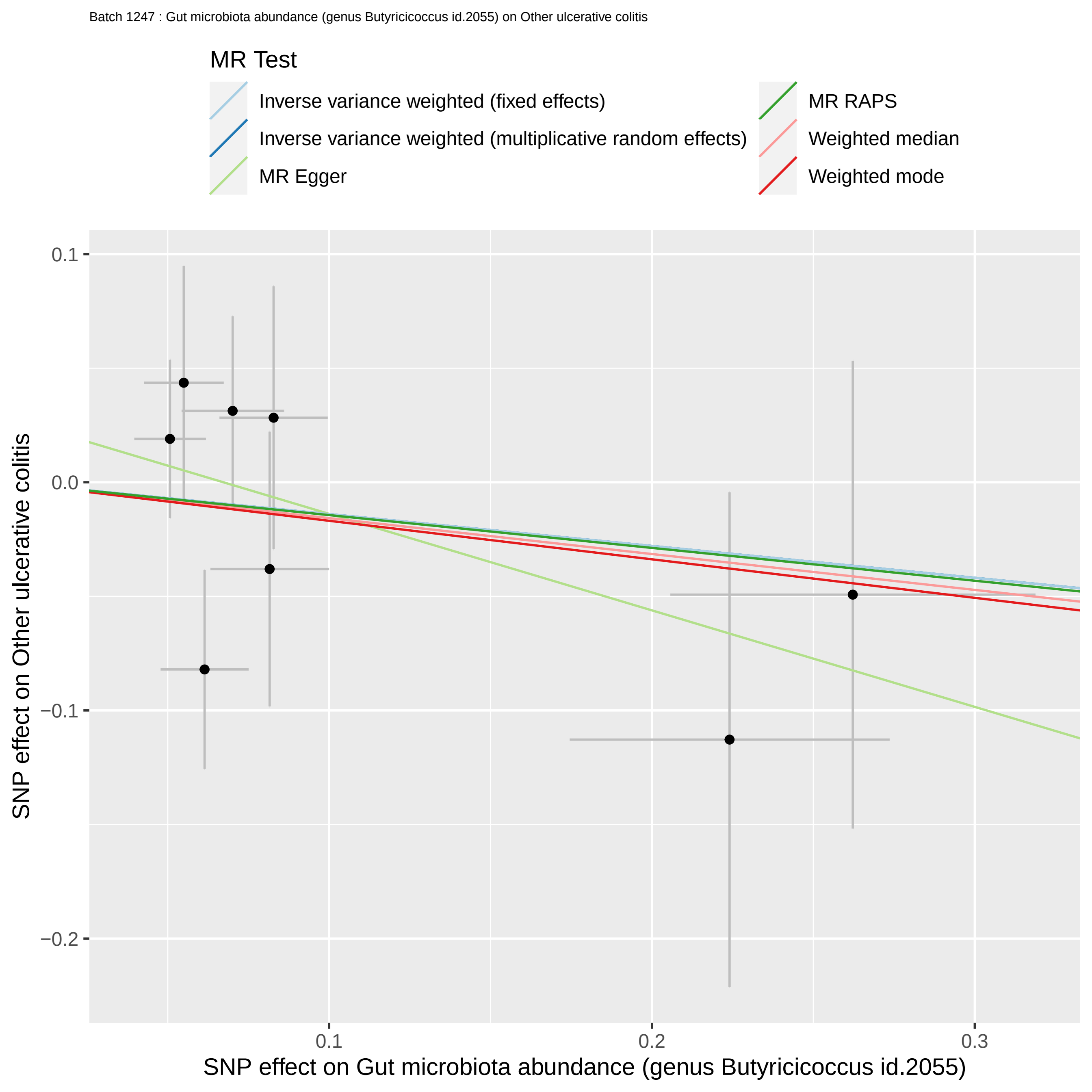
Batch 1247 : Gut microbiota abundance (genus Butyricicoccus id.2055) on Other ulcerative colitis
MR Test
Inverse variance weighted (fixed effects)
Inverse variance weighted (multiplicative random effects)
MR Egger
MR RAPS
Weighted median
Weighted mode
0.1
0.0
−0.1
−0.2
0.1
0.2
0.3
SNP effect on Other ulcerative colitis
SNP effect on Gut microbiota abundance (genus Butyricicoccus id.2055)

Batch 1248 : Gut microbiota abundance (genus Butyricimonas id.945) on Other ulcerative colitis

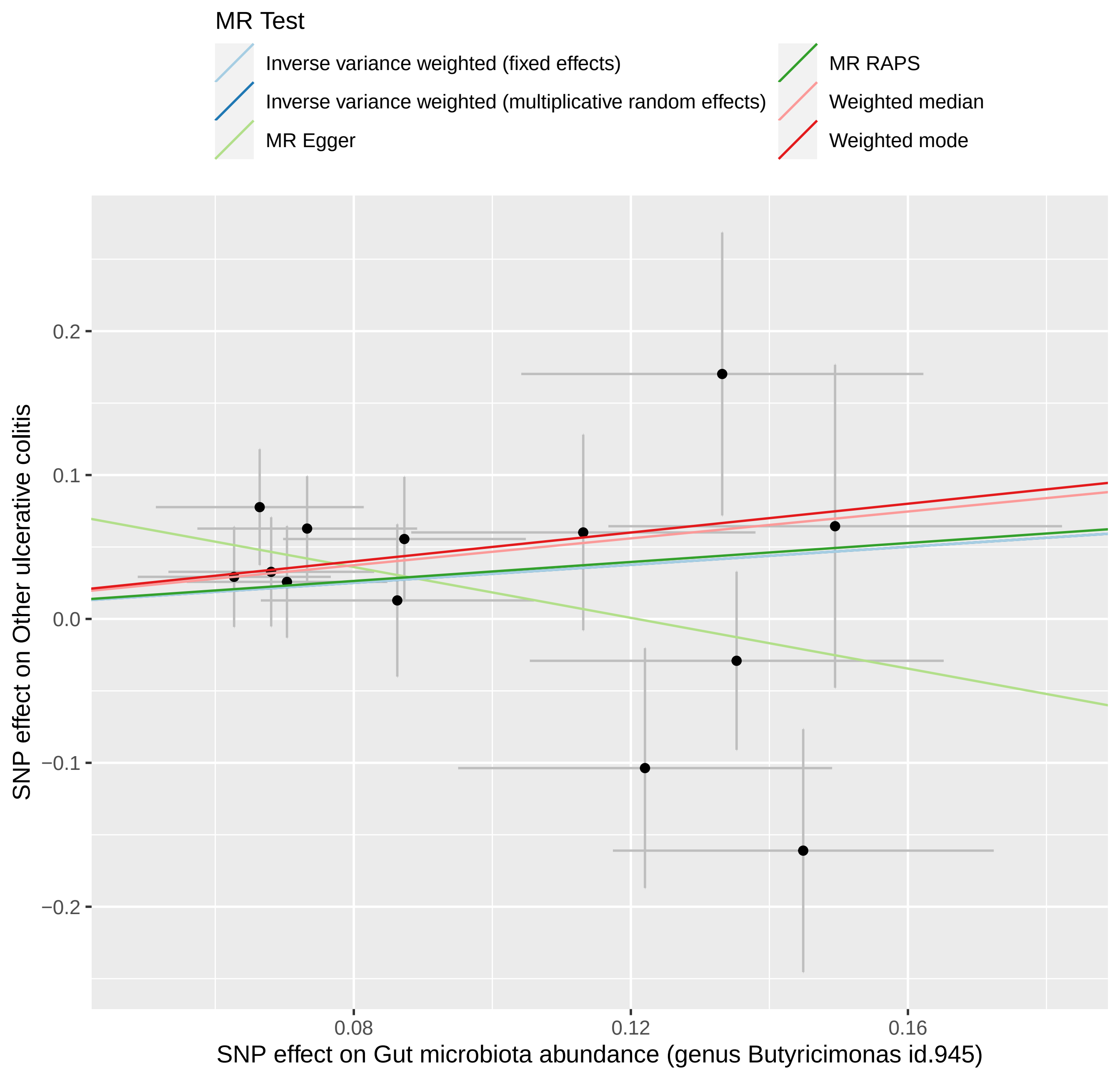

Batch 1249 : Gut microbiota abundance (genus Butyrivibrio id.1993) on Other ulcerative colitis

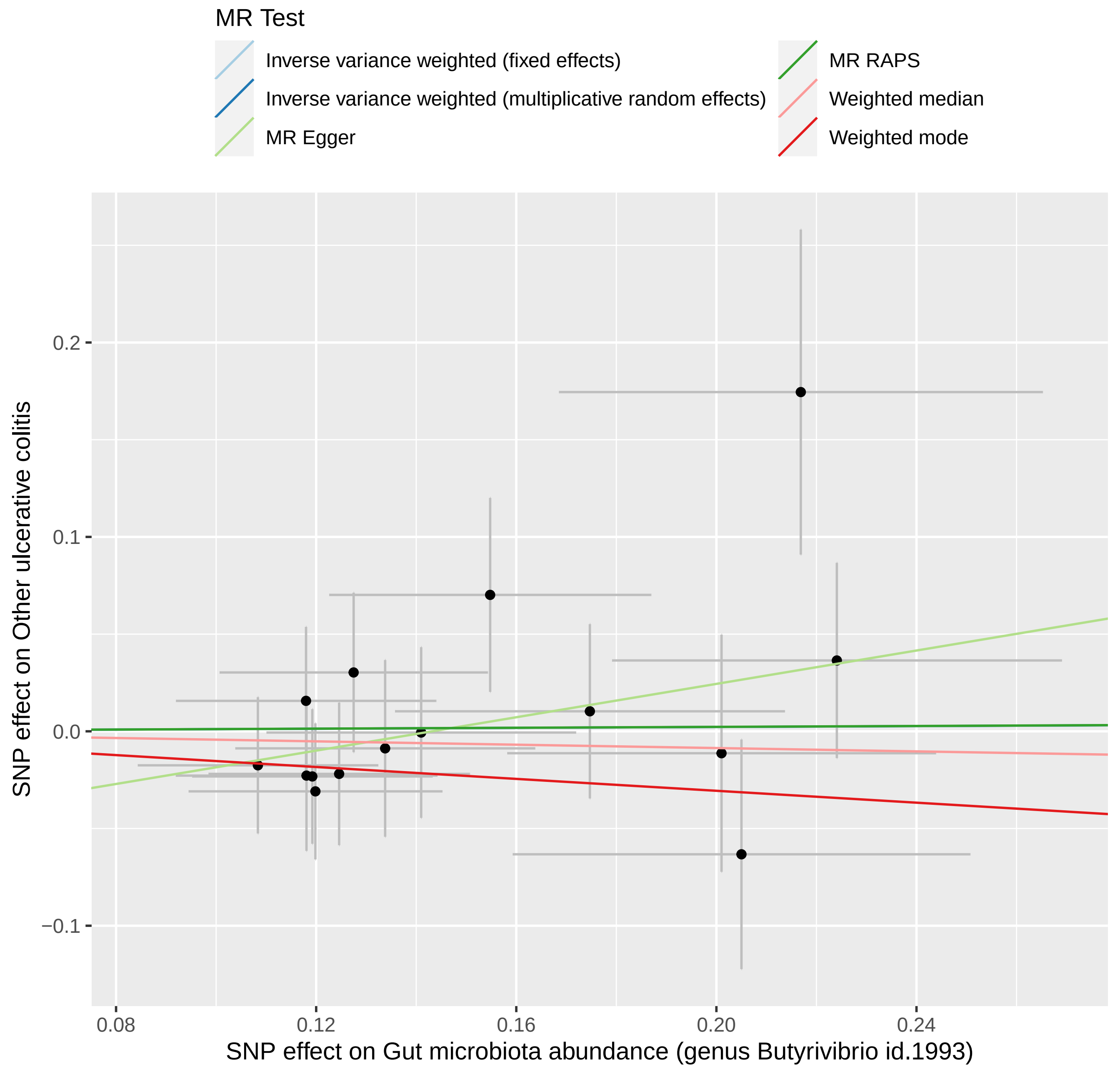

Batch 1250 : Gut microbiota abundance (genus Candidatus Soleaferrea id.11350) on Other ulcerative colitis

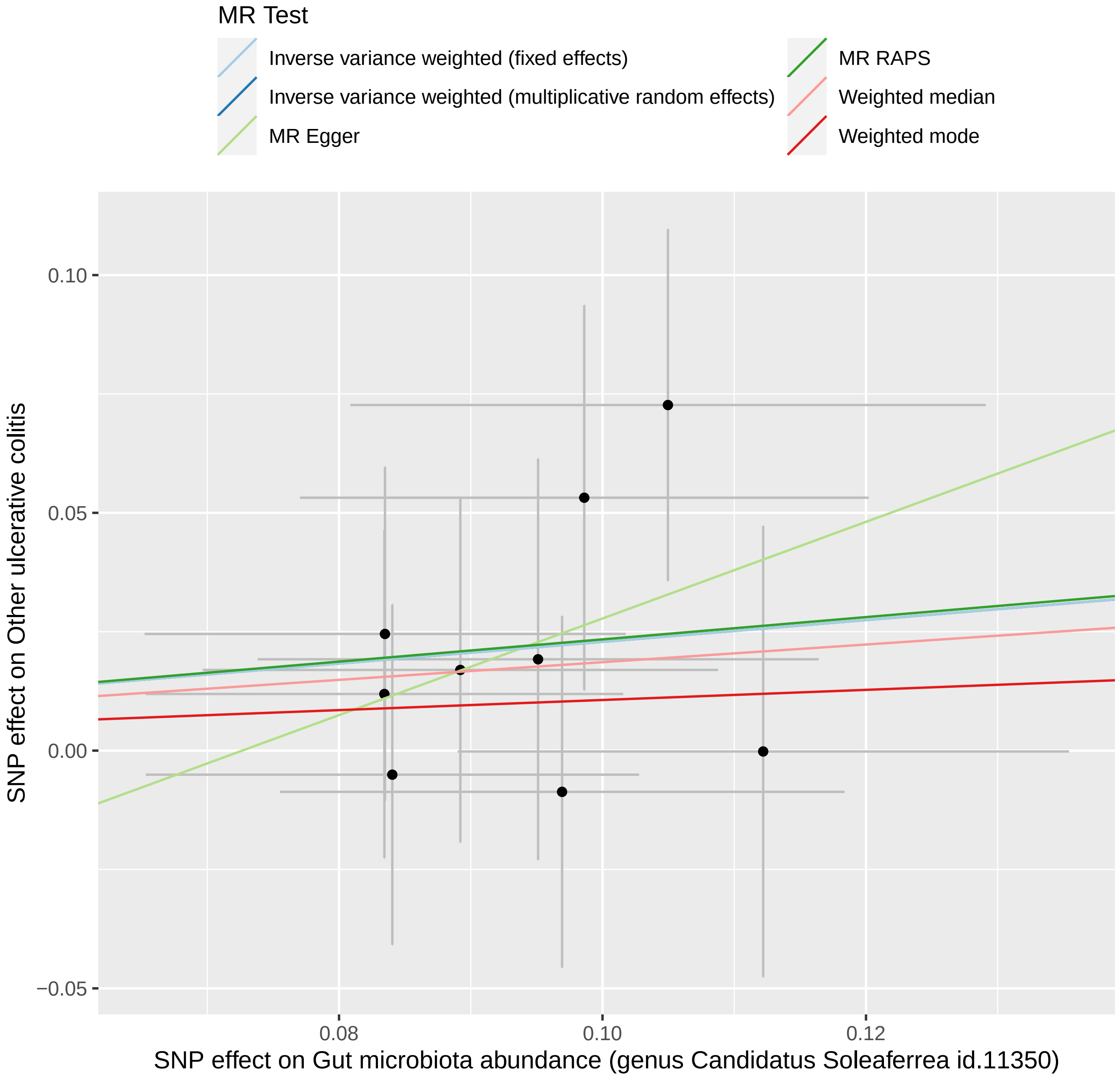

Batch 1251 : Gut microbiota abundance (genus Catenibacterium id.2153) on Other ulcerative colitis

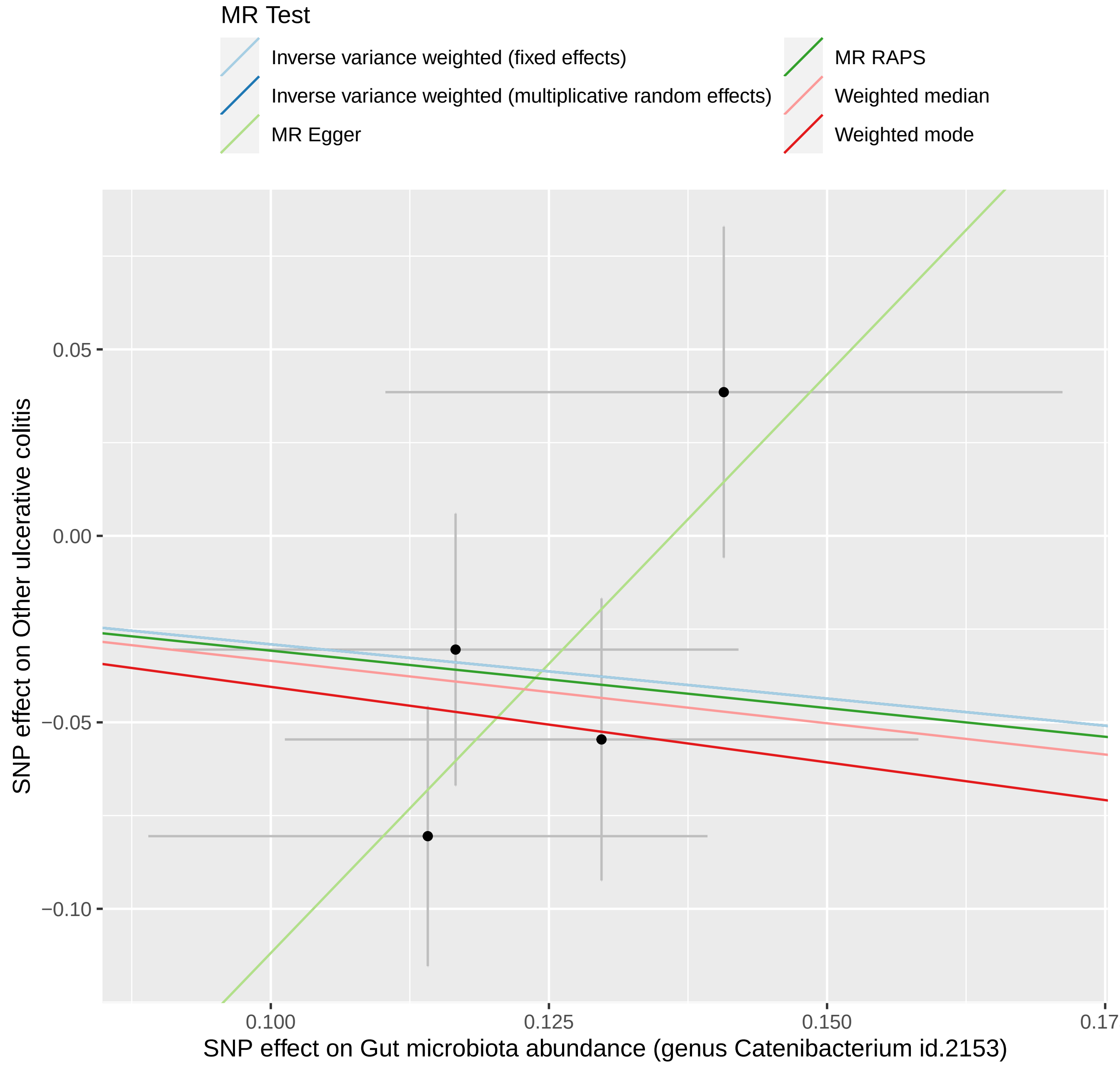

Batch 1252 : Gut microbiota abundance (genus Christensenellaceae R 7group id.11283) on Other ulcerative colitis

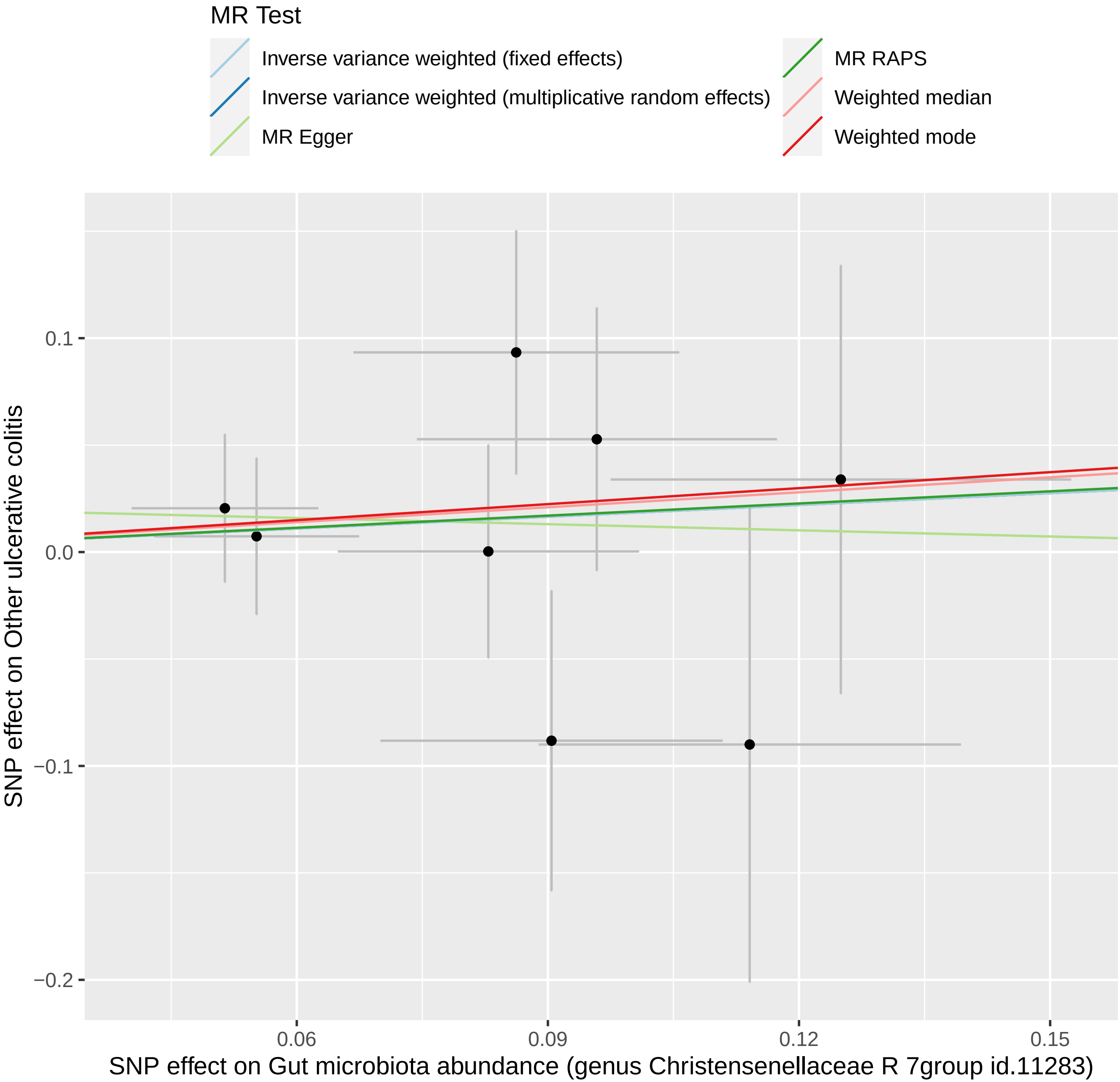

Batch 1253 : Gut microbiota abundance (genus Clostridium innocuum group id.14397) on Other ulcerative colitis

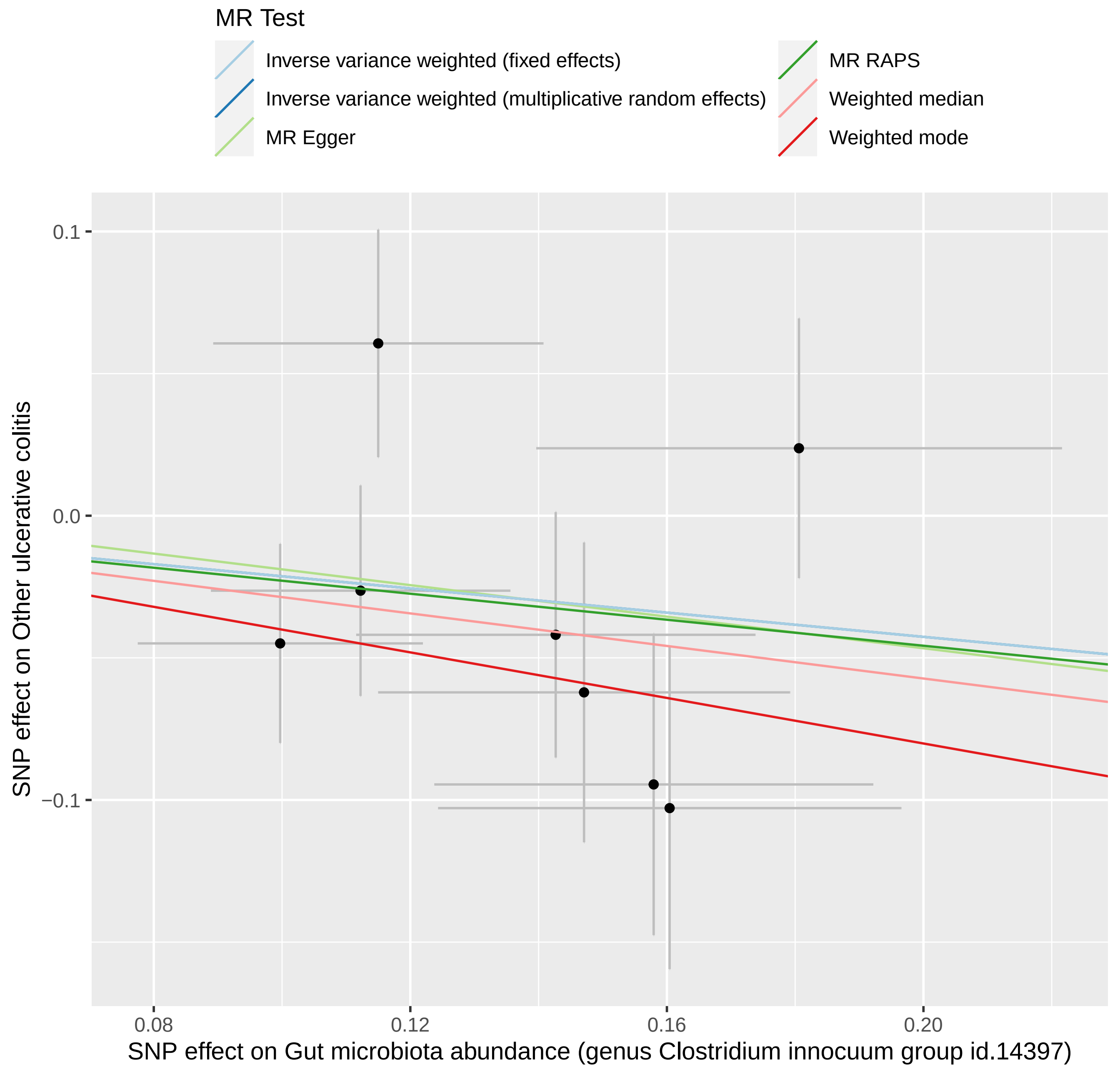

Batch 1254 : Gut microbiota abundance (genus Clostridium sensustricto1 id.1873) on Other ulcerative colitis

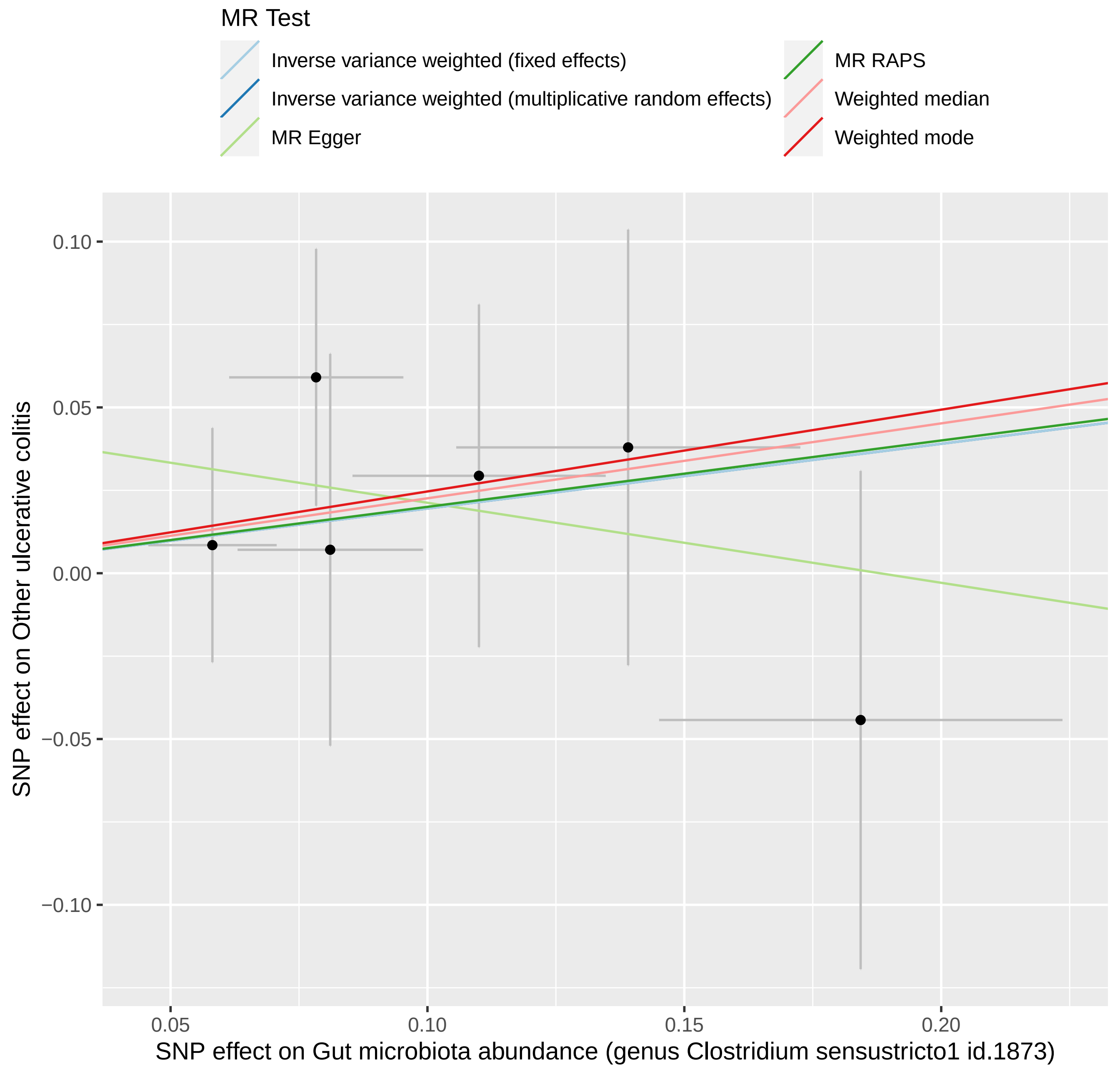

Batch 1255 : Gut microbiota abundance (genus Collinsella id.815) on Other ulcerative colitis

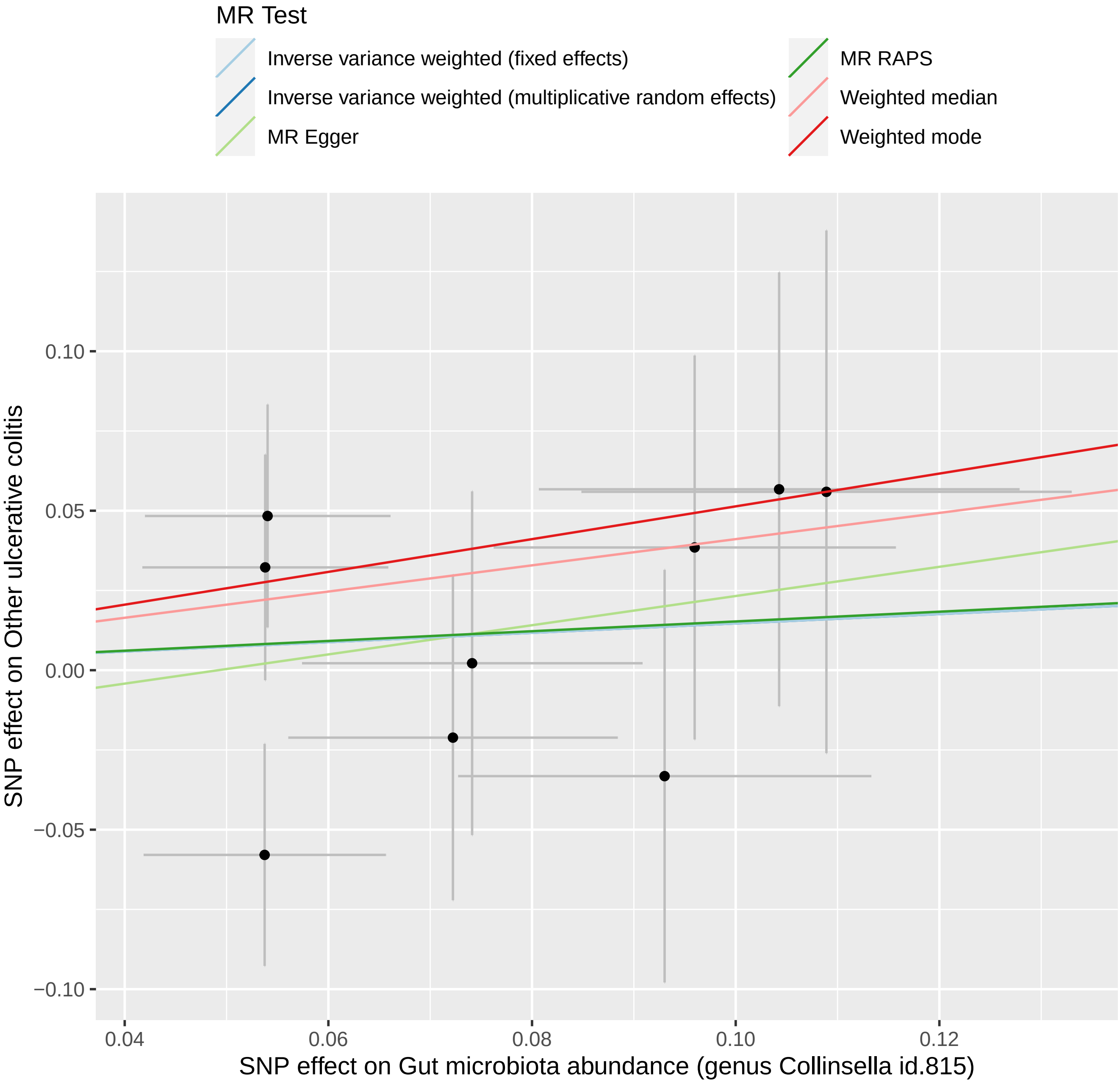

Batch 1256 : Gut microbiota abundance (genus Coprobacter id.949) on Other ulcerative colitis

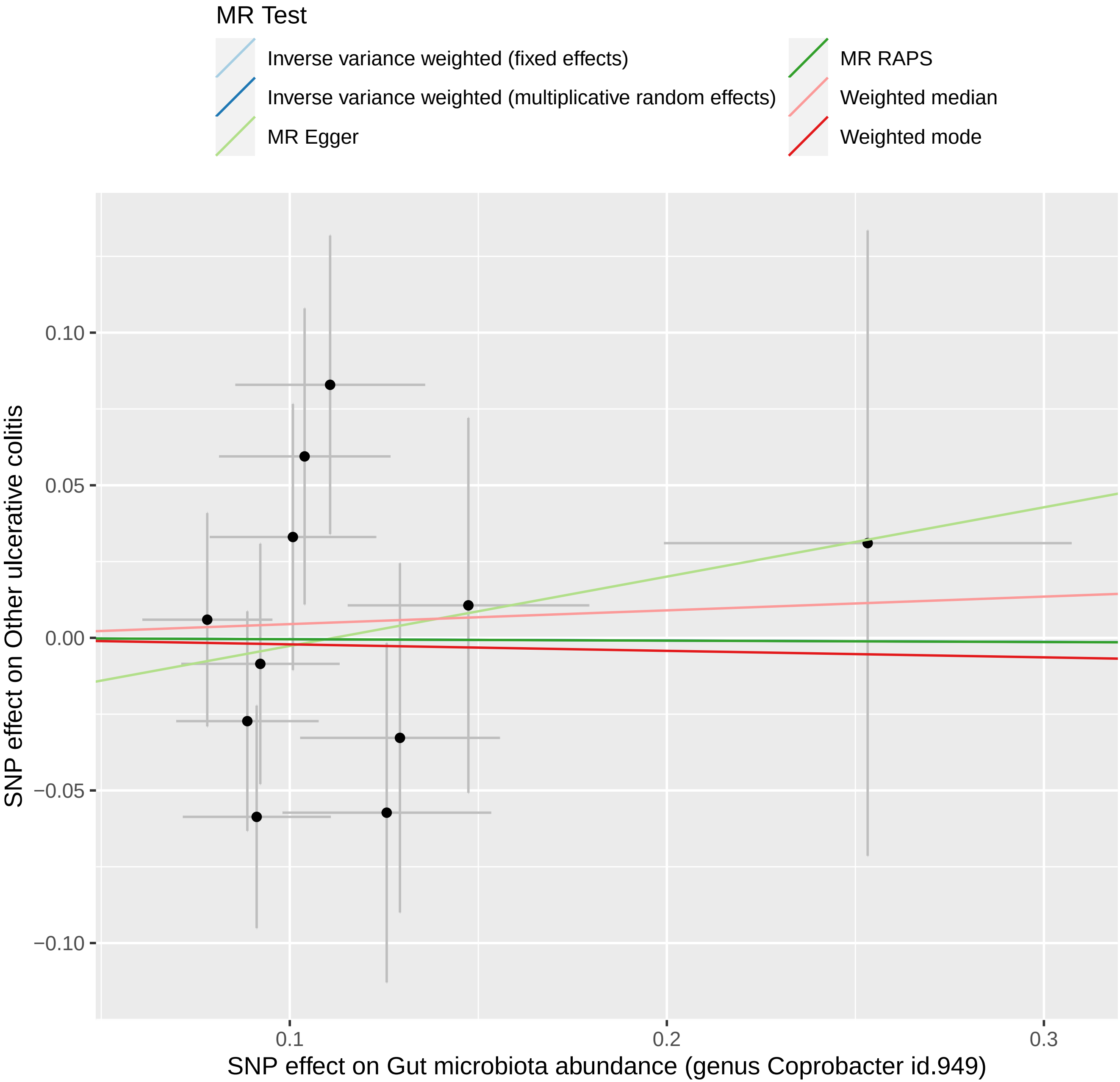

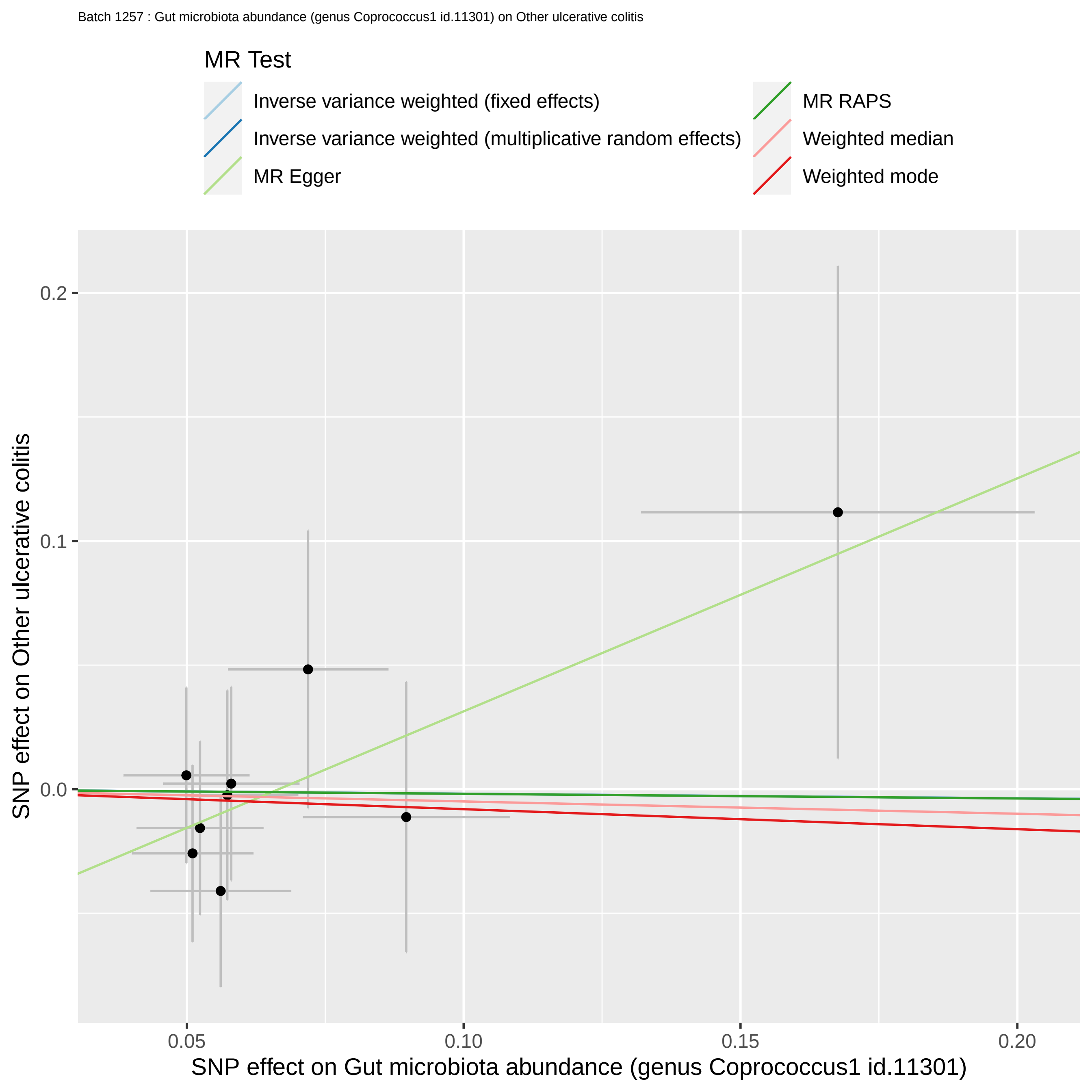
Batch 1257 : Gut microbiota abundance (genus Coprococcus1 id.11301) on Other ulcerative colitis
MR Test
Inverse variance weighted (fixed effects)
Inverse variance weighted (multiplicative random effects)
MR Egger
MR RAPS
Weighted median
Weighted mode
0.2
0.1
0.0
0.05
0.10
0.15
0.20
SNP effect on Other ulcerative colitis
SNP effect on Gut microbiota abundance (genus Coprococcus1 id.11301)

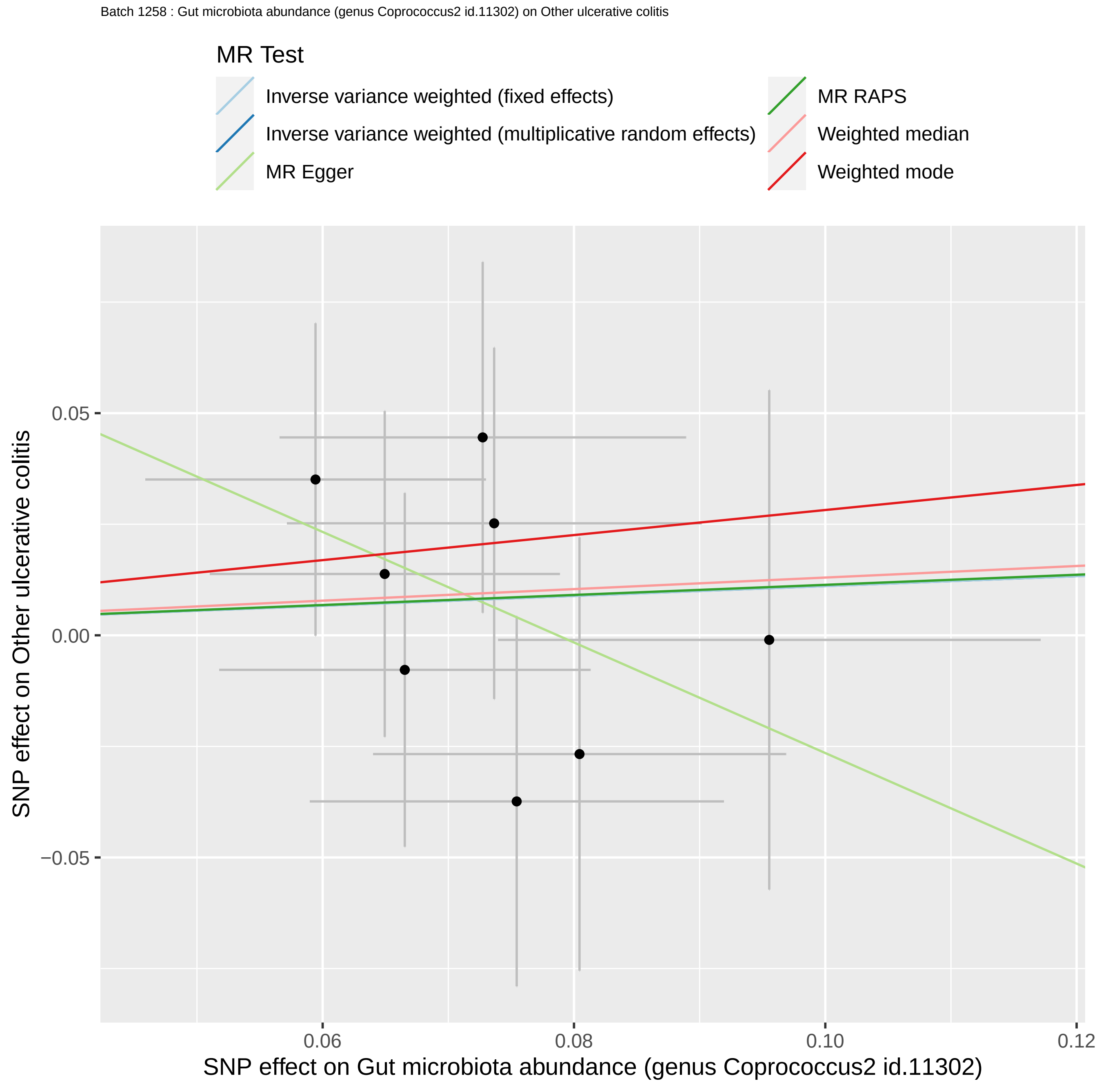
Batch 1258 : Gut microbiota abundance (genus Coprococcus2 id.11302) on Other ulcerative colitis
MR Test
Inverse variance weighted (fixed effects)
Inverse variance weighted (multiplicative random effects)
MR Egger
MR RAPS
Weighted median
Weighted mode
0.05
0.00
−0.05
0.06
0.08
0.10
0.12
SNP effect on Other ulcerative colitis
SNP effect on Gut microbiota abundance (genus Coprococcus2 id.11302)

Batch 1259 : Gut microbiota abundance (genus Coprococcus3 id.11303) on Other ulcerative colitis

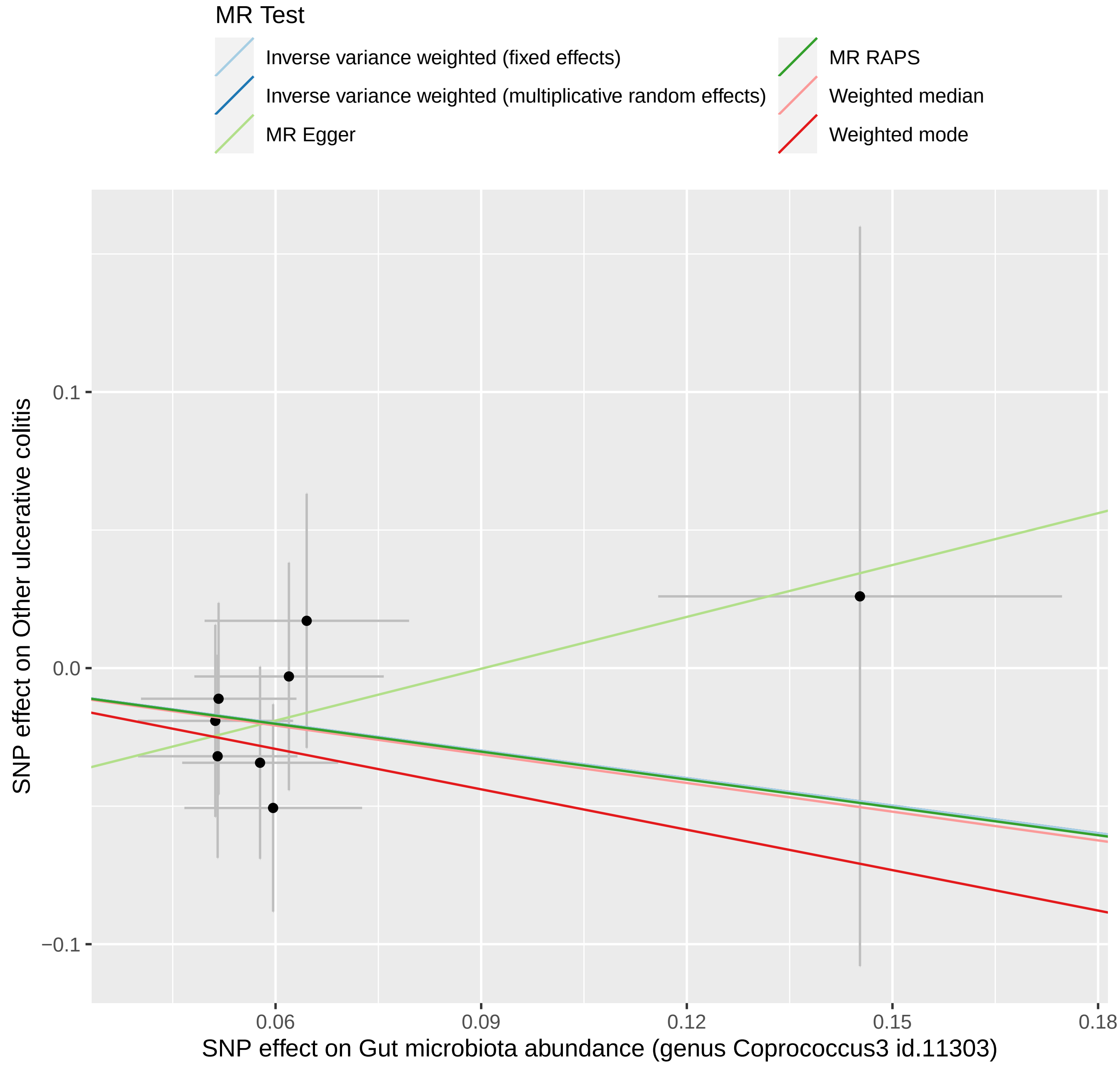

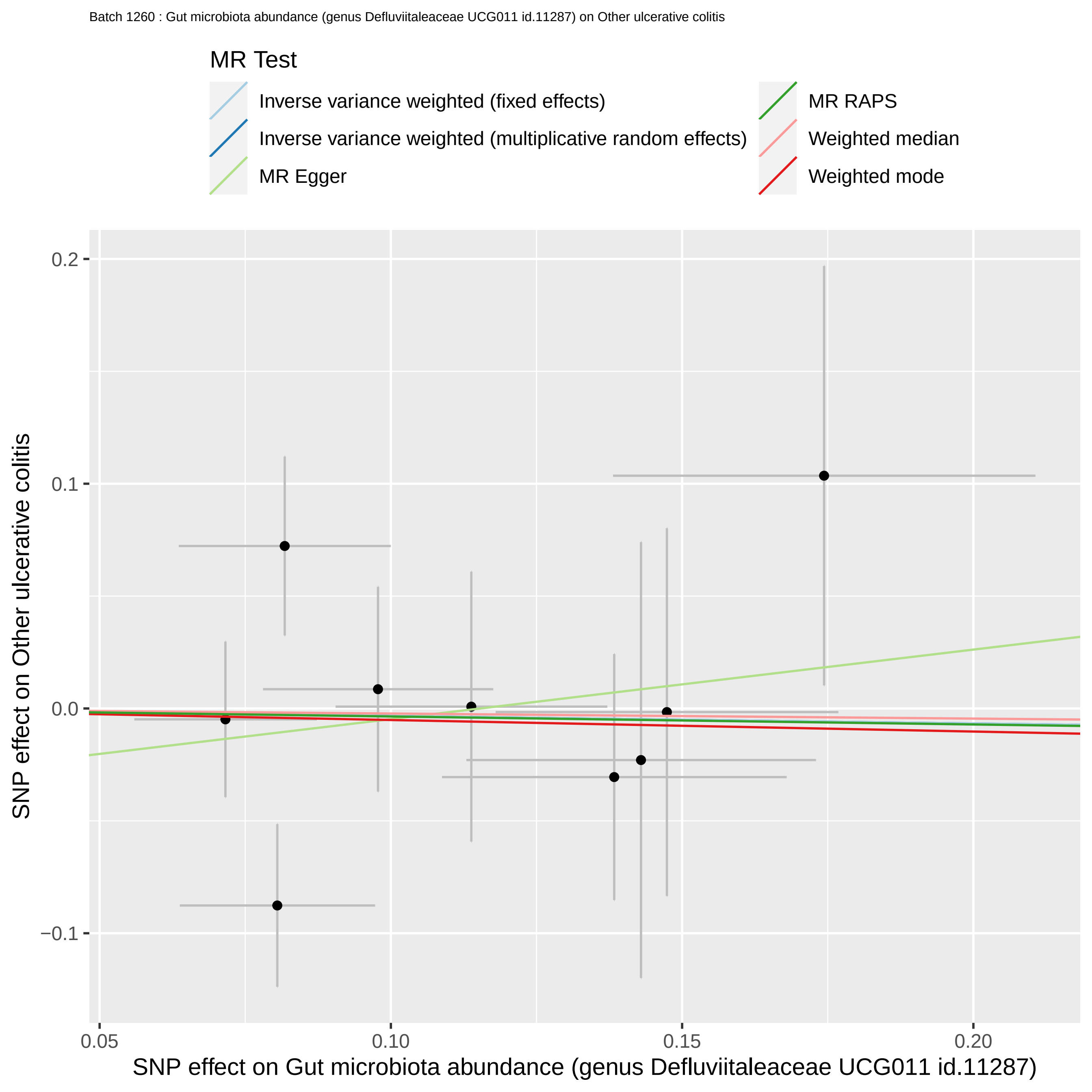
Batch 1260 : Gut microbiota abundance (genus Defluviitaleaceae UCG011 id.11287) on Other ulcerative colitis
MR Test
Inverse variance weighted (fixed effects)
Inverse variance weighted (multiplicative random effects)
MR Egger
MR RAPS
Weighted median
Weighted mode
0.2
0.1
0.0
−0.1
0.05
0.10
0.15
0.20
SNP effect on Other ulcerative colitis
SNP effect on Gut microbiota abundance (genus Defluviitaleaceae UCG011 id.11287)

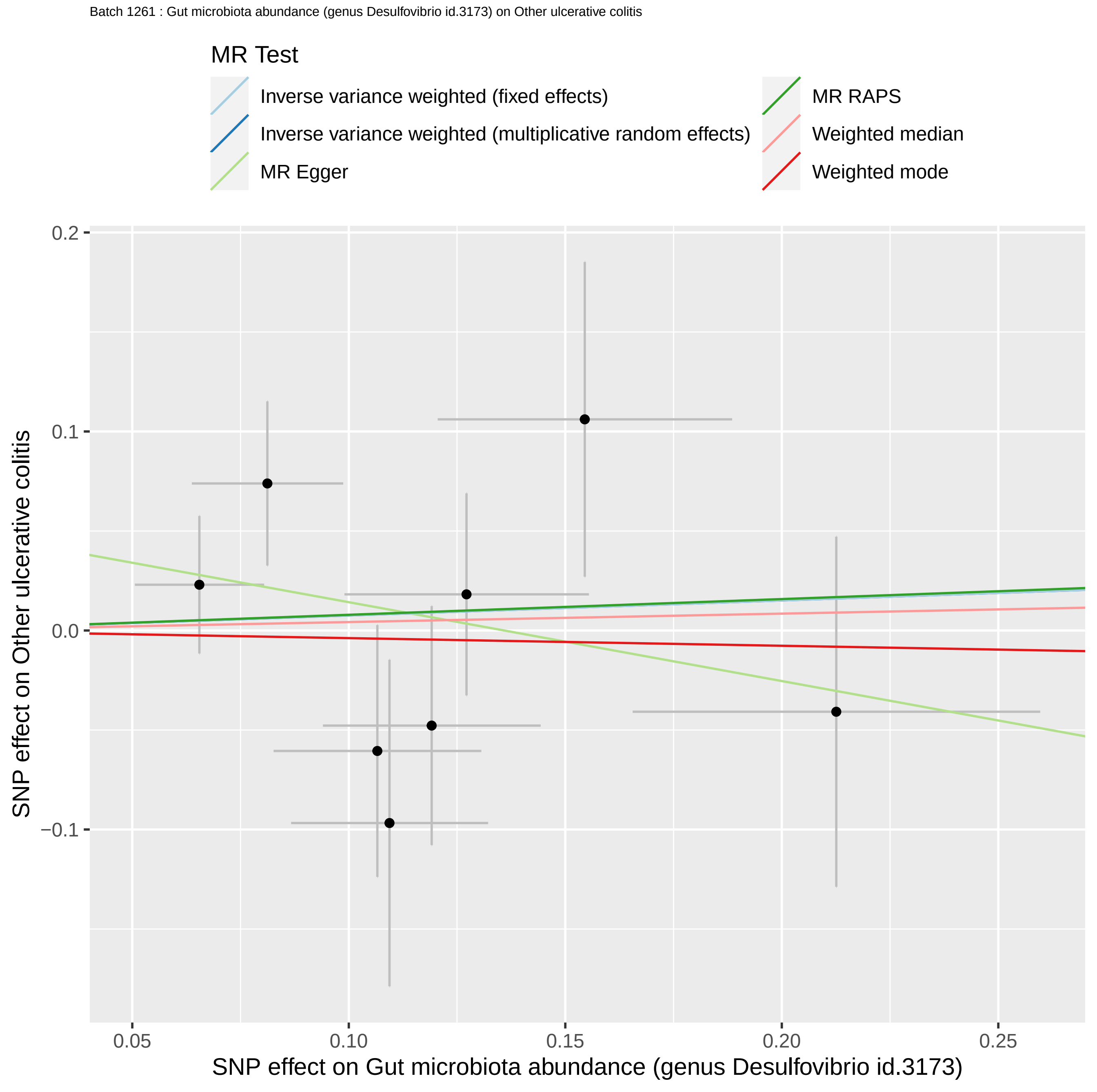
Batch 1261 : Gut microbiota abundance (genus Desulfovibrio id.3173) on Other ulcerative colitis
MR Test
Inverse variance weighted (fixed effects)
Inverse variance weighted (multiplicative random effects)
MR Egger
MR RAPS
Weighted median
Weighted mode
0.2
0.1
0.0
−0.1
0.05
0.10
0.15
0.20
0.25
SNP effect on Other ulcerative colitis
SNP effect on Gut microbiota abundance (genus Desulfovibrio id.3173)

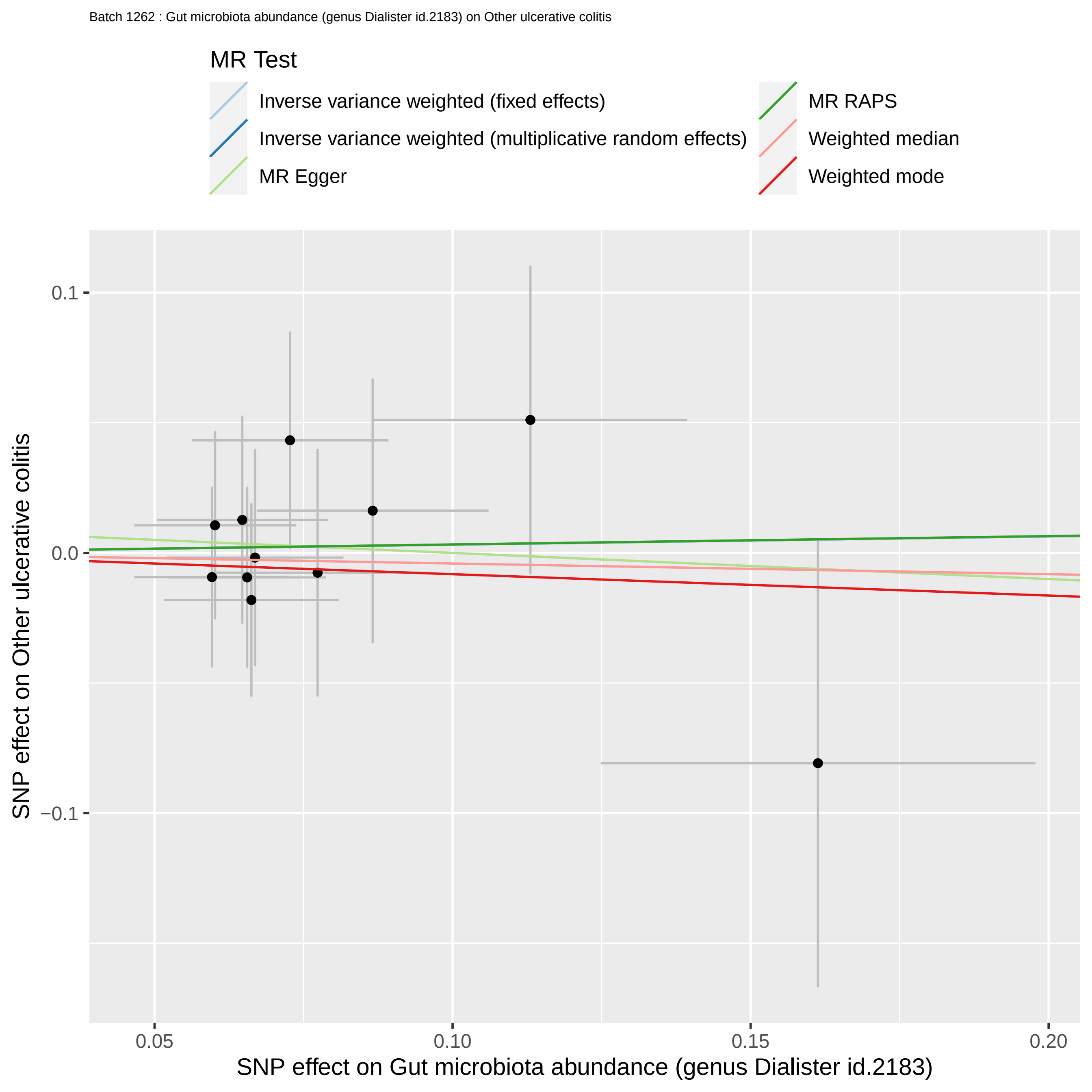

Batch 1262 : Gut microbiota abundance (genus Dialister id.2183) on Other ulcerative colitis
MR Test
Inverse variance weighted (fixed effects)
Inverse variance weighted (multiplicative random effects)
MR Egger
MR RAPS
Weighted median
Weighted mode
0.1
0.0
−0.1
0.05
0.10
0.15
0.20
SNP effect on Other ulcerative colitis
SNP effect on Gut microbiota abundance (genus Dialister id.2183)

Batch 1263 : Gut microbiota abundance (genus Dorea id.1997) on Other ulcerative colitis

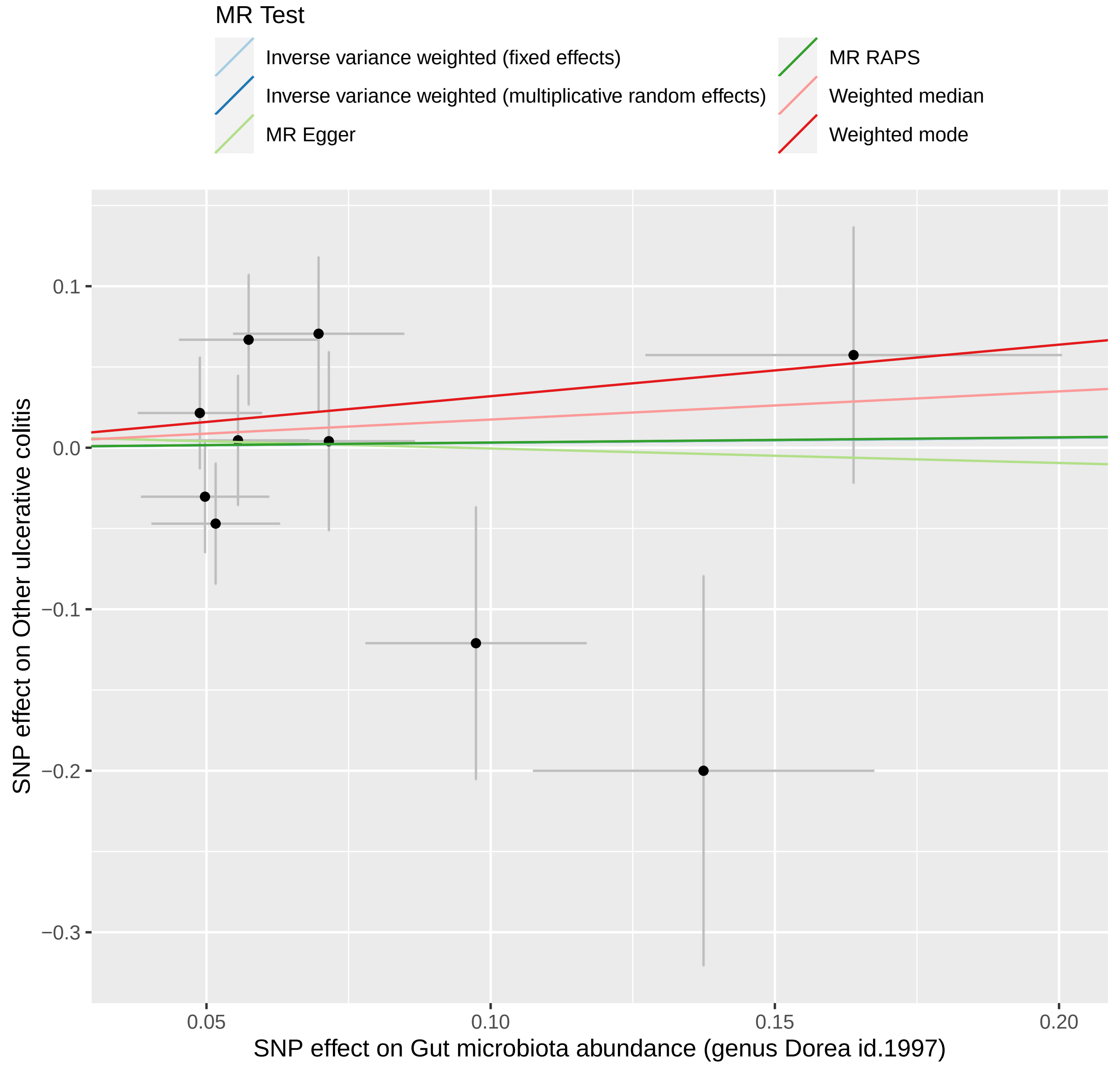

Batch 1264 : Gut microbiota abundance (genus Eggerthella id.819) on Other ulcerative colitis

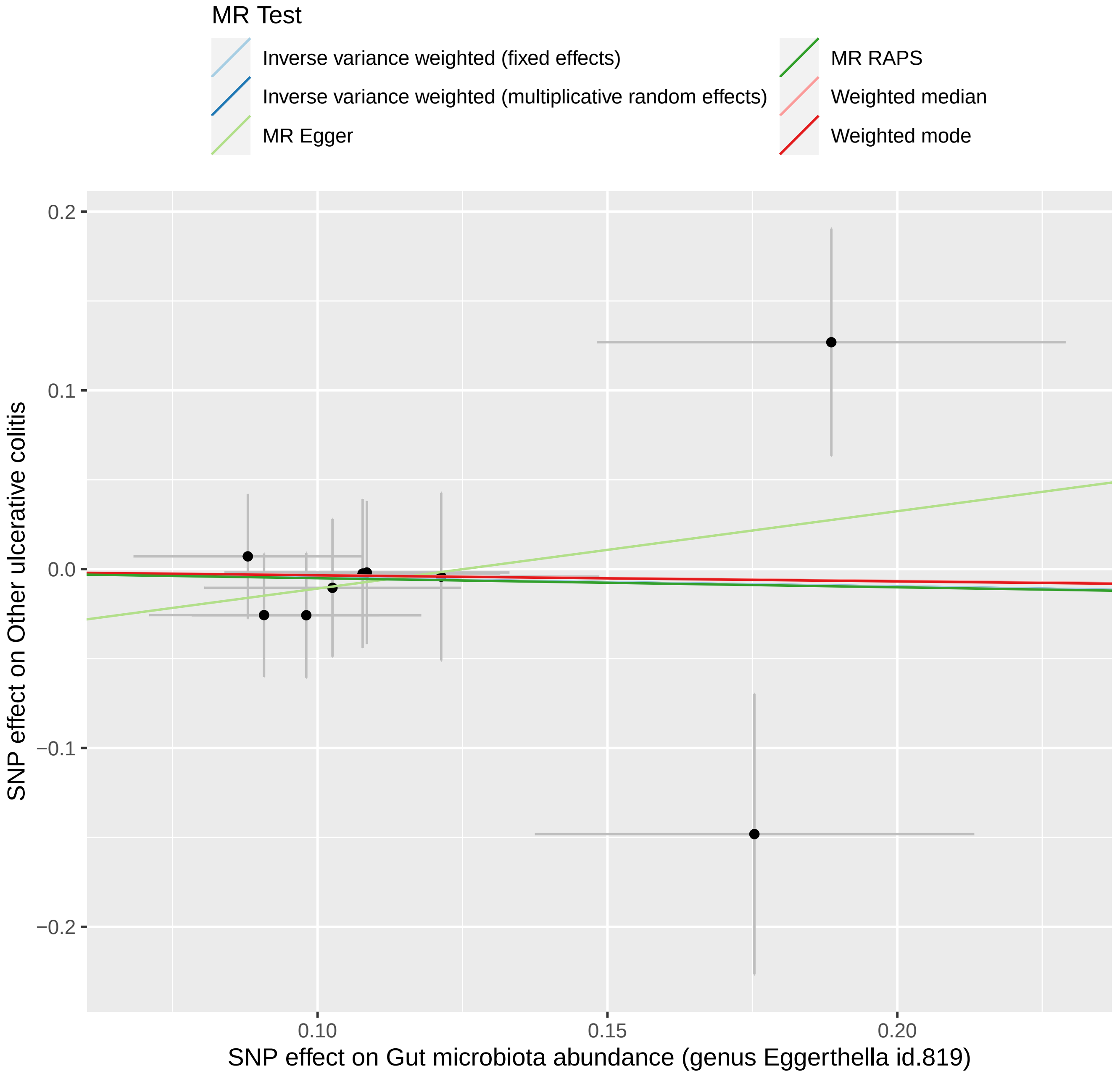

Batch 1265 : Gut microbiota abundance (genus Eisenbergiella id.11304) on Other ulcerative colitis

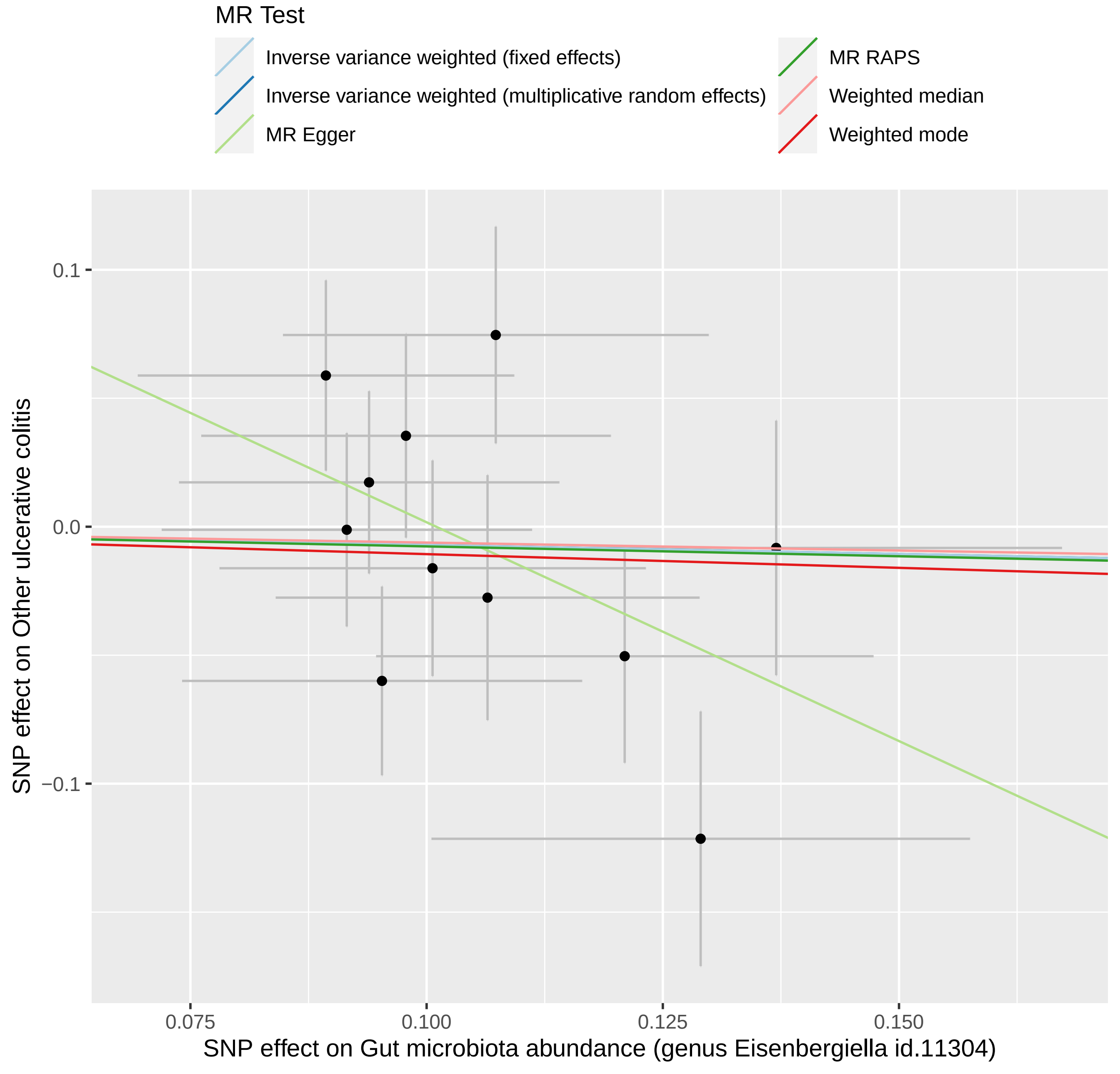

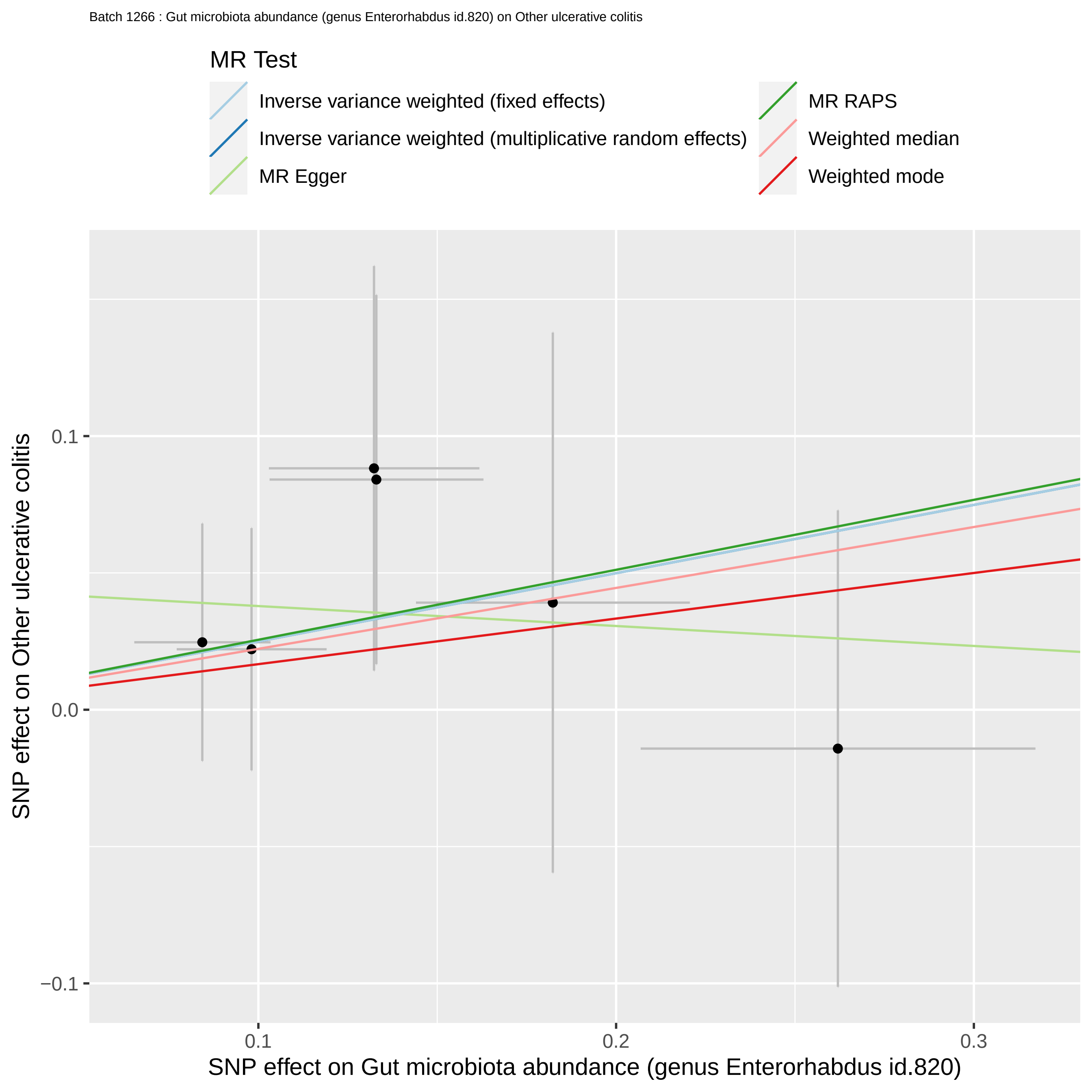

Batch 1266 : Gut microbiota abundance (genus Enterorhabdus id.820) on Other ulcerative colitis
MR Test
Inverse variance weighted (fixed effects)
Inverse variance weighted (multiplicative random effects)
MR Egger
MR RAPS
Weighted median
Weighted mode
0.1
0.0
−0.1
0.1
0.2
0.3
SNP effect on Other ulcerative colitis
SNP effect on Gut microbiota abundance (genus Enterorhabdus id.820)

Batch 1267 : Gut microbiota abundance (genus Erysipelatoclostridium id.11381) on Other ulcerative colitis

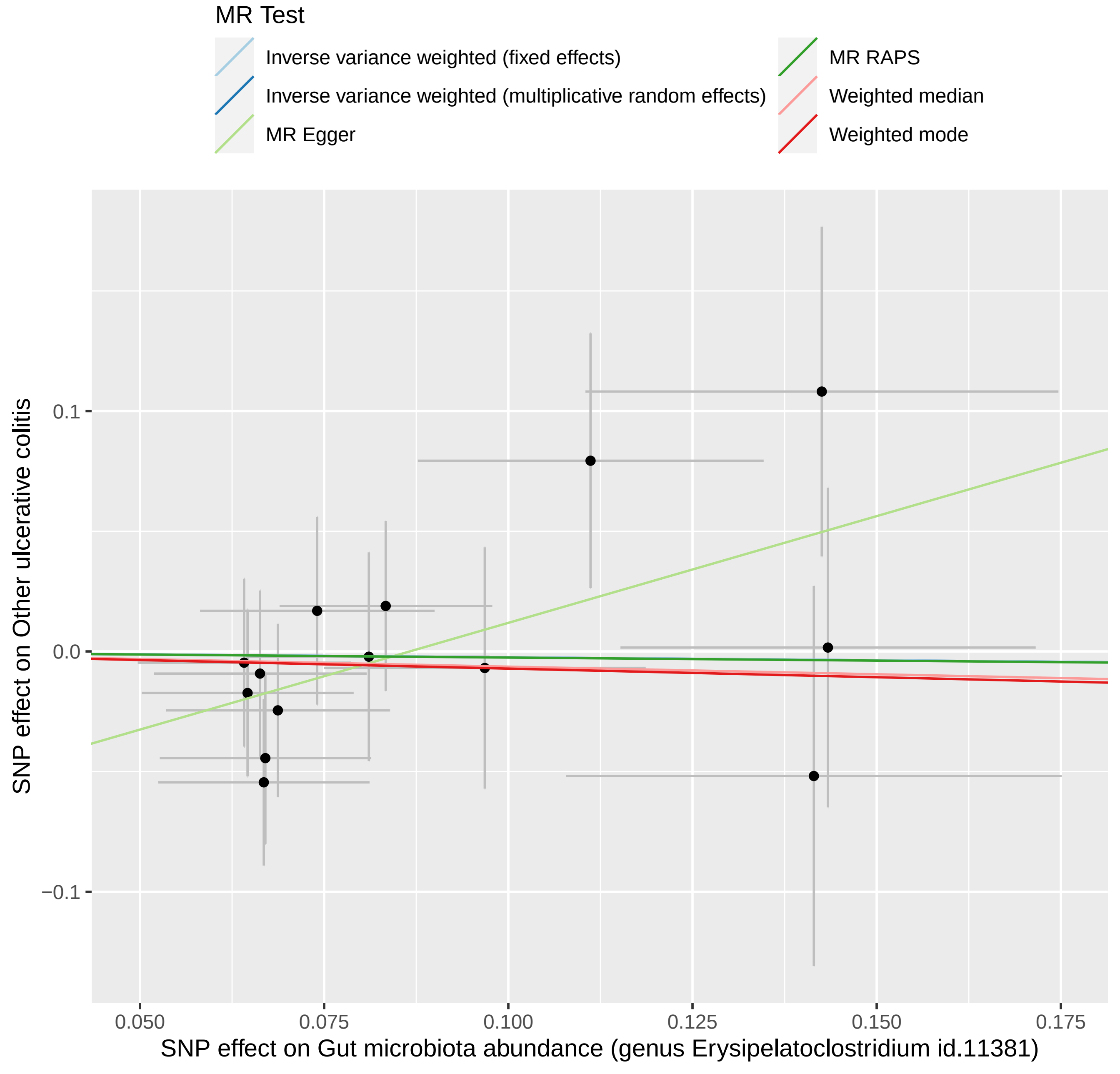

Insufficient number of SNPs

Batch 1269 : Gut microbiota abundance (genus Escherichia Shigella id.3504) on Other ulcerative colitis

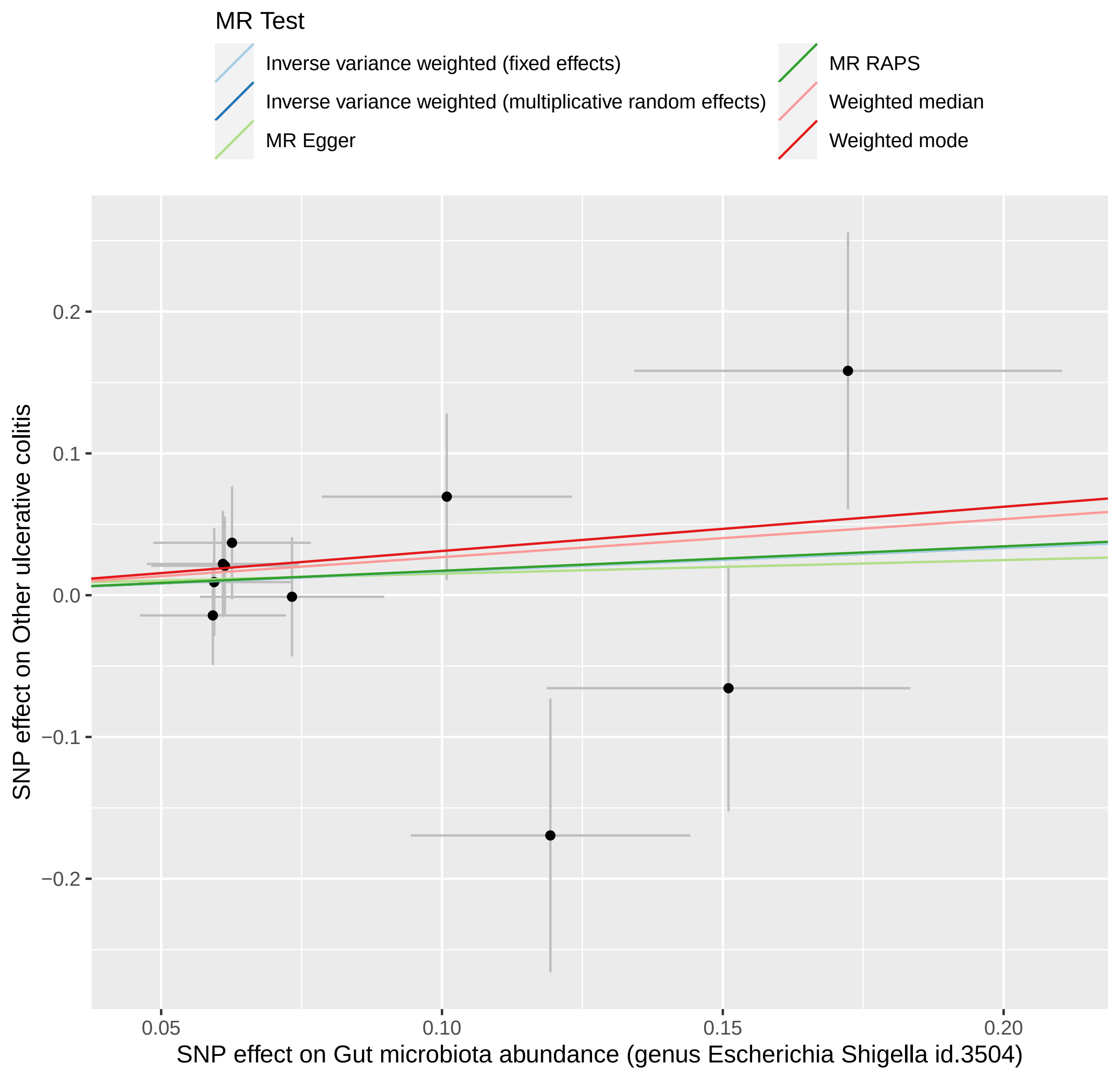

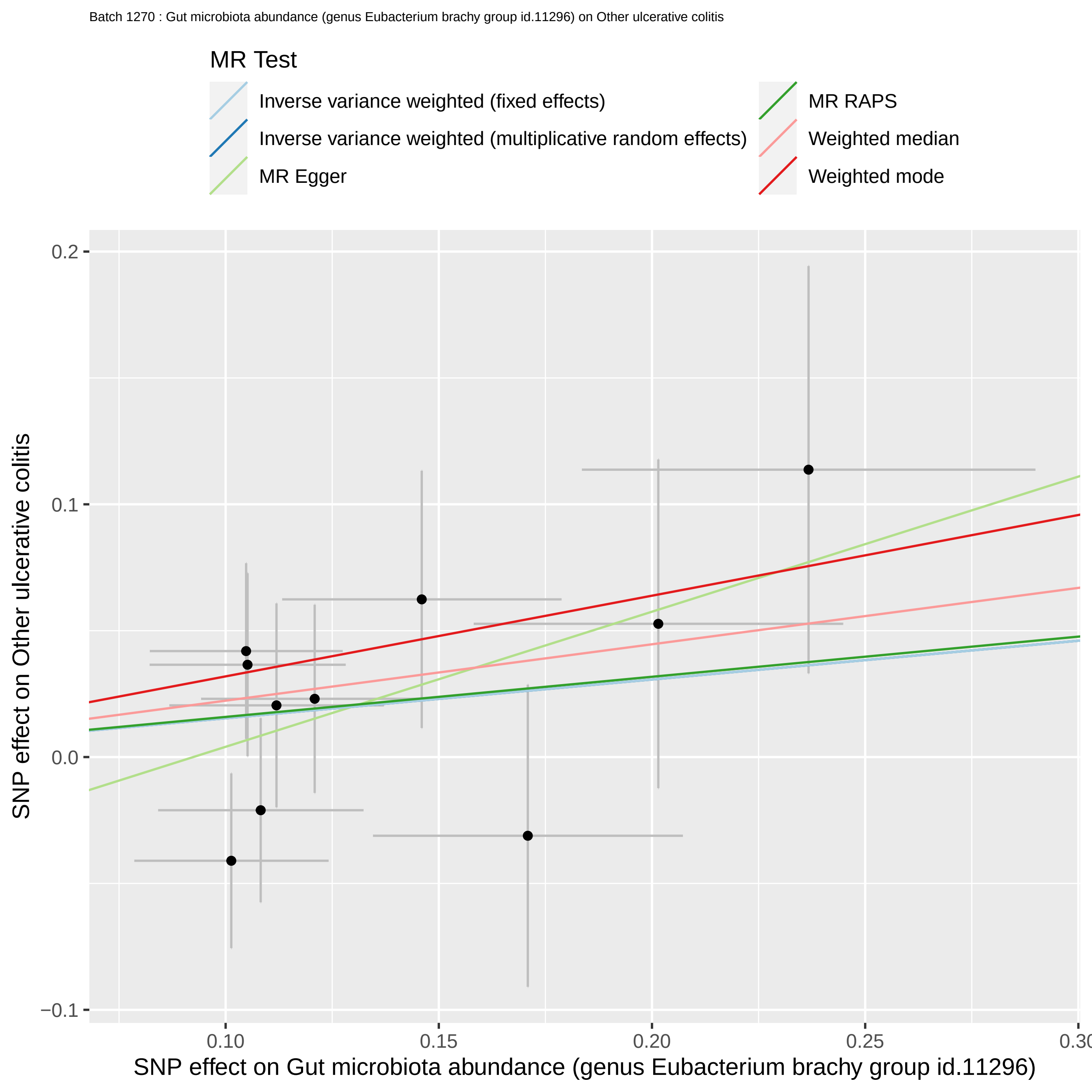
Batch 1270 : Gut microbiota abundance (genus Eubacterium brachy group id.11296) on Other ulcerative colitis
MR Test
Inverse variance weighted (fixed effects)
Inverse variance weighted (multiplicative random effects)
MR Egger
MR RAPS
Weighted median
Weighted mode
0.2
0.1
0.0
−0.1
0.10
0.15
0.20
0.25
0.30
SNP effect on Other ulcerative colitis
SNP effect on Gut microbiota abundance (genus Eubacterium brachy group id.11296)

Batch 1271 : Gut microbiota abundance (genus Eubacterium coprostanoligenes group id.11375) on Other ulcerative colitis

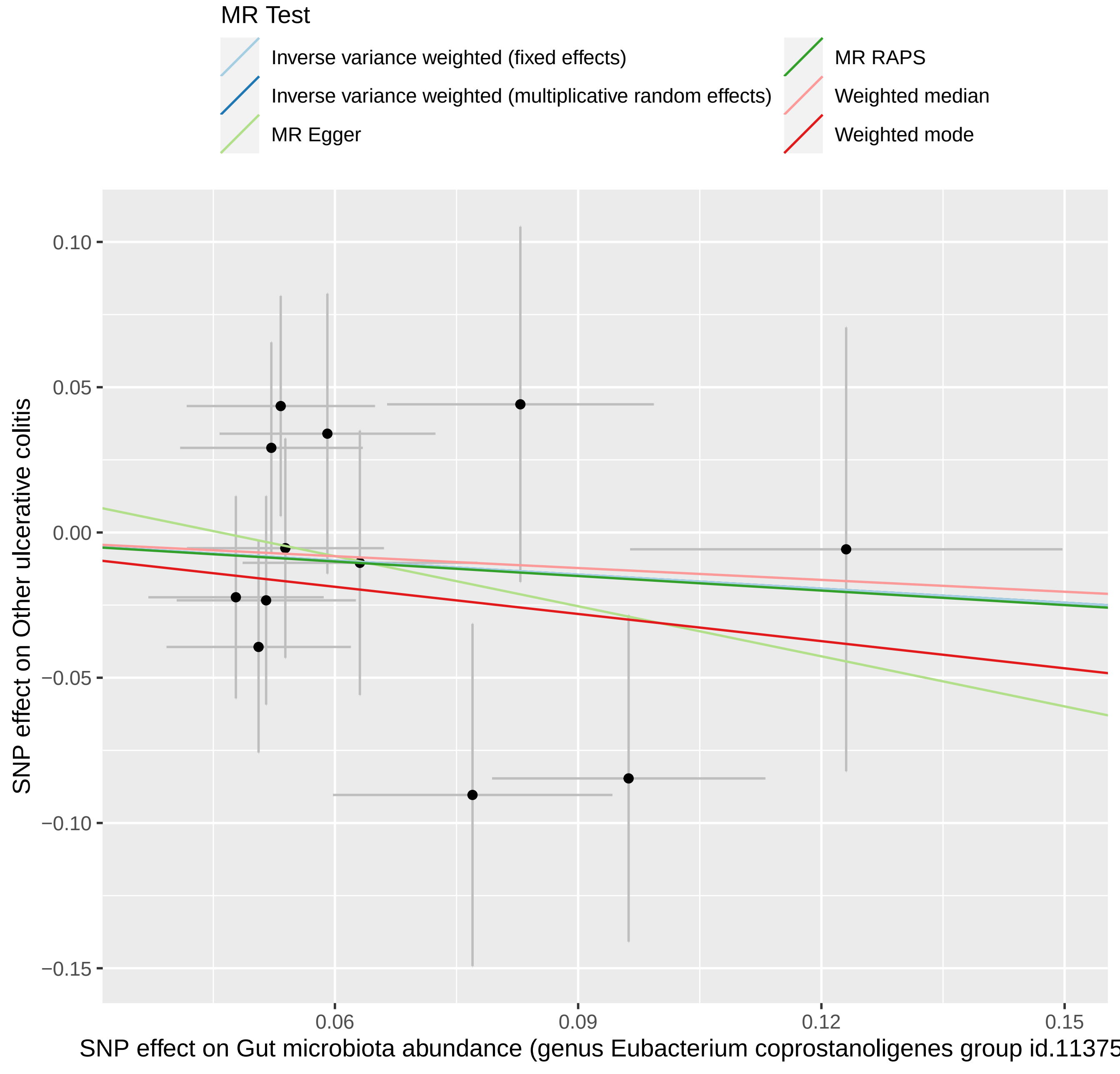

Batch 1272 : Gut microbiota abundance (genus Eubacterium eligens group id.14372) on Other ulcerative colitis

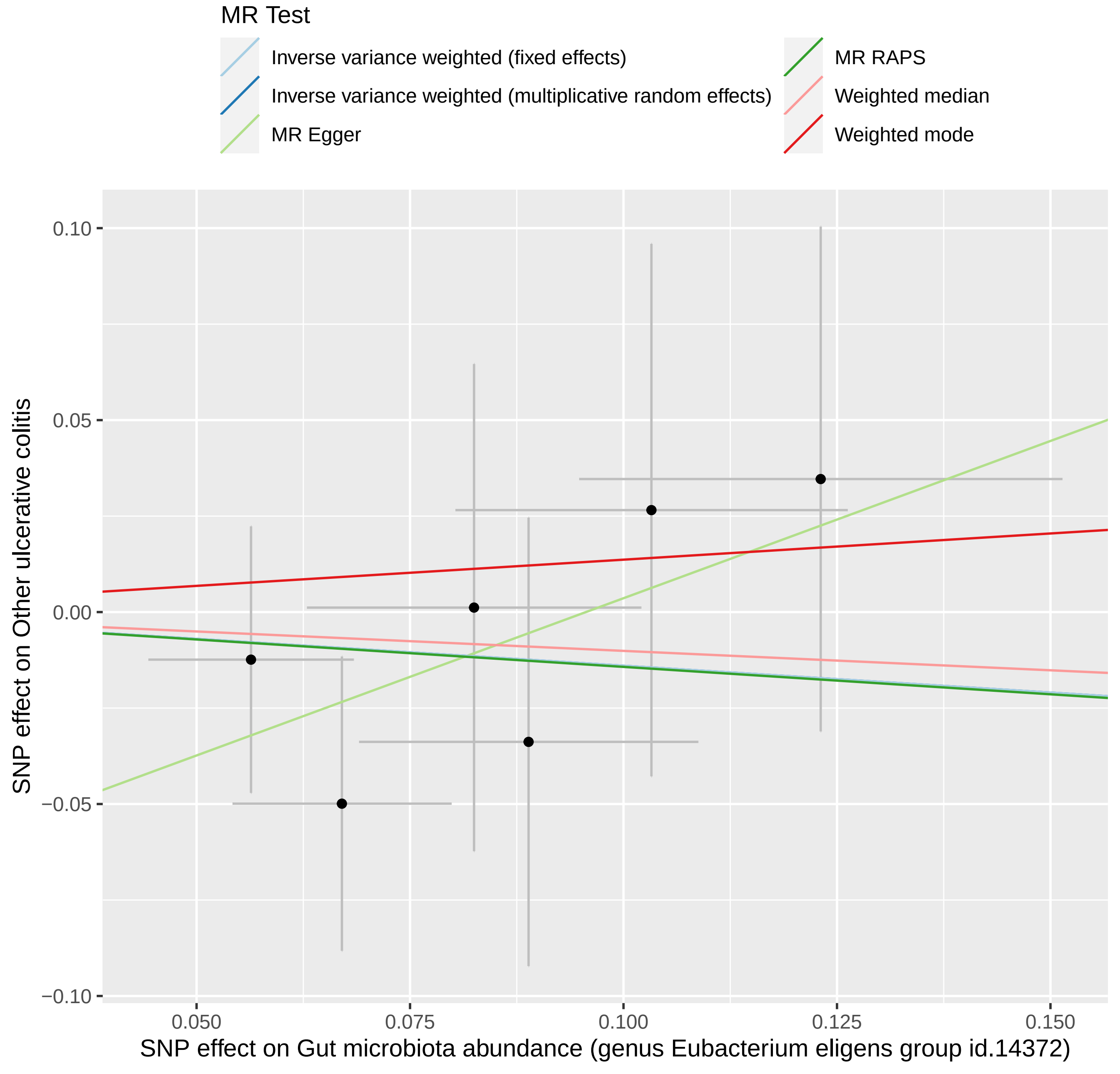

Batch 1273 : Gut microbiota abundance (genus Eubacterium fissicatena group id.14373) on Other ulcerative colitis

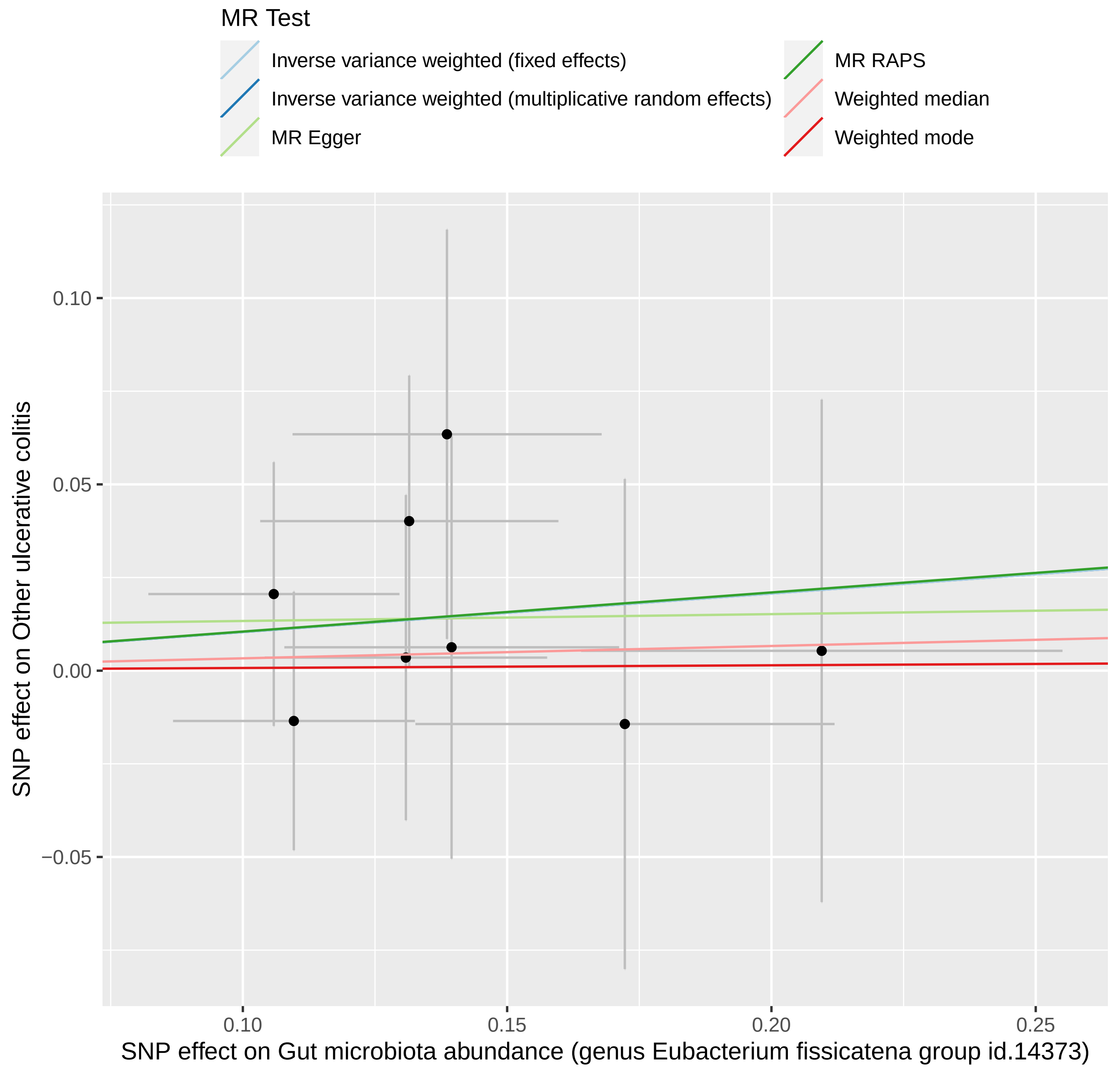

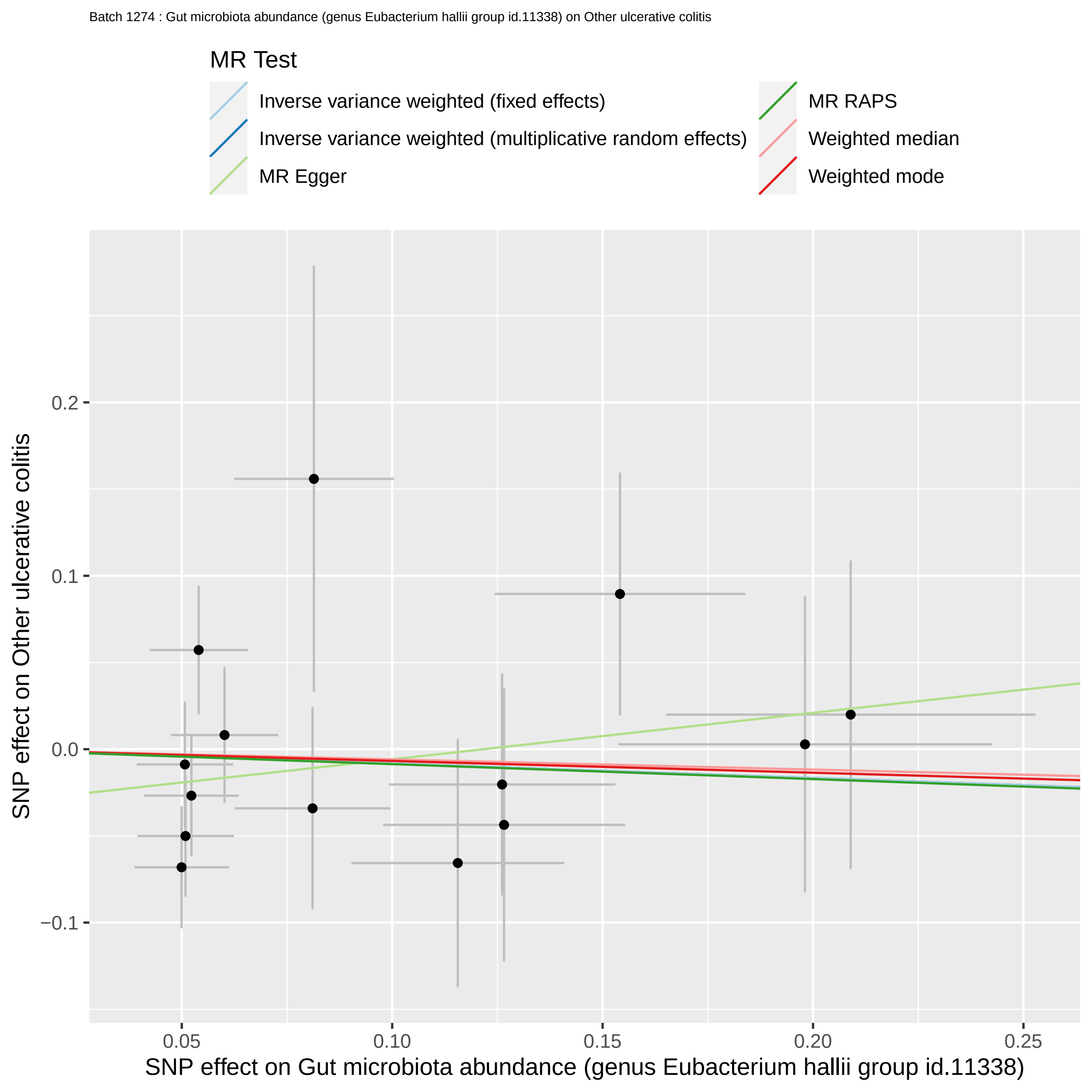

Batch 1274 : Gut microbiota abundance (genus Eubacterium hallii group id.11338) on Other ulcerative colitis
MR Test
Inverse variance weighted (fixed effects)
Inverse variance weighted (multiplicative random effects)
MR Egger
MR RAPS
Weighted median
Weighted mode
0.2
0.1
0.0
−0.1
0.05
0.10
0.15
0.20
0.25
SNP effect on Other ulcerative colitis
SNP effect on Gut microbiota abundance (genus Eubacterium hallii group id.11338)

Batch 1275 : Gut microbiota abundance (genus Eubacterium nodatum group id.11297) on Other ulcerative colitis

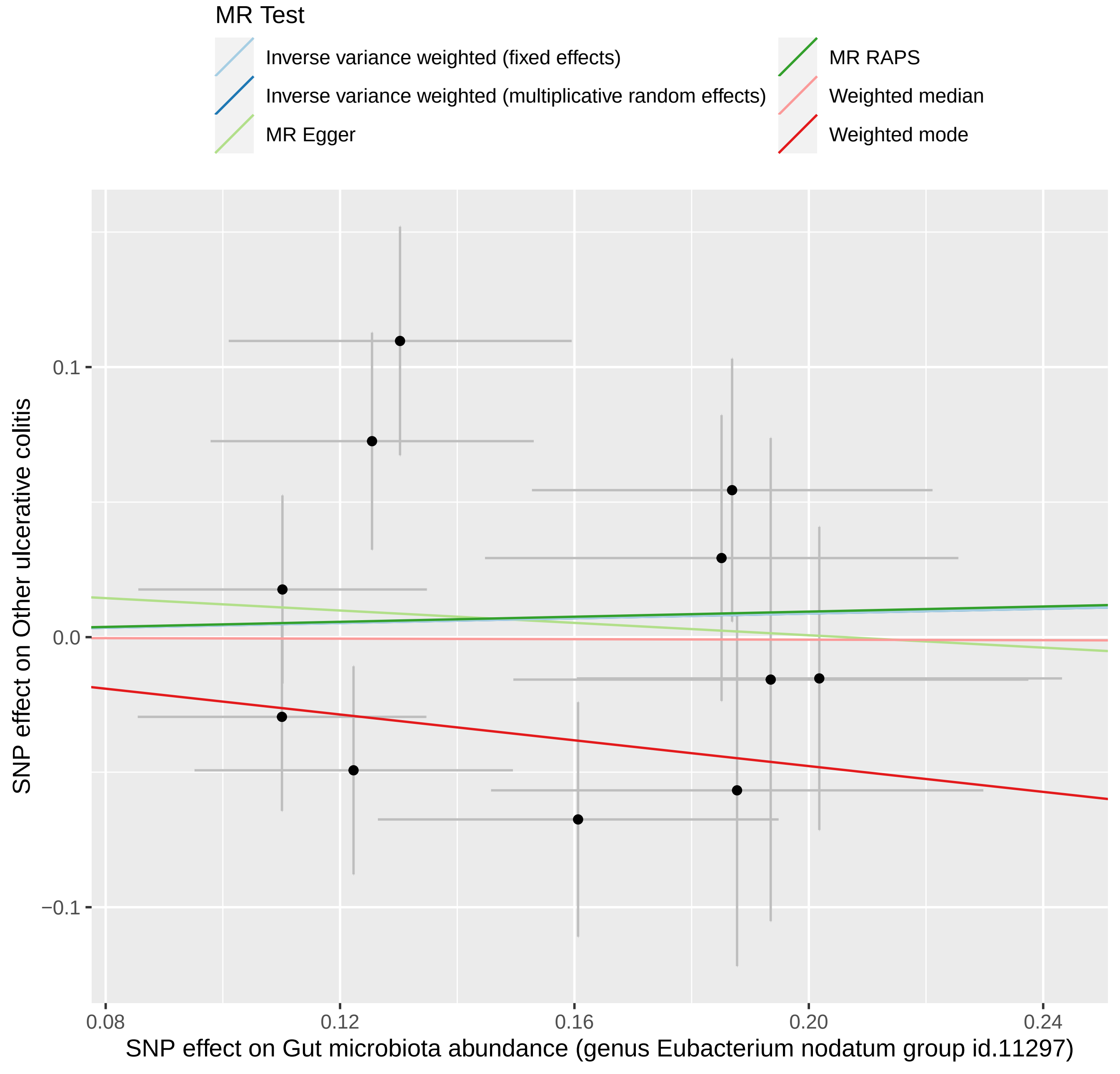

Batch 1276 : Gut microbiota abundance (genus Eubacterium oxidoreducens group id.11339) on Other ulcerative colitis

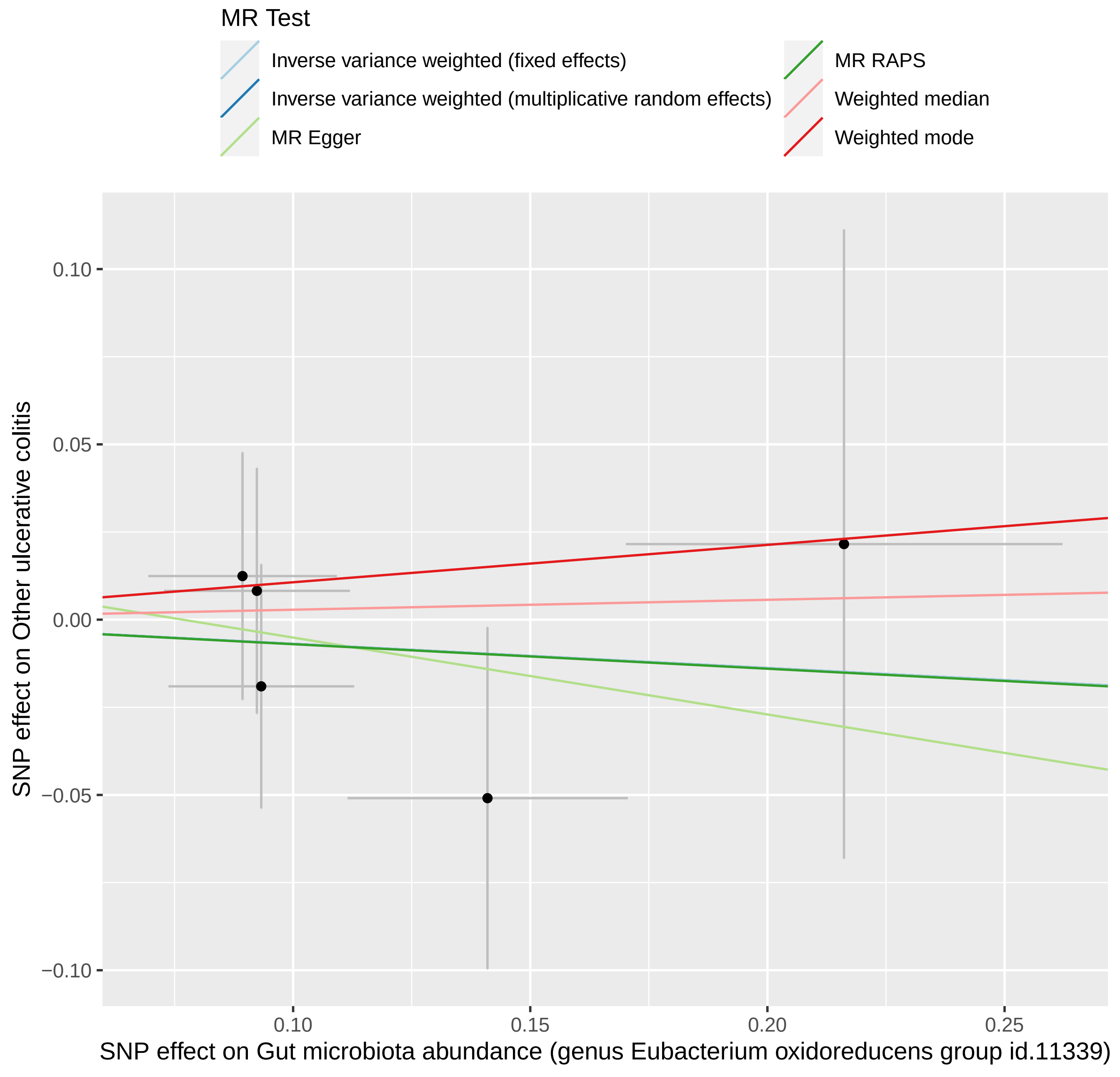

Batch 1277 : Gut microbiota abundance (genus Eubacterium rectale group id.14374) on Other ulcerative colitis

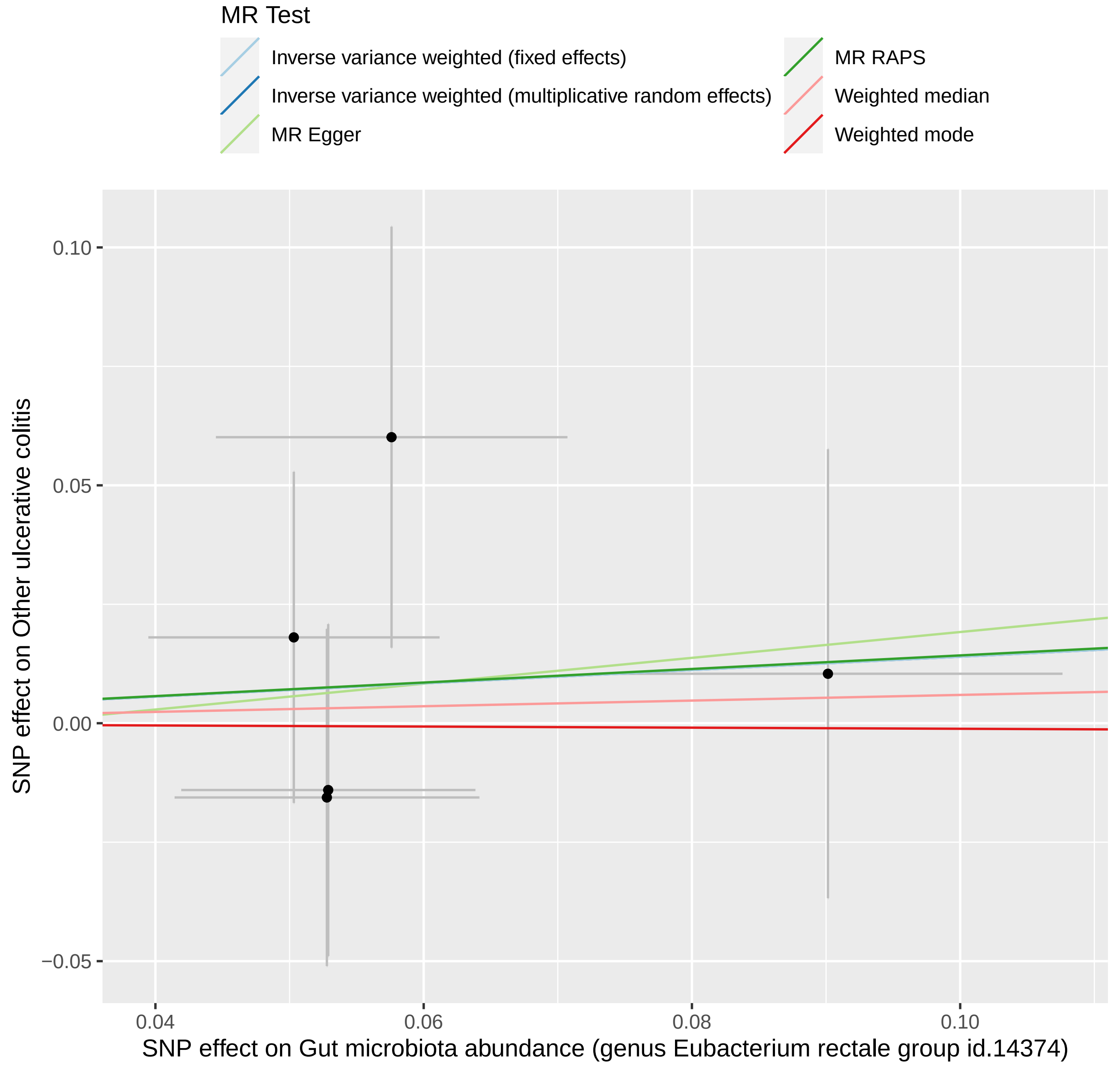

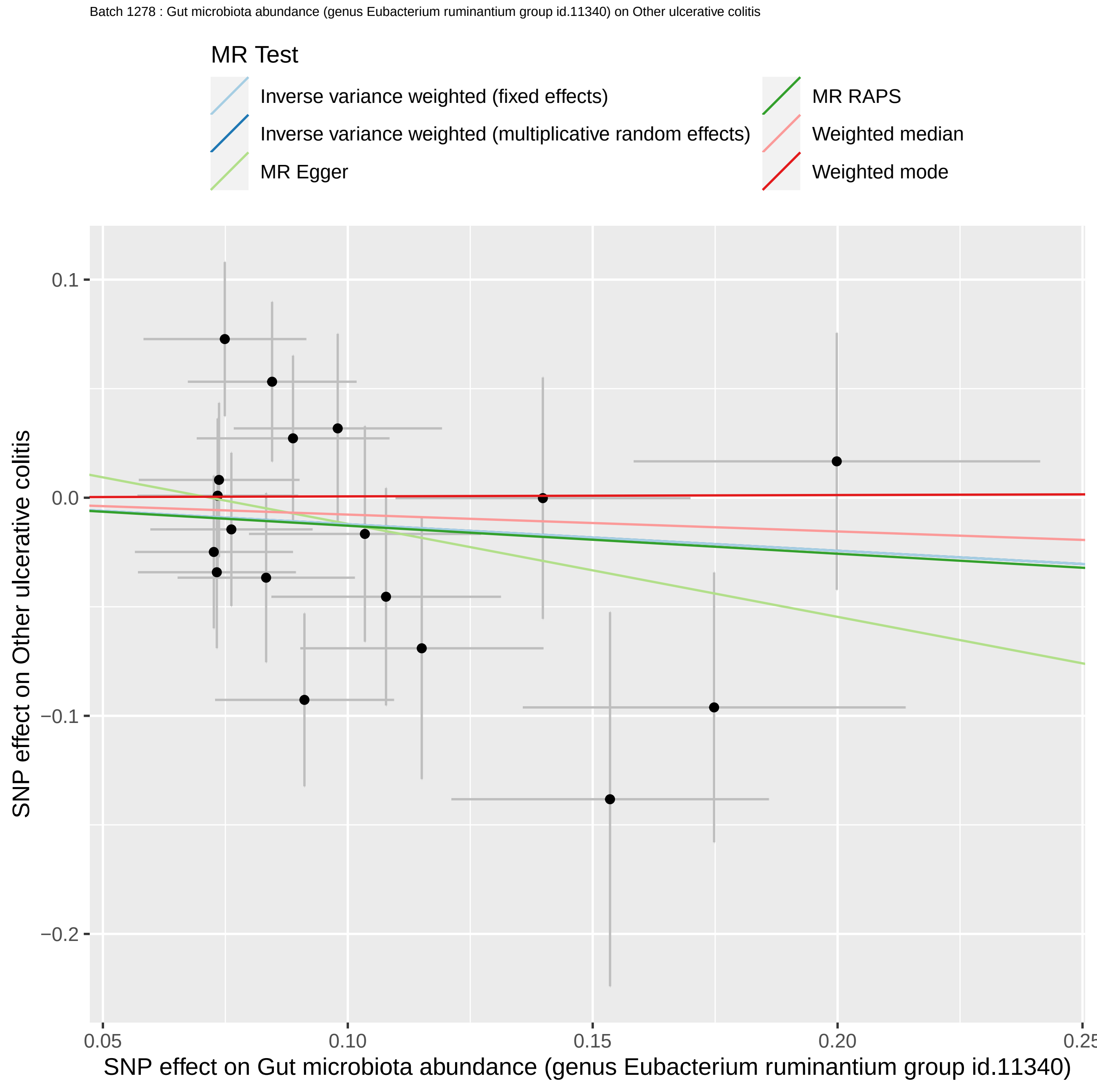

Batch 1278 : Gut microbiota abundance (genus Eubacterium ruminantium group id.11340) on Other ulcerative colitis
MR Test
Inverse variance weighted (fixed effects)
Inverse variance weighted (multiplicative random effects)
MR Egger
MR RAPS
Weighted median
Weighted mode
0.1
0.0
−0.1
−0.2
0.05
0.10
0.15
0.20
0.25
SNP effect on Other ulcerative colitis
SNP effect on Gut microbiota abundance (genus Eubacterium ruminantium group id.11340)

Batch 1279 : Gut microbiota abundance (genus Eubacterium ventriosum group id.11341) on Other ulcerative colitis

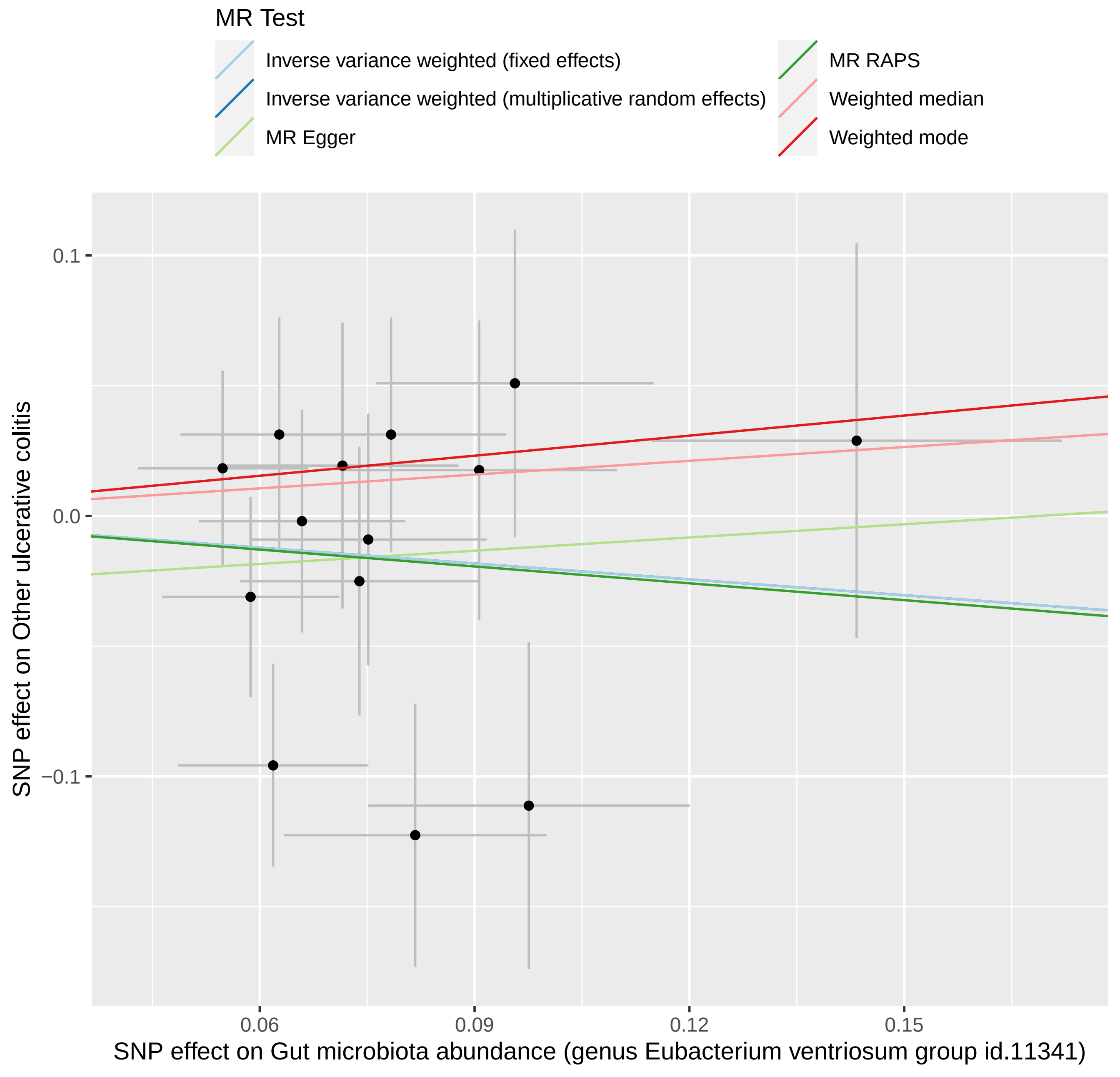

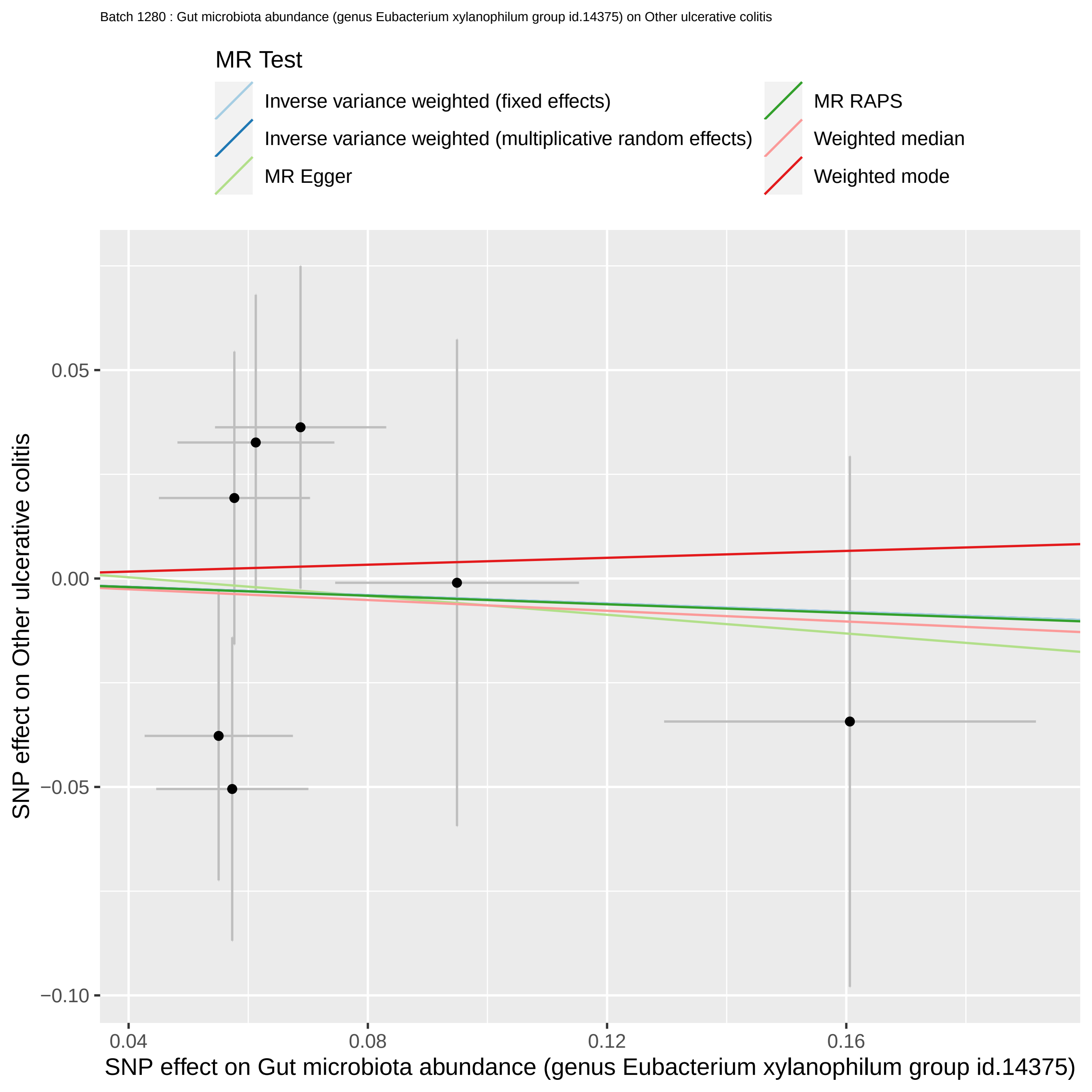

Batch 1280 : Gut microbiota abundance (genus Eubacterium xylanophilum group id.14375) on Other ulcerative colitis
MR Test
Inverse variance weighted (fixed effects)
Inverse variance weighted (multiplicative random effects)
MR Egger
MR RAPS
Weighted median
Weighted mode
SNP effect on Other ulcerative colitis
0.05
0.00
−0.05
−0.10
0.04
0.08
0.12
0.16
SNP effect on Gut microbiota abundance (genus Eubacterium xylanophilum group id.14375)

Batch 1281 : Gut microbiota abundance (genus Faecalibacterium id.2057) on Other ulcerative colitis

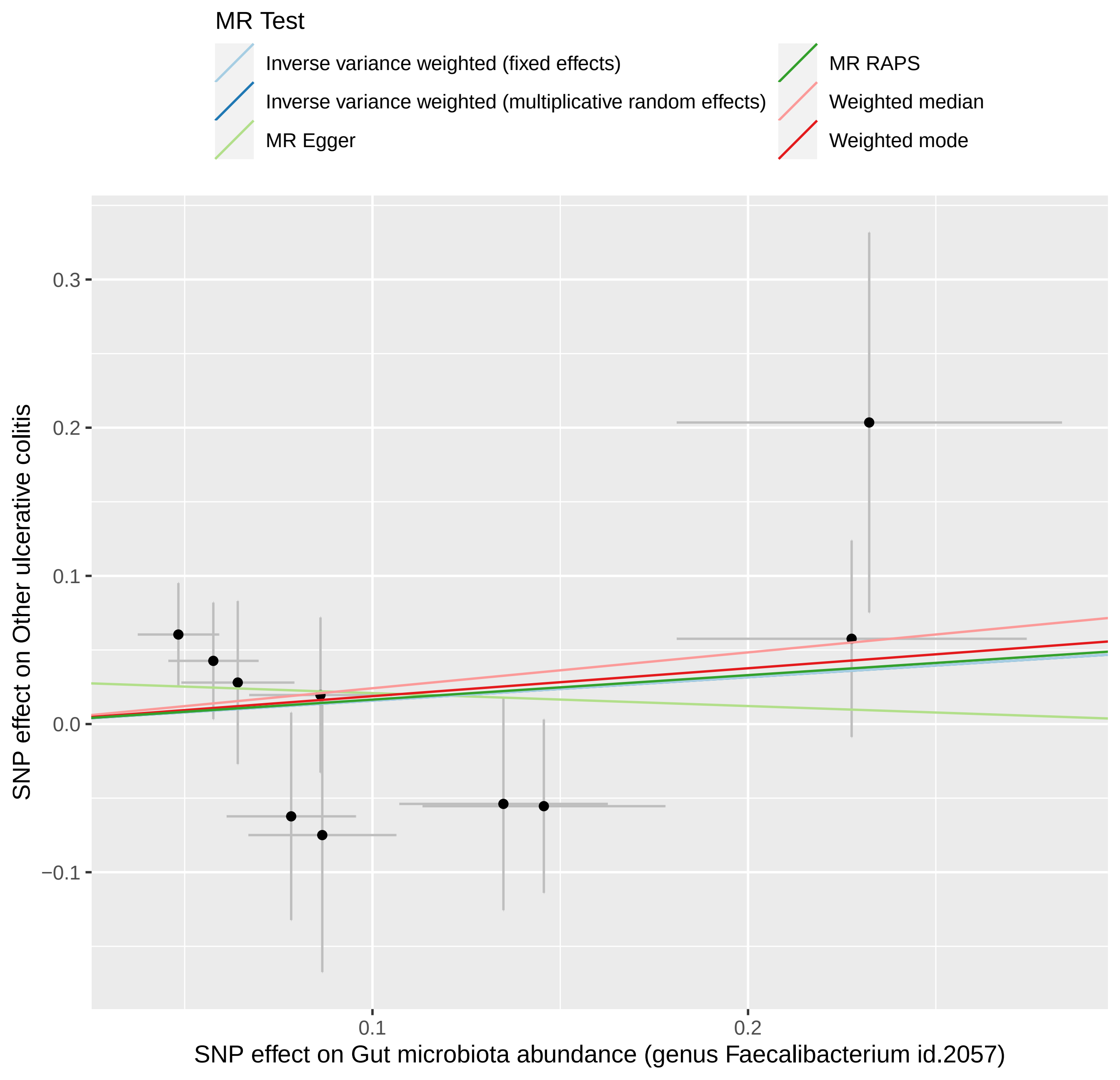

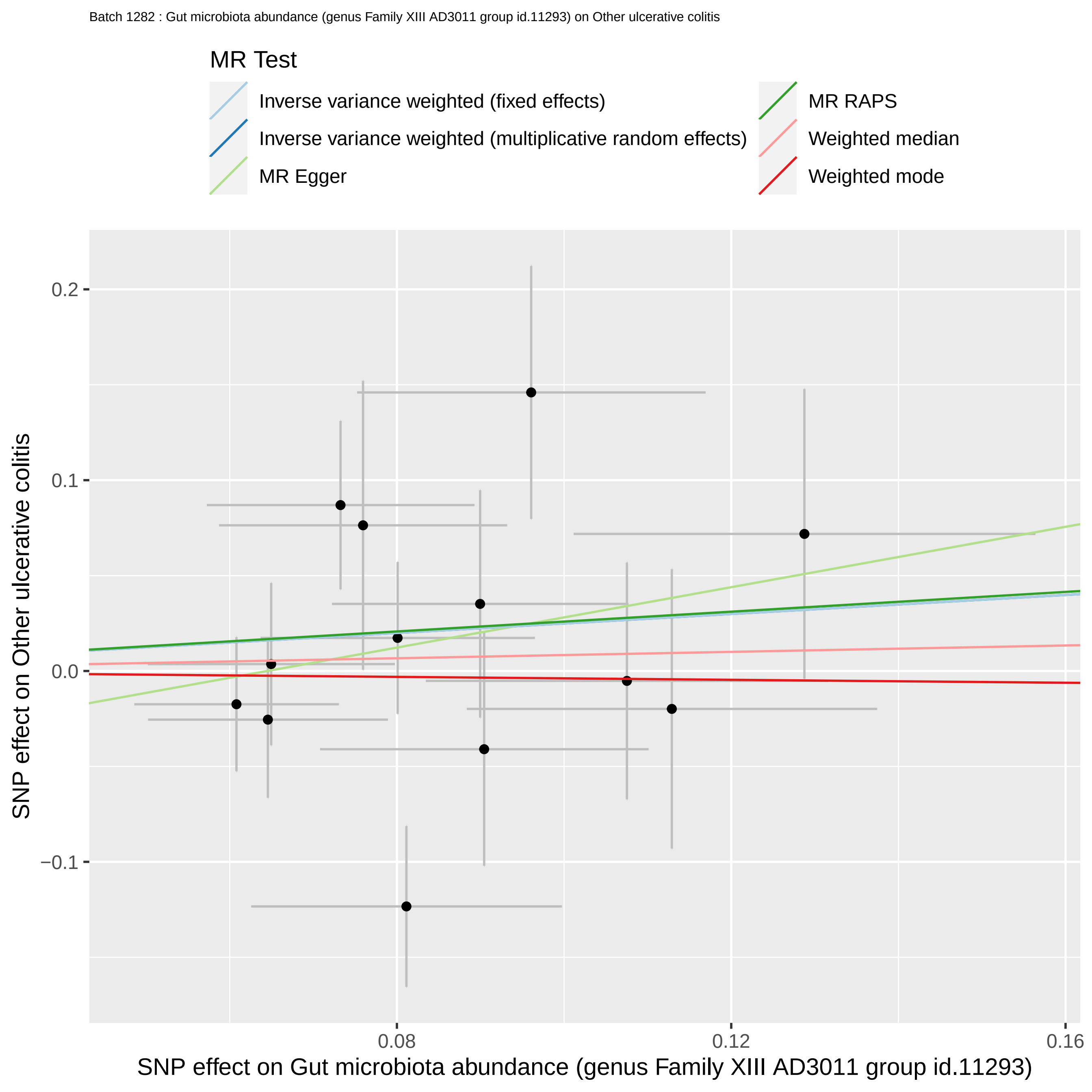

Batch 1282 : Gut microbiota abundance (genus Family XIII AD3011 group id.11293) on Other ulcerative colitis
MR Test
Inverse variance weighted (fixed effects)
Inverse variance weighted (multiplicative random effects)
MR Egger
MR RAPS
Weighted median
Weighted mode
SNP effect on Other ulcerative colitis
0.2
0.1
0.0
−0.1
0.08
0.12
0.16
SNP effect on Gut microbiota abundance (genus Family XIII AD3011 group id.11293)

Batch 1283 : Gut microbiota abundance (genus Family XIII UCG001 id.11294) on Other ulcerative colitis

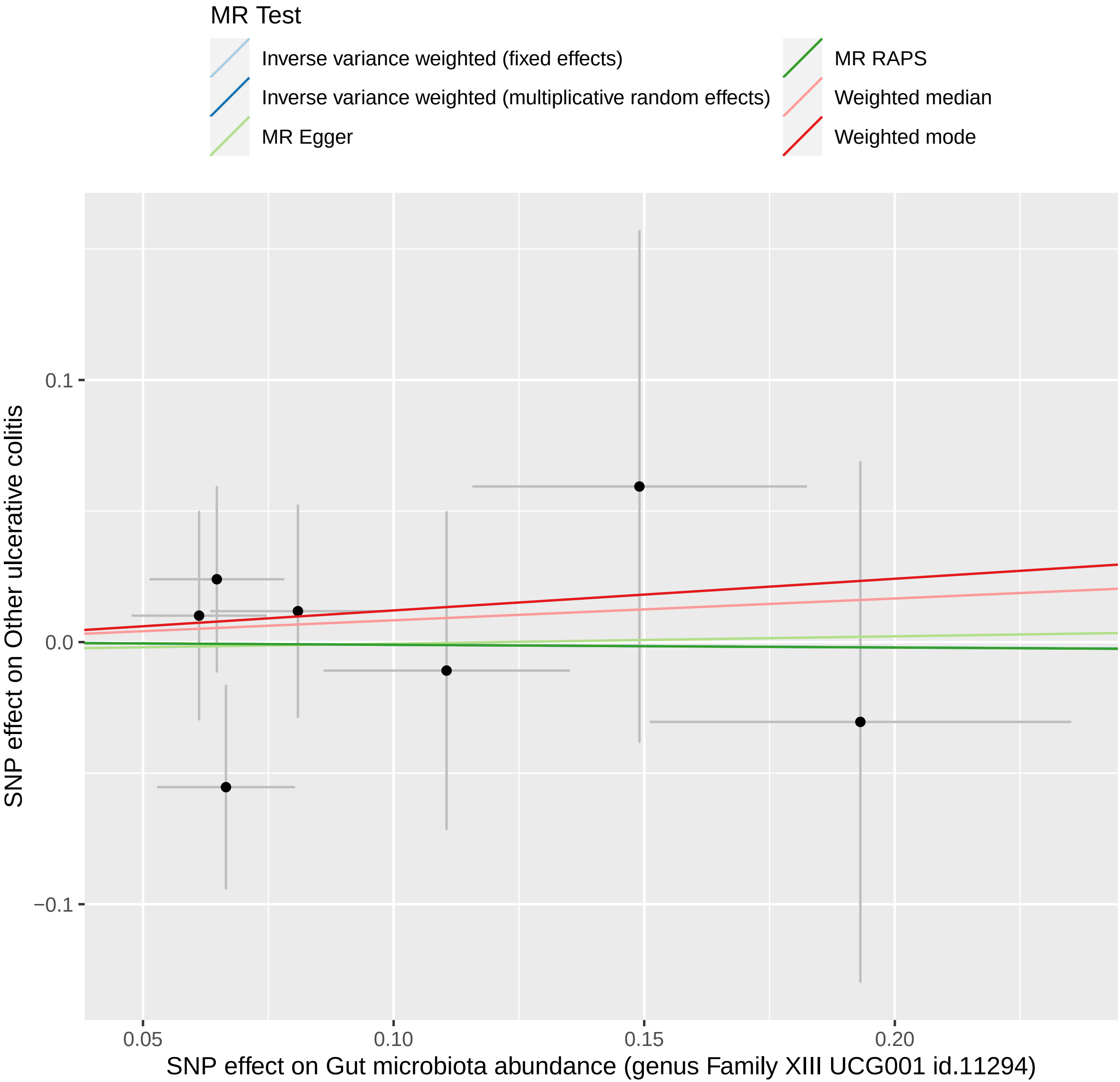

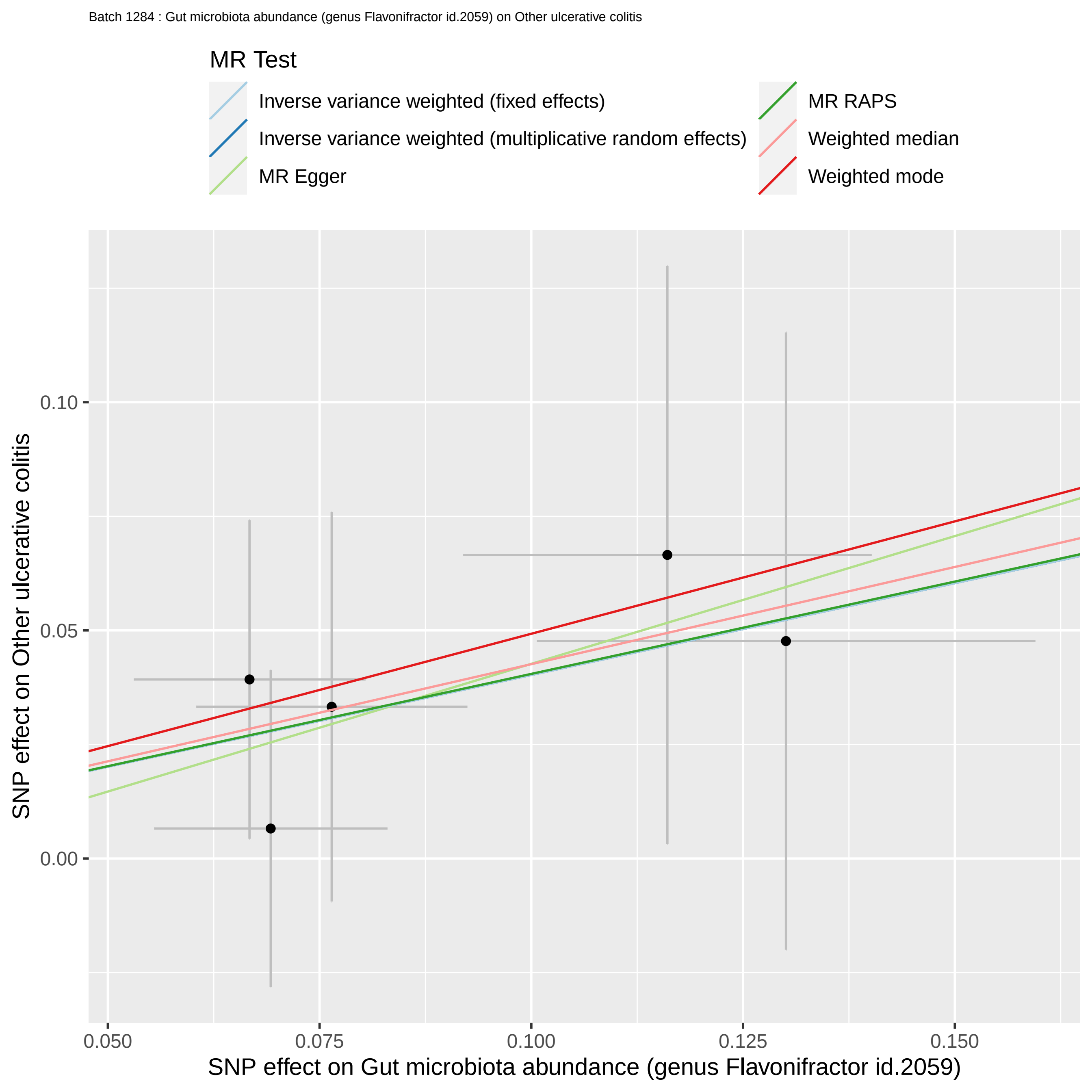

Batch 1284 : Gut microbiota abundance (genus Flavonifractor id.2059) on Other ulcerative colitis
MR Test
Inverse variance weighted (fixed effects)
Inverse variance weighted (multiplicative random effects)
MR Egger
MR RAPS
Weighted median
Weighted mode
0.10
0.05
0.00
0.050
0.075
0.100
0.125
0.150
SNP effect on Other ulcerative colitis
SNP effect on Gut microbiota abundance (genus Flavonifractor id.2059)

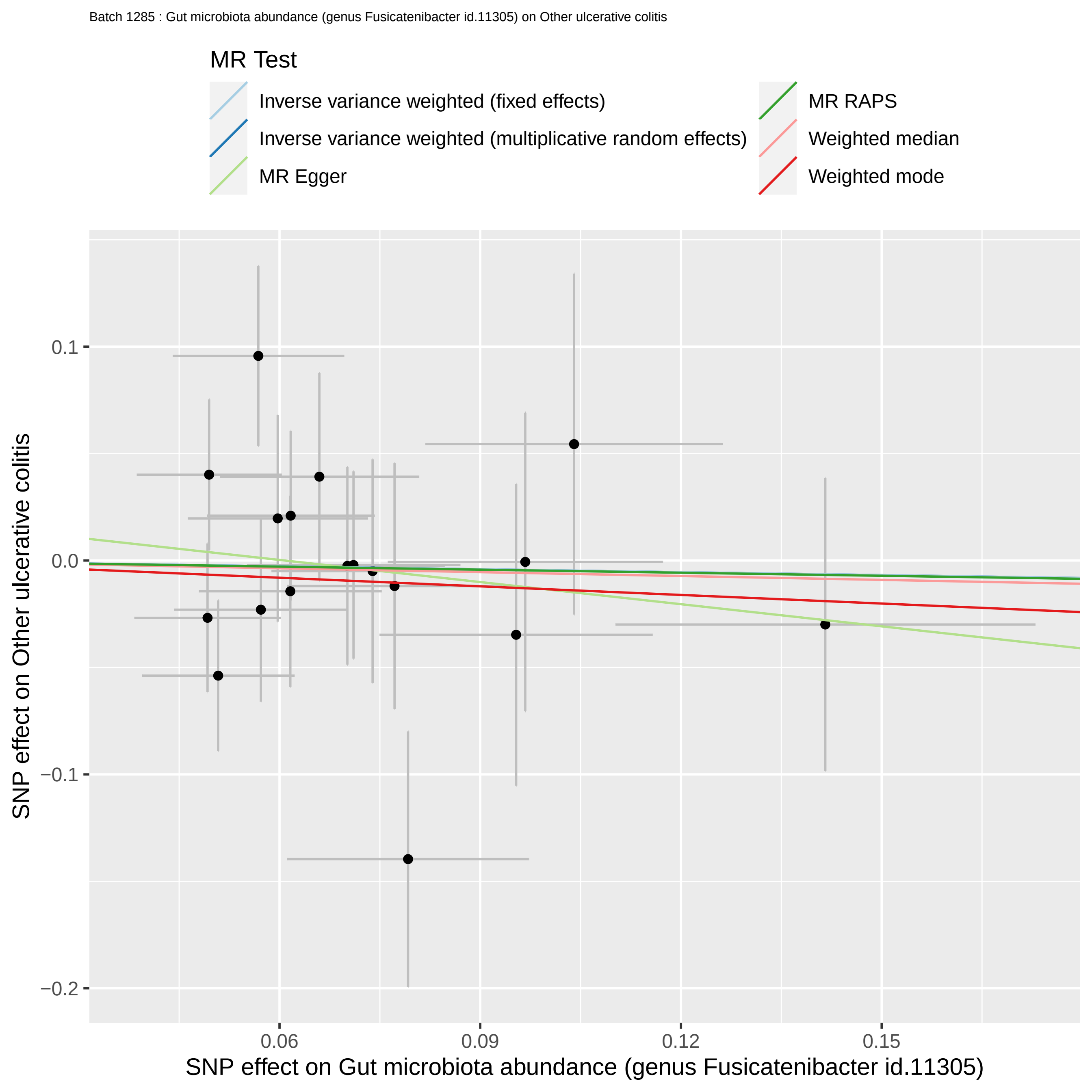

Batch 1285 : Gut microbiota abundance (genus Fusicatenibacter id.11305) on Other ulcerative colitis
MR Test
Inverse variance weighted (fixed effects)
Inverse variance weighted (multiplicative random effects)
MR Egger
MR RAPS
Weighted median
Weighted mode
0.1
0.0
−0.1
−0.2
0.06
0.09
0.12
0.15
SNP effect on Other ulcerative colitis
SNP effect on Gut microbiota abundance (genus Fusicatenibacter id.11305)

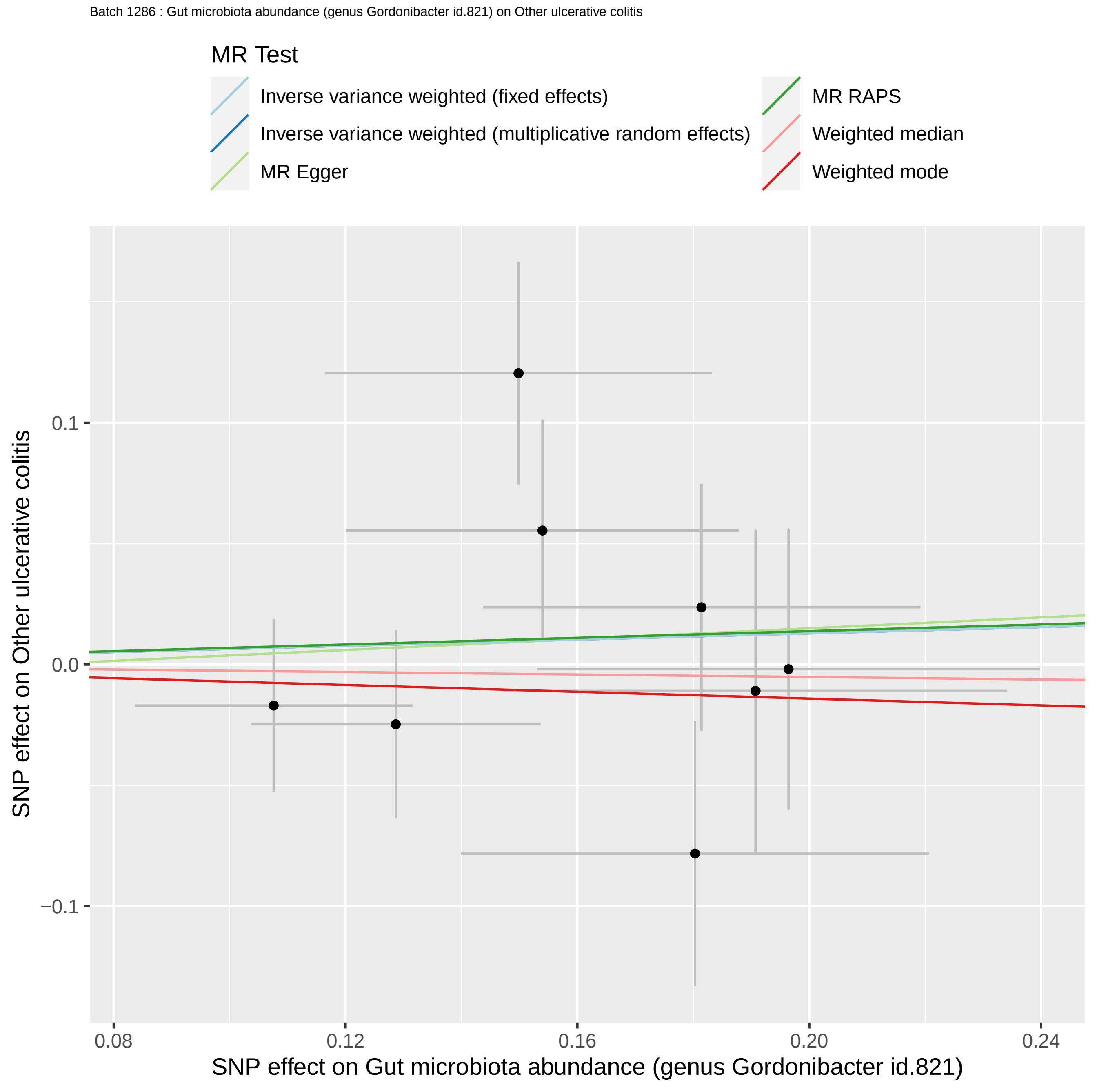
Batch 1286 : Gut microbiota abundance (genus Gordonibacter id.821) on Other ulcerative colitis
MR Test
Inverse variance weighted (fixed effects)
Inverse variance weighted (multiplicative random effects)
MR Egger
MR RAPS
Weighted median
Weighted mode
0.1
0.0
−0.1
0.08
0.12
0.16
0.20
0.24
SNP effect on Other ulcerative colitis
SNP effect on Gut microbiota abundance (genus Gordonibacter id.821)

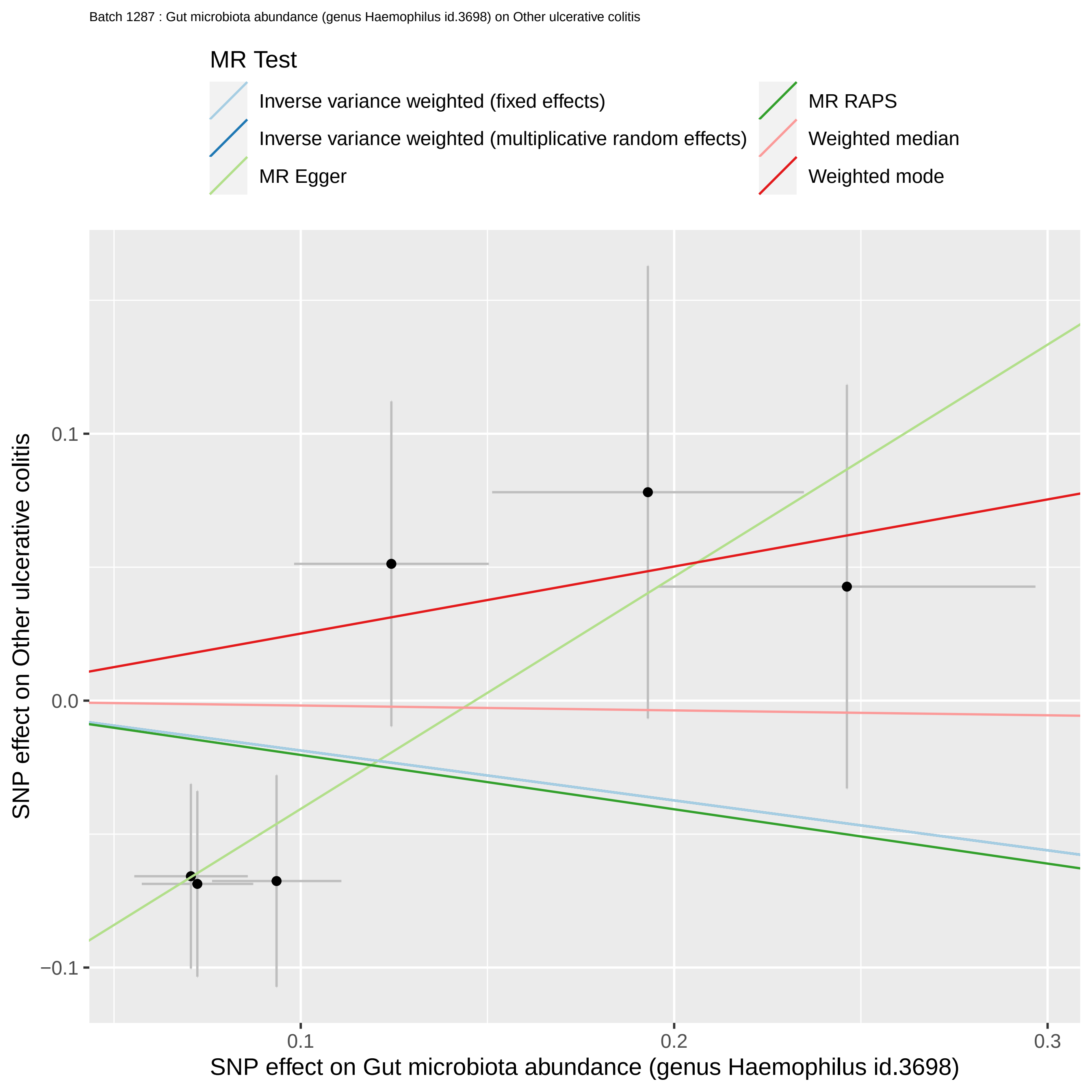
Batch 1287 : Gut microbiota abundance (genus Haemophilus id.3698) on Other ulcerative colitis
MR Test
Inverse variance weighted (fixed effects)
Inverse variance weighted (multiplicative random effects)
MR Egger
MR RAPS
Weighted median
Weighted mode
0.1
0.0
−0.1
0.1
0.2
0.3
SNP effect on Other ulcerative colitis
SNP effect on Gut microbiota abundance (genus Haemophilus id.3698)

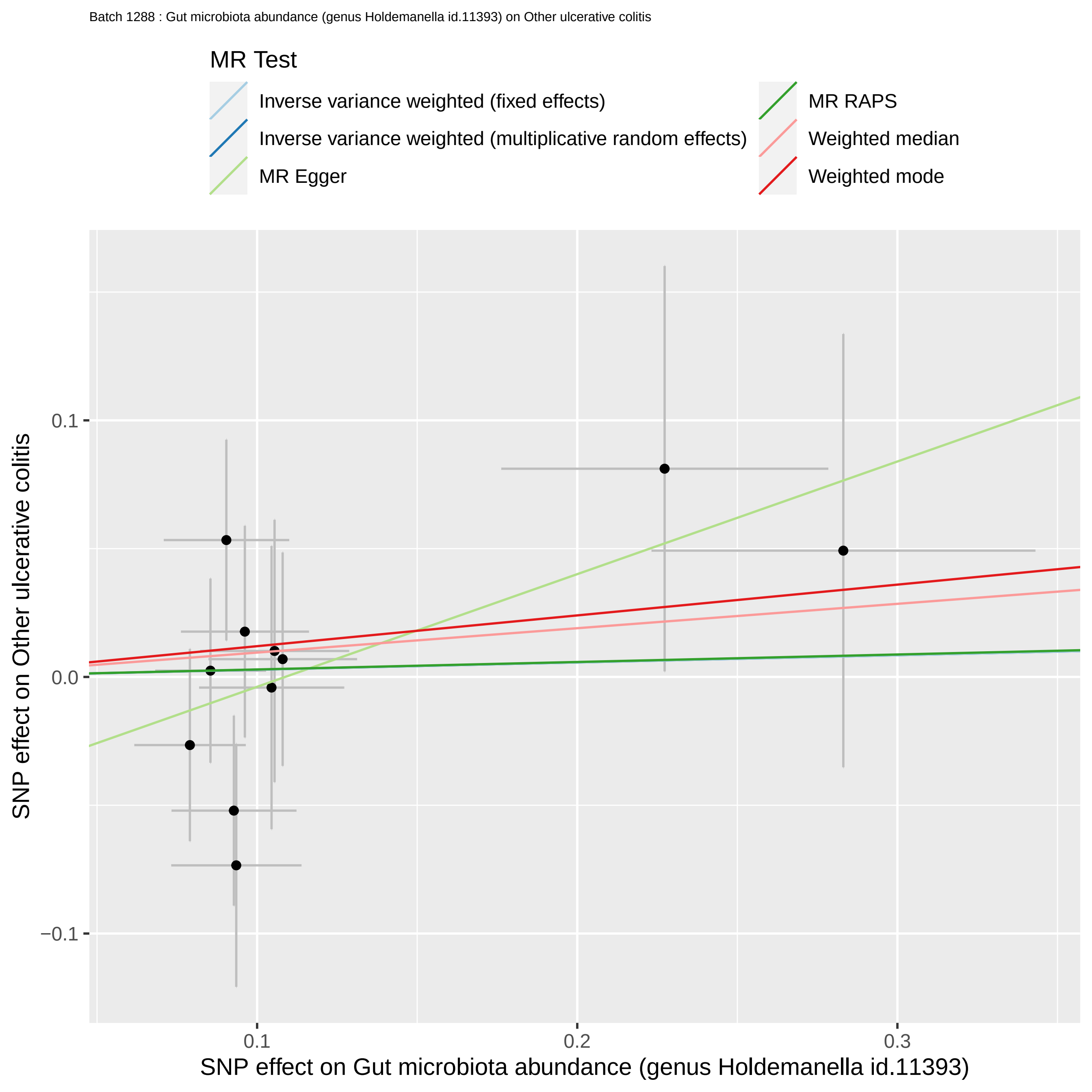

Batch 1288 : Gut microbiota abundance (genus Holdemanella id.11393) on Other ulcerative colitis
MR Test
Inverse variance weighted (fixed effects)
Inverse variance weighted (multiplicative random effects)
MR Egger
MR RAPS
Weighted median
Weighted mode
0.1
0.0
−0.1
0.1
0.2
0.3
SNP effect on Other ulcerative colitis
SNP effect on Gut microbiota abundance (genus Holdemanella id.11393)

Batch 1289 : Gut microbiota abundance (genus Holdemania id.2157) on Other ulcerative colitis

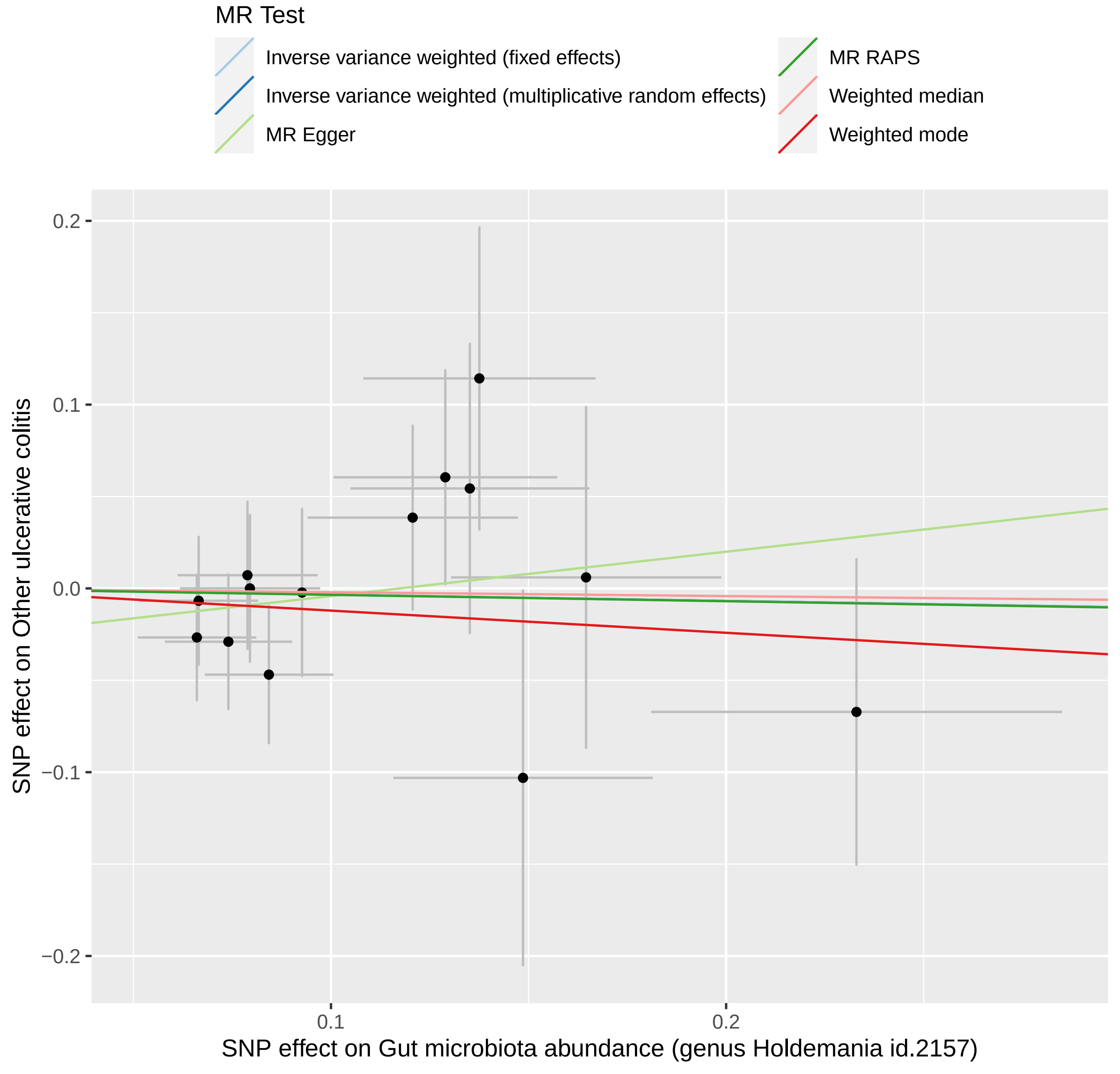

Batch 1290 : Gut microbiota abundance (genus Howardella id.2000) on Other ulcerative colitis

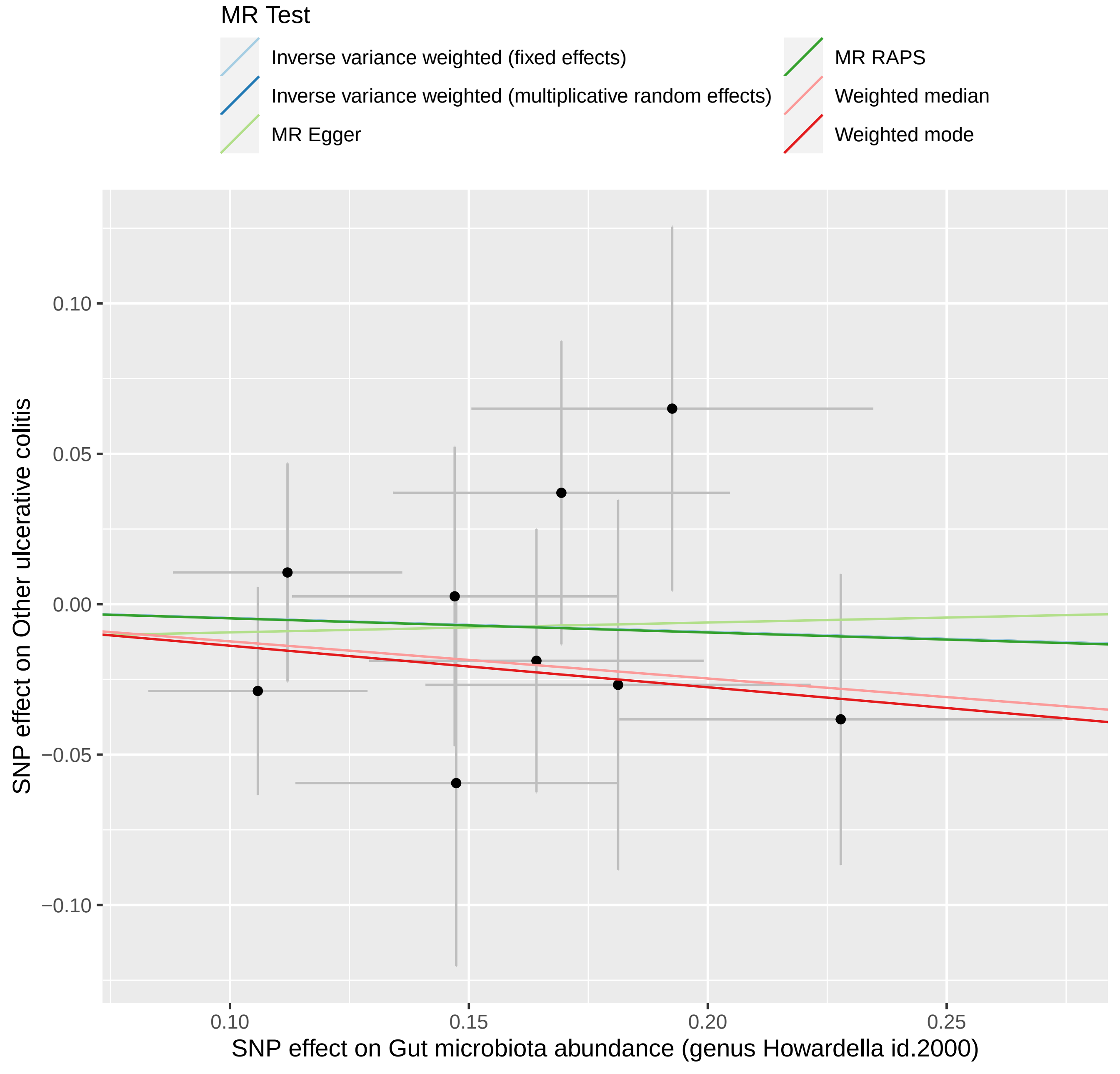

Batch 1291 : Gut microbiota abundance (genus Hungatella id.11306) on Other ulcerative colitis

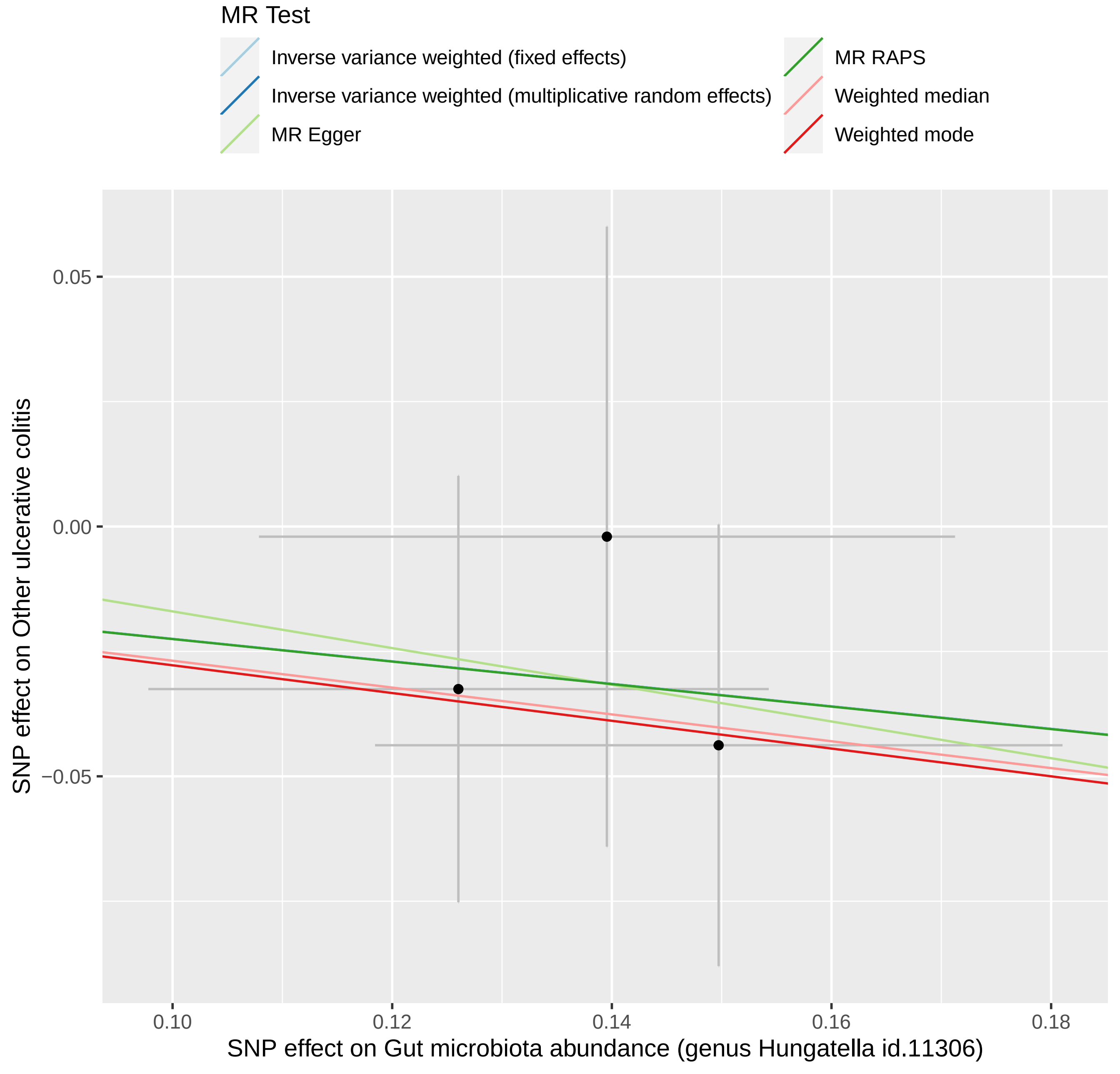

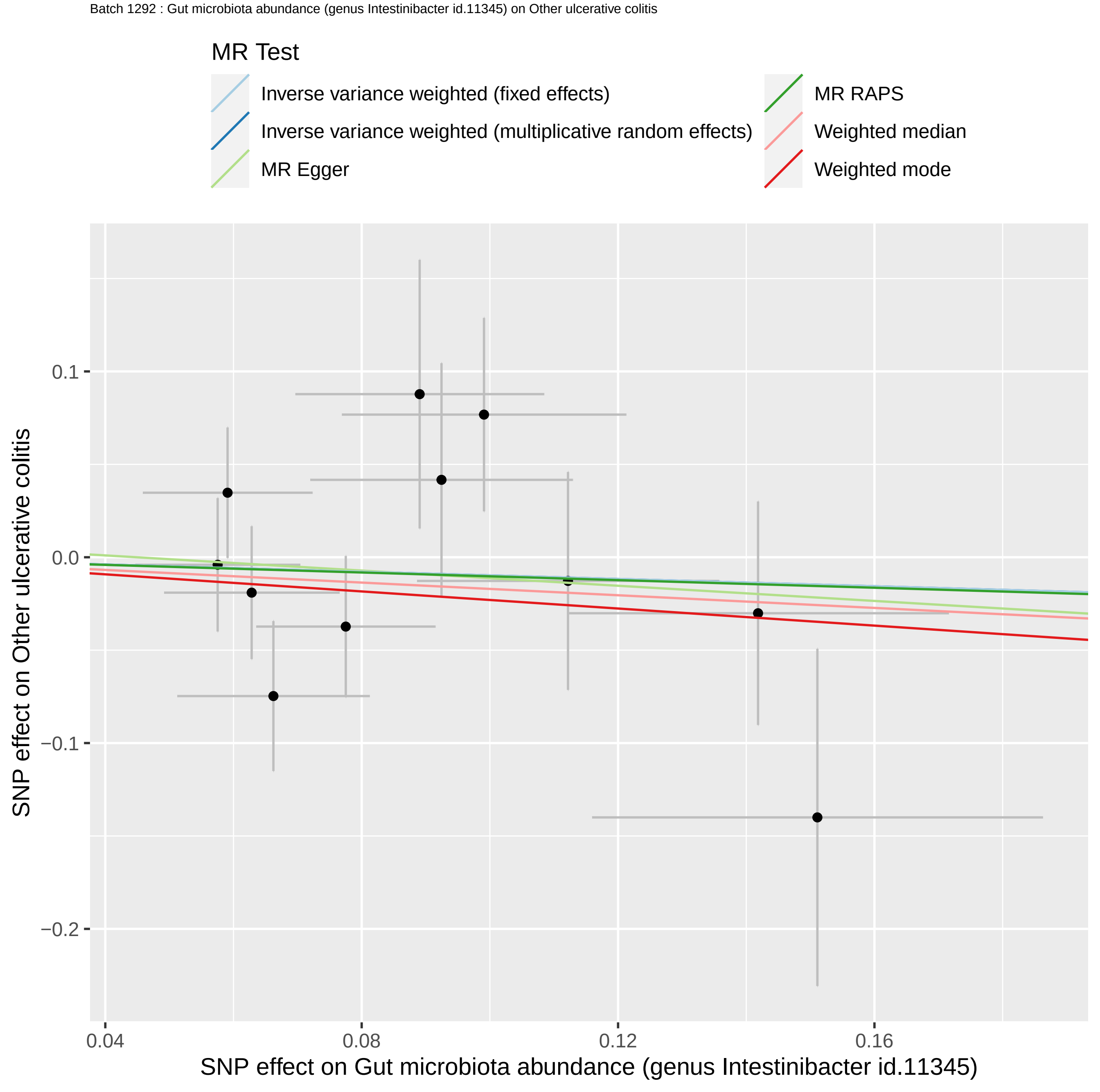
Batch 1292 : Gut microbiota abundance (genus Intestinibacter id.11345) on Other ulcerative colitis
MR Test
Inverse variance weighted (fixed effects)
Inverse variance weighted (multiplicative random effects)
MR Egger
MR RAPS
Weighted median
Weighted mode
0.1
0.0
−0.1
−0.2
0.04
0.08
0.12
0.16
SNP effect on Other ulcerative colitis
SNP effect on Gut microbiota abundance (genus Intestinibacter id.11345)

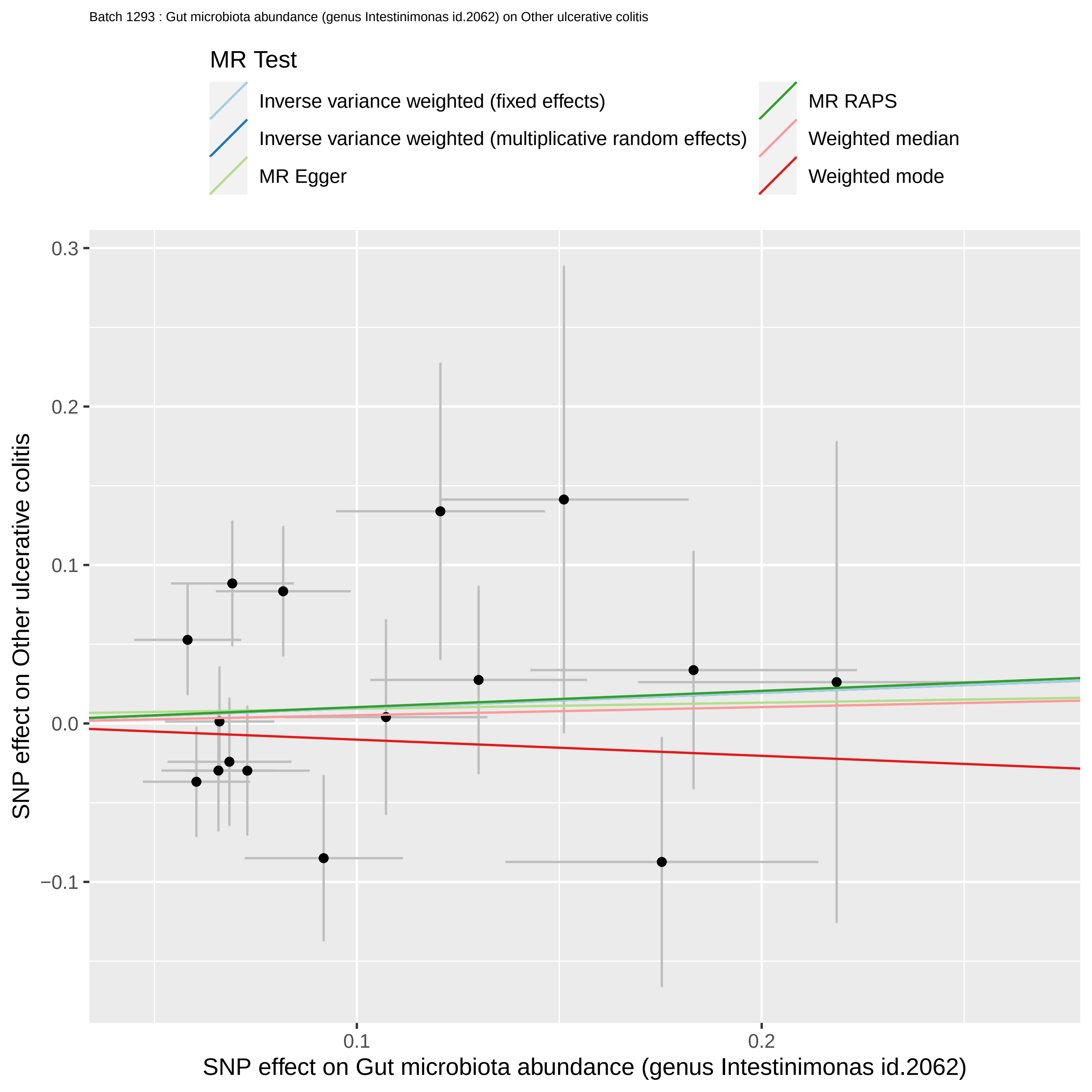

Batch 1293 : Gut microbiota abundance (genus Intestinimonas id.2062) on Other ulcerative colitis
MR Test
Inverse variance weighted (fixed effects)
Inverse variance weighted (multiplicative random effects)
MR Egger
MR RAPS
Weighted median
Weighted mode
0.3
0.2
0.1
0.0
−0.1
0.1
0.2
SNP effect on Other ulcerative colitis
SNP effect on Gut microbiota abundance (genus Intestinimonas id.2062)

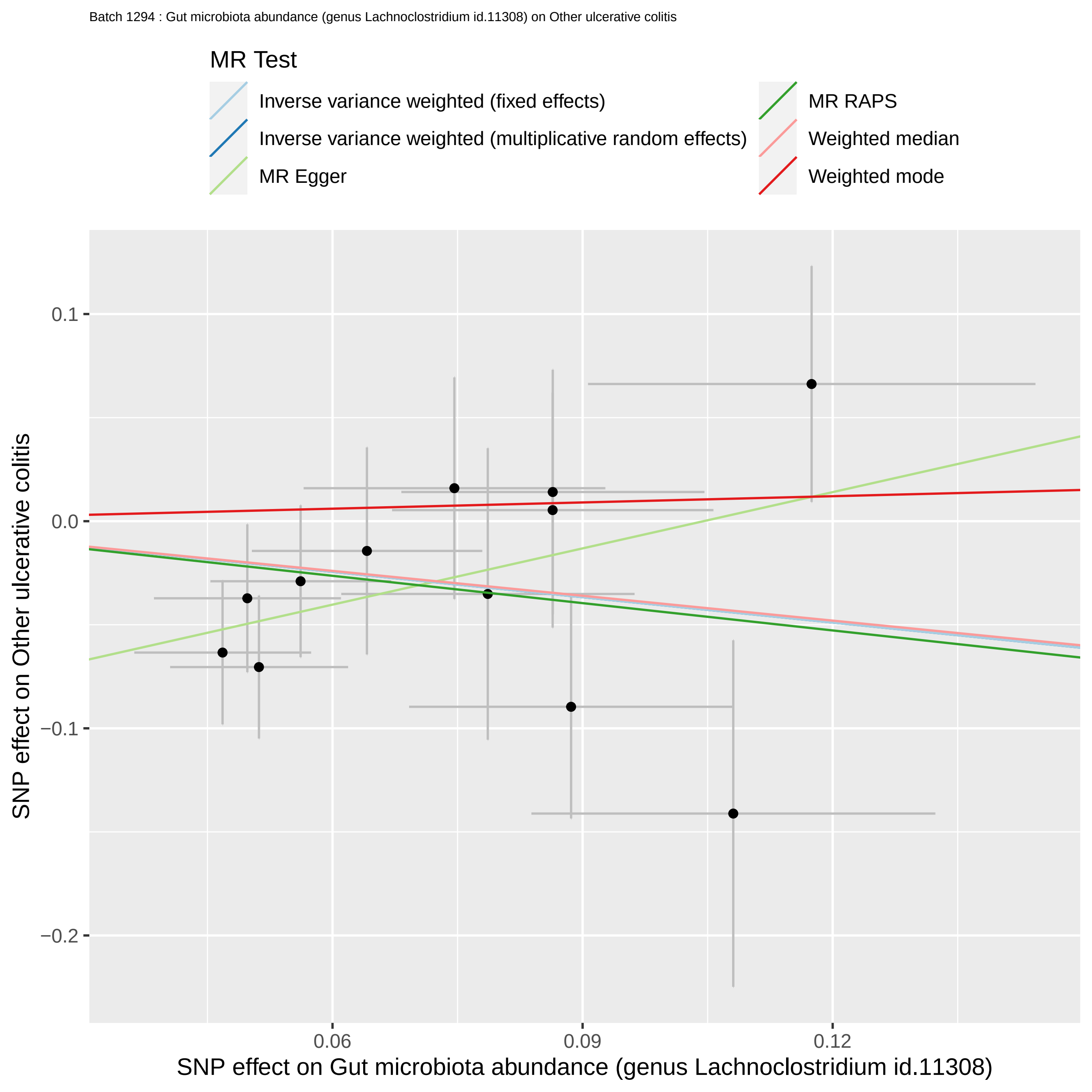
Batch 1294 : Gut microbiota abundance (genus Lachnoclostridium id.11308) on Other ulcerative colitis
MR Test
Inverse variance weighted (fixed effects)
Inverse variance weighted (multiplicative random effects)
MR Egger
MR RAPS
Weighted median
Weighted mode
0.1
0.0
−0.1
−0.2
0.06
0.09
0.12
SNP effect on Other ulcerative colitis
SNP effect on Gut microbiota abundance (genus Lachnoclostridium id.11308)

Batch 1295 : Gut microbiota abundance (genus Lachnospiraceae FCS020 group id.11314) on Other ulcerative colitis

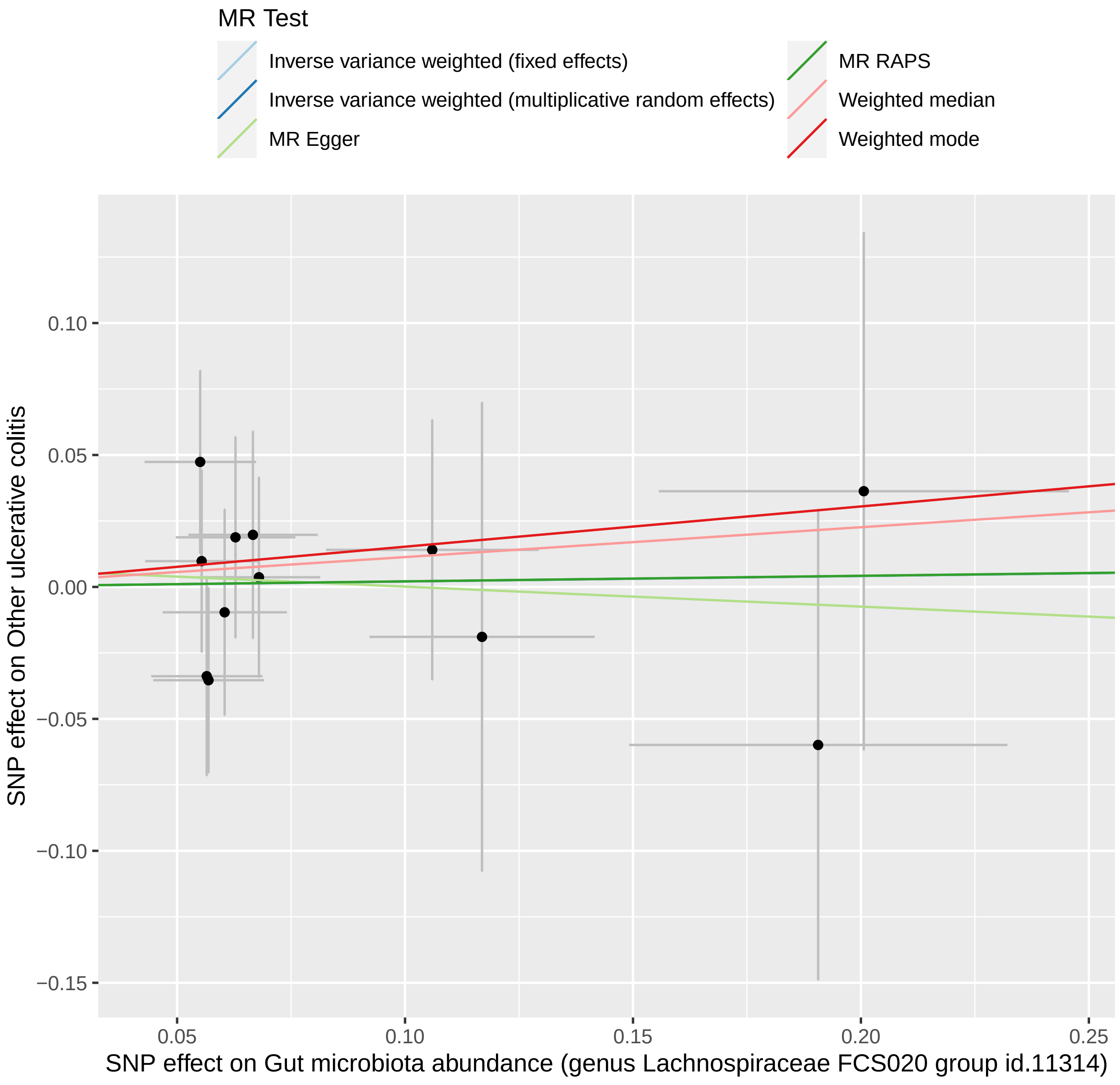

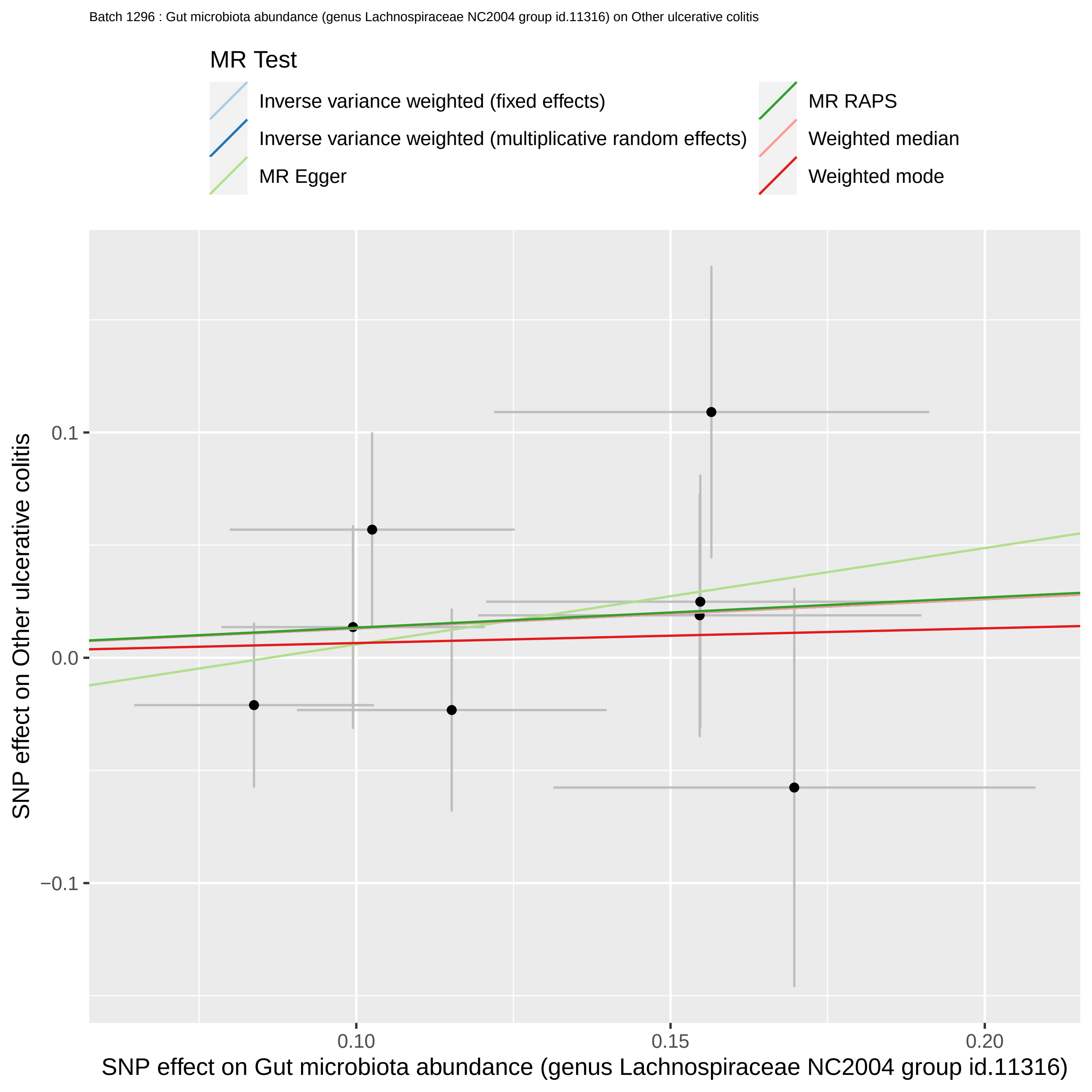

Batch 1296 : Gut microbiota abundance (genus Lachnospiraceae NC2004 group id.11316) on Other ulcerative colitis
MR Test
Inverse variance weighted (fixed effects)
Inverse variance weighted (multiplicative random effects)
MR Egger
MR RAPS
Weighted median
Weighted mode
0.1
0.0
−0.1
0.10
0.15
0.20
SNP effect on Other ulcerative colitis
SNP effect on Gut microbiota abundance (genus Lachnospiraceae NC2004 group id.11316)

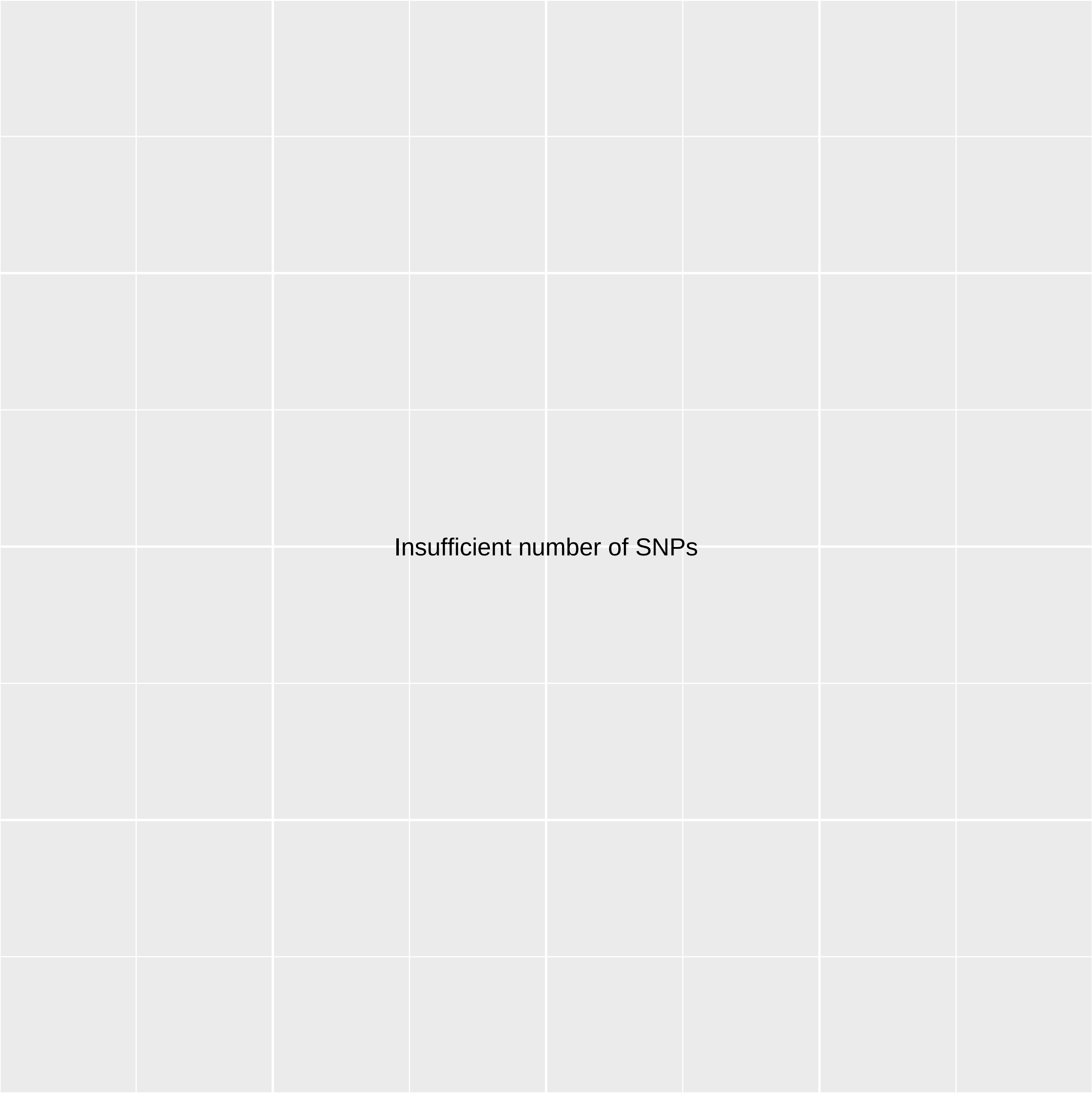
Insufficient number of SNPs

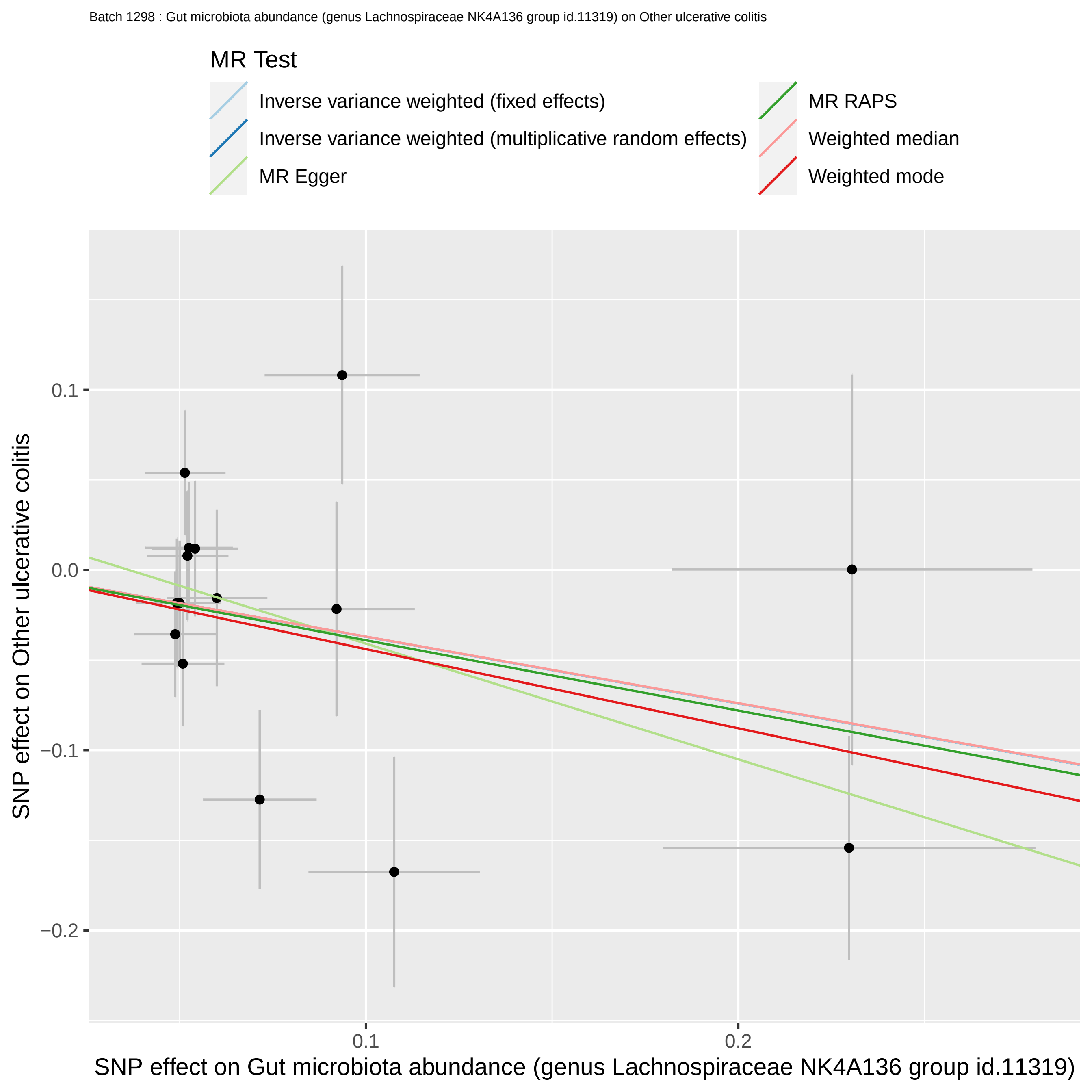
Batch 1298 : Gut microbiota abundance (genus Lachnospiraceae NK4A136 group id.11319) on Other ulcerative colitis
MR Test
Inverse variance weighted (fixed effects)
Inverse variance weighted (multiplicative random effects)
MR Egger
MR RAPS
Weighted median
Weighted mode
SNP effect on Other ulcerative colitis
0.1
0.0
−0.1
−0.2
0.1
0.2
SNP effect on Gut microbiota abundance (genus Lachnospiraceae NK4A136 group id.11319)

Batch 1299 : Gut microbiota abundance (genus Lachnospiraceae UCG001 id.11321) on Other ulcerative colitis

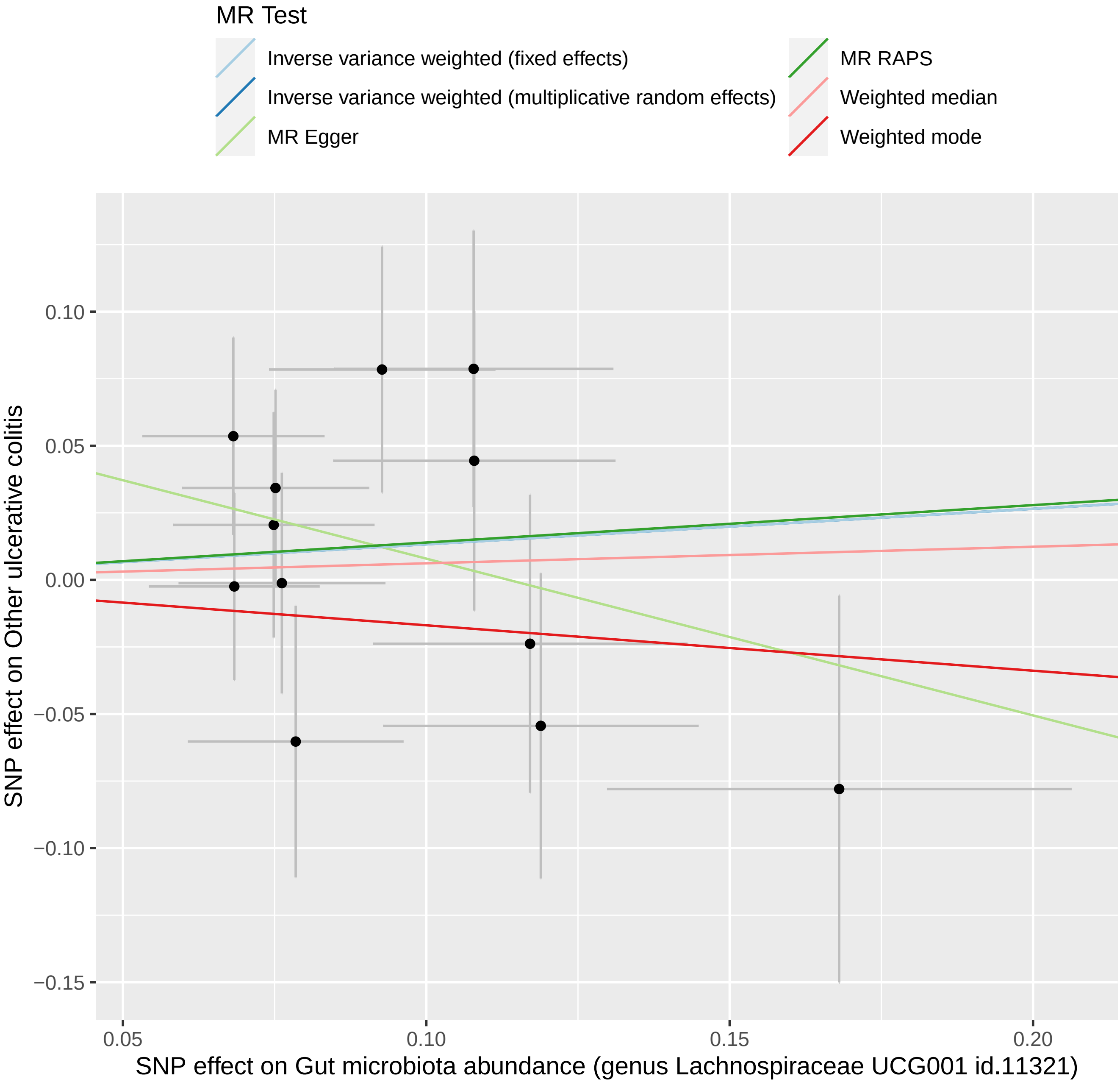

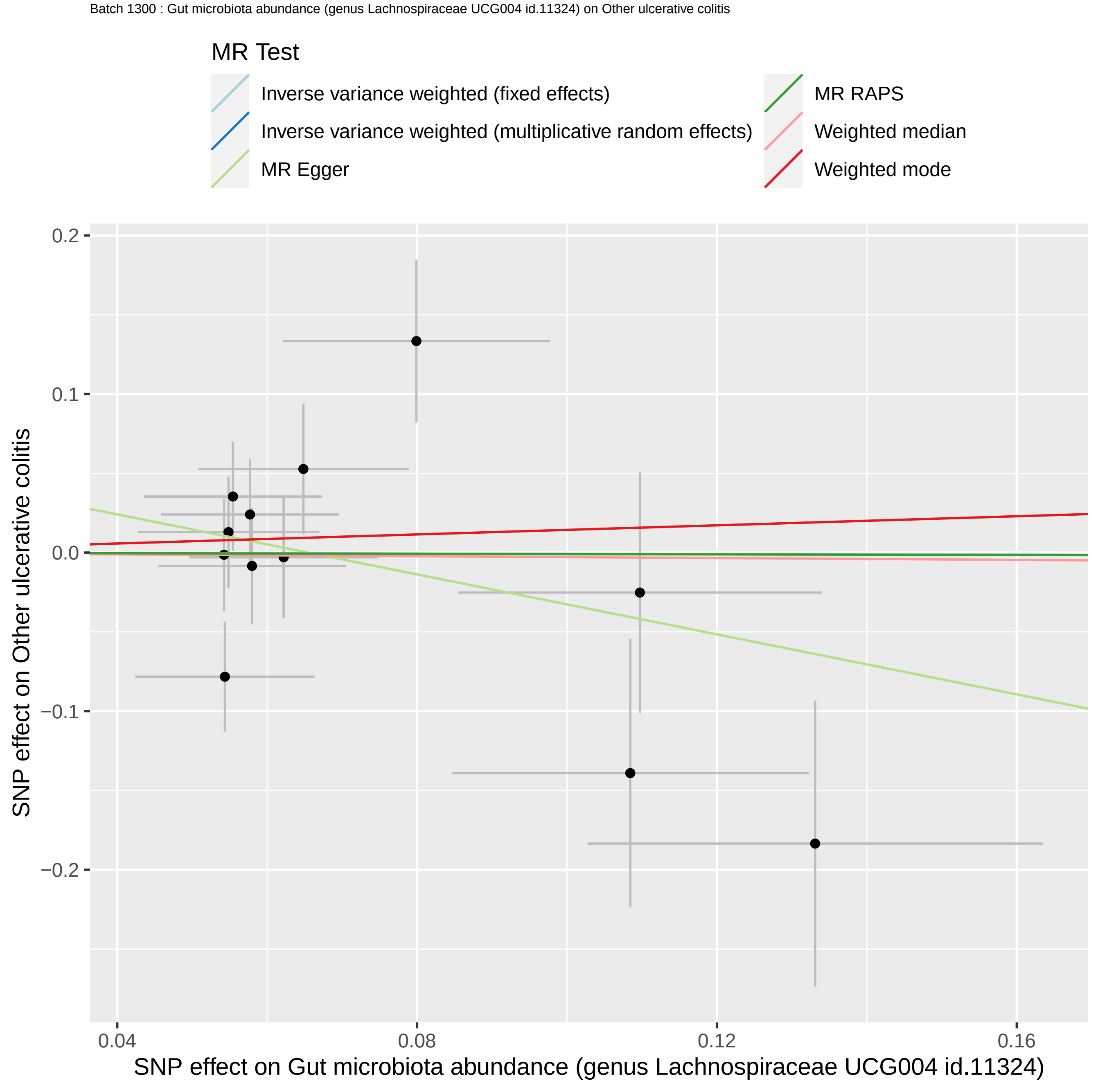

Batch 1300 : Gut microbiota abundance (genus Lachnospiraceae UCG004 id.11324) on Other ulcerative colitis
MR Test
Inverse variance weighted (fixed effects)
Inverse variance weighted (multiplicative random effects)
MR Egger
MR RAPS
Weighted median
Weighted mode
0.2
0.1
0.0
−0.1
−0.2
0.04
0.08
0.12
0.16
SNP effect on Other ulcerative colitis
SNP effect on Gut microbiota abundance (genus Lachnospiraceae UCG004 id.11324)

Batch 1301 : Gut microbiota abundance (genus Lachnospiraceae UCG008 id.11328) on Other ulcerative colitis

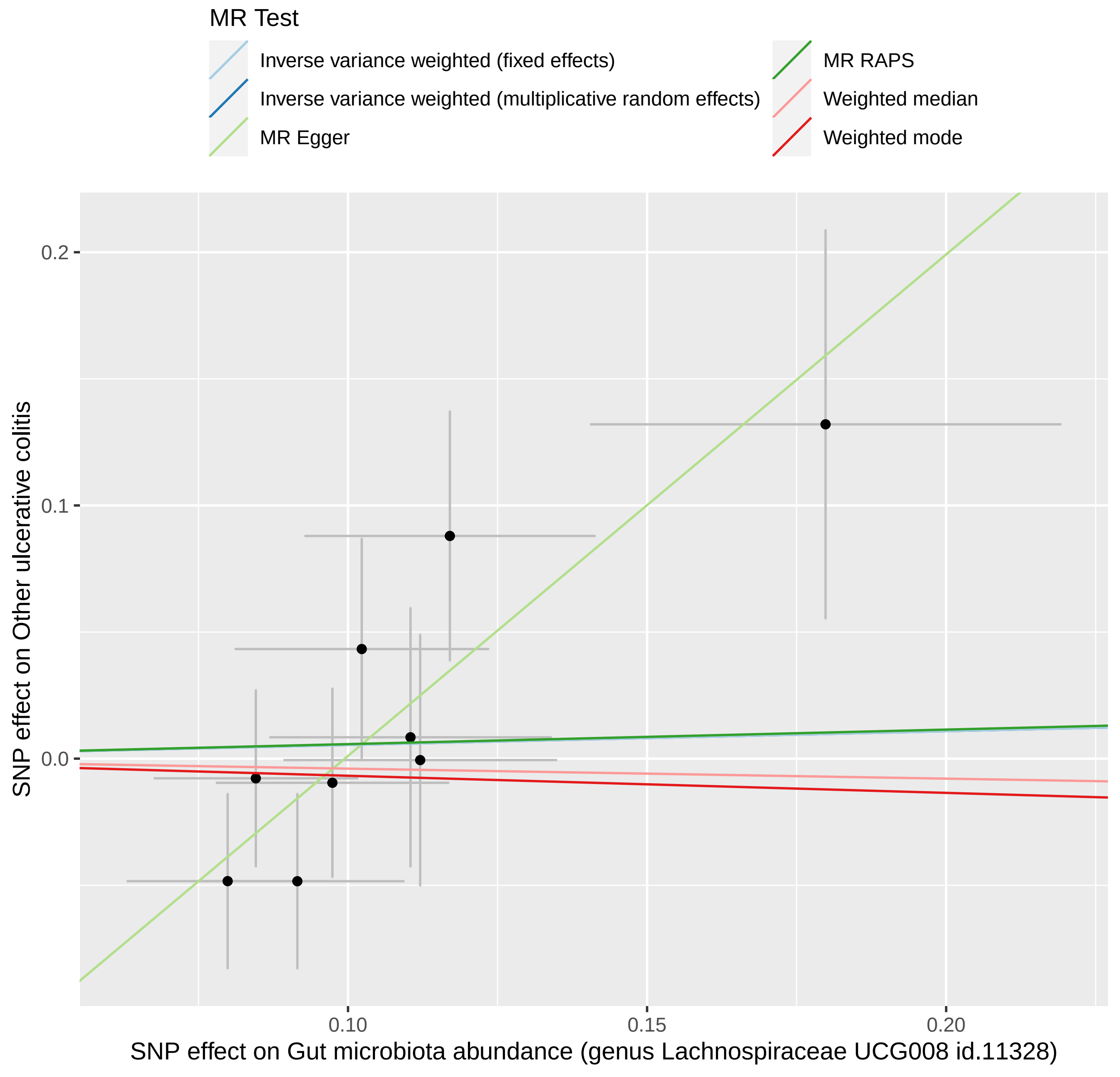

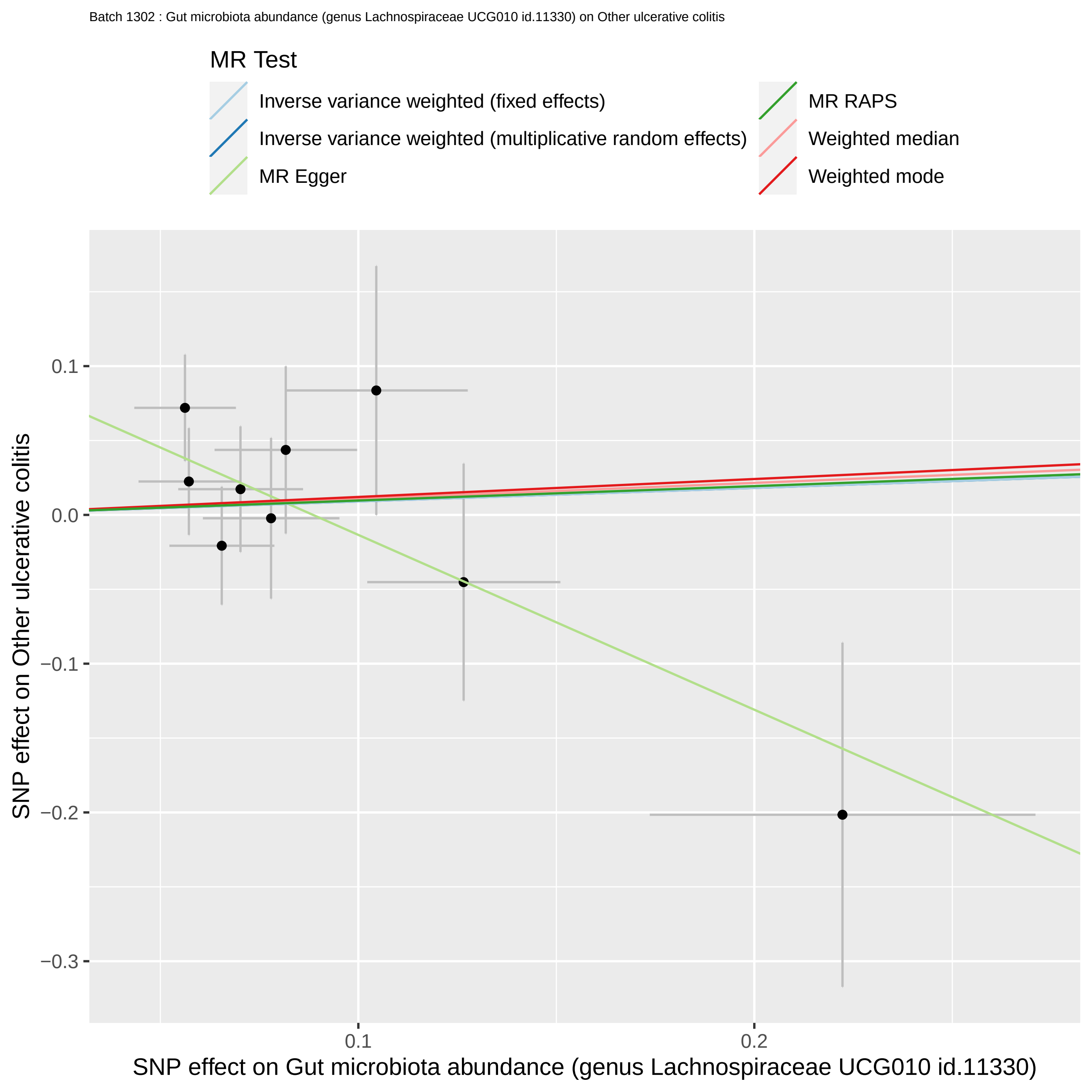
Batch 1302 : Gut microbiota abundance (genus Lachnospiraceae UCG010 id.11330) on Other ulcerative colitis
MR Test
Inverse variance weighted (fixed effects)
Inverse variance weighted (multiplicative random effects)
MR Egger
MR RAPS
Weighted median
Weighted mode
SNP effect on Other ulcerative colitis
0.1
0.0
−0.1
−0.2
−0.3
0.1
0.2
SNP effect on Gut microbiota abundance (genus Lachnospiraceae UCG010 id.11330)

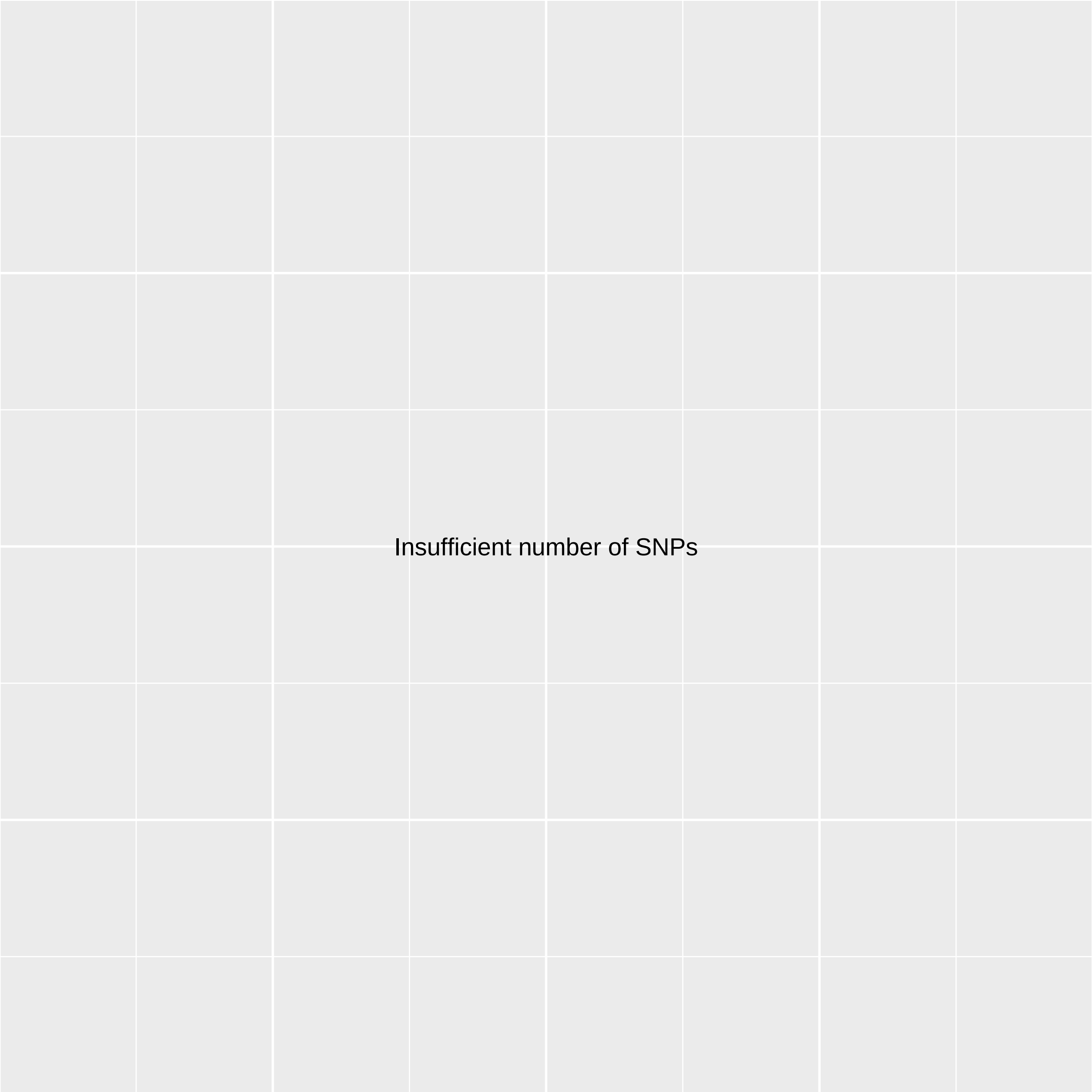
Insufficient number of SNPs

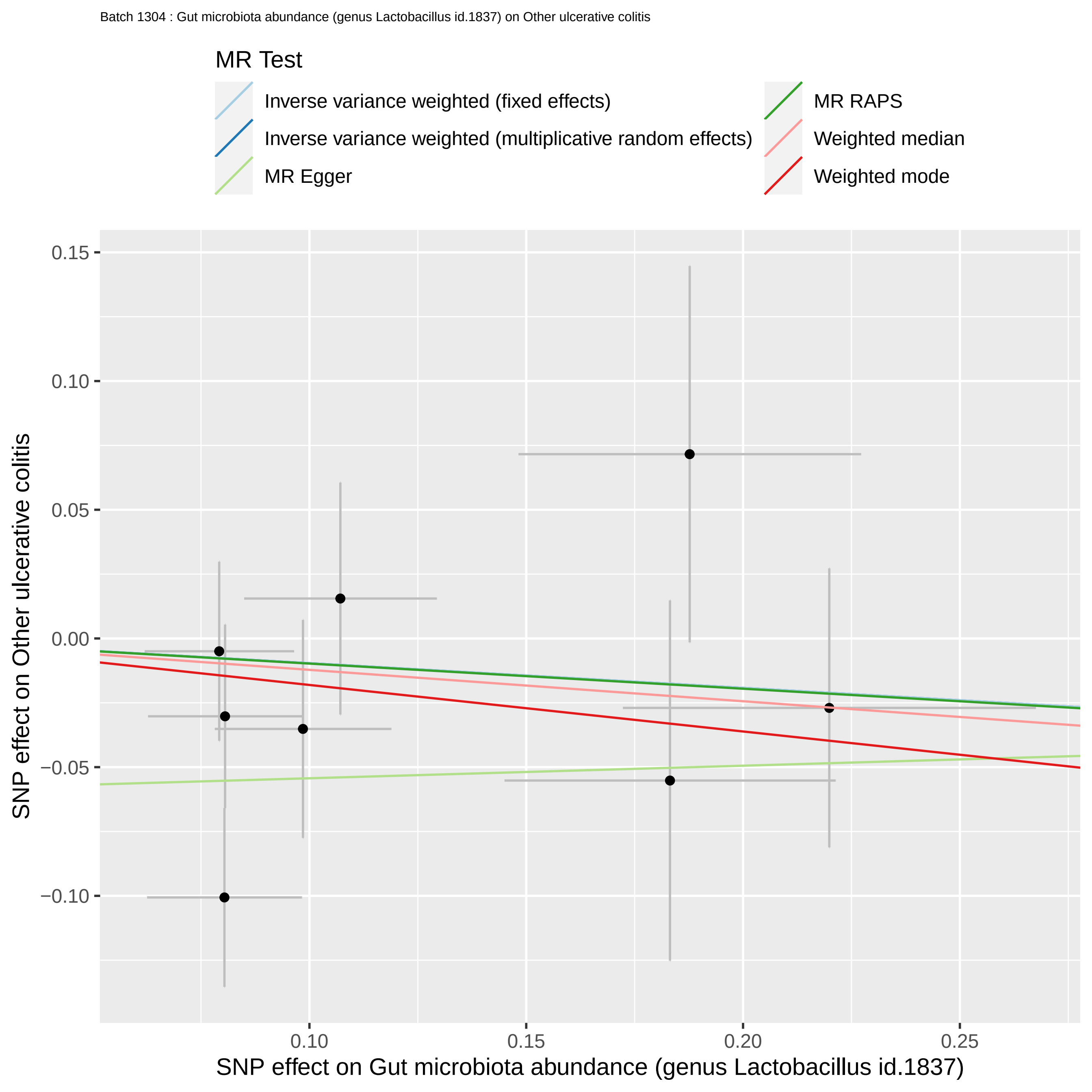
Batch 1304 : Gut microbiota abundance (genus Lactobacillus id.1837) on Other ulcerative colitis
MR Test
Inverse variance weighted (fixed effects)
Inverse variance weighted (multiplicative random effects)
MR Egger
MR RAPS
Weighted median
Weighted mode
0.15
0.10
0.05
0.00
−0.05
−0.10
0.10
0.15
0.20
0.25
SNP effect on Other ulcerative colitis
SNP effect on Gut microbiota abundance (genus Lactobacillus id.1837)

Batch 1305 : Gut microbiota abundance (genus Lactococcus id.1851) on Other ulcerative colitis

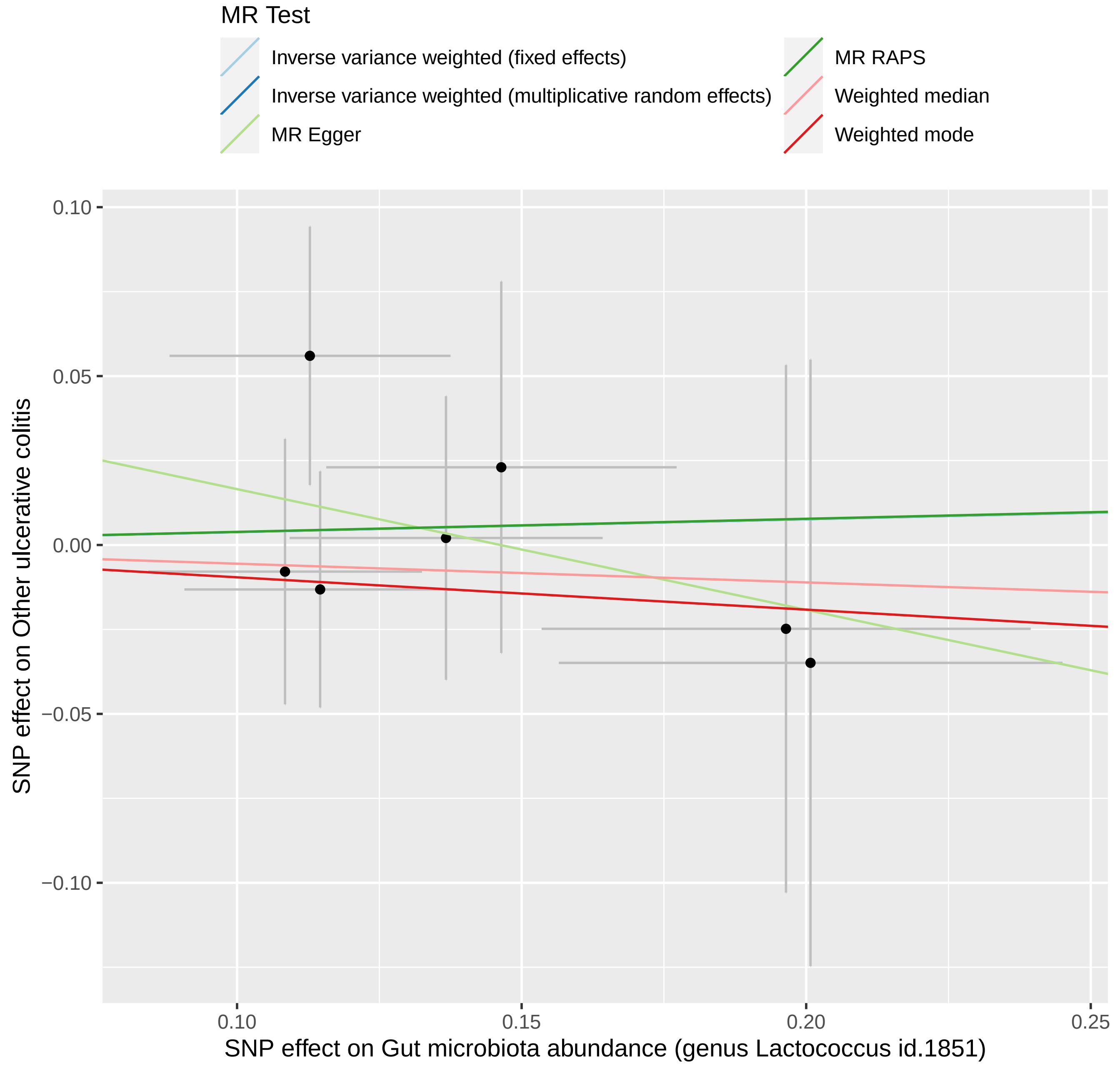

Batch 1306 : Gut microbiota abundance (genus Marvinbryantia id.2005) on Other ulcerative colitis

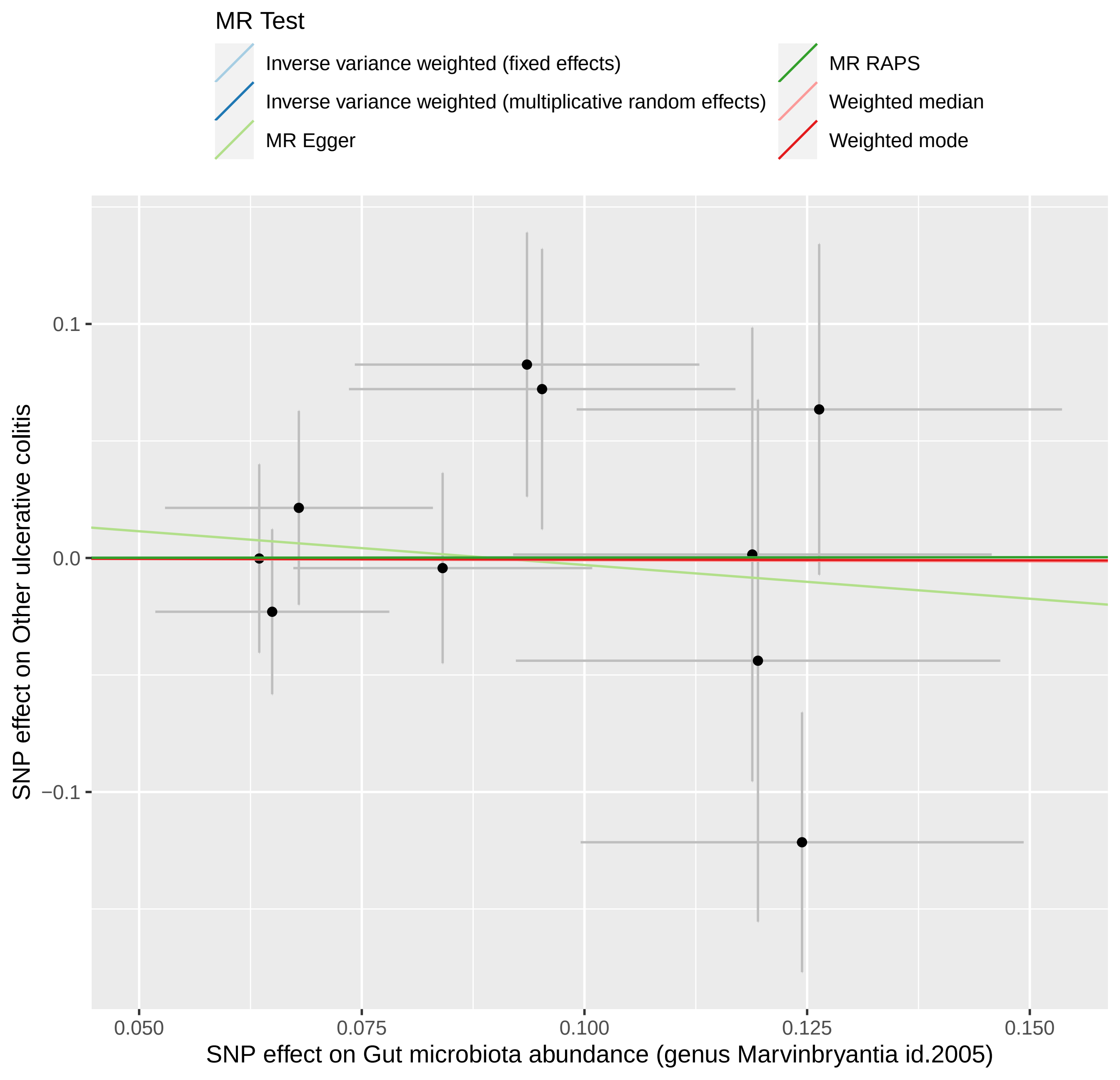

Batch 1307 : Gut microbiota abundance (genus Methanobrevibacter id.123) on Other ulcerative colitis

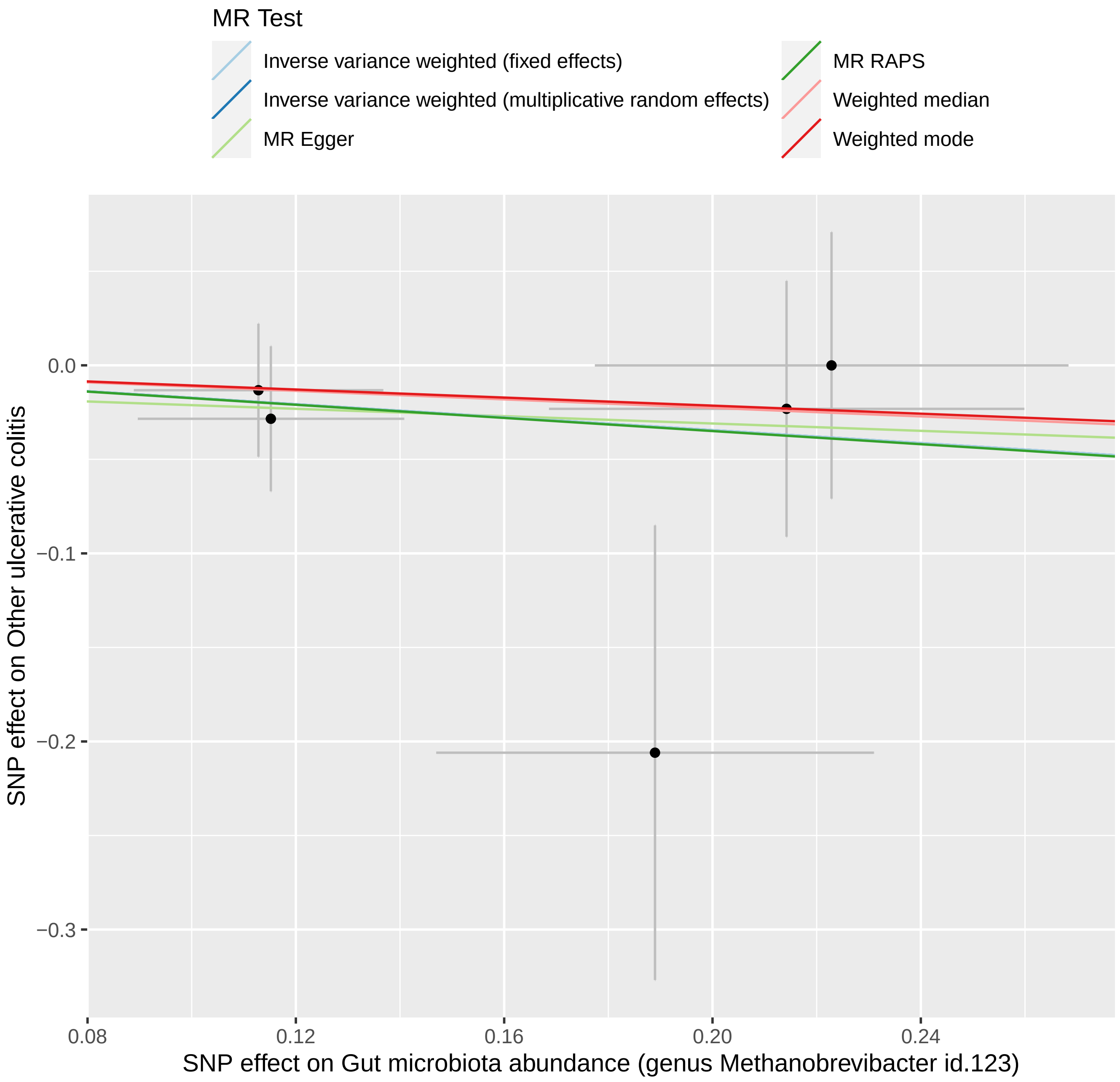

Batch 1308 : Gut microbiota abundance (genus Odoribacter id.952) on Other ulcerative colitis

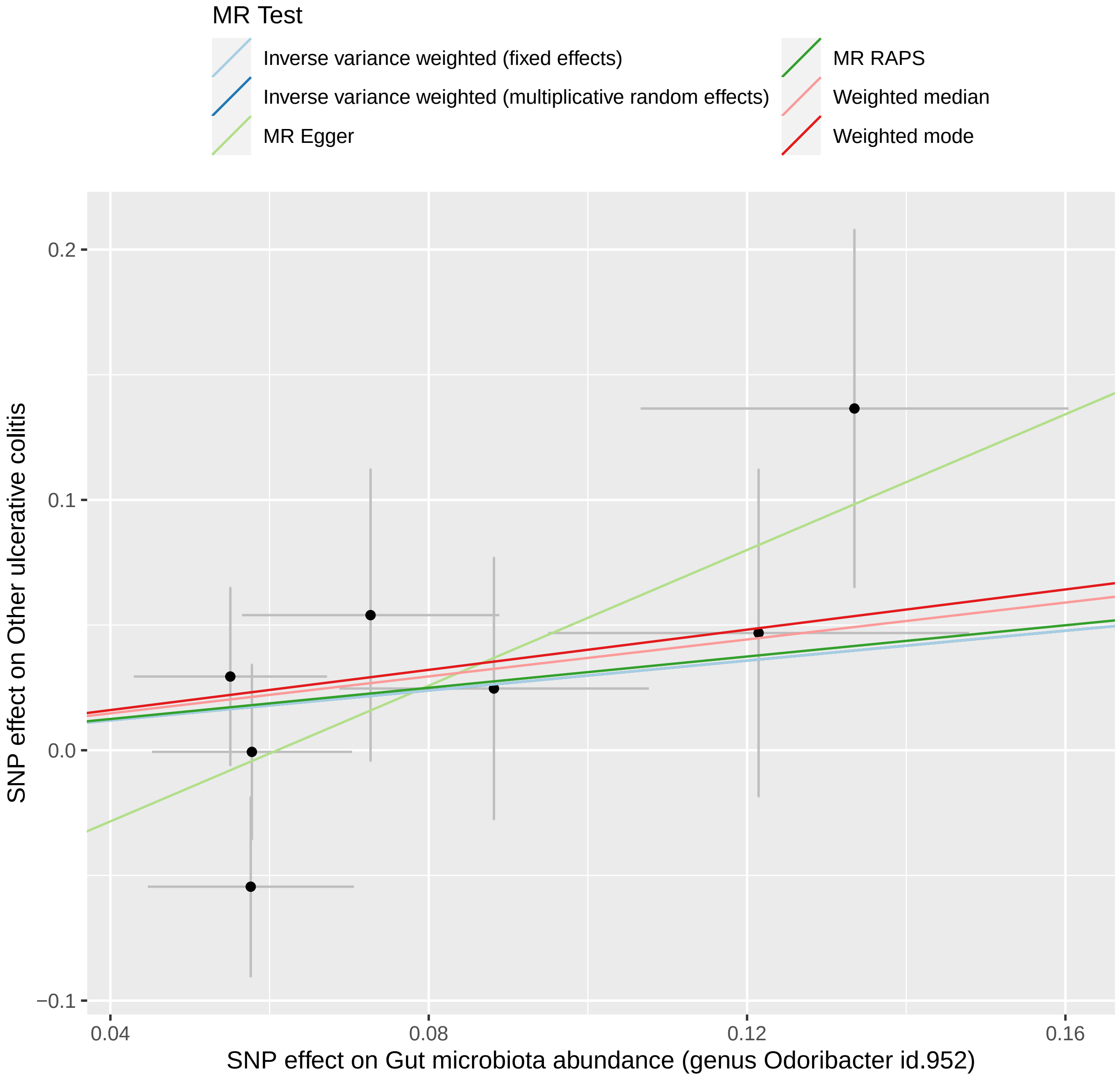

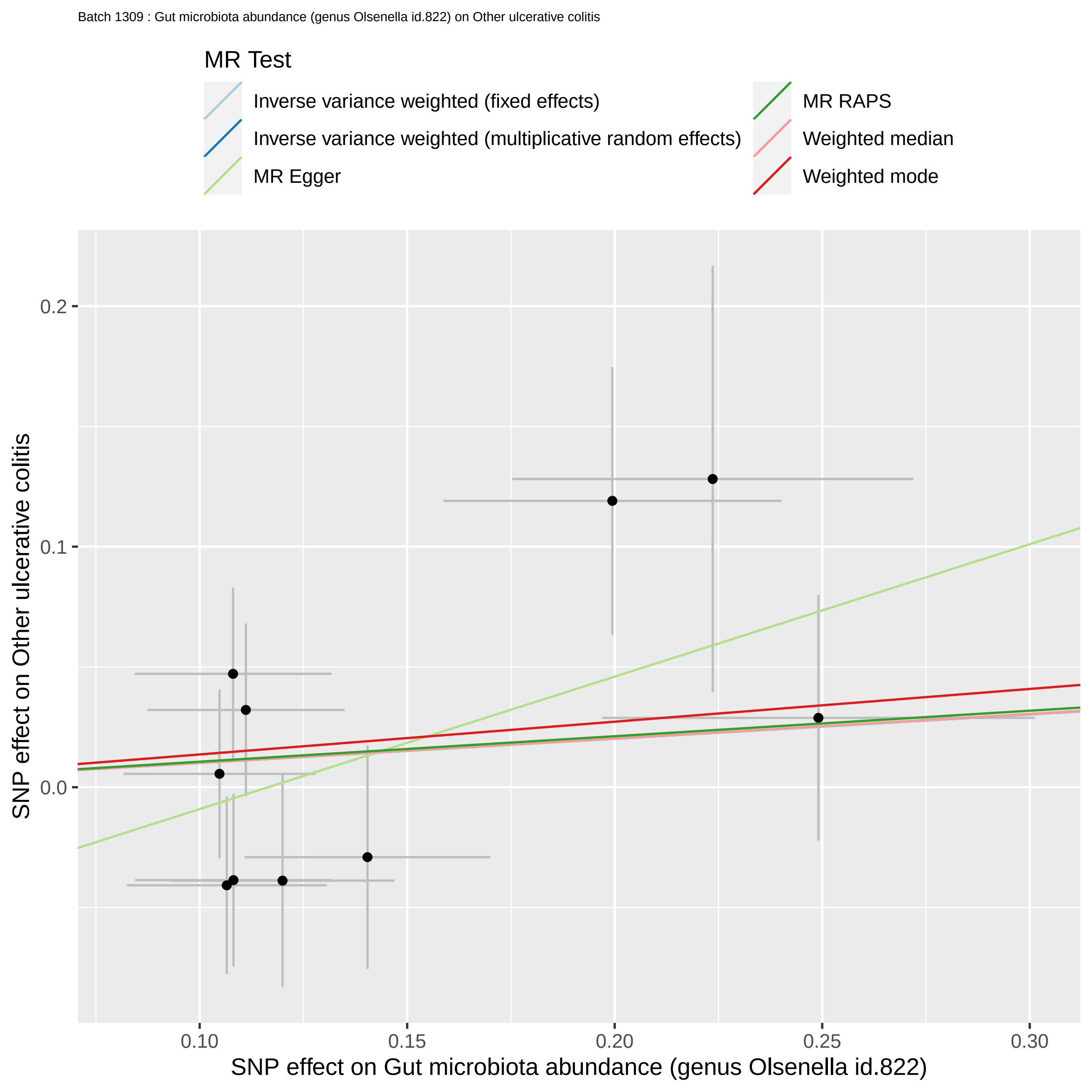

Batch 1309 : Gut microbiota abundance (genus Olsenella id.822) on Other ulcerative colitis
MR Test
Inverse variance weighted (fixed effects)
Inverse variance weighted (multiplicative random effects)
MR Egger
MR RAPS
Weighted median
Weighted mode
SNP effect on Other ulcerative colitis
0.2
0.1
0.0
0.10
0.15
0.20
0.25
0.30
SNP effect on Gut microbiota abundance (genus Olsenella id.822)

Batch 1310 : Gut microbiota abundance (genus Oscillibacter id.2063) on Other ulcerative colitis

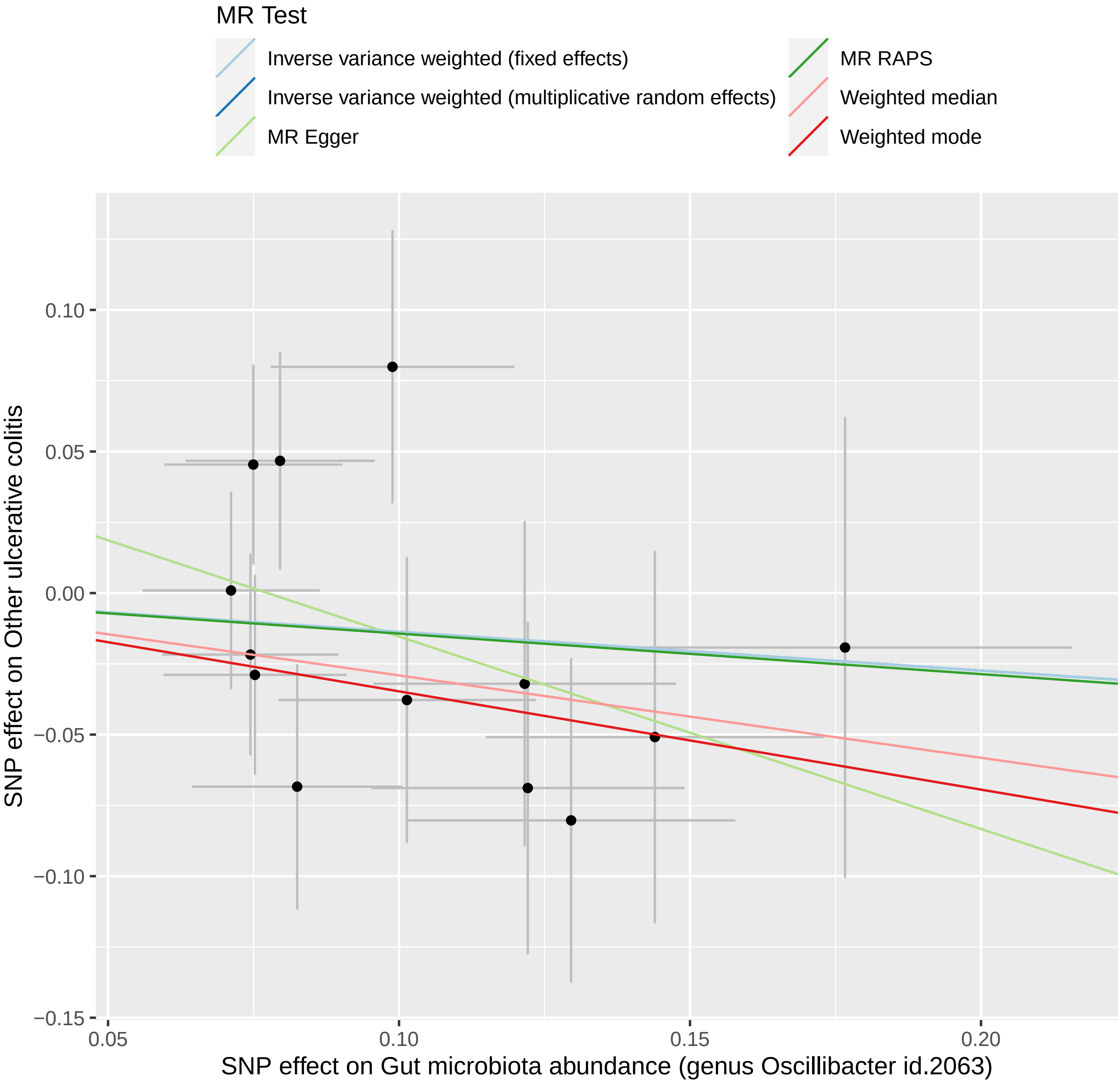

Batch 1311 : Gut microbiota abundance (genus Oscillospira id.2064) on Other ulcerative colitis

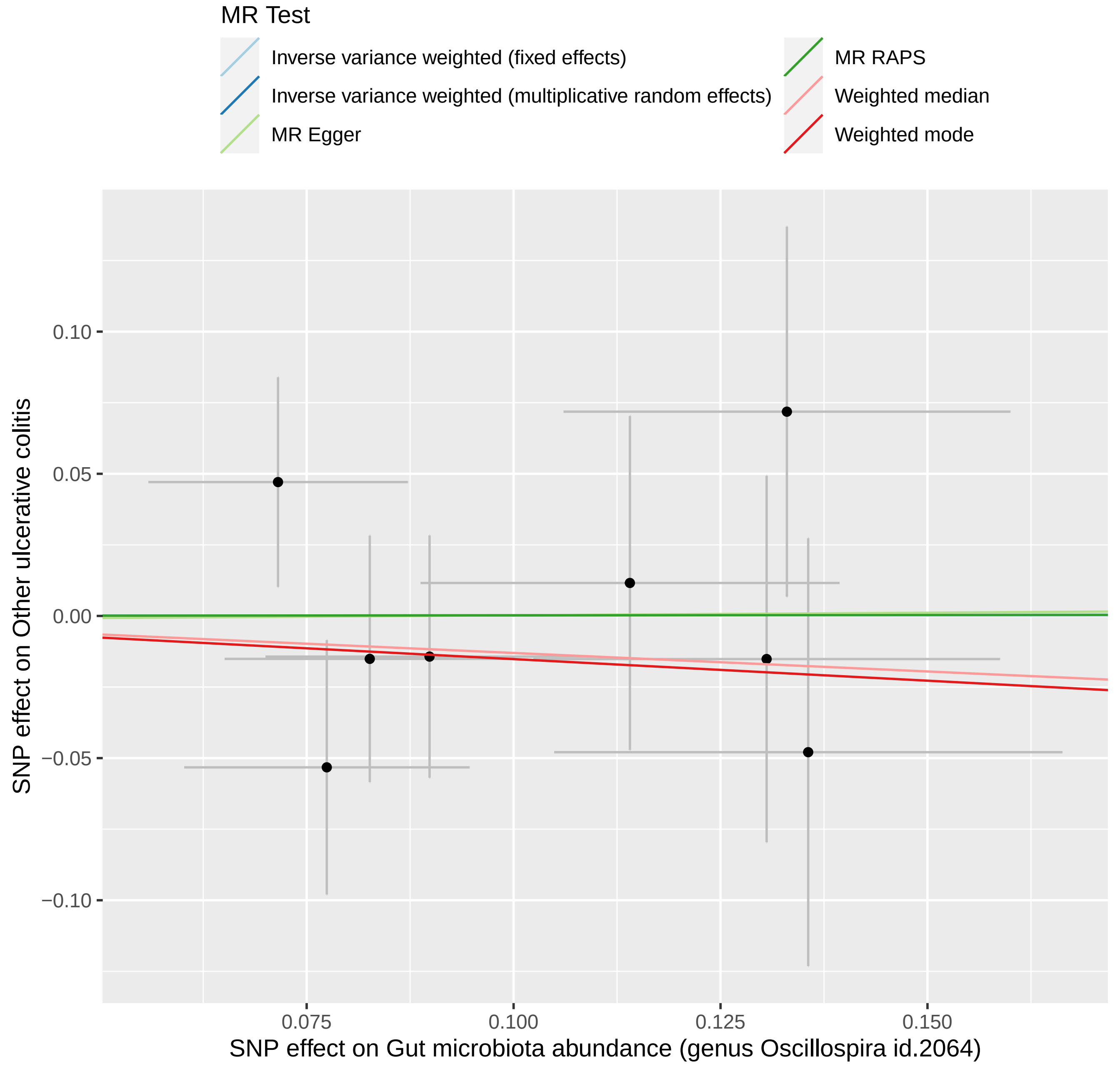

Batch 1312 : Gut microbiota abundance (genus Oxalobacter id.2978) on Other ulcerative colitis

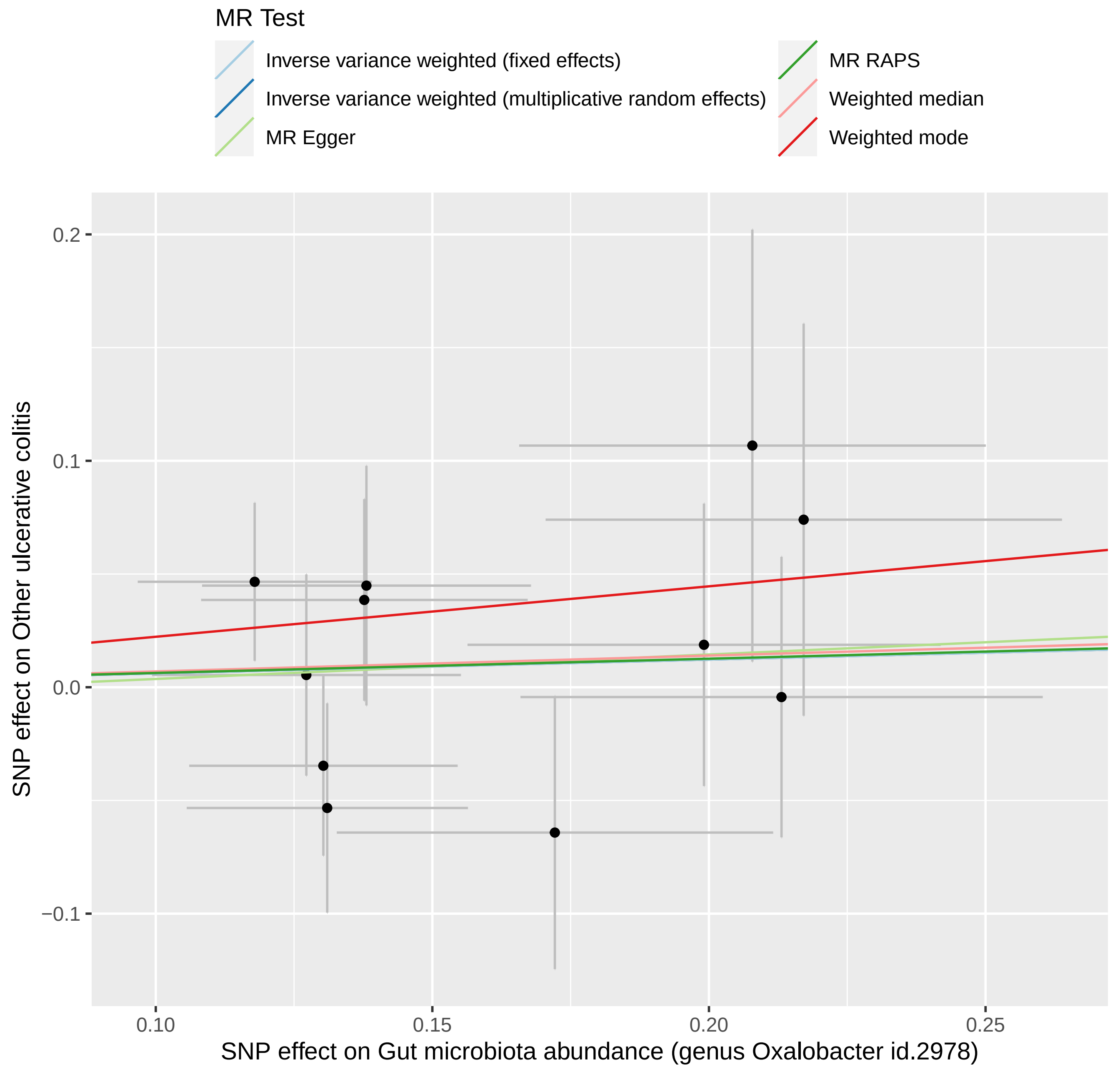

Batch 1313 : Gut microbiota abundance (genus Parabacteroides id.954) on Other ulcerative colitis

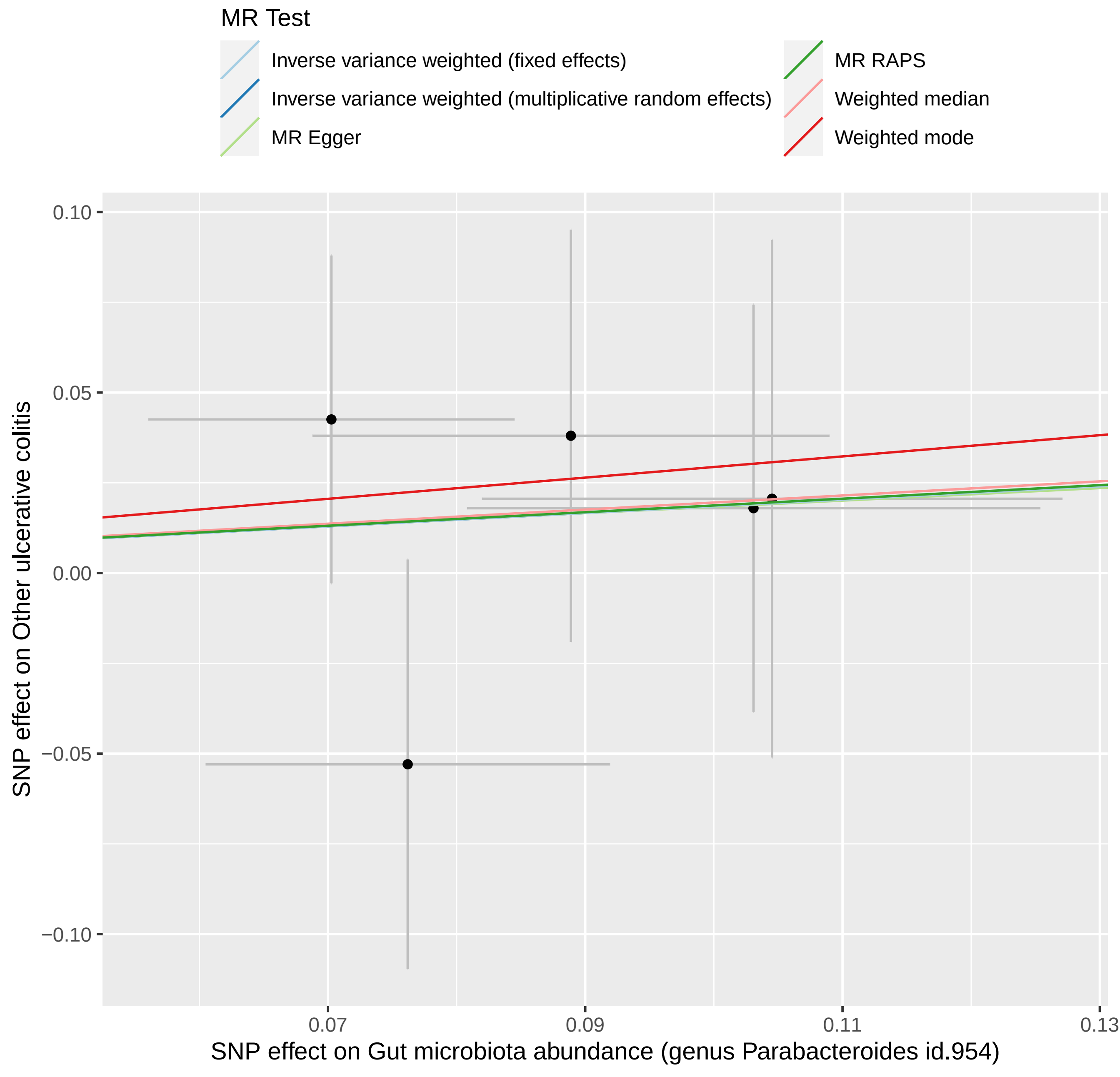

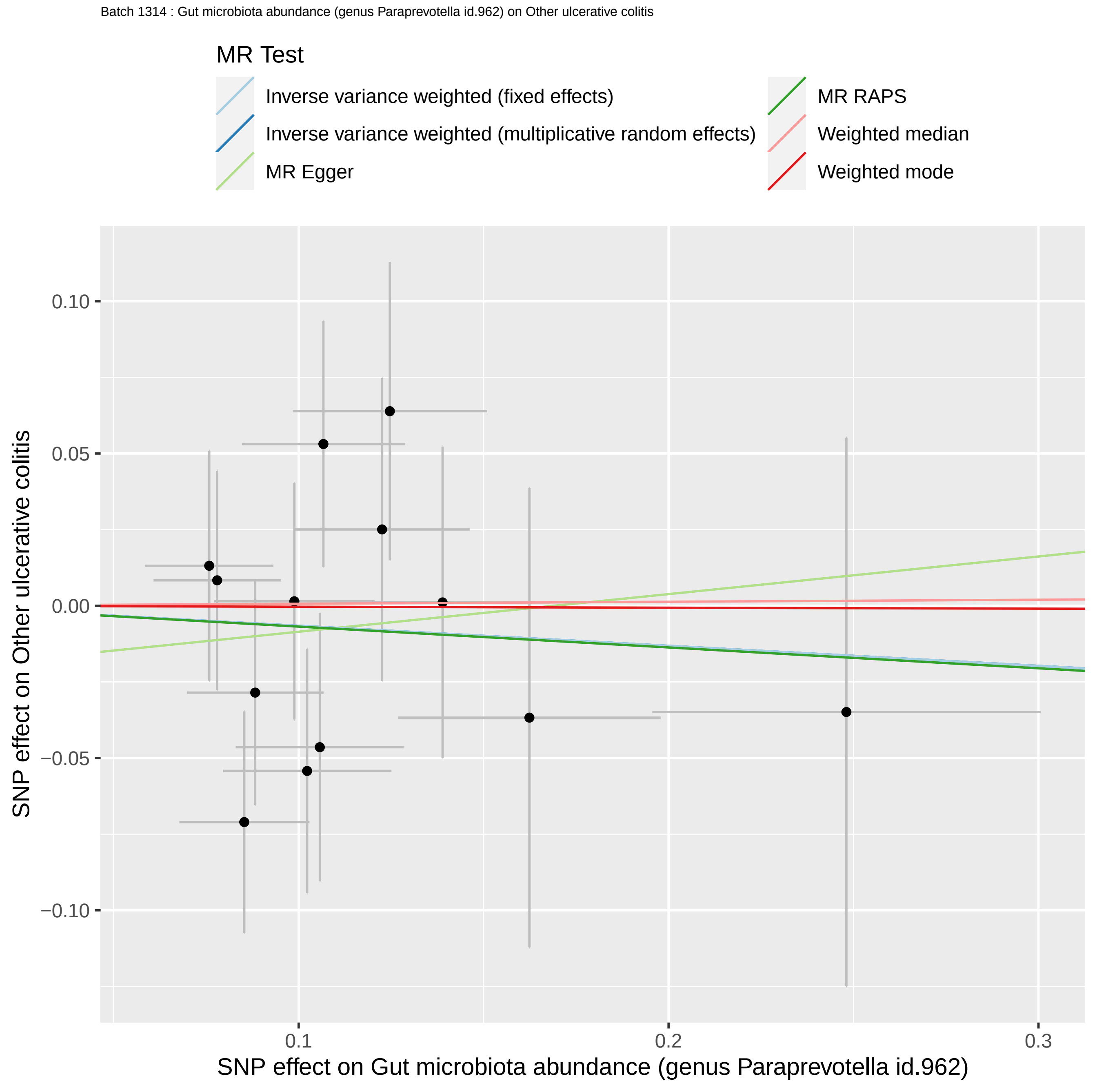
Batch 1314 : Gut microbiota abundance (genus Paraprevotella id.962) on Other ulcerative colitis
MR Test
Inverse variance weighted (fixed effects)
Inverse variance weighted (multiplicative random effects)
MR Egger
MR RAPS
Weighted median
Weighted mode
0.10
0.05
0.00
−0.05
−0.10
0.1
0.2
0.3
SNP effect on Other ulcerative colitis
SNP effect on Gut microbiota abundance (genus Paraprevotella id.962)

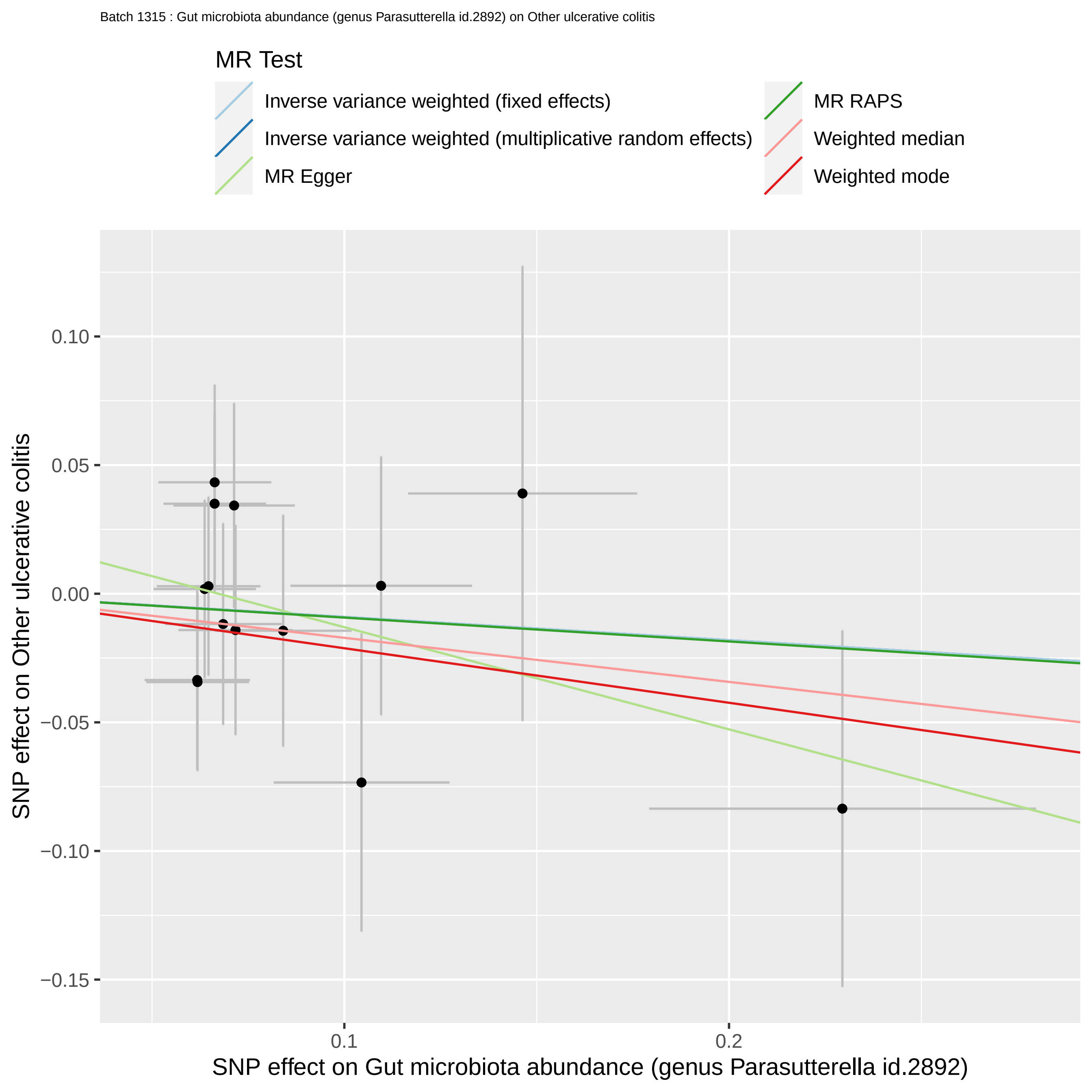
Batch 1315 : Gut microbiota abundance (genus Parasutterella id.2892) on Other ulcerative colitis
MR Test
Inverse variance weighted (fixed effects)
Inverse variance weighted (multiplicative random effects)
MR Egger
MR RAPS
Weighted median
Weighted mode
0.10
0.05
0.00
−0.05
−0.10
−0.15
0.1
0.2
SNP effect on Other ulcerative colitis
SNP effect on Gut microbiota abundance (genus Parasutterella id.2892)

Batch 1316 : Gut microbiota abundance (genus Peptococcus id.2037) on Other ulcerative colitis

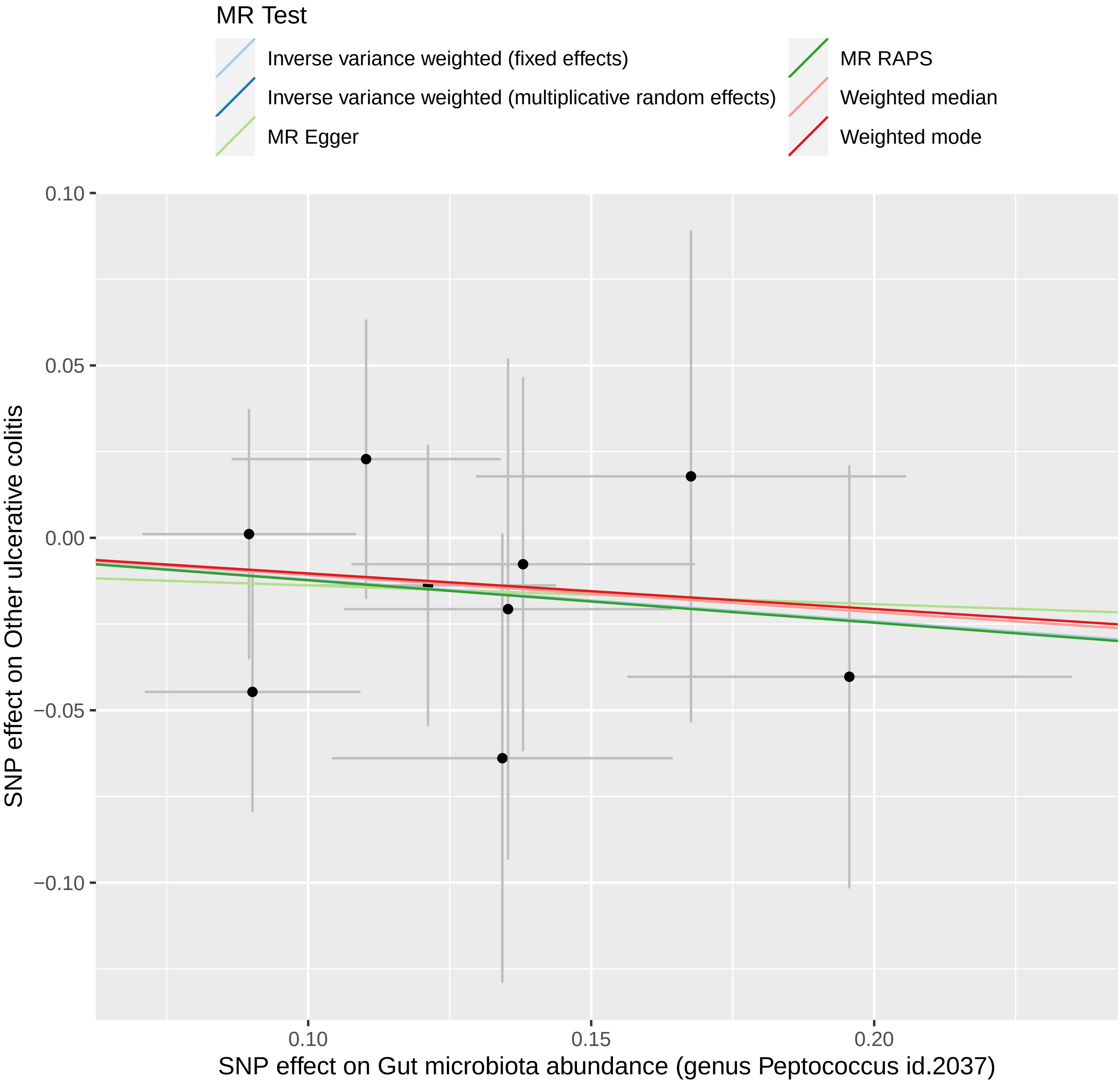

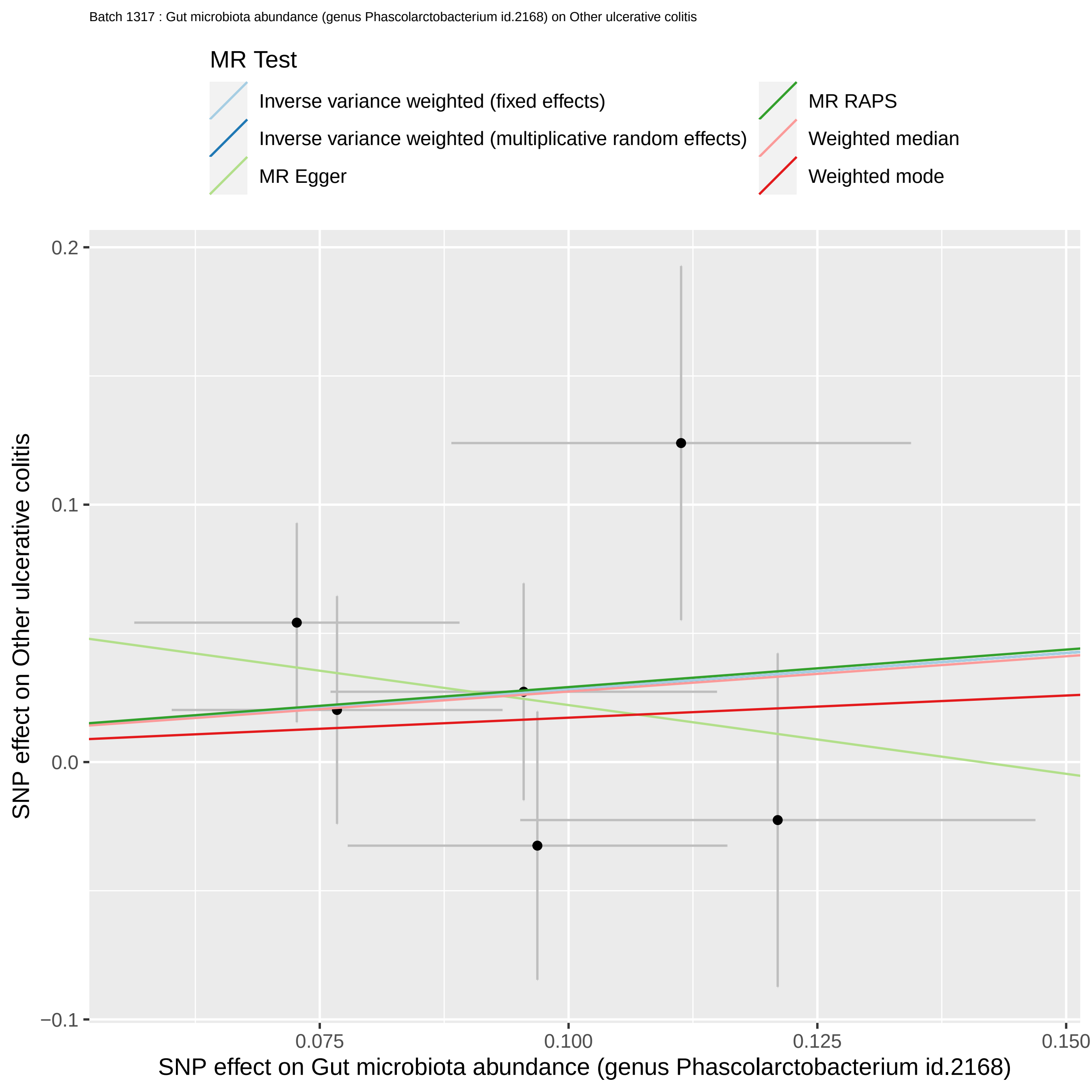
Batch 1317 : Gut microbiota abundance (genus Phascolarctobacterium id.2168) on Other ulcerative colitis
MR Test
Inverse variance weighted (fixed effects)
Inverse variance weighted (multiplicative random effects)
MR Egger
MR RAPS
Weighted median
Weighted mode
0.2
0.1
0.0
−0.1
0.075
0.100
0.125
0.150
SNP effect on Other ulcerative colitis
SNP effect on Gut microbiota abundance (genus Phascolarctobacterium id.2168)

Batch 1318 : Gut microbiota abundance (genus Prevotella7 id.11182) on Other ulcerative colitis

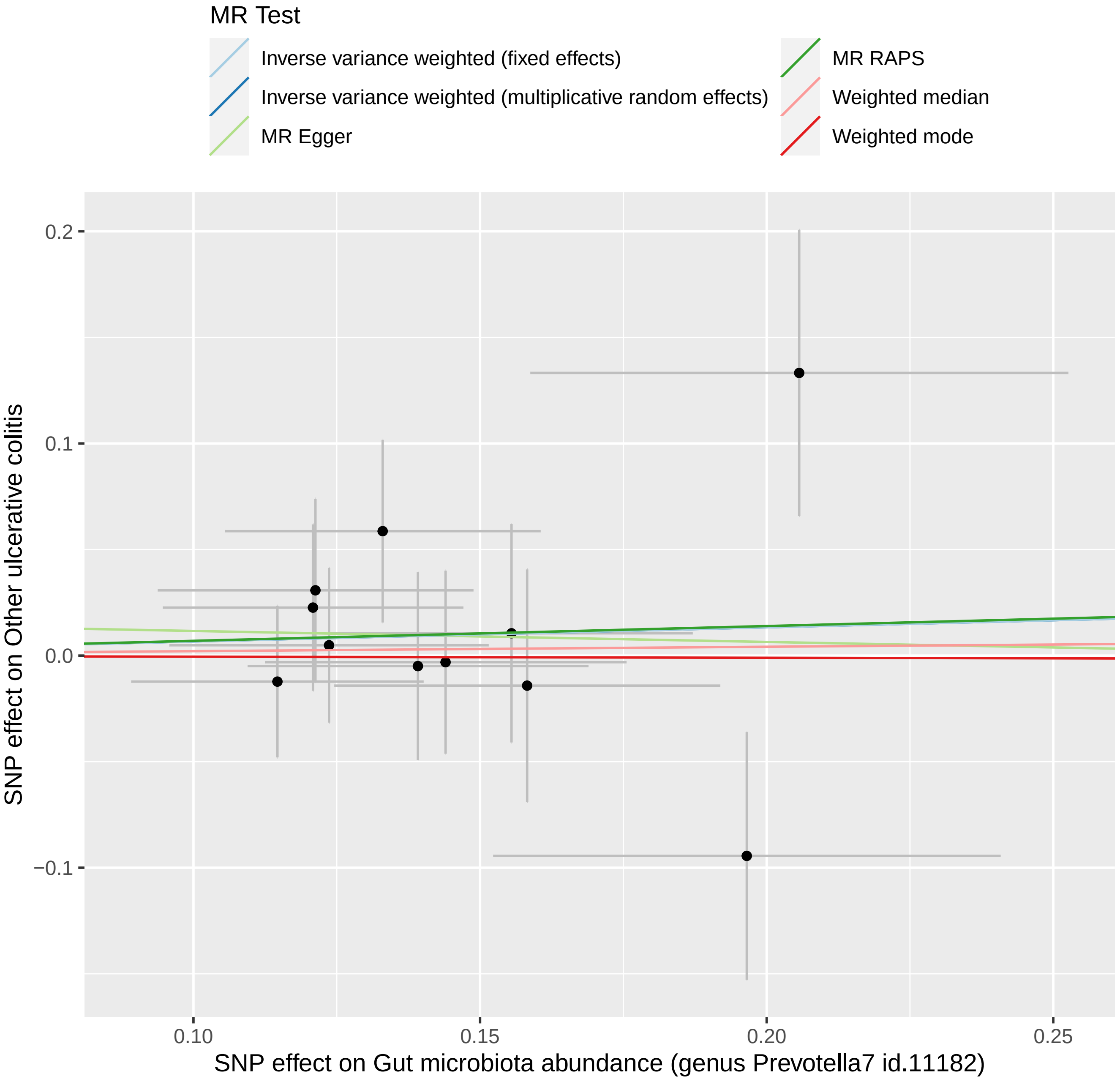

Batch 1319 : Gut microbiota abundance (genus Prevotella9 id.11183) on Other ulcerative colitis

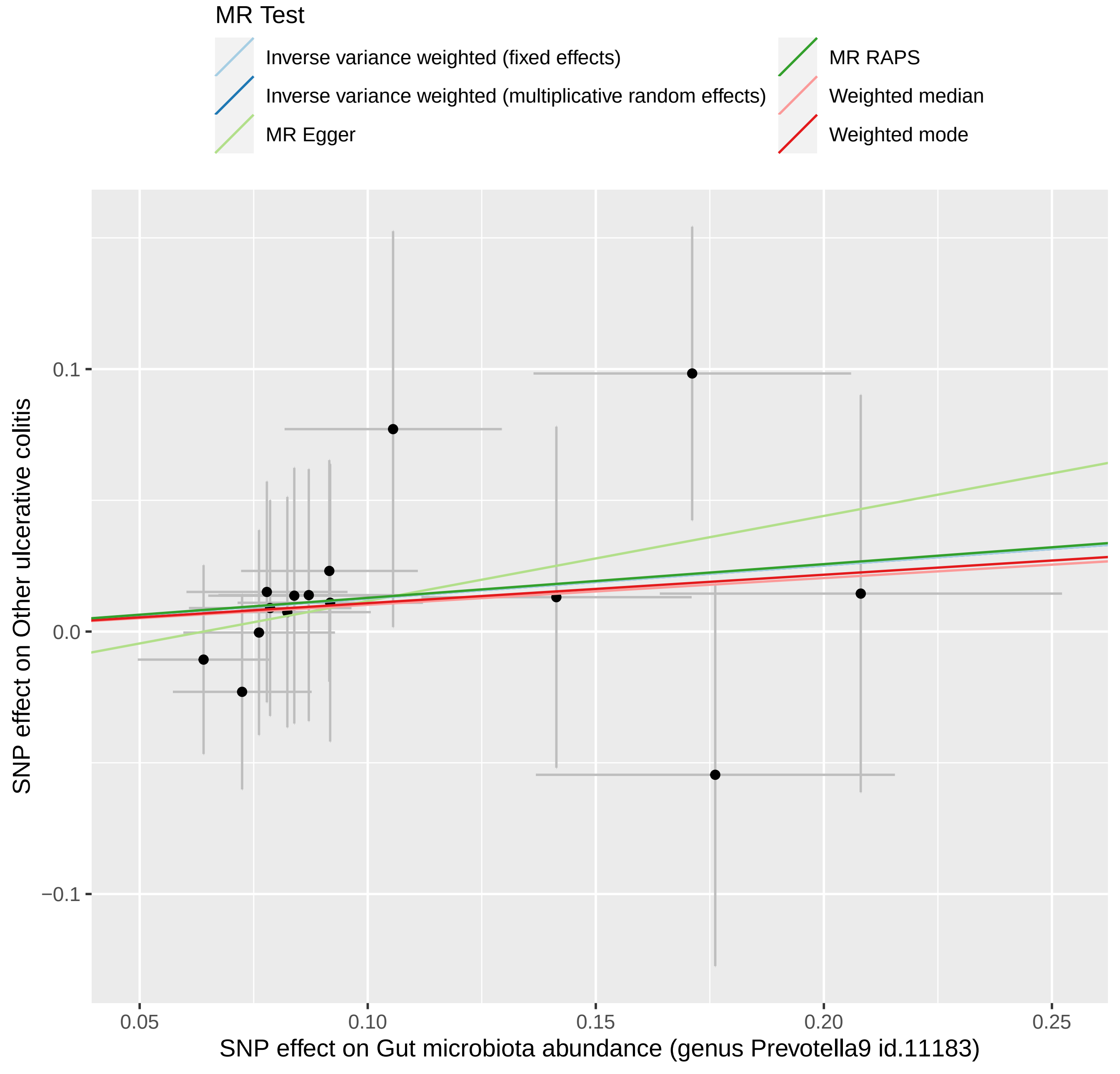

Batch 1320 : Gut microbiota abundance (genus Rikenellaceae RC9 gut group id.11191) on Other ulcerative colitis

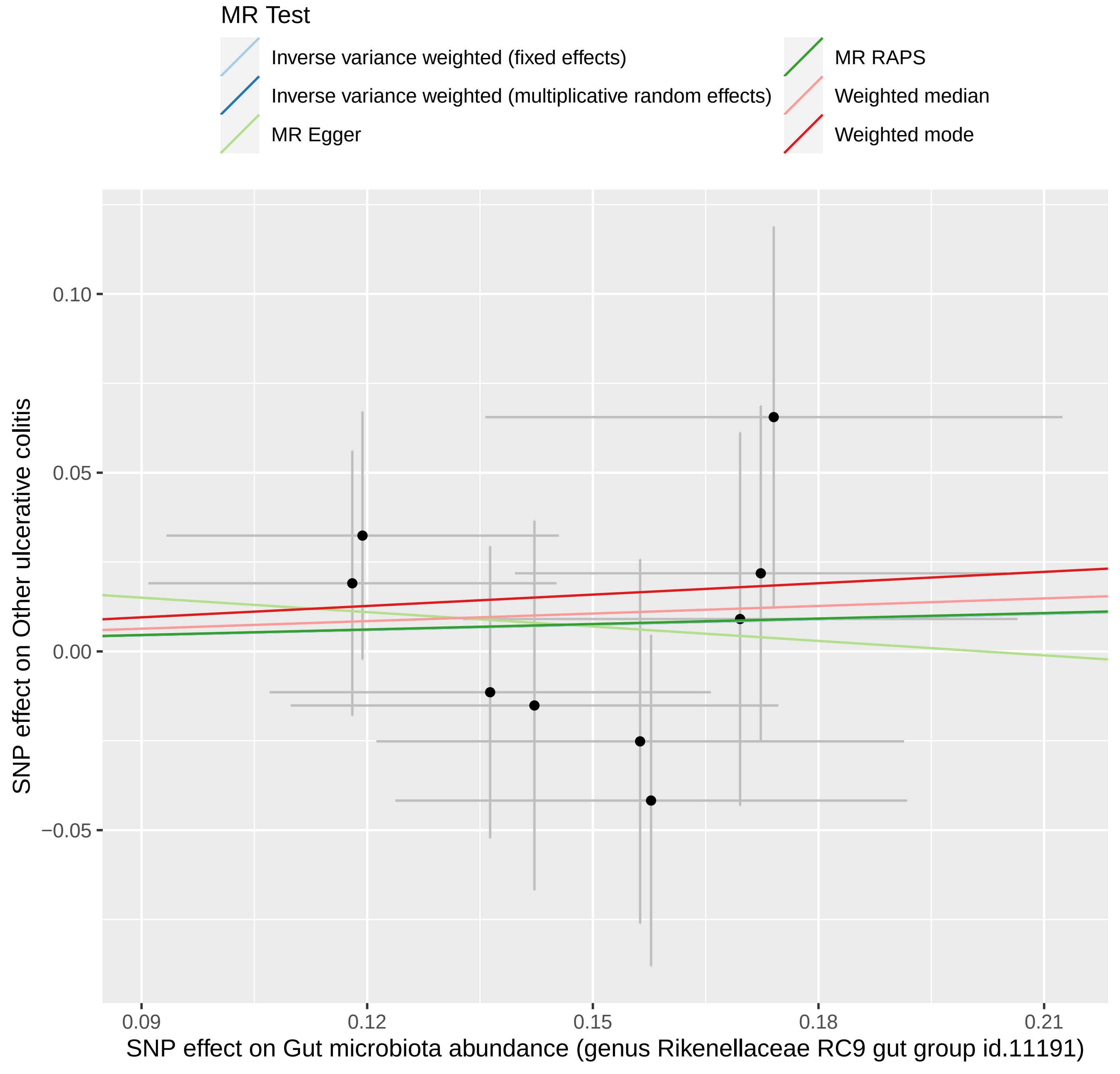

Batch 1321 : Gut microbiota abundance (genus Romboutsia id.11347) on Other ulcerative colitis

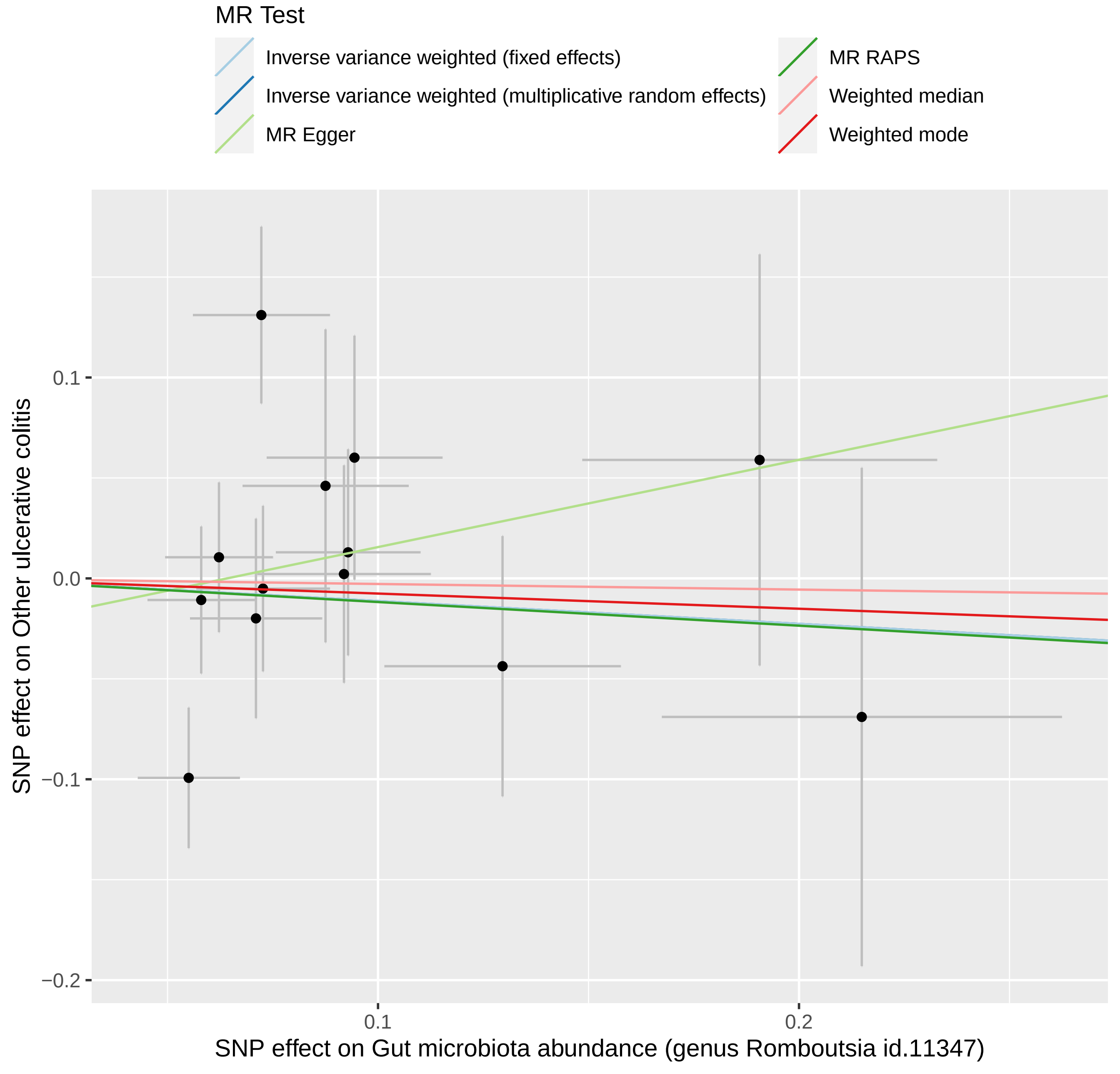

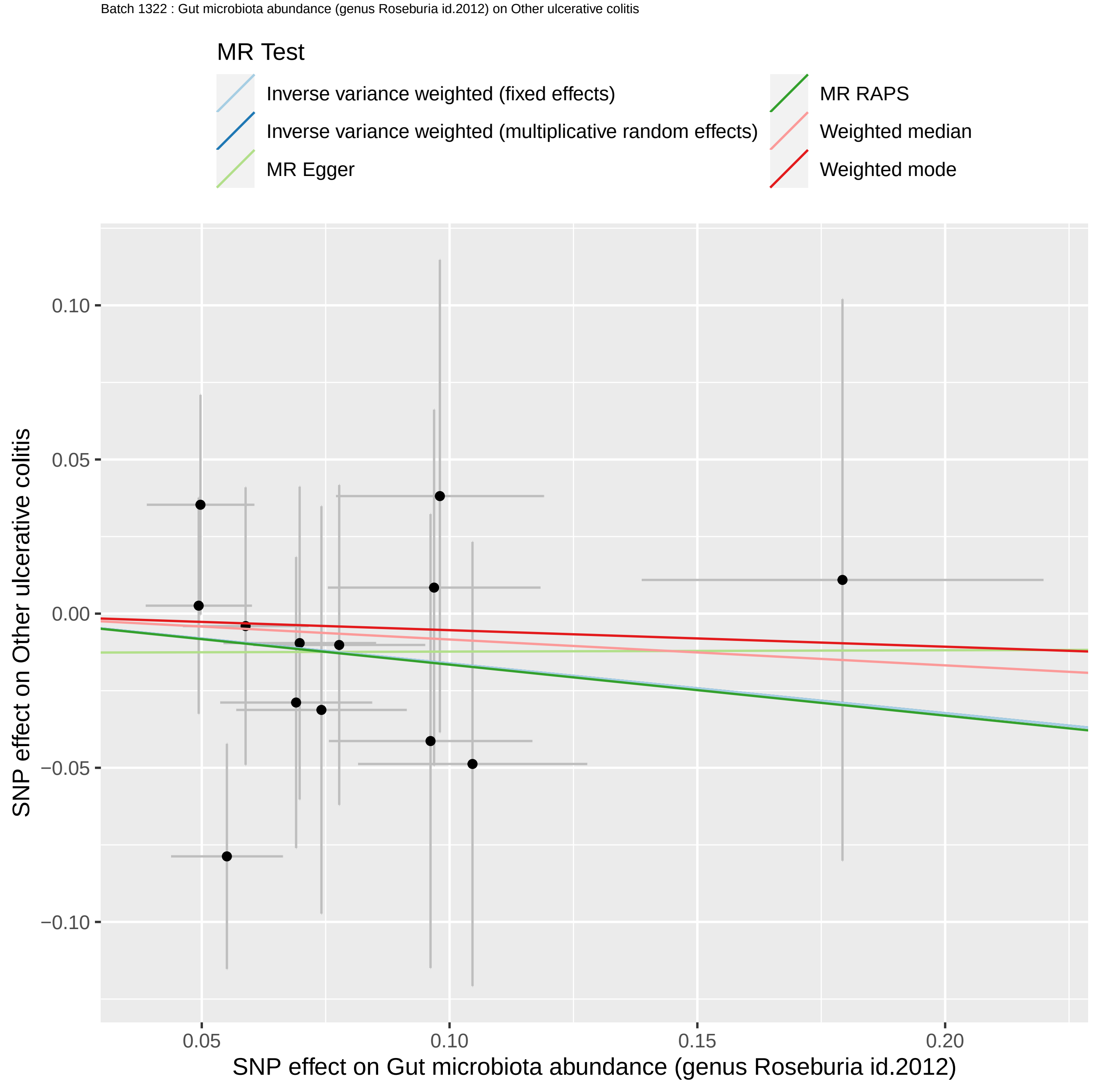
Batch 1322 : Gut microbiota abundance (genus Roseburia id.2012) on Other ulcerative colitis
MR Test
Inverse variance weighted (fixed effects)
Inverse variance weighted (multiplicative random effects)
MR Egger
MR RAPS
Weighted median
Weighted mode
0.10
0.05
0.00
−0.05
−0.10
SNP effect on Other ulcerative colitis
0.05
0.10
0.15
0.20
SNP effect on Gut microbiota abundance (genus Roseburia id.2012)

Batch 1323 : Gut microbiota abundance (genus Ruminiclostridium5 id.11355) on Other ulcerative colitis

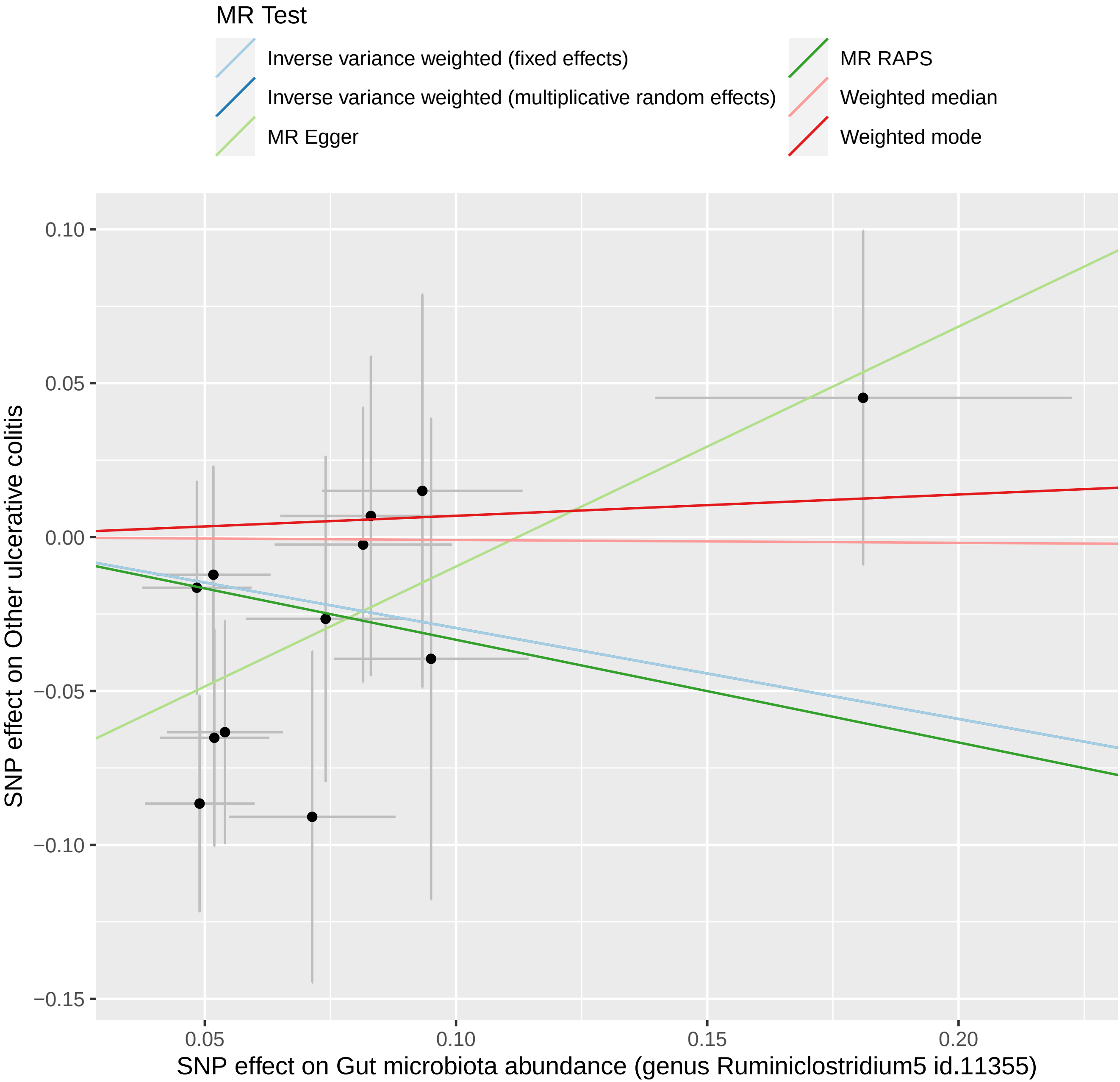

Batch 1324 : Gut microbiota abundance (genus Ruminiclostridium6 id.11356) on Other ulcerative colitis

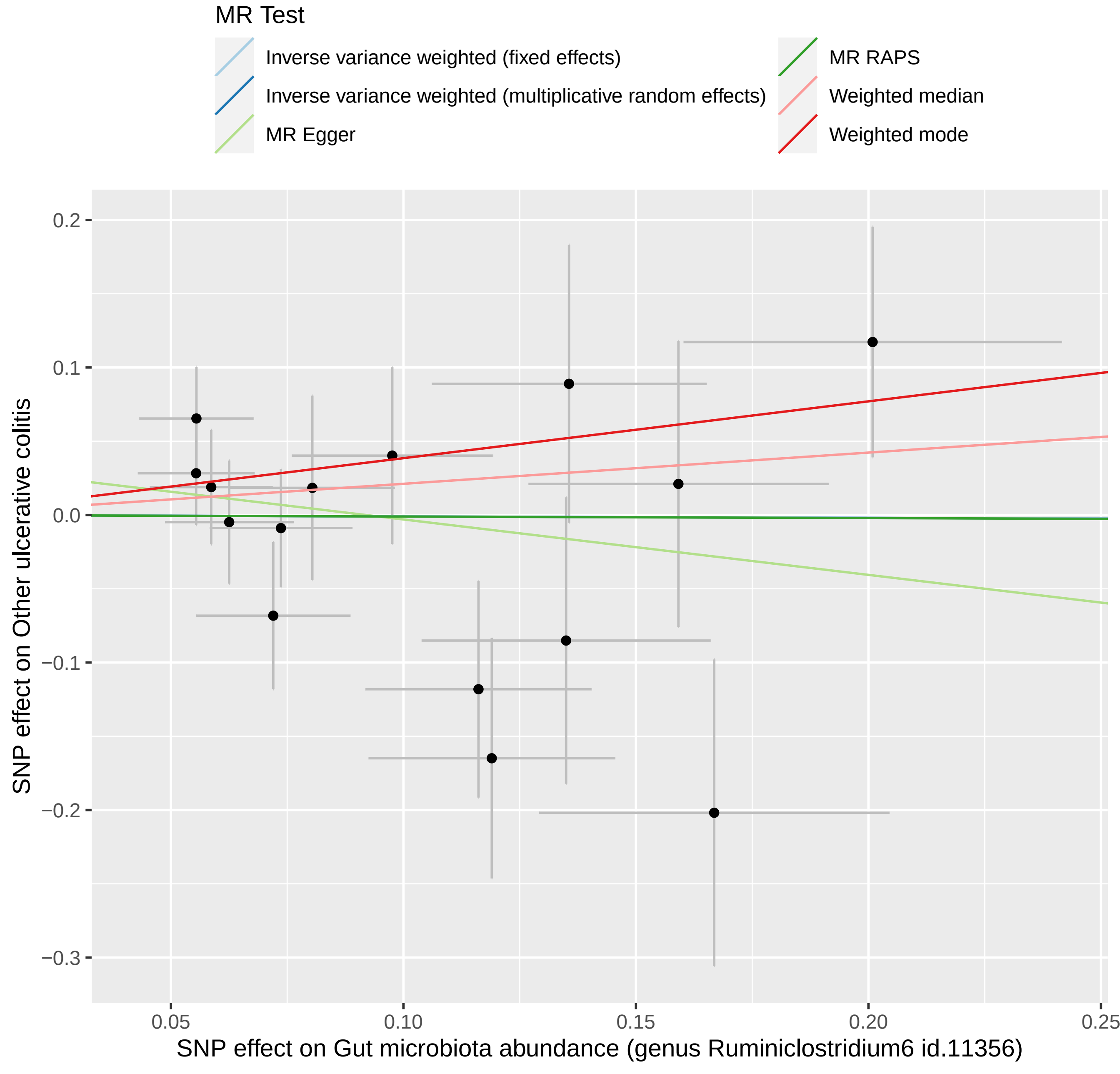

Batch 1325 : Gut microbiota abundance (genus Ruminiclostridium9 id.11357) on Other ulcerative colitis

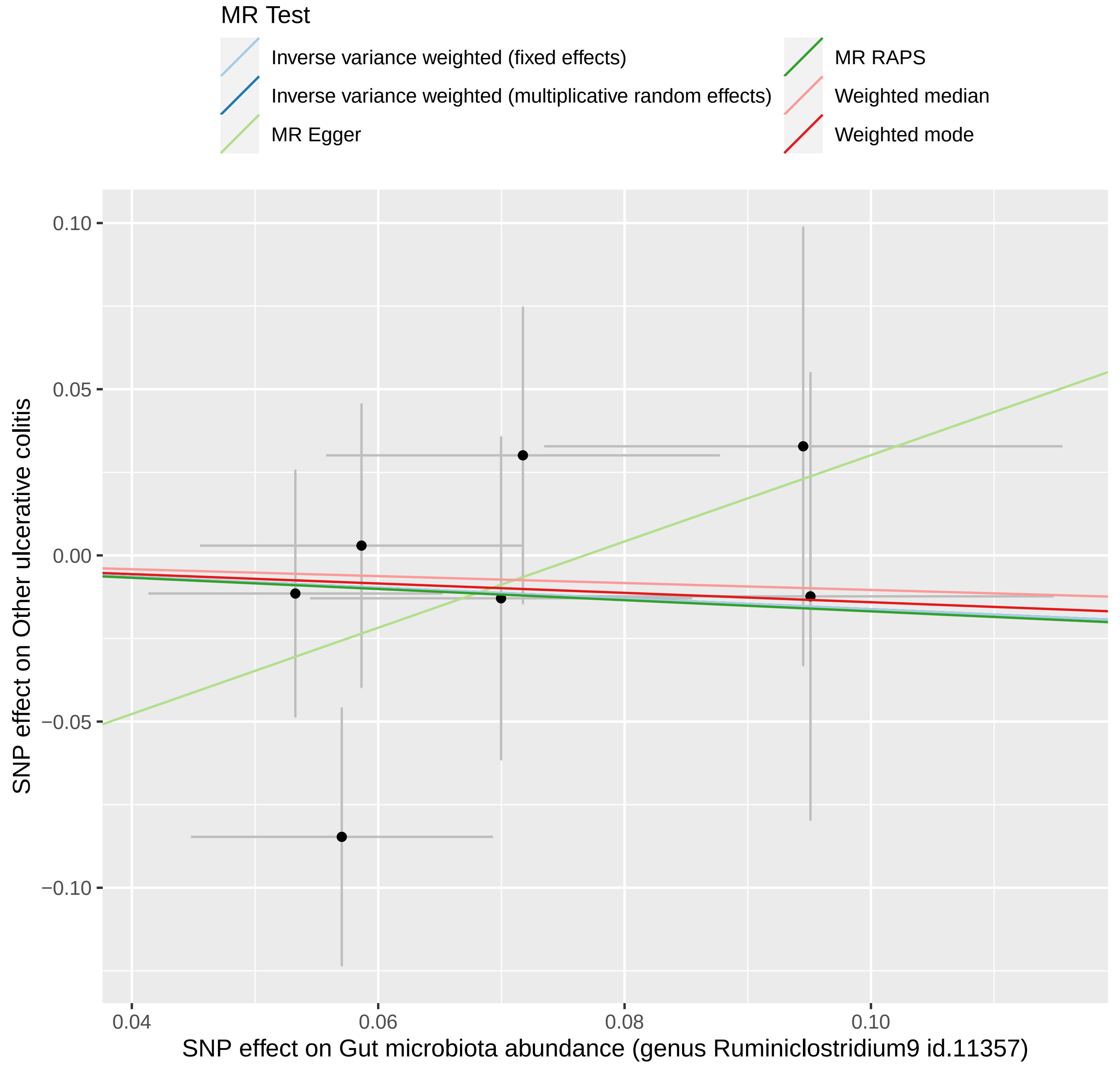

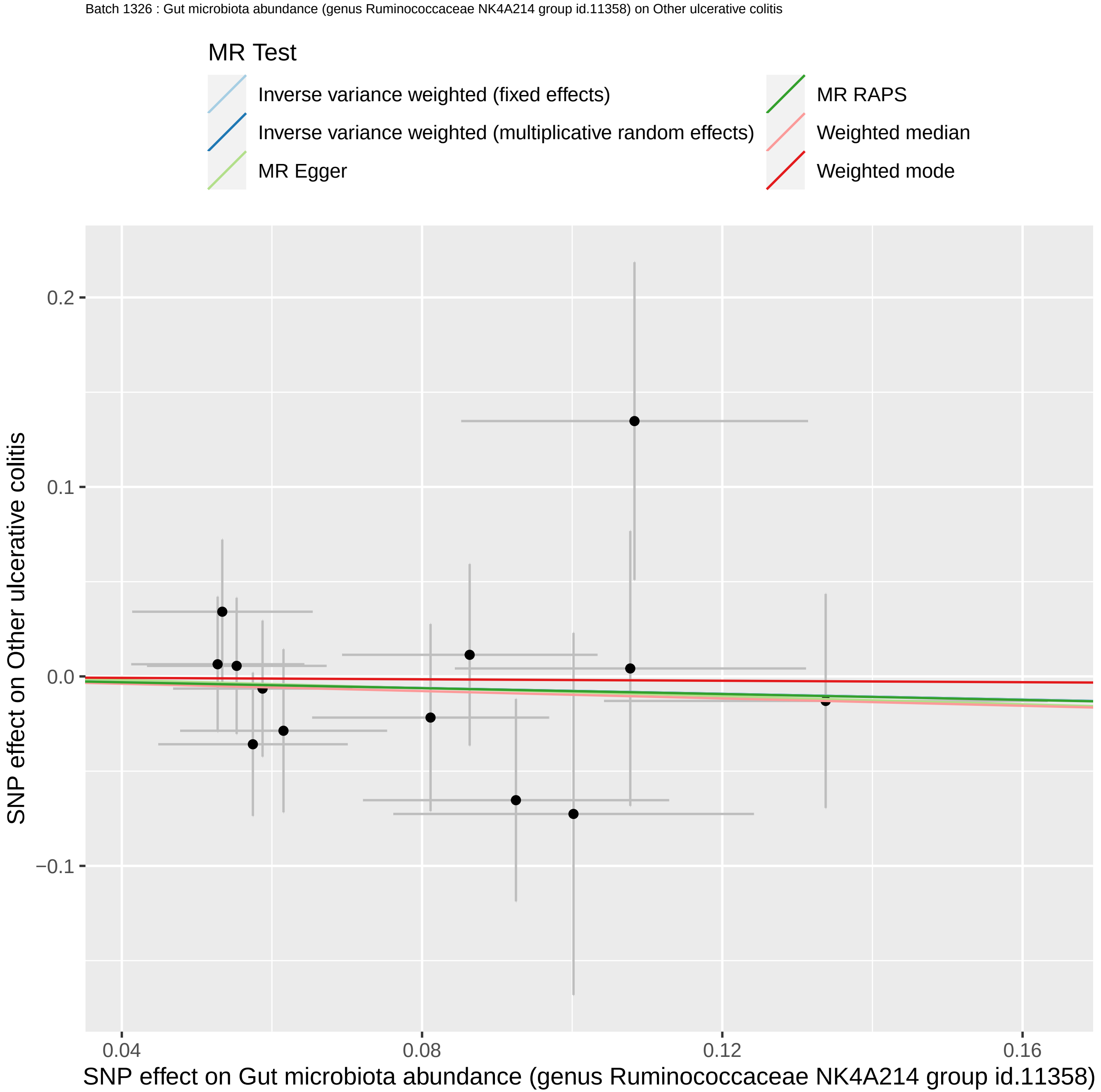

Batch 1326 : Gut microbiota abundance (genus Ruminococcaceae NK4A214 group id.11358) on Other ulcerative colitis
MR Test
Inverse variance weighted (fixed effects)
Inverse variance weighted (multiplicative random effects)
MR Egger
MR RAPS
Weighted median
Weighted mode
0.2
0.1
0.0
−0.1
0.04
0.08
0.12
0.16
SNP effect on Other ulcerative colitis
SNP effect on Gut microbiota abundance (genus Ruminococcaceae NK4A214 group id.11358)

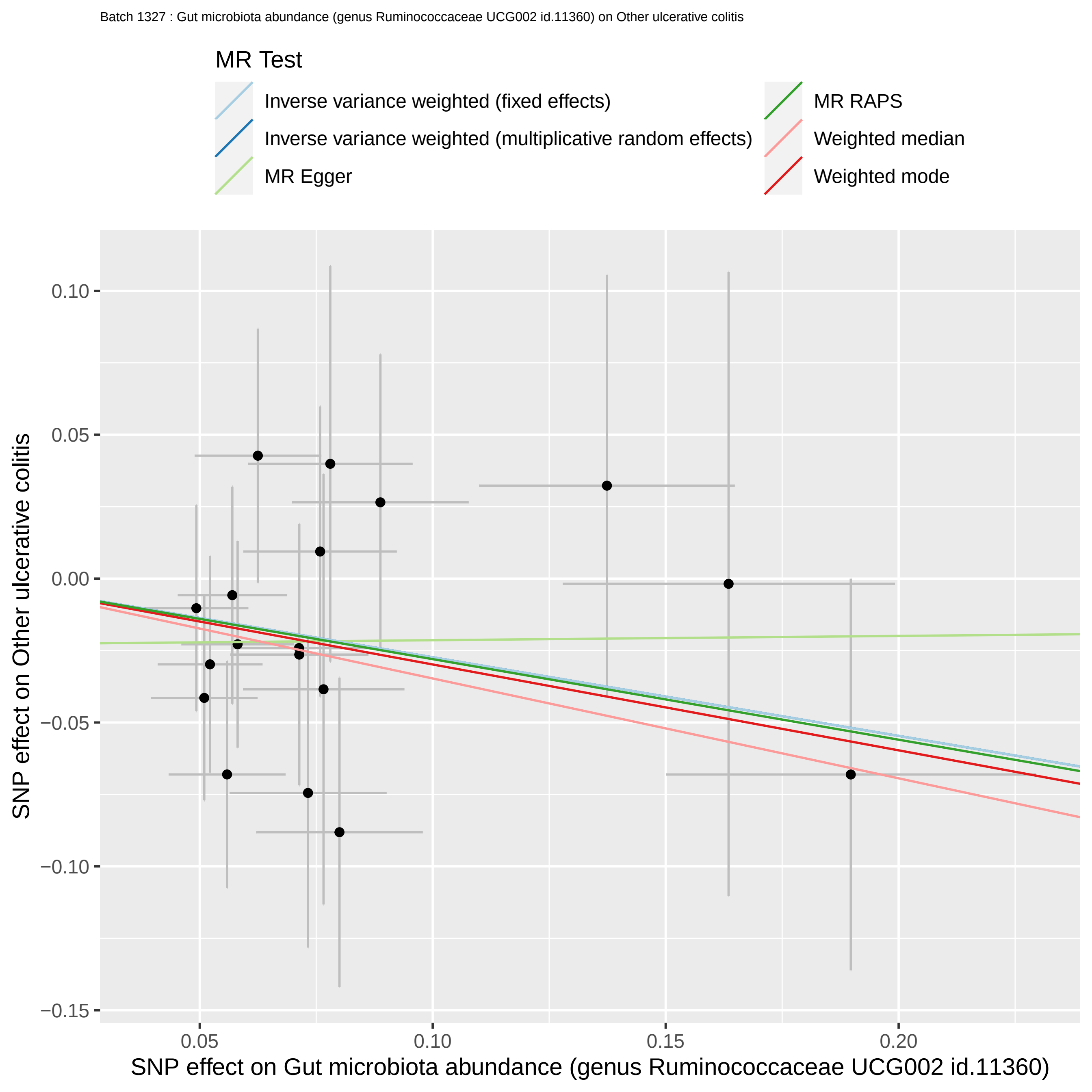
Batch 1327 : Gut microbiota abundance (genus Ruminococcaceae UCG002 id.11360) on Other ulcerative colitis
MR Test
Inverse variance weighted (fixed effects)
Inverse variance weighted (multiplicative random effects)
MR Egger
MR RAPS
Weighted median
Weighted mode
0.10
0.05
0.00
−0.05
−0.10
−0.15
0.05
0.10
0.15
0.20
SNP effect on Other ulcerative colitis
SNP effect on Gut microbiota abundance (genus Ruminococcaceae UCG002 id.11360)

Batch 1328 : Gut microbiota abundance (genus Ruminococcaceae UCG003 id.11361) on Other ulcerative colitis

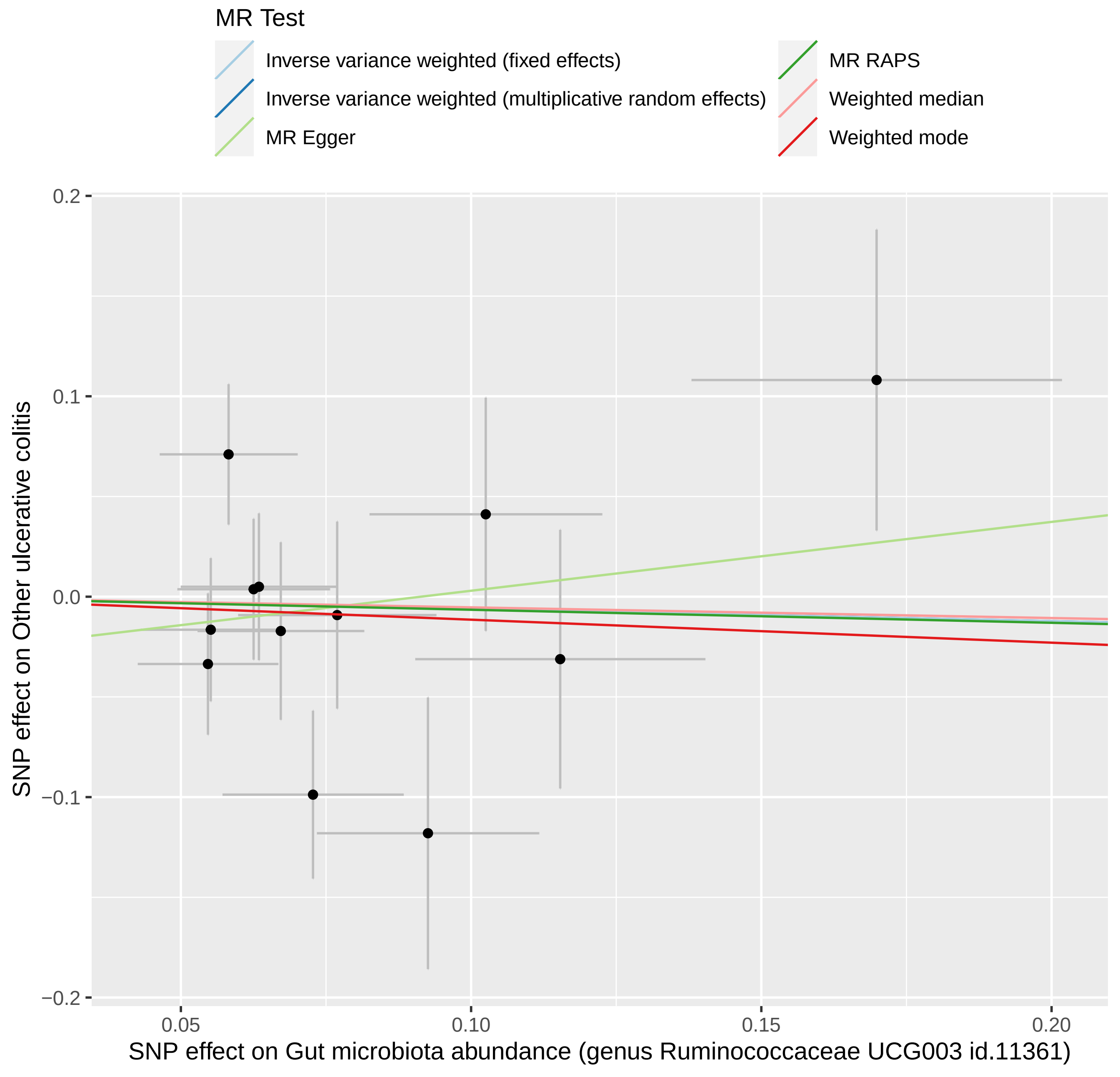

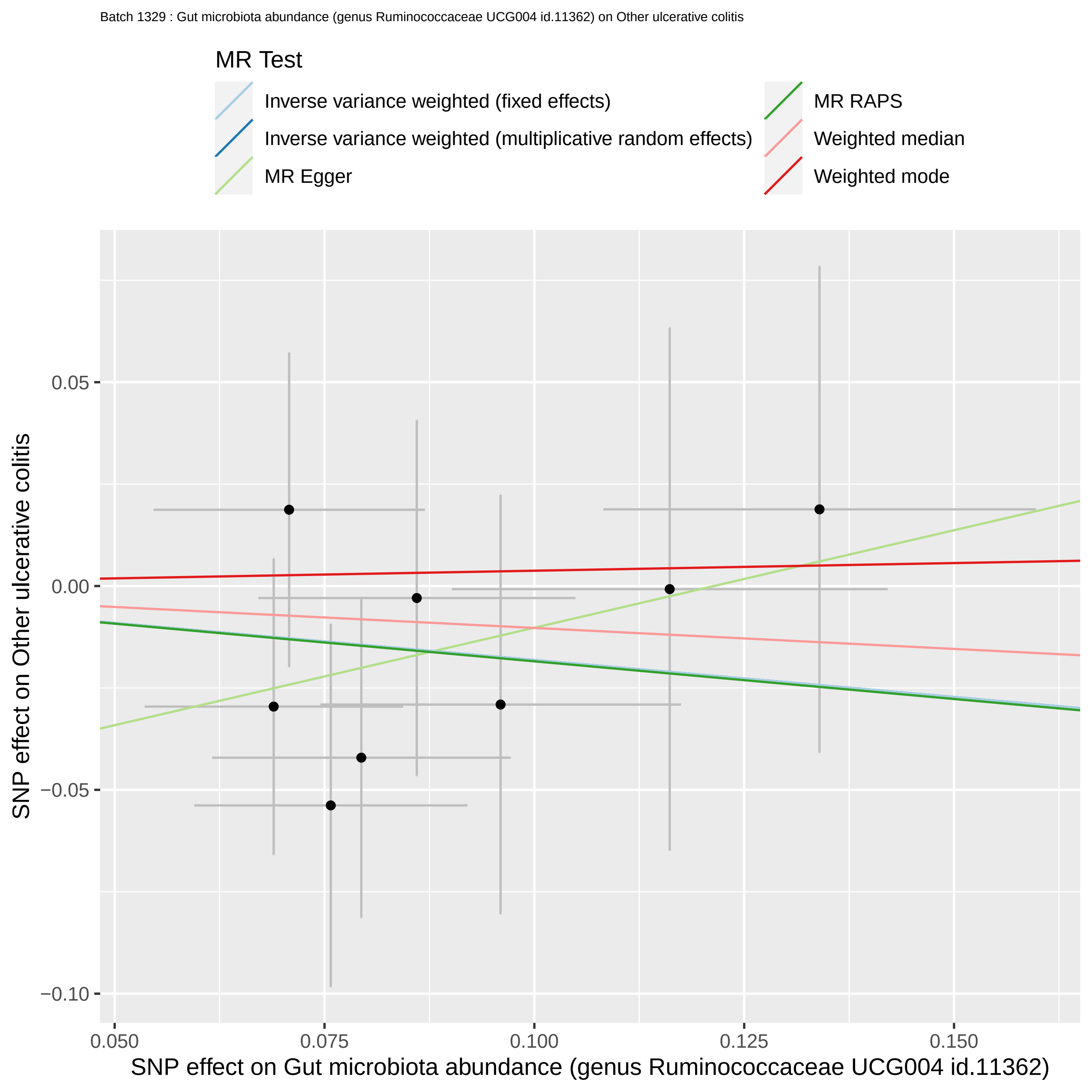
Batch 1329 : Gut microbiota abundance (genus Ruminococcaceae UCG004 id.11362) on Other ulcerative colitis
MR Test
Inverse variance weighted (fixed effects)
Inverse variance weighted (multiplicative random effects)
MR Egger
MR RAPS
Weighted median
Weighted mode
0.05
0.00
−0.05
−0.10
0.050
0.075
0.100
0.125
0.150
SNP effect on Other ulcerative colitis
SNP effect on Gut microbiota abundance (genus Ruminococcaceae UCG004 id.11362)

Batch 1330 : Gut microbiota abundance (genus Ruminococcaceae UCG005 id.11363) on Other ulcerative colitis

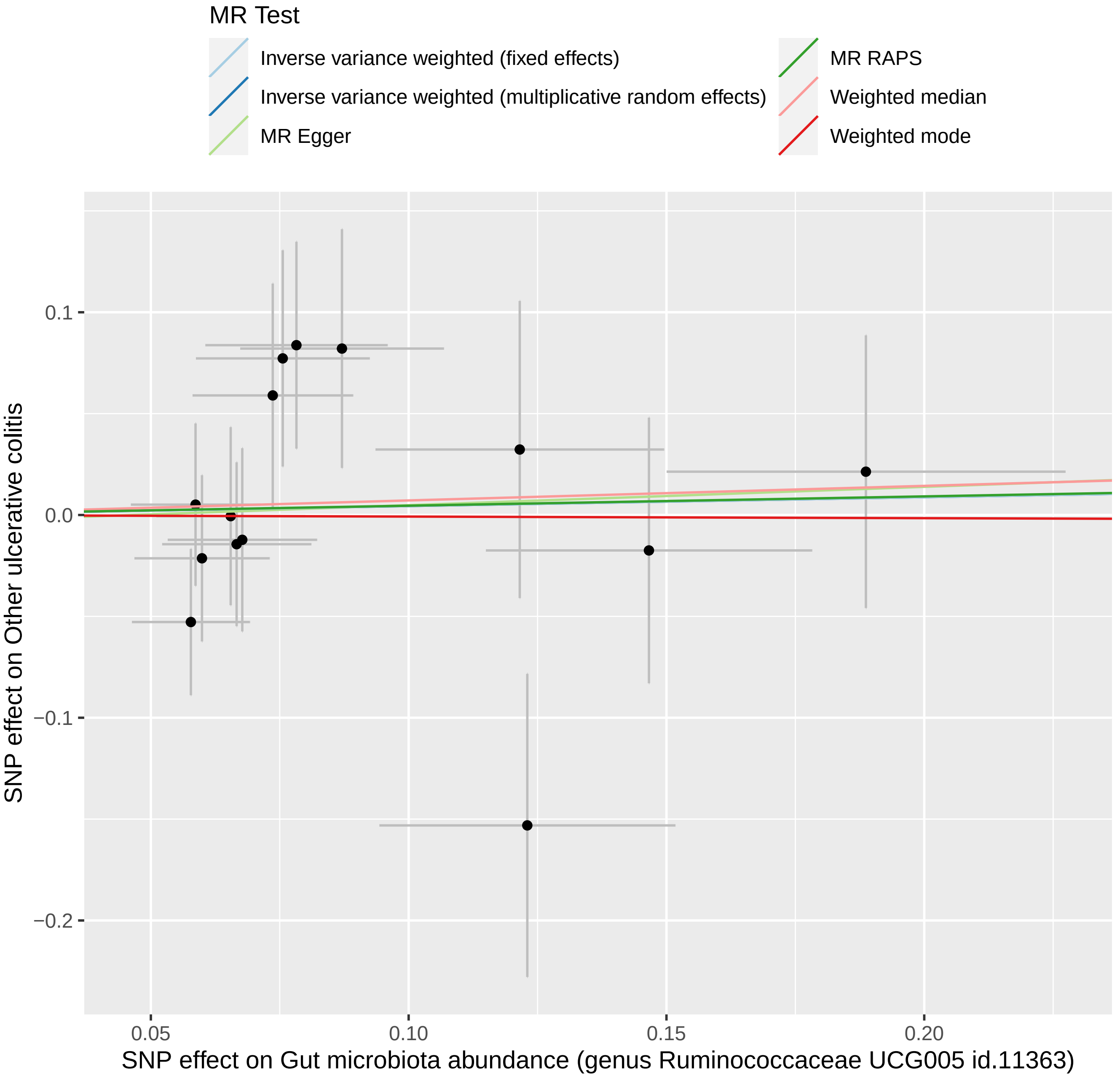

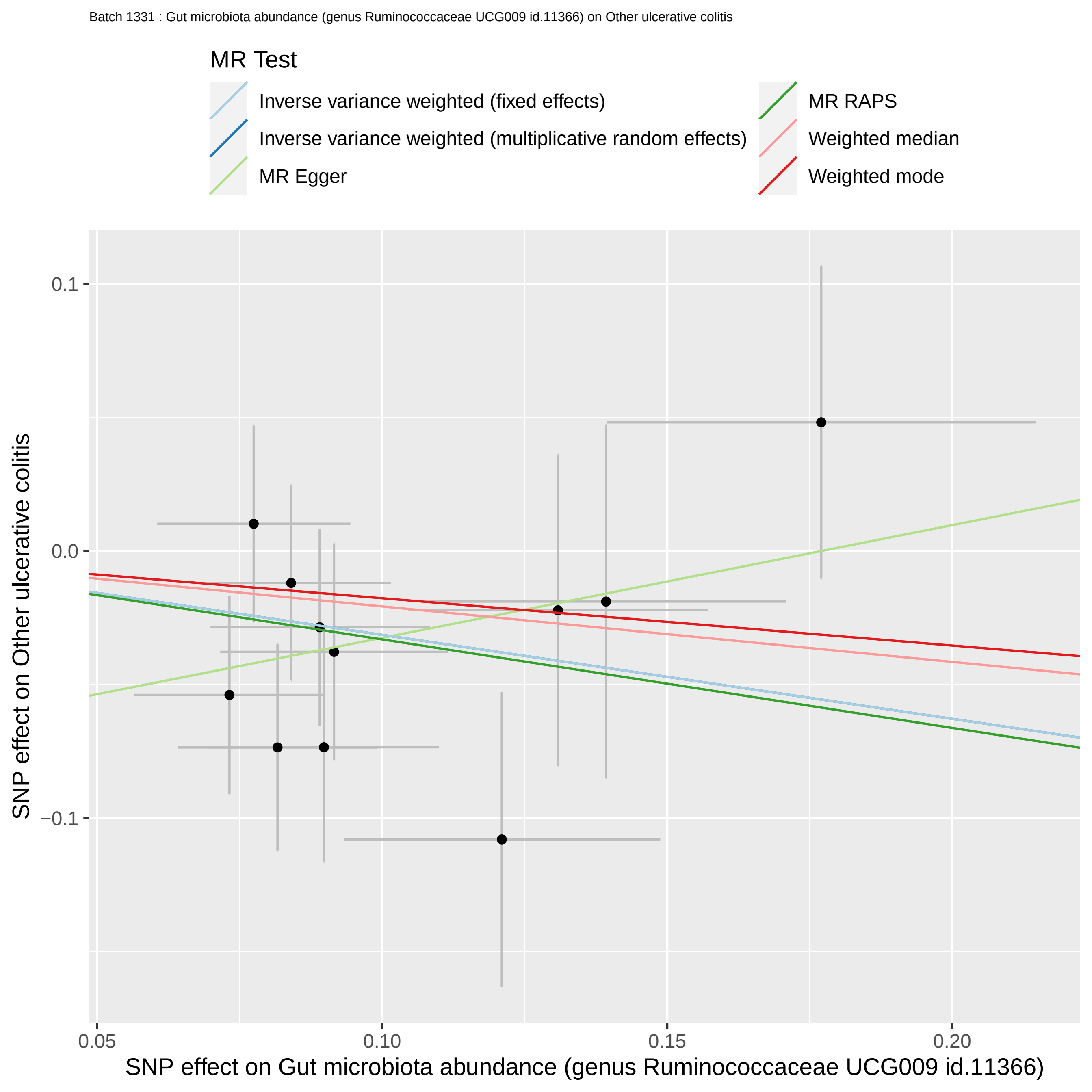

Batch 1331 : Gut microbiota abundance (genus Ruminococcaceae UCG009 id.11366) on Other ulcerative colitis
MR Test
Inverse variance weighted (fixed effects)
Inverse variance weighted (multiplicative random effects)
MR Egger
MR RAPS
Weighted median
Weighted mode
0.1
0.0
−0.1
0.05
0.10
0.15
0.20
SNP effect on Other ulcerative colitis
SNP effect on Gut microbiota abundance (genus Ruminococcaceae UCG009 id.11366)

Batch 1332 : Gut microbiota abundance (genus Ruminococcaceae UCG010 id.11367) on Other ulcerative colitis

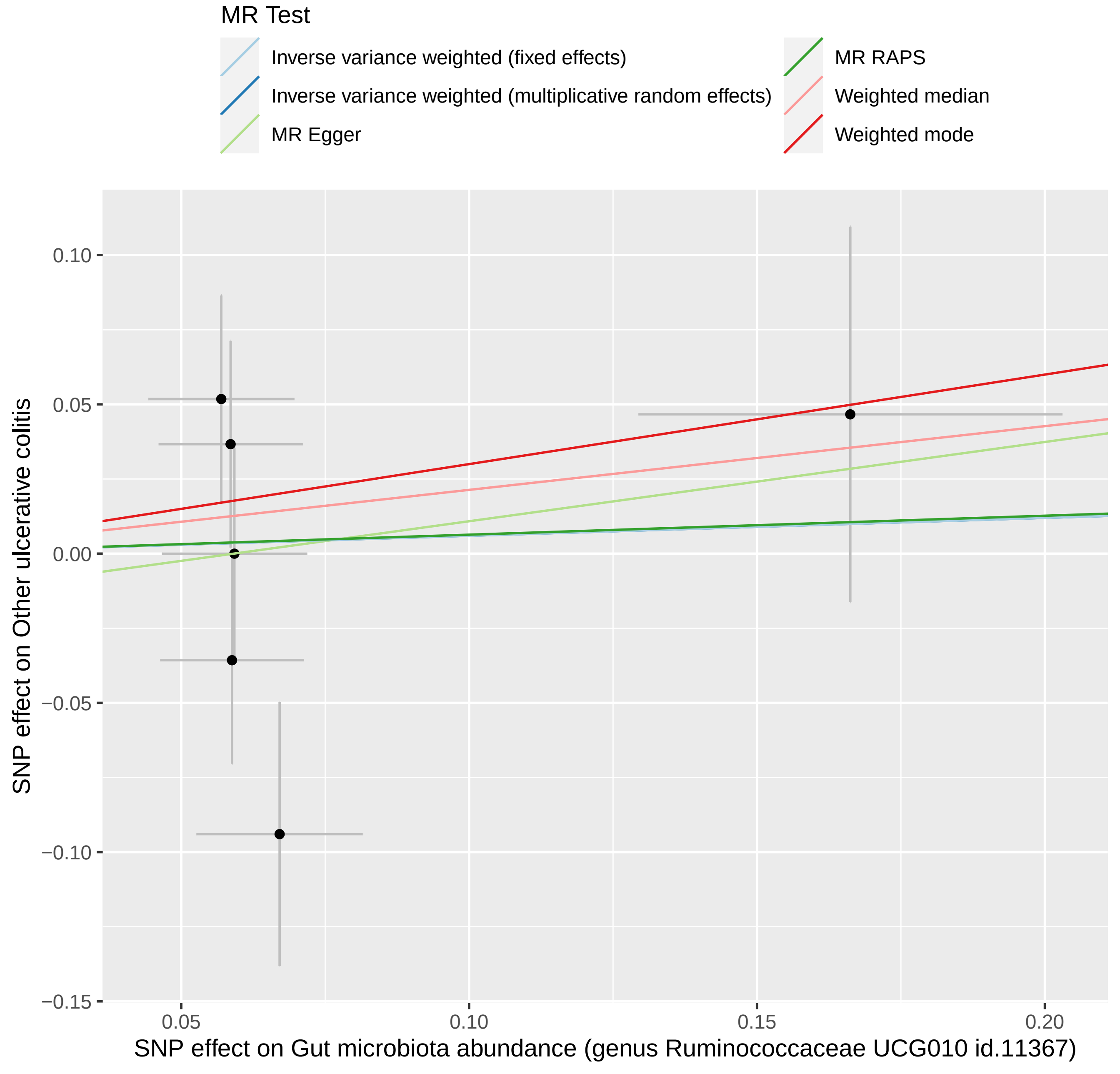

Batch 1333 : Gut microbiota abundance (genus Ruminococcaceae UCG011 id.11368) on Other ulcerative colitis

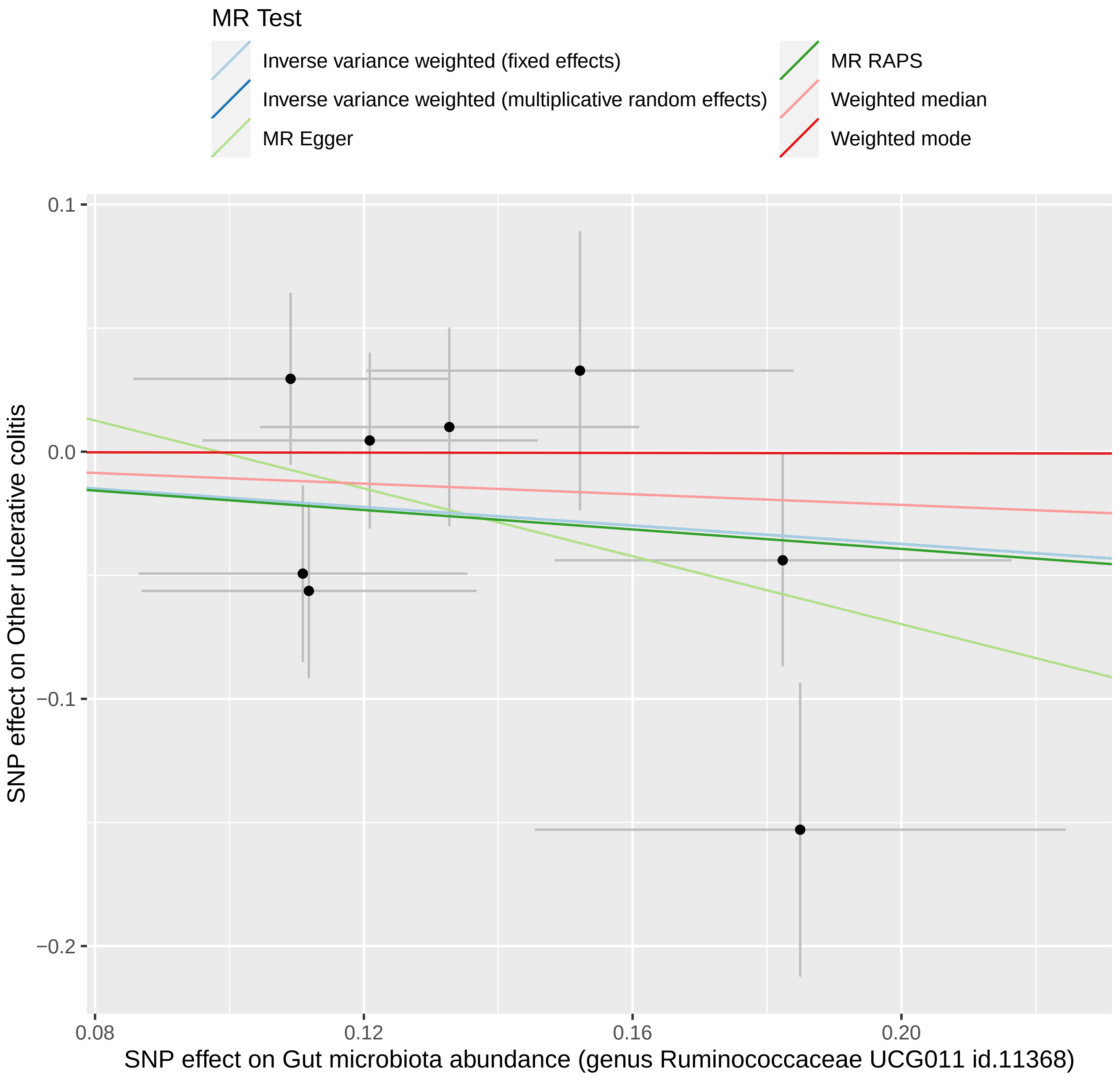

Batch 1334 : Gut microbiota abundance (genus Ruminococcaceae UCG013 id.11370) on Other ulcerative colitis

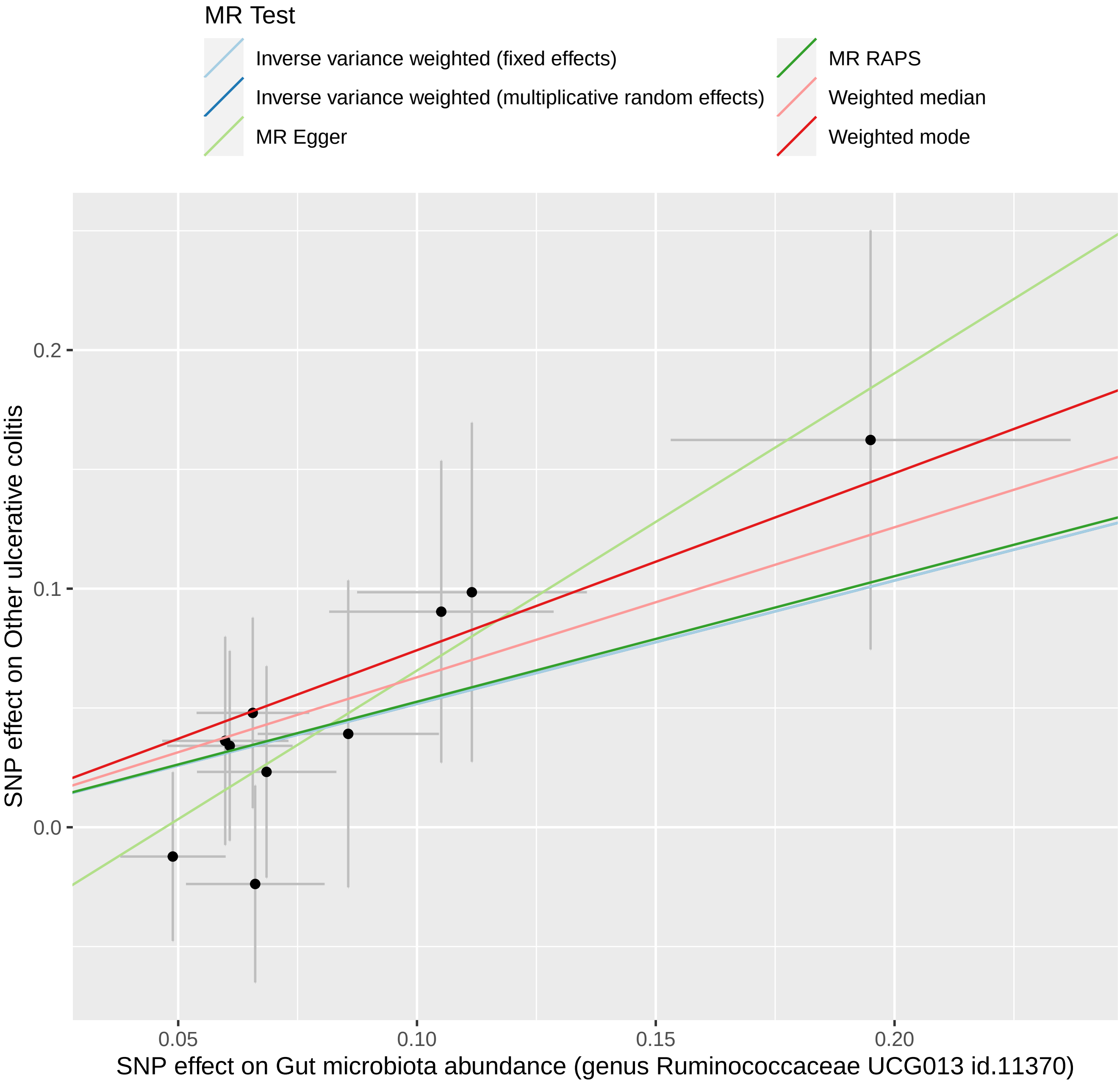

Batch 1335 : Gut microbiota abundance (genus Ruminococcaceae UCG014 id.11371) on Other ulcerative colitis

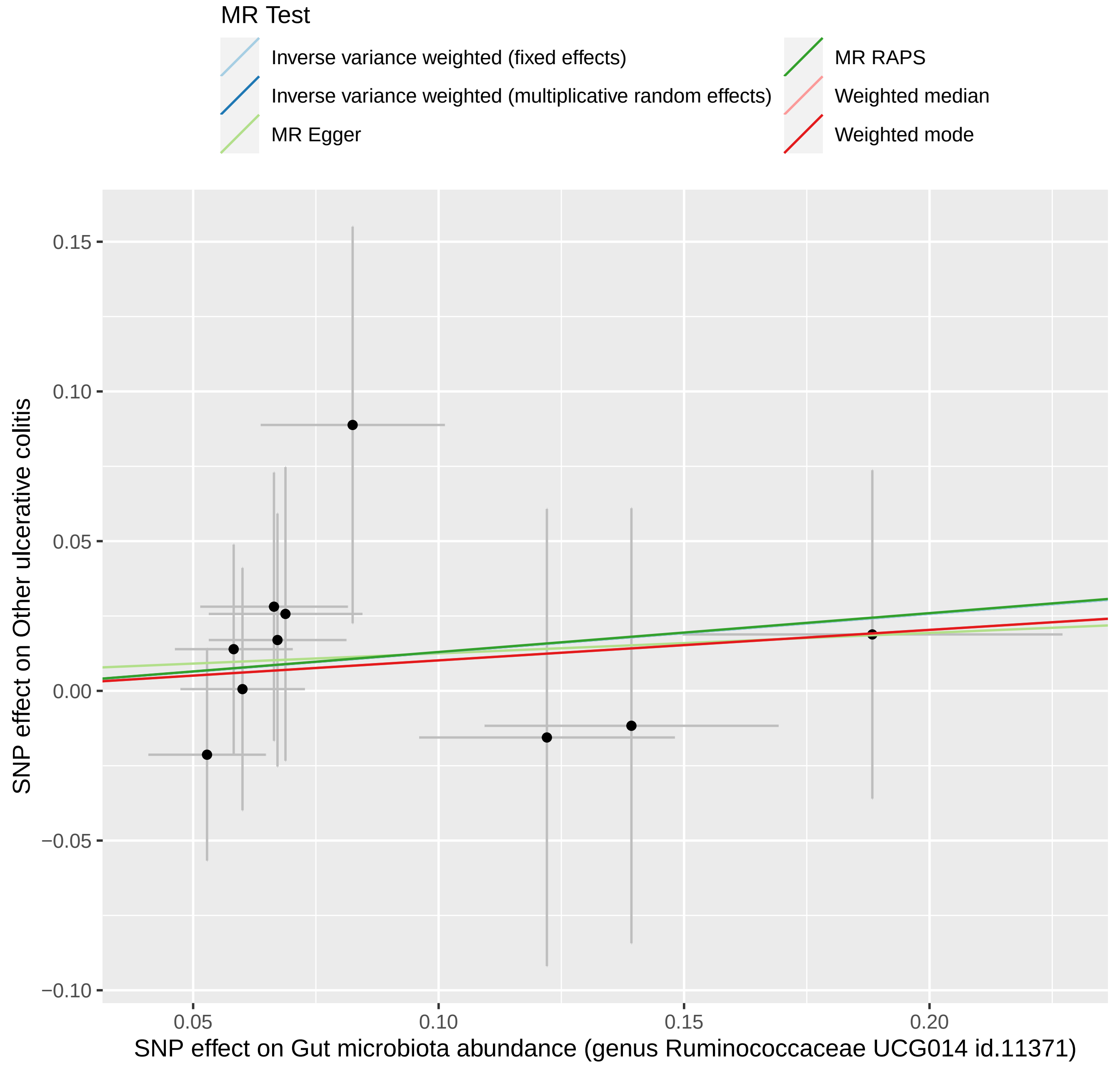

Batch 1336 : Gut microbiota abundance (genus Ruminococcus1 id.11373) on Other ulcerative colitis

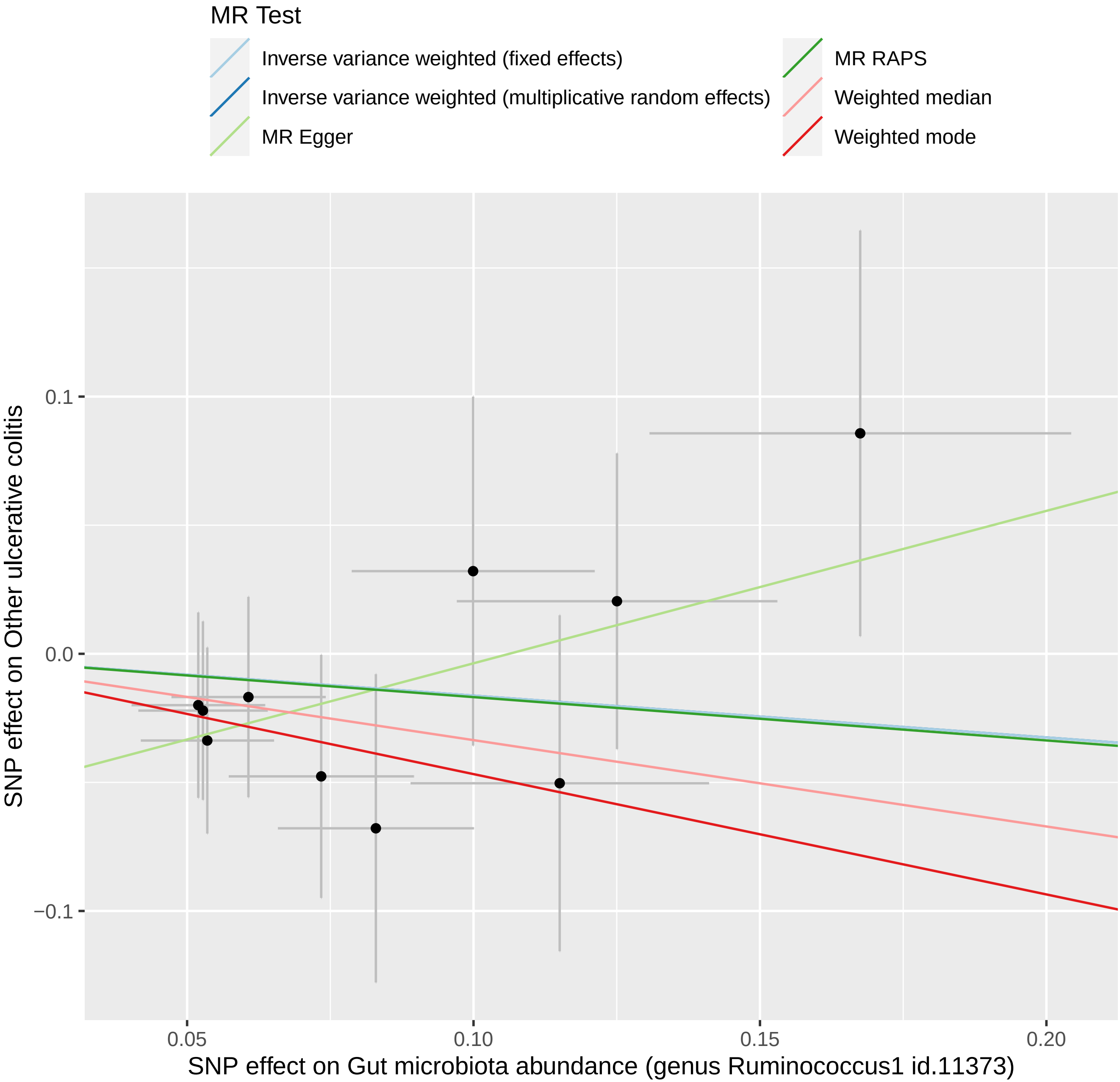

Batch 1337 : Gut microbiota abundance (genus Ruminococcus2 id.11374) on Other ulcerative colitis

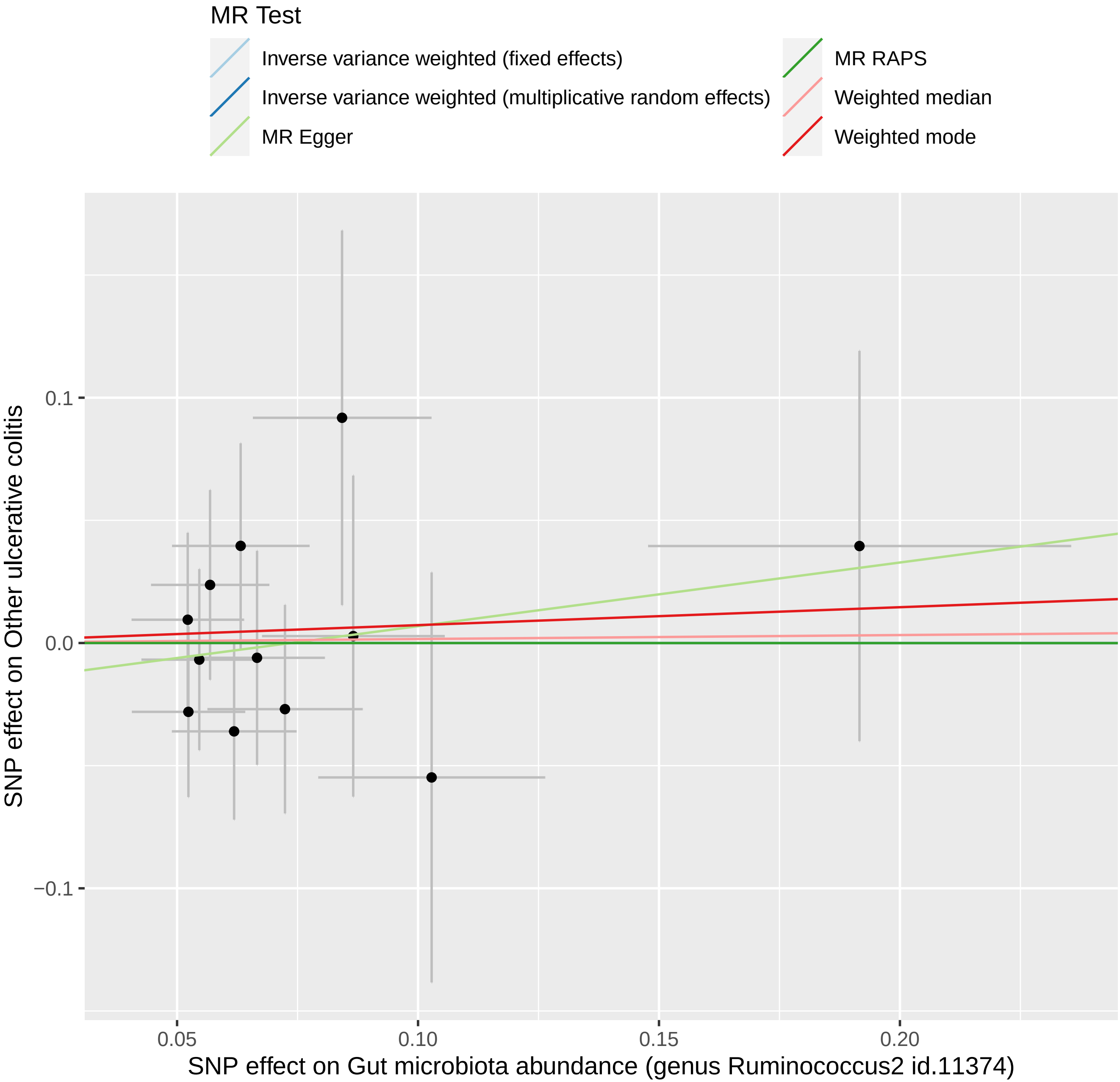

Batch 1338 : Gut microbiota abundance (genus Ruminococcus gauvreauii group id.11342) on Other ulcerative colitis

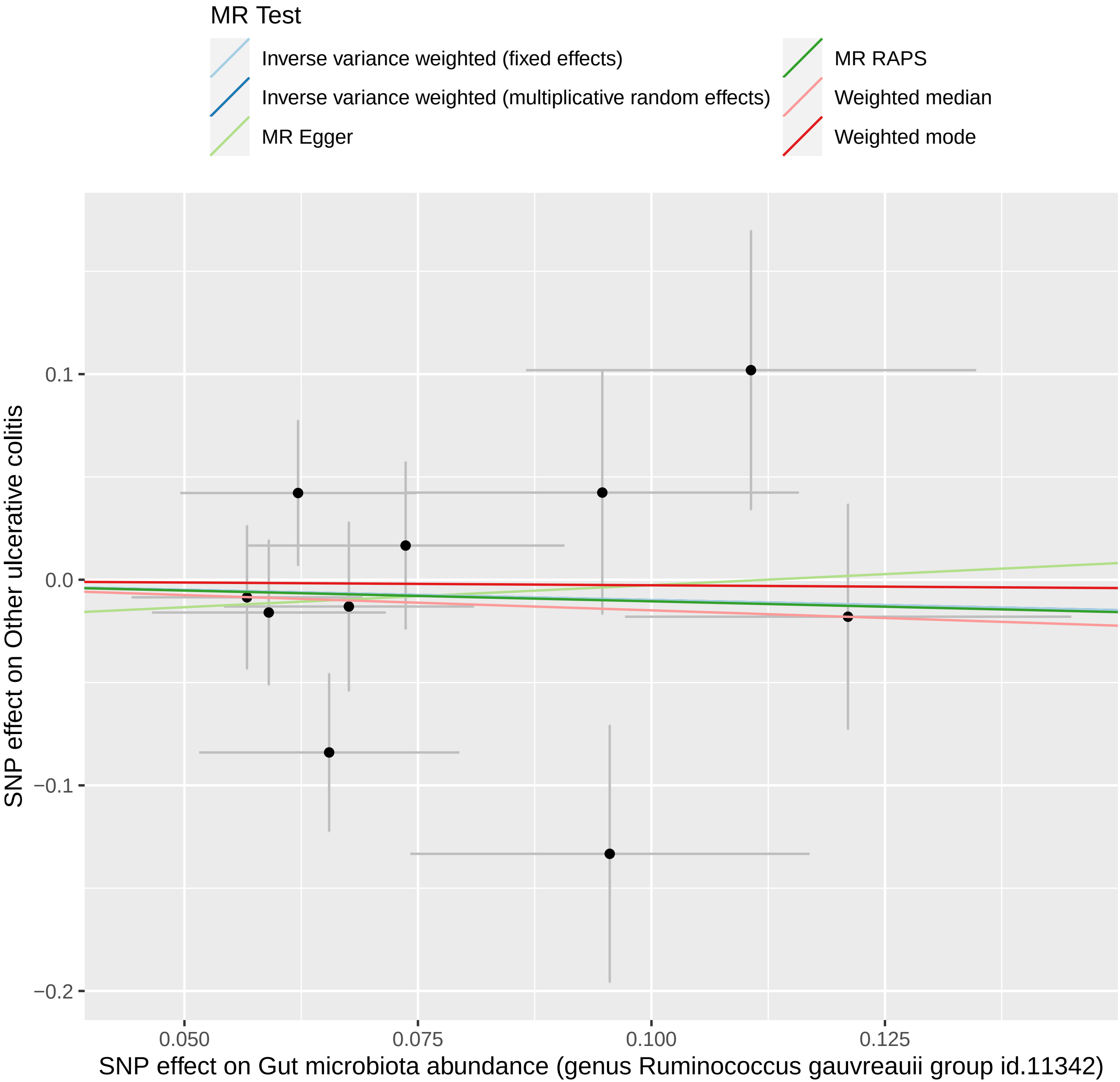

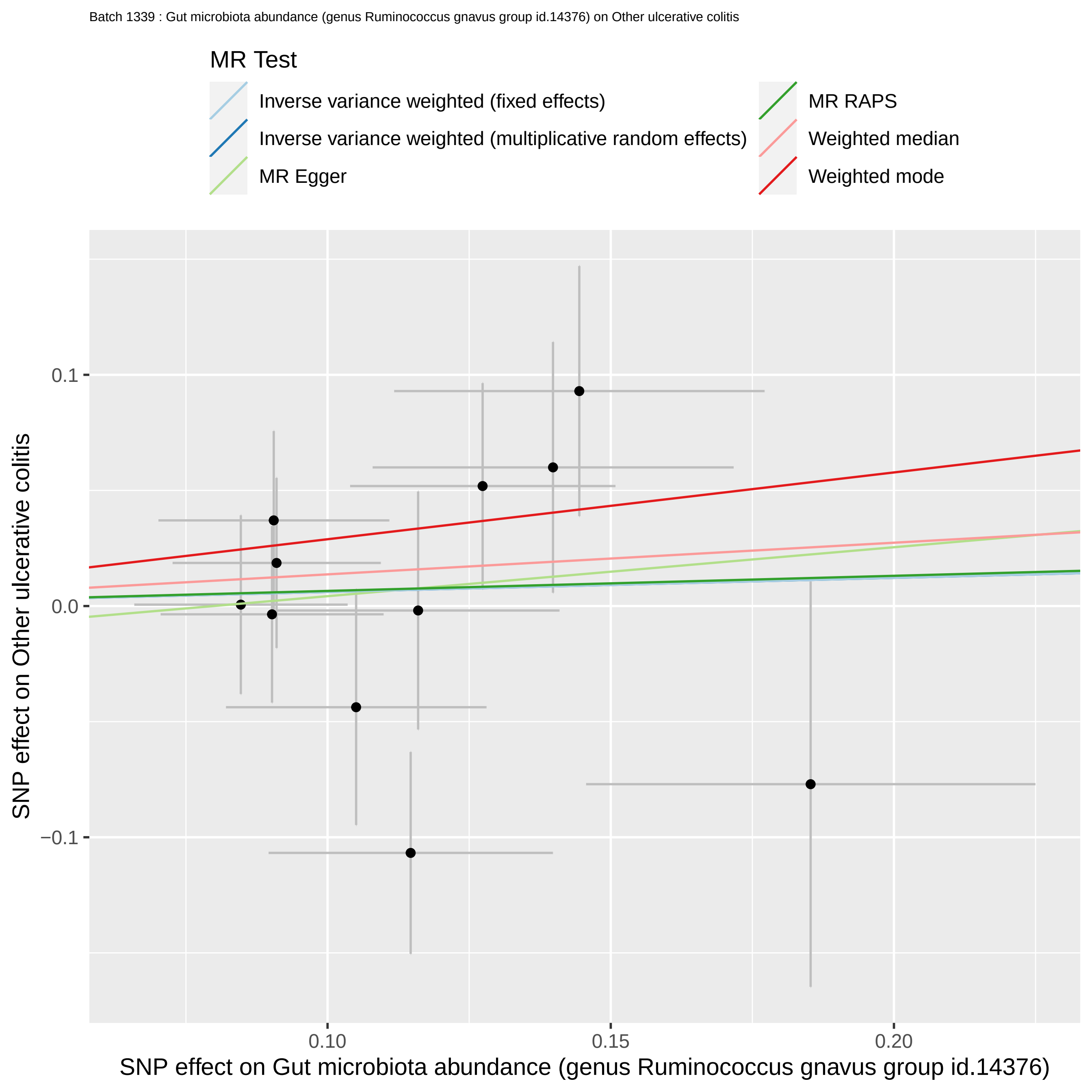

Batch 1339 : Gut microbiota abundance (genus Ruminococcus gnavus group id.14376) on Other ulcerative colitis
MR Test
Inverse variance weighted (fixed effects)
Inverse variance weighted (multiplicative random effects)
MR Egger
MR RAPS
Weighted median
Weighted mode
0.1
0.0
−0.1
0.10
0.15
0.20
SNP effect on Other ulcerative colitis
SNP effect on Gut microbiota abundance (genus Ruminococcus gnavus group id.14376)

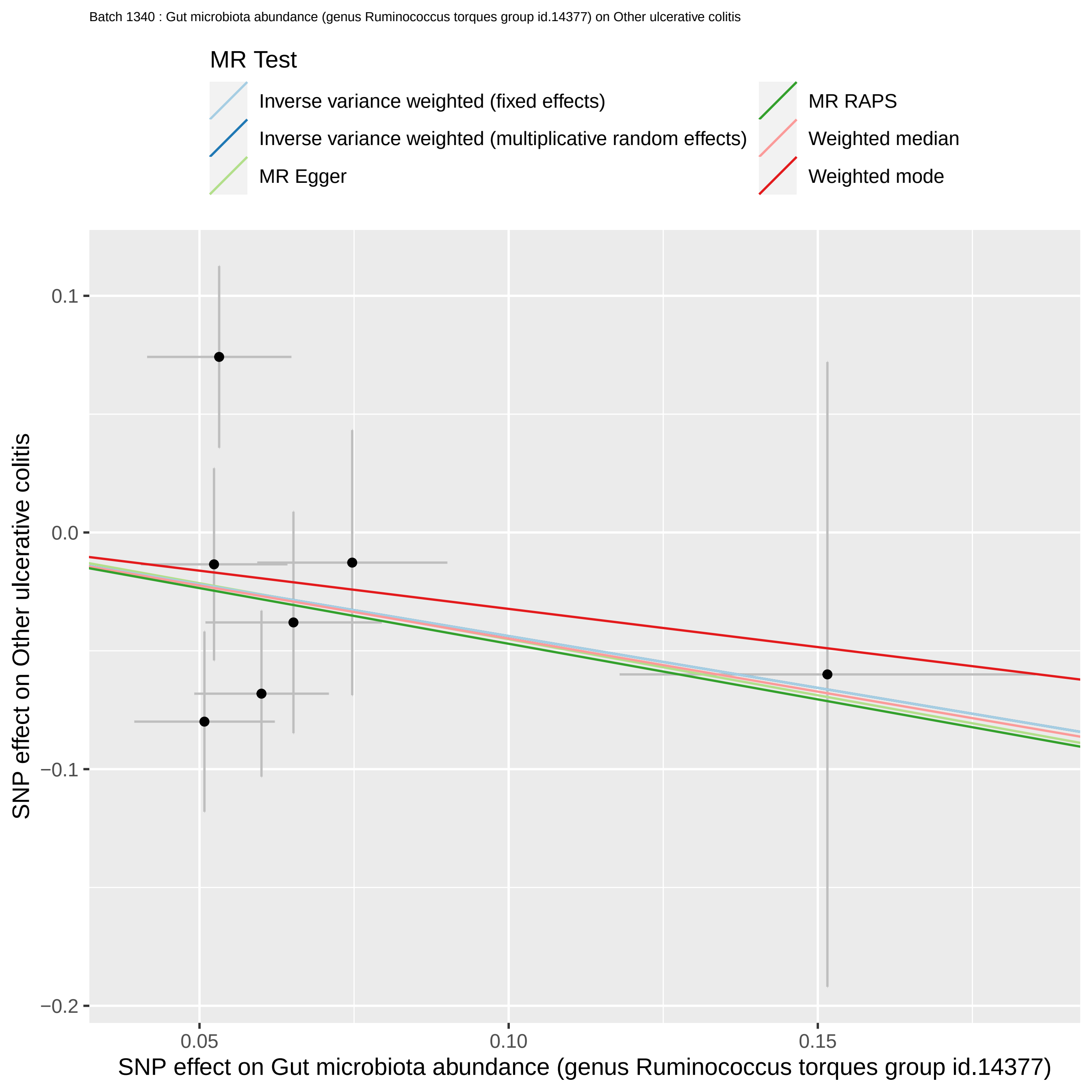

Batch 1340 : Gut microbiota abundance (genus Ruminococcus torques group id.14377) on Other ulcerative colitis
MR Test
Inverse variance weighted (fixed effects)
Inverse variance weighted (multiplicative random effects)
MR Egger
MR RAPS
Weighted median
Weighted mode
0.1
0.0
−0.1
−0.2
0.05
0.10
0.15
SNP effect on Other ulcerative colitis
SNP effect on Gut microbiota abundance (genus Ruminococcus torques group id.14377)

Batch 1341 : Gut microbiota abundance (genus Sellimonas id.14369) on Other ulcerative colitis

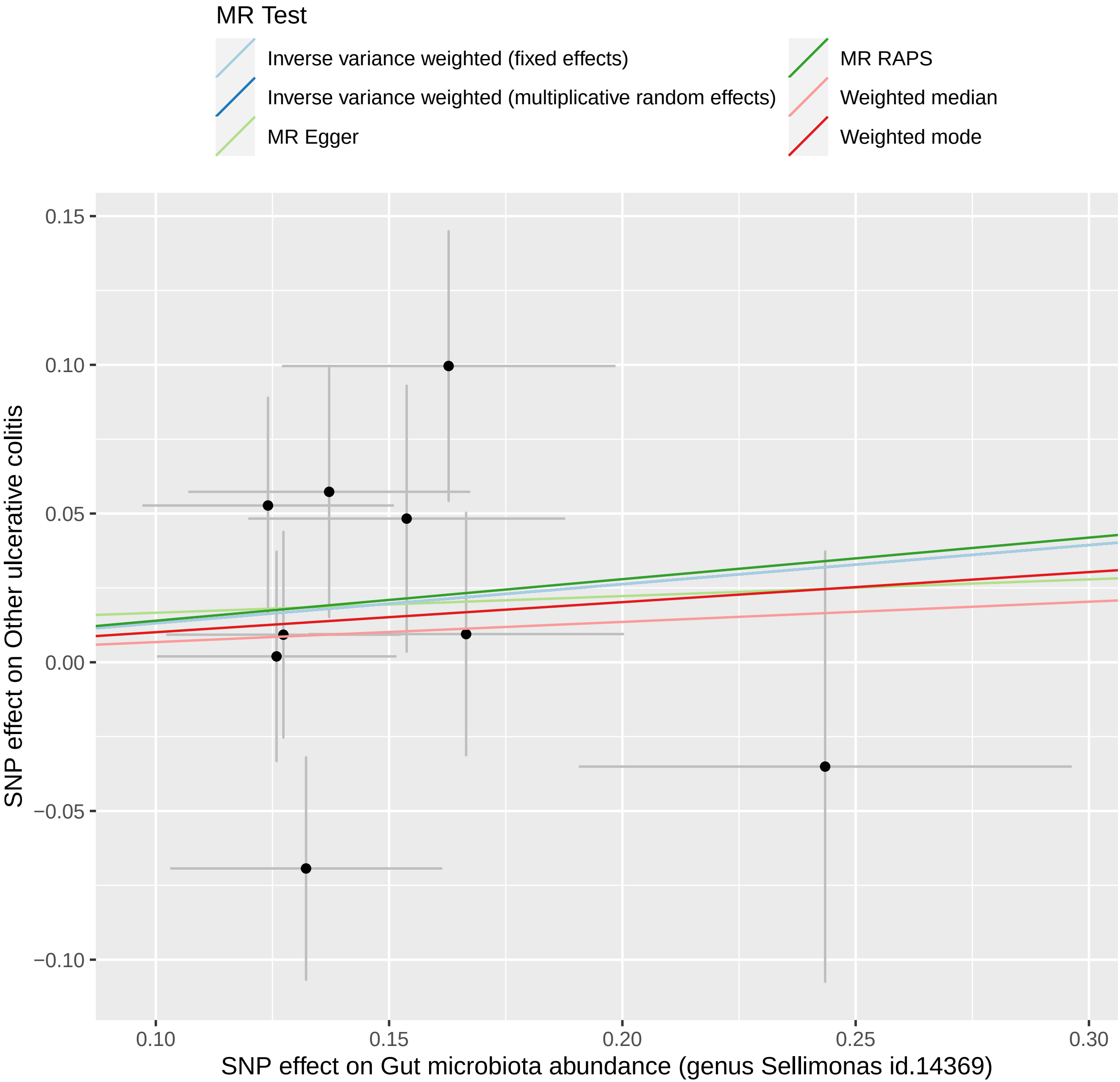

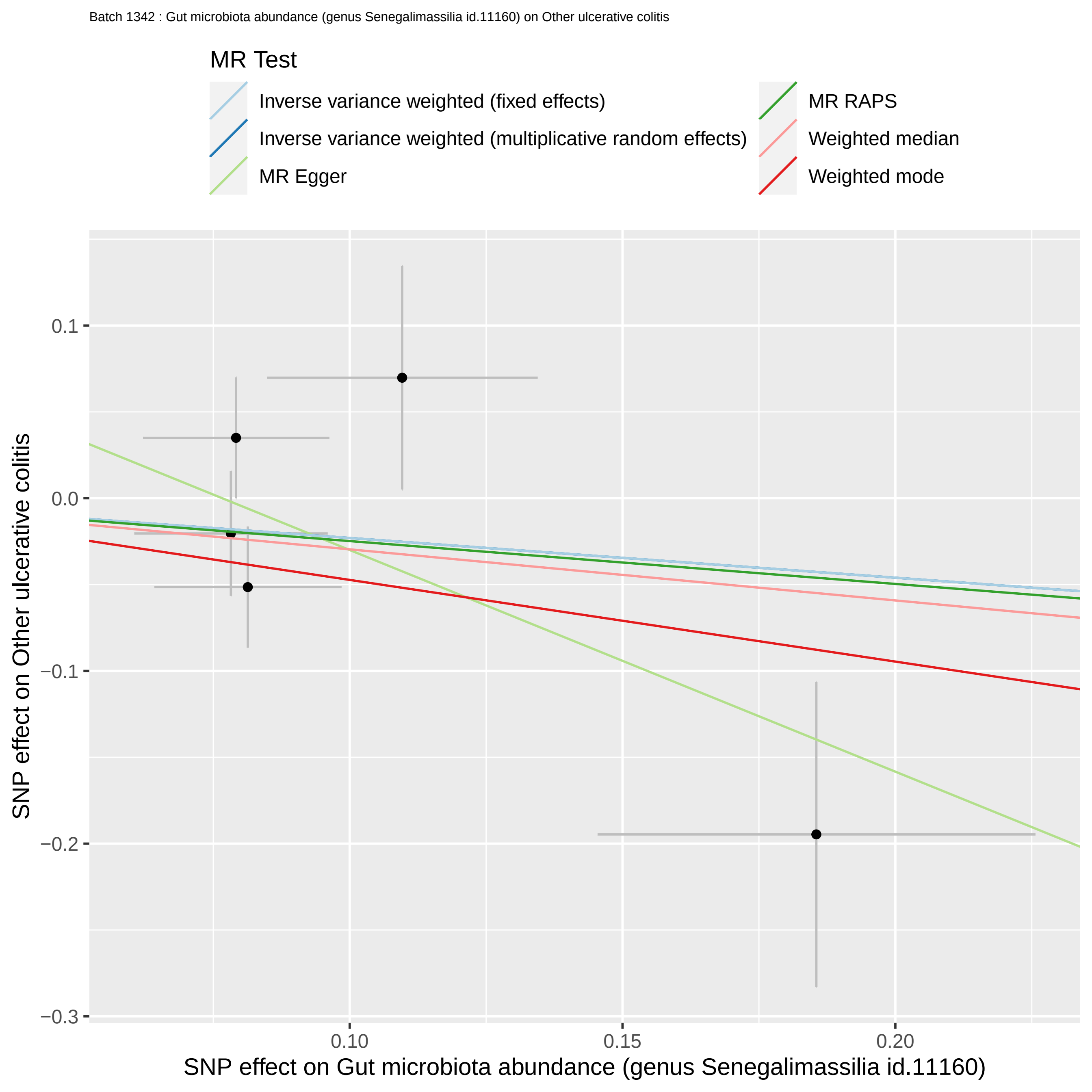
Batch 1342 : Gut microbiota abundance (genus Senegalimassilia id.11160) on Other ulcerative colitis
MR Test
Inverse variance weighted (fixed effects)
Inverse variance weighted (multiplicative random effects)
MR Egger
MR RAPS
Weighted median
Weighted mode
0.1
0.0
−0.1
−0.2
−0.3
0.10
0.15
0.20
SNP effect on Other ulcerative colitis
SNP effect on Gut microbiota abundance (genus Senegalimassilia id.11160)

Batch 1343 : Gut microbiota abundance (genus Slackia id.825) on Other ulcerative colitis

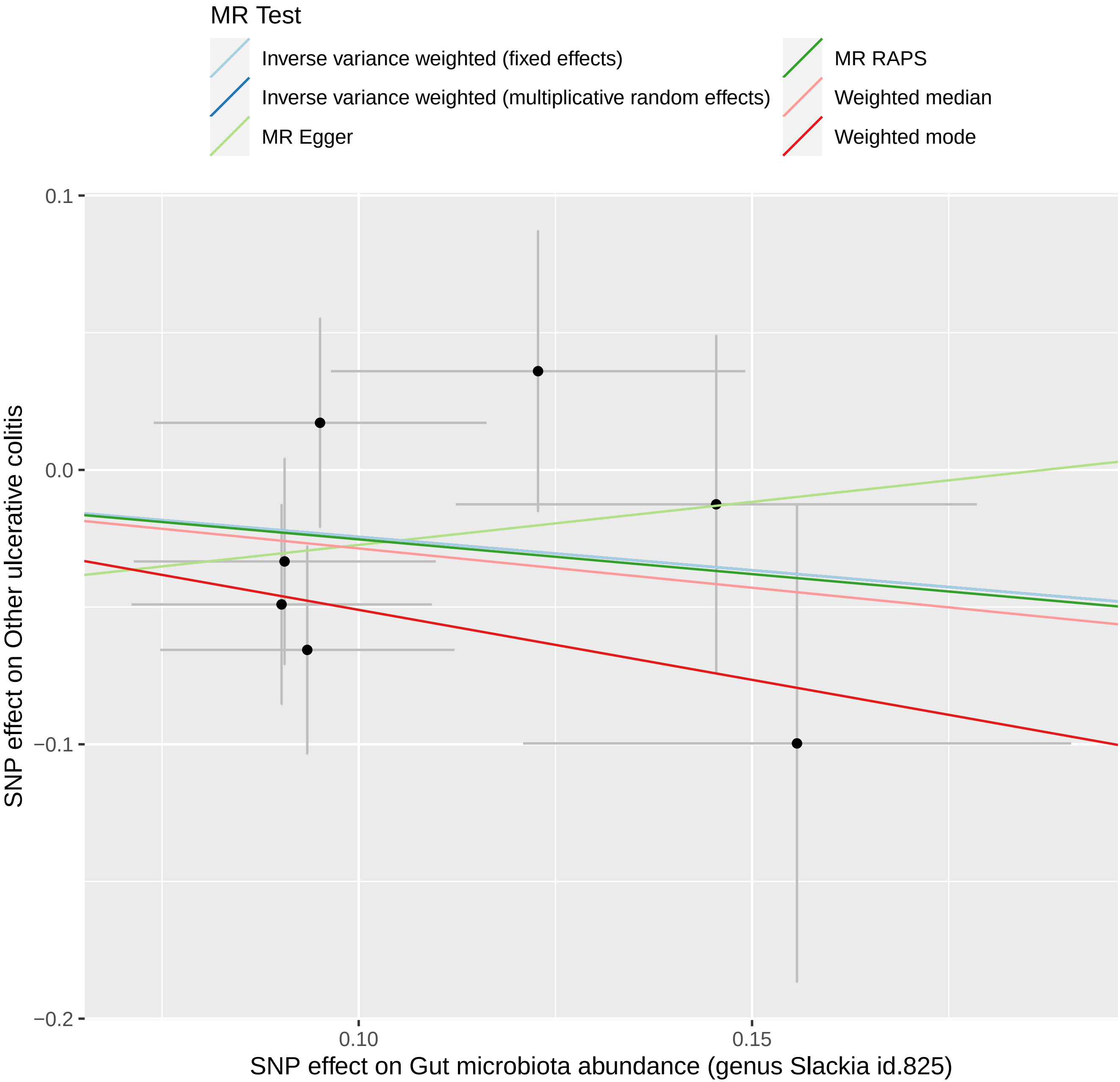

Batch 1344 : Gut microbiota abundance (genus Streptococcus id.1853) on Other ulcerative colitis

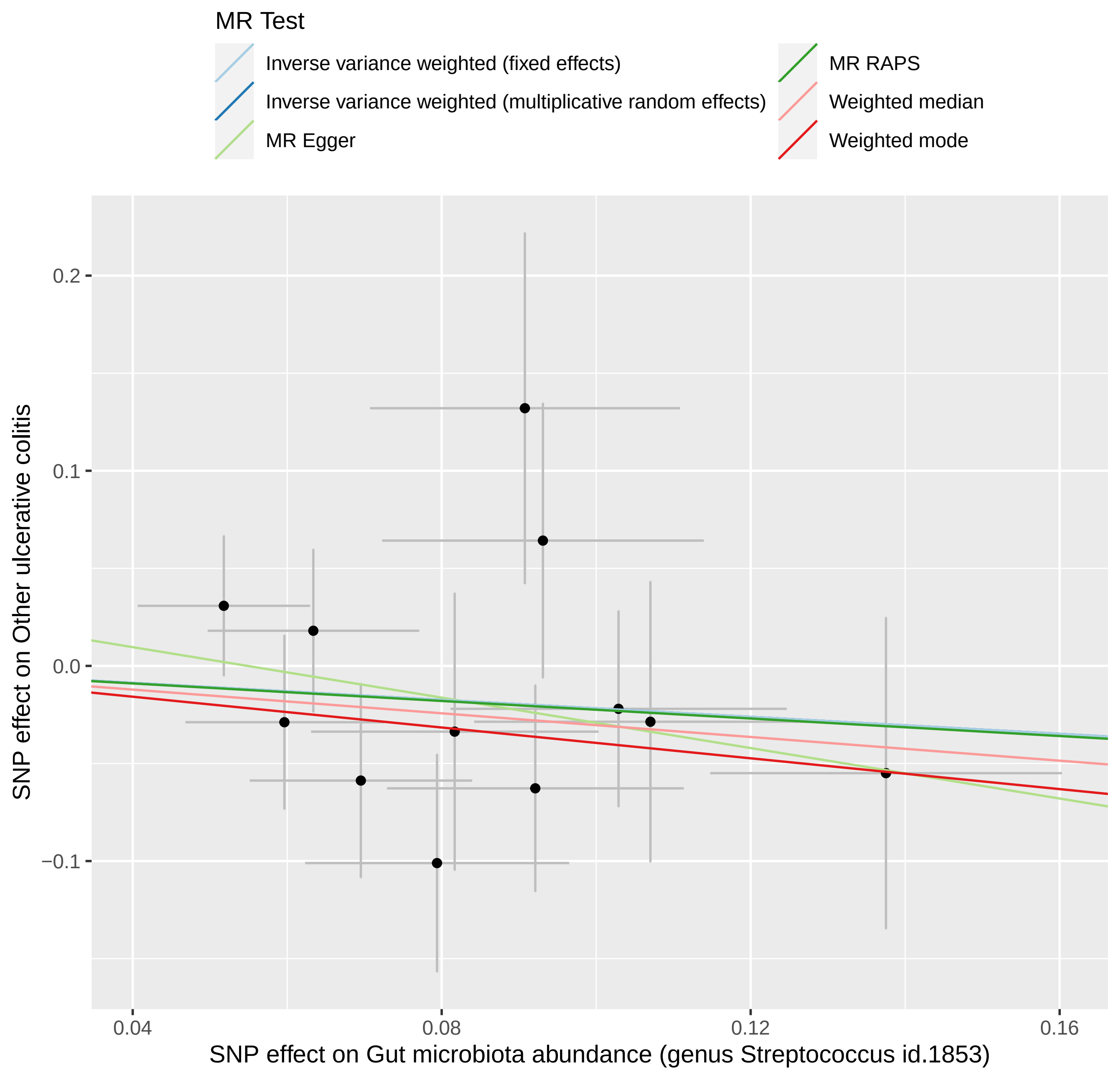

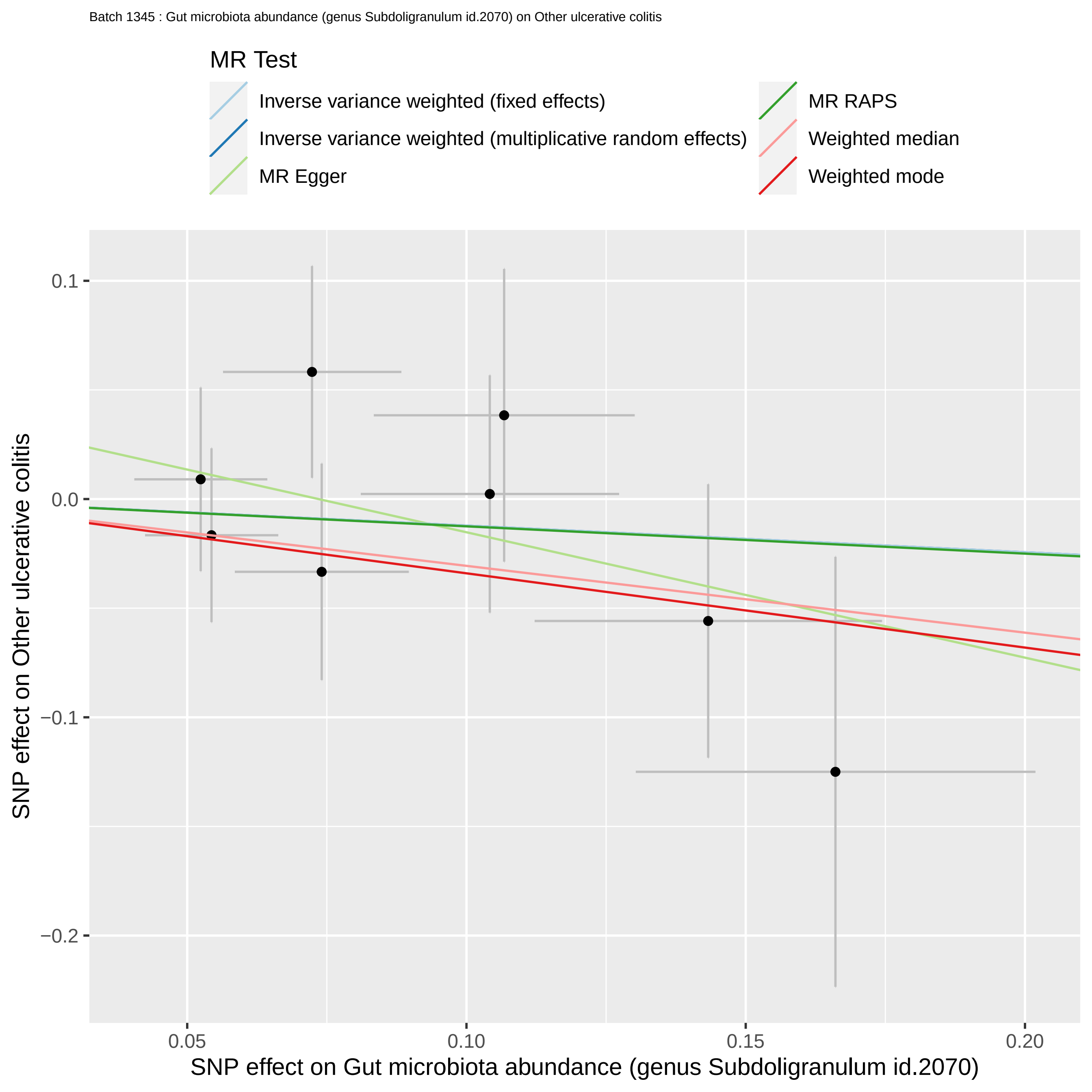

Batch 1345 : Gut microbiota abundance (genus Subdoligranulum id.2070) on Other ulcerative colitis
MR Test
Inverse variance weighted (fixed effects)
Inverse variance weighted (multiplicative random effects)
MR Egger
MR RAPS
Weighted median
Weighted mode
0.1
0.0
−0.1
−0.2
0.05
0.10
0.15
0.20
SNP effect on Other ulcerative colitis
SNP effect on Gut microbiota abundance (genus Subdoligranulum id.2070)

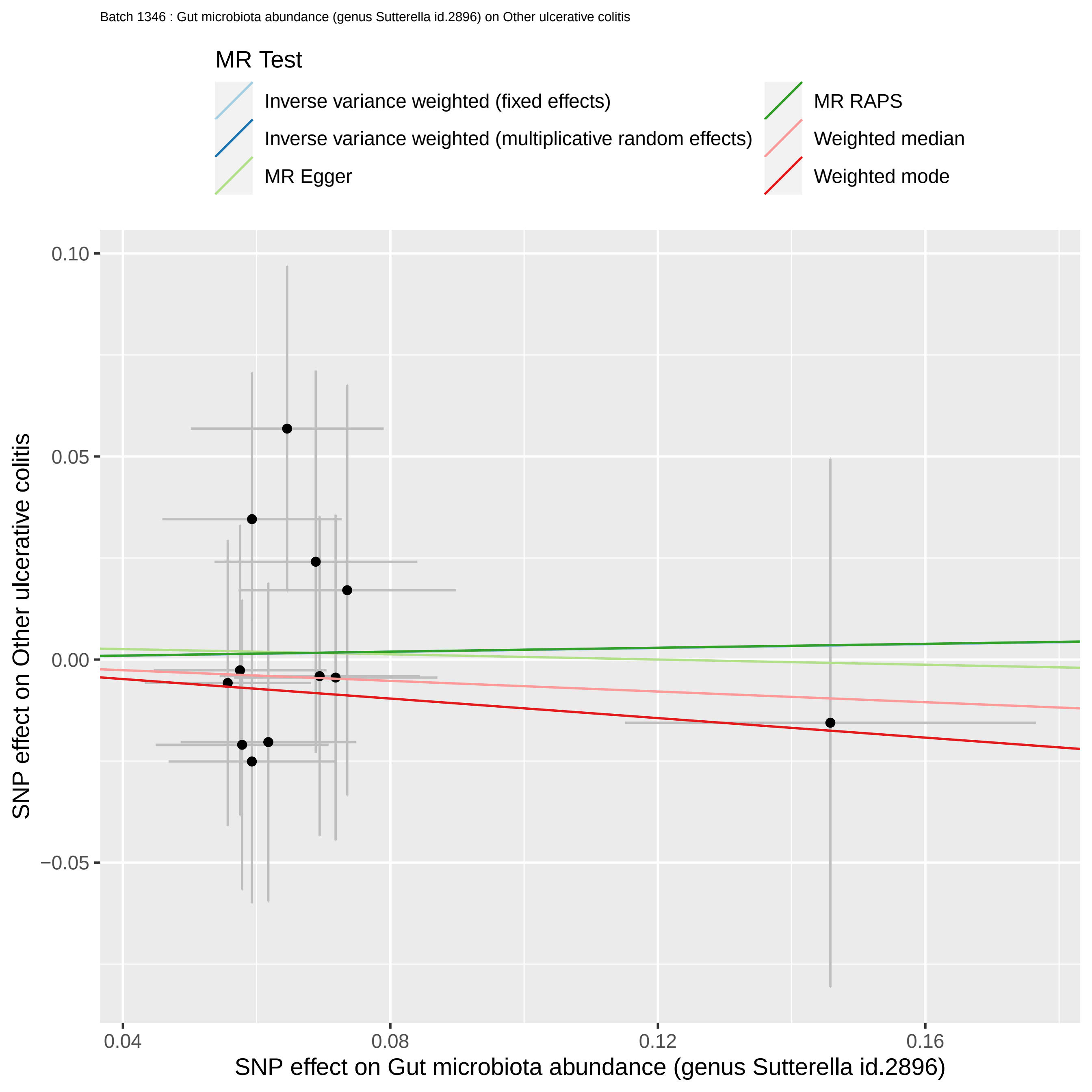

Batch 1346 : Gut microbiota abundance (genus Sutterella id.2896) on Other ulcerative colitis
MR Test
Inverse variance weighted (fixed effects)
Inverse variance weighted (multiplicative random effects)
MR Egger
MR RAPS
Weighted median
Weighted mode
0.10
0.05
0.00
−0.05
0.04
0.08
0.12
0.16
SNP effect on Other ulcerative colitis
SNP effect on Gut microbiota abundance (genus Sutterella id.2896)

Batch 1347 : Gut microbiota abundance (genus Terrisporobacter id.11348) on Other ulcerative colitis

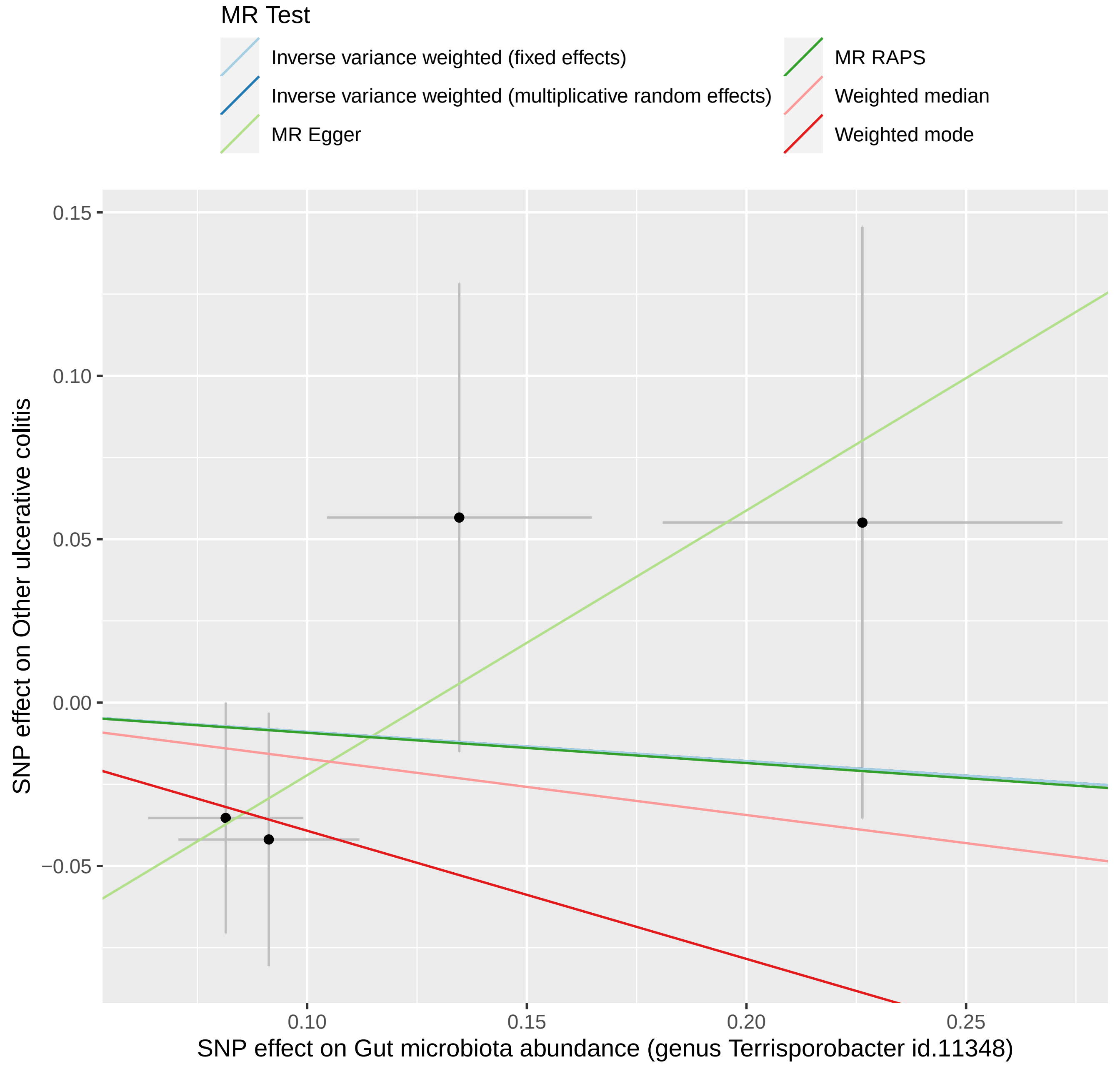

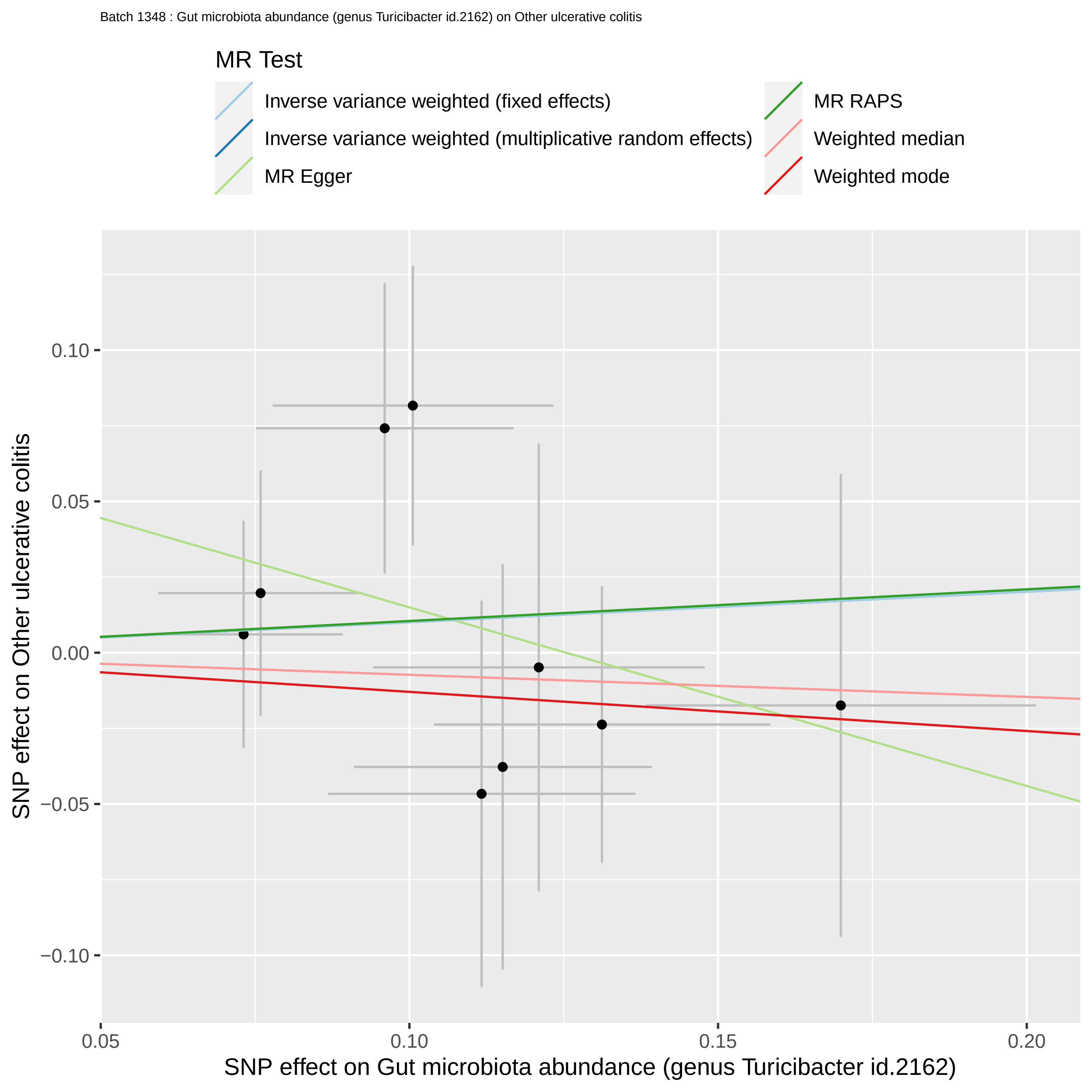
Batch 1348 : Gut microbiota abundance (genus Turicibacter id.2162) on Other ulcerative colitis
MR Test
Inverse variance weighted (fixed effects)
Inverse variance weighted (multiplicative random effects)
MR Egger
MR RAPS
Weighted median
Weighted mode
SNP effect on Other ulcerative colitis
0.10
0.05
0.00
−0.05
−0.10
0.05
0.10
0.15
0.20
SNP effect on Gut microbiota abundance (genus Turicibacter id.2162)

Batch 1349 : Gut microbiota abundance (genus Tyzzerella3 id.11335) on Other ulcerative colitis

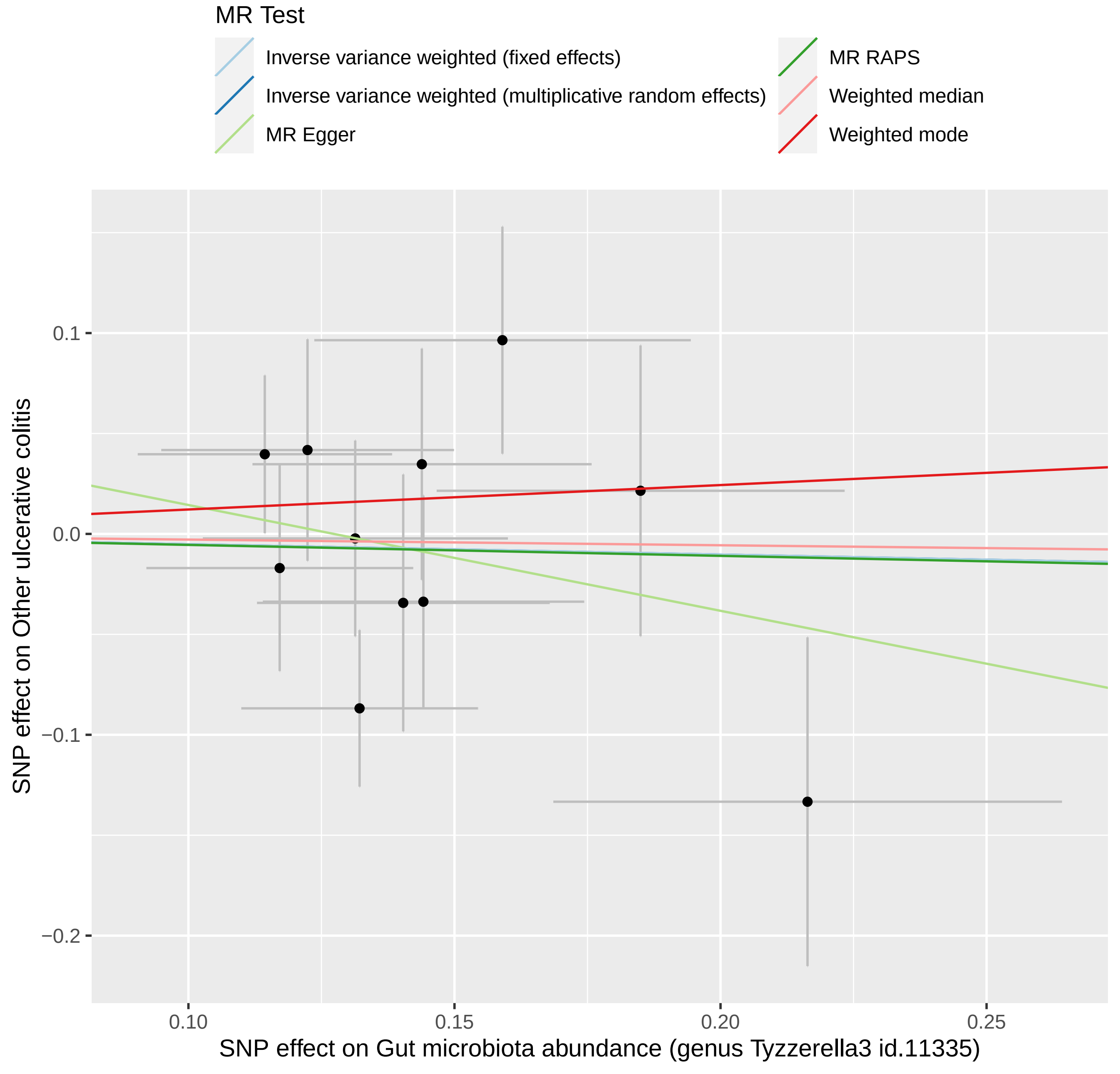

Batch 1350 : Gut microbiota abundance (genus Veillonella id.2198) on Other ulcerative colitis

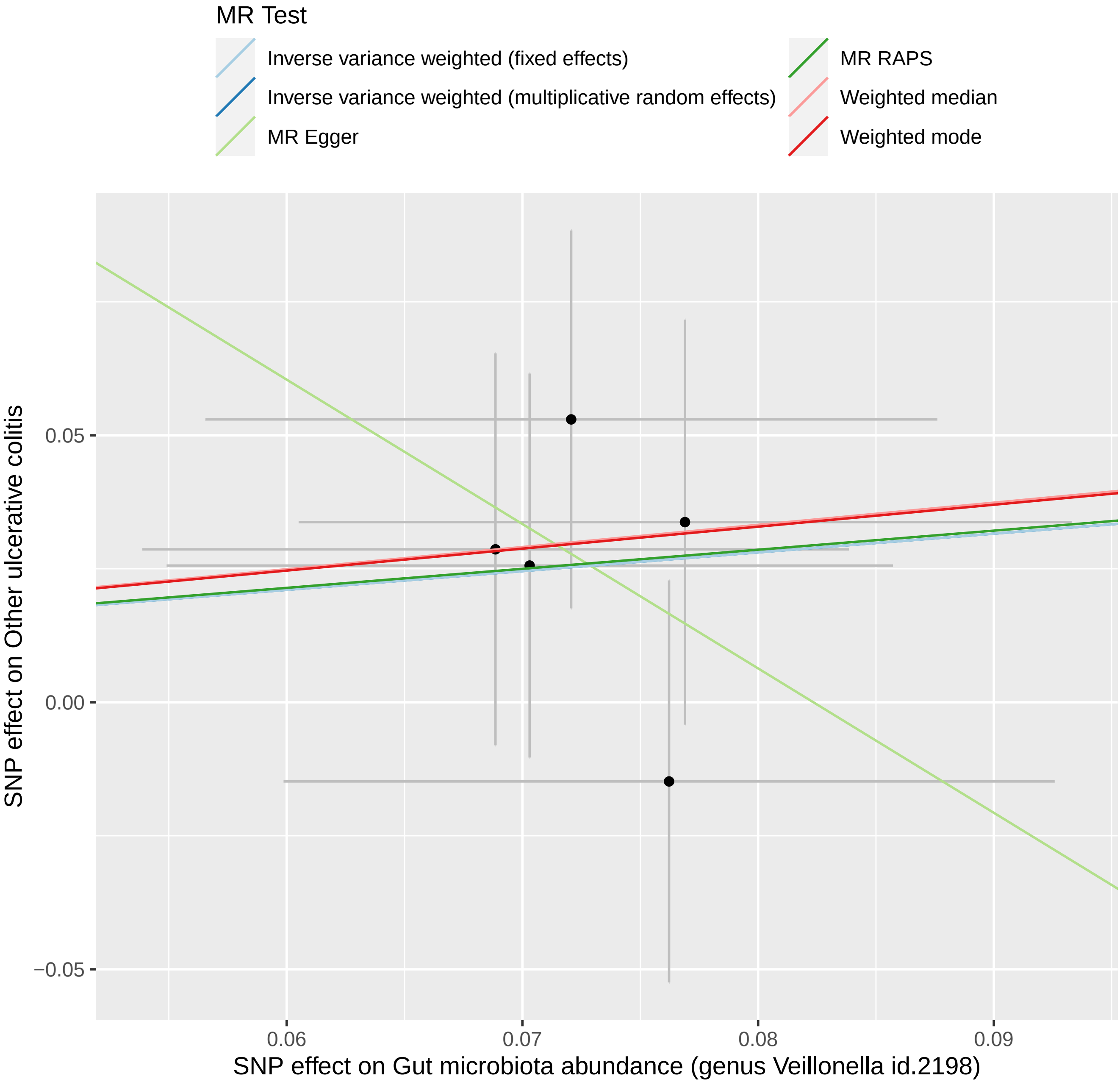

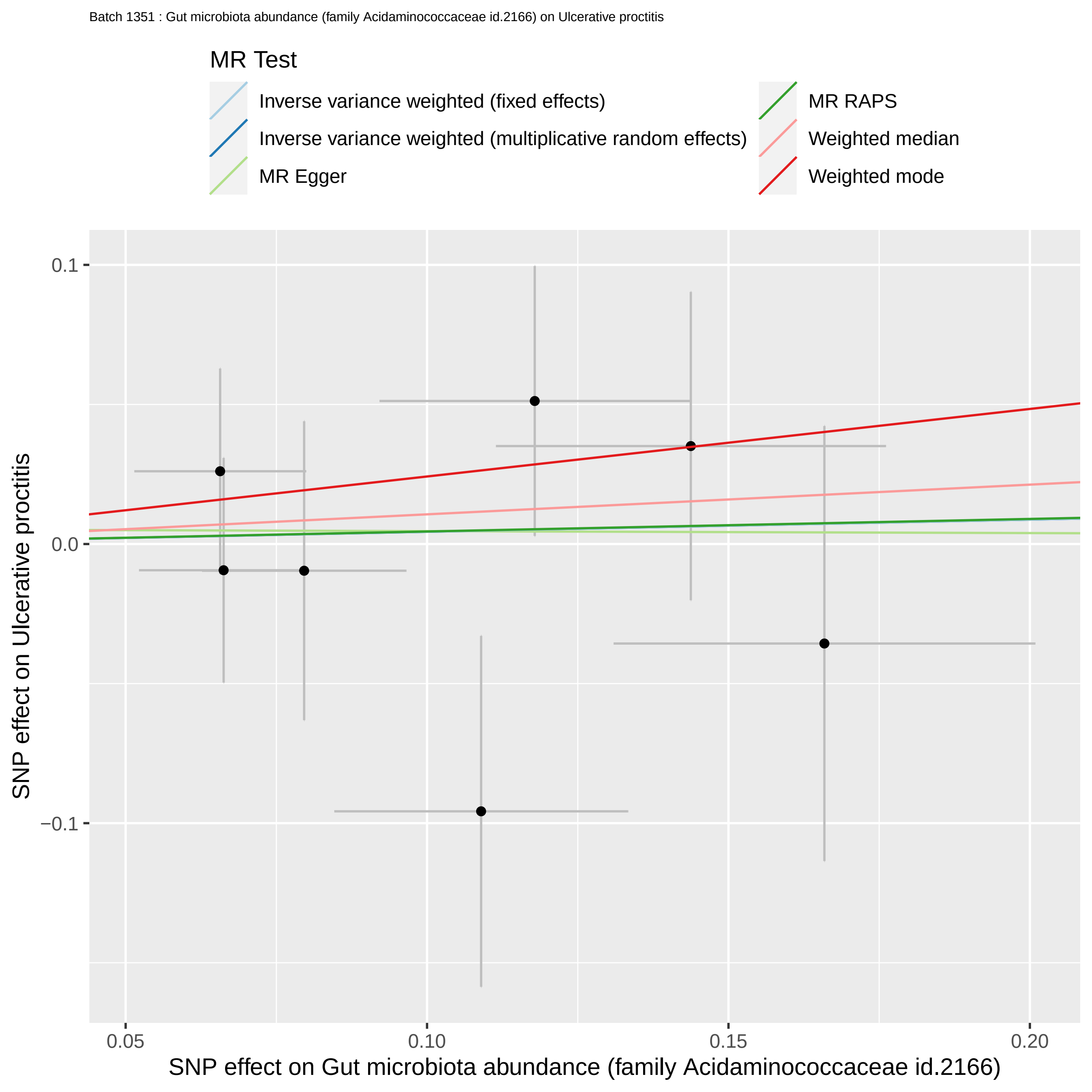
Batch 1351 : Gut microbiota abundance (family Acidaminococcaceae id.2166) on Ulcerative proctitis
MR Test
Inverse variance weighted (fixed effects)
Inverse variance weighted (multiplicative random effects)
MR Egger
MR RAPS
Weighted median
Weighted mode
0.1
0.0
−0.1
0.05
0.10
0.15
0.20
SNP effect on Ulcerative proctitis
SNP effect on Gut microbiota abundance (family Acidaminococcaceae id.2166)

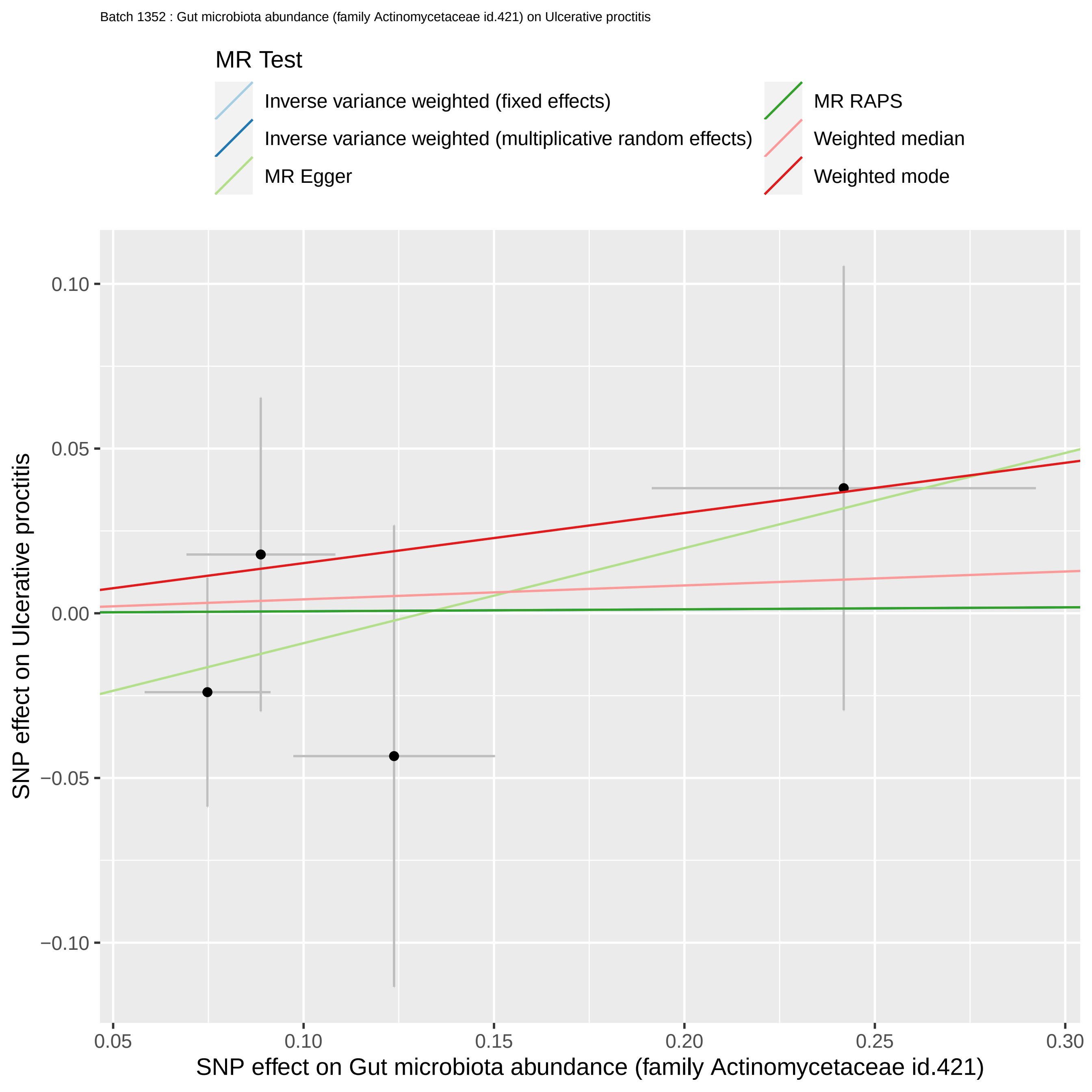
Batch 1352 : Gut microbiota abundance (family Actinomycetaceae id.421) on Ulcerative proctitis
MR Test
Inverse variance weighted (fixed effects)
Inverse variance weighted (multiplicative random effects)
MR Egger
MR RAPS
Weighted median
Weighted mode
0.10
0.05
0.00
−0.05
−0.10
0.05
0.10
0.15
0.20
0.25
0.30
SNP effect on Ulcerative proctitis
SNP effect on Gut microbiota abundance (family Actinomycetaceae id.421)

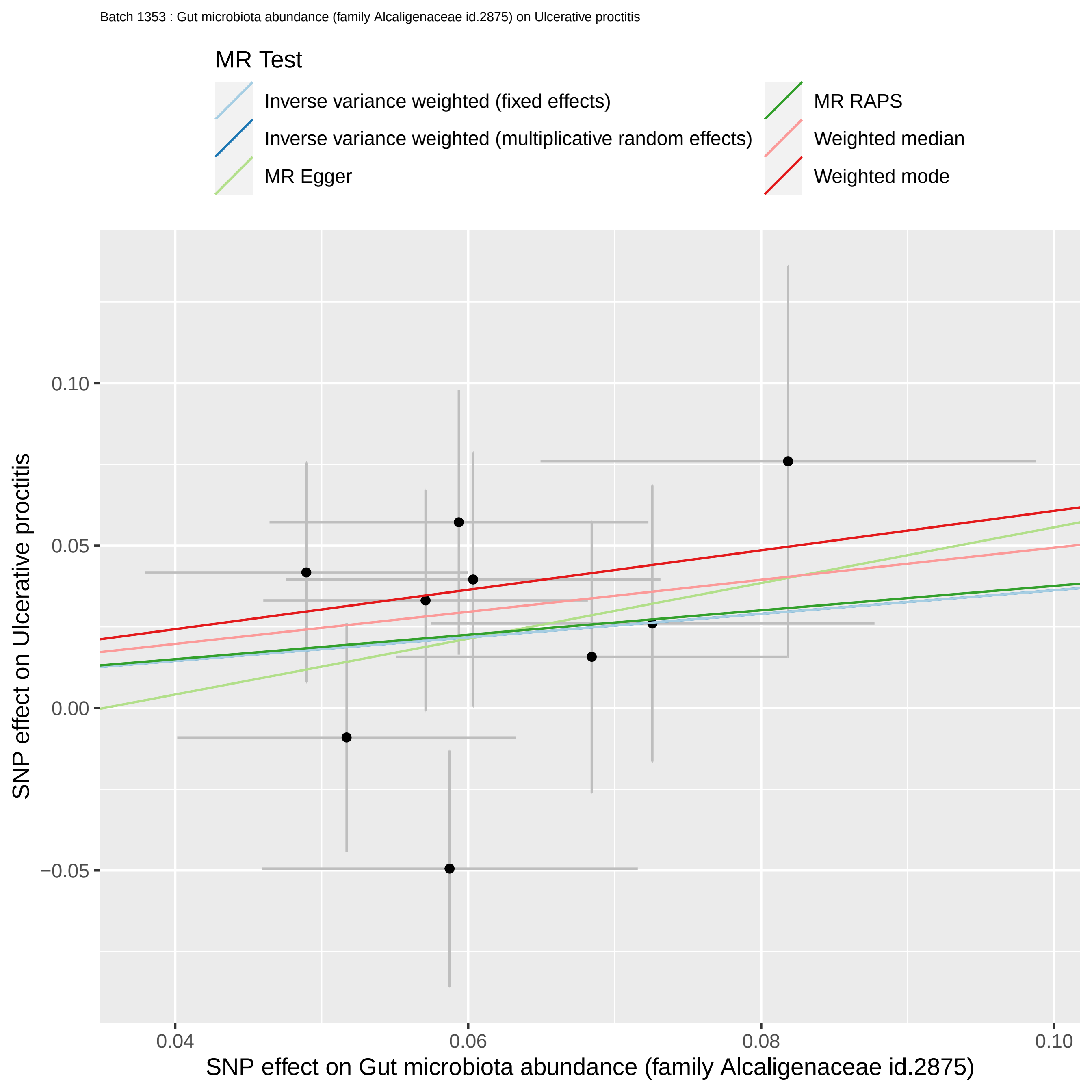

Batch 1353 : Gut microbiota abundance (family Alcaligenaceae id.2875) on Ulcerative proctitis
MR Test
Inverse variance weighted (fixed effects)
Inverse variance weighted (multiplicative random effects)
MR Egger
MR RAPS
Weighted median
Weighted mode
0.10
0.05
0.00
−0.05
0.04
0.06
0.08
0.10
SNP effect on Ulcerative proctitis
SNP effect on Gut microbiota abundance (family Alcaligenaceae id.2875)

Batch 1354 : Gut microbiota abundance (family Bacteroidaceae id.917) on Ulcerative proctitis

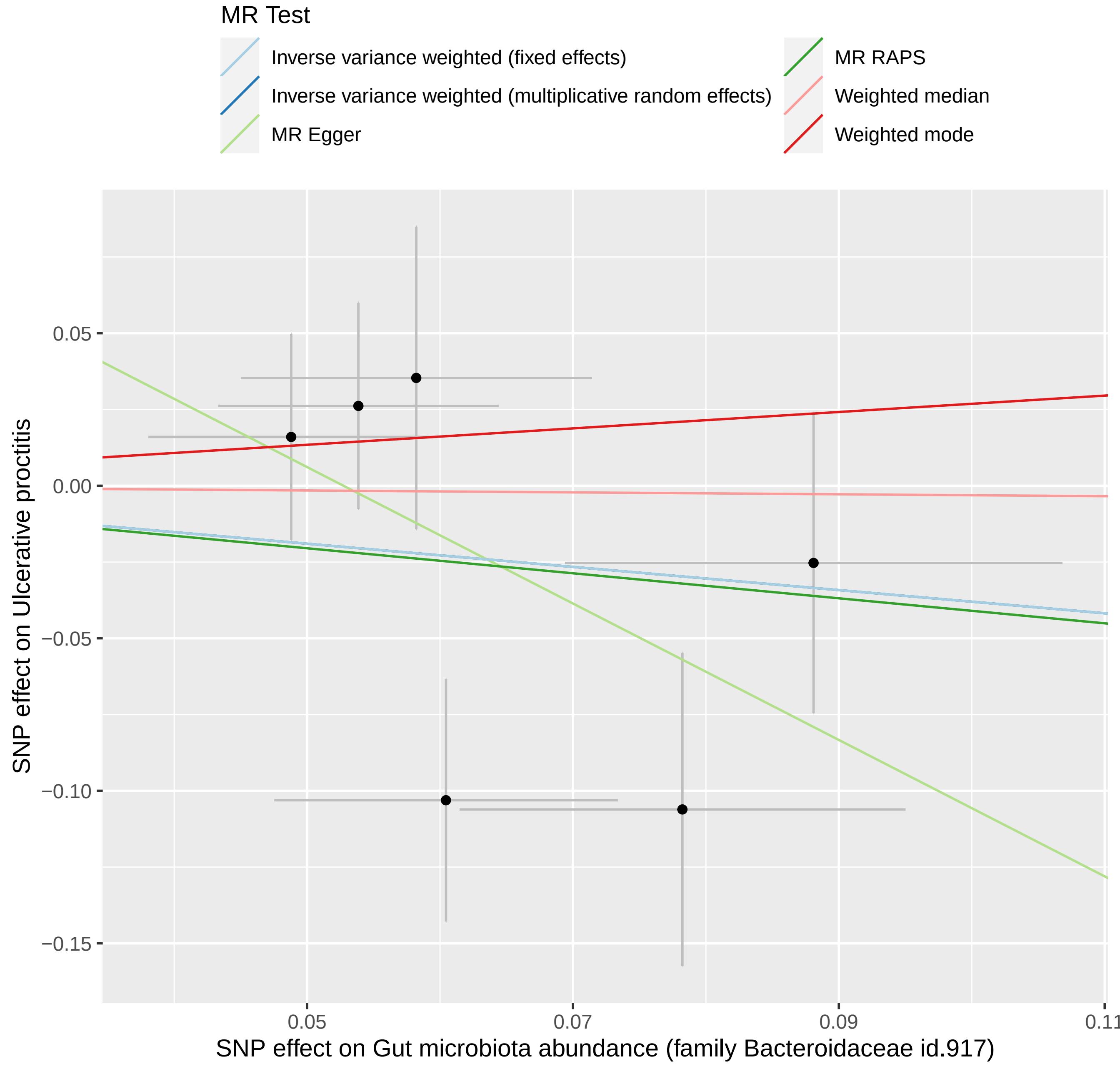

Batch 1355 : Gut microbiota abundance (family Bacteroidales S24 7group id.11173) on Ulcerative proctitis

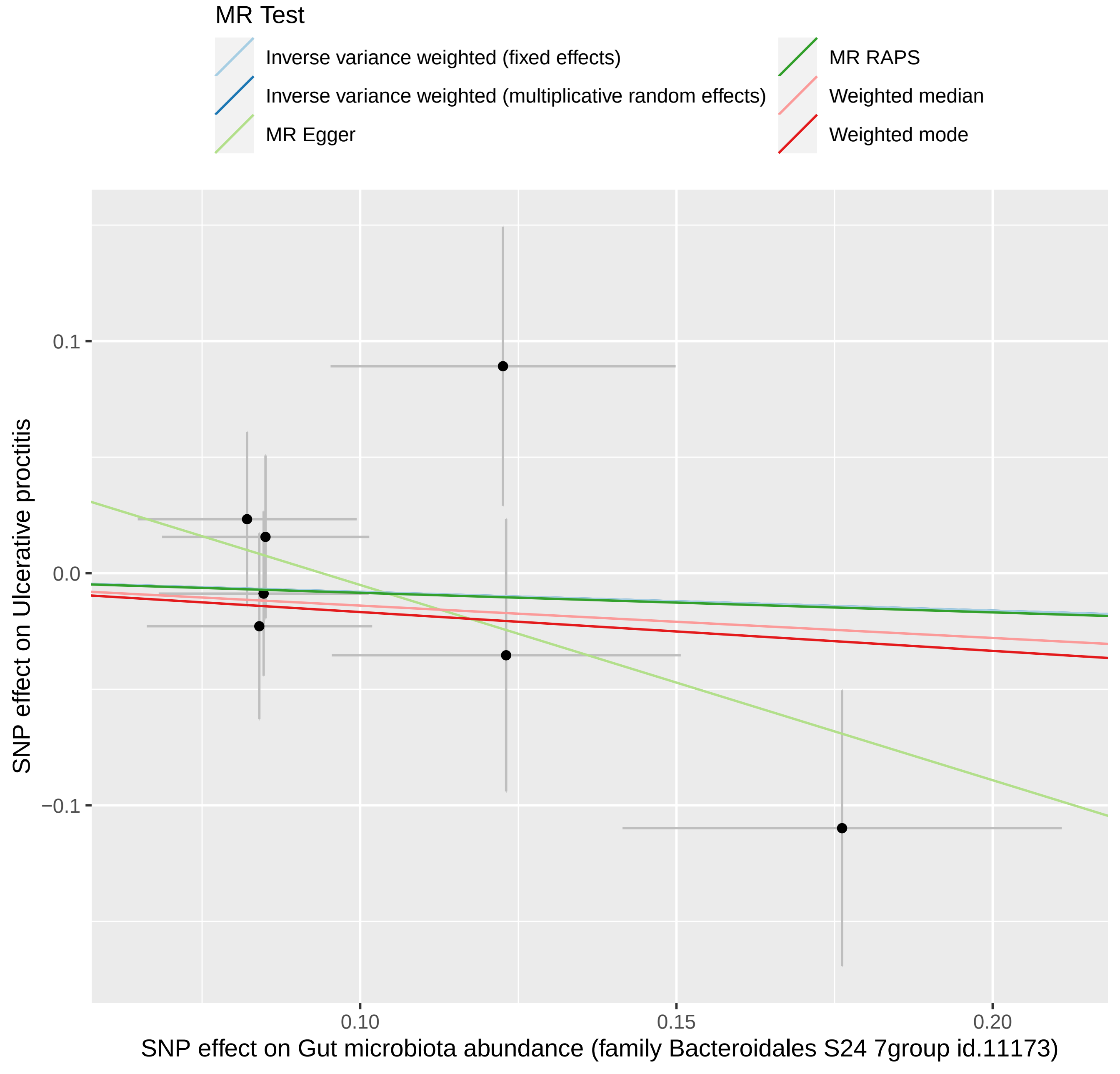

Batch 1356 : Gut microbiota abundance (family Bifidobacteriaceae id.433) on Ulcerative proctitis

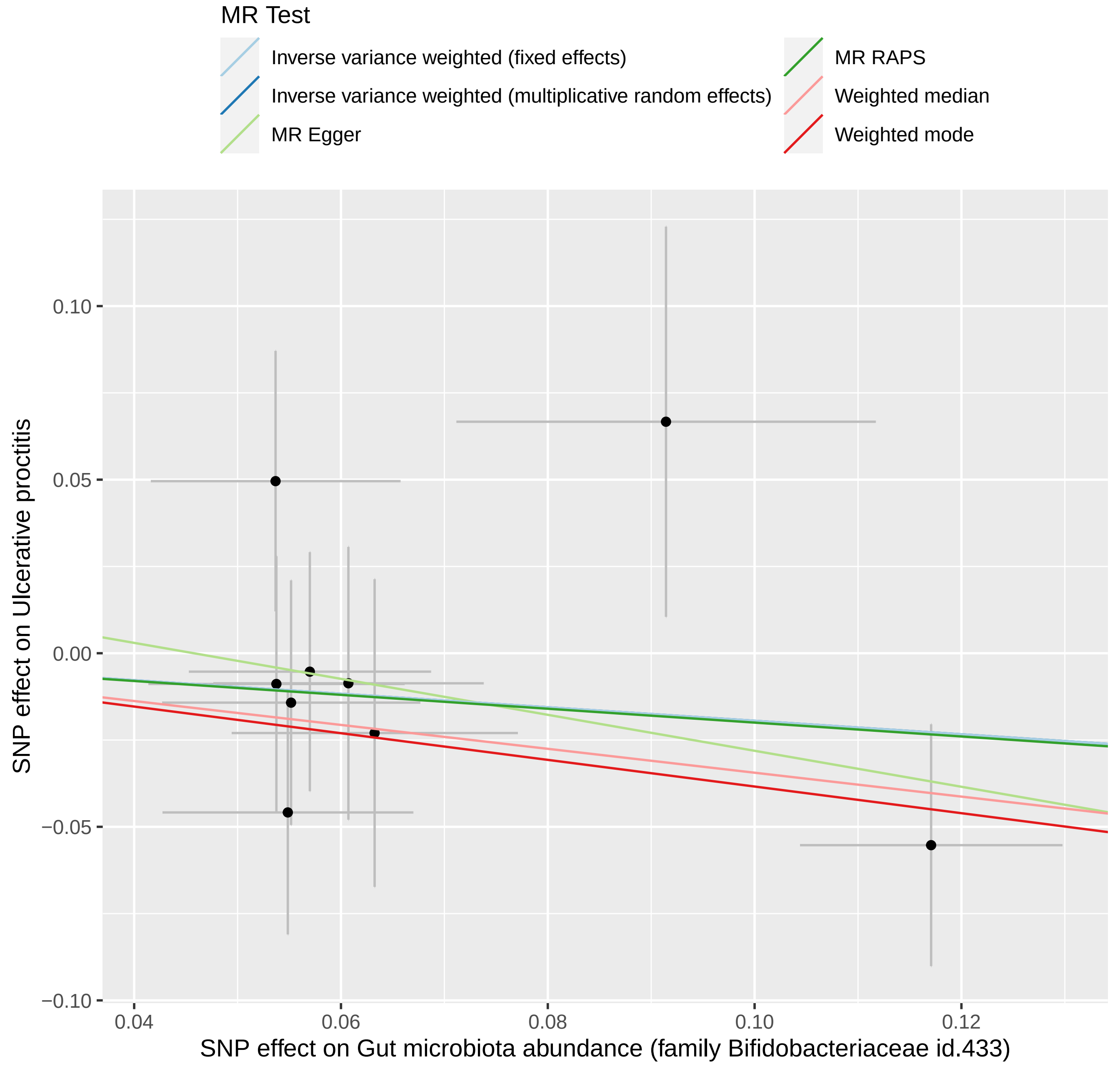

Batch 1358 : Gut microbiota abundance (family Clostridiaceae1 id.1869) on Ulcerative proctitis

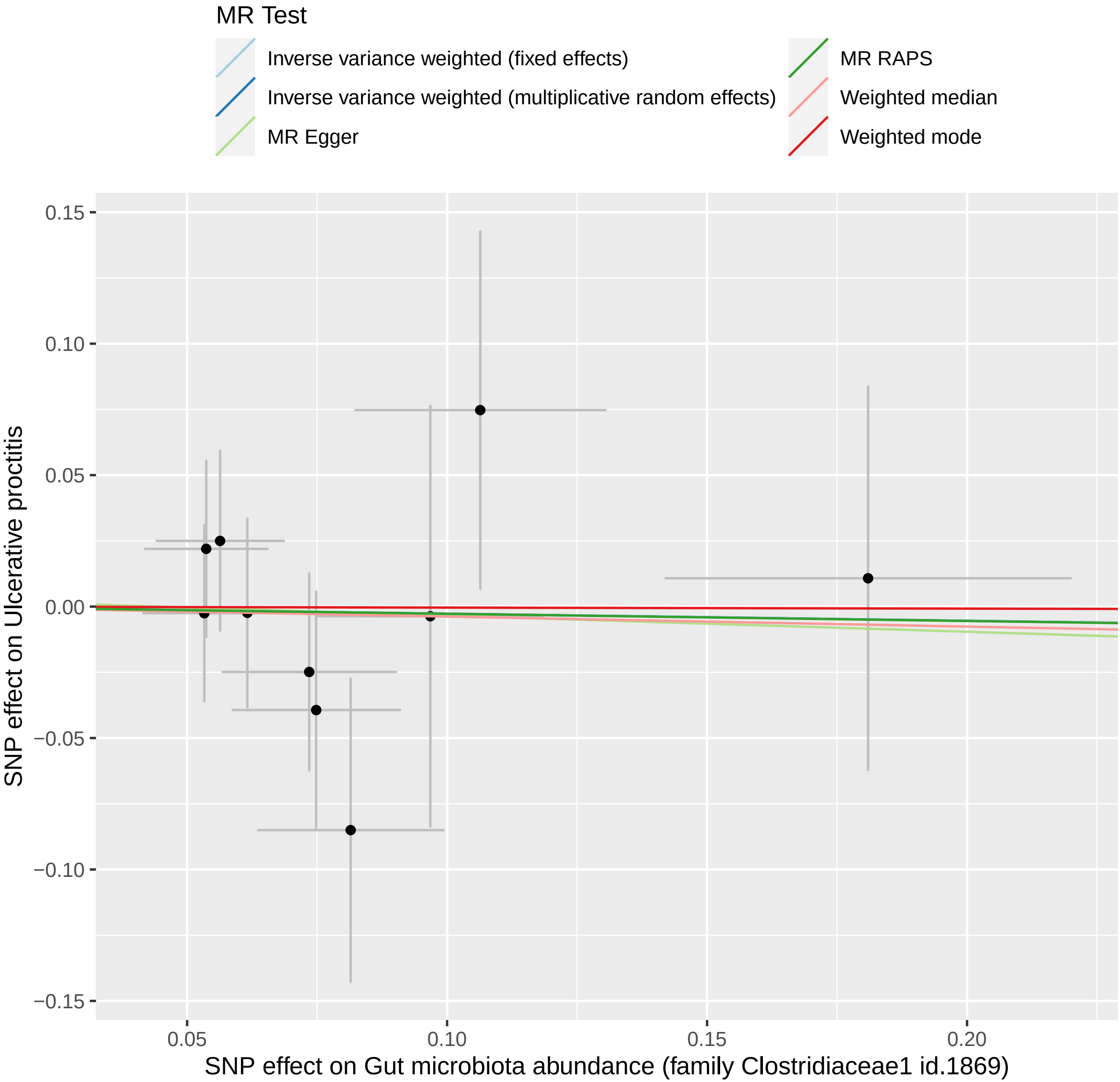

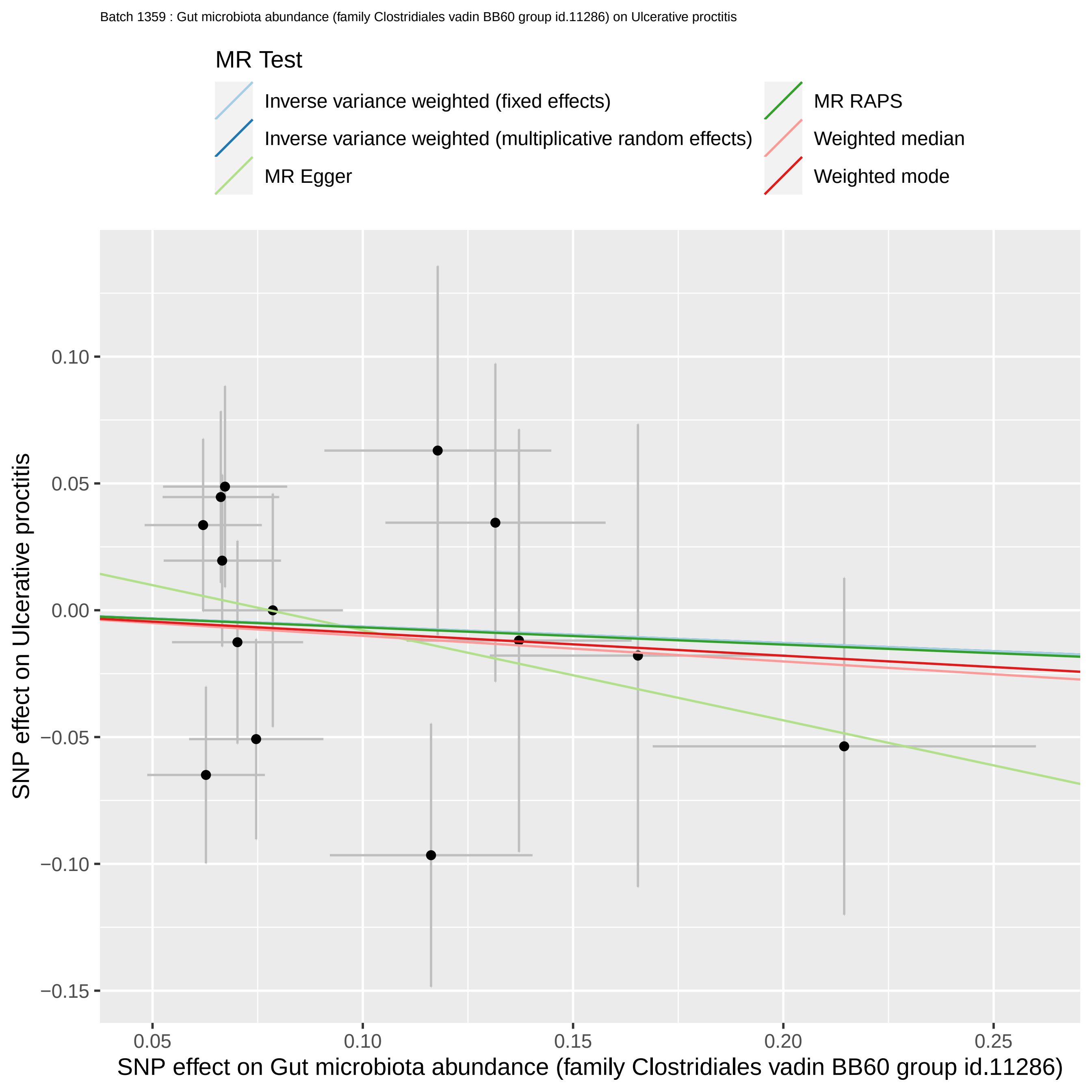
Batch 1359 : Gut microbiota abundance (family Clostridiales vadin BB60 group id.11286) on Ulcerative proctitis
MR Test
Inverse variance weighted (fixed effects)
Inverse variance weighted (multiplicative random effects)
MR Egger
MR RAPS
Weighted median
Weighted mode
0.10
0.05
0.00
−0.05
−0.10
−0.15
0.05
0.10
0.15
0.20
0.25
SNP effect on Ulcerative proctitis
SNP effect on Gut microbiota abundance (family Clostridiales vadin BB60 group id.11286)

Batch 1360 : Gut microbiota abundance (family Coriobacteriaceae id.811) on Ulcerative proctitis

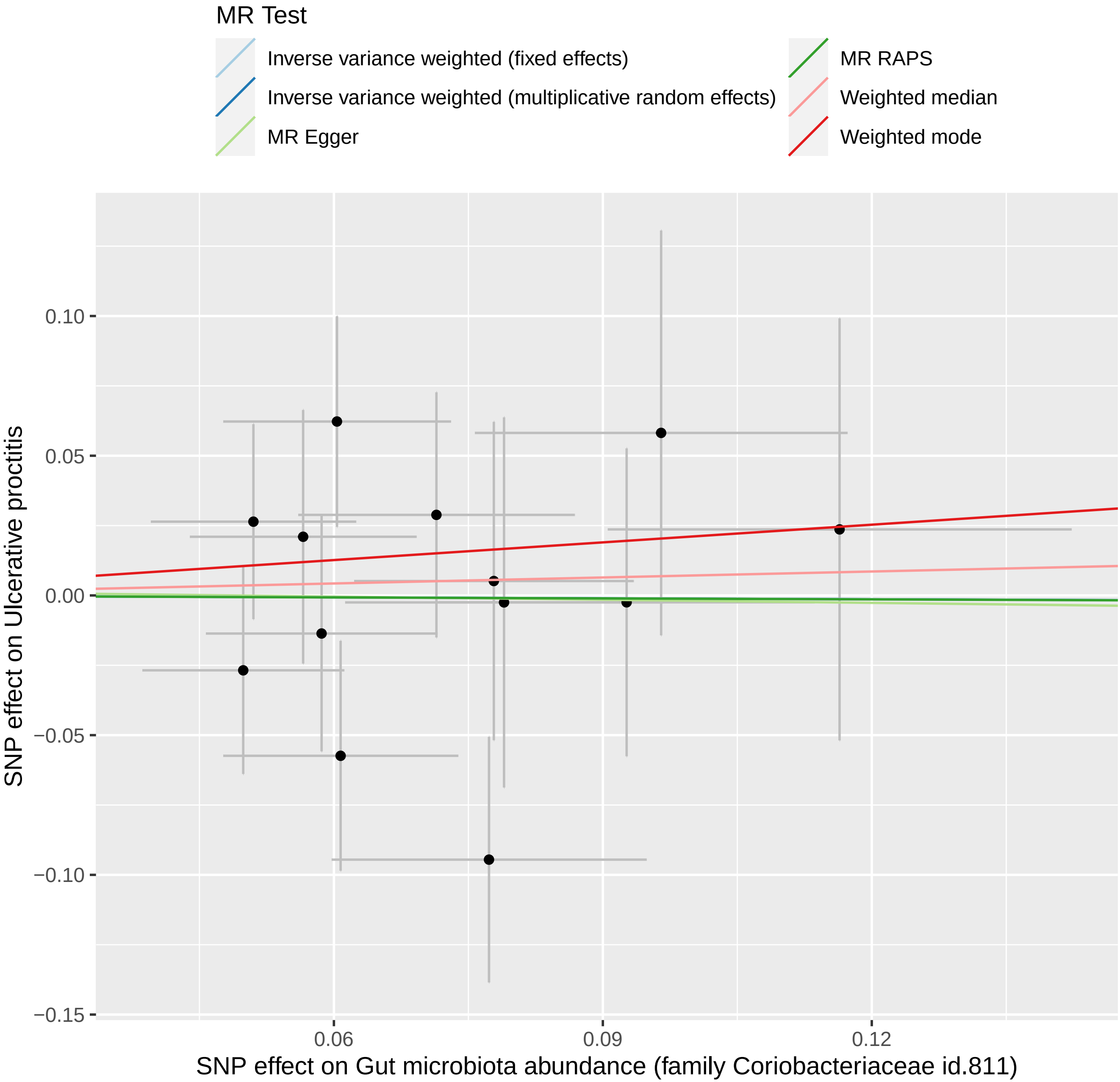

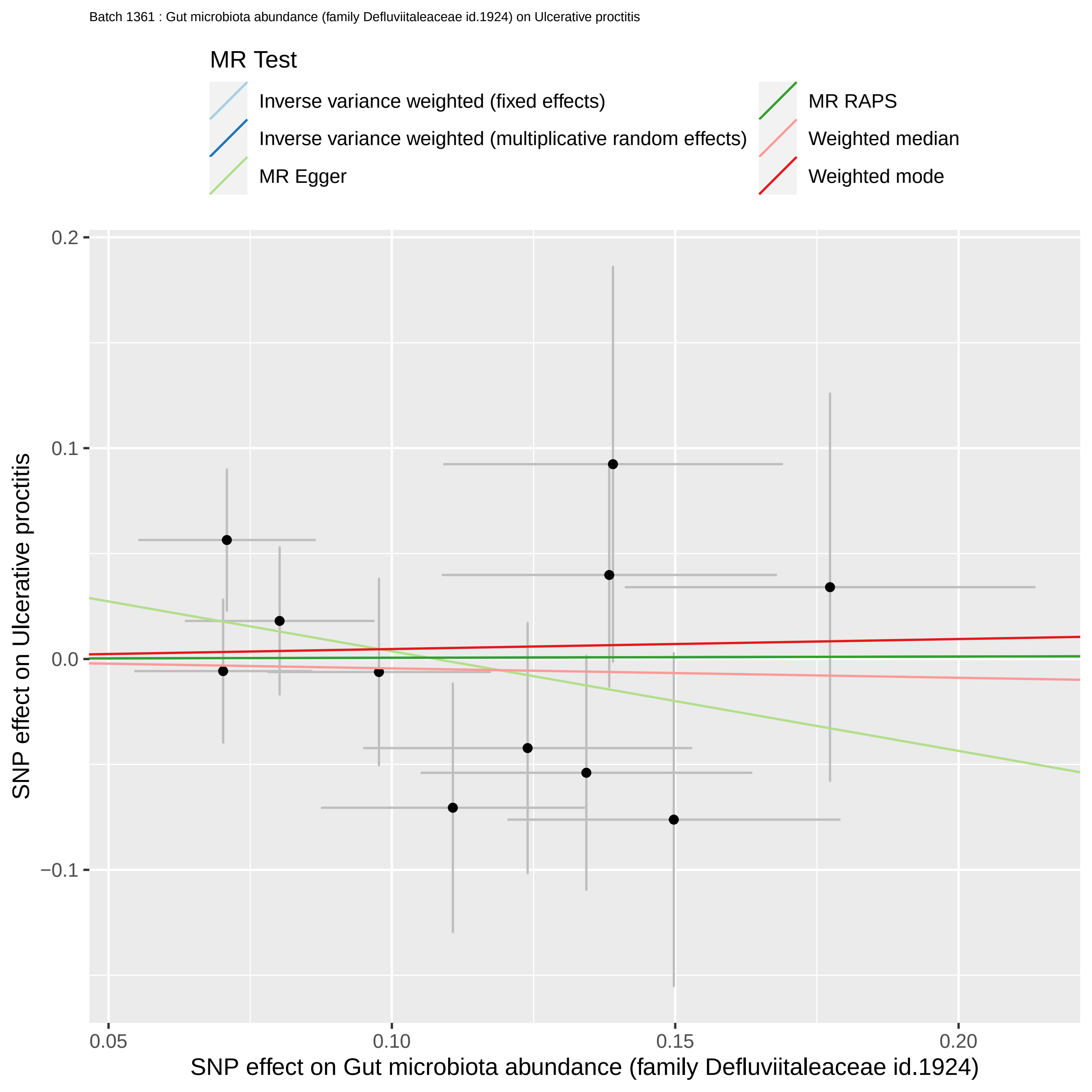

Batch 1361 : Gut microbiota abundance (family Defluviitaleaceae id.1924) on Ulcerative proctitis
MR Test
Inverse variance weighted (fixed effects)
Inverse variance weighted (multiplicative random effects)
MR Egger
MR RAPS
Weighted median
Weighted mode
0.2
0.1
0.0
−0.1
0.05
0.10
0.15
0.20
SNP effect on Ulcerative proctitis
SNP effect on Gut microbiota abundance (family Defluviitaleaceae id.1924)

Batch 1362 : Gut microbiota abundance (family Desulfovibrionaceae id.3169) on Ulcerative proctitis

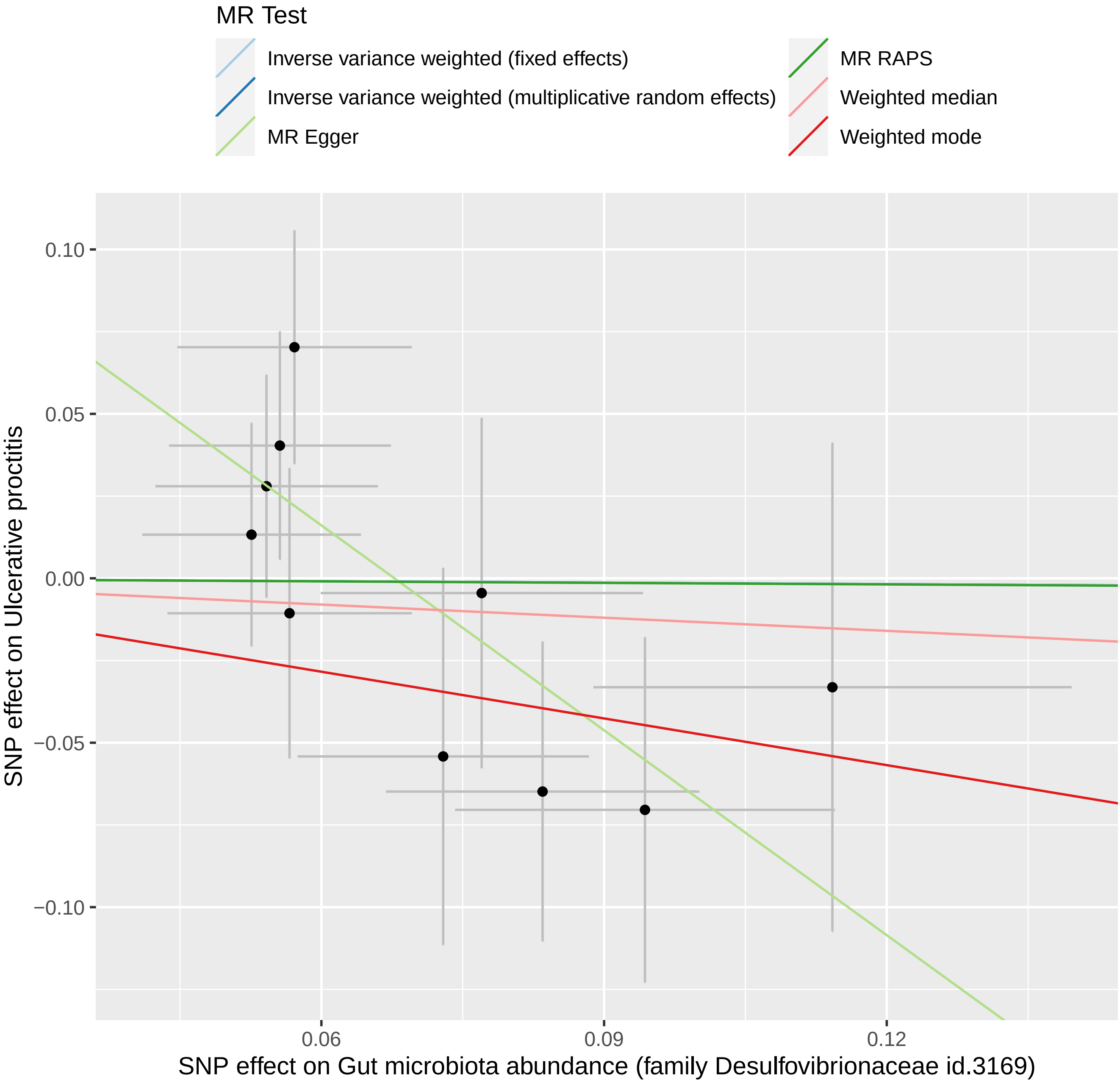

Batch 1363 : Gut microbiota abundance (family Enterobacteriaceae id.3469) on Ulcerative proctitis

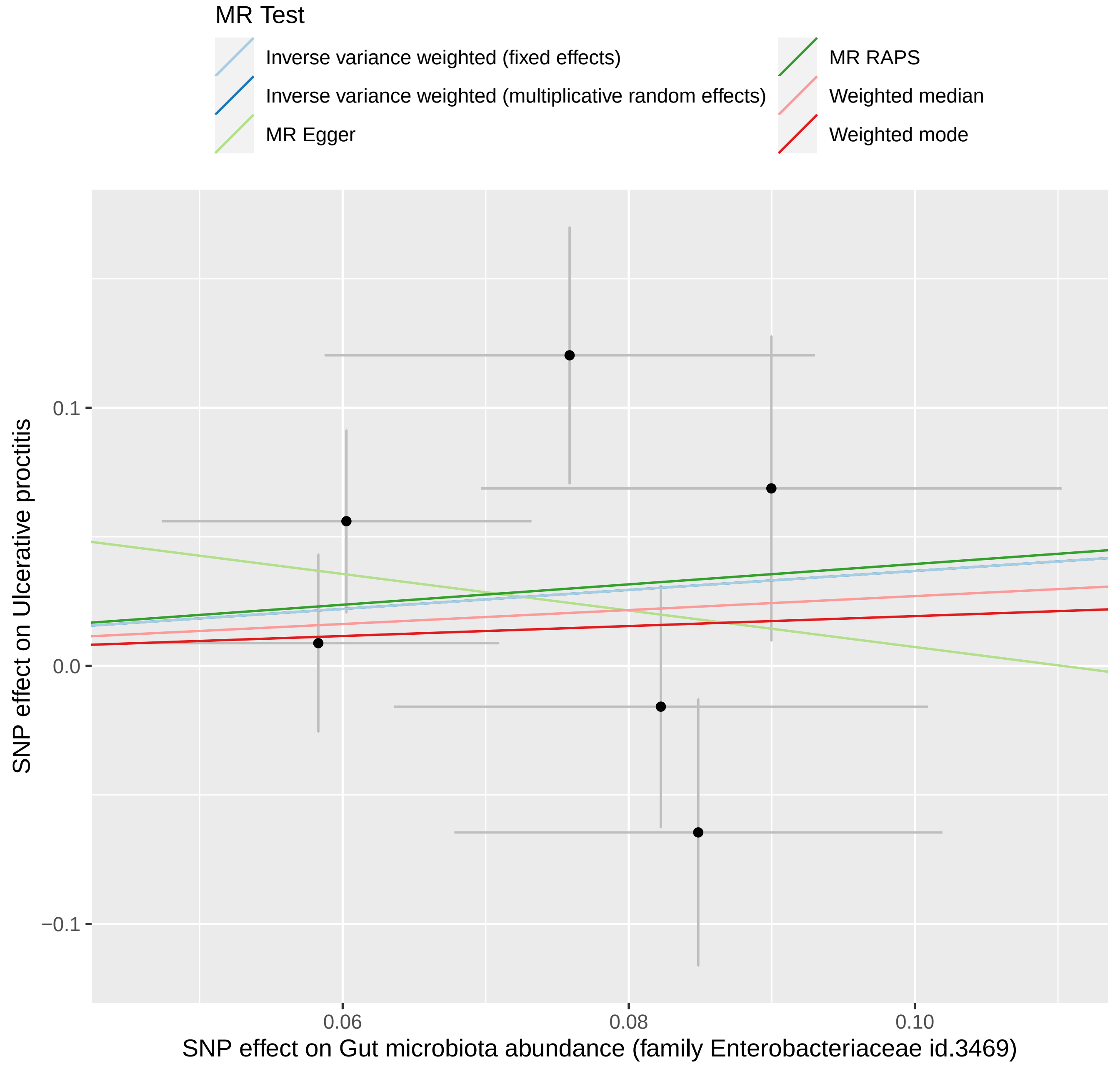

Batch 1364 : Gut microbiota abundance (family Erysipelotrichaceae id.2149) on Ulcerative proctitis

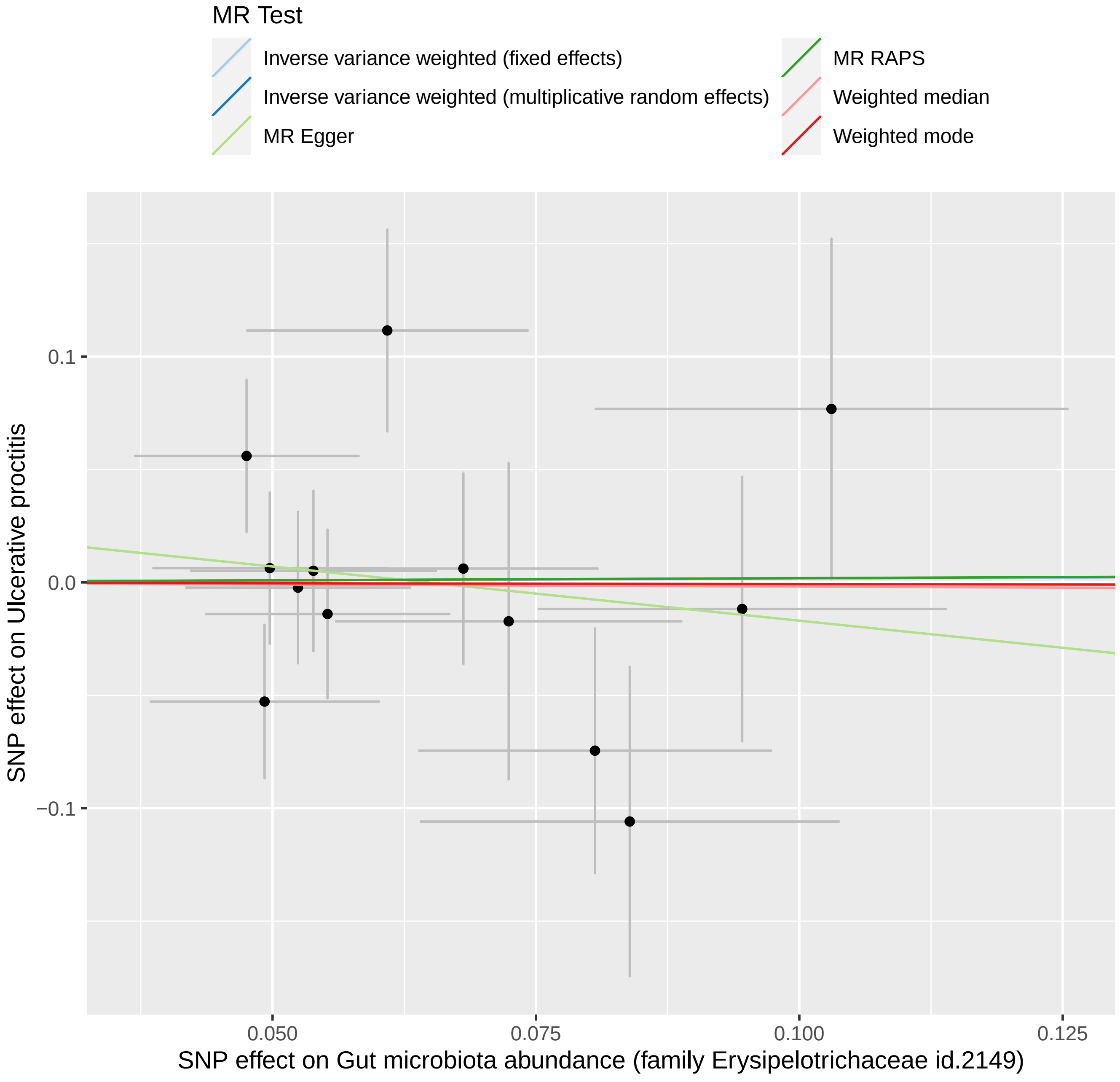

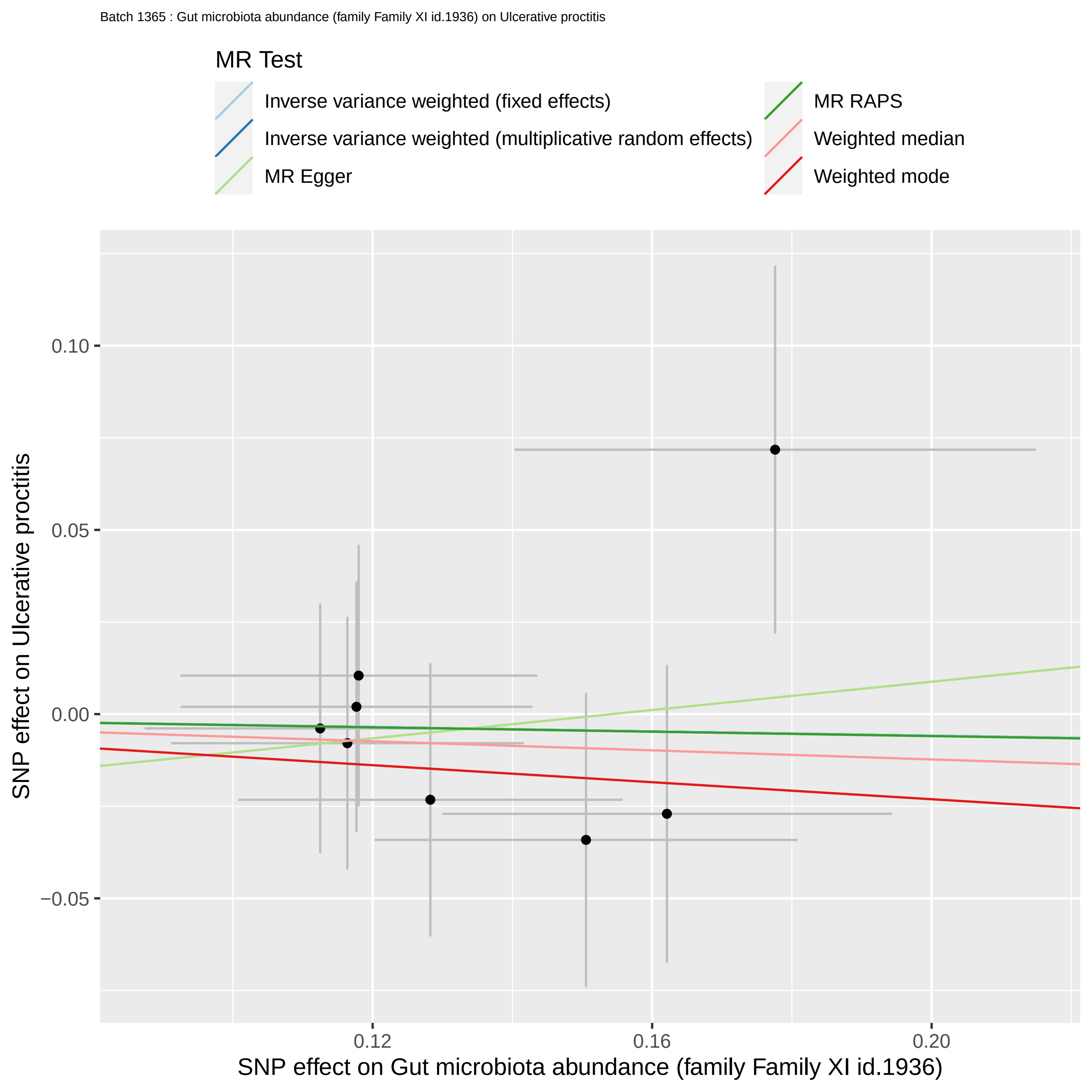
Batch 1365 : Gut microbiota abundance (family Family XI id.1936) on Ulcerative proctitis
MR Test
Inverse variance weighted (fixed effects)
Inverse variance weighted (multiplicative random effects)
MR Egger
MR RAPS
Weighted median
Weighted mode
0.10
0.05
0.00
−0.05
0.12
0.16
0.20
SNP effect on Ulcerative proctitis
SNP effect on Gut microbiota abundance (family Family XI id.1936)

Batch 1366 : Gut microbiota abundance (family Family XIII id.1957) on Ulcerative proctitis

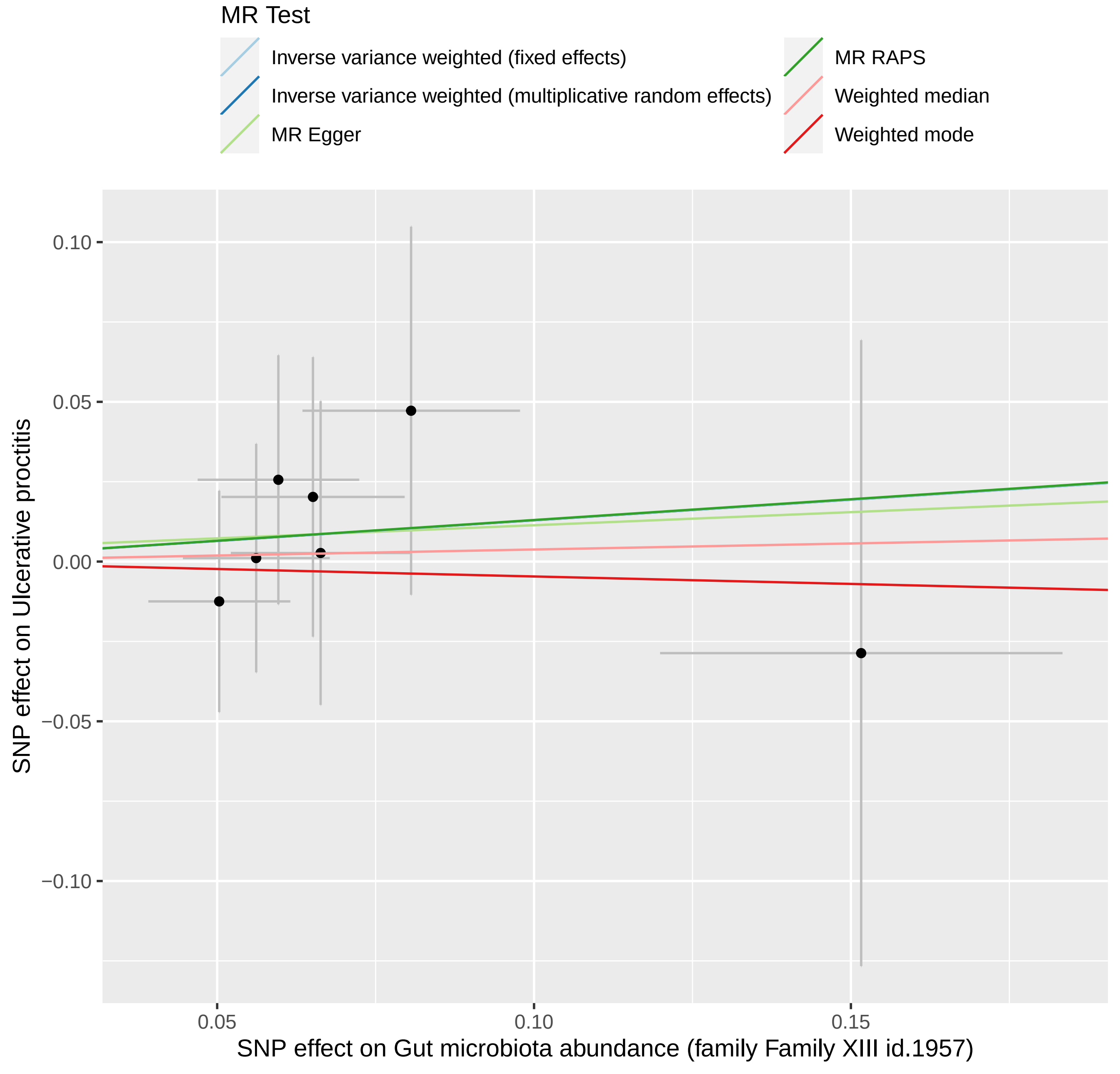

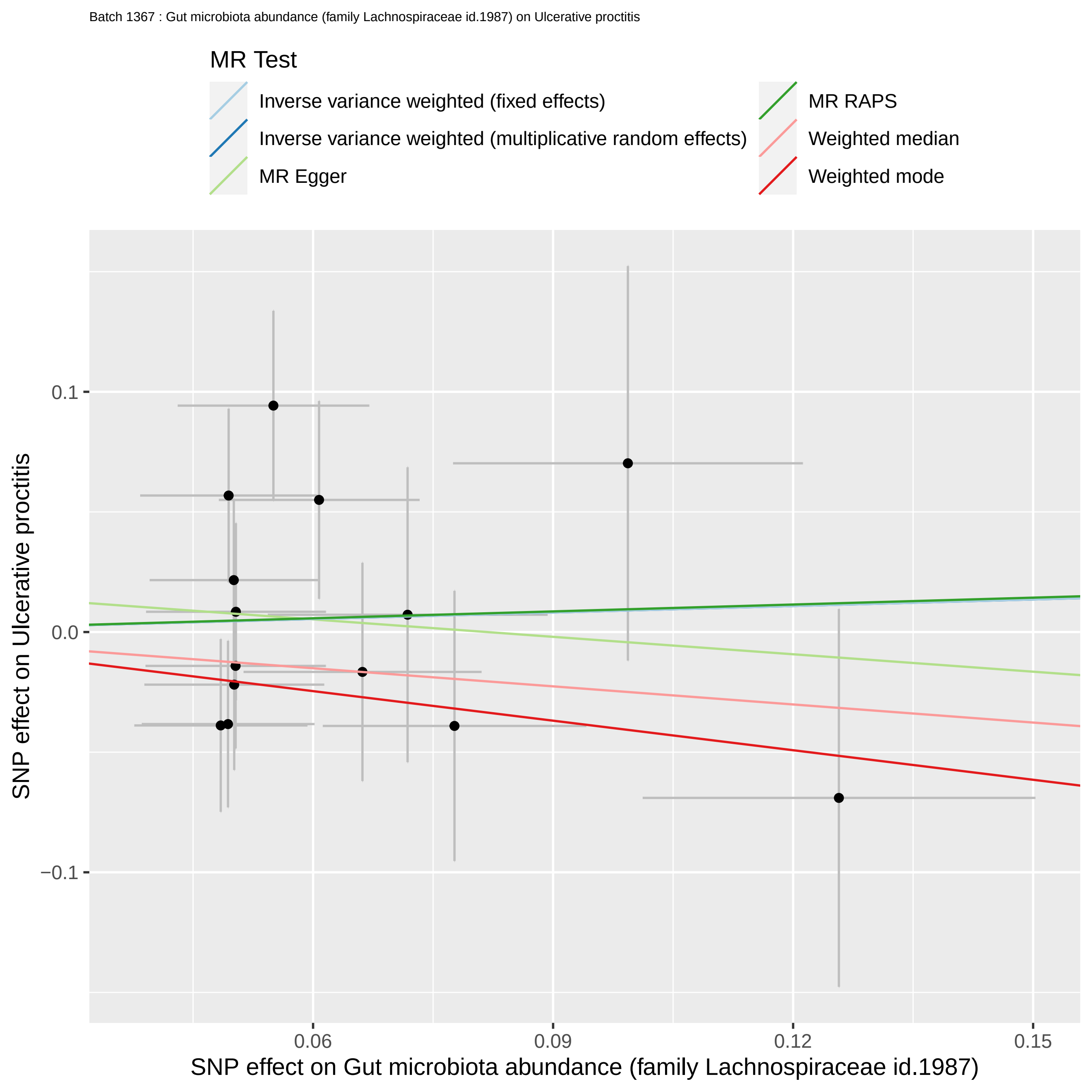

Batch 1367 : Gut microbiota abundance (family Lachnospiraceae id.1987) on Ulcerative proctitis
MR Test
Inverse variance weighted (fixed effects)
Inverse variance weighted (multiplicative random effects)
MR Egger
MR RAPS
Weighted median
Weighted mode
SNP effect on Ulcerative proctitis
0.1
0.0
−0.1
0.06
0.09
0.12
0.15
SNP effect on Gut microbiota abundance (family Lachnospiraceae id.1987)

Batch 1368 : Gut microbiota abundance (family Lactobacillaceae id.1836) on Ulcerative proctitis

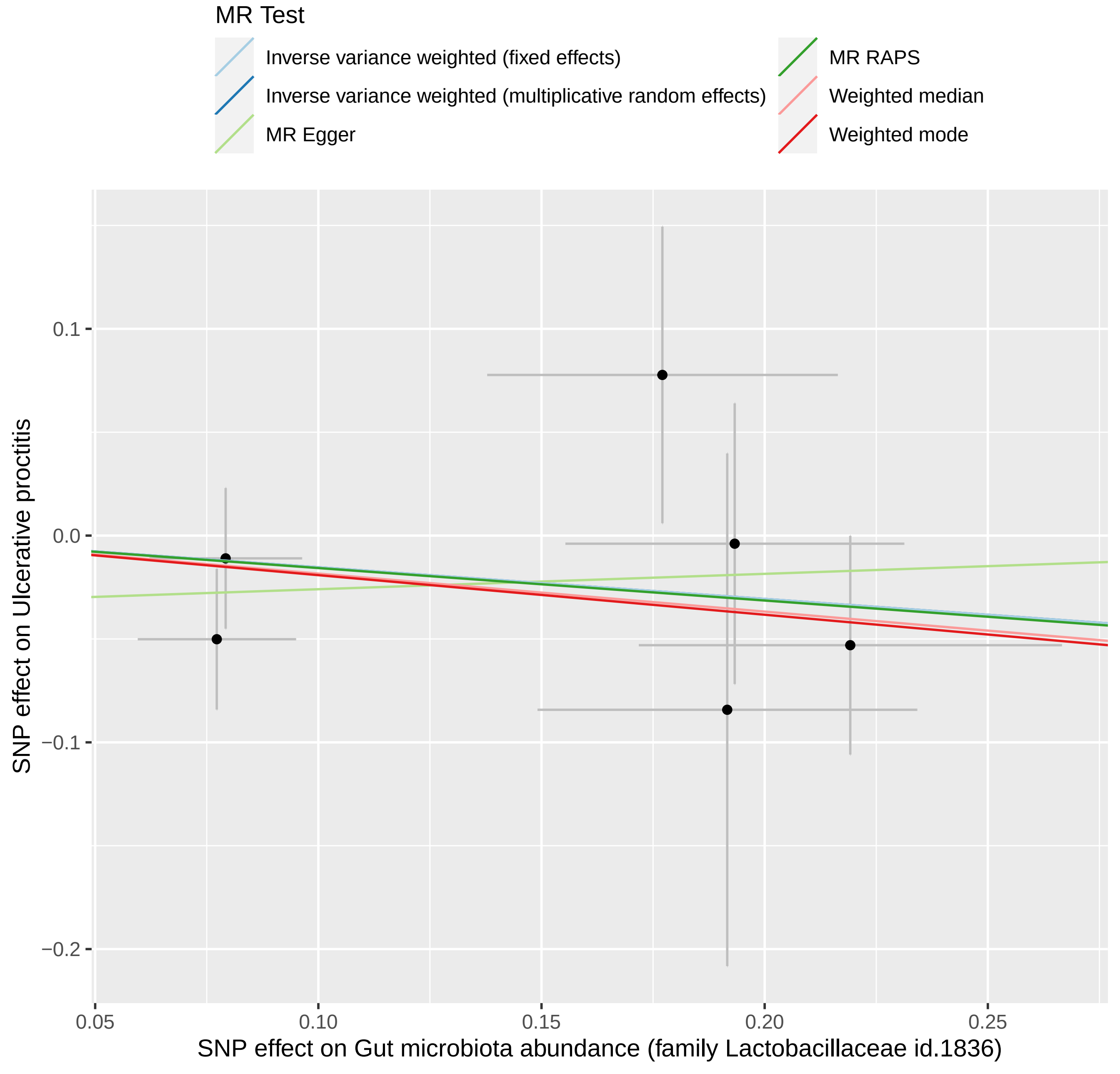

Batch 1369 : Gut microbiota abundance (family Methanobacteriaceae id.121) on Ulcerative proctitis

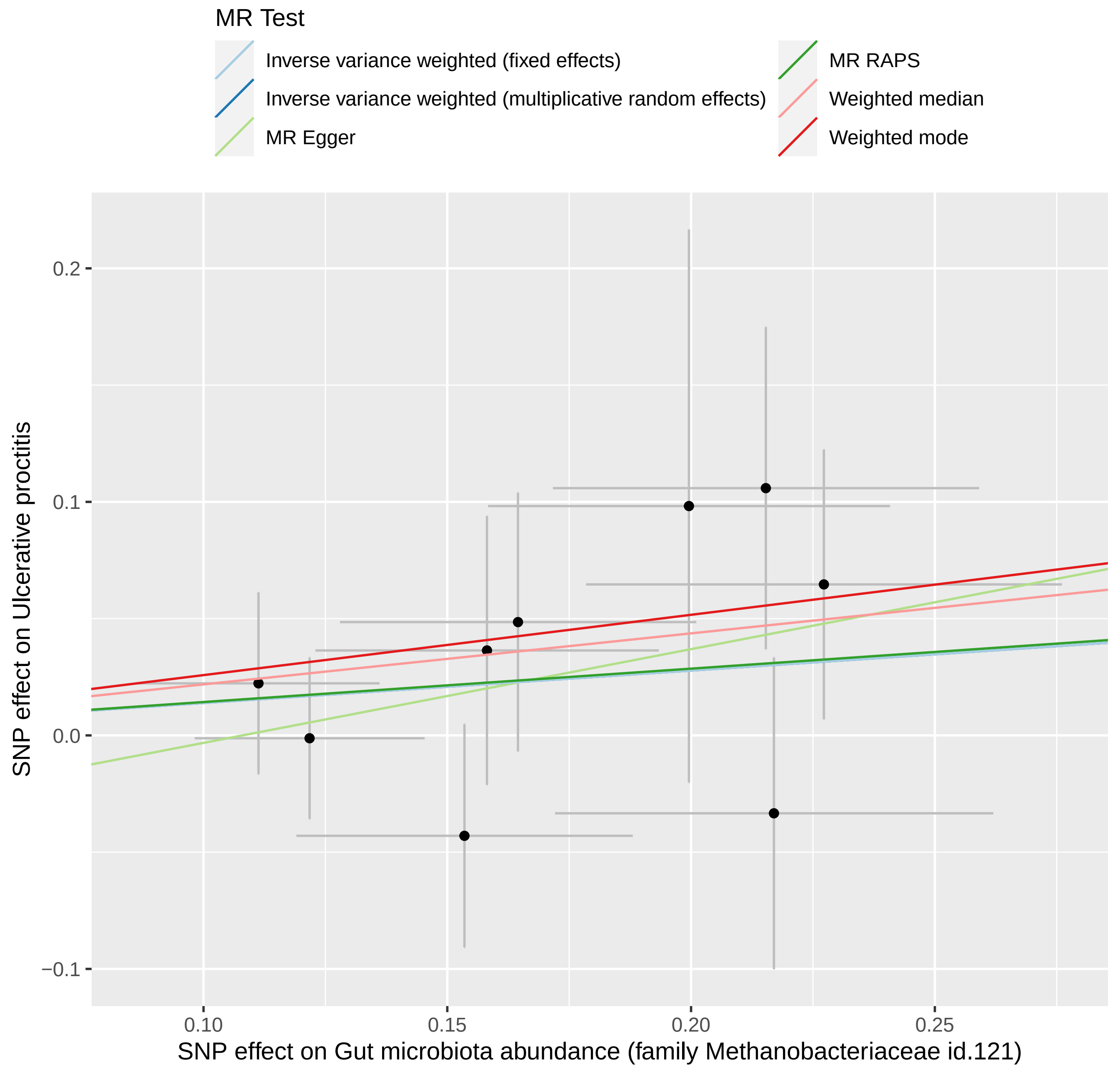

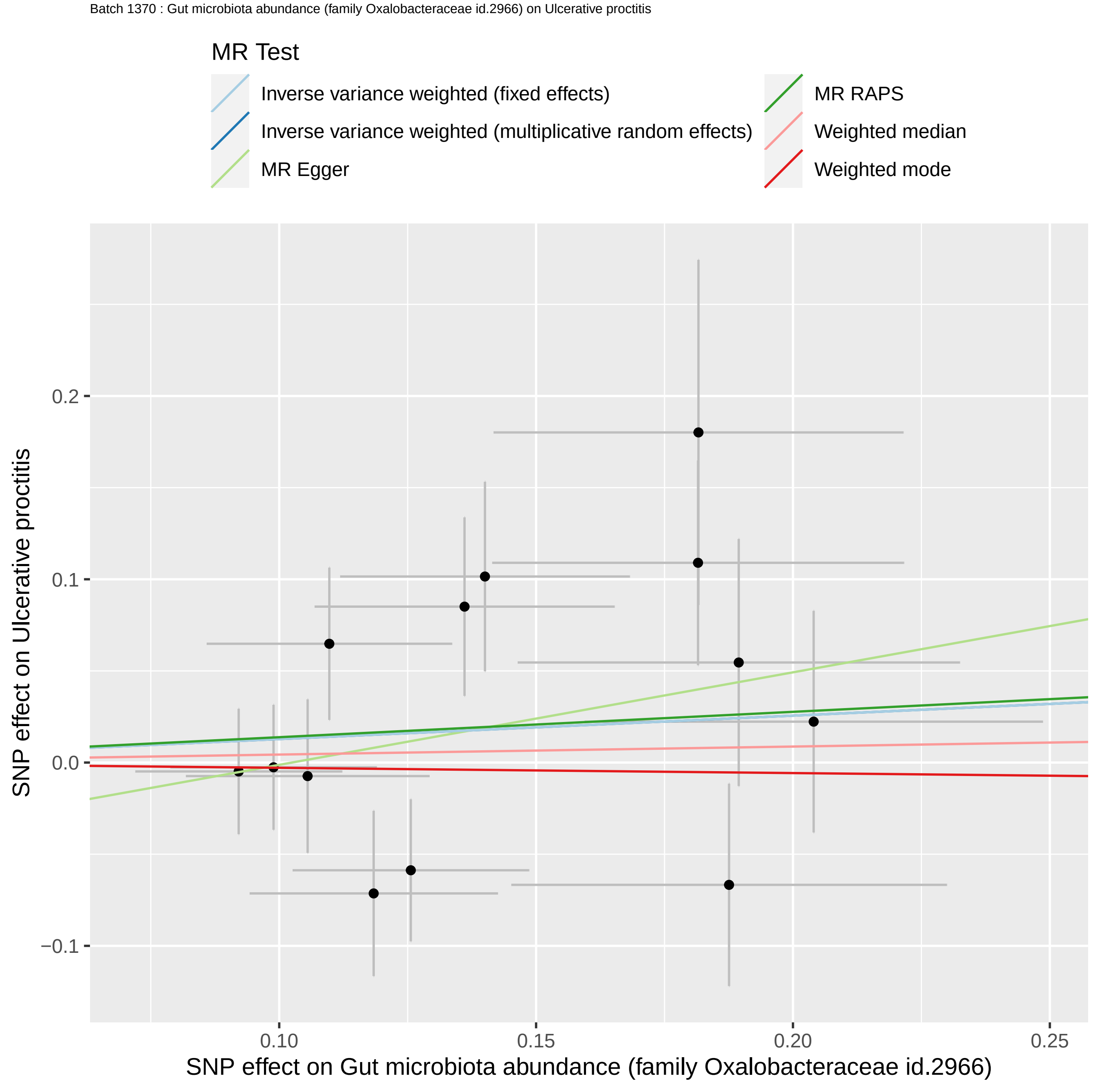
Batch 1370 : Gut microbiota abundance (family Oxalobacteraceae id.2966) on Ulcerative proctitis
MR Test
Inverse variance weighted (fixed effects)
Inverse variance weighted (multiplicative random effects)
MR Egger
MR RAPS
Weighted median
Weighted mode
0.2
0.1
0.0
−0.1
0.10
0.15
0.20
0.25
SNP effect on Ulcerative proctitis
SNP effect on Gut microbiota abundance (family Oxalobacteraceae id.2966)

Batch 1371 : Gut microbiota abundance (family Pasteurellaceae id.3689) on Ulcerative proctitis

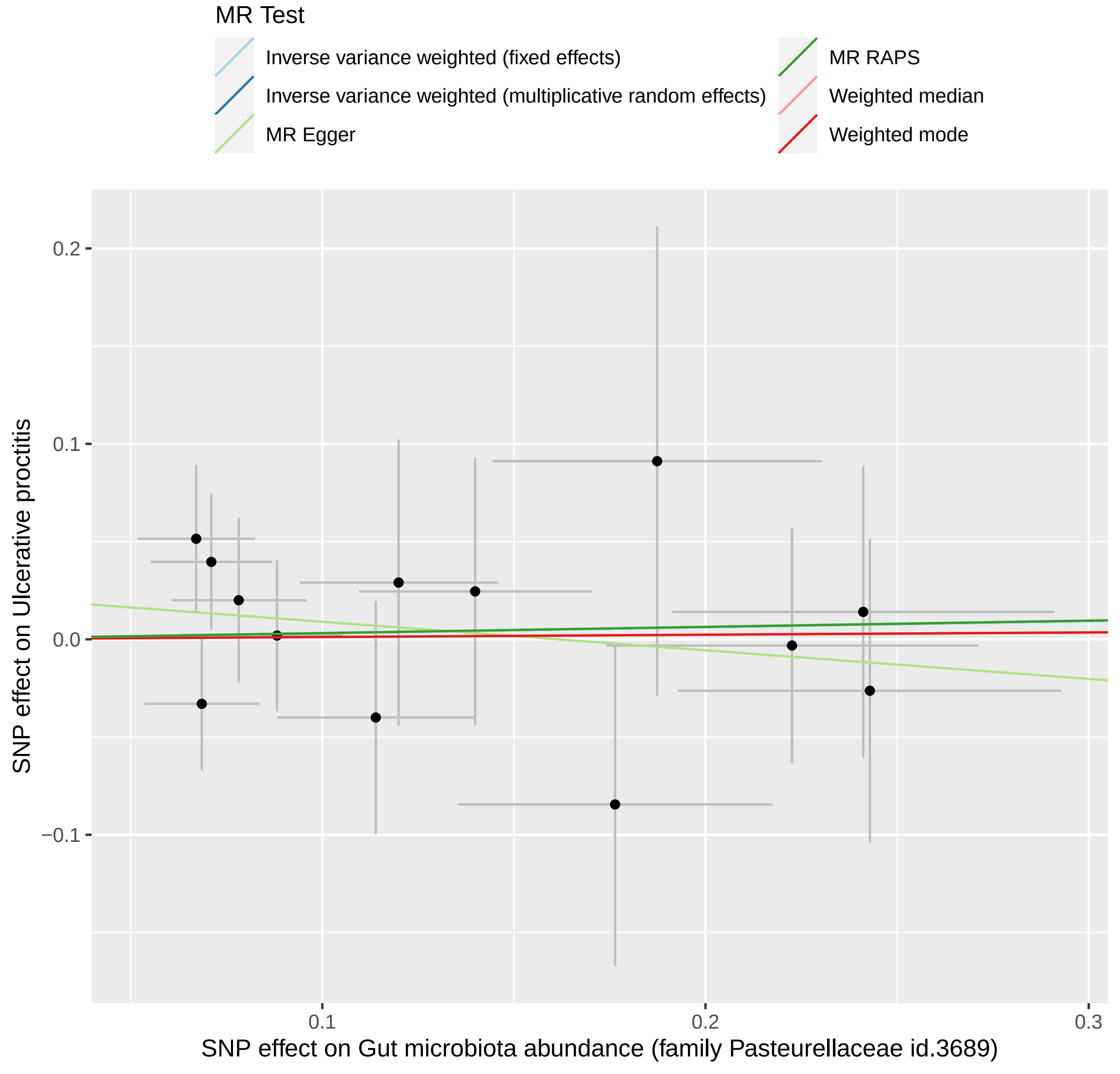

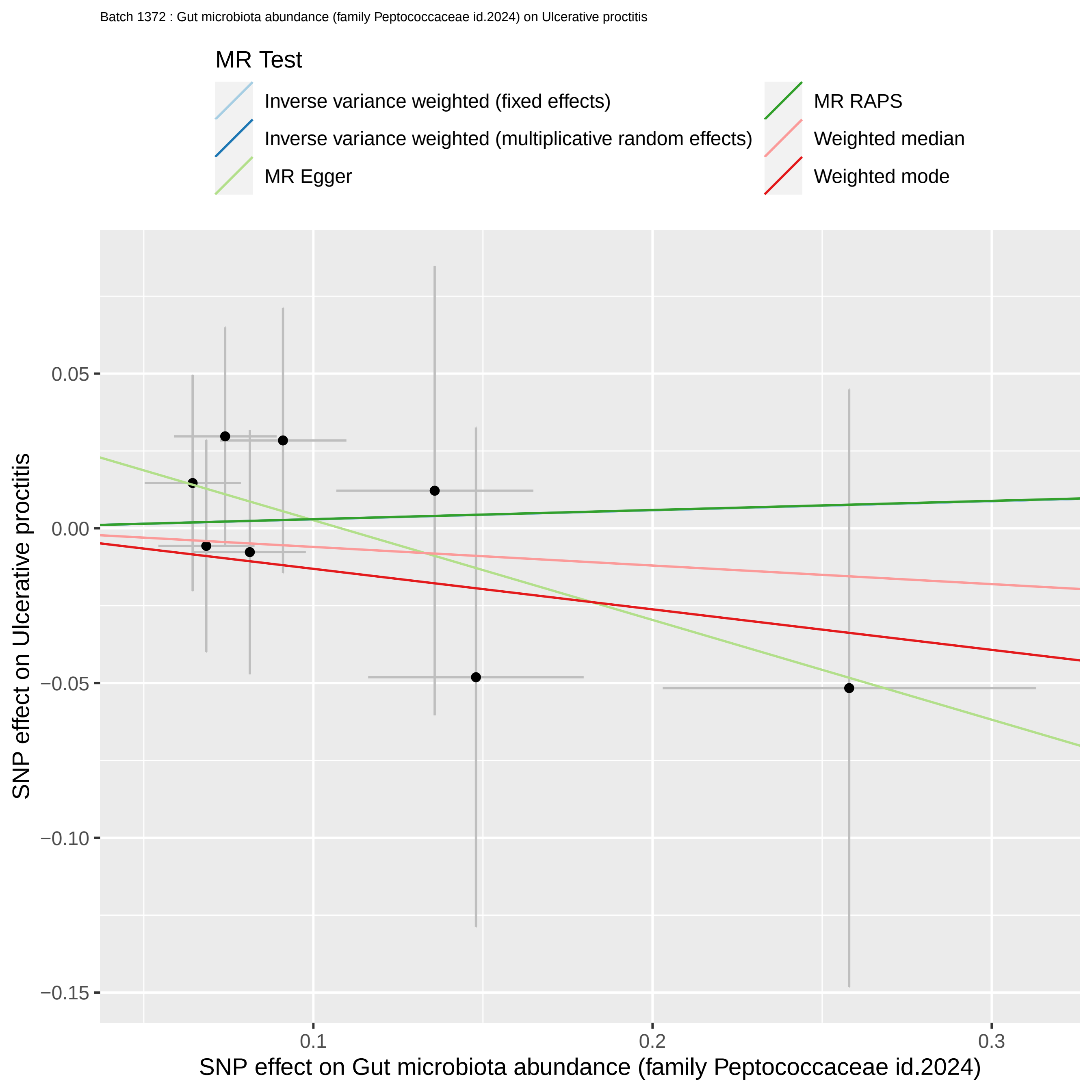
Batch 1372 : Gut microbiota abundance (family Peptococcaceae id.2024) on Ulcerative proctitis
MR Test
Inverse variance weighted (fixed effects)
Inverse variance weighted (multiplicative random effects)
MR Egger
MR RAPS
Weighted median
Weighted mode
0.05
0.00
−0.05
−0.10
−0.15
SNP effect on Ulcerative proctitis
0.1
0.2
0.3
SNP effect on Gut microbiota abundance (family Peptococcaceae id.2024)

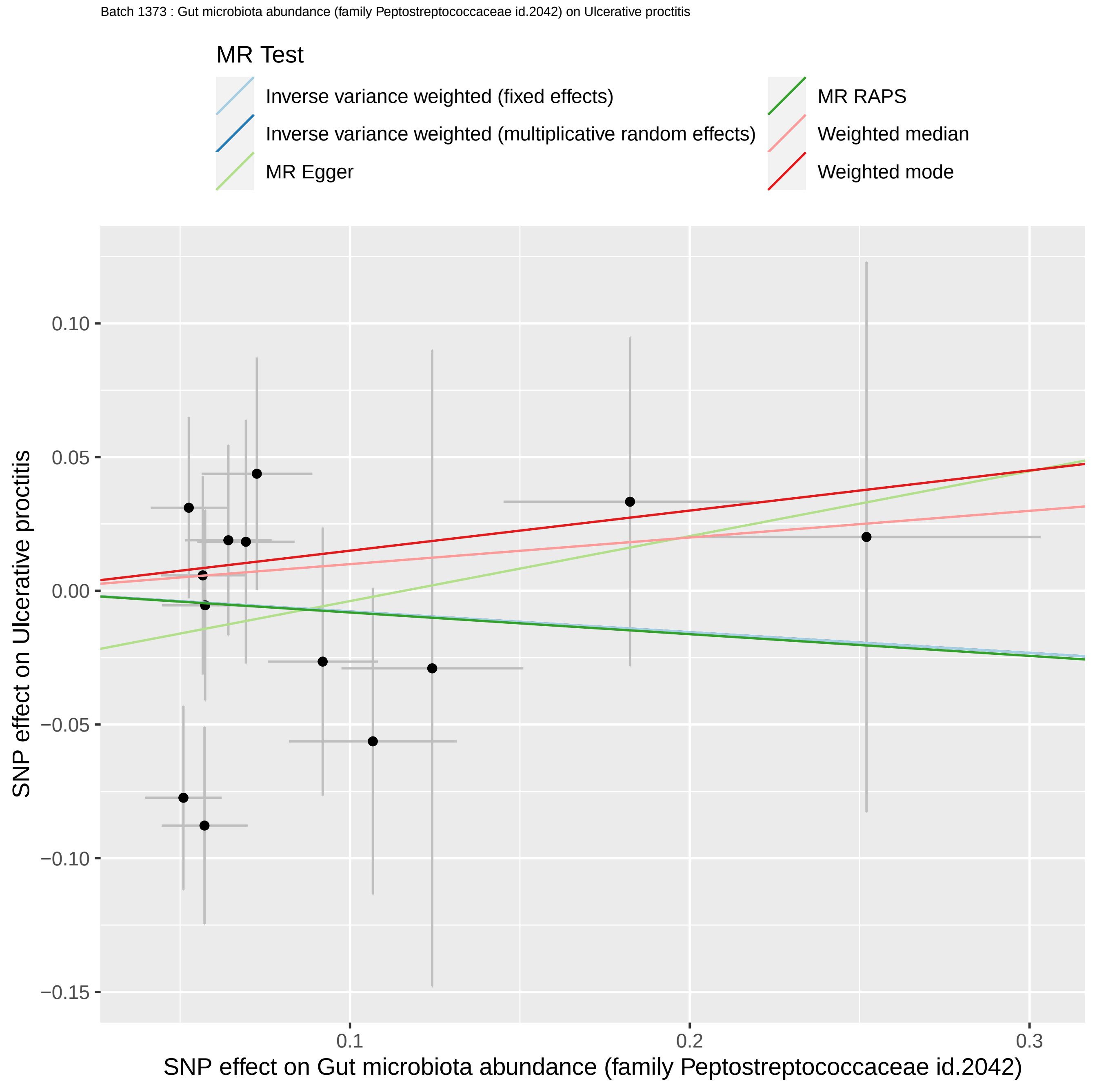

Batch 1373 : Gut microbiota abundance (family Peptostreptococcaceae id.2042) on Ulcerative proctitis
MR Test
Inverse variance weighted (fixed effects)
Inverse variance weighted (multiplicative random effects)
MR Egger
MR RAPS
Weighted median
Weighted mode
0.10
0.05
0.00
−0.05
−0.10
−0.15
0.1
0.2
0.3
SNP effect on Ulcerative proctitis
SNP effect on Gut microbiota abundance (family Peptostreptococcaceae id.2042)

Batch 1374 : Gut microbiota abundance (family Porphyromonadaceae id.943) on Ulcerative proctitis

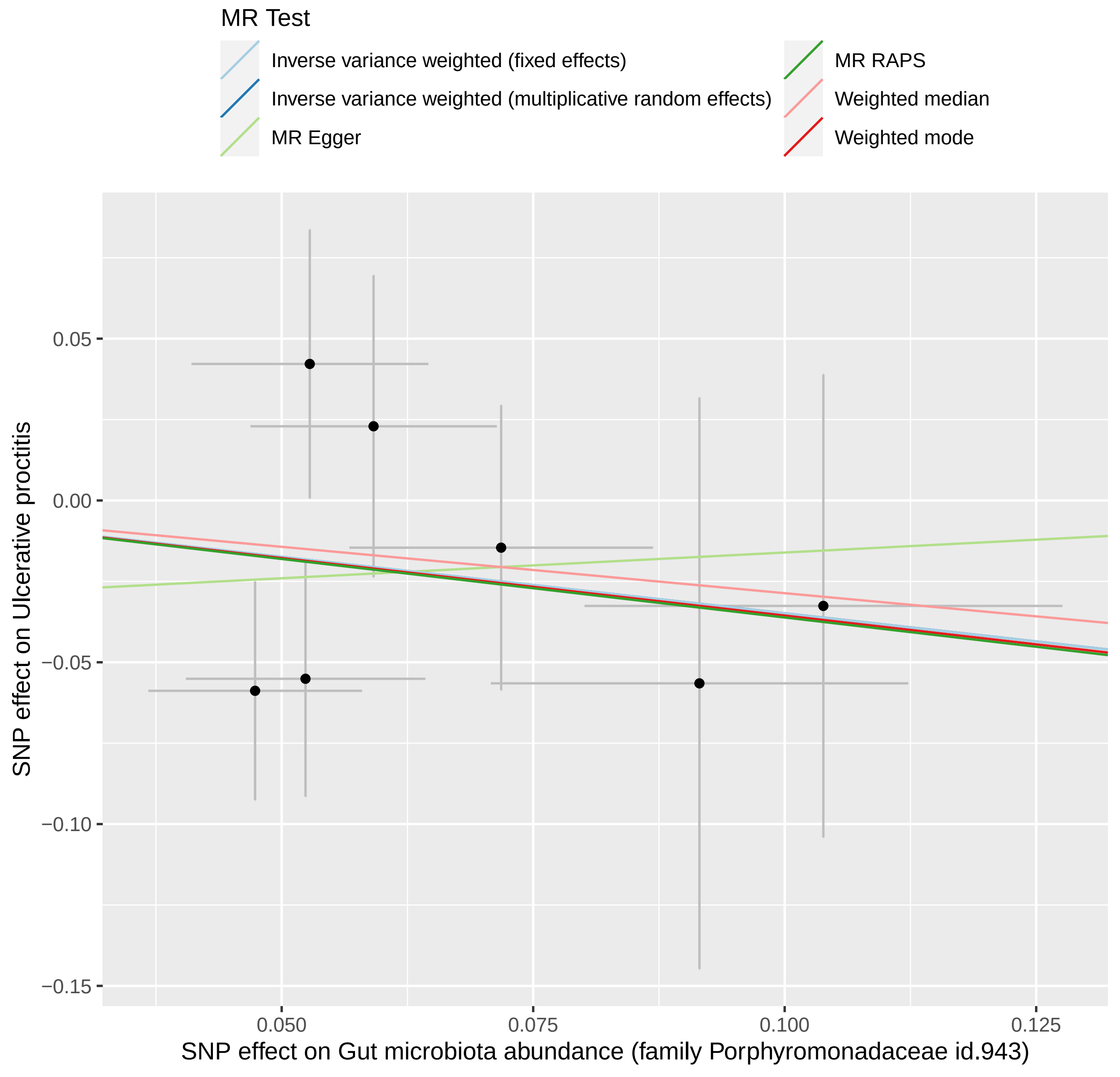

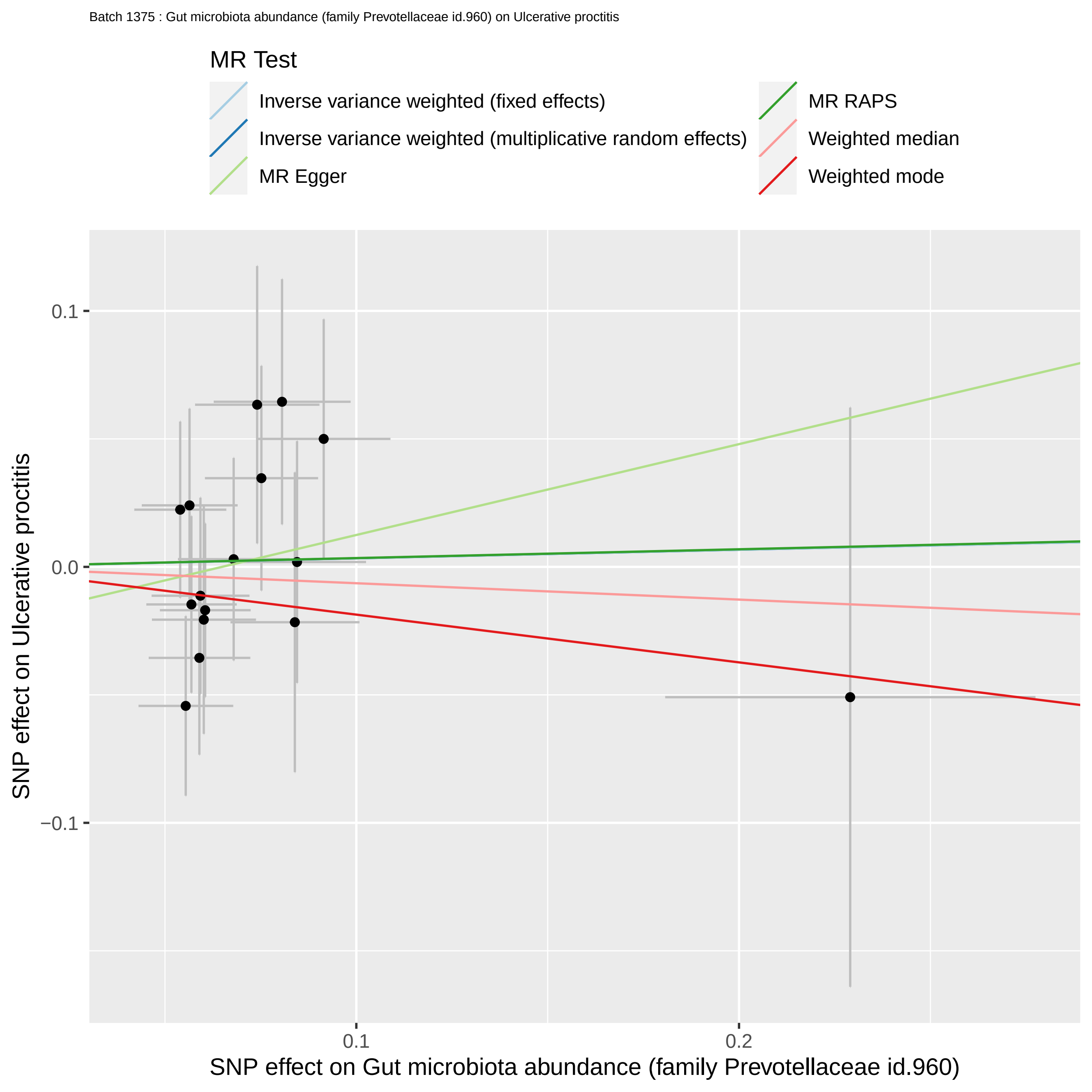

Batch 1375 : Gut microbiota abundance (family Prevotellaceae id.960) on Ulcerative proctitis
MR Test
Inverse variance weighted (fixed effects)
Inverse variance weighted (multiplicative random effects)
MR Egger
MR RAPS
Weighted median
Weighted mode
0.1
0.0
−0.1
0.1
0.2
SNP effect on Ulcerative proctitis
SNP effect on Gut microbiota abundance (family Prevotellaceae id.960)

Batch 1376 : Gut microbiota abundance (family Rhodospirillaceae id.2717) on Ulcerative proctitis

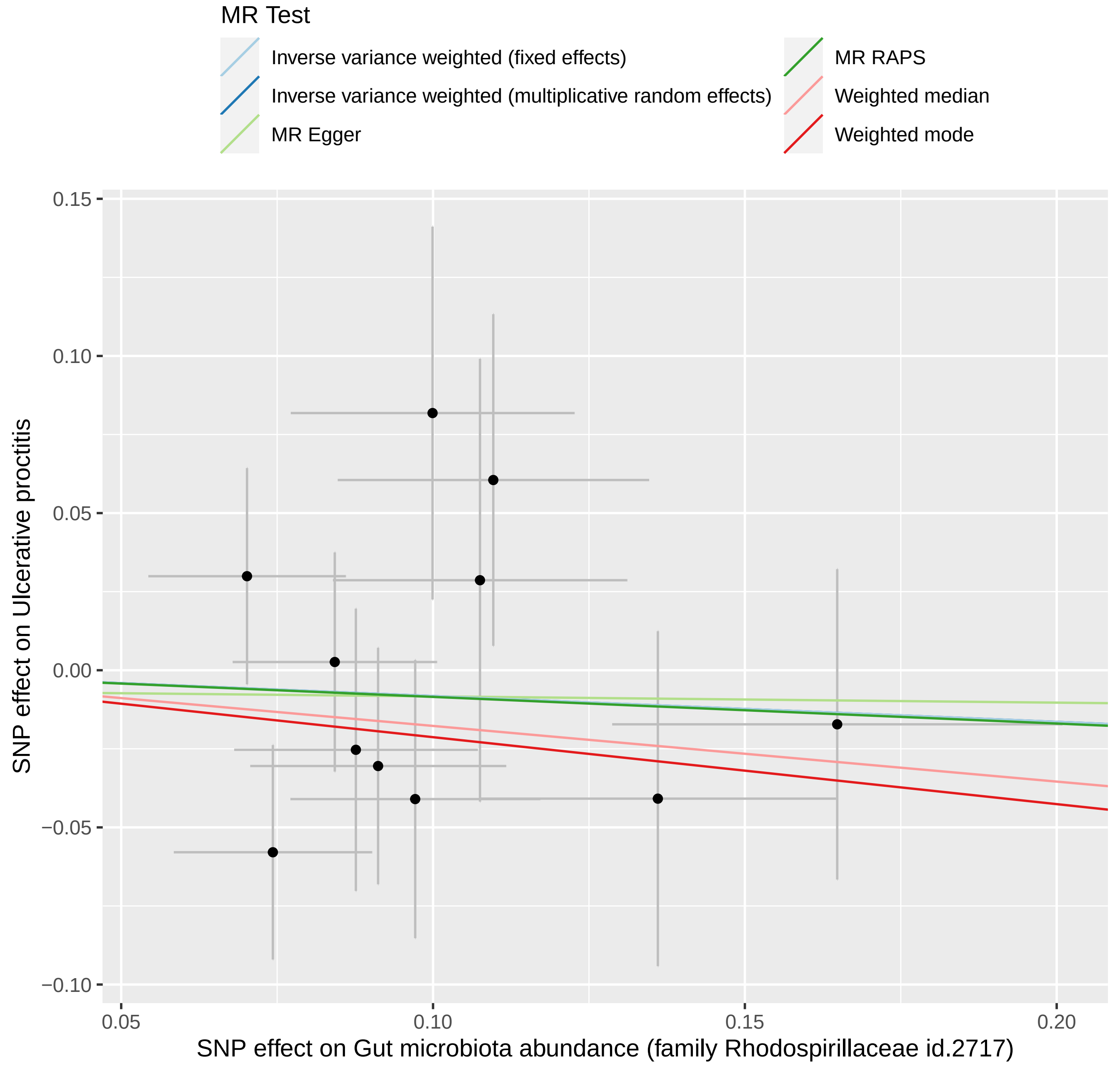

Batch 1377 : Gut microbiota abundance (family Rikenellaceae id.967) on Ulcerative proctitis

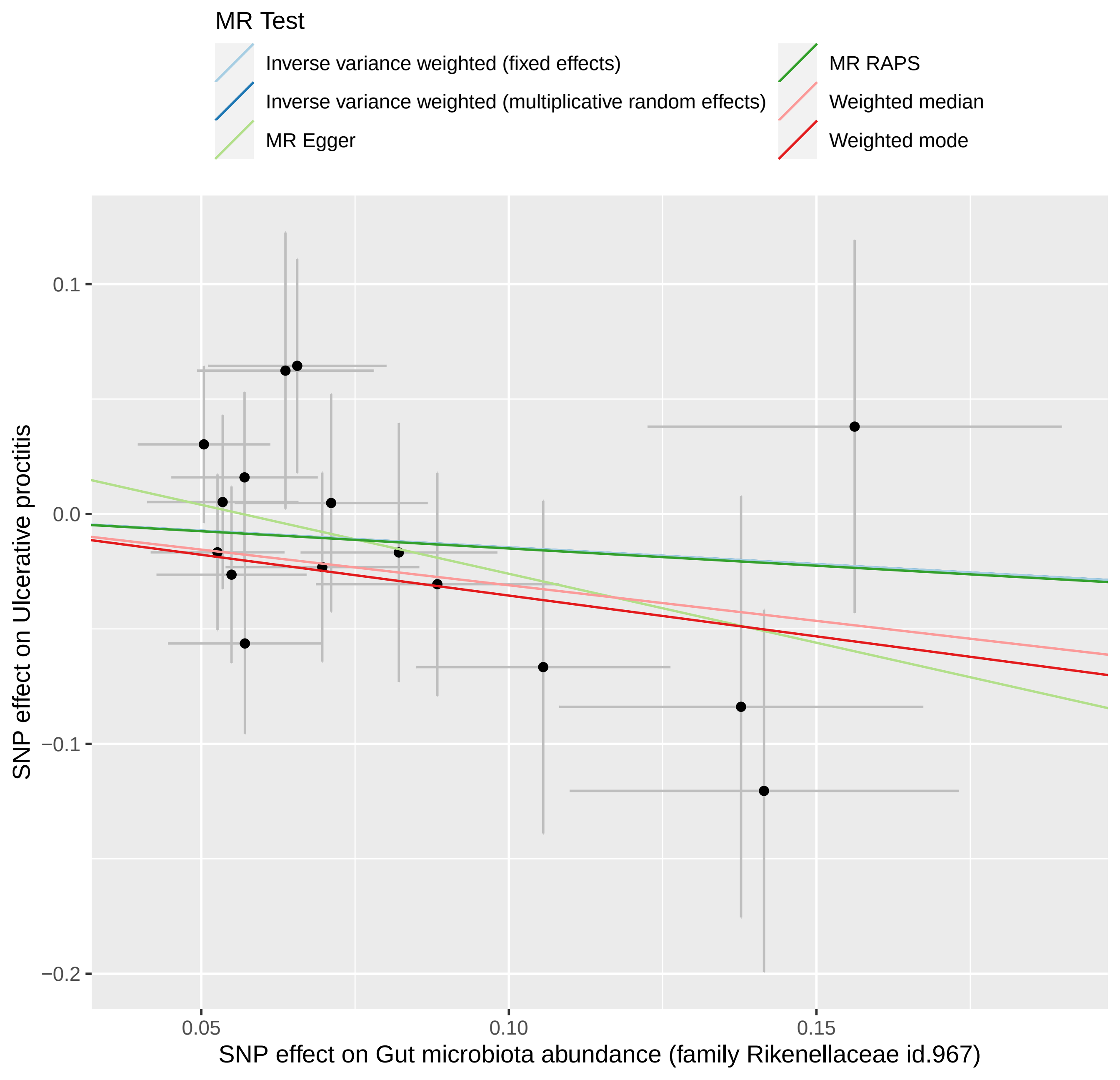

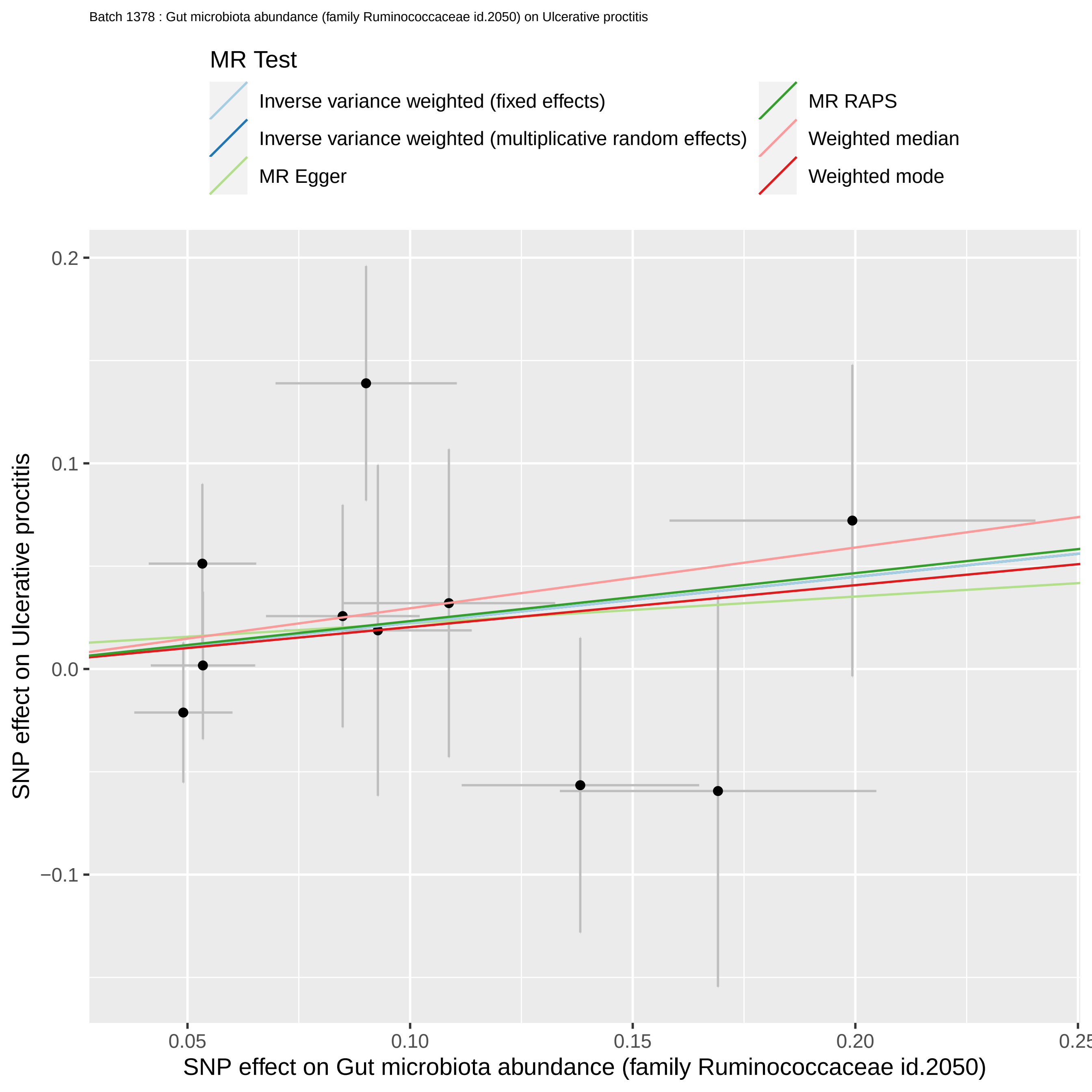
Batch 1378 : Gut microbiota abundance (family Ruminococcaceae id.2050) on Ulcerative proctitis
MR Test
Inverse variance weighted (fixed effects)
Inverse variance weighted (multiplicative random effects)
MR Egger
MR RAPS
Weighted median
Weighted mode
0.2
0.1
0.0
−0.1
0.05
0.10
0.15
0.20
0.25
SNP effect on Ulcerative proctitis
SNP effect on Gut microbiota abundance (family Ruminococcaceae id.2050)

Batch 1379 : Gut microbiota abundance (family Streptococcaceae id.1850) on Ulcerative proctitis

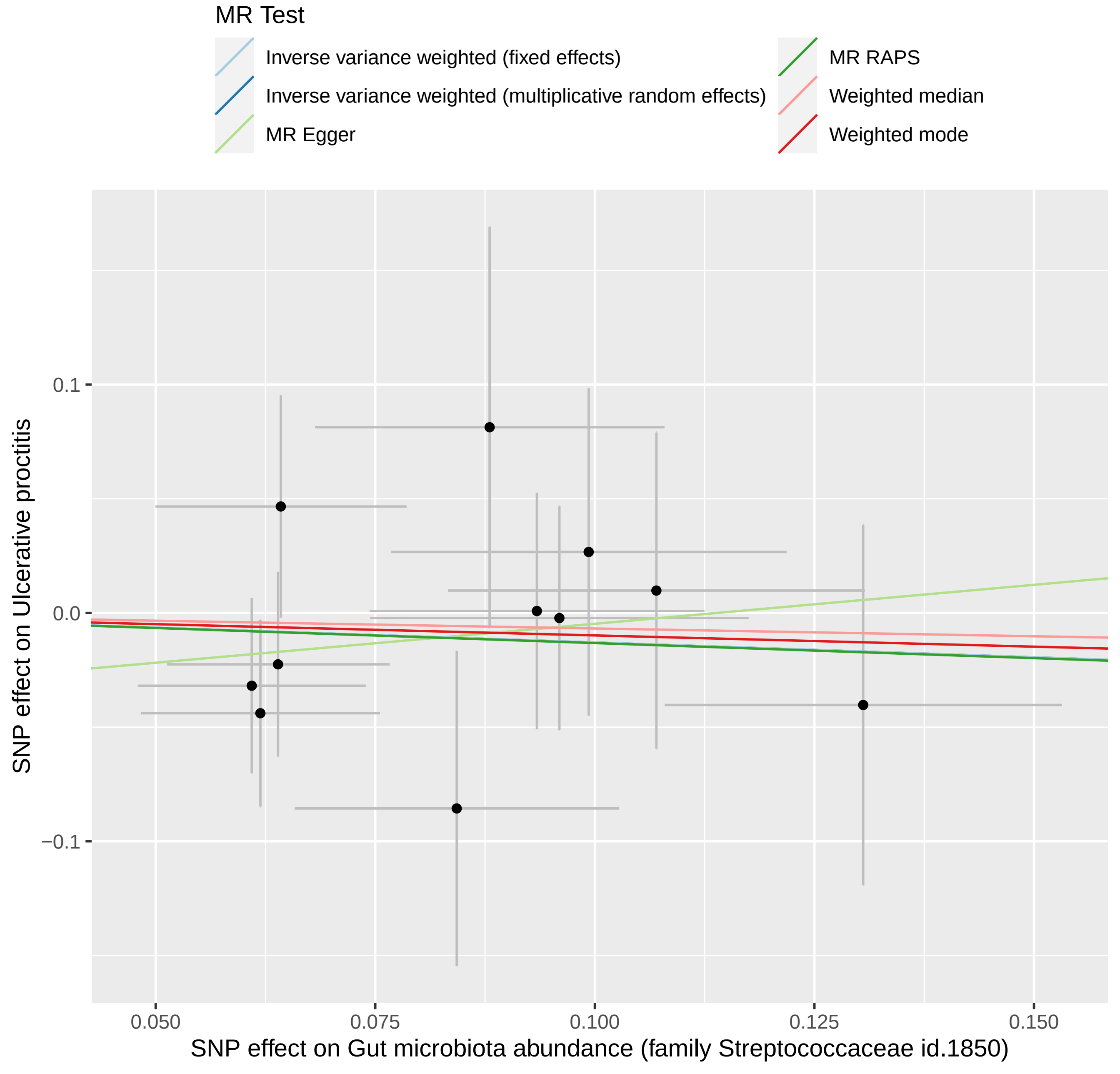

Batch 1380 : Gut microbiota abundance (family Veillonellaceae id.2172) on Ulcerative proctitis

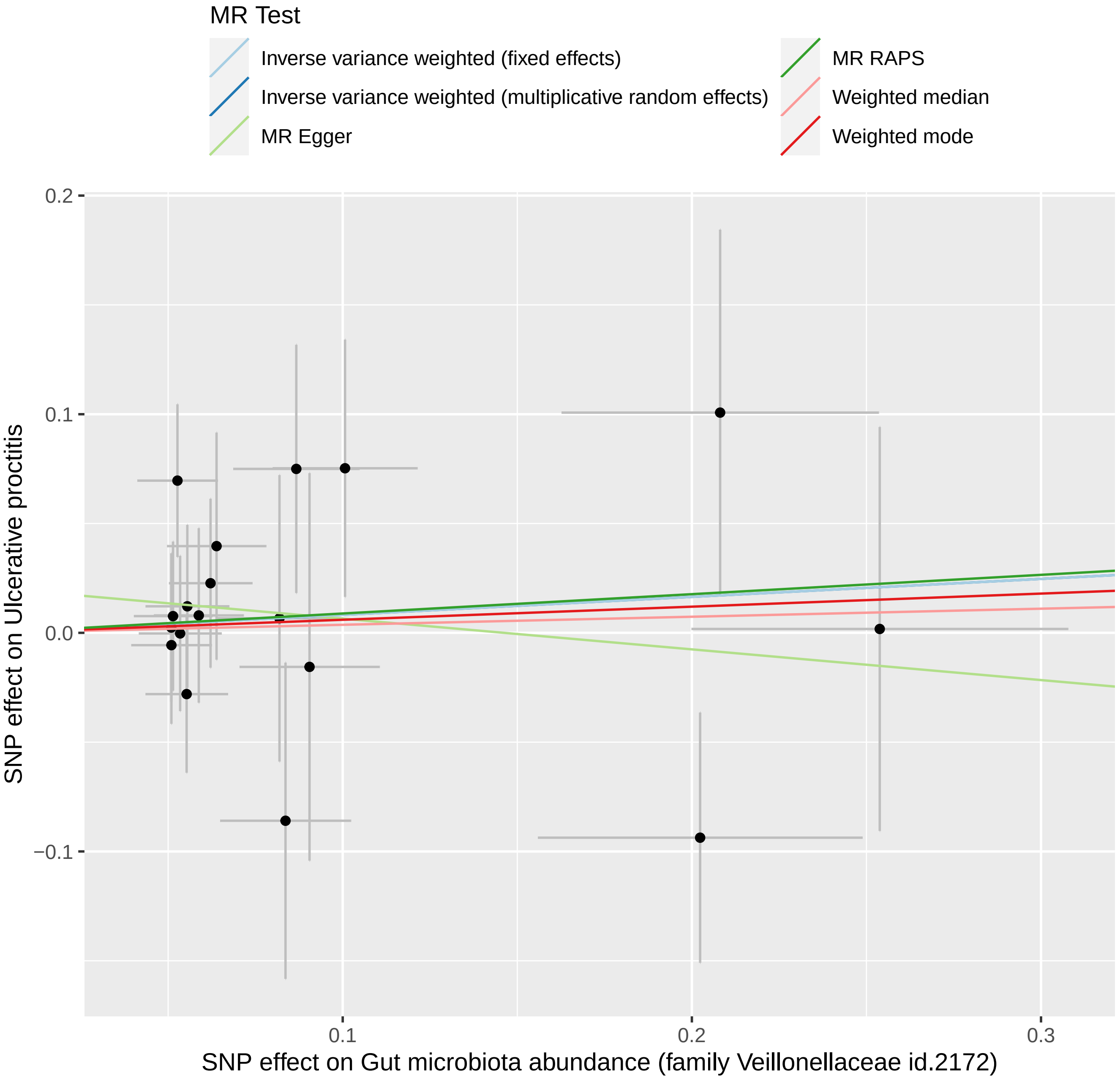

Batch 1381 : Gut microbiota abundance (family Verrucomicrobiaceae id.4036) on Ulcerative proctitis

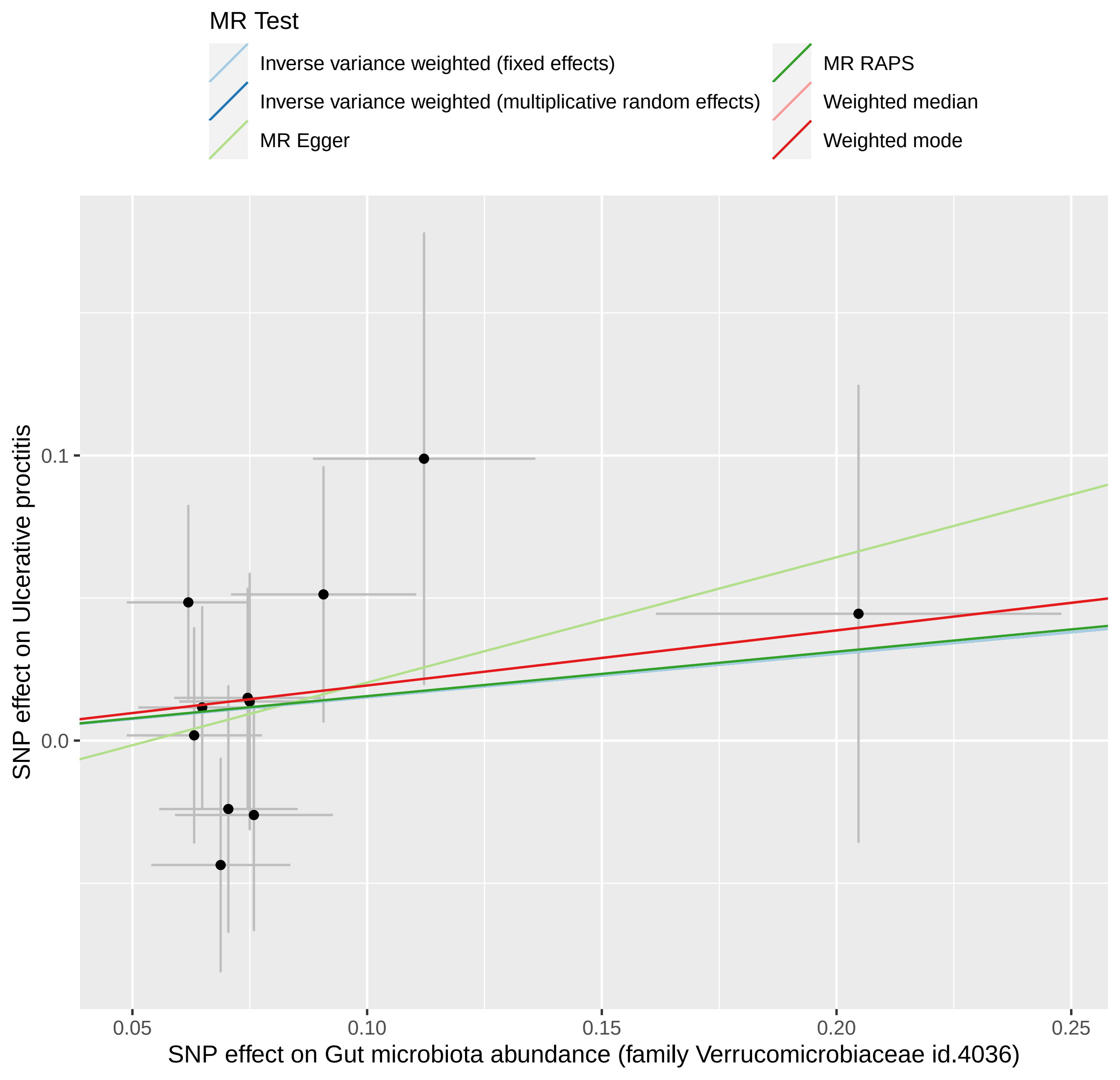

Batch 1382 : Gut microbiota abundance (family Victivallaceae id.2255) on Ulcerative proctitis

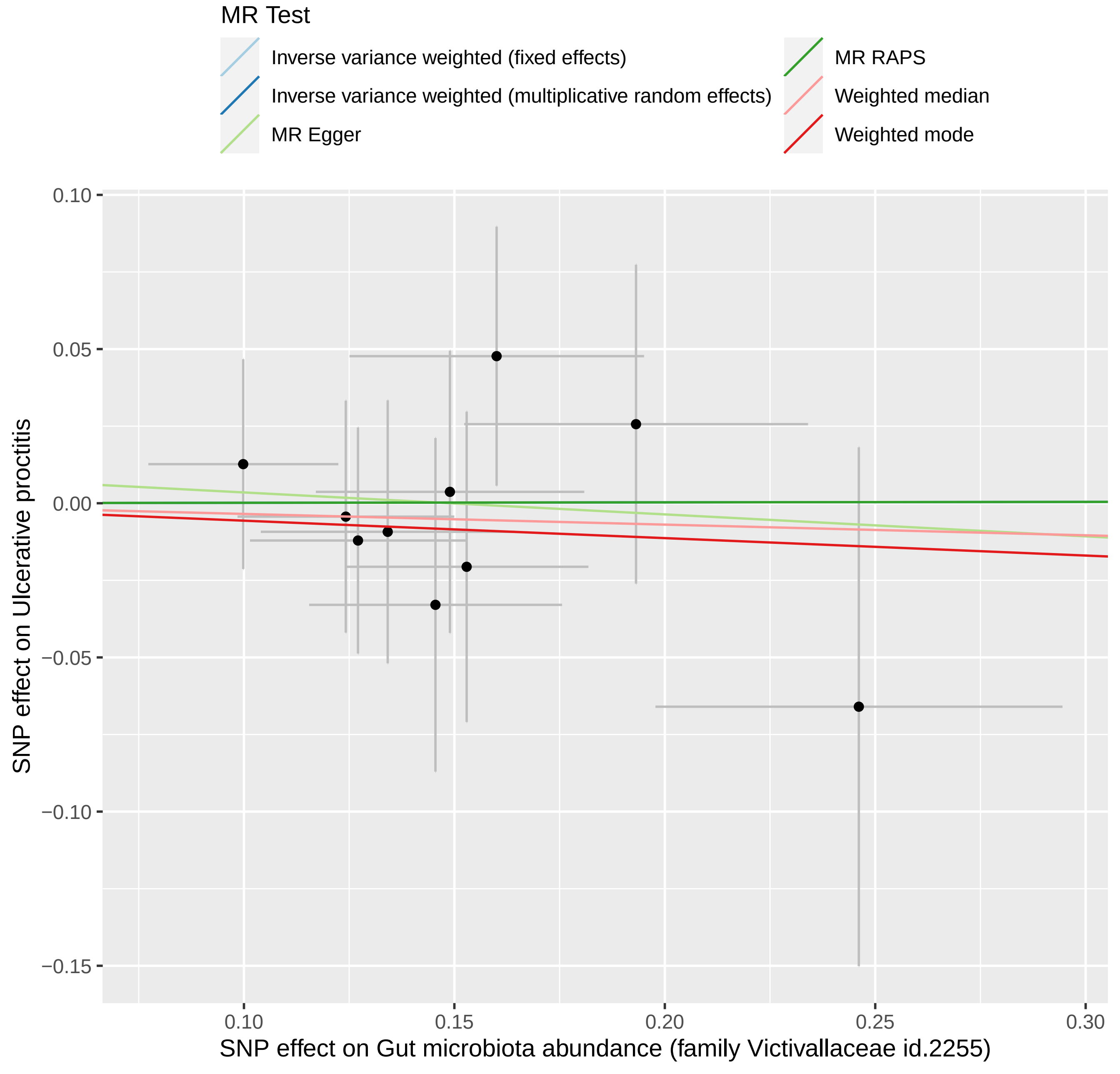

Batch 1383 : Gut microbiota abundance (genus Actinomyces id.423) on Ulcerative proctitis

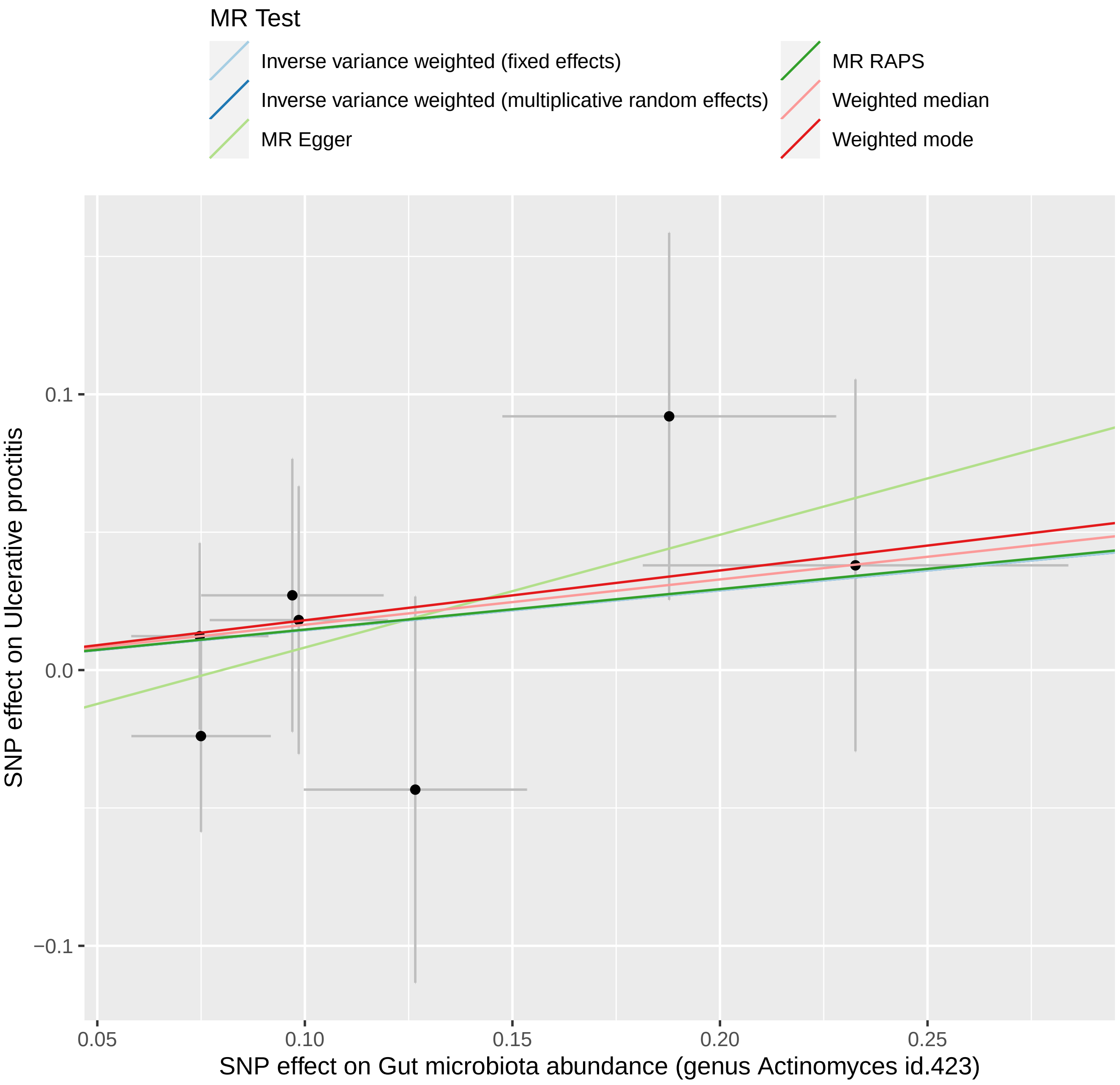

Batch 1384 : Gut microbiota abundance (genus Adlercreutzia id.812) on Ulcerative proctitis

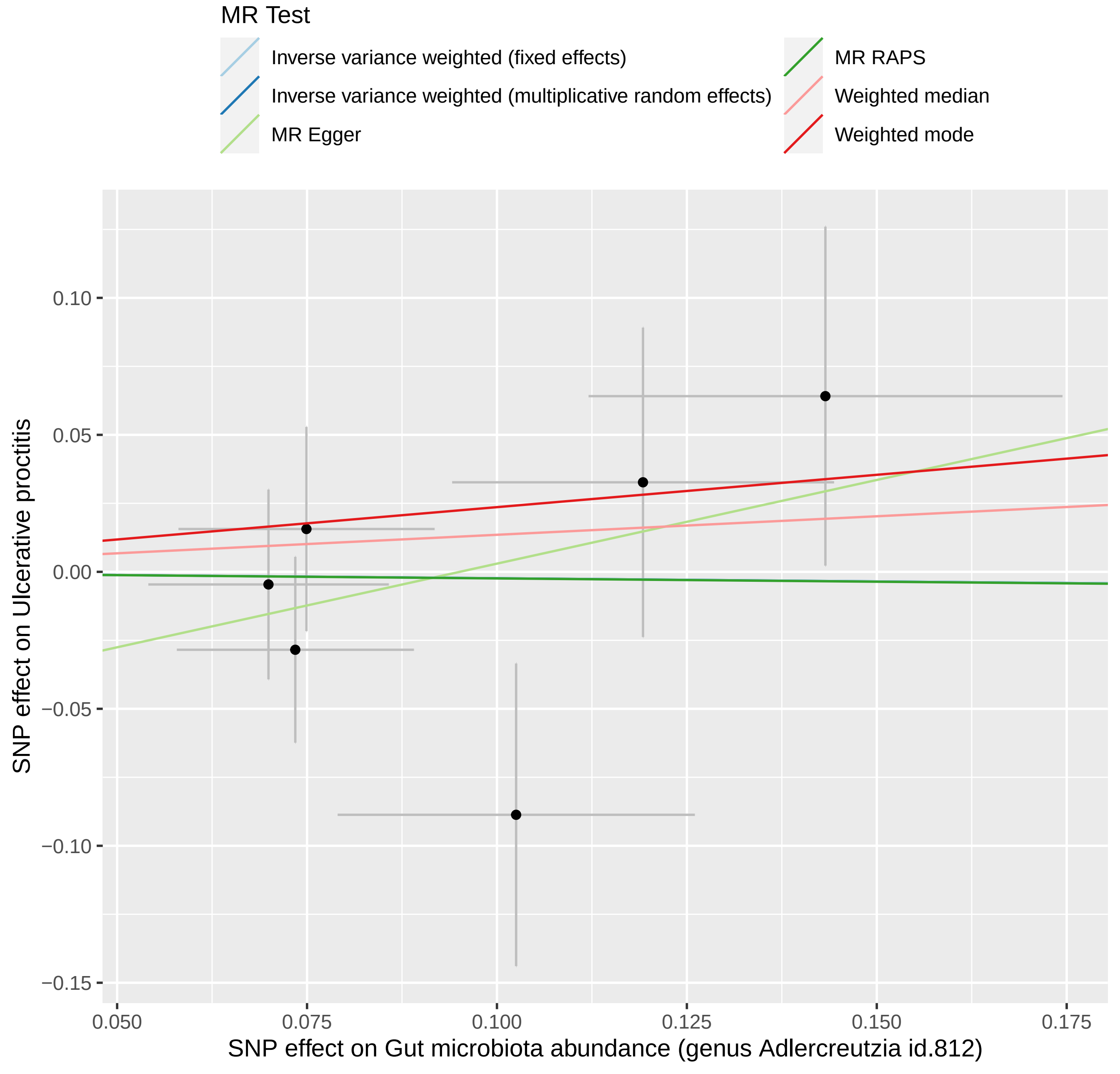

Batch 1385 : Gut microbiota abundance (genus Akkermansia id.4037) on Ulcerative proctitis

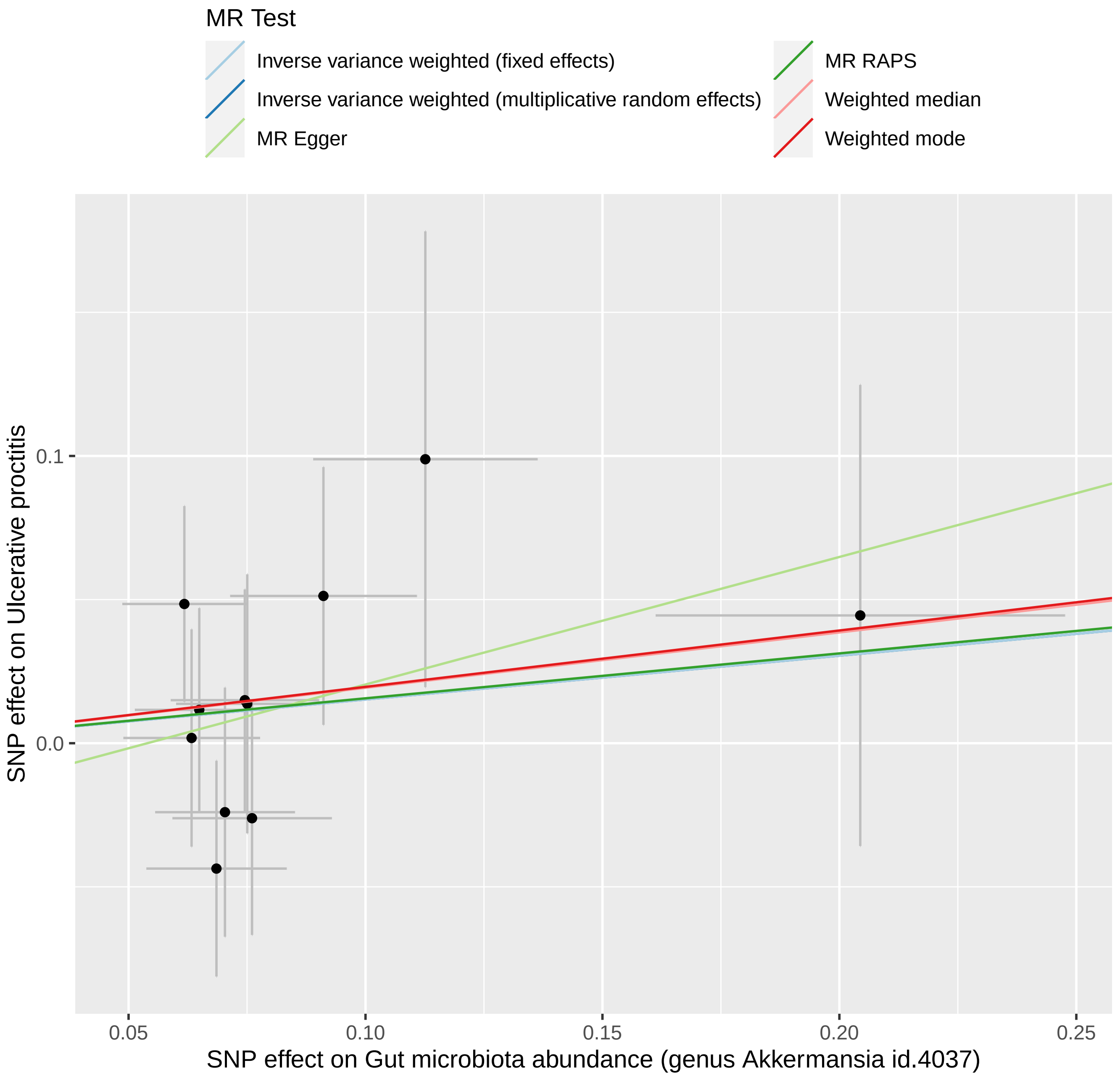

Batch 1386 : Gut microbiota abundance (genus Alistipes id.968) on Ulcerative proctitis

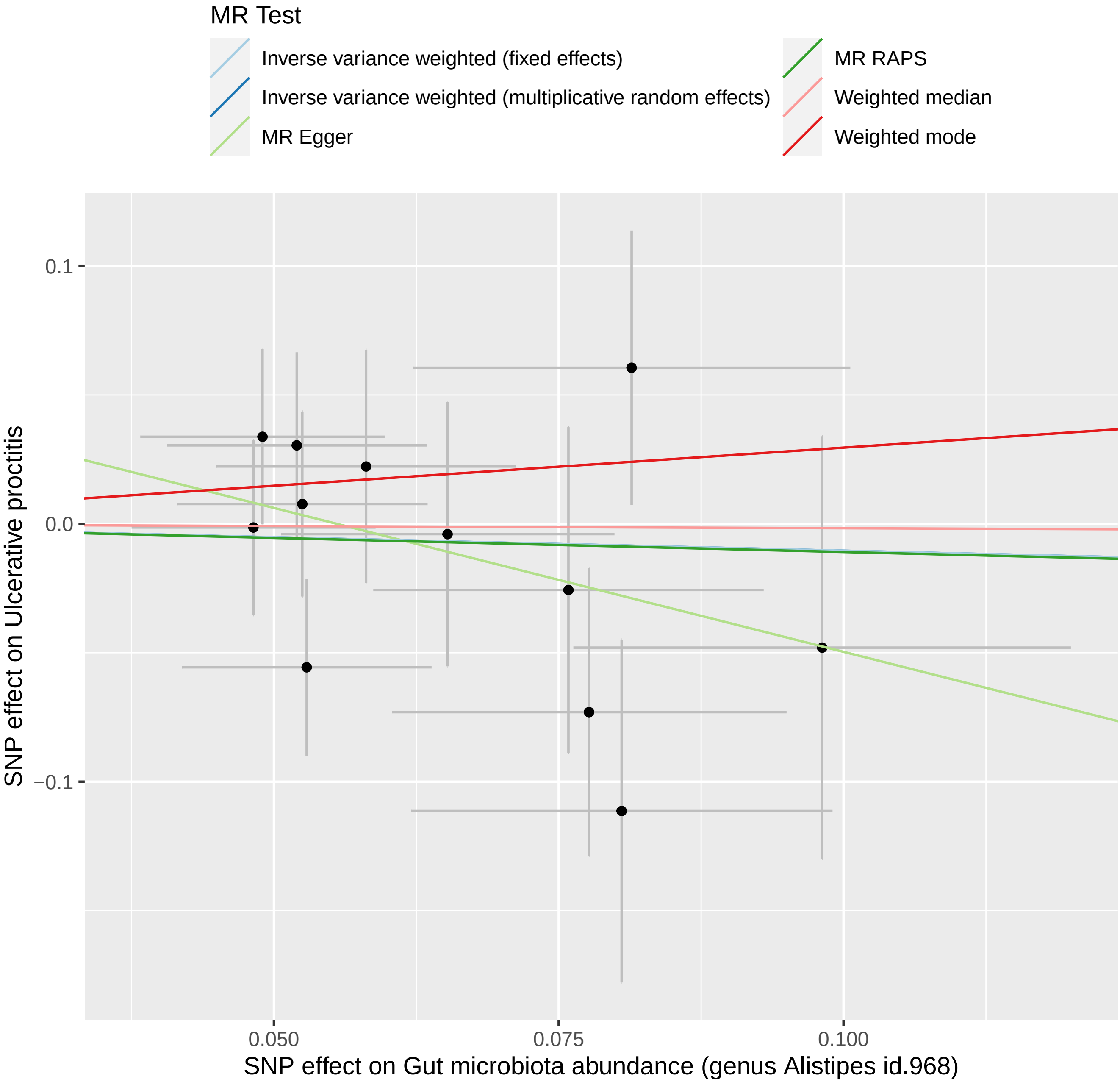

Batch 1387 : Gut microbiota abundance (genus Allisonella id.2174) on Ulcerative proctitis

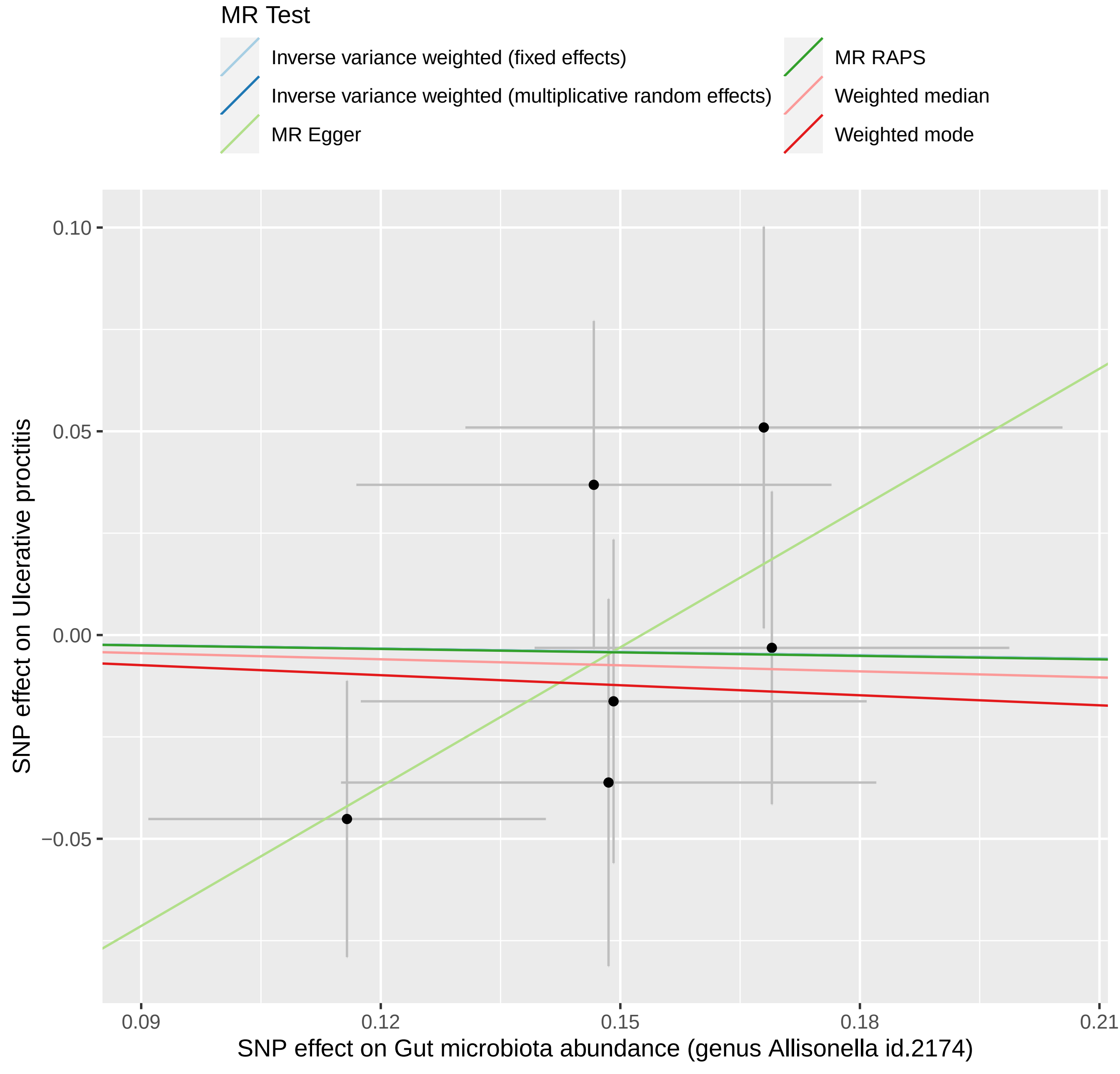

Batch 1388 : Gut microbiota abundance (genus Alloprevotella id.961) on Ulcerative proctitis

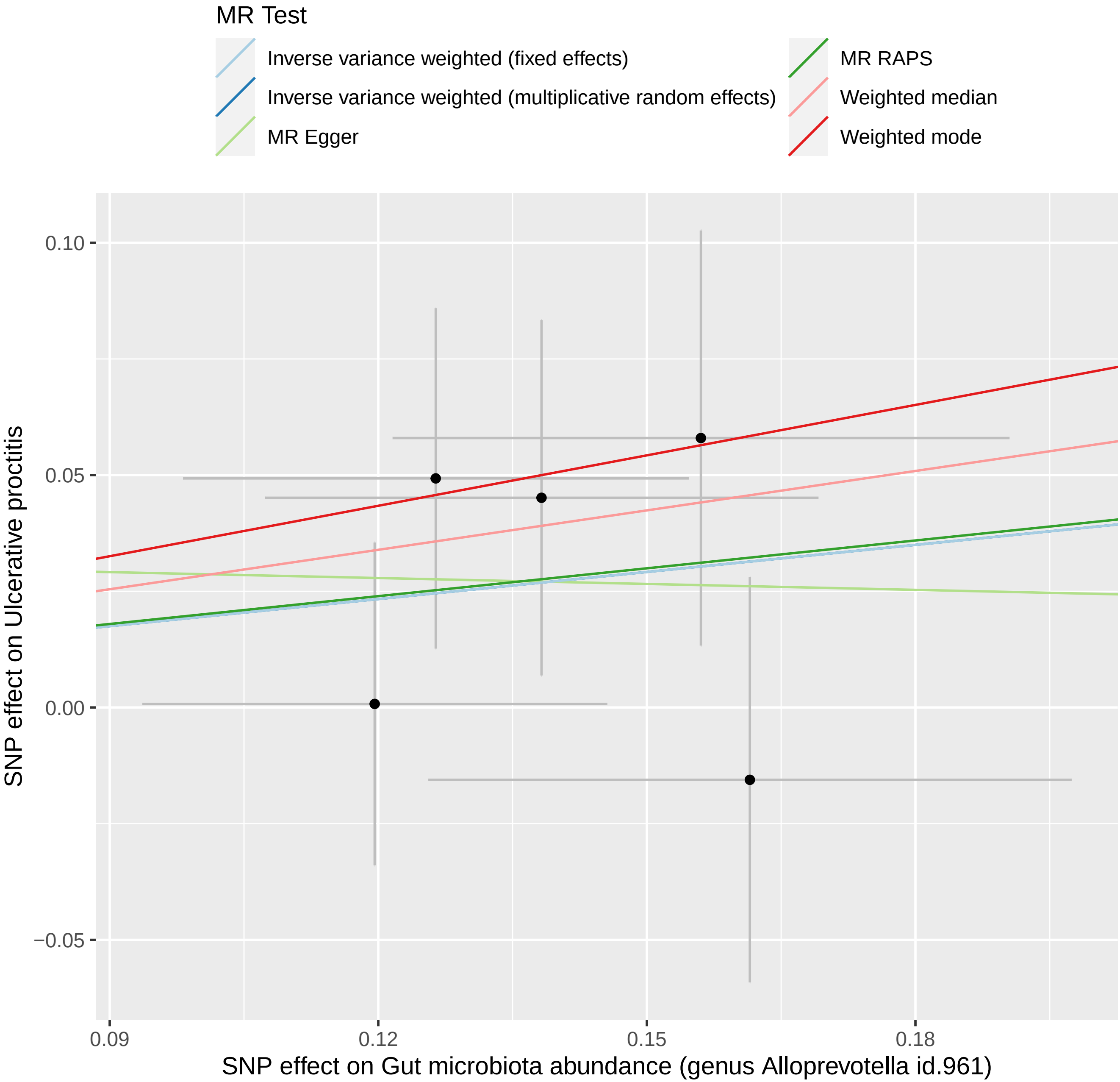

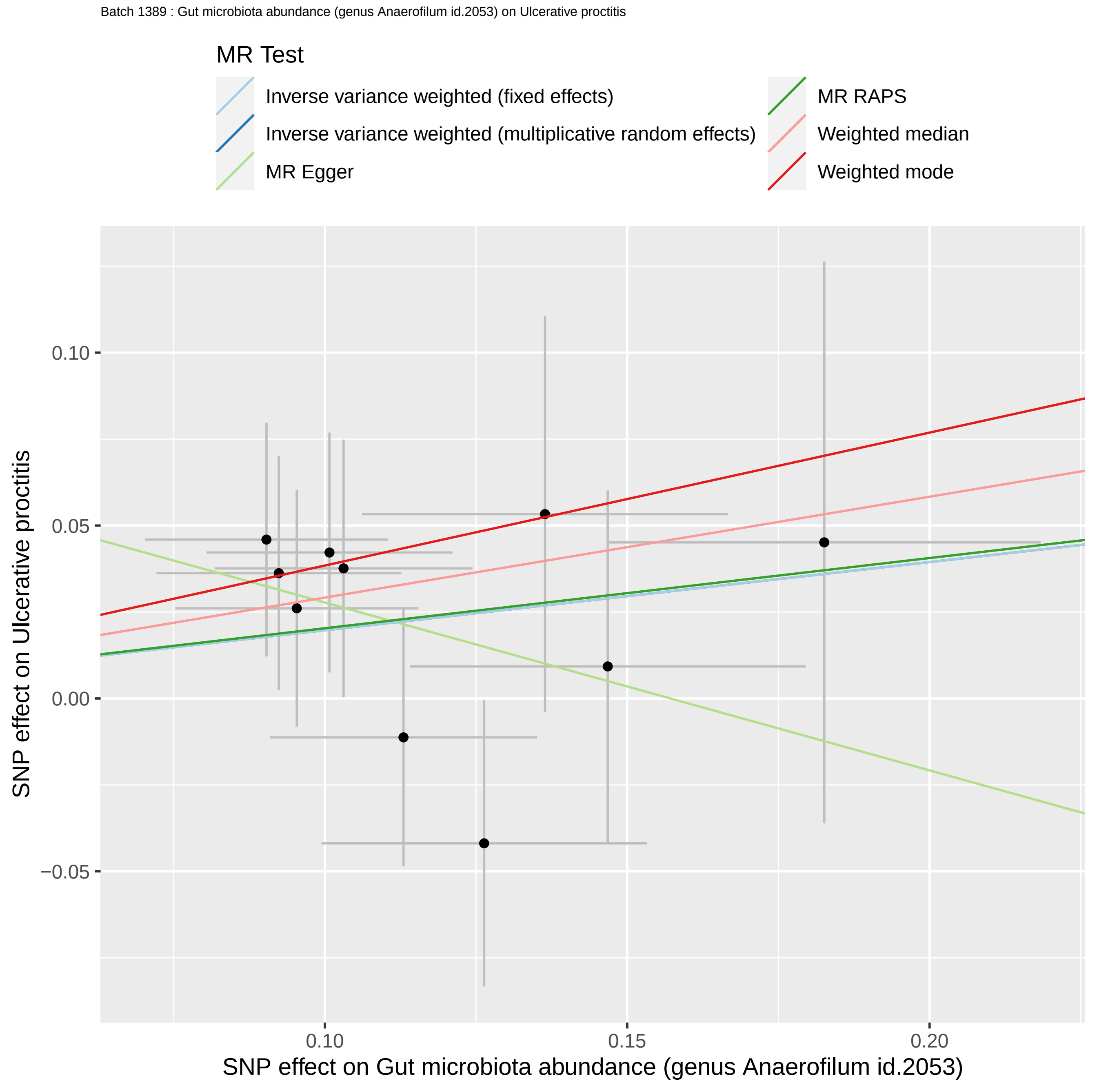
Batch 1389 : Gut microbiota abundance (genus Anaerofilum id.2053) on Ulcerative proctitis
MR Test
Inverse variance weighted (fixed effects)
Inverse variance weighted (multiplicative random effects)
MR Egger
MR RAPS
Weighted median
Weighted mode
0.10
0.05
0.00
−0.05
0.10
0.15
0.20
SNP effect on Ulcerative proctitis
SNP effect on Gut microbiota abundance (genus Anaerofilum id.2053)

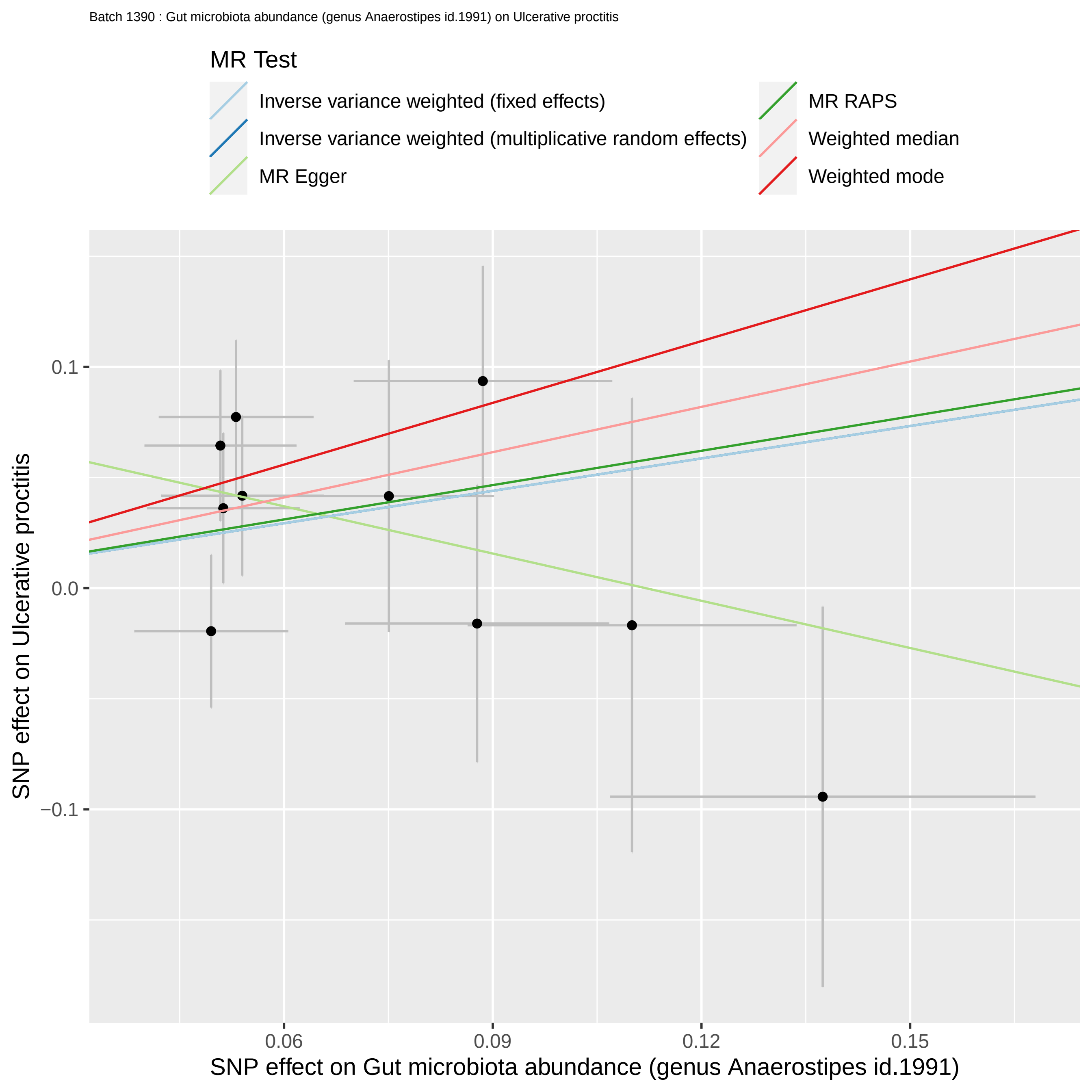

Batch 1390 : Gut microbiota abundance (genus Anaerostipes id.1991) on Ulcerative proctitis
MR Test
Inverse variance weighted (fixed effects)
Inverse variance weighted (multiplicative random effects)
MR Egger
MR RAPS
Weighted median
Weighted mode
0.1
0.0
−0.1
0.06
0.09
0.12
0.15
SNP effect on Ulcerative proctitis
SNP effect on Gut microbiota abundance (genus Anaerostipes id.1991)

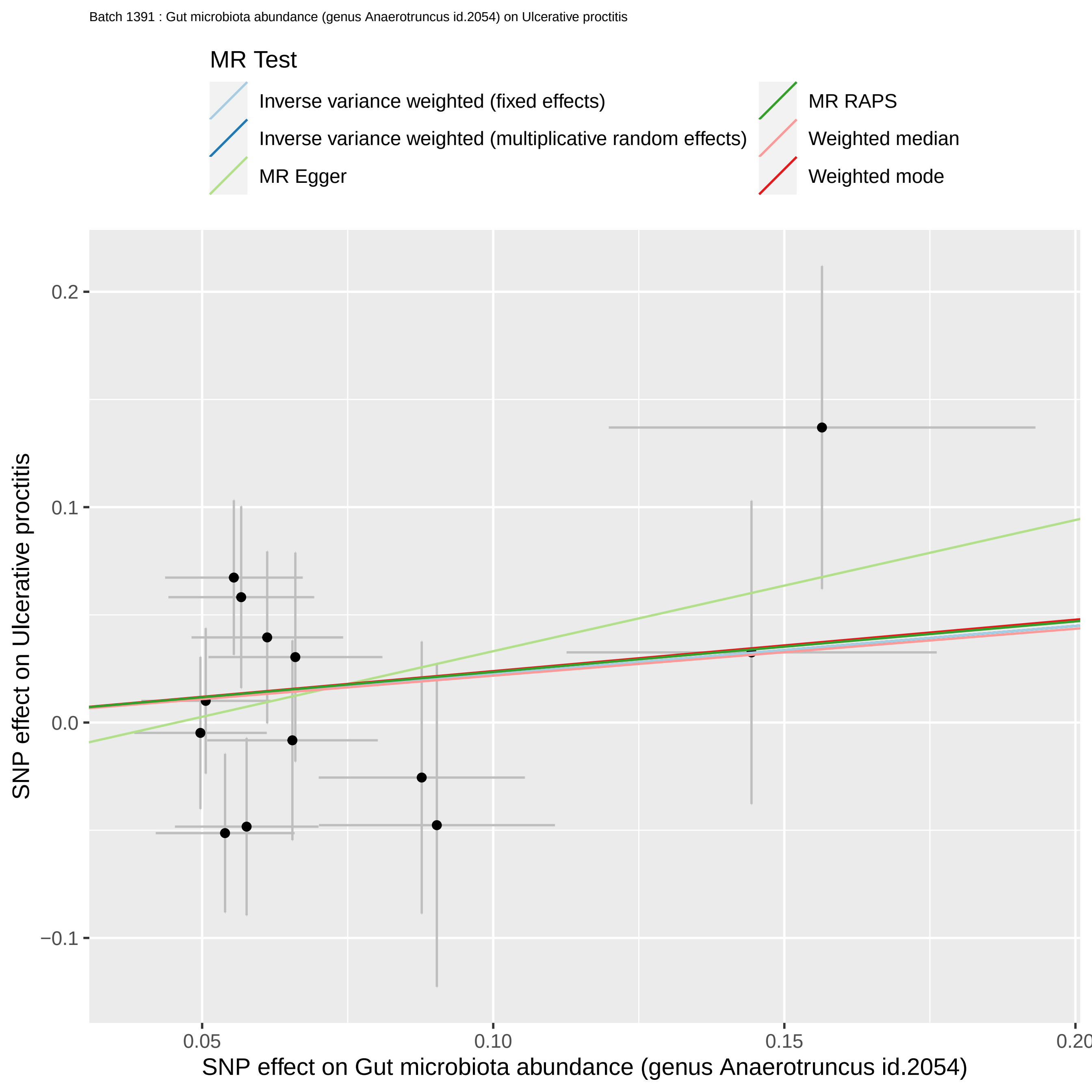

Batch 1391 : Gut microbiota abundance (genus Anaerotruncus id.2054) on Ulcerative proctitis
MR Test
Inverse variance weighted (fixed effects)
Inverse variance weighted (multiplicative random effects)
MR Egger
MR RAPS
Weighted median
Weighted mode
0.2
0.1
0.0
−0.1
0.05
0.10
0.15
0.20
SNP effect on Ulcerative proctitis
SNP effect on Gut microbiota abundance (genus Anaerotruncus id.2054)

Batch 1392 : Gut microbiota abundance (genus Bacteroides id.918) on Ulcerative proctitis

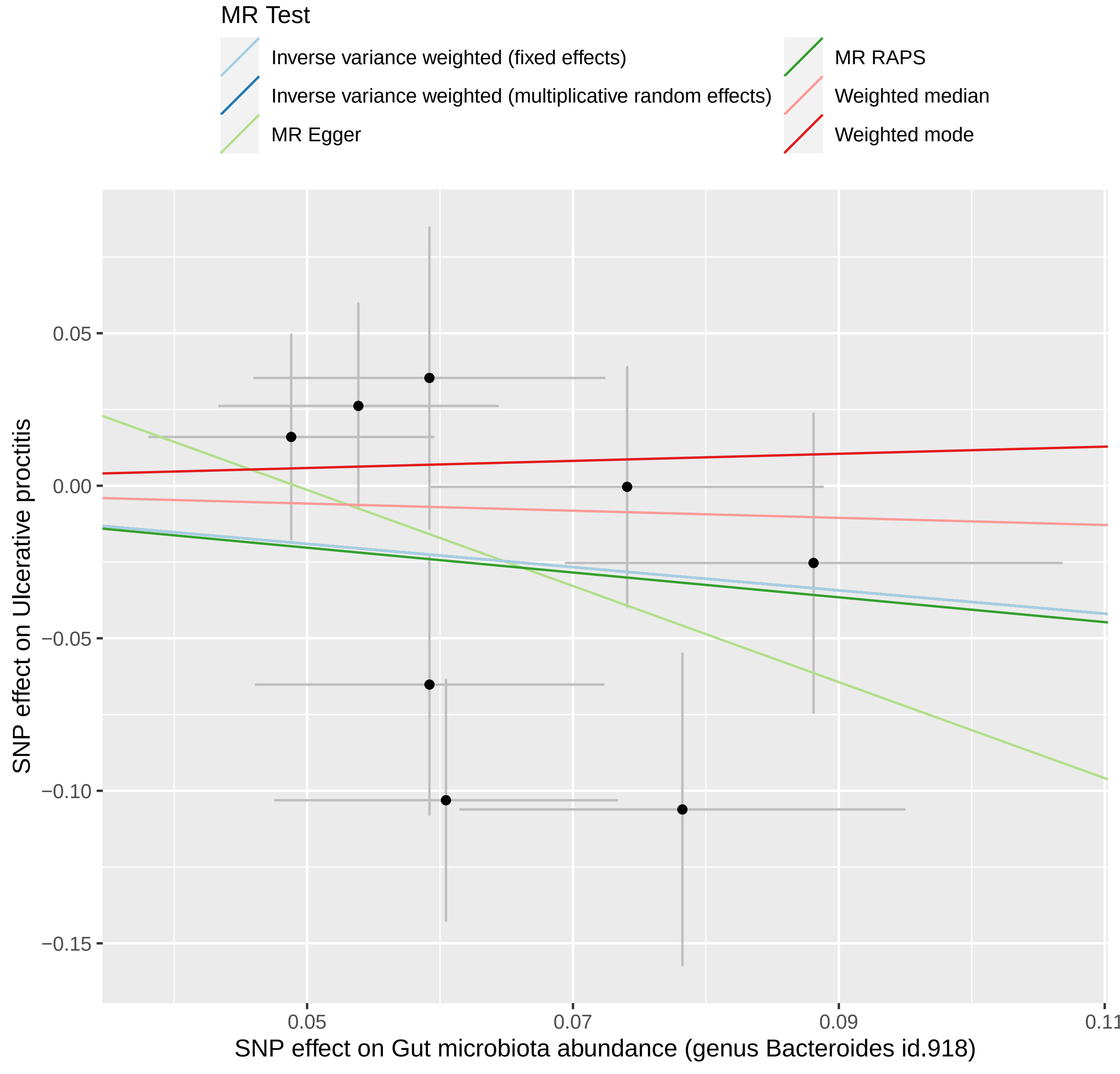

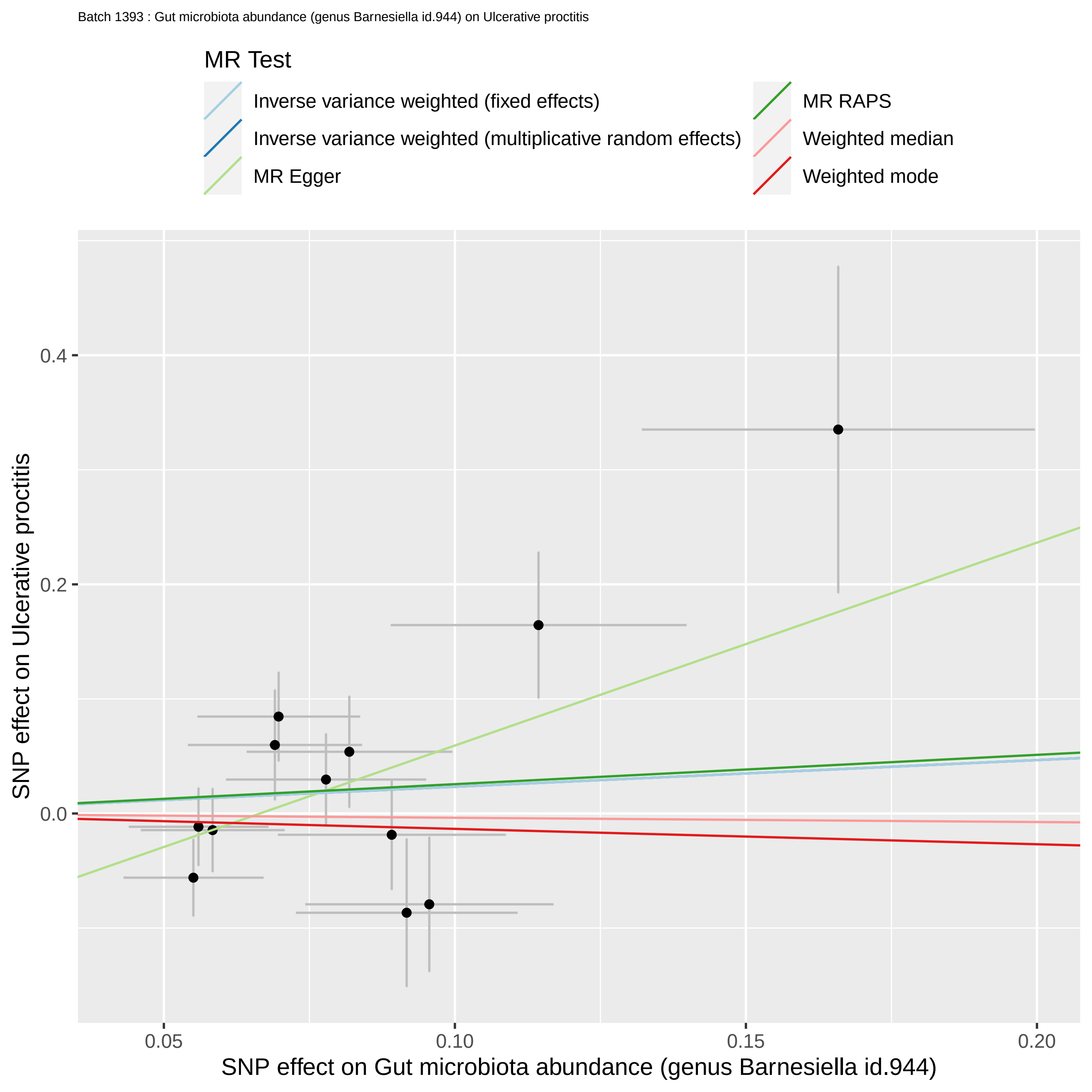
Batch 1393 : Gut microbiota abundance (genus Barnesiella id.944) on Ulcerative proctitis
MR Test
Inverse variance weighted (fixed effects)
Inverse variance weighted (multiplicative random effects)
MR Egger
MR RAPS
Weighted median
Weighted mode
0.4
0.2
0.0
0.05
0.10
0.15
0.20
SNP effect on Ulcerative proctitis
SNP effect on Gut microbiota abundance (genus Barnesiella id.944)

Batch 1394 : Gut microbiota abundance (genus Bifidobacterium id.436) on Ulcerative proctitis

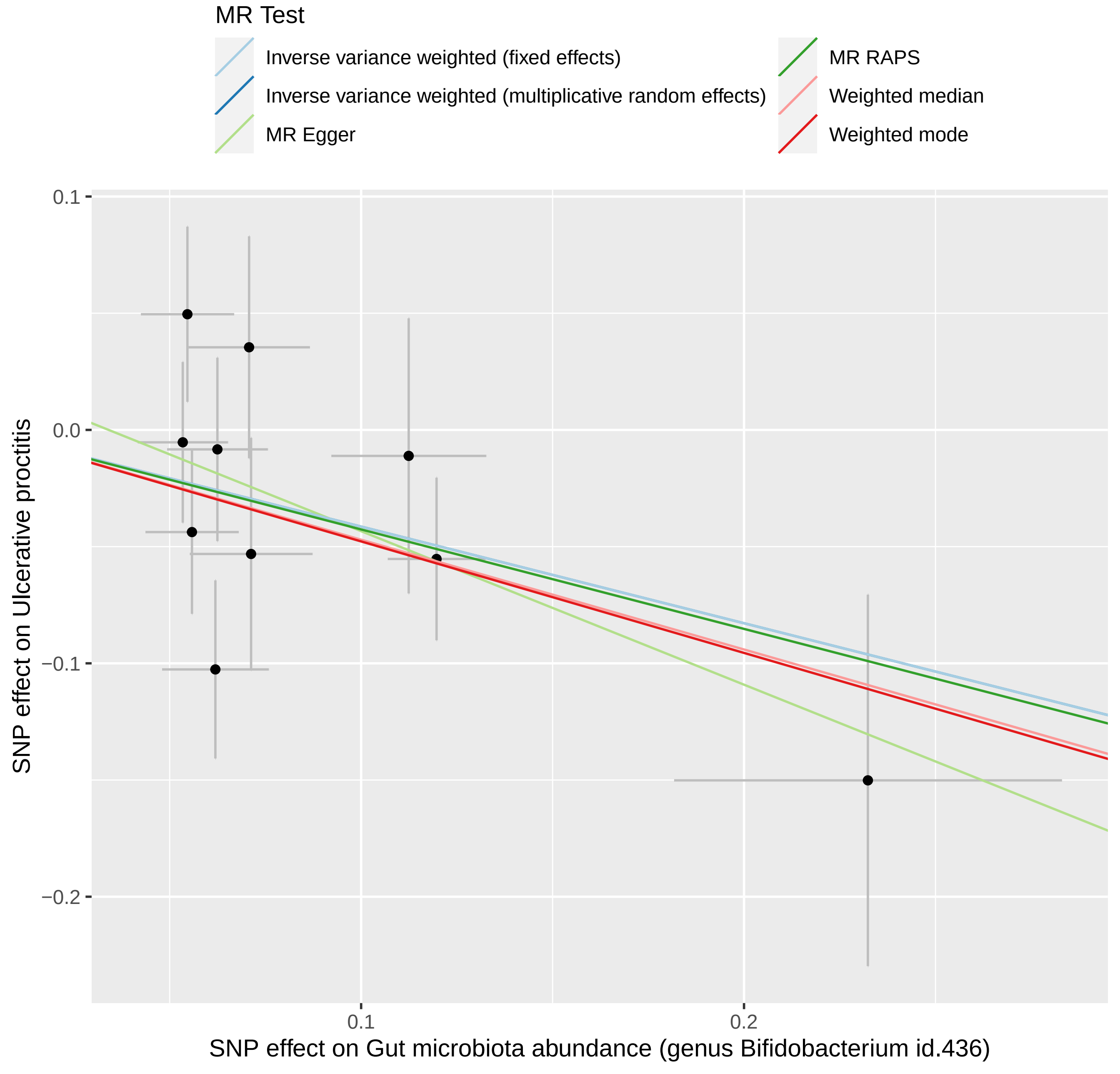

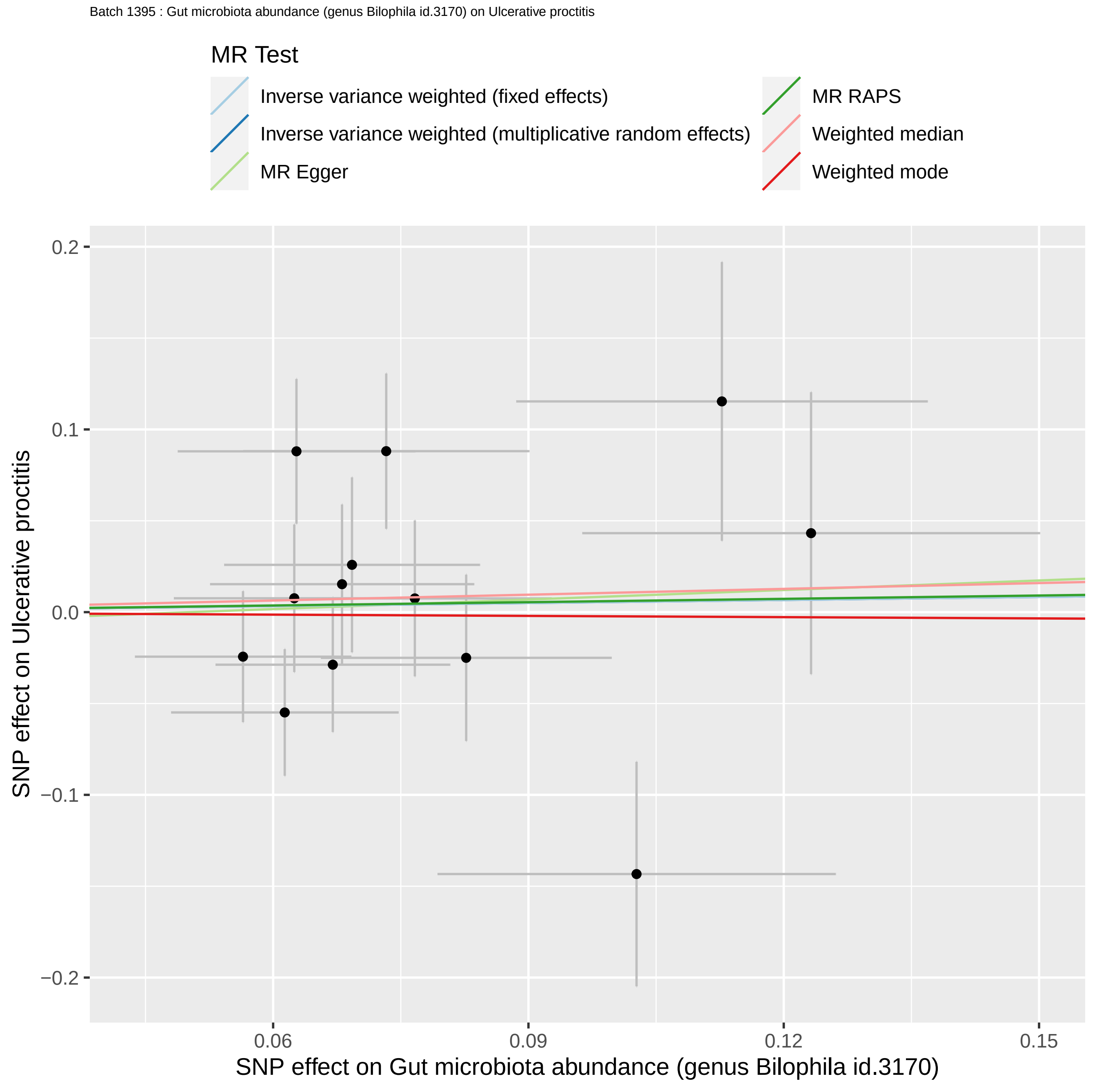
Batch 1395 : Gut microbiota abundance (genus Bilophila id.3170) on Ulcerative proctitis
MR Test
Inverse variance weighted (fixed effects)
Inverse variance weighted (multiplicative random effects)
MR Egger
MR RAPS
Weighted median
Weighted mode
0.2
0.1
0.0
−0.1
−0.2
0.06
0.09
0.12
0.15
SNP effect on Ulcerative proctitis
SNP effect on Gut microbiota abundance (genus Bilophila id.3170)

Batch 1396 : Gut microbiota abundance (genus Blautia id.1992) on Ulcerative proctitis

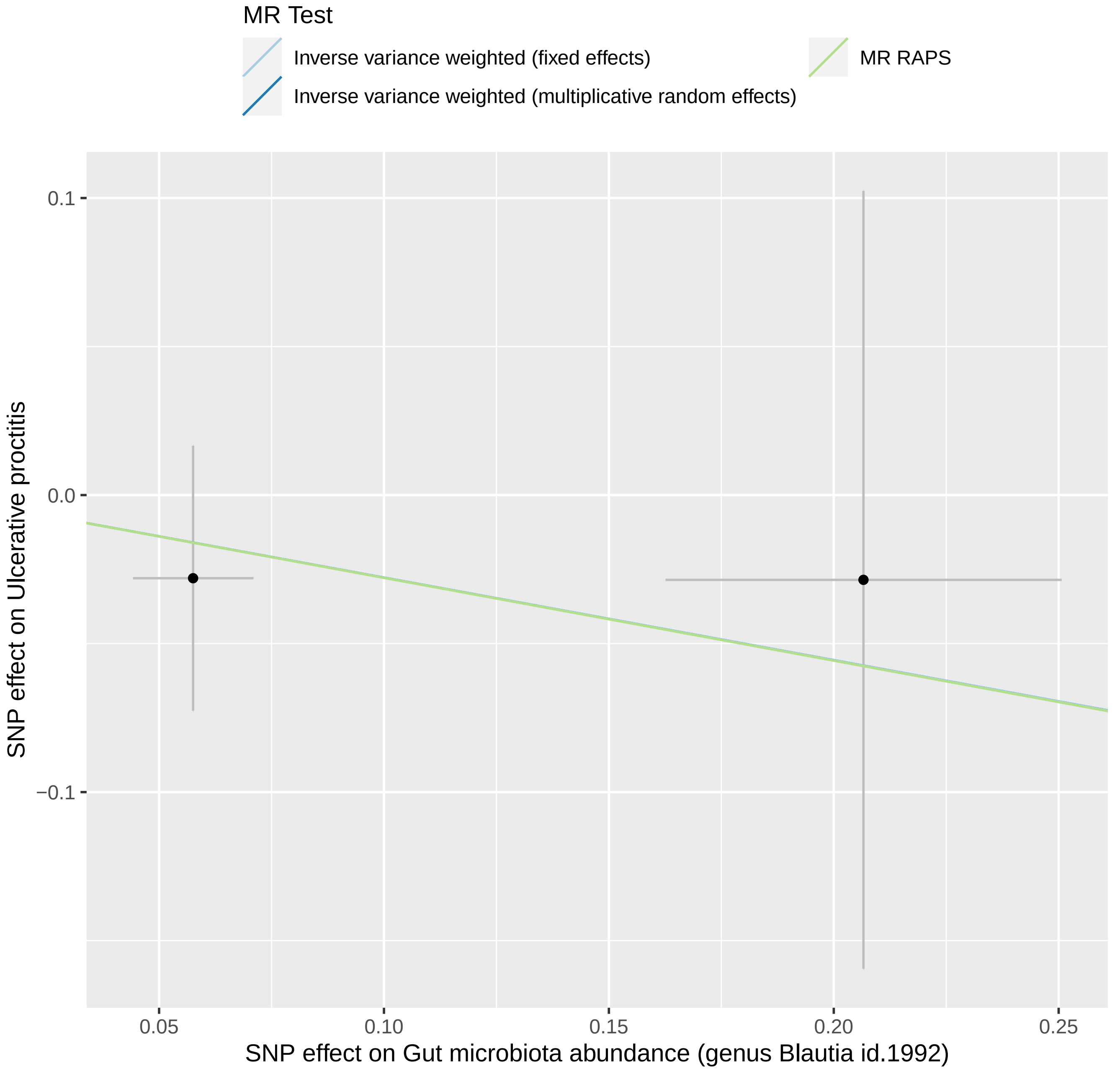

Batch 1397 : Gut microbiota abundance (genus Butyricicoccus id.2055) on Ulcerative proctitis

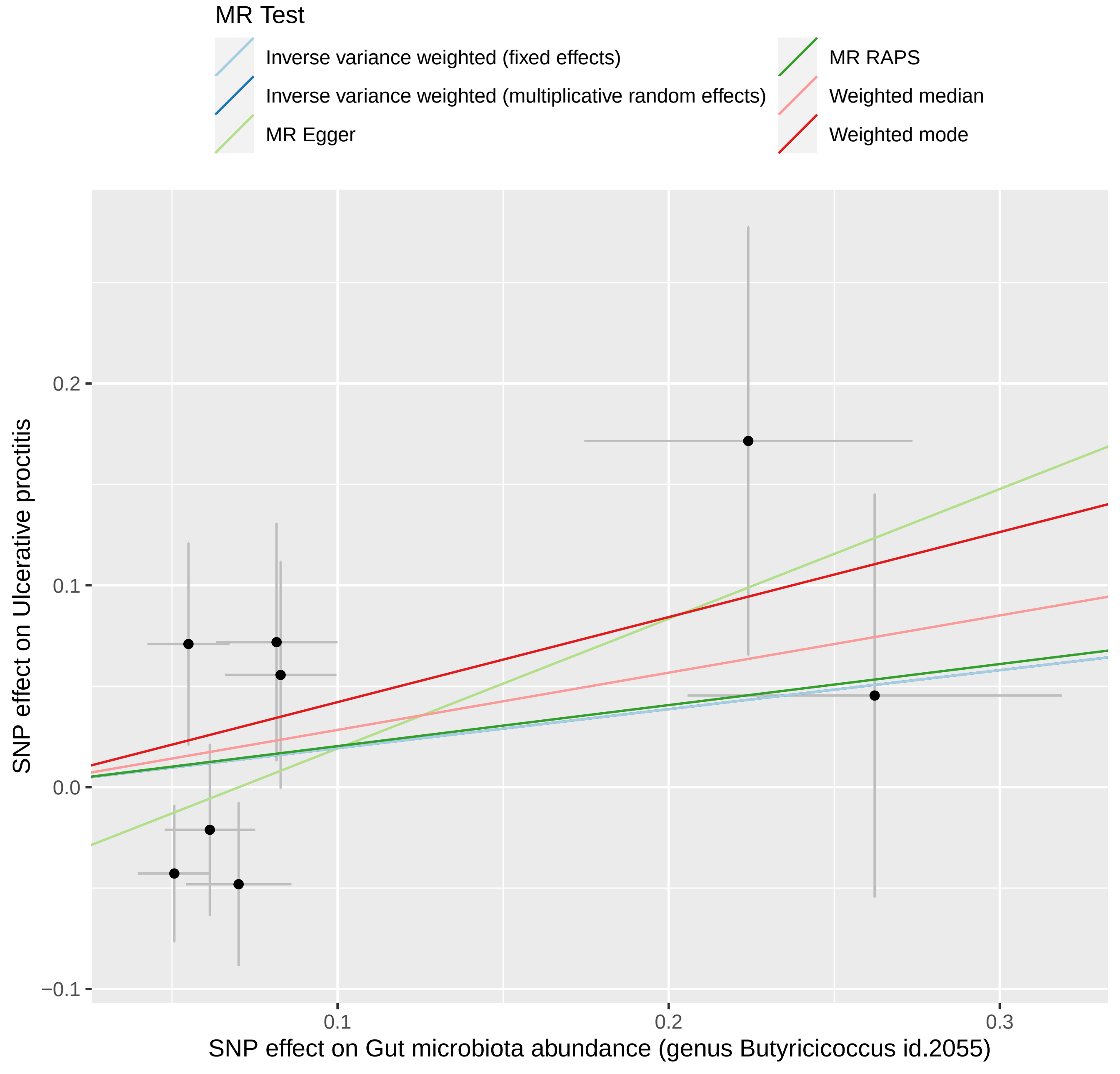

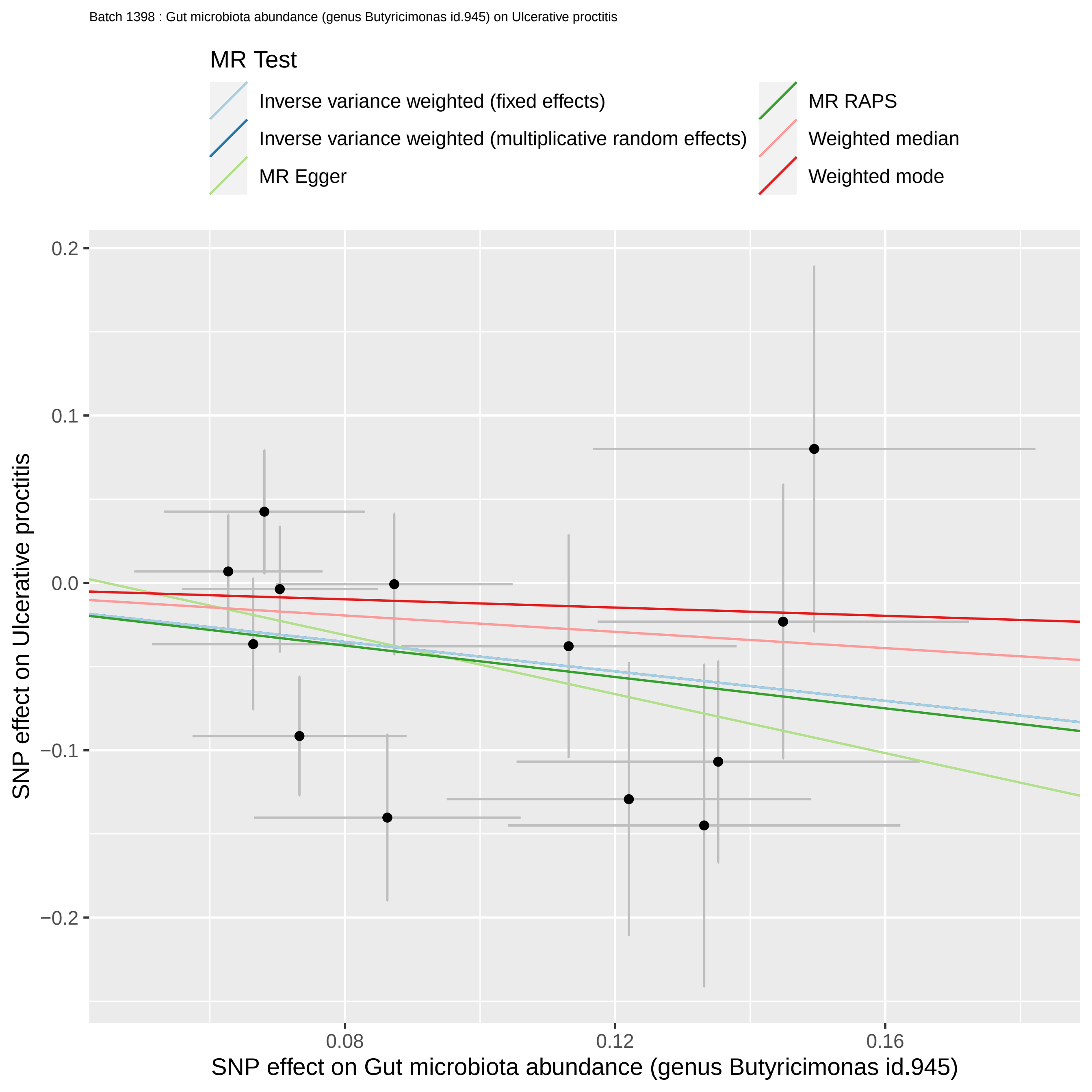

Batch 1398 : Gut microbiota abundance (genus Butyricimonas id.945) on Ulcerative proctitis
MR Test
Inverse variance weighted (fixed effects)
Inverse variance weighted (multiplicative random effects)
MR Egger
MR RAPS
Weighted median
Weighted mode
0.2
0.1
0.0
−0.1
−0.2
0.08
0.12
0.16
SNP effect on Ulcerative proctitis
SNP effect on Gut microbiota abundance (genus Butyricimonas id.945)

Batch 1399 : Gut microbiota abundance (genus Butyrivibrio id.1993) on Ulcerative proctitis

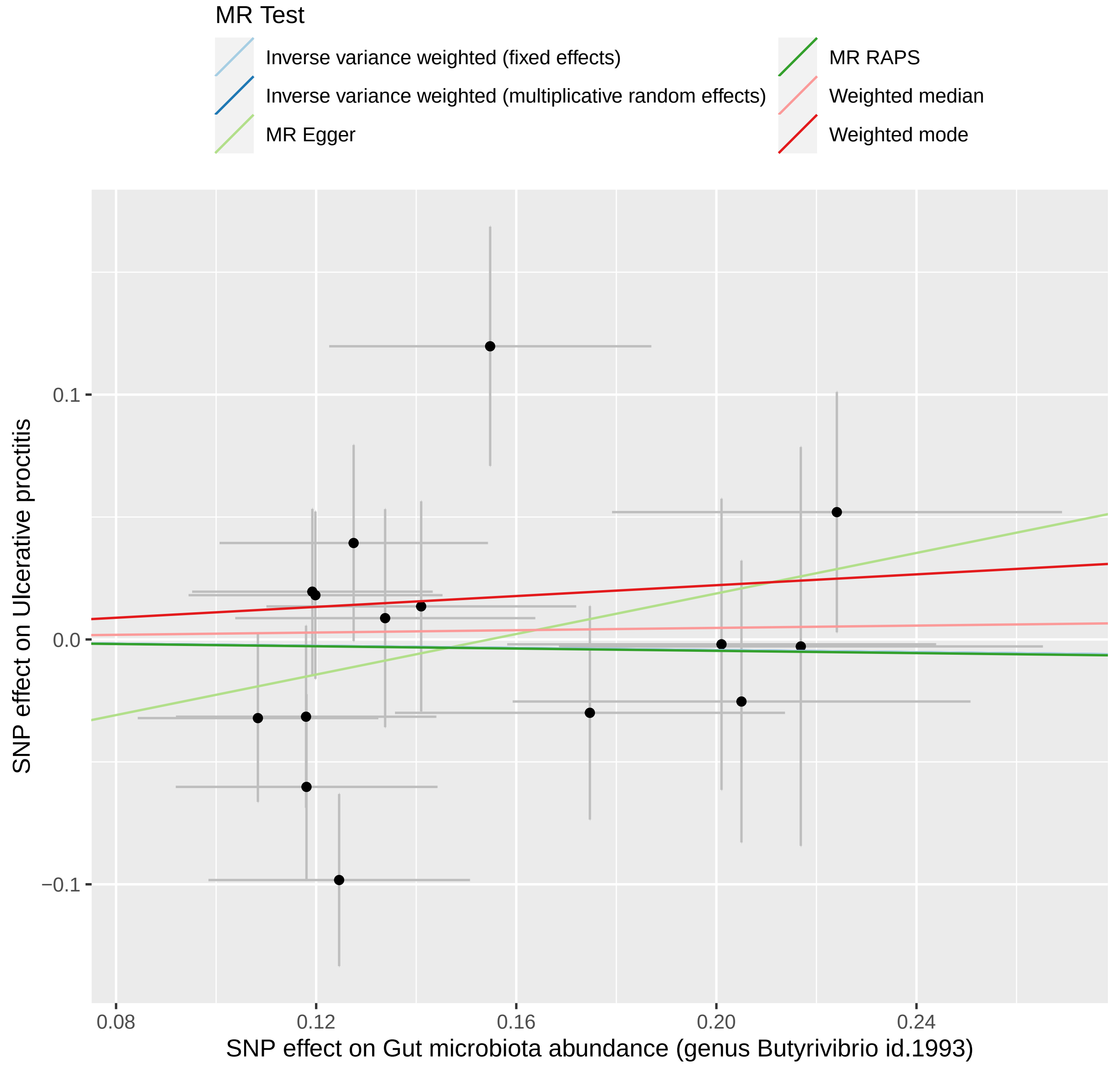

Batch 1400 : Gut microbiota abundance (genus Candidatus Soleaferrea id.11350) on Ulcerative proctitis

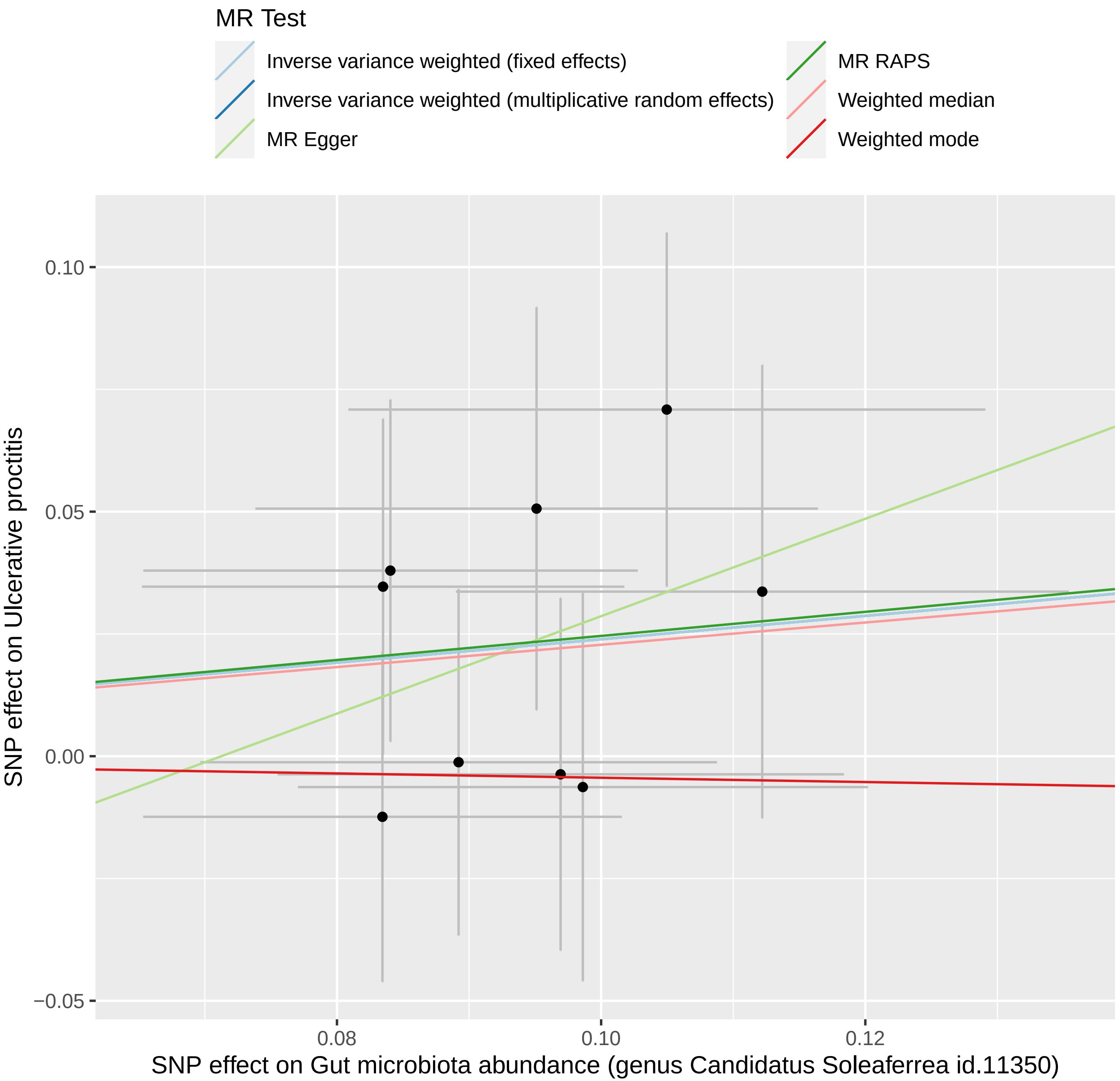

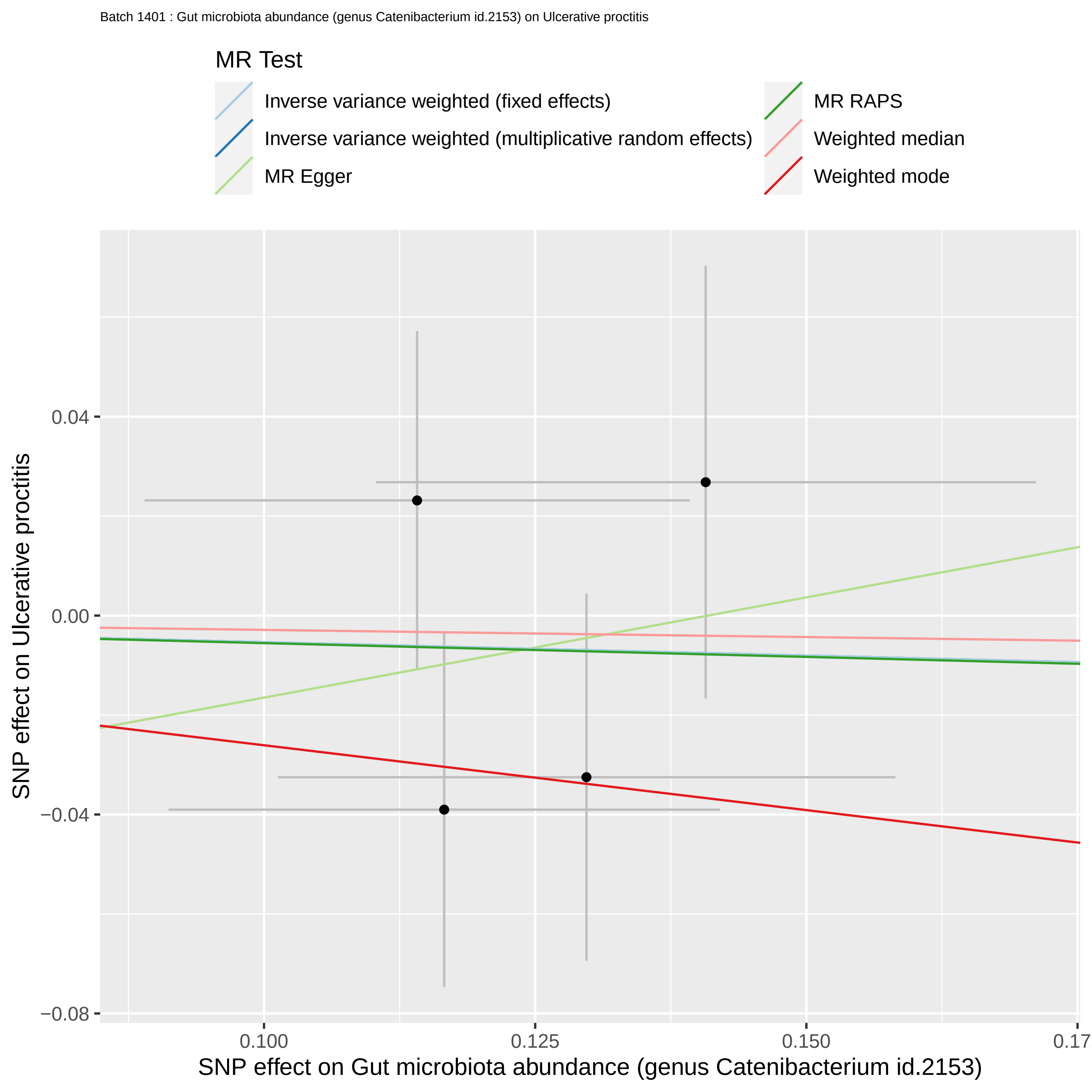

Batch 1401 : Gut microbiota abundance (genus Catenibacterium id.2153) on Ulcerative proctitis
MR Test
Inverse variance weighted (fixed effects)
Inverse variance weighted (multiplicative random effects)
MR Egger
MR RAPS
Weighted median
Weighted mode
0.04
0.00
−0.04
−0.08
0.100
0.125
0.150
0.17
SNP effect on Ulcerative proctitis
SNP effect on Gut microbiota abundance (genus Catenibacterium id.2153)

Batch 1402 : Gut microbiota abundance (genus Christensenellaceae R 7group id.11283) on Ulcerative proctitis

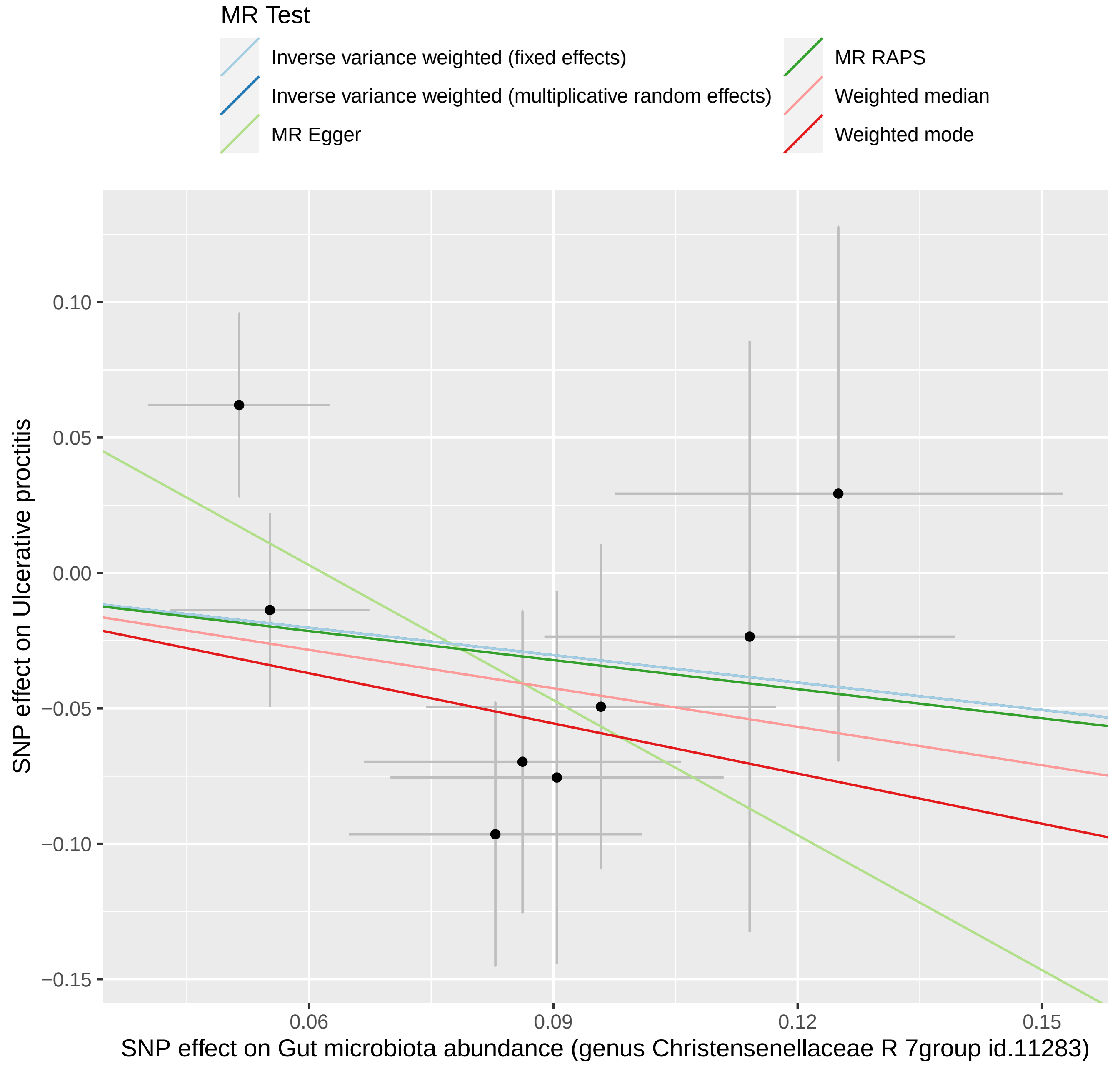

Batch 1403 : Gut microbiota abundance (genus Clostridium innocuum group id.14397) on Ulcerative proctitis

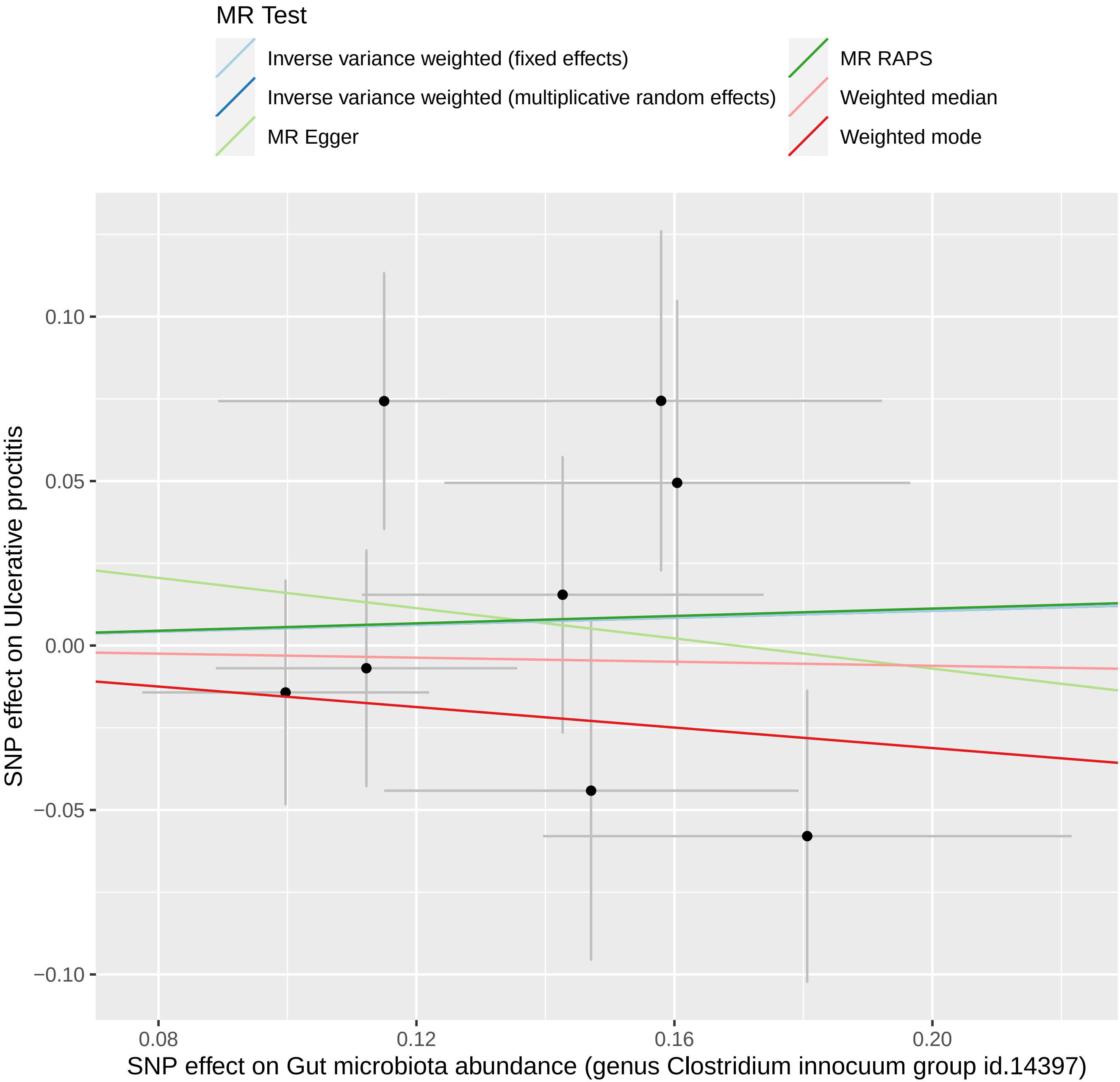

Batch 1404 : Gut microbiota abundance (genus Clostridium sensustricto1 id.1873) on Ulcerative proctitis

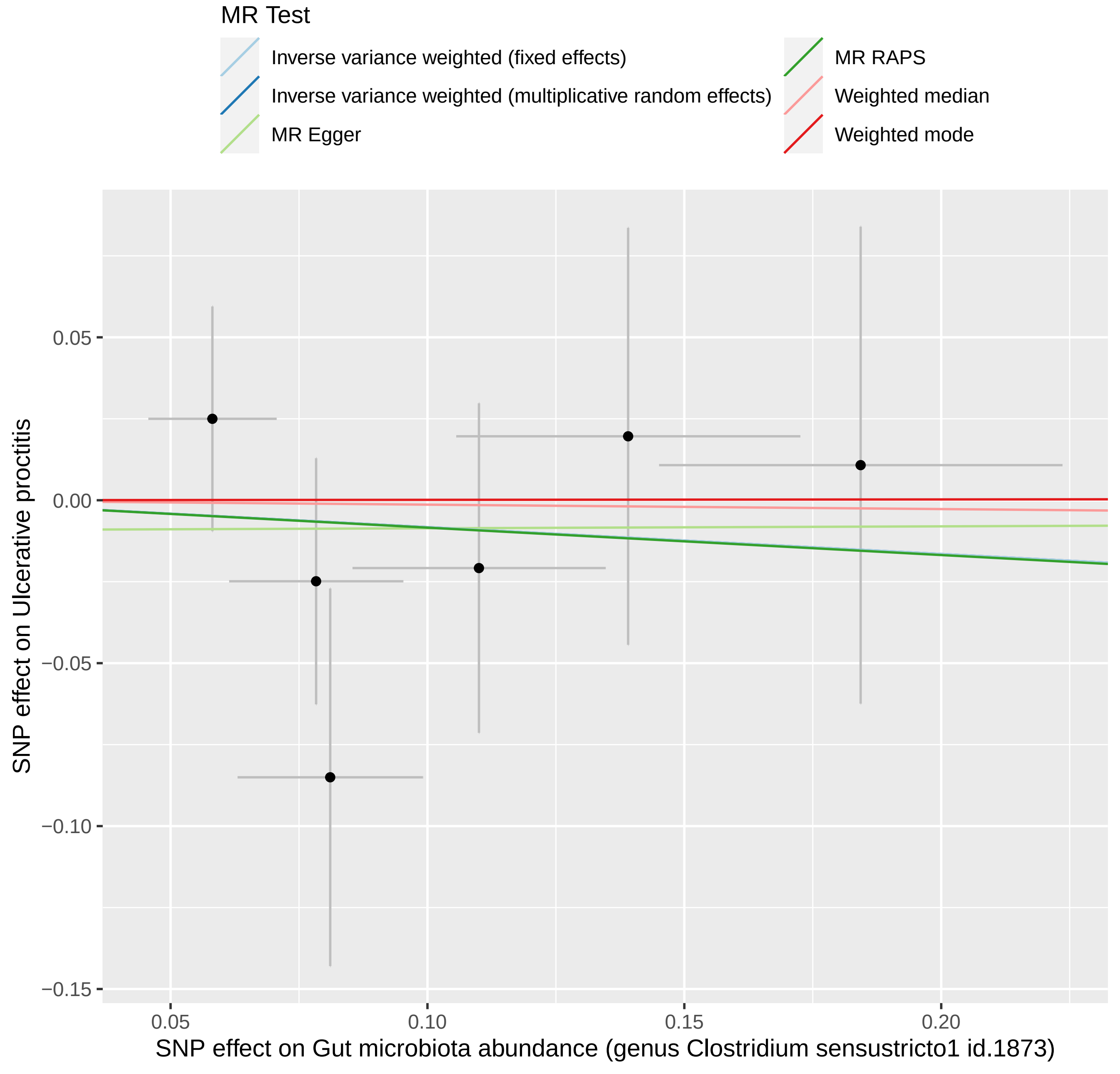

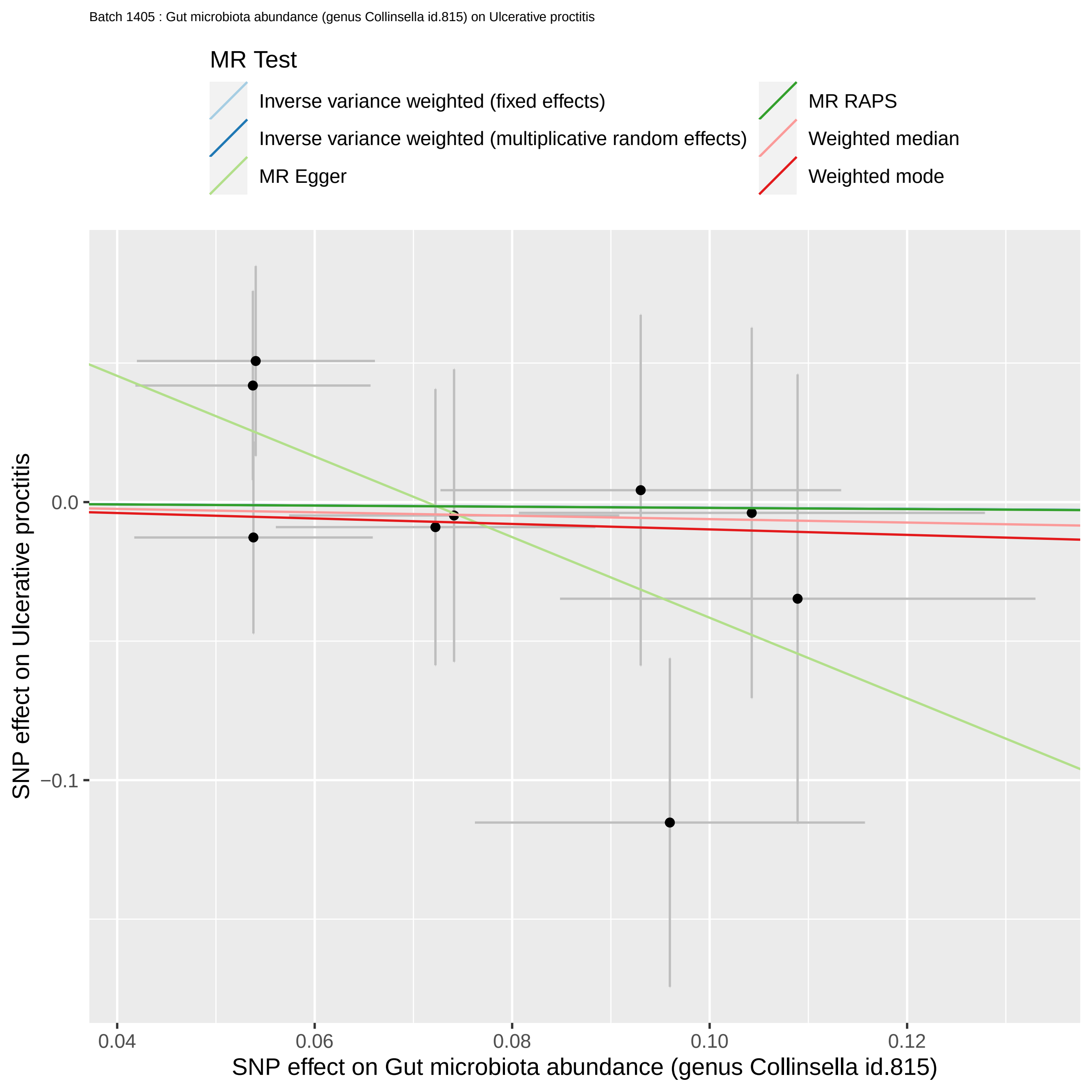

Batch 1405 : Gut microbiota abundance (genus Collinsella id.815) on Ulcerative proctitis
MR Test
Inverse variance weighted (fixed effects)
Inverse variance weighted (multiplicative random effects)
MR Egger
MR RAPS
Weighted median
Weighted mode
SNP effect on Ulcerative proctitis
0.0
−0.1
0.04
0.06
0.08
0.10
0.12
SNP effect on Gut microbiota abundance (genus Collinsella id.815)

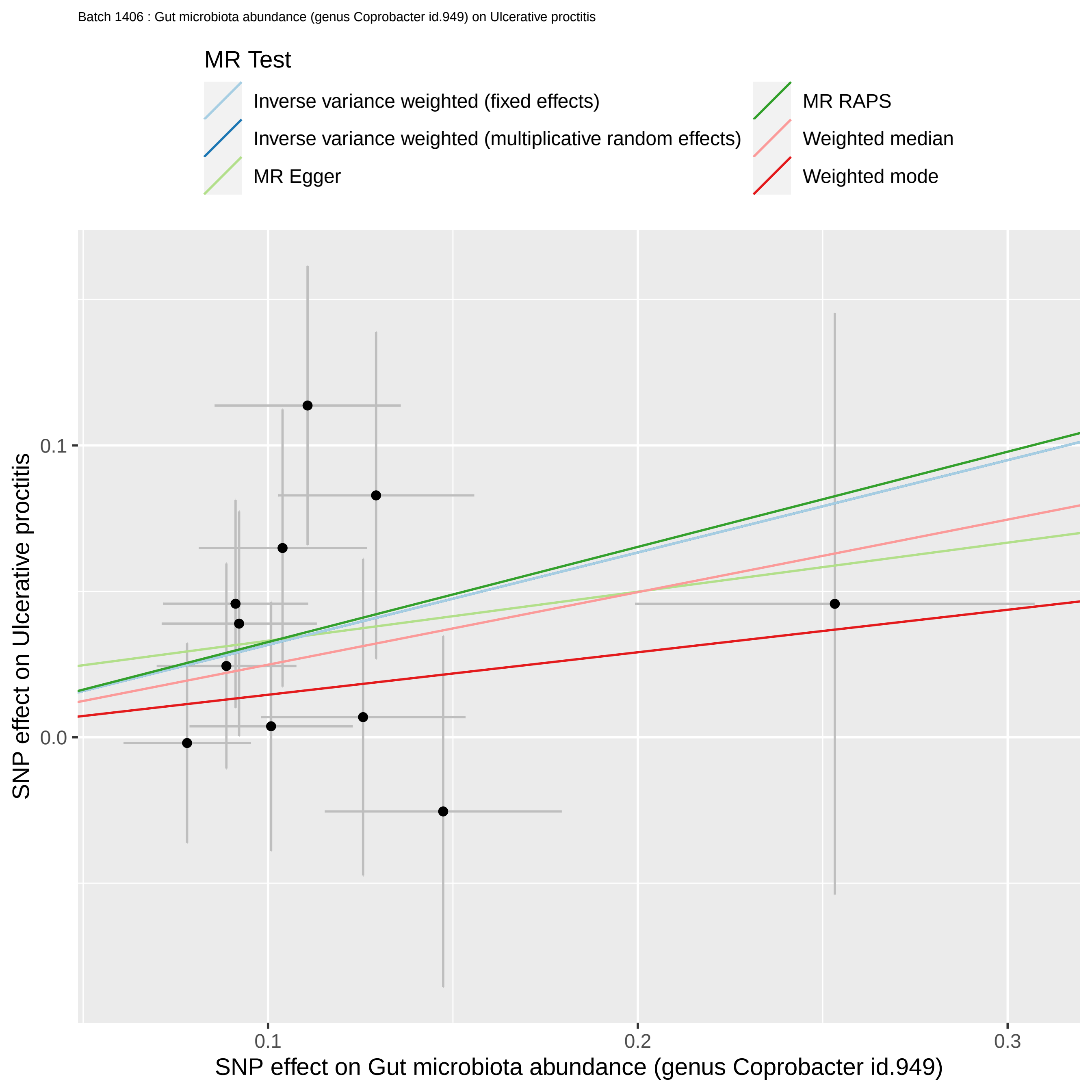

Batch 1406 : Gut microbiota abundance (genus Coprobacter id.949) on Ulcerative proctitis
MR Test
Inverse variance weighted (fixed effects)
Inverse variance weighted (multiplicative random effects)
MR Egger
MR RAPS
Weighted median
Weighted mode
SNP effect on Ulcerative proctitis
0.1
0.0
0.1
0.2
0.3
SNP effect on Gut microbiota abundance (genus Coprobacter id.949)

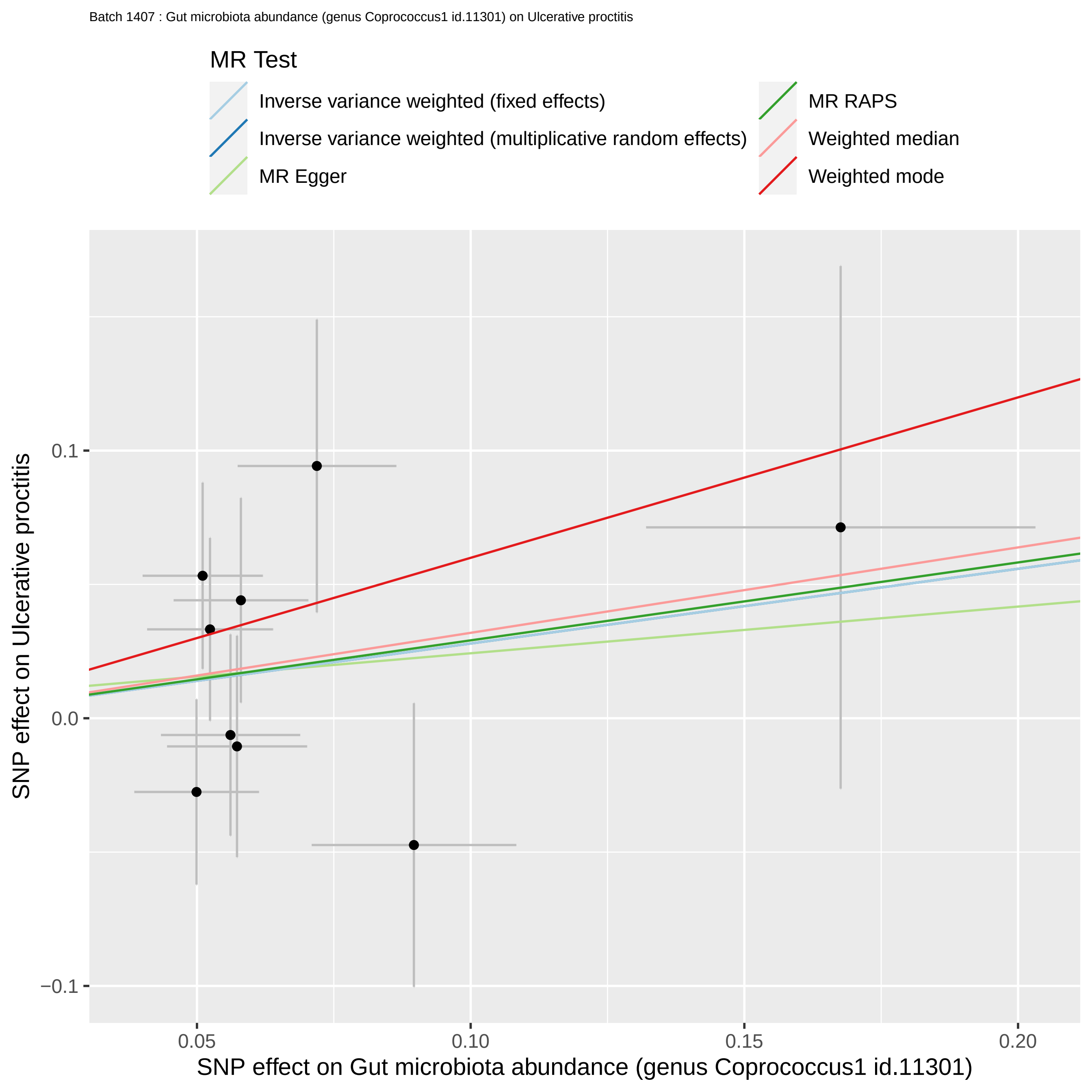
Batch 1407 : Gut microbiota abundance (genus Coprococcus1 id.11301) on Ulcerative proctitis
MR Test
Inverse variance weighted (fixed effects)
Inverse variance weighted (multiplicative random effects)
MR Egger
MR RAPS
Weighted median
Weighted mode
0.1
0.0
−0.1
0.05
0.10
0.15
0.20
SNP effect on Ulcerative proctitis
SNP effect on Gut microbiota abundance (genus Coprococcus1 id.11301)

Batch 1408 : Gut microbiota abundance (genus Coprococcus2 id.11302) on Ulcerative proctitis

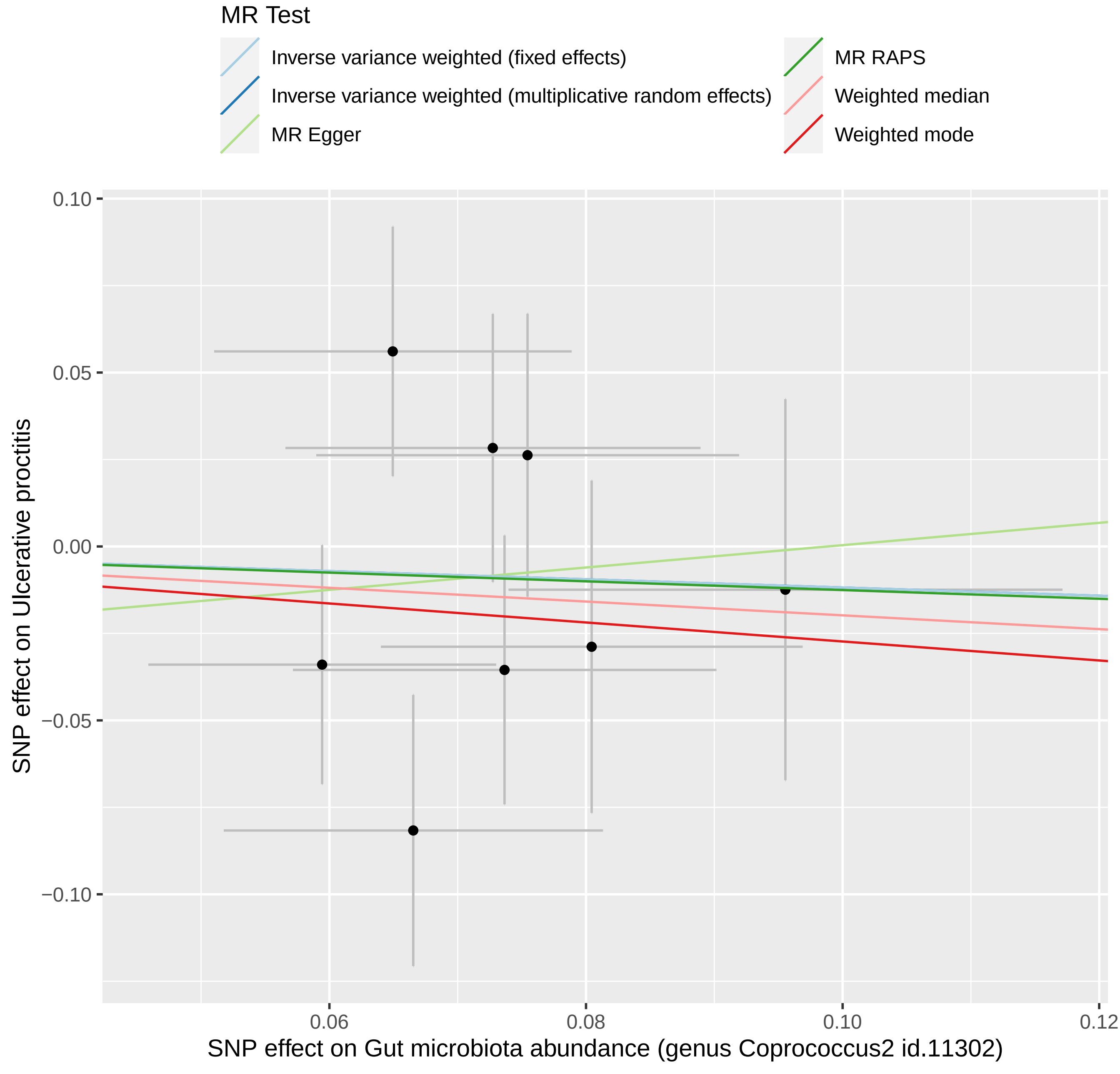

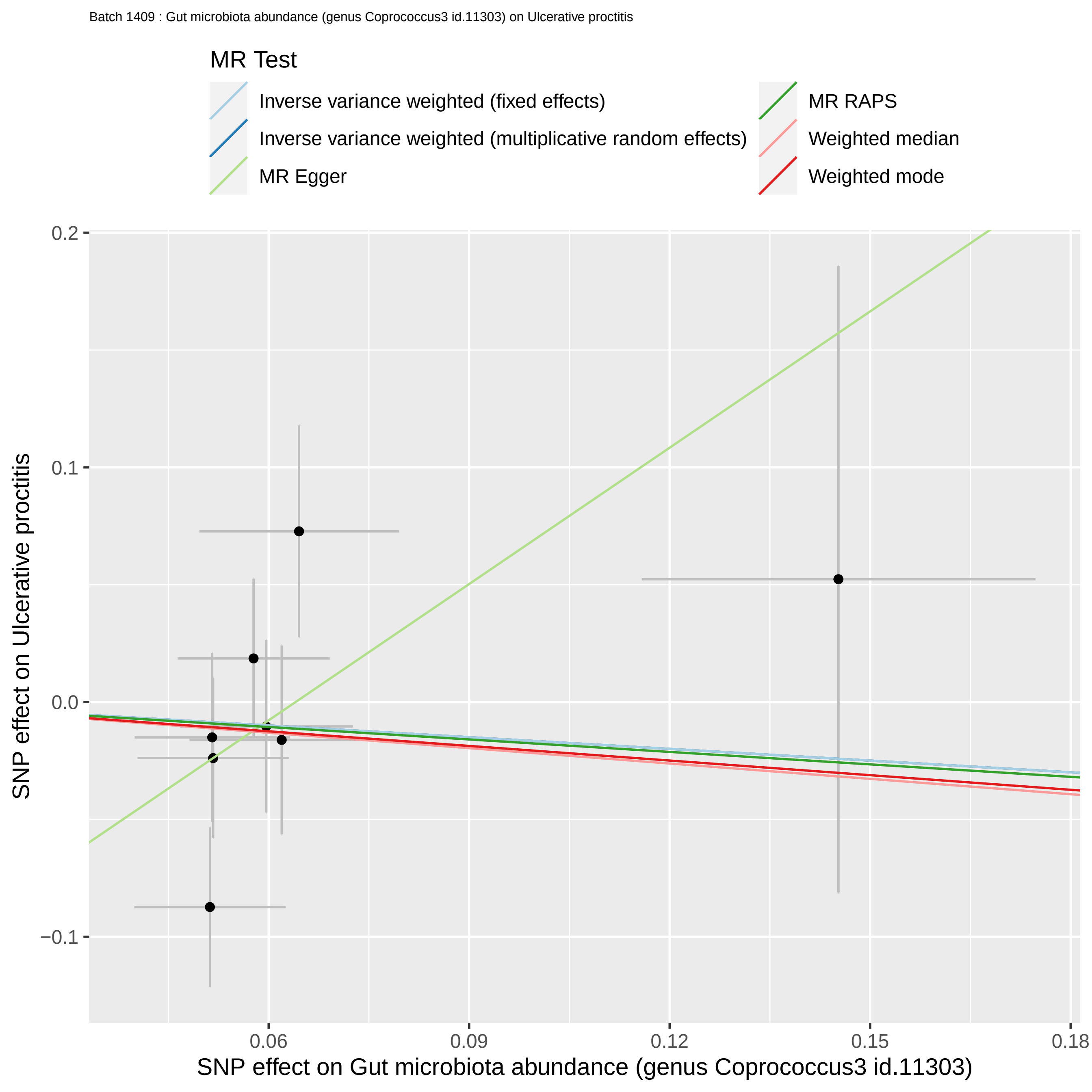
Batch 1409 : Gut microbiota abundance (genus Coprococcus3 id.11303) on Ulcerative proctitis
MR Test
Inverse variance weighted (fixed effects)
Inverse variance weighted (multiplicative random effects)
MR Egger
MR RAPS
Weighted median
Weighted mode
0.2
0.1
0.0
−0.1
0.06
0.09
0.12
0.15
0.18
SNP effect on Ulcerative proctitis
SNP effect on Gut microbiota abundance (genus Coprococcus3 id.11303)

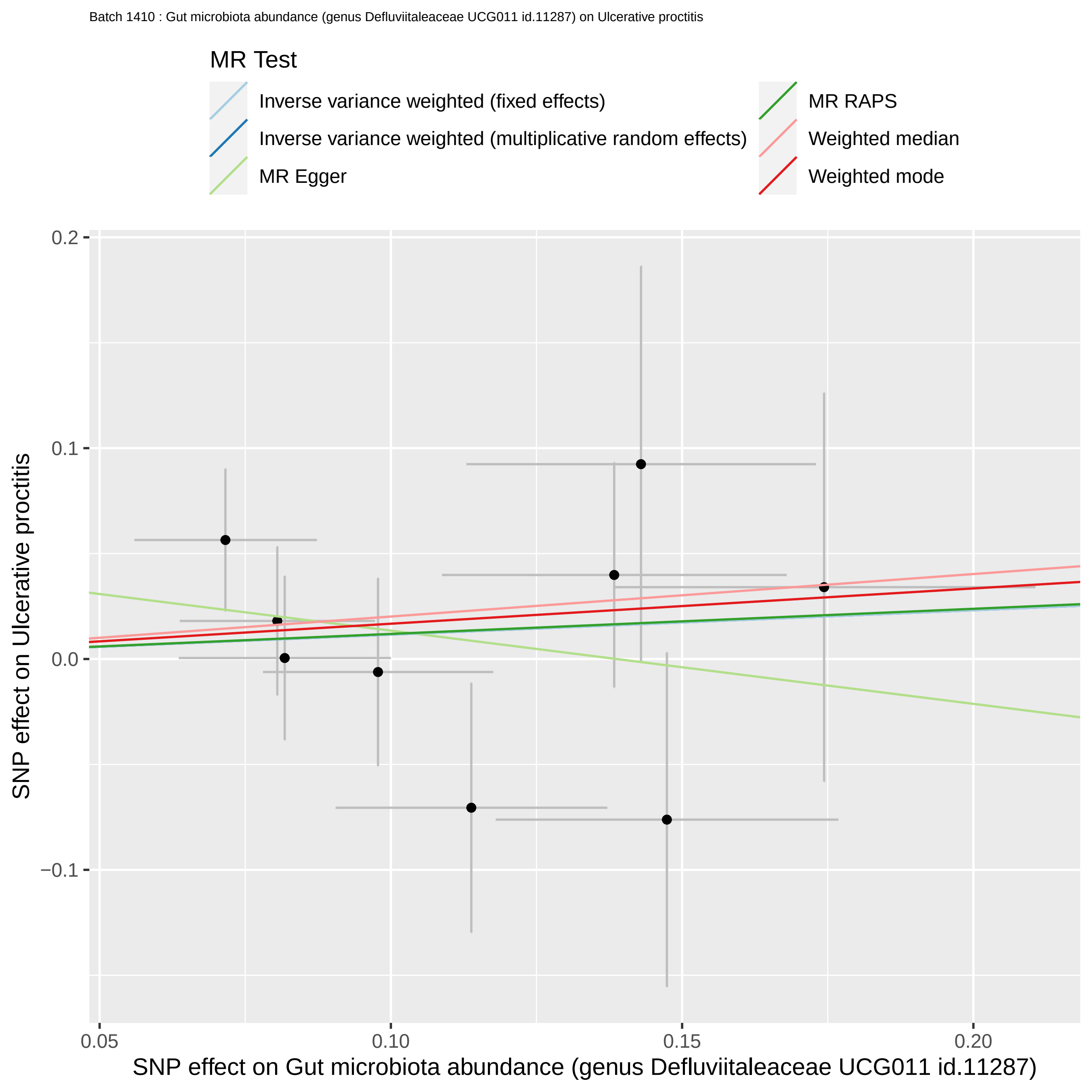
Batch 1410 : Gut microbiota abundance (genus Defluviitaleaceae UCG011 id.11287) on Ulcerative proctitis
MR Test
Inverse variance weighted (fixed effects)
Inverse variance weighted (multiplicative random effects)
MR Egger
MR RAPS
Weighted median
Weighted mode
0.2
0.1
0.0
−0.1
0.05
0.10
0.15
0.20
SNP effect on Ulcerative proctitis
SNP effect on Gut microbiota abundance (genus Defluviitaleaceae UCG011 id.11287)

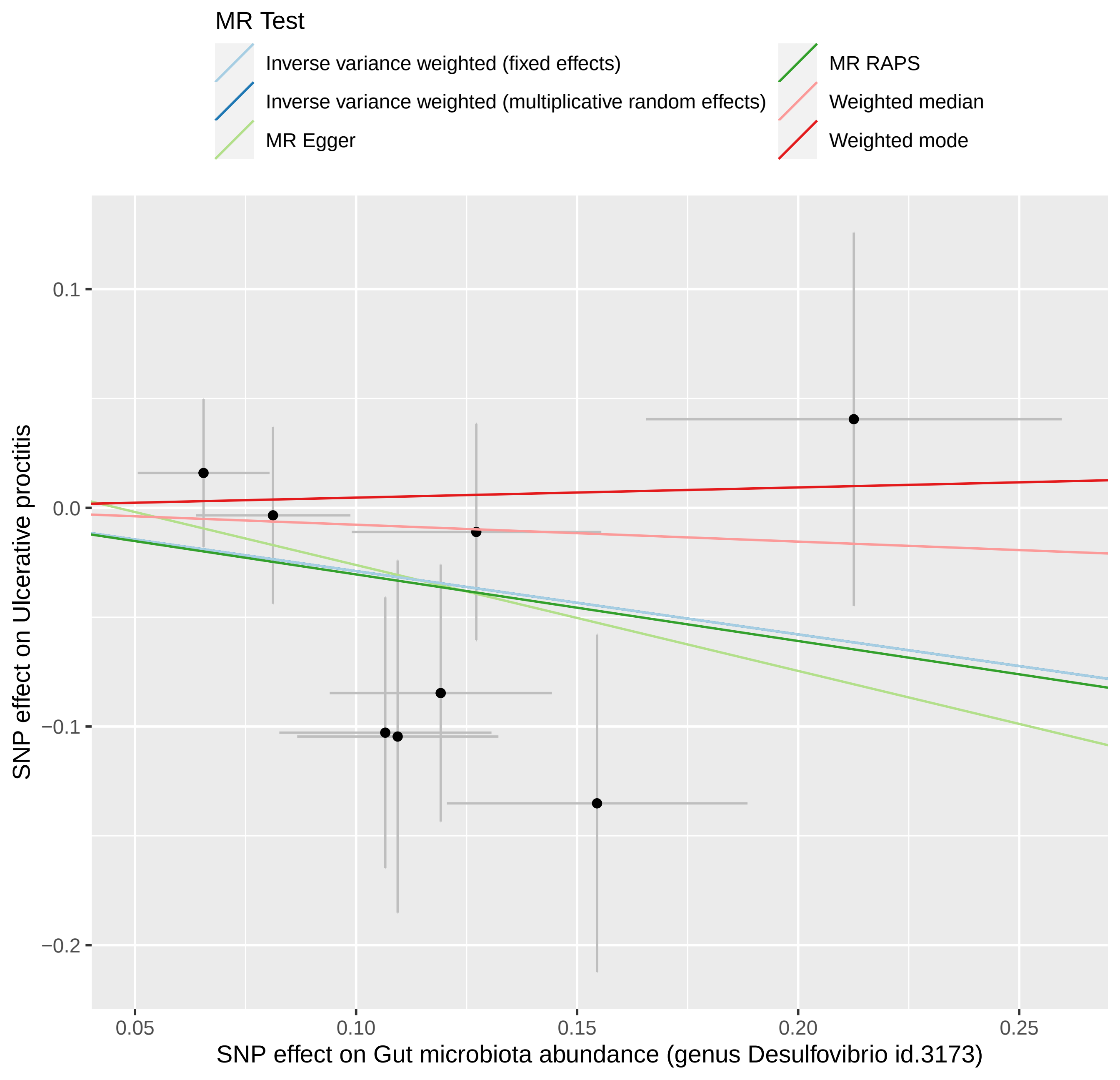
MR Test
Inverse variance weighted (fixed effects)
Inverse variance weighted (multiplicative random effects)
MR Egger
MR RAPS
Weighted median
Weighted mode
0.1
0.0
−0.1
−0.2
SNP effect on Ulcerative proctitis
0.05
0.10
0.15
0.20
0.25
SNP effect on Gut microbiota abundance (genus Desulfovibrio id.3173)

Batch 1412 : Gut microbiota abundance (genus Dialister id.2183) on Ulcerative proctitis

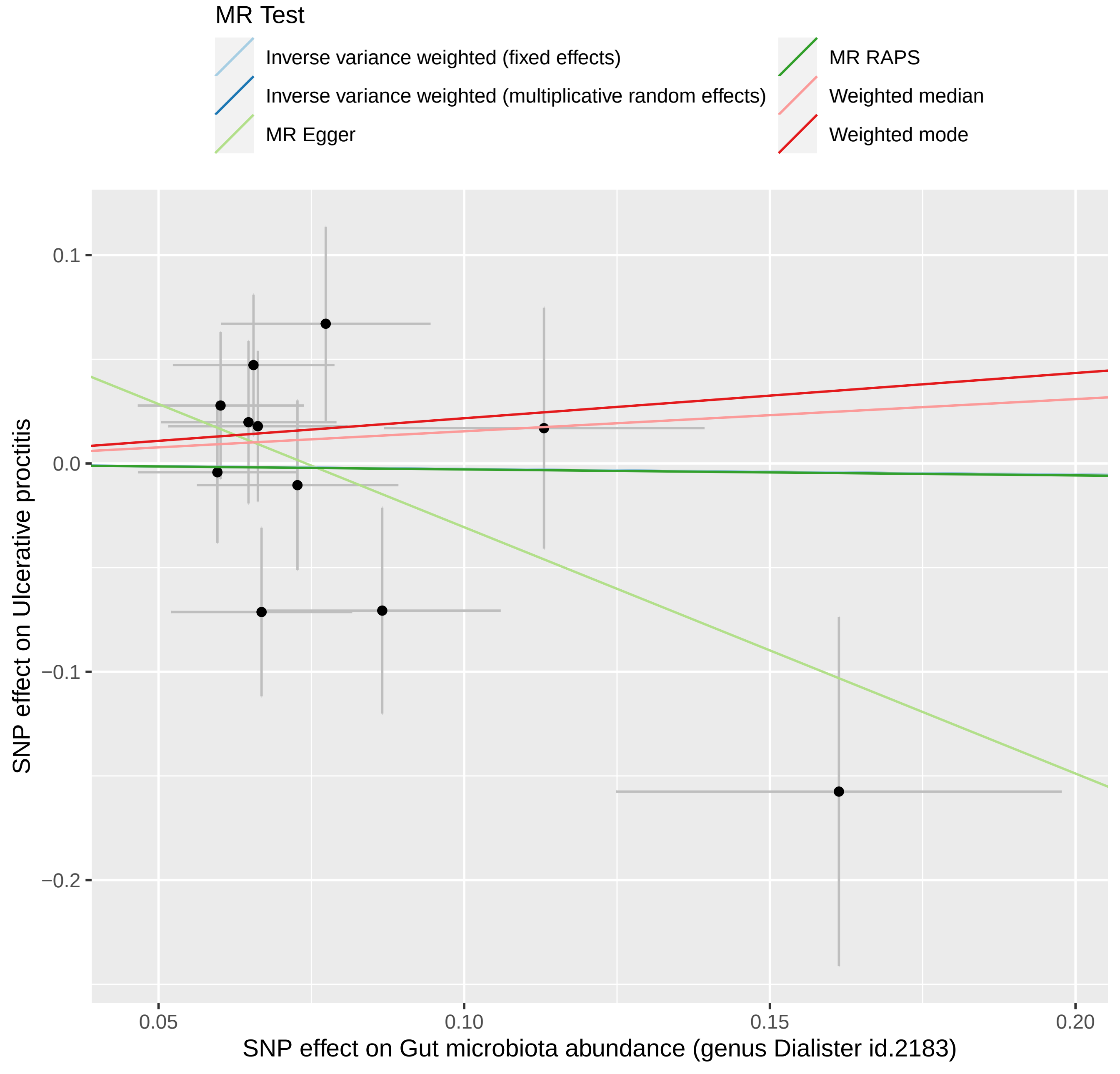

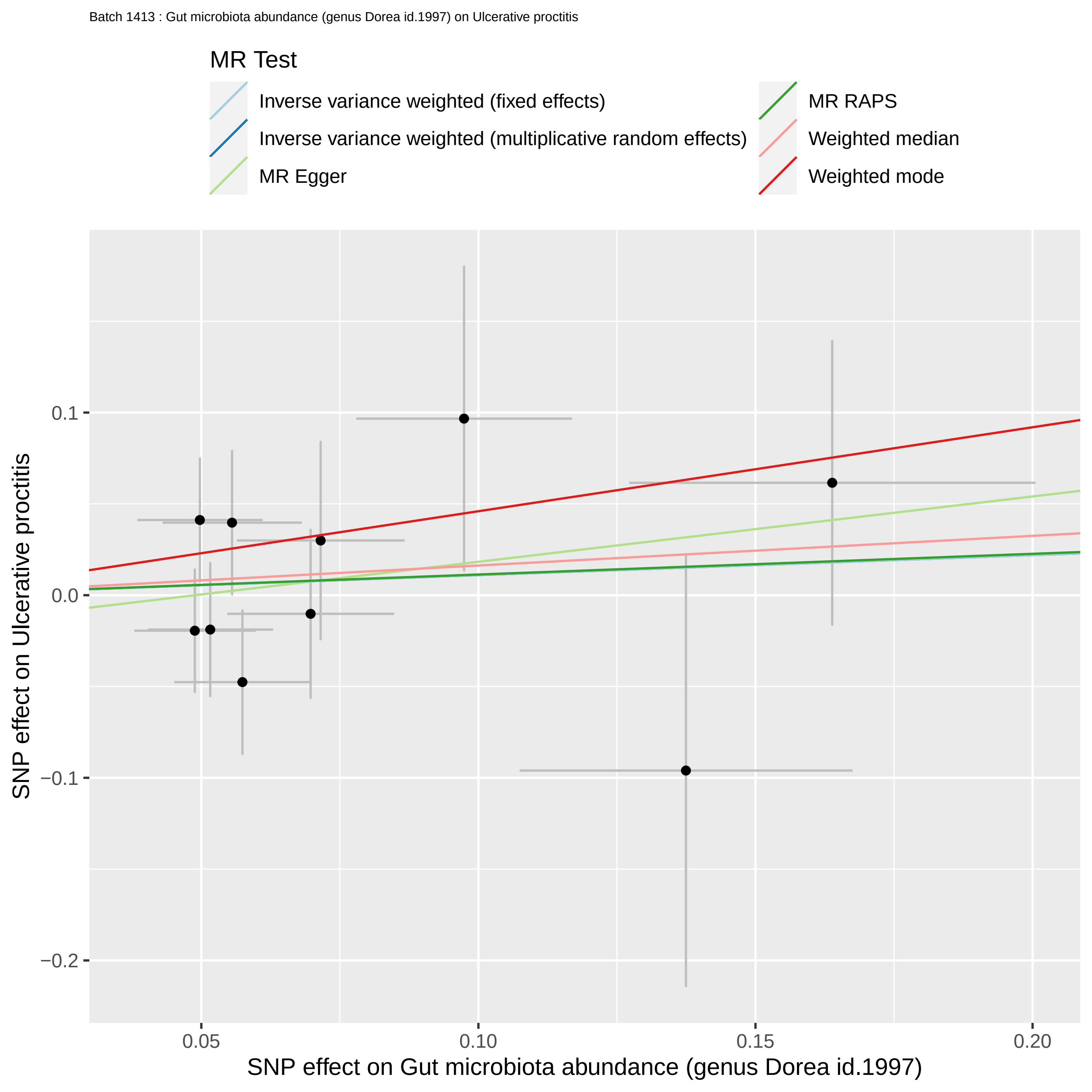

Batch 1413 : Gut microbiota abundance (genus Dorea id.1997) on Ulcerative proctitis
MR Test
Inverse variance weighted (fixed effects)
Inverse variance weighted (multiplicative random effects)
MR Egger
MR RAPS
Weighted median
Weighted mode
SNP effect on Ulcerative proctitis
0.1
0.0
−0.1
−0.2
0.05
0.10
0.15
0.20
SNP effect on Gut microbiota abundance (genus Dorea id.1997)

Batch 1414 : Gut microbiota abundance (genus Eggerthella id.819) on Ulcerative proctitis

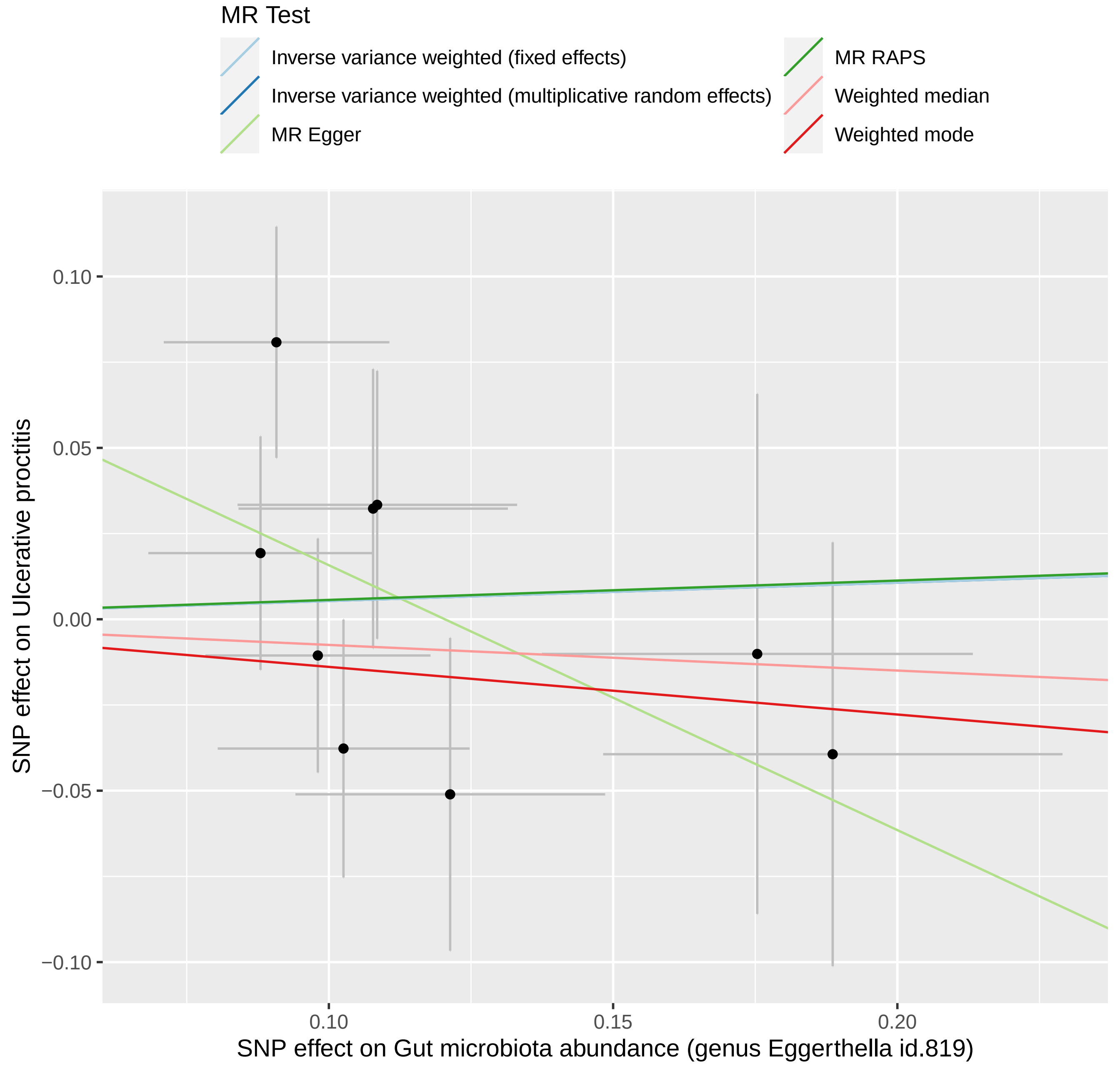

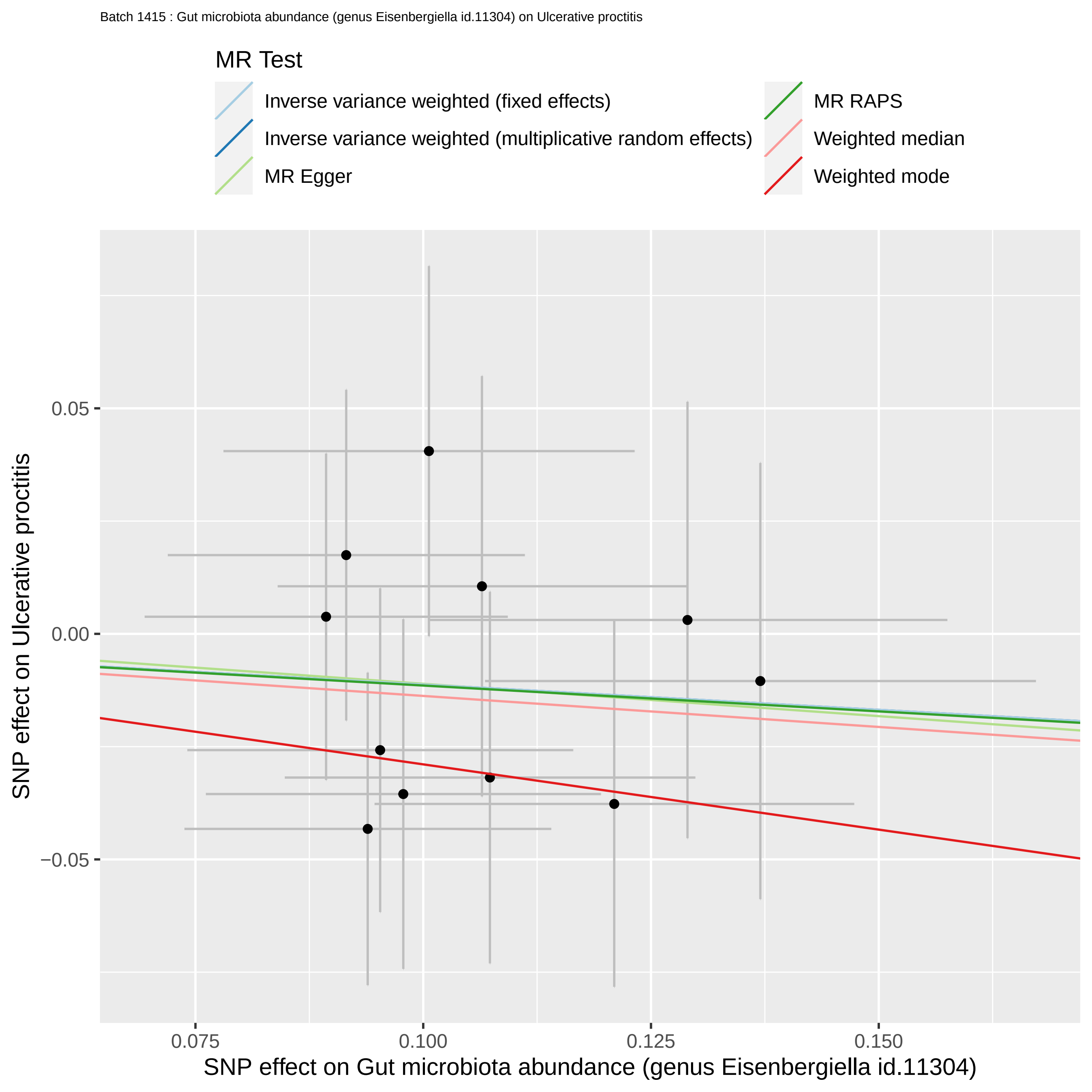

Batch 1415 : Gut microbiota abundance (genus Eisenbergiella id.11304) on Ulcerative proctitis
MR Test
Inverse variance weighted (fixed effects)
Inverse variance weighted (multiplicative random effects)
MR Egger
MR RAPS
Weighted median
Weighted mode
0.05
0.00
−0.05
0.075
0.100
0.125
0.150
SNP effect on Ulcerative proctitis
SNP effect on Gut microbiota abundance (genus Eisenbergiella id.11304)

Batch 1416 : Gut microbiota abundance (genus Enterorhabdus id.820) on Ulcerative proctitis

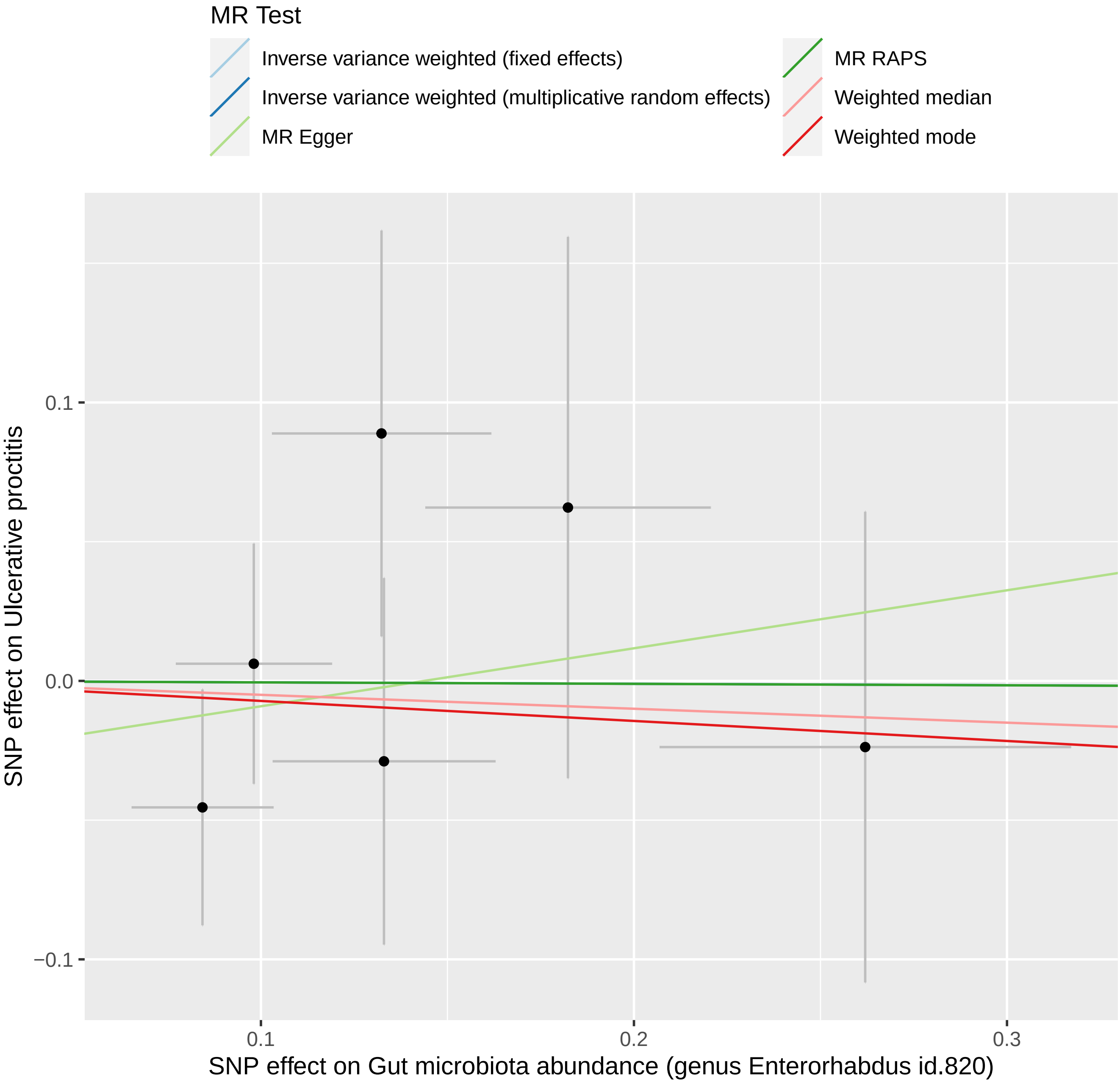

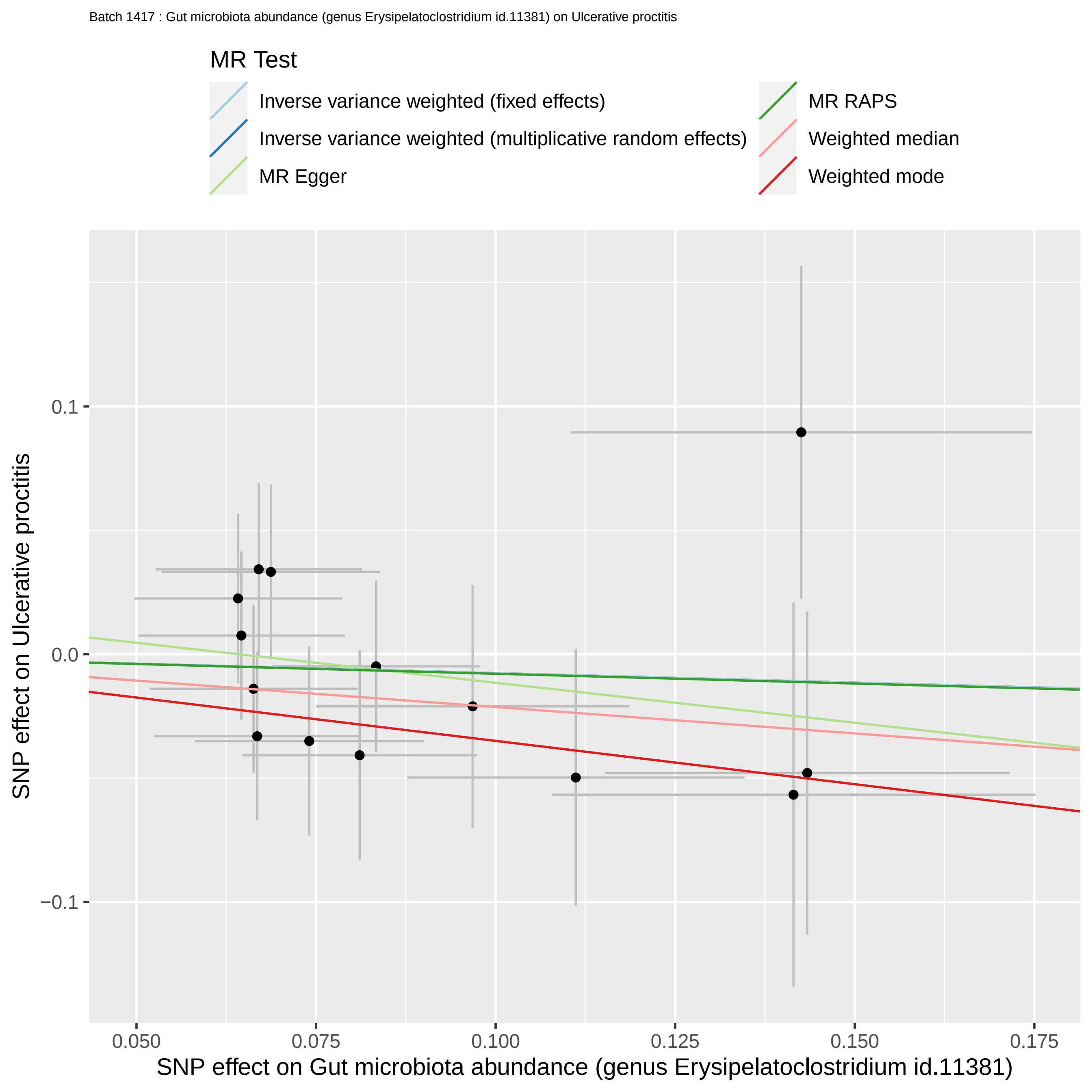

Batch 1417 : Gut microbiota abundance (genus Erysipelatoclostridium id.11381) on Ulcerative proctitis
MR Test
Inverse variance weighted (fixed effects)
Inverse variance weighted (multiplicative random effects)
MR Egger
MR RAPS
Weighted median
Weighted mode
SNP effect on Ulcerative proctitis
0.1
0.0
−0.1
0.050
0.075
0.100
0.125
0.150
0.175
SNP effect on Gut microbiota abundance (genus Erysipelatoclostridium id.11381)

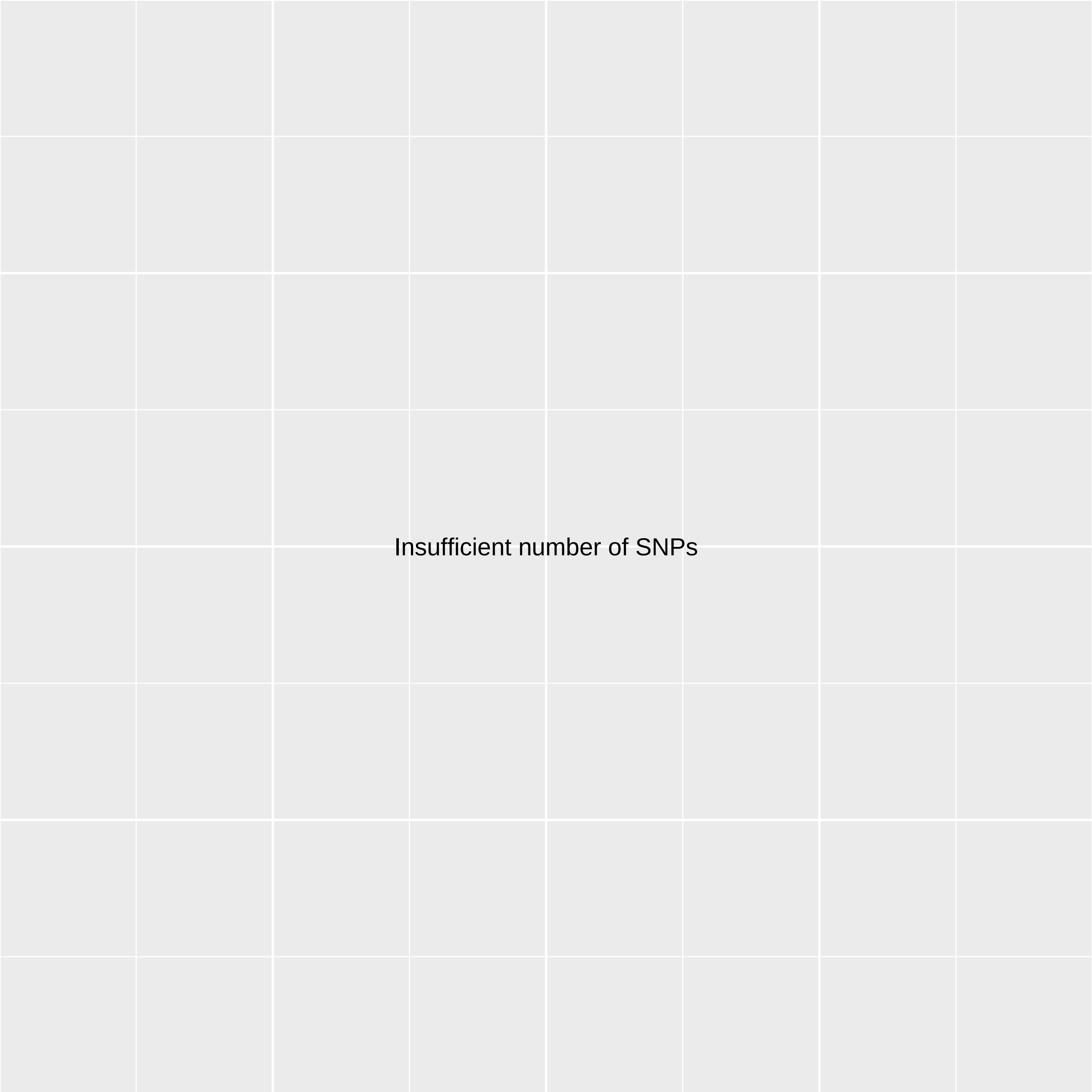
Insufficient number of SNPs

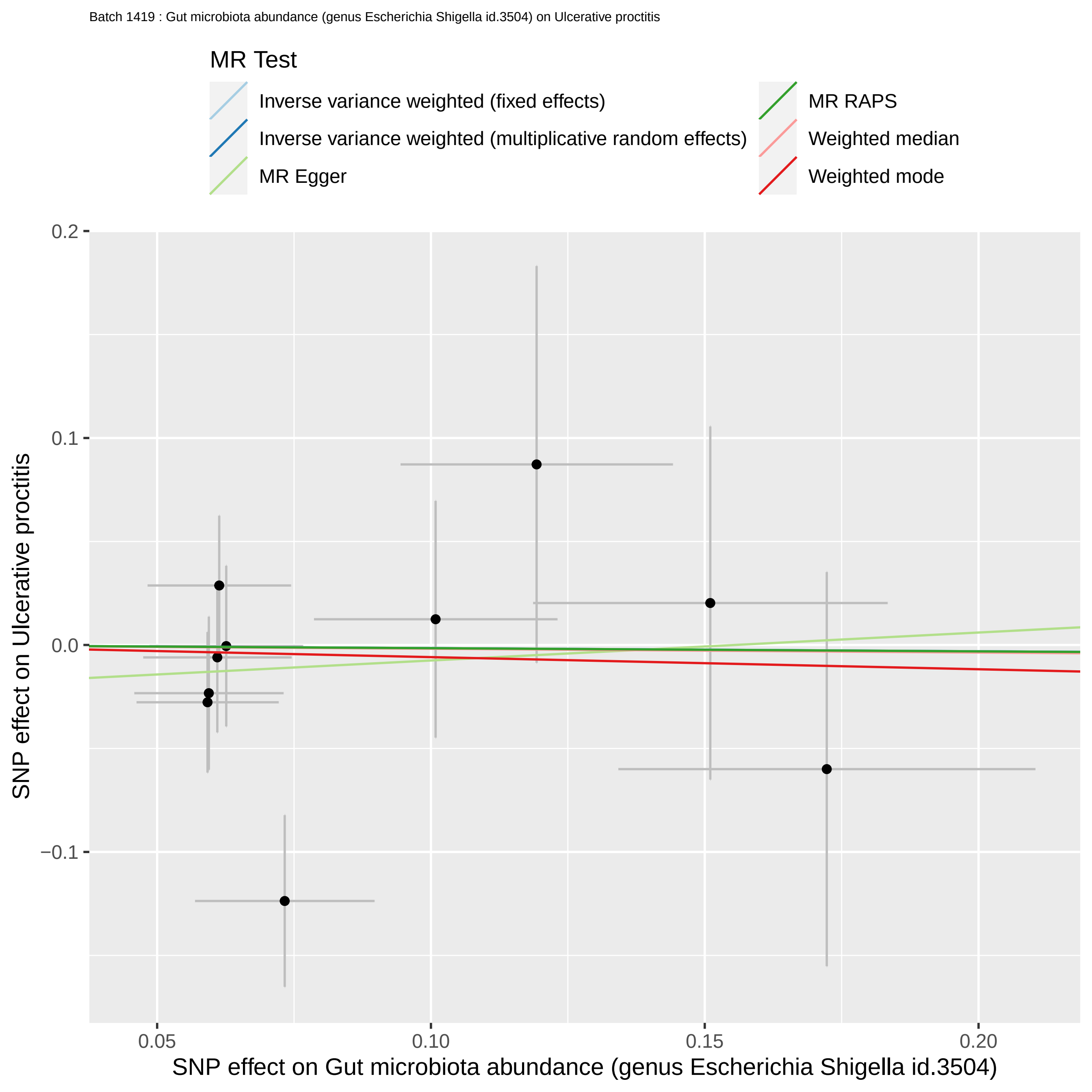

Batch 1419 : Gut microbiota abundance (genus Escherichia Shigella id.3504) on Ulcerative proctitis
MR Test
Inverse variance weighted (fixed effects)
Inverse variance weighted (multiplicative random effects)
MR Egger
MR RAPS
Weighted median
Weighted mode
0.2
0.1
0.0
−0.1
0.05
0.10
0.15
0.20
SNP effect on Ulcerative proctitis
SNP effect on Gut microbiota abundance (genus Escherichia Shigella id.3504)

Batch 1420 : Gut microbiota abundance (genus Eubacterium brachy group id.11296) on Ulcerative proctitis

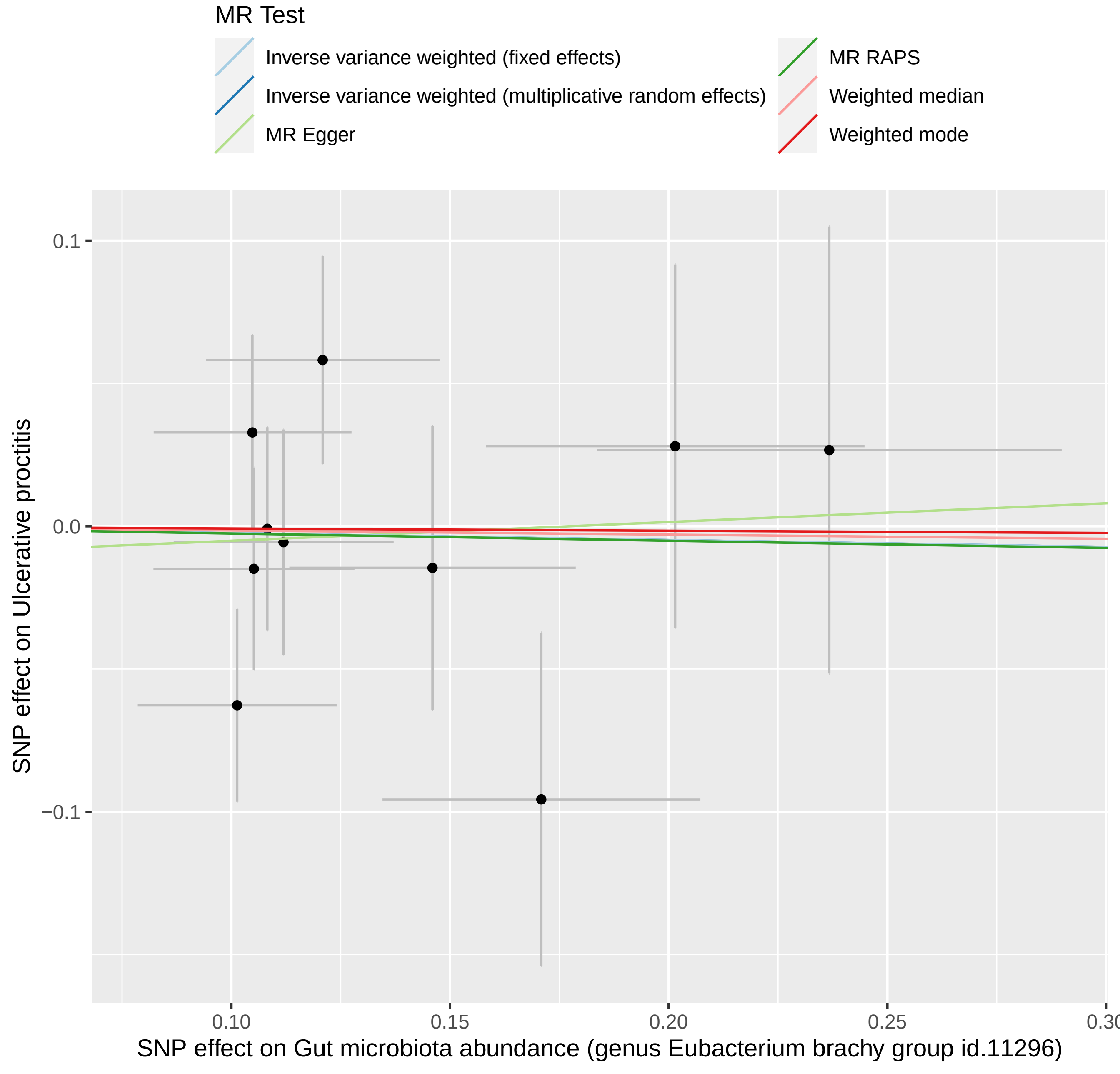

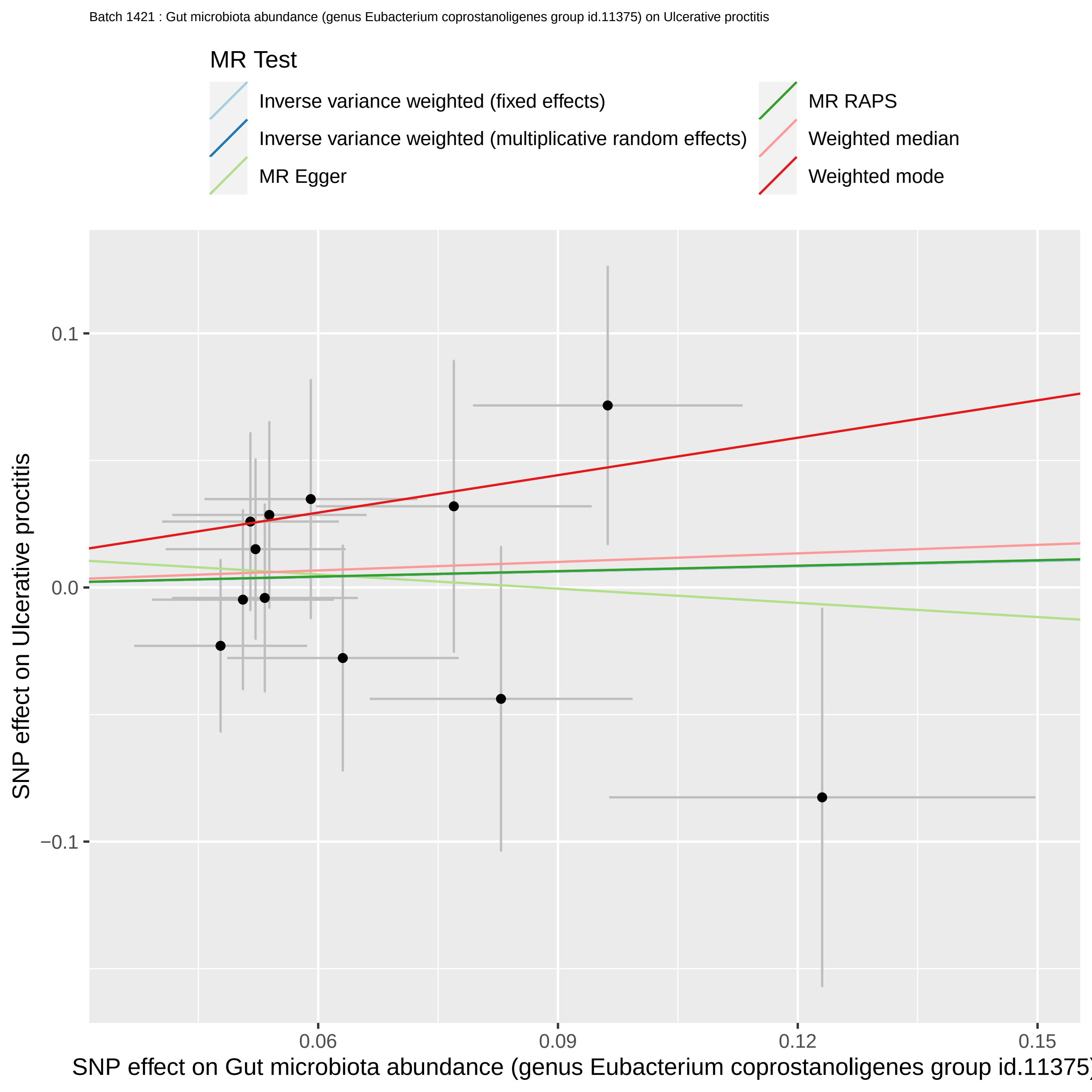
Batch 1421 : Gut microbiota abundance (genus Eubacterium coprostanoligenes group id.11375) on Ulcerative proctitis
MR Test
Inverse variance weighted (fixed effects)
Inverse variance weighted (multiplicative random effects)
MR Egger
MR RAPS
Weighted median
Weighted mode
0.1
0.0
−0.1
0.06
0.09
0.12
0.15
SNP effect on Ulcerative proctitis
SNP effect on Gut microbiota abundance (genus Eubacterium coprostanoligenes group id.11375)

Batch 1422 : Gut microbiota abundance (genus Eubacterium eligens group id.14372) on Ulcerative proctitis

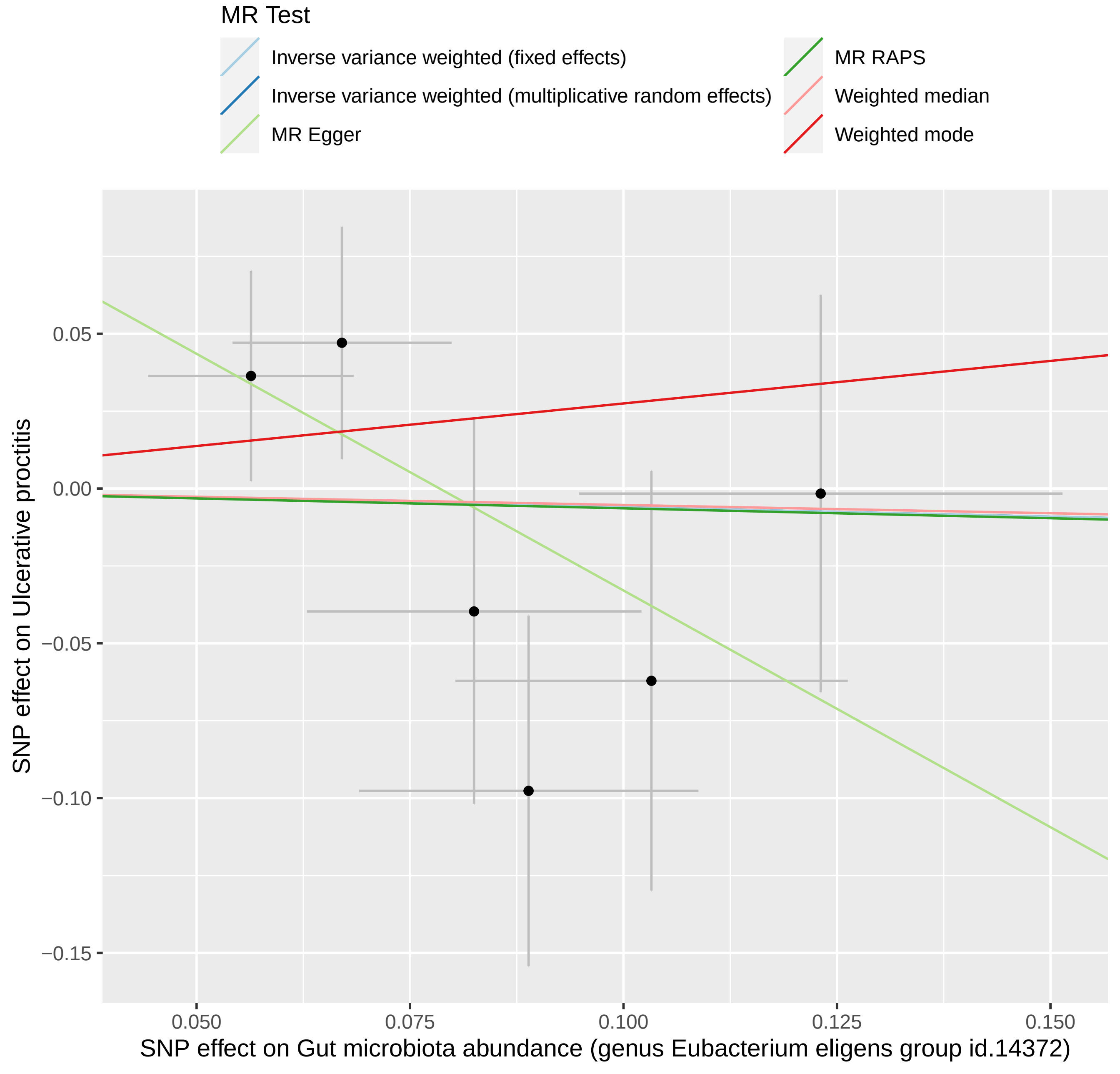

Batch 1423 : Gut microbiota abundance (genus Eubacterium fissicatena group id.14373) on Ulcerative proctitis

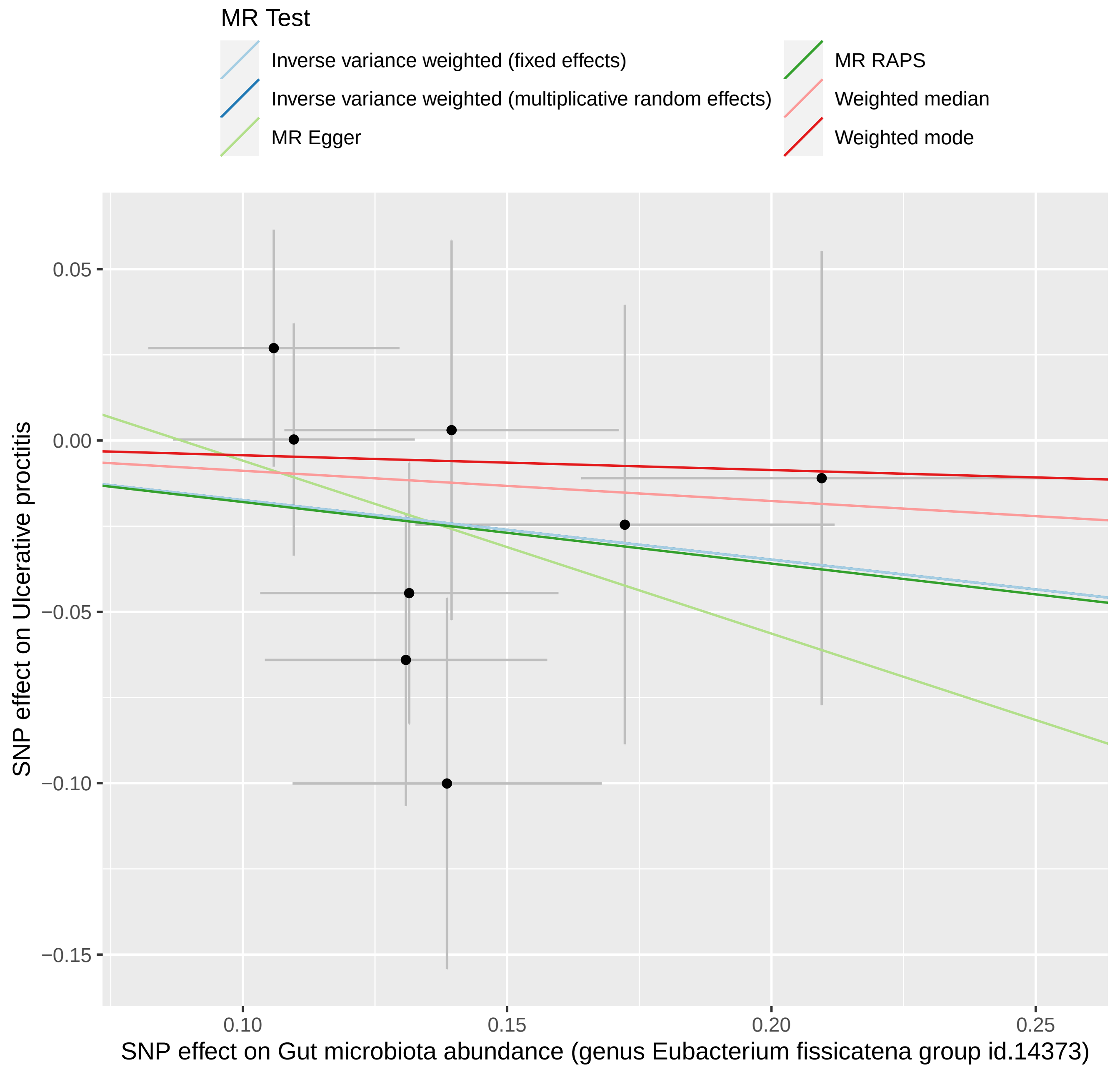

Batch 1424 : Gut microbiota abundance (genus Eubacterium hallii group id.11338) on Ulcerative proctitis

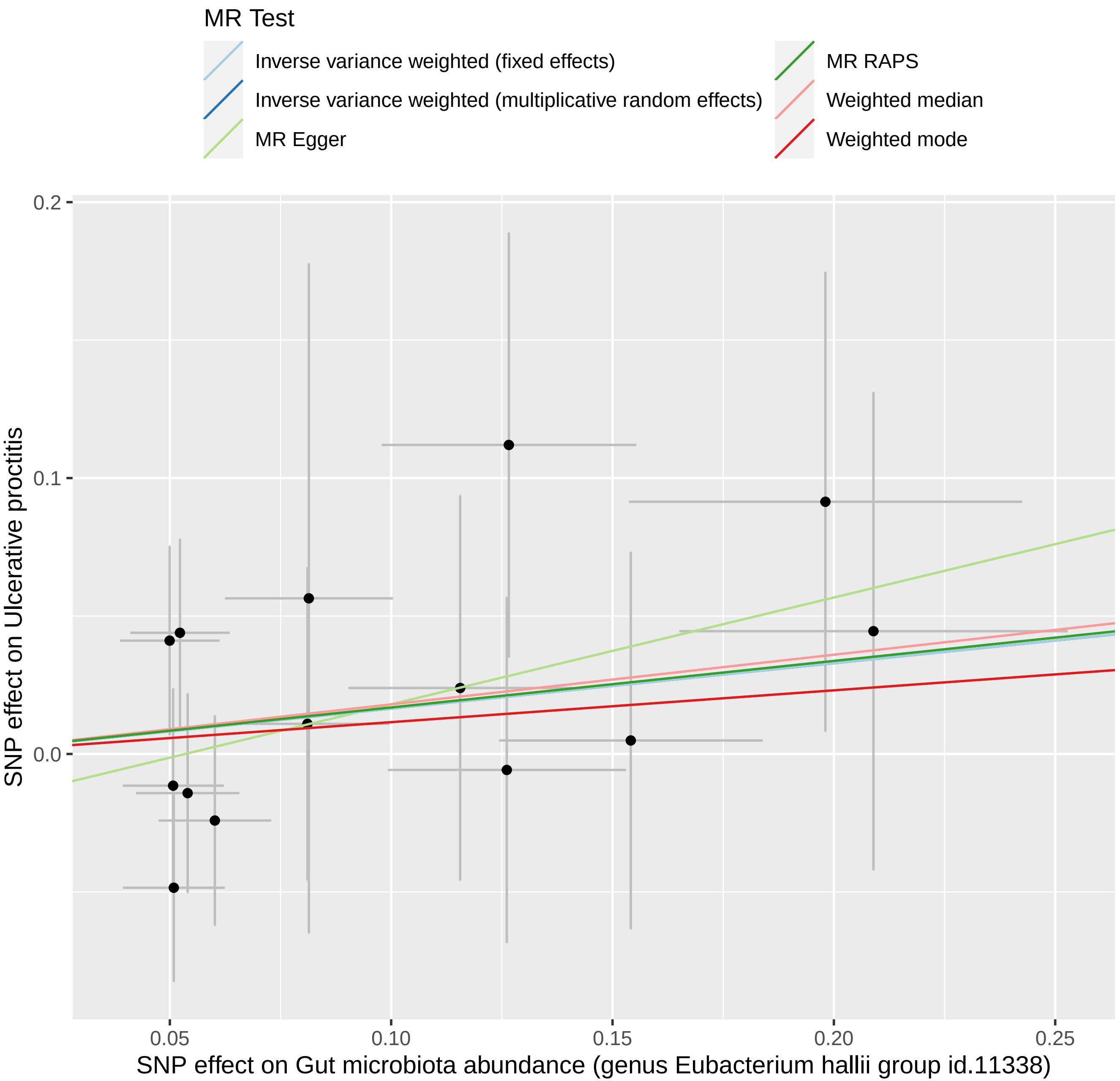

Batch 1425 : Gut microbiota abundance (genus Eubacterium nodatum group id.11297) on Ulcerative proctitis

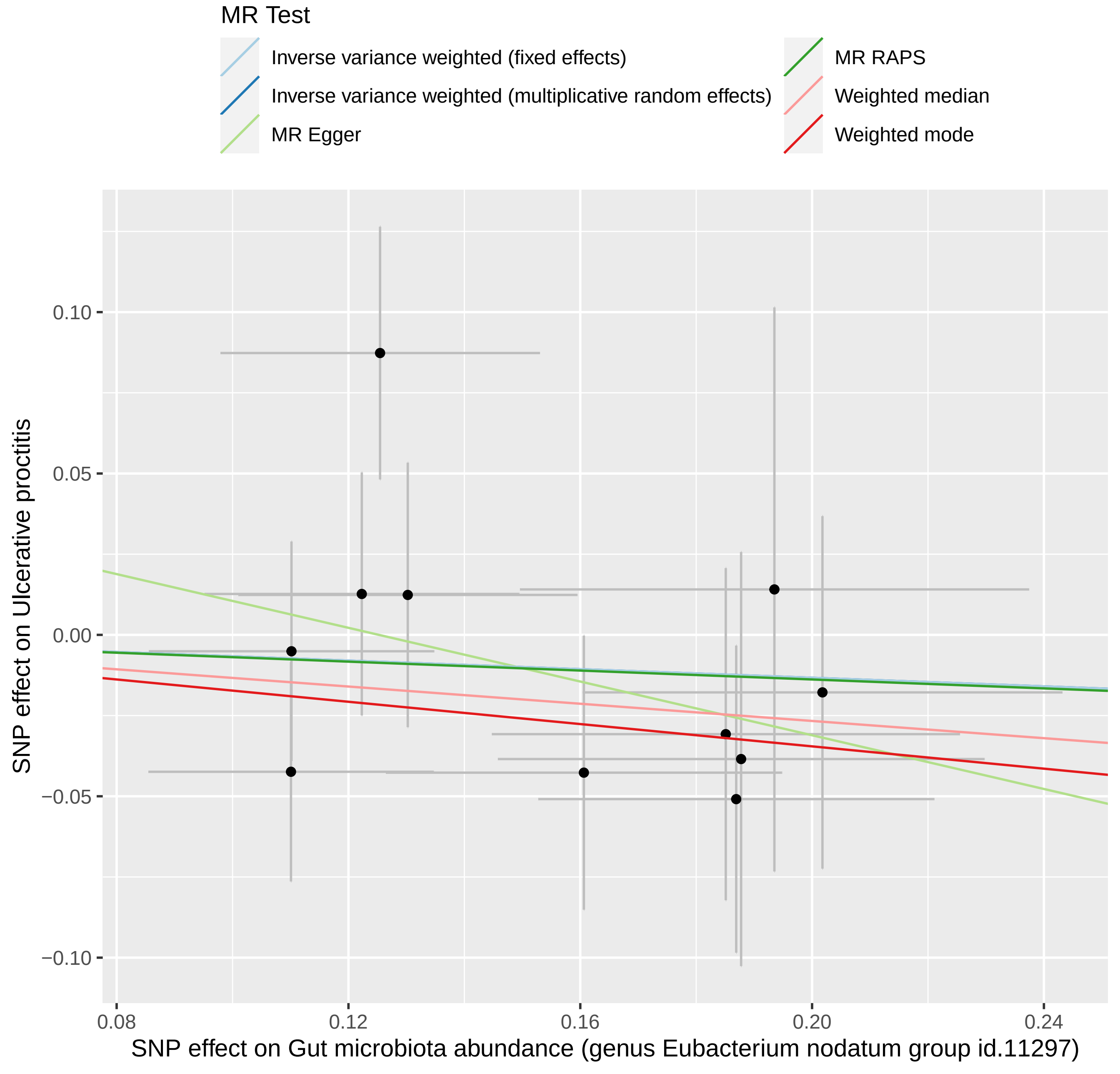

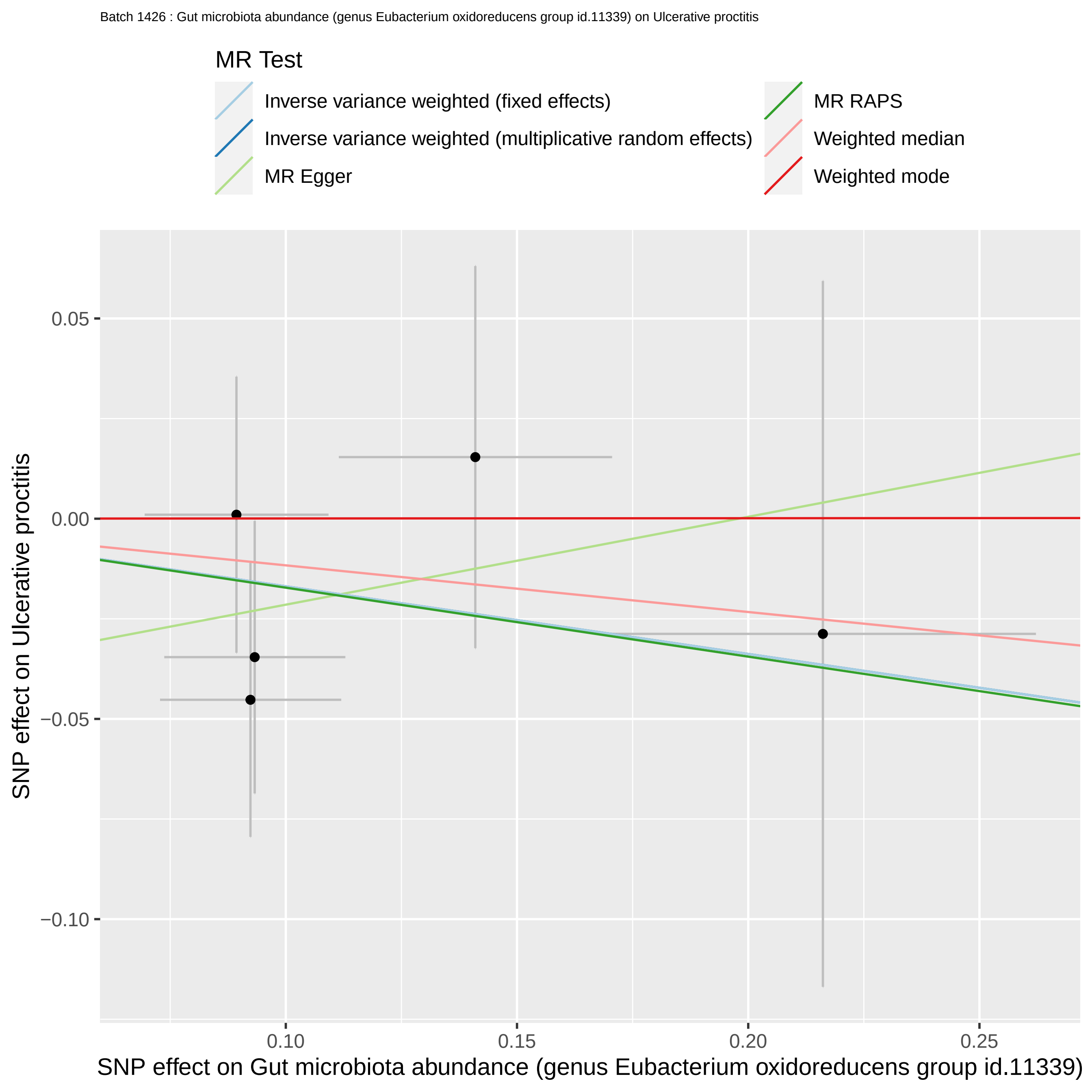

Batch 1426 : Gut microbiota abundance (genus Eubacterium oxidoreducens group id.11339) on Ulcerative proctitis
MR Test
Inverse variance weighted (fixed effects)
Inverse variance weighted (multiplicative random effects)
MR Egger
MR RAPS
Weighted median
Weighted mode
0.05
0.00
−0.05
−0.10
0.10
0.15
0.20
0.25
SNP effect on Ulcerative proctitis
SNP effect on Gut microbiota abundance (genus Eubacterium oxidoreducens group id.11339)

Batch 1427 : Gut microbiota abundance (genus Eubacterium rectale group id.14374) on Ulcerative proctitis

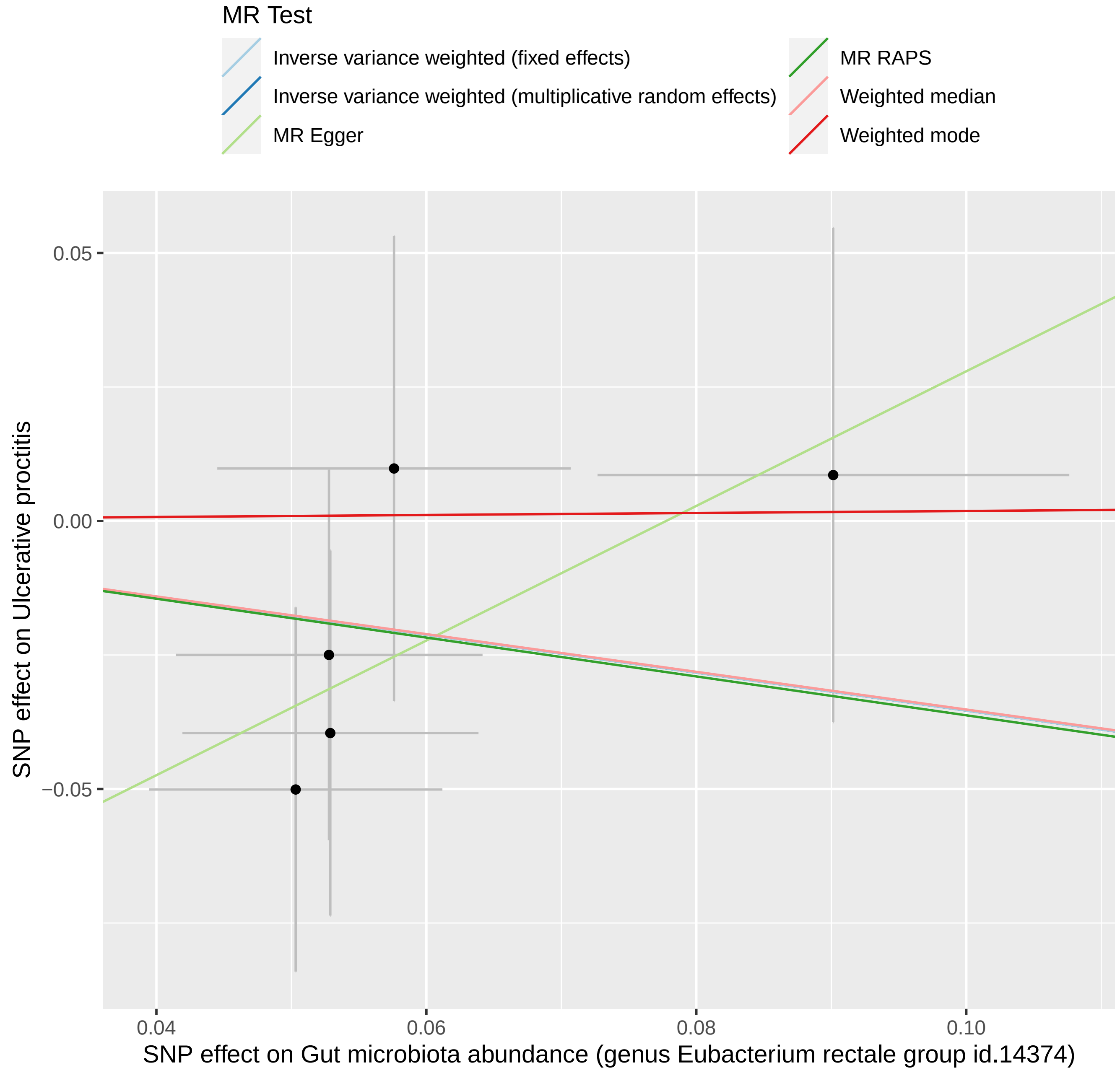

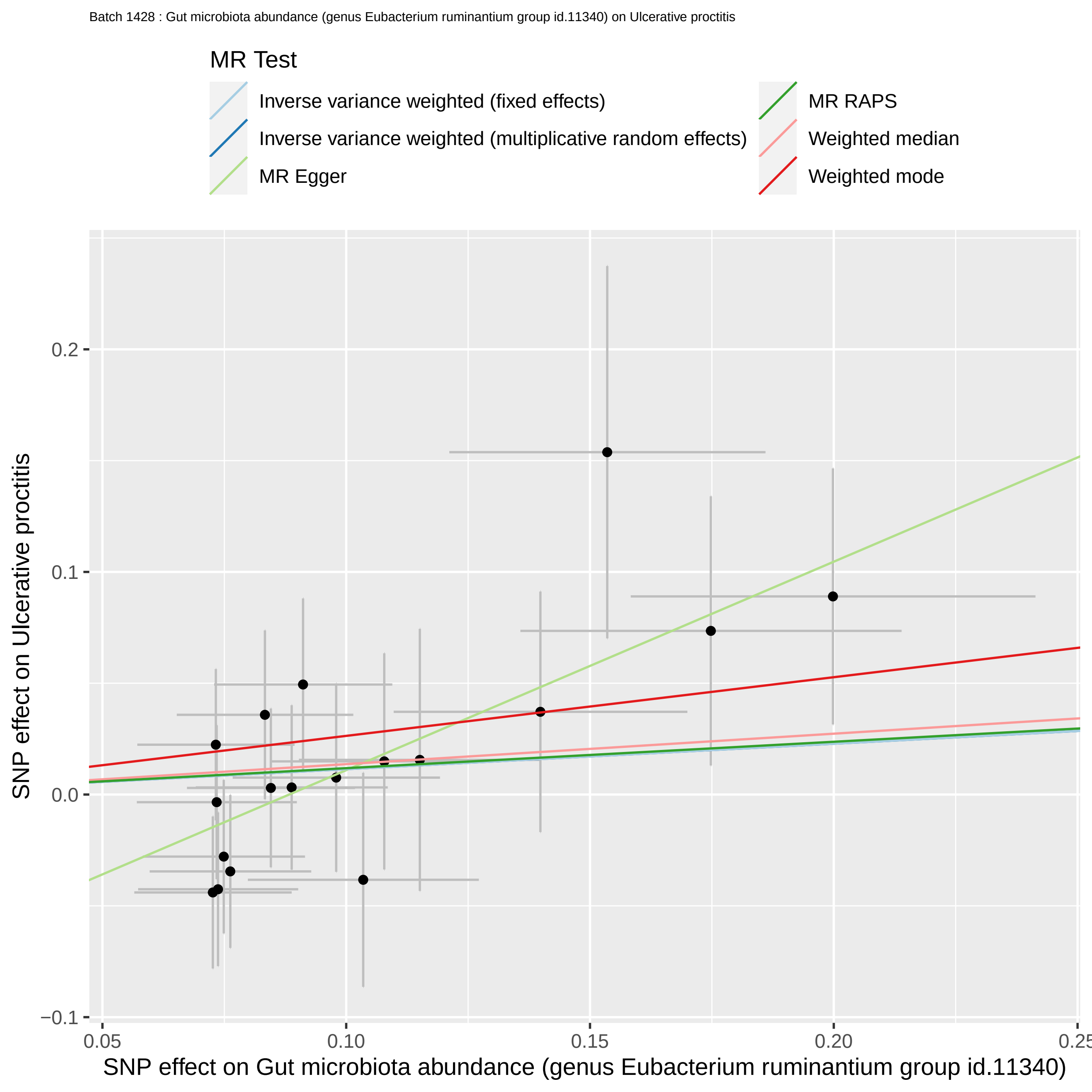

Batch 1428 : Gut microbiota abundance (genus Eubacterium ruminantium group id.11340) on Ulcerative proctitis
MR Test
Inverse variance weighted (fixed effects)
Inverse variance weighted (multiplicative random effects)
MR Egger
MR RAPS
Weighted median
Weighted mode
SNP effect on Ulcerative proctitis
0.2
0.1
0.0
−0.1
0.05
0.10
0.15
0.20
0.25
SNP effect on Gut microbiota abundance (genus Eubacterium ruminantium group id.11340)

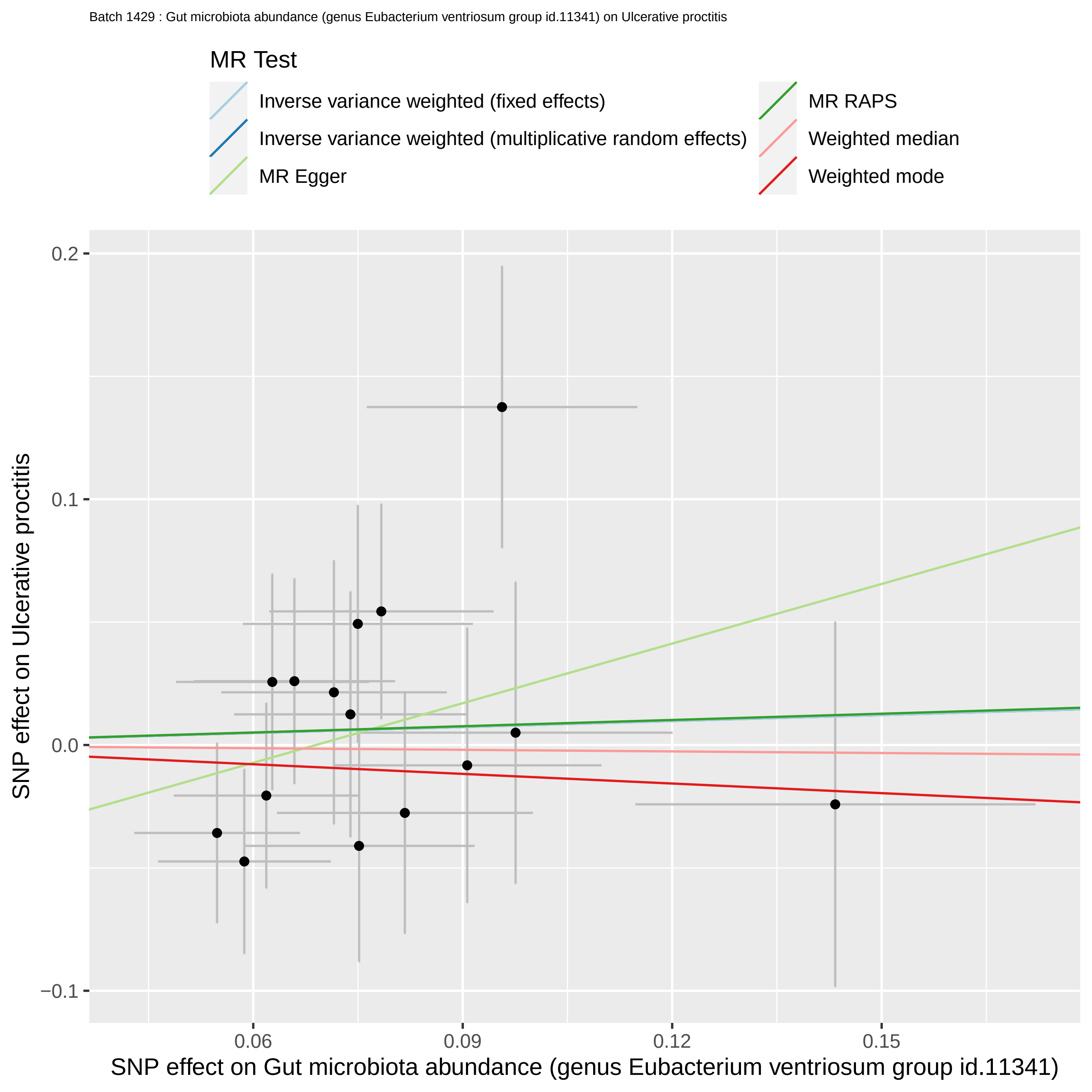

Batch 1429 : Gut microbiota abundance (genus Eubacterium ventriosum group id.11341) on Ulcerative proctitis
MR Test
Inverse variance weighted (fixed effects)
Inverse variance weighted (multiplicative random effects)
MR Egger
MR RAPS
Weighted median
Weighted mode
0.2
0.1
0.0
−0.1
0.06
0.09
0.12
0.15
SNP effect on Ulcerative proctitis
SNP effect on Gut microbiota abundance (genus Eubacterium ventriosum group id.11341)

Batch 1430 : Gut microbiota abundance (genus Eubacterium xylanophilum group id.14375) on Ulcerative proctitis

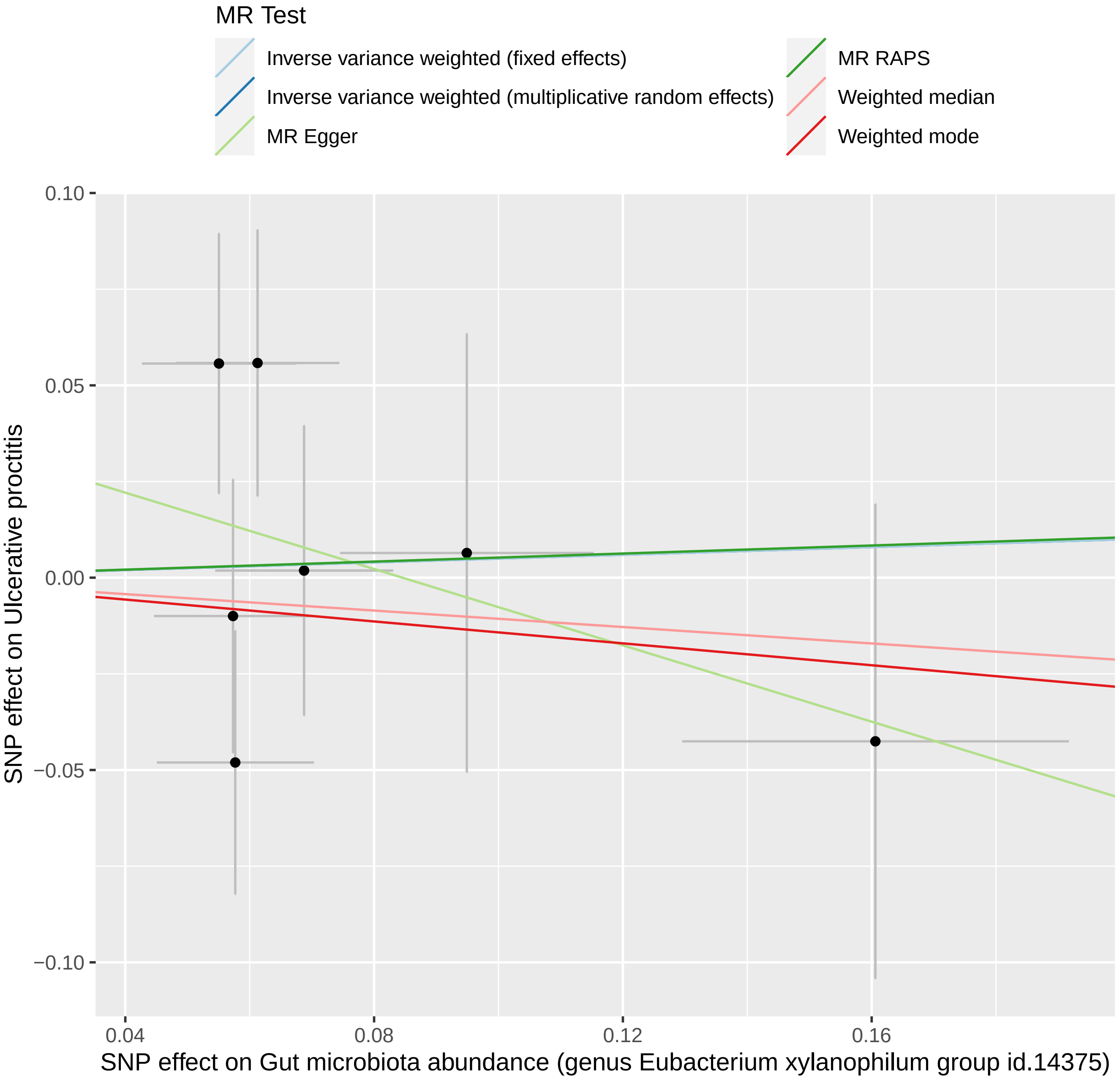

Batch 1431 : Gut microbiota abundance (genus Faecalibacterium id.2057) on Ulcerative proctitis

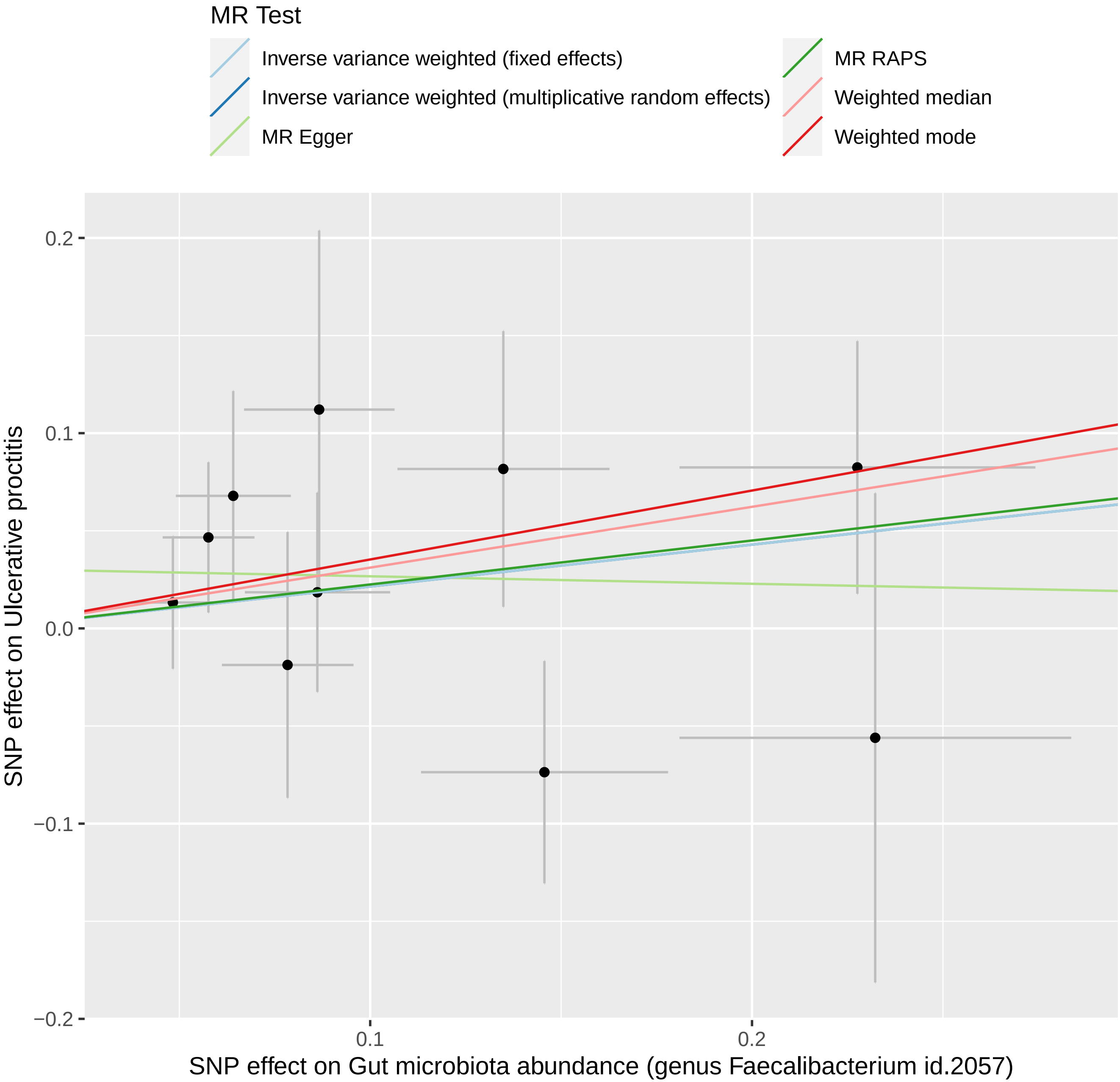

Batch 1432 : Gut microbiota abundance (genus Family XIII AD3011 group id.11293) on Ulcerative proctitis

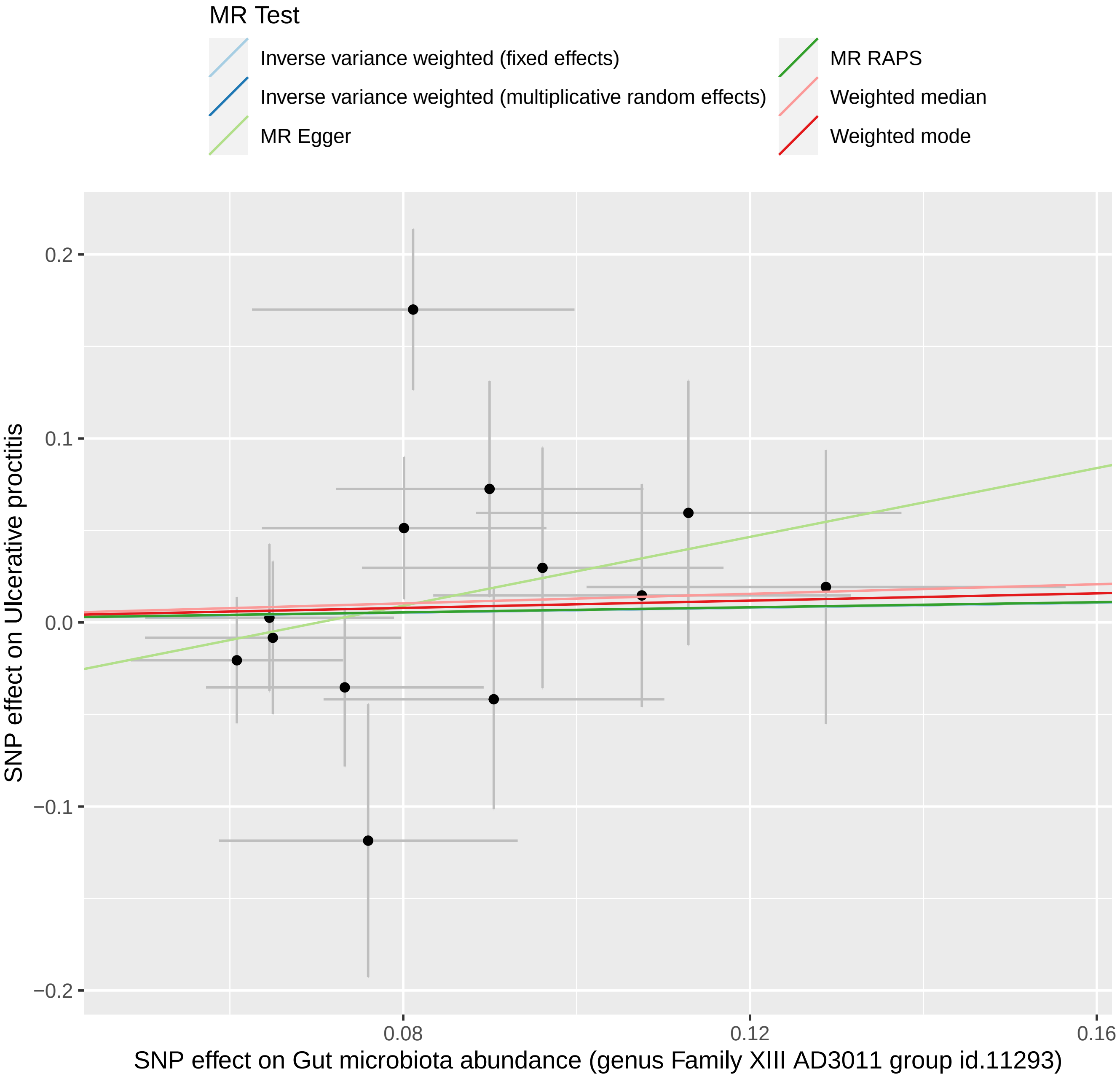

Batch 1433 : Gut microbiota abundance (genus Family XIII UCG001 id.11294) on Ulcerative proctitis

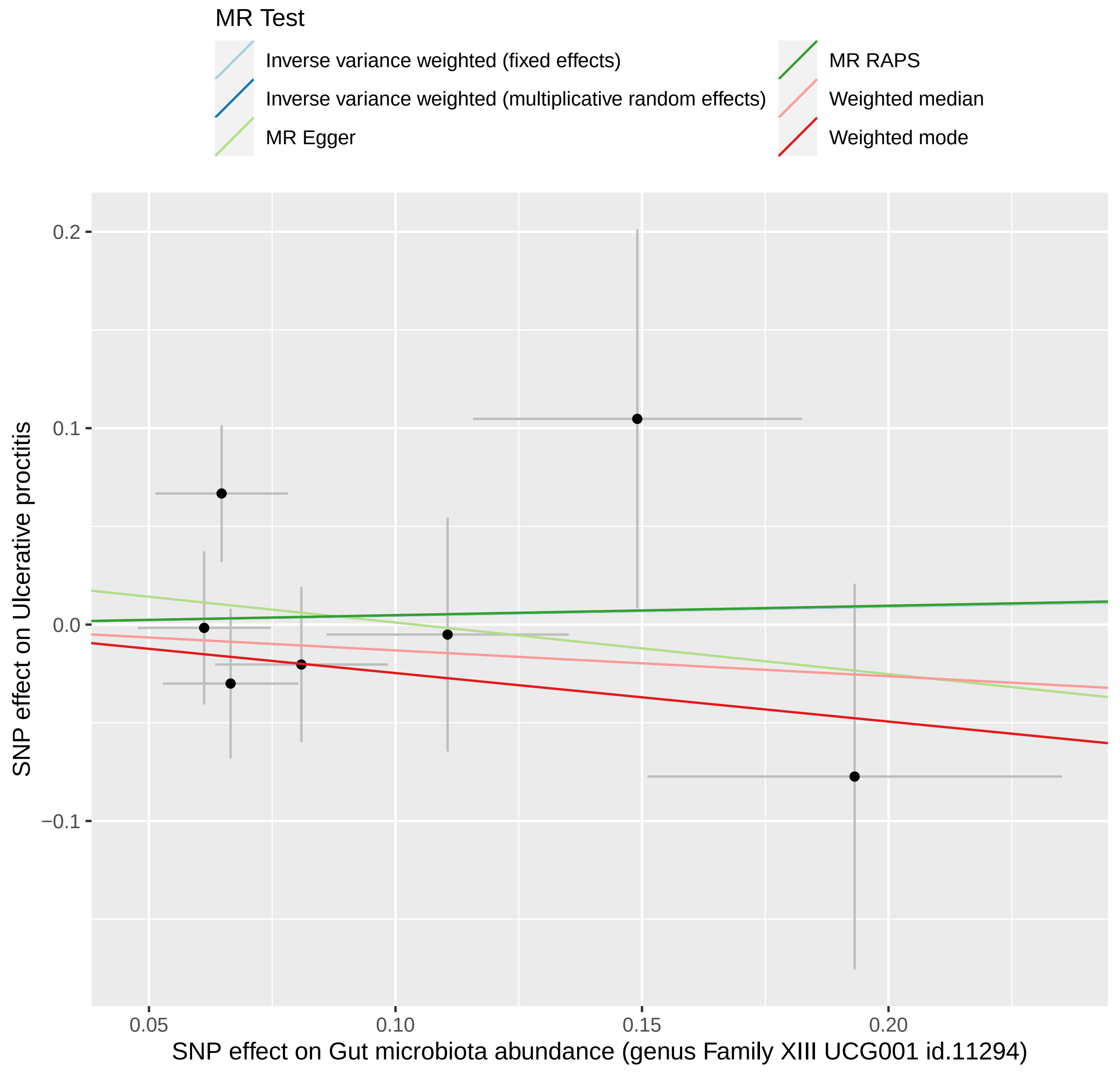

Batch 1434 : Gut microbiota abundance (genus Flavonifractor id.2059) on Ulcerative proctitis

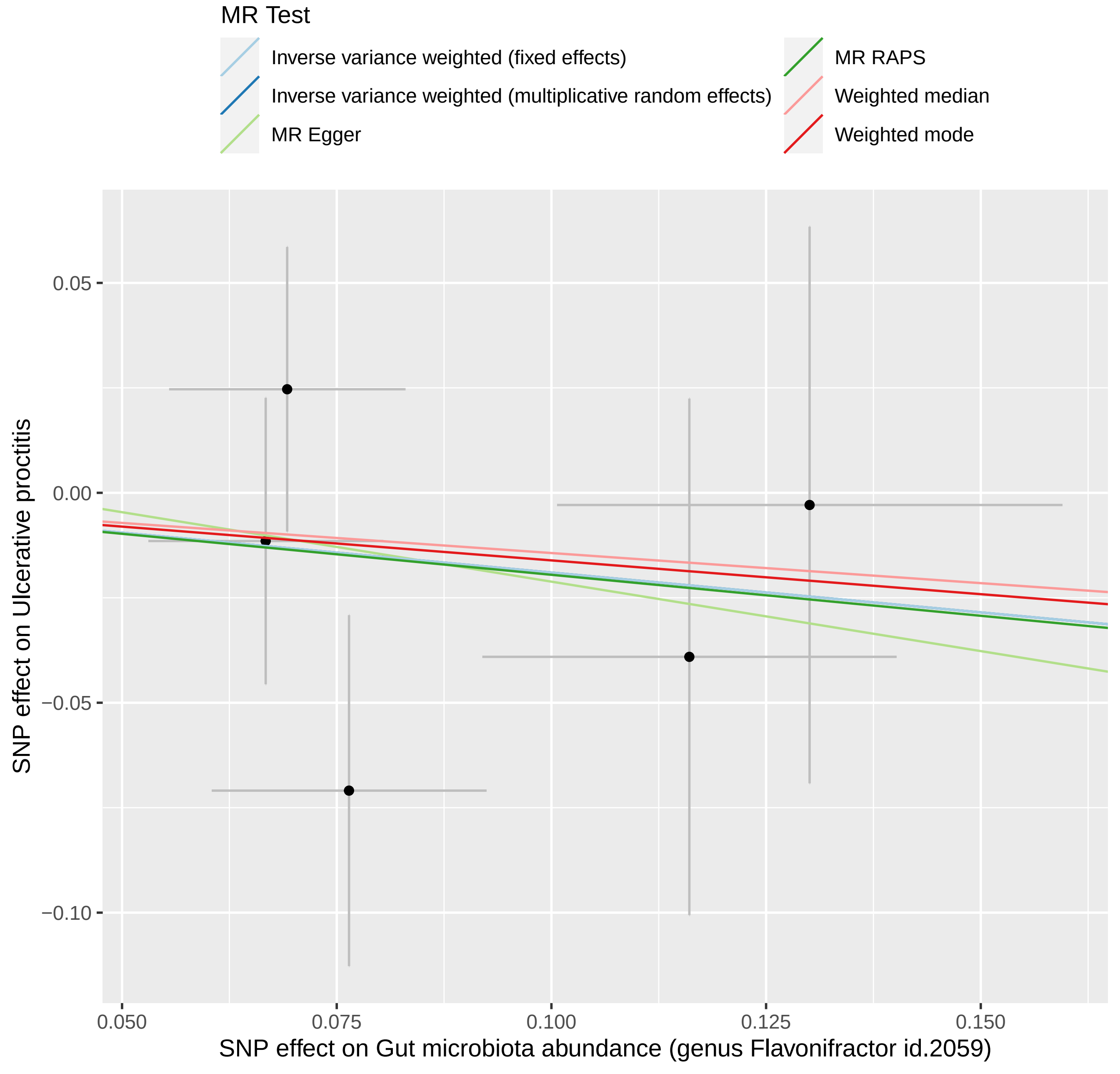

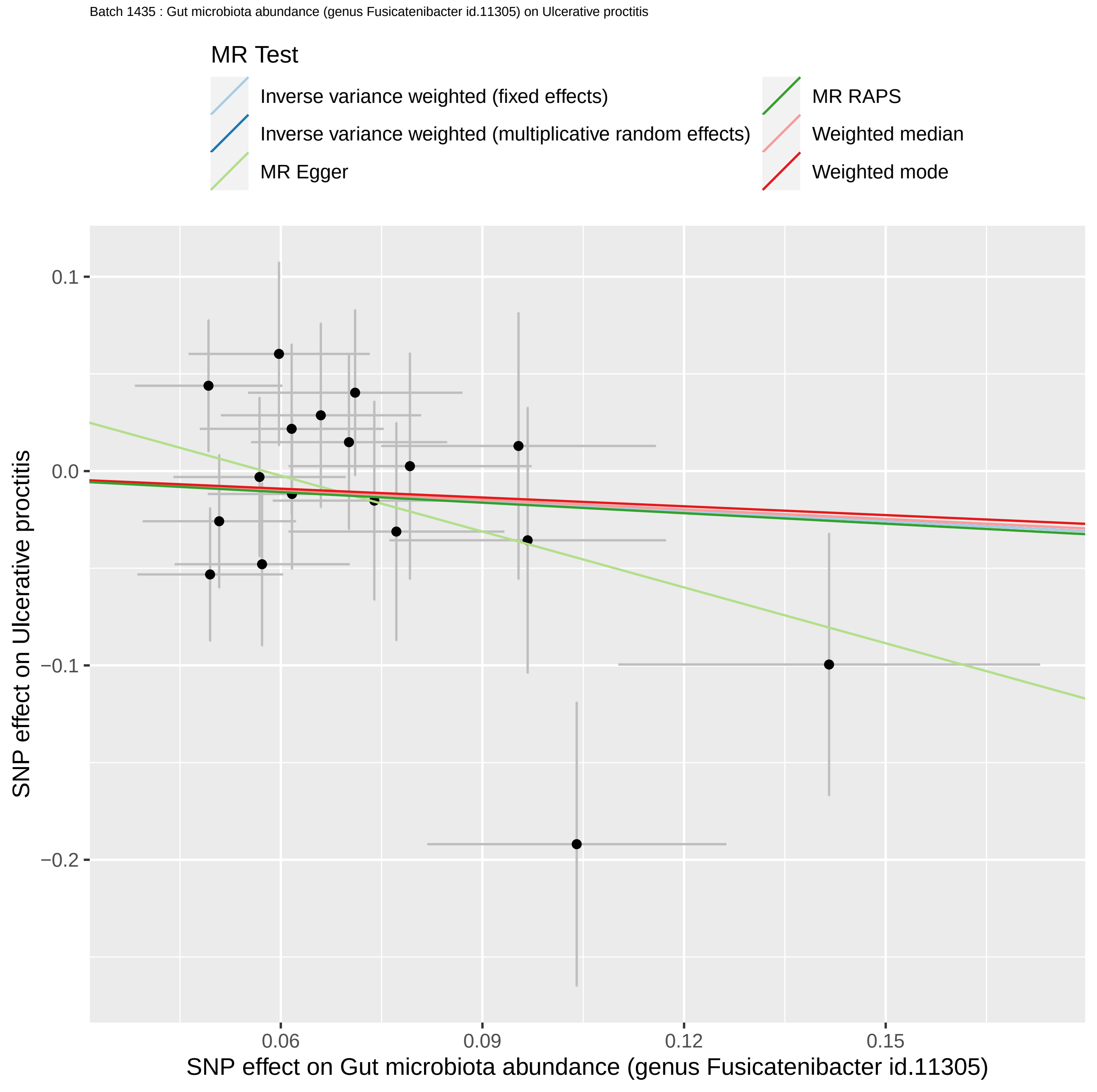
Batch 1435 : Gut microbiota abundance (genus Fusicatenibacter id.11305) on Ulcerative proctitis
MR Test
Inverse variance weighted (fixed effects)
Inverse variance weighted (multiplicative random effects)
MR Egger
MR RAPS
Weighted median
Weighted mode
0.1
0.0
−0.1
−0.2
0.06
0.09
0.12
0.15
SNP effect on Gut microbiota abundance (genus Fusicatenibacter id.11305)
SNP effect on Ulcerative proctitis

Batch 1436 : Gut microbiota abundance (genus Gordonibacter id.821) on Ulcerative proctitis

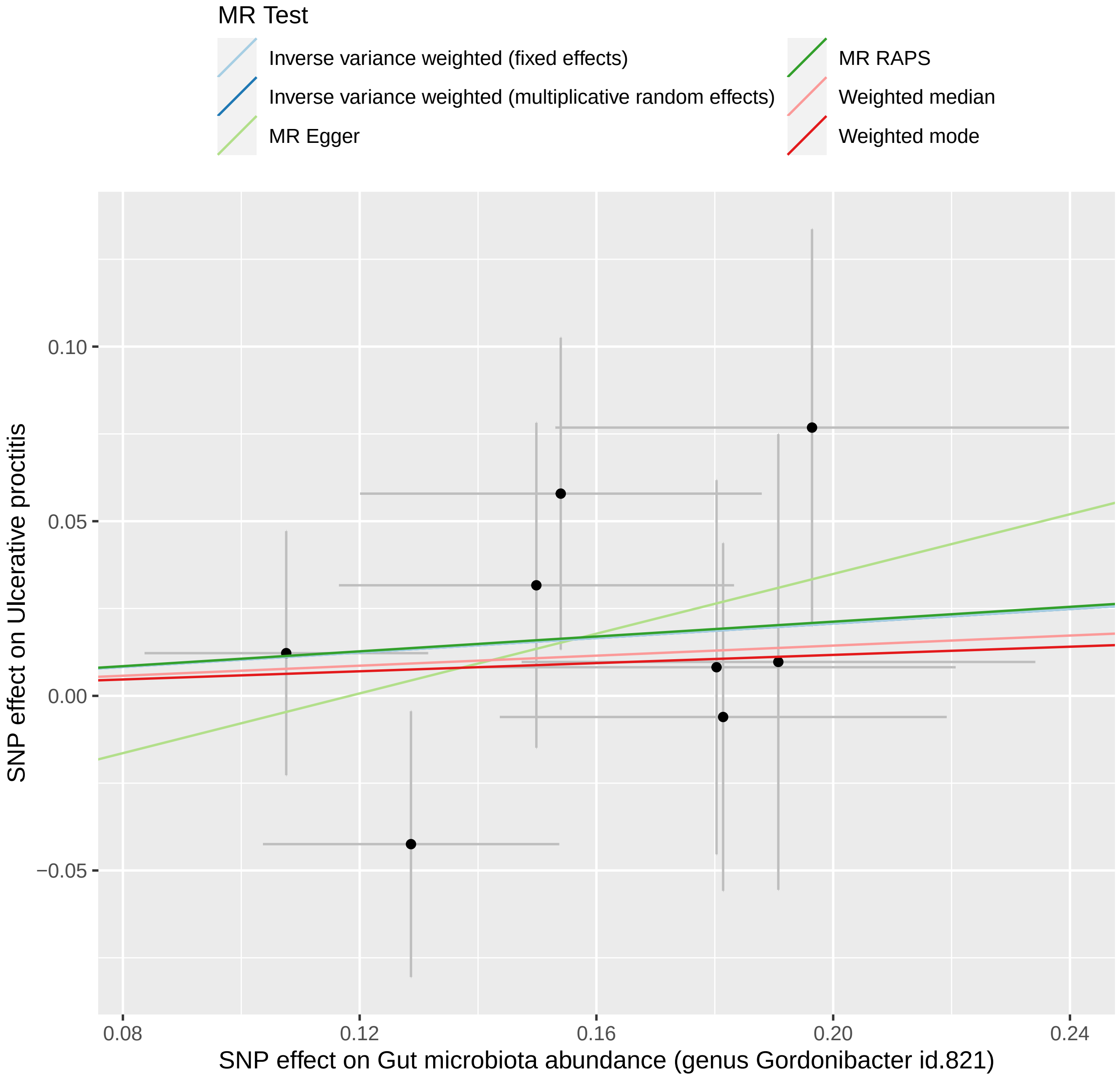

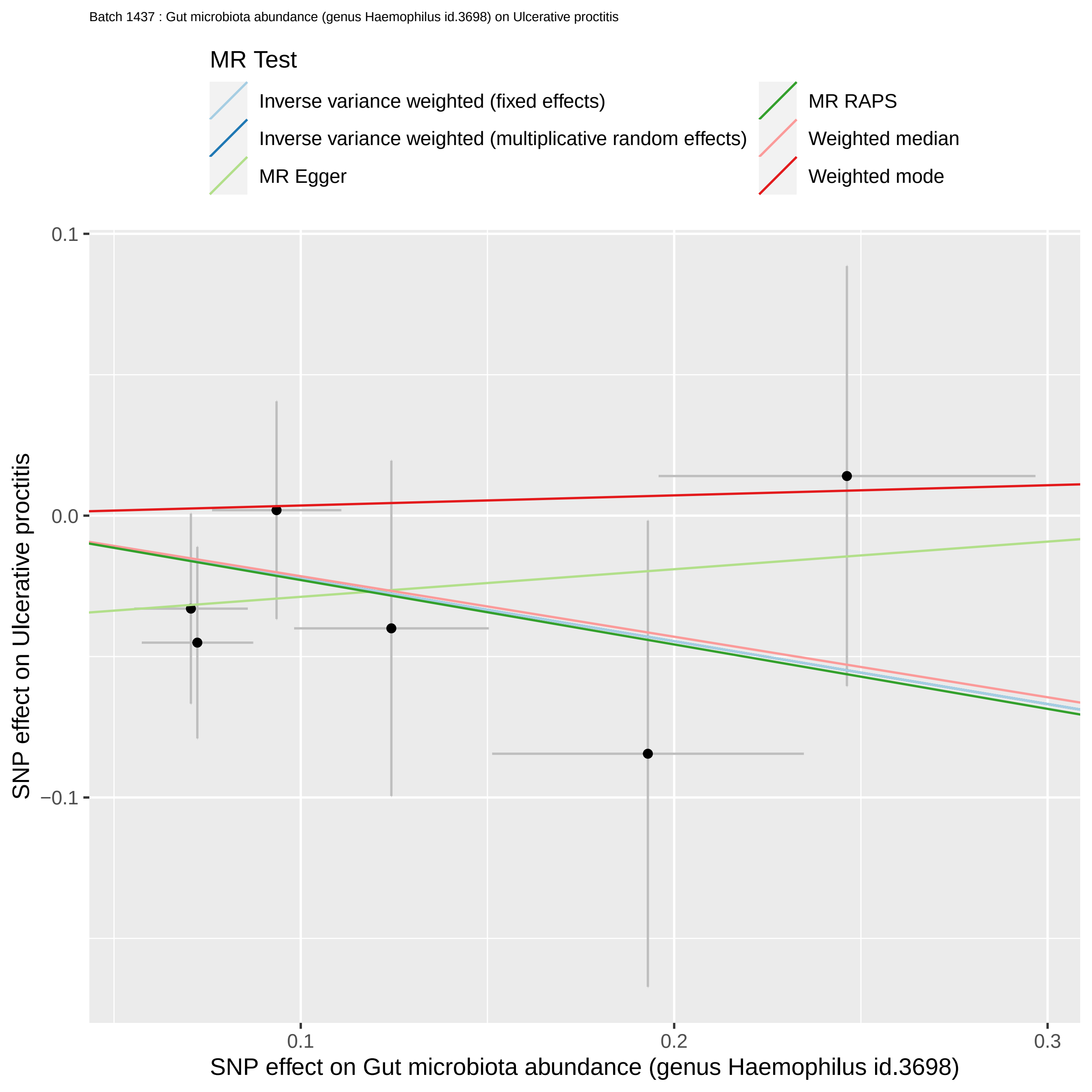
Batch 1437 : Gut microbiota abundance (genus Haemophilus id.3698) on Ulcerative proctitis
MR Test
Inverse variance weighted (fixed effects)
Inverse variance weighted (multiplicative random effects)
MR Egger
MR RAPS
Weighted median
Weighted mode
0.1
0.0
−0.1
0.1
0.2
0.3
SNP effect on Ulcerative proctitis
SNP effect on Gut microbiota abundance (genus Haemophilus id.3698)

Batch 1438 : Gut microbiota abundance (genus Holdemanella id.11393) on Ulcerative proctitis

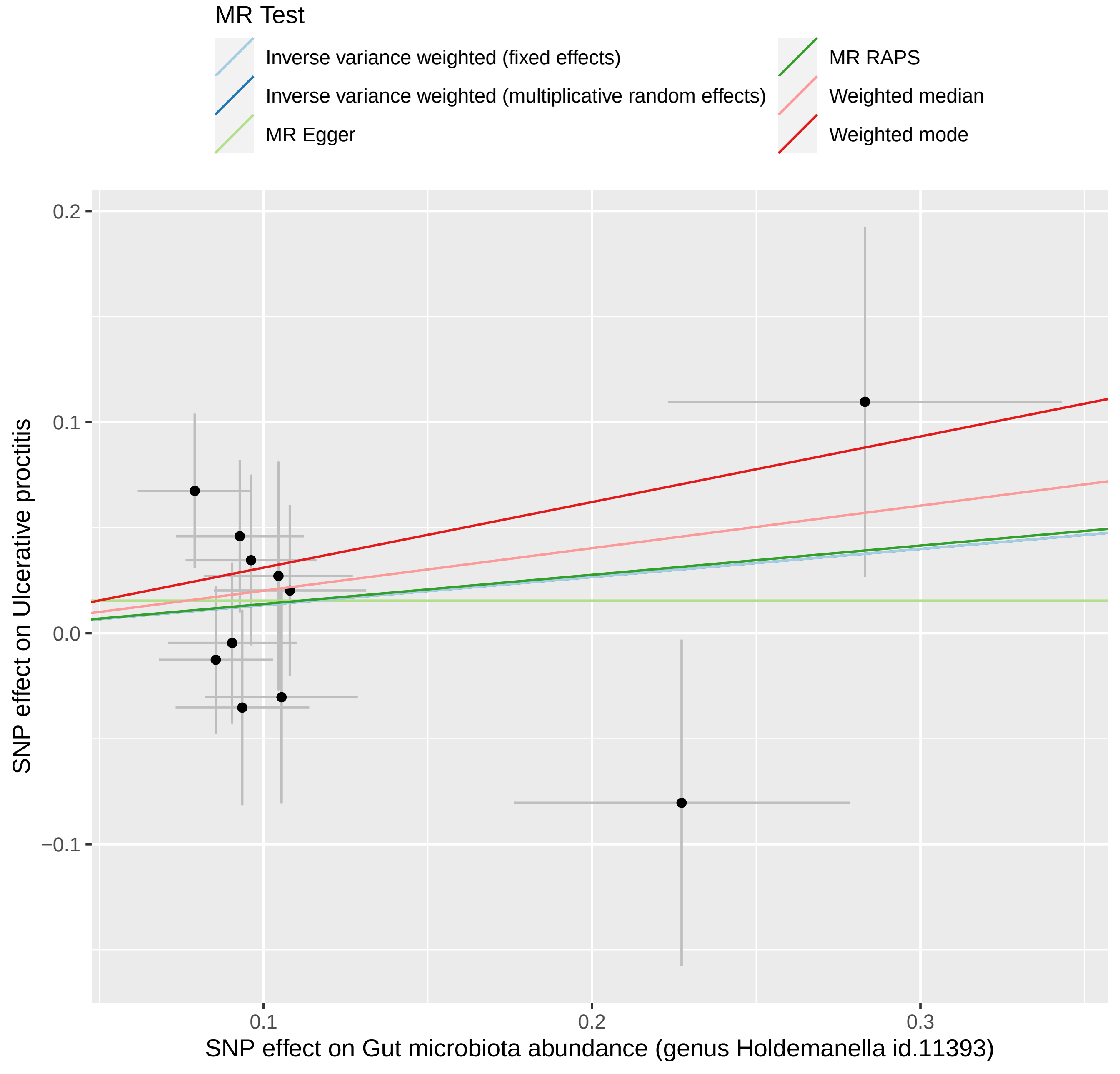

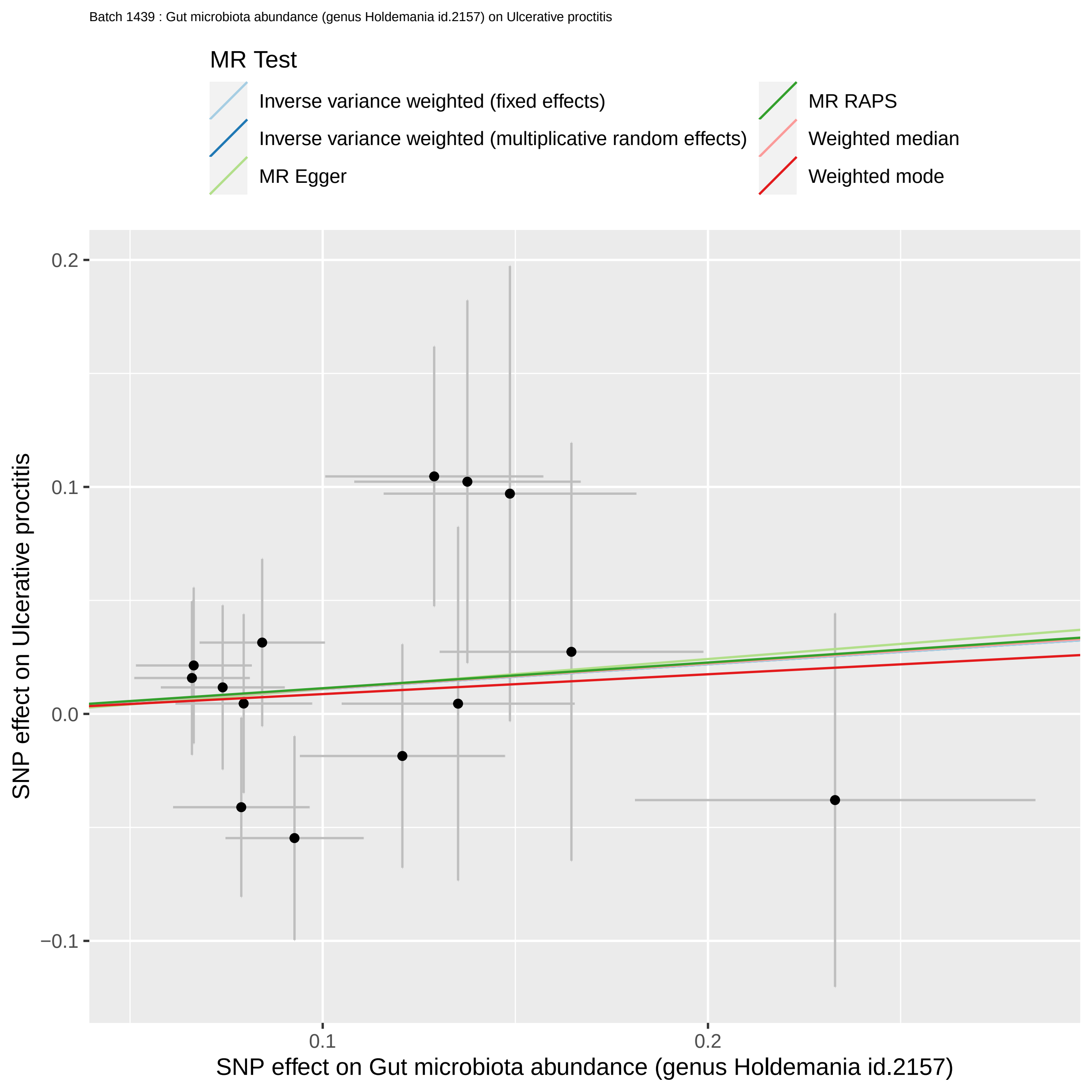
Batch 1439 : Gut microbiota abundance (genus Holdemania id.2157) on Ulcerative proctitis
MR Test
Inverse variance weighted (fixed effects)
Inverse variance weighted (multiplicative random effects)
MR Egger
MR RAPS
Weighted median
Weighted mode
0.2
0.1
0.0
−0.1
0.1
0.2
SNP effect on Ulcerative proctitis
SNP effect on Gut microbiota abundance (genus Holdemania id.2157)

Batch 1440 : Gut microbiota abundance (genus Howardella id.2000) on Ulcerative proctitis

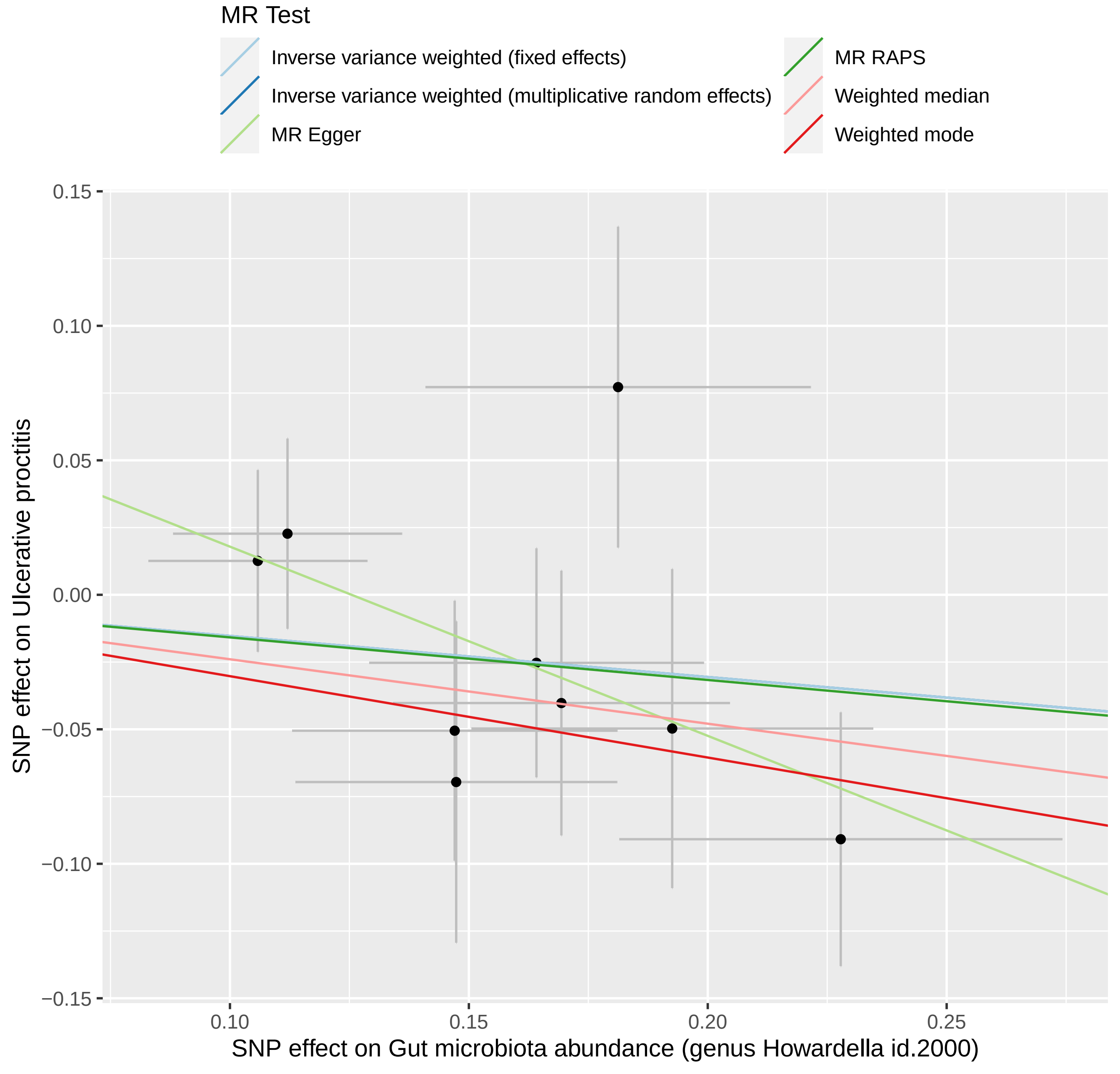

Batch 1441 : Gut microbiota abundance (genus Hungatella id.11306) on Ulcerative proctitis

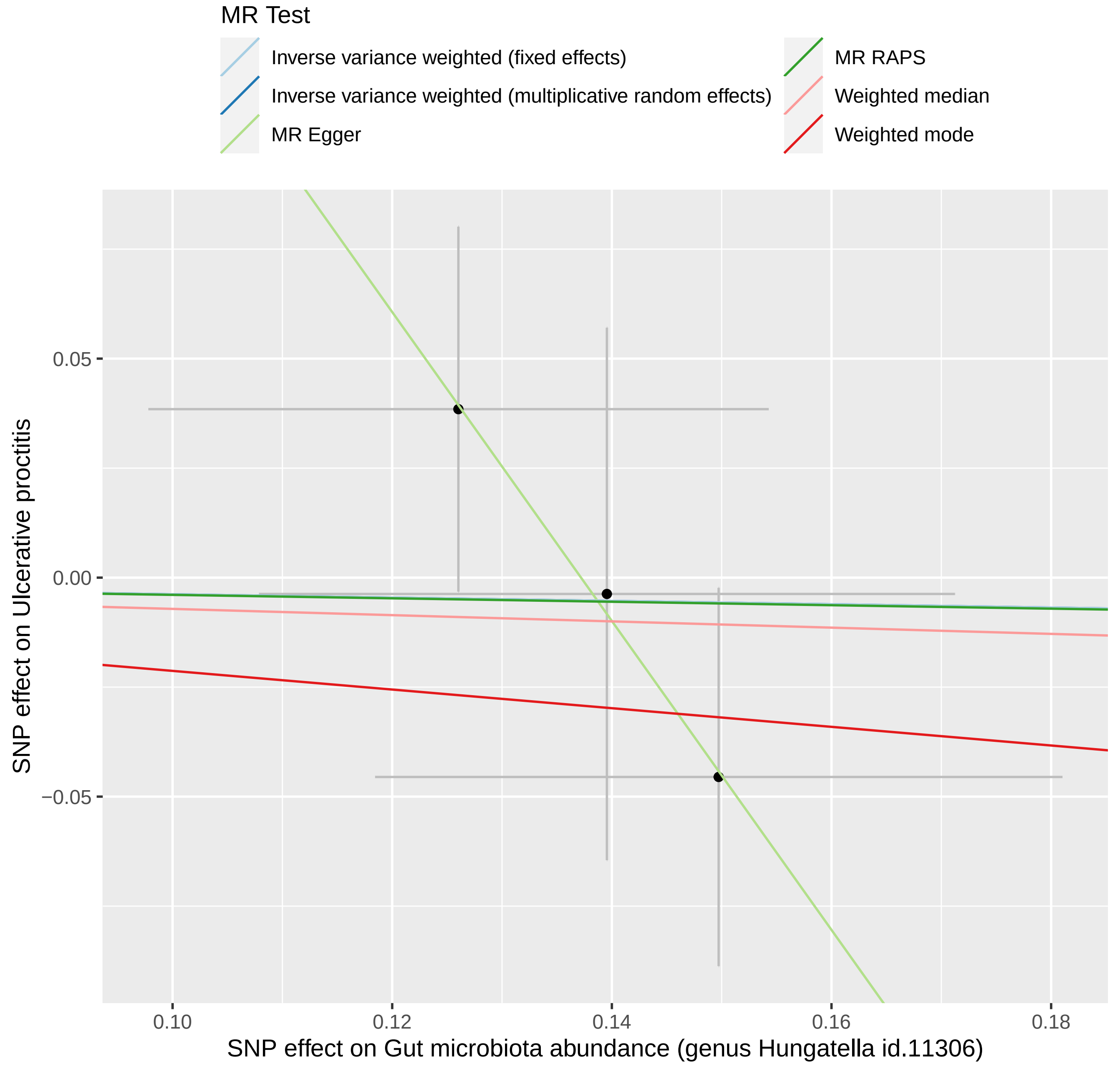

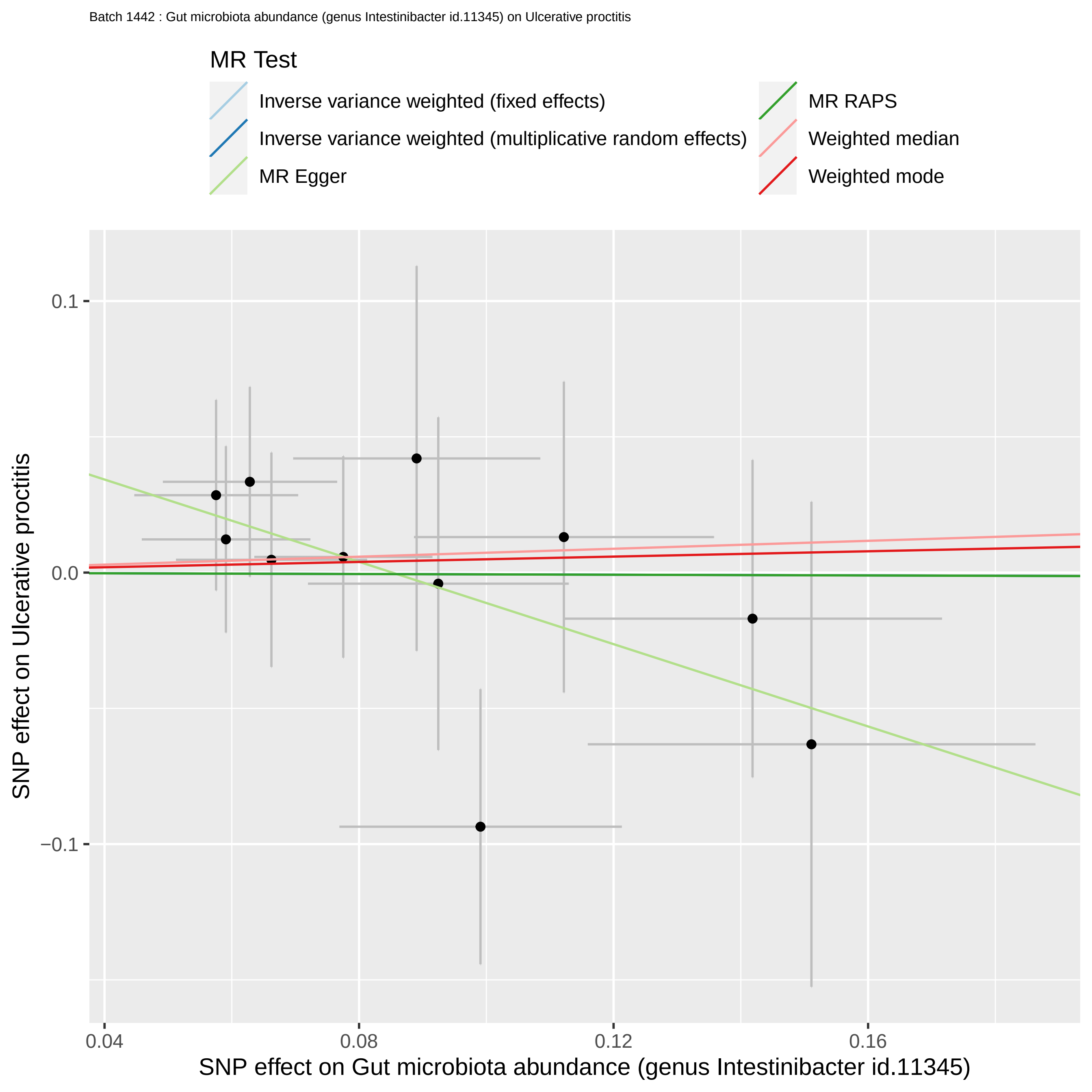

Batch 1442 : Gut microbiota abundance (genus Intestinibacter id.11345) on Ulcerative proctitis
MR Test
Inverse variance weighted (fixed effects)
Inverse variance weighted (multiplicative random effects)
MR Egger
MR RAPS
Weighted median
Weighted mode
0.1
0.0
−0.1
0.04
0.08
0.12
0.16
SNP effect on Ulcerative proctitis
SNP effect on Gut microbiota abundance (genus Intestinibacter id.11345)

Batch 1443 : Gut microbiota abundance (genus Intestinimonas id.2062) on Ulcerative proctitis

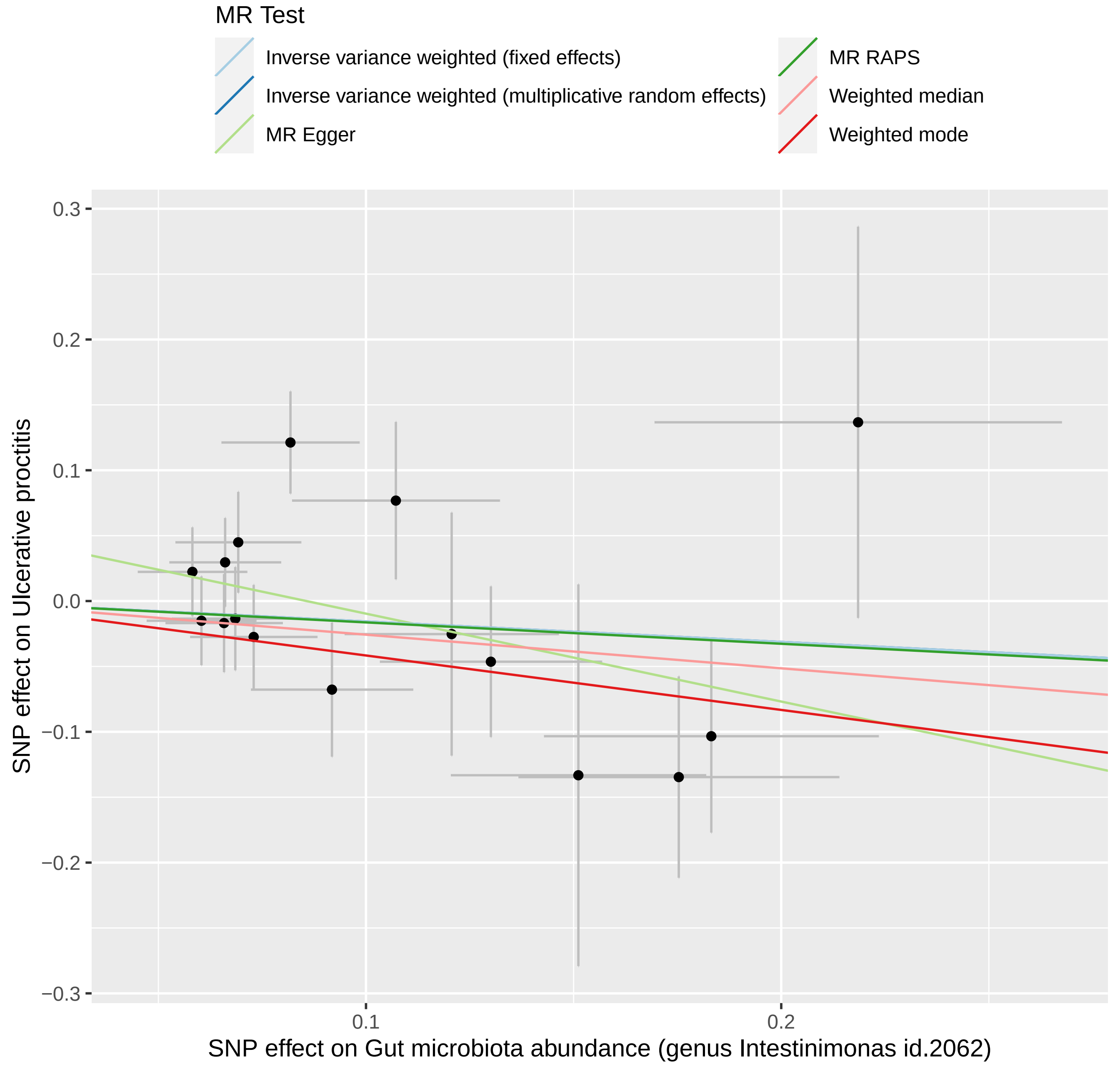

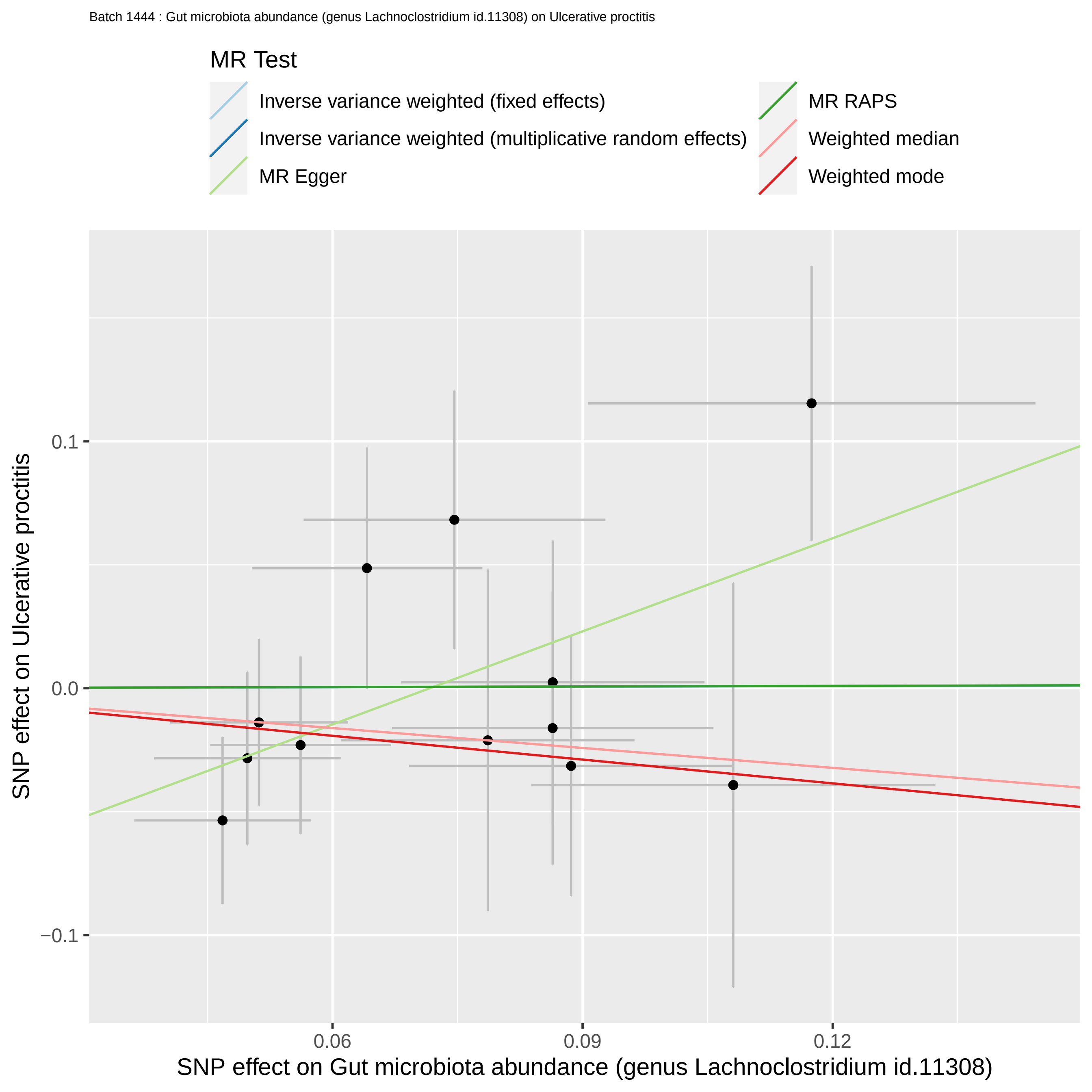
Batch 1444 : Gut microbiota abundance (genus Lachnoclostridium id.11308) on Ulcerative proctitis
MR Test
Inverse variance weighted (fixed effects)
Inverse variance weighted (multiplicative random effects)
MR Egger
MR RAPS
Weighted median
Weighted mode
0.1
0.0
−0.1
0.06
0.09
0.12
SNP effect on Ulcerative proctitis
SNP effect on Gut microbiota abundance (genus Lachnoclostridium id.11308)

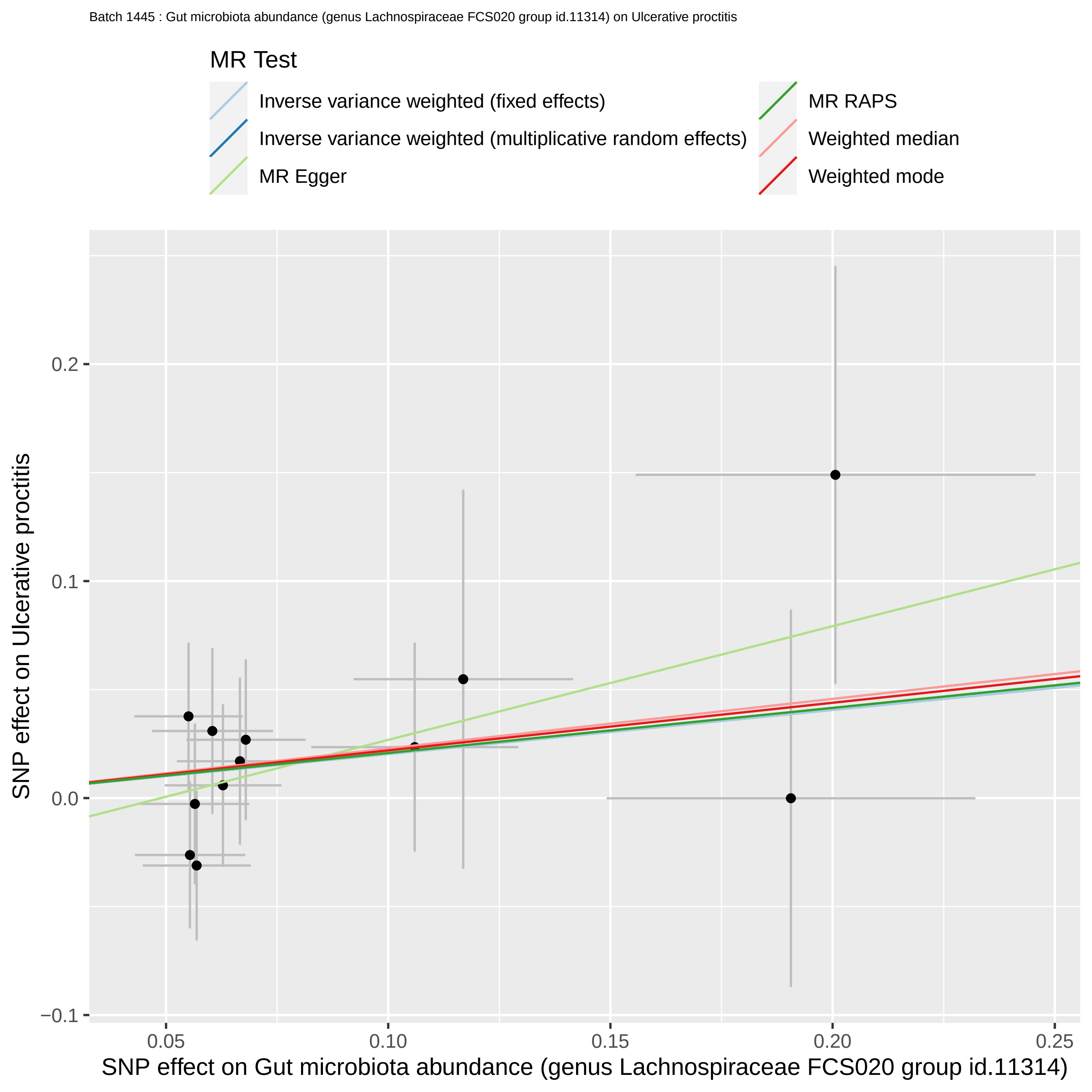

Batch 1445 : Gut microbiota abundance (genus Lachnospiraceae FCS020 group id.11314) on Ulcerative proctitis
MR Test
Inverse variance weighted (fixed effects)
Inverse variance weighted (multiplicative random effects)
MR Egger
MR RAPS
Weighted median
Weighted mode
0.2
0.1
0.0
−0.1
0.05
0.10
0.15
0.20
0.25
SNP effect on Ulcerative proctitis
SNP effect on Gut microbiota abundance (genus Lachnospiraceae FCS020 group id.11314)

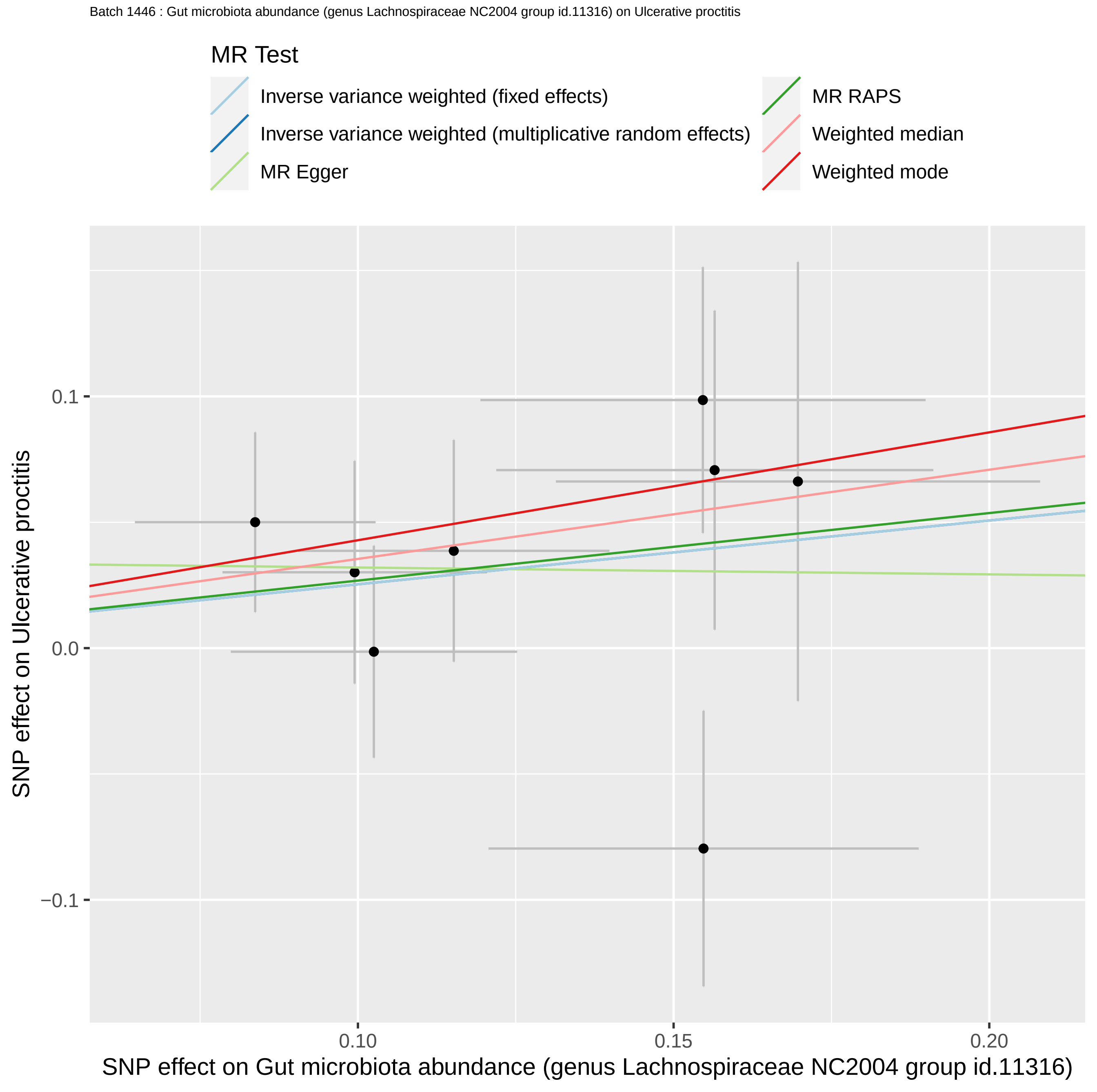
Batch 1446 : Gut microbiota abundance (genus Lachnospiraceae NC2004 group id.11316) on Ulcerative proctitis
MR Test
Inverse variance weighted (fixed effects)
Inverse variance weighted (multiplicative random effects)
MR Egger
MR RAPS
Weighted median
Weighted mode
SNP effect on Ulcerative proctitis
0.1
0.0
−0.1
0.10
0.15
0.20
SNP effect on Gut microbiota abundance (genus Lachnospiraceae NC2004 group id.11316)

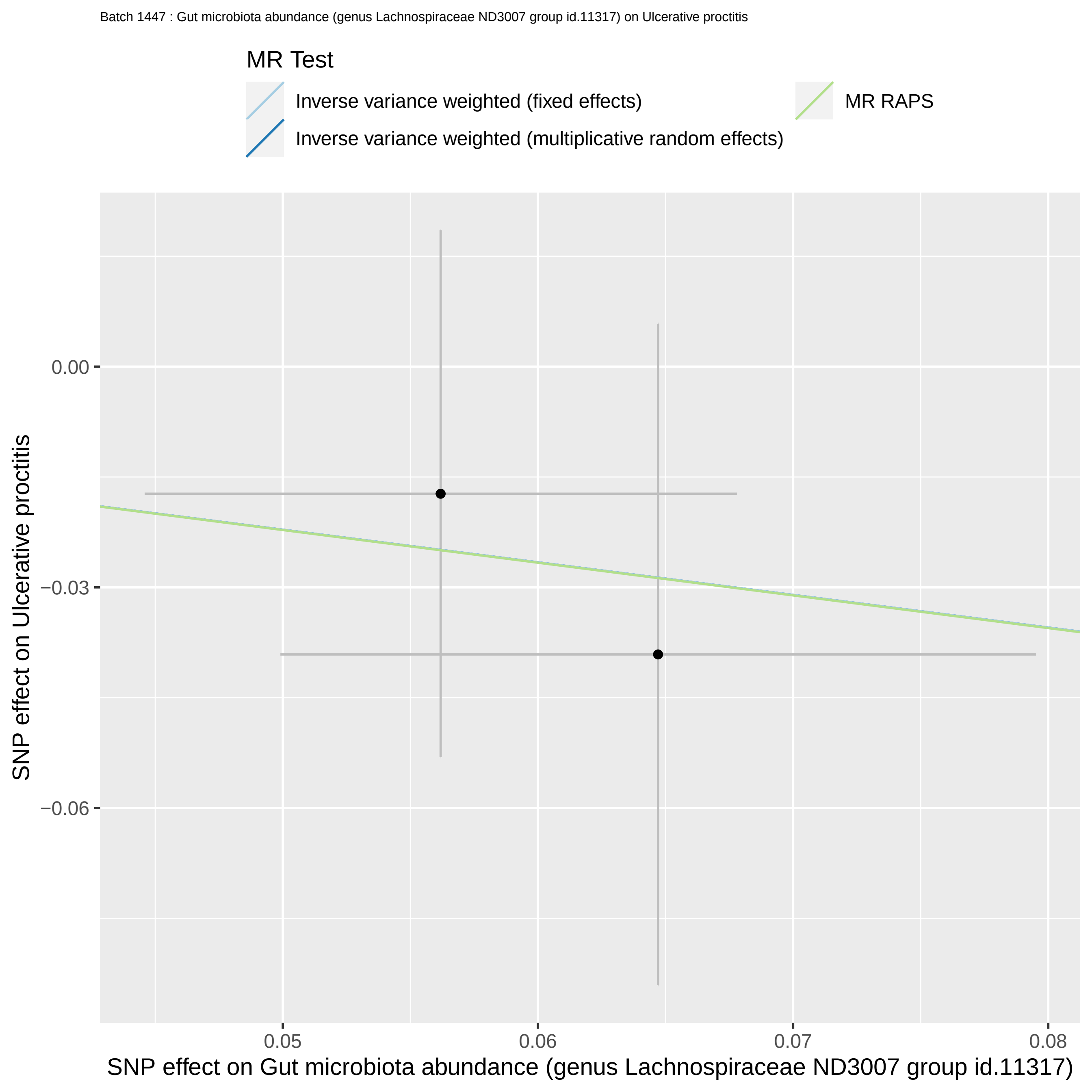

Batch 1447 : Gut microbiota abundance (genus Lachnospiraceae ND3007 group id.11317) on Ulcerative proctitis
MR Test
Inverse variance weighted (fixed effects)
Inverse variance weighted (multiplicative random effects)
MR RAPS
0.00
−0.03
−0.06
0.05
0.06
0.07
0.08
SNP effect on Ulcerative proctitis
SNP effect on Gut microbiota abundance (genus Lachnospiraceae ND3007 group id.11317)

Batch 1448 : Gut microbiota abundance (genus Lachnospiraceae NK4A136 group id.11319) on Ulcerative proctitis

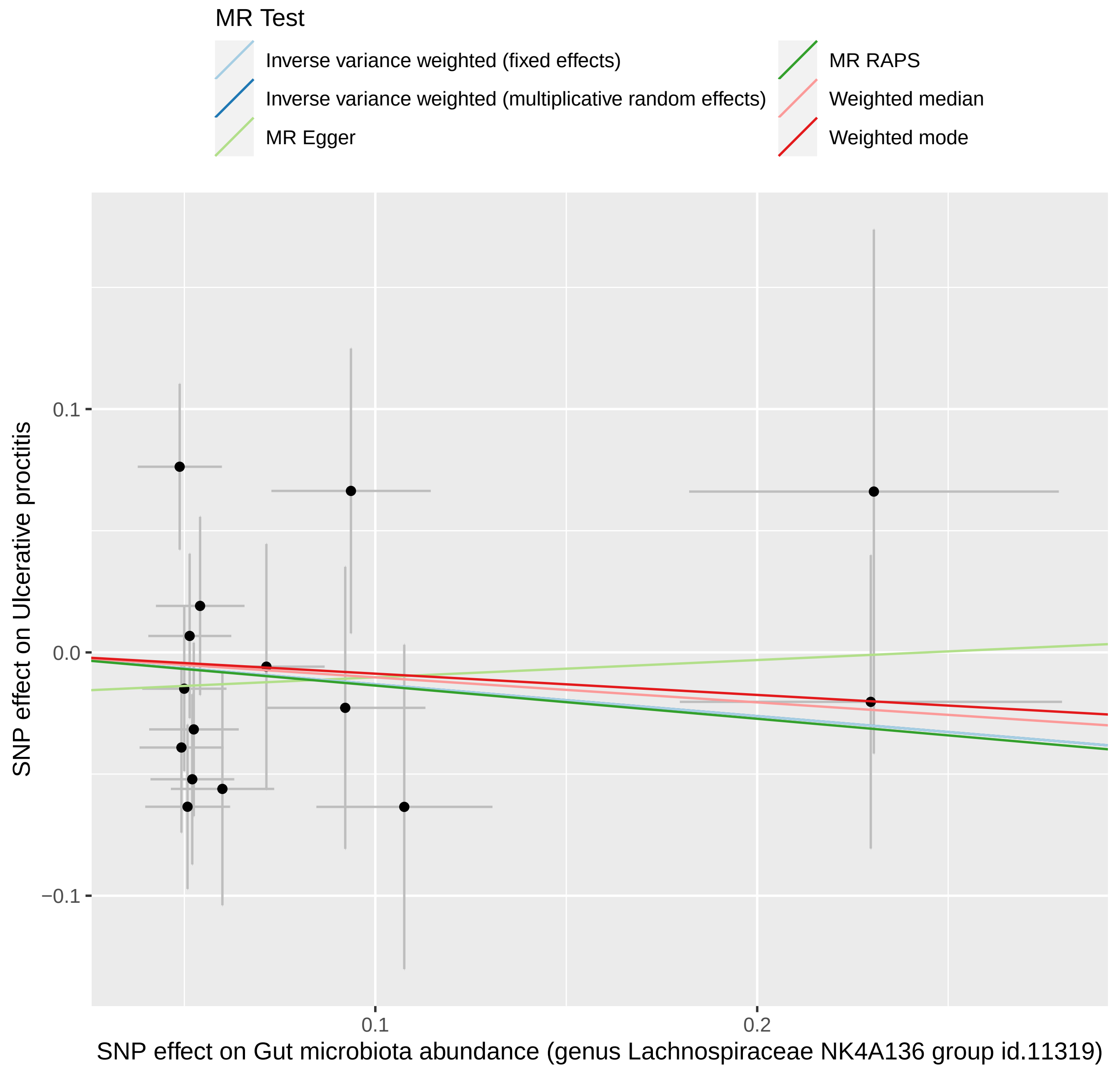

Batch 1449 : Gut microbiota abundance (genus Lachnospiraceae UCG001 id.11321) on Ulcerative proctitis

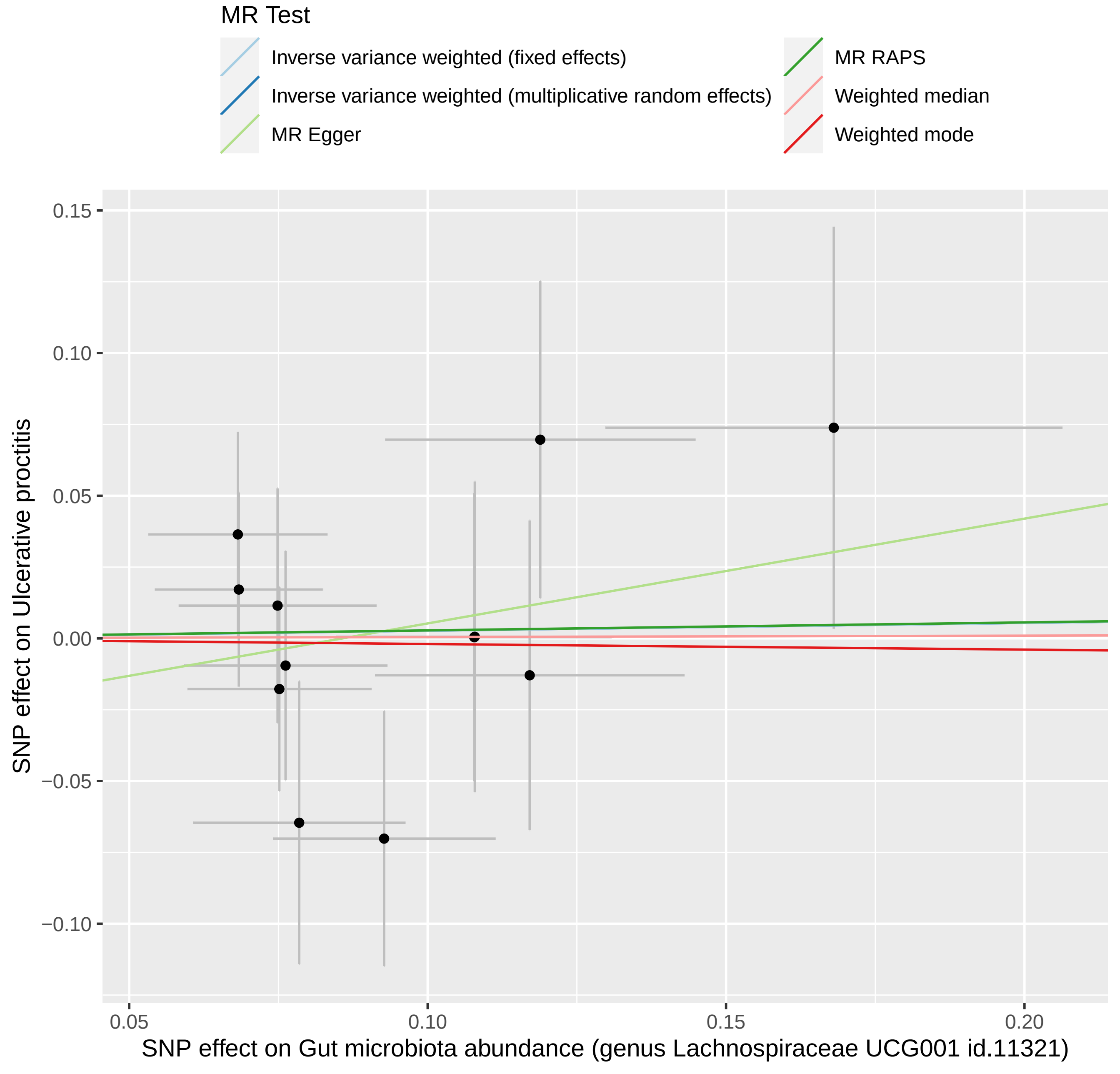

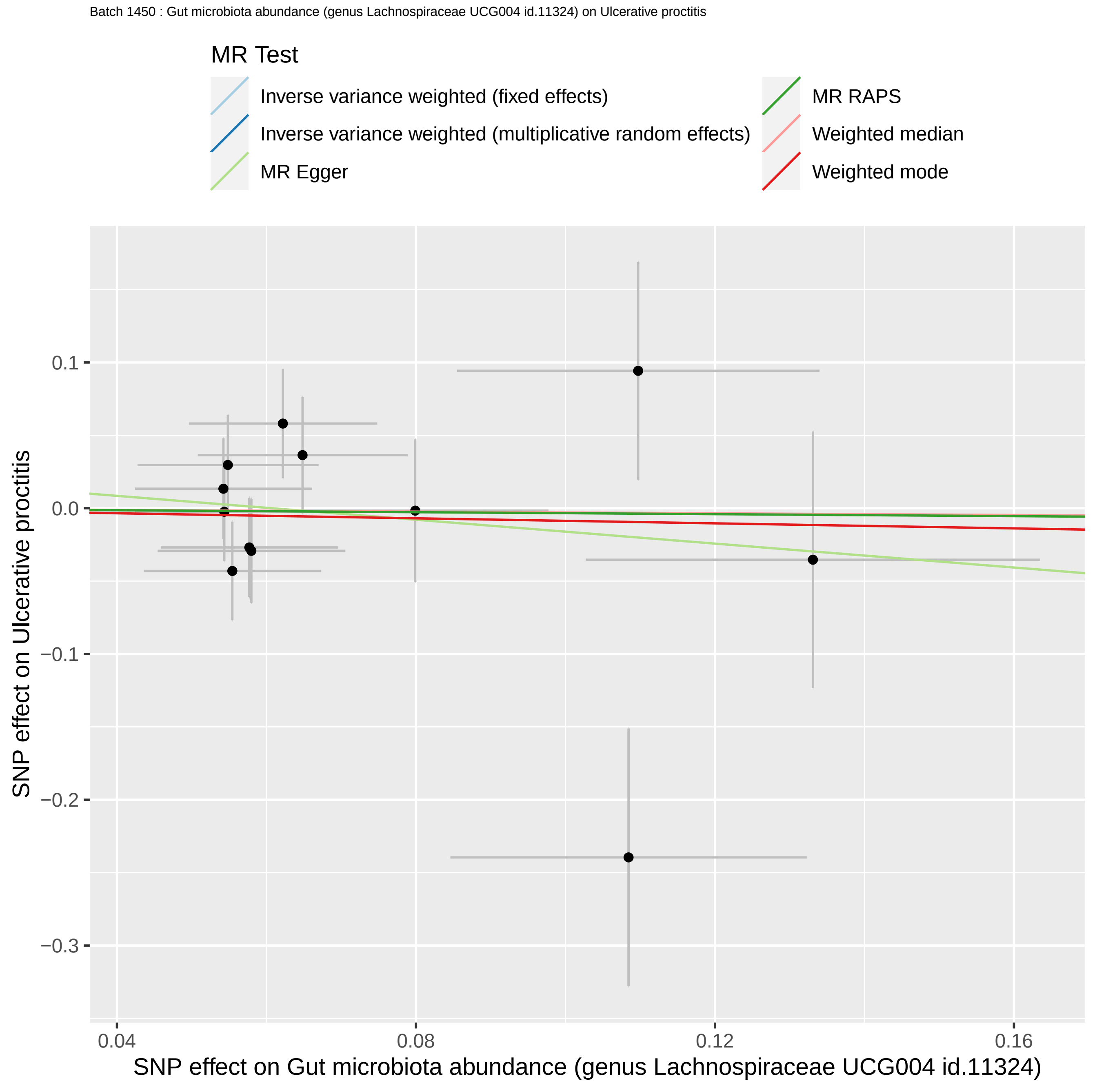

Batch 1450 : Gut microbiota abundance (genus Lachnospiraceae UCG004 id.11324) on Ulcerative proctitis
MR Test
Inverse variance weighted (fixed effects)
Inverse variance weighted (multiplicative random effects)
MR Egger
MR RAPS
Weighted median
Weighted mode
SNP effect on Ulcerative proctitis
SNP effect on Gut microbiota abundance (genus Lachnospiraceae UCG004 id.11324)
0.1
0.0
−0.1
−0.2
−0.3
0.04
0.08
0.12
0.16

Batch 1451 : Gut microbiota abundance (genus Lachnospiraceae UCG008 id.11328) on Ulcerative proctitis

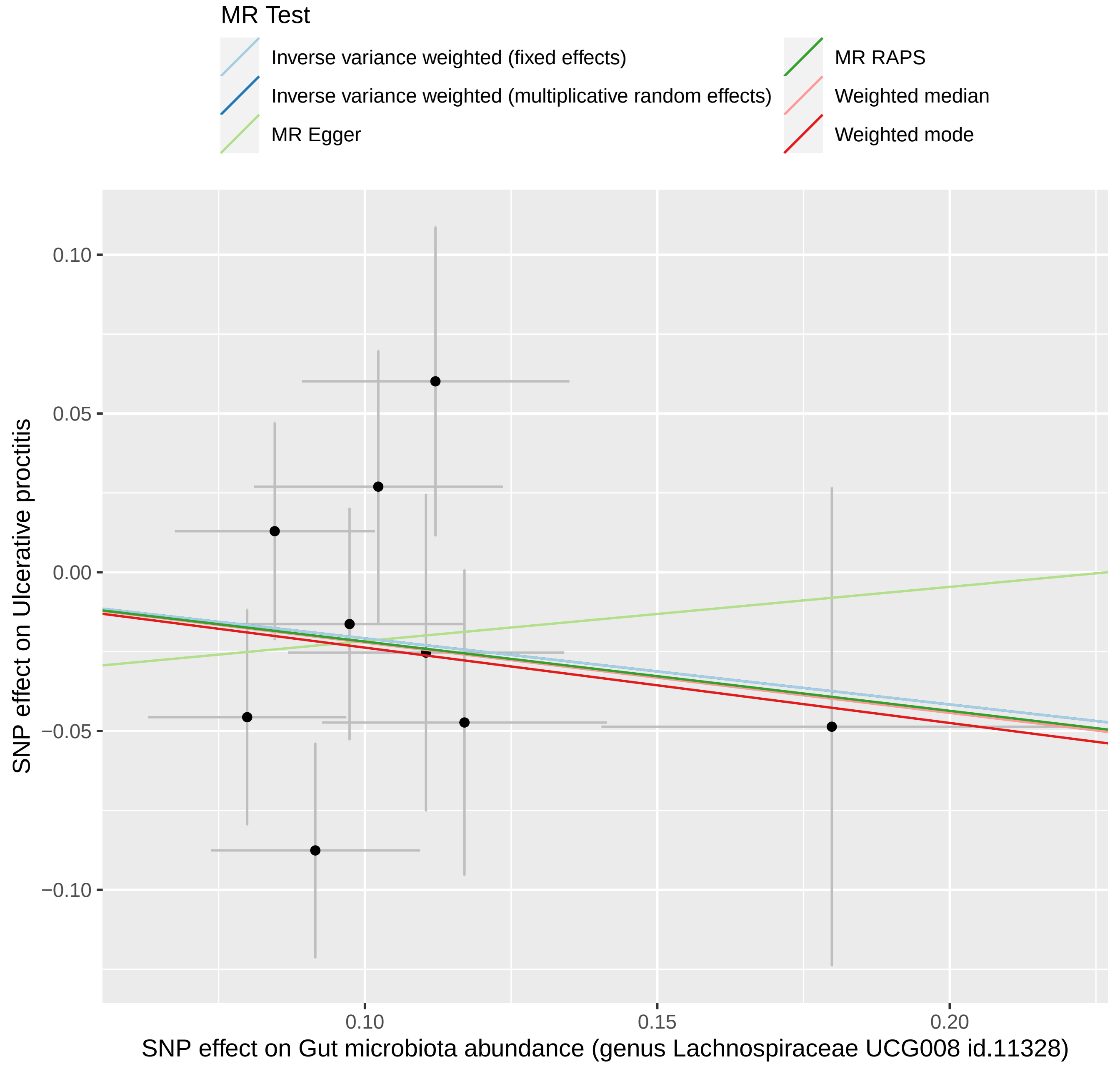

Batch 1452 : Gut microbiota abundance (genus Lachnospiraceae UCG010 id.11330) on Ulcerative proctitis

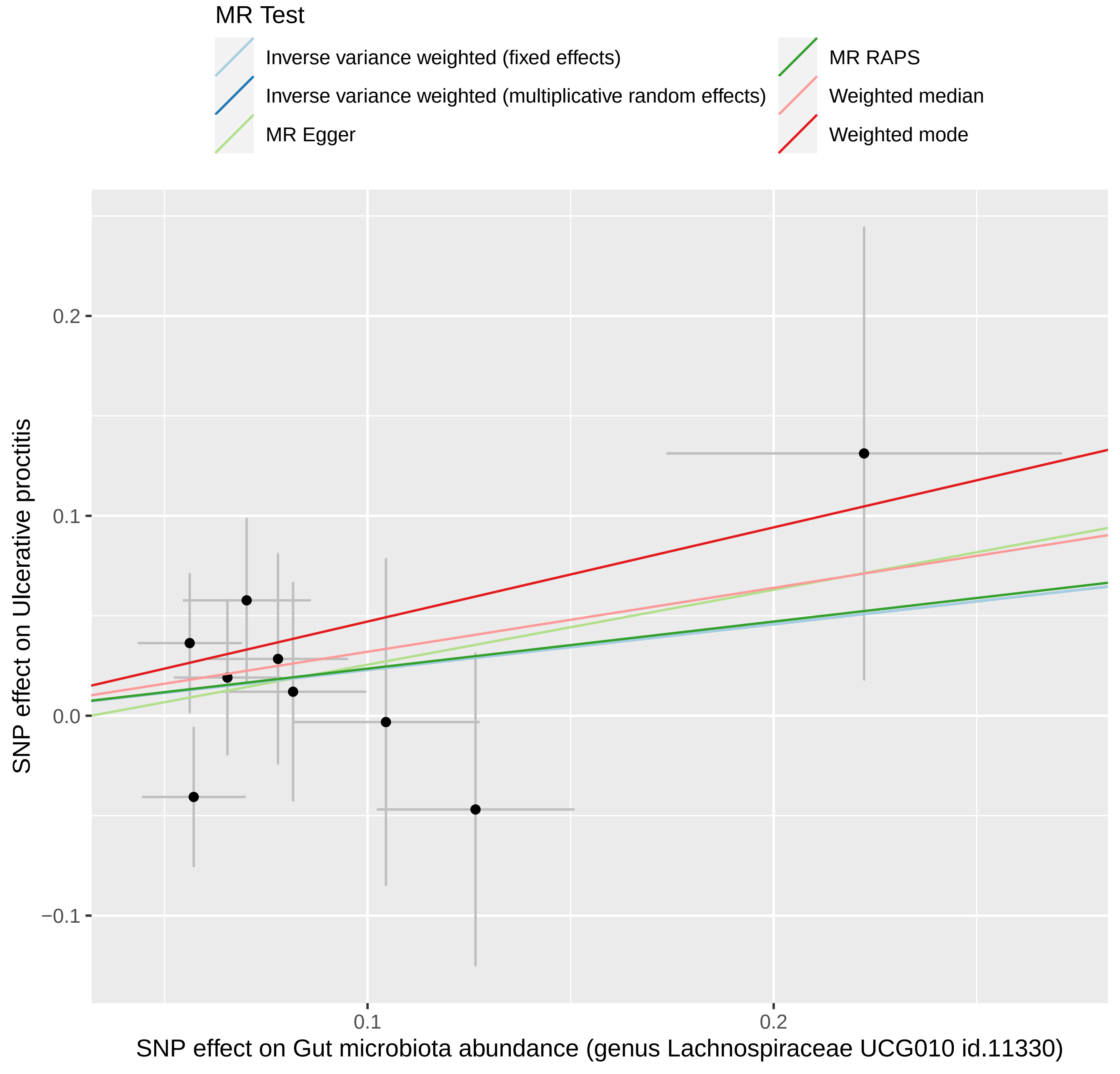

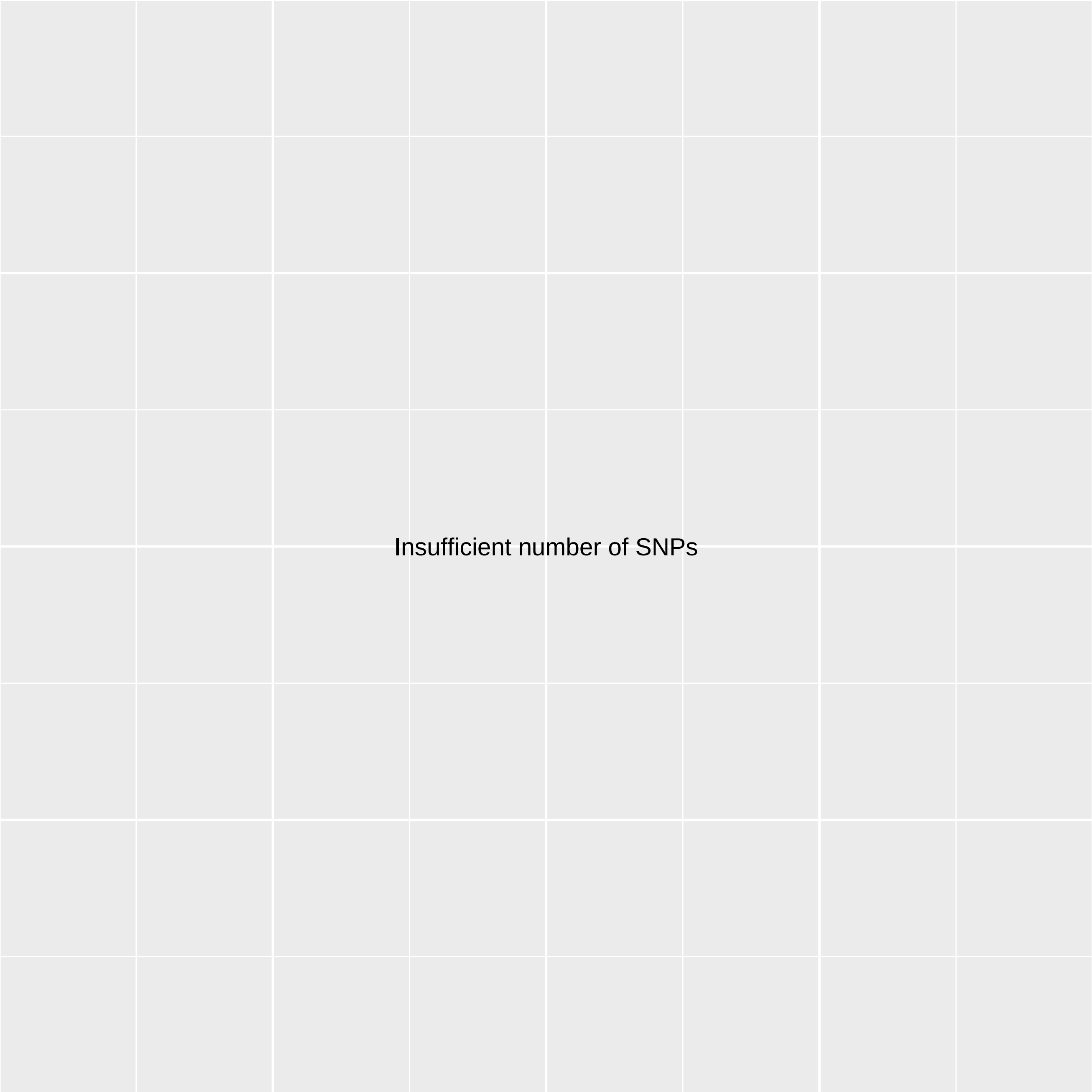
Insufficient number of SNPs

Batch 1454 : Gut microbiota abundance (genus Lactobacillus id.1837) on Ulcerative proctitis

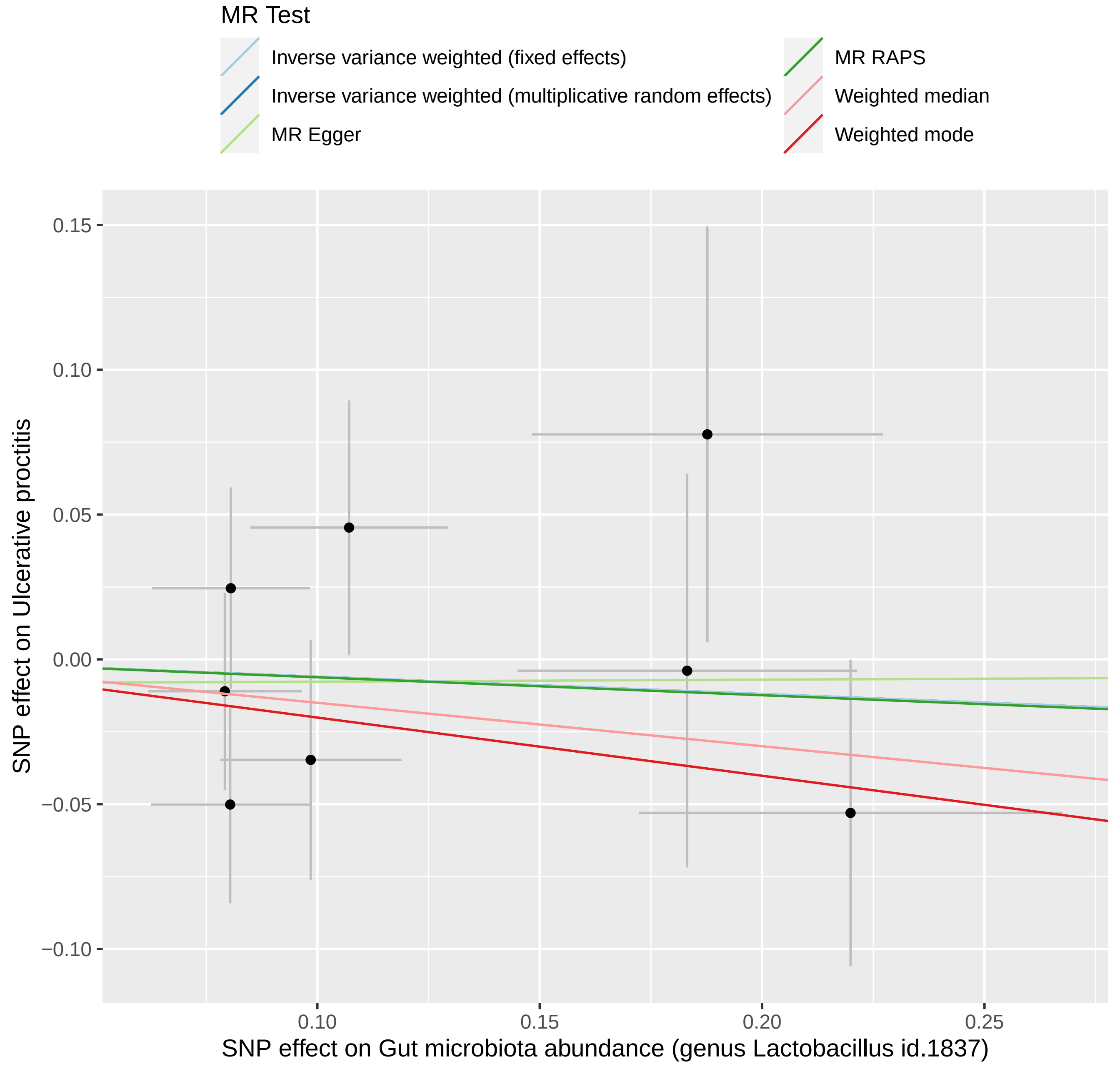

Batch 1455 : Gut microbiota abundance (genus Lactococcus id.1851) on Ulcerative proctitis

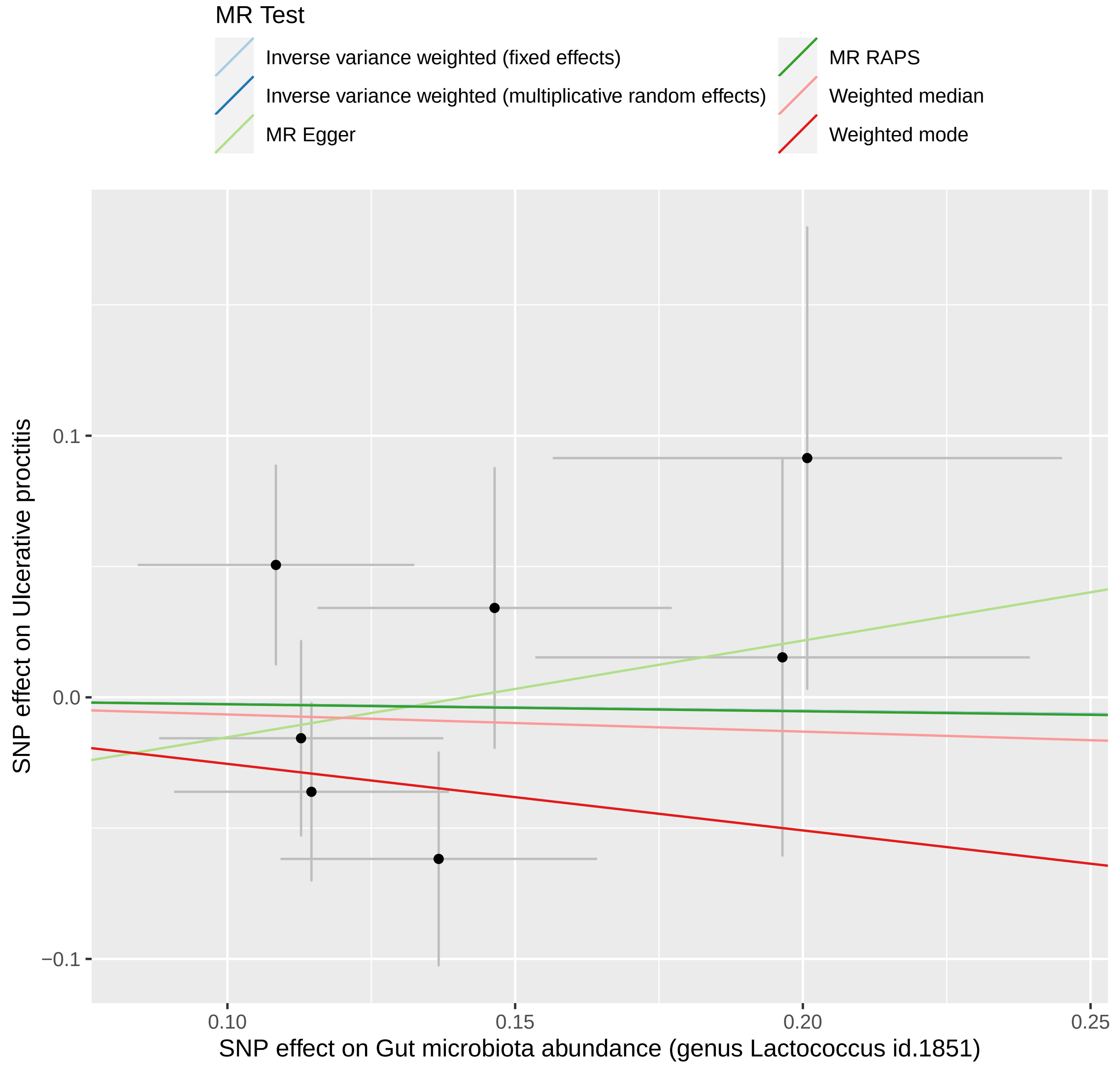

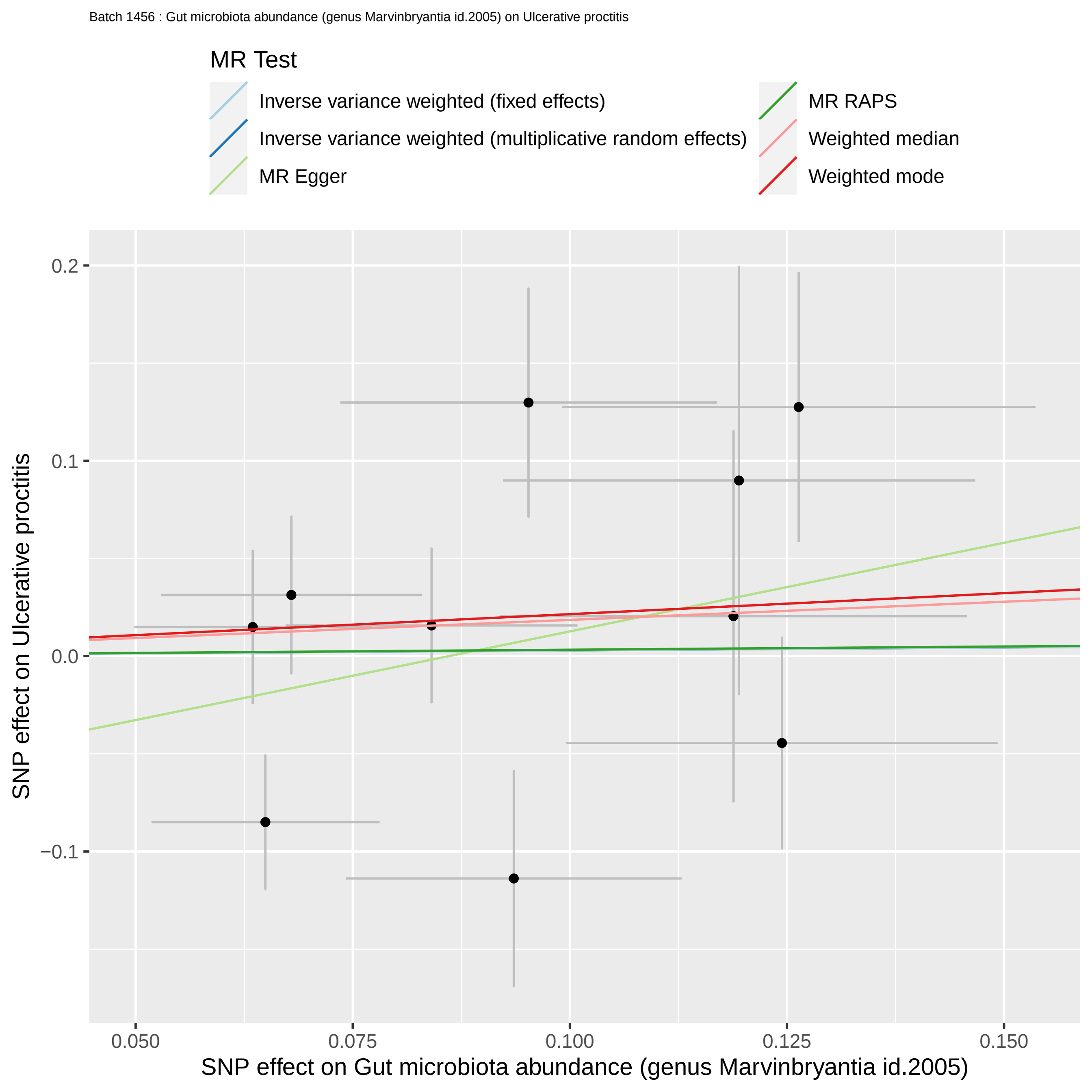
Batch 1456 : Gut microbiota abundance (genus Marvinbryantia id.2005) on Ulcerative proctitis
MR Test
Inverse variance weighted (fixed effects)
Inverse variance weighted (multiplicative random effects)
MR Egger
MR RAPS
Weighted median
Weighted mode
0.2
0.1
0.0
−0.1
0.050
0.075
0.100
0.125
0.150
SNP effect on Ulcerative proctitis
SNP effect on Gut microbiota abundance (genus Marvinbryantia id.2005)

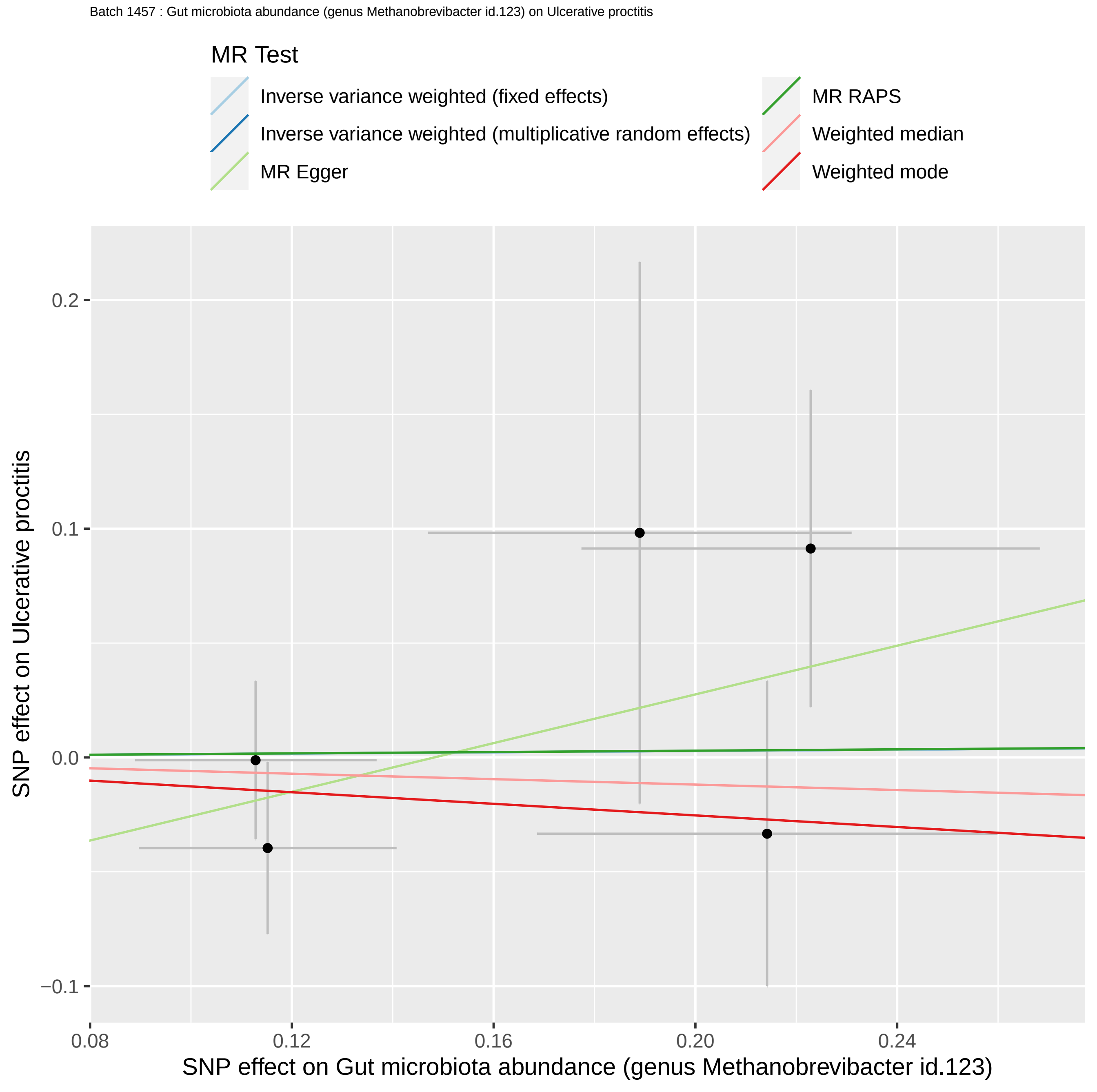
Batch 1457 : Gut microbiota abundance (genus Methanobrevibacter id.123) on Ulcerative proctitis
MR Test
Inverse variance weighted (fixed effects)
Inverse variance weighted (multiplicative random effects)
MR Egger
MR RAPS
Weighted median
Weighted mode
0.2
0.1
0.0
−0.1
0.08
0.12
0.16
0.20
0.24
SNP effect on Ulcerative proctitis
SNP effect on Gut microbiota abundance (genus Methanobrevibacter id.123)

Batch 1458 : Gut microbiota abundance (genus Odoribacter id.952) on Ulcerative proctitis

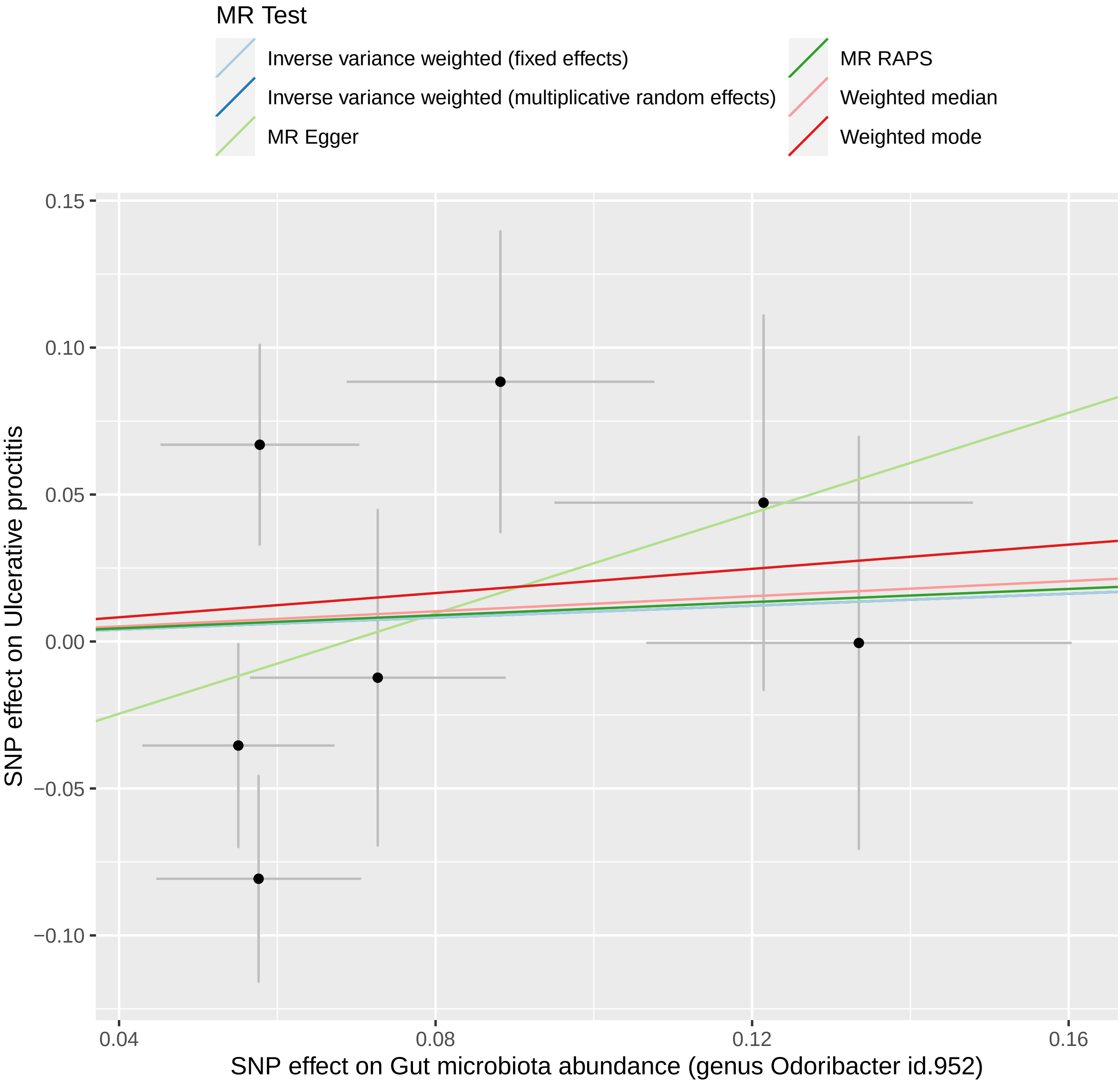

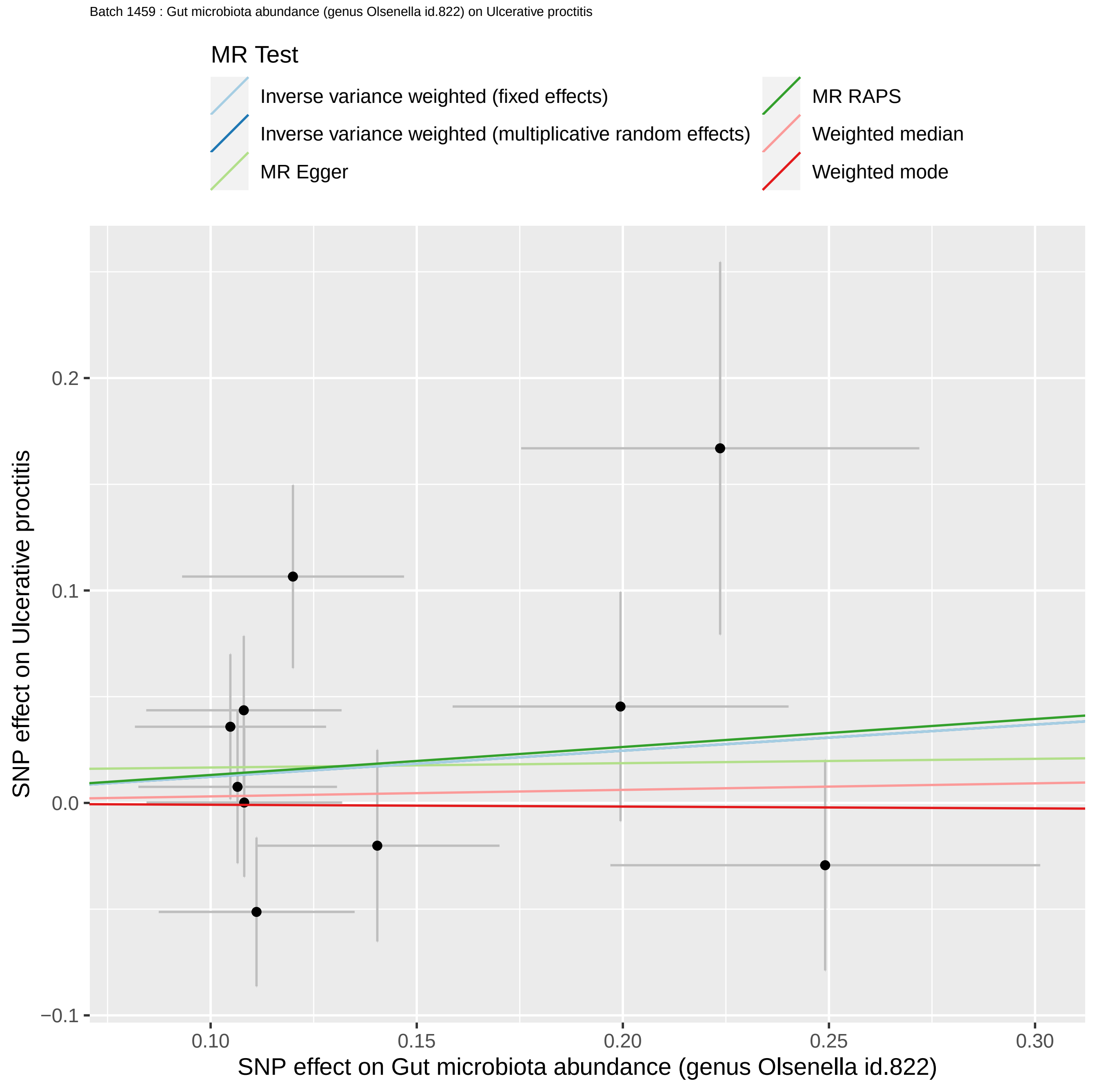

Batch 1459 : Gut microbiota abundance (genus Olsenella id.822) on Ulcerative proctitis
MR Test
Inverse variance weighted (fixed effects)
Inverse variance weighted (multiplicative random effects)
MR Egger
MR RAPS
Weighted median
Weighted mode
SNP effect on Ulcerative proctitis
0.2
0.1
0.0
−0.1
0.10
0.15
0.20
0.25
0.30
SNP effect on Gut microbiota abundance (genus Olsenella id.822)

Batch 1460 : Gut microbiota abundance (genus Oscillibacter id.2063) on Ulcerative proctitis

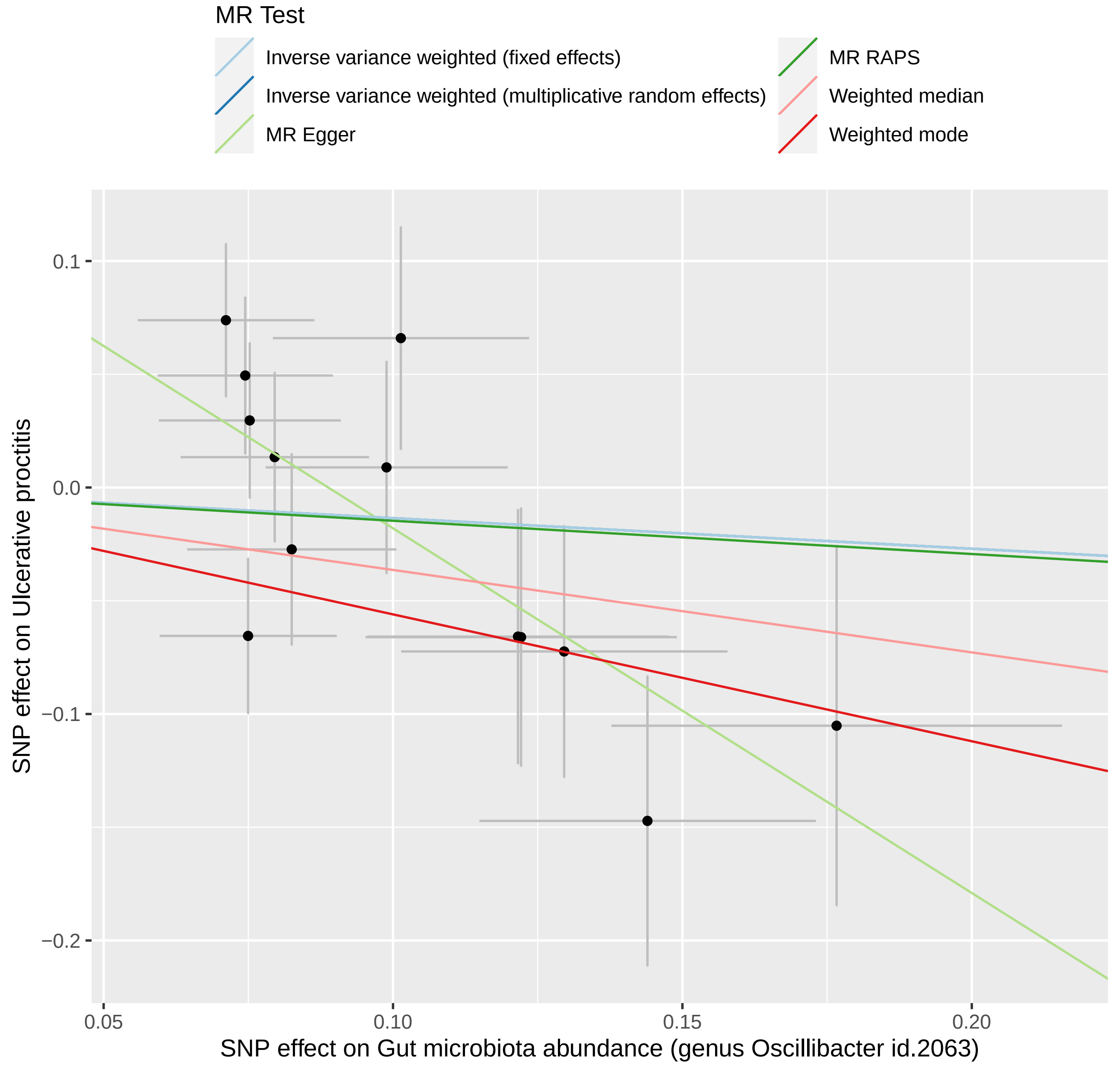

Batch 1461 : Gut microbiota abundance (genus Oscillospira id.2064) on Ulcerative proctitis

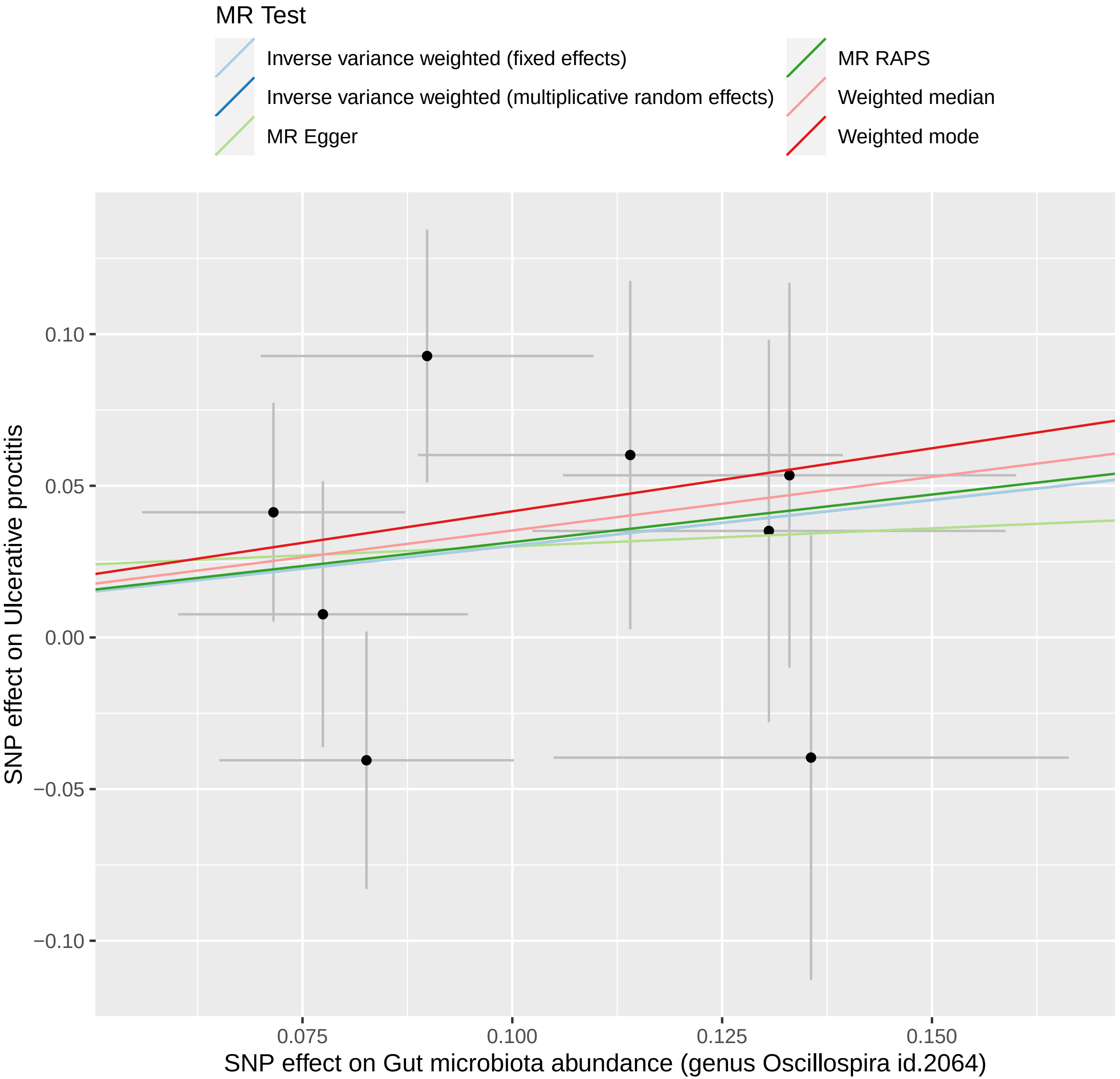

Batch 1462 : Gut microbiota abundance (genus Oxalobacter id.2978) on Ulcerative proctitis

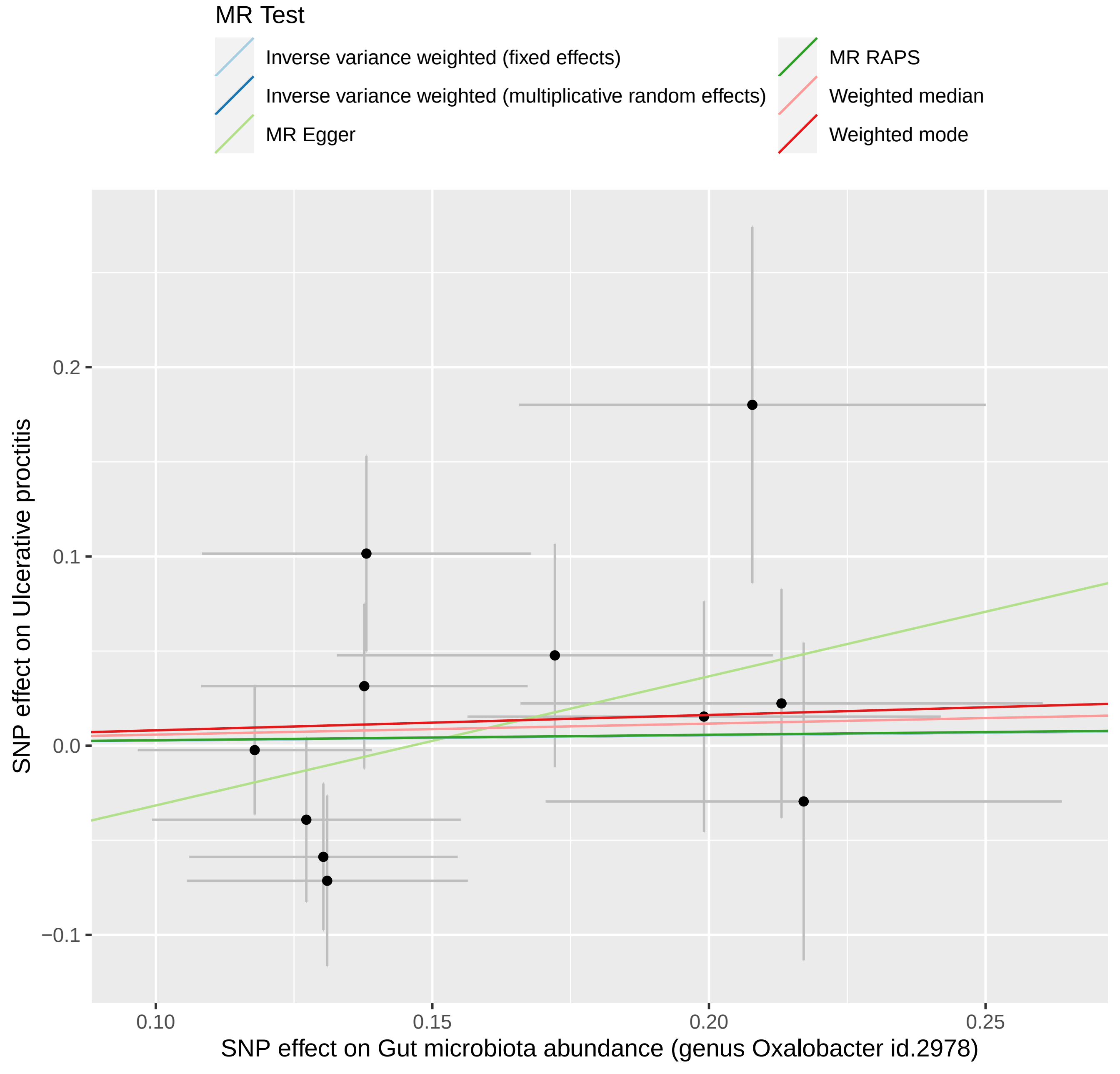

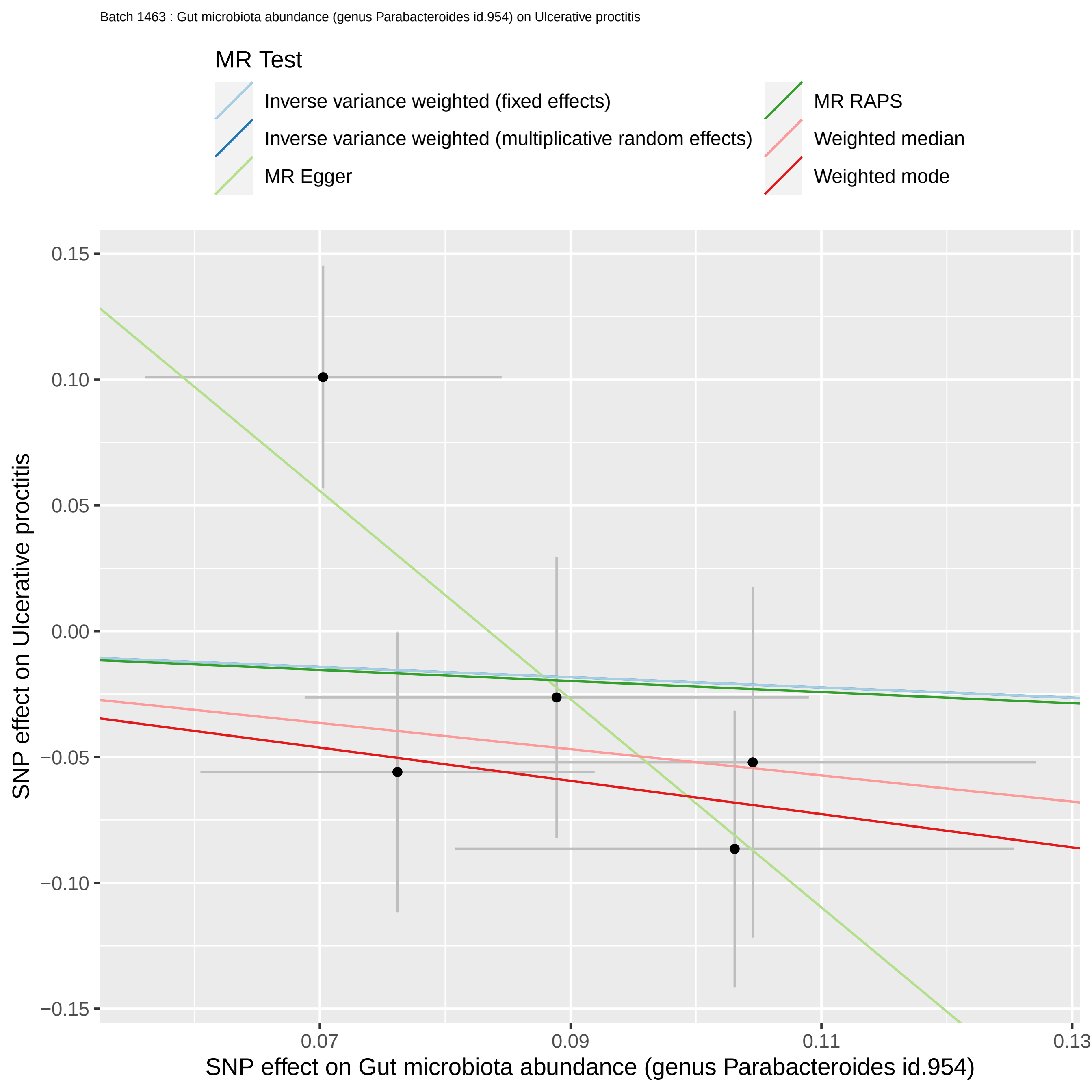
Batch 1463 : Gut microbiota abundance (genus Parabacteroides id.954) on Ulcerative proctitis
MR Test
Inverse variance weighted (fixed effects)
Inverse variance weighted (multiplicative random effects)
MR Egger
MR RAPS
Weighted median
Weighted mode
0.15
0.10
0.05
0.00
−0.05
−0.10
−0.15
SNP effect on Ulcerative proctitis
0.07
0.09
0.11
0.13
SNP effect on Gut microbiota abundance (genus Parabacteroides id.954)

Batch 1464 : Gut microbiota abundance (genus Paraprevotella id.962) on Ulcerative proctitis

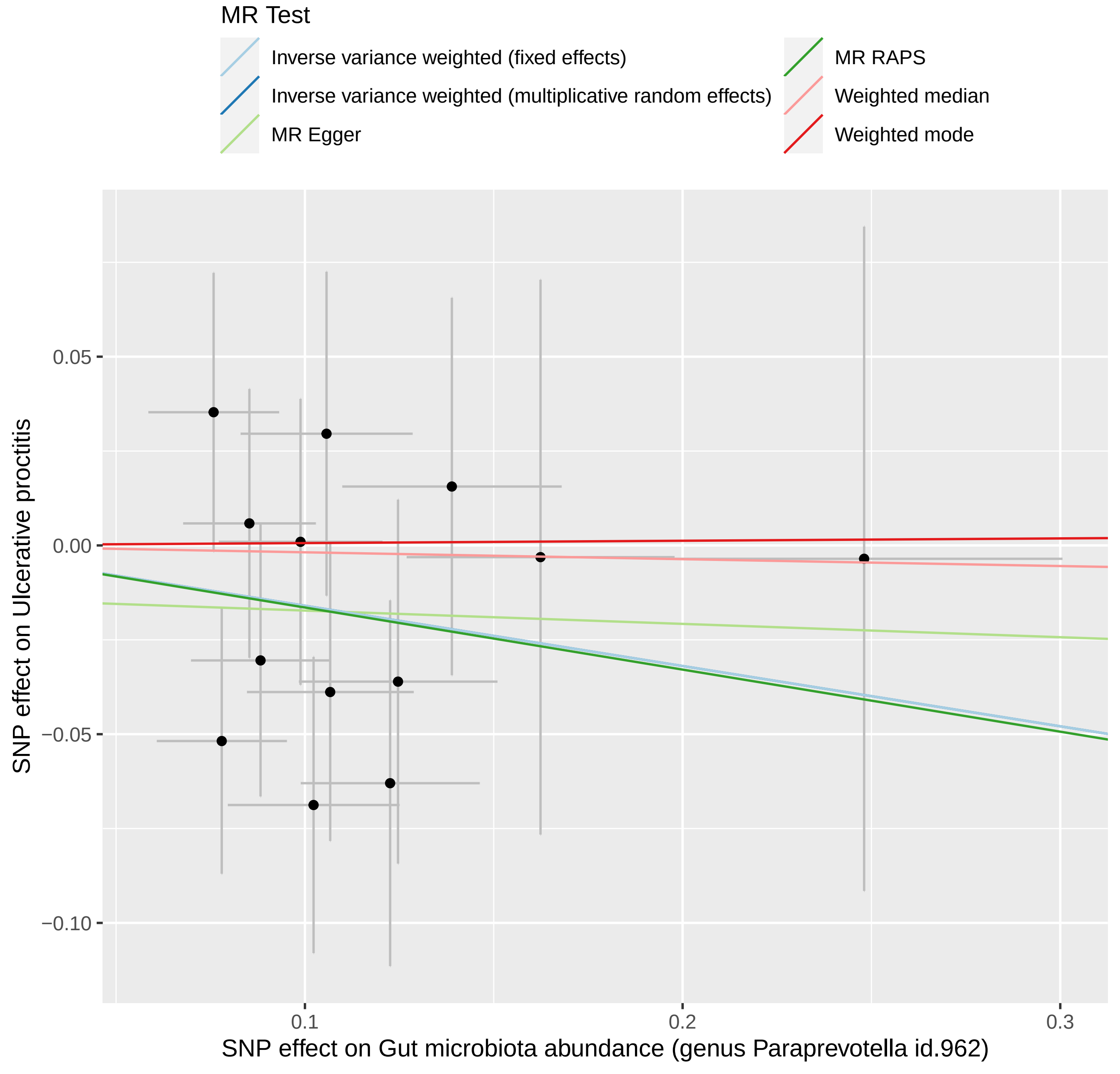

Batch 1465 : Gut microbiota abundance (genus Parasutterella id.2892) on Ulcerative proctitis

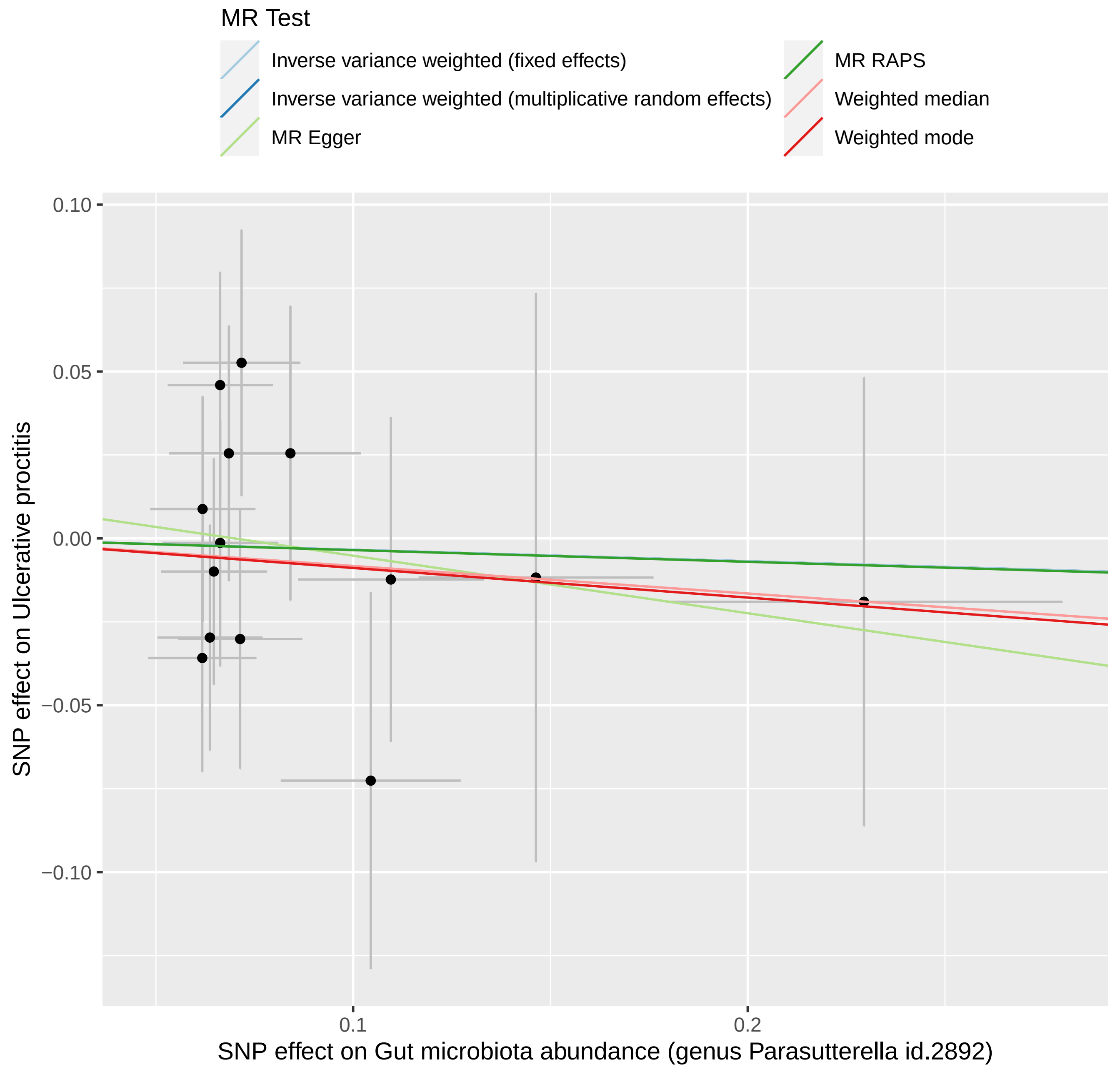

Batch 1466 : Gut microbiota abundance (genus Peptococcus id.2037) on Ulcerative proctitis

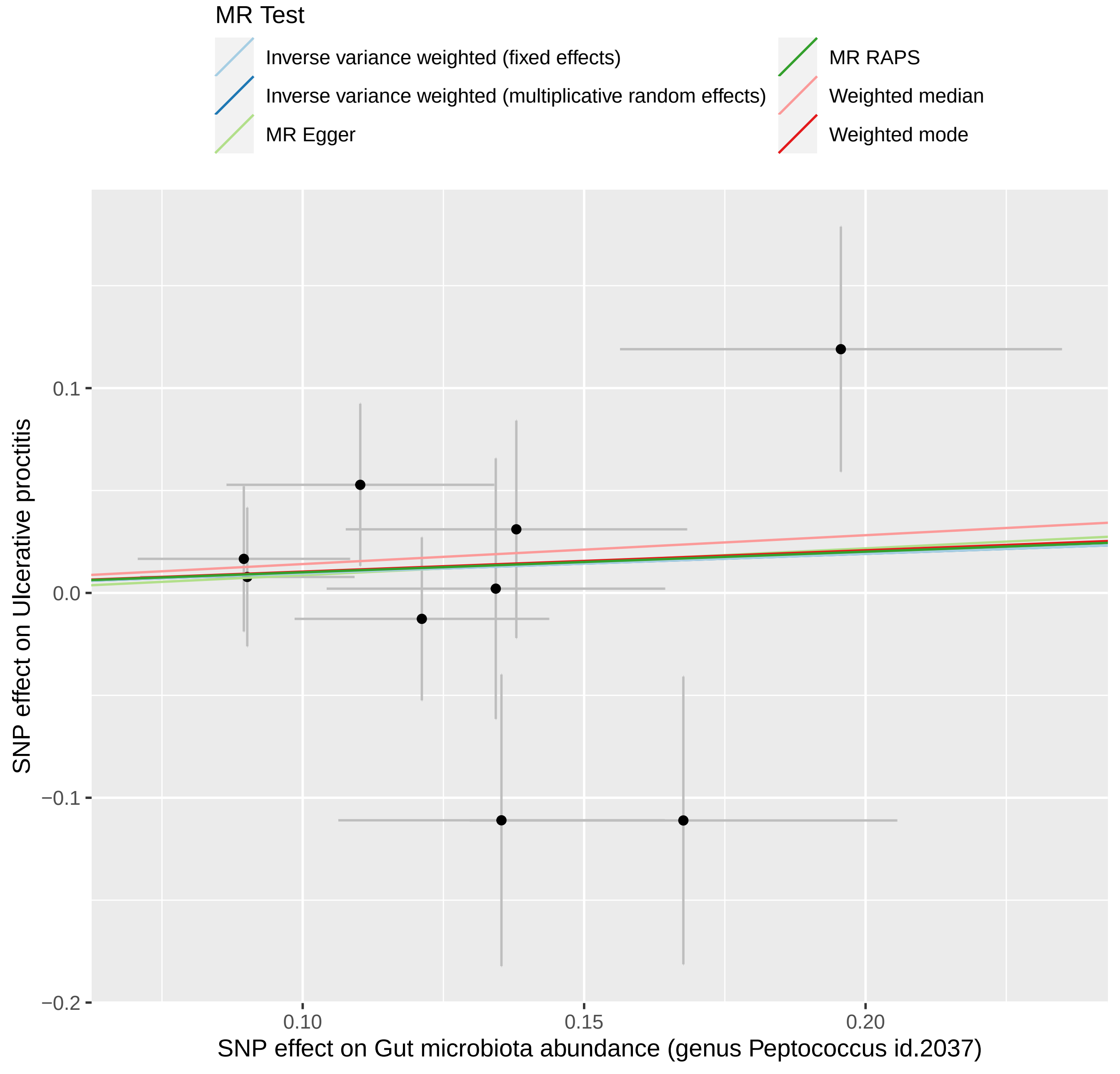

Batch 1467 : Gut microbiota abundance (genus Phascolarctobacterium id.2168) on Ulcerative proctitis

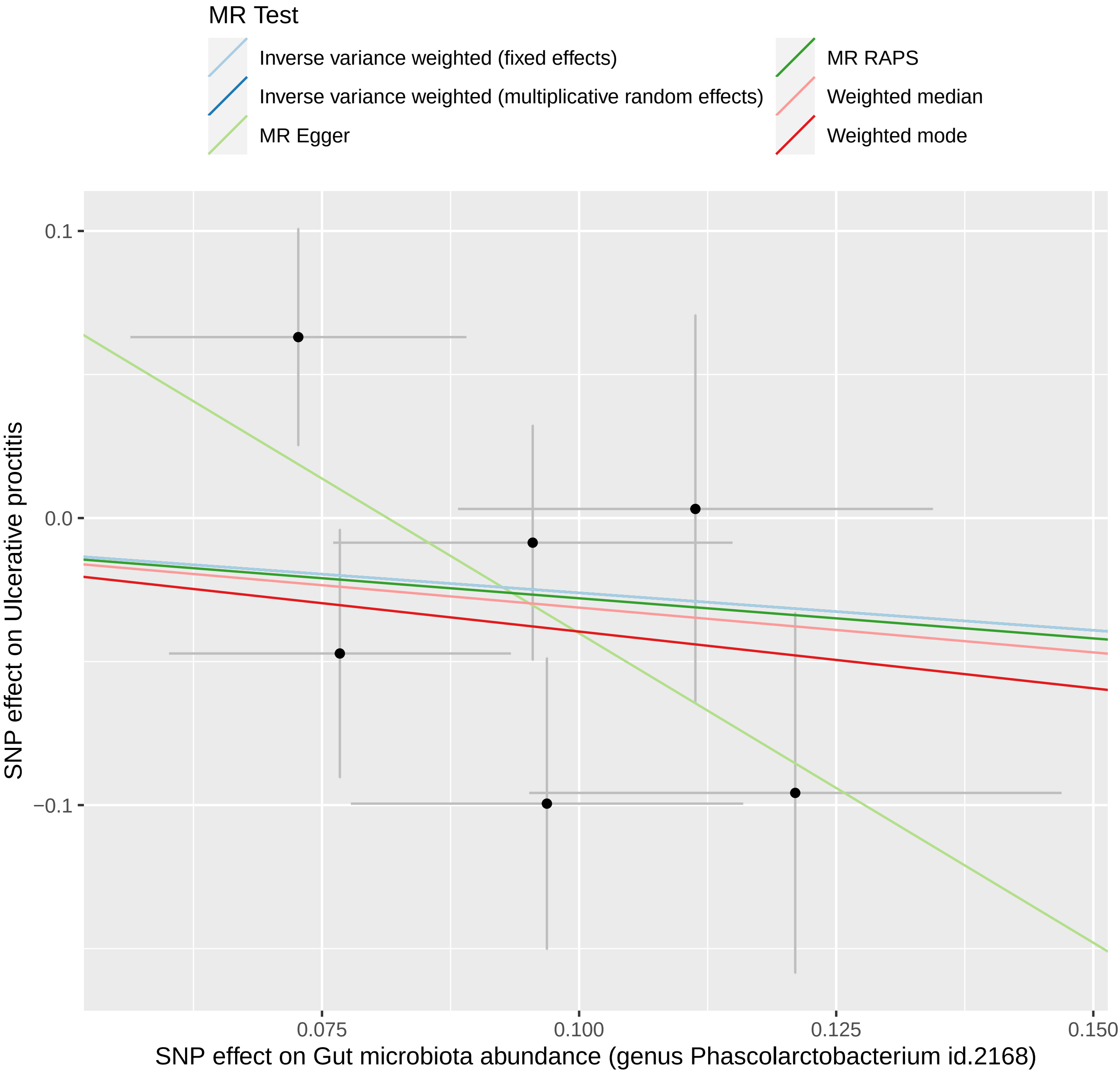

Batch 1468 : Gut microbiota abundance (genus Prevotella7 id.11182) on Ulcerative proctitis

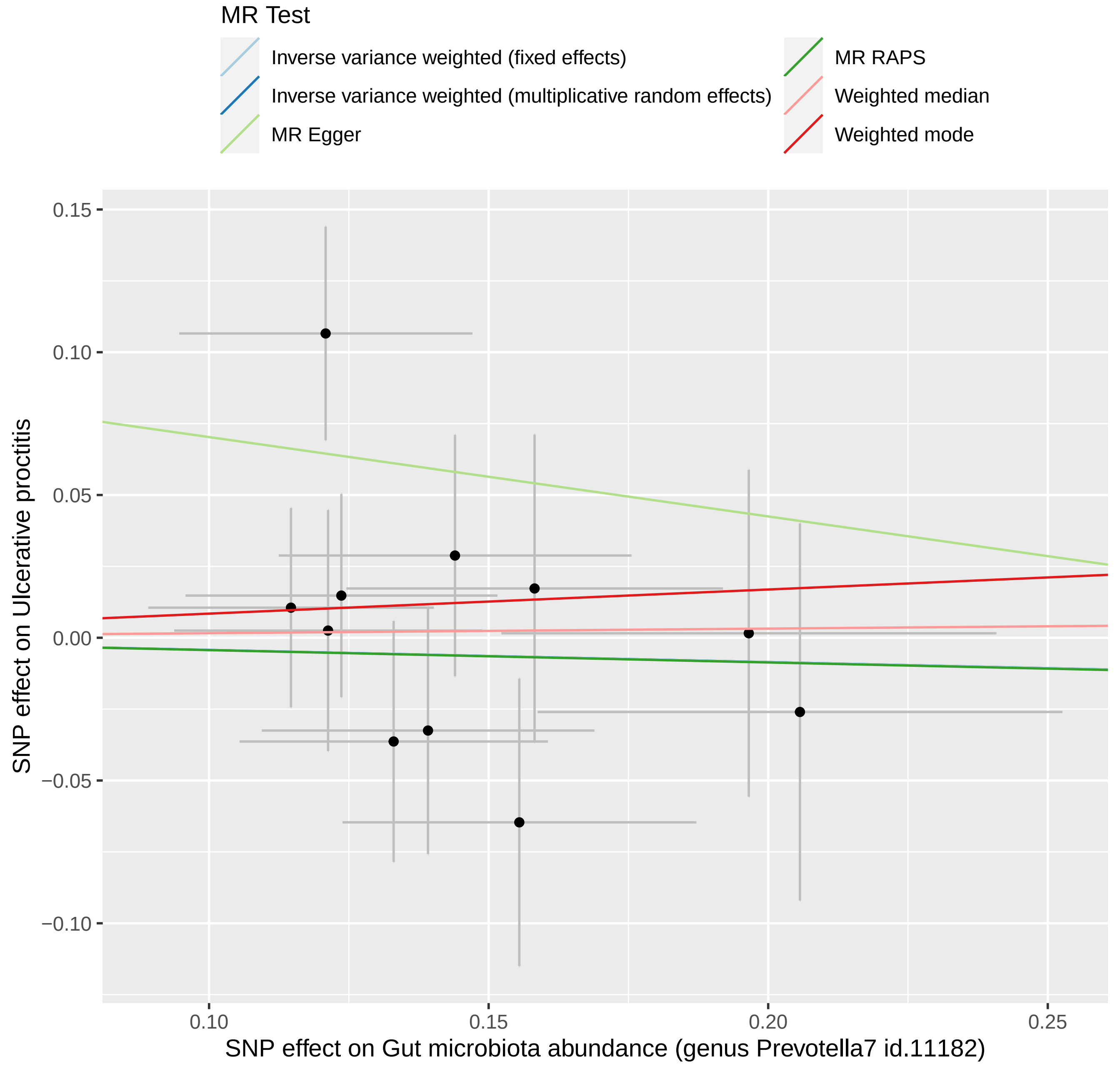

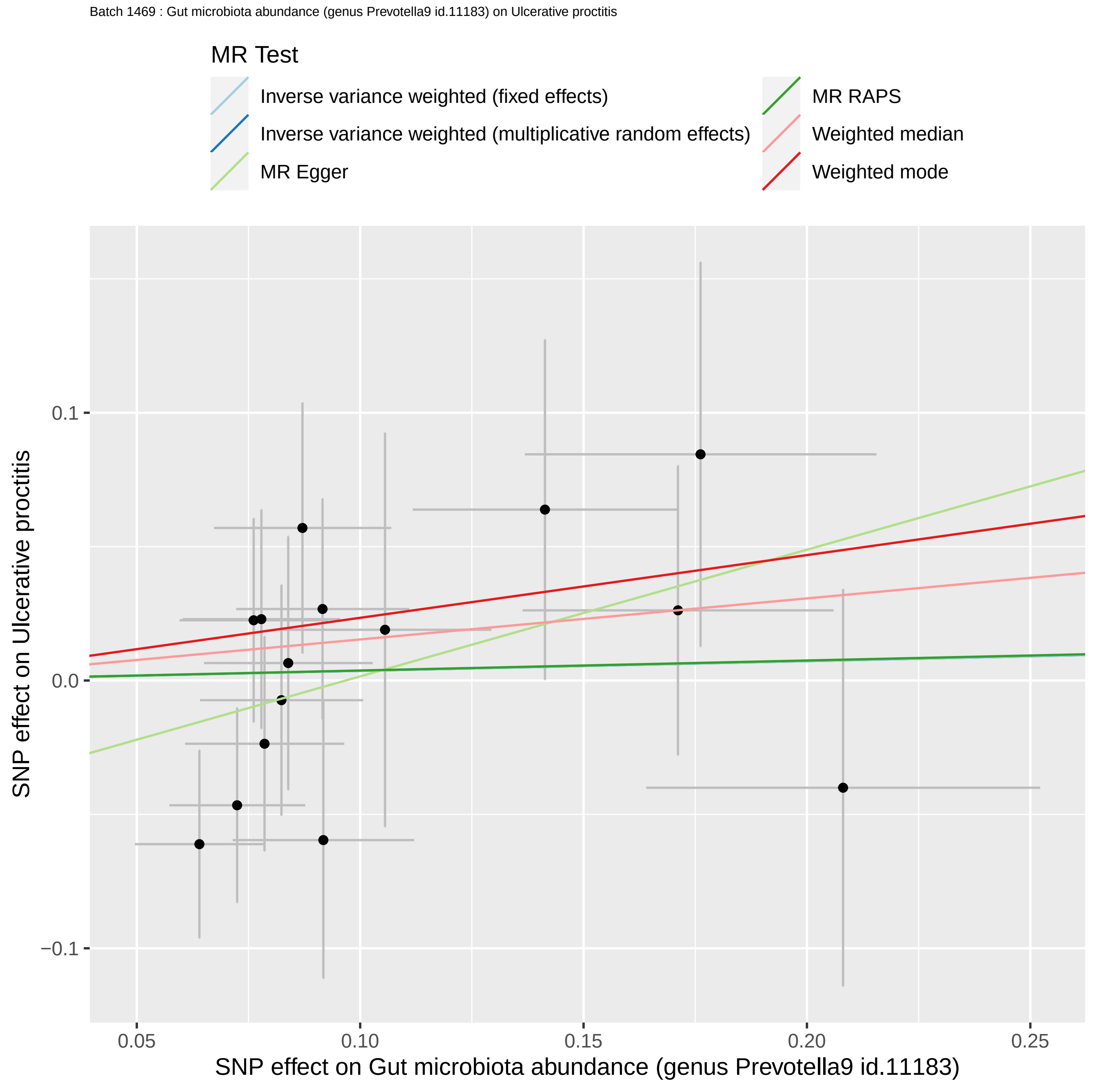

Batch 1469 : Gut microbiota abundance (genus Prevotella9 id.11183) on Ulcerative proctitis
MR Test
Inverse variance weighted (fixed effects)
Inverse variance weighted (multiplicative random effects)
MR Egger
MR RAPS
Weighted median
Weighted mode
SNP effect on Ulcerative proctitis
0.1
0.0
−0.1
0.05
0.10
0.15
0.20
0.25
SNP effect on Gut microbiota abundance (genus Prevotella9 id.11183)

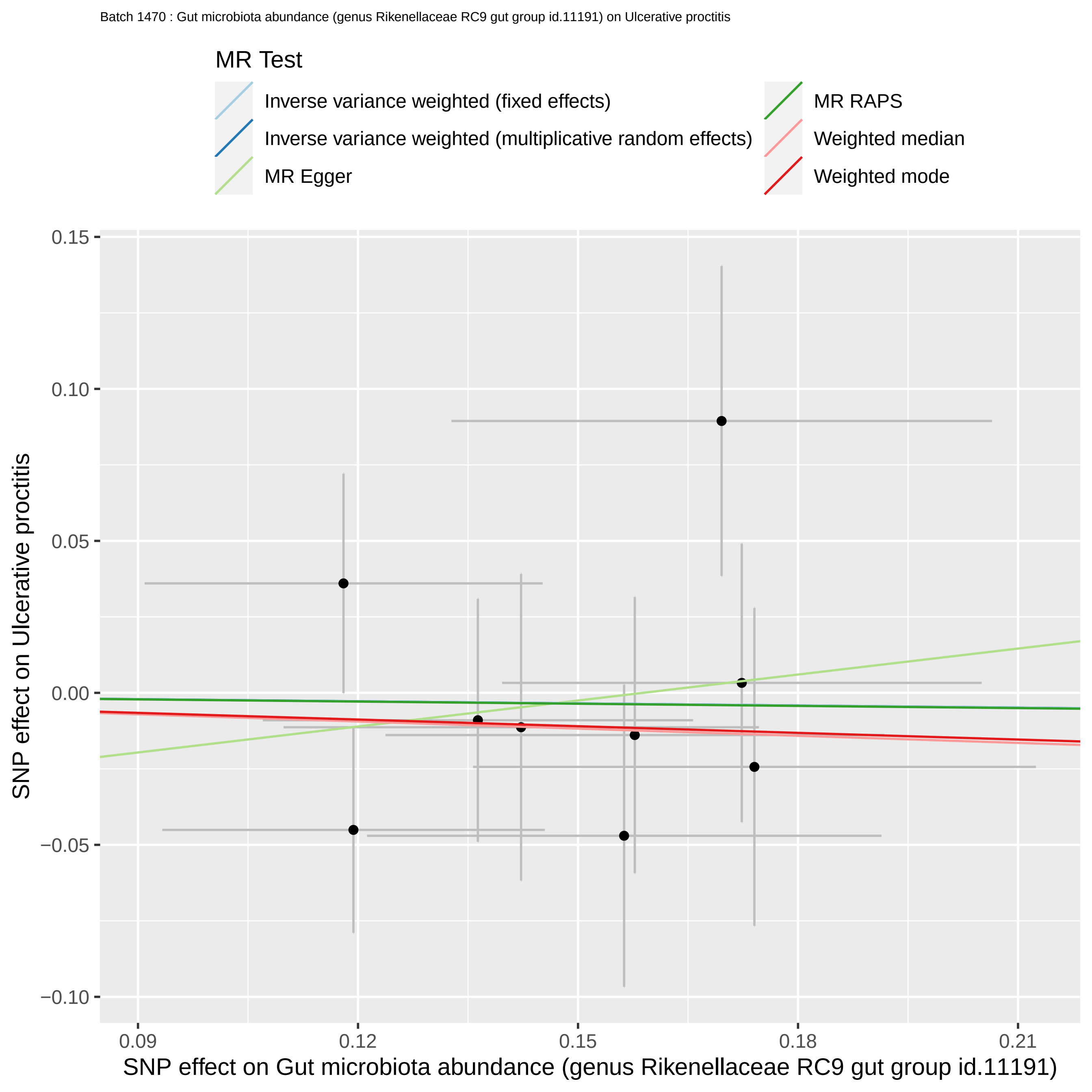
Batch 1470 : Gut microbiota abundance (genus Rikenellaceae RC9 gut group id.11191) on Ulcerative proctitis
MR Test
Inverse variance weighted (fixed effects)
Inverse variance weighted (multiplicative random effects)
MR Egger
MR RAPS
Weighted median
Weighted mode
0.15
0.10
0.05
0.00
−0.05
−0.10
0.09
0.12
0.15
0.18
0.21
SNP effect on Ulcerative proctitis
SNP effect on Gut microbiota abundance (genus Rikenellaceae RC9 gut group id.11191)

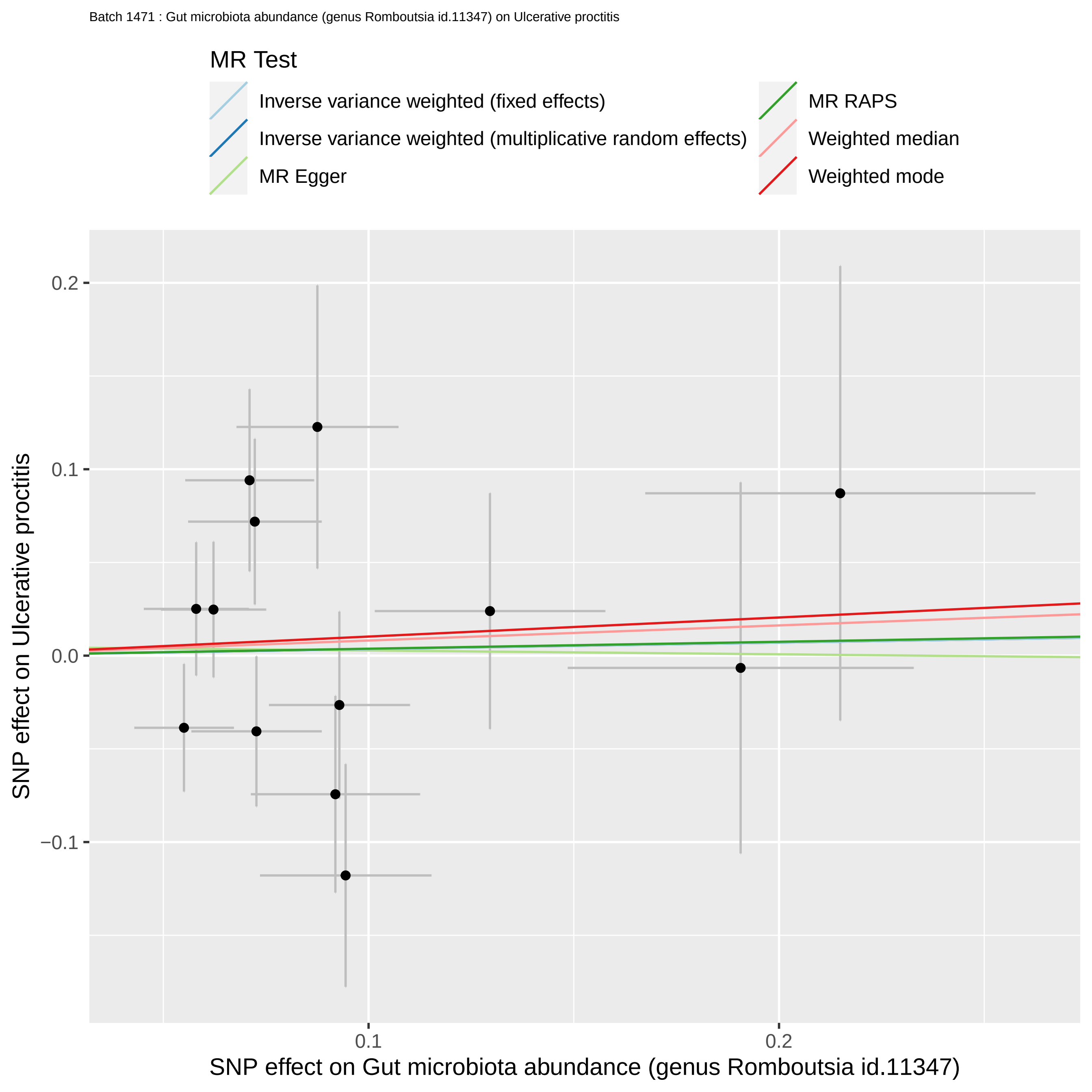

Batch 1471 : Gut microbiota abundance (genus Romboutsia id.11347) on Ulcerative proctitis
MR Test
Inverse variance weighted (fixed effects)
Inverse variance weighted (multiplicative random effects)
MR Egger
MR RAPS
Weighted median
Weighted mode
0.2
0.1
0.0
−0.1
0.1
0.2
SNP effect on Ulcerative proctitis
SNP effect on Gut microbiota abundance (genus Romboutsia id.11347)

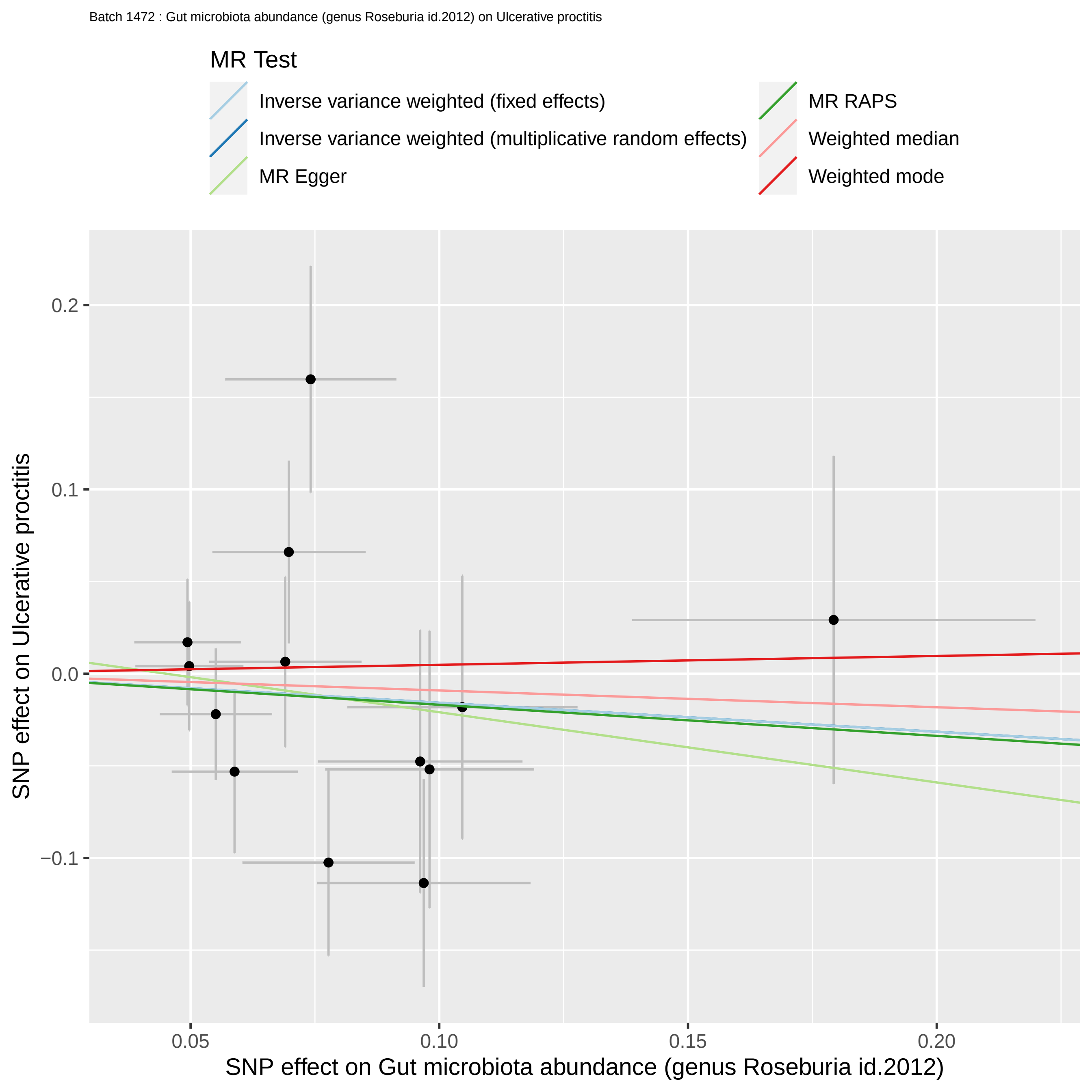

Batch 1472 : Gut microbiota abundance (genus Roseburia id.2012) on Ulcerative proctitis
MR Test
Inverse variance weighted (fixed effects)
Inverse variance weighted (multiplicative random effects)
MR Egger
MR RAPS
Weighted median
Weighted mode
0.2
0.1
0.0
−0.1
0.05
0.10
0.15
0.20
SNP effect on Ulcerative proctitis
SNP effect on Gut microbiota abundance (genus Roseburia id.2012)

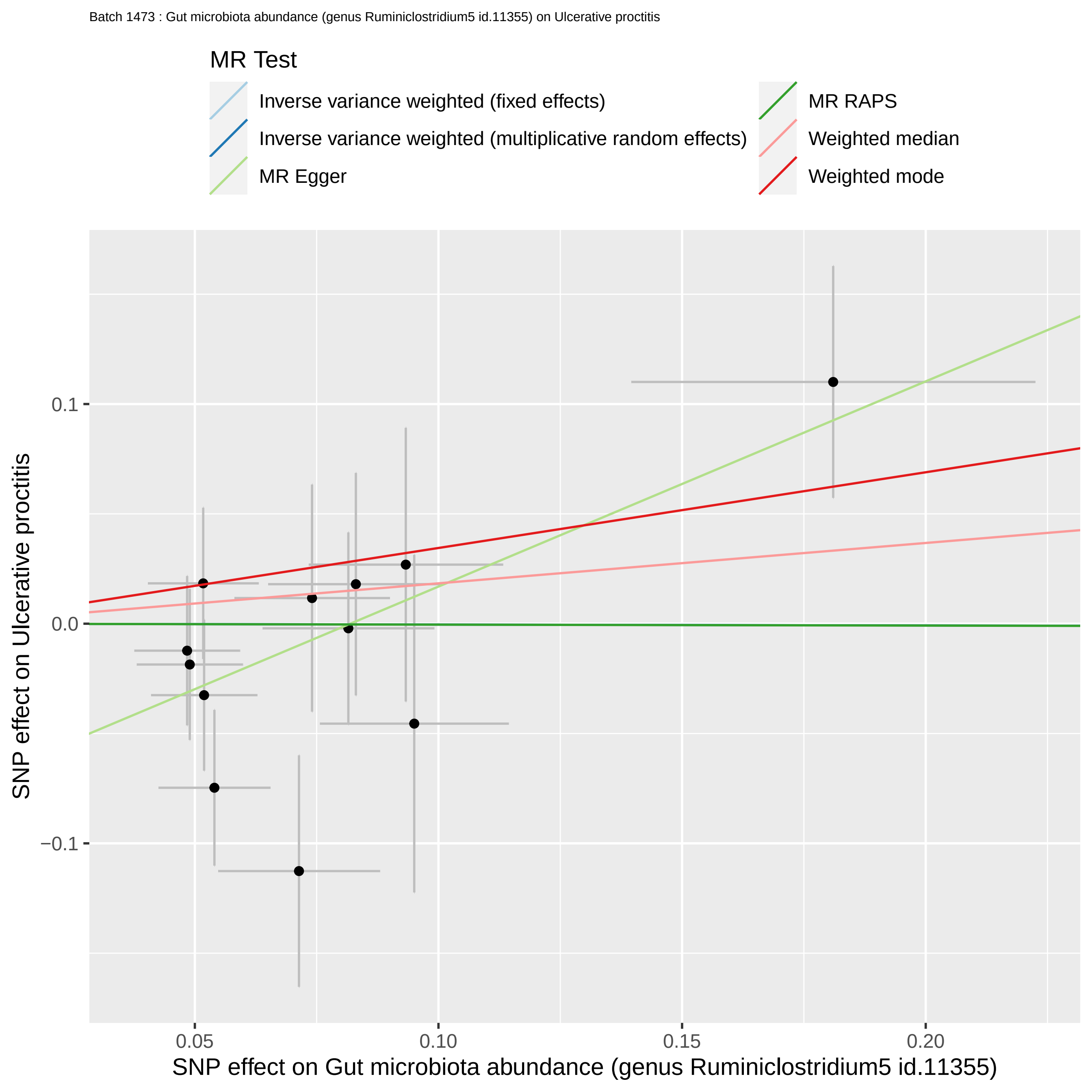

Batch 1473 : Gut microbiota abundance (genus Ruminiclostridium5 id.11355) on Ulcerative proctitis
MR Test
Inverse variance weighted (fixed effects)
Inverse variance weighted (multiplicative random effects)
MR Egger
MR RAPS
Weighted median
Weighted mode
SNP effect on Ulcerative proctitis
0.1
0.0
−0.1
0.05
0.10
0.15
0.20
SNP effect on Gut microbiota abundance (genus Ruminiclostridium5 id.11355)

Batch 1474 : Gut microbiota abundance (genus Ruminiclostridium6 id.11356) on Ulcerative proctitis

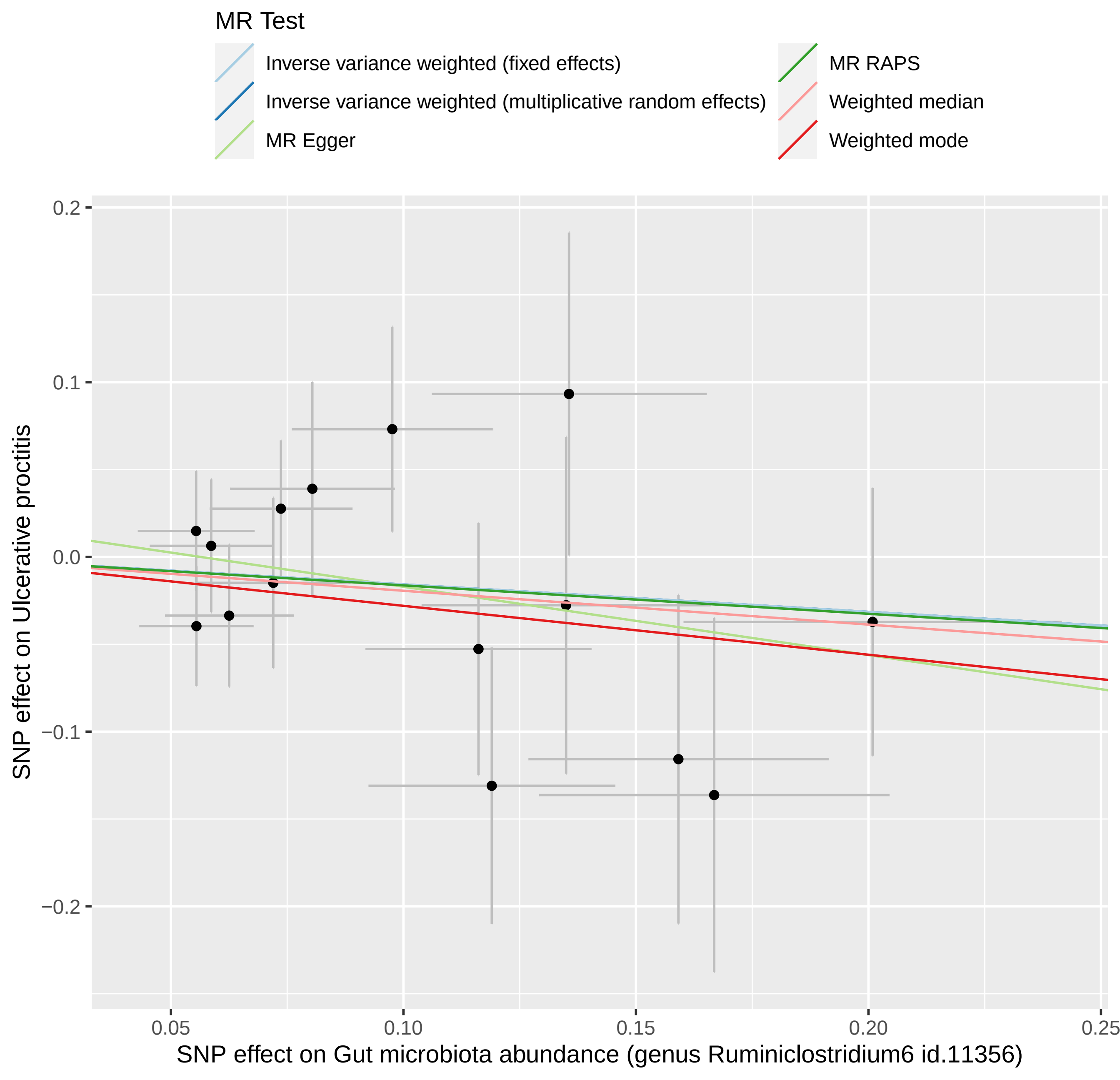

Batch 1475 : Gut microbiota abundance (genus Ruminiclostridium9 id.11357) on Ulcerative proctitis

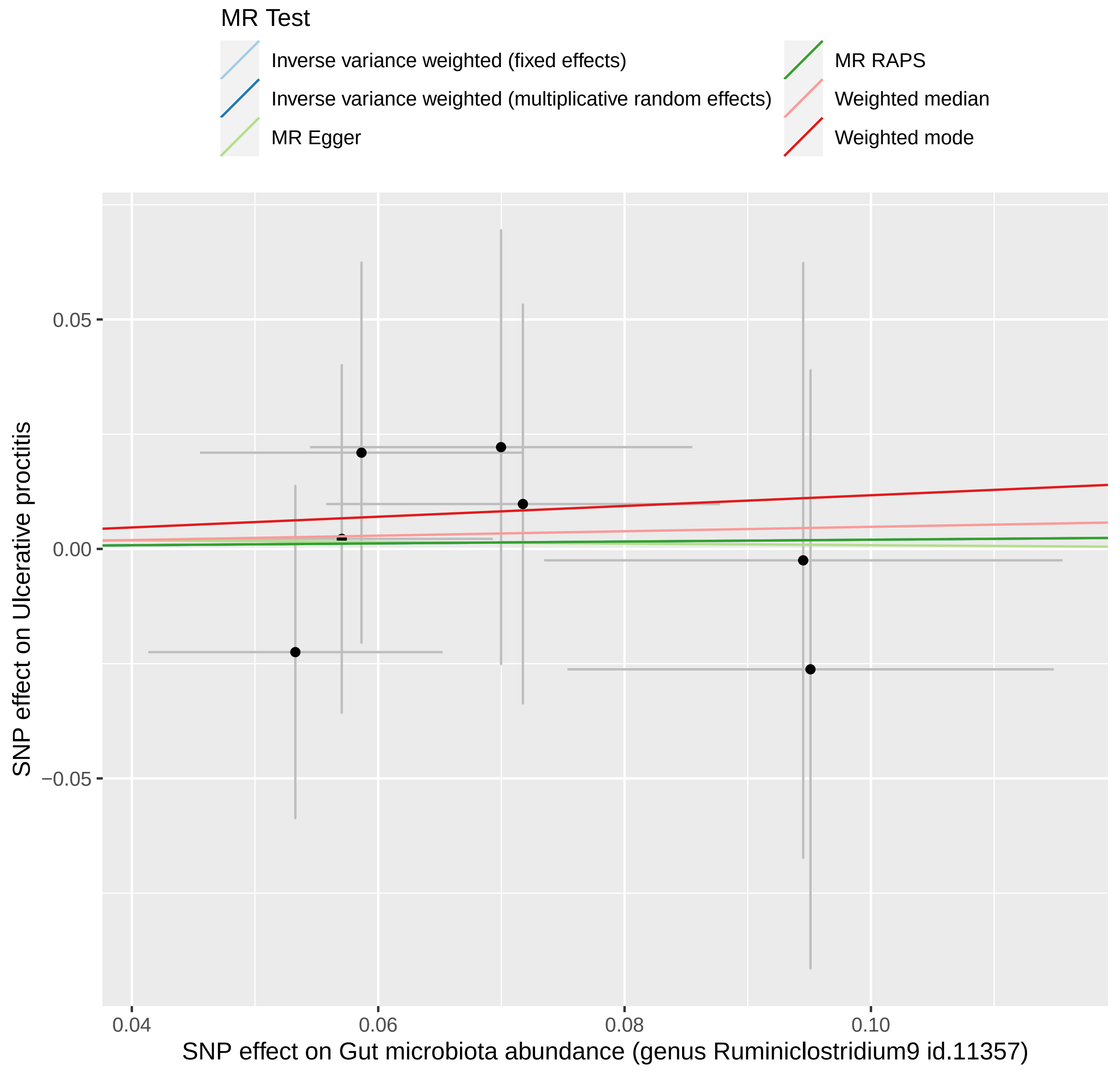

Batch 1476 : Gut microbiota abundance (genus Ruminococcaceae NK4A214 group id.11358) on Ulcerative proctitis

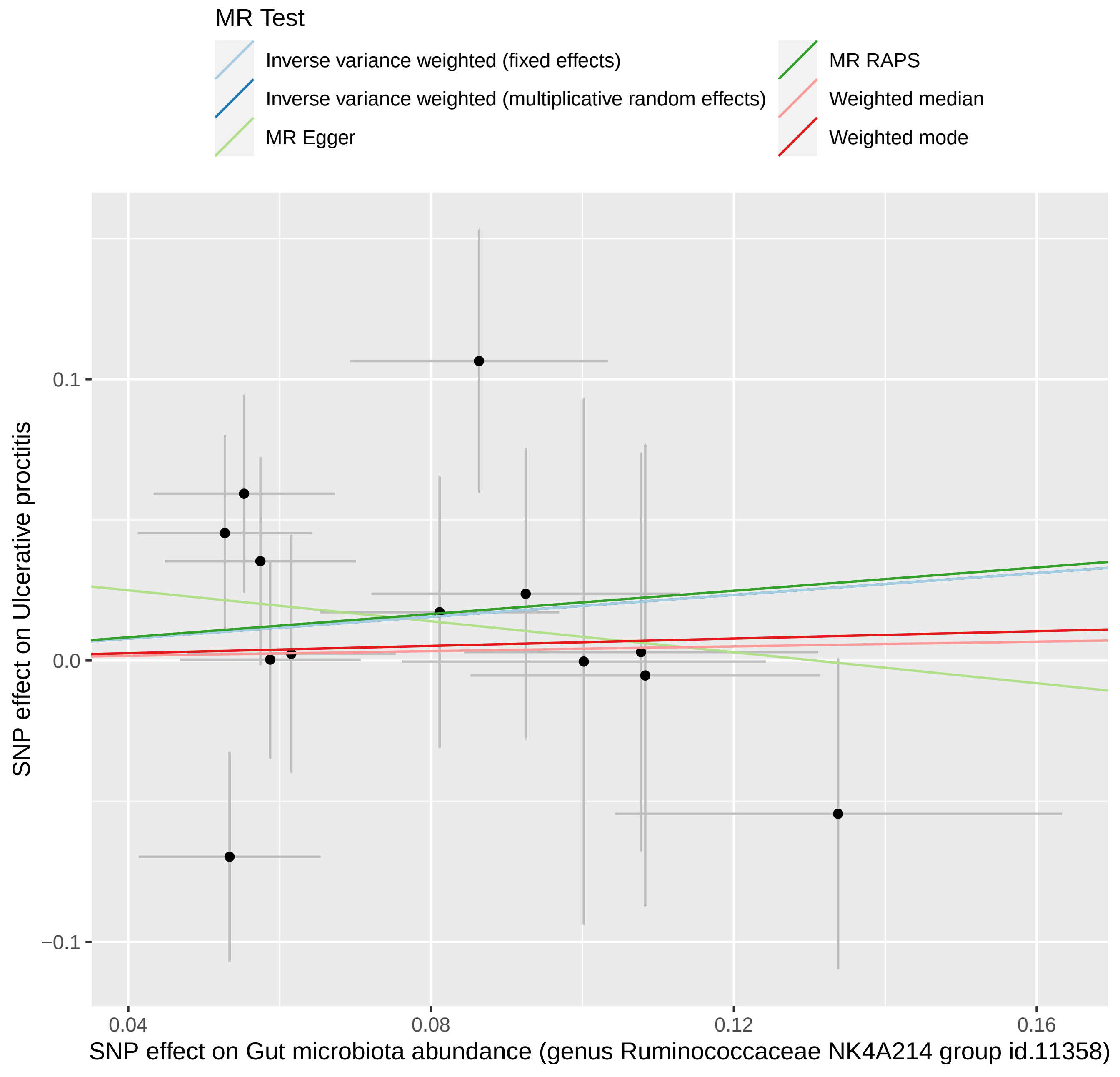

Batch 1477 : Gut microbiota abundance (genus Ruminococcaceae UCG002 id.11360) on Ulcerative proctitis

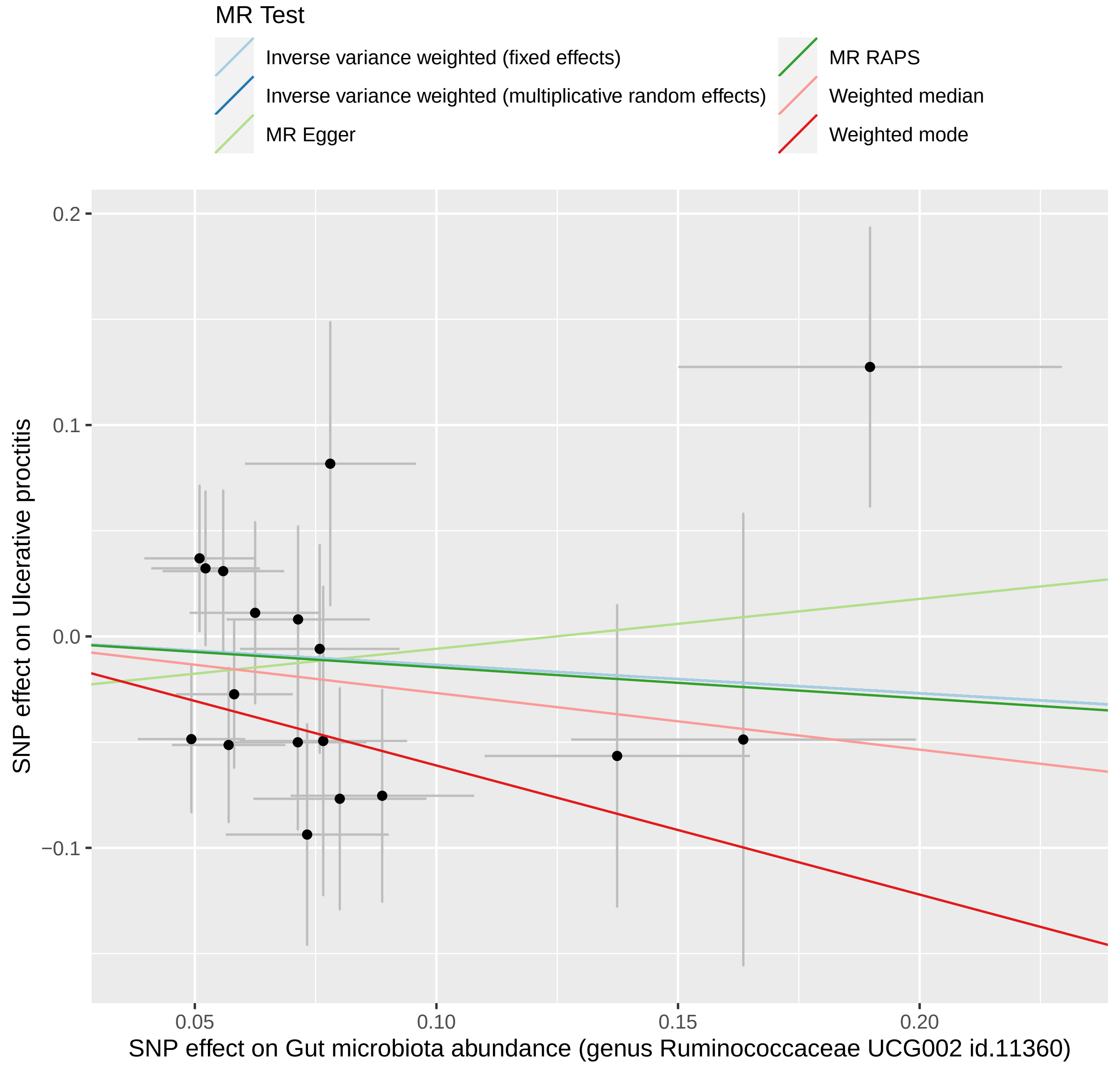

Batch 1478 : Gut microbiota abundance (genus Ruminococcaceae UCG003 id.11361) on Ulcerative proctitis

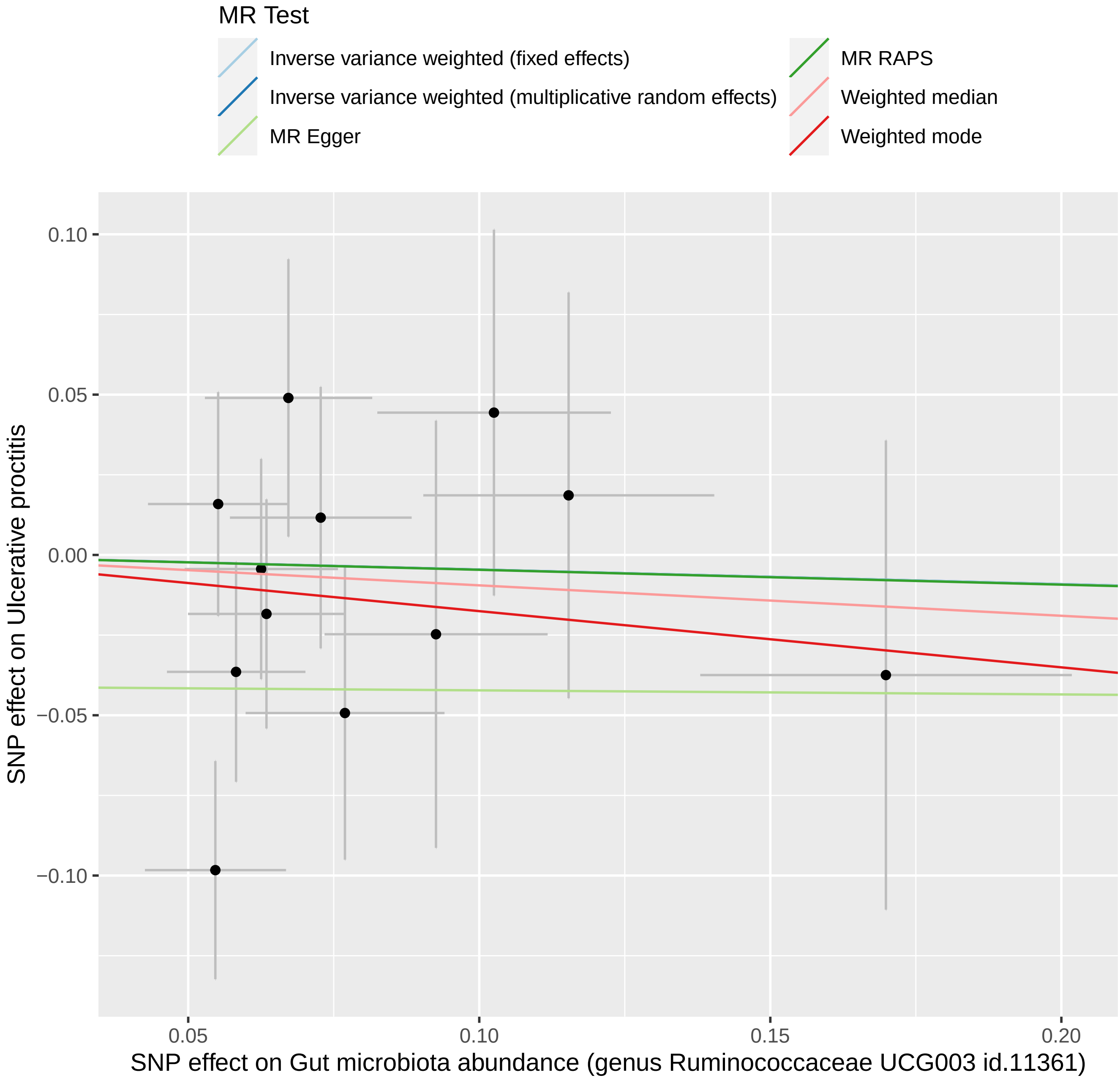

Batch 1479 : Gut microbiota abundance (genus Ruminococcaceae UCG004 id.11362) on Ulcerative proctitis

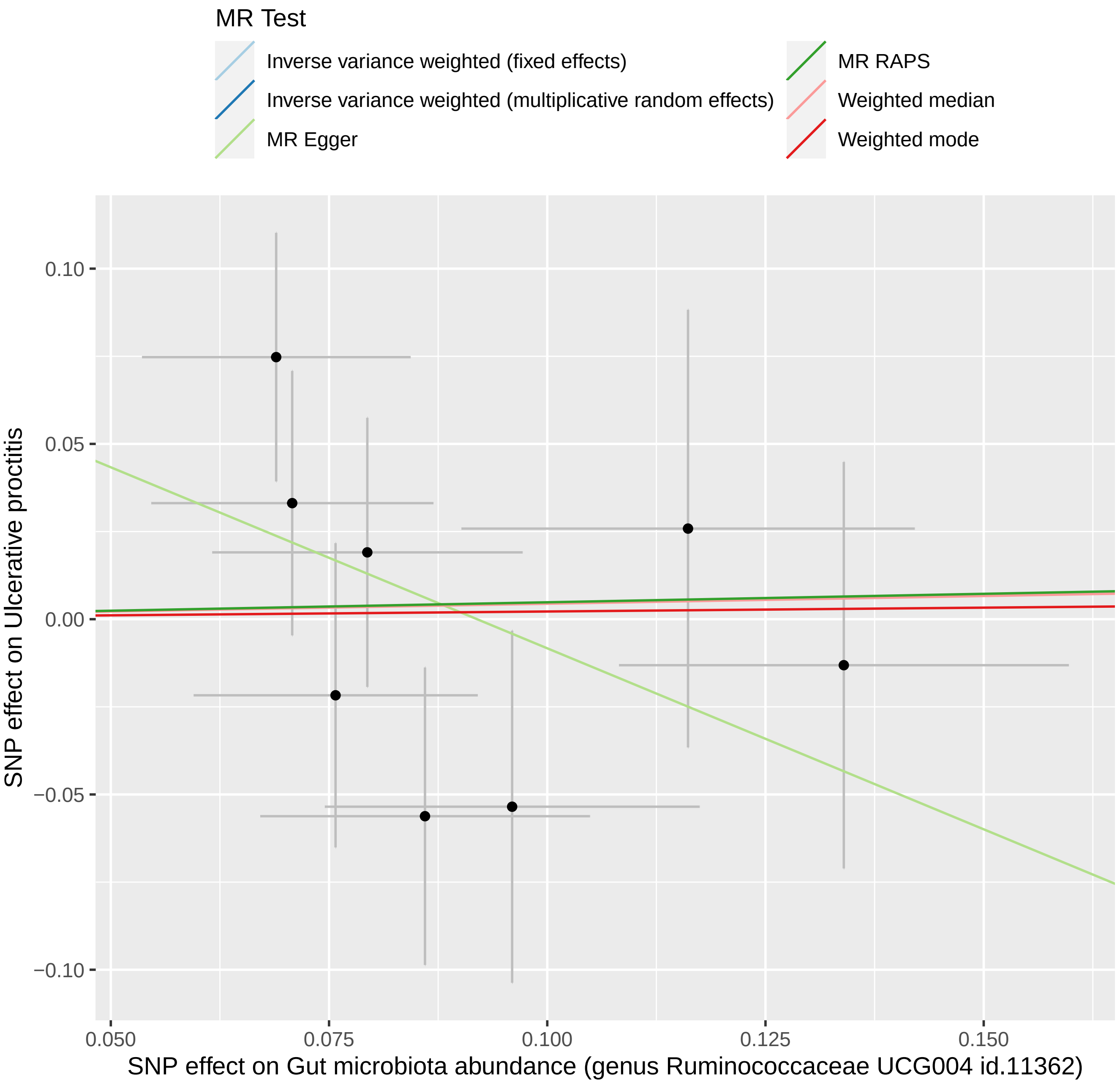

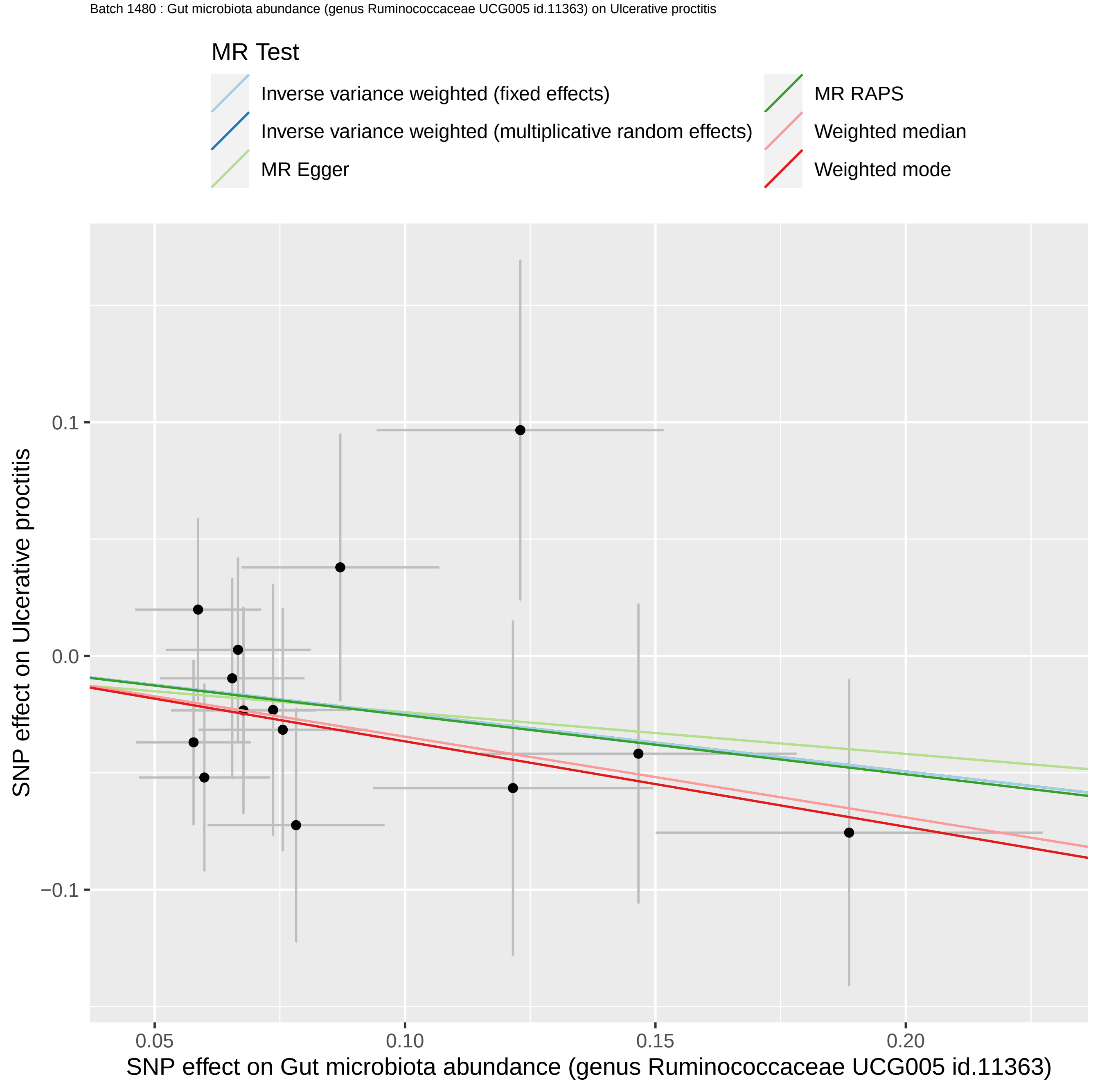

Batch 1480 : Gut microbiota abundance (genus Ruminococcaceae UCG005 id.11363) on Ulcerative proctitis
MR Test
Inverse variance weighted (fixed effects)
Inverse variance weighted (multiplicative random effects)
MR Egger
MR RAPS
Weighted median
Weighted mode
0.1
0.0
−0.1
0.05
0.10
0.15
0.20
SNP effect on Ulcerative proctitis
SNP effect on Gut microbiota abundance (genus Ruminococcaceae UCG005 id.11363)

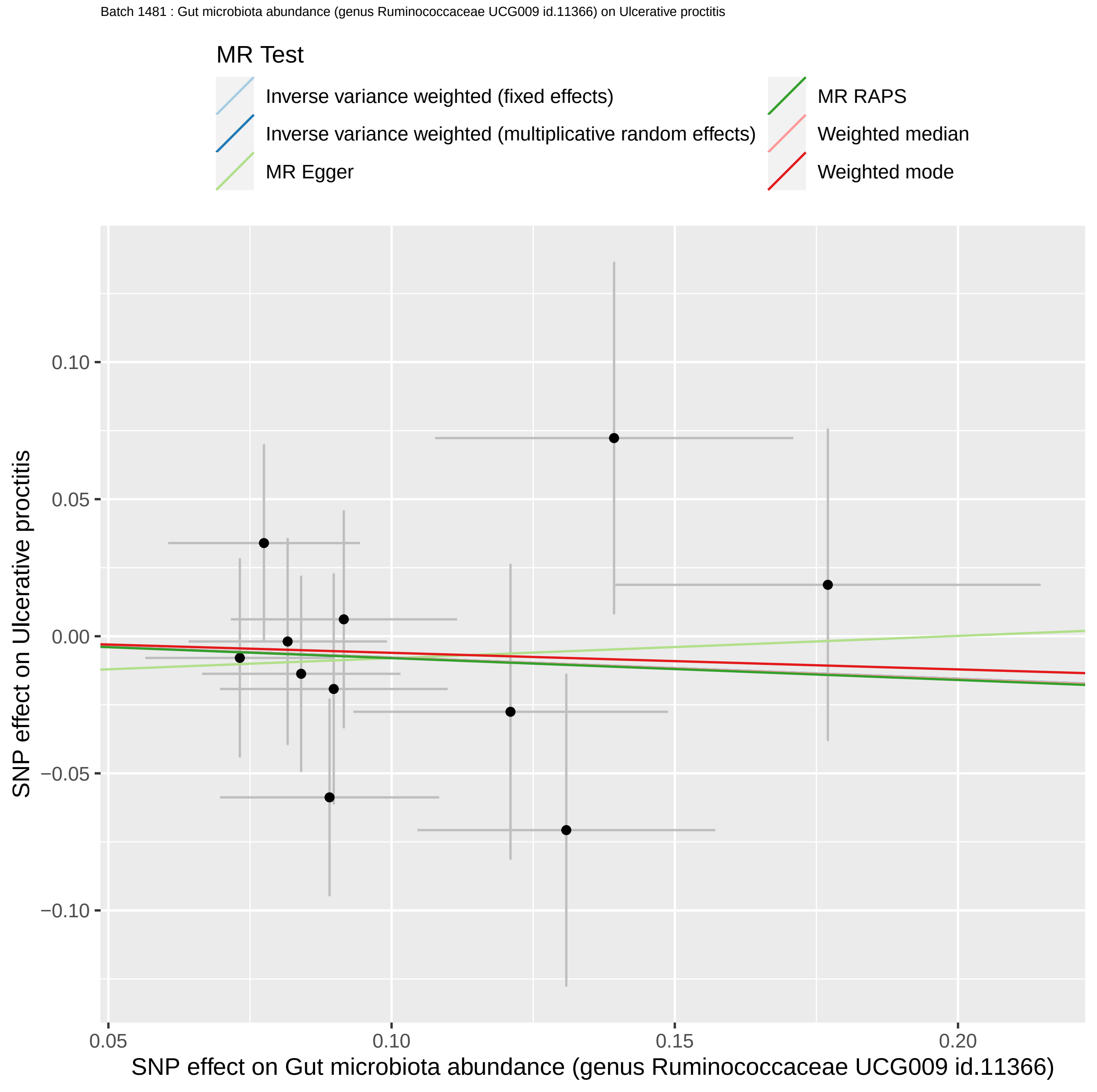

Batch 1481 : Gut microbiota abundance (genus Ruminococcaceae UCG009 id.11366) on Ulcerative proctitis
MR Test
Inverse variance weighted (fixed effects)
Inverse variance weighted (multiplicative random effects)
MR Egger
MR RAPS
Weighted median
Weighted mode
0.10
0.05
0.00
−0.05
−0.10
SNP effect on Ulcerative proctitis
0.05
0.10
0.15
0.20
SNP effect on Gut microbiota abundance (genus Ruminococcaceae UCG009 id.11366)

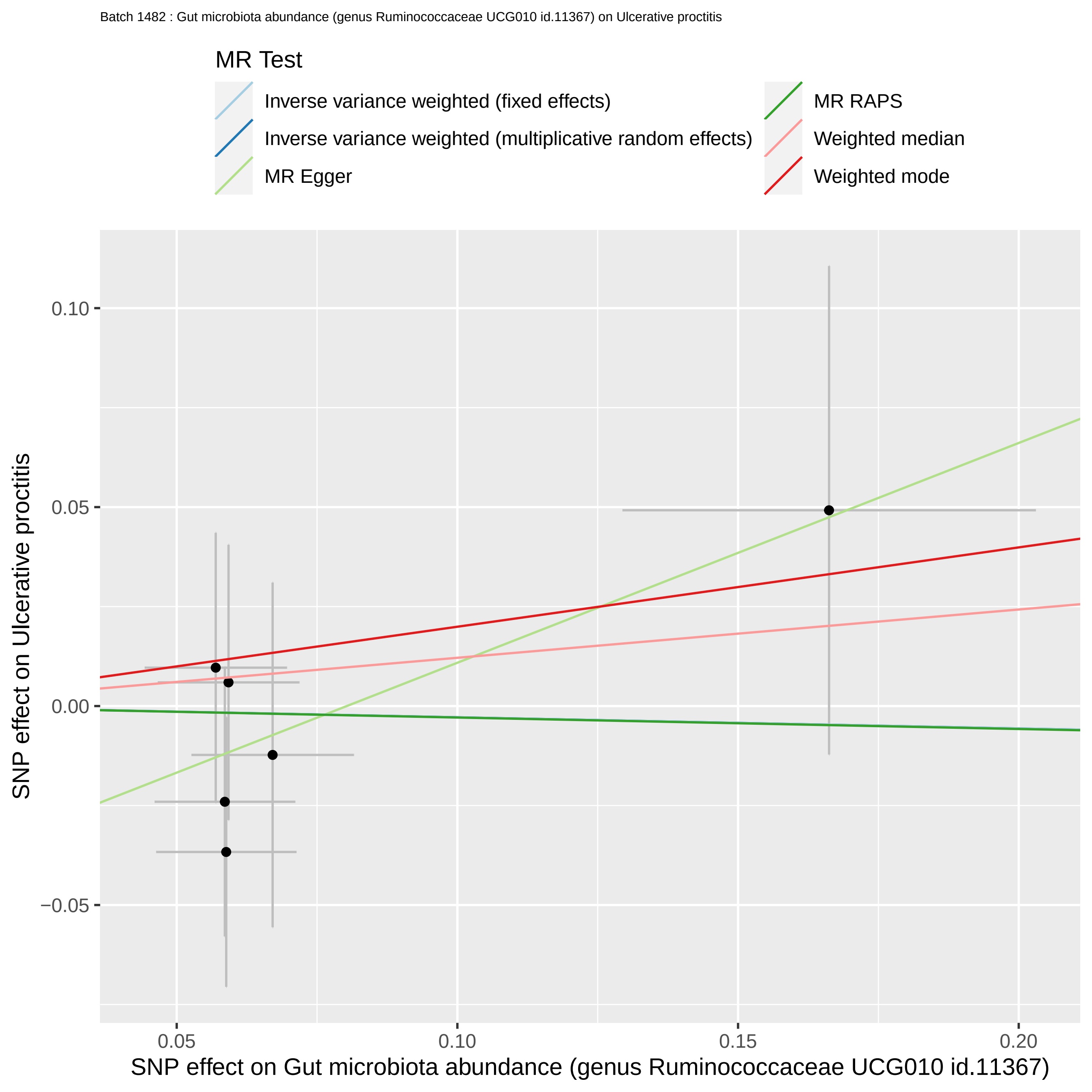

Batch 1482 : Gut microbiota abundance (genus Ruminococcaceae UCG010 id.11367) on Ulcerative proctitis
MR Test
Inverse variance weighted (fixed effects)
Inverse variance weighted (multiplicative random effects)
MR Egger
MR RAPS
Weighted median
Weighted mode
SNP effect on Ulcerative proctitis
0.10
0.05
0.00
−0.05
0.05
0.10
0.15
0.20
SNP effect on Gut microbiota abundance (genus Ruminococcaceae UCG010 id.11367)

Batch 1483 : Gut microbiota abundance (genus Ruminococcaceae UCG011 id.11368) on Ulcerative proctitis

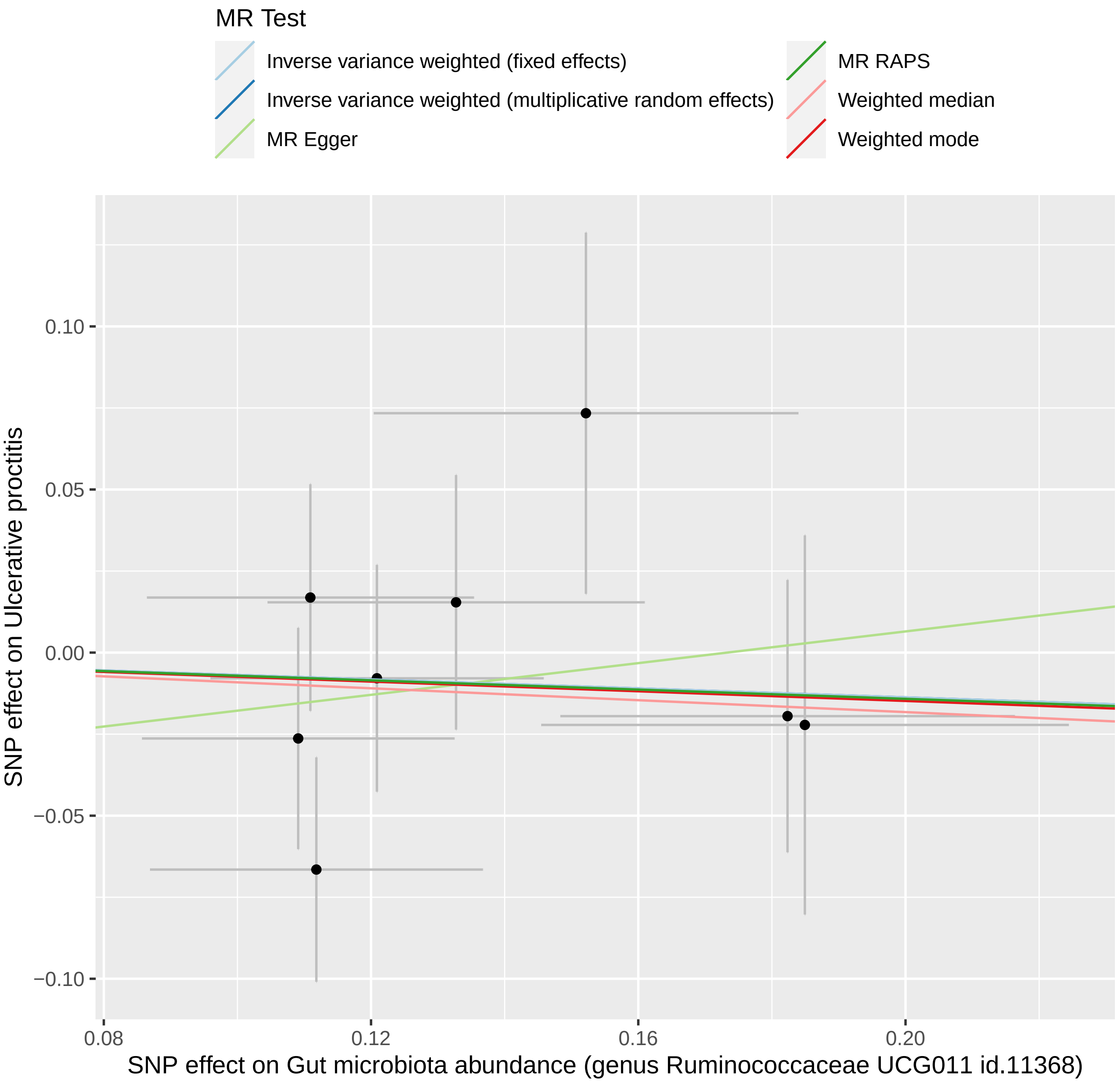

Batch 1484 : Gut microbiota abundance (genus Ruminococcaceae UCG013 id.11370) on Ulcerative proctitis

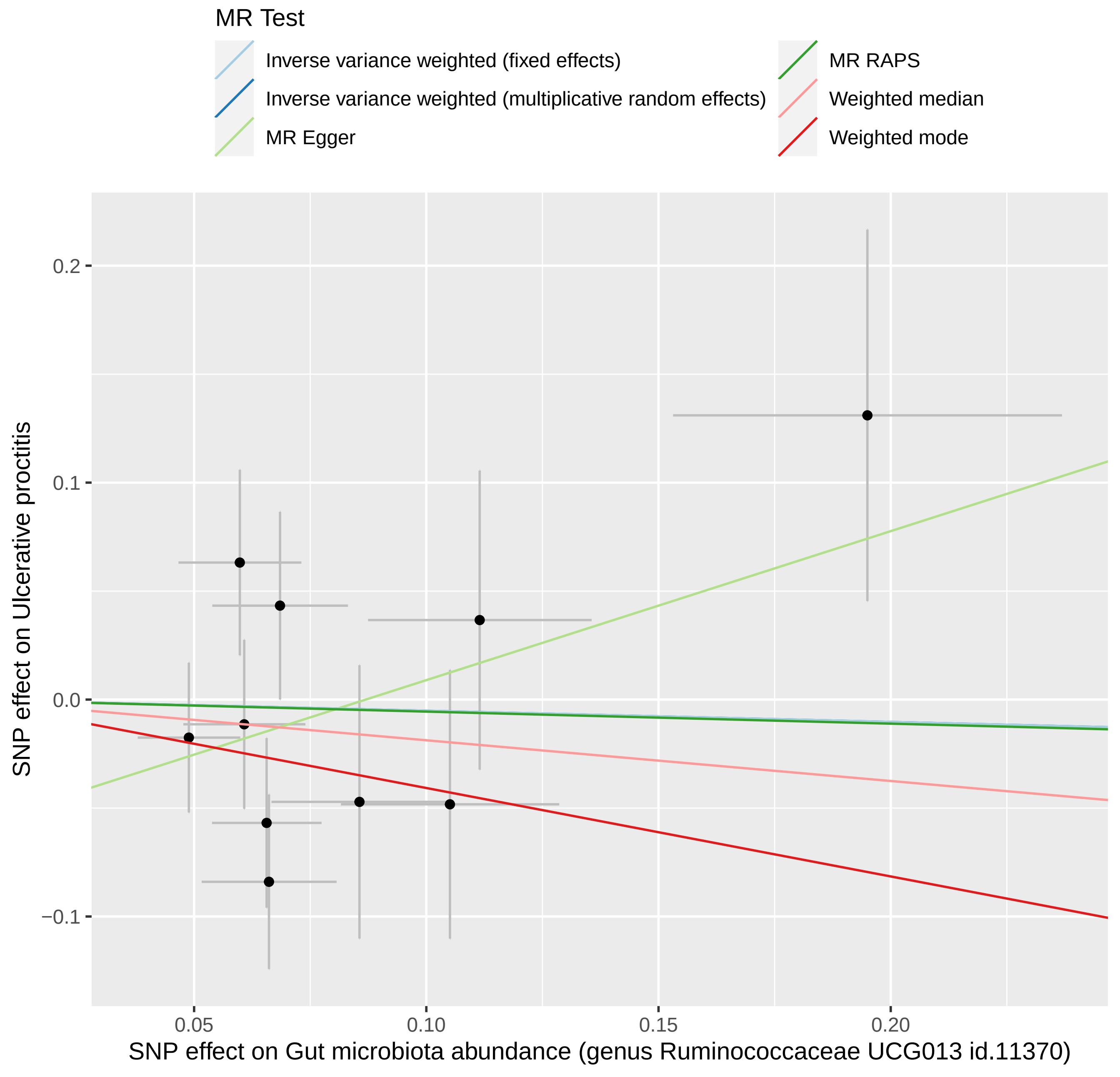

Batch 1485 : Gut microbiota abundance (genus Ruminococcaceae UCG014 id.11371) on Ulcerative proctitis

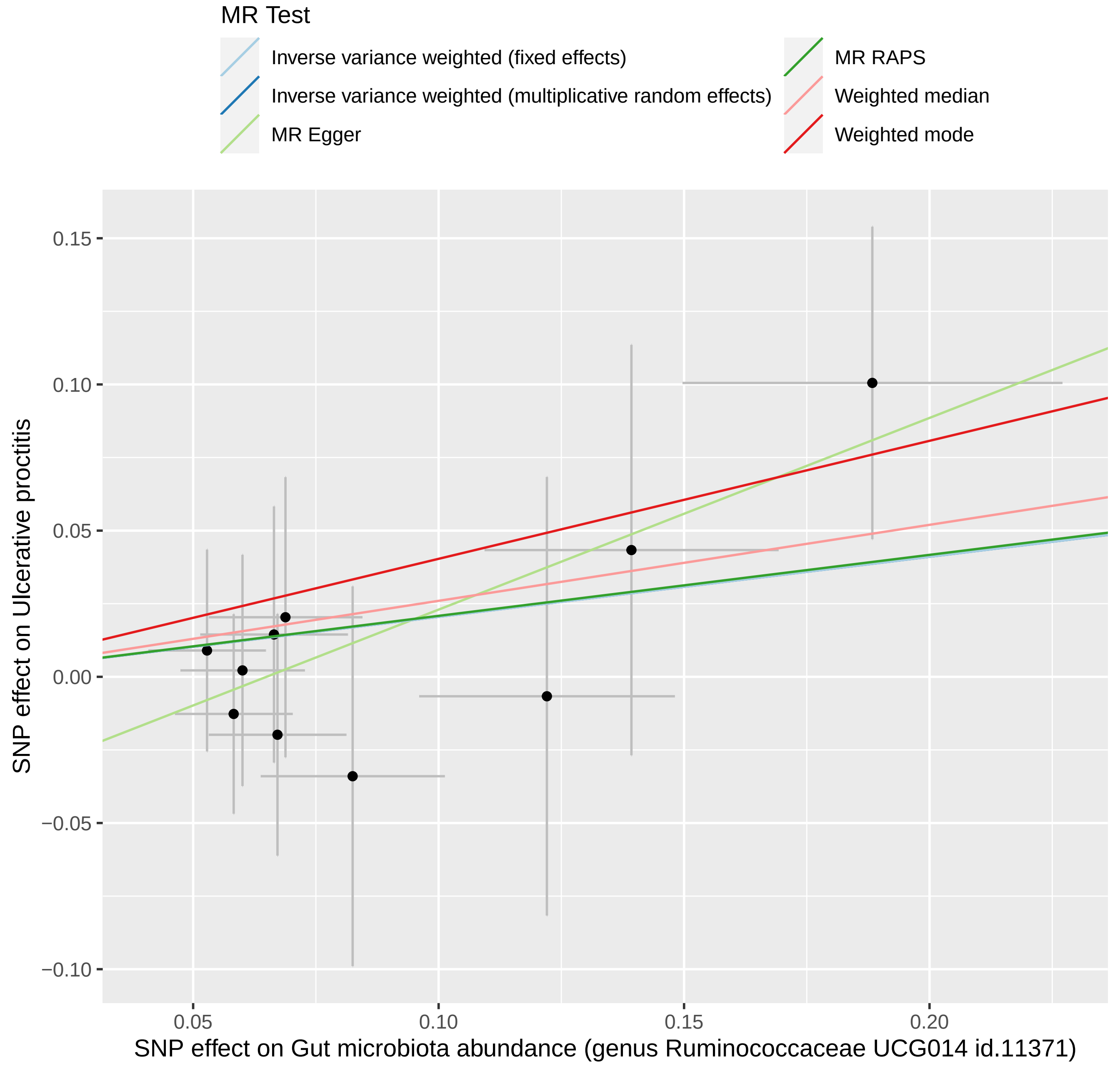

Batch 1486 : Gut microbiota abundance (genus Ruminococcus1 id.11373) on Ulcerative proctitis

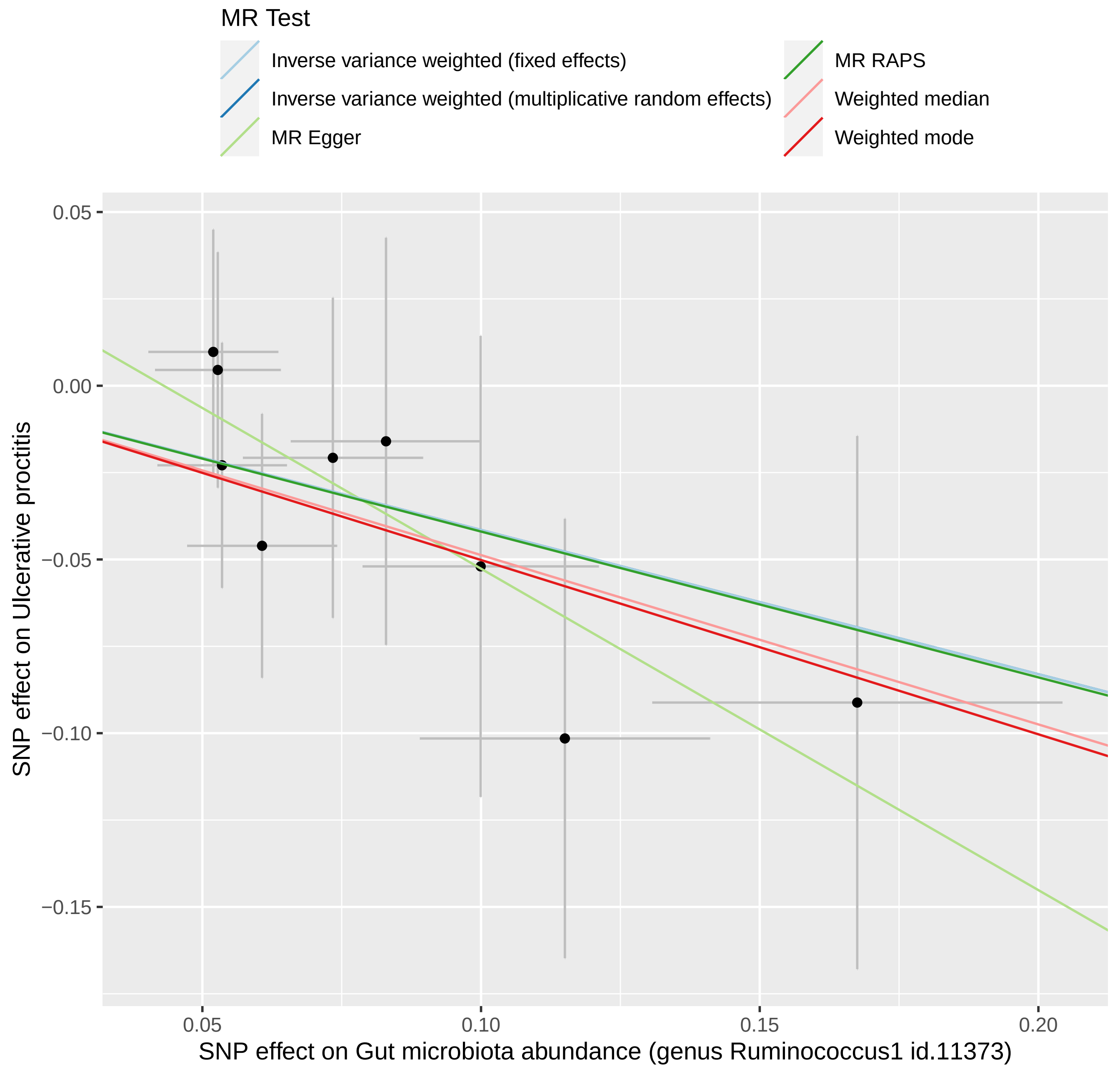

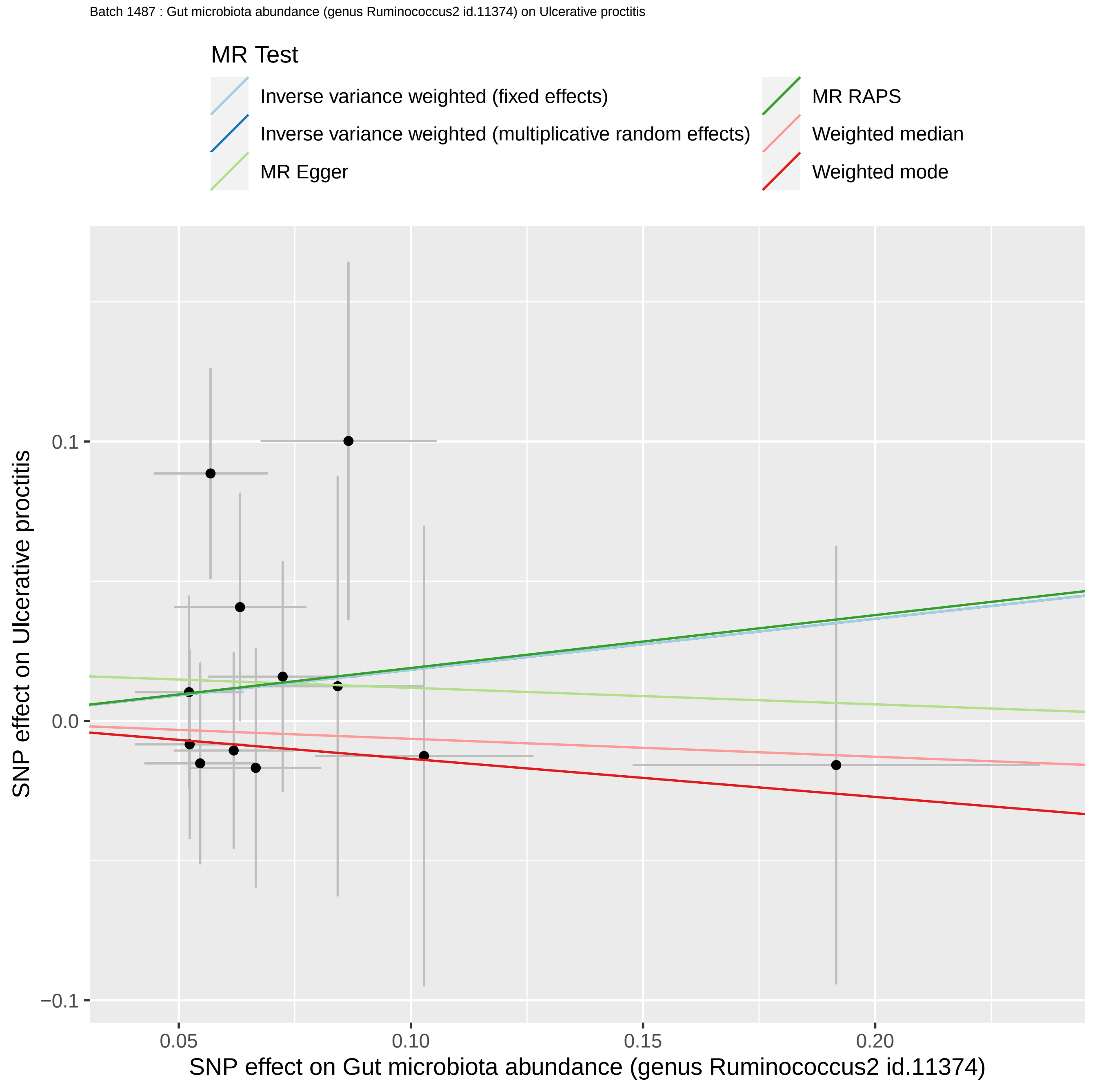
Batch 1487 : Gut microbiota abundance (genus Ruminococcus2 id.11374) on Ulcerative proctitis
MR Test
Inverse variance weighted (fixed effects)
Inverse variance weighted (multiplicative random effects)
MR Egger
MR RAPS
Weighted median
Weighted mode
SNP effect on Ulcerative proctitis
0.1
0.0
−0.1
0.05
0.10
0.15
0.20
SNP effect on Gut microbiota abundance (genus Ruminococcus2 id.11374)

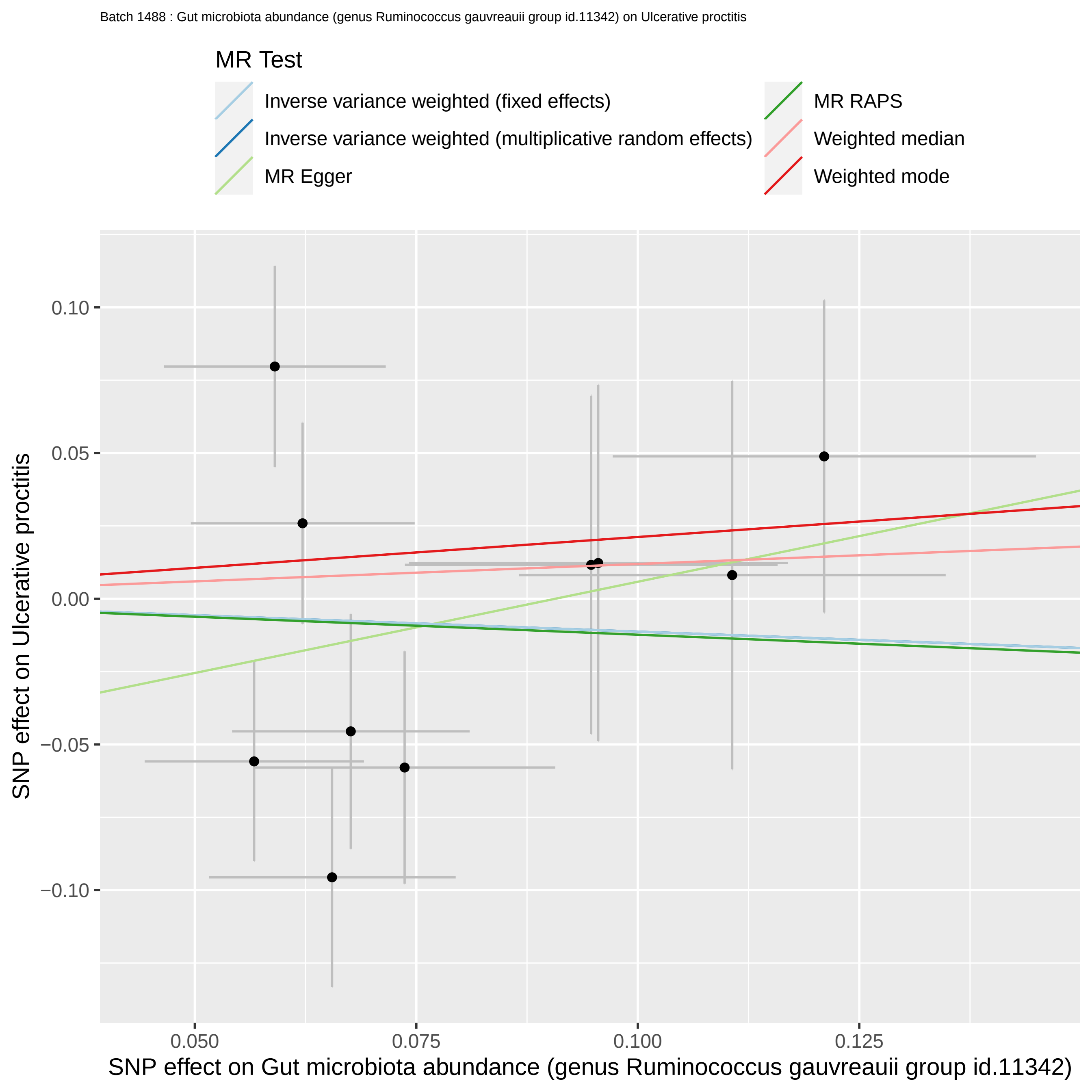
Batch 1488 : Gut microbiota abundance (genus Ruminococcus gauvreauii group id.11342) on Ulcerative proctitis
MR Test
Inverse variance weighted (fixed effects)
Inverse variance weighted (multiplicative random effects)
MR Egger
MR RAPS
Weighted median
Weighted mode
0.10
0.05
0.00
−0.05
−0.10
0.050
0.075
0.100
0.125
SNP effect on Ulcerative proctitis
SNP effect on Gut microbiota abundance (genus Ruminococcus gauvreauii group id.11342)

Batch 1489 : Gut microbiota abundance (genus Ruminococcus gnavus group id.14376) on Ulcerative proctitis

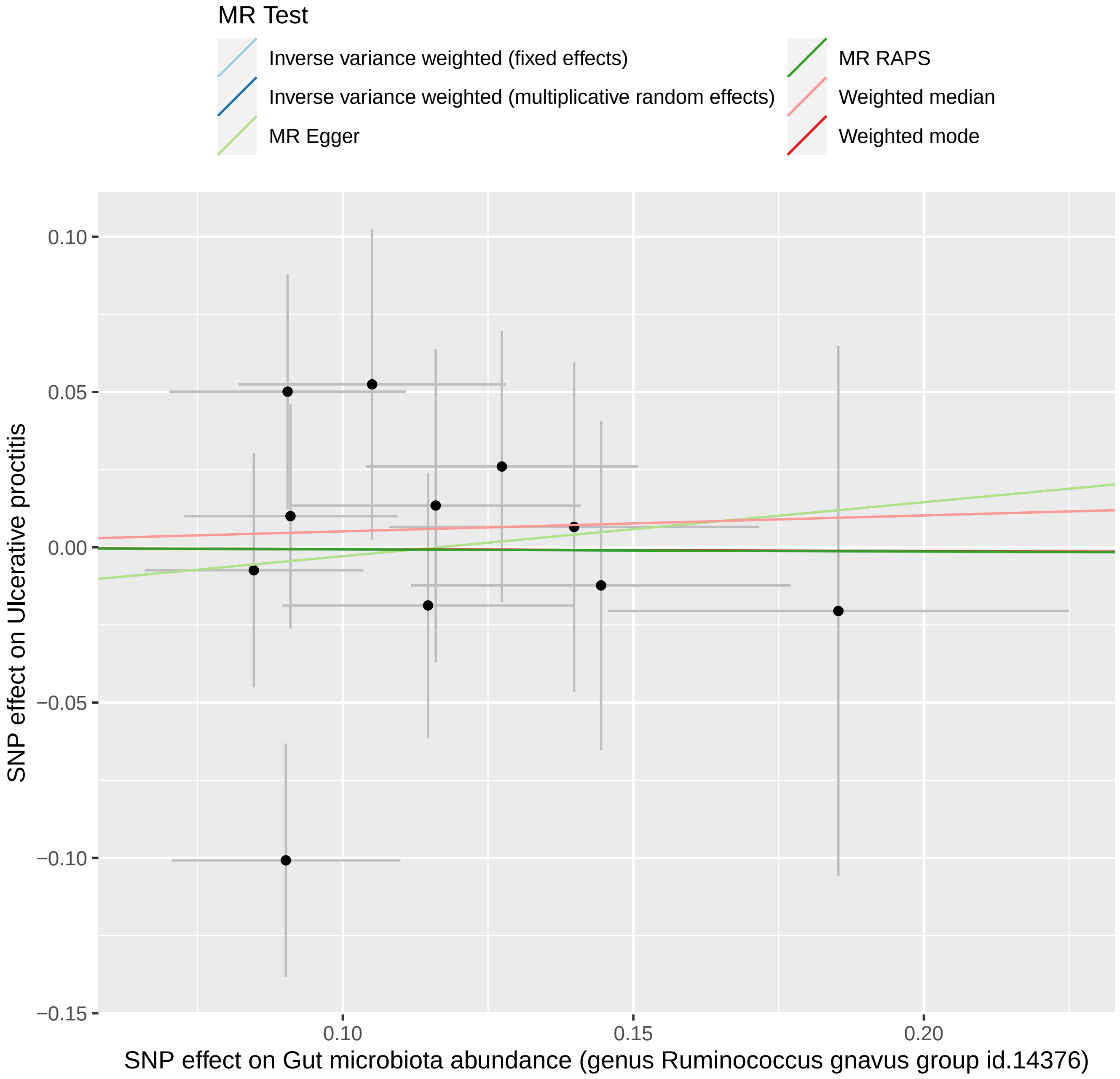

Batch 1490 : Gut microbiota abundance (genus Ruminococcus torques group id.14377) on Ulcerative proctitis

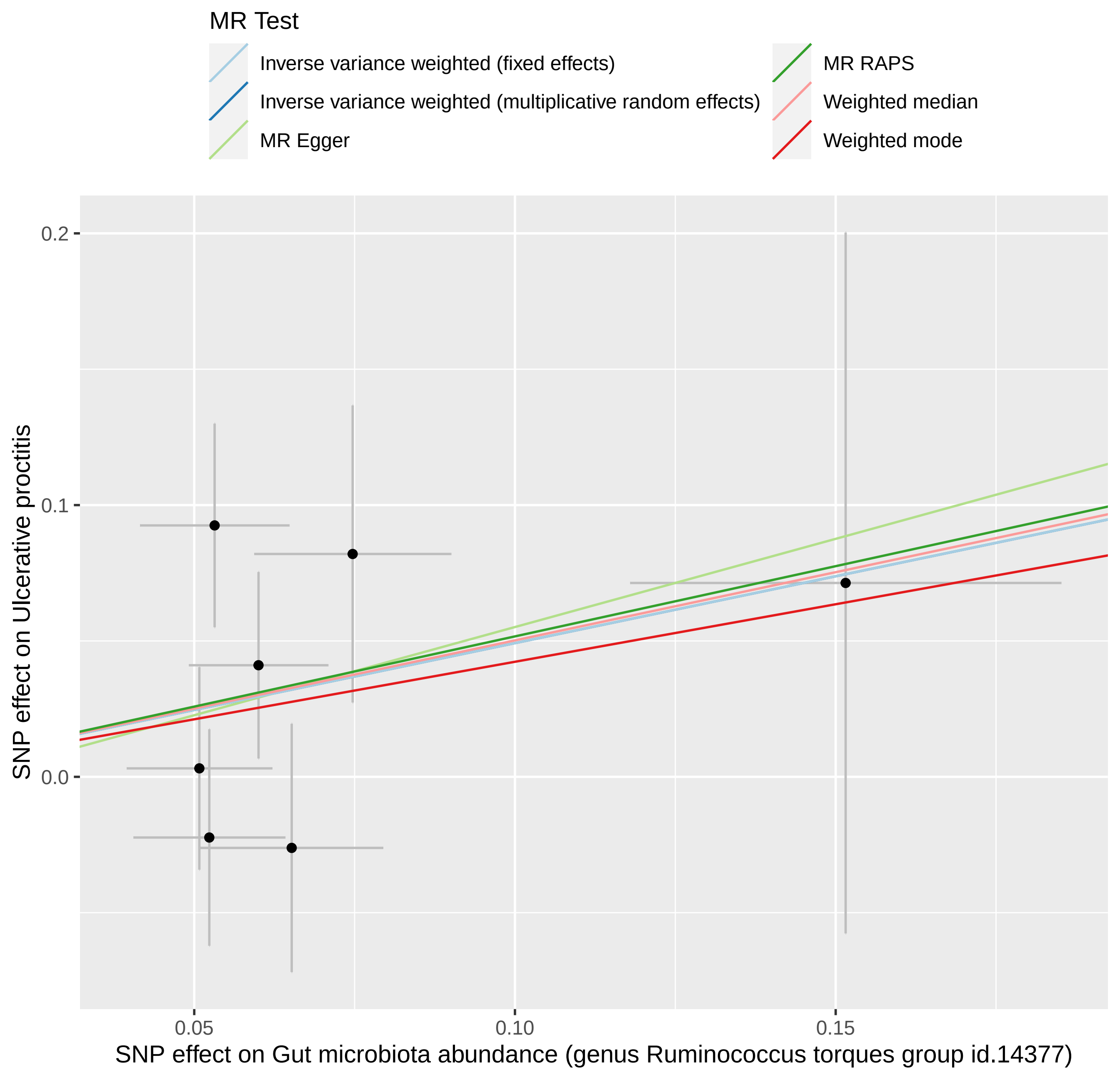

Batch 1491 : Gut microbiota abundance (genus Sellimonas id.14369) on Ulcerative proctitis

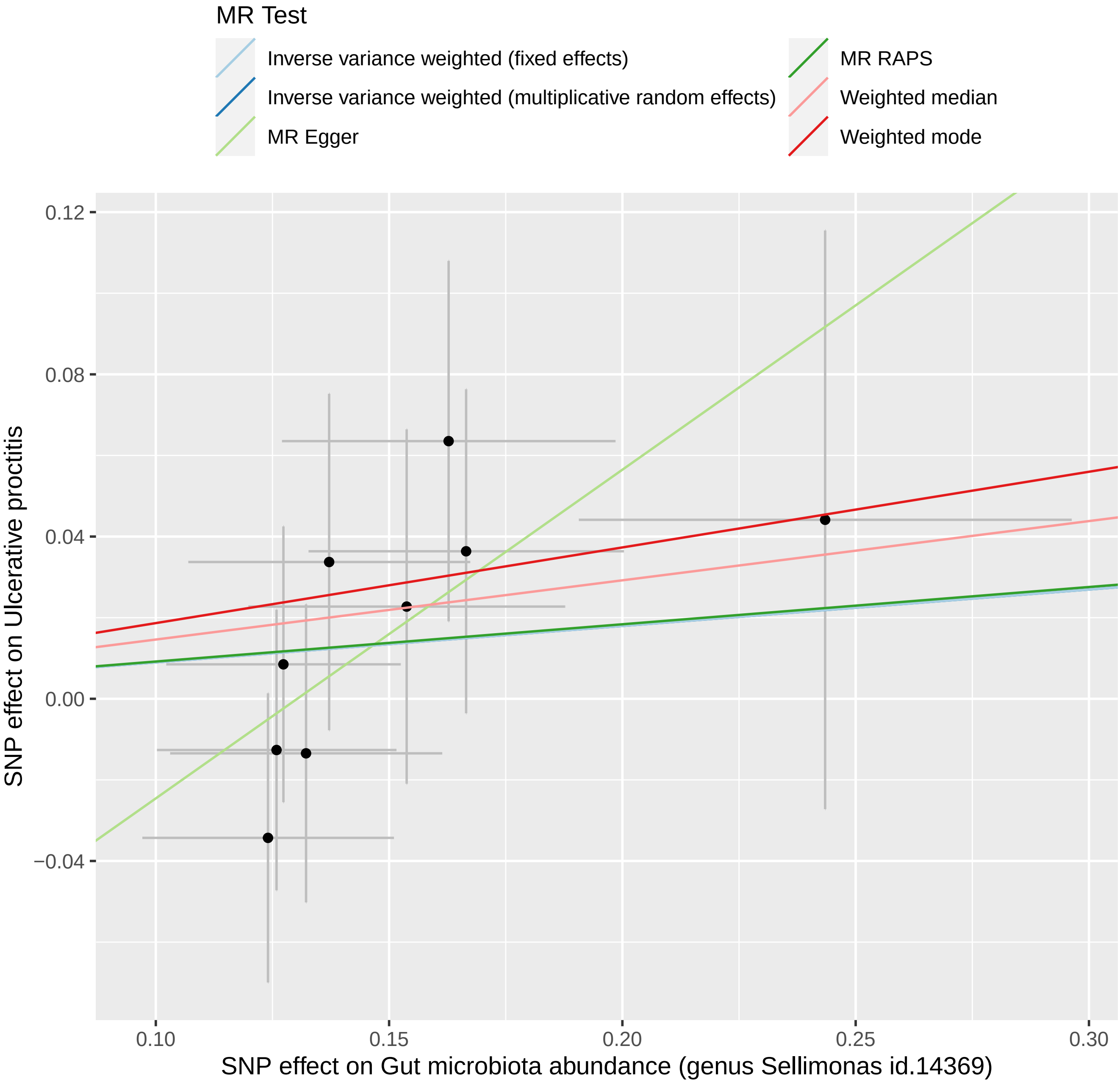

Batch 1492 : Gut microbiota abundance (genus Senegalimassilia id.11160) on Ulcerative proctitis

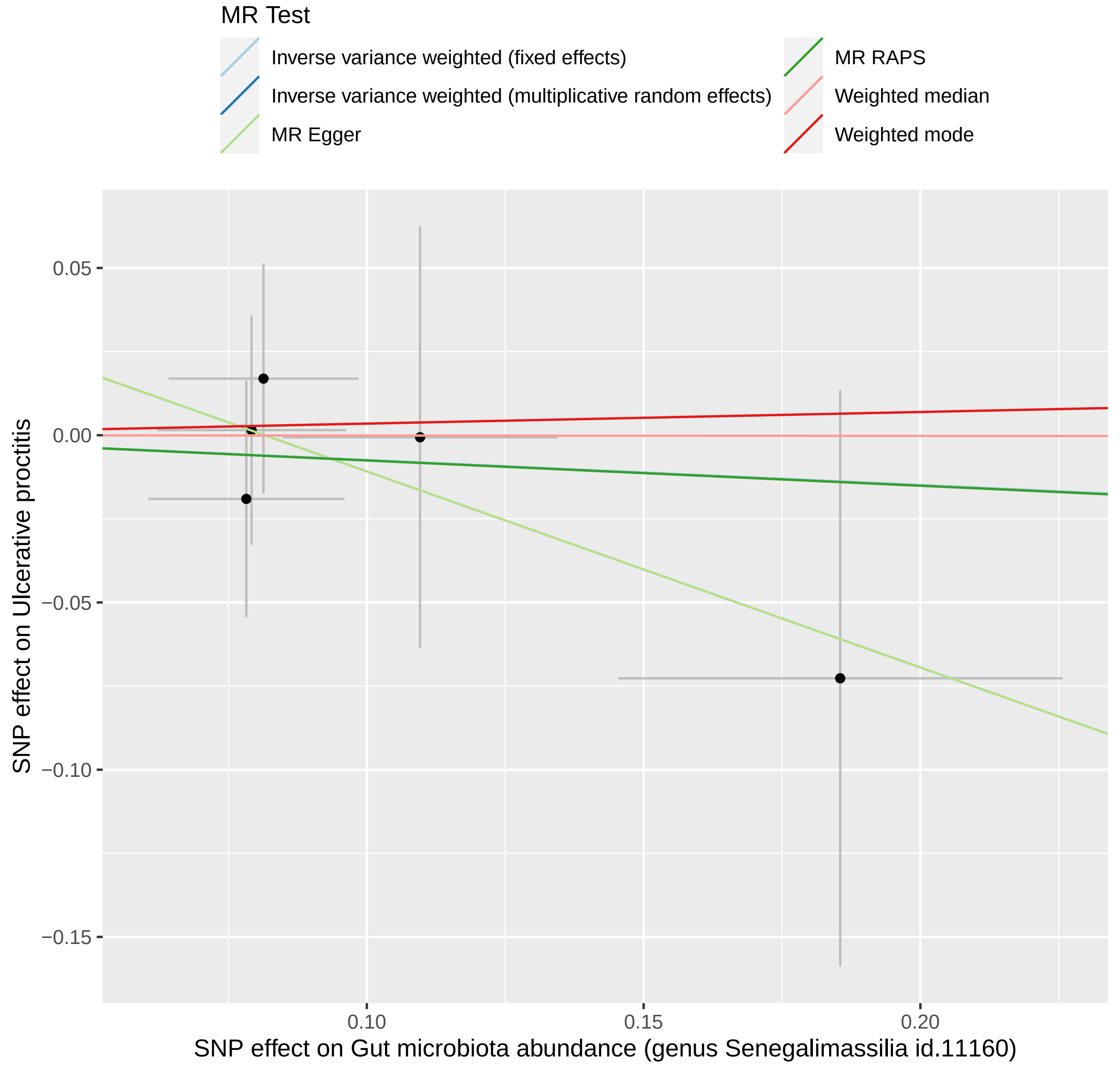

Batch 1493 : Gut microbiota abundance (genus Slackia id.825) on Ulcerative proctitis

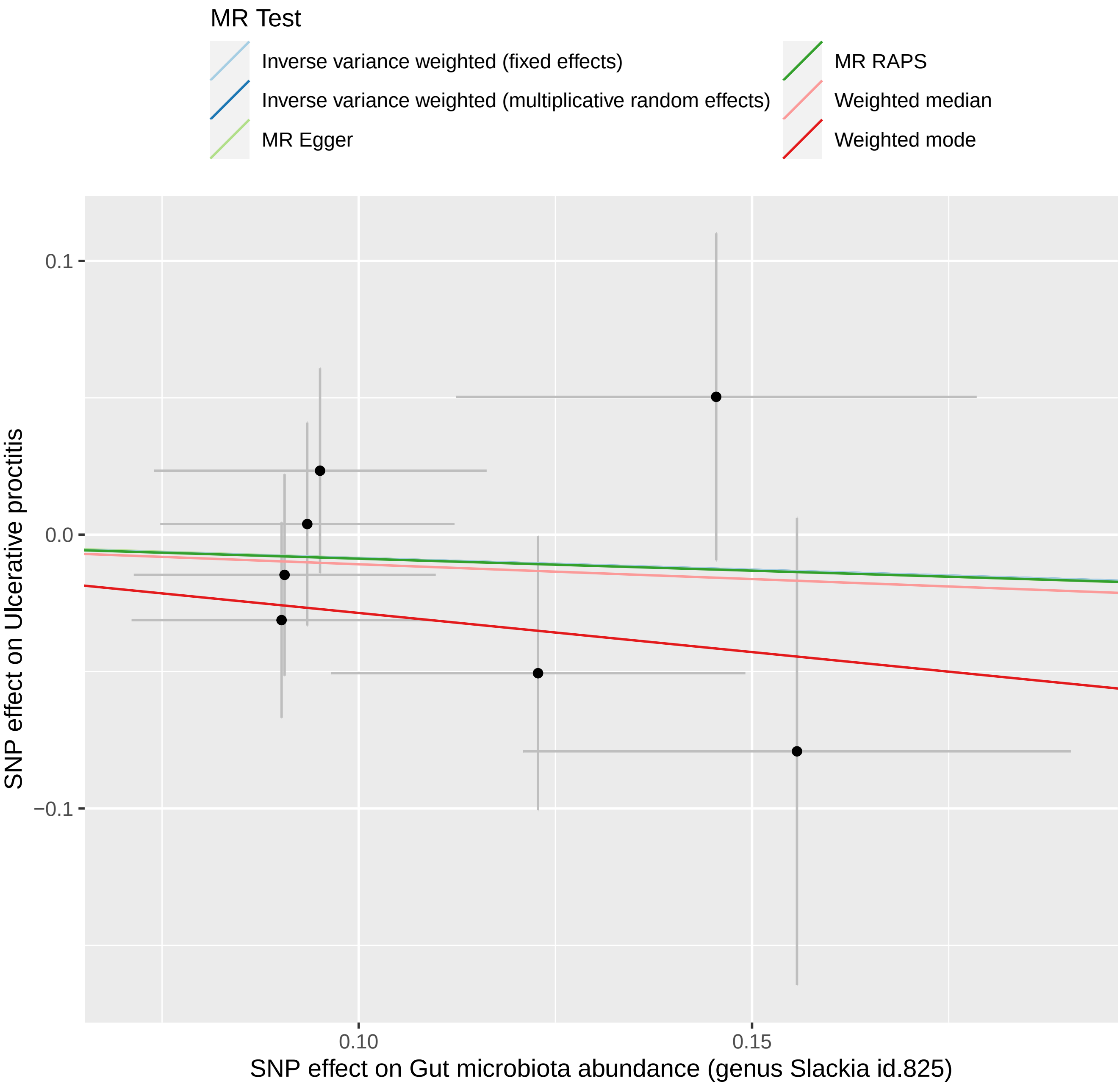

Batch 1494 : Gut microbiota abundance (genus Streptococcus id.1853) on Ulcerative proctitis

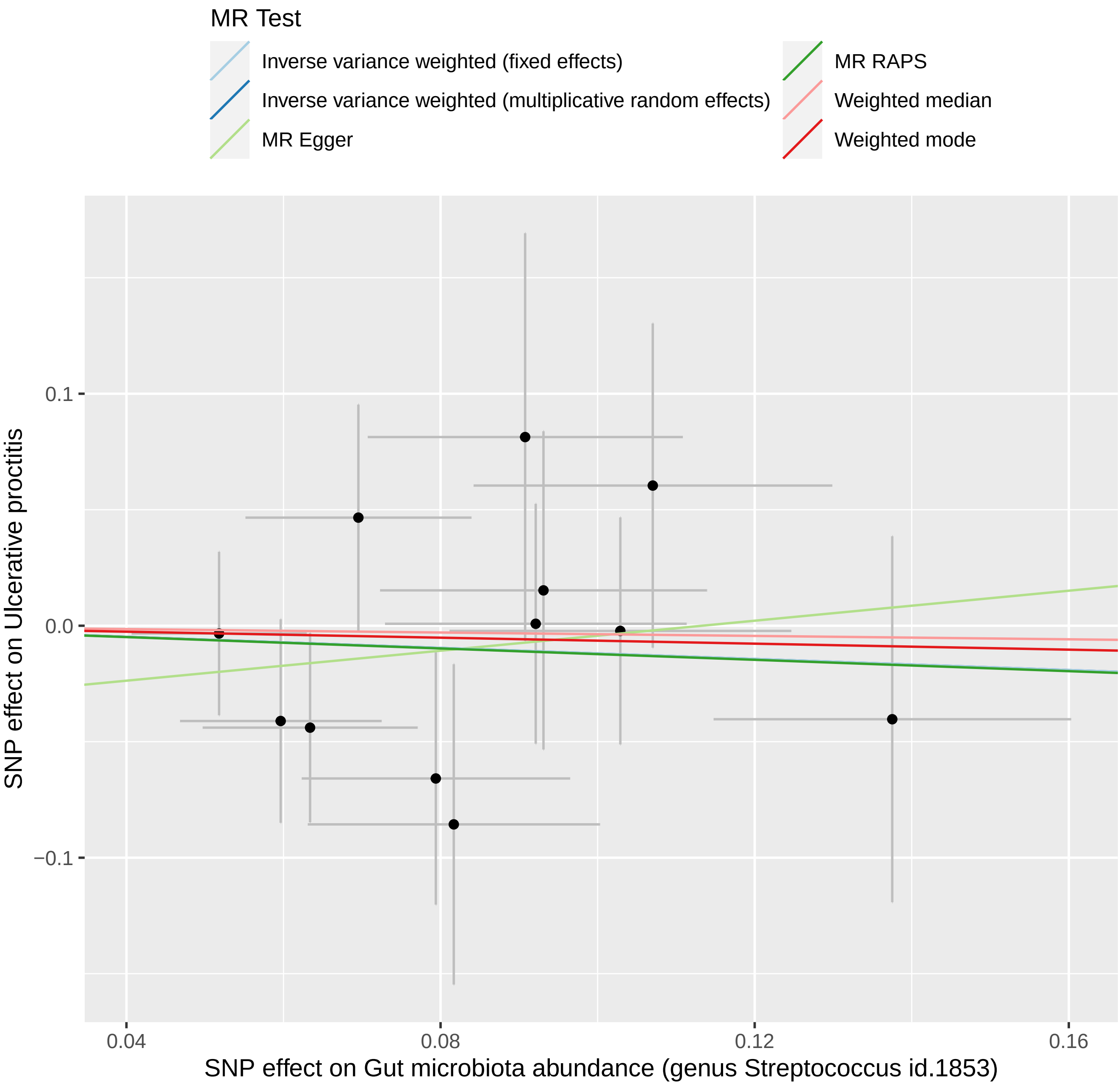

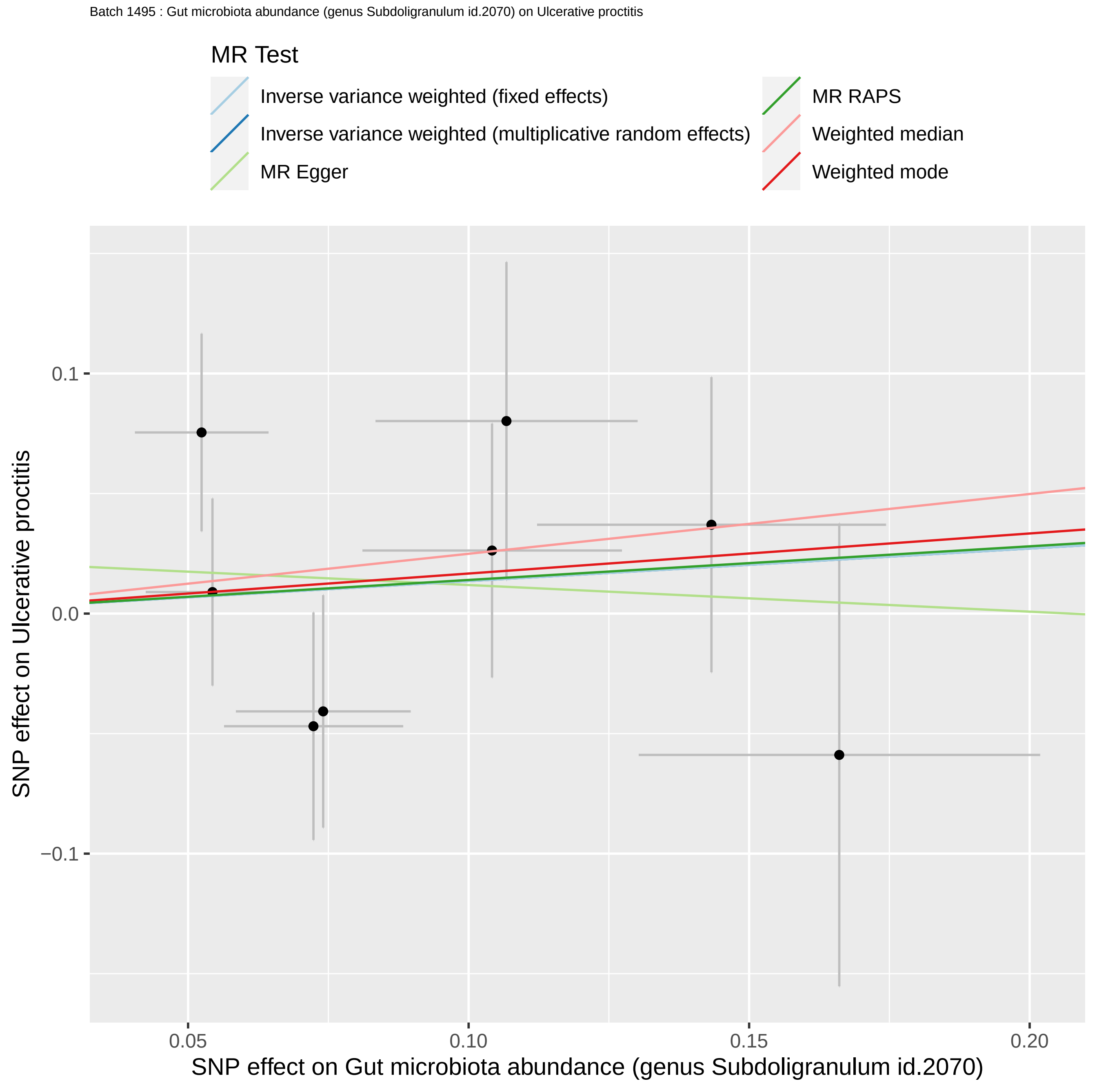

Batch 1495 : Gut microbiota abundance (genus Subdoligranulum id.2070) on Ulcerative proctitis
MR Test
Inverse variance weighted (fixed effects)
Inverse variance weighted (multiplicative random effects)
MR Egger
MR RAPS
Weighted median
Weighted mode
0.1
0.0
−0.1
0.05
0.10
0.15
0.20
SNP effect on Ulcerative proctitis
SNP effect on Gut microbiota abundance (genus Subdoligranulum id.2070)

Batch 1496 : Gut microbiota abundance (genus Sutterella id.2896) on Ulcerative proctitis

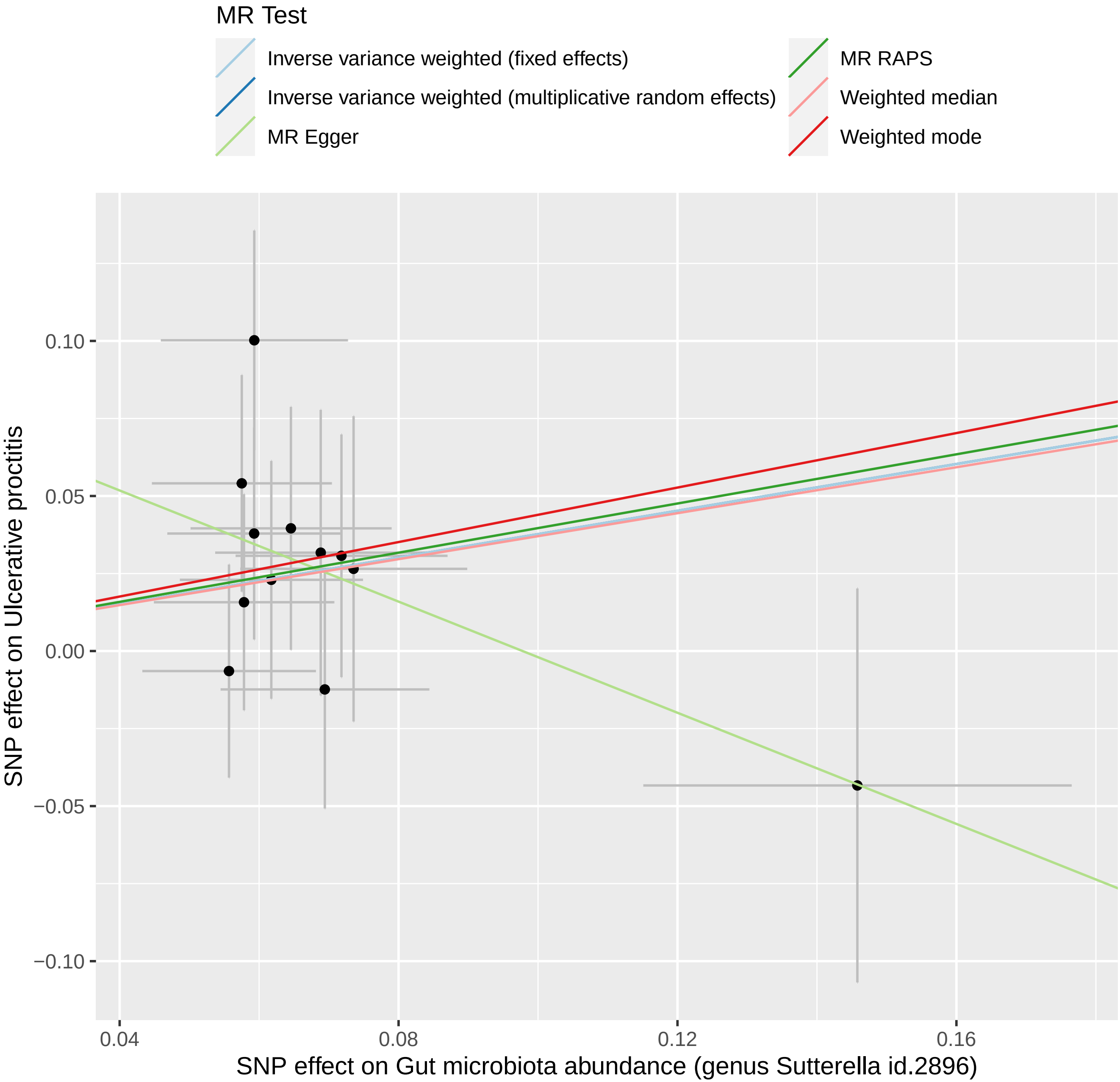

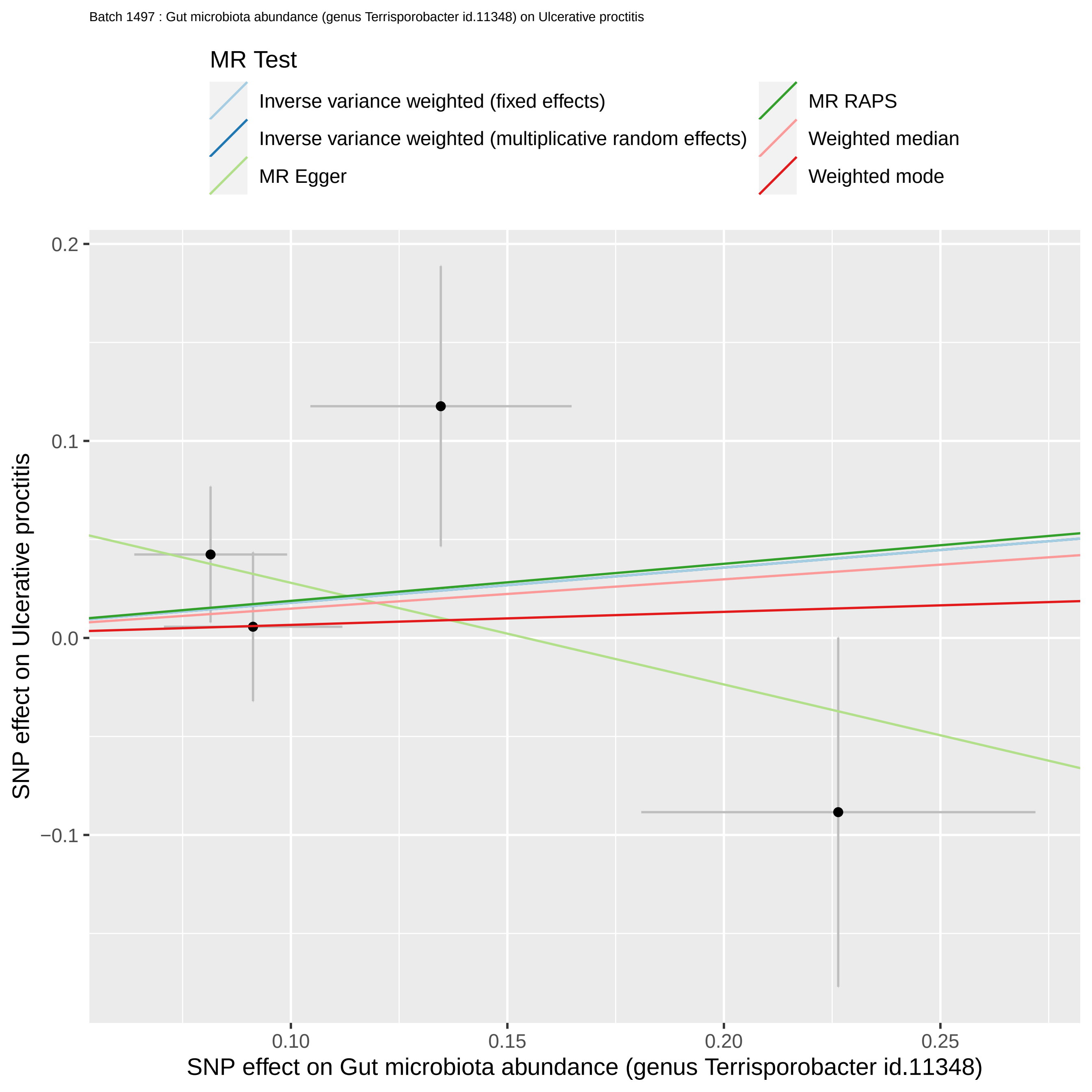
Batch 1497 : Gut microbiota abundance (genus Terrisporobacter id.11348) on Ulcerative proctitis
MR Test
Inverse variance weighted (fixed effects)
Inverse variance weighted (multiplicative random effects)
MR Egger
MR RAPS
Weighted median
Weighted mode
0.2
0.1
0.0
−0.1
0.10
0.15
0.20
0.25
SNP effect on Ulcerative proctitis
SNP effect on Gut microbiota abundance (genus Terrisporobacter id.11348)

Batch 1498 : Gut microbiota abundance (genus Turicibacter id.2162) on Ulcerative proctitis

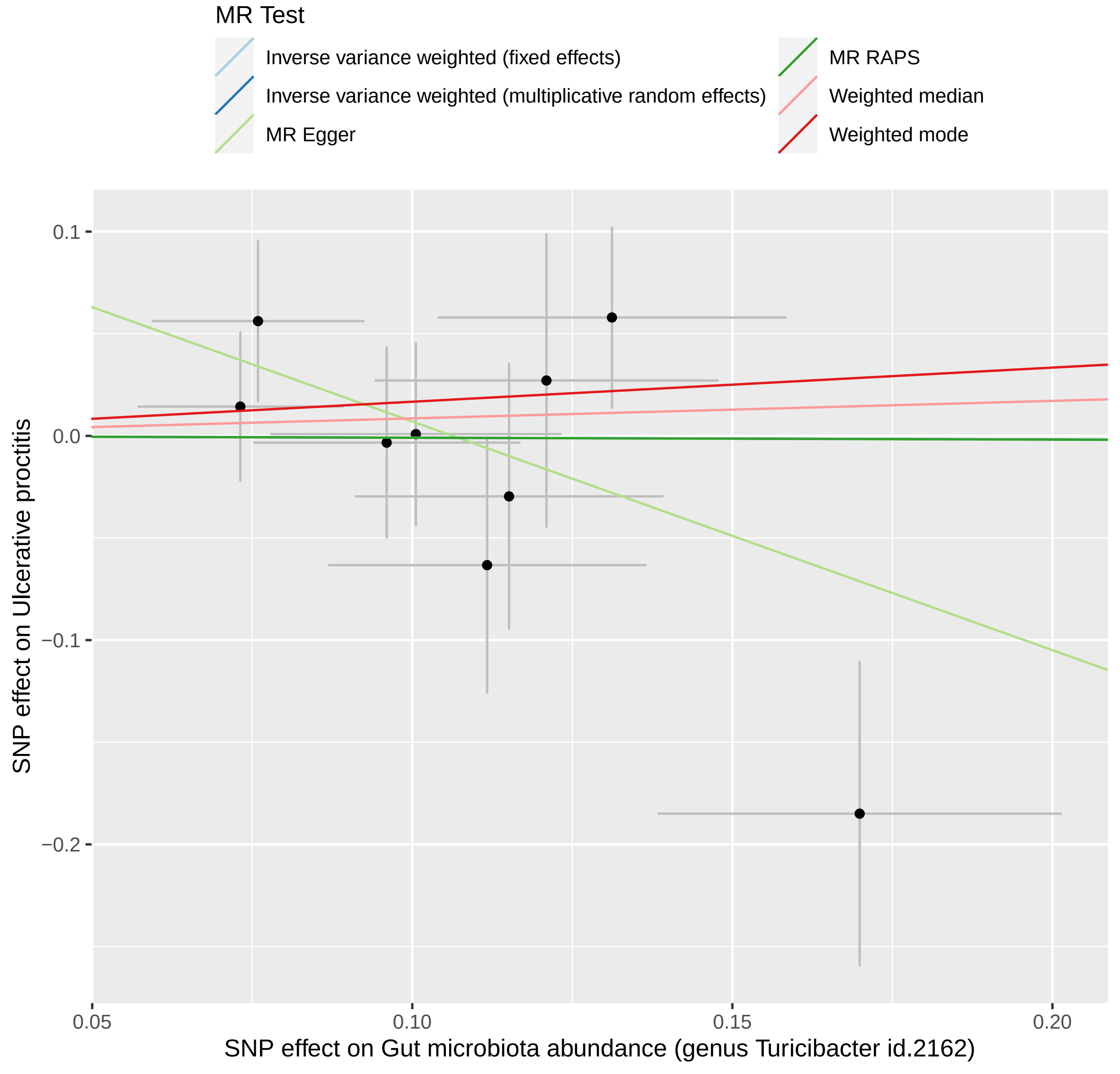

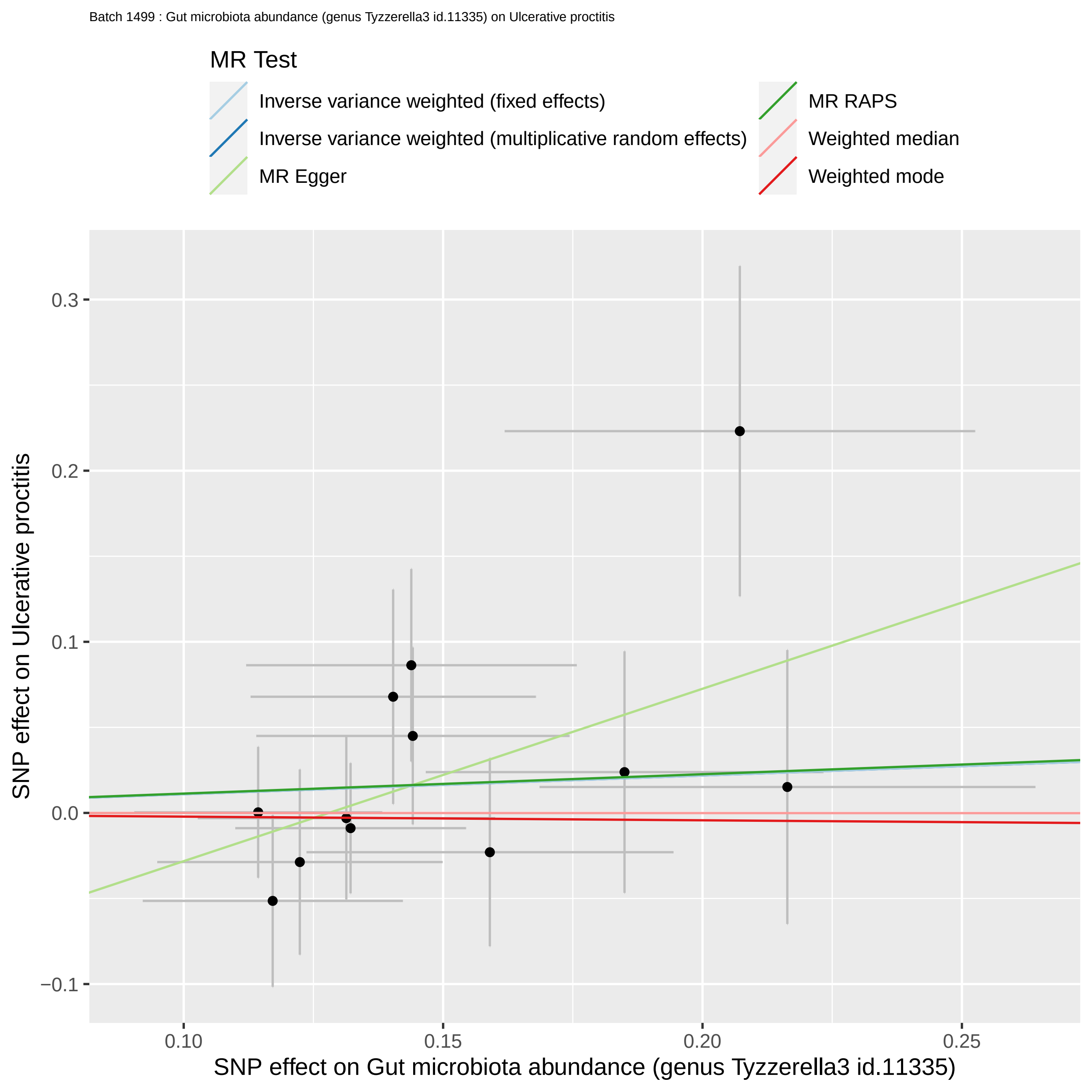

Batch 1499 : Gut microbiota abundance (genus Tyzzerella3 id.11335) on Ulcerative proctitis
MR Test
Inverse variance weighted (fixed effects)
Inverse variance weighted (multiplicative random effects)
MR Egger
MR RAPS
Weighted median
Weighted mode
0.3
0.2
0.1
0.0
−0.1
0.10
0.15
0.20
0.25
SNP effect on Ulcerative proctitis
SNP effect on Gut microbiota abundance (genus Tyzzerella3 id.11335)

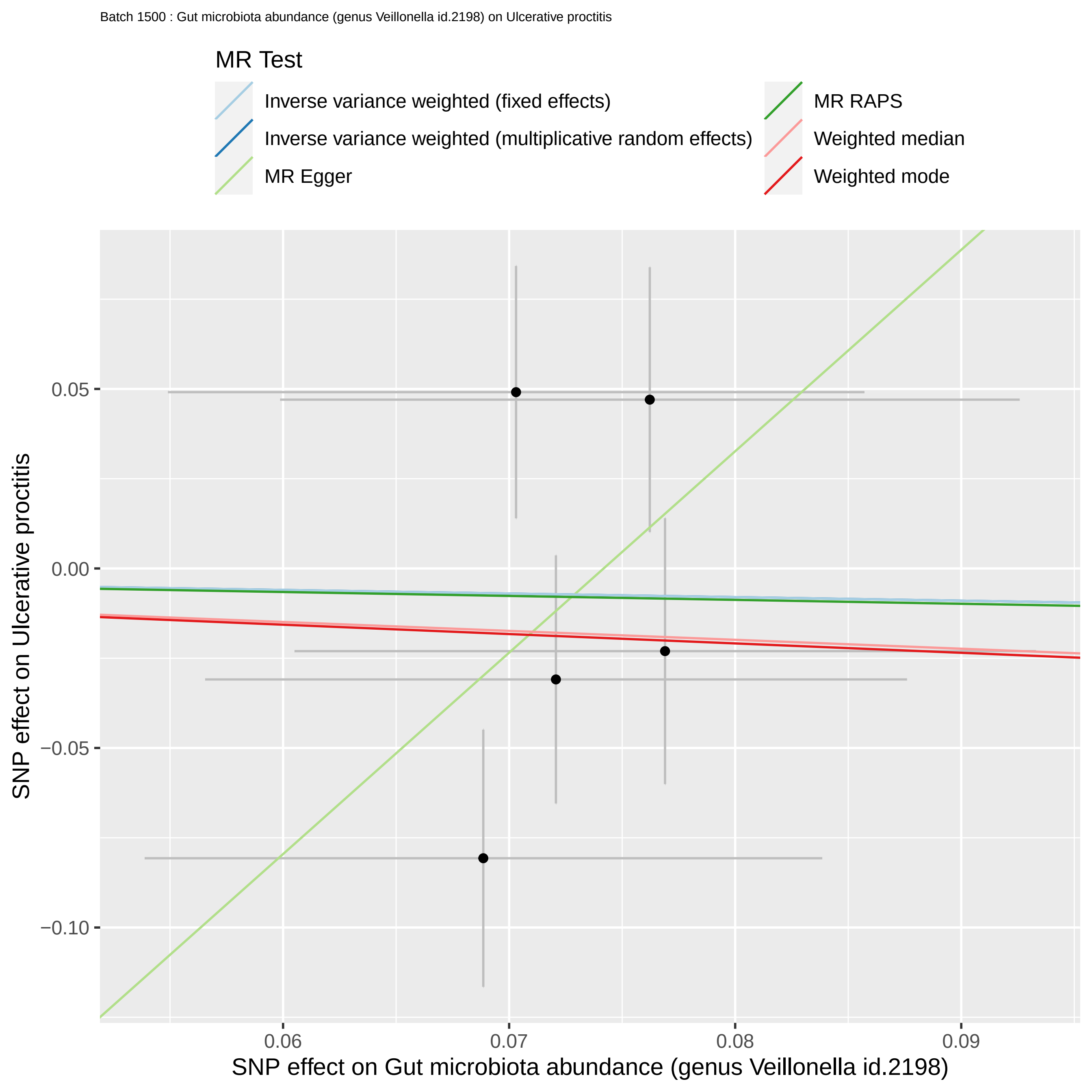

Batch 1500 : Gut microbiota abundance (genus Veillonella id.2198) on Ulcerative proctitis
MR Test
Inverse variance weighted (fixed effects)
Inverse variance weighted (multiplicative random effects)
MR Egger
MR RAPS
Weighted median
Weighted mode
0.05
0.00
−0.05
−0.10
0.06
0.07
0.08
0.09
SNP effect on Ulcerative proctitis
SNP effect on Gut microbiota abundance (genus Veillonella id.2198)

Batch 1501 : Gut microbiota abundance (family Acidaminococcaceae id.2166) on Ulcerative rectosigmoiditis

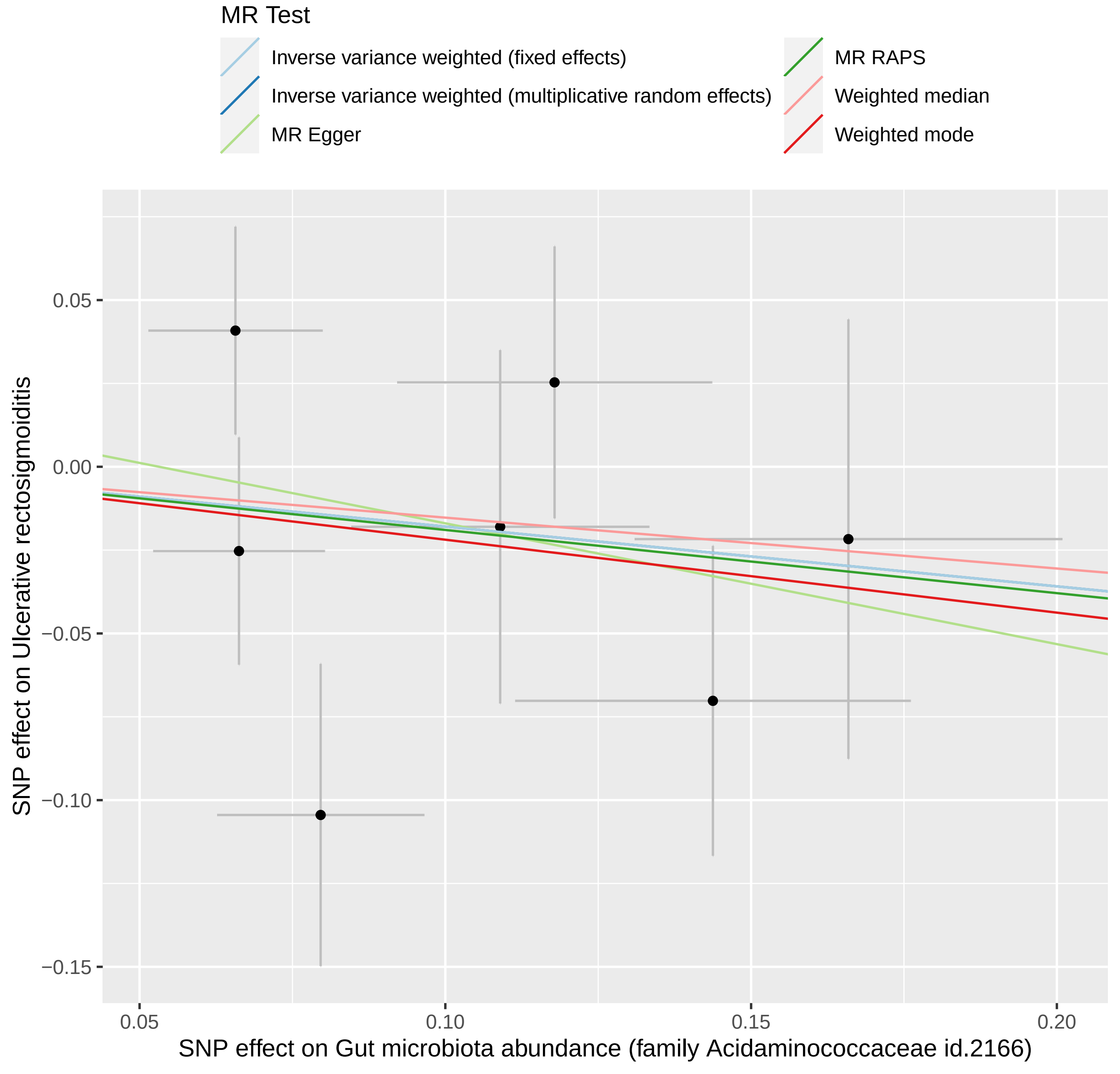

Batch 1502 : Gut microbiota abundance (family Actinomycetaceae id.421) on Ulcerative rectosigmoiditis

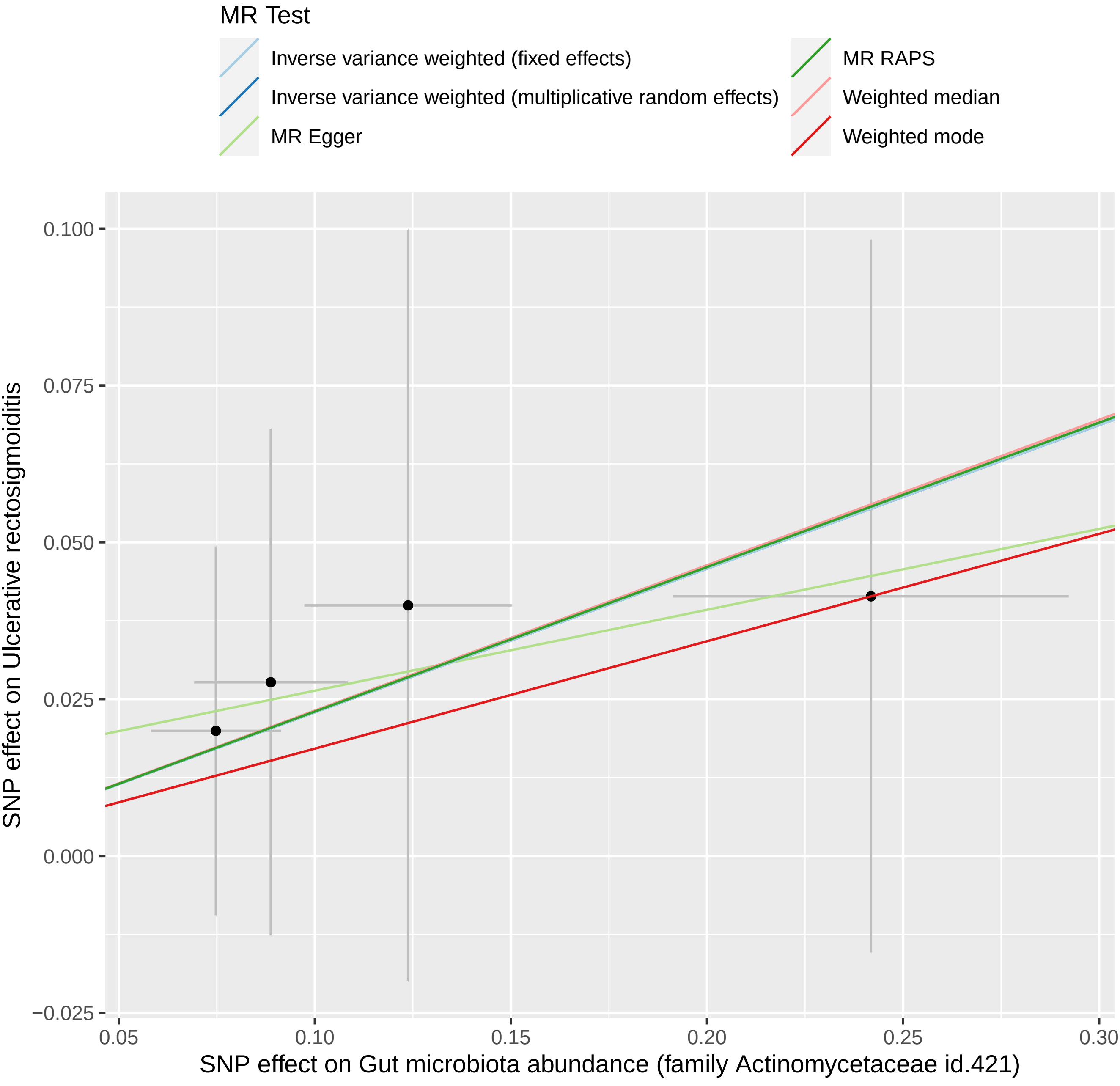

Batch 1503 : Gut microbiota abundance (family Alcaligenaceae id.2875) on Ulcerative rectosigmoiditis

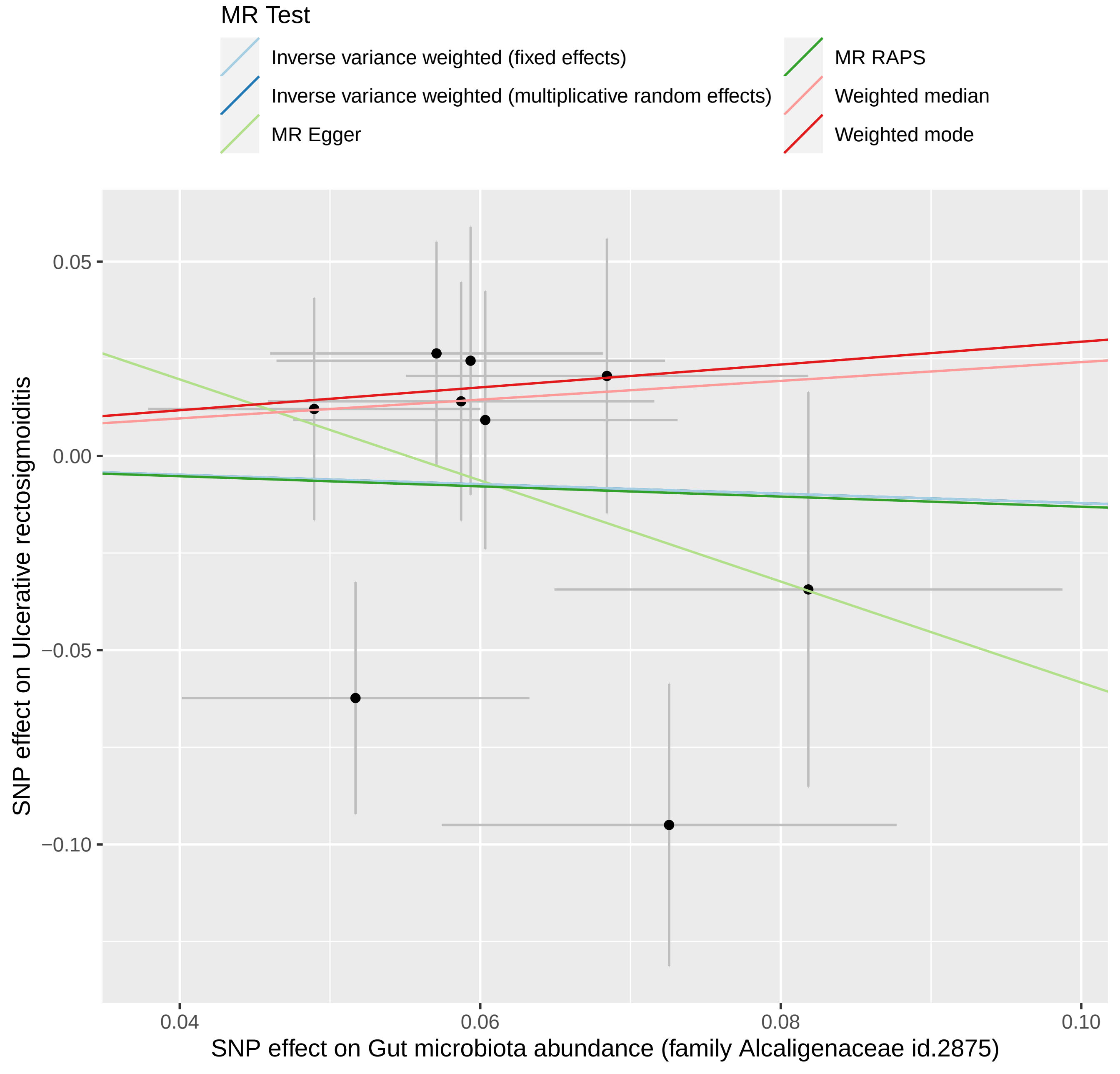

Batch 1504 : Gut microbiota abundance (family Bacteroidaceae id.917) on Ulcerative rectosigmoiditis

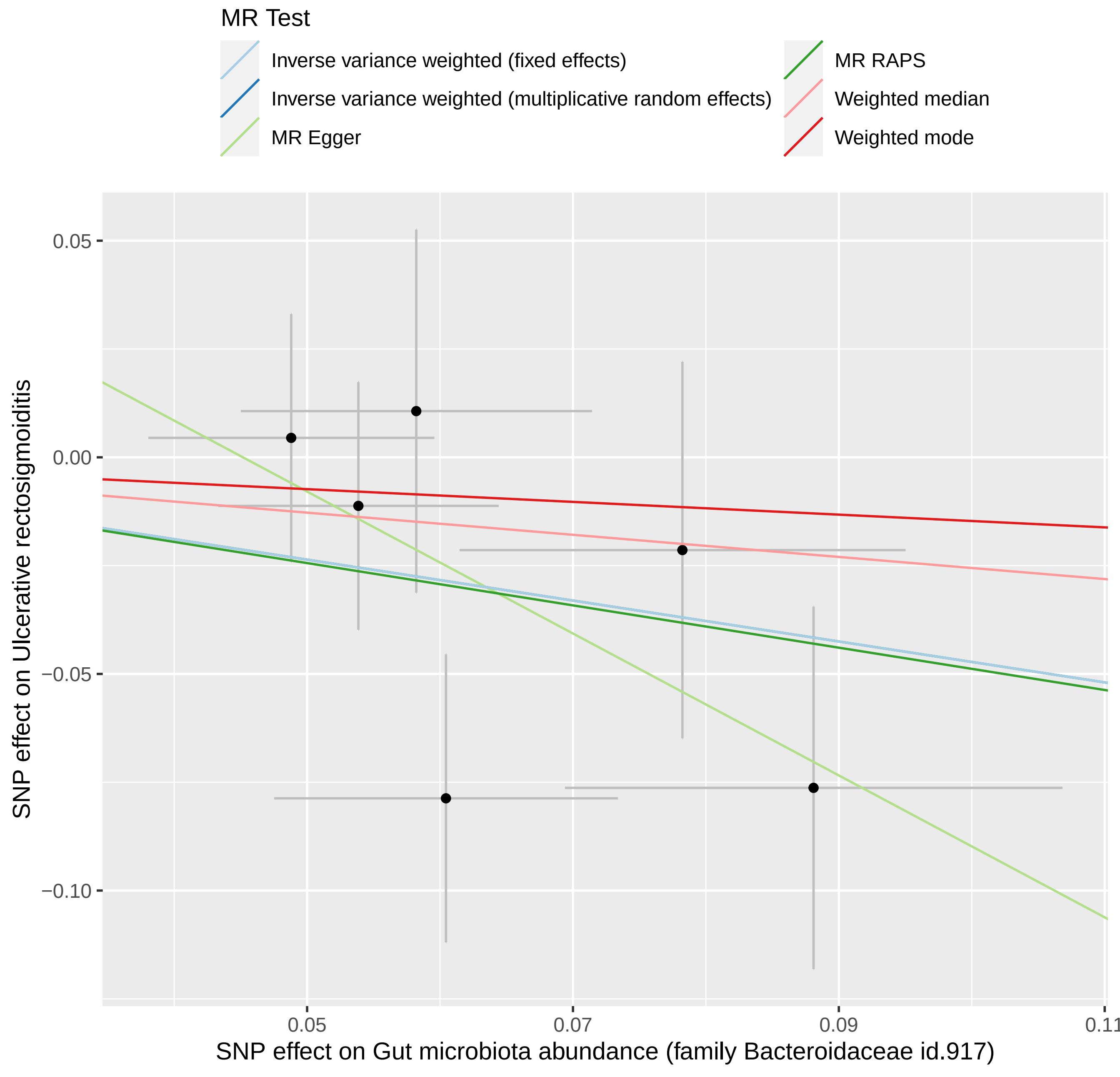

Batch 1505 : Gut microbiota abundance (family Bacteroidales S24 7group id.11173) on Ulcerative rectosigmoiditis

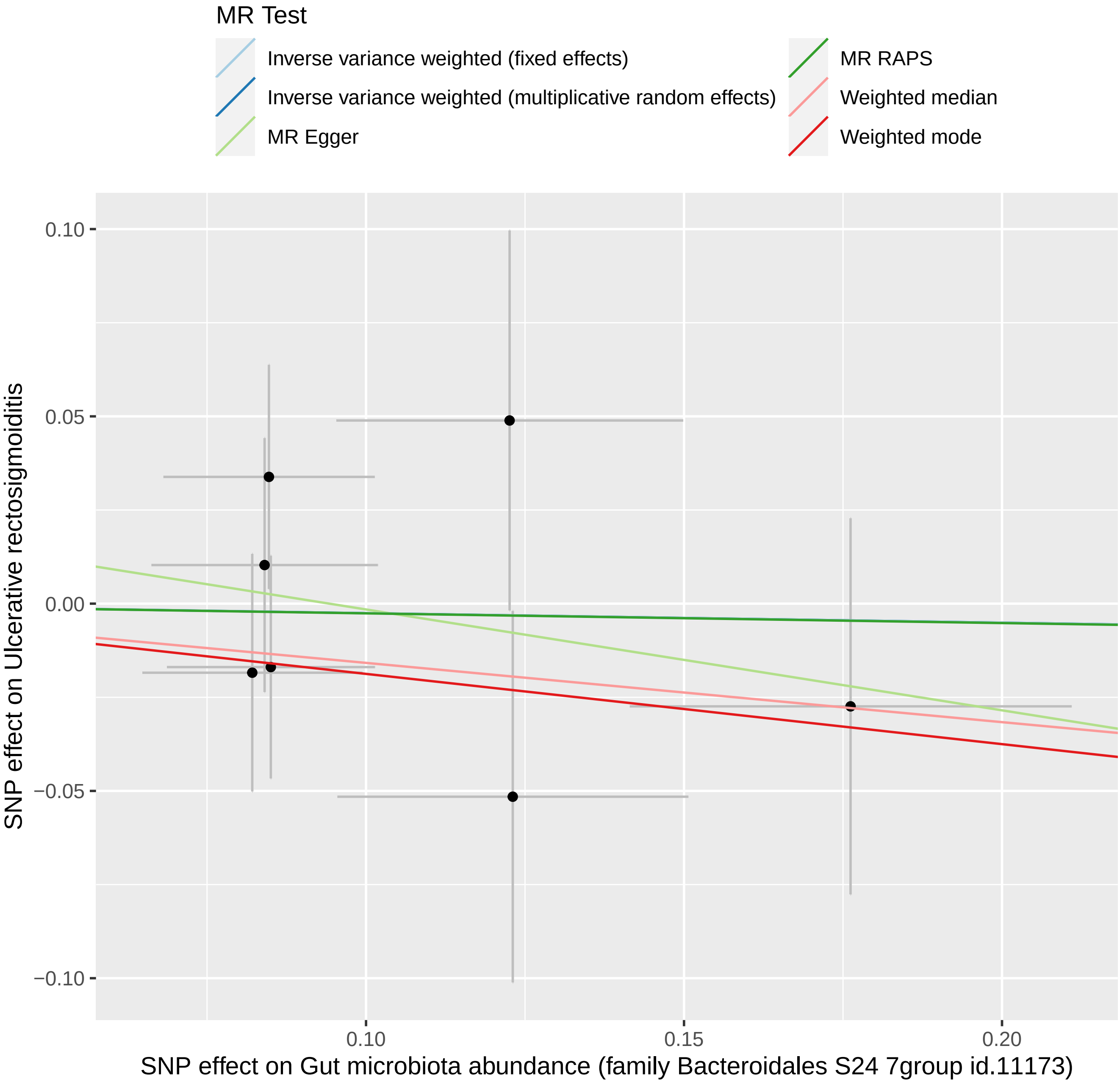

Batch 1506 : Gut microbiota abundance (family Bifidobacteriaceae id.433) on Ulcerative rectosigmoiditis

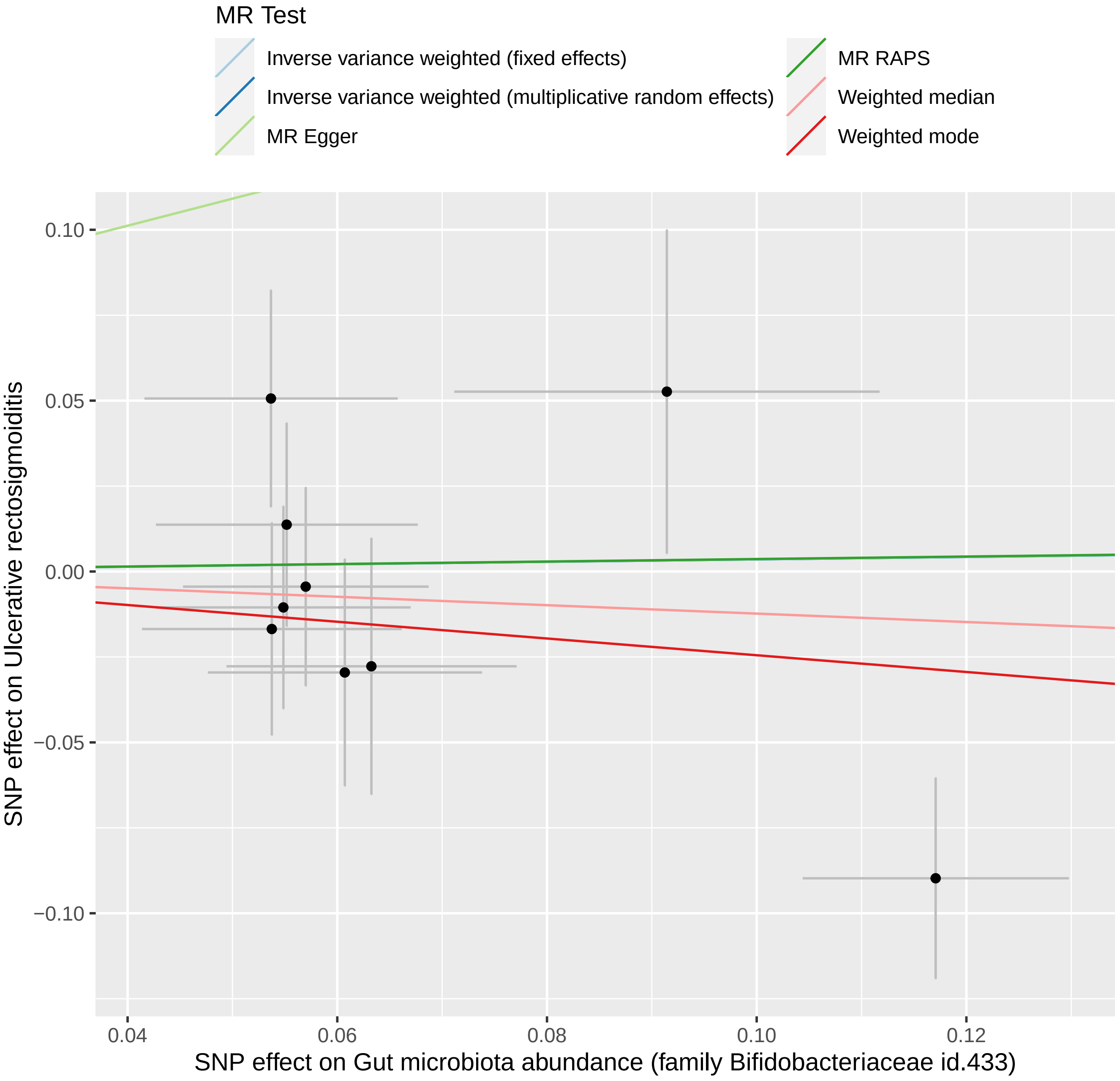

Batch 1508 : Gut microbiota abundance (family Clostridiaceae1 id.1869) on Ulcerative rectosigmoiditis

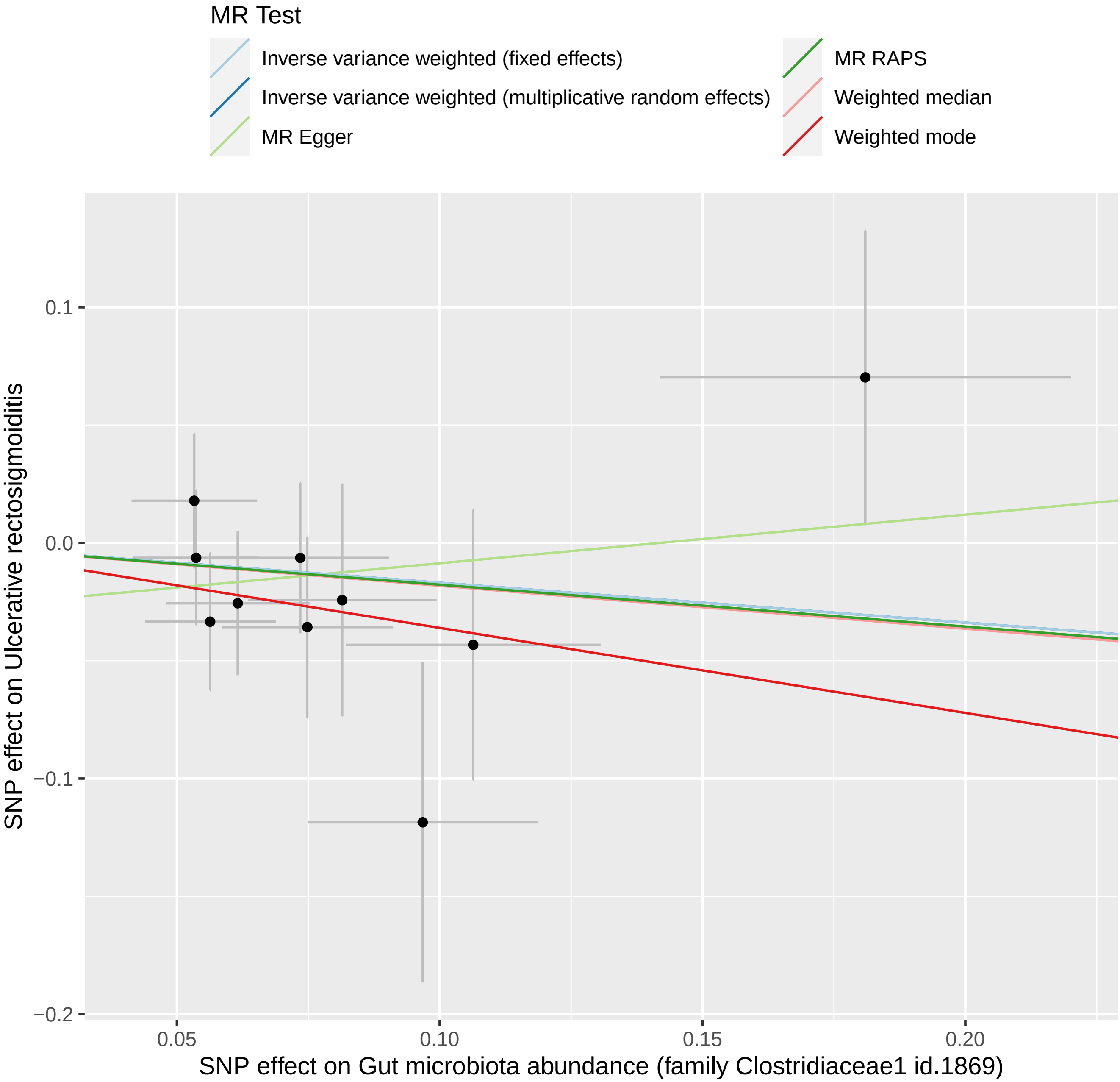

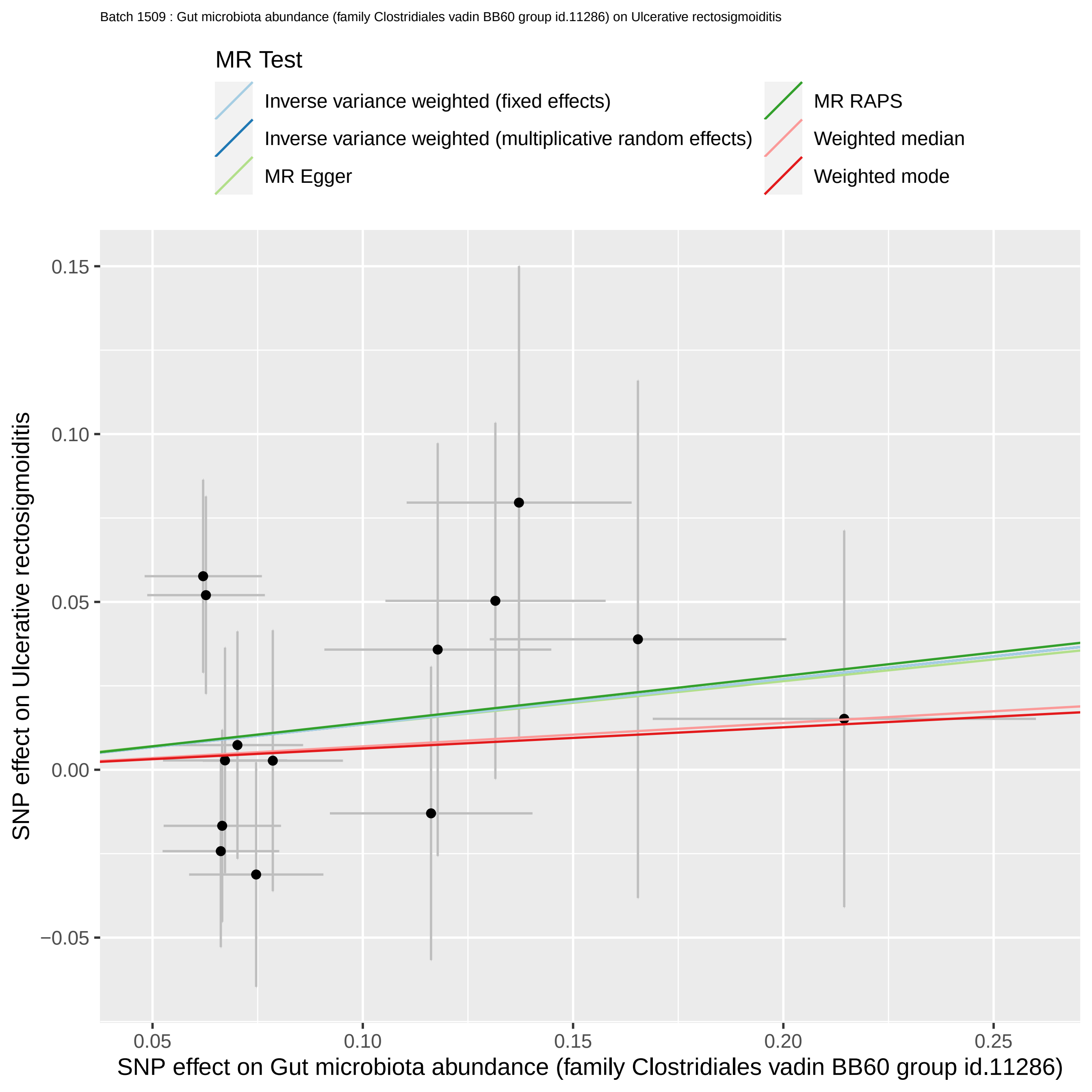
Batch 1509 : Gut microbiota abundance (family Clostridiales vadin BB60 group id.11286) on Ulcerative rectosigmoiditis
MR Test
Inverse variance weighted (fixed effects)
Inverse variance weighted (multiplicative random effects)
MR Egger
MR RAPS
Weighted median
Weighted mode
0.15
0.10
0.05
0.00
−0.05
0.05
0.10
0.15
0.20
0.25
SNP effect on Ulcerative rectosigmoiditis
SNP effect on Gut microbiota abundance (family Clostridiales vadin BB60 group id.11286)

Batch 1510 : Gut microbiota abundance (family Coriobacteriaceae id.811) on Ulcerative rectosigmoiditis

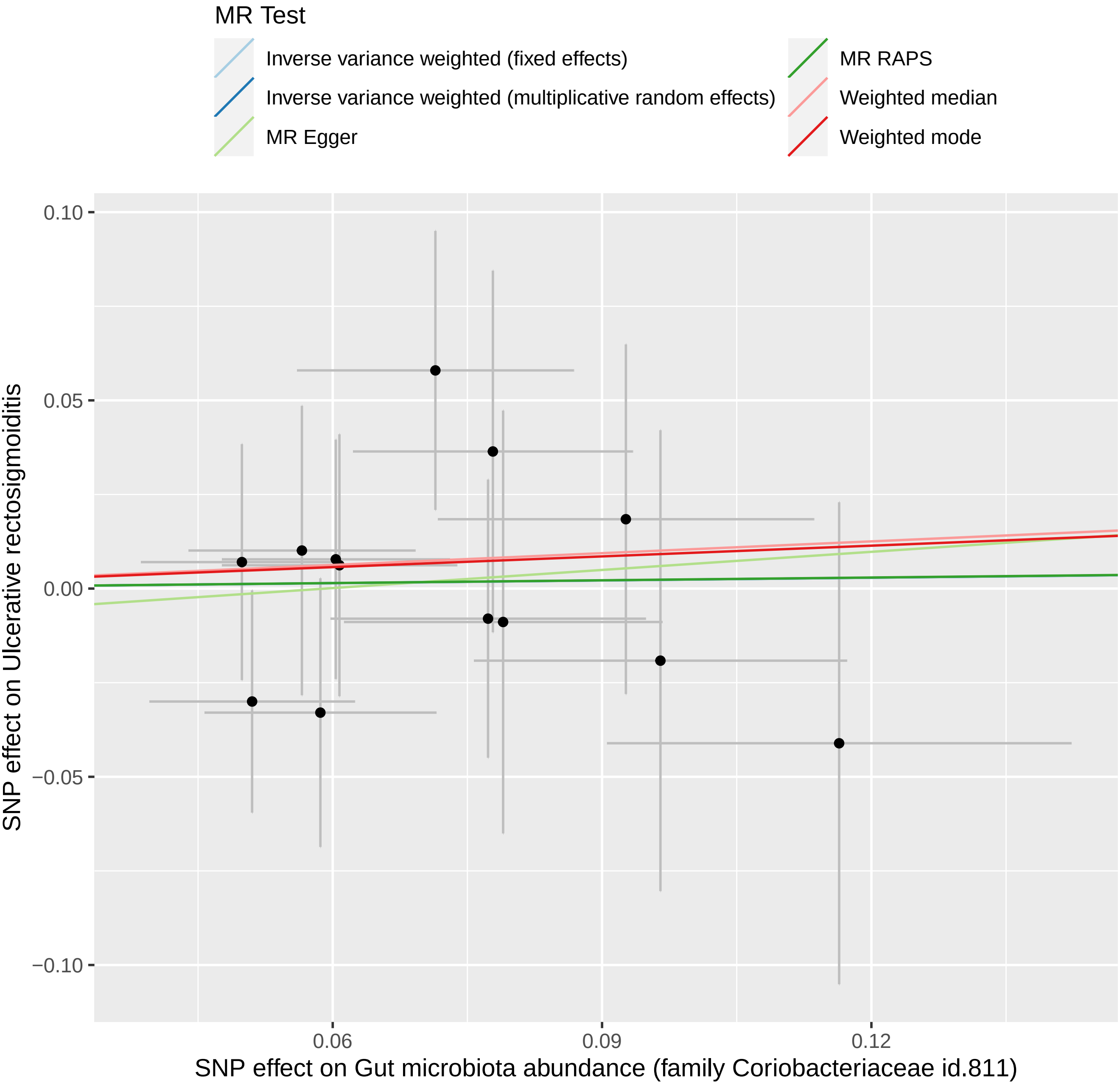

Batch 1511 : Gut microbiota abundance (family Defluviitaleaceae id.1924) on Ulcerative rectosigmoiditis

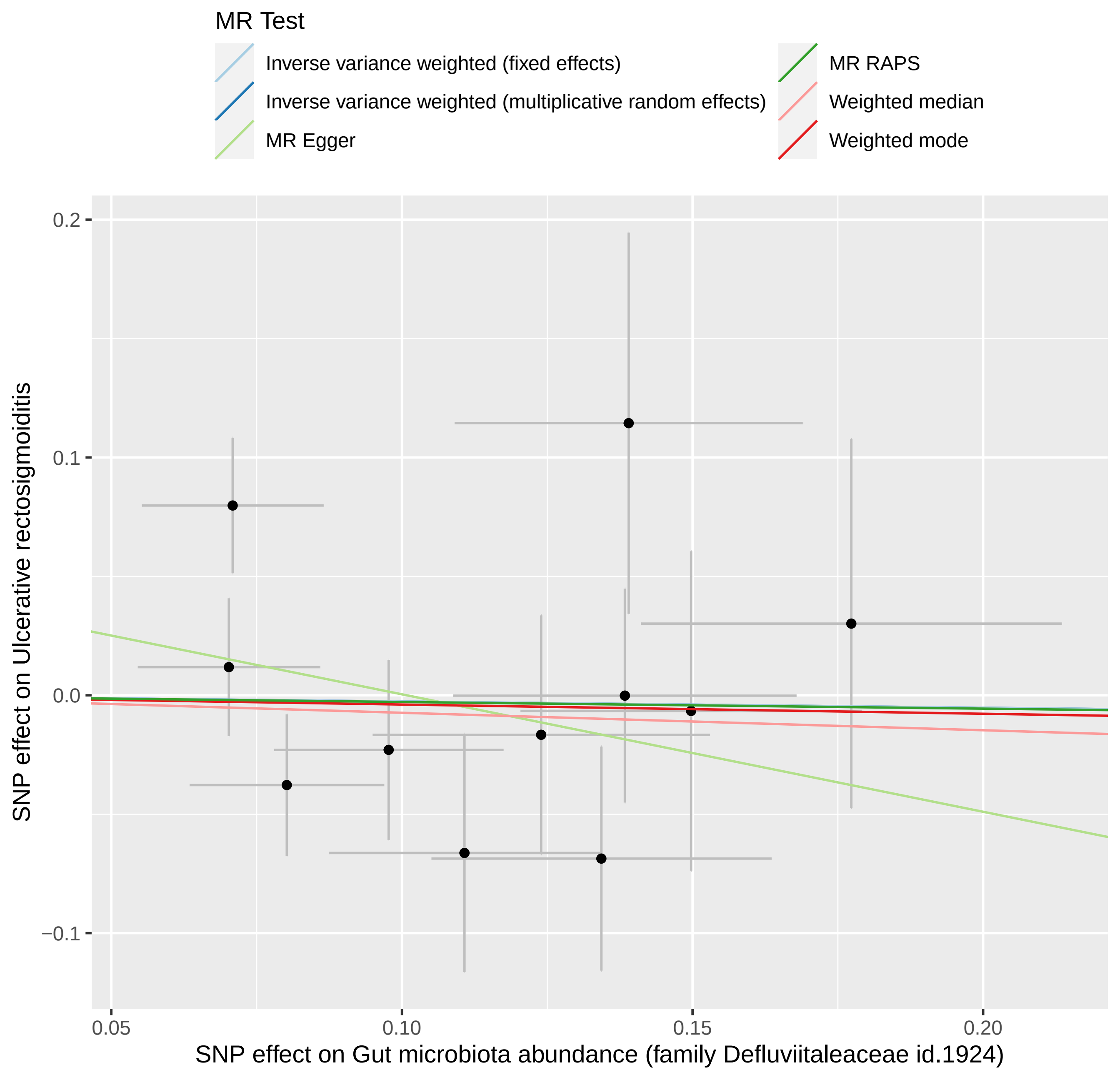

Batch 1512 : Gut microbiota abundance (family Desulfovibrionaceae id.3169) on Ulcerative rectosigmoiditis

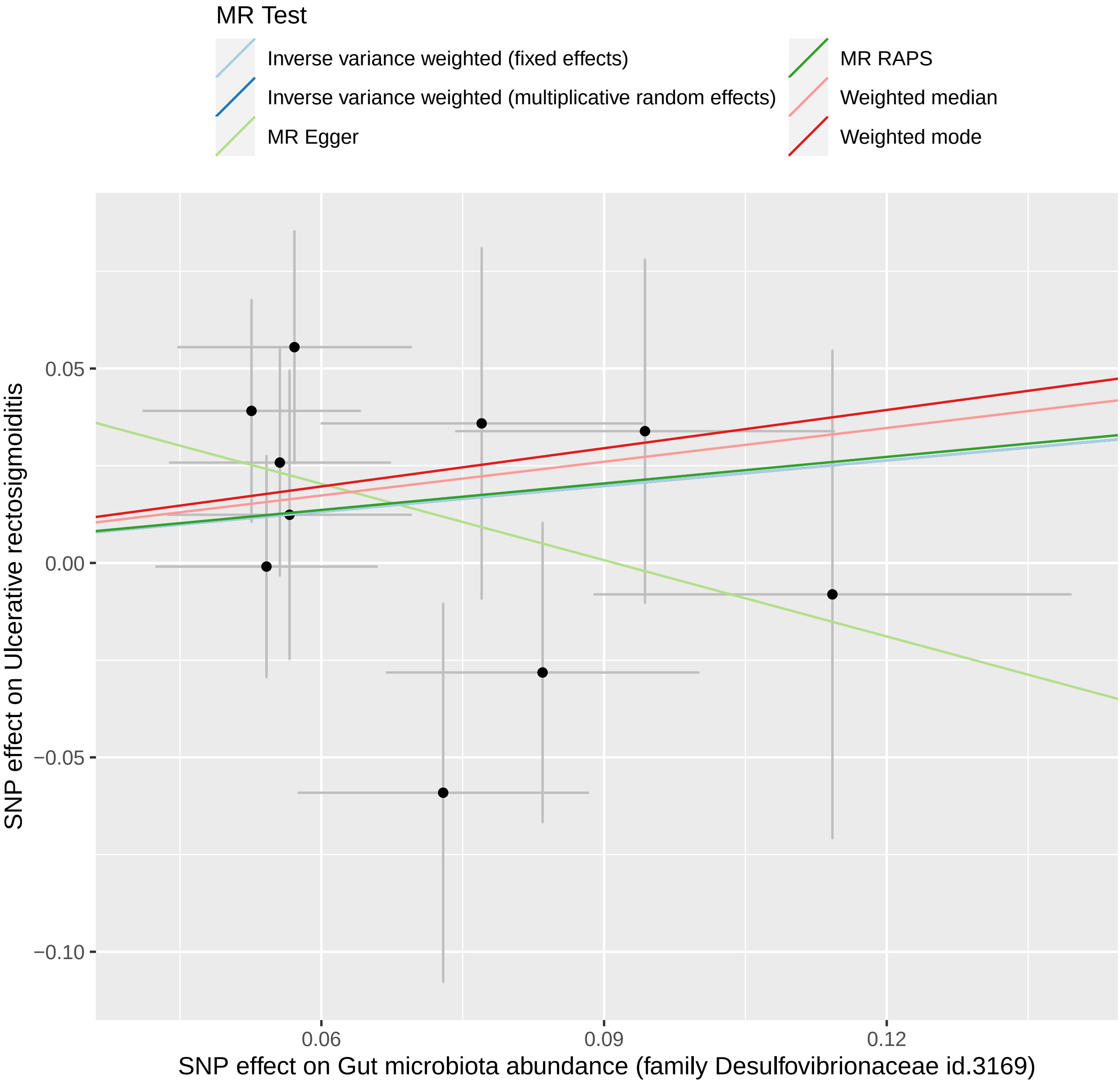

Batch 1513 : Gut microbiota abundance (family Enterobacteriaceae id.3469) on Ulcerative rectosigmoiditis

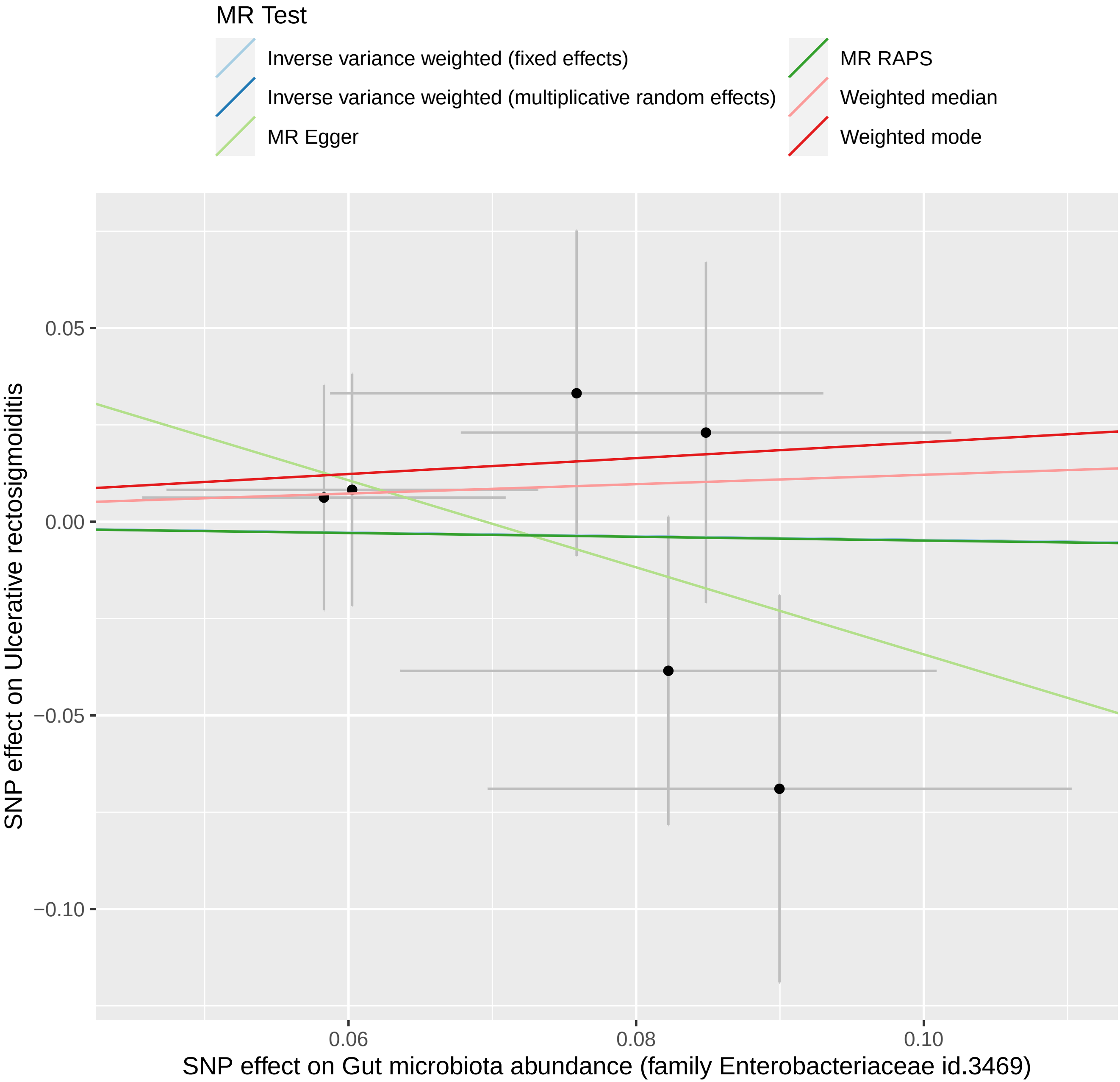

Batch 1514 : Gut microbiota abundance (family Erysipelotrichaceae id.2149) on Ulcerative rectosigmoiditis

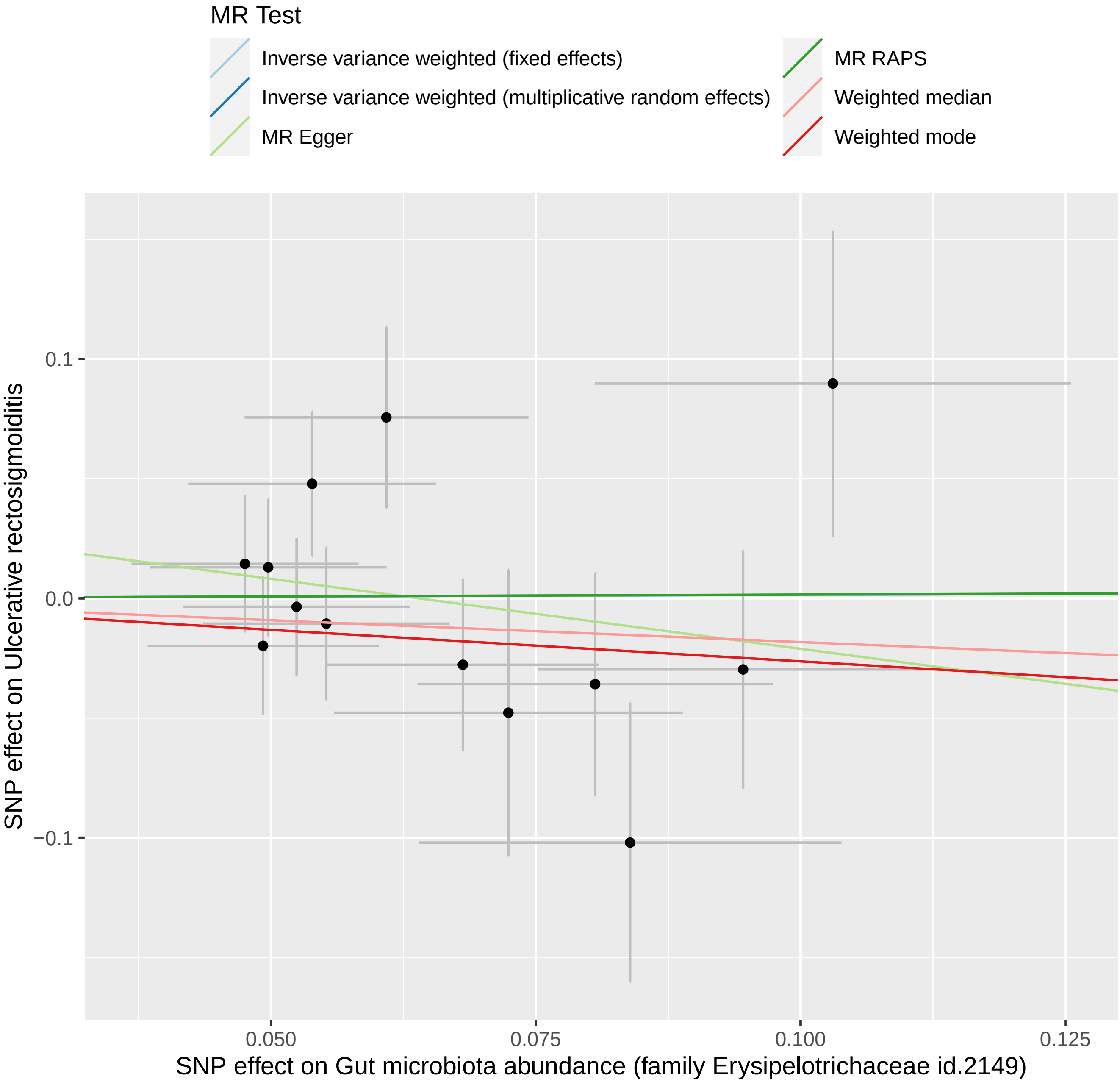

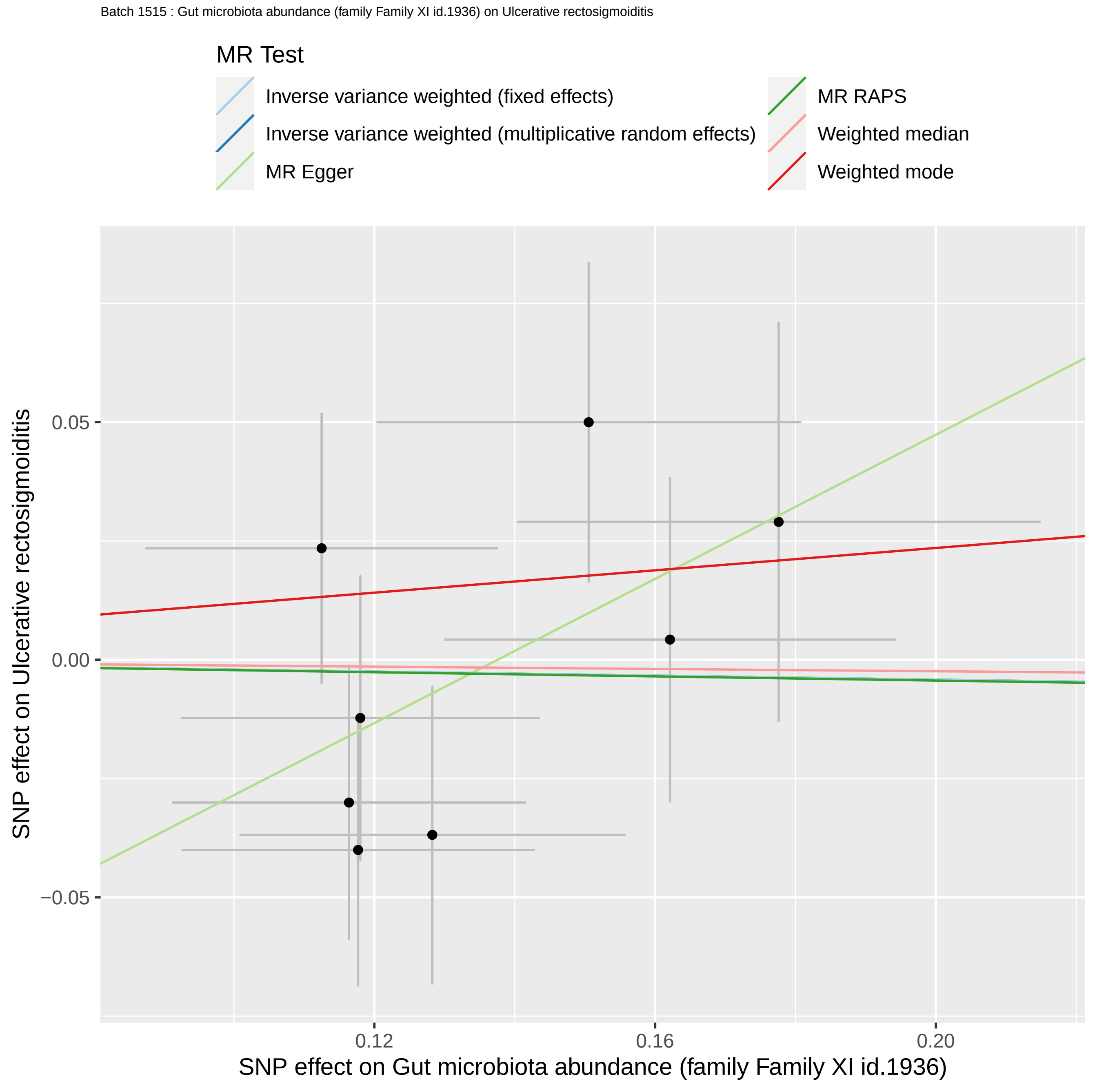

Batch 1515 : Gut microbiota abundance (family Family XI id.1936) on Ulcerative rectosigmoiditis
MR Test
Inverse variance weighted (fixed effects)
Inverse variance weighted (multiplicative random effects)
MR Egger
MR RAPS
Weighted median
Weighted mode
0.05
0.00
−0.05
0.12
0.16
0.20
SNP effect on Ulcerative rectosigmoiditis
SNP effect on Gut microbiota abundance (family Family XI id.1936)

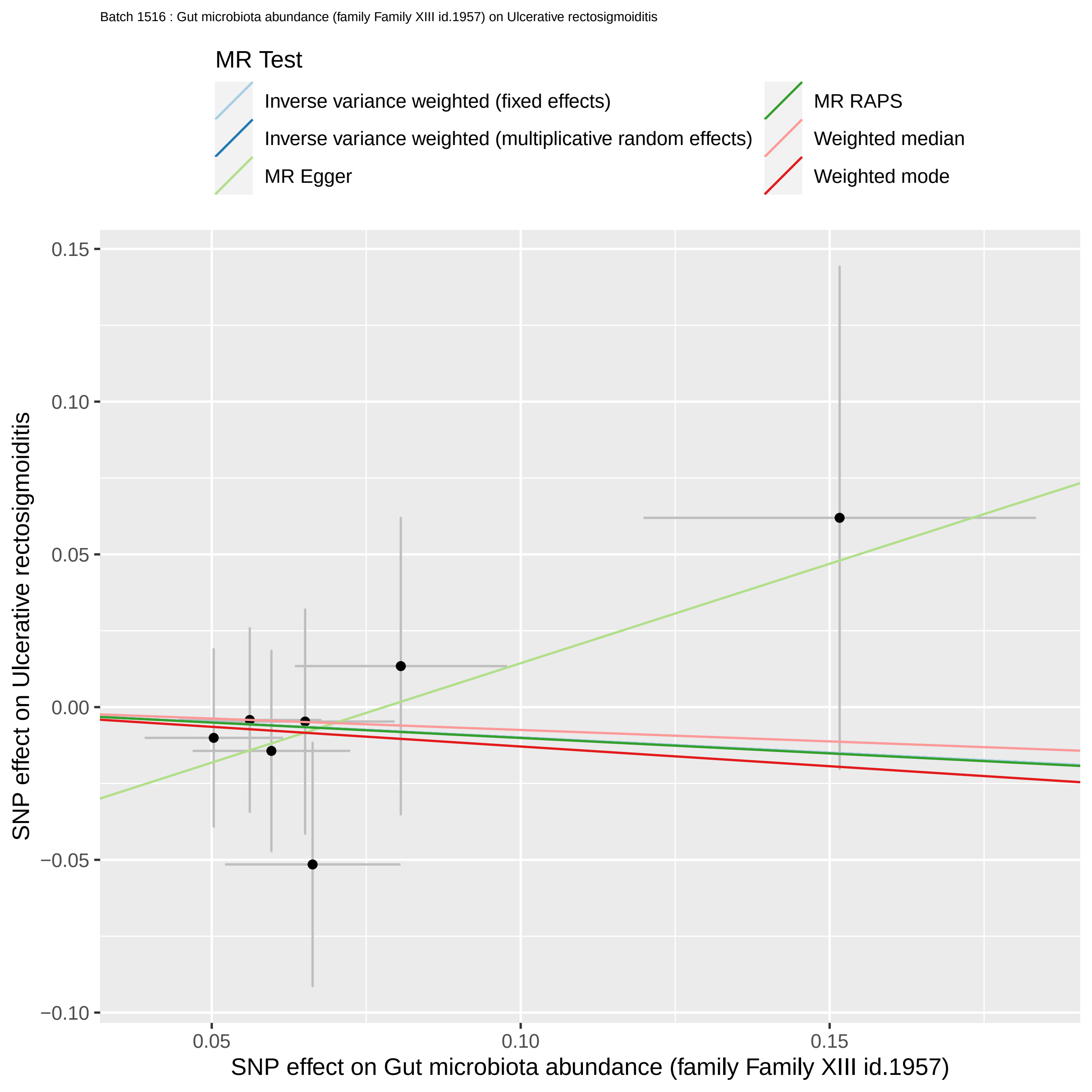

Batch 1516 : Gut microbiota abundance (family Family XIII id.1957) on Ulcerative rectosigmoiditis
MR Test
Inverse variance weighted (fixed effects)
Inverse variance weighted (multiplicative random effects)
MR Egger
MR RAPS
Weighted median
Weighted mode
0.15
0.10
0.05
0.00
−0.05
−0.10
0.05
0.10
0.15
SNP effect on Ulcerative rectosigmoiditis
SNP effect on Gut microbiota abundance (family Family XIII id.1957)

Batch 1517 : Gut microbiota abundance (family Lachnospiraceae id.1987) on Ulcerative rectosigmoiditis

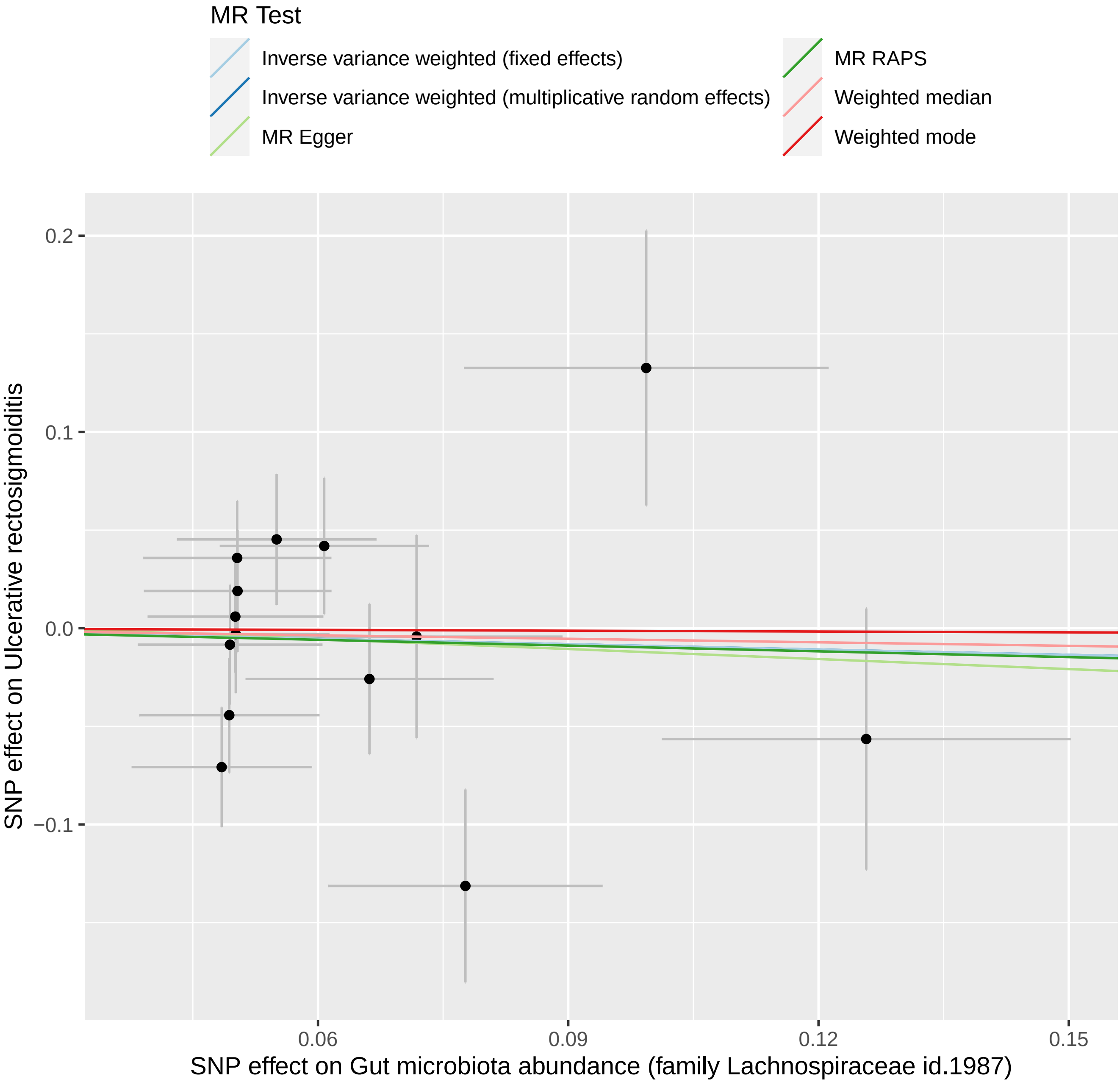

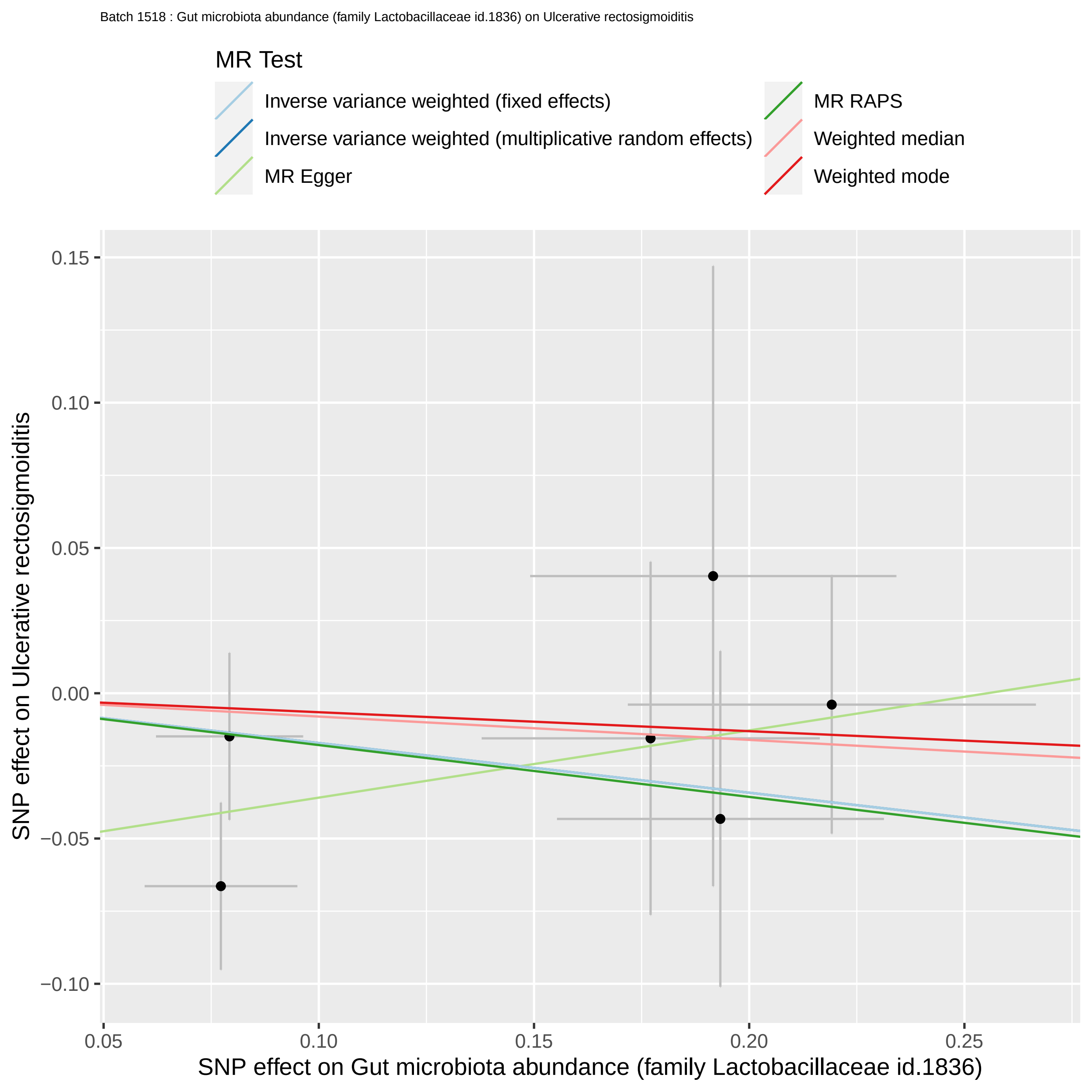

Batch 1518 : Gut microbiota abundance (family Lactobacillaceae id.1836) on Ulcerative rectosigmoiditis
MR Test
Inverse variance weighted (fixed effects)
Inverse variance weighted (multiplicative random effects)
MR Egger
MR RAPS
Weighted median
Weighted mode
SNP effect on Ulcerative rectosigmoiditis
0.15
0.10
0.05
0.00
−0.05
−0.10
0.05
0.10
0.15
0.20
0.25
SNP effect on Gut microbiota abundance (family Lactobacillaceae id.1836)

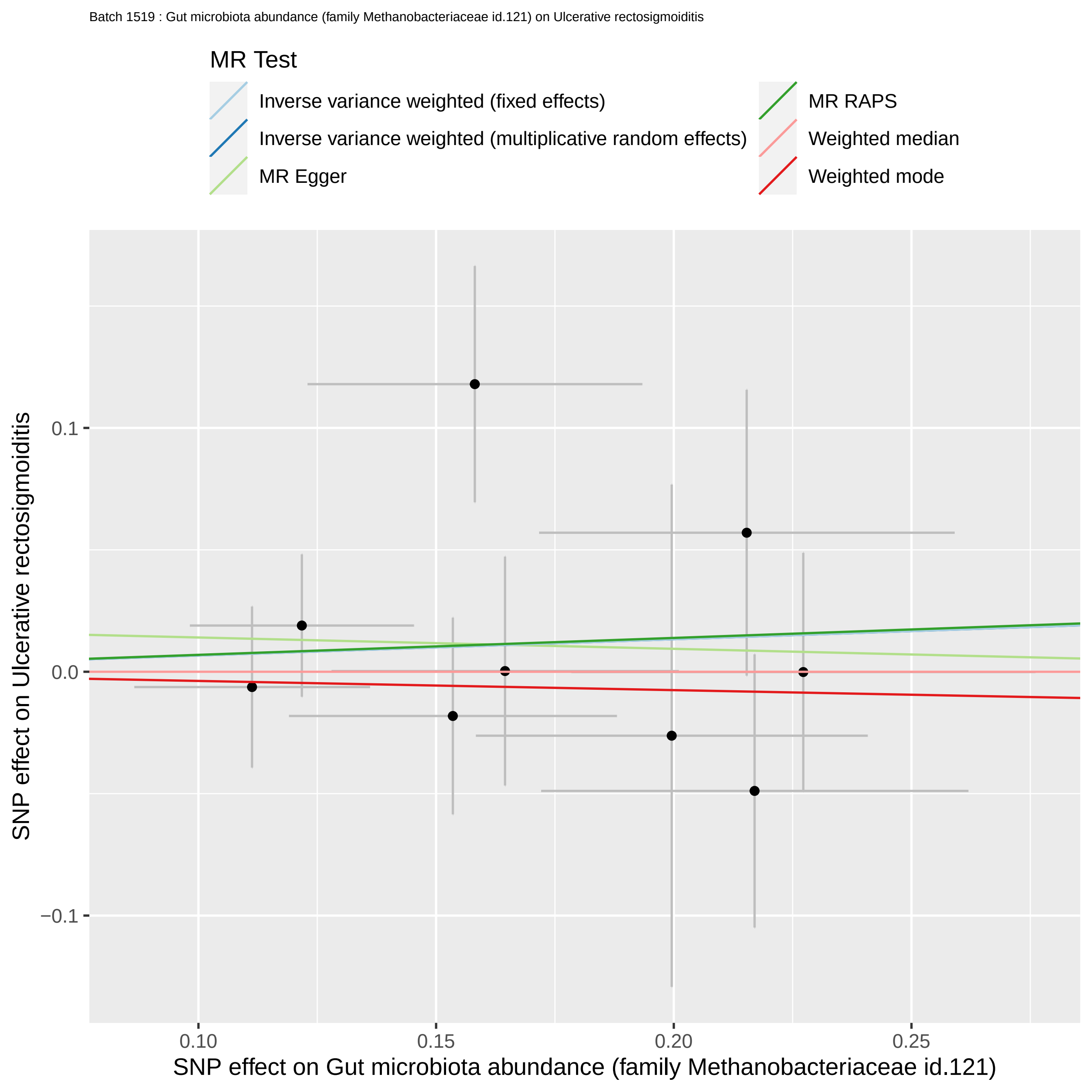
Batch 1519 : Gut microbiota abundance (family Methanobacteriaceae id.121) on Ulcerative rectosigmoiditis
MR Test
Inverse variance weighted (fixed effects)
Inverse variance weighted (multiplicative random effects)
MR Egger
MR RAPS
Weighted median
Weighted mode
0.1
0.0
−0.1
0.10
0.15
0.20
0.25
SNP effect on Ulcerative rectosigmoiditis
SNP effect on Gut microbiota abundance (family Methanobacteriaceae id.121)

Batch 1520 : Gut microbiota abundance (family Oxalobacteraceae id.2966) on Ulcerative rectosigmoiditis

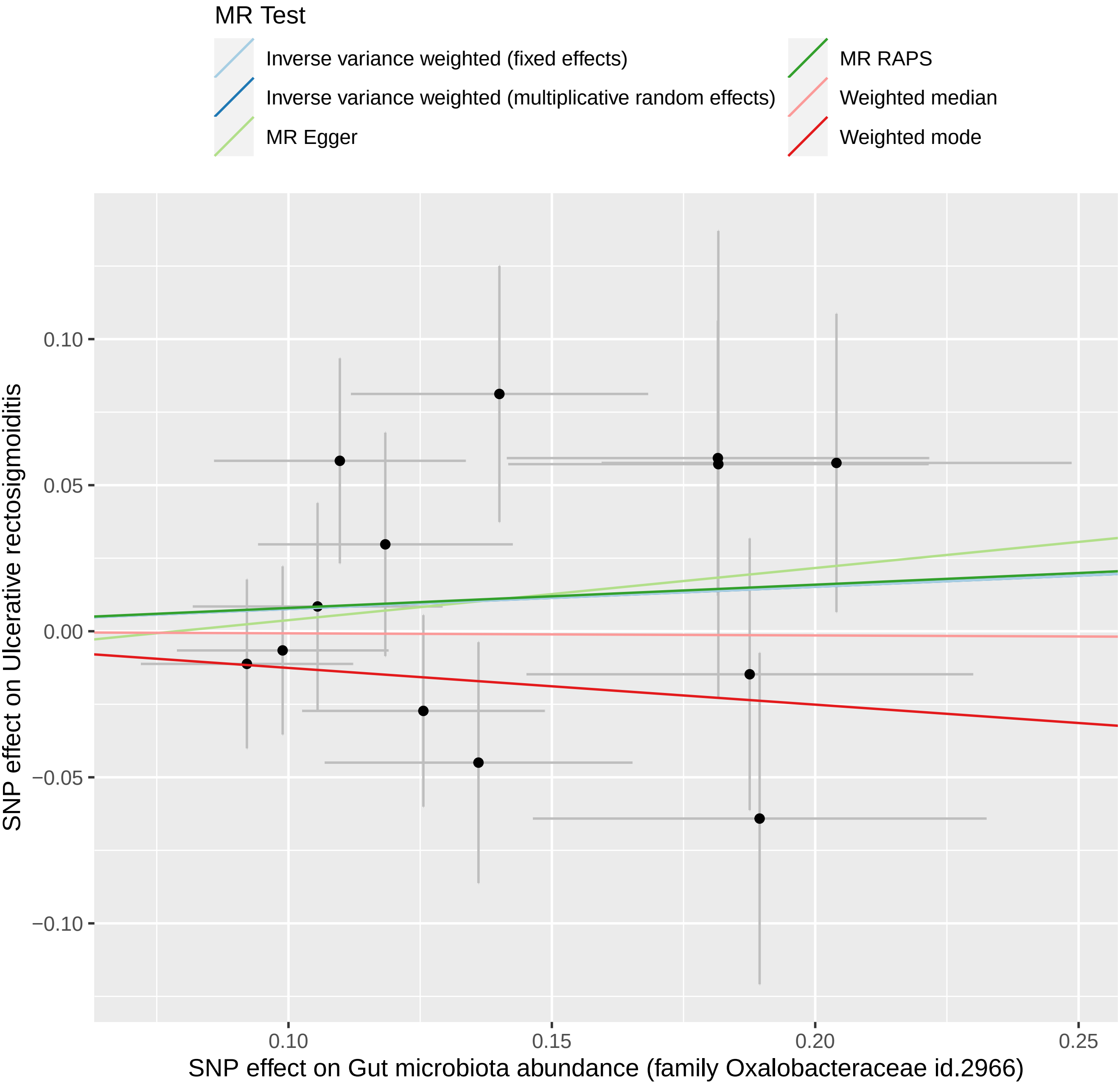

Batch 1521 : Gut microbiota abundance (family Pasteurellaceae id.3689) on Ulcerative rectosigmoiditis

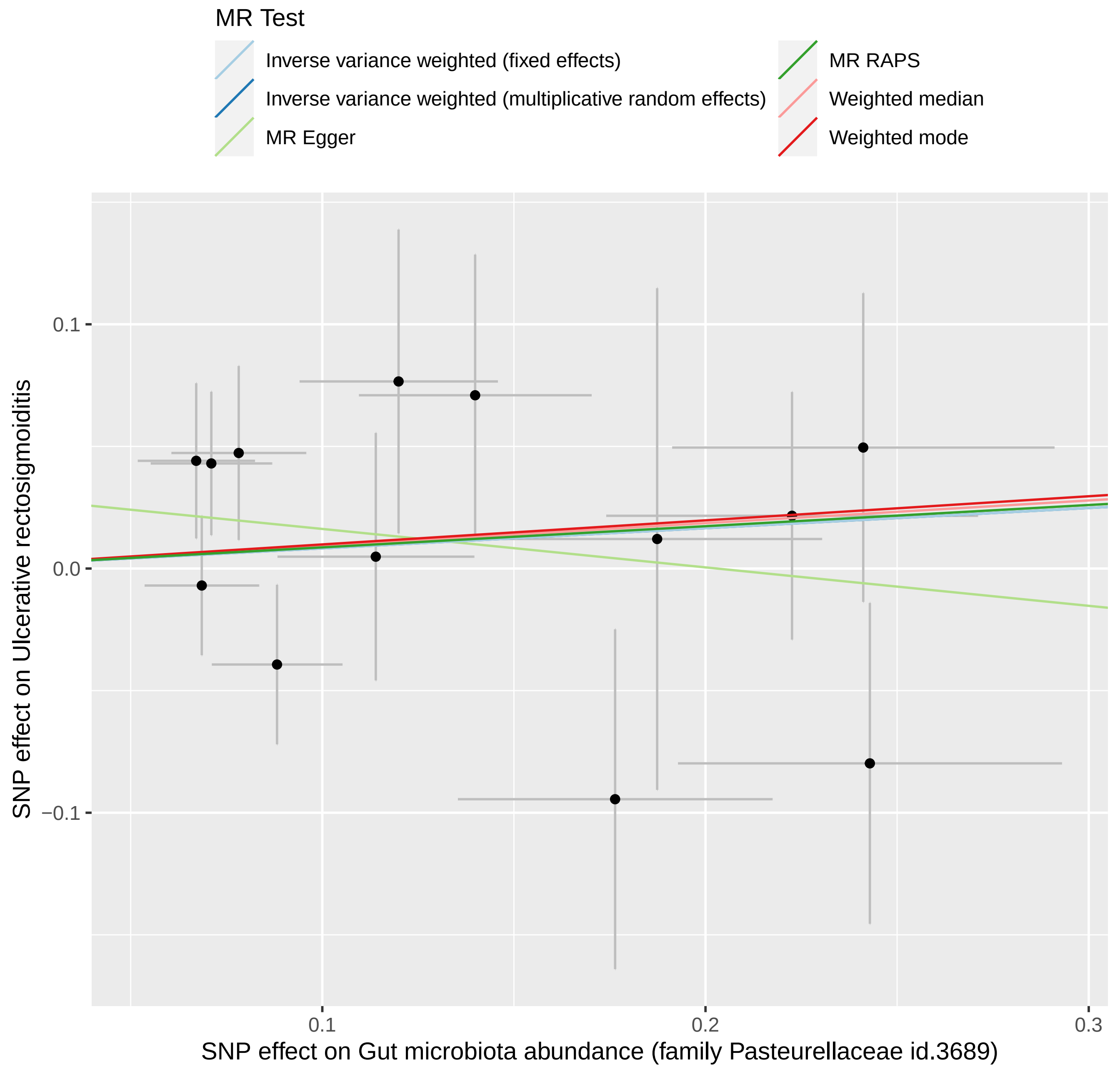

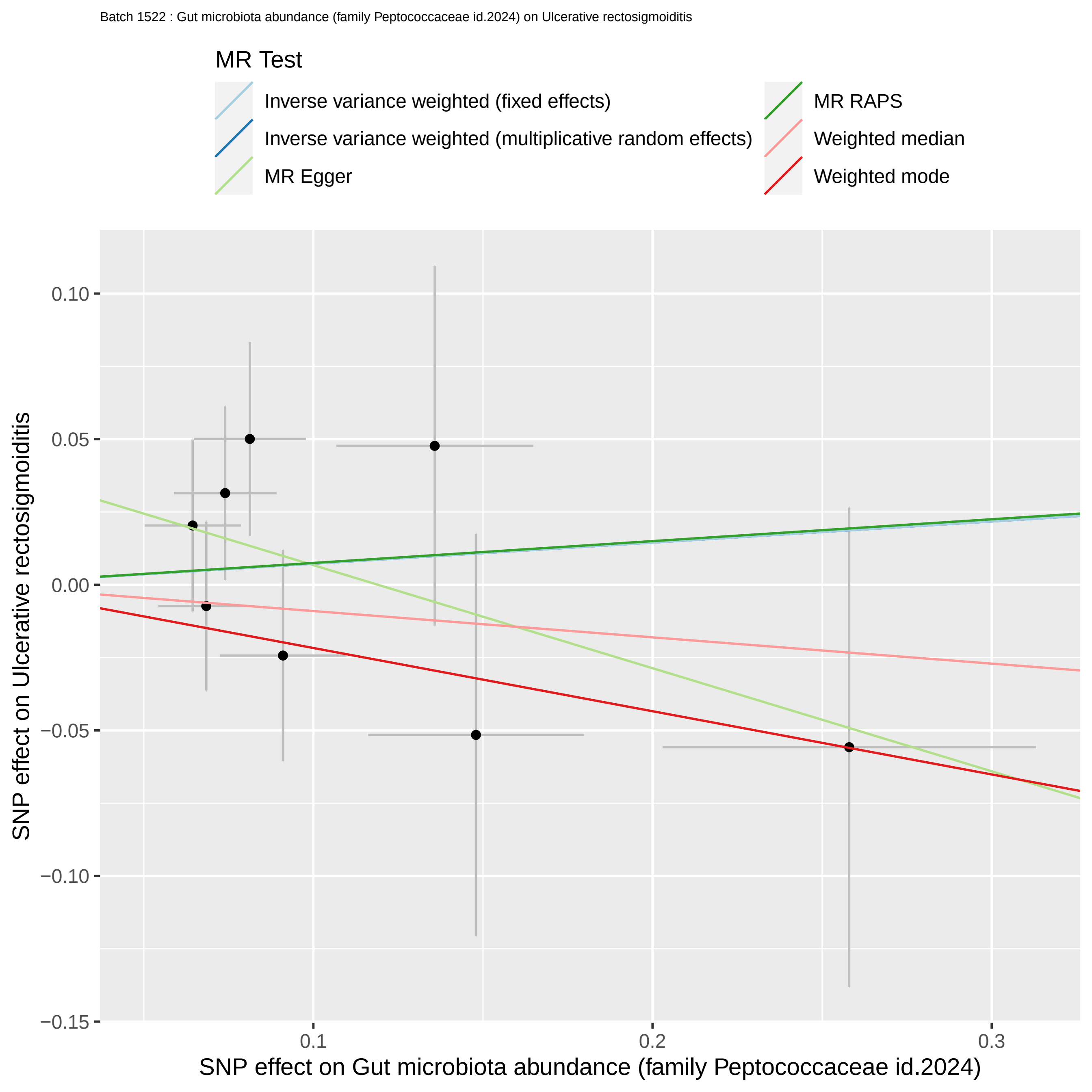
Batch 1522 : Gut microbiota abundance (family Peptococcaceae id.2024) on Ulcerative rectosigmoiditis
MR Test
Inverse variance weighted (fixed effects)
Inverse variance weighted (multiplicative random effects)
MR Egger
MR RAPS
Weighted median
Weighted mode
SNP effect on Ulcerative rectosigmoiditis
0.10
0.05
0.00
−0.05
−0.10
−0.15
0.1
0.2
0.3
SNP effect on Gut microbiota abundance (family Peptococcaceae id.2024)

Batch 1523 : Gut microbiota abundance (family Peptostreptococcaceae id.2042) on Ulcerative rectosigmoiditis

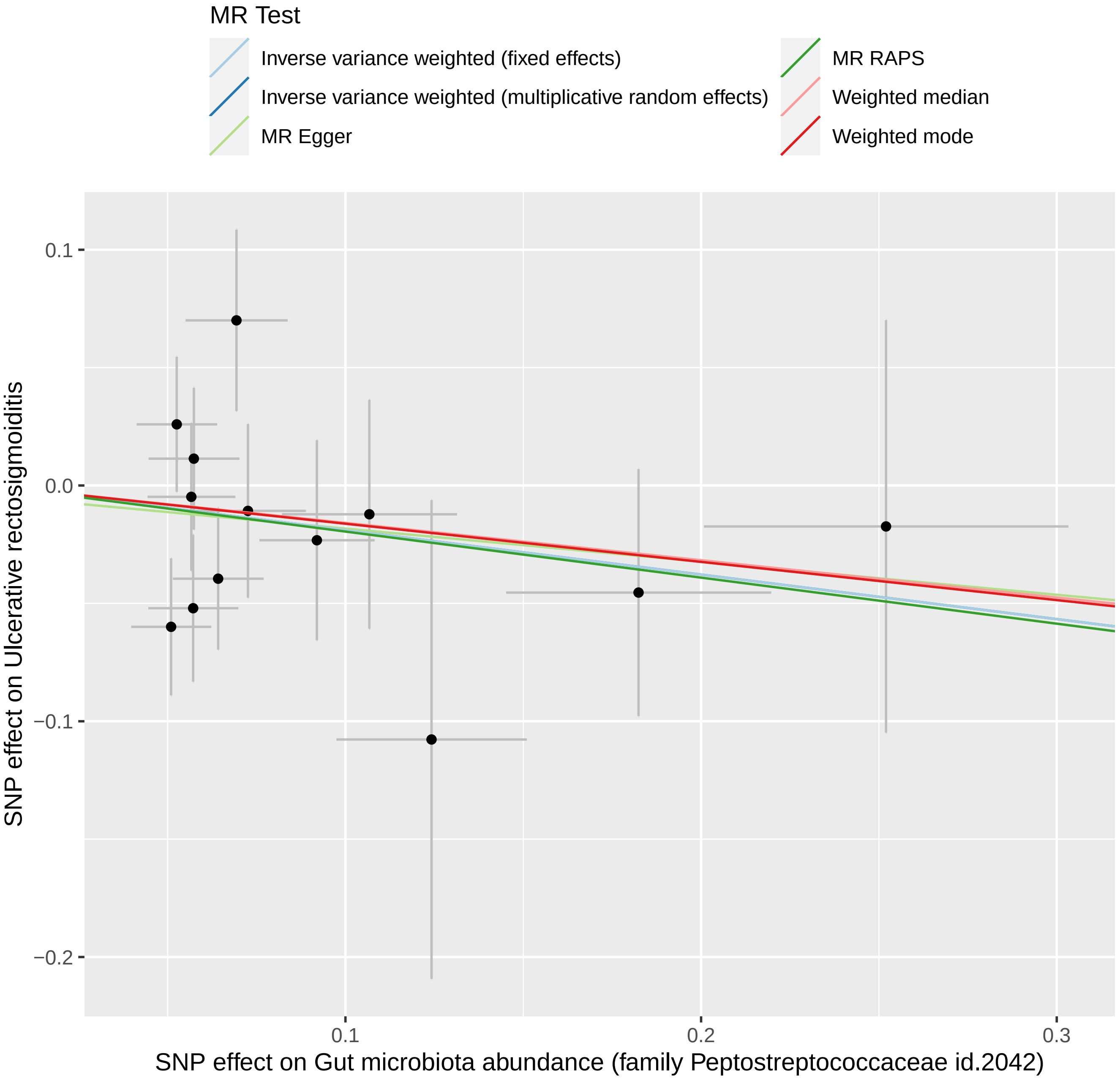

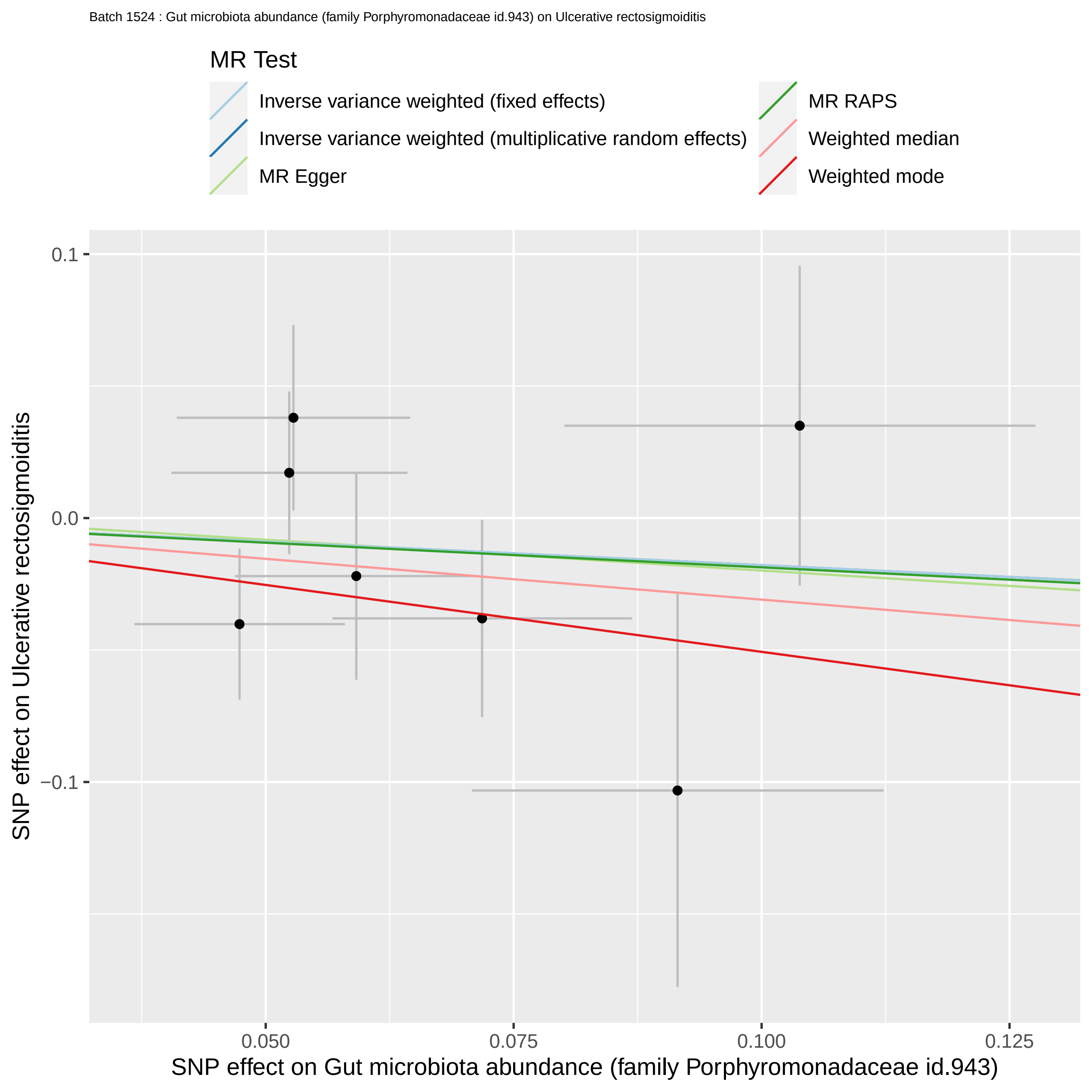

Batch 1524 : Gut microbiota abundance (family Porphyromonadaceae id.943) on Ulcerative rectosigmoiditis
MR Test
Inverse variance weighted (fixed effects)
Inverse variance weighted (multiplicative random effects)
MR Egger
MR RAPS
Weighted median
Weighted mode
0.1
0.0
−0.1
0.050
0.075
0.100
0.125
SNP effect on Ulcerative rectosigmoiditis
SNP effect on Gut microbiota abundance (family Porphyromonadaceae id.943)

Batch 1525 : Gut microbiota abundance (family Prevotellaceae id.960) on Ulcerative rectosigmoiditis

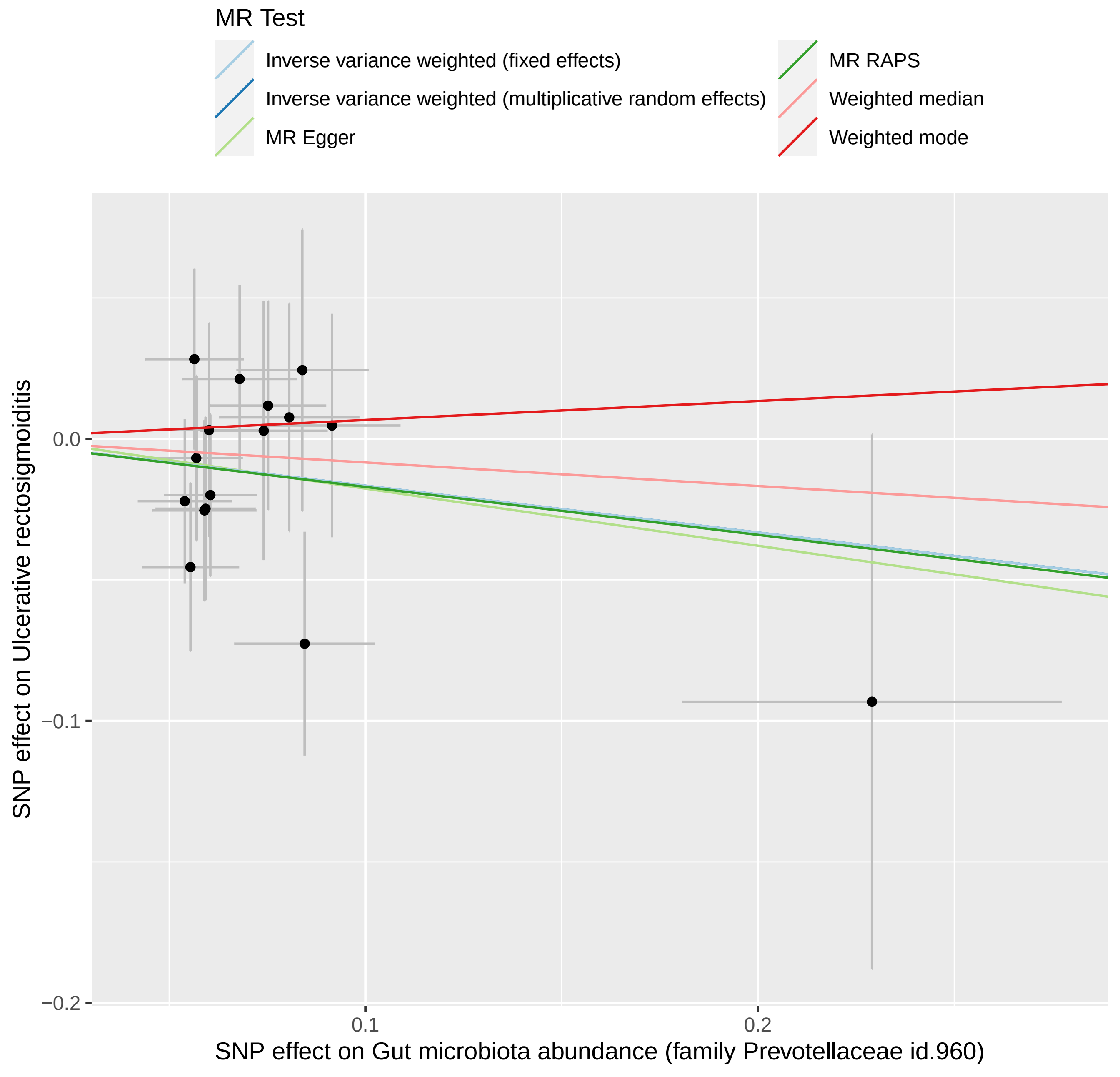

Batch 1526 : Gut microbiota abundance (family Rhodospirillaceae id.2717) on Ulcerative rectosigmoiditis

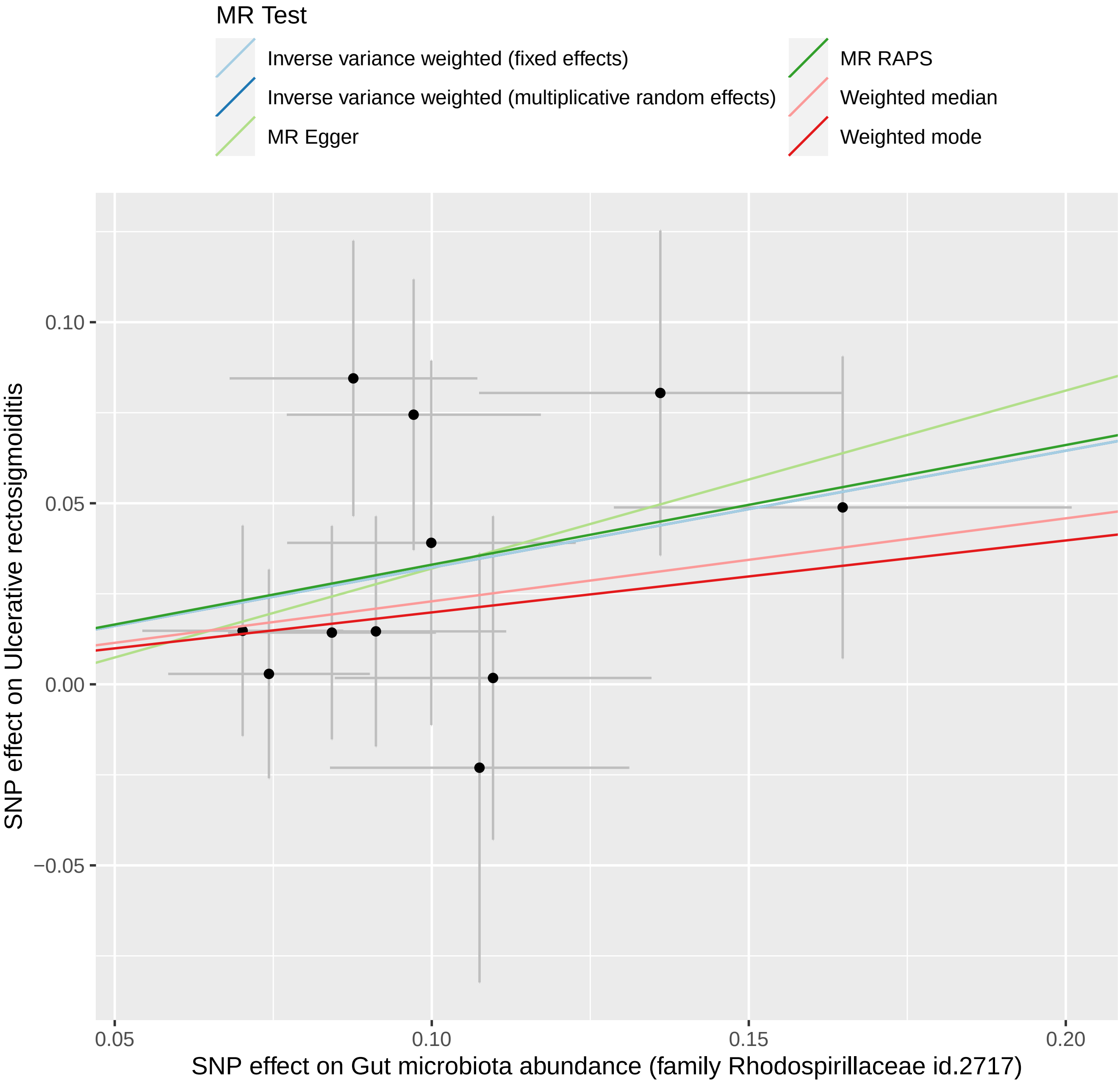

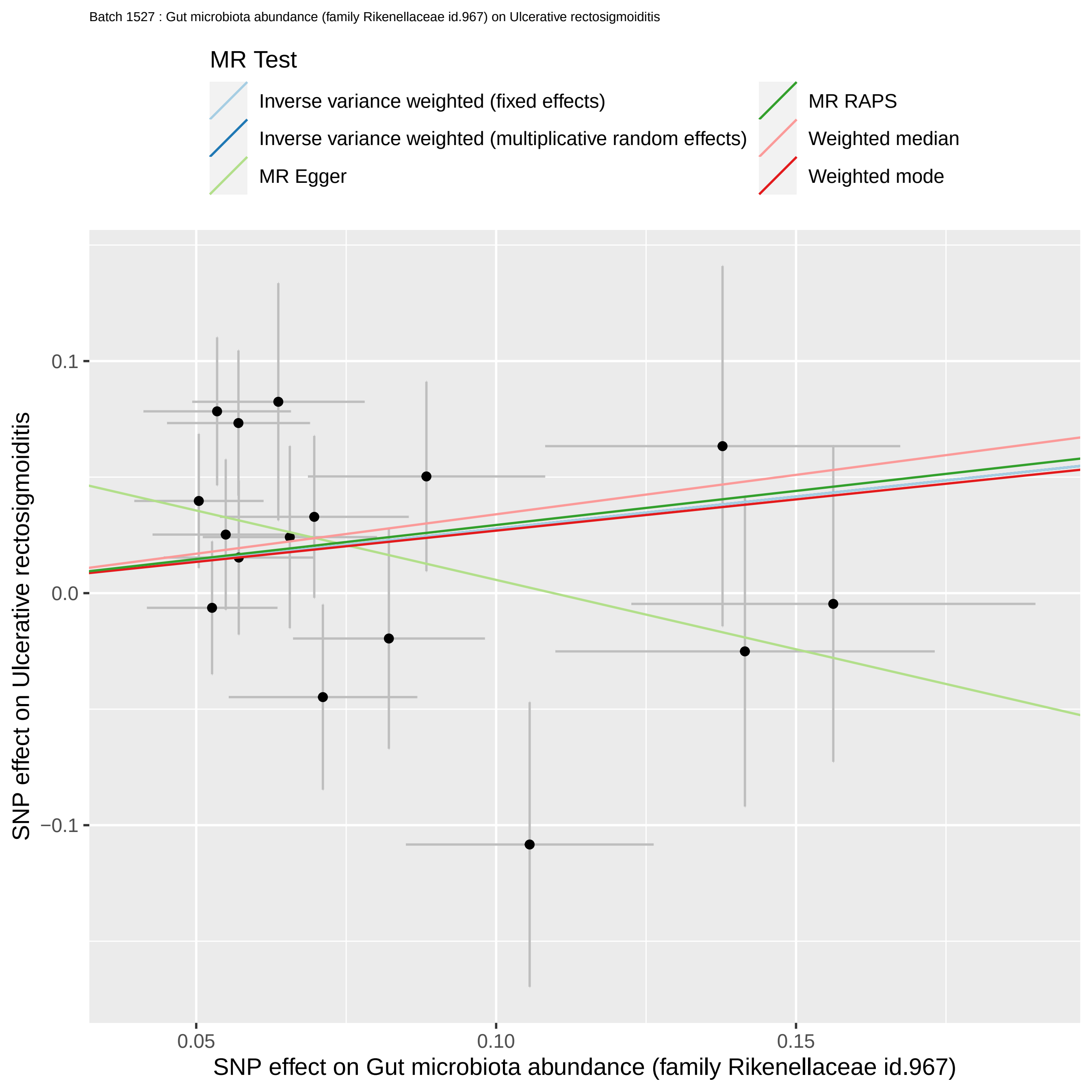

Batch 1527 : Gut microbiota abundance (family Rikenellaceae id.967) on Ulcerative rectosigmoiditis
MR Test
Inverse variance weighted (fixed effects)
Inverse variance weighted (multiplicative random effects)
MR Egger
MR RAPS
Weighted median
Weighted mode
0.1
0.0
−0.1
0.05
0.10
0.15
SNP effect on Ulcerative rectosigmoiditis
SNP effect on Gut microbiota abundance (family Rikenellaceae id.967)

Batch 1528 : Gut microbiota abundance (family Ruminococcaceae id.2050) on Ulcerative rectosigmoiditis

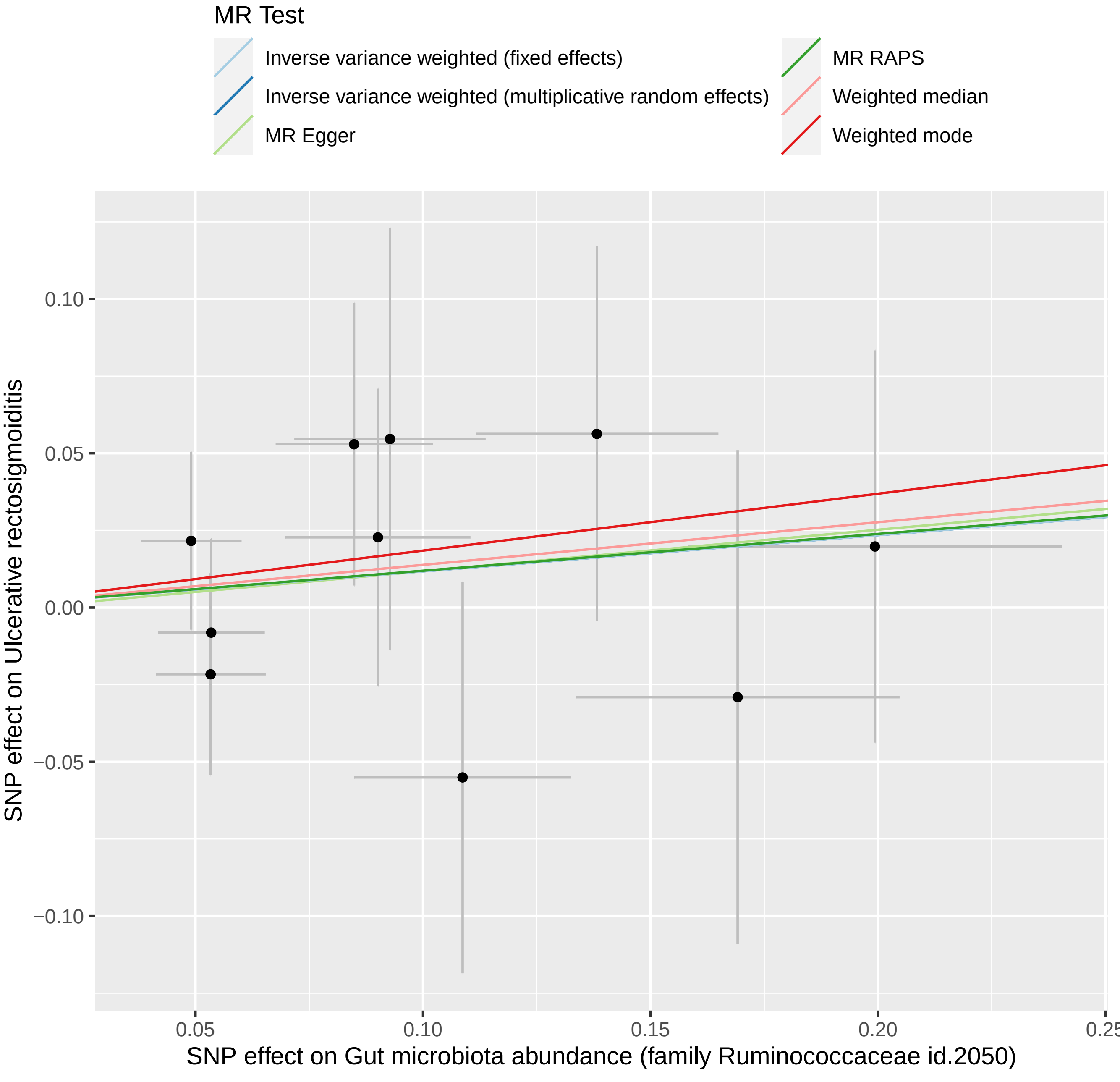

Batch 1529 : Gut microbiota abundance (family Streptococcaceae id.1850) on Ulcerative rectosigmoiditis

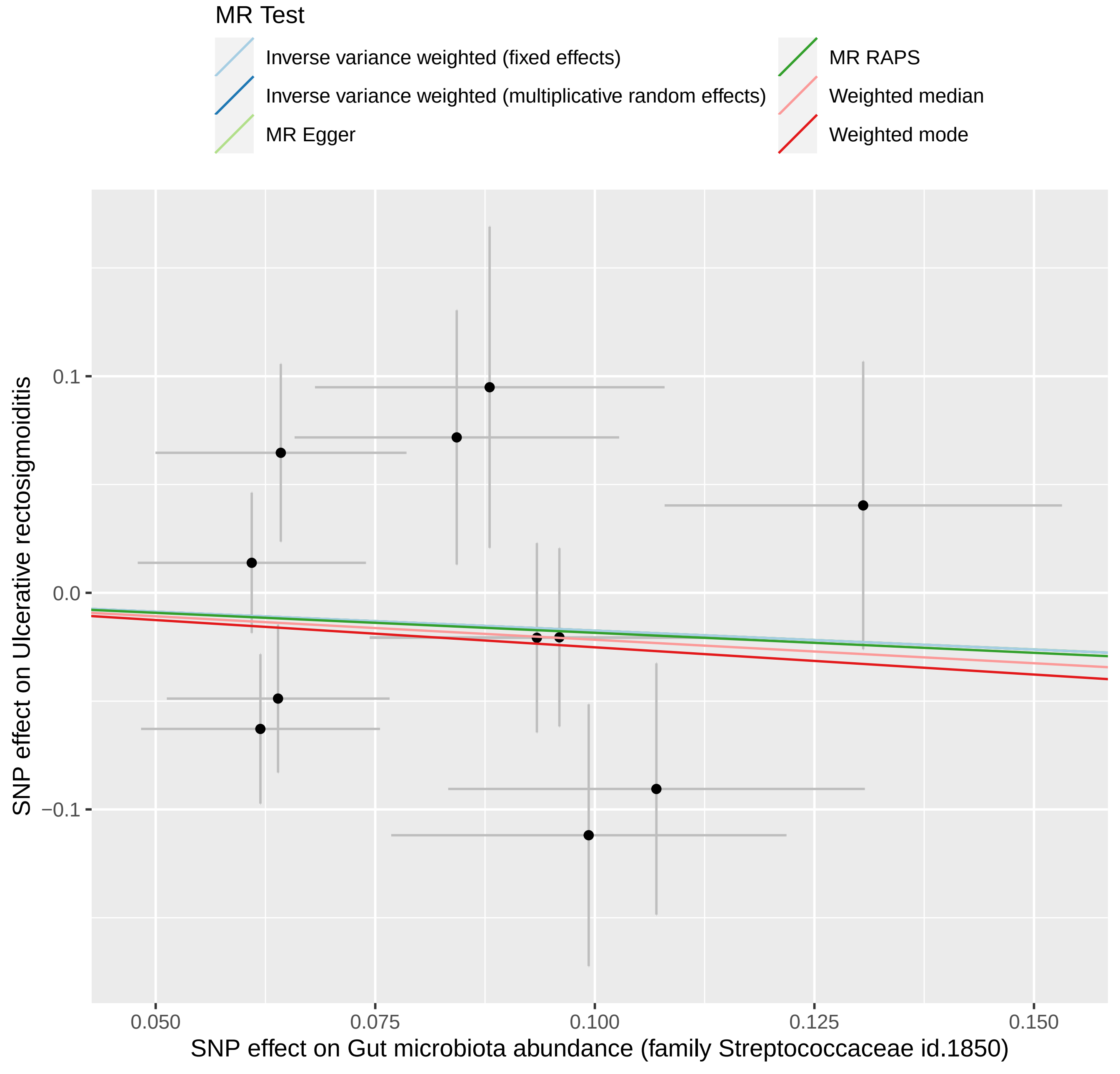

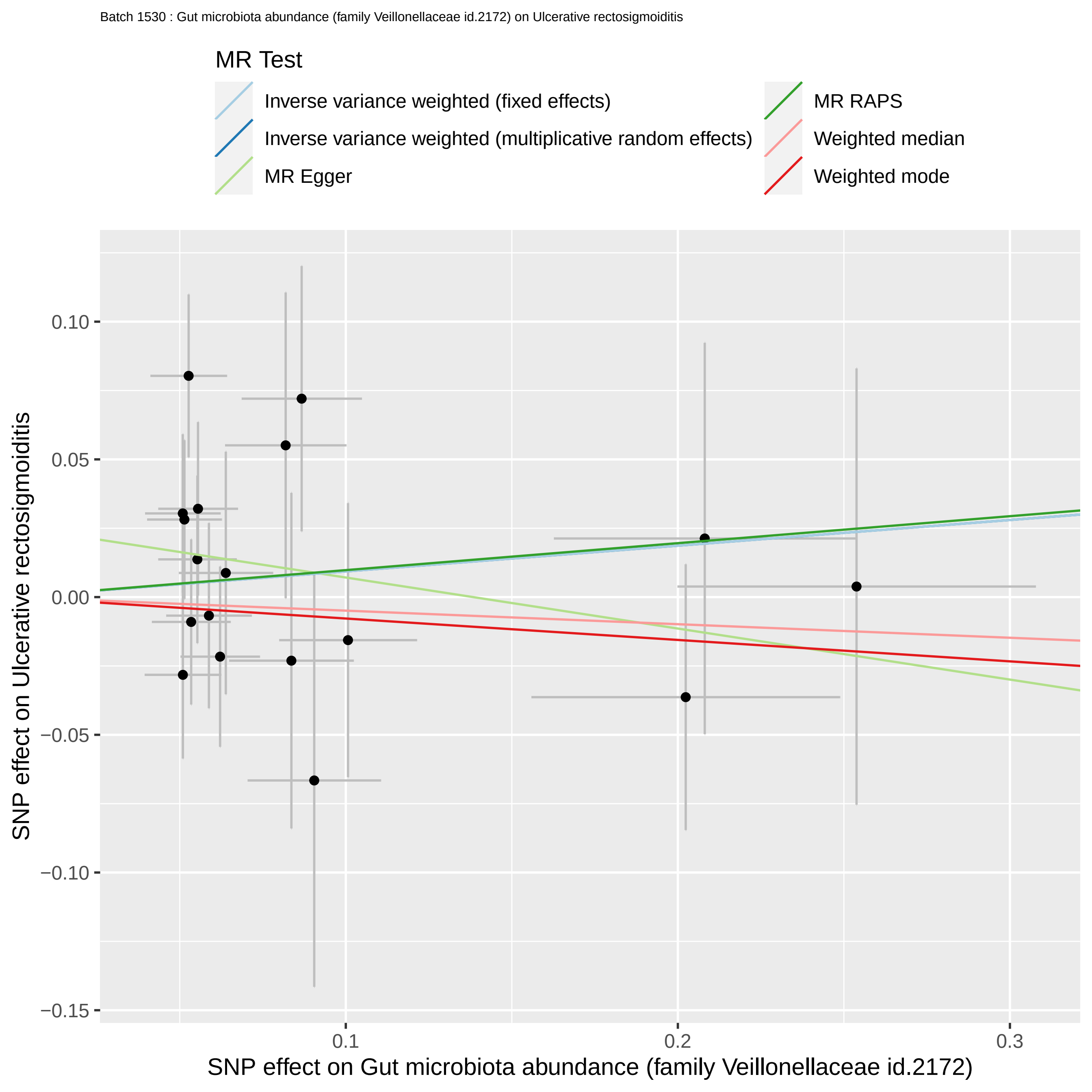

Batch 1530 : Gut microbiota abundance (family Veillonellaceae id.2172) on Ulcerative rectosigmoiditis
MR Test
Inverse variance weighted (fixed effects)
Inverse variance weighted (multiplicative random effects)
MR Egger
MR RAPS
Weighted median
Weighted mode
SNP effect on Ulcerative rectosigmoiditis
0.10
0.05
0.00
−0.05
−0.10
−0.15
0.1
0.2
0.3
SNP effect on Gut microbiota abundance (family Veillonellaceae id.2172)

Batch 1531 : Gut microbiota abundance (family Verrucomicrobiaceae id.4036) on Ulcerative rectosigmoiditis

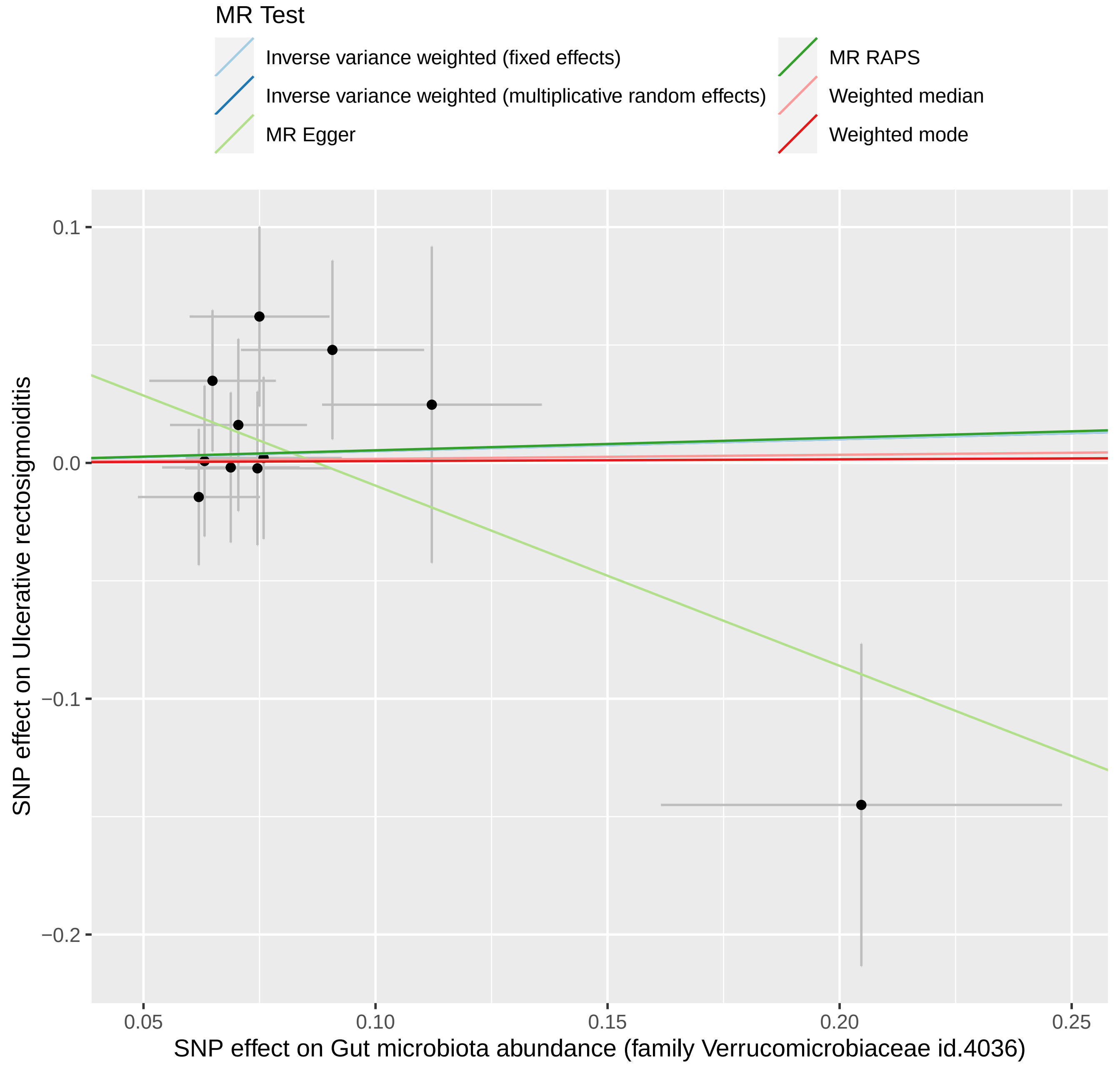

Batch 1532 : Gut microbiota abundance (family Victivallaceae id.2255) on Ulcerative rectosigmoiditis

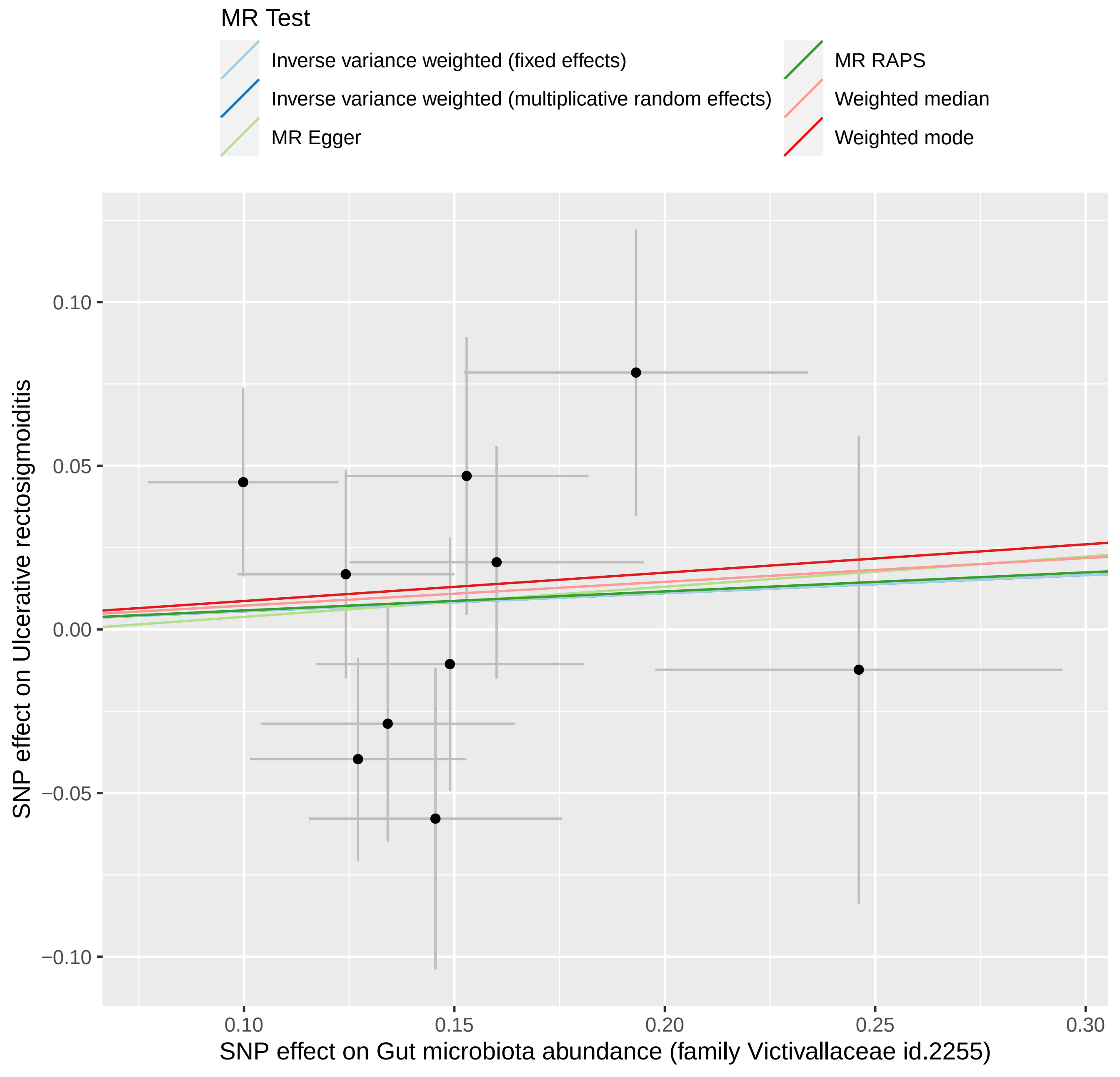

Batch 1533 : Gut microbiota abundance (genus Actinomyces id.423) on Ulcerative rectosigmoiditis

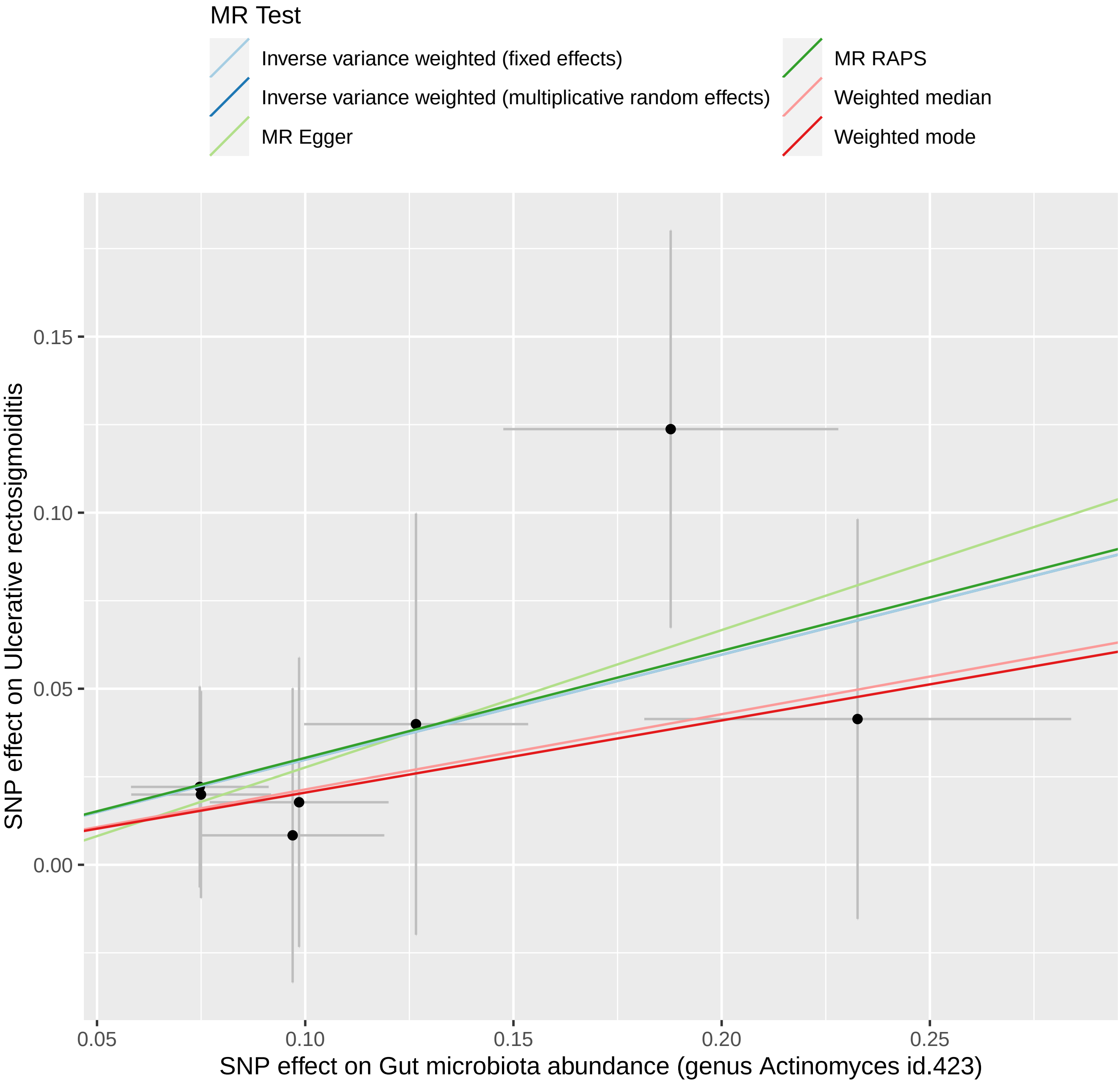

Batch 1534 : Gut microbiota abundance (genus Adlercreutzia id.812) on Ulcerative rectosigmoiditis

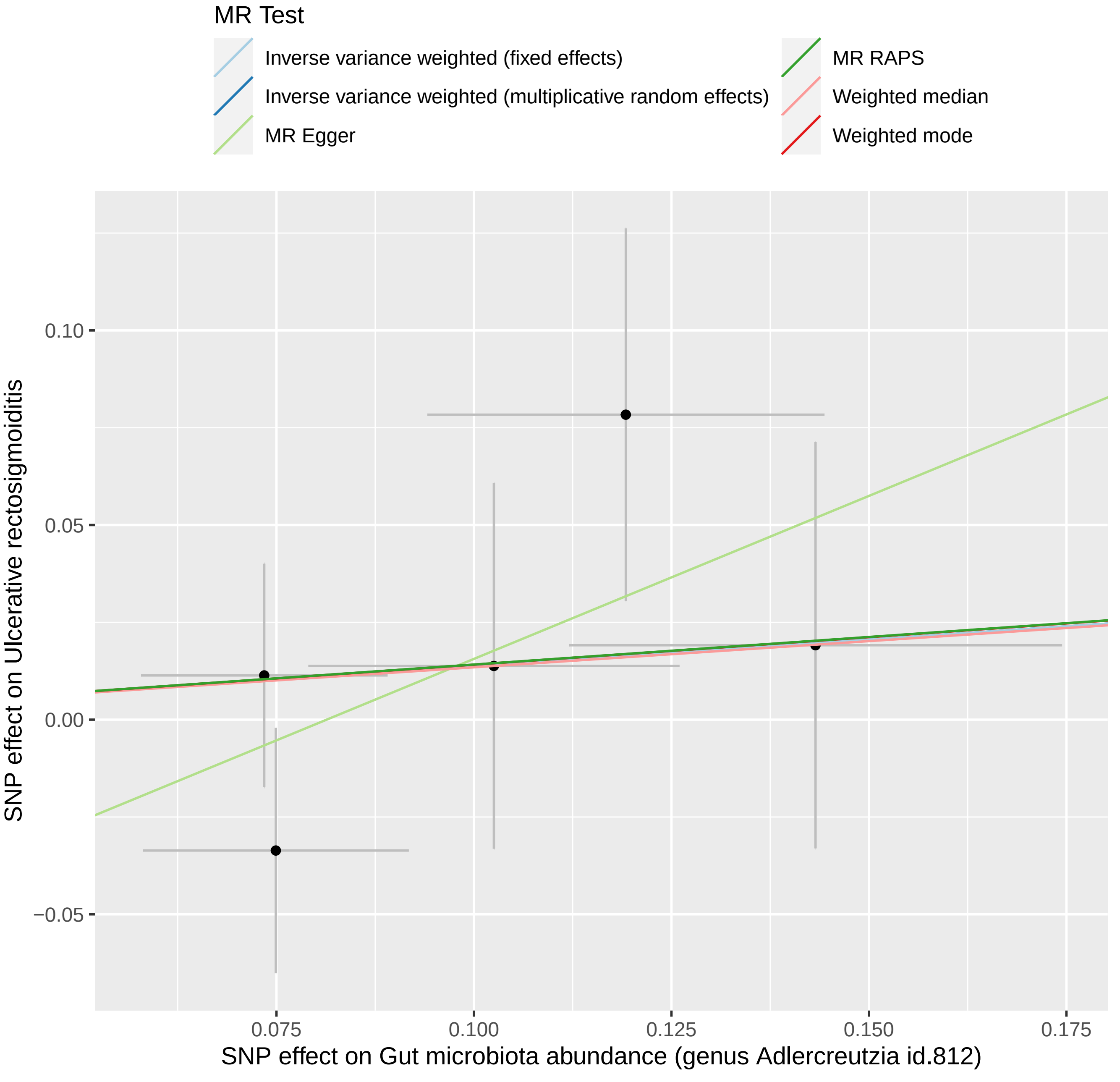

Batch 1535 : Gut microbiota abundance (genus Akkermansia id.4037) on Ulcerative rectosigmoiditis

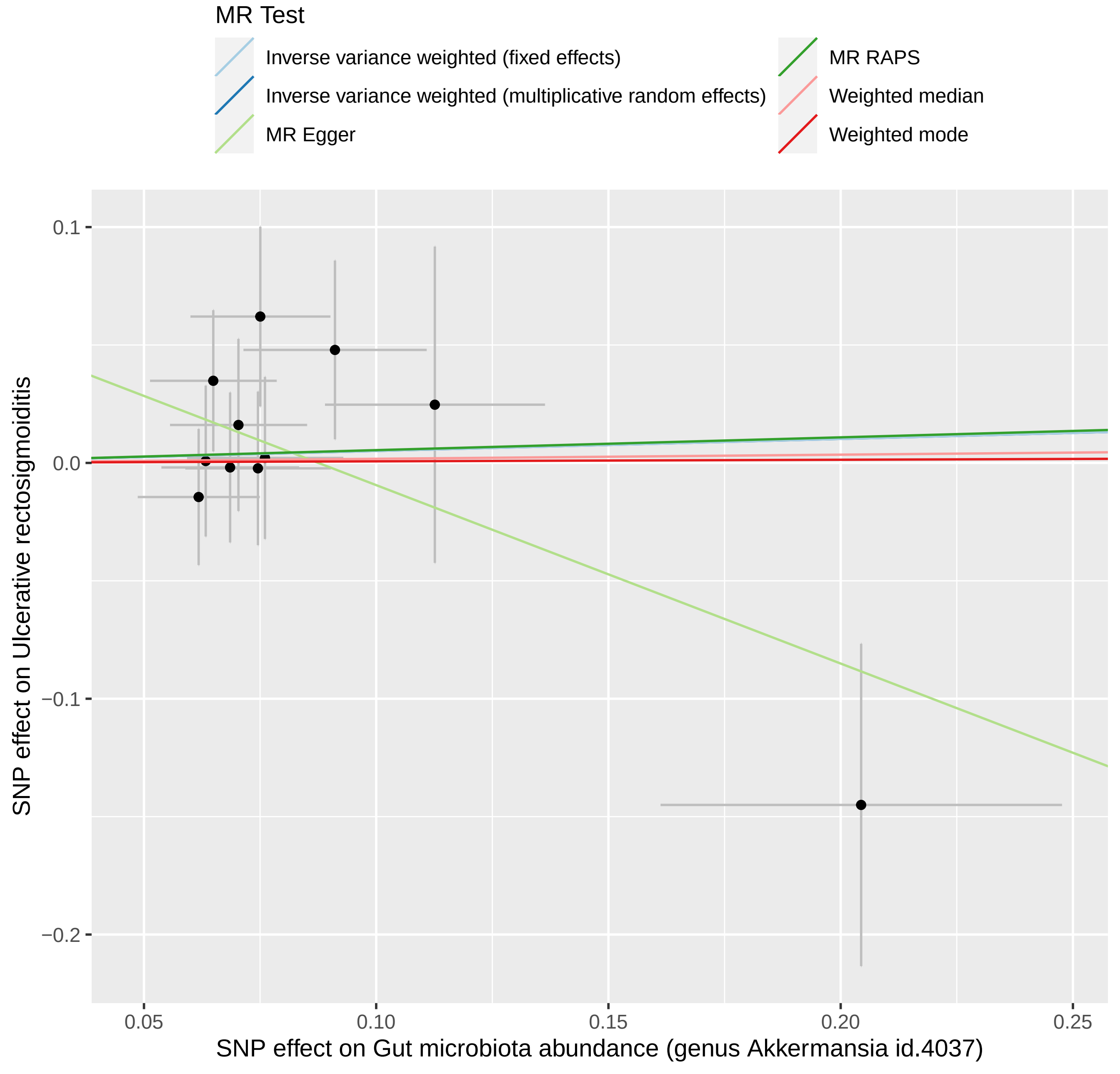

Batch 1536 : Gut microbiota abundance (genus Alistipes id.968) on Ulcerative rectosigmoiditis

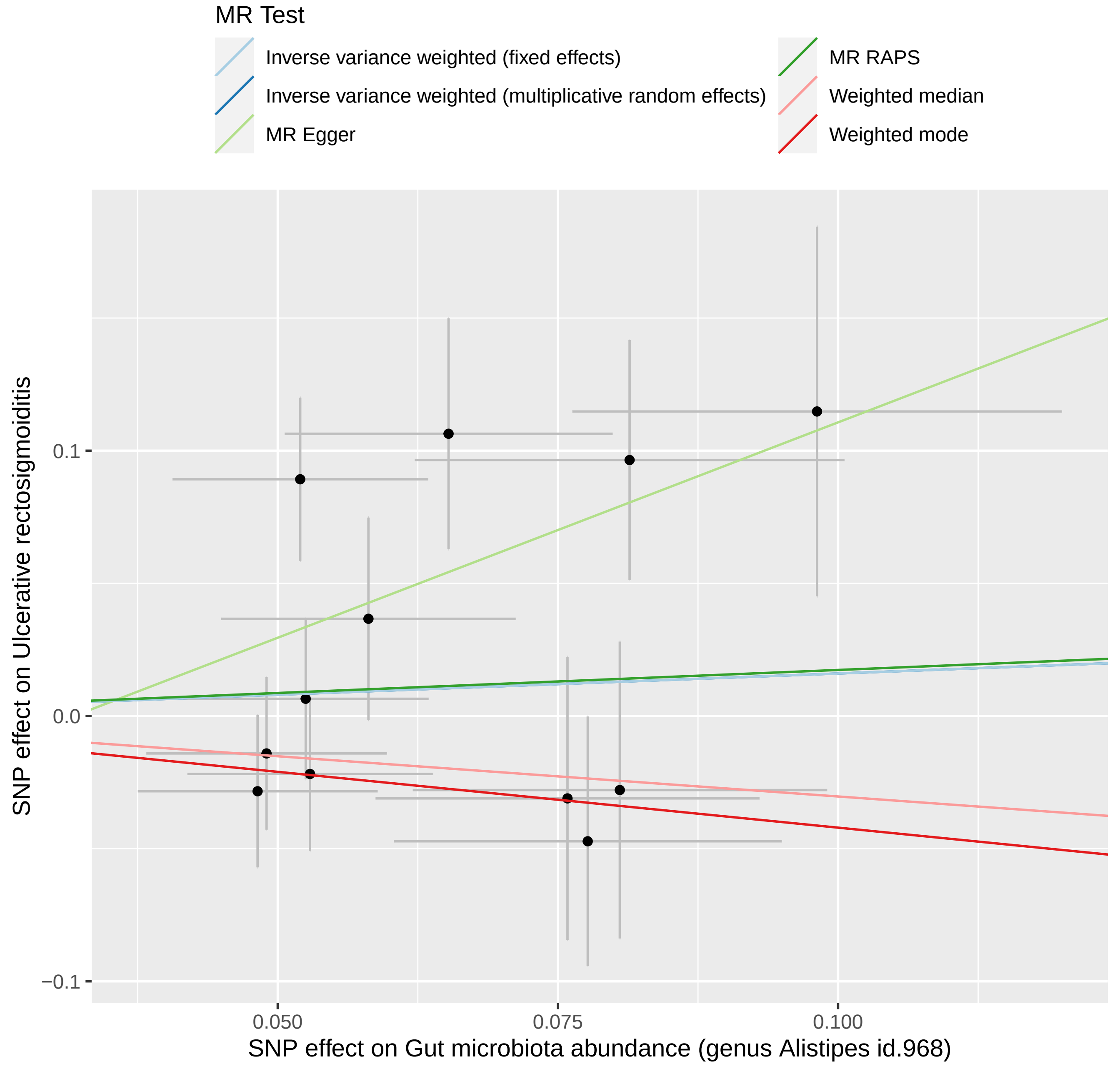

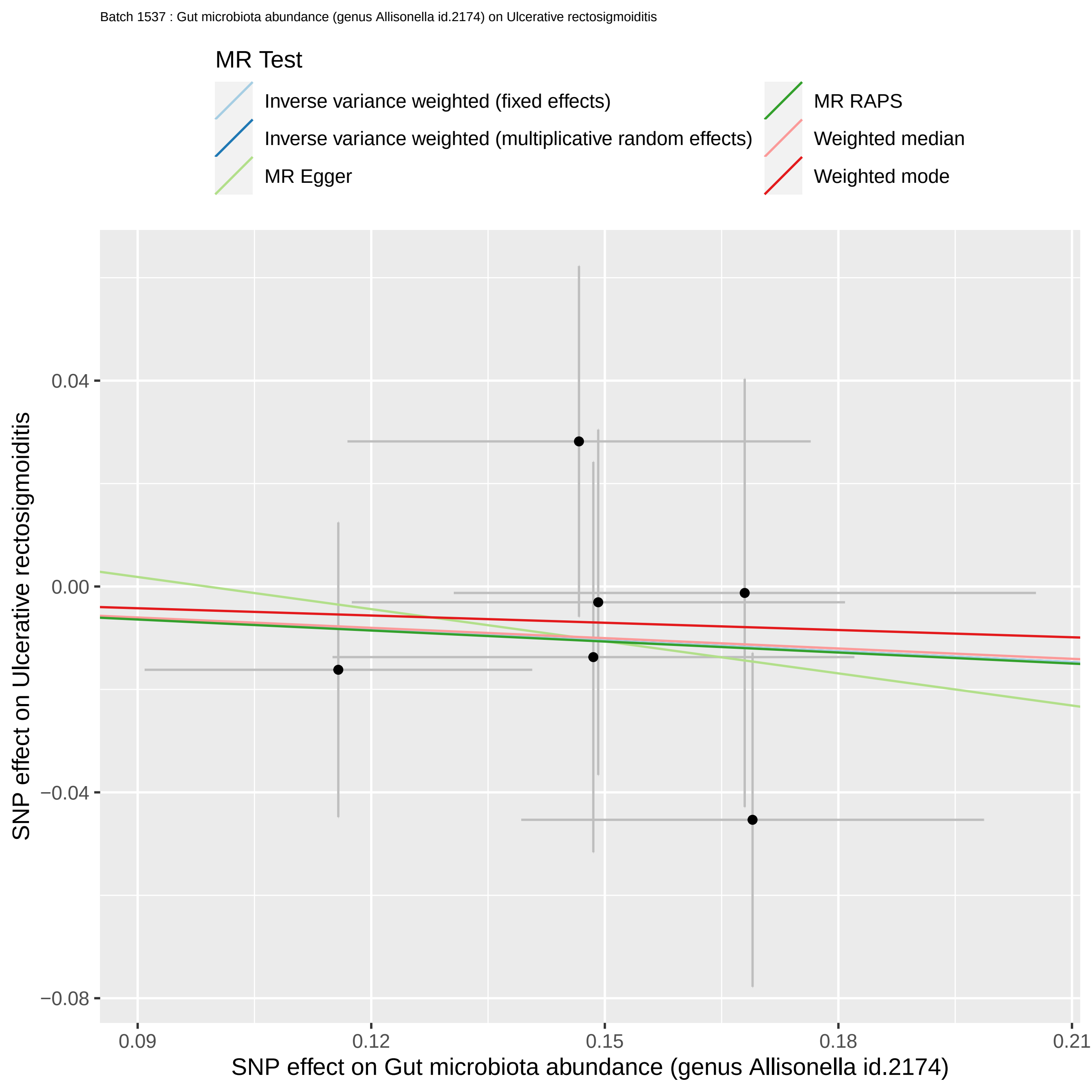

Batch 1537 : Gut microbiota abundance (genus Allisonella id.2174) on Ulcerative rectosigmoiditis
MR Test
Inverse variance weighted (fixed effects)
Inverse variance weighted (multiplicative random effects)
MR Egger
MR RAPS
Weighted median
Weighted mode
0.04
0.00
−0.04
−0.08
0.09
0.12
0.15
0.18
0.21
SNP effect on Ulcerative rectosigmoiditis
SNP effect on Gut microbiota abundance (genus Allisonella id.2174)

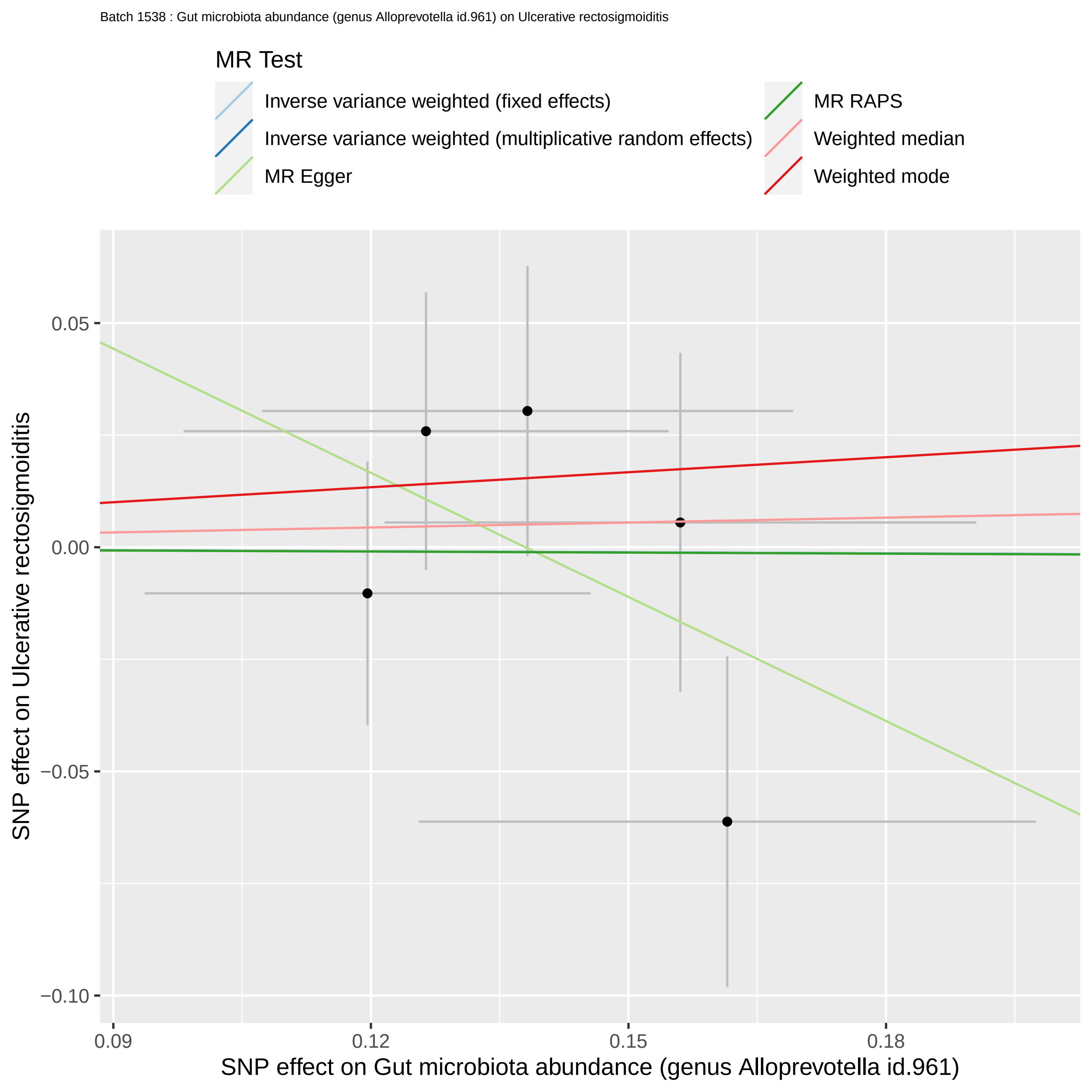

Batch 1538 : Gut microbiota abundance (genus Alloprevotella id.961) on Ulcerative rectosigmoiditis
MR Test
Inverse variance weighted (fixed effects)
Inverse variance weighted (multiplicative random effects)
MR Egger
MR RAPS
Weighted median
Weighted mode
0.05
0.00
−0.05
−0.10
0.09
0.12
0.15
0.18
SNP effect on Ulcerative rectosigmoiditis
SNP effect on Gut microbiota abundance (genus Alloprevotella id.961)

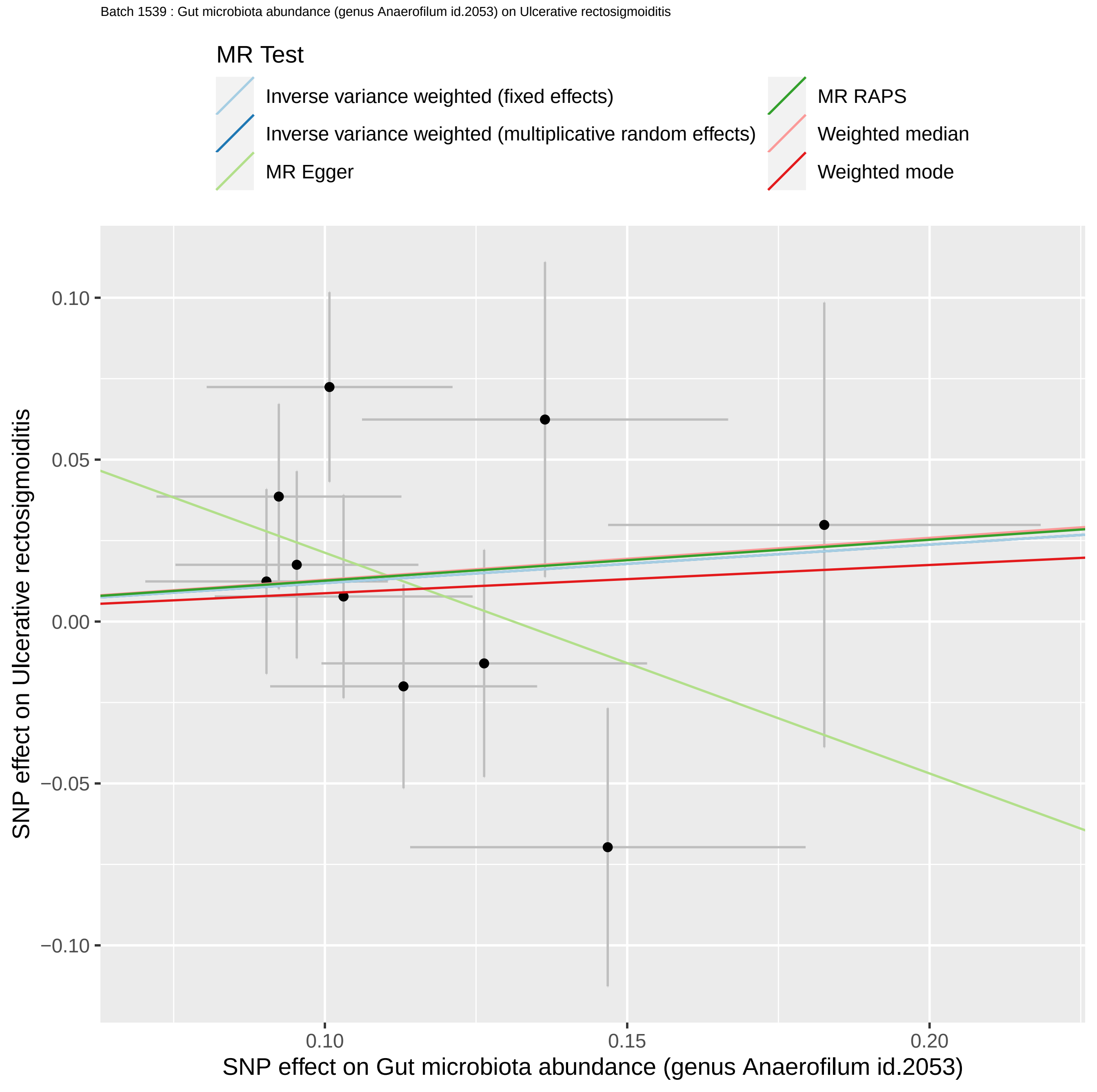

Batch 1539 : Gut microbiota abundance (genus Anaerofilum id.2053) on Ulcerative rectosigmoiditis
MR Test
Inverse variance weighted (fixed effects)
Inverse variance weighted (multiplicative random effects)
MR Egger
MR RAPS
Weighted median
Weighted mode
0.10
0.05
0.00
−0.05
−0.10
0.10
0.15
0.20
SNP effect on Ulcerative rectosigmoiditis
SNP effect on Gut microbiota abundance (genus Anaerofilum id.2053)

Batch 1540 : Gut microbiota abundance (genus Anaerostipes id.1991) on Ulcerative rectosigmoiditis

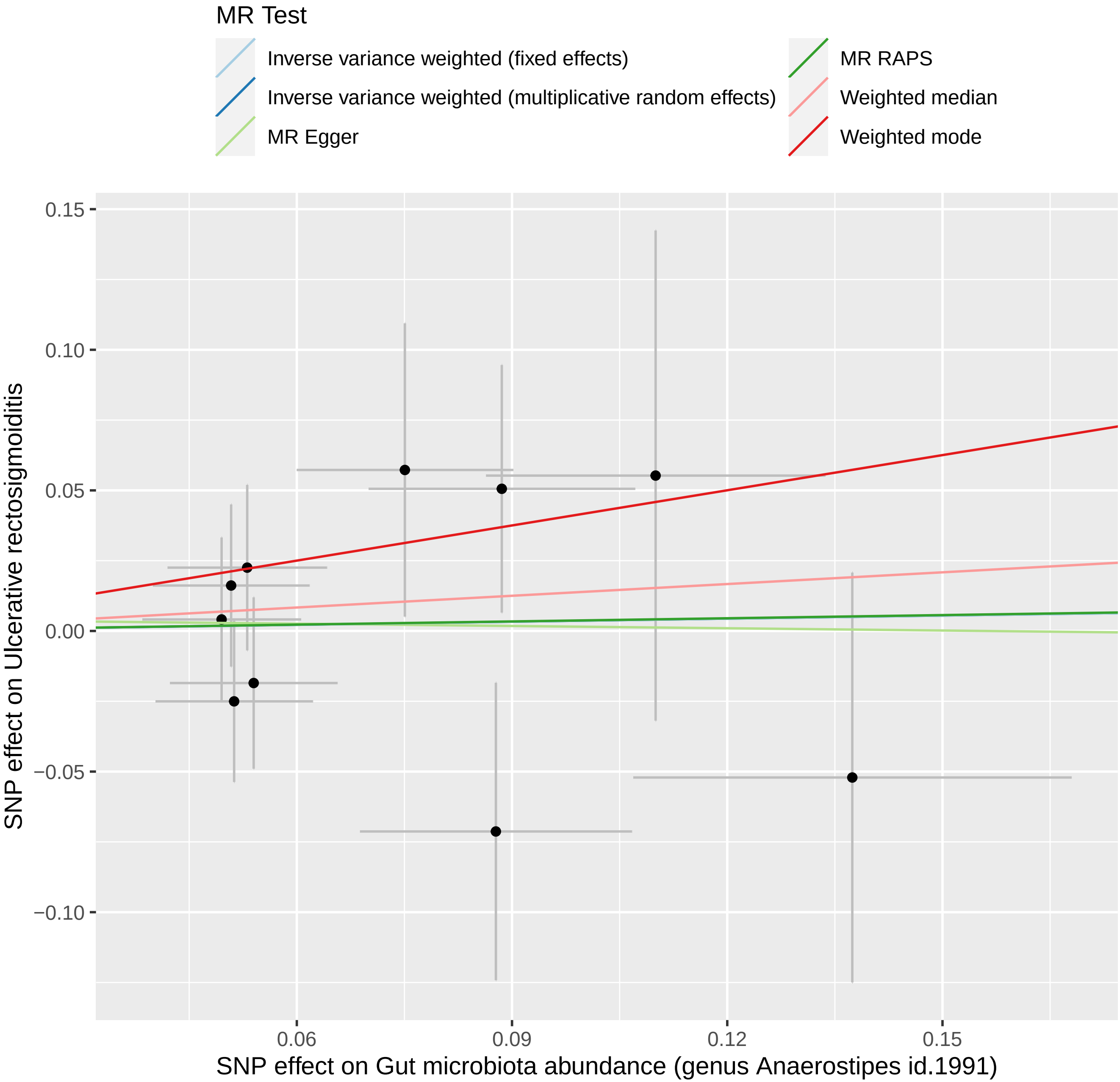

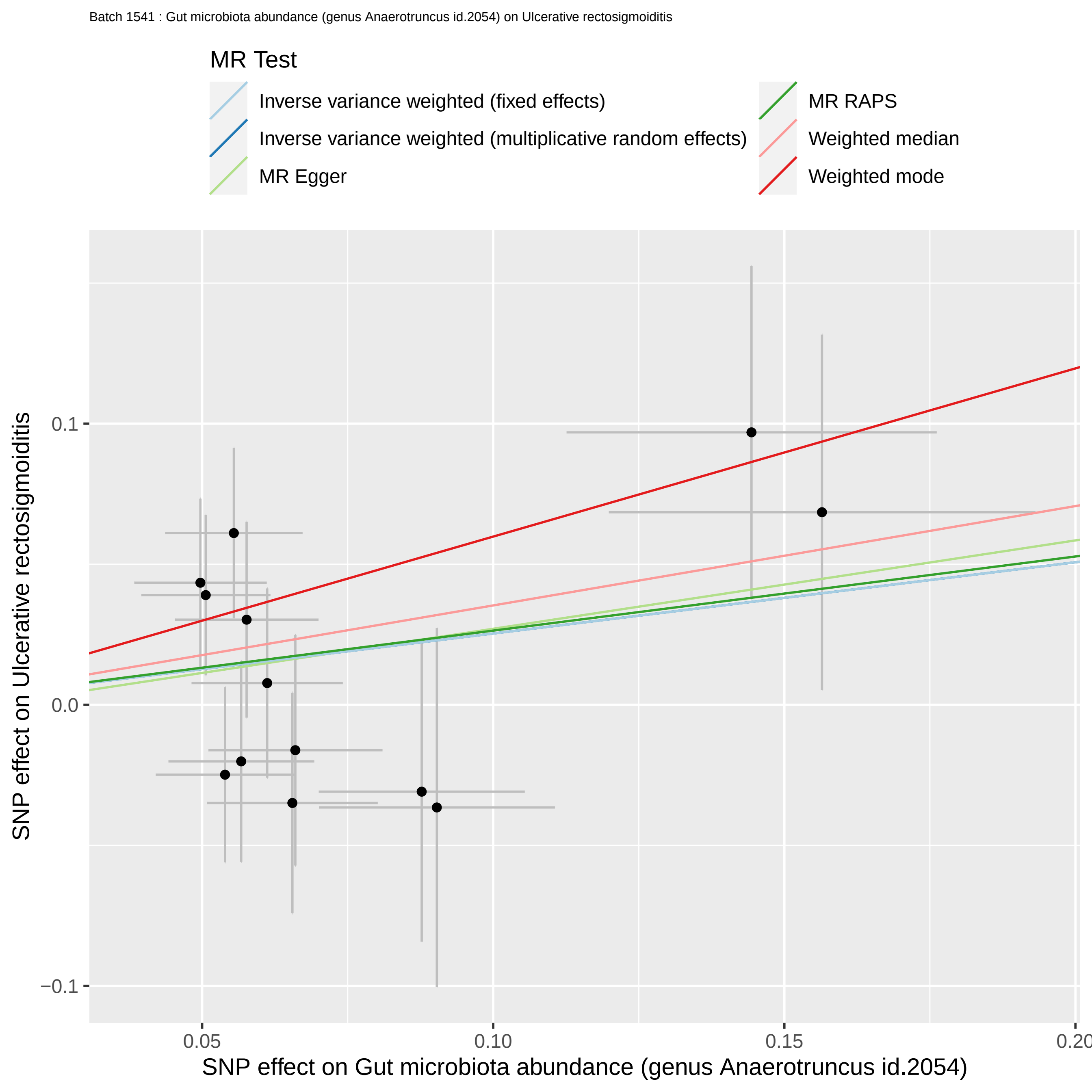
Batch 1541 : Gut microbiota abundance (genus Anaerotruncus id.2054) on Ulcerative rectosigmoiditis
MR Test
Inverse variance weighted (fixed effects)
Inverse variance weighted (multiplicative random effects)
MR Egger
MR RAPS
Weighted median
Weighted mode
0.1
0.0
−0.1
0.05
0.10
0.15
0.20
SNP effect on Ulcerative rectosigmoiditis
SNP effect on Gut microbiota abundance (genus Anaerotruncus id.2054)

Batch 1542 : Gut microbiota abundance (genus Bacteroides id.918) on Ulcerative rectosigmoiditis

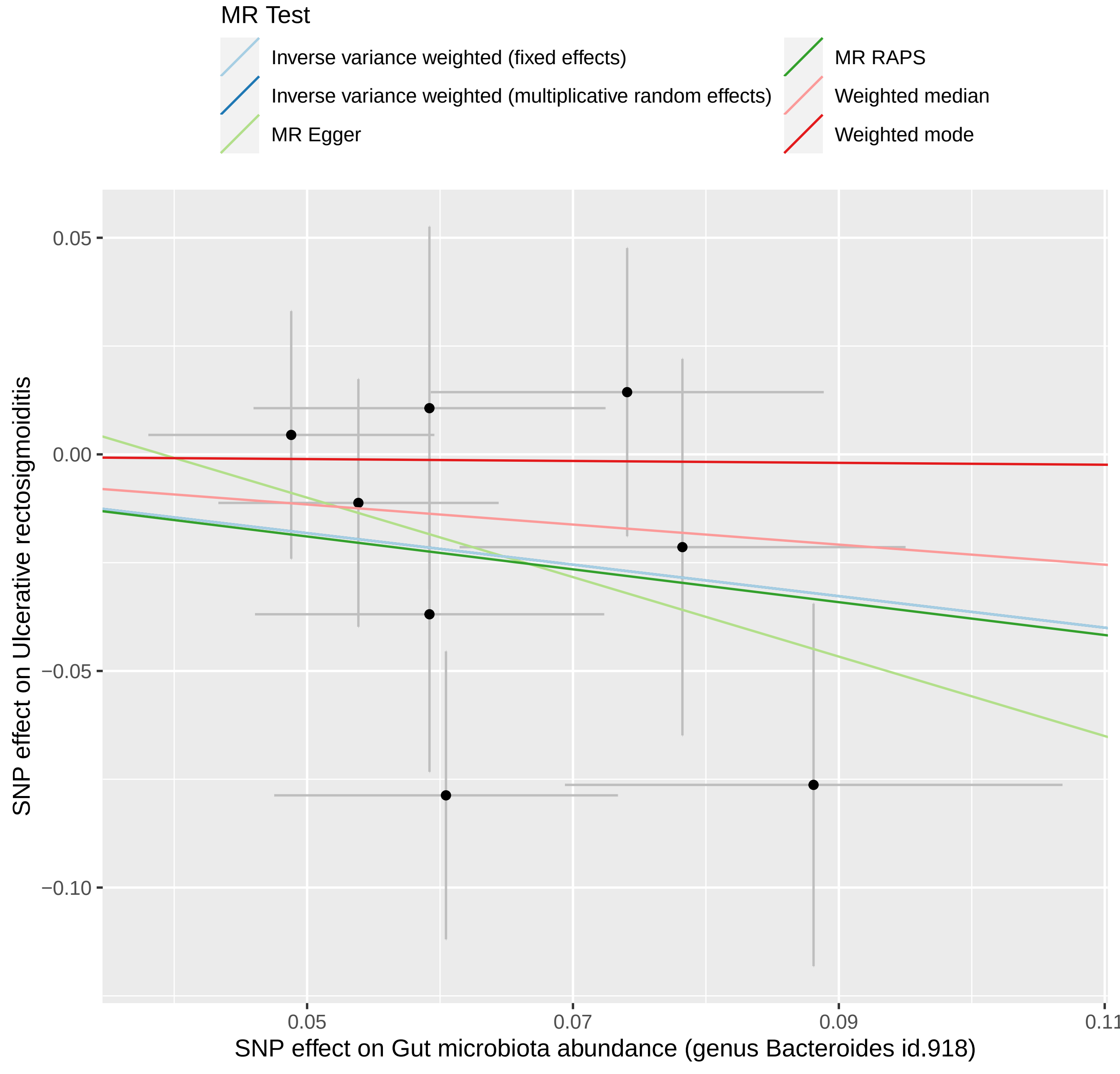

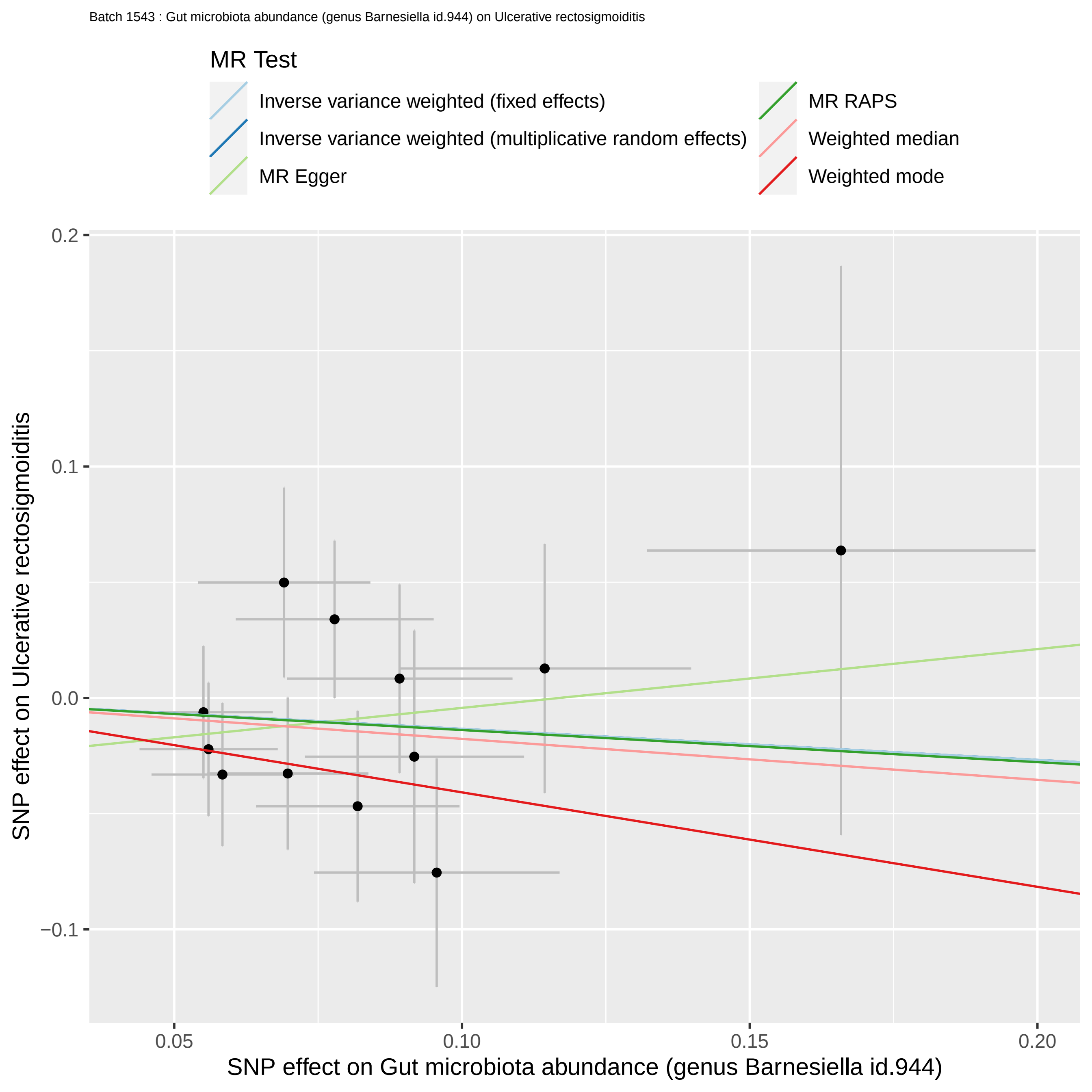
Batch 1543 : Gut microbiota abundance (genus Barnesiella id.944) on Ulcerative rectosigmoiditis
MR Test
Inverse variance weighted (fixed effects)
Inverse variance weighted (multiplicative random effects)
MR Egger
MR RAPS
Weighted median
Weighted mode
0.2
0.1
0.0
−0.1
0.05
0.10
0.15
0.20
SNP effect on Ulcerative rectosigmoiditis
SNP effect on Gut microbiota abundance (genus Barnesiella id.944)

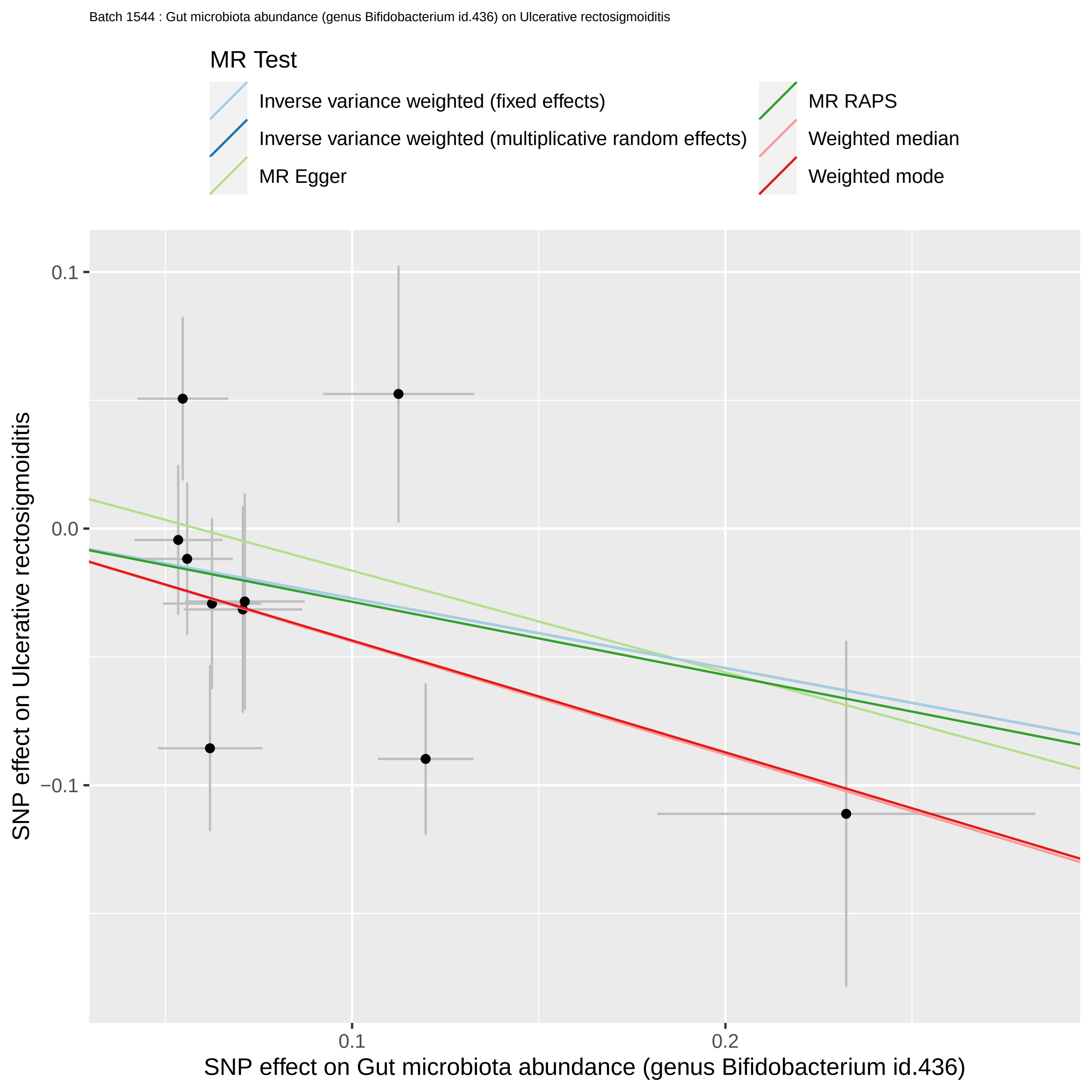
Batch 1544 : Gut microbiota abundance (genus Bifidobacterium id.436) on Ulcerative rectosigmoiditis
MR Test
Inverse variance weighted (fixed effects)
Inverse variance weighted (multiplicative random effects)
MR Egger
MR RAPS
Weighted median
Weighted mode
0.1
0.0
−0.1
0.1
0.2
SNP effect on Ulcerative rectosigmoiditis
SNP effect on Gut microbiota abundance (genus Bifidobacterium id.436)

Batch 1545 : Gut microbiota abundance (genus Bilophila id.3170) on Ulcerative rectosigmoiditis

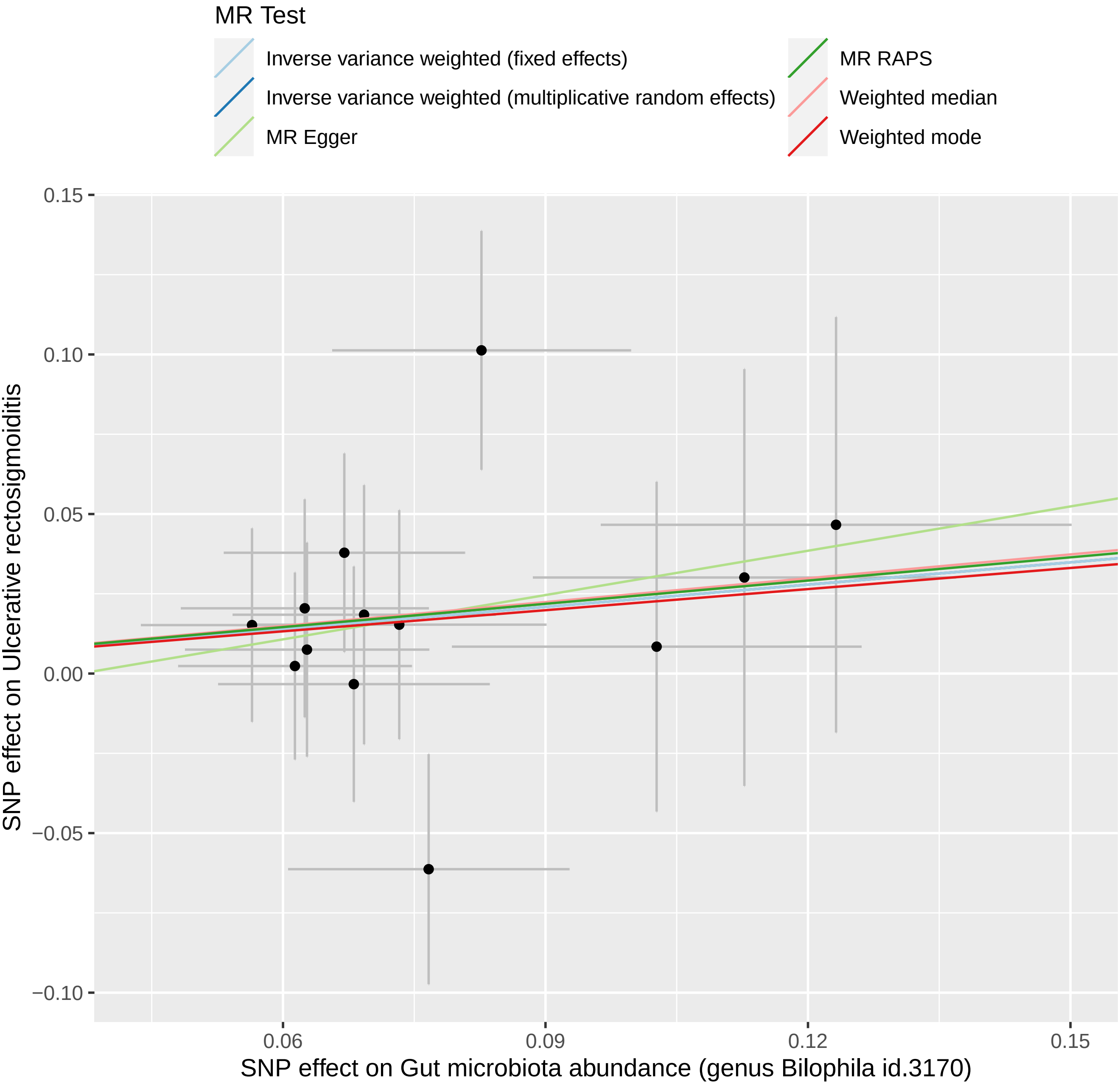

Batch 1546 : Gut microbiota abundance (genus Blautia id.1992) on Ulcerative rectosigmoiditis

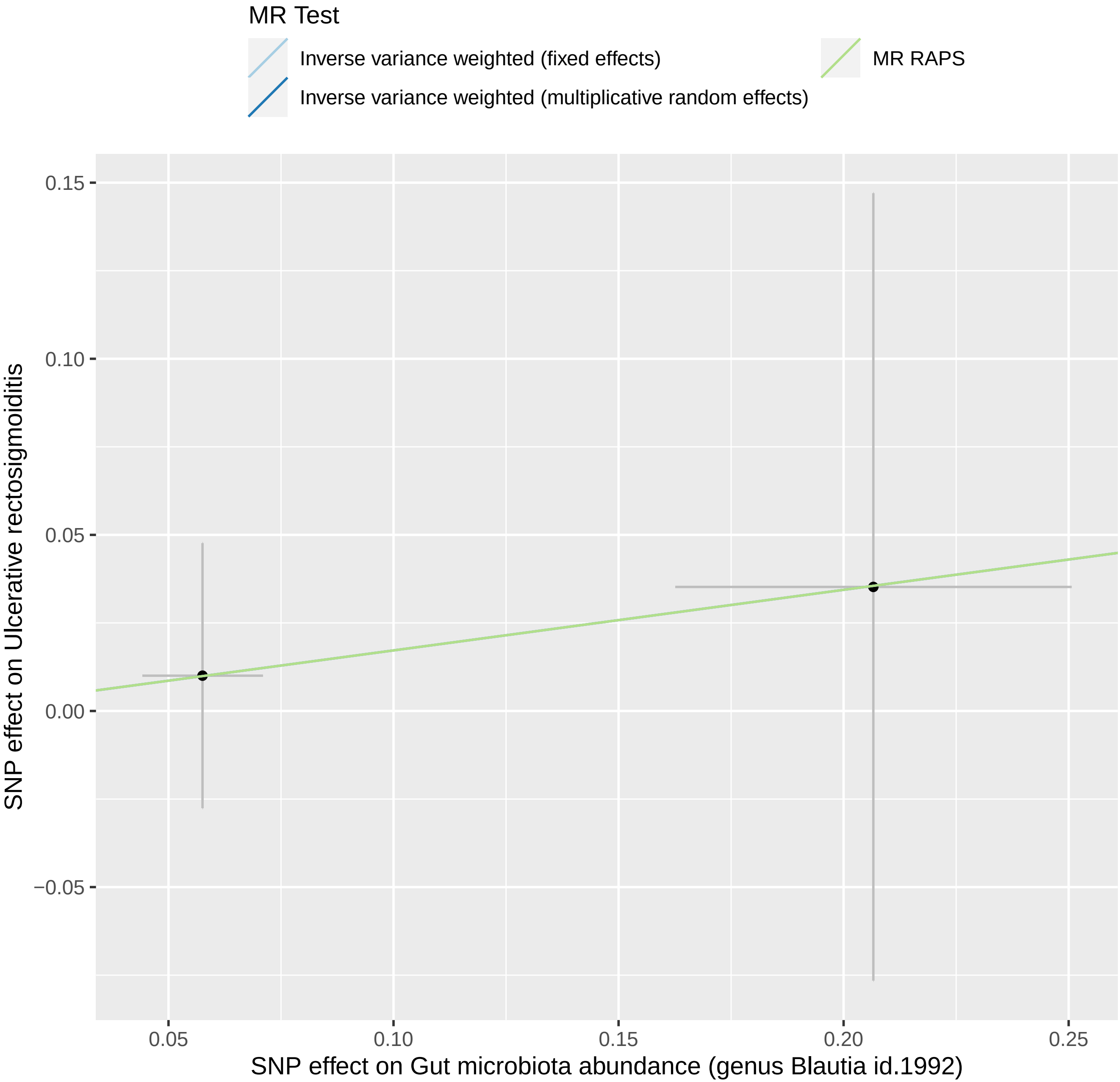

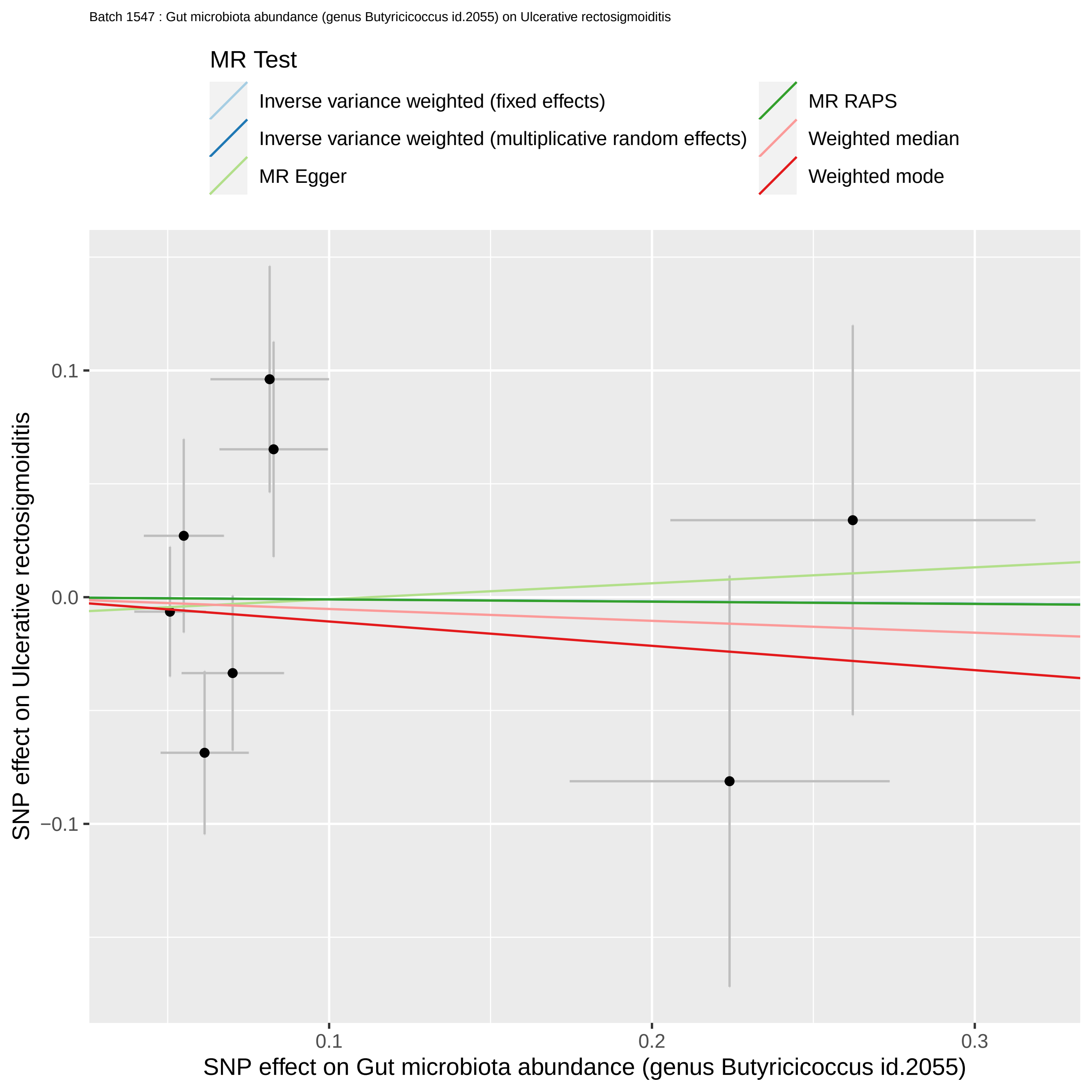

Batch 1547 : Gut microbiota abundance (genus Butyricicoccus id.2055) on Ulcerative rectosigmoiditis
MR Test
Inverse variance weighted (fixed effects)
Inverse variance weighted (multiplicative random effects)
MR Egger
MR RAPS
Weighted median
Weighted mode
0.1
0.0
−0.1
0.1
0.2
0.3
SNP effect on Ulcerative rectosigmoiditis
SNP effect on Gut microbiota abundance (genus Butyricicoccus id.2055)

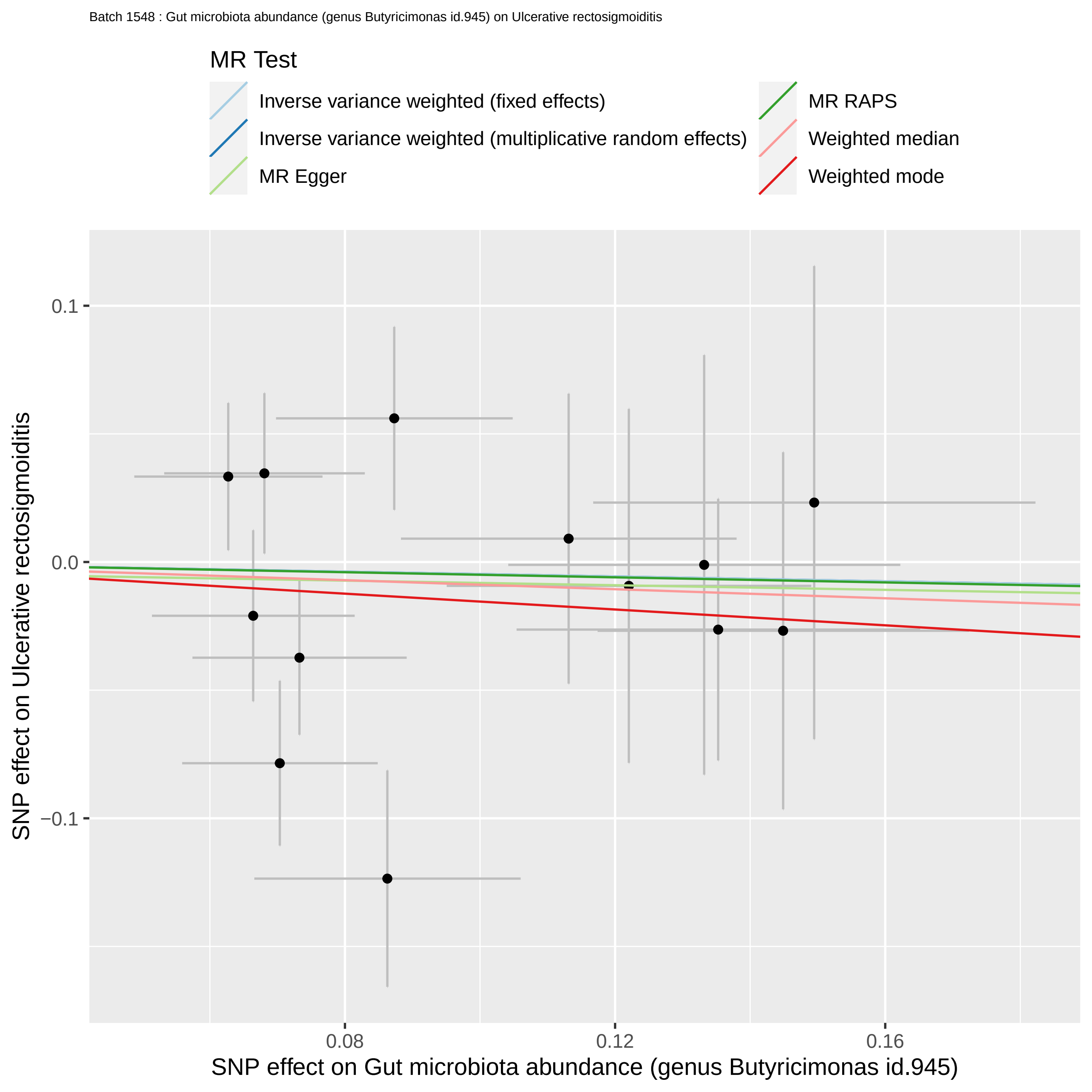

Batch 1548 : Gut microbiota abundance (genus Butyricimonas id.945) on Ulcerative rectosigmoiditis
MR Test
Inverse variance weighted (fixed effects)
Inverse variance weighted (multiplicative random effects)
MR Egger
MR RAPS
Weighted median
Weighted mode
0.1
0.0
−0.1
0.08
0.12
0.16
SNP effect on Ulcerative rectosigmoiditis
SNP effect on Gut microbiota abundance (genus Butyricimonas id.945)

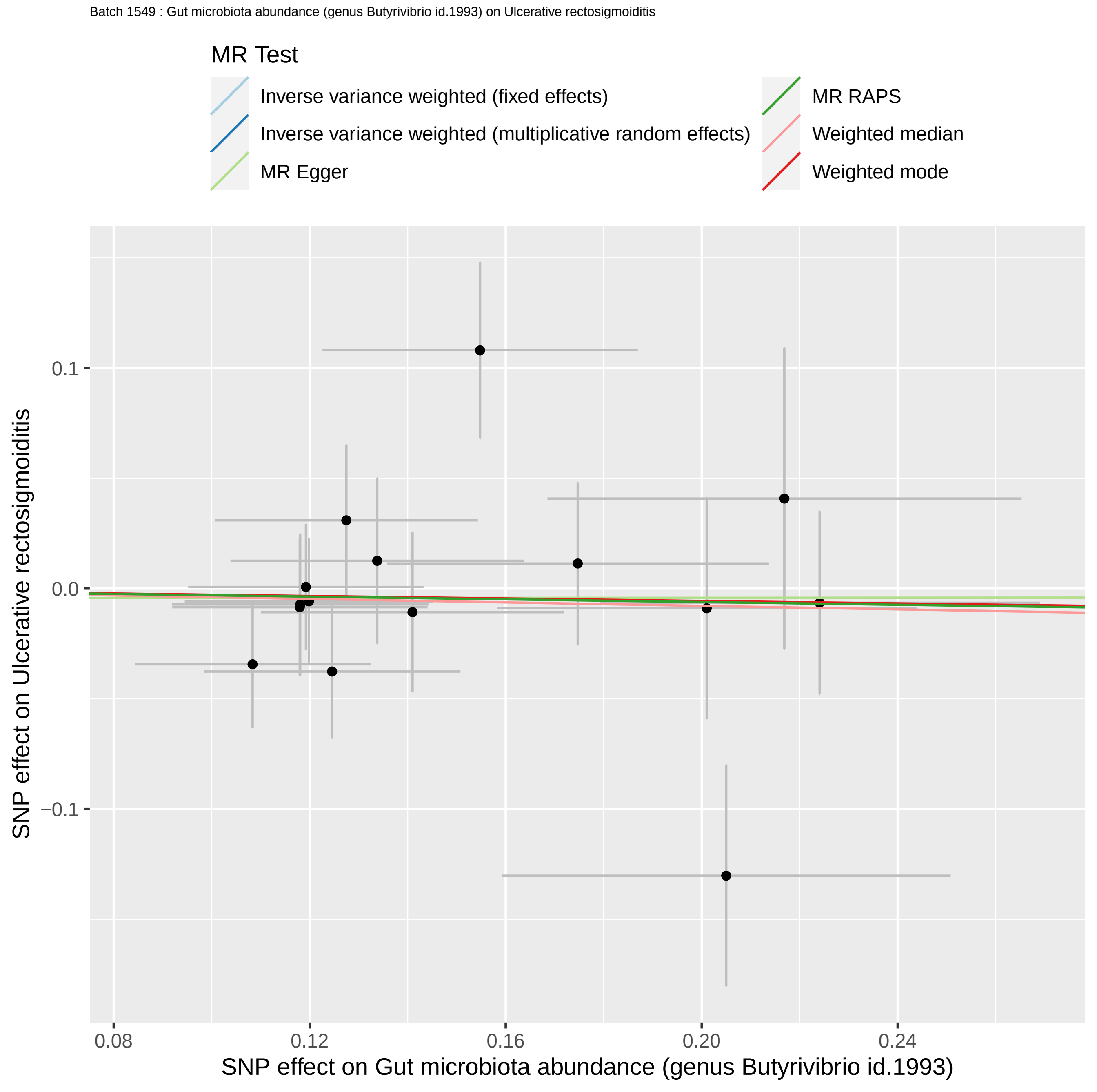
Batch 1549 : Gut microbiota abundance (genus Butyrivibrio id.1993) on Ulcerative rectosigmoiditis
MR Test
Inverse variance weighted (fixed effects)
Inverse variance weighted (multiplicative random effects)
MR Egger
MR RAPS
Weighted median
Weighted mode
0.1
0.0
−0.1
0.08
0.12
0.16
0.20
0.24
SNP effect on Ulcerative rectosigmoiditis
SNP effect on Gut microbiota abundance (genus Butyrivibrio id.1993)

Batch 1550 : Gut microbiota abundance (genus Candidatus Soleaferrea id.11350) on Ulcerative rectosigmoiditis

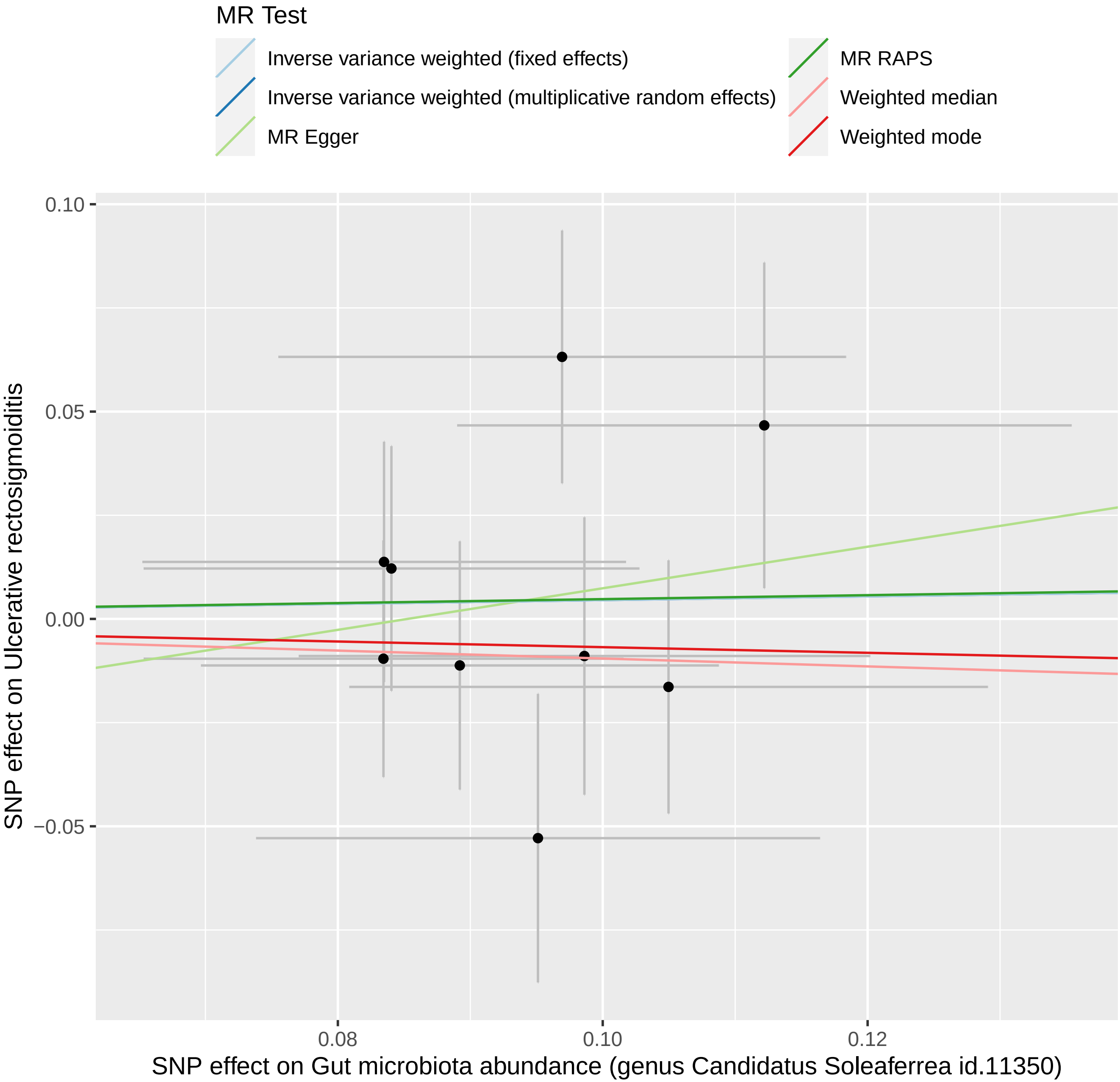

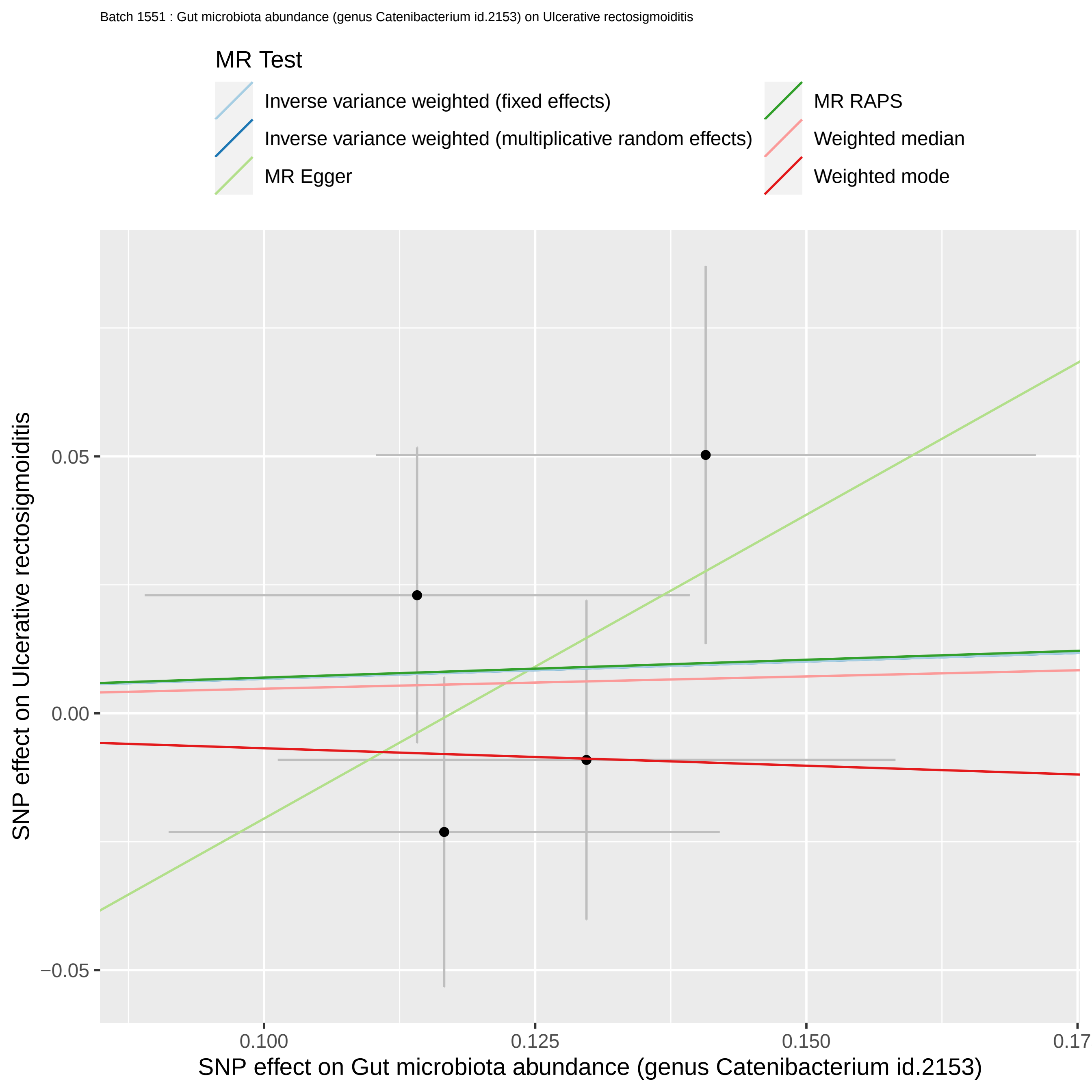
Batch 1551 : Gut microbiota abundance (genus Catenibacterium id.2153) on Ulcerative rectosigmoiditis
MR Test
Inverse variance weighted (fixed effects)
Inverse variance weighted (multiplicative random effects)
MR Egger
MR RAPS
Weighted median
Weighted mode
0.05
0.00
−0.05
0.100
0.125
0.150
0.17
SNP effect on Ulcerative rectosigmoiditis
SNP effect on Gut microbiota abundance (genus Catenibacterium id.2153)

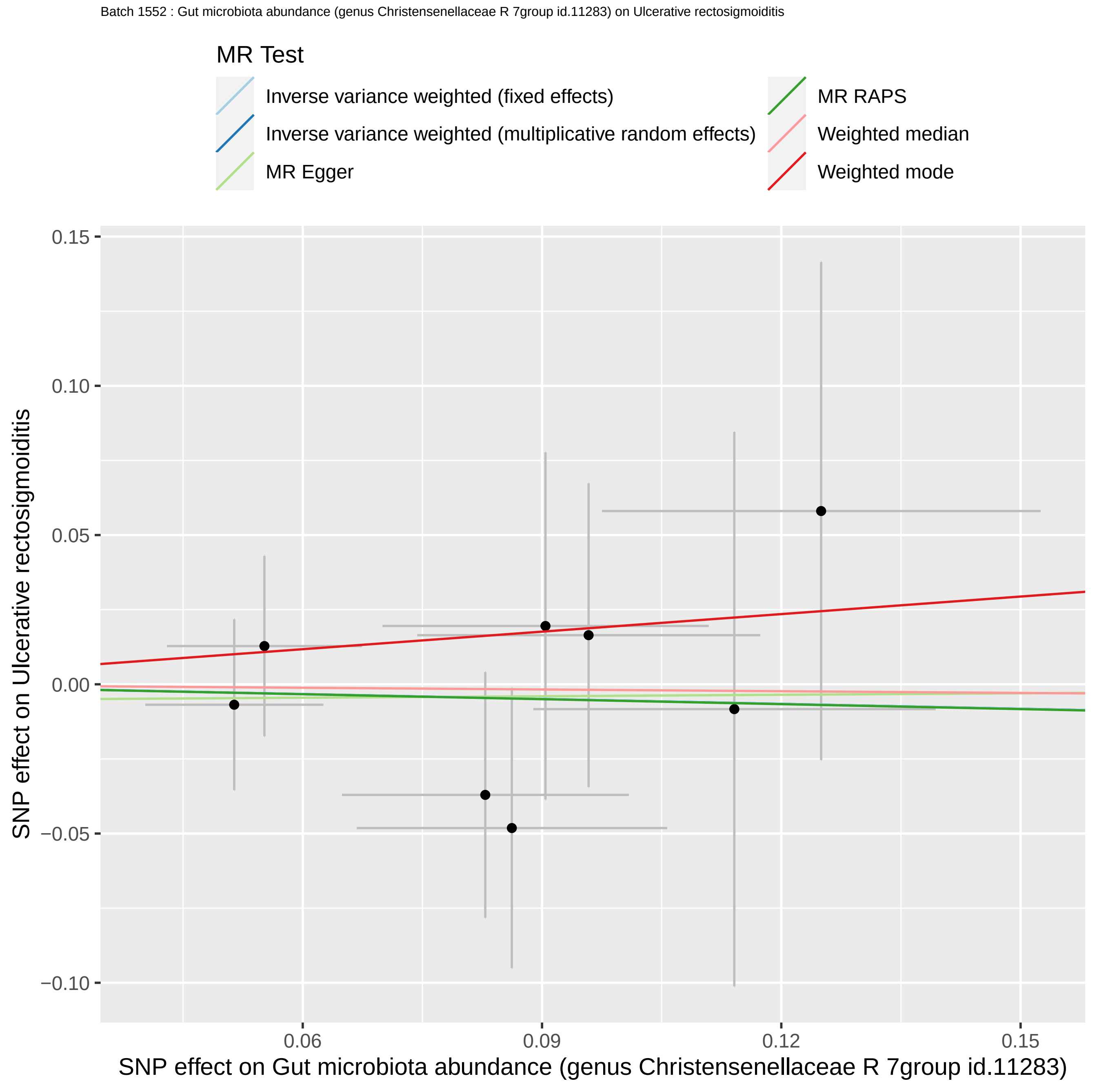

Batch 1552 : Gut microbiota abundance (genus Christensenellaceae R 7group id.11283) on Ulcerative rectosigmoiditis
MR Test
Inverse variance weighted (fixed effects)
Inverse variance weighted (multiplicative random effects)
MR Egger
MR RAPS
Weighted median
Weighted mode
0.15
0.10
0.05
0.00
−0.05
−0.10
0.06
0.09
0.12
0.15
SNP effect on Ulcerative rectosigmoiditis
SNP effect on Gut microbiota abundance (genus Christensenellaceae R 7group id.11283)

Batch 1553 : Gut microbiota abundance (genus Clostridium innocuum group id.14397) on Ulcerative rectosigmoiditis

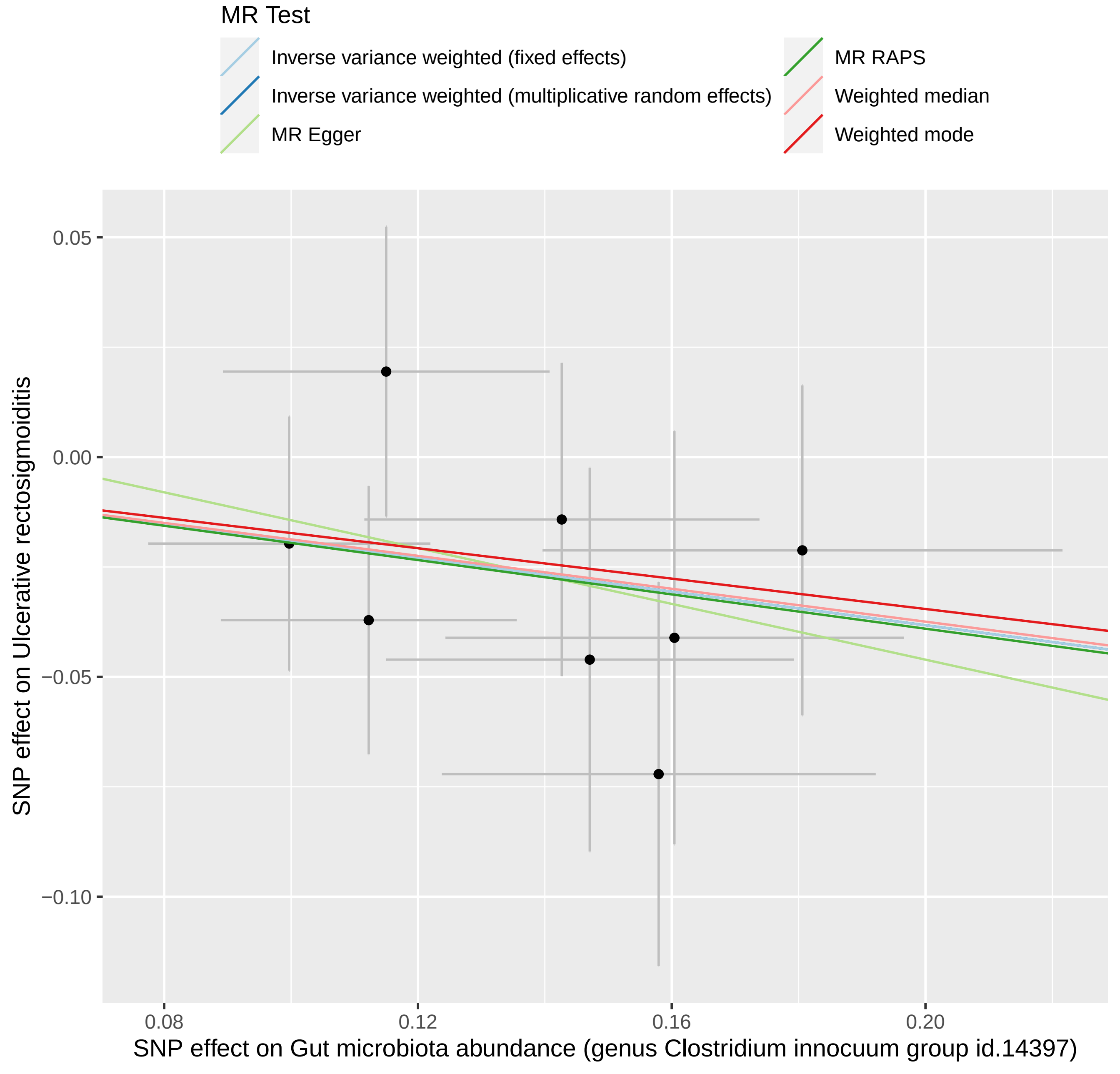

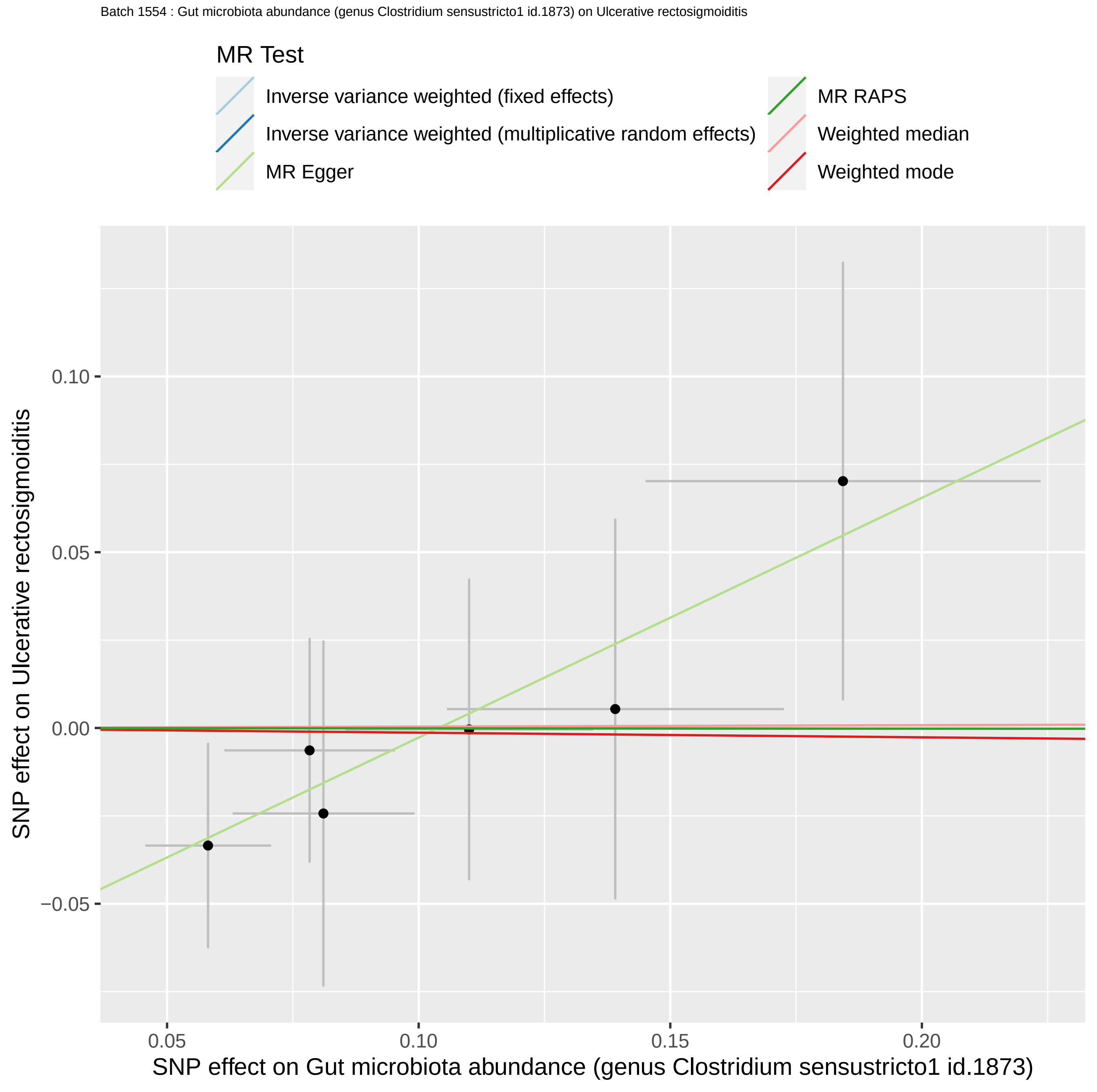

Batch 1554 : Gut microbiota abundance (genus Clostridium sensustricto1 id.1873) on Ulcerative rectosigmoiditis
MR Test
Inverse variance weighted (fixed effects)
Inverse variance weighted (multiplicative random effects)
MR Egger
MR RAPS
Weighted median
Weighted mode
0.10
0.05
0.00
−0.05
SNP effect on Ulcerative rectosigmoiditis
0.05
0.10
0.15
0.20
SNP effect on Gut microbiota abundance (genus Clostridium sensustricto1 id.1873)

Batch 1555 : Gut microbiota abundance (genus Collinsella id.815) on Ulcerative rectosigmoiditis

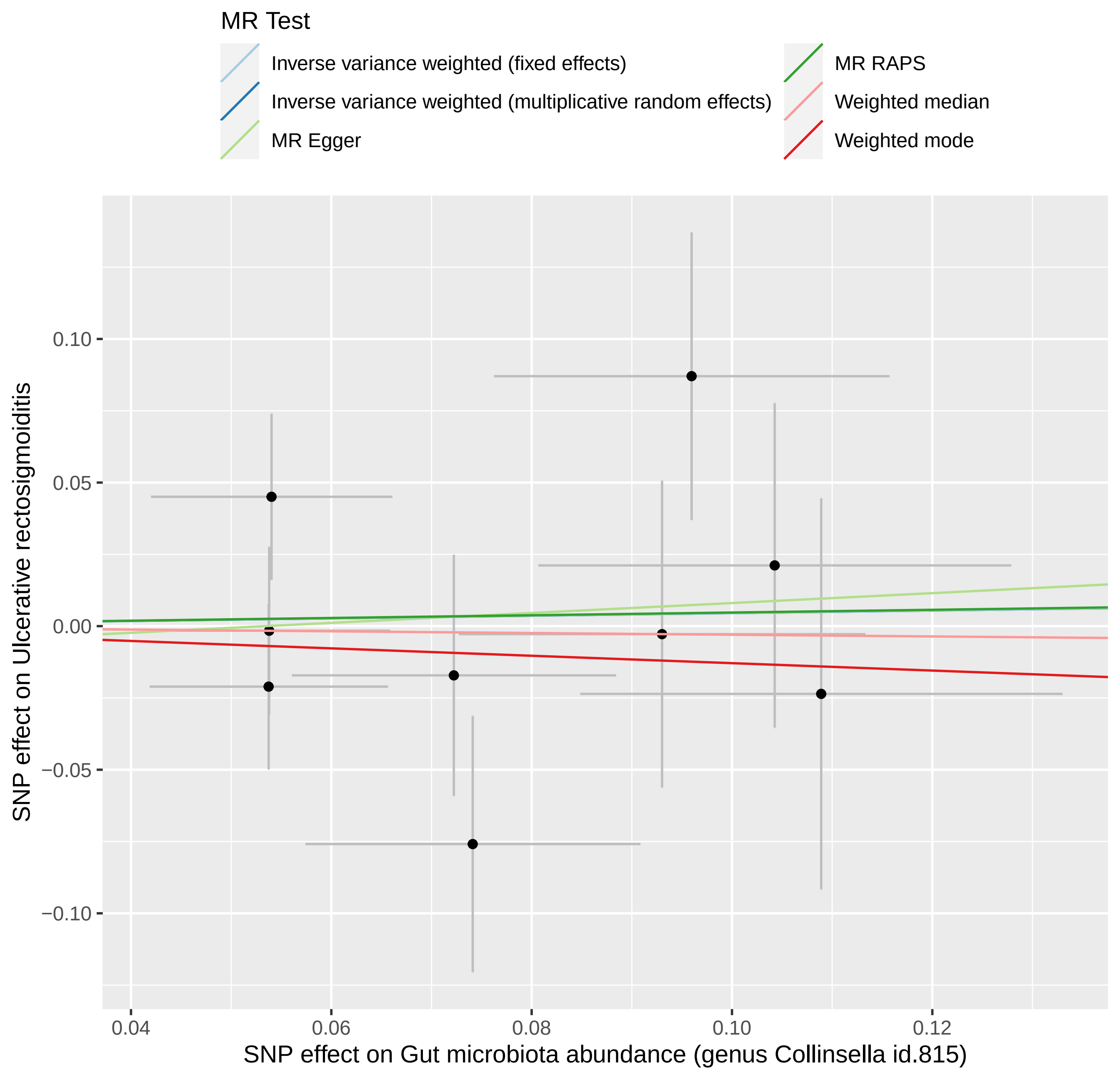

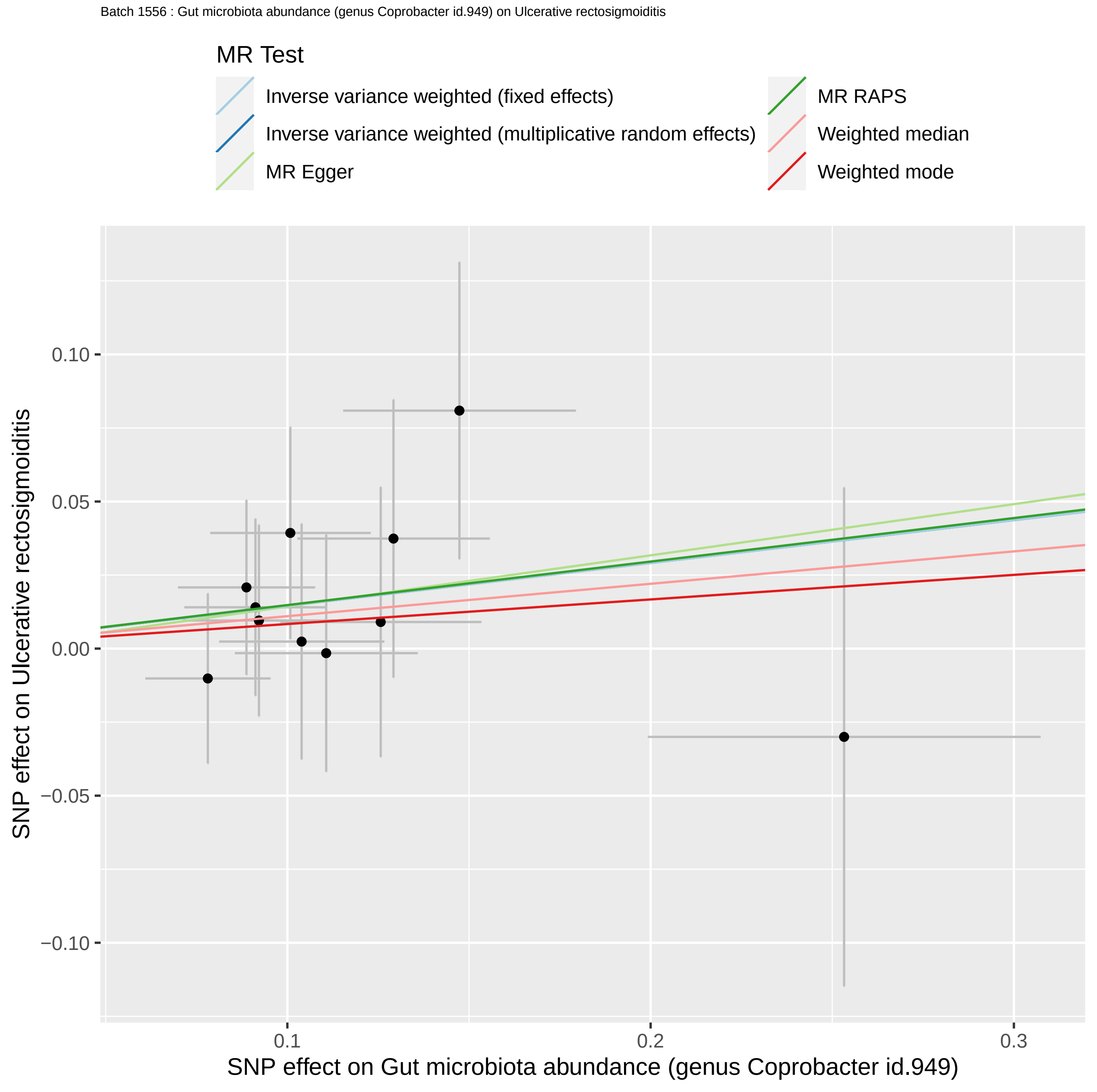

Batch 1556 : Gut microbiota abundance (genus Coprobacter id.949) on Ulcerative rectosigmoiditis
MR Test
Inverse variance weighted (fixed effects)
Inverse variance weighted (multiplicative random effects)
MR Egger
MR RAPS
Weighted median
Weighted mode
0.10
0.05
0.00
−0.05
−0.10
0.1
0.2
0.3
SNP effect on Ulcerative rectosigmoiditis
SNP effect on Gut microbiota abundance (genus Coprobacter id.949)

Batch 1557 : Gut microbiota abundance (genus Coprococcus1 id.11301) on Ulcerative rectosigmoiditis

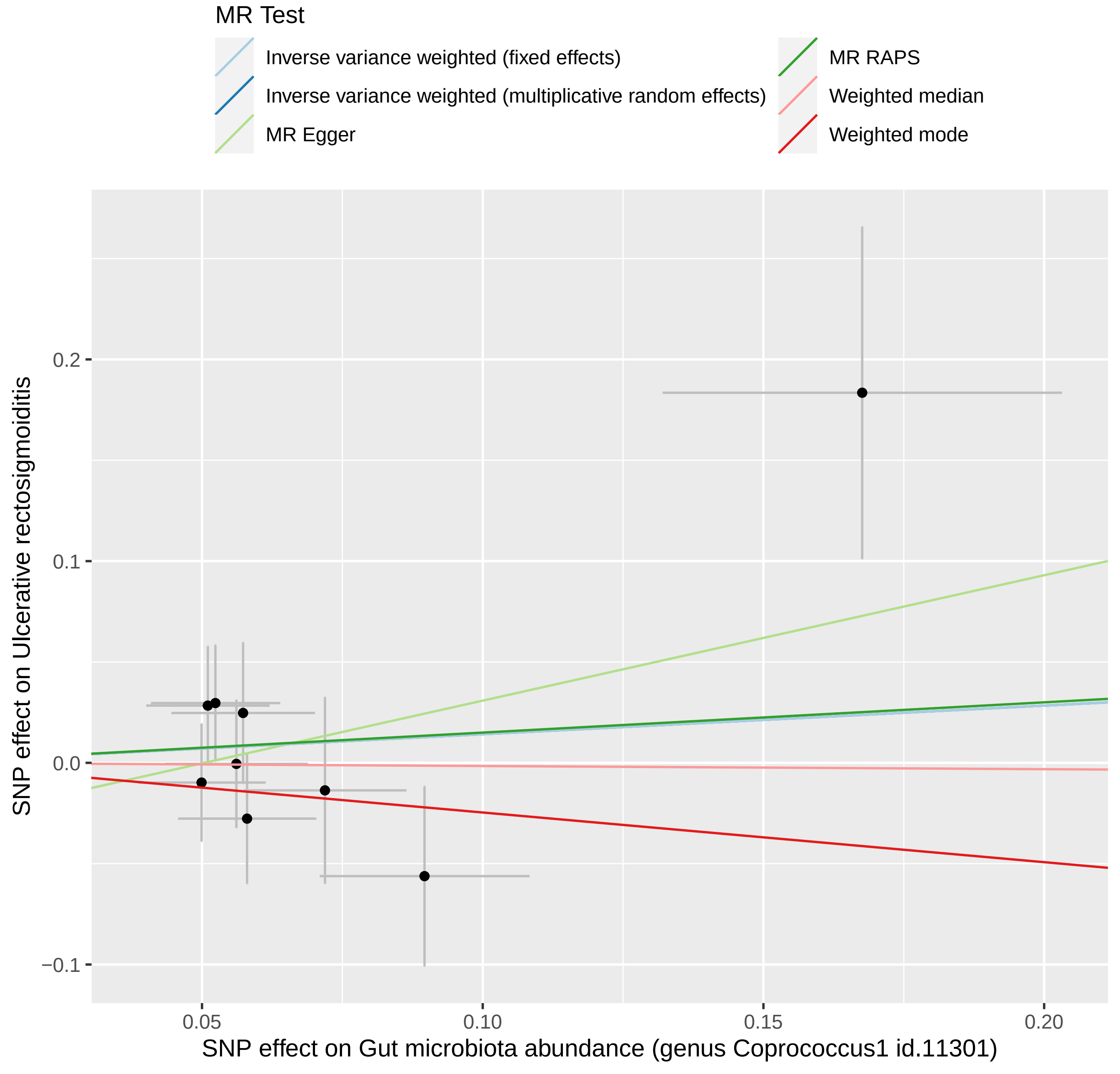

Batch 1558 : Gut microbiota abundance (genus Coprococcus2 id.11302) on Ulcerative rectosigmoiditis

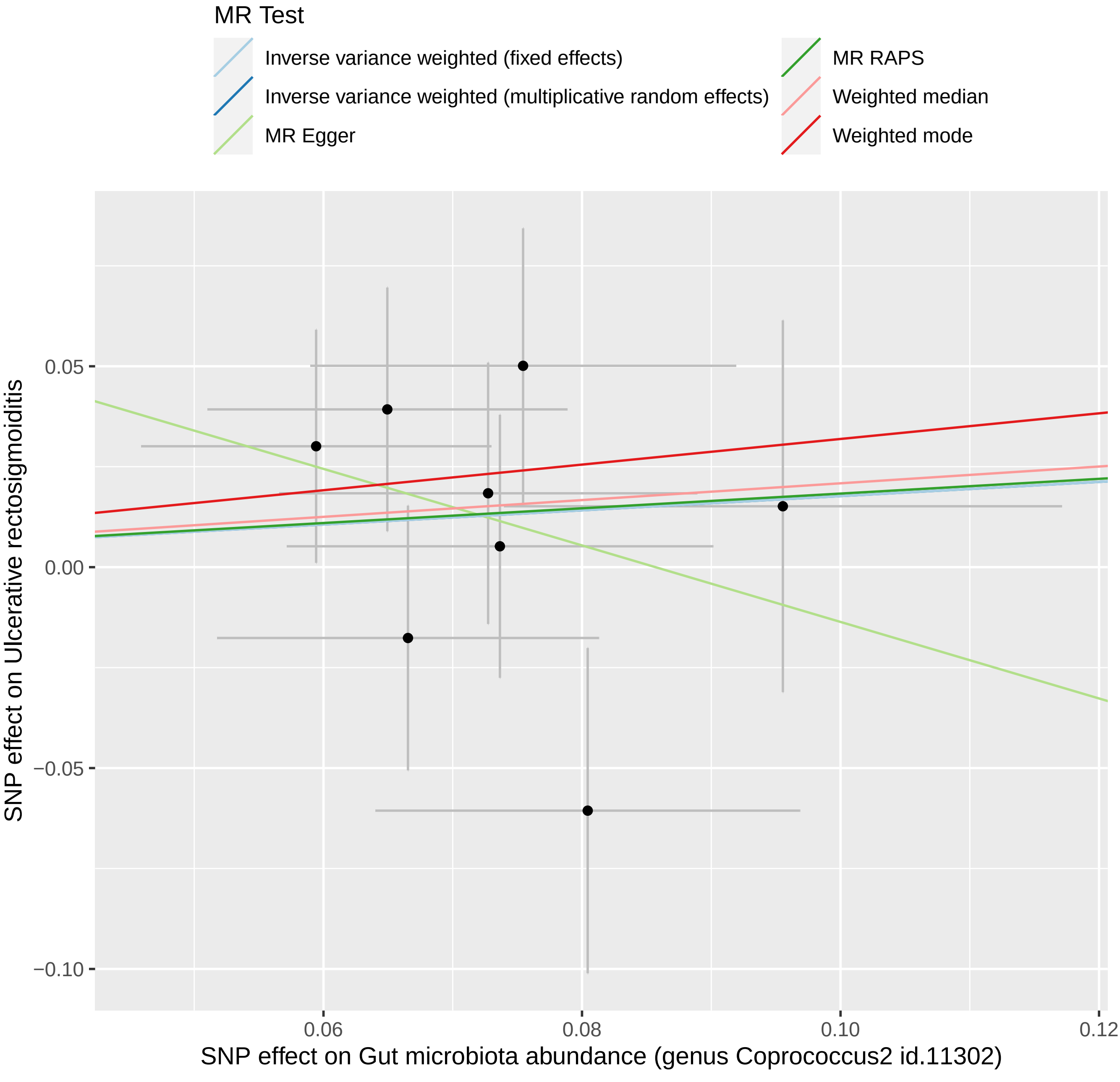

Batch 1559 : Gut microbiota abundance (genus Coprococcus3 id.11303) on Ulcerative rectosigmoiditis

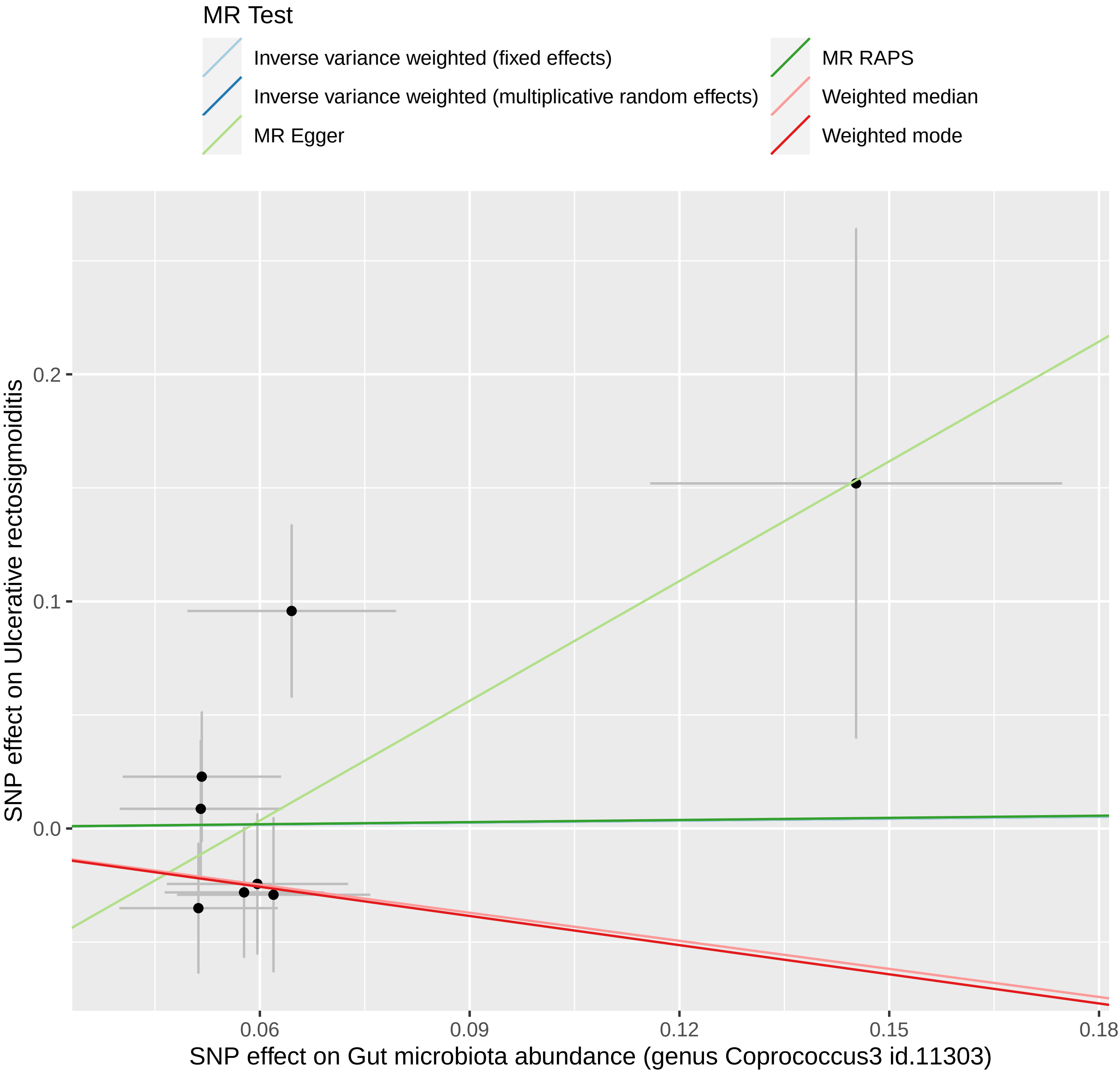

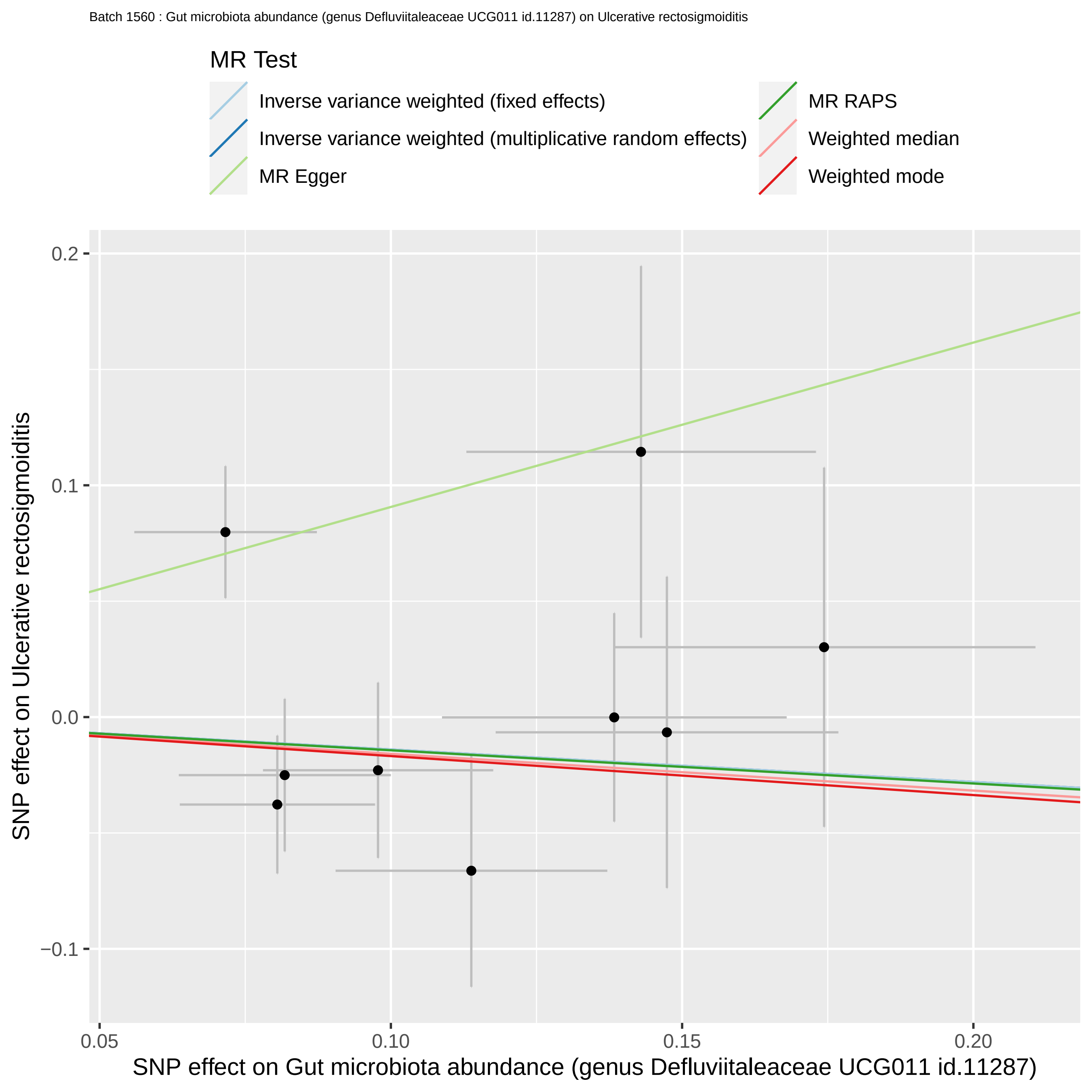
Batch 1560 : Gut microbiota abundance (genus Defluviitaleaceae UCG011 id.11287) on Ulcerative rectosigmoiditis
MR Test
Inverse variance weighted (fixed effects)
Inverse variance weighted (multiplicative random effects)
MR Egger
MR RAPS
Weighted median
Weighted mode
0.2
0.1
0.0
−0.1
0.05
0.10
0.15
0.20
SNP effect on Ulcerative rectosigmoiditis
SNP effect on Gut microbiota abundance (genus Defluviitaleaceae UCG011 id.11287)

Batch 1561 : Gut microbiota abundance (genus Desulfovibrio id.3173) on Ulcerative rectosigmoiditis

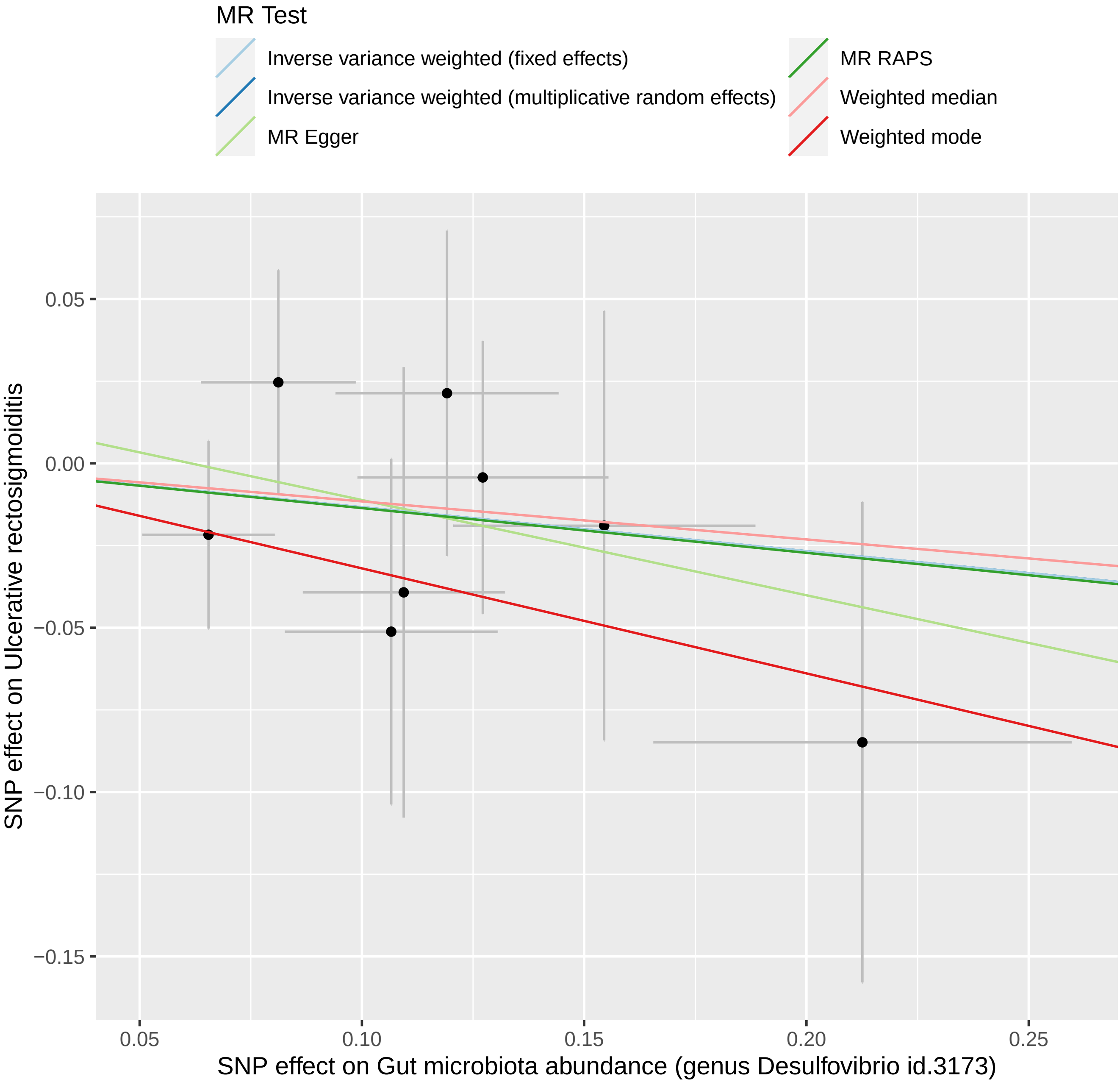

Batch 1562 : Gut microbiota abundance (genus Dialister id.2183) on Ulcerative rectosigmoiditis

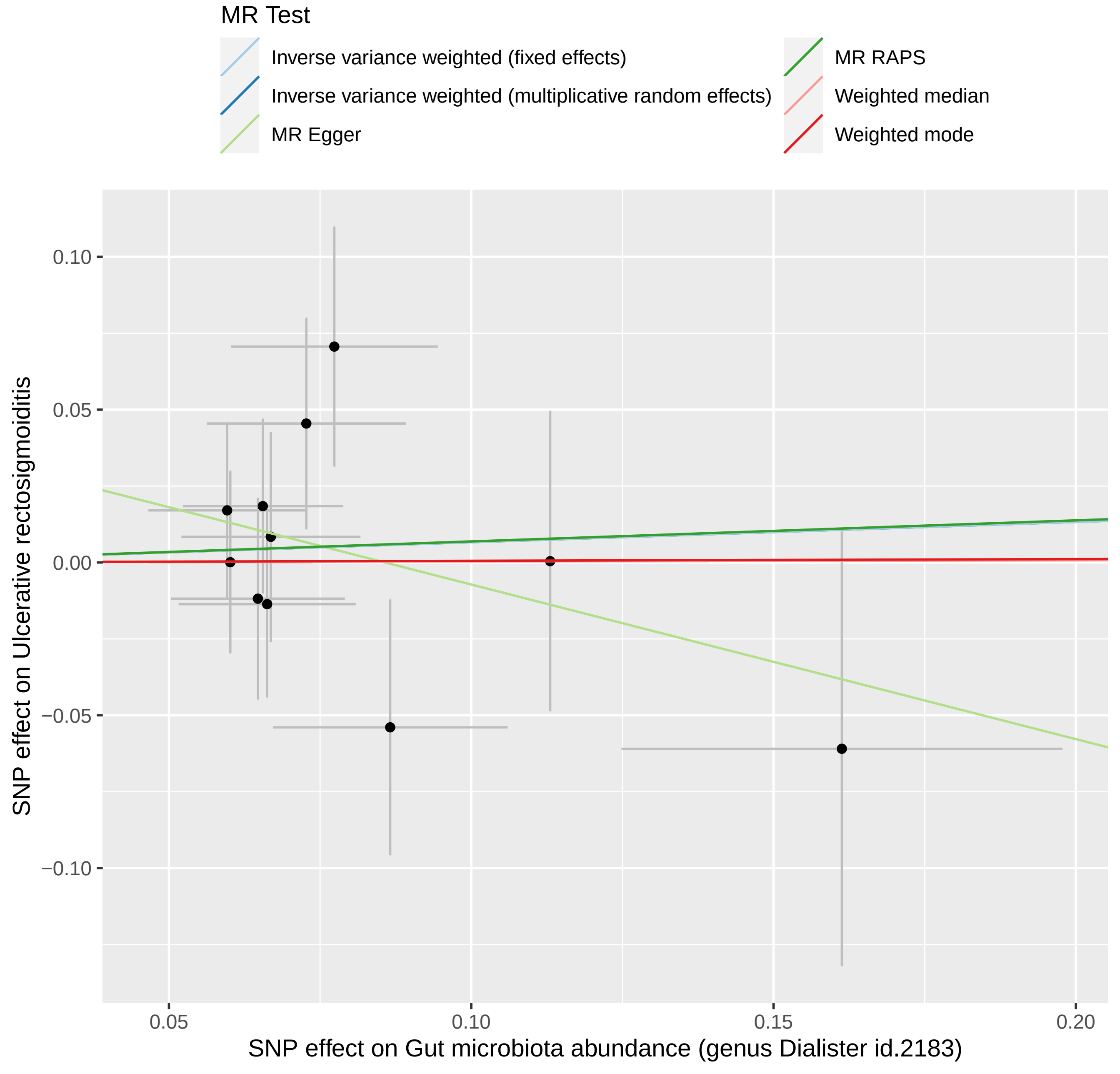

Batch 1563 : Gut microbiota abundance (genus Dorea id.1997) on Ulcerative rectosigmoiditis

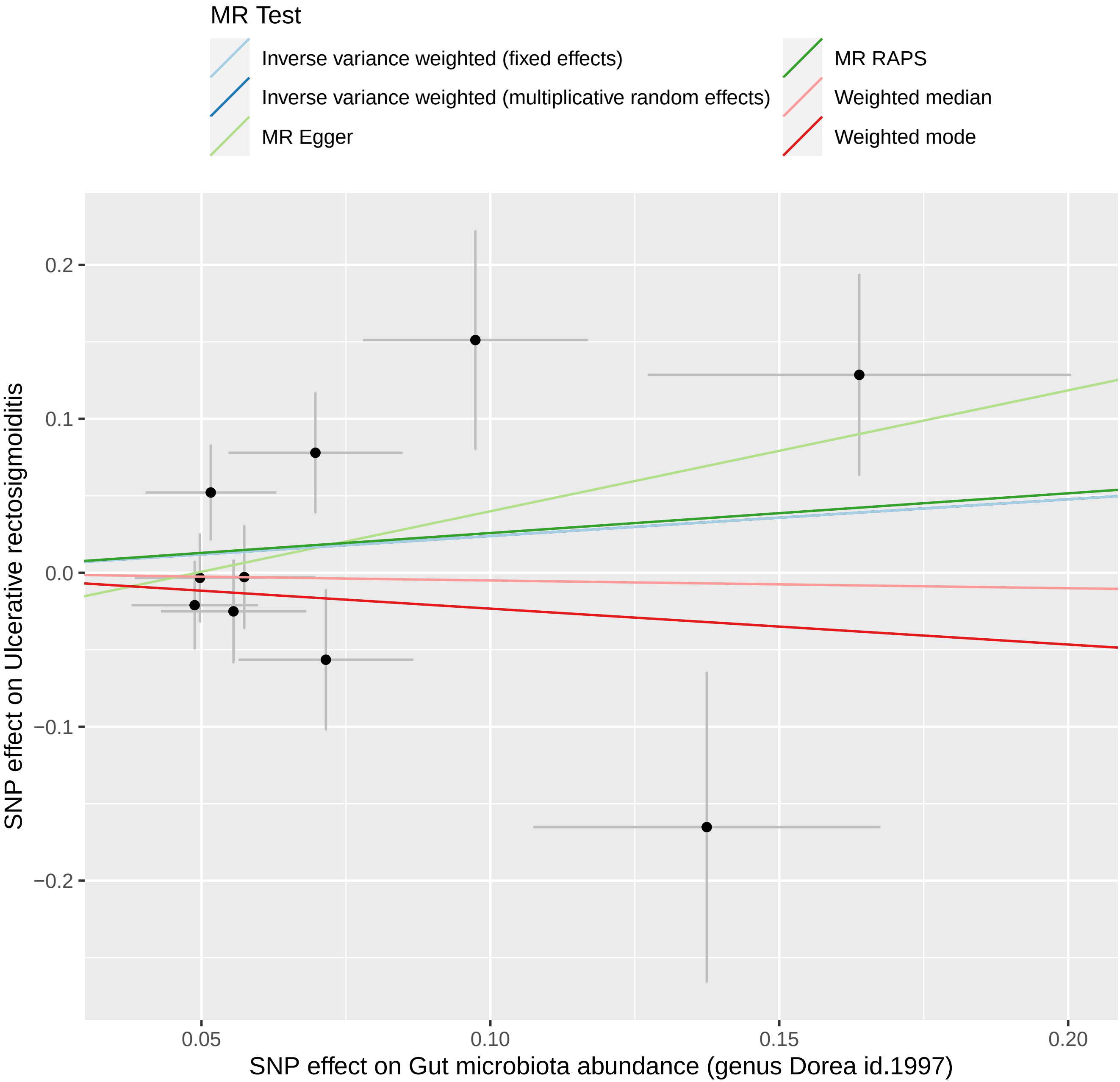

Batch 1564 : Gut microbiota abundance (genus Eggerthella id.819) on Ulcerative rectosigmoiditis

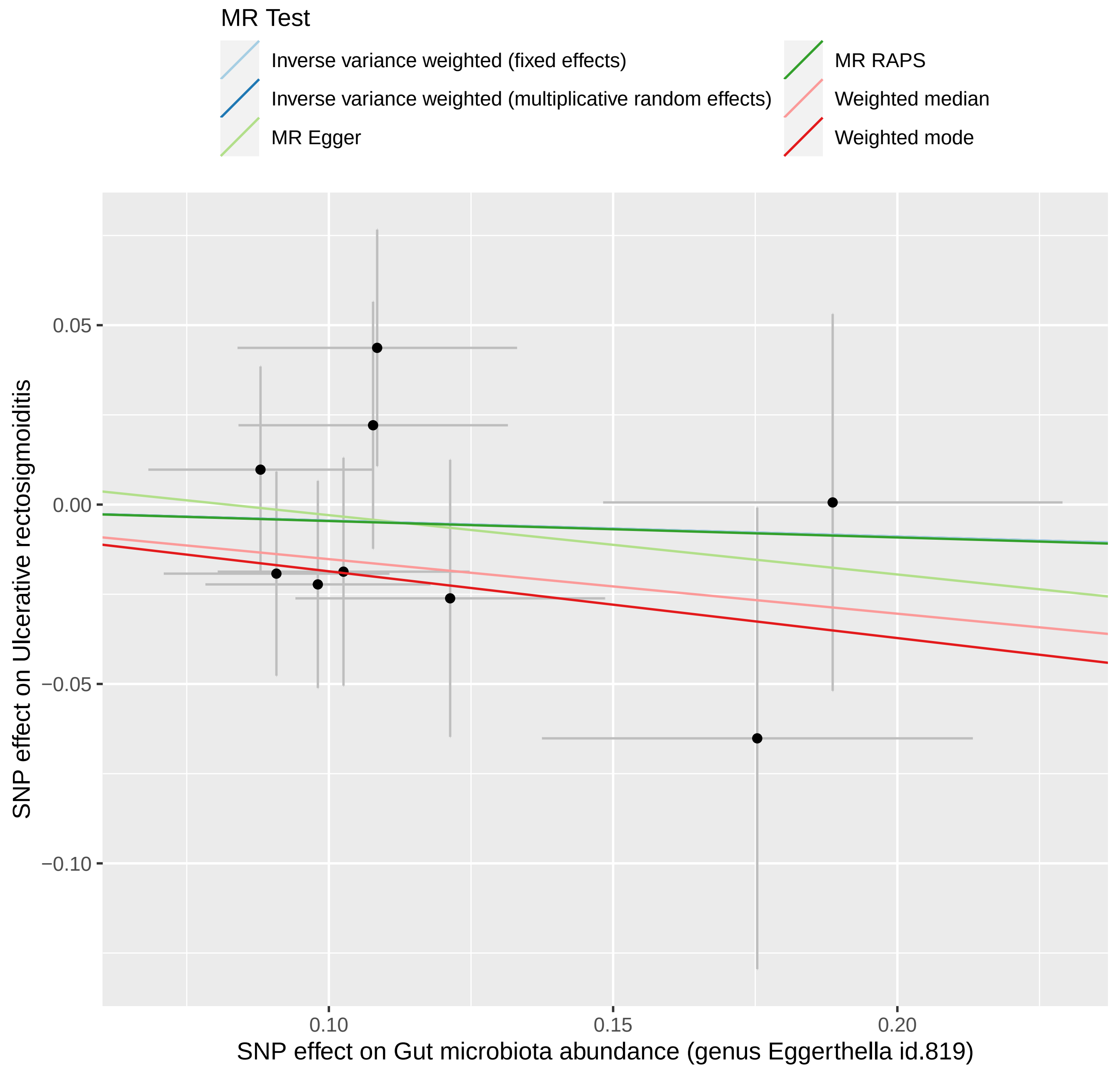

Batch 1565 : Gut microbiota abundance (genus Eisenbergiella id.11304) on Ulcerative rectosigmoiditis

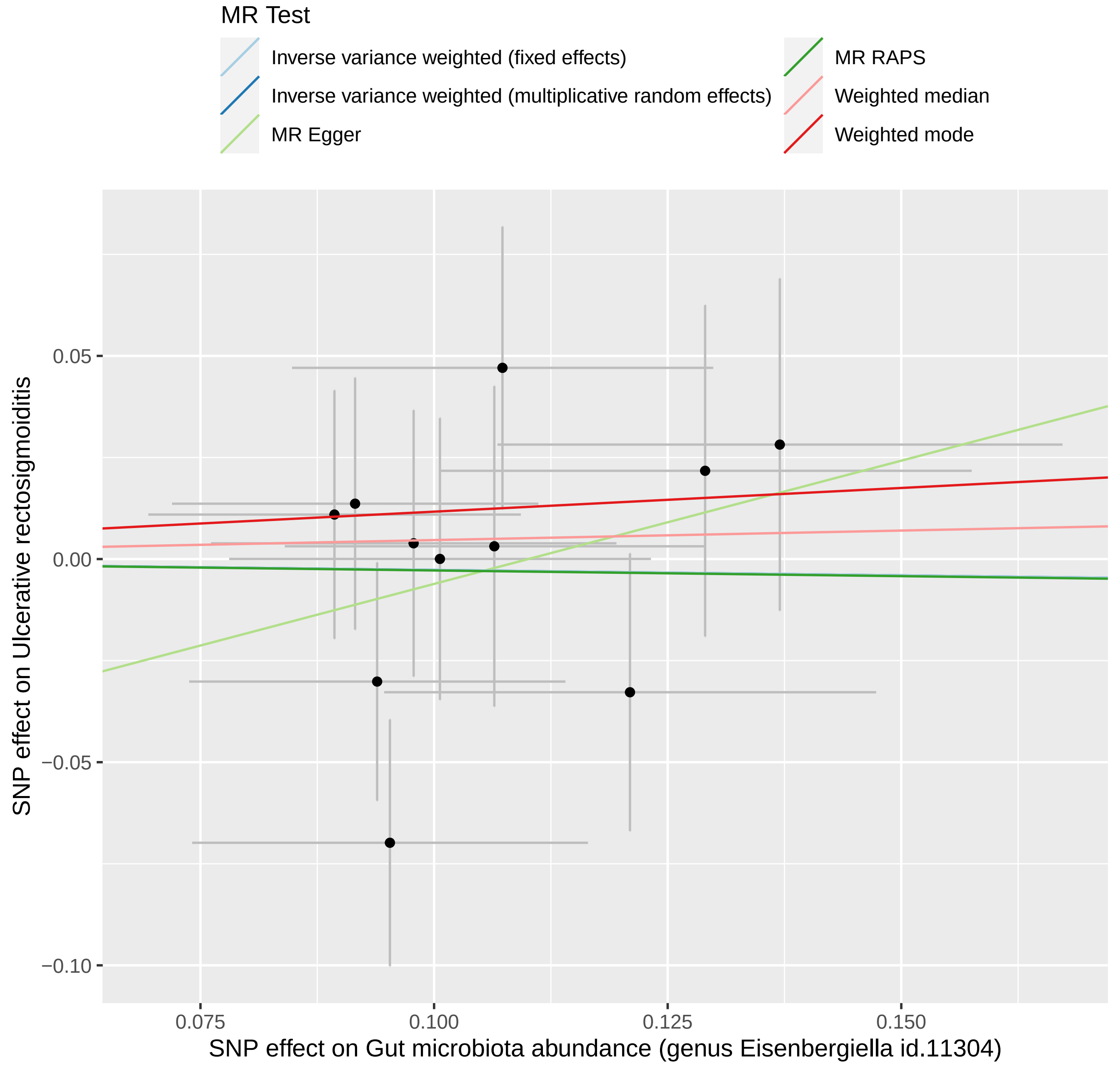

Batch 1566 : Gut microbiota abundance (genus Enterorhabdus id.820) on Ulcerative rectosigmoiditis

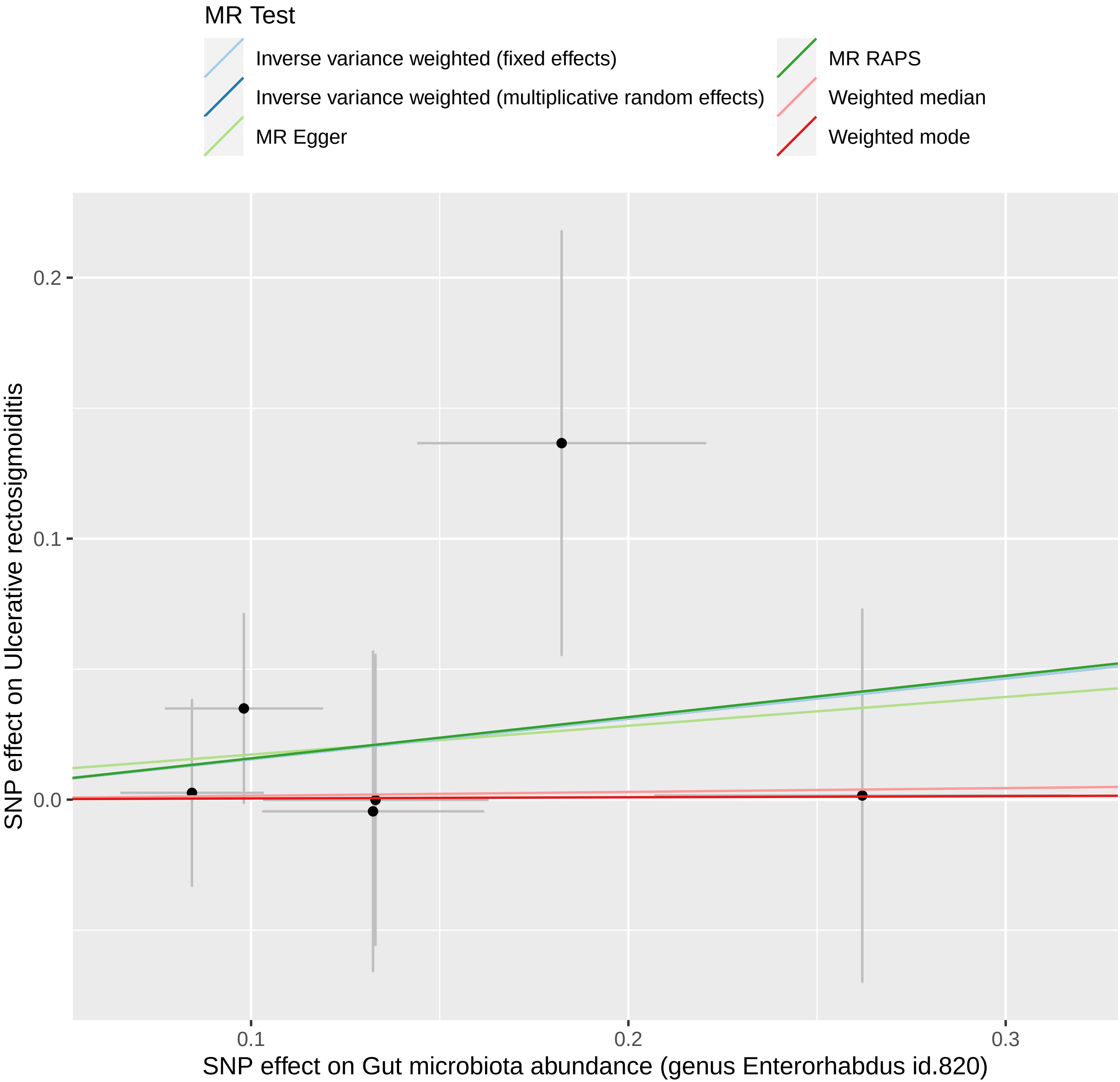

Batch 1567 : Gut microbiota abundance (genus Erysipelatoclostridium id.11381) on Ulcerative rectosigmoiditis

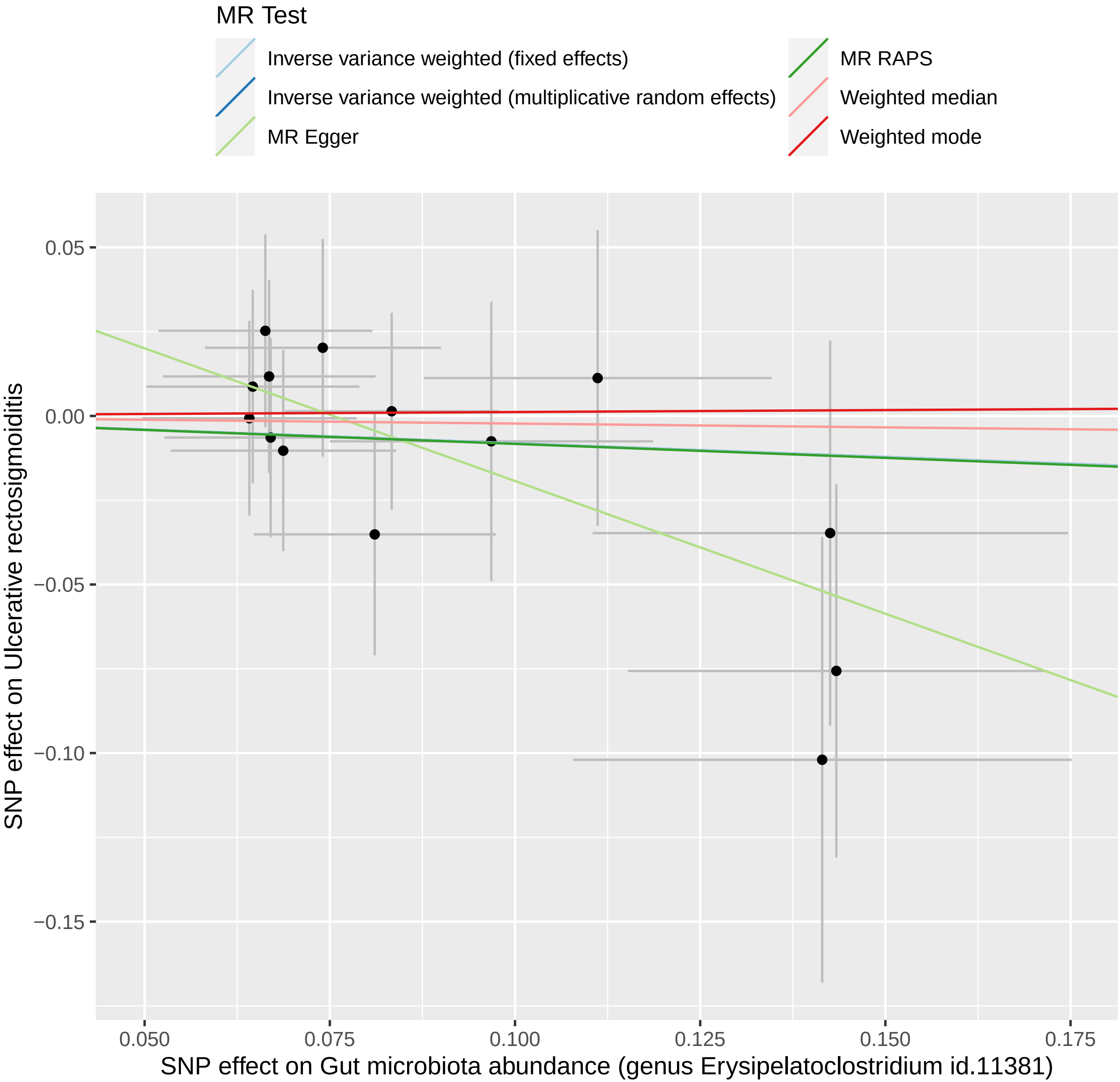

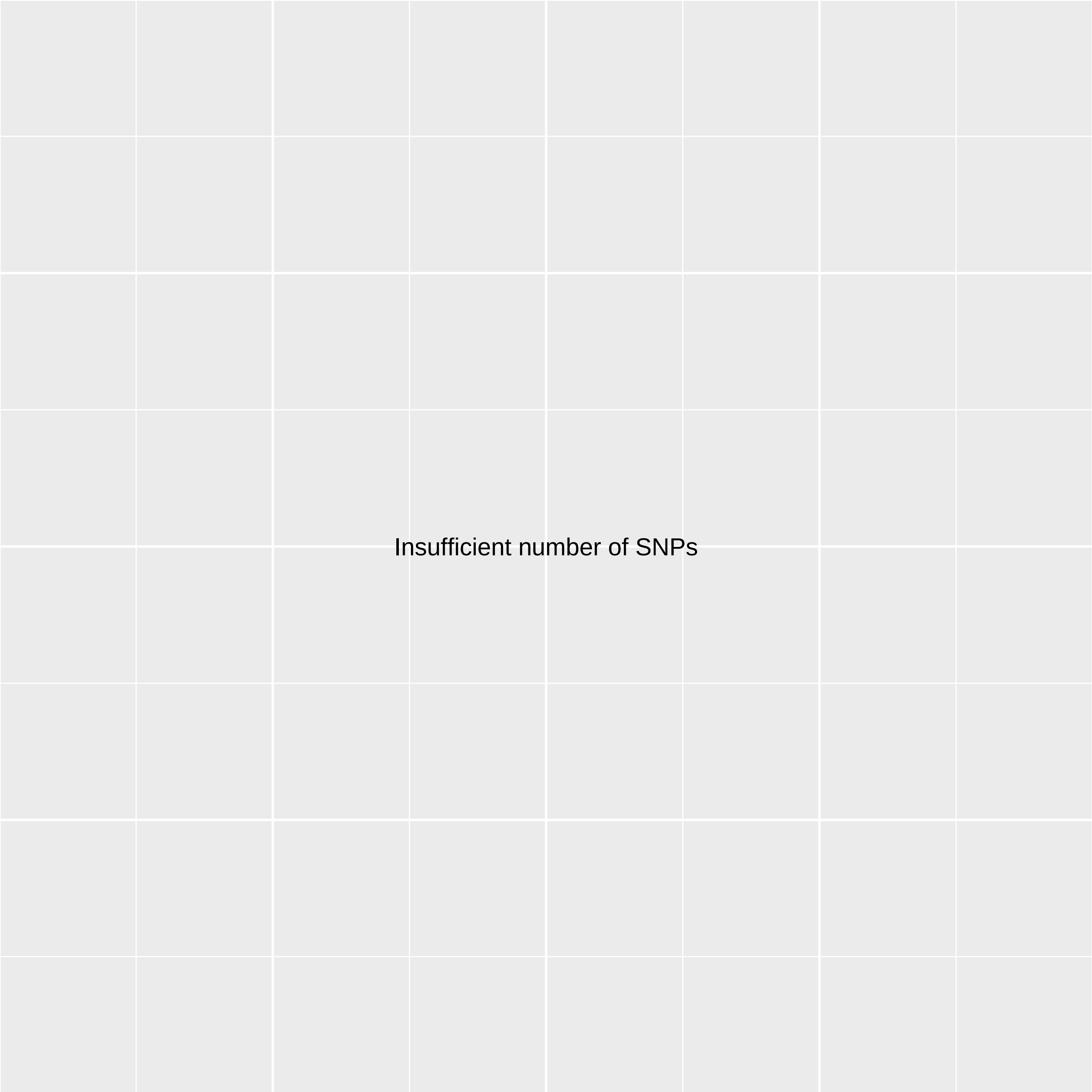
Insufficient number of SNPs

Batch 1569 : Gut microbiota abundance (genus Escherichia Shigella id.3504) on Ulcerative rectosigmoiditis

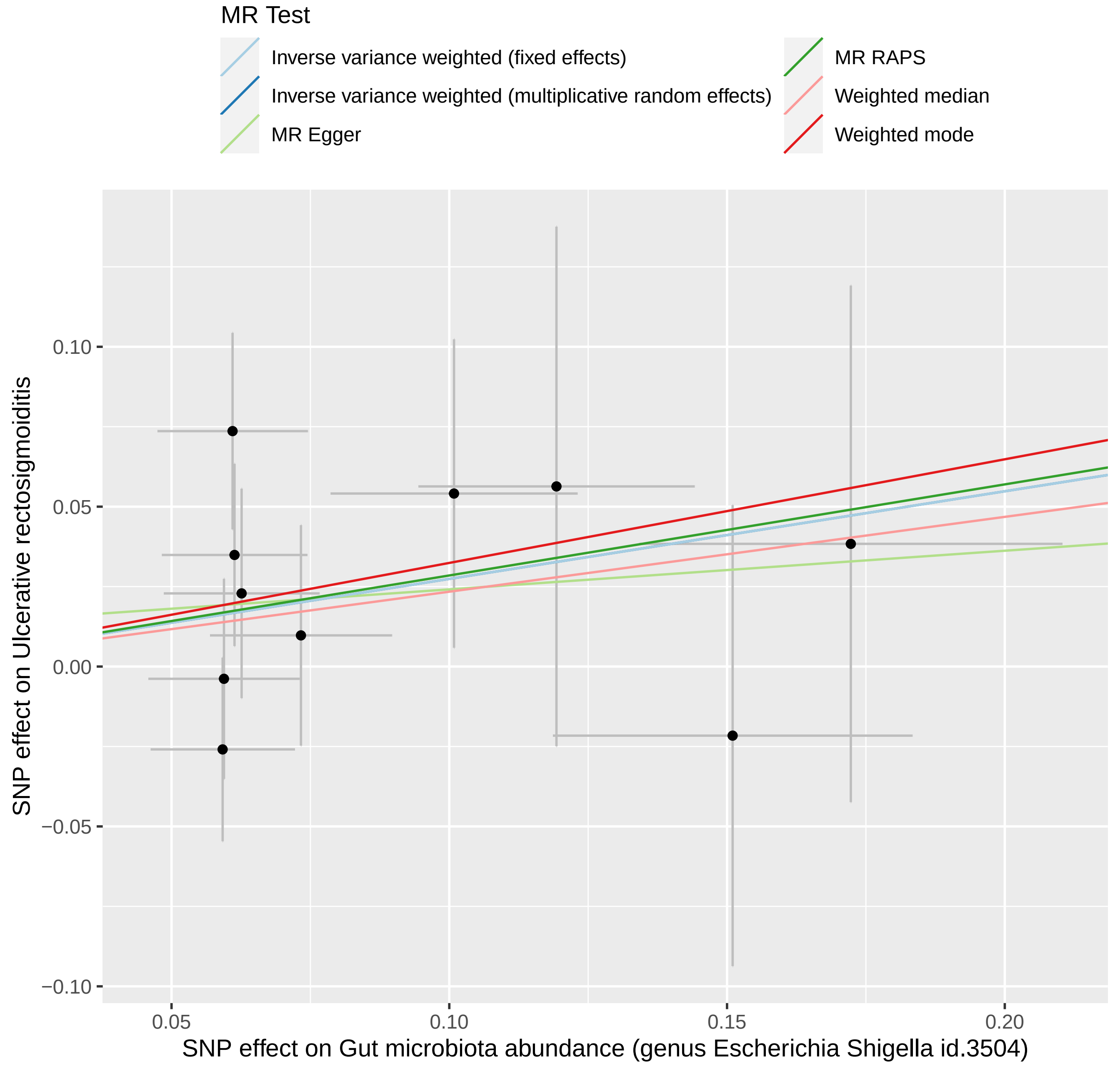

Batch 1570 : Gut microbiota abundance (genus Eubacterium brachy group id.11296) on Ulcerative rectosigmoiditis

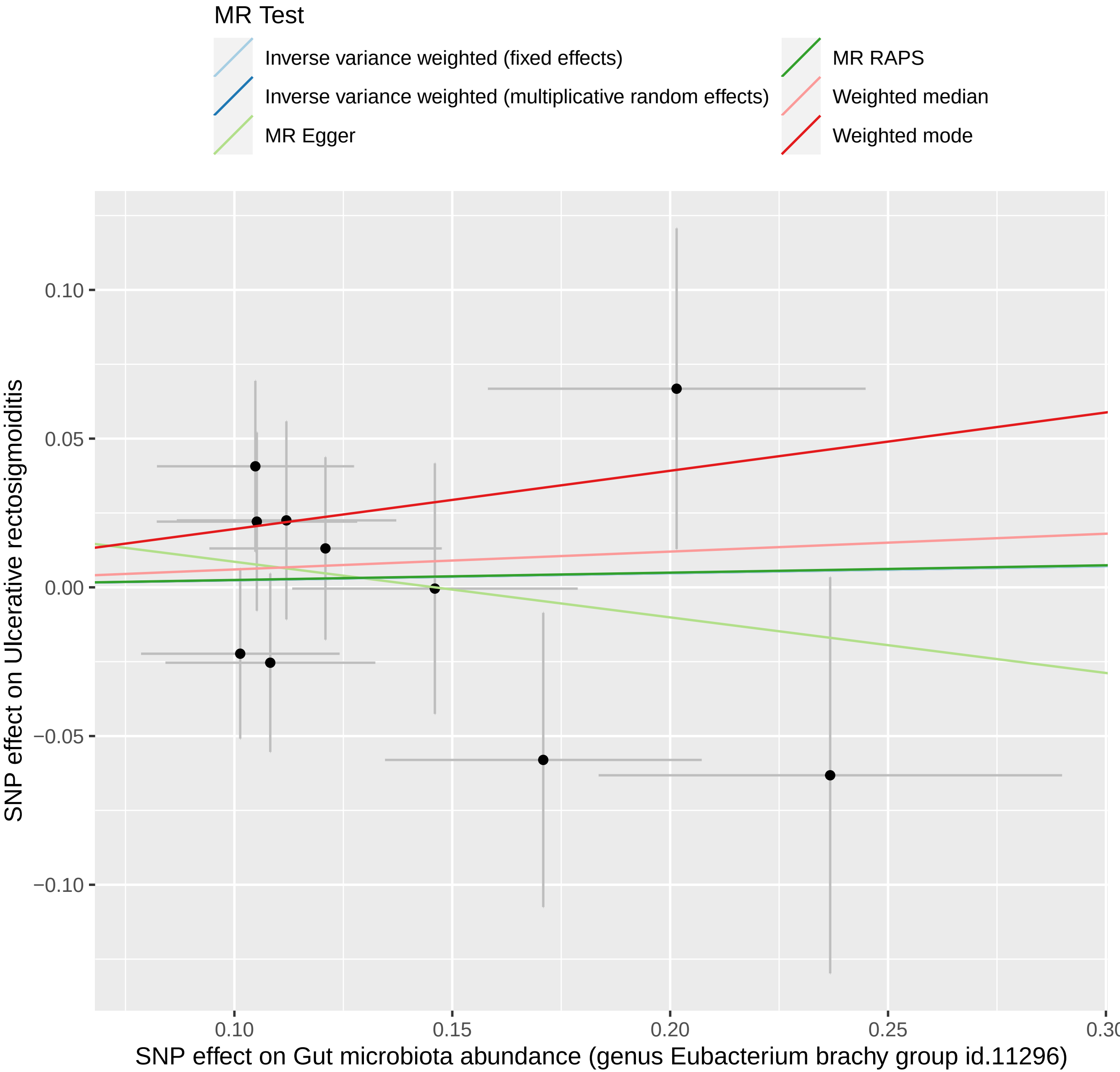

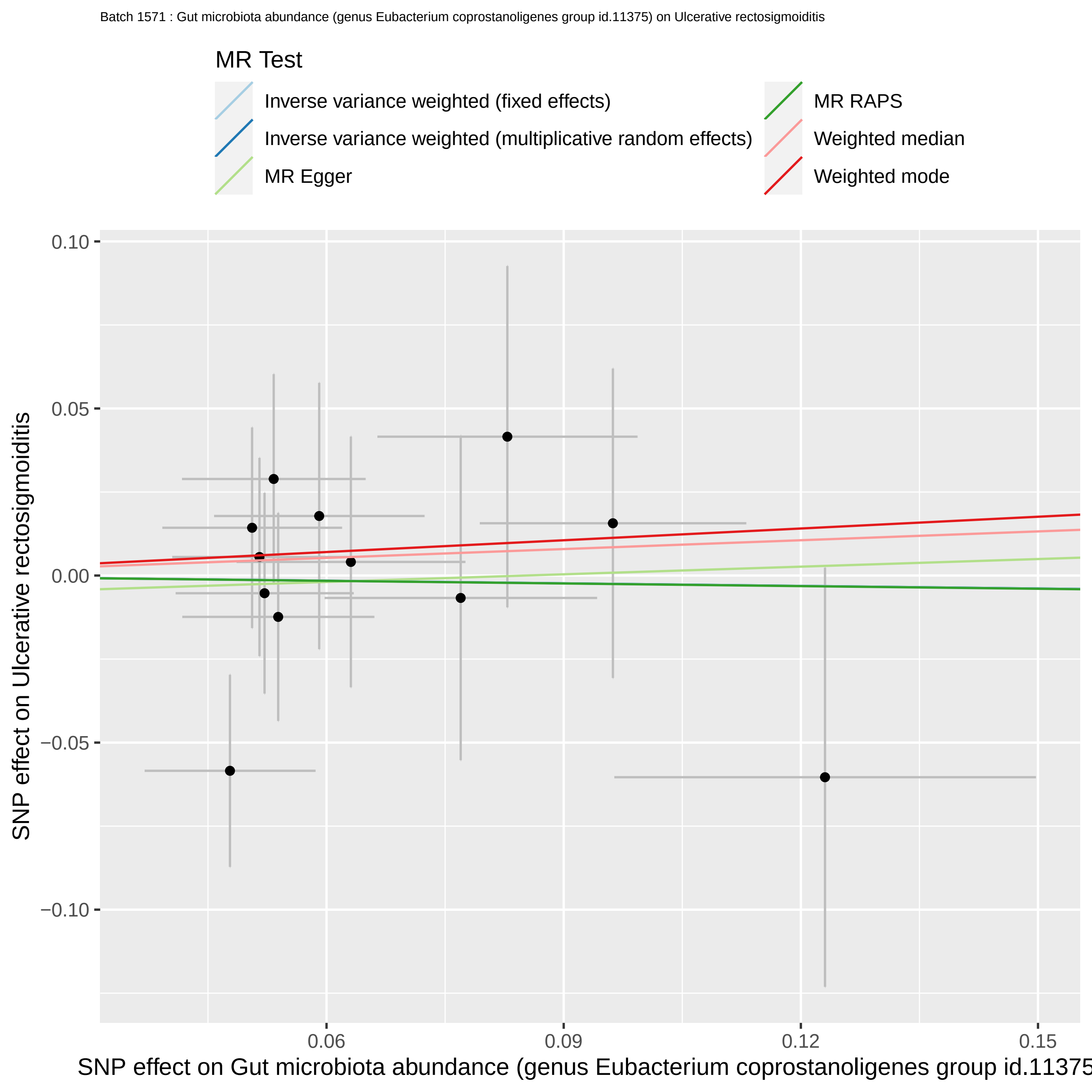

Batch 1571 : Gut microbiota abundance (genus Eubacterium coprostanoligenes group id.11375) on Ulcerative rectosigmoiditis
MR Test
Inverse variance weighted (fixed effects)
Inverse variance weighted (multiplicative random effects)
MR Egger
MR RAPS
Weighted median
Weighted mode
0.10
0.05
0.00
−0.05
−0.10
SNP effect on Ulcerative rectosigmoiditis
0.06
0.09
0.12
0.15
SNP effect on Gut microbiota abundance (genus Eubacterium coprostanoligenes group id.11375

Batch 1572 : Gut microbiota abundance (genus Eubacterium eligens group id.14372) on Ulcerative rectosigmoiditis

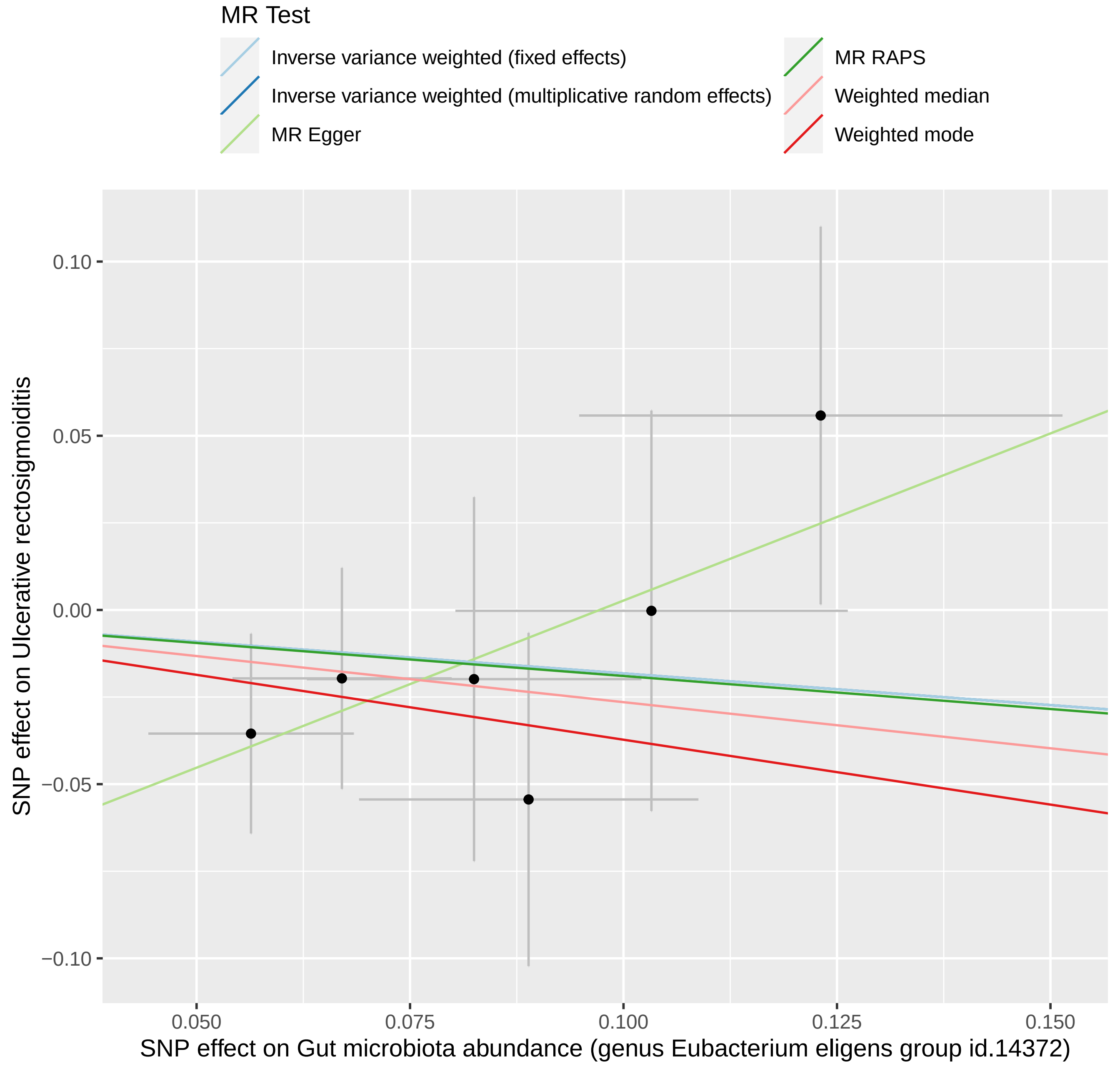

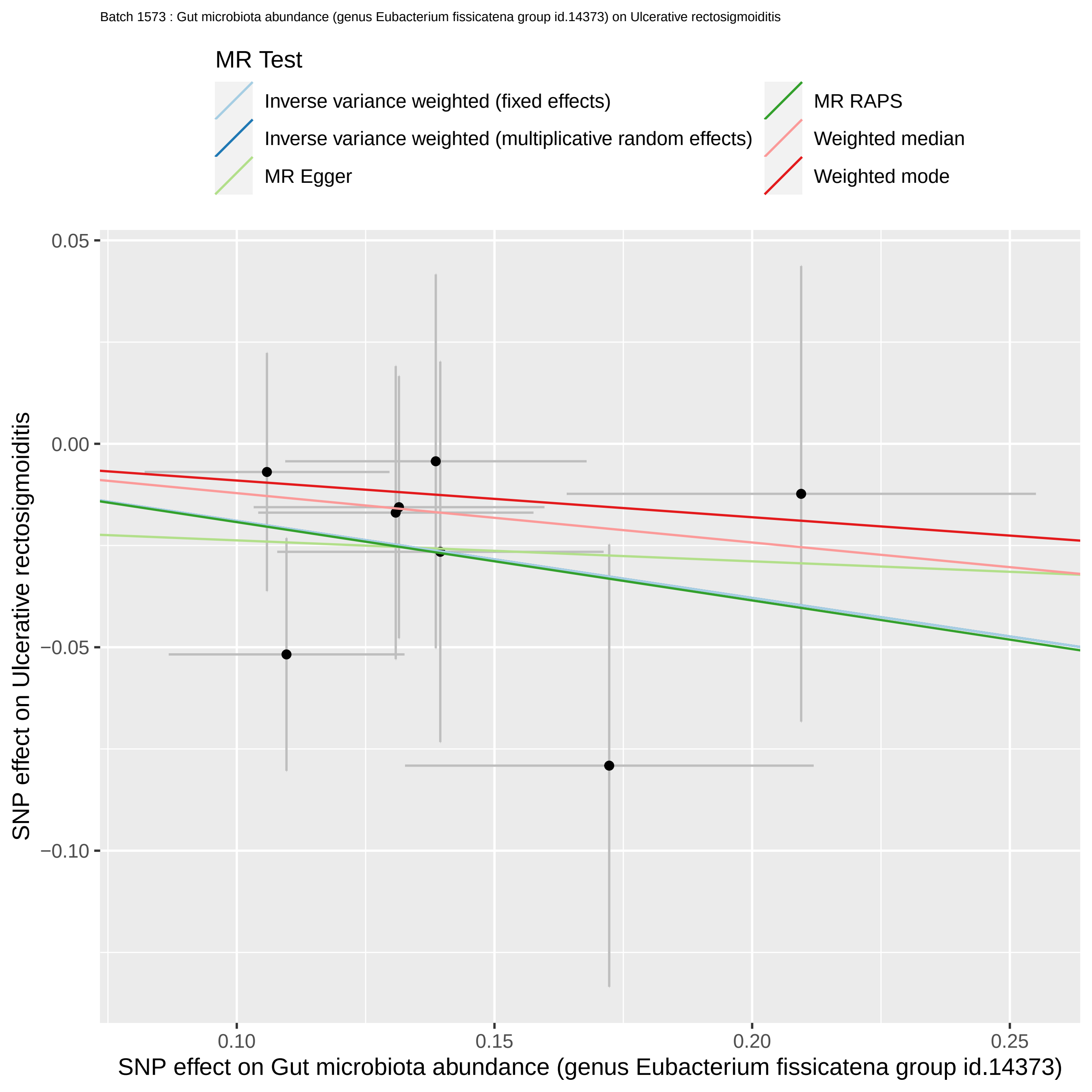

Batch 1573 : Gut microbiota abundance (genus Eubacterium fissicatena group id.14373) on Ulcerative rectosigmoiditis
MR Test
Inverse variance weighted (fixed effects)
Inverse variance weighted (multiplicative random effects)
MR Egger
MR RAPS
Weighted median
Weighted mode
SNP effect on Ulcerative rectosigmoiditis
SNP effect on Gut microbiota abundance (genus Eubacterium fissicatena group id.14373)
0.05
0.00
−0.05
−0.10
0.10
0.15
0.20
0.25

Batch 1574 : Gut microbiota abundance (genus Eubacterium hallii group id.11338) on Ulcerative rectosigmoiditis

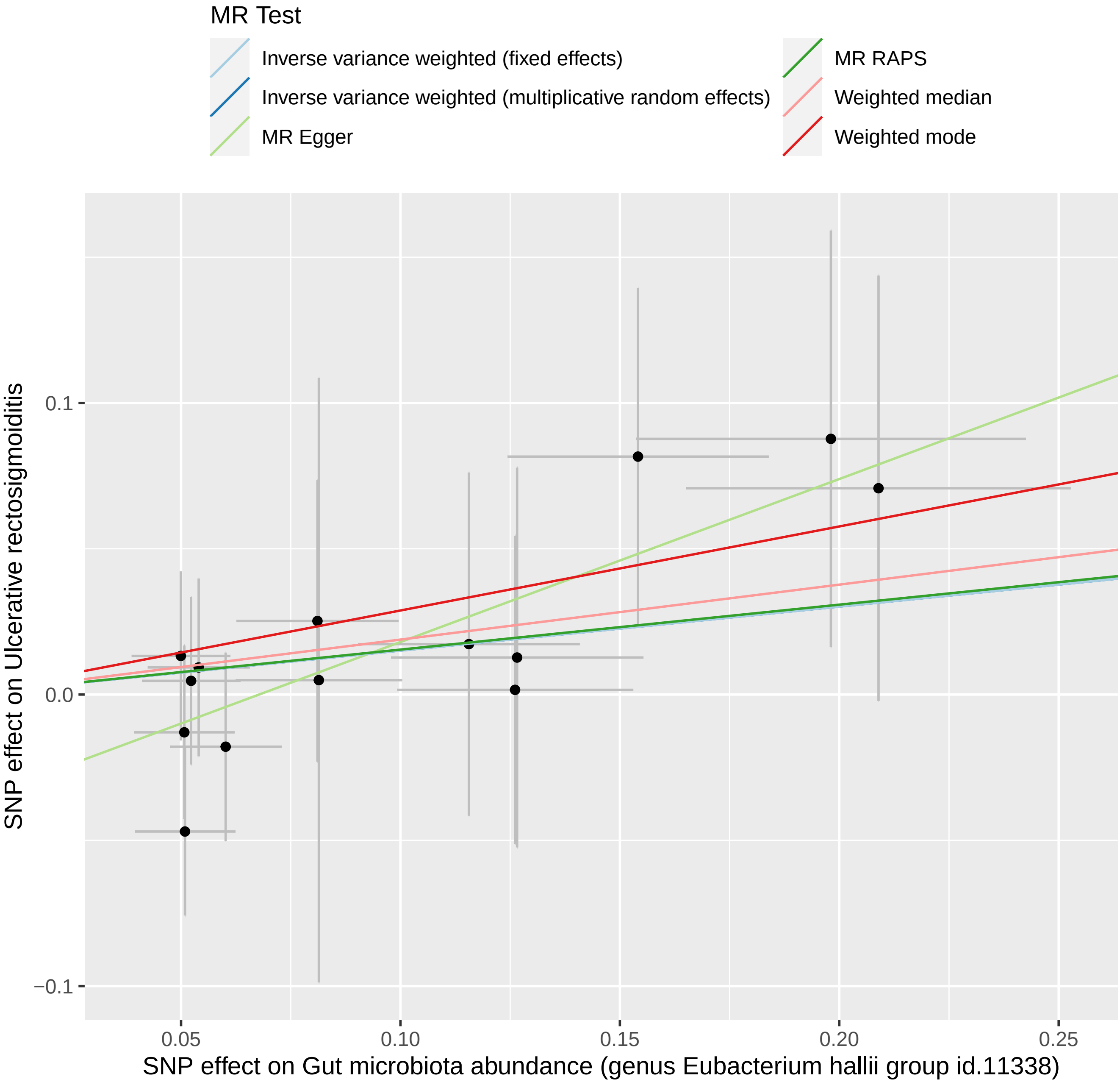

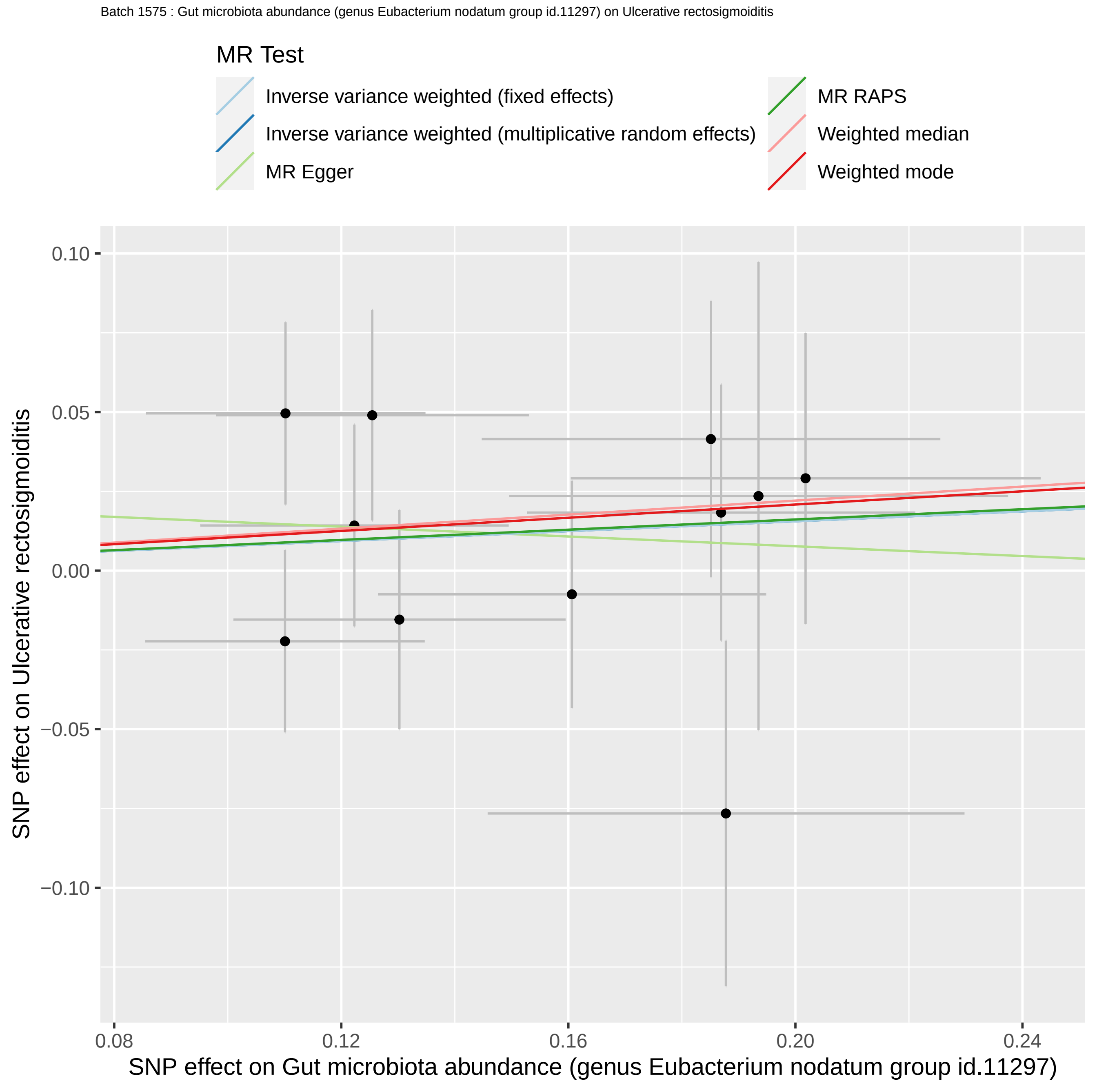
Batch 1575 : Gut microbiota abundance (genus Eubacterium nodatum group id.11297) on Ulcerative rectosigmoiditis
MR Test
Inverse variance weighted (fixed effects)
Inverse variance weighted (multiplicative random effects)
MR Egger
MR RAPS
Weighted median
Weighted mode
0.10
0.05
0.00
−0.05
−0.10
0.08
0.12
0.16
0.20
0.24
SNP effect on Ulcerative rectosigmoiditis
SNP effect on Gut microbiota abundance (genus Eubacterium nodatum group id.11297)

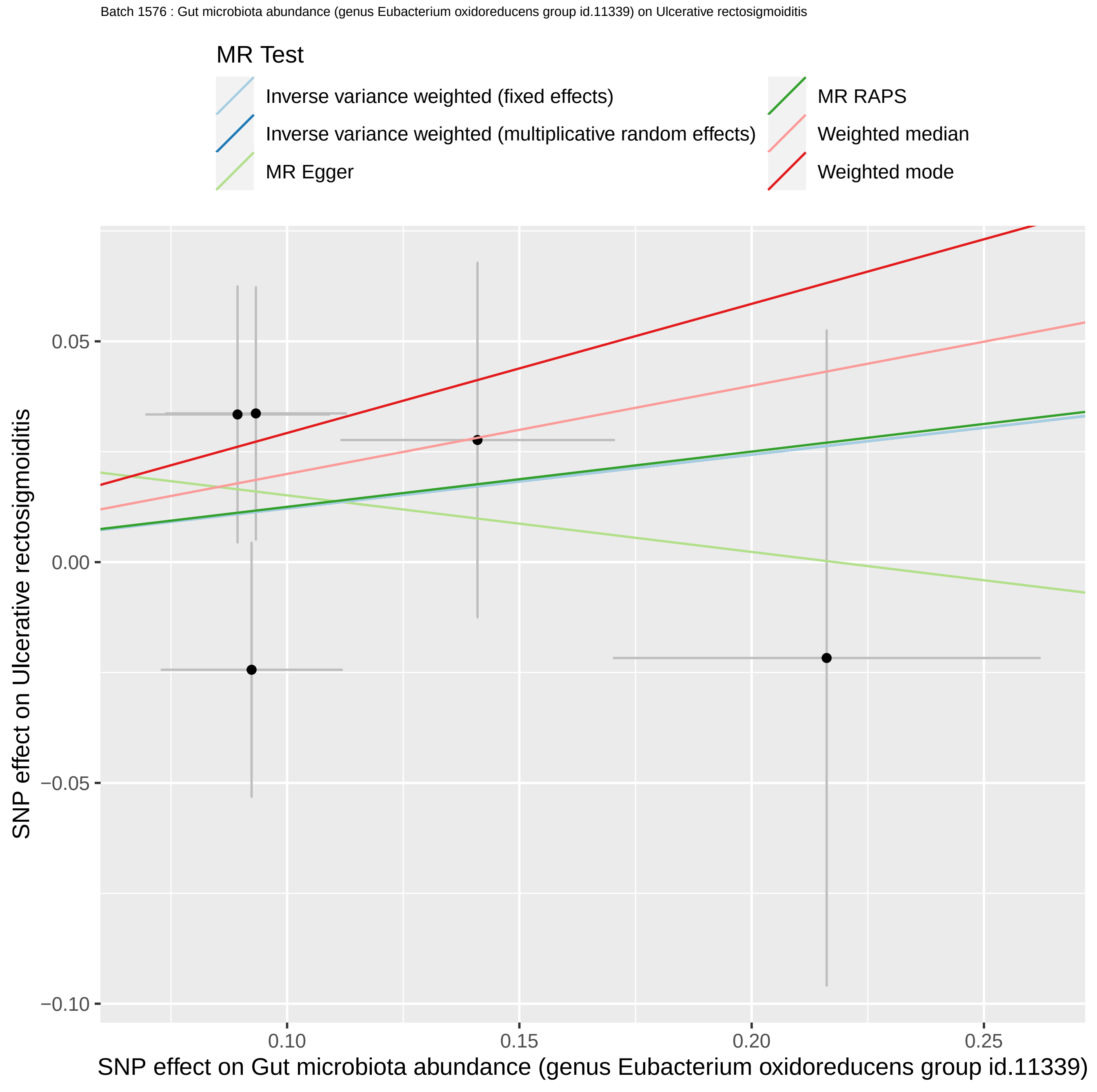
Batch 1576 : Gut microbiota abundance (genus Eubacterium oxidoreducens group id.11339) on Ulcerative rectosigmoiditis
MR Test
Inverse variance weighted (fixed effects)
Inverse variance weighted (multiplicative random effects)
MR Egger
MR RAPS
Weighted median
Weighted mode
0.05
0.00
−0.05
−0.10
0.10
0.15
0.20
0.25
SNP effect on Ulcerative rectosigmoiditis
SNP effect on Gut microbiota abundance (genus Eubacterium oxidoreducens group id.11339)

Batch 1577 : Gut microbiota abundance (genus Eubacterium rectale group id.14374) on Ulcerative rectosigmoiditis

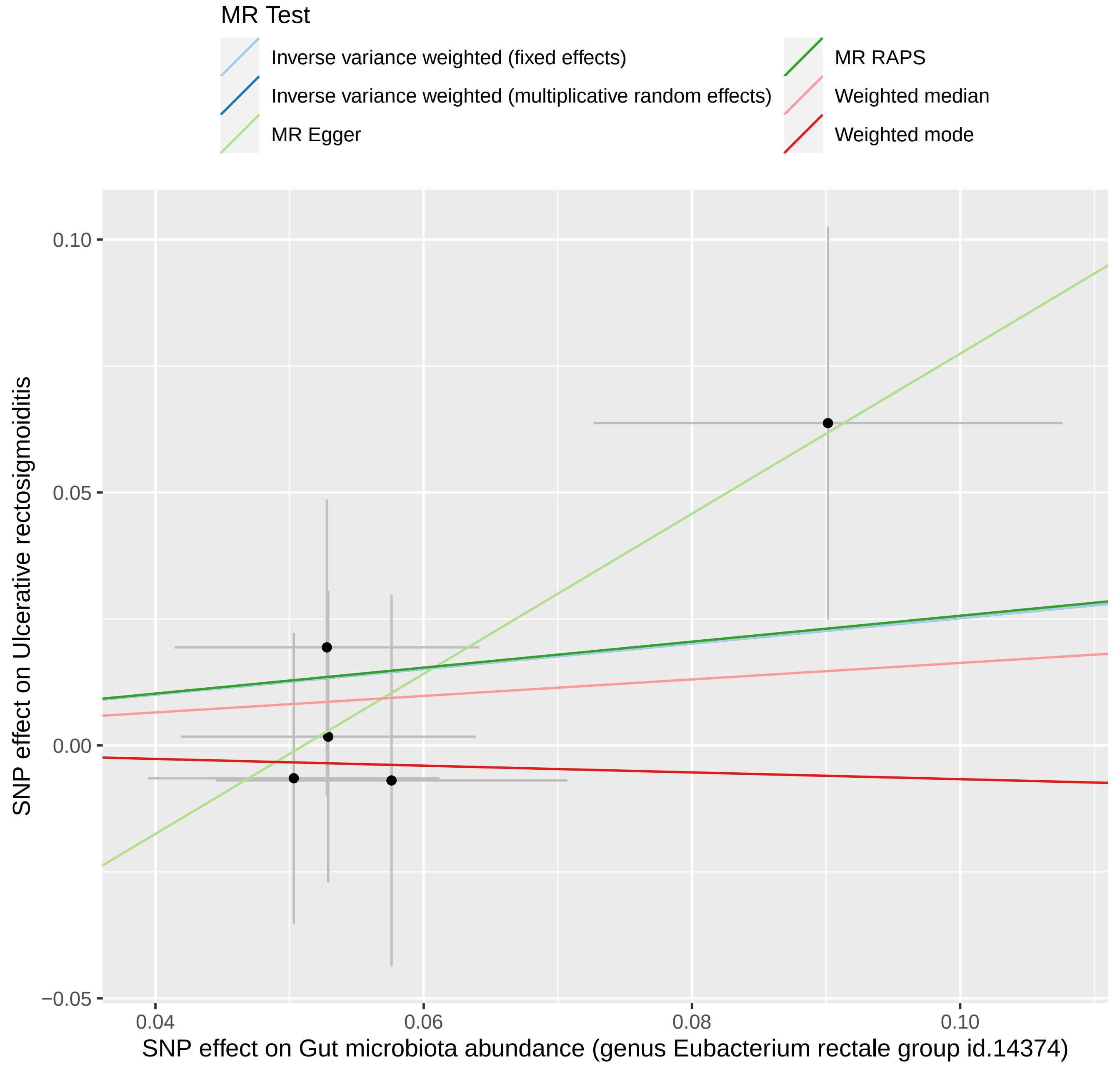

Batch 1578 : Gut microbiota abundance (genus Eubacterium ruminantium group id.11340) on Ulcerative rectosigmoiditis

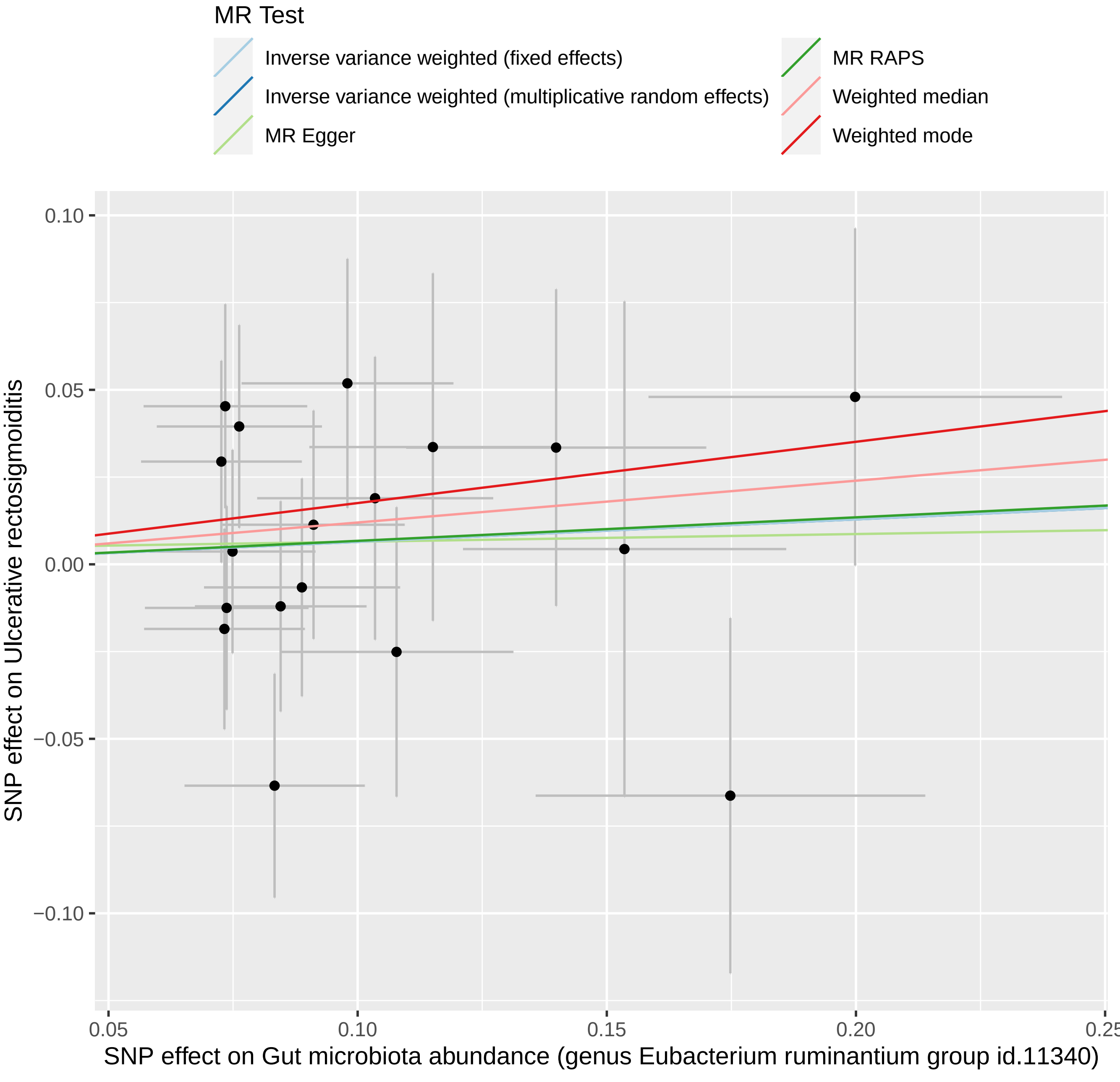

Batch 1579 : Gut microbiota abundance (genus Eubacterium ventriosum group id.11341) on Ulcerative rectosigmoiditis

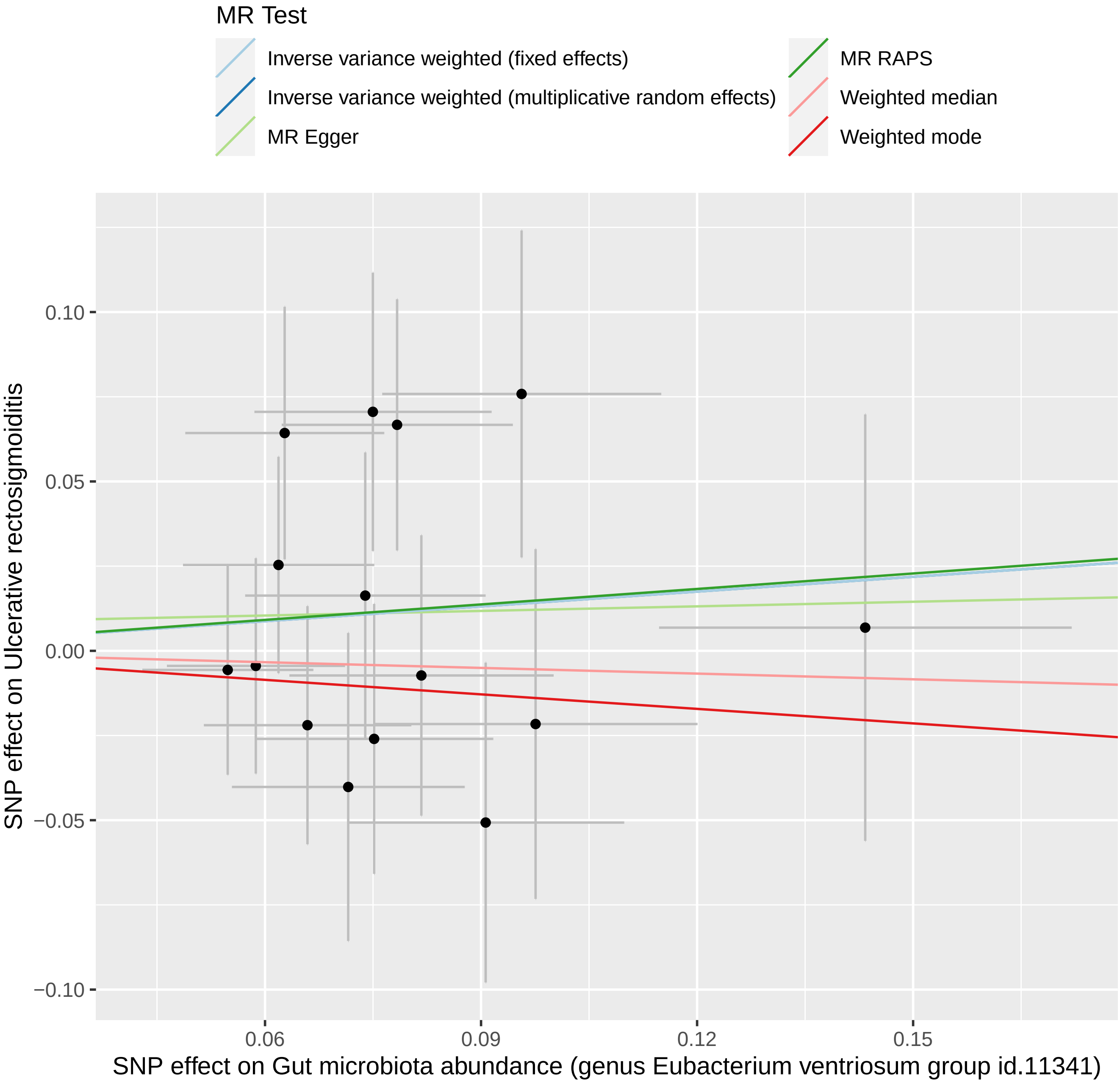

Batch 1580 : Gut microbiota abundance (genus Eubacterium xylanophilum group id.14375) on Ulcerative rectosigmoiditis

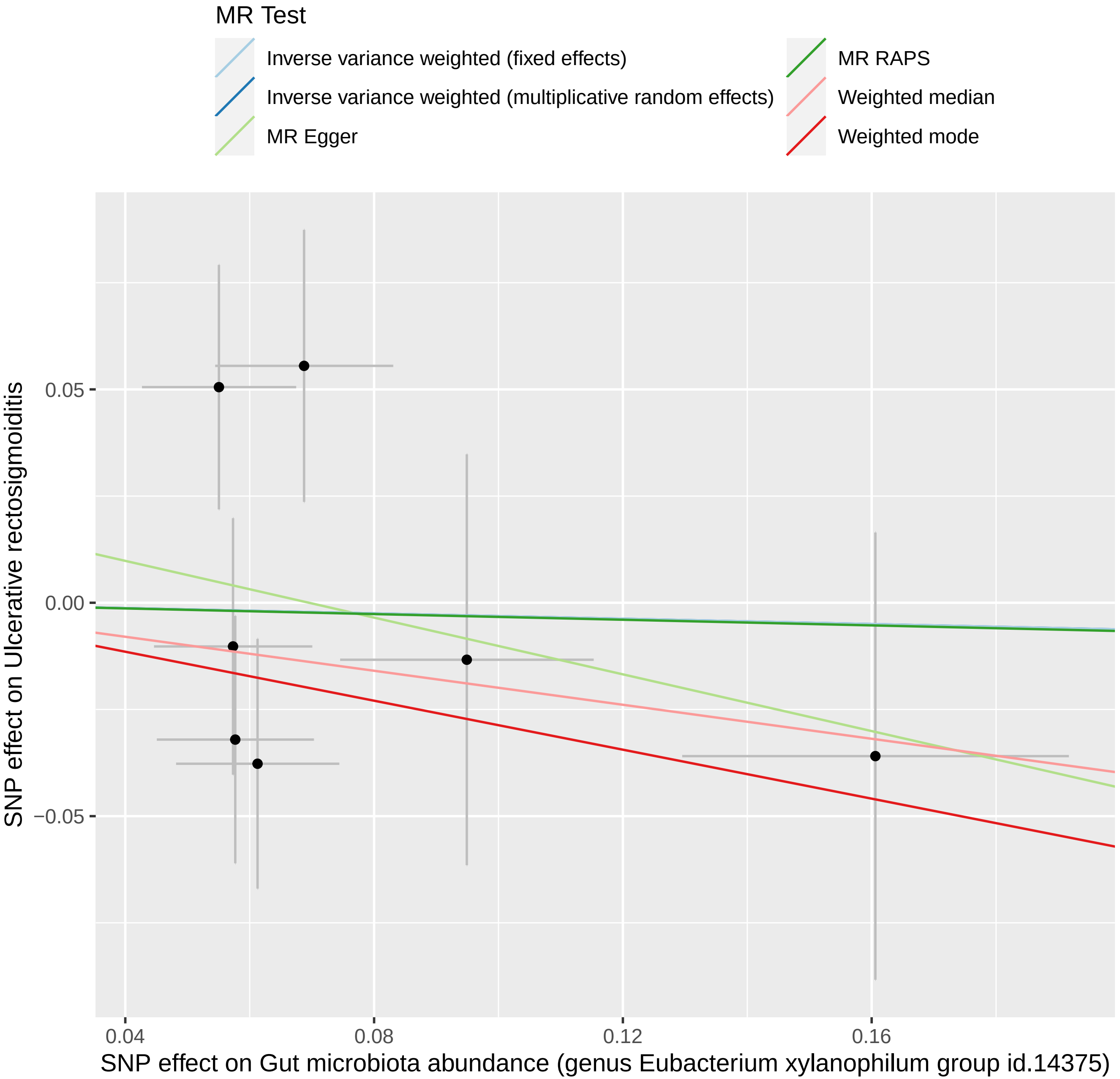

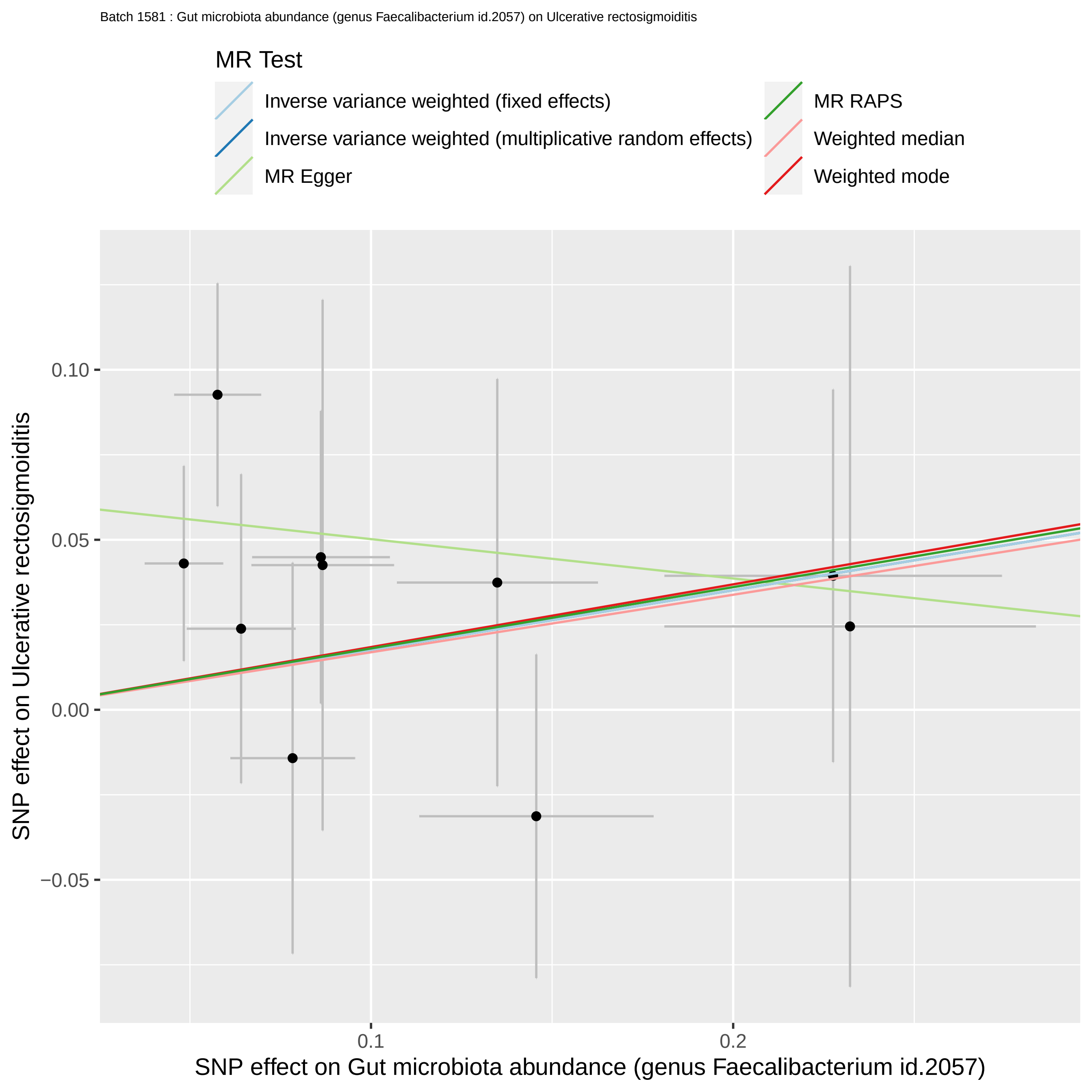

Batch 1581 : Gut microbiota abundance (genus Faecalibacterium id.2057) on Ulcerative rectosigmoiditis
MR Test
Inverse variance weighted (fixed effects)
Inverse variance weighted (multiplicative random effects)
MR Egger
MR RAPS
Weighted median
Weighted mode
0.10
0.05
0.00
−0.05
0.1
0.2
SNP effect on Ulcerative rectosigmoiditis
SNP effect on Gut microbiota abundance (genus Faecalibacterium id.2057)

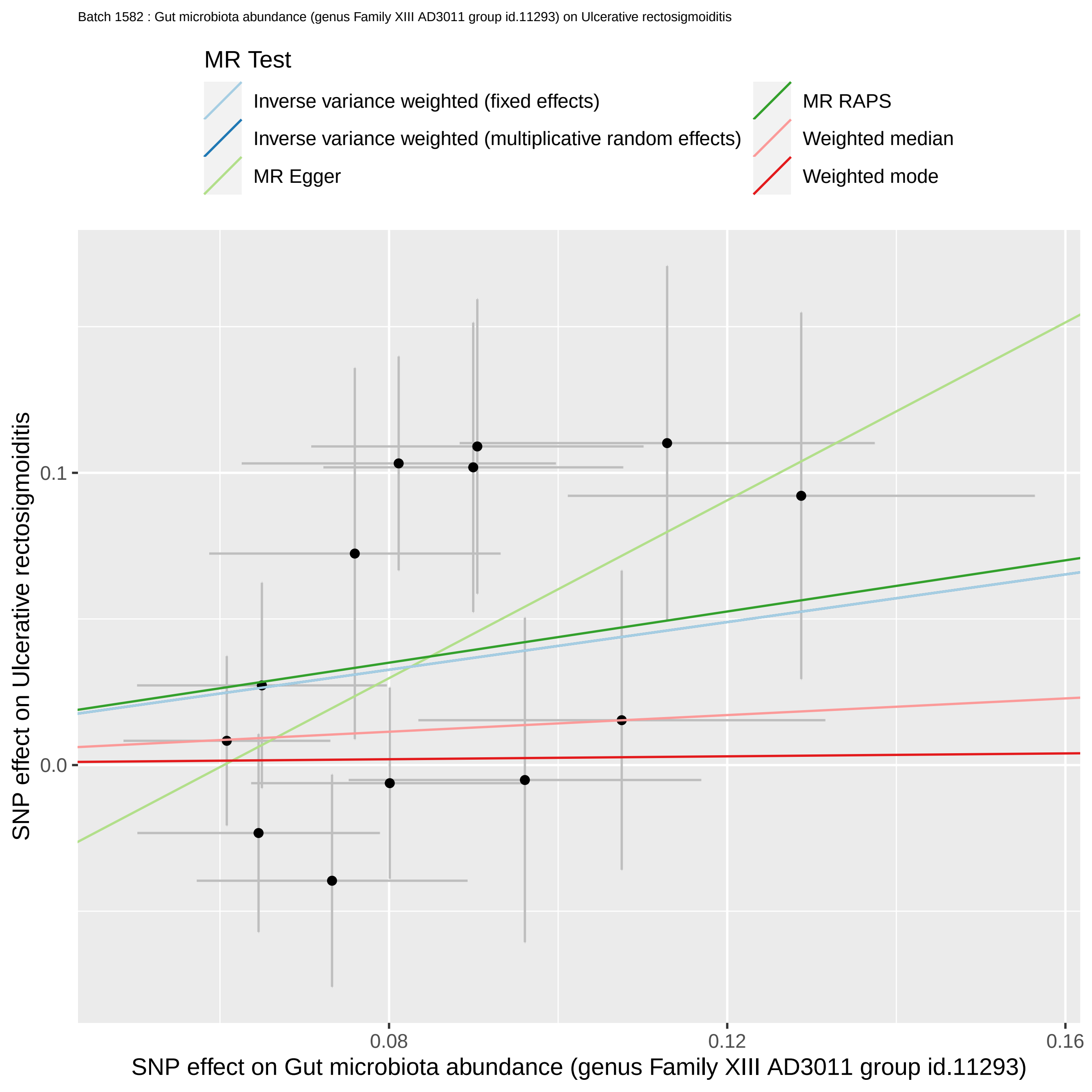

Batch 1582 : Gut microbiota abundance (genus Family XIII AD3011 group id.11293) on Ulcerative rectosigmoiditis
MR Test
Inverse variance weighted (fixed effects)
Inverse variance weighted (multiplicative random effects)
MR Egger
MR RAPS
Weighted median
Weighted mode
SNP effect on Ulcerative rectosigmoiditis
0.1
0.0
0.08
0.12
0.16
SNP effect on Gut microbiota abundance (genus Family XIII AD3011 group id.11293)

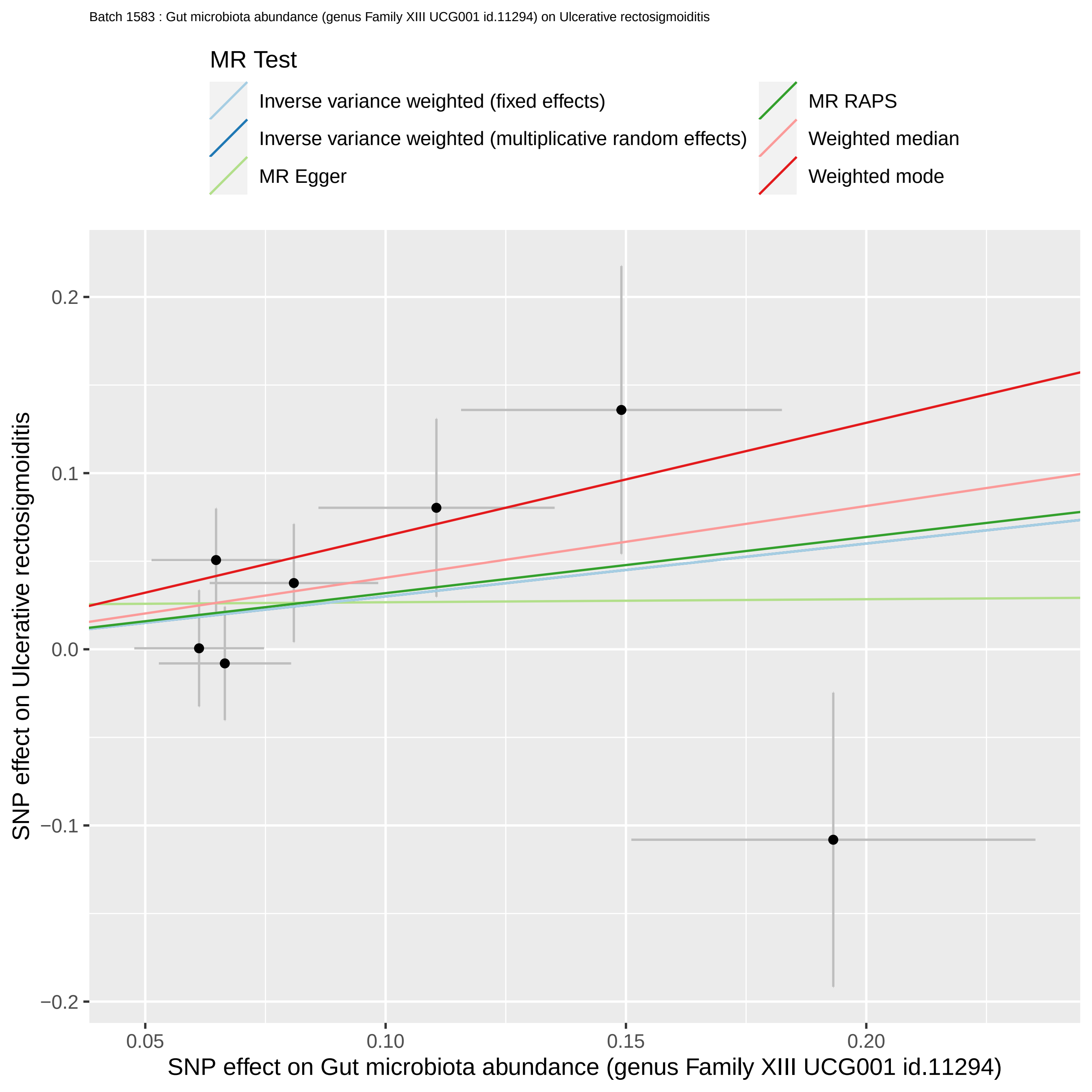

Batch 1583 : Gut microbiota abundance (genus Family XIII UCG001 id.11294) on Ulcerative rectosigmoiditis
MR Test
Inverse variance weighted (fixed effects)
Inverse variance weighted (multiplicative random effects)
MR Egger
MR RAPS
Weighted median
Weighted mode
0.2
0.1
0.0
−0.1
−0.2
0.05
0.10
0.15
0.20
SNP effect on Ulcerative rectosigmoiditis
SNP effect on Gut microbiota abundance (genus Family XIII UCG001 id.11294)

Batch 1584 : Gut microbiota abundance (genus Flavonifractor id.2059) on Ulcerative rectosigmoiditis

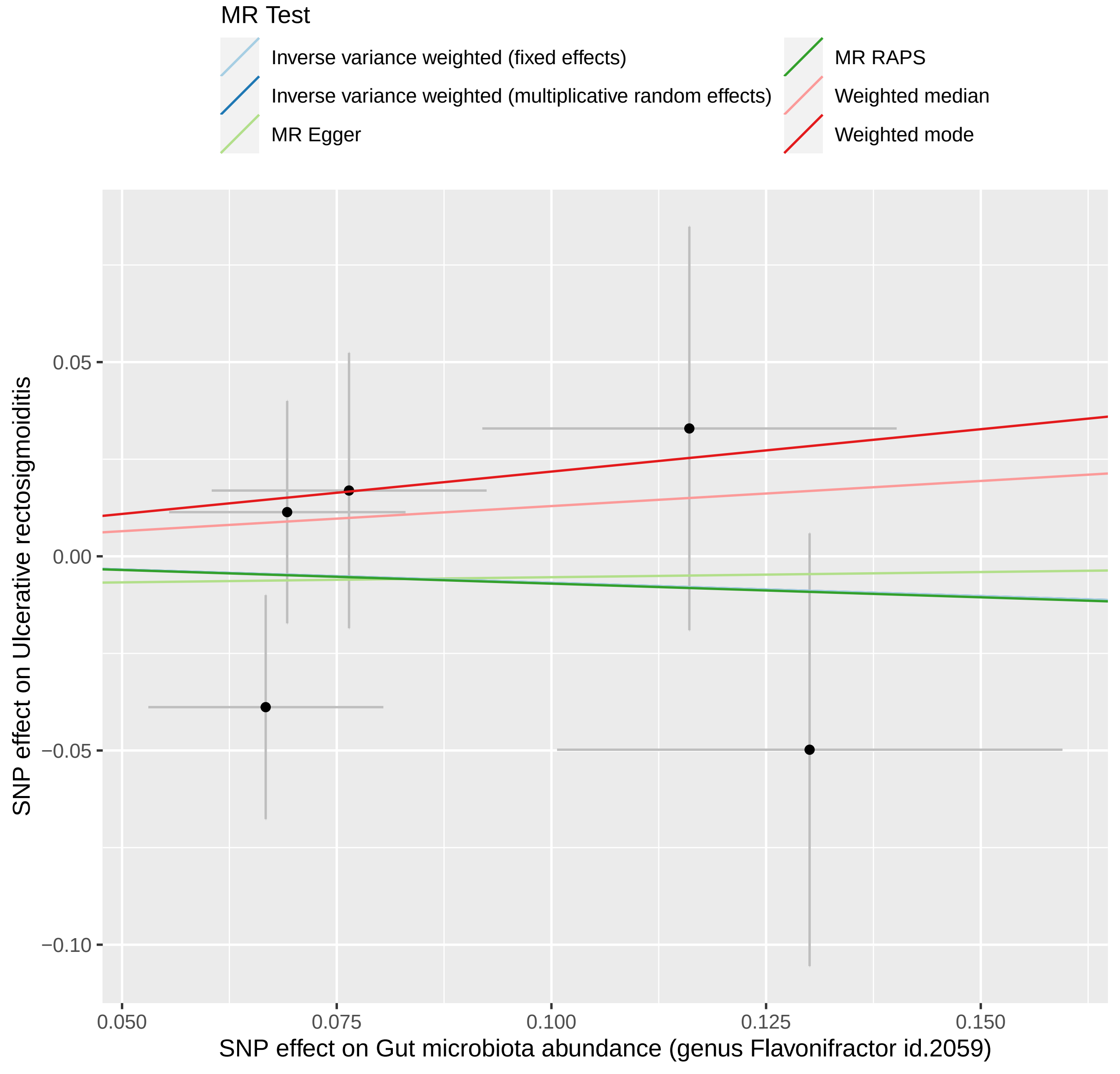

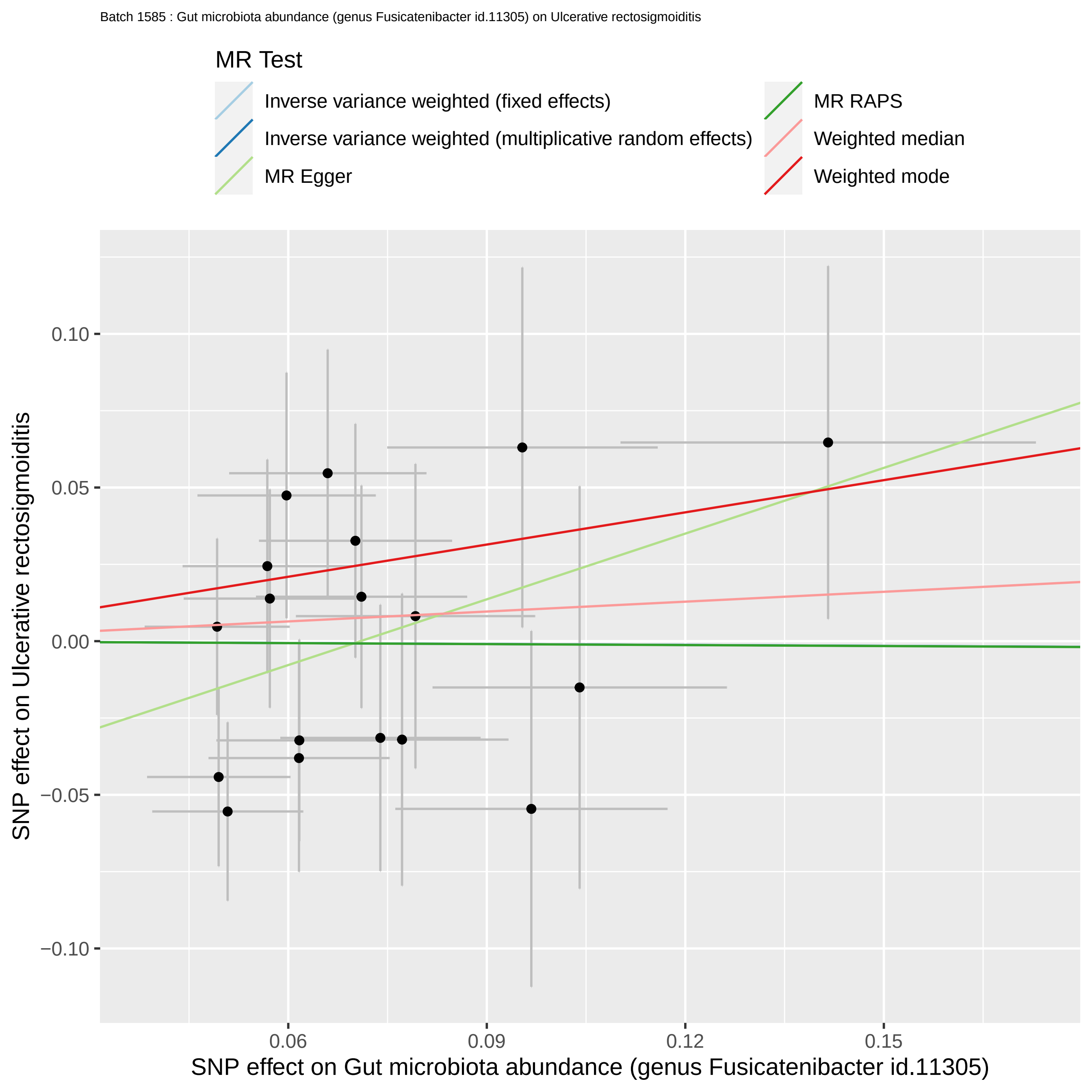
Batch 1585 : Gut microbiota abundance (genus Fusicatenibacter id.11305) on Ulcerative rectosigmoiditis
MR Test
Inverse variance weighted (fixed effects)
Inverse variance weighted (multiplicative random effects)
MR Egger
MR RAPS
Weighted median
Weighted mode
0.10
0.05
0.00
−0.05
−0.10
0.06
0.09
0.12
0.15
SNP effect on Ulcerative rectosigmoiditis
SNP effect on Gut microbiota abundance (genus Fusicatenibacter id.11305)

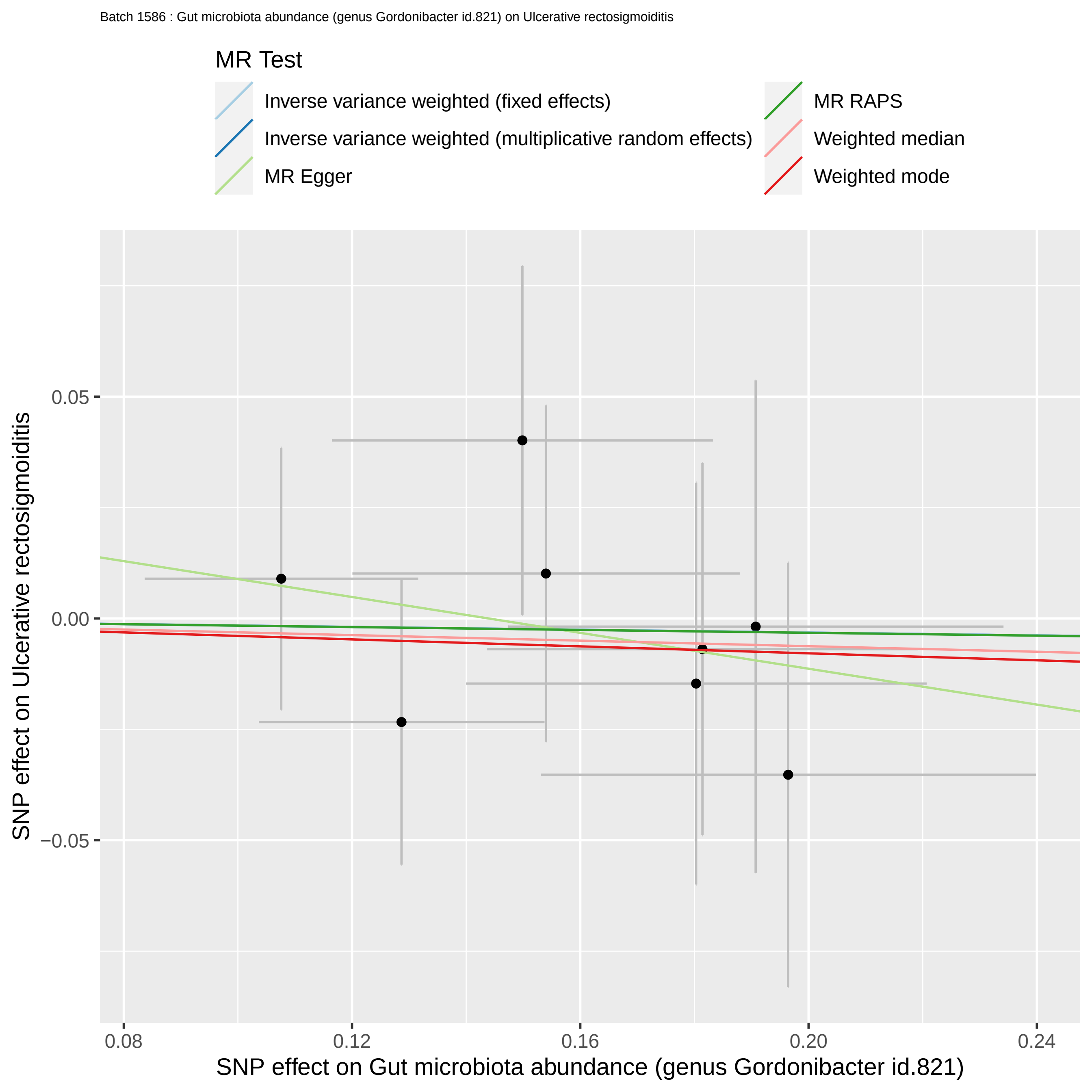
Batch 1586 : Gut microbiota abundance (genus Gordonibacter id.821) on Ulcerative rectosigmoiditis
MR Test
Inverse variance weighted (fixed effects)
Inverse variance weighted (multiplicative random effects)
MR Egger
MR RAPS
Weighted median
Weighted mode
0.05
0.00
−0.05
0.08
0.12
0.16
0.20
0.24
SNP effect on Ulcerative rectosigmoiditis
SNP effect on Gut microbiota abundance (genus Gordonibacter id.821)

Batch 1587 : Gut microbiota abundance (genus Haemophilus id.3698) on Ulcerative rectosigmoiditis

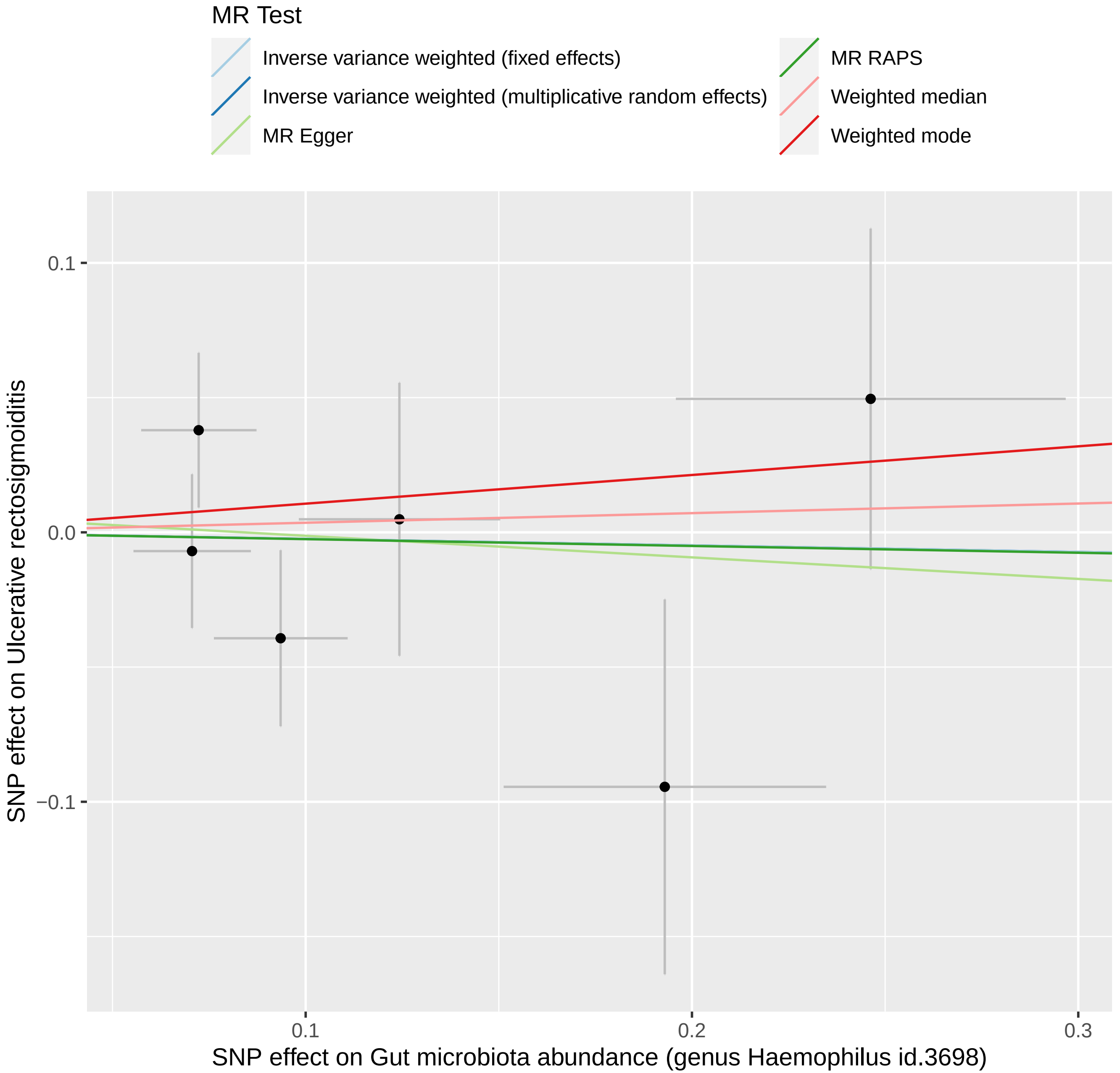

Batch 1588 : Gut microbiota abundance (genus Holdemanella id.11393) on Ulcerative rectosigmoiditis

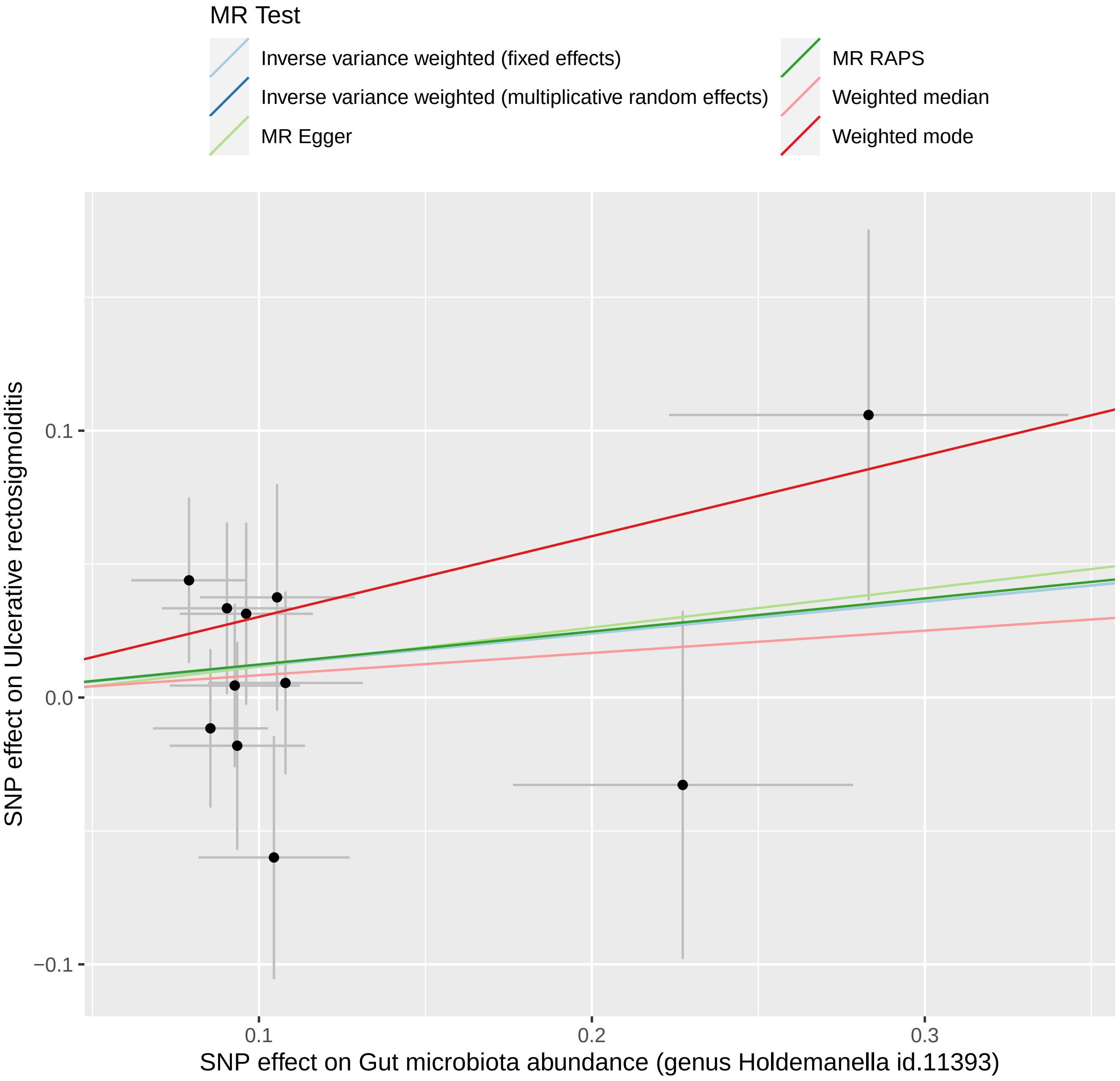

Batch 1589 : Gut microbiota abundance (genus Holdemania id.2157) on Ulcerative rectosigmoiditis

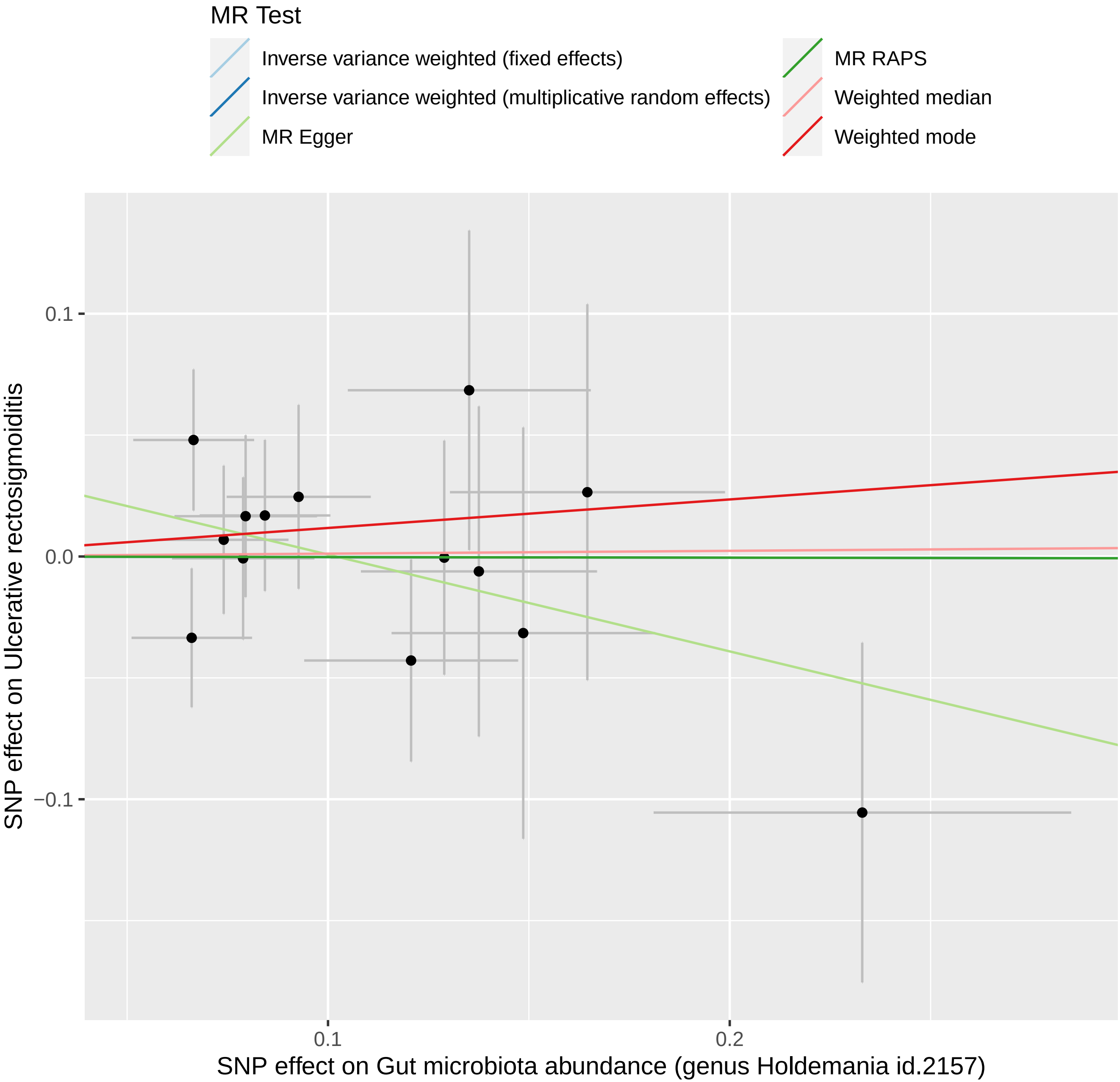

Batch 1590 : Gut microbiota abundance (genus Howardella id.2000) on Ulcerative rectosigmoiditis

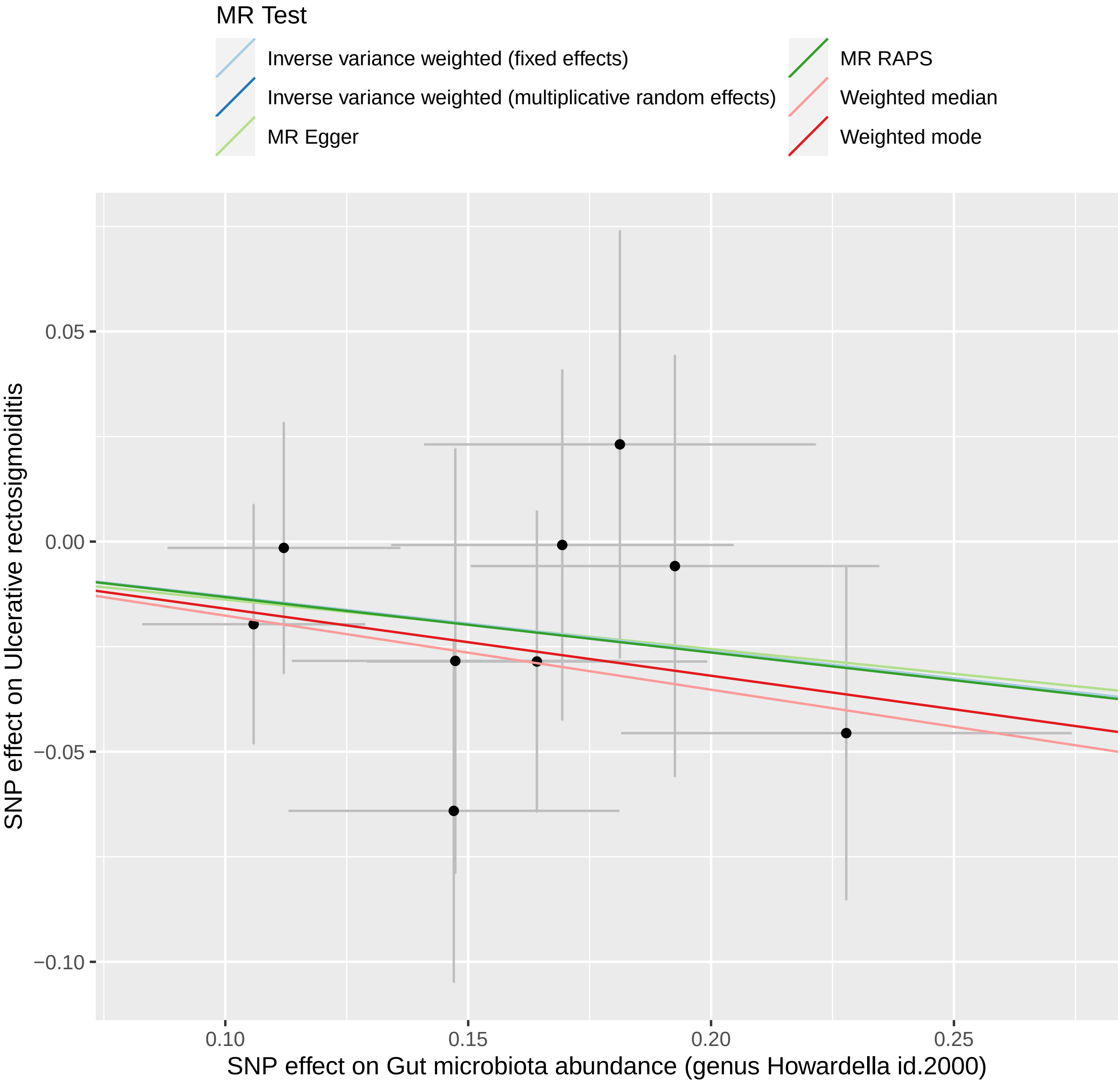

Batch 1591 : Gut microbiota abundance (genus Hungatella id.11306) on Ulcerative rectosigmoiditis

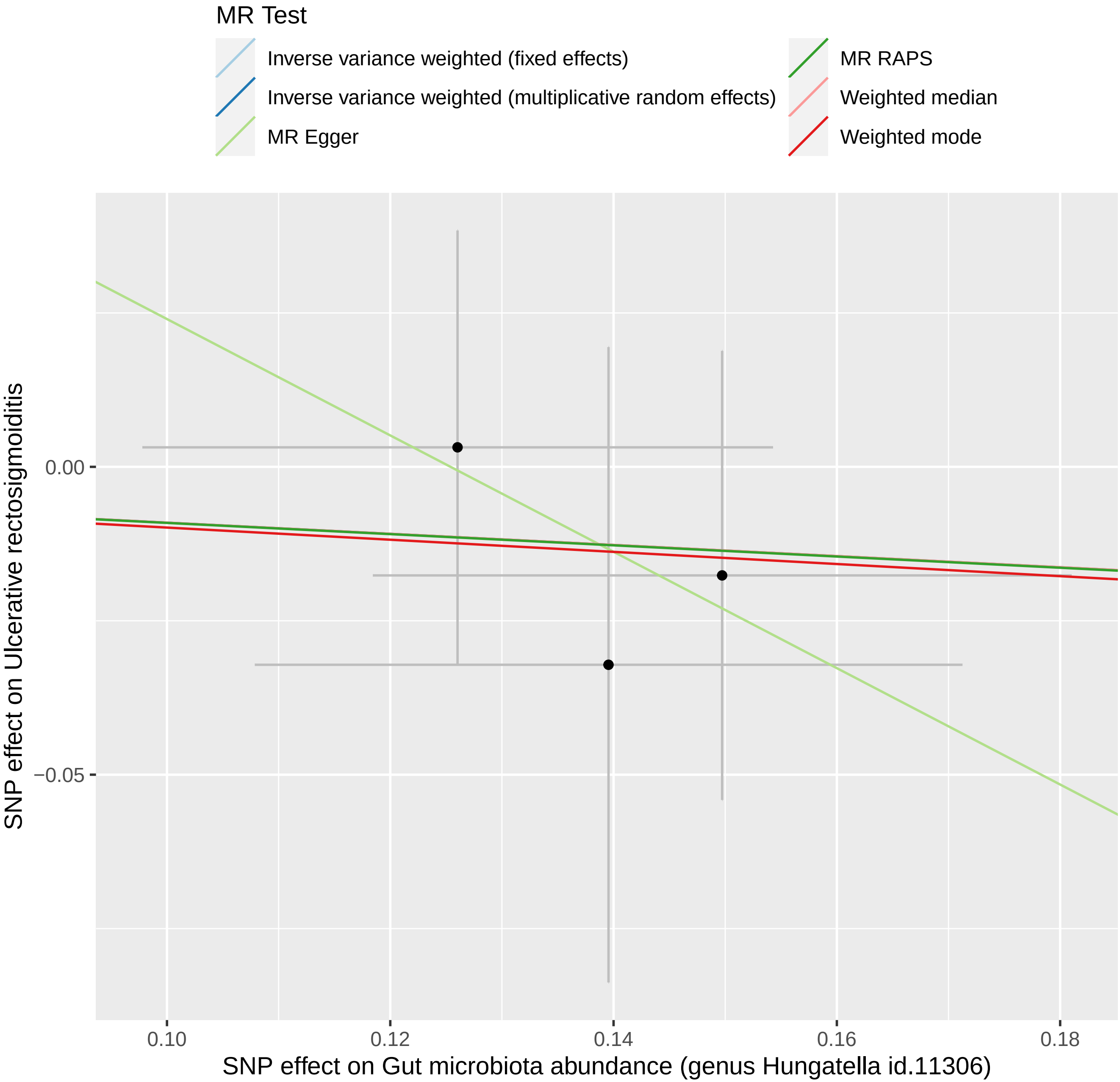

Batch 1592 : Gut microbiota abundance (genus Intestinibacter id.11345) on Ulcerative rectosigmoiditis

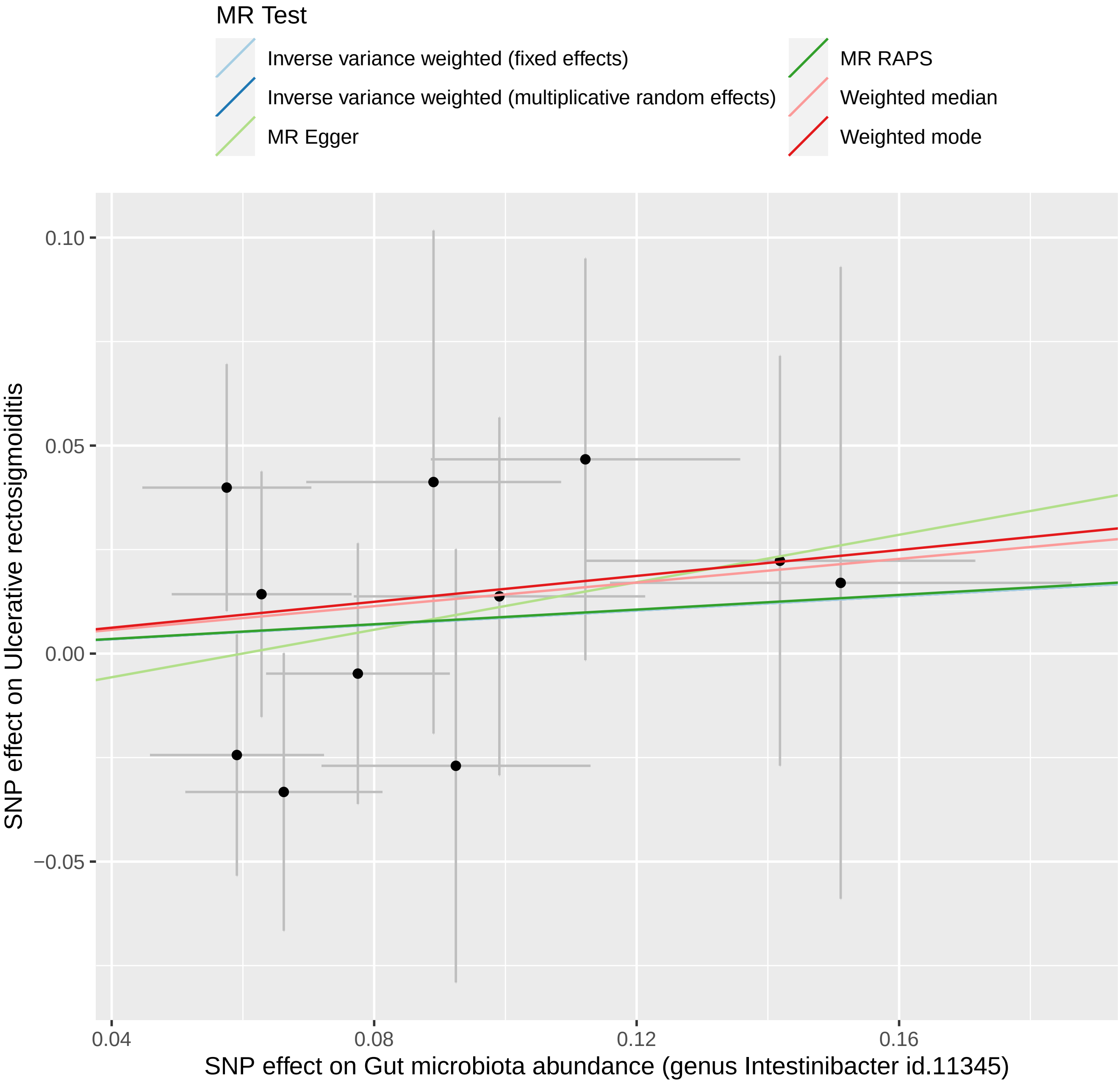

Batch 1593 : Gut microbiota abundance (genus Intestinimonas id.2062) on Ulcerative rectosigmoiditis

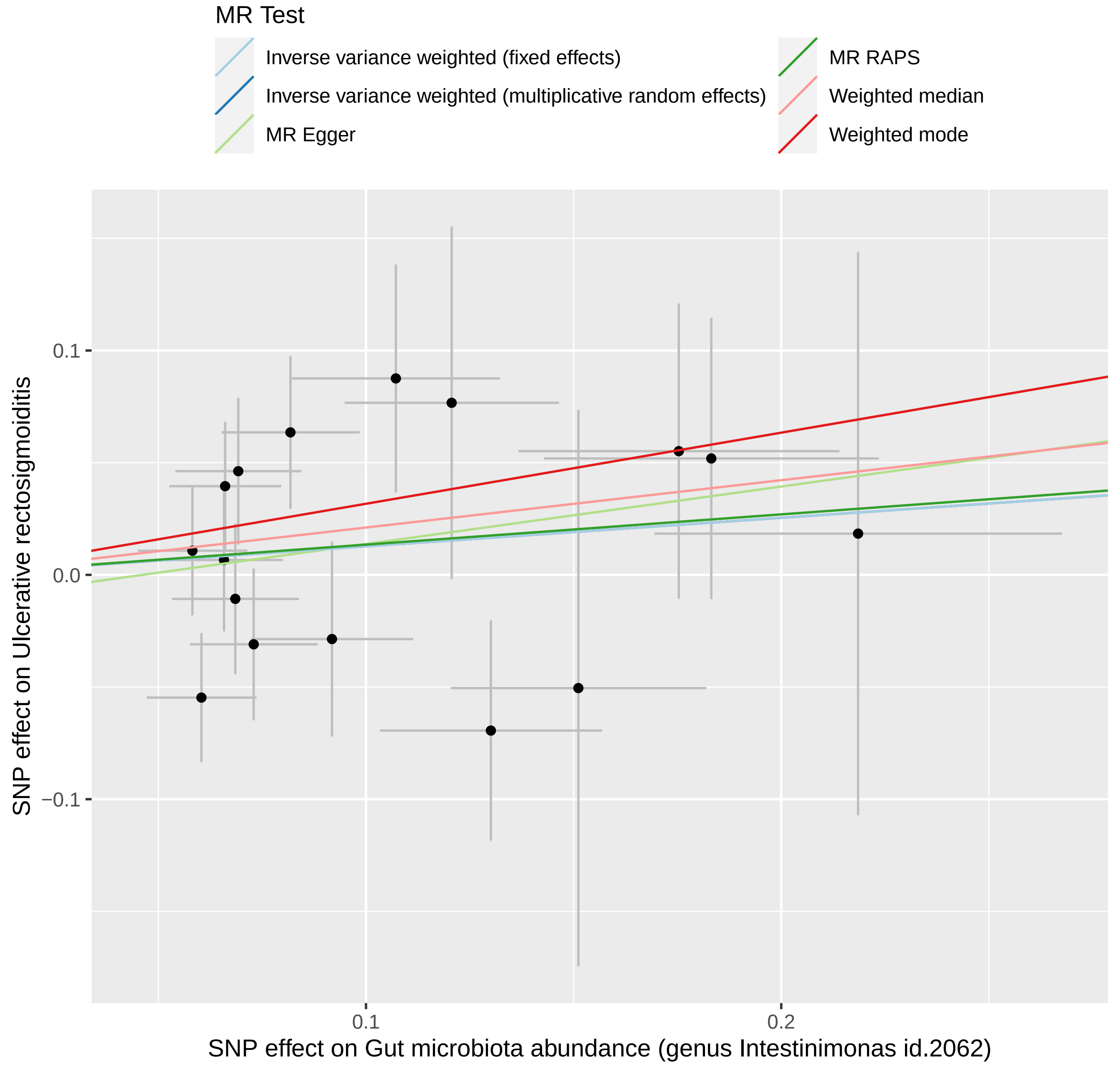

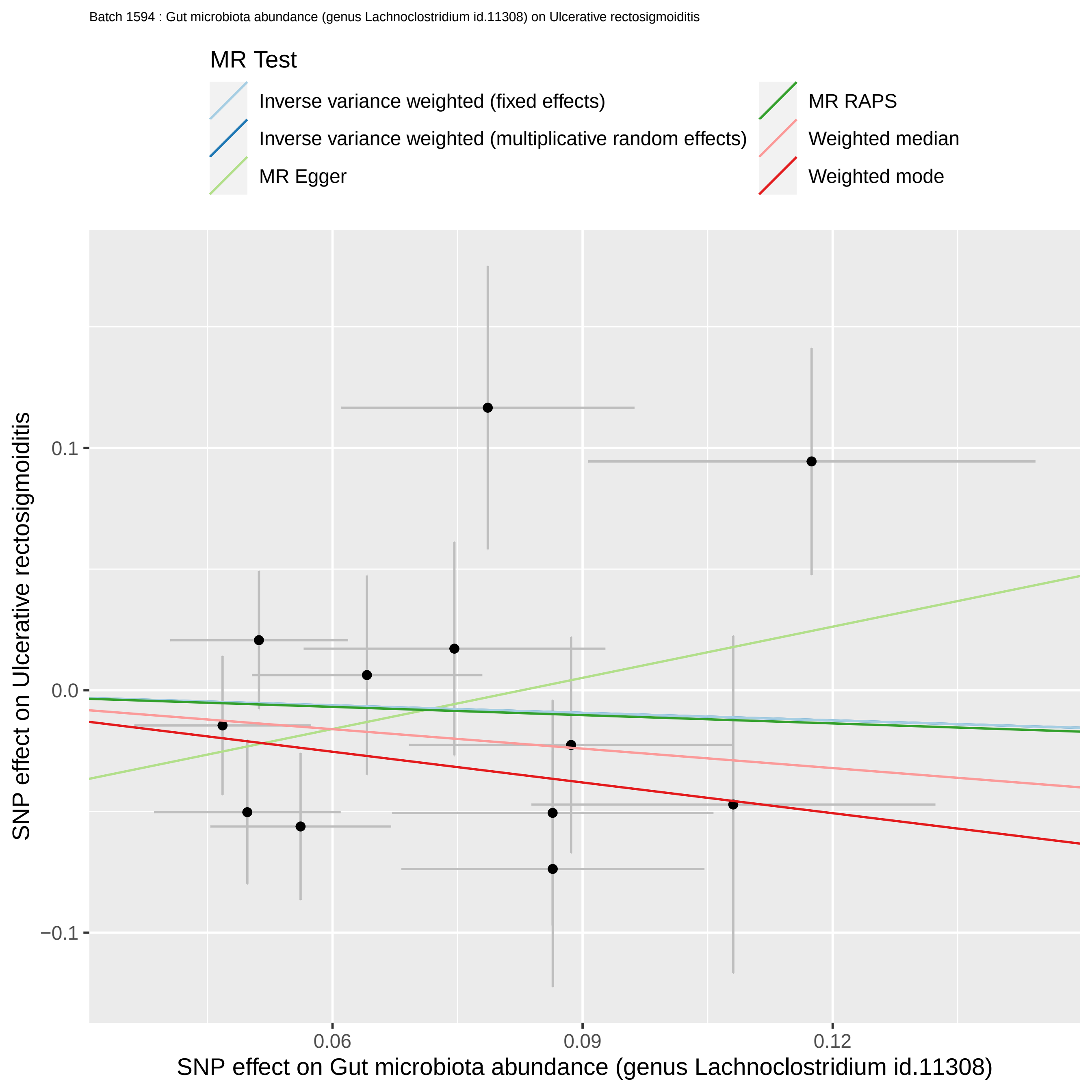
Batch 1594 : Gut microbiota abundance (genus Lachnoclostridium id.11308) on Ulcerative rectosigmoiditis
MR Test
Inverse variance weighted (fixed effects)
Inverse variance weighted (multiplicative random effects)
MR Egger
MR RAPS
Weighted median
Weighted mode
0.1
0.0
−0.1
0.06
0.09
0.12
SNP effect on Ulcerative rectosigmoiditis
SNP effect on Gut microbiota abundance (genus Lachnoclostridium id.11308)

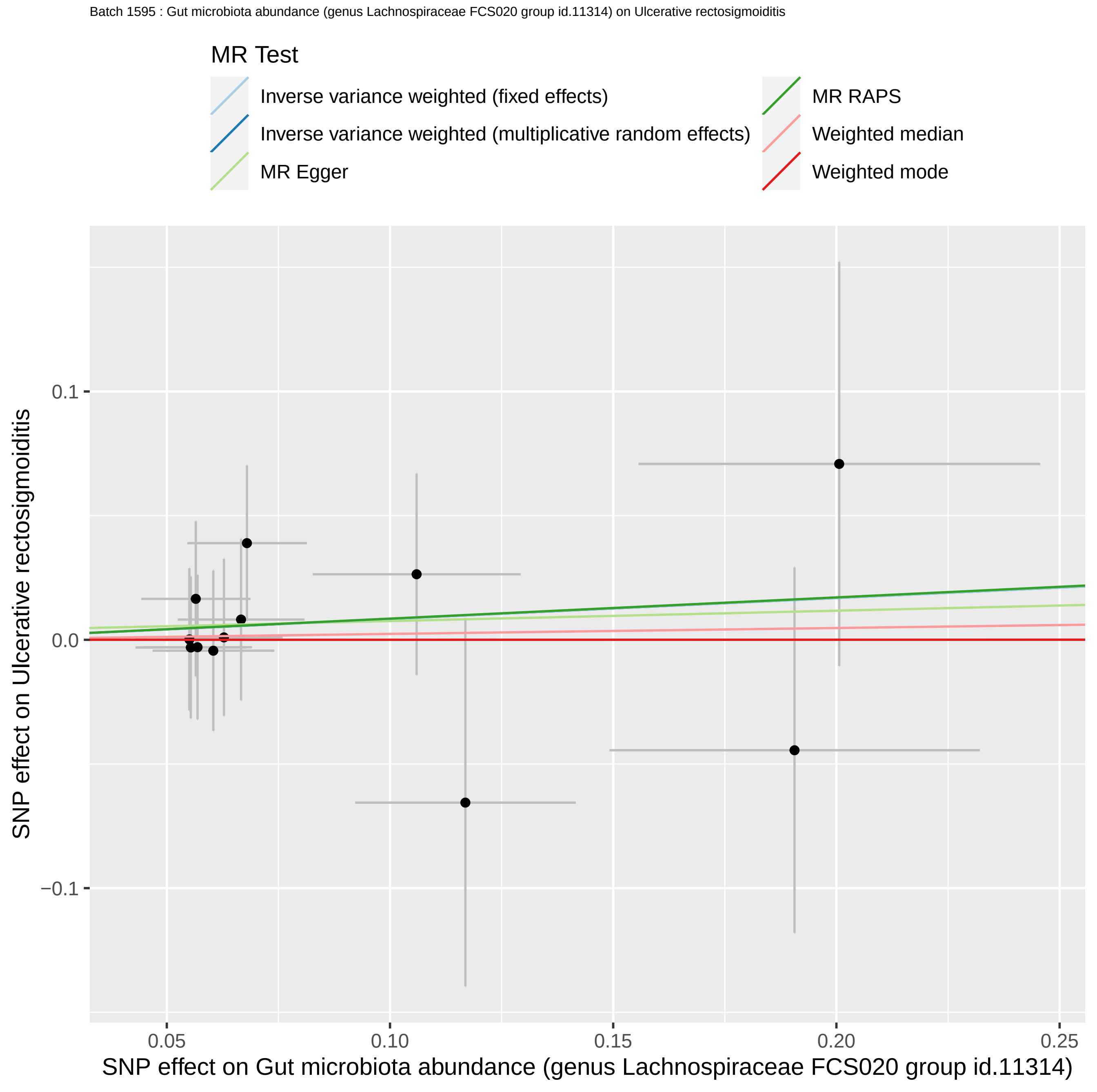

Batch 1595 : Gut microbiota abundance (genus Lachnospiraceae FCS020 group id.11314) on Ulcerative rectosigmoiditis
MR Test
Inverse variance weighted (fixed effects)
Inverse variance weighted (multiplicative random effects)
MR Egger
MR RAPS
Weighted median
Weighted mode
SNP effect on Ulcerative rectosigmoiditis
0.1
0.0
−0.1
0.05
0.10
0.15
0.20
0.25
SNP effect on Gut microbiota abundance (genus Lachnospiraceae FCS020 group id.11314)

Batch 1596 : Gut microbiota abundance (genus Lachnospiraceae NC2004 group id.11316) on Ulcerative rectosigmoiditis

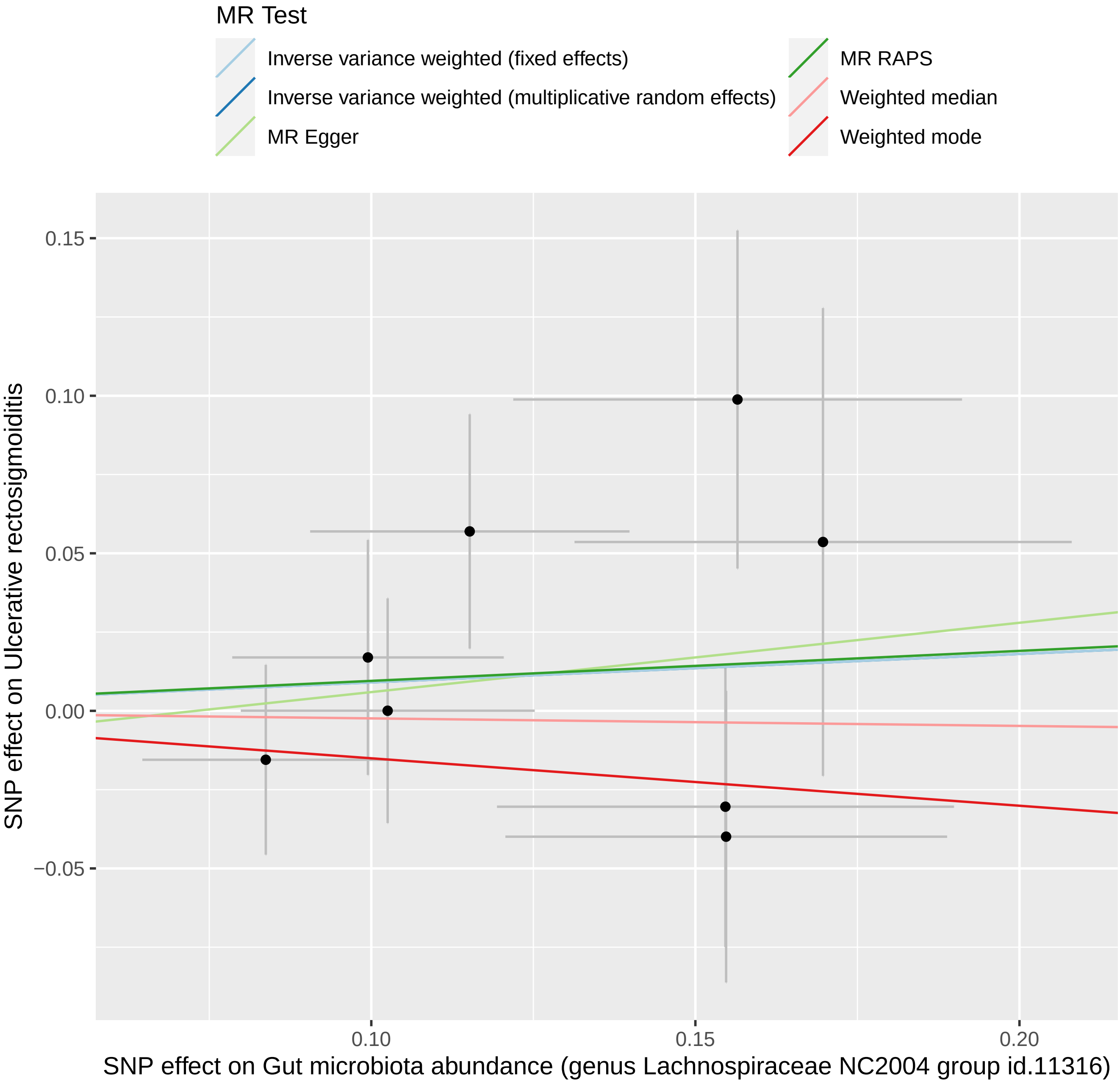

Batch 1597 : Gut microbiota abundance (genus Lachnospiraceae ND3007 group id.11317) on Ulcerative rectosigmoiditis

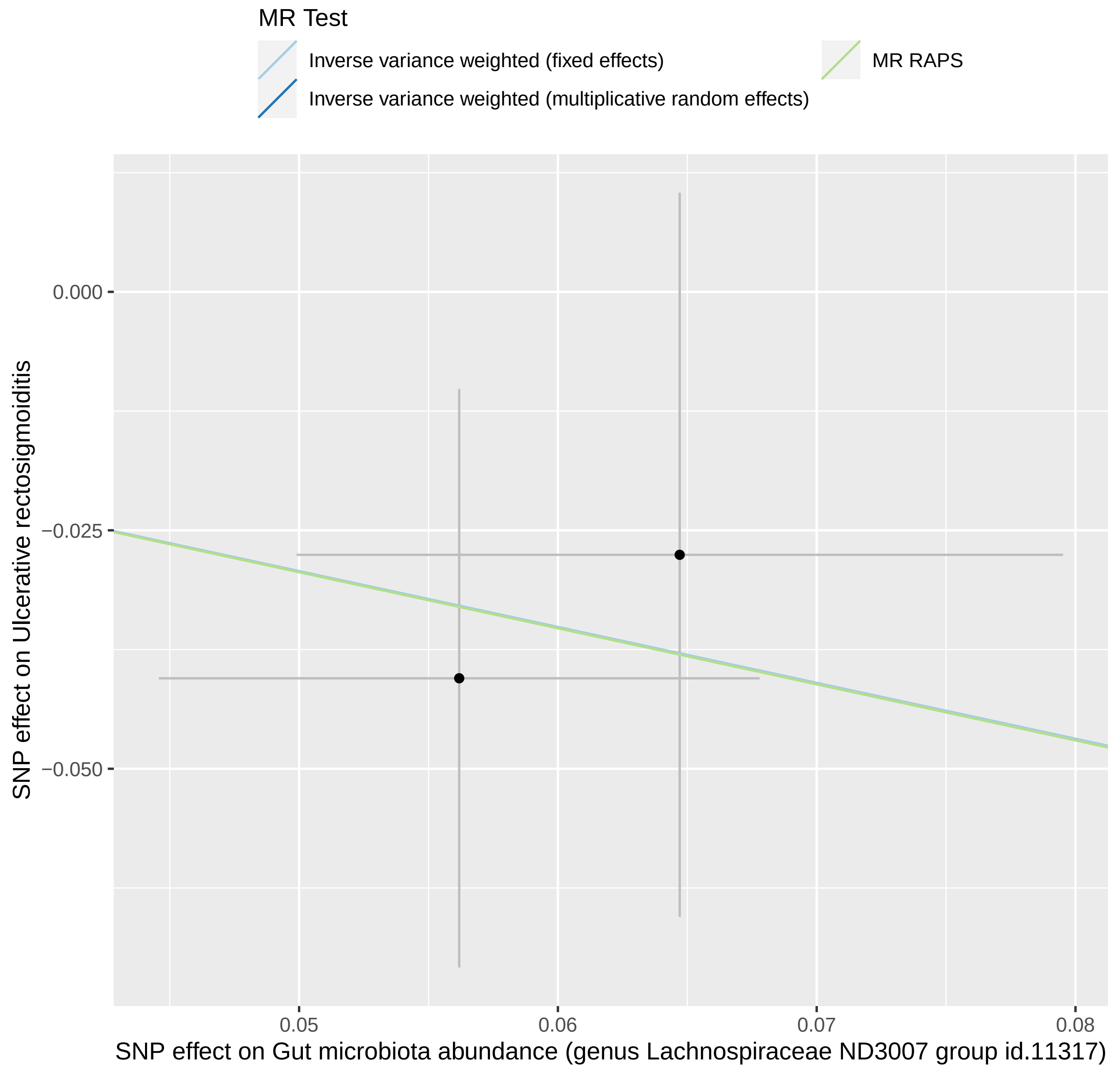

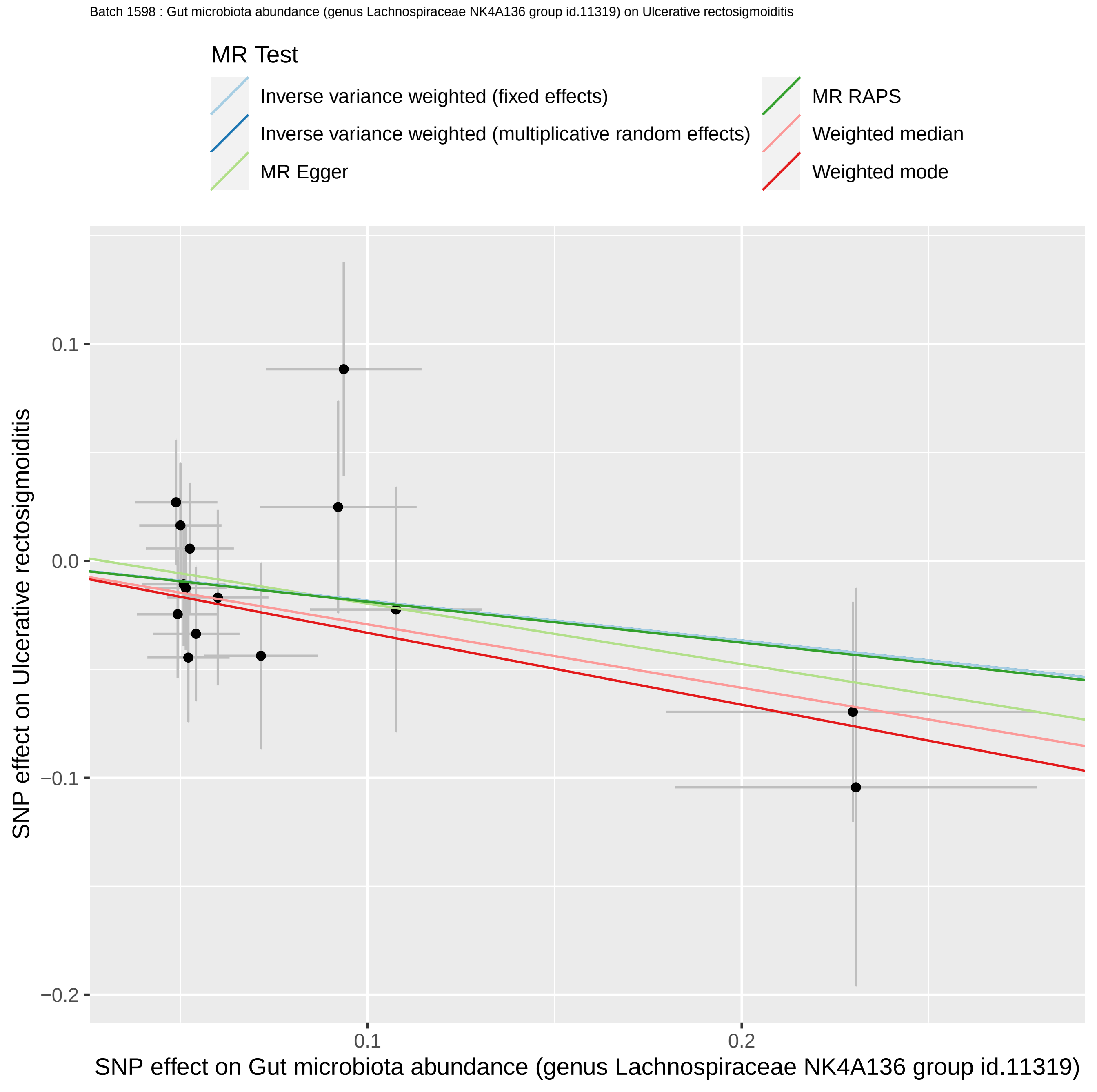

Batch 1598 : Gut microbiota abundance (genus Lachnospiraceae NK4A136 group id.11319) on Ulcerative rectosigmoiditis
MR Test
Inverse variance weighted (fixed effects)
Inverse variance weighted (multiplicative random effects)
MR Egger
MR RAPS
Weighted median
Weighted mode
0.1
0.0
−0.1
−0.2
0.1
0.2
SNP effect on Ulcerative rectosigmoiditis
SNP effect on Gut microbiota abundance (genus Lachnospiraceae NK4A136 group id.11319)

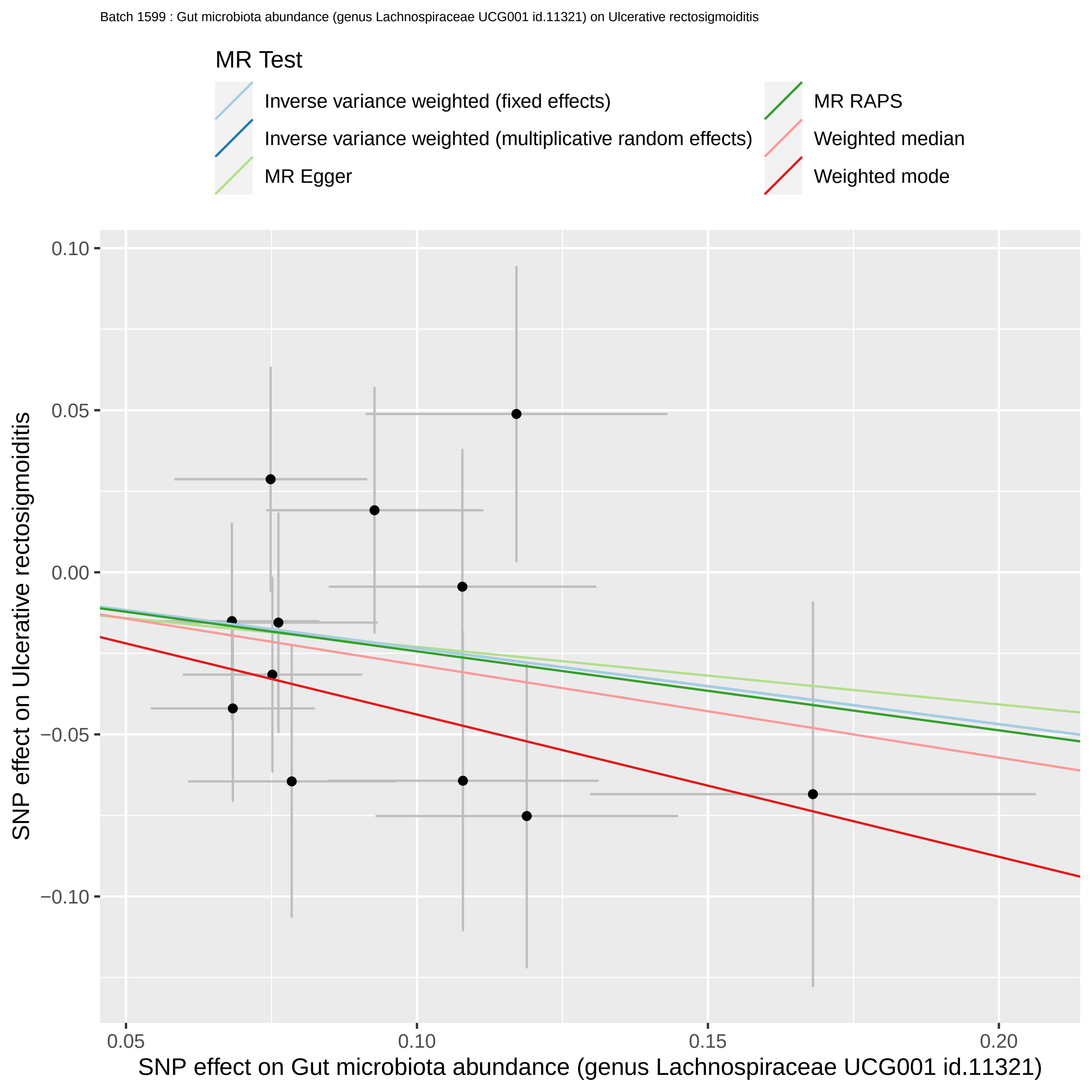

Batch 1599 : Gut microbiota abundance (genus Lachnospiraceae UCG001 id.11321) on Ulcerative rectosigmoiditis
MR Test
Inverse variance weighted (fixed effects)
Inverse variance weighted (multiplicative random effects)
MR Egger
MR RAPS
Weighted median
Weighted mode
0.10
0.05
0.00
−0.05
−0.10
0.05
0.10
0.15
0.20
SNP effect on Ulcerative rectosigmoiditis
SNP effect on Gut microbiota abundance (genus Lachnospiraceae UCG001 id.11321)

Batch 1600 : Gut microbiota abundance (genus Lachnospiraceae UCG004 id.11324) on Ulcerative rectosigmoiditis

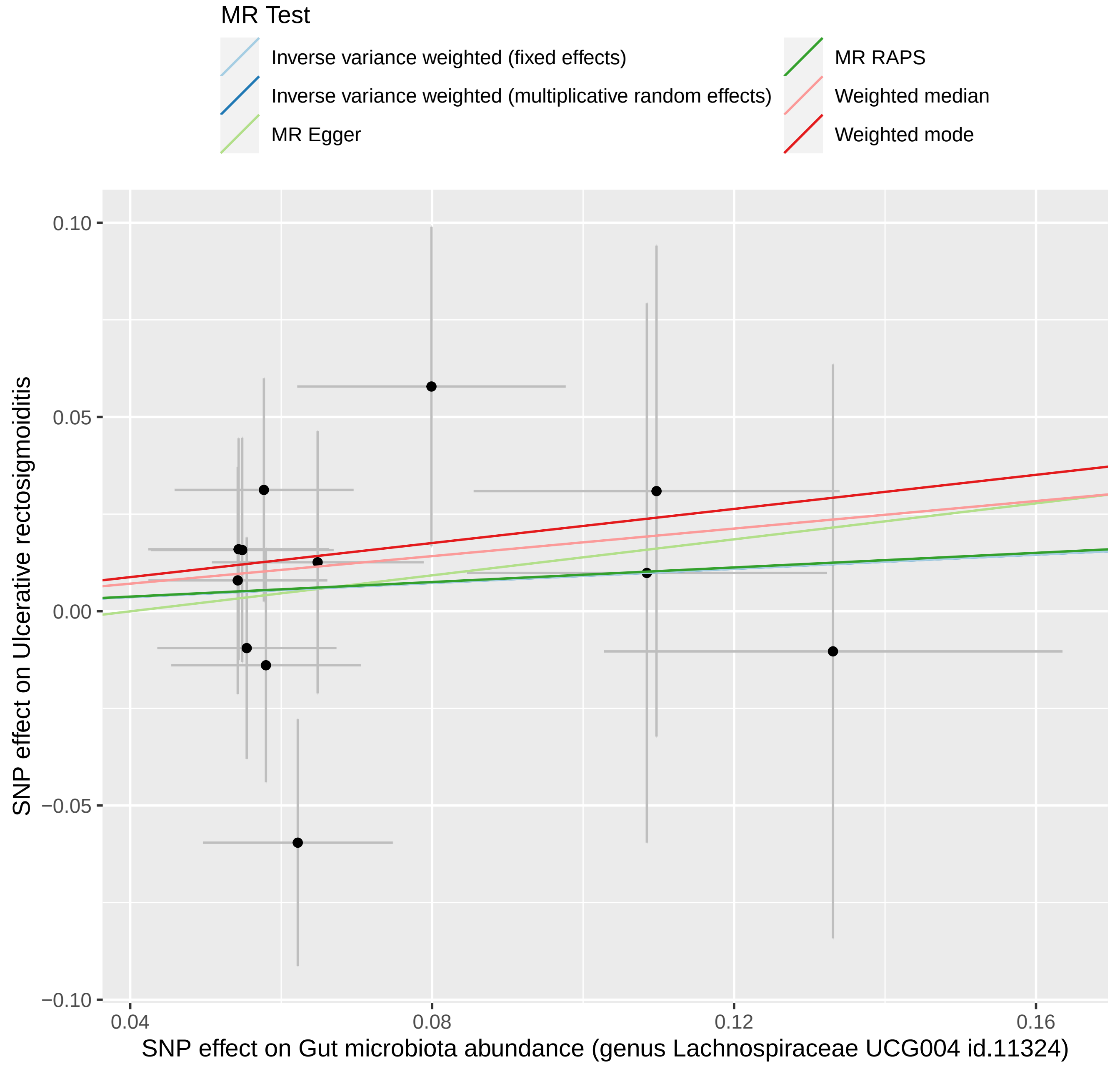

Batch 1601 : Gut microbiota abundance (genus Lachnospiraceae UCG008 id.11328) on Ulcerative rectosigmoiditis

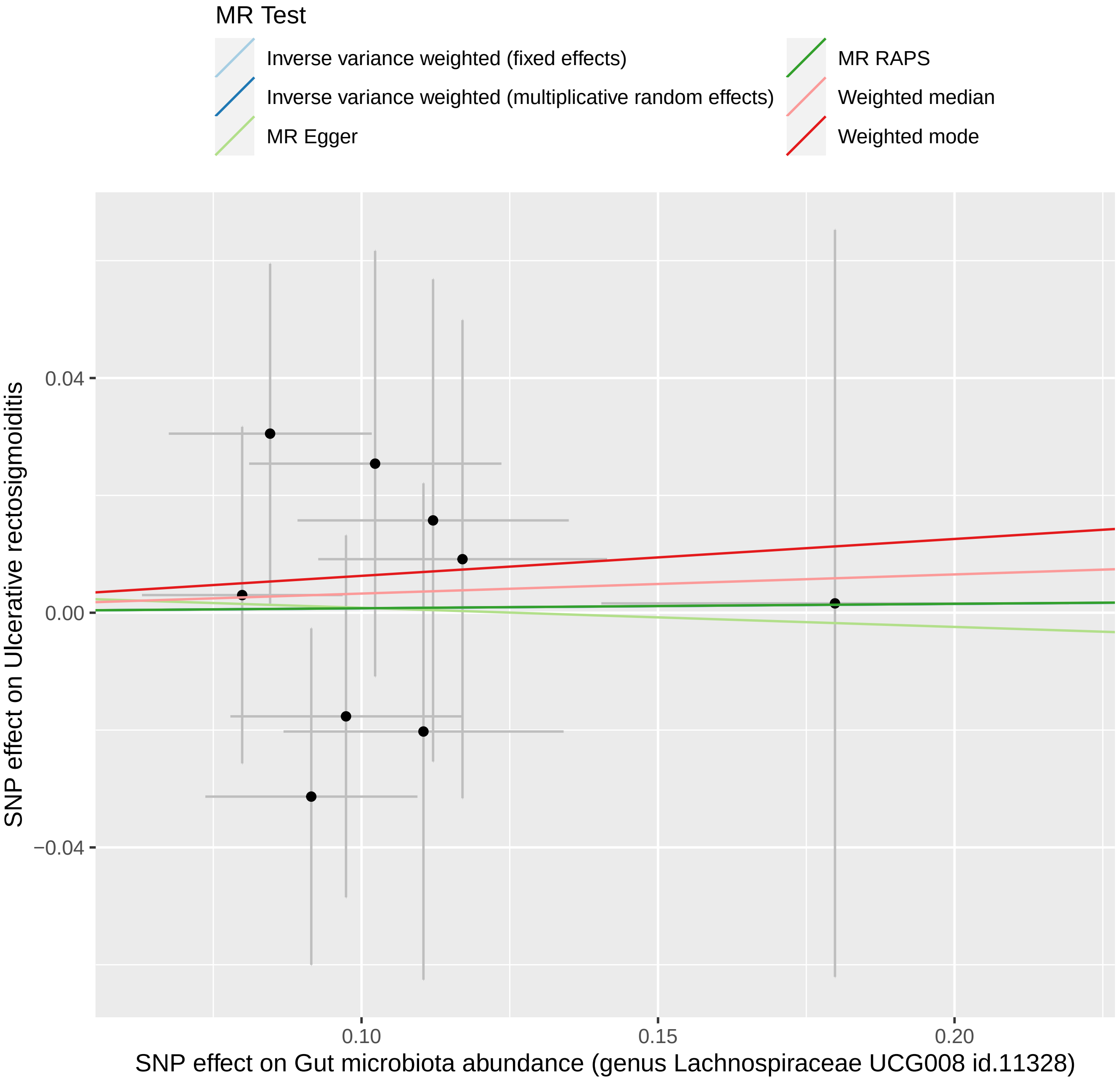

Batch 1602 : Gut microbiota abundance (genus Lachnospiraceae UCG010 id.11330) on Ulcerative rectosigmoiditis

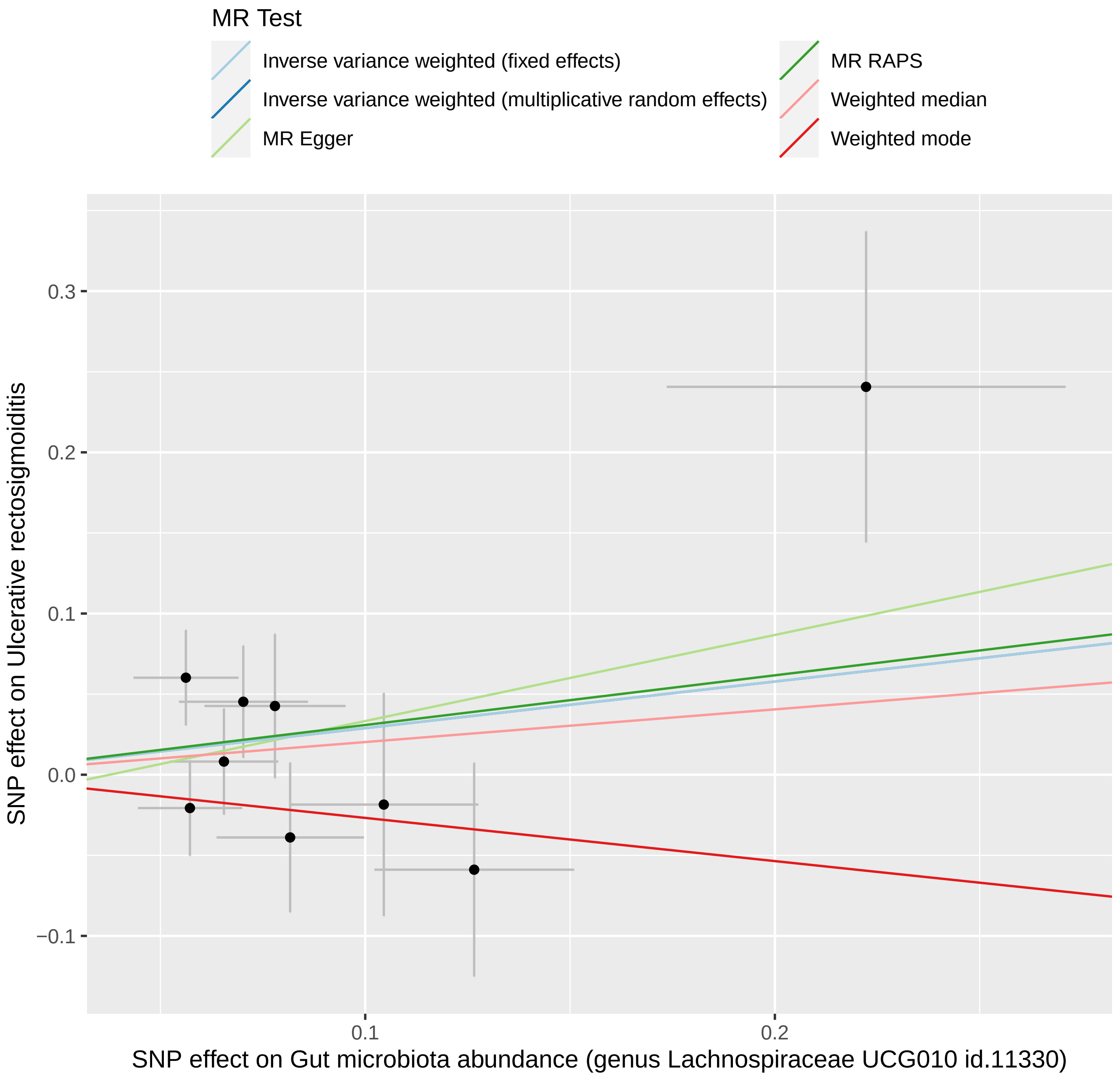

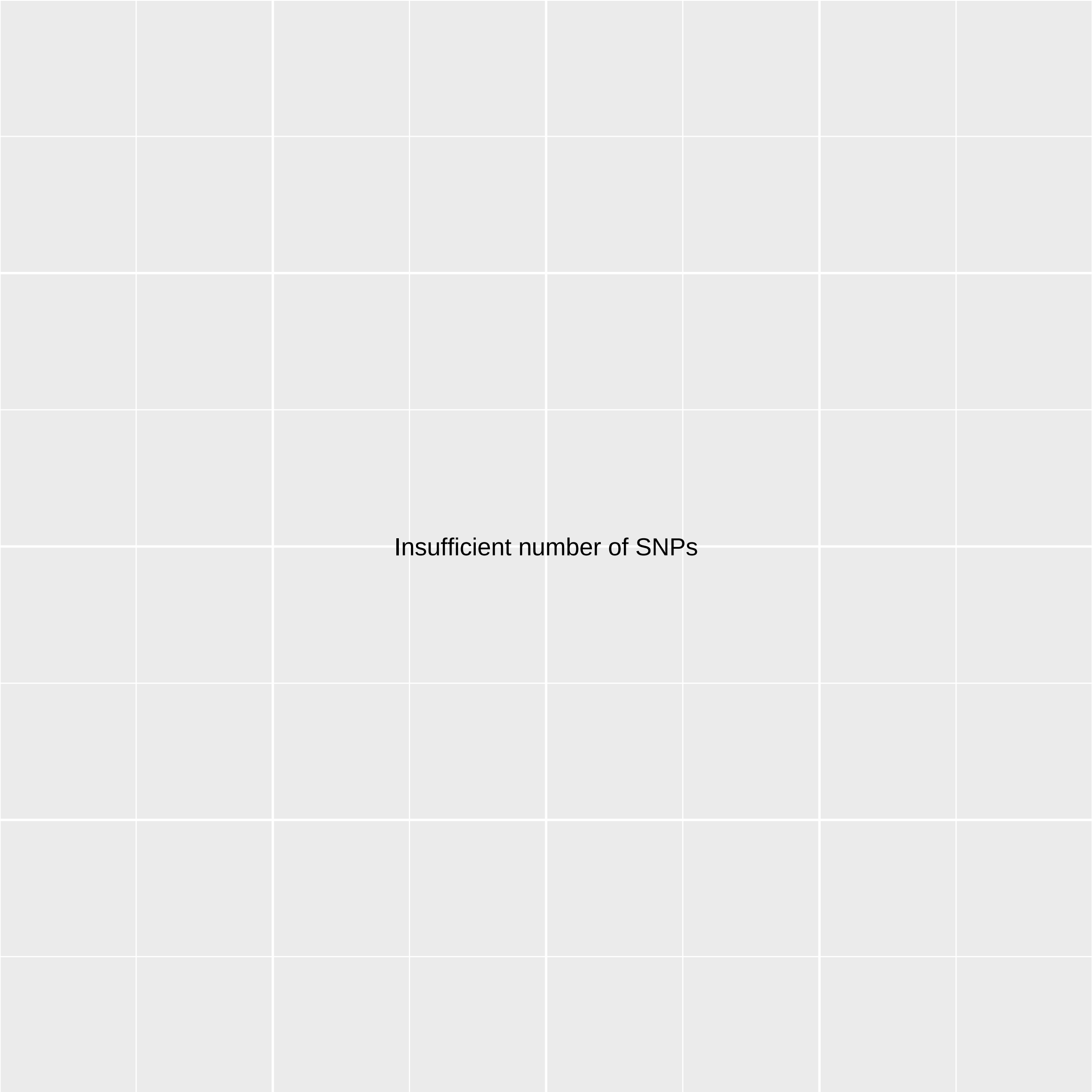
Insufficient number of SNPs

Batch 1604 : Gut microbiota abundance (genus Lactobacillus id.1837) on Ulcerative rectosigmoiditis

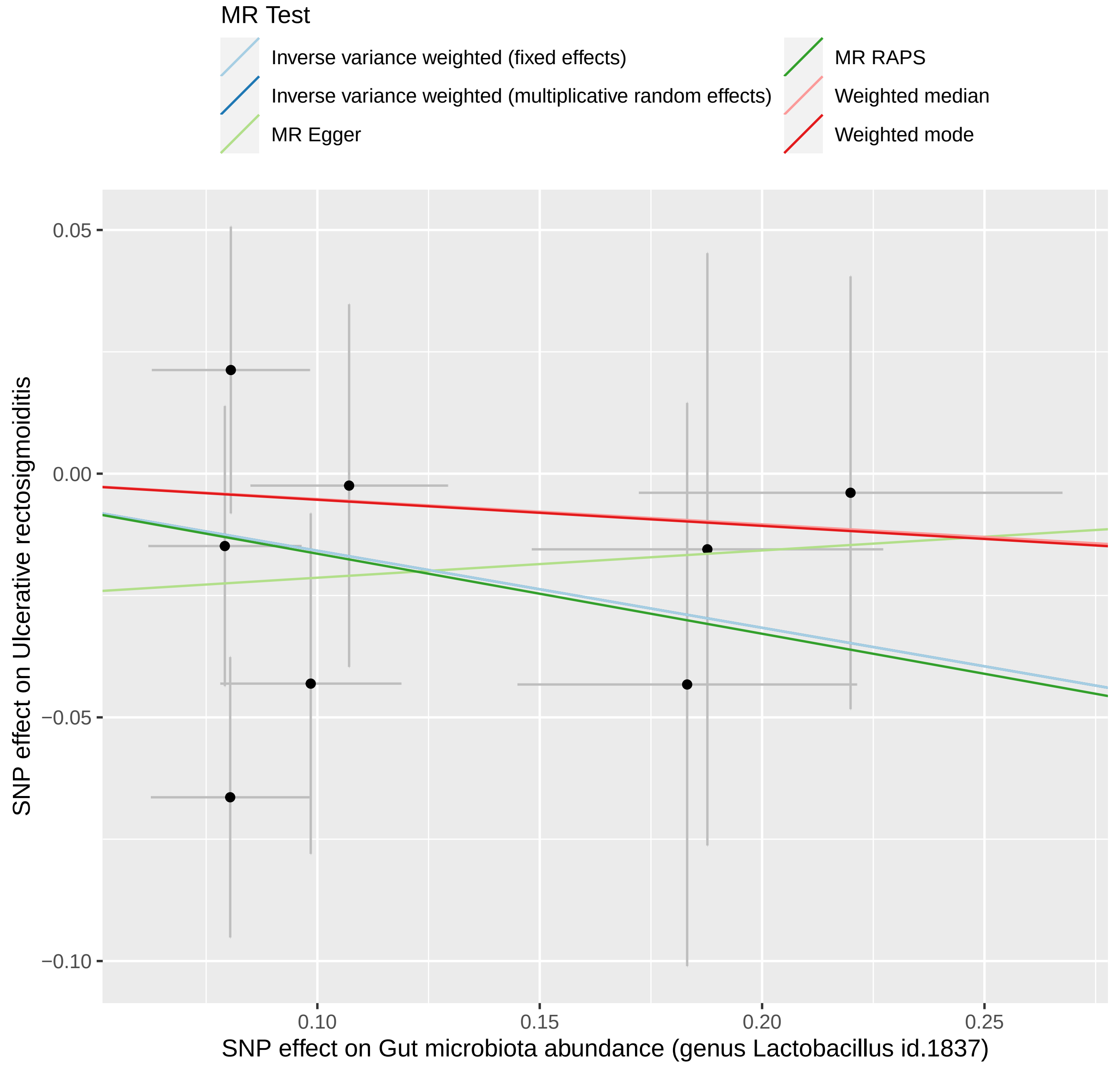

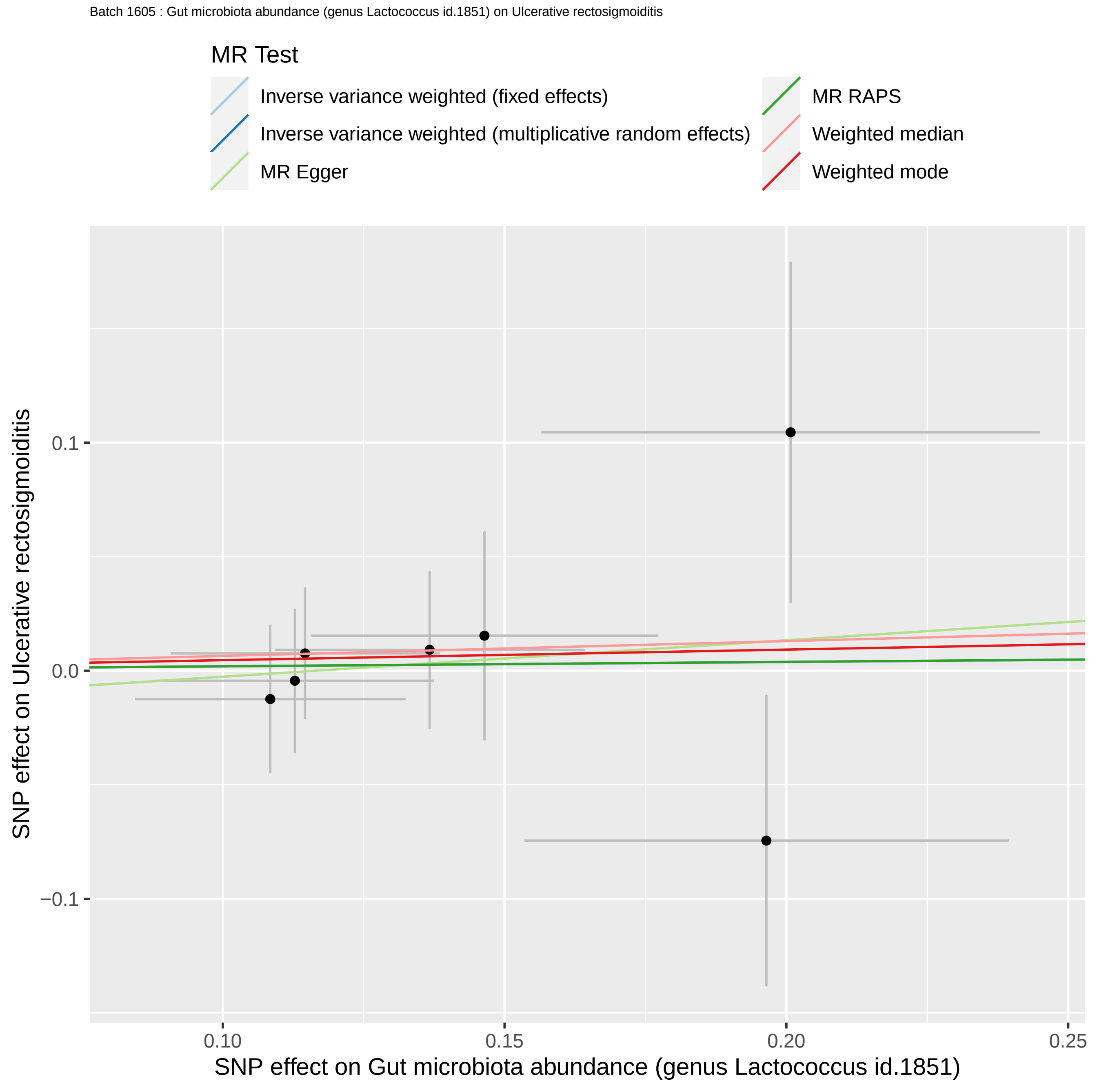
Batch 1605 : Gut microbiota abundance (genus Lactococcus id.1851) on Ulcerative rectosigmoiditis
MR Test
Inverse variance weighted (fixed effects)
Inverse variance weighted (multiplicative random effects)
MR Egger
MR RAPS
Weighted median
Weighted mode
SNP effect on Ulcerative rectosigmoiditis
0.1
0.0
−0.1
0.10
0.15
0.20
0.25
SNP effect on Gut microbiota abundance (genus Lactococcus id.1851)

Batch 1606 : Gut microbiota abundance (genus Marvinbryantia id.2005) on Ulcerative rectosigmoiditis

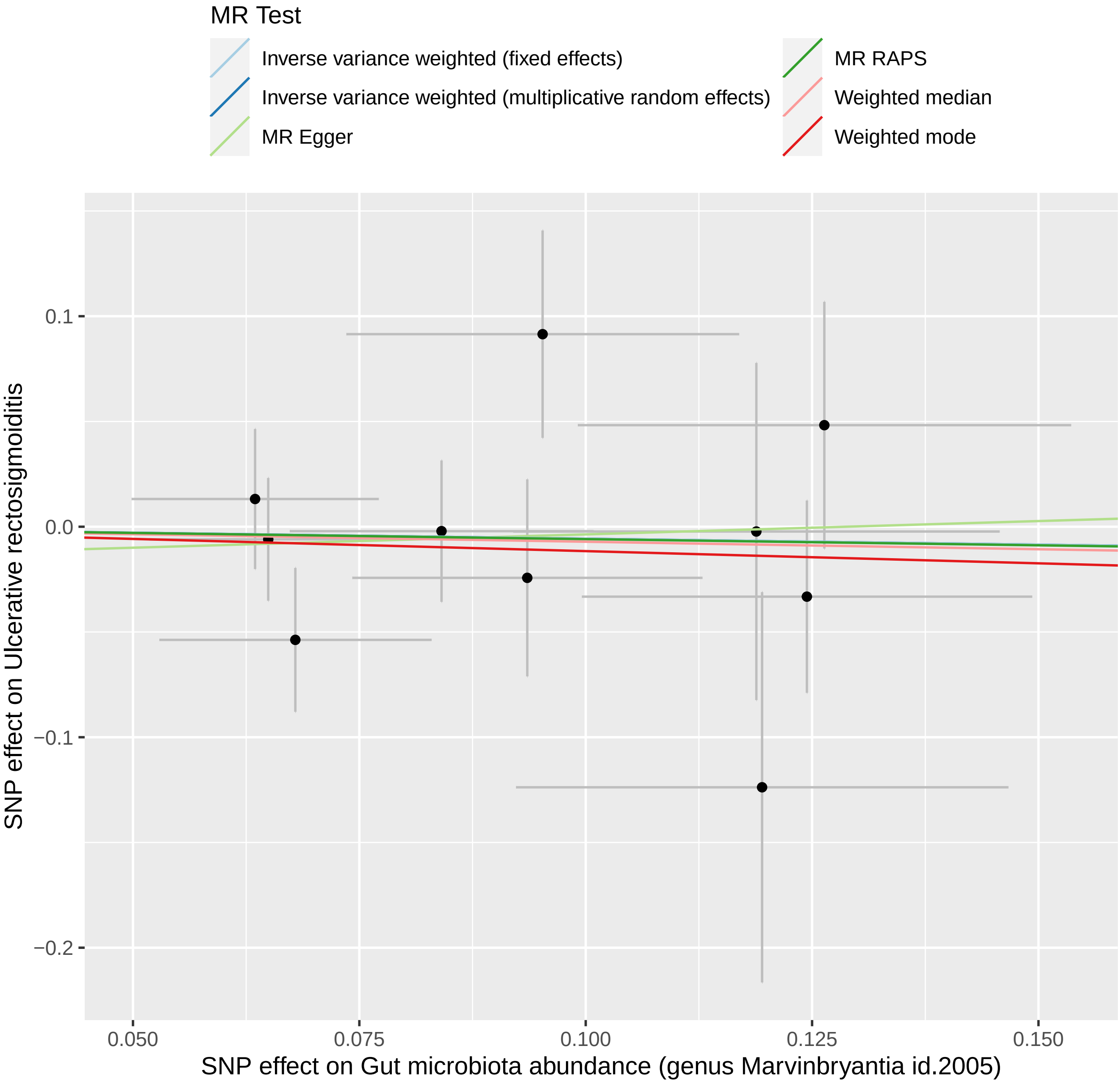

Batch 1607 : Gut microbiota abundance (genus Methanobrevibacter id.123) on Ulcerative rectosigmoiditis

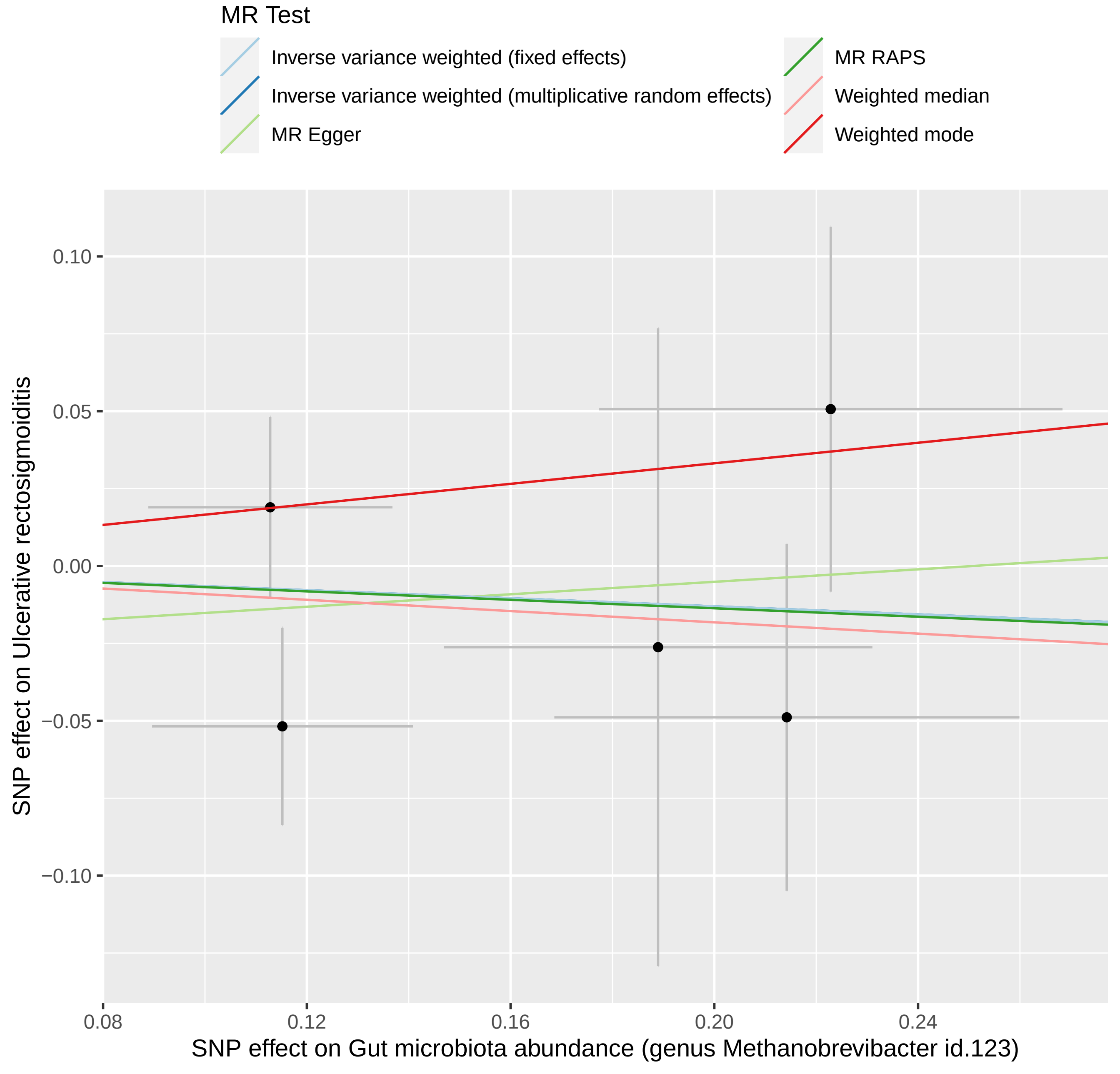

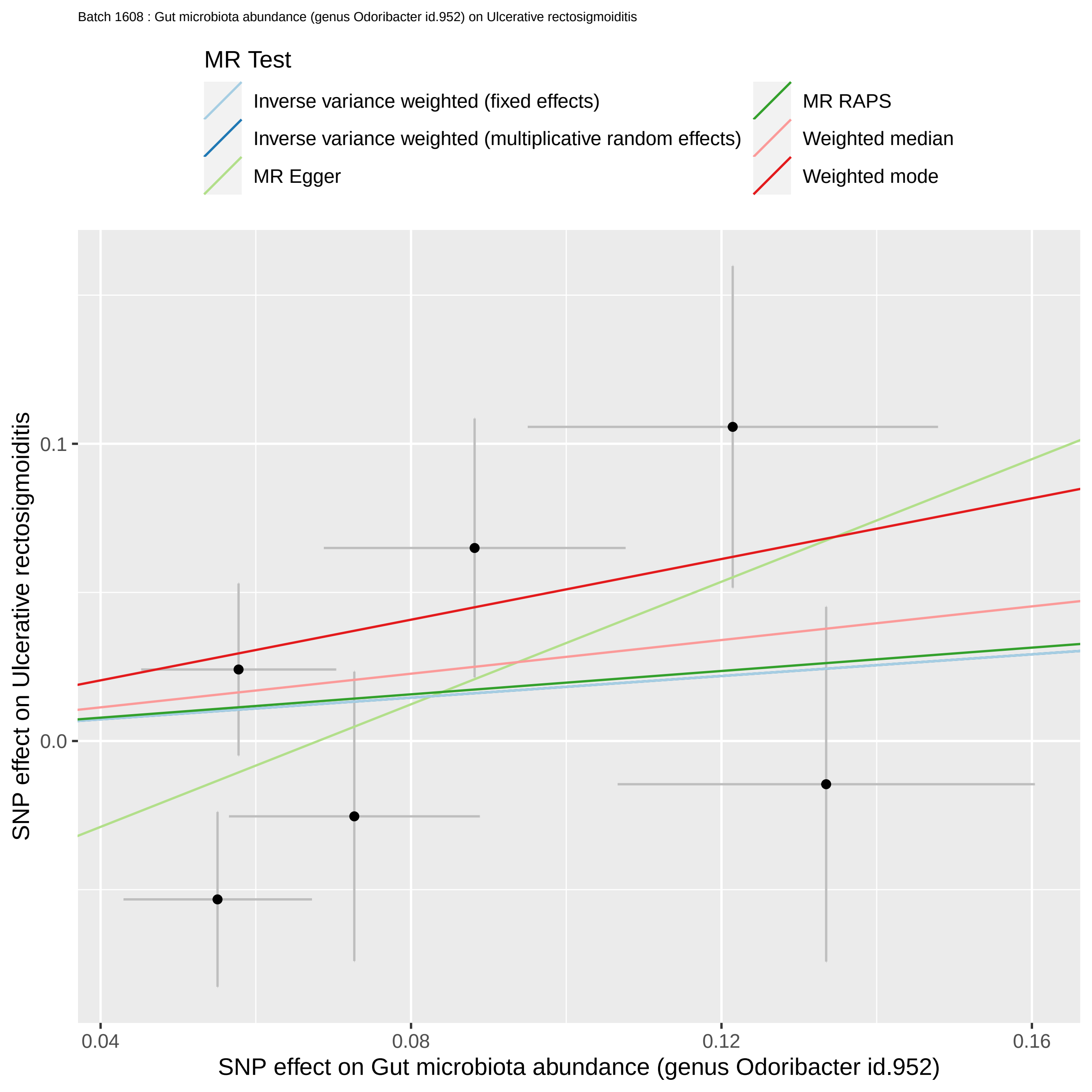

Batch 1608 : Gut microbiota abundance (genus Odoribacter id.952) on Ulcerative rectosigmoiditis
MR Test
Inverse variance weighted (fixed effects)
Inverse variance weighted (multiplicative random effects)
MR Egger
MR RAPS
Weighted median
Weighted mode
SNP effect on Ulcerative rectosigmoiditis
0.1
0.0
0.04
0.08
0.12
0.16
SNP effect on Gut microbiota abundance (genus Odoribacter id.952)

Batch 1609 : Gut microbiota abundance (genus Olsenella id.822) on Ulcerative rectosigmoiditis

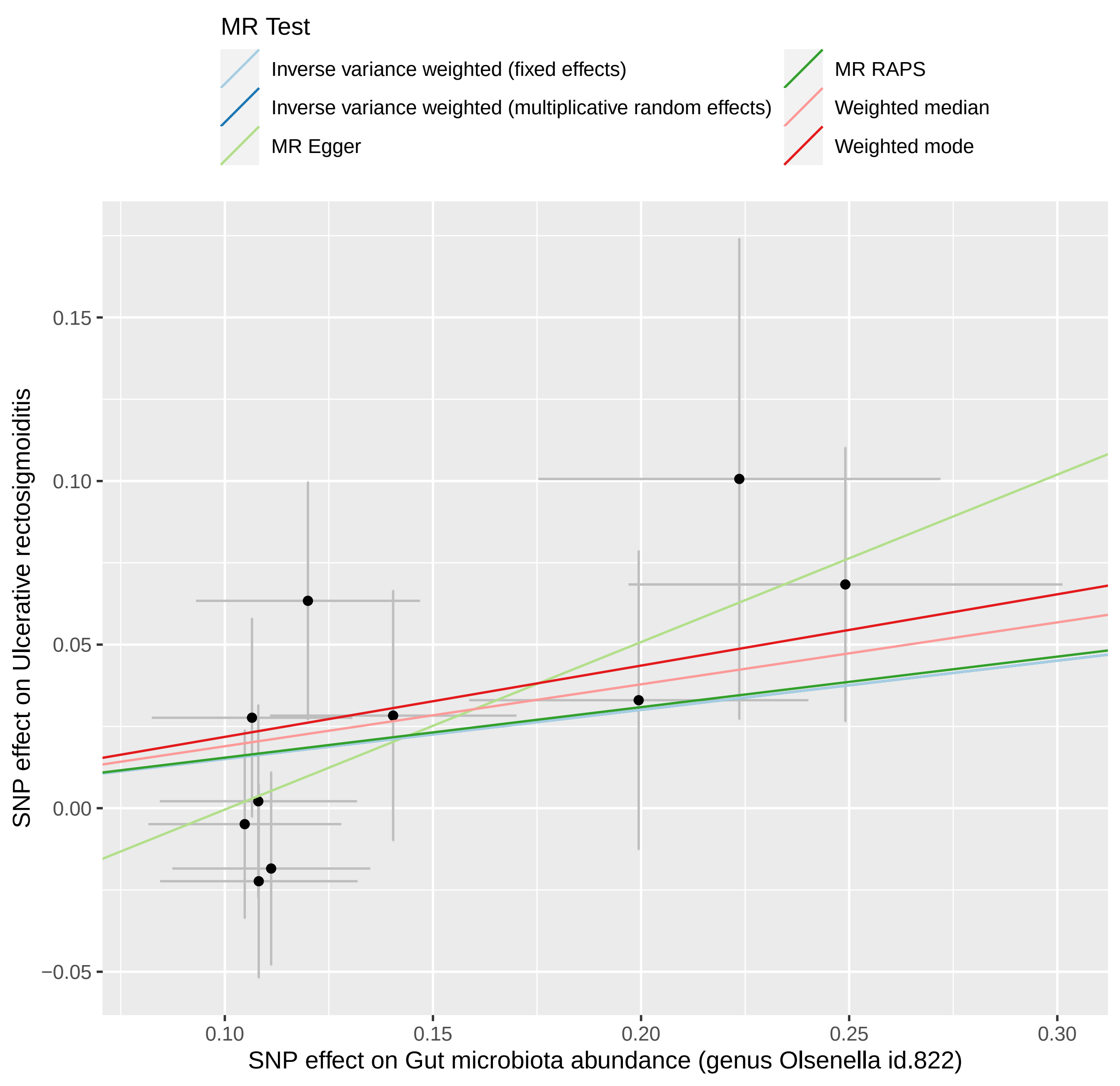

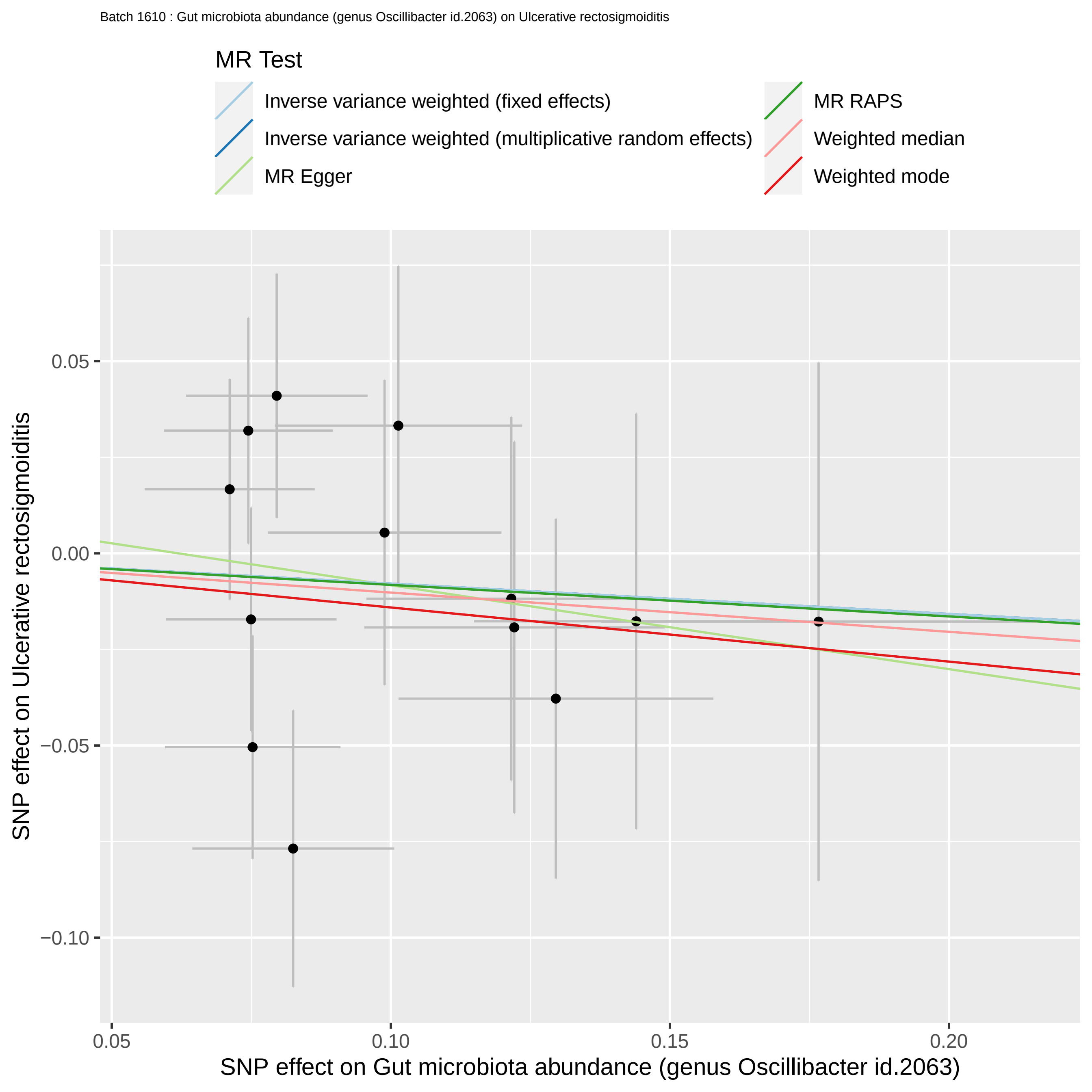

Batch 1610 : Gut microbiota abundance (genus Oscillibacter id.2063) on Ulcerative rectosigmoiditis
MR Test
Inverse variance weighted (fixed effects)
Inverse variance weighted (multiplicative random effects)
MR Egger
MR RAPS
Weighted median
Weighted mode
0.05
0.00
−0.05
−0.10
0.05
0.10
0.15
0.20
SNP effect on Ulcerative rectosigmoiditis
SNP effect on Gut microbiota abundance (genus Oscillibacter id.2063)

Batch 1611 : Gut microbiota abundance (genus Oscillospira id.2064) on Ulcerative rectosigmoiditis

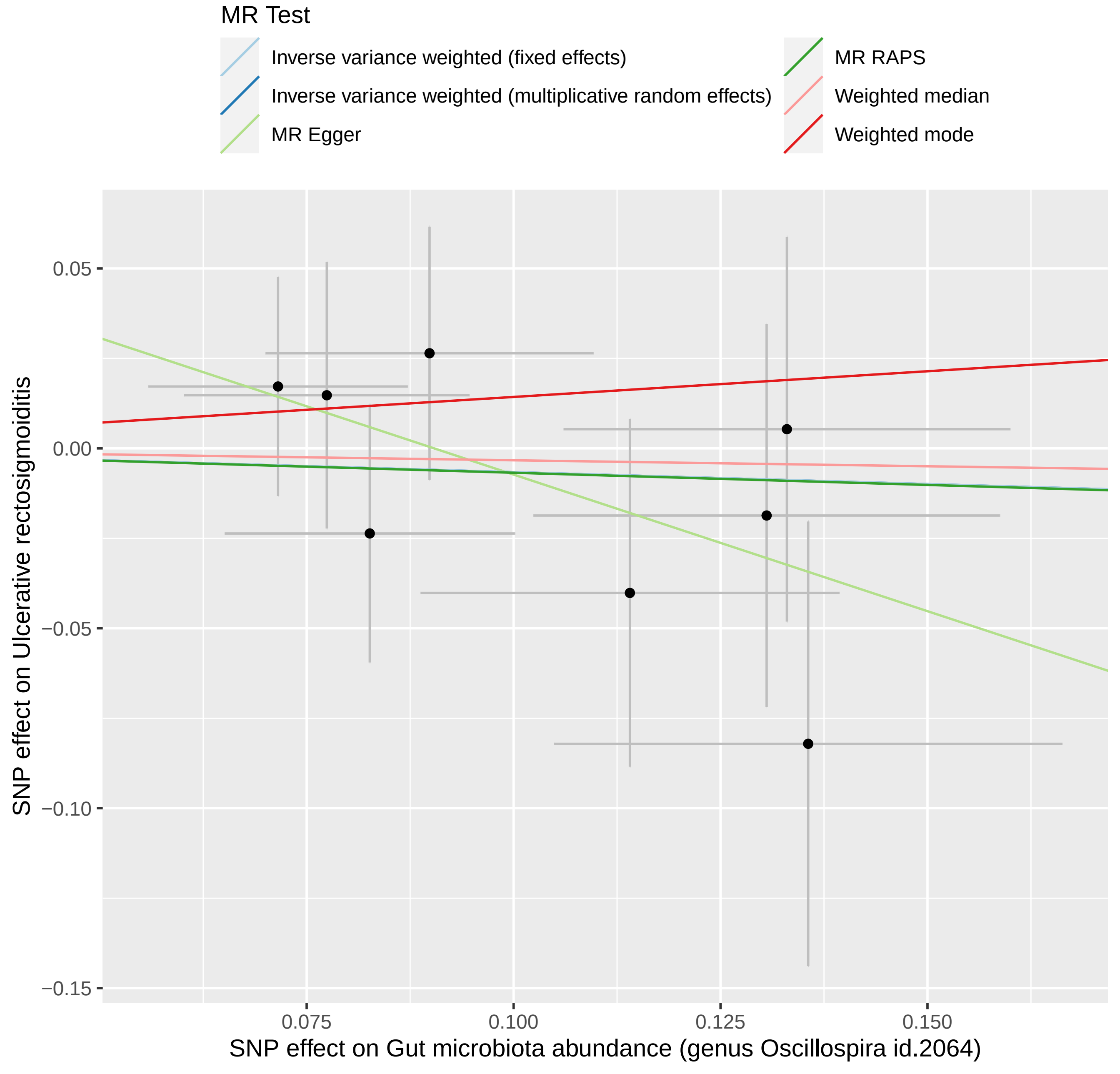

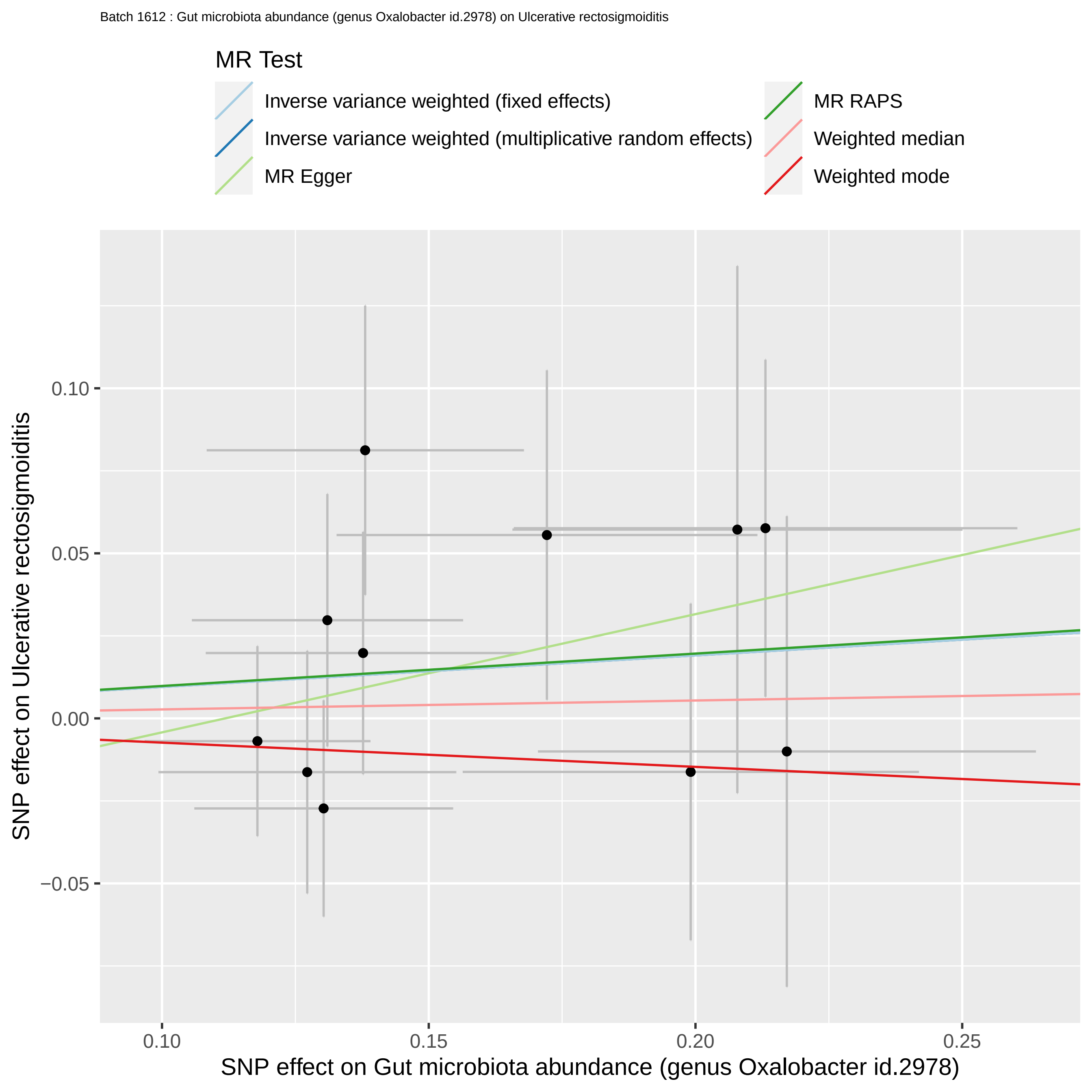
Batch 1612 : Gut microbiota abundance (genus Oxalobacter id.2978) on Ulcerative rectosigmoiditis
MR Test
Inverse variance weighted (fixed effects)
Inverse variance weighted (multiplicative random effects)
MR Egger
MR RAPS
Weighted median
Weighted mode
0.10
0.05
0.00
−0.05
0.10
0.15
0.20
0.25
SNP effect on Ulcerative rectosigmoiditis
SNP effect on Gut microbiota abundance (genus Oxalobacter id.2978)

Batch 1613 : Gut microbiota abundance (genus Parabacteroides id.954) on Ulcerative rectosigmoiditis

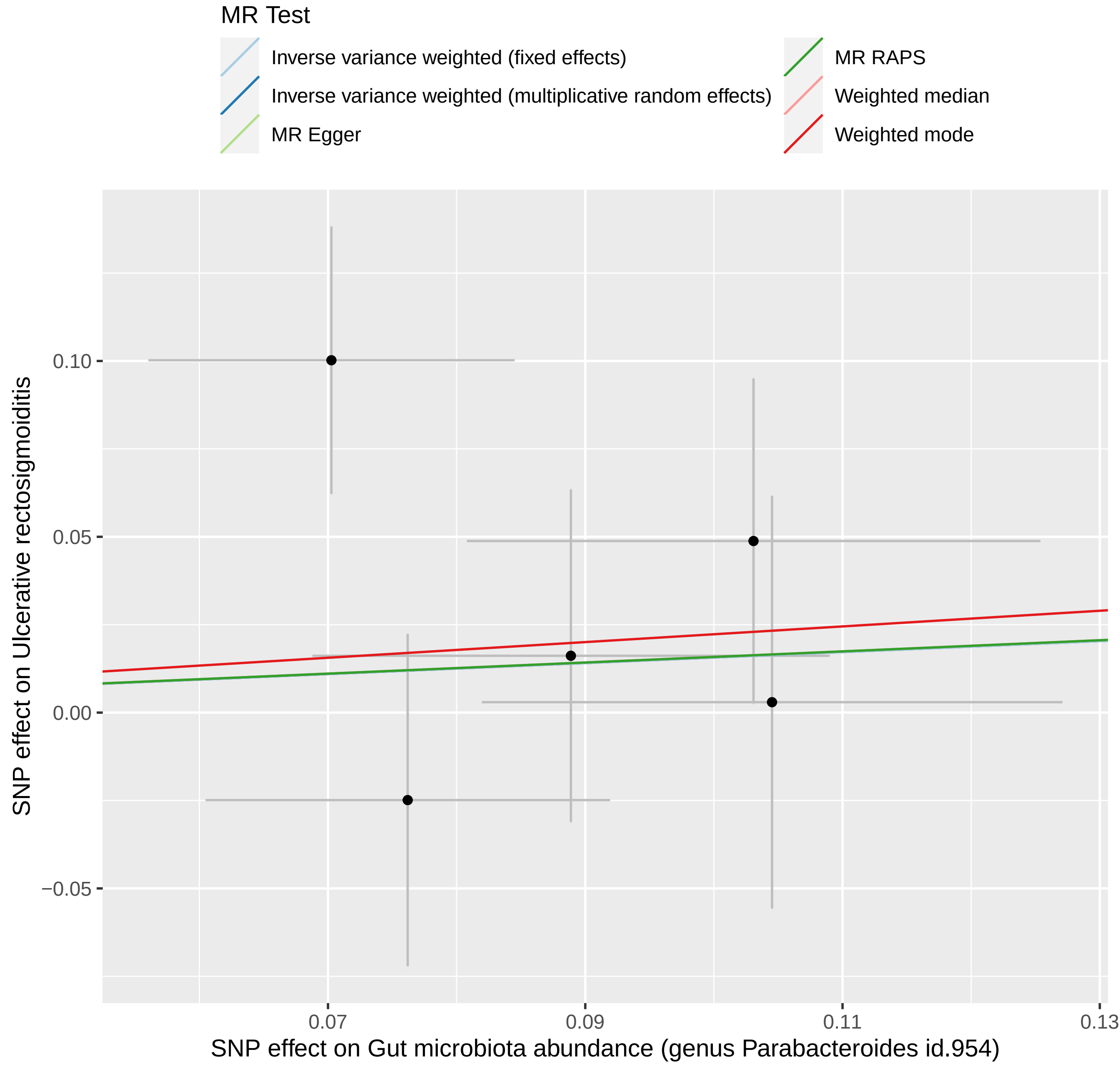

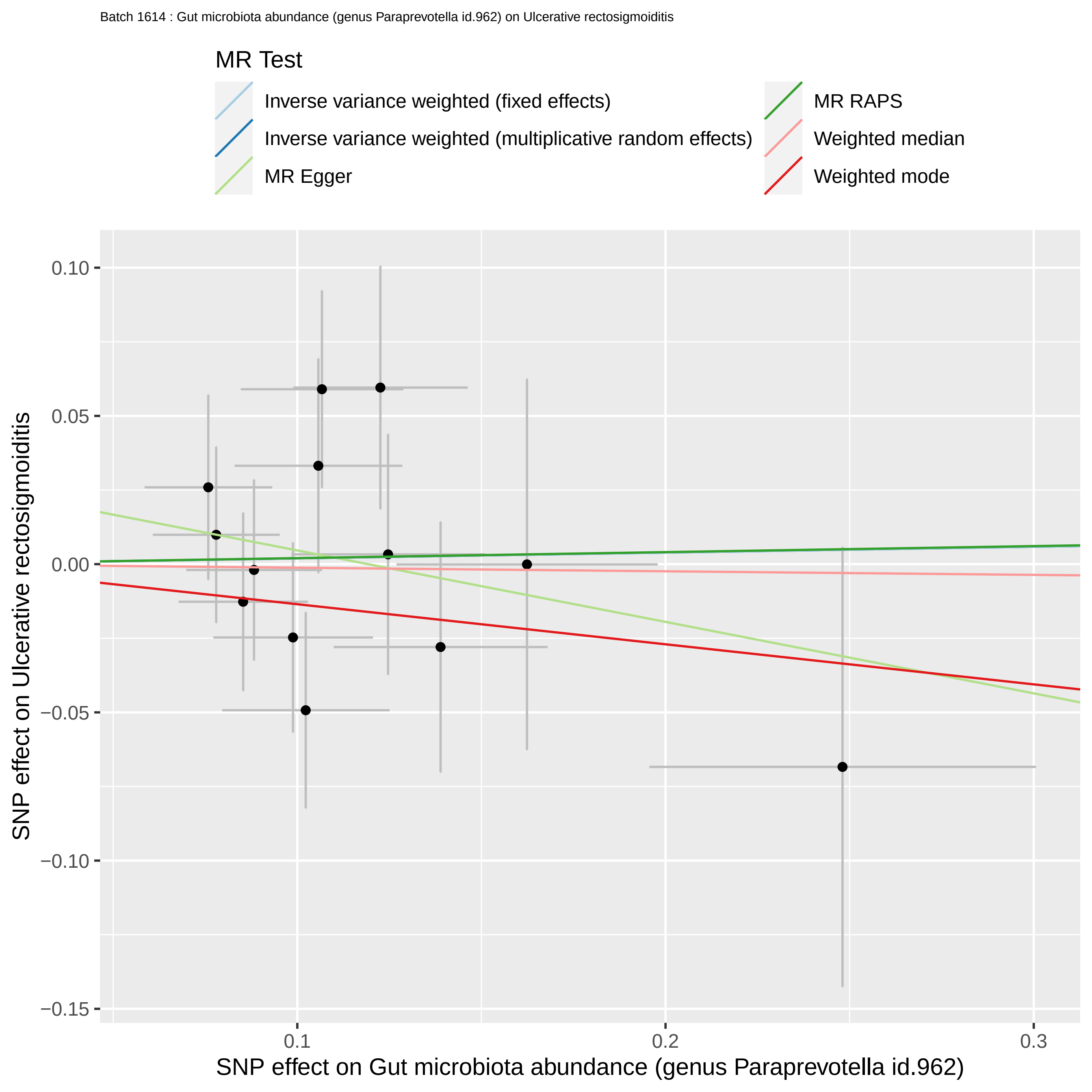
Batch 1614 : Gut microbiota abundance (genus Paraprevotella id.962) on Ulcerative rectosigmoiditis
MR Test
Inverse variance weighted (fixed effects)
Inverse variance weighted (multiplicative random effects)
MR Egger
MR RAPS
Weighted median
Weighted mode
SNP effect on Ulcerative rectosigmoiditis
0.10
0.05
0.00
−0.05
−0.10
−0.15
0.1
0.2
0.3
SNP effect on Gut microbiota abundance (genus Paraprevotella id.962)

Batch 1615 : Gut microbiota abundance (genus Parasutterella id.2892) on Ulcerative rectosigmoiditis

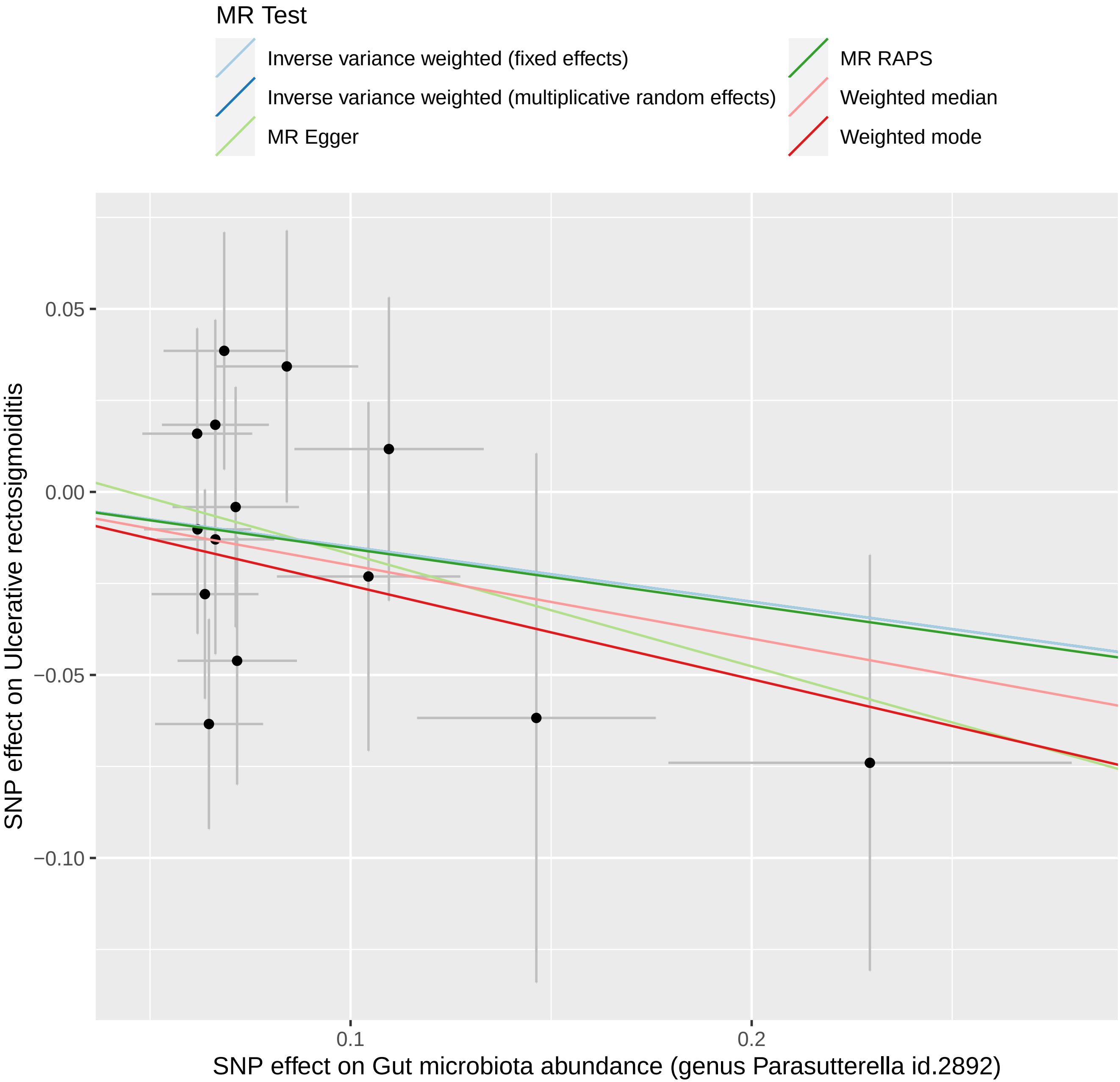

Batch 1616 : Gut microbiota abundance (genus Peptococcus id.2037) on Ulcerative rectosigmoiditis

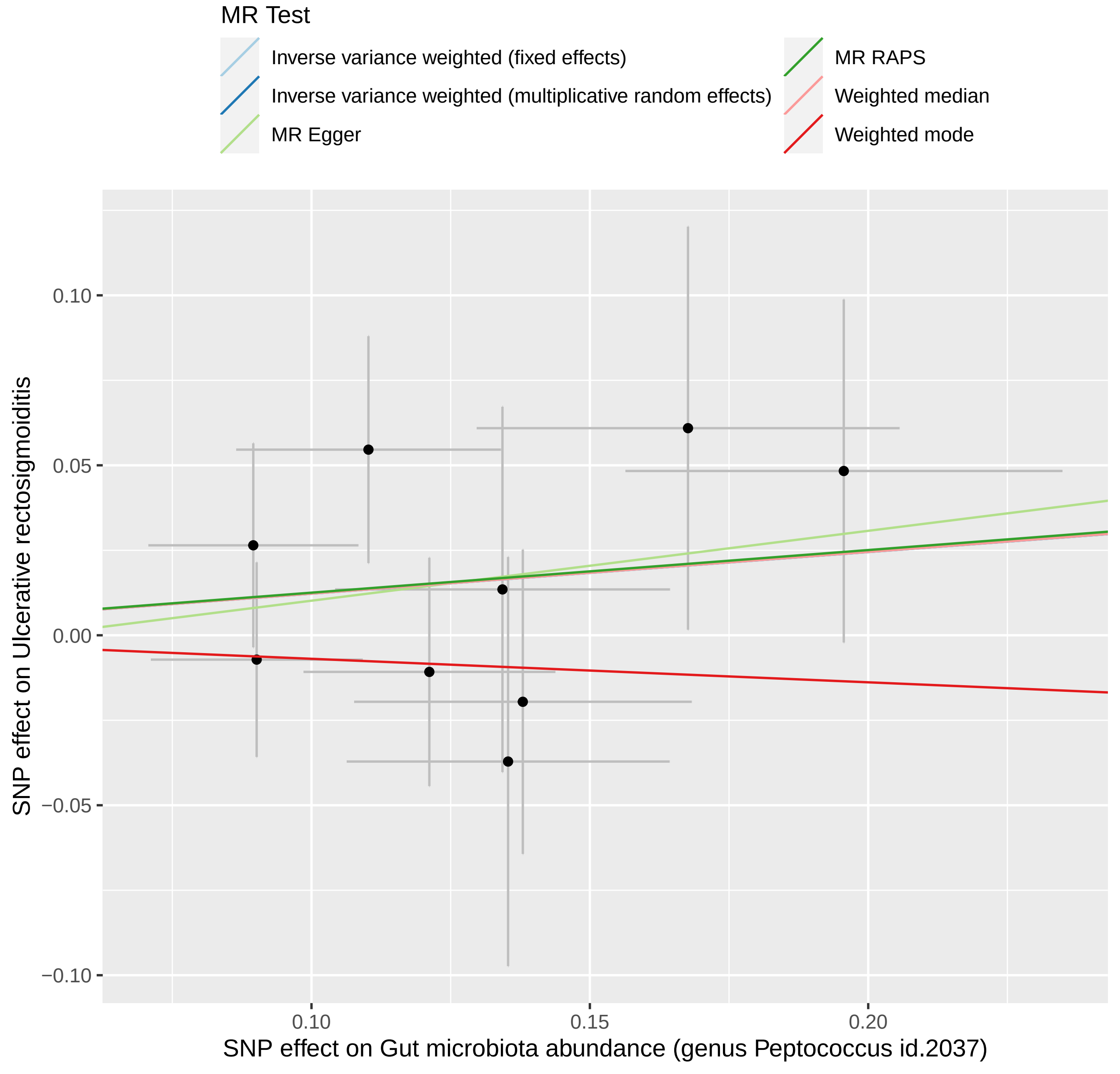

Batch 1617 : Gut microbiota abundance (genus Phascolarctobacterium id.2168) on Ulcerative rectosigmoiditis

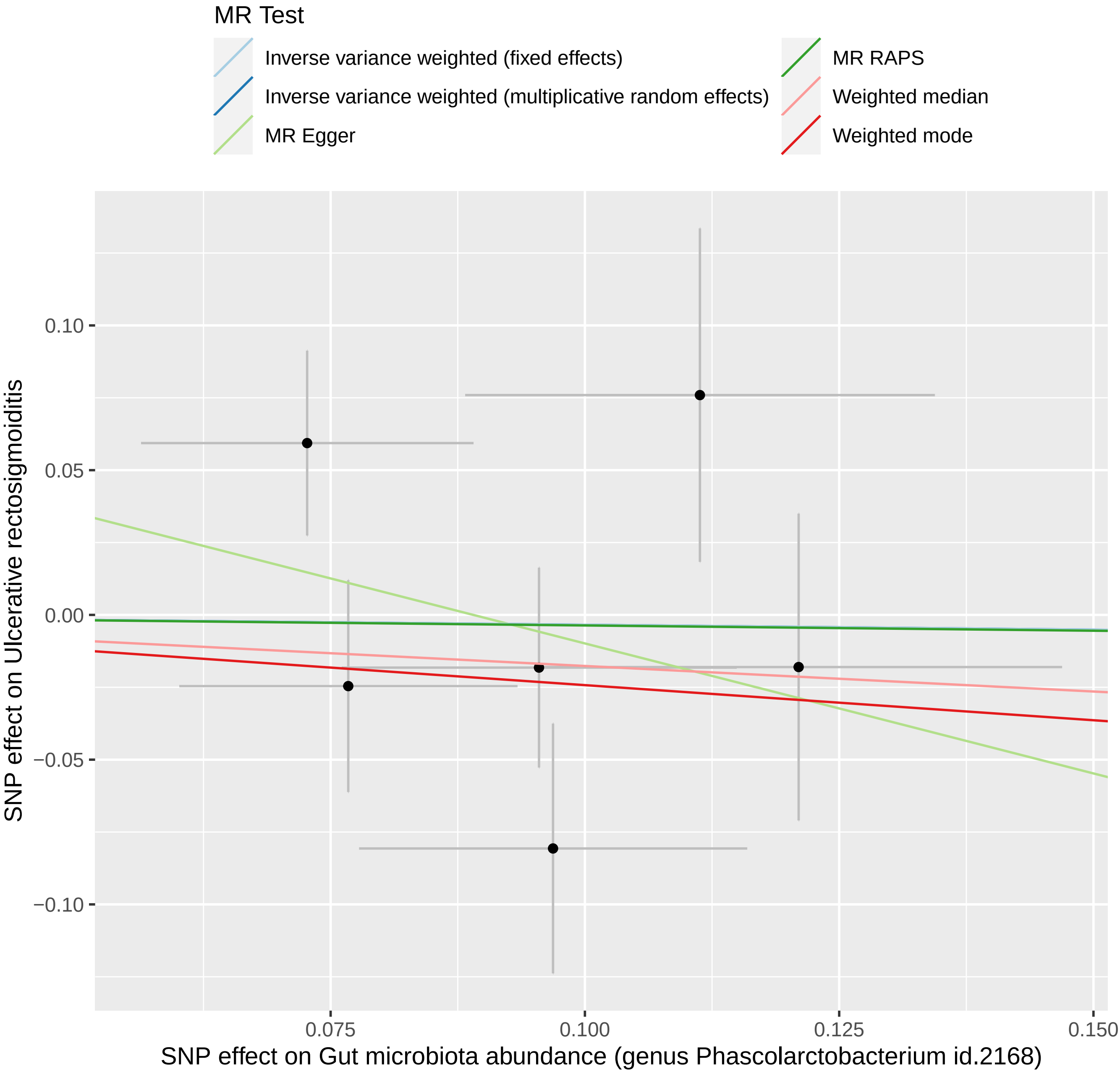

Batch 1618 : Gut microbiota abundance (genus Prevotella7 id.11182) on Ulcerative rectosigmoiditis

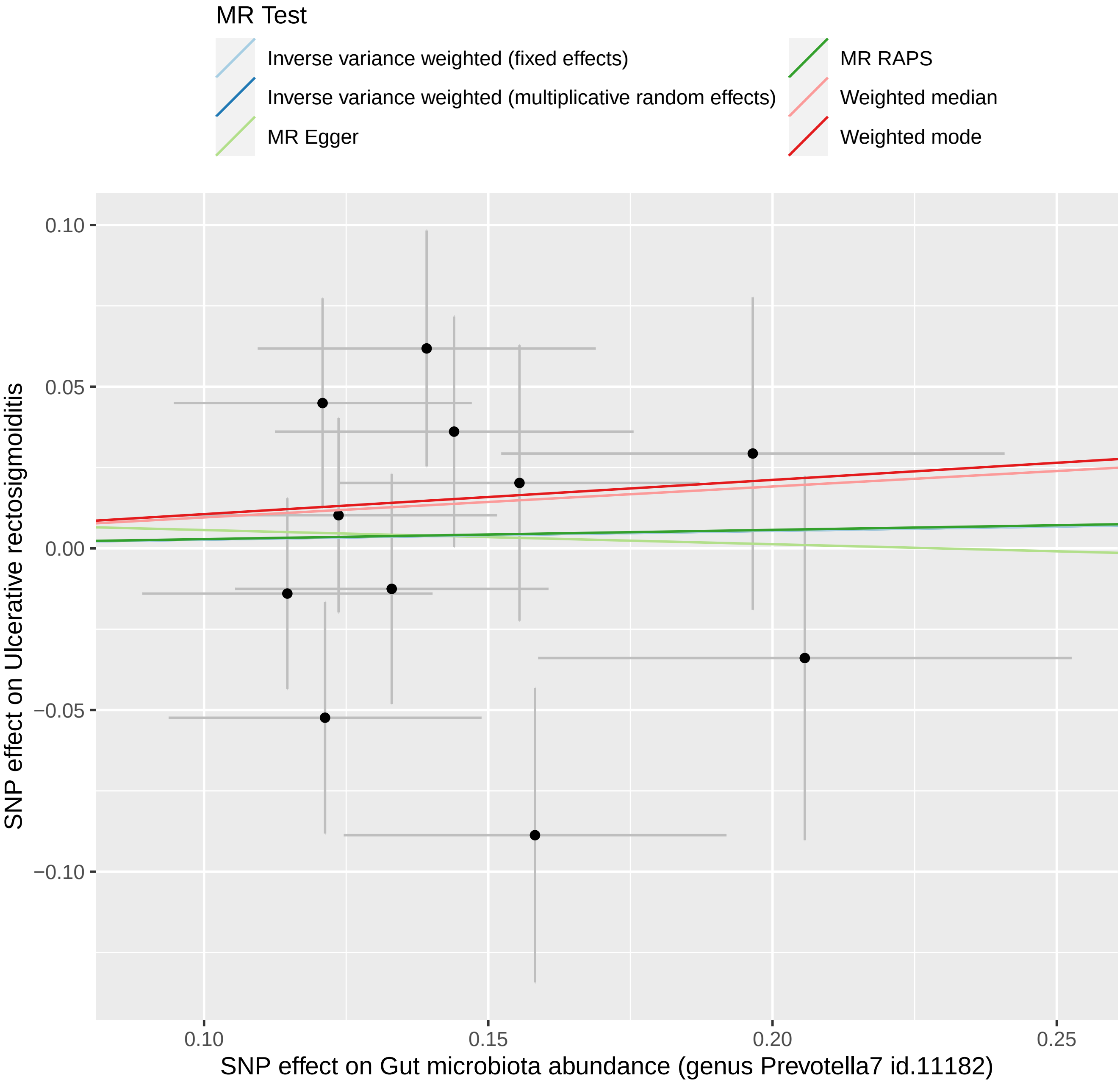

Batch 1619 : Gut microbiota abundance (genus Prevotella9 id.11183) on Ulcerative rectosigmoiditis

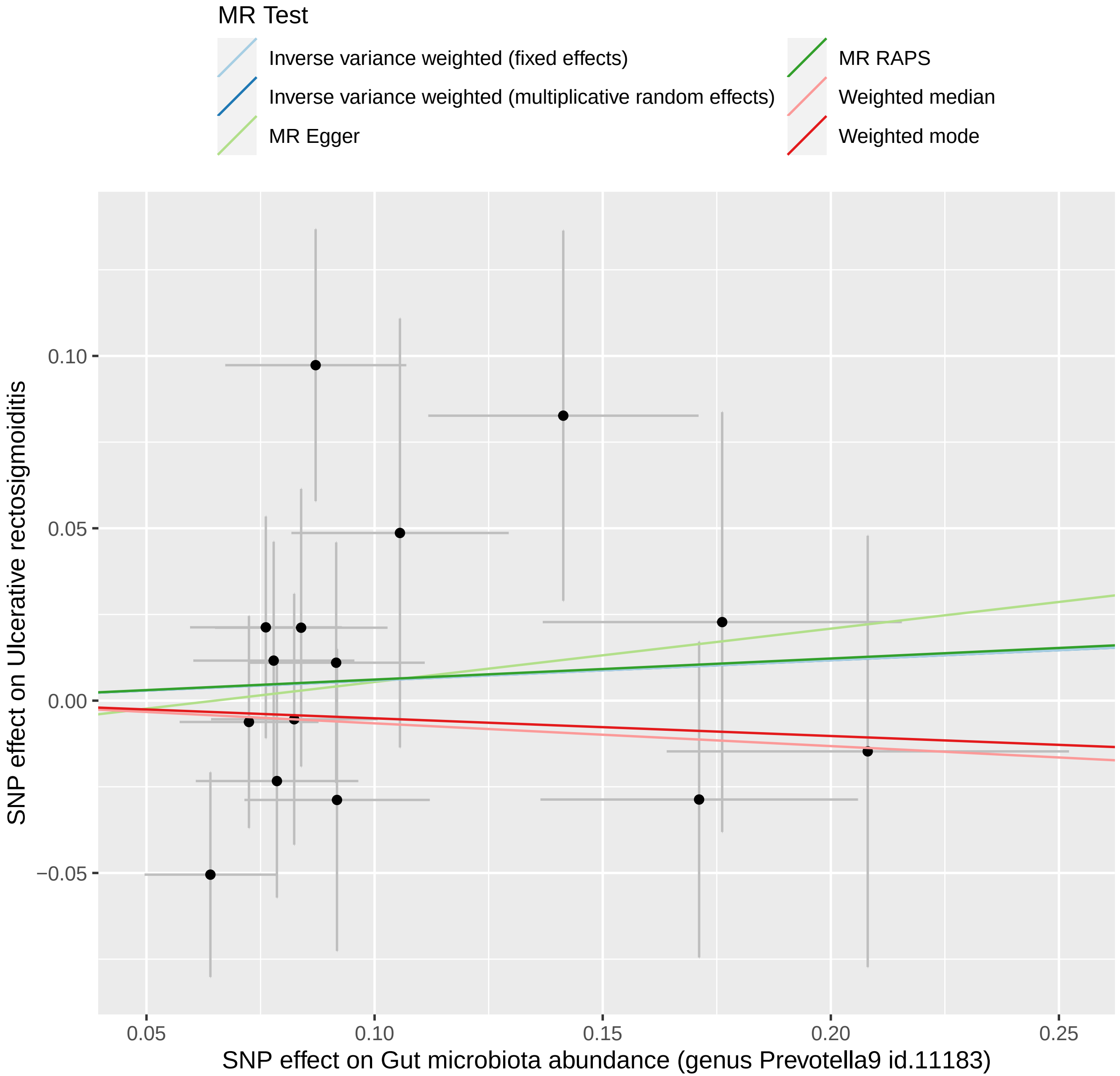

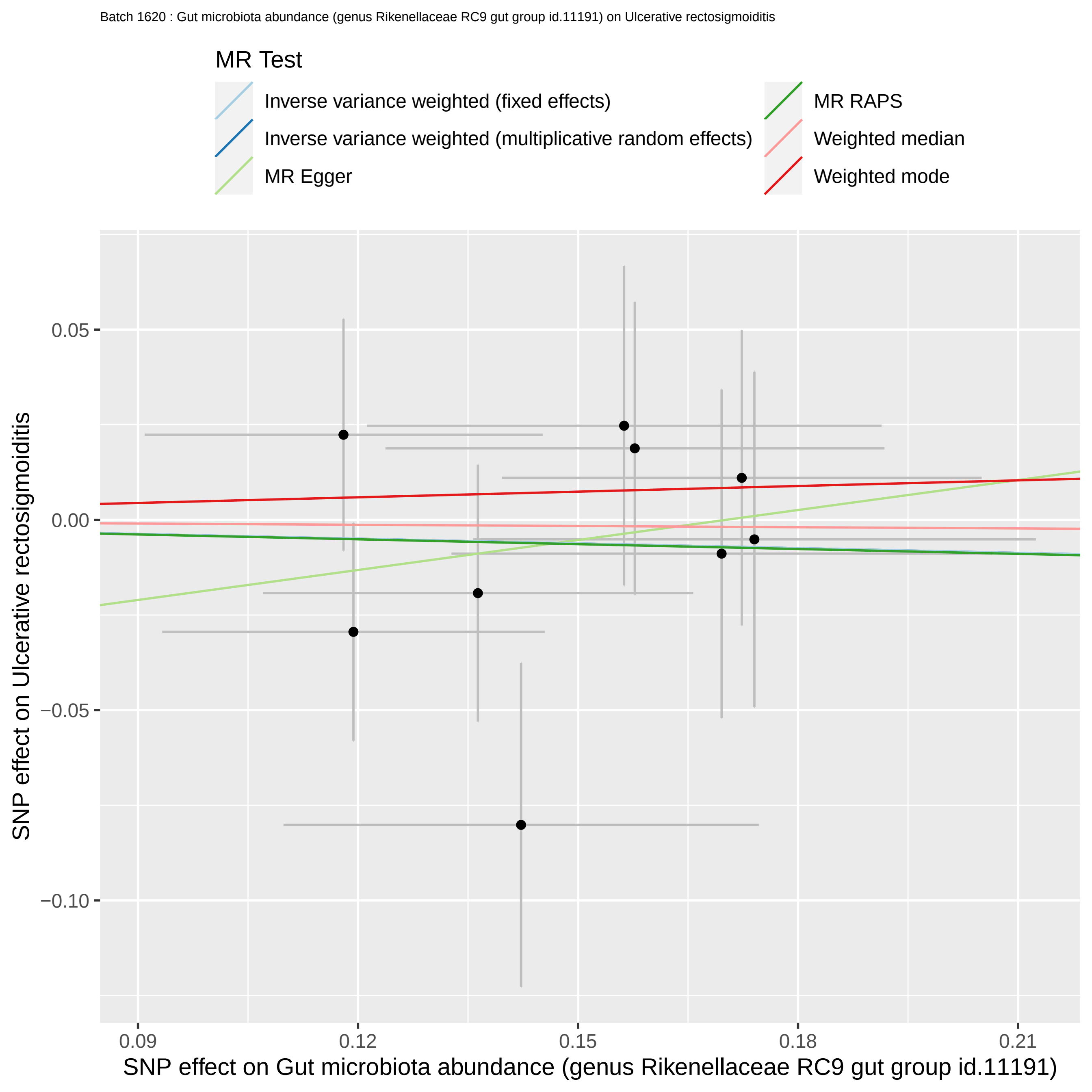

Batch 1620 : Gut microbiota abundance (genus Rikenellaceae RC9 gut group id.11191) on Ulcerative rectosigmoiditis
MR Test
Inverse variance weighted (fixed effects)
Inverse variance weighted (multiplicative random effects)
MR Egger
MR RAPS
Weighted median
Weighted mode
0.05
0.00
−0.05
−0.10
0.09
0.12
0.15
0.18
0.21
SNP effect on Ulcerative rectosigmoiditis
SNP effect on Gut microbiota abundance (genus Rikenellaceae RC9 gut group id.11191)

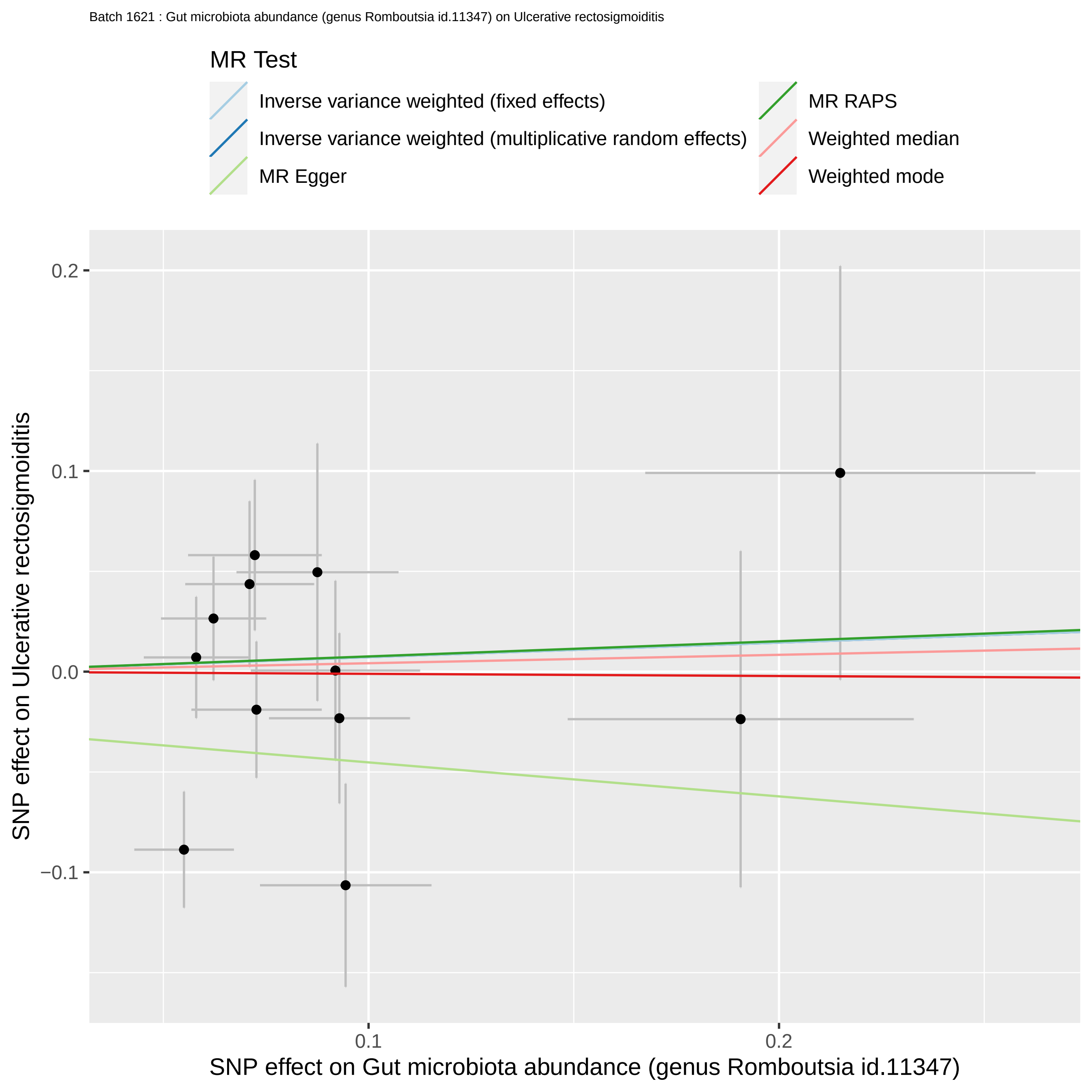

Batch 1621 : Gut microbiota abundance (genus Romboutsia id.11347) on Ulcerative rectosigmoiditis
MR Test
Inverse variance weighted (fixed effects)
Inverse variance weighted (multiplicative random effects)
MR Egger
MR RAPS
Weighted median
Weighted mode
SNP effect on Ulcerative rectosigmoiditis
0.2
0.1
0.0
−0.1
0.1
0.2
SNP effect on Gut microbiota abundance (genus Romboutsia id.11347)

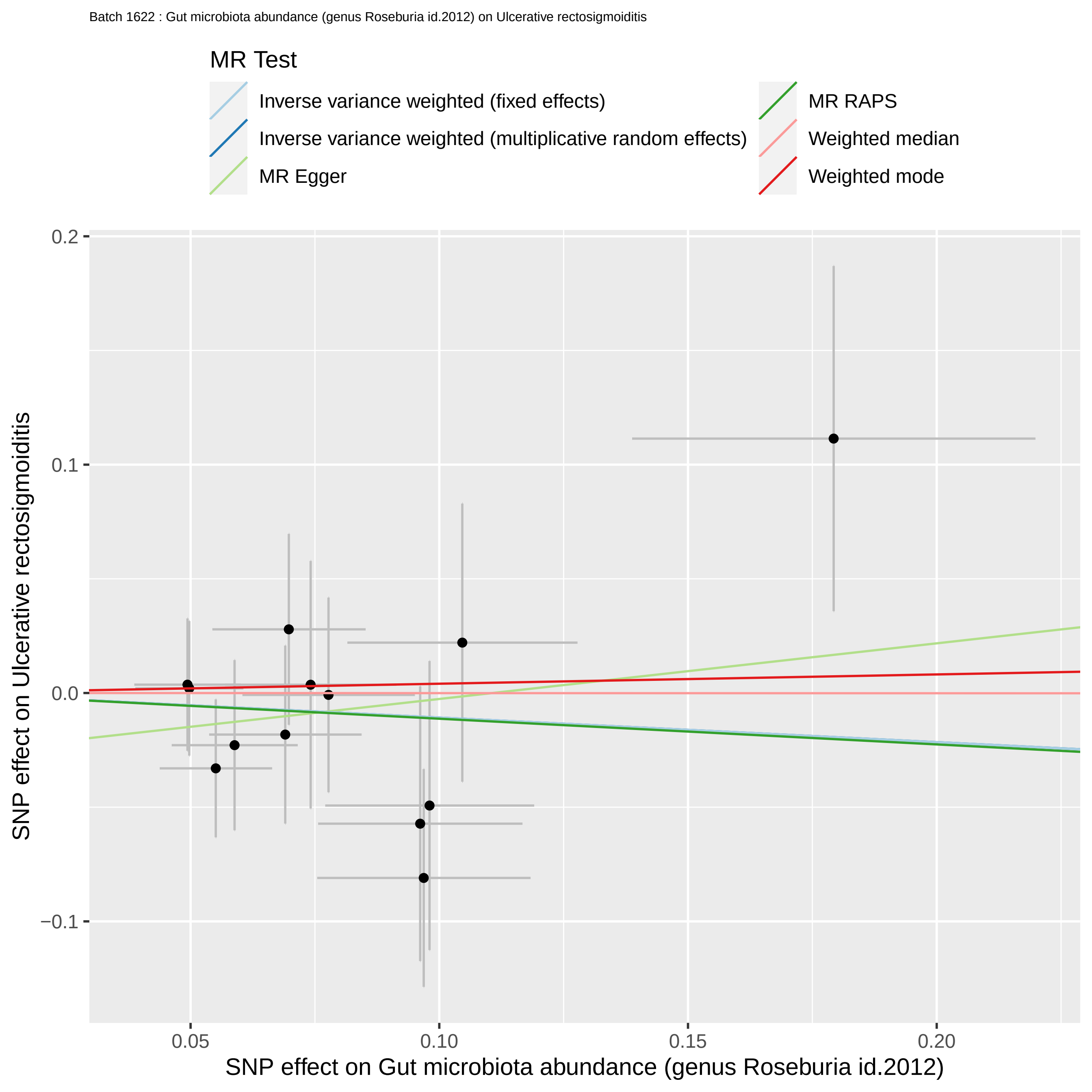

Batch 1622 : Gut microbiota abundance (genus Roseburia id.2012) on Ulcerative rectosigmoiditis
MR Test
Inverse variance weighted (fixed effects)
Inverse variance weighted (multiplicative random effects)
MR Egger
MR RAPS
Weighted median
Weighted mode
0.2
0.1
0.0
−0.1
0.05
0.10
0.15
0.20
SNP effect on Ulcerative rectosigmoiditis
SNP effect on Gut microbiota abundance (genus Roseburia id.2012)

Batch 1623 : Gut microbiota abundance (genus Ruminiclostridium5 id.11355) on Ulcerative rectosigmoiditis

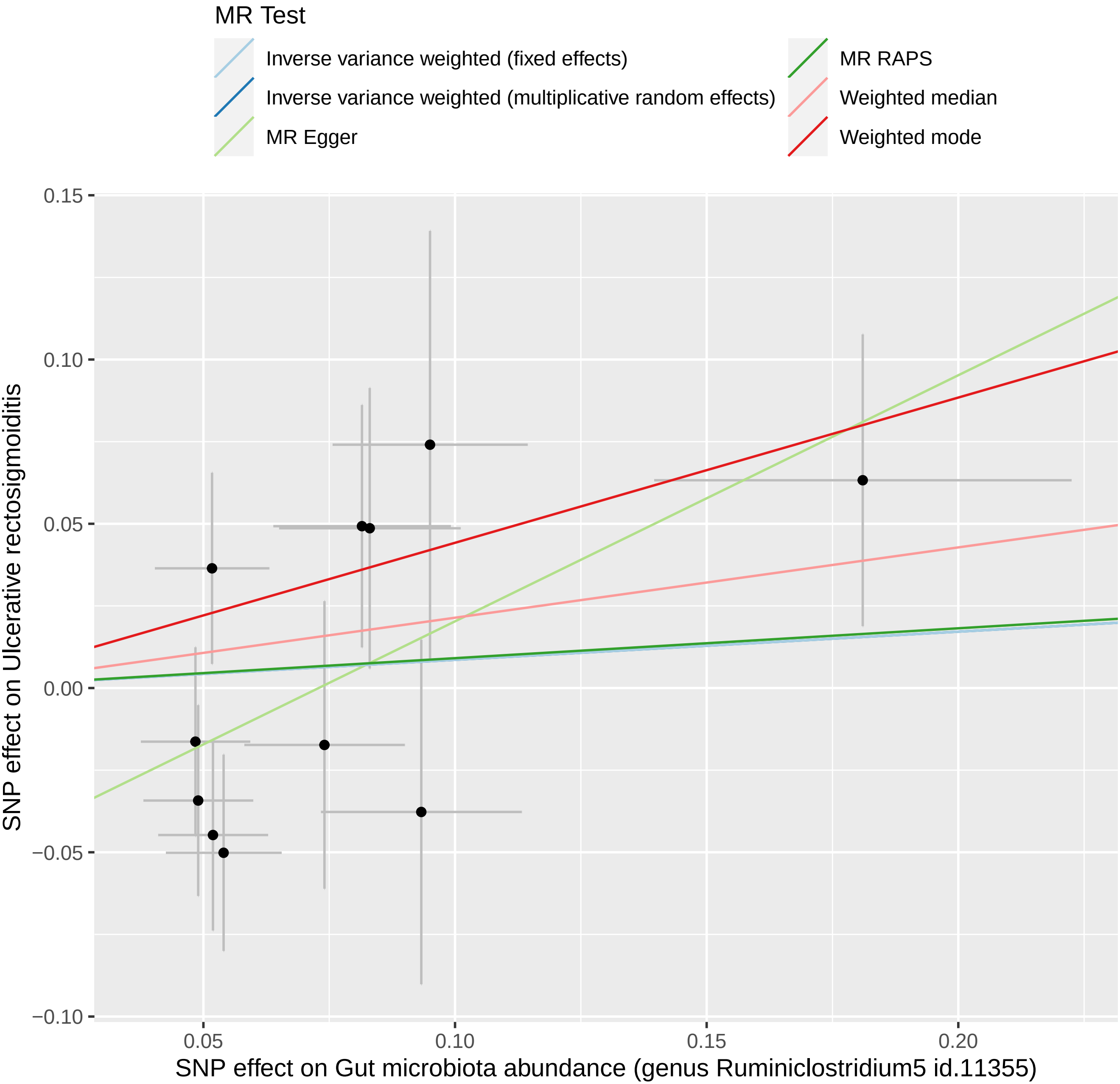

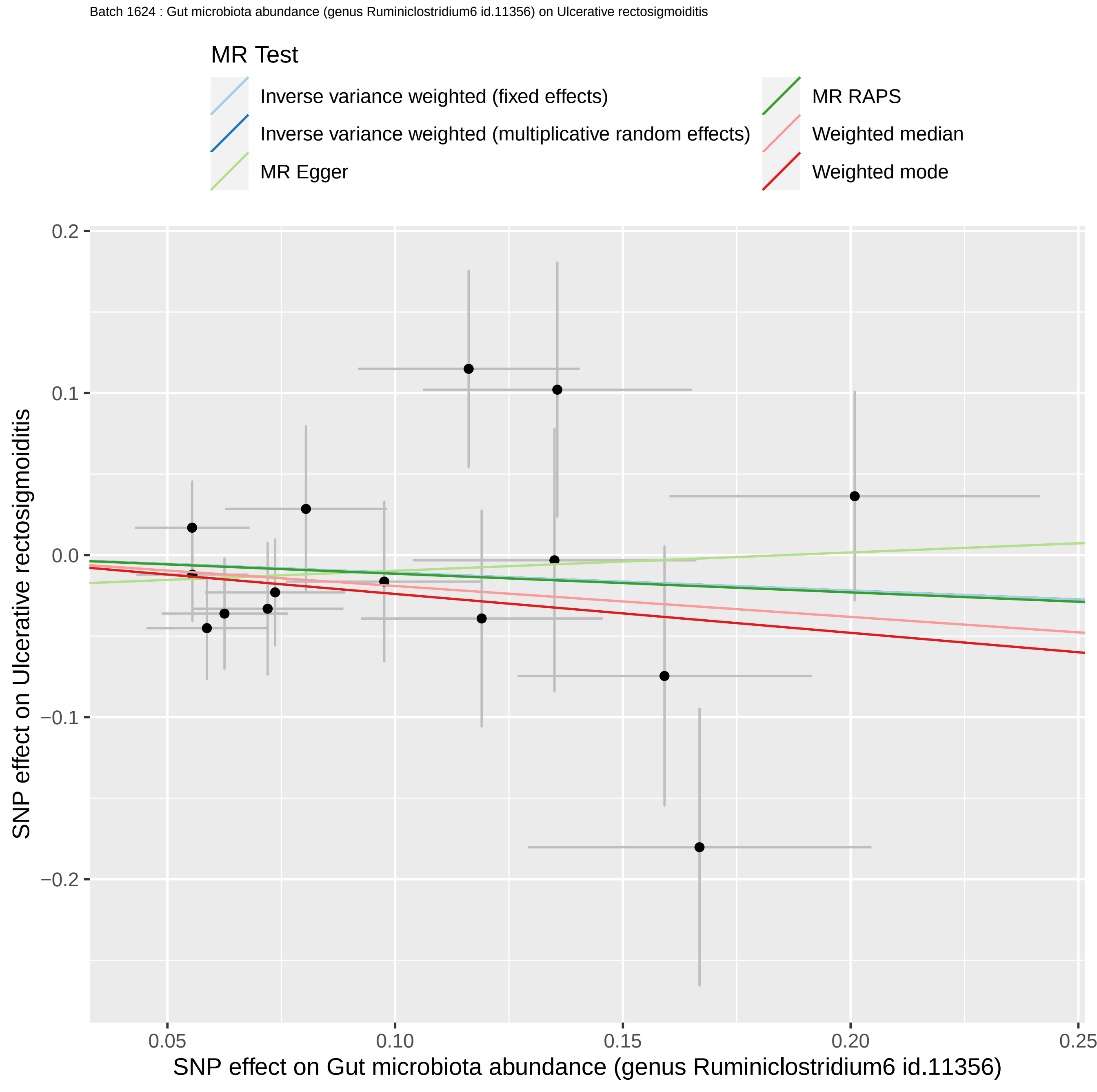
Batch 1624 : Gut microbiota abundance (genus Ruminiclostridium6 id.11356) on Ulcerative rectosigmoiditis
MR Test
Inverse variance weighted (fixed effects)
Inverse variance weighted (multiplicative random effects)
MR Egger
MR RAPS
Weighted median
Weighted mode
0.2
0.1
0.0
−0.1
−0.2
0.05
0.10
0.15
0.20
0.25
SNP effect on Ulcerative rectosigmoiditis
SNP effect on Gut microbiota abundance (genus Ruminiclostridium6 id.11356)

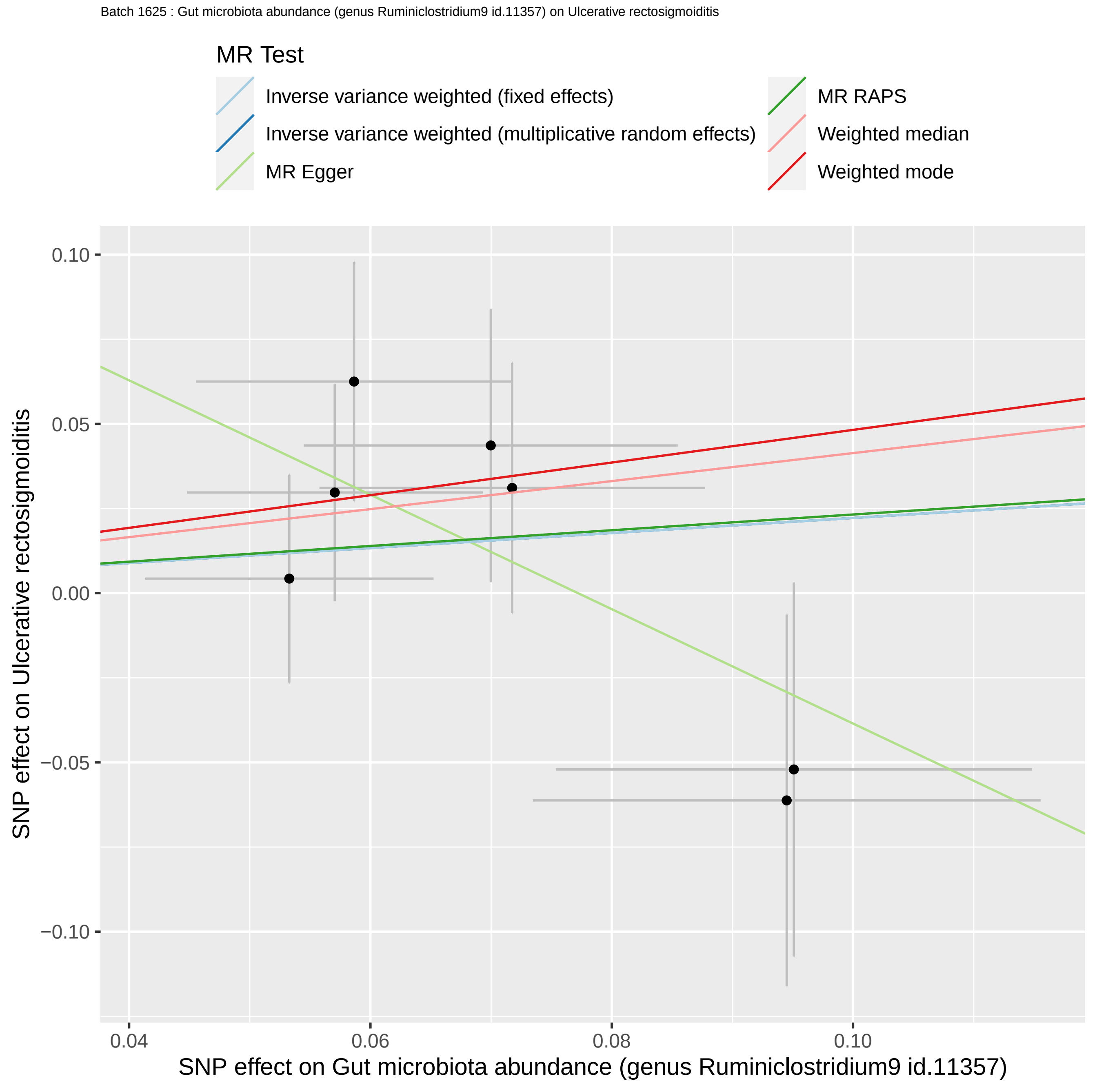

Batch 1625 : Gut microbiota abundance (genus Ruminiclostridium9 id.11357) on Ulcerative rectosigmoiditis
MR Test
Inverse variance weighted (fixed effects)
Inverse variance weighted (multiplicative random effects)
MR Egger
MR RAPS
Weighted median
Weighted mode
0.10
0.05
0.00
−0.05
−0.10
0.04
0.06
0.08
0.10
SNP effect on Ulcerative rectosigmoiditis
SNP effect on Gut microbiota abundance (genus Ruminiclostridium9 id.11357)

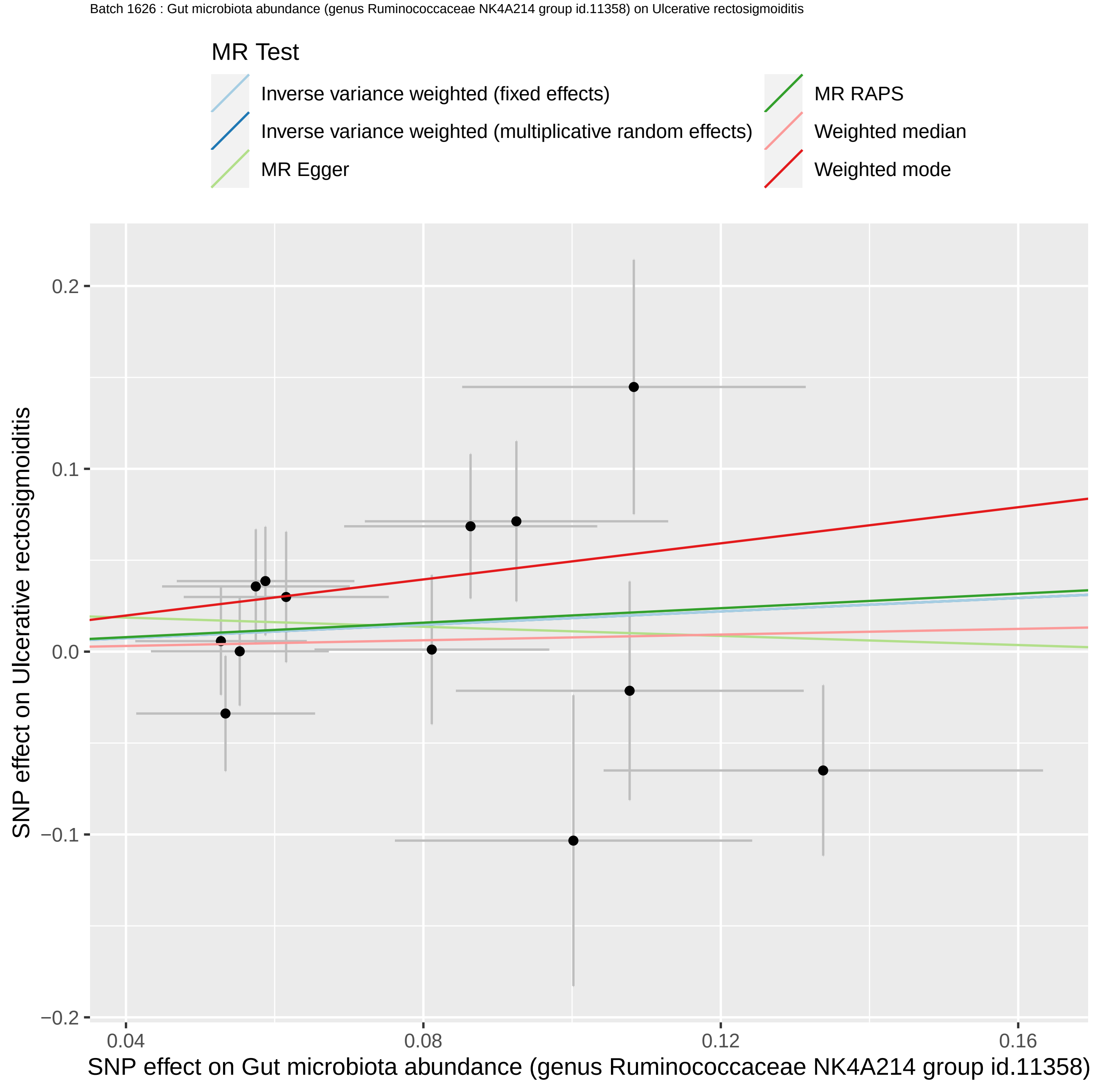
Batch 1626 : Gut microbiota abundance (genus Ruminococcaceae NK4A214 group id.11358) on Ulcerative rectosigmoiditis
MR Test
Inverse variance weighted (fixed effects)
Inverse variance weighted (multiplicative random effects)
MR Egger
MR RAPS
Weighted median
Weighted mode
0.2
0.1
0.0
−0.1
−0.2
0.04
0.08
0.12
0.16
SNP effect on Ulcerative rectosigmoiditis
SNP effect on Gut microbiota abundance (genus Ruminococcaceae NK4A214 group id.11358)

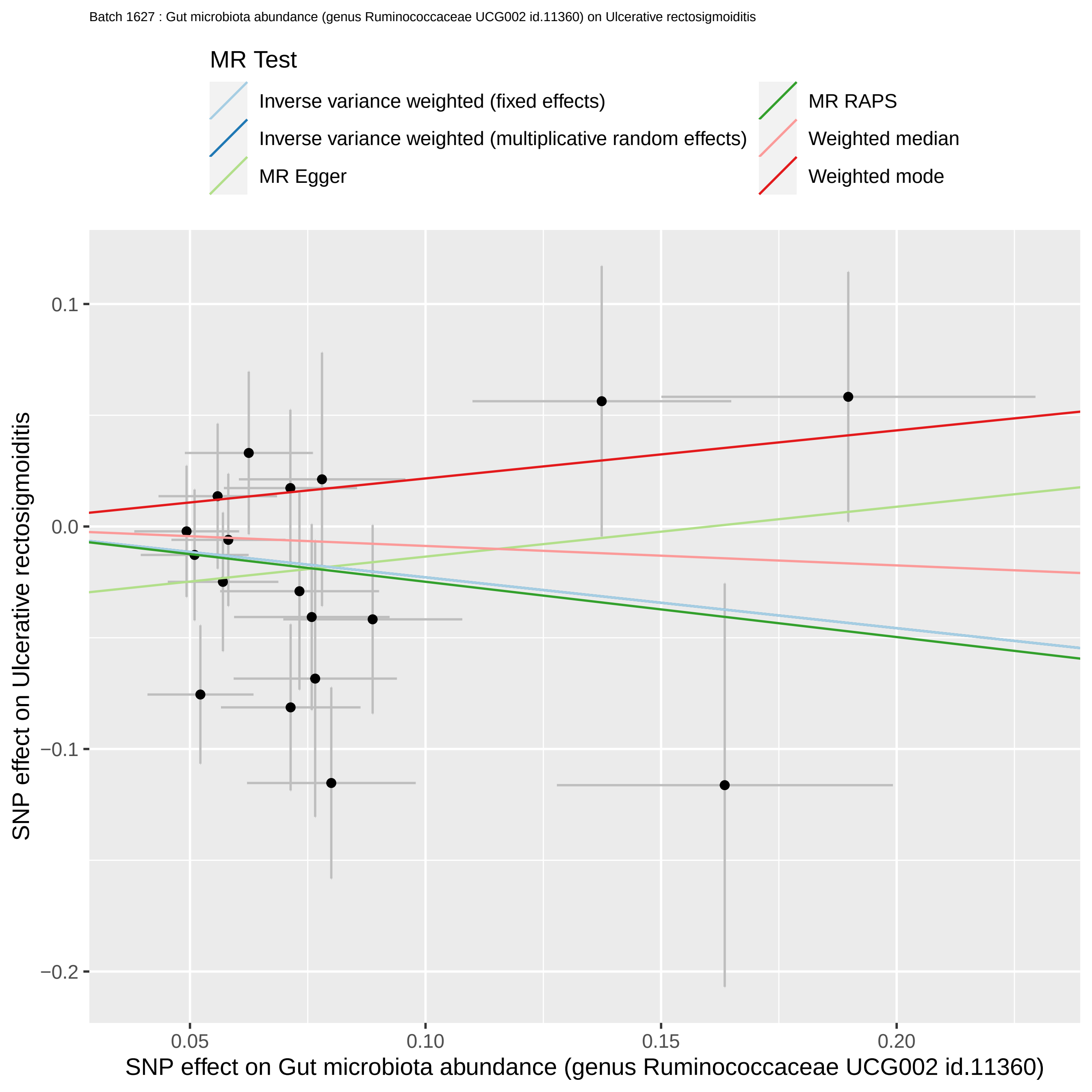
Batch 1627 : Gut microbiota abundance (genus Ruminococcaceae UCG002 id.11360) on Ulcerative rectosigmoiditis
MR Test
Inverse variance weighted (fixed effects)
Inverse variance weighted (multiplicative random effects)
MR Egger
MR RAPS
Weighted median
Weighted mode
0.1
0.0
−0.1
−0.2
0.05
0.10
0.15
0.20
SNP effect on Ulcerative rectosigmoiditis
SNP effect on Gut microbiota abundance (genus Ruminococcaceae UCG002 id.11360)

Batch 1628 : Gut microbiota abundance (genus Ruminococcaceae UCG003 id.11361) on Ulcerative rectosigmoiditis

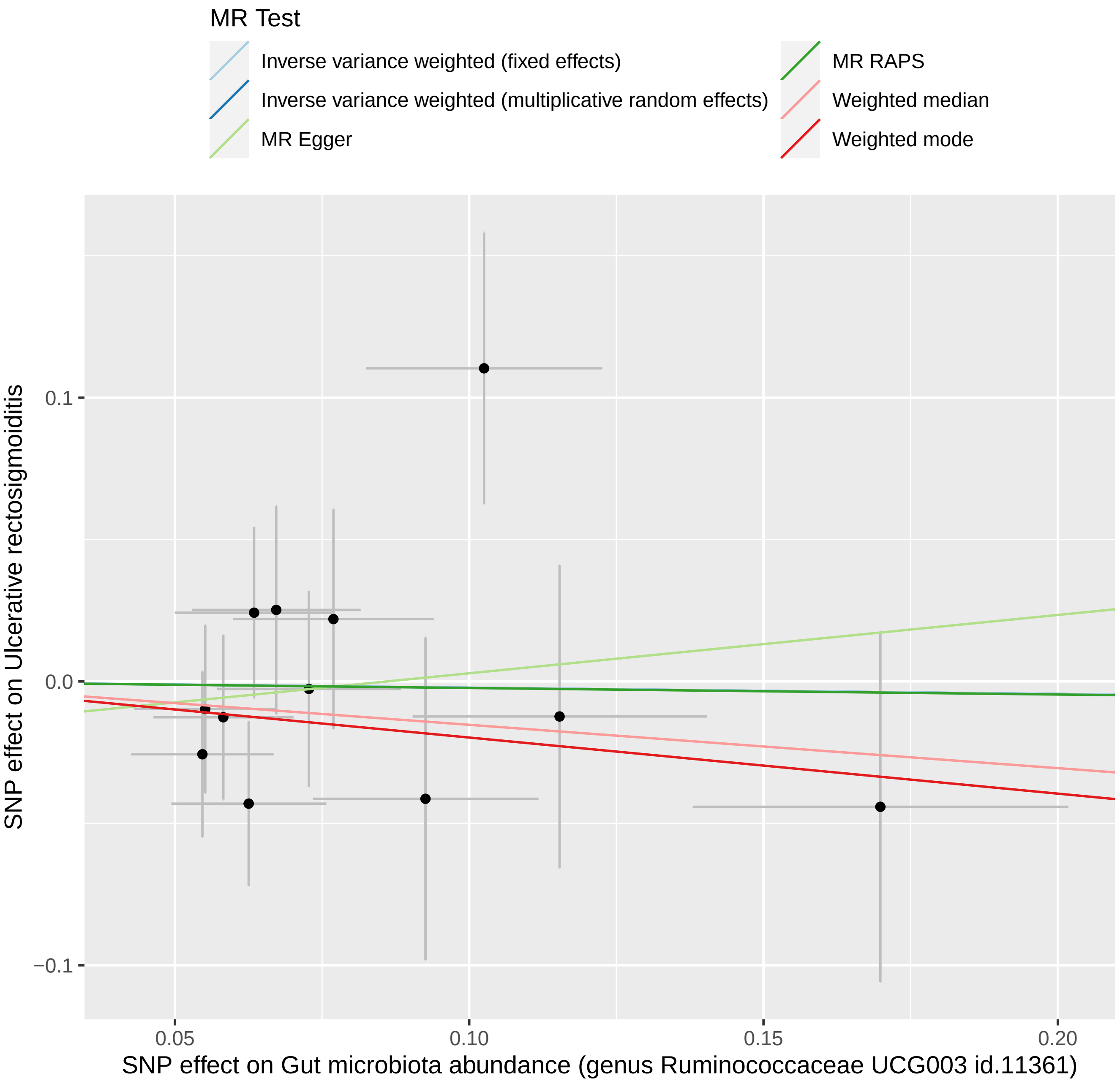

Batch 1629 : Gut microbiota abundance (genus Ruminococcaceae UCG004 id.11362) on Ulcerative rectosigmoiditis

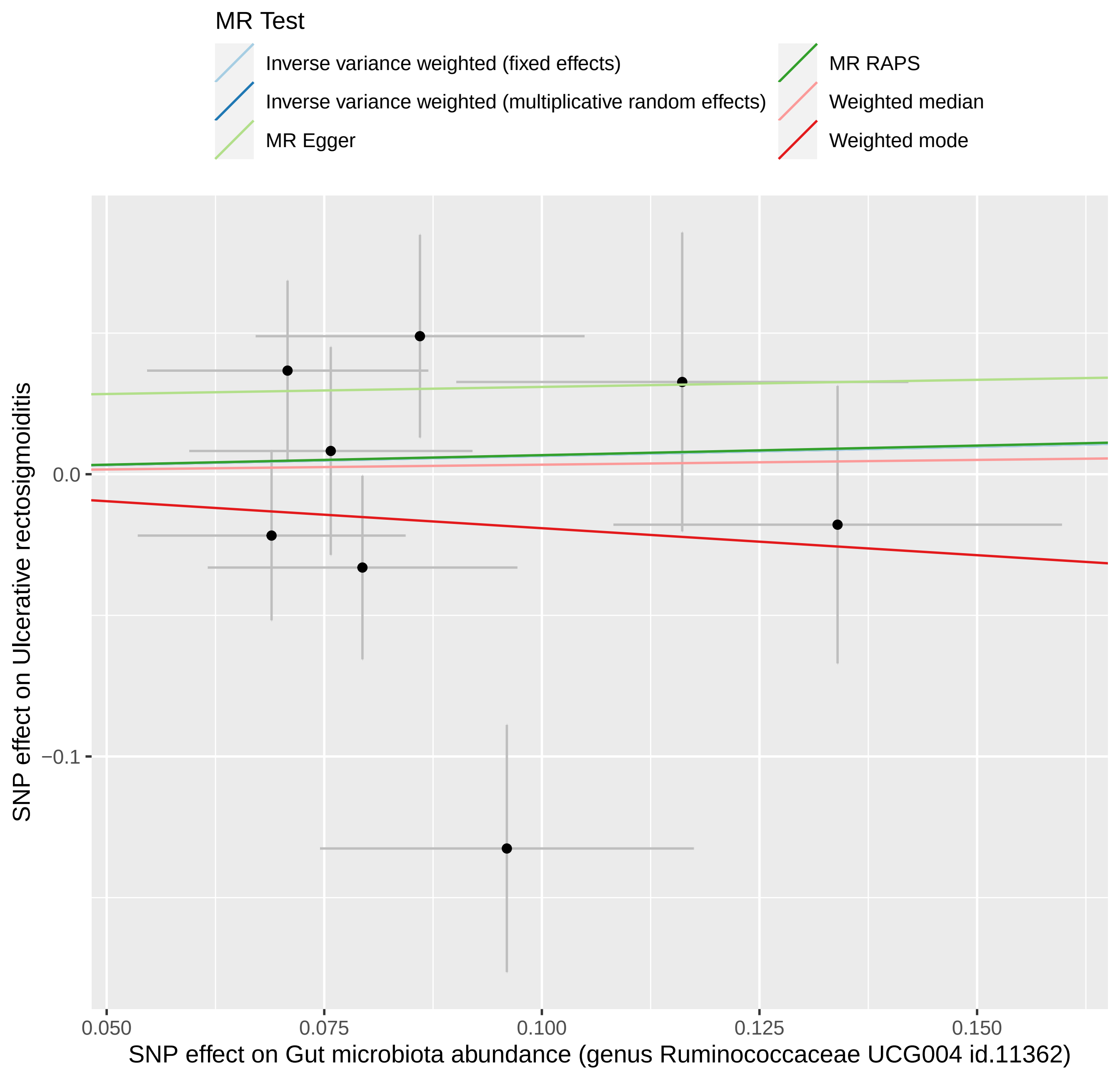

Batch 1630 : Gut microbiota abundance (genus Ruminococcaceae UCG005 id.11363) on Ulcerative rectosigmoiditis

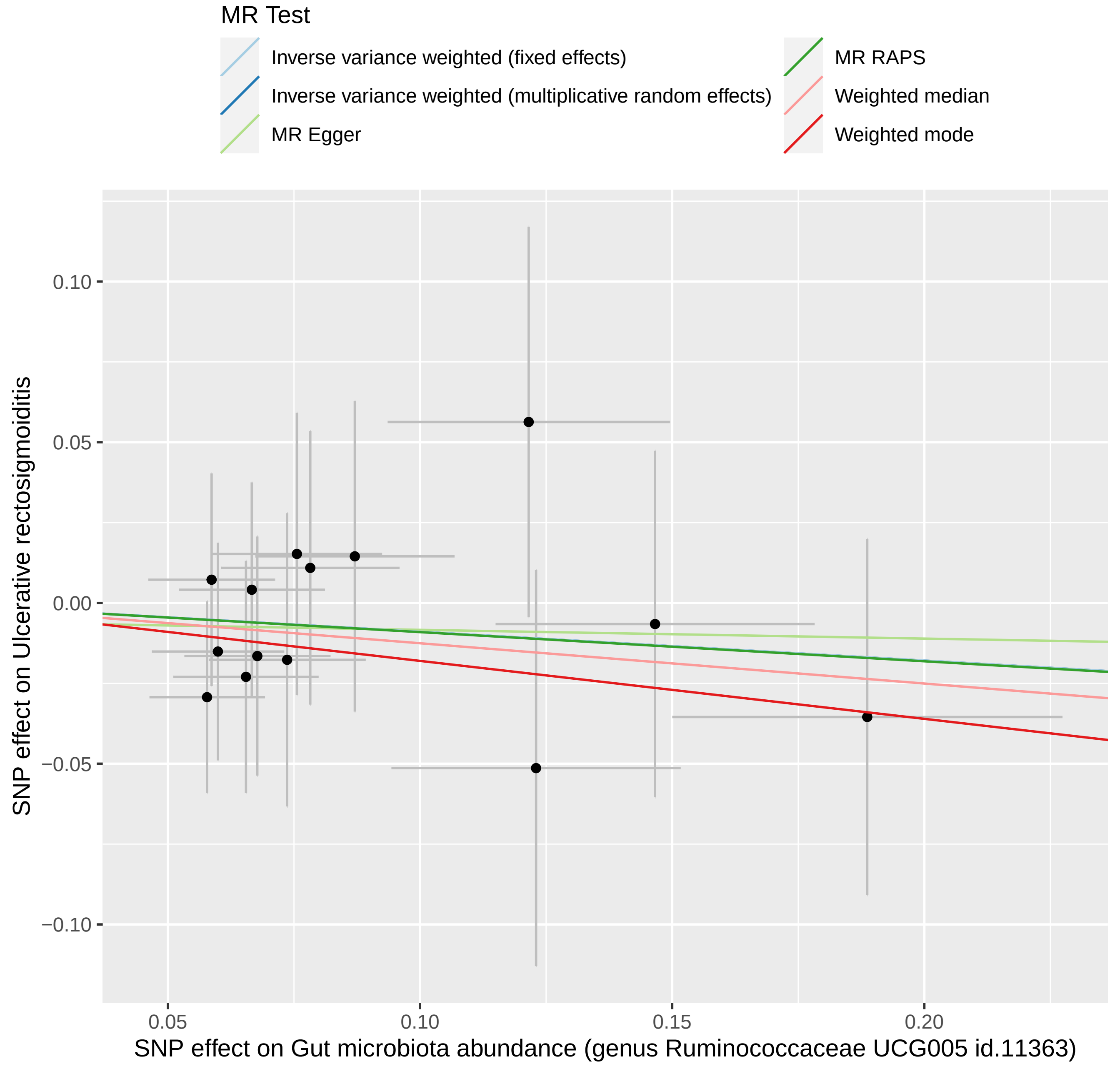

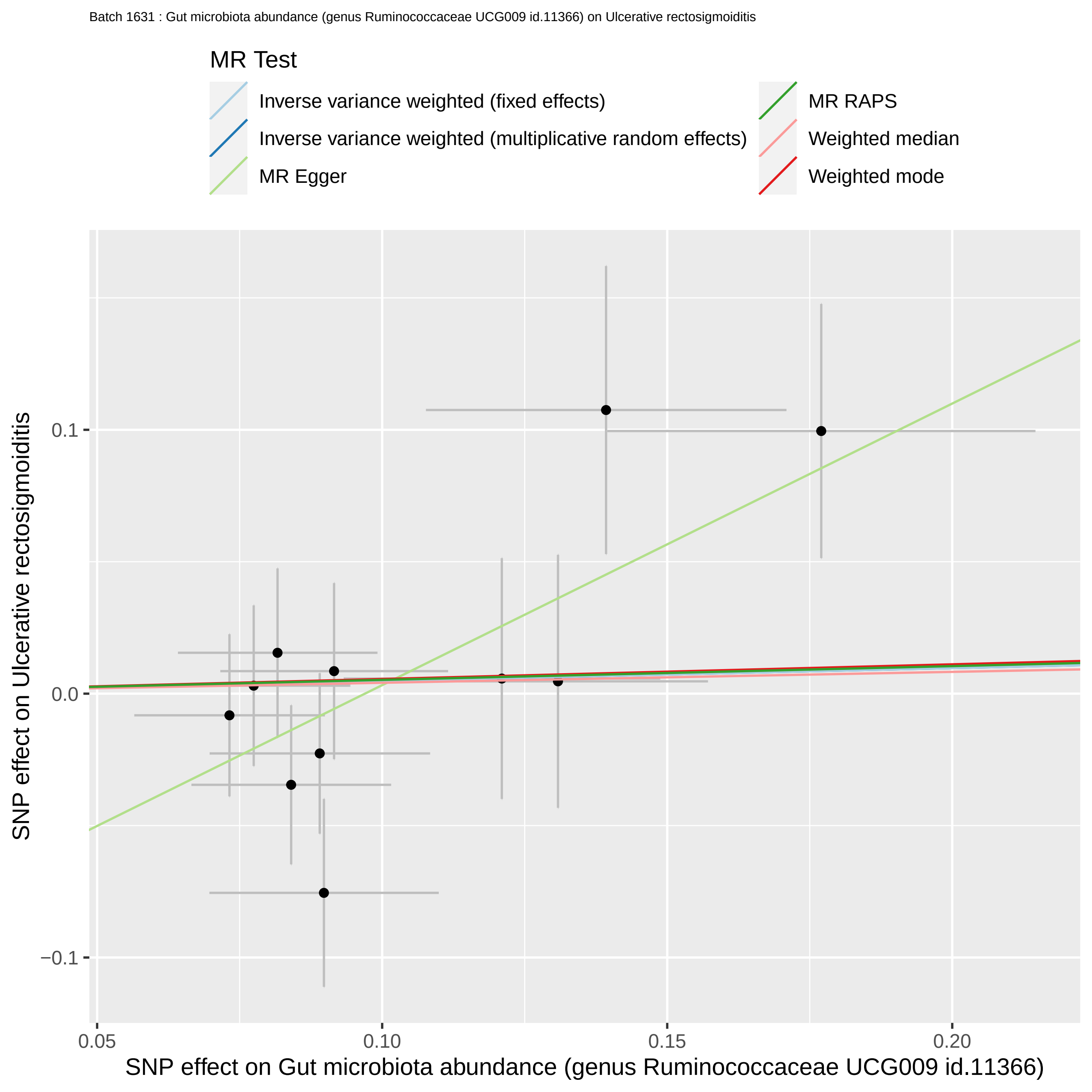
Batch 1631 : Gut microbiota abundance (genus Ruminococcaceae UCG009 id.11366) on Ulcerative rectosigmoiditis
MR Test
Inverse variance weighted (fixed effects)
Inverse variance weighted (multiplicative random effects)
MR Egger
MR RAPS
Weighted median
Weighted mode
SNP effect on Ulcerative rectosigmoiditis
0.1
0.0
−0.1
0.05
0.10
0.15
0.20
SNP effect on Gut microbiota abundance (genus Ruminococcaceae UCG009 id.11366)

Batch 1632 : Gut microbiota abundance (genus Ruminococcaceae UCG010 id.11367) on Ulcerative rectosigmoiditis

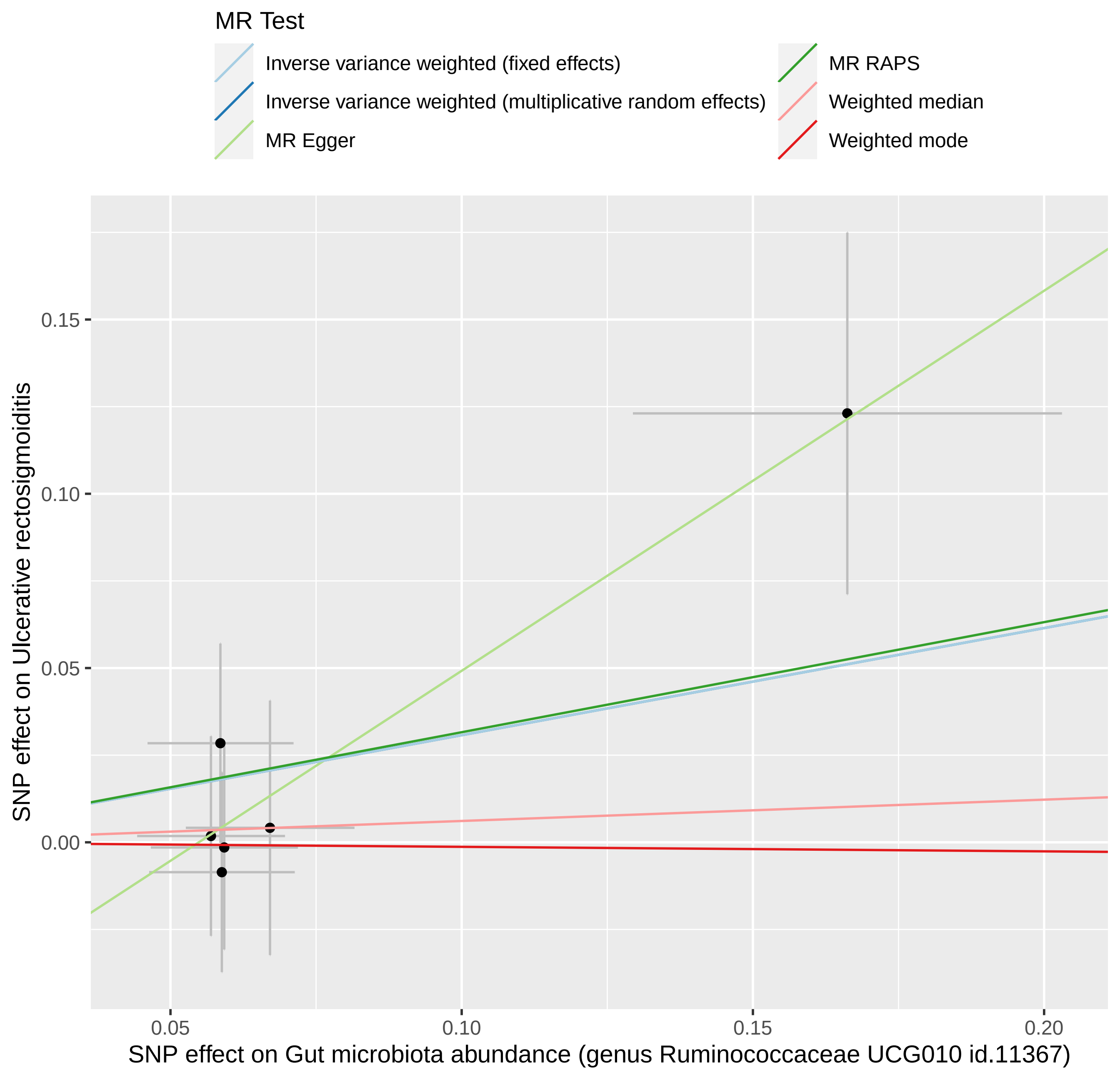

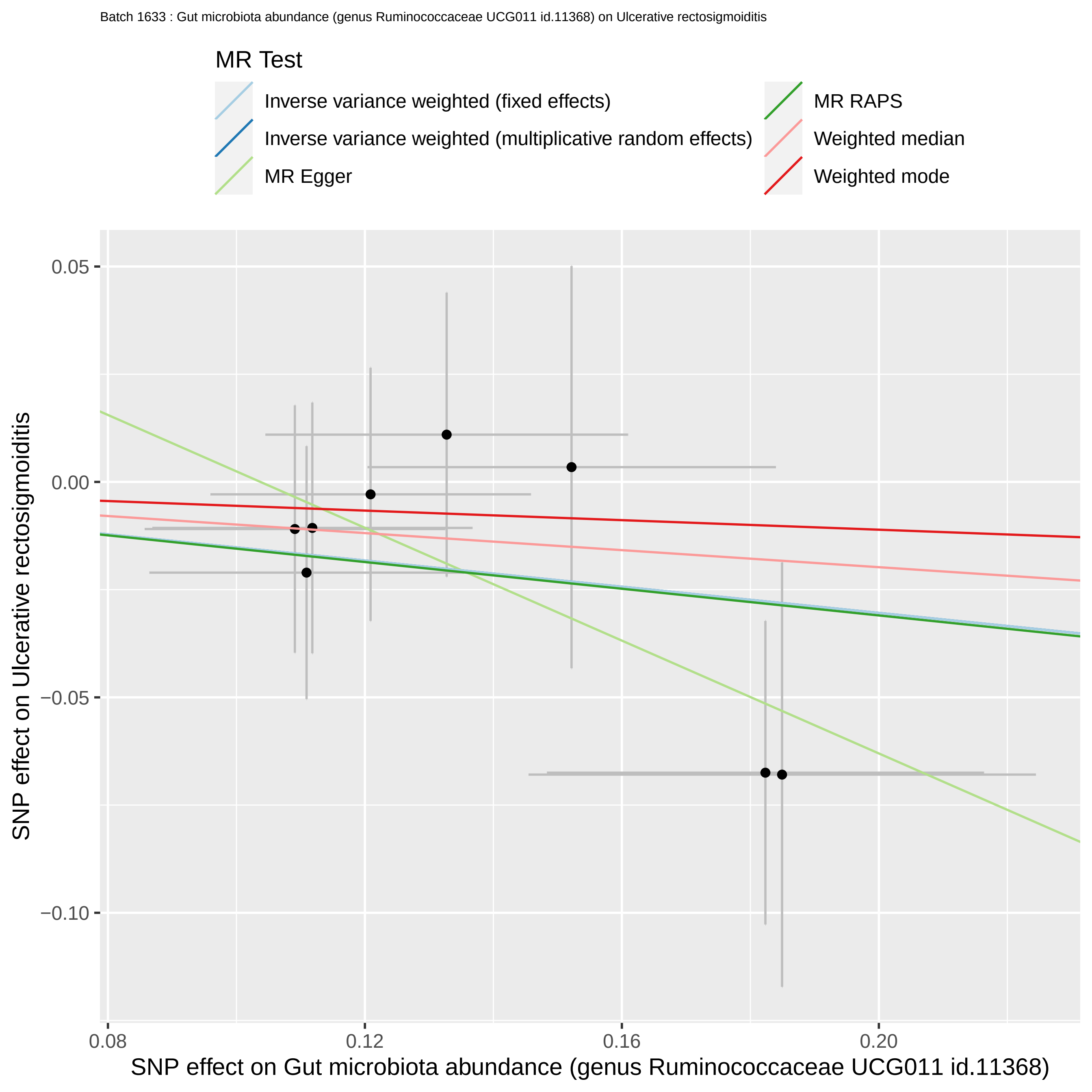
Batch 1633 : Gut microbiota abundance (genus Ruminococcaceae UCG011 id.11368) on Ulcerative rectosigmoiditis
MR Test
Inverse variance weighted (fixed effects)
Inverse variance weighted (multiplicative random effects)
MR Egger
MR RAPS
Weighted median
Weighted mode
0.05
0.00
−0.05
−0.10
0.08
0.12
0.16
0.20
SNP effect on Ulcerative rectosigmoiditis
SNP effect on Gut microbiota abundance (genus Ruminococcaceae UCG011 id.11368)

Batch 1634 : Gut microbiota abundance (genus Ruminococcaceae UCG013 id.11370) on Ulcerative rectosigmoiditis

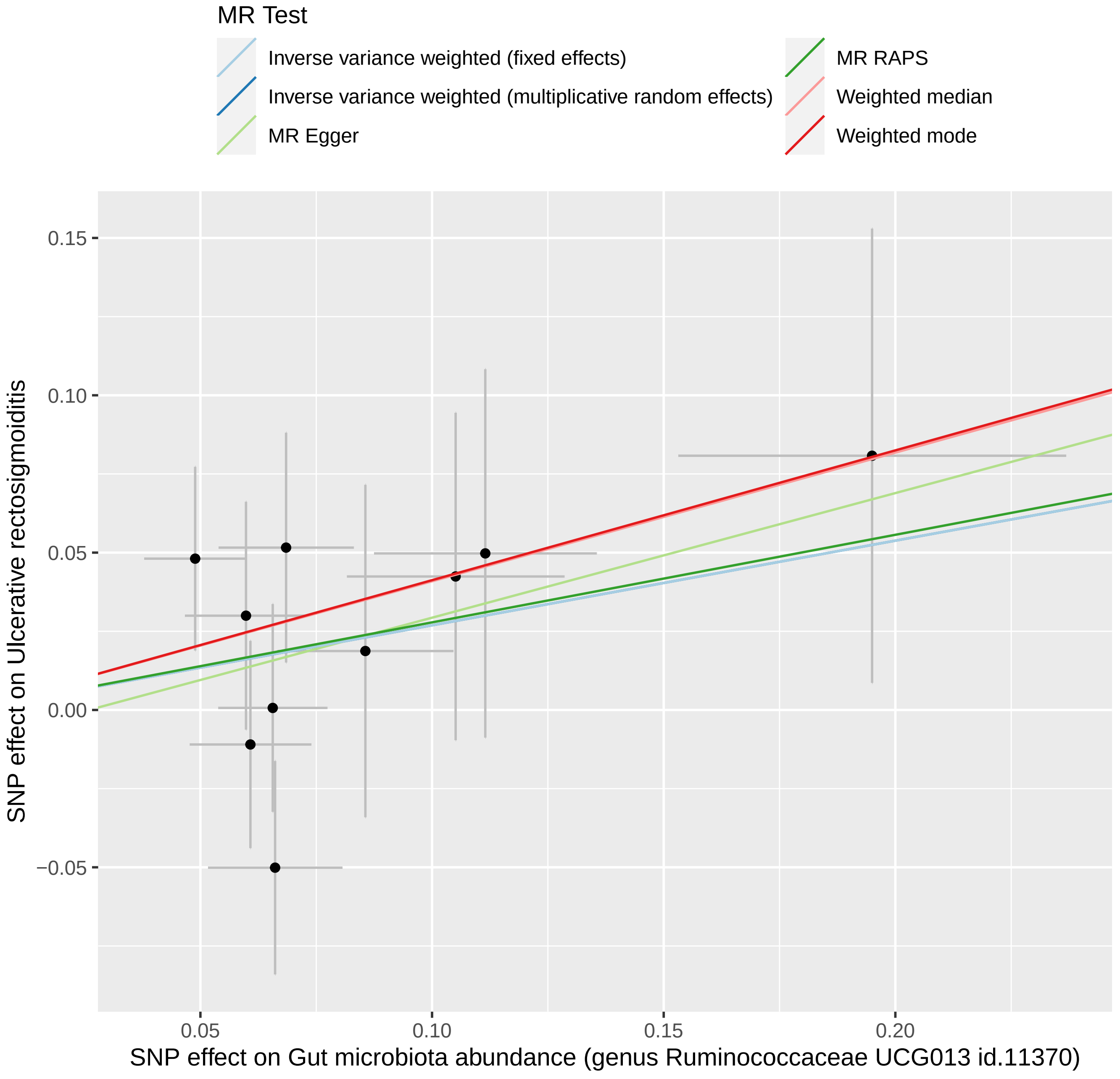

Batch 1635 : Gut microbiota abundance (genus Ruminococcaceae UCG014 id.11371) on Ulcerative rectosigmoiditis

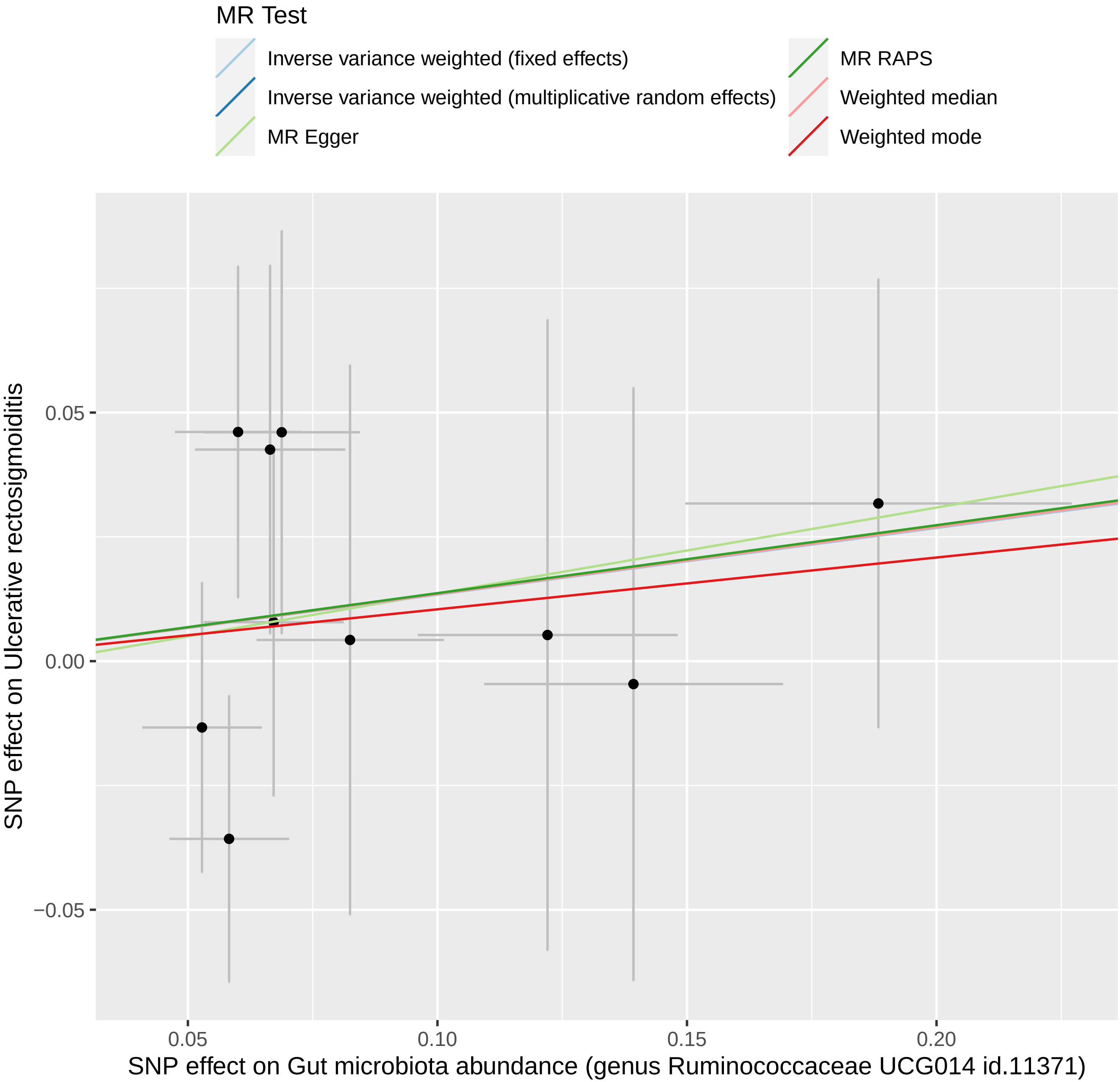

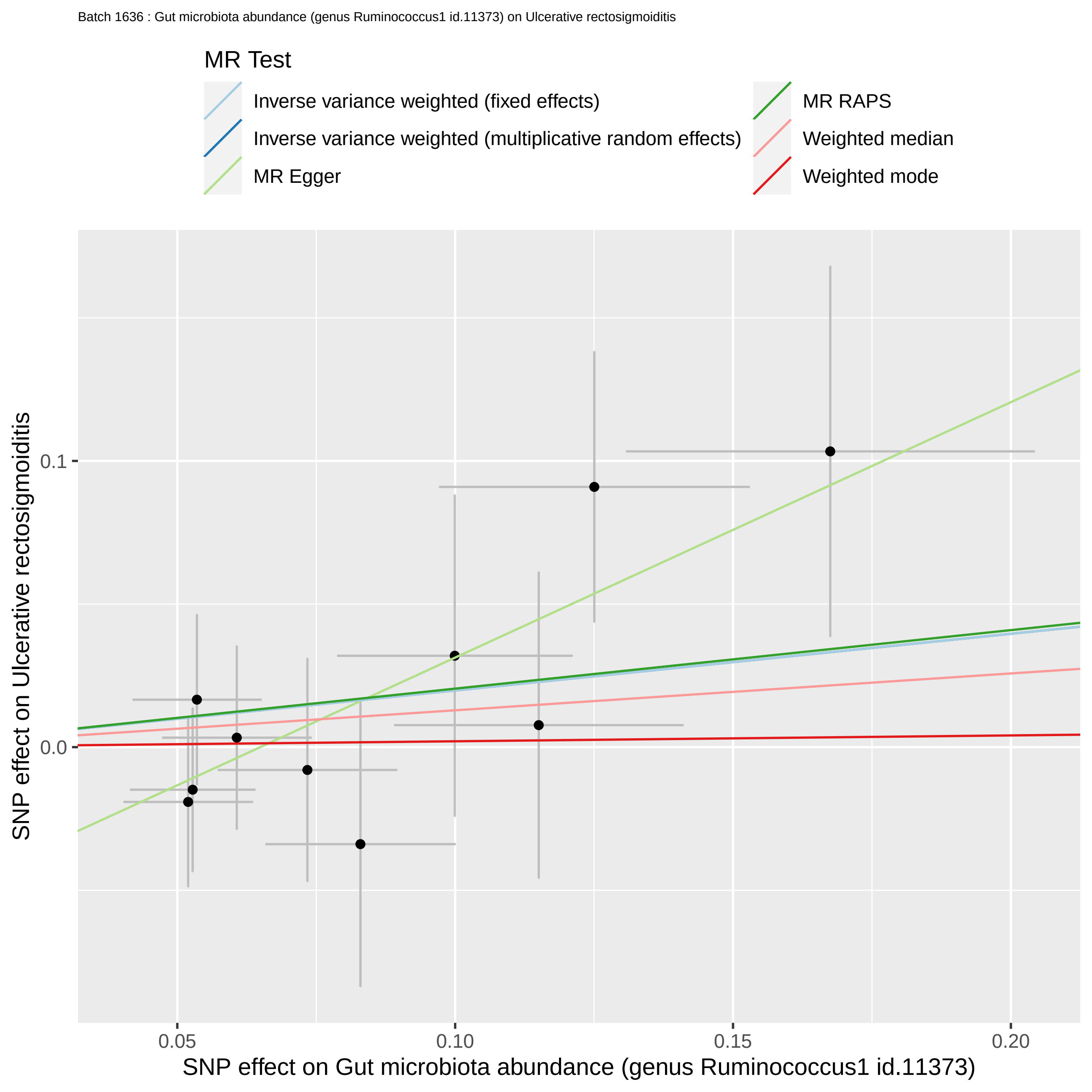

Batch 1636 : Gut microbiota abundance (genus Ruminococcus1 id.11373) on Ulcerative rectosigmoiditis
MR Test
Inverse variance weighted (fixed effects)
Inverse variance weighted (multiplicative random effects)
MR Egger
MR RAPS
Weighted median
Weighted mode
SNP effect on Ulcerative rectosigmoiditis
0.1
0.0
0.05
0.10
0.15
0.20
SNP effect on Gut microbiota abundance (genus Ruminococcus1 id.11373)

Batch 1637 : Gut microbiota abundance (genus Ruminococcus2 id.11374) on Ulcerative rectosigmoiditis

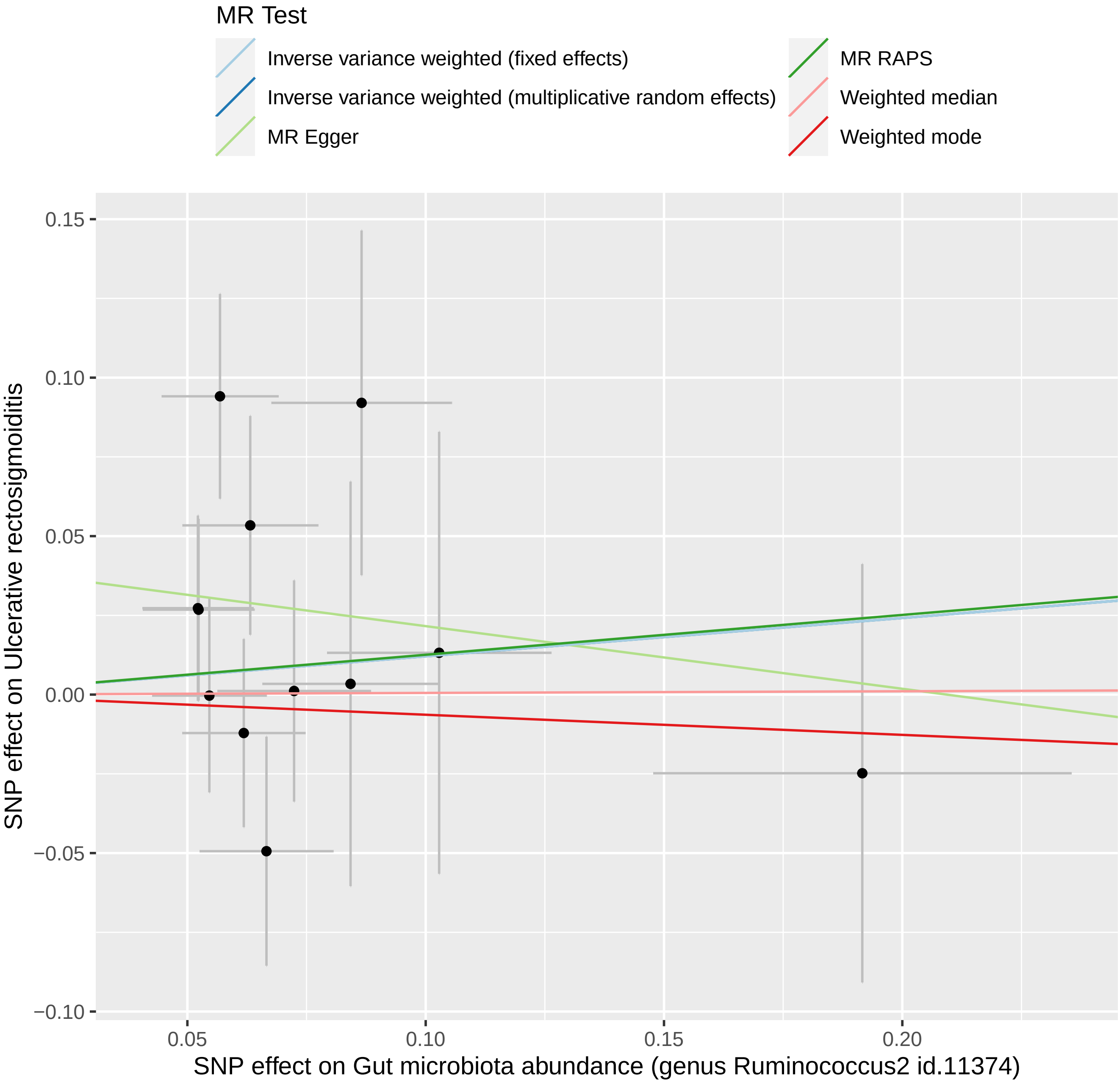

Batch 1638 : Gut microbiota abundance (genus Ruminococcus gauvreauii group id.11342) on Ulcerative rectosigmoiditis

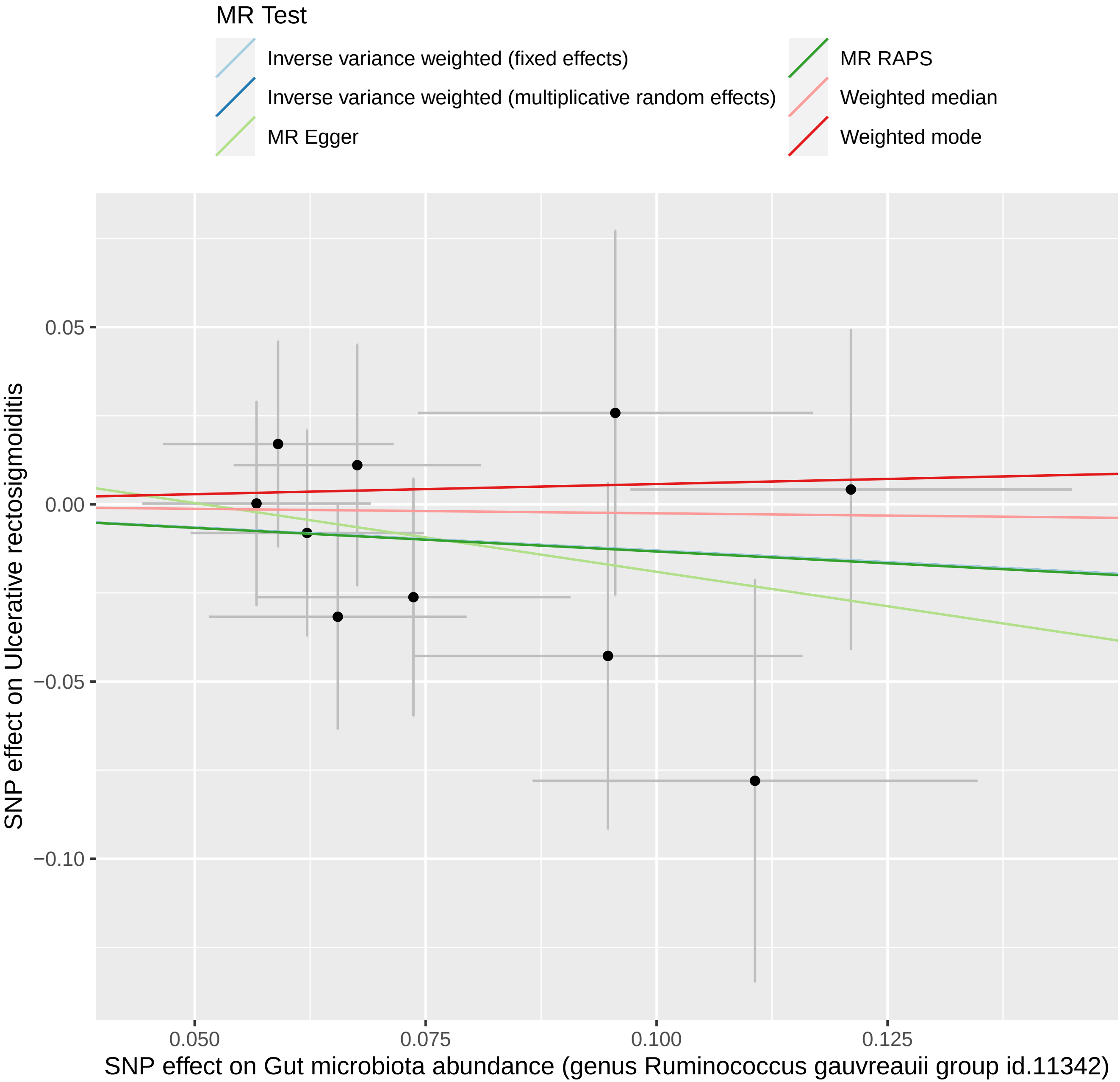

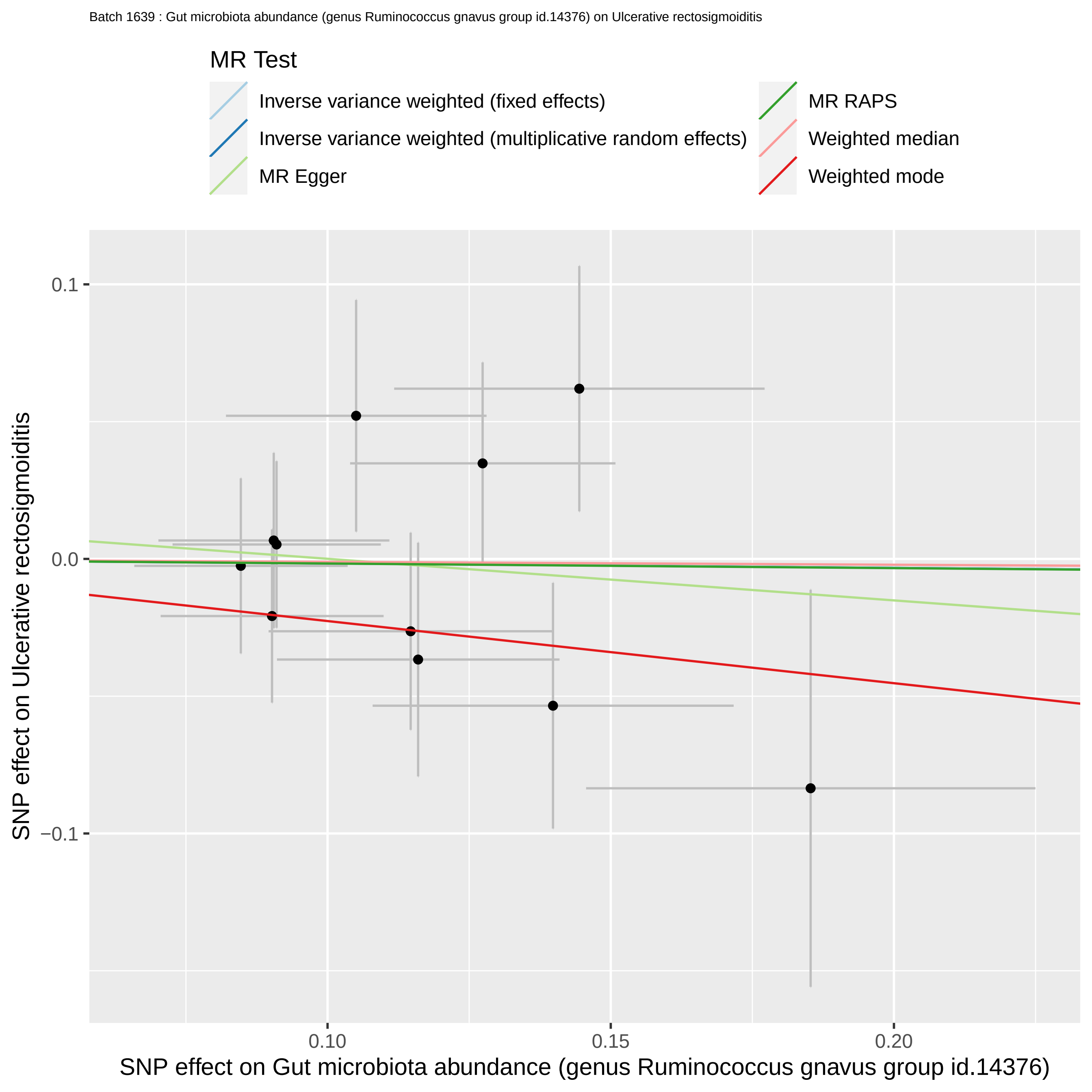
Batch 1639 : Gut microbiota abundance (genus Ruminococcus gnavus group id.14376) on Ulcerative rectosigmoiditis
MR Test
Inverse variance weighted (fixed effects)
Inverse variance weighted (multiplicative random effects)
MR Egger
MR RAPS
Weighted median
Weighted mode
0.1
0.0
−0.1
0.10
0.15
0.20
SNP effect on Ulcerative rectosigmoiditis
SNP effect on Gut microbiota abundance (genus Ruminococcus gnavus group id.14376)

Batch 1640 : Gut microbiota abundance (genus Ruminococcus torques group id.14377) on Ulcerative rectosigmoiditis

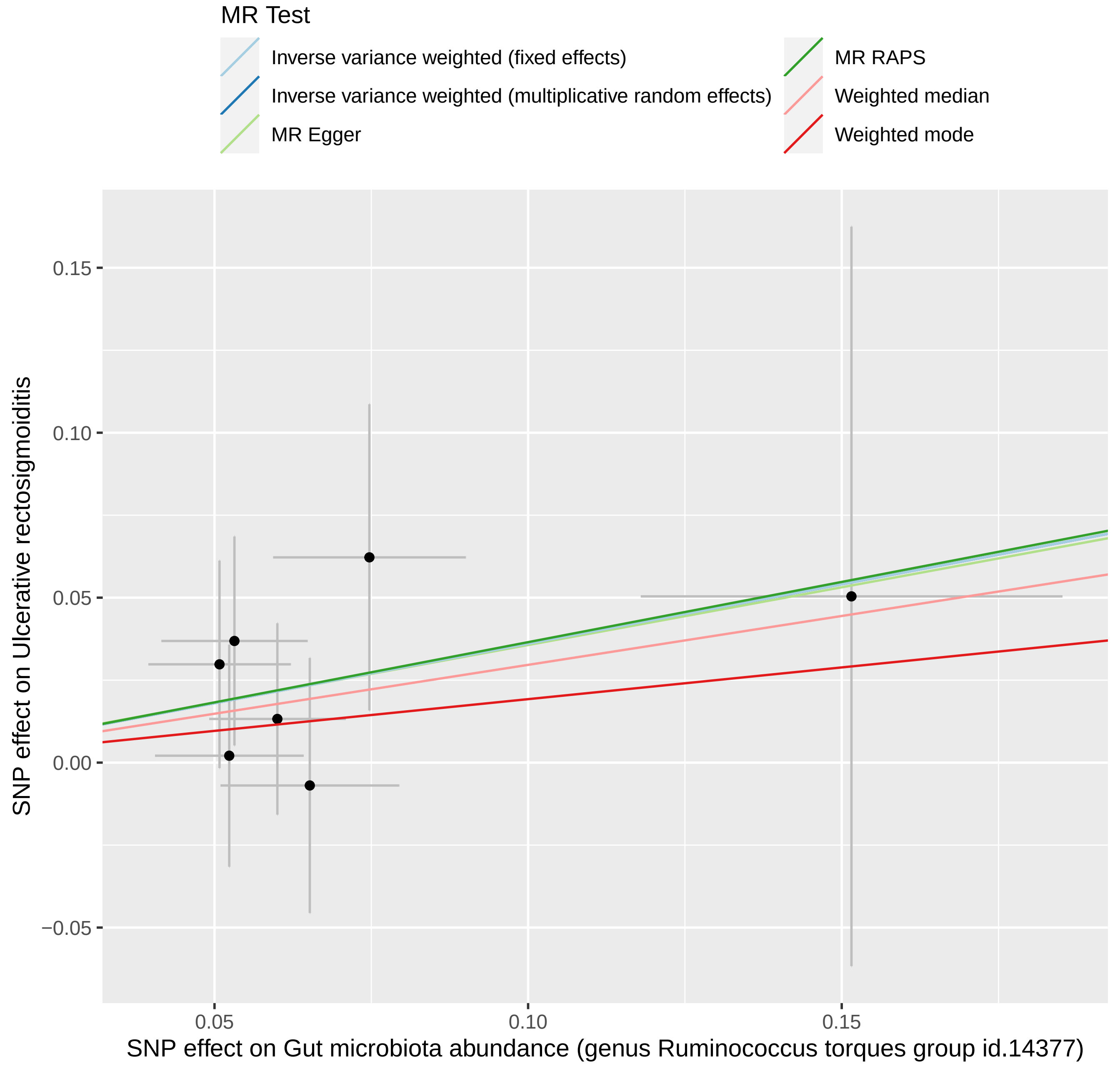

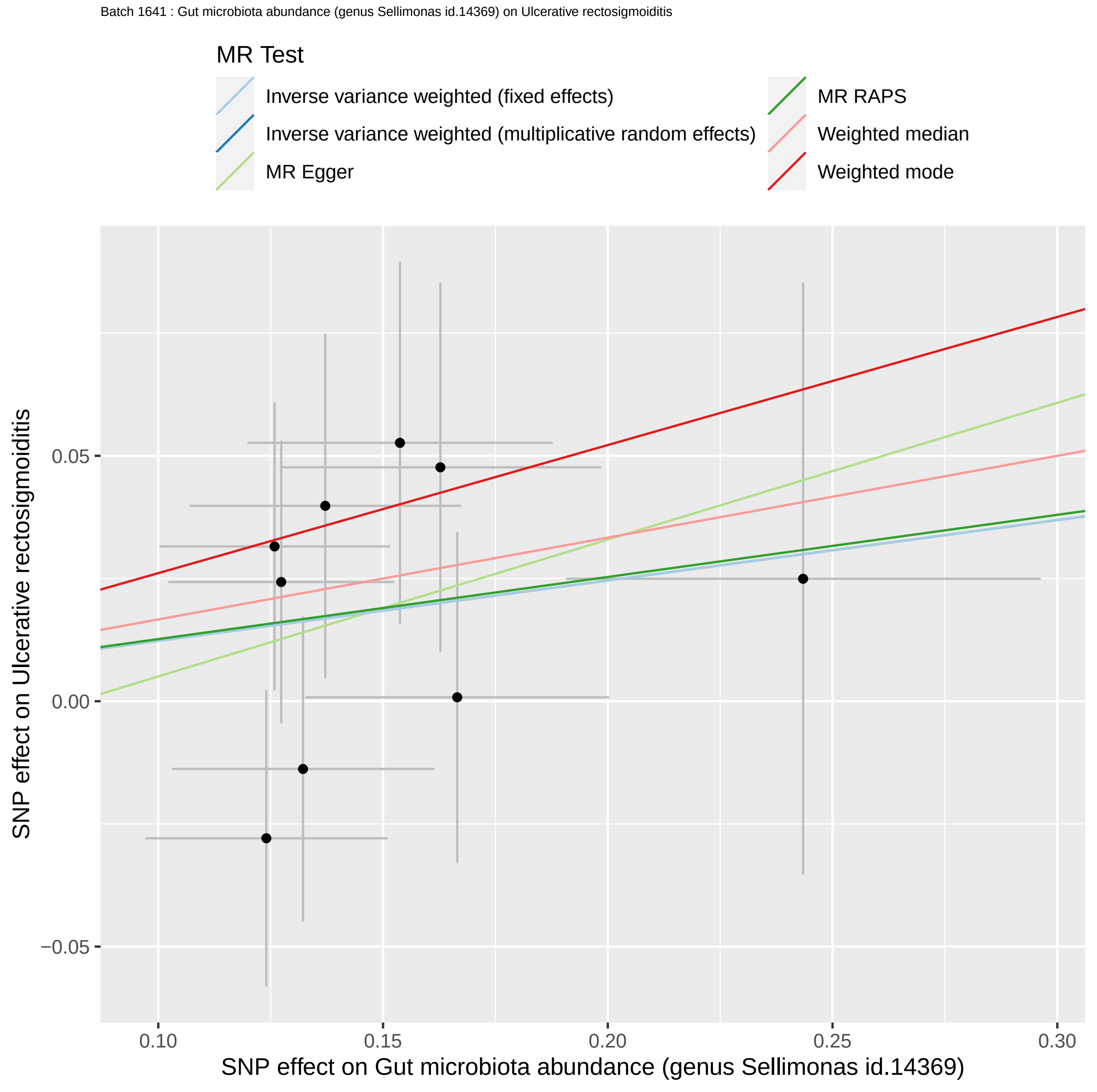

Batch 1641 : Gut microbiota abundance (genus Sellimonas id.14369) on Ulcerative rectosigmoiditis
MR Test
Inverse variance weighted (fixed effects)
Inverse variance weighted (multiplicative random effects)
MR Egger
MR RAPS
Weighted median
Weighted mode
SNP effect on Ulcerative rectosigmoiditis
0.05
0.00
−0.05
0.10
0.15
0.20
0.25
0.30
SNP effect on Gut microbiota abundance (genus Sellimonas id.14369)

Batch 1642 : Gut microbiota abundance (genus Senegalimassilia id.11160) on Ulcerative rectosigmoiditis

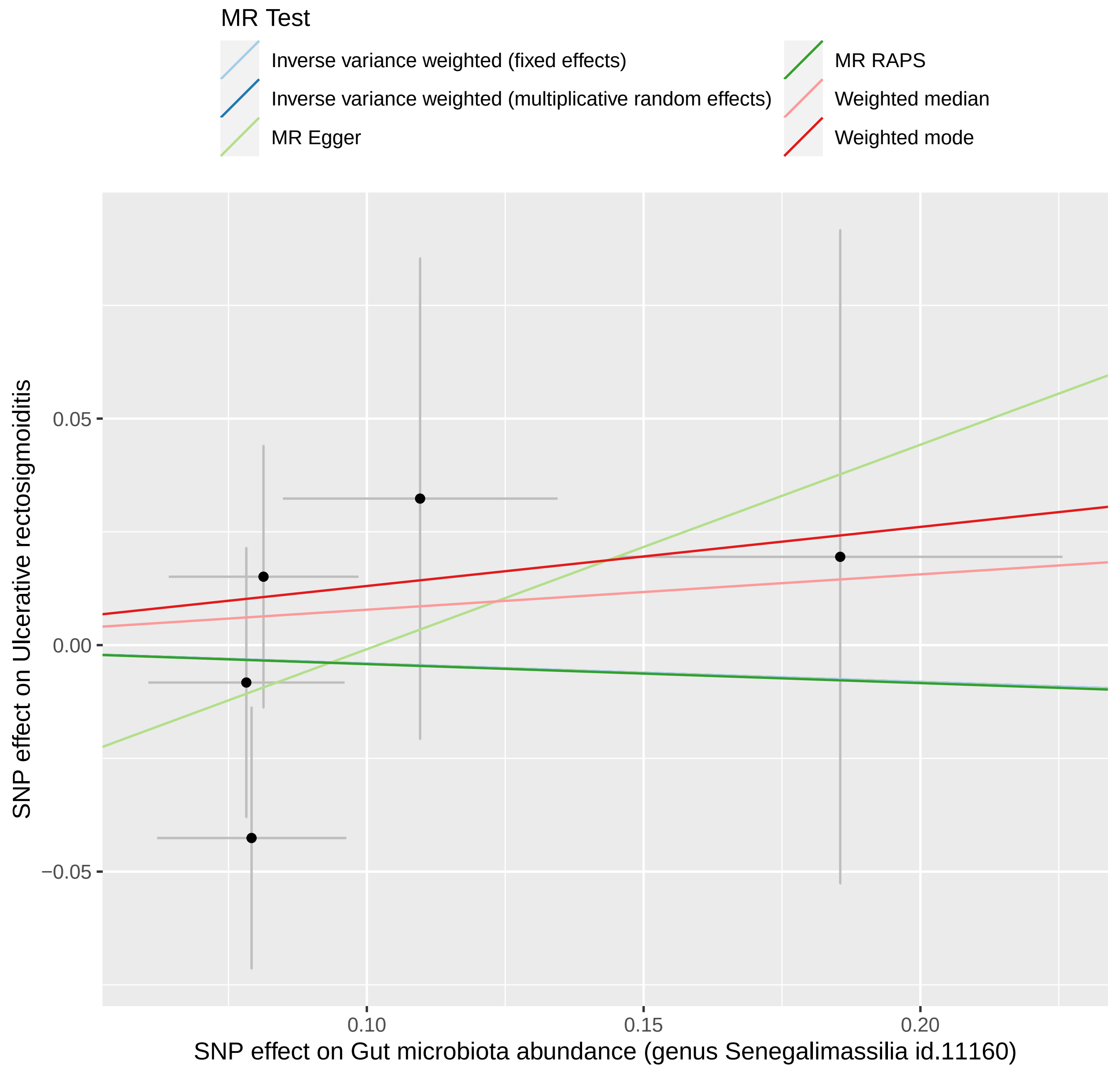

Batch 1643 : Gut microbiota abundance (genus Slackia id.825) on Ulcerative rectosigmoiditis

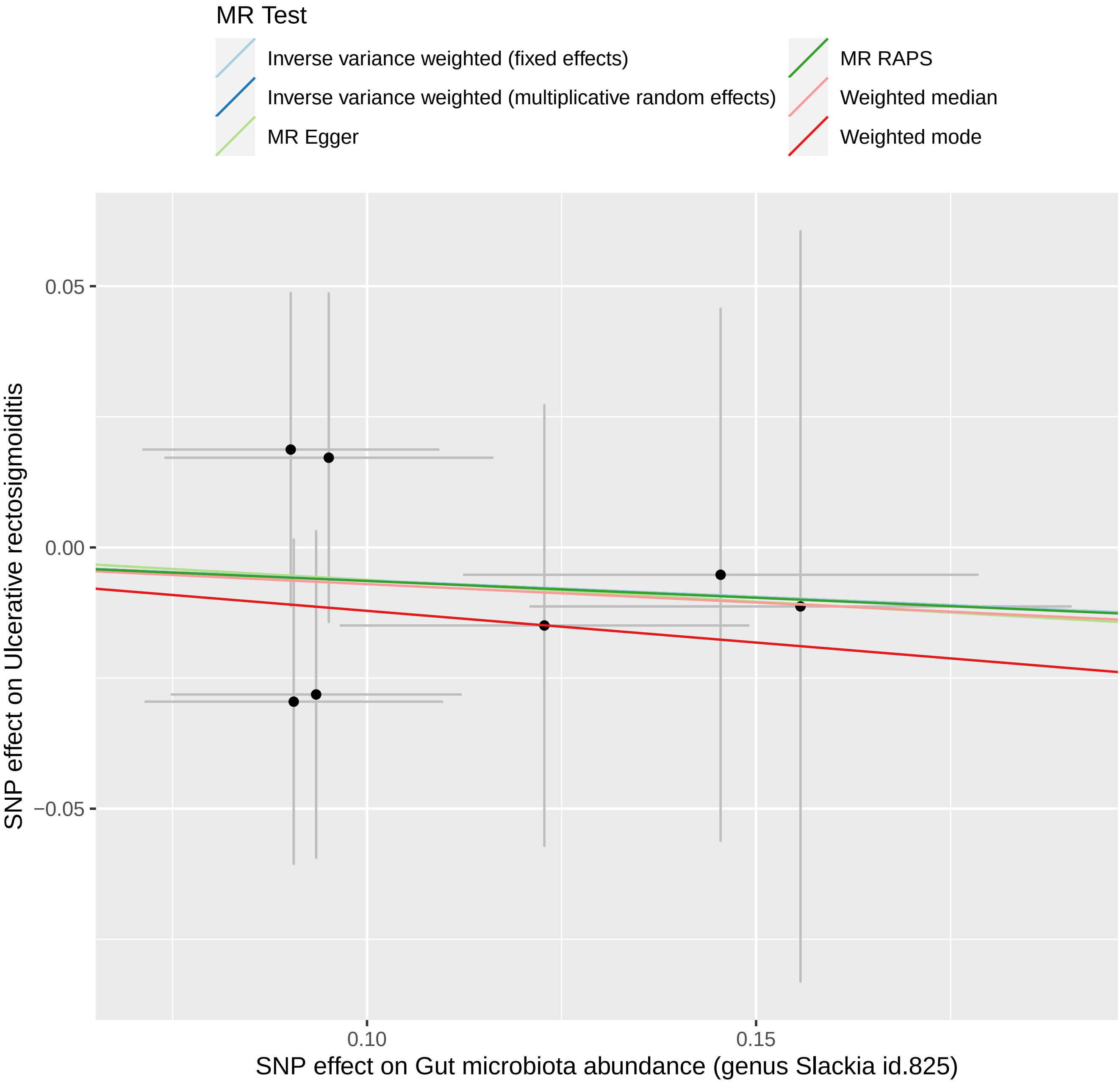

Batch 1644 : Gut microbiota abundance (genus Streptococcus id.1853) on Ulcerative rectosigmoiditis

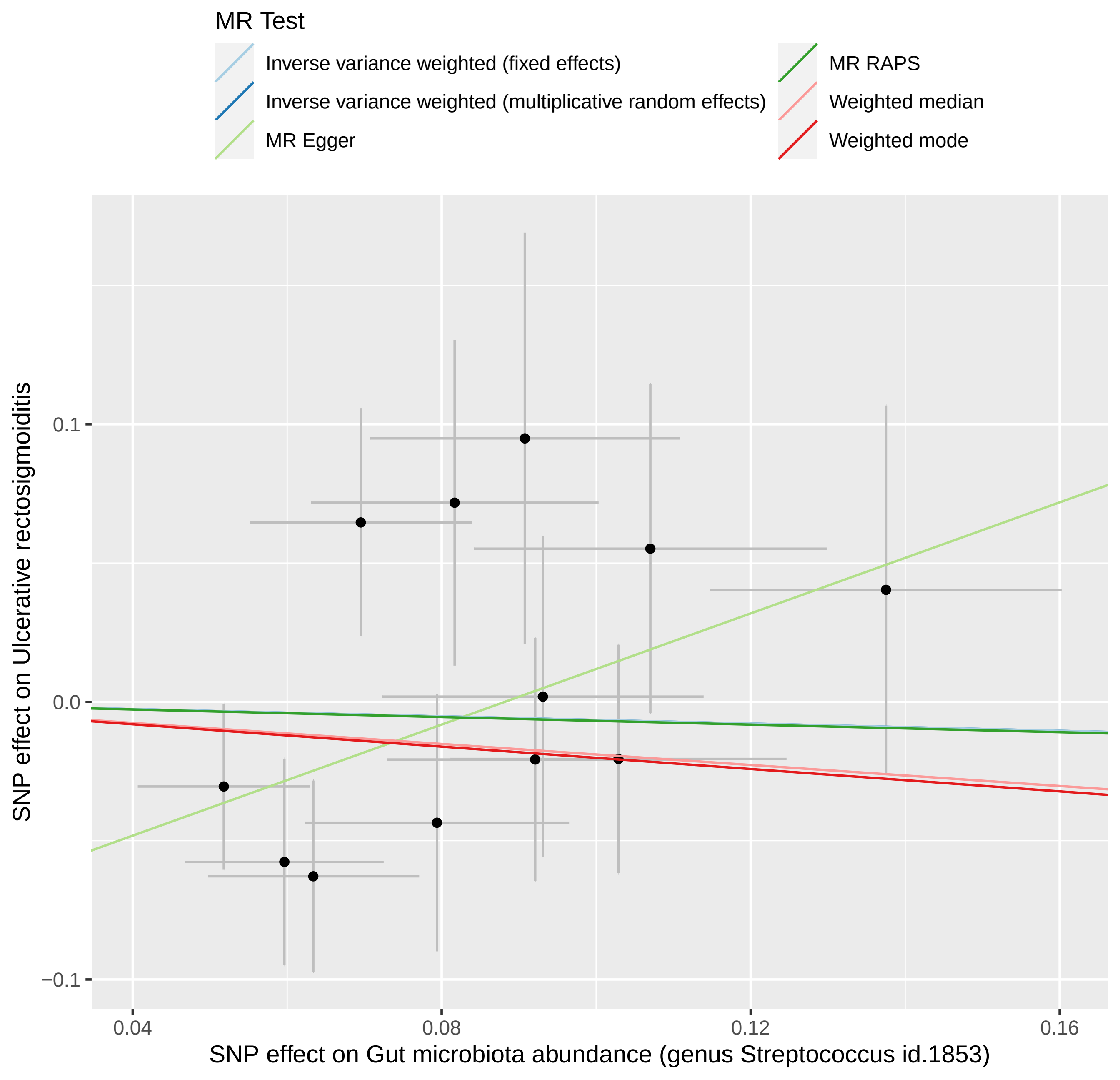

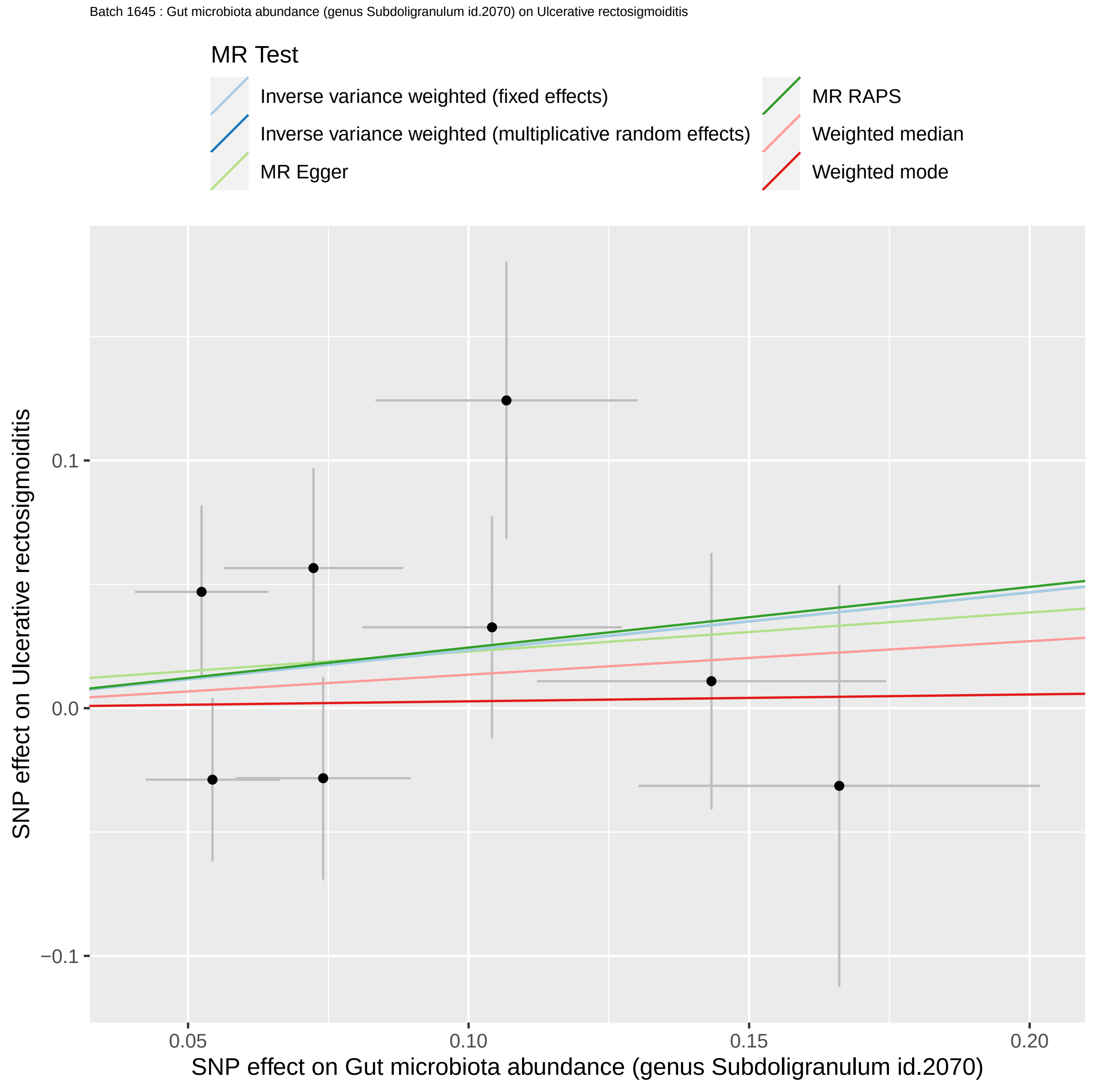

Batch 1645 : Gut microbiota abundance (genus Subdoligranulum id.2070) on Ulcerative rectosigmoiditis
MR Test
Inverse variance weighted (fixed effects)
Inverse variance weighted (multiplicative random effects)
MR Egger
MR RAPS
Weighted median
Weighted mode
0.1
0.0
−0.1
0.05
0.10
0.15
0.20
SNP effect on Ulcerative rectosigmoiditis
SNP effect on Gut microbiota abundance (genus Subdoligranulum id.2070)

Batch 1646 : Gut microbiota abundance (genus Sutterella id.2896) on Ulcerative rectosigmoiditis

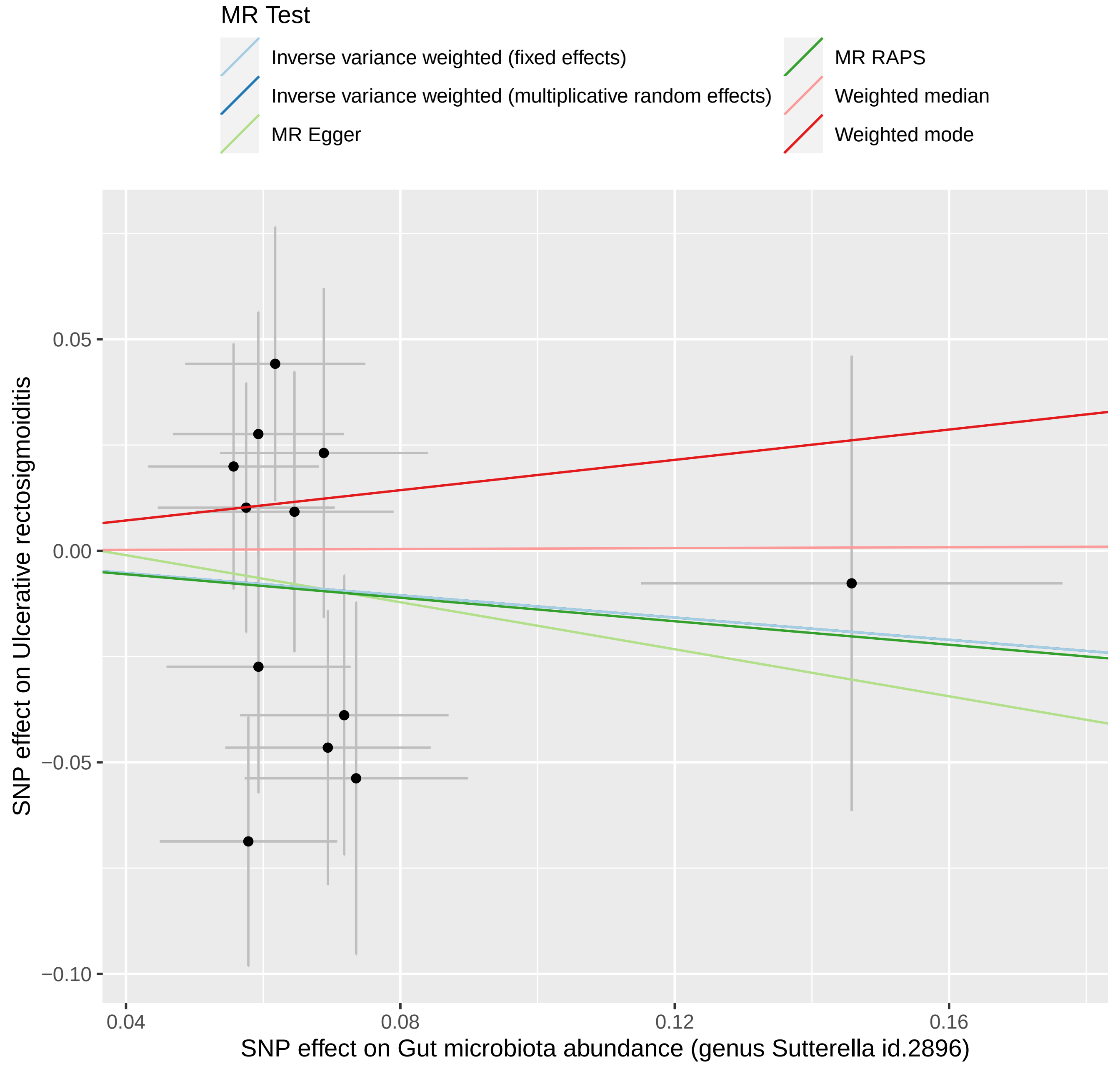

Batch 1647 : Gut microbiota abundance (genus Terrisporobacter id.11348) on Ulcerative rectosigmoiditis

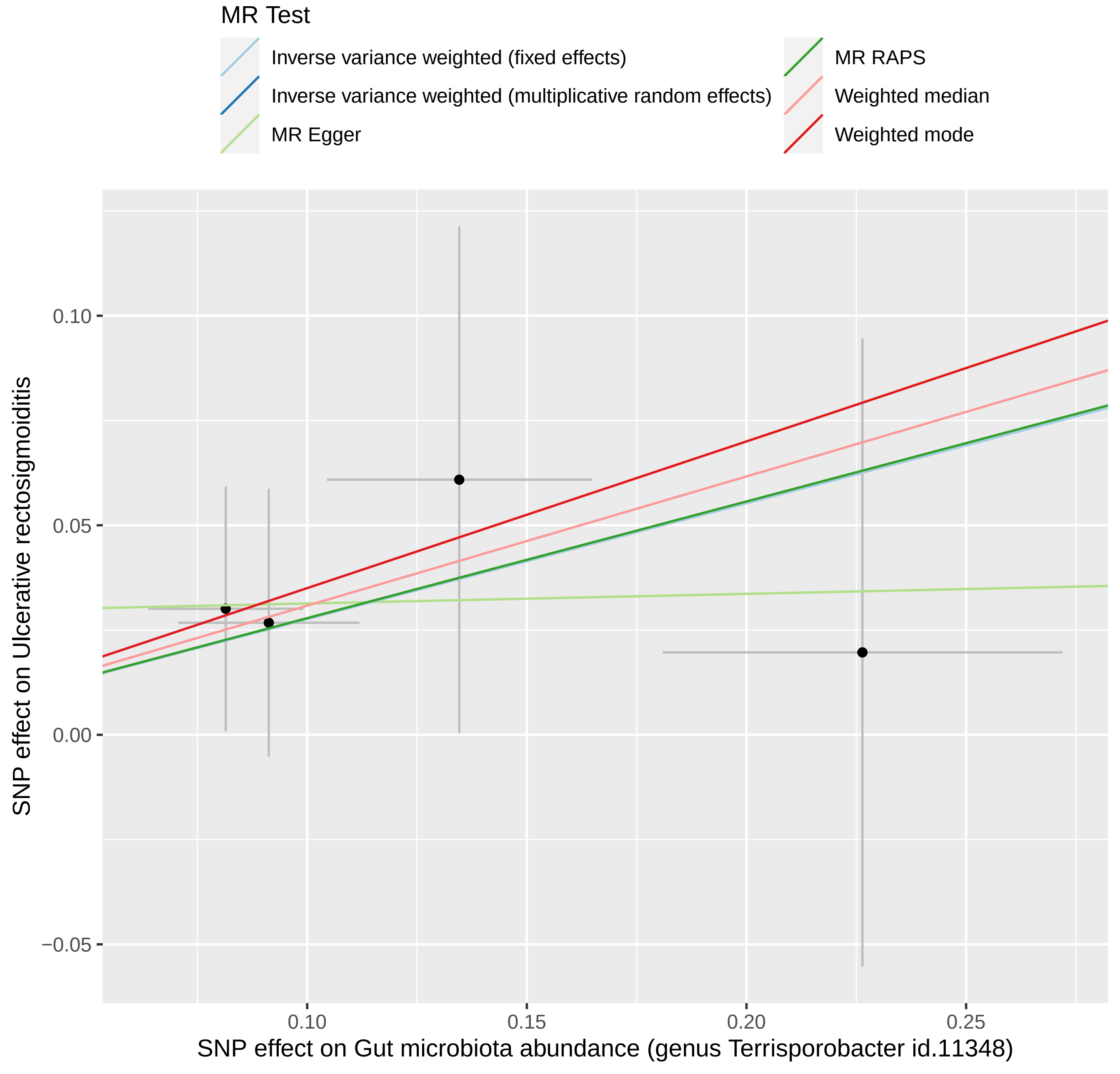

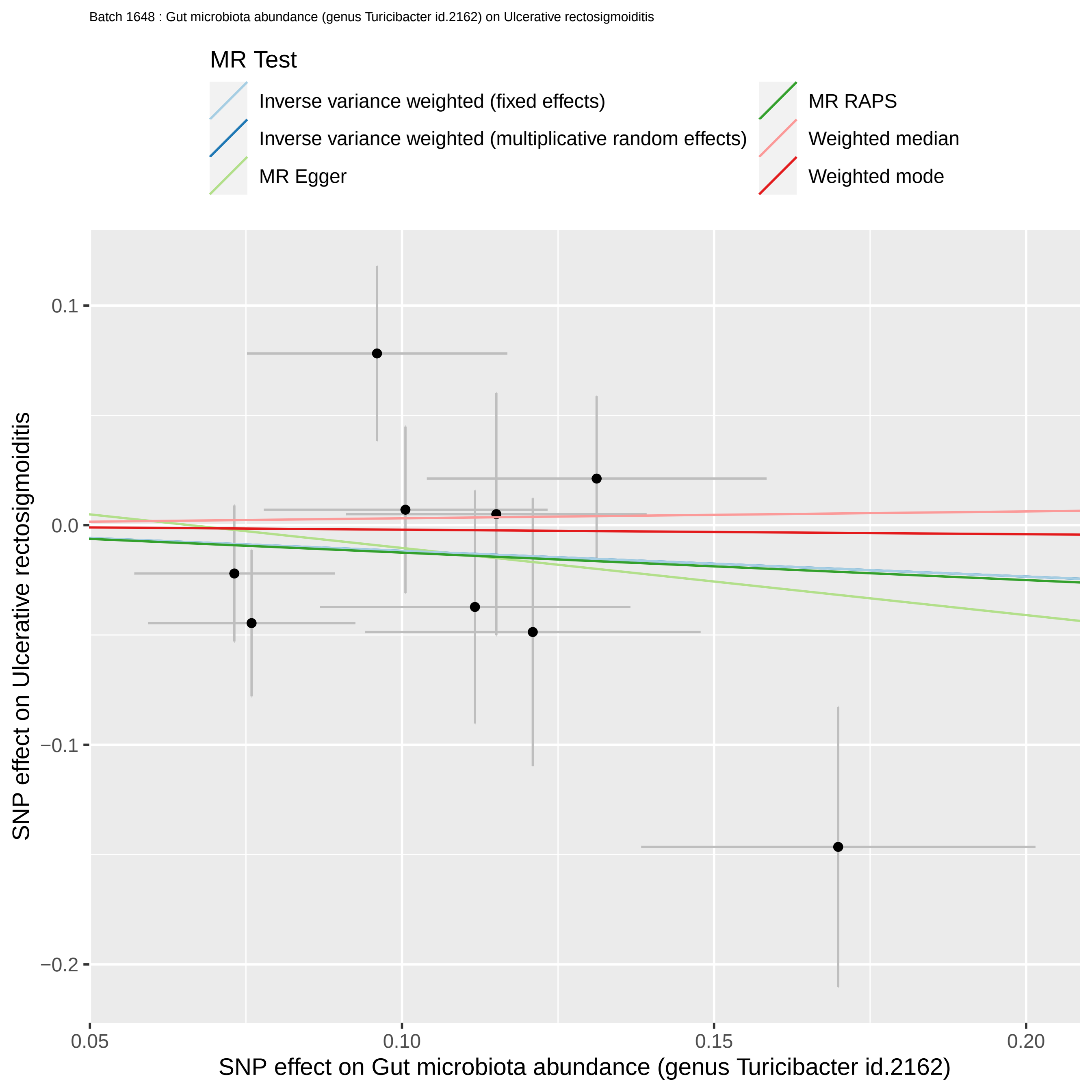
Batch 1648 : Gut microbiota abundance (genus Turicibacter id.2162) on Ulcerative rectosigmoiditis
MR Test
Inverse variance weighted (fixed effects)
Inverse variance weighted (multiplicative random effects)
MR Egger
MR RAPS
Weighted median
Weighted mode
0.1
0.0
−0.1
−0.2
SNP effect on Ulcerative rectosigmoiditis
0.05
0.10
0.15
0.20
SNP effect on Gut microbiota abundance (genus Turicibacter id.2162)

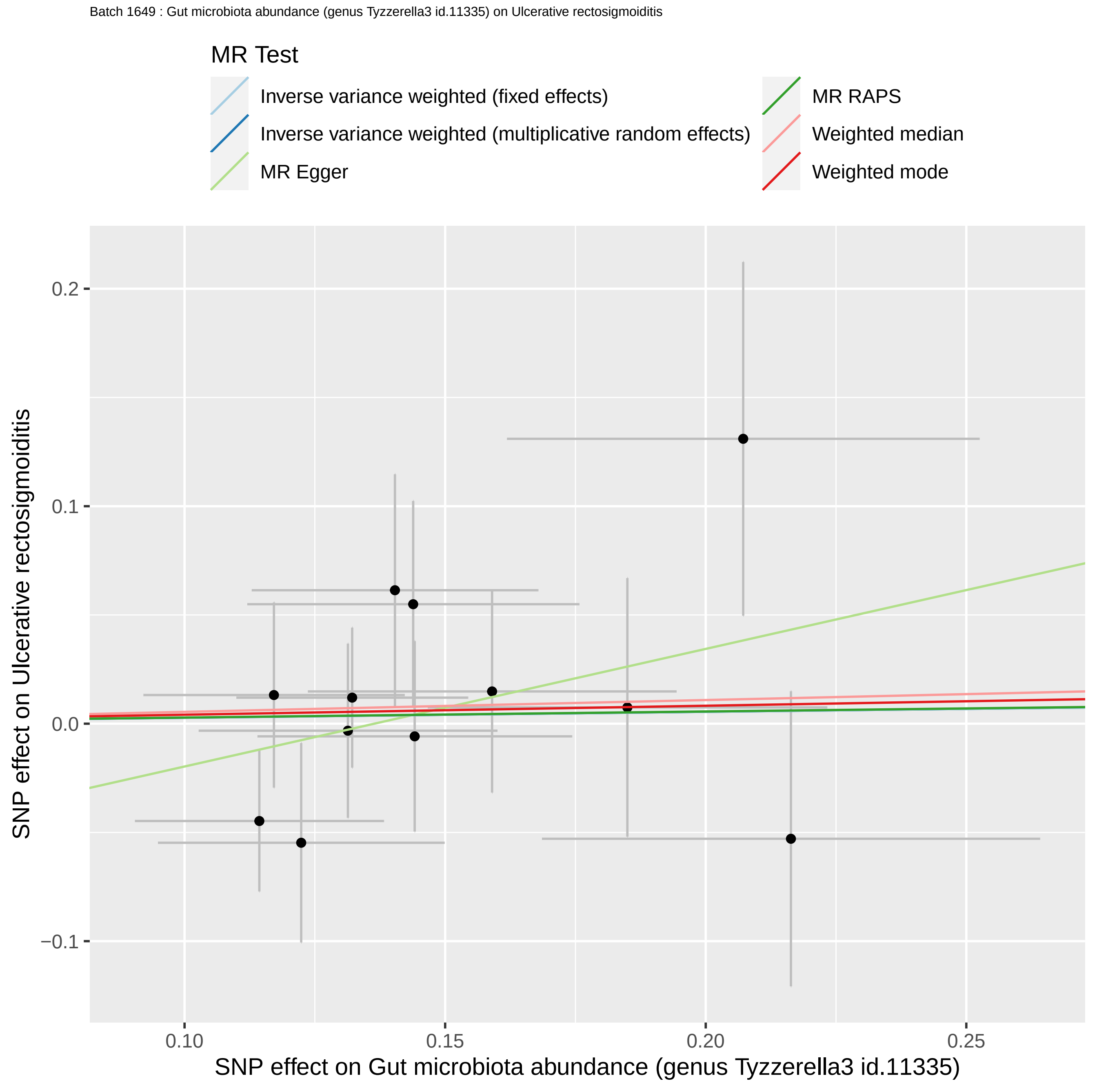
Batch 1649 : Gut microbiota abundance (genus Tyzzerella3 id.11335) on Ulcerative rectosigmoiditis
MR Test
Inverse variance weighted (fixed effects)
Inverse variance weighted (multiplicative random effects)
MR Egger
MR RAPS
Weighted median
Weighted mode
0.2
0.1
0.0
−0.1
0.10
0.15
0.20
0.25
SNP effect on Ulcerative rectosigmoiditis
SNP effect on Gut microbiota abundance (genus Tyzzerella3 id.11335)

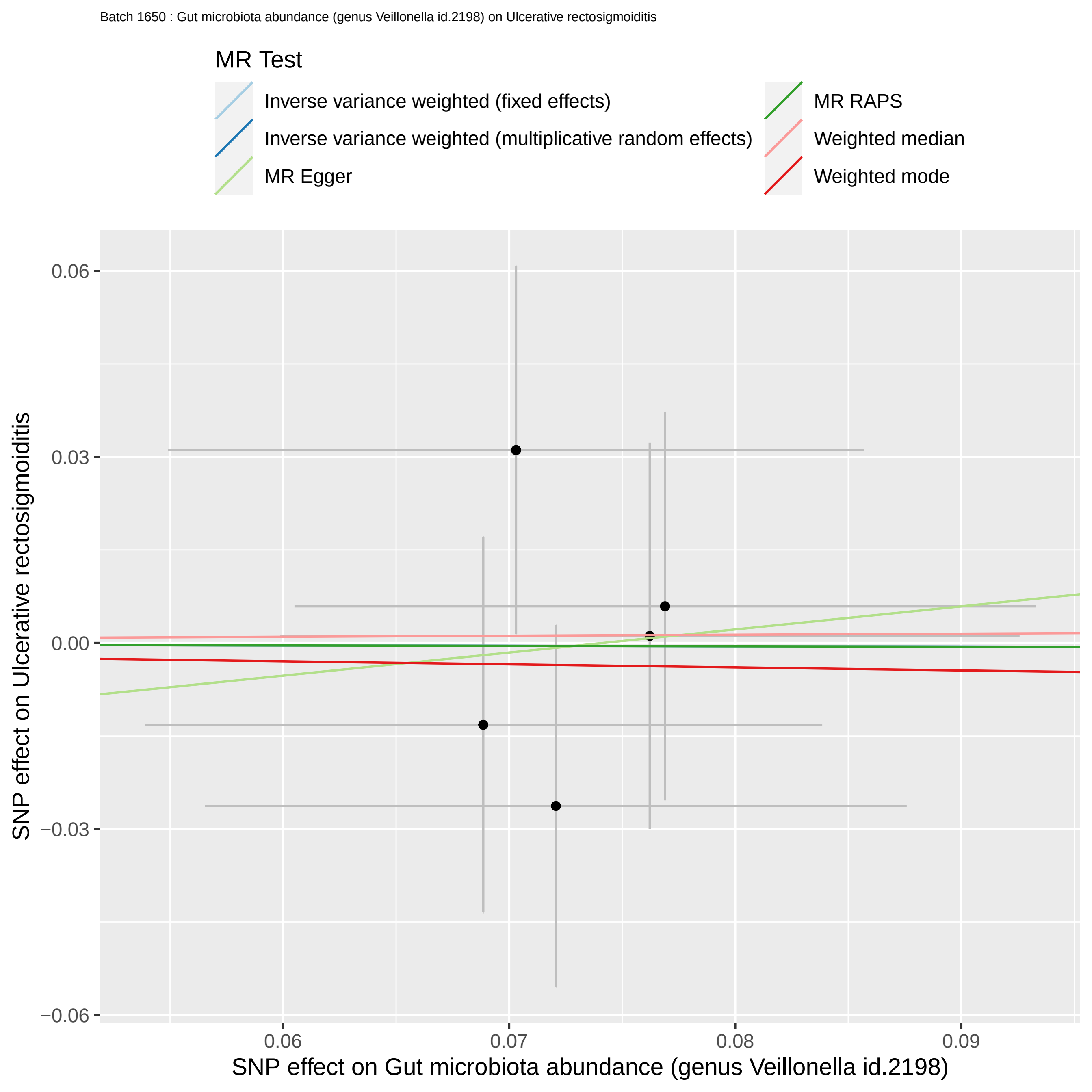

Batch 1650 : Gut microbiota abundance (genus Veillonella id.2198) on Ulcerative rectosigmoiditis
MR Test
Inverse variance weighted (fixed effects)
Inverse variance weighted (multiplicative random effects)
MR Egger
MR RAPS
Weighted median
Weighted mode
0.06
0.03
0.00
−0.03
−0.06
0.06
0.07
0.08
0.09
SNP effect on Ulcerative rectosigmoiditis
SNP effect on Gut microbiota abundance (genus Veillonella id.2198)
