# Supplementary material for: Exploring the intestinal ecosystem: from gut microbiota to associations with subtypes of inflammatory bowel disease
Source: Front Cell Infect Microbiol. 2024 Jan 4;13:1304858. doi: 10.3389/fcimb.2023.1304858 (PMC10794348; doi:10.3389/fcimb.2023.1304858)
Supplement: Supplementary file 3 [file Image_3.pdf]

Batch 1 : Gut microbiota abundance (family Acidaminococcaceae id.2166) on Crohn's disease of large intestine

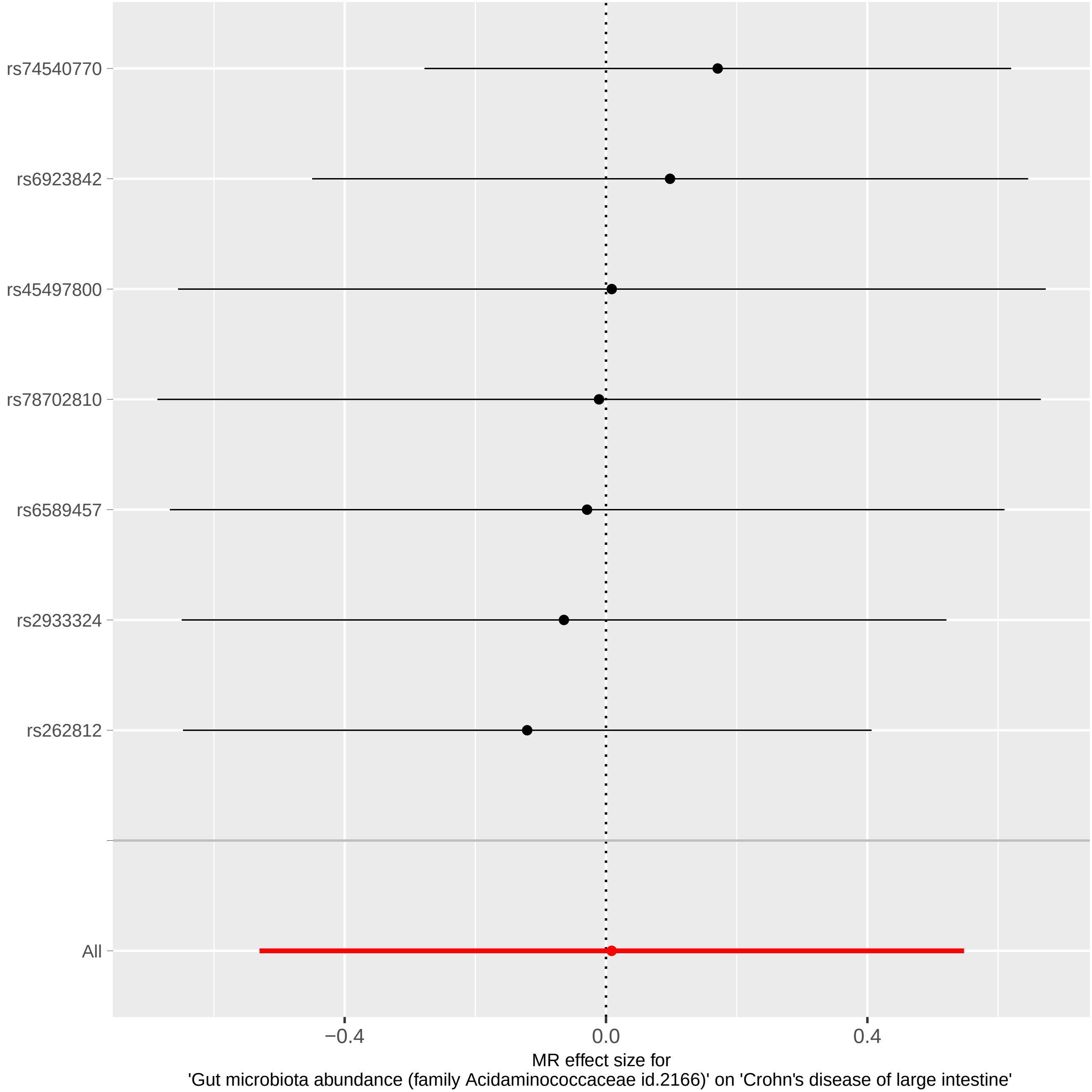

Batch 2 : Gut microbiota abundance (family Actinomycetaceae id.421) on Crohn's disease of large intestine

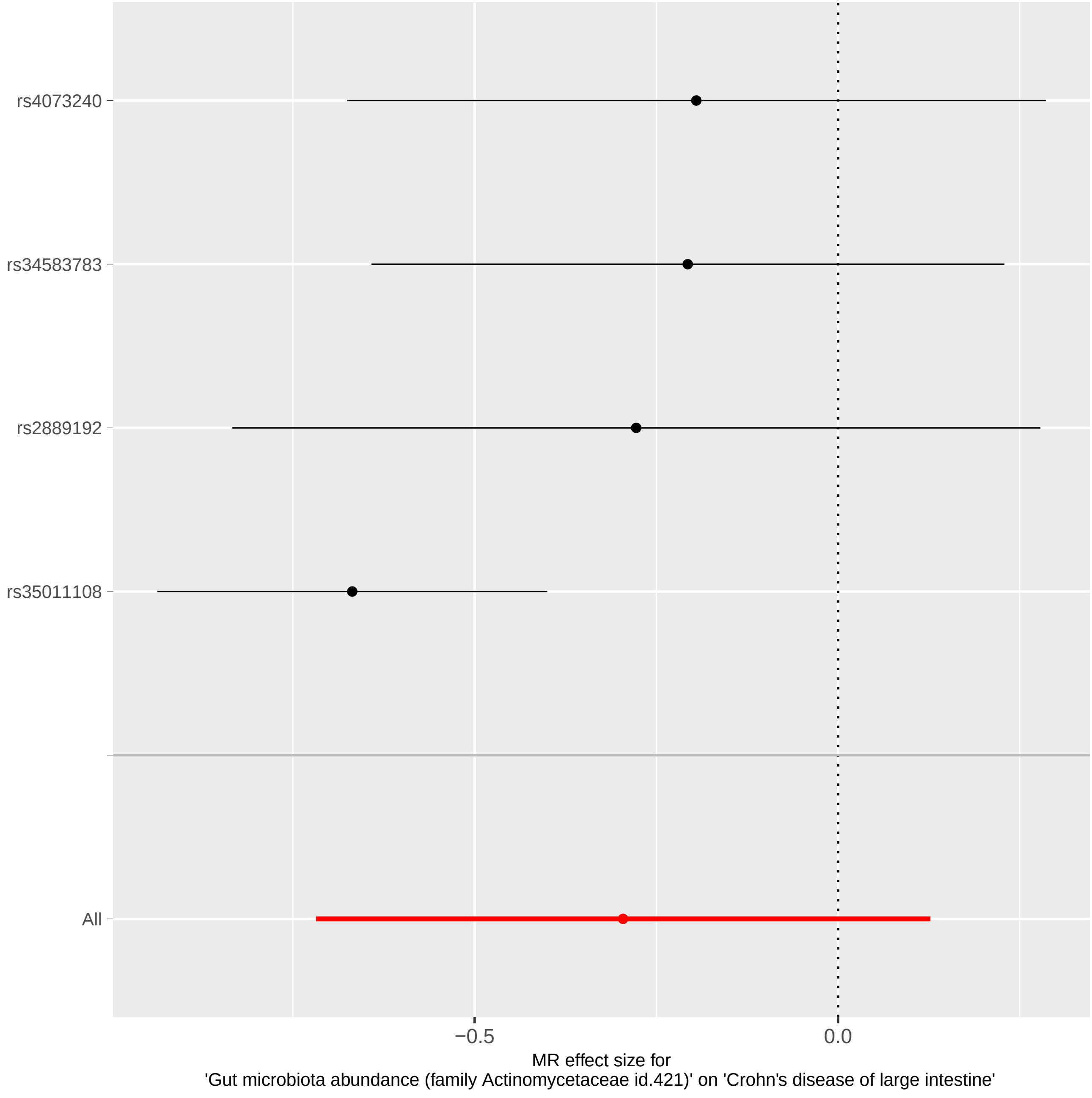

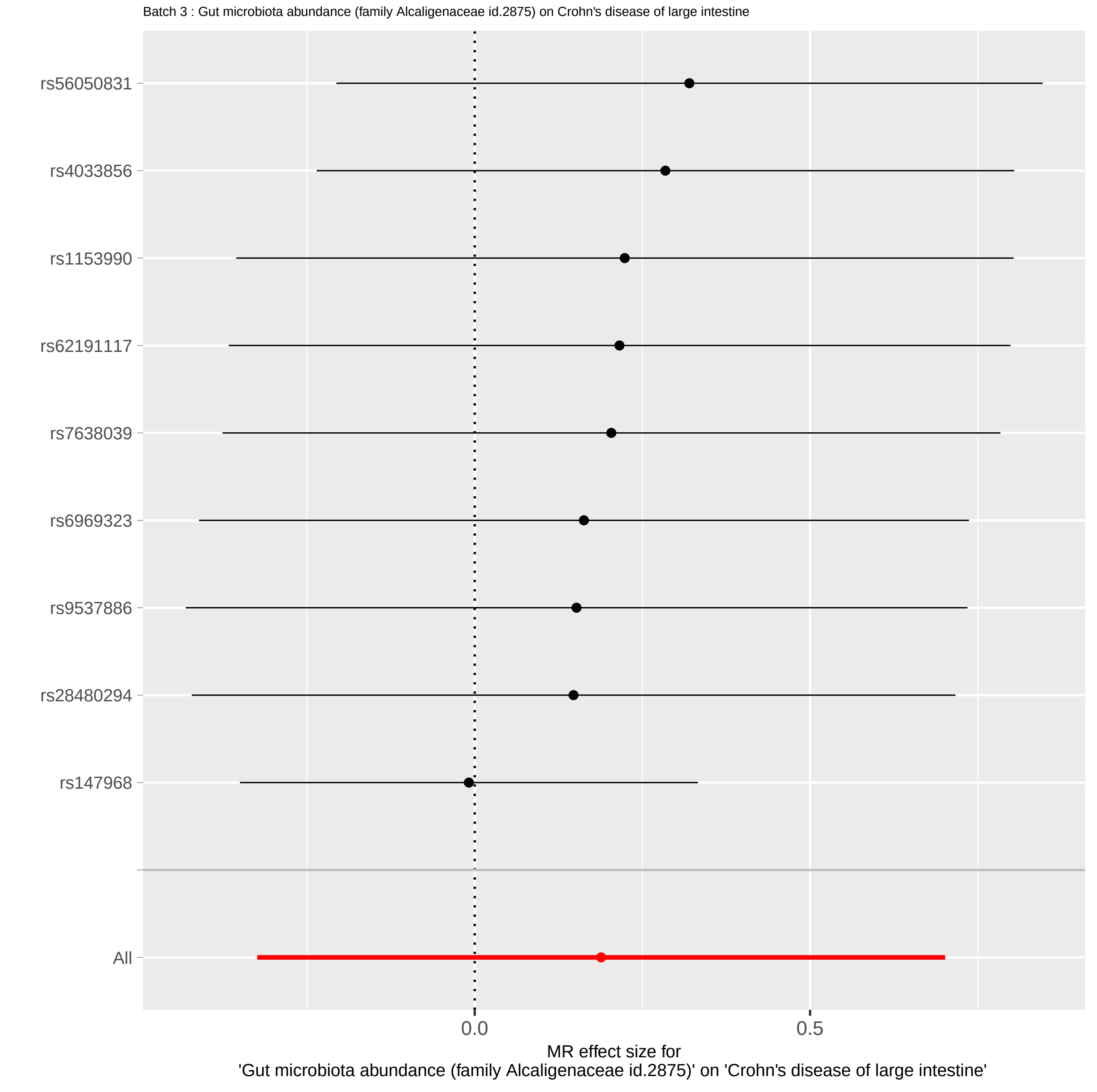
Batch 3 : Gut microbiota abundance (family Alcaligenaceae id.2875) on Crohn's disease of large intestine
rs56050831
rs4033856
rs1153990
rs62191117
rs7638039
rs6969323
rs9537886
rs28480294
rs147968
All
0.0
0.5
MR effect size for
'Gut microbiota abundance (family Alcaligenaceae id.2875)' on 'Crohn's disease of large intestine'

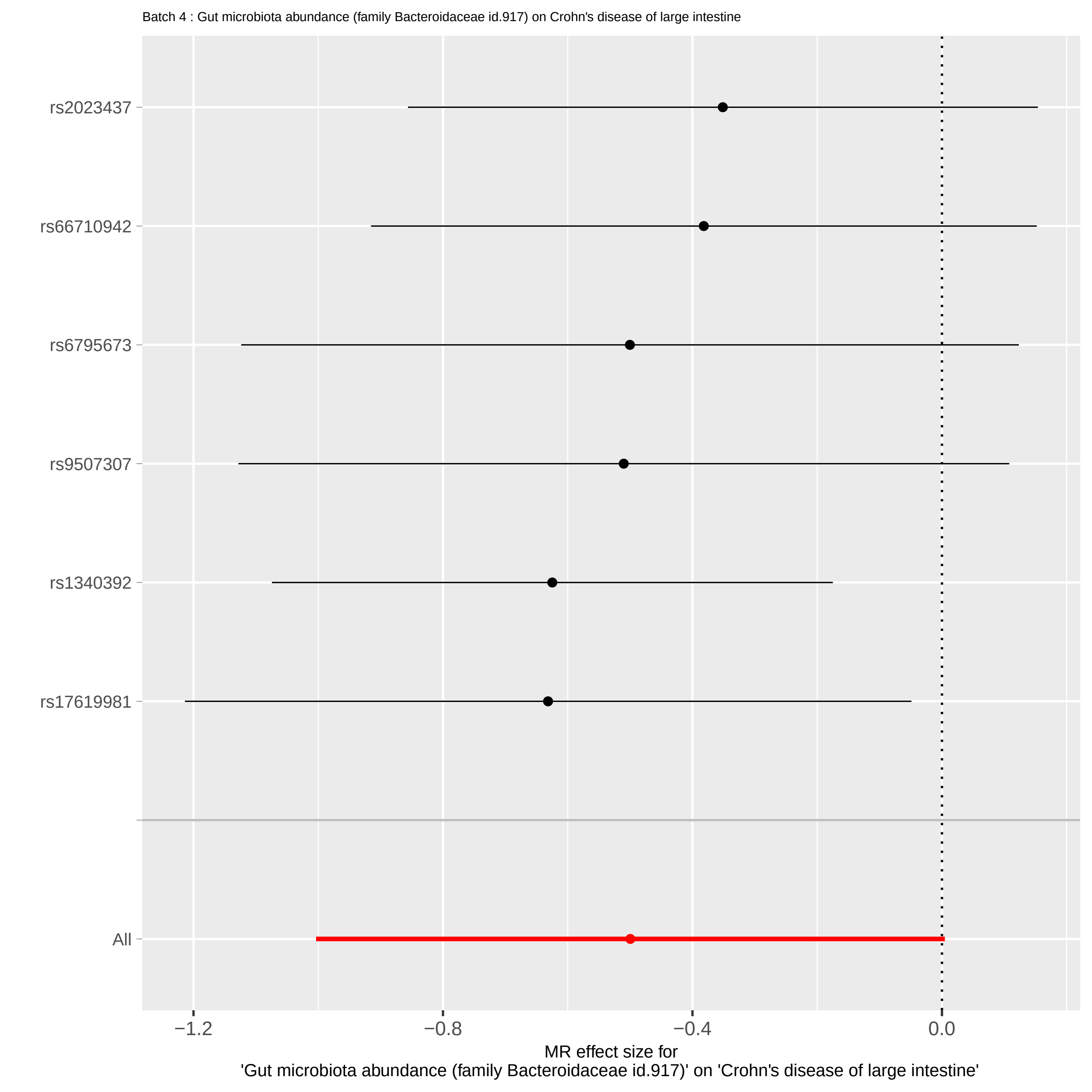
Batch 4 : Gut microbiota abundance (family Bacteroidaceae id.917) on Crohn's disease of large intestine
rs2023437
rs66710942
rs6795673
rs9507307
rs1340392
rs17619981
All
−1.2
−0.8
−0.4
0.0
MR effect size for
'Gut microbiota abundance (family Bacteroidaceae id.917)' on 'Crohn's disease of large intestine'

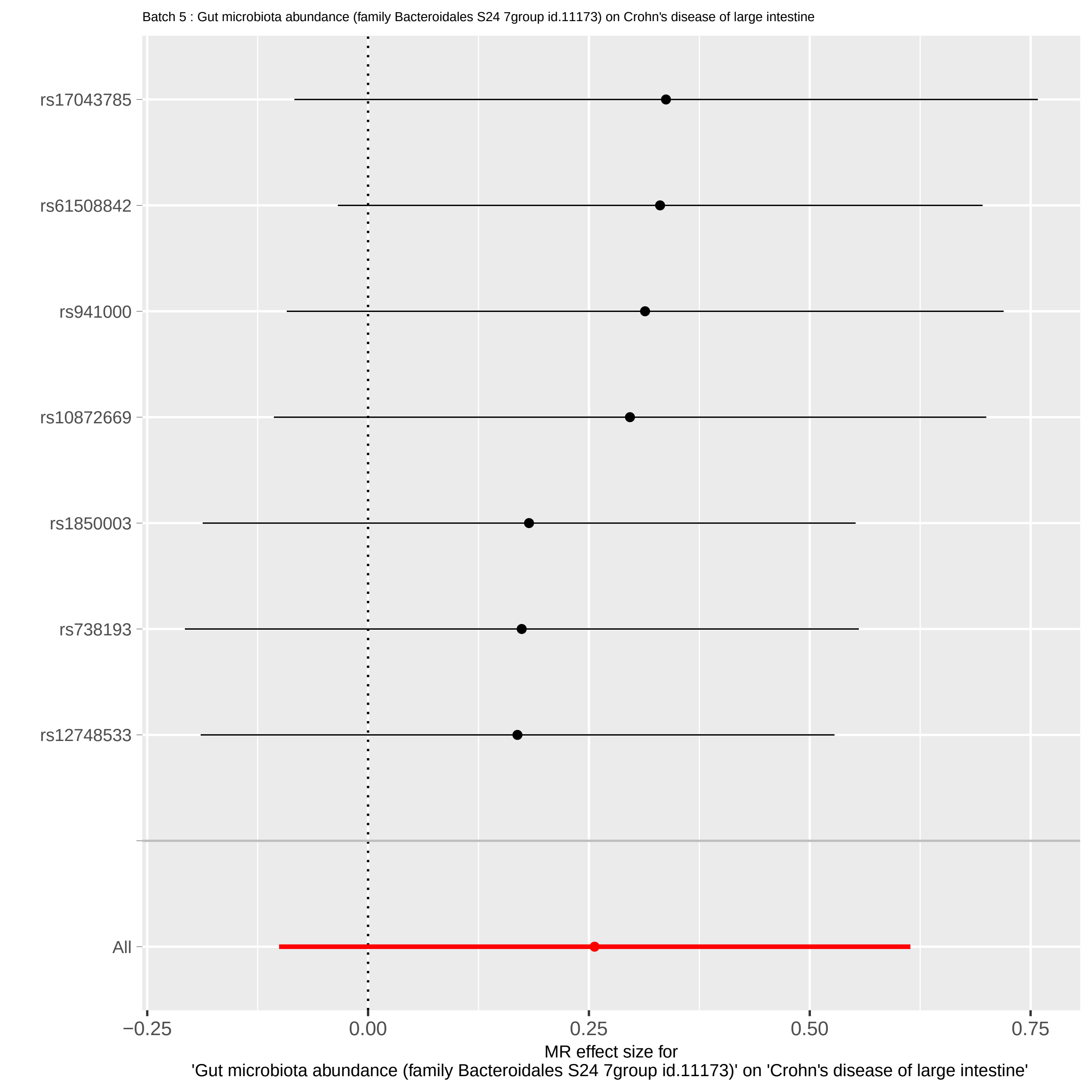

Batch 5 : Gut microbiota abundance (family Bacteroidales S24 7group id.11173) on Crohn's disease of large intestine
rs17043785
rs61508842
rs941000
rs10872669
rs1850003
rs738193
rs12748533
All
−0.25
0.00
0.25
0.50
0.75
MR effect size for
'Gut microbiota abundance (family Bacteroidales S24 7group id.11173)' on 'Crohn's disease of large intestine'

Batch 6 : Gut microbiota abundance (family Bifidobacteriaceae id.433) on Crohn's disease of large intestine

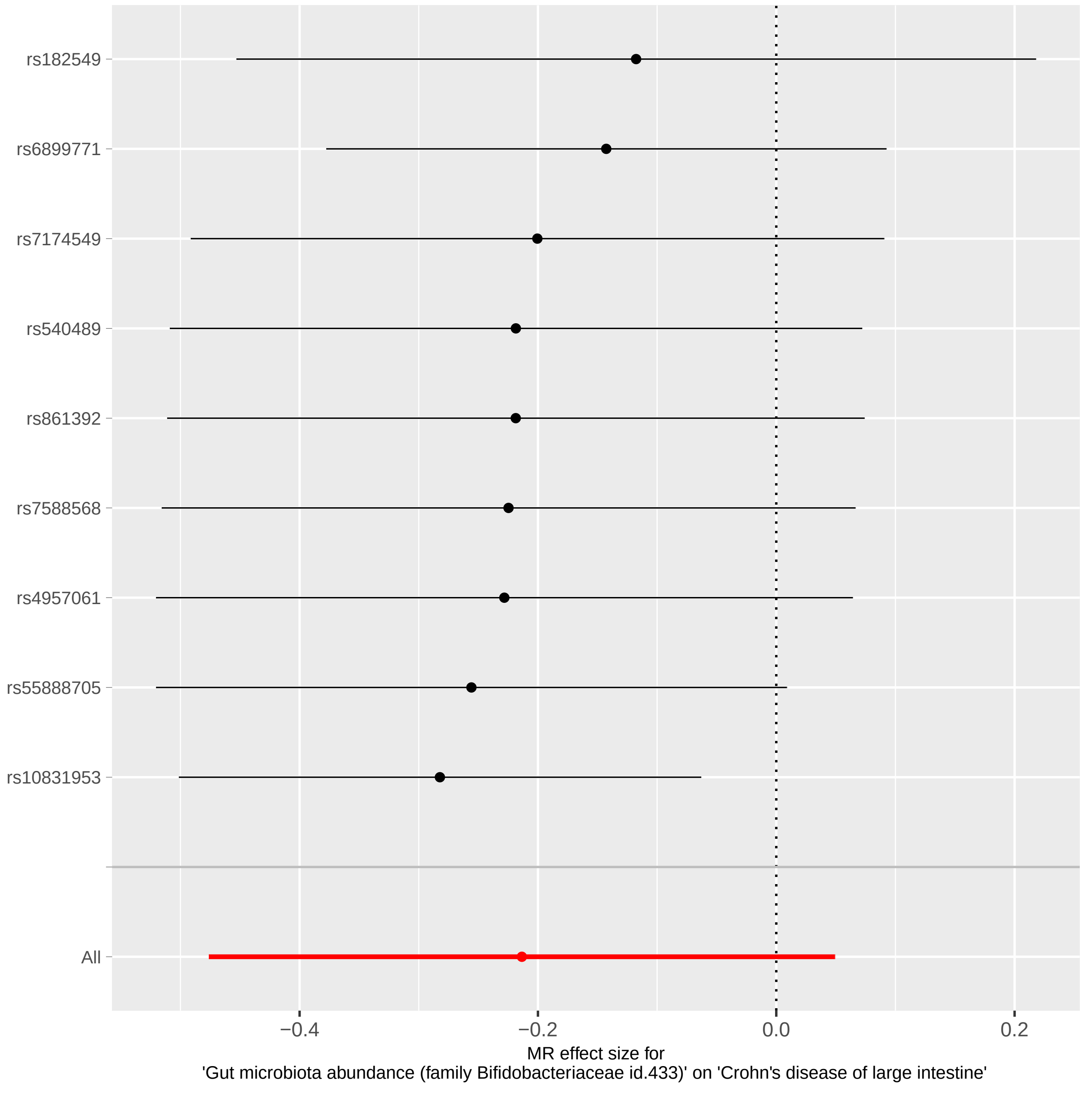

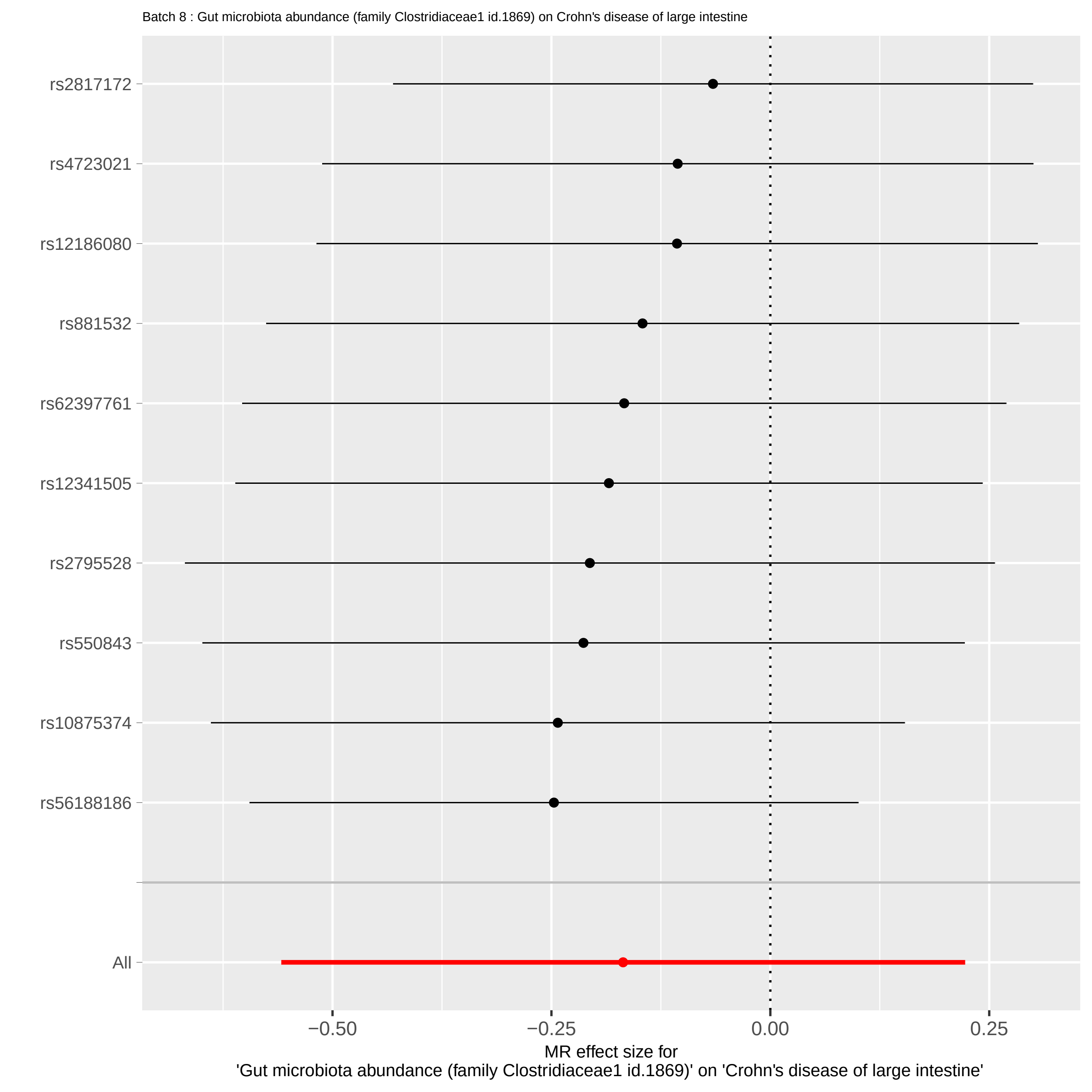
Batch 8 : Gut microbiota abundance (family Clostridiaceae1 id.1869) on Crohn's disease of large intestine
rs2817172
rs4723021
rs12186080
rs881532
rs62397761
rs12341505
rs2795528
rs550843
rs10875374
rs56188186
All
−0.50
−0.25
0.00
0.25
MR effect size for
'Gut microbiota abundance (family Clostridiaceae1 id.1869)' on 'Crohn's disease of large intestine'

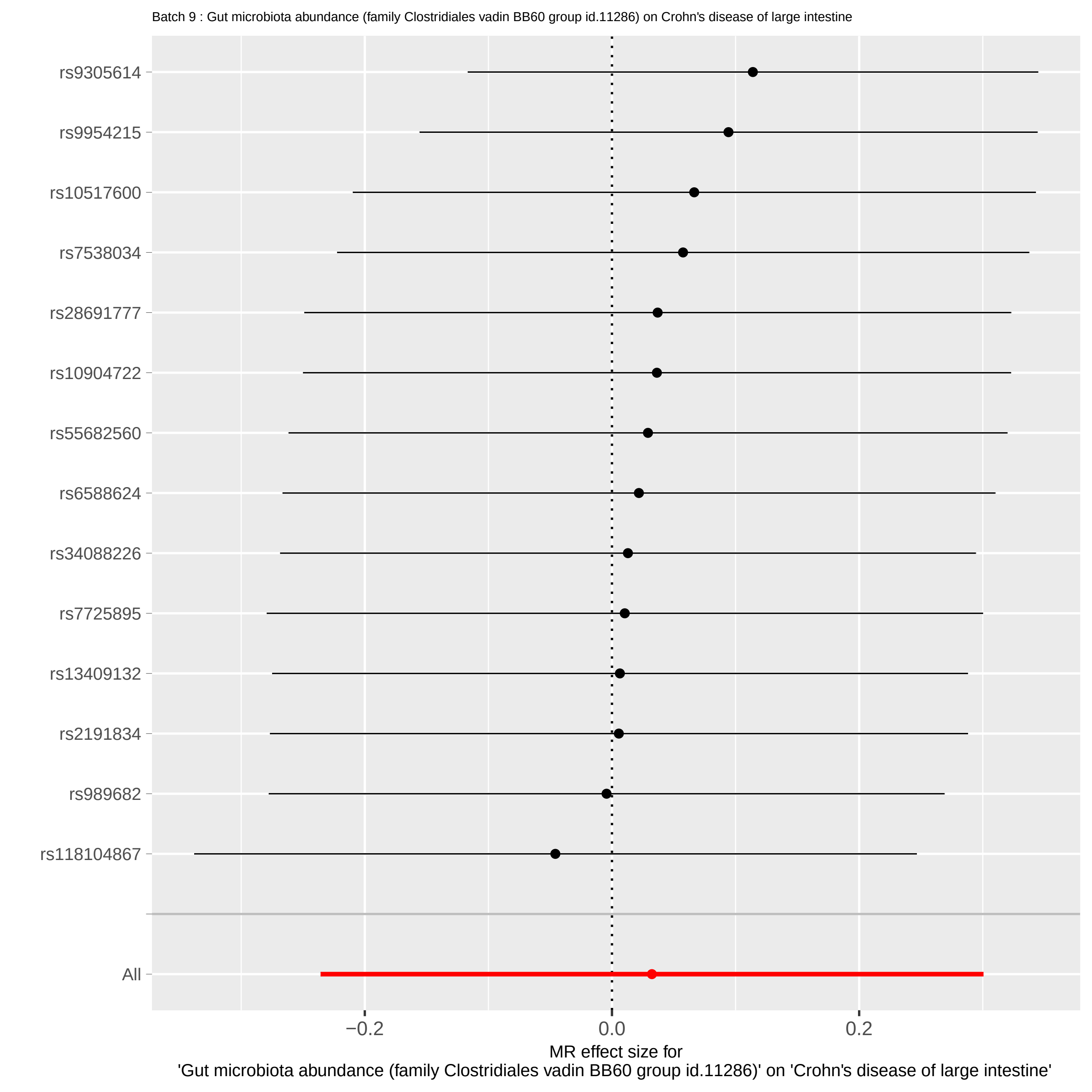

Batch 9 : Gut microbiota abundance (family Clostridiales vadin BB60 group id.11286) on Crohn's disease of large intestine
rs9305614
rs9954215
rs10517600
rs7538034
rs28691777
rs10904722
rs55682560
rs6588624
rs34088226
rs7725895
rs13409132
rs2191834
rs989682
rs118104867
All
−0.2
0.0
0.2
MR effect size for
'Gut microbiota abundance (family Clostridiales vadin BB60 group id.11286)' on 'Crohn's disease of large intestine'

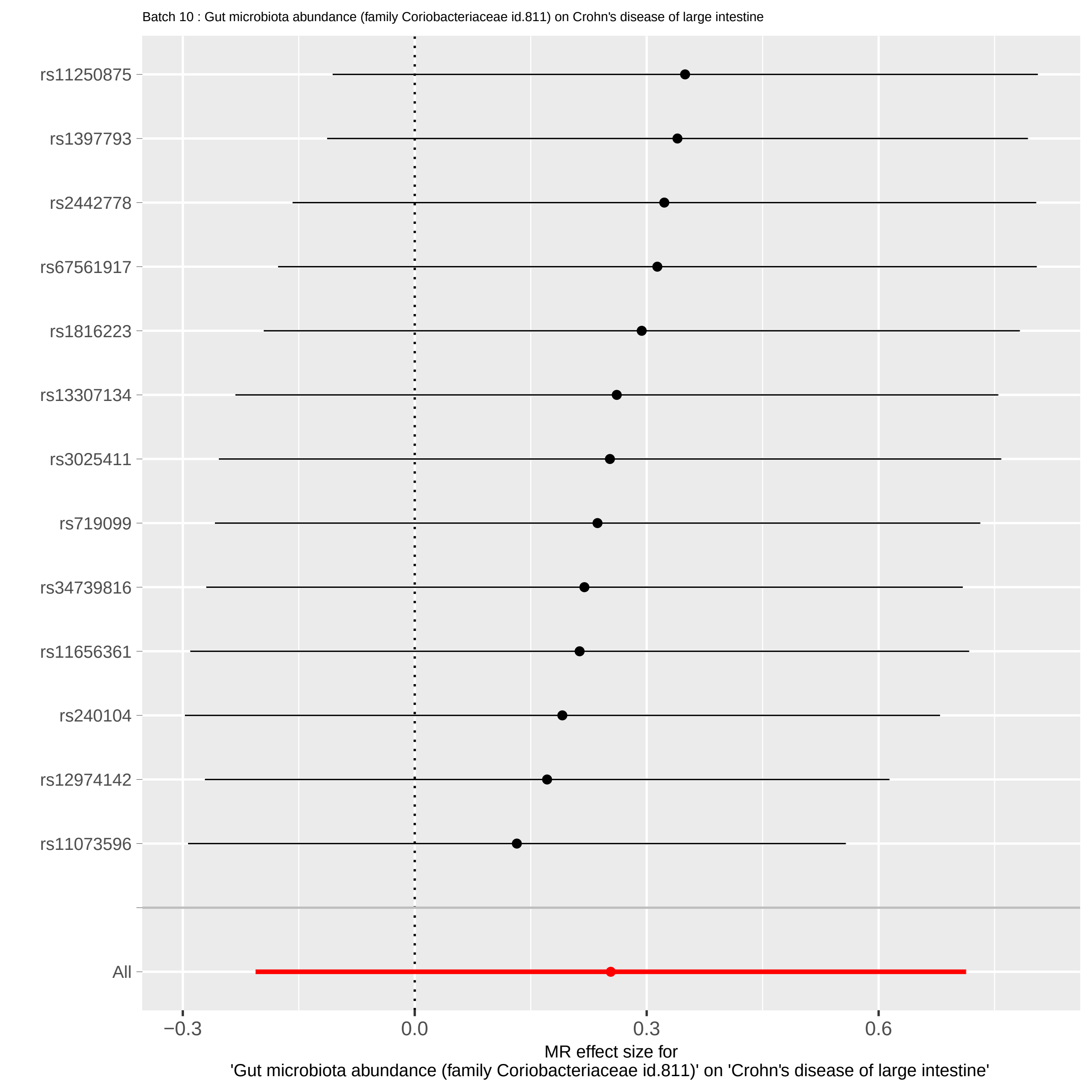
Batch 10 : Gut microbiota abundance (family Coriobacteriaceae id.811) on Crohn's disease of large intestine
rs11250875
rs1397793
rs2442778
rs67561917
rs1816223
rs13307134
rs3025411
rs719099
rs34739816
rs11656361
rs240104
rs12974142
rs11073596
All
−0.3
0.0
0.3
0.6
MR effect size for
'Gut microbiota abundance (family Coriobacteriaceae id.811)' on 'Crohn's disease of large intestine'

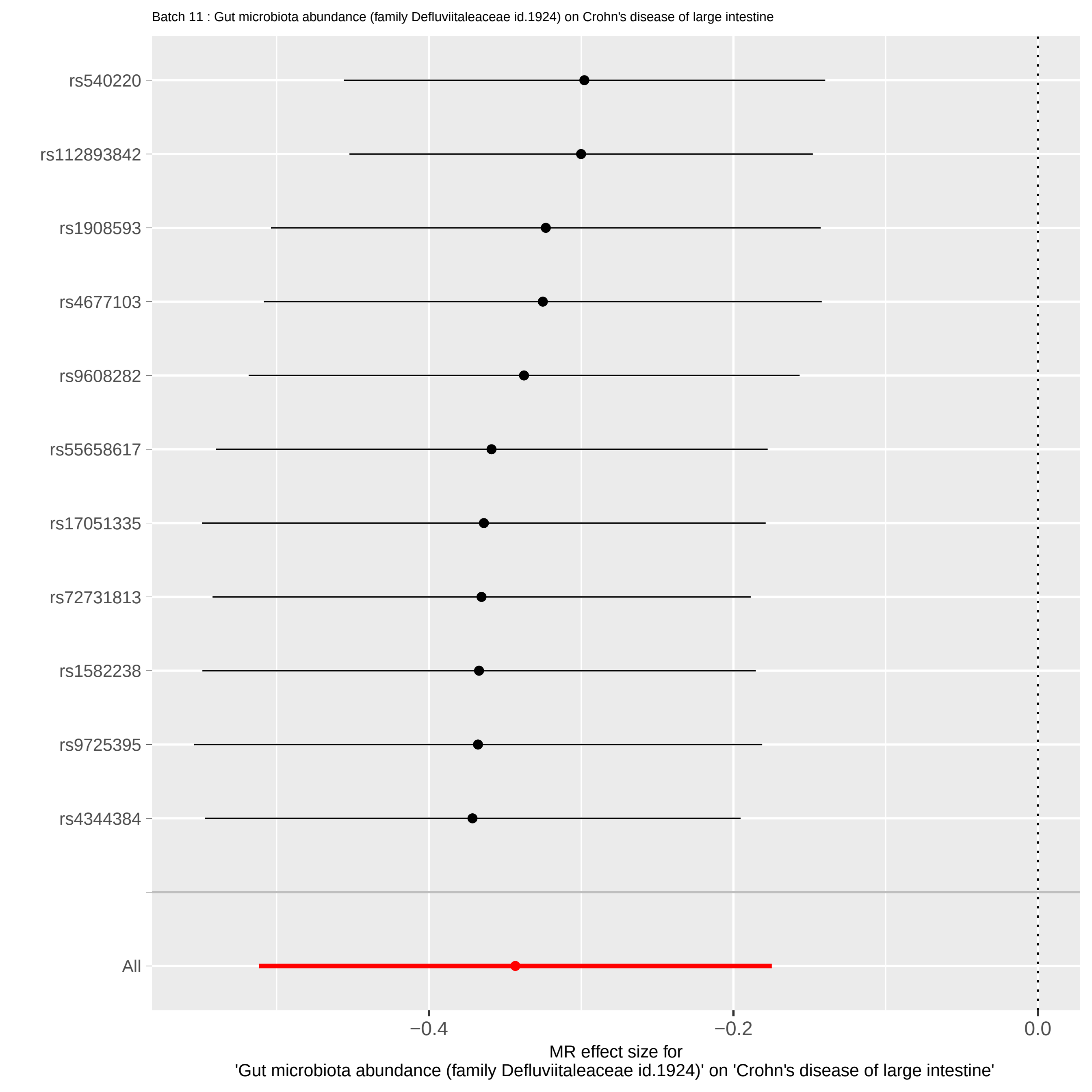
Batch 11 : Gut microbiota abundance (family Defluviitaleaceae id.1924) on Crohn's disease of large intestine
rs540220
rs112893842
rs1908593
rs4677103
rs9608282
rs55658617
rs17051335
rs72731813
rs1582238
rs9725395
rs4344384
All
−0.4
−0.2
0.0
MR effect size for
'Gut microbiota abundance (family Defluviitaleaceae id.1924)' on 'Crohn's disease of large intestine'

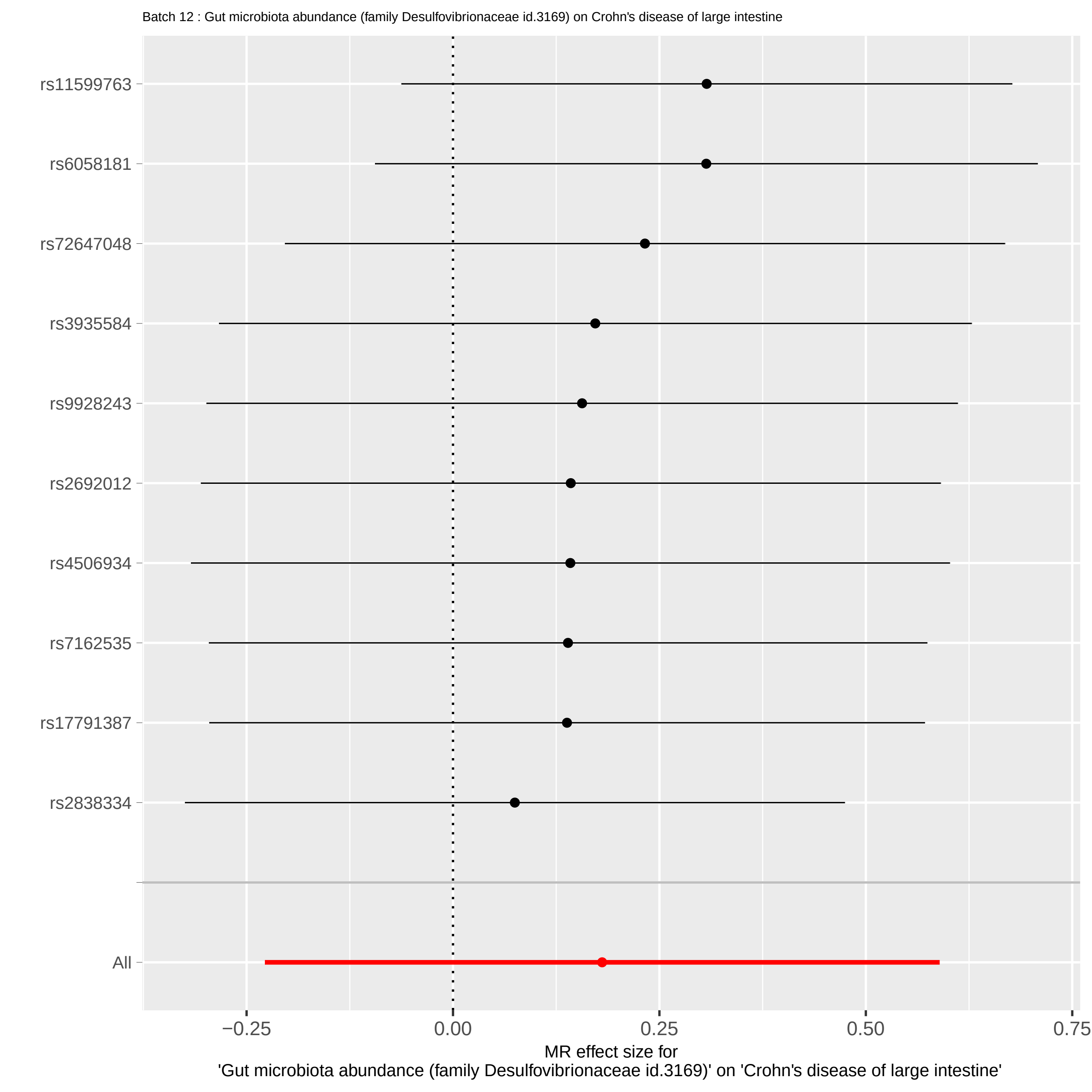
Batch 12 : Gut microbiota abundance (family Desulfovibrionaceae id.3169) on Crohn's disease of large intestine
rs11599763
rs6058181
rs72647048
rs3935584
rs9928243
rs2692012
rs4506934
rs7162535
rs17791387
rs2838334
All
−0.25
0.00
0.25
0.50
0.75
MR effect size for
'Gut microbiota abundance (family Desulfovibrionaceae id.3169)' on 'Crohn's disease of large intestine'

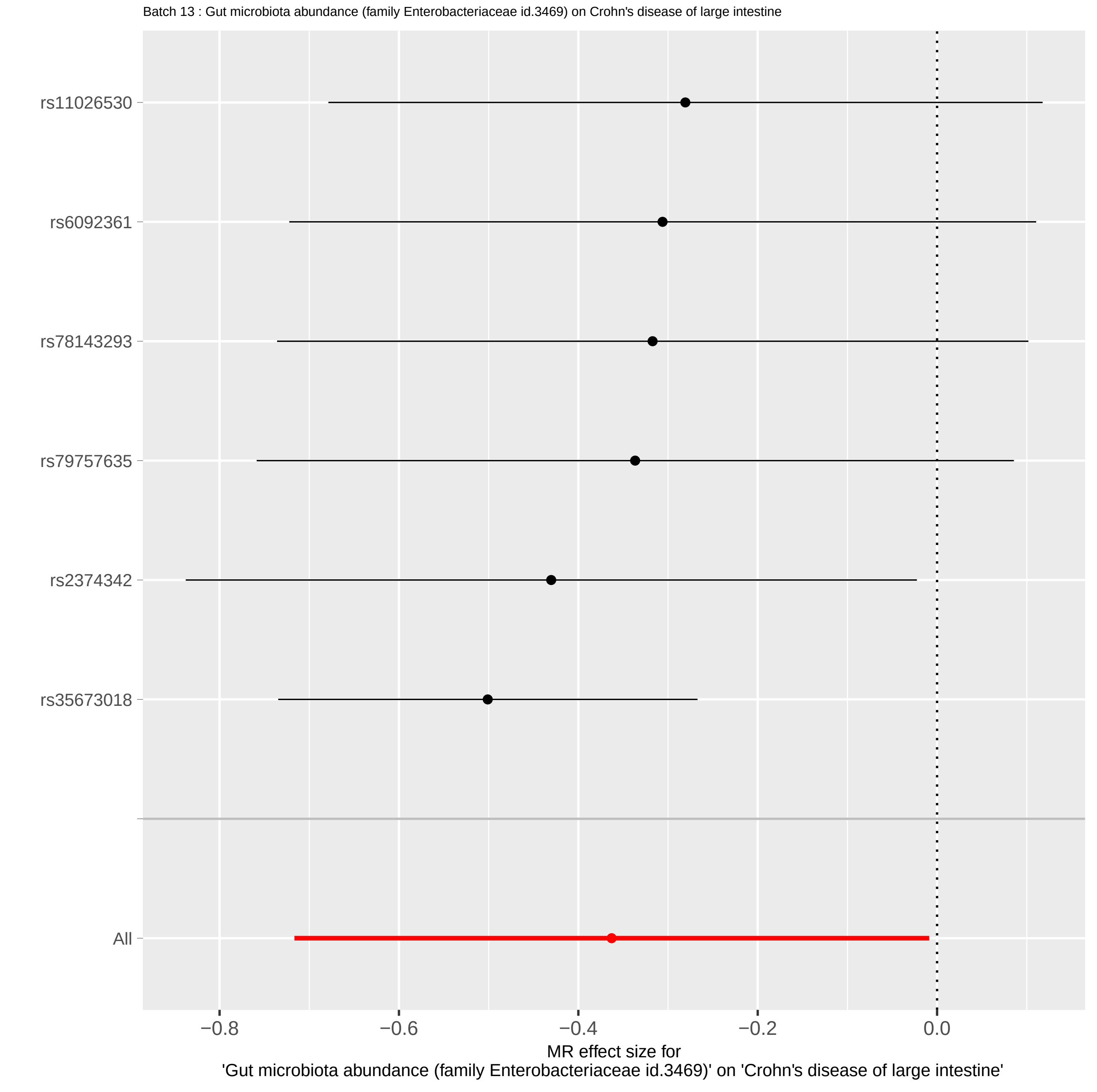
Batch 13 : Gut microbiota abundance (family Enterobacteriaceae id.3469) on Crohn's disease of large intestine
rs11026530
rs6092361
rs78143293
rs79757635
rs2374342
rs35673018
All
−0.8
−0.6
−0.4
−0.2
0.0
MR effect size for
'Gut microbiota abundance (family Enterobacteriaceae id.3469)' on 'Crohn's disease of large intestine'

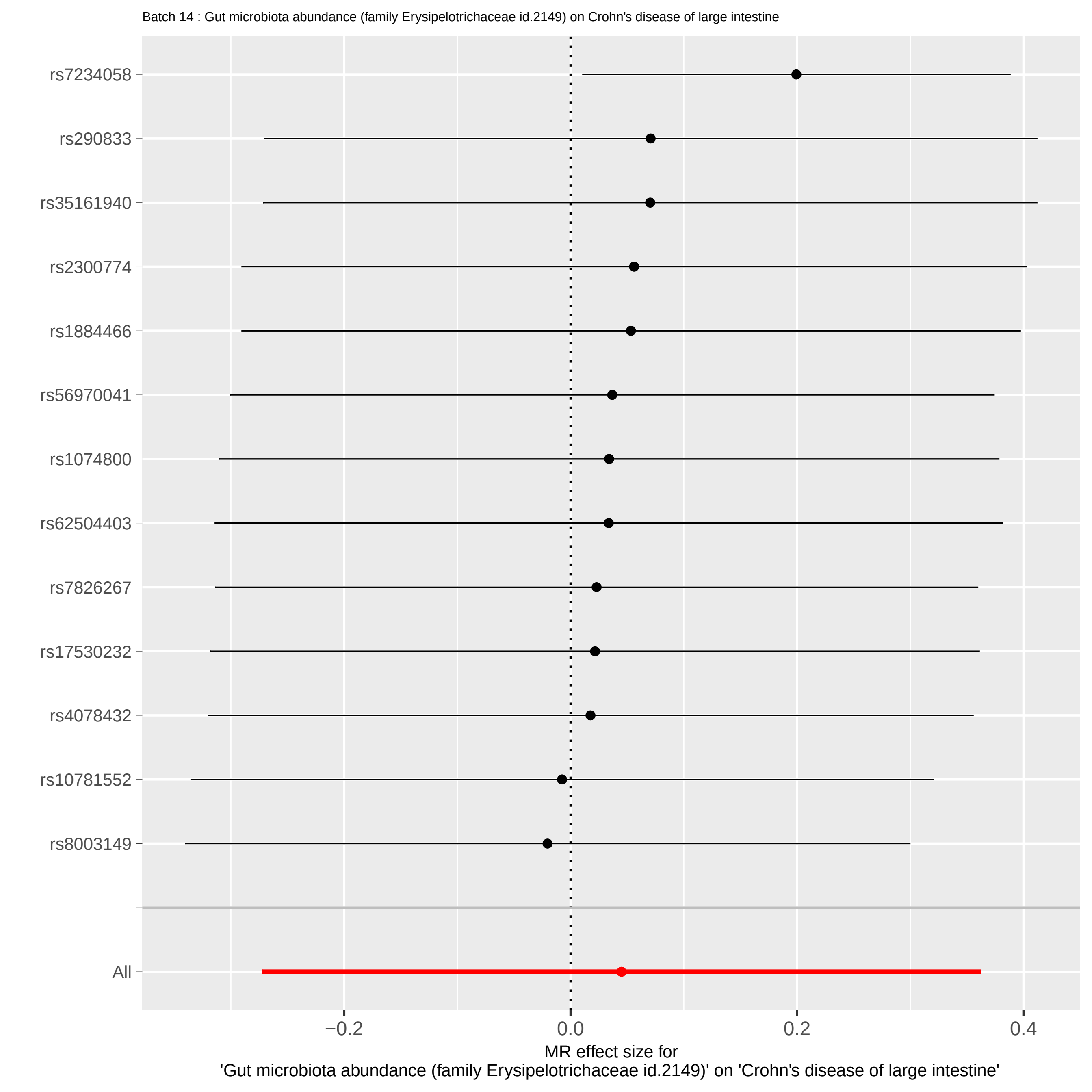
Batch 14 : Gut microbiota abundance (family Erysipelotrichaceae id.2149) on Crohn's disease of large intestine
rs7234058
rs290833
rs35161940
rs2300774
rs1884466
rs56970041
rs1074800
rs62504403
rs7826267
rs17530232
rs4078432
rs10781552
rs8003149
All
−0.2
0.0
0.2
0.4
MR effect size for
'Gut microbiota abundance (family Erysipelotrichaceae id.2149)' on 'Crohn's disease of large intestine'

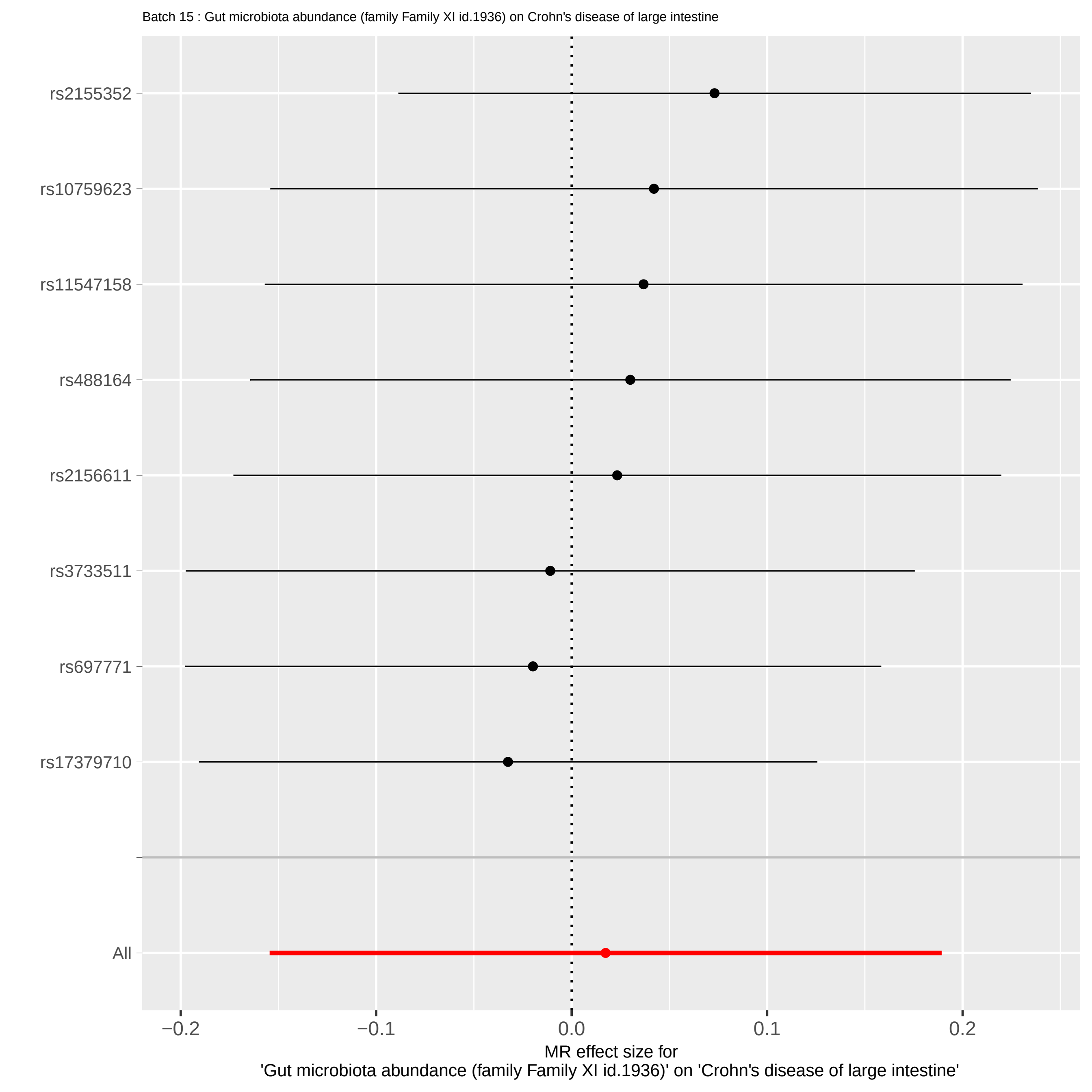
Batch 15 : Gut microbiota abundance (family Family XI id.1936) on Crohn's disease of large intestine
rs2155352
rs10759623
rs11547158
rs488164
rs2156611
rs3733511
rs697771
rs17379710
All
−0.2
−0.1
0.0
0.1
0.2
MR effect size for
'Gut microbiota abundance (family Family XI id.1936)' on 'Crohn's disease of large intestine'

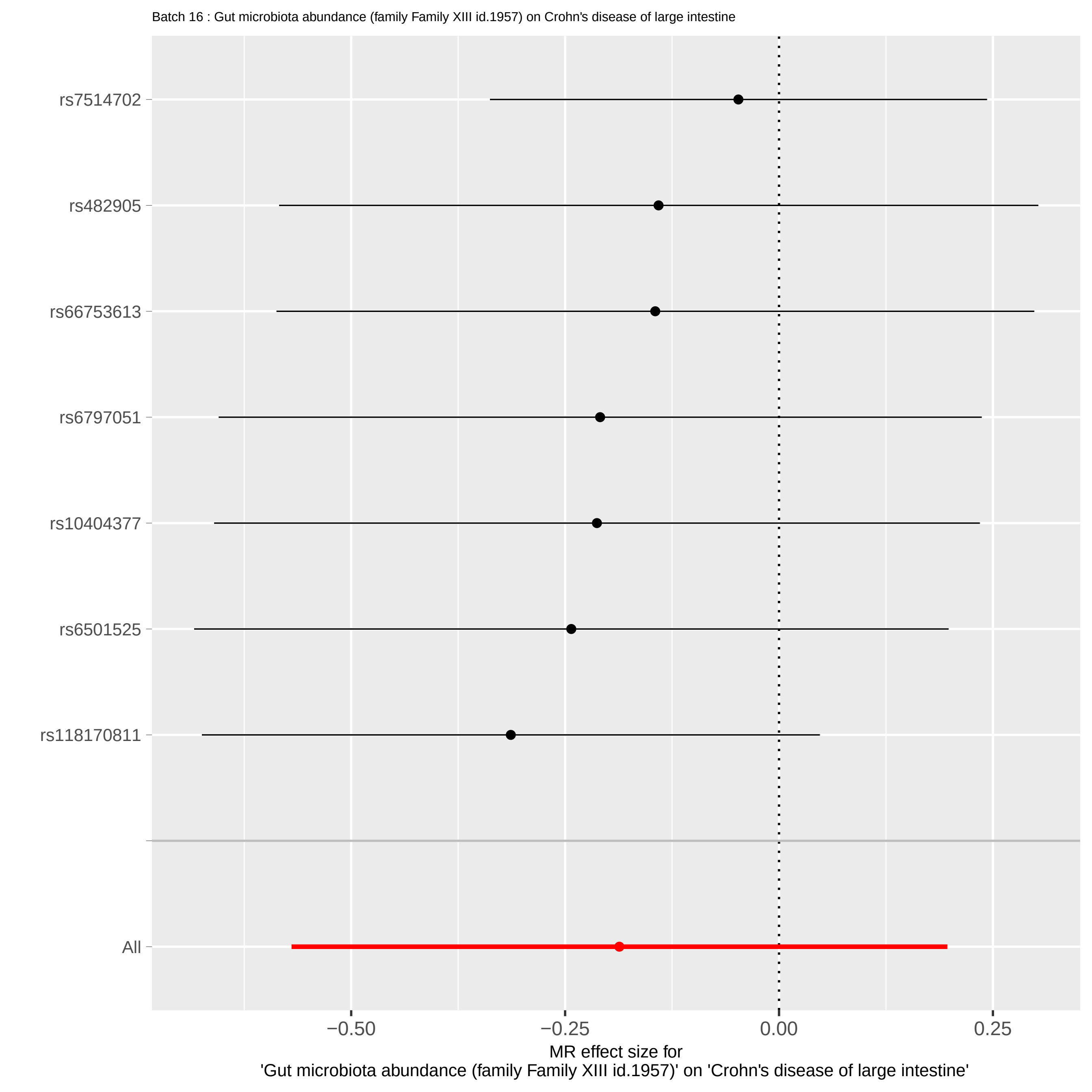
Batch 16 : Gut microbiota abundance (family Family XIII id.1957) on Crohn's disease of large intestine
rs7514702
rs482905
rs66753613
rs6797051
rs10404377
rs6501525
rs118170811
All
−0.50
−0.25
0.00
0.25
MR effect size for
'Gut microbiota abundance (family Family XIII id.1957)' on 'Crohn's disease of large intestine'

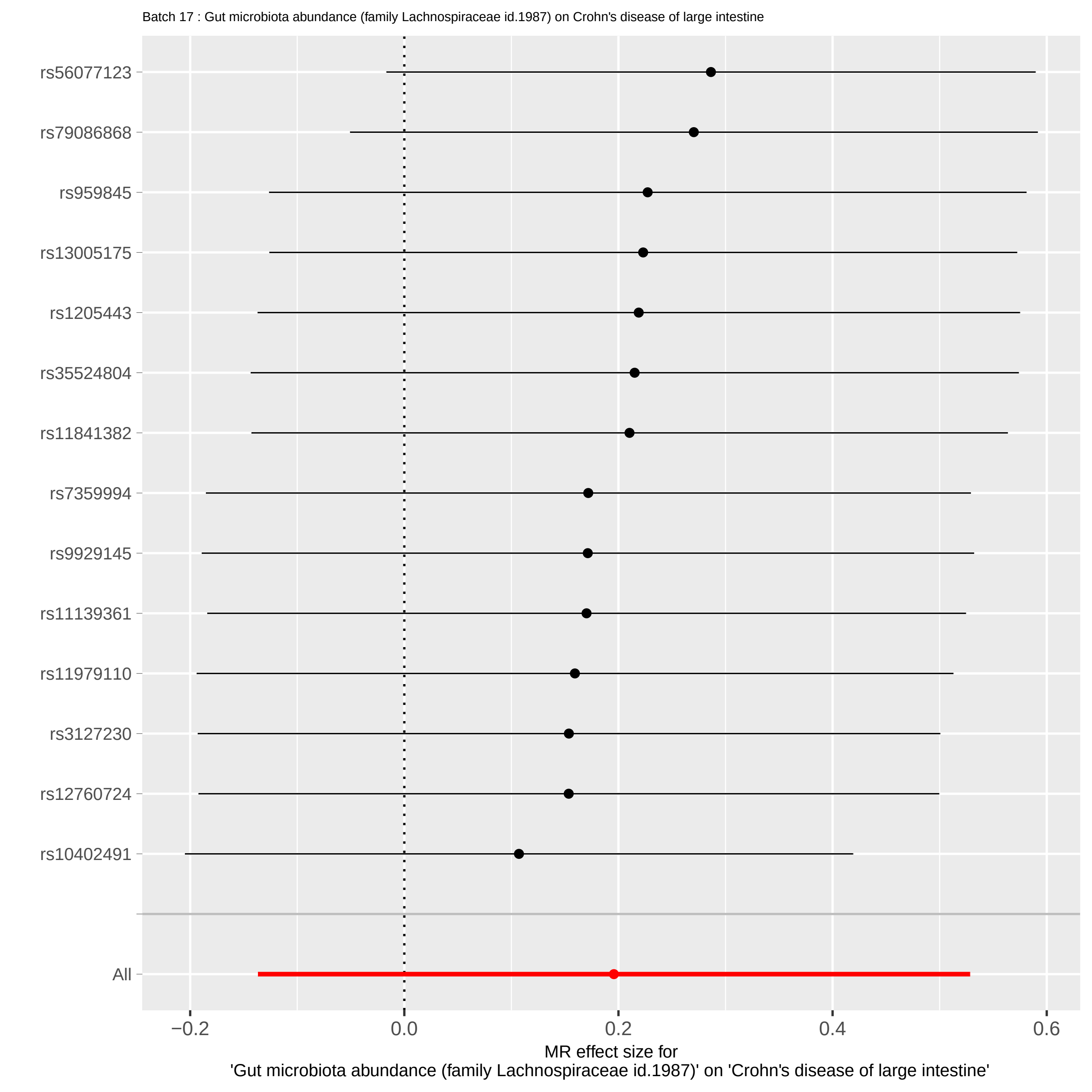
Batch 17 : Gut microbiota abundance (family Lachnospiraceae id.1987) on Crohn's disease of large intestine
rs56077123
rs79086868
rs959845
rs13005175
rs1205443
rs35524804
rs11841382
rs7359994
rs9929145
rs11139361
rs11979110
rs3127230
rs12760724
rs10402491
All
−0.2
0.0
0.2
0.4
0.6
MR effect size for
'Gut microbiota abundance (family Lachnospiraceae id.1987)' on 'Crohn's disease of large intestine'

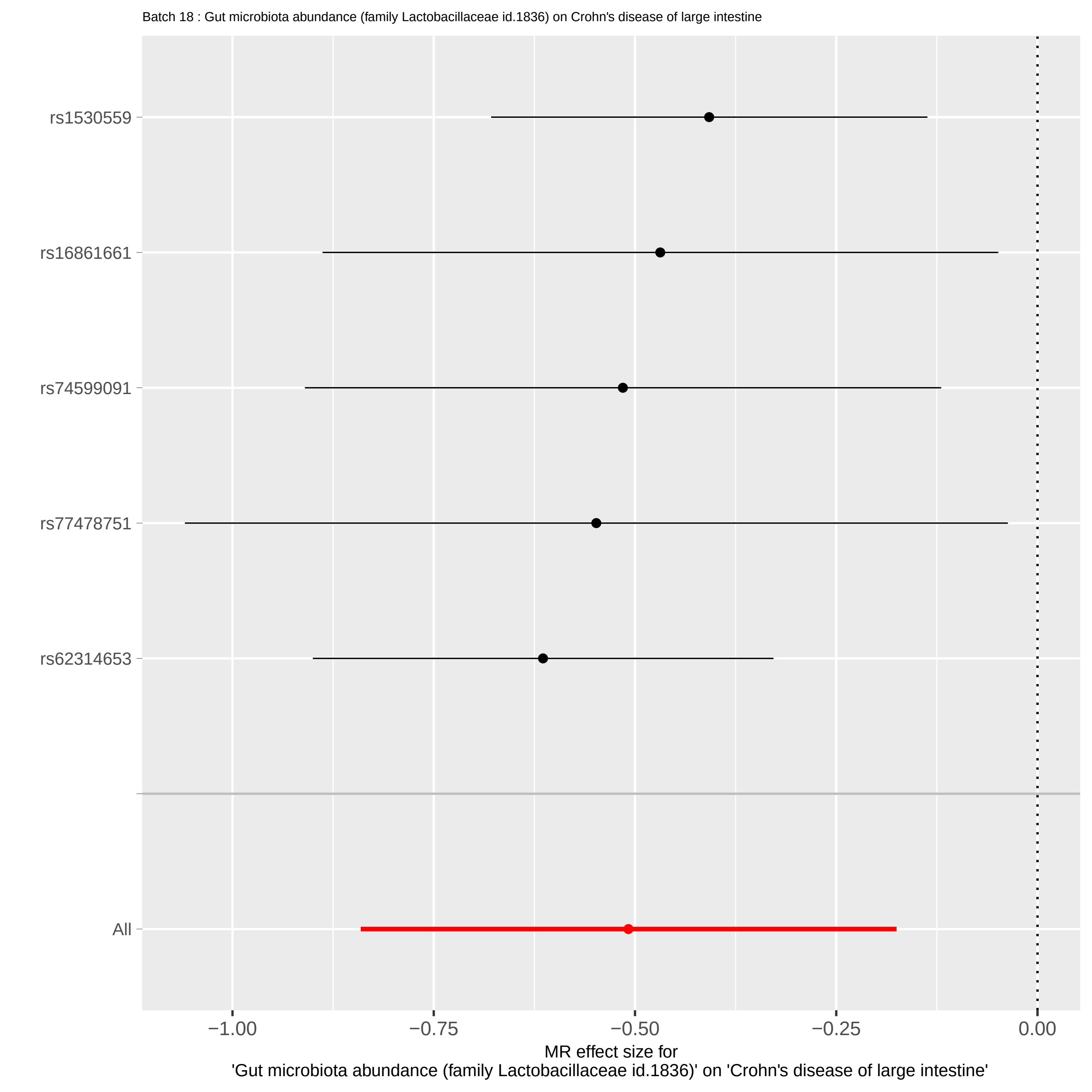
Batch 18 : Gut microbiota abundance (family Lactobacillaceae id.1836) on Crohn's disease of large intestine
rs1530559
rs16861661
rs74599091
rs77478751
rs62314653
All
−1.00
−0.75
−0.50
−0.25
0.00
MR effect size for
'Gut microbiota abundance (family Lactobacillaceae id.1836)' on 'Crohn's disease of large intestine'

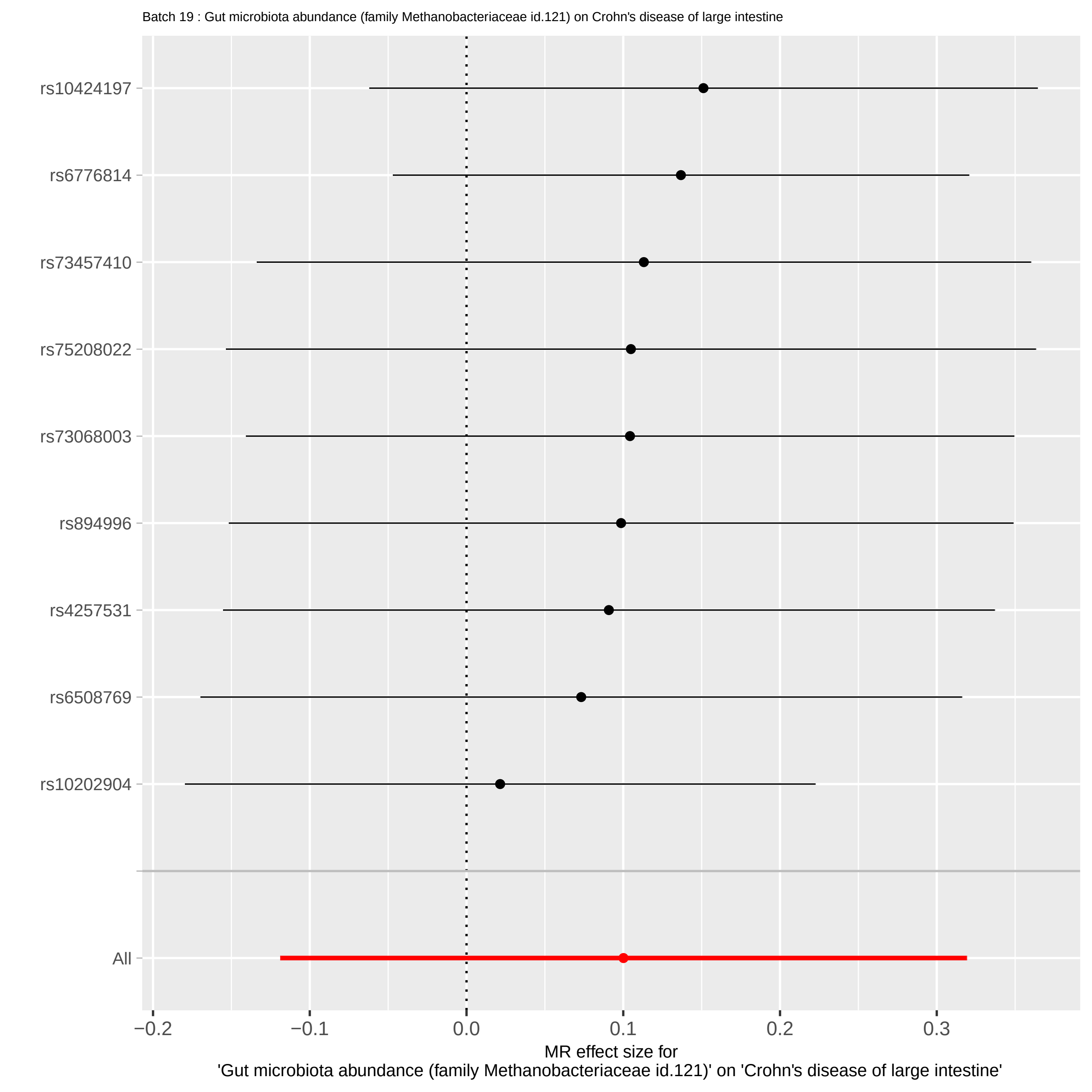

Batch 19 : Gut microbiota abundance (family Methanobacteriaceae id.121) on Crohn's disease of large intestine
rs10424197
rs6776814
rs73457410
rs75208022
rs73068003
rs894996
rs4257531
rs6508769
rs10202904
All
−0.2
−0.1
0.0
0.1
0.2
0.3
MR effect size for
'Gut microbiota abundance (family Methanobacteriaceae id.121)' on 'Crohn's disease of large intestine'

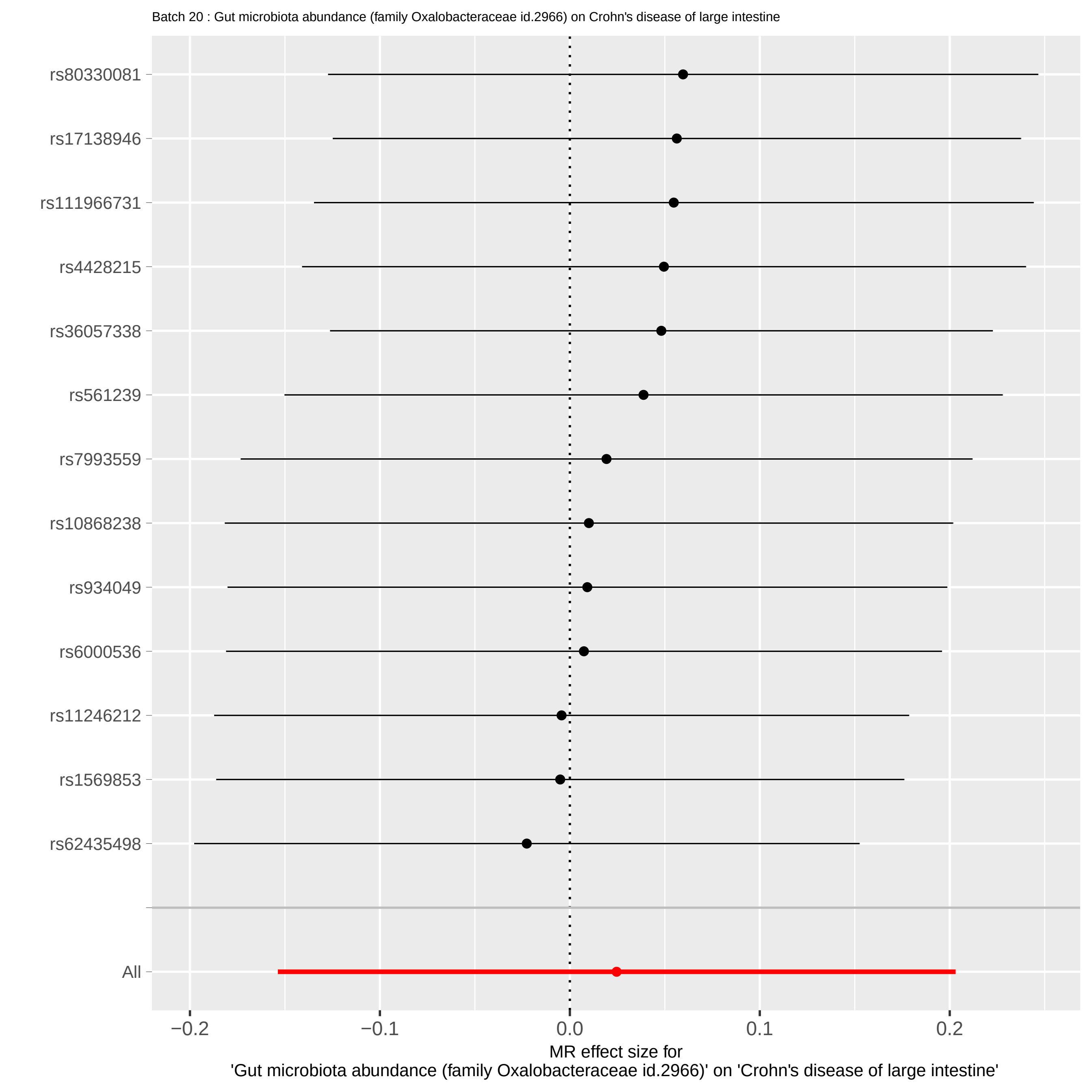
Batch 20 : Gut microbiota abundance (family Oxalobacteraceae id.2966) on Crohn's disease of large intestine
rs80330081
rs17138946
rs111966731
rs4428215
rs36057338
rs561239
rs7993559
rs10868238
rs934049
rs6000536
rs11246212
rs1569853
rs62435498
All
−0.2
−0.1
0.0
0.1
0.2
MR effect size for
'Gut microbiota abundance (family Oxalobacteraceae id.2966)' on 'Crohn's disease of large intestine'

Batch 21 : Gut microbiota abundance (family Pasteurellaceae id.3689) on Crohn's disease of large intestine

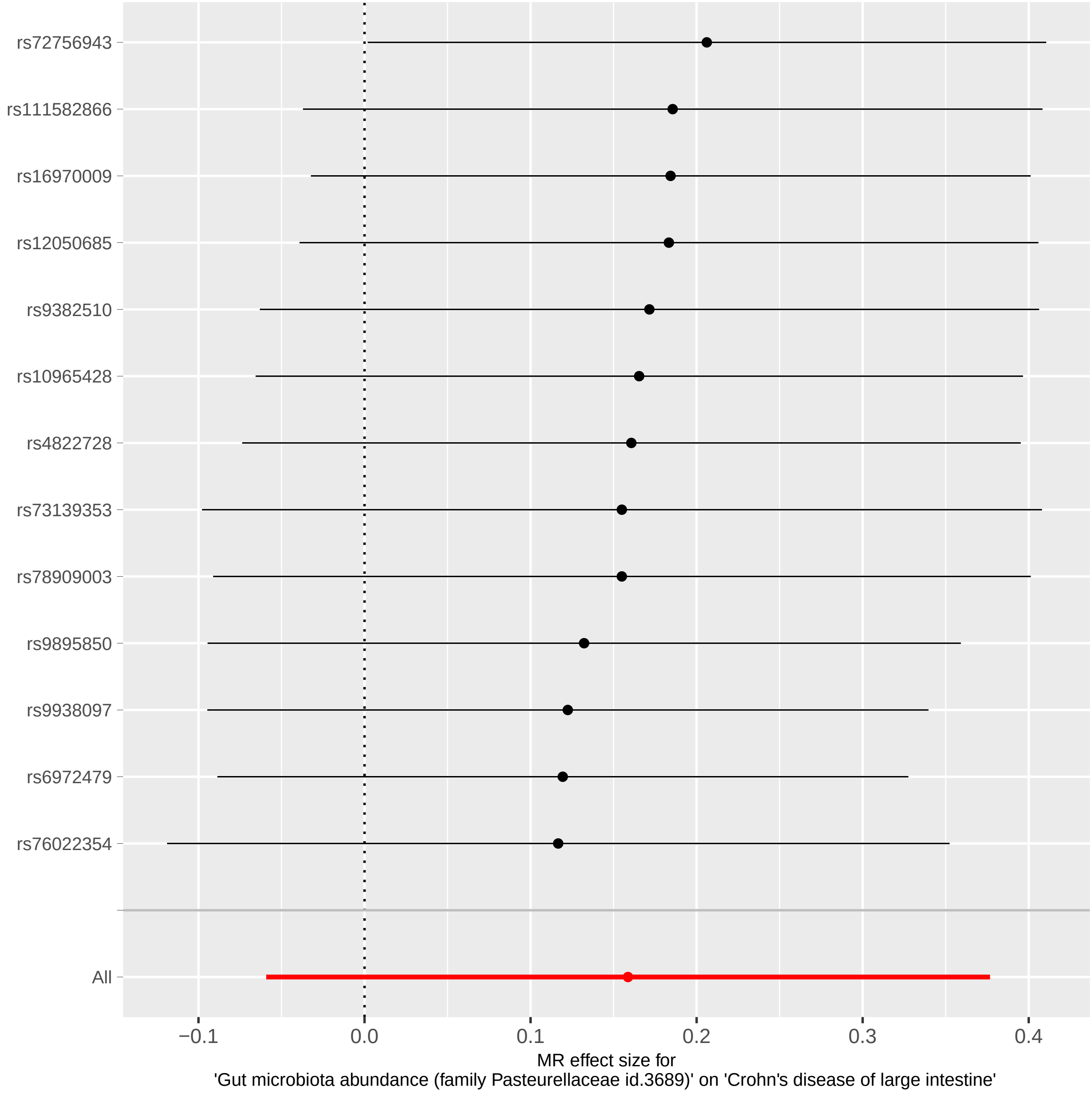

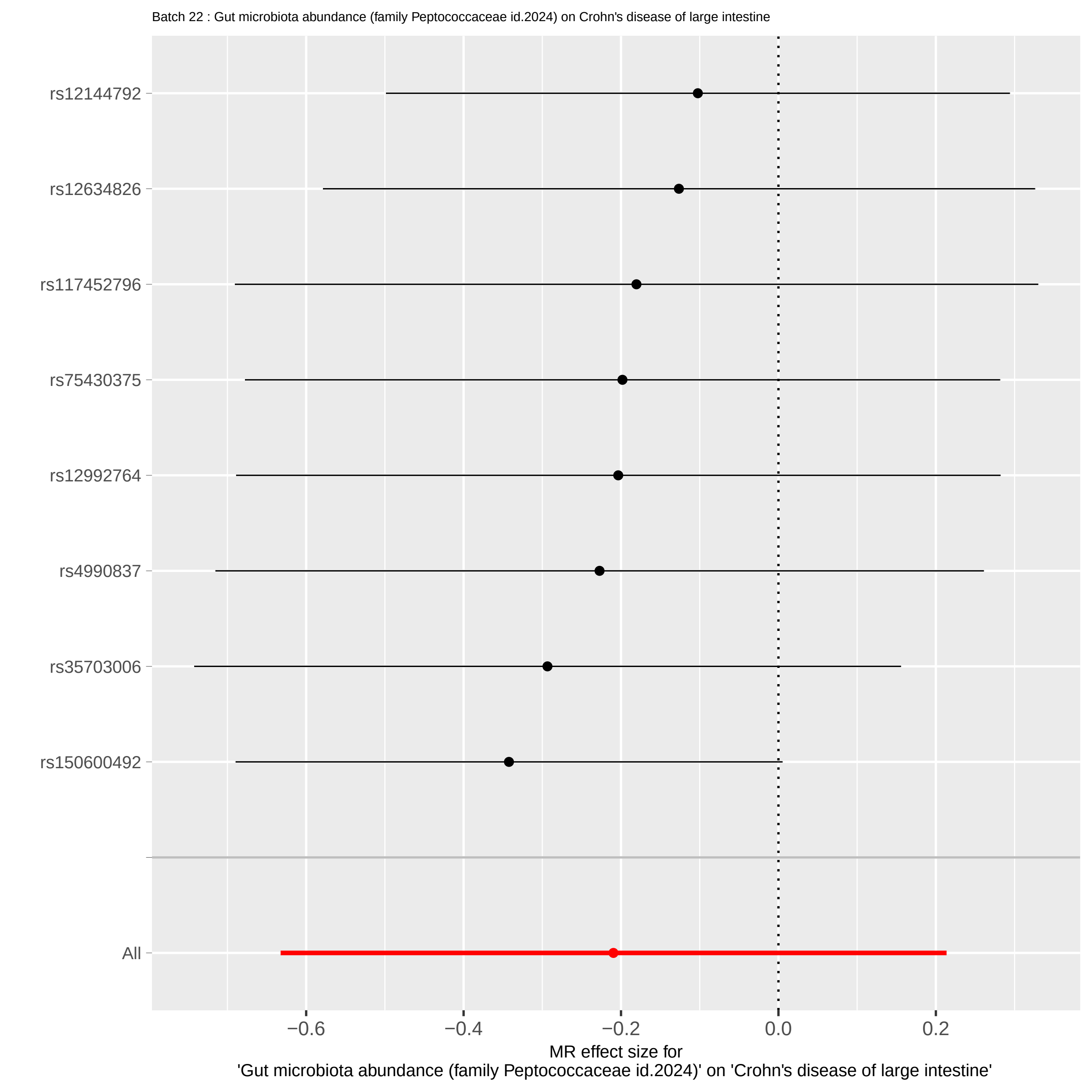

Batch 22 : Gut microbiota abundance (family Peptococcaceae id.2024) on Crohn's disease of large intestine
rs12144792
rs12634826
rs117452796
rs75430375
rs12992764
rs4990837
rs35703006
rs150600492
All
−0.6
−0.4
−0.2
0.0
0.2
MR effect size for
'Gut microbiota abundance (family Peptococcaceae id.2024)' on 'Crohn's disease of large intestine'

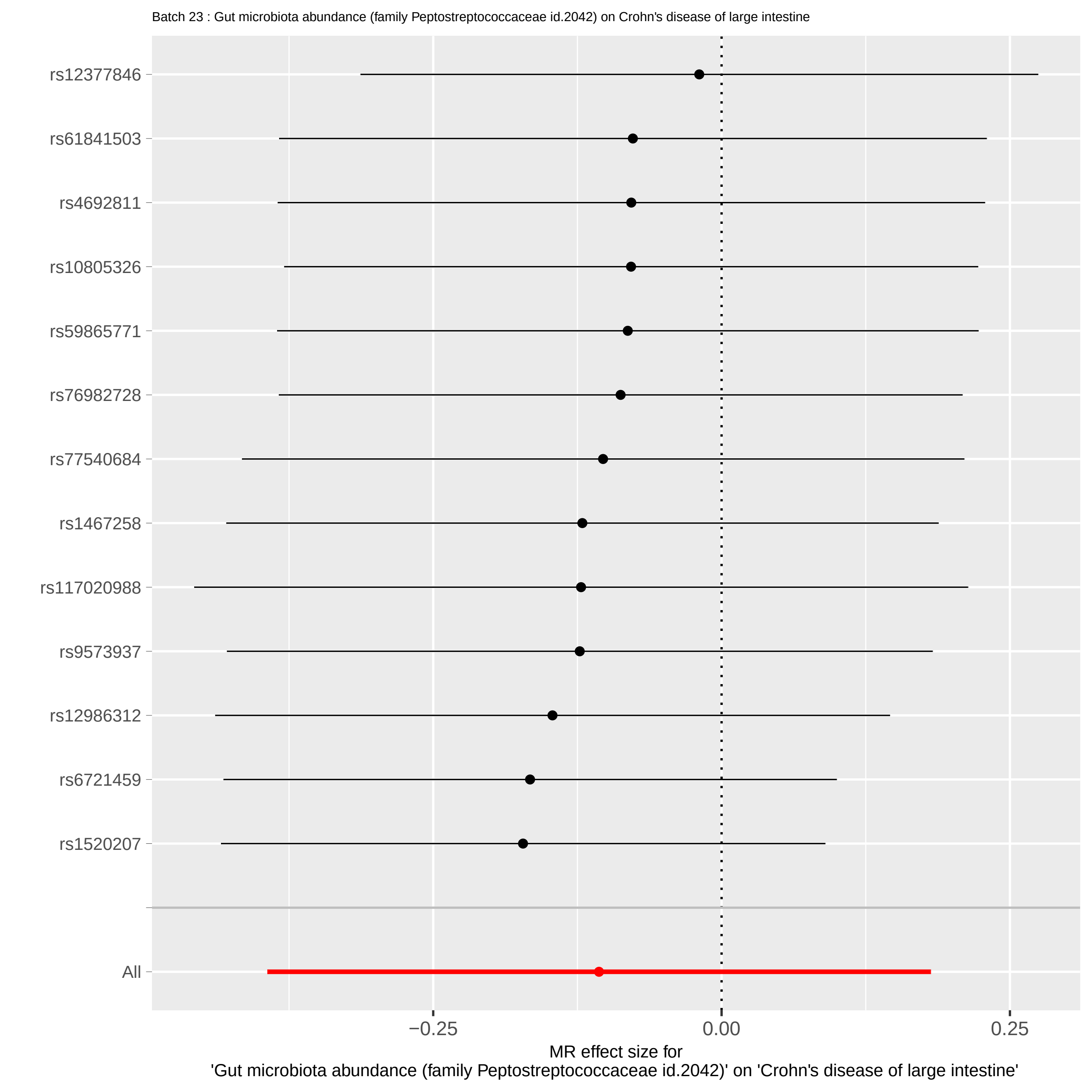
Batch 23 : Gut microbiota abundance (family Peptostreptococcaceae id.2042) on Crohn's disease of large intestine
rs12377846
rs61841503
rs4692811
rs10805326
rs59865771
rs76982728
rs77540684
rs1467258
rs117020988
rs9573937
rs12986312
rs6721459
rs1520207
All
−0.25
0.00
0.25
MR effect size for
'Gut microbiota abundance (family Peptostreptococcaceae id.2042)' on 'Crohn's disease of large intestine'

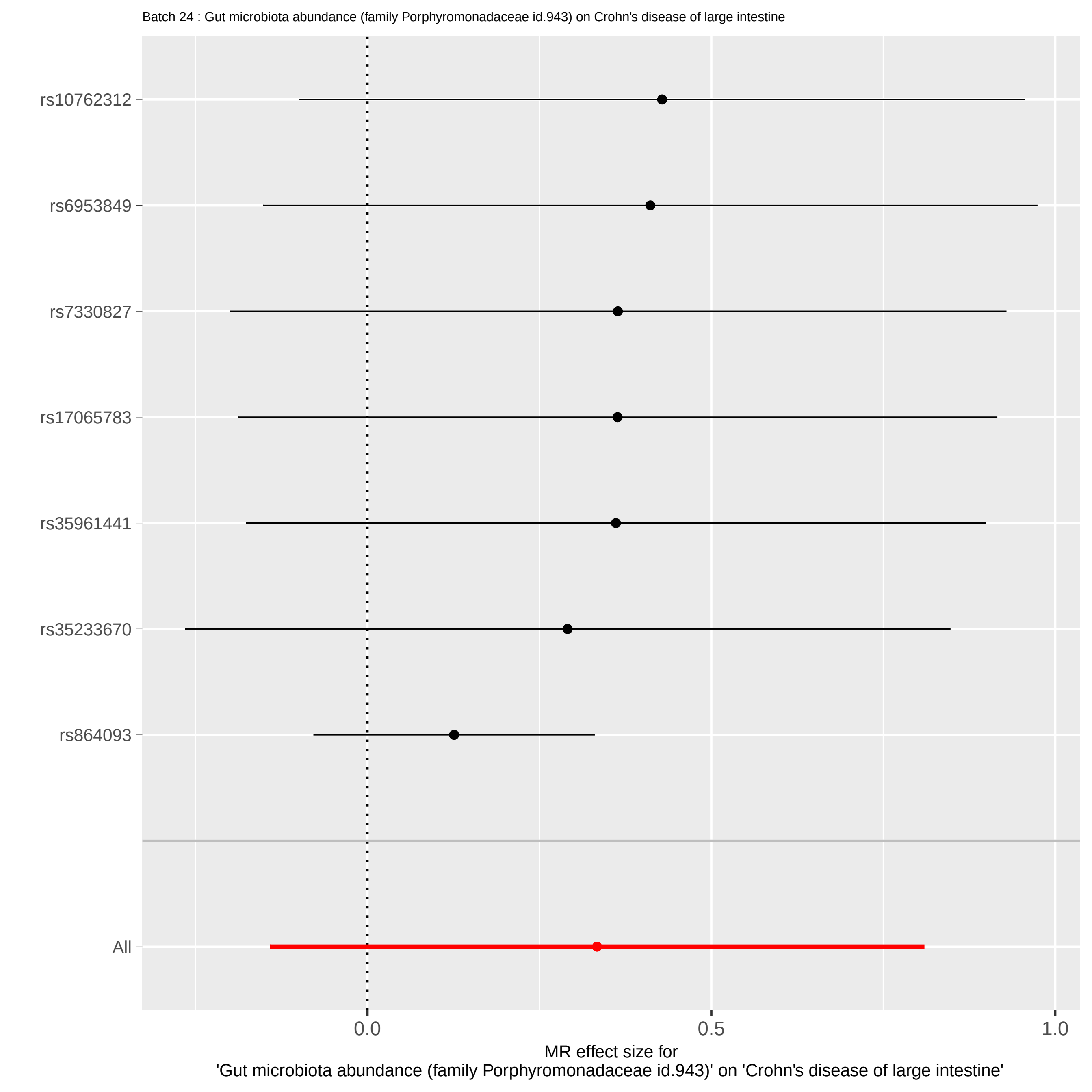

Batch 24 : Gut microbiota abundance (family Porphyromonadaceae id.943) on Crohn's disease of large intestine
rs10762312
rs6953849
rs7330827
rs17065783
rs35961441
rs35233670
rs864093
All
0.0
0.5
1.0
MR effect size for
'Gut microbiota abundance (family Porphyromonadaceae id.943)' on 'Crohn's disease of large intestine'

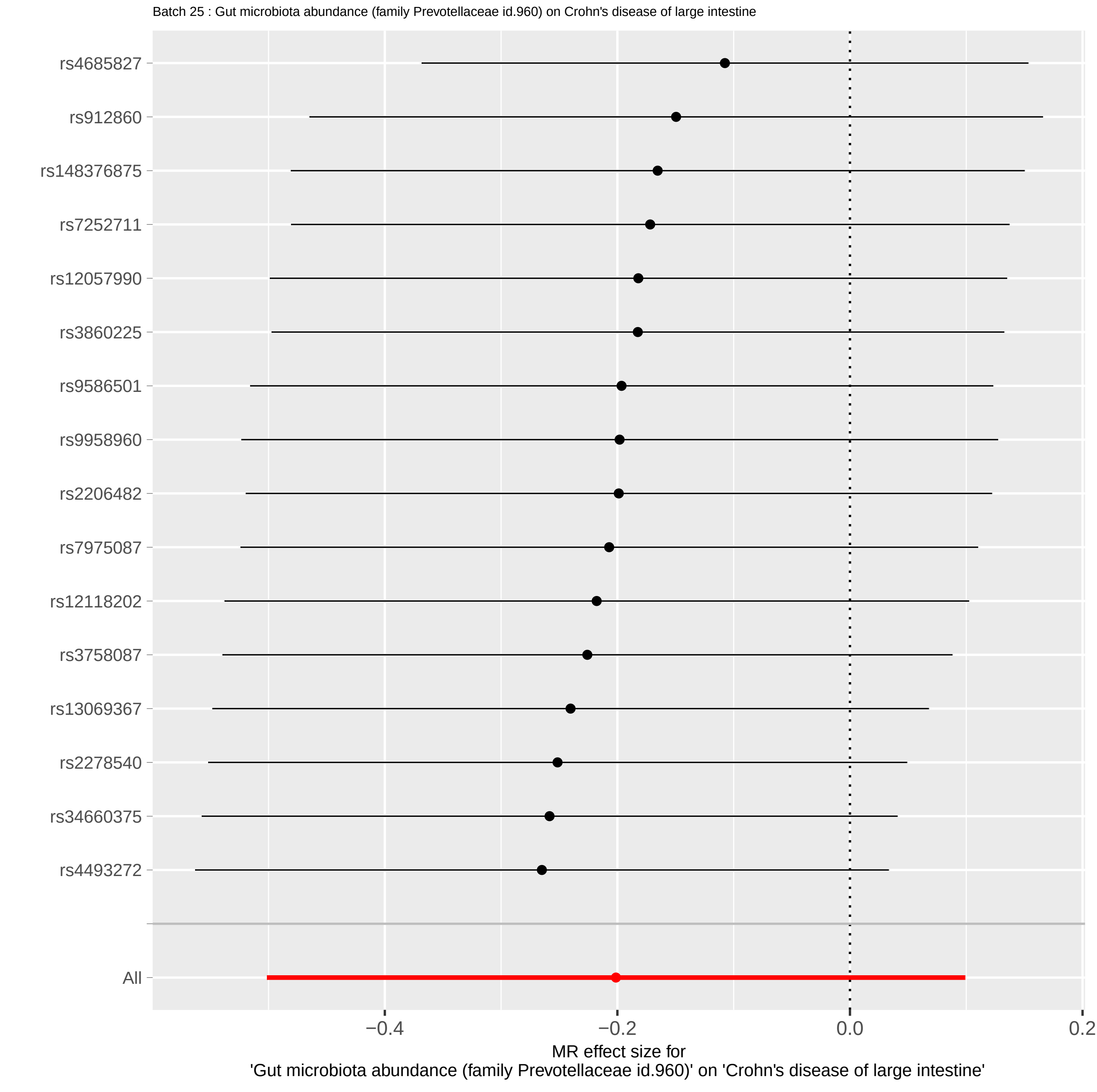
Batch 25 : Gut microbiota abundance (family Prevotellaceae id.960) on Crohn's disease of large intestine
rs4685827
rs912860
rs148376875
rs7252711
rs12057990
rs3860225
rs9586501
rs9958960
rs2206482
rs7975087
rs12118202
rs3758087
rs13069367
rs2278540
rs34660375
rs4493272
All
−0.4
−0.2
0.0
0.2
MR effect size for
'Gut microbiota abundance (family Prevotellaceae id.960)' on 'Crohn's disease of large intestine'

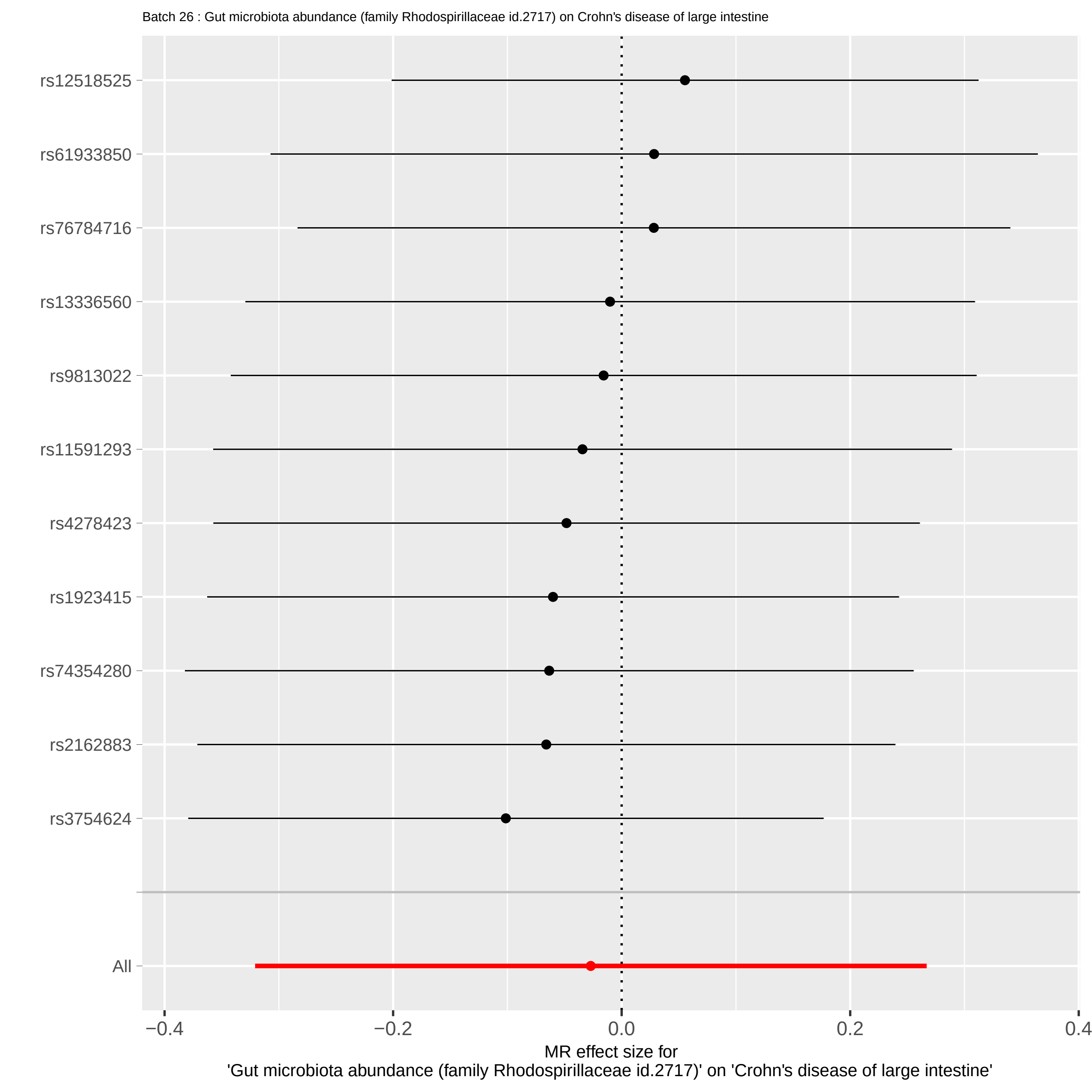

Batch 26 : Gut microbiota abundance (family Rhodospirillaceae id.2717) on Crohn's disease of large intestine
rs12518525
rs61933850
rs76784716
rs13336560
rs9813022
rs11591293
rs4278423
rs1923415
rs74354280
rs2162883
rs3754624
All
−0.4
−0.2
0.0
0.2
0.4
MR effect size for
'Gut microbiota abundance (family Rhodospirillaceae id.2717)' on 'Crohn's disease of large intestine'

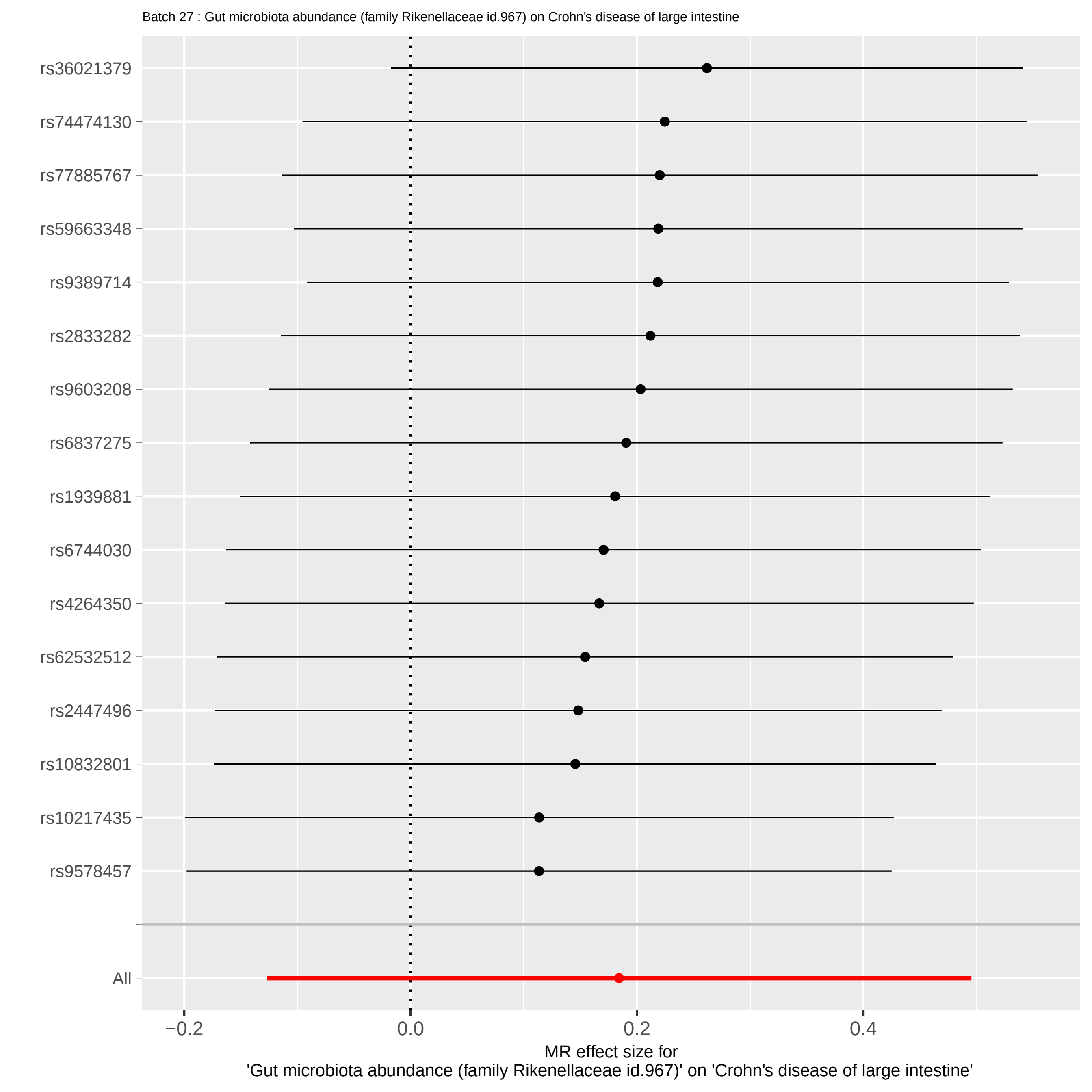
Batch 27 : Gut microbiota abundance (family Rikenellaceae id.967) on Crohn's disease of large intestine
rs36021379
rs74474130
rs77885767
rs59663348
rs9389714
rs2833282
rs9603208
rs6837275
rs1939881
rs6744030
rs4264350
rs62532512
rs2447496
rs10832801
rs10217435
rs9578457
All
−0.2
0.0
0.2
0.4
MR effect size for
'Gut microbiota abundance (family Rikenellaceae id.967)' on 'Crohn's disease of large intestine'

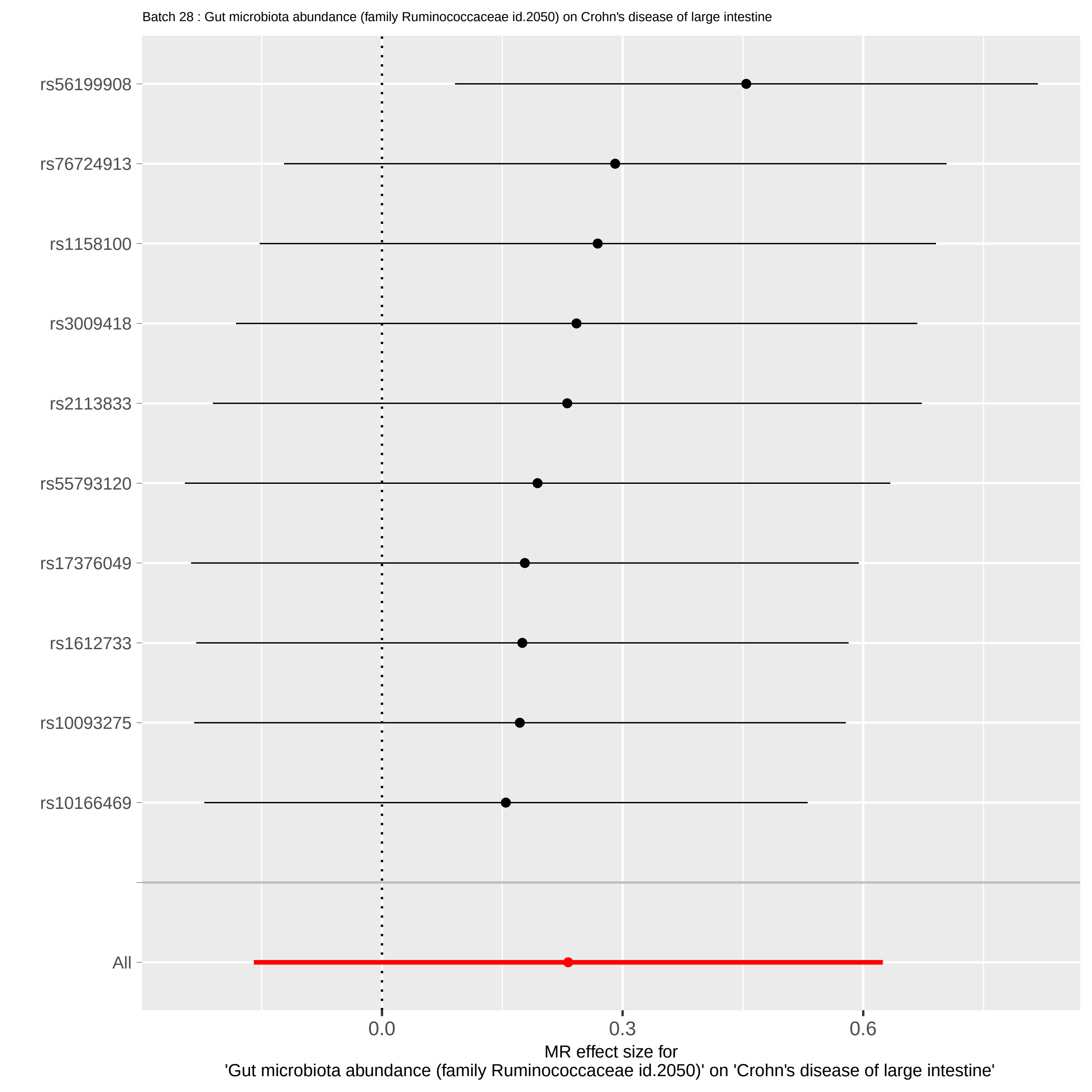

Batch 28 : Gut microbiota abundance (family Ruminococcaceae id.2050) on Crohn's disease of large intestine
rs56199908
rs76724913
rs1158100
rs3009418
rs2113833
rs55793120
rs17376049
rs1612733
rs10093275
rs10166469
All
0.0
0.3
0.6
MR effect size for
'Gut microbiota abundance (family Ruminococcaceae id.2050)' on 'Crohn's disease of large intestine'

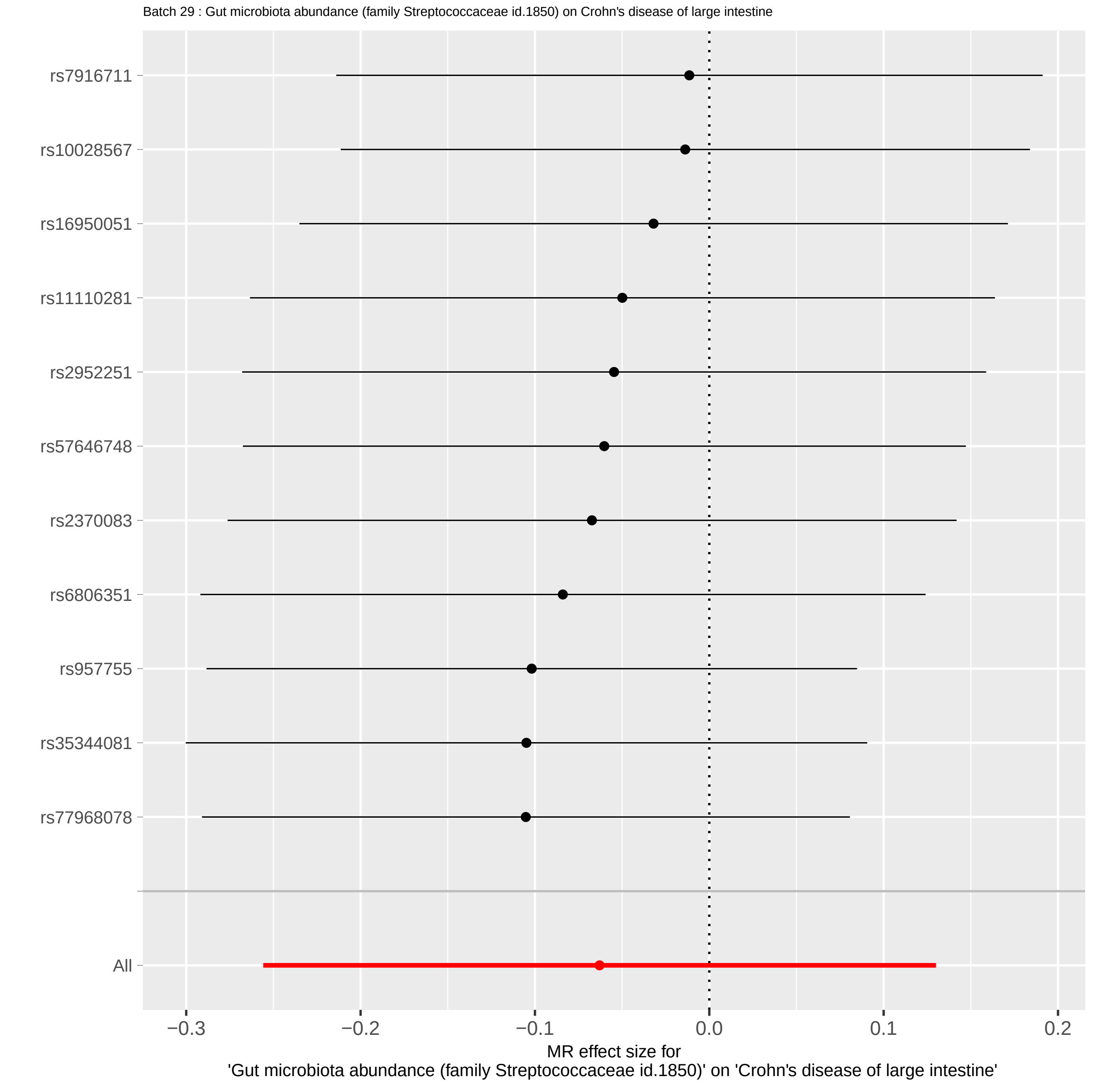

Batch 29 : Gut microbiota abundance (family Streptococcaceae id.1850) on Crohn's disease of large intestine
rs7916711
rs10028567
rs16950051
rs11110281
rs2952251
rs57646748
rs2370083
rs6806351
rs957755
rs35344081
rs77968078
All
−0.3
−0.2
−0.1
0.0
0.1
0.2
MR effect size for
'Gut microbiota abundance (family Streptococcaceae id.1850)' on 'Crohn's disease of large intestine'

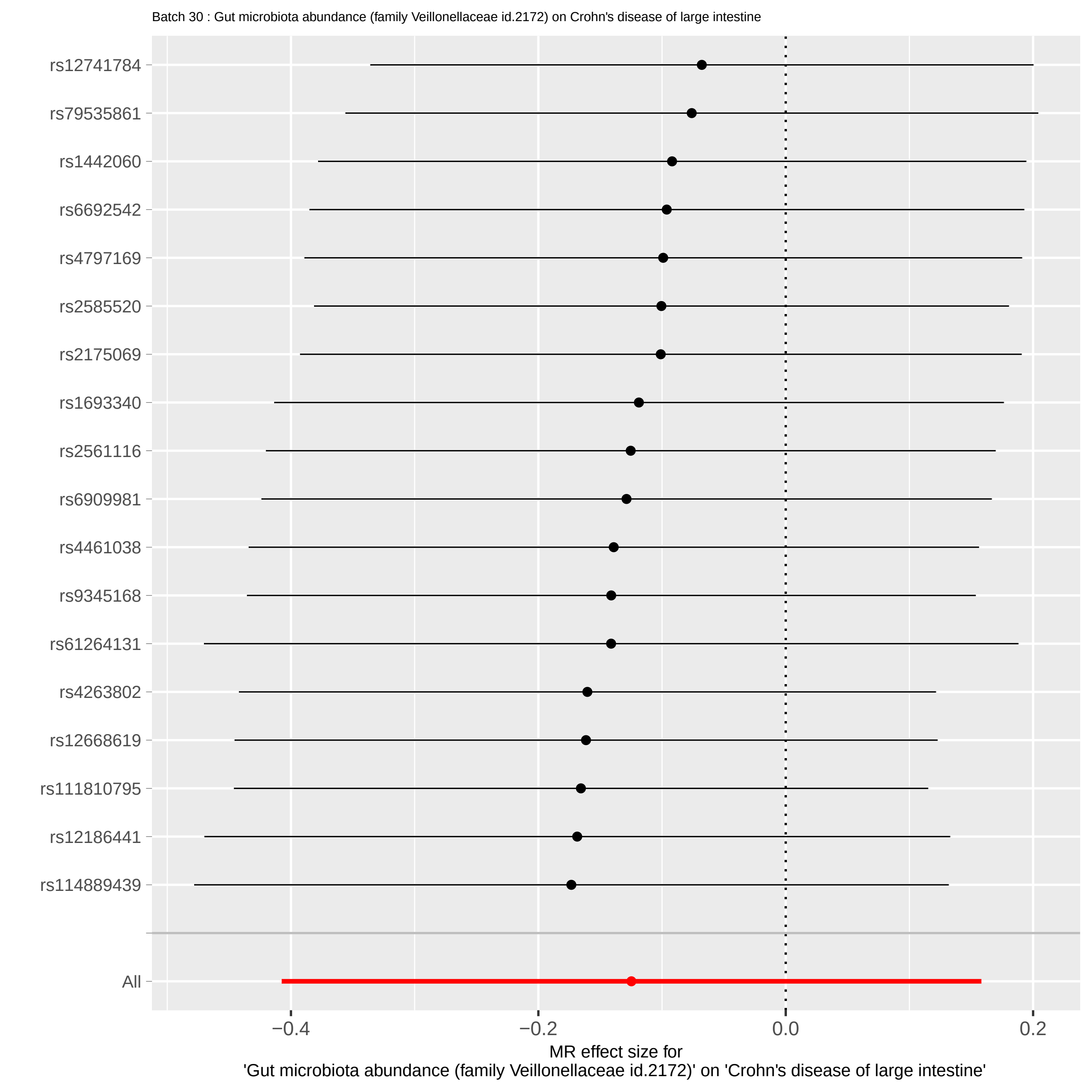

Batch 30 : Gut microbiota abundance (family Veillonellaceae id.2172) on Crohn's disease of large intestine
rs12741784
rs79535861
rs1442060
rs6692542
rs4797169
rs2585520
rs2175069
rs1693340
rs2561116
rs6909981
rs4461038
rs9345168
rs61264131
rs4263802
rs12668619
rs111810795
rs12186441
rs114889439
All
−0.4
−0.2
0.0
0.2
MR effect size for
'Gut microbiota abundance (family Veillonellaceae id.2172)' on 'Crohn's disease of large intestine'

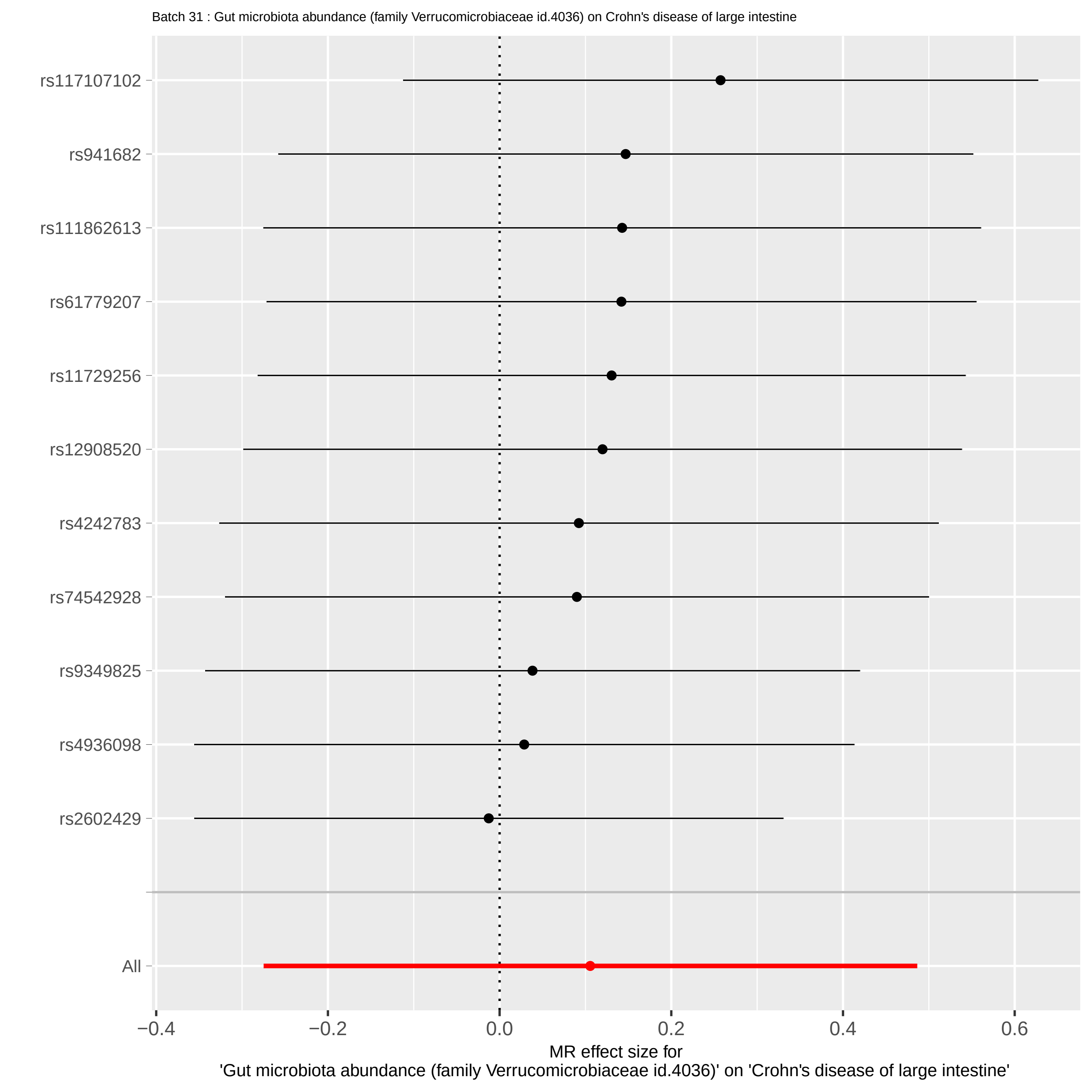
Batch 31 : Gut microbiota abundance (family Verrucomicrobiaceae id.4036) on Crohn's disease of large intestine
rs117107102
rs941682
rs111862613
rs61779207
rs11729256
rs12908520
rs4242783
rs74542928
rs9349825
rs4936098
rs2602429
All
−0.4
−0.2
0.0
0.2
0.4
0.6
MR effect size for
'Gut microbiota abundance (family Verrucomicrobiaceae id.4036)' on 'Crohn's disease of large intestine'

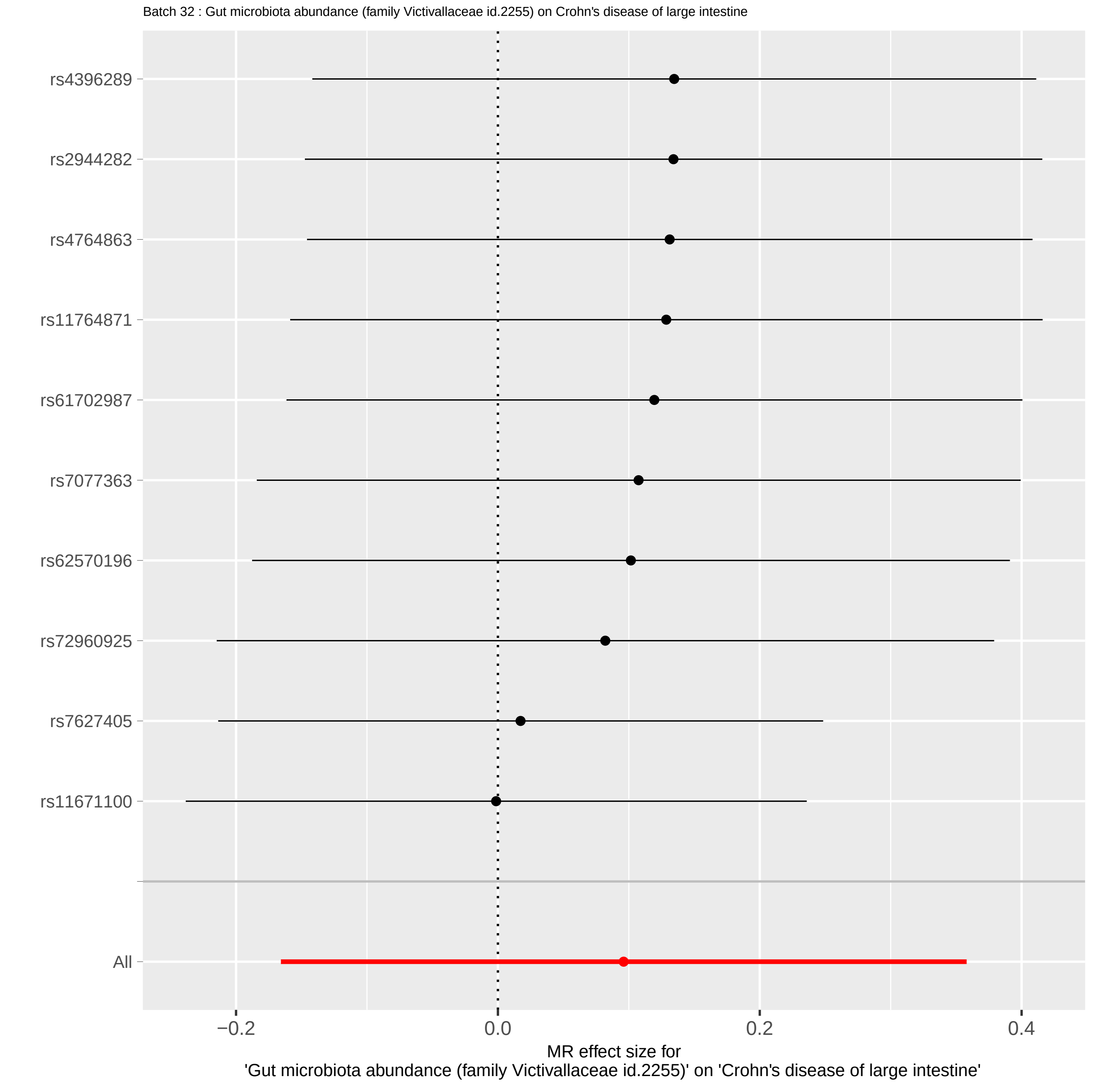

Batch 32 : Gut microbiota abundance (family Victivallaceae id.2255) on Crohn's disease of large intestine
rs4396289
rs2944282
rs4764863
rs11764871
rs61702987
rs7077363
rs62570196
rs72960925
rs7627405
rs11671100
All
−0.2
0.0
0.2
0.4
MR effect size for
'Gut microbiota abundance (family Victivallaceae id.2255)' on 'Crohn's disease of large intestine'

Batch 33 : Gut microbiota abundance (genus Actinomyces id.423) on Crohn's disease of large intestine

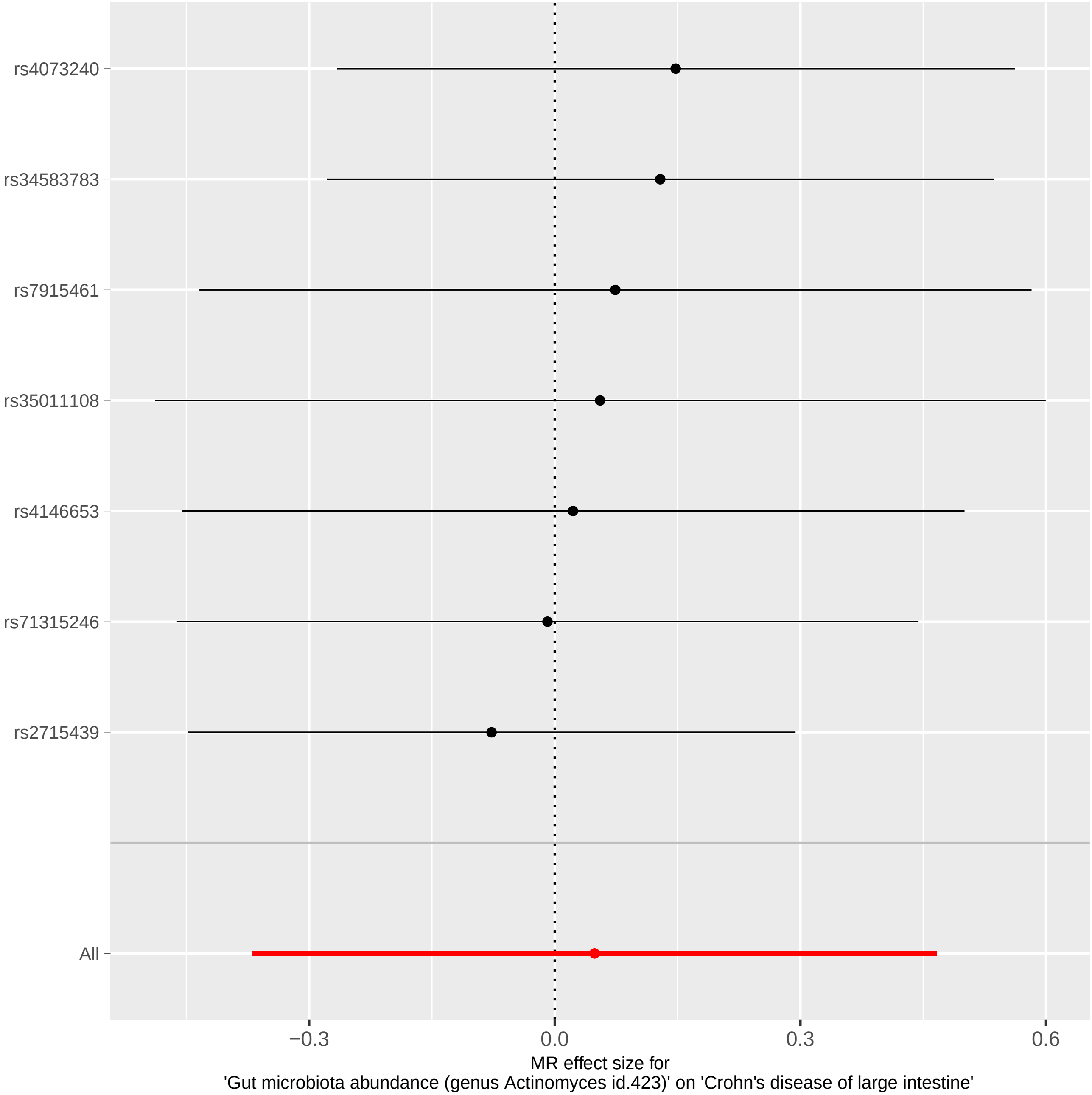

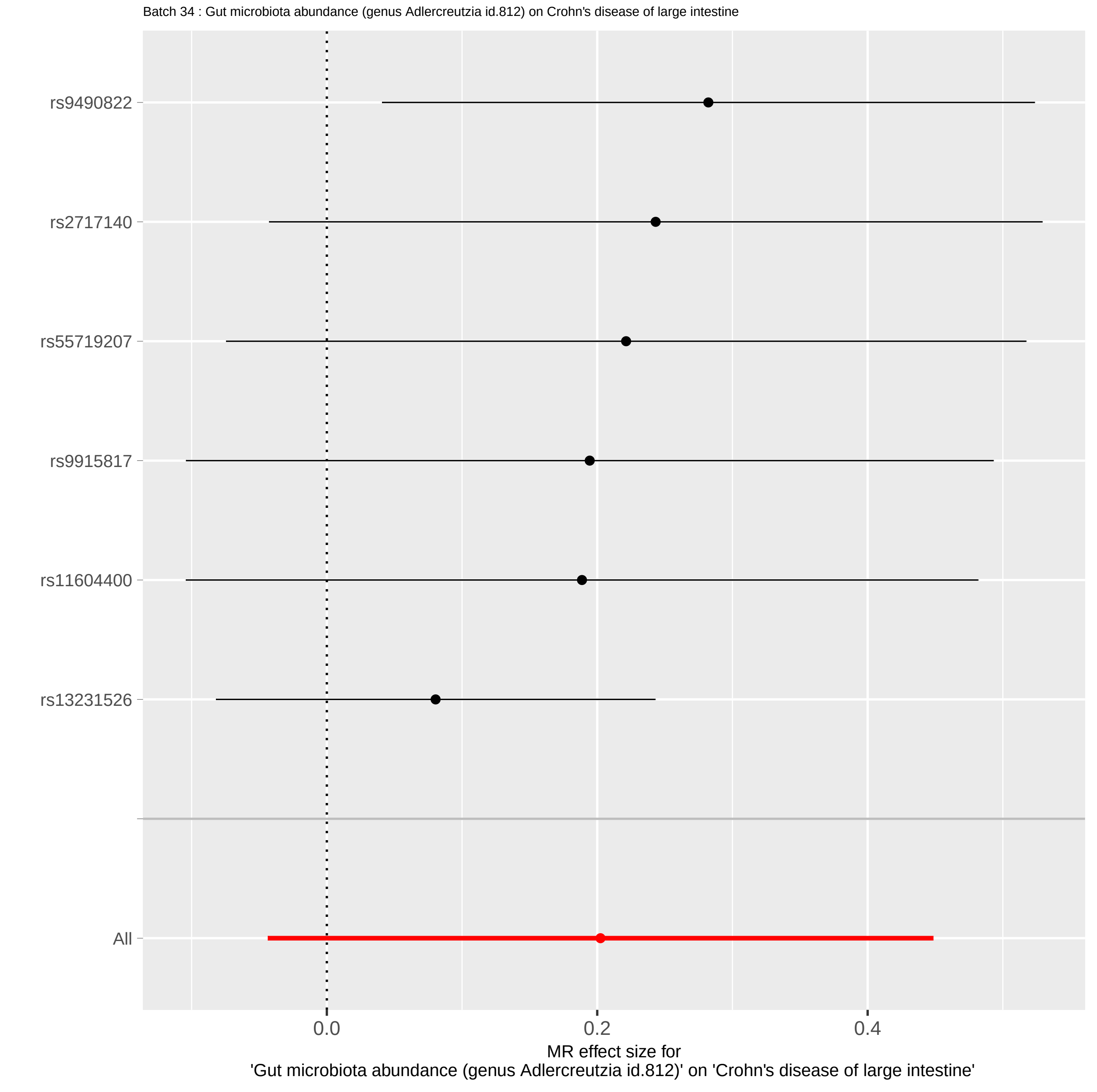
Batch 34 : Gut microbiota abundance (genus Adlercreutzia id.812) on Crohn's disease of large intestine
rs9490822
rs2717140
rs55719207
rs9915817
rs11604400
rs13231526
All
0.0
0.2
0.4
MR effect size for
'Gut microbiota abundance (genus Adlercreutzia id.812)' on 'Crohn's disease of large intestine'

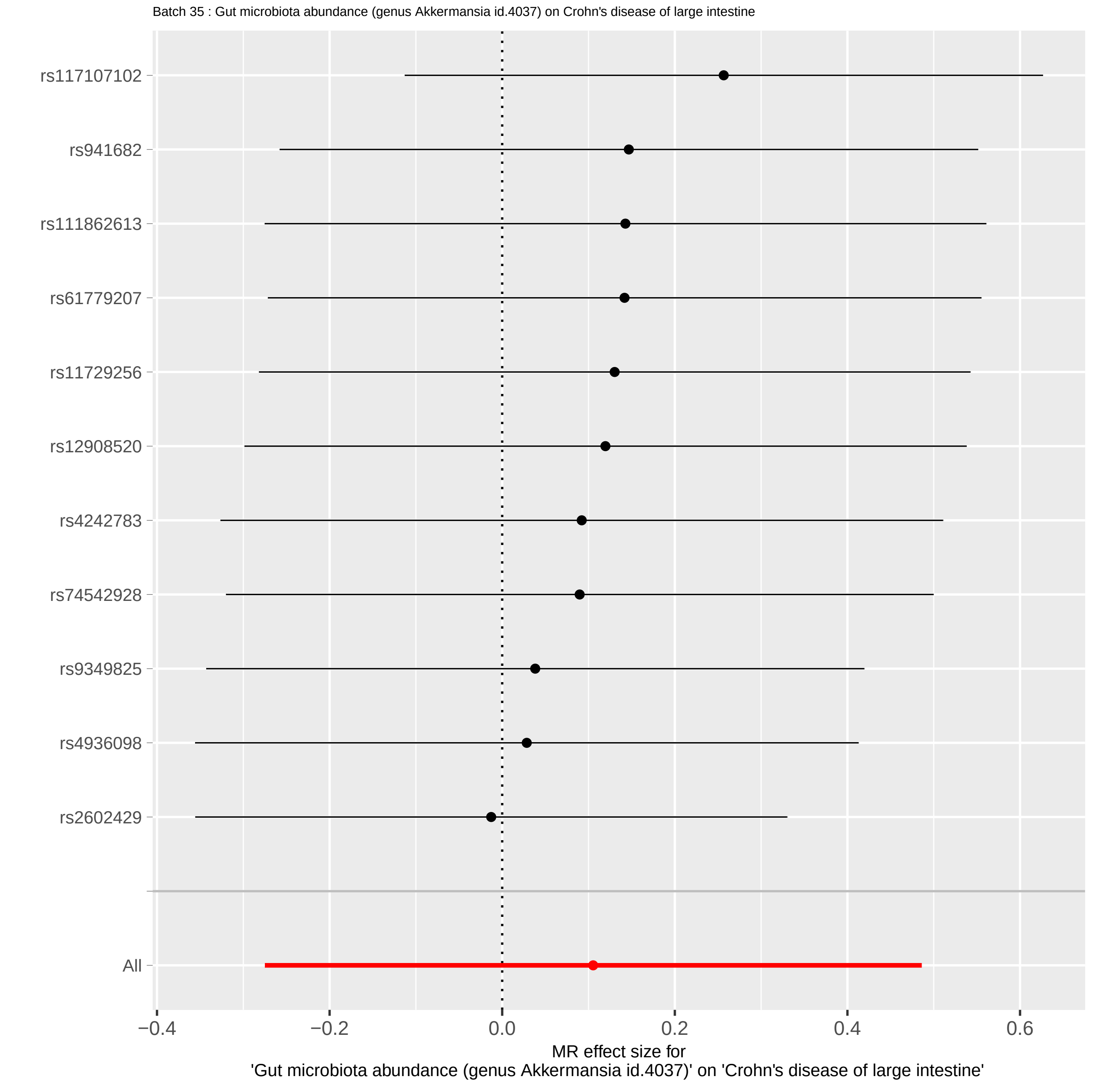

Batch 35 : Gut microbiota abundance (genus Akkermansia id.4037) on Crohn's disease of large intestine
rs117107102
rs941682
rs111862613
rs61779207
rs11729256
rs12908520
rs4242783
rs74542928
rs9349825
rs4936098
rs2602429
All
−0.4
−0.2
0.0
0.2
0.4
0.6
MR effect size for
'Gut microbiota abundance (genus Akkermansia id.4037)' on 'Crohn's disease of large intestine'

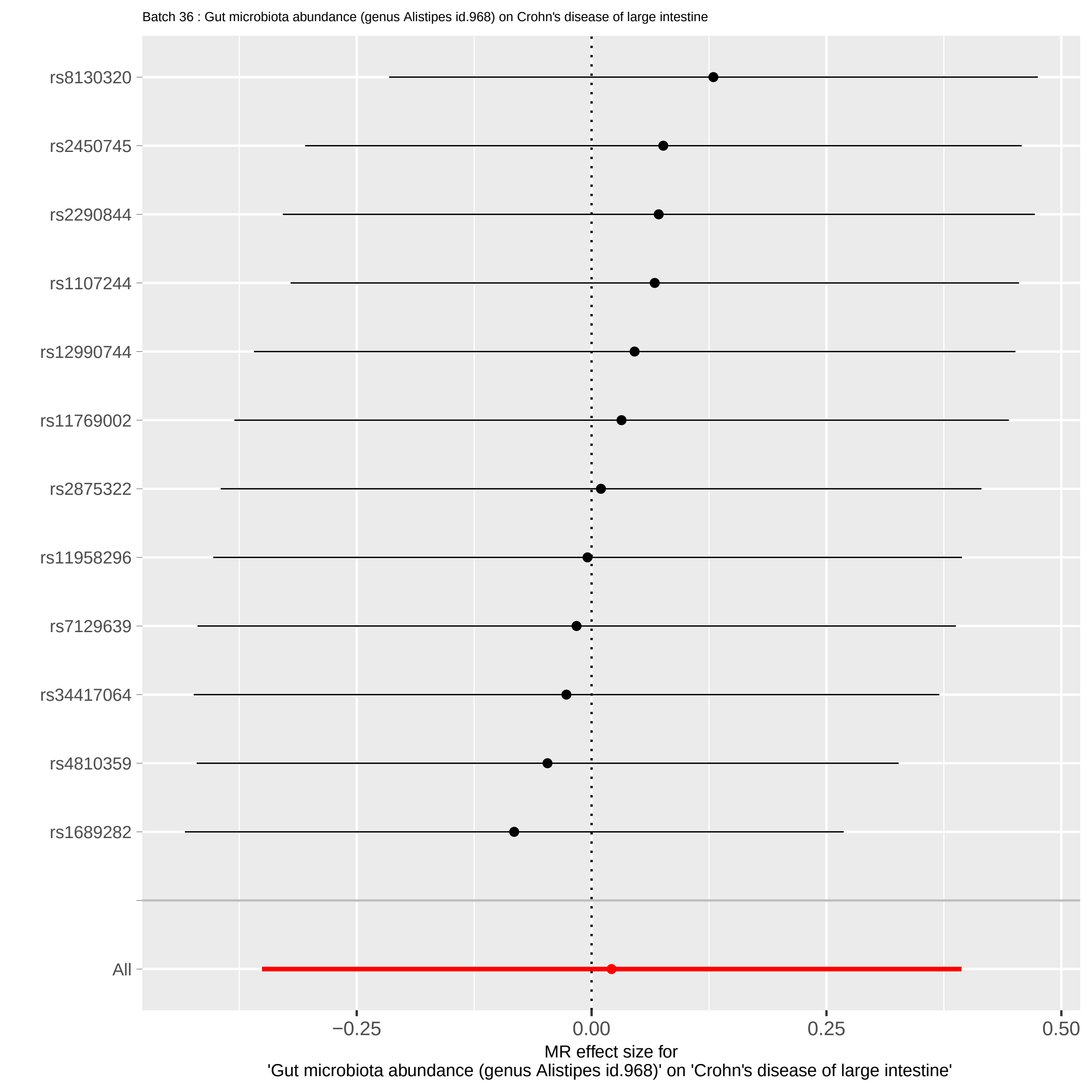
Batch 36 : Gut microbiota abundance (genus Alistipes id.968) on Crohn's disease of large intestine
rs8130320
rs2450745
rs2290844
rs1107244
rs12990744
rs11769002
rs2875322
rs11958296
rs7129639
rs34417064
rs4810359
rs1689282
All
−0.25
0.00
0.25
0.50
MR effect size for
'Gut microbiota abundance (genus Alistipes id.968)' on 'Crohn's disease of large intestine'

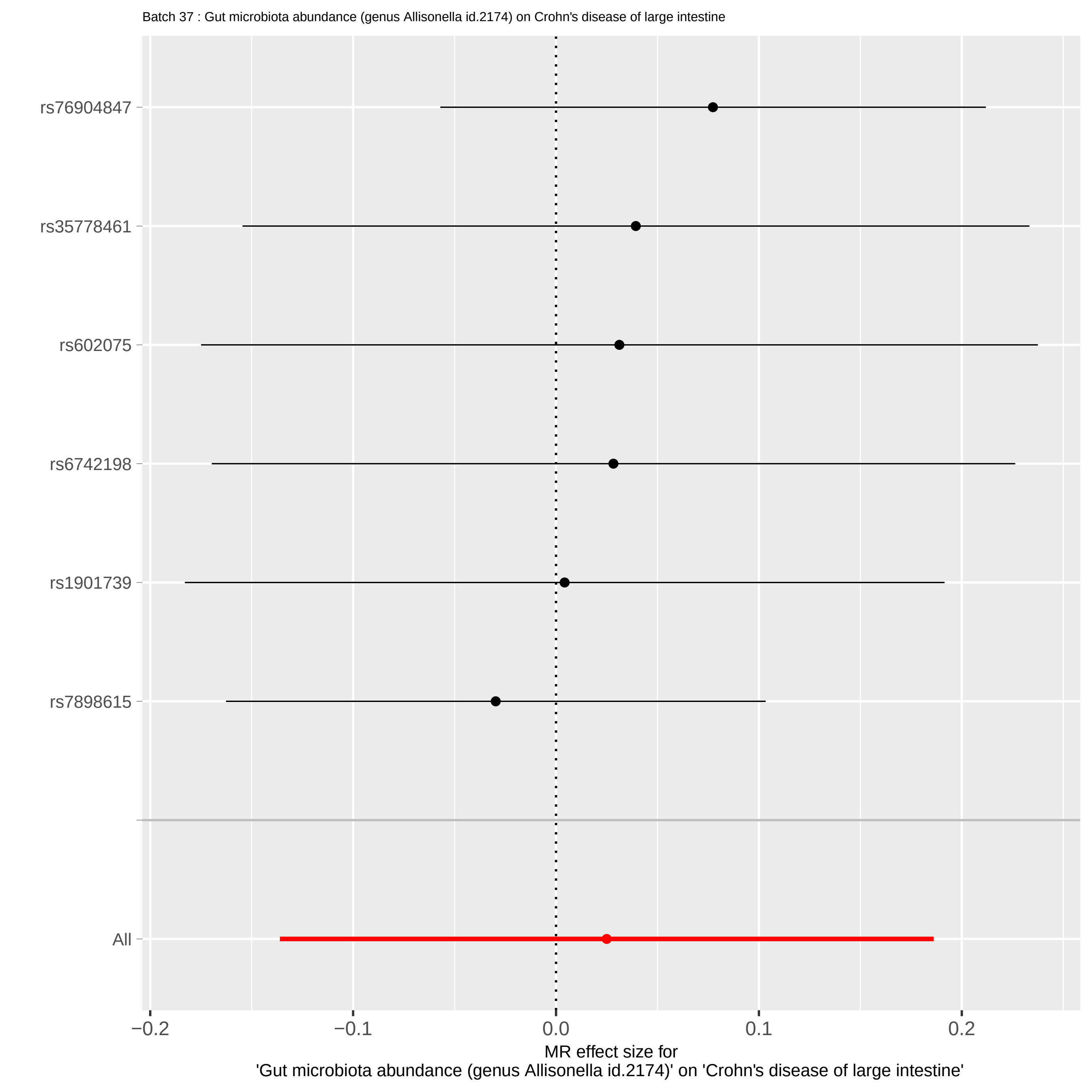
Batch 37 : Gut microbiota abundance (genus Allisonella id.2174) on Crohn's disease of large intestine
rs76904847
rs35778461
rs602075
rs6742198
rs1901739
rs7898615
All
−0.2
−0.1
0.0
0.1
0.2
MR effect size for
'Gut microbiota abundance (genus Allisonella id.2174)' on 'Crohn's disease of large intestine'

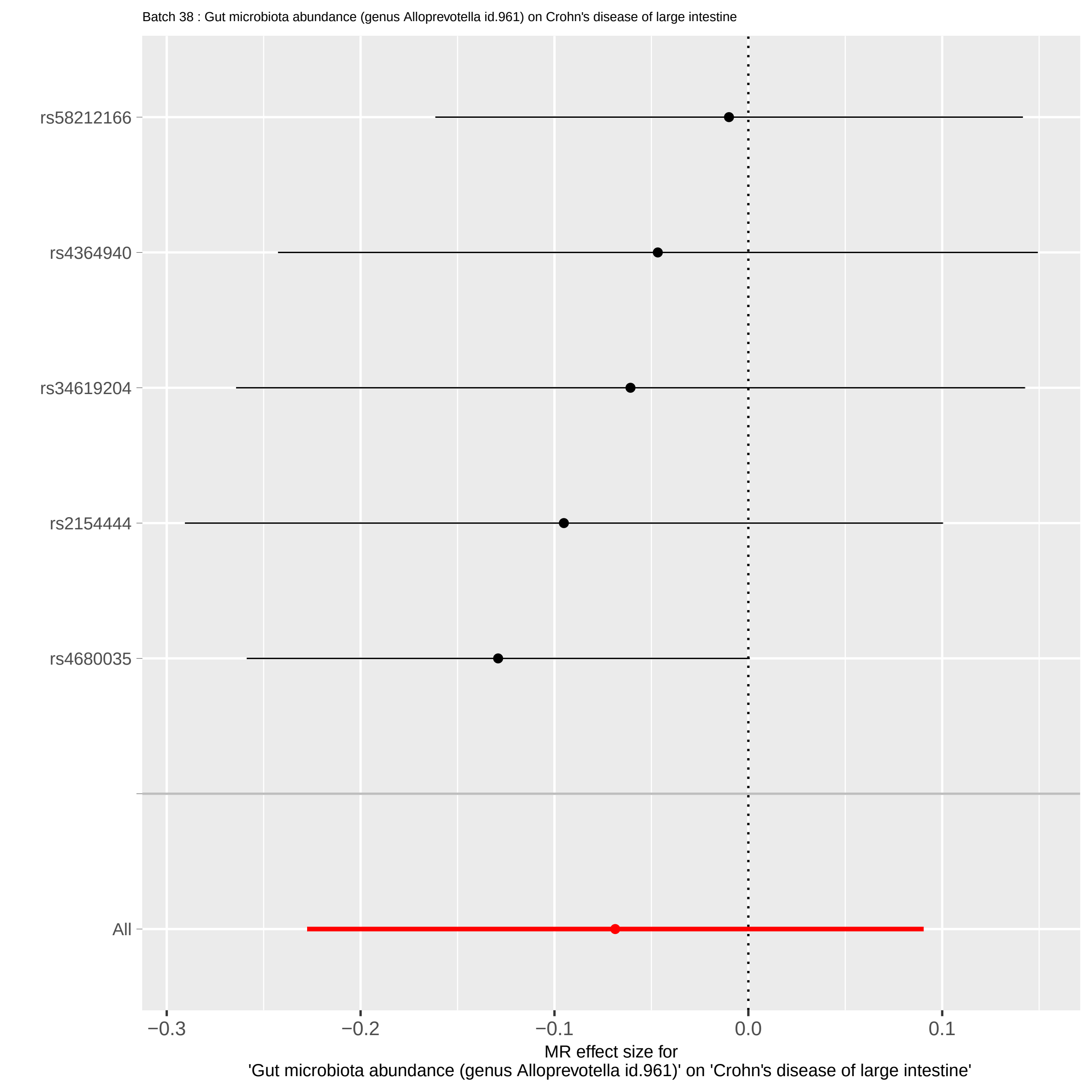
Batch 38 : Gut microbiota abundance (genus Alloprevotella id.961) on Crohn's disease of large intestine
rs58212166
rs4364940
rs34619204
rs2154444
rs4680035
All
−0.3
−0.2
−0.1
0.0
0.1
MR effect size for
'Gut microbiota abundance (genus Alloprevotella id.961)' on 'Crohn's disease of large intestine'

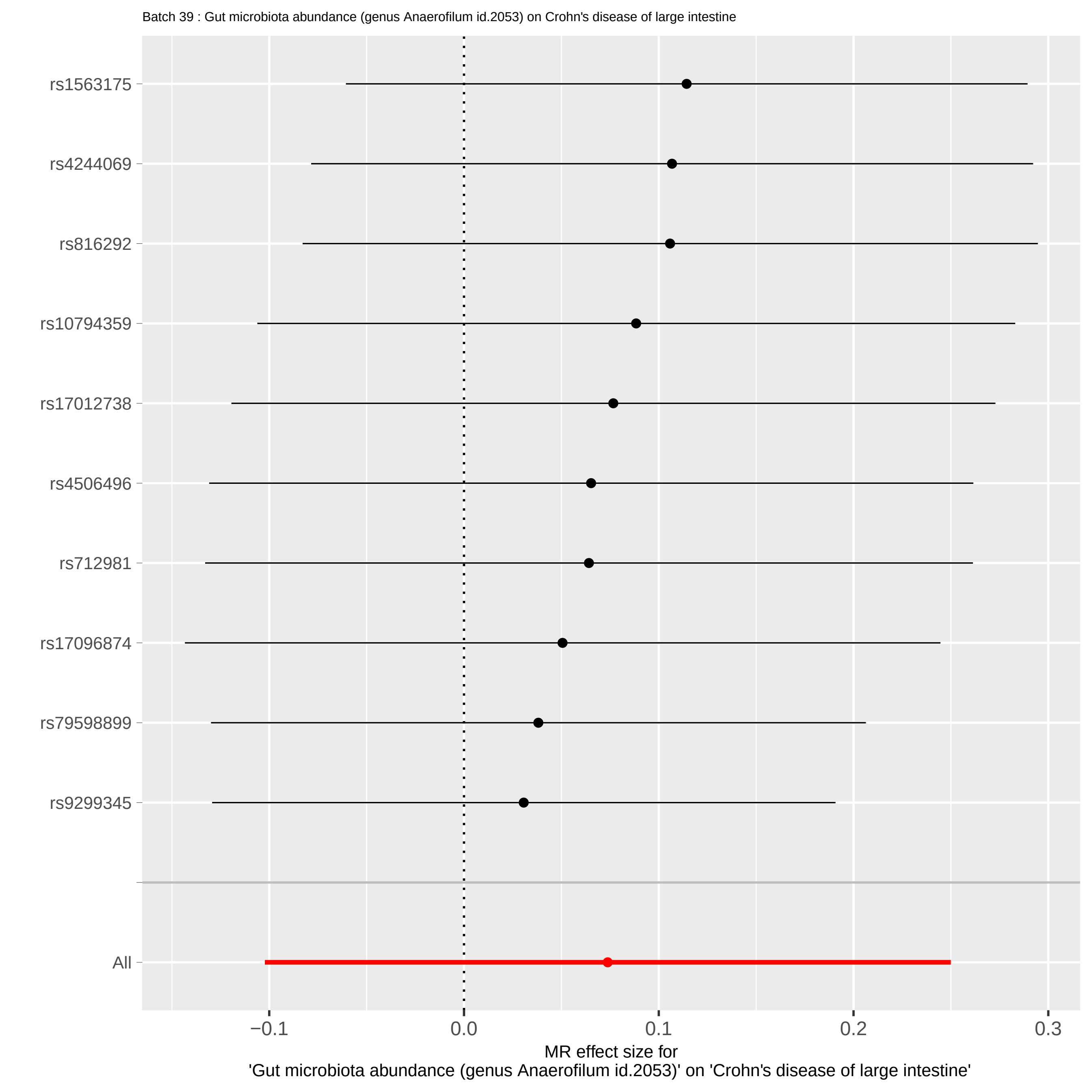
Batch 39 : Gut microbiota abundance (genus Anaerofilum id.2053) on Crohn's disease of large intestine
rs1563175
rs4244069
rs816292
rs10794359
rs17012738
rs4506496
rs712981
rs17096874
rs79598899
rs9299345
All
−0.1
0.0
0.1
0.2
0.3
MR effect size for
'Gut microbiota abundance (genus Anaerofilum id.2053)' on 'Crohn's disease of large intestine'

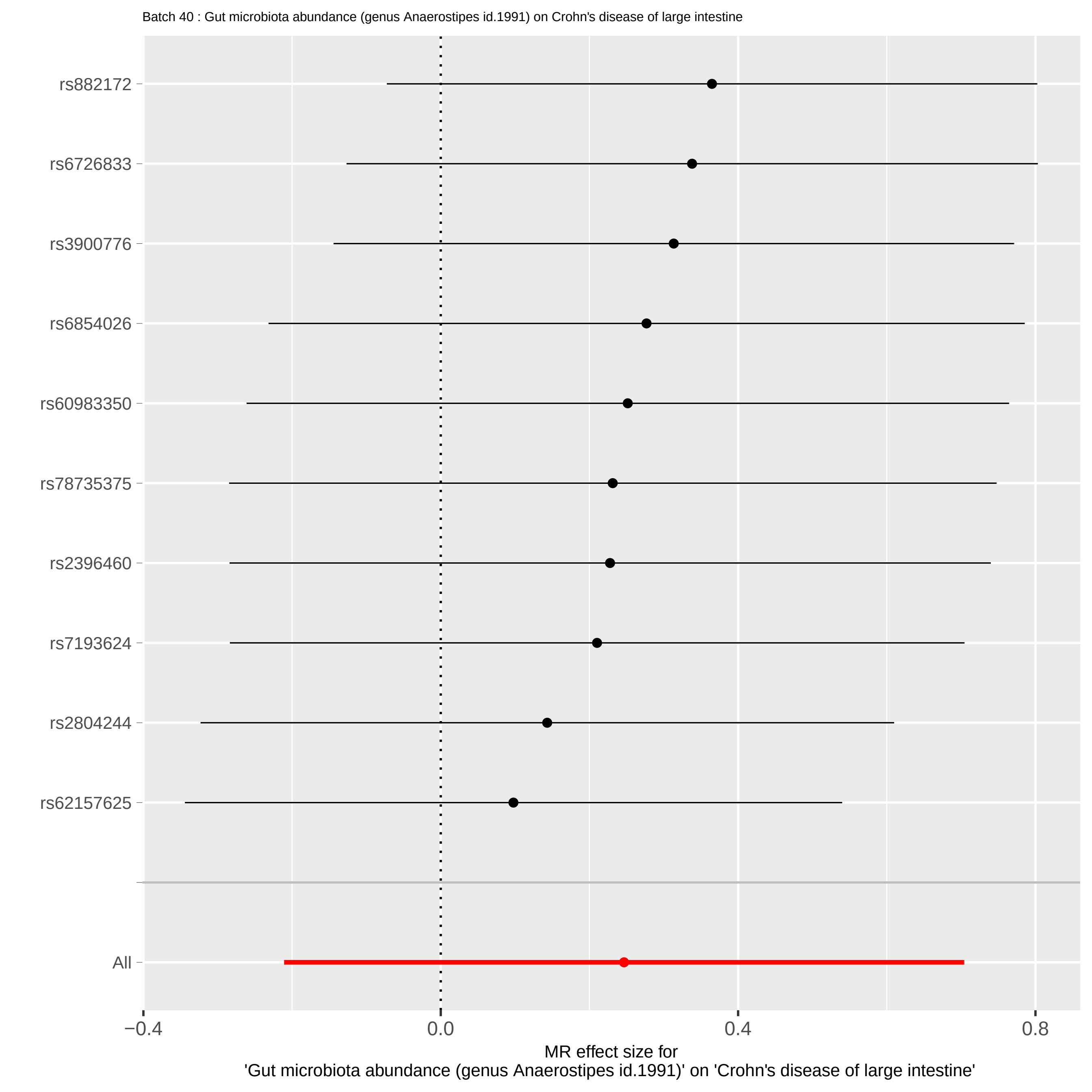
Batch 40 : Gut microbiota abundance (genus Anaerostipes id.1991) on Crohn's disease of large intestine
rs882172
rs6726833
rs3900776
rs6854026
rs60983350
rs78735375
rs2396460
rs7193624
rs2804244
rs62157625
All
−0.4
0.0
0.4
0.8
MR effect size for
'Gut microbiota abundance (genus Anaerostipes id.1991)' on 'Crohn's disease of large intestine'

Batch 41 : Gut microbiota abundance (genus Anaerotruncus id.2054) on Crohn's disease of large intestine

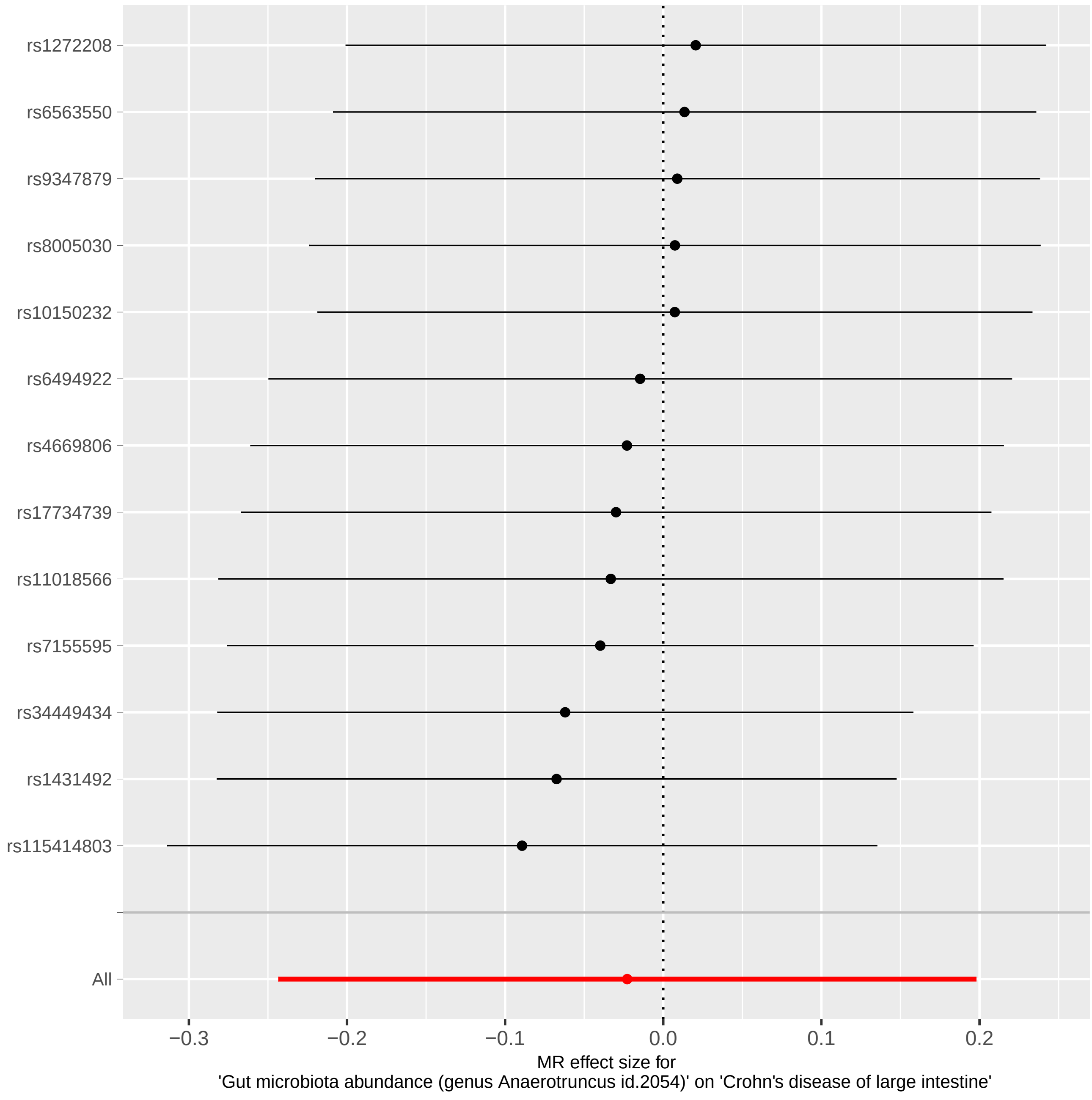

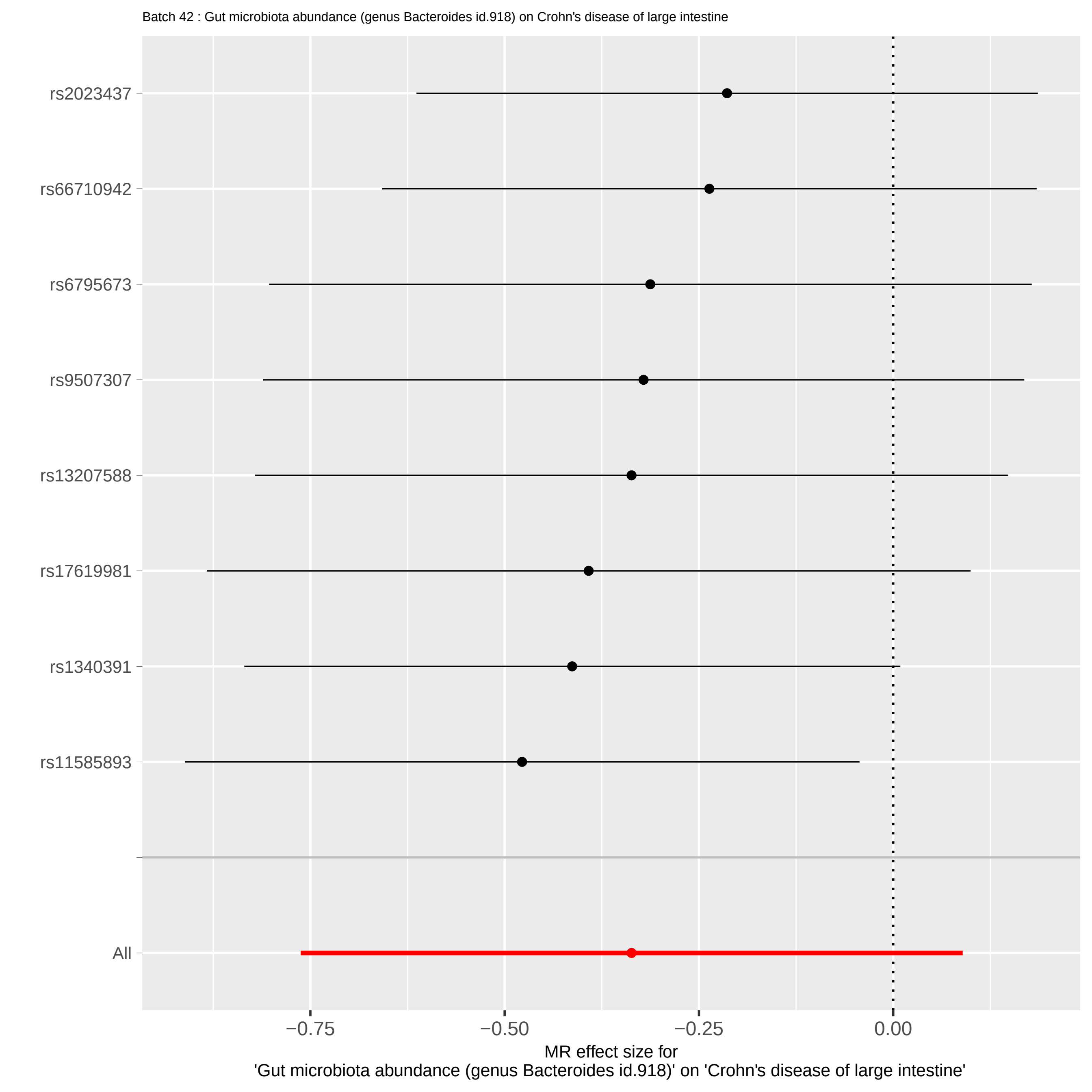

Batch 42 : Gut microbiota abundance (genus Bacteroides id.918) on Crohn's disease of large intestine
rs2023437
rs66710942
rs6795673
rs9507307
rs13207588
rs17619981
rs1340391
rs11585893
All
−0.75
−0.50
−0.25
0.00
MR effect size for
'Gut microbiota abundance (genus Bacteroides id.918)' on 'Crohn's disease of large intestine'

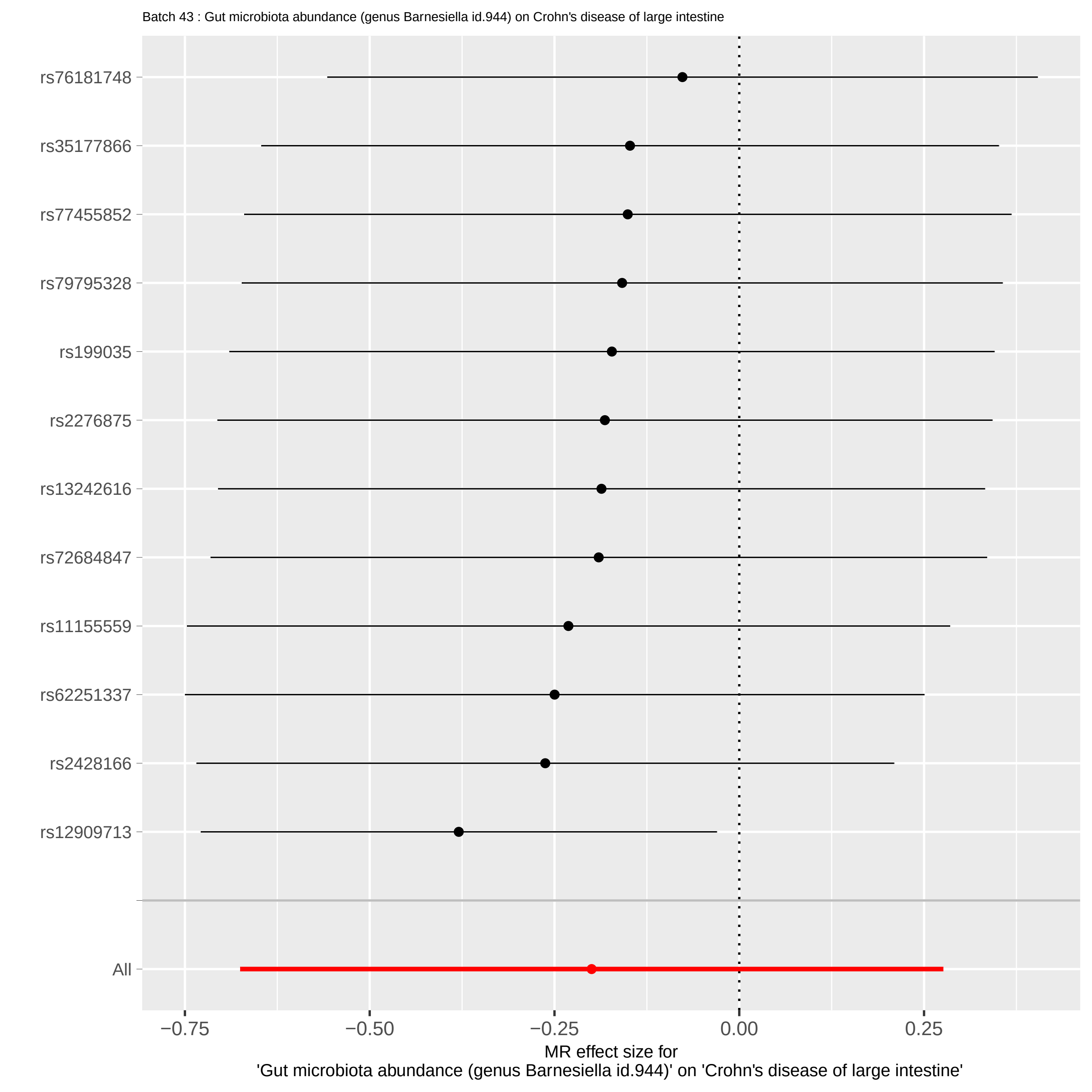
Batch 43 : Gut microbiota abundance (genus Barnesiella id.944) on Crohn's disease of large intestine
rs76181748
rs35177866
rs77455852
rs79795328
rs199035
rs2276875
rs13242616
rs72684847
rs11155559
rs62251337
rs2428166
rs12909713
All
−0.75
−0.50
−0.25
0.00
0.25
MR effect size for
'Gut microbiota abundance (genus Barnesiella id.944)' on 'Crohn's disease of large intestine'

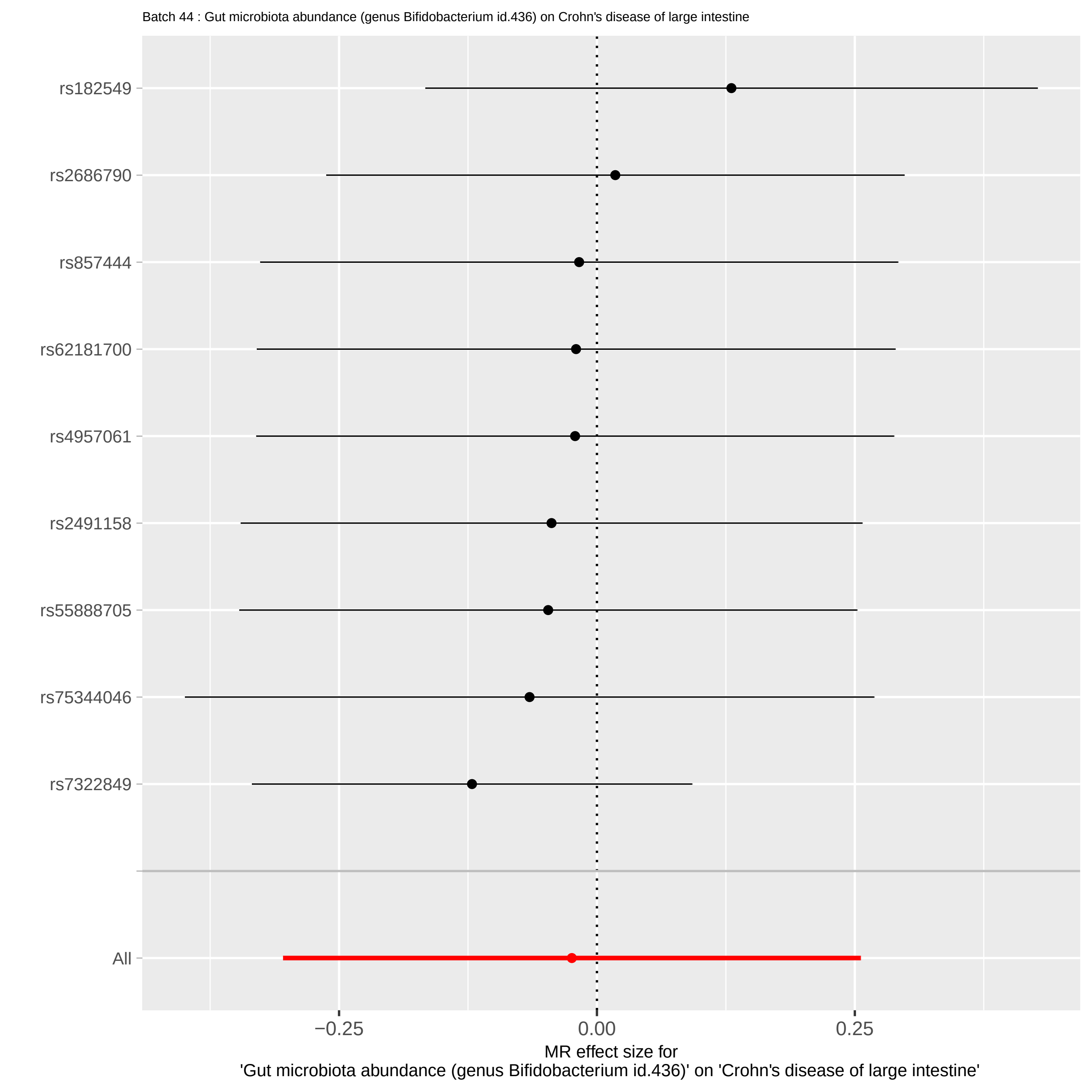
Batch 44 : Gut microbiota abundance (genus Bifidobacterium id.436) on Crohn's disease of large intestine
rs182549
rs2686790
rs857444
rs62181700
rs4957061
rs2491158
rs55888705
rs75344046
rs7322849
All
−0.25
0.00
0.25
MR effect size for
'Gut microbiota abundance (genus Bifidobacterium id.436)' on 'Crohn's disease of large intestine'

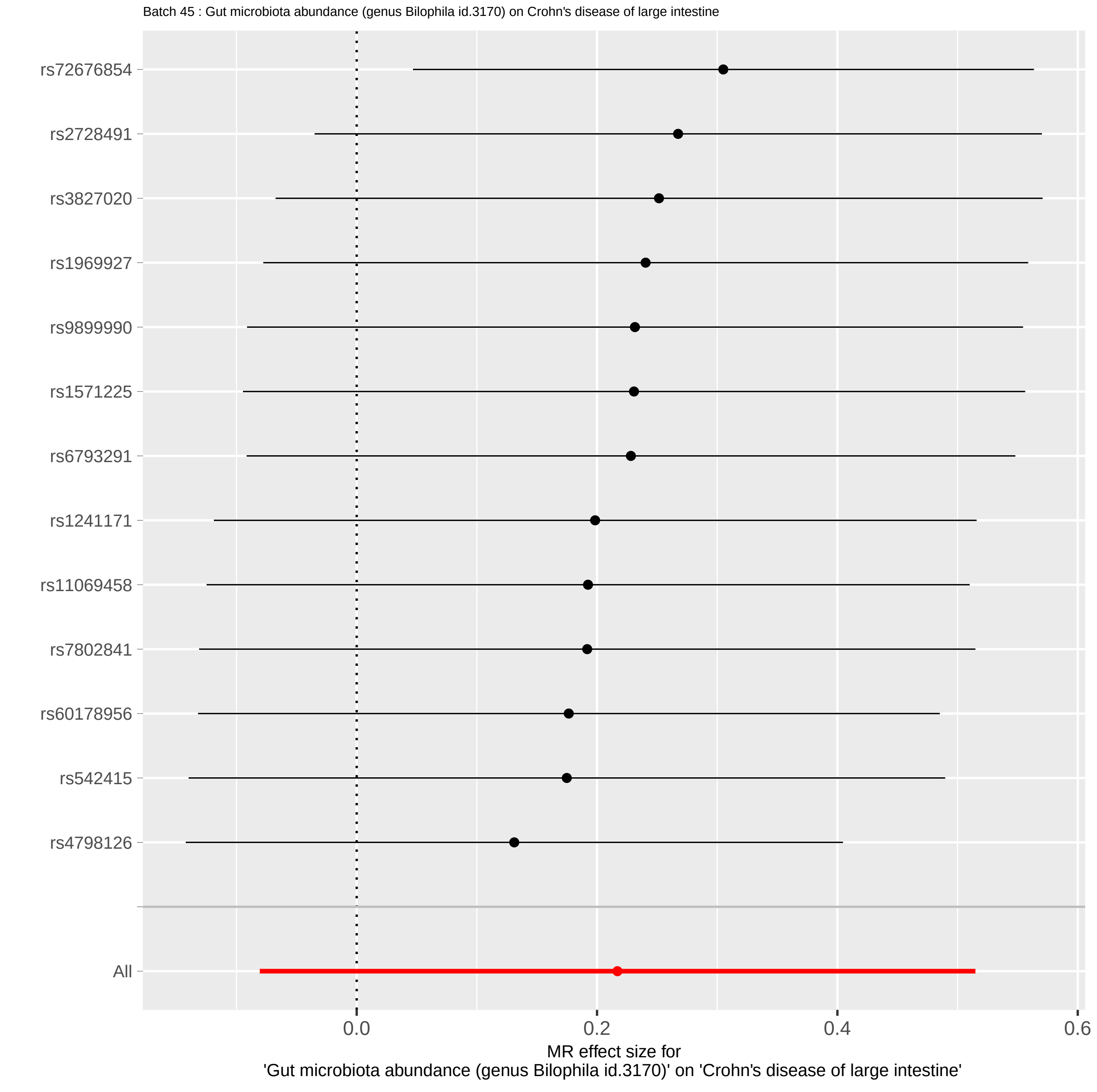
Batch 45 : Gut microbiota abundance (genus Bilophila id.3170) on Crohn's disease of large intestine
rs72676854
rs2728491
rs3827020
rs1969927
rs9899990
rs1571225
rs6793291
rs1241171
rs11069458
rs7802841
rs60178956
rs542415
rs4798126
All
0.0
0.2
0.4
0.6
MR effect size for
'Gut microbiota abundance (genus Bilophila id.3170)' on 'Crohn's disease of large intestine'

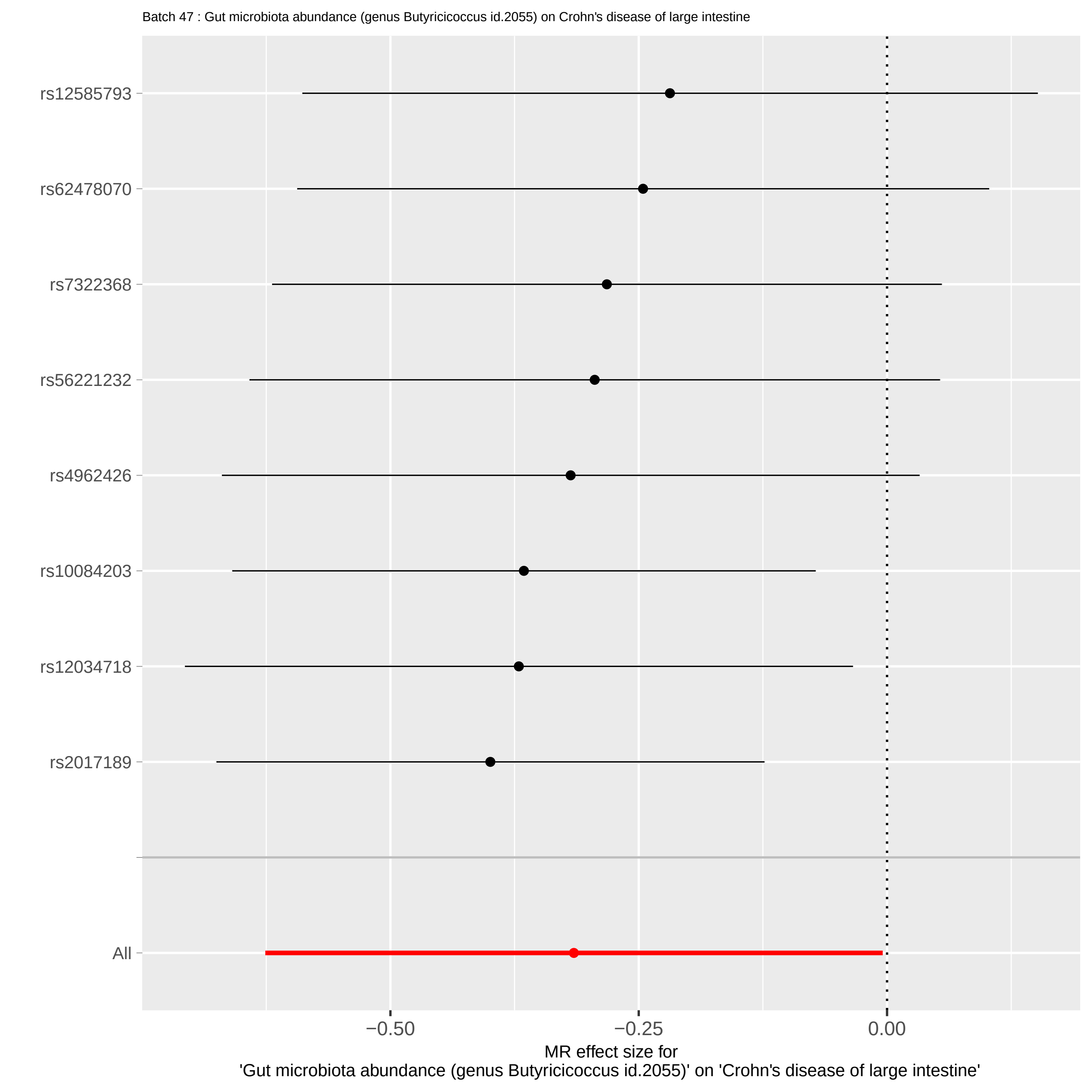

Batch 47 : Gut microbiota abundance (genus Butyricicoccus id.2055) on Crohn's disease of large intestine
rs12585793
rs62478070
rs7322368
rs56221232
rs4962426
rs10084203
rs12034718
rs2017189
All
−0.50
−0.25
0.00
MR effect size for
'Gut microbiota abundance (genus Butyricicoccus id.2055)' on 'Crohn's disease of large intestine'

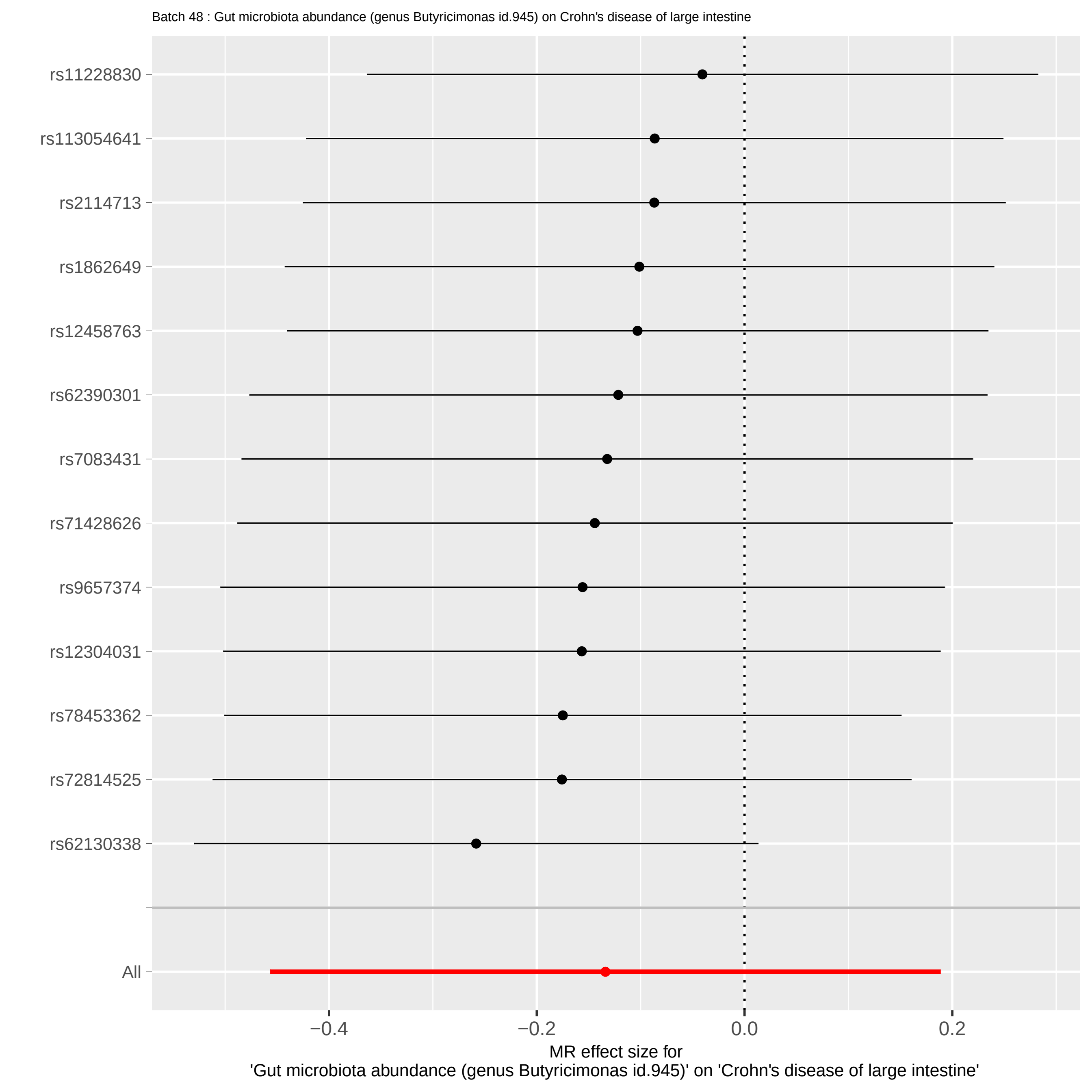
Batch 48 : Gut microbiota abundance (genus Butyricimonas id.945) on Crohn's disease of large intestine
rs11228830
rs113054641
rs2114713
rs1862649
rs12458763
rs62390301
rs7083431
rs71428626
rs9657374
rs12304031
rs78453362
rs72814525
rs62130338
All
−0.4
−0.2
0.0
0.2
MR effect size for
'Gut microbiota abundance (genus Butyricimonas id.945)' on 'Crohn's disease of large intestine'

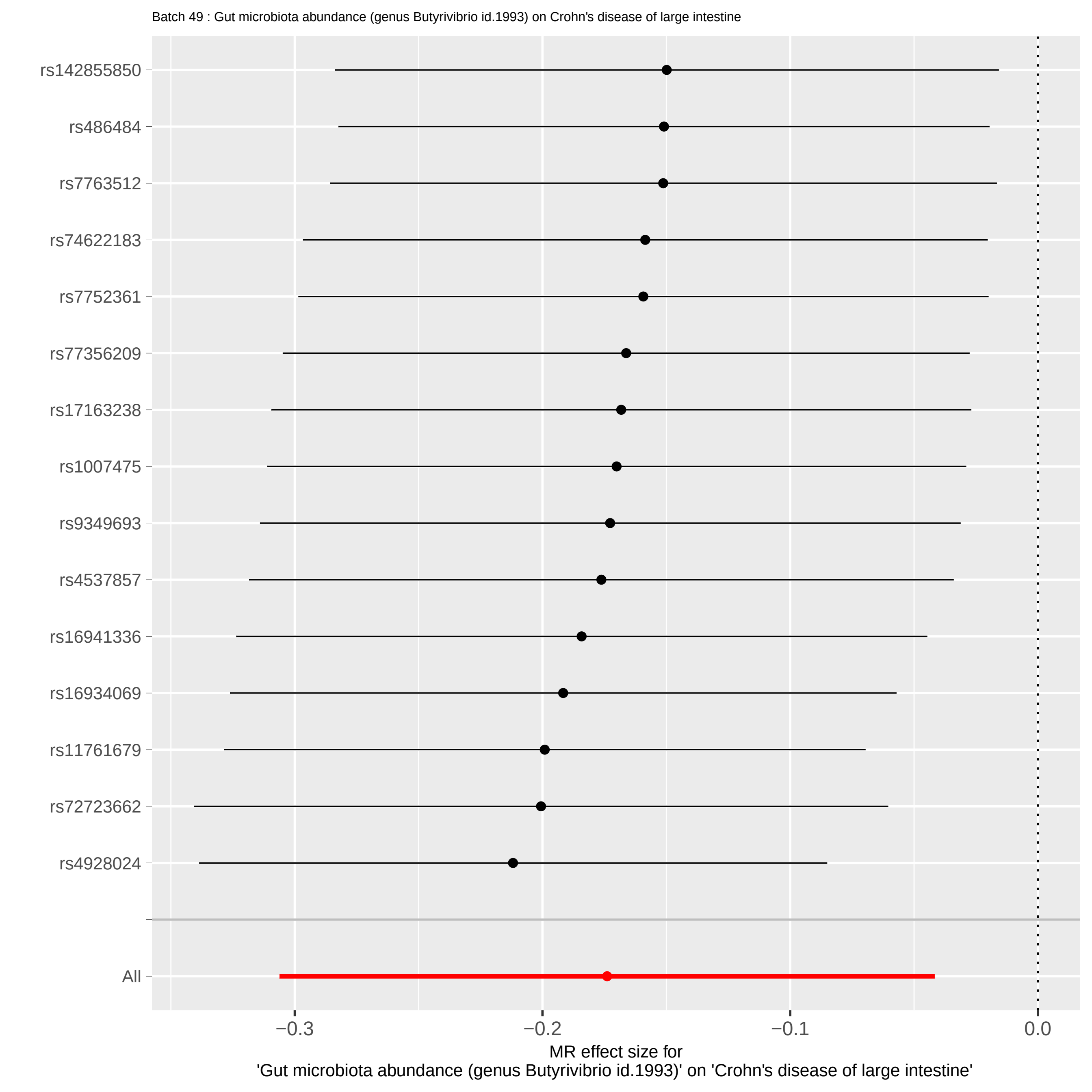

Batch 49 : Gut microbiota abundance (genus Butyrivibrio id.1993) on Crohn's disease of large intestine
rs142855850
rs486484
rs7763512
rs74622183
rs7752361
rs77356209
rs17163238
rs1007475
rs9349693
rs4537857
rs16941336
rs16934069
rs11761679
rs72723662
rs4928024
All
−0.3
−0.2
−0.1
0.0
MR effect size for
'Gut microbiota abundance (genus Butyrivibrio id.1993)' on 'Crohn's disease of large intestine'

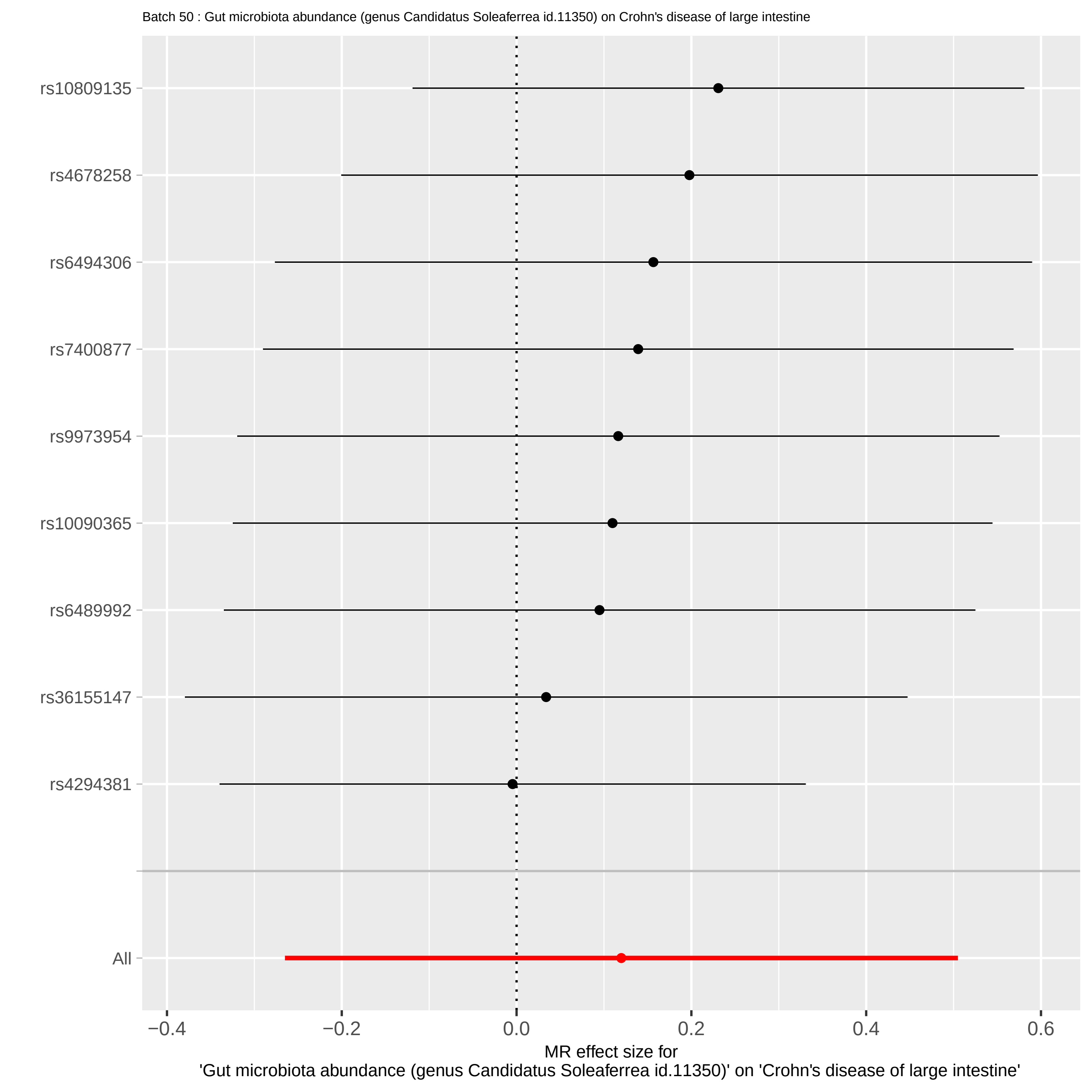
Batch 50 : Gut microbiota abundance (genus Candidatus Soleaferrea id.11350) on Crohn's disease of large intestine
rs10809135
rs4678258
rs6494306
rs7400877
rs9973954
rs10090365
rs6489992
rs36155147
rs4294381
All
−0.4
−0.2
0.0
0.2
0.4
0.6
MR effect size for
'Gut microbiota abundance (genus Candidatus Soleaferrea id.11350)' on 'Crohn's disease of large intestine'

Batch 51 : Gut microbiota abundance (genus Catenibacterium id.2153) on Crohn's disease of large intestine

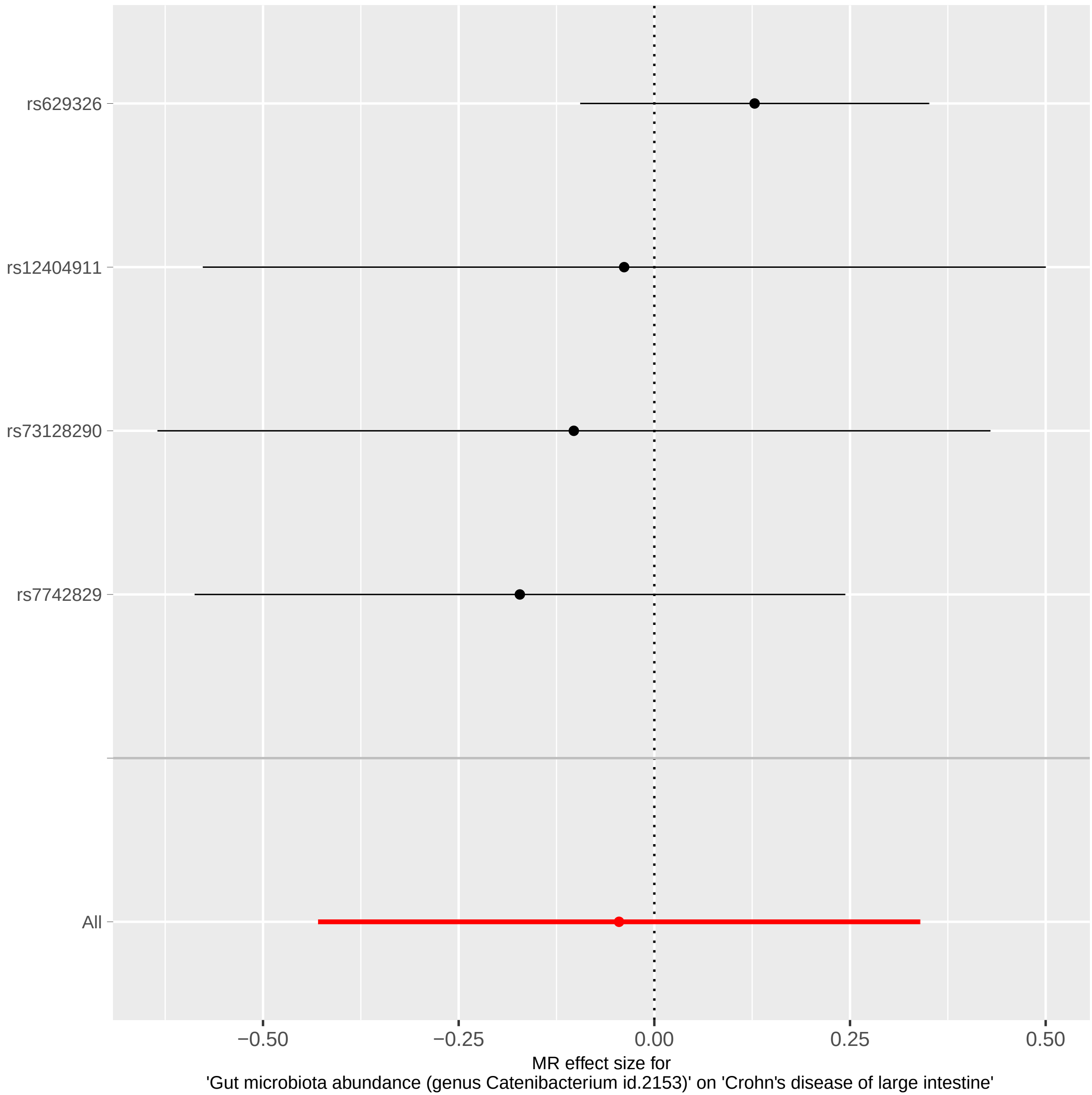

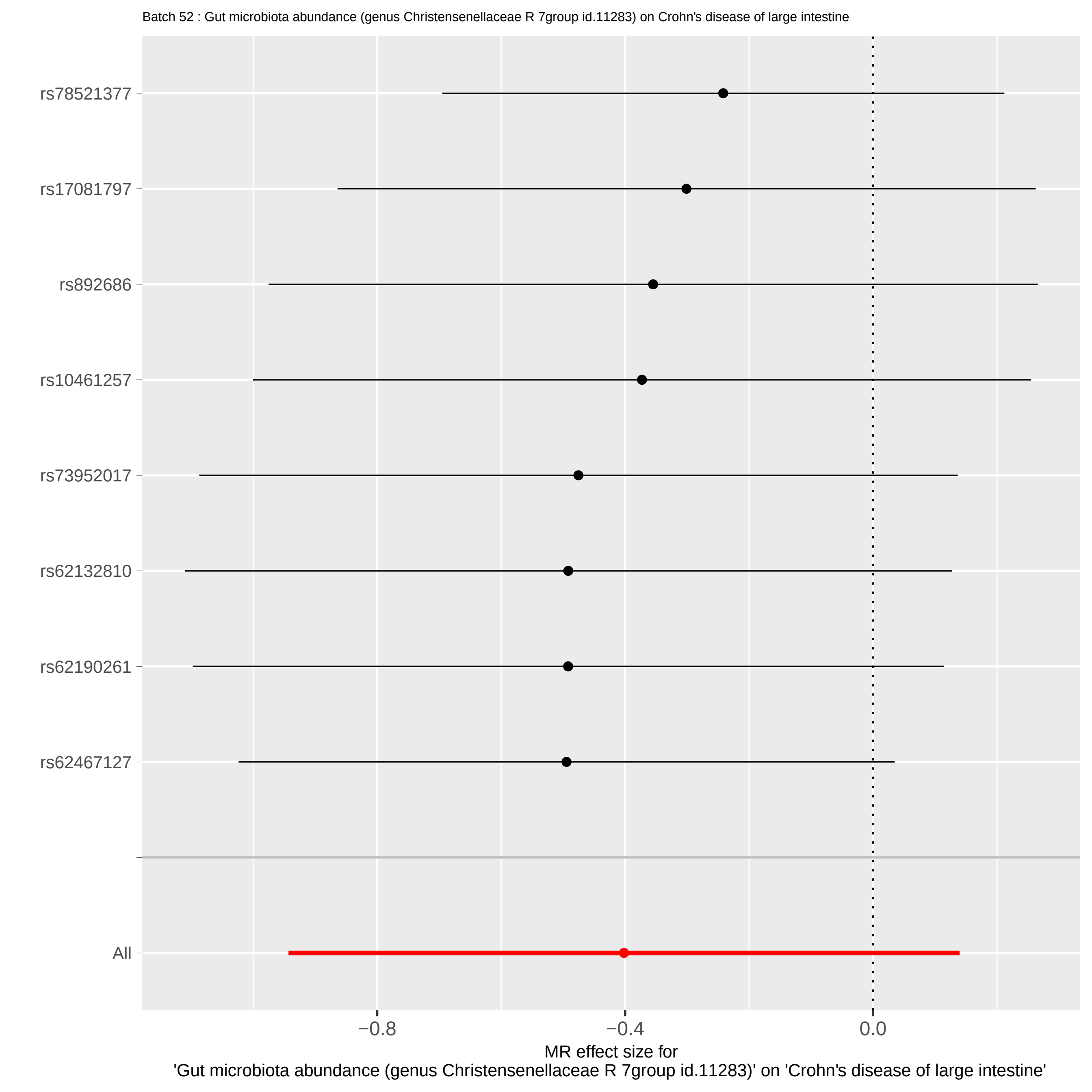
Batch 52 : Gut microbiota abundance (genus Christensenellaceae R 7group id.11283) on Crohn's disease of large intestine
rs78521377
rs17081797
rs892686
rs10461257
rs73952017
rs62132810
rs62190261
rs62467127
All
−0.8
−0.4
0.0
MR effect size for
'Gut microbiota abundance (genus Christensenellaceae R 7group id.11283)' on 'Crohn's disease of large intestine'

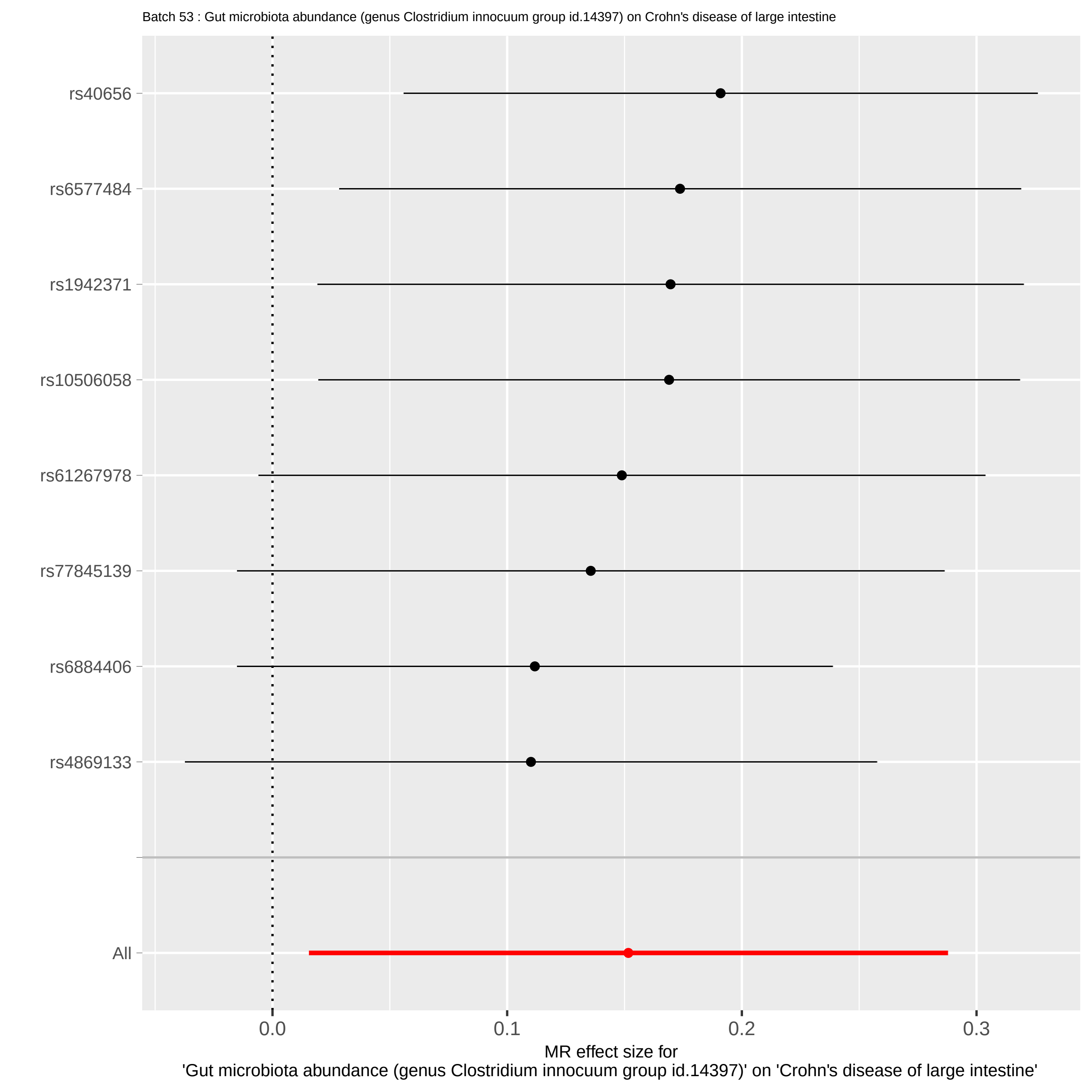
Batch 53 : Gut microbiota abundance (genus Clostridium innocuum group id.14397) on Crohn's disease of large intestine
rs40656
rs6577484
rs1942371
rs10506058
rs61267978
rs77845139
rs6884406
rs4869133
All
0.0
0.1
0.2
0.3
MR effect size for
'Gut microbiota abundance (genus Clostridium innocuum group id.14397)' on 'Crohn's disease of large intestine'

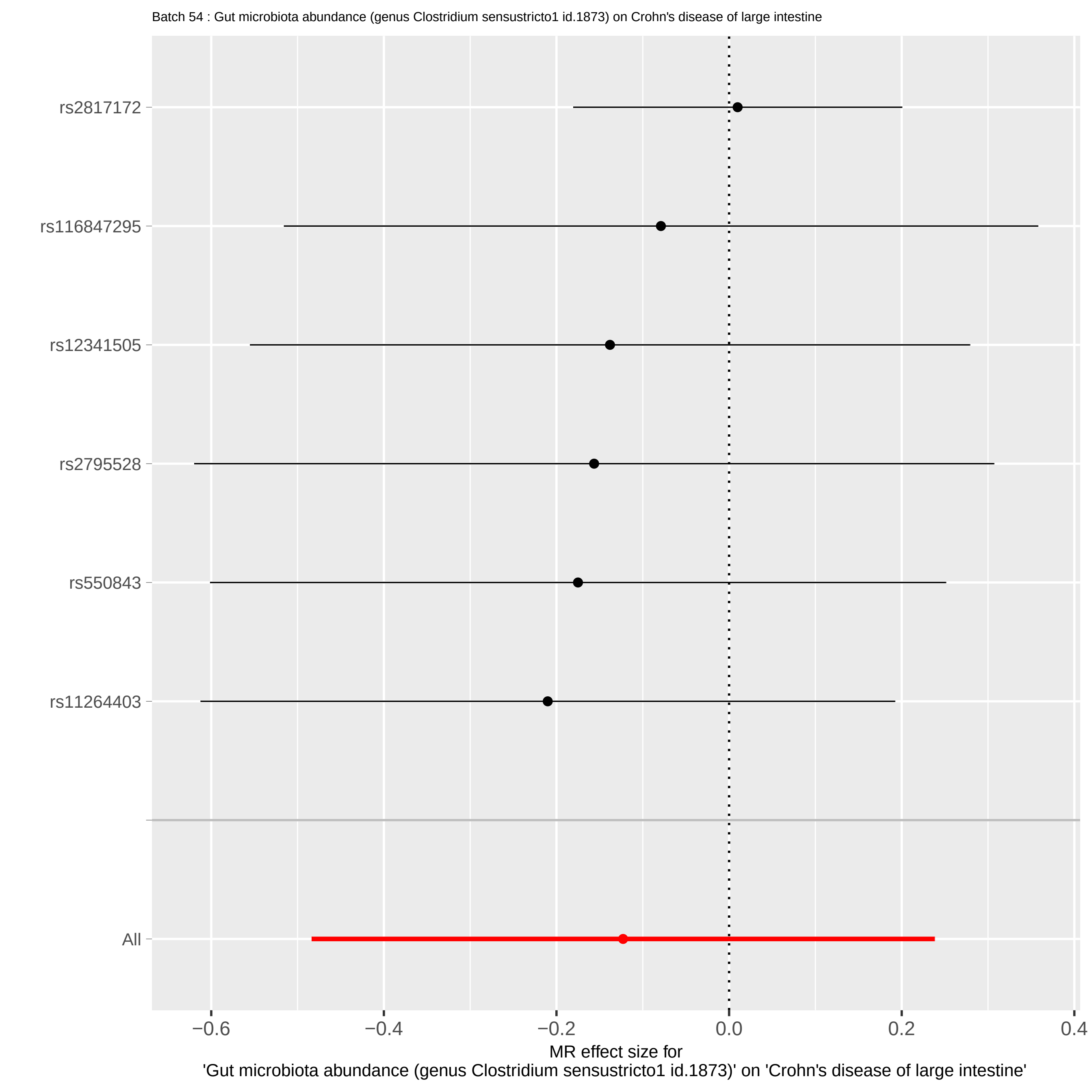
Batch 54 : Gut microbiota abundance (genus Clostridium sensustricto1 id.1873) on Crohn's disease of large intestine
rs2817172
rs116847295
rs12341505
rs2795528
rs550843
rs11264403
All
−0.6
−0.4
−0.2
0.0
0.2
0.4
MR effect size for
'Gut microbiota abundance (genus Clostridium sensustricto1 id.1873)' on 'Crohn's disease of large intestine'

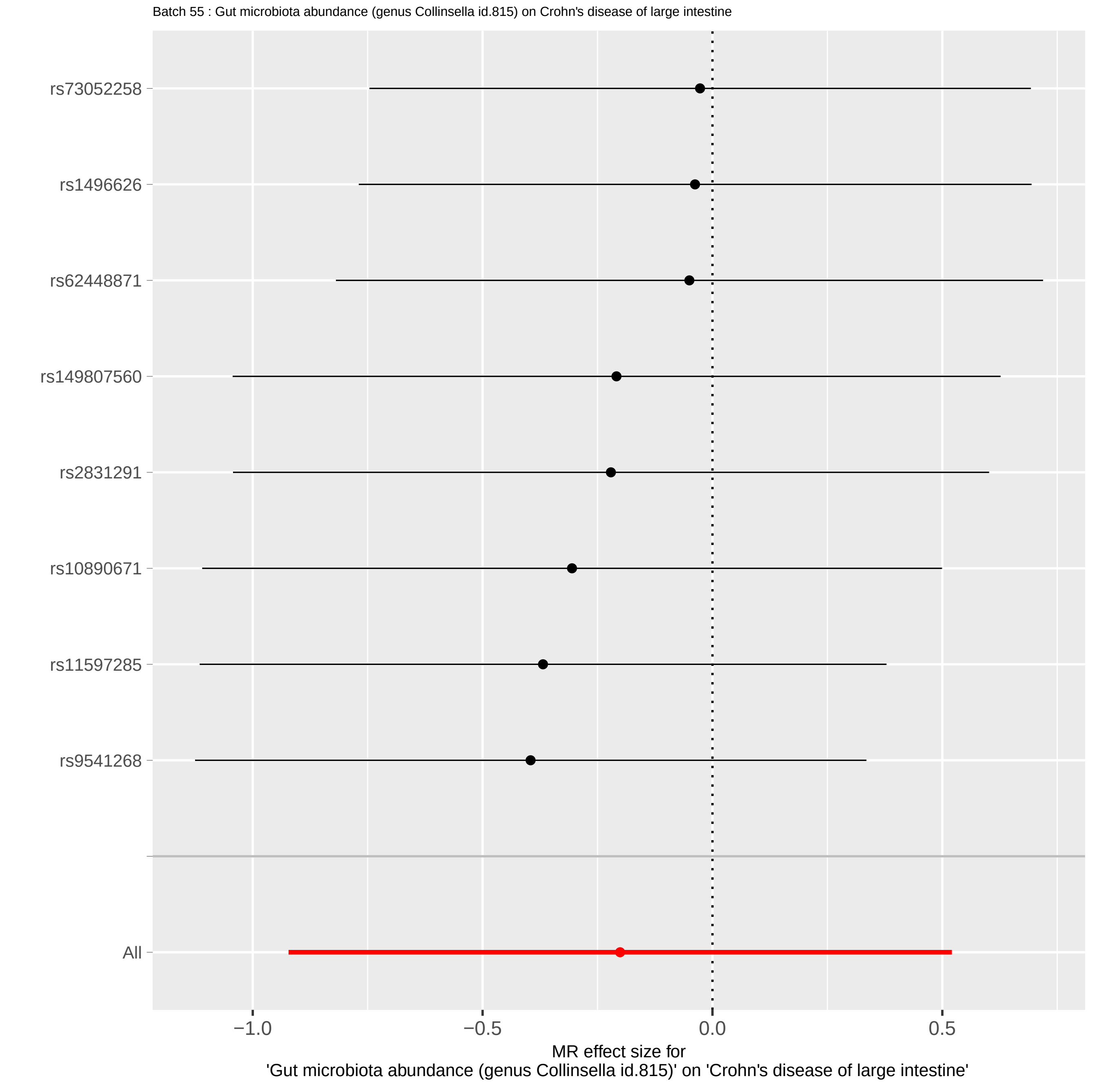
Batch 55 : Gut microbiota abundance (genus Collinsella id.815) on Crohn's disease of large intestine
rs73052258
rs1496626
rs62448871
rs149807560
rs2831291
rs10890671
rs11597285
rs9541268
All
−1.0
−0.5
0.0
0.5
MR effect size for
'Gut microbiota abundance (genus Collinsella id.815)' on 'Crohn's disease of large intestine'

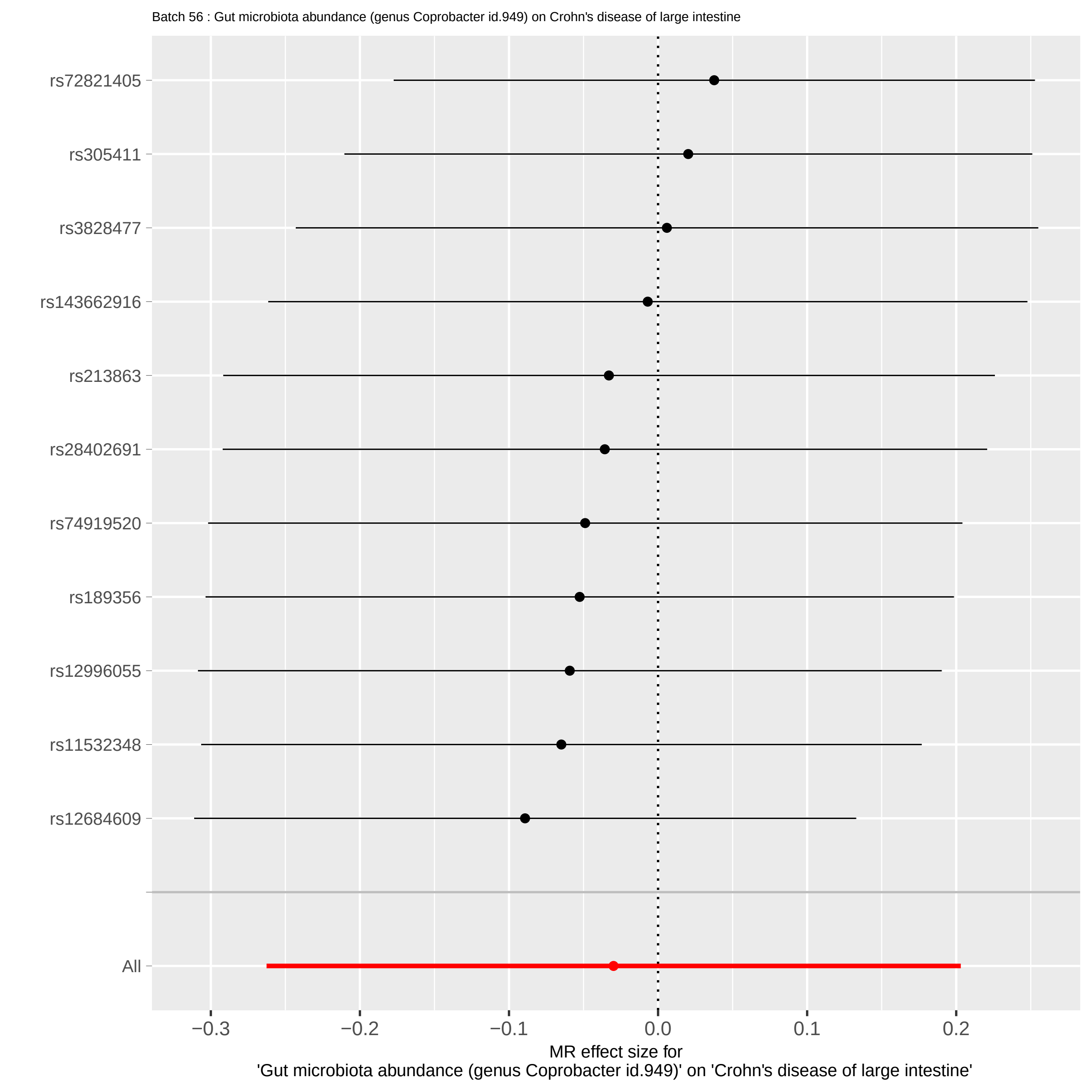
Batch 56 : Gut microbiota abundance (genus Coprobacter id.949) on Crohn's disease of large intestine
rs72821405
rs305411
rs3828477
rs143662916
rs213863
rs28402691
rs74919520
rs189356
rs12996055
rs11532348
rs12684609
All
−0.3
−0.2
−0.1
0.0
0.1
0.2
MR effect size for
'Gut microbiota abundance (genus Coprobacter id.949)' on 'Crohn's disease of large intestine'

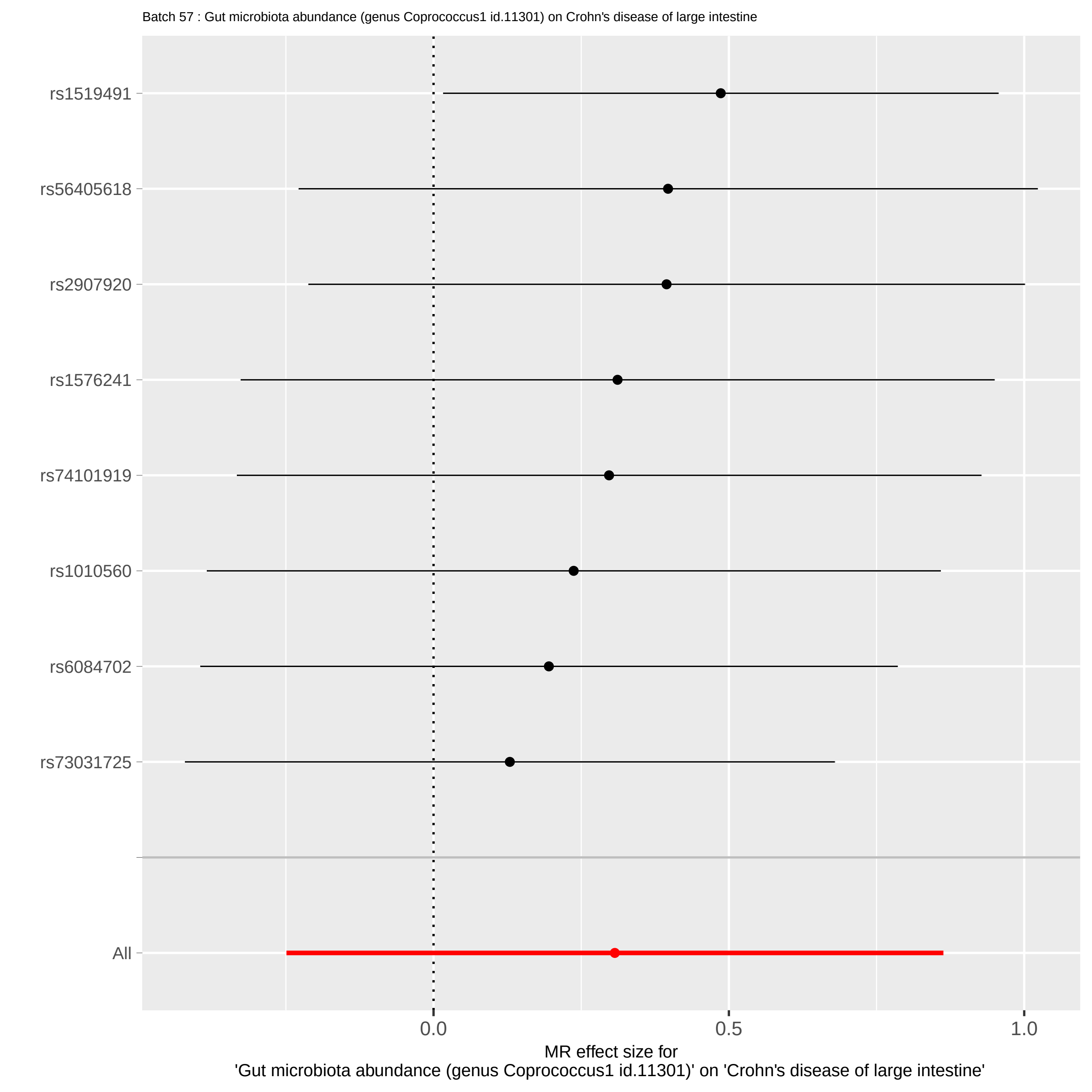
Batch 57 : Gut microbiota abundance (genus Coprococcus1 id.11301) on Crohn's disease of large intestine
rs1519491
rs56405618
rs2907920
rs1576241
rs74101919
rs1010560
rs6084702
rs73031725
All
0.0
0.5
1.0
MR effect size for
'Gut microbiota abundance (genus Coprococcus1 id.11301)' on 'Crohn's disease of large intestine'

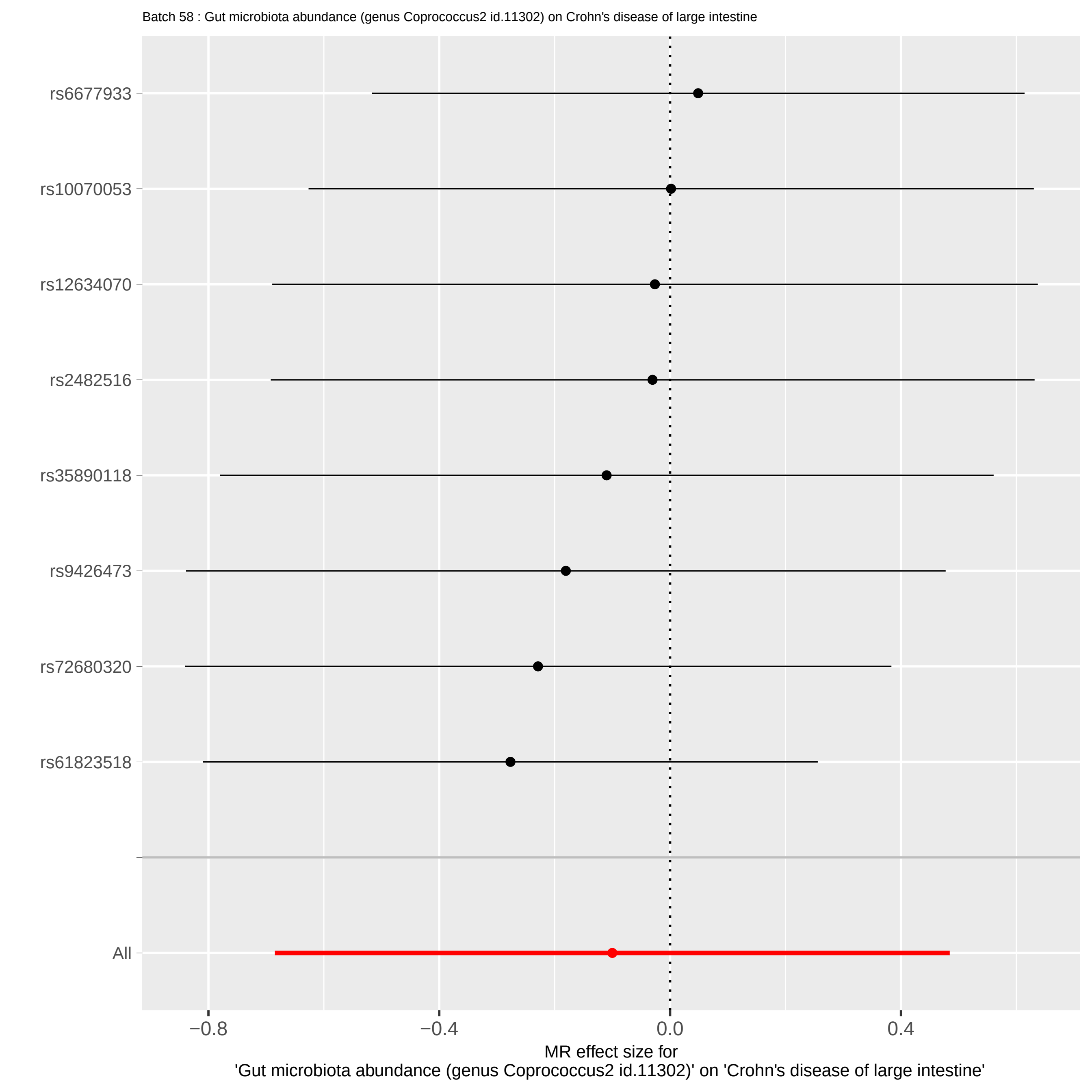

Batch 58 : Gut microbiota abundance (genus Coprococcus2 id.11302) on Crohn's disease of large intestine
rs6677933
rs10070053
rs12634070
rs2482516
rs35890118
rs9426473
rs72680320
rs61823518
All
−0.8
−0.4
0.0
0.4
MR effect size for
'Gut microbiota abundance (genus Coprococcus2 id.11302)' on 'Crohn's disease of large intestine'

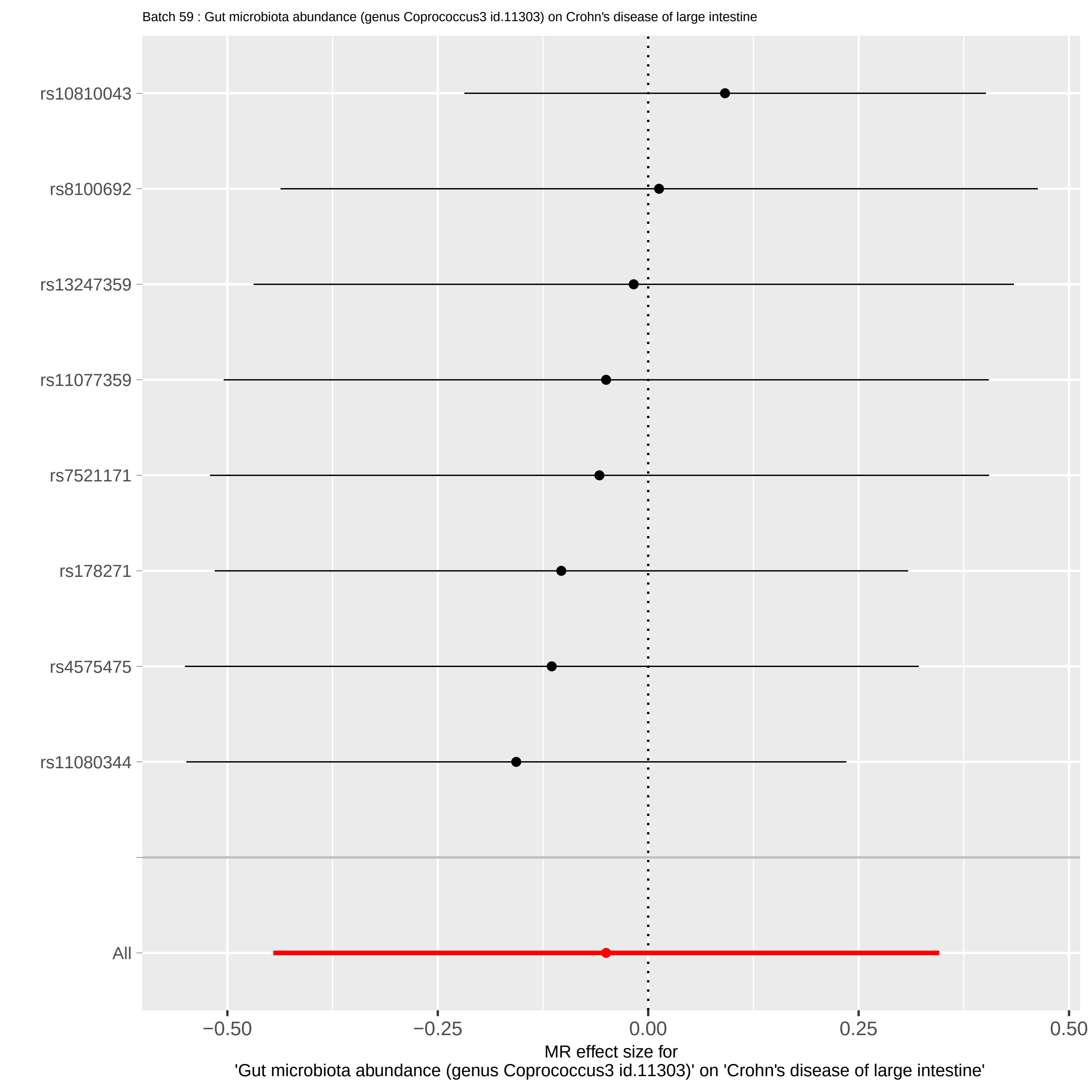
Batch 59 : Gut microbiota abundance (genus Coprococcus3 id.11303) on Crohn's disease of large intestine
rs10810043
rs8100692
rs13247359
rs11077359
rs7521171
rs178271
rs4575475
rs11080344
All
−0.50
−0.25
0.00
0.25
0.50
MR effect size for
'Gut microbiota abundance (genus Coprococcus3 id.11303)' on 'Crohn's disease of large intestine'

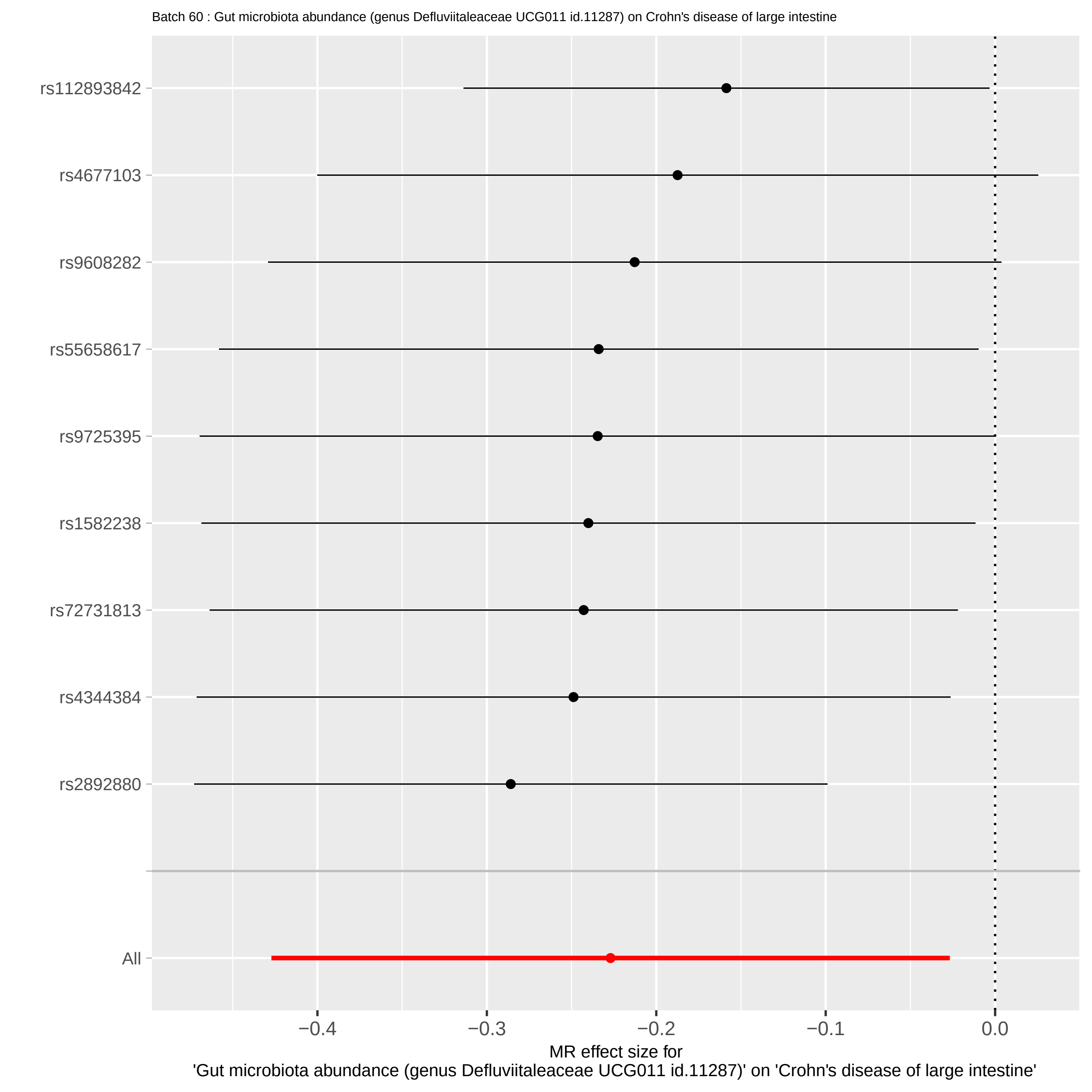
Batch 60 : Gut microbiota abundance (genus Defluviitaleaceae UCG011 id.11287) on Crohn's disease of large intestine
rs112893842
rs4677103
rs9608282
rs55658617
rs9725395
rs1582238
rs72731813
rs4344384
rs2892880
All
−0.4
−0.3
−0.2
−0.1
0.0
MR effect size for
'Gut microbiota abundance (genus Defluviitaleaceae UCG011 id.11287)' on 'Crohn's disease of large intestine'

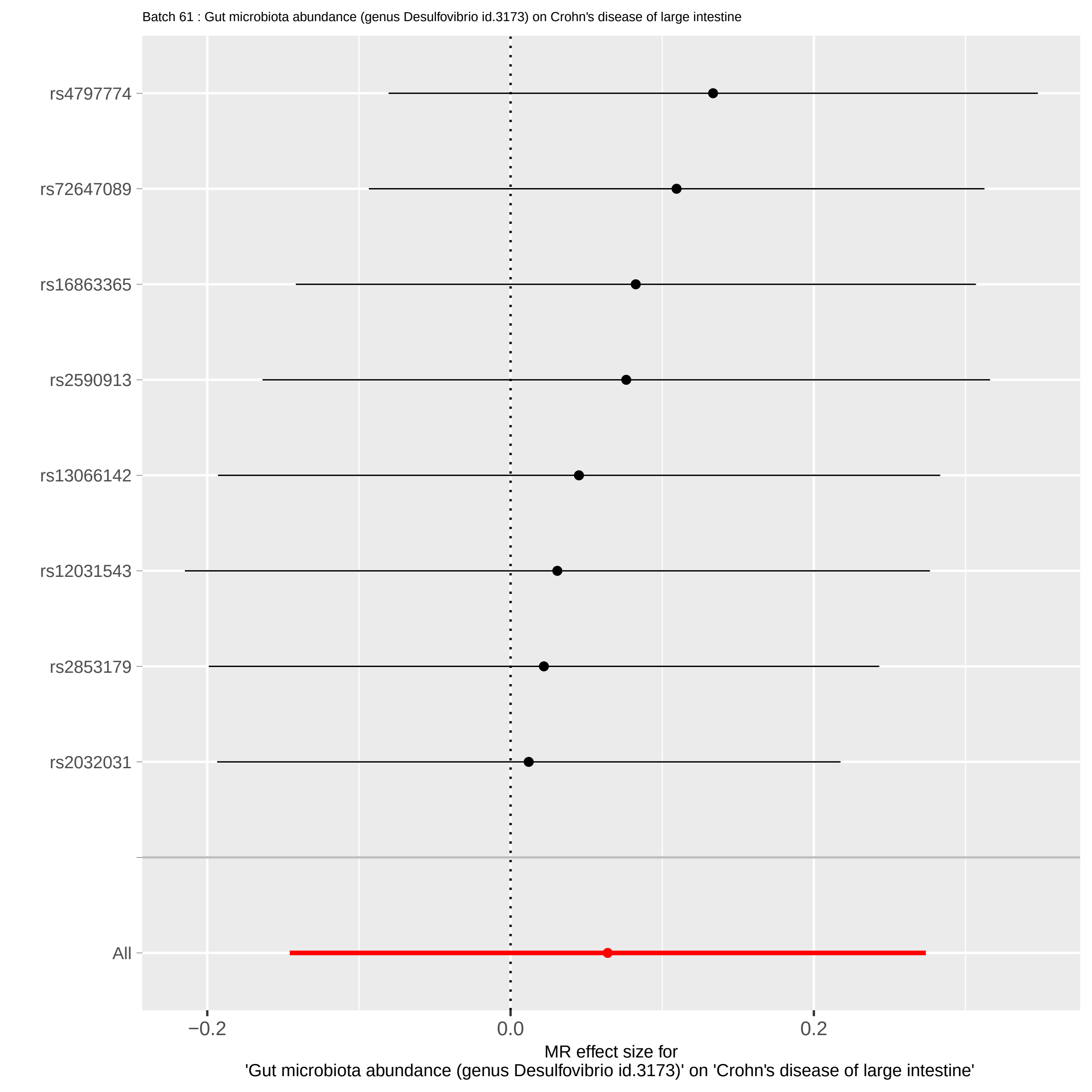
Batch 61 : Gut microbiota abundance (genus Desulfovibrio id.3173) on Crohn's disease of large intestine
rs4797774
rs72647089
rs16863365
rs2590913
rs13066142
rs12031543
rs2853179
rs2032031
All
−0.2
0.0
0.2
MR effect size for
'Gut microbiota abundance (genus Desulfovibrio id.3173)' on 'Crohn's disease of large intestine'

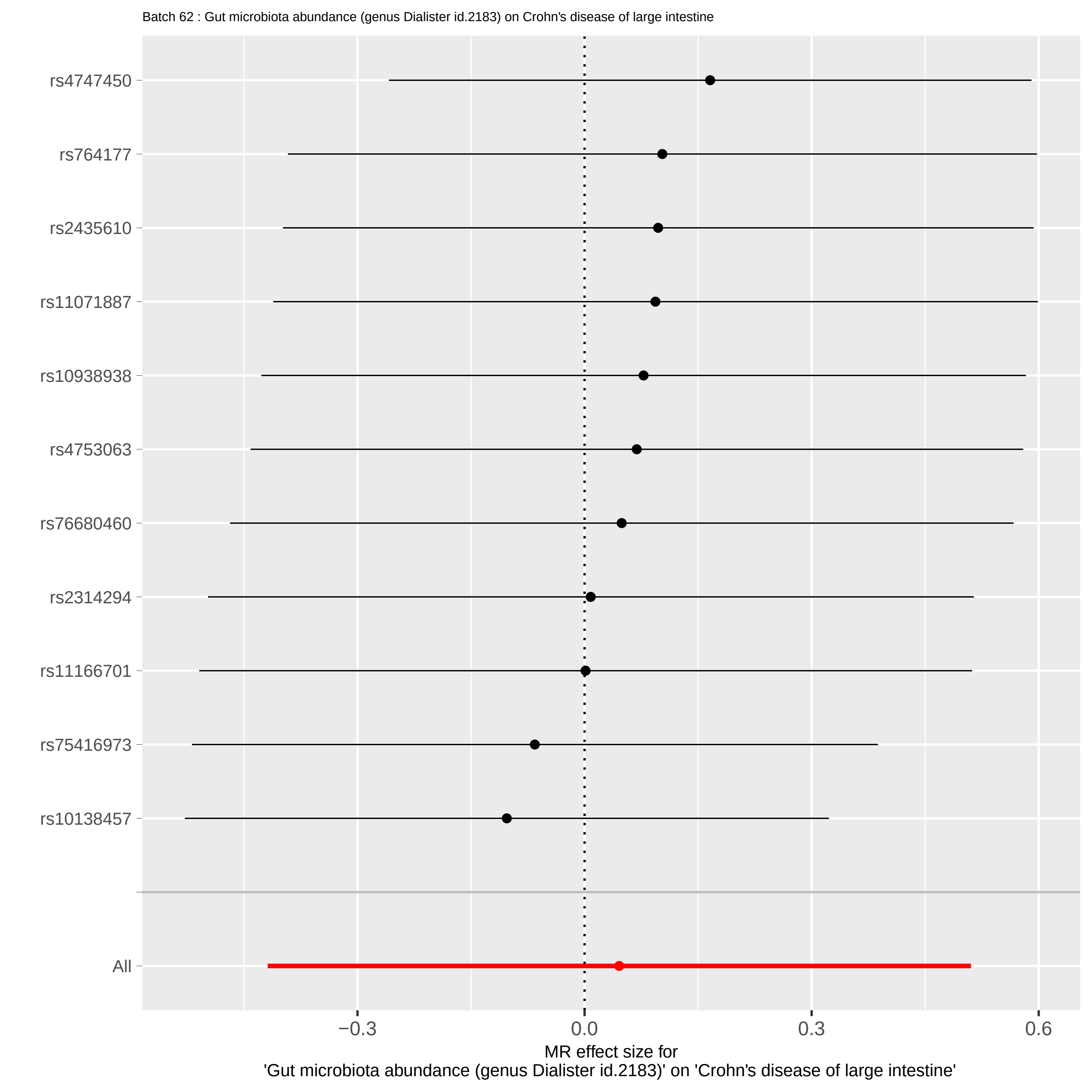
Batch 62 : Gut microbiota abundance (genus Dialister id.2183) on Crohn's disease of large intestine
rs4747450
rs764177
rs2435610
rs11071887
rs10938938
rs4753063
rs76680460
rs2314294
rs11166701
rs75416973
rs10138457
All
−0.3
0.0
0.3
0.6
MR effect size for
'Gut microbiota abundance (genus Dialister id.2183)' on 'Crohn's disease of large intestine'

Batch 63 : Gut microbiota abundance (genus Dorea id.1997) on Crohn's disease of large intestine

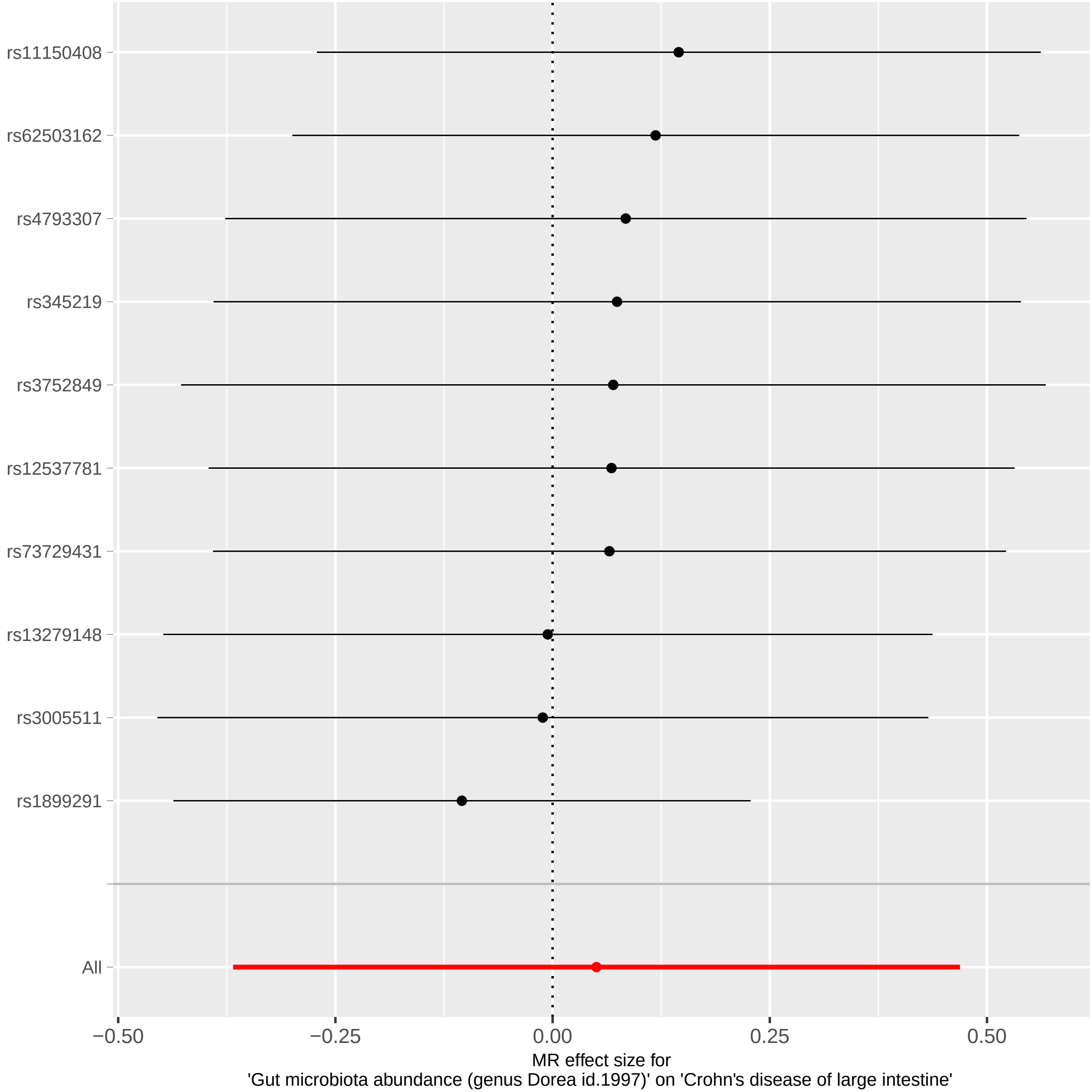

Batch 64 : Gut microbiota abundance (genus Eggerthella id.819) on Crohn's disease of large intestine

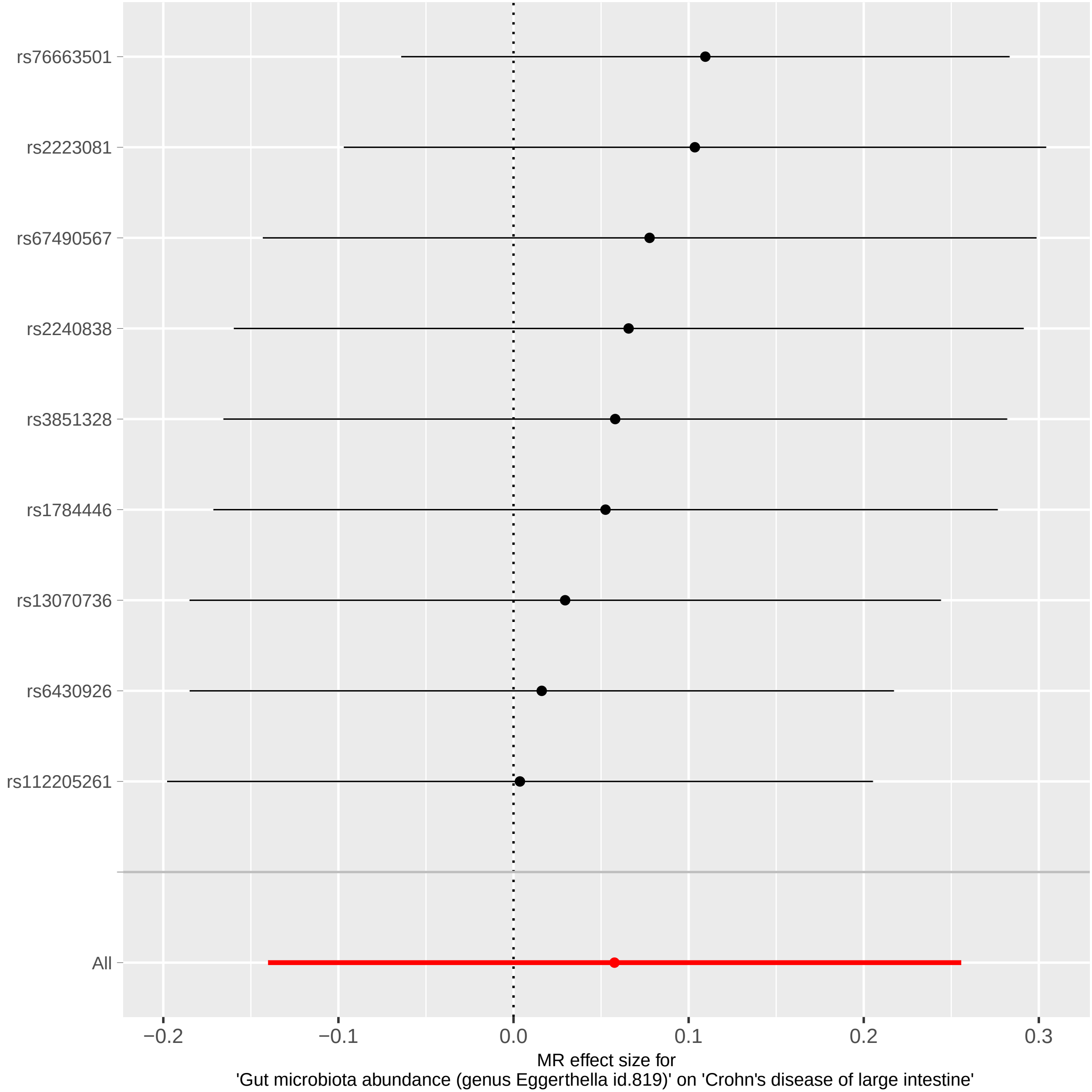

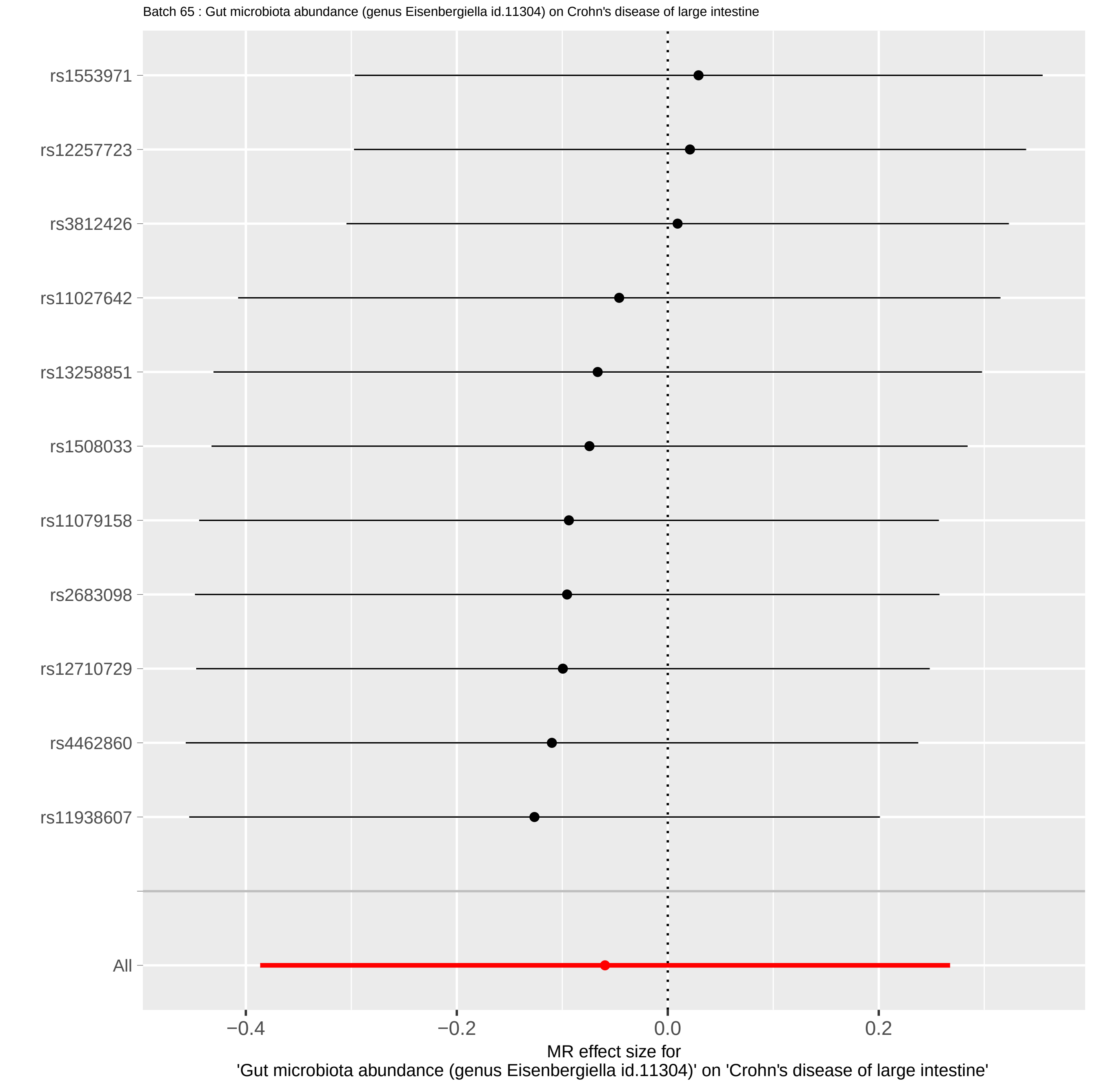

Batch 65 : Gut microbiota abundance (genus Eisenbergiella id.11304) on Crohn's disease of large intestine
rs1553971
rs12257723
rs3812426
rs11027642
rs13258851
rs1508033
rs11079158
rs2683098
rs12710729
rs4462860
rs11938607
All
−0.4
−0.2
0.0
0.2
MR effect size for
'Gut microbiota abundance (genus Eisenbergiella id.11304)' on 'Crohn's disease of large intestine'

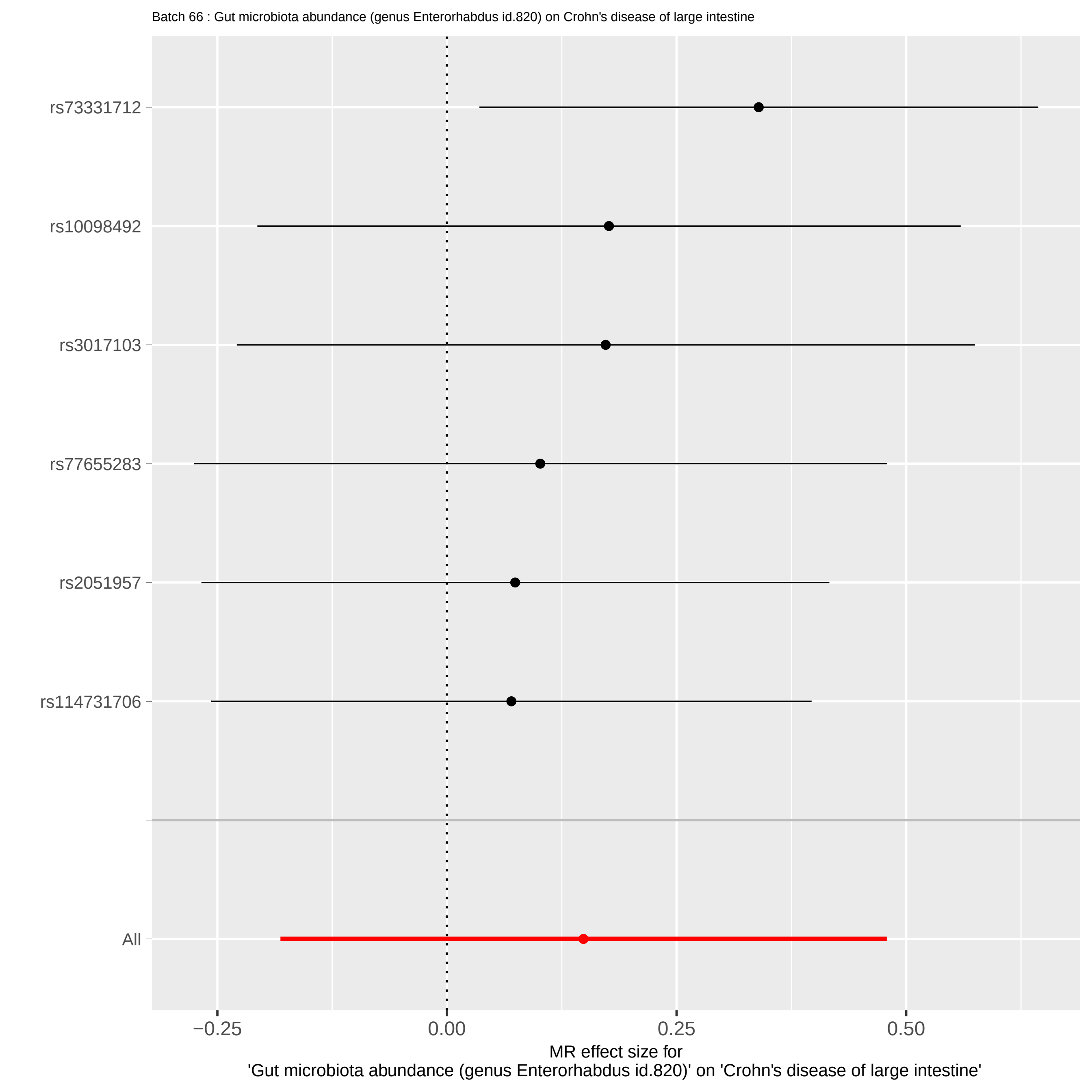
Batch 66 : Gut microbiota abundance (genus Enterorhabdus id.820) on Crohn's disease of large intestine
rs73331712
rs10098492
rs3017103
rs77655283
rs2051957
rs114731706
All
−0.25
0.00
0.25
0.50
MR effect size for
'Gut microbiota abundance (genus Enterorhabdus id.820)' on 'Crohn's disease of large intestine'

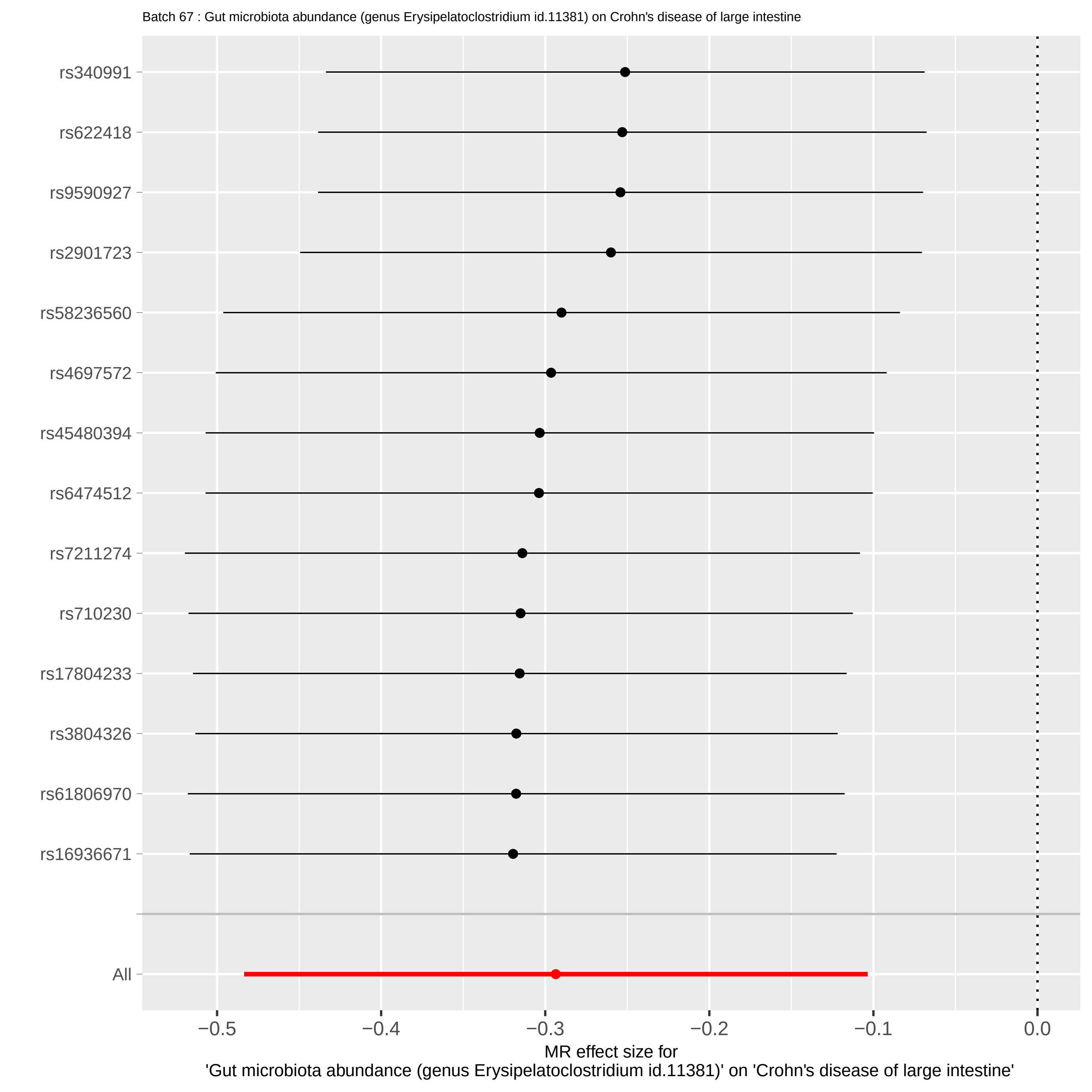

Batch 67 : Gut microbiota abundance (genus Erysipelatoclostridium id.11381) on Crohn's disease of large intestine
rs340991
rs622418
rs9590927
rs2901723
rs58236560
rs4697572
rs45480394
rs6474512
rs7211274
rs710230
rs17804233
rs3804326
rs61806970
rs16936671
All
−0.5
−0.4
−0.3
−0.2
−0.1
0.0
MR effect size for
'Gut microbiota abundance (genus Erysipelatoclostridium id.11381)' on 'Crohn's disease of large intestine'

Batch 69 : Gut microbiota abundance (genus Escherichia Shigella id.3504) on Crohn's disease of large intestine

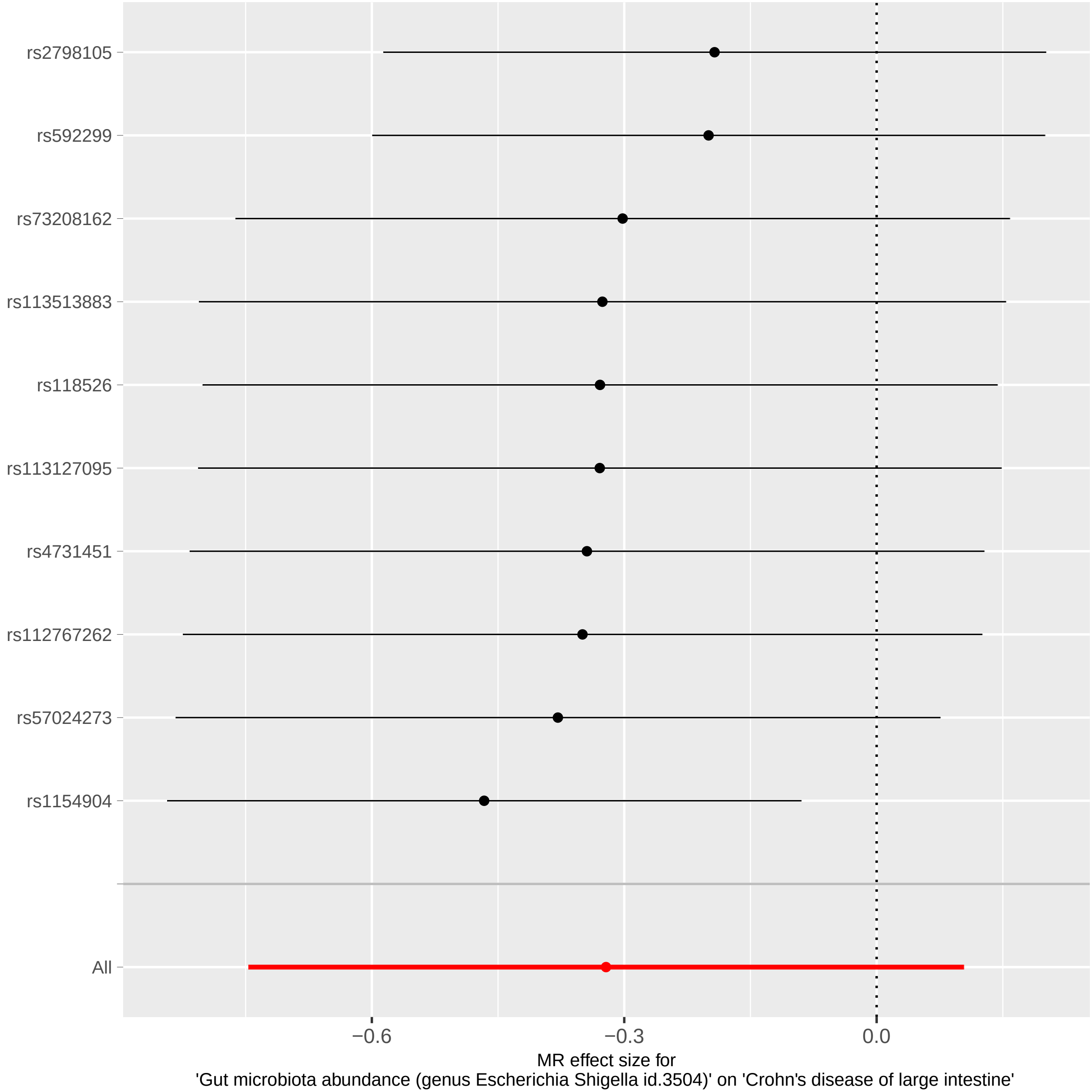

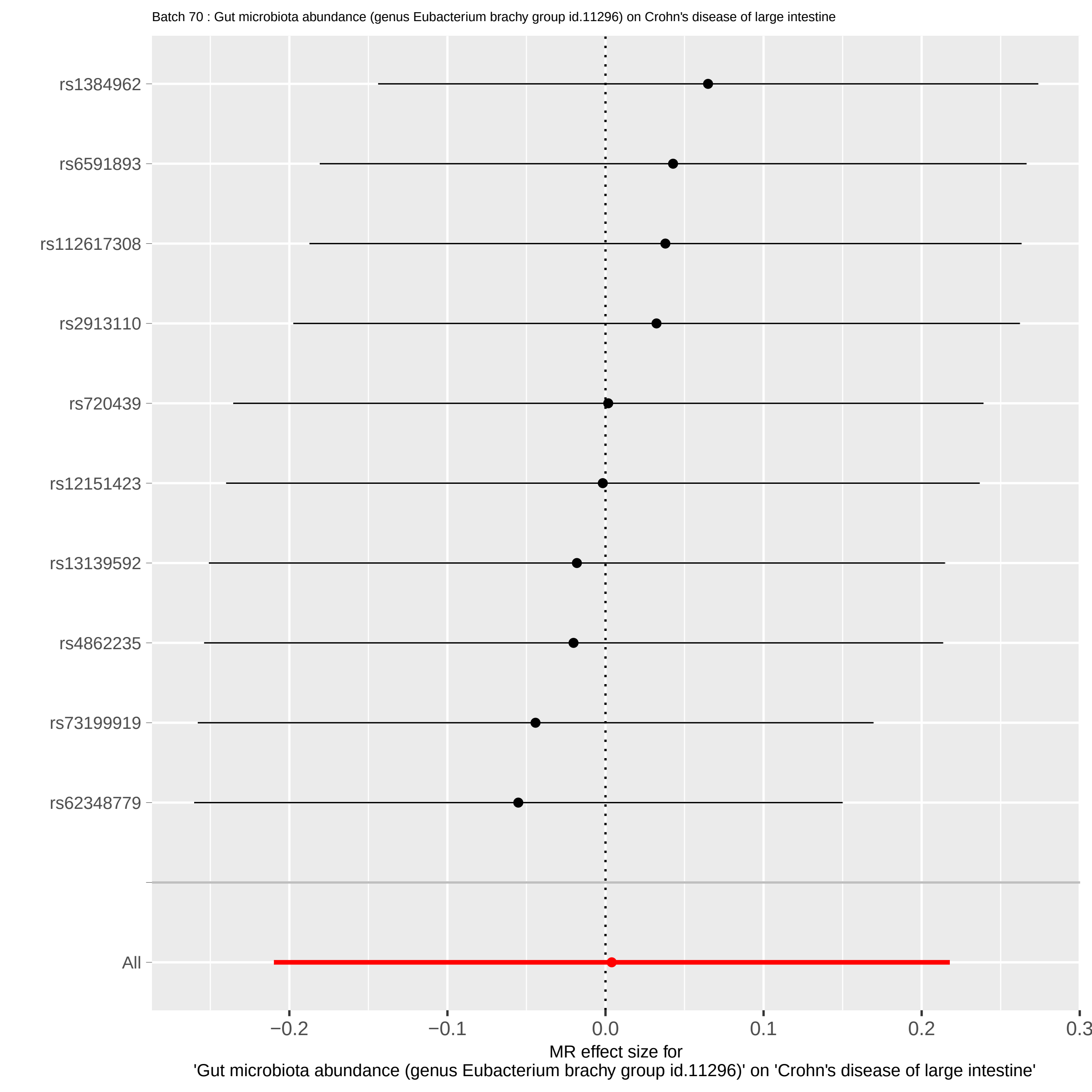
Batch 70 : Gut microbiota abundance (genus Eubacterium brachy group id.11296) on Crohn's disease of large intestine
rs1384962
rs6591893
rs112617308
rs2913110
rs720439
rs12151423
rs13139592
rs4862235
rs73199919
rs62348779
All
−0.2
−0.1
0.0
0.1
0.2
0.3
MR effect size for
'Gut microbiota abundance (genus Eubacterium brachy group id.11296)' on 'Crohn's disease of large intestine'

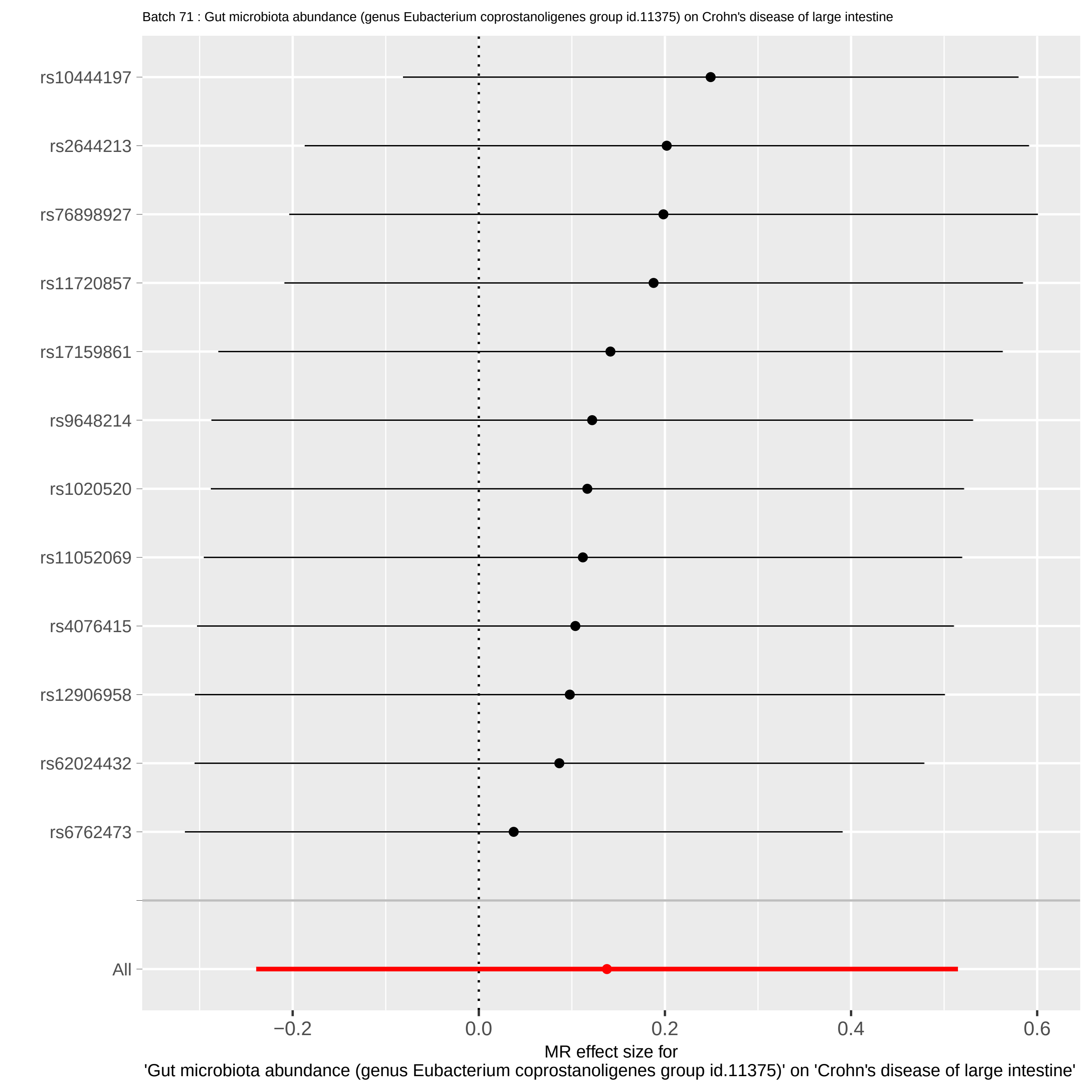
Batch 71 : Gut microbiota abundance (genus Eubacterium coprostanoligenes group id.11375) on Crohn's disease of large intestine
rs10444197
rs2644213
rs76898927
rs11720857
rs17159861
rs9648214
rs1020520
rs11052069
rs4076415
rs12906958
rs62024432
rs6762473
All
−0.2
0.0
0.2
0.4
0.6
MR effect size for
'Gut microbiota abundance (genus Eubacterium coprostanoligenes group id.11375)' on 'Crohn's disease of large intestine'

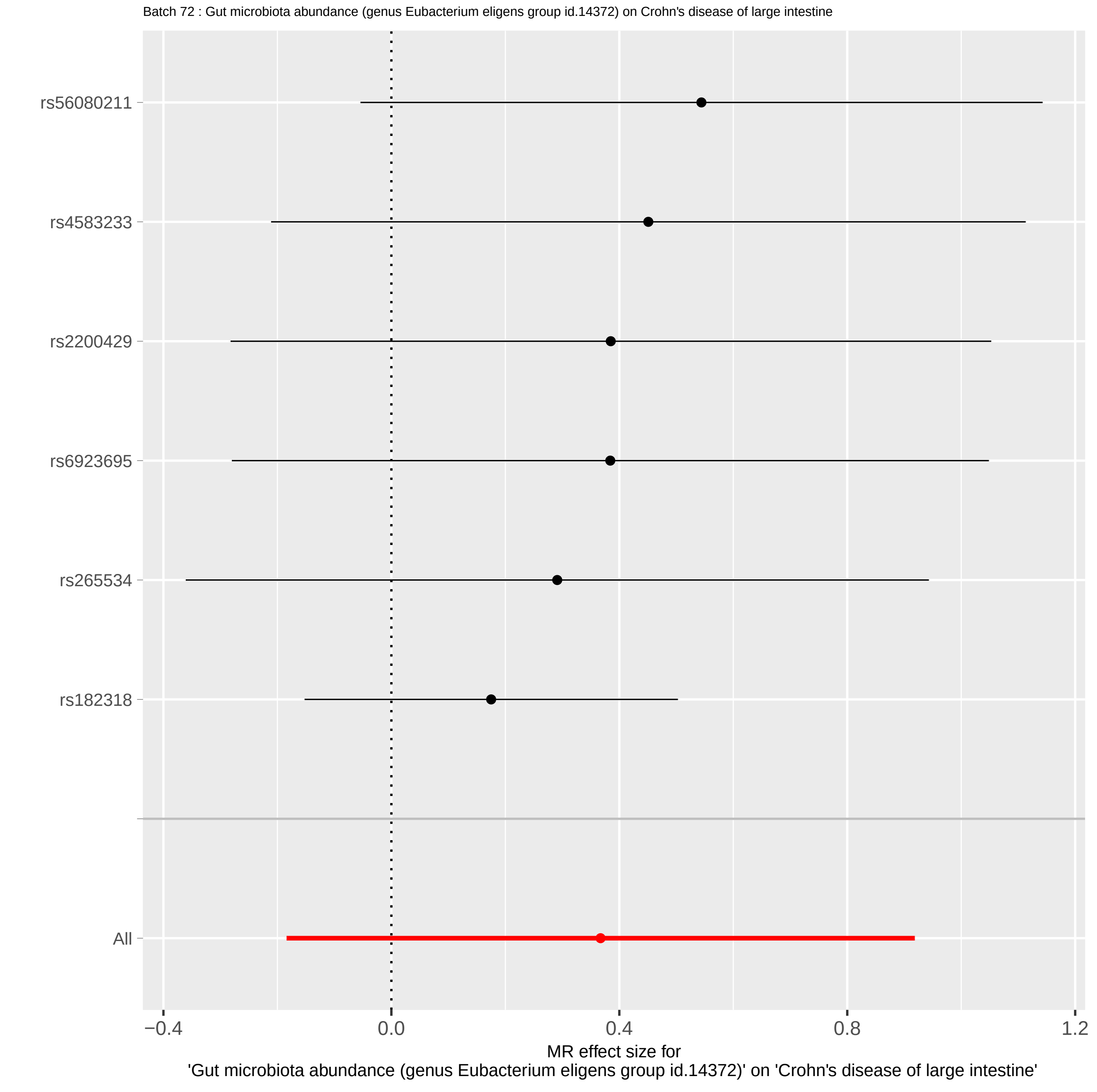
Batch 72 : Gut microbiota abundance (genus Eubacterium eligens group id.14372) on Crohn's disease of large intestine
rs56080211
rs4583233
rs2200429
rs6923695
rs265534
rs182318
All
−0.4
0.0
0.4
0.8
1.2
MR effect size for
'Gut microbiota abundance (genus Eubacterium eligens group id.14372)' on 'Crohn's disease of large intestine'

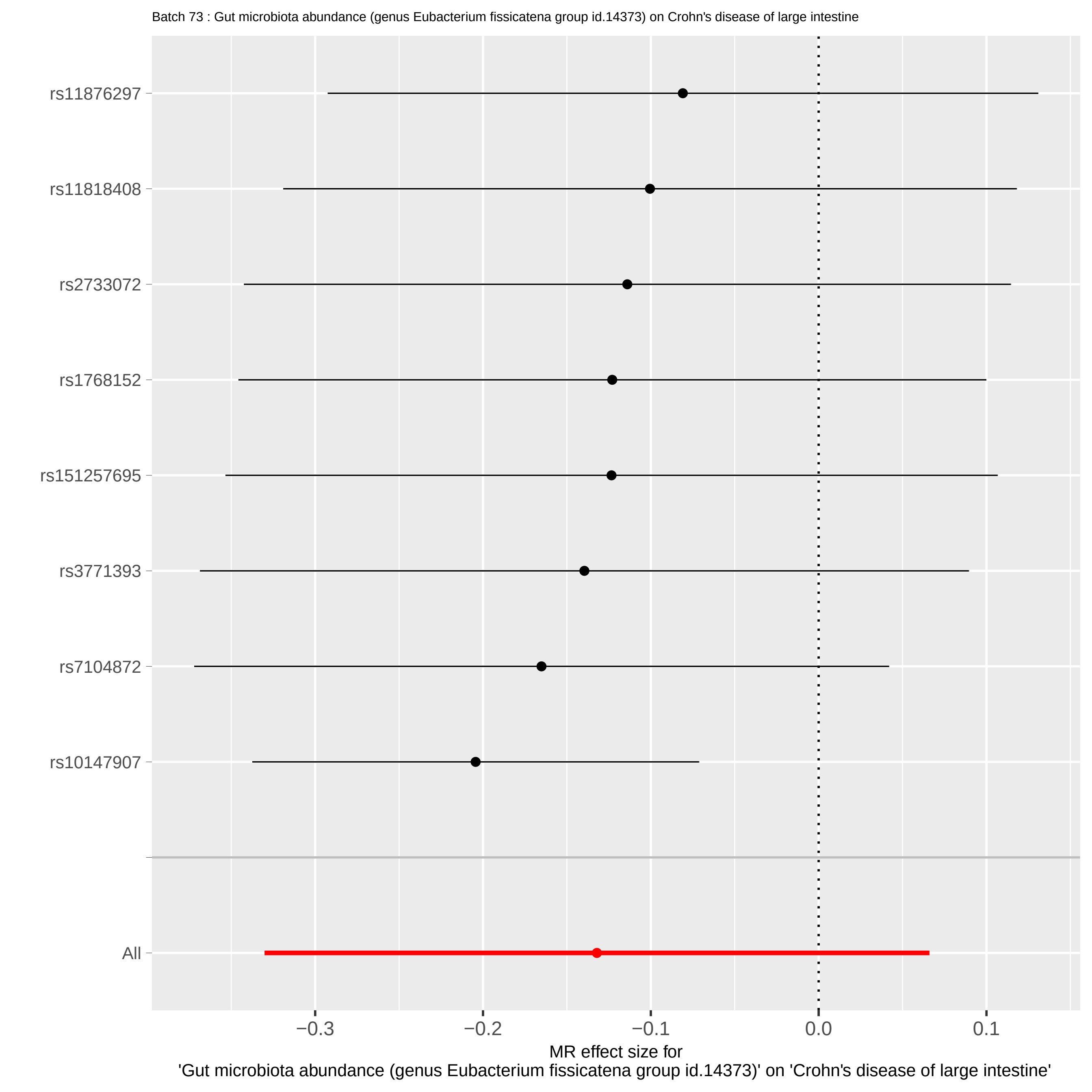
Batch 73 : Gut microbiota abundance (genus Eubacterium fissicatena group id.14373) on Crohn's disease of large intestine
rs11876297
rs11818408
rs2733072
rs1768152
rs151257695
rs3771393
rs7104872
rs10147907
All
−0.3
−0.2
−0.1
0.0
0.1
MR effect size for
'Gut microbiota abundance (genus Eubacterium fissicatena group id.14373)' on 'Crohn's disease of large intestine'

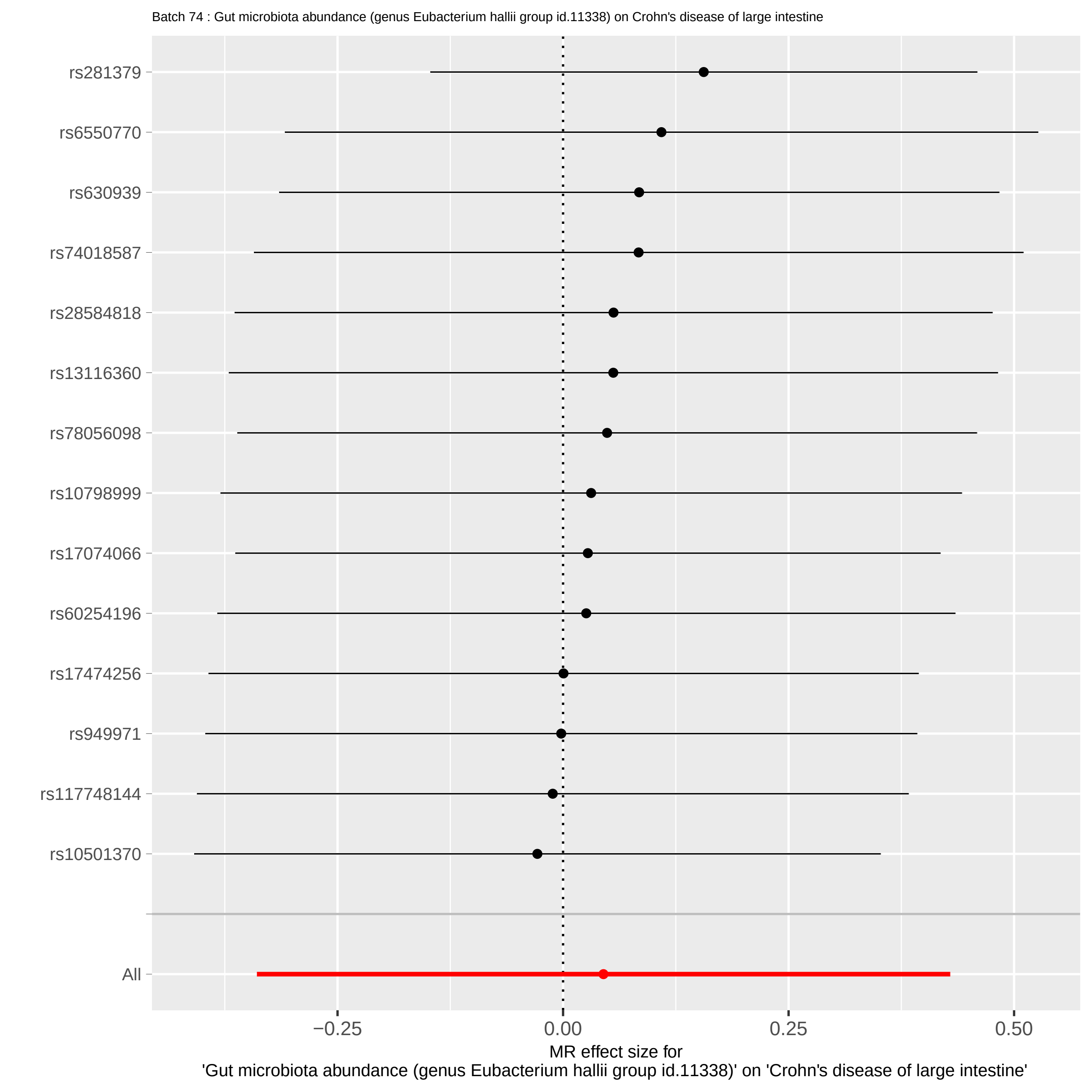

Batch 74 : Gut microbiota abundance (genus Eubacterium hallii group id.11338) on Crohn's disease of large intestine
rs281379
rs6550770
rs630939
rs74018587
rs28584818
rs13116360
rs78056098
rs10798999
rs17074066
rs60254196
rs17474256
rs949971
rs117748144
rs10501370
All
−0.25
0.00
0.25
0.50
MR effect size for
'Gut microbiota abundance (genus Eubacterium hallii group id.11338)' on 'Crohn's disease of large intestine'

Batch 75 : Gut microbiota abundance (genus Eubacterium nodatum group id.11297) on Crohn's disease of large intestine

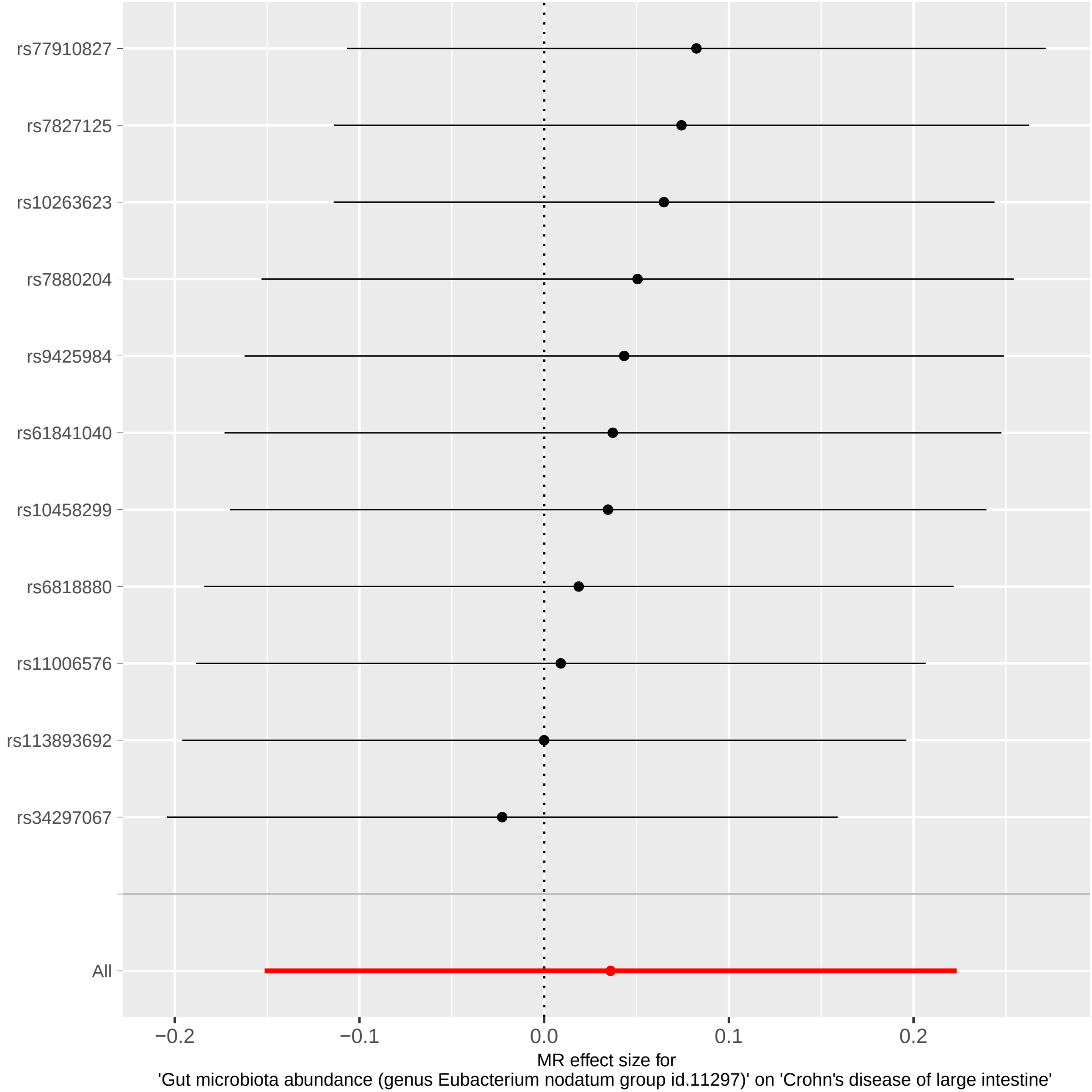

Batch 76 : Gut microbiota abundance (genus Eubacterium oxidoreducens group id.11339) on Crohn's disease of large intestine

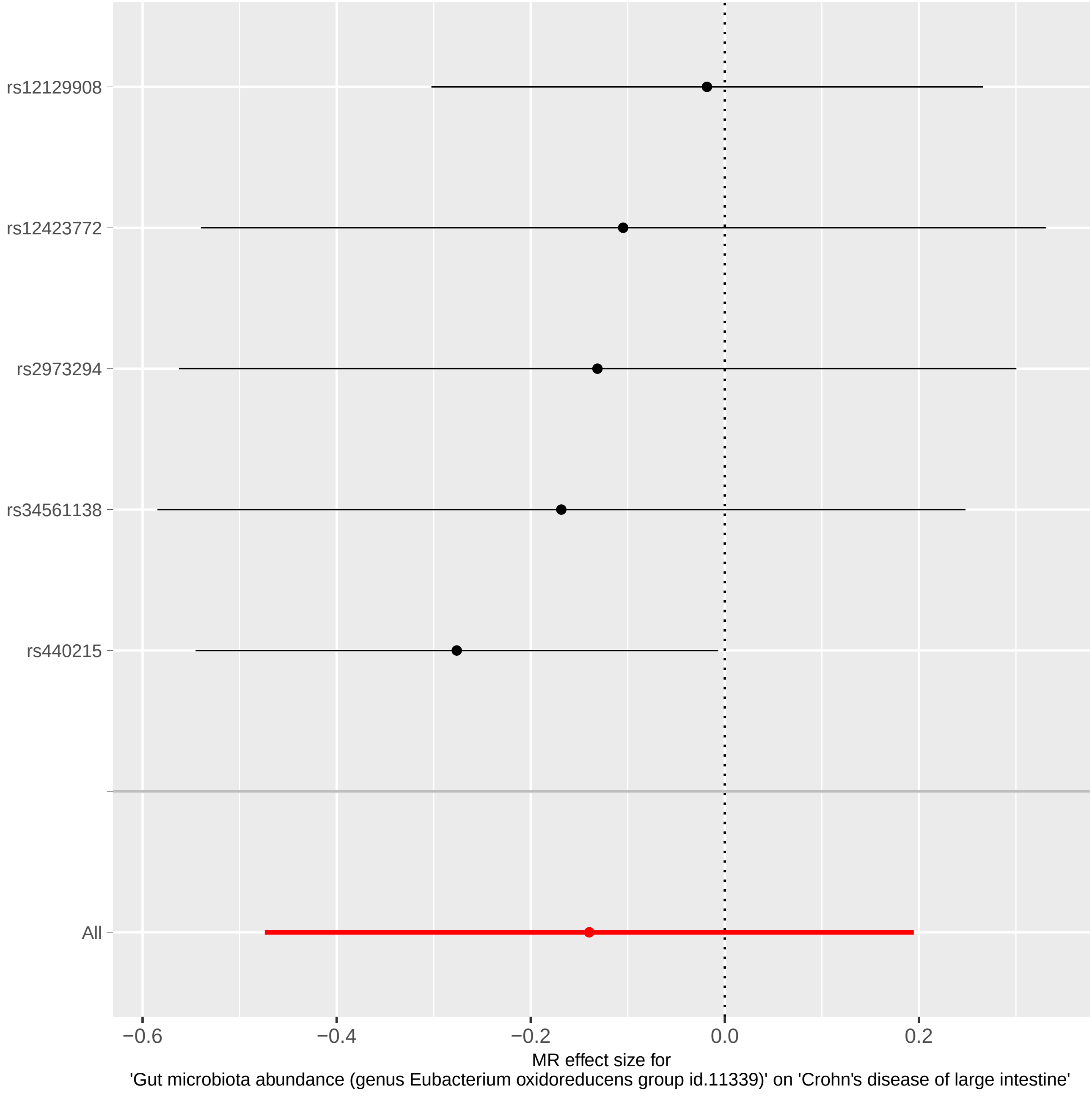

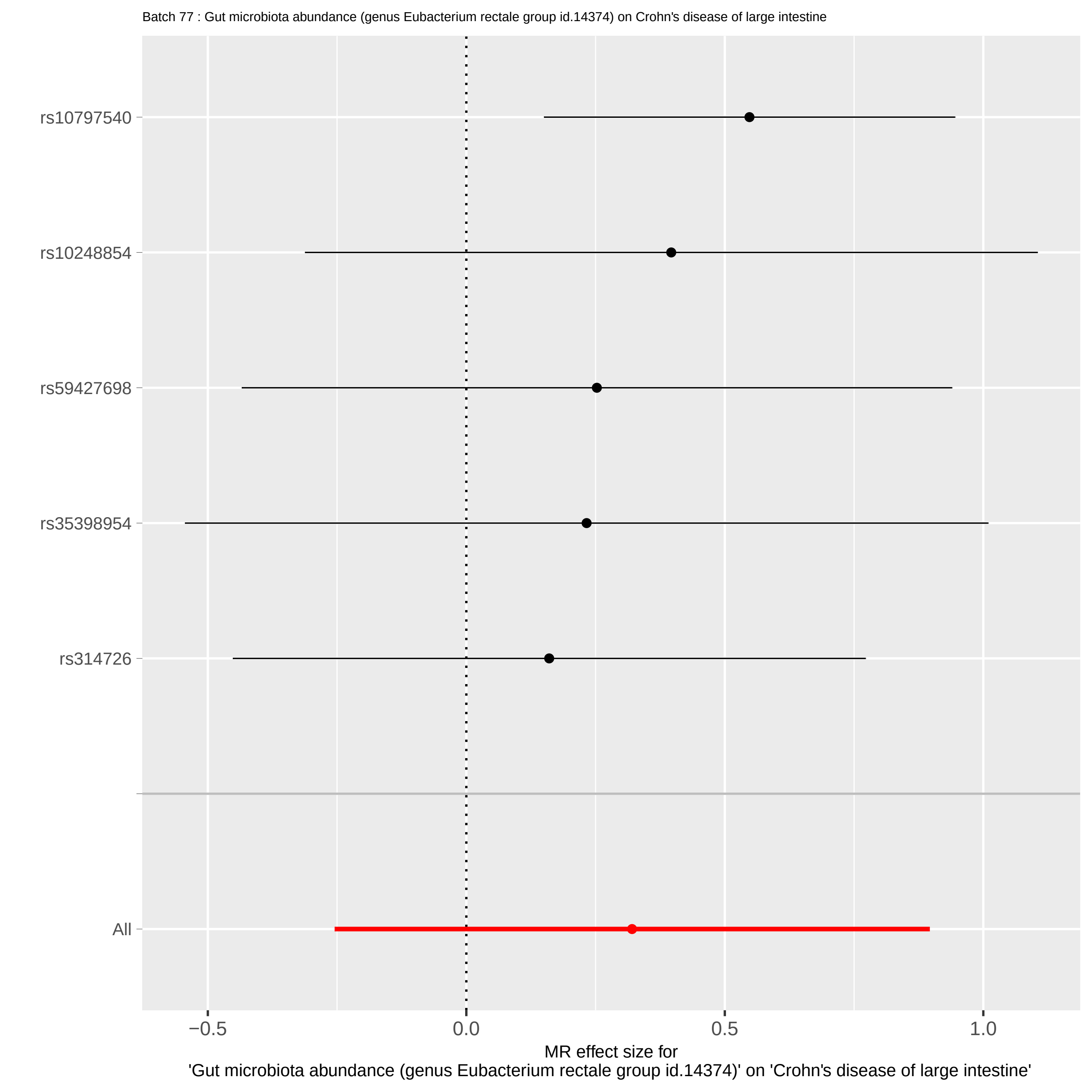

Batch 77 : Gut microbiota abundance (genus Eubacterium rectale group id.14374) on Crohn's disease of large intestine
rs10797540
rs10248854
rs59427698
rs35398954
rs314726
All
−0.5
0.0
0.5
1.0
MR effect size for
'Gut microbiota abundance (genus Eubacterium rectale group id.14374)' on 'Crohn's disease of large intestine'

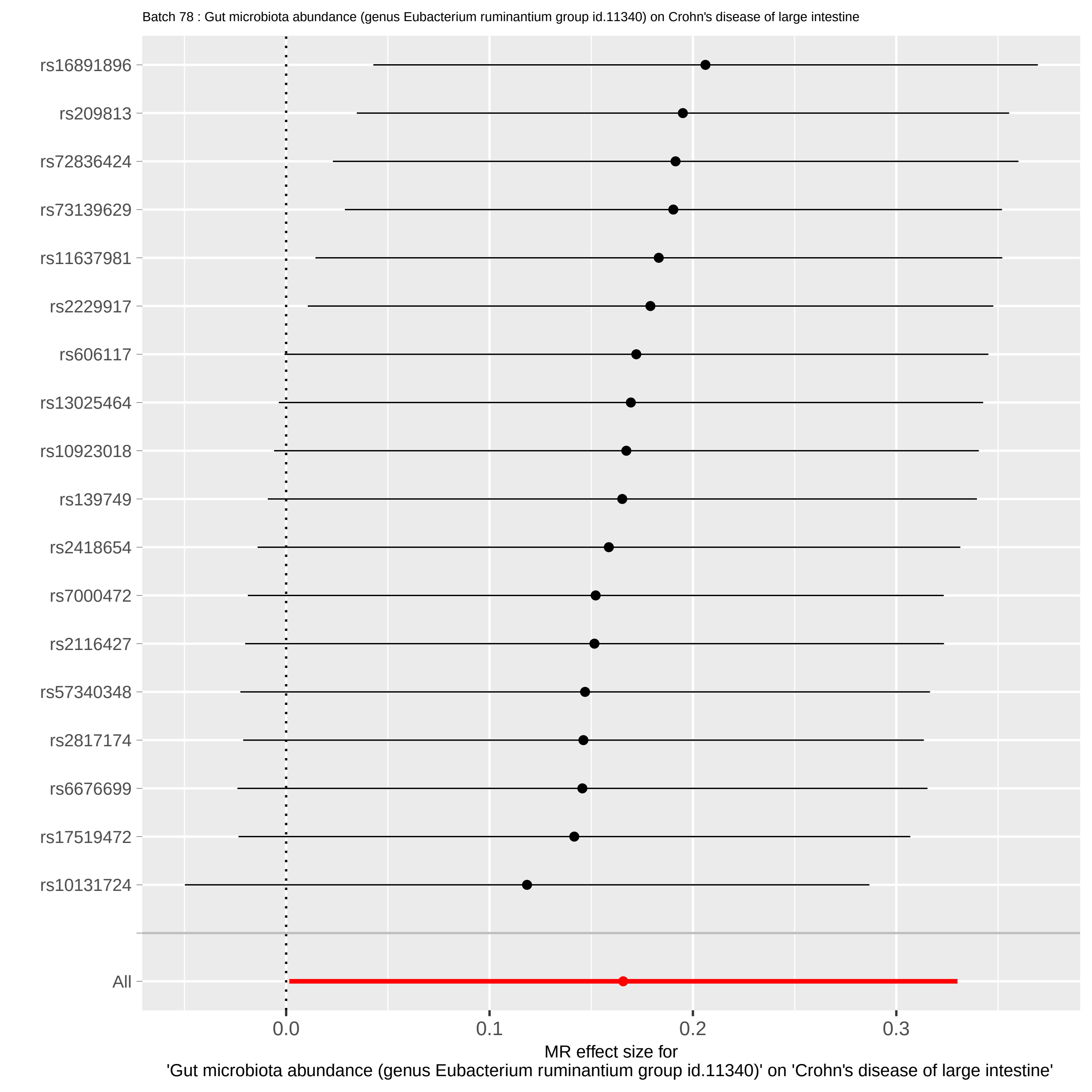
Batch 78 : Gut microbiota abundance (genus Eubacterium ruminantium group id.11340) on Crohn's disease of large intestine
rs16891896
rs209813
rs72836424
rs73139629
rs11637981
rs2229917
rs606117
rs13025464
rs10923018
rs139749
rs2418654
rs7000472
rs2116427
rs57340348
rs2817174
rs6676699
rs17519472
rs10131724
All
0.0
0.1
0.2
0.3
MR effect size for
'Gut microbiota abundance (genus Eubacterium ruminantium group id.11340)' on 'Crohn's disease of large intestine'

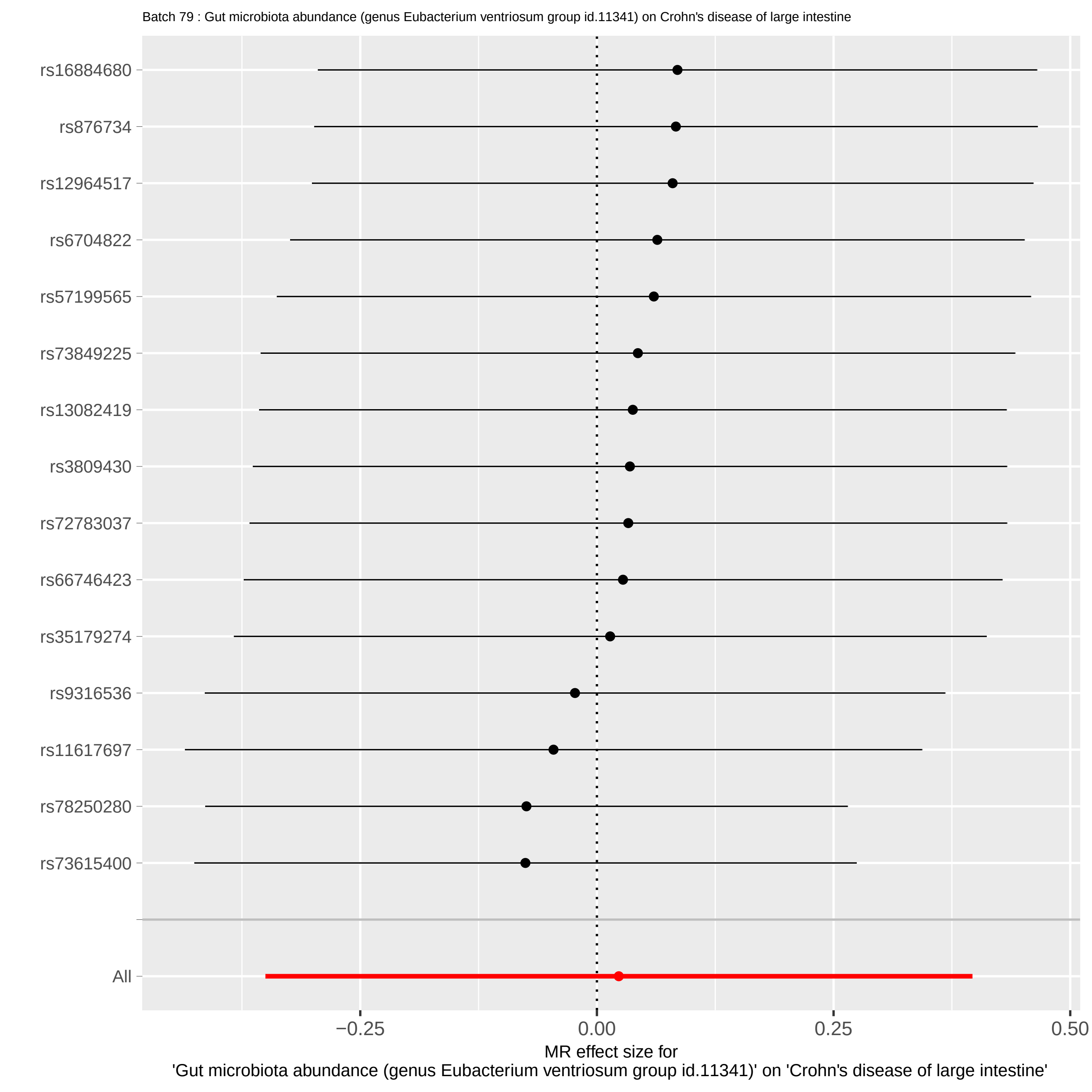
Batch 79 : Gut microbiota abundance (genus Eubacterium ventriosum group id.11341) on Crohn's disease of large intestine
rs16884680
rs876734
rs12964517
rs6704822
rs57199565
rs73849225
rs13082419
rs3809430
rs72783037
rs66746423
rs35179274
rs9316536
rs11617697
rs78250280
rs73615400
All
−0.25
0.00
0.25
0.50
MR effect size for
'Gut microbiota abundance (genus Eubacterium ventriosum group id.11341)' on 'Crohn's disease of large intestine'

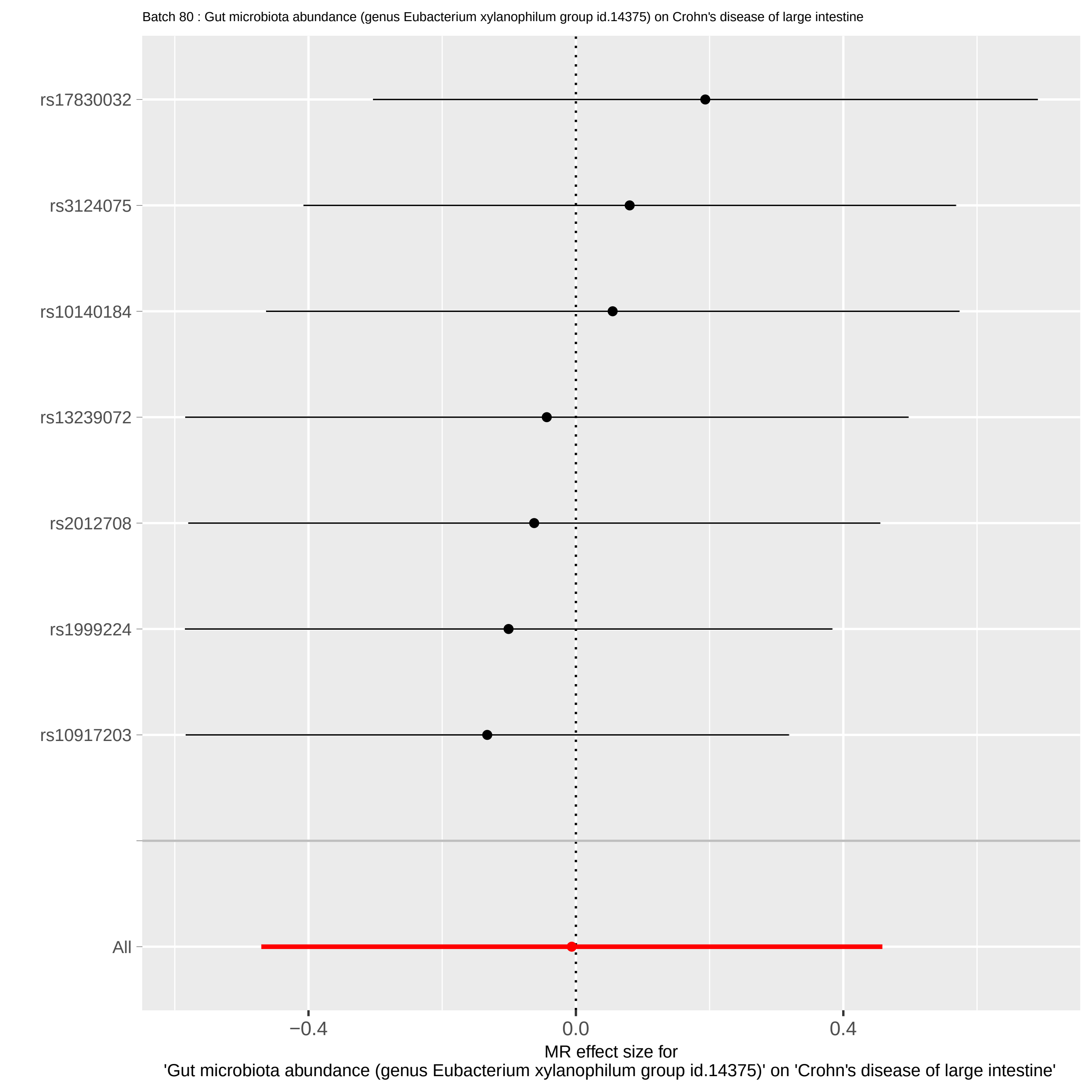

Batch 80 : Gut microbiota abundance (genus Eubacterium xylanophilum group id.14375) on Crohn's disease of large intestine
rs17830032
rs3124075
rs10140184
rs13239072
rs2012708
rs1999224
rs10917203
All
−0.4
0.0
0.4
MR effect size for
'Gut microbiota abundance (genus Eubacterium xylanophilum group id.14375)' on 'Crohn's disease of large intestine'

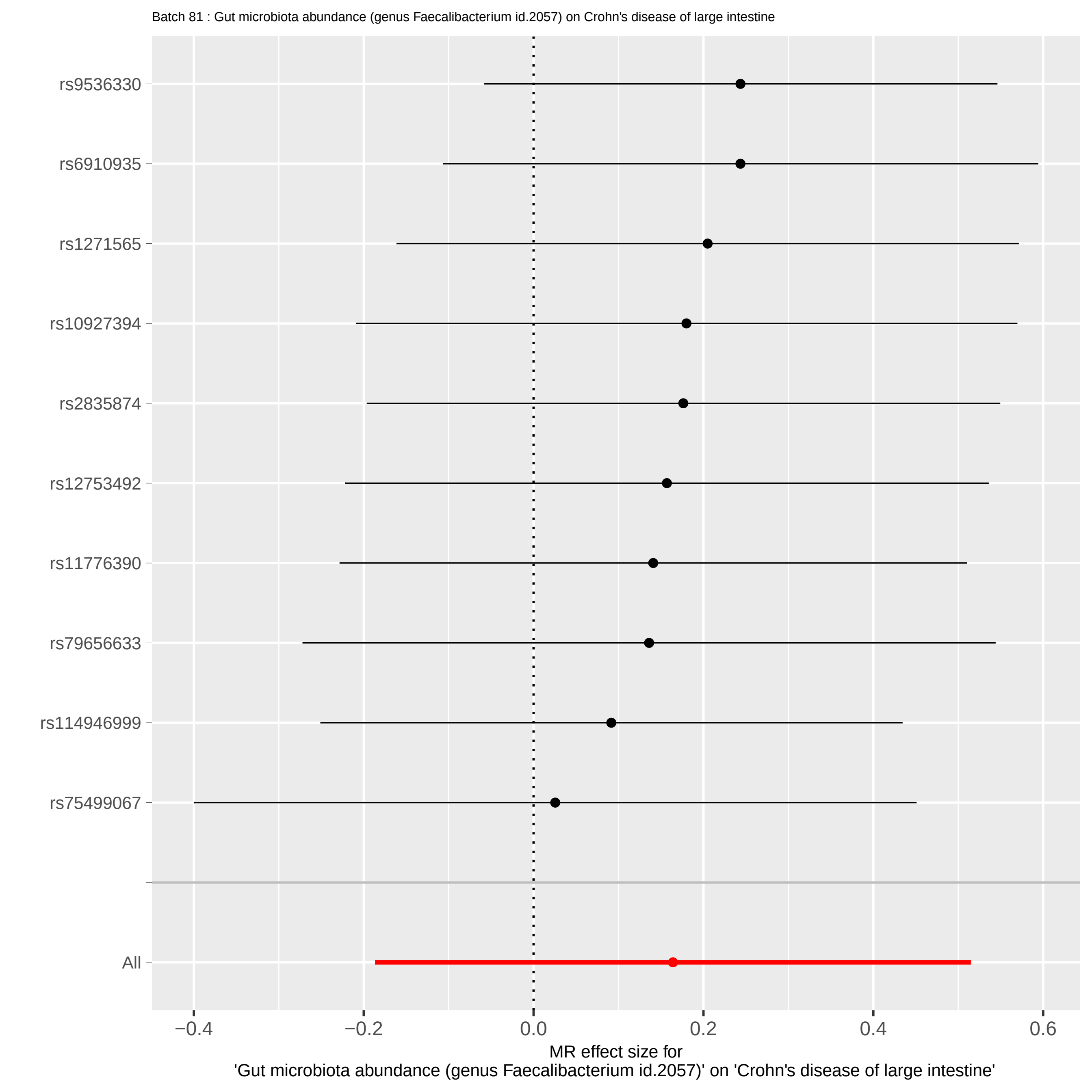

Batch 81 : Gut microbiota abundance (genus Faecalibacterium id.2057) on Crohn's disease of large intestine
rs9536330
rs6910935
rs1271565
rs10927394
rs2835874
rs12753492
rs11776390
rs79656633
rs114946999
rs75499067
All
−0.4
−0.2
0.0
0.2
0.4
0.6
MR effect size for
'Gut microbiota abundance (genus Faecalibacterium id.2057)' on 'Crohn's disease of large intestine'

Batch 82 : Gut microbiota abundance (genus Family XIII AD3011 group id.11293) on Crohn's disease of large intestine

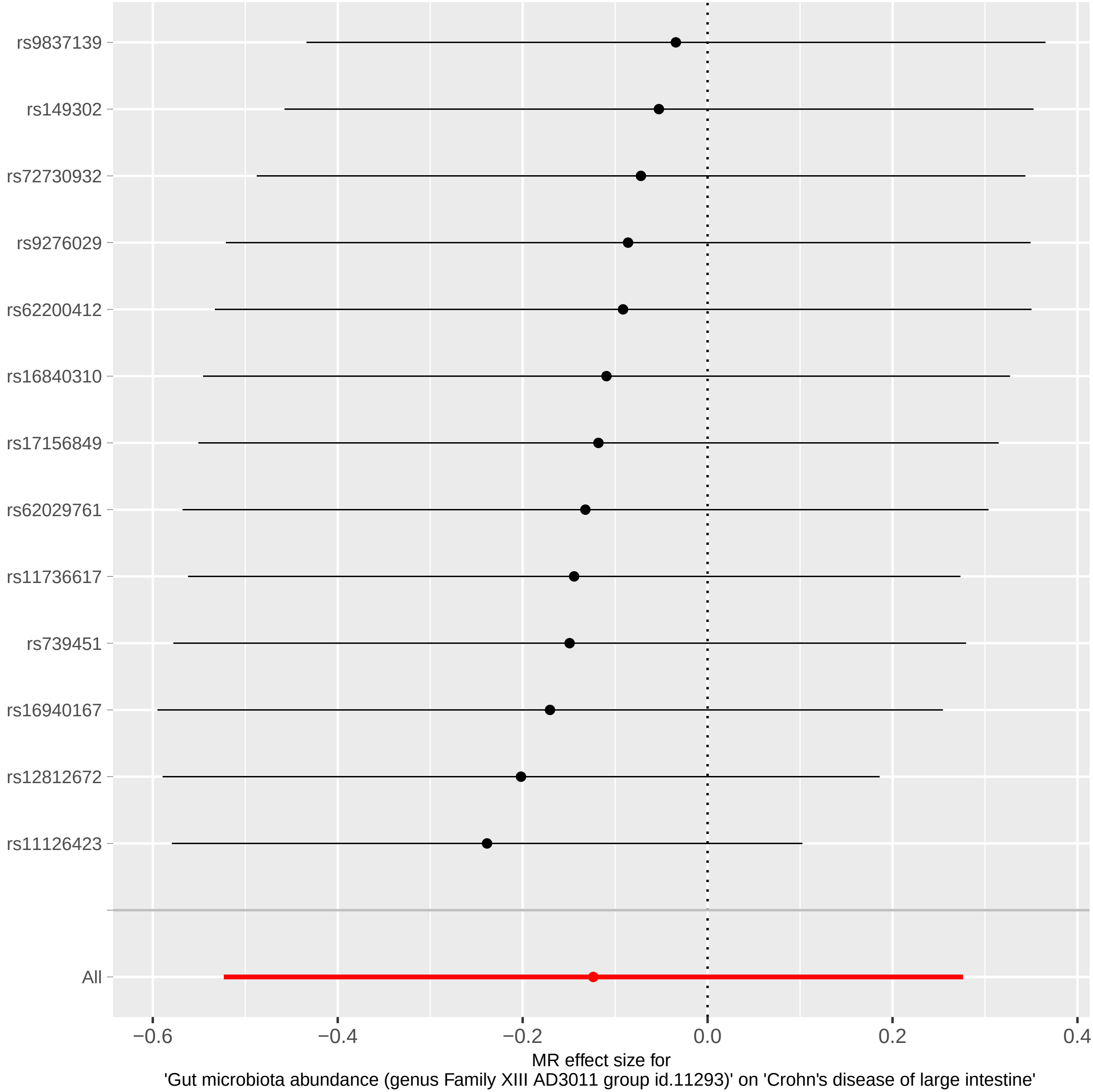

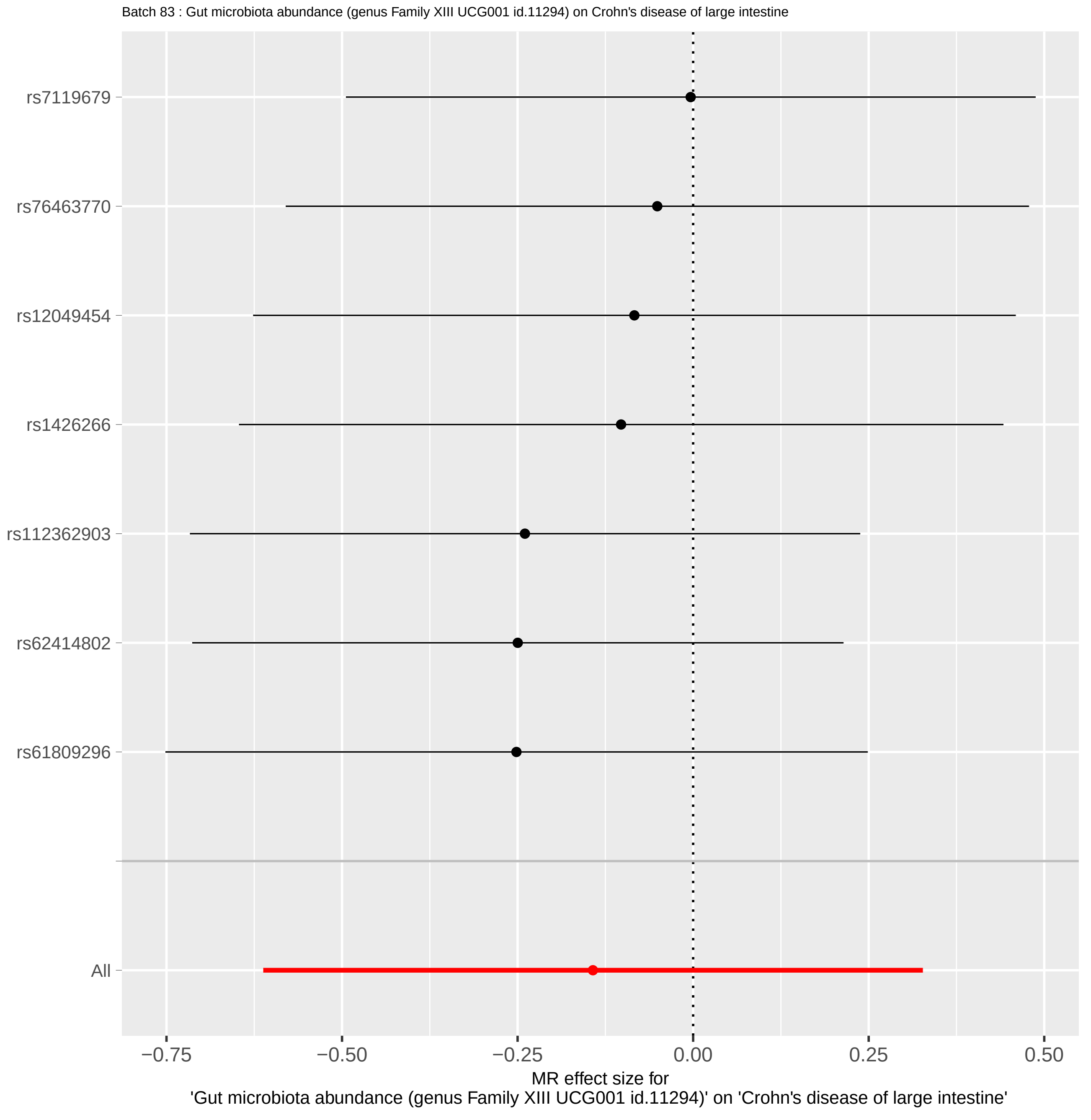
Batch 83 : Gut microbiota abundance (genus Family XIII UCG001 id.11294) on Crohn's disease of large intestine
rs7119679
rs76463770
rs12049454
rs1426266
rs112362903
rs62414802
rs61809296
All
−0.75
−0.50
−0.25
0.00
0.25
0.50
MR effect size for
'Gut microbiota abundance (genus Family XIII UCG001 id.11294)' on 'Crohn's disease of large intestine'

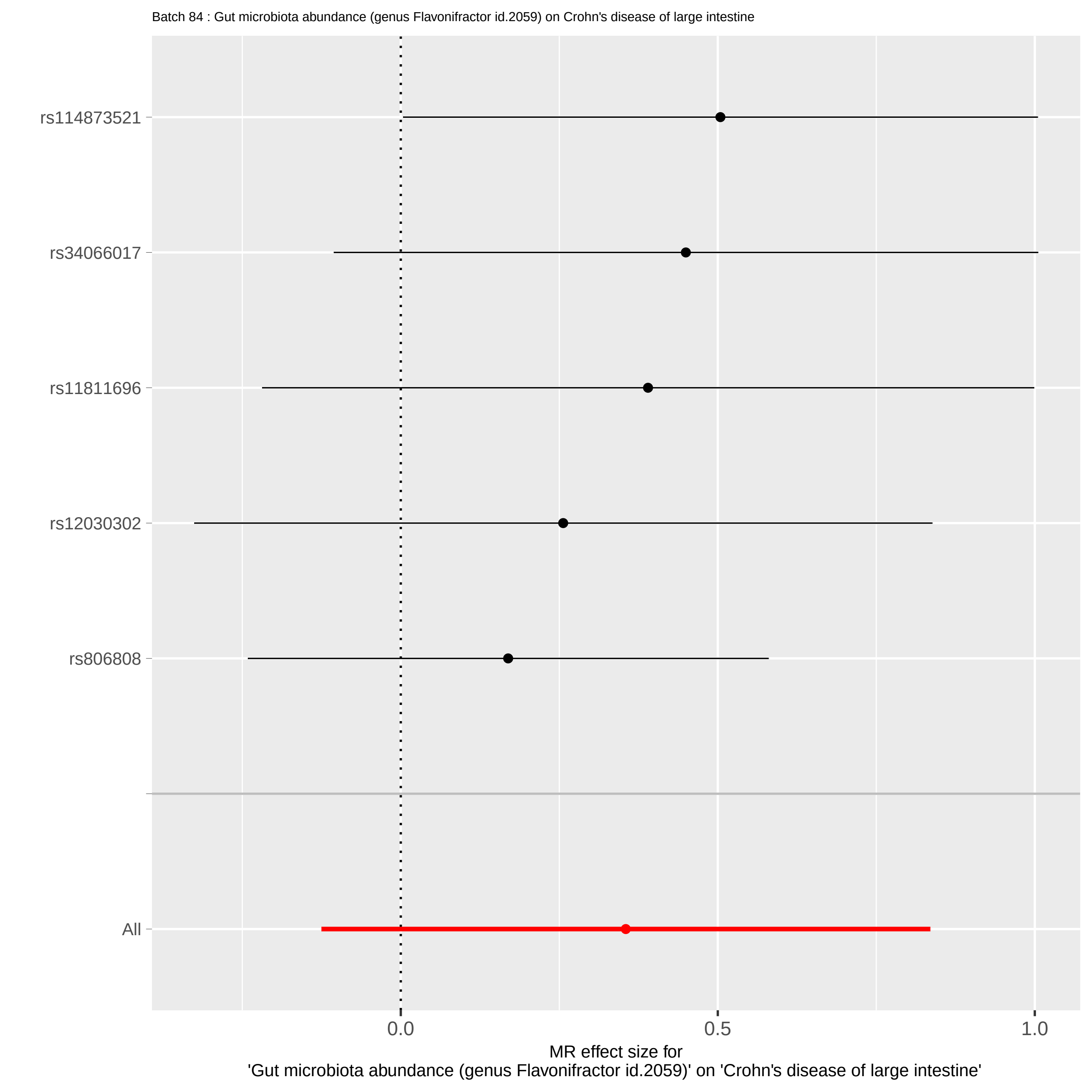
Batch 84 : Gut microbiota abundance (genus Flavonifractor id.2059) on Crohn's disease of large intestine
rs114873521
rs34066017
rs11811696
rs12030302
rs806808
All
0.0
0.5
1.0
MR effect size for
'Gut microbiota abundance (genus Flavonifractor id.2059)' on 'Crohn's disease of large intestine'

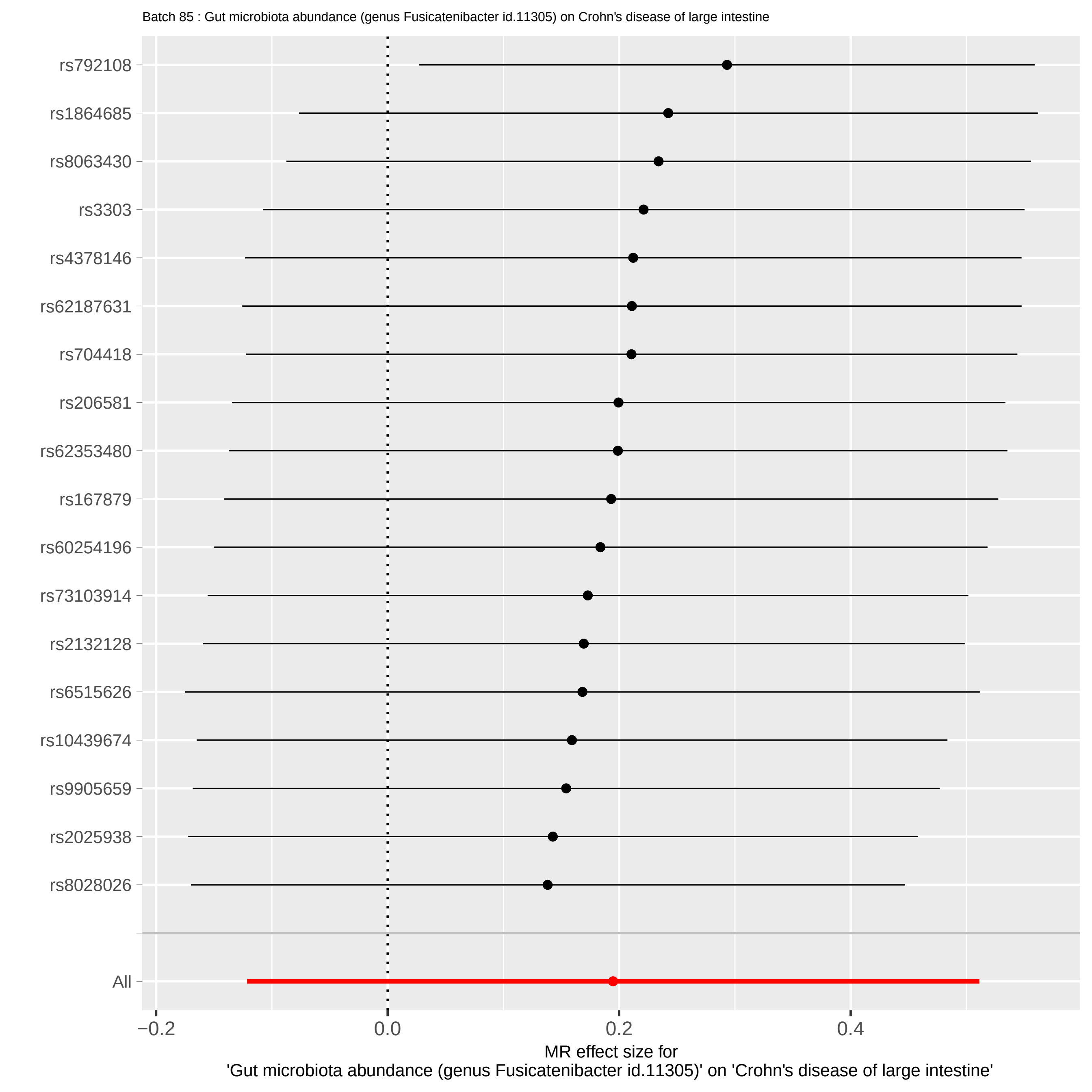

Batch 85 : Gut microbiota abundance (genus Fusicatenibacter id.11305) on Crohn's disease of large intestine
rs792108
rs1864685
rs8063430
rs3303
rs4378146
rs62187631
rs704418
rs206581
rs62353480
rs167879
rs60254196
rs73103914
rs2132128
rs6515626
rs10439674
rs9905659
rs2025938
rs8028026
All
−0.2
0.0
0.2
0.4
MR effect size for
'Gut microbiota abundance (genus Fusicatenibacter id.11305)' on 'Crohn's disease of large intestine'

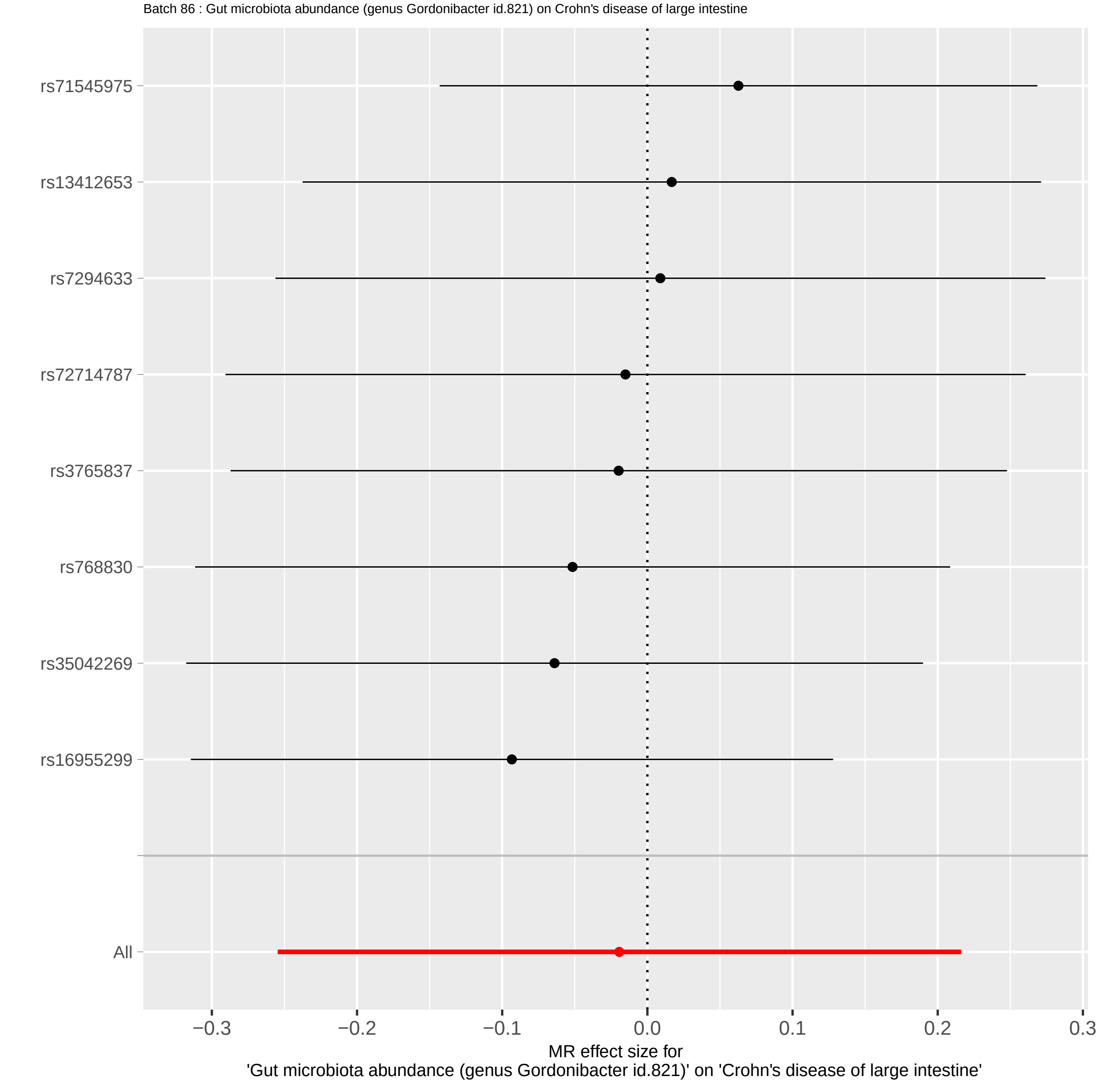
Batch 86 : Gut microbiota abundance (genus Gordonibacter id.821) on Crohn's disease of large intestine
rs71545975
rs13412653
rs7294633
rs72714787
rs3765837
rs768830
rs35042269
rs16955299
All
−0.3
−0.2
−0.1
0.0
0.1
0.2
0.3
MR effect size for
'Gut microbiota abundance (genus Gordonibacter id.821)' on 'Crohn's disease of large intestine'

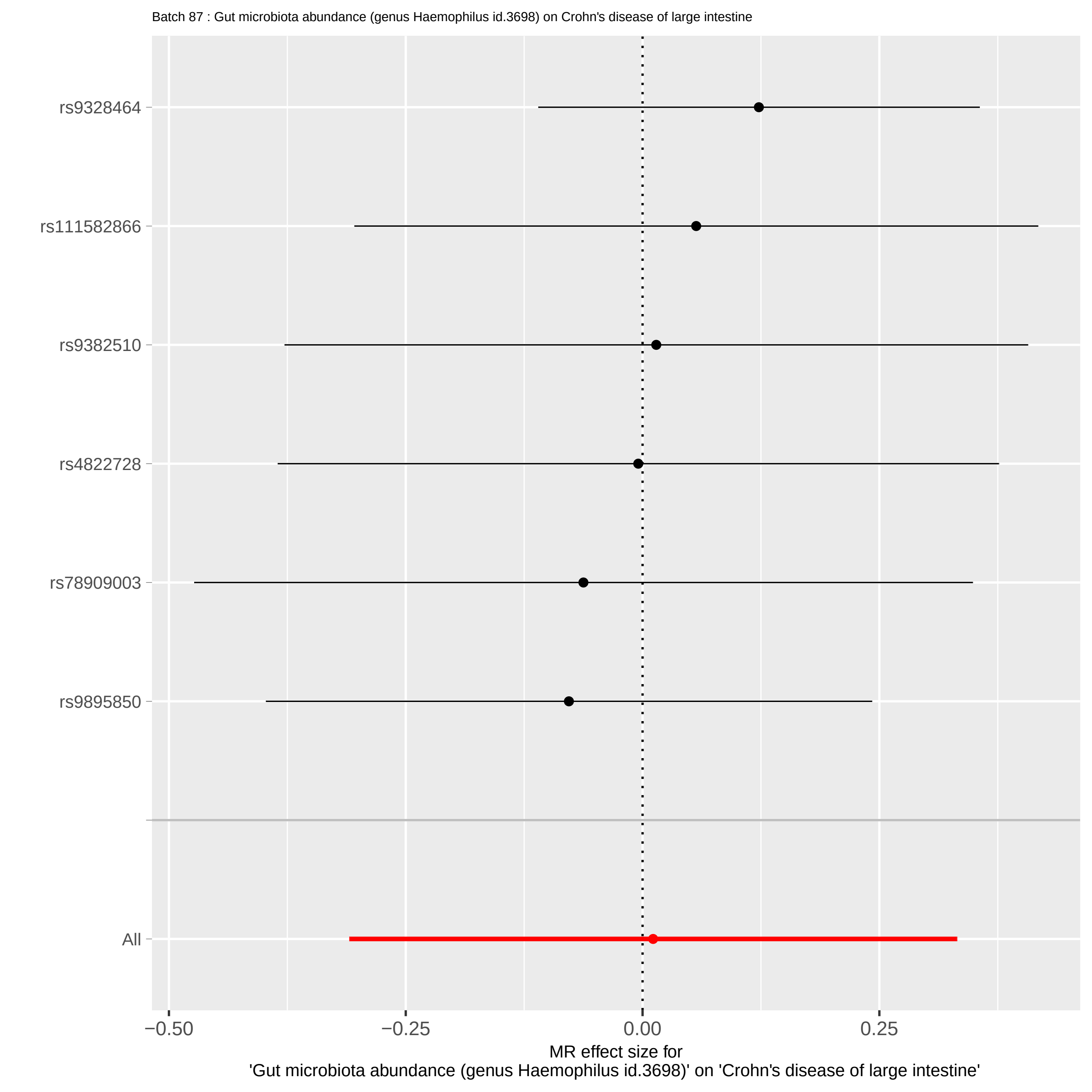

Batch 87 : Gut microbiota abundance (genus Haemophilus id.3698) on Crohn's disease of large intestine
rs9328464
rs111582866
rs9382510
rs4822728
rs78909003
rs9895850
All
−0.50
−0.25
0.00
0.25
MR effect size for
'Gut microbiota abundance (genus Haemophilus id.3698)' on 'Crohn's disease of large intestine'

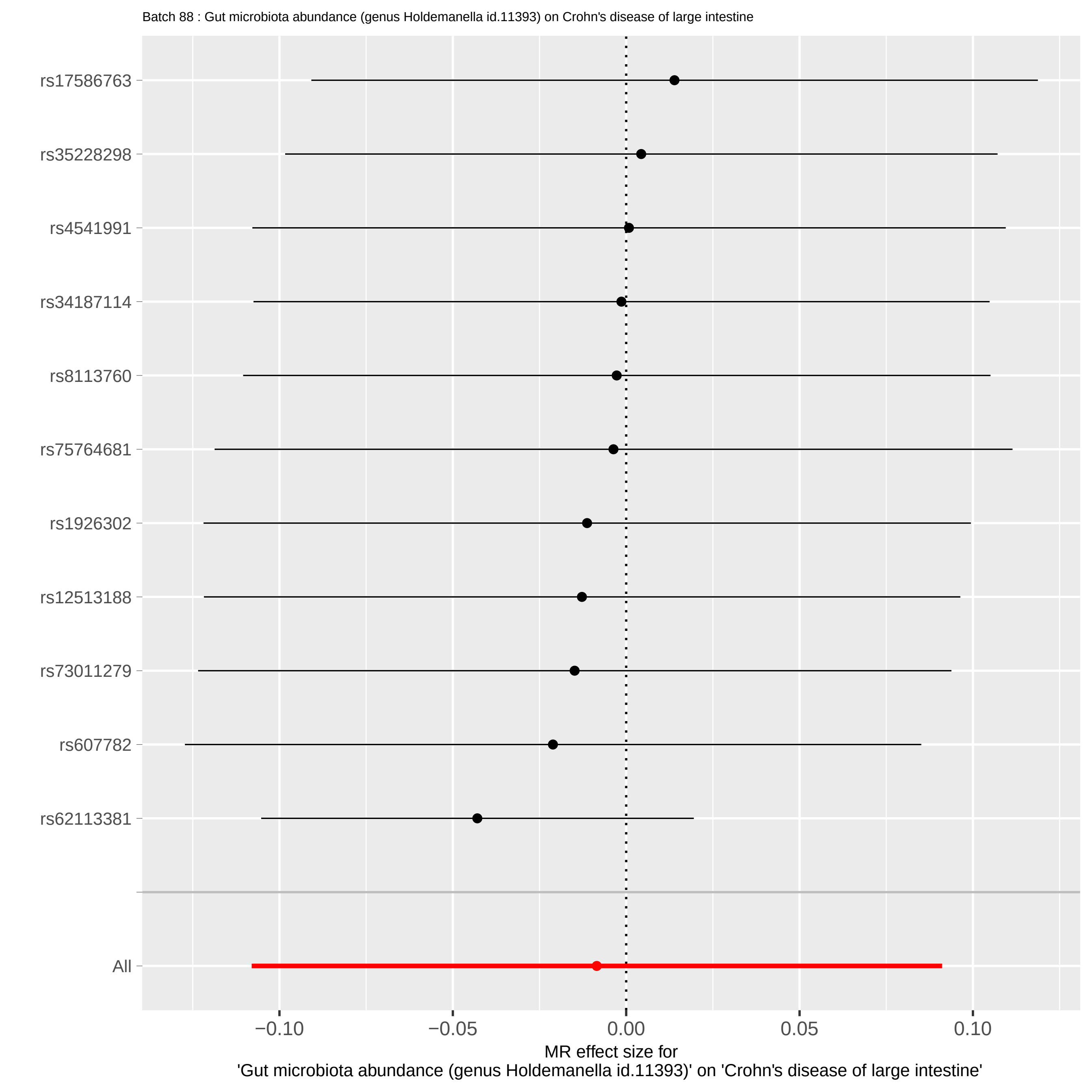
Batch 88 : Gut microbiota abundance (genus Holdemanella id.11393) on Crohn's disease of large intestine
rs17586763
rs35228298
rs4541991
rs34187114
rs8113760
rs75764681
rs1926302
rs12513188
rs73011279
rs607782
rs62113381
All
−0.10
−0.05
0.00
0.05
0.10
MR effect size for
'Gut microbiota abundance (genus Holdemanella id.11393)' on 'Crohn's disease of large intestine'

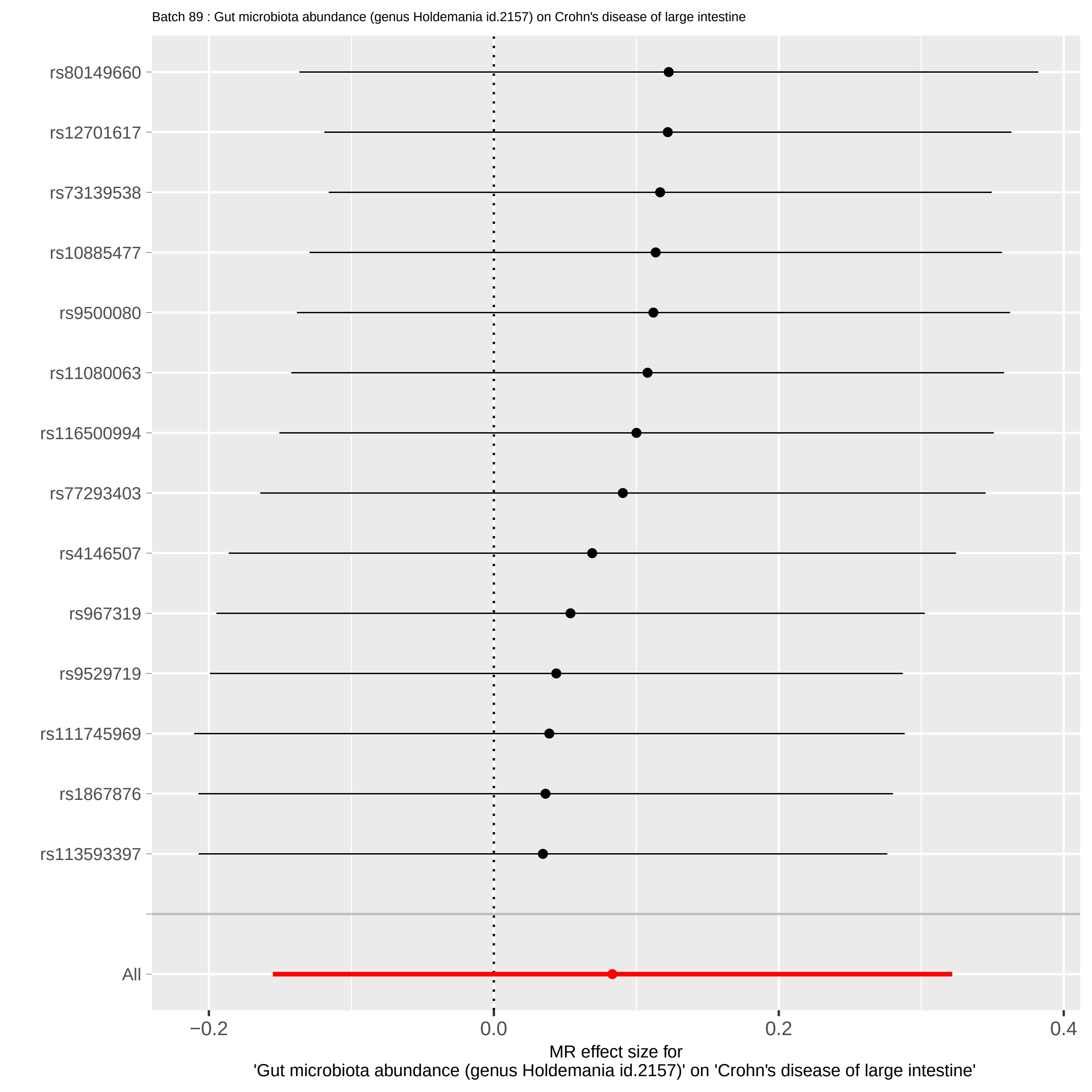

Batch 89 : Gut microbiota abundance (genus Holdemania id.2157) on Crohn's disease of large intestine
rs80149660
rs12701617
rs73139538
rs10885477
rs9500080
rs11080063
rs116500994
rs77293403
rs4146507
rs967319
rs9529719
rs111745969
rs1867876
rs113593397
All
−0.2
0.0
0.2
0.4
MR effect size for
'Gut microbiota abundance (genus Holdemania id.2157)' on 'Crohn's disease of large intestine'

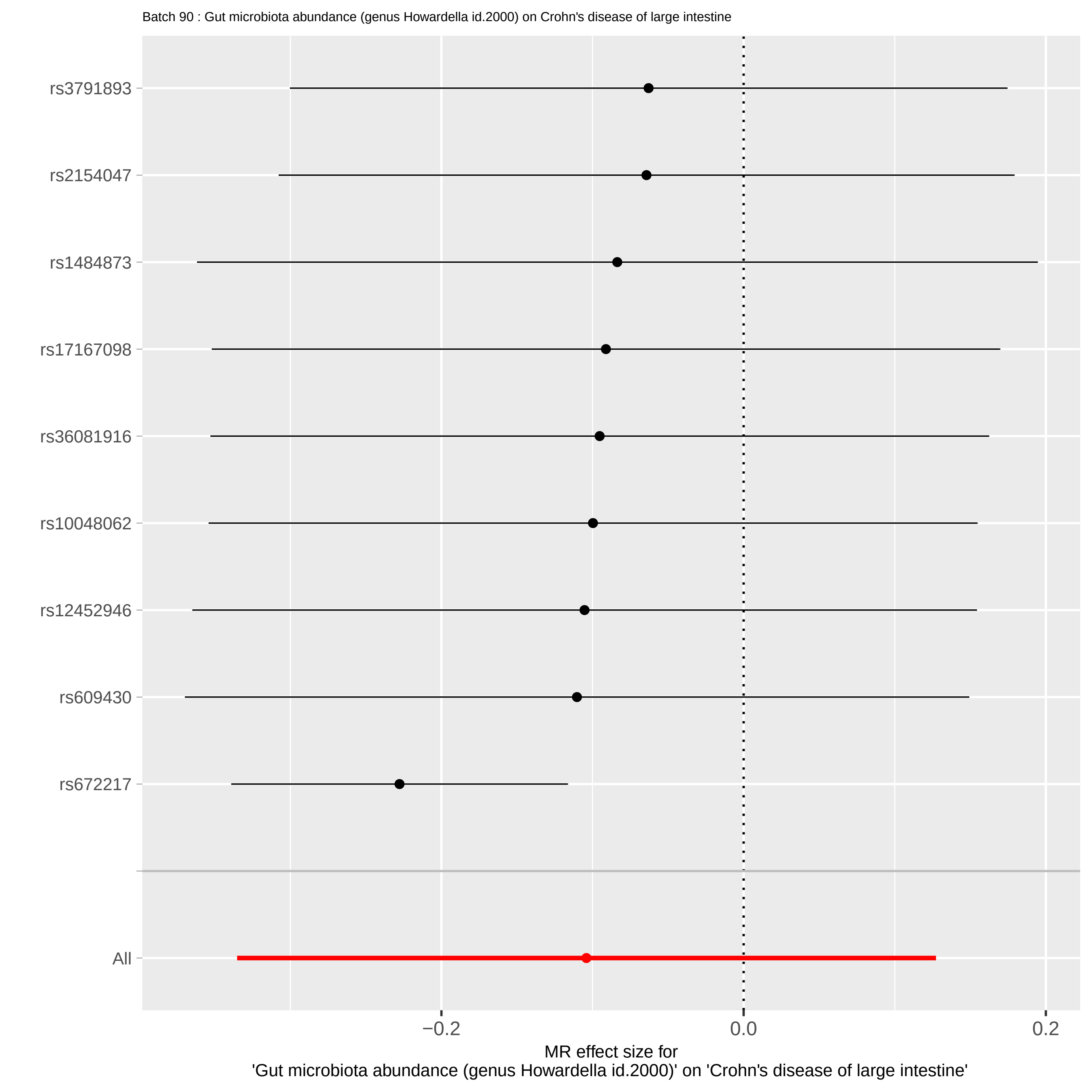
Batch 90 : Gut microbiota abundance (genus Howardella id.2000) on Crohn's disease of large intestine
rs3791893
rs2154047
rs1484873
rs17167098
rs36081916
rs10048062
rs12452946
rs609430
rs672217
All
−0.2
0.0
0.2
MR effect size for
'Gut microbiota abundance (genus Howardella id.2000)' on 'Crohn's disease of large intestine'

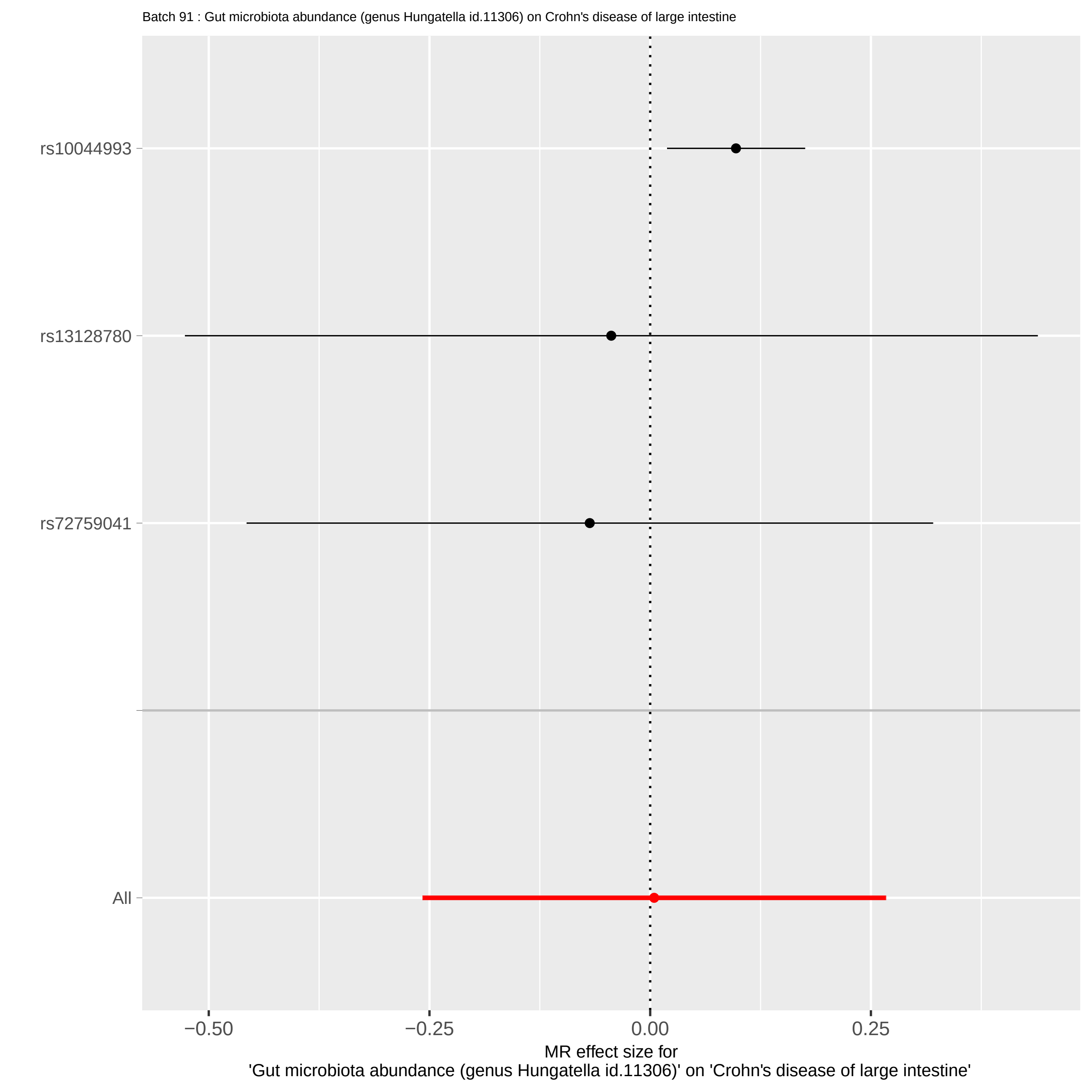

Batch 91 : Gut microbiota abundance (genus Hungatella id.11306) on Crohn's disease of large intestine
rs10044993
rs13128780
rs72759041
All
−0.50
−0.25
0.00
0.25
MR effect size for
'Gut microbiota abundance (genus Hungatella id.11306)' on 'Crohn's disease of large intestine'

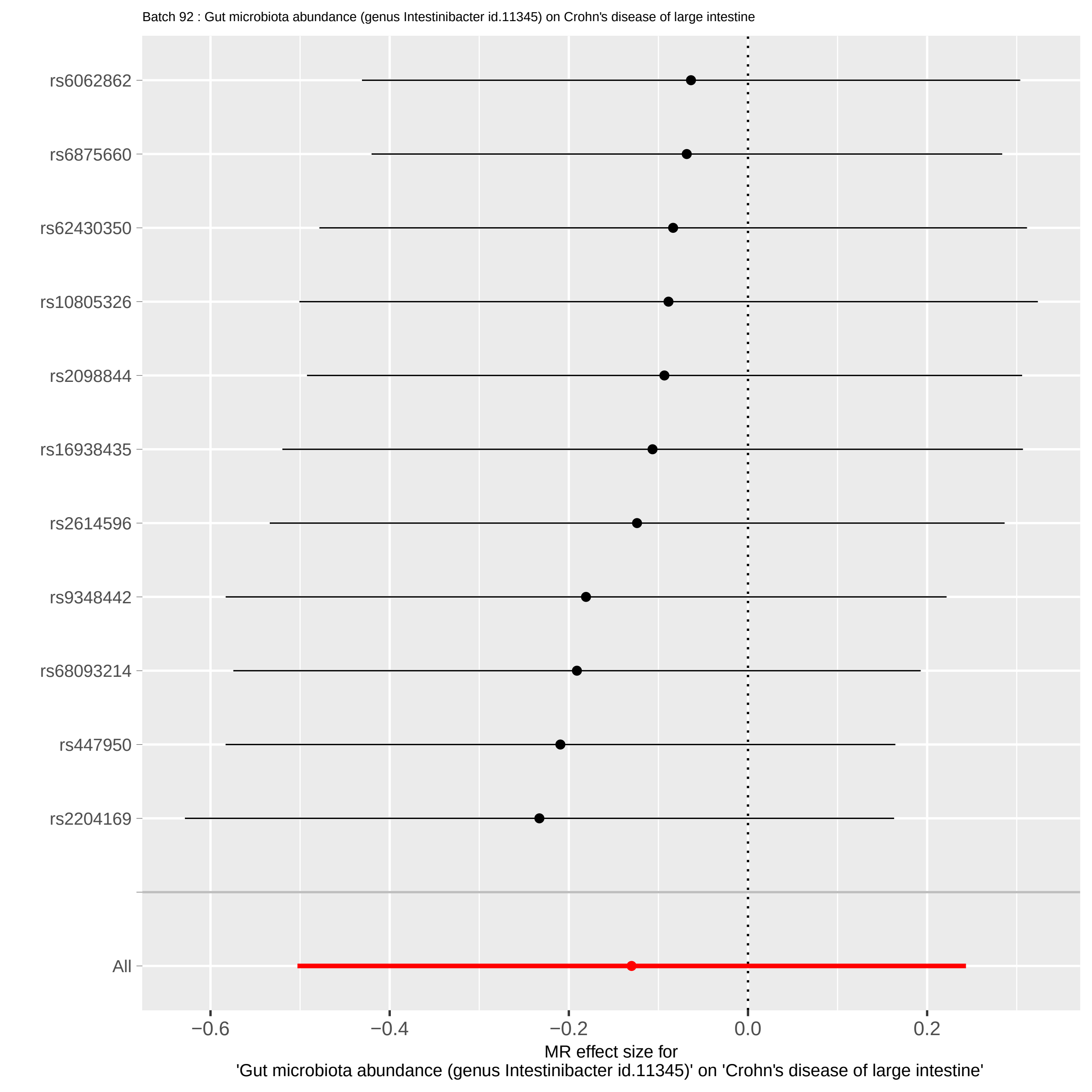
Batch 92 : Gut microbiota abundance (genus Intestinibacter id.11345) on Crohn's disease of large intestine
rs6062862
rs6875660
rs62430350
rs10805326
rs2098844
rs16938435
rs2614596
rs9348442
rs68093214
rs447950
rs2204169
All
−0.6
−0.4
−0.2
0.0
0.2
MR effect size for
'Gut microbiota abundance (genus Intestinibacter id.11345)' on 'Crohn's disease of large intestine'

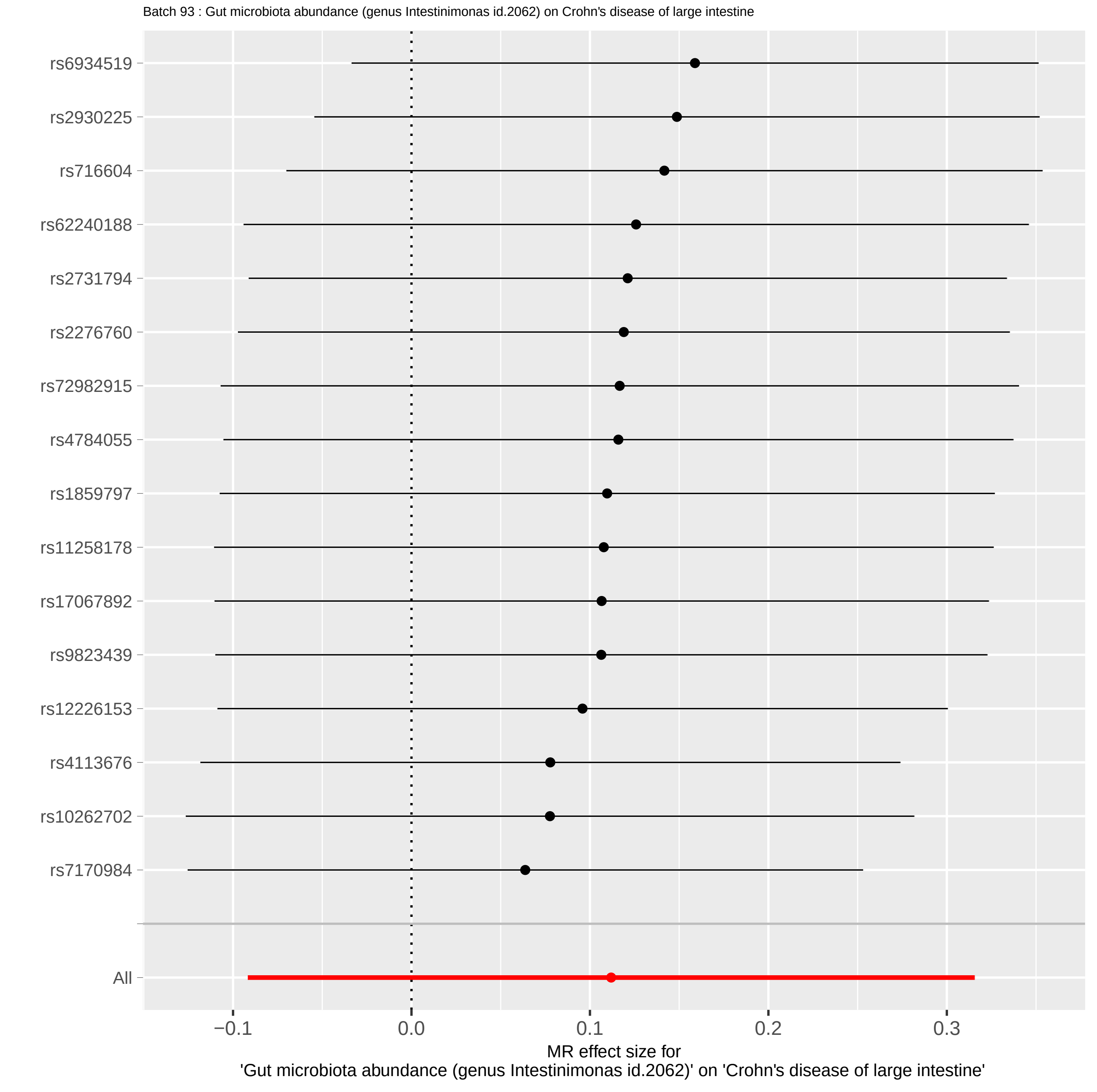

Batch 93 : Gut microbiota abundance (genus Intestinimonas id.2062) on Crohn's disease of large intestine
rs6934519
rs2930225
rs716604
rs62240188
rs2731794
rs2276760
rs72982915
rs4784055
rs1859797
rs11258178
rs17067892
rs9823439
rs12226153
rs4113676
rs10262702
rs7170984
All
−0.1
0.0
0.1
0.2
0.3
MR effect size for
'Gut microbiota abundance (genus Intestinimonas id.2062)' on 'Crohn's disease of large intestine'

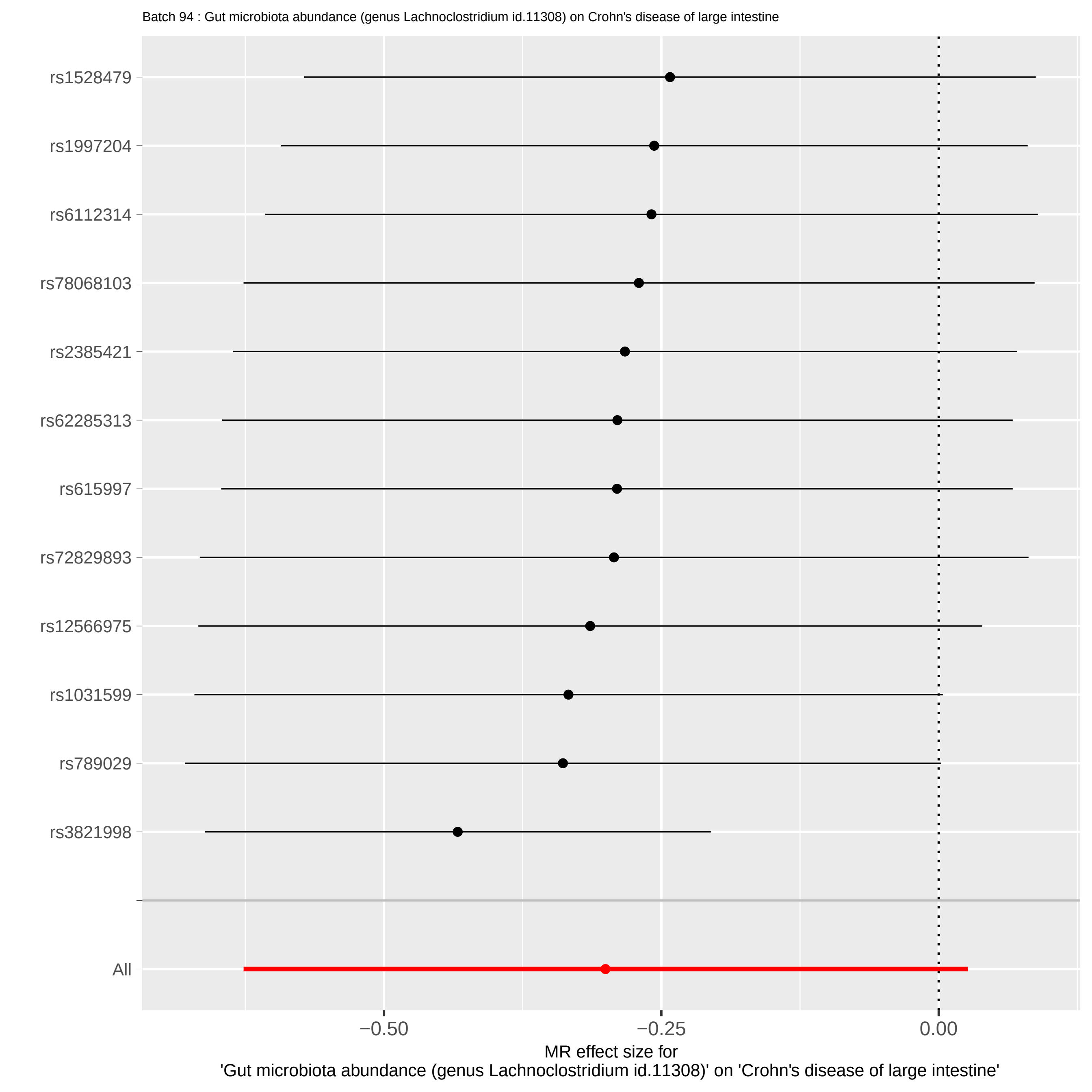

Batch 94 : Gut microbiota abundance (genus Lachnoclostridium id.11308) on Crohn's disease of large intestine
rs1528479
rs1997204
rs6112314
rs78068103
rs2385421
rs62285313
rs615997
rs72829893
rs12566975
rs1031599
rs789029
rs3821998
All
−0.50
−0.25
0.00
MR effect size for
'Gut microbiota abundance (genus Lachnoclostridium id.11308)' on 'Crohn's disease of large intestine'

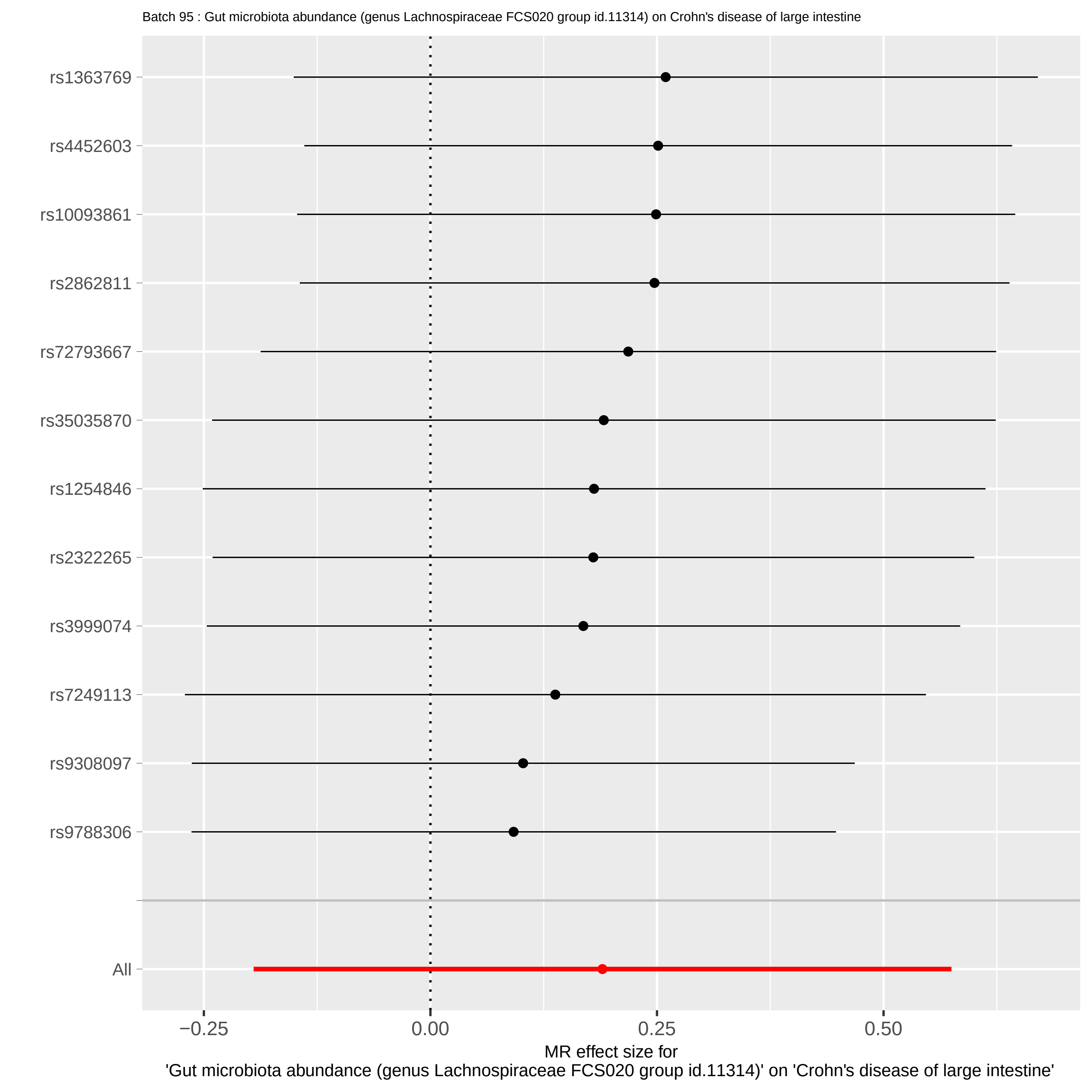

Batch 95 : Gut microbiota abundance (genus Lachnospiraceae FCS020 group id.11314) on Crohn's disease of large intestine
rs1363769
rs4452603
rs10093861
rs2862811
rs72793667
rs35035870
rs1254846
rs2322265
rs3999074
rs7249113
rs9308097
rs9788306
All
−0.25
0.00
0.25
0.50
MR effect size for
'Gut microbiota abundance (genus Lachnospiraceae FCS020 group id.11314)' on 'Crohn's disease of large intestine'

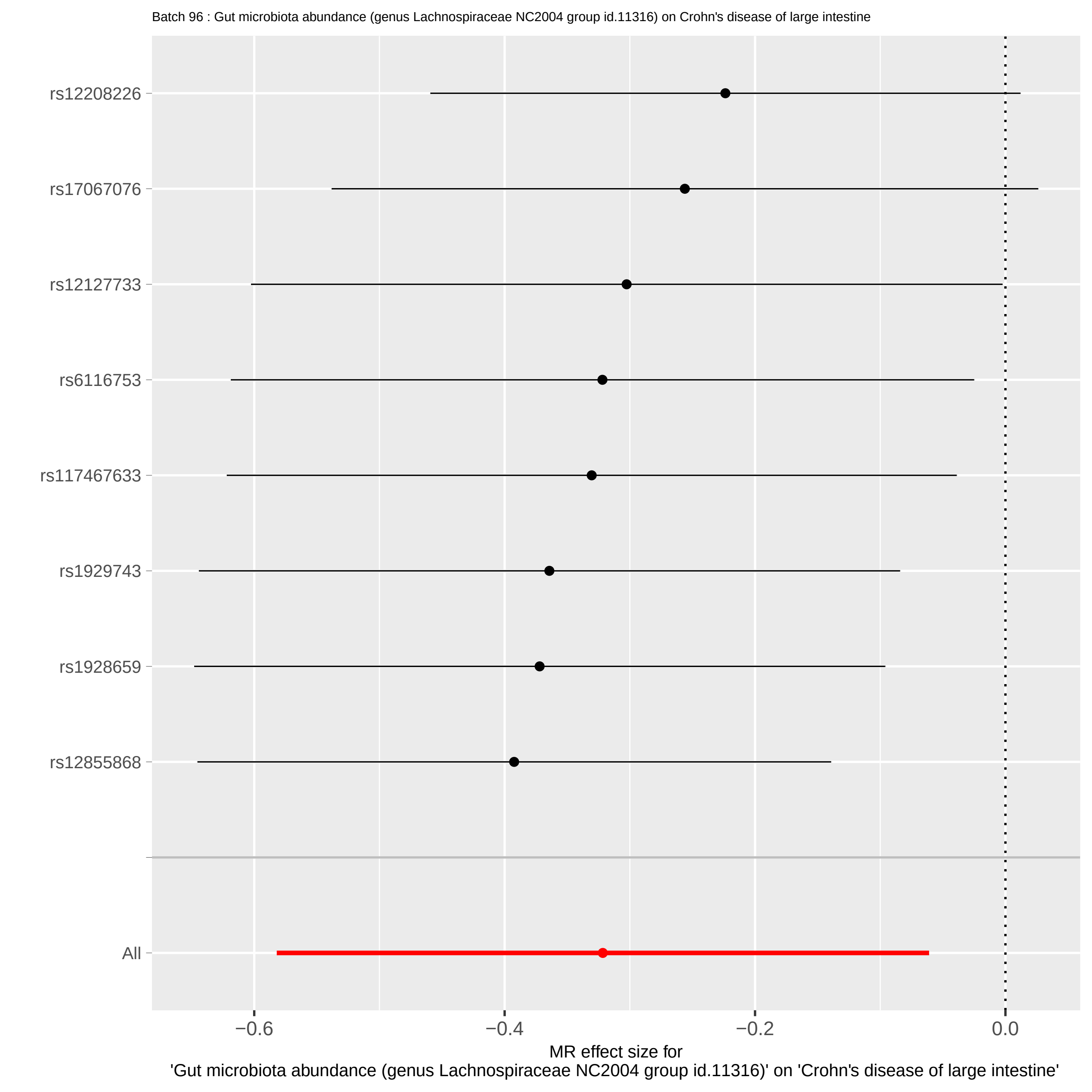

Batch 96 : Gut microbiota abundance (genus Lachnospiraceae NC2004 group id.11316) on Crohn's disease of large intestine
rs12208226
rs17067076
rs12127733
rs6116753
rs117467633
rs1929743
rs1928659
rs12855868
All
−0.6
−0.4
−0.2
0.0
MR effect size for
'Gut microbiota abundance (genus Lachnospiraceae NC2004 group id.11316)' on 'Crohn's disease of large intestine'

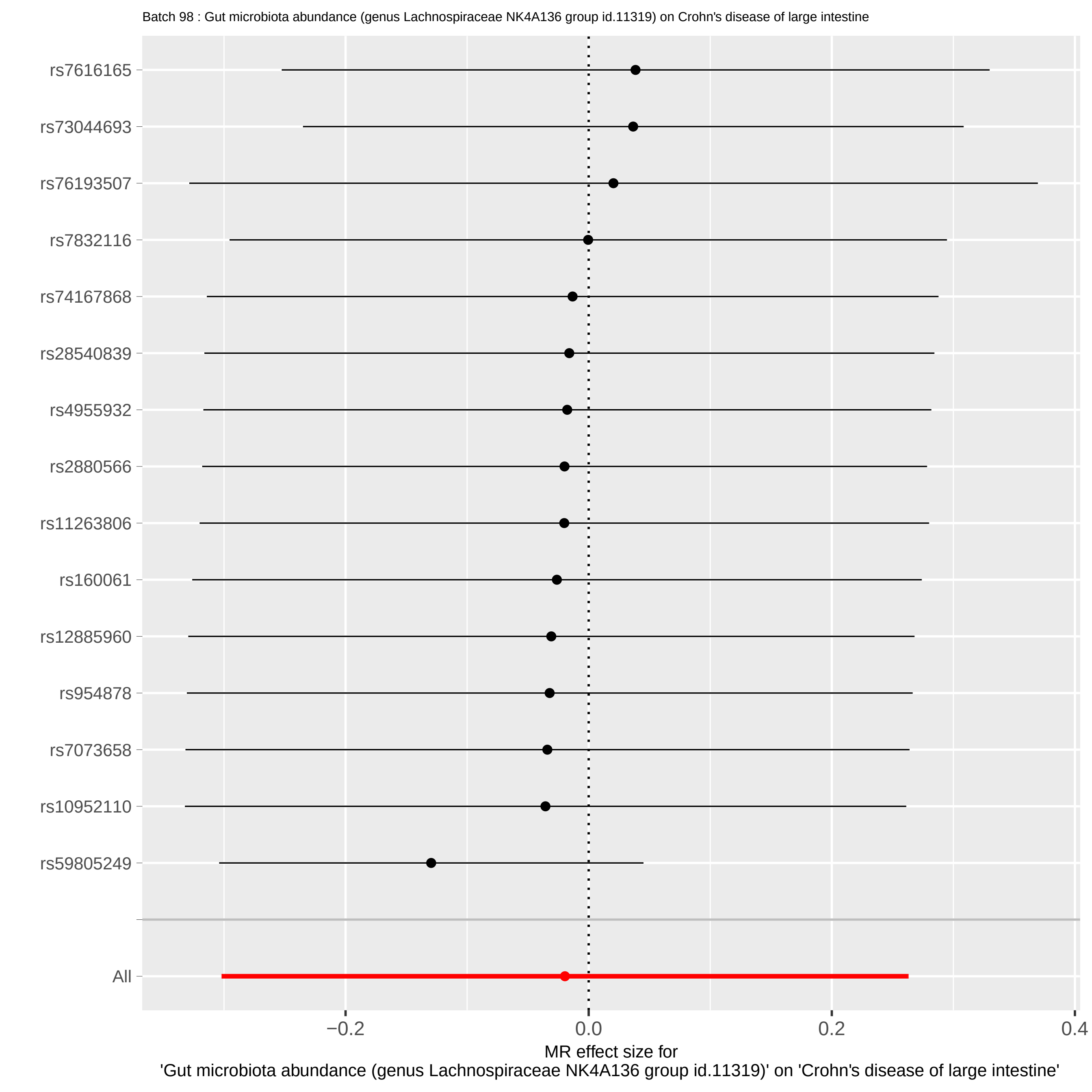
Batch 98 : Gut microbiota abundance (genus Lachnospiraceae NK4A136 group id.11319) on Crohn's disease of large intestine
rs7616165
rs73044693
rs76193507
rs7832116
rs74167868
rs28540839
rs4955932
rs2880566
rs11263806
rs160061
rs12885960
rs954878
rs7073658
rs10952110
rs59805249
All
−0.2
0.0
0.2
0.4
MR effect size for
'Gut microbiota abundance (genus Lachnospiraceae NK4A136 group id.11319)' on 'Crohn's disease of large intestine'

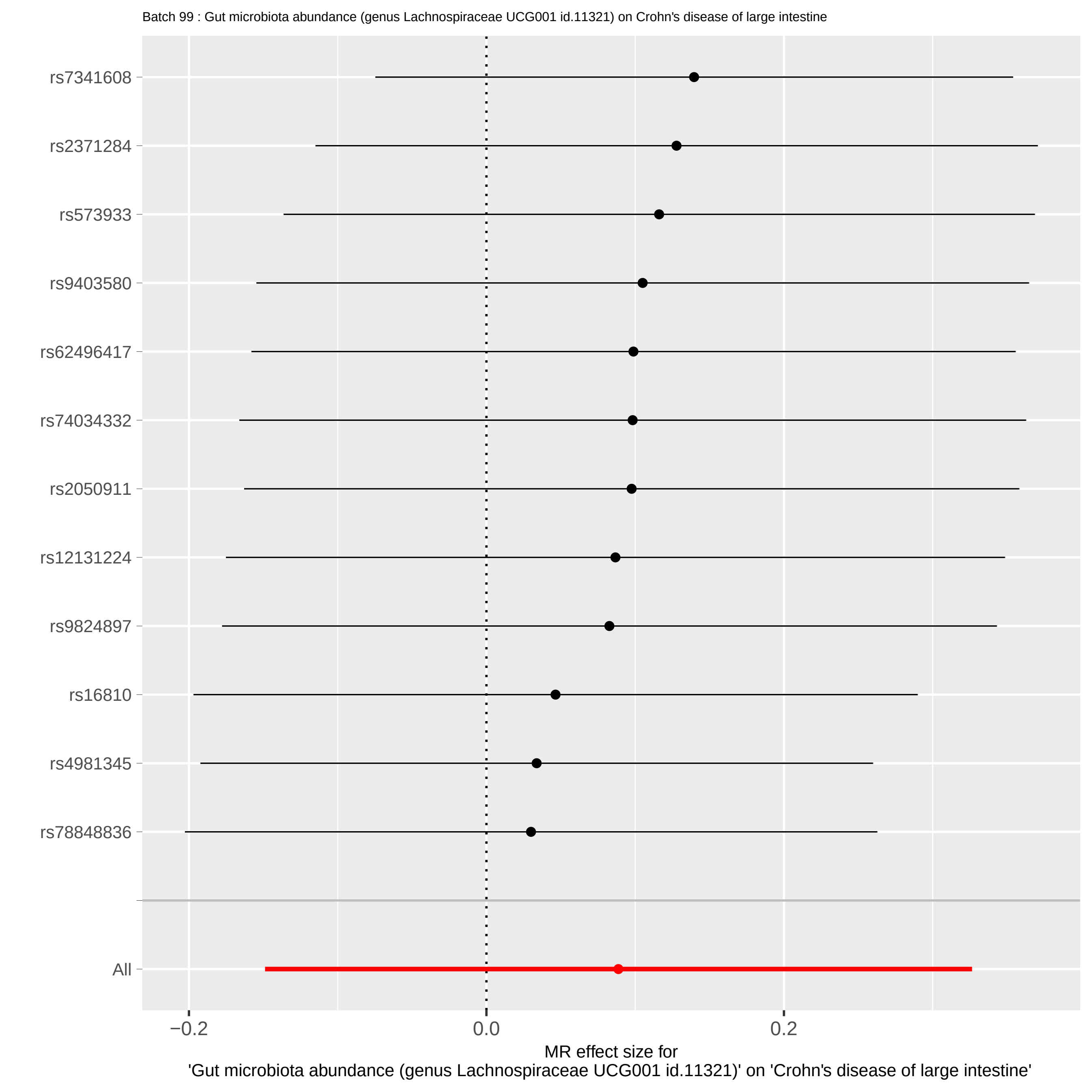

Batch 99 : Gut microbiota abundance (genus Lachnospiraceae UCG001 id.11321) on Crohn's disease of large intestine
rs7341608
rs2371284
rs573933
rs9403580
rs62496417
rs74034332
rs2050911
rs12131224
rs9824897
rs16810
rs4981345
rs78848836
All
−0.2
0.0
0.2
MR effect size for
'Gut microbiota abundance (genus Lachnospiraceae UCG001 id.11321)' on 'Crohn's disease of large intestine'

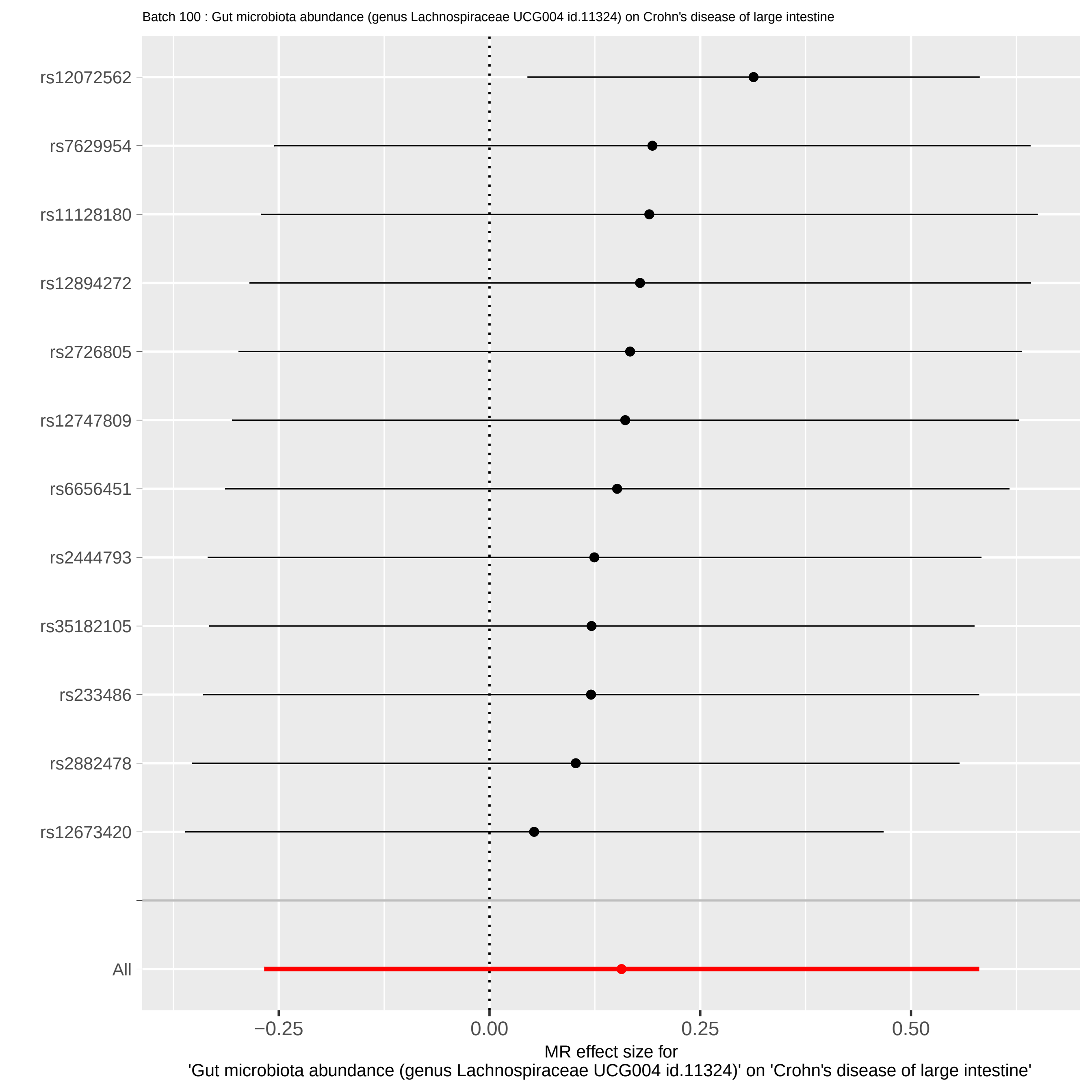
Batch 100 : Gut microbiota abundance (genus Lachnospiraceae UCG004 id.11324) on Crohn's disease of large intestine
rs12072562
rs7629954
rs11128180
rs12894272
rs2726805
rs12747809
rs6656451
rs2444793
rs35182105
rs233486
rs2882478
rs12673420
All
−0.25
0.00
0.25
0.50
MR effect size for
'Gut microbiota abundance (genus Lachnospiraceae UCG004 id.11324)' on 'Crohn's disease of large intestine'

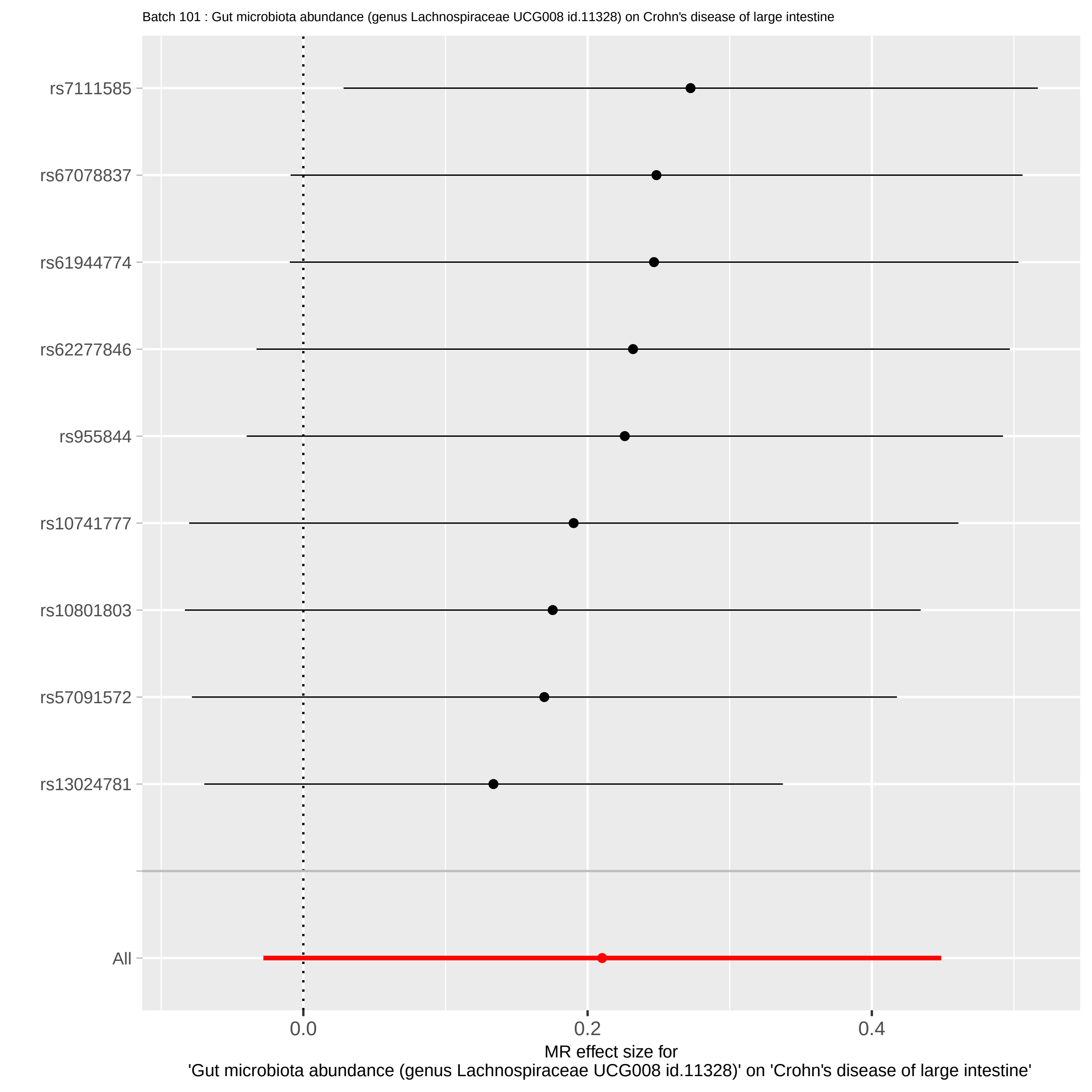

Batch 101 : Gut microbiota abundance (genus Lachnospiraceae UCG008 id.11328) on Crohn's disease of large intestine
rs7111585
rs67078837
rs61944774
rs62277846
rs955844
rs10741777
rs10801803
rs57091572
rs13024781
All
0.0
0.2
0.4
MR effect size for
'Gut microbiota abundance (genus Lachnospiraceae UCG008 id.11328)' on 'Crohn's disease of large intestine'

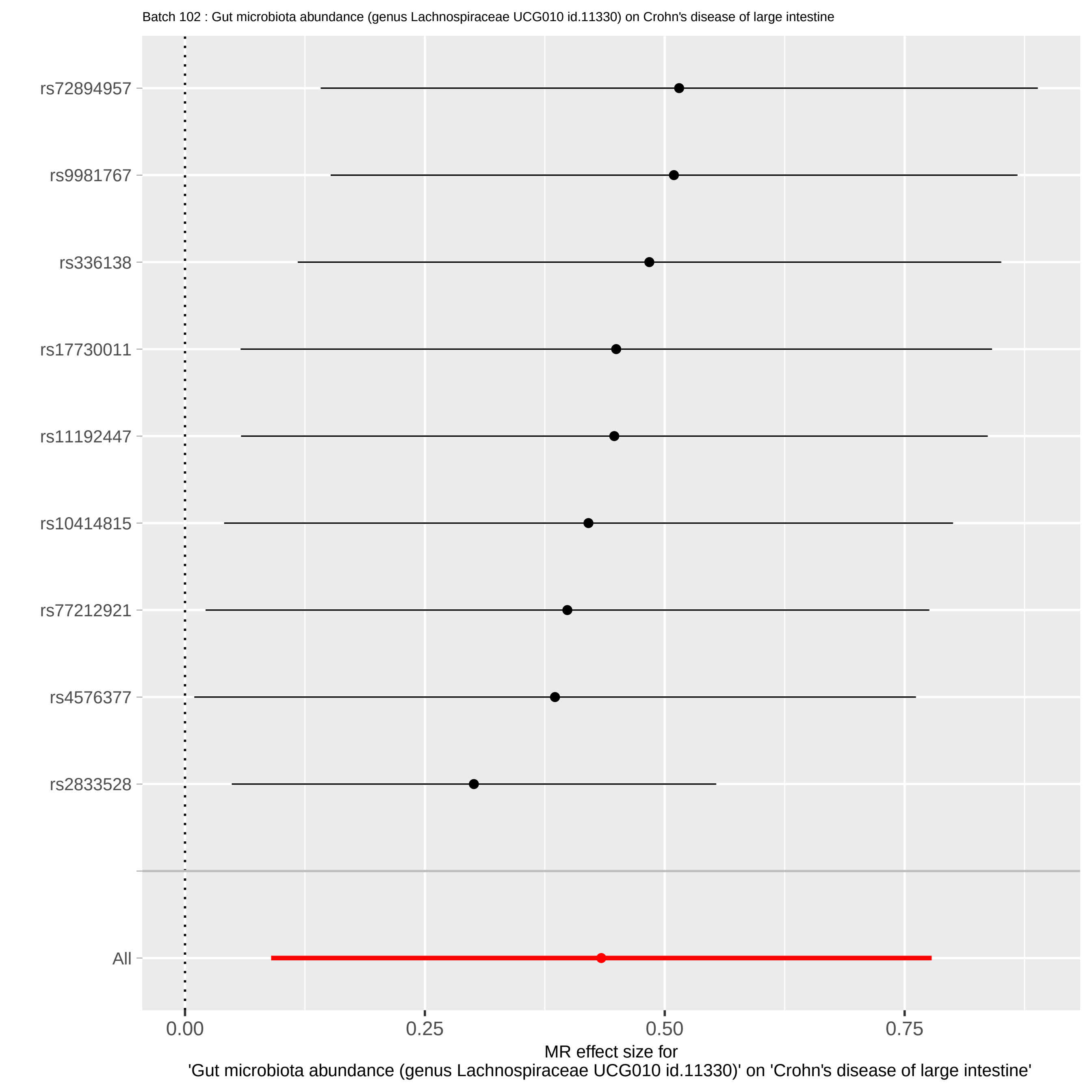

Batch 102 : Gut microbiota abundance (genus Lachnospiraceae UCG010 id.11330) on Crohn's disease of large intestine
rs72894957
rs9981767
rs336138
rs17730011
rs11192447
rs10414815
rs77212921
rs4576377
rs2833528
All
0.00
0.25
0.50
0.75
MR effect size for
'Gut microbiota abundance (genus Lachnospiraceae UCG010 id.11330)' on 'Crohn's disease of large intestine'

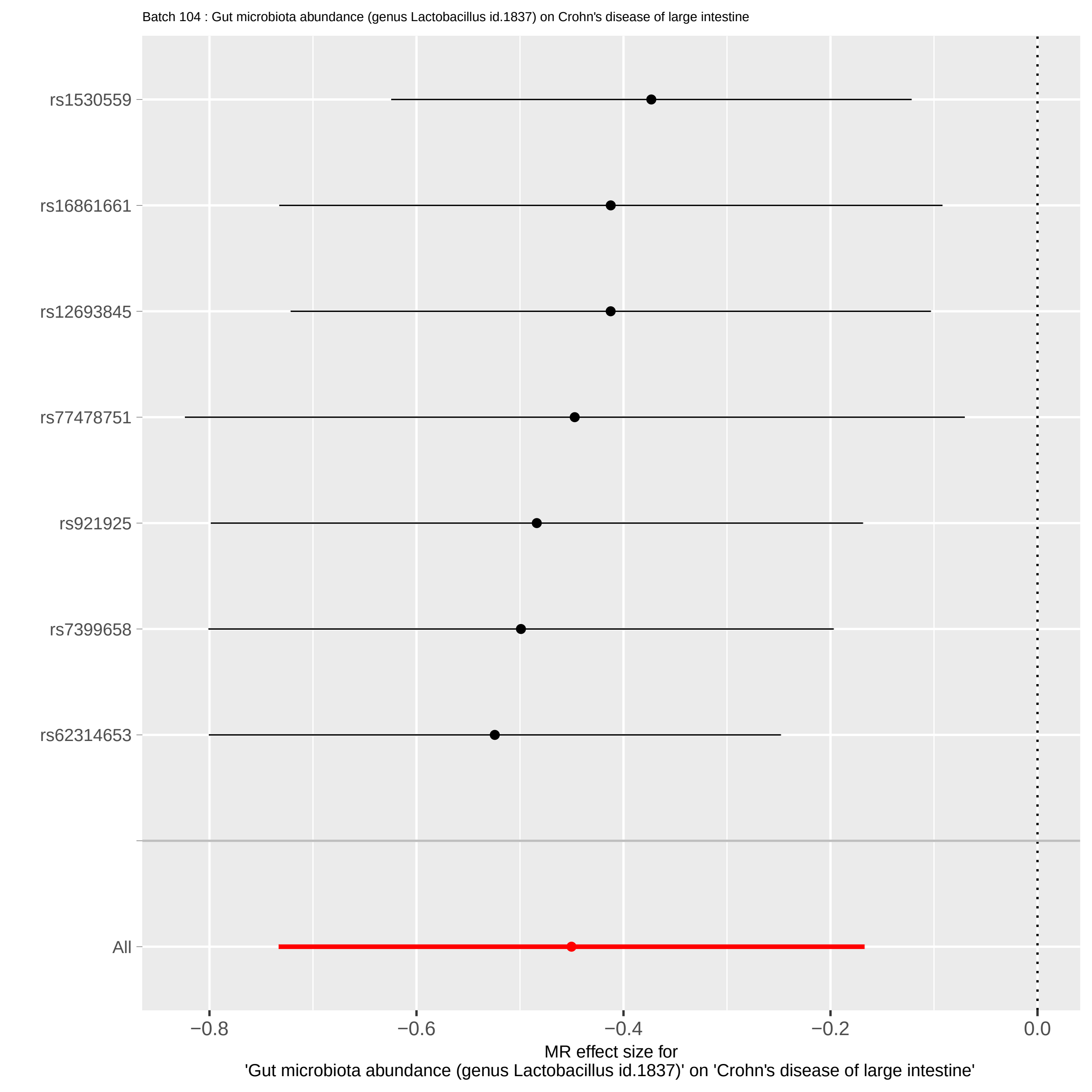
Batch 104 : Gut microbiota abundance (genus Lactobacillus id.1837) on Crohn's disease of large intestine
rs1530559
rs16861661
rs12693845
rs77478751
rs921925
rs7399658
rs62314653
All
−0.8
−0.6
−0.4
−0.2
0.0
MR effect size for
'Gut microbiota abundance (genus Lactobacillus id.1837)' on 'Crohn's disease of large intestine'

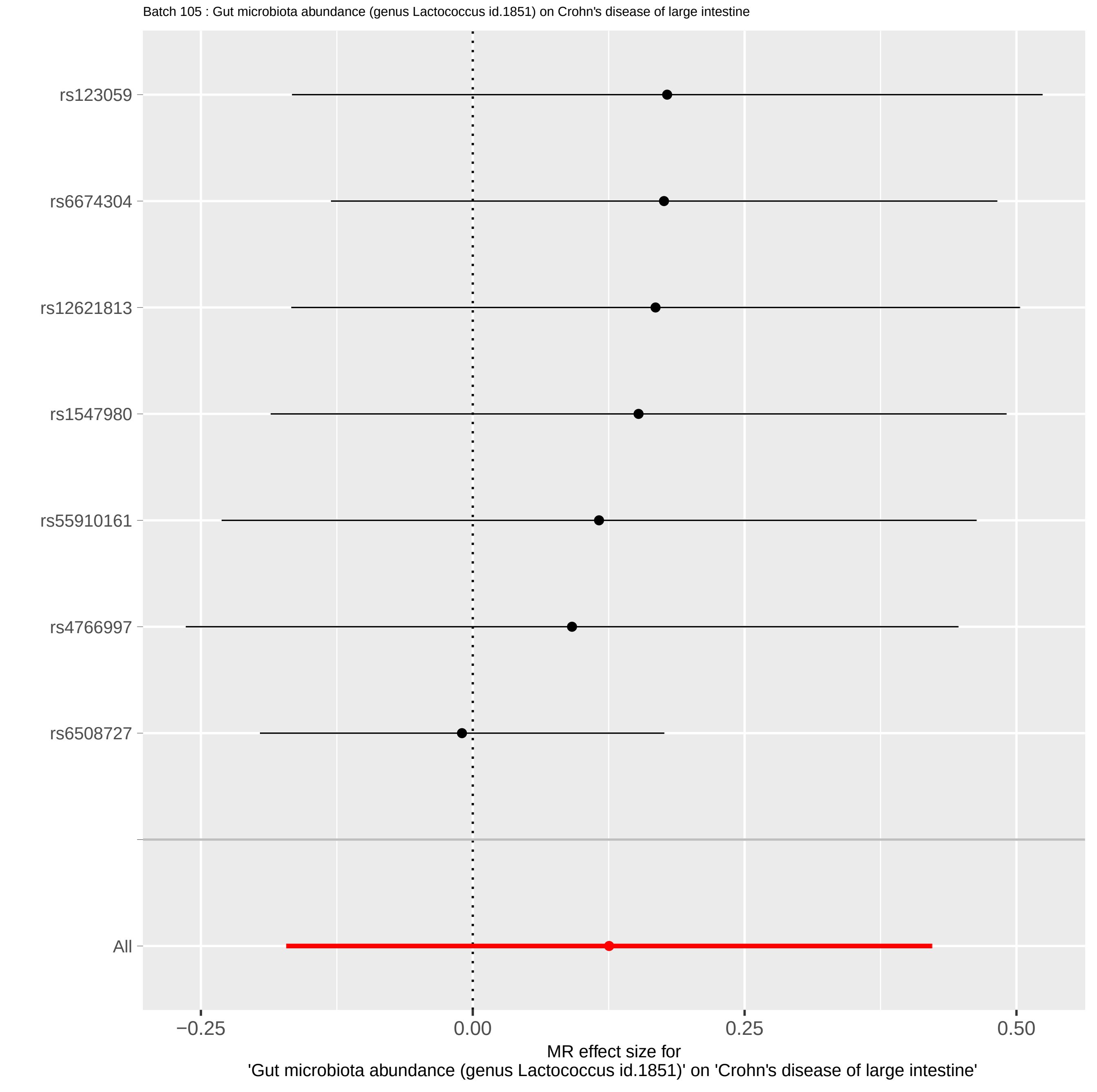

Batch 105 : Gut microbiota abundance (genus Lactococcus id.1851) on Crohn's disease of large intestine
rs123059
rs6674304
rs12621813
rs1547980
rs55910161
rs4766997
rs6508727
All
−0.25
0.00
0.25
0.50
MR effect size for
'Gut microbiota abundance (genus Lactococcus id.1851)' on 'Crohn's disease of large intestine'

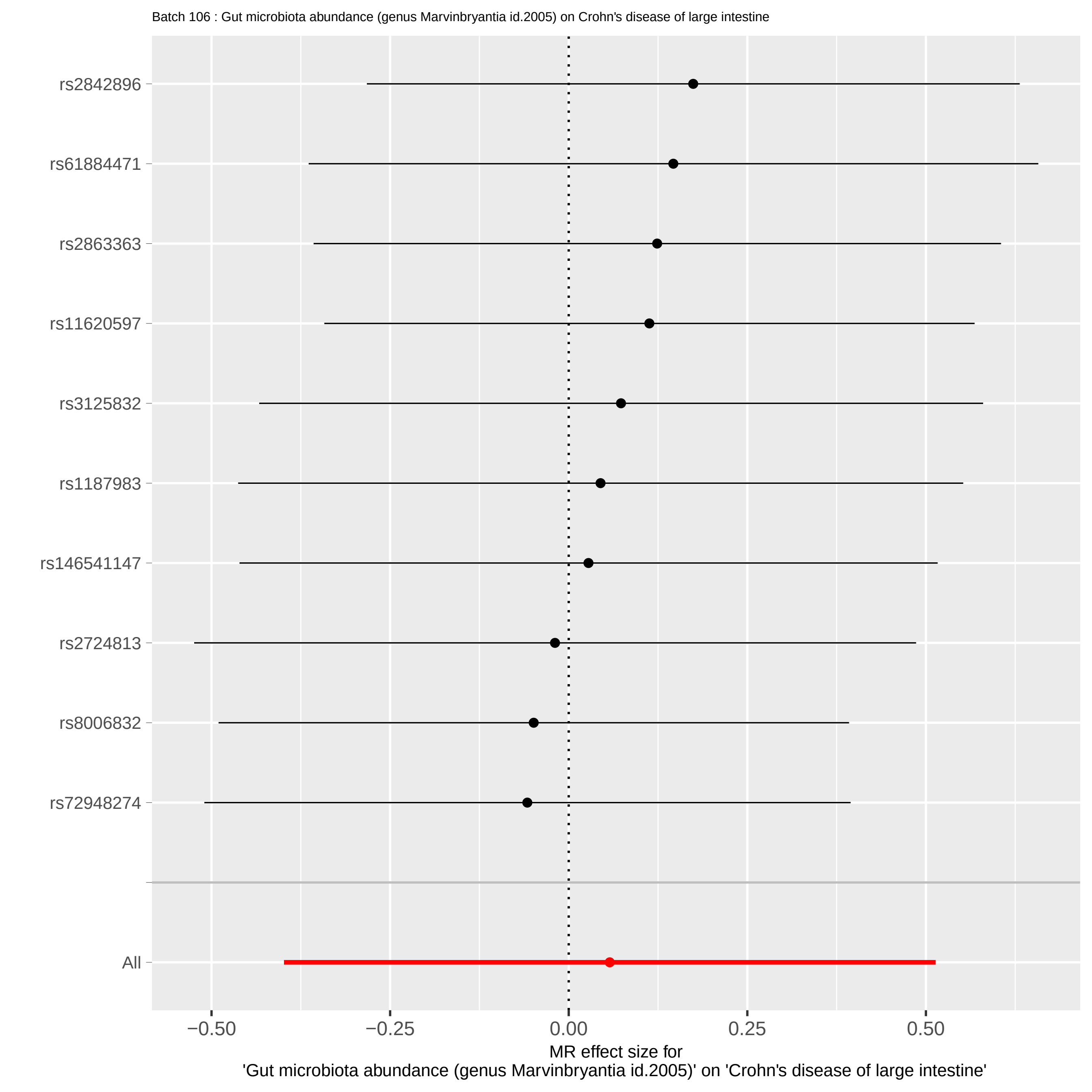

Batch 106 : Gut microbiota abundance (genus Marvinbryantia id.2005) on Crohn's disease of large intestine
rs2842896
rs61884471
rs2863363
rs11620597
rs3125832
rs1187983
rs146541147
rs2724813
rs8006832
rs72948274
All
−0.50
−0.25
0.00
0.25
0.50
MR effect size for
'Gut microbiota abundance (genus Marvinbryantia id.2005)' on 'Crohn's disease of large intestine'

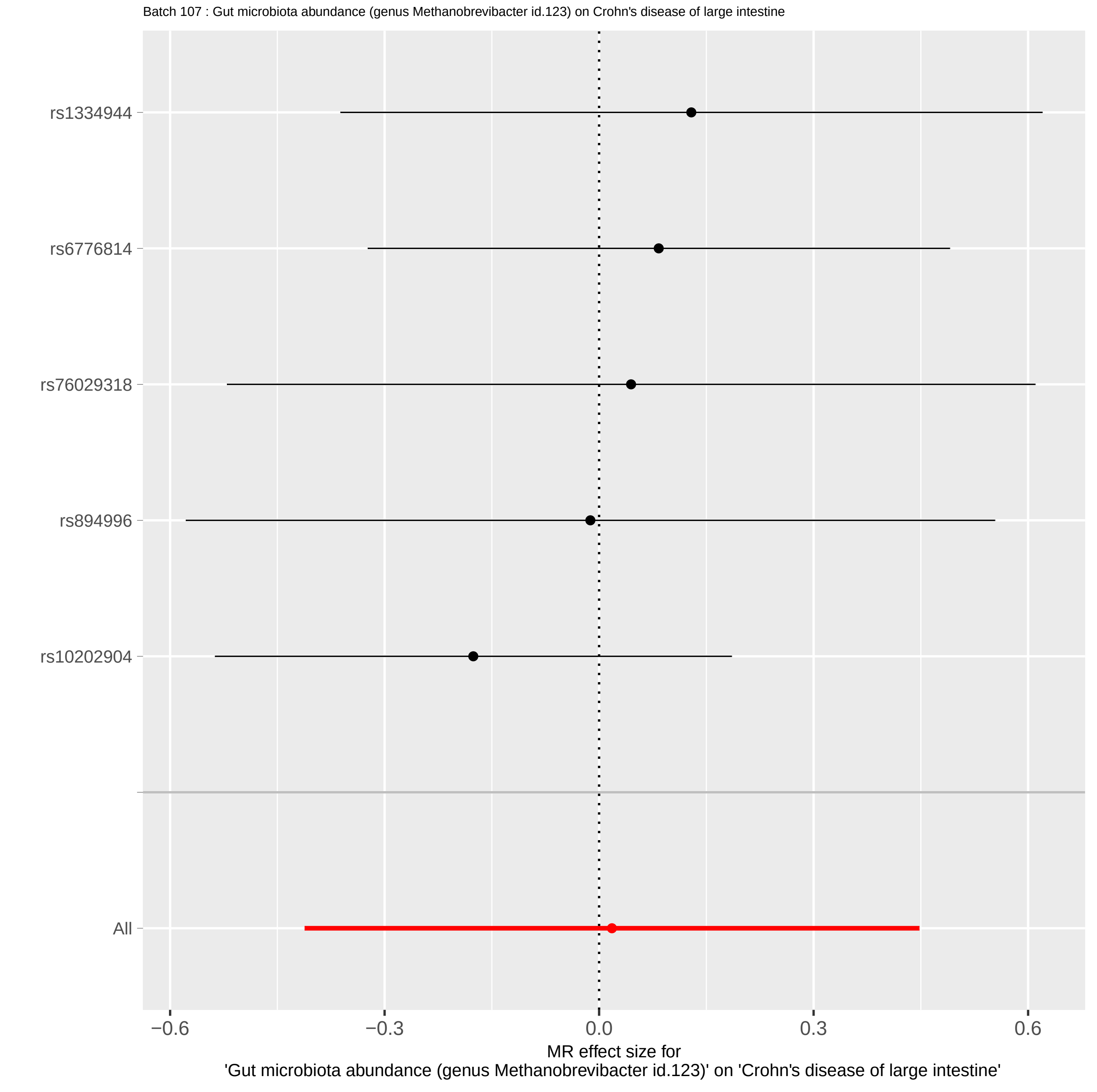

Batch 107 : Gut microbiota abundance (genus Methanobrevibacter id.123) on Crohn's disease of large intestine
rs1334944
rs6776814
rs76029318
rs894996
rs10202904
All
−0.6
−0.3
0.0
0.3
0.6
MR effect size for
'Gut microbiota abundance (genus Methanobrevibacter id.123)' on 'Crohn's disease of large intestine'

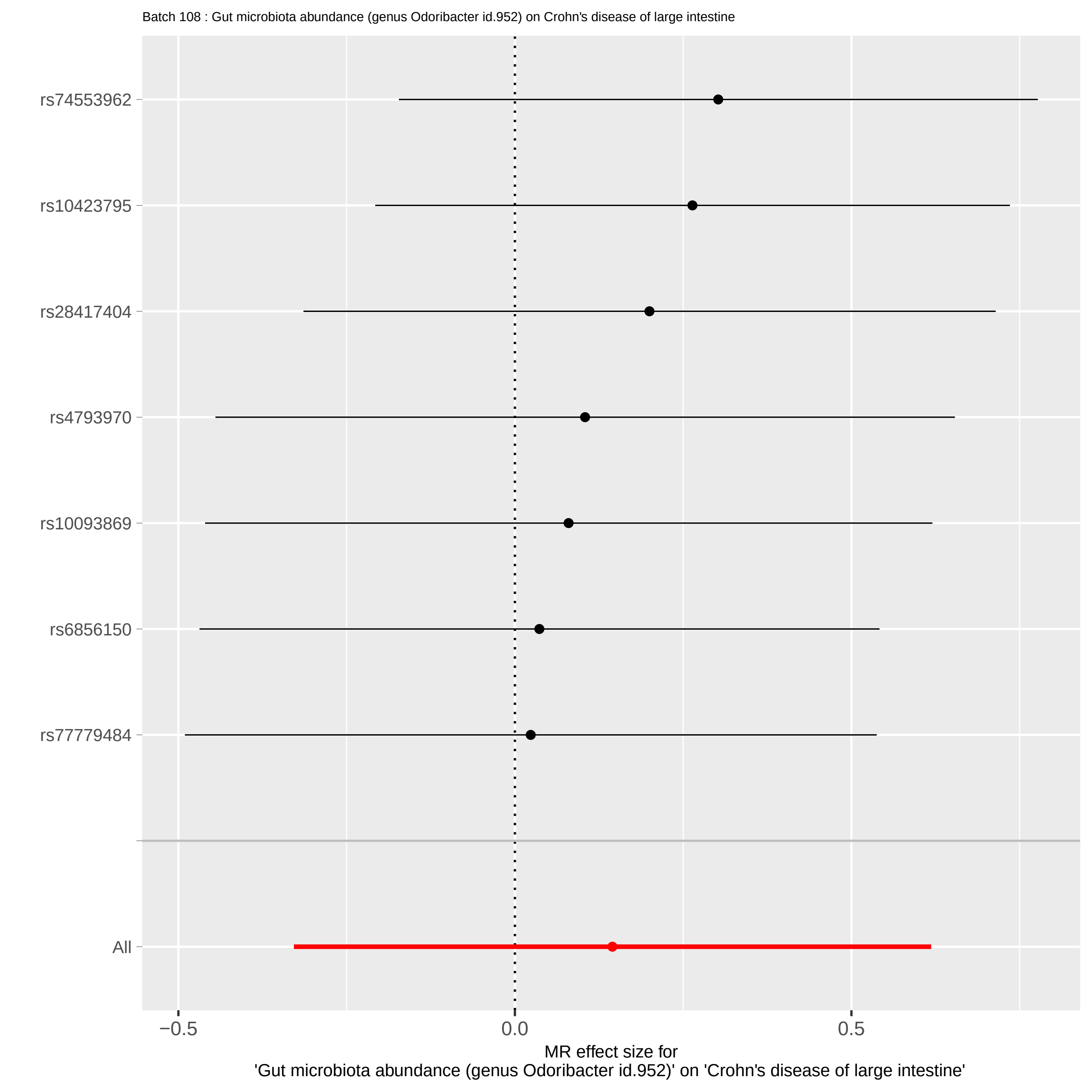

Batch 108 : Gut microbiota abundance (genus Odoribacter id.952) on Crohn's disease of large intestine
rs74553962
rs10423795
rs28417404
rs4793970
rs10093869
rs6856150
rs77779484
All
−0.5
0.0
0.5
MR effect size for
'Gut microbiota abundance (genus Odoribacter id.952)' on 'Crohn's disease of large intestine'

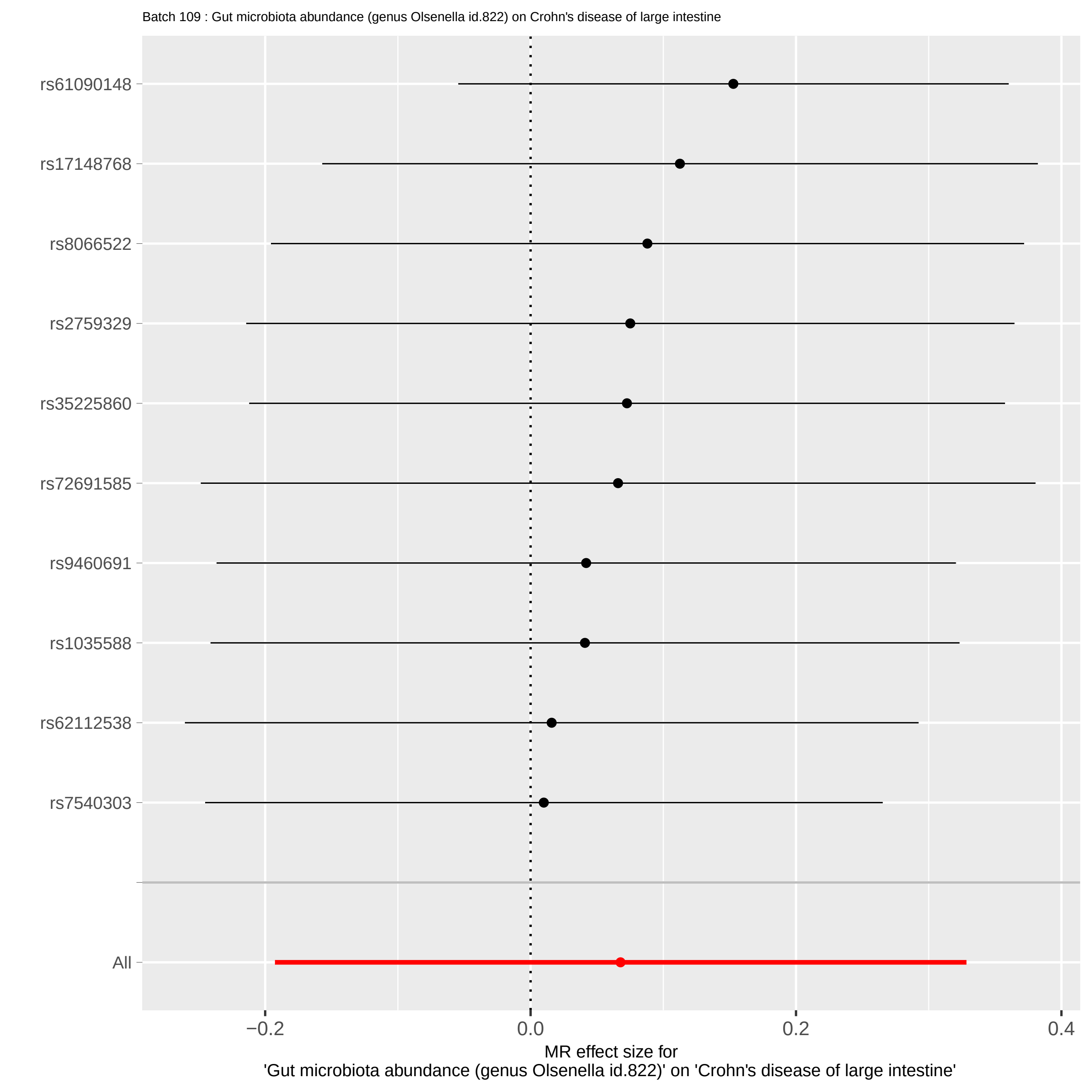

Batch 109 : Gut microbiota abundance (genus Olsenella id.822) on Crohn's disease of large intestine
rs61090148
rs17148768
rs8066522
rs2759329
rs35225860
rs72691585
rs9460691
rs1035588
rs62112538
rs7540303
All
−0.2
0.0
0.2
0.4
MR effect size for
'Gut microbiota abundance (genus Olsenella id.822)' on 'Crohn's disease of large intestine'

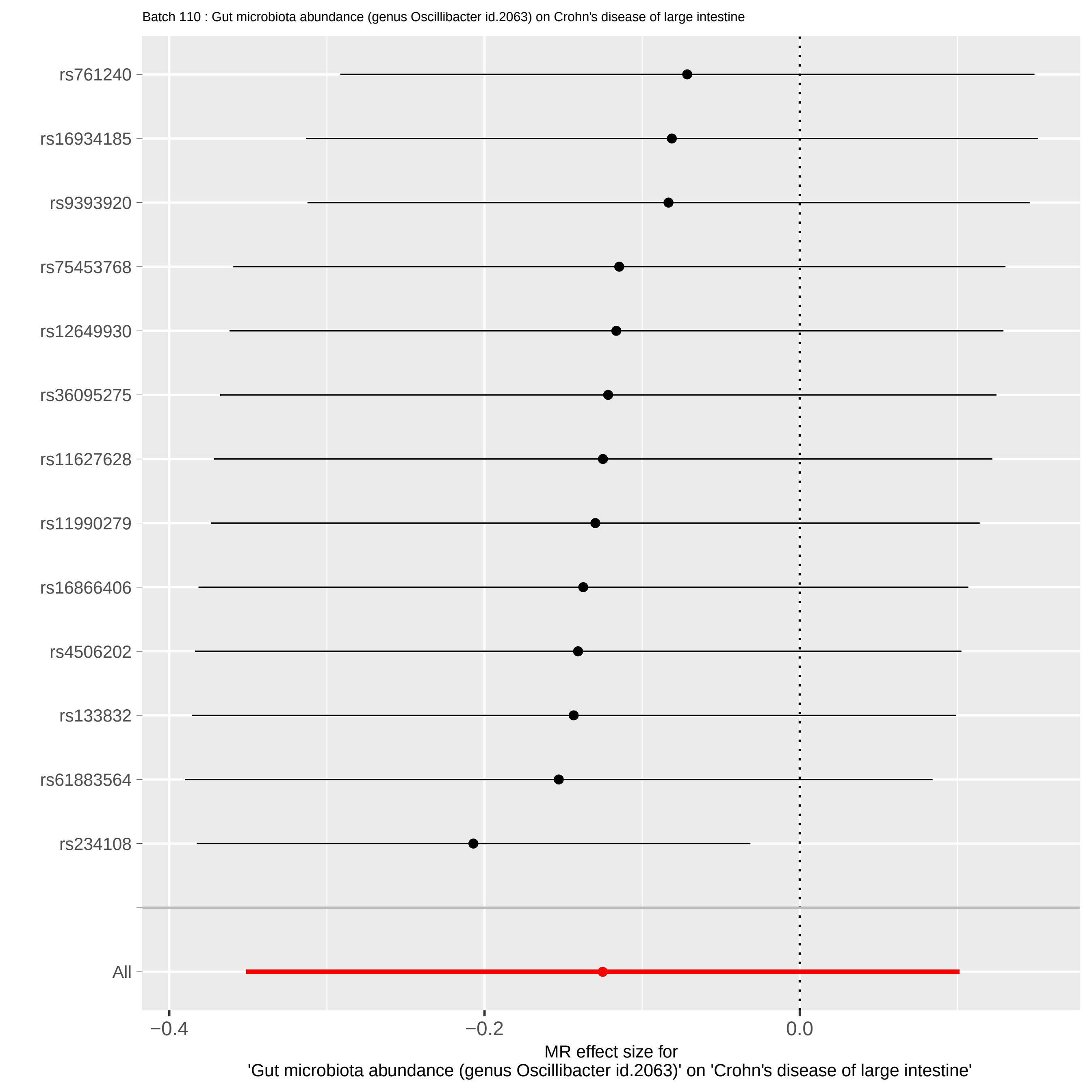

Batch 110 : Gut microbiota abundance (genus Oscillibacter id.2063) on Crohn's disease of large intestine
rs761240
rs16934185
rs9393920
rs75453768
rs12649930
rs36095275
rs11627628
rs11990279
rs16866406
rs4506202
rs133832
rs61883564
rs234108
All
−0.4
−0.2
0.0
MR effect size for
'Gut microbiota abundance (genus Oscillibacter id.2063)' on 'Crohn's disease of large intestine'

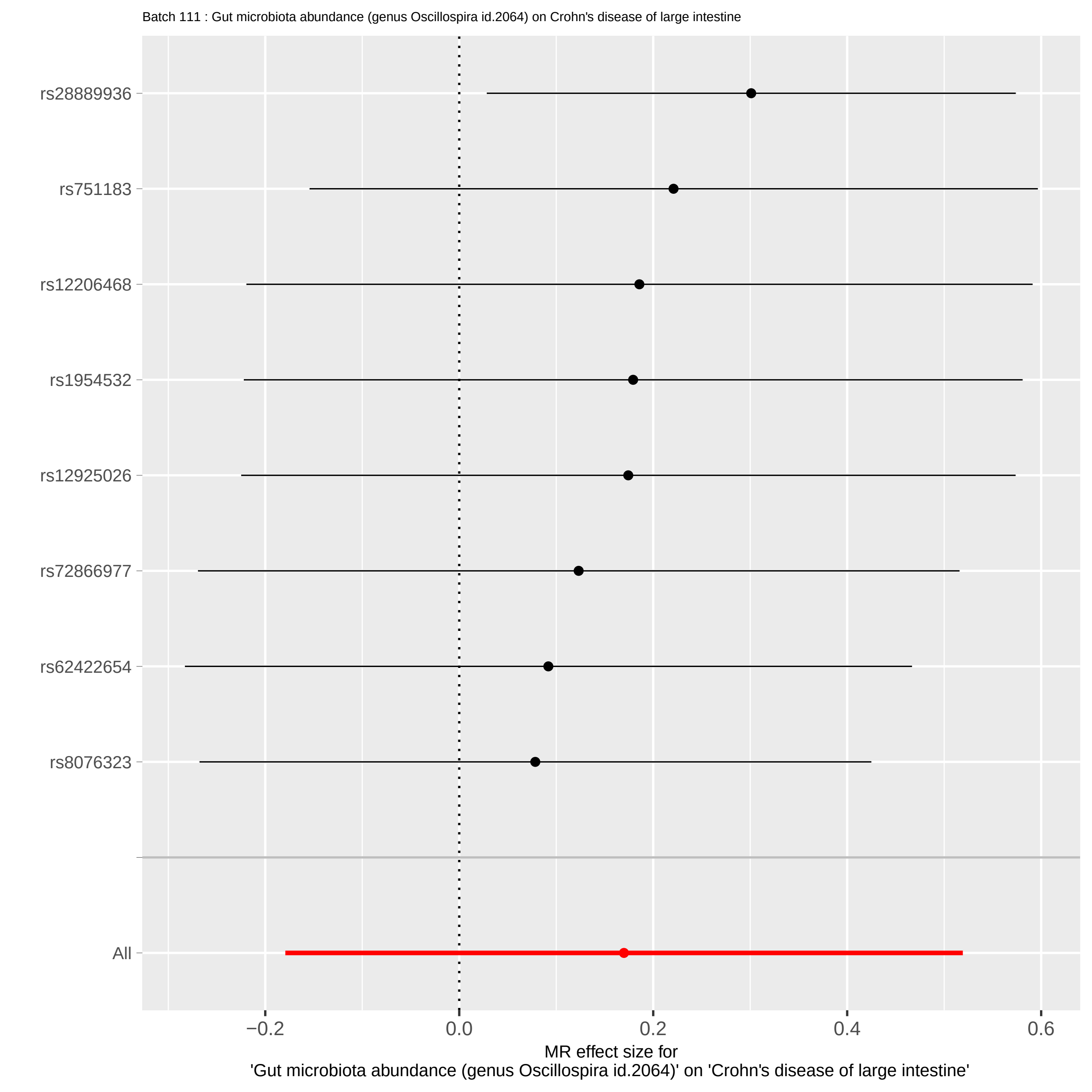

Batch 111 : Gut microbiota abundance (genus Oscillospira id.2064) on Crohn's disease of large intestine
rs28889936
rs751183
rs12206468
rs1954532
rs12925026
rs72866977
rs62422654
rs8076323
All
−0.2
0.0
0.2
0.4
0.6
MR effect size for
'Gut microbiota abundance (genus Oscillospira id.2064)' on 'Crohn's disease of large intestine'

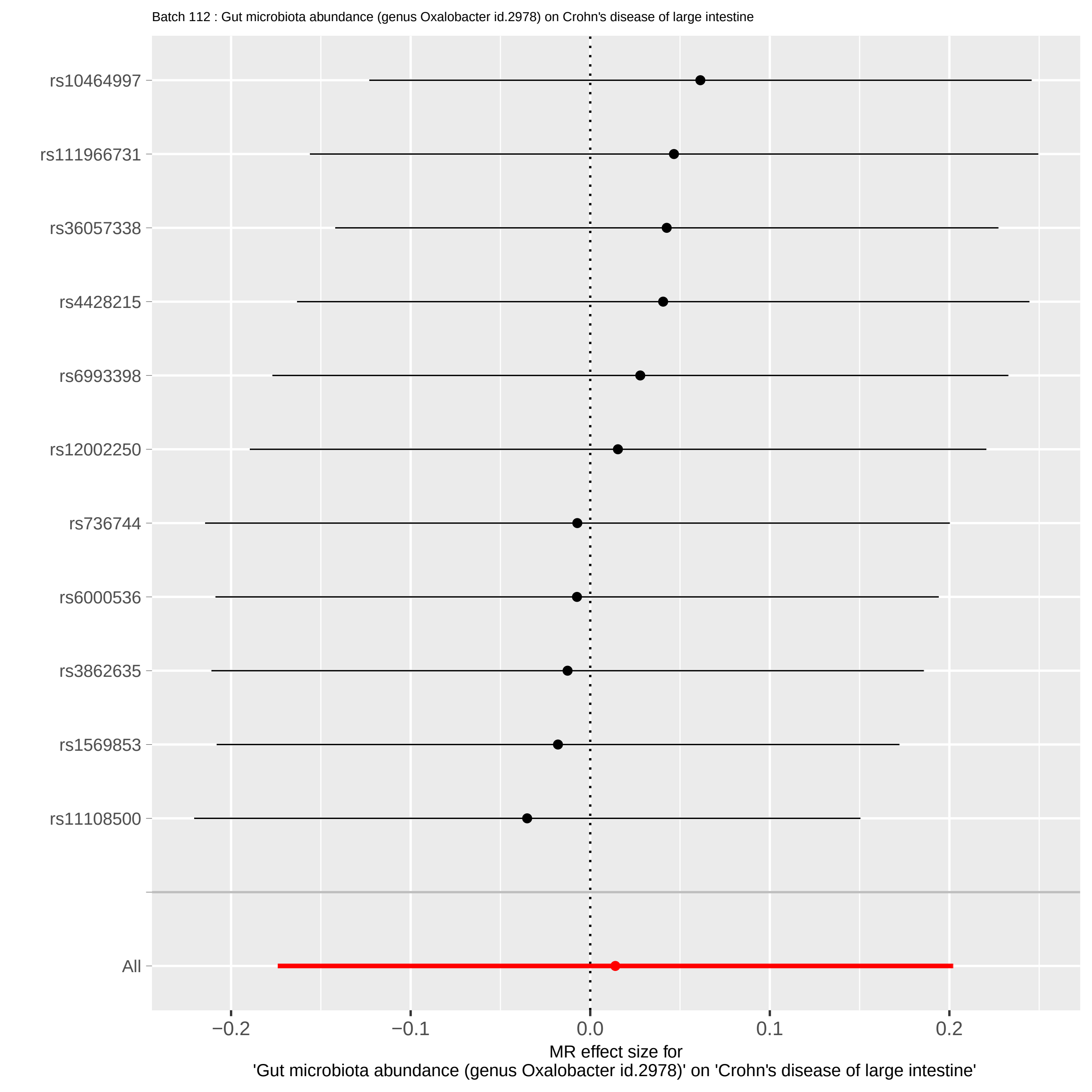

Batch 112 : Gut microbiota abundance (genus Oxalobacter id.2978) on Crohn's disease of large intestine
rs10464997
rs111966731
rs36057338
rs4428215
rs6993398
rs12002250
rs736744
rs6000536
rs3862635
rs1569853
rs11108500
All
−0.2
−0.1
0.0
0.1
0.2
MR effect size for
'Gut microbiota abundance (genus Oxalobacter id.2978)' on 'Crohn's disease of large intestine'

Batch 113 : Gut microbiota abundance (genus Parabacteroides id.954) on Crohn's disease of large intestine

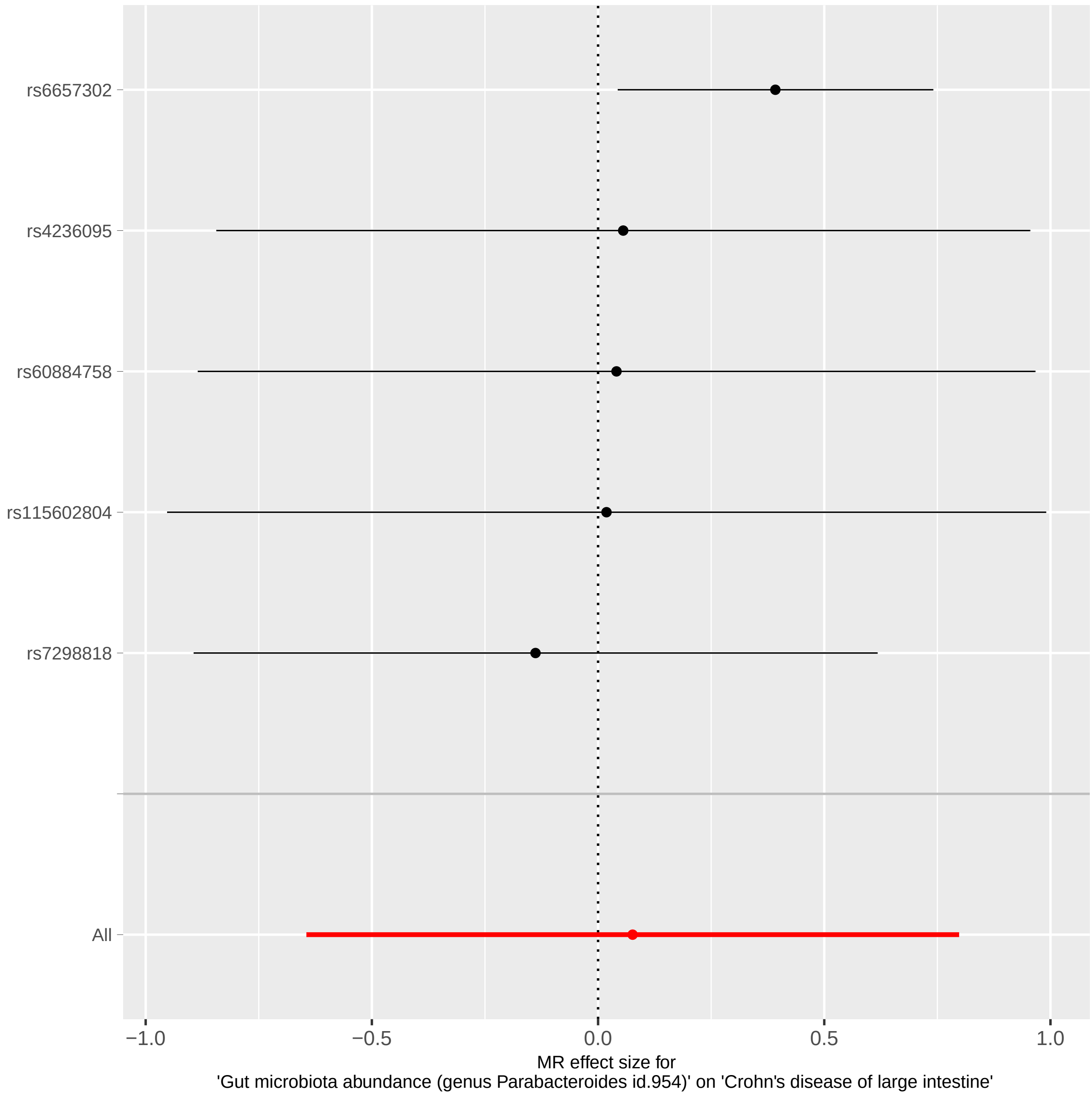

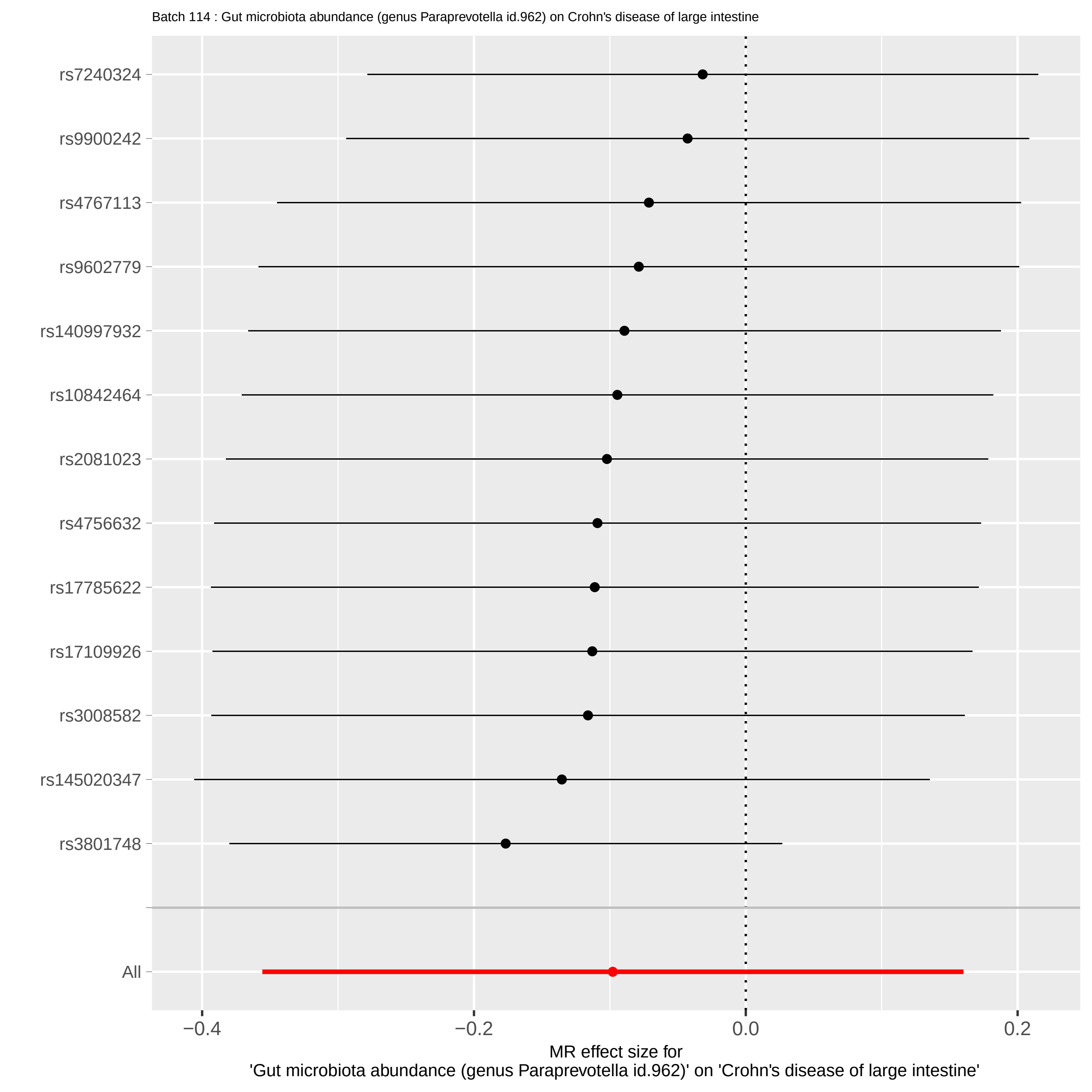
Batch 114 : Gut microbiota abundance (genus Paraprevotella id.962) on Crohn's disease of large intestine
rs7240324
rs9900242
rs4767113
rs9602779
rs140997932
rs10842464
rs2081023
rs4756632
rs17785622
rs17109926
rs3008582
rs145020347
rs3801748
All
−0.4
−0.2
0.0
0.2
MR effect size for
'Gut microbiota abundance (genus Paraprevotella id.962)' on 'Crohn's disease of large intestine'

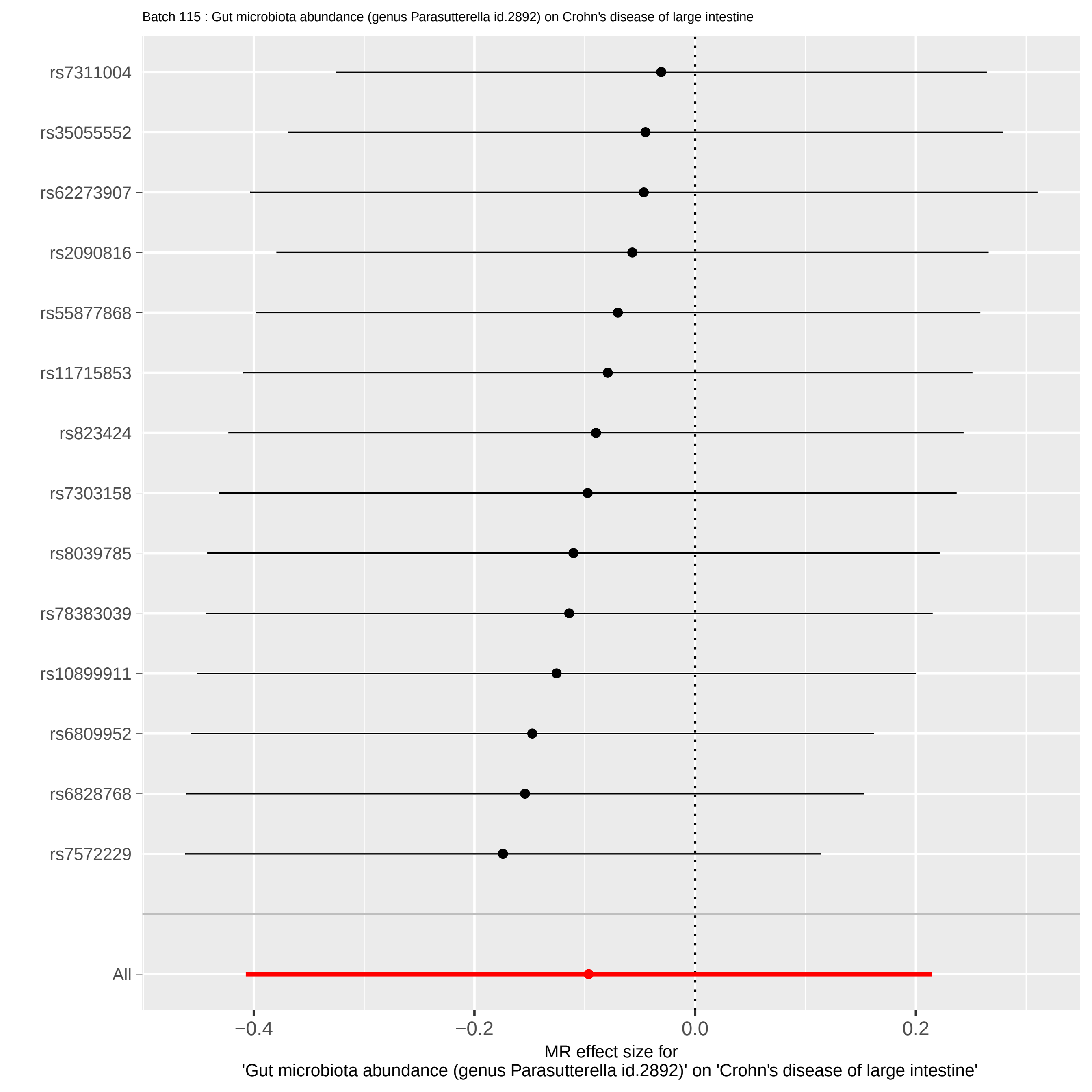
Batch 115 : Gut microbiota abundance (genus Parasutterella id.2892) on Crohn's disease of large intestine
rs7311004
rs35055552
rs62273907
rs2090816
rs55877868
rs11715853
rs823424
rs7303158
rs8039785
rs78383039
rs10899911
rs6809952
rs6828768
rs7572229
All
−0.4
−0.2
0.0
0.2
MR effect size for
'Gut microbiota abundance (genus Parasutterella id.2892)' on 'Crohn's disease of large intestine'

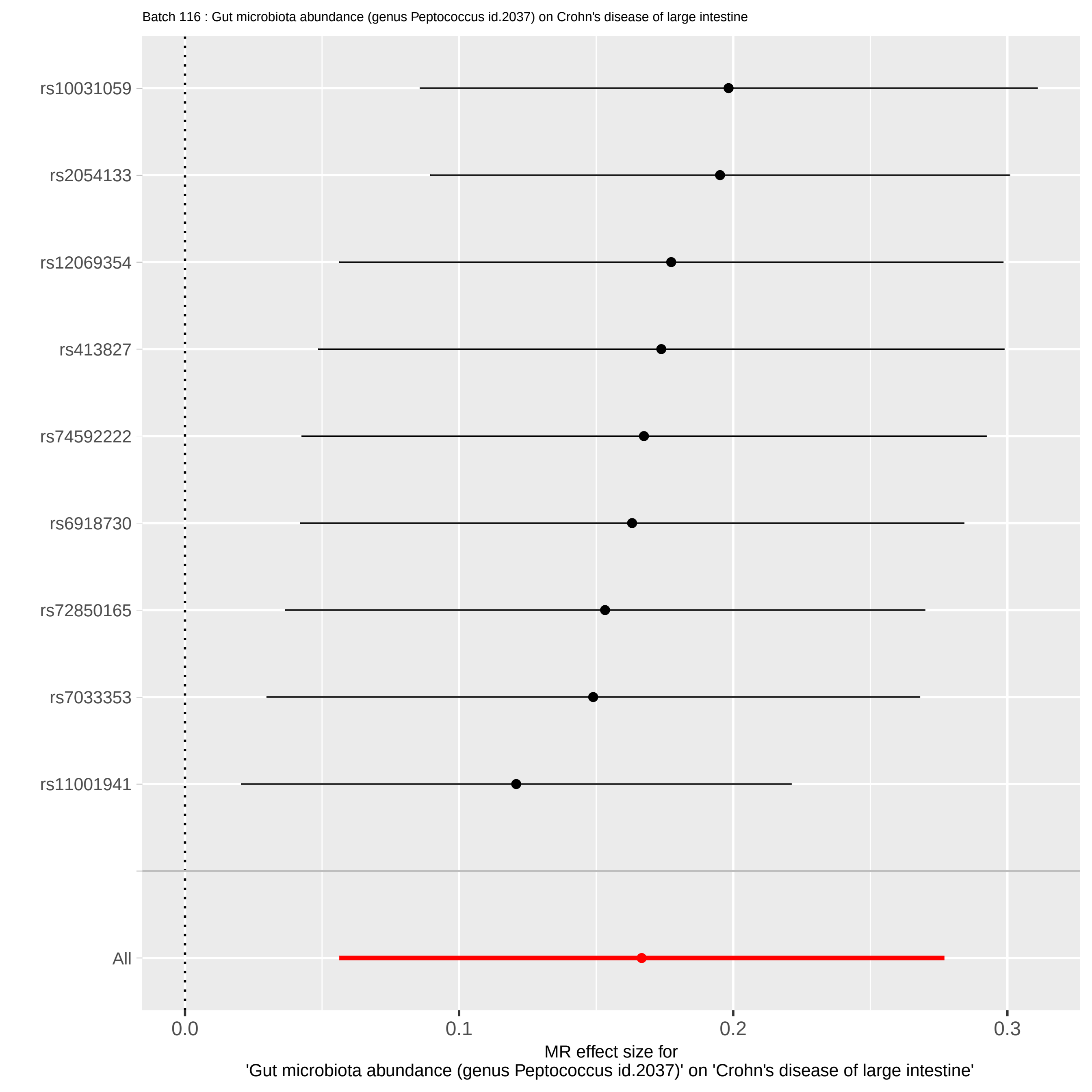
Batch 116 : Gut microbiota abundance (genus Peptococcus id.2037) on Crohn's disease of large intestine
rs10031059
rs2054133
rs12069354
rs413827
rs74592222
rs6918730
rs72850165
rs7033353
rs11001941
All
0.0
0.1
0.2
0.3
MR effect size for
'Gut microbiota abundance (genus Peptococcus id.2037)' on 'Crohn's disease of large intestine'

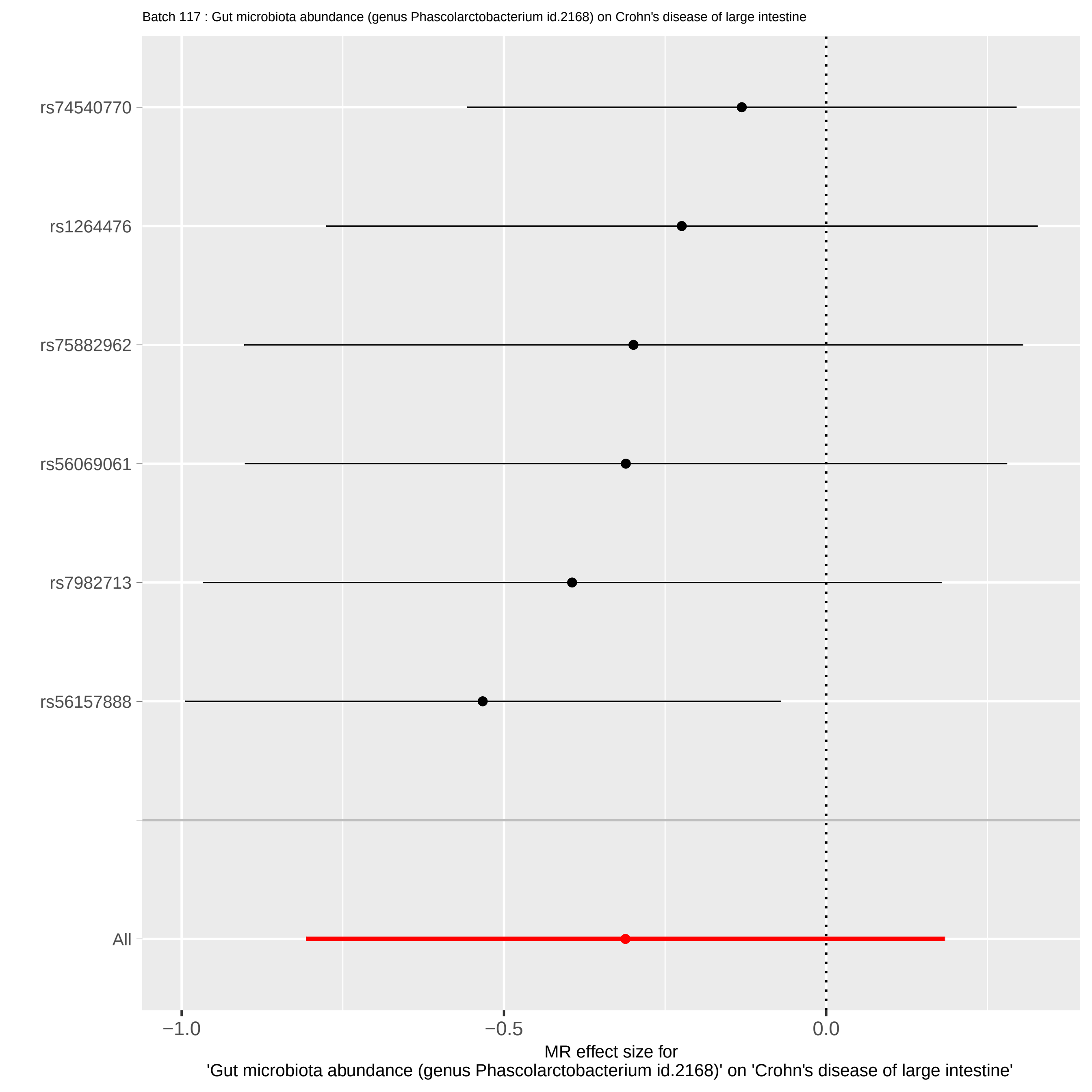
Batch 117 : Gut microbiota abundance (genus Phascolarctobacterium id.2168) on Crohn's disease of large intestine
rs74540770
rs1264476
rs75882962
rs56069061
rs7982713
rs56157888
All
−1.0
−0.5
0.0
MR effect size for
'Gut microbiota abundance (genus Phascolarctobacterium id.2168)' on 'Crohn's disease of large intestine'

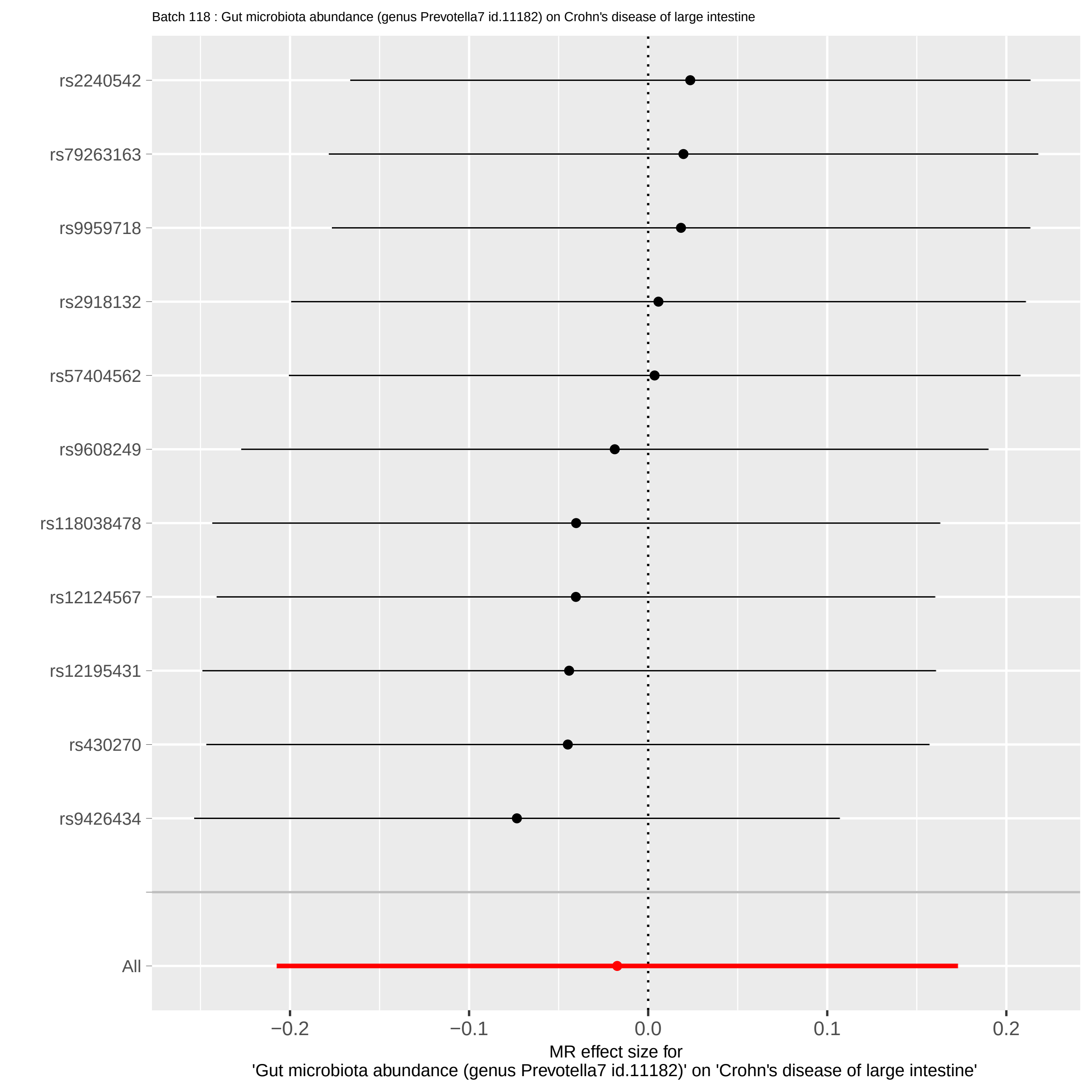

Batch 118 : Gut microbiota abundance (genus Prevotella7 id.11182) on Crohn's disease of large intestine
rs2240542
rs79263163
rs9959718
rs2918132
rs57404562
rs9608249
rs118038478
rs12124567
rs12195431
rs430270
rs9426434
All
−0.2
−0.1
0.0
0.1
0.2
MR effect size for
'Gut microbiota abundance (genus Prevotella7 id.11182)' on 'Crohn's disease of large intestine'

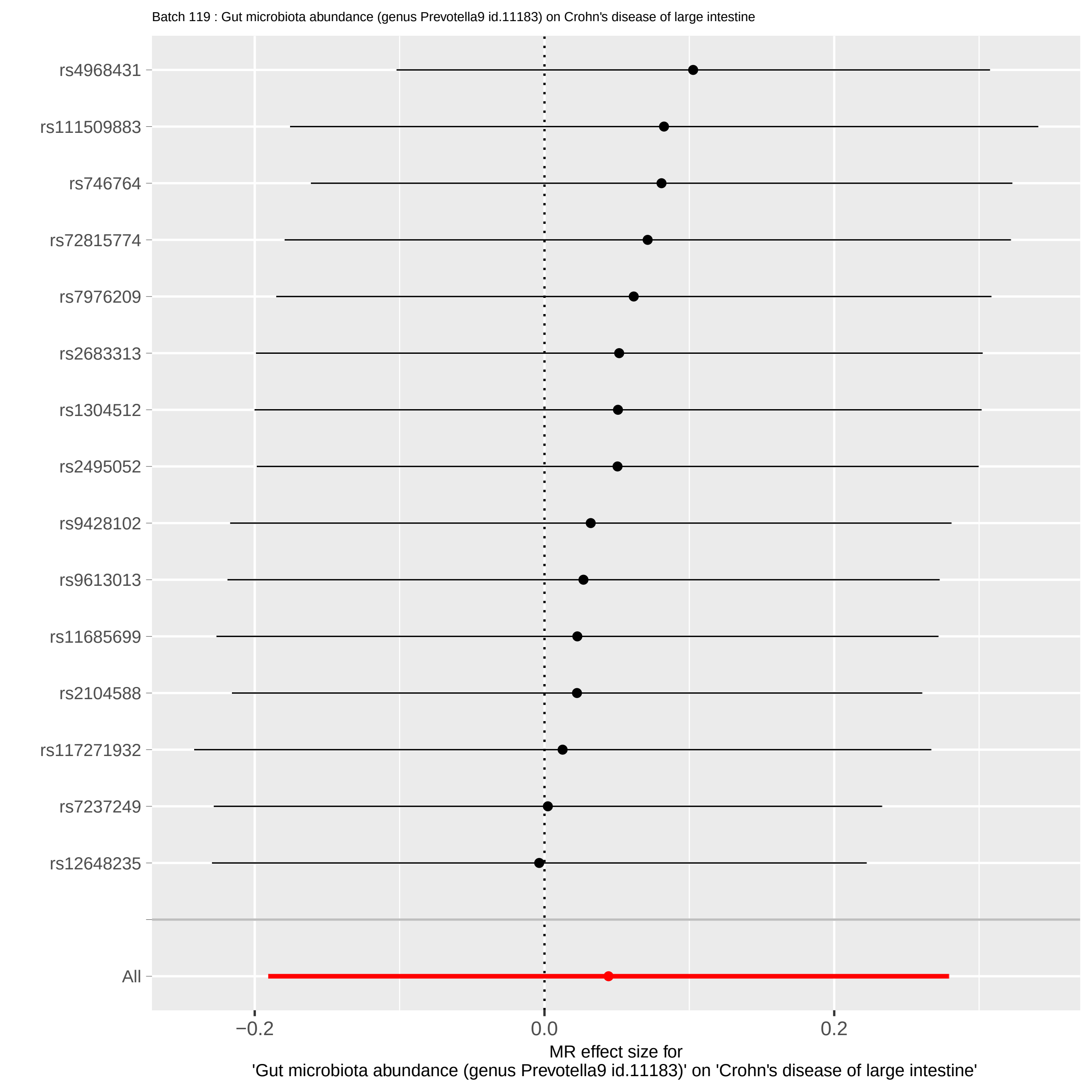

Batch 119 : Gut microbiota abundance (genus Prevotella9 id.11183) on Crohn's disease of large intestine
rs4968431
rs111509883
rs746764
rs72815774
rs7976209
rs2683313
rs1304512
rs2495052
rs9428102
rs9613013
rs11685699
rs2104588
rs117271932
rs7237249
rs12648235
All
−0.2
0.0
0.2
MR effect size for
'Gut microbiota abundance (genus Prevotella9 id.11183)' on 'Crohn's disease of large intestine'

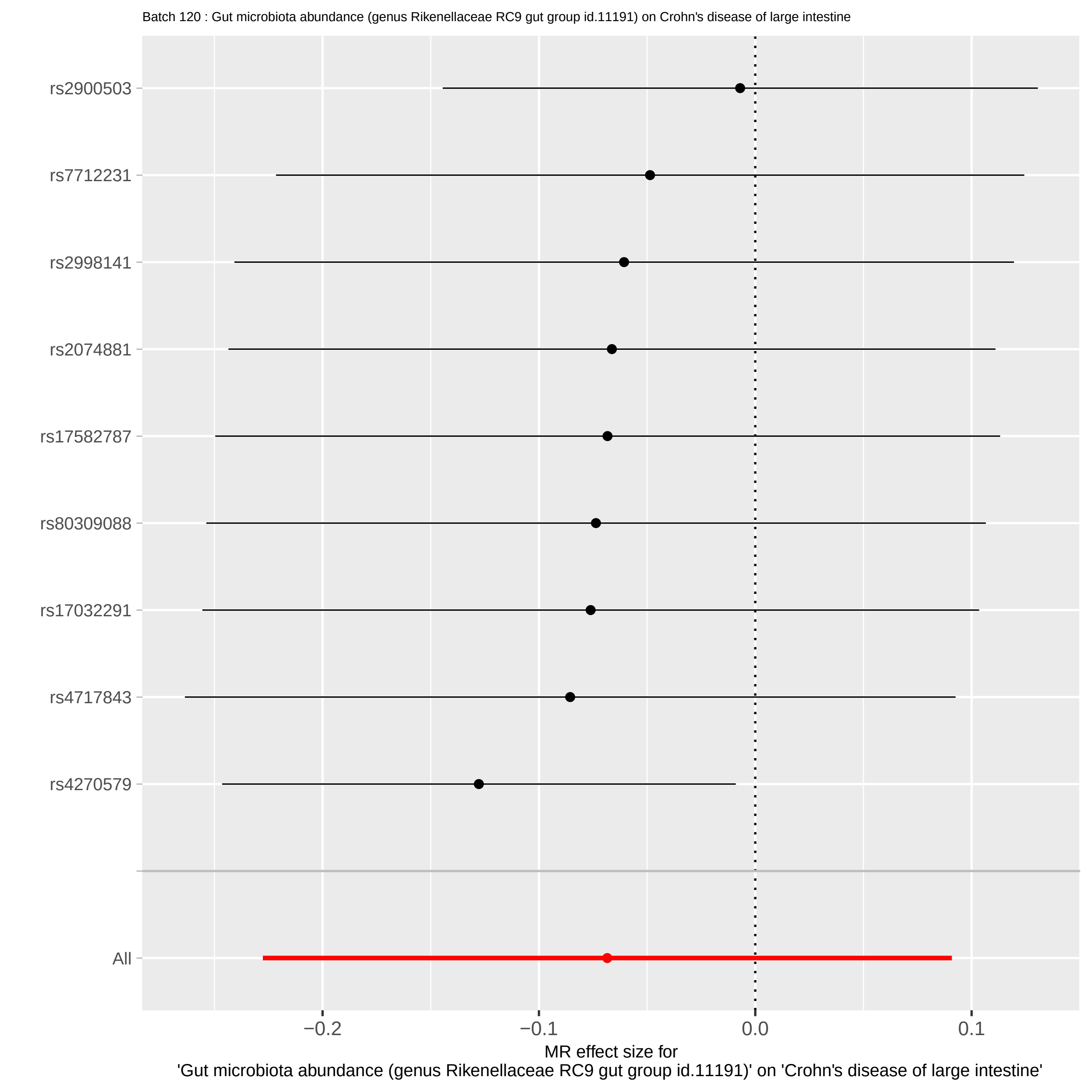
Batch 120 : Gut microbiota abundance (genus Rikenellaceae RC9 gut group id.11191) on Crohn's disease of large intestine
rs2900503
rs7712231
rs2998141
rs2074881
rs17582787
rs80309088
rs17032291
rs4717843
rs4270579
All
−0.2
−0.1
0.0
0.1
MR effect size for
'Gut microbiota abundance (genus Rikenellaceae RC9 gut group id.11191)' on 'Crohn's disease of large intestine'

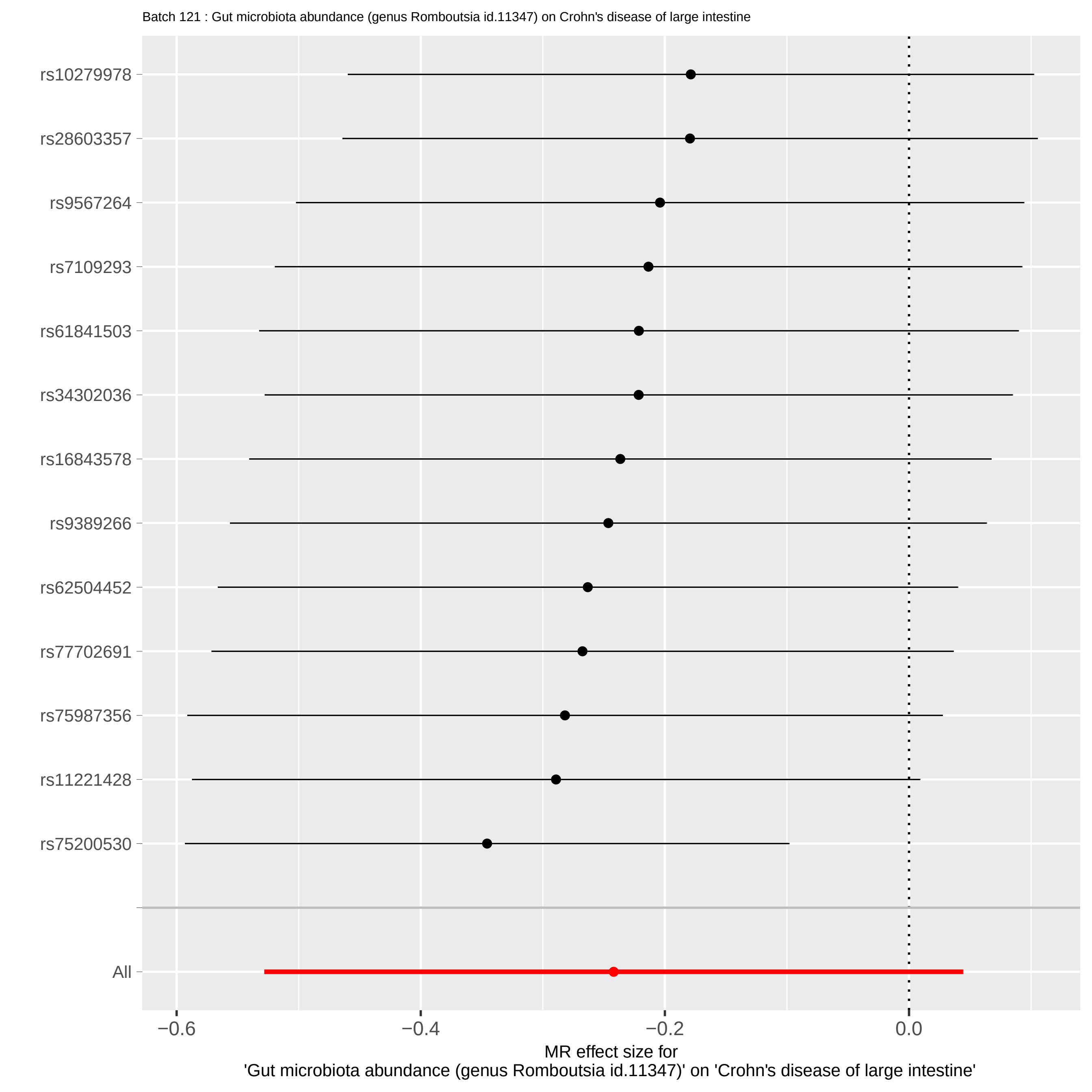

Batch 121 : Gut microbiota abundance (genus Romboutsia id.11347) on Crohn's disease of large intestine
rs10279978
rs28603357
rs9567264
rs7109293
rs61841503
rs34302036
rs16843578
rs9389266
rs62504452
rs77702691
rs75987356
rs11221428
rs75200530
All
−0.6
−0.4
−0.2
0.0
MR effect size for
'Gut microbiota abundance (genus Romboutsia id.11347)' on 'Crohn's disease of large intestine'

Batch 122 : Gut microbiota abundance (genus Roseburia id.2012) on Crohn's disease of large intestine

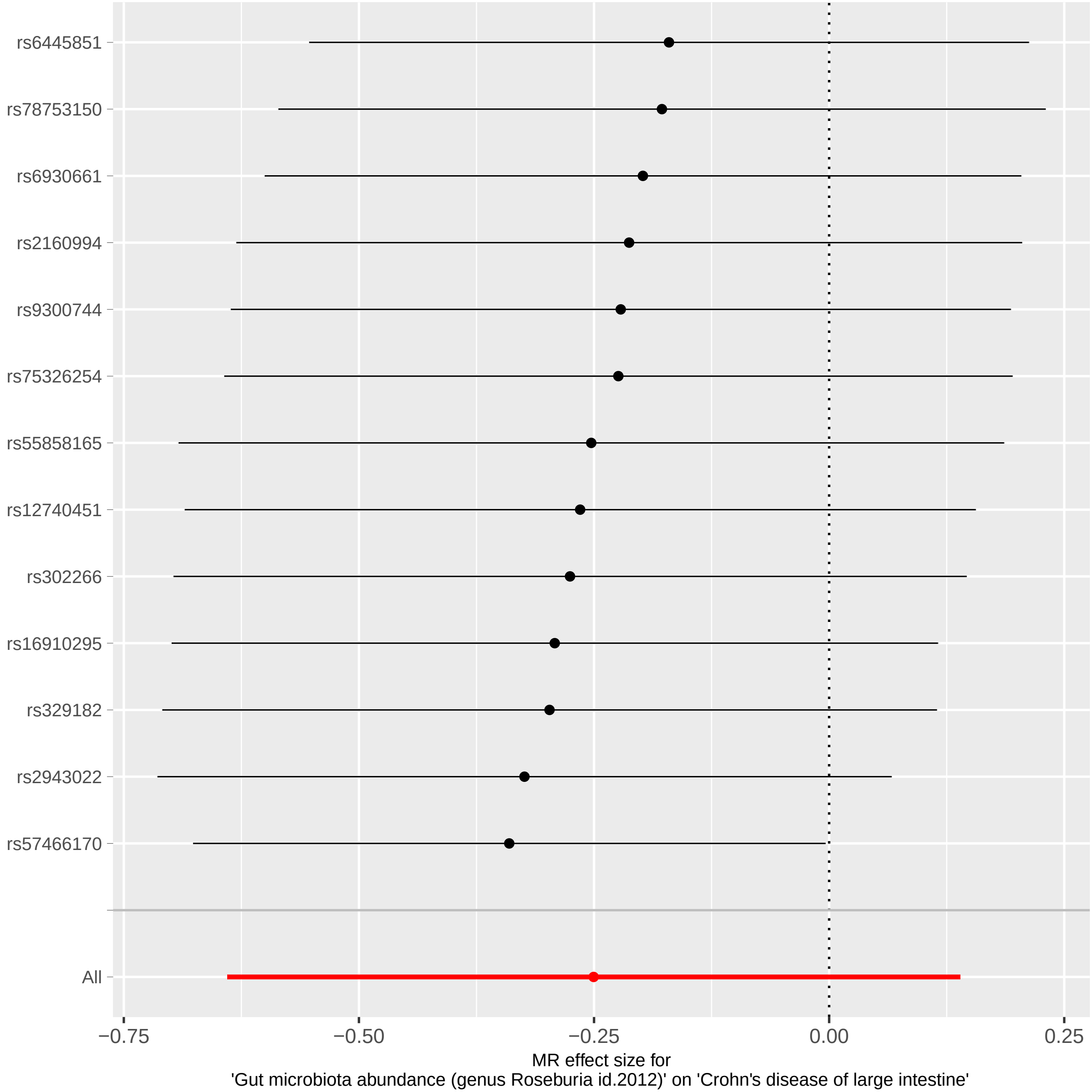

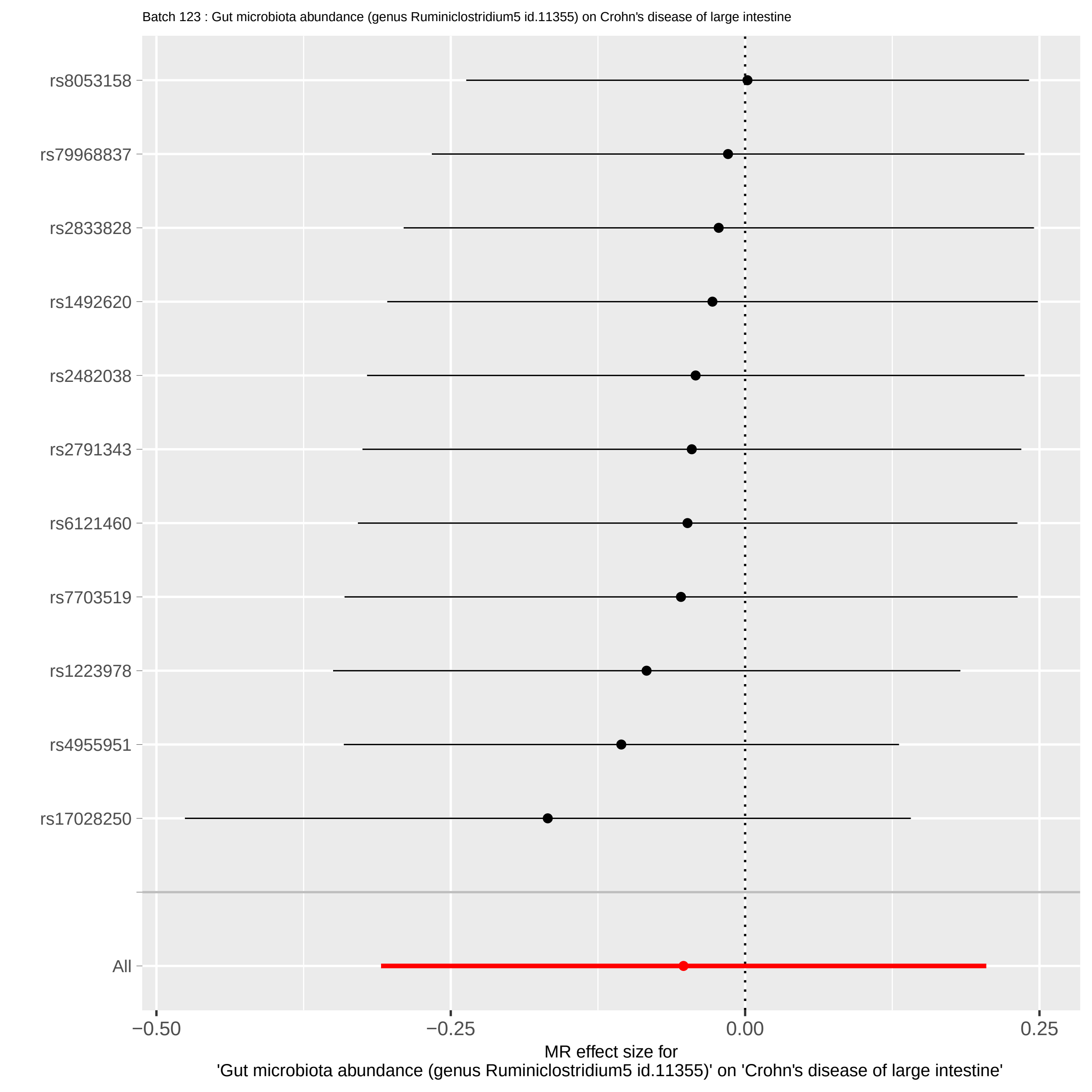
Batch 123 : Gut microbiota abundance (genus Ruminiclostridium5 id.11355) on Crohn's disease of large intestine
rs8053158
rs79968837
rs2833828
rs1492620
rs2482038
rs2791343
rs6121460
rs7703519
rs1223978
rs4955951
rs17028250
All
−0.50
−0.25
0.00
0.25
MR effect size for
'Gut microbiota abundance (genus Ruminiclostridium5 id.11355)' on 'Crohn's disease of large intestine'

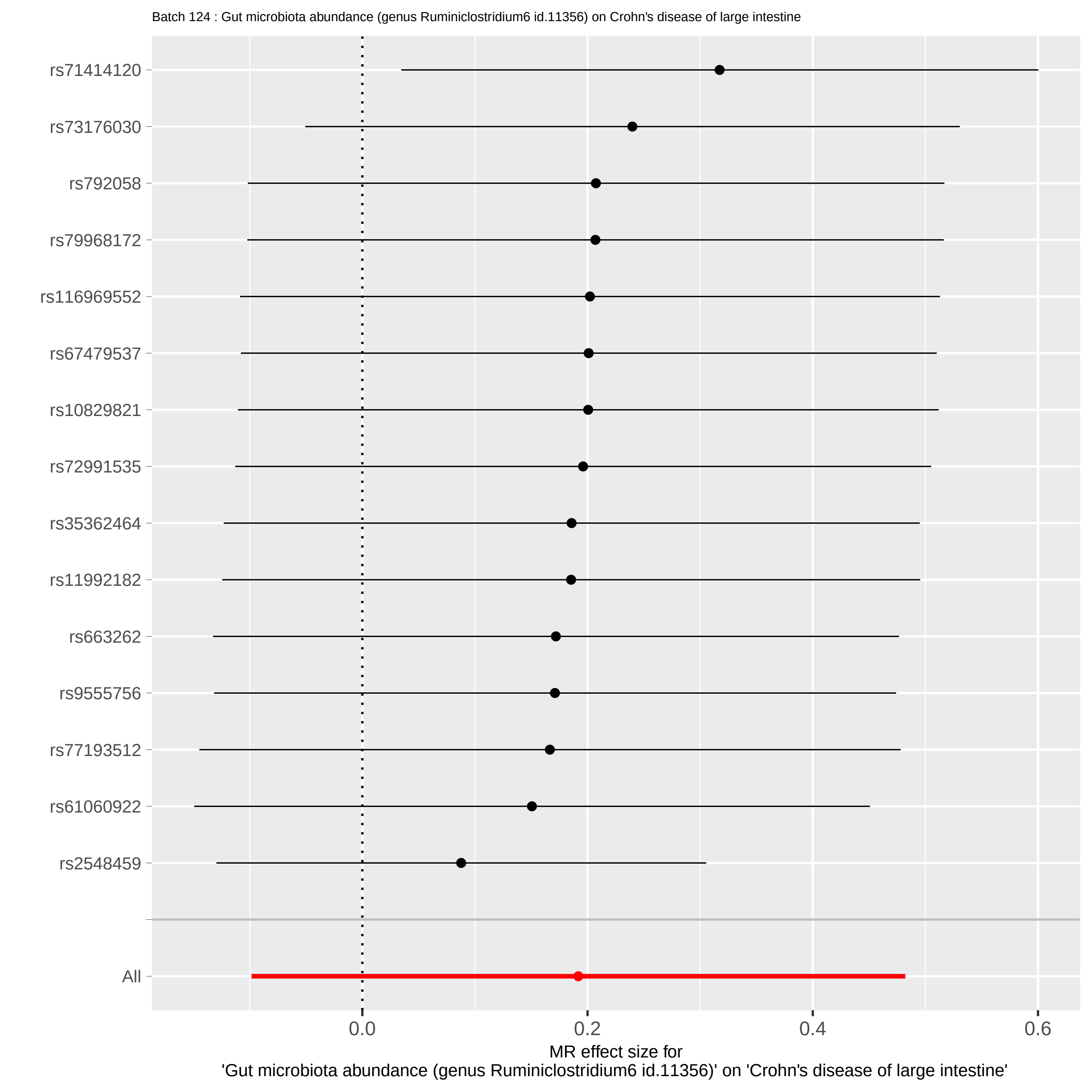
Batch 124 : Gut microbiota abundance (genus Ruminiclostridium6 id.11356) on Crohn's disease of large intestine
rs71414120
rs73176030
rs792058
rs79968172
rs116969552
rs67479537
rs10829821
rs72991535
rs35362464
rs11992182
rs663262
rs9555756
rs77193512
rs61060922
rs2548459
All
0.0
0.2
0.4
0.6
MR effect size for
'Gut microbiota abundance (genus Ruminiclostridium6 id.11356)' on 'Crohn's disease of large intestine'

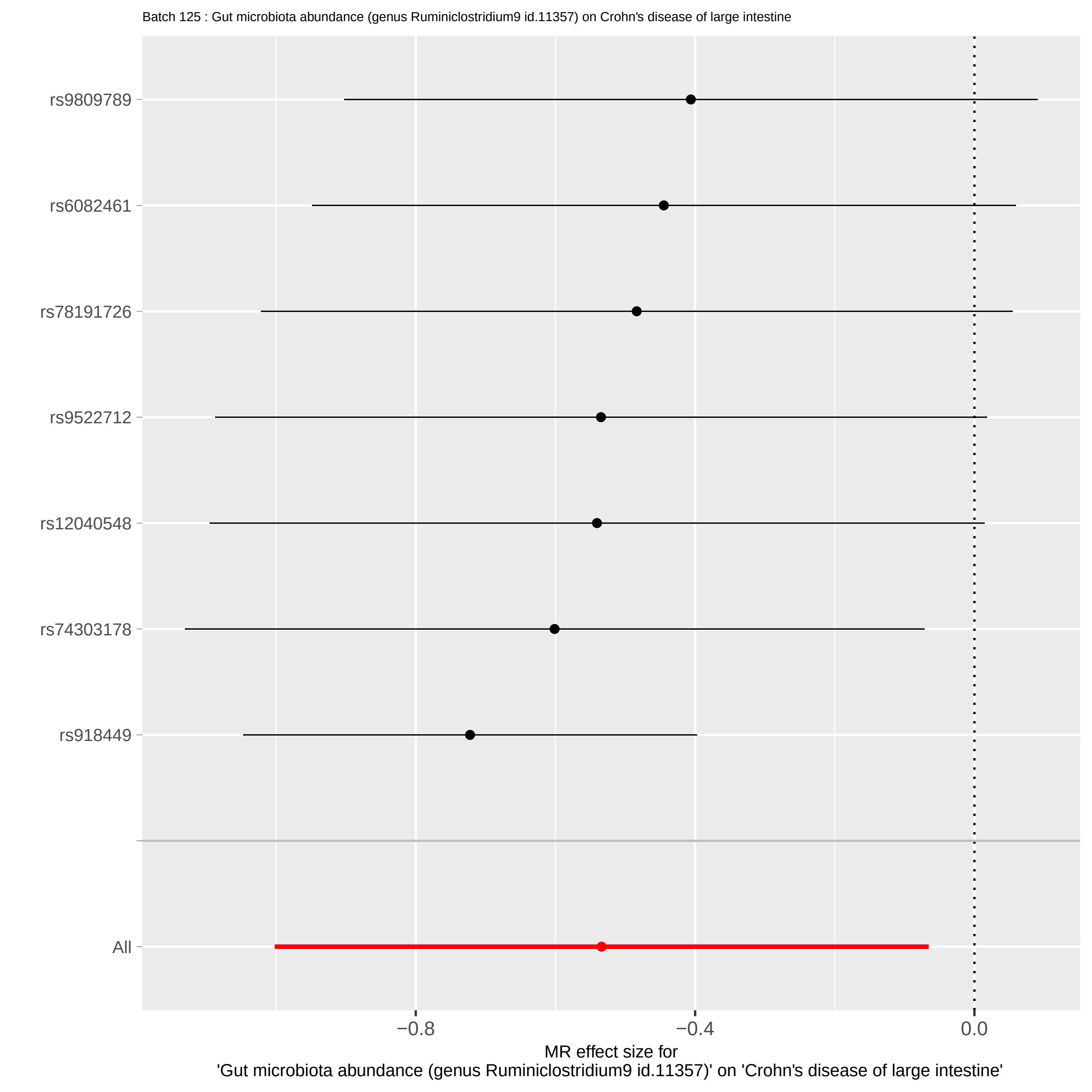
Batch 125 : Gut microbiota abundance (genus Ruminiclostridium9 id.11357) on Crohn's disease of large intestine
rs9809789
rs6082461
rs78191726
rs9522712
rs12040548
rs74303178
rs918449
All
−0.8
−0.4
0.0
MR effect size for
'Gut microbiota abundance (genus Ruminiclostridium9 id.11357)' on 'Crohn's disease of large intestine'

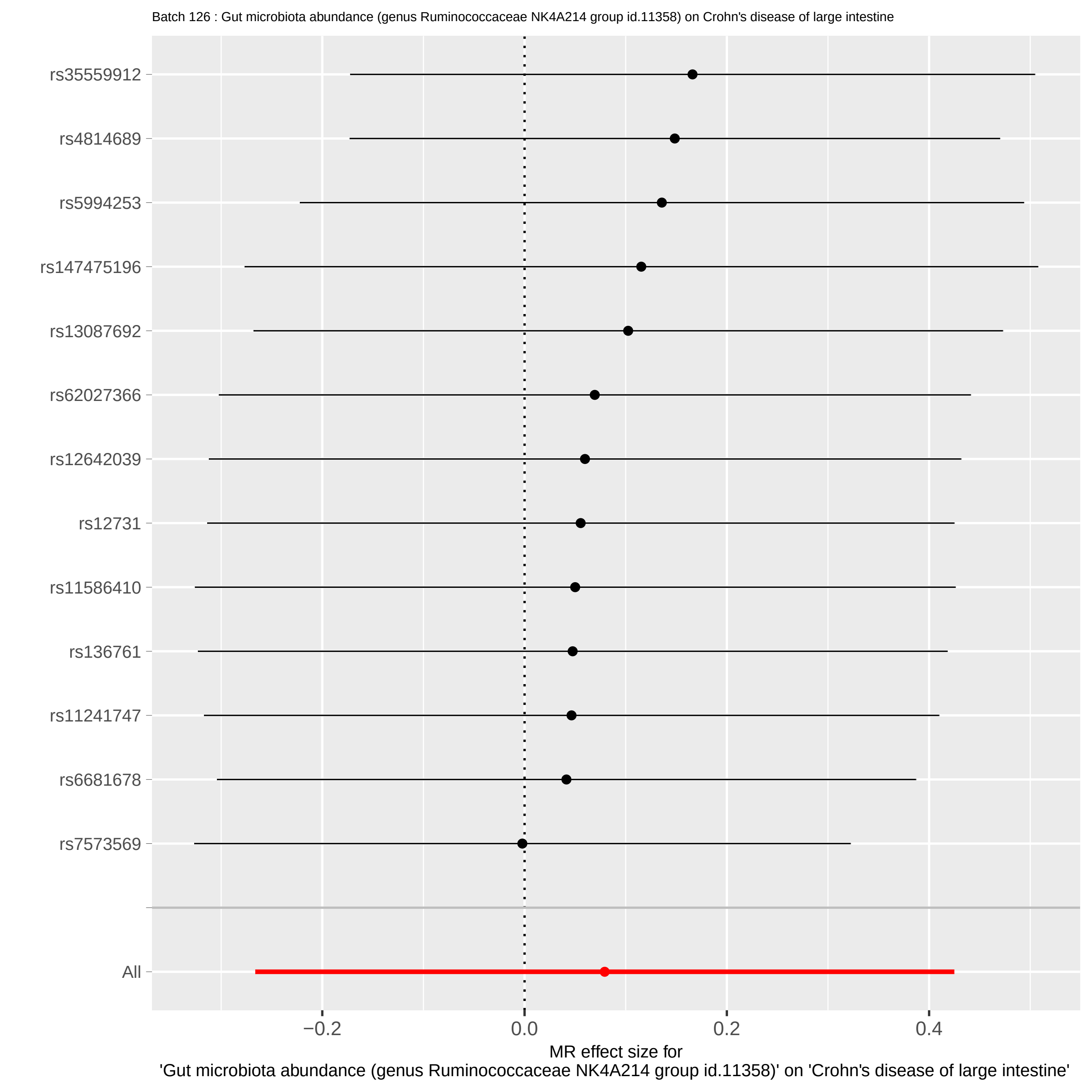

Batch 126 : Gut microbiota abundance (genus Ruminococcaceae NK4A214 group id.11358) on Crohn's disease of large intestine
rs35559912
rs4814689
rs5994253
rs147475196
rs13087692
rs62027366
rs12642039
rs12731
rs11586410
rs136761
rs11241747
rs6681678
rs7573569
All
−0.2
0.0
0.2
0.4
MR effect size for
'Gut microbiota abundance (genus Ruminococcaceae NK4A214 group id.11358)' on 'Crohn's disease of large intestine'

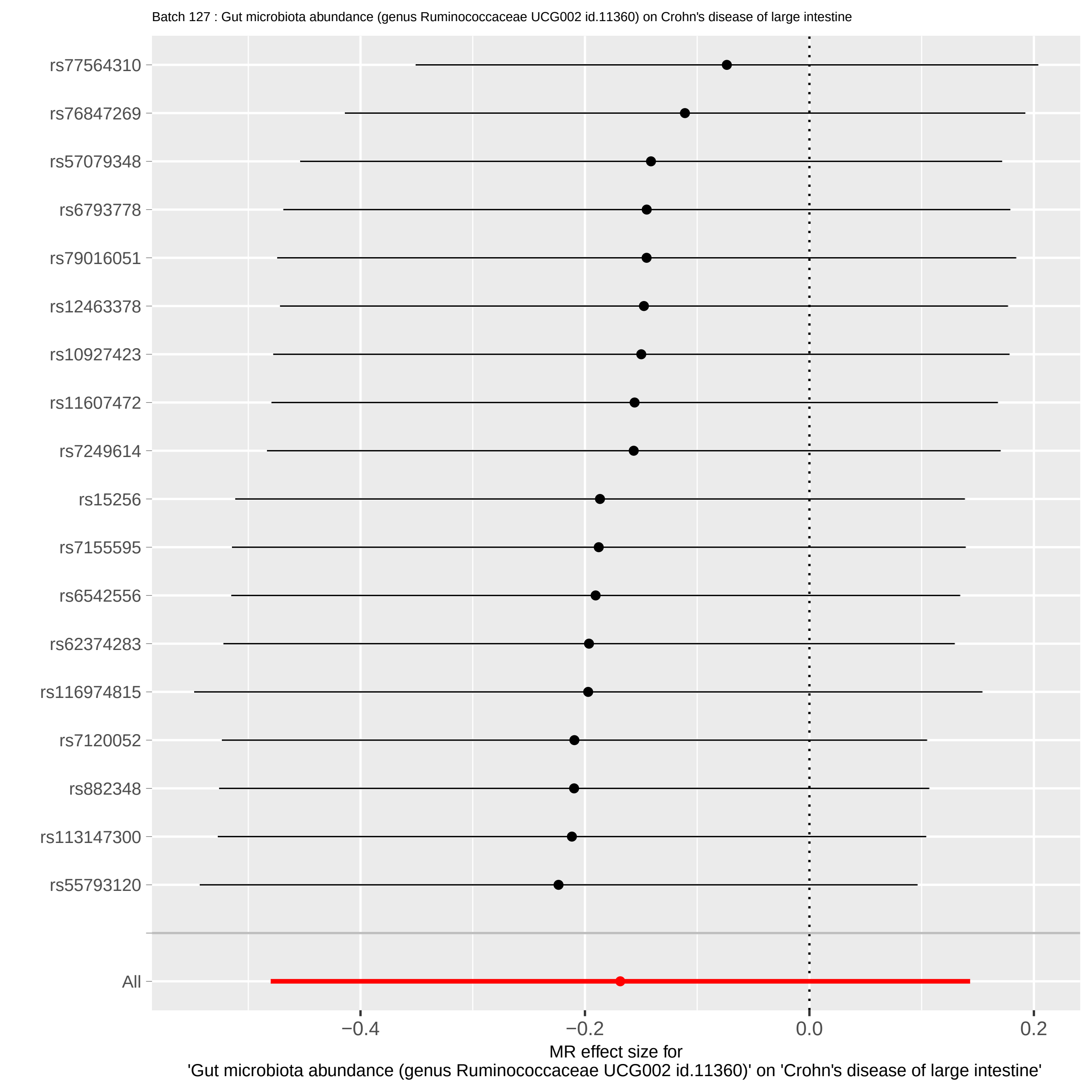
Batch 127 : Gut microbiota abundance (genus Ruminococcaceae UCG002 id.11360) on Crohn's disease of large intestine
rs77564310
rs76847269
rs57079348
rs6793778
rs79016051
rs12463378
rs10927423
rs11607472
rs7249614
rs15256
rs7155595
rs6542556
rs62374283
rs116974815
rs7120052
rs882348
rs113147300
rs55793120
All
−0.4
−0.2
0.0
0.2
MR effect size for
'Gut microbiota abundance (genus Ruminococcaceae UCG002 id.11360)' on 'Crohn's disease of large intestine'

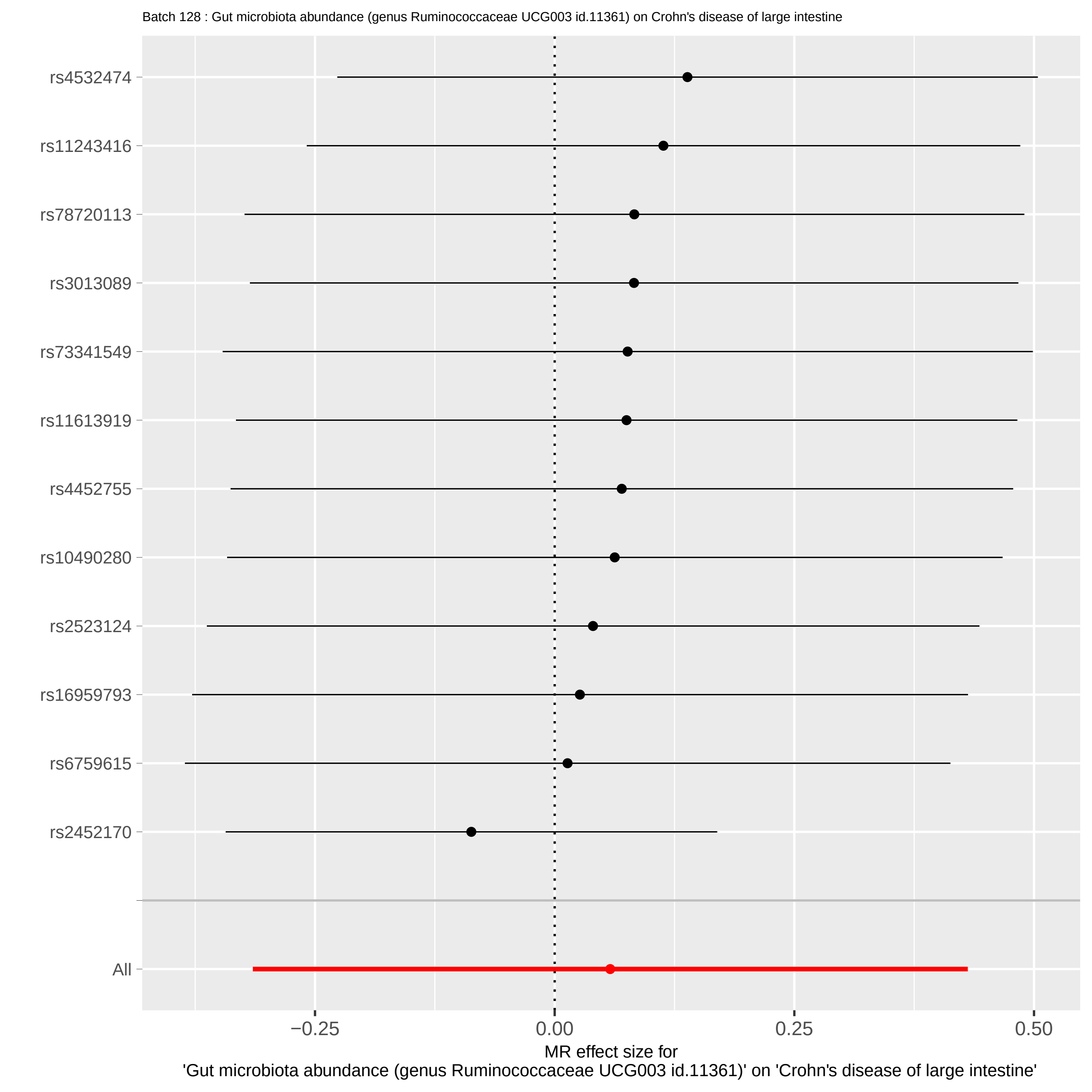
Batch 128 : Gut microbiota abundance (genus Ruminococcaceae UCG003 id.11361) on Crohn's disease of large intestine
rs4532474
rs11243416
rs78720113
rs3013089
rs73341549
rs11613919
rs4452755
rs10490280
rs2523124
rs16959793
rs6759615
rs2452170
All
−0.25
0.00
0.25
0.50
MR effect size for
'Gut microbiota abundance (genus Ruminococcaceae UCG003 id.11361)' on 'Crohn's disease of large intestine'

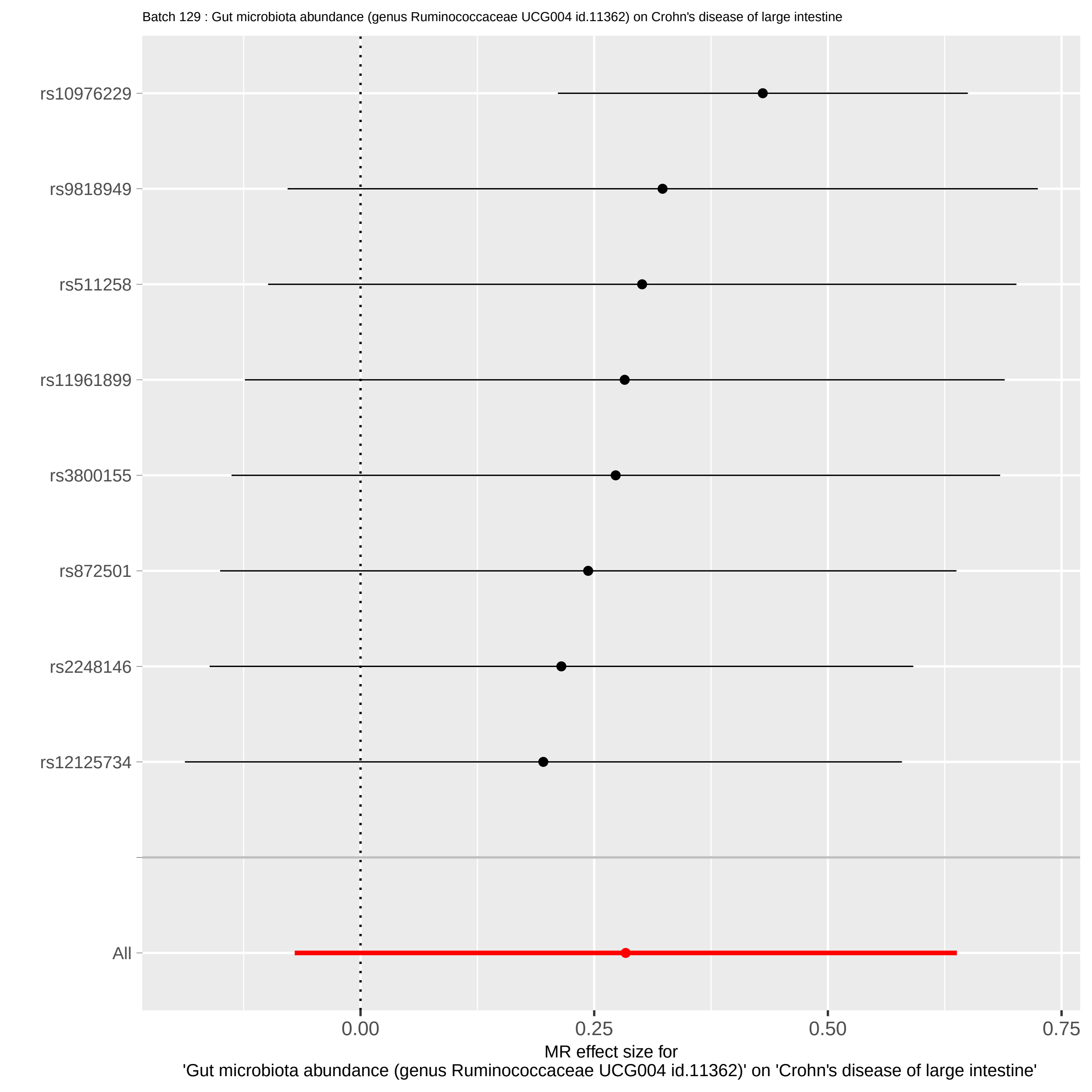
Batch 129 : Gut microbiota abundance (genus Ruminococcaceae UCG004 id.11362) on Crohn's disease of large intestine
rs10976229
rs9818949
rs511258
rs11961899
rs3800155
rs872501
rs2248146
rs12125734
All
0.00
0.25
0.50
0.75
MR effect size for
'Gut microbiota abundance (genus Ruminococcaceae UCG004 id.11362)' on 'Crohn's disease of large intestine'

Batch 130 : Gut microbiota abundance (genus Ruminococcaceae UCG005 id.11363) on Crohn's disease of large intestine

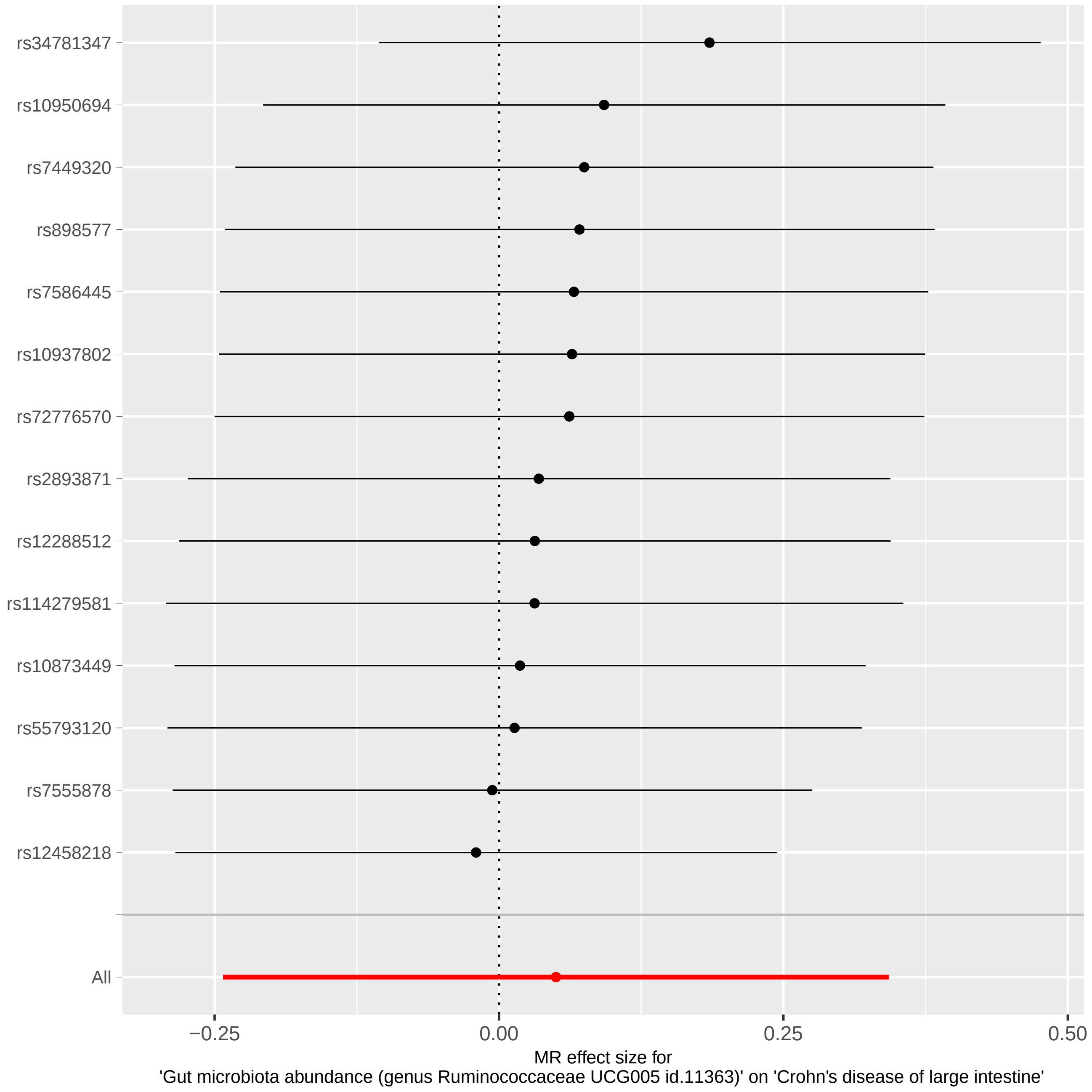

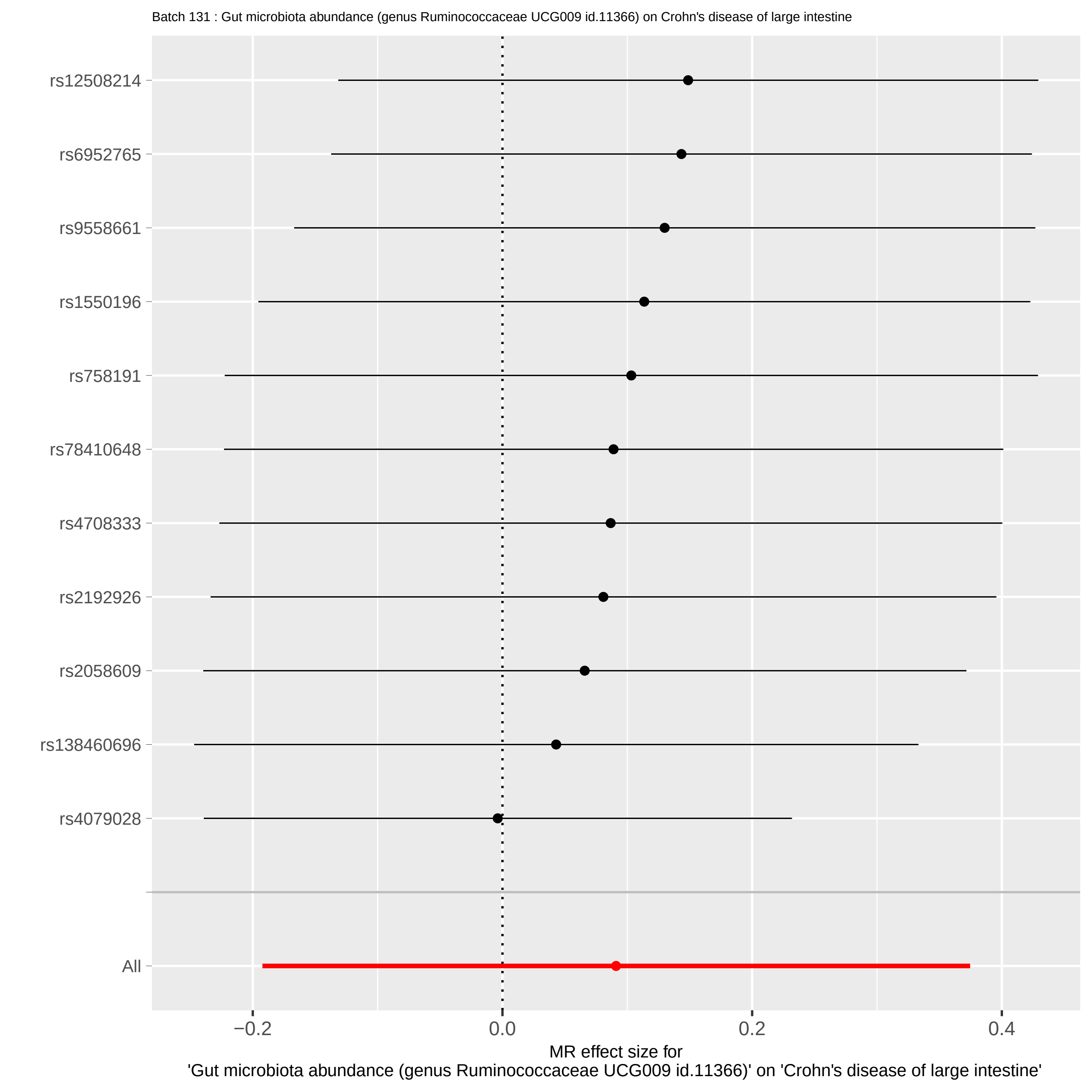
Batch 131 : Gut microbiota abundance (genus Ruminococcaceae UCG009 id.11366) on Crohn's disease of large intestine
rs12508214
rs6952765
rs9558661
rs1550196
rs758191
rs78410648
rs4708333
rs2192926
rs2058609
rs138460696
rs4079028
All
−0.2
0.0
0.2
0.4
MR effect size for
'Gut microbiota abundance (genus Ruminococcaceae UCG009 id.11366)' on 'Crohn's disease of large intestine'

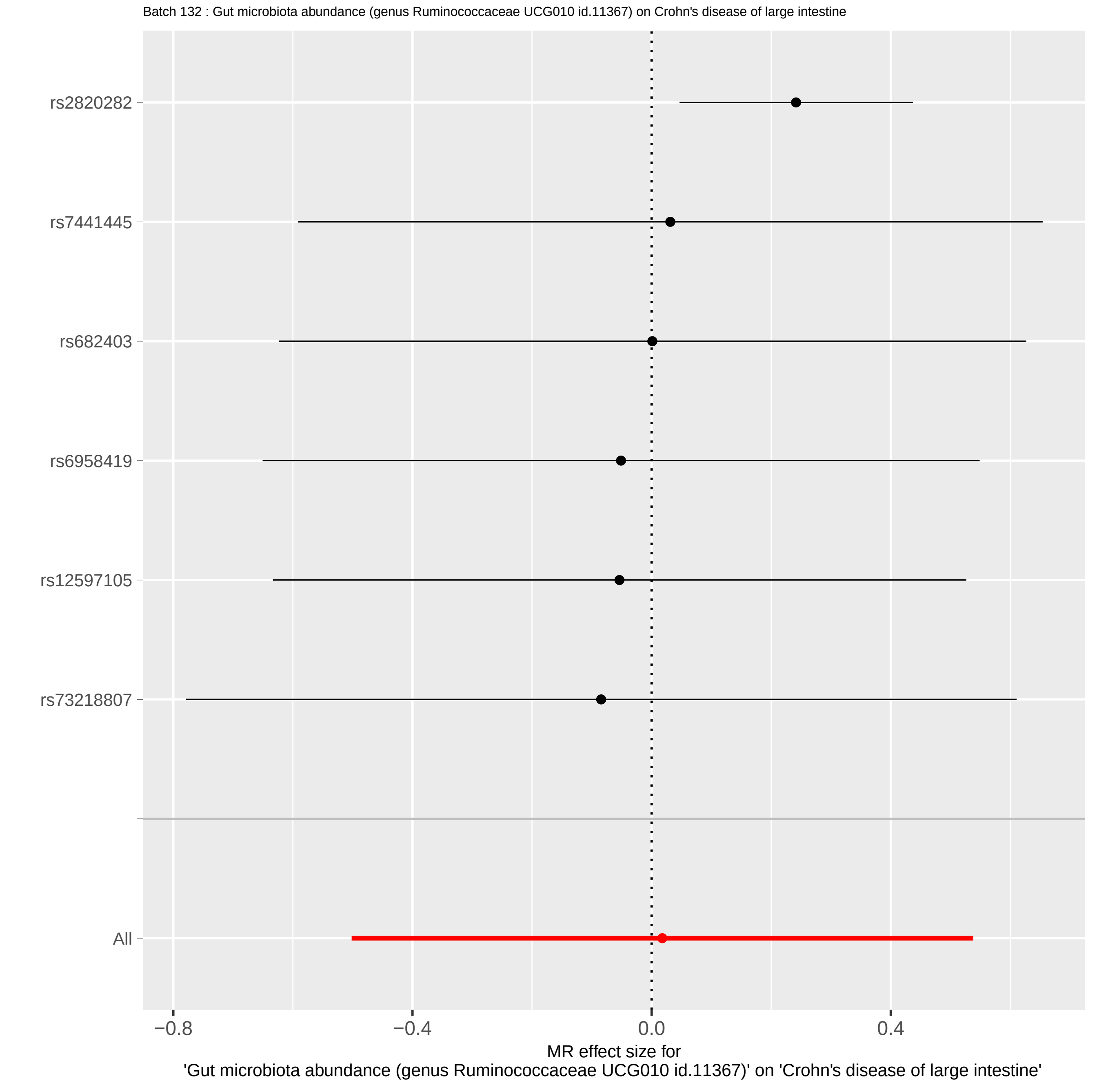
Batch 132 : Gut microbiota abundance (genus Ruminococcaceae UCG010 id.11367) on Crohn's disease of large intestine
rs2820282
rs7441445
rs682403
rs6958419
rs12597105
rs73218807
All
−0.8
−0.4
0.0
0.4
MR effect size for
'Gut microbiota abundance (genus Ruminococcaceae UCG010 id.11367)' on 'Crohn's disease of large intestine'

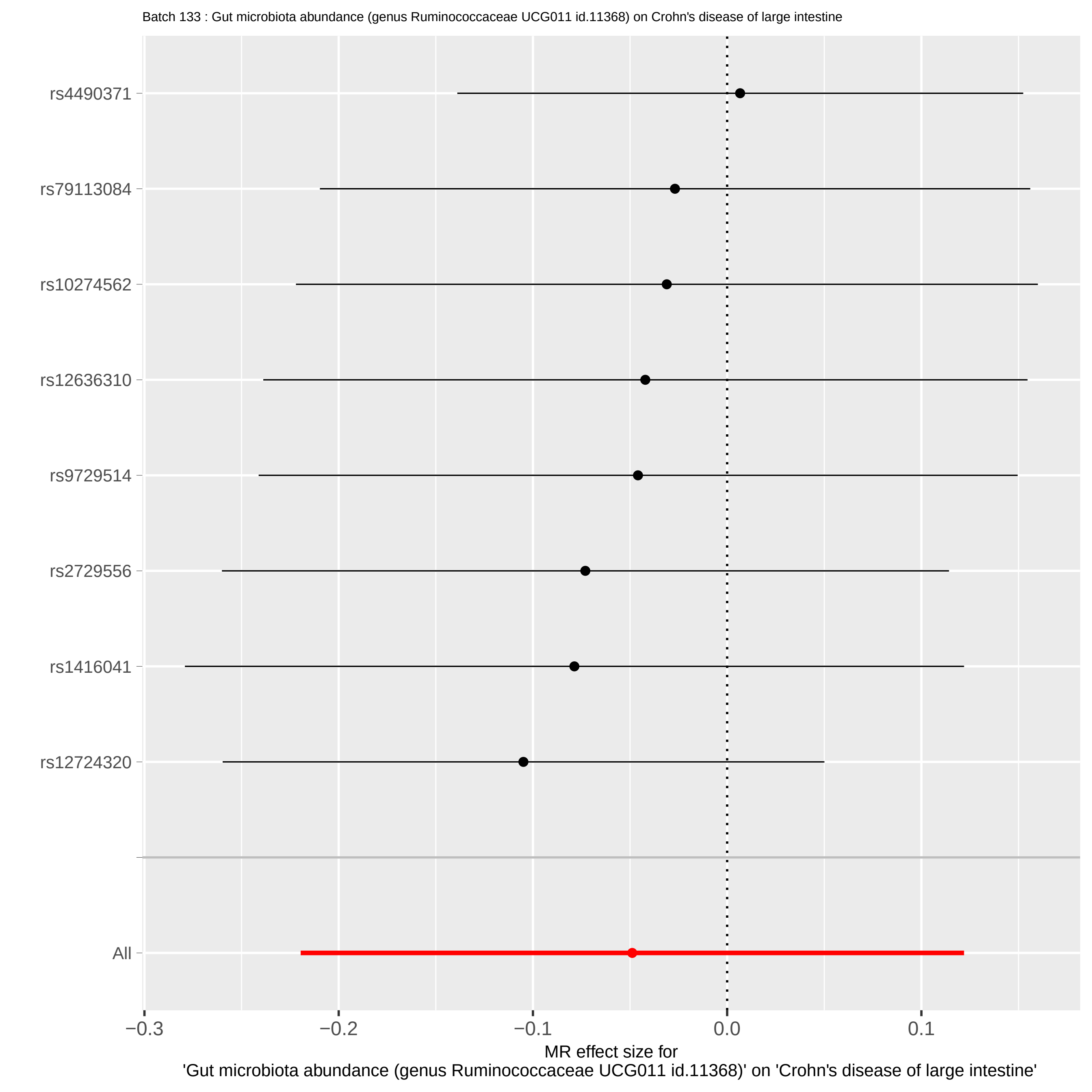
Batch 133 : Gut microbiota abundance (genus Ruminococcaceae UCG011 id.11368) on Crohn's disease of large intestine
rs4490371
rs79113084
rs10274562
rs12636310
rs9729514
rs2729556
rs1416041
rs12724320
All
−0.3
−0.2
−0.1
0.0
0.1
MR effect size for
'Gut microbiota abundance (genus Ruminococcaceae UCG011 id.11368)' on 'Crohn's disease of large intestine'

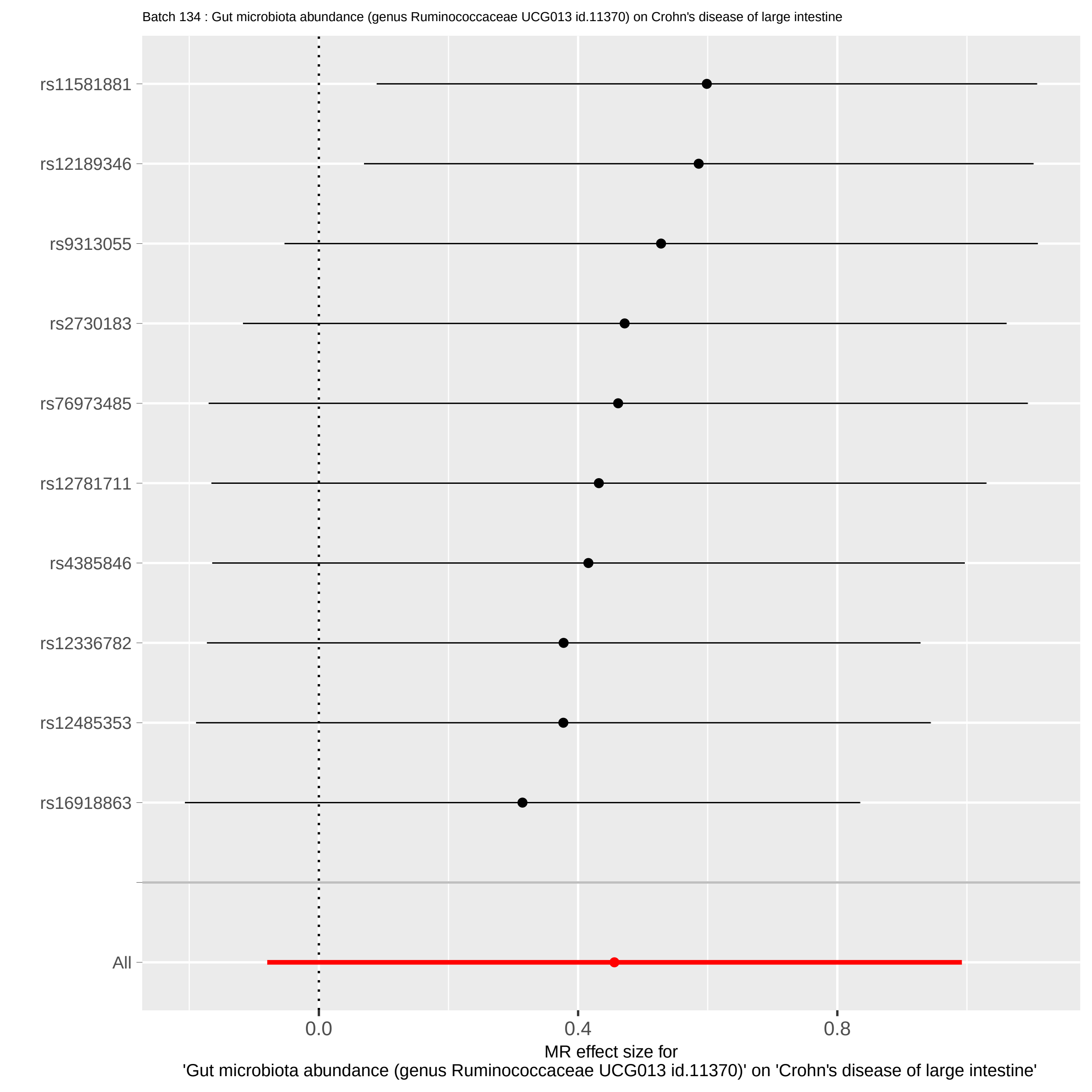

Batch 134 : Gut microbiota abundance (genus Ruminococcaceae UCG013 id.11370) on Crohn's disease of large intestine
rs11581881
rs12189346
rs9313055
rs2730183
rs76973485
rs12781711
rs4385846
rs12336782
rs12485353
rs16918863
All
0.0
0.4
0.8
MR effect size for
'Gut microbiota abundance (genus Ruminococcaceae UCG013 id.11370)' on 'Crohn's disease of large intestine'

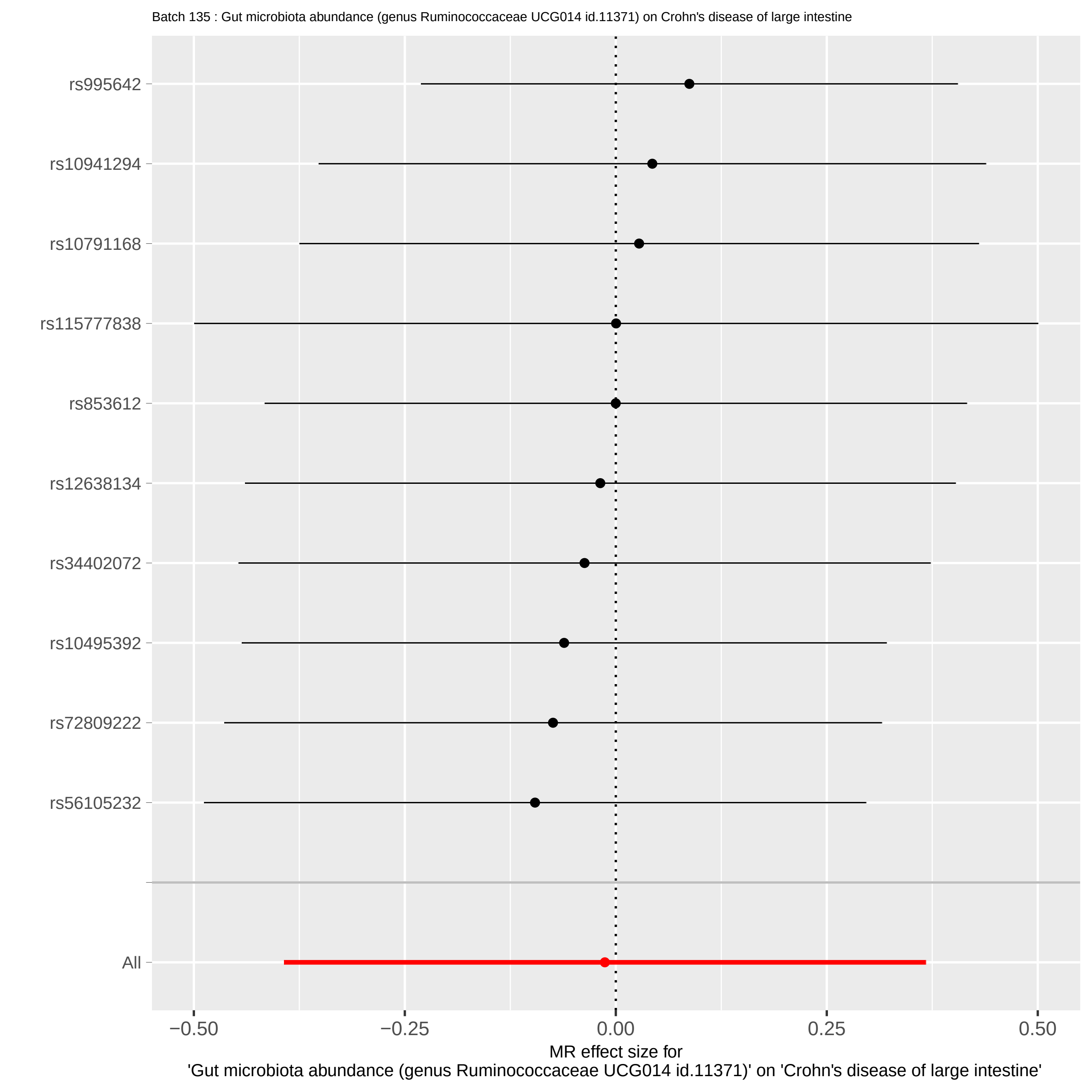
Batch 135 : Gut microbiota abundance (genus Ruminococcaceae UCG014 id.11371) on Crohn's disease of large intestine
rs995642
rs10941294
rs10791168
rs115777838
rs853612
rs12638134
rs34402072
rs10495392
rs72809222
rs56105232
All
−0.50
−0.25
0.00
0.25
0.50
MR effect size for
'Gut microbiota abundance (genus Ruminococcaceae UCG014 id.11371)' on 'Crohn's disease of large intestine'

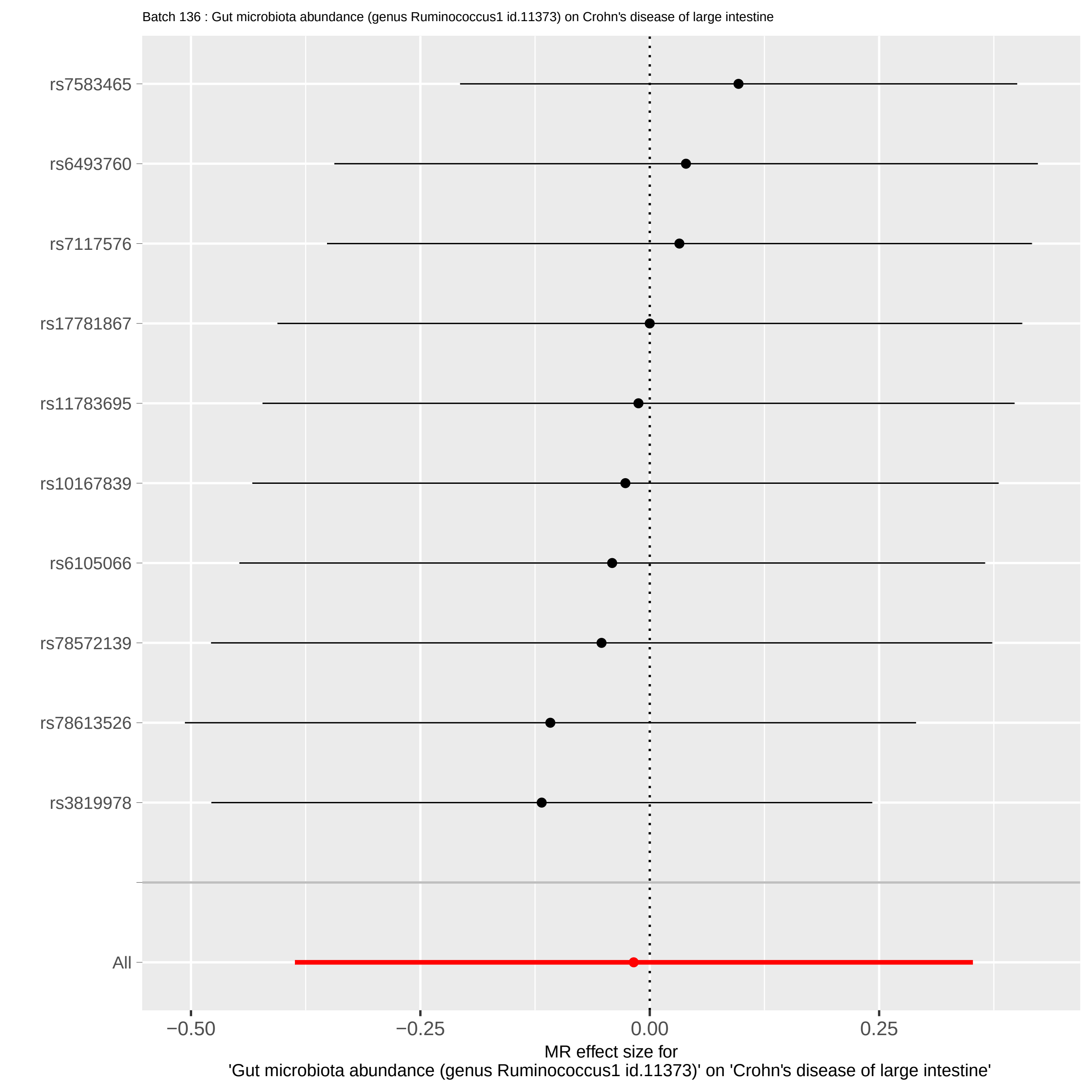

Batch 136 : Gut microbiota abundance (genus Ruminococcus1 id.11373) on Crohn's disease of large intestine
rs7583465
rs6493760
rs7117576
rs17781867
rs11783695
rs10167839
rs6105066
rs78572139
rs78613526
rs3819978
All
−0.50
−0.25
0.00
0.25
MR effect size for
'Gut microbiota abundance (genus Ruminococcus1 id.11373)' on 'Crohn's disease of large intestine'

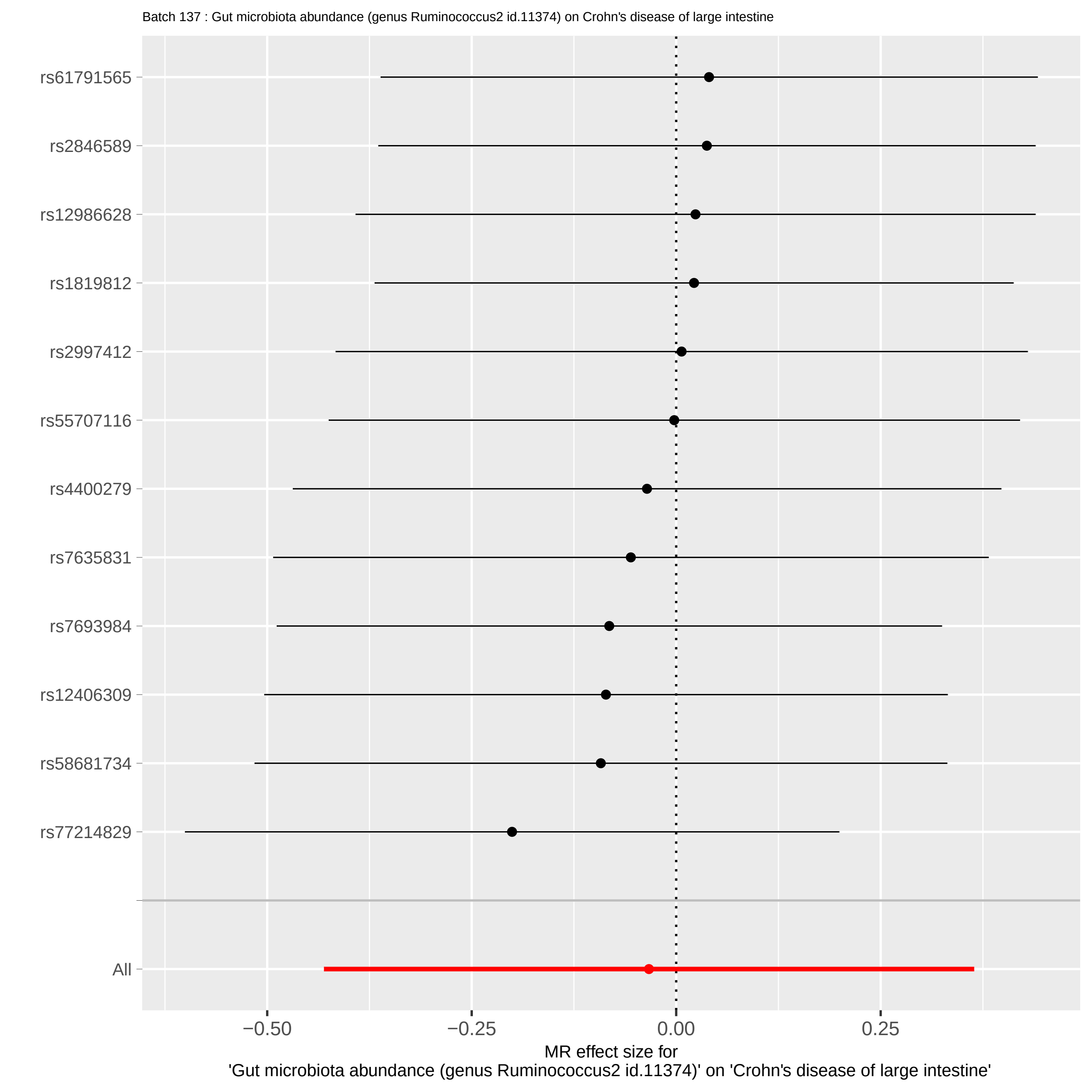

Batch 137 : Gut microbiota abundance (genus Ruminococcus2 id.11374) on Crohn's disease of large intestine
rs61791565
rs2846589
rs12986628
rs1819812
rs2997412
rs55707116
rs4400279
rs7635831
rs7693984
rs12406309
rs58681734
rs77214829
All
−0.50
−0.25
0.00
0.25
MR effect size for
'Gut microbiota abundance (genus Ruminococcus2 id.11374)' on 'Crohn's disease of large intestine'

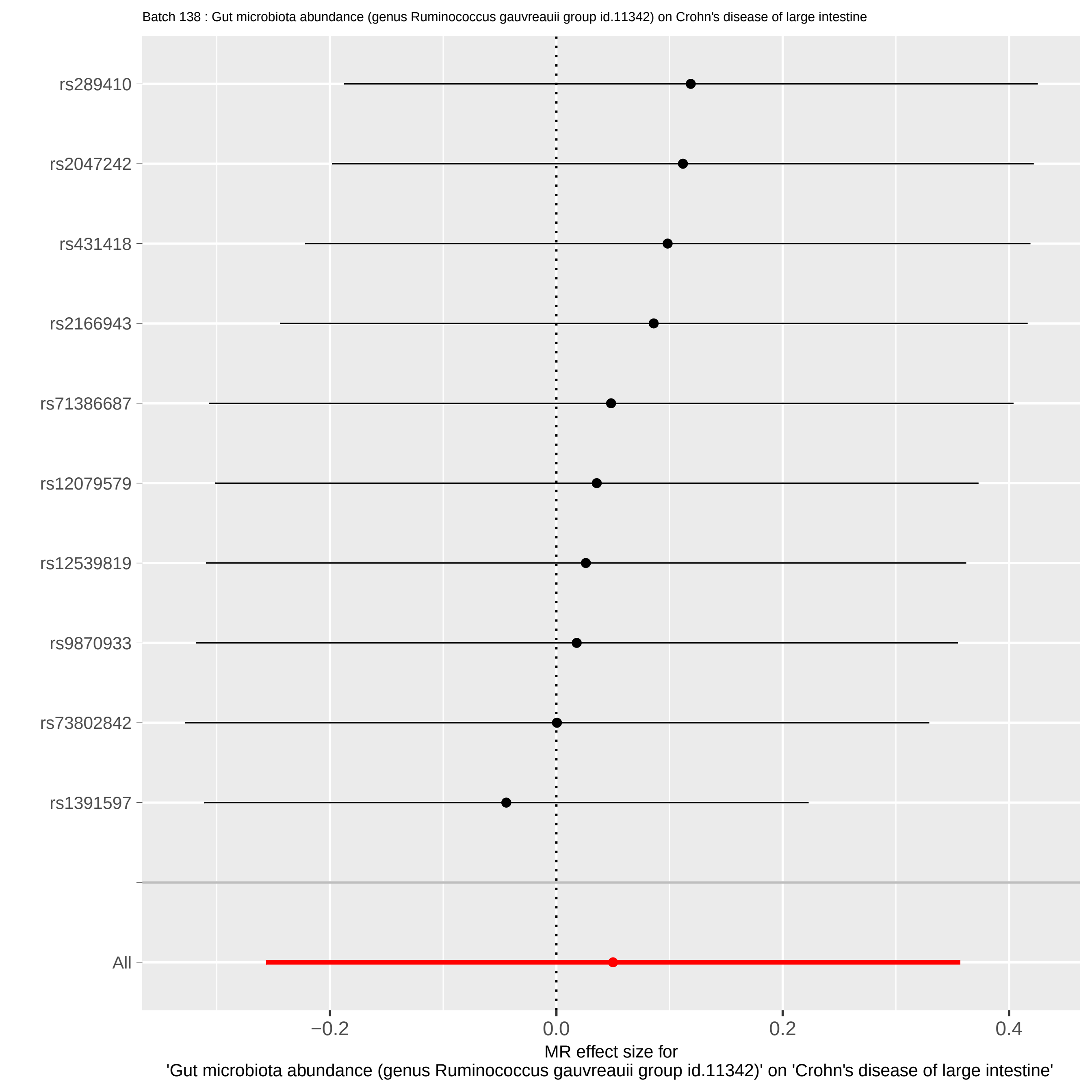
Batch 138 : Gut microbiota abundance (genus Ruminococcus gauvreauii group id.11342) on Crohn's disease of large intestine
rs289410
rs2047242
rs431418
rs2166943
rs71386687
rs12079579
rs12539819
rs9870933
rs73802842
rs1391597
All
−0.2
0.0
0.2
0.4
MR effect size for
'Gut microbiota abundance (genus Ruminococcus gauvreauii group id.11342)' on 'Crohn's disease of large intestine'

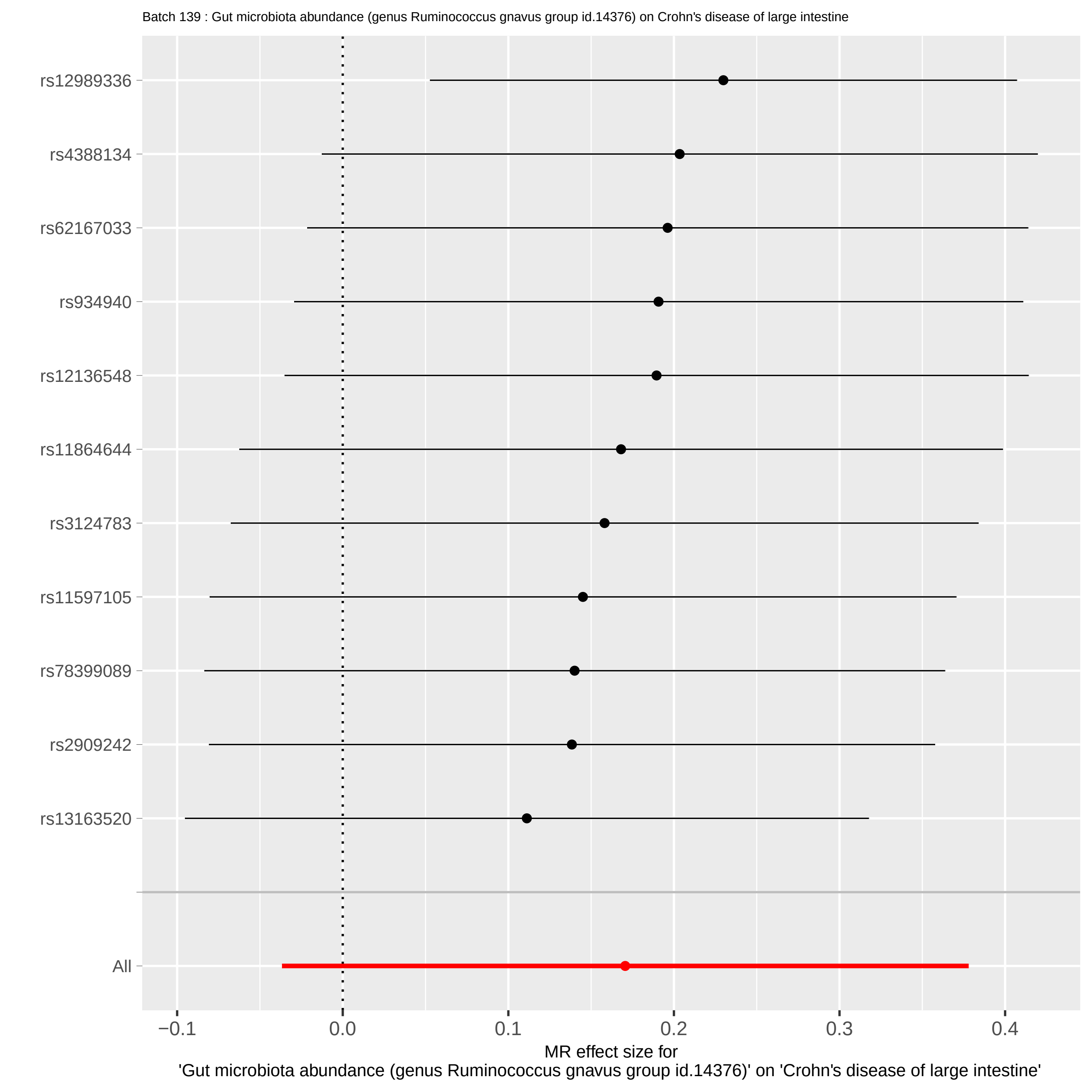
Batch 139 : Gut microbiota abundance (genus Ruminococcus gnavus group id.14376) on Crohn's disease of large intestine
rs12989336
rs4388134
rs62167033
rs934940
rs12136548
rs11864644
rs3124783
rs11597105
rs78399089
rs2909242
rs13163520
All
−0.1
0.0
0.1
0.2
0.3
0.4
MR effect size for
'Gut microbiota abundance (genus Ruminococcus gnavus group id.14376)' on 'Crohn's disease of large intestine'

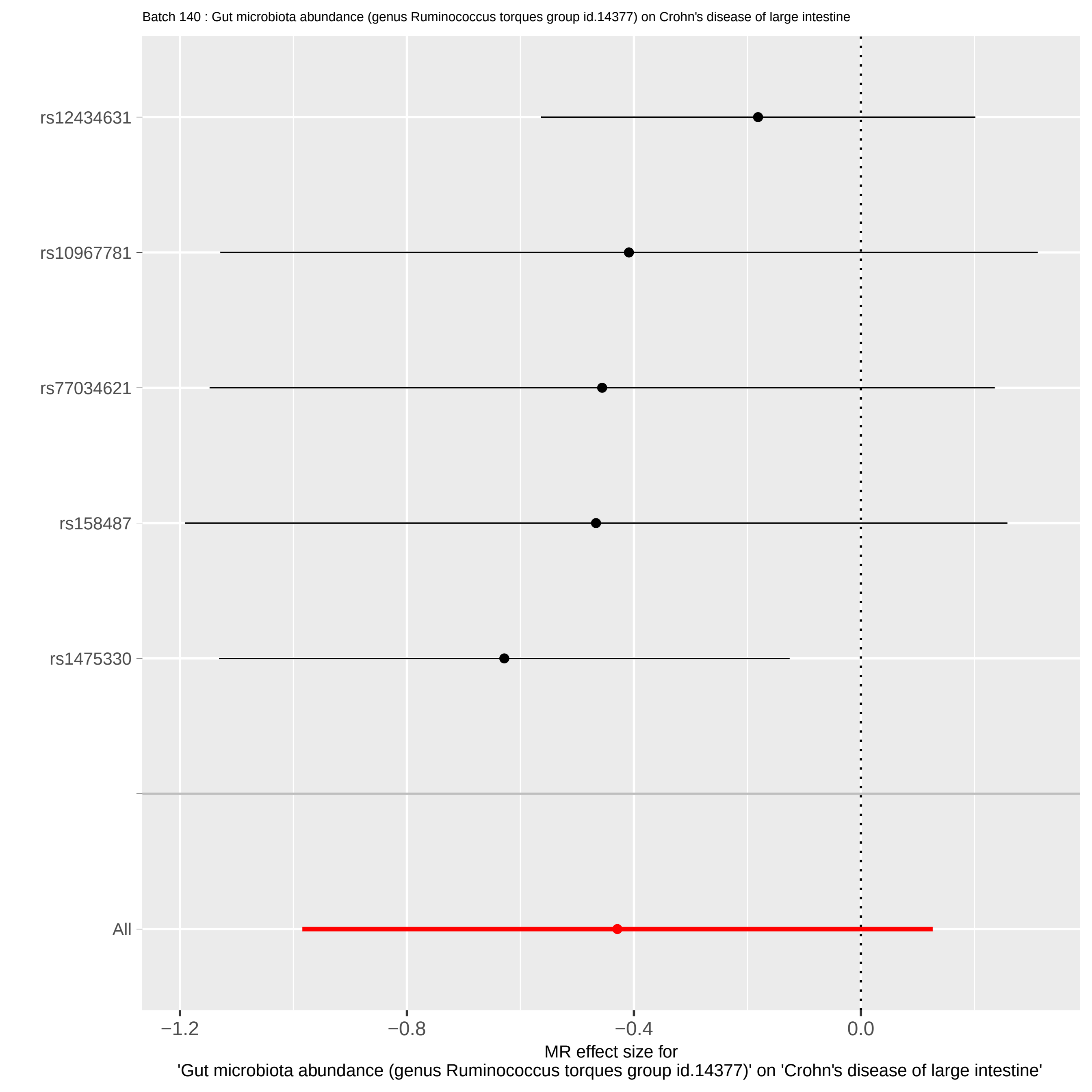
Batch 140 : Gut microbiota abundance (genus Ruminococcus torques group id.14377) on Crohn's disease of large intestine
rs12434631
rs10967781
rs77034621
rs158487
rs1475330
All
−1.2
−0.8
−0.4
0.0
MR effect size for
'Gut microbiota abundance (genus Ruminococcus torques group id.14377)' on 'Crohn's disease of large intestine'

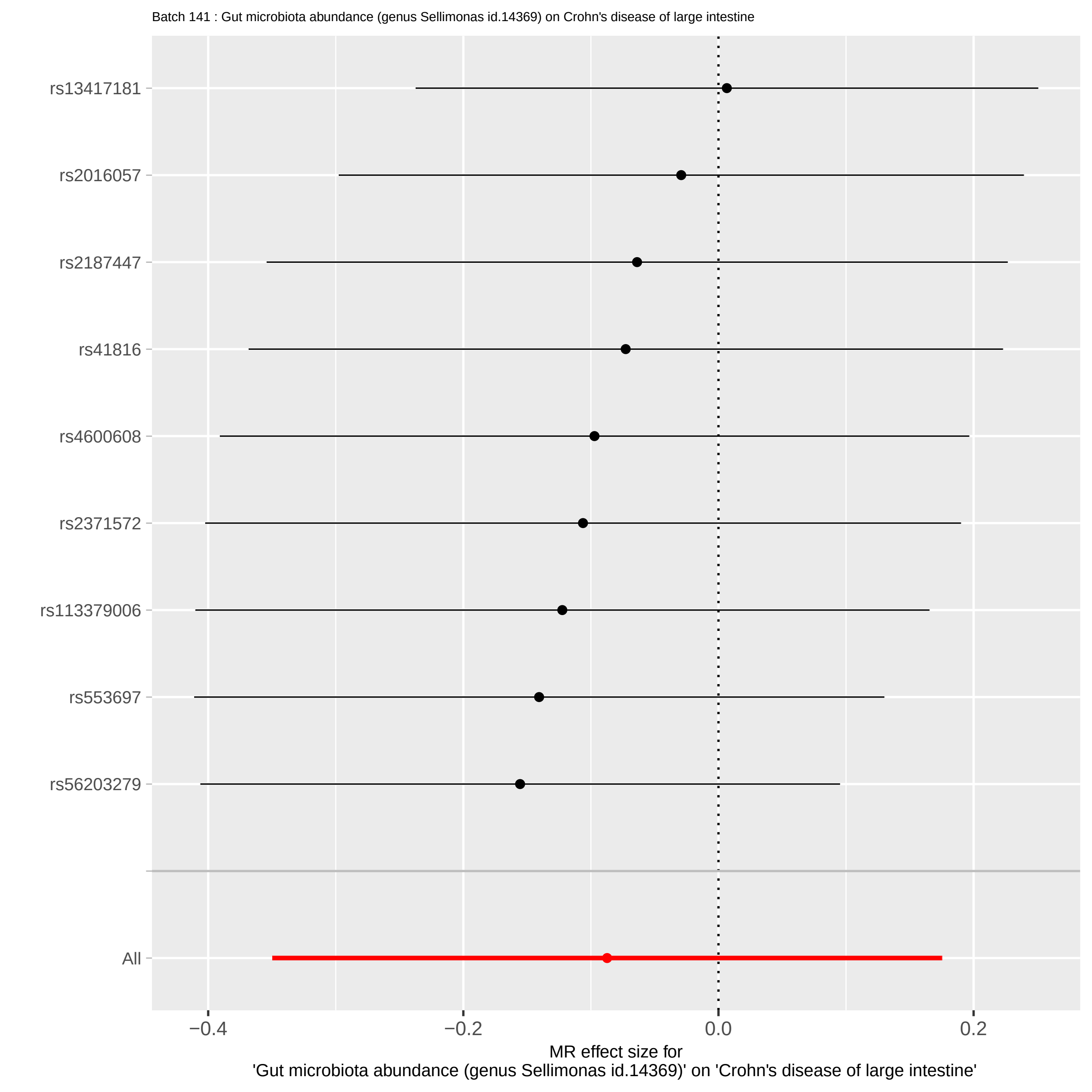
Batch 141 : Gut microbiota abundance (genus Sellimonas id.14369) on Crohn's disease of large intestine
rs13417181
rs2016057
rs2187447
rs41816
rs4600608
rs2371572
rs113379006
rs553697
rs56203279
All
−0.4
−0.2
0.0
0.2
MR effect size for
'Gut microbiota abundance (genus Sellimonas id.14369)' on 'Crohn's disease of large intestine'

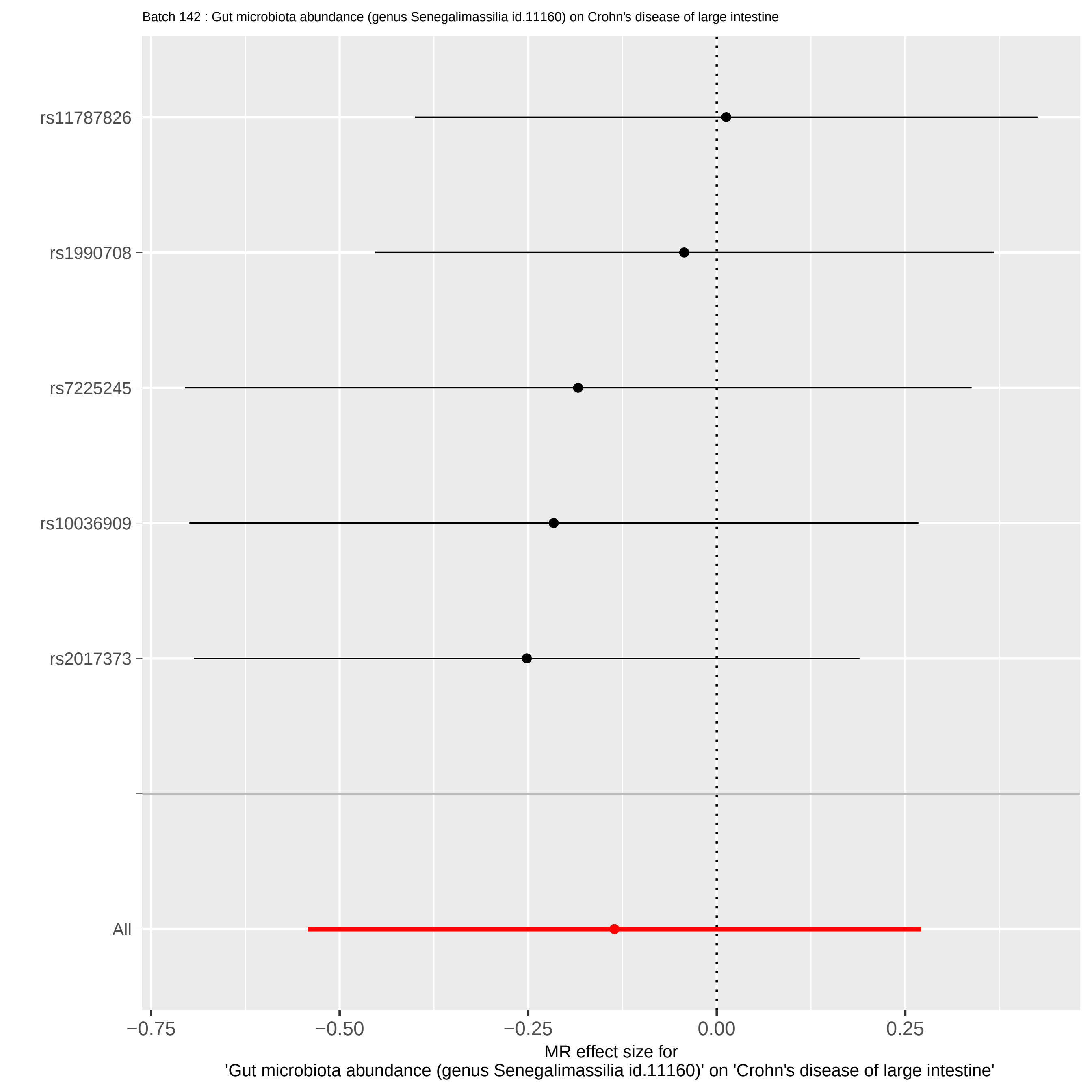

Batch 142 : Gut microbiota abundance (genus Senegalimassilia id.11160) on Crohn's disease of large intestine
rs11787826
rs1990708
rs7225245
rs10036909
rs2017373
All
−0.75
−0.50
−0.25
0.00
0.25
MR effect size for
'Gut microbiota abundance (genus Senegalimassilia id.11160)' on 'Crohn's disease of large intestine'

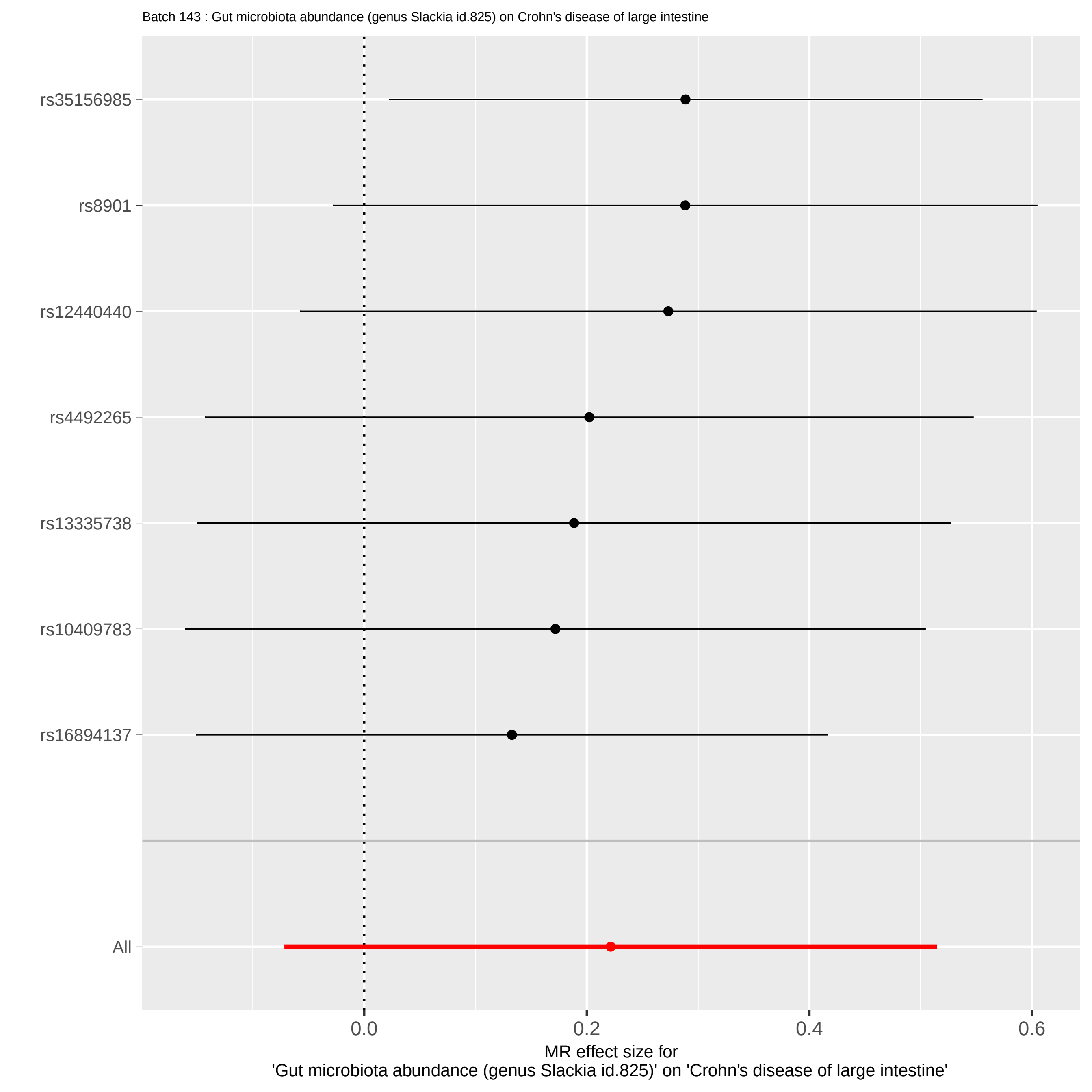
Batch 143 : Gut microbiota abundance (genus Slackia id.825) on Crohn's disease of large intestine
rs35156985
rs8901
rs12440440
rs4492265
rs13335738
rs10409783
rs16894137
All
0.0
0.2
0.4
0.6
MR effect size for
'Gut microbiota abundance (genus Slackia id.825)' on 'Crohn's disease of large intestine'

Batch 144 : Gut microbiota abundance (genus Streptococcus id.1853) on Crohn's disease of large intestine

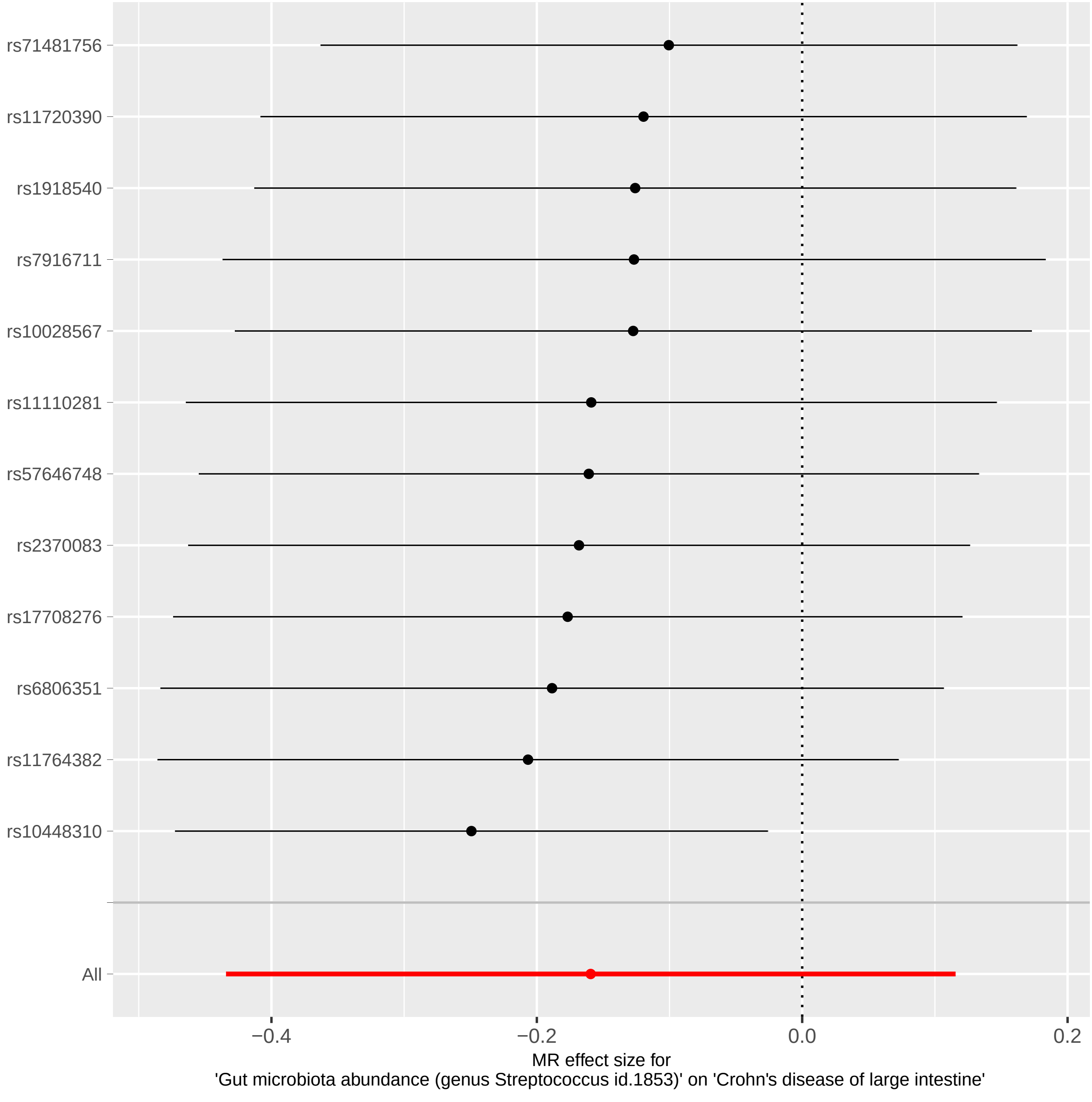

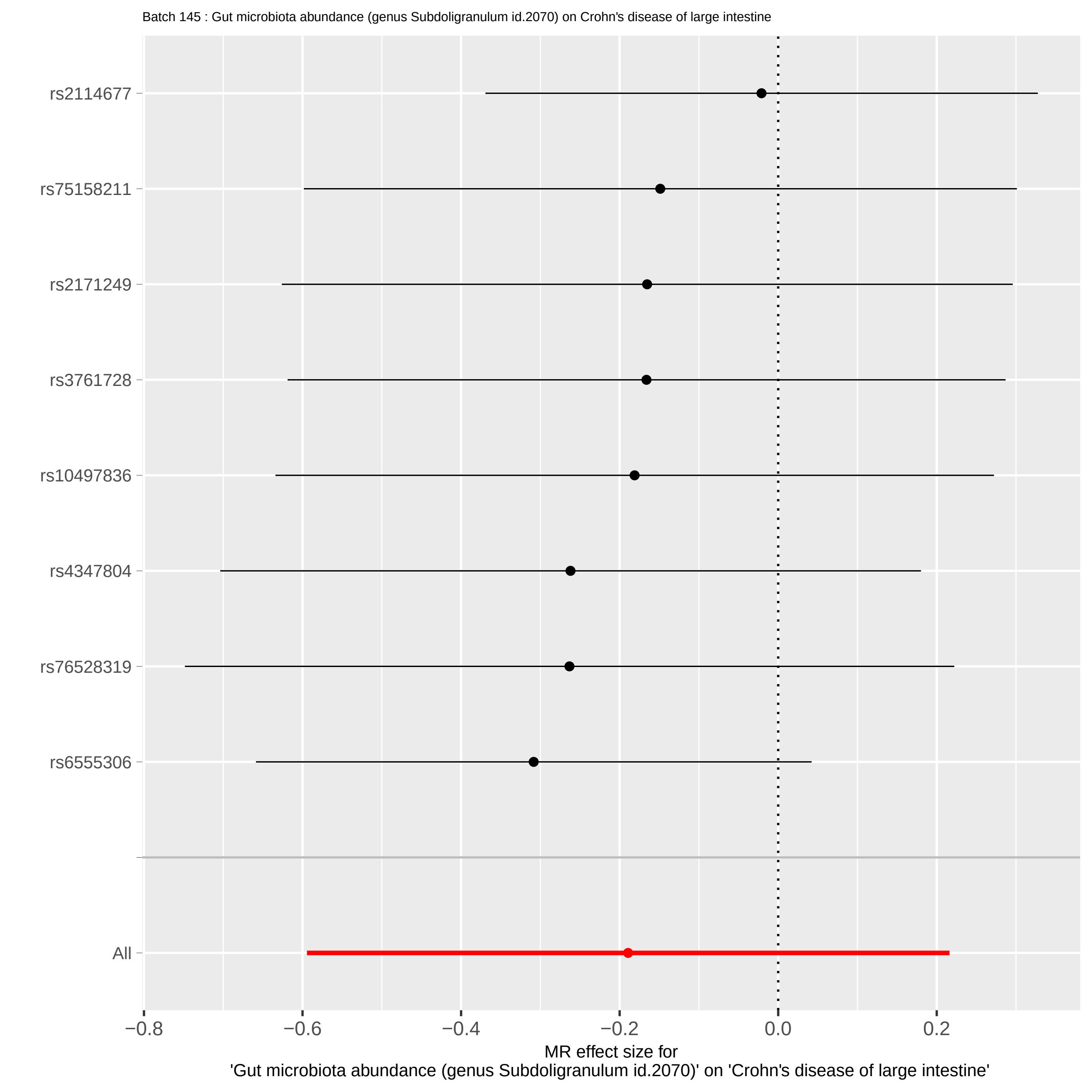
Batch 145 : Gut microbiota abundance (genus Subdoligranulum id.2070) on Crohn's disease of large intestine
rs2114677
rs75158211
rs2171249
rs3761728
rs10497836
rs4347804
rs76528319
rs6555306
All
−0.8
−0.6
−0.4
−0.2
0.0
0.2
MR effect size for
'Gut microbiota abundance (genus Subdoligranulum id.2070)' on 'Crohn's disease of large intestine'

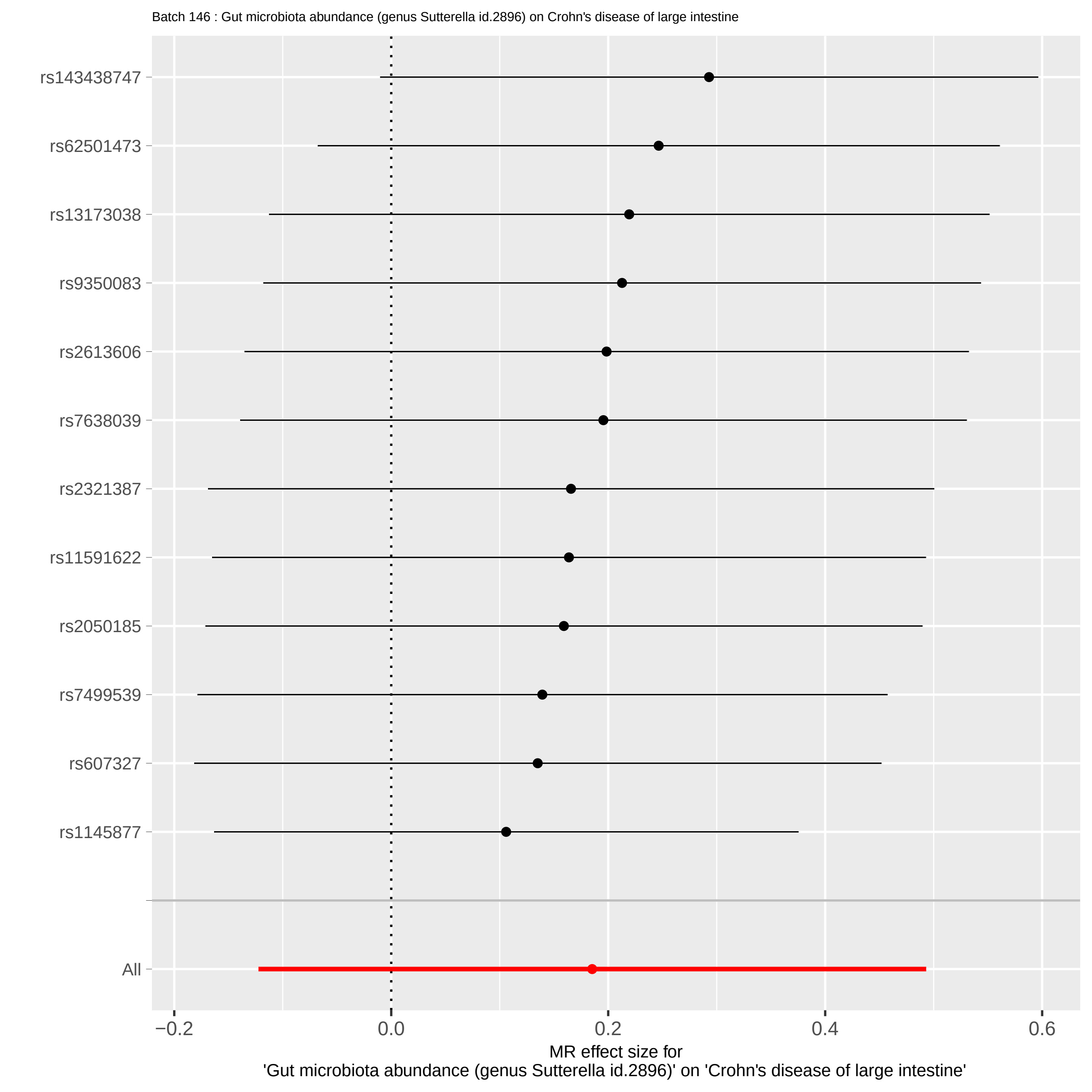
Batch 146 : Gut microbiota abundance (genus Sutterella id.2896) on Crohn's disease of large intestine
rs143438747
rs62501473
rs13173038
rs9350083
rs2613606
rs7638039
rs2321387
rs11591622
rs2050185
rs7499539
rs607327
rs1145877
All
−0.2
0.0
0.2
0.4
0.6
MR effect size for
'Gut microbiota abundance (genus Sutterella id.2896)' on 'Crohn's disease of large intestine'

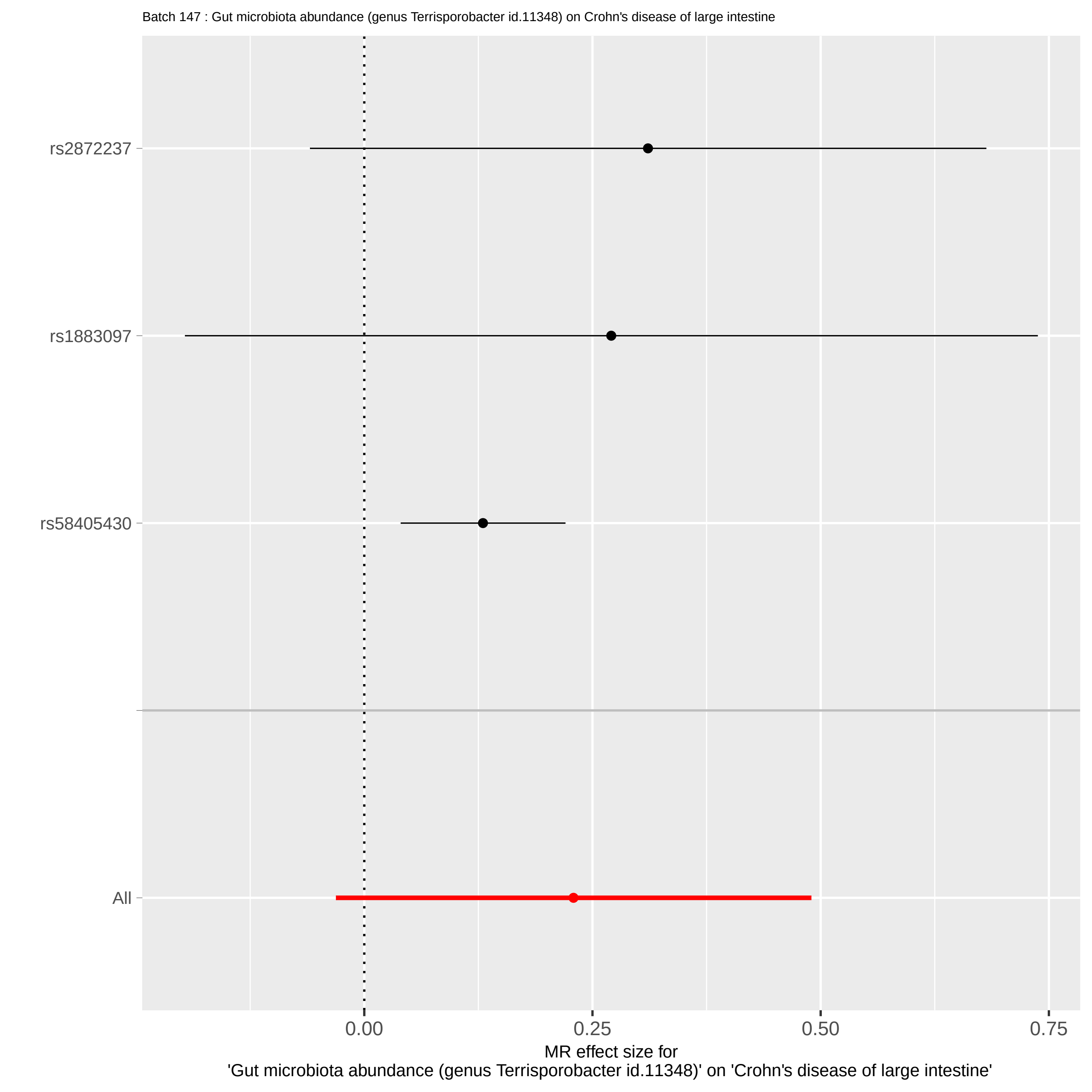
Batch 147 : Gut microbiota abundance (genus Terrisporobacter id.11348) on Crohn's disease of large intestine
rs2872237
rs1883097
rs58405430
All
0.00
0.25
0.50
0.75
MR effect size for
'Gut microbiota abundance (genus Terrisporobacter id.11348)' on 'Crohn's disease of large intestine'

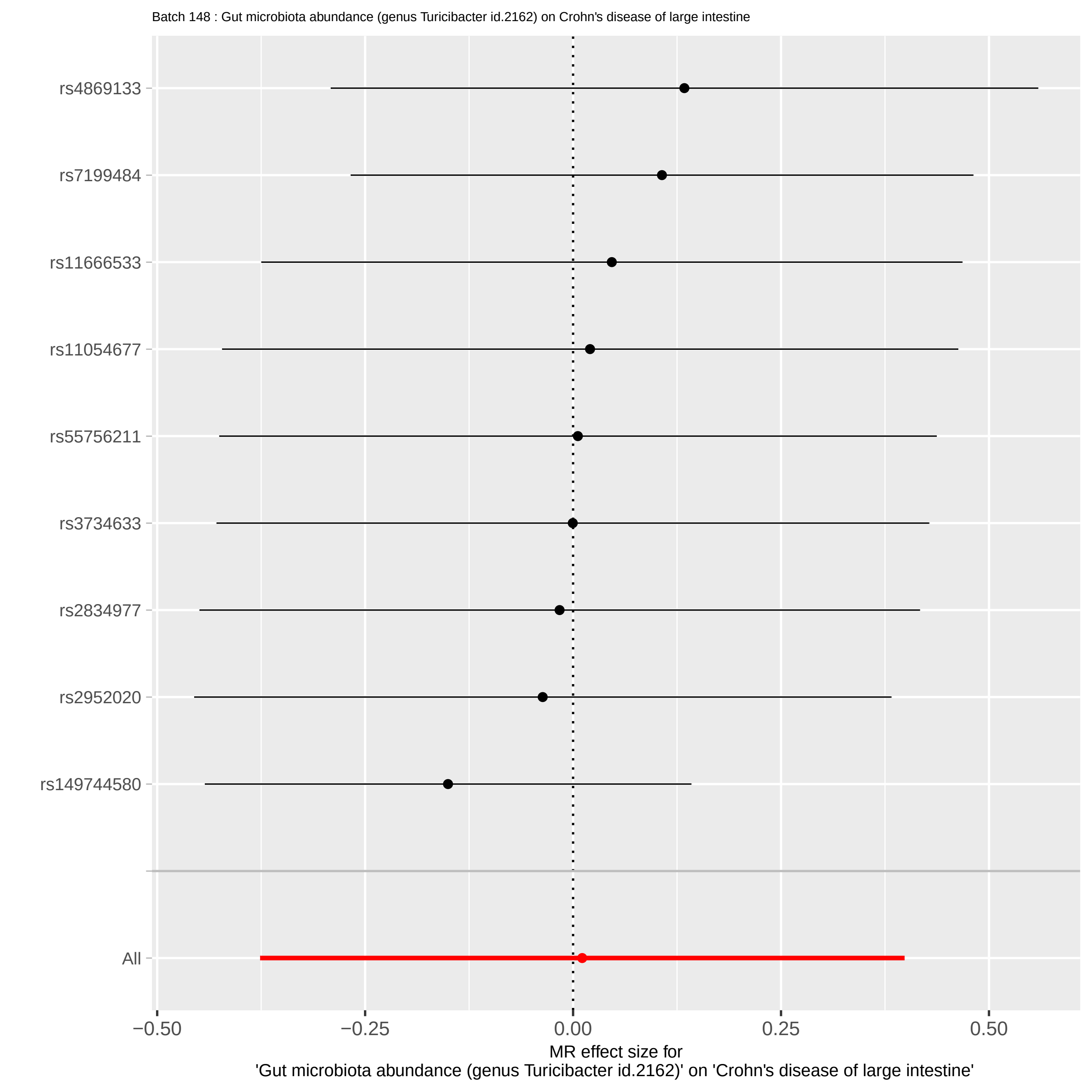
Batch 148 : Gut microbiota abundance (genus Turicibacter id.2162) on Crohn's disease of large intestine
rs4869133
rs7199484
rs11666533
rs11054677
rs55756211
rs3734633
rs2834977
rs2952020
rs149744580
All
−0.50
−0.25
0.00
0.25
0.50
MR effect size for
'Gut microbiota abundance (genus Turicibacter id.2162)' on 'Crohn's disease of large intestine'

Batch 149 : Gut microbiota abundance (genus Tyzzerella3 id.11335) on Crohn's disease of large intestine

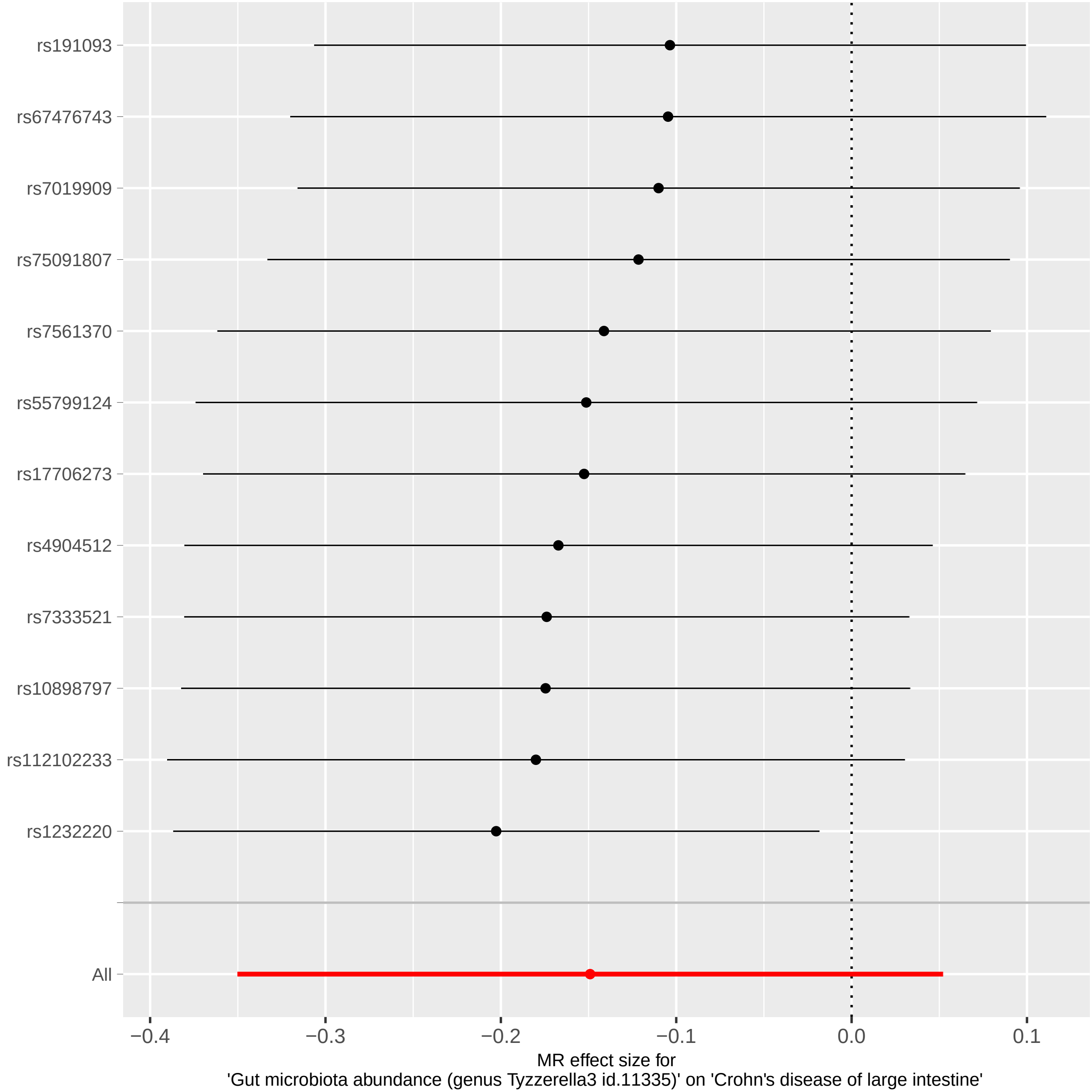

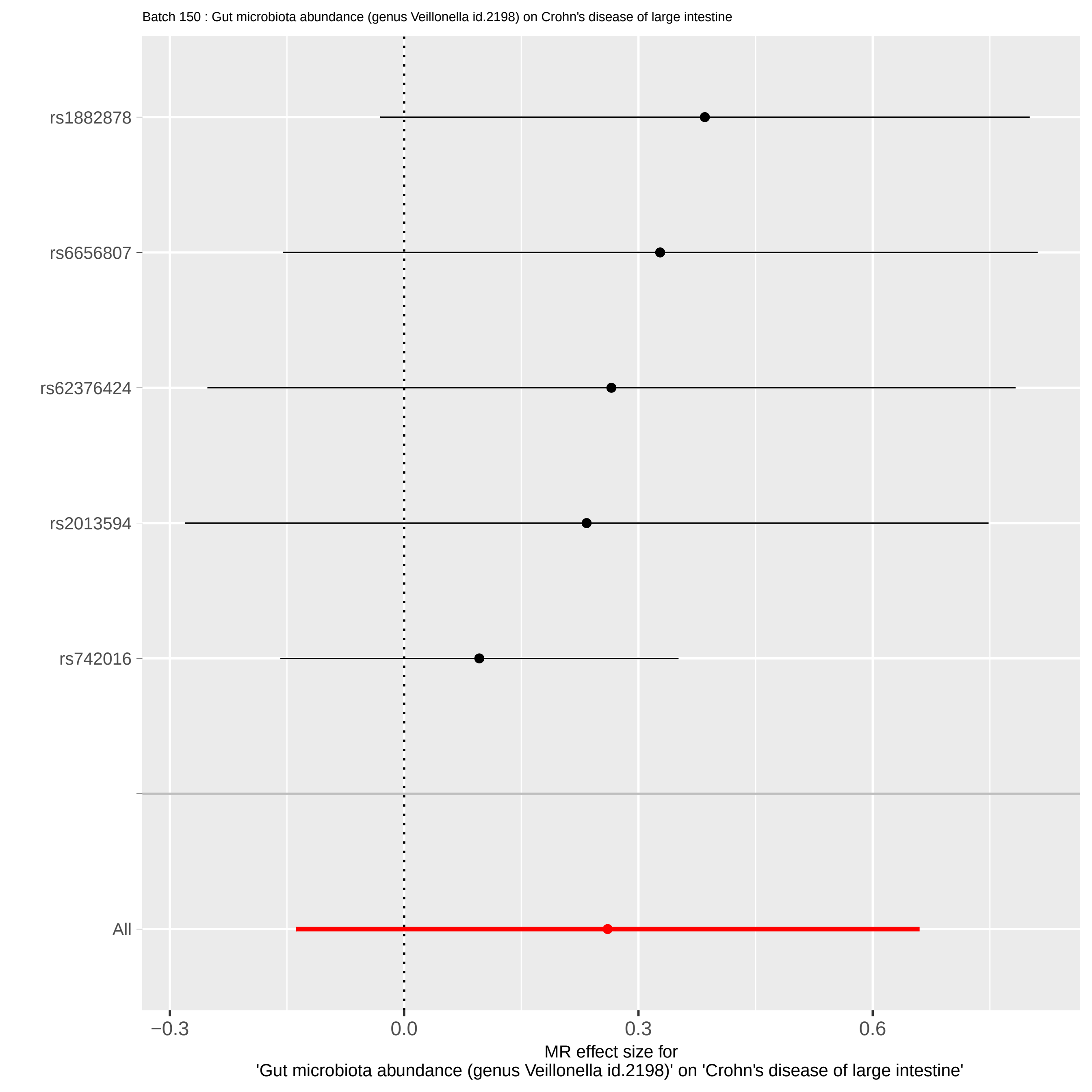
Batch 150 : Gut microbiota abundance (genus Veillonella id.2198) on Crohn's disease of large intestine
rs1882878
rs6656807
rs62376424
rs2013594
rs742016
All
−0.3
0.0
0.3
0.6
MR effect size for
'Gut microbiota abundance (genus Veillonella id.2198)' on 'Crohn's disease of large intestine'

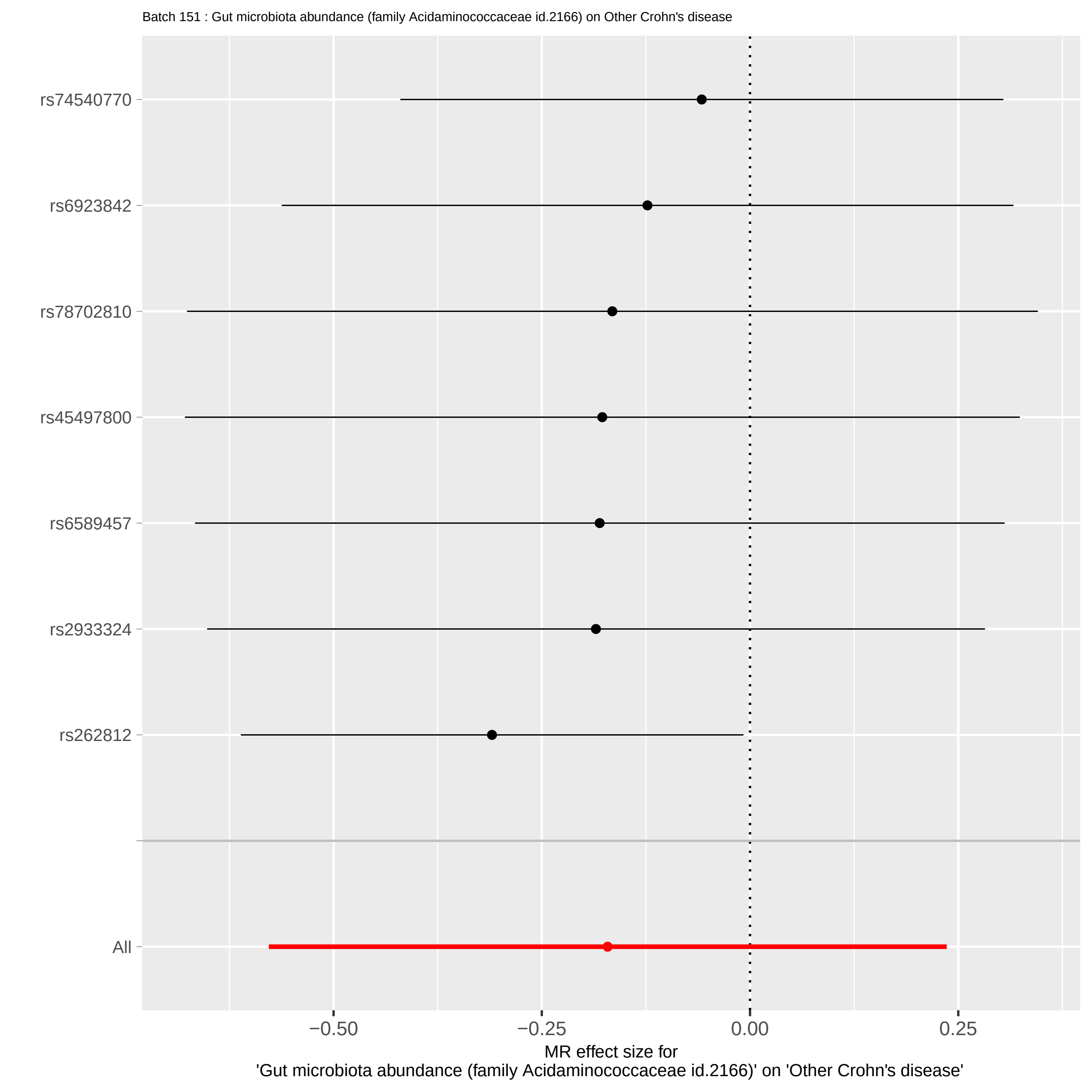

Batch 151 : Gut microbiota abundance (family Acidaminococcaceae id.2166) on Other Crohn's disease
rs74540770
rs6923842
rs78702810
rs45497800
rs6589457
rs2933324
rs262812
All
−0.50
−0.25
0.00
0.25
MR effect size for
'Gut microbiota abundance (family Acidaminococcaceae id.2166)' on 'Other Crohn's disease'

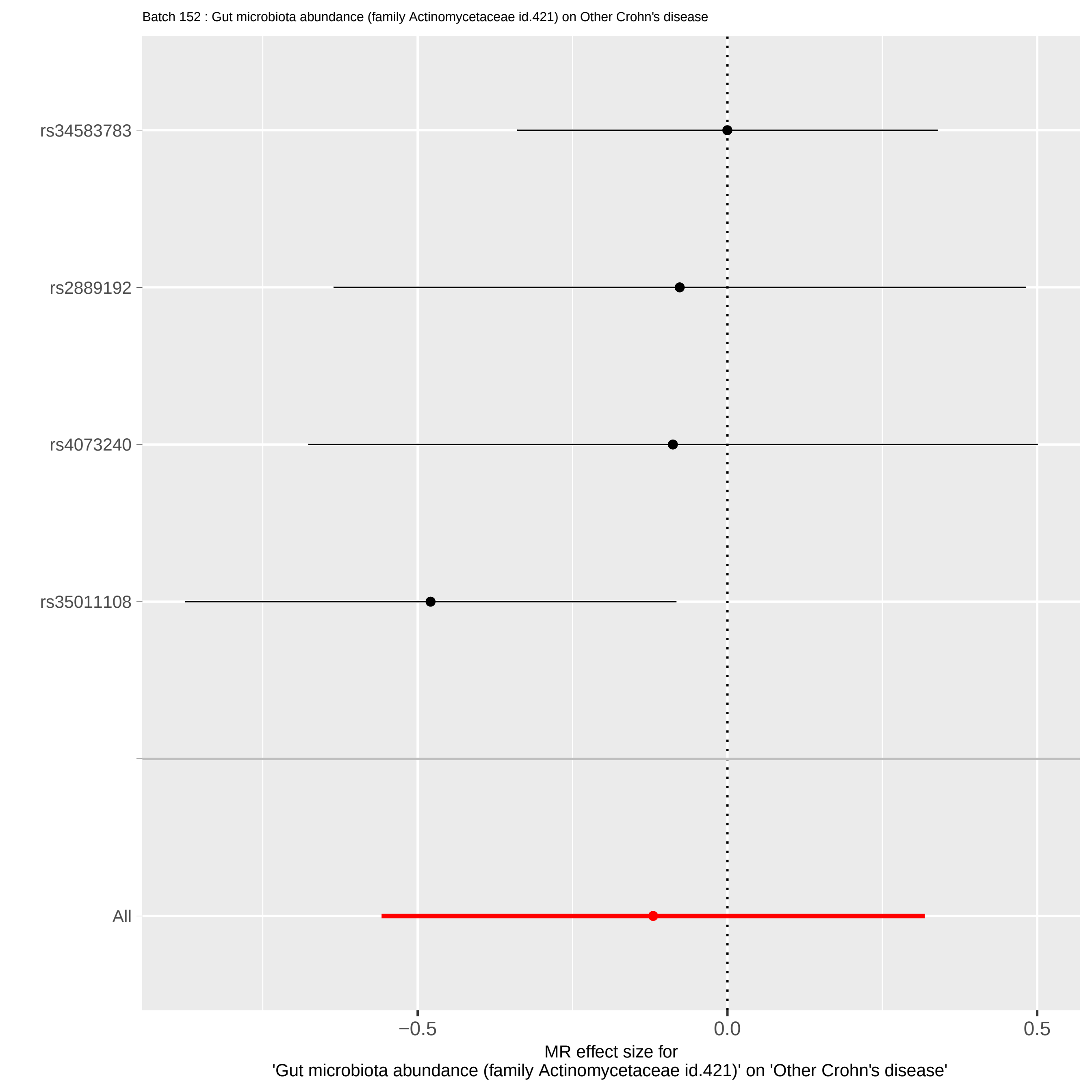
Batch 152 : Gut microbiota abundance (family Actinomycetaceae id.421) on Other Crohn's disease
rs34583783
rs2889192
rs4073240
rs35011108
All
−0.5
0.0
0.5
MR effect size for
'Gut microbiota abundance (family Actinomycetaceae id.421)' on 'Other Crohn's disease'

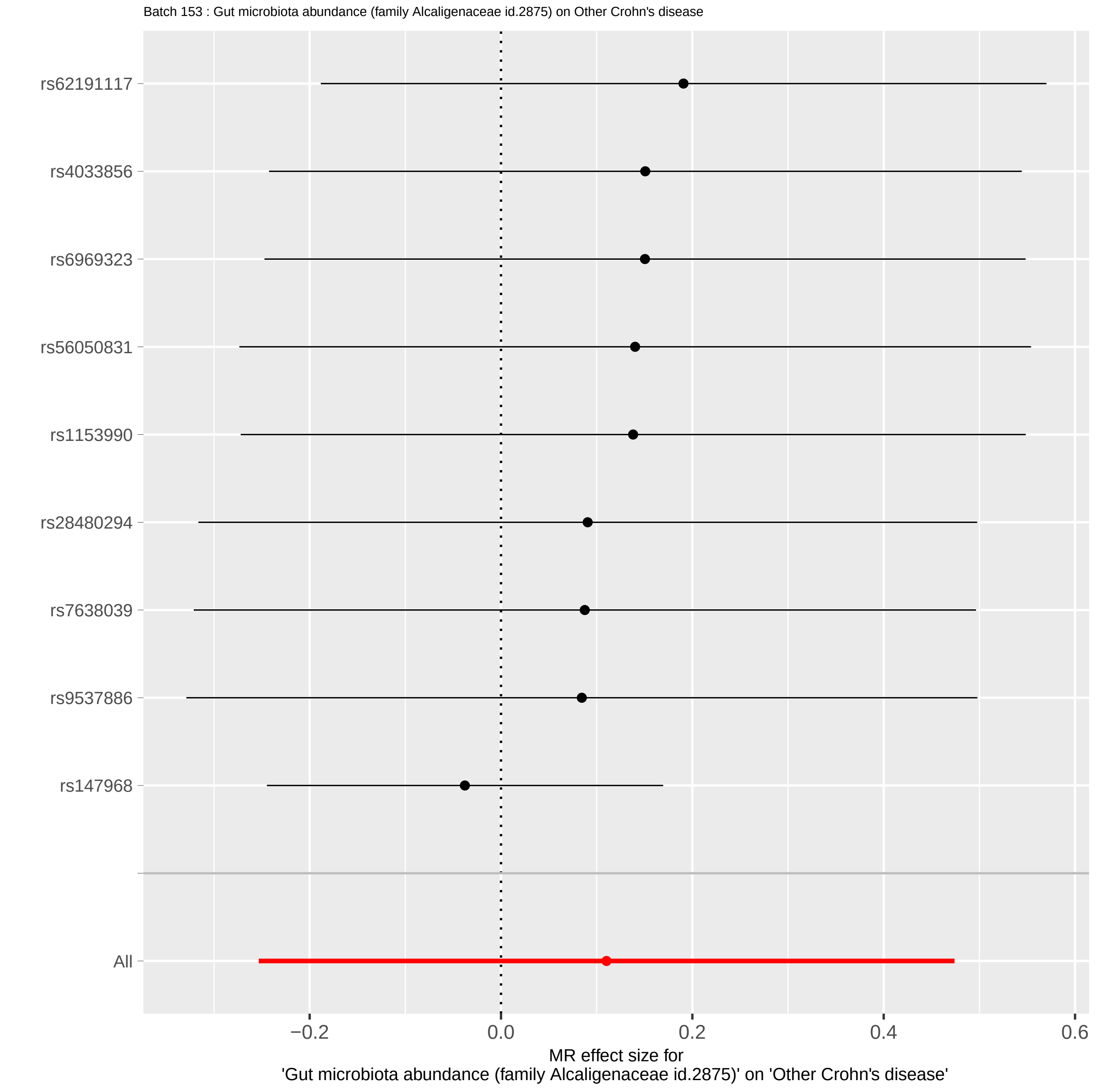
Batch 153 : Gut microbiota abundance (family Alcaligenaceae id.2875) on Other Crohn's disease
rs62191117
rs4033856
rs6969323
rs56050831
rs1153990
rs28480294
rs7638039
rs9537886
rs147968
All
−0.2
0.0
0.2
0.4
0.6
MR effect size for
'Gut microbiota abundance (family Alcaligenaceae id.2875)' on 'Other Crohn's disease'

Batch 154 : Gut microbiota abundance (family Bacteroidaceae id.917) on Other Crohn's disease

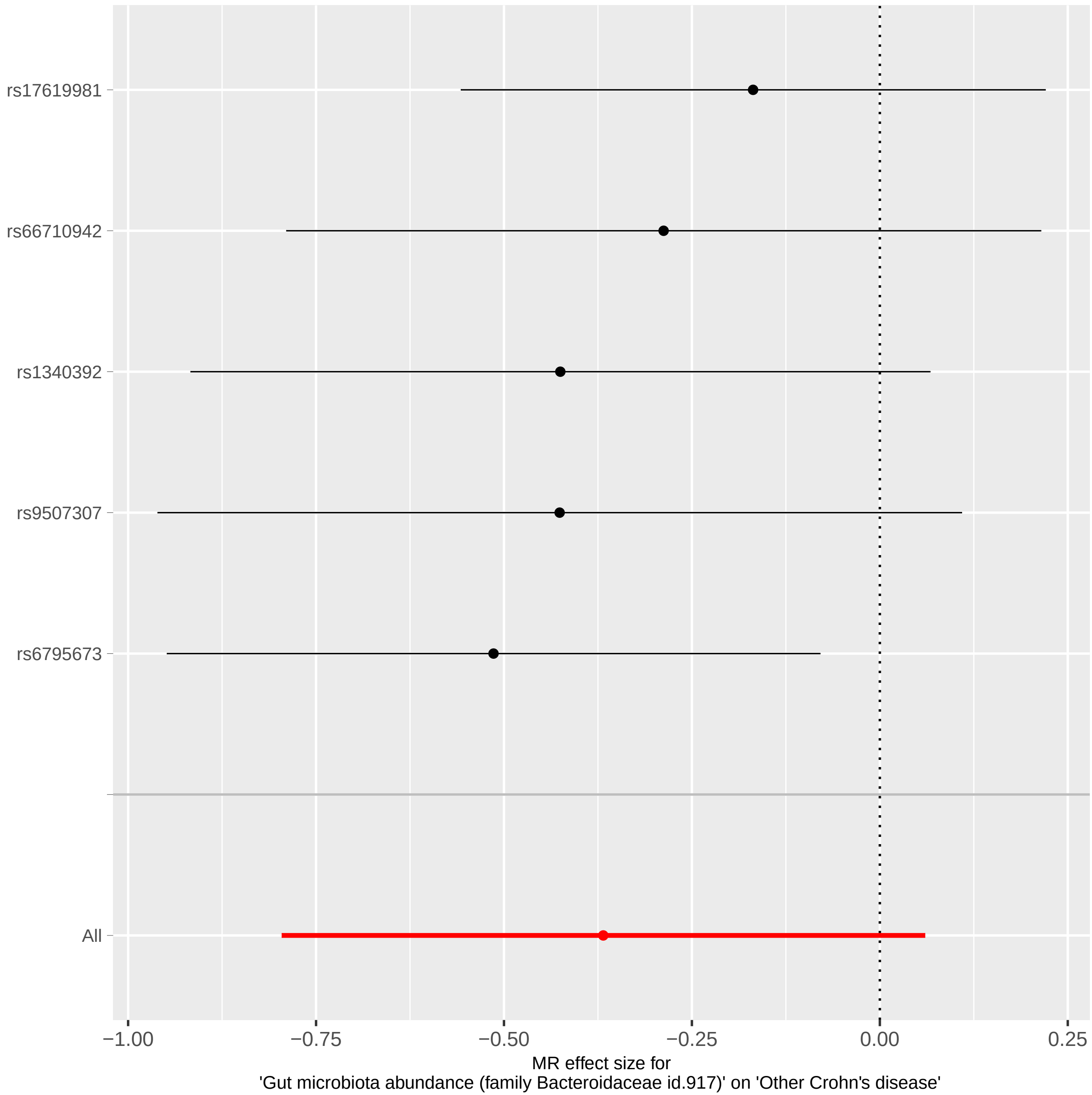

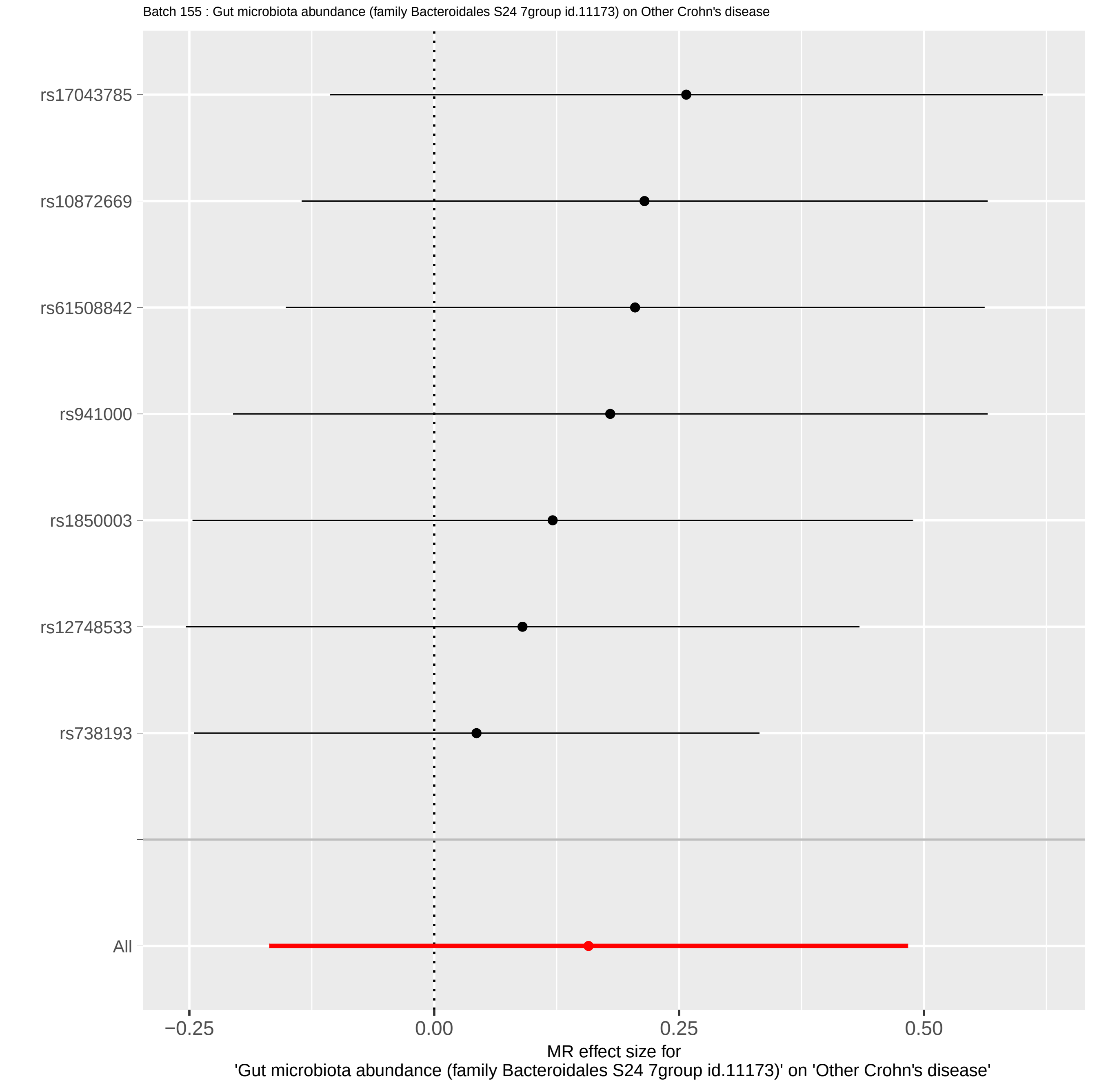
Batch 155 : Gut microbiota abundance (family Bacteroidales S24 7group id.11173) on Other Crohn's disease
rs17043785
rs10872669
rs61508842
rs941000
rs1850003
rs12748533
rs738193
All
−0.25
0.00
0.25
0.50
MR effect size for
'Gut microbiota abundance (family Bacteroidales S24 7group id.11173)' on 'Other Crohn's disease'

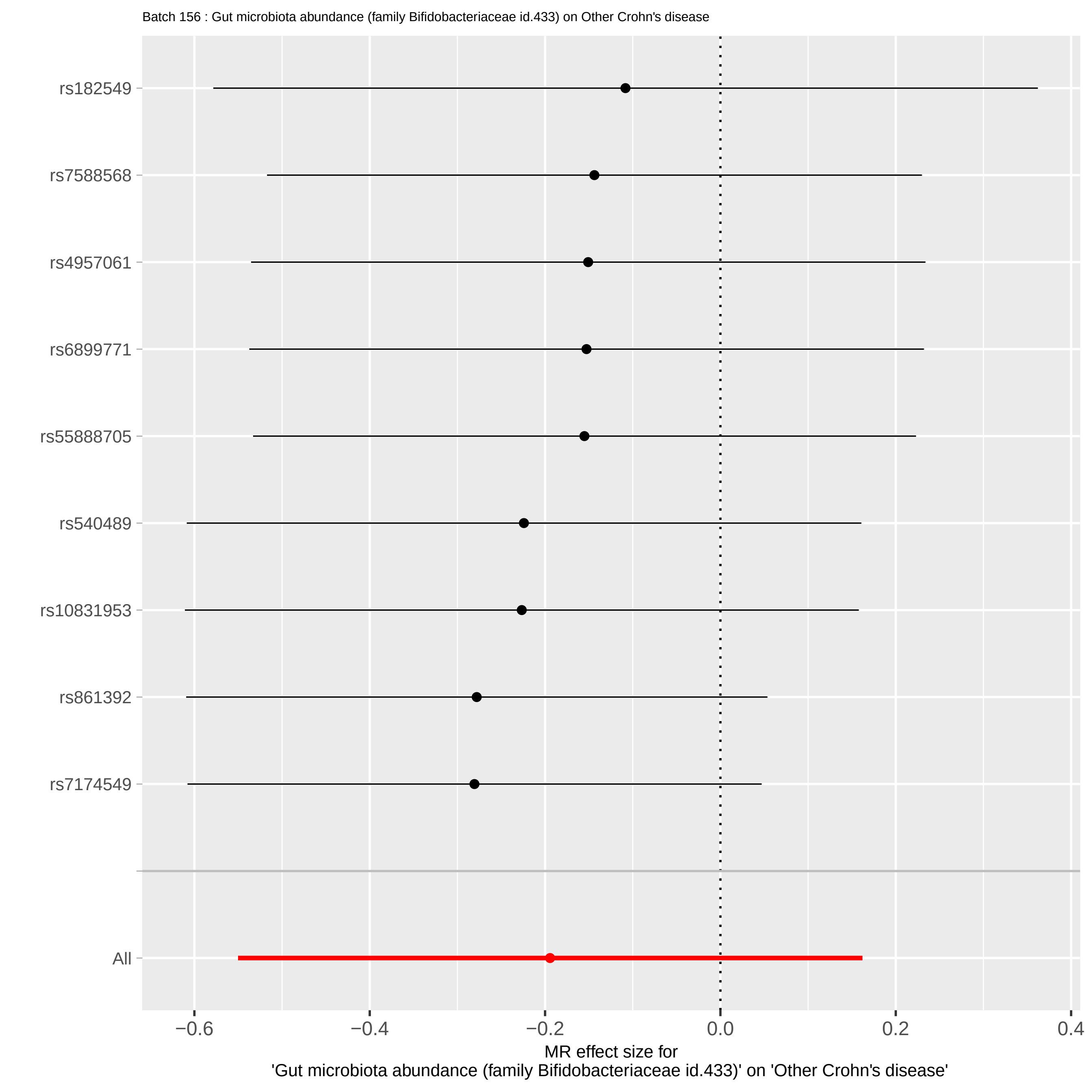
Batch 156 : Gut microbiota abundance (family Bifidobacteriaceae id.433) on Other Crohn's disease
rs182549
rs7588568
rs4957061
rs6899771
rs55888705
rs540489
rs10831953
rs861392
rs7174549
All
−0.6
−0.4
−0.2
0.0
0.2
0.4
MR effect size for
'Gut microbiota abundance (family Bifidobacteriaceae id.433)' on 'Other Crohn's disease'

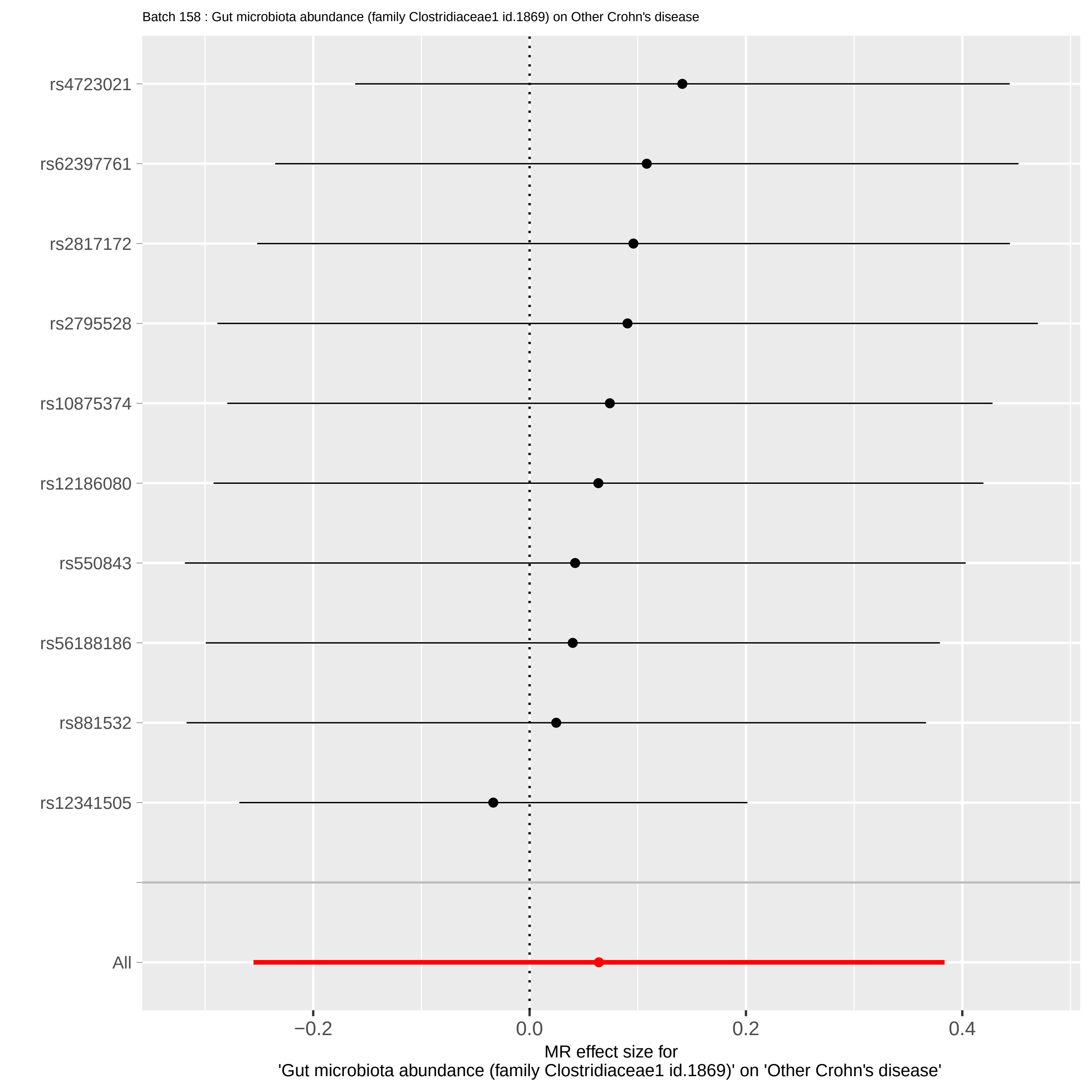
Batch 158 : Gut microbiota abundance (family Clostridiaceae1 id.1869) on Other Crohn's disease
rs4723021
rs62397761
rs2817172
rs2795528
rs10875374
rs12186080
rs550843
rs56188186
rs881532
rs12341505
All
−0.2
0.0
0.2
0.4
MR effect size for
'Gut microbiota abundance (family Clostridiaceae1 id.1869)' on 'Other Crohn's disease'

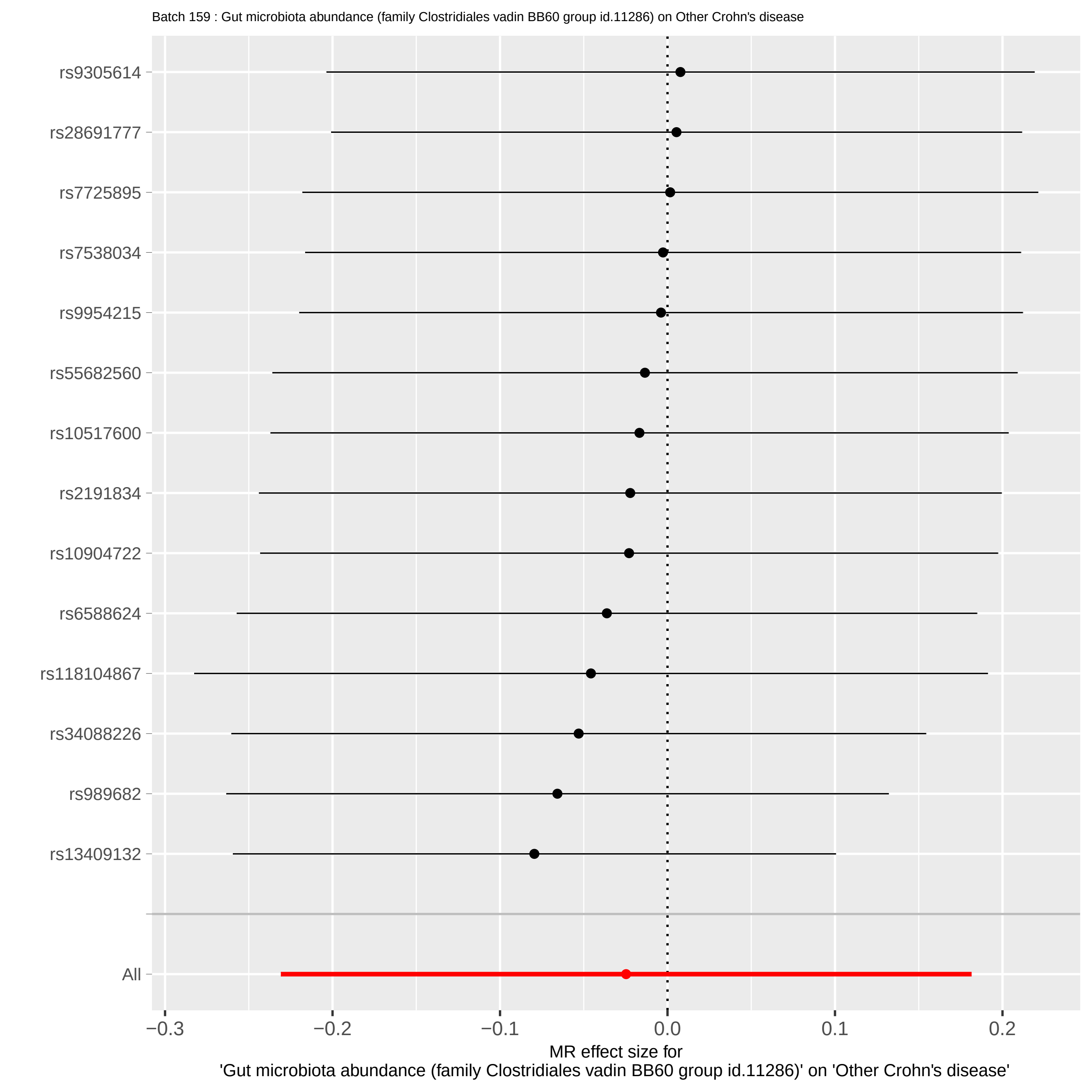
Batch 159 : Gut microbiota abundance (family Clostridiales vadin BB60 group id.11286) on Other Crohn's disease
rs9305614
rs28691777
rs7725895
rs7538034
rs9954215
rs55682560
rs10517600
rs2191834
rs10904722
rs6588624
rs118104867
rs34088226
rs989682
rs13409132
All
−0.3
−0.2
−0.1
0.0
0.1
0.2
MR effect size for
'Gut microbiota abundance (family Clostridiales vadin BB60 group id.11286)' on 'Other Crohn's disease'

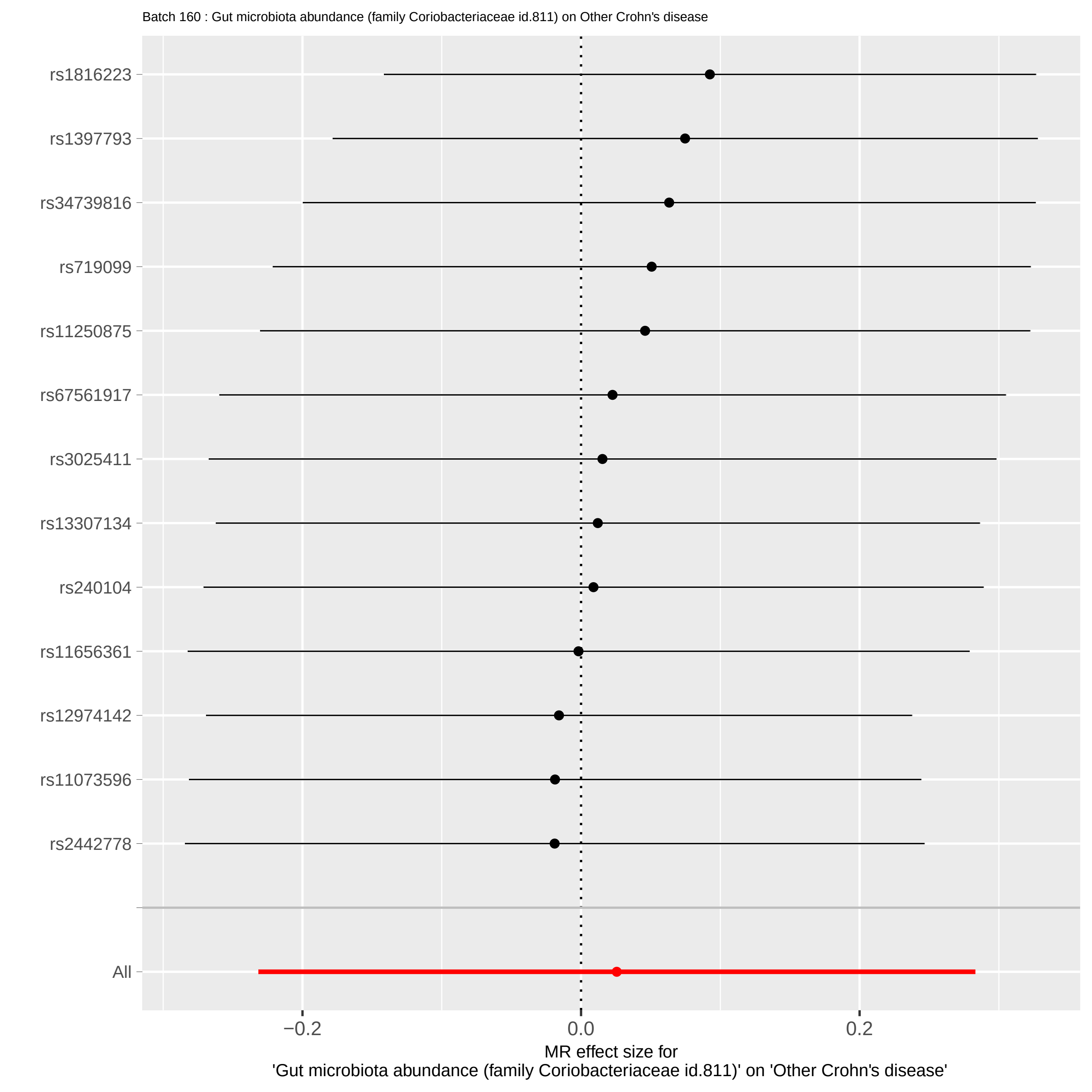
Batch 160 : Gut microbiota abundance (family Coriobacteriaceae id.811) on Other Crohn's disease
rs1816223
rs1397793
rs34739816
rs719099
rs11250875
rs67561917
rs3025411
rs13307134
rs240104
rs11656361
rs12974142
rs11073596
rs2442778
All
−0.2
0.0
0.2
MR effect size for
'Gut microbiota abundance (family Coriobacteriaceae id.811)' on 'Other Crohn's disease'

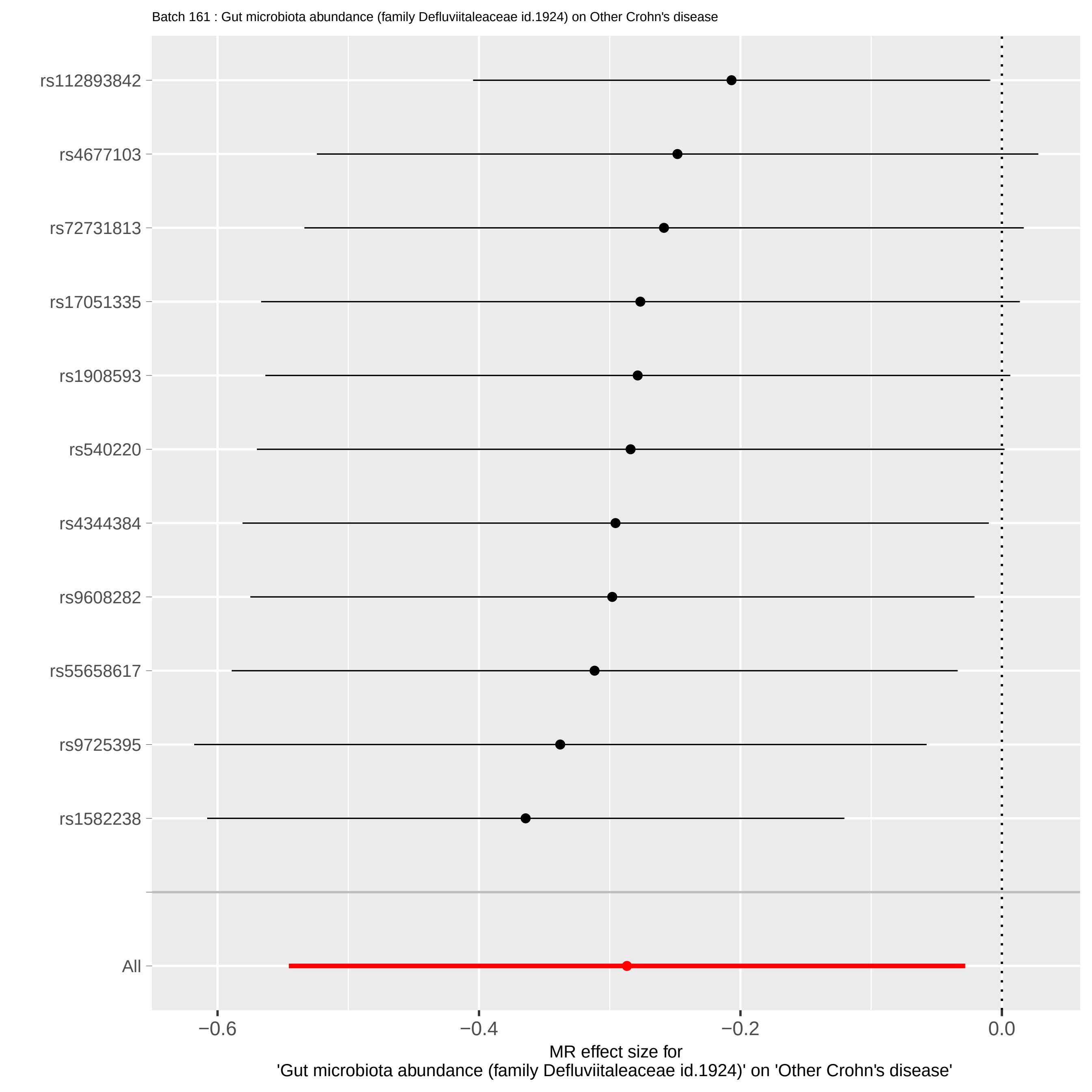
Batch 161 : Gut microbiota abundance (family Defluviitaleaceae id.1924) on Other Crohn's disease
rs112893842
rs4677103
rs72731813
rs17051335
rs1908593
rs540220
rs4344384
rs9608282
rs55658617
rs9725395
rs1582238
All
−0.6
−0.4
−0.2
0.0
MR effect size for
'Gut microbiota abundance (family Defluviitaleaceae id.1924)' on 'Other Crohn's disease'

Batch 162 : Gut microbiota abundance (family Desulfovibrionaceae id.3169) on Other Crohn's disease

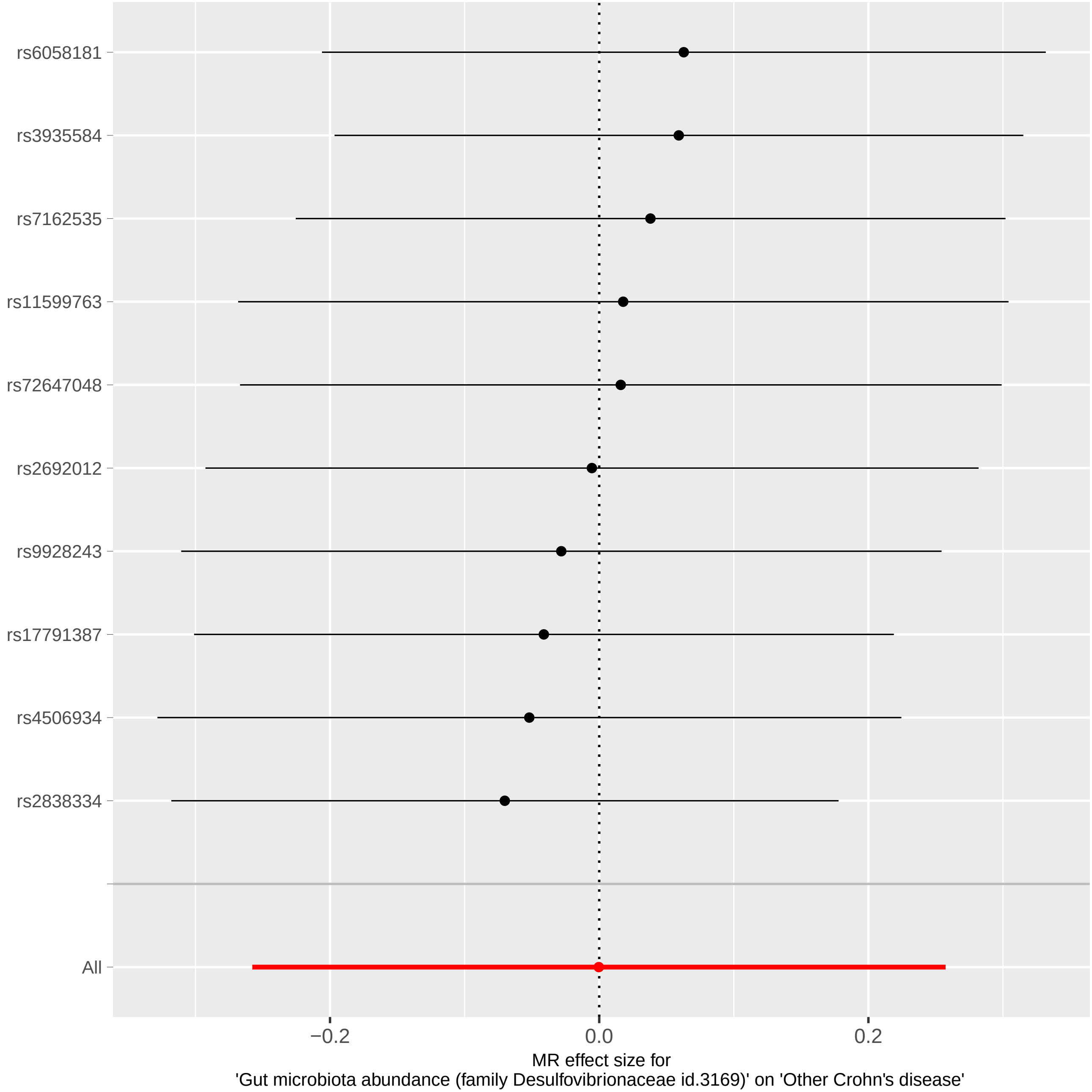

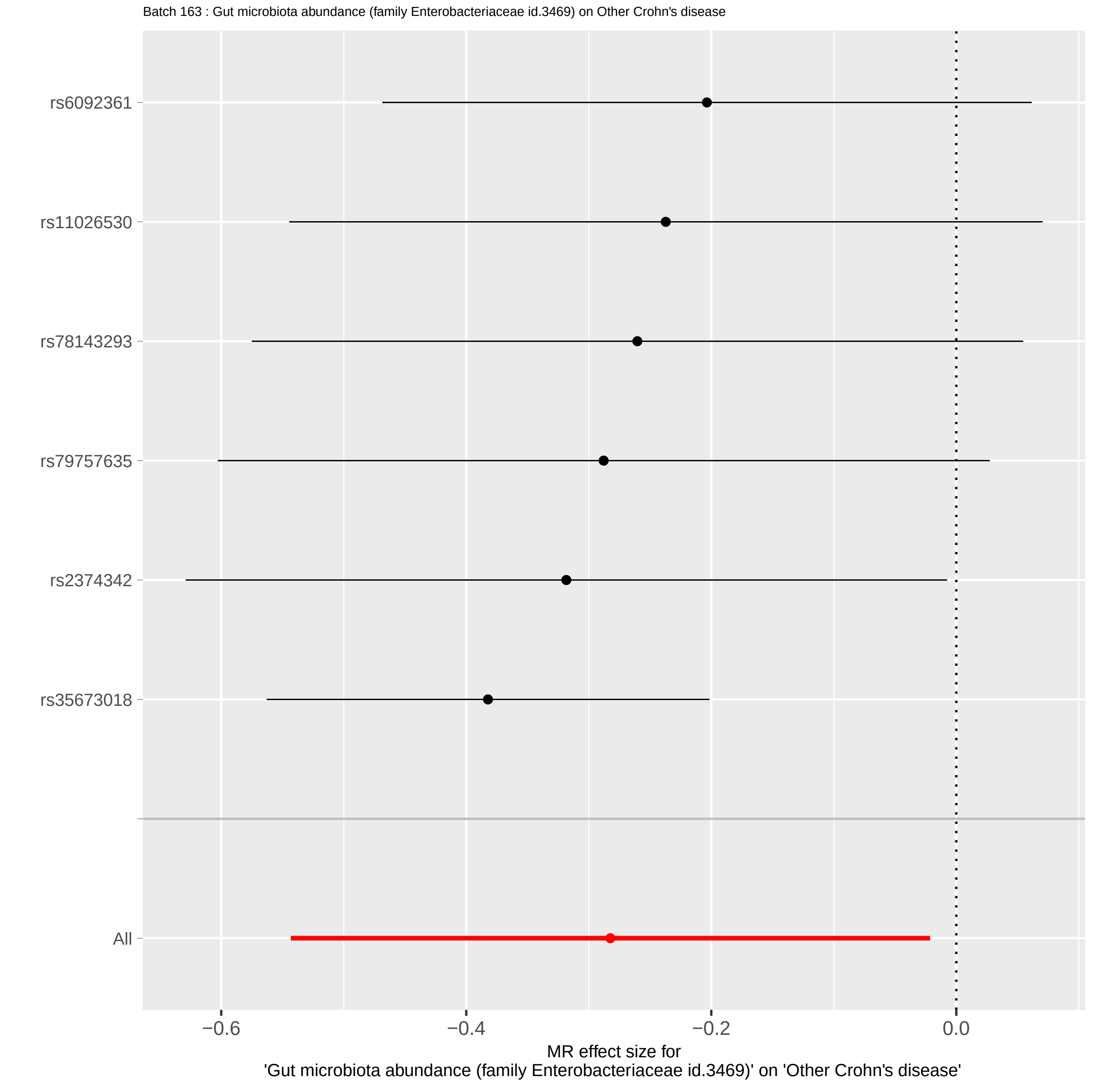
Batch 163 : Gut microbiota abundance (family Enterobacteriaceae id.3469) on Other Crohn's disease
rs6092361
rs11026530
rs78143293
rs79757635
rs2374342
rs35673018
All
−0.6
−0.4
−0.2
0.0
MR effect size for
'Gut microbiota abundance (family Enterobacteriaceae id.3469)' on 'Other Crohn's disease'

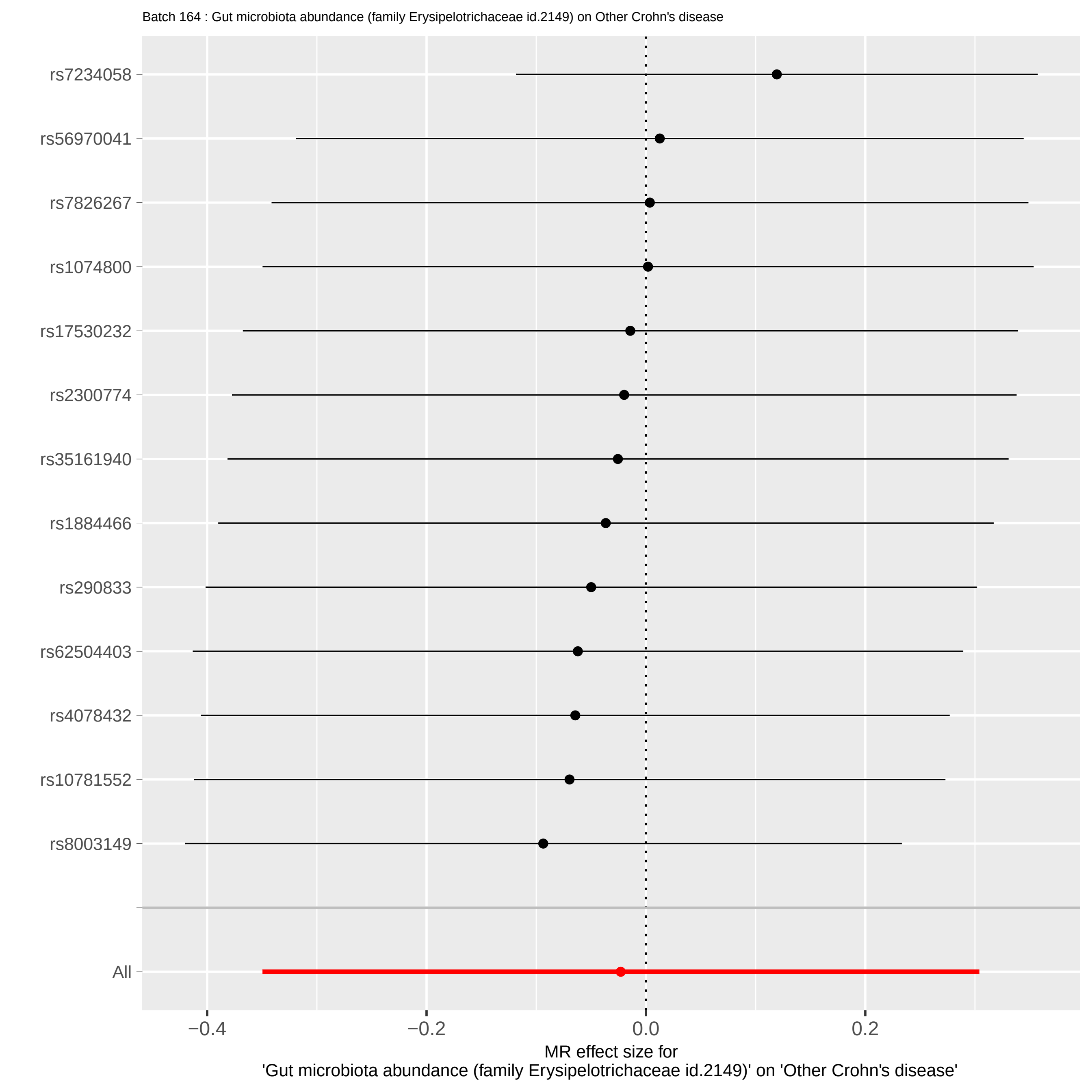
Batch 164 : Gut microbiota abundance (family Erysipelotrichaceae id.2149) on Other Crohn's disease
rs7234058
rs56970041
rs7826267
rs1074800
rs17530232
rs2300774
rs35161940
rs1884466
rs290833
rs62504403
rs4078432
rs10781552
rs8003149
All
−0.4
−0.2
0.0
0.2
MR effect size for
'Gut microbiota abundance (family Erysipelotrichaceae id.2149)' on 'Other Crohn's disease'

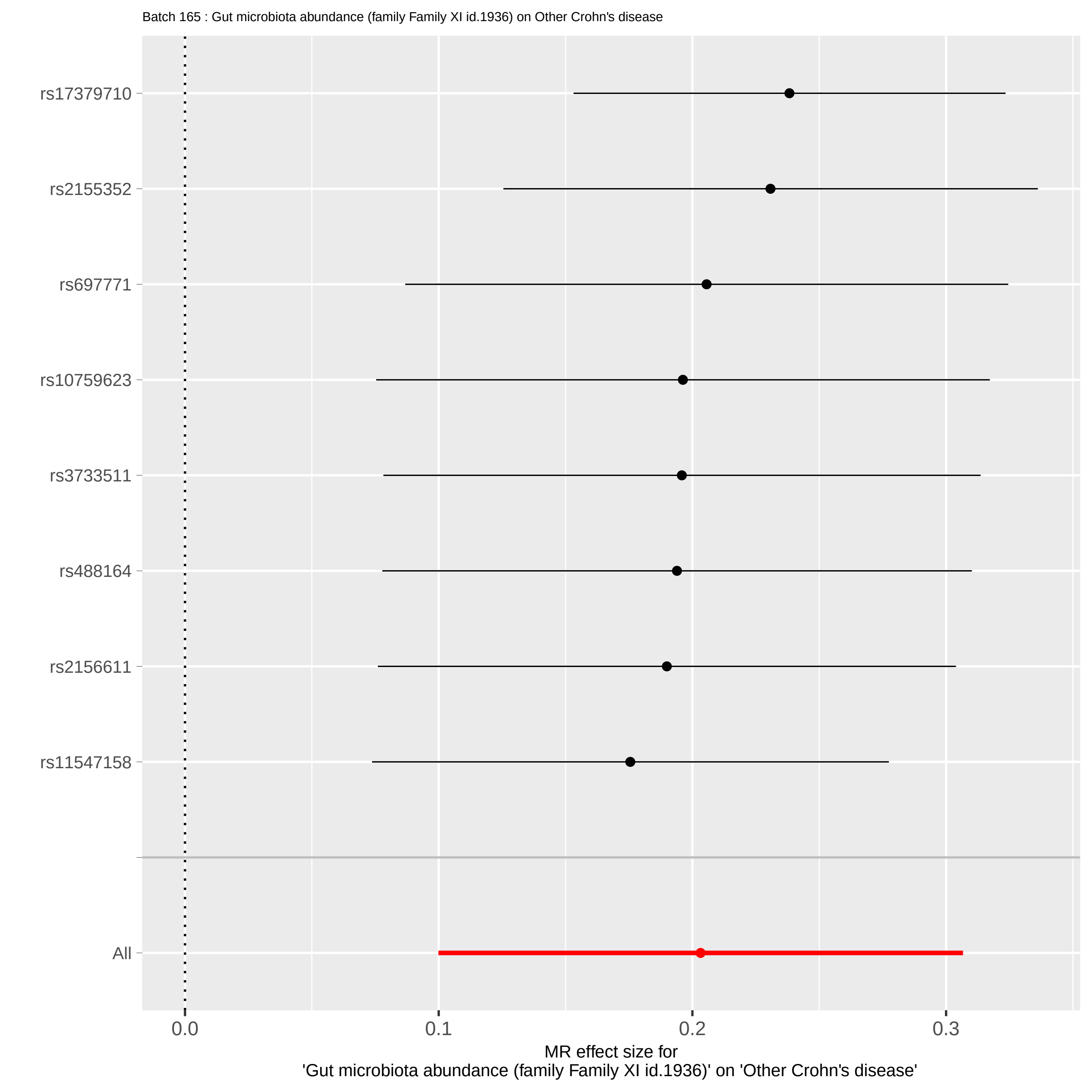
Batch 165 : Gut microbiota abundance (family Family XI id.1936) on Other Crohn's disease
rs17379710
rs2155352
rs697771
rs10759623
rs3733511
rs488164
rs2156611
rs11547158
All
0.0
0.1
0.2
0.3
MR effect size for
'Gut microbiota abundance (family Family XI id.1936)' on 'Other Crohn's disease'

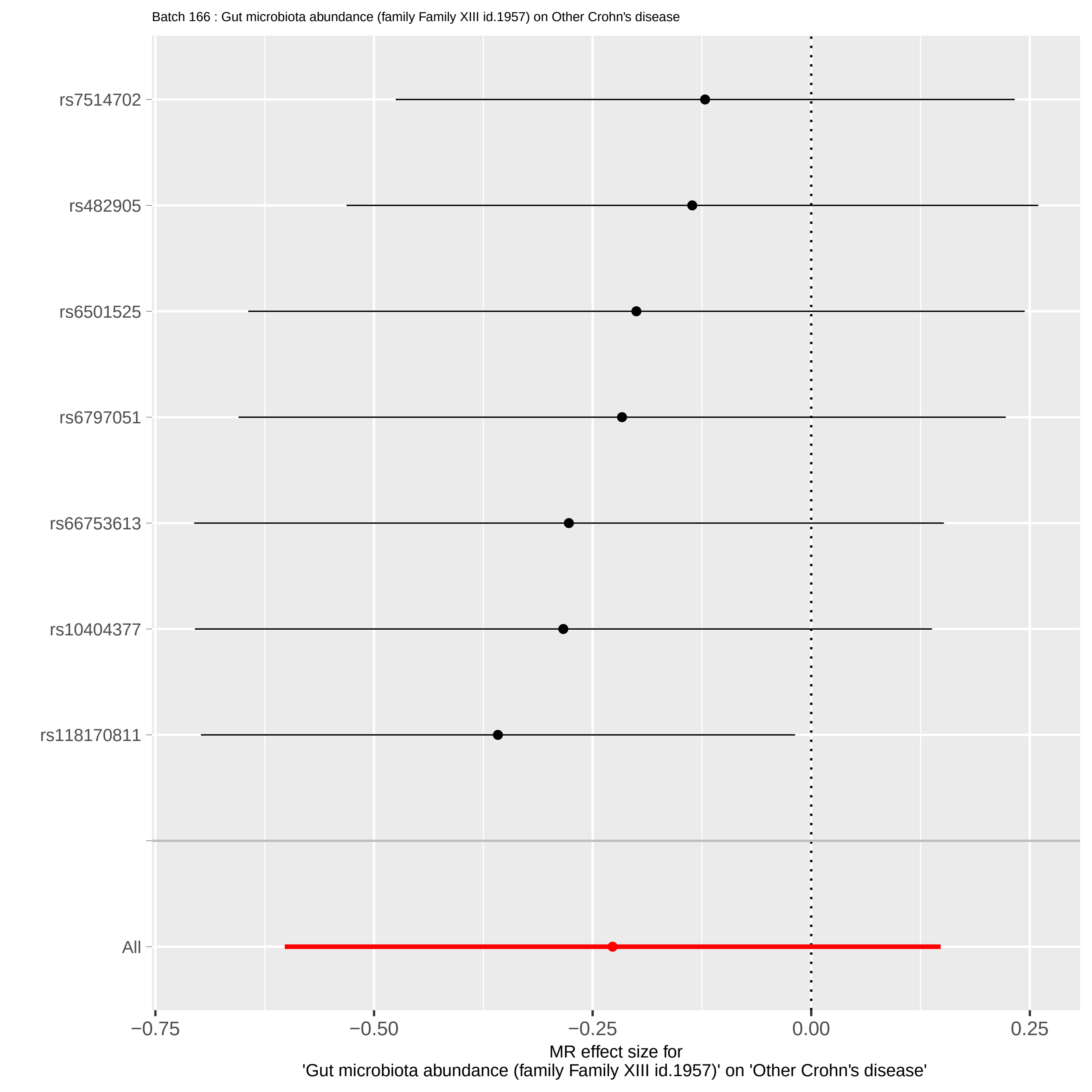
Batch 166 : Gut microbiota abundance (family Family XIII id.1957) on Other Crohn's disease
rs7514702
rs482905
rs6501525
rs6797051
rs66753613
rs10404377
rs118170811
All
−0.75
−0.50
−0.25
0.00
0.25
MR effect size for
'Gut microbiota abundance (family Family XIII id.1957)' on 'Other Crohn's disease'

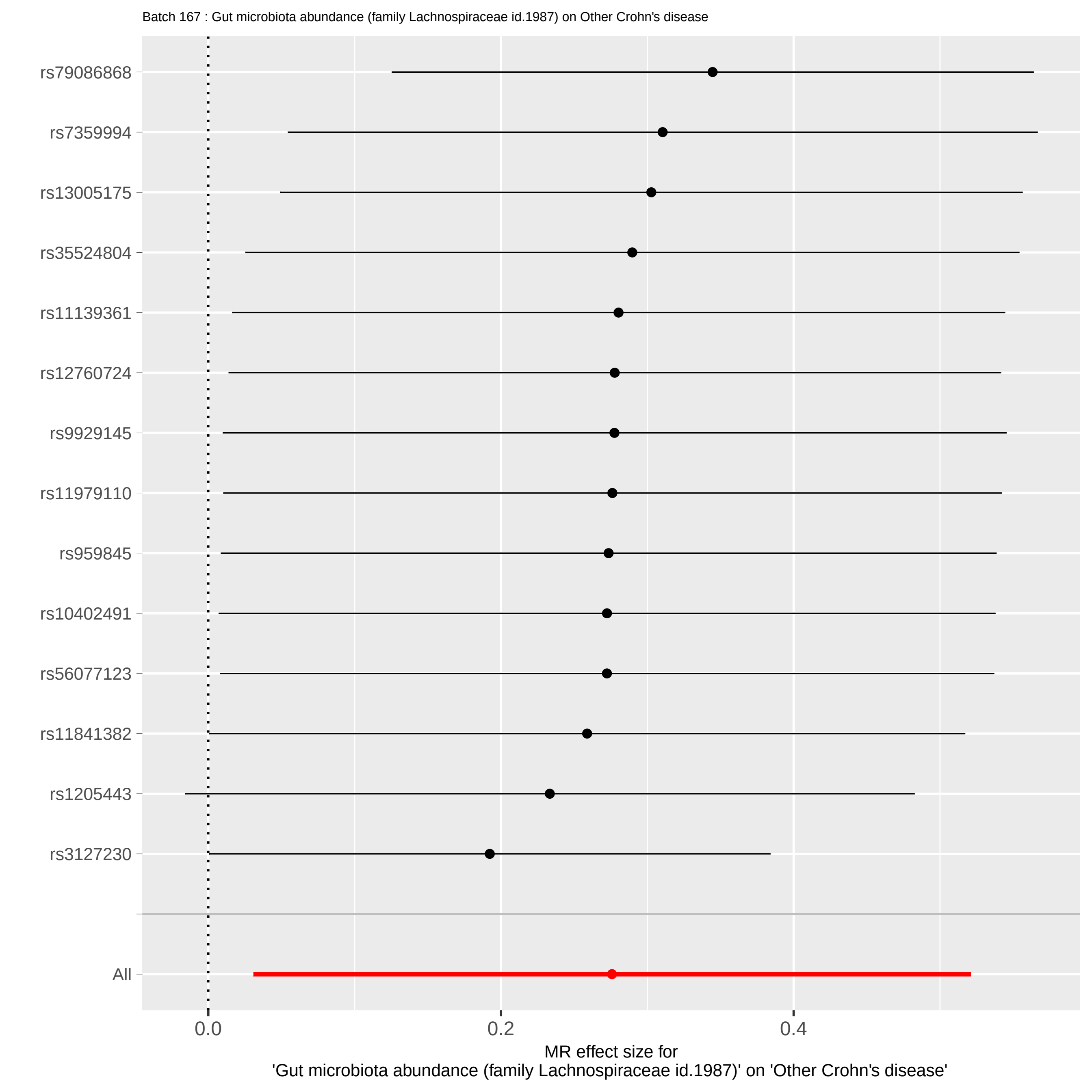

Batch 167 : Gut microbiota abundance (family Lachnospiraceae id.1987) on Other Crohn's disease
rs79086868
rs7359994
rs13005175
rs35524804
rs11139361
rs12760724
rs9929145
rs11979110
rs959845
rs10402491
rs56077123
rs11841382
rs1205443
rs3127230
All
0.0
0.2
0.4
MR effect size for
'Gut microbiota abundance (family Lachnospiraceae id.1987)' on 'Other Crohn's disease'

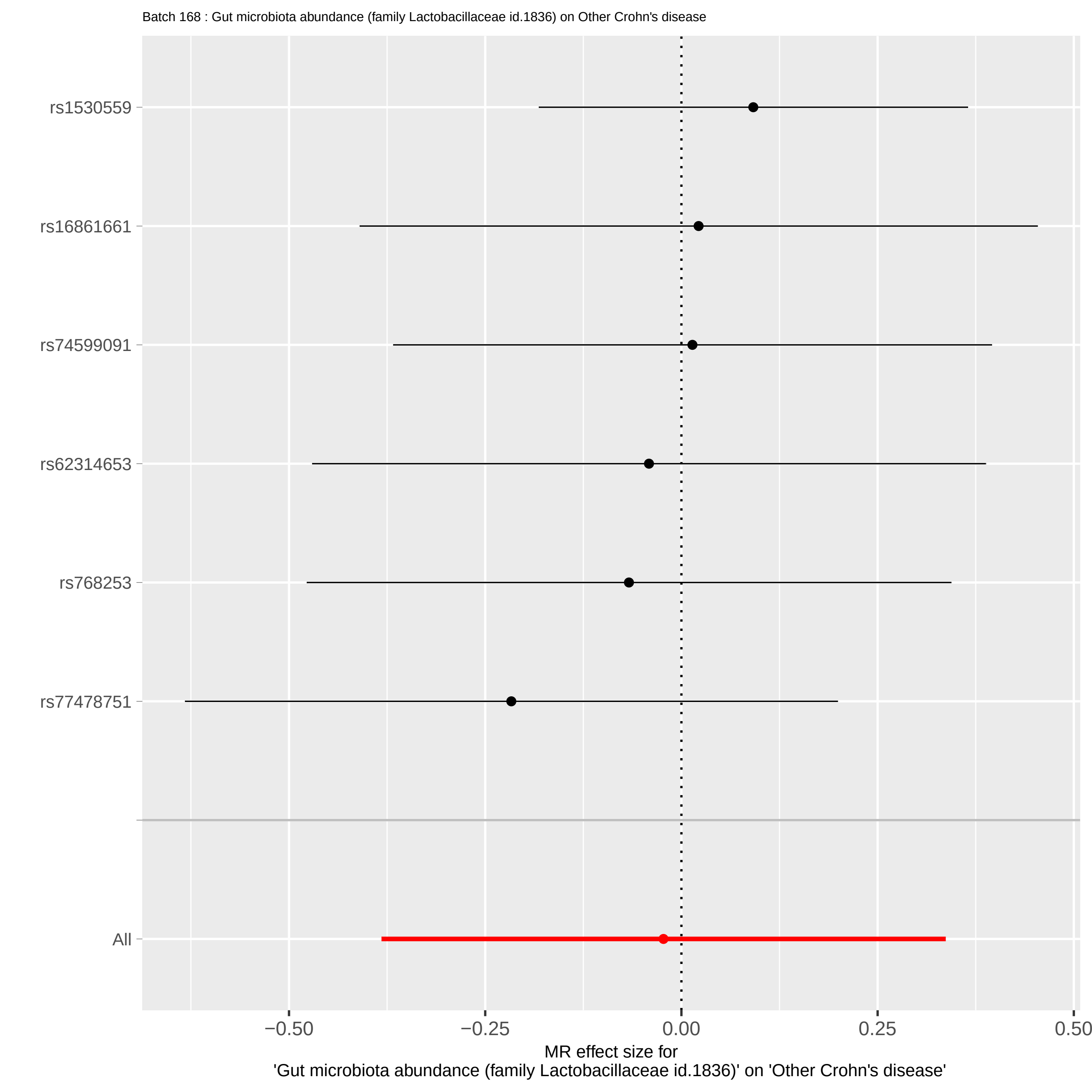
Batch 168 : Gut microbiota abundance (family Lactobacillaceae id.1836) on Other Crohn's disease
rs1530559
rs16861661
rs74599091
rs62314653
rs768253
rs77478751
All
−0.50
−0.25
0.00
0.25
0.50
MR effect size for
'Gut microbiota abundance (family Lactobacillaceae id.1836)' on 'Other Crohn's disease'

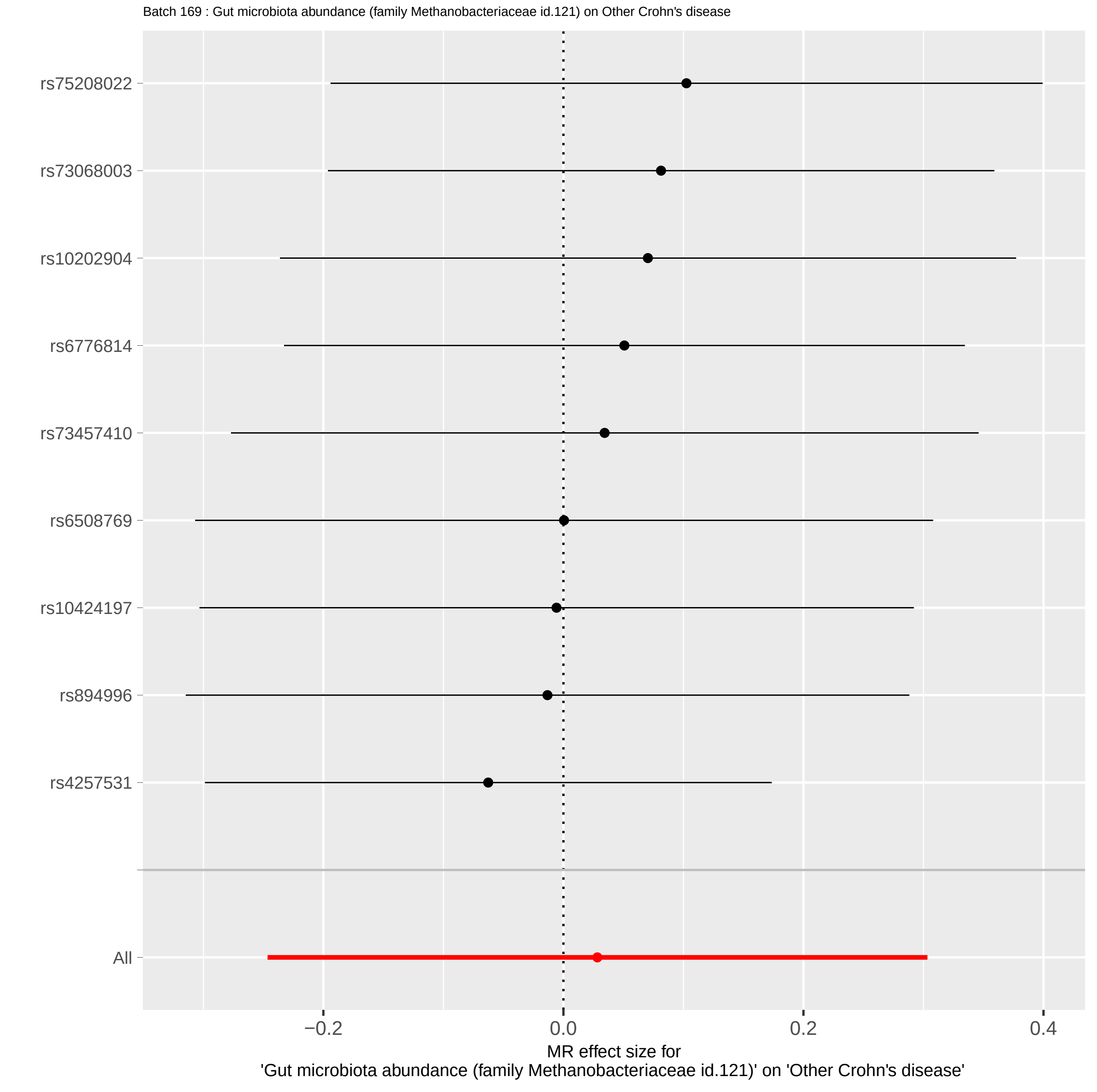

Batch 169 : Gut microbiota abundance (family Methanobacteriaceae id.121) on Other Crohn's disease
rs75208022
rs73068003
rs10202904
rs6776814
rs73457410
rs6508769
rs10424197
rs894996
rs4257531
All
−0.2
0.0
0.2
0.4
MR effect size for
'Gut microbiota abundance (family Methanobacteriaceae id.121)' on 'Other Crohn's disease'

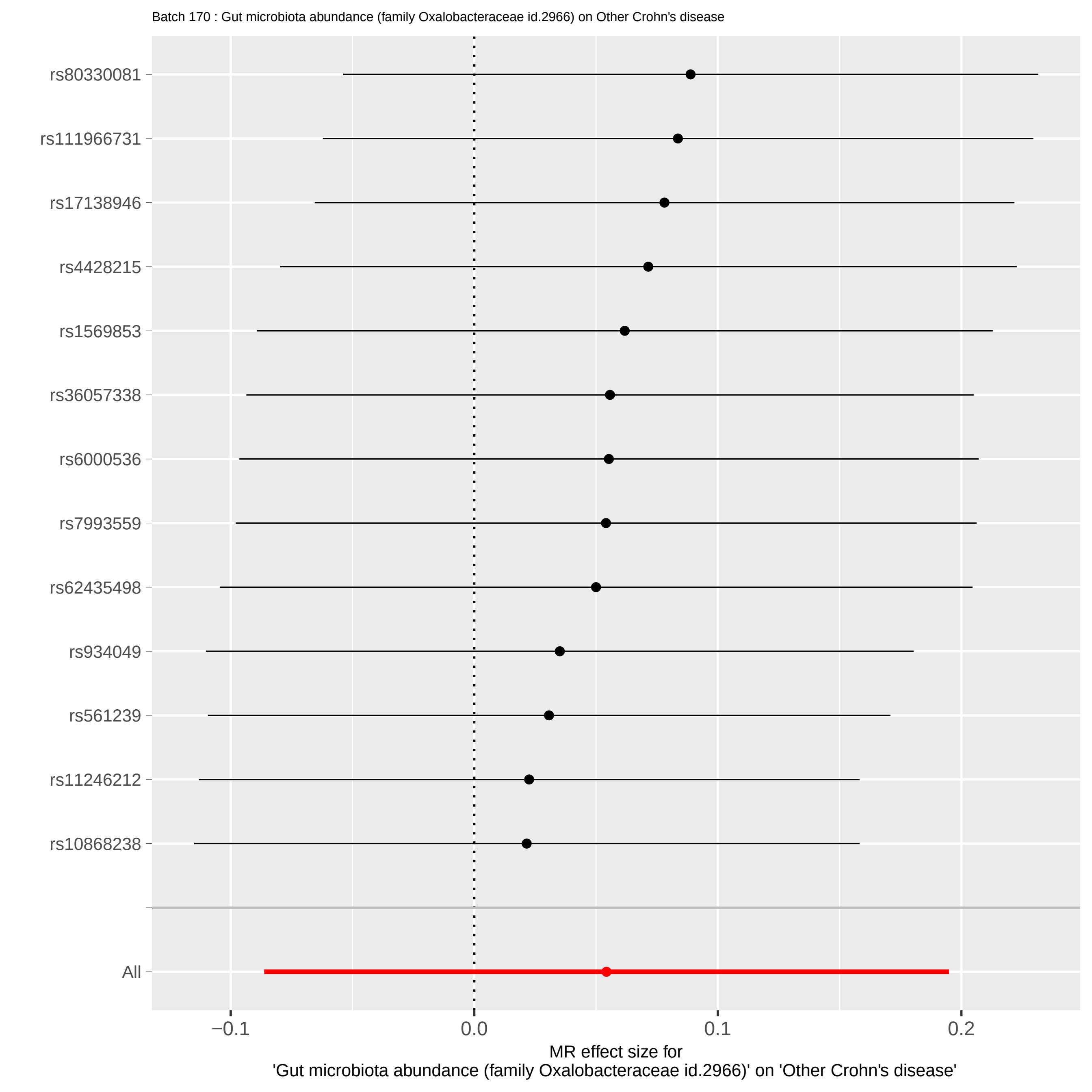

Batch 170 : Gut microbiota abundance (family Oxalobacteraceae id.2966) on Other Crohn's disease
rs80330081
rs111966731
rs17138946
rs4428215
rs1569853
rs36057338
rs6000536
rs7993559
rs62435498
rs934049
rs561239
rs11246212
rs10868238
All
−0.1
0.0
0.1
0.2
MR effect size for
'Gut microbiota abundance (family Oxalobacteraceae id.2966)' on 'Other Crohn's disease'

Batch 171 : Gut microbiota abundance (family Pasteurellaceae id.3689) on Other Crohn's disease

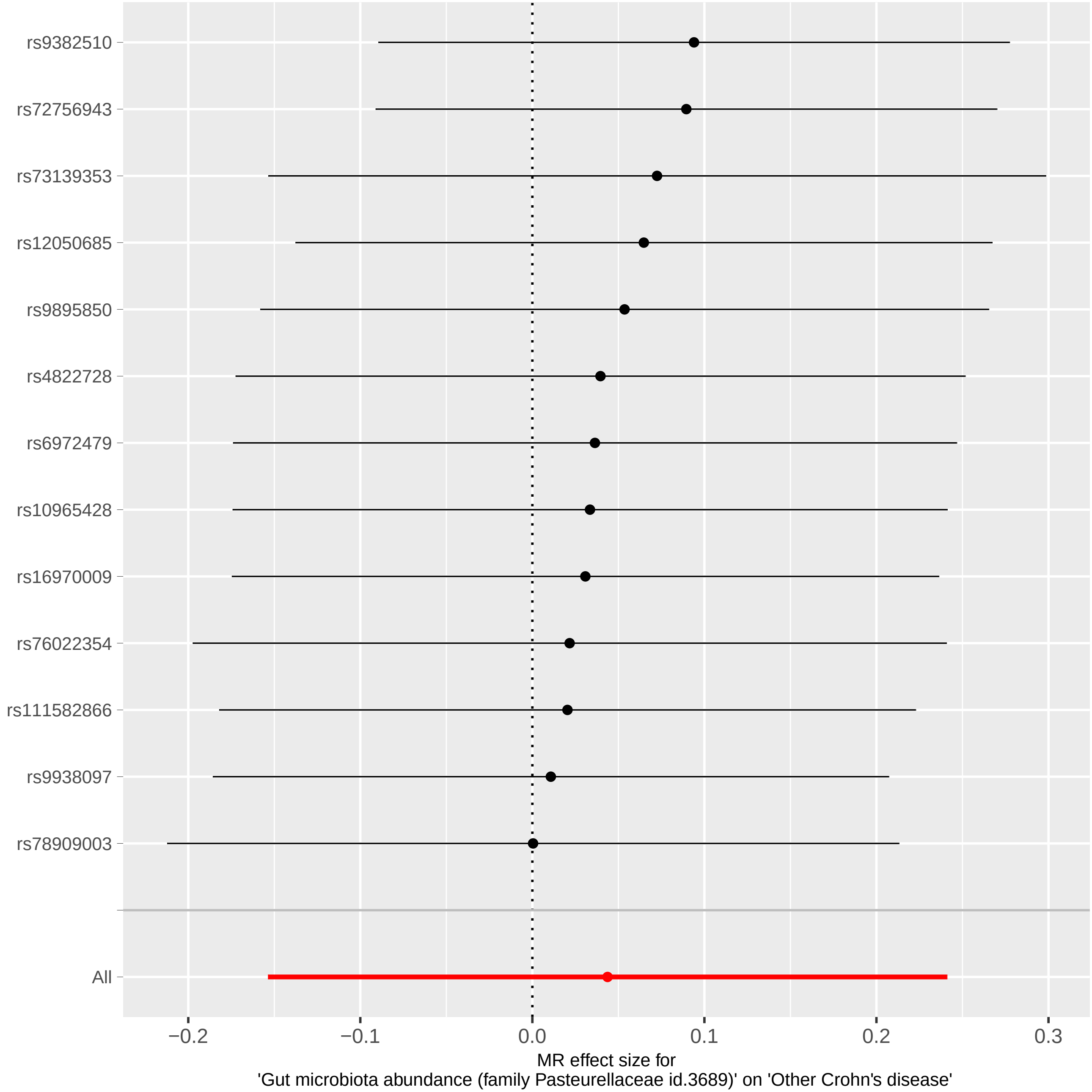

Batch 172 : Gut microbiota abundance (family Peptococcaceae id.2024) on Other Crohn's disease

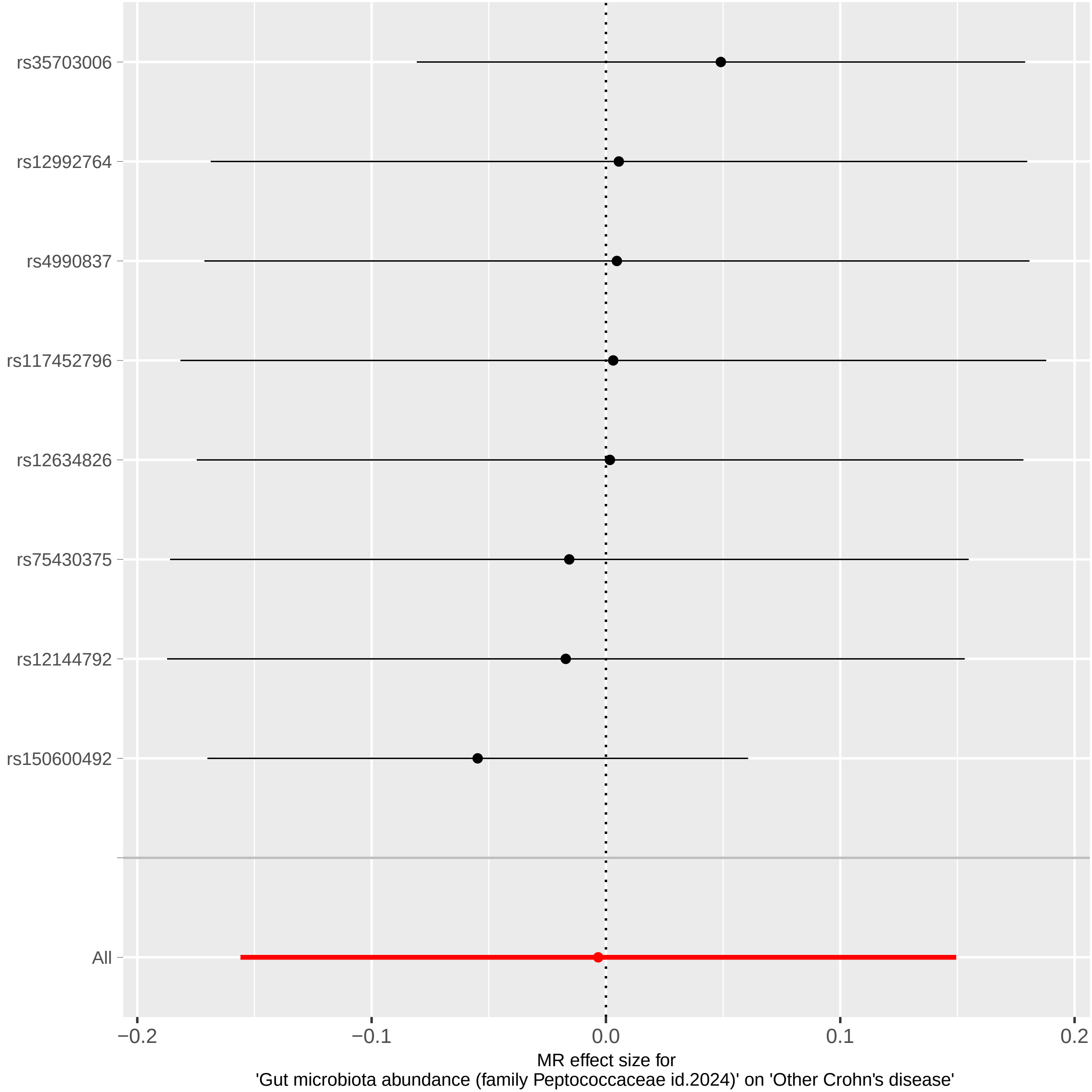

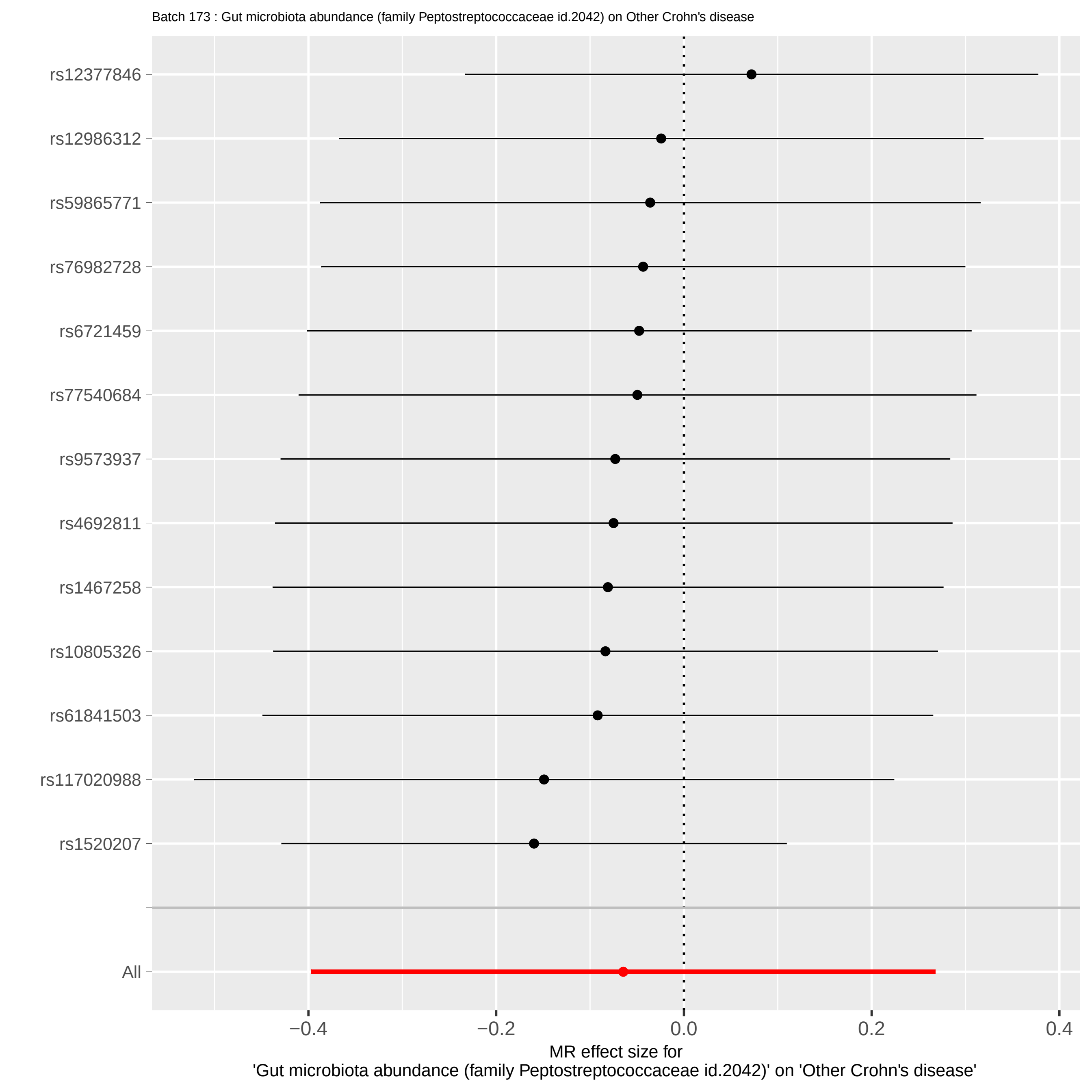

Batch 173 : Gut microbiota abundance (family Peptostreptococcaceae id.2042) on Other Crohn's disease
rs12377846
rs12986312
rs59865771
rs76982728
rs6721459
rs77540684
rs9573937
rs4692811
rs1467258
rs10805326
rs61841503
rs117020988
rs1520207
All
−0.4
−0.2
0.0
0.2
0.4
MR effect size for
'Gut microbiota abundance (family Peptostreptococcaceae id.2042)' on 'Other Crohn's disease'

Batch 174 : Gut microbiota abundance (family Porphyromonadaceae id.943) on Other Crohn's disease

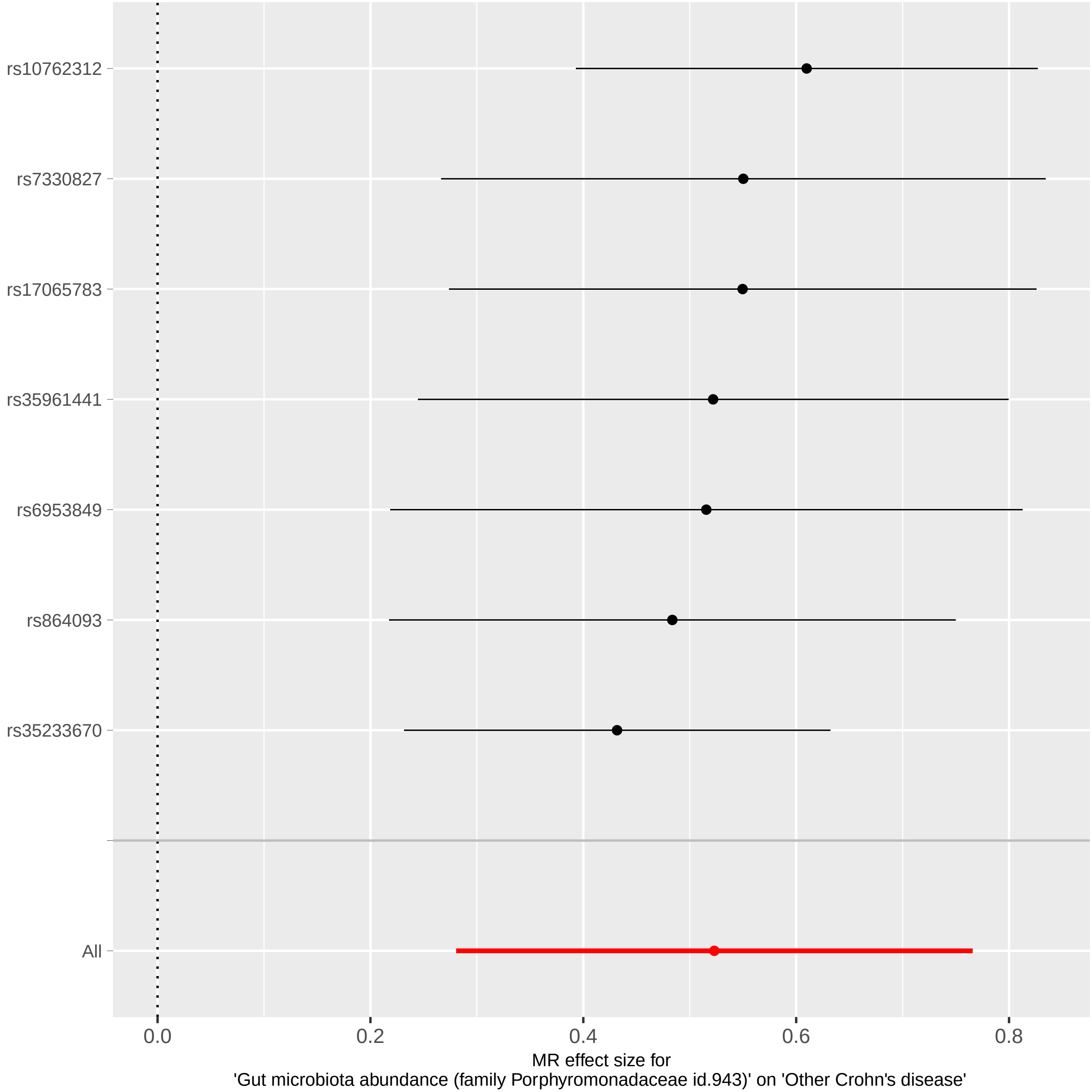

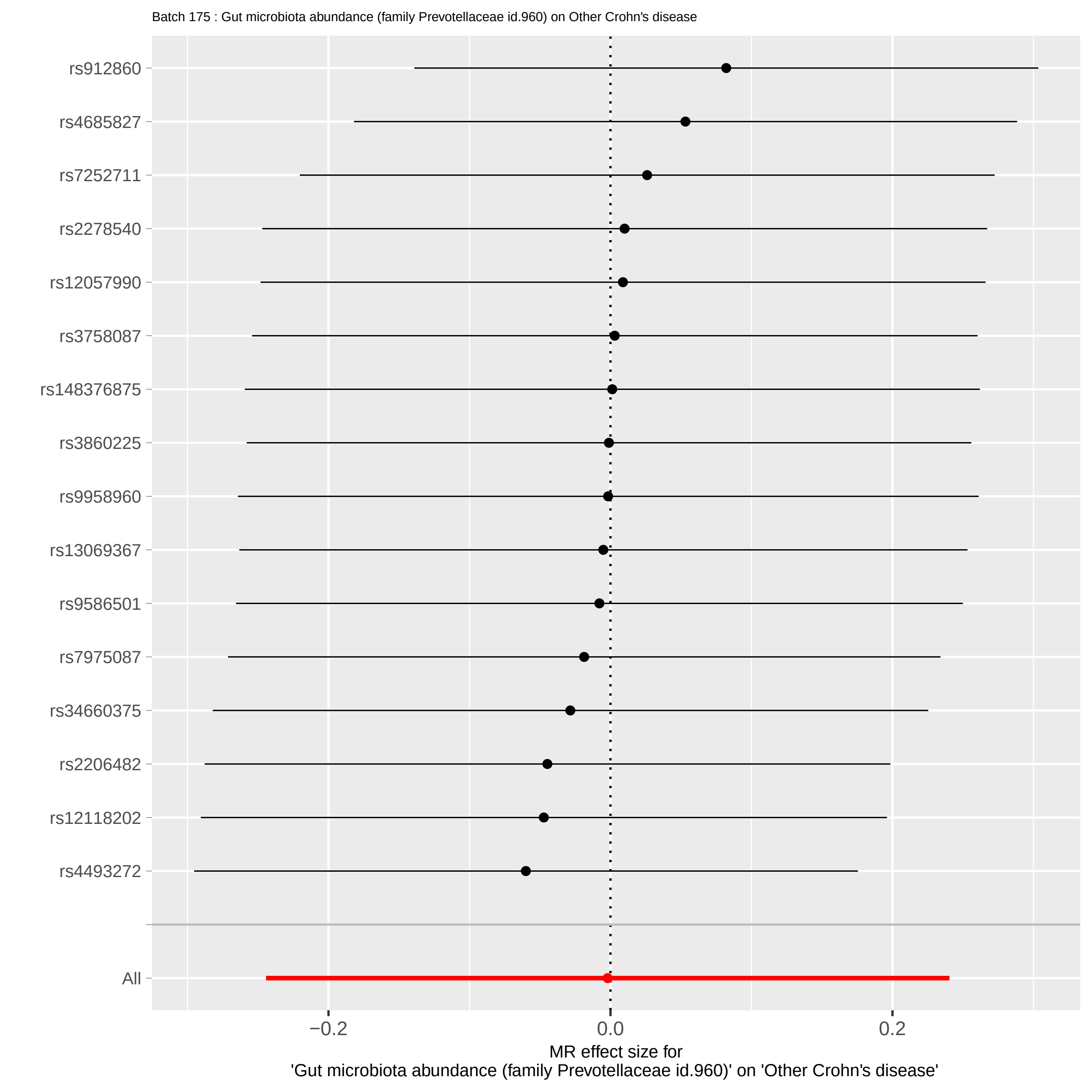
Batch 175 : Gut microbiota abundance (family Prevotellaceae id.960) on Other Crohn's disease
rs912860
rs4685827
rs7252711
rs2278540
rs12057990
rs3758087
rs148376875
rs3860225
rs9958960
rs13069367
rs9586501
rs7975087
rs34660375
rs2206482
rs12118202
rs4493272
All
−0.2
0.0
0.2
MR effect size for
'Gut microbiota abundance (family Prevotellaceae id.960)' on 'Other Crohn's disease'

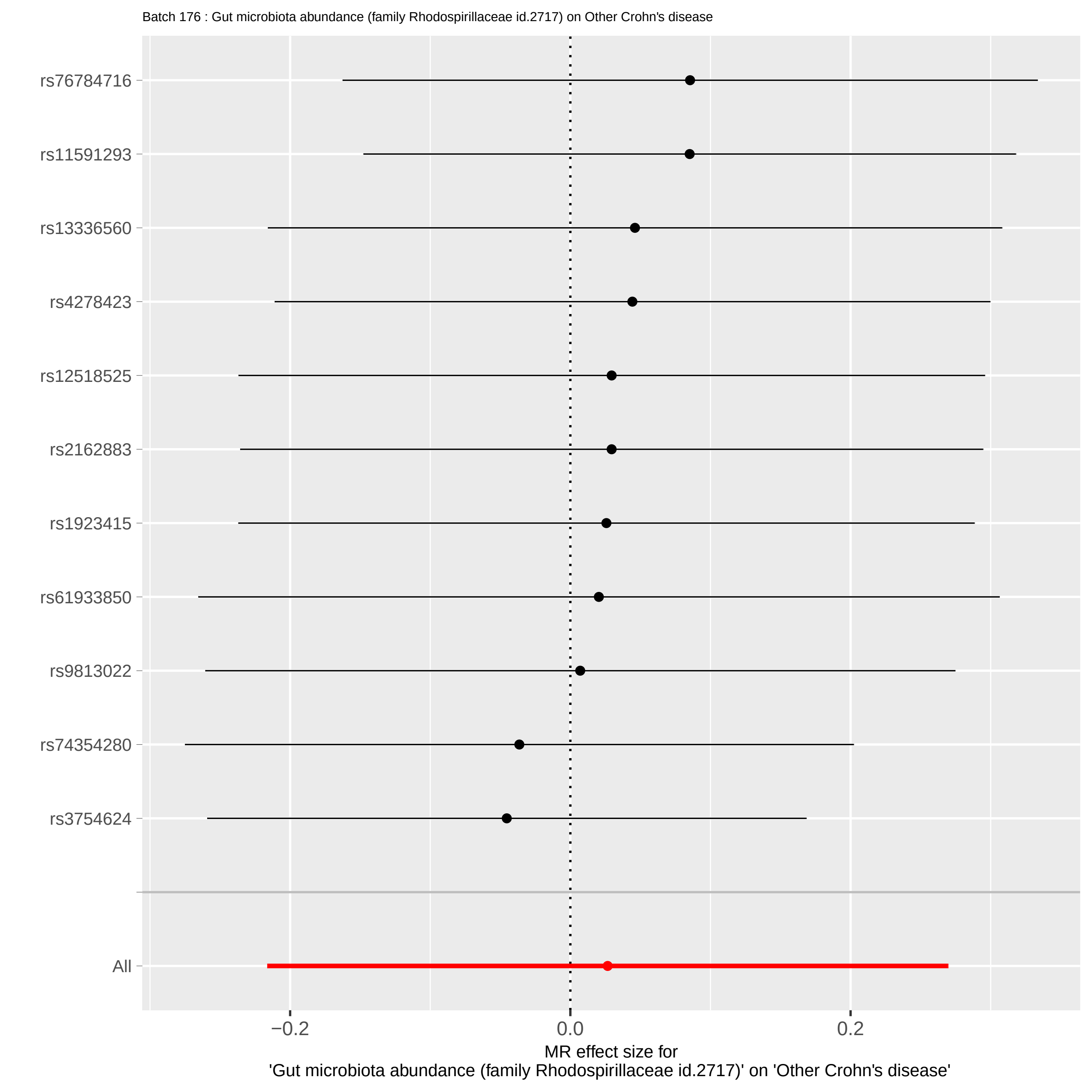

Batch 176 : Gut microbiota abundance (family Rhodospirillaceae id.2717) on Other Crohn's disease
rs76784716
rs11591293
rs13336560
rs4278423
rs12518525
rs2162883
rs1923415
rs61933850
rs9813022
rs74354280
rs3754624
All
−0.2
0.0
0.2
MR effect size for
'Gut microbiota abundance (family Rhodospirillaceae id.2717)' on 'Other Crohn's disease'

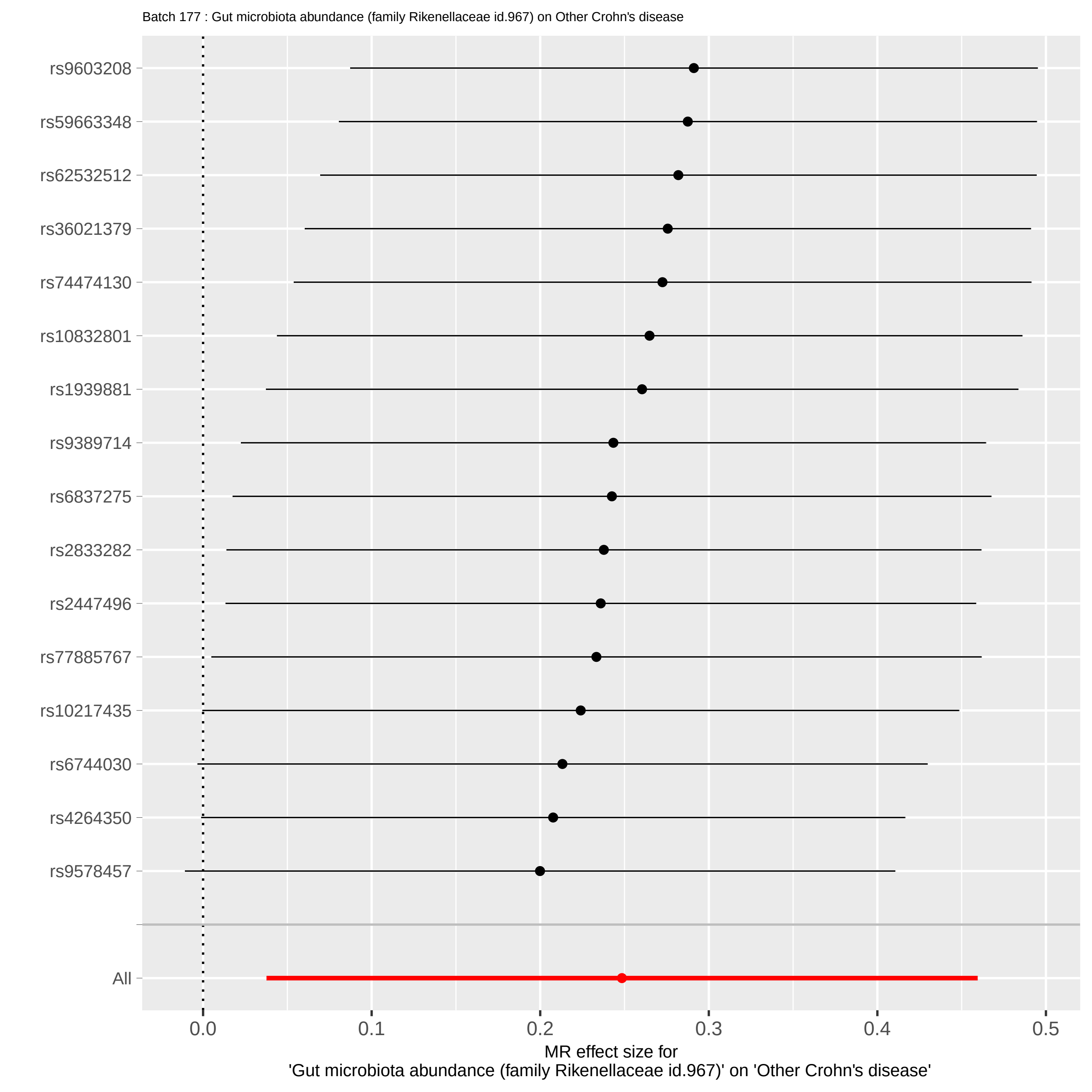

Batch 177 : Gut microbiota abundance (family Rikenellaceae id.967) on Other Crohn's disease
rs9603208
rs59663348
rs62532512
rs36021379
rs74474130
rs10832801
rs1939881
rs9389714
rs6837275
rs2833282
rs2447496
rs77885767
rs10217435
rs6744030
rs4264350
rs9578457
All
0.0
0.1
0.2
0.3
0.4
0.5
MR effect size for
'Gut microbiota abundance (family Rikenellaceae id.967)' on 'Other Crohn's disease'

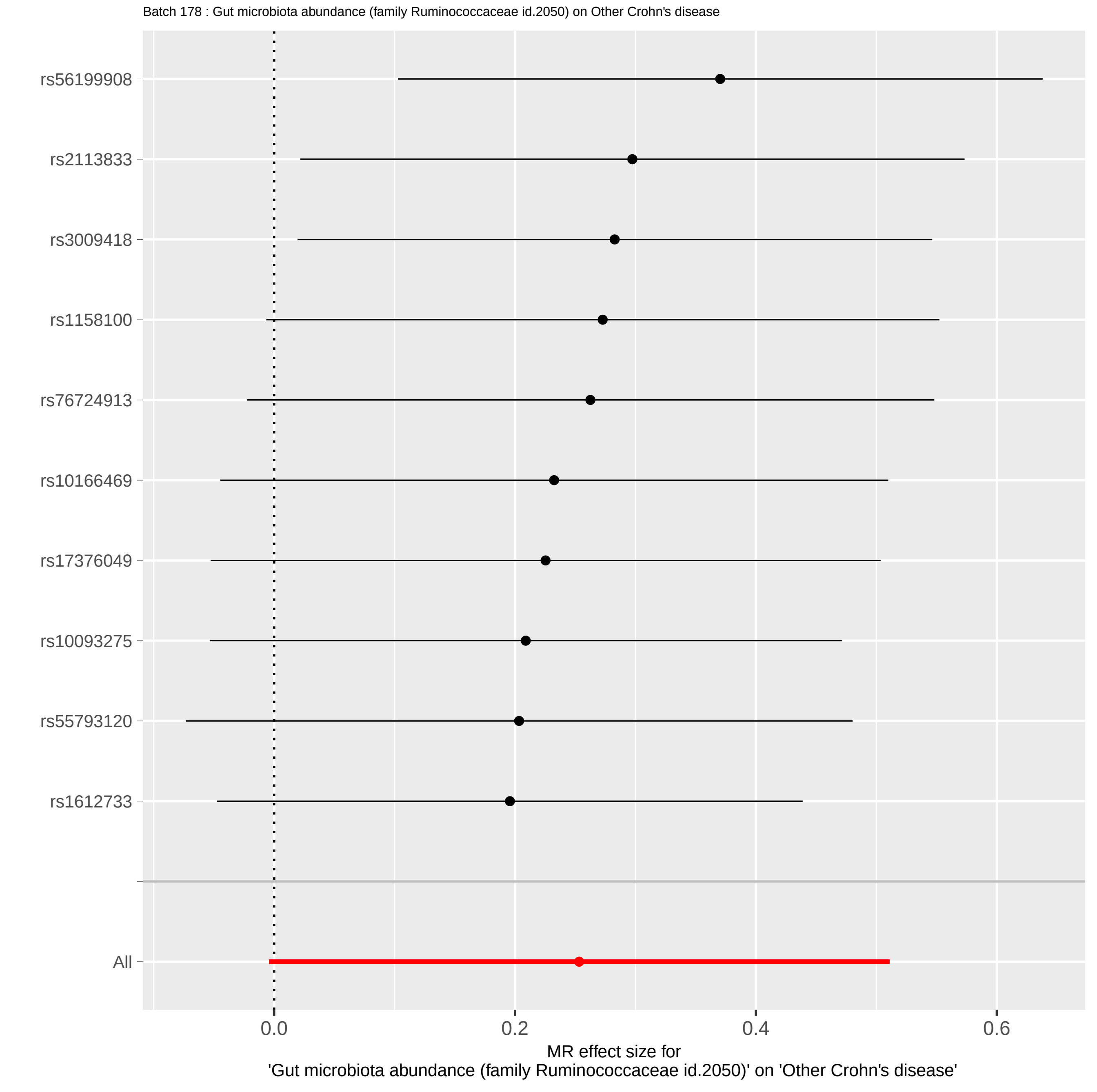
Batch 178 : Gut microbiota abundance (family Ruminococcaceae id.2050) on Other Crohn's disease
rs56199908
rs2113833
rs3009418
rs1158100
rs76724913
rs10166469
rs17376049
rs10093275
rs55793120
rs1612733
All
0.0
0.2
0.4
0.6
MR effect size for
'Gut microbiota abundance (family Ruminococcaceae id.2050)' on 'Other Crohn's disease'

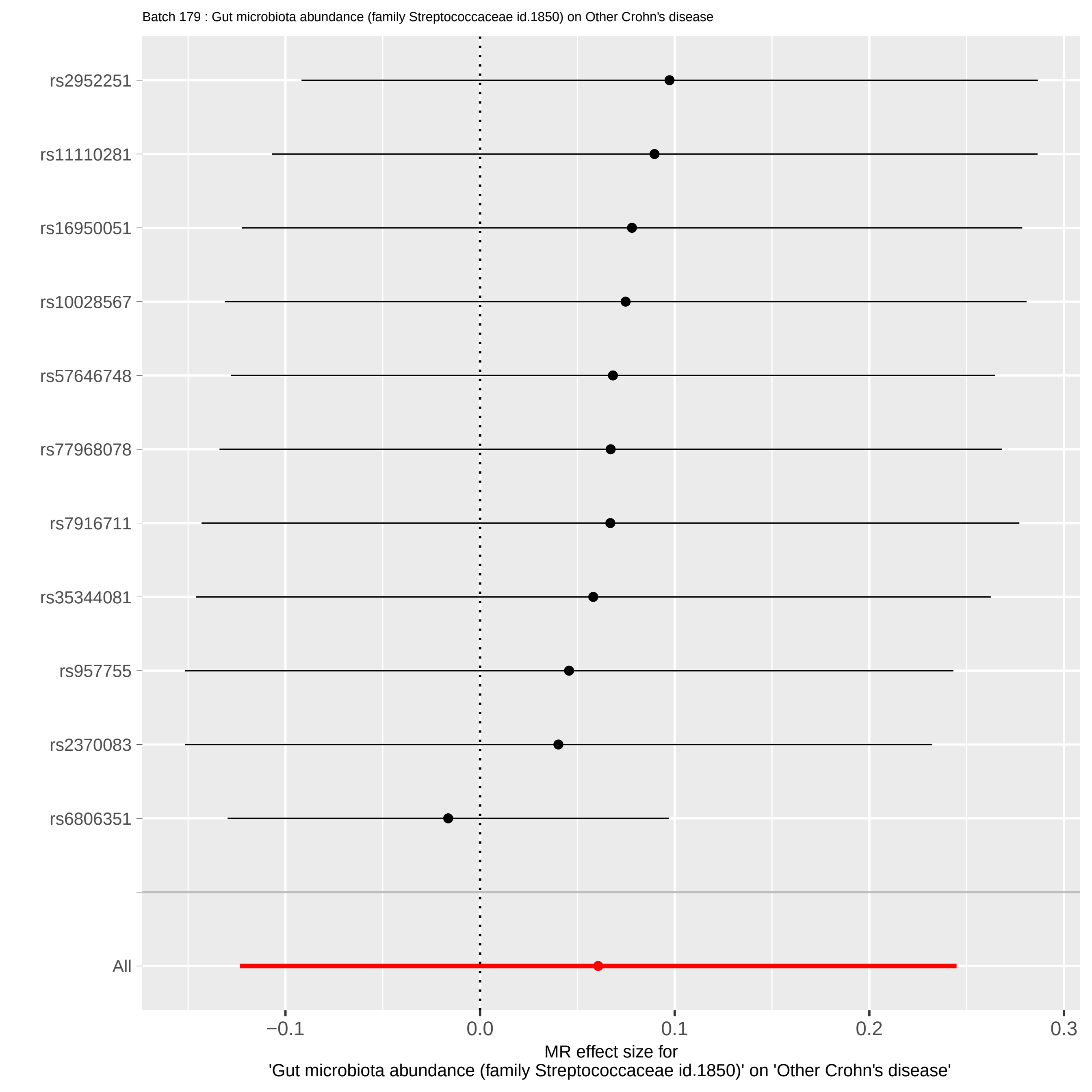
Batch 179 : Gut microbiota abundance (family Streptococcaceae id.1850) on Other Crohn's disease
rs2952251
rs11110281
rs16950051
rs10028567
rs57646748
rs77968078
rs7916711
rs35344081
rs957755
rs2370083
rs6806351
All
−0.1
0.0
0.1
0.2
0.3
MR effect size for
'Gut microbiota abundance (family Streptococcaceae id.1850)' on 'Other Crohn's disease'

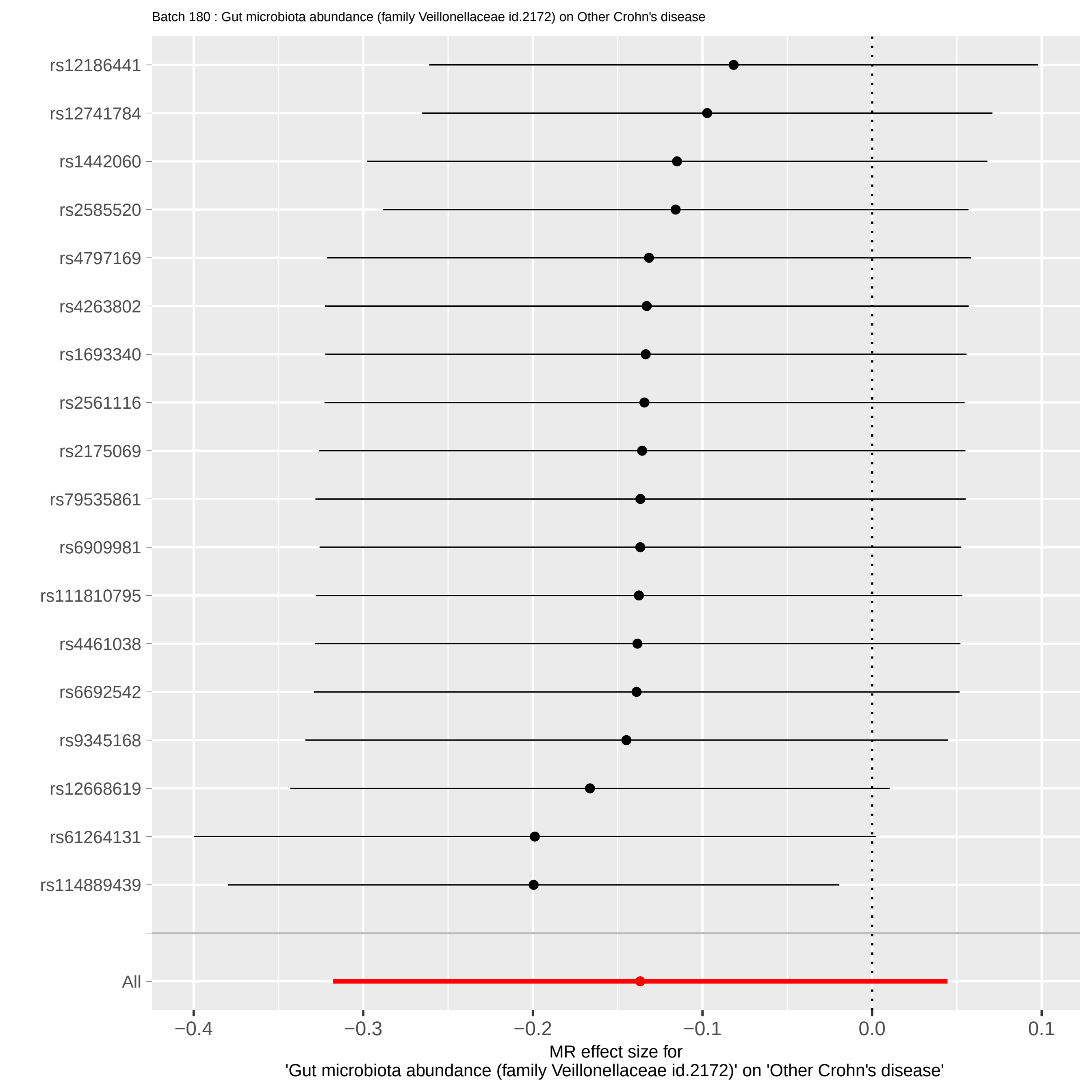

Batch 180 : Gut microbiota abundance (family Veillonellaceae id.2172) on Other Crohn's disease
rs12186441
rs12741784
rs1442060
rs2585520
rs4797169
rs4263802
rs1693340
rs2561116
rs2175069
rs79535861
rs6909981
rs111810795
rs4461038
rs6692542
rs9345168
rs12668619
rs61264131
rs114889439
All
−0.4
−0.3
−0.2
−0.1
0.0
0.1
MR effect size for
'Gut microbiota abundance (family Veillonellaceae id.2172)' on 'Other Crohn's disease'

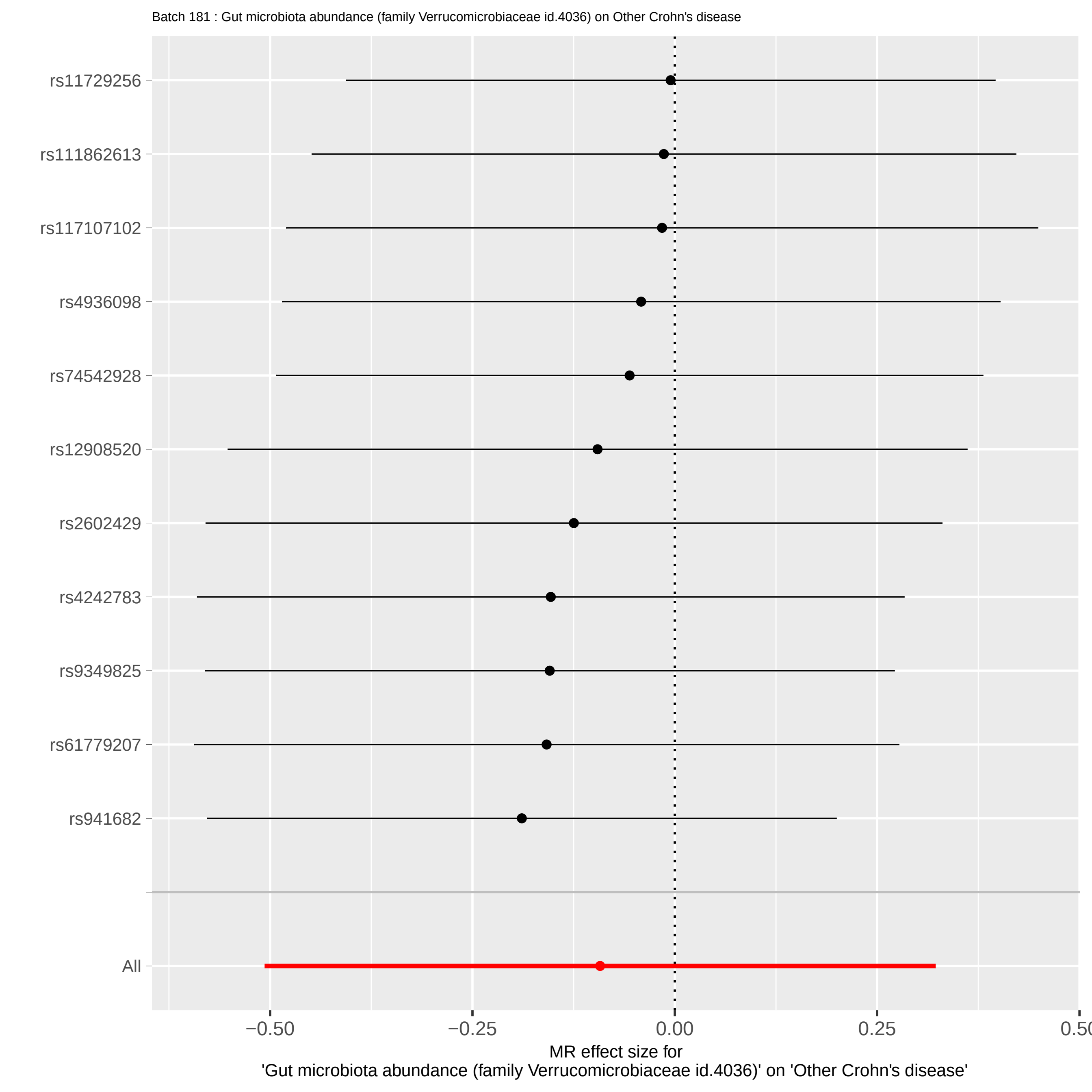
Batch 181 : Gut microbiota abundance (family Verrucomicrobiaceae id.4036) on Other Crohn's disease
rs11729256
rs111862613
rs117107102
rs4936098
rs74542928
rs12908520
rs2602429
rs4242783
rs9349825
rs61779207
rs941682
All
−0.50
−0.25
0.00
0.25
0.50
MR effect size for
'Gut microbiota abundance (family Verrucomicrobiaceae id.4036)' on 'Other Crohn's disease'

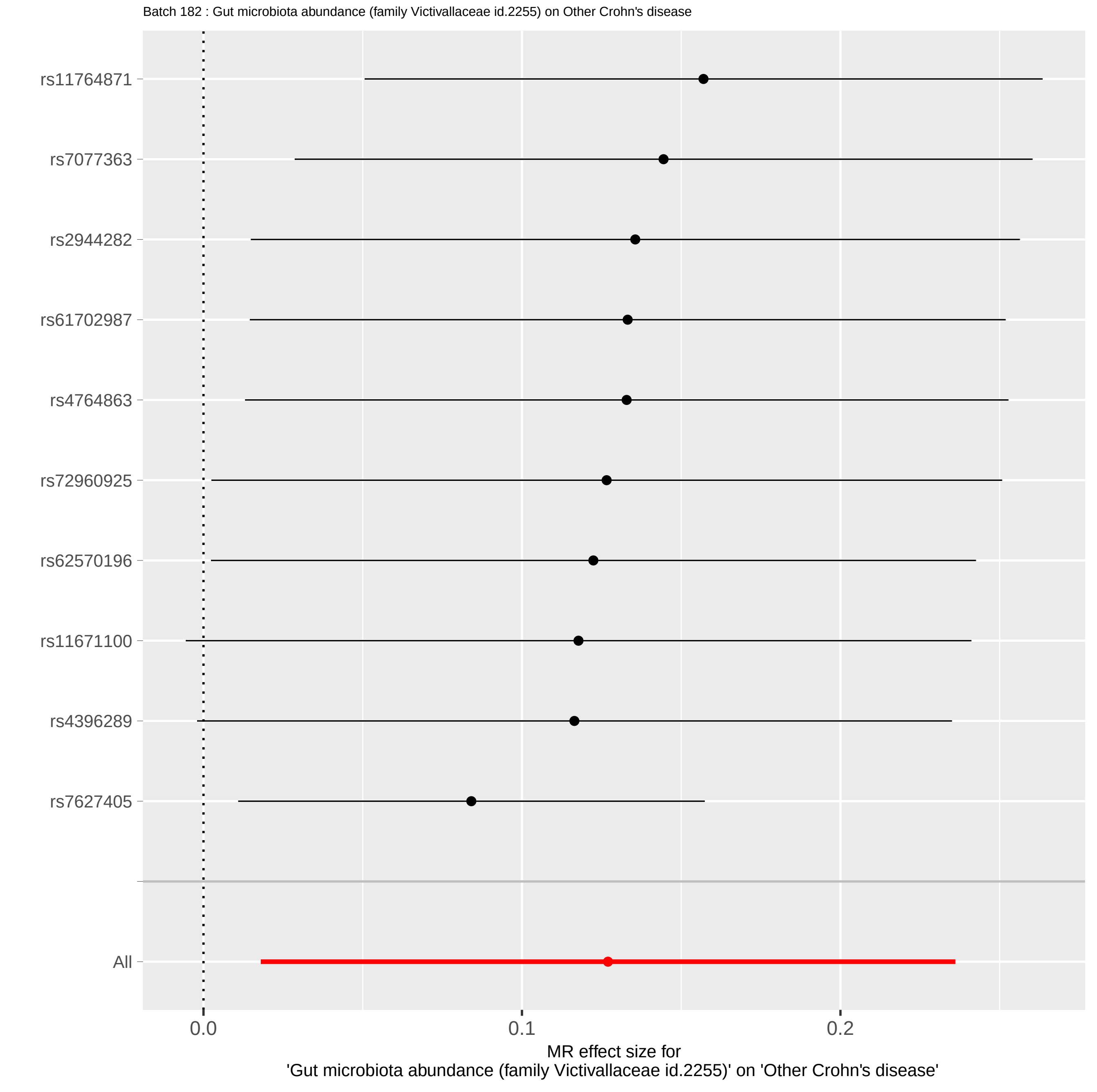

Batch 182 : Gut microbiota abundance (family Victivallaceae id.2255) on Other Crohn's disease
rs11764871
rs7077363
rs2944282
rs61702987
rs4764863
rs72960925
rs62570196
rs11671100
rs4396289
rs7627405
All
0.0
0.1
0.2
MR effect size for
'Gut microbiota abundance (family Victivallaceae id.2255)' on 'Other Crohn's disease'

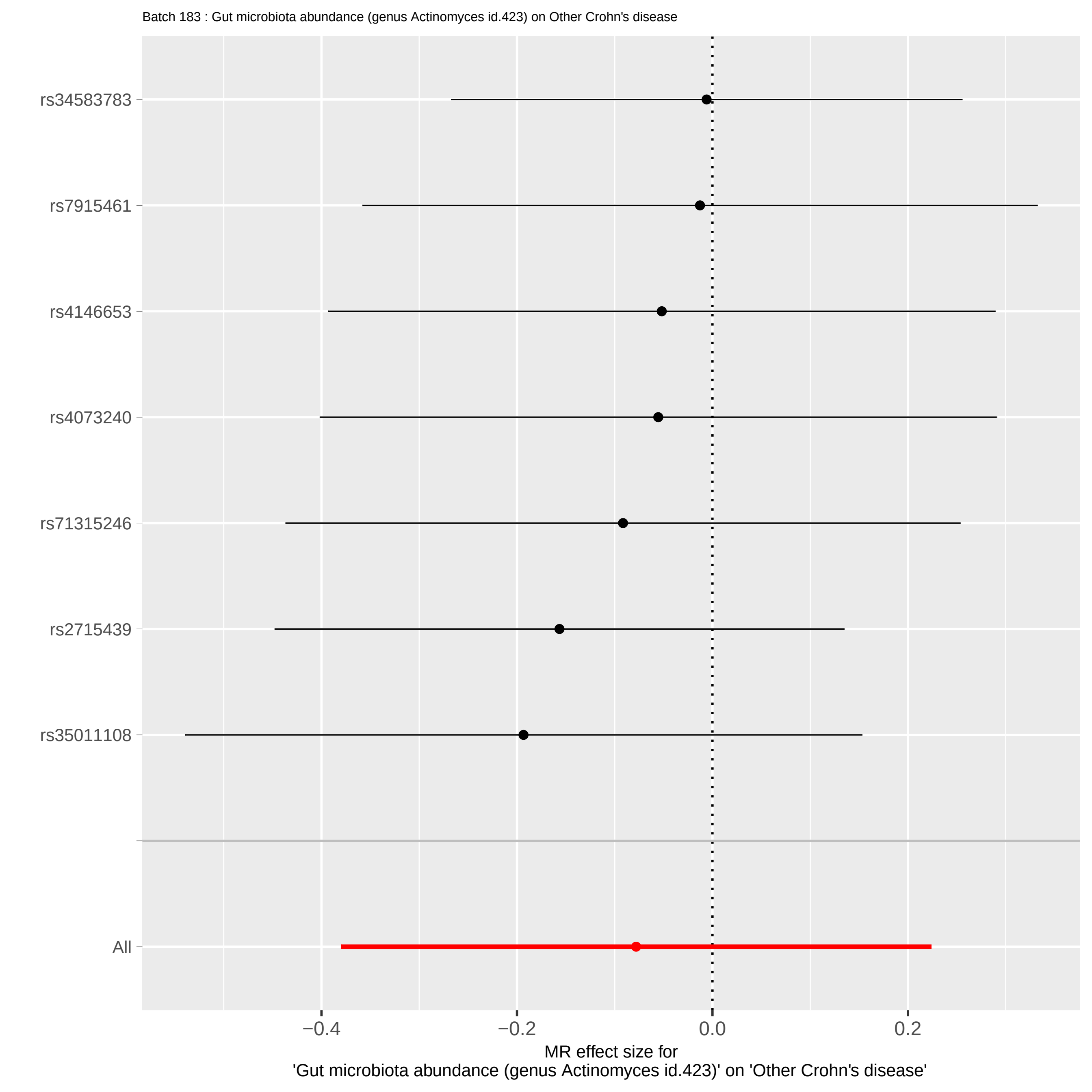

Batch 183 : Gut microbiota abundance (genus Actinomyces id.423) on Other Crohn's disease
rs34583783
rs7915461
rs4146653
rs4073240
rs71315246
rs2715439
rs35011108
All
−0.4
−0.2
0.0
0.2
MR effect size for
'Gut microbiota abundance (genus Actinomyces id.423)' on 'Other Crohn's disease'

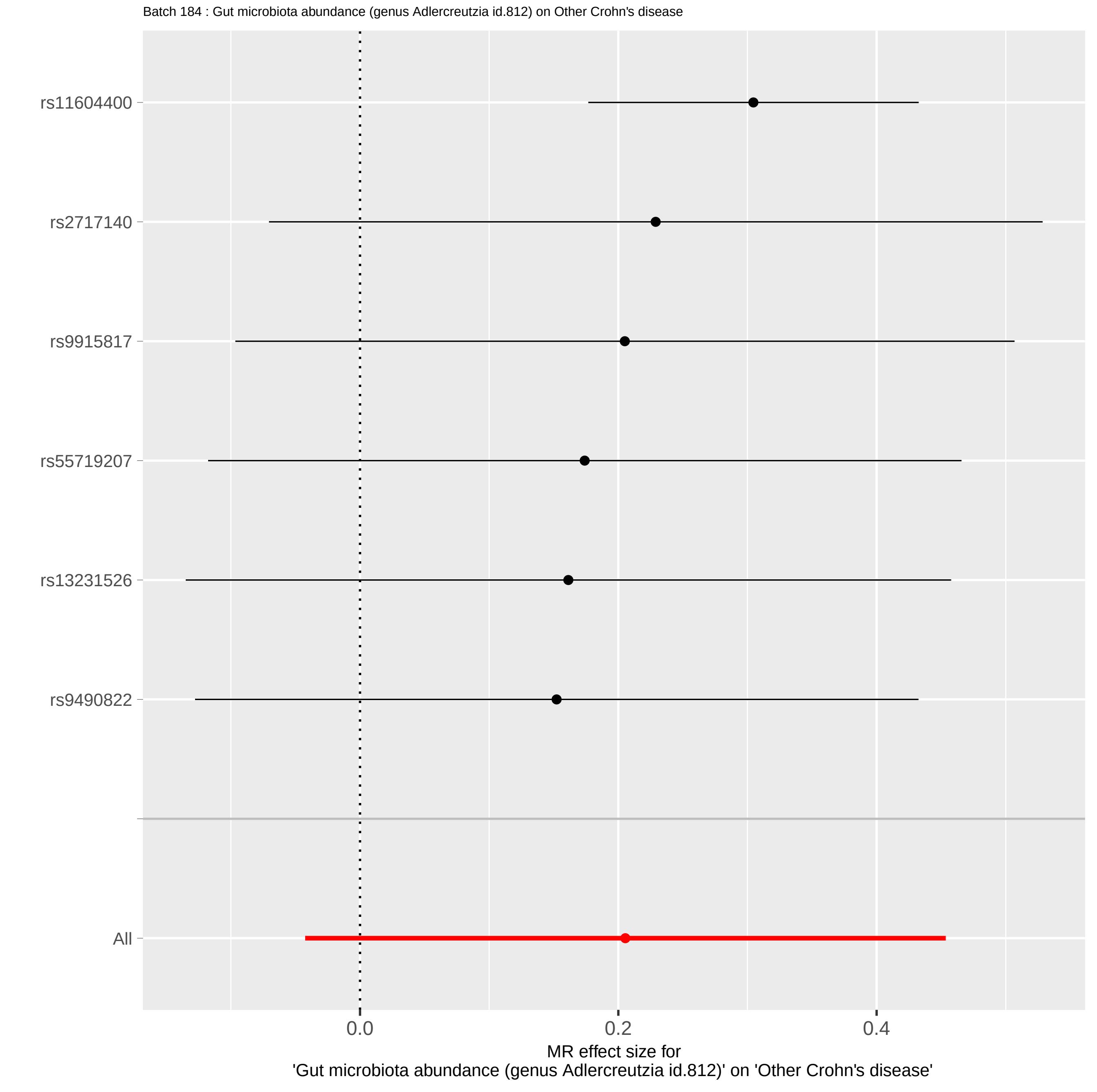
Batch 184 : Gut microbiota abundance (genus Adlercreutzia id.812) on Other Crohn's disease
rs11604400
rs2717140
rs9915817
rs55719207
rs13231526
rs9490822
All
0.0
0.2
0.4
MR effect size for
'Gut microbiota abundance (genus Adlercreutzia id.812)' on 'Other Crohn's disease'

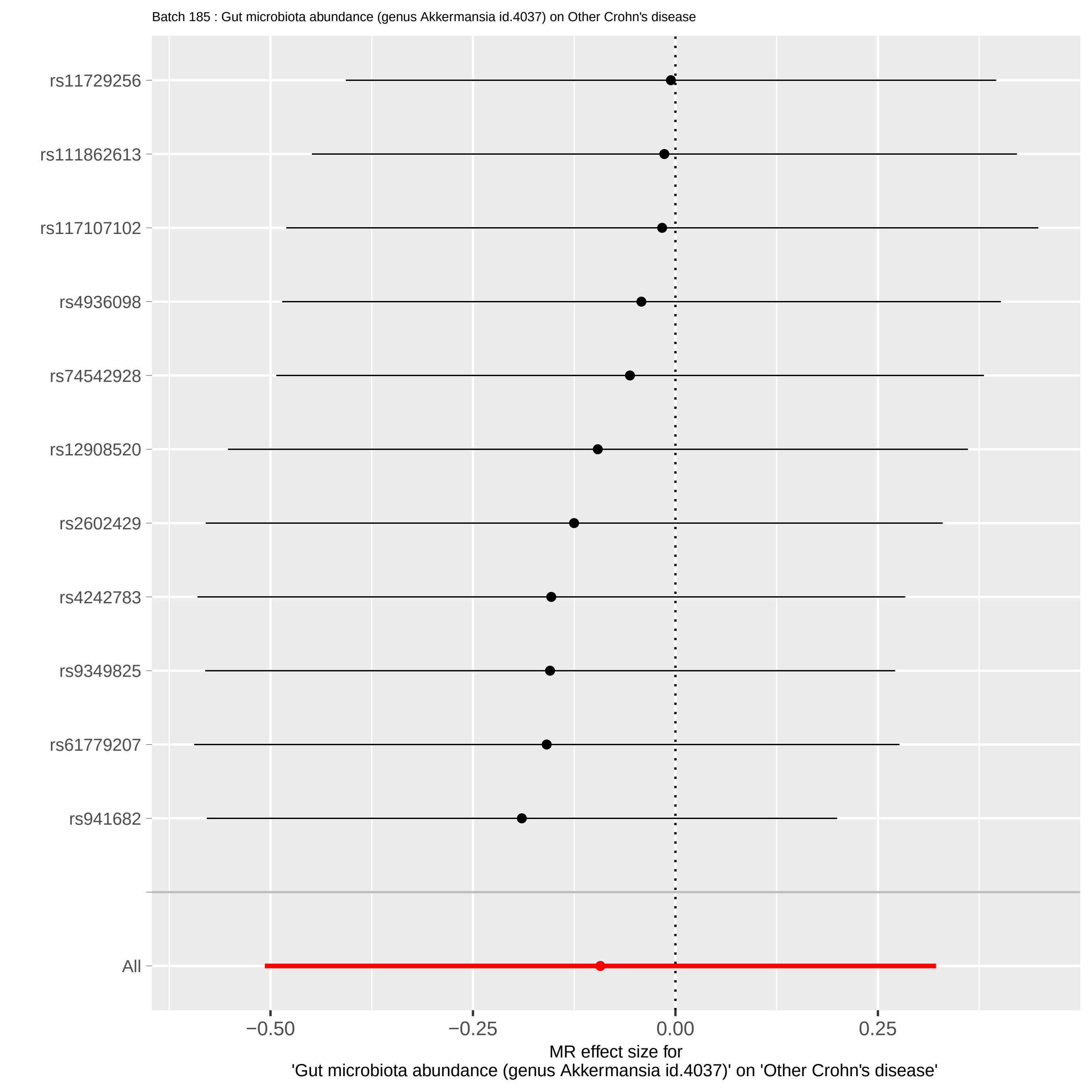

Batch 185 : Gut microbiota abundance (genus Akkermansia id.4037) on Other Crohn's disease
rs11729256
rs111862613
rs117107102
rs4936098
rs74542928
rs12908520
rs2602429
rs4242783
rs9349825
rs61779207
rs941682
All
−0.50
−0.25
0.00
0.25
MR effect size for
'Gut microbiota abundance (genus Akkermansia id.4037)' on 'Other Crohn's disease'

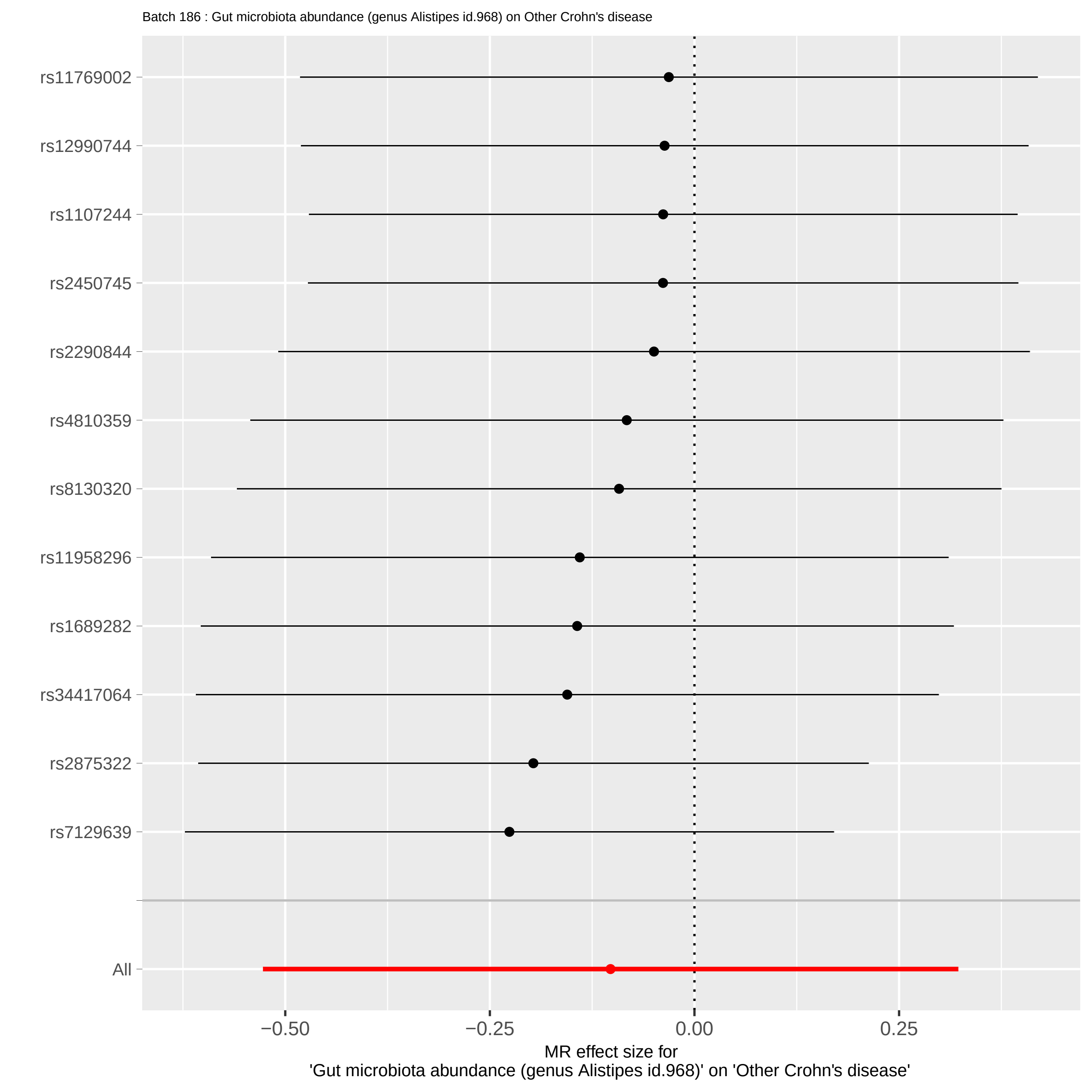

Batch 186 : Gut microbiota abundance (genus Alistipes id.968) on Other Crohn's disease
rs11769002
rs12990744
rs1107244
rs2450745
rs2290844
rs4810359
rs8130320
rs11958296
rs1689282
rs34417064
rs2875322
rs7129639
All
−0.50
−0.25
0.00
0.25
MR effect size for
'Gut microbiota abundance (genus Alistipes id.968)' on 'Other Crohn's disease'

Batch 187 : Gut microbiota abundance (genus Allisonella id.2174) on Other Crohn's disease

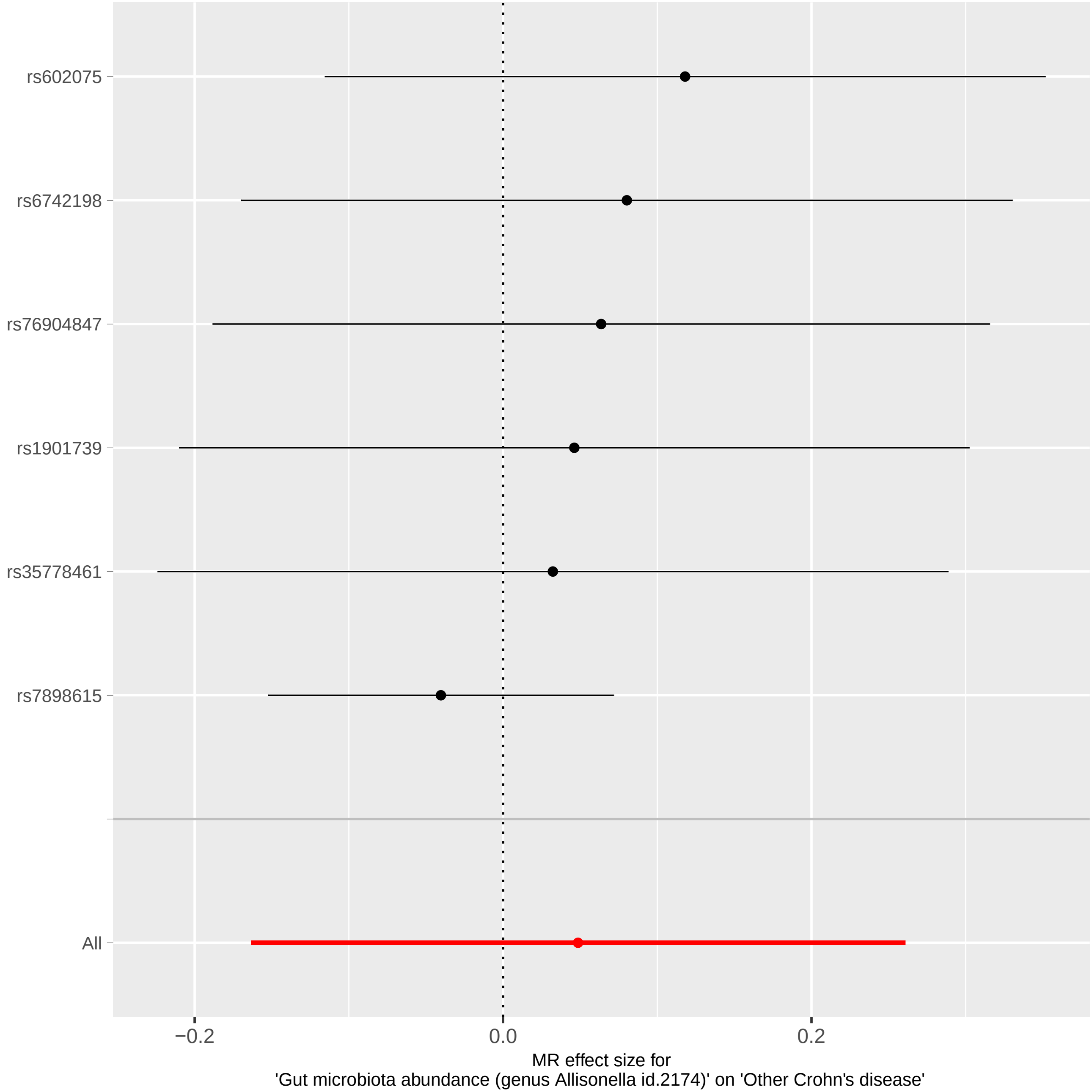

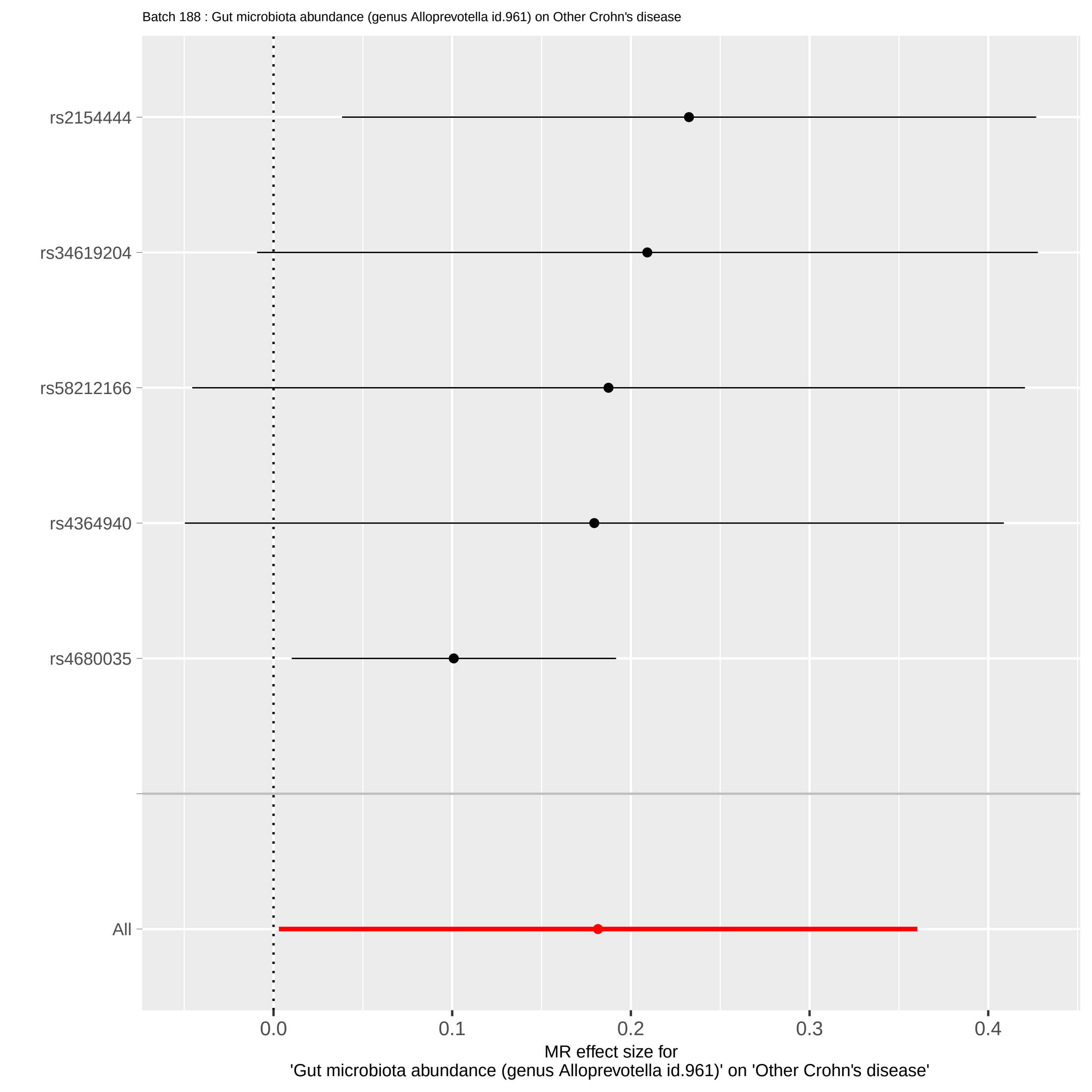
Batch 188 : Gut microbiota abundance (genus Alloprevotella id.961) on Other Crohn's disease
rs2154444
rs34619204
rs58212166
rs4364940
rs4680035
All
0.0
0.1
0.2
0.3
0.4
MR effect size for
'Gut microbiota abundance (genus Alloprevotella id.961)' on 'Other Crohn's disease'

Batch 189 : Gut microbiota abundance (genus Anaerofilum id.2053) on Other Crohn's disease

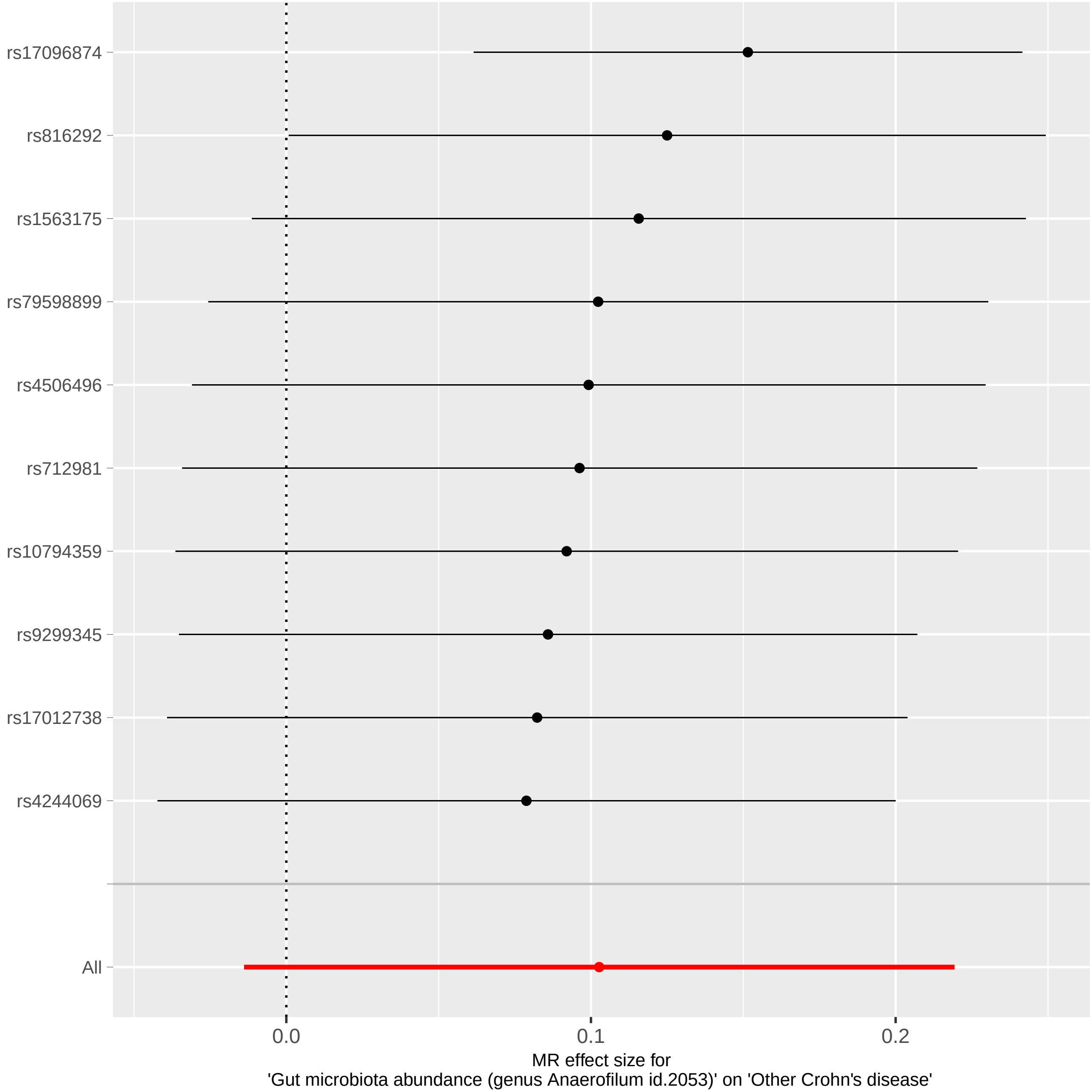

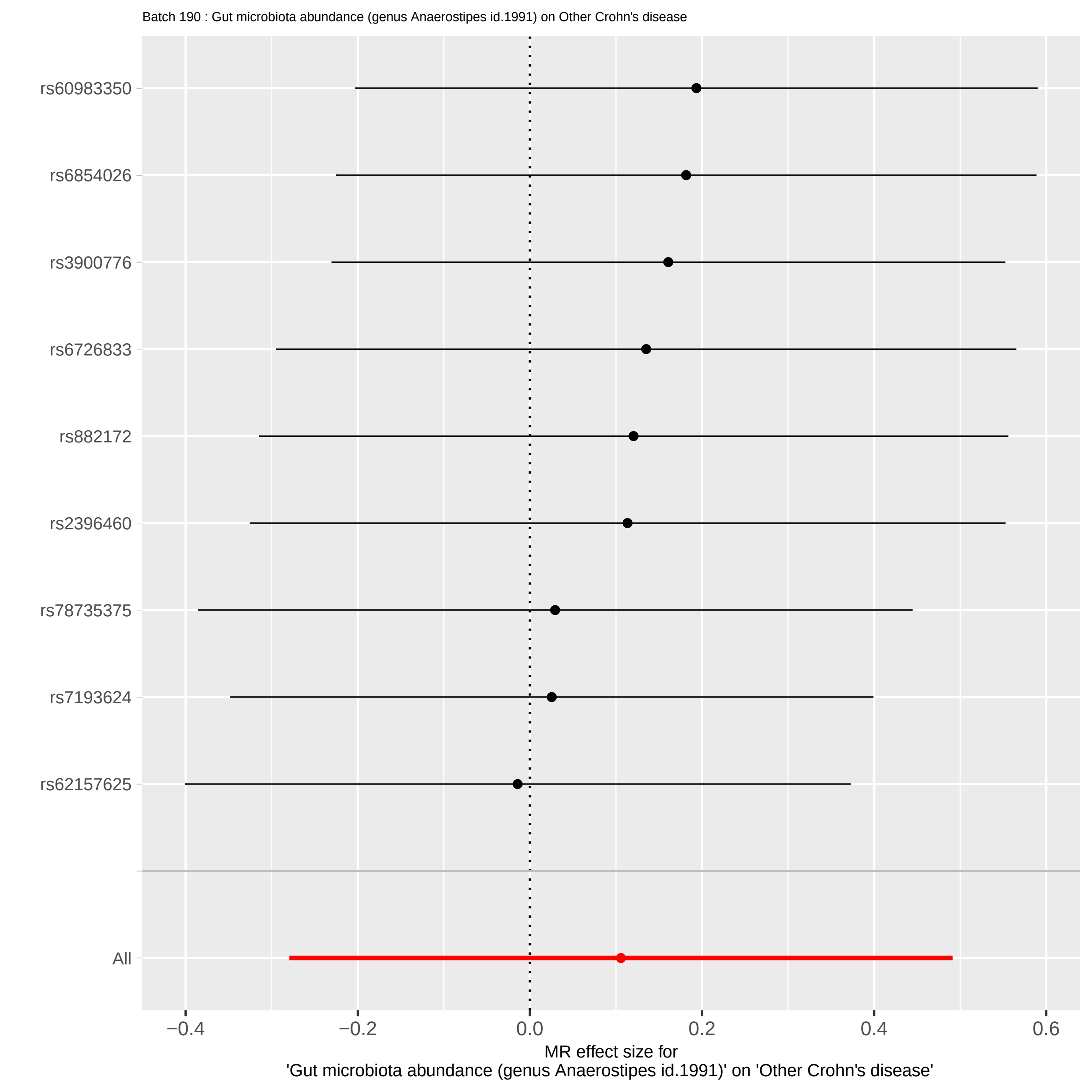

Batch 190 : Gut microbiota abundance (genus Anaerostipes id.1991) on Other Crohn's disease
rs60983350
rs6854026
rs3900776
rs6726833
rs882172
rs2396460
rs78735375
rs7193624
rs62157625
All
−0.4
−0.2
0.0
0.2
0.4
0.6
MR effect size for
'Gut microbiota abundance (genus Anaerostipes id.1991)' on 'Other Crohn's disease'

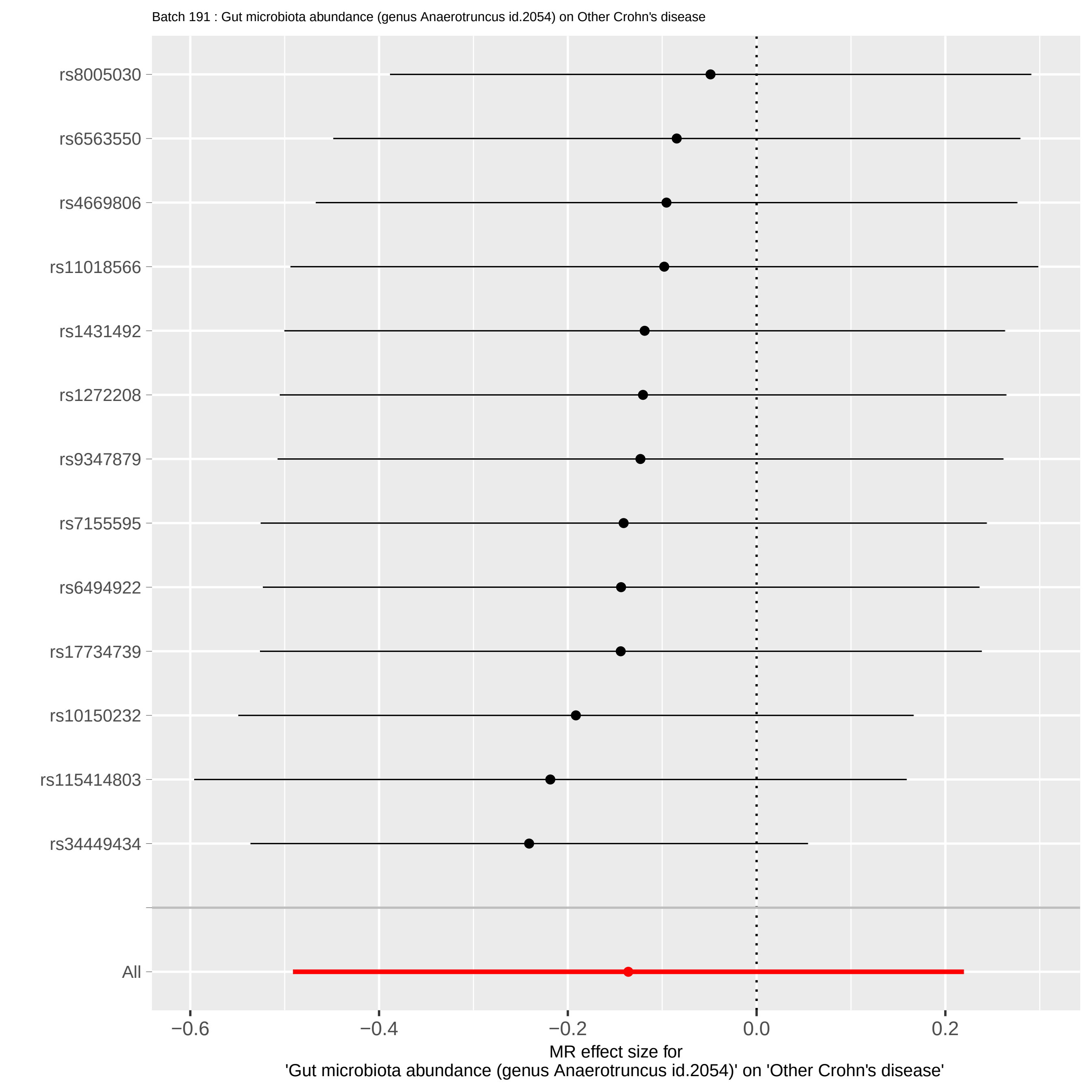
Batch 191 : Gut microbiota abundance (genus Anaerotruncus id.2054) on Other Crohn's disease
rs8005030
rs6563550
rs4669806
rs11018566
rs1431492
rs1272208
rs9347879
rs7155595
rs6494922
rs17734739
rs10150232
rs115414803
rs34449434
All
−0.6
−0.4
−0.2
0.0
0.2
MR effect size for
'Gut microbiota abundance (genus Anaerotruncus id.2054)' on 'Other Crohn's disease'

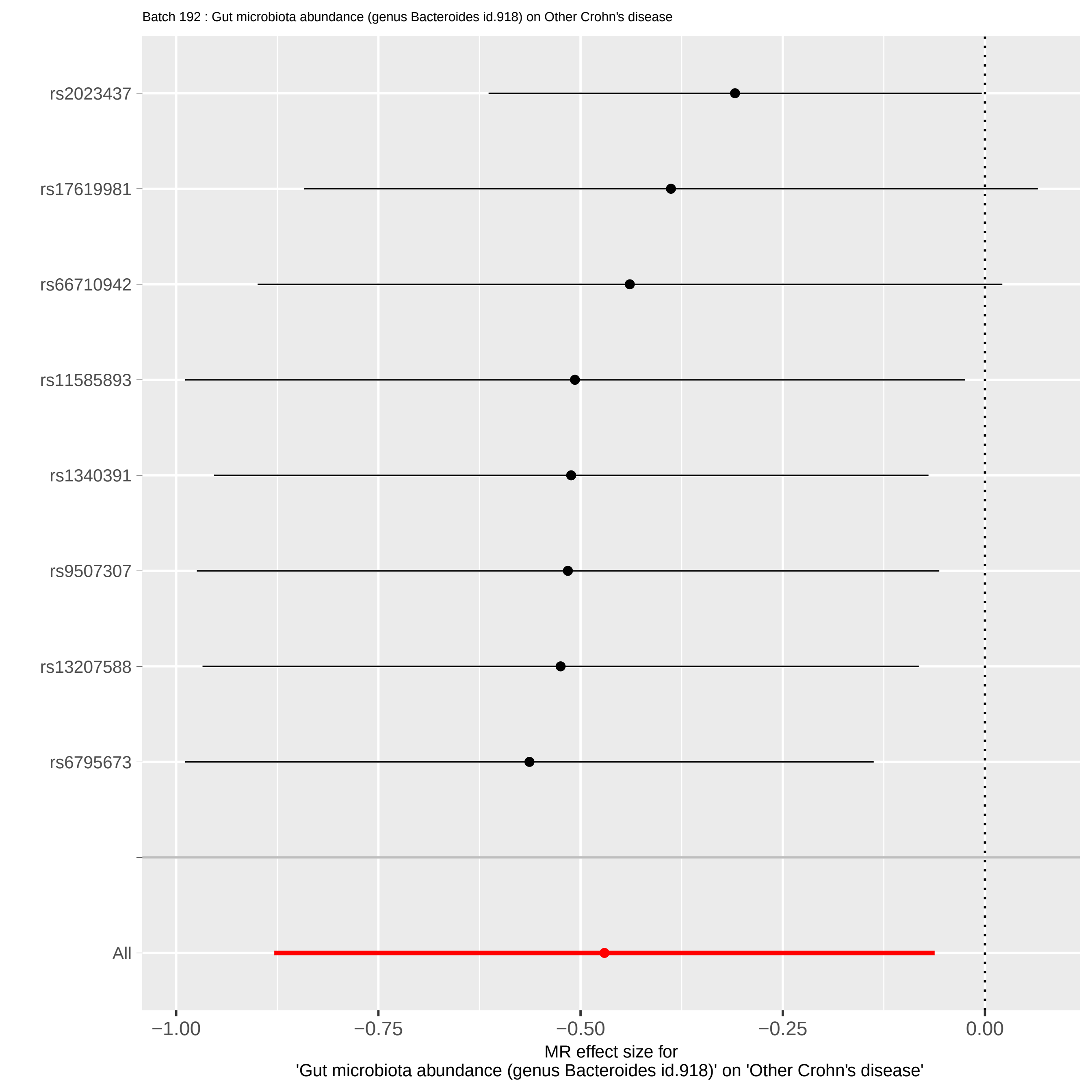

Batch 192 : Gut microbiota abundance (genus Bacteroides id.918) on Other Crohn's disease
rs2023437
rs17619981
rs66710942
rs11585893
rs1340391
rs9507307
rs13207588
rs6795673
All
−1.00
−0.75
−0.50
−0.25
0.00
MR effect size for
'Gut microbiota abundance (genus Bacteroides id.918)' on 'Other Crohn's disease'

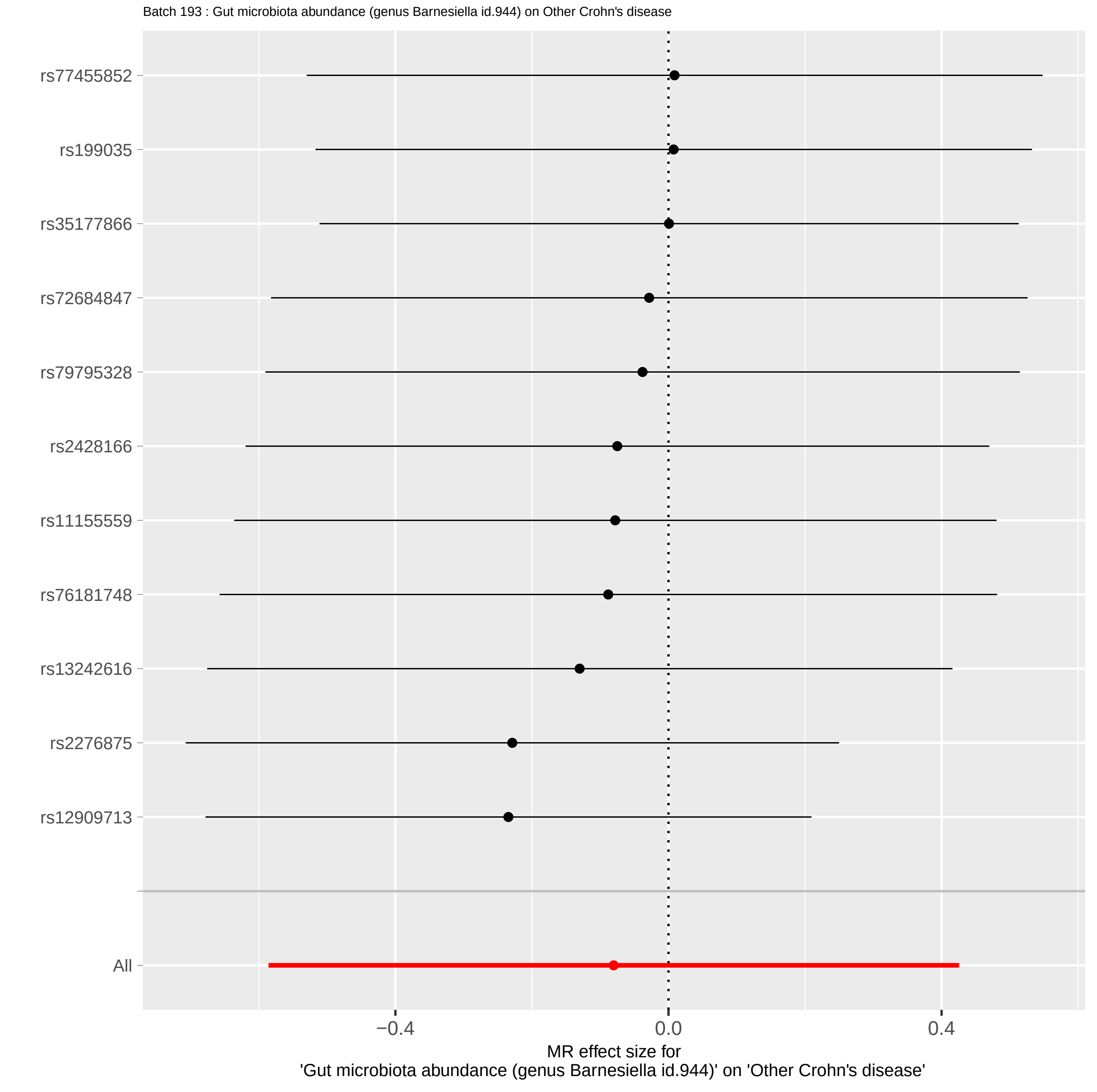
Batch 193 : Gut microbiota abundance (genus Barnesiella id.944) on Other Crohn's disease
rs77455852
rs199035
rs35177866
rs72684847
rs79795328
rs2428166
rs11155559
rs76181748
rs13242616
rs2276875
rs12909713
All
−0.4
0.0
0.4
MR effect size for
'Gut microbiota abundance (genus Barnesiella id.944)' on 'Other Crohn's disease'

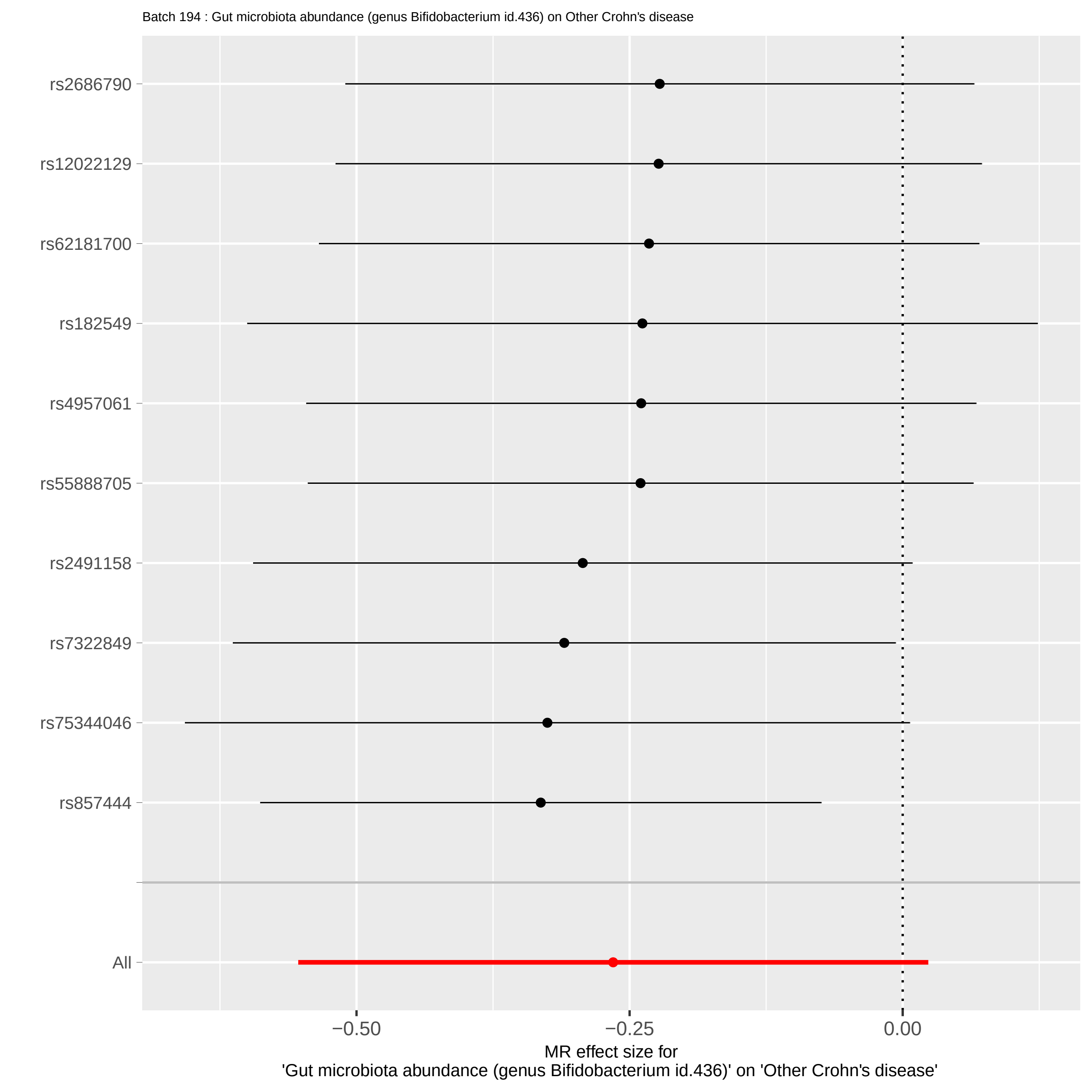
Batch 194 : Gut microbiota abundance (genus Bifidobacterium id.436) on Other Crohn's disease
rs2686790
rs12022129
rs62181700
rs182549
rs4957061
rs55888705
rs2491158
rs7322849
rs75344046
rs857444
All
−0.50
−0.25
0.00
MR effect size for
'Gut microbiota abundance (genus Bifidobacterium id.436)' on 'Other Crohn's disease'

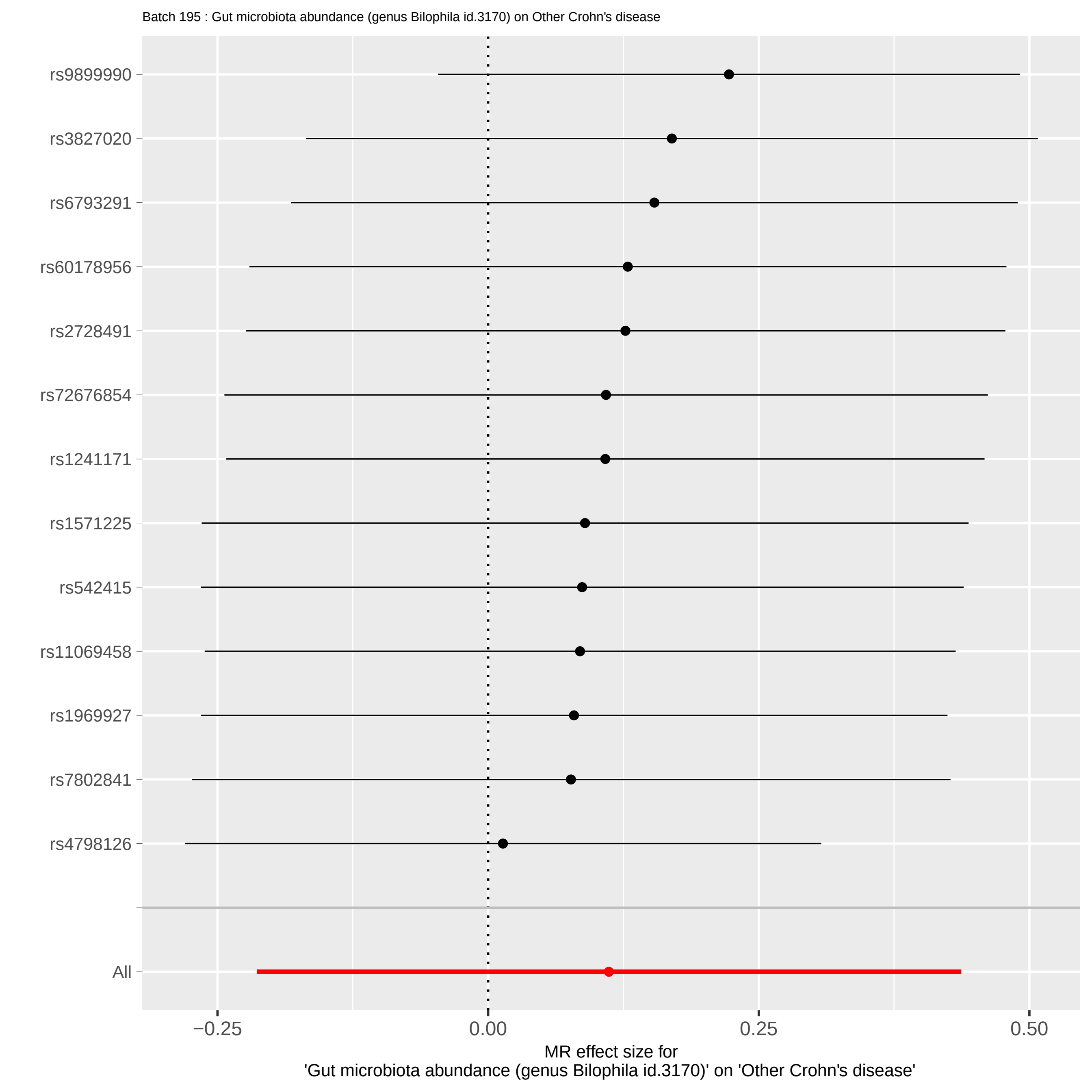

Batch 195 : Gut microbiota abundance (genus Bilophila id.3170) on Other Crohn's disease
rs9899990
rs3827020
rs6793291
rs60178956
rs2728491
rs72676854
rs1241171
rs1571225
rs542415
rs11069458
rs1969927
rs7802841
rs4798126
All
−0.25
0.00
0.25
0.50
MR effect size for
'Gut microbiota abundance (genus Bilophila id.3170)' on 'Other Crohn's disease'

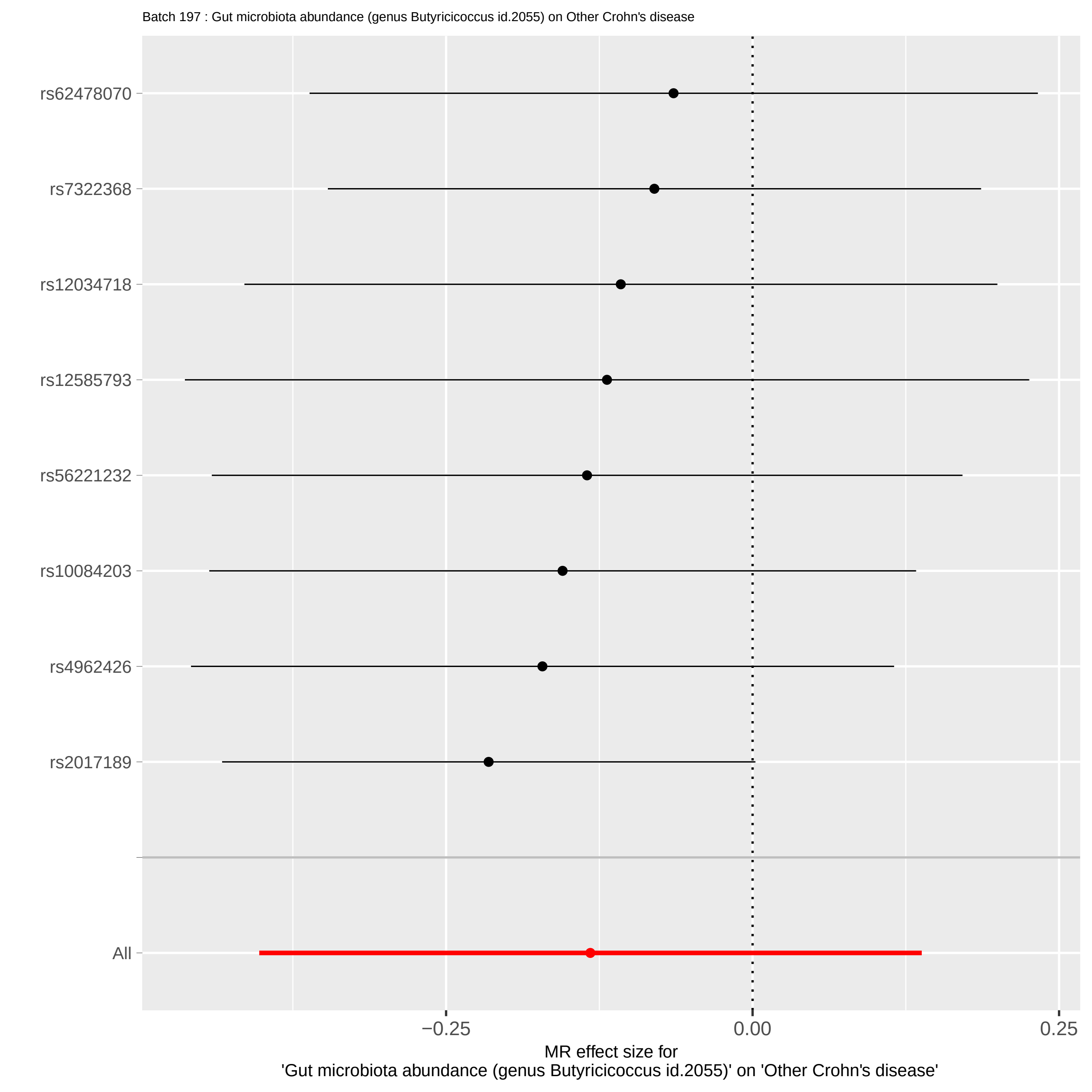
Batch 197 : Gut microbiota abundance (genus Butyricicoccus id.2055) on Other Crohn's disease
rs62478070
rs7322368
rs12034718
rs12585793
rs56221232
rs10084203
rs4962426
rs2017189
All
−0.25
0.00
0.25
MR effect size for
'Gut microbiota abundance (genus Butyricicoccus id.2055)' on 'Other Crohn's disease'

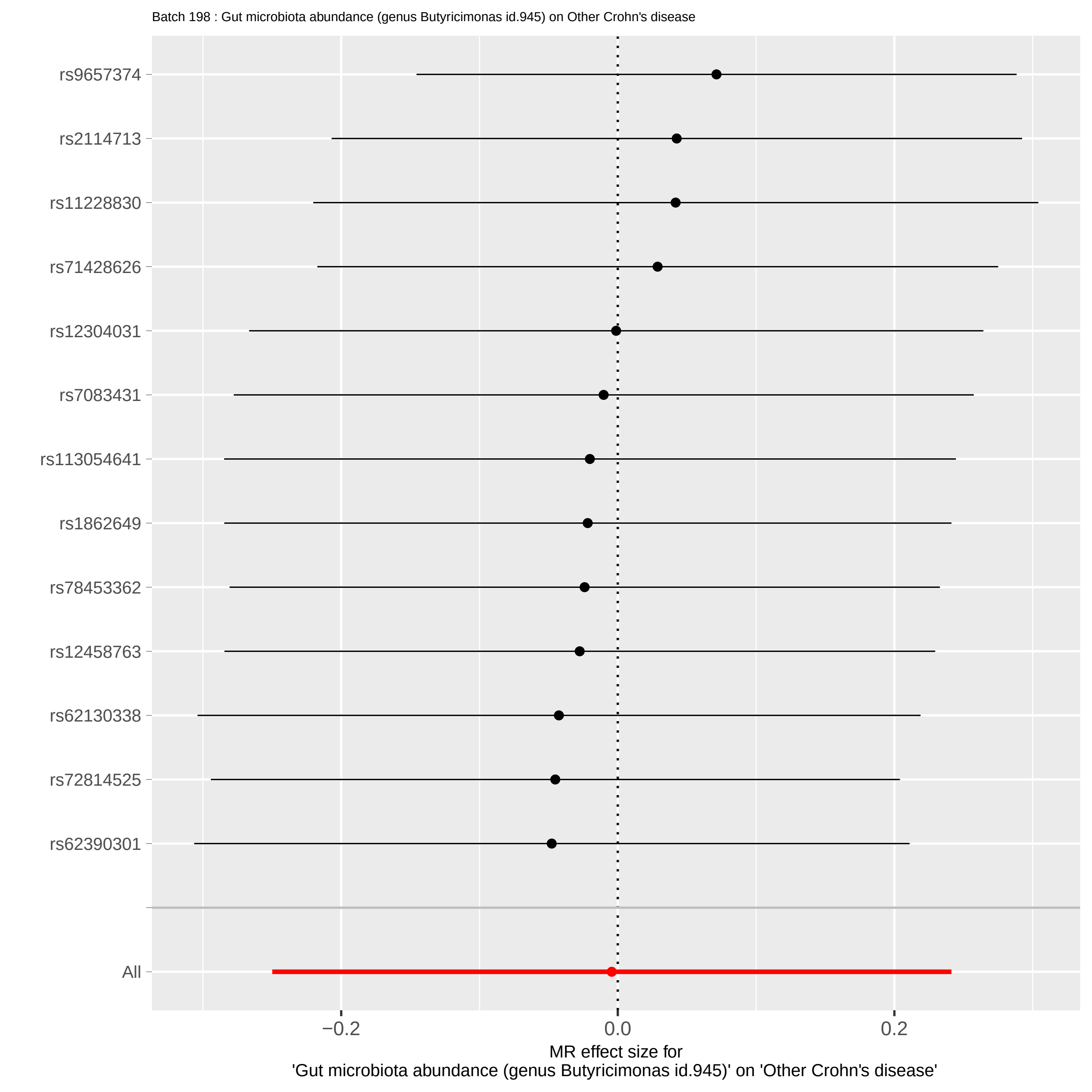

Batch 198 : Gut microbiota abundance (genus Butyricimonas id.945) on Other Crohn's disease
rs9657374
rs2114713
rs11228830
rs71428626
rs12304031
rs7083431
rs113054641
rs1862649
rs78453362
rs12458763
rs62130338
rs72814525
rs62390301
All
−0.2
0.0
0.2
MR effect size for
'Gut microbiota abundance (genus Butyricimonas id.945)' on 'Other Crohn's disease'

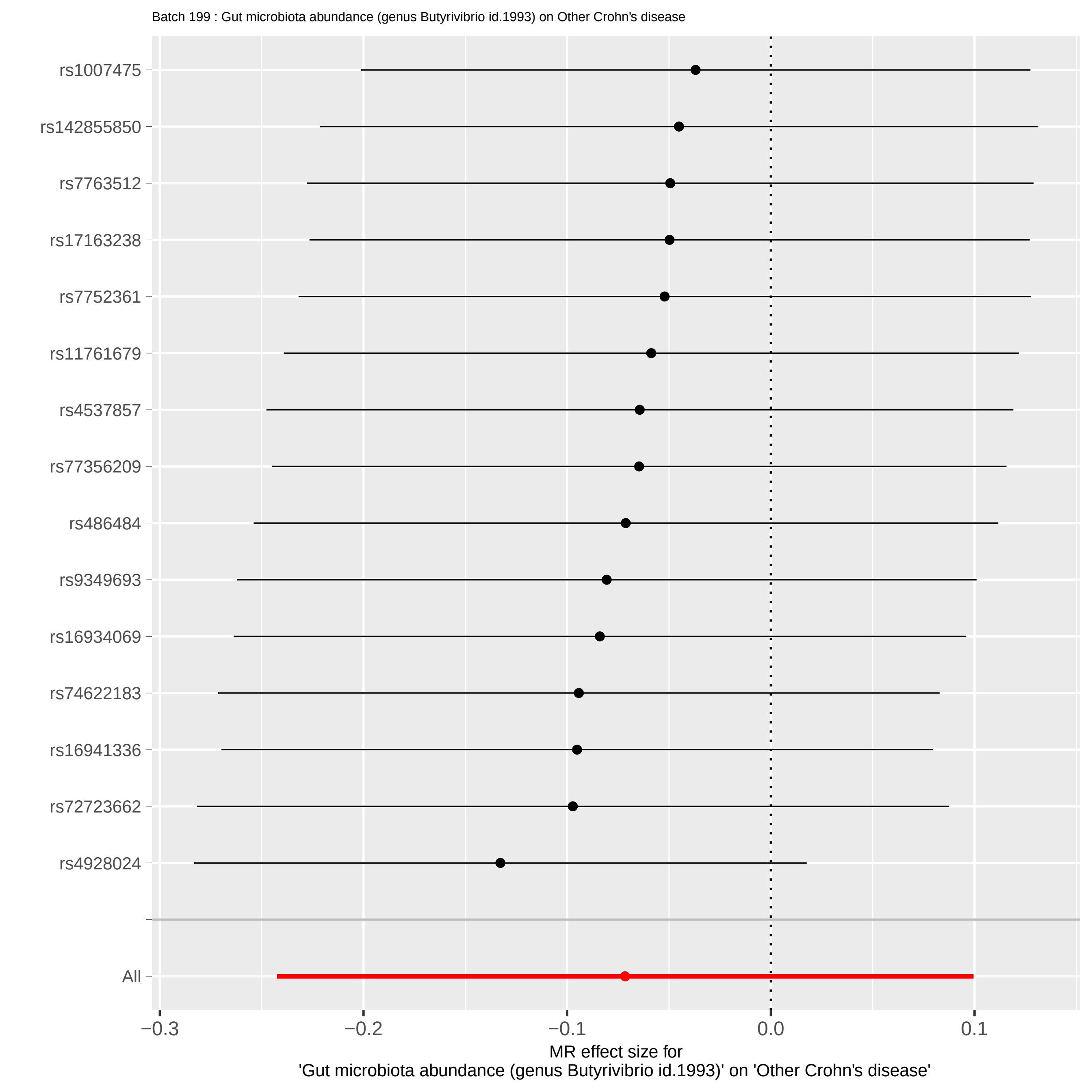

Batch 199 : Gut microbiota abundance (genus Butyrivibrio id.1993) on Other Crohn's disease
rs1007475
rs142855850
rs7763512
rs17163238
rs7752361
rs11761679
rs4537857
rs77356209
rs486484
rs9349693
rs16934069
rs74622183
rs16941336
rs72723662
rs4928024
All
−0.3
−0.2
−0.1
0.0
0.1
MR effect size for
'Gut microbiota abundance (genus Butyrivibrio id.1993)' on 'Other Crohn's disease'

Batch 200 : Gut microbiota abundance (genus Candidatus Soleaferrea id.11350) on Other Crohn's disease

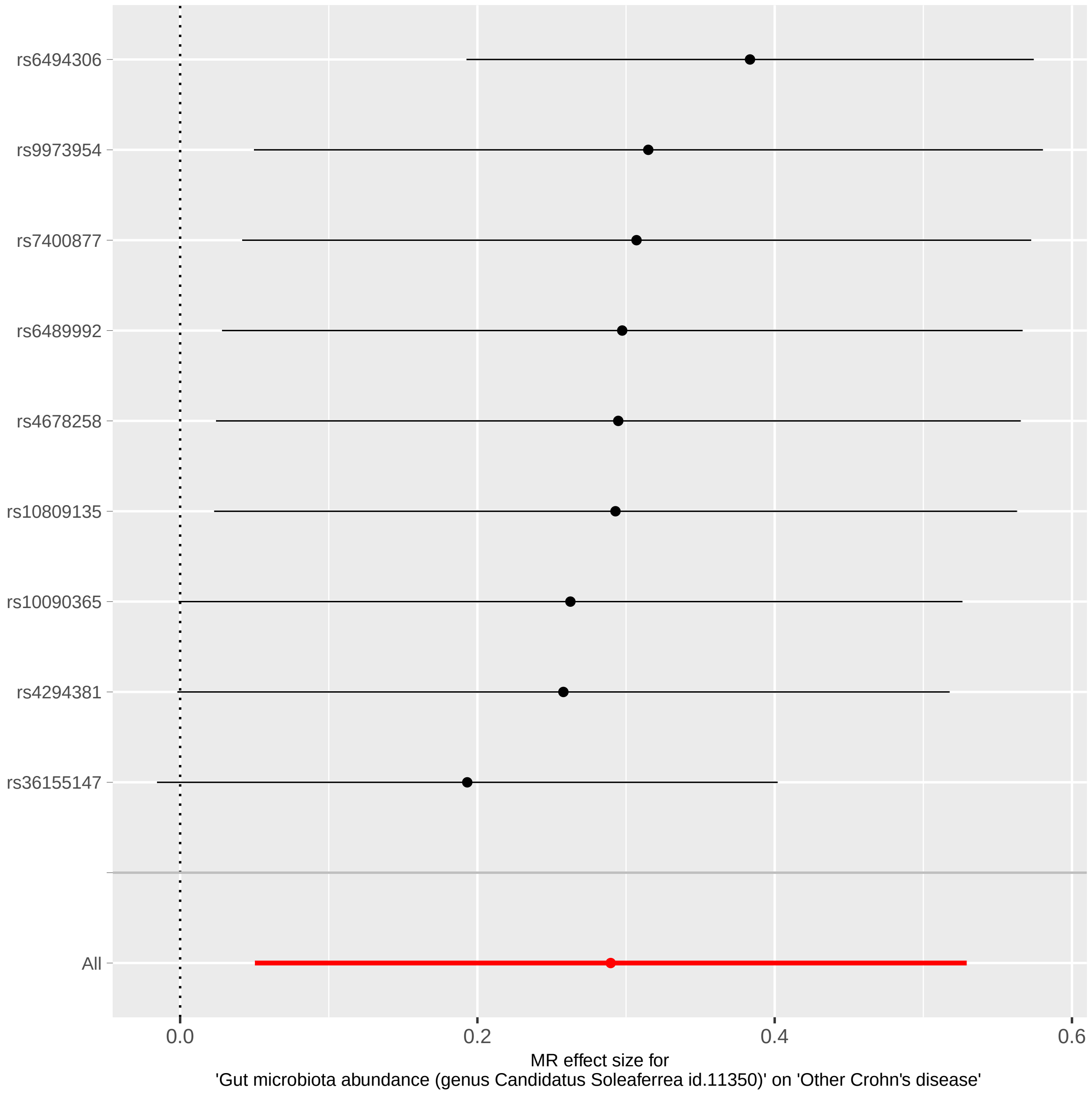

Batch 201 : Gut microbiota abundance (genus Catenibacterium id.2153) on Other Crohn's disease

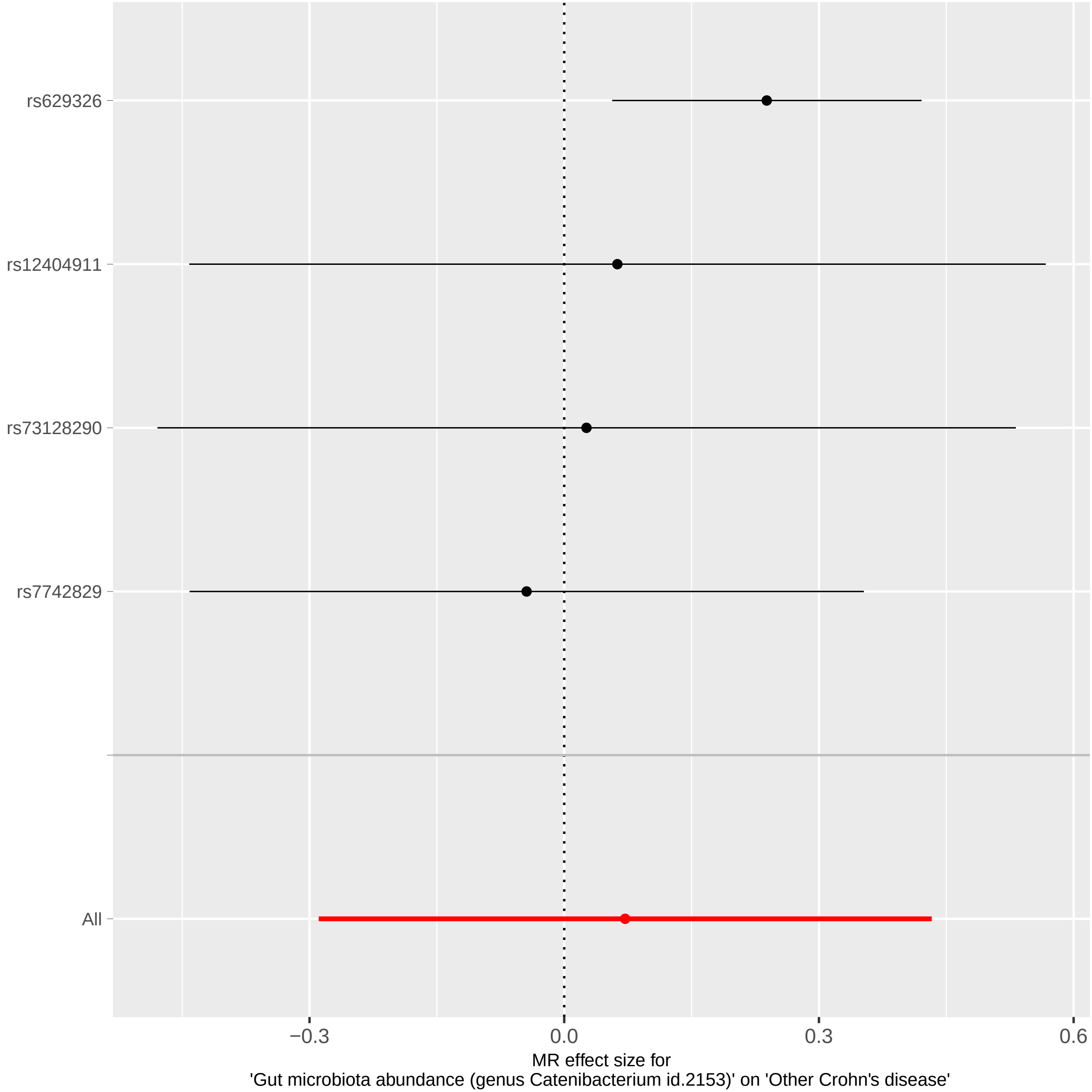

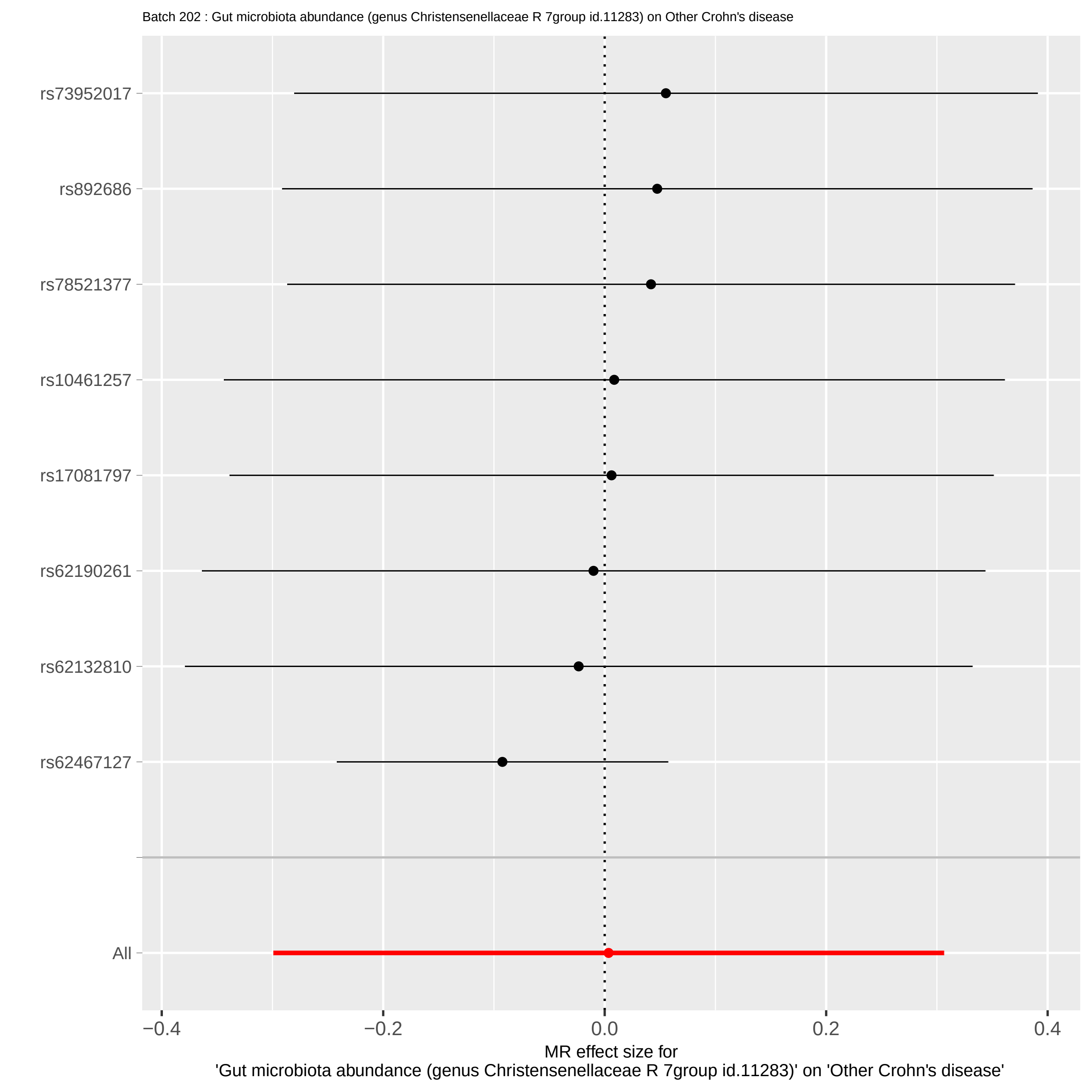

Batch 202 : Gut microbiota abundance (genus Christensenellaceae R 7group id.11283) on Other Crohn's disease
rs73952017
rs892686
rs78521377
rs10461257
rs17081797
rs62190261
rs62132810
rs62467127
All
−0.4
−0.2
0.0
0.2
0.4
MR effect size for
'Gut microbiota abundance (genus Christensenellaceae R 7group id.11283)' on 'Other Crohn's disease'

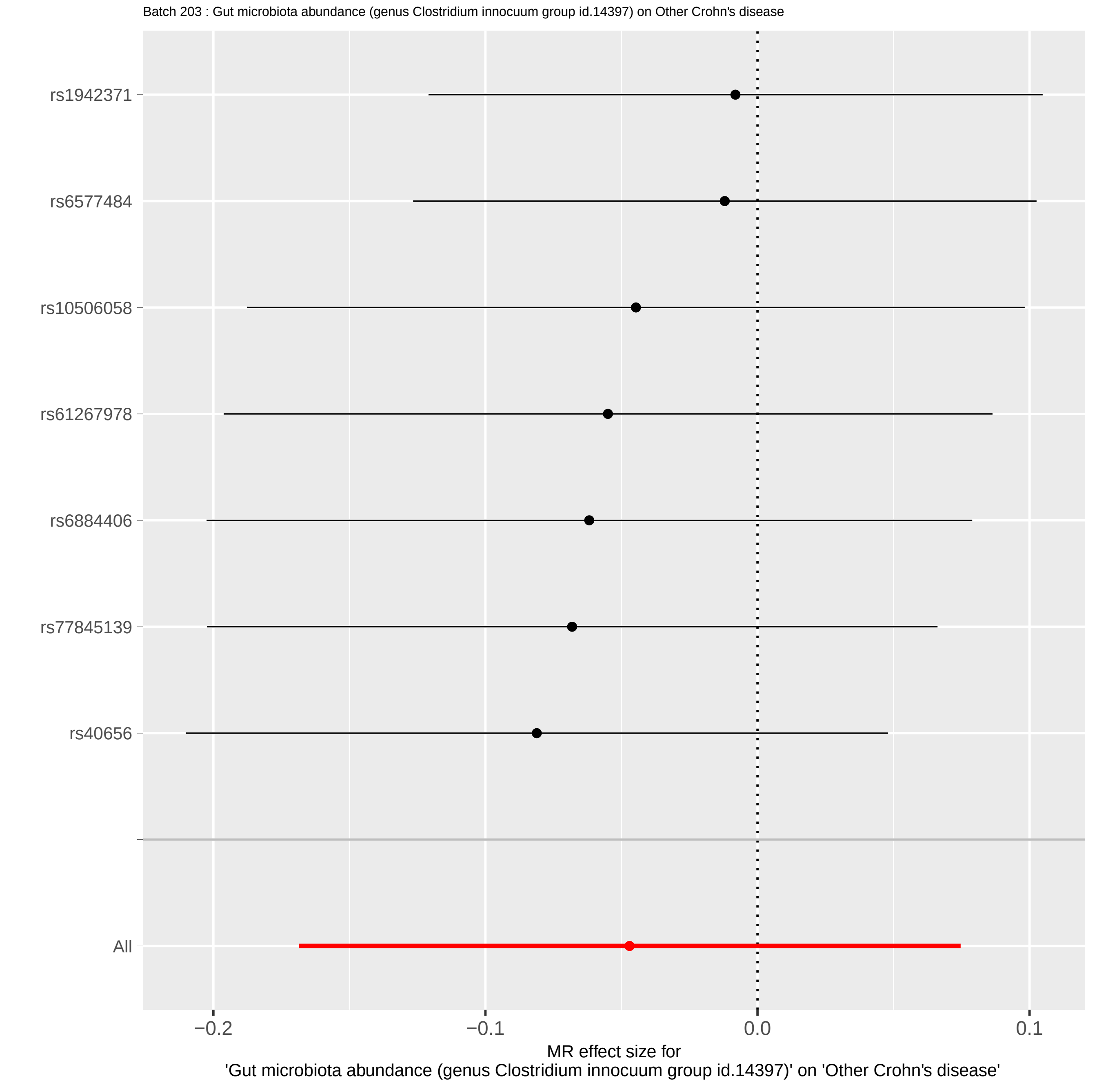

Batch 203 : Gut microbiota abundance (genus Clostridium innocuum group id.14397) on Other Crohn's disease
rs1942371
rs6577484
rs10506058
rs61267978
rs6884406
rs77845139
rs40656
All
−0.2
−0.1
0.0
0.1
MR effect size for
'Gut microbiota abundance (genus Clostridium innocuum group id.14397)' on 'Other Crohn's disease'

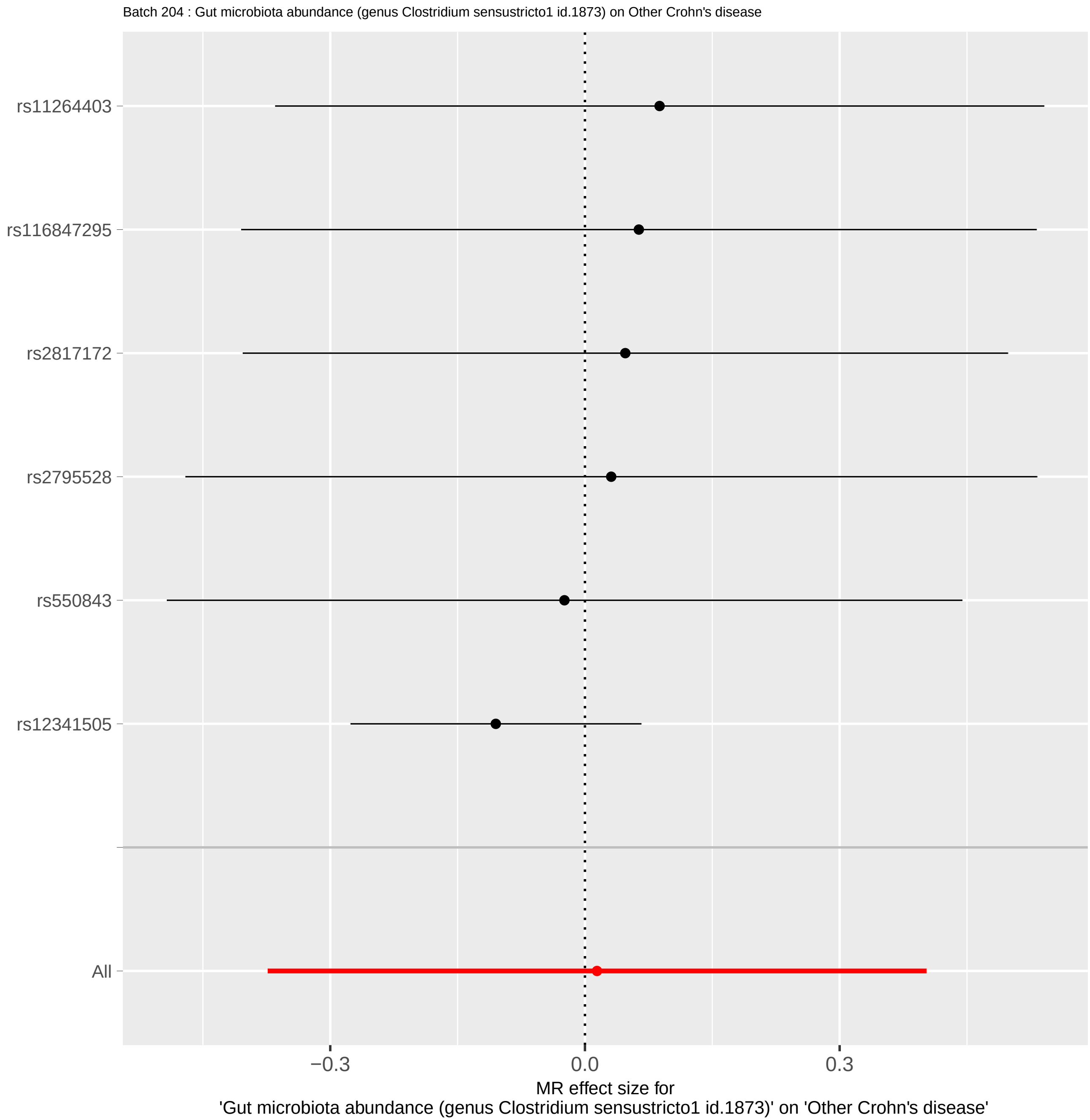

Batch 204 : Gut microbiota abundance (genus Clostridium sensustricto1 id.1873) on Other Crohn's disease
rs11264403
rs116847295
rs2817172
rs2795528
rs550843
rs12341505
All
−0.3
0.0
0.3
MR effect size for
'Gut microbiota abundance (genus Clostridium sensustricto1 id.1873)' on 'Other Crohn's disease'

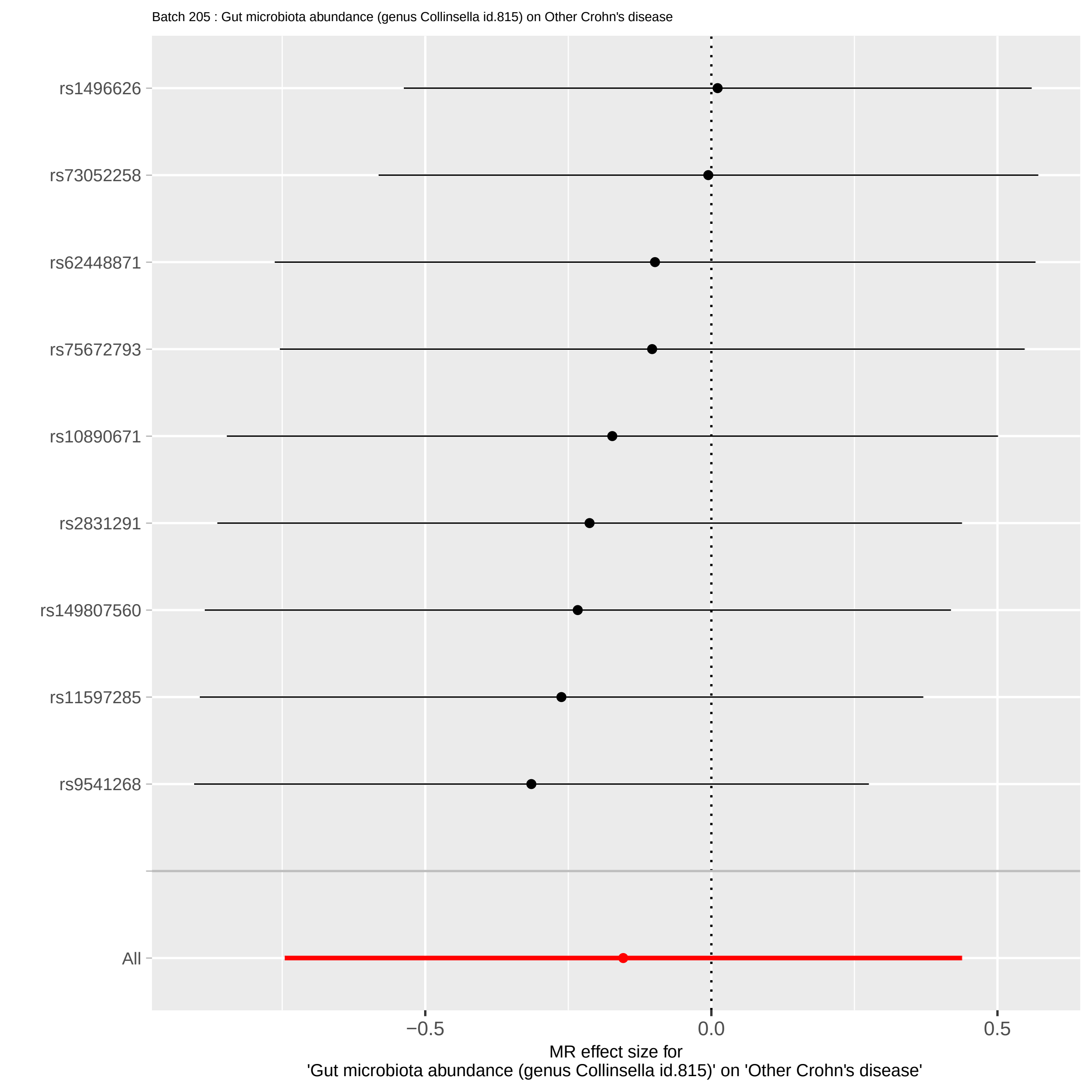
Batch 205 : Gut microbiota abundance (genus Collinsella id.815) on Other Crohn's disease
rs1496626
rs73052258
rs62448871
rs75672793
rs10890671
rs2831291
rs149807560
rs11597285
rs9541268
All
−0.5
0.0
0.5
MR effect size for
'Gut microbiota abundance (genus Collinsella id.815)' on 'Other Crohn's disease'

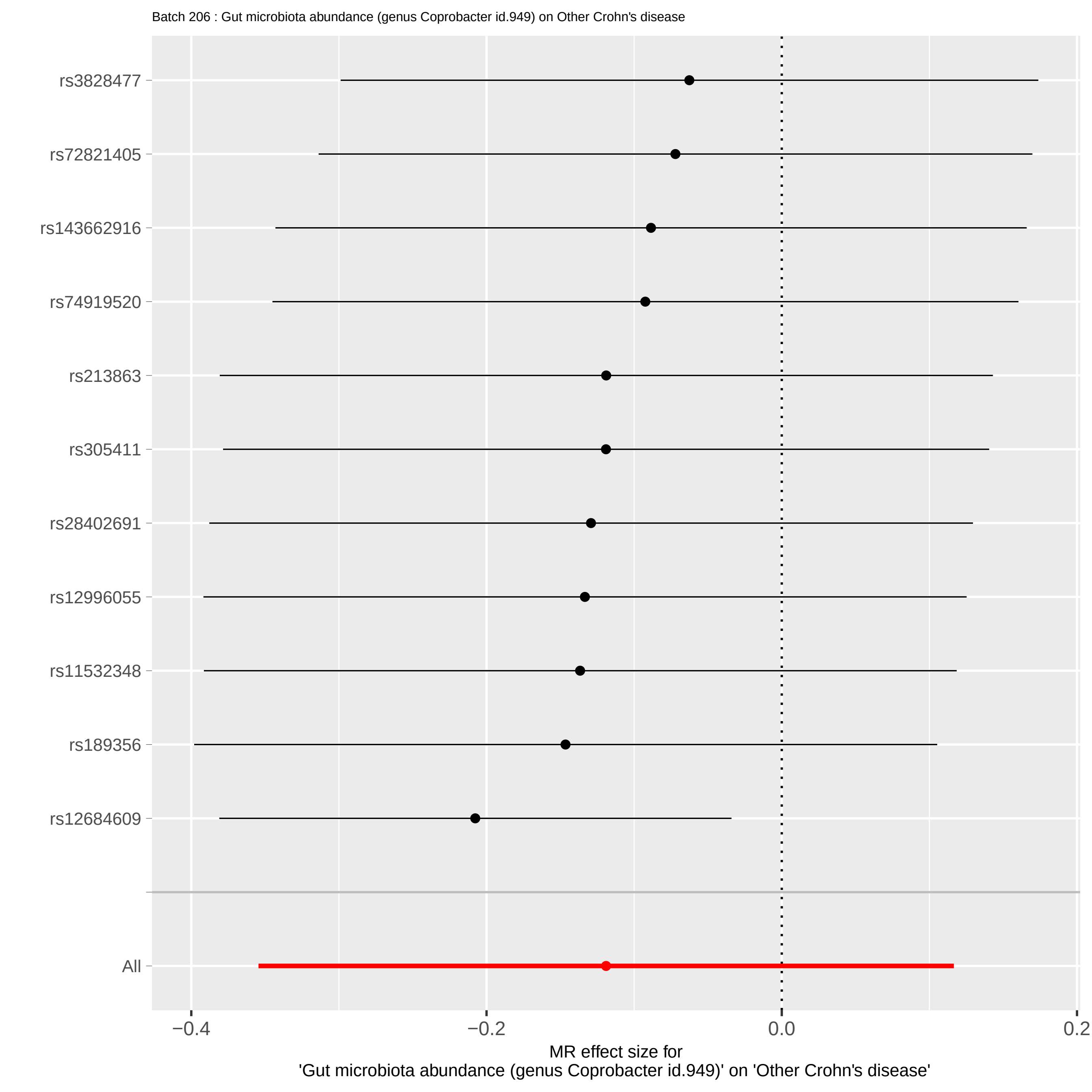

Batch 206 : Gut microbiota abundance (genus Coprobacter id.949) on Other Crohn's disease
rs3828477
rs72821405
rs143662916
rs74919520
rs213863
rs305411
rs28402691
rs12996055
rs11532348
rs189356
rs12684609
All
−0.4
−0.2
0.0
0.2
MR effect size for
'Gut microbiota abundance (genus Coprobacter id.949)' on 'Other Crohn's disease'

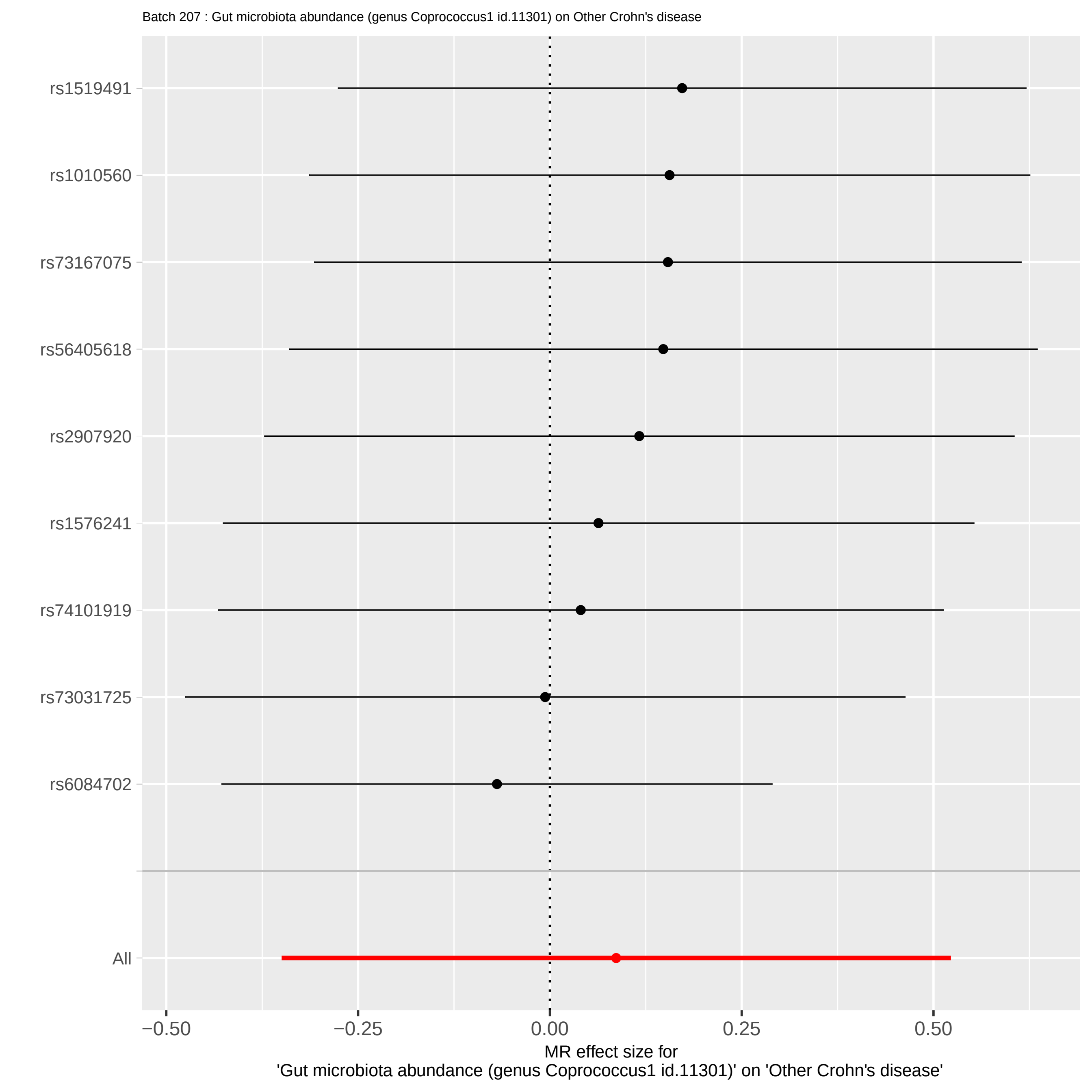

Batch 207 : Gut microbiota abundance (genus Coprococcus1 id.11301) on Other Crohn's disease
rs1519491
rs1010560
rs73167075
rs56405618
rs2907920
rs1576241
rs74101919
rs73031725
rs6084702
All
−0.50
−0.25
0.00
0.25
0.50
MR effect size for
'Gut microbiota abundance (genus Coprococcus1 id.11301)' on 'Other Crohn's disease'

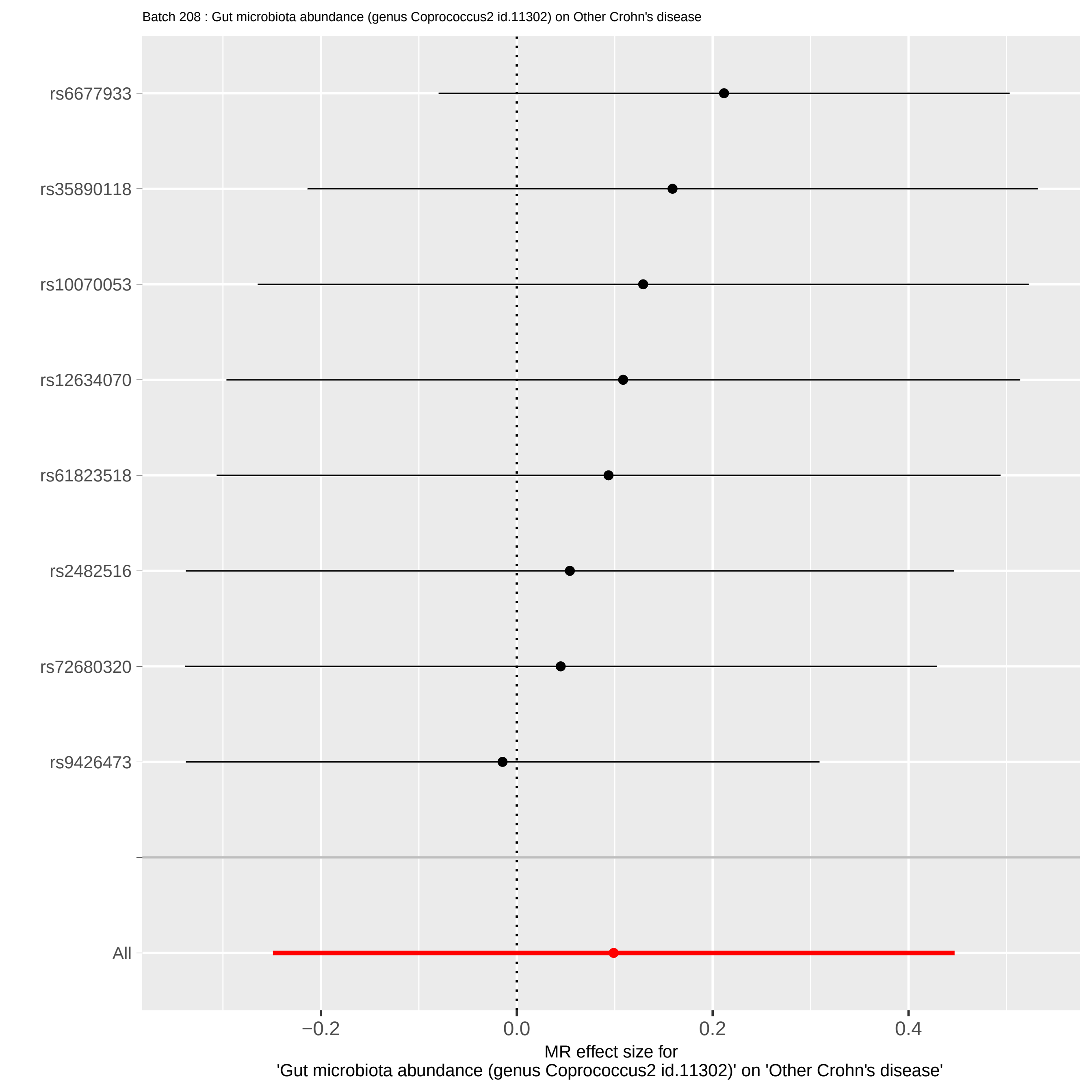
Batch 208 : Gut microbiota abundance (genus Coprococcus2 id.11302) on Other Crohn's disease
rs6677933
rs35890118
rs10070053
rs12634070
rs61823518
rs2482516
rs72680320
rs9426473
All
−0.2
0.0
0.2
0.4
MR effect size for
'Gut microbiota abundance (genus Coprococcus2 id.11302)' on 'Other Crohn's disease'

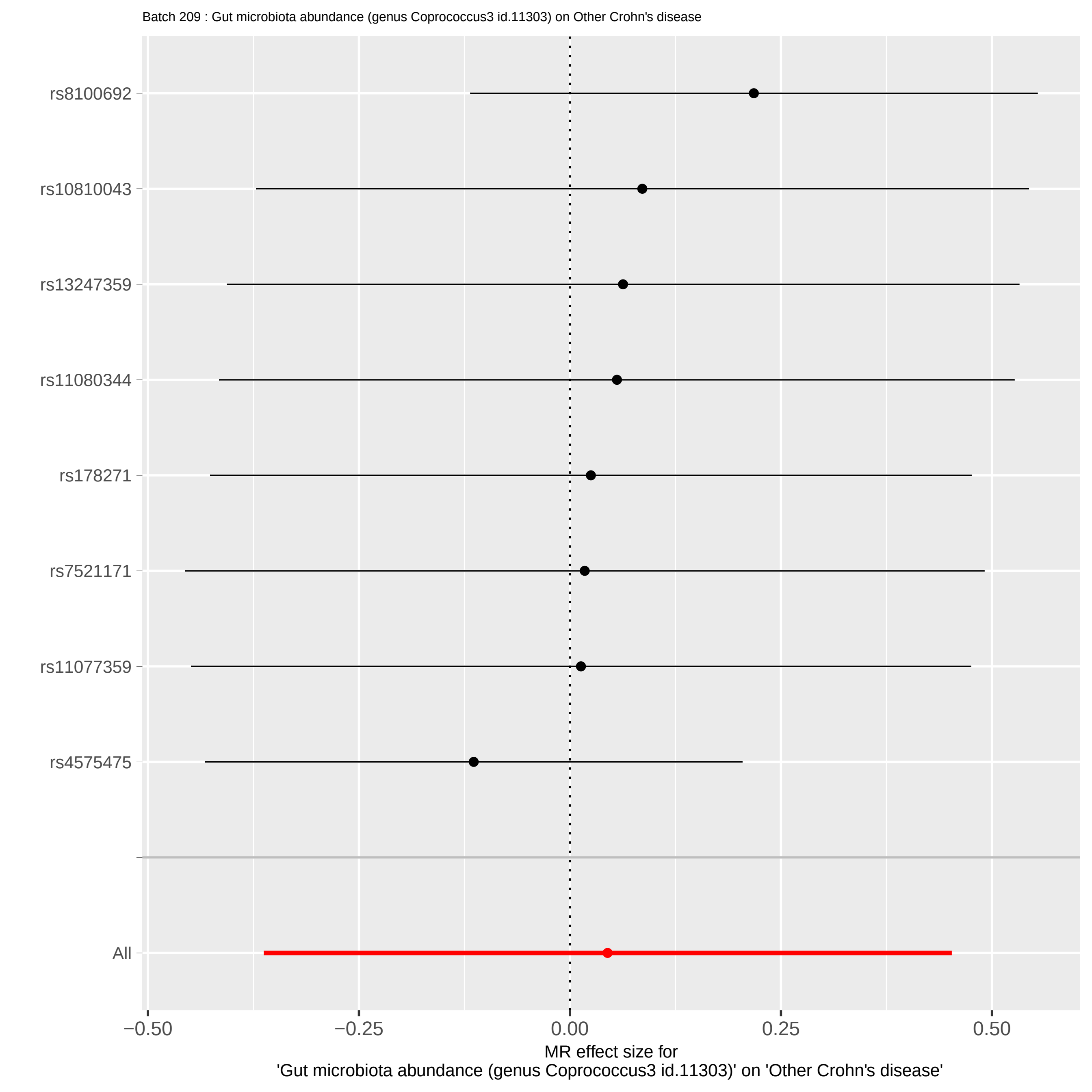

Batch 209 : Gut microbiota abundance (genus Coprococcus3 id.11303) on Other Crohn's disease
rs8100692
rs10810043
rs13247359
rs11080344
rs178271
rs7521171
rs11077359
rs4575475
All
−0.50
−0.25
0.00
0.25
0.50
MR effect size for
'Gut microbiota abundance (genus Coprococcus3 id.11303)' on 'Other Crohn's disease'

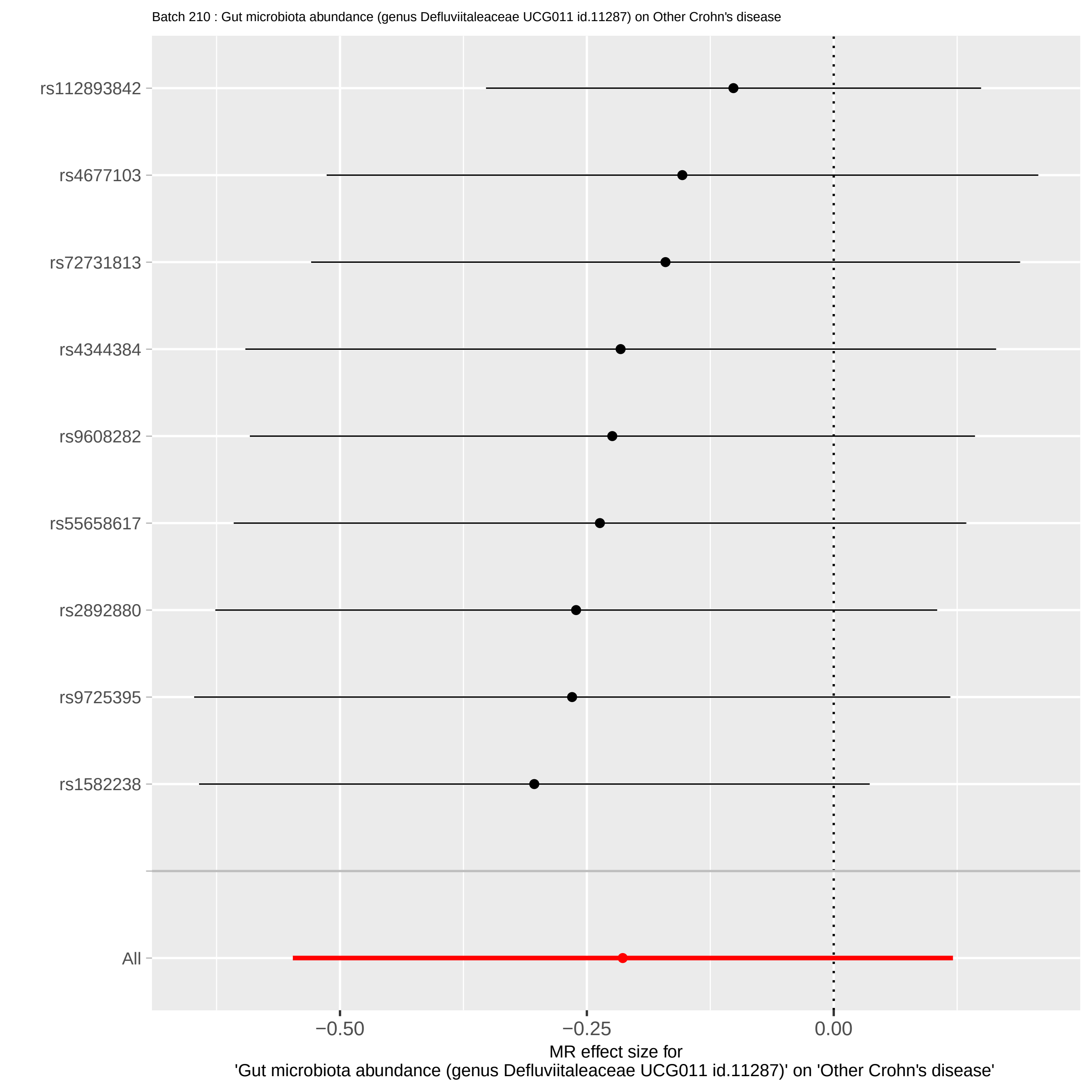

Batch 210 : Gut microbiota abundance (genus Defluviitaleaceae UCG011 id.11287) on Other Crohn's disease
rs112893842
rs4677103
rs72731813
rs4344384
rs9608282
rs55658617
rs2892880
rs9725395
rs1582238
All
−0.50
−0.25
0.00
MR effect size for
'Gut microbiota abundance (genus Defluviitaleaceae UCG011 id.11287)' on 'Other Crohn's disease'

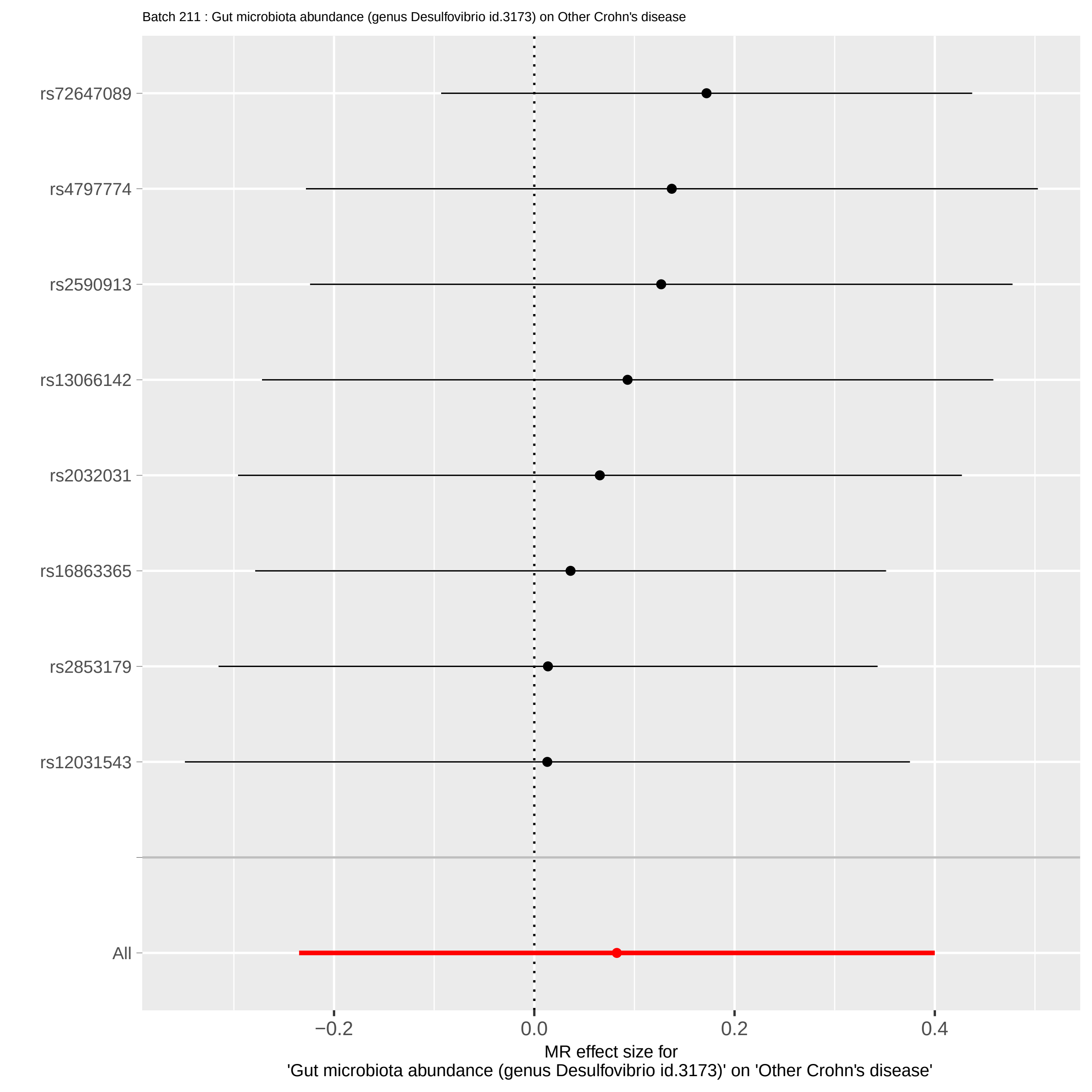

Batch 211 : Gut microbiota abundance (genus Desulfovibrio id.3173) on Other Crohn's disease
rs72647089
rs4797774
rs2590913
rs13066142
rs2032031
rs16863365
rs2853179
rs12031543
All
−0.2
0.0
0.2
0.4
MR effect size for
'Gut microbiota abundance (genus Desulfovibrio id.3173)' on 'Other Crohn's disease'

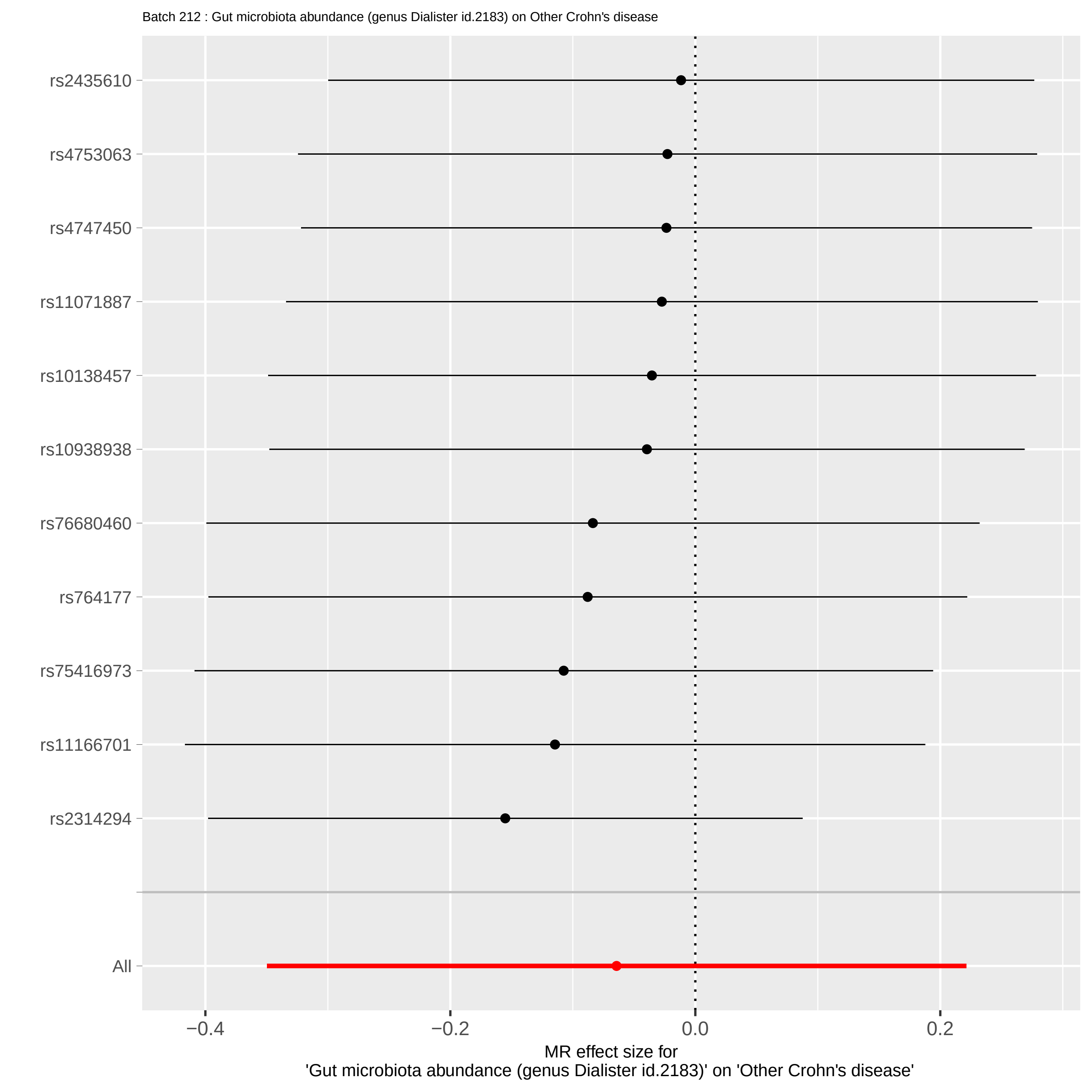
Batch 212 : Gut microbiota abundance (genus Dialister id.2183) on Other Crohn's disease
rs2435610
rs4753063
rs4747450
rs11071887
rs10138457
rs10938938
rs76680460
rs764177
rs75416973
rs11166701
rs2314294
All
−0.4
−0.2
0.0
0.2
MR effect size for
'Gut microbiota abundance (genus Dialister id.2183)' on 'Other Crohn's disease'

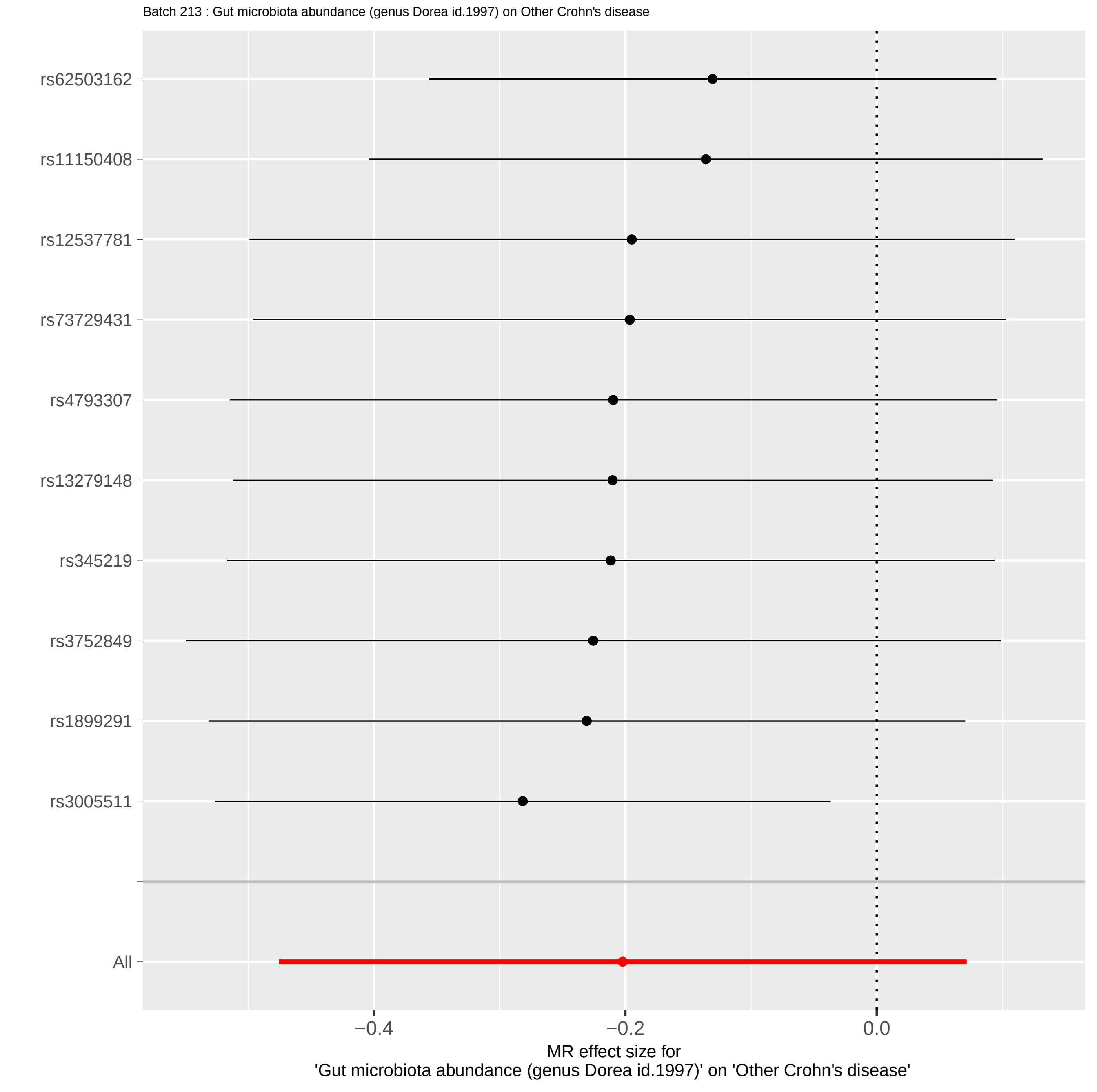
Batch 213 : Gut microbiota abundance (genus Dorea id.1997) on Other Crohn's disease
rs62503162
rs11150408
rs12537781
rs73729431
rs4793307
rs13279148
rs345219
rs3752849
rs1899291
rs3005511
All
−0.4
−0.2
0.0
MR effect size for
'Gut microbiota abundance (genus Dorea id.1997)' on 'Other Crohn's disease'

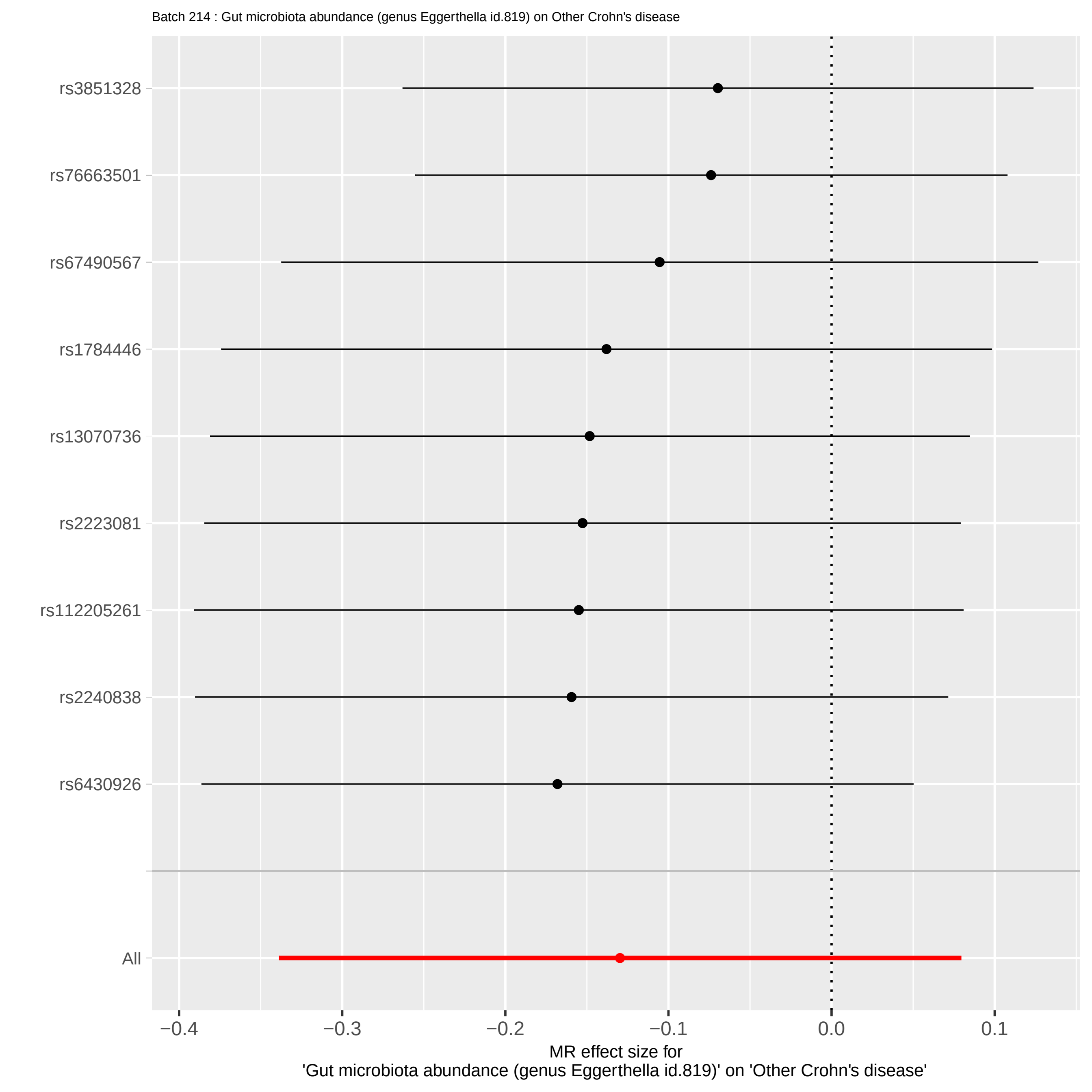
Batch 214 : Gut microbiota abundance (genus Eggerthella id.819) on Other Crohn's disease
rs3851328
rs76663501
rs67490567
rs1784446
rs13070736
rs2223081
rs112205261
rs2240838
rs6430926
All
−0.4
−0.3
−0.2
−0.1
0.0
0.1
MR effect size for
'Gut microbiota abundance (genus Eggerthella id.819)' on 'Other Crohn's disease'

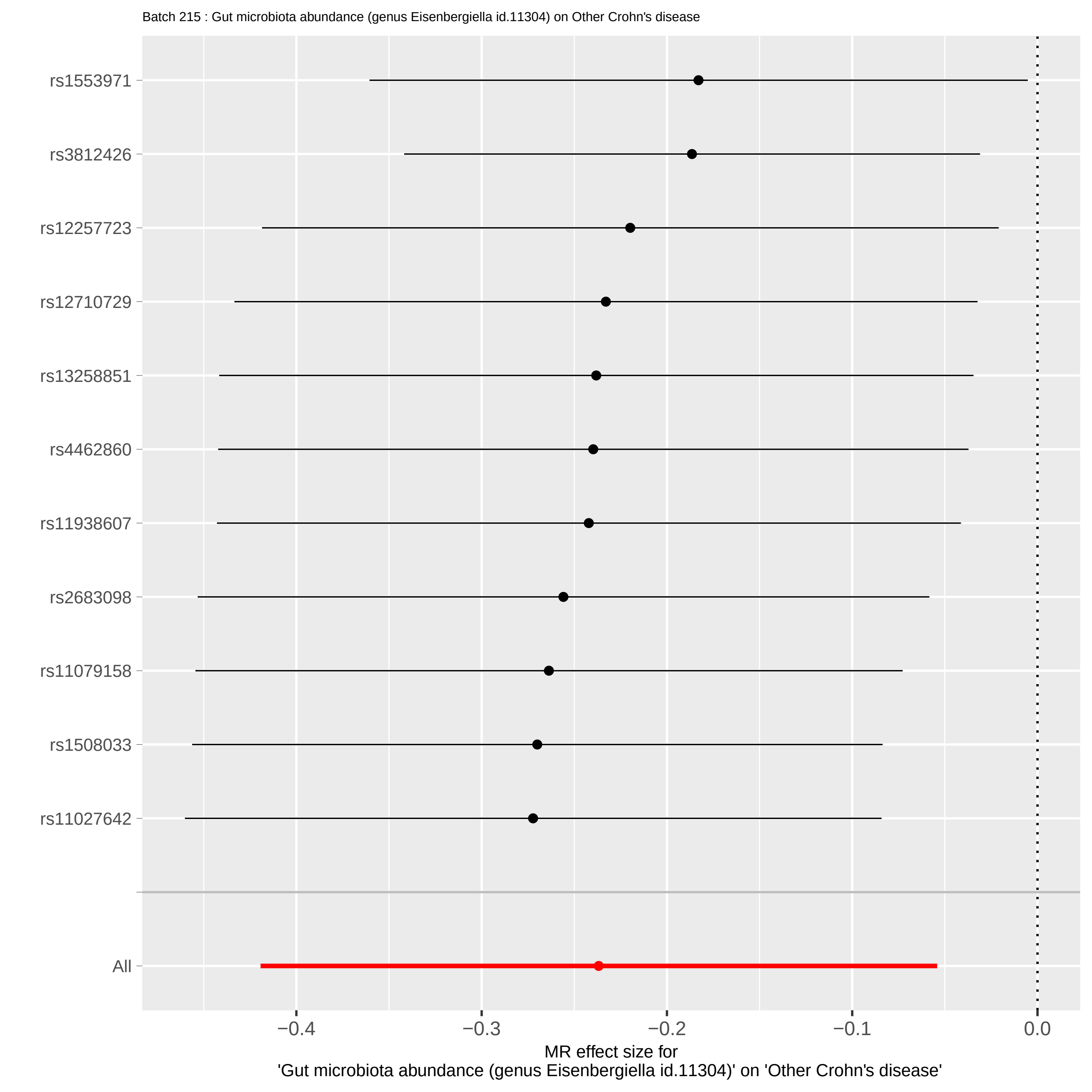
Batch 215 : Gut microbiota abundance (genus Eisenbergiella id.11304) on Other Crohn's disease
rs1553971
rs3812426
rs12257723
rs12710729
rs13258851
rs4462860
rs11938607
rs2683098
rs11079158
rs1508033
rs11027642
All
−0.4
−0.3
−0.2
−0.1
0.0
MR effect size for
'Gut microbiota abundance (genus Eisenbergiella id.11304)' on 'Other Crohn's disease'

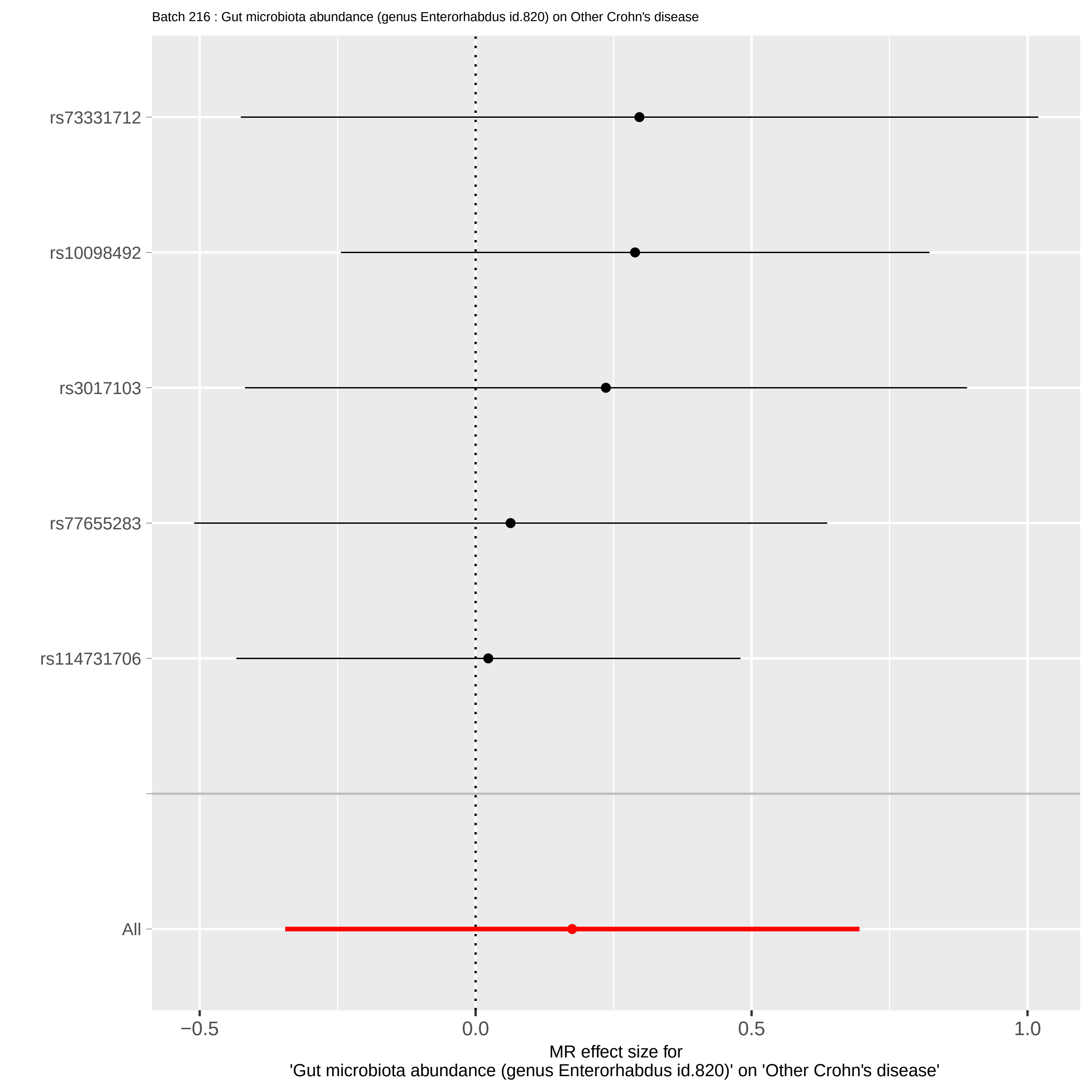

Batch 216 : Gut microbiota abundance (genus Enterorhabdus id.820) on Other Crohn's disease
rs73331712
rs10098492
rs3017103
rs77655283
rs114731706
All
−0.5
0.0
0.5
1.0
MR effect size for
'Gut microbiota abundance (genus Enterorhabdus id.820)' on 'Other Crohn's disease'

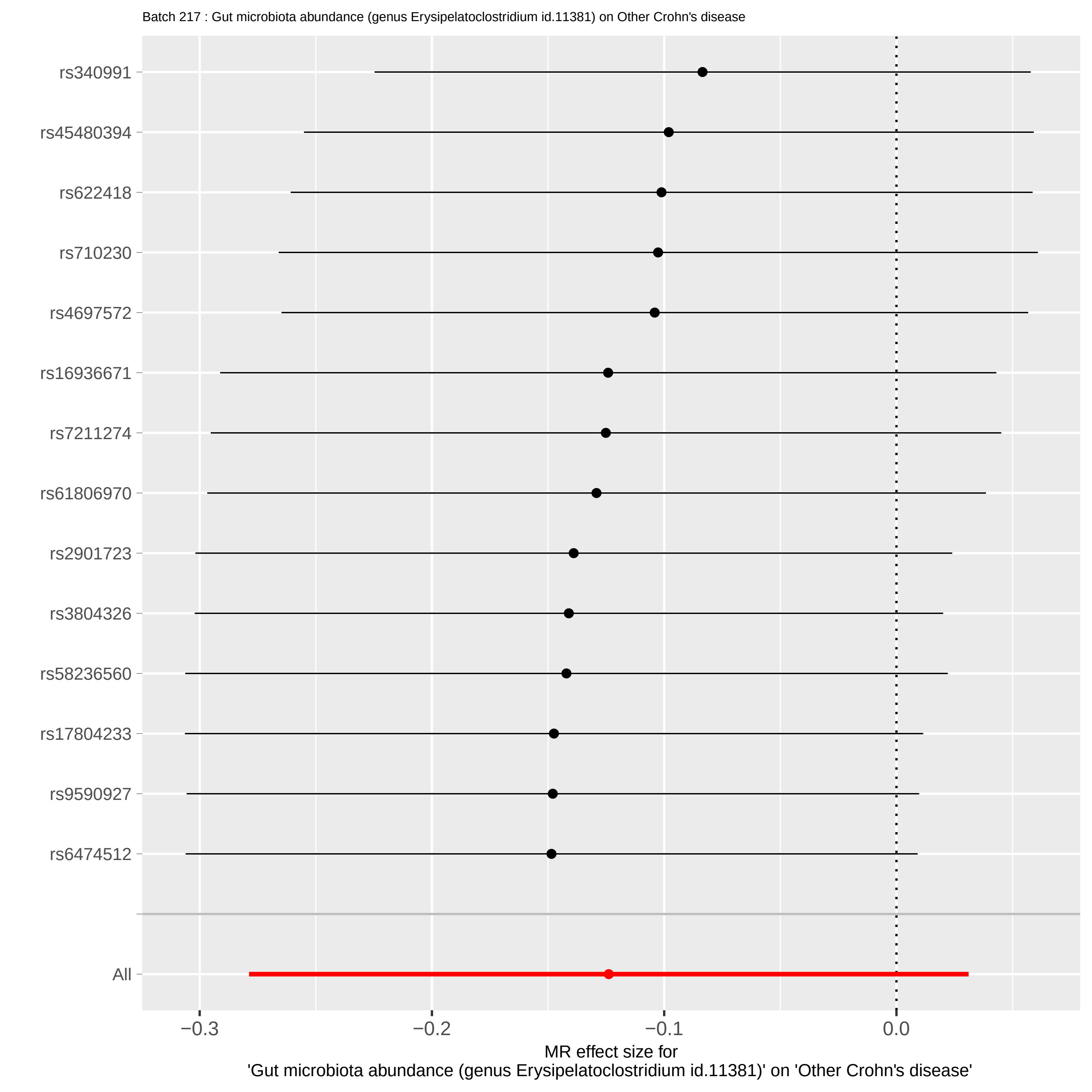

Batch 217 : Gut microbiota abundance (genus Erysipelatoclostridium id.11381) on Other Crohn's disease
rs340991
rs45480394
rs622418
rs710230
rs4697572
rs16936671
rs7211274
rs61806970
rs2901723
rs3804326
rs58236560
rs17804233
rs9590927
rs6474512
All
−0.3
−0.2
−0.1
0.0
MR effect size for
'Gut microbiota abundance (genus Erysipelatoclostridium id.11381)' on 'Other Crohn's disease'

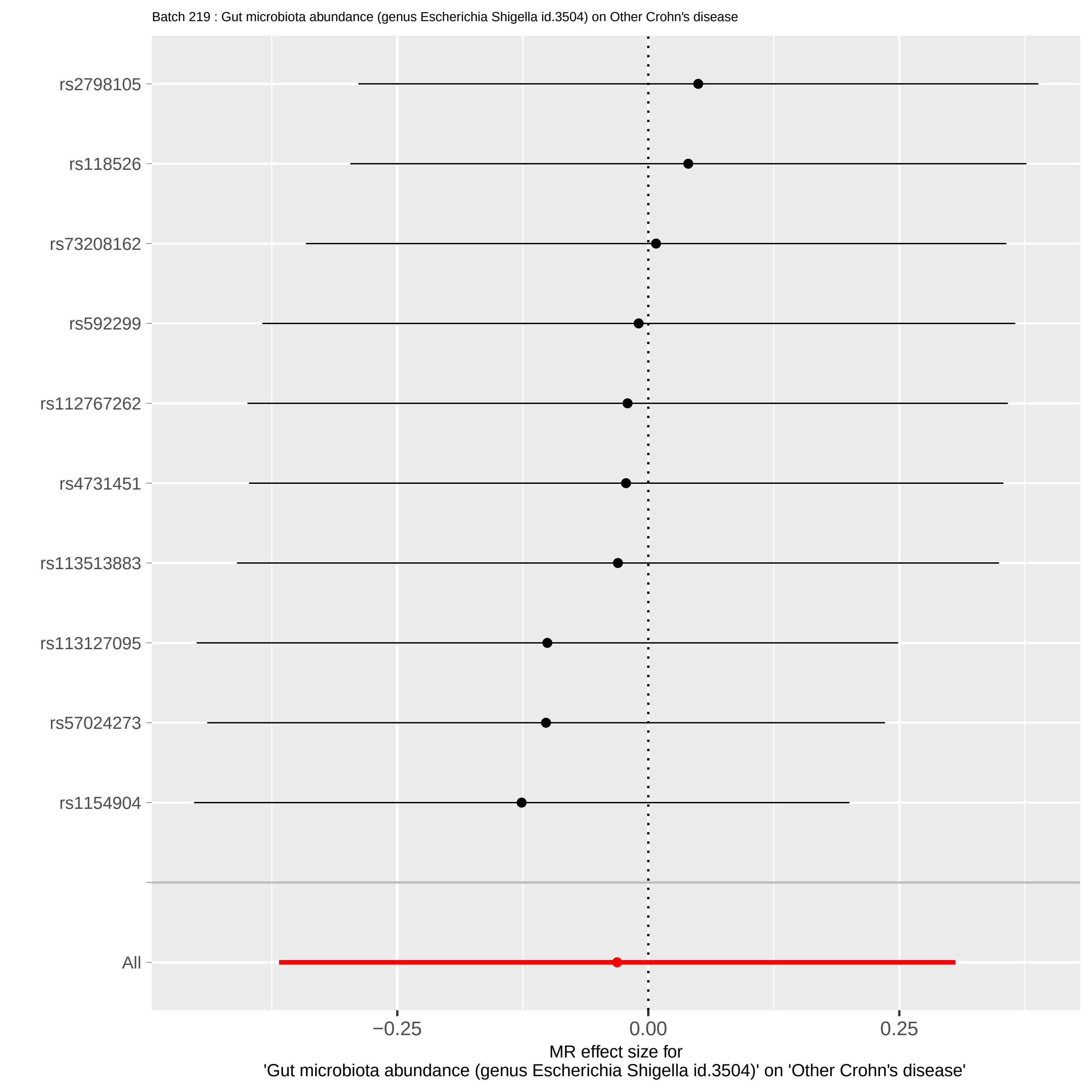

Batch 219 : Gut microbiota abundance (genus Escherichia Shigella id.3504) on Other Crohn's disease
rs2798105
rs118526
rs73208162
rs592299
rs112767262
rs4731451
rs113513883
rs113127095
rs57024273
rs1154904
All
−0.25
0.00
0.25
MR effect size for
'Gut microbiota abundance (genus Escherichia Shigella id.3504)' on 'Other Crohn's disease'

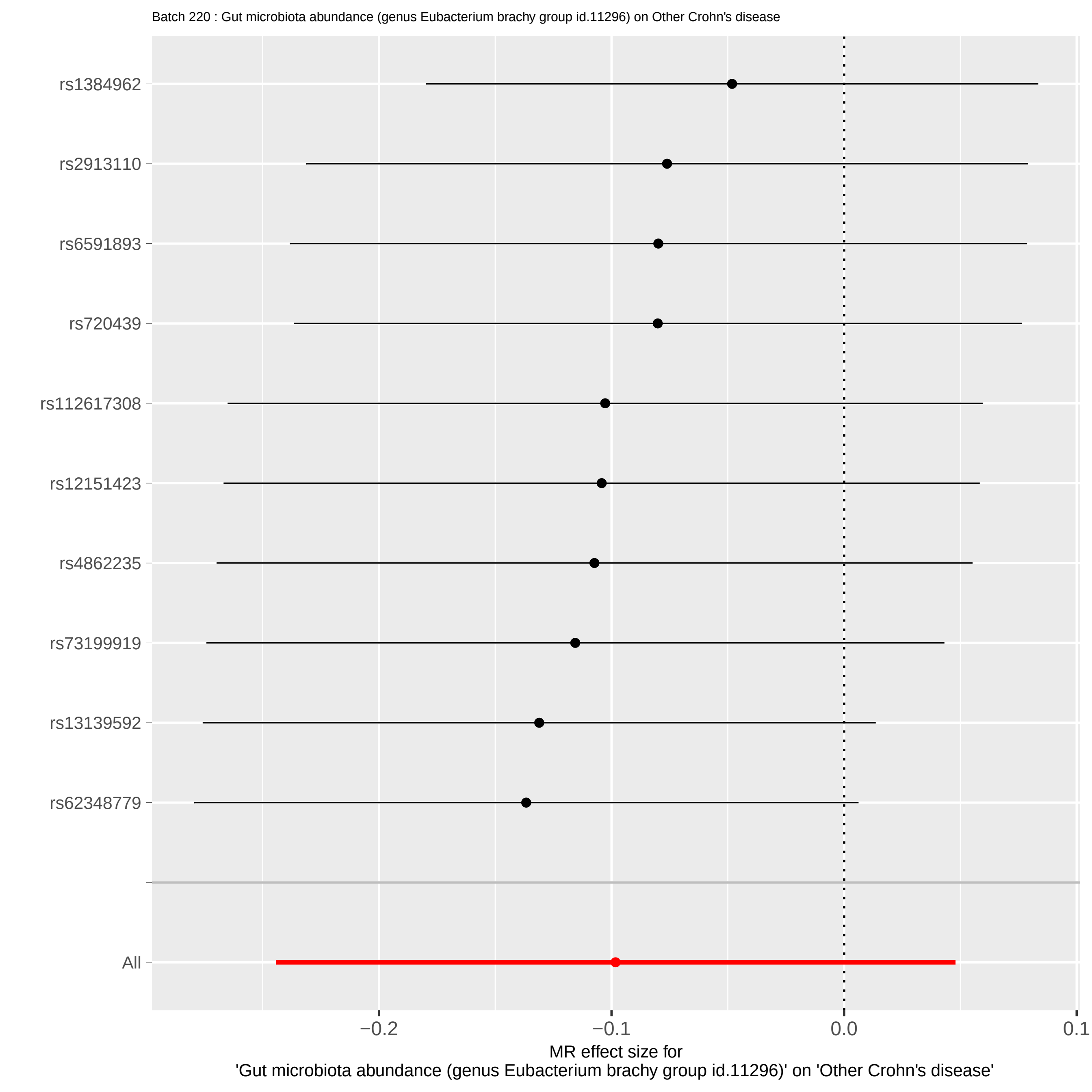

Batch 220 : Gut microbiota abundance (genus Eubacterium brachy group id.11296) on Other Crohn's disease
rs1384962
rs2913110
rs6591893
rs720439
rs112617308
rs12151423
rs4862235
rs73199919
rs13139592
rs62348779
All
−0.2
−0.1
0.0
0.1
MR effect size for
'Gut microbiota abundance (genus Eubacterium brachy group id.11296)' on 'Other Crohn's disease'

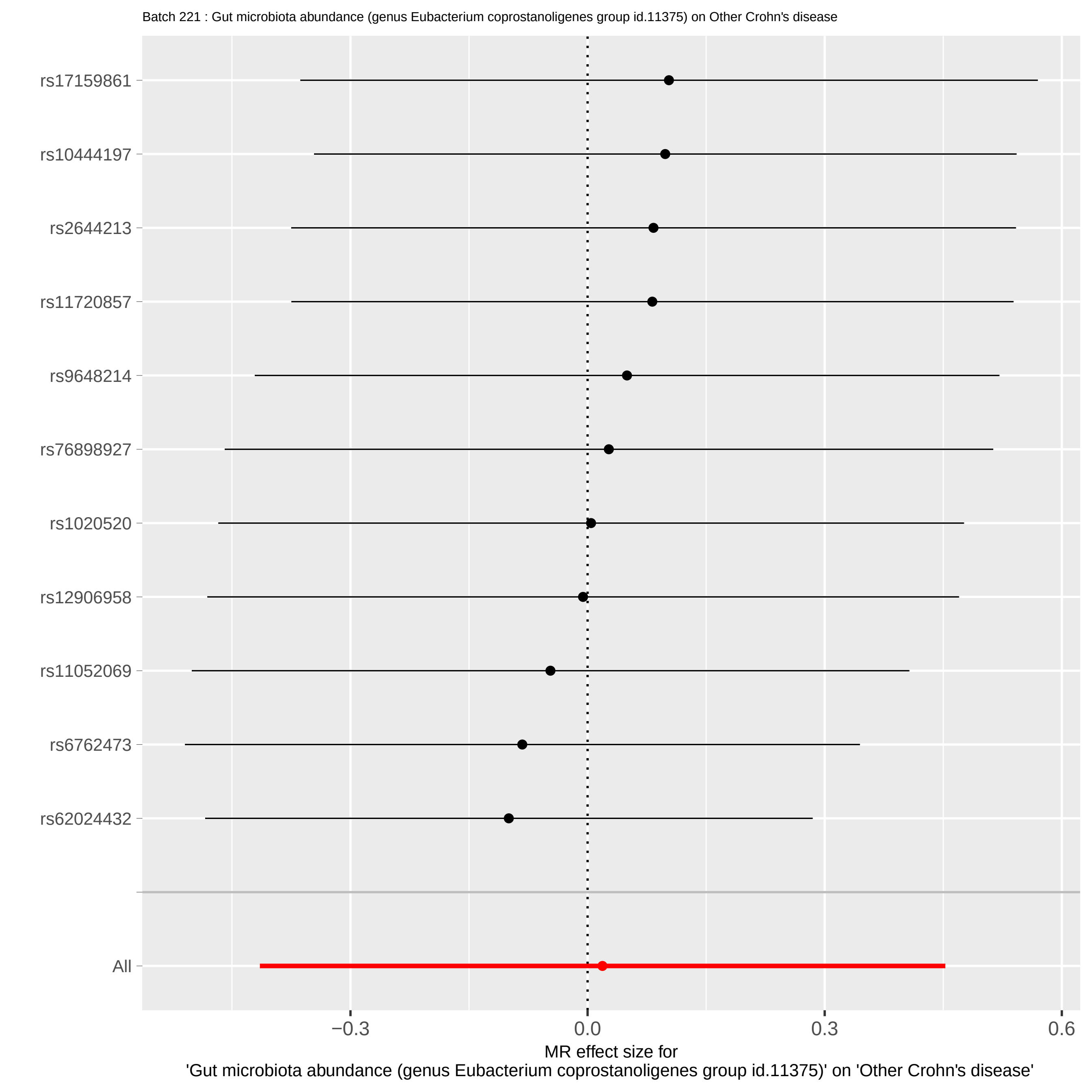

Batch 221 : Gut microbiota abundance (genus Eubacterium coprostanoligenes group id.11375) on Other Crohn's disease
rs17159861
rs10444197
rs2644213
rs11720857
rs9648214
rs76898927
rs1020520
rs12906958
rs11052069
rs6762473
rs62024432
All
−0.3
0.0
0.3
0.6
MR effect size for
'Gut microbiota abundance (genus Eubacterium coprostanoligenes group id.11375)' on 'Other Crohn's disease'

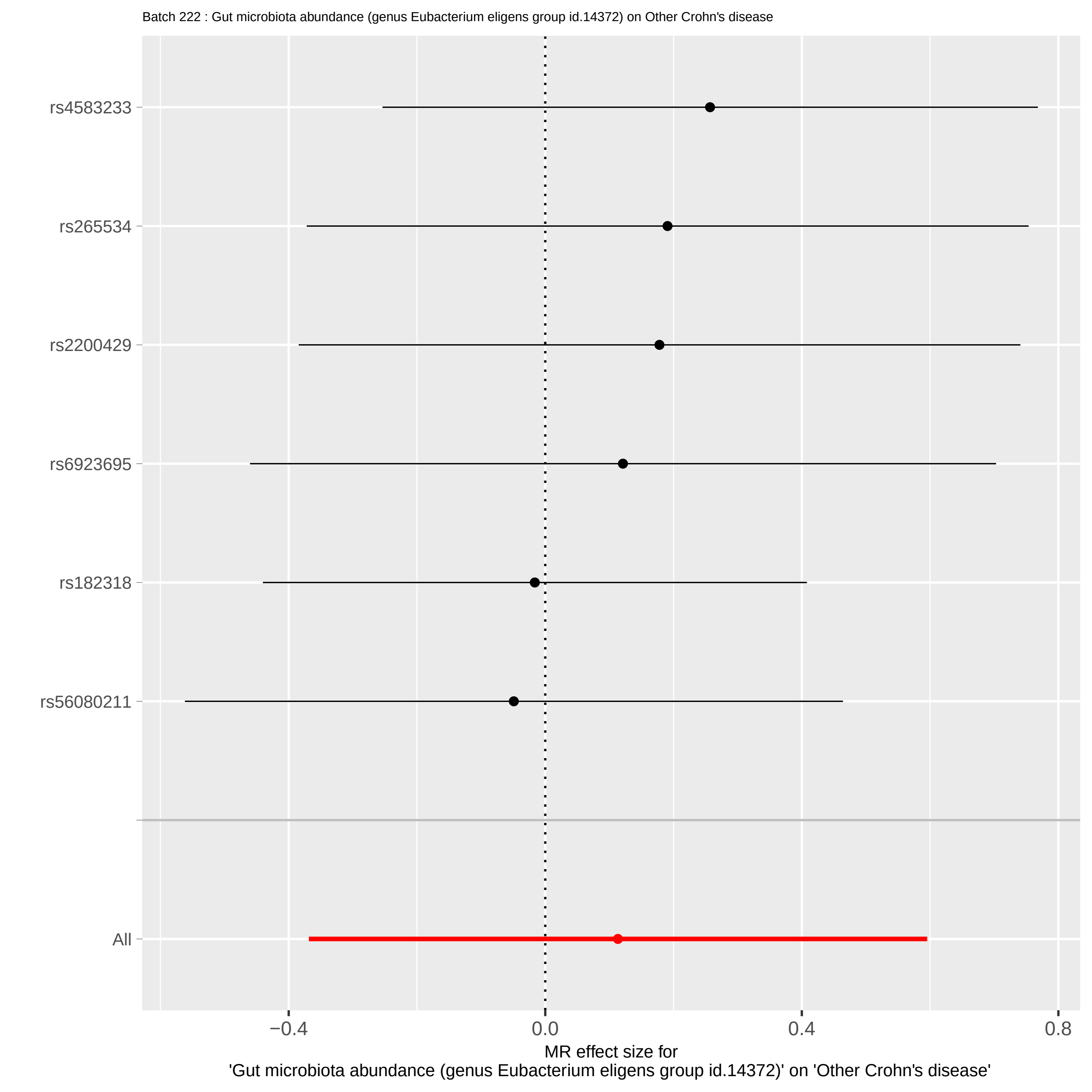
Batch 222 : Gut microbiota abundance (genus Eubacterium eligens group id.14372) on Other Crohn's disease
rs4583233
rs265534
rs2200429
rs6923695
rs182318
rs56080211
All
−0.4
0.0
0.4
0.8
MR effect size for
'Gut microbiota abundance (genus Eubacterium eligens group id.14372)' on 'Other Crohn's disease'

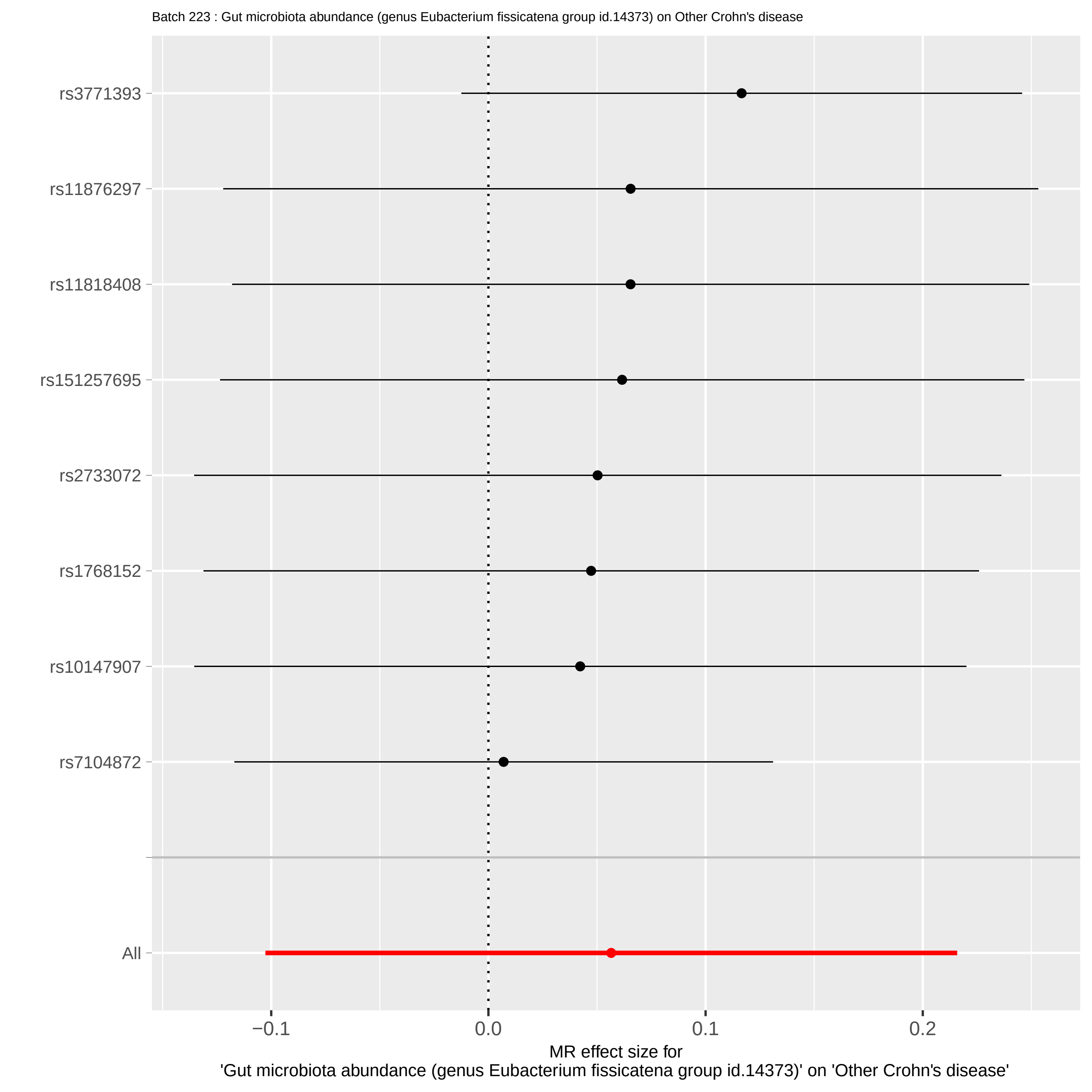

Batch 223 : Gut microbiota abundance (genus Eubacterium fissicatena group id.14373) on Other Crohn's disease
rs3771393
rs11876297
rs11818408
rs151257695
rs2733072
rs1768152
rs10147907
rs7104872
All
−0.1
0.0
0.1
0.2
MR effect size for
'Gut microbiota abundance (genus Eubacterium fissicatena group id.14373)' on 'Other Crohn's disease'

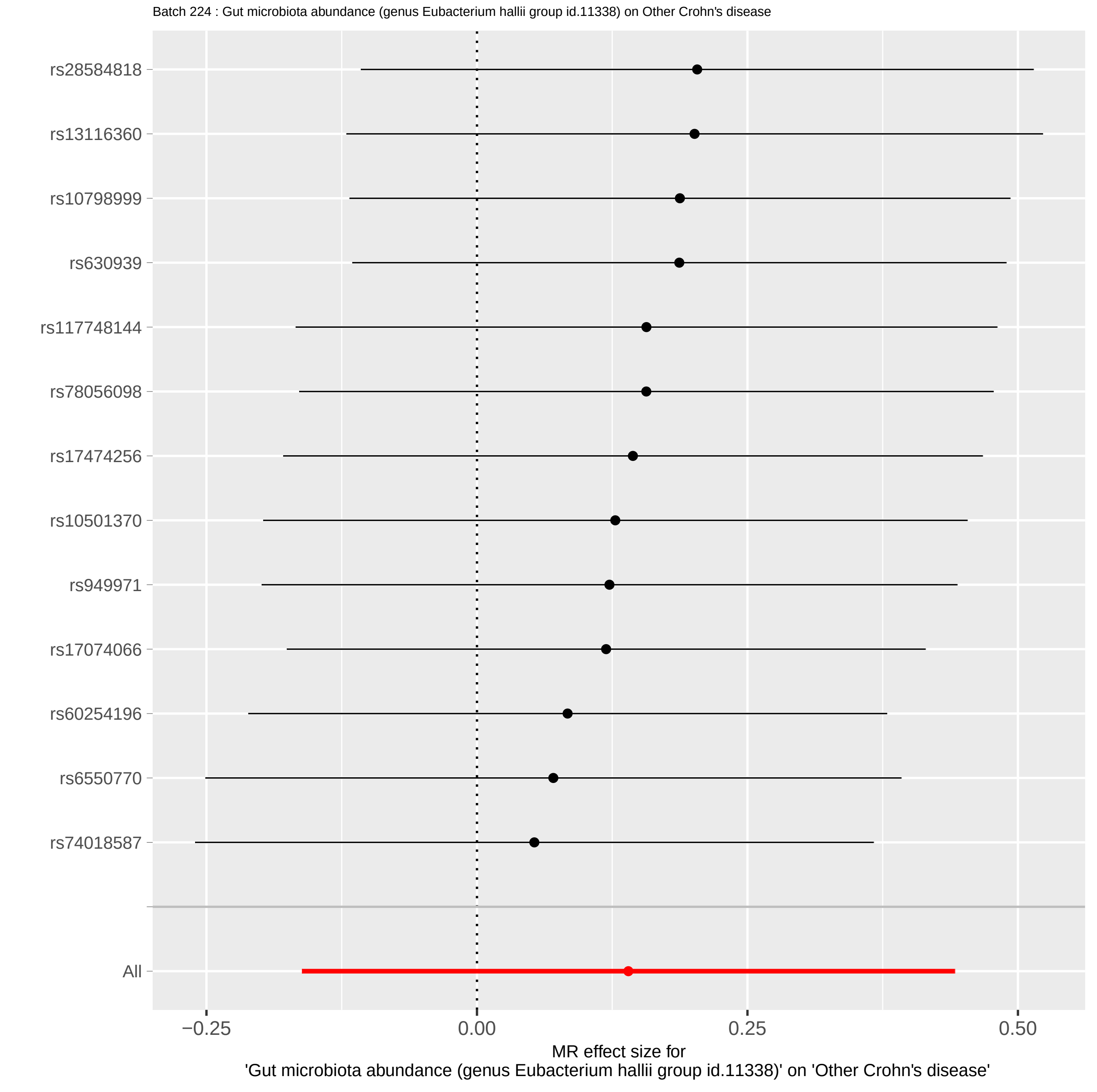
Batch 224 : Gut microbiota abundance (genus Eubacterium hallii group id.11338) on Other Crohn's disease
rs28584818
rs13116360
rs10798999
rs630939
rs117748144
rs78056098
rs17474256
rs10501370
rs949971
rs17074066
rs60254196
rs6550770
rs74018587
All
−0.25
0.00
0.25
0.50
MR effect size for
'Gut microbiota abundance (genus Eubacterium hallii group id.11338)' on 'Other Crohn's disease'

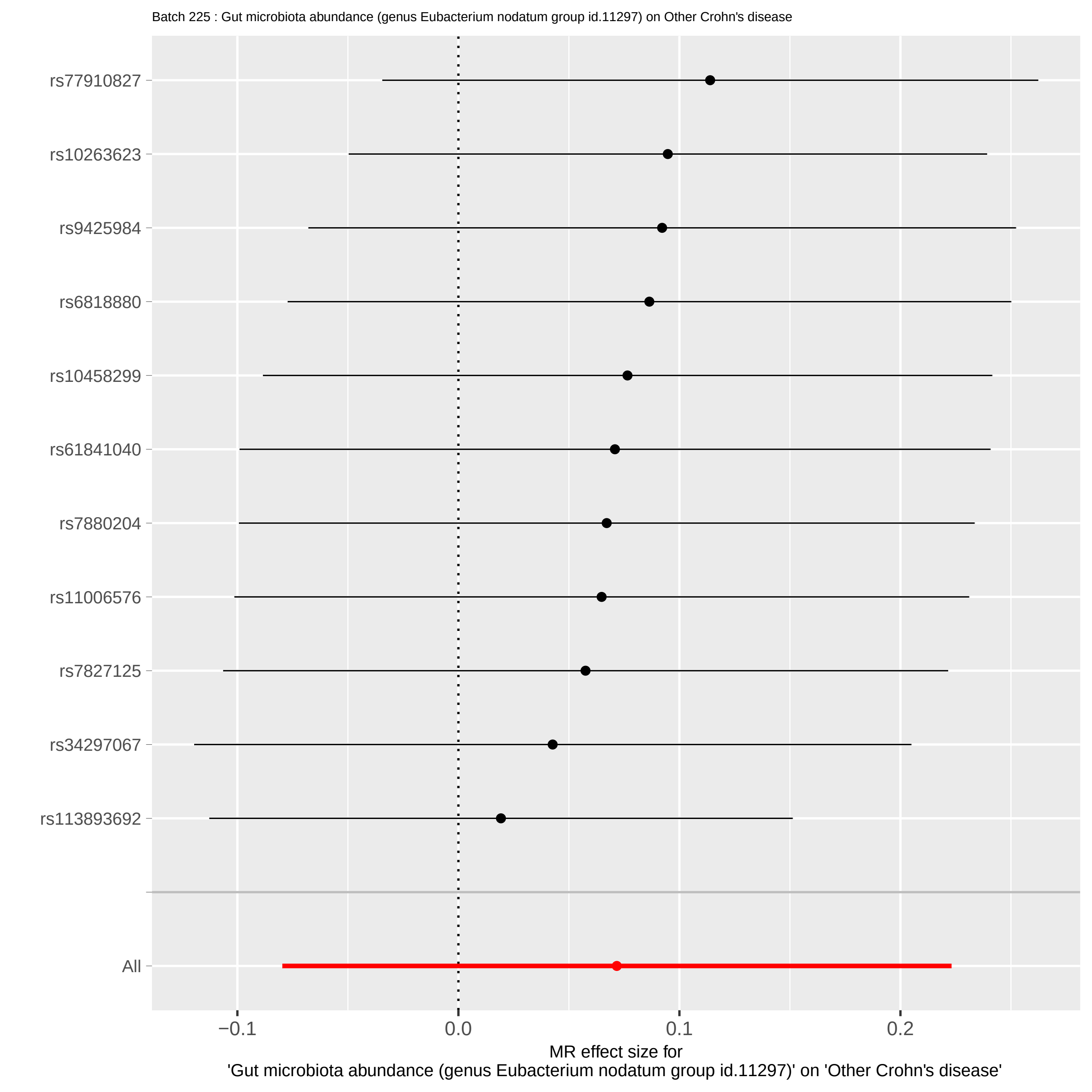
Batch 225 : Gut microbiota abundance (genus Eubacterium nodatum group id.11297) on Other Crohn's disease
rs77910827
rs10263623
rs9425984
rs6818880
rs10458299
rs61841040
rs7880204
rs11006576
rs7827125
rs34297067
rs113893692
All
−0.1
0.0
0.1
0.2
MR effect size for
'Gut microbiota abundance (genus Eubacterium nodatum group id.11297)' on 'Other Crohn's disease'

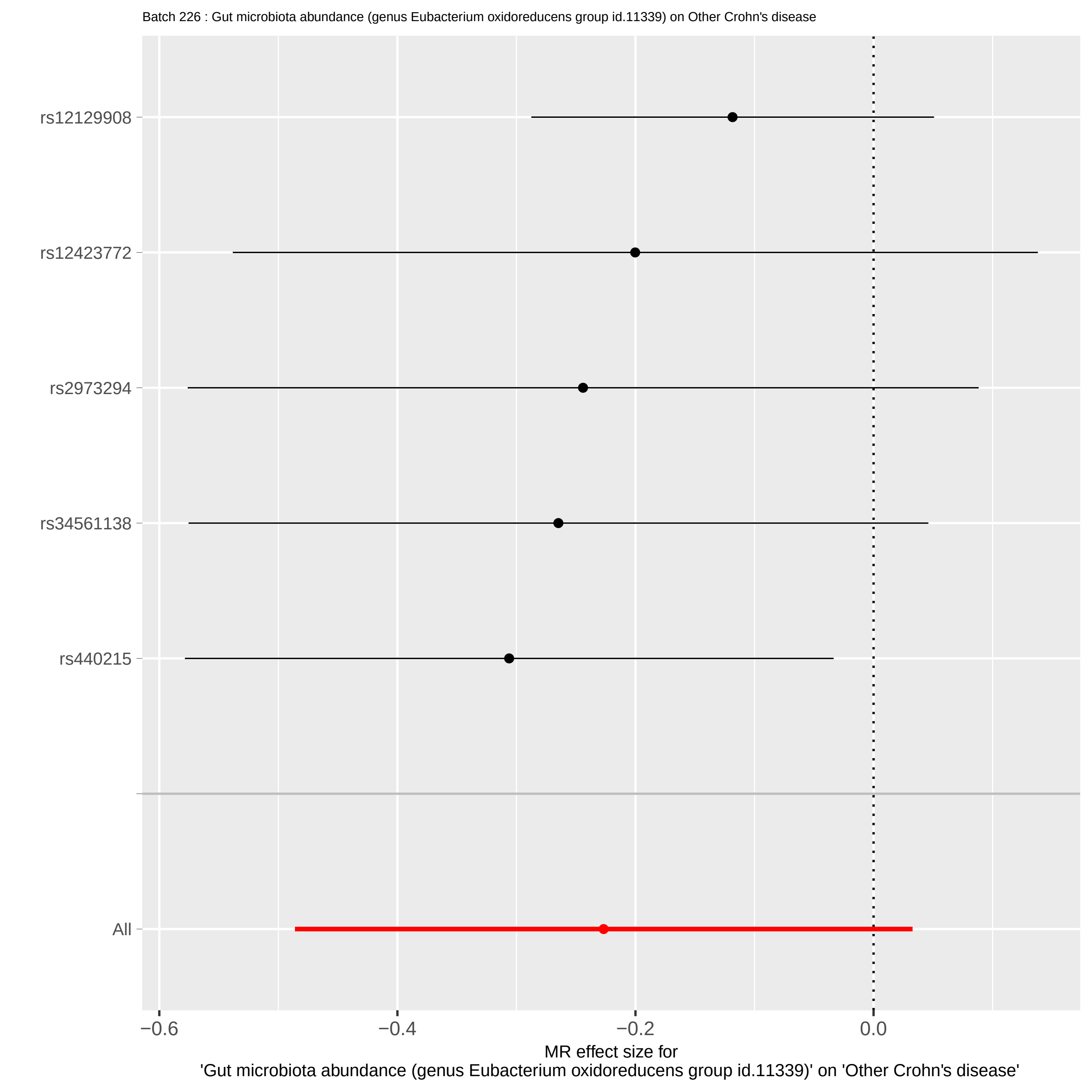

Batch 226 : Gut microbiota abundance (genus Eubacterium oxidoreducens group id.11339) on Other Crohn's disease
rs12129908
rs12423772
rs2973294
rs34561138
rs440215
All
−0.6
−0.4
−0.2
0.0
MR effect size for
'Gut microbiota abundance (genus Eubacterium oxidoreducens group id.11339)' on 'Other Crohn's disease'

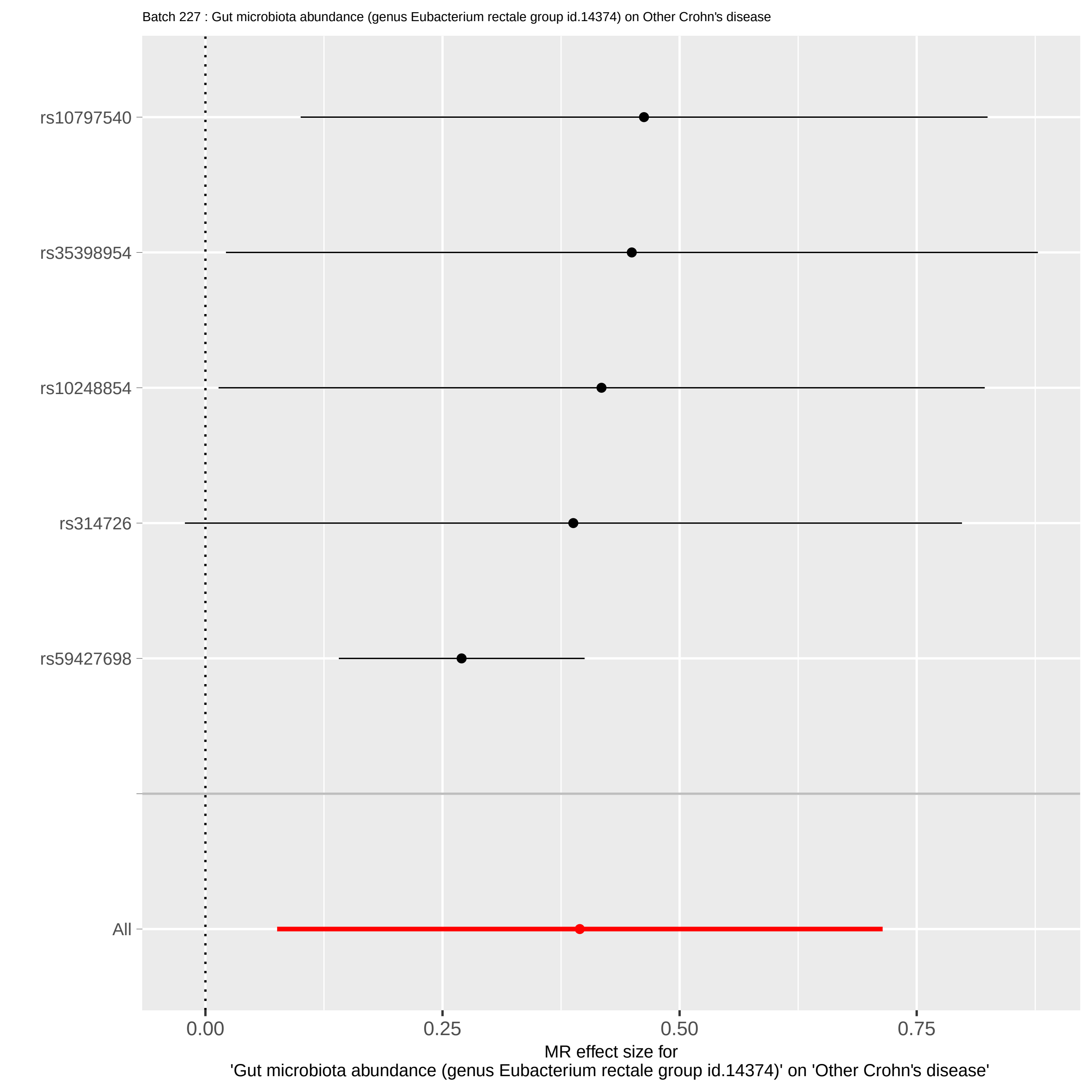

Batch 227 : Gut microbiota abundance (genus Eubacterium rectale group id.14374) on Other Crohn's disease
rs10797540
rs35398954
rs10248854
rs314726
rs59427698
All
0.00
0.25
0.50
0.75
MR effect size for
'Gut microbiota abundance (genus Eubacterium rectale group id.14374)' on 'Other Crohn's disease'

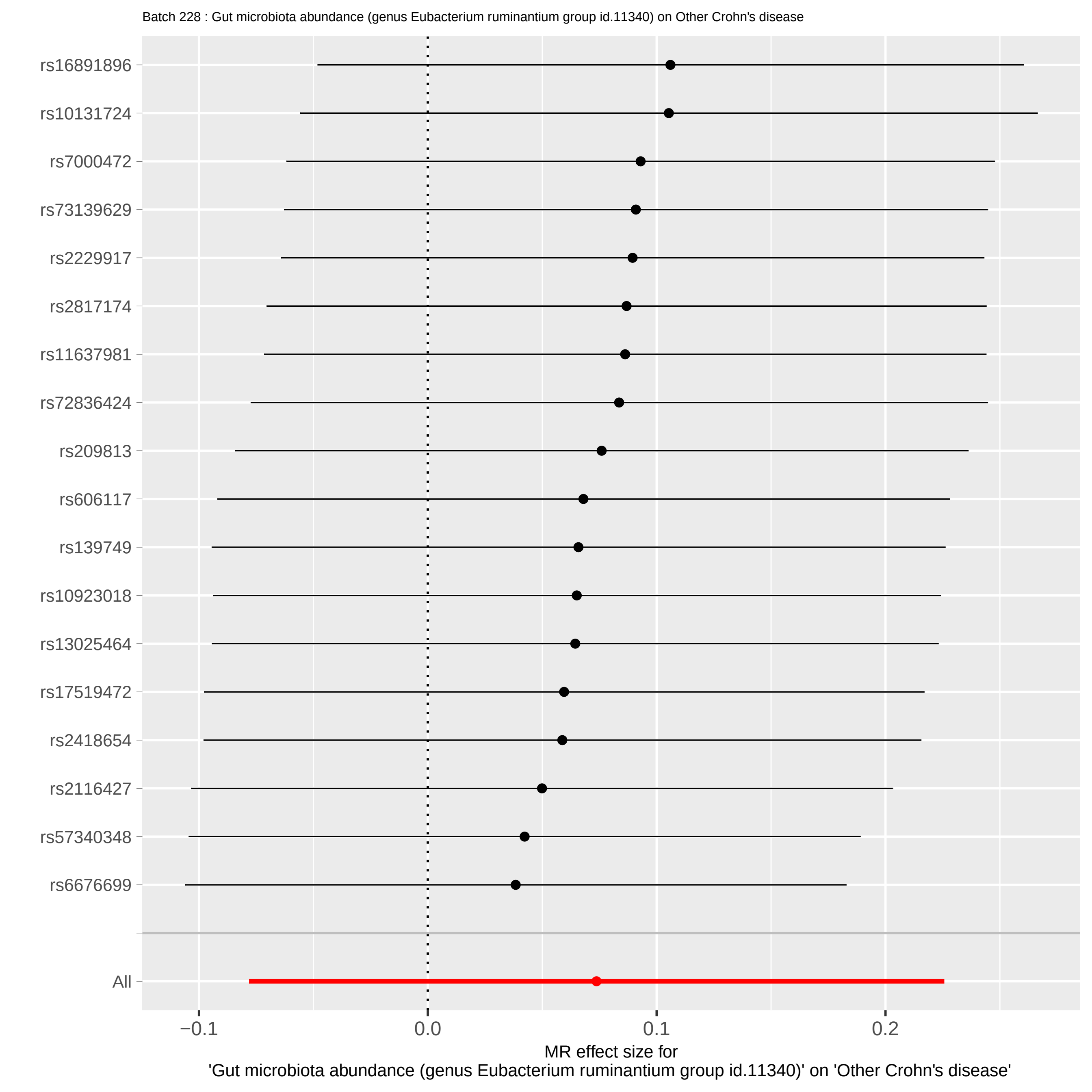

Batch 228 : Gut microbiota abundance (genus Eubacterium ruminantium group id.11340) on Other Crohn's disease
rs16891896
rs10131724
rs7000472
rs73139629
rs2229917
rs2817174
rs11637981
rs72836424
rs209813
rs606117
rs139749
rs10923018
rs13025464
rs17519472
rs2418654
rs2116427
rs57340348
rs6676699
All
−0.1
0.0
0.1
0.2
MR effect size for
'Gut microbiota abundance (genus Eubacterium ruminantium group id.11340)' on 'Other Crohn's disease'

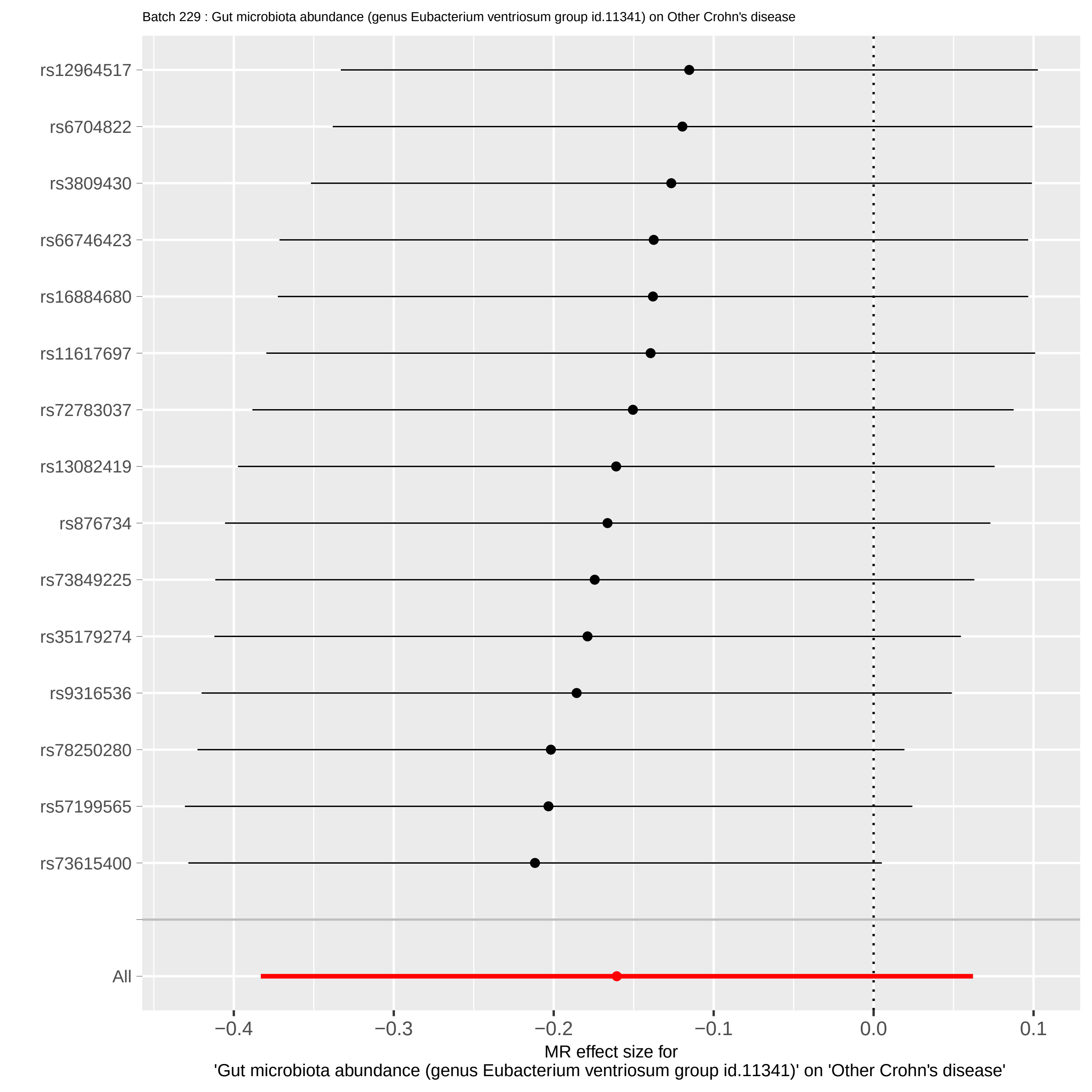

Batch 229 : Gut microbiota abundance (genus Eubacterium ventriosum group id.11341) on Other Crohn's disease
rs12964517
rs6704822
rs3809430
rs66746423
rs16884680
rs11617697
rs72783037
rs13082419
rs876734
rs73849225
rs35179274
rs9316536
rs78250280
rs57199565
rs73615400
All
−0.4
−0.3
−0.2
−0.1
0.0
0.1
MR effect size for
'Gut microbiota abundance (genus Eubacterium ventriosum group id.11341)' on 'Other Crohn's disease'

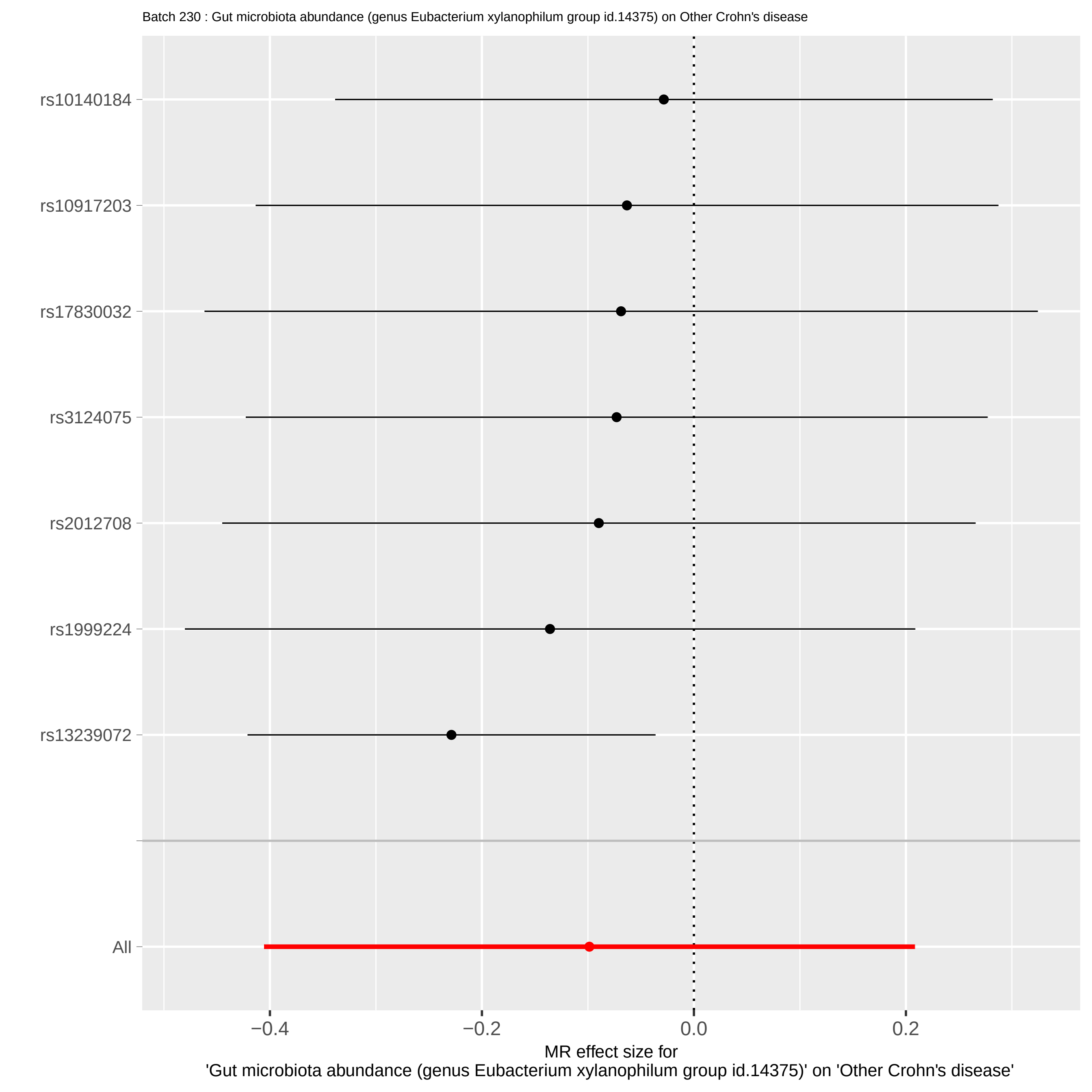
Batch 230 : Gut microbiota abundance (genus Eubacterium xylanophilum group id.14375) on Other Crohn's disease
rs10140184
rs10917203
rs17830032
rs3124075
rs2012708
rs1999224
rs13239072
All
−0.4
−0.2
0.0
0.2
MR effect size for
'Gut microbiota abundance (genus Eubacterium xylanophilum group id.14375)' on 'Other Crohn's disease'

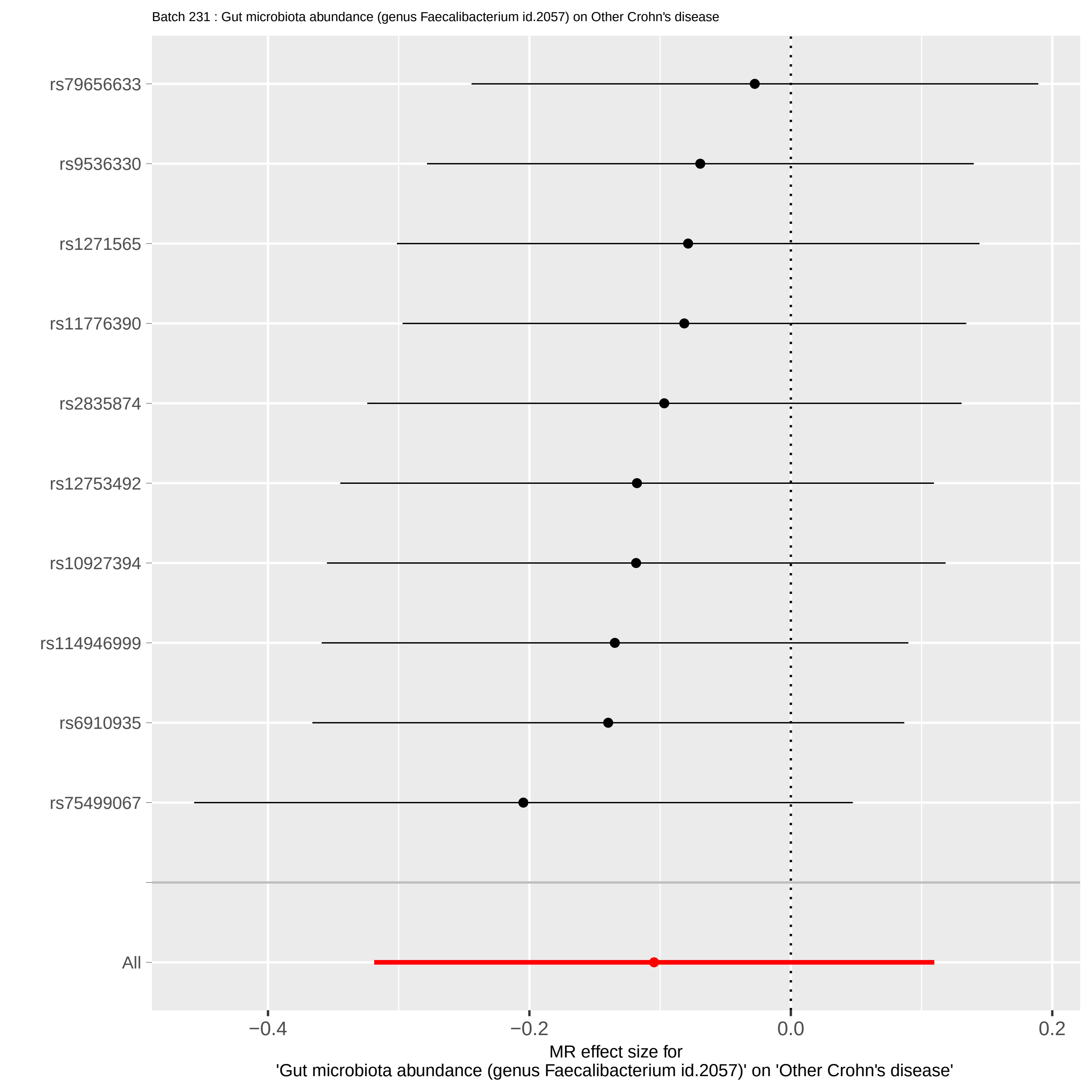
Batch 231 : Gut microbiota abundance (genus Faecalibacterium id.2057) on Other Crohn's disease
rs79656633
rs9536330
rs1271565
rs11776390
rs2835874
rs12753492
rs10927394
rs114946999
rs6910935
rs75499067
All
−0.4
−0.2
0.0
0.2
MR effect size for
'Gut microbiota abundance (genus Faecalibacterium id.2057)' on 'Other Crohn's disease'

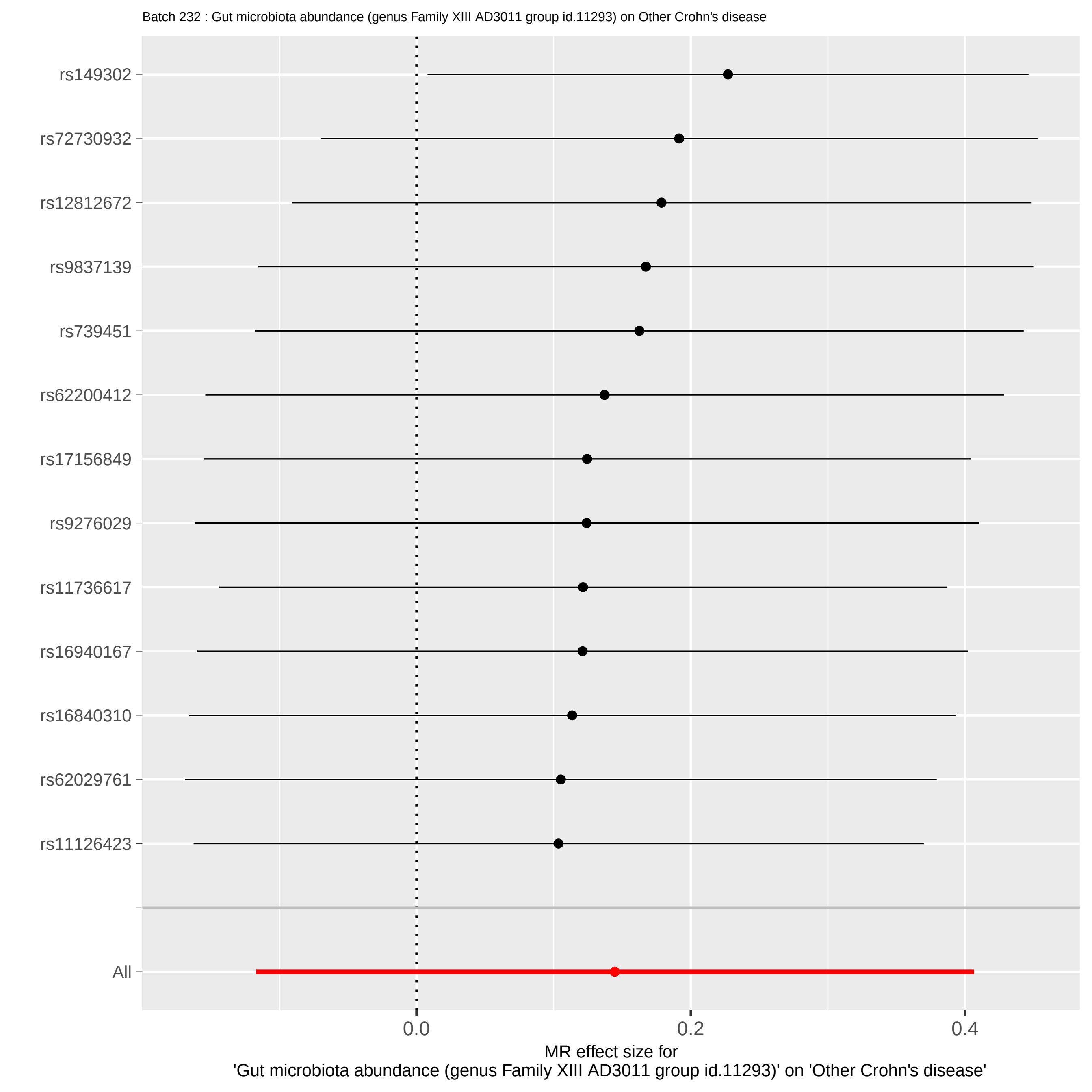

Batch 232 : Gut microbiota abundance (genus Family XIII AD3011 group id.11293) on Other Crohn's disease
rs149302
rs72730932
rs12812672
rs9837139
rs739451
rs62200412
rs17156849
rs9276029
rs11736617
rs16940167
rs16840310
rs62029761
rs11126423
All
0.0
0.2
0.4
MR effect size for
'Gut microbiota abundance (genus Family XIII AD3011 group id.11293)' on 'Other Crohn's disease'

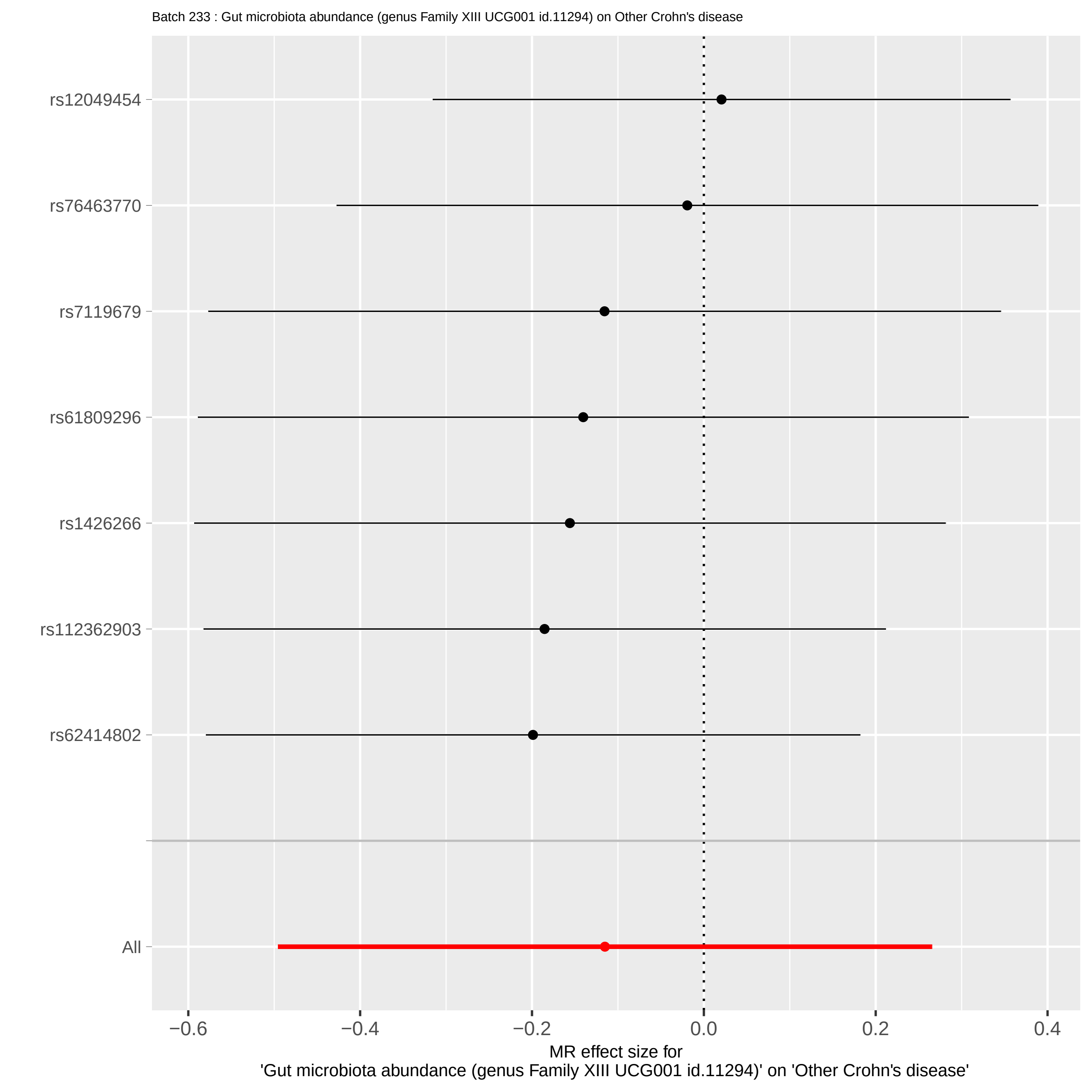
Batch 233 : Gut microbiota abundance (genus Family XIII UCG001 id.11294) on Other Crohn's disease
rs12049454
rs76463770
rs7119679
rs61809296
rs1426266
rs112362903
rs62414802
All
−0.6
−0.4
−0.2
0.0
0.2
0.4
MR effect size for
'Gut microbiota abundance (genus Family XIII UCG001 id.11294)' on 'Other Crohn's disease'

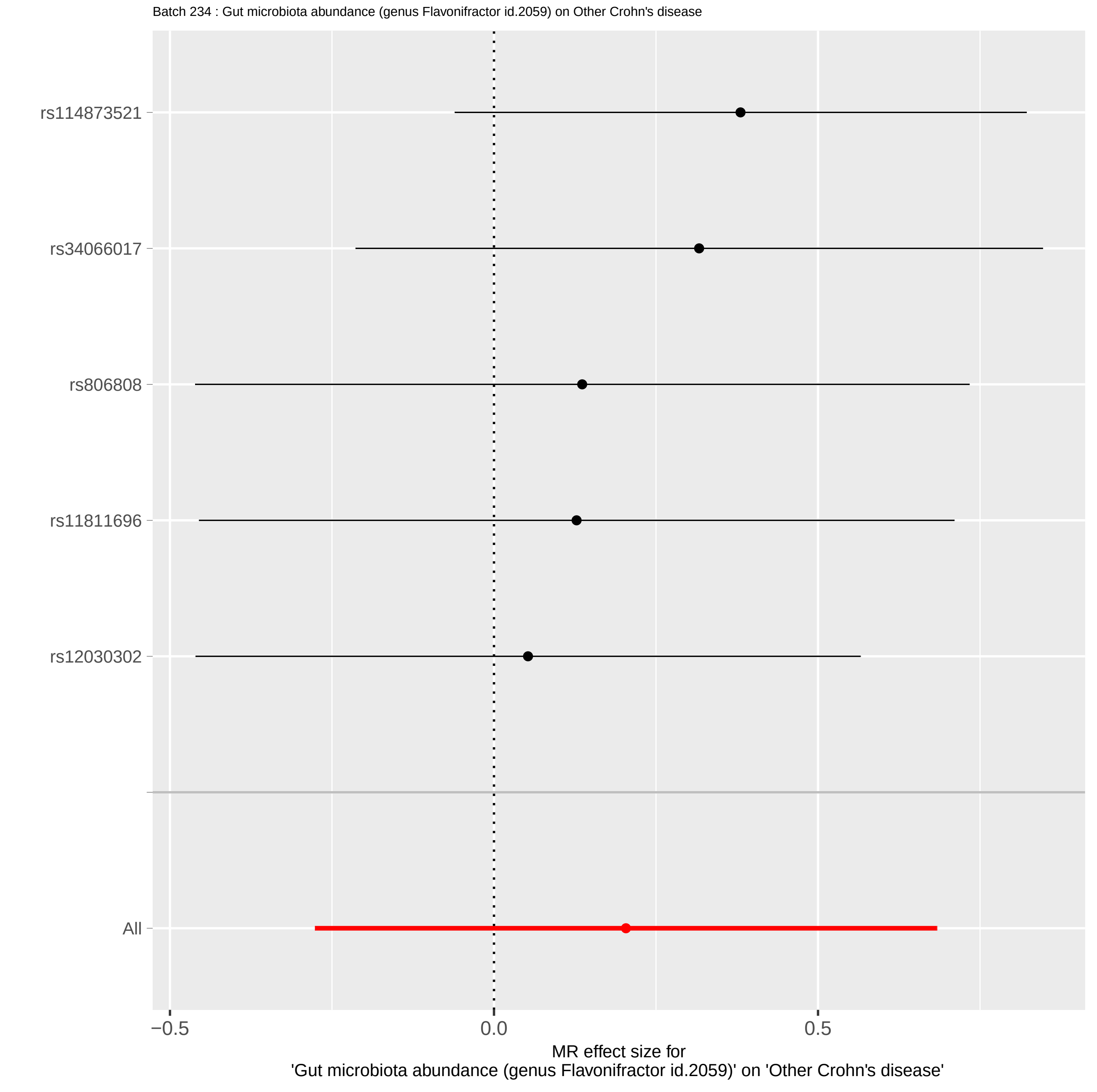
Batch 234 : Gut microbiota abundance (genus Flavonifractor id.2059) on Other Crohn's disease
rs114873521
rs34066017
rs806808
rs11811696
rs12030302
All
−0.5
0.0
0.5
MR effect size for
'Gut microbiota abundance (genus Flavonifractor id.2059)' on 'Other Crohn's disease'

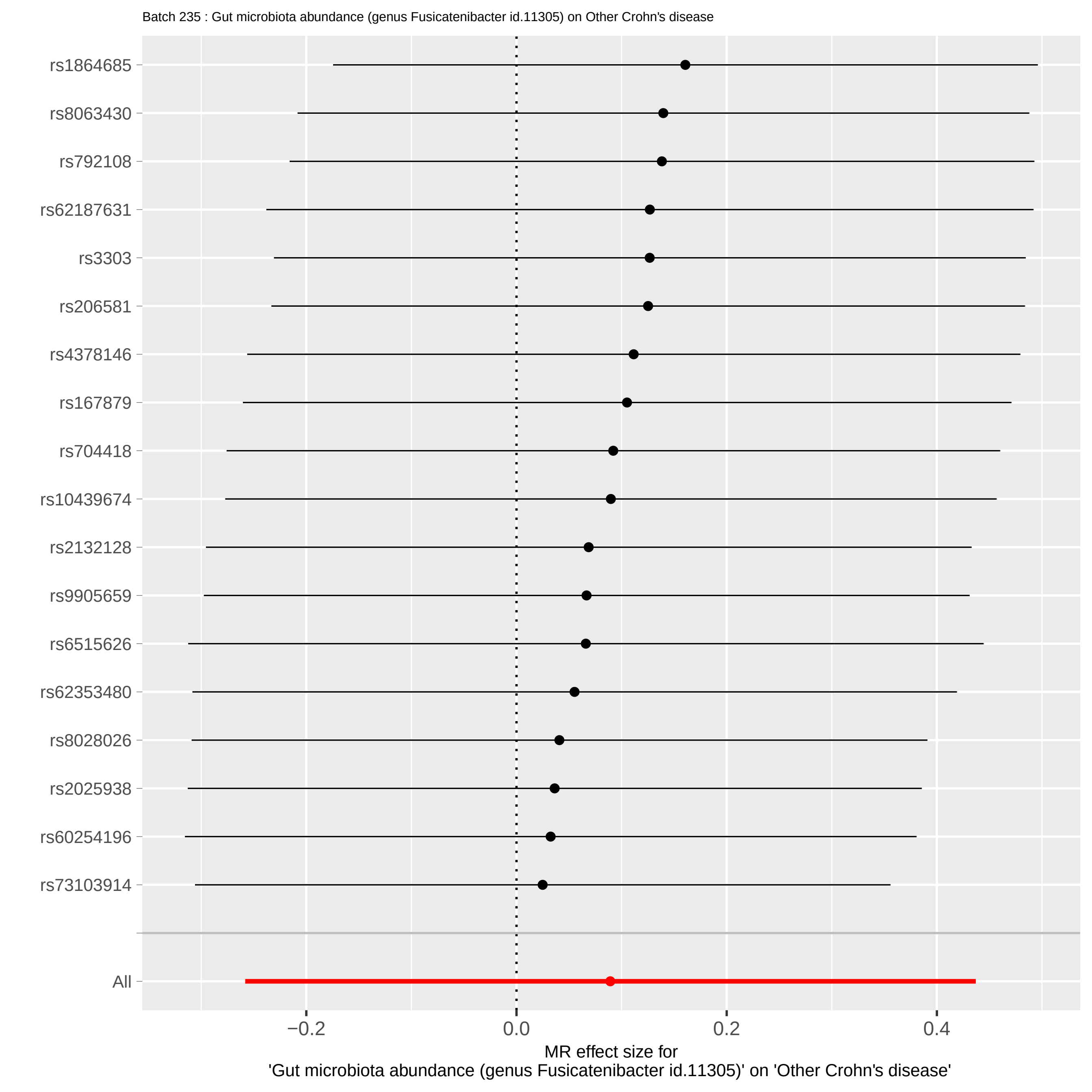
Batch 235 : Gut microbiota abundance (genus Fusicatenibacter id.11305) on Other Crohn's disease
rs1864685
rs8063430
rs792108
rs62187631
rs3303
rs206581
rs4378146
rs167879
rs704418
rs10439674
rs2132128
rs9905659
rs6515626
rs62353480
rs8028026
rs2025938
rs60254196
rs73103914
All
−0.2
0.0
0.2
0.4
MR effect size for
'Gut microbiota abundance (genus Fusicatenibacter id.11305)' on 'Other Crohn's disease'

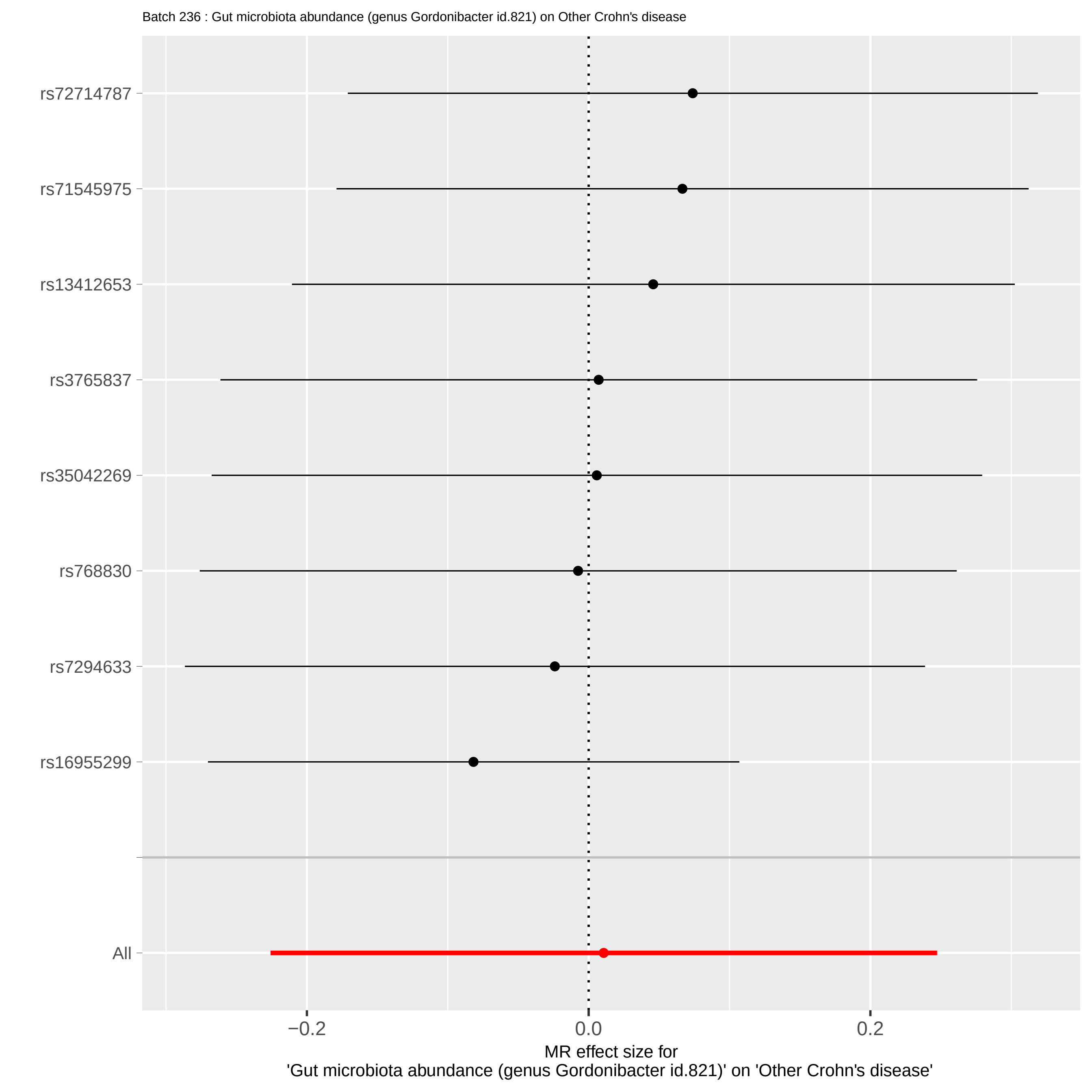

Batch 236 : Gut microbiota abundance (genus Gordonibacter id.821) on Other Crohn's disease
rs72714787
rs71545975
rs13412653
rs3765837
rs35042269
rs768830
rs7294633
rs16955299
All
−0.2
0.0
0.2
MR effect size for
'Gut microbiota abundance (genus Gordonibacter id.821)' on 'Other Crohn's disease'

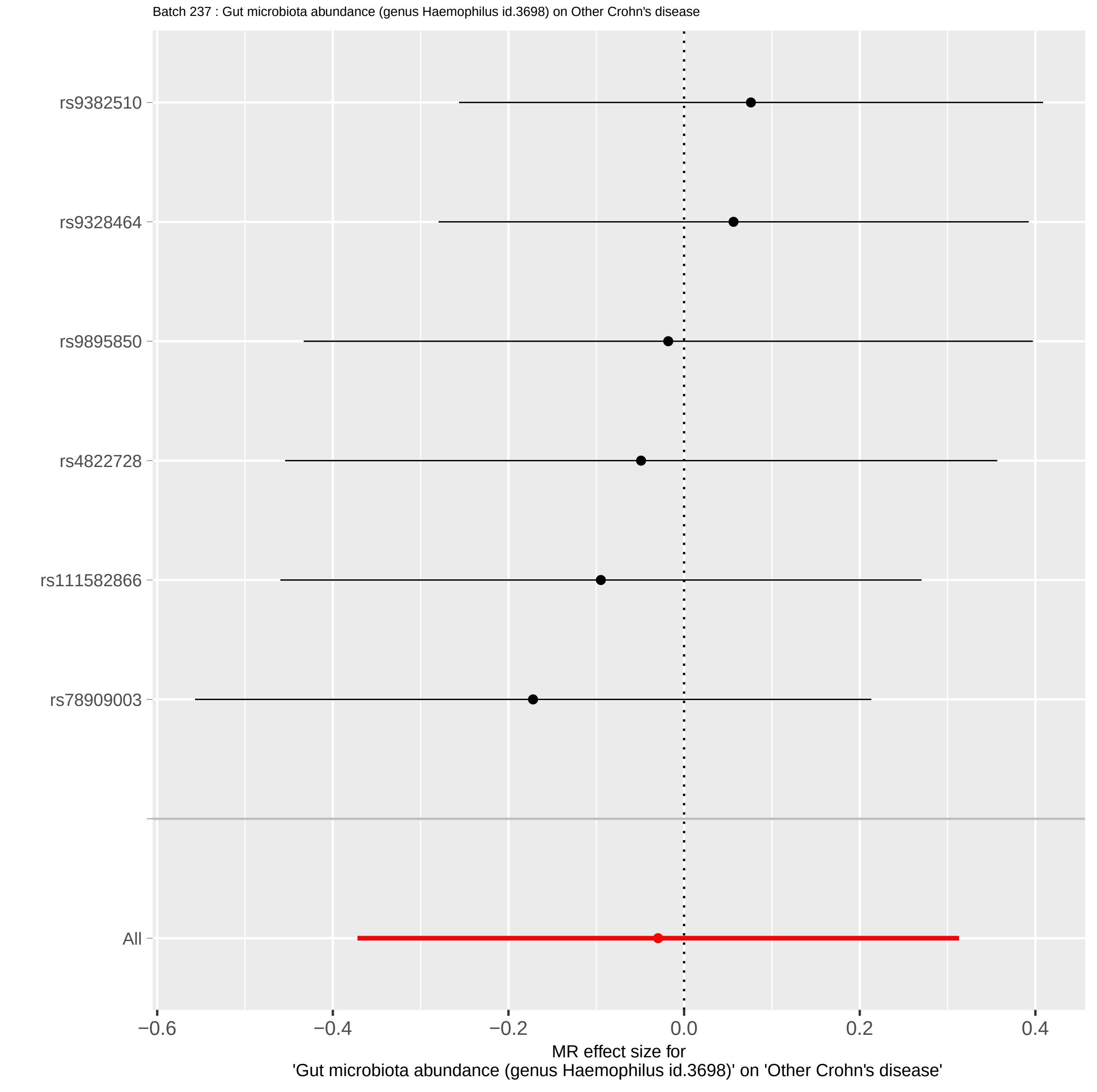
Batch 237 : Gut microbiota abundance (genus Haemophilus id.3698) on Other Crohn's disease
rs9382510
rs9328464
rs9895850
rs4822728
rs111582866
rs78909003
All
−0.6
−0.4
−0.2
0.0
0.2
0.4
MR effect size for
'Gut microbiota abundance (genus Haemophilus id.3698)' on 'Other Crohn's disease'

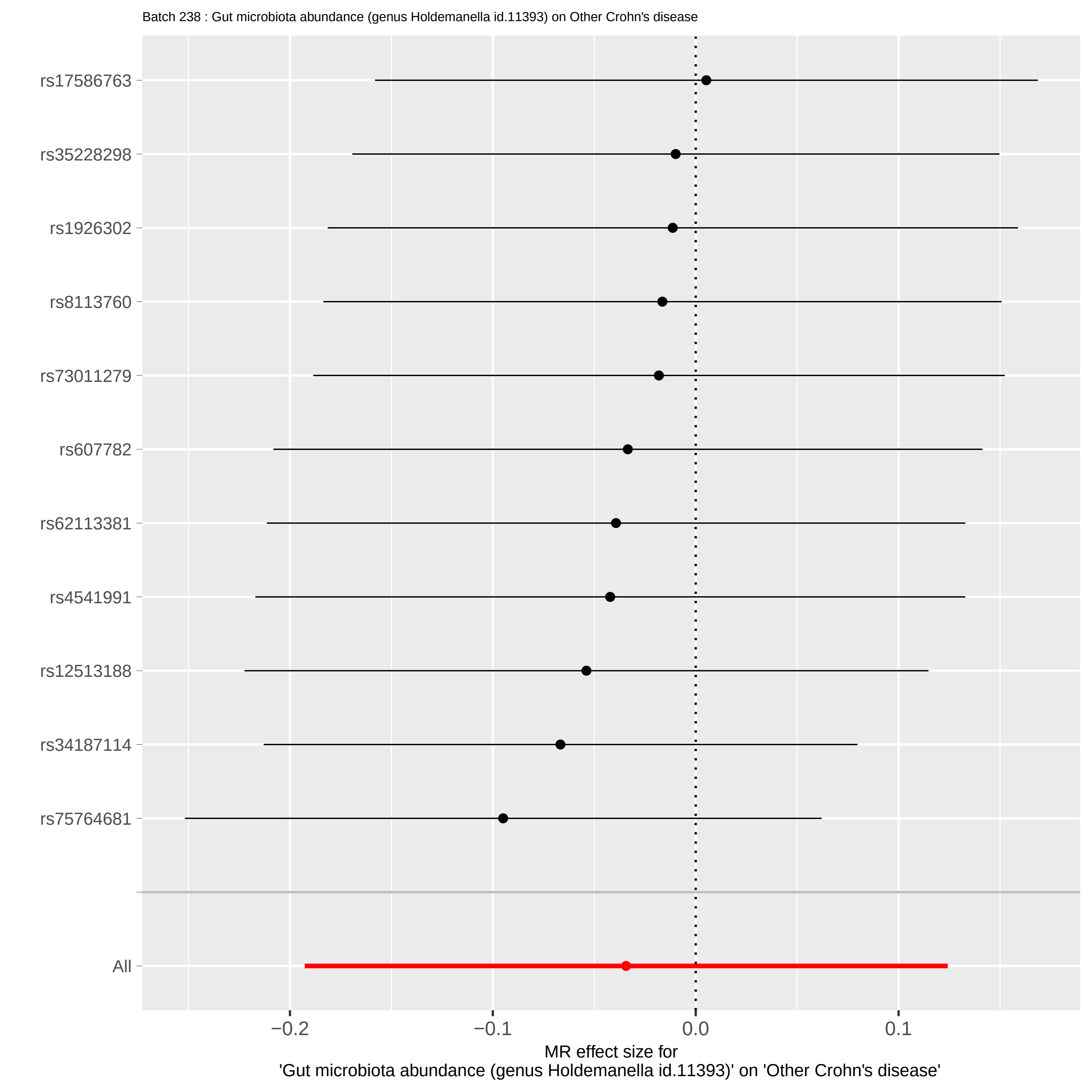

Batch 238 : Gut microbiota abundance (genus Holdemanella id.11393) on Other Crohn's disease
rs17586763
rs35228298
rs1926302
rs8113760
rs73011279
rs607782
rs62113381
rs4541991
rs12513188
rs34187114
rs75764681
All
−0.2
−0.1
0.0
0.1
MR effect size for
'Gut microbiota abundance (genus Holdemanella id.11393)' on 'Other Crohn's disease'

Batch 239 : Gut microbiota abundance (genus Holdemania id.2157) on Other Crohn's disease

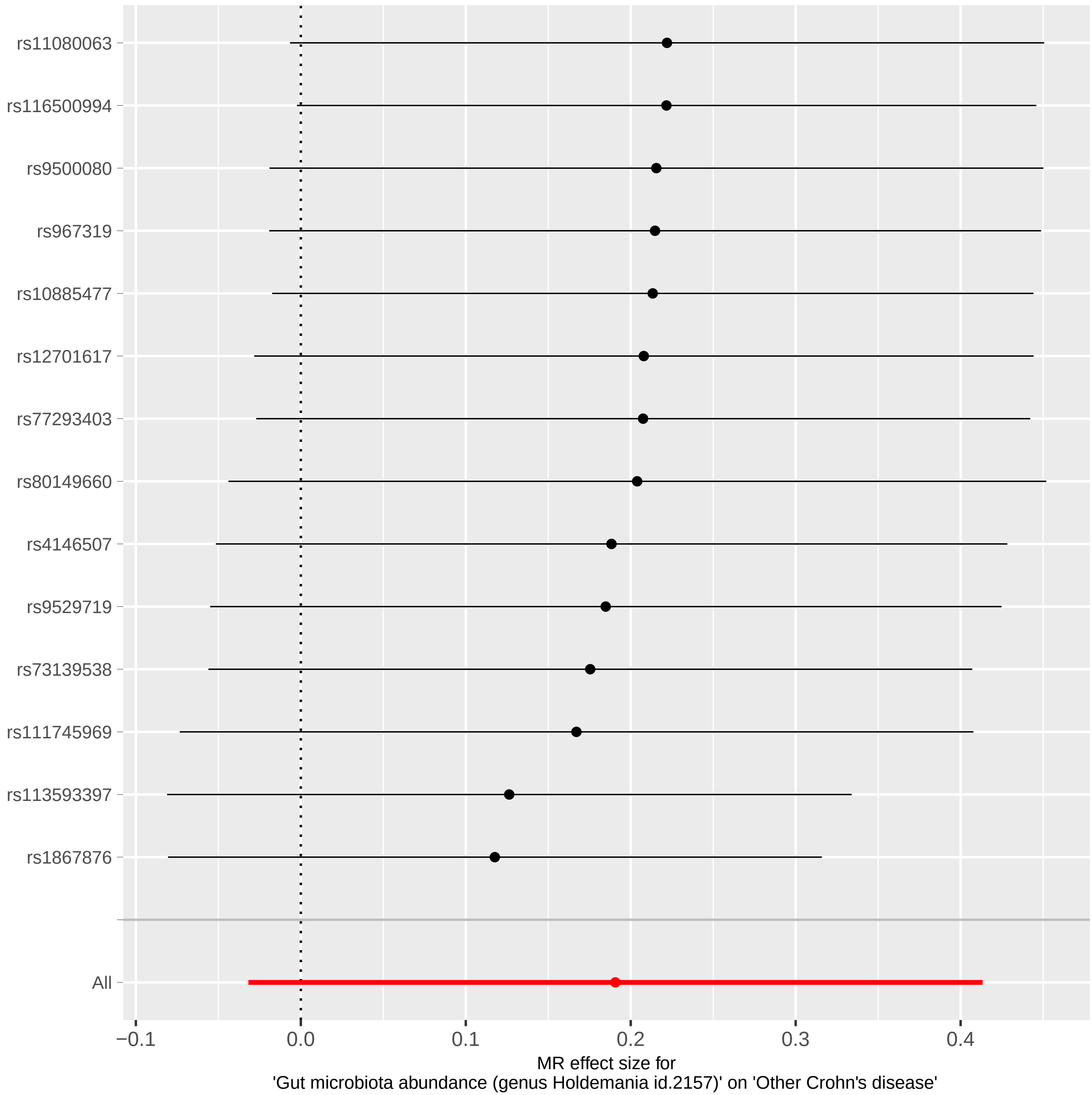

Batch 240 : Gut microbiota abundance (genus Howardella id.2000) on Other Crohn's disease

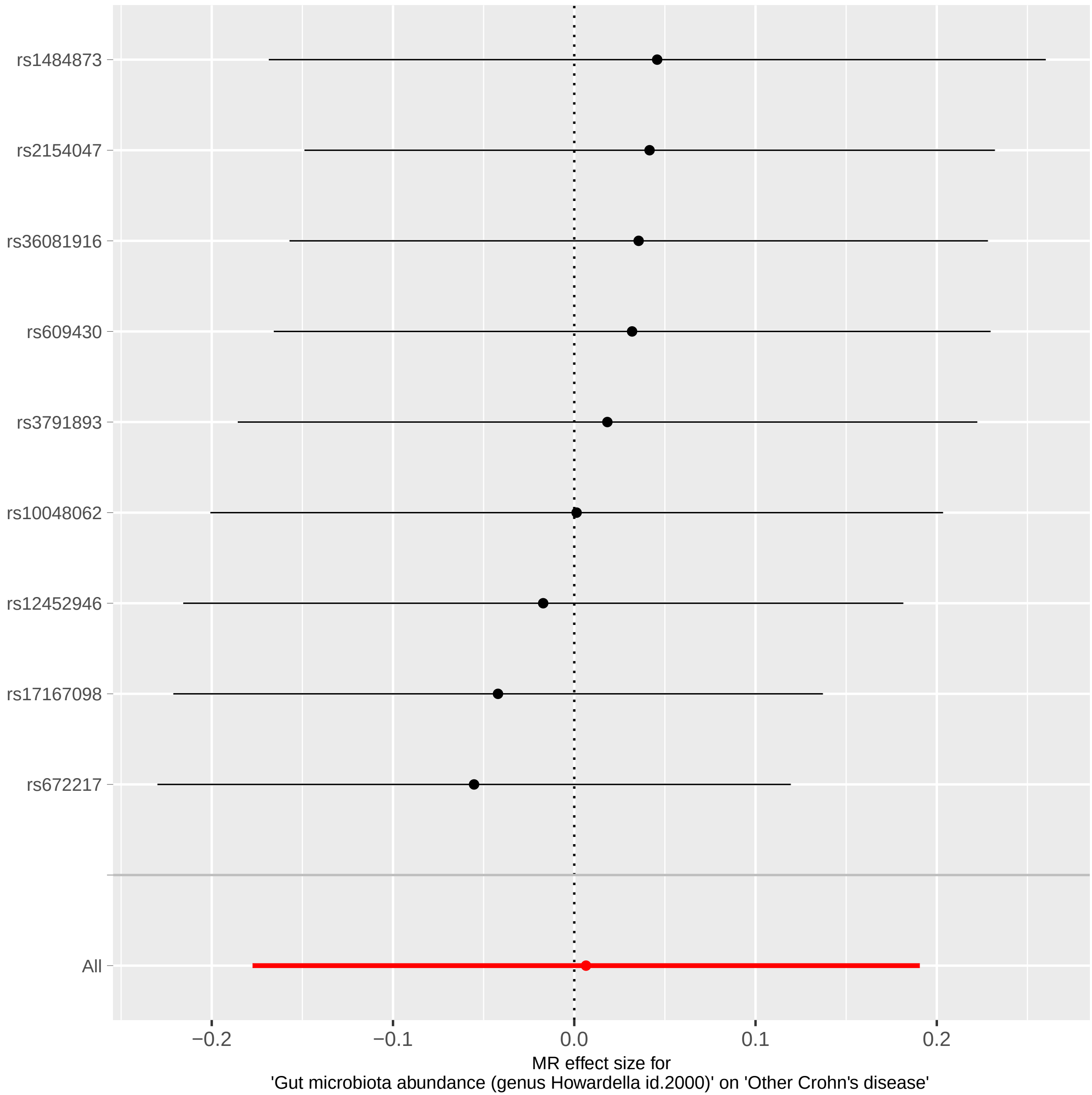

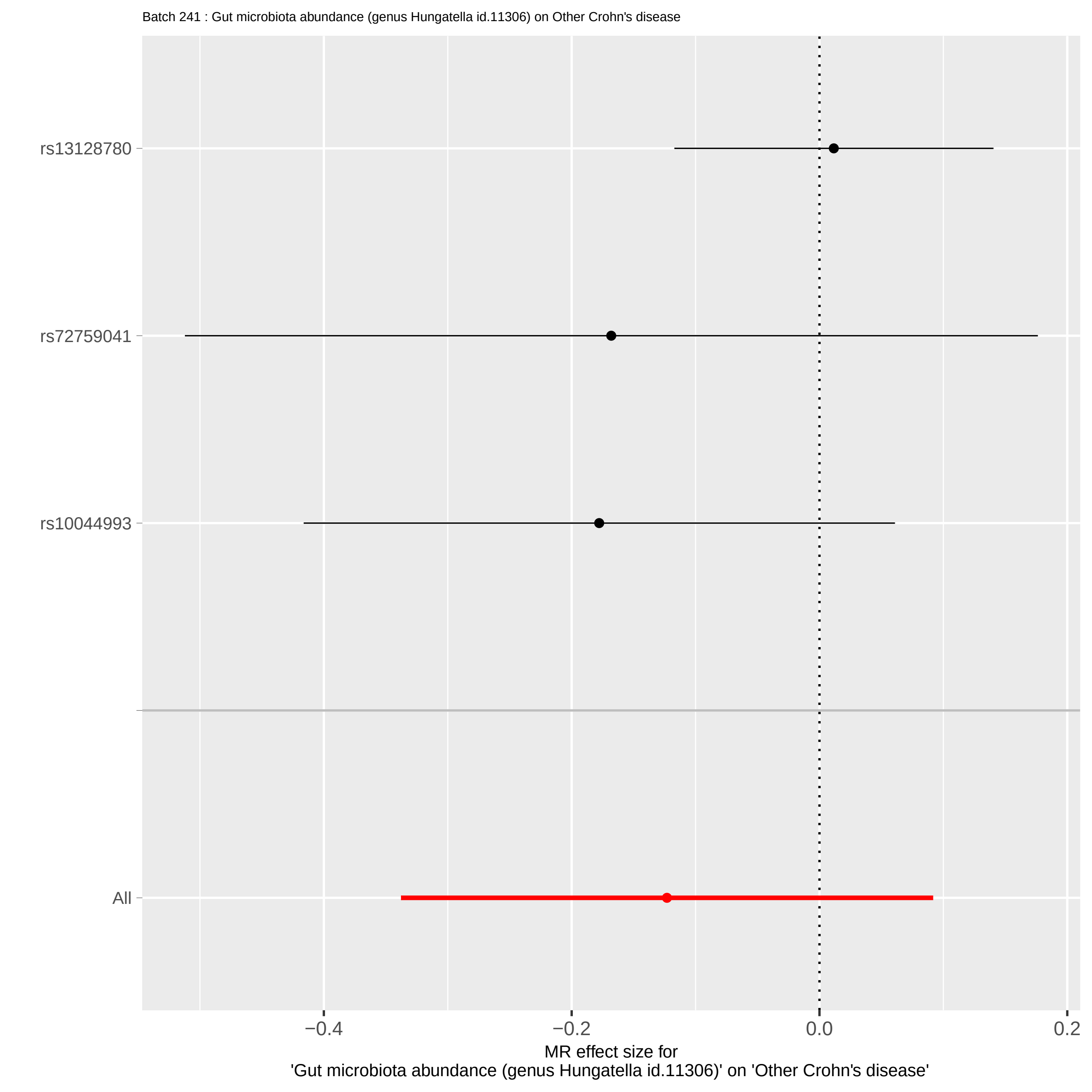
Batch 241 : Gut microbiota abundance (genus Hungatella id.11306) on Other Crohn's disease
rs13128780
rs72759041
rs10044993
All
−0.4
−0.2
0.0
0.2
MR effect size for
'Gut microbiota abundance (genus Hungatella id.11306)' on 'Other Crohn's disease'

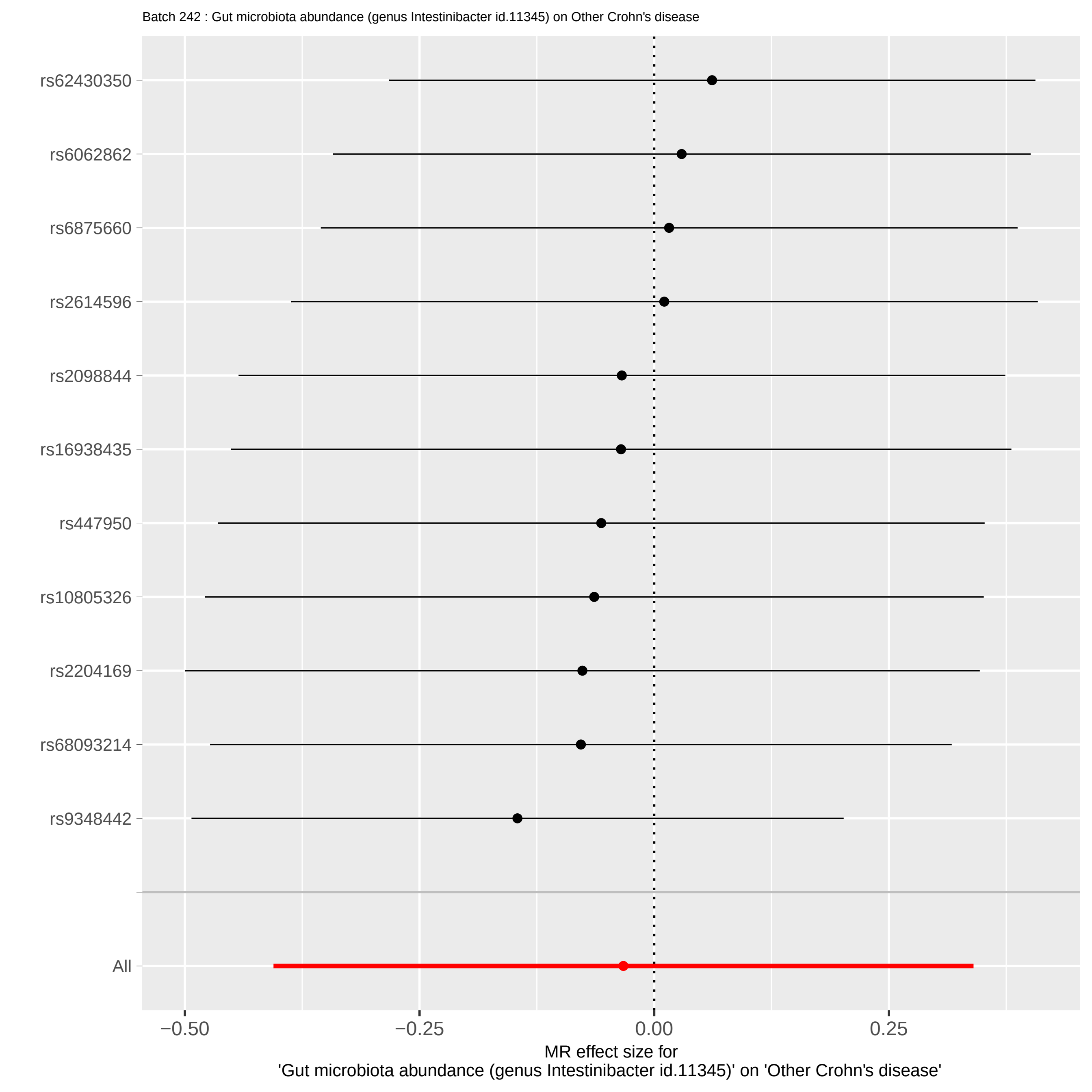
Batch 242 : Gut microbiota abundance (genus Intestinibacter id.11345) on Other Crohn's disease
rs62430350
rs6062862
rs6875660
rs2614596
rs2098844
rs16938435
rs447950
rs10805326
rs2204169
rs68093214
rs9348442
All
−0.50
−0.25
0.00
0.25
MR effect size for
'Gut microbiota abundance (genus Intestinibacter id.11345)' on 'Other Crohn's disease'

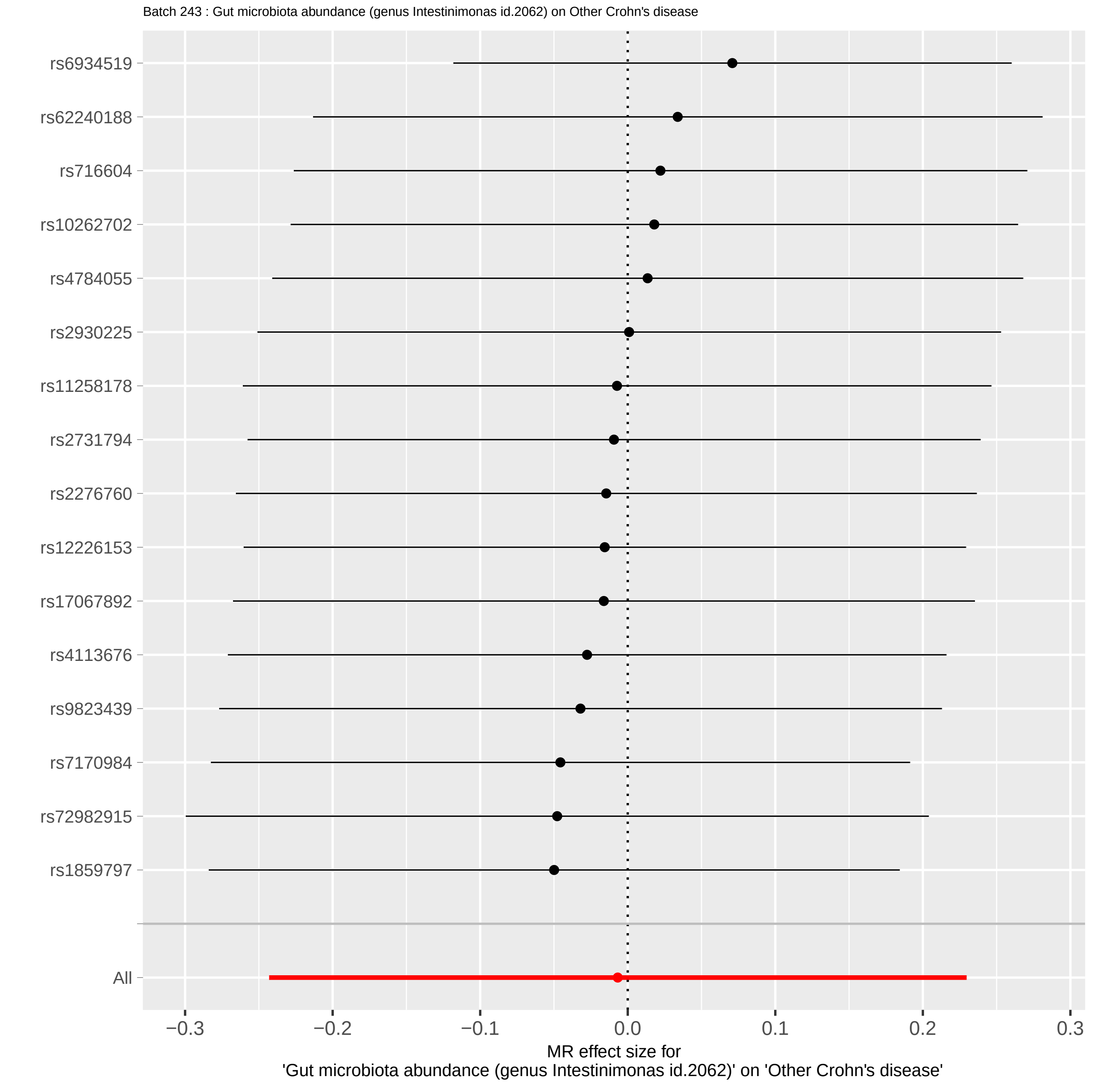

Batch 243 : Gut microbiota abundance (genus Intestinimonas id.2062) on Other Crohn's disease
rs6934519
rs62240188
rs716604
rs10262702
rs4784055
rs2930225
rs11258178
rs2731794
rs2276760
rs12226153
rs17067892
rs4113676
rs9823439
rs7170984
rs72982915
rs1859797
All
−0.3
−0.2
−0.1
0.0
0.1
0.2
0.3
MR effect size for
'Gut microbiota abundance (genus Intestinimonas id.2062)' on 'Other Crohn's disease'

Batch 244 : Gut microbiota abundance (genus Lachnoclostridium id.11308) on Other Crohn's disease

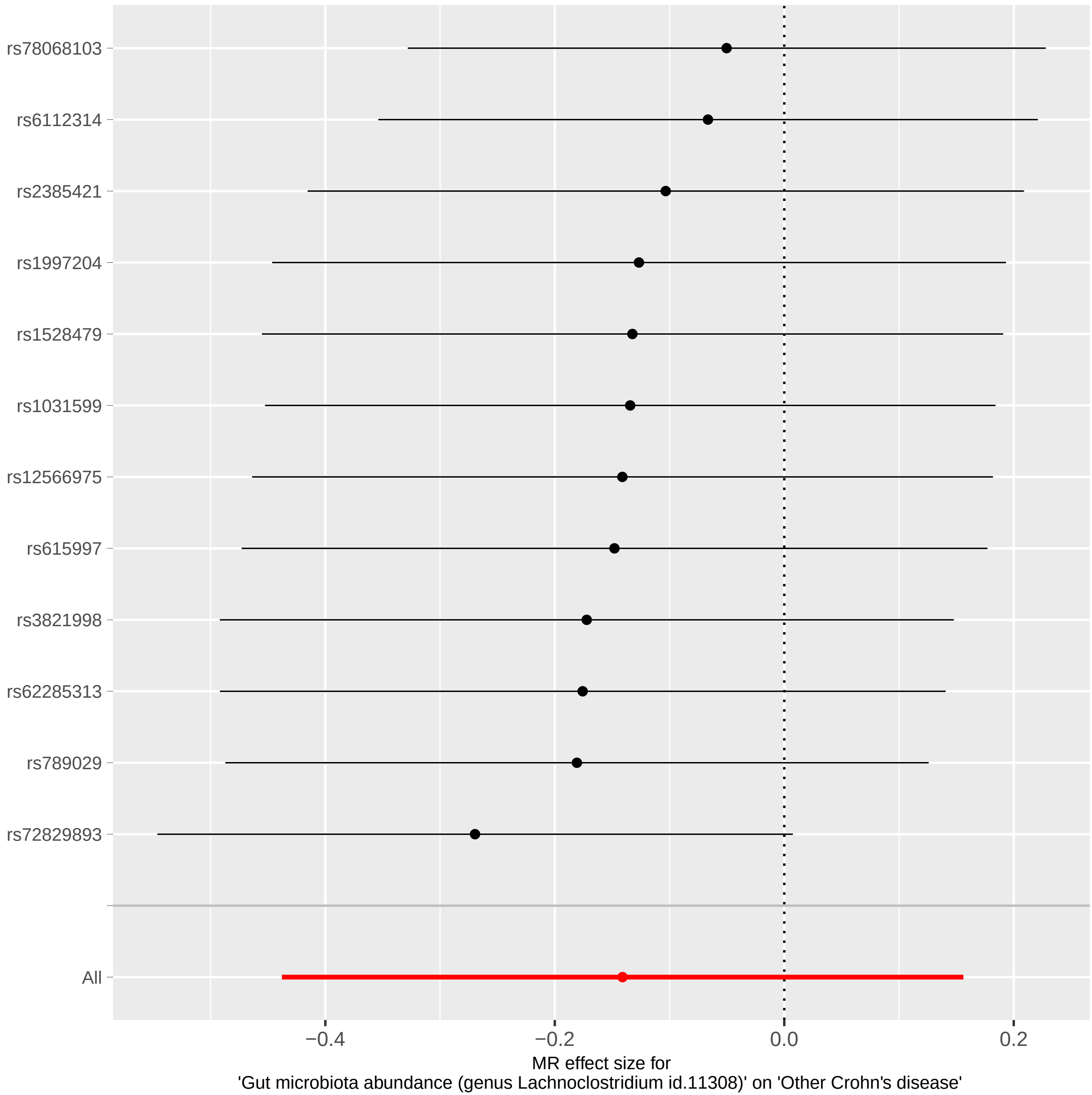

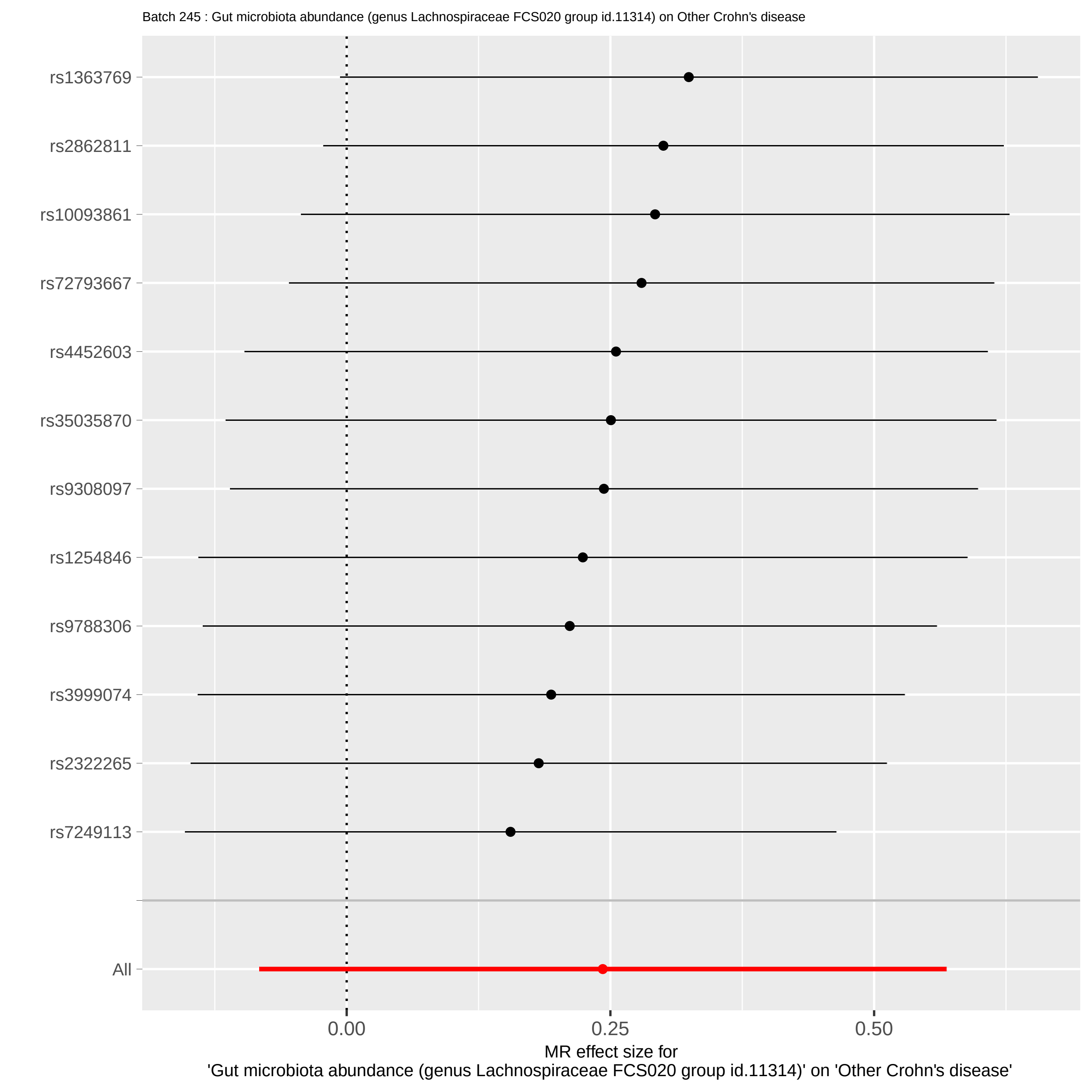
Batch 245 : Gut microbiota abundance (genus Lachnospiraceae FCS020 group id.11314) on Other Crohn's disease
rs1363769
rs2862811
rs10093861
rs72793667
rs4452603
rs35035870
rs9308097
rs1254846
rs9788306
rs3999074
rs2322265
rs7249113
All
0.00
0.25
0.50
MR effect size for
'Gut microbiota abundance (genus Lachnospiraceae FCS020 group id.11314)' on 'Other Crohn's disease'

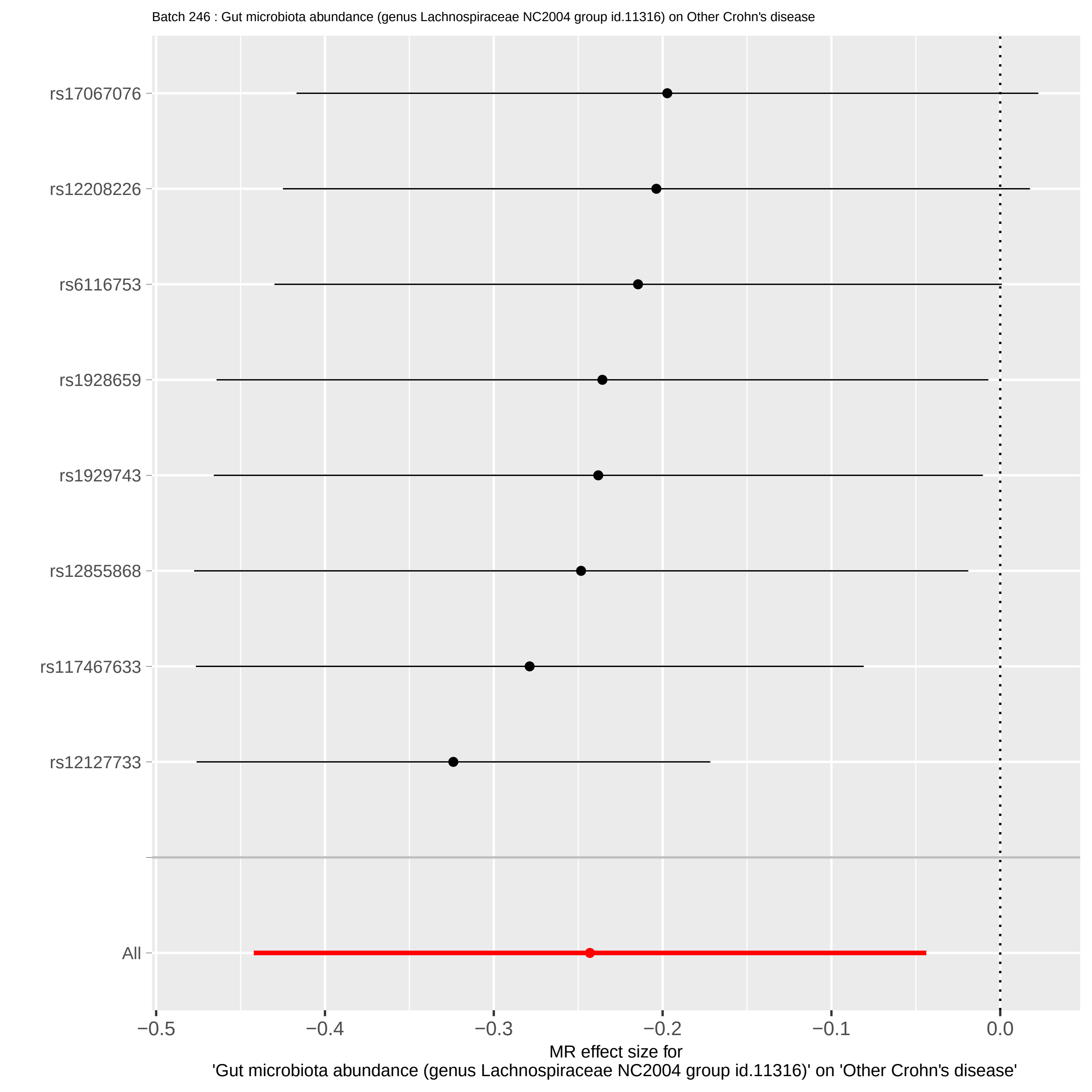

Batch 246 : Gut microbiota abundance (genus Lachnospiraceae NC2004 group id.11316) on Other Crohn's disease
rs17067076
rs12208226
rs6116753
rs1928659
rs1929743
rs12855868
rs117467633
rs12127733
All
−0.5
−0.4
−0.3
−0.2
−0.1
0.0
MR effect size for
'Gut microbiota abundance (genus Lachnospiraceae NC2004 group id.11316)' on 'Other Crohn's disease'

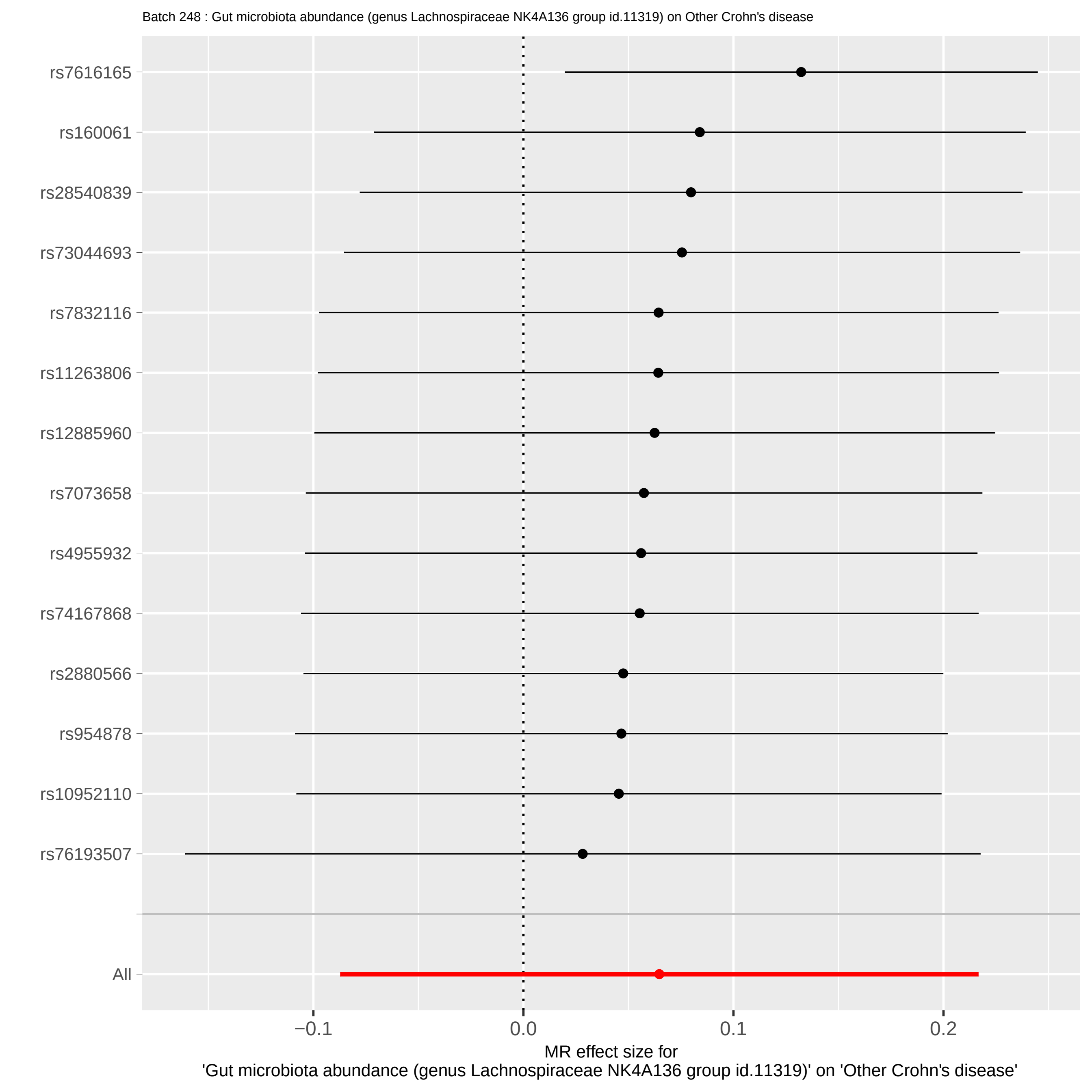

Batch 248 : Gut microbiota abundance (genus Lachnospiraceae NK4A136 group id.11319) on Other Crohn's disease
rs7616165
rs160061
rs28540839
rs73044693
rs7832116
rs11263806
rs12885960
rs7073658
rs4955932
rs74167868
rs2880566
rs954878
rs10952110
rs76193507
All
−0.1
0.0
0.1
0.2
MR effect size for
'Gut microbiota abundance (genus Lachnospiraceae NK4A136 group id.11319)' on 'Other Crohn's disease'

Batch 249 : Gut microbiota abundance (genus Lachnospiraceae UCG001 id.11321) on Other Crohn's disease

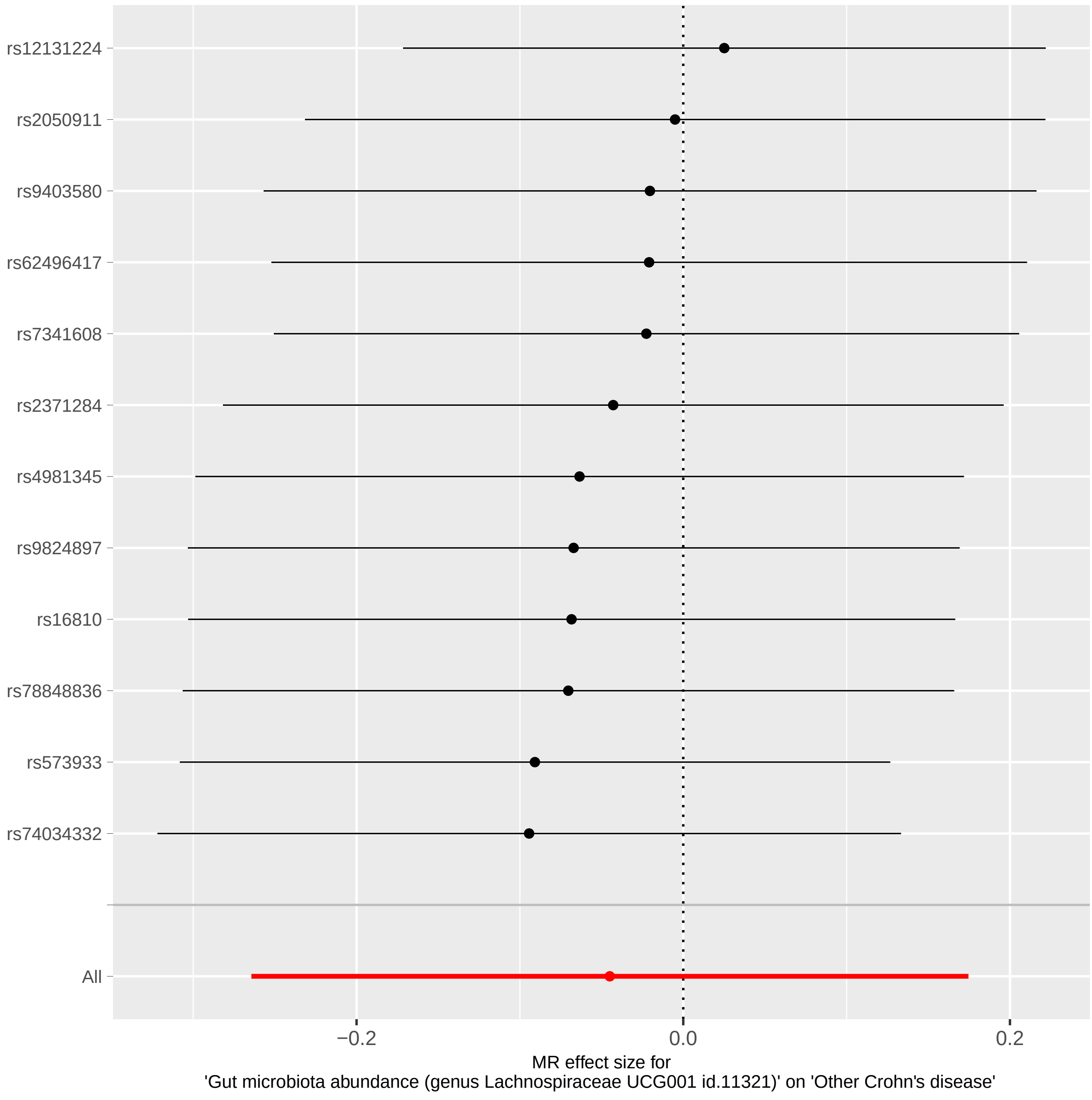

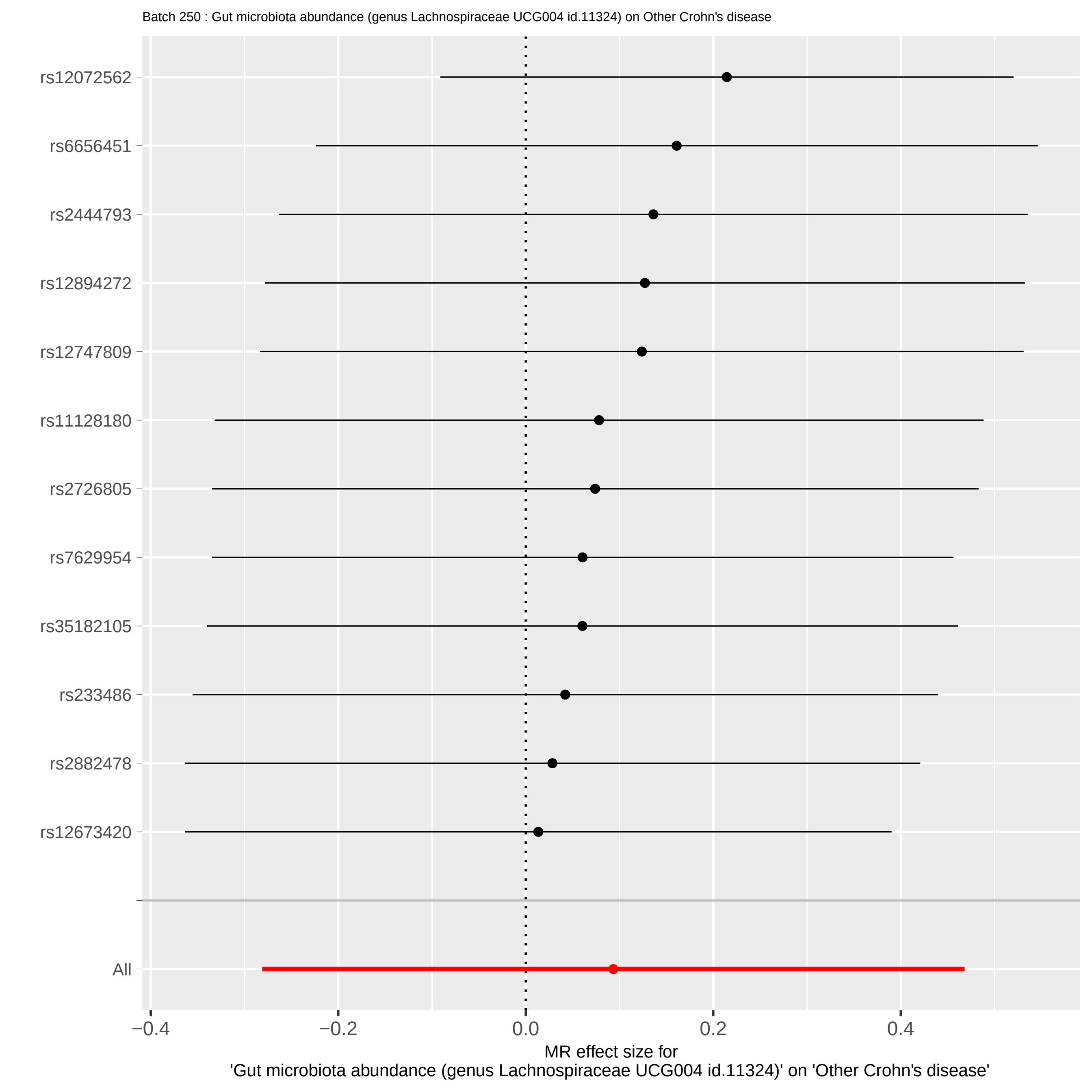

Batch 250 : Gut microbiota abundance (genus Lachnospiraceae UCG004 id.11324) on Other Crohn's disease
rs12072562
rs6656451
rs2444793
rs12894272
rs12747809
rs11128180
rs2726805
rs7629954
rs35182105
rs233486
rs2882478
rs12673420
All
−0.4
−0.2
0.0
0.2
0.4
MR effect size for
'Gut microbiota abundance (genus Lachnospiraceae UCG004 id.11324)' on 'Other Crohn's disease'

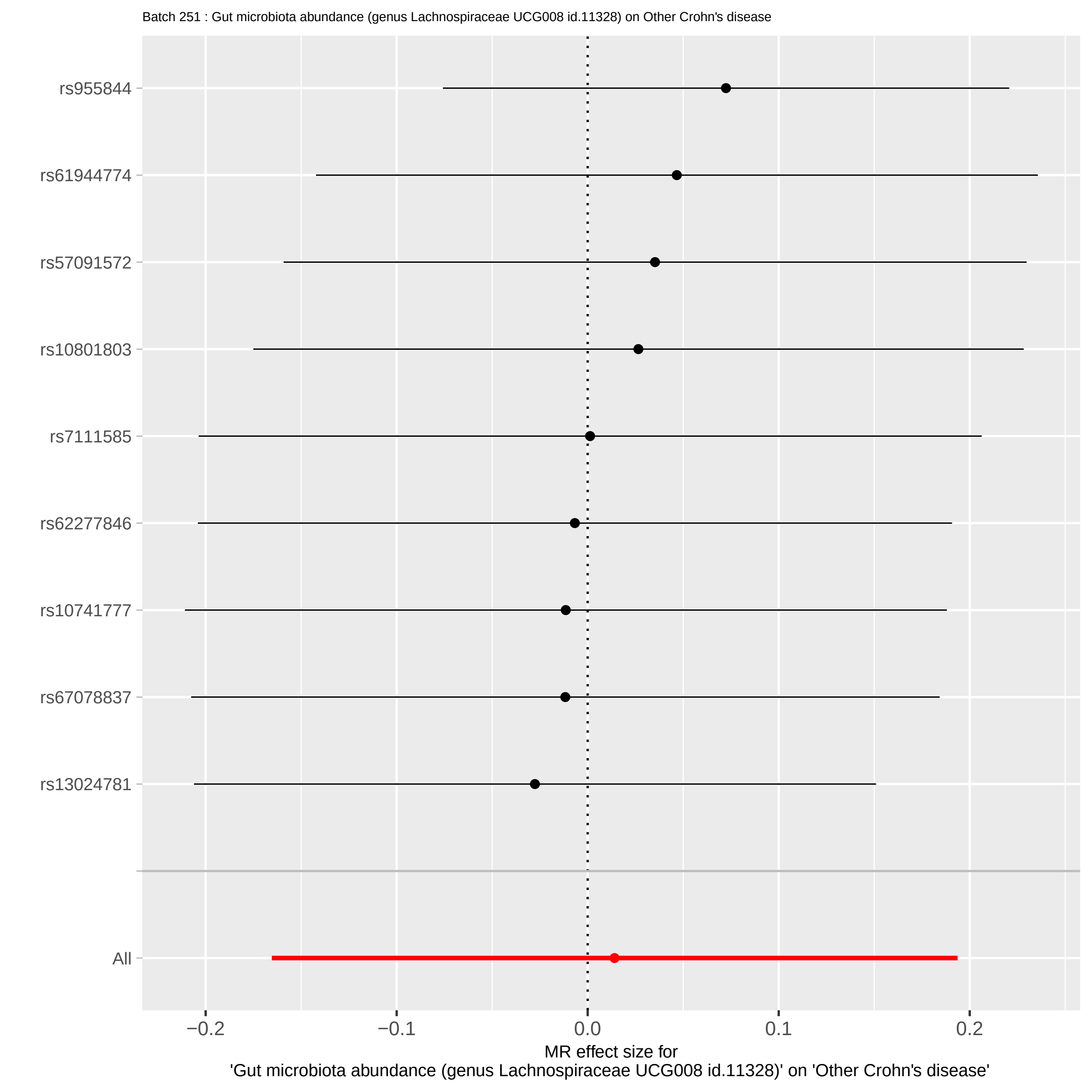
Batch 251 : Gut microbiota abundance (genus Lachnospiraceae UCG008 id.11328) on Other Crohn's disease
rs955844
rs61944774
rs57091572
rs10801803
rs7111585
rs62277846
rs10741777
rs67078837
rs13024781
All
−0.2
−0.1
0.0
0.1
0.2
MR effect size for
'Gut microbiota abundance (genus Lachnospiraceae UCG008 id.11328)' on 'Other Crohn's disease'

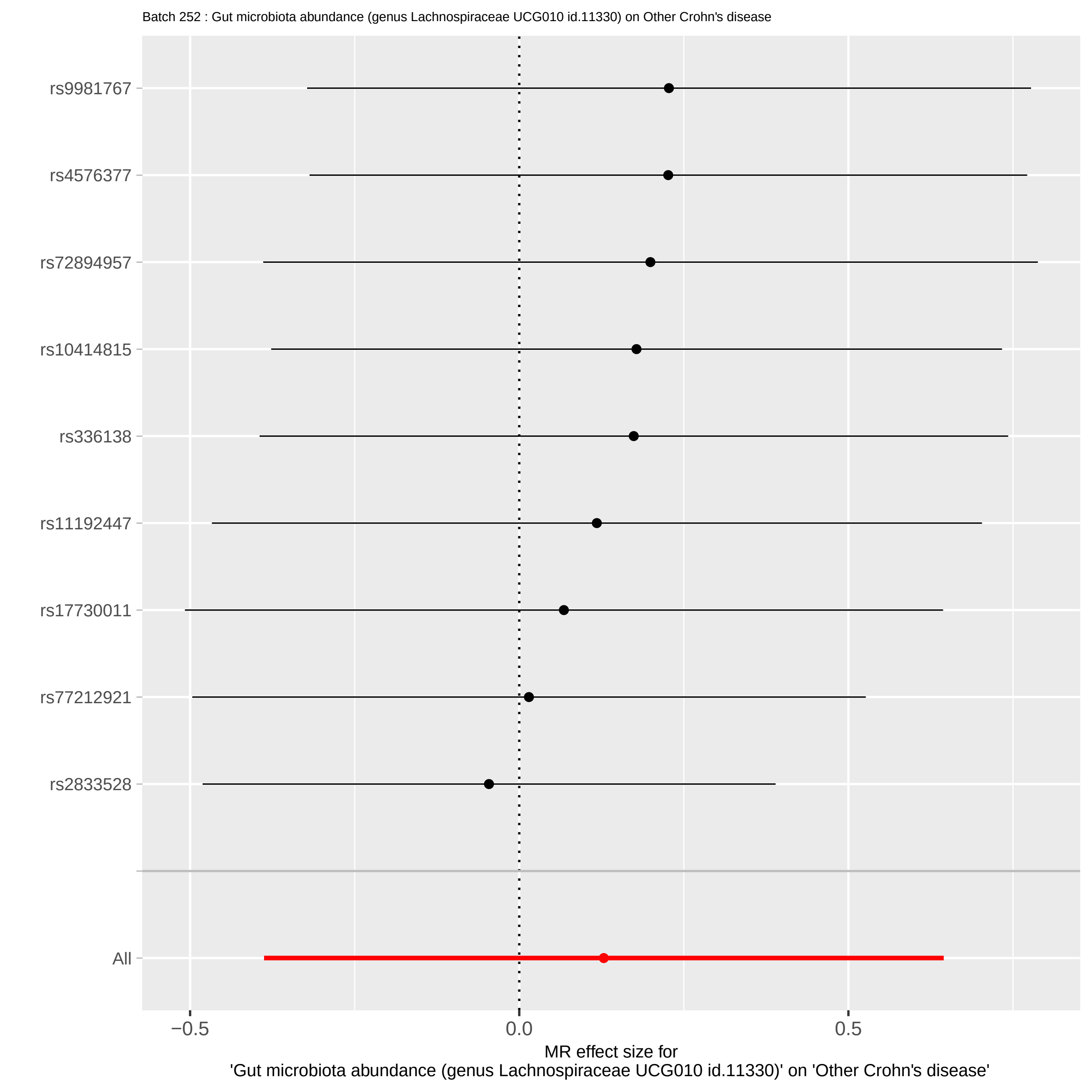
Batch 252 : Gut microbiota abundance (genus Lachnospiraceae UCG010 id.11330) on Other Crohn's disease
rs9981767
rs4576377
rs72894957
rs10414815
rs336138
rs11192447
rs17730011
rs77212921
rs2833528
All
−0.5
0.0
0.5
MR effect size for
'Gut microbiota abundance (genus Lachnospiraceae UCG010 id.11330)' on 'Other Crohn's disease'

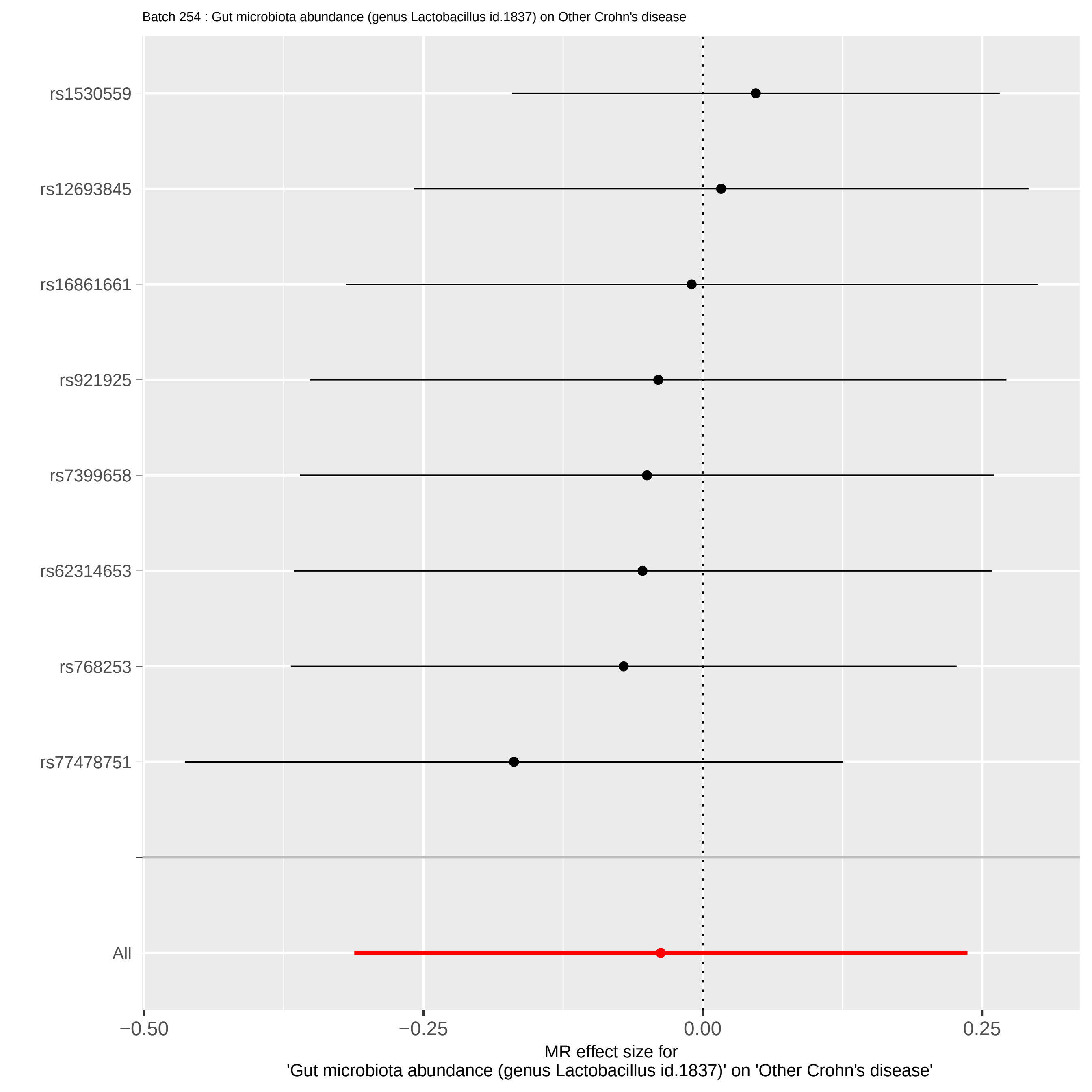
Batch 254 : Gut microbiota abundance (genus Lactobacillus id.1837) on Other Crohn's disease
rs1530559
rs12693845
rs16861661
rs921925
rs7399658
rs62314653
rs768253
rs77478751
All
−0.50
−0.25
0.00
0.25
MR effect size for
'Gut microbiota abundance (genus Lactobacillus id.1837)' on 'Other Crohn's disease'

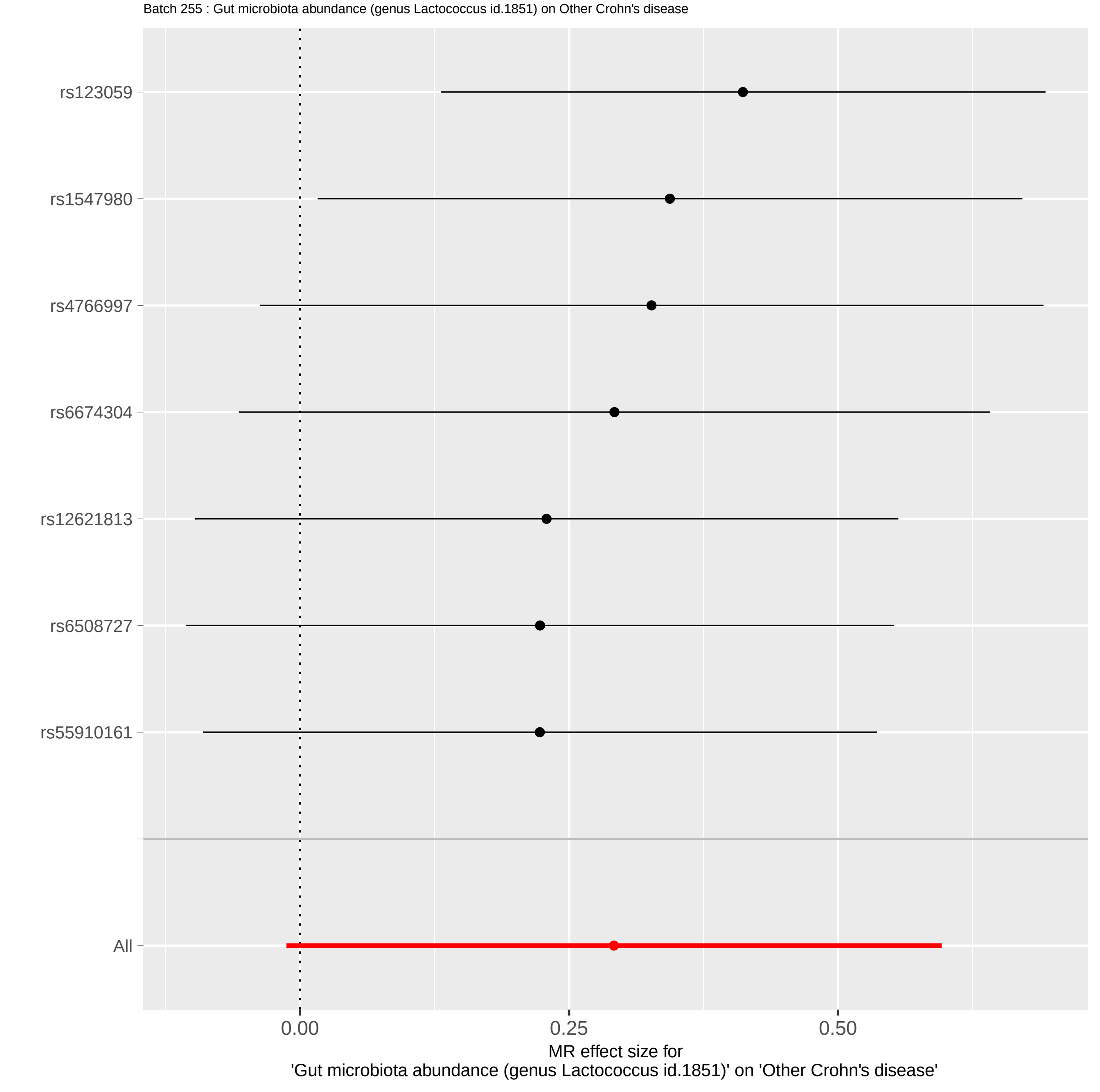
Batch 255 : Gut microbiota abundance (genus Lactococcus id.1851) on Other Crohn's disease
rs123059
rs1547980
rs4766997
rs6674304
rs12621813
rs6508727
rs55910161
All
0.00
0.25
0.50
MR effect size for
'Gut microbiota abundance (genus Lactococcus id.1851)' on 'Other Crohn's disease'

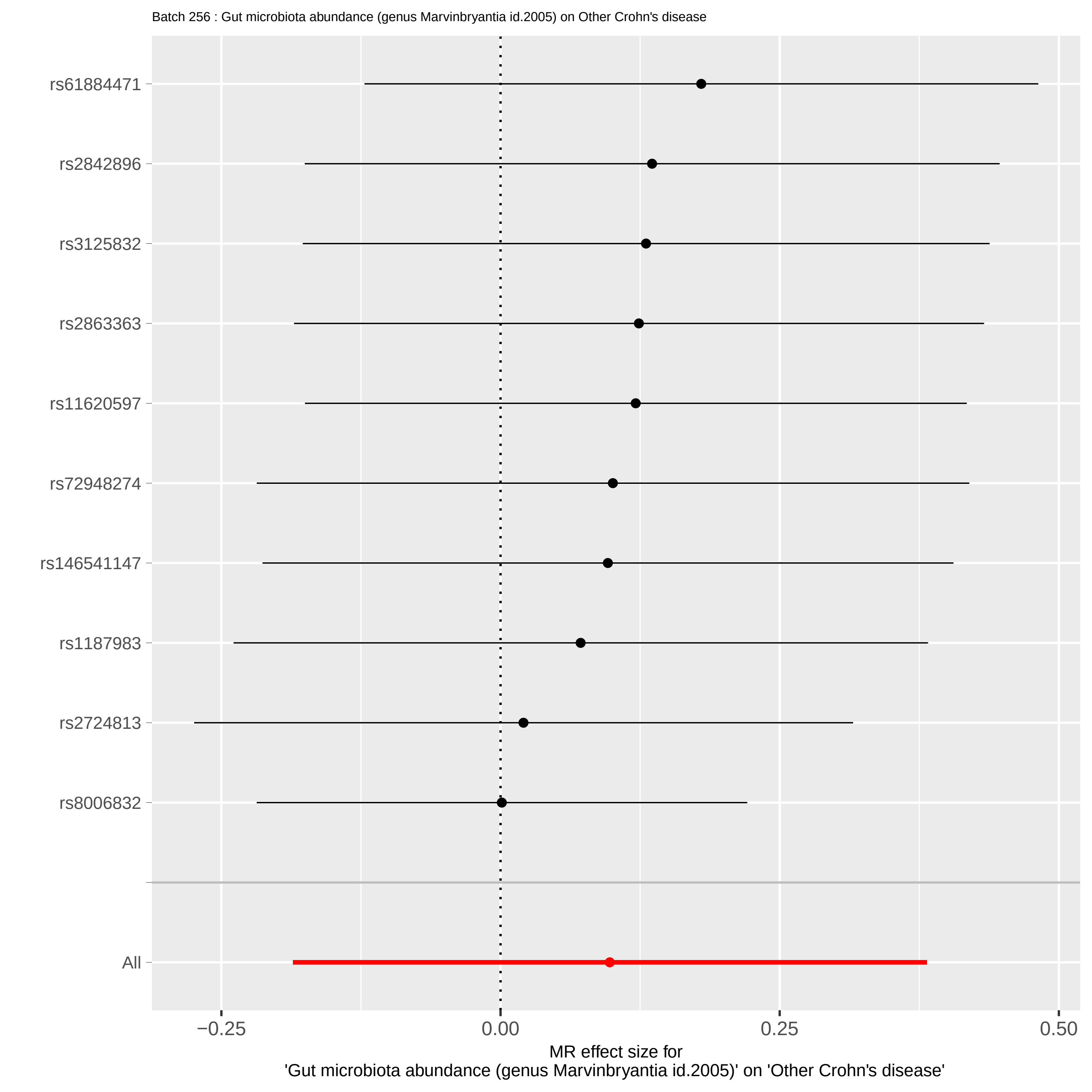

Batch 256 : Gut microbiota abundance (genus Marvinbryantia id.2005) on Other Crohn's disease
rs61884471
rs2842896
rs3125832
rs2863363
rs11620597
rs72948274
rs146541147
rs1187983
rs2724813
rs8006832
All
−0.25
0.00
0.25
0.50
MR effect size for
'Gut microbiota abundance (genus Marvinbryantia id.2005)' on 'Other Crohn's disease'

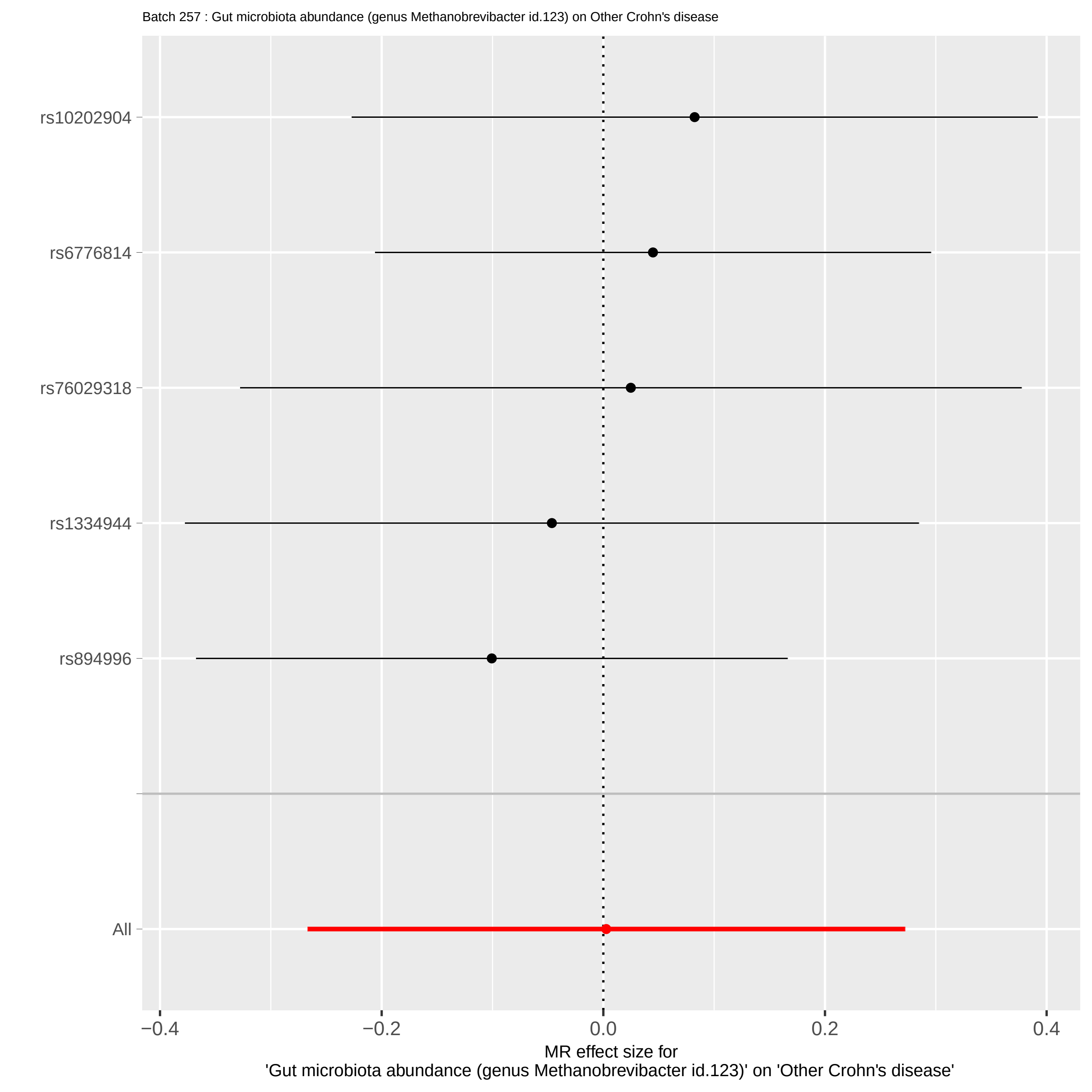
Batch 257 : Gut microbiota abundance (genus Methanobrevibacter id.123) on Other Crohn's disease
rs10202904
rs6776814
rs76029318
rs1334944
rs894996
All
−0.4
−0.2
0.0
0.2
0.4
MR effect size for
'Gut microbiota abundance (genus Methanobrevibacter id.123)' on 'Other Crohn's disease'

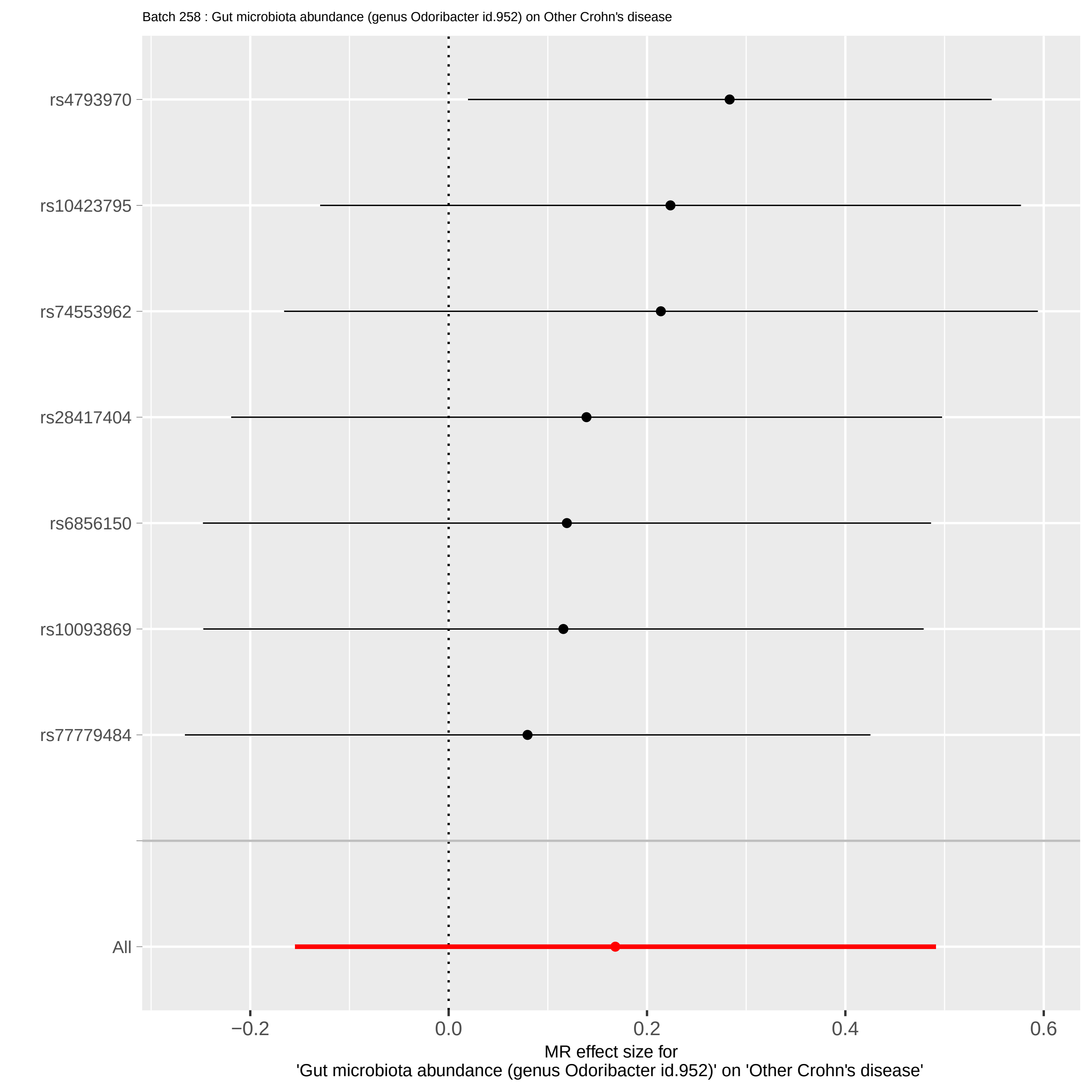
Batch 258 : Gut microbiota abundance (genus Odoribacter id.952) on Other Crohn's disease
rs4793970
rs10423795
rs74553962
rs28417404
rs6856150
rs10093869
rs77779484
All
−0.2
0.0
0.2
0.4
0.6
MR effect size for
'Gut microbiota abundance (genus Odoribacter id.952)' on 'Other Crohn's disease'

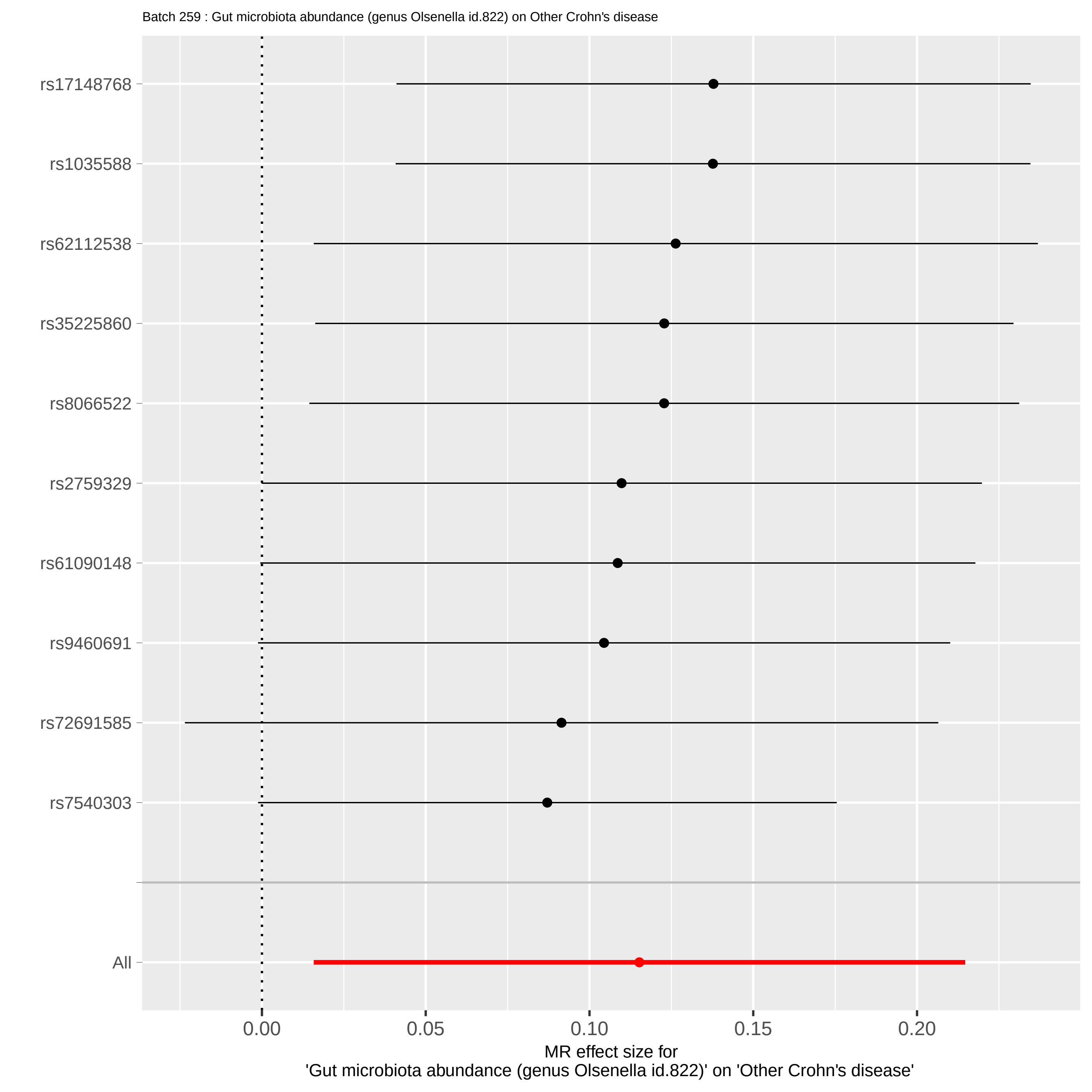
Batch 259 : Gut microbiota abundance (genus Olsenella id.822) on Other Crohn's disease
rs17148768
rs1035588
rs62112538
rs35225860
rs8066522
rs2759329
rs61090148
rs9460691
rs72691585
rs7540303
All
0.00
0.05
0.10
0.15
0.20
MR effect size for
'Gut microbiota abundance (genus Olsenella id.822)' on 'Other Crohn's disease'

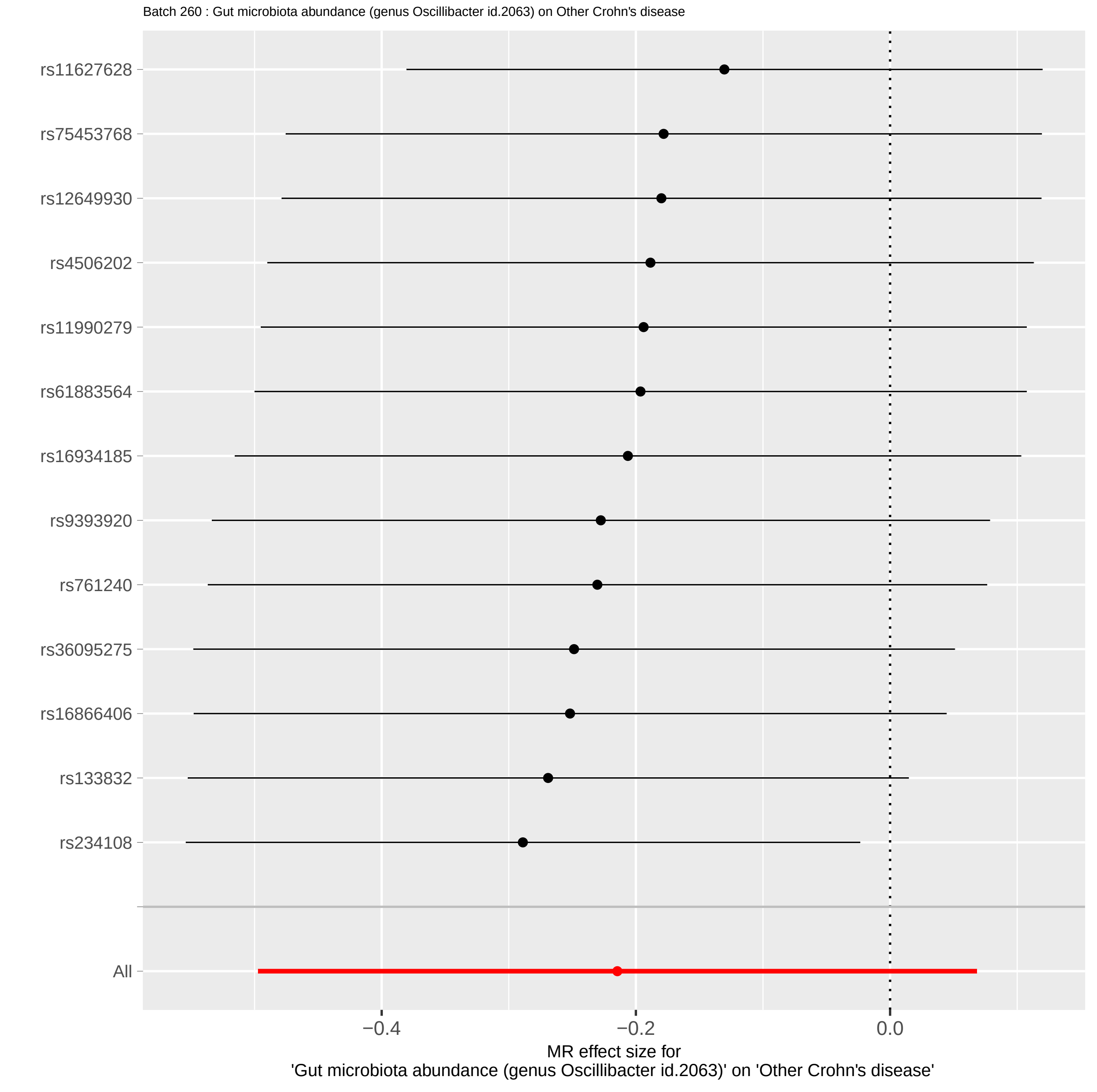
Batch 260 : Gut microbiota abundance (genus Oscillibacter id.2063) on Other Crohn's disease
rs11627628
rs75453768
rs12649930
rs4506202
rs11990279
rs61883564
rs16934185
rs9393920
rs761240
rs36095275
rs16866406
rs133832
rs234108
All
−0.4
−0.2
0.0
MR effect size for
'Gut microbiota abundance (genus Oscillibacter id.2063)' on 'Other Crohn's disease'

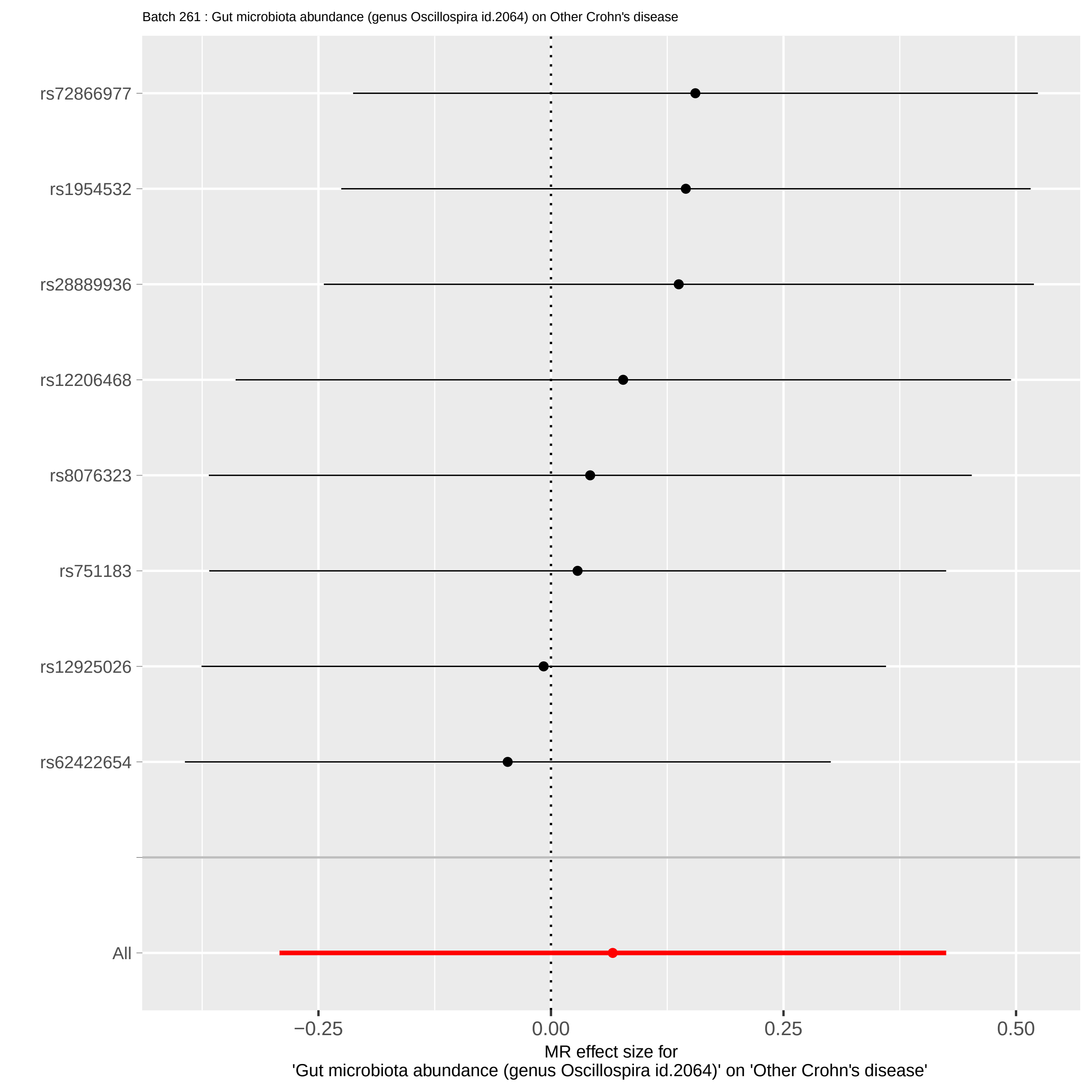
Batch 261 : Gut microbiota abundance (genus Oscillospira id.2064) on Other Crohn's disease
rs72866977
rs1954532
rs28889936
rs12206468
rs8076323
rs751183
rs12925026
rs62422654
All
−0.25
0.00
0.25
0.50
MR effect size for
'Gut microbiota abundance (genus Oscillospira id.2064)' on 'Other Crohn's disease'

Batch 262 : Gut microbiota abundance (genus Oxalobacter id.2978) on Other Crohn's disease

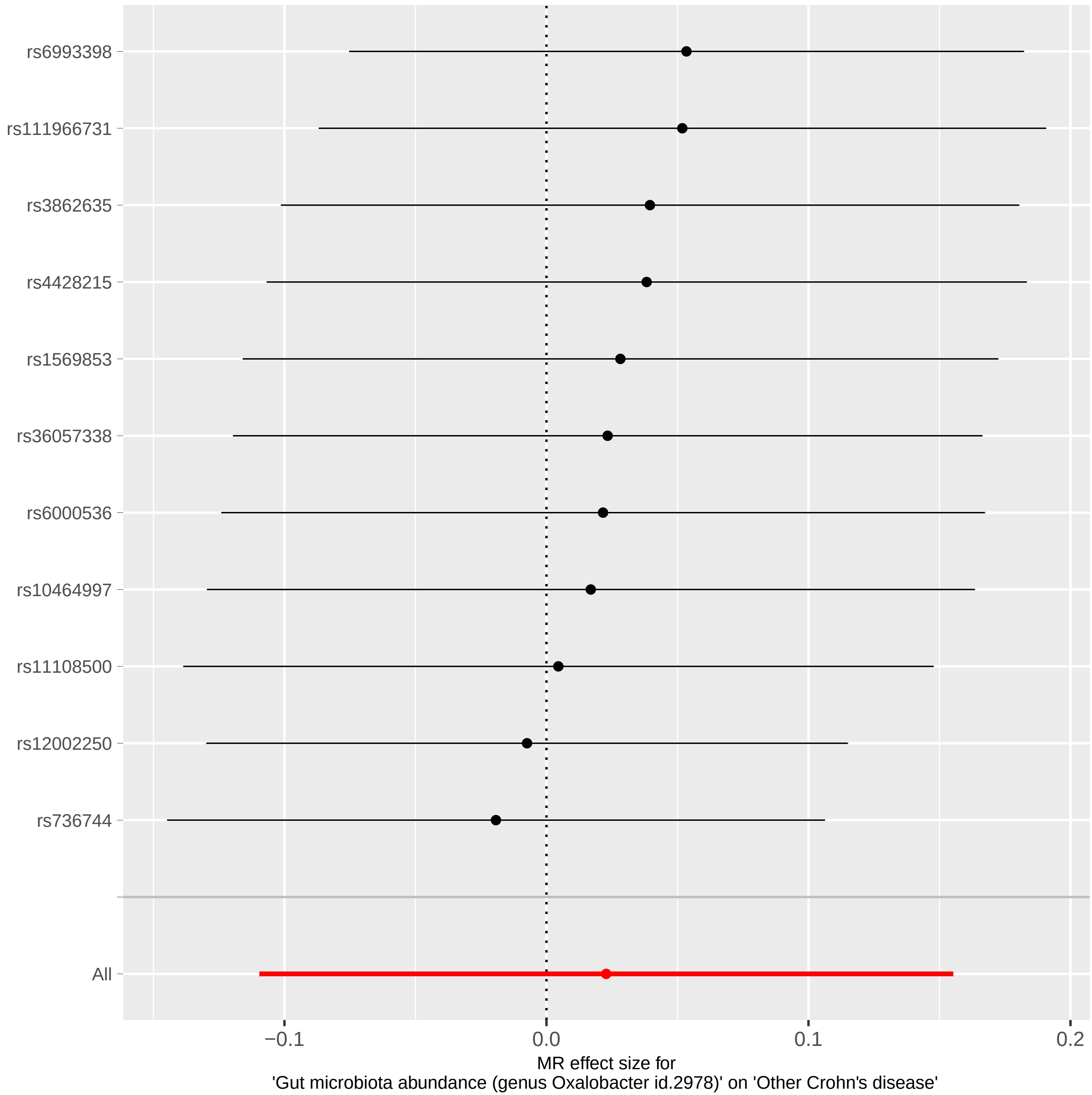

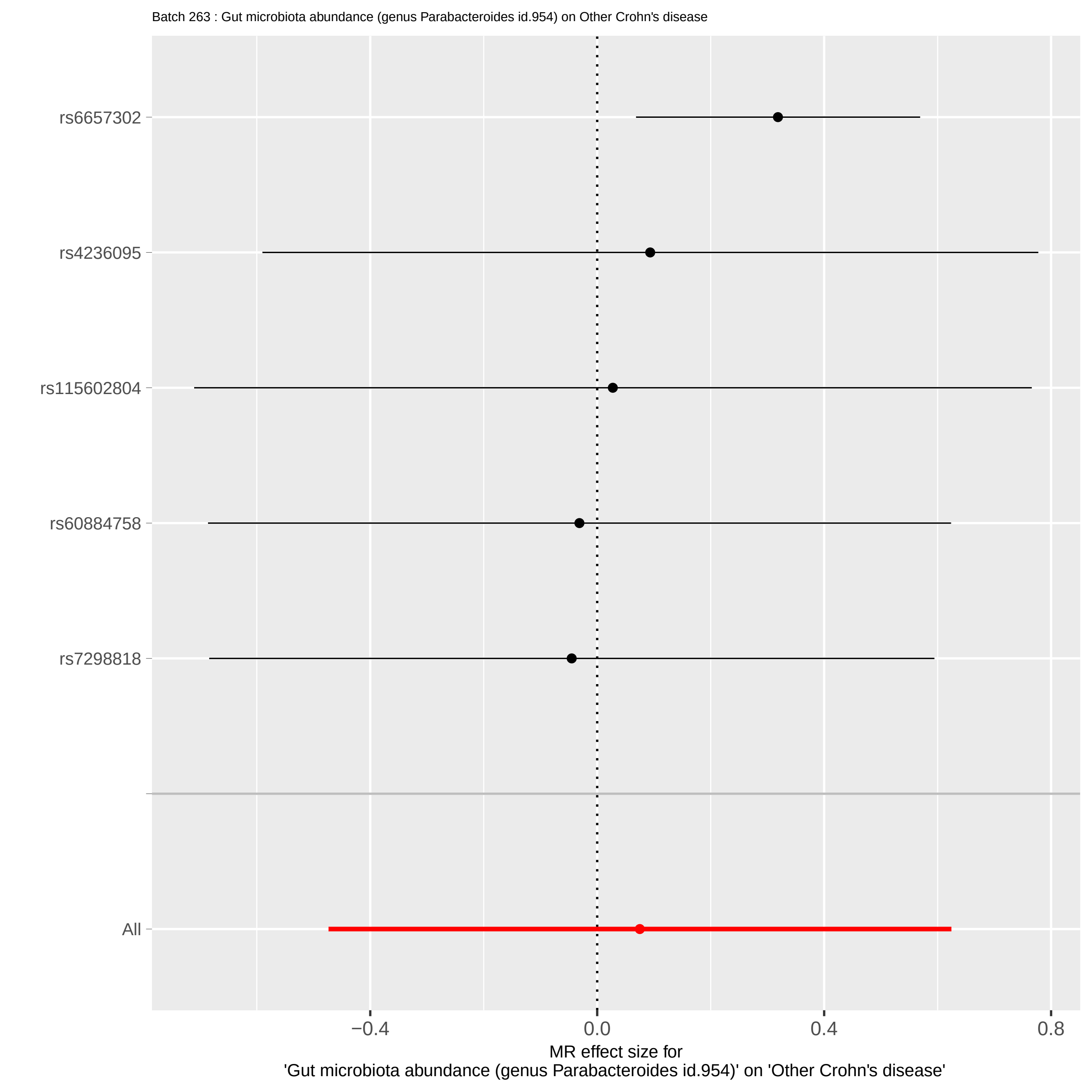
Batch 263 : Gut microbiota abundance (genus Parabacteroides id.954) on Other Crohn's disease
rs6657302
rs4236095
rs115602804
rs60884758
rs7298818
All
−0.4
0.0
0.4
0.8
MR effect size for
'Gut microbiota abundance (genus Parabacteroides id.954)' on 'Other Crohn's disease'

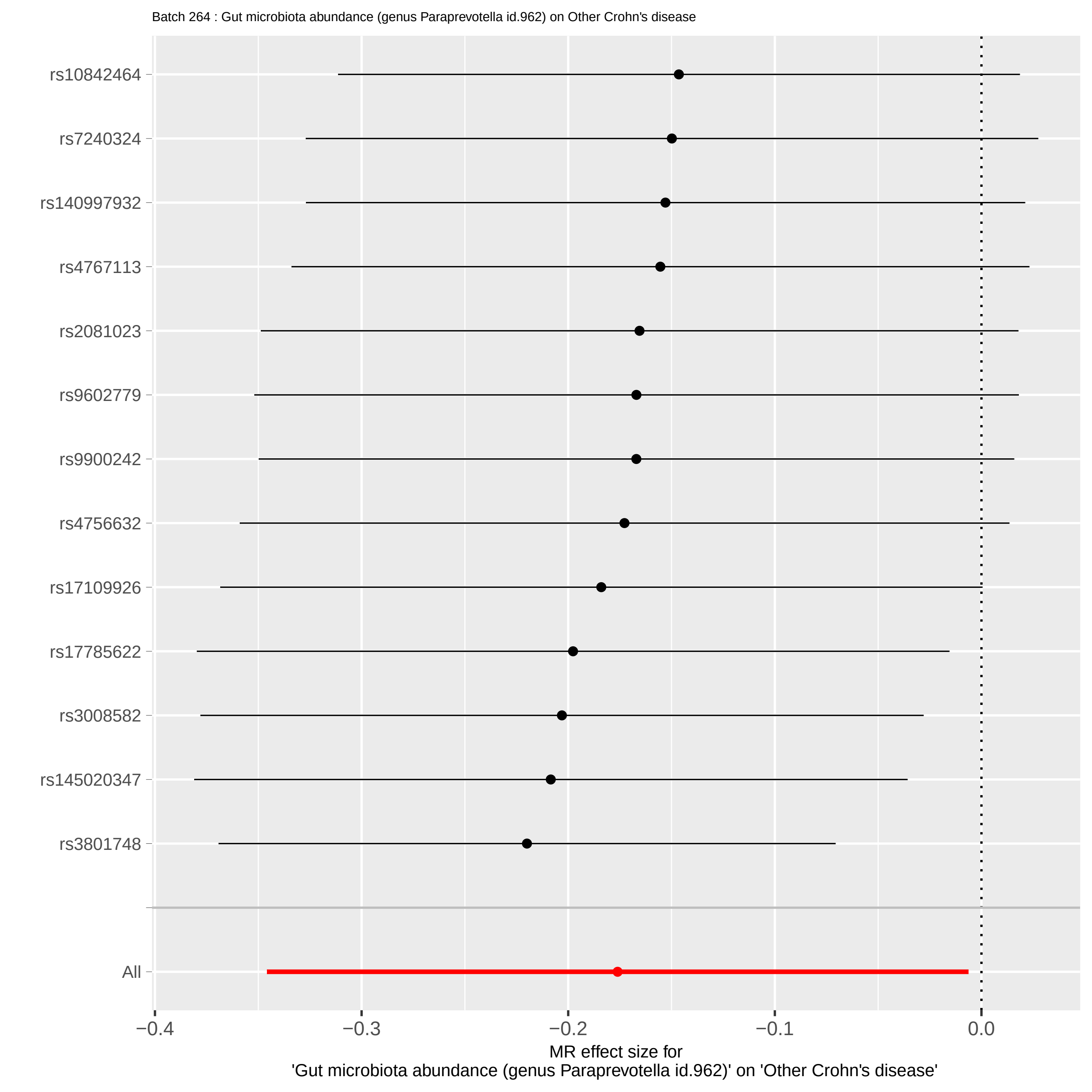
Batch 264 : Gut microbiota abundance (genus Paraprevotella id.962) on Other Crohn's disease
rs10842464
rs7240324
rs140997932
rs4767113
rs2081023
rs9602779
rs9900242
rs4756632
rs17109926
rs17785622
rs3008582
rs145020347
rs3801748
All
−0.4
−0.3
−0.2
−0.1
0.0
MR effect size for
'Gut microbiota abundance (genus Paraprevotella id.962)' on 'Other Crohn's disease'

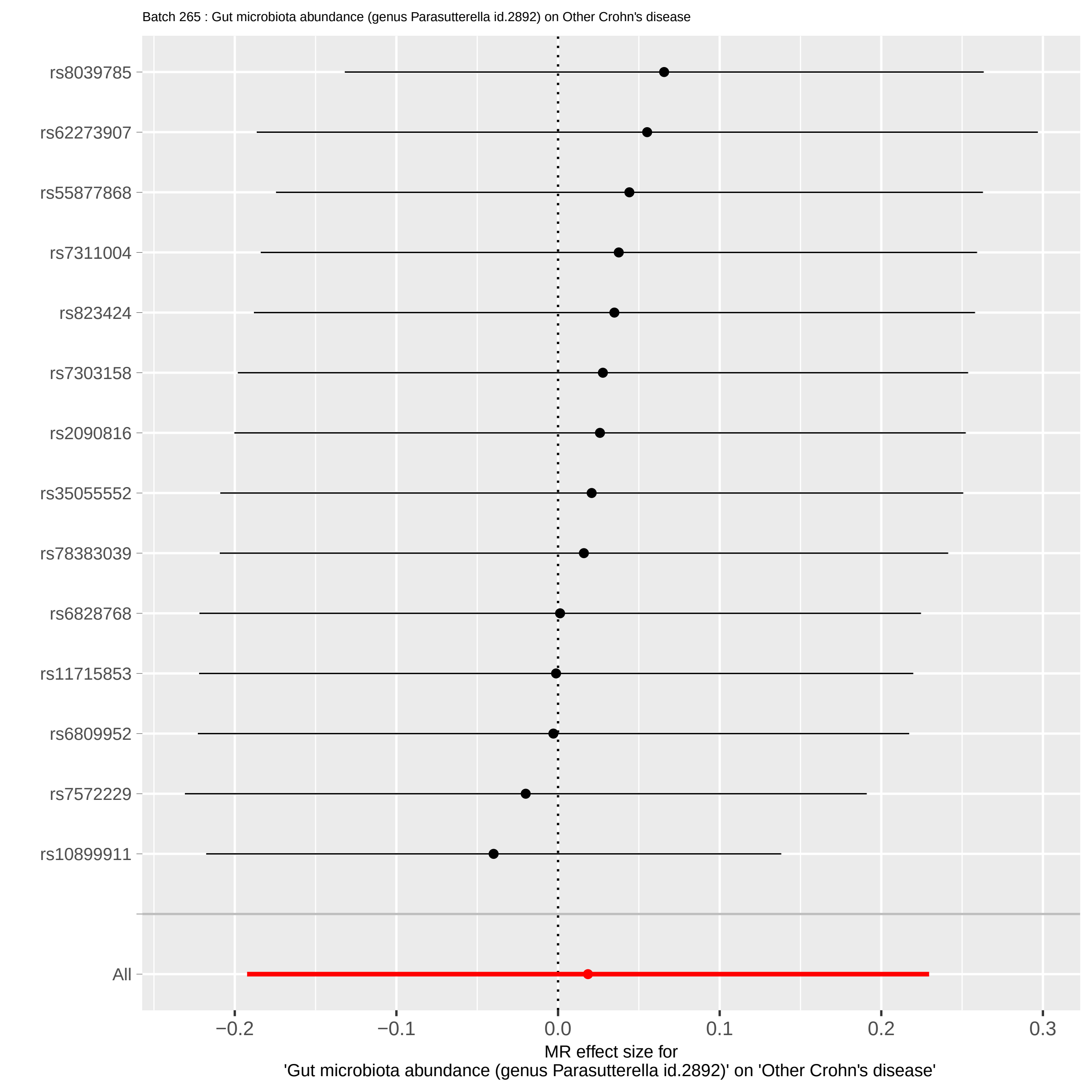

Batch 265 : Gut microbiota abundance (genus Parasutterella id.2892) on Other Crohn's disease
rs8039785
rs62273907
rs55877868
rs7311004
rs823424
rs7303158
rs2090816
rs35055552
rs78383039
rs6828768
rs11715853
rs6809952
rs7572229
rs10899911
All
−0.2
−0.1
0.0
0.1
0.2
0.3
MR effect size for
'Gut microbiota abundance (genus Parasutterella id.2892)' on 'Other Crohn's disease'

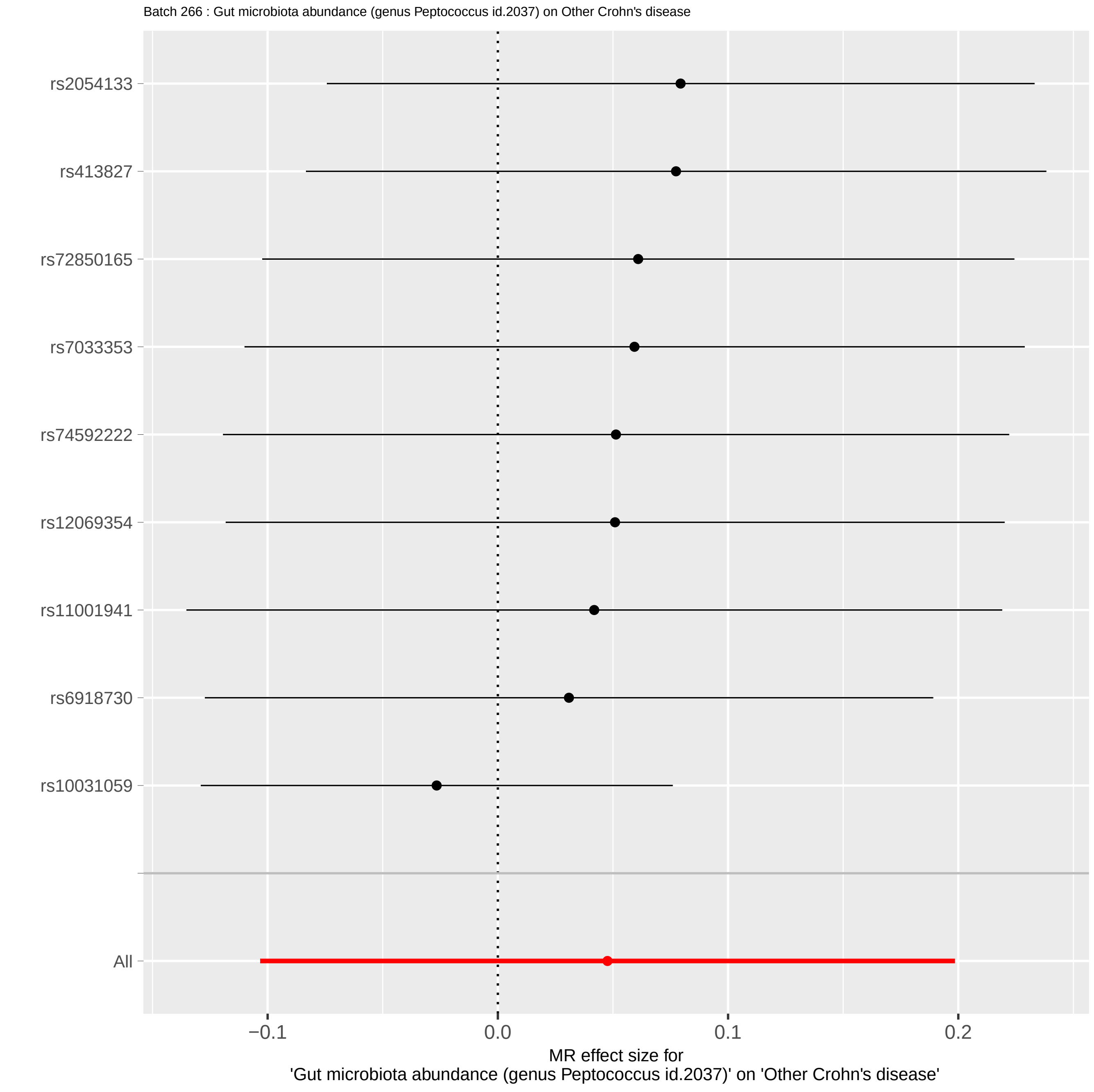
Batch 266 : Gut microbiota abundance (genus Peptococcus id.2037) on Other Crohn's disease
rs2054133
rs413827
rs72850165
rs7033353
rs74592222
rs12069354
rs11001941
rs6918730
rs10031059
All
−0.1
0.0
0.1
0.2
MR effect size for
'Gut microbiota abundance (genus Peptococcus id.2037)' on 'Other Crohn's disease'

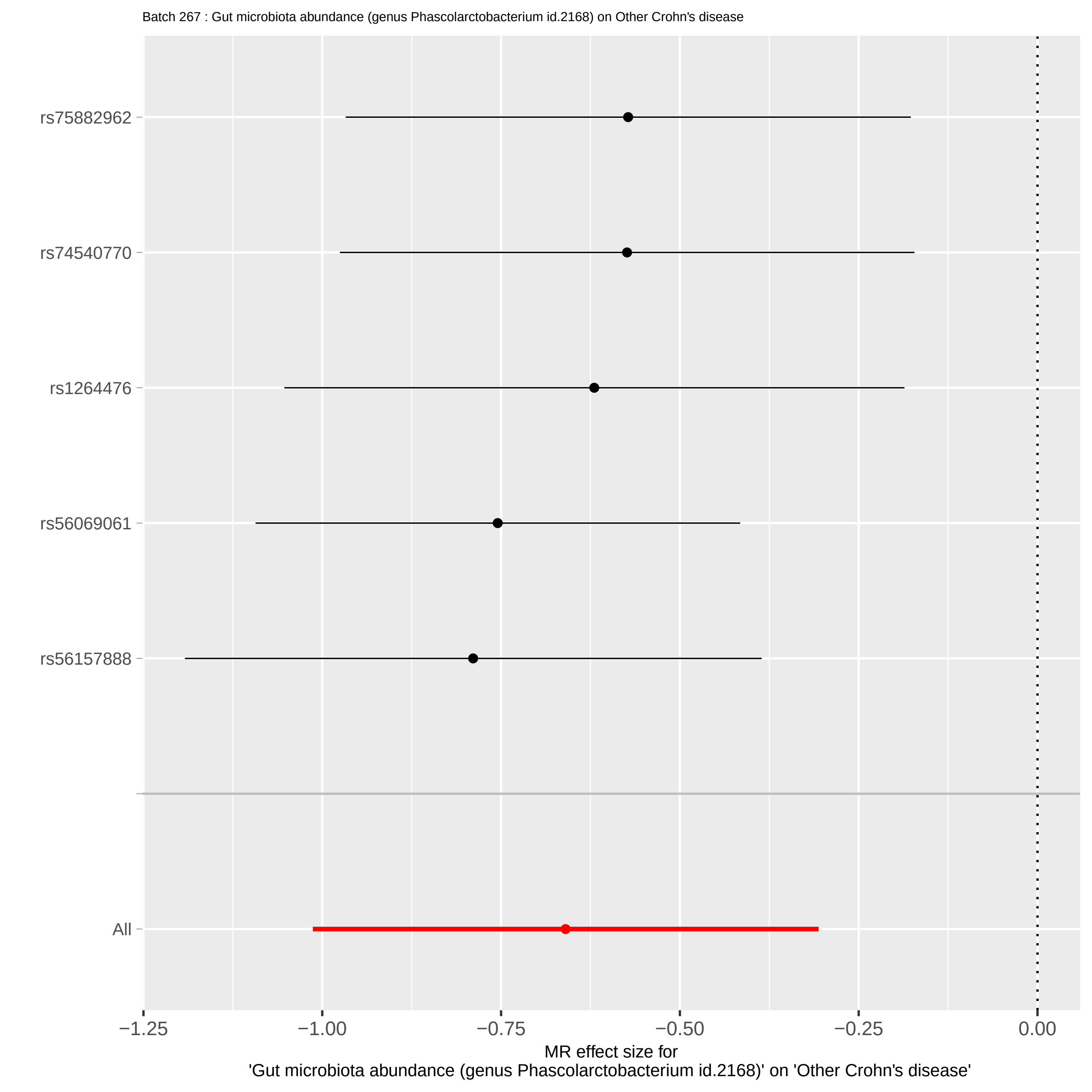

Batch 267 : Gut microbiota abundance (genus Phascolarctobacterium id.2168) on Other Crohn's disease
rs75882962
rs74540770
rs1264476
rs56069061
rs56157888
All
−1.25
−1.00
−0.75
−0.50
−0.25
0.00
MR effect size for
'Gut microbiota abundance (genus Phascolarctobacterium id.2168)' on 'Other Crohn's disease'

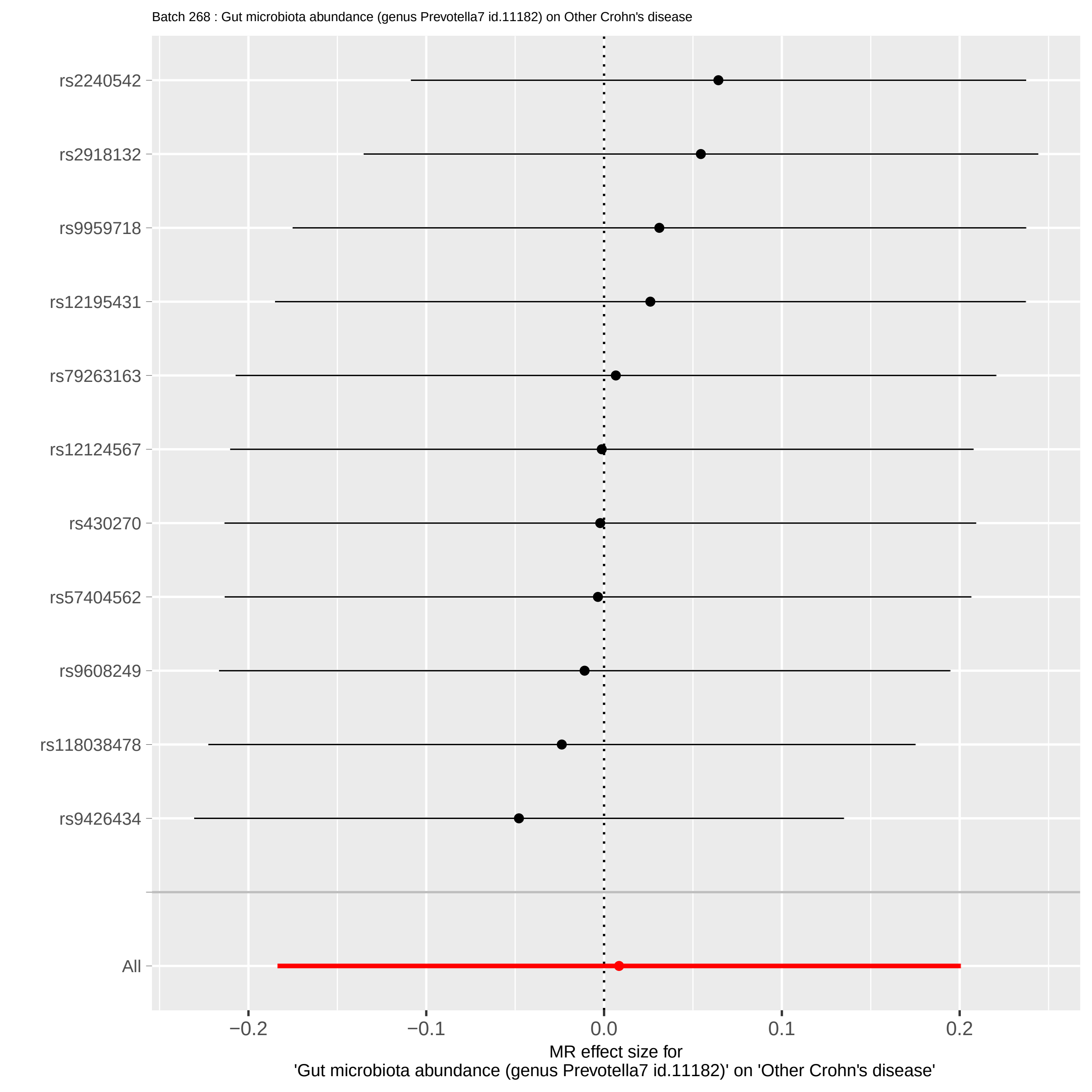
Batch 268 : Gut microbiota abundance (genus Prevotella7 id.11182) on Other Crohn's disease
rs2240542
rs2918132
rs9959718
rs12195431
rs79263163
rs12124567
rs430270
rs57404562
rs9608249
rs118038478
rs9426434
All
−0.2
−0.1
0.0
0.1
0.2
MR effect size for
'Gut microbiota abundance (genus Prevotella7 id.11182)' on 'Other Crohn's disease'

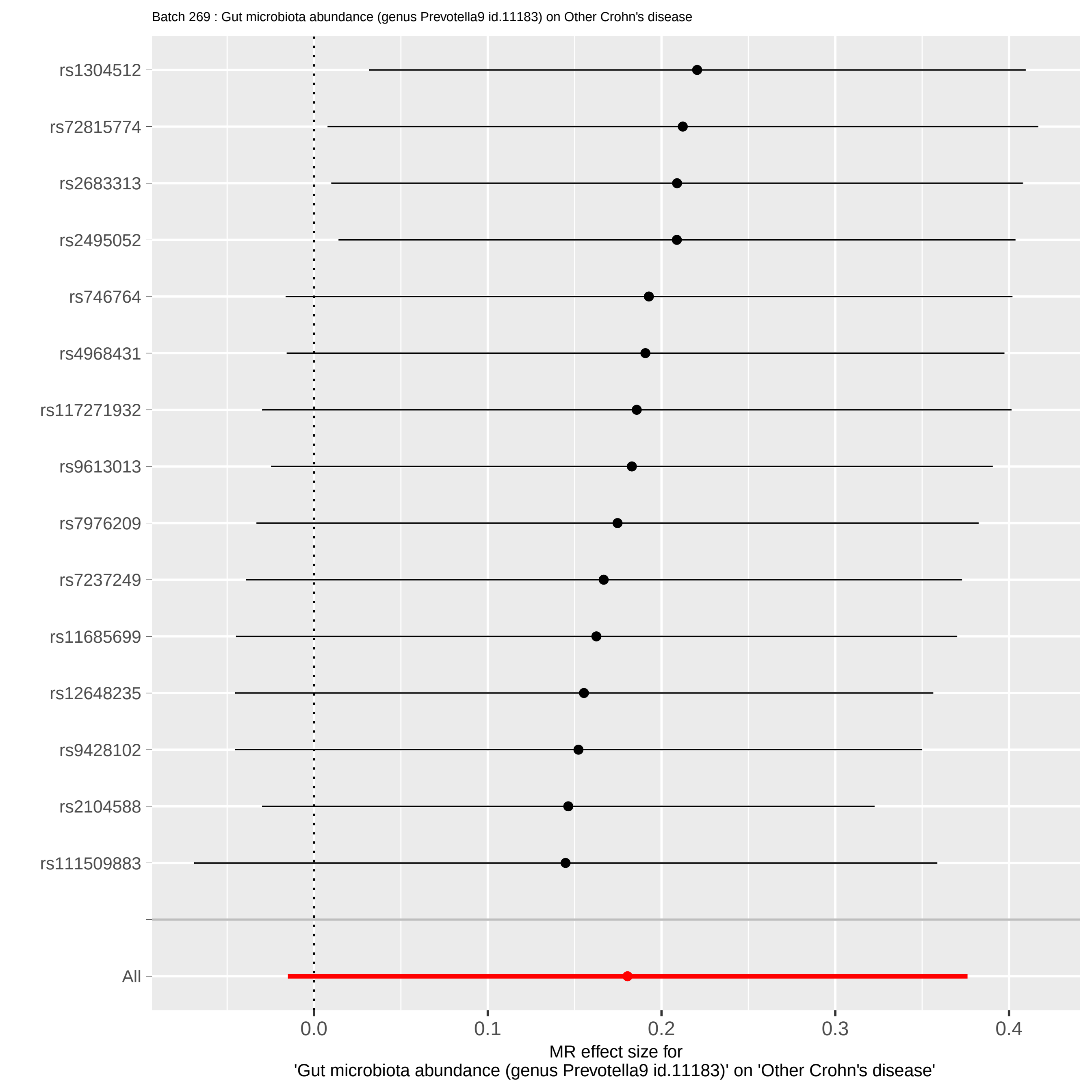

Batch 269 : Gut microbiota abundance (genus Prevotella9 id.11183) on Other Crohn's disease
rs1304512
rs72815774
rs2683313
rs2495052
rs746764
rs4968431
rs117271932
rs9613013
rs7976209
rs7237249
rs11685699
rs12648235
rs9428102
rs2104588
rs111509883
All
0.0
0.1
0.2
0.3
0.4
MR effect size for
'Gut microbiota abundance (genus Prevotella9 id.11183)' on 'Other Crohn's disease'

Batch 270 : Gut microbiota abundance (genus Rikenellaceae RC9 gut group id.11191) on Other Crohn's disease

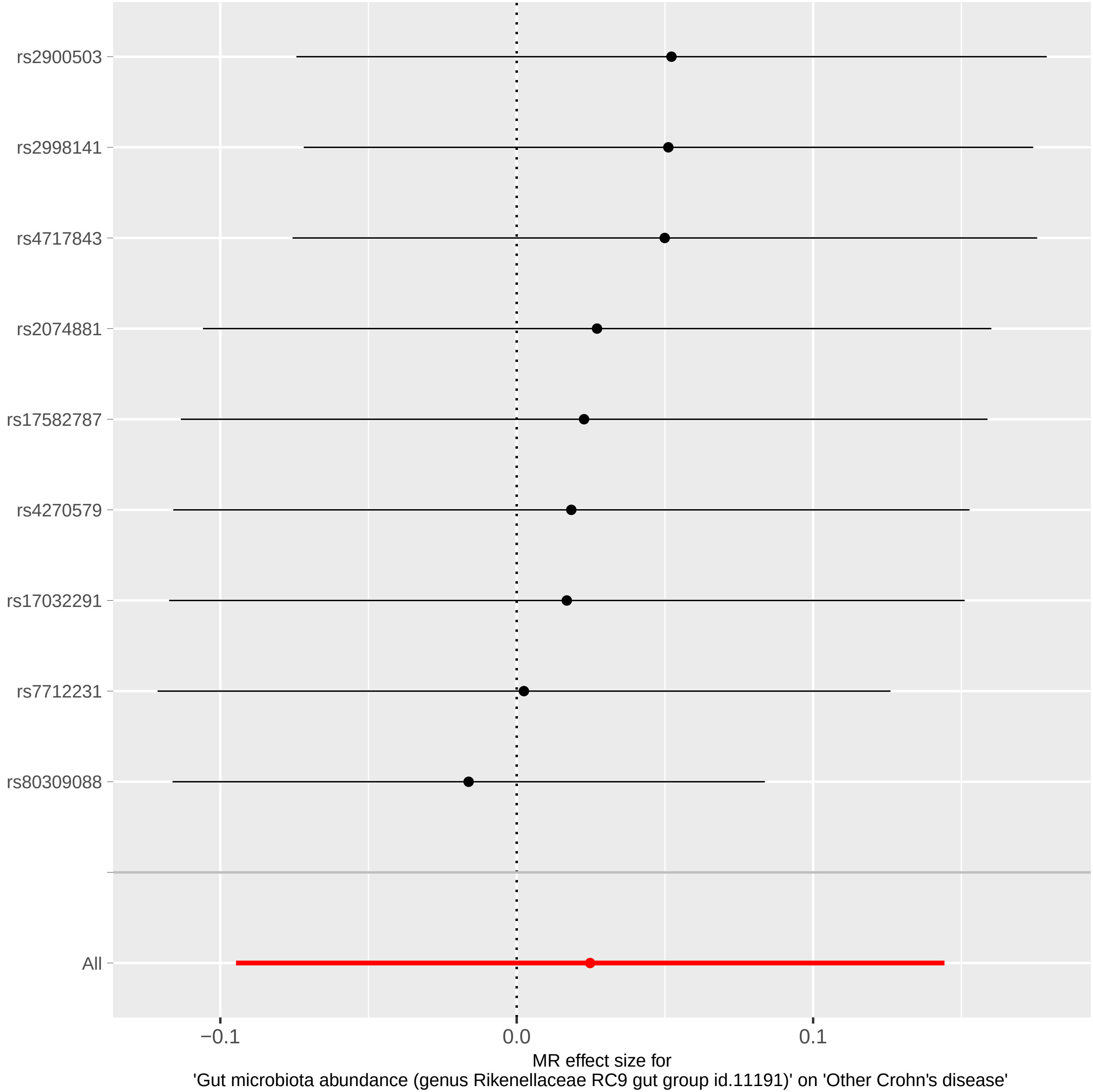

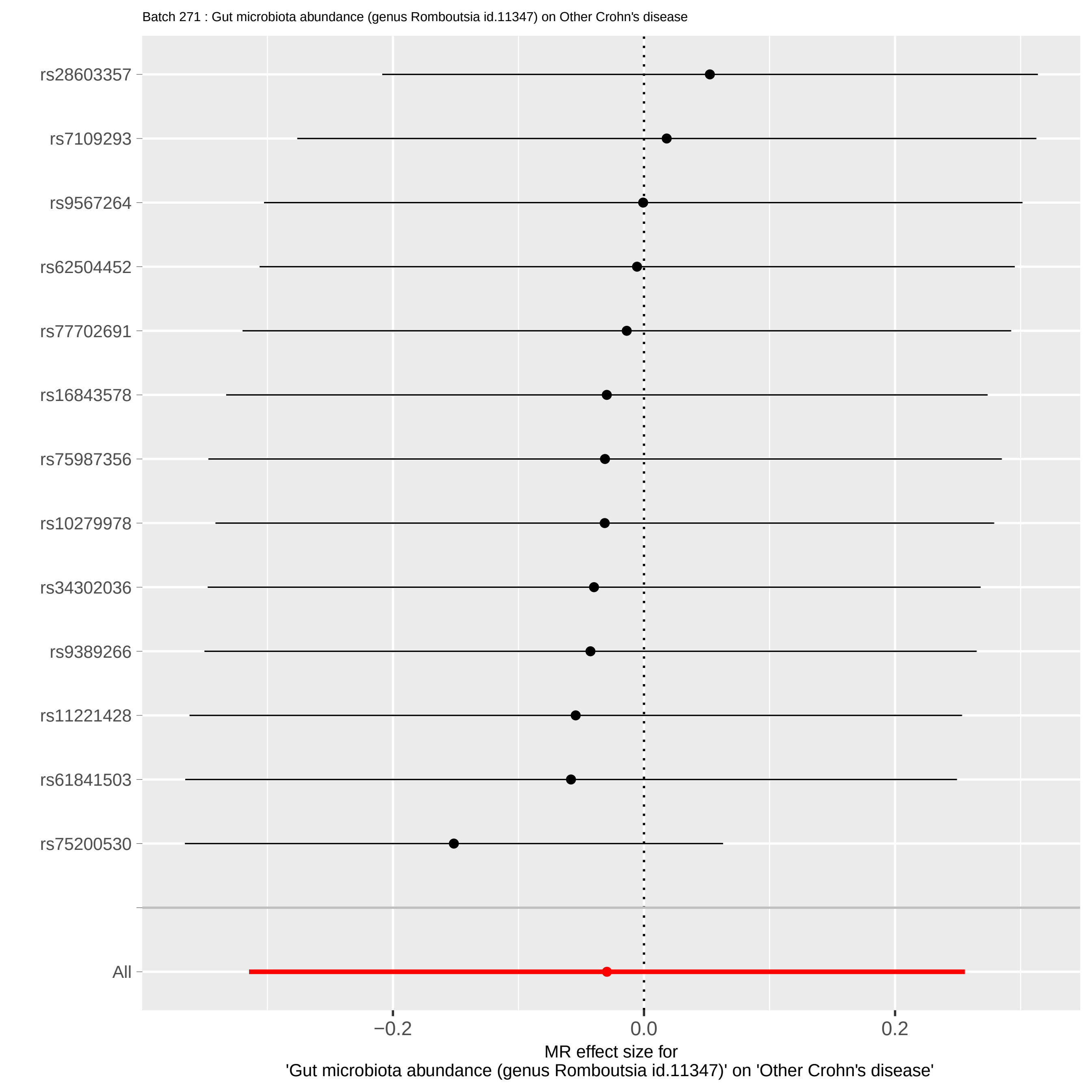
Batch 271 : Gut microbiota abundance (genus Romboutsia id.11347) on Other Crohn's disease
rs28603357
rs7109293
rs9567264
rs62504452
rs77702691
rs16843578
rs75987356
rs10279978
rs34302036
rs9389266
rs11221428
rs61841503
rs75200530
All
−0.2
0.0
0.2
MR effect size for
'Gut microbiota abundance (genus Romboutsia id.11347)' on 'Other Crohn's disease'

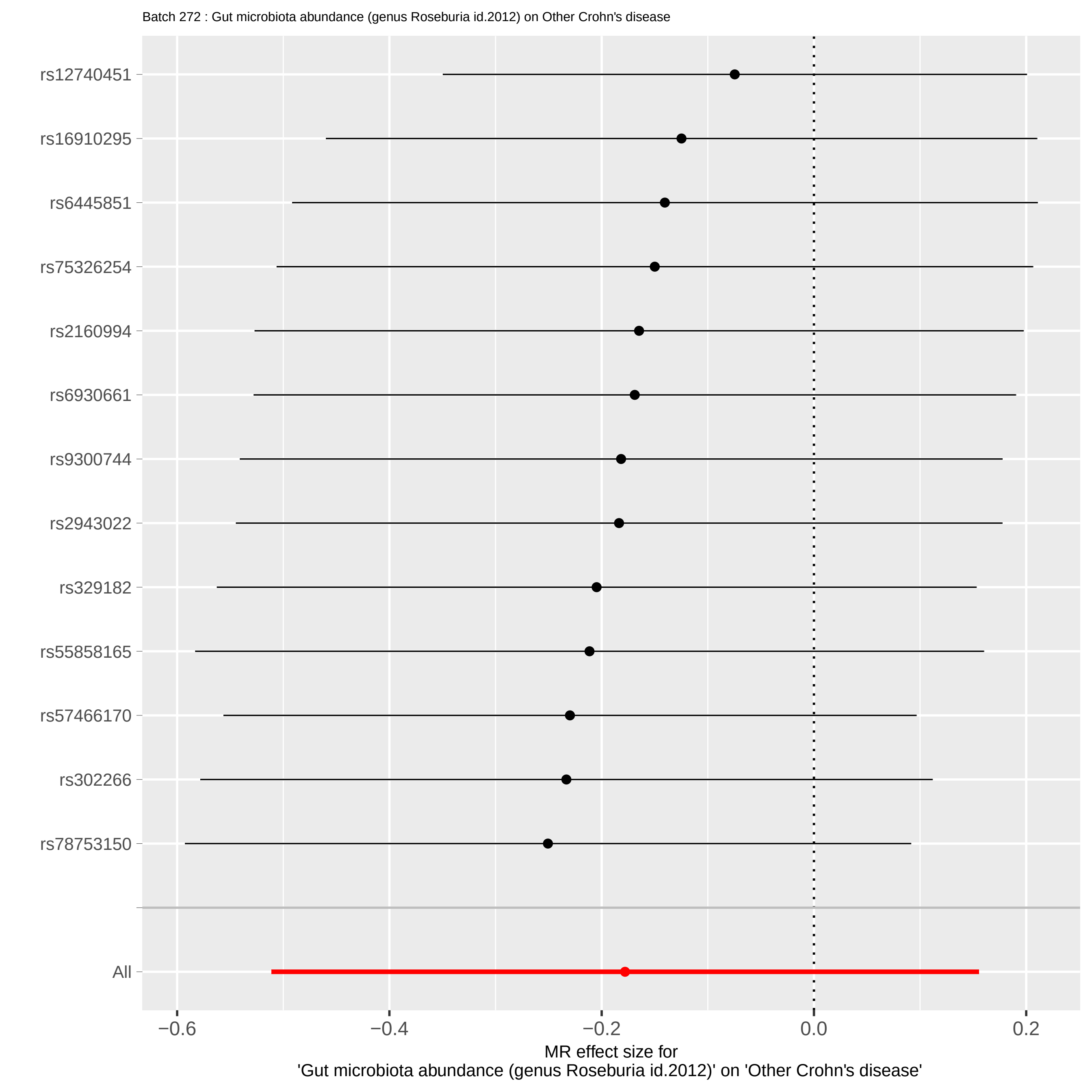
Batch 272 : Gut microbiota abundance (genus Roseburia id.2012) on Other Crohn's disease
rs12740451
rs16910295
rs6445851
rs75326254
rs2160994
rs6930661
rs9300744
rs2943022
rs329182
rs55858165
rs57466170
rs302266
rs78753150
All
−0.6
−0.4
−0.2
0.0
0.2
MR effect size for
'Gut microbiota abundance (genus Roseburia id.2012)' on 'Other Crohn's disease'

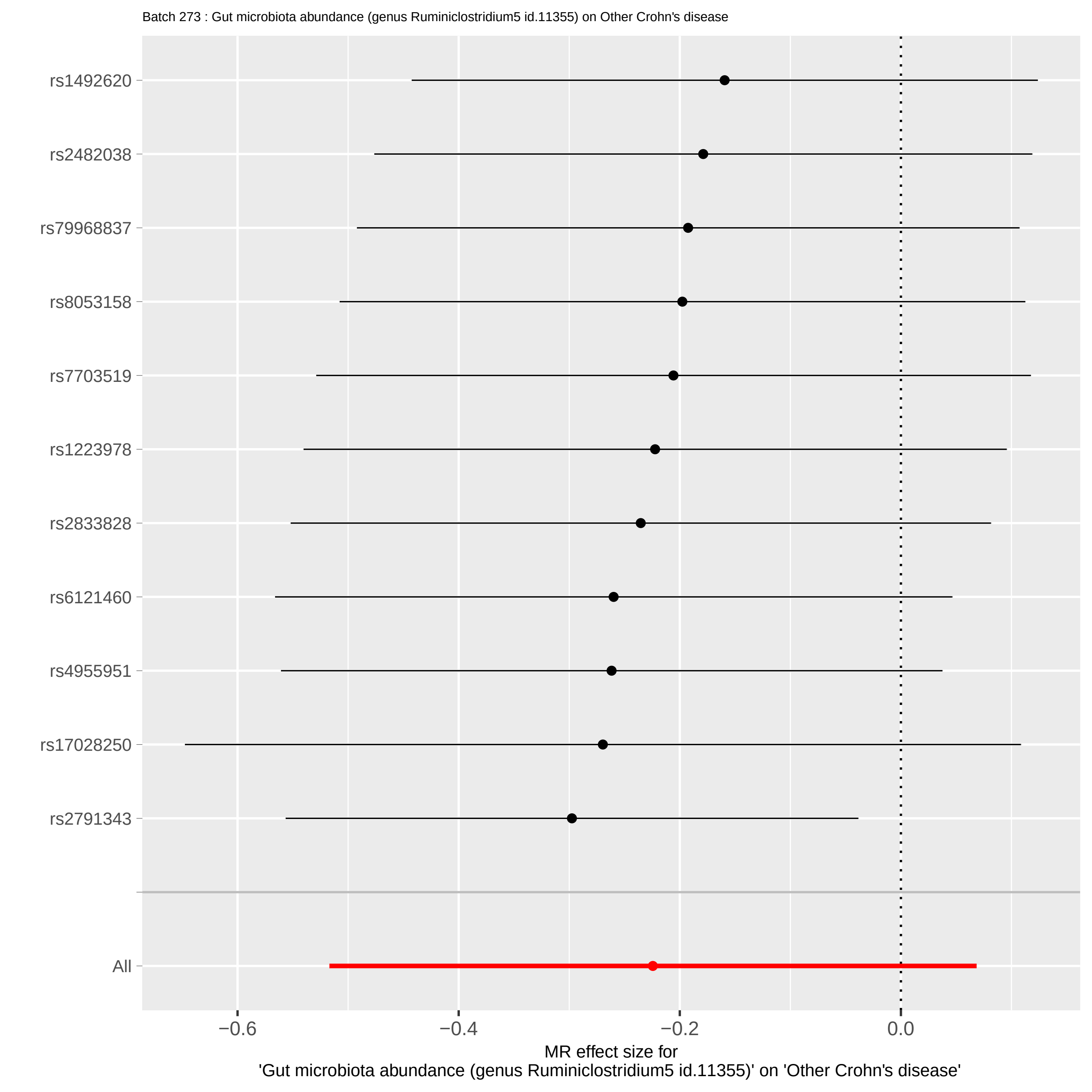
Batch 273 : Gut microbiota abundance (genus Ruminiclostridium5 id.11355) on Other Crohn's disease
rs1492620
rs2482038
rs79968837
rs8053158
rs7703519
rs1223978
rs2833828
rs6121460
rs4955951
rs17028250
rs2791343
All
−0.6
−0.4
−0.2
0.0
MR effect size for
'Gut microbiota abundance (genus Ruminiclostridium5 id.11355)' on 'Other Crohn's disease'

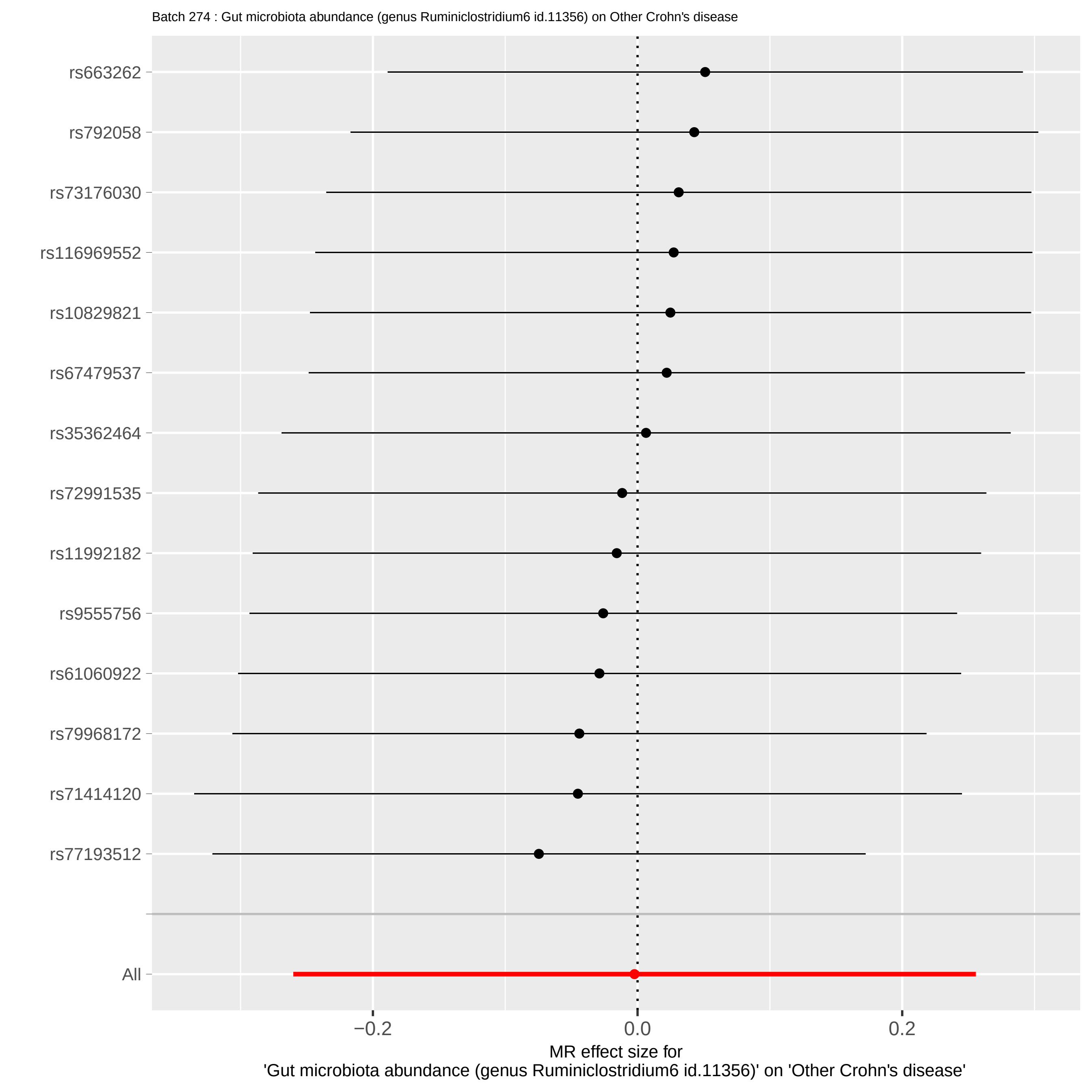

Batch 274 : Gut microbiota abundance (genus Ruminiclostridium6 id.11356) on Other Crohn's disease
rs663262
rs792058
rs73176030
rs116969552
rs10829821
rs67479537
rs35362464
rs72991535
rs11992182
rs9555756
rs61060922
rs79968172
rs71414120
rs77193512
All
−0.2
0.0
0.2
MR effect size for
'Gut microbiota abundance (genus Ruminiclostridium6 id.11356)' on 'Other Crohn's disease'

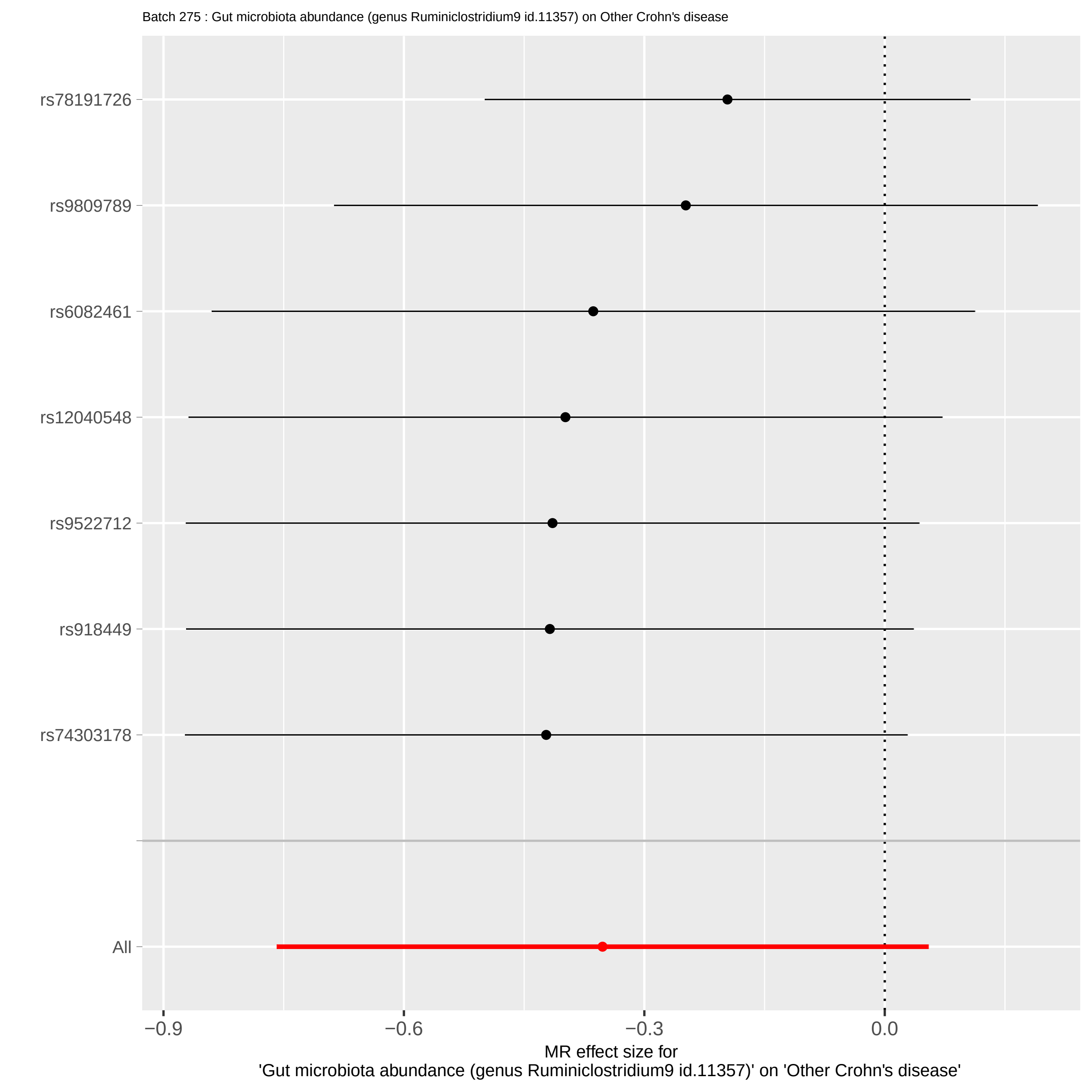

Batch 275 : Gut microbiota abundance (genus Ruminiclostridium9 id.11357) on Other Crohn's disease
rs78191726
rs9809789
rs6082461
rs12040548
rs9522712
rs918449
rs74303178
All
−0.9
−0.6
−0.3
0.0
MR effect size for
'Gut microbiota abundance (genus Ruminiclostridium9 id.11357)' on 'Other Crohn's disease'

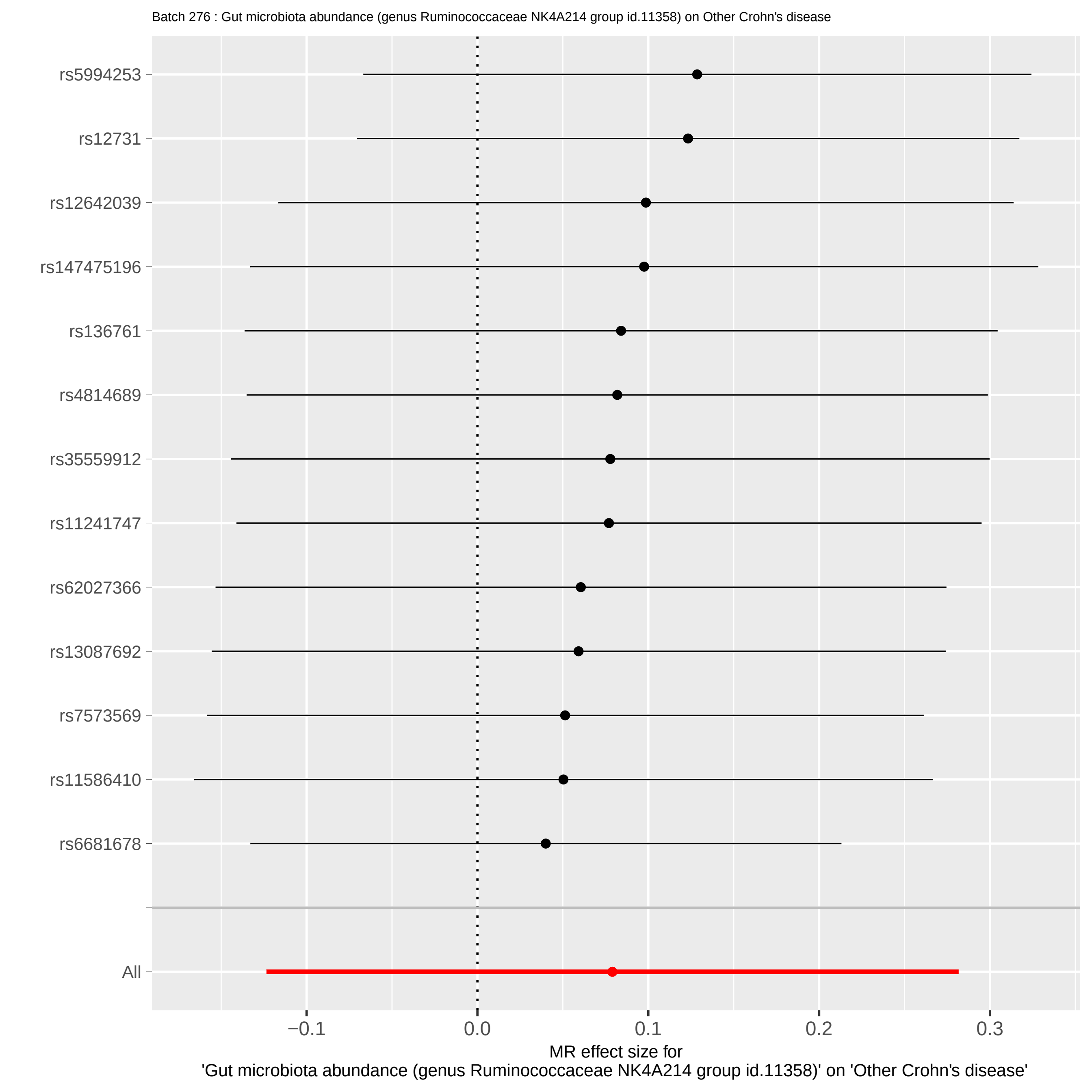

Batch 276 : Gut microbiota abundance (genus Ruminococcaceae NK4A214 group id.11358) on Other Crohn's disease
rs5994253
rs12731
rs12642039
rs147475196
rs136761
rs4814689
rs35559912
rs11241747
rs62027366
rs13087692
rs7573569
rs11586410
rs6681678
All
−0.1
0.0
0.1
0.2
0.3
MR effect size for
'Gut microbiota abundance (genus Ruminococcaceae NK4A214 group id.11358)' on 'Other Crohn's disease'

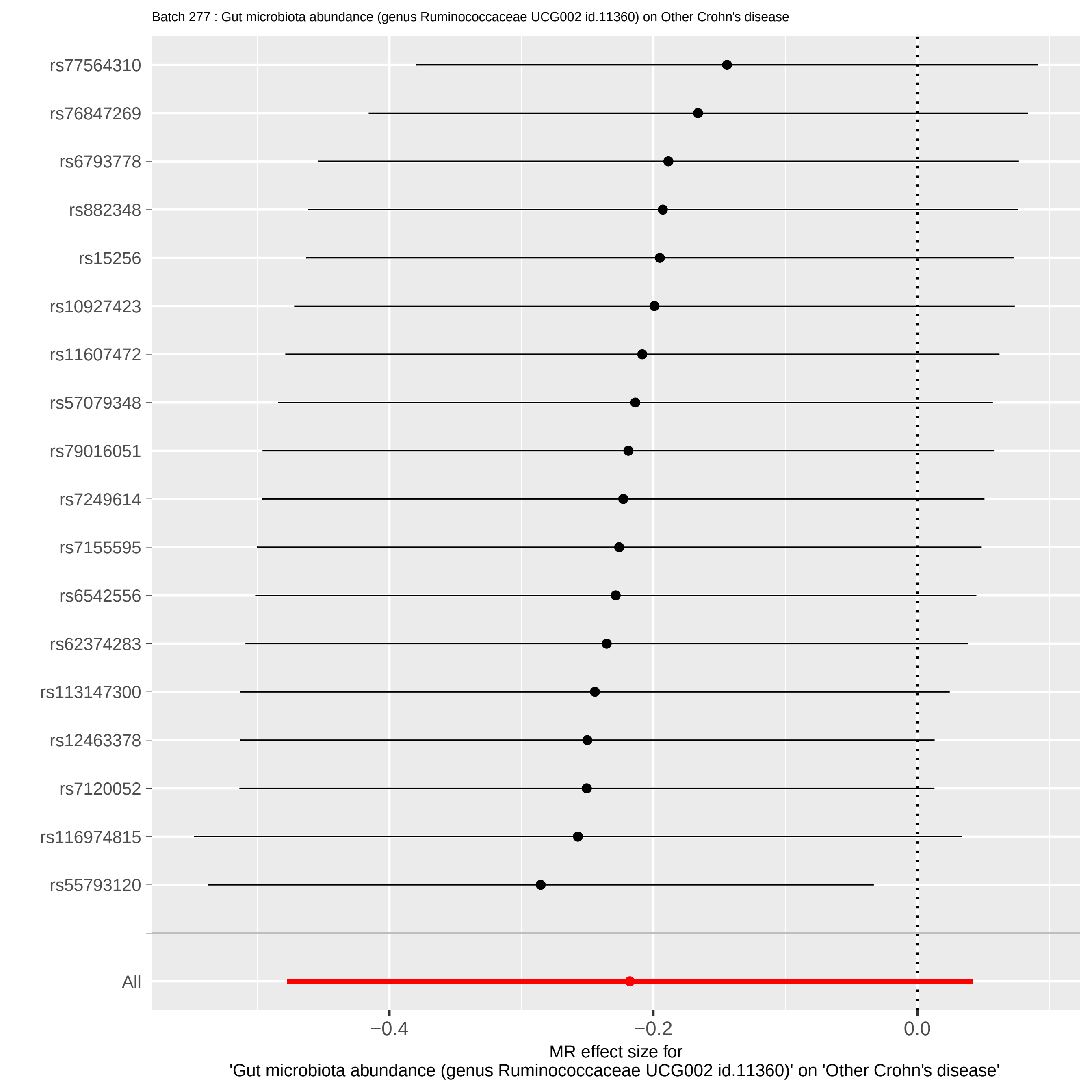
Batch 277 : Gut microbiota abundance (genus Ruminococcaceae UCG002 id.11360) on Other Crohn's disease
rs77564310
rs76847269
rs6793778
rs882348
rs15256
rs10927423
rs11607472
rs57079348
rs79016051
rs7249614
rs7155595
rs6542556
rs62374283
rs113147300
rs12463378
rs7120052
rs116974815
rs55793120
All
−0.4
−0.2
0.0
MR effect size for
'Gut microbiota abundance (genus Ruminococcaceae UCG002 id.11360)' on 'Other Crohn's disease'

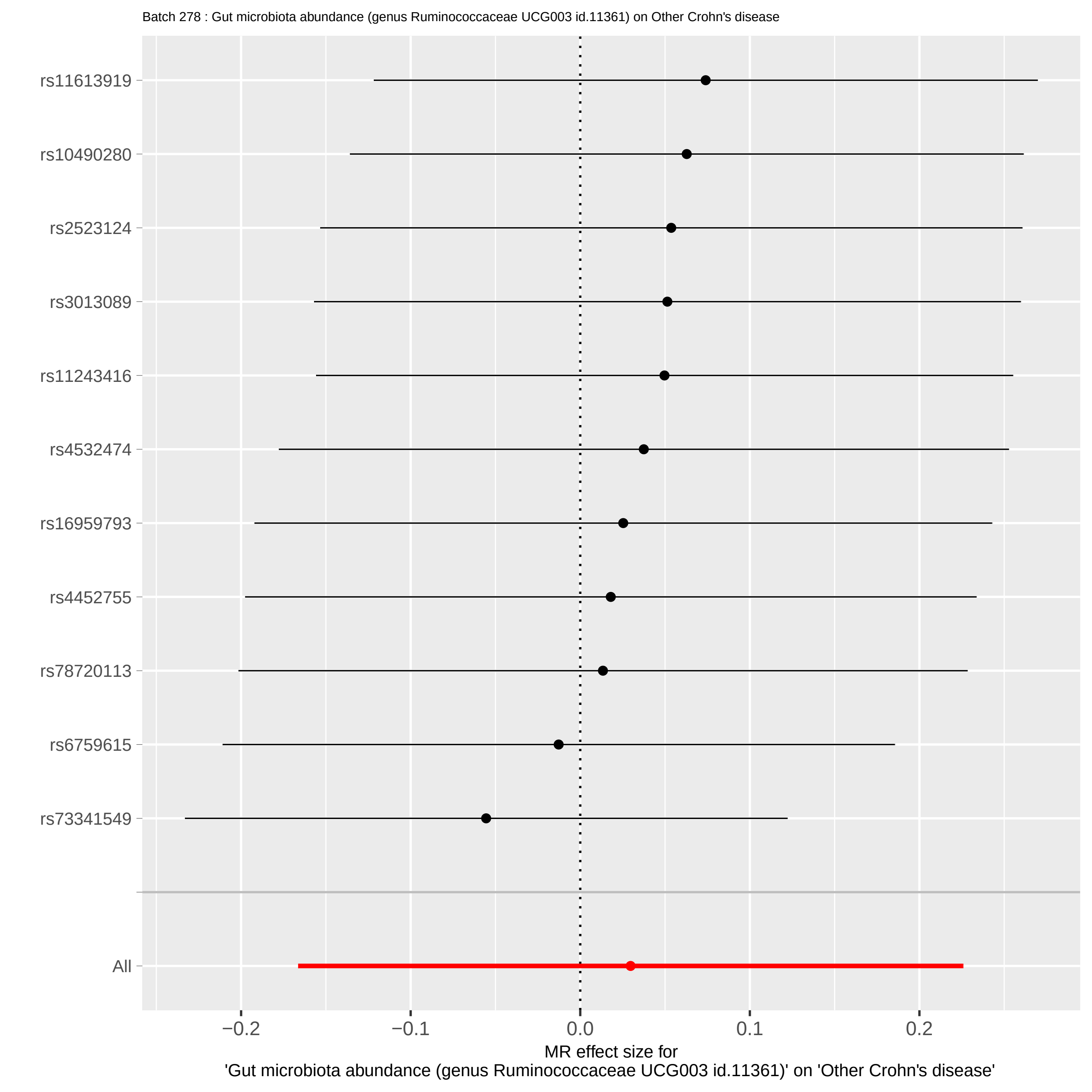

Batch 278 : Gut microbiota abundance (genus Ruminococcaceae UCG003 id.11361) on Other Crohn's disease
rs11613919
rs10490280
rs2523124
rs3013089
rs11243416
rs4532474
rs16959793
rs4452755
rs78720113
rs6759615
rs73341549
All
−0.2
−0.1
0.0
0.1
0.2
MR effect size for
'Gut microbiota abundance (genus Ruminococcaceae UCG003 id.11361)' on 'Other Crohn's disease'

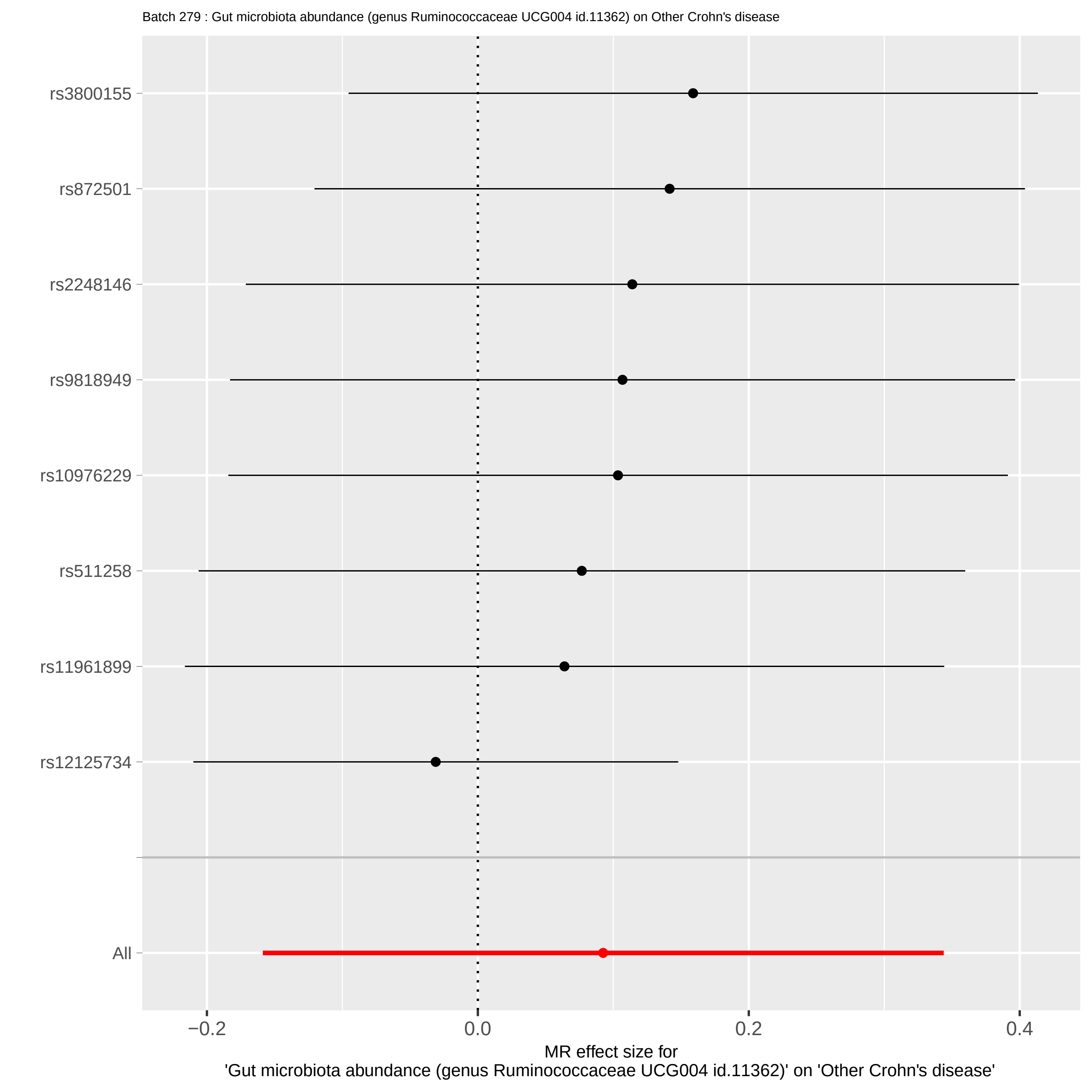
Batch 279 : Gut microbiota abundance (genus Ruminococcaceae UCG004 id.11362) on Other Crohn's disease
rs3800155
rs872501
rs2248146
rs9818949
rs10976229
rs511258
rs11961899
rs12125734
All
−0.2
0.0
0.2
0.4
MR effect size for
'Gut microbiota abundance (genus Ruminococcaceae UCG004 id.11362)' on 'Other Crohn's disease'

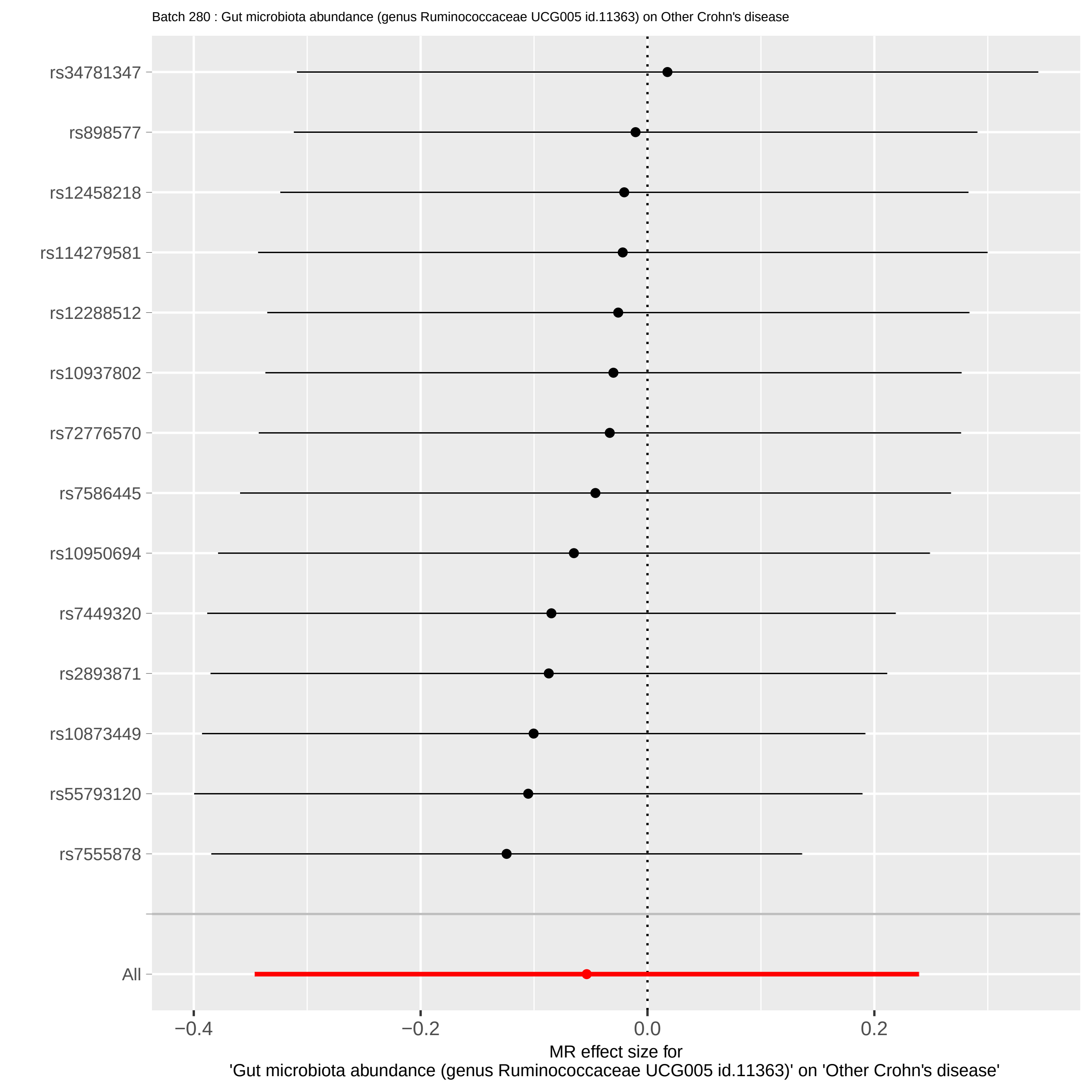

Batch 280 : Gut microbiota abundance (genus Ruminococcaceae UCG005 id.11363) on Other Crohn's disease
rs34781347
rs898577
rs12458218
rs114279581
rs12288512
rs10937802
rs72776570
rs7586445
rs10950694
rs7449320
rs2893871
rs10873449
rs55793120
rs7555878
All
−0.4
−0.2
0.0
0.2
MR effect size for
'Gut microbiota abundance (genus Ruminococcaceae UCG005 id.11363)' on 'Other Crohn's disease'

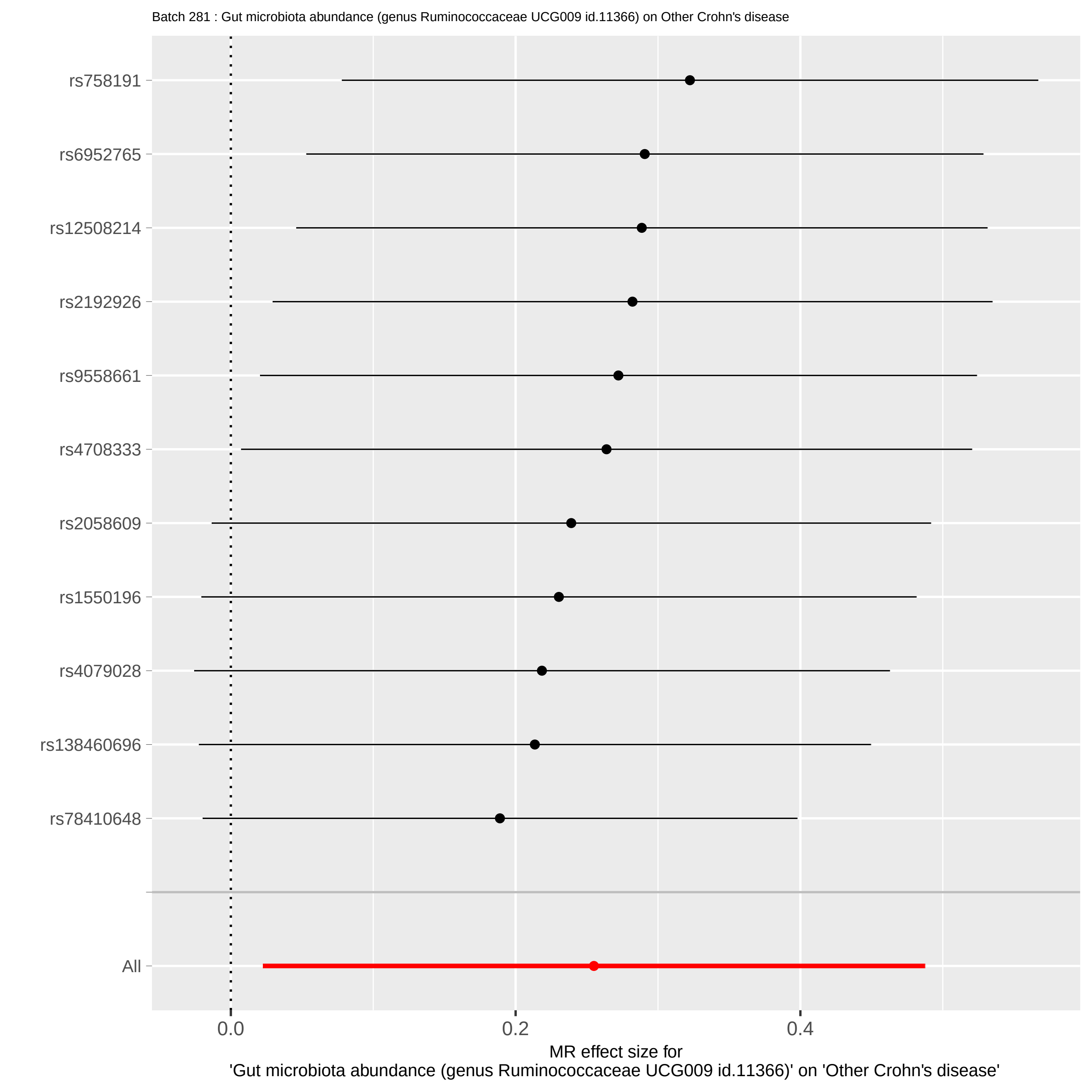

Batch 281 : Gut microbiota abundance (genus Ruminococcaceae UCG009 id.11366) on Other Crohn's disease
rs758191
rs6952765
rs12508214
rs2192926
rs9558661
rs4708333
rs2058609
rs1550196
rs4079028
rs138460696
rs78410648
All
0.0
0.2
0.4
MR effect size for
'Gut microbiota abundance (genus Ruminococcaceae UCG009 id.11366)' on 'Other Crohn's disease'

Batch 282 : Gut microbiota abundance (genus Ruminococcaceae UCG010 id.11367) on Other Crohn's disease

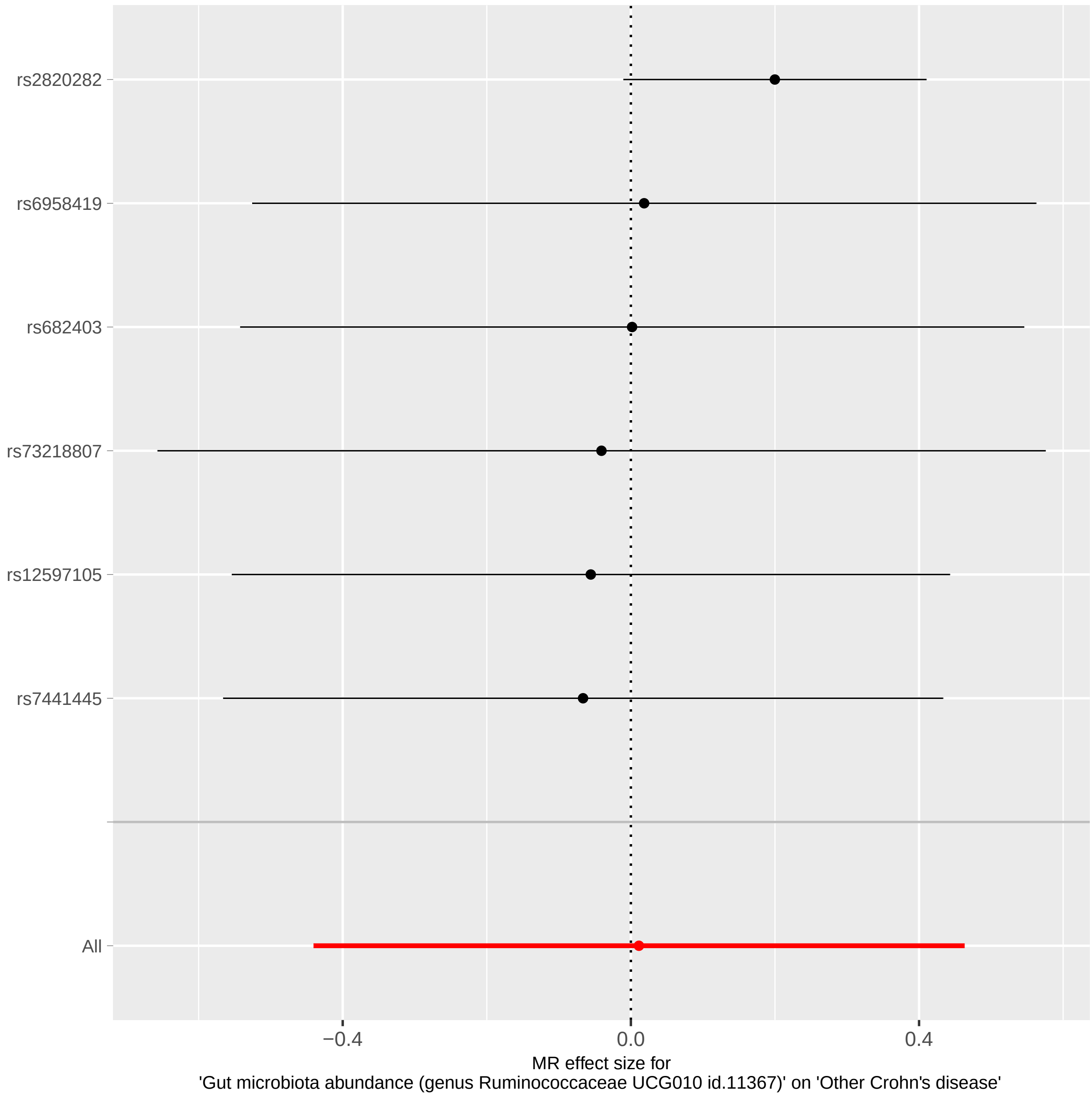

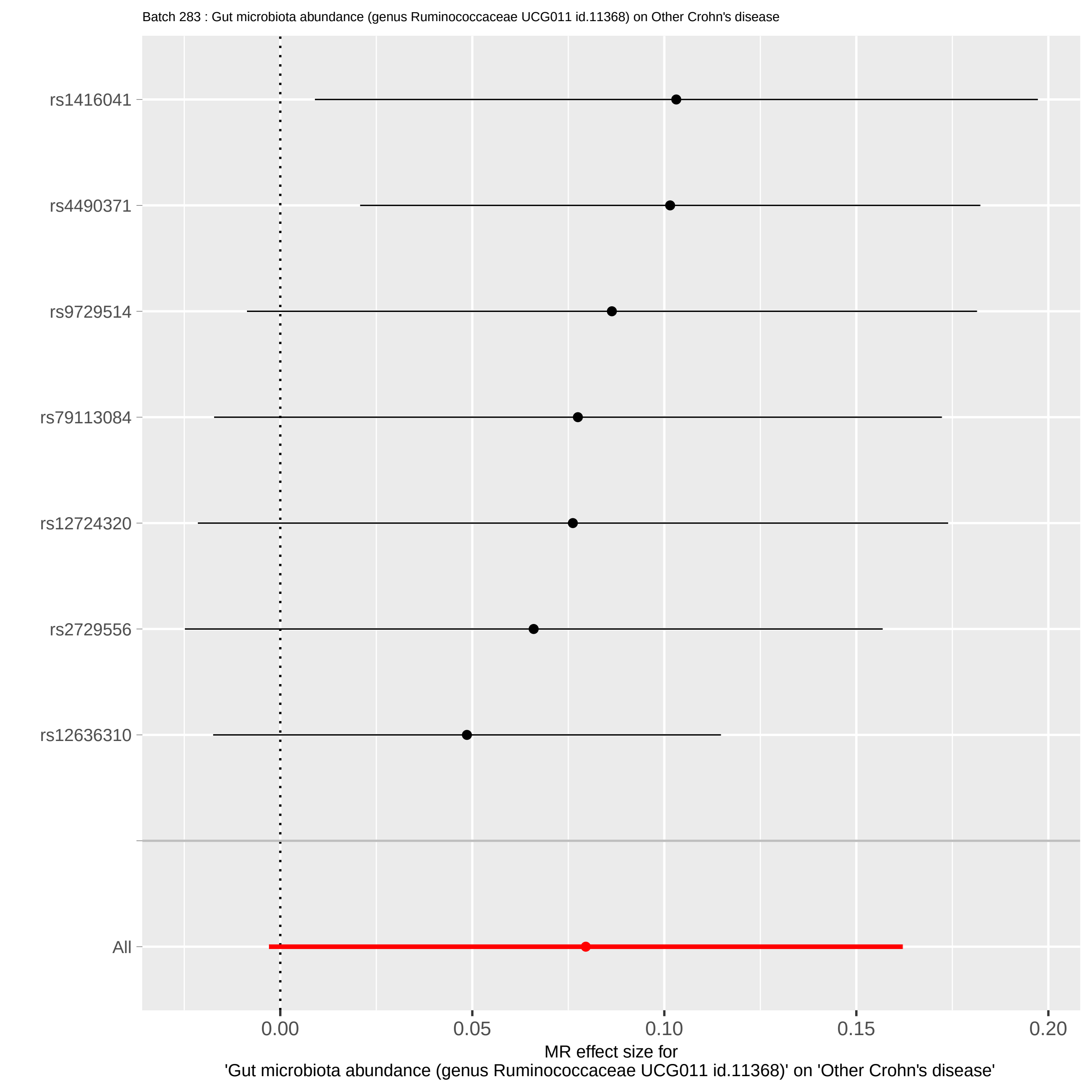

Batch 283 : Gut microbiota abundance (genus Ruminococcaceae UCG011 id.11368) on Other Crohn's disease
rs1416041
rs4490371
rs9729514
rs79113084
rs12724320
rs2729556
rs12636310
All
0.00
0.05
0.10
0.15
0.20
MR effect size for
'Gut microbiota abundance (genus Ruminococcaceae UCG011 id.11368)' on 'Other Crohn's disease'

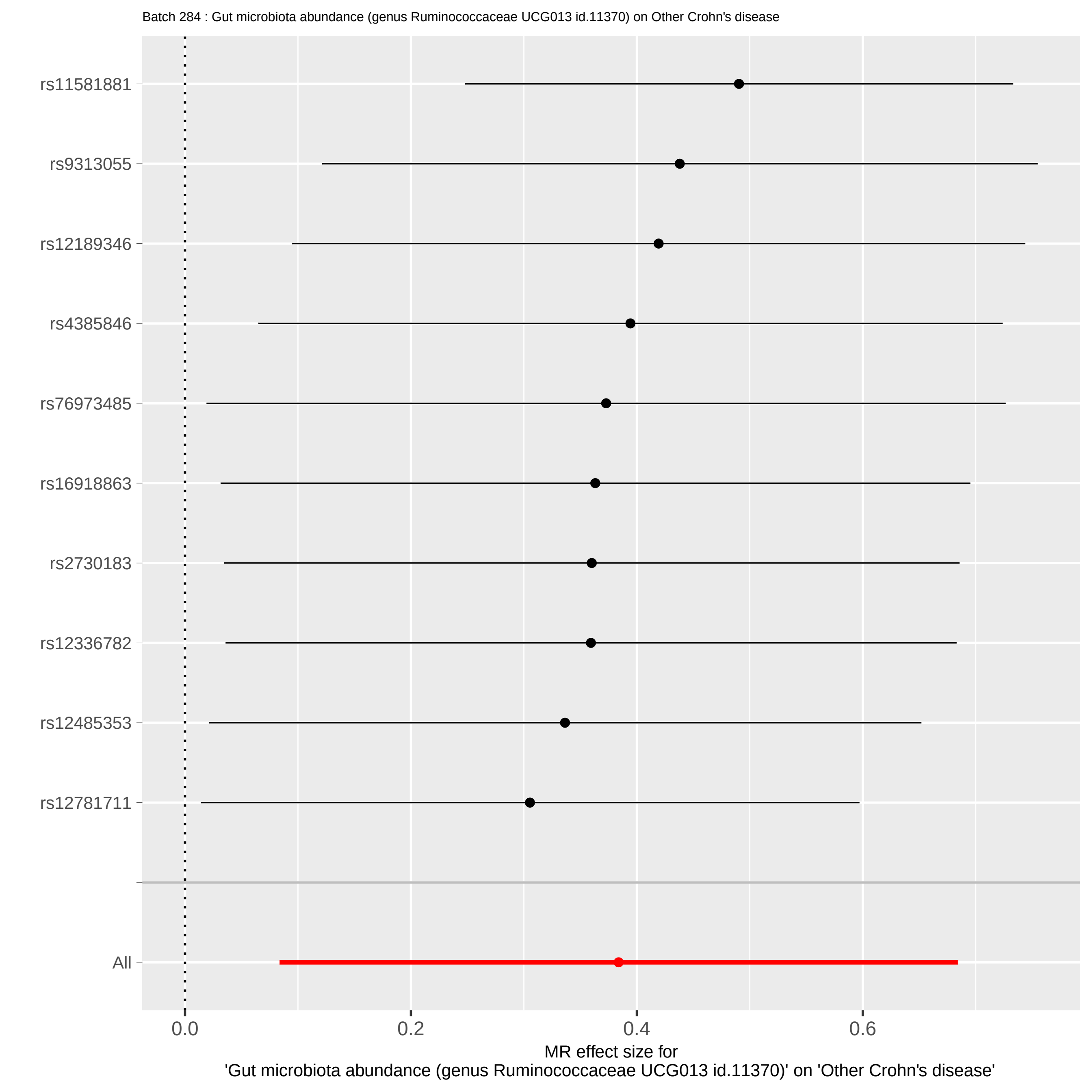

Batch 284 : Gut microbiota abundance (genus Ruminococcaceae UCG013 id.11370) on Other Crohn's disease
rs11581881
rs9313055
rs12189346
rs4385846
rs76973485
rs16918863
rs2730183
rs12336782
rs12485353
rs12781711
All
0.0
0.2
0.4
0.6
MR effect size for
'Gut microbiota abundance (genus Ruminococcaceae UCG013 id.11370)' on 'Other Crohn's disease'

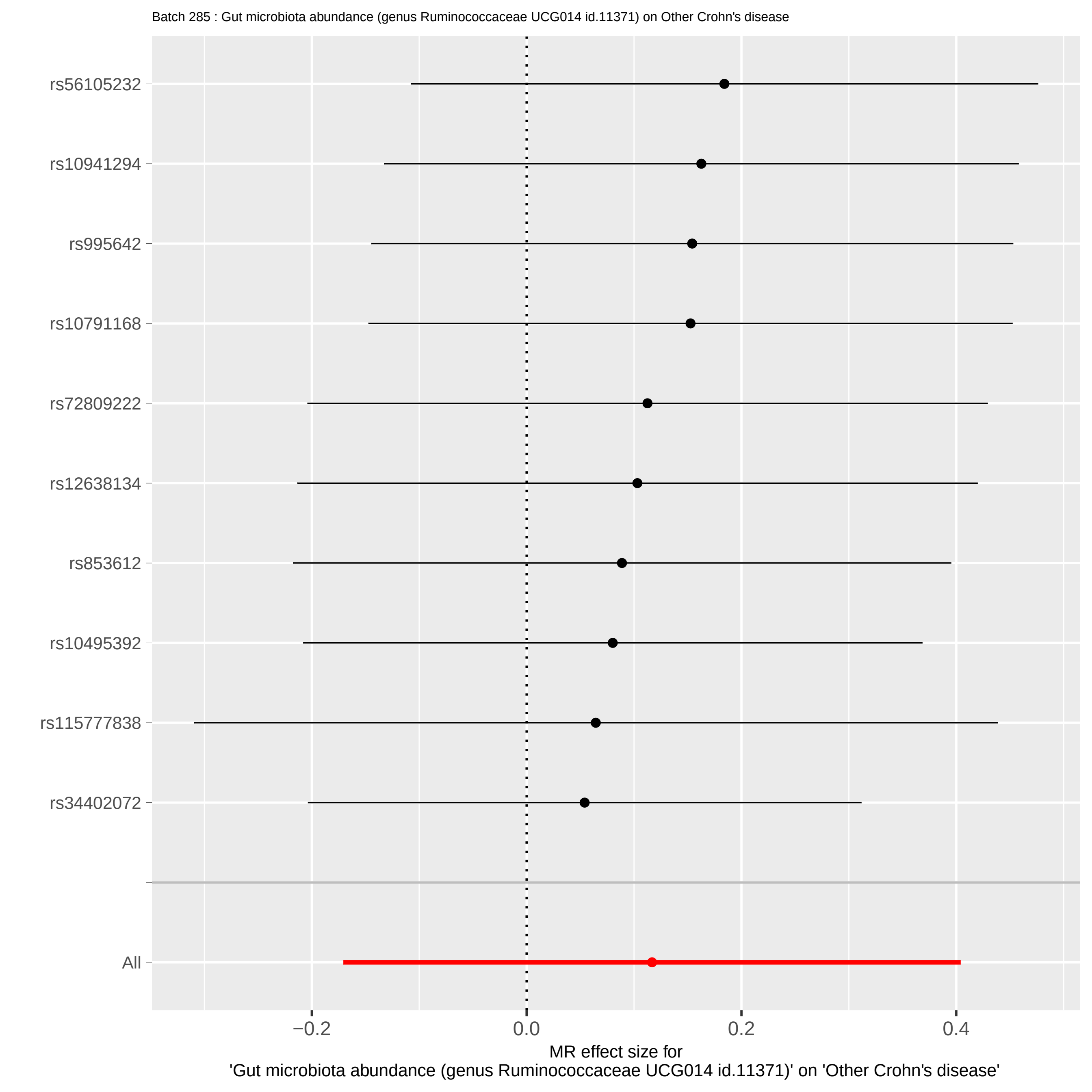
Batch 285 : Gut microbiota abundance (genus Ruminococcaceae UCG014 id.11371) on Other Crohn's disease
rs56105232
rs10941294
rs995642
rs10791168
rs72809222
rs12638134
rs853612
rs10495392
rs115777838
rs34402072
All
−0.2
0.0
0.2
0.4
MR effect size for
'Gut microbiota abundance (genus Ruminococcaceae UCG014 id.11371)' on 'Other Crohn's disease'

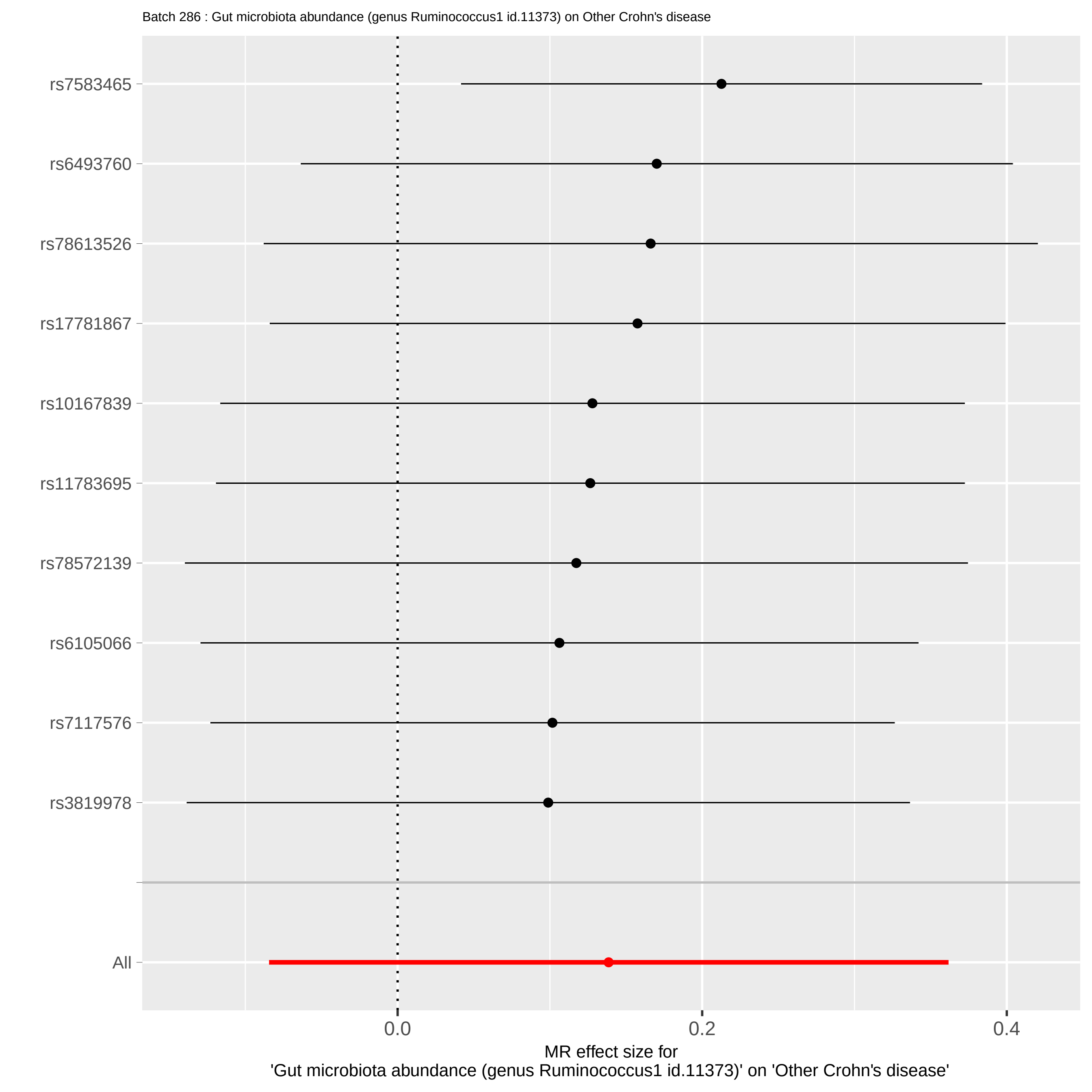

Batch 286 : Gut microbiota abundance (genus Ruminococcus1 id.11373) on Other Crohn's disease
rs7583465
rs6493760
rs78613526
rs17781867
rs10167839
rs11783695
rs78572139
rs6105066
rs7117576
rs3819978
All
0.0
0.2
0.4
MR effect size for
'Gut microbiota abundance (genus Ruminococcus1 id.11373)' on 'Other Crohn's disease'

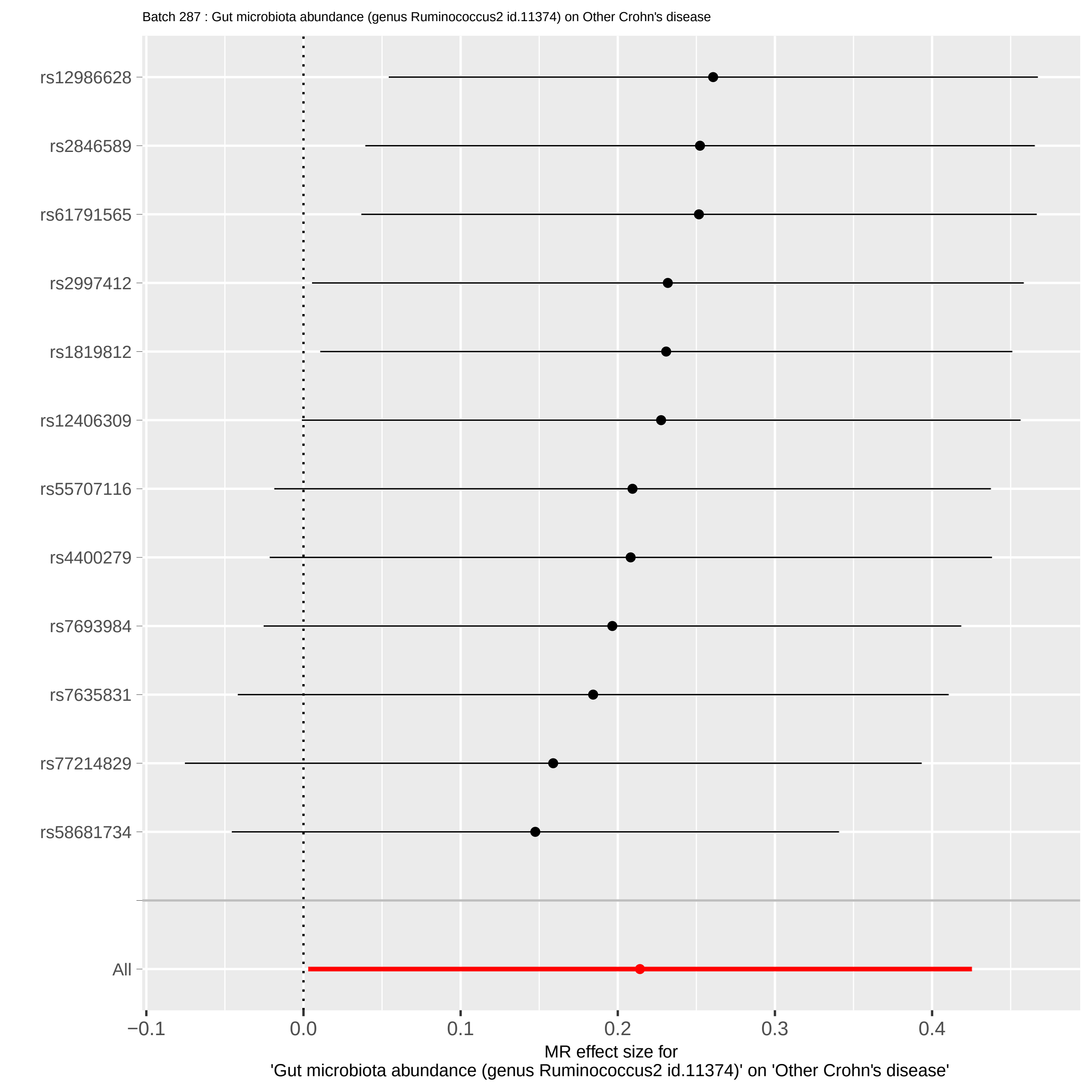
Batch 287 : Gut microbiota abundance (genus Ruminococcus2 id.11374) on Other Crohn's disease
rs12986628
rs2846589
rs61791565
rs2997412
rs1819812
rs12406309
rs55707116
rs4400279
rs7693984
rs7635831
rs77214829
rs58681734
All
−0.1
0.0
0.1
0.2
0.3
0.4
MR effect size for
'Gut microbiota abundance (genus Ruminococcus2 id.11374)' on 'Other Crohn's disease'

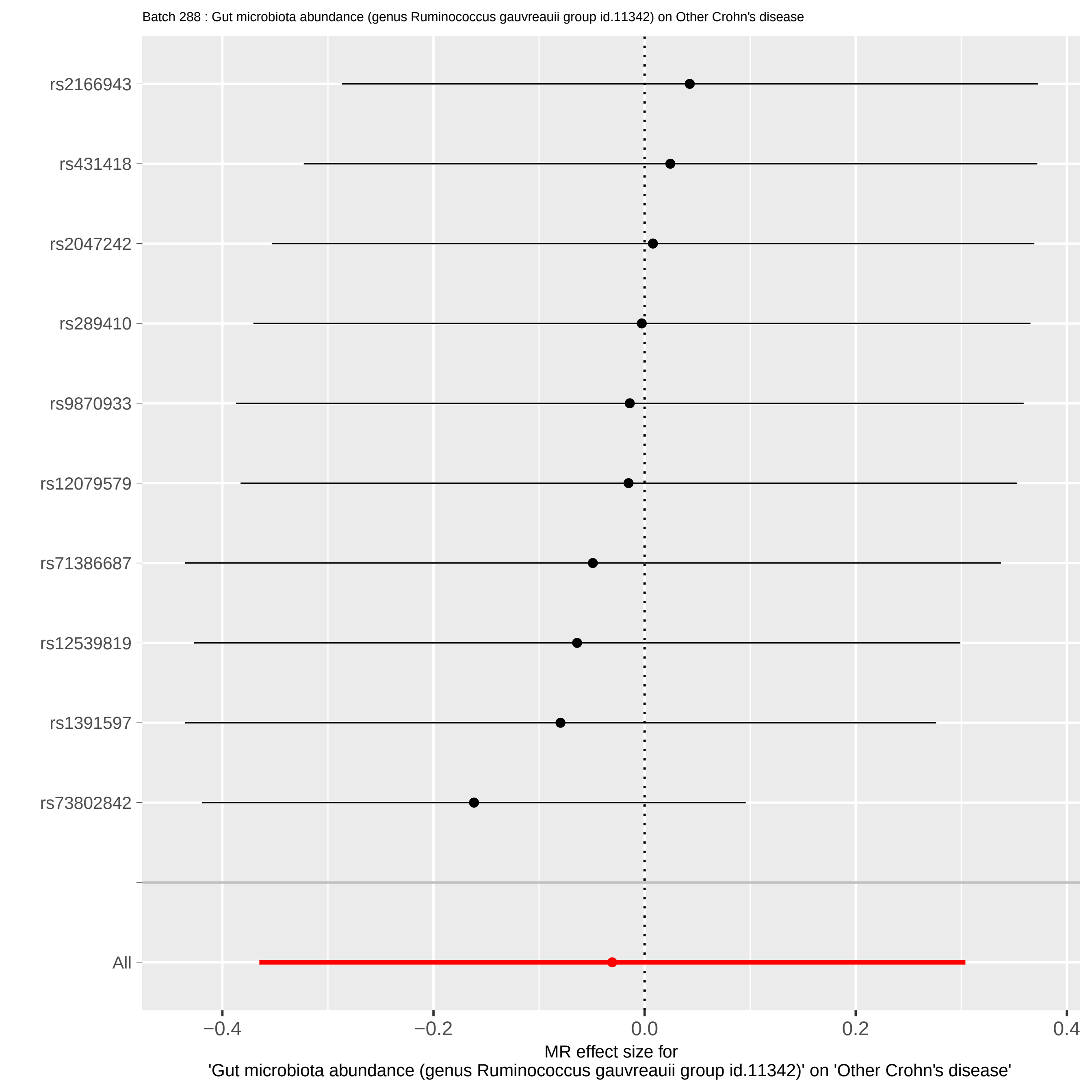

Batch 288 : Gut microbiota abundance (genus Ruminococcus gauvreauii group id.11342) on Other Crohn's disease
rs2166943
rs431418
rs2047242
rs289410
rs9870933
rs12079579
rs71386687
rs12539819
rs1391597
rs73802842
All
−0.4
−0.2
0.0
0.2
0.4
MR effect size for
'Gut microbiota abundance (genus Ruminococcus gauvreauii group id.11342)' on 'Other Crohn's disease'

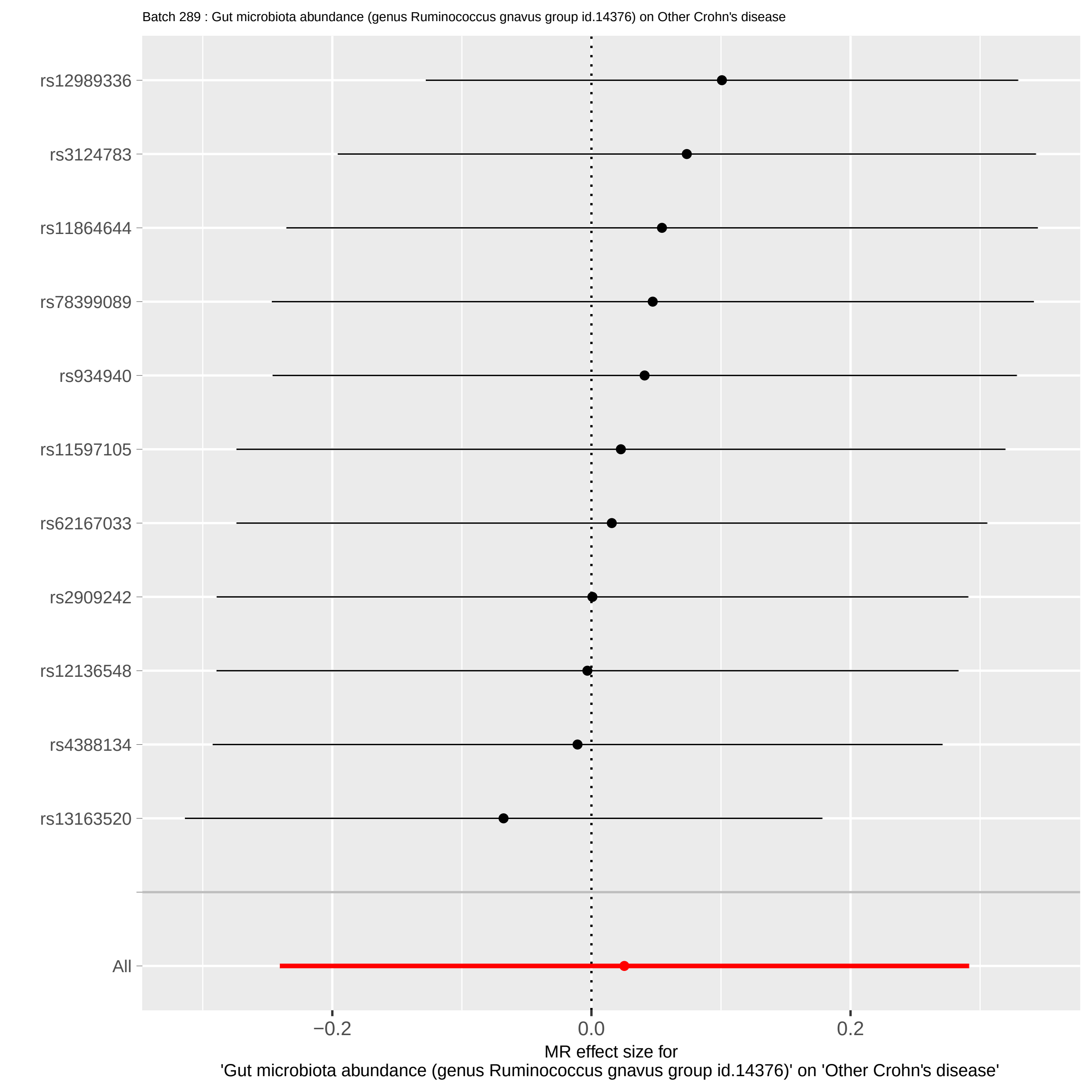
Batch 289 : Gut microbiota abundance (genus Ruminococcus gnavus group id.14376) on Other Crohn's disease
rs12989336
rs3124783
rs11864644
rs78399089
rs934940
rs11597105
rs62167033
rs2909242
rs12136548
rs4388134
rs13163520
All
−0.2
0.0
0.2
MR effect size for
'Gut microbiota abundance (genus Ruminococcus gnavus group id.14376)' on 'Other Crohn's disease'

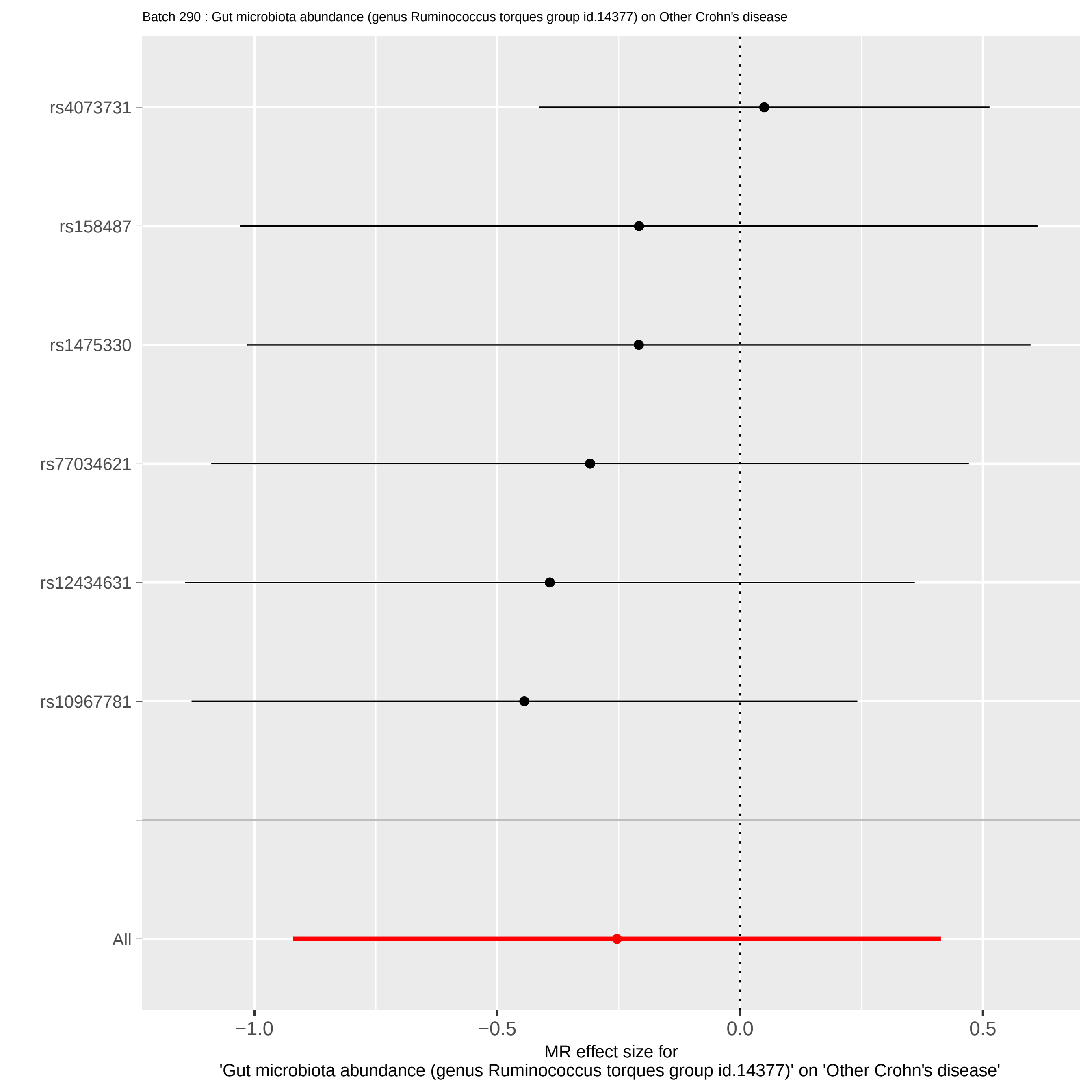

Batch 290 : Gut microbiota abundance (genus Ruminococcus torques group id.14377) on Other Crohn's disease
rs4073731
rs158487
rs1475330
rs77034621
rs12434631
rs10967781
All
−1.0
−0.5
0.0
0.5
MR effect size for
'Gut microbiota abundance (genus Ruminococcus torques group id.14377)' on 'Other Crohn's disease'

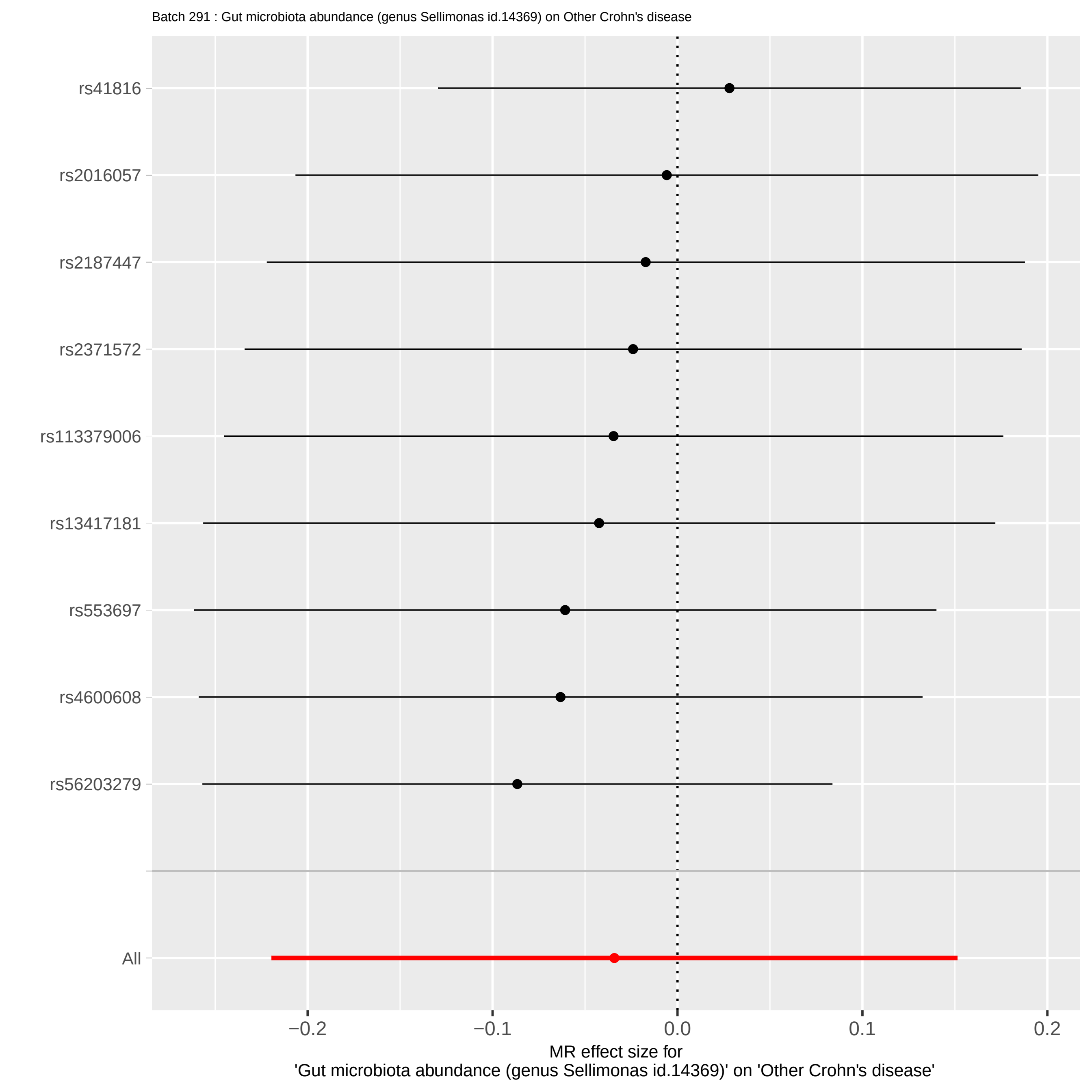

Batch 291 : Gut microbiota abundance (genus Sellimonas id.14369) on Other Crohn's disease
rs41816
rs2016057
rs2187447
rs2371572
rs113379006
rs13417181
rs553697
rs4600608
rs56203279
All
−0.2
−0.1
0.0
0.1
0.2
MR effect size for
'Gut microbiota abundance (genus Sellimonas id.14369)' on 'Other Crohn's disease'

Batch 292 : Gut microbiota abundance (genus Senegalimassilia id.11160) on Other Crohn's disease

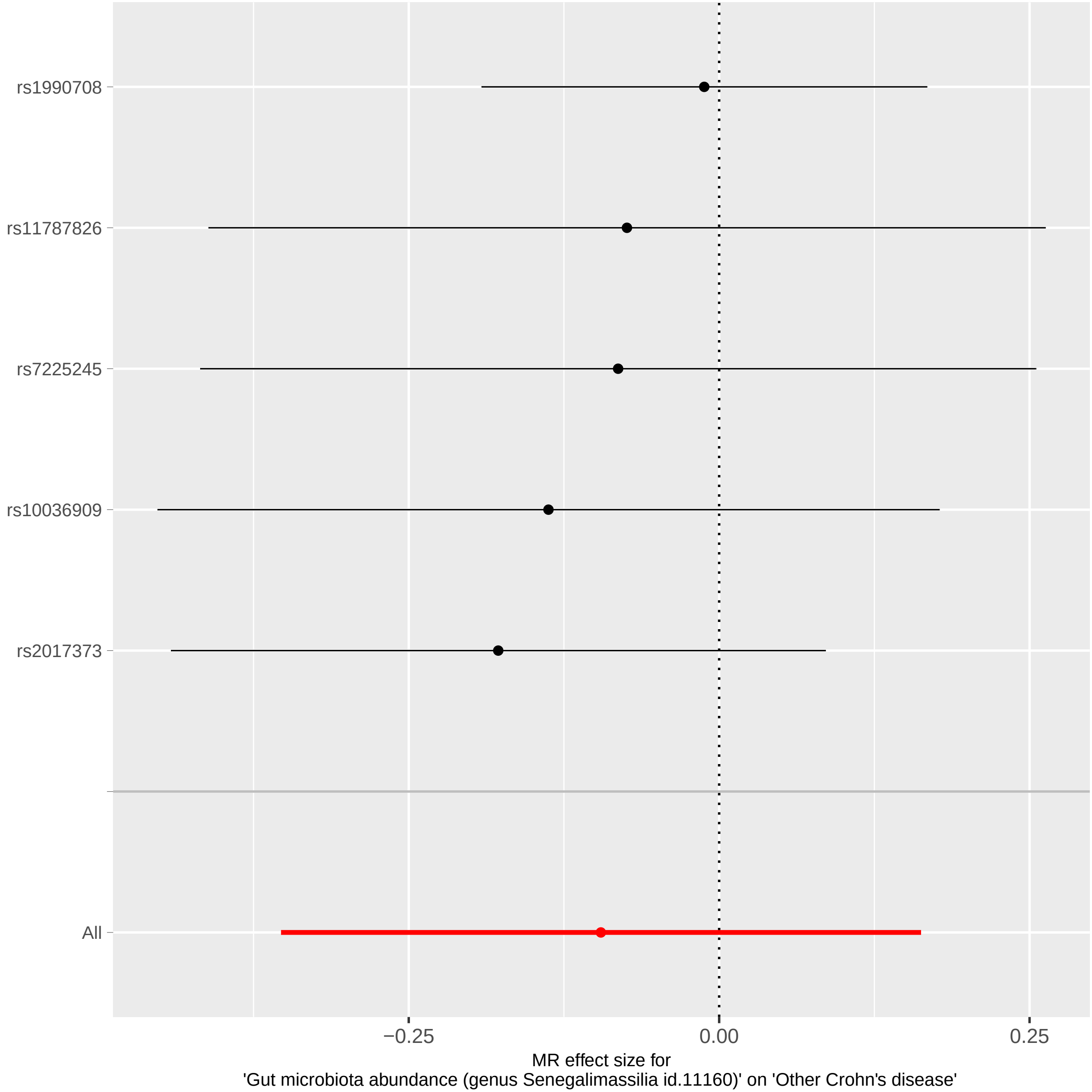

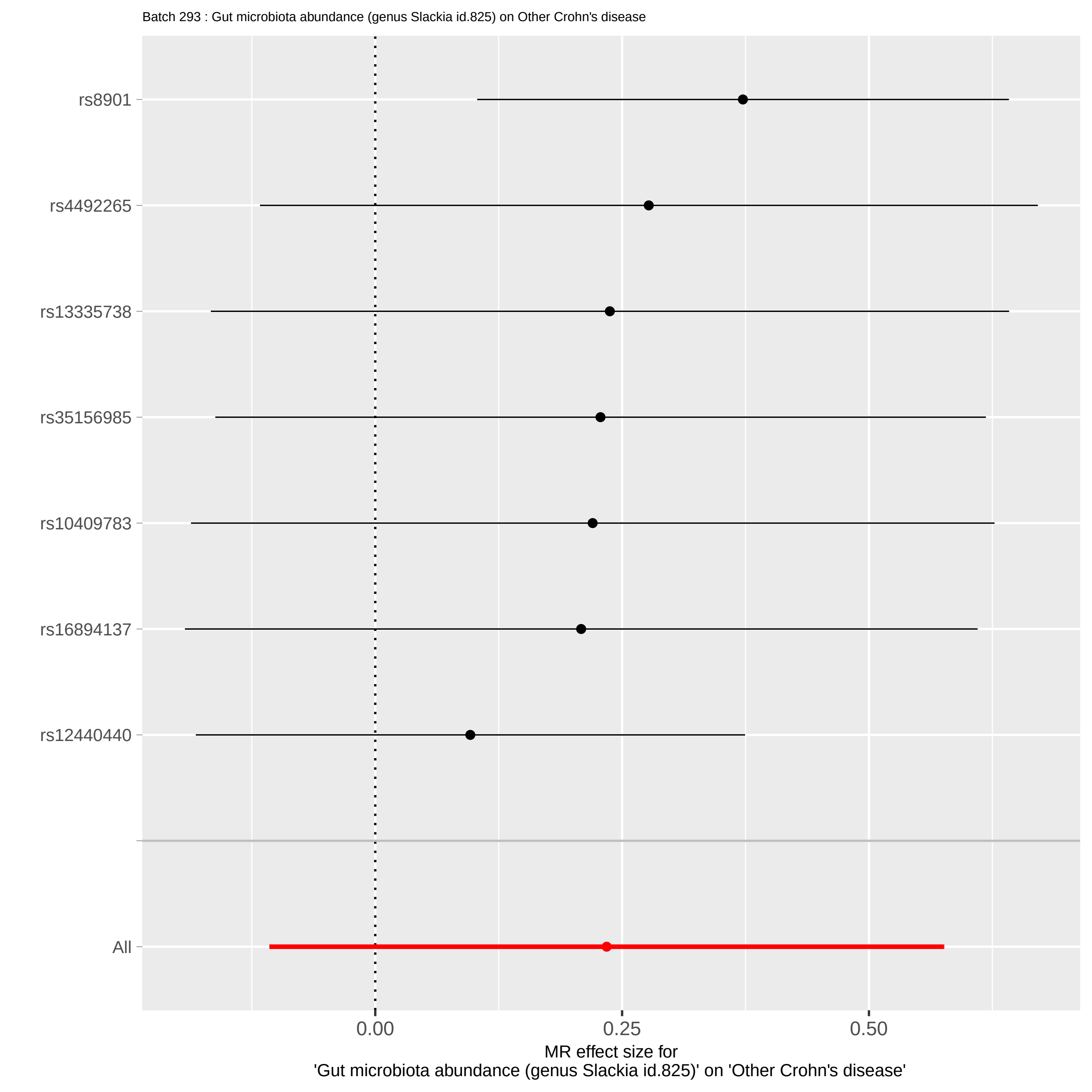
Batch 293 : Gut microbiota abundance (genus Slackia id.825) on Other Crohn's disease
rs8901
rs4492265
rs13335738
rs35156985
rs10409783
rs16894137
rs12440440
All
0.00
0.25
0.50
MR effect size for
'Gut microbiota abundance (genus Slackia id.825)' on 'Other Crohn's disease'

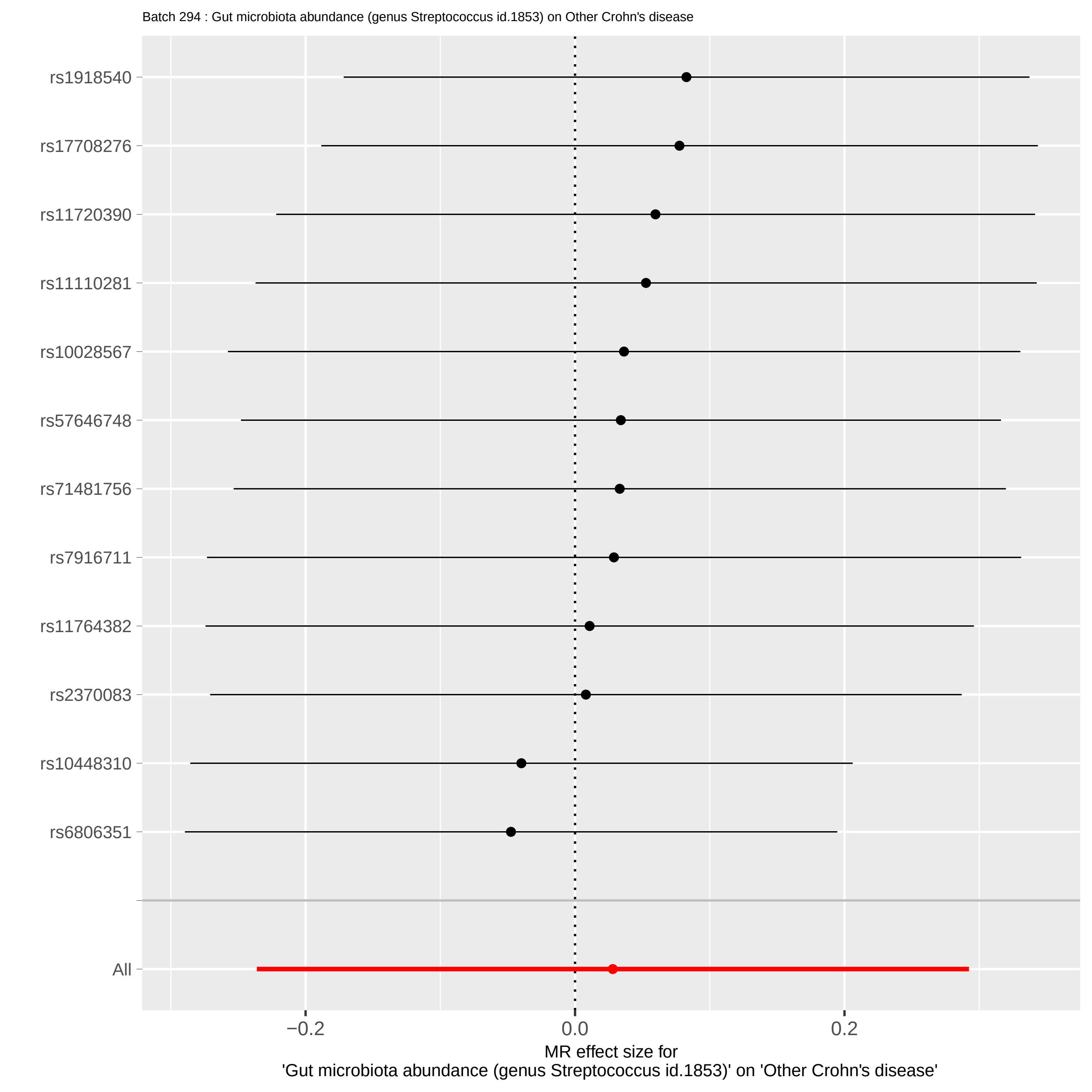

Batch 294 : Gut microbiota abundance (genus Streptococcus id.1853) on Other Crohn's disease
rs1918540
rs17708276
rs11720390
rs11110281
rs10028567
rs57646748
rs71481756
rs7916711
rs11764382
rs2370083
rs10448310
rs6806351
All
−0.2
0.0
0.2
MR effect size for
'Gut microbiota abundance (genus Streptococcus id.1853)' on 'Other Crohn's disease'

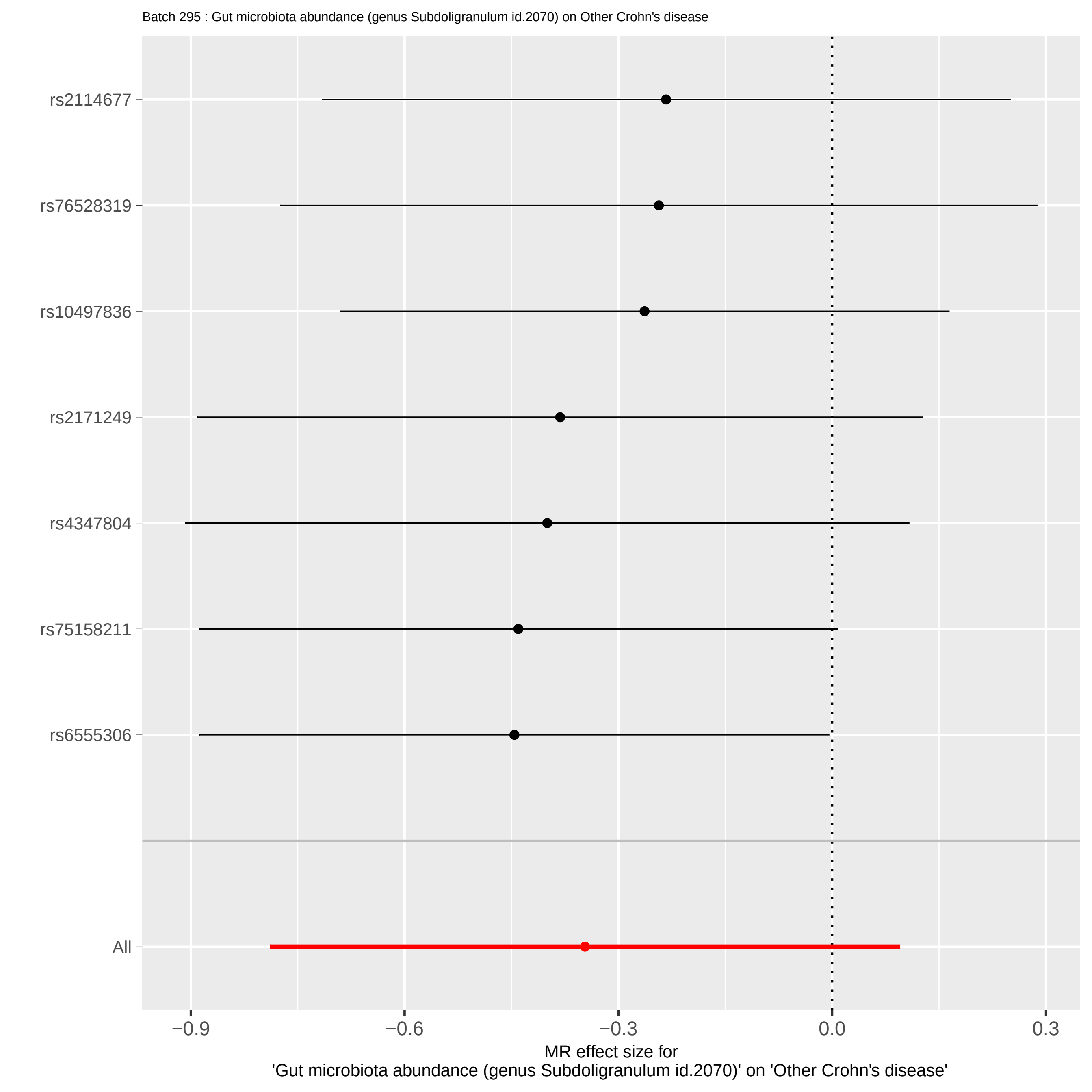

Batch 295 : Gut microbiota abundance (genus Subdoligranulum id.2070) on Other Crohn's disease
rs2114677
rs76528319
rs10497836
rs2171249
rs4347804
rs75158211
rs6555306
All
−0.9
−0.6
−0.3
0.0
0.3
MR effect size for
'Gut microbiota abundance (genus Subdoligranulum id.2070)' on 'Other Crohn's disease'

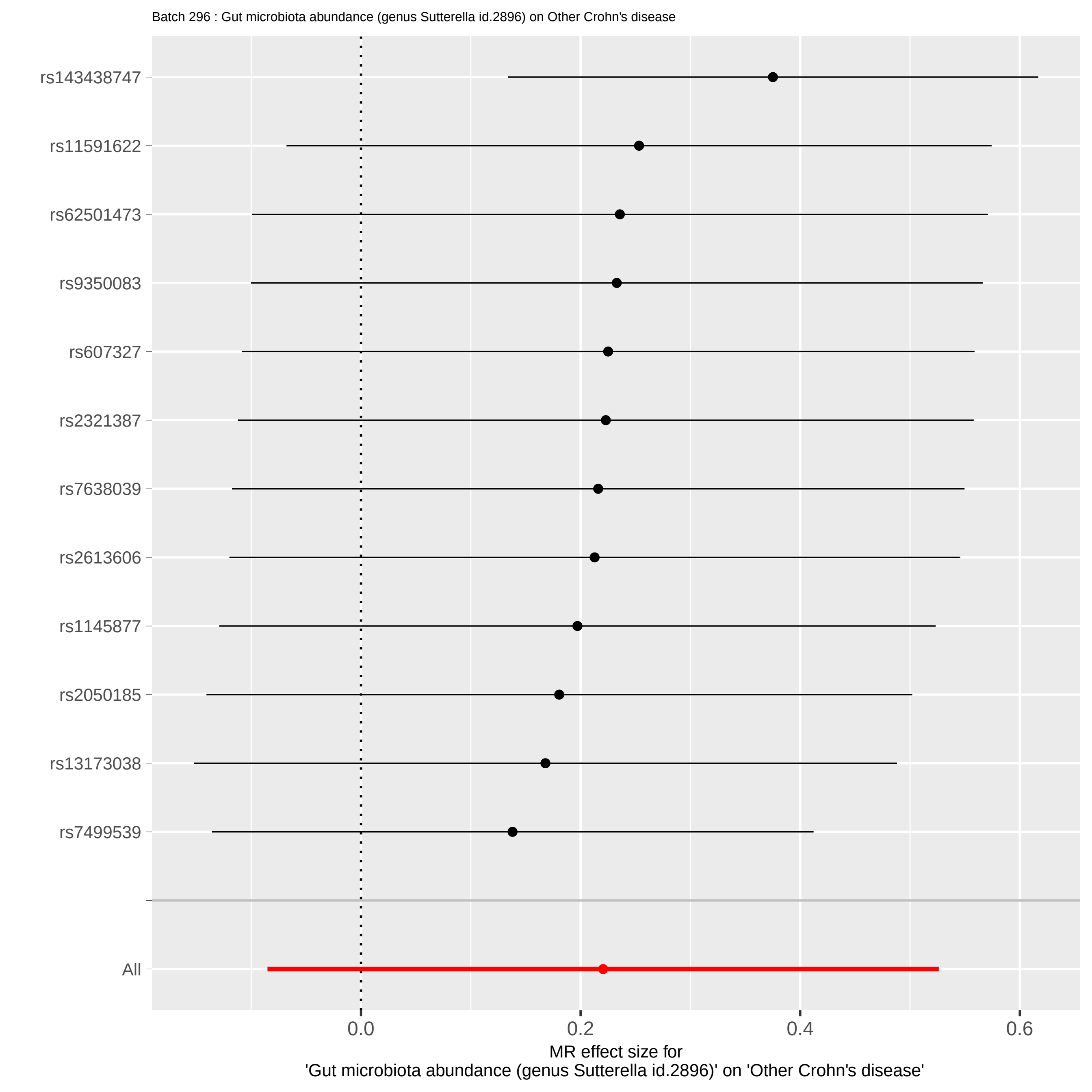

Batch 296 : Gut microbiota abundance (genus Sutterella id.2896) on Other Crohn's disease
rs143438747
rs11591622
rs62501473
rs9350083
rs607327
rs2321387
rs7638039
rs2613606
rs1145877
rs2050185
rs13173038
rs7499539
All
0.0
0.2
0.4
0.6
MR effect size for
'Gut microbiota abundance (genus Sutterella id.2896)' on 'Other Crohn's disease'

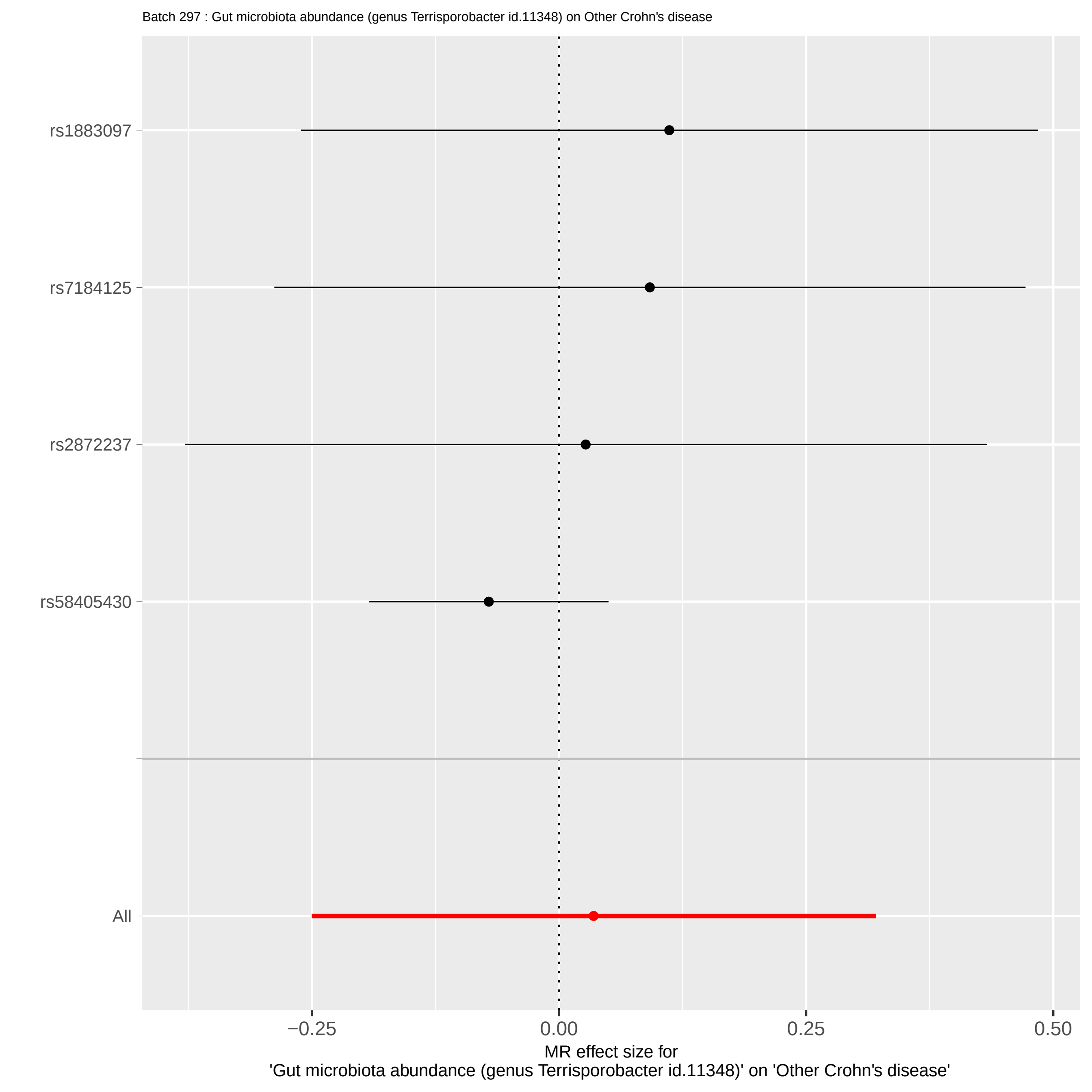

Batch 297 : Gut microbiota abundance (genus Terrisporobacter id.11348) on Other Crohn's disease
rs1883097
rs7184125
rs2872237
rs58405430
All
−0.25
0.00
0.25
0.50
MR effect size for
'Gut microbiota abundance (genus Terrisporobacter id.11348)' on 'Other Crohn's disease'

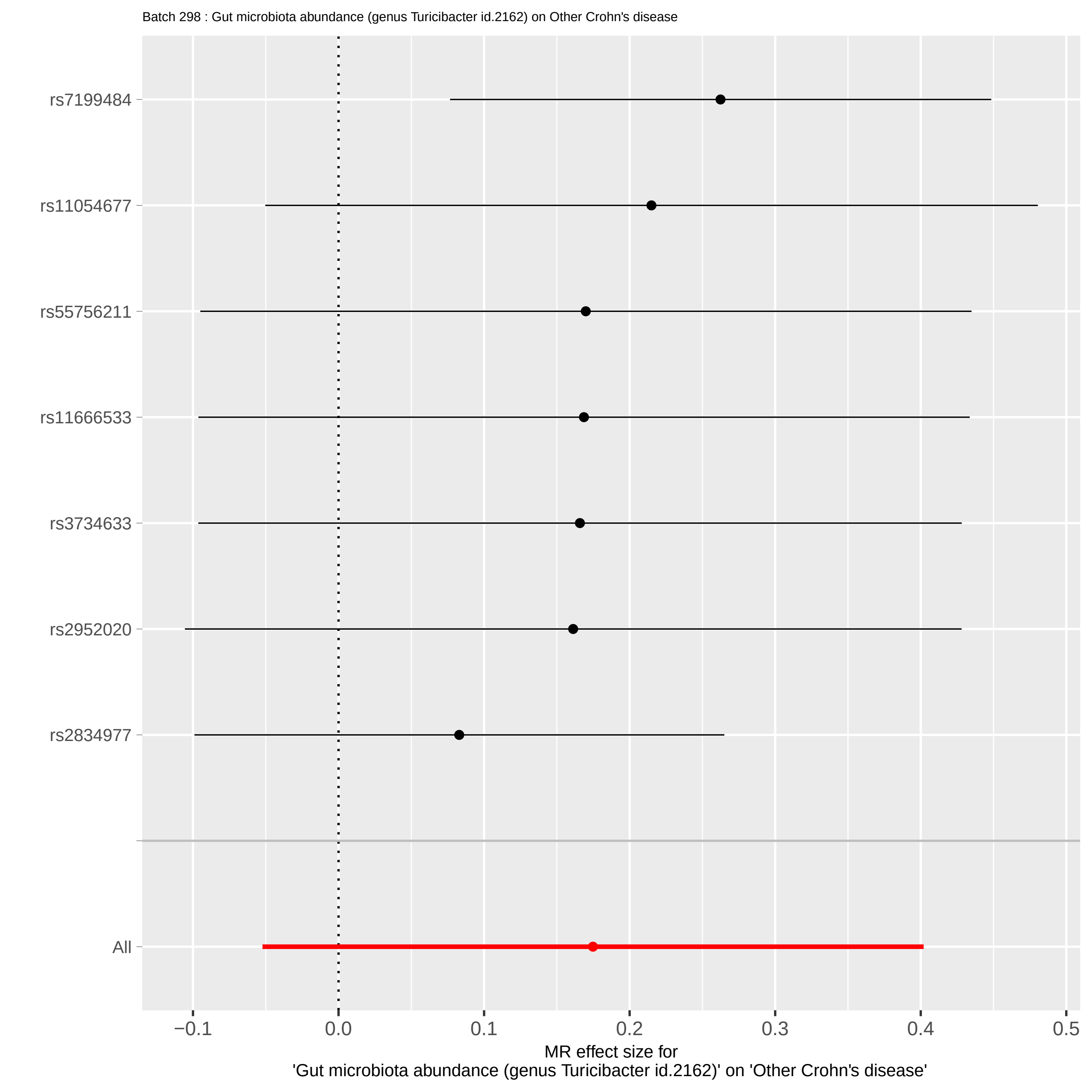

Batch 298 : Gut microbiota abundance (genus Turicibacter id.2162) on Other Crohn's disease
rs7199484
rs11054677
rs55756211
rs11666533
rs3734633
rs2952020
rs2834977
All
−0.1
0.0
0.1
0.2
0.3
0.4
0.5
MR effect size for
'Gut microbiota abundance (genus Turicibacter id.2162)' on 'Other Crohn's disease'

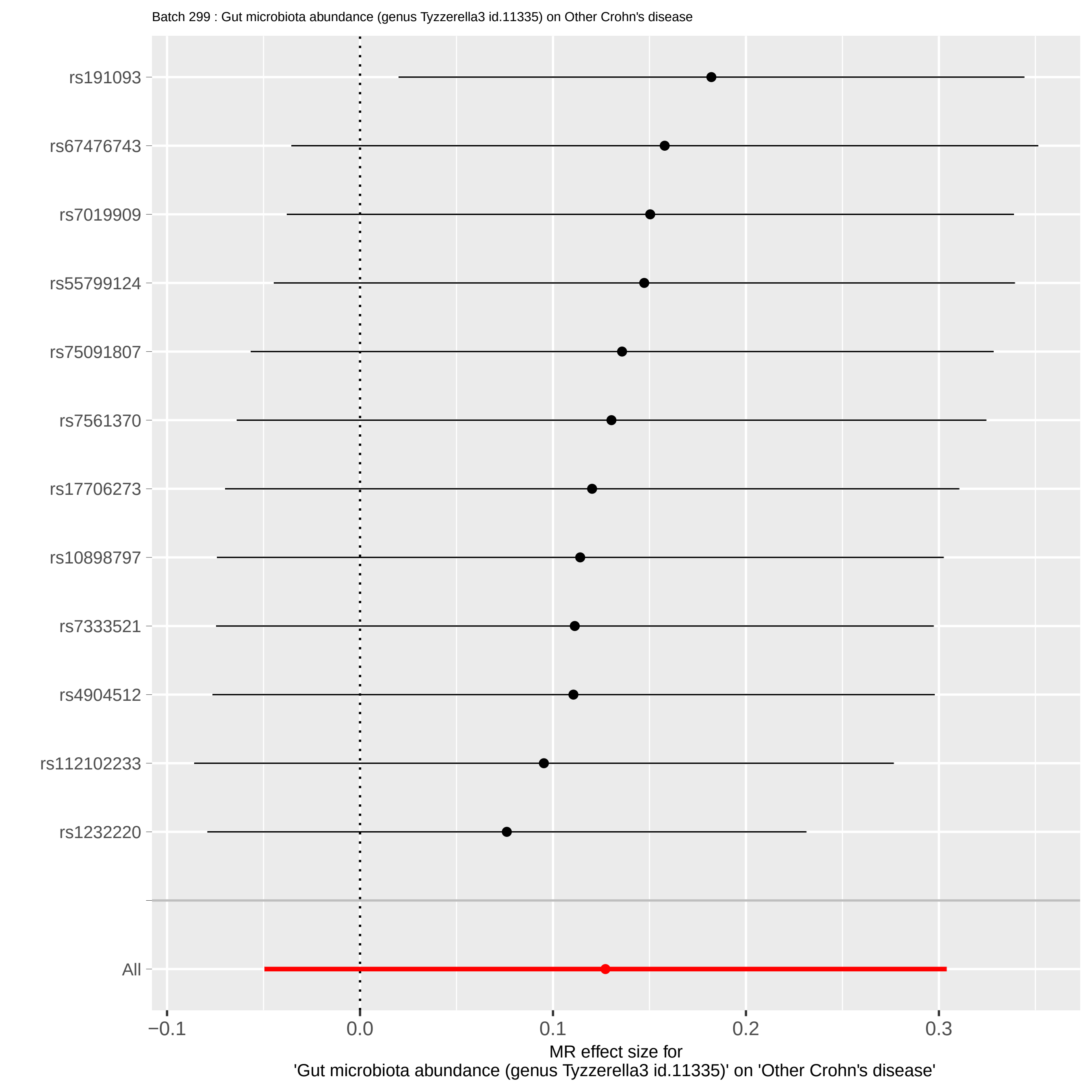
Batch 299 : Gut microbiota abundance (genus Tyzzerella3 id.11335) on Other Crohn's disease
rs191093
rs67476743
rs7019909
rs55799124
rs75091807
rs7561370
rs17706273
rs10898797
rs7333521
rs4904512
rs112102233
rs1232220
All
−0.1
0.0
0.1
0.2
0.3
MR effect size for
'Gut microbiota abundance (genus Tyzzerella3 id.11335)' on 'Other Crohn's disease'

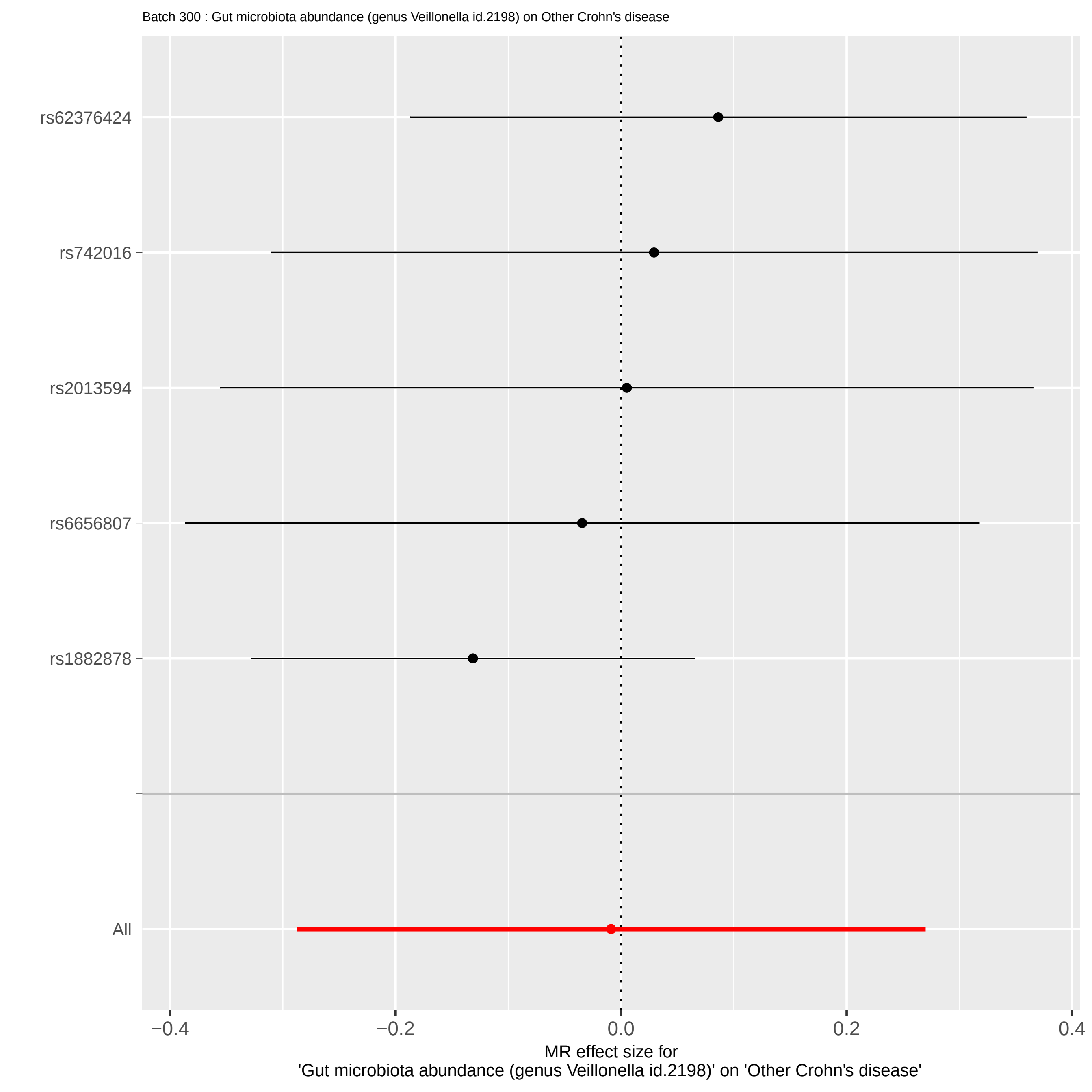

Batch 300 : Gut microbiota abundance (genus Veillonella id.2198) on Other Crohn's disease
rs62376424
rs742016
rs2013594
rs6656807
rs1882878
All
−0.4
−0.2
0.0
0.2
0.4
MR effect size for
'Gut microbiota abundance (genus Veillonella id.2198)' on 'Other Crohn's disease'

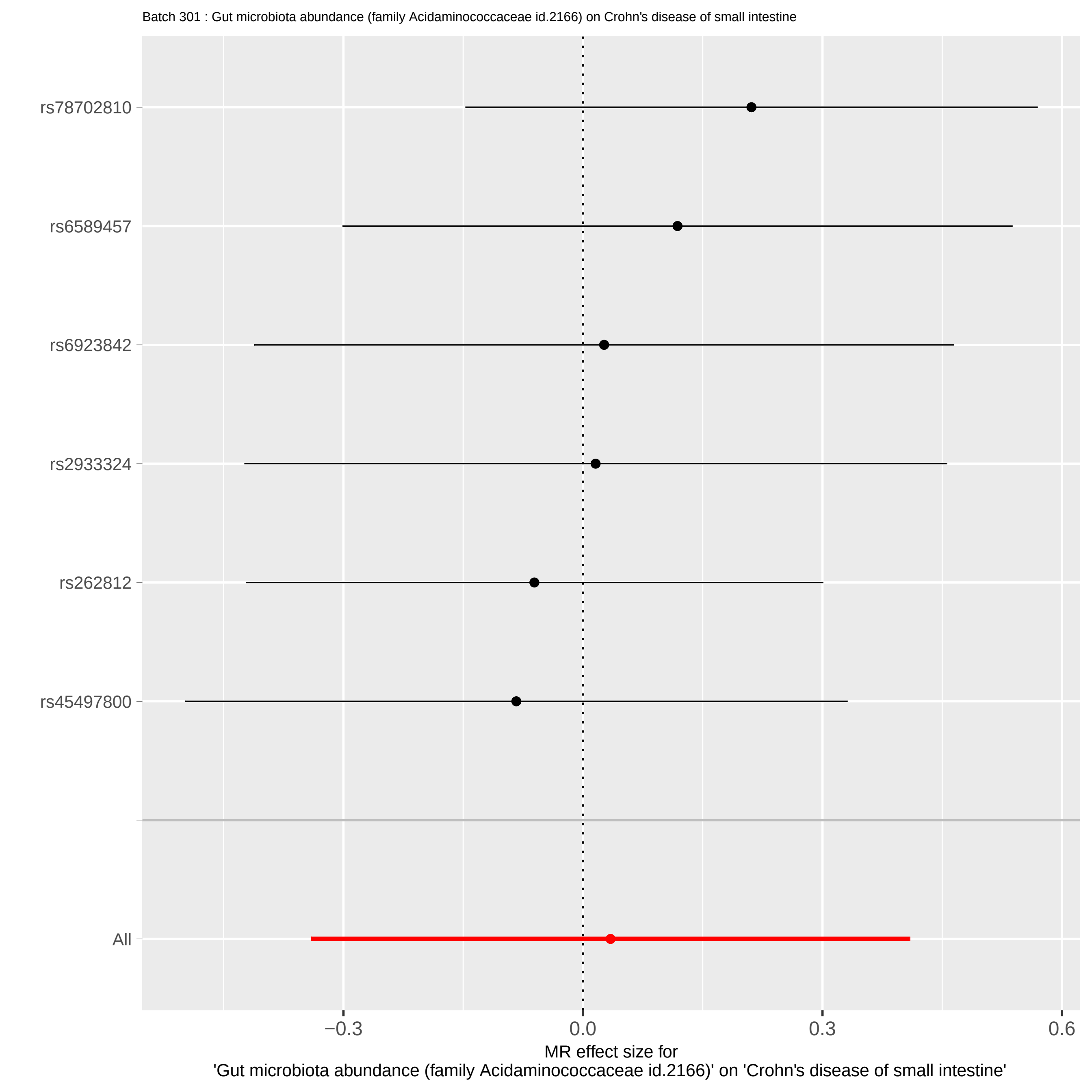
Batch 301 : Gut microbiota abundance (family Acidaminococcaceae id.2166) on Crohn's disease of small intestine
rs78702810
rs6589457
rs6923842
rs2933324
rs262812
rs45497800
All
−0.3
0.0
0.3
0.6
MR effect size for
'Gut microbiota abundance (family Acidaminococcaceae id.2166)' on 'Crohn's disease of small intestine'

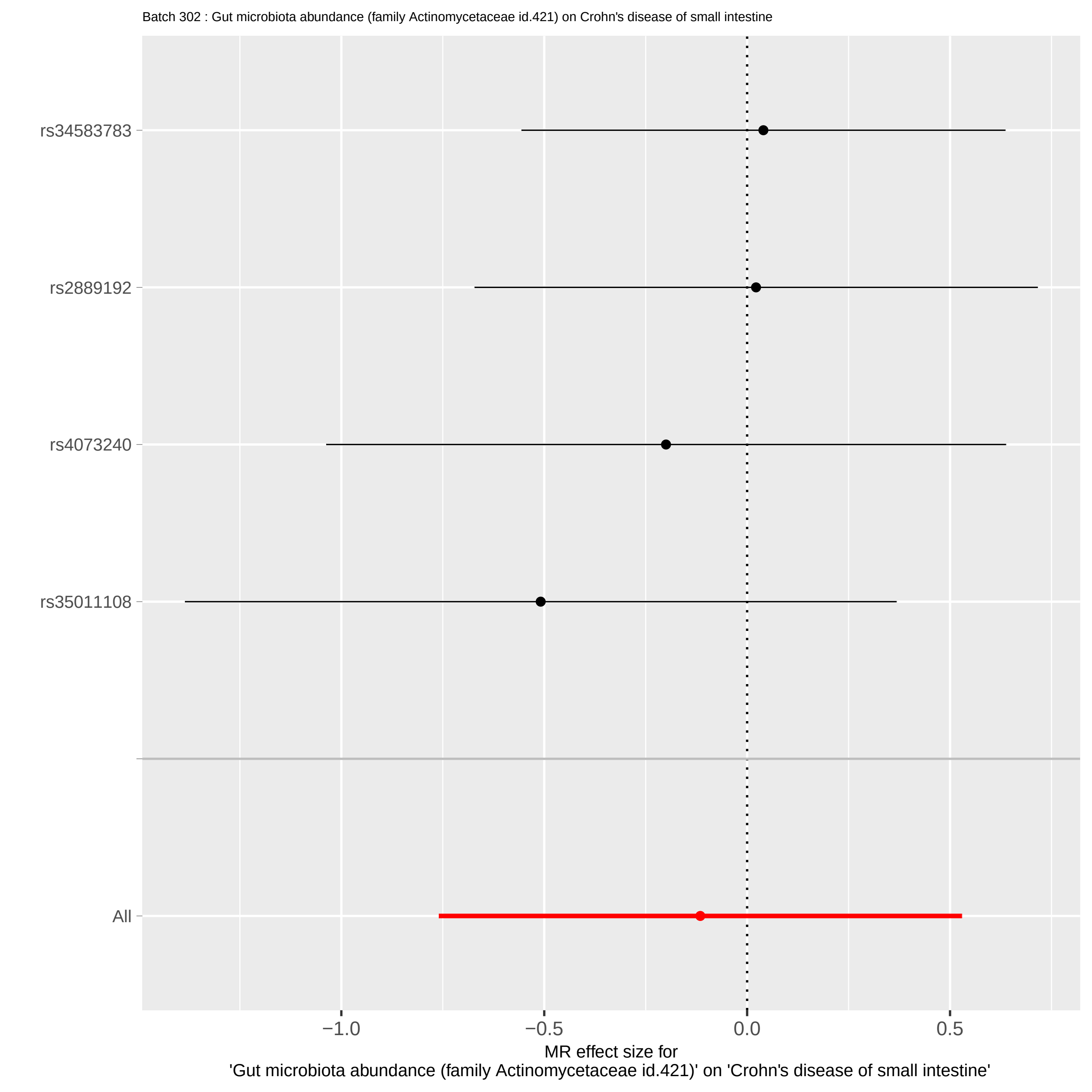

Batch 302 : Gut microbiota abundance (family Actinomycetaceae id.421) on Crohn's disease of small intestine
rs34583783
rs2889192
rs4073240
rs35011108
All
−1.0
−0.5
0.0
0.5
MR effect size for
'Gut microbiota abundance (family Actinomycetaceae id.421)' on 'Crohn's disease of small intestine'

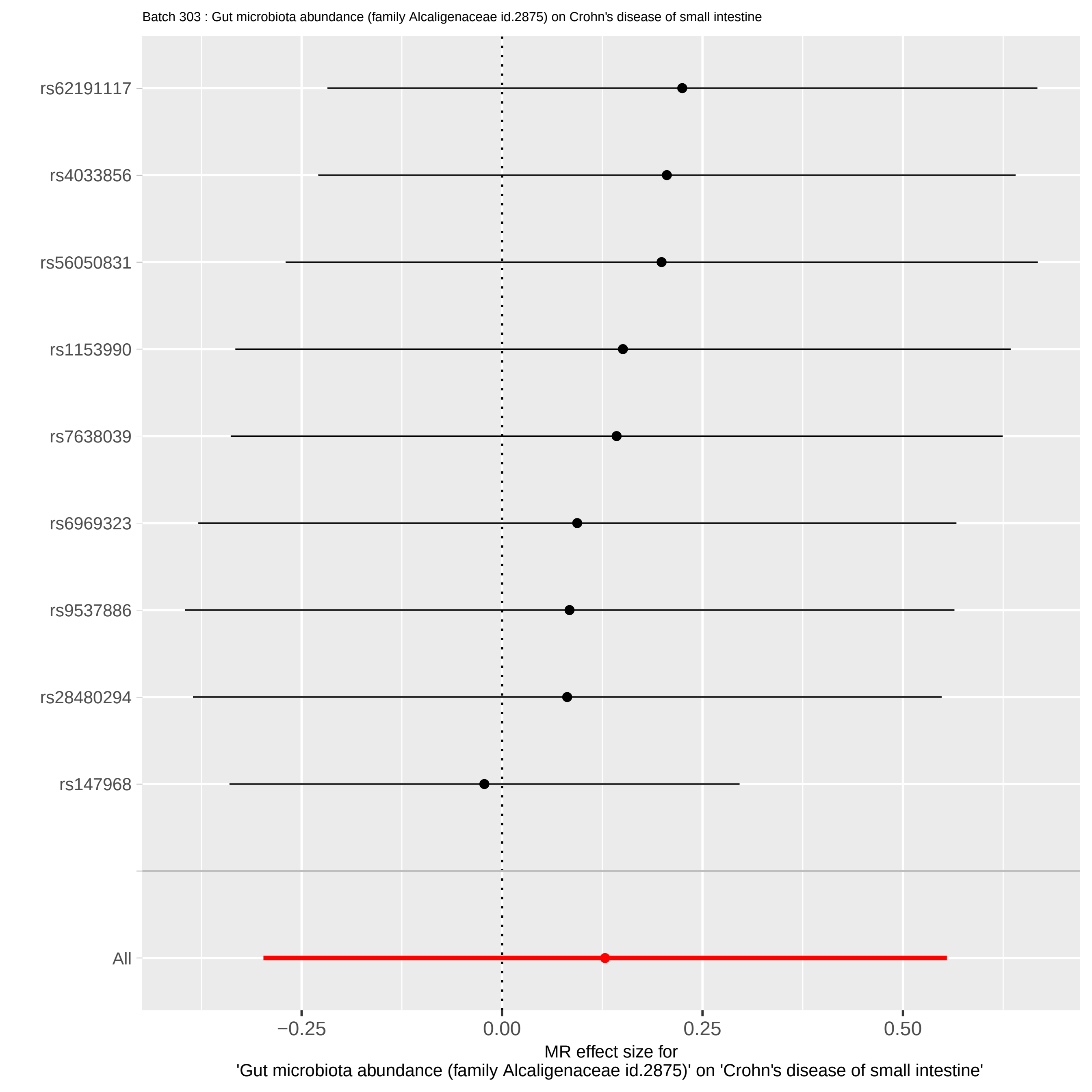

Batch 303 : Gut microbiota abundance (family Alcaligenaceae id.2875) on Crohn's disease of small intestine
rs62191117
rs4033856
rs56050831
rs1153990
rs7638039
rs6969323
rs9537886
rs28480294
rs147968
All
−0.25
0.00
0.25
0.50
MR effect size for
'Gut microbiota abundance (family Alcaligenaceae id.2875)' on 'Crohn's disease of small intestine'

Batch 304 : Gut microbiota abundance (family Bacteroidaceae id.917) on Crohn's disease of small intestine

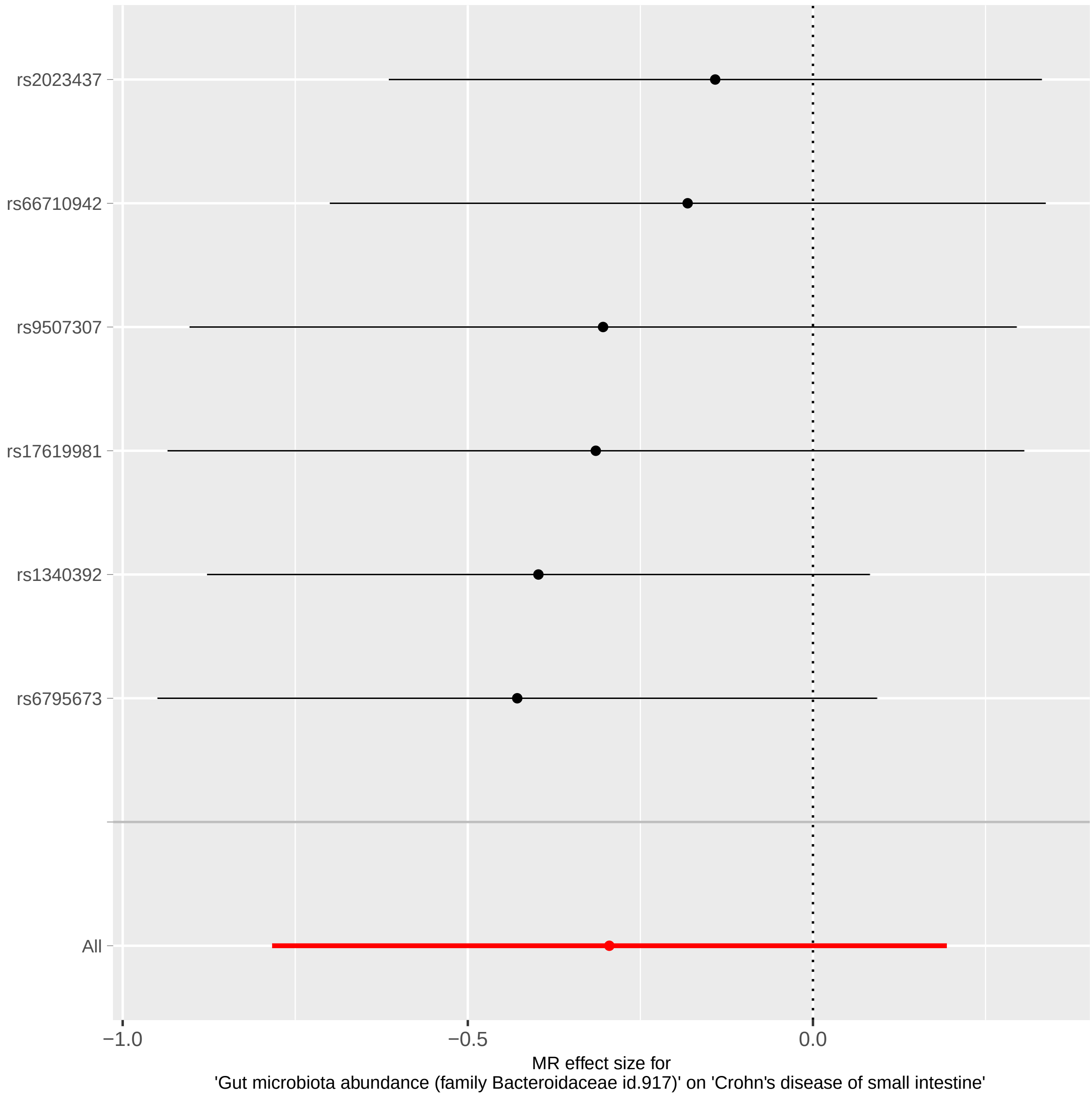

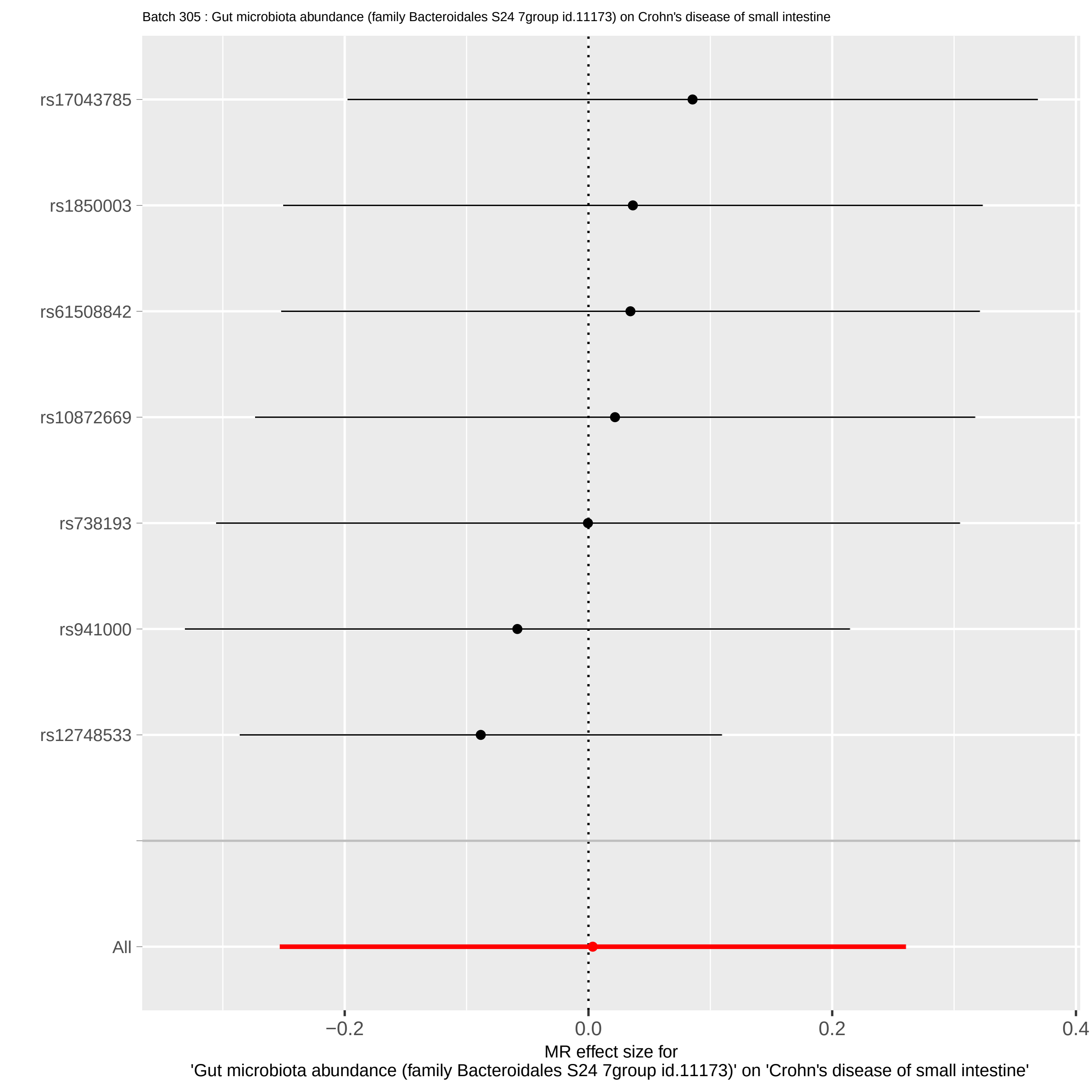
Batch 305 : Gut microbiota abundance (family Bacteroidales S24 7group id.11173) on Crohn's disease of small intestine
rs17043785
rs1850003
rs61508842
rs10872669
rs738193
rs941000
rs12748533
All
−0.2
0.0
0.2
0.4
MR effect size for
'Gut microbiota abundance (family Bacteroidales S24 7group id.11173)' on 'Crohn's disease of small intestine'

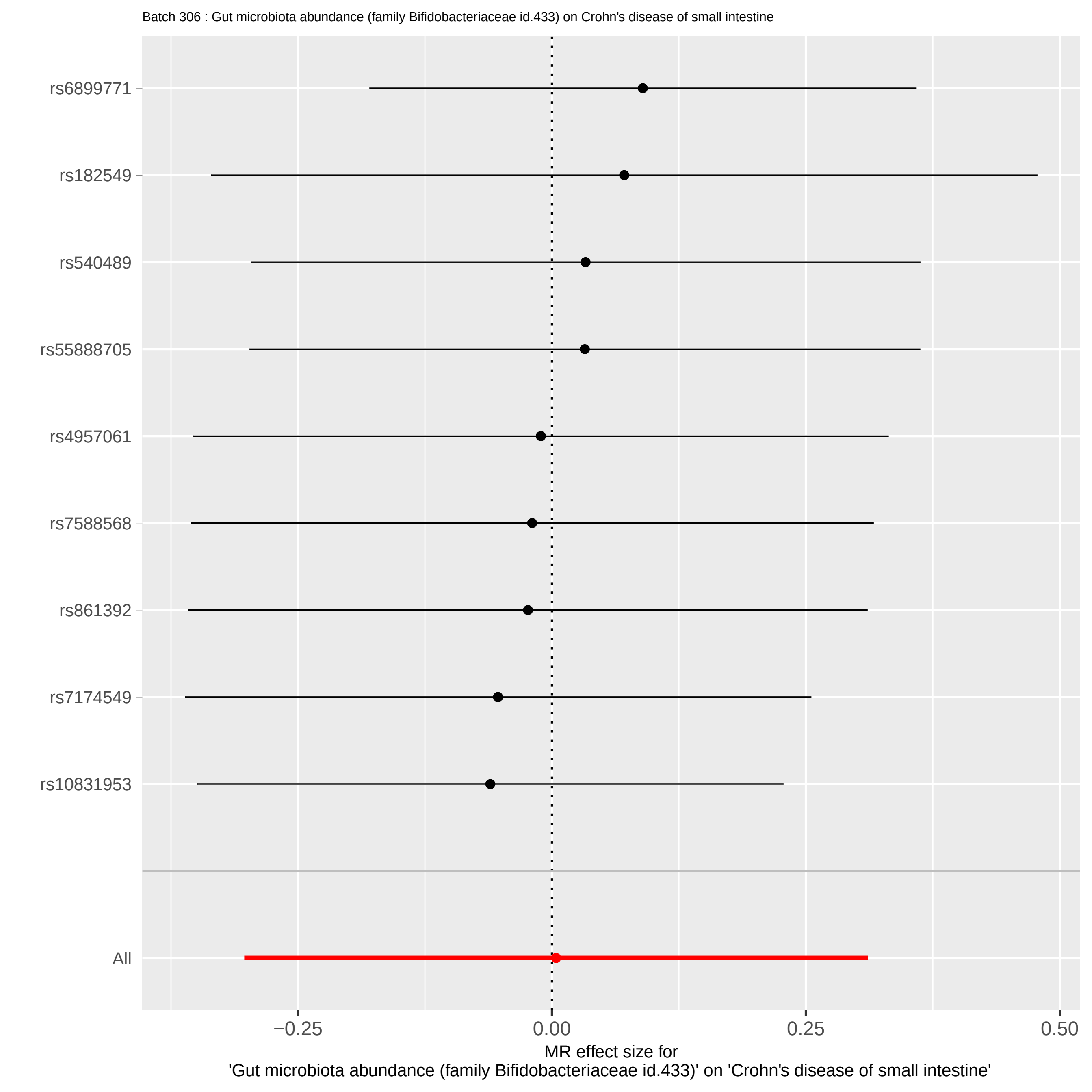
Batch 306 : Gut microbiota abundance (family Bifidobacteriaceae id.433) on Crohn's disease of small intestine
rs6899771
rs182549
rs540489
rs55888705
rs4957061
rs7588568
rs861392
rs7174549
rs10831953
All
−0.25
0.00
0.25
0.50
MR effect size for
'Gut microbiota abundance (family Bifidobacteriaceae id.433)' on 'Crohn's disease of small intestine'

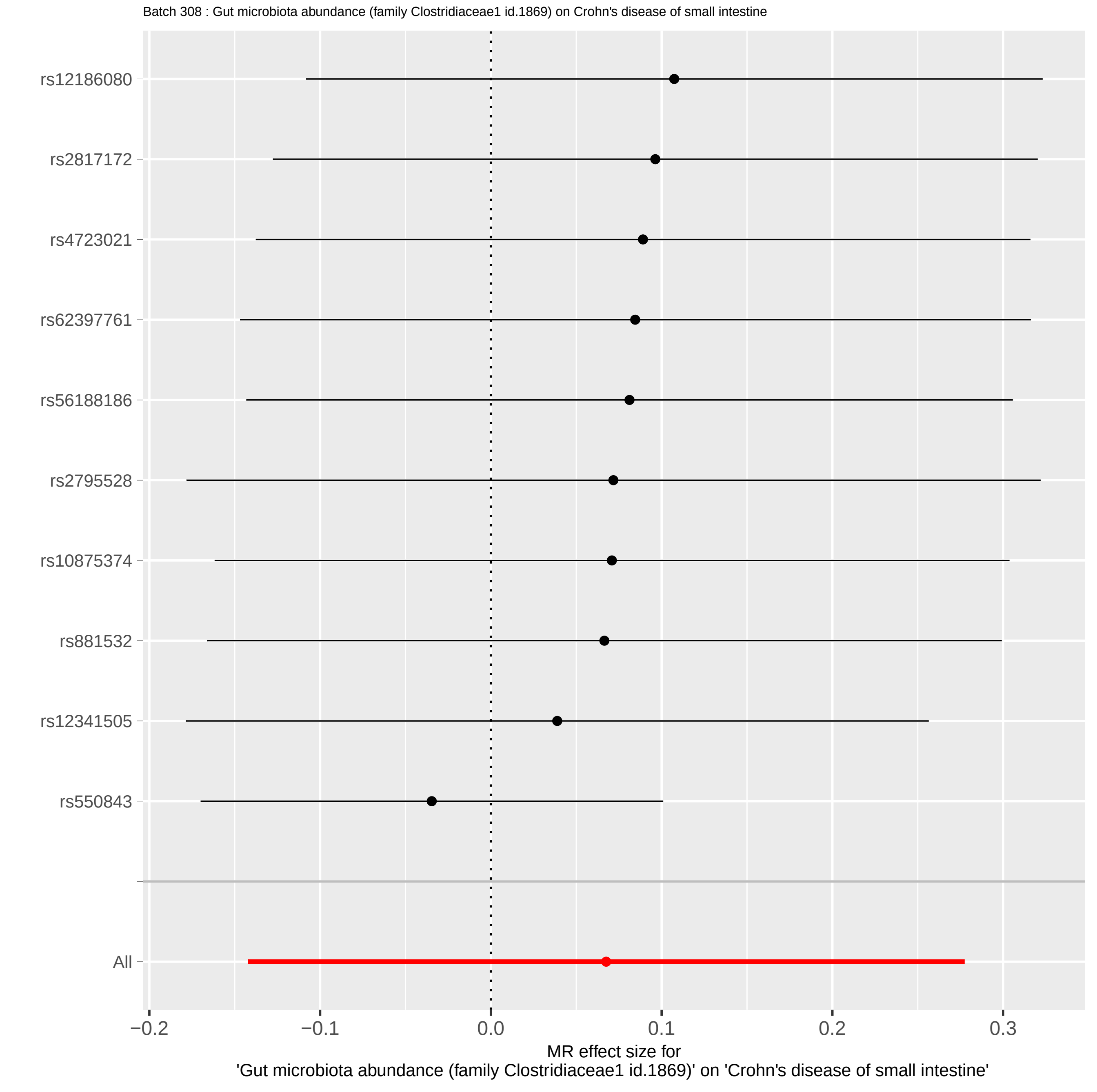

Batch 308 : Gut microbiota abundance (family Clostridiaceae1 id.1869) on Crohn's disease of small intestine
rs12186080
rs2817172
rs4723021
rs62397761
rs56188186
rs2795528
rs10875374
rs881532
rs12341505
rs550843
All
−0.2
−0.1
0.0
0.1
0.2
0.3
MR effect size for
'Gut microbiota abundance (family Clostridiaceae1 id.1869)' on 'Crohn's disease of small intestine'

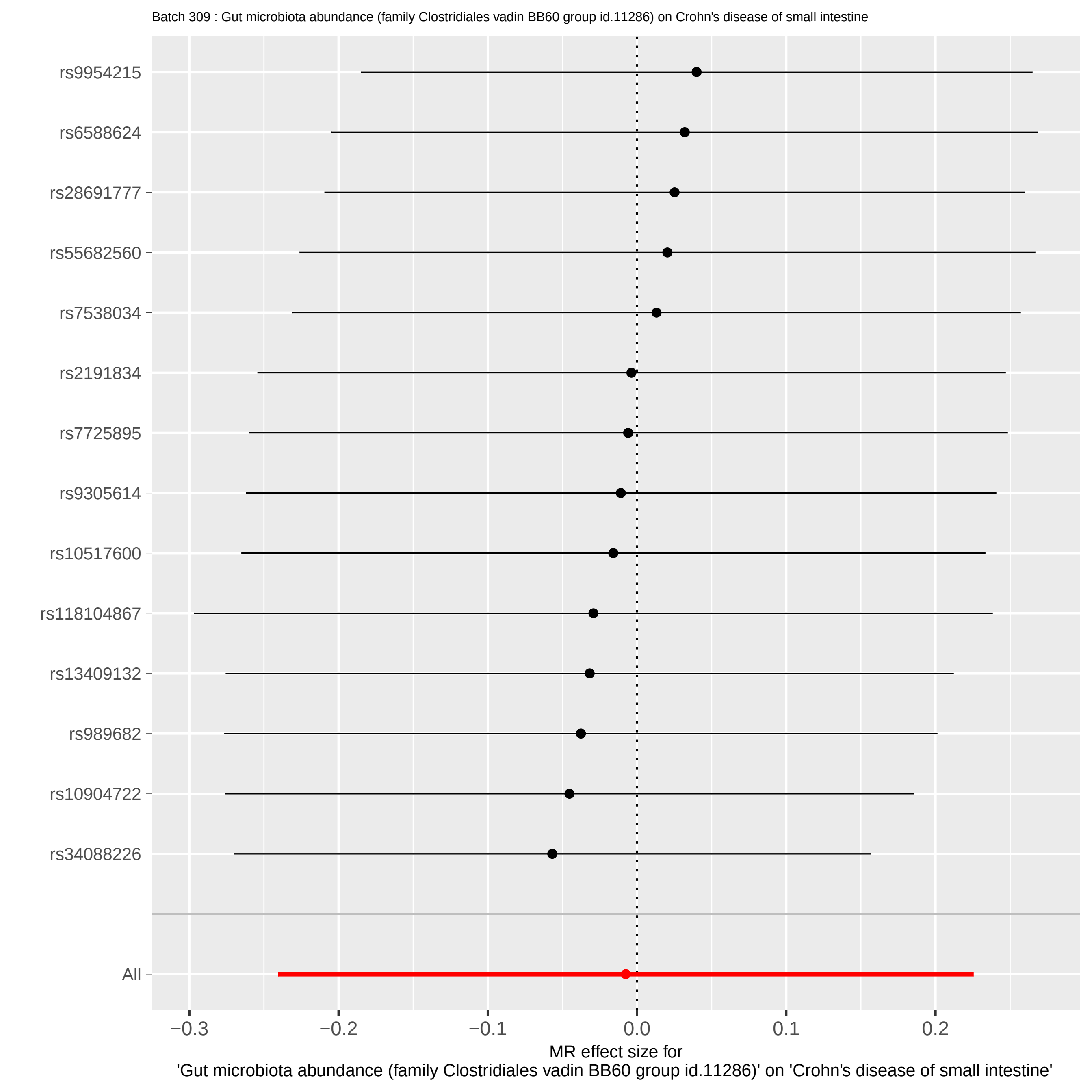

Batch 309 : Gut microbiota abundance (family Clostridiales vadin BB60 group id.11286) on Crohn's disease of small intestine
rs9954215
rs6588624
rs28691777
rs55682560
rs7538034
rs2191834
rs7725895
rs9305614
rs10517600
rs118104867
rs13409132
rs989682
rs10904722
rs34088226
All
−0.3
−0.2
−0.1
0.0
0.1
0.2
MR effect size for
'Gut microbiota abundance (family Clostridiales vadin BB60 group id.11286)' on 'Crohn's disease of small intestine'

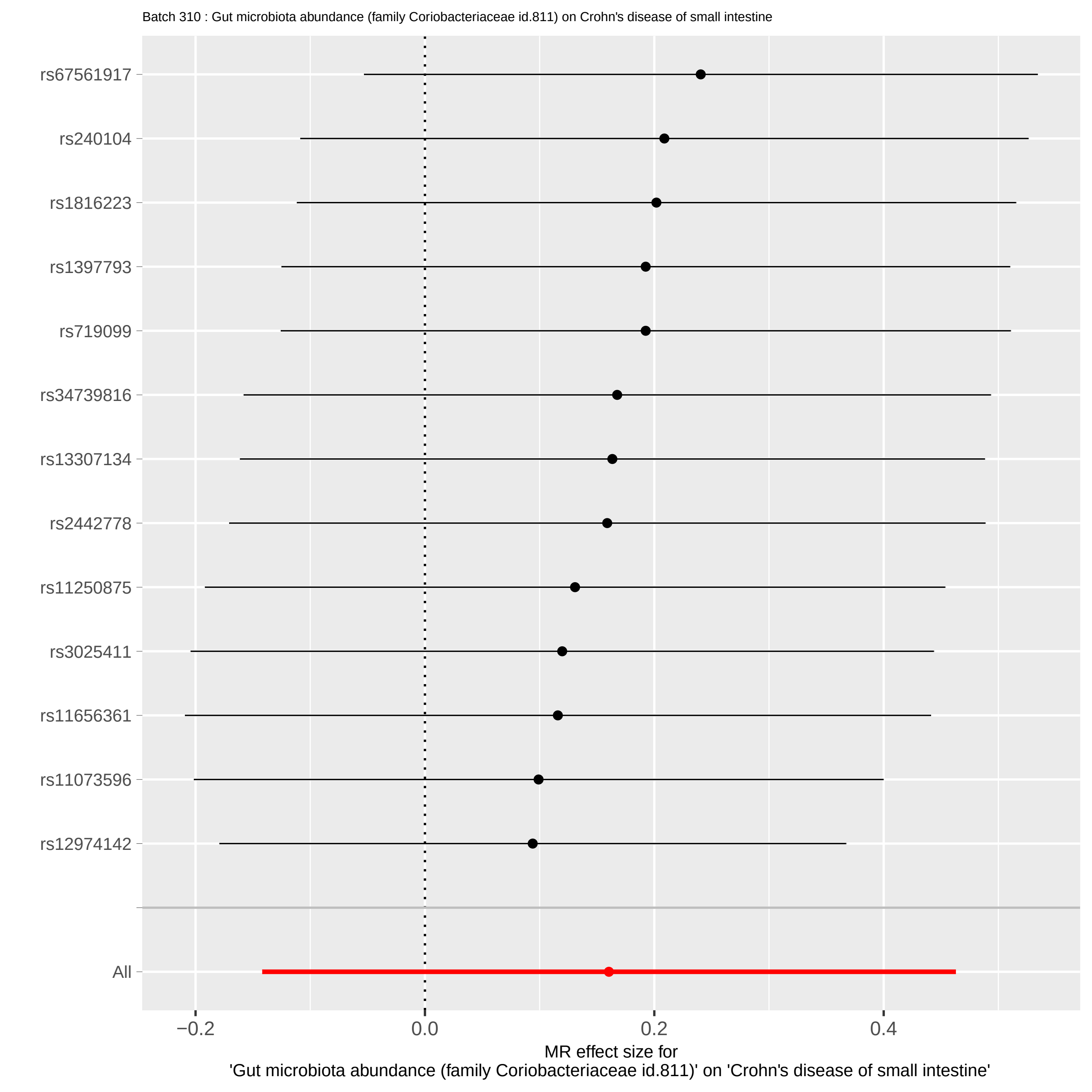
Batch 310 : Gut microbiota abundance (family Coriobacteriaceae id.811) on Crohn's disease of small intestine
rs67561917
rs240104
rs1816223
rs1397793
rs719099
rs34739816
rs13307134
rs2442778
rs11250875
rs3025411
rs11656361
rs11073596
rs12974142
All
−0.2
0.0
0.2
0.4
MR effect size for
'Gut microbiota abundance (family Coriobacteriaceae id.811)' on 'Crohn's disease of small intestine'

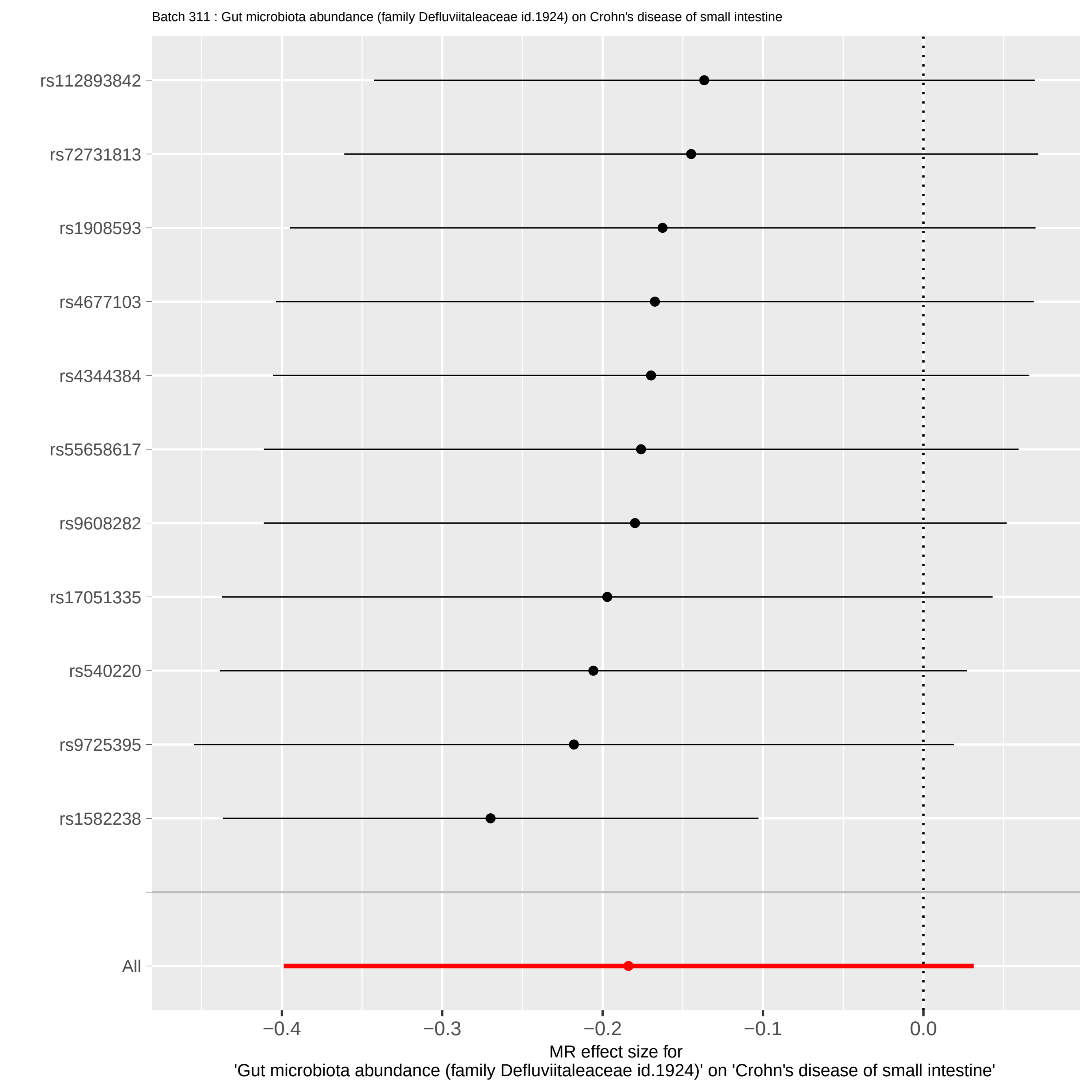
Batch 311 : Gut microbiota abundance (family Defluviitaleaceae id.1924) on Crohn's disease of small intestine
rs112893842
rs72731813
rs1908593
rs4677103
rs4344384
rs55658617
rs9608282
rs17051335
rs540220
rs9725395
rs1582238
All
−0.4
−0.3
−0.2
−0.1
0.0
MR effect size for
'Gut microbiota abundance (family Defluviitaleaceae id.1924)' on 'Crohn's disease of small intestine'

Batch 312 : Gut microbiota abundance (family Desulfovibrionaceae id.3169) on Crohn's disease of small intestine

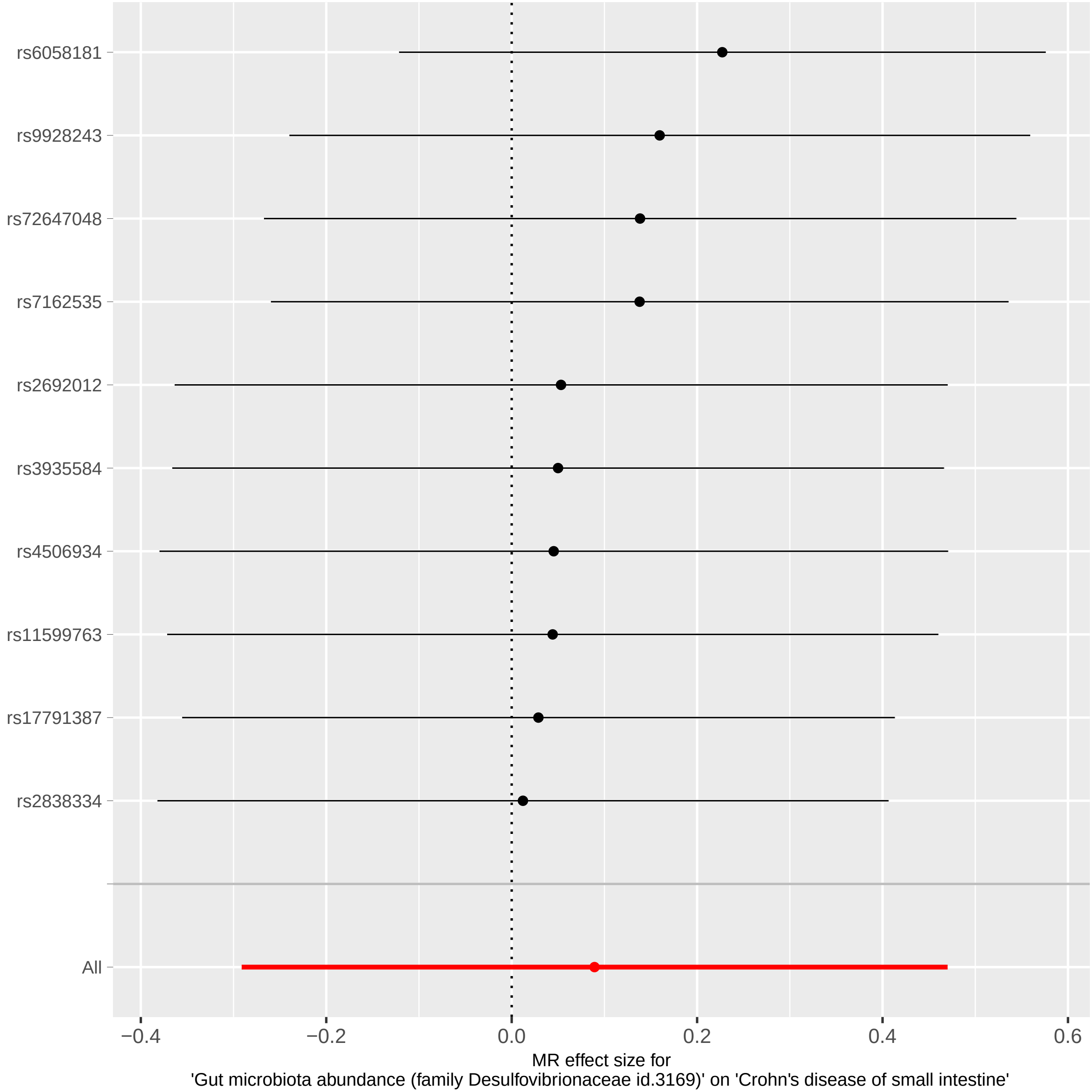

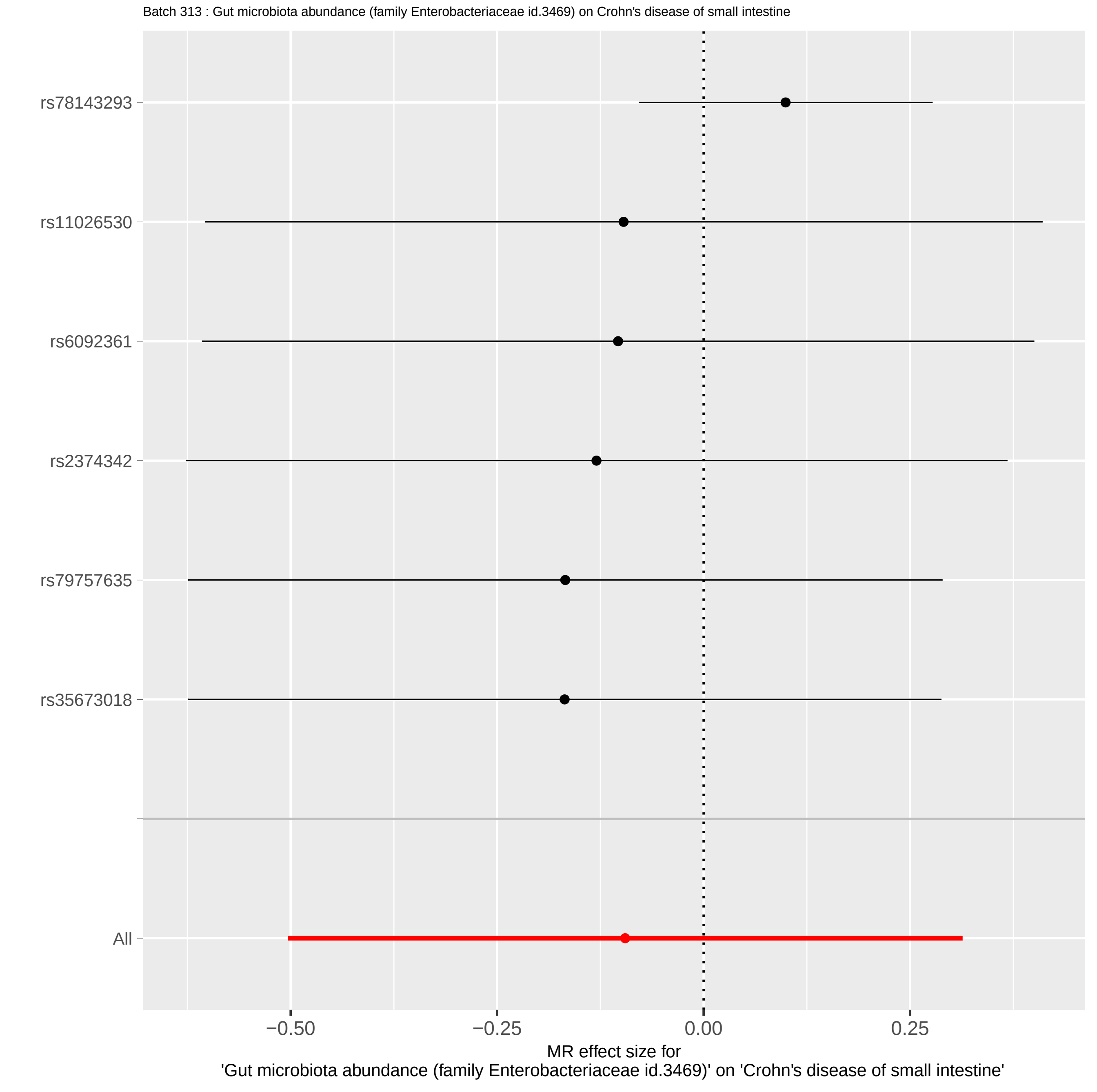

Batch 313 : Gut microbiota abundance (family Enterobacteriaceae id.3469) on Crohn's disease of small intestine
rs78143293
rs11026530
rs6092361
rs2374342
rs79757635
rs35673018
All
−0.50
−0.25
0.00
0.25
MR effect size for
'Gut microbiota abundance (family Enterobacteriaceae id.3469)' on 'Crohn's disease of small intestine'

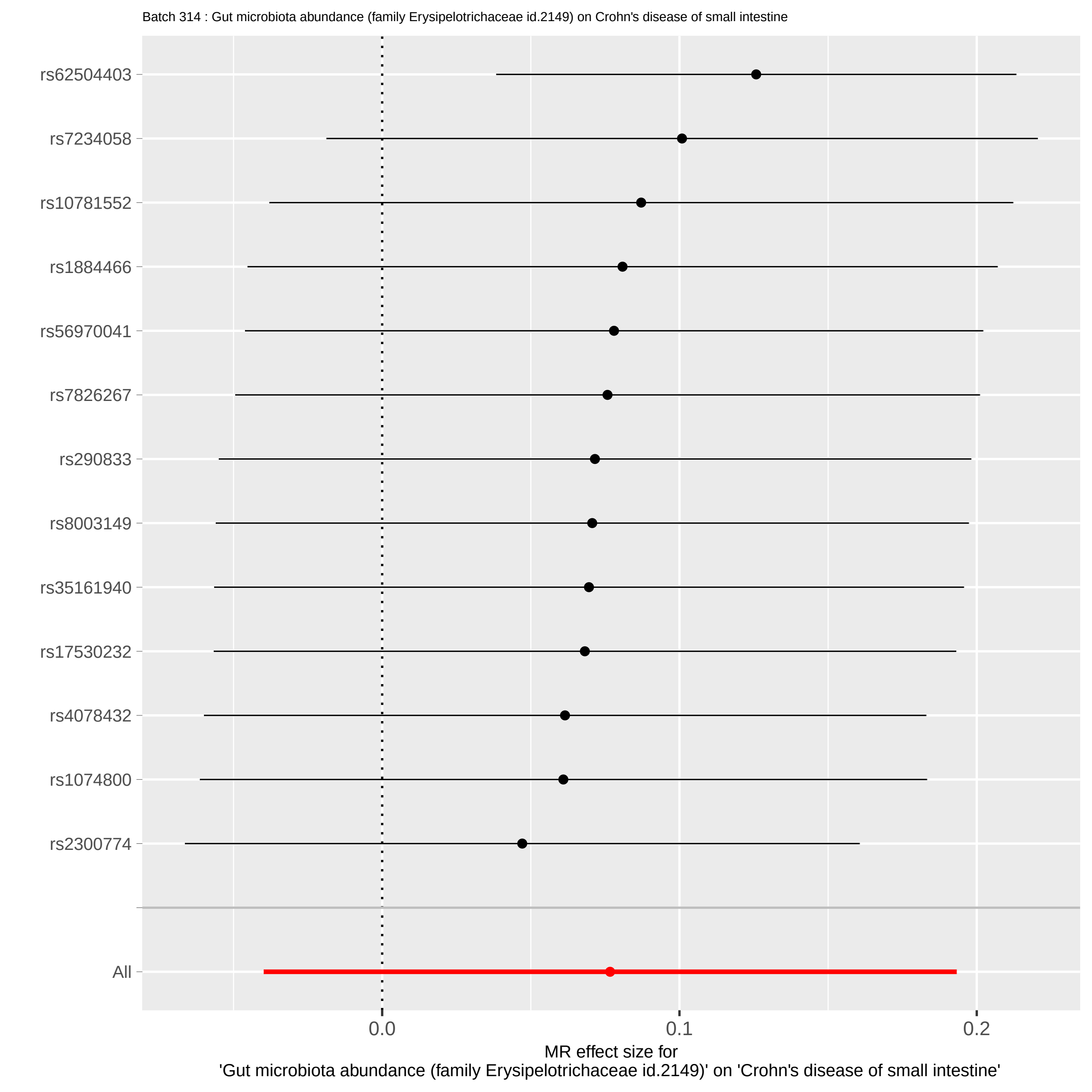

Batch 314 : Gut microbiota abundance (family Erysipelotrichaceae id.2149) on Crohn's disease of small intestine
rs62504403
rs7234058
rs10781552
rs1884466
rs56970041
rs7826267
rs290833
rs8003149
rs35161940
rs17530232
rs4078432
rs1074800
rs2300774
All
0.0
0.1
0.2
MR effect size for
'Gut microbiota abundance (family Erysipelotrichaceae id.2149)' on 'Crohn's disease of small intestine'

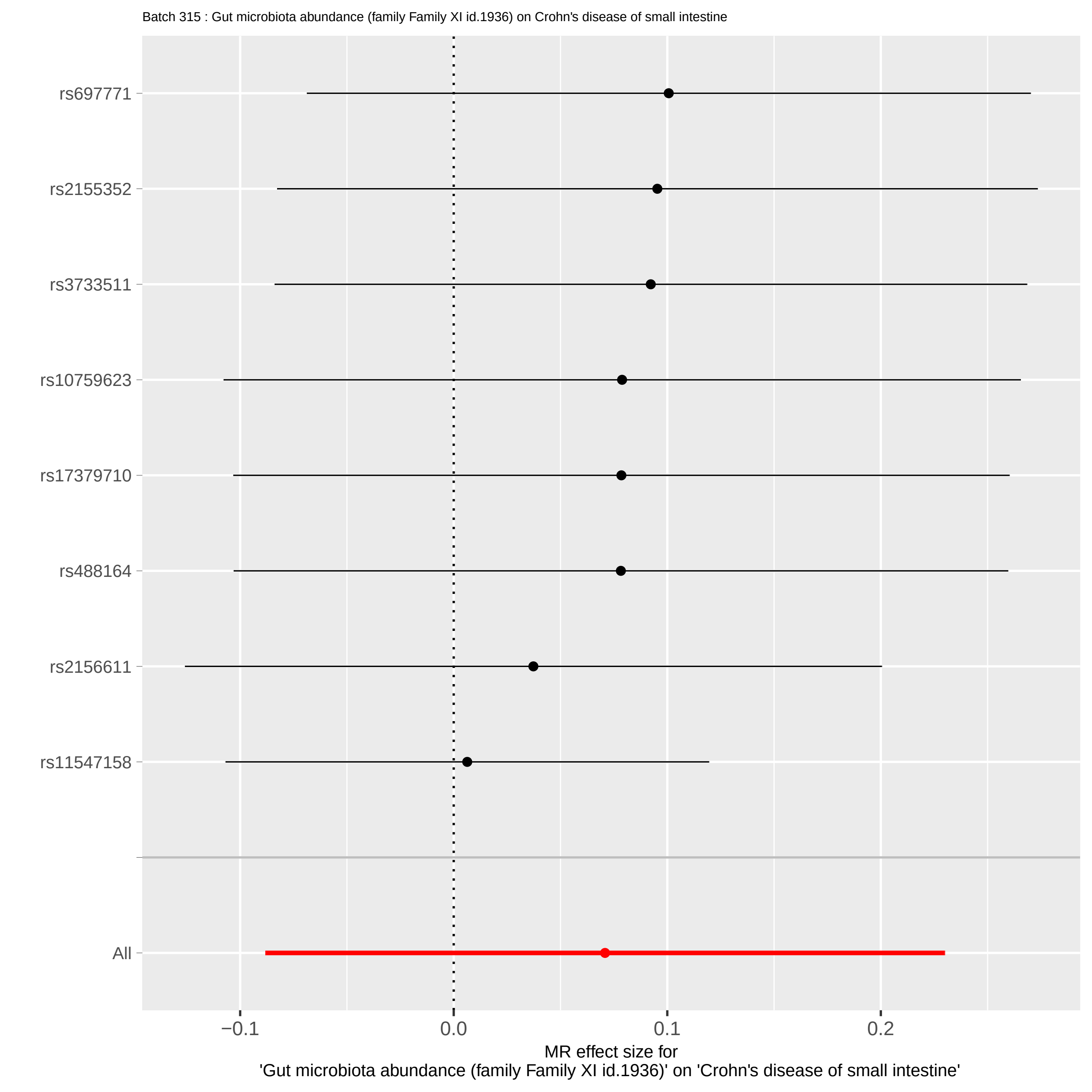
Batch 315 : Gut microbiota abundance (family Family XI id.1936) on Crohn's disease of small intestine
rs697771
rs2155352
rs3733511
rs10759623
rs17379710
rs488164
rs2156611
rs11547158
All
−0.1
0.0
0.1
0.2
MR effect size for
'Gut microbiota abundance (family Family XI id.1936)' on 'Crohn's disease of small intestine'

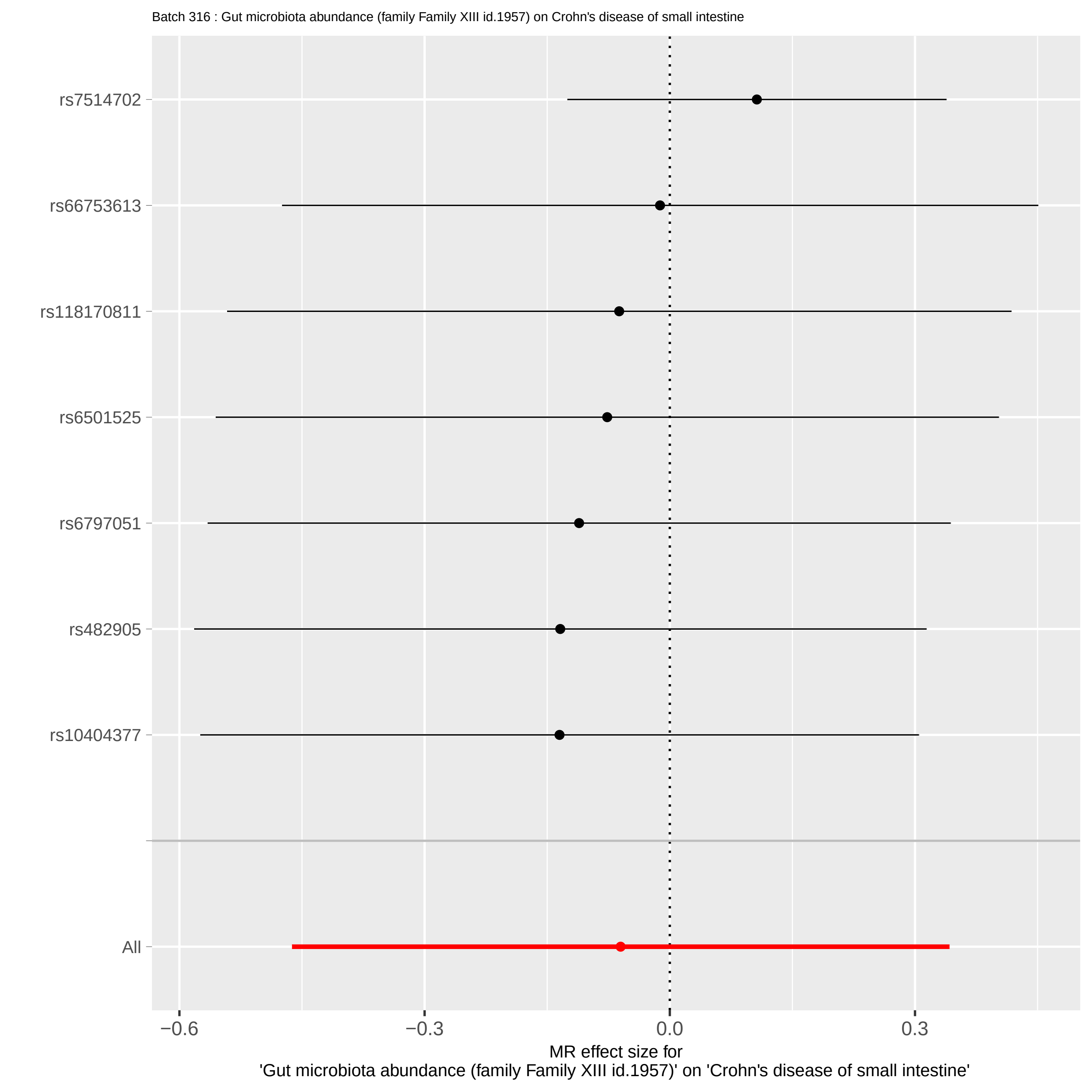

Batch 316 : Gut microbiota abundance (family Family XIII id.1957) on Crohn's disease of small intestine
rs7514702
rs66753613
rs118170811
rs6501525
rs6797051
rs482905
rs10404377
All
−0.6
−0.3
0.0
0.3
MR effect size for
'Gut microbiota abundance (family Family XIII id.1957)' on 'Crohn's disease of small intestine'

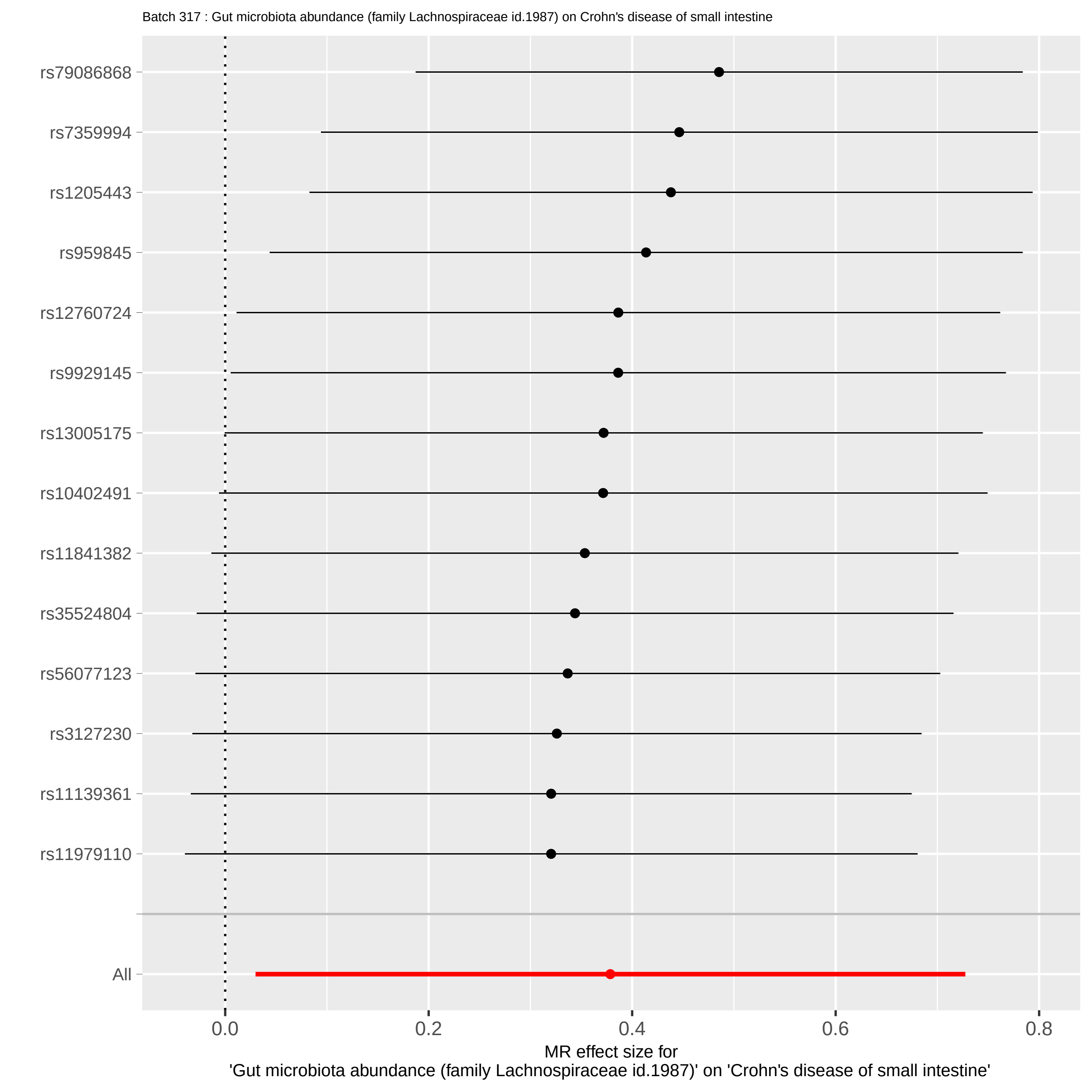

Batch 317 : Gut microbiota abundance (family Lachnospiraceae id.1987) on Crohn's disease of small intestine
rs79086868
rs7359994
rs1205443
rs959845
rs12760724
rs9929145
rs13005175
rs10402491
rs11841382
rs35524804
rs56077123
rs3127230
rs11139361
rs11979110
All
0.0
0.2
0.4
0.6
0.8
MR effect size for
'Gut microbiota abundance (family Lachnospiraceae id.1987)' on 'Crohn's disease of small intestine'

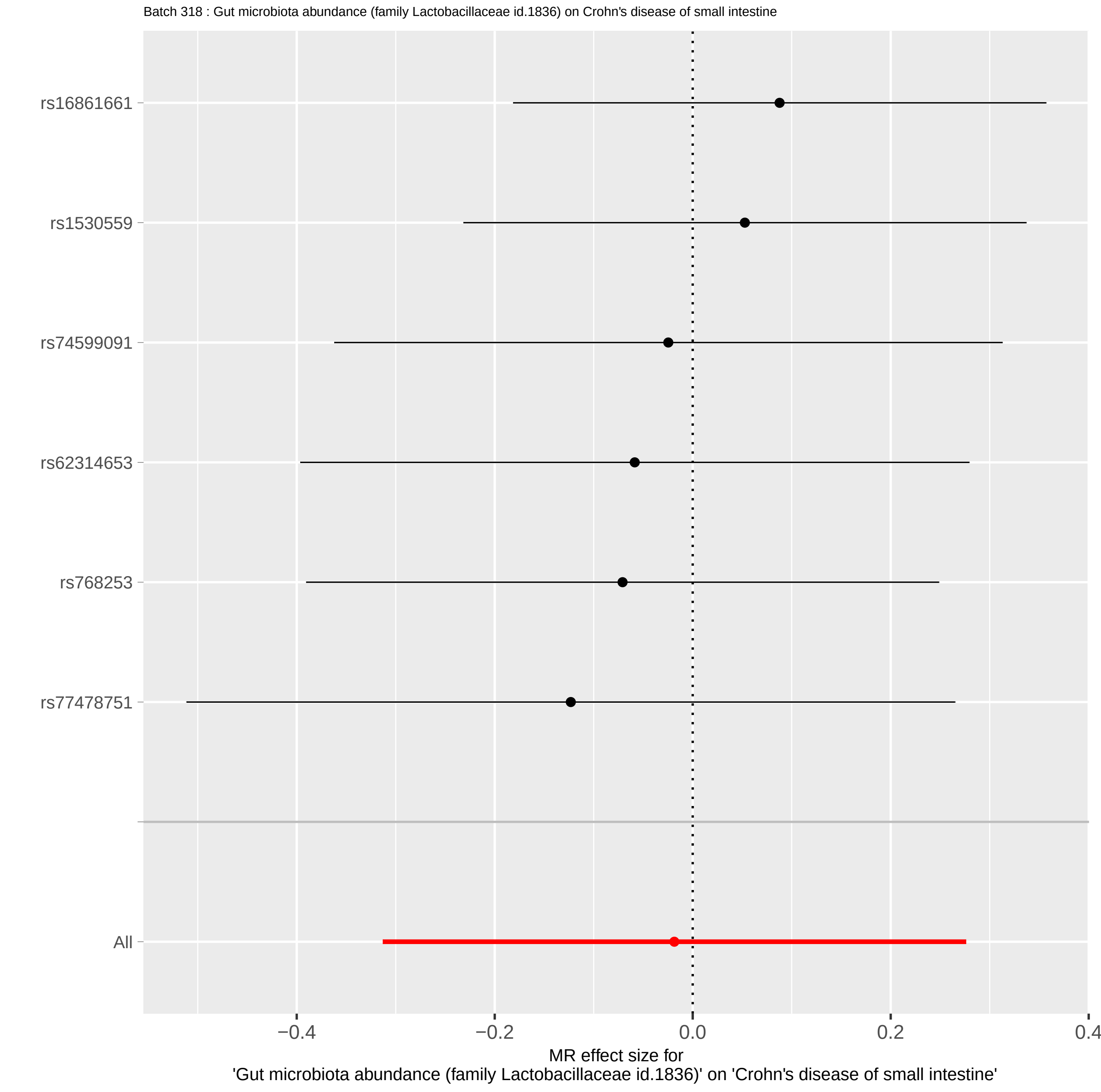
Batch 318 : Gut microbiota abundance (family Lactobacillaceae id.1836) on Crohn's disease of small intestine
rs16861661
rs1530559
rs74599091
rs62314653
rs768253
rs77478751
All
−0.4
−0.2
0.0
0.2
0.4
MR effect size for
'Gut microbiota abundance (family Lactobacillaceae id.1836)' on 'Crohn's disease of small intestine'

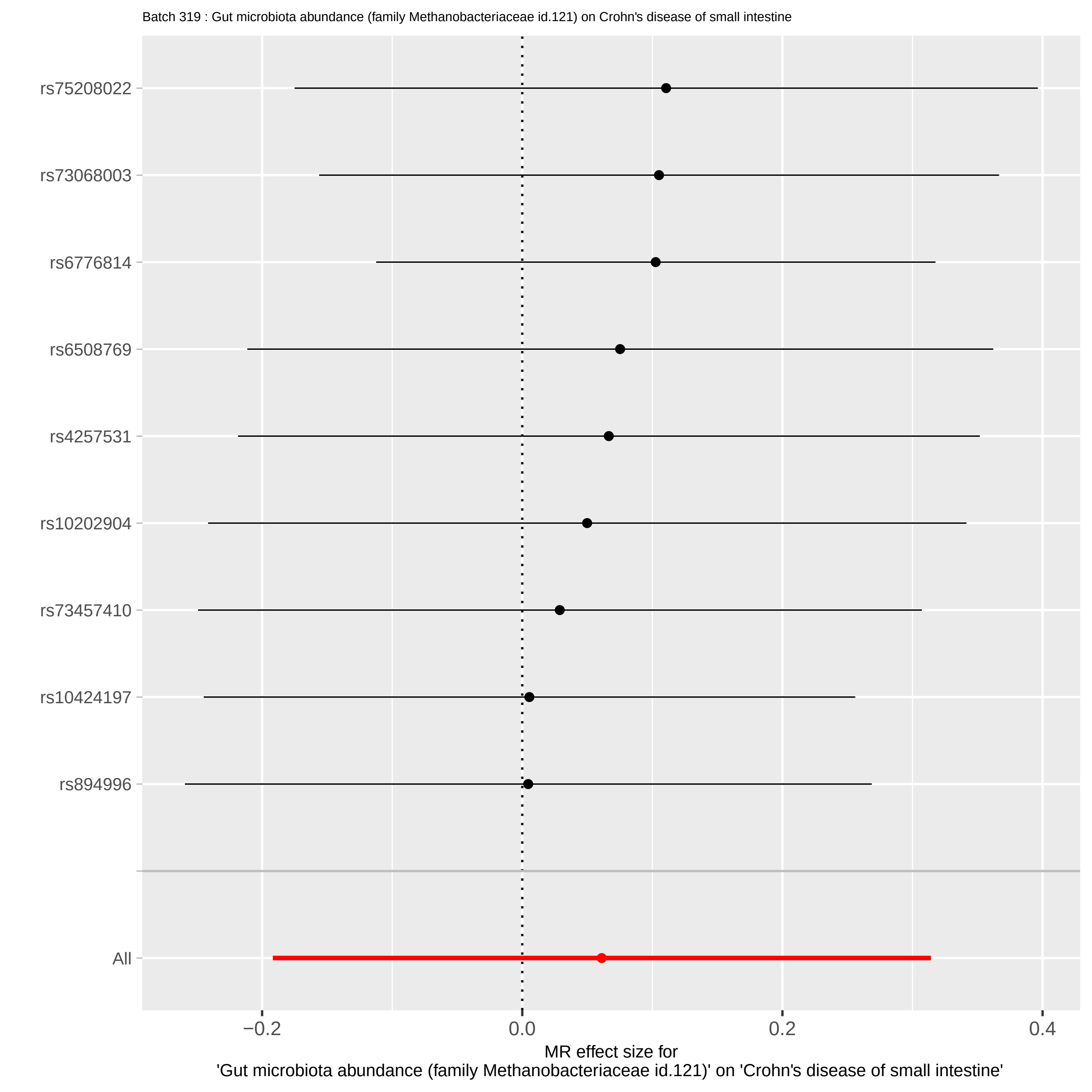

Batch 319 : Gut microbiota abundance (family Methanobacteriaceae id.121) on Crohn's disease of small intestine
rs75208022
rs73068003
rs6776814
rs6508769
rs4257531
rs10202904
rs73457410
rs10424197
rs894996
All
−0.2
0.0
0.2
0.4
MR effect size for
'Gut microbiota abundance (family Methanobacteriaceae id.121)' on 'Crohn's disease of small intestine'

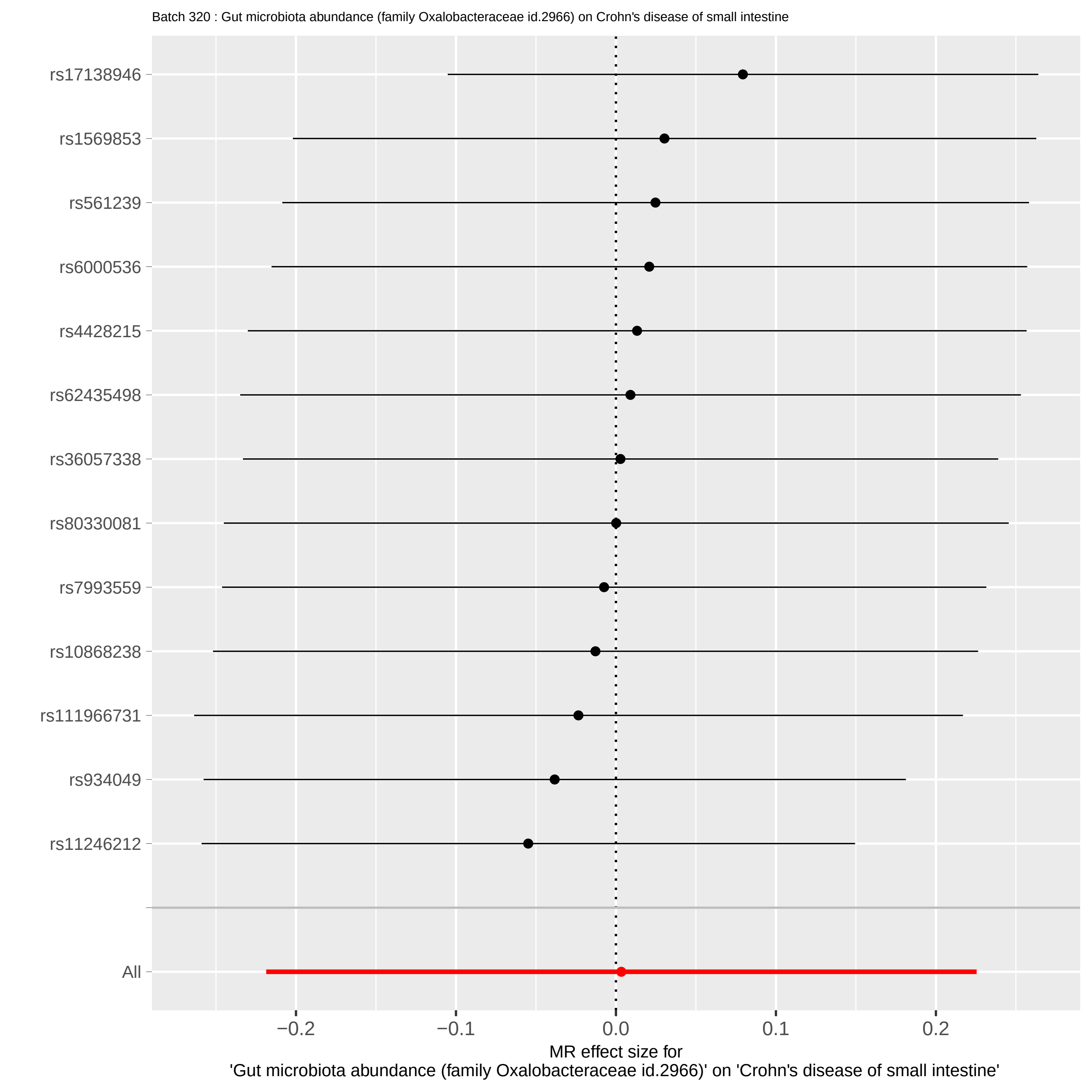

Batch 320 : Gut microbiota abundance (family Oxalobacteraceae id.2966) on Crohn's disease of small intestine
rs17138946
rs1569853
rs561239
rs6000536
rs4428215
rs62435498
rs36057338
rs80330081
rs7993559
rs10868238
rs111966731
rs934049
rs11246212
All
−0.2
−0.1
0.0
0.1
0.2
MR effect size for
'Gut microbiota abundance (family Oxalobacteraceae id.2966)' on 'Crohn's disease of small intestine'

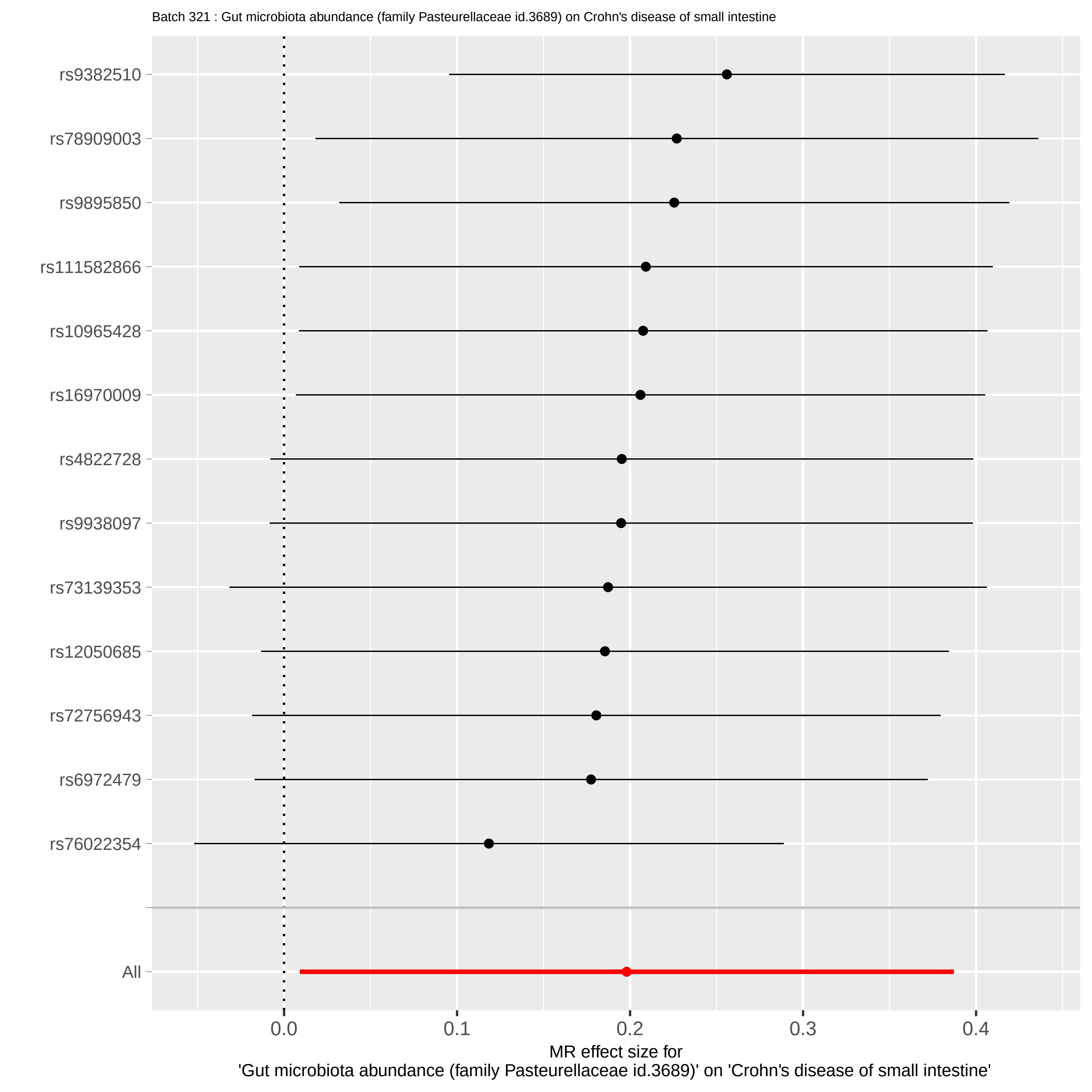

Batch 321 : Gut microbiota abundance (family Pasteurellaceae id.3689) on Crohn's disease of small intestine
rs9382510
rs78909003
rs9895850
rs111582866
rs10965428
rs16970009
rs4822728
rs9938097
rs73139353
rs12050685
rs72756943
rs6972479
rs76022354
All
0.0
0.1
0.2
0.3
0.4
MR effect size for
'Gut microbiota abundance (family Pasteurellaceae id.3689)' on 'Crohn's disease of small intestine'

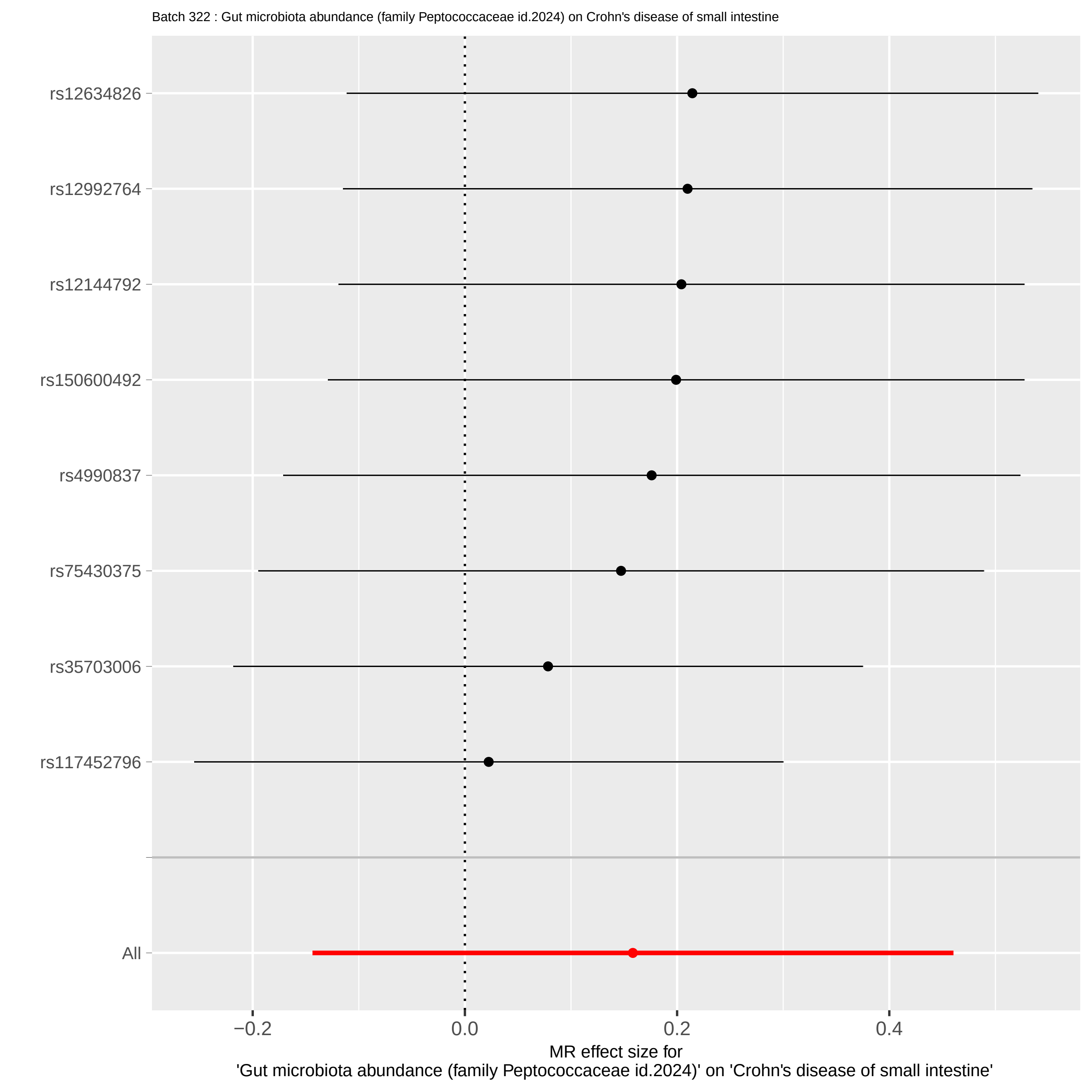

Batch 322 : Gut microbiota abundance (family Peptococcaceae id.2024) on Crohn's disease of small intestine
rs12634826
rs12992764
rs12144792
rs150600492
rs4990837
rs75430375
rs35703006
rs117452796
All
−0.2
0.0
0.2
0.4
MR effect size for
'Gut microbiota abundance (family Peptococcaceae id.2024)' on 'Crohn's disease of small intestine'

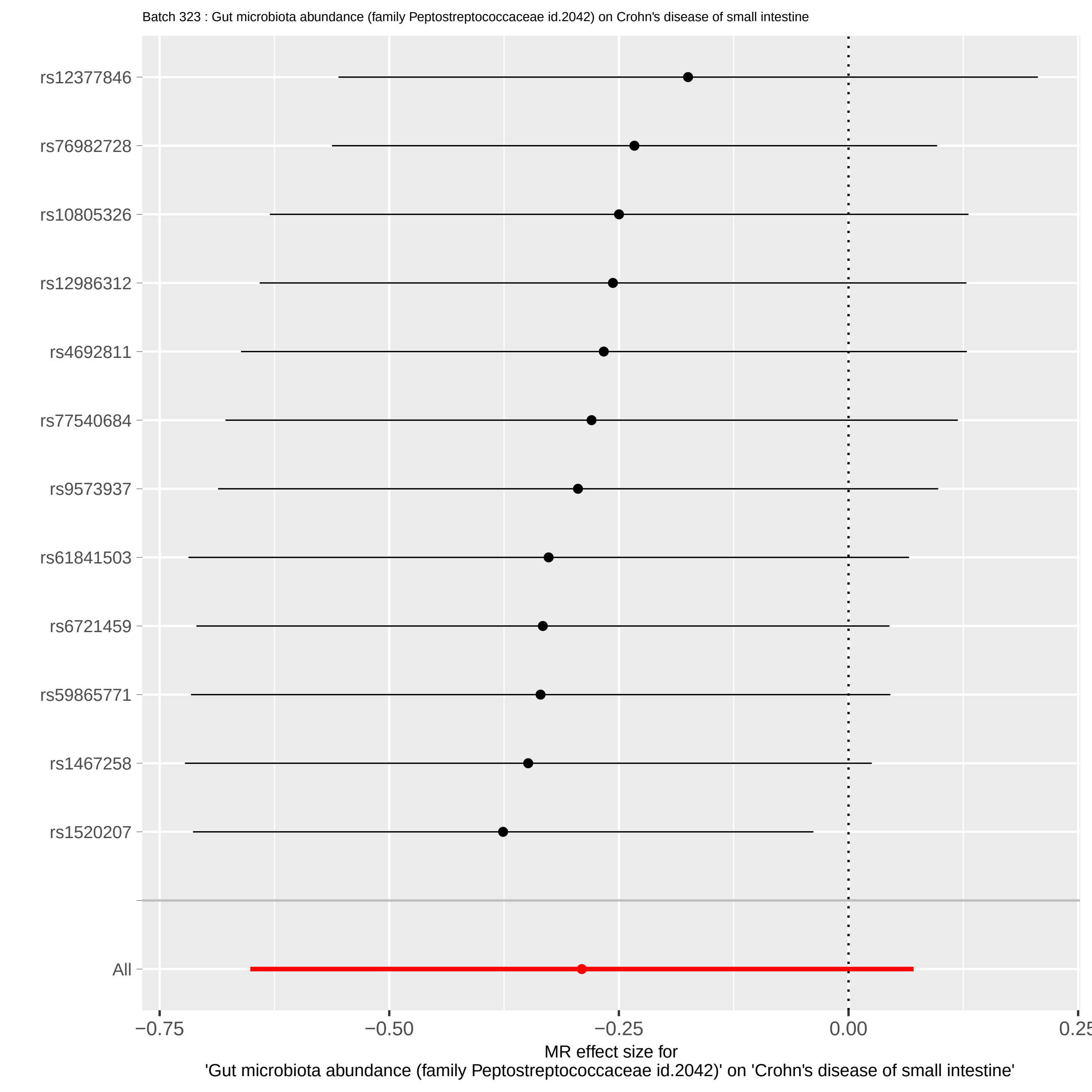

Batch 323 : Gut microbiota abundance (family Peptostreptococcaceae id.2042) on Crohn's disease of small intestine
rs12377846
rs76982728
rs10805326
rs12986312
rs4692811
rs77540684
rs9573937
rs61841503
rs6721459
rs59865771
rs1467258
rs1520207
All
−0.75
−0.50
−0.25
0.00
0.25
MR effect size for
'Gut microbiota abundance (family Peptostreptococcaceae id.2042)' on 'Crohn's disease of small intestine'

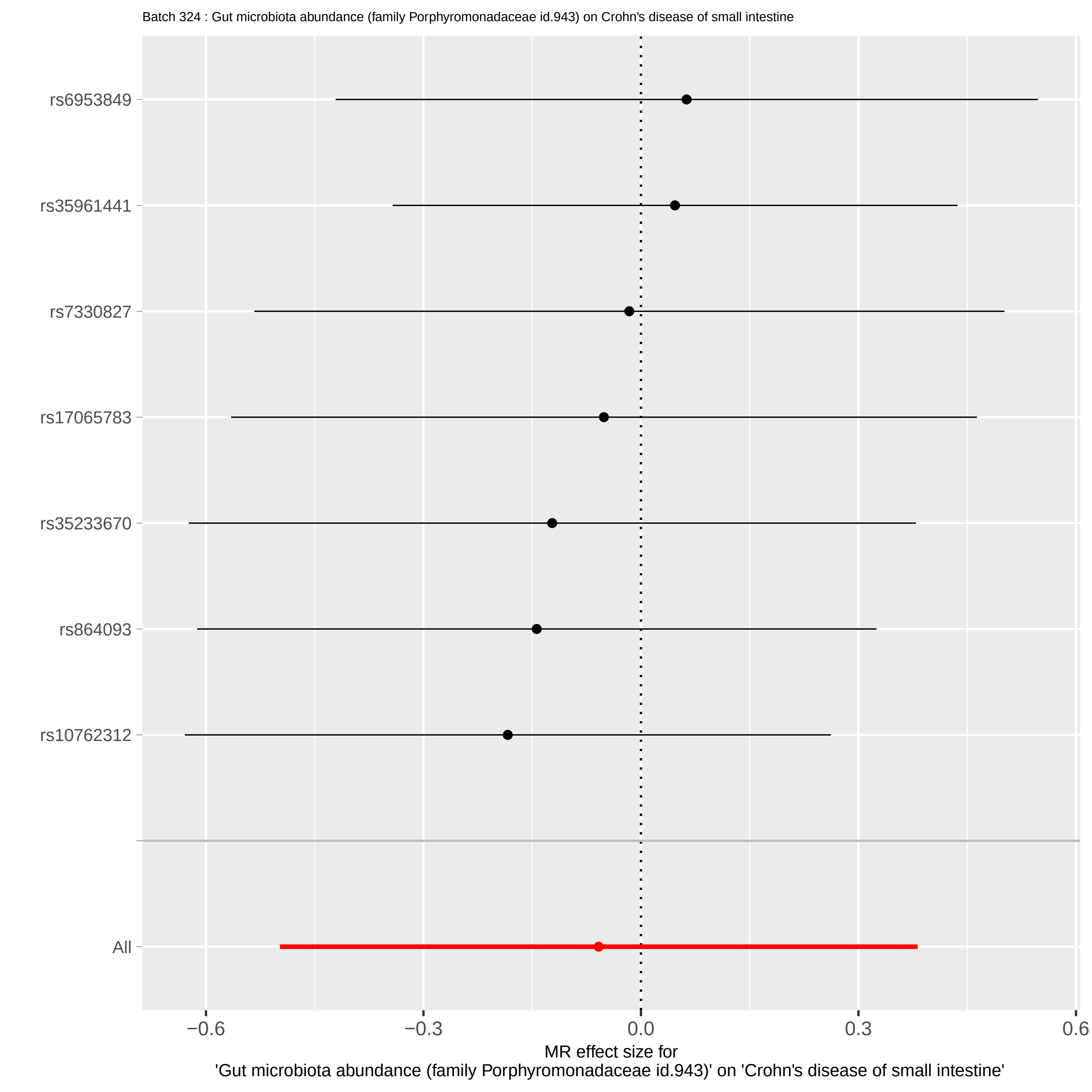
Batch 324 : Gut microbiota abundance (family Porphyromonadaceae id.943) on Crohn's disease of small intestine
rs6953849
rs35961441
rs7330827
rs17065783
rs35233670
rs864093
rs10762312
All
−0.6
−0.3
0.0
0.3
0.6
MR effect size for
'Gut microbiota abundance (family Porphyromonadaceae id.943)' on 'Crohn's disease of small intestine'

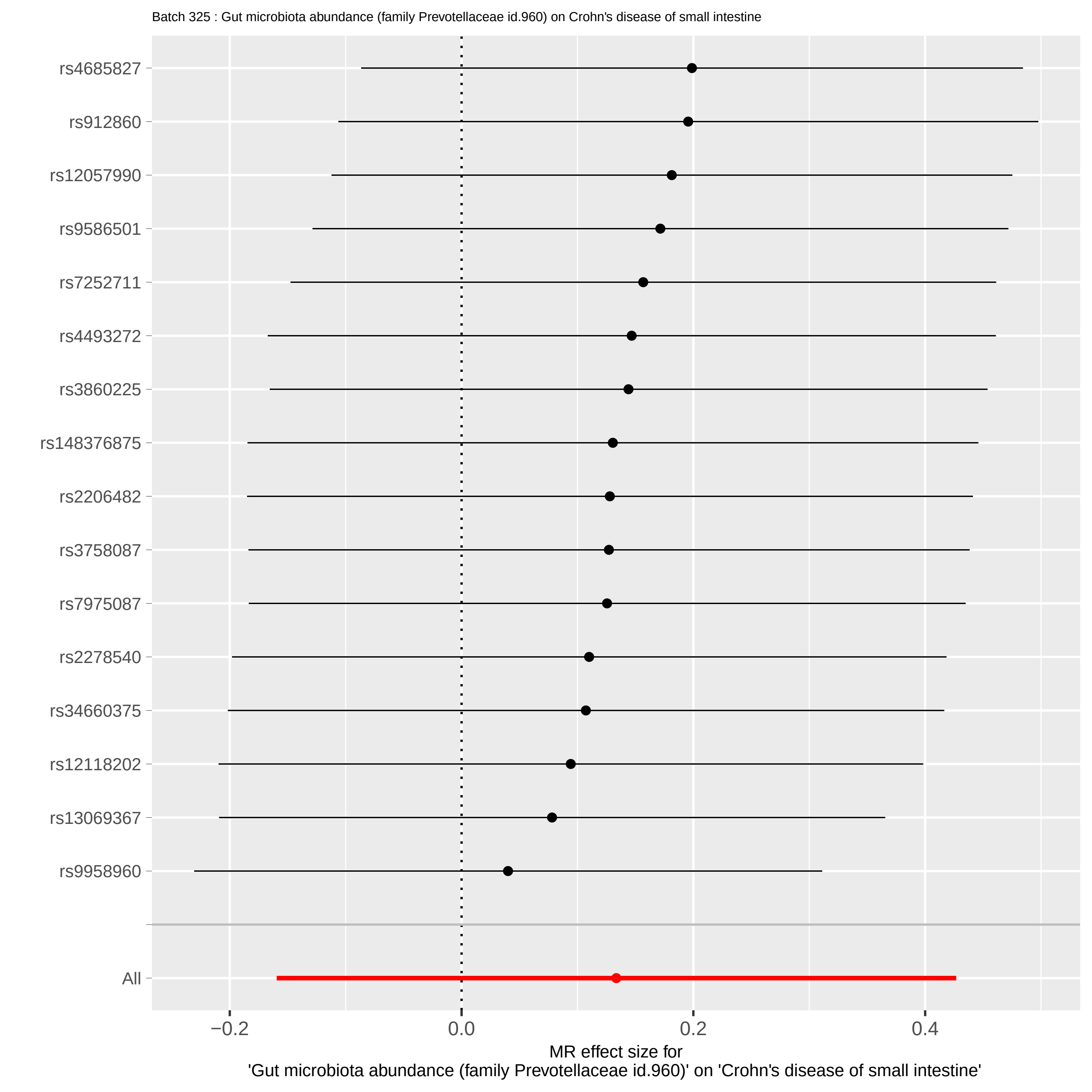
Batch 325 : Gut microbiota abundance (family Prevotellaceae id.960) on Crohn's disease of small intestine
rs4685827
rs912860
rs12057990
rs9586501
rs7252711
rs4493272
rs3860225
rs148376875
rs2206482
rs3758087
rs7975087
rs2278540
rs34660375
rs12118202
rs13069367
rs9958960
All
−0.2
0.0
0.2
0.4
MR effect size for
'Gut microbiota abundance (family Prevotellaceae id.960)' on 'Crohn's disease of small intestine'

Batch 326 : Gut microbiota abundance (family Rhodospirillaceae id.2717) on Crohn's disease of small intestine

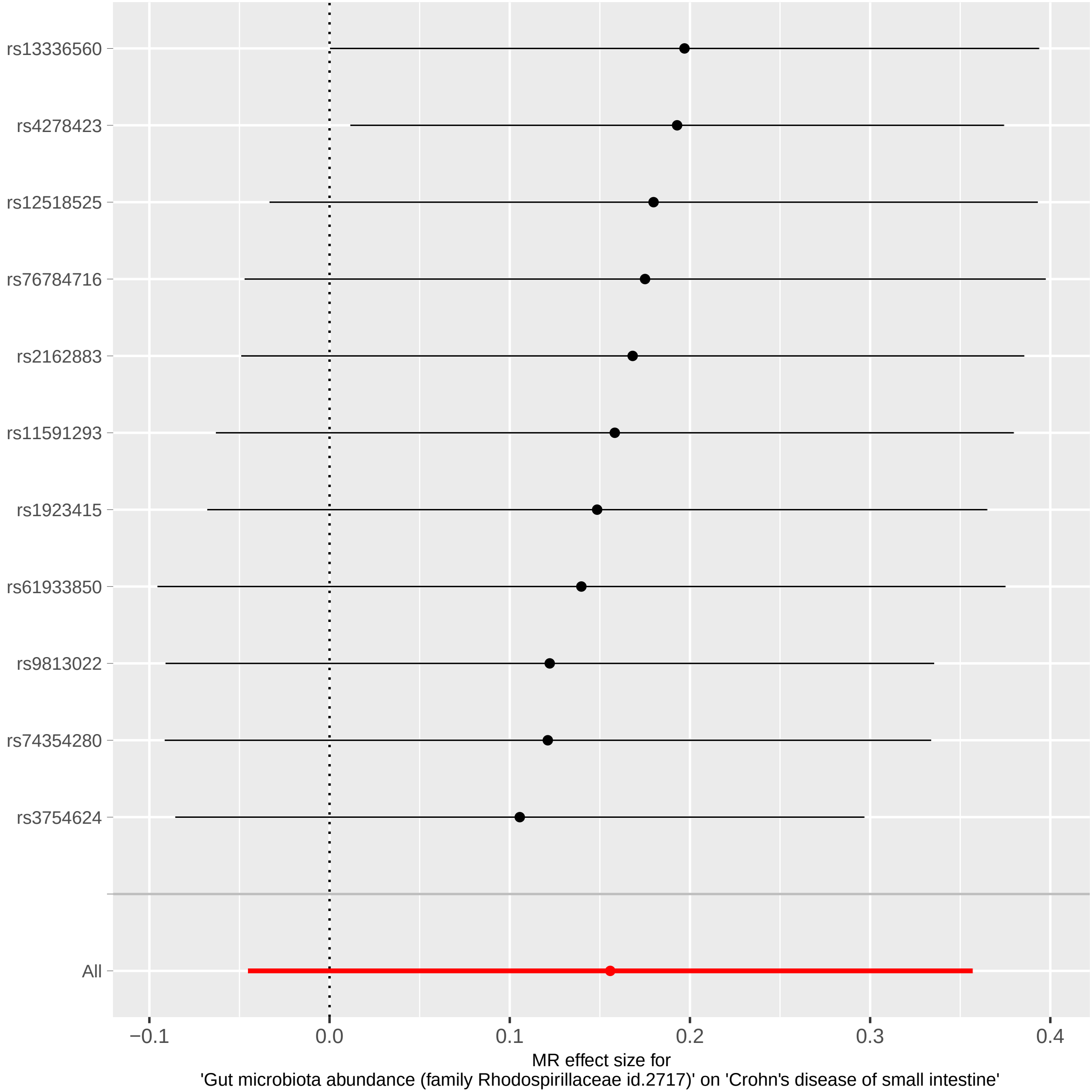

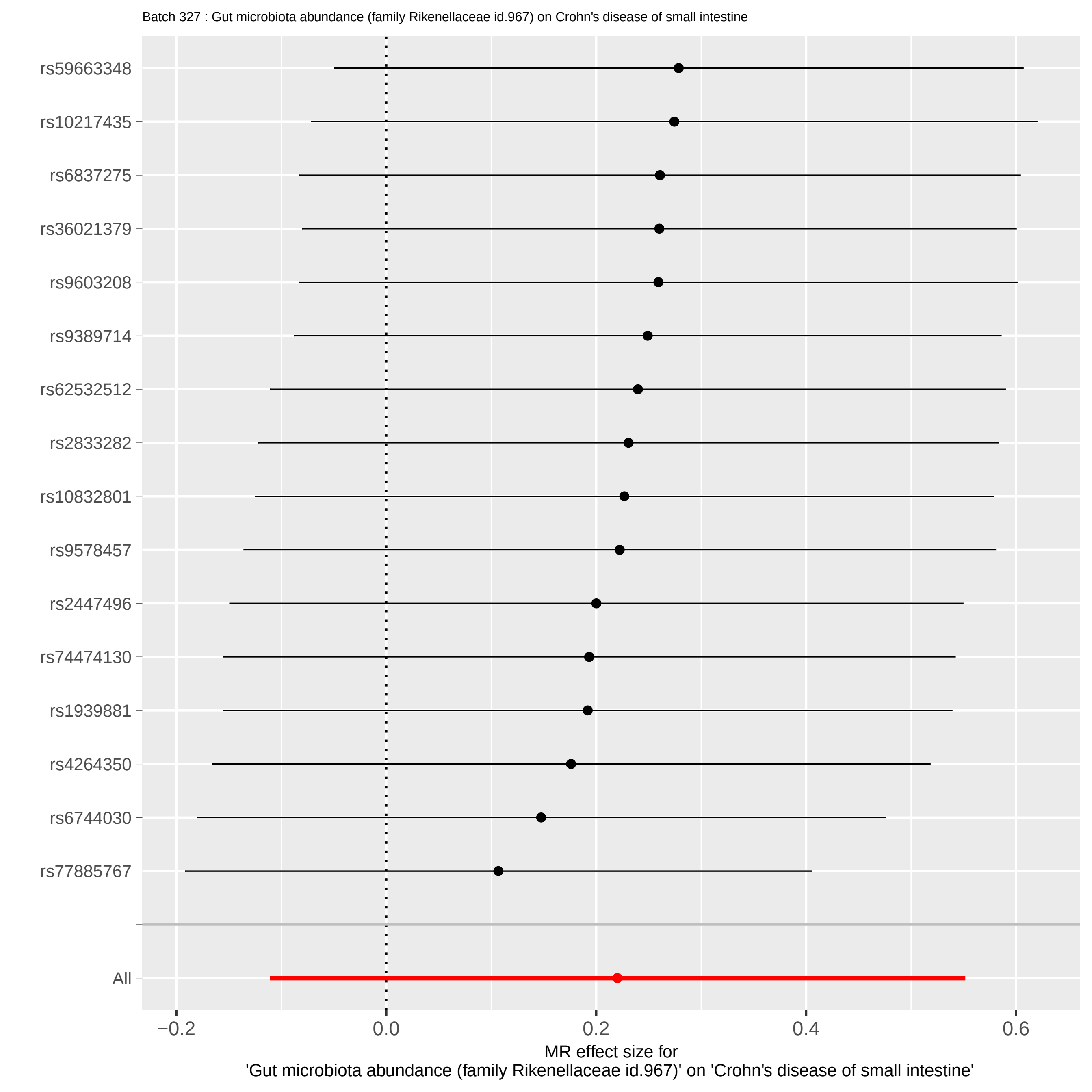

Batch 327 : Gut microbiota abundance (family Rikenellaceae id.967) on Crohn's disease of small intestine
rs59663348
rs10217435
rs6837275
rs36021379
rs9603208
rs9389714
rs62532512
rs2833282
rs10832801
rs9578457
rs2447496
rs74474130
rs1939881
rs4264350
rs6744030
rs77885767
All
−0.2
0.0
0.2
0.4
0.6
MR effect size for
'Gut microbiota abundance (family Rikenellaceae id.967)' on 'Crohn's disease of small intestine'

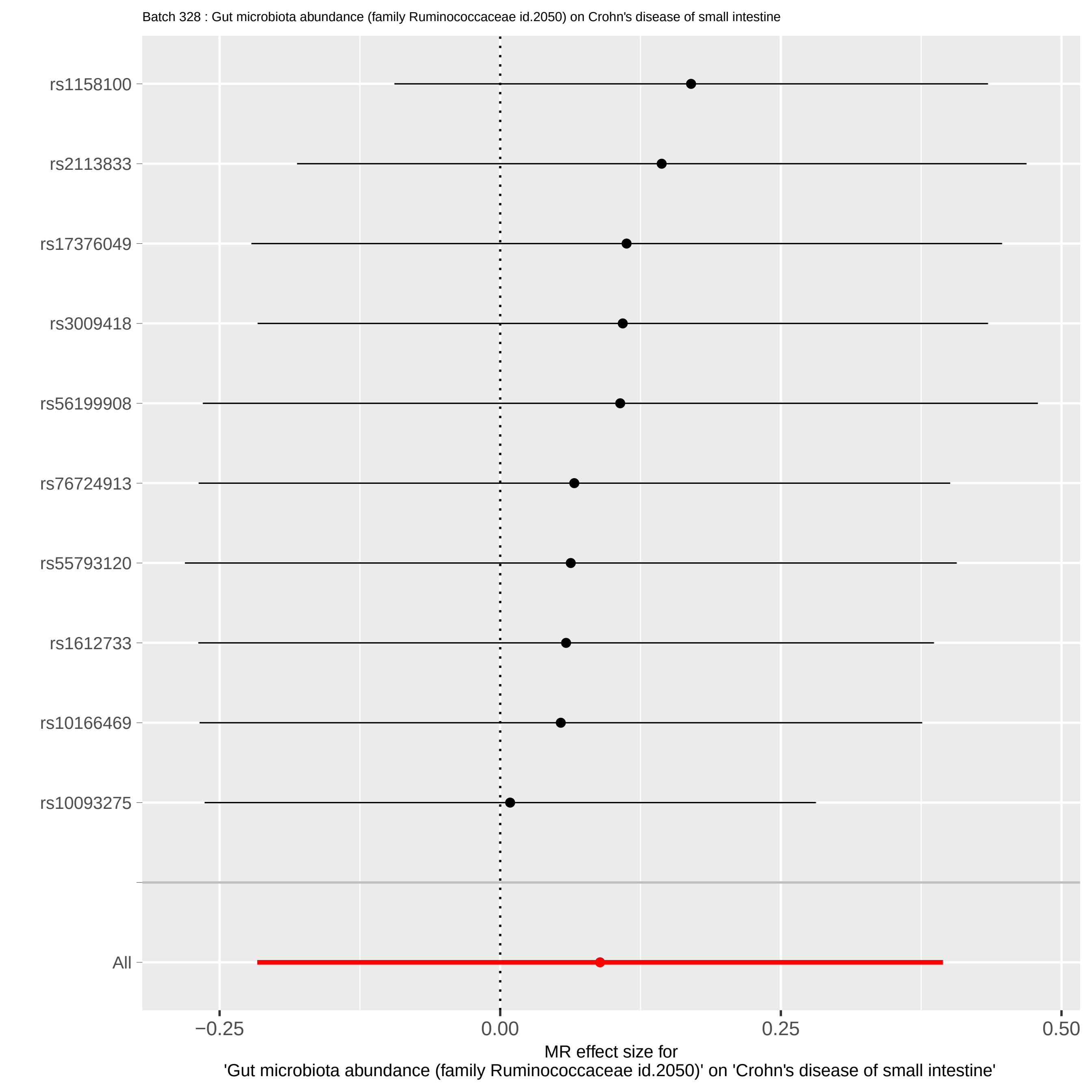

Batch 328 : Gut microbiota abundance (family Ruminococcaceae id.2050) on Crohn's disease of small intestine
rs1158100
rs2113833
rs17376049
rs3009418
rs56199908
rs76724913
rs55793120
rs1612733
rs10166469
rs10093275
All
−0.25
0.00
0.25
0.50
MR effect size for
'Gut microbiota abundance (family Ruminococcaceae id.2050)' on 'Crohn's disease of small intestine'

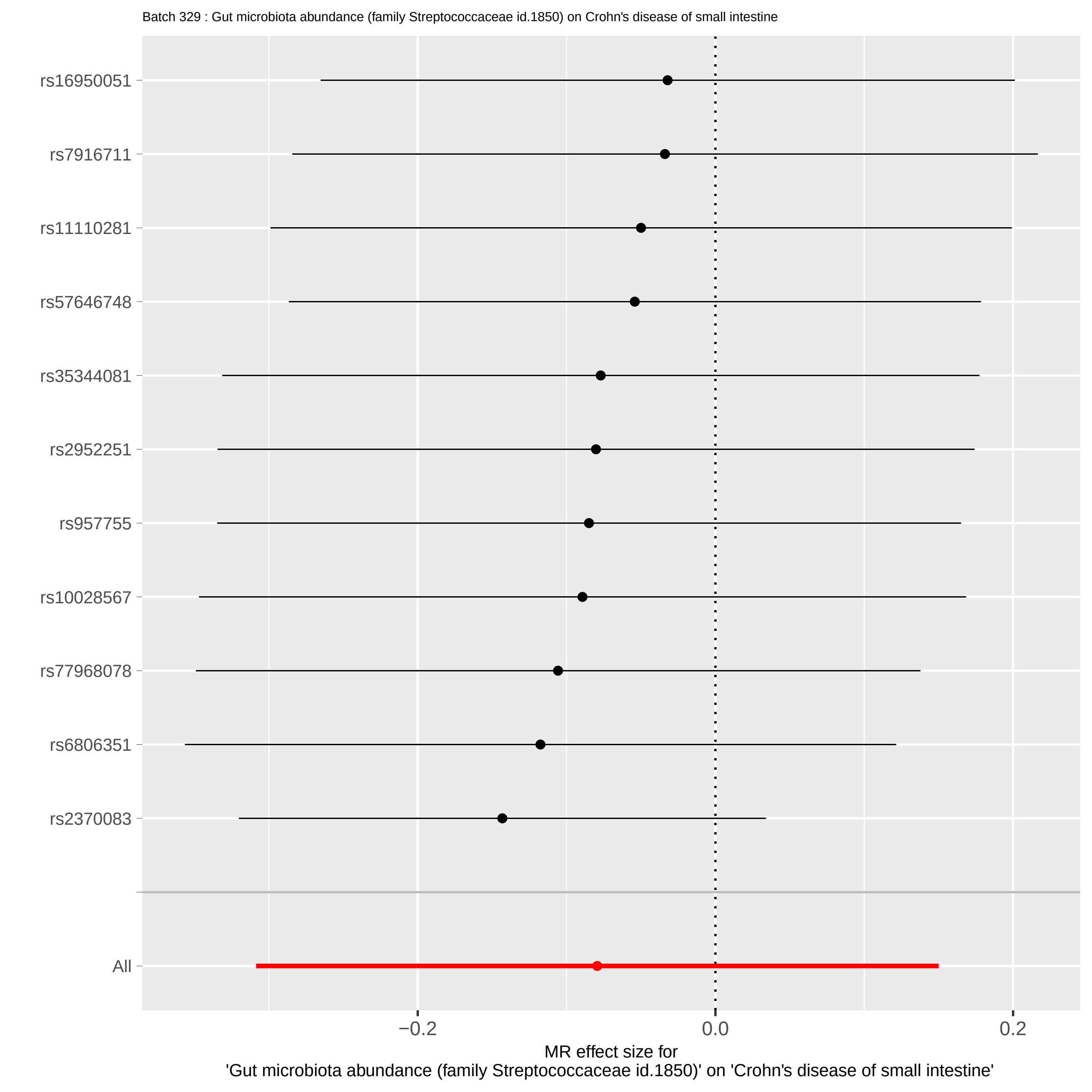
Batch 329 : Gut microbiota abundance (family Streptococcaceae id.1850) on Crohn's disease of small intestine
rs16950051
rs7916711
rs11110281
rs57646748
rs35344081
rs2952251
rs957755
rs10028567
rs77968078
rs6806351
rs2370083
All
−0.2
0.0
0.2
MR effect size for
'Gut microbiota abundance (family Streptococcaceae id.1850)' on 'Crohn's disease of small intestine'

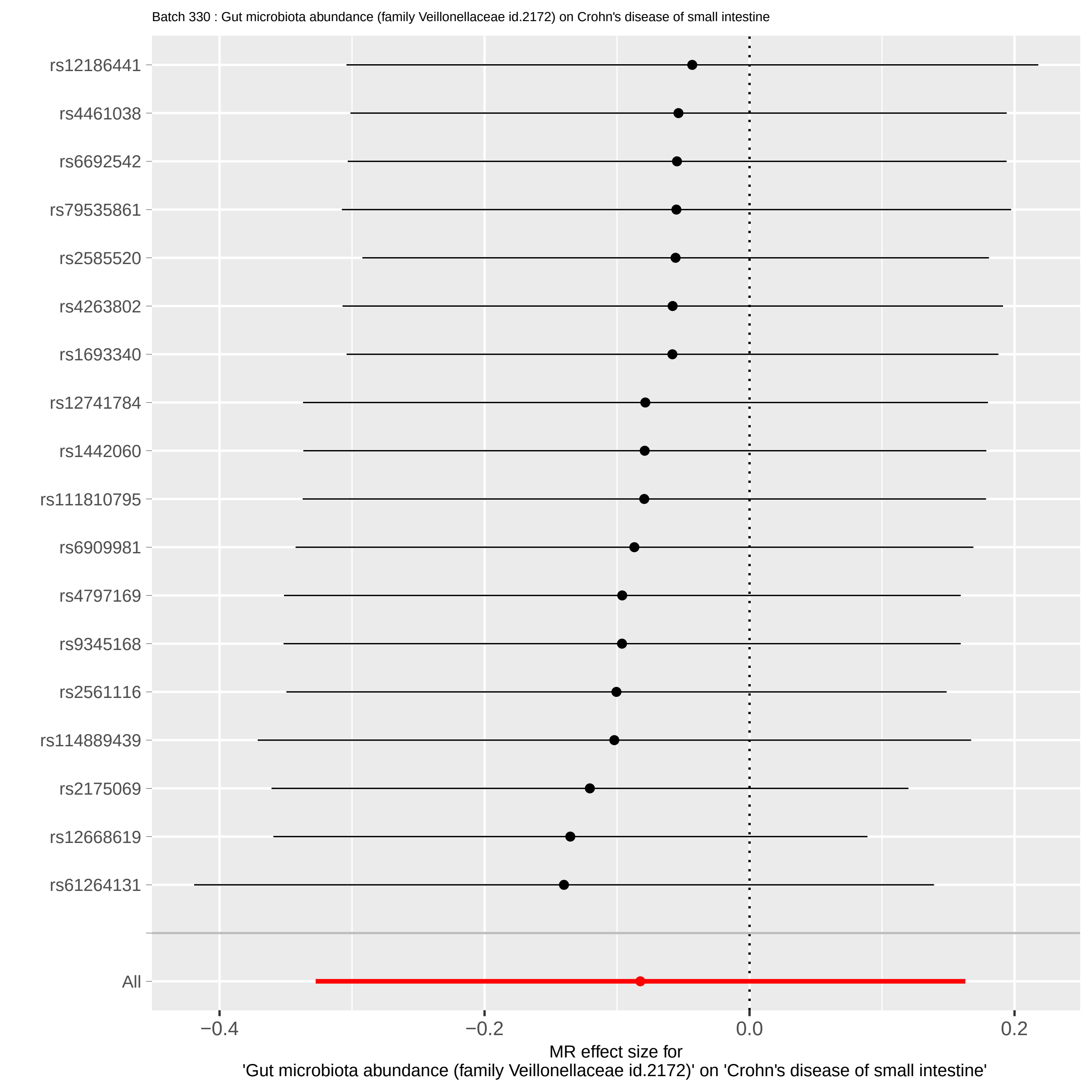

Batch 330 : Gut microbiota abundance (family Veillonellaceae id.2172) on Crohn's disease of small intestine
rs12186441
rs4461038
rs6692542
rs79535861
rs2585520
rs4263802
rs1693340
rs12741784
rs1442060
rs111810795
rs6909981
rs4797169
rs9345168
rs2561116
rs114889439
rs2175069
rs12668619
rs61264131
All
−0.4
−0.2
0.0
0.2
MR effect size for
'Gut microbiota abundance (family Veillonellaceae id.2172)' on 'Crohn's disease of small intestine'

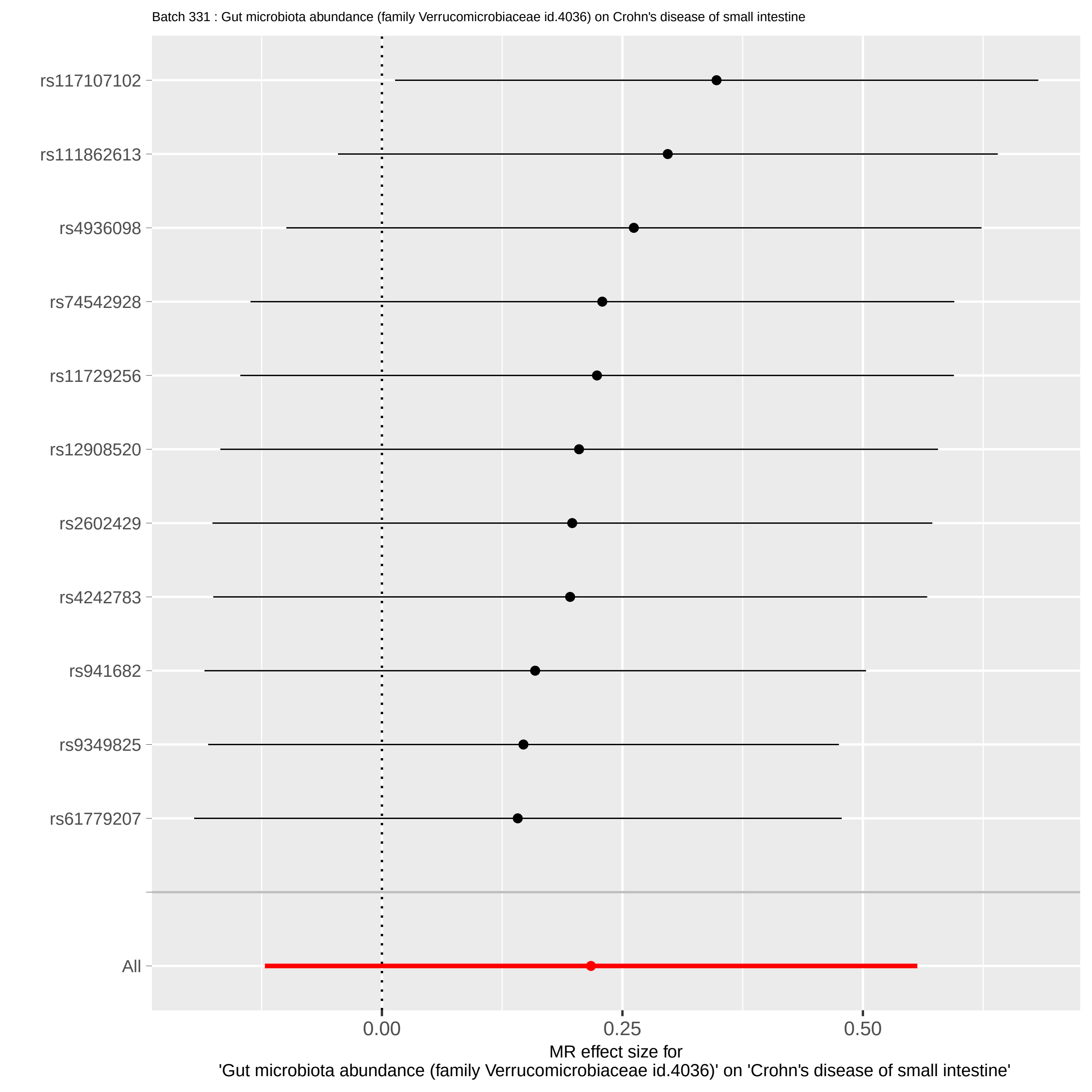
Batch 331 : Gut microbiota abundance (family Verrucomicrobiaceae id.4036) on Crohn's disease of small intestine
rs117107102
rs111862613
rs4936098
rs74542928
rs11729256
rs12908520
rs2602429
rs4242783
rs941682
rs9349825
rs61779207
All
0.00
0.25
0.50
MR effect size for
'Gut microbiota abundance (family Verrucomicrobiaceae id.4036)' on 'Crohn's disease of small intestine'

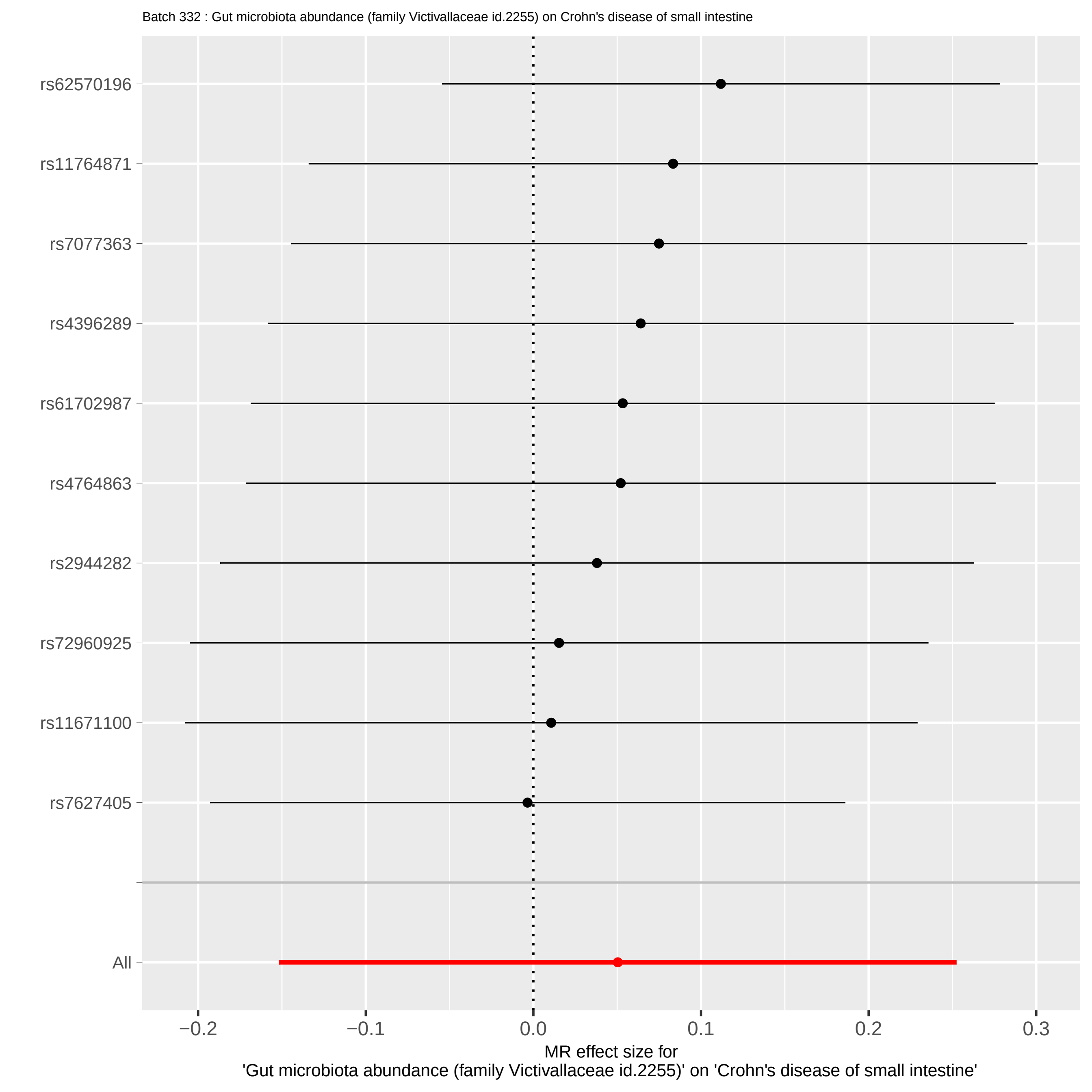

Batch 332 : Gut microbiota abundance (family Victivallaceae id.2255) on Crohn's disease of small intestine
rs62570196
rs11764871
rs7077363
rs4396289
rs61702987
rs4764863
rs2944282
rs72960925
rs11671100
rs7627405
All
−0.2
−0.1
0.0
0.1
0.2
0.3
MR effect size for
'Gut microbiota abundance (family Victivallaceae id.2255)' on 'Crohn's disease of small intestine'

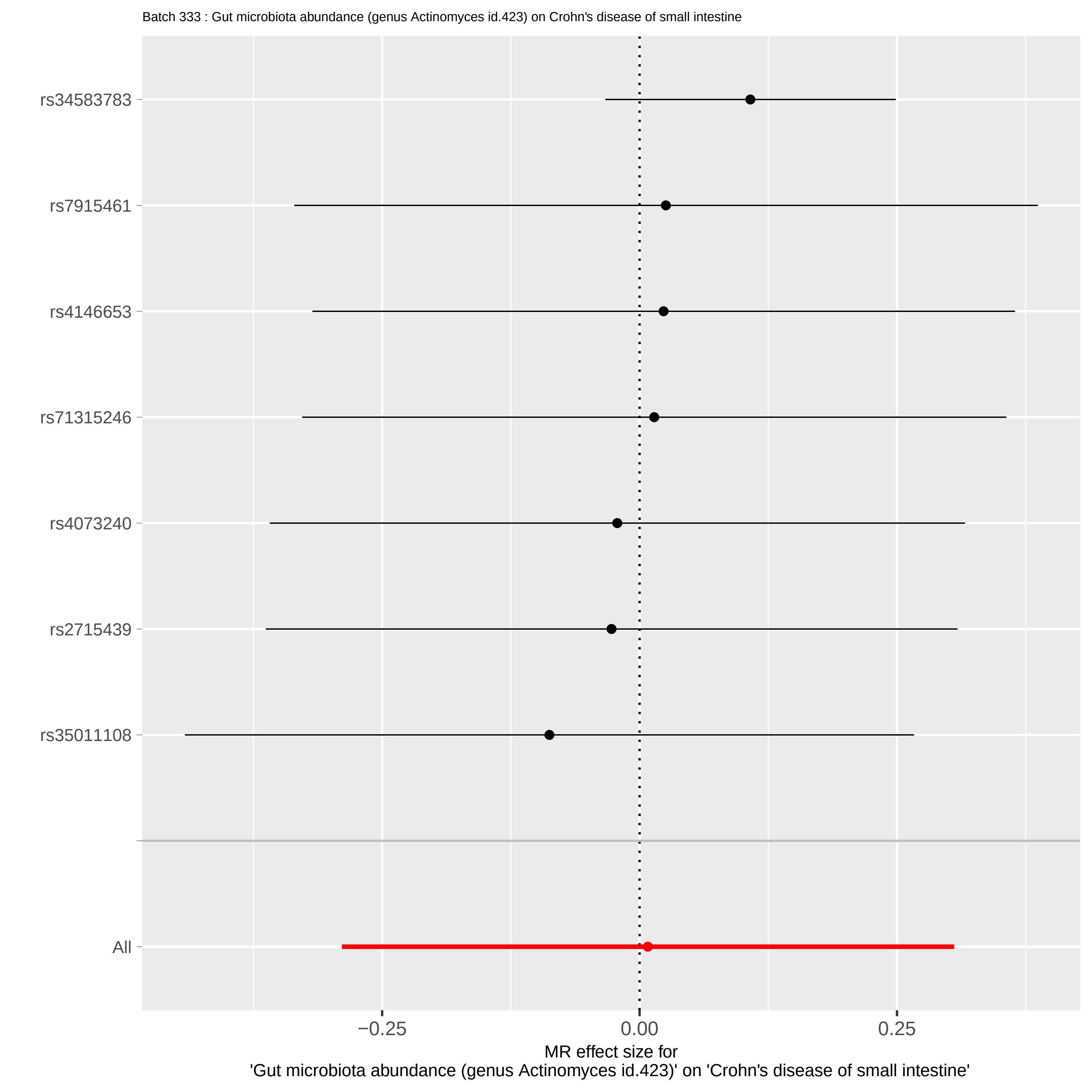

Batch 333 : Gut microbiota abundance (genus Actinomyces id.423) on Crohn's disease of small intestine
rs34583783
rs7915461
rs4146653
rs71315246
rs4073240
rs2715439
rs35011108
All
−0.25
0.00
0.25
MR effect size for
'Gut microbiota abundance (genus Actinomyces id.423)' on 'Crohn's disease of small intestine'

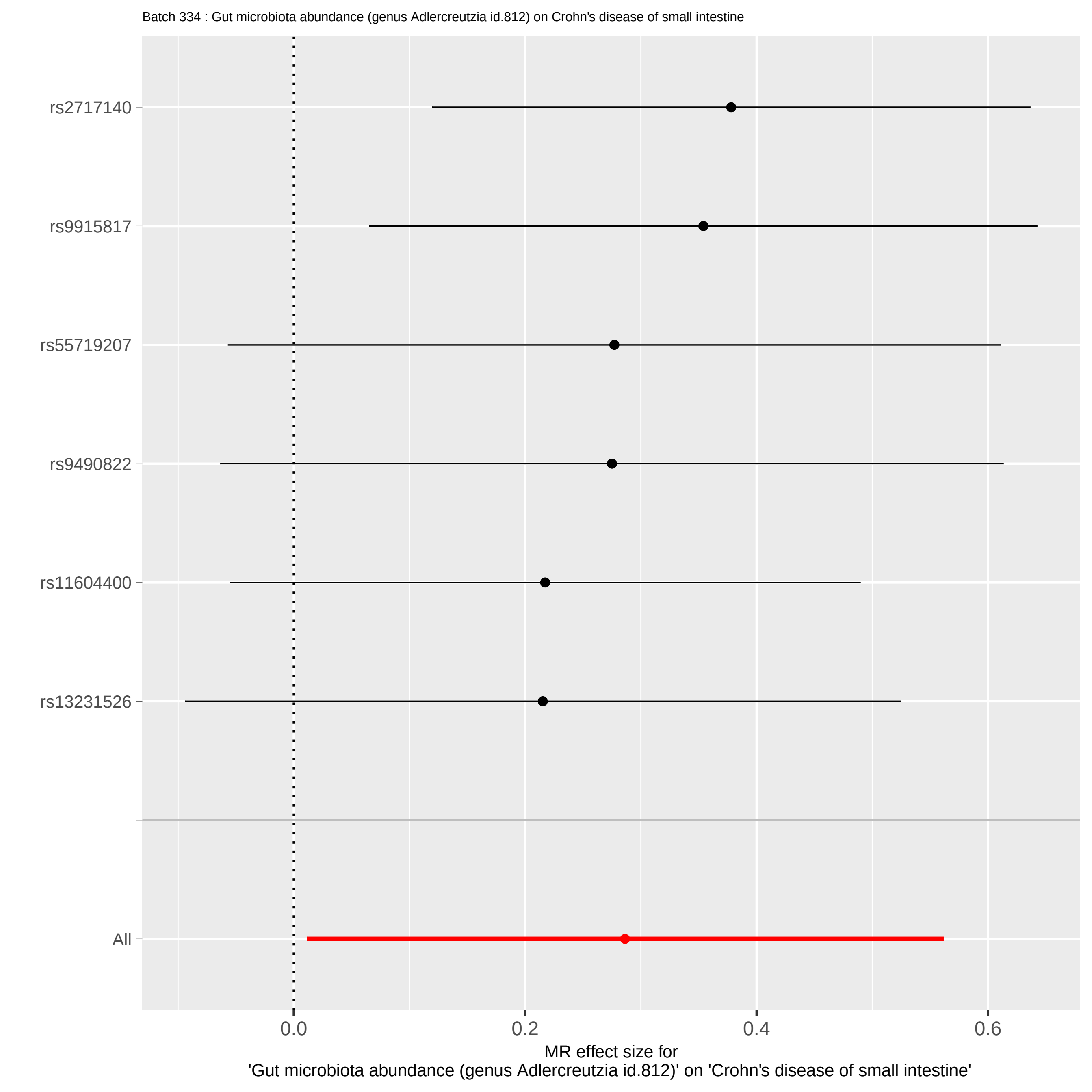

Batch 334 : Gut microbiota abundance (genus Adlercreutzia id.812) on Crohn's disease of small intestine
rs2717140
rs9915817
rs55719207
rs9490822
rs11604400
rs13231526
All
0.0
0.2
0.4
0.6
MR effect size for
'Gut microbiota abundance (genus Adlercreutzia id.812)' on 'Crohn's disease of small intestine'

Batch 335 : Gut microbiota abundance (genus Akkermansia id.4037) on Crohn's disease of small intestine

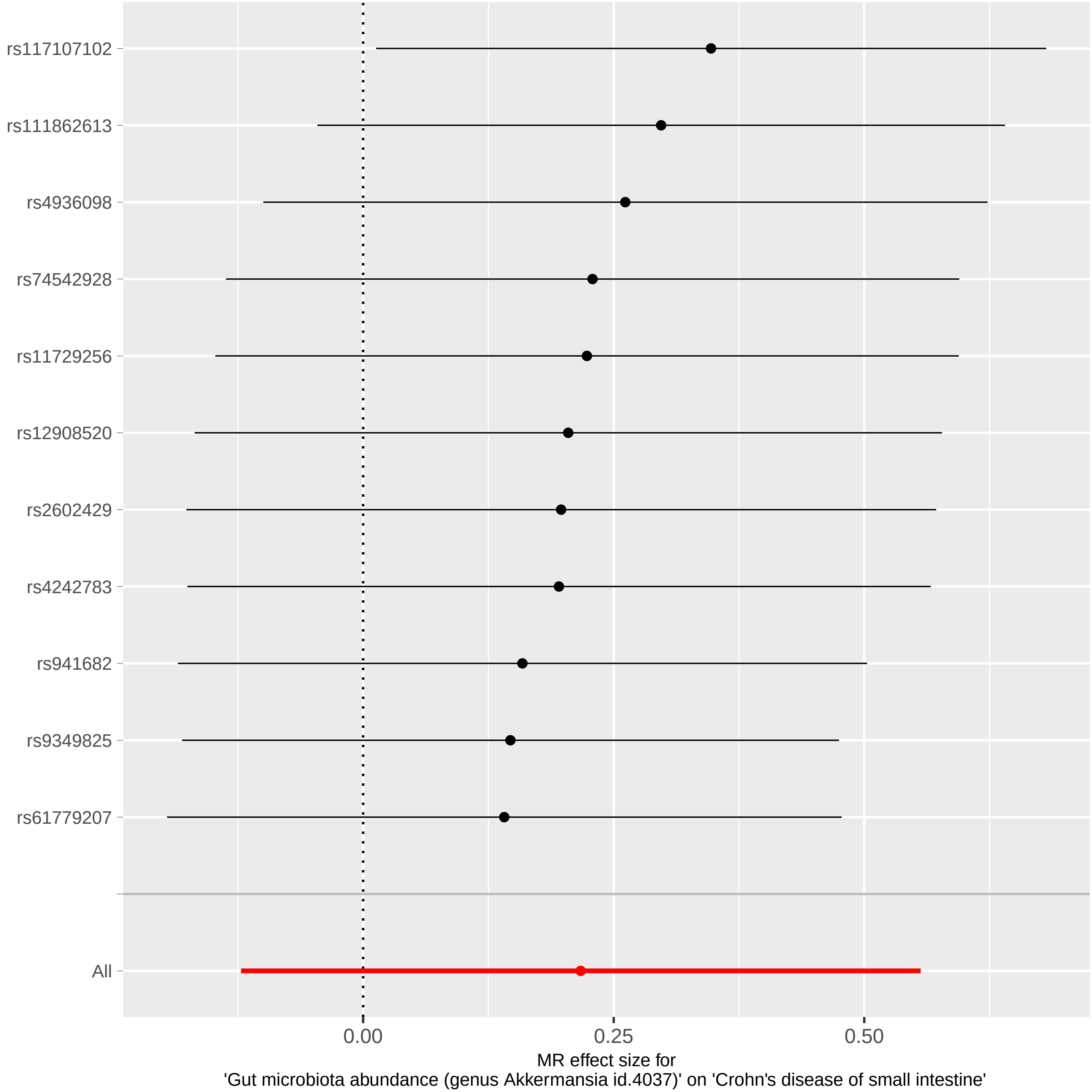

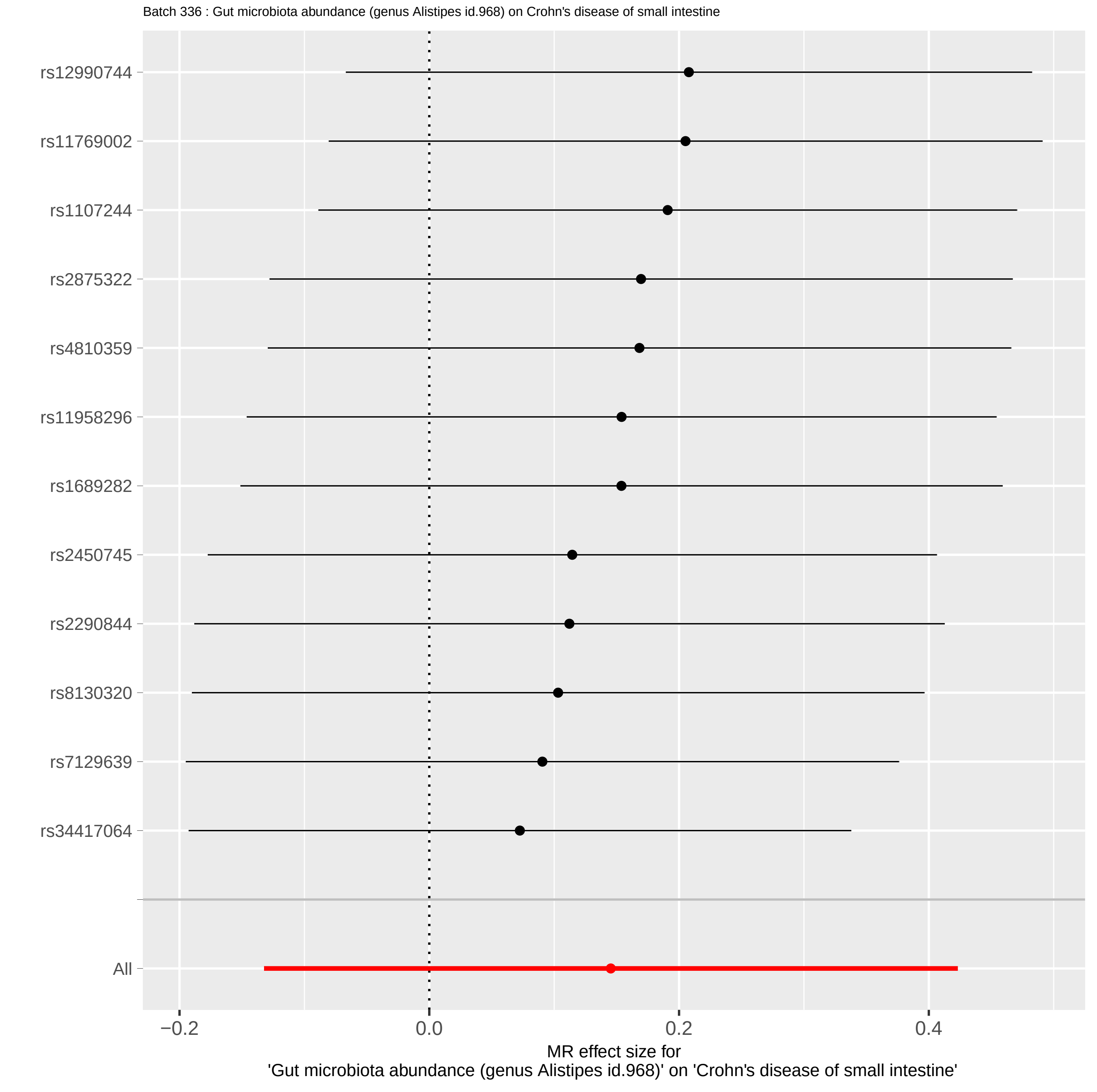

Batch 336 : Gut microbiota abundance (genus Alistipes id.968) on Crohn's disease of small intestine
rs12990744
rs11769002
rs1107244
rs2875322
rs4810359
rs11958296
rs1689282
rs2450745
rs2290844
rs8130320
rs7129639
rs34417064
All
−0.2
0.0
0.2
0.4
MR effect size for
'Gut microbiota abundance (genus Alistipes id.968)' on 'Crohn's disease of small intestine'

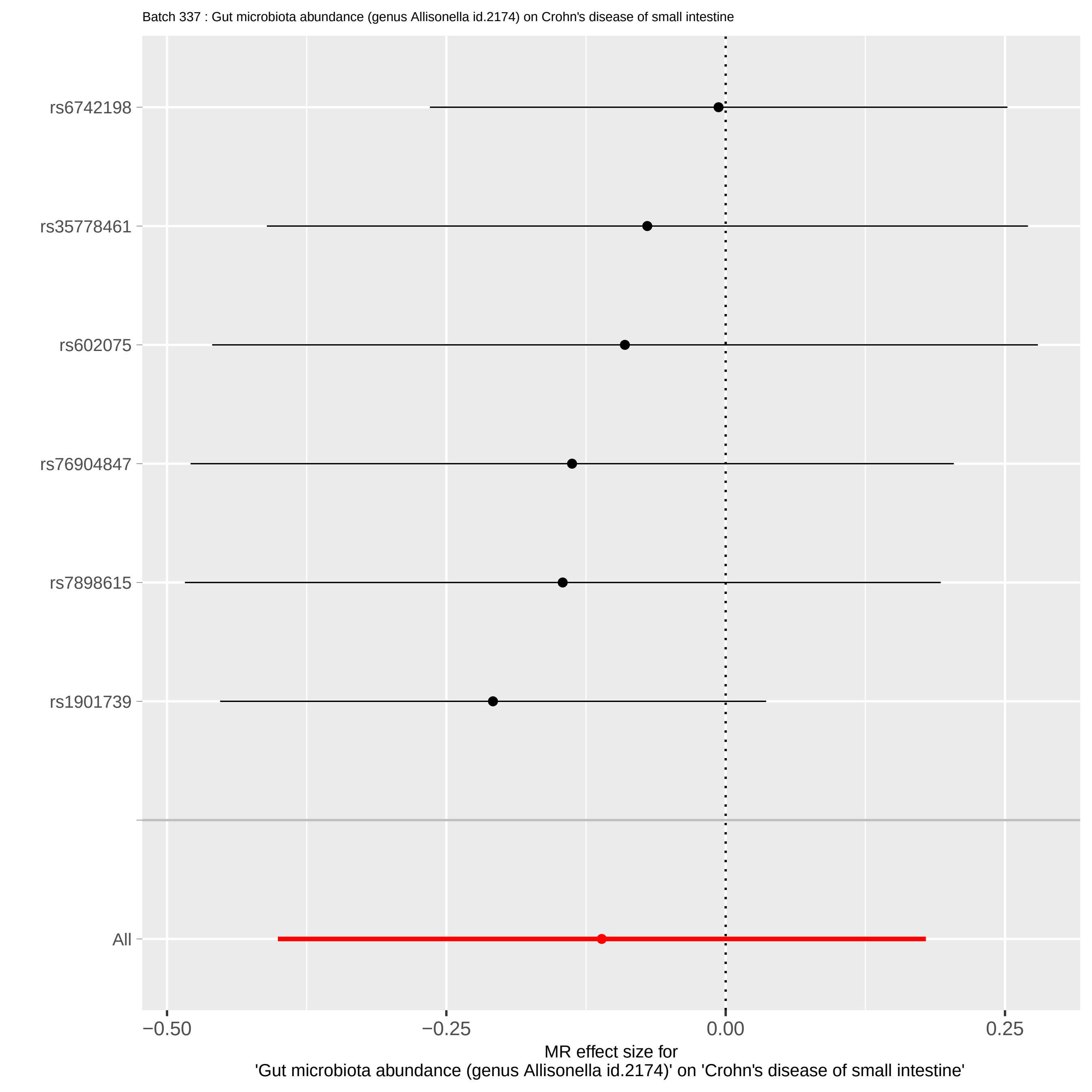

Batch 337 : Gut microbiota abundance (genus Allisonella id.2174) on Crohn's disease of small intestine
rs6742198
rs35778461
rs602075
rs76904847
rs7898615
rs1901739
All
−0.50
−0.25
0.00
0.25
MR effect size for
'Gut microbiota abundance (genus Allisonella id.2174)' on 'Crohn's disease of small intestine'

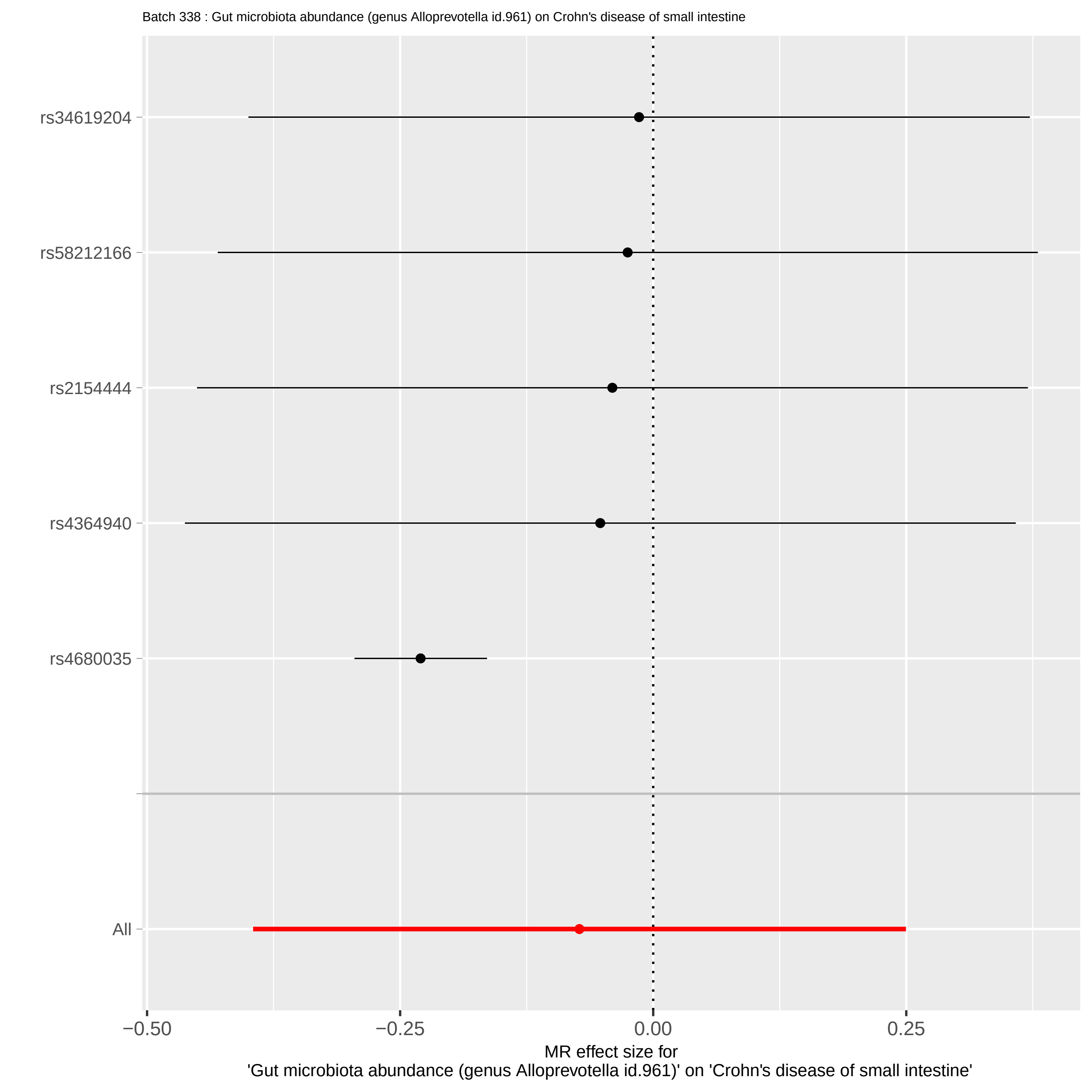

Batch 338 : Gut microbiota abundance (genus Alloprevotella id.961) on Crohn's disease of small intestine
rs34619204
rs58212166
rs2154444
rs4364940
rs4680035
All
−0.50
−0.25
0.00
0.25
MR effect size for
'Gut microbiota abundance (genus Alloprevotella id.961)' on 'Crohn's disease of small intestine'

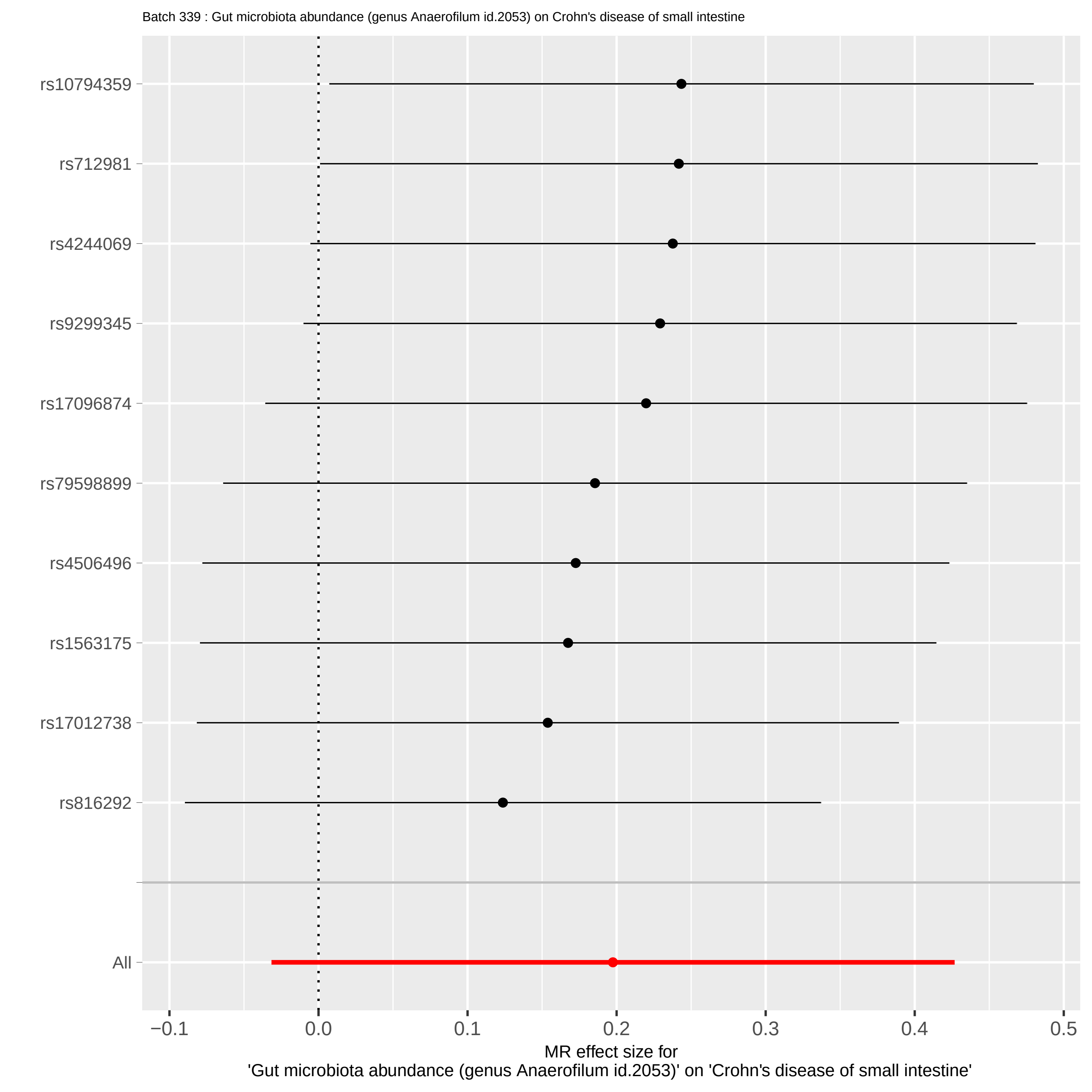
Batch 339 : Gut microbiota abundance (genus Anaerofilum id.2053) on Crohn's disease of small intestine
rs10794359
rs712981
rs4244069
rs9299345
rs17096874
rs79598899
rs4506496
rs1563175
rs17012738
rs816292
All
−0.1
0.0
0.1
0.2
0.3
0.4
0.5
MR effect size for
'Gut microbiota abundance (genus Anaerofilum id.2053)' on 'Crohn's disease of small intestine'

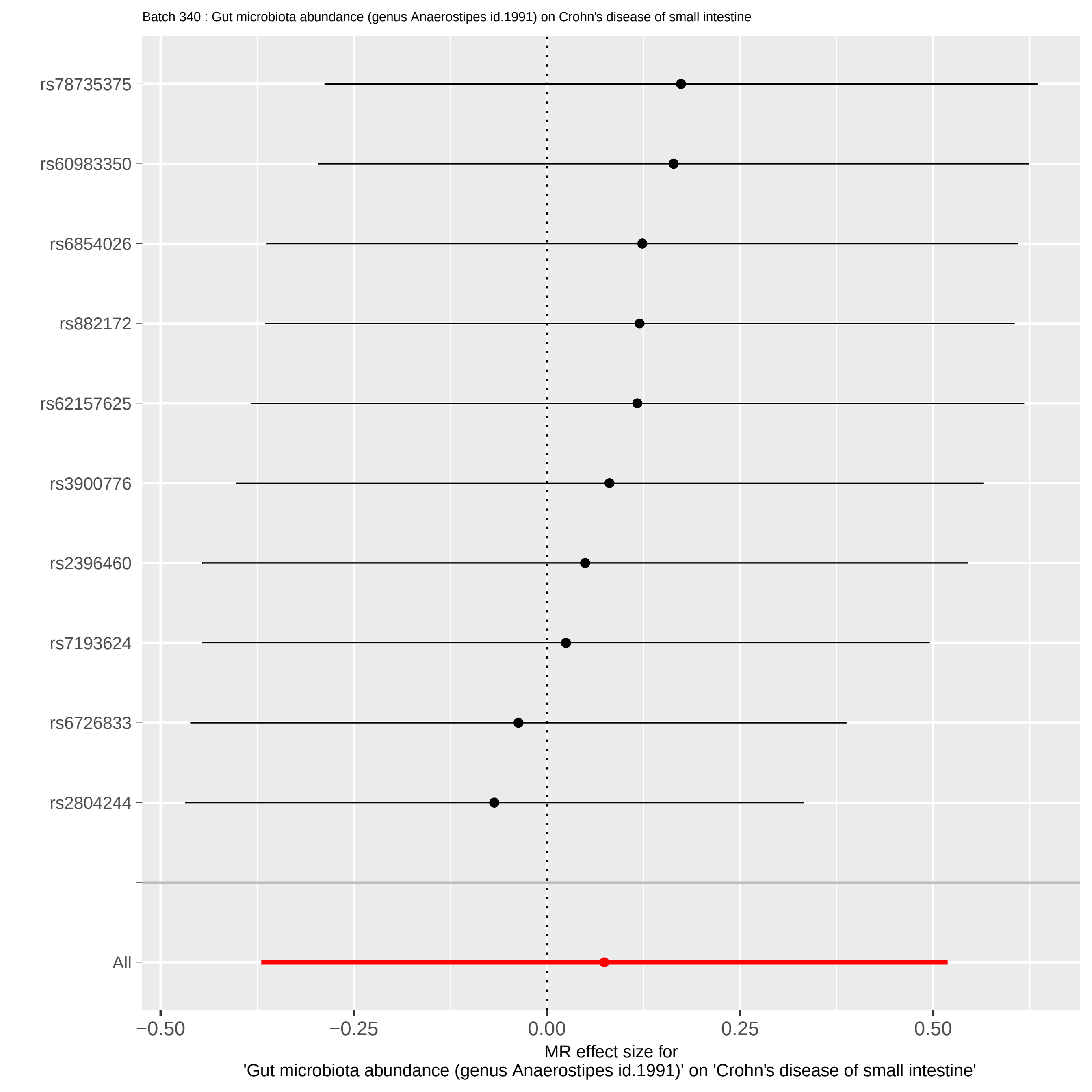

Batch 340 : Gut microbiota abundance (genus Anaerostipes id.1991) on Crohn's disease of small intestine
rs78735375
rs60983350
rs6854026
rs882172
rs62157625
rs3900776
rs2396460
rs7193624
rs6726833
rs2804244
All
−0.50
−0.25
0.00
0.25
0.50
MR effect size for
'Gut microbiota abundance (genus Anaerostipes id.1991)' on 'Crohn's disease of small intestine'

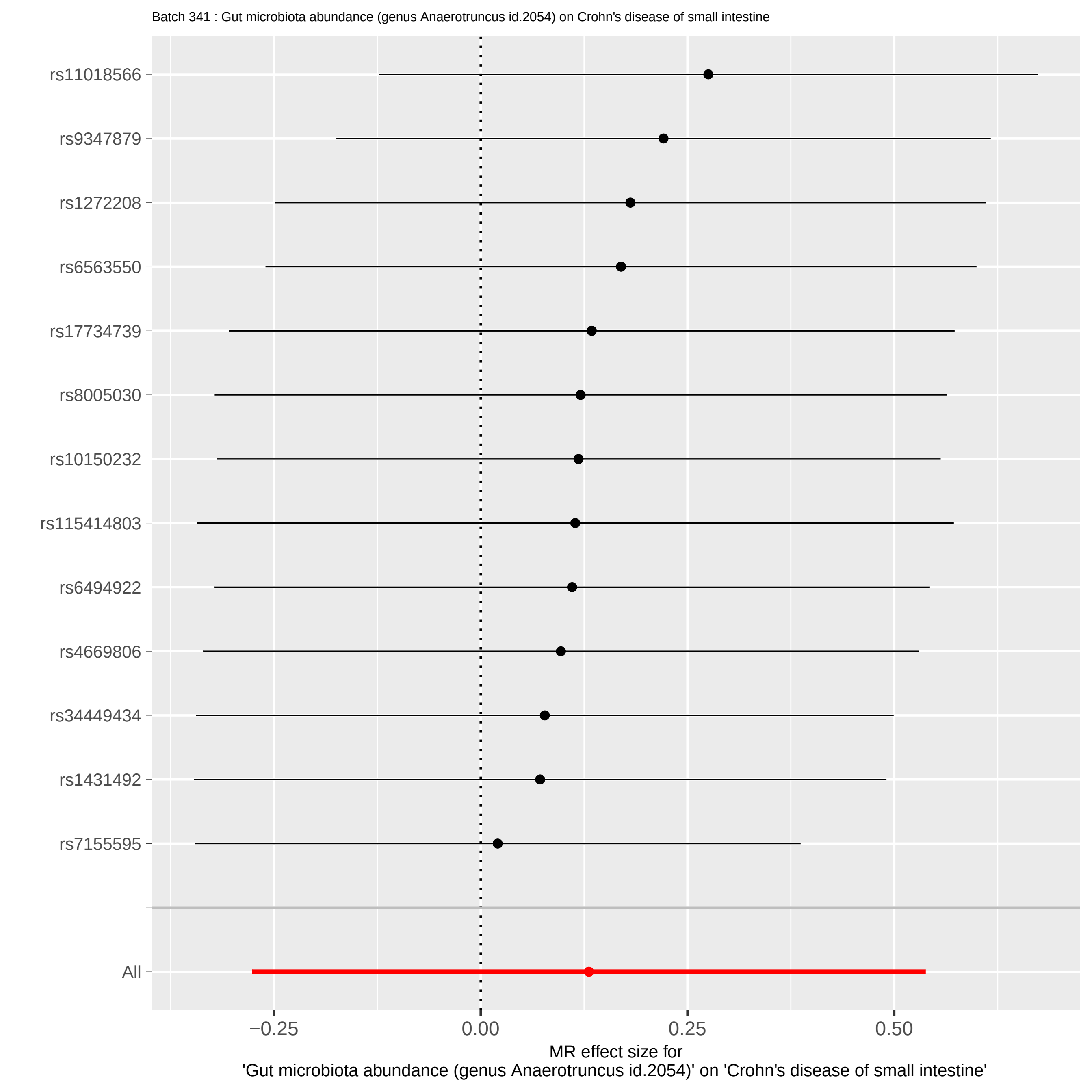

Batch 341 : Gut microbiota abundance (genus Anaerotruncus id.2054) on Crohn's disease of small intestine
rs11018566
rs9347879
rs1272208
rs6563550
rs17734739
rs8005030
rs10150232
rs115414803
rs6494922
rs4669806
rs34449434
rs1431492
rs7155595
All
−0.25
0.00
0.25
0.50
MR effect size for
'Gut microbiota abundance (genus Anaerotruncus id.2054)' on 'Crohn's disease of small intestine'

Batch 342 : Gut microbiota abundance (genus Bacteroides id.918) on Crohn's disease of small intestine

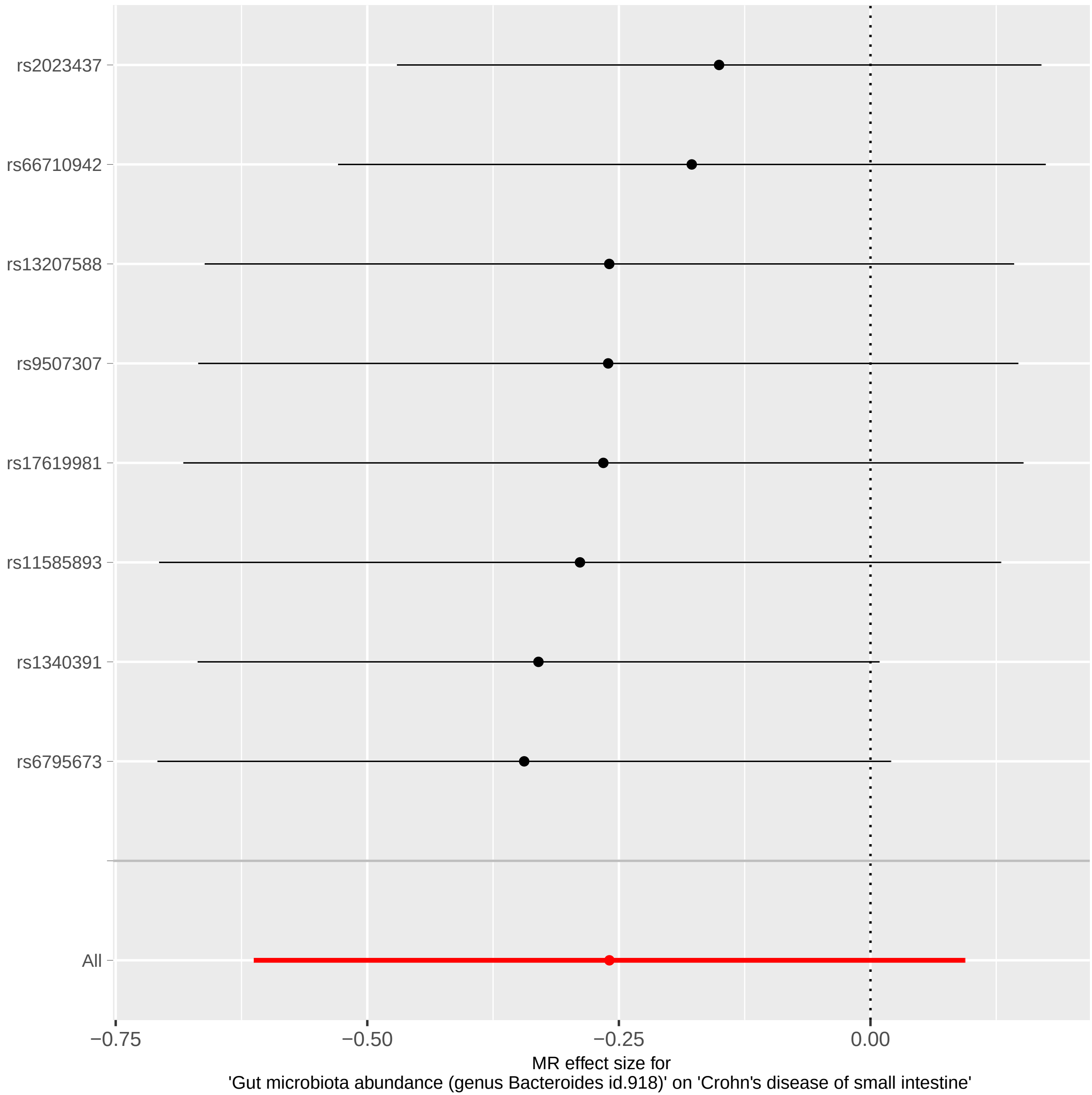

Batch 343 : Gut microbiota abundance (genus Barnesiella id.944) on Crohn's disease of small intestine

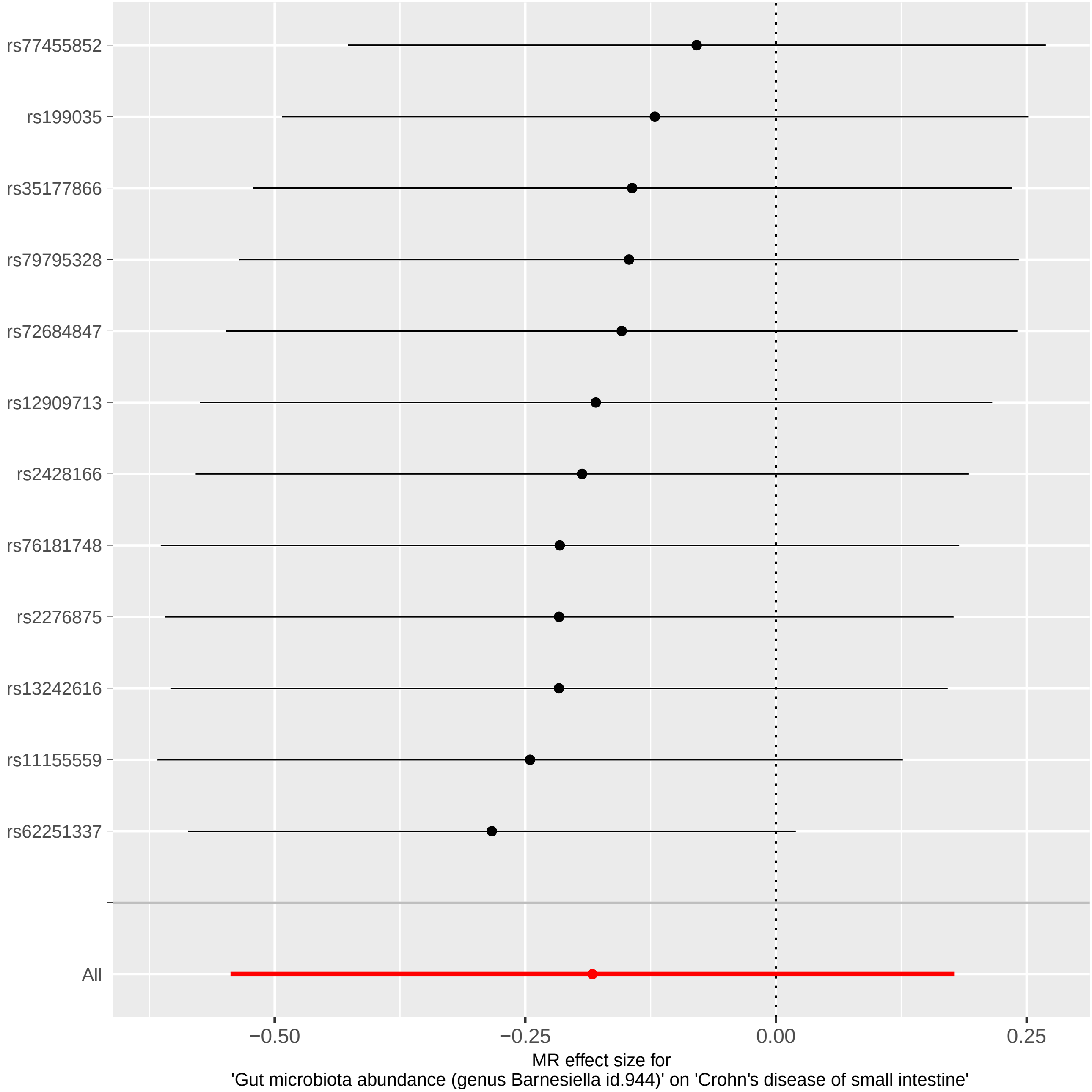

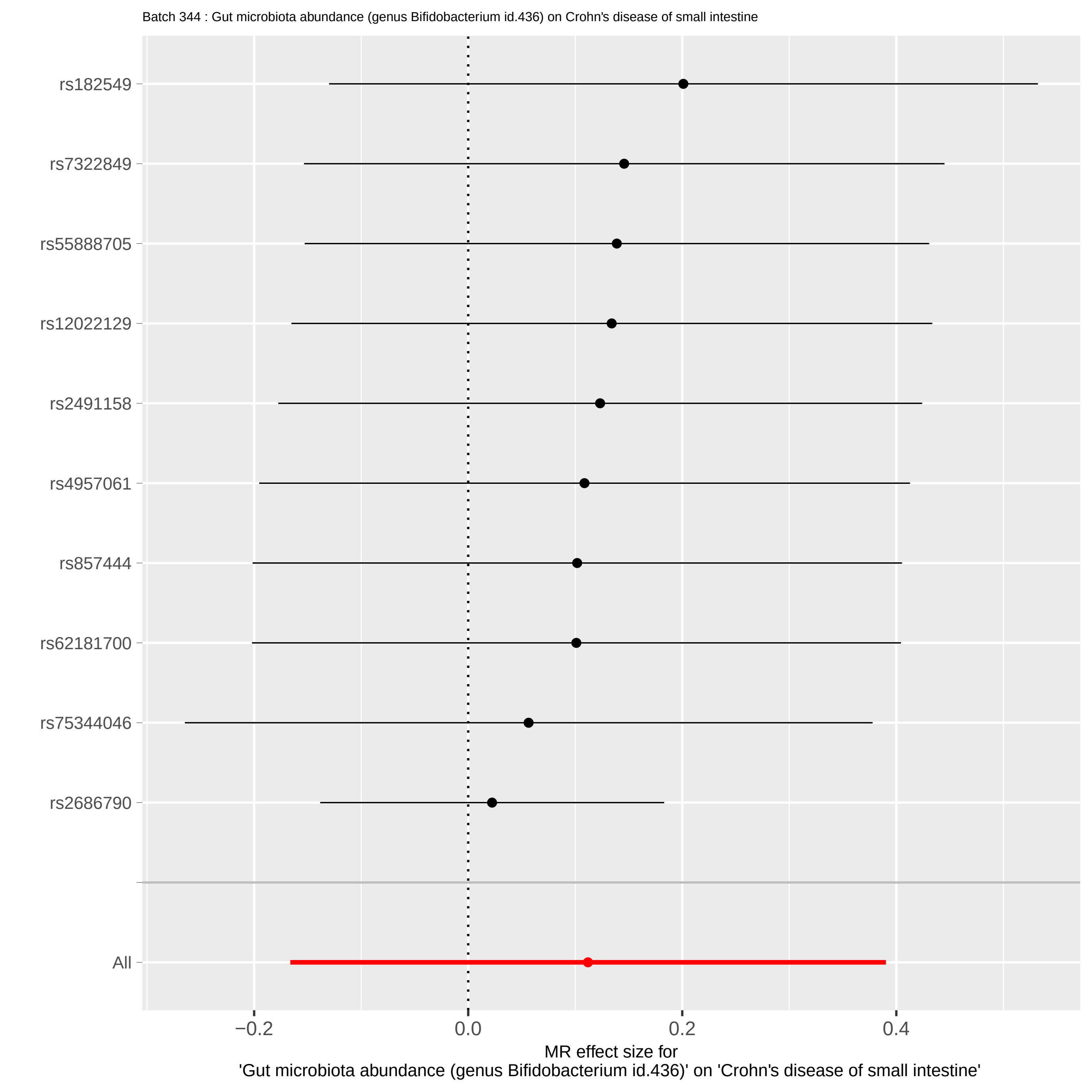
Batch 344 : Gut microbiota abundance (genus Bifidobacterium id.436) on Crohn's disease of small intestine
rs182549
rs7322849
rs55888705
rs12022129
rs2491158
rs4957061
rs857444
rs62181700
rs75344046
rs2686790
All
−0.2
0.0
0.2
0.4
MR effect size for
'Gut microbiota abundance (genus Bifidobacterium id.436)' on 'Crohn's disease of small intestine'

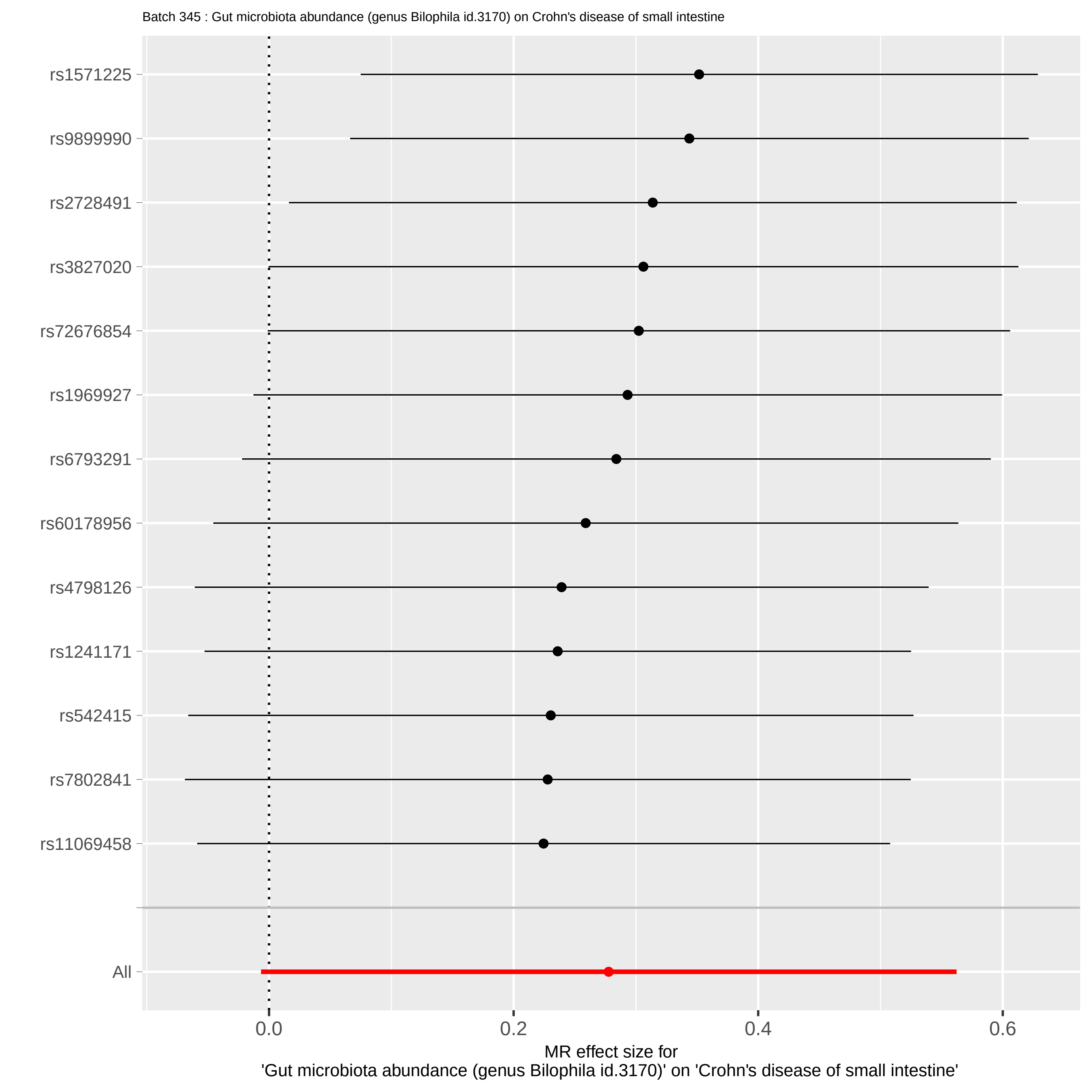
Batch 345 : Gut microbiota abundance (genus Bilophila id.3170) on Crohn's disease of small intestine
rs1571225
rs9899990
rs2728491
rs3827020
rs72676854
rs1969927
rs6793291
rs60178956
rs4798126
rs1241171
rs542415
rs7802841
rs11069458
All
0.0
0.2
0.4
0.6
MR effect size for
'Gut microbiota abundance (genus Bilophila id.3170)' on 'Crohn's disease of small intestine'

Batch 347 : Gut microbiota abundance (genus Butyricicoccus id.2055) on Crohn's disease of small intestine

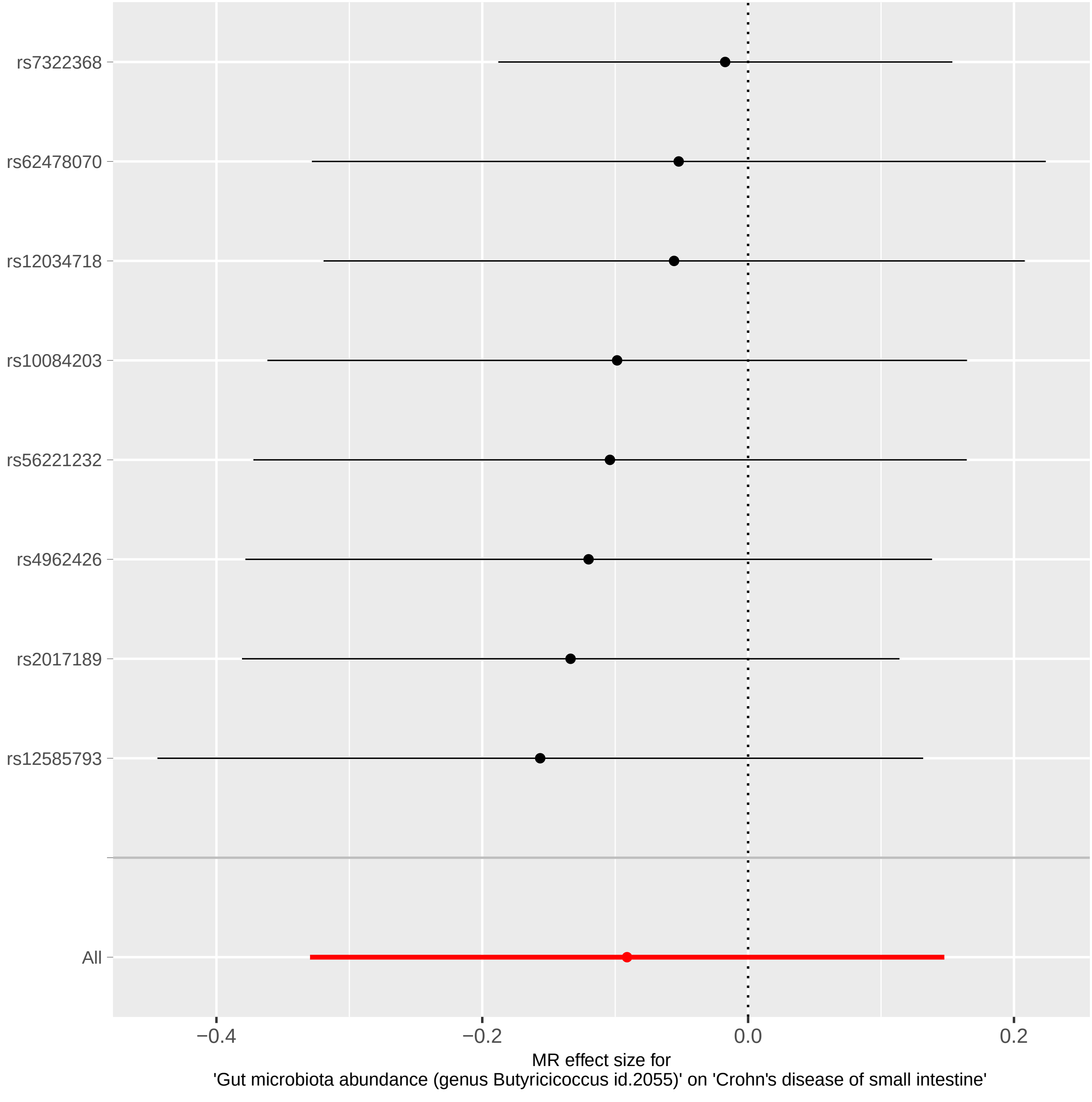

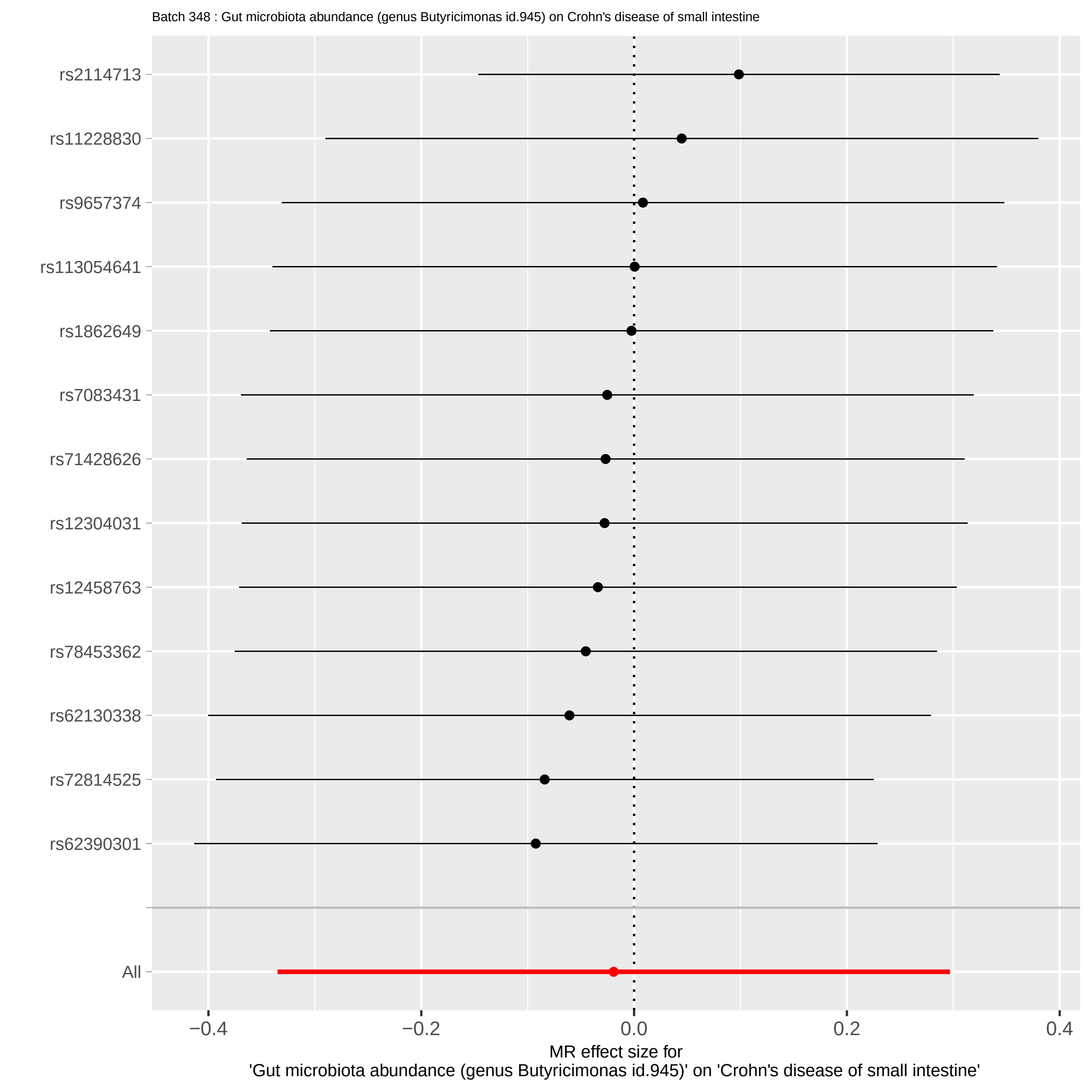
Batch 348 : Gut microbiota abundance (genus Butyricimonas id.945) on Crohn's disease of small intestine
rs2114713
rs11228830
rs9657374
rs113054641
rs1862649
rs7083431
rs71428626
rs12304031
rs12458763
rs78453362
rs62130338
rs72814525
rs62390301
All
−0.4
−0.2
0.0
0.2
0.4
MR effect size for
'Gut microbiota abundance (genus Butyricimonas id.945)' on 'Crohn's disease of small intestine'

Batch 349 : Gut microbiota abundance (genus Butyrivibrio id.1993) on Crohn's disease of small intestine

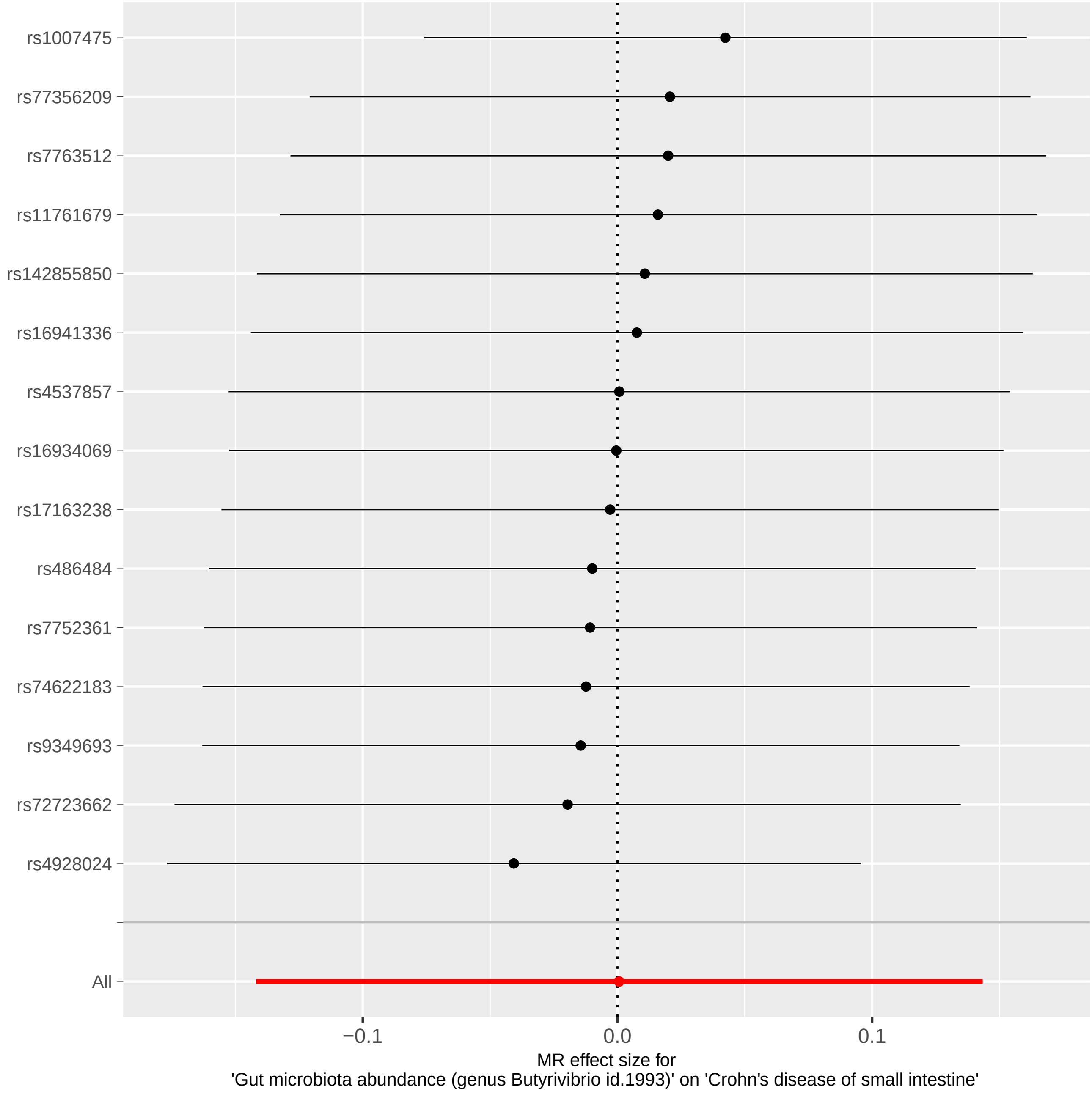

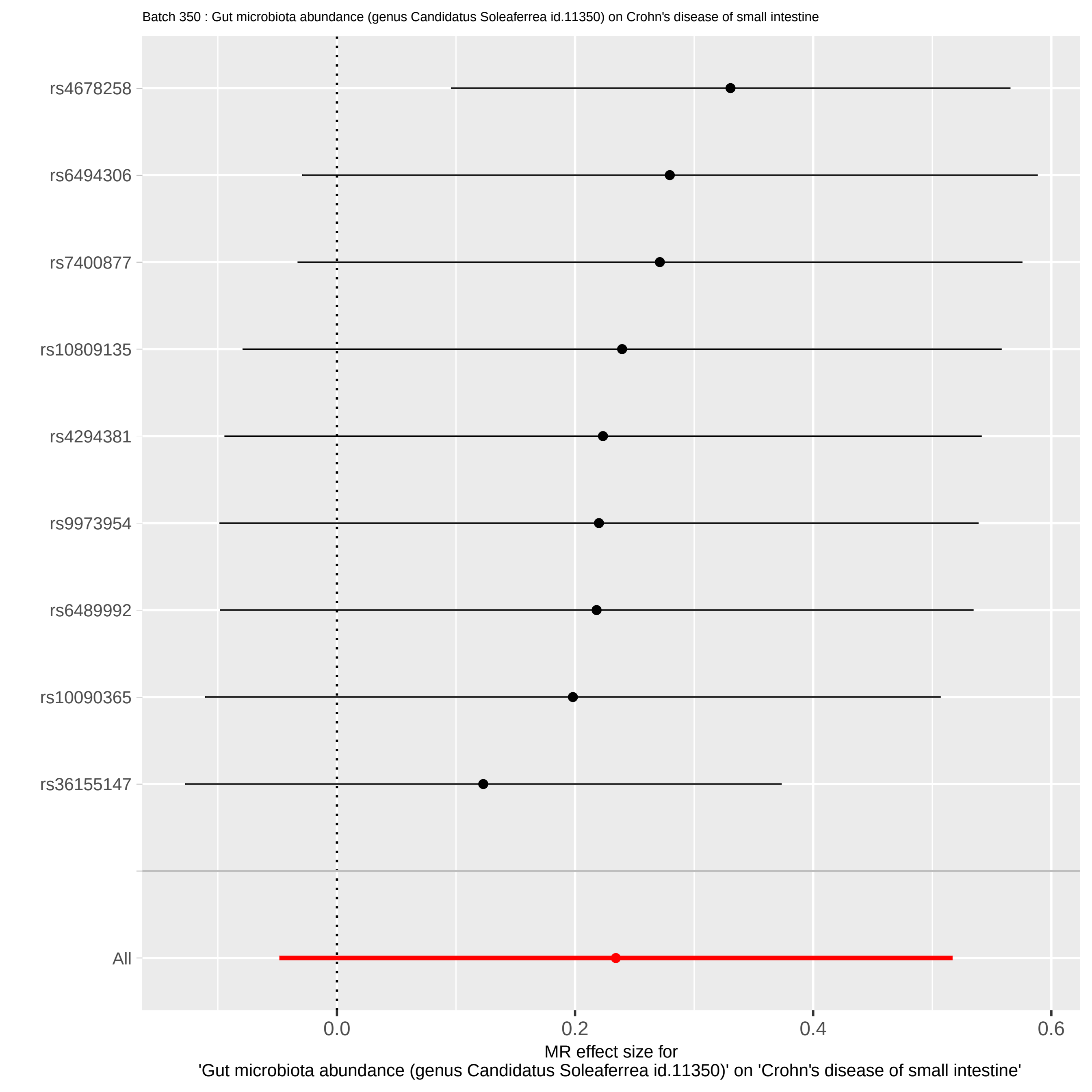

Batch 350 : Gut microbiota abundance (genus Candidatus Soleaferrea id.11350) on Crohn's disease of small intestine
rs4678258
rs6494306
rs7400877
rs10809135
rs4294381
rs9973954
rs6489992
rs10090365
rs36155147
All
0.0
0.2
0.4
0.6
MR effect size for
'Gut microbiota abundance (genus Candidatus Soleaferrea id.11350)' on 'Crohn's disease of small intestine'

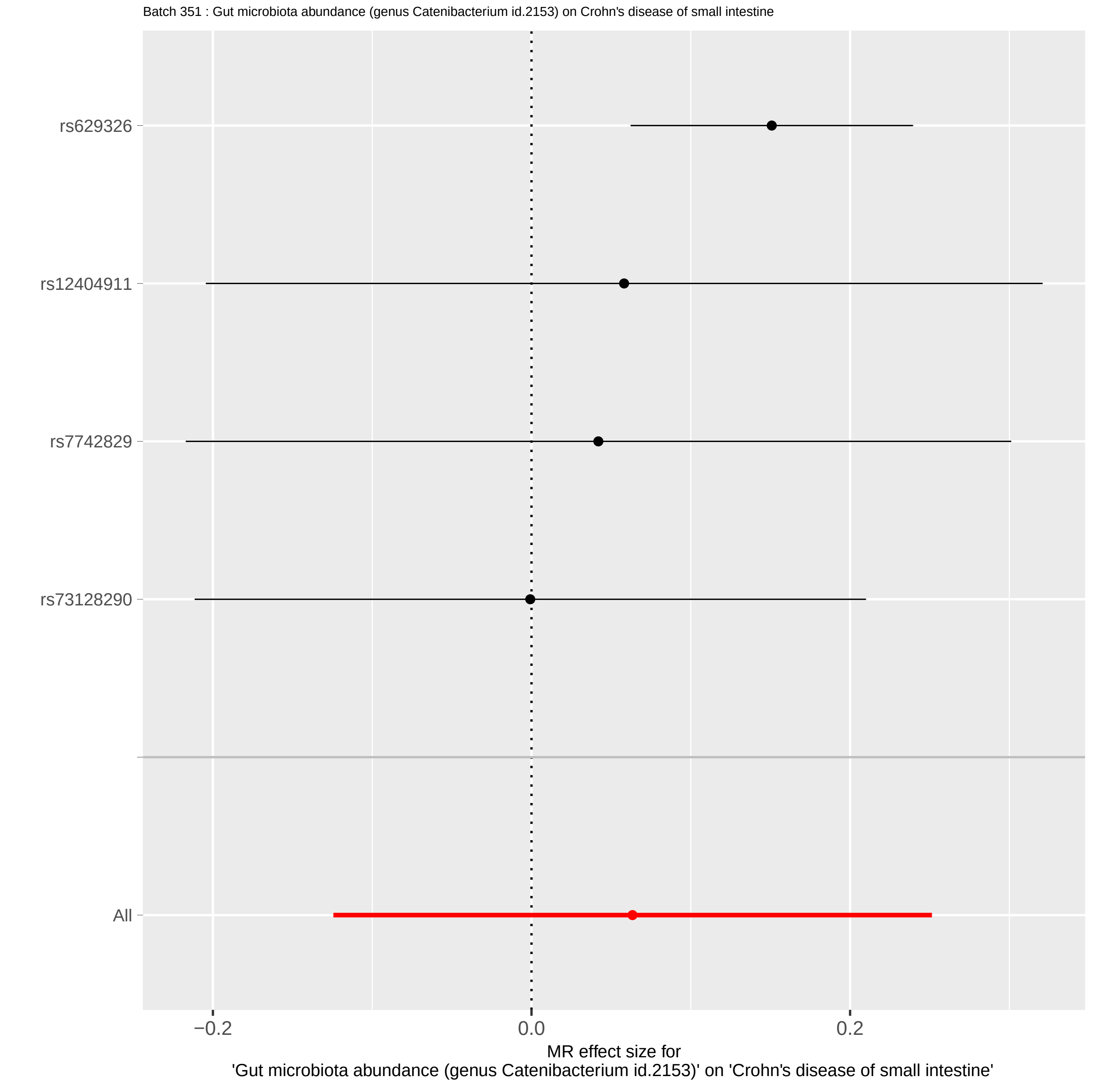
Batch 351 : Gut microbiota abundance (genus Catenibacterium id.2153) on Crohn's disease of small intestine
rs629326
rs12404911
rs7742829
rs73128290
All
−0.2
0.0
0.2
MR effect size for
'Gut microbiota abundance (genus Catenibacterium id.2153)' on 'Crohn's disease of small intestine'

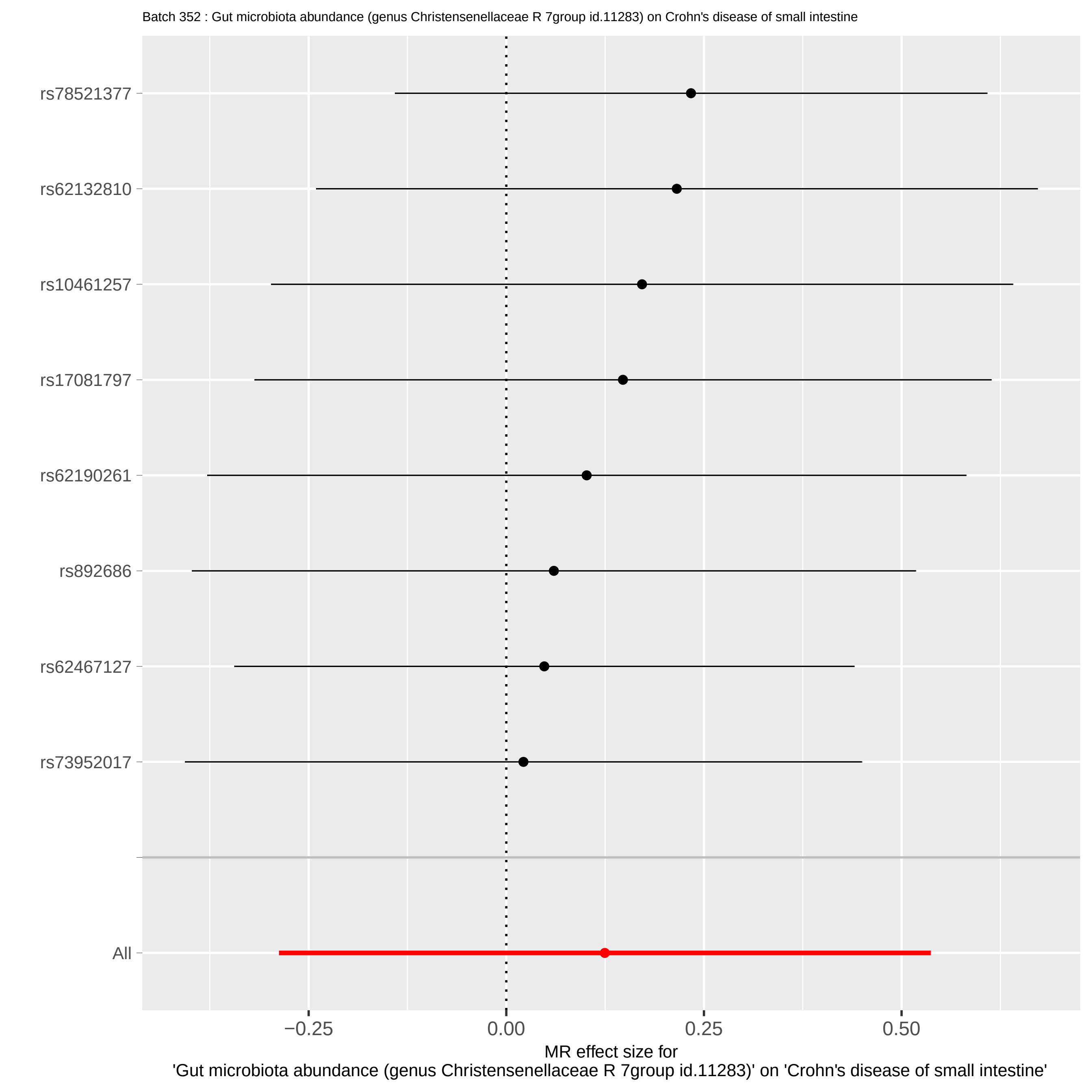

Batch 352 : Gut microbiota abundance (genus Christensenellaceae R 7group id.11283) on Crohn's disease of small intestine
rs78521377
rs62132810
rs10461257
rs17081797
rs62190261
rs892686
rs62467127
rs73952017
All
−0.25
0.00
0.25
0.50
MR effect size for
'Gut microbiota abundance (genus Christensenellaceae R 7group id.11283)' on 'Crohn's disease of small intestine'

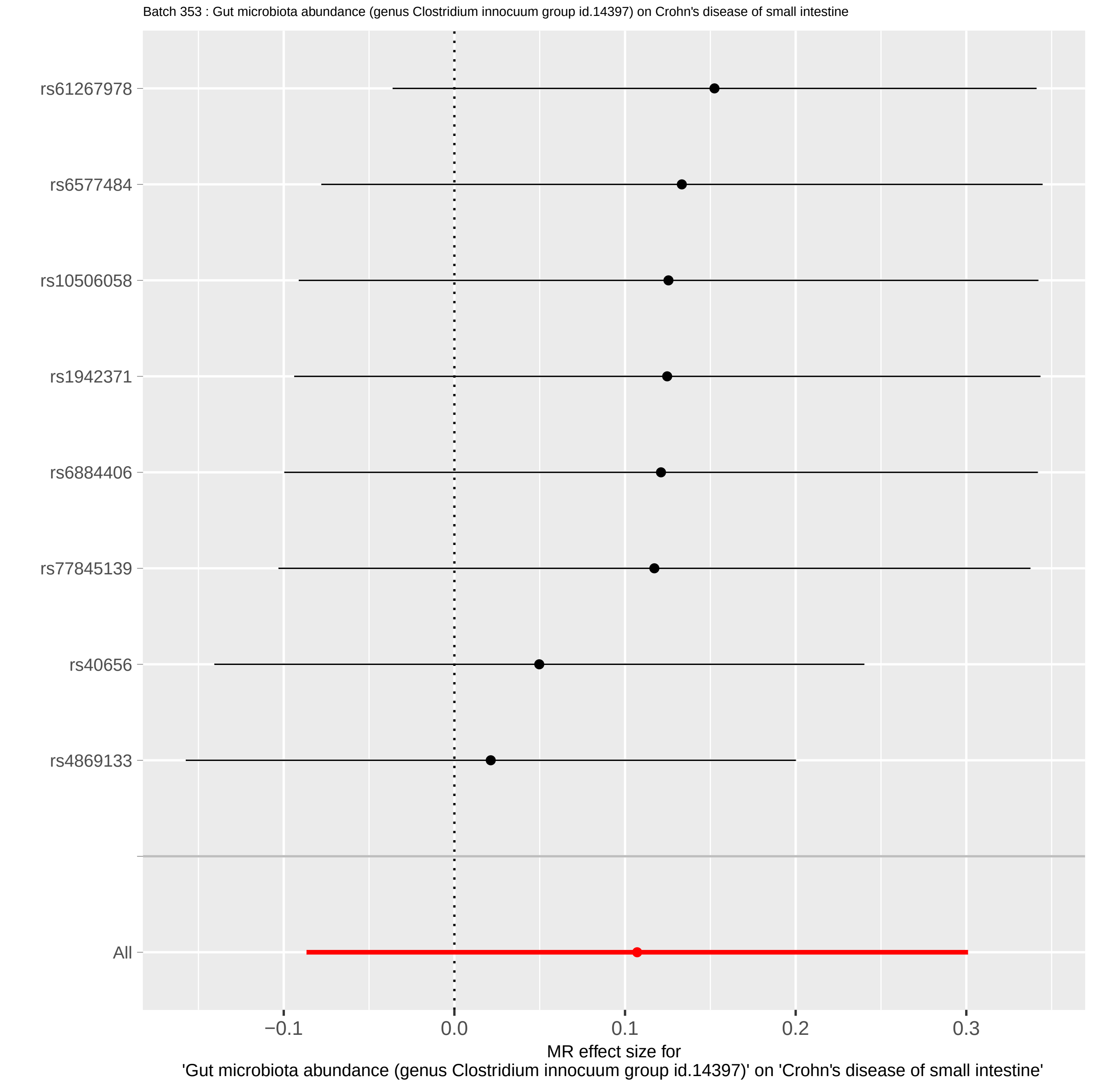

Batch 353 : Gut microbiota abundance (genus Clostridium innocuum group id.14397) on Crohn's disease of small intestine
rs61267978
rs6577484
rs10506058
rs1942371
rs6884406
rs77845139
rs40656
rs4869133
All
−0.1
0.0
0.1
0.2
0.3
MR effect size for
'Gut microbiota abundance (genus Clostridium innocuum group id.14397)' on 'Crohn's disease of small intestine'

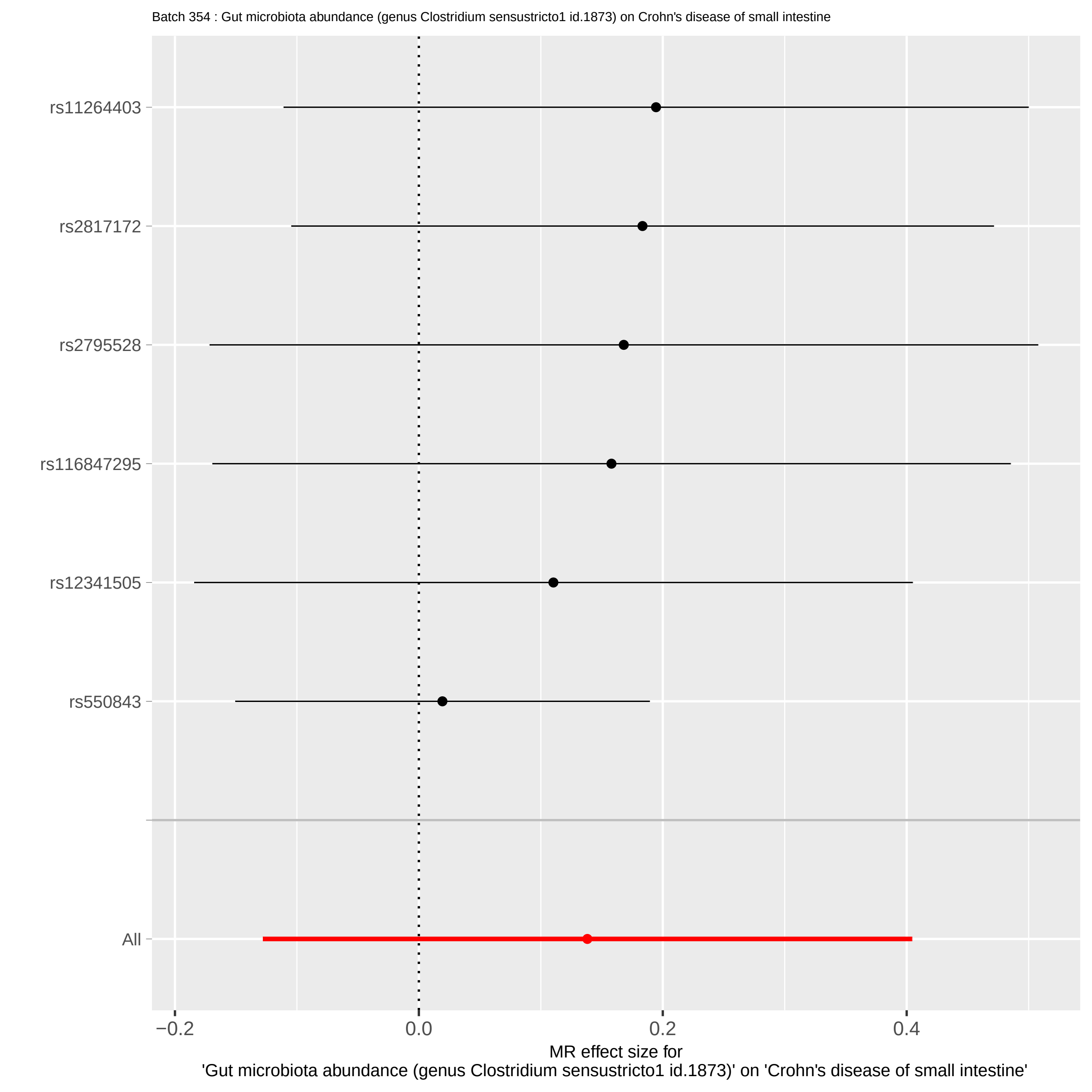

Batch 354 : Gut microbiota abundance (genus Clostridium sensustricto1 id.1873) on Crohn's disease of small intestine
rs11264403
rs2817172
rs2795528
rs116847295
rs12341505
rs550843
All
−0.2
0.0
0.2
0.4
MR effect size for
'Gut microbiota abundance (genus Clostridium sensustricto1 id.1873)' on 'Crohn's disease of small intestine'

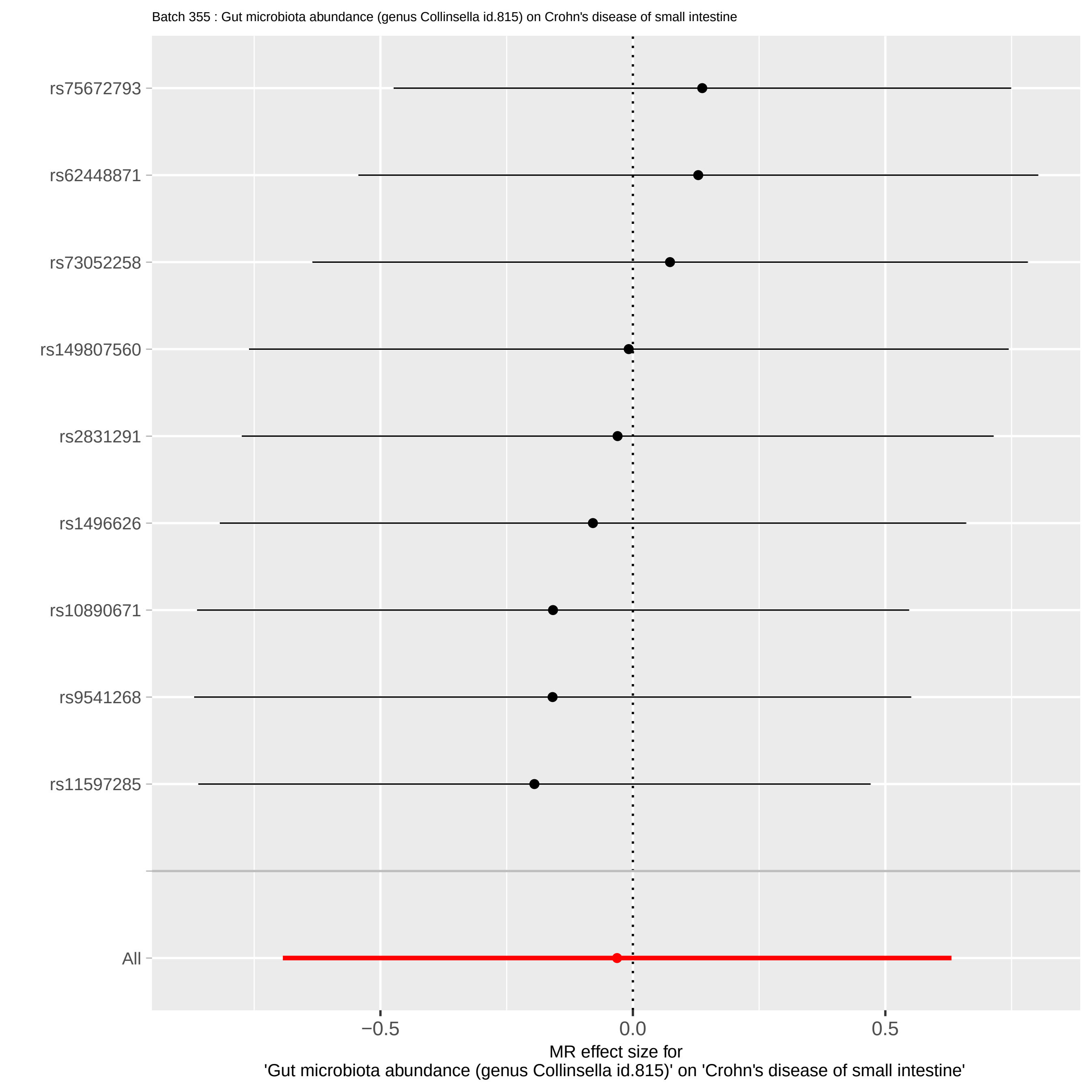

Batch 355 : Gut microbiota abundance (genus Collinsella id.815) on Crohn's disease of small intestine
rs75672793
rs62448871
rs73052258
rs149807560
rs2831291
rs1496626
rs10890671
rs9541268
rs11597285
All
−0.5
0.0
0.5
MR effect size for
'Gut microbiota abundance (genus Collinsella id.815)' on 'Crohn's disease of small intestine'

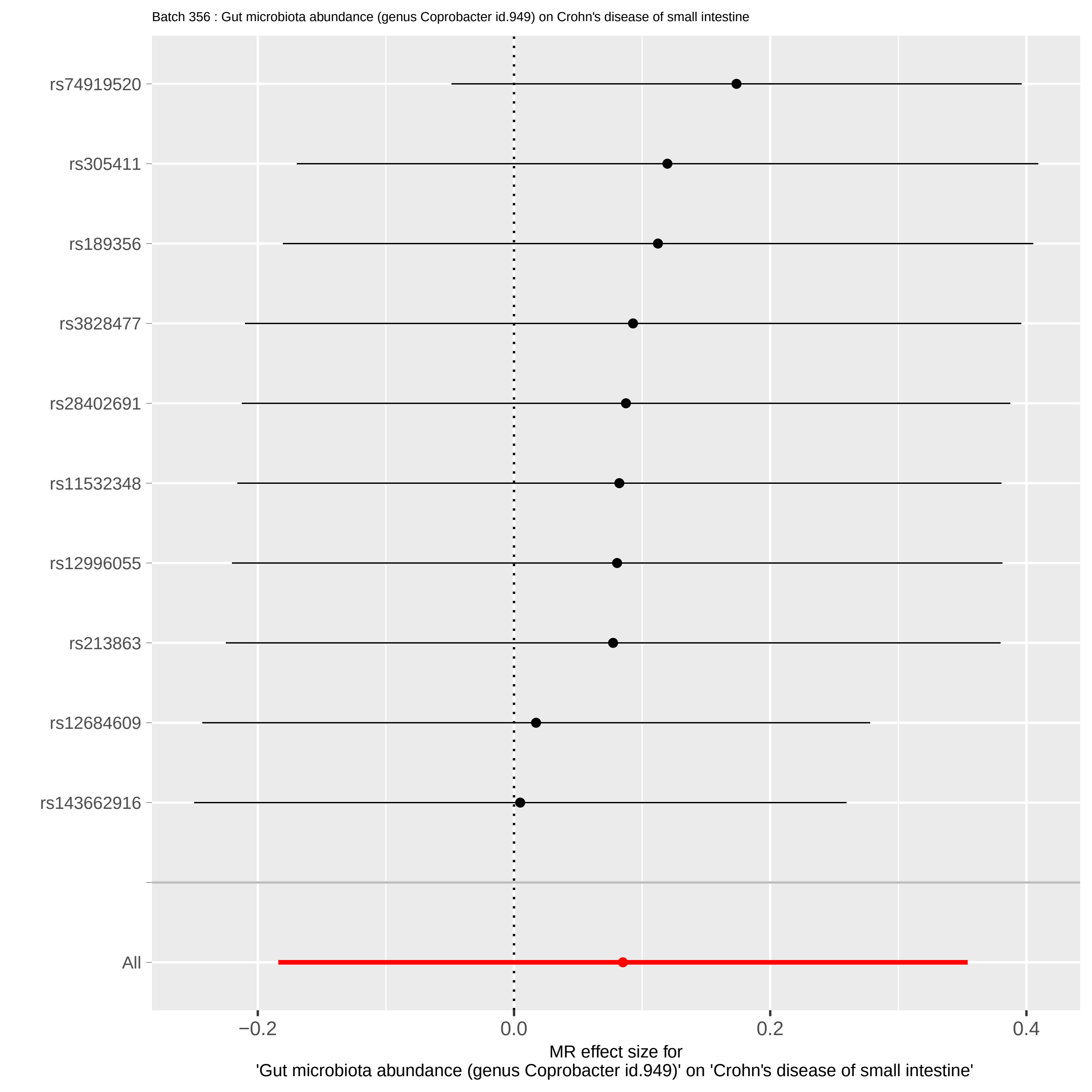
Batch 356 : Gut microbiota abundance (genus Coprobacter id.949) on Crohn's disease of small intestine
rs74919520
rs305411
rs189356
rs3828477
rs28402691
rs11532348
rs12996055
rs213863
rs12684609
rs143662916
All
−0.2
0.0
0.2
0.4
MR effect size for
'Gut microbiota abundance (genus Coprobacter id.949)' on 'Crohn's disease of small intestine'

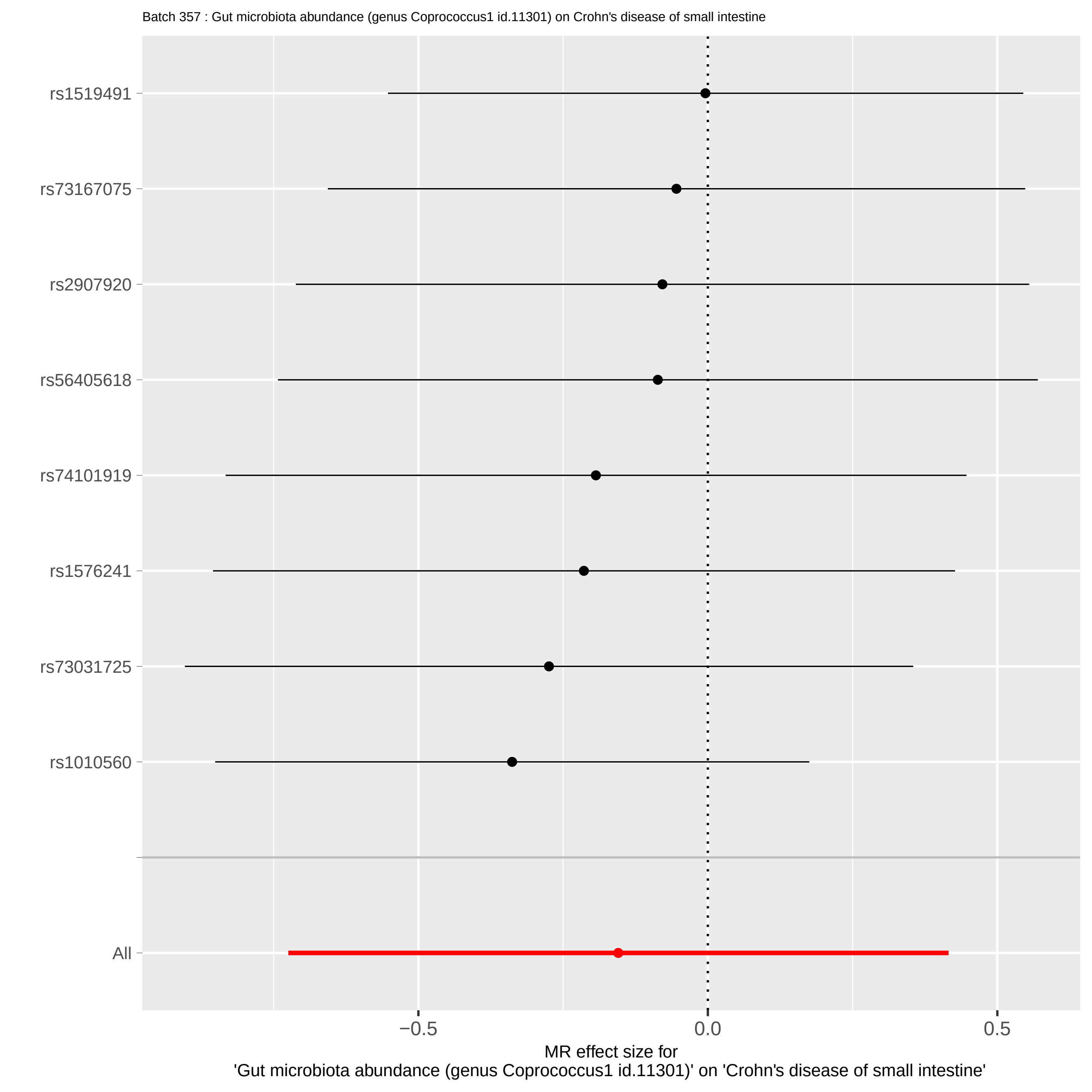
Batch 357 : Gut microbiota abundance (genus Coprococcus1 id.11301) on Crohn's disease of small intestine
rs1519491
rs73167075
rs2907920
rs56405618
rs74101919
rs1576241
rs73031725
rs1010560
All
−0.5
0.0
0.5
MR effect size for
'Gut microbiota abundance (genus Coprococcus1 id.11301)' on 'Crohn's disease of small intestine'

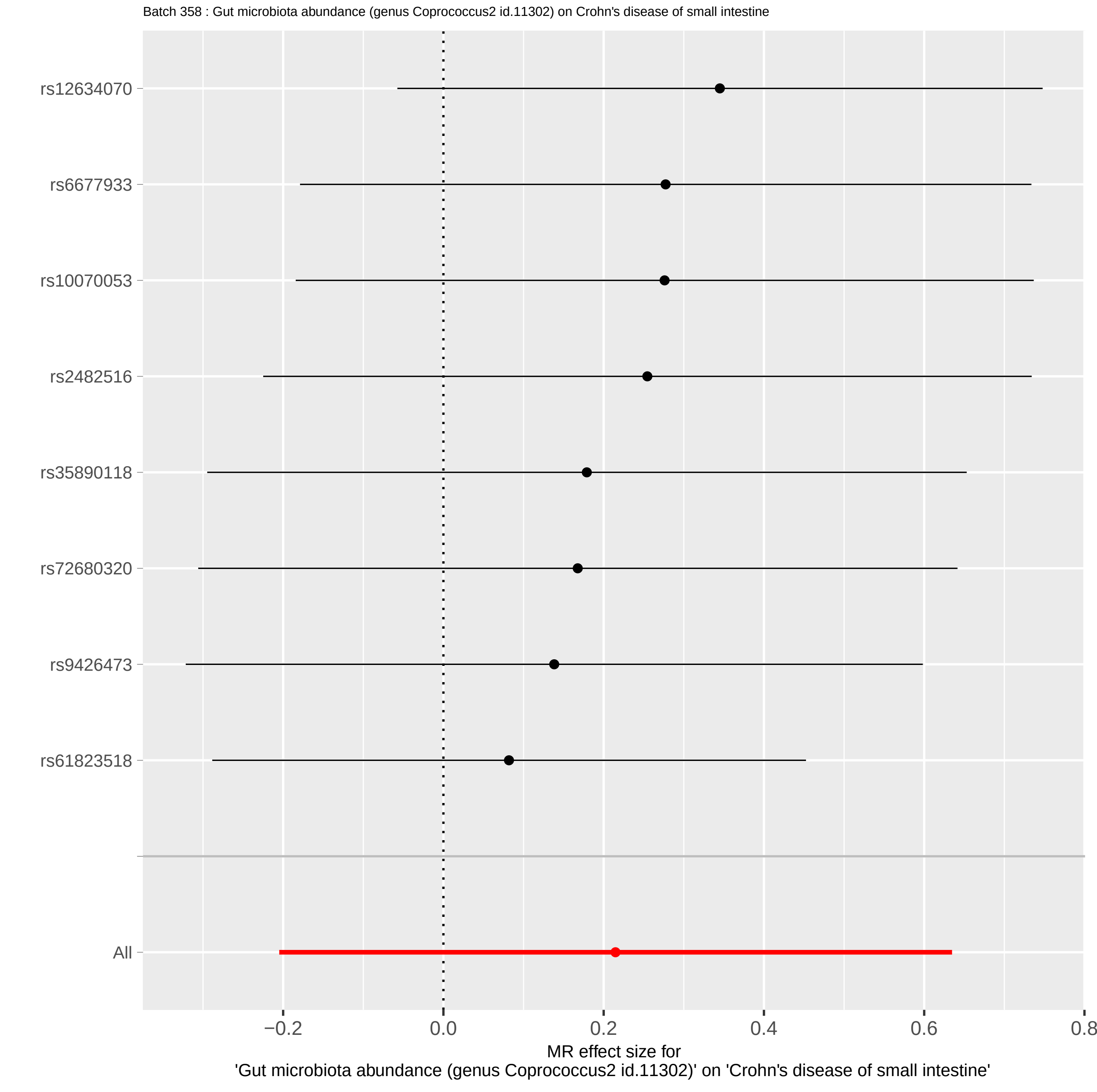
Batch 358 : Gut microbiota abundance (genus Coprococcus2 id.11302) on Crohn's disease of small intestine
rs12634070
rs6677933
rs10070053
rs2482516
rs35890118
rs72680320
rs9426473
rs61823518
All
−0.2
0.0
0.2
0.4
0.6
0.8
MR effect size for
'Gut microbiota abundance (genus Coprococcus2 id.11302)' on 'Crohn's disease of small intestine'

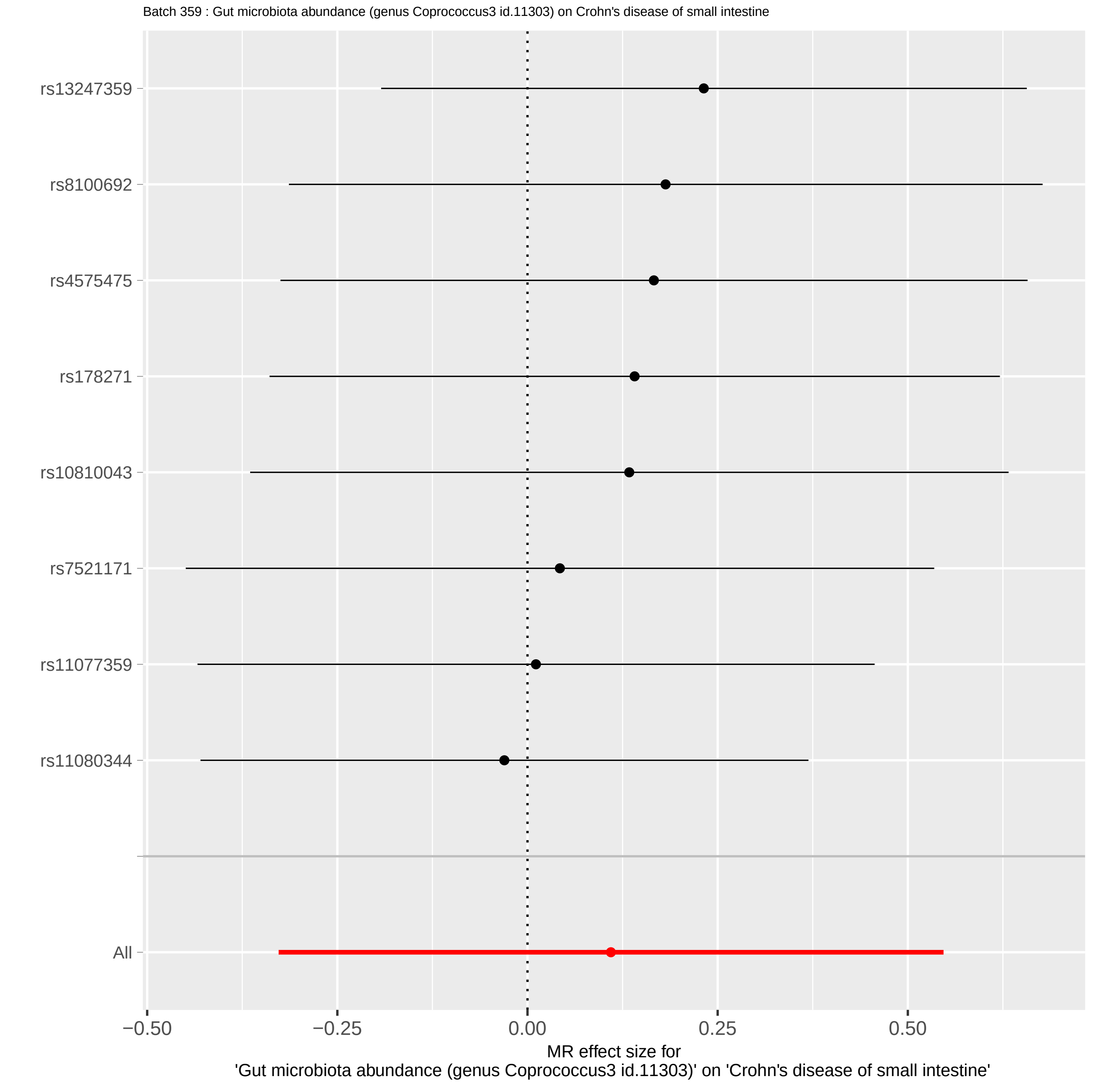
Batch 359 : Gut microbiota abundance (genus Coprococcus3 id.11303) on Crohn's disease of small intestine
rs13247359
rs8100692
rs4575475
rs178271
rs10810043
rs7521171
rs11077359
rs11080344
All
−0.50
−0.25
0.00
0.25
0.50
MR effect size for
'Gut microbiota abundance (genus Coprococcus3 id.11303)' on 'Crohn's disease of small intestine'

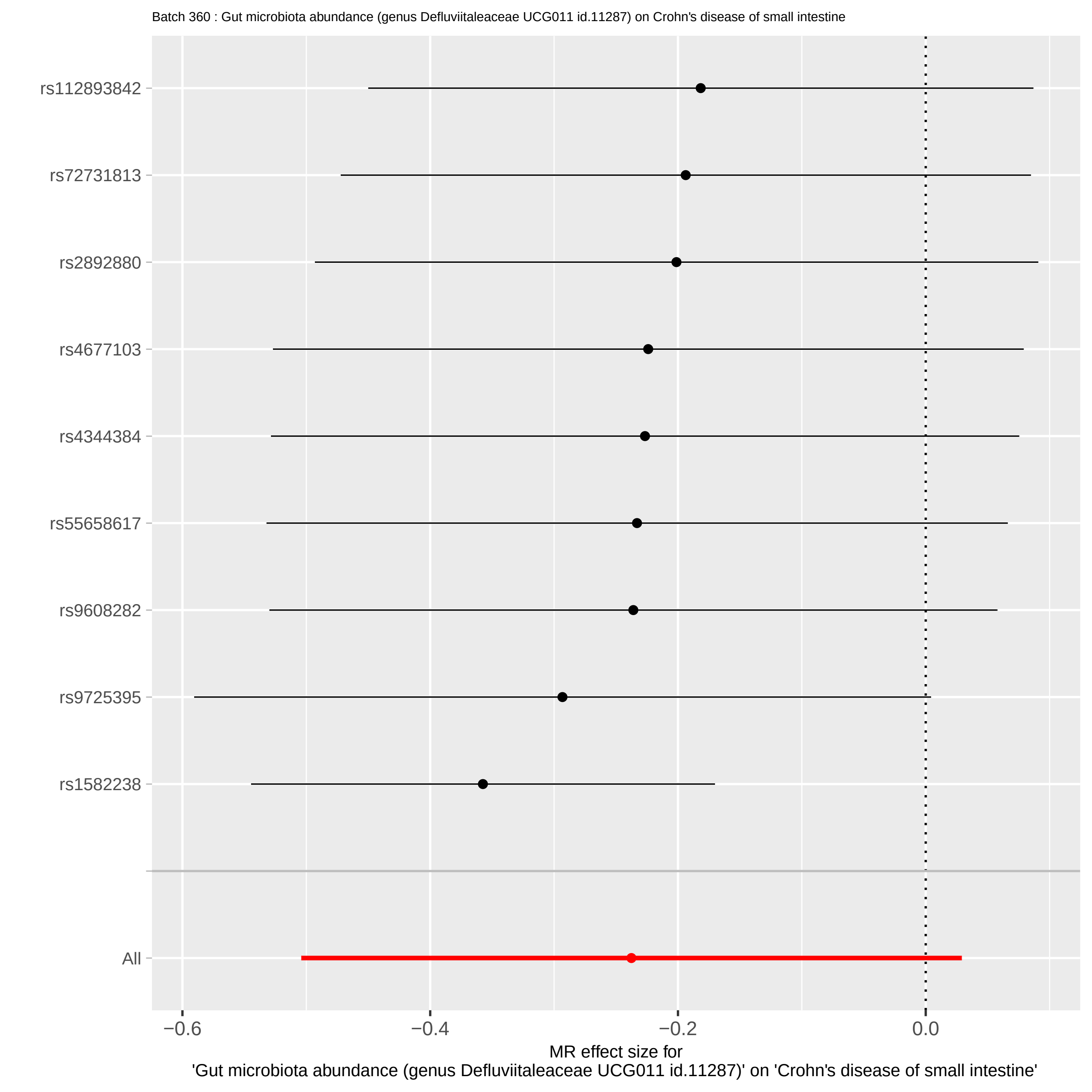

Batch 360 : Gut microbiota abundance (genus Defluviitaleaceae UCG011 id.11287) on Crohn's disease of small intestine
rs112893842
rs72731813
rs2892880
rs4677103
rs4344384
rs55658617
rs9608282
rs9725395
rs1582238
All
−0.6
−0.4
−0.2
0.0
MR effect size for
'Gut microbiota abundance (genus Defluviitaleaceae UCG011 id.11287)' on 'Crohn's disease of small intestine'

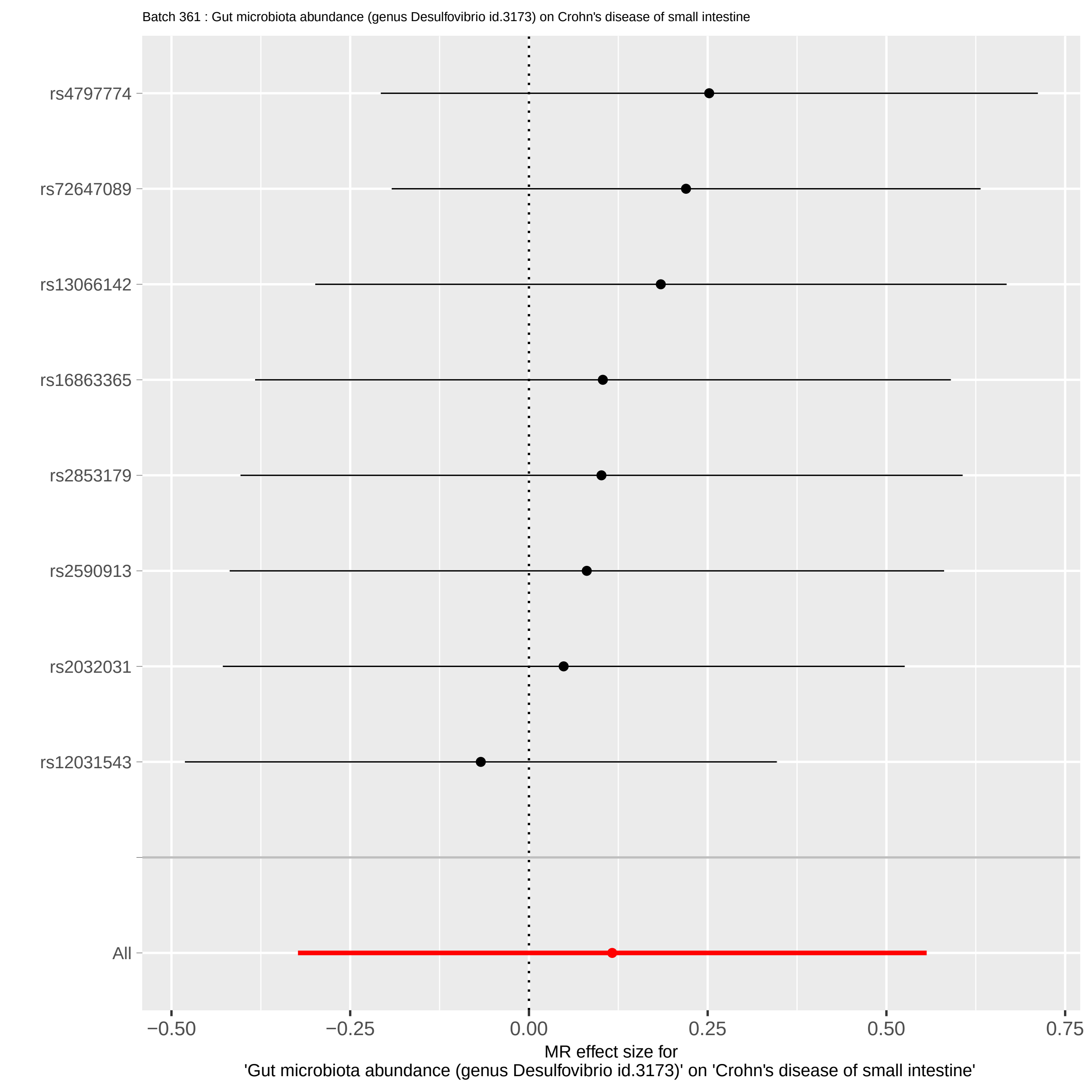
Batch 361 : Gut microbiota abundance (genus Desulfovibrio id.3173) on Crohn's disease of small intestine
rs4797774
rs72647089
rs13066142
rs16863365
rs2853179
rs2590913
rs2032031
rs12031543
All
−0.50
−0.25
0.00
0.25
0.50
0.75
MR effect size for
'Gut microbiota abundance (genus Desulfovibrio id.3173)' on 'Crohn's disease of small intestine'

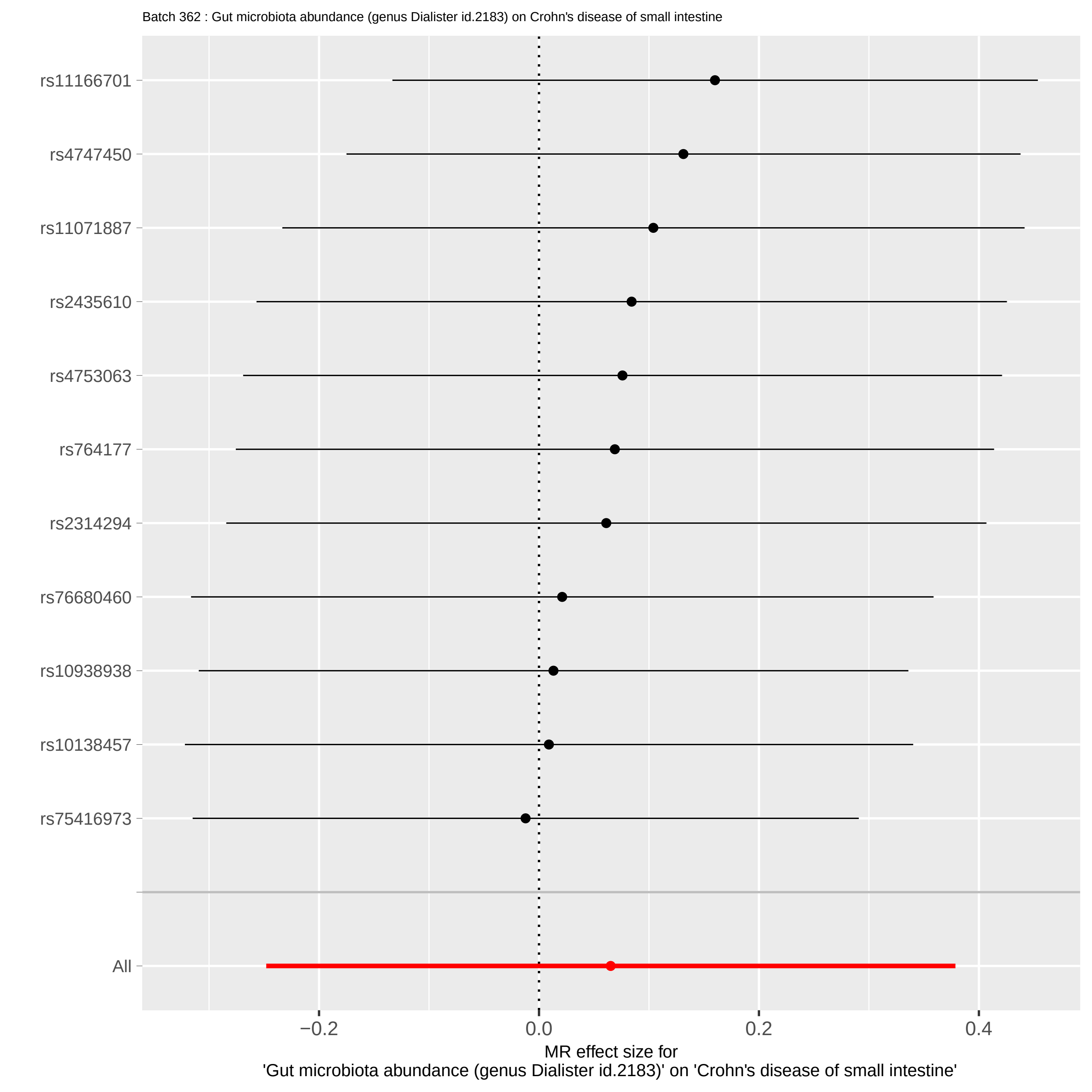
Batch 362 : Gut microbiota abundance (genus Dialister id.2183) on Crohn's disease of small intestine
rs11166701
rs4747450
rs11071887
rs2435610
rs4753063
rs764177
rs2314294
rs76680460
rs10938938
rs10138457
rs75416973
All
−0.2
0.0
0.2
0.4
MR effect size for
'Gut microbiota abundance (genus Dialister id.2183)' on 'Crohn's disease of small intestine'

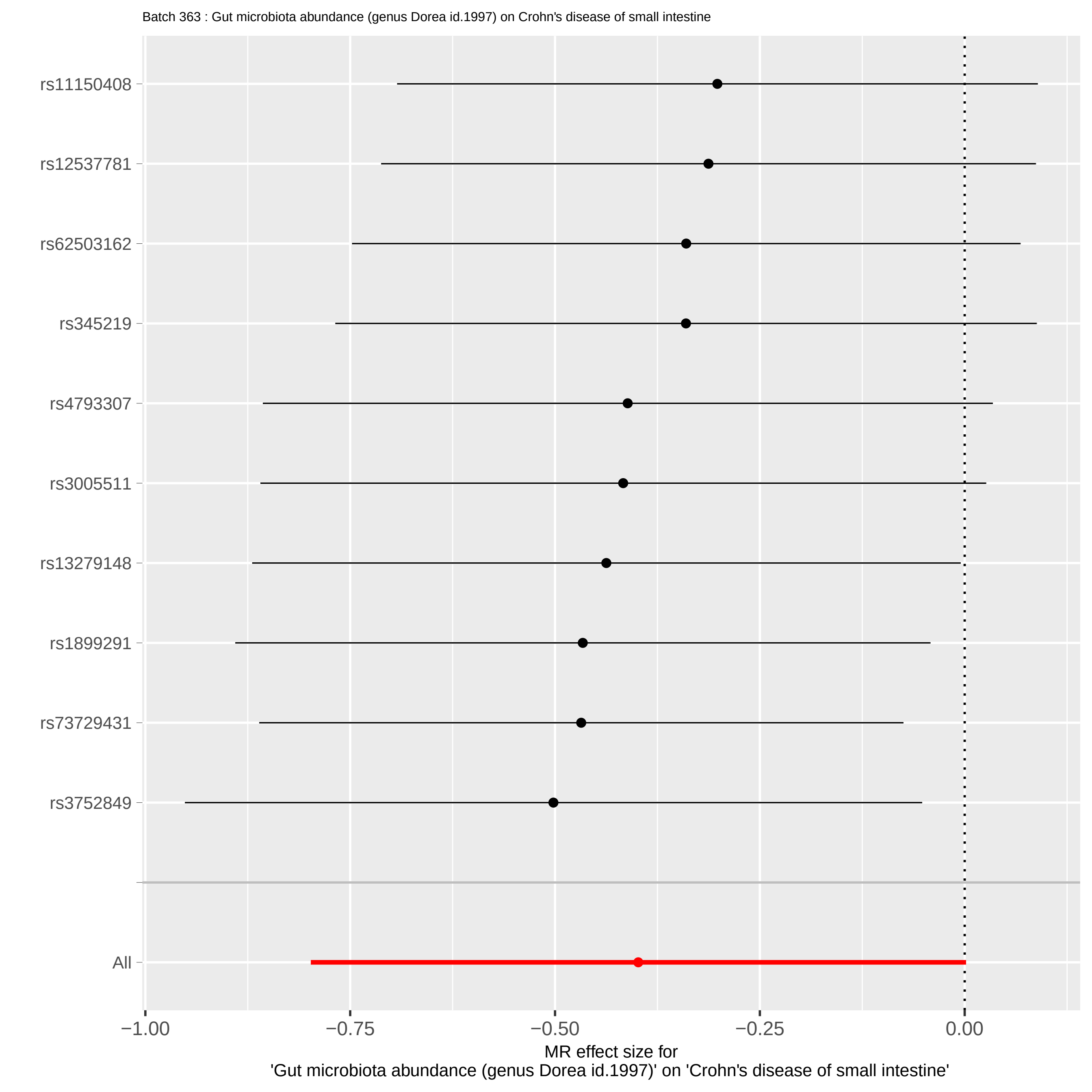
Batch 363 : Gut microbiota abundance (genus Dorea id.1997) on Crohn's disease of small intestine
rs11150408
rs12537781
rs62503162
rs345219
rs4793307
rs3005511
rs13279148
rs1899291
rs73729431
rs3752849
All
−1.00
−0.75
−0.50
−0.25
0.00
MR effect size for
'Gut microbiota abundance (genus Dorea id.1997)' on 'Crohn's disease of small intestine'

Batch 364 : Gut microbiota abundance (genus Eggerthella id.819) on Crohn's disease of small intestine

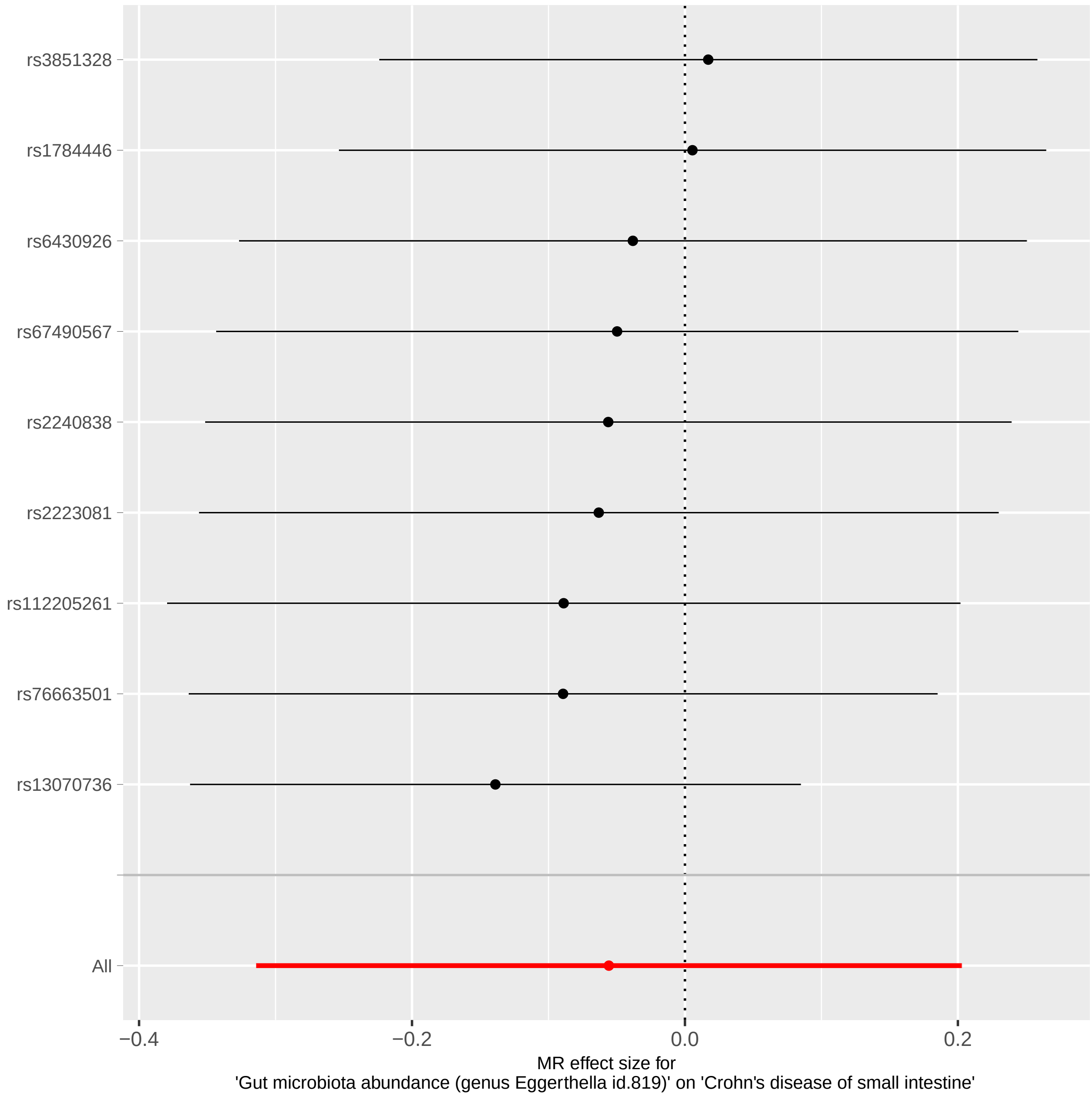

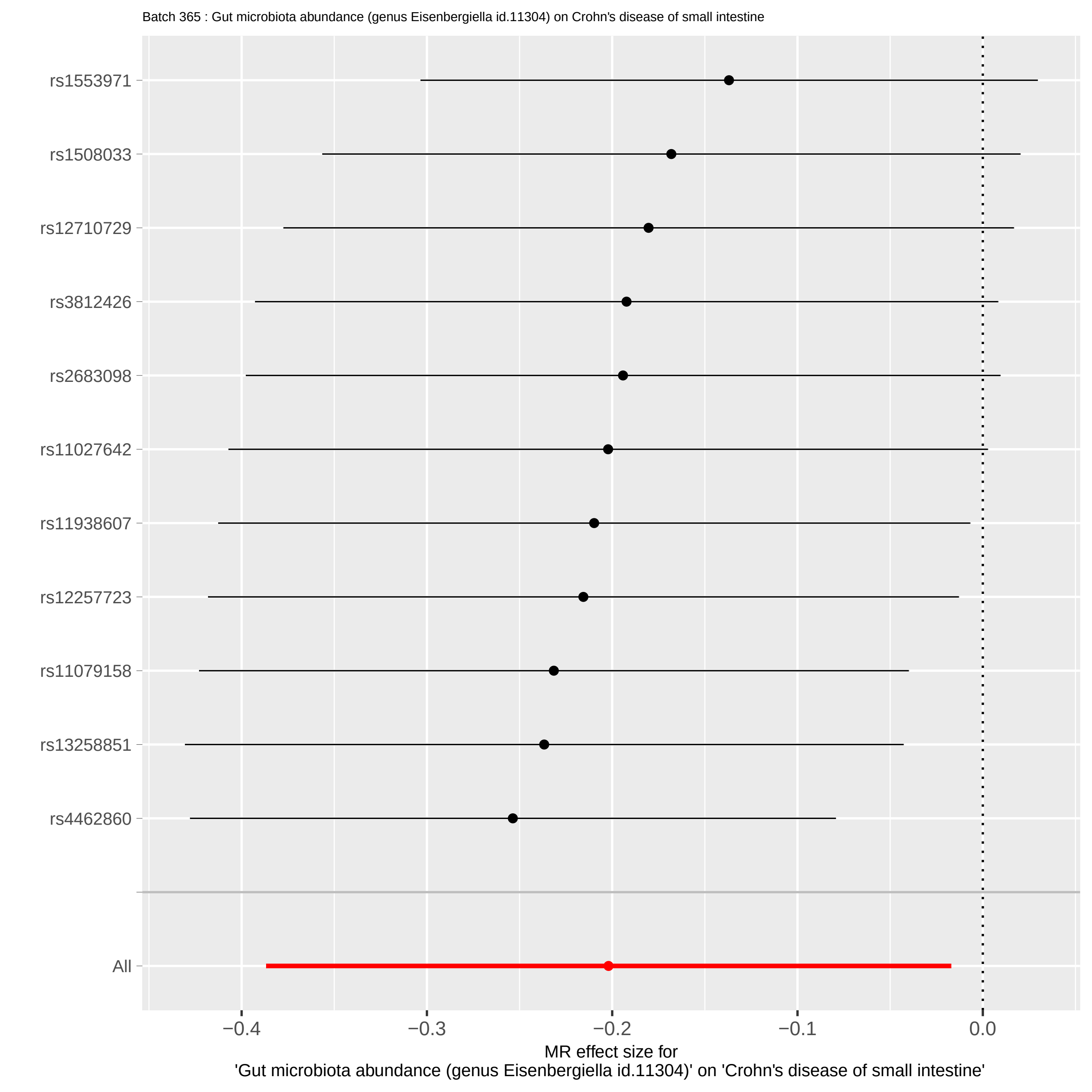

Batch 365 : Gut microbiota abundance (genus Eisenbergiella id.11304) on Crohn's disease of small intestine
rs1553971
rs1508033
rs12710729
rs3812426
rs2683098
rs11027642
rs11938607
rs12257723
rs11079158
rs13258851
rs4462860
All
−0.4
−0.3
−0.2
−0.1
0.0
MR effect size for
'Gut microbiota abundance (genus Eisenbergiella id.11304)' on 'Crohn's disease of small intestine'

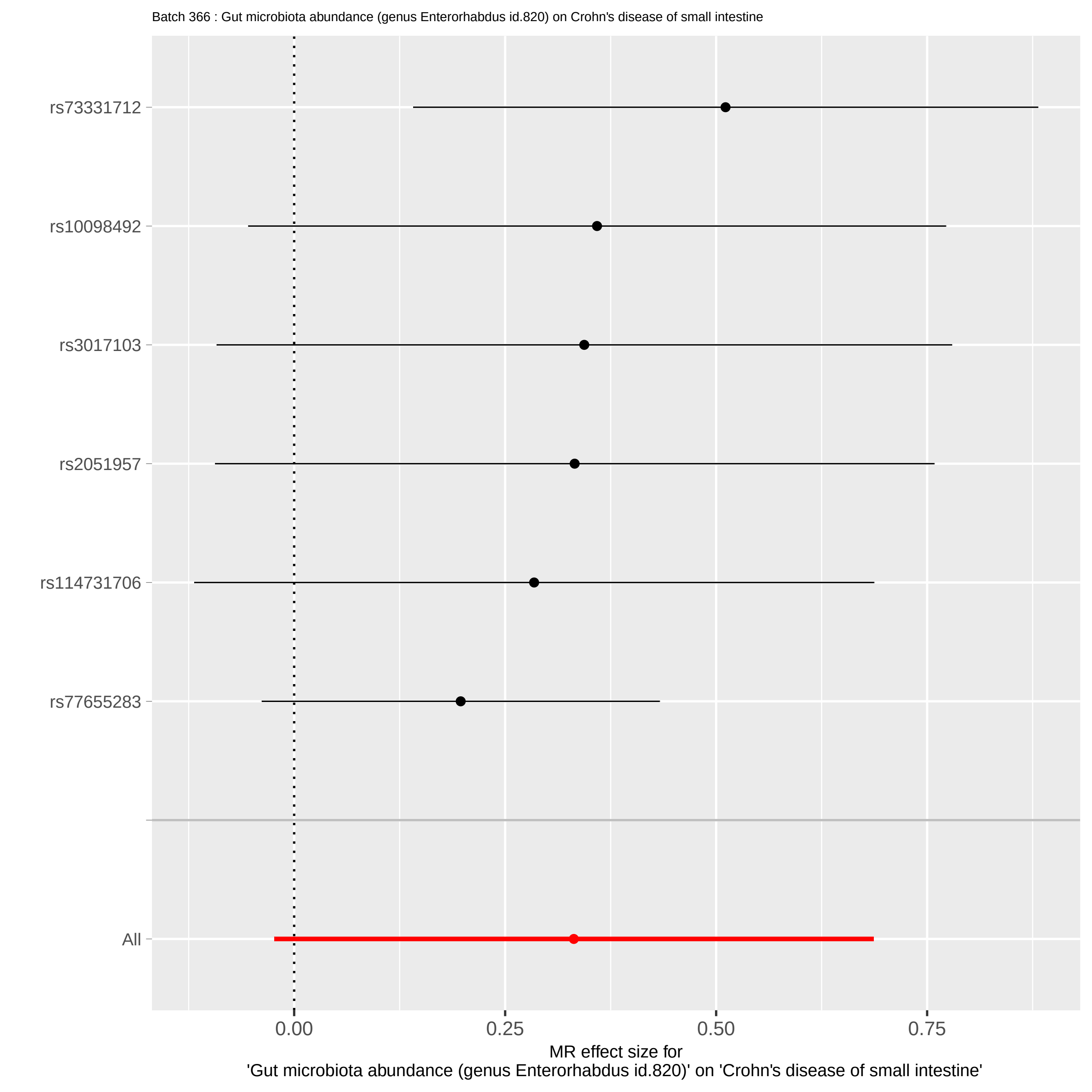

Batch 366 : Gut microbiota abundance (genus Enterorhabdus id.820) on Crohn's disease of small intestine
rs73331712
rs10098492
rs3017103
rs2051957
rs114731706
rs77655283
All
0.00
0.25
0.50
0.75
MR effect size for
'Gut microbiota abundance (genus Enterorhabdus id.820)' on 'Crohn's disease of small intestine'

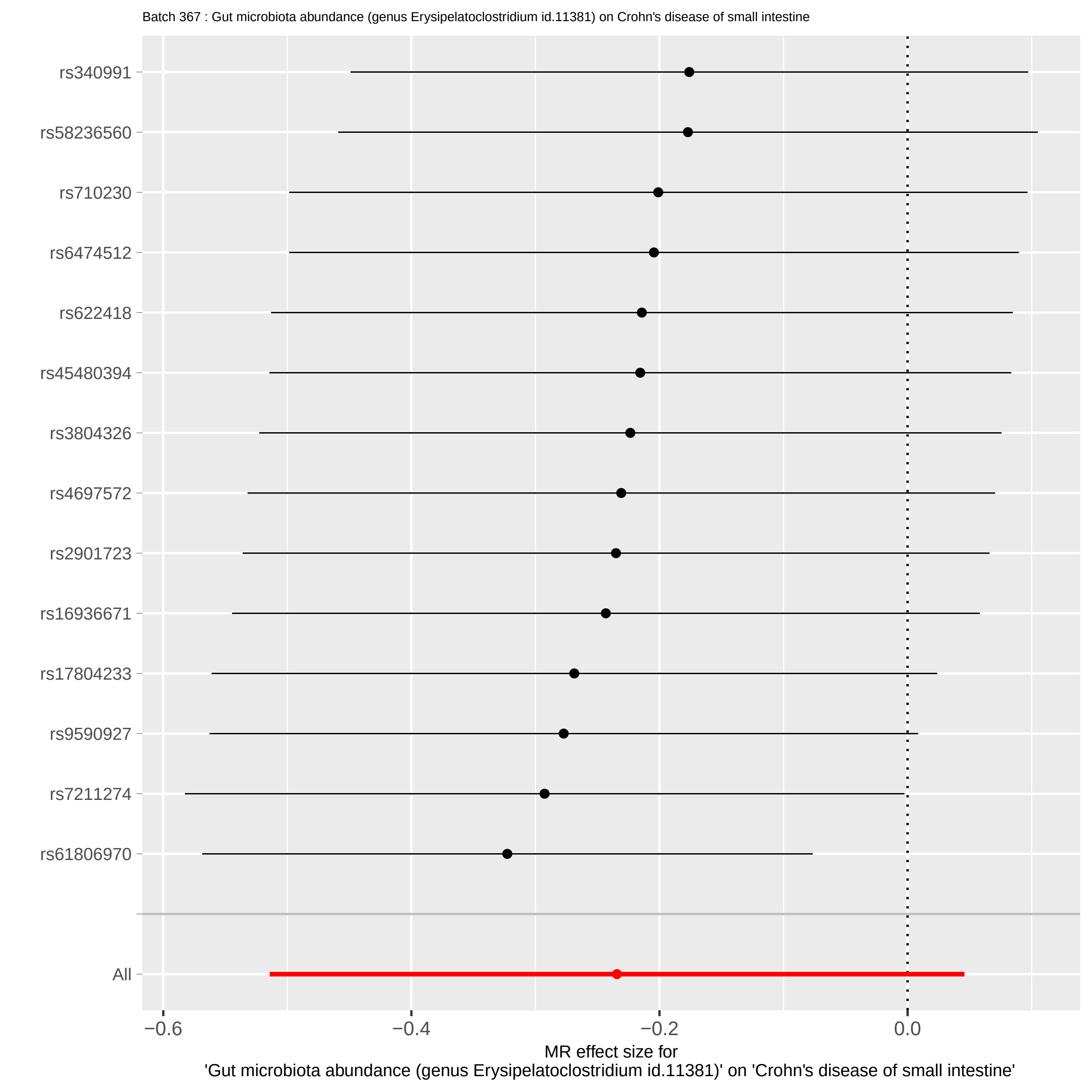

Batch 367 : Gut microbiota abundance (genus Erysipelatoclostridium id.11381) on Crohn's disease of small intestine
rs340991
rs58236560
rs710230
rs6474512
rs622418
rs45480394
rs3804326
rs4697572
rs2901723
rs16936671
rs17804233
rs9590927
rs7211274
rs61806970
All
−0.6
−0.4
−0.2
0.0
MR effect size for
'Gut microbiota abundance (genus Erysipelatoclostridium id.11381)' on 'Crohn's disease of small intestine'

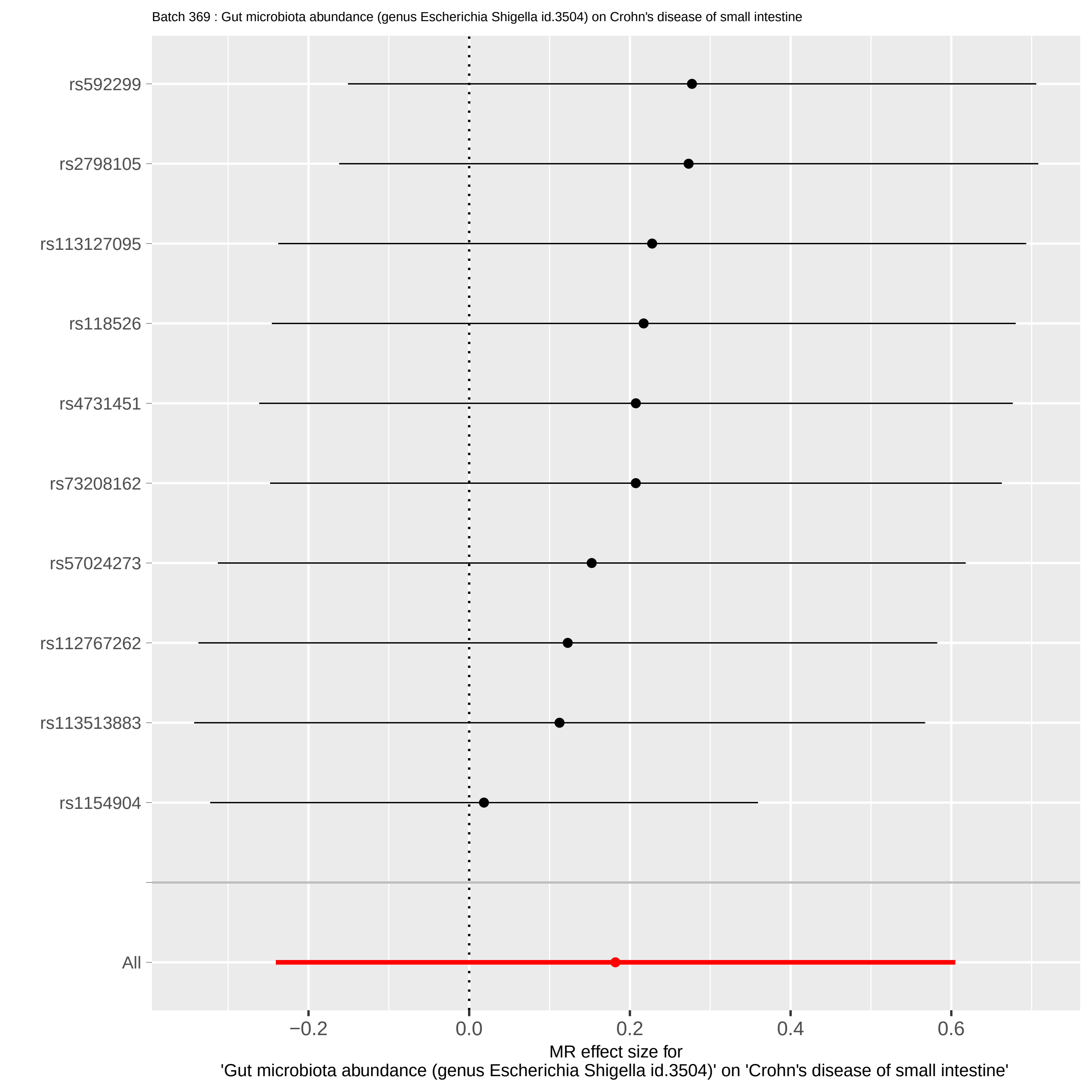
Batch 369 : Gut microbiota abundance (genus Escherichia Shigella id.3504) on Crohn's disease of small intestine
rs592299
rs2798105
rs113127095
rs118526
rs4731451
rs73208162
rs57024273
rs112767262
rs113513883
rs1154904
All
−0.2
0.0
0.2
0.4
0.6
MR effect size for
'Gut microbiota abundance (genus Escherichia Shigella id.3504)' on 'Crohn's disease of small intestine'

Batch 370 : Gut microbiota abundance (genus Eubacterium brachy group id.11296) on Crohn's disease of small intestine

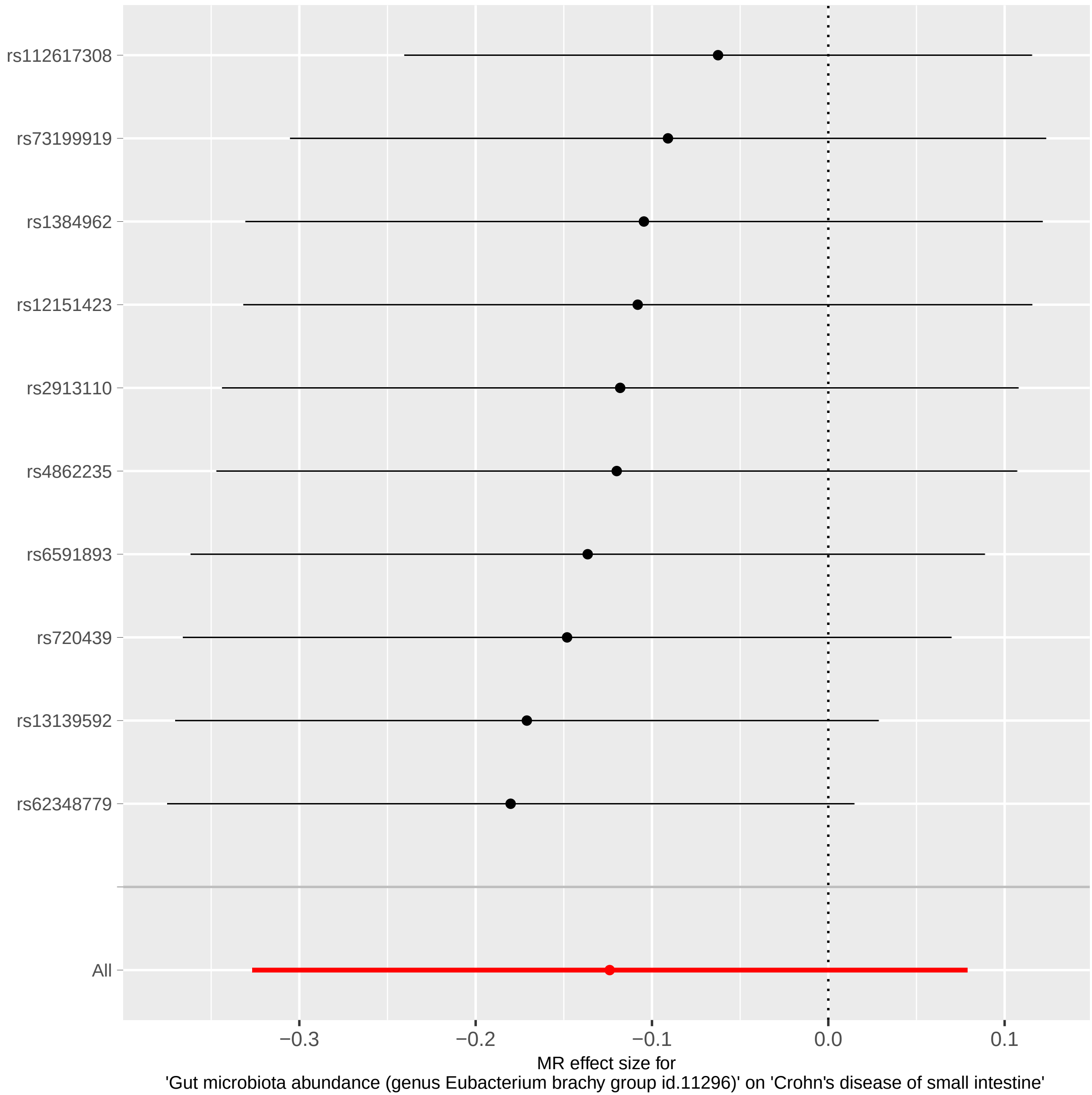

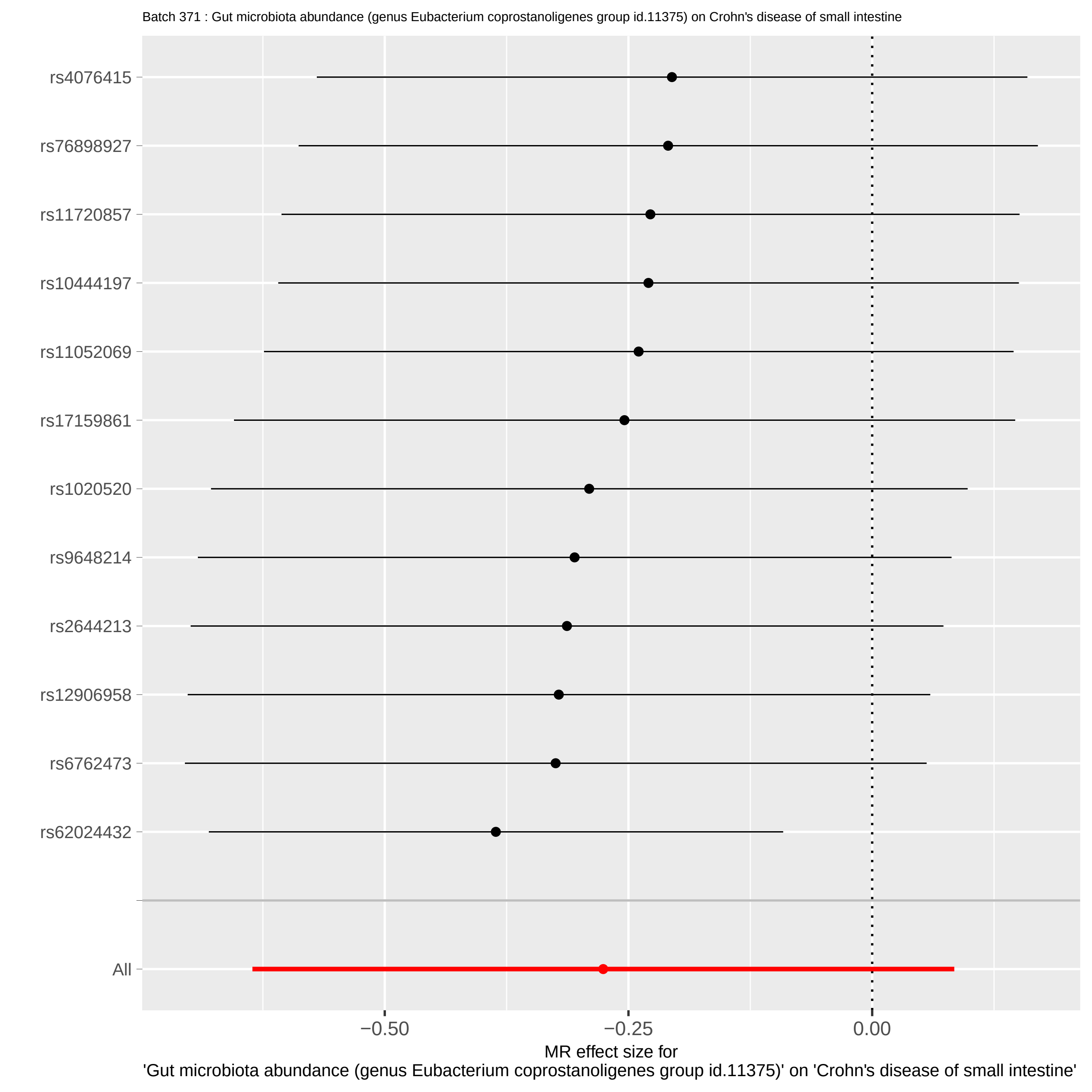

Batch 371 : Gut microbiota abundance (genus Eubacterium coprostanoligenes group id.11375) on Crohn's disease of small intestine
rs4076415
rs76898927
rs11720857
rs10444197
rs11052069
rs17159861
rs1020520
rs9648214
rs2644213
rs12906958
rs6762473
rs62024432
All
−0.50
−0.25
0.00
MR effect size for
'Gut microbiota abundance (genus Eubacterium coprostanoligenes group id.11375)' on 'Crohn's disease of small intestine'

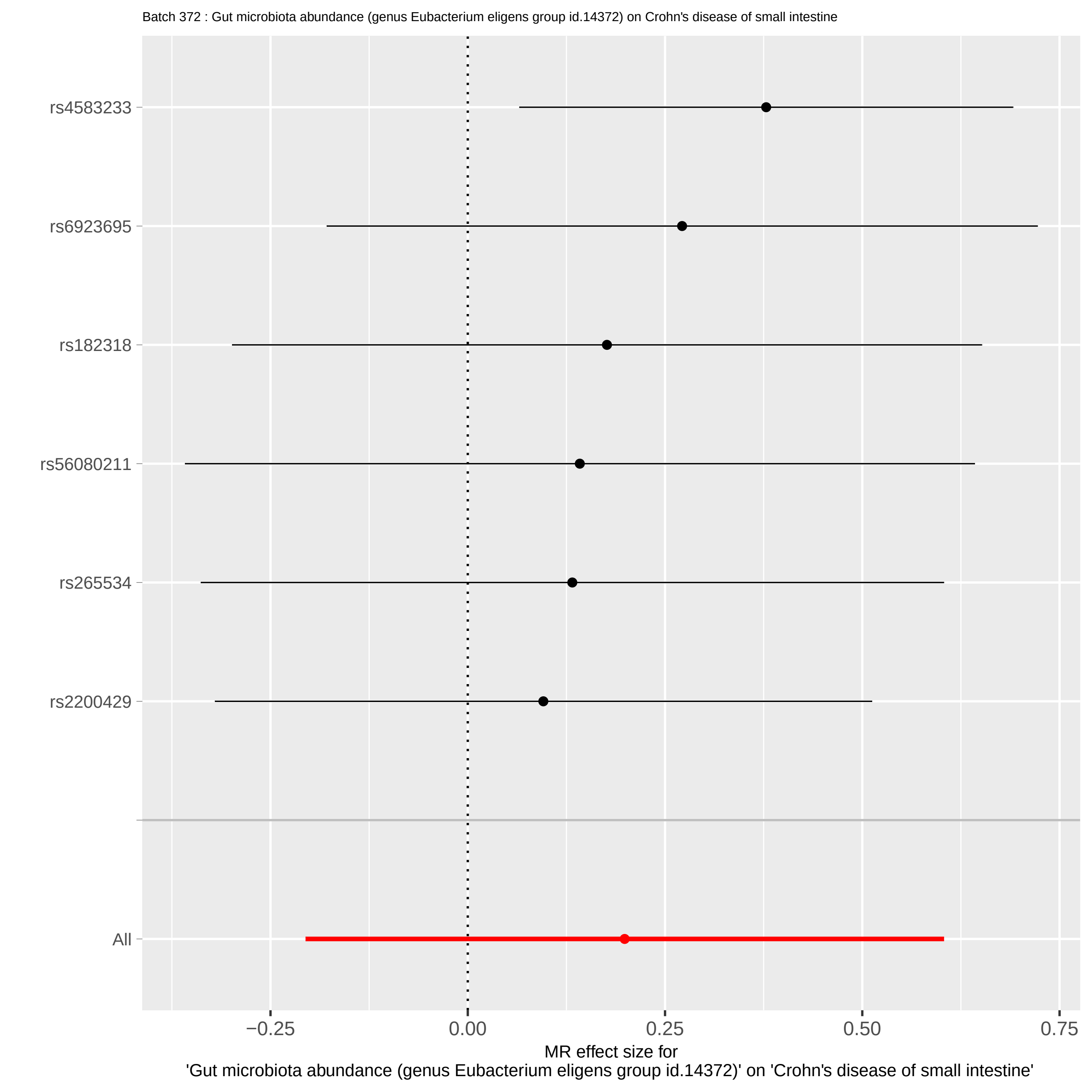
Batch 372 : Gut microbiota abundance (genus Eubacterium eligens group id.14372) on Crohn's disease of small intestine
rs4583233
rs6923695
rs182318
rs56080211
rs265534
rs2200429
All
−0.25
0.00
0.25
0.50
0.75
MR effect size for
'Gut microbiota abundance (genus Eubacterium eligens group id.14372)' on 'Crohn's disease of small intestine'

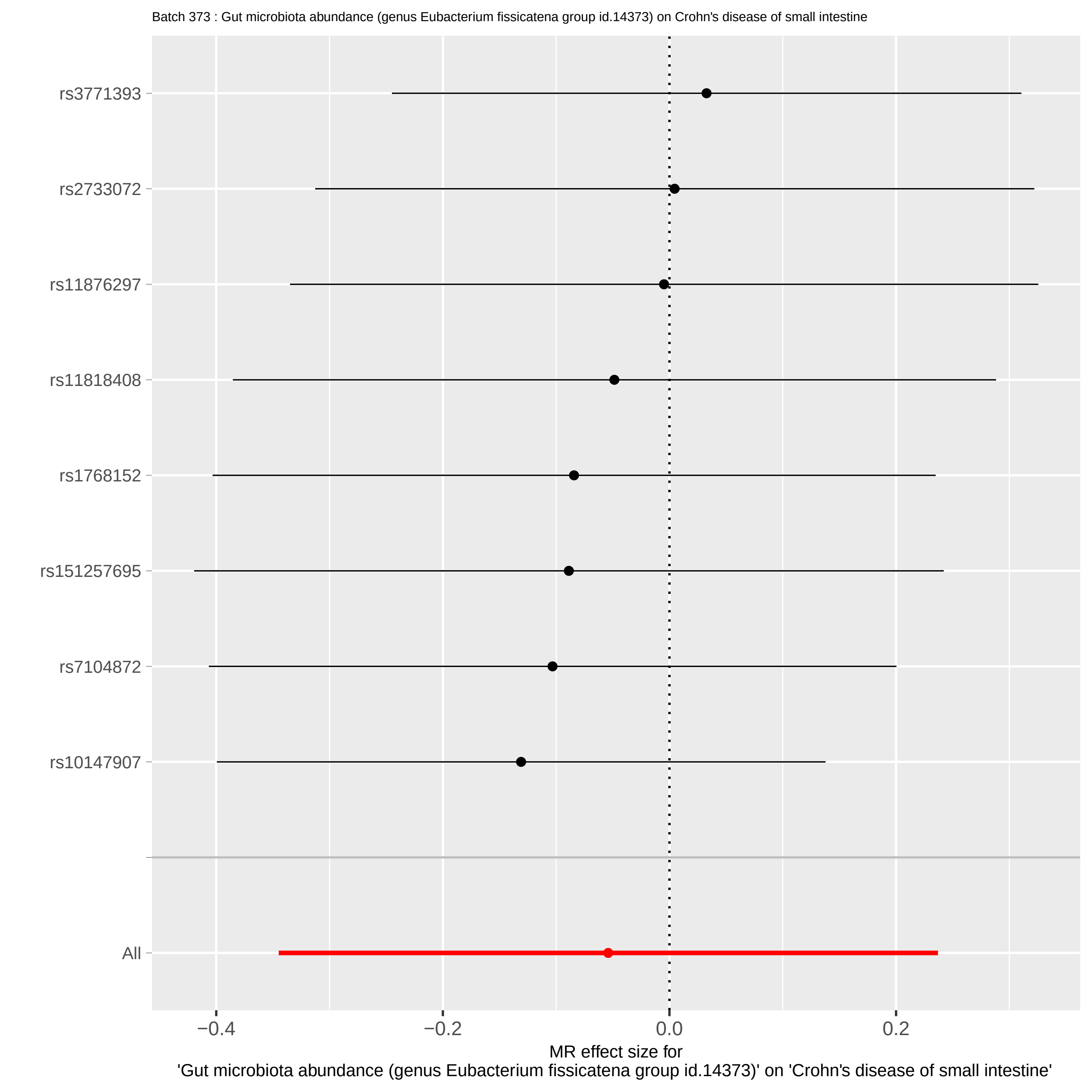
Batch 373 : Gut microbiota abundance (genus Eubacterium fissicatena group id.14373) on Crohn's disease of small intestine
rs3771393
rs2733072
rs11876297
rs11818408
rs1768152
rs151257695
rs7104872
rs10147907
All
−0.4
−0.2
0.0
0.2
MR effect size for
'Gut microbiota abundance (genus Eubacterium fissicatena group id.14373)' on 'Crohn's disease of small intestine'

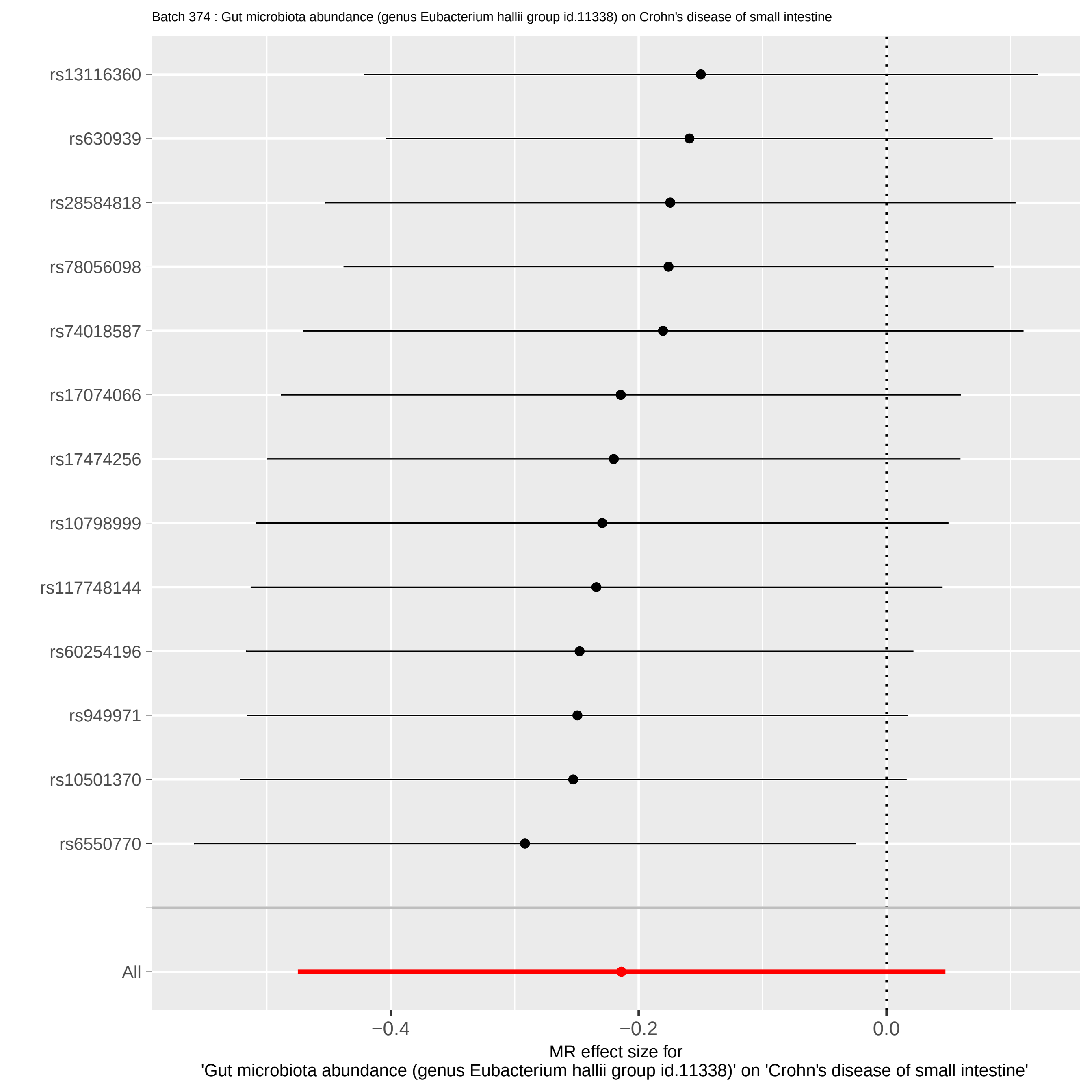

Batch 374 : Gut microbiota abundance (genus Eubacterium hallii group id.11338) on Crohn's disease of small intestine
rs13116360
rs630939
rs28584818
rs78056098
rs74018587
rs17074066
rs17474256
rs10798999
rs117748144
rs60254196
rs949971
rs10501370
rs6550770
All
−0.4
−0.2
0.0
MR effect size for
'Gut microbiota abundance (genus Eubacterium hallii group id.11338)' on 'Crohn's disease of small intestine'

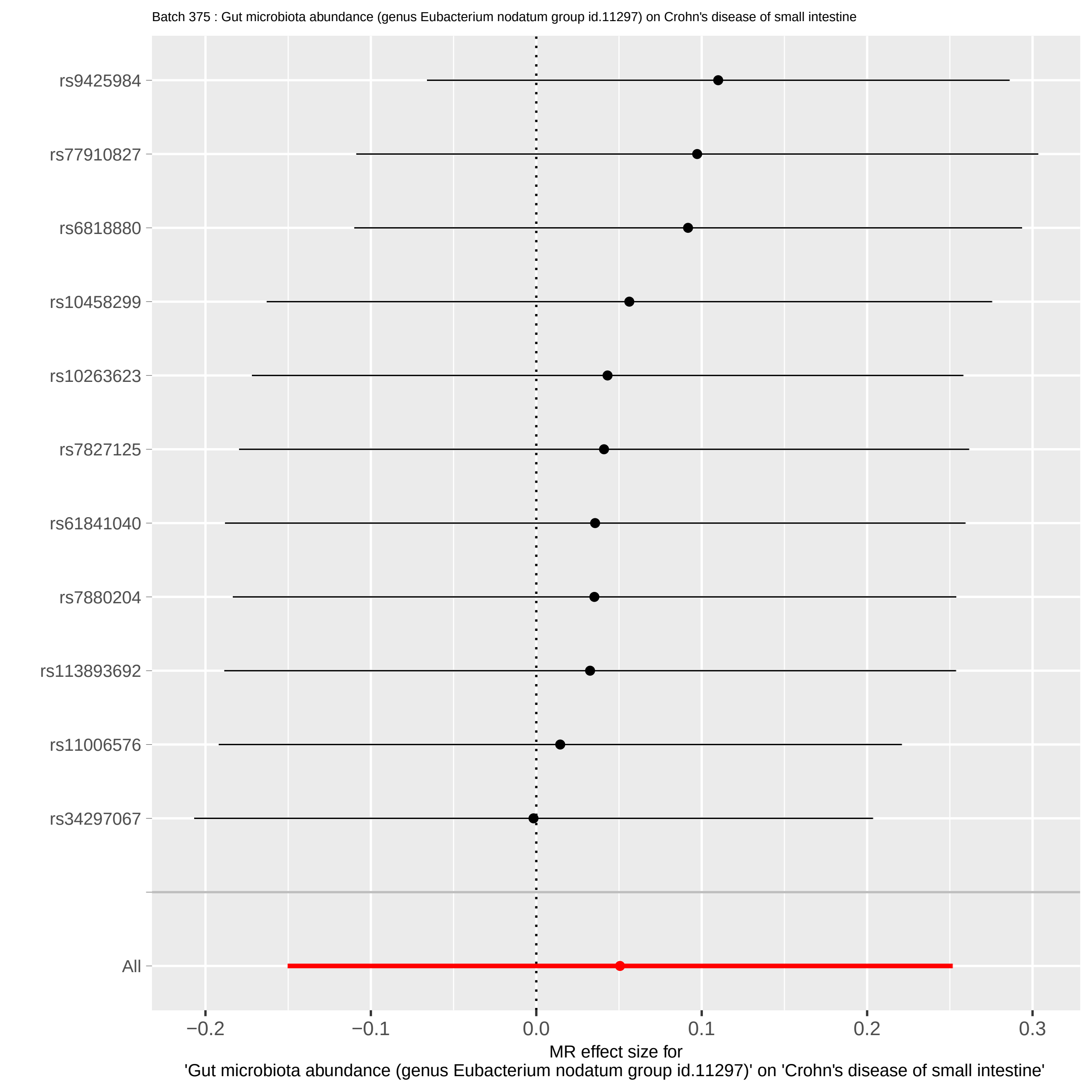
Batch 375 : Gut microbiota abundance (genus Eubacterium nodatum group id.11297) on Crohn's disease of small intestine
rs9425984
rs77910827
rs6818880
rs10458299
rs10263623
rs7827125
rs61841040
rs7880204
rs113893692
rs11006576
rs34297067
All
−0.2
−0.1
0.0
0.1
0.2
0.3
MR effect size for
'Gut microbiota abundance (genus Eubacterium nodatum group id.11297)' on 'Crohn's disease of small intestine'

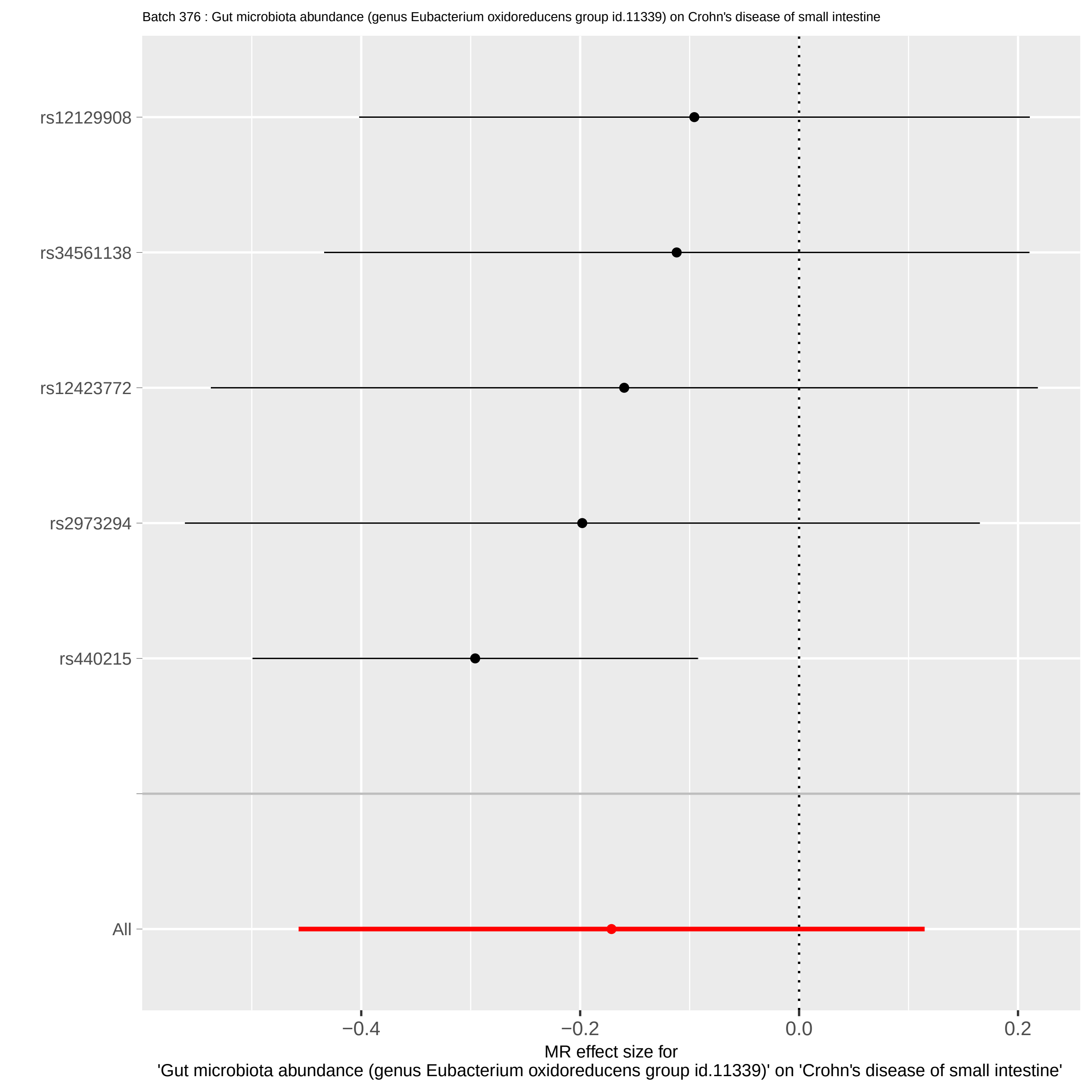

Batch 376 : Gut microbiota abundance (genus Eubacterium oxidoreducens group id.11339) on Crohn's disease of small intestine
rs12129908
rs34561138
rs12423772
rs2973294
rs440215
All
−0.4
−0.2
0.0
0.2
MR effect size for
'Gut microbiota abundance (genus Eubacterium oxidoreducens group id.11339)' on 'Crohn's disease of small intestine'

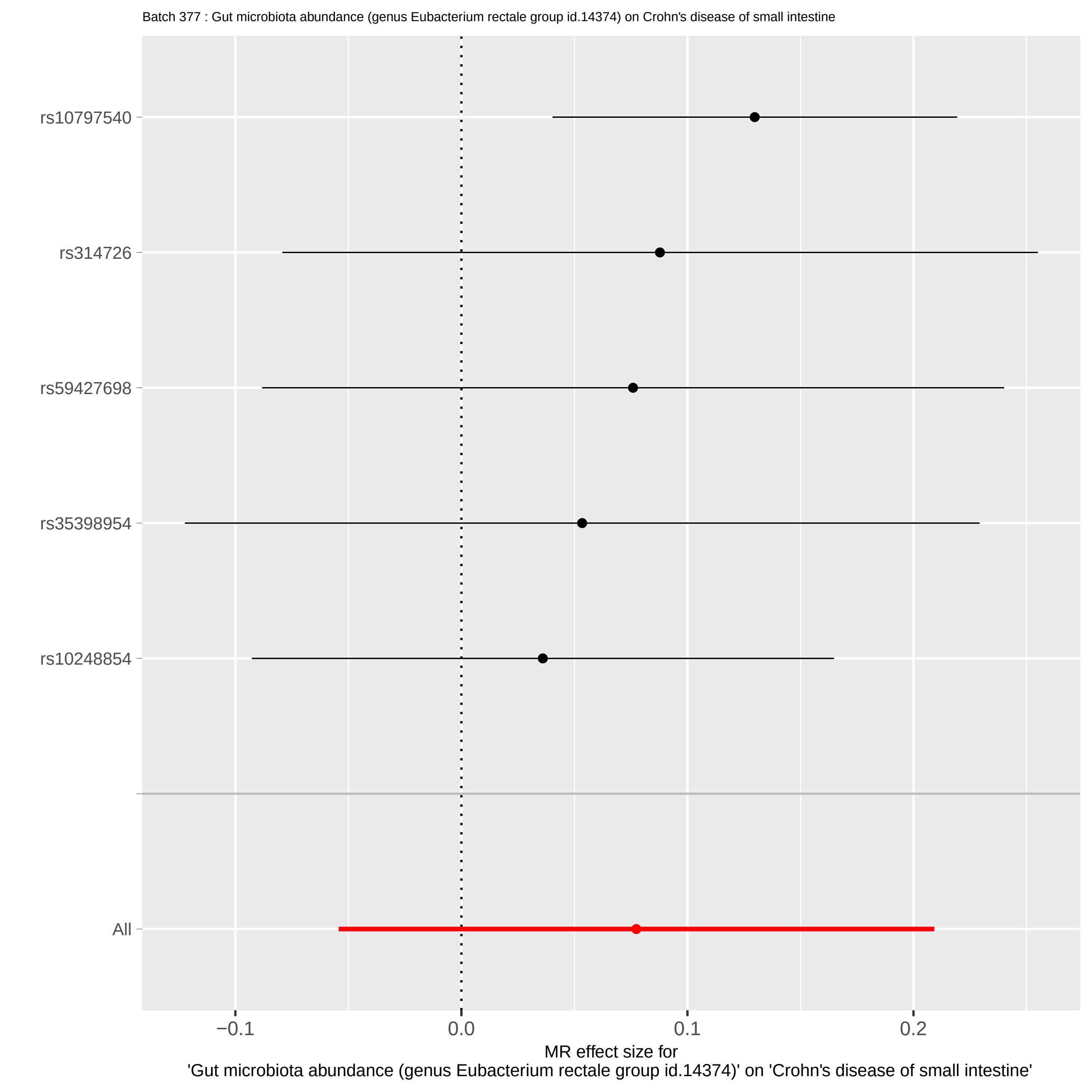

Batch 377 : Gut microbiota abundance (genus Eubacterium rectale group id.14374) on Crohn's disease of small intestine
rs10797540
rs314726
rs59427698
rs35398954
rs10248854
All
−0.1
0.0
0.1
0.2
MR effect size for
'Gut microbiota abundance (genus Eubacterium rectale group id.14374)' on 'Crohn's disease of small intestine'

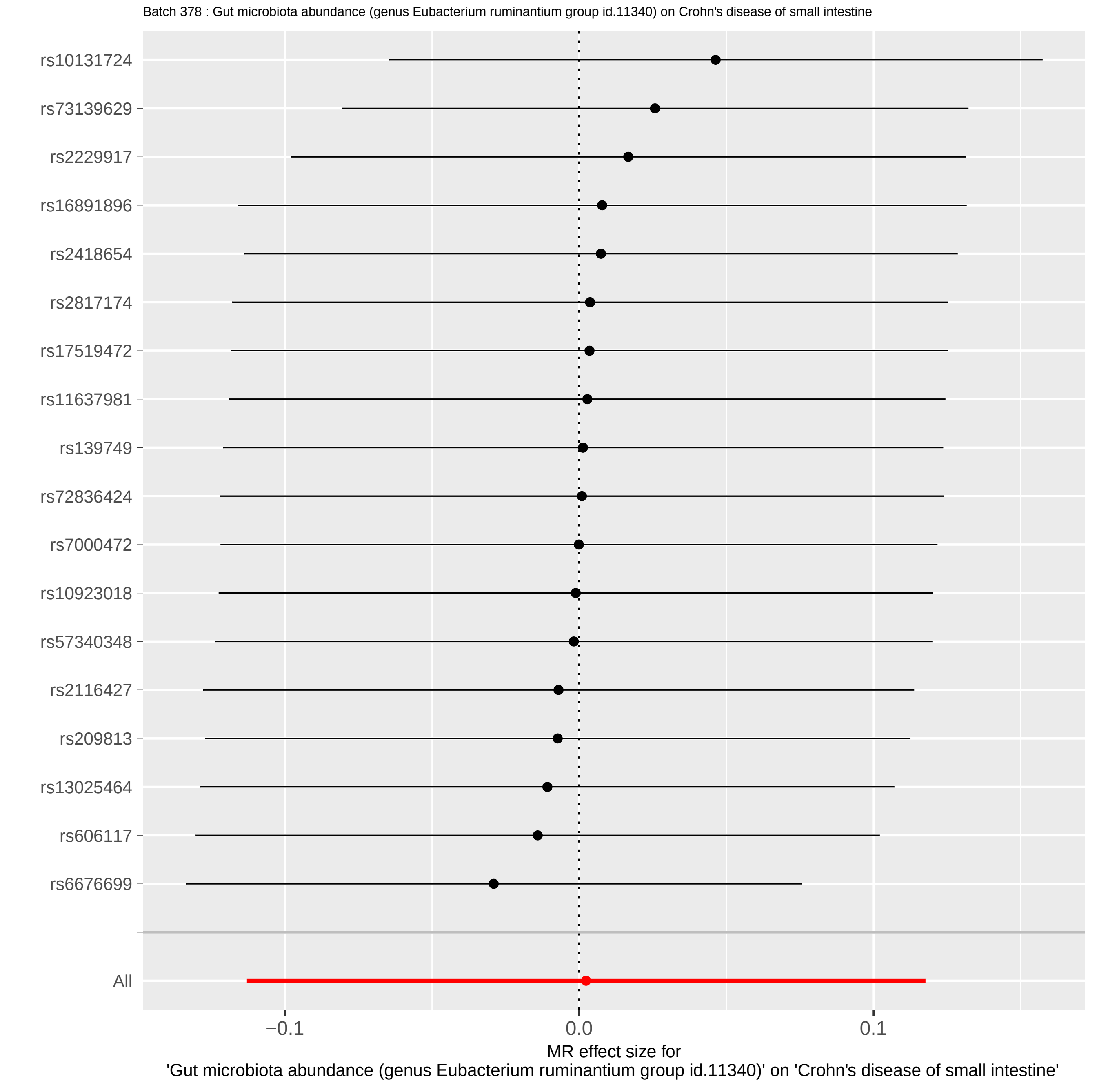
Batch 378 : Gut microbiota abundance (genus Eubacterium ruminantium group id.11340) on Crohn's disease of small intestine
rs10131724
rs73139629
rs2229917
rs16891896
rs2418654
rs2817174
rs17519472
rs11637981
rs139749
rs72836424
rs7000472
rs10923018
rs57340348
rs2116427
rs209813
rs13025464
rs606117
rs6676699
All
−0.1
0.0
0.1
MR effect size for
'Gut microbiota abundance (genus Eubacterium ruminantium group id.11340)' on 'Crohn's disease of small intestine'

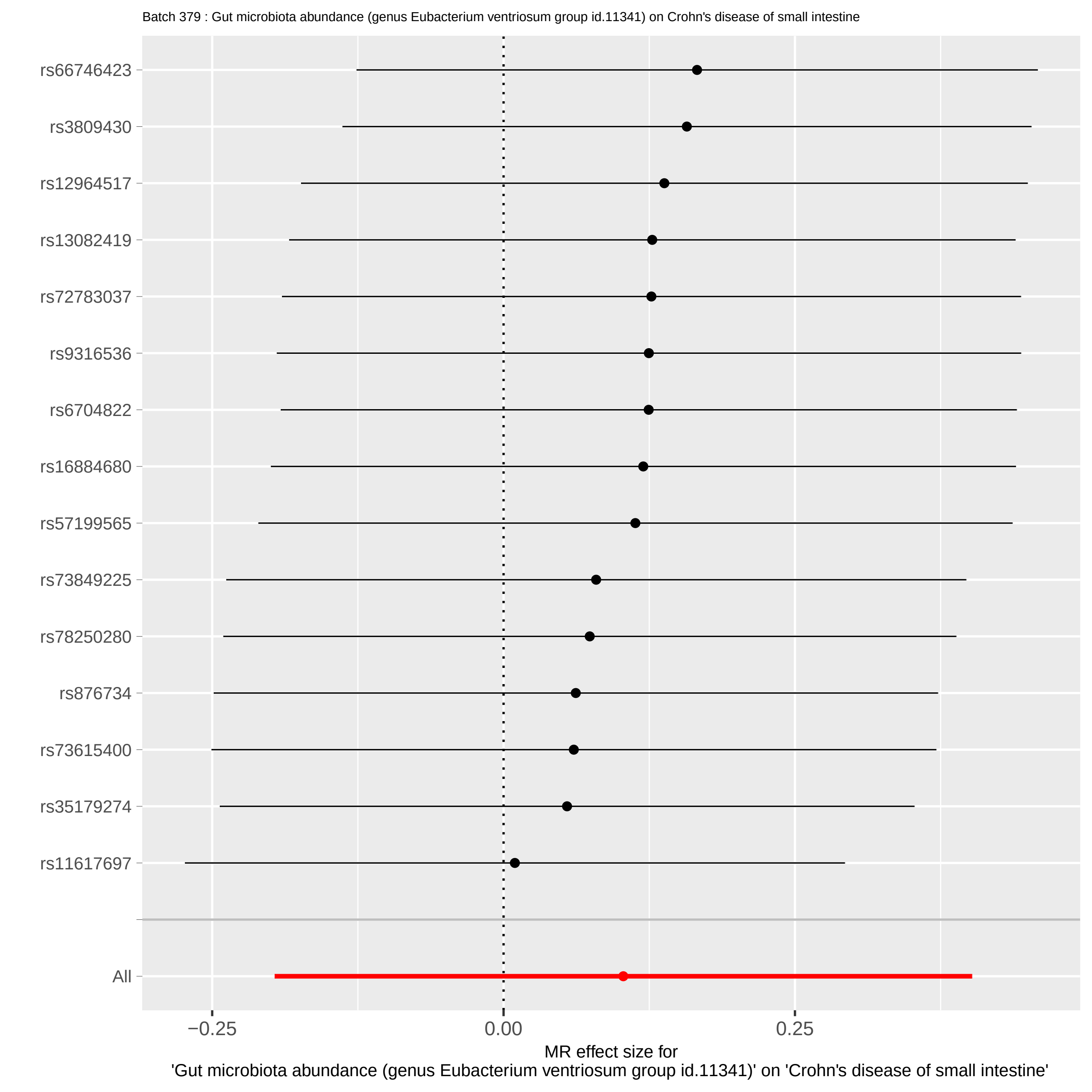

Batch 379 : Gut microbiota abundance (genus Eubacterium ventriosum group id.11341) on Crohn's disease of small intestine
rs66746423
rs3809430
rs12964517
rs13082419
rs72783037
rs9316536
rs6704822
rs16884680
rs57199565
rs73849225
rs78250280
rs876734
rs73615400
rs35179274
rs11617697
All
−0.25
0.00
0.25
MR effect size for
'Gut microbiota abundance (genus Eubacterium ventriosum group id.11341)' on 'Crohn's disease of small intestine'

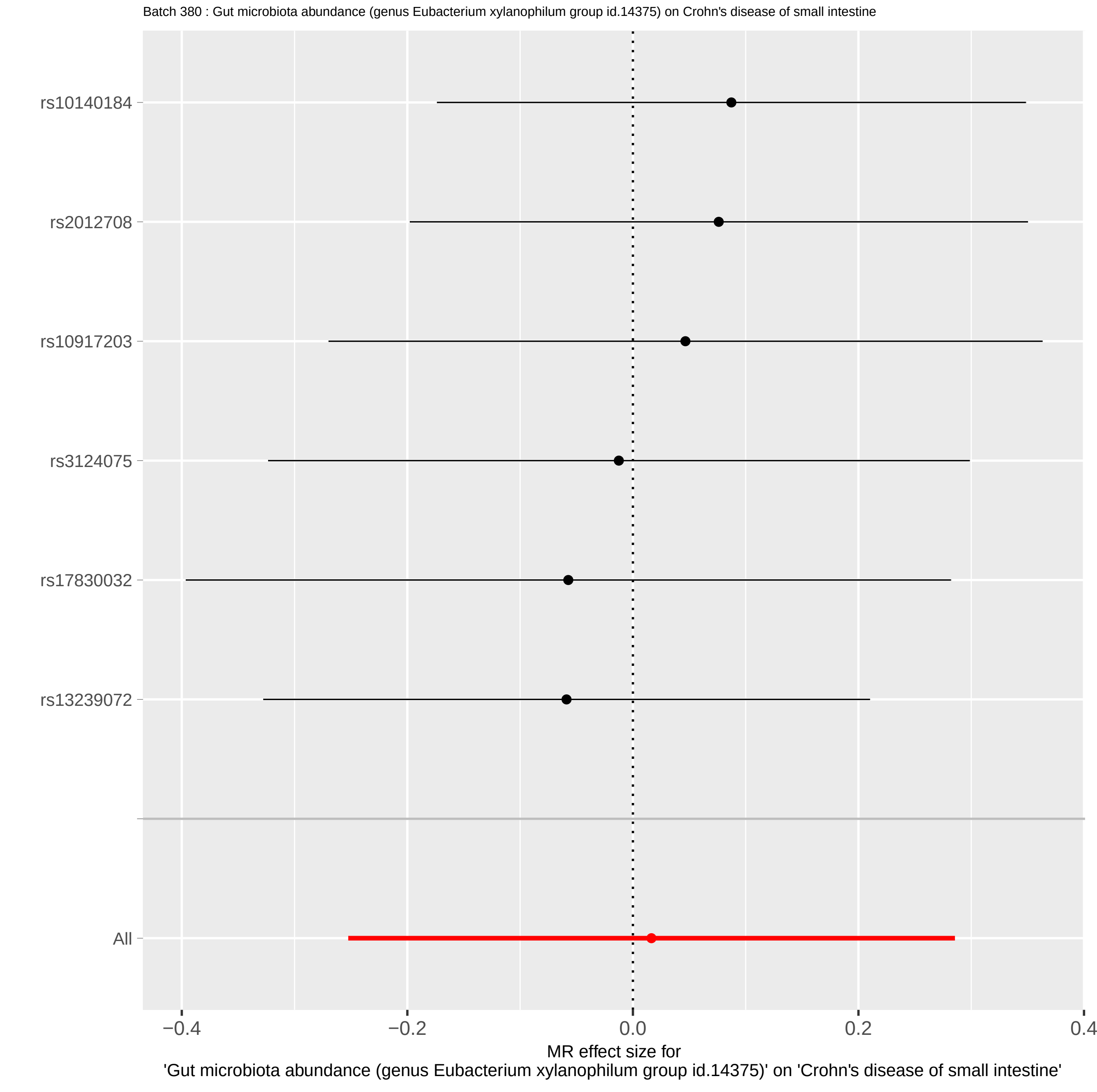
Batch 380 : Gut microbiota abundance (genus Eubacterium xylanophilum group id.14375) on Crohn's disease of small intestine
rs10140184
rs2012708
rs10917203
rs3124075
rs17830032
rs13239072
All
−0.4
−0.2
0.0
0.2
0.4
MR effect size for
'Gut microbiota abundance (genus Eubacterium xylanophilum group id.14375)' on 'Crohn's disease of small intestine'

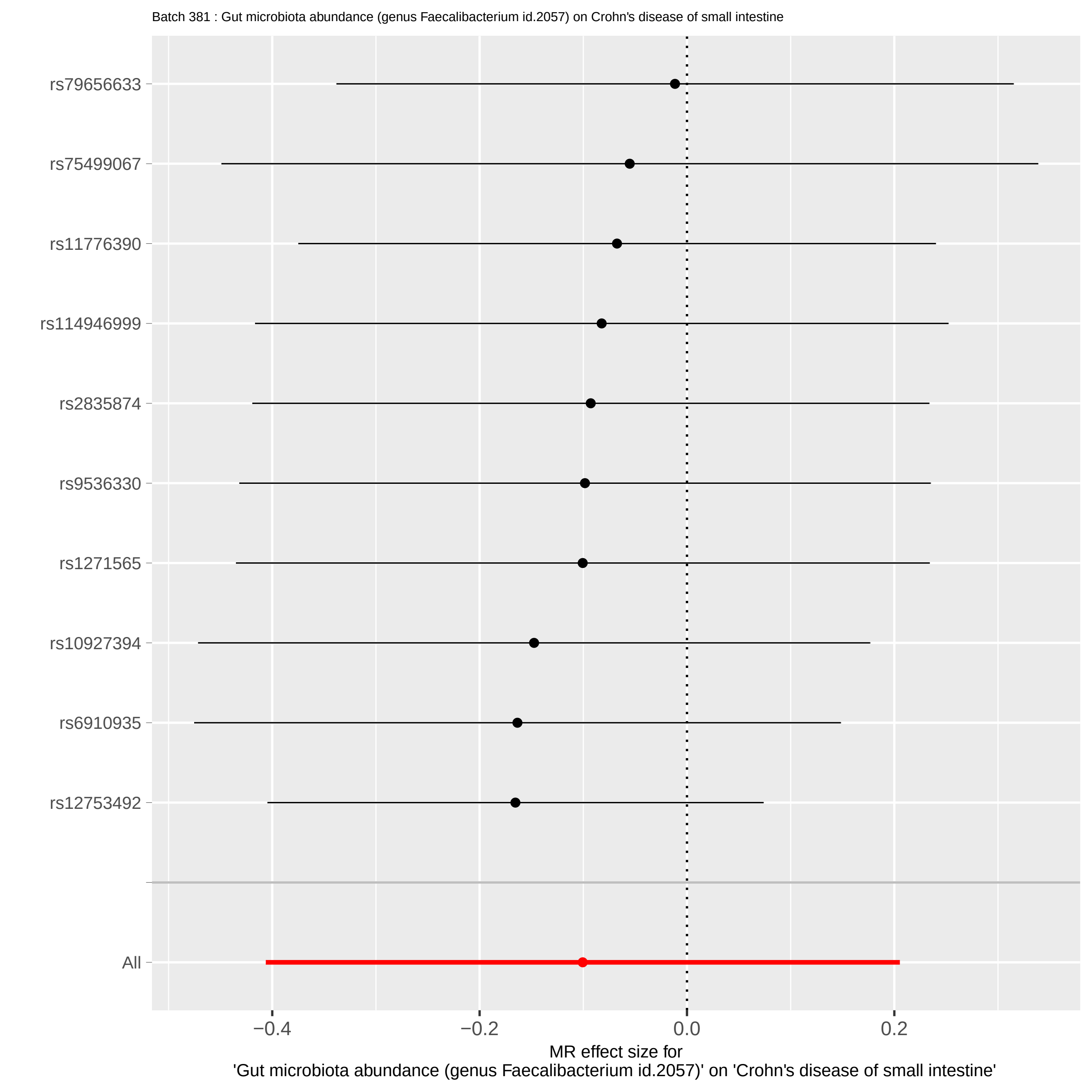
Batch 381 : Gut microbiota abundance (genus Faecalibacterium id.2057) on Crohn's disease of small intestine
rs79656633
rs75499067
rs11776390
rs114946999
rs2835874
rs9536330
rs1271565
rs10927394
rs6910935
rs12753492
All
−0.4
−0.2
0.0
0.2
MR effect size for
'Gut microbiota abundance (genus Faecalibacterium id.2057)' on 'Crohn's disease of small intestine'

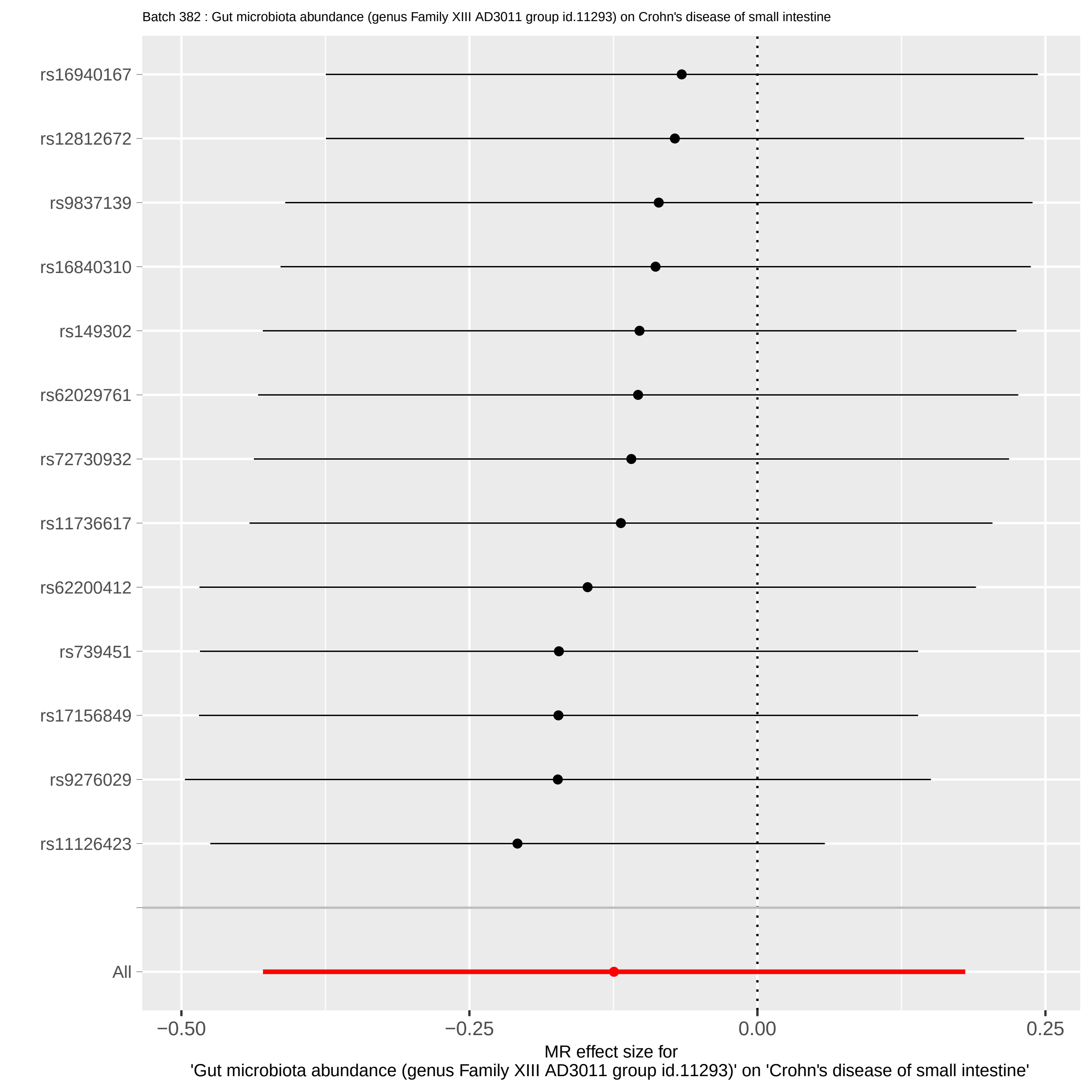

Batch 382 : Gut microbiota abundance (genus Family XIII AD3011 group id.11293) on Crohn's disease of small intestine
rs16940167
rs12812672
rs9837139
rs16840310
rs149302
rs62029761
rs72730932
rs11736617
rs62200412
rs739451
rs17156849
rs9276029
rs11126423
All
−0.50
−0.25
0.00
0.25
MR effect size for
'Gut microbiota abundance (genus Family XIII AD3011 group id.11293)' on 'Crohn's disease of small intestine'

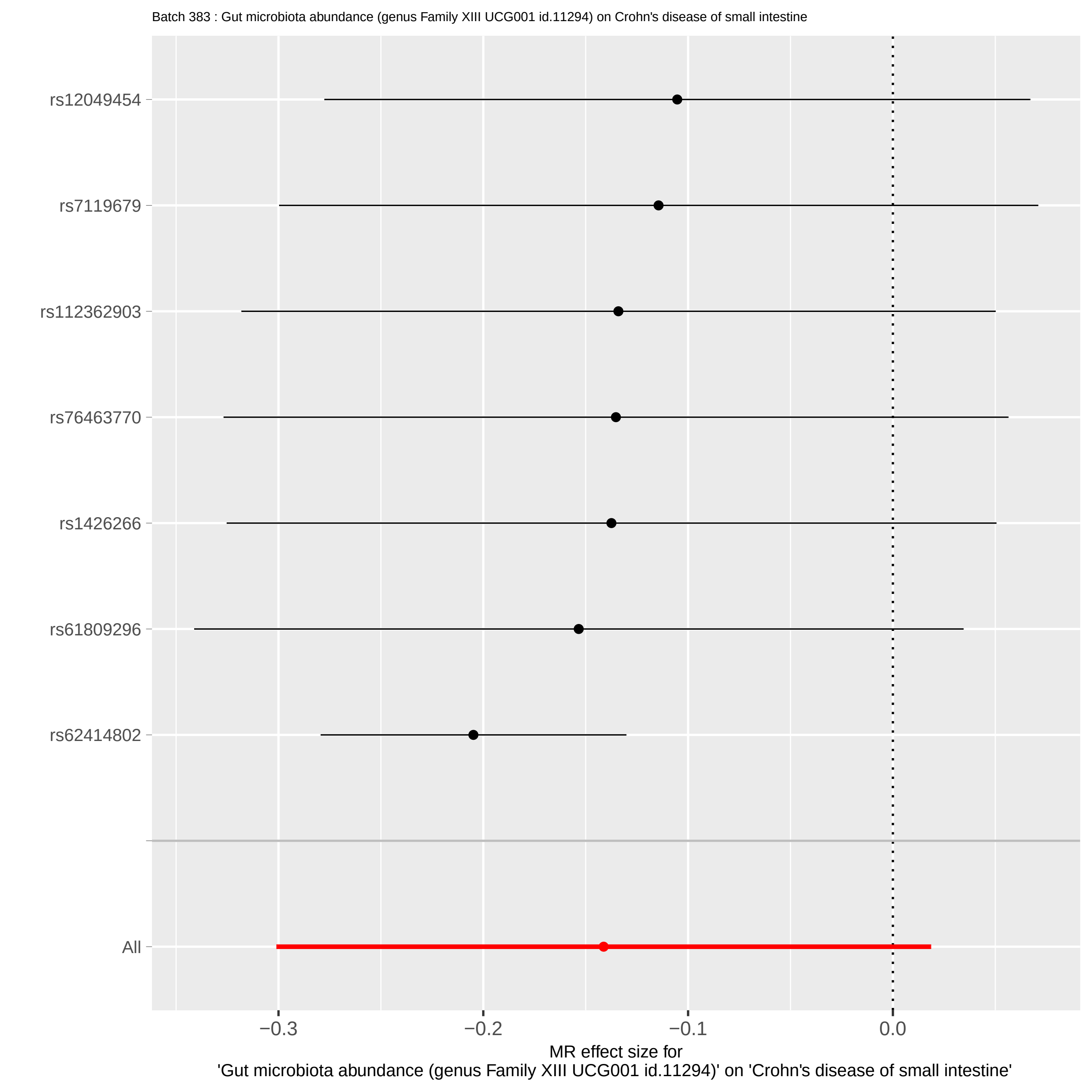
Batch 383 : Gut microbiota abundance (genus Family XIII UCG001 id.11294) on Crohn's disease of small intestine
rs12049454
rs7119679
rs112362903
rs76463770
rs1426266
rs61809296
rs62414802
All
−0.3
−0.2
−0.1
0.0
MR effect size for
'Gut microbiota abundance (genus Family XIII UCG001 id.11294)' on 'Crohn's disease of small intestine'

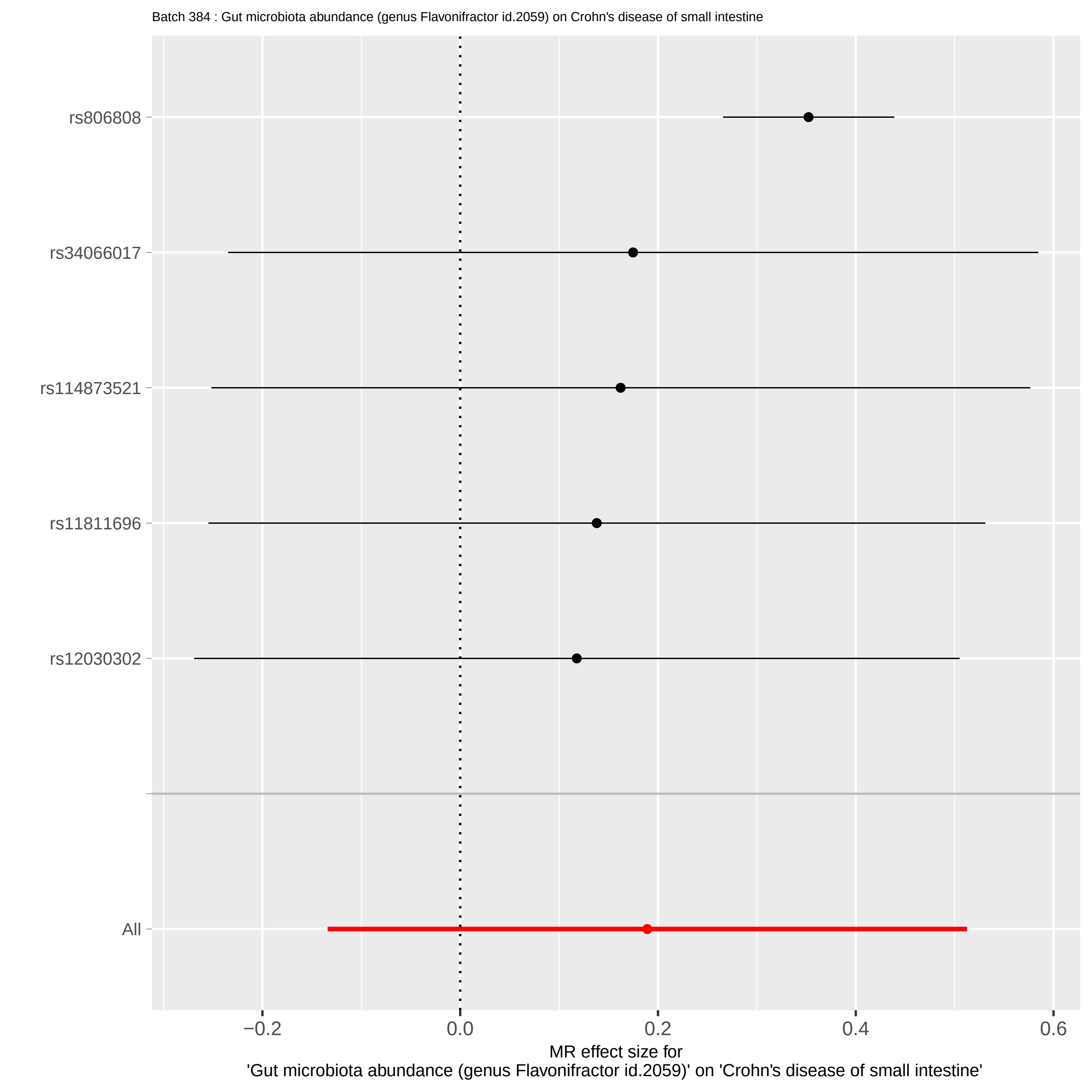

Batch 384 : Gut microbiota abundance (genus Flavonifractor id.2059) on Crohn's disease of small intestine
rs806808
rs34066017
rs114873521
rs11811696
rs12030302
All
−0.2
0.0
0.2
0.4
0.6
MR effect size for
'Gut microbiota abundance (genus Flavonifractor id.2059)' on 'Crohn's disease of small intestine'

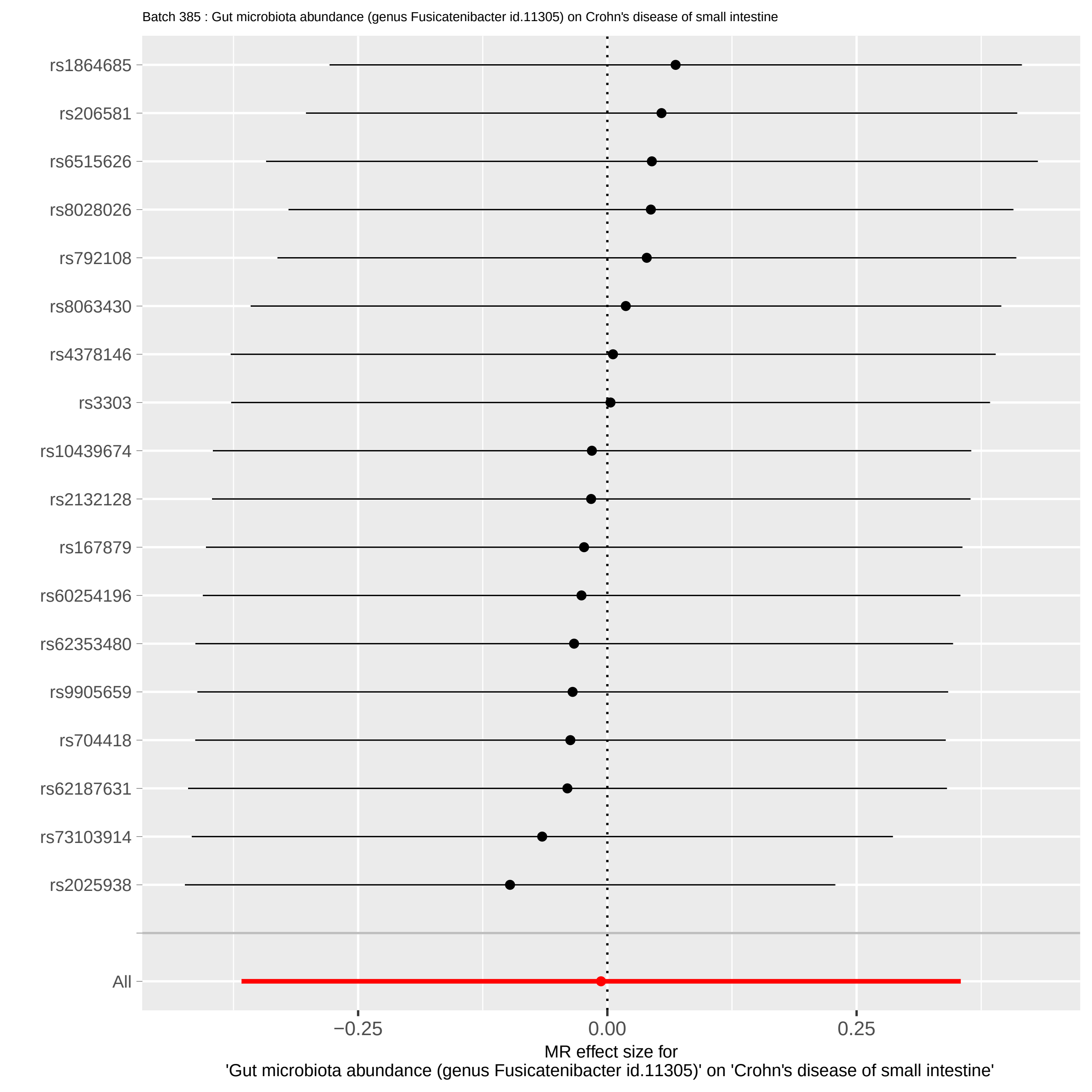
Batch 385 : Gut microbiota abundance (genus Fusicatenibacter id.11305) on Crohn's disease of small intestine
rs1864685
rs206581
rs6515626
rs8028026
rs792108
rs8063430
rs4378146
rs3303
rs10439674
rs2132128
rs167879
rs60254196
rs62353480
rs9905659
rs704418
rs62187631
rs73103914
rs2025938
All
−0.25
0.00
0.25
MR effect size for
'Gut microbiota abundance (genus Fusicatenibacter id.11305)' on 'Crohn's disease of small intestine'

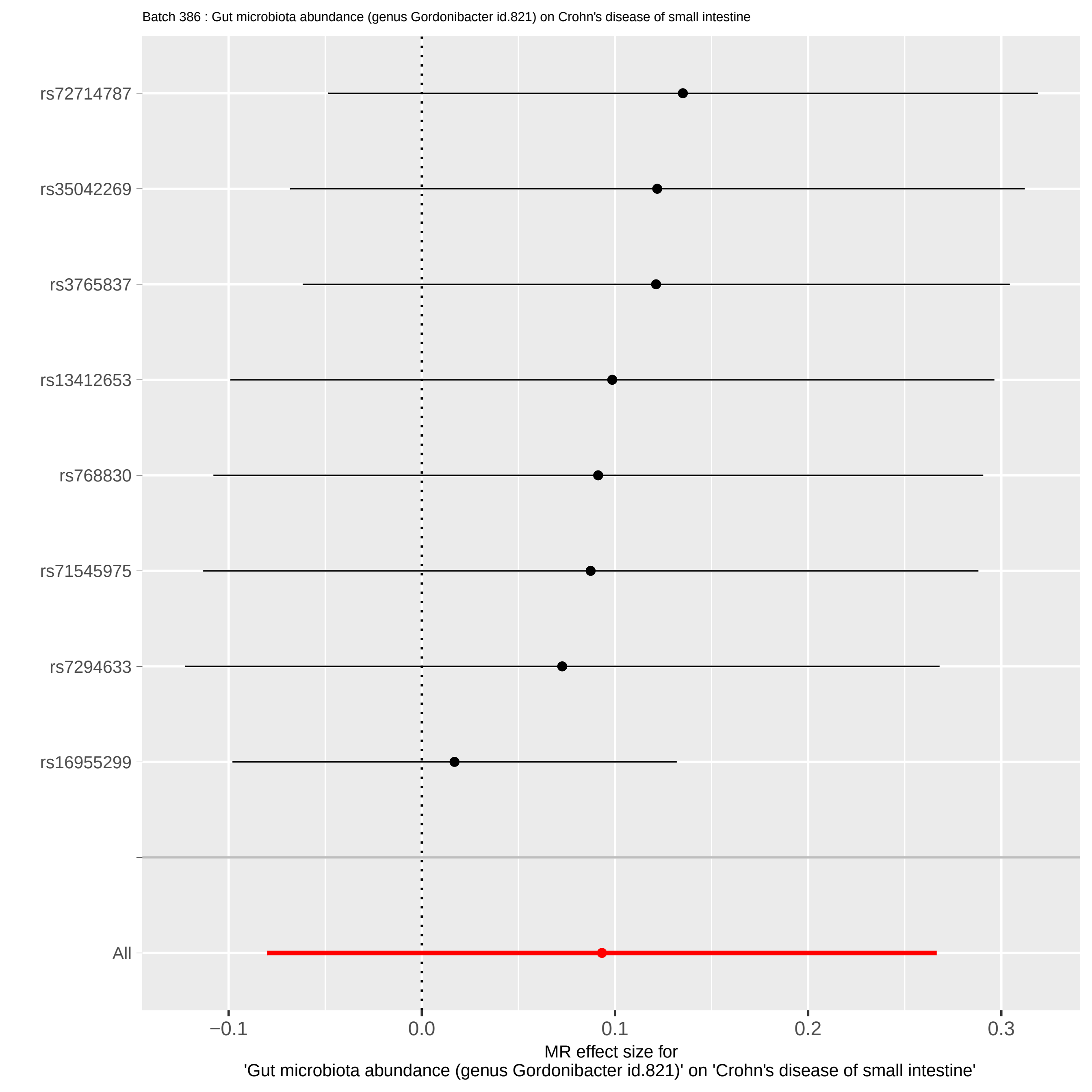

Batch 386 : Gut microbiota abundance (genus Gordonibacter id.821) on Crohn's disease of small intestine
rs72714787
rs35042269
rs3765837
rs13412653
rs768830
rs71545975
rs7294633
rs16955299
All
−0.1
0.0
0.1
0.2
0.3
MR effect size for
'Gut microbiota abundance (genus Gordonibacter id.821)' on 'Crohn's disease of small intestine'

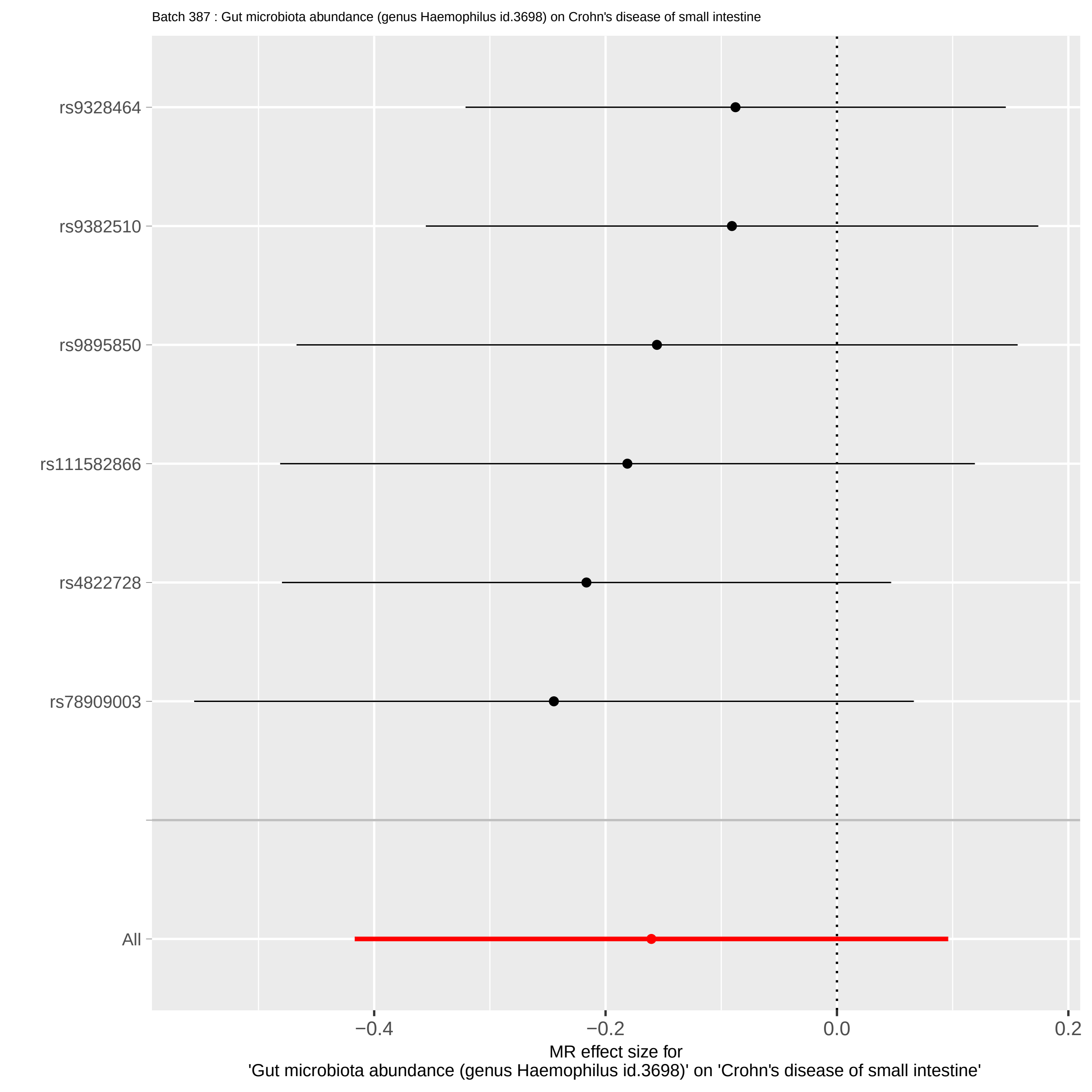

Batch 387 : Gut microbiota abundance (genus Haemophilus id.3698) on Crohn's disease of small intestine
rs9328464
rs9382510
rs9895850
rs111582866
rs4822728
rs78909003
All
−0.4
−0.2
0.0
0.2
MR effect size for
'Gut microbiota abundance (genus Haemophilus id.3698)' on 'Crohn's disease of small intestine'

Batch 388 : Gut microbiota abundance (genus Holdemanella id.11393) on Crohn's disease of small intestine

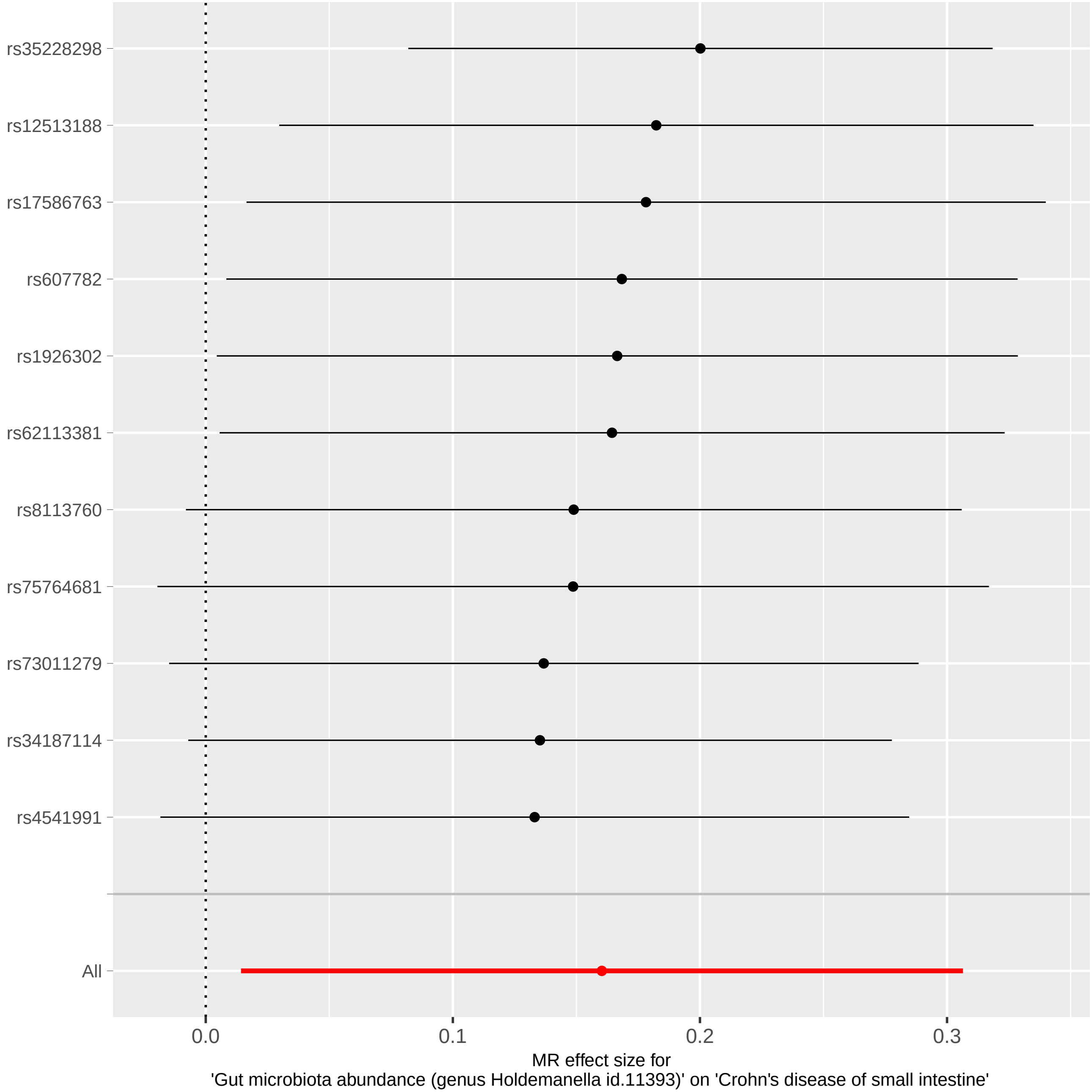

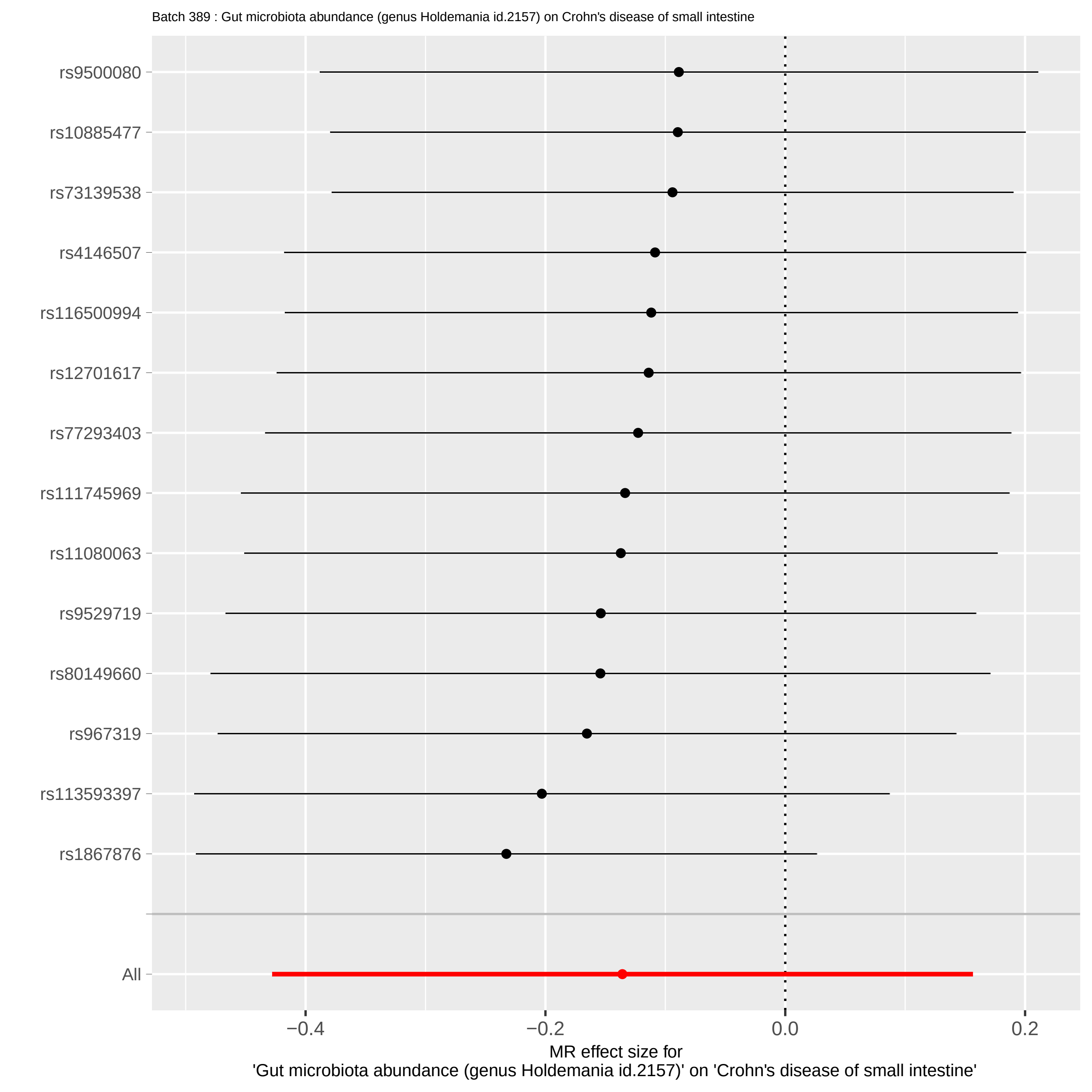
Batch 389 : Gut microbiota abundance (genus Holdemania id.2157) on Crohn's disease of small intestine
rs9500080
rs10885477
rs73139538
rs4146507
rs116500994
rs12701617
rs77293403
rs111745969
rs11080063
rs9529719
rs80149660
rs967319
rs113593397
rs1867876
All
−0.4
−0.2
0.0
0.2
MR effect size for
'Gut microbiota abundance (genus Holdemania id.2157)' on 'Crohn's disease of small intestine'

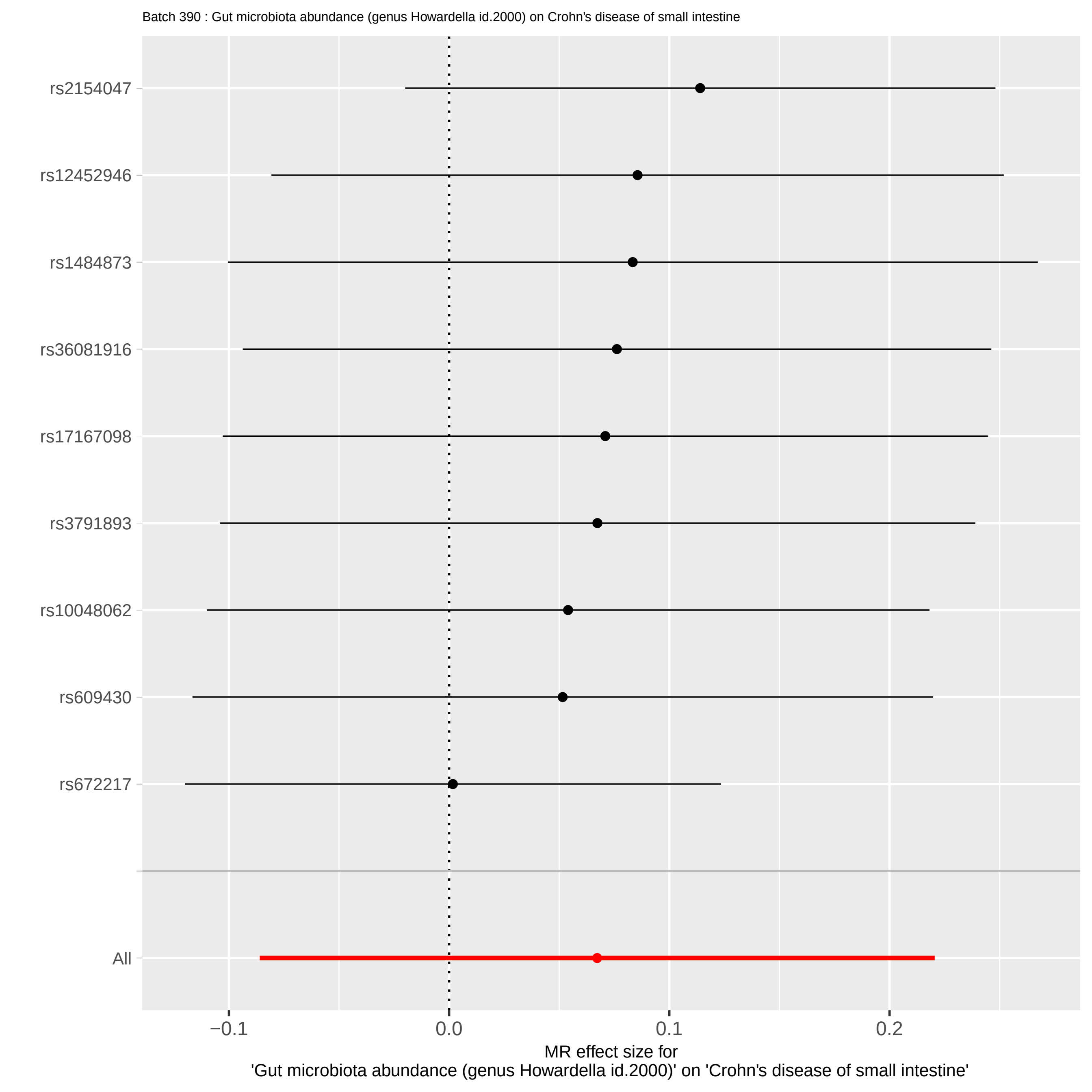
Batch 390 : Gut microbiota abundance (genus Howardella id.2000) on Crohn's disease of small intestine
rs2154047
rs12452946
rs1484873
rs36081916
rs17167098
rs3791893
rs10048062
rs609430
rs672217
All
−0.1
0.0
0.1
0.2
MR effect size for
'Gut microbiota abundance (genus Howardella id.2000)' on 'Crohn's disease of small intestine'

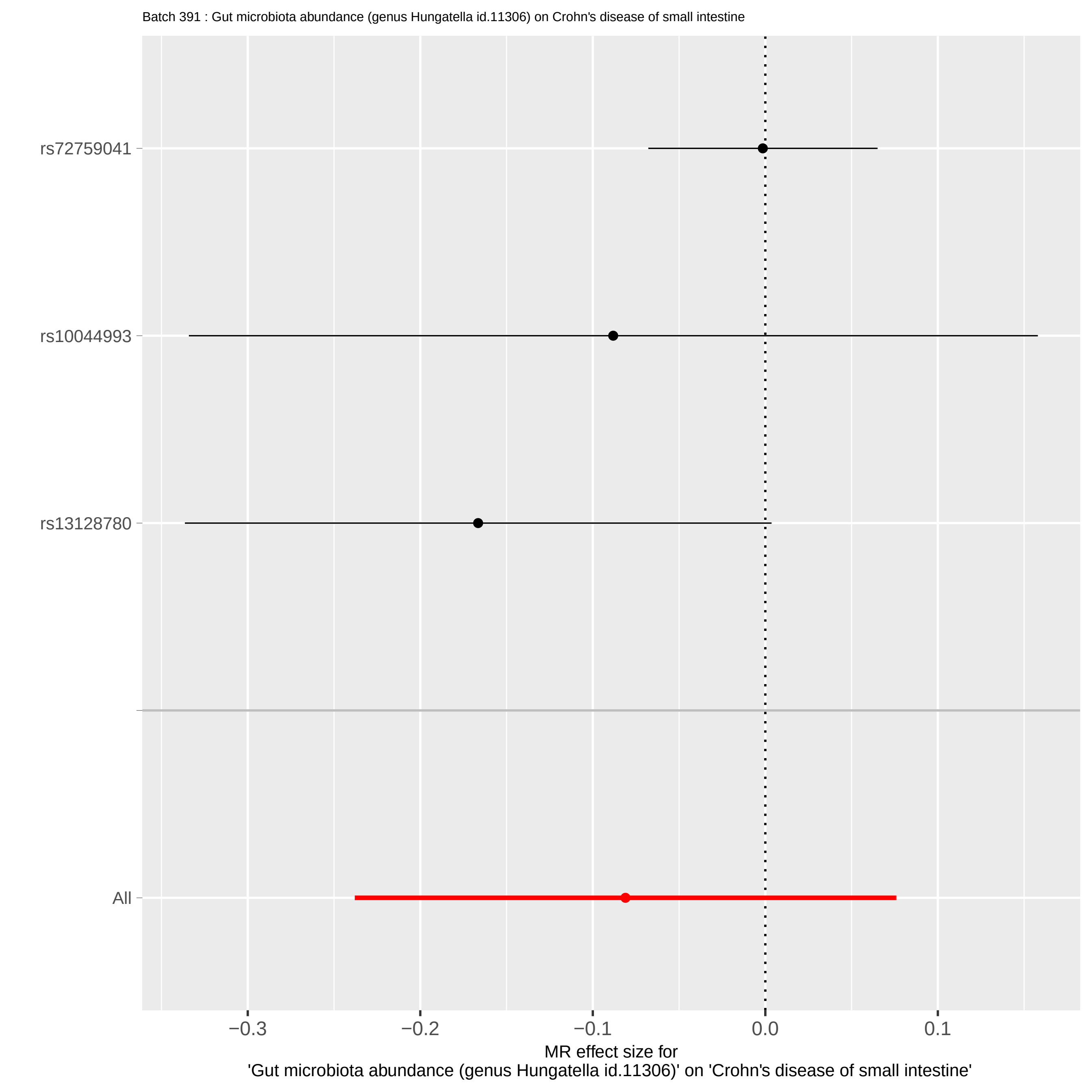

Batch 391 : Gut microbiota abundance (genus Hungatella id.11306) on Crohn's disease of small intestine
rs72759041
rs10044993
rs13128780
All
−0.3
−0.2
−0.1
0.0
0.1
MR effect size for
'Gut microbiota abundance (genus Hungatella id.11306)' on 'Crohn's disease of small intestine'

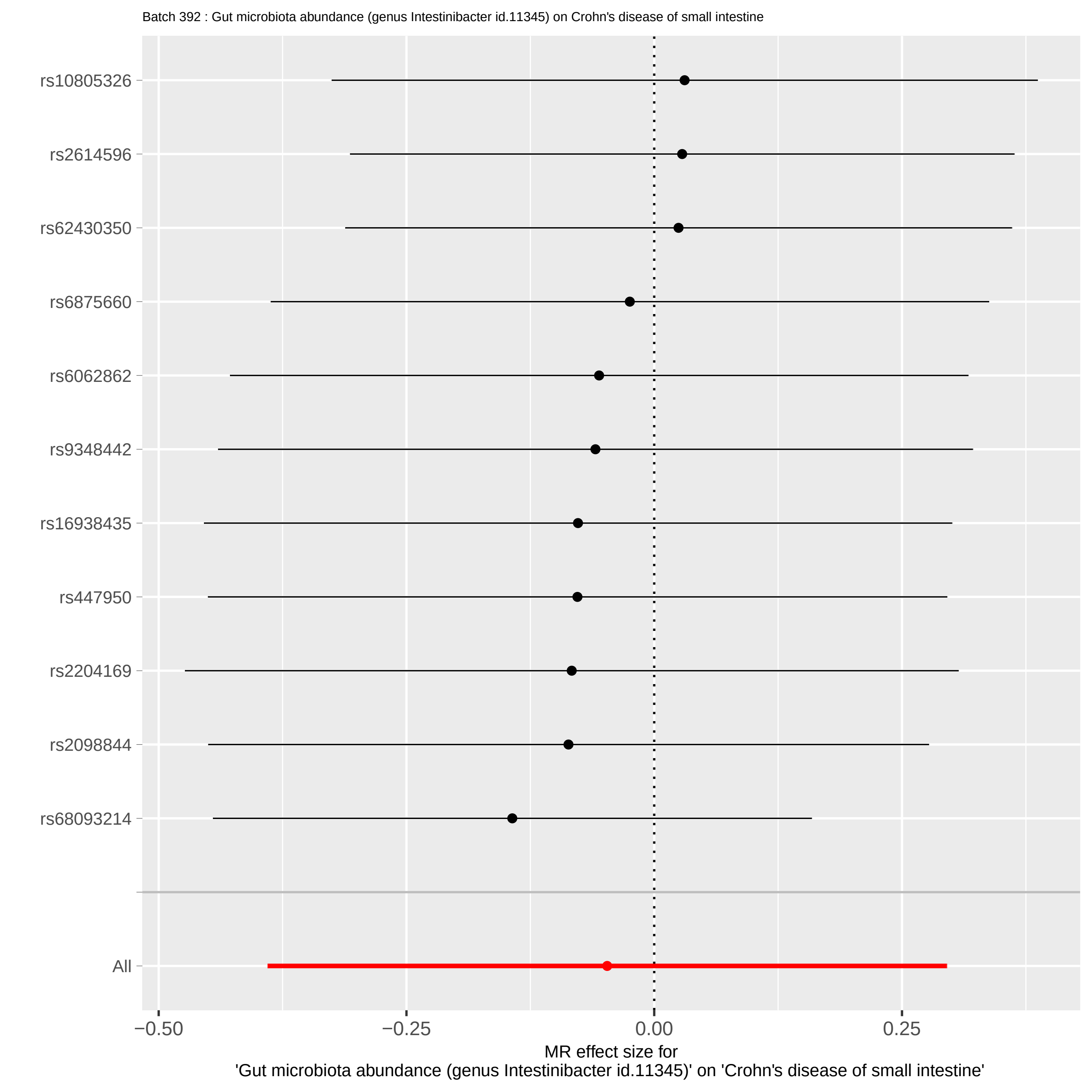
Batch 392 : Gut microbiota abundance (genus Intestinibacter id.11345) on Crohn's disease of small intestine
rs10805326
rs2614596
rs62430350
rs6875660
rs6062862
rs9348442
rs16938435
rs447950
rs2204169
rs2098844
rs68093214
All
−0.50
−0.25
0.00
0.25
MR effect size for
'Gut microbiota abundance (genus Intestinibacter id.11345)' on 'Crohn's disease of small intestine'

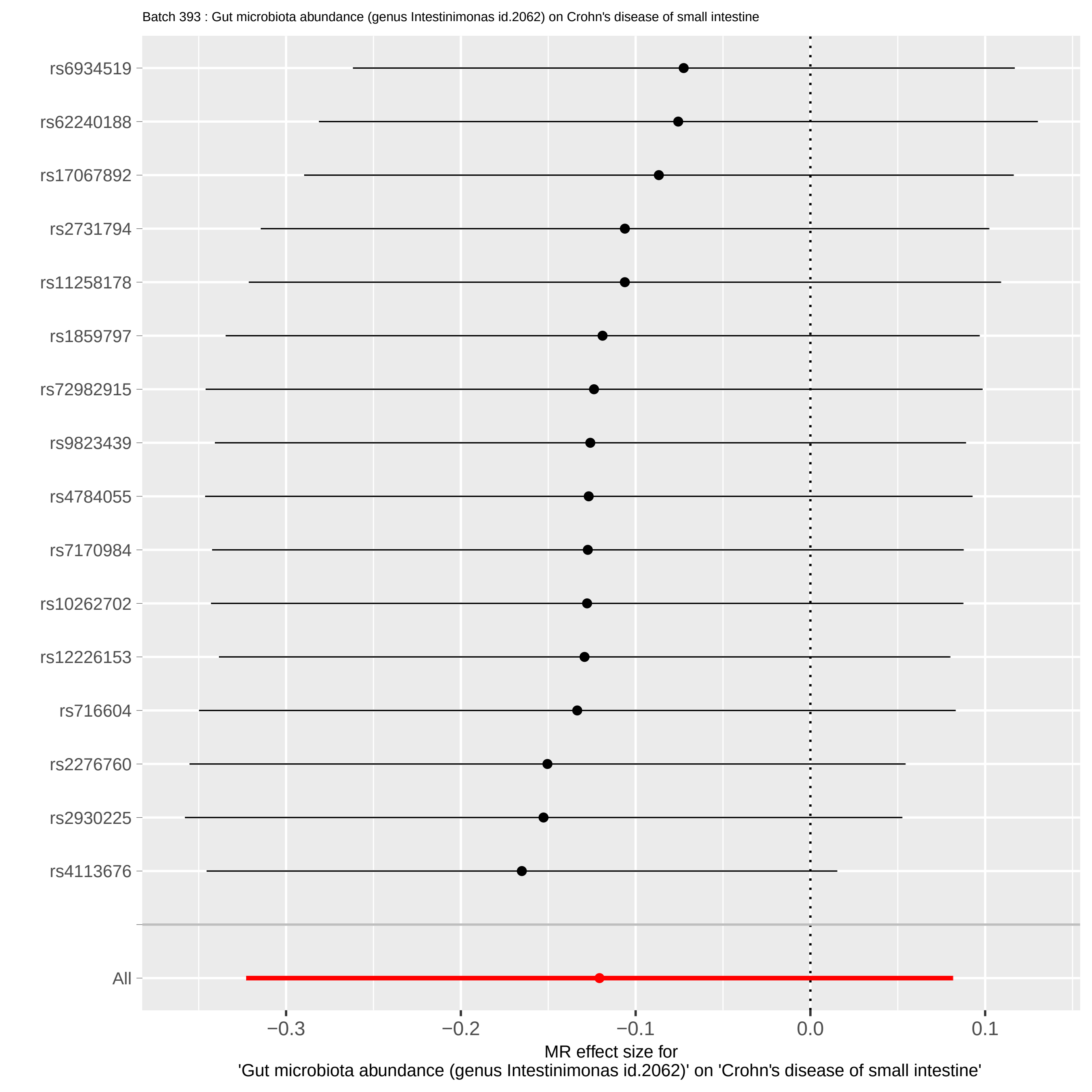
Batch 393 : Gut microbiota abundance (genus Intestinimonas id.2062) on Crohn's disease of small intestine
rs6934519
rs62240188
rs17067892
rs2731794
rs11258178
rs1859797
rs72982915
rs9823439
rs4784055
rs7170984
rs10262702
rs12226153
rs716604
rs2276760
rs2930225
rs4113676
All
−0.3
−0.2
−0.1
0.0
0.1
MR effect size for
'Gut microbiota abundance (genus Intestinimonas id.2062)' on 'Crohn's disease of small intestine'

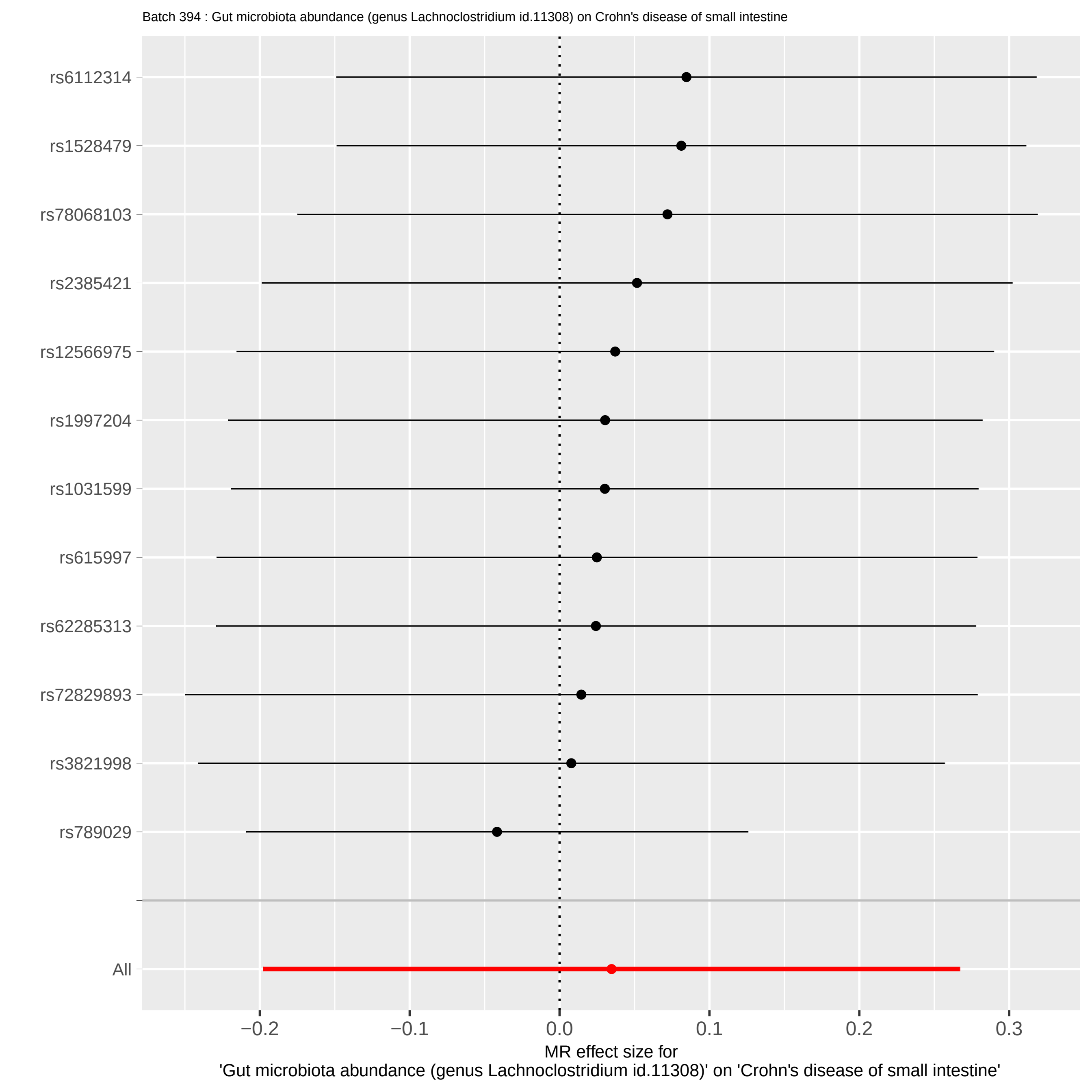
Batch 394 : Gut microbiota abundance (genus Lachnoclostridium id.11308) on Crohn's disease of small intestine
rs6112314
rs1528479
rs78068103
rs2385421
rs12566975
rs1997204
rs1031599
rs615997
rs62285313
rs72829893
rs3821998
rs789029
All
−0.2
−0.1
0.0
0.1
0.2
0.3
MR effect size for
'Gut microbiota abundance (genus Lachnoclostridium id.11308)' on 'Crohn's disease of small intestine'

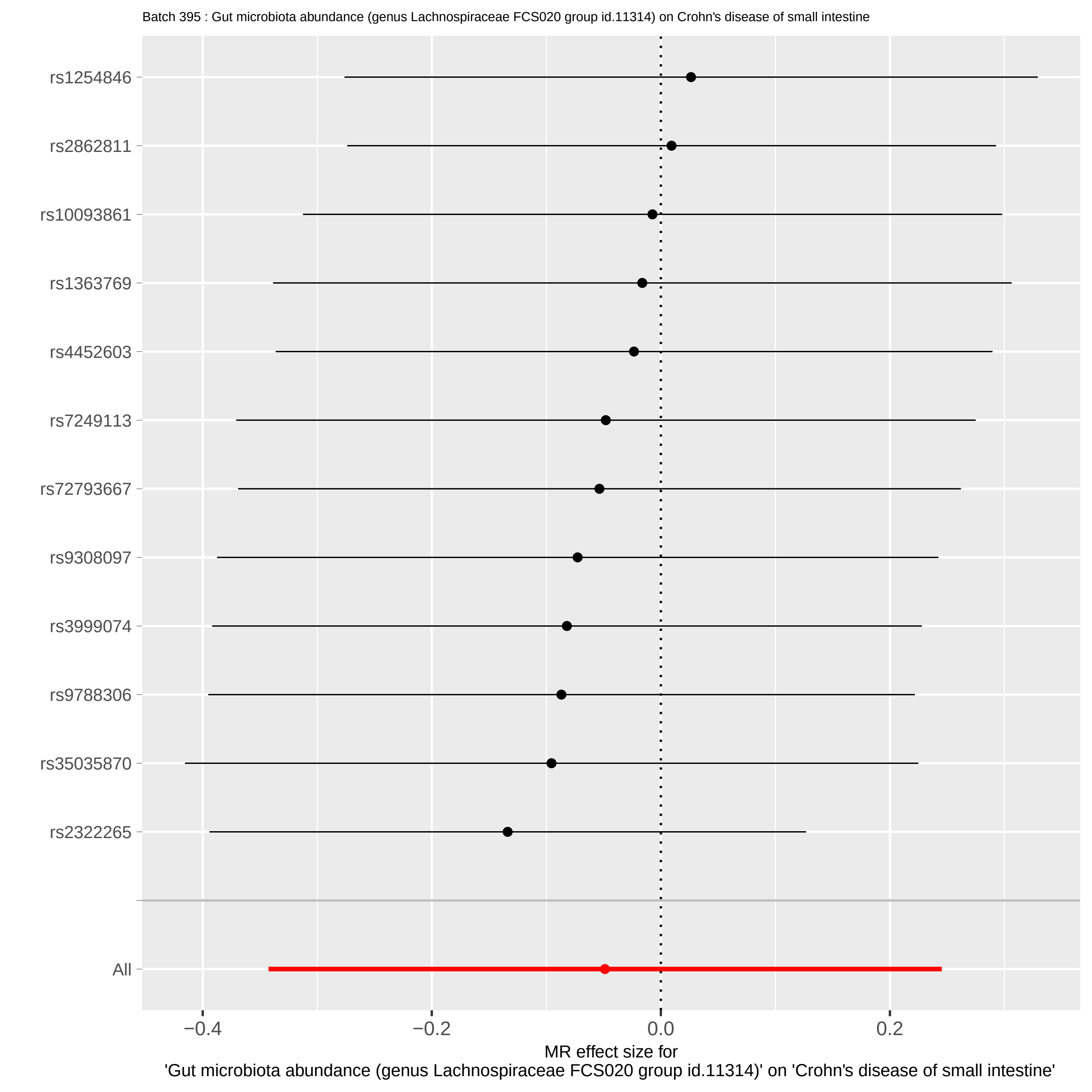

Batch 395 : Gut microbiota abundance (genus Lachnospiraceae FCS020 group id.11314) on Crohn's disease of small intestine
rs1254846
rs2862811
rs10093861
rs1363769
rs4452603
rs7249113
rs72793667
rs9308097
rs3999074
rs9788306
rs35035870
rs2322265
All
−0.4
−0.2
0.0
0.2
MR effect size for
'Gut microbiota abundance (genus Lachnospiraceae FCS020 group id.11314)' on 'Crohn's disease of small intestine'

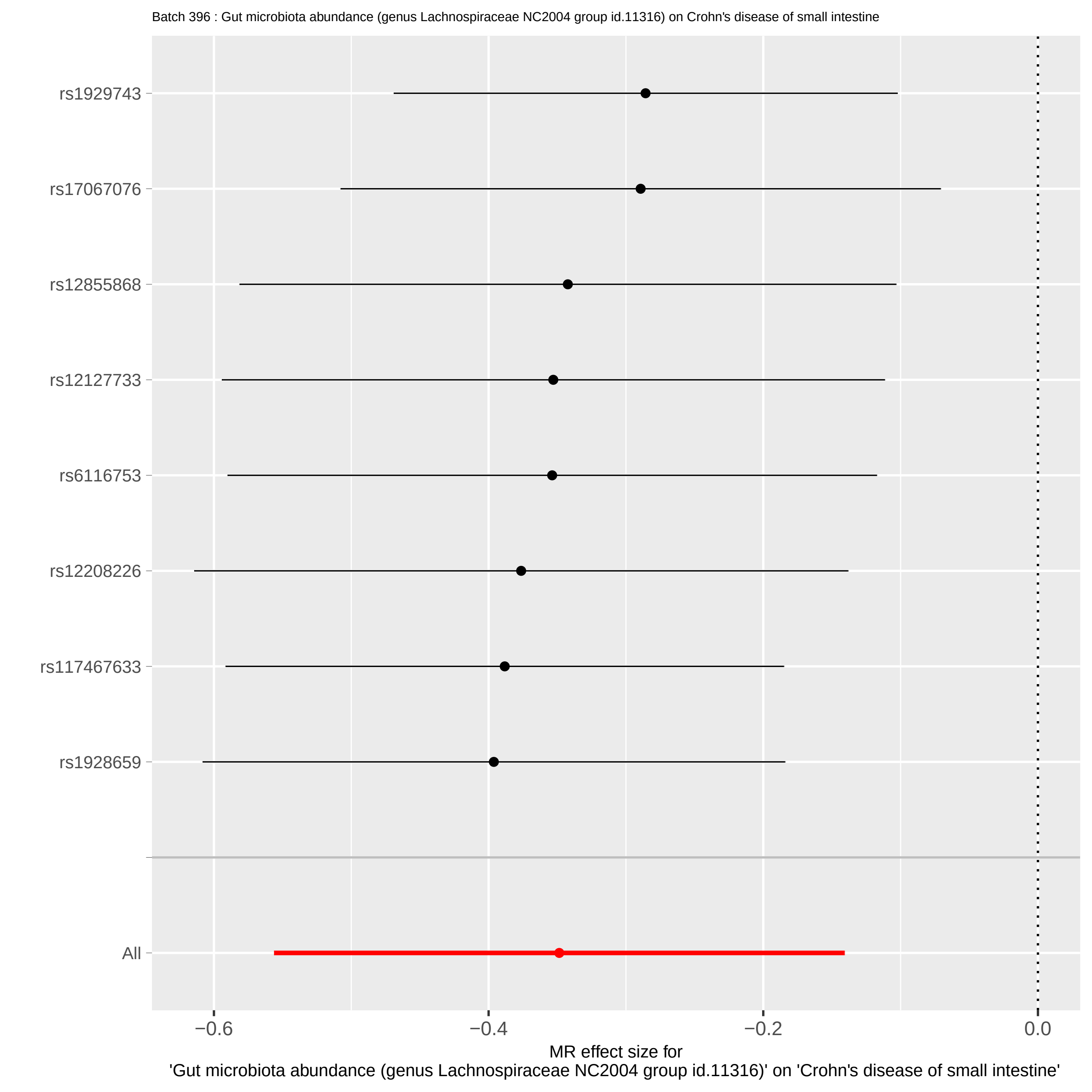

Batch 396 : Gut microbiota abundance (genus Lachnospiraceae NC2004 group id.11316) on Crohn's disease of small intestine
rs1929743
rs17067076
rs12855868
rs12127733
rs6116753
rs12208226
rs117467633
rs1928659
All
−0.6
−0.4
−0.2
0.0
MR effect size for
'Gut microbiota abundance (genus Lachnospiraceae NC2004 group id.11316)' on 'Crohn's disease of small intestine'

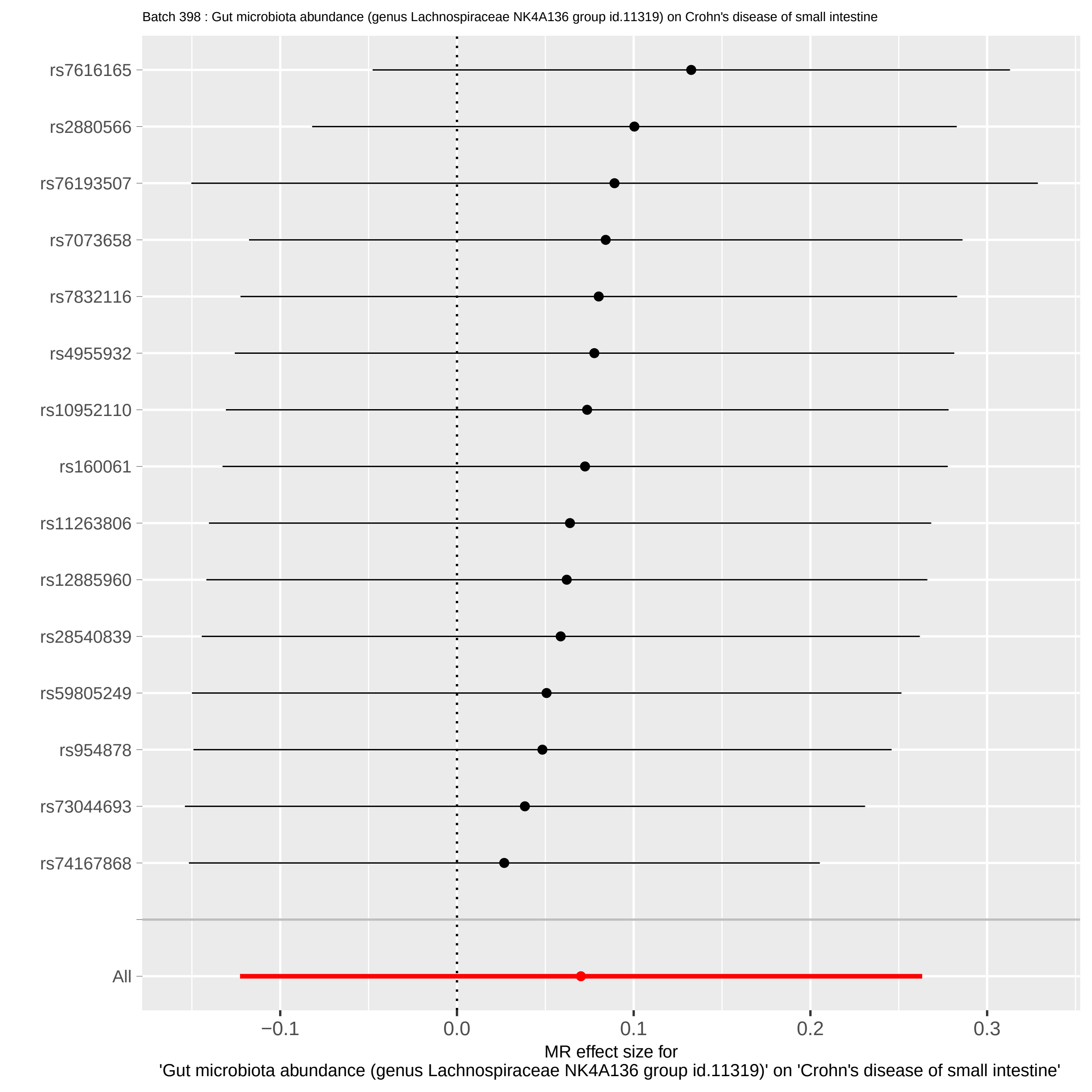
Batch 398 : Gut microbiota abundance (genus Lachnospiraceae NK4A136 group id.11319) on Crohn's disease of small intestine
rs7616165
rs2880566
rs76193507
rs7073658
rs7832116
rs4955932
rs10952110
rs160061
rs11263806
rs12885960
rs28540839
rs59805249
rs954878
rs73044693
rs74167868
All
−0.1
0.0
0.1
0.2
0.3
MR effect size for
'Gut microbiota abundance (genus Lachnospiraceae NK4A136 group id.11319)' on 'Crohn's disease of small intestine'

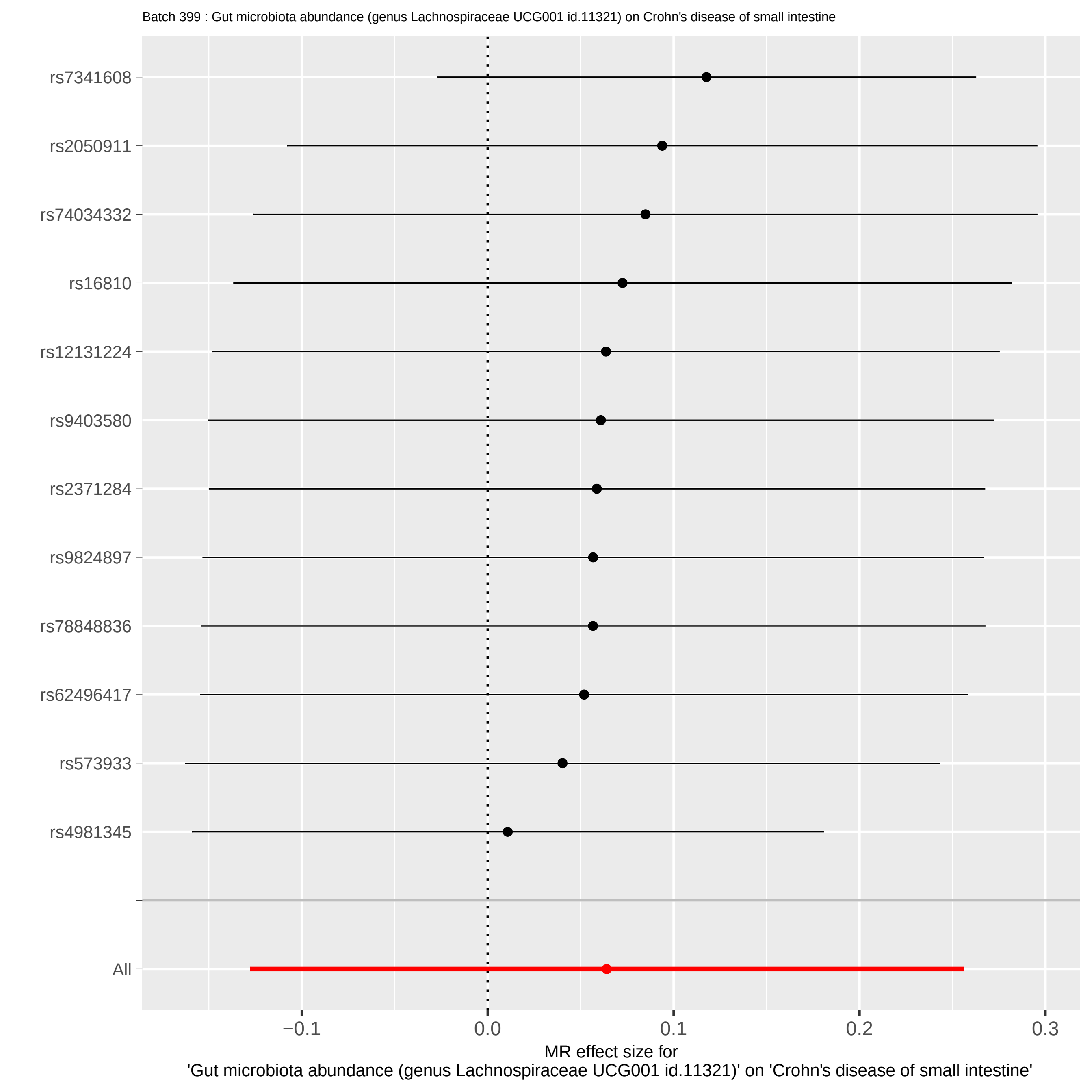

Batch 399 : Gut microbiota abundance (genus Lachnospiraceae UCG001 id.11321) on Crohn's disease of small intestine
rs7341608
rs2050911
rs74034332
rs16810
rs12131224
rs9403580
rs2371284
rs9824897
rs78848836
rs62496417
rs573933
rs4981345
All
−0.1
0.0
0.1
0.2
0.3
MR effect size for
'Gut microbiota abundance (genus Lachnospiraceae UCG001 id.11321)' on 'Crohn's disease of small intestine'

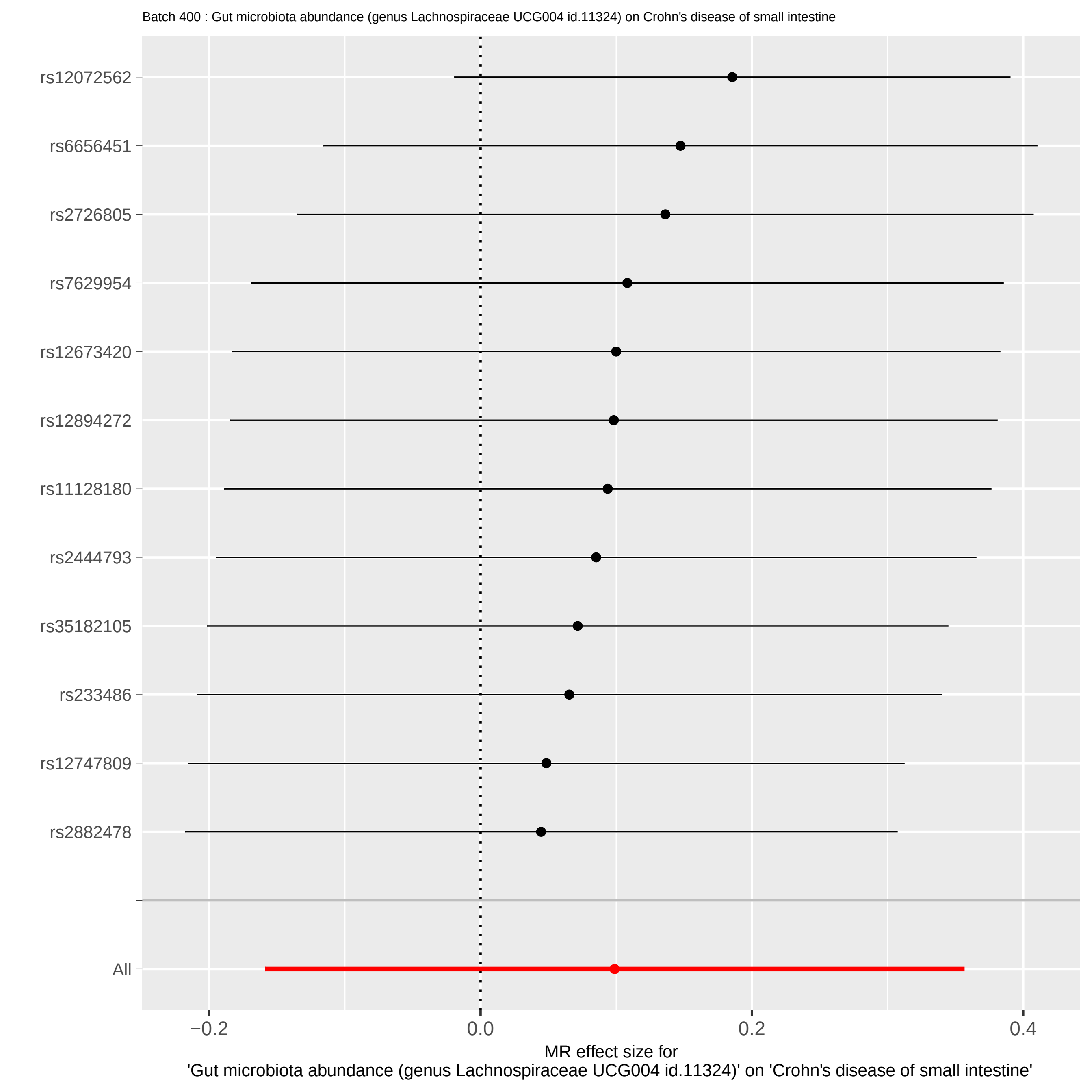

Batch 400 : Gut microbiota abundance (genus Lachnospiraceae UCG004 id.11324) on Crohn's disease of small intestine
rs12072562
rs6656451
rs2726805
rs7629954
rs12673420
rs12894272
rs11128180
rs2444793
rs35182105
rs233486
rs12747809
rs2882478
All
−0.2
0.0
0.2
0.4
MR effect size for
'Gut microbiota abundance (genus Lachnospiraceae UCG004 id.11324)' on 'Crohn's disease of small intestine'

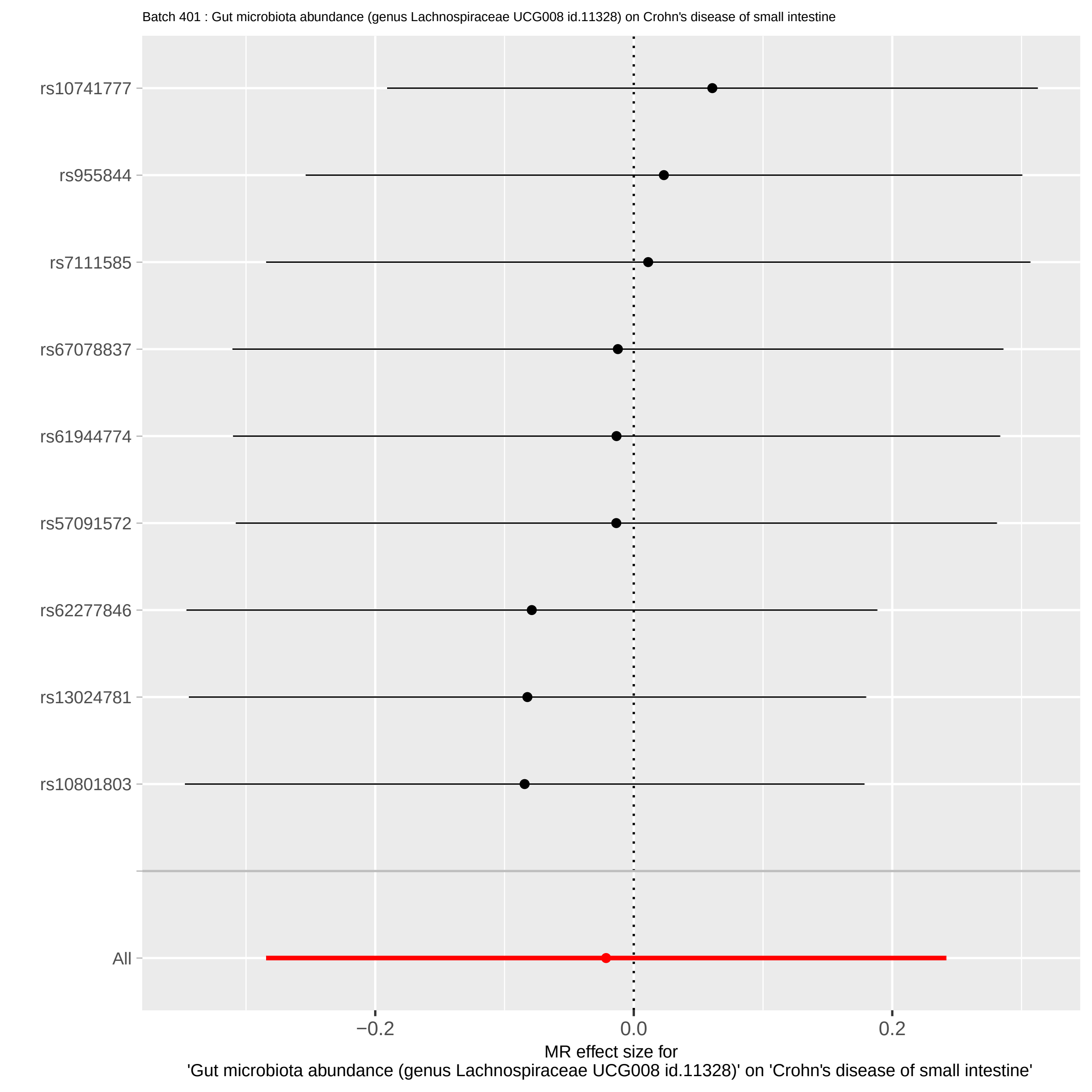
Batch 401 : Gut microbiota abundance (genus Lachnospiraceae UCG008 id.11328) on Crohn's disease of small intestine
rs10741777
rs955844
rs7111585
rs67078837
rs61944774
rs57091572
rs62277846
rs13024781
rs10801803
All
−0.2
0.0
0.2
MR effect size for
'Gut microbiota abundance (genus Lachnospiraceae UCG008 id.11328)' on 'Crohn's disease of small intestine'

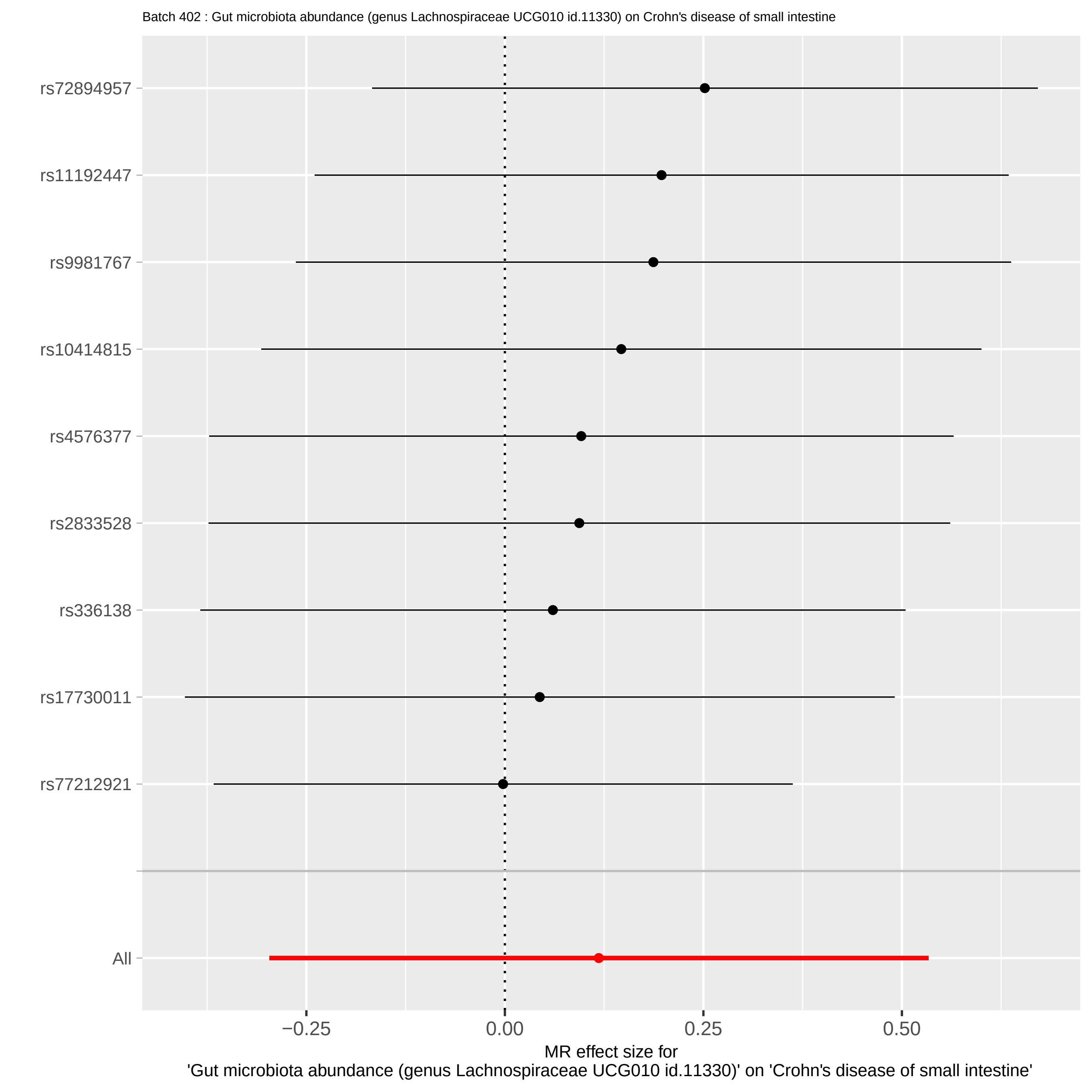
Batch 402 : Gut microbiota abundance (genus Lachnospiraceae UCG010 id.11330) on Crohn's disease of small intestine
rs72894957
rs11192447
rs9981767
rs10414815
rs4576377
rs2833528
rs336138
rs17730011
rs77212921
All
−0.25
0.00
0.25
0.50
MR effect size for
'Gut microbiota abundance (genus Lachnospiraceae UCG010 id.11330)' on 'Crohn's disease of small intestine'

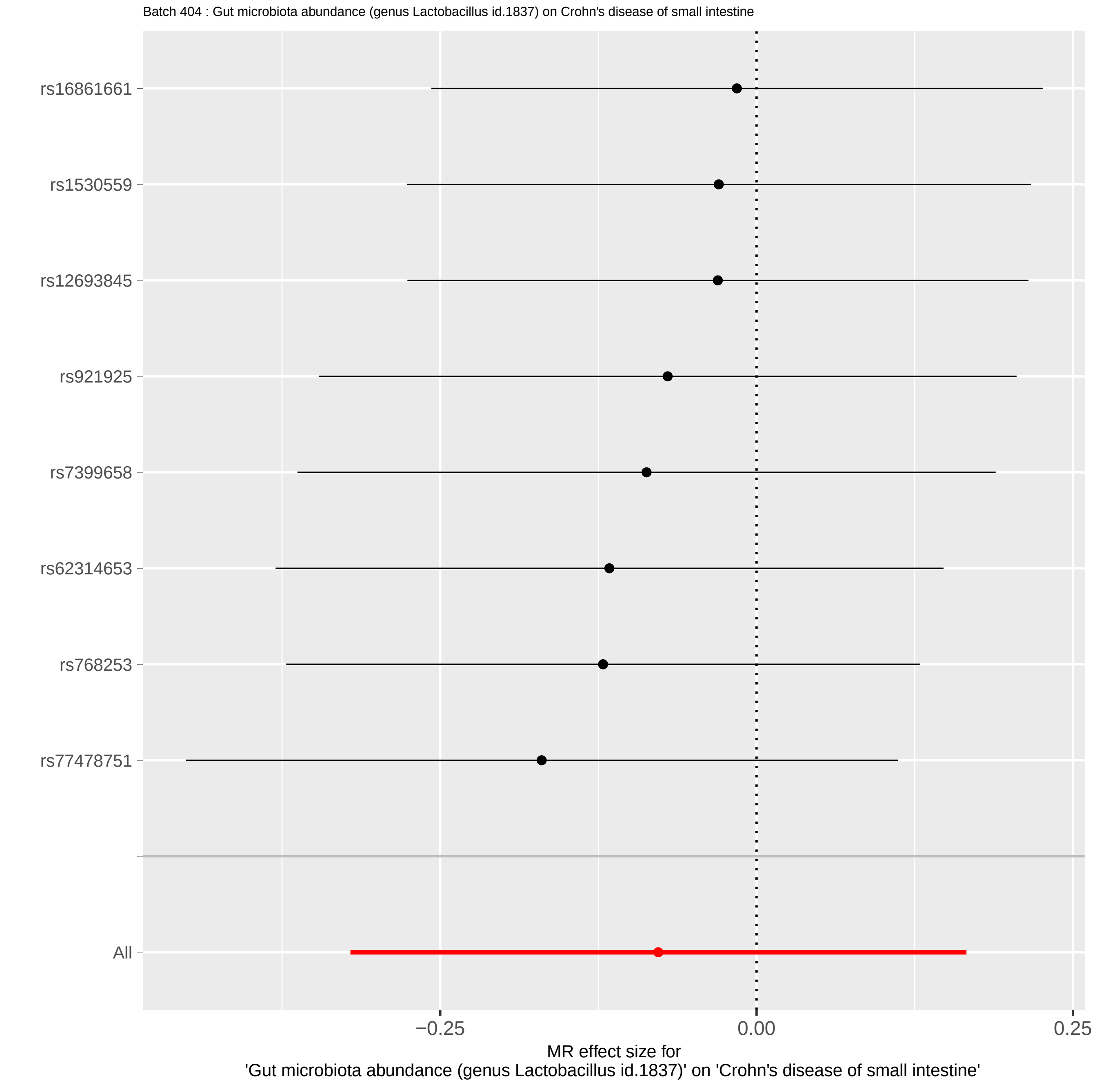
Batch 404 : Gut microbiota abundance (genus Lactobacillus id.1837) on Crohn's disease of small intestine
rs16861661
rs1530559
rs12693845
rs921925
rs7399658
rs62314653
rs768253
rs77478751
All
−0.25
0.00
0.25
MR effect size for
'Gut microbiota abundance (genus Lactobacillus id.1837)' on 'Crohn's disease of small intestine'

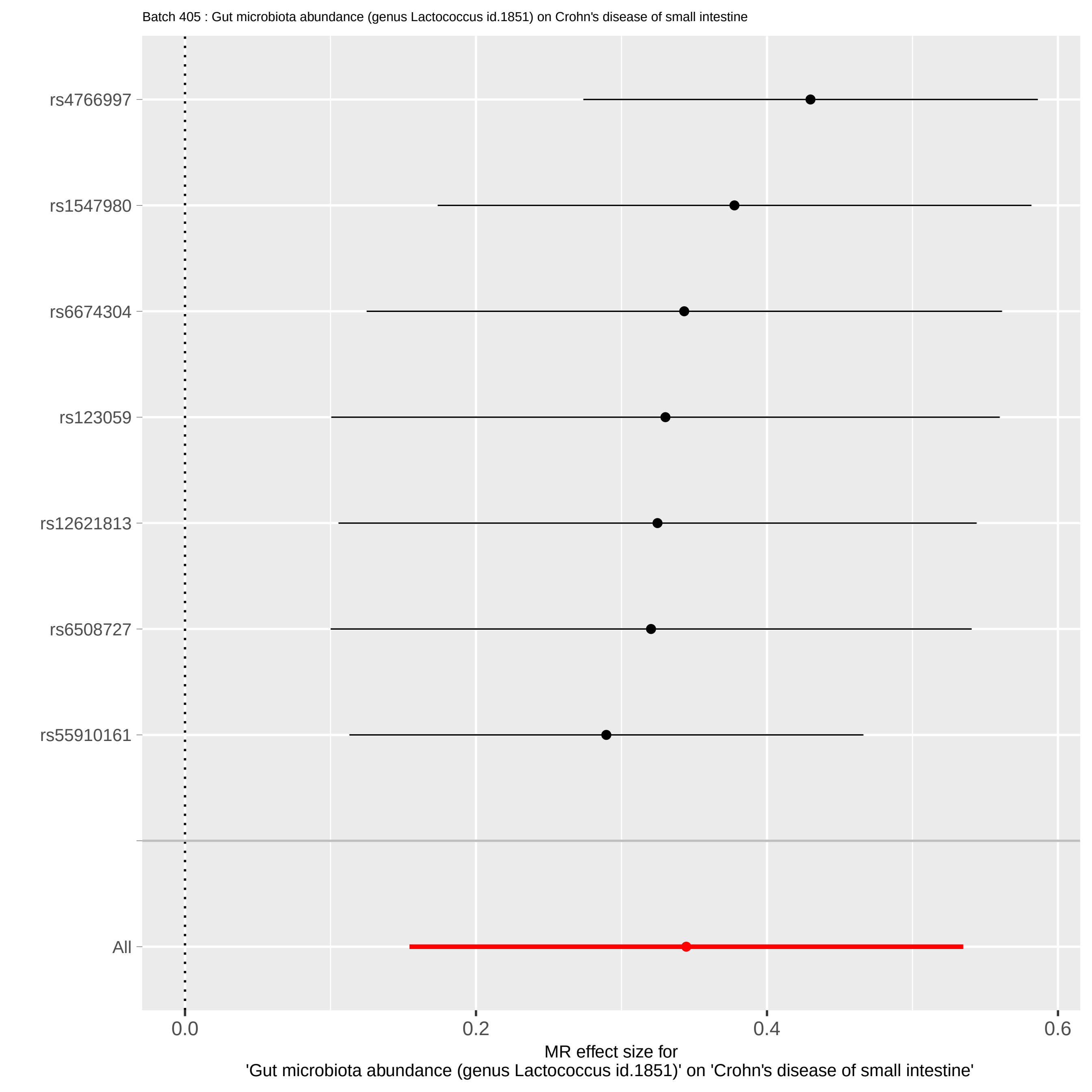
Batch 405 : Gut microbiota abundance (genus Lactococcus id.1851) on Crohn's disease of small intestine
rs4766997
rs1547980
rs6674304
rs123059
rs12621813
rs6508727
rs55910161
All
0.0
0.2
0.4
0.6
MR effect size for
'Gut microbiota abundance (genus Lactococcus id.1851)' on 'Crohn's disease of small intestine'

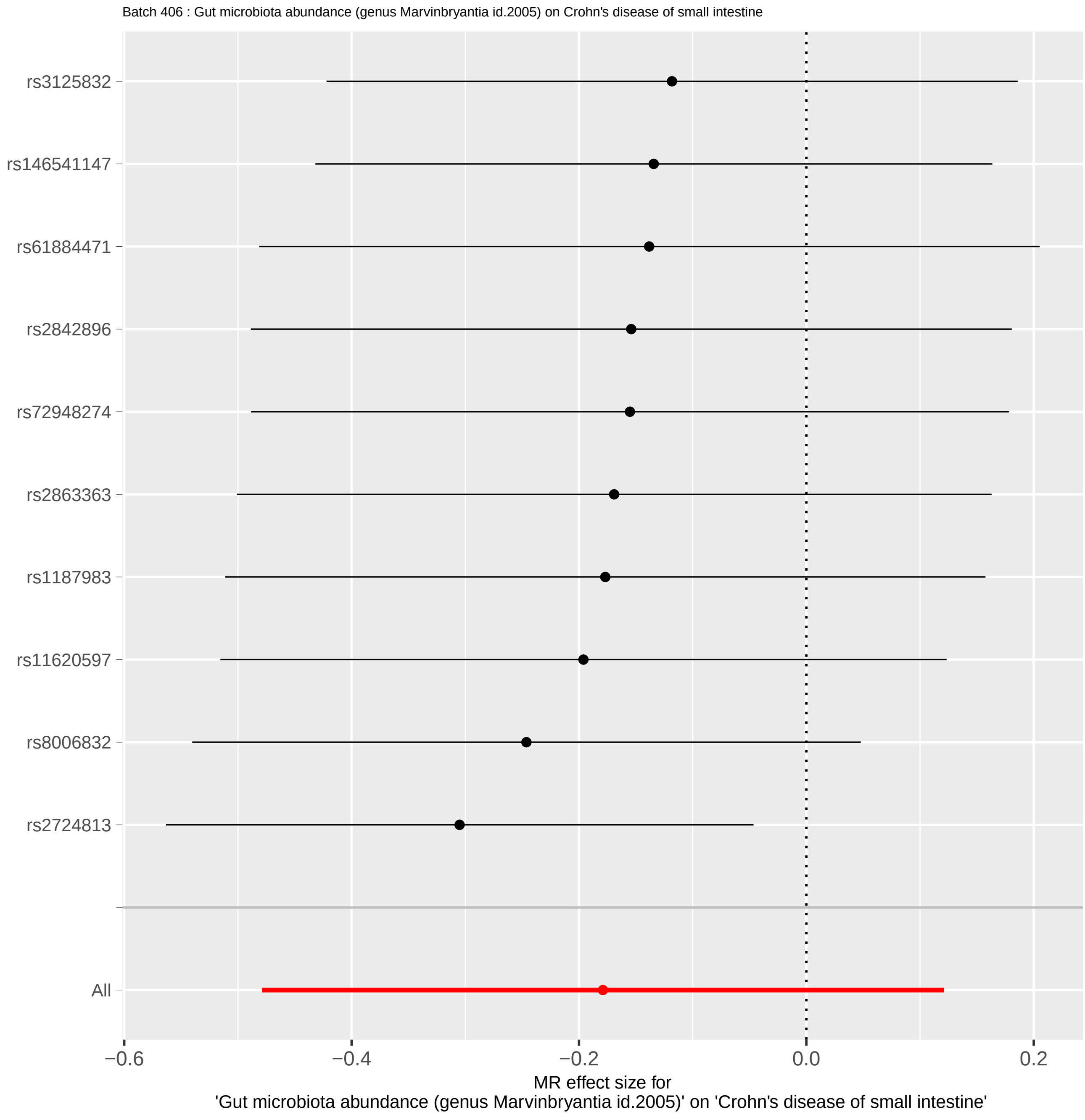

Batch 406 : Gut microbiota abundance (genus Marvinbryantia id.2005) on Crohn's disease of small intestine
rs3125832
rs146541147
rs61884471
rs2842896
rs72948274
rs2863363
rs1187983
rs11620597
rs8006832
rs2724813
All
−0.6
−0.4
−0.2
0.0
0.2
MR effect size for
'Gut microbiota abundance (genus Marvinbryantia id.2005)' on 'Crohn's disease of small intestine'

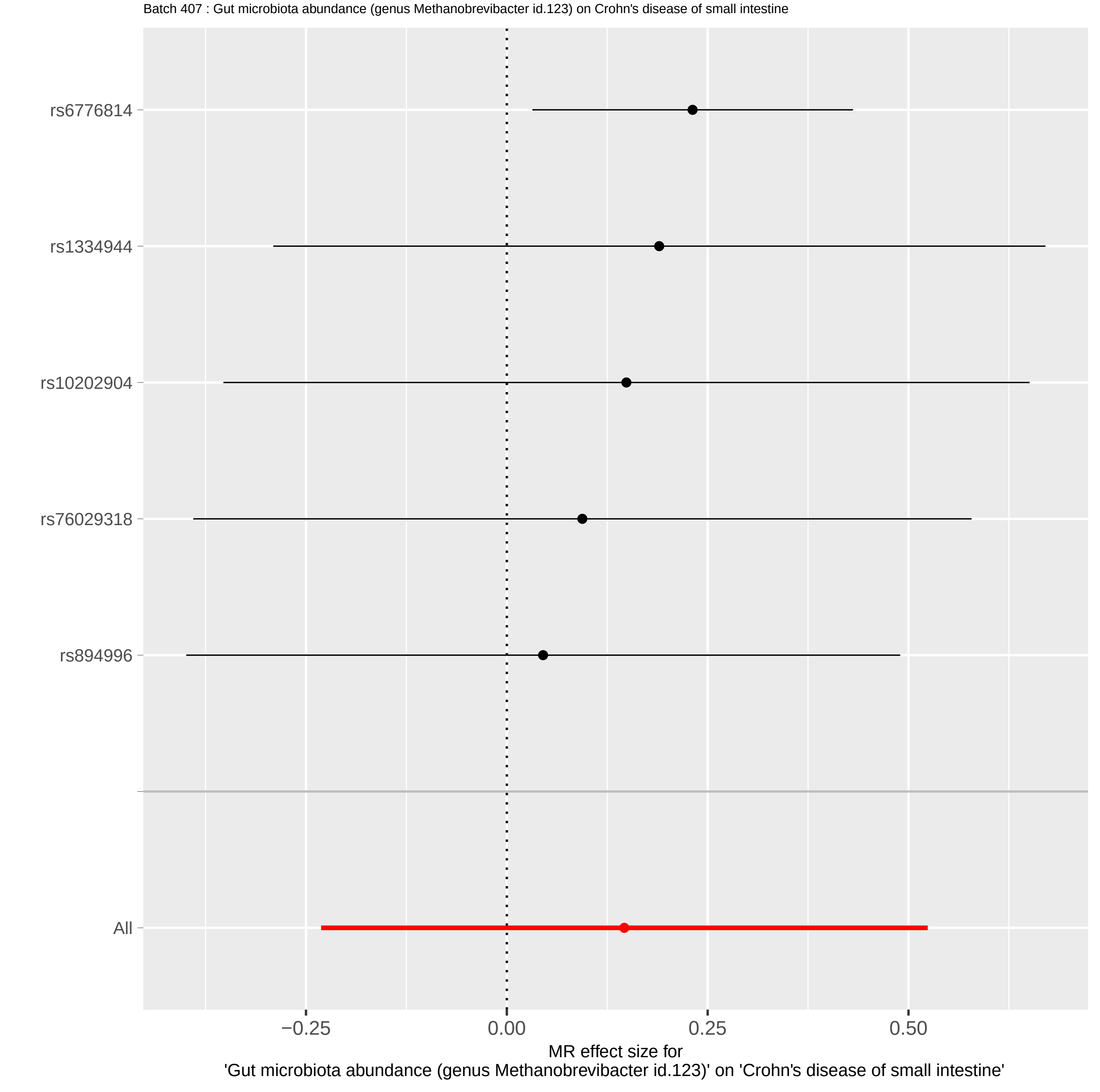
Batch 407 : Gut microbiota abundance (genus Methanobrevibacter id.123) on Crohn's disease of small intestine
rs6776814
rs1334944
rs10202904
rs76029318
rs894996
All
−0.25
0.00
0.25
0.50
MR effect size for
'Gut microbiota abundance (genus Methanobrevibacter id.123)' on 'Crohn's disease of small intestine'

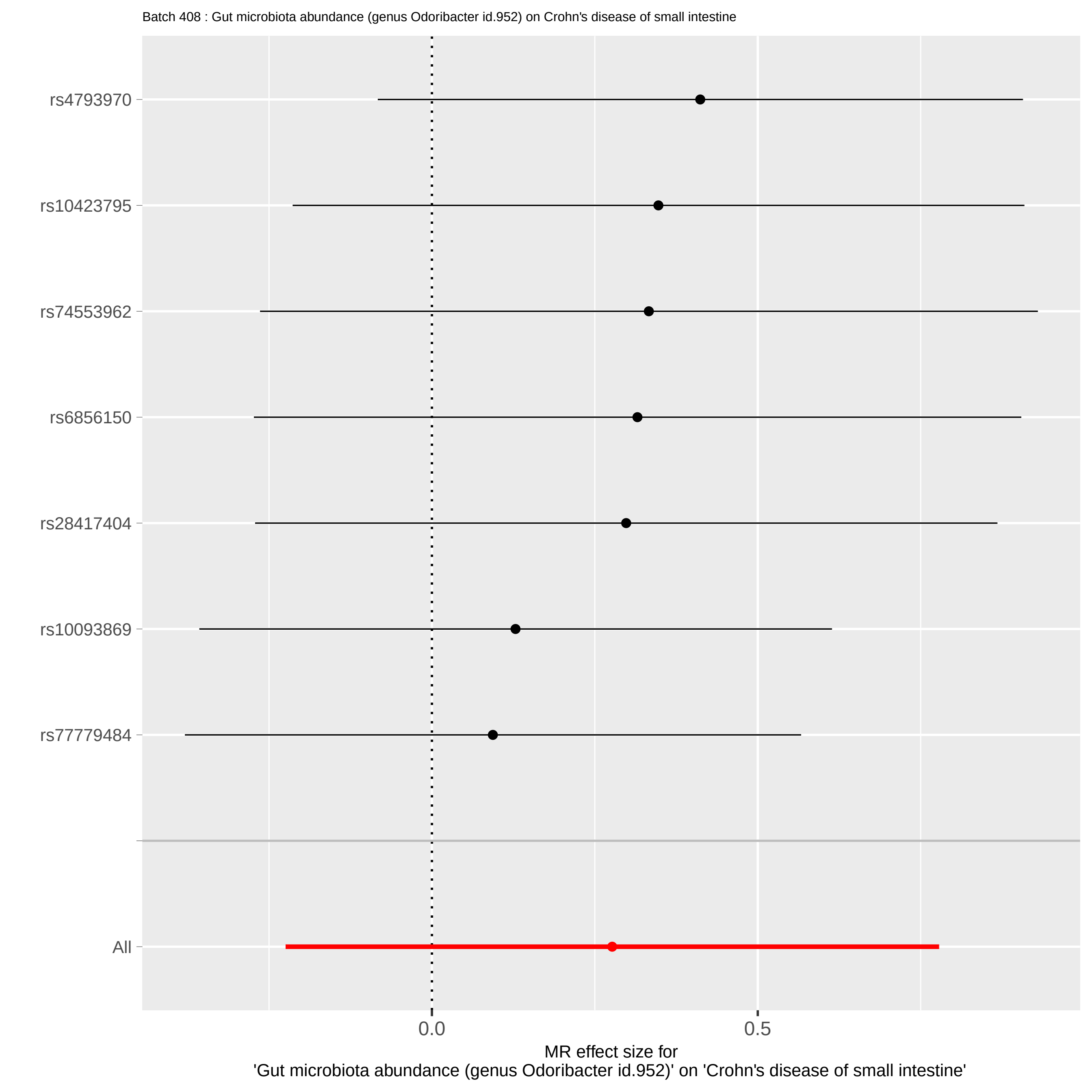

Batch 408 : Gut microbiota abundance (genus Odoribacter id.952) on Crohn's disease of small intestine
rs4793970
rs10423795
rs74553962
rs6856150
rs28417404
rs10093869
rs77779484
All
0.0
0.5
MR effect size for
'Gut microbiota abundance (genus Odoribacter id.952)' on 'Crohn's disease of small intestine'

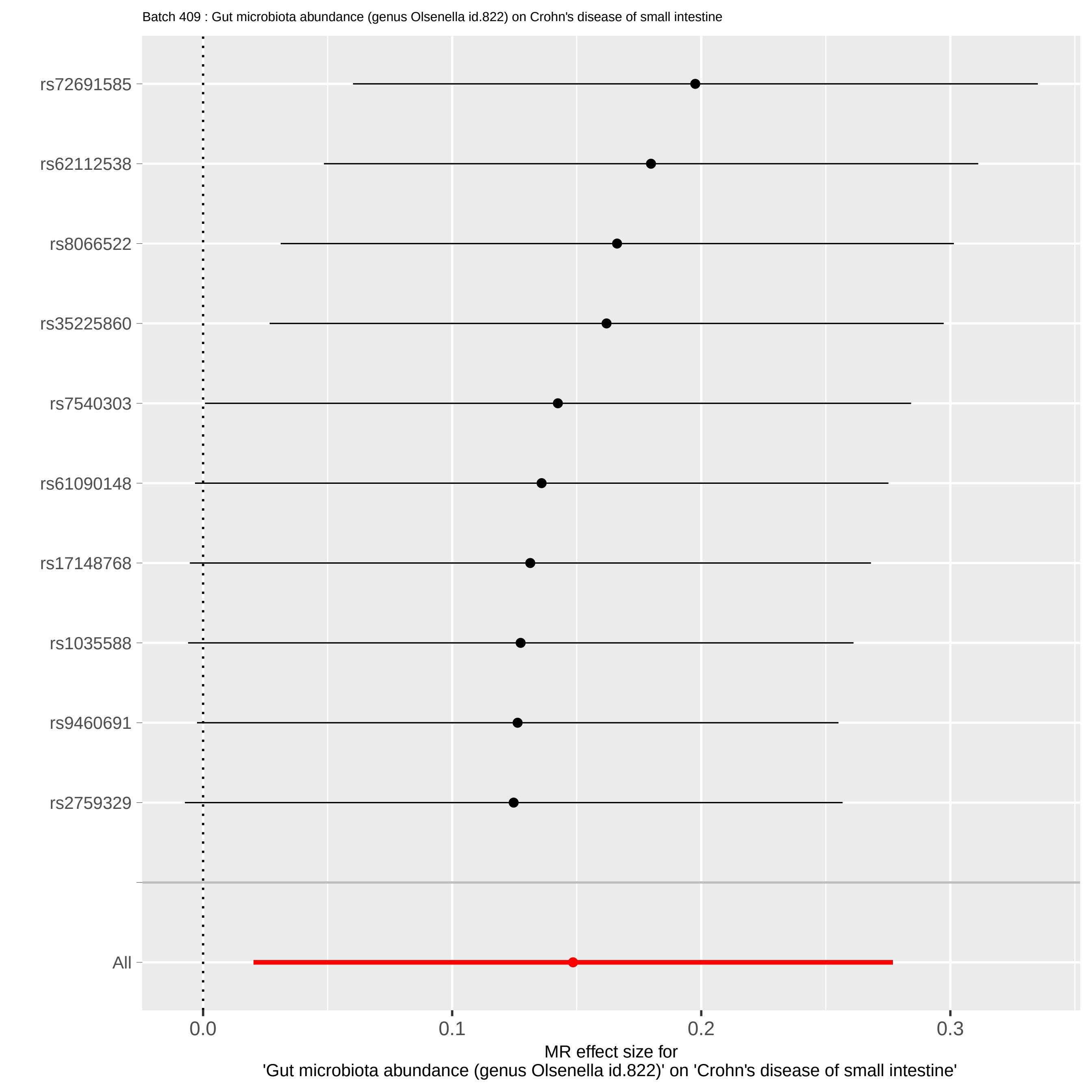

Batch 409 : Gut microbiota abundance (genus Olsenella id.822) on Crohn's disease of small intestine
rs72691585
rs62112538
rs8066522
rs35225860
rs7540303
rs61090148
rs17148768
rs1035588
rs9460691
rs2759329
All
0.0
0.1
0.2
0.3
MR effect size for
'Gut microbiota abundance (genus Olsenella id.822)' on 'Crohn's disease of small intestine'

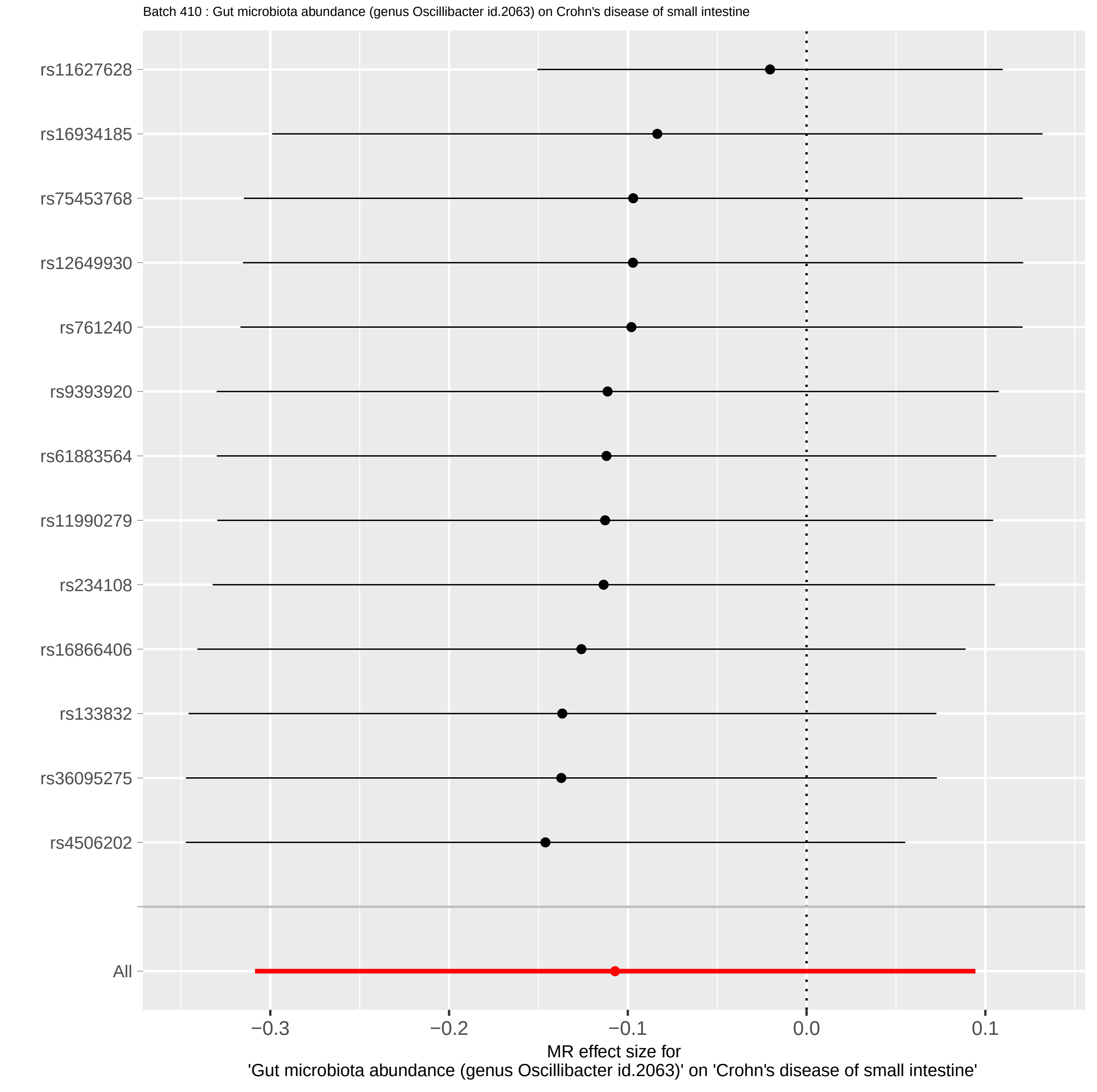

Batch 410 : Gut microbiota abundance (genus Oscillibacter id.2063) on Crohn's disease of small intestine
rs11627628
rs16934185
rs75453768
rs12649930
rs761240
rs9393920
rs61883564
rs11990279
rs234108
rs16866406
rs133832
rs36095275
rs4506202
All
−0.3
−0.2
−0.1
0.0
0.1
MR effect size for
'Gut microbiota abundance (genus Oscillibacter id.2063)' on 'Crohn's disease of small intestine'

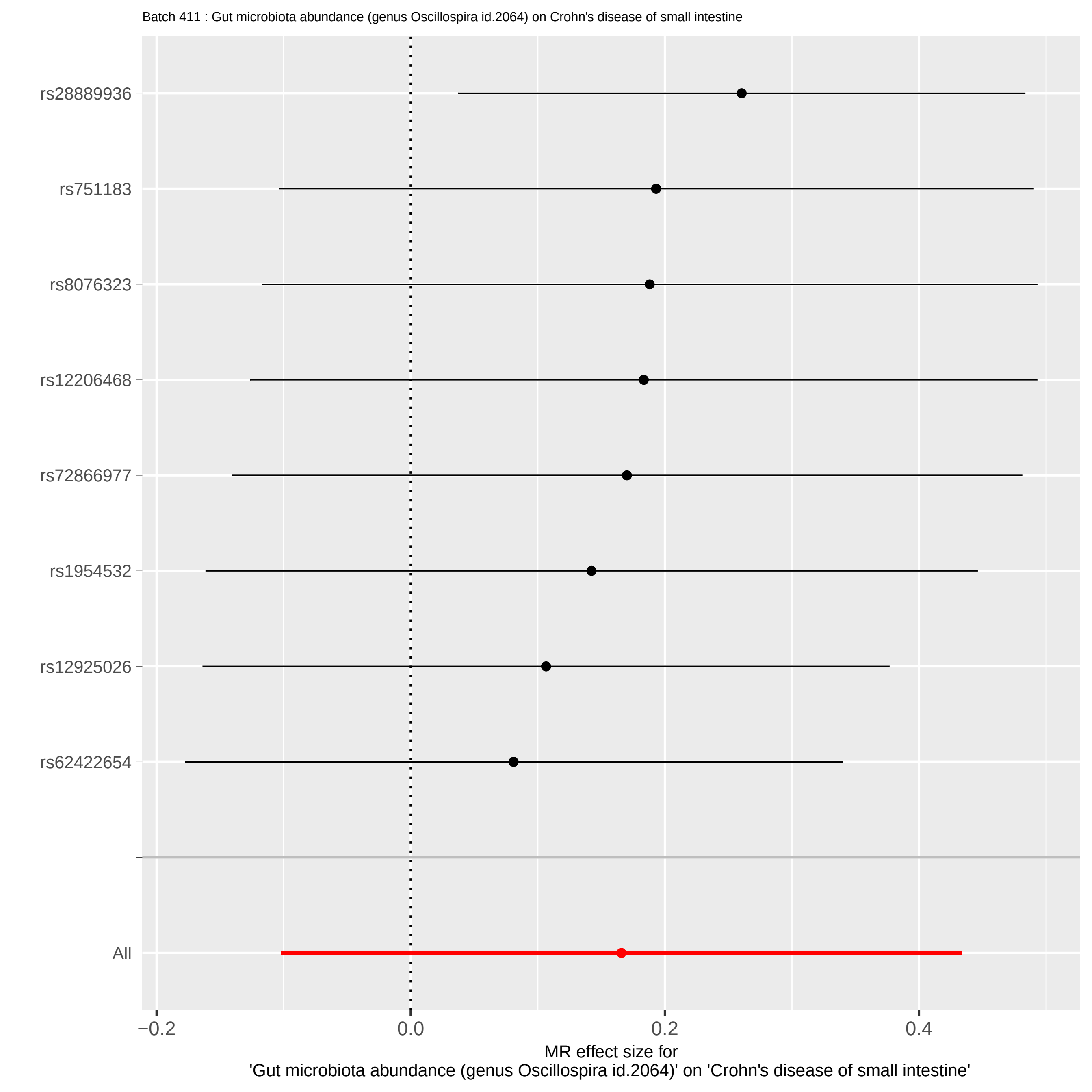
Batch 411 : Gut microbiota abundance (genus Oscillospira id.2064) on Crohn's disease of small intestine
rs28889936
rs751183
rs8076323
rs12206468
rs72866977
rs1954532
rs12925026
rs62422654
All
−0.2
0.0
0.2
0.4
MR effect size for
'Gut microbiota abundance (genus Oscillospira id.2064)' on 'Crohn's disease of small intestine'

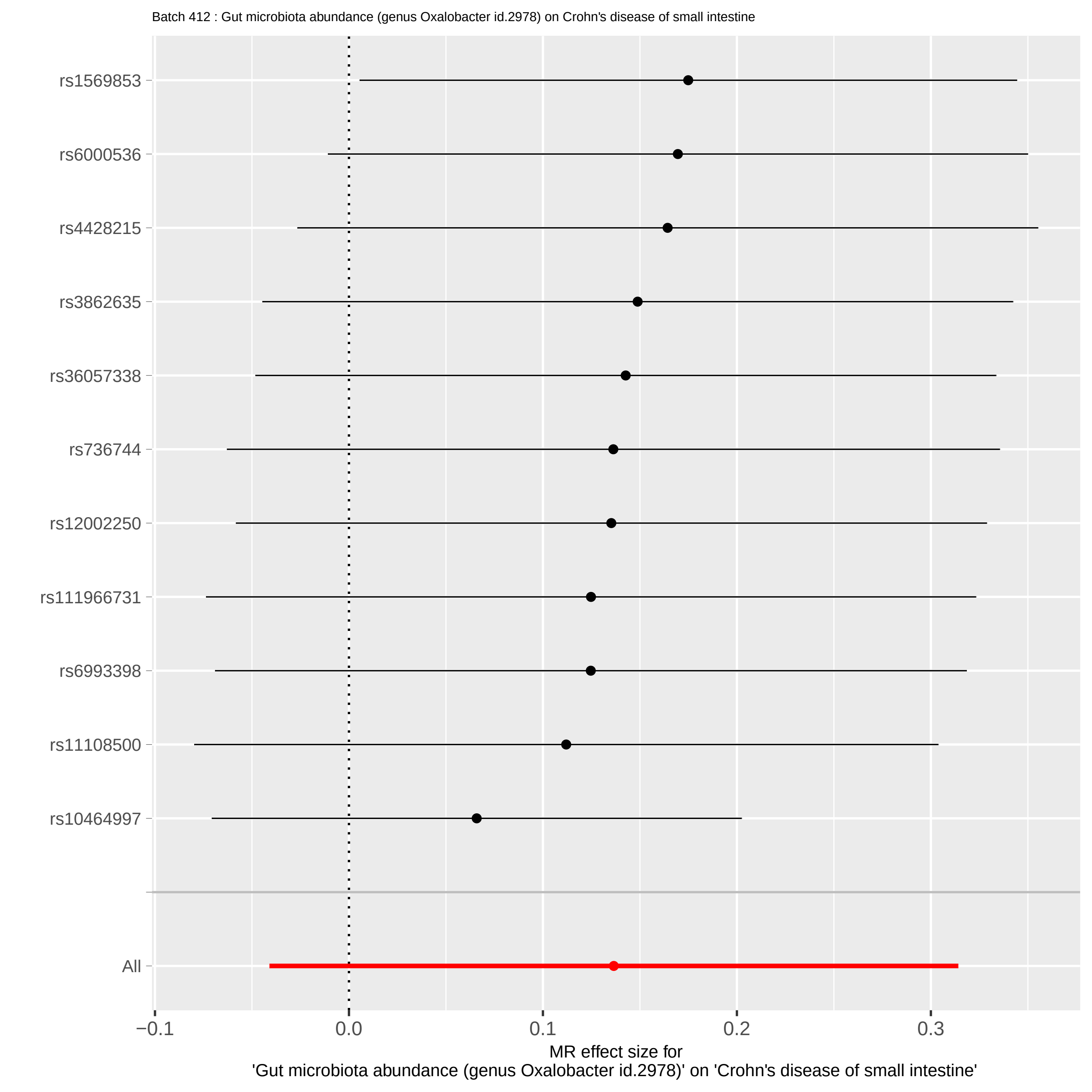
Batch 412 : Gut microbiota abundance (genus Oxalobacter id.2978) on Crohn's disease of small intestine
rs1569853
rs6000536
rs4428215
rs3862635
rs36057338
rs736744
rs12002250
rs111966731
rs6993398
rs11108500
rs10464997
All
−0.1
0.0
0.1
0.2
0.3
MR effect size for
'Gut microbiota abundance (genus Oxalobacter id.2978)' on 'Crohn's disease of small intestine'

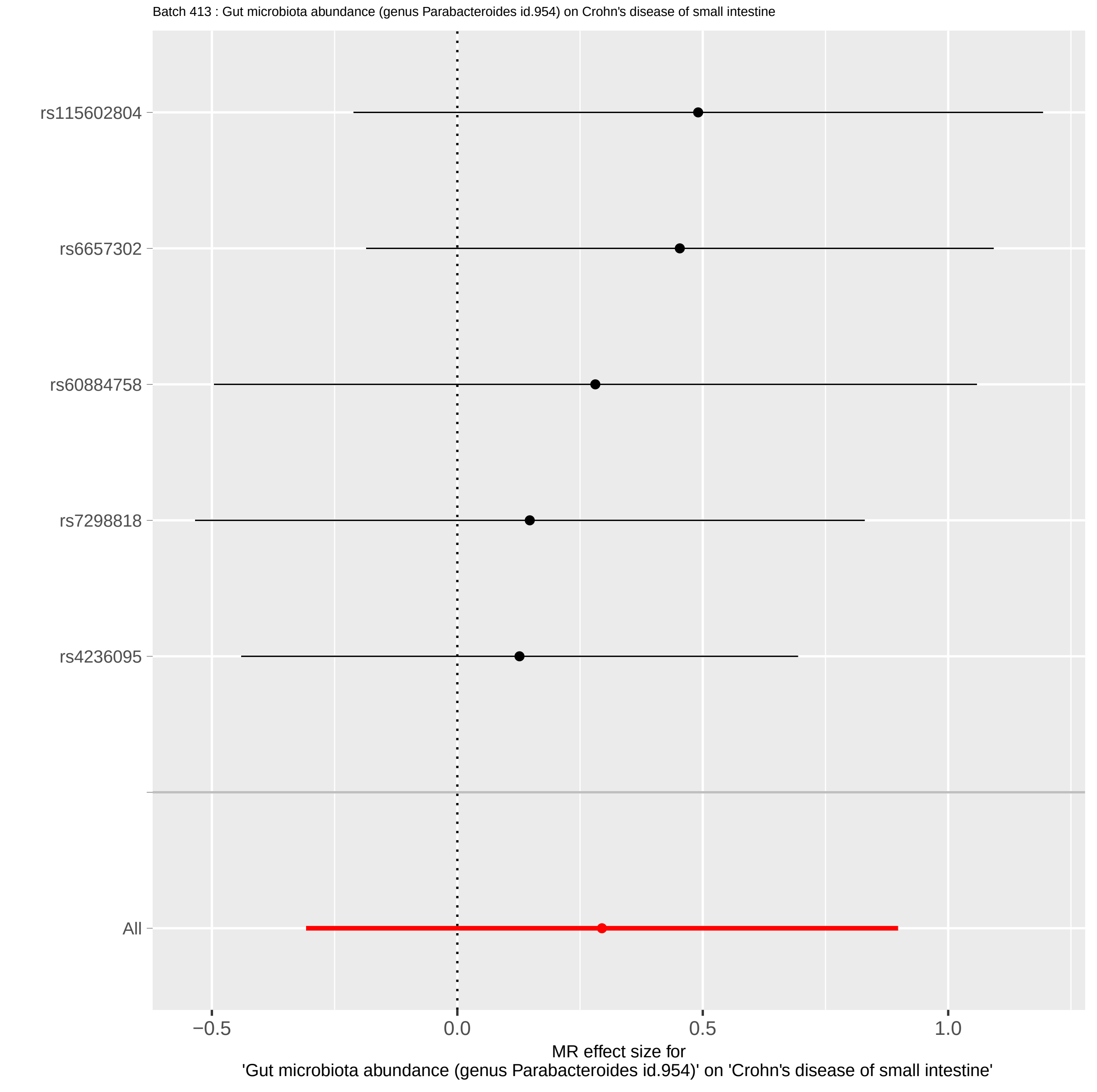
Batch 413 : Gut microbiota abundance (genus Parabacteroides id.954) on Crohn's disease of small intestine
rs115602804
rs6657302
rs60884758
rs7298818
rs4236095
All
−0.5
0.0
0.5
1.0
MR effect size for
'Gut microbiota abundance (genus Parabacteroides id.954)' on 'Crohn's disease of small intestine'

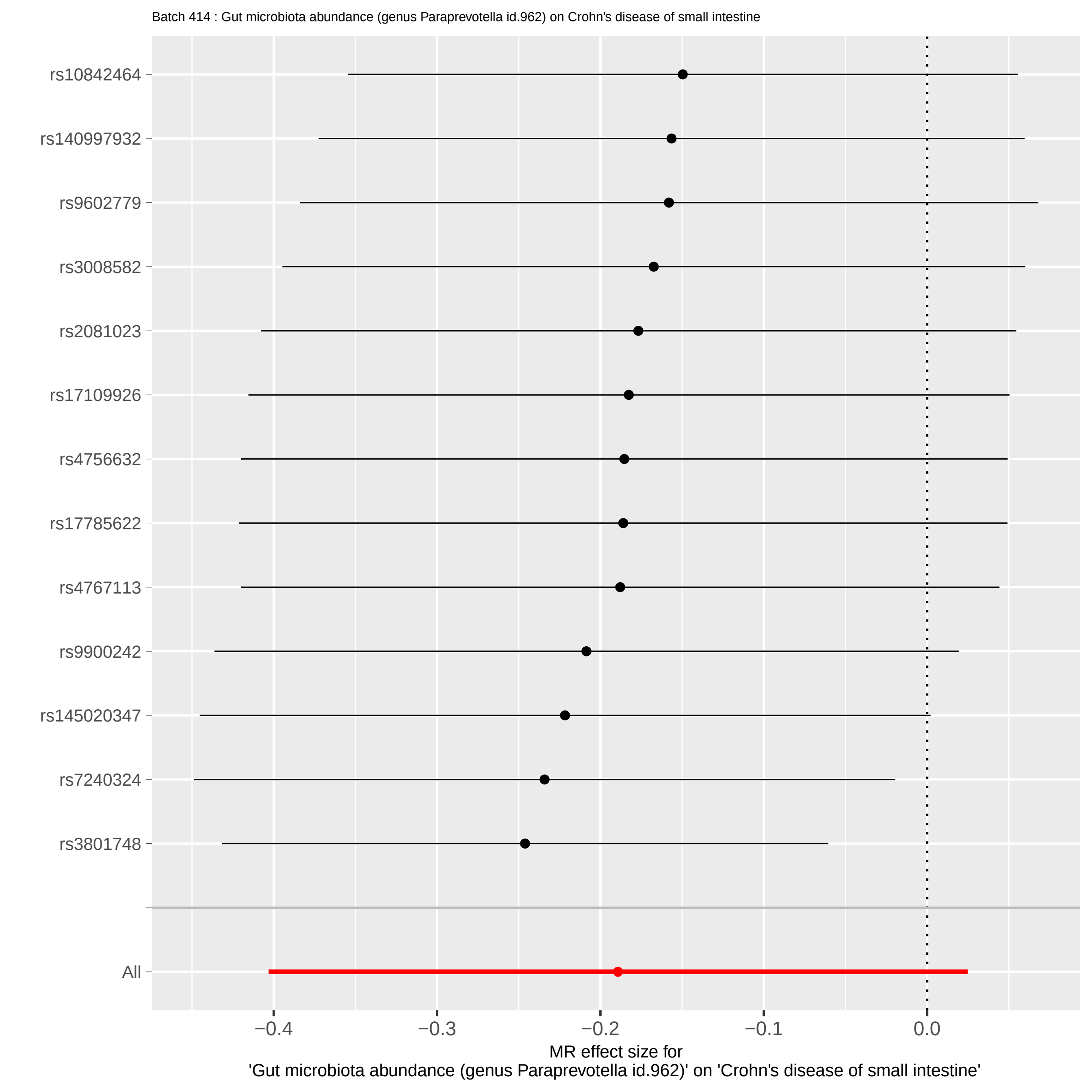
Batch 414 : Gut microbiota abundance (genus Paraprevotella id.962) on Crohn's disease of small intestine
rs10842464
rs140997932
rs9602779
rs3008582
rs2081023
rs17109926
rs4756632
rs17785622
rs4767113
rs9900242
rs145020347
rs7240324
rs3801748
All
−0.4
−0.3
−0.2
−0.1
0.0
MR effect size for
'Gut microbiota abundance (genus Paraprevotella id.962)' on 'Crohn's disease of small intestine'

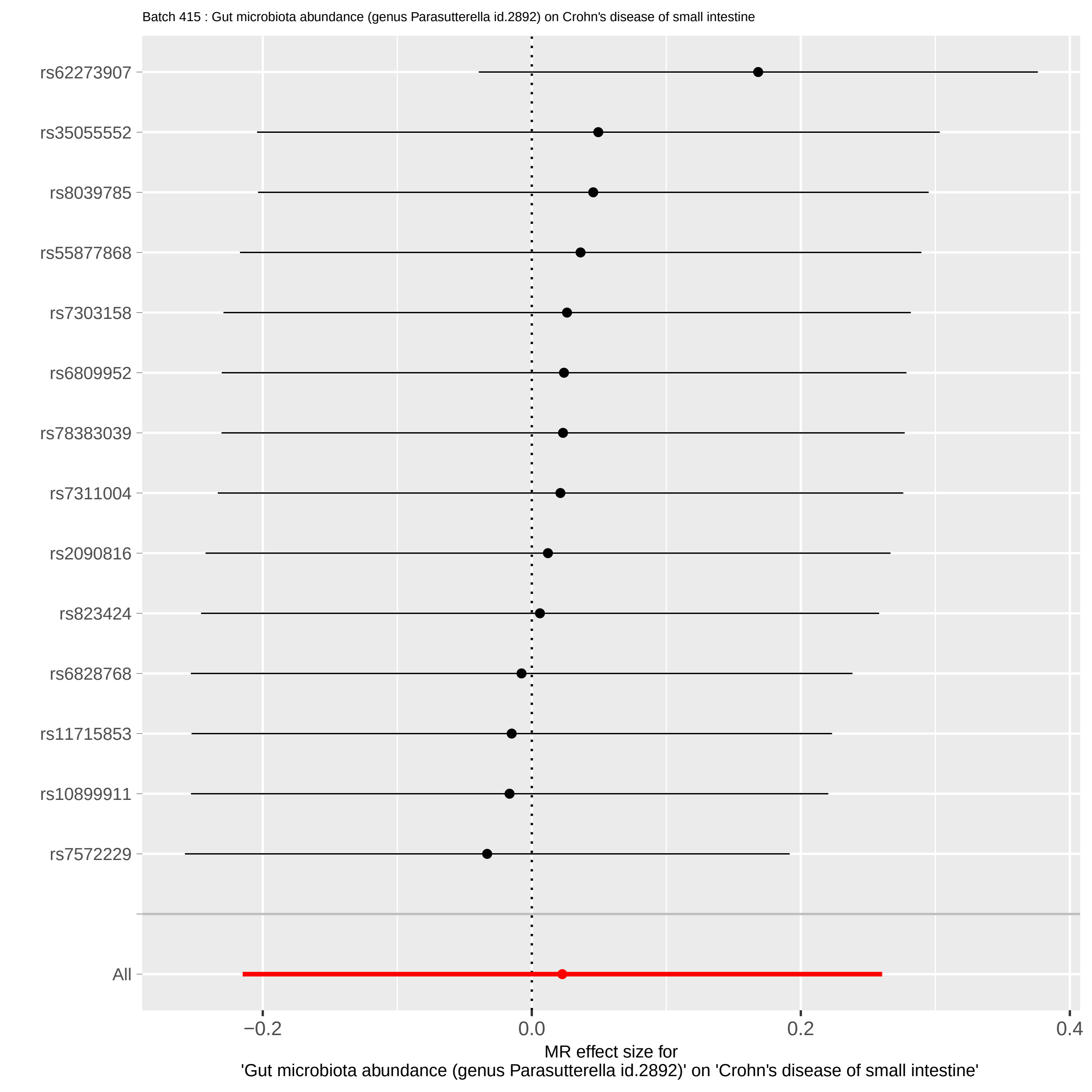

Batch 415 : Gut microbiota abundance (genus Parasutterella id.2892) on Crohn's disease of small intestine
rs62273907
rs35055552
rs8039785
rs55877868
rs7303158
rs6809952
rs78383039
rs7311004
rs2090816
rs823424
rs6828768
rs11715853
rs10899911
rs7572229
All
−0.2
0.0
0.2
0.4
MR effect size for
'Gut microbiota abundance (genus Parasutterella id.2892)' on 'Crohn's disease of small intestine'

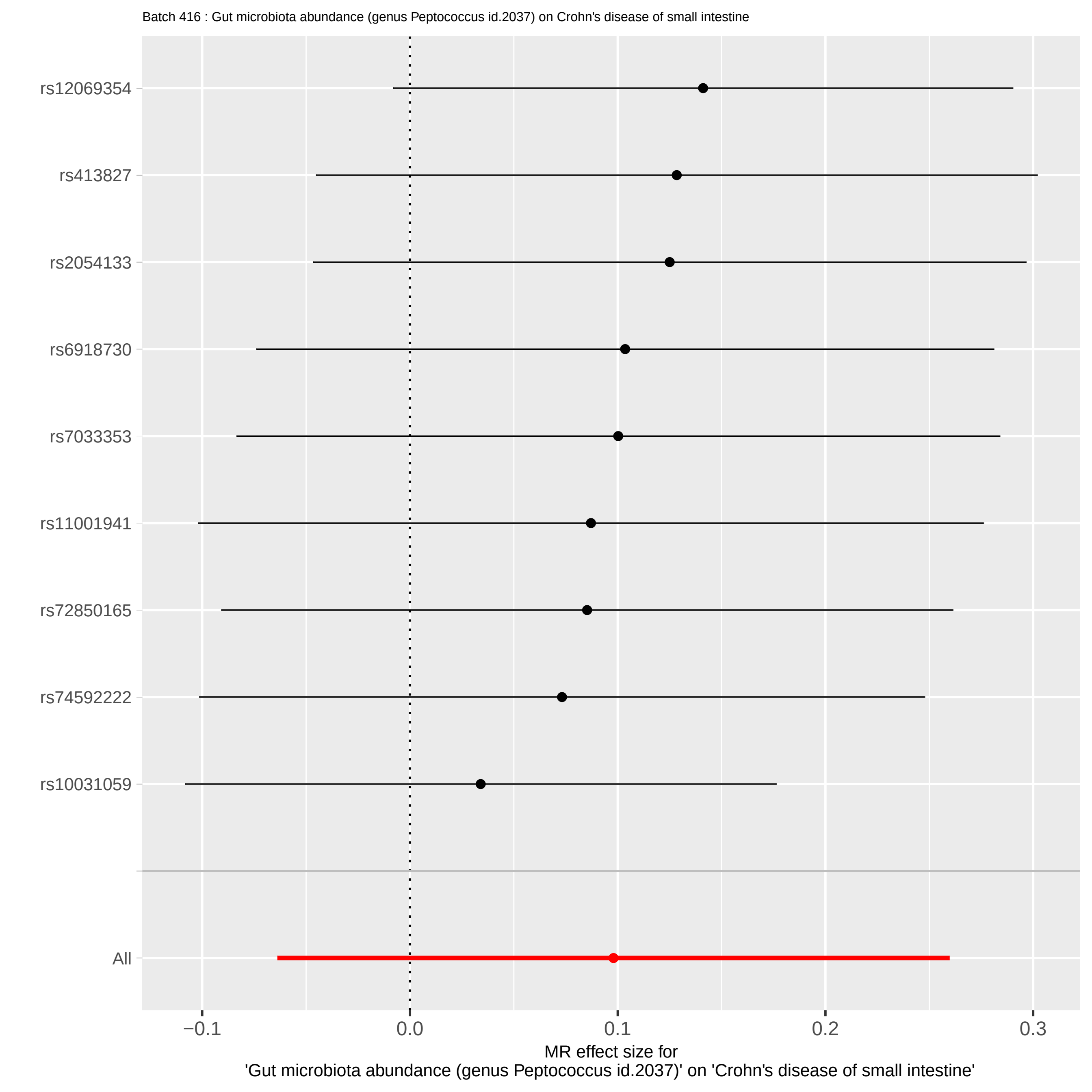
Batch 416 : Gut microbiota abundance (genus Peptococcus id.2037) on Crohn's disease of small intestine
rs12069354
rs413827
rs2054133
rs6918730
rs7033353
rs11001941
rs72850165
rs74592222
rs10031059
All
−0.1
0.0
0.1
0.2
0.3
MR effect size for
'Gut microbiota abundance (genus Peptococcus id.2037)' on 'Crohn's disease of small intestine'

Batch 417 : Gut microbiota abundance (genus Phascolarctobacterium id.2168) on Crohn's disease of small intestine

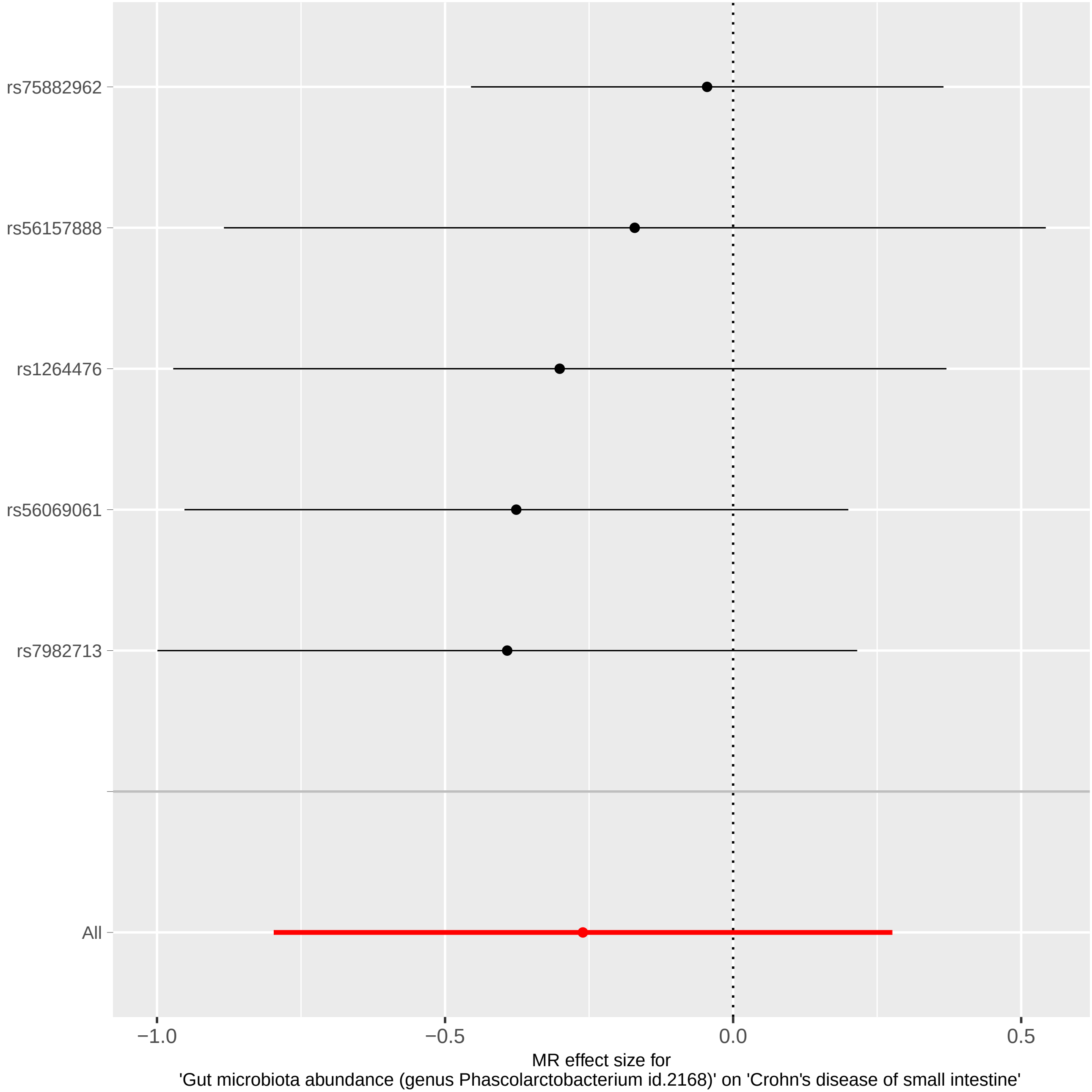

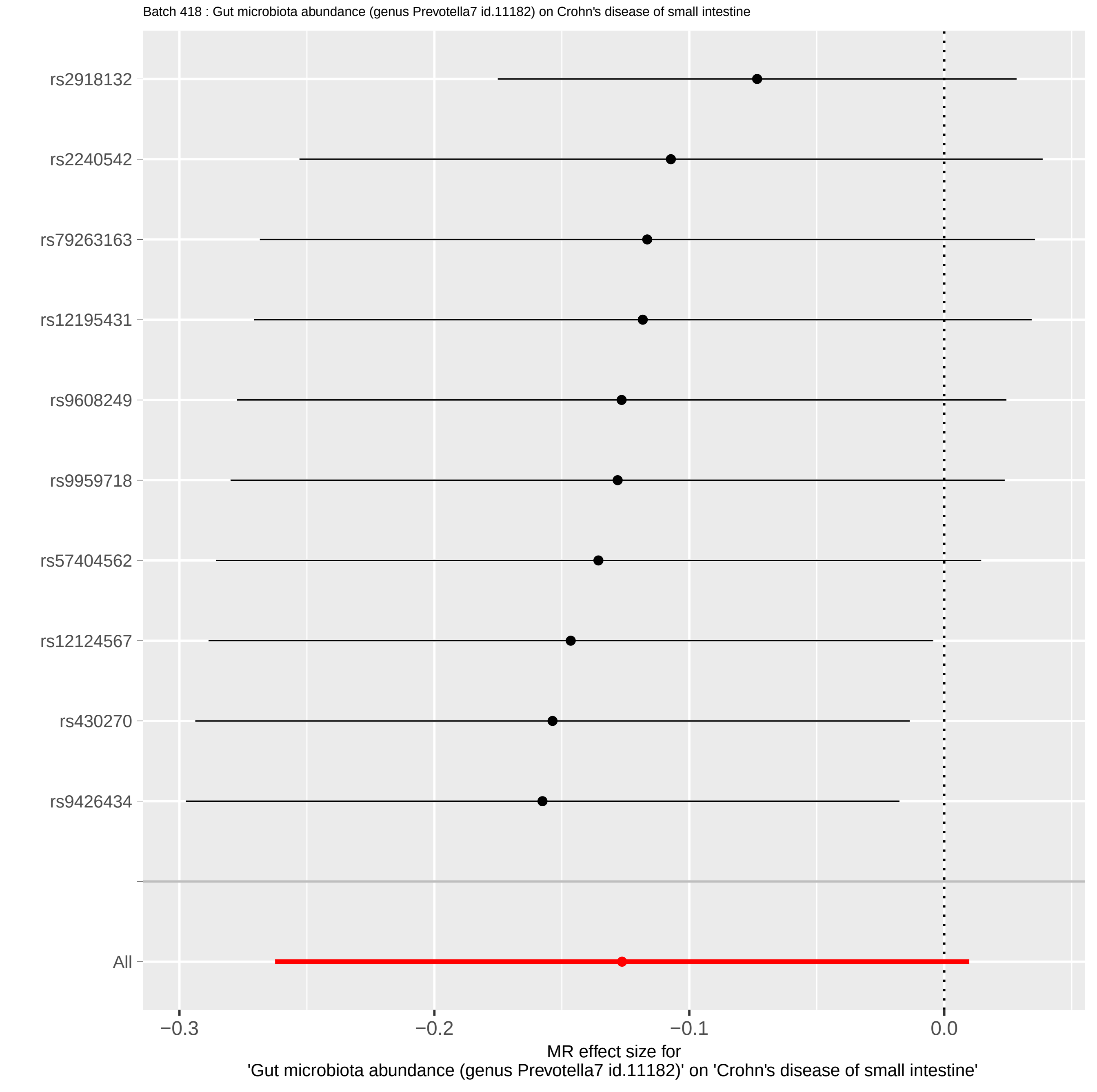

Batch 418 : Gut microbiota abundance (genus Prevotella7 id.11182) on Crohn's disease of small intestine
rs2918132
rs2240542
rs79263163
rs12195431
rs9608249
rs9959718
rs57404562
rs12124567
rs430270
rs9426434
All
−0.3
−0.2
−0.1
0.0
MR effect size for
'Gut microbiota abundance (genus Prevotella7 id.11182)' on 'Crohn's disease of small intestine'

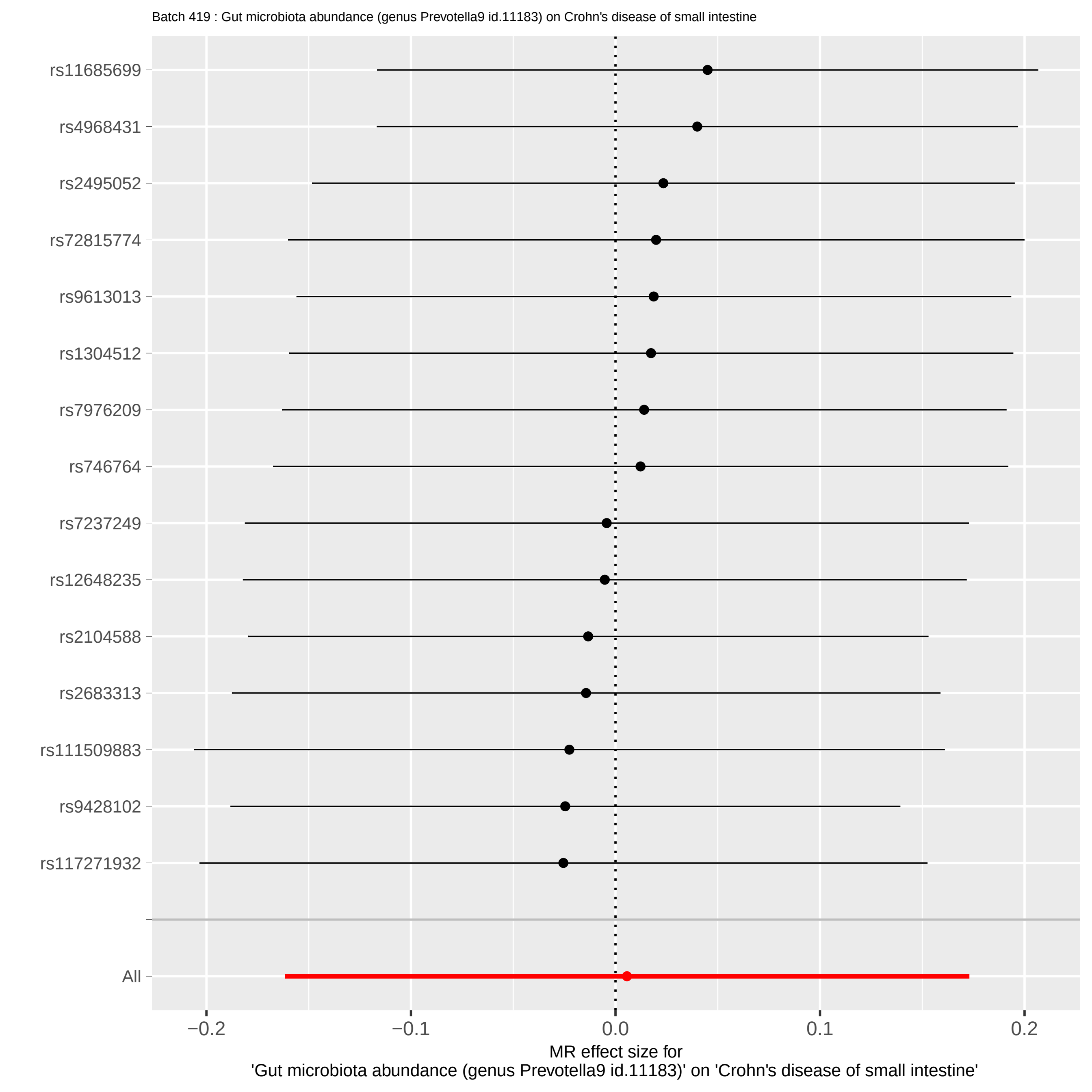

Batch 419 : Gut microbiota abundance (genus Prevotella9 id.11183) on Crohn's disease of small intestine
rs11685699
rs4968431
rs2495052
rs72815774
rs9613013
rs1304512
rs7976209
rs746764
rs7237249
rs12648235
rs2104588
rs2683313
rs111509883
rs9428102
rs117271932
All
−0.2
−0.1
0.0
0.1
0.2
MR effect size for
'Gut microbiota abundance (genus Prevotella9 id.11183)' on 'Crohn's disease of small intestine'

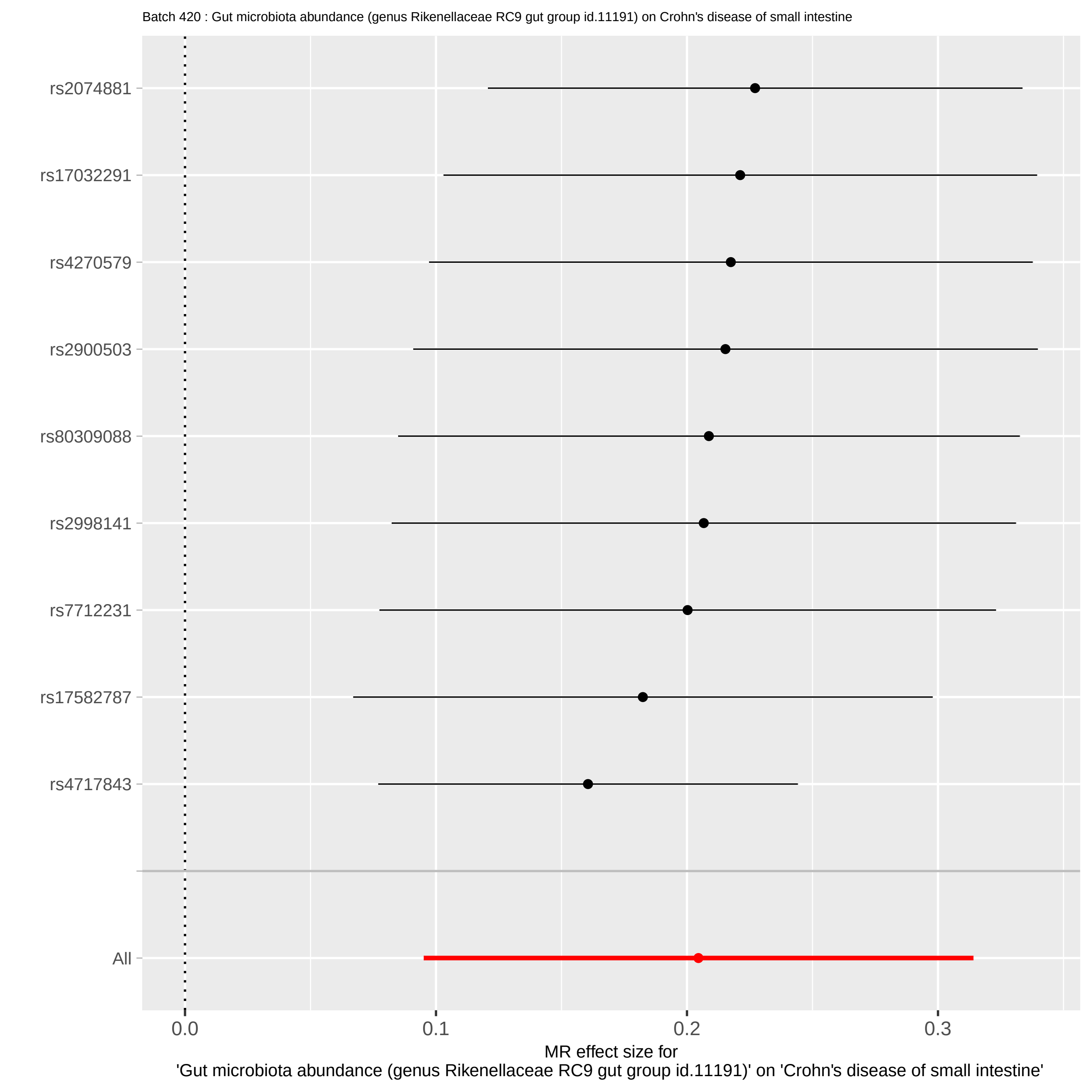
Batch 420 : Gut microbiota abundance (genus Rikenellaceae RC9 gut group id.11191) on Crohn's disease of small intestine
rs2074881
rs17032291
rs4270579
rs2900503
rs80309088
rs2998141
rs7712231
rs17582787
rs4717843
All
0.0
0.1
0.2
0.3
MR effect size for
'Gut microbiota abundance (genus Rikenellaceae RC9 gut group id.11191)' on 'Crohn's disease of small intestine'

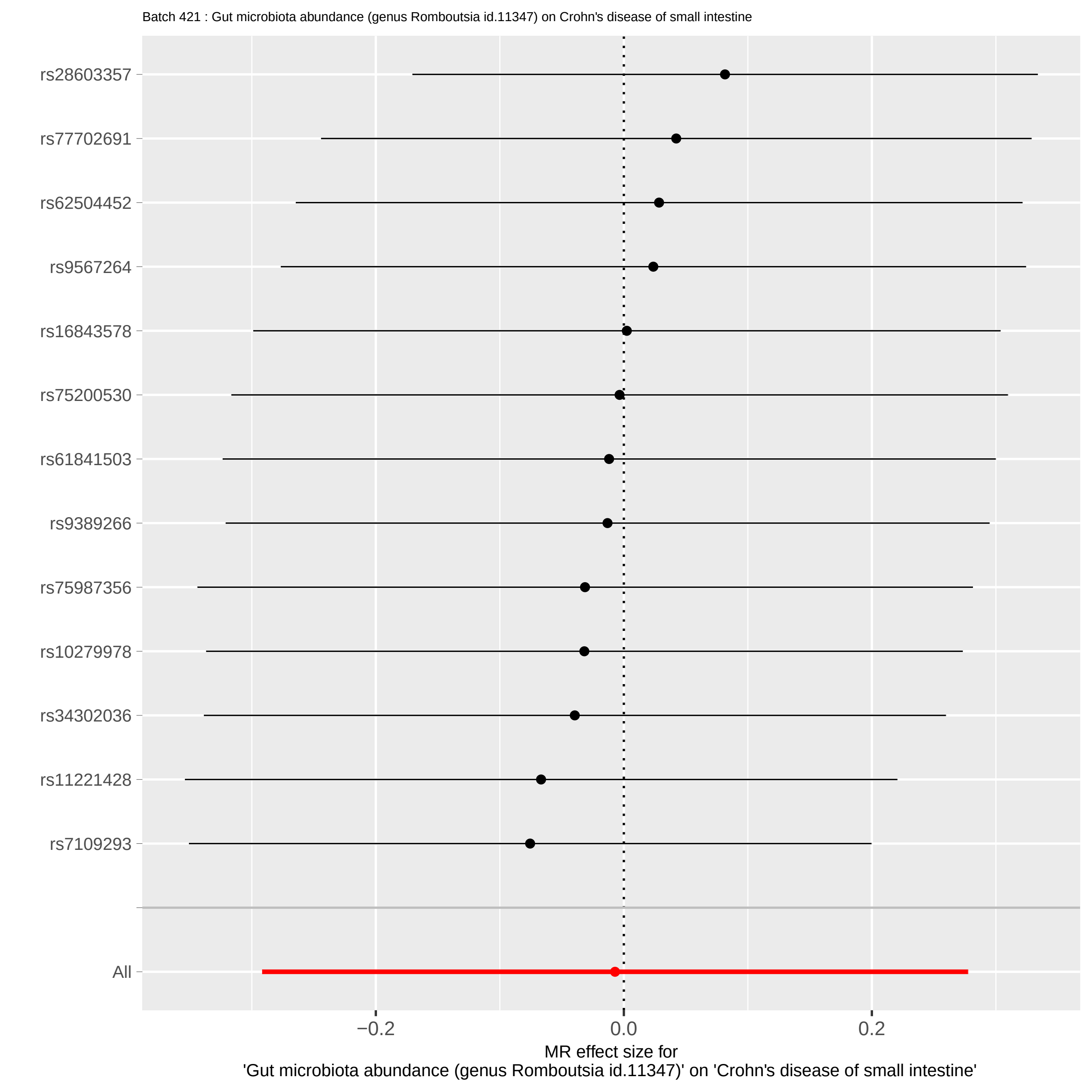
Batch 421 : Gut microbiota abundance (genus Romboutsia id.11347) on Crohn's disease of small intestine
rs28603357
rs77702691
rs62504452
rs9567264
rs16843578
rs75200530
rs61841503
rs9389266
rs75987356
rs10279978
rs34302036
rs11221428
rs7109293
All
−0.2
0.0
0.2
MR effect size for
'Gut microbiota abundance (genus Romboutsia id.11347)' on 'Crohn's disease of small intestine'

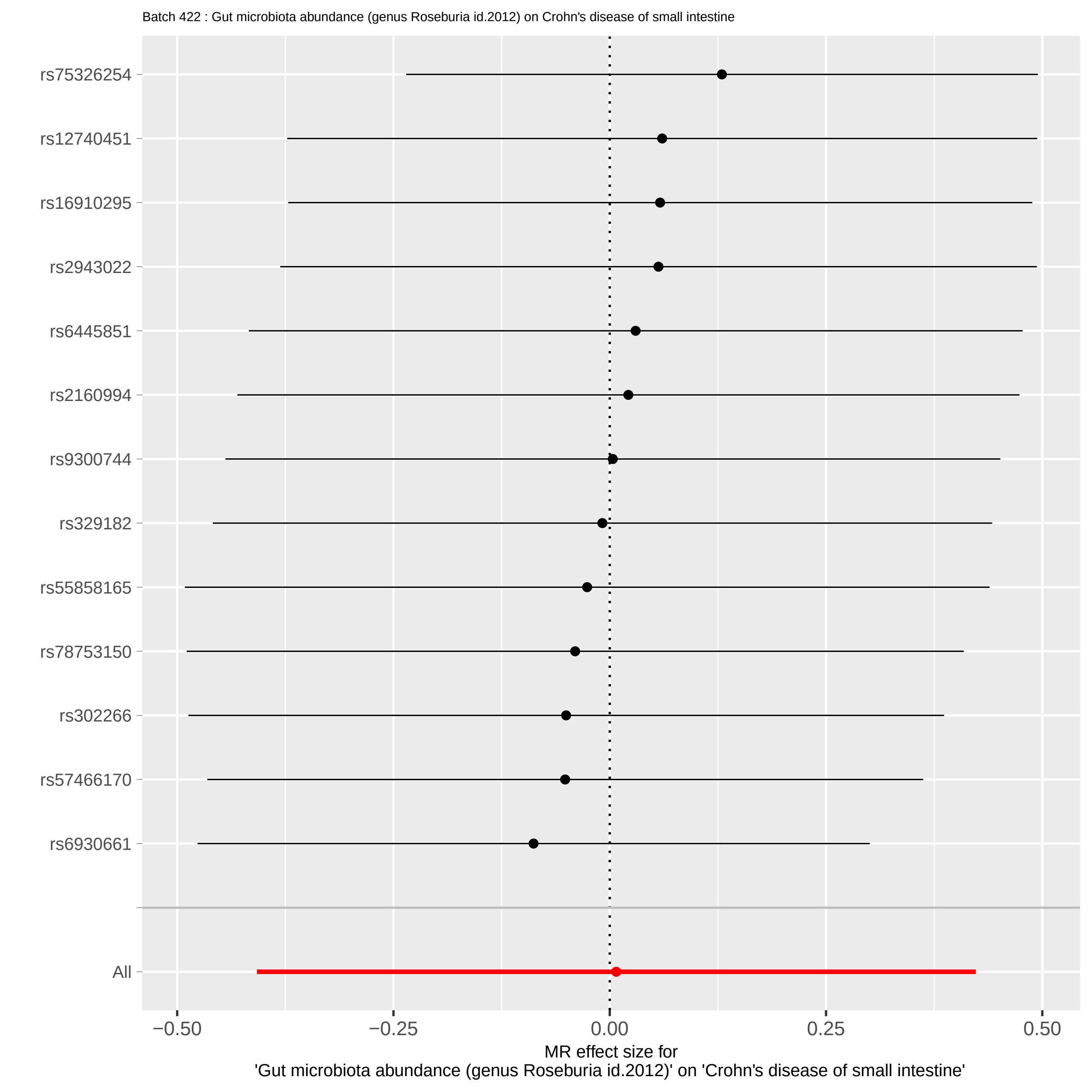

Batch 422 : Gut microbiota abundance (genus Roseburia id.2012) on Crohn's disease of small intestine
rs75326254
rs12740451
rs16910295
rs2943022
rs6445851
rs2160994
rs9300744
rs329182
rs55858165
rs78753150
rs302266
rs57466170
rs6930661
All
−0.50
−0.25
0.00
0.25
0.50
MR effect size for
'Gut microbiota abundance (genus Roseburia id.2012)' on 'Crohn's disease of small intestine'

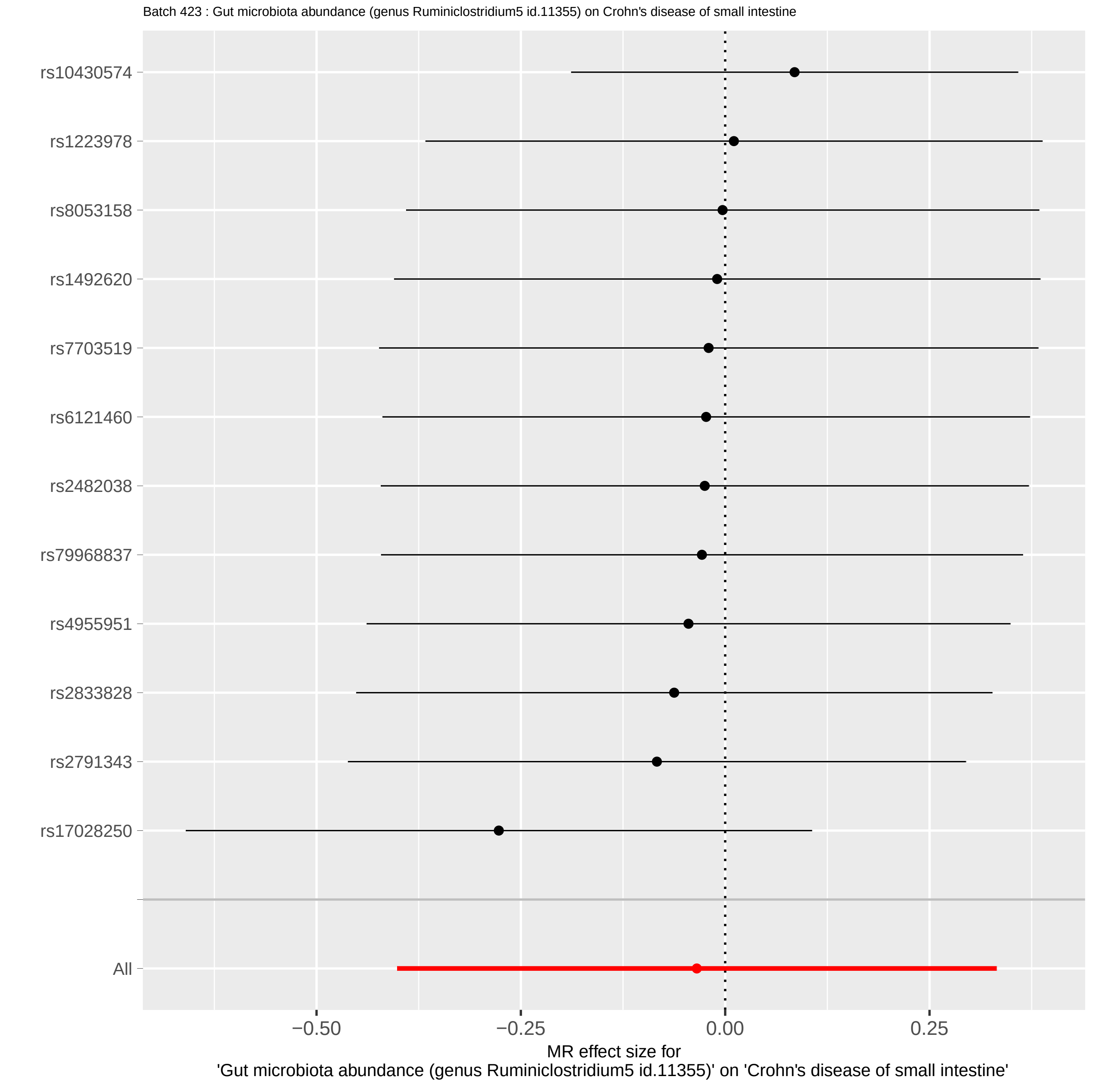
Batch 423 : Gut microbiota abundance (genus Ruminiclostridium5 id.11355) on Crohn's disease of small intestine
rs10430574
rs1223978
rs8053158
rs1492620
rs7703519
rs6121460
rs2482038
rs79968837
rs4955951
rs2833828
rs2791343
rs17028250
All
−0.50
−0.25
0.00
0.25
MR effect size for
'Gut microbiota abundance (genus Ruminiclostridium5 id.11355)' on 'Crohn's disease of small intestine'

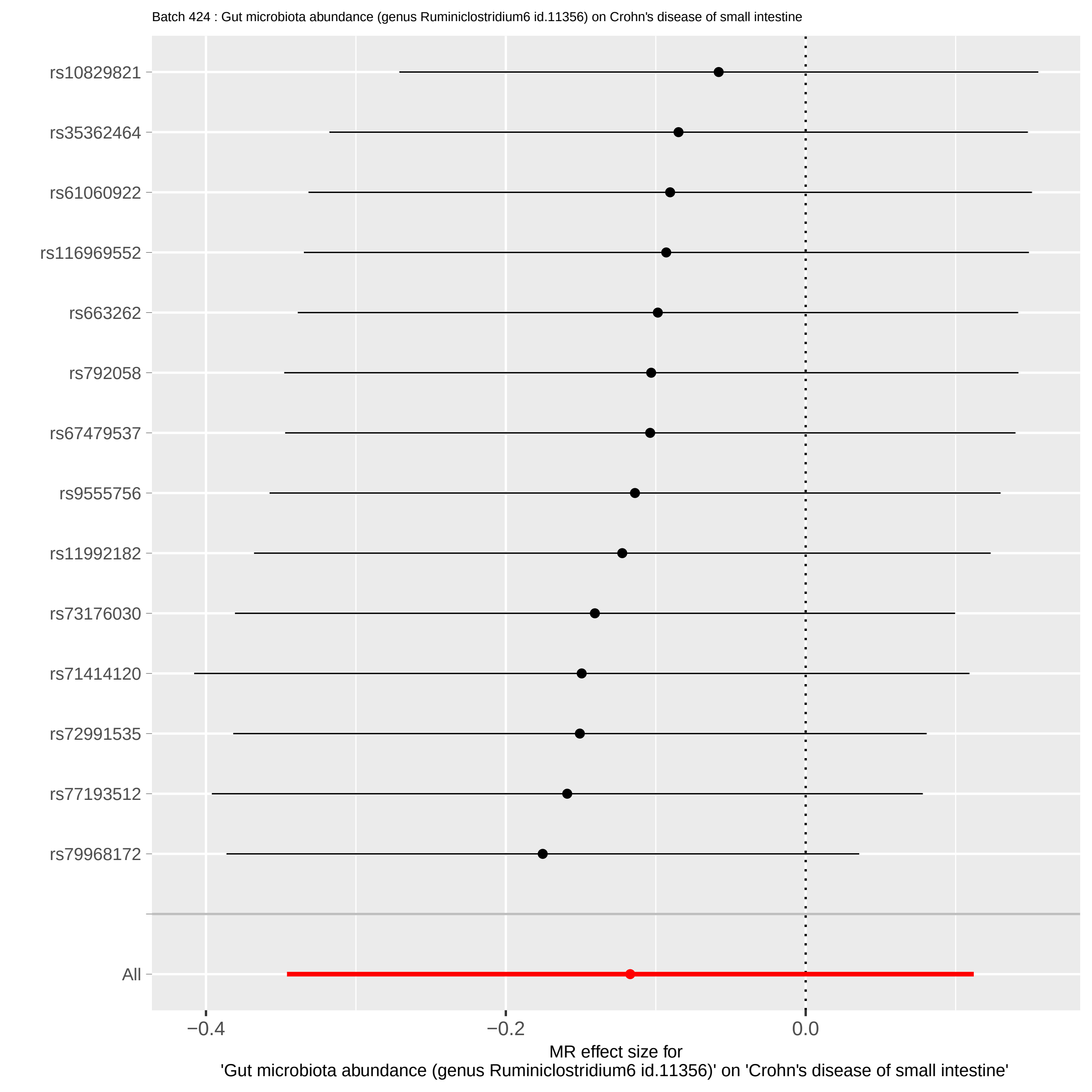
Batch 424 : Gut microbiota abundance (genus Ruminiclostridium6 id.11356) on Crohn's disease of small intestine
rs10829821
rs35362464
rs61060922
rs116969552
rs663262
rs792058
rs67479537
rs9555756
rs11992182
rs73176030
rs71414120
rs72991535
rs77193512
rs79968172
All
−0.4
−0.2
0.0
MR effect size for
'Gut microbiota abundance (genus Ruminiclostridium6 id.11356)' on 'Crohn's disease of small intestine'

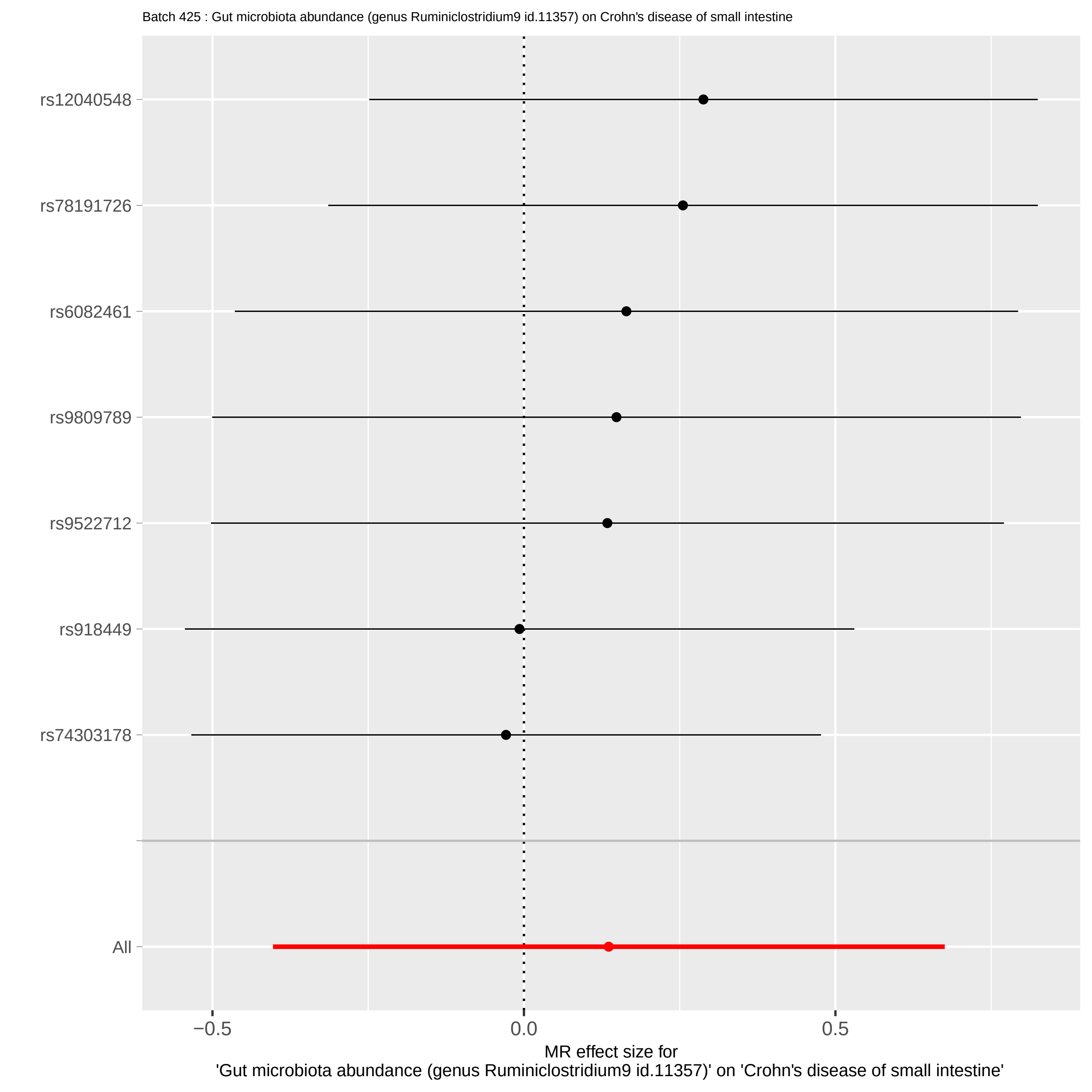

Batch 425 : Gut microbiota abundance (genus Ruminiclostridium9 id.11357) on Crohn's disease of small intestine
rs12040548
rs78191726
rs6082461
rs9809789
rs9522712
rs918449
rs74303178
All
−0.5
0.0
0.5
MR effect size for
'Gut microbiota abundance (genus Ruminiclostridium9 id.11357)' on 'Crohn's disease of small intestine'

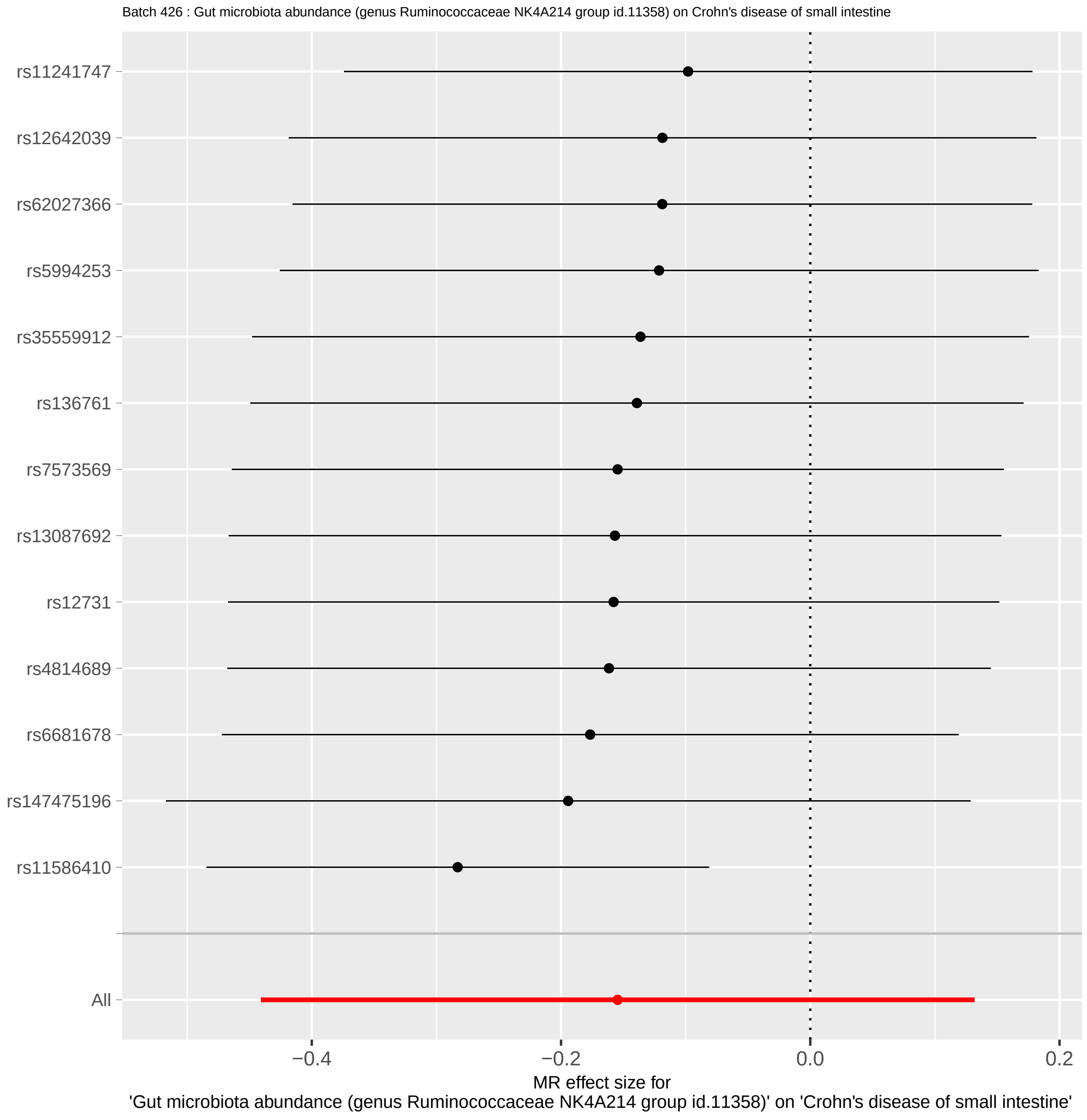

Batch 426 : Gut microbiota abundance (genus Ruminococcaceae NK4A214 group id.11358) on Crohn's disease of small intestine
rs11241747
rs12642039
rs62027366
rs5994253
rs35559912
rs136761
rs7573569
rs13087692
rs12731
rs4814689
rs6681678
rs147475196
rs11586410
All
−0.4
−0.2
0.0
0.2
MR effect size for
'Gut microbiota abundance (genus Ruminococcaceae NK4A214 group id.11358)' on 'Crohn's disease of small intestine'

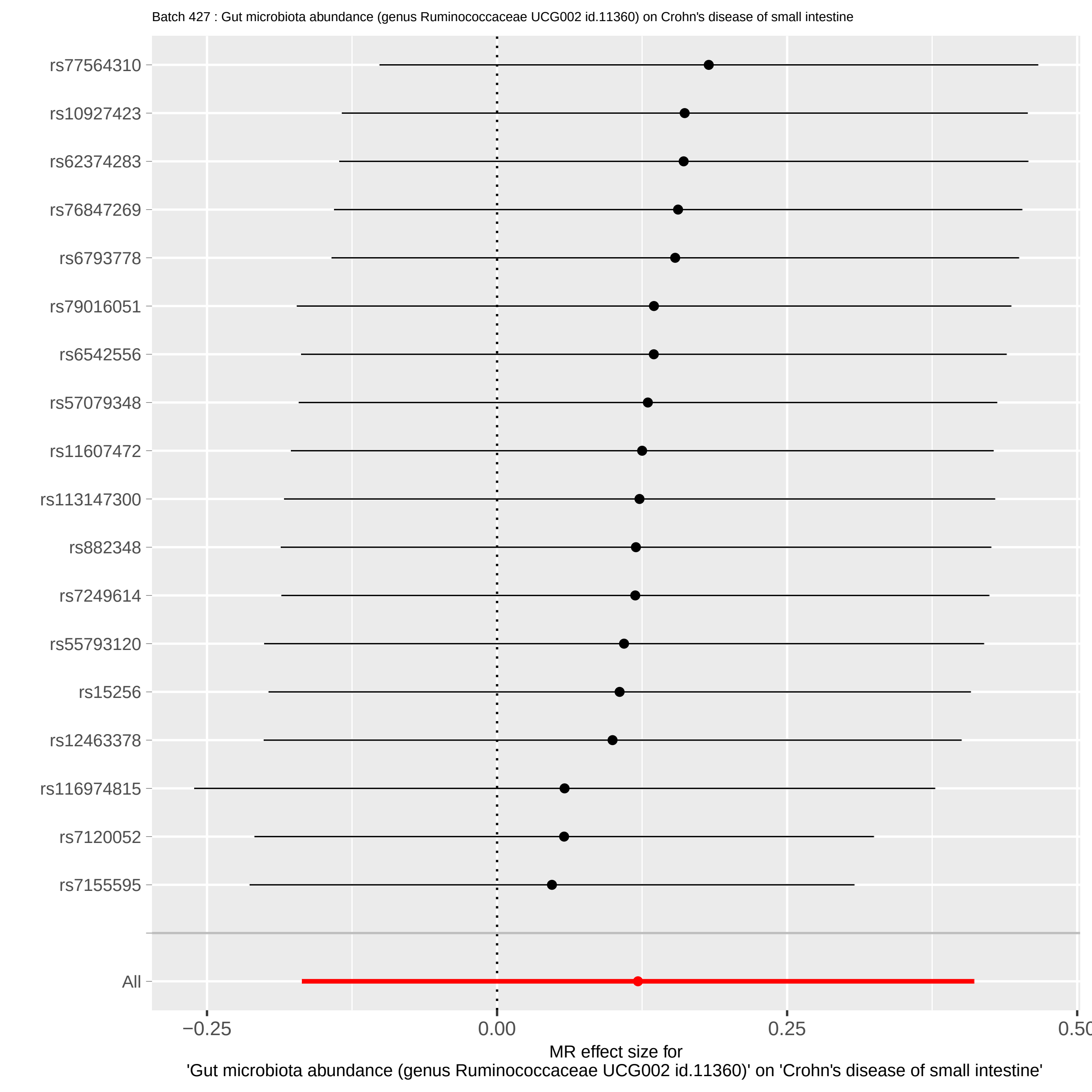

Batch 427 : Gut microbiota abundance (genus Ruminococcaceae UCG002 id.11360) on Crohn's disease of small intestine
rs77564310
rs10927423
rs62374283
rs76847269
rs6793778
rs79016051
rs6542556
rs57079348
rs11607472
rs113147300
rs882348
rs7249614
rs55793120
rs15256
rs12463378
rs116974815
rs7120052
rs7155595
All
−0.25
0.00
0.25
0.50
MR effect size for
'Gut microbiota abundance (genus Ruminococcaceae UCG002 id.11360)' on 'Crohn's disease of small intestine'

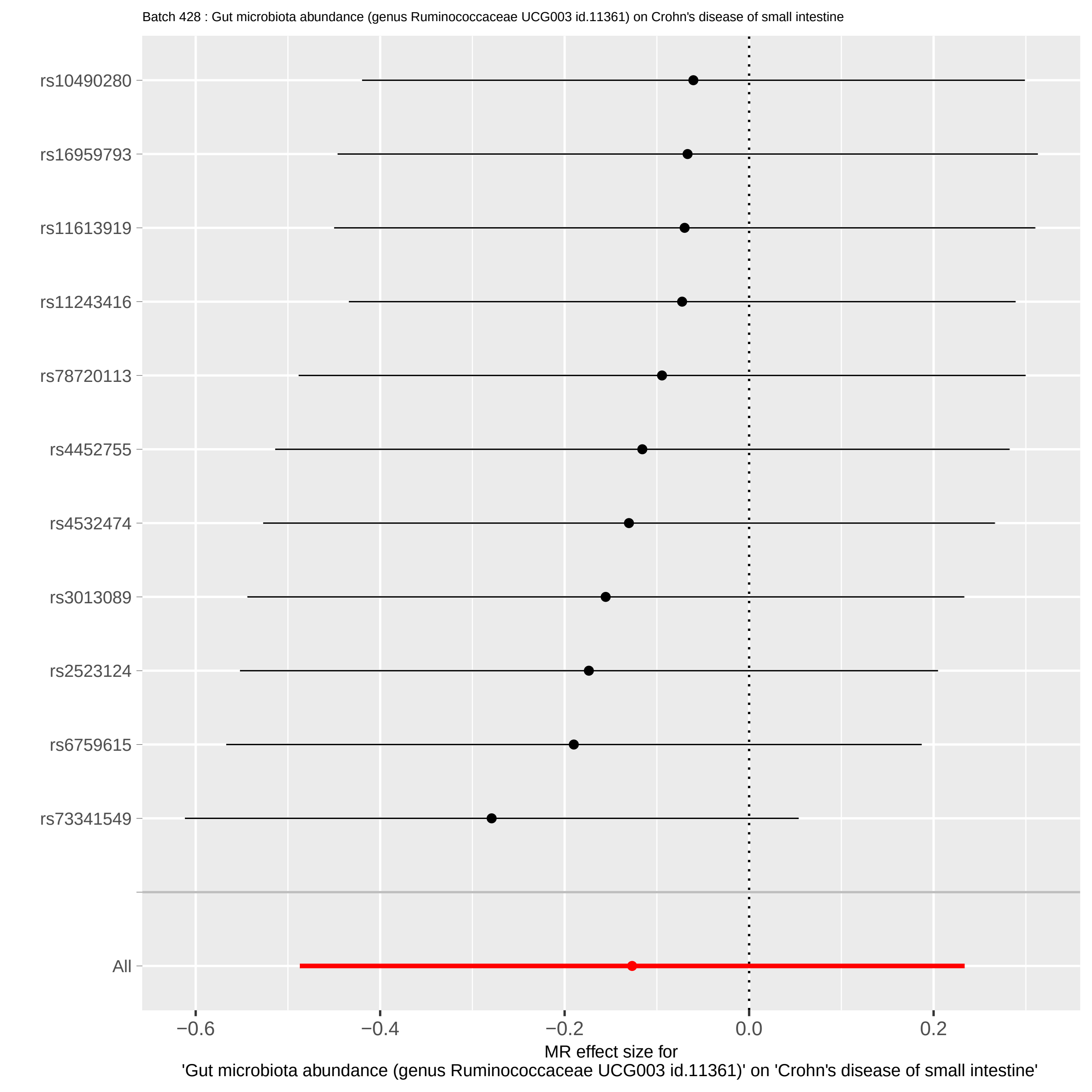
Batch 428 : Gut microbiota abundance (genus Ruminococcaceae UCG003 id.11361) on Crohn's disease of small intestine
rs10490280
rs16959793
rs11613919
rs11243416
rs78720113
rs4452755
rs4532474
rs3013089
rs2523124
rs6759615
rs73341549
All
−0.6
−0.4
−0.2
0.0
0.2
MR effect size for
'Gut microbiota abundance (genus Ruminococcaceae UCG003 id.11361)' on 'Crohn's disease of small intestine'

Batch 429 : Gut microbiota abundance (genus Ruminococcaceae UCG004 id.11362) on Crohn's disease of small intestine

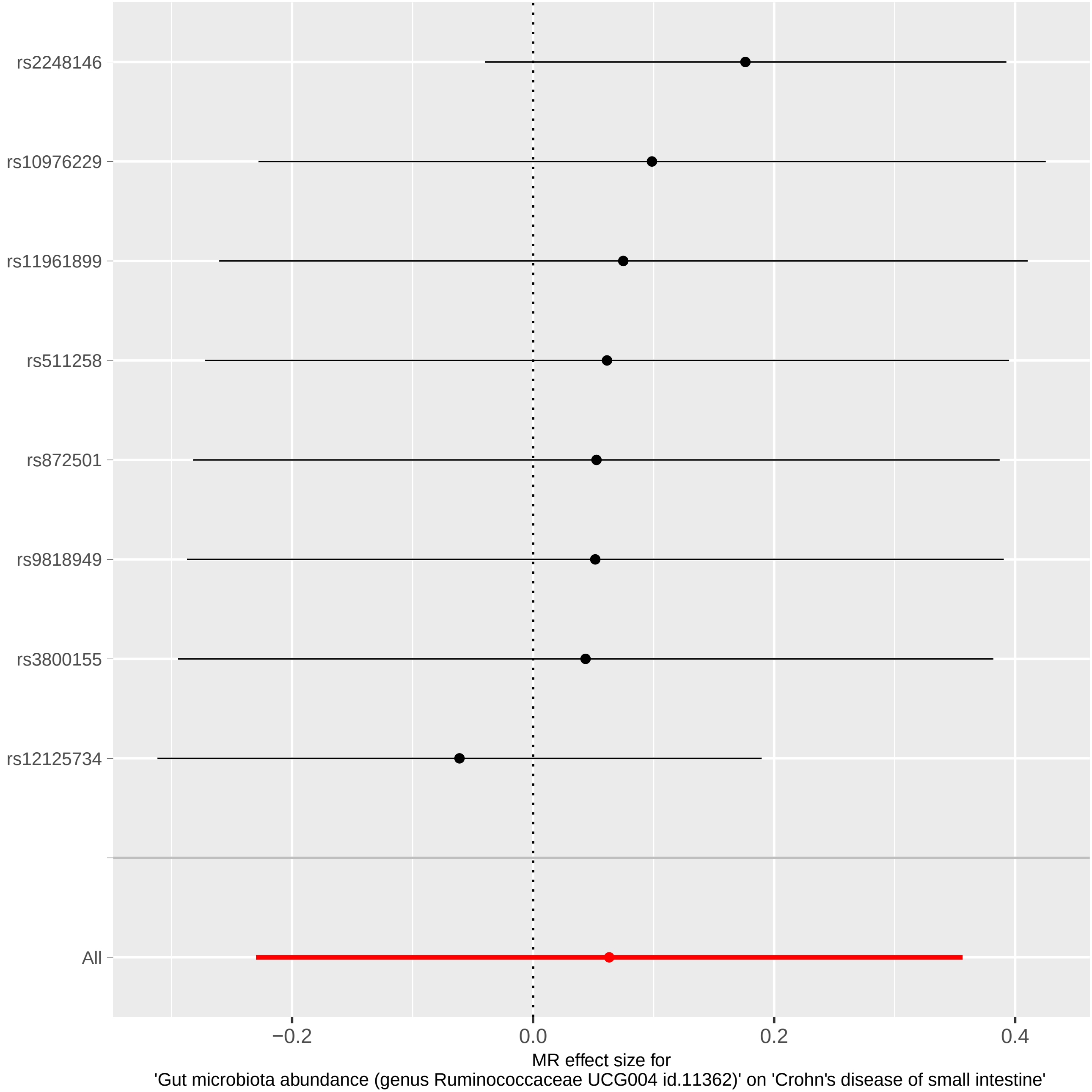

Batch 430 : Gut microbiota abundance (genus Ruminococcaceae UCG005 id.11363) on Crohn's disease of small intestine

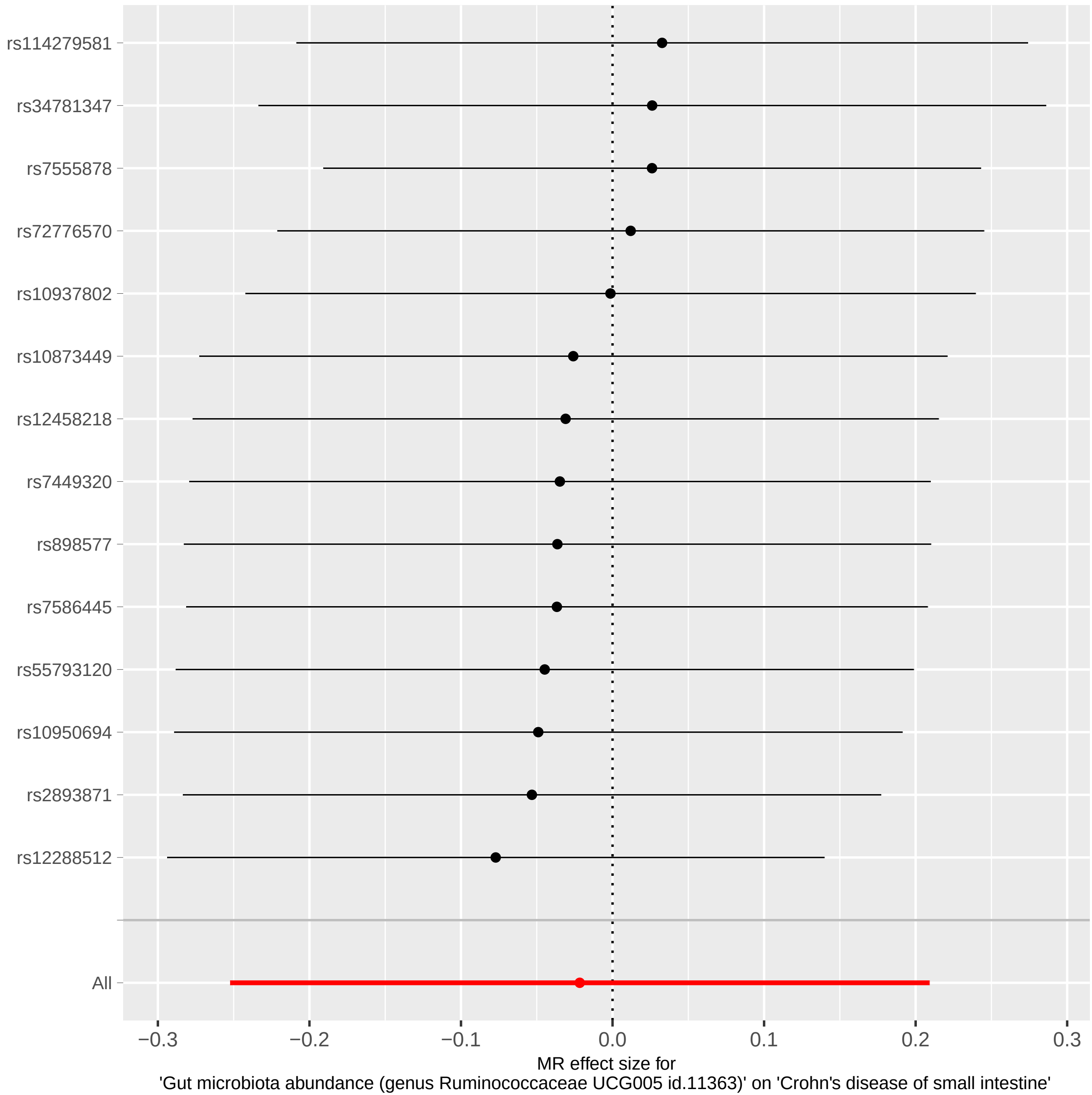

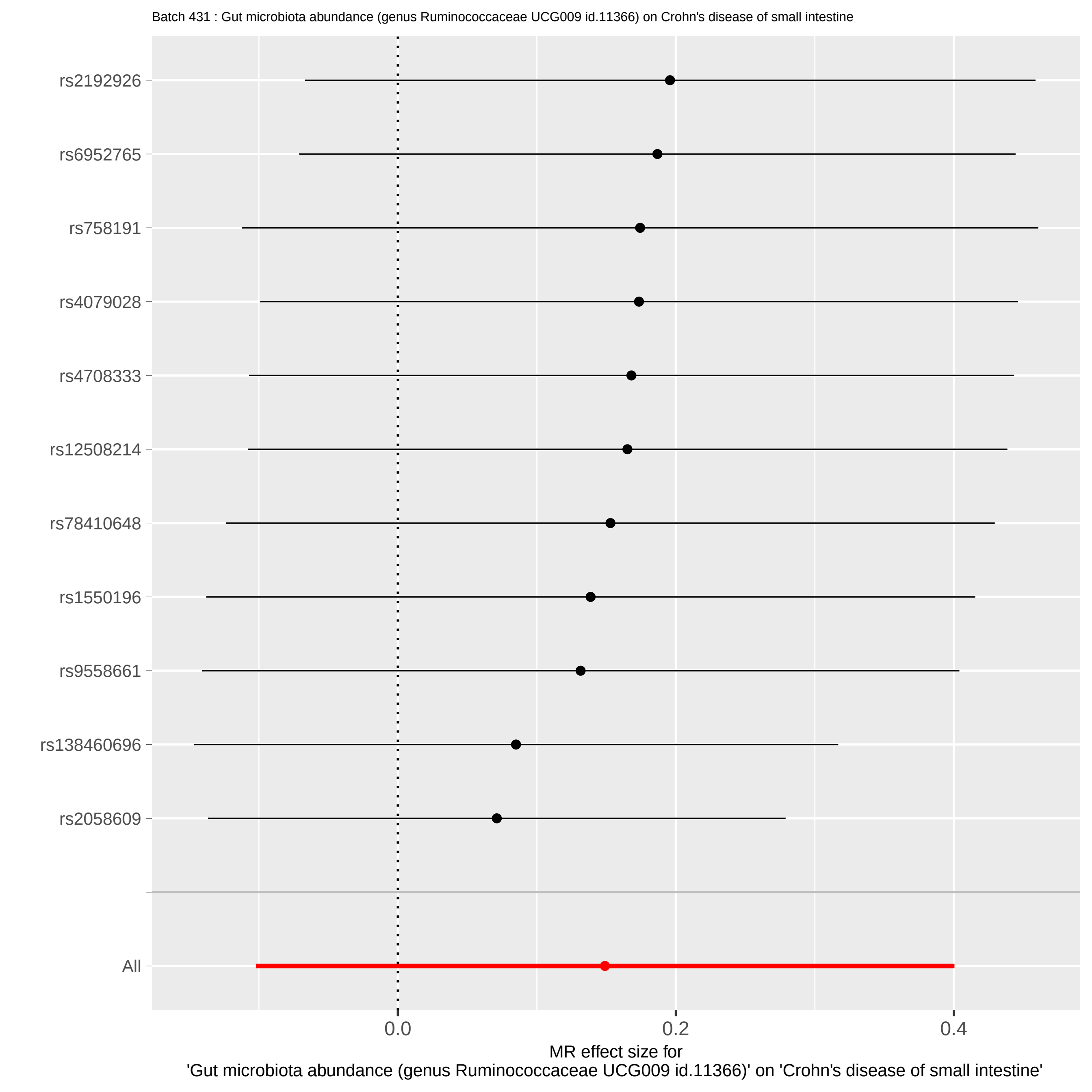

Batch 431 : Gut microbiota abundance (genus Ruminococcaceae UCG009 id.11366) on Crohn's disease of small intestine
rs2192926
rs6952765
rs758191
rs4079028
rs4708333
rs12508214
rs78410648
rs1550196
rs9558661
rs138460696
rs2058609
All
0.0
0.2
0.4
MR effect size for
'Gut microbiota abundance (genus Ruminococcaceae UCG009 id.11366)' on 'Crohn's disease of small intestine'

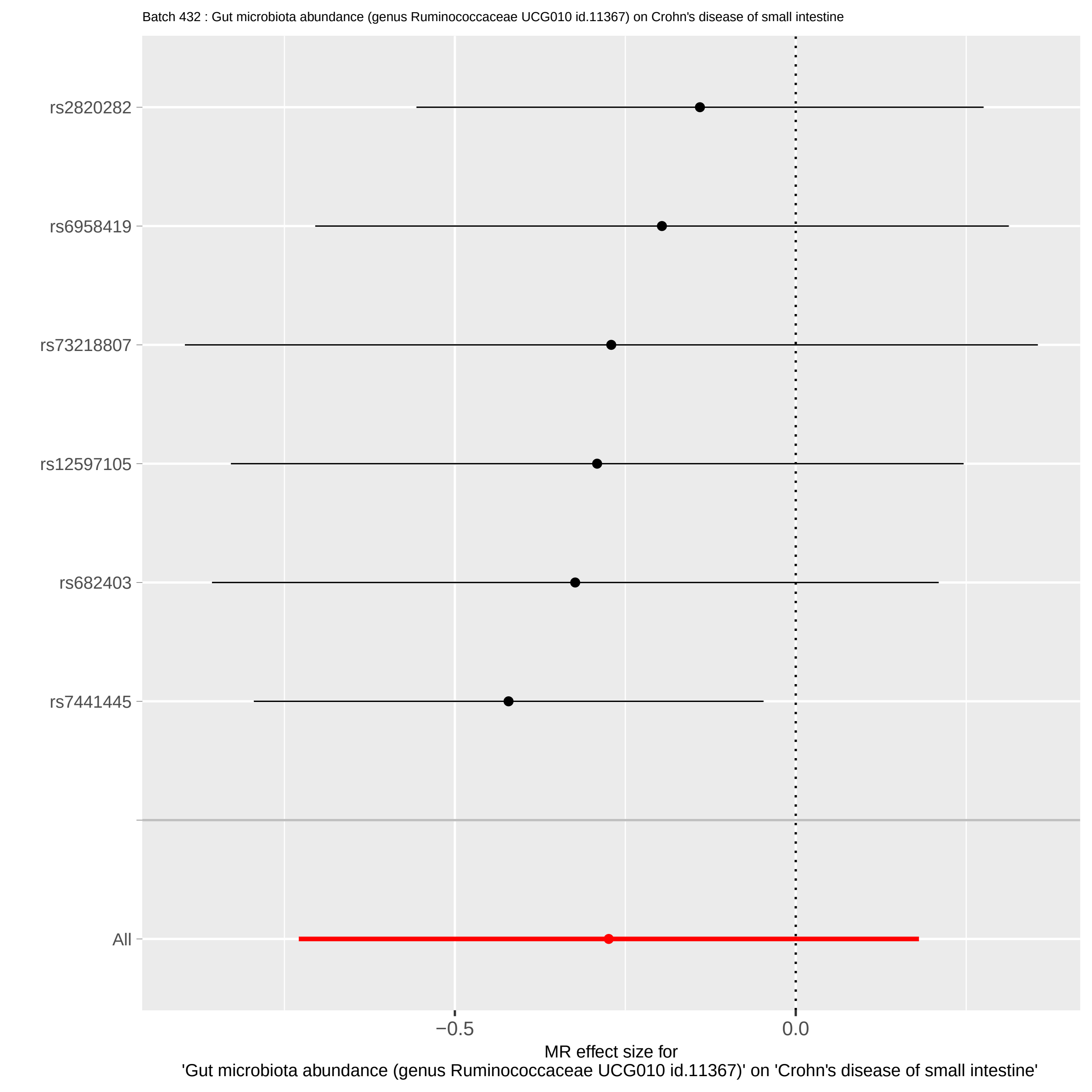

Batch 432 : Gut microbiota abundance (genus Ruminococcaceae UCG010 id.11367) on Crohn's disease of small intestine
rs2820282
rs6958419
rs73218807
rs12597105
rs682403
rs7441445
All
−0.5
0.0
MR effect size for
'Gut microbiota abundance (genus Ruminococcaceae UCG010 id.11367)' on 'Crohn's disease of small intestine'

Batch 433 : Gut microbiota abundance (genus Ruminococcaceae UCG011 id.11368) on Crohn's disease of small intestine

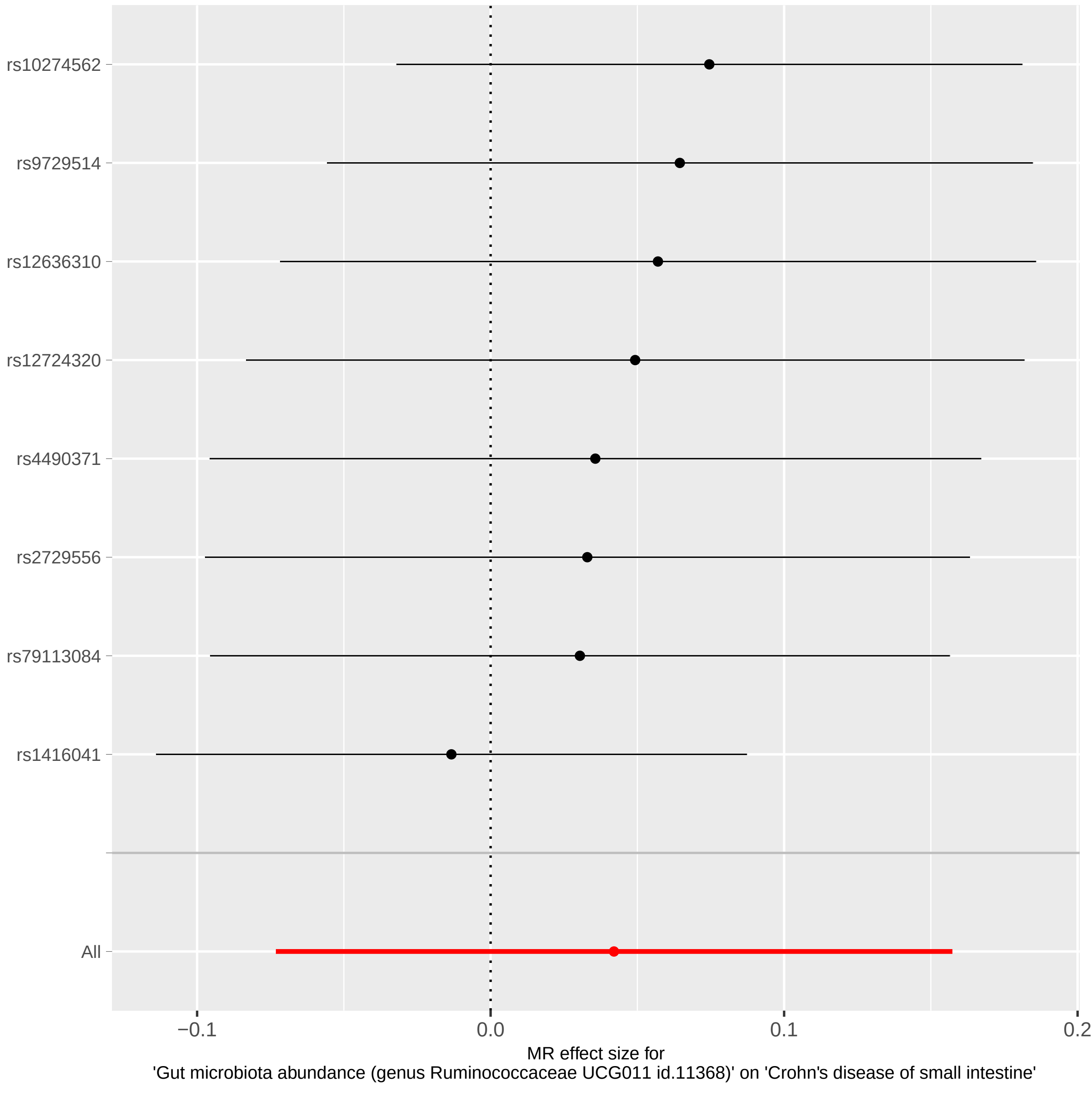

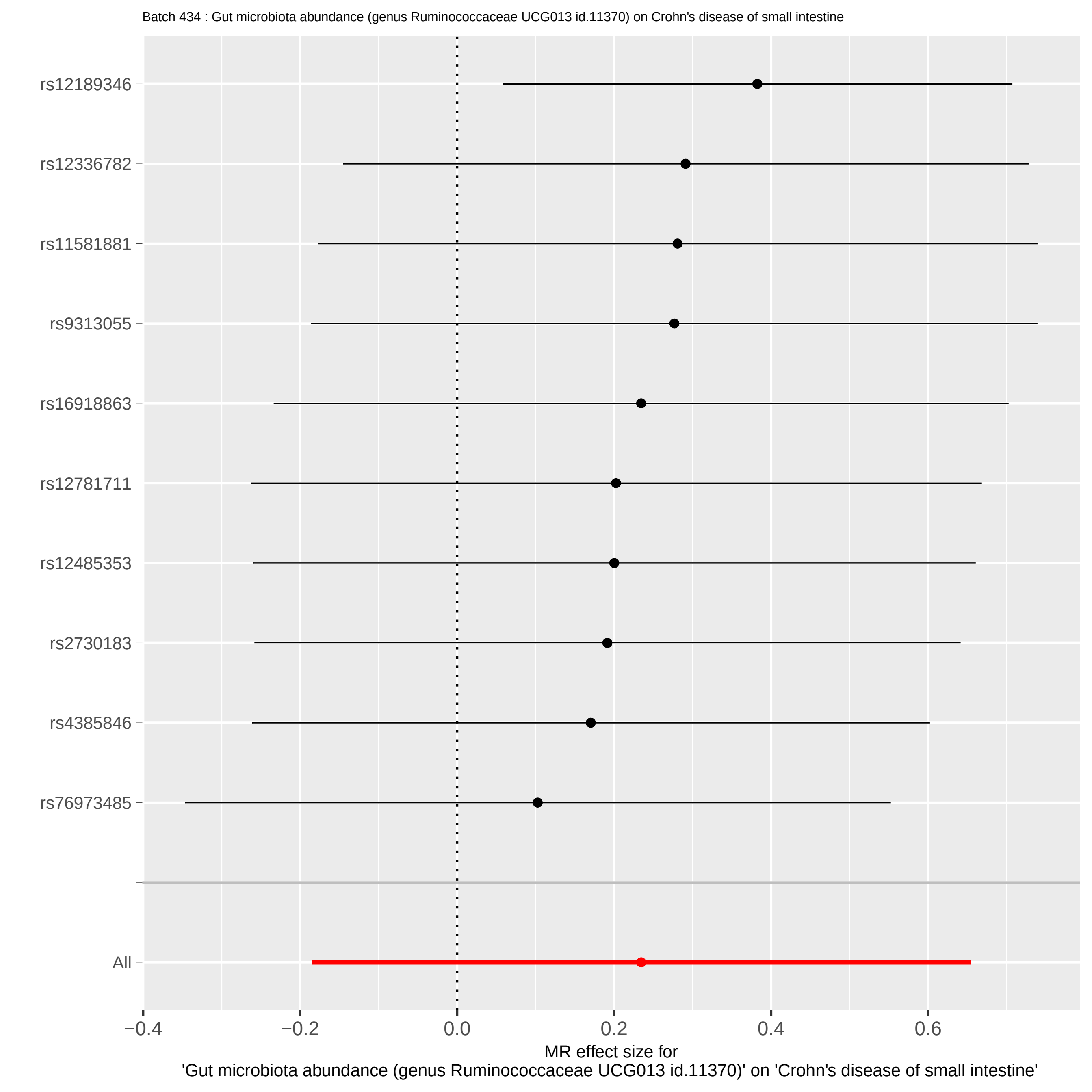

Batch 434 : Gut microbiota abundance (genus Ruminococcaceae UCG013 id.11370) on Crohn's disease of small intestine
rs12189346
rs12336782
rs11581881
rs9313055
rs16918863
rs12781711
rs12485353
rs2730183
rs4385846
rs76973485
All
−0.4
−0.2
0.0
0.2
0.4
0.6
MR effect size for
'Gut microbiota abundance (genus Ruminococcaceae UCG013 id.11370)' on 'Crohn's disease of small intestine'

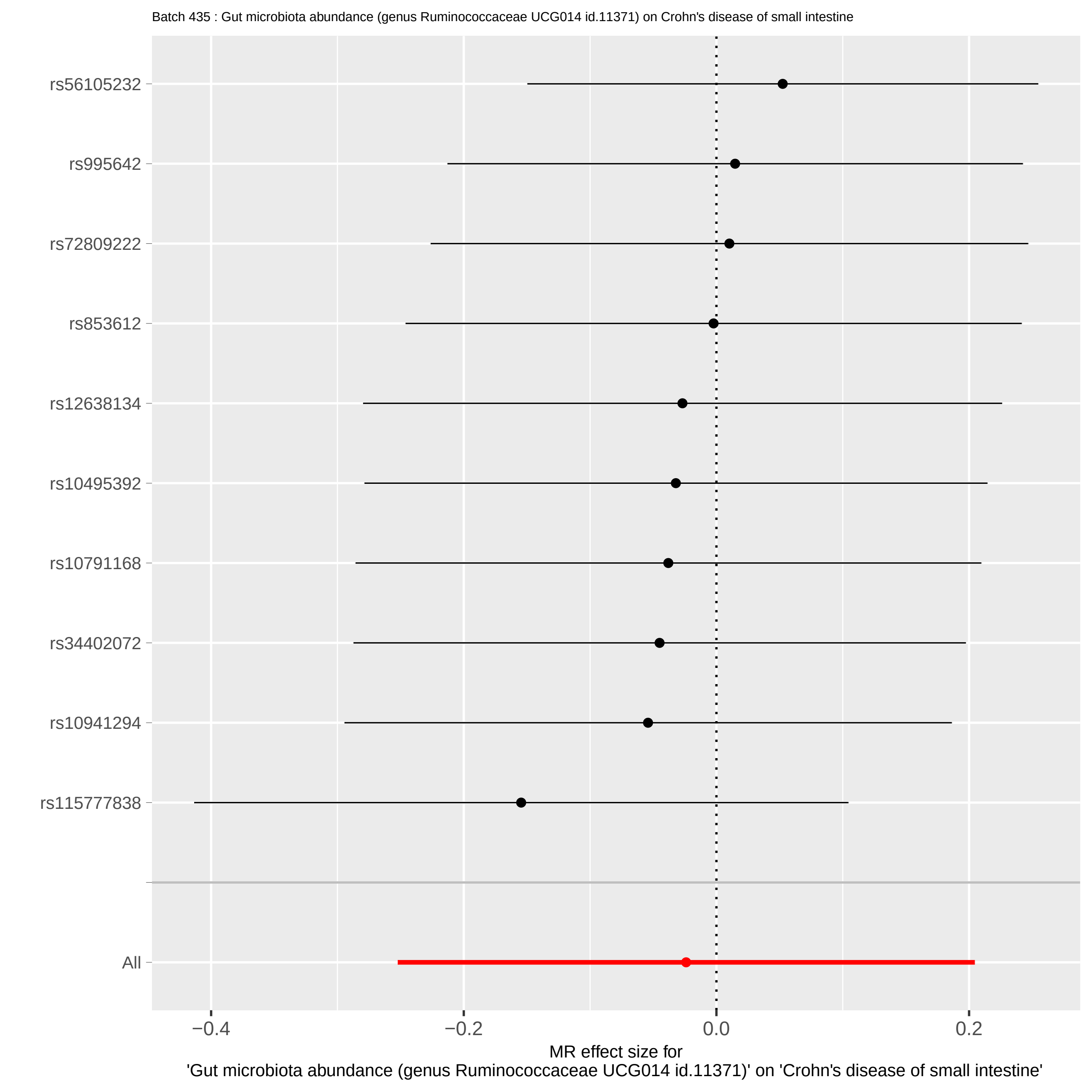

Batch 435 : Gut microbiota abundance (genus Ruminococcaceae UCG014 id.11371) on Crohn's disease of small intestine
rs56105232
rs995642
rs72809222
rs853612
rs12638134
rs10495392
rs10791168
rs34402072
rs10941294
rs115777838
All
−0.4
−0.2
0.0
0.2
MR effect size for
'Gut microbiota abundance (genus Ruminococcaceae UCG014 id.11371)' on 'Crohn's disease of small intestine'

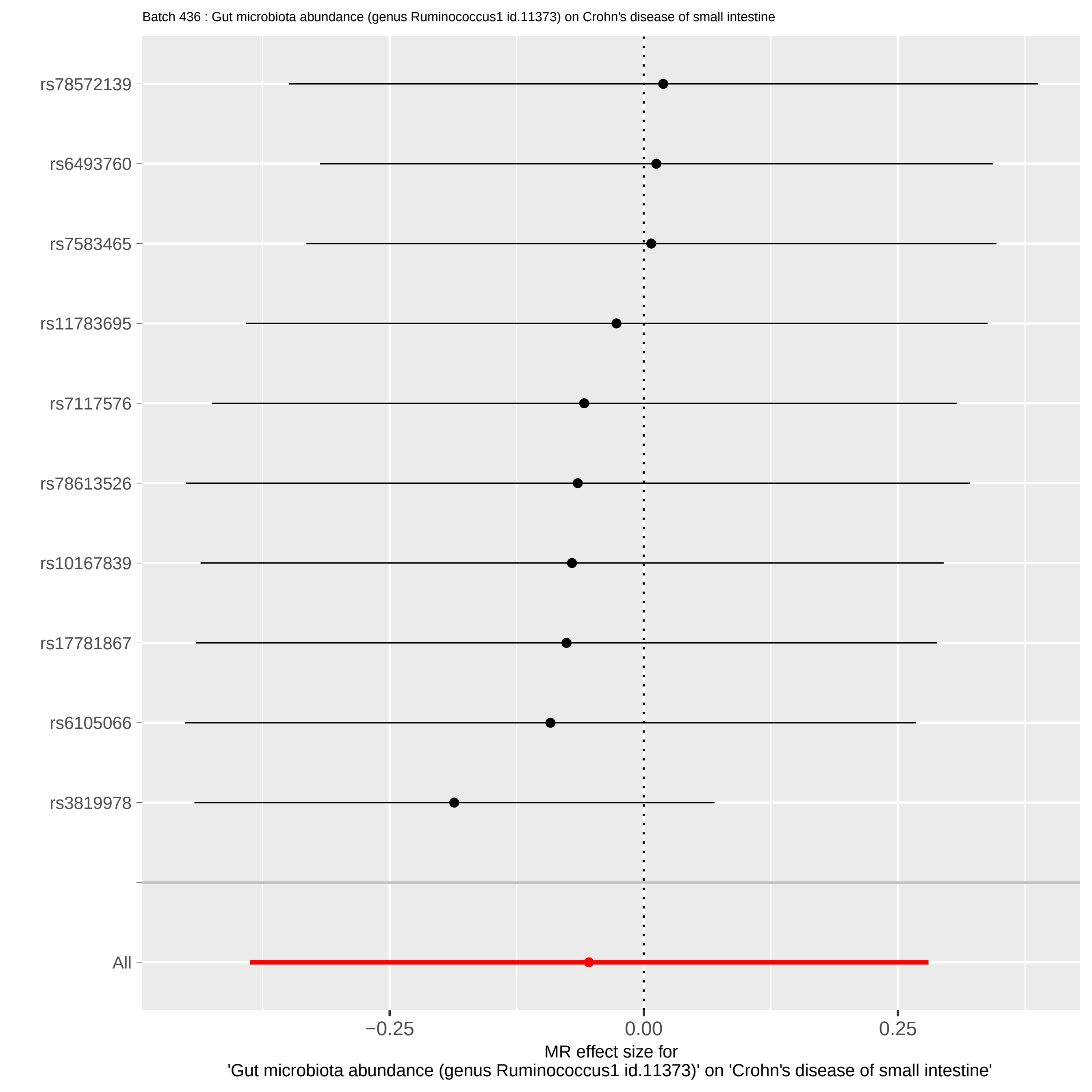
Batch 436 : Gut microbiota abundance (genus Ruminococcus1 id.11373) on Crohn's disease of small intestine
rs78572139
rs6493760
rs7583465
rs11783695
rs7117576
rs78613526
rs10167839
rs17781867
rs6105066
rs3819978
All
−0.25
0.00
0.25
MR effect size for
'Gut microbiota abundance (genus Ruminococcus1 id.11373)' on 'Crohn's disease of small intestine'

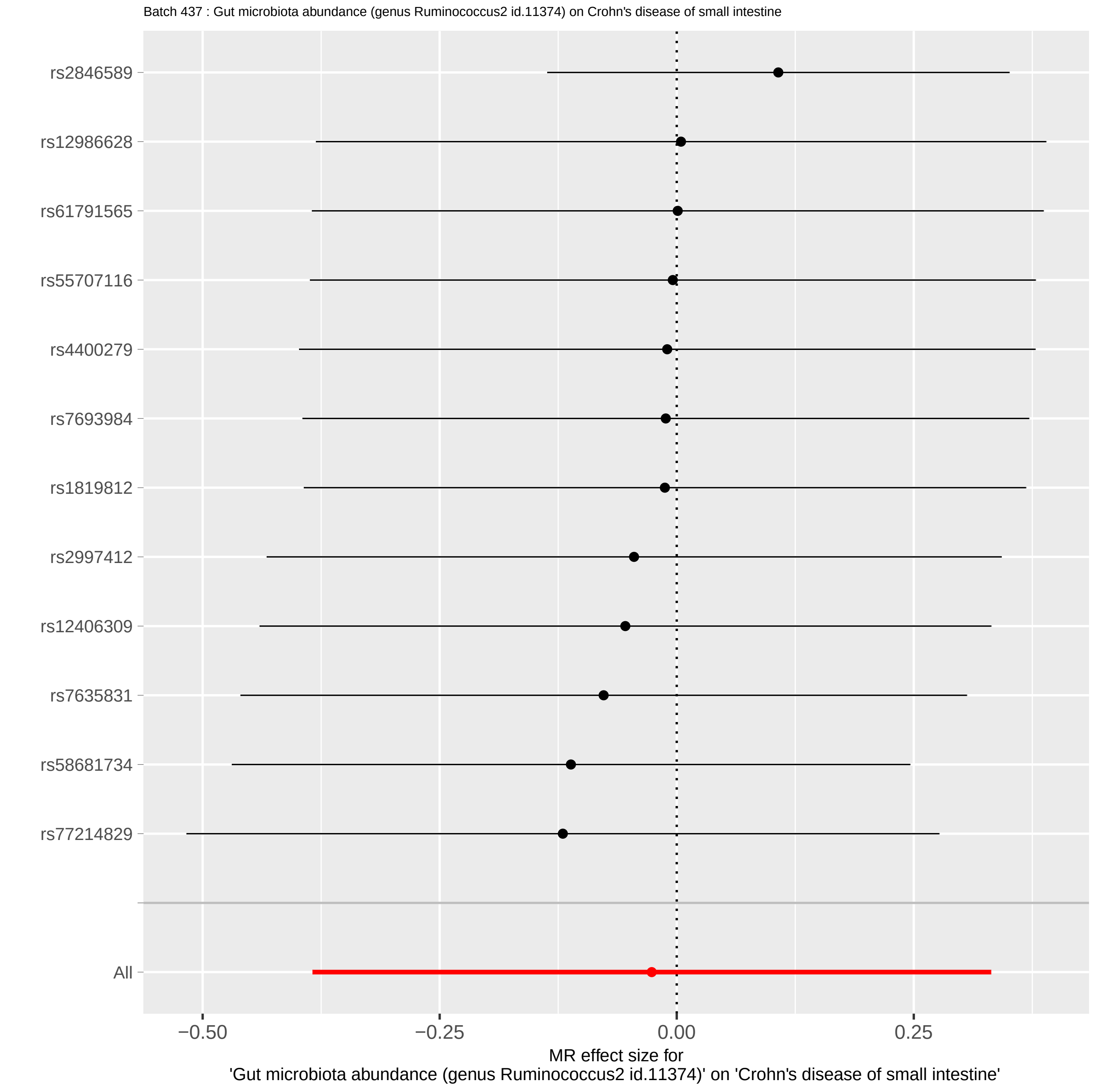

Batch 437 : Gut microbiota abundance (genus Ruminococcus2 id.11374) on Crohn's disease of small intestine
rs2846589
rs12986628
rs61791565
rs55707116
rs4400279
rs7693984
rs1819812
rs2997412
rs12406309
rs7635831
rs58681734
rs77214829
All
−0.50
−0.25
0.00
0.25
MR effect size for
'Gut microbiota abundance (genus Ruminococcus2 id.11374)' on 'Crohn's disease of small intestine'

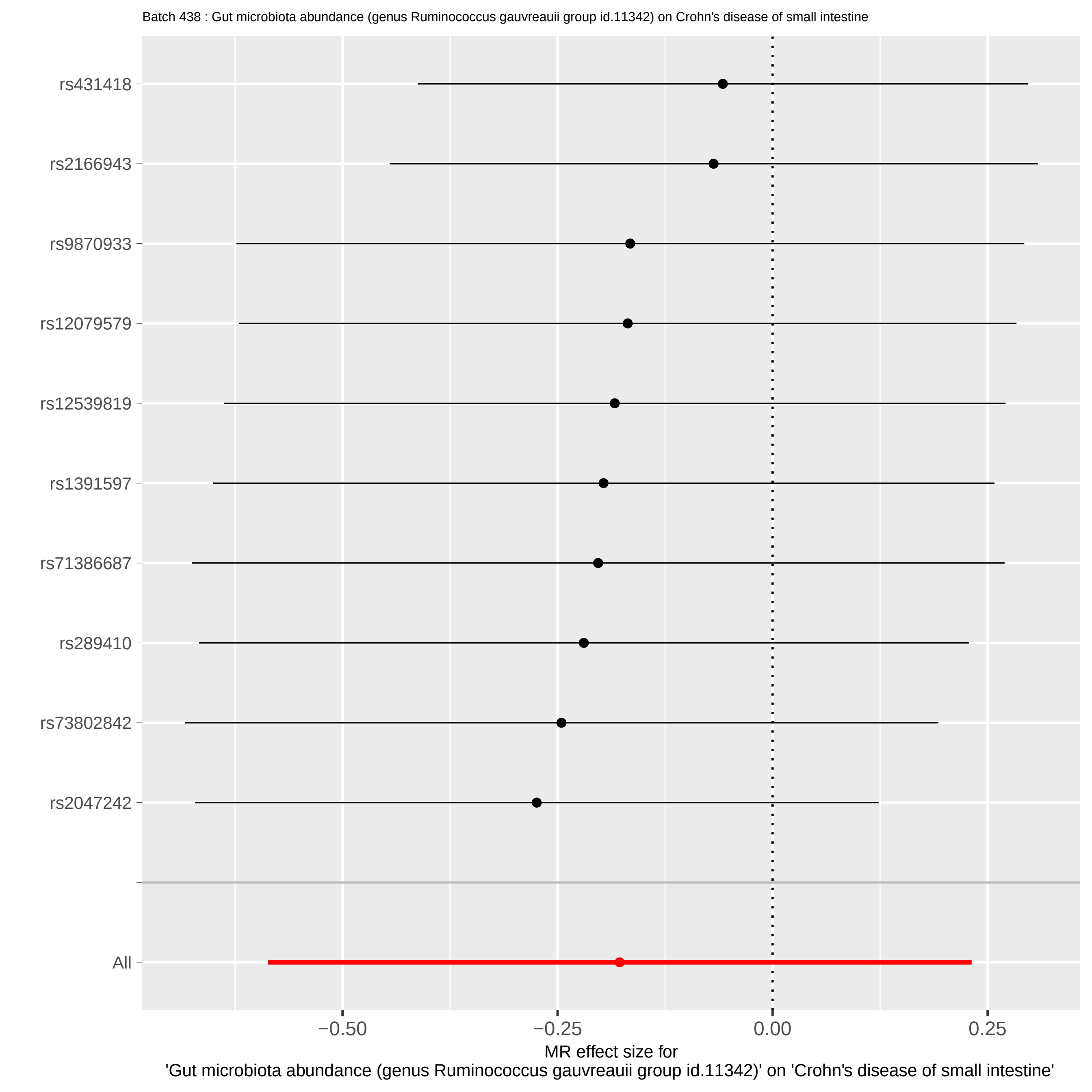
Batch 438 : Gut microbiota abundance (genus Ruminococcus gauvreauii group id.11342) on Crohn's disease of small intestine
rs431418
rs2166943
rs9870933
rs12079579
rs12539819
rs1391597
rs71386687
rs289410
rs73802842
rs2047242
All
−0.50
−0.25
0.00
0.25
MR effect size for
'Gut microbiota abundance (genus Ruminococcus gauvreauii group id.11342)' on 'Crohn's disease of small intestine'

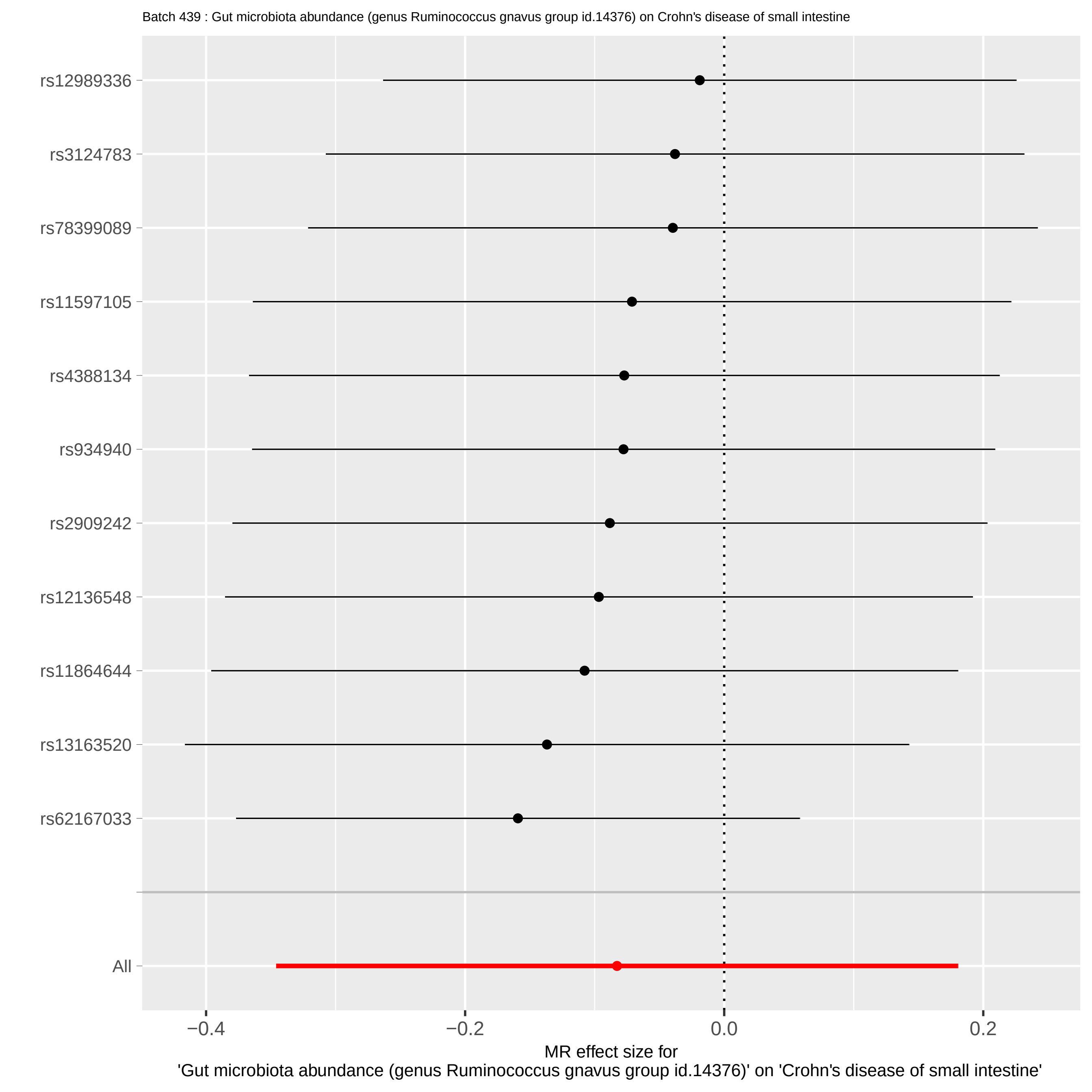
Batch 439 : Gut microbiota abundance (genus Ruminococcus gnavus group id.14376) on Crohn's disease of small intestine
rs12989336
rs3124783
rs78399089
rs11597105
rs4388134
rs934940
rs2909242
rs12136548
rs11864644
rs13163520
rs62167033
All
−0.4
−0.2
0.0
0.2
MR effect size for
'Gut microbiota abundance (genus Ruminococcus gnavus group id.14376)' on 'Crohn's disease of small intestine'

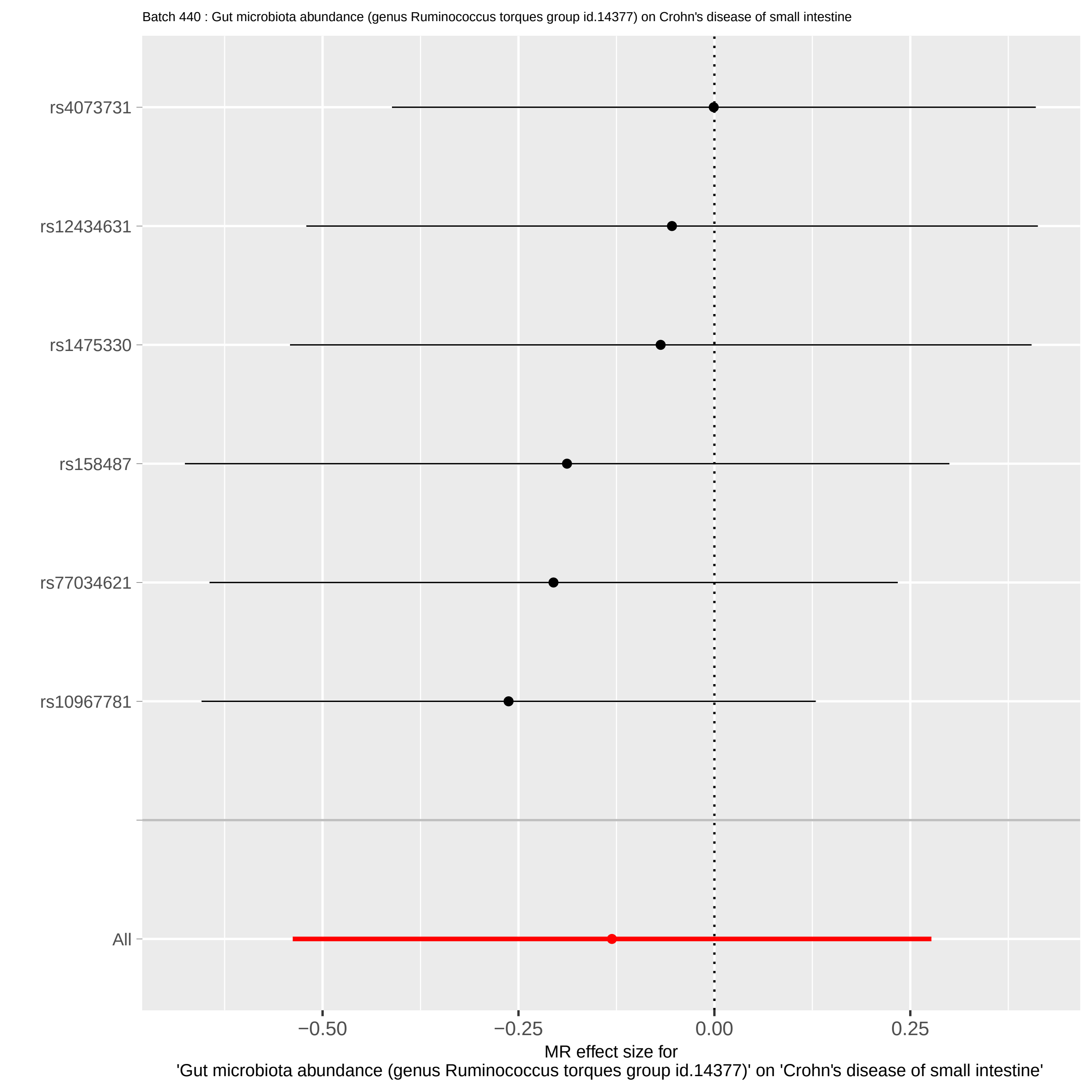
Batch 440 : Gut microbiota abundance (genus Ruminococcus torques group id.14377) on Crohn's disease of small intestine
rs4073731
rs12434631
rs1475330
rs158487
rs77034621
rs10967781
All
−0.50
−0.25
0.00
0.25
MR effect size for
'Gut microbiota abundance (genus Ruminococcus torques group id.14377)' on 'Crohn's disease of small intestine'

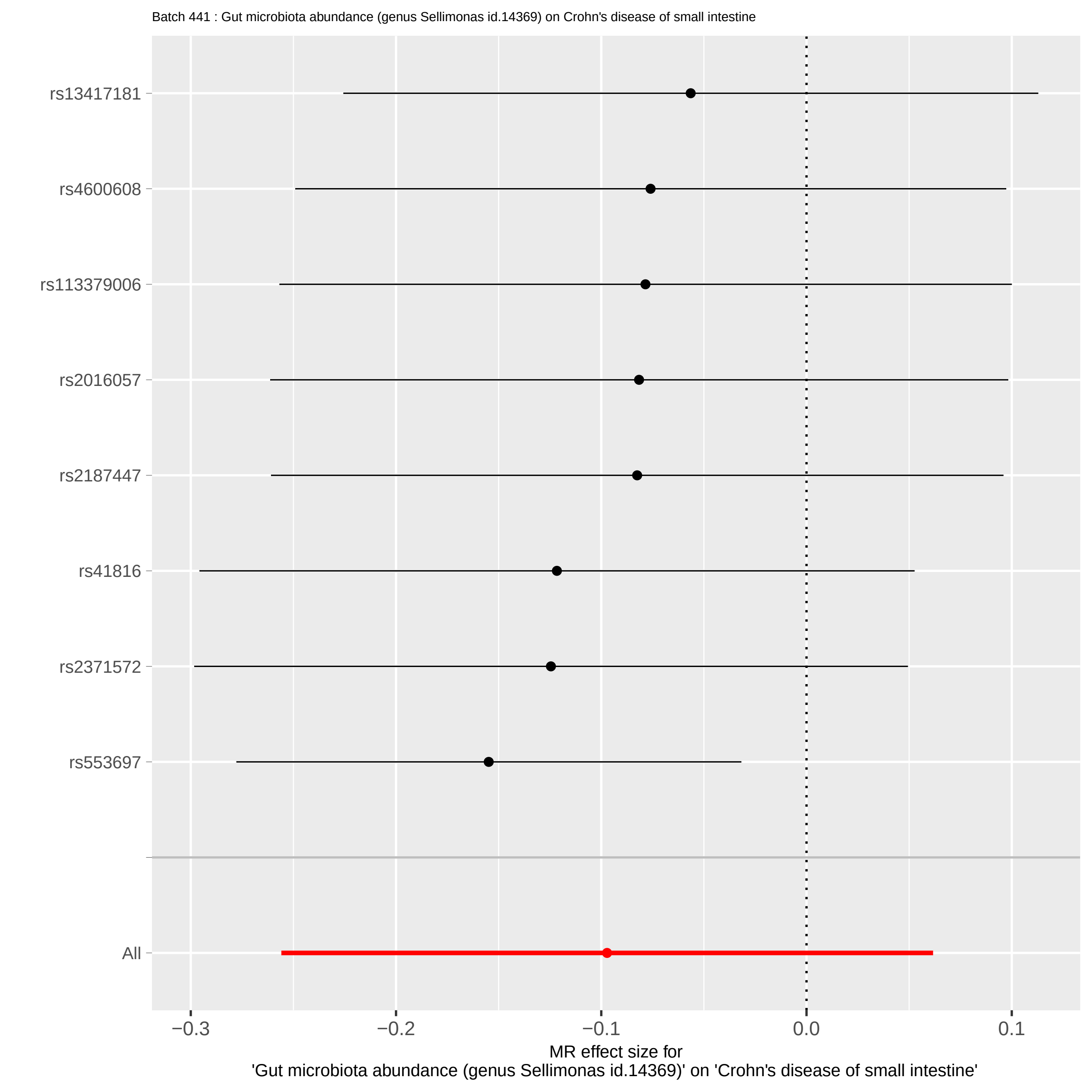
Batch 441 : Gut microbiota abundance (genus Sellimonas id.14369) on Crohn's disease of small intestine
rs13417181
rs4600608
rs113379006
rs2016057
rs2187447
rs41816
rs2371572
rs553697
All
−0.3
−0.2
−0.1
0.0
0.1
MR effect size for
'Gut microbiota abundance (genus Sellimonas id.14369)' on 'Crohn's disease of small intestine'

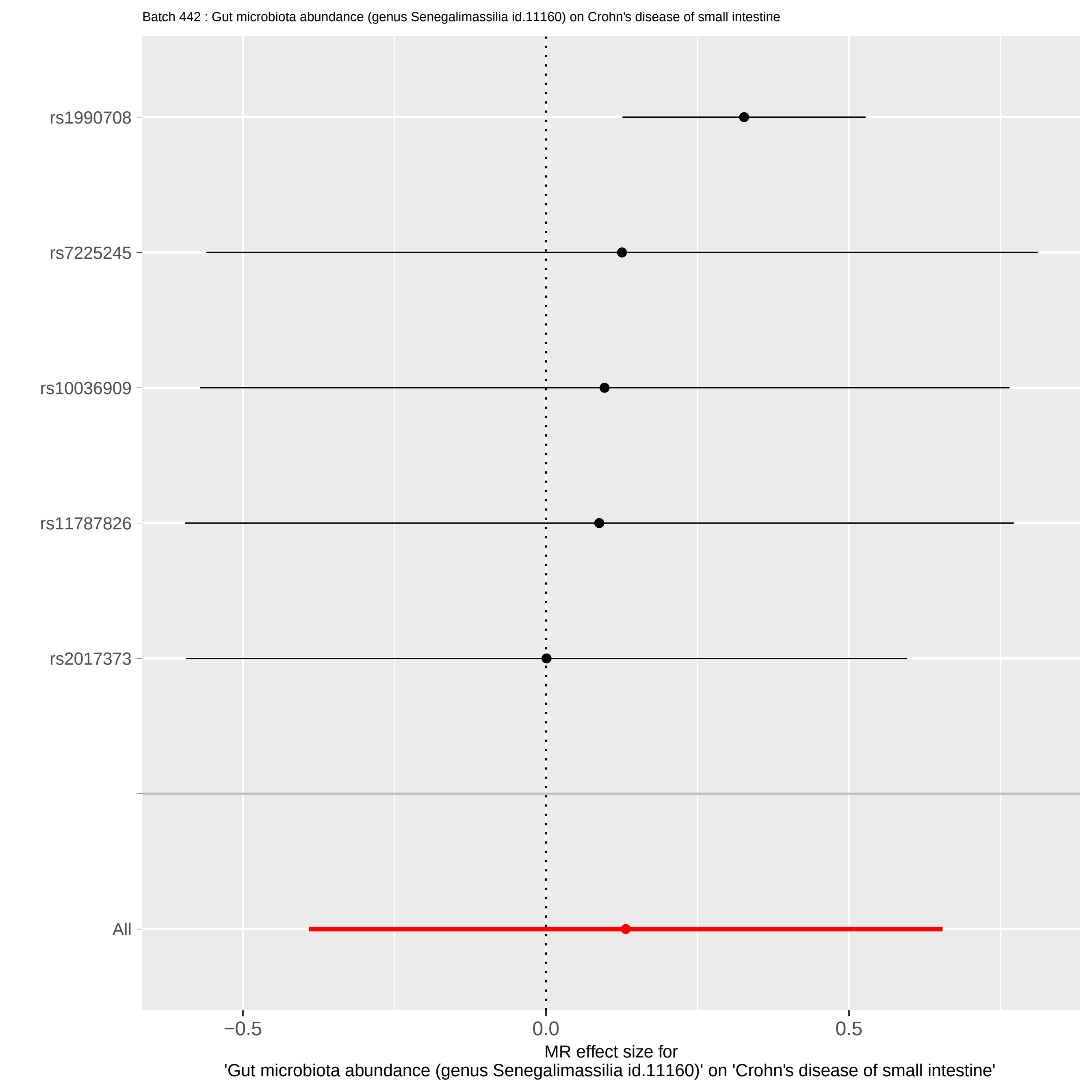
Batch 442 : Gut microbiota abundance (genus Senegalimassilia id.11160) on Crohn's disease of small intestine
rs1990708
rs7225245
rs10036909
rs11787826
rs2017373
All
−0.5
0.0
0.5
MR effect size for
'Gut microbiota abundance (genus Senegalimassilia id.11160)' on 'Crohn's disease of small intestine'

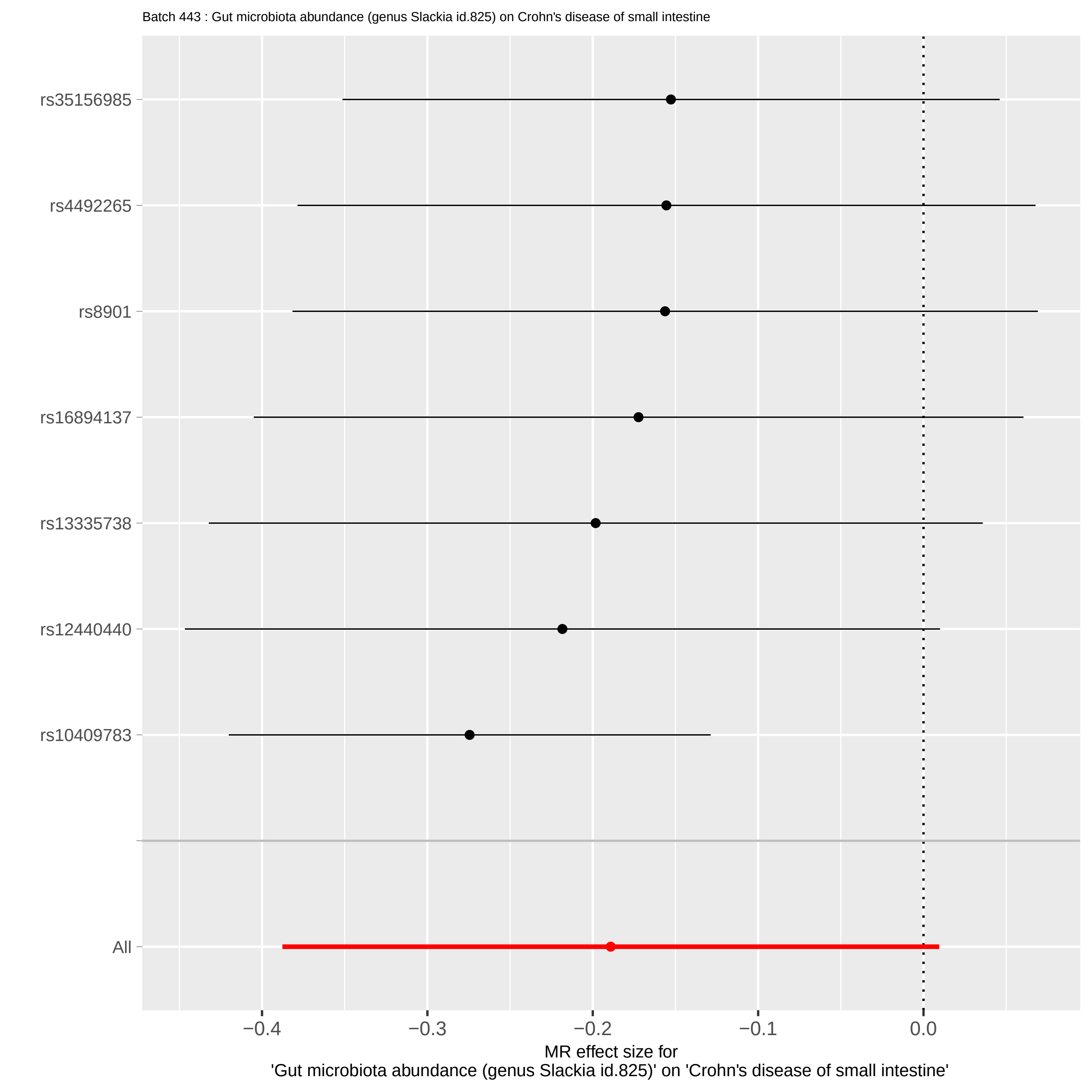

Batch 443 : Gut microbiota abundance (genus Slackia id.825) on Crohn's disease of small intestine
rs35156985
rs4492265
rs8901
rs16894137
rs13335738
rs12440440
rs10409783
All
−0.4
−0.3
−0.2
−0.1
0.0
MR effect size for
'Gut microbiota abundance (genus Slackia id.825)' on 'Crohn's disease of small intestine'

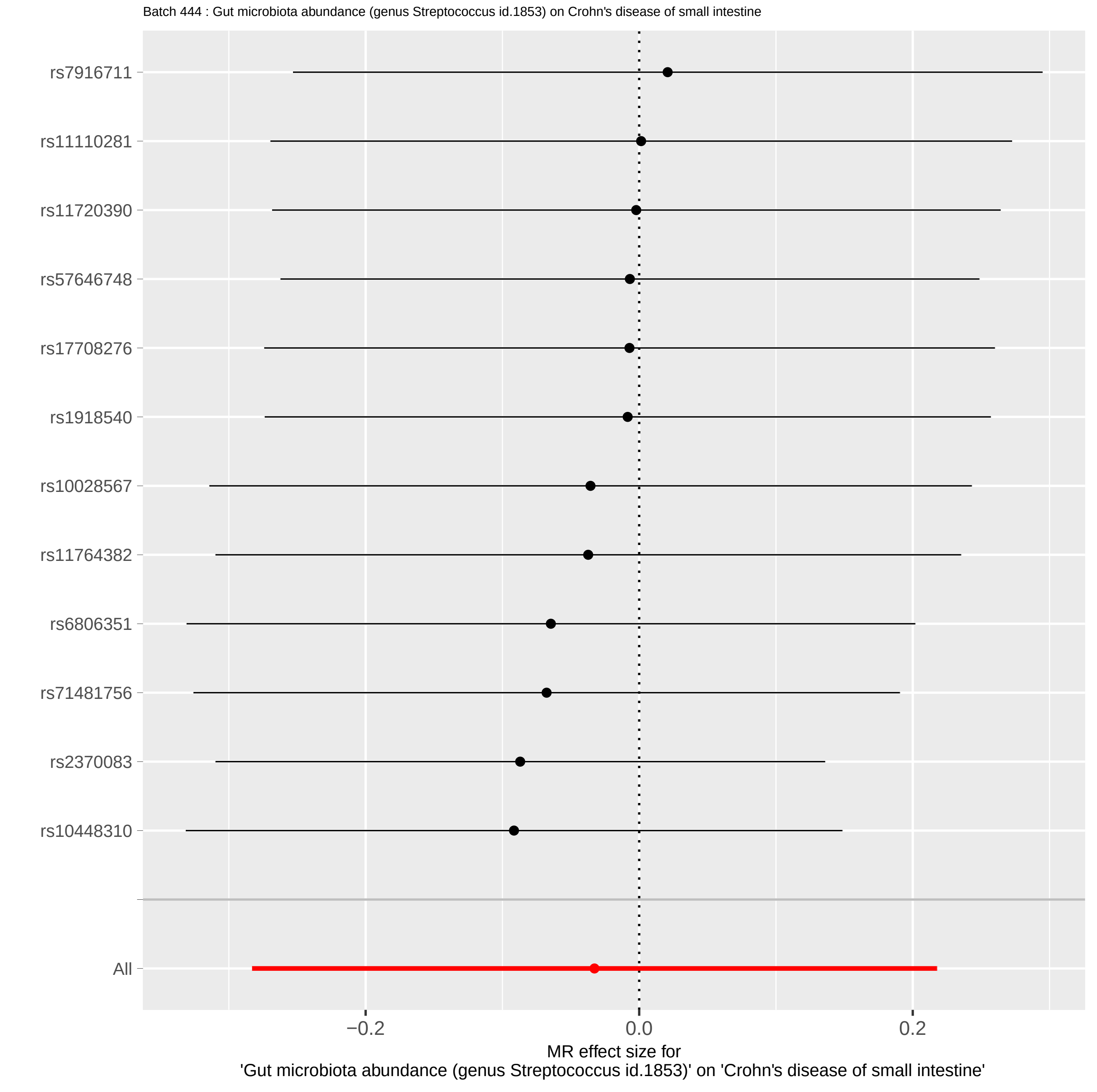

Batch 444 : Gut microbiota abundance (genus Streptococcus id.1853) on Crohn's disease of small intestine
rs7916711
rs11110281
rs11720390
rs57646748
rs17708276
rs1918540
rs10028567
rs11764382
rs6806351
rs71481756
rs2370083
rs10448310
All
−0.2
0.0
0.2
MR effect size for
'Gut microbiota abundance (genus Streptococcus id.1853)' on 'Crohn's disease of small intestine'

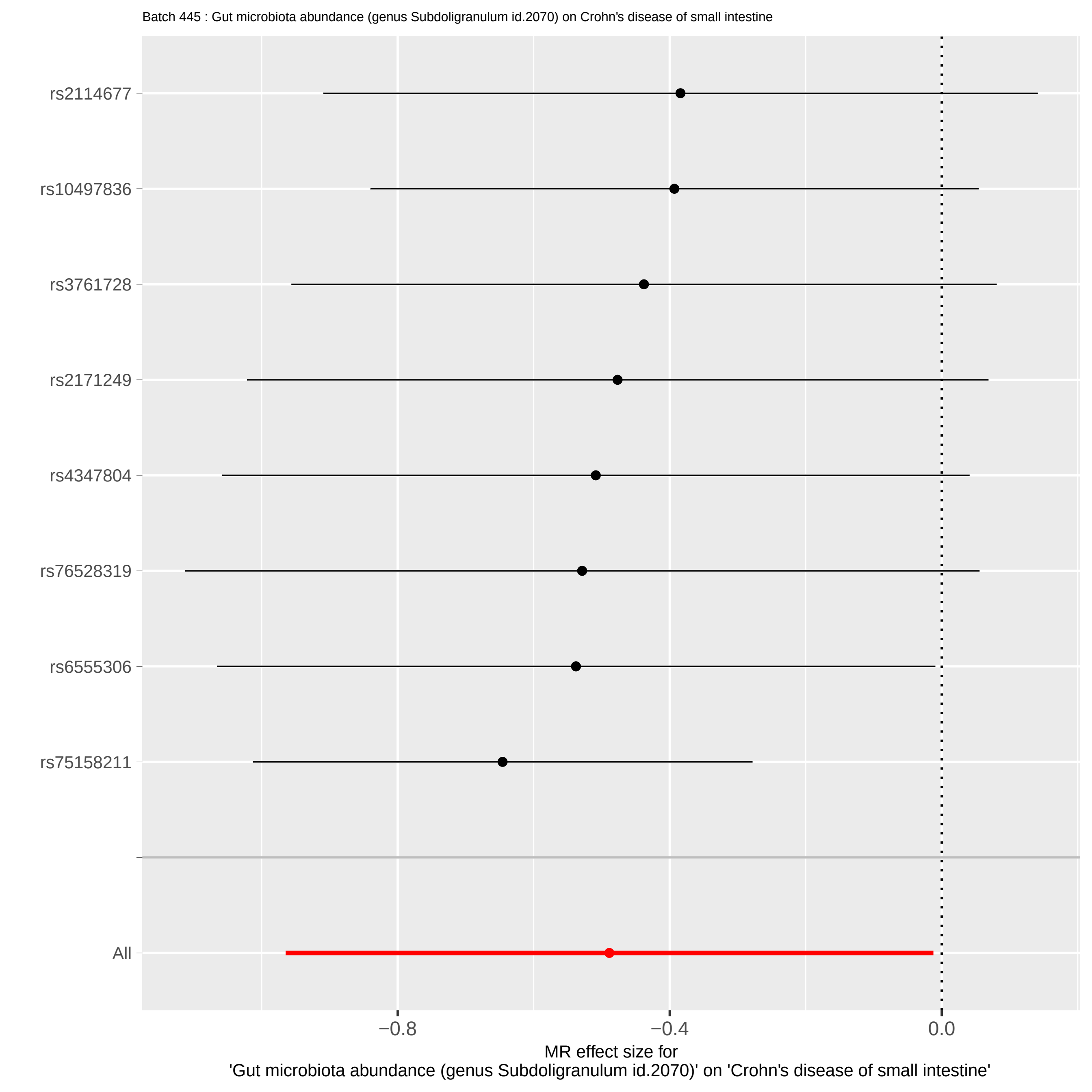

Batch 445 : Gut microbiota abundance (genus Subdoligranulum id.2070) on Crohn's disease of small intestine
rs2114677
rs10497836
rs3761728
rs2171249
rs4347804
rs76528319
rs6555306
rs75158211
All
−0.8
−0.4
0.0
MR effect size for
'Gut microbiota abundance (genus Subdoligranulum id.2070)' on 'Crohn's disease of small intestine'

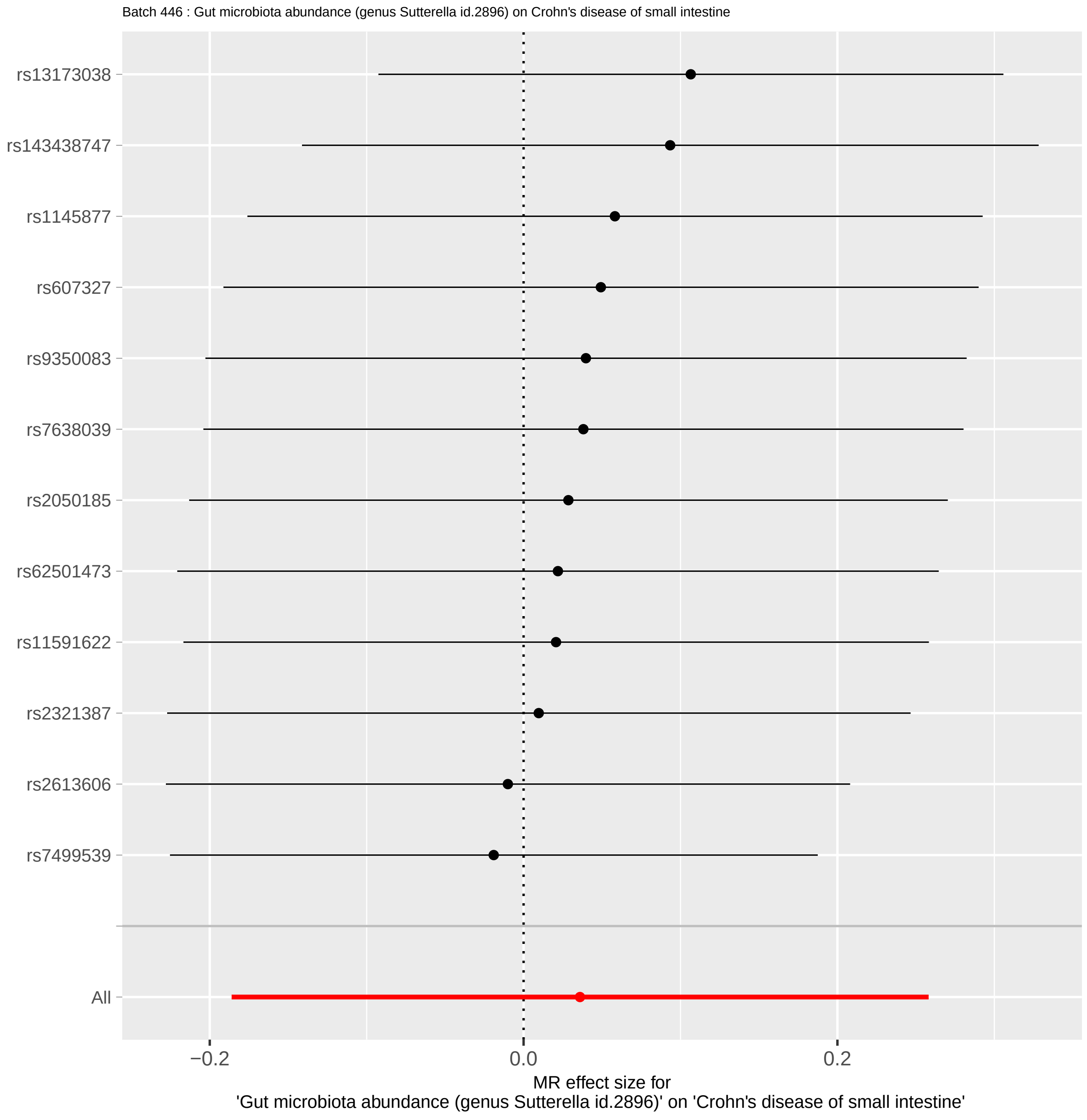

Batch 446 : Gut microbiota abundance (genus Sutterella id.2896) on Crohn's disease of small intestine
rs13173038
rs143438747
rs1145877
rs607327
rs9350083
rs7638039
rs2050185
rs62501473
rs11591622
rs2321387
rs2613606
rs7499539
All
−0.2
0.0
0.2
MR effect size for
'Gut microbiota abundance (genus Sutterella id.2896)' on 'Crohn's disease of small intestine'

Batch 447 : Gut microbiota abundance (genus Terrisporobacter id.11348) on Crohn's disease of small intestine

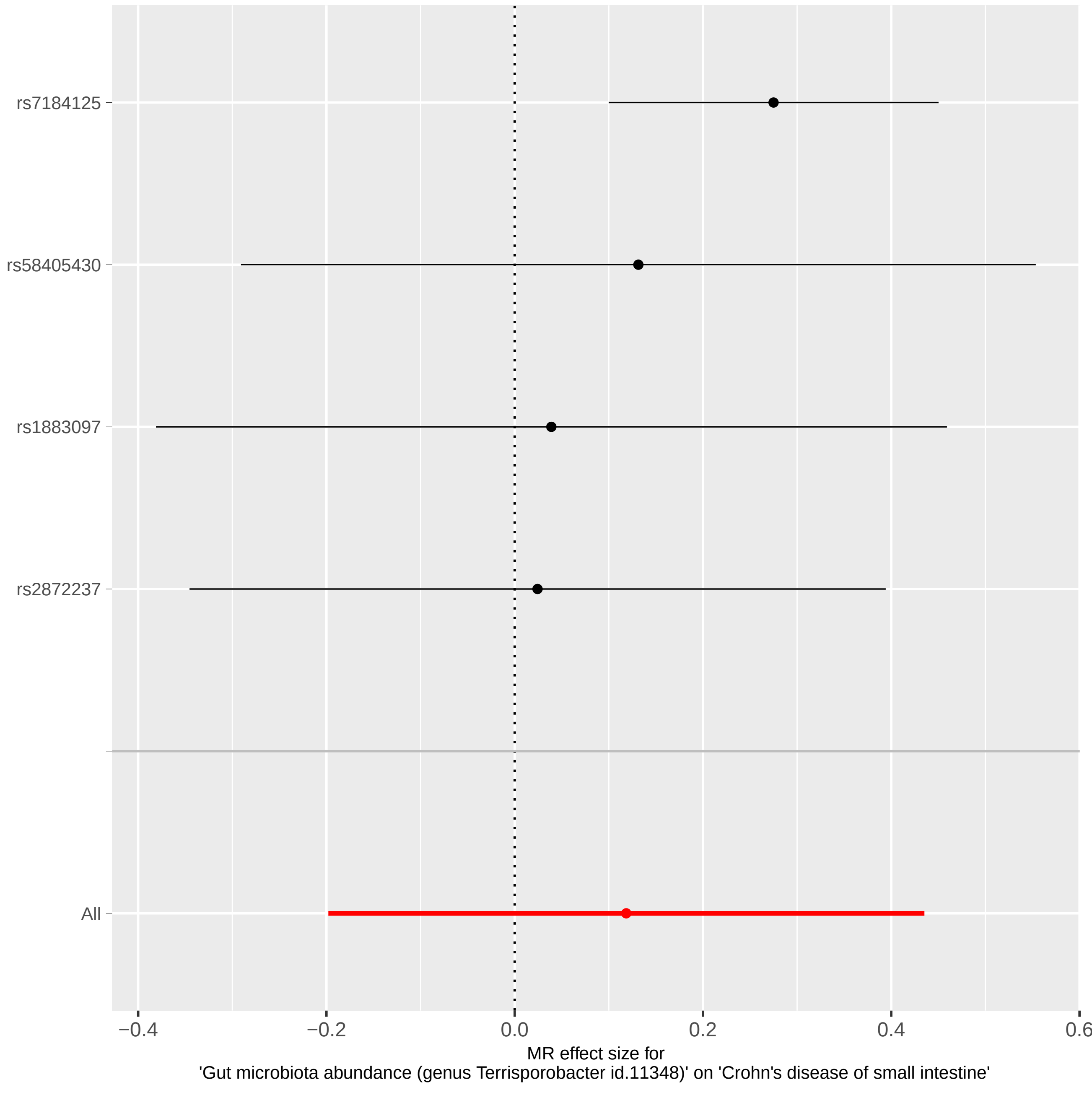

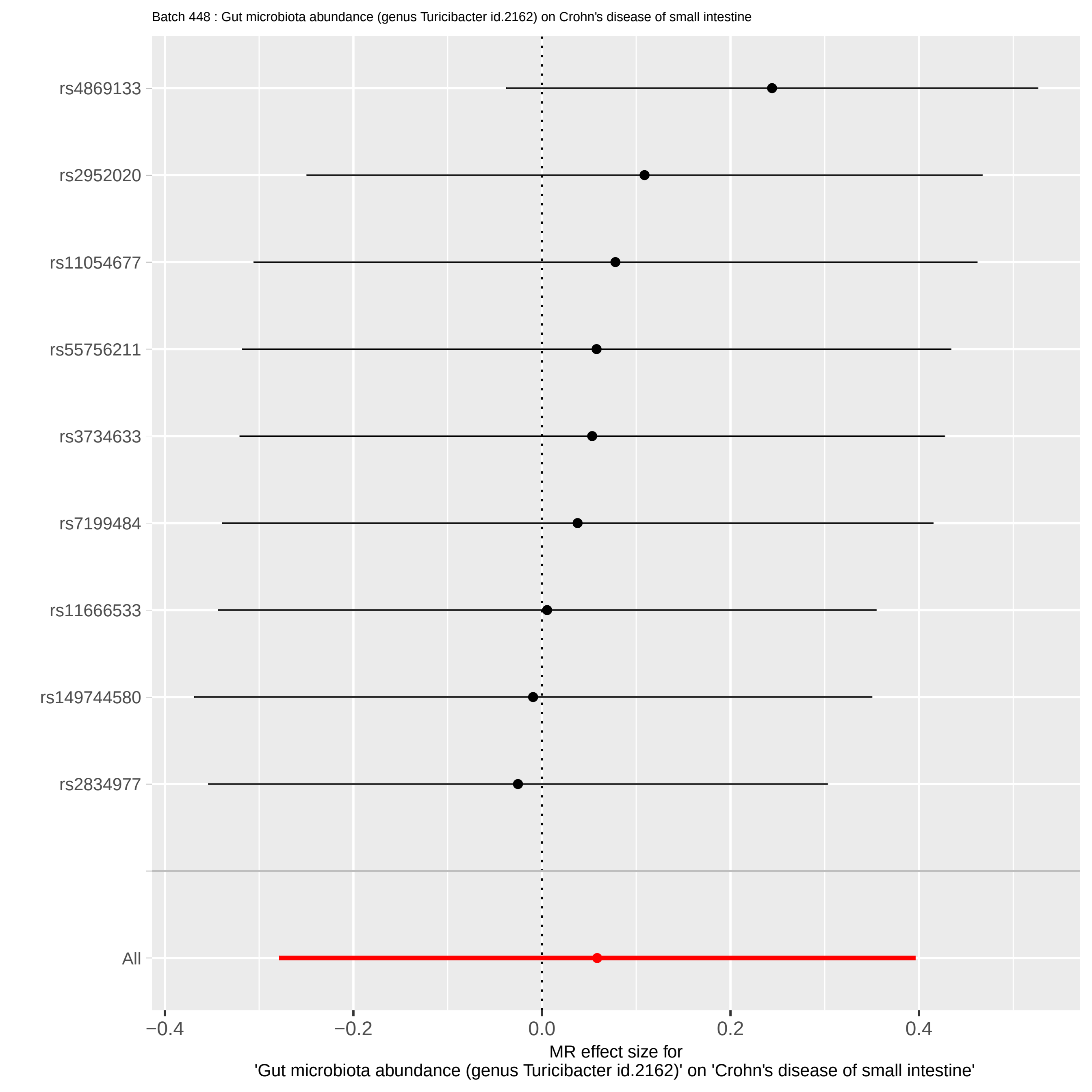

Batch 448 : Gut microbiota abundance (genus Turicibacter id.2162) on Crohn's disease of small intestine
rs4869133
rs2952020
rs11054677
rs55756211
rs3734633
rs7199484
rs11666533
rs149744580
rs2834977
All
−0.4
−0.2
0.0
0.2
0.4
MR effect size for
'Gut microbiota abundance (genus Turicibacter id.2162)' on 'Crohn's disease of small intestine'

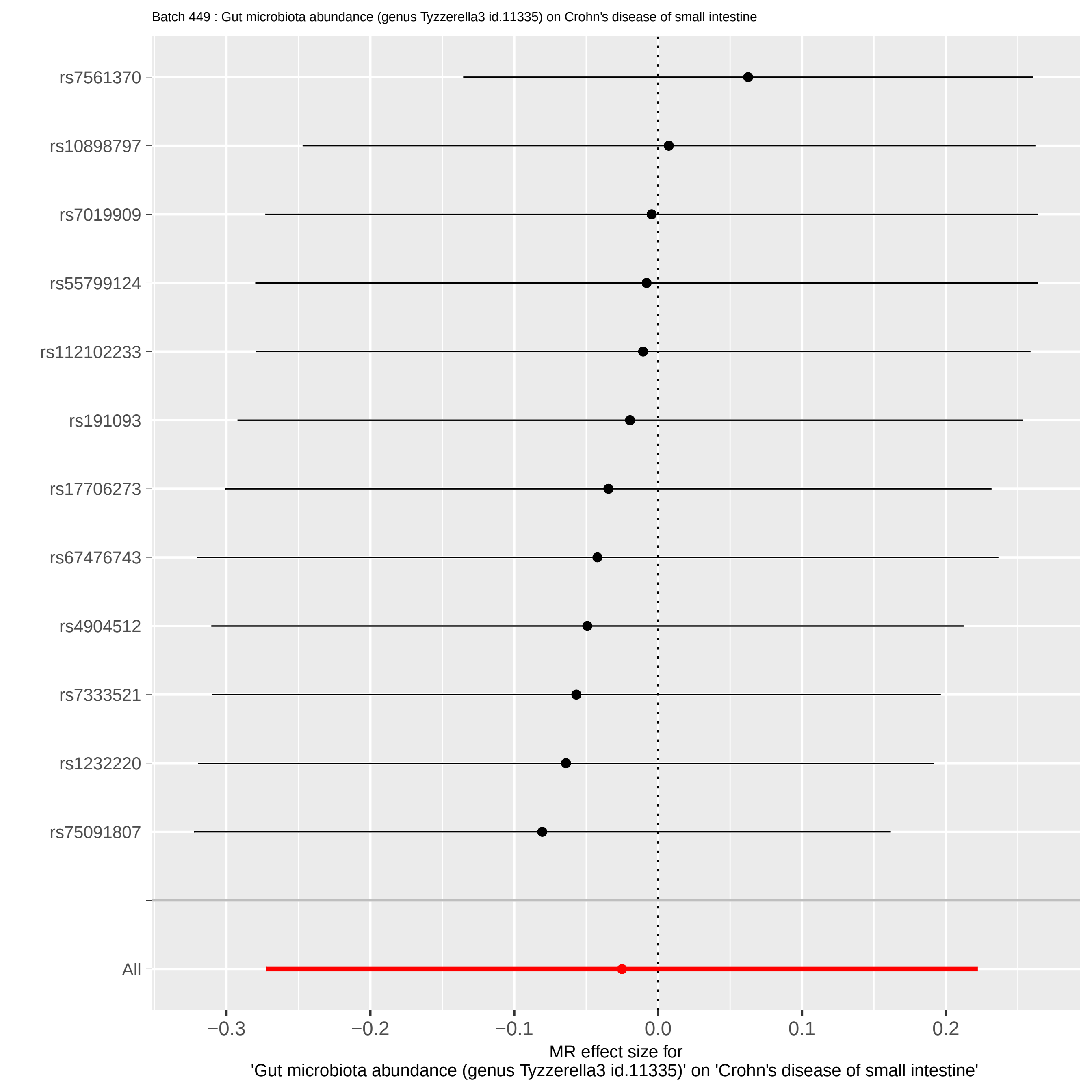

Batch 449 : Gut microbiota abundance (genus Tyzzerella3 id.11335) on Crohn's disease of small intestine
rs7561370
rs10898797
rs7019909
rs55799124
rs112102233
rs191093
rs17706273
rs67476743
rs4904512
rs7333521
rs1232220
rs75091807
All
−0.3
−0.2
−0.1
0.0
0.1
0.2
MR effect size for
'Gut microbiota abundance (genus Tyzzerella3 id.11335)' on 'Crohn's disease of small intestine'

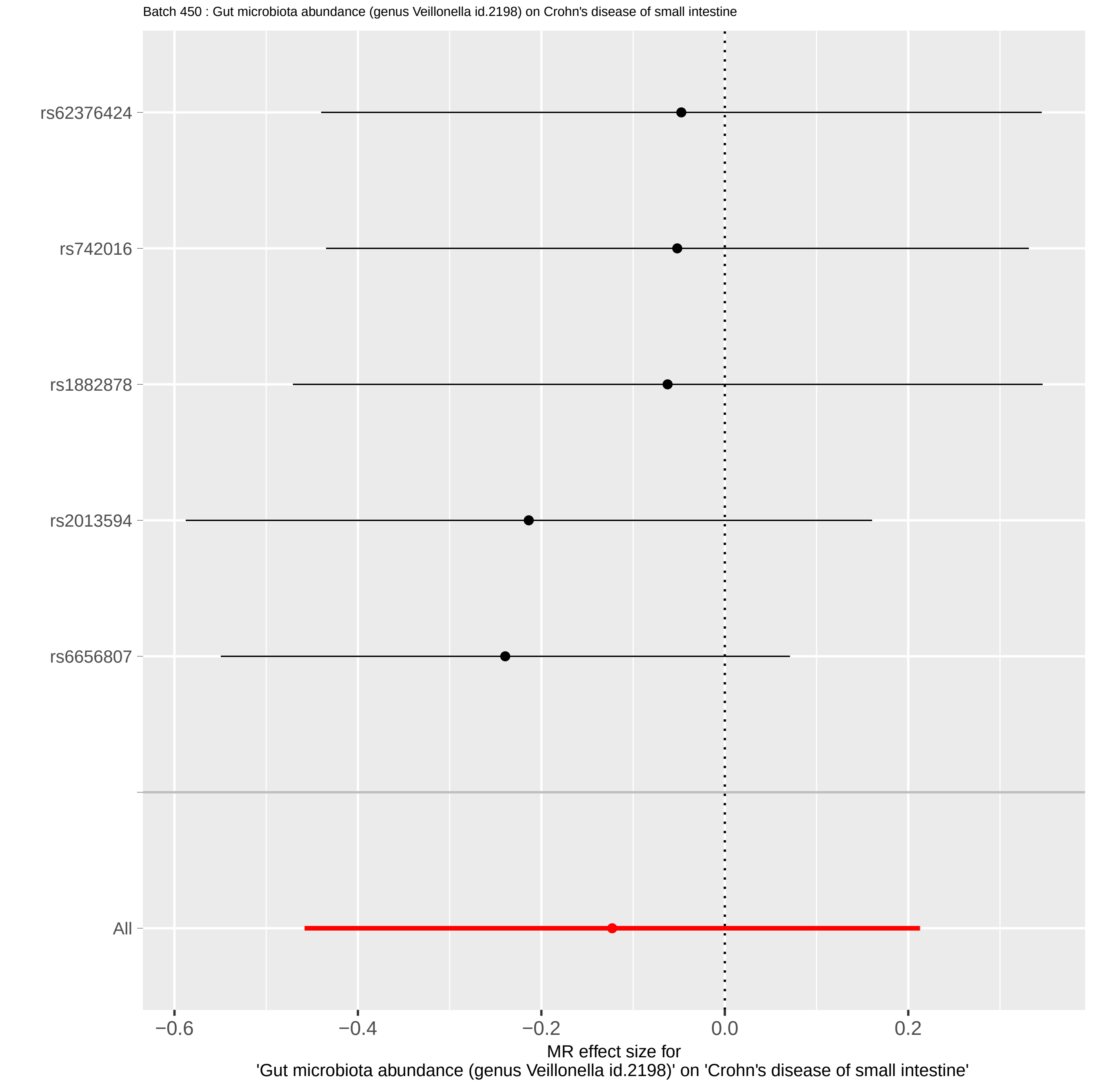

Batch 450 : Gut microbiota abundance (genus Veillonella id.2198) on Crohn's disease of small intestine
rs62376424
rs742016
rs1882878
rs2013594
rs6656807
All
−0.6
−0.4
−0.2
0.0
0.2
MR effect size for
'Gut microbiota abundance (genus Veillonella id.2198)' on 'Crohn's disease of small intestine'

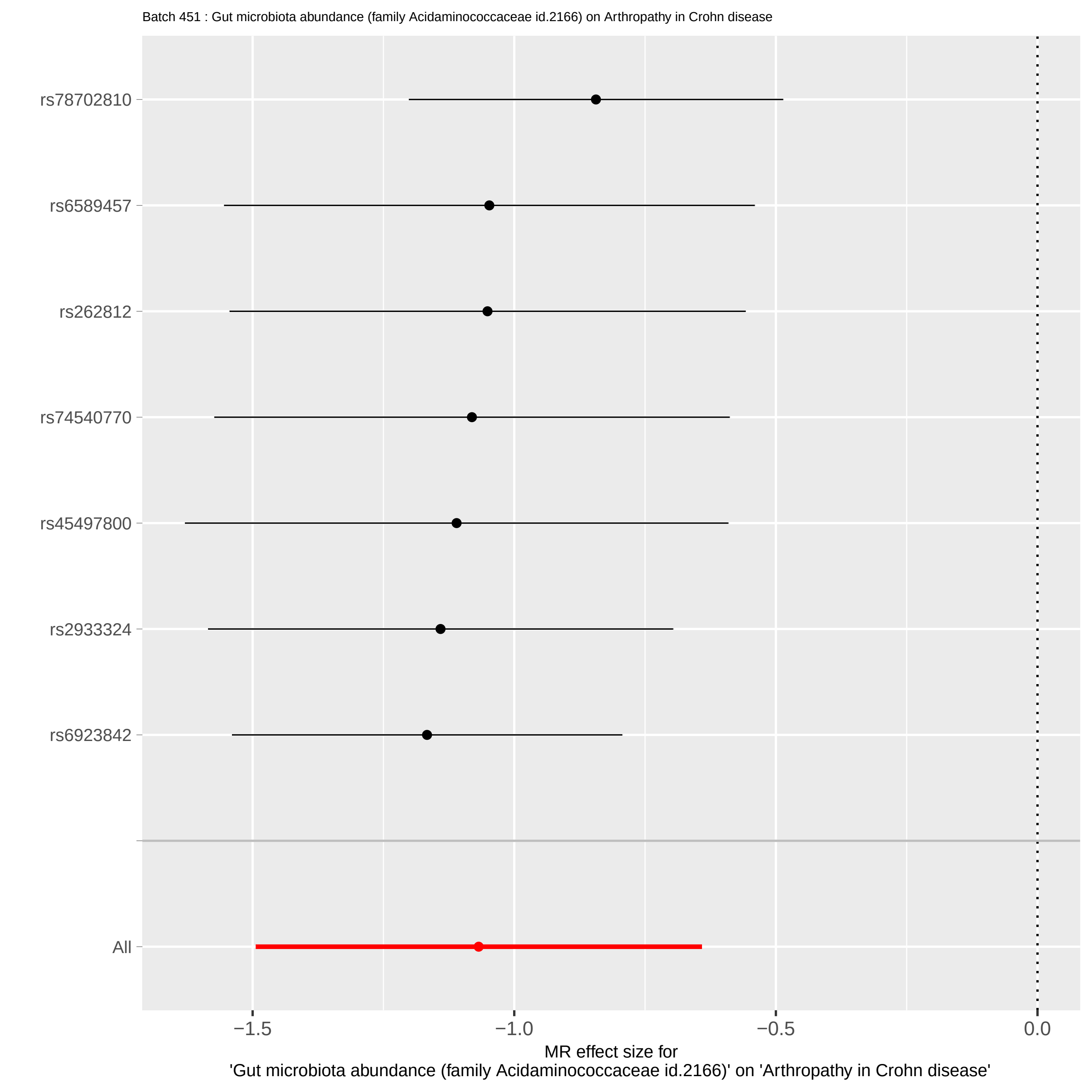

Batch 451 : Gut microbiota abundance (family Acidaminococcaceae id.2166) on Arthropathy in Crohn disease
rs78702810
rs6589457
rs262812
rs74540770
rs45497800
rs2933324
rs6923842
All
−1.5
−1.0
−0.5
0.0
MR effect size for
'Gut microbiota abundance (family Acidaminococcaceae id.2166)' on 'Arthropathy in Crohn disease'

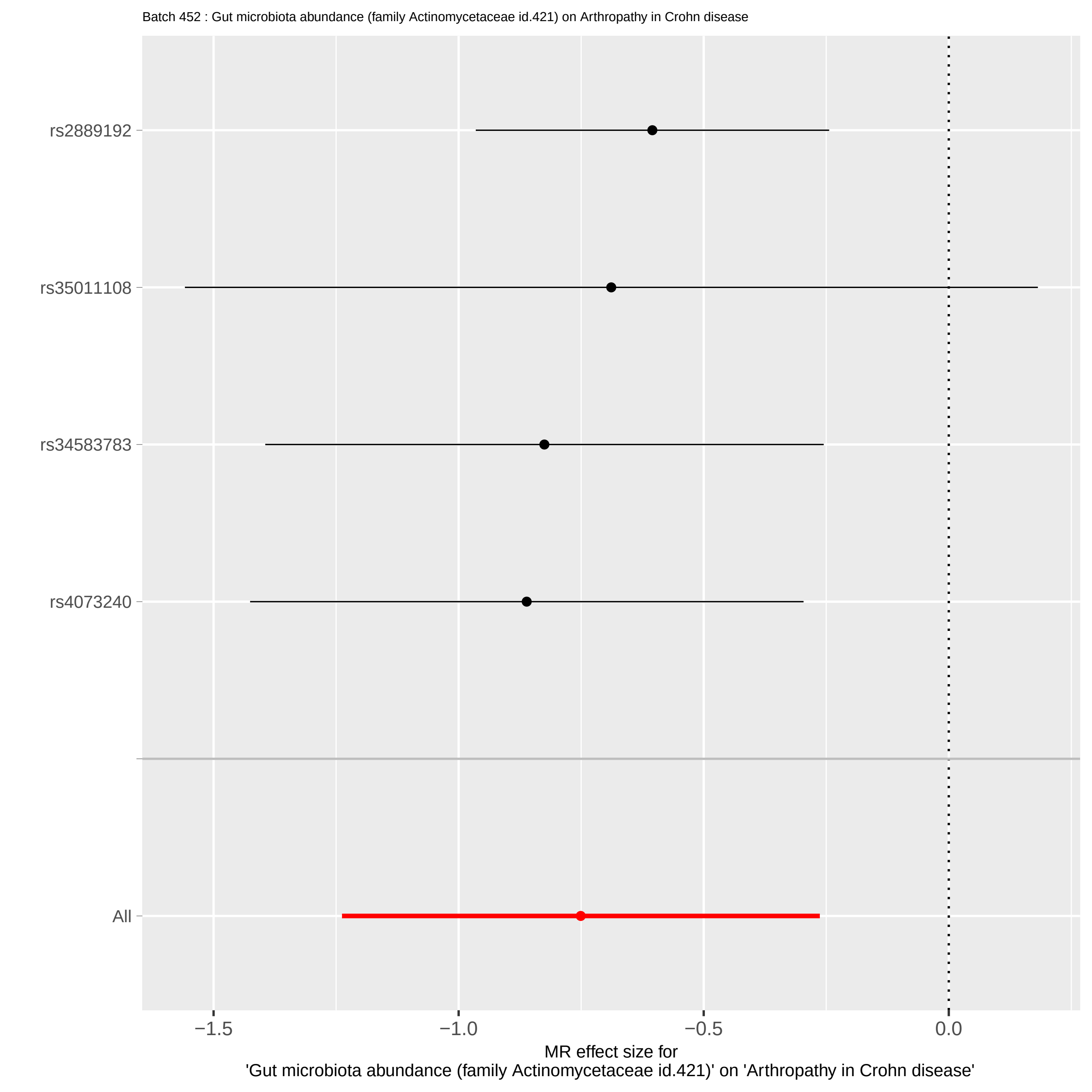
Batch 452 : Gut microbiota abundance (family Actinomycetaceae id.421) on Arthropathy in Crohn disease
rs2889192
rs35011108
rs34583783
rs4073240
All
−1.5
−1.0
−0.5
0.0
MR effect size for
'Gut microbiota abundance (family Actinomycetaceae id.421)' on 'Arthropathy in Crohn disease'

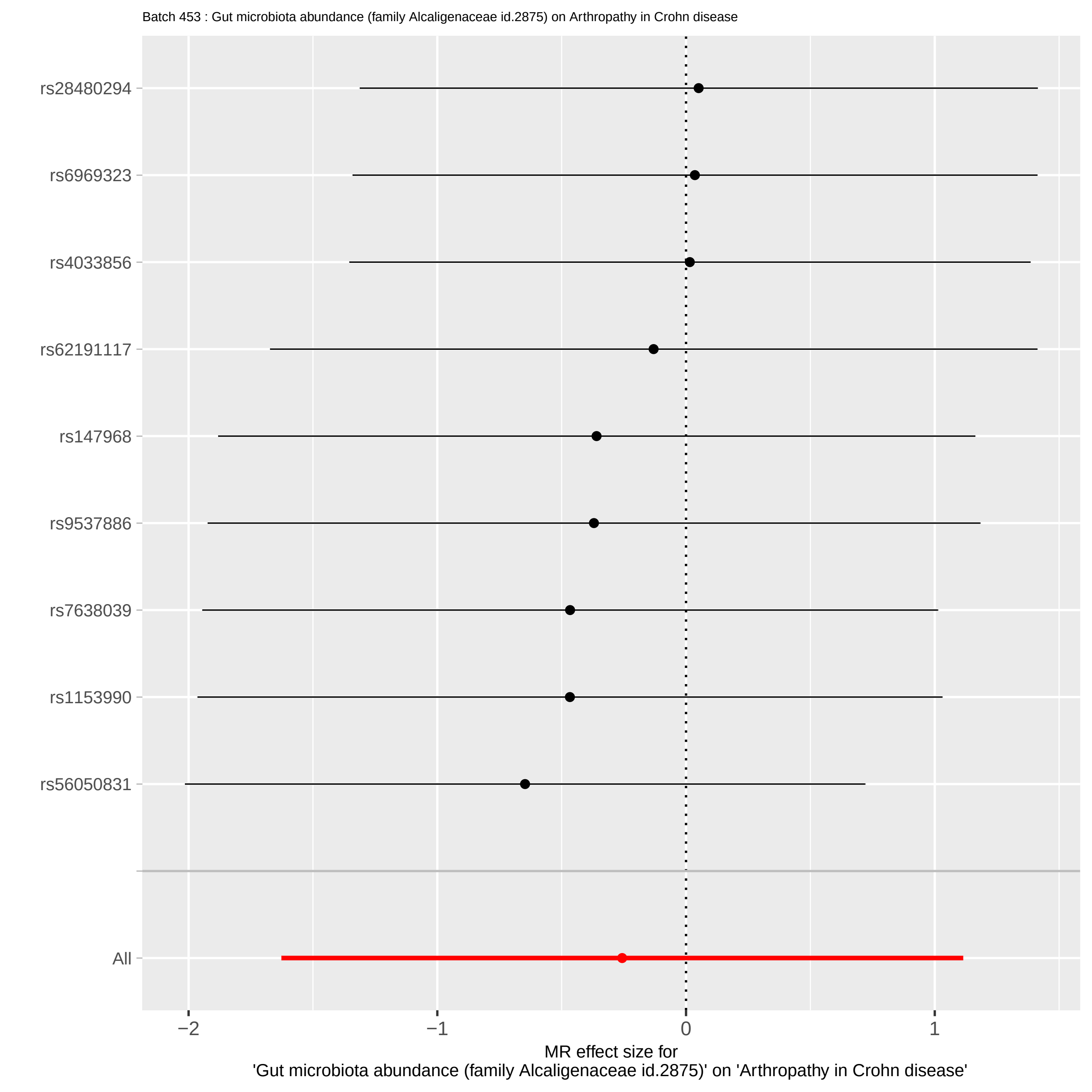
Batch 453 : Gut microbiota abundance (family Alcaligenaceae id.2875) on Arthropathy in Crohn disease
rs28480294
rs6969323
rs4033856
rs62191117
rs147968
rs9537886
rs7638039
rs1153990
rs56050831
All
−2
−1
0
1
MR effect size for
'Gut microbiota abundance (family Alcaligenaceae id.2875)' on 'Arthropathy in Crohn disease'

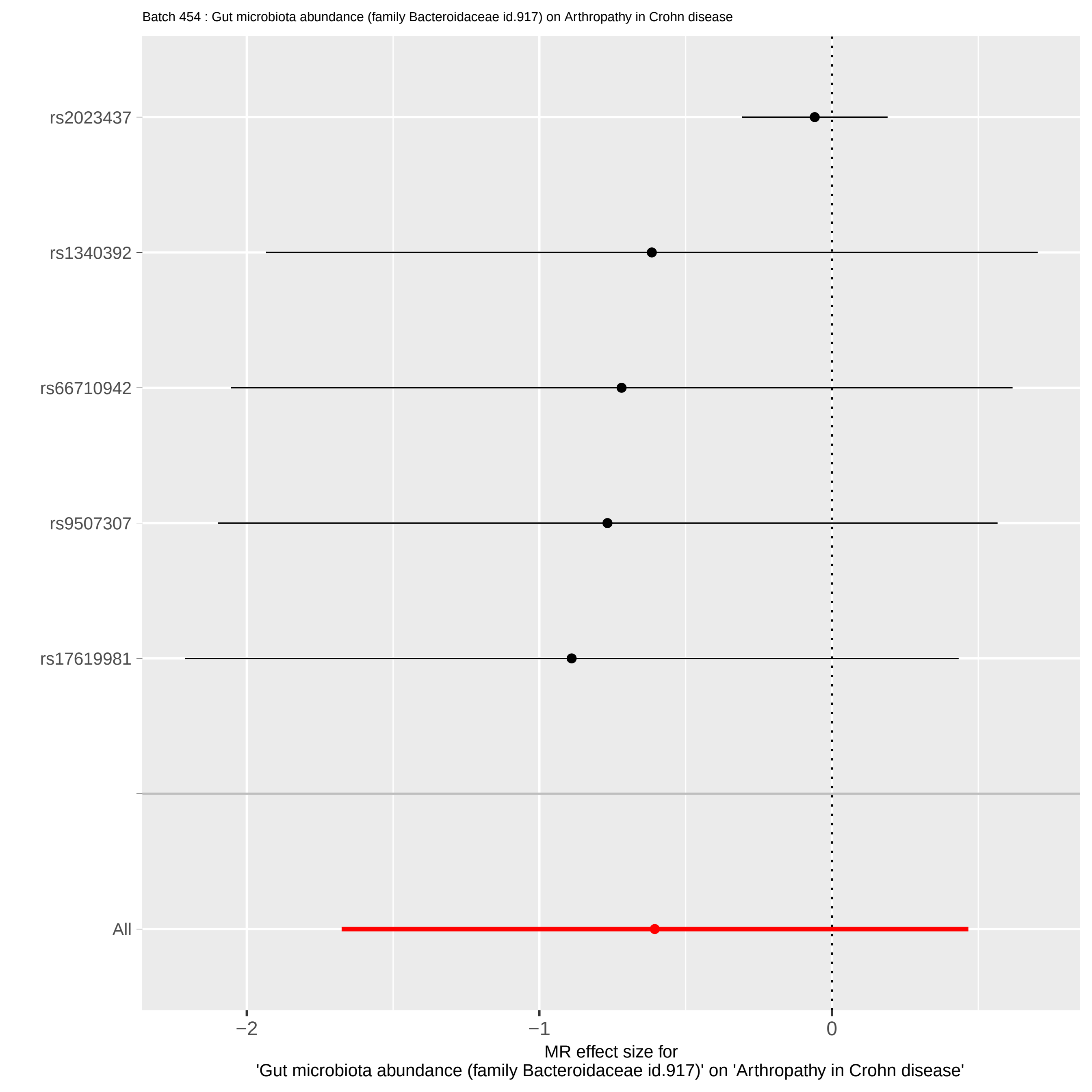

Batch 454 : Gut microbiota abundance (family Bacteroidaceae id.917) on Arthropathy in Crohn disease
rs2023437
rs1340392
rs66710942
rs9507307
rs17619981
All
−2
−1
0
MR effect size for
'Gut microbiota abundance (family Bacteroidaceae id.917)' on 'Arthropathy in Crohn disease'

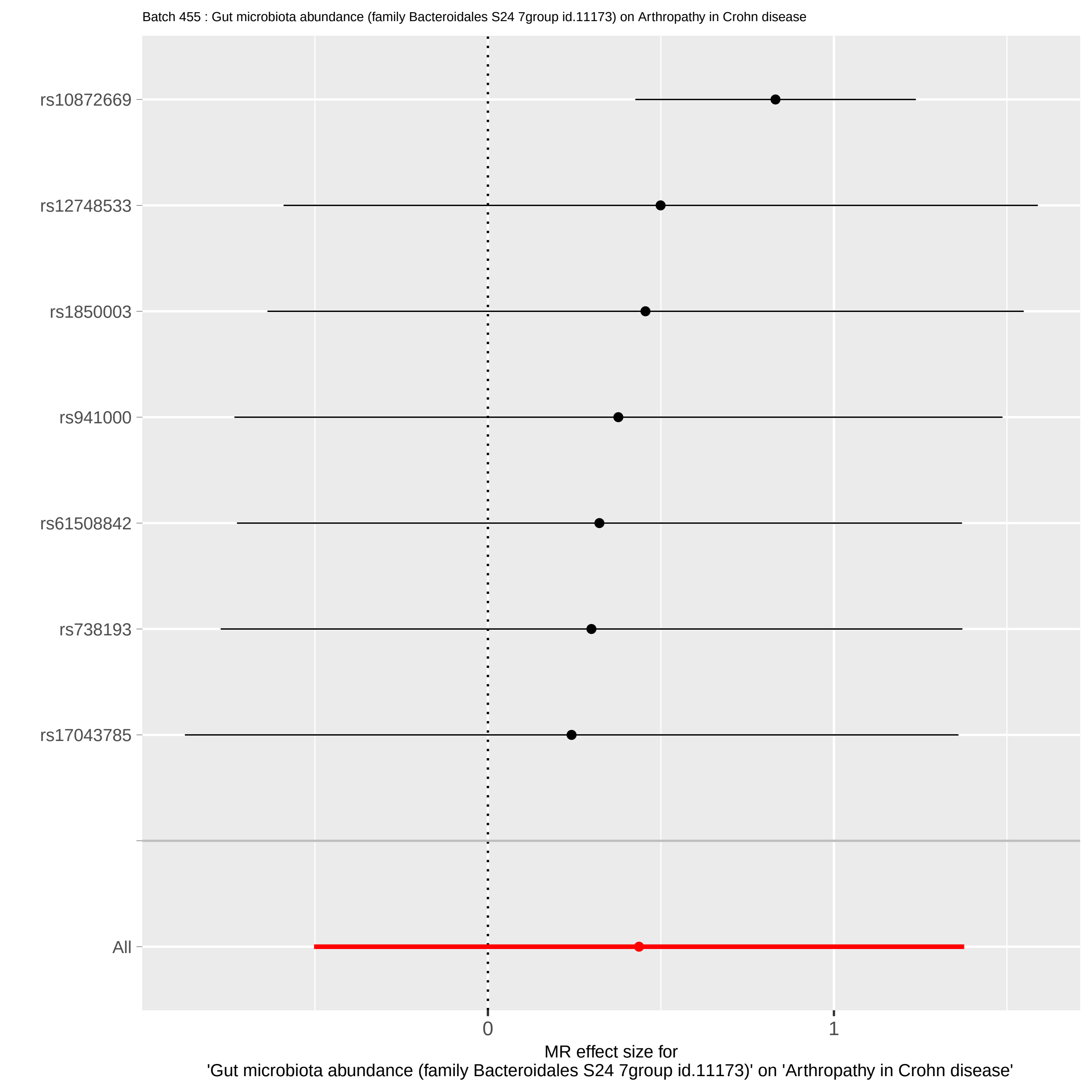
Batch 455 : Gut microbiota abundance (family Bacteroidales S24 7group id.11173) on Arthropathy in Crohn disease
rs10872669
rs12748533
rs1850003
rs941000
rs61508842
rs738193
rs17043785
All
0
1
MR effect size for
'Gut microbiota abundance (family Bacteroidales S24 7group id.11173)' on 'Arthropathy in Crohn disease'

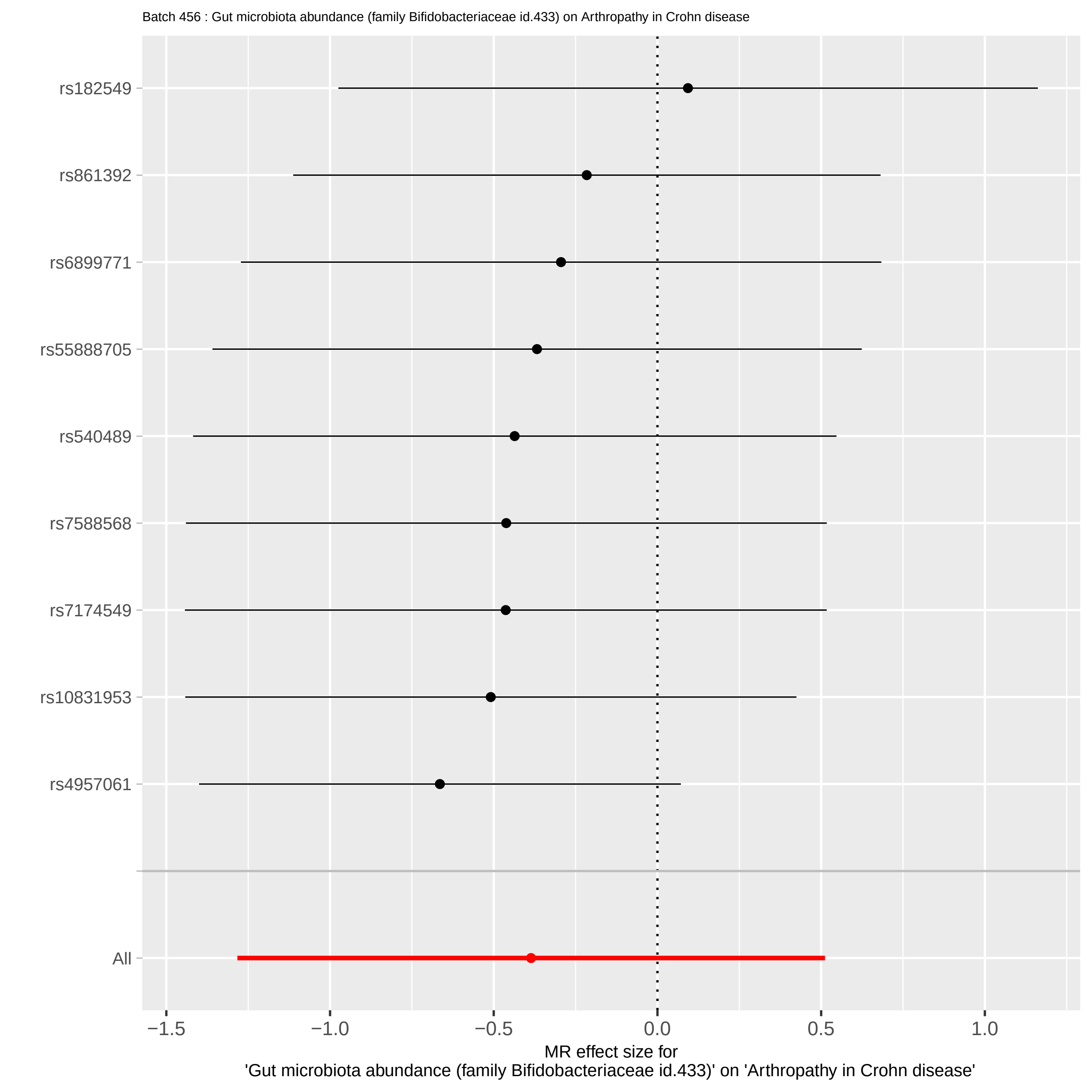

Batch 456 : Gut microbiota abundance (family Bifidobacteriaceae id.433) on Arthropathy in Crohn disease
rs182549
rs861392
rs6899771
rs55888705
rs540489
rs7588568
rs7174549
rs10831953
rs4957061
All
−1.5
−1.0
−0.5
0.0
0.5
1.0
MR effect size for
'Gut microbiota abundance (family Bifidobacteriaceae id.433)' on 'Arthropathy in Crohn disease'

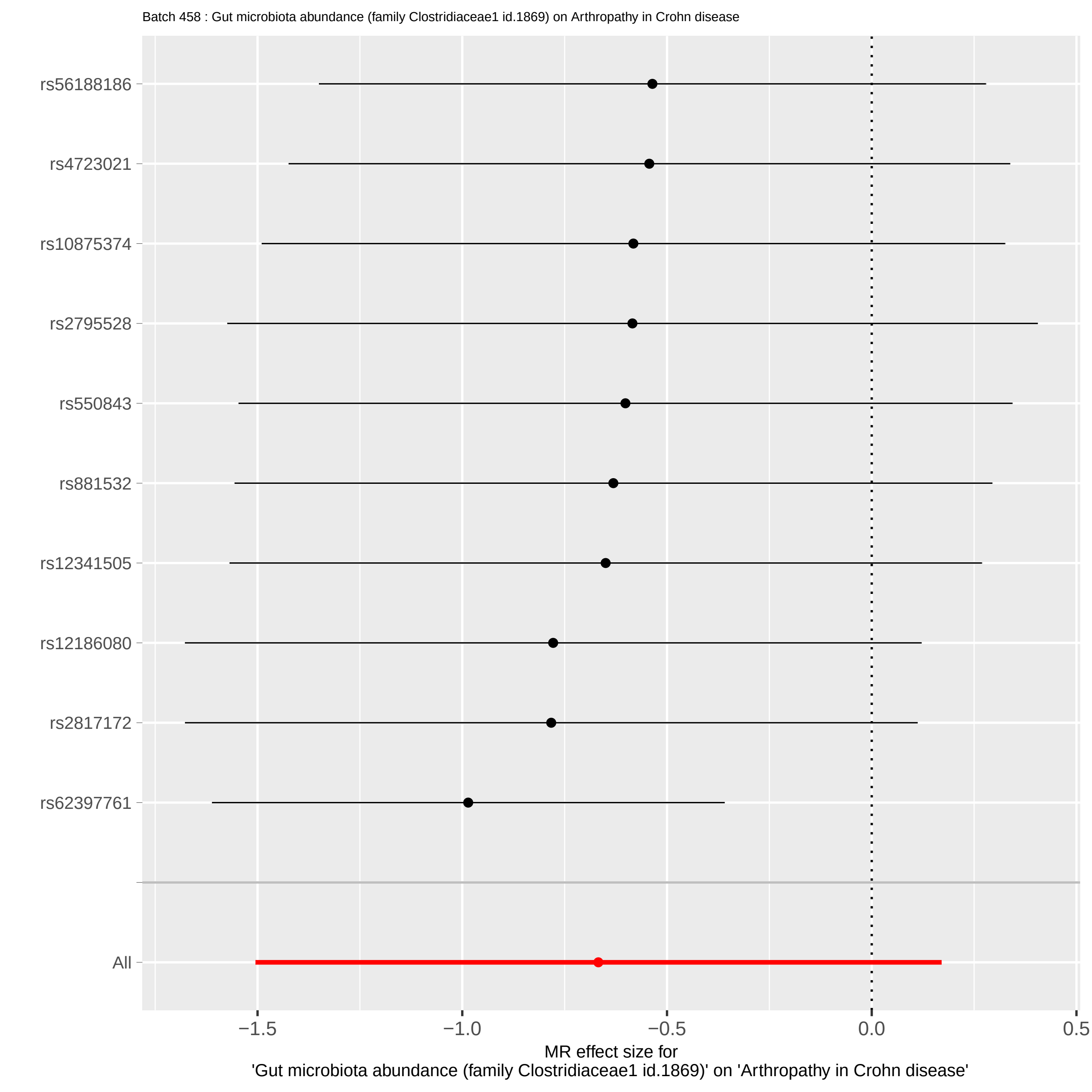

Batch 458 : Gut microbiota abundance (family Clostridiaceae1 id.1869) on Arthropathy in Crohn disease
rs56188186
rs4723021
rs10875374
rs2795528
rs550843
rs881532
rs12341505
rs12186080
rs2817172
rs62397761
All
−1.5
−1.0
−0.5
0.0
0.5
MR effect size for
'Gut microbiota abundance (family Clostridiaceae1 id.1869)' on 'Arthropathy in Crohn disease'

Batch 459 : Gut microbiota abundance (family Clostridiales vadin BB60 group id.11286) on Arthropathy in Crohn disease

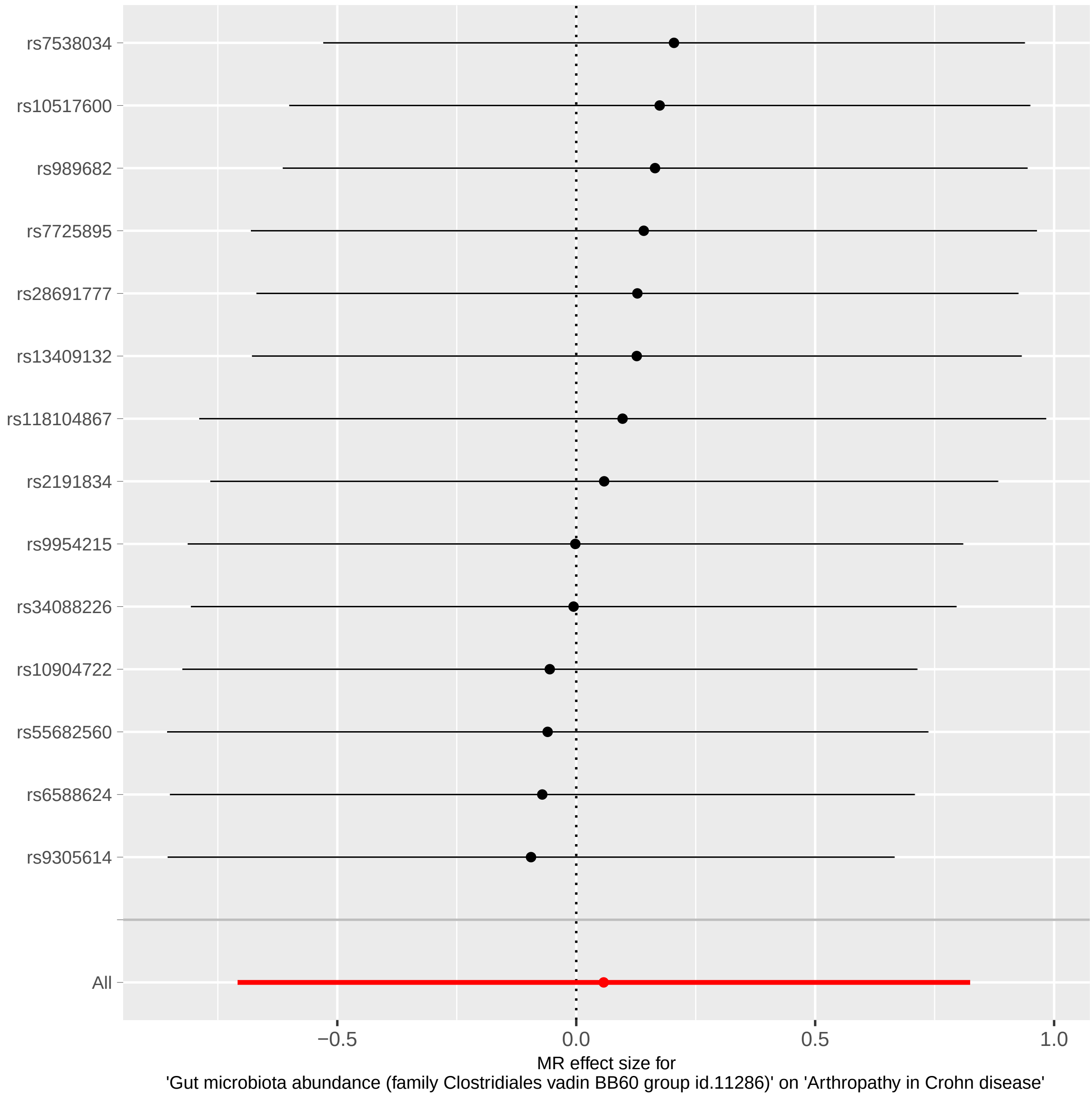

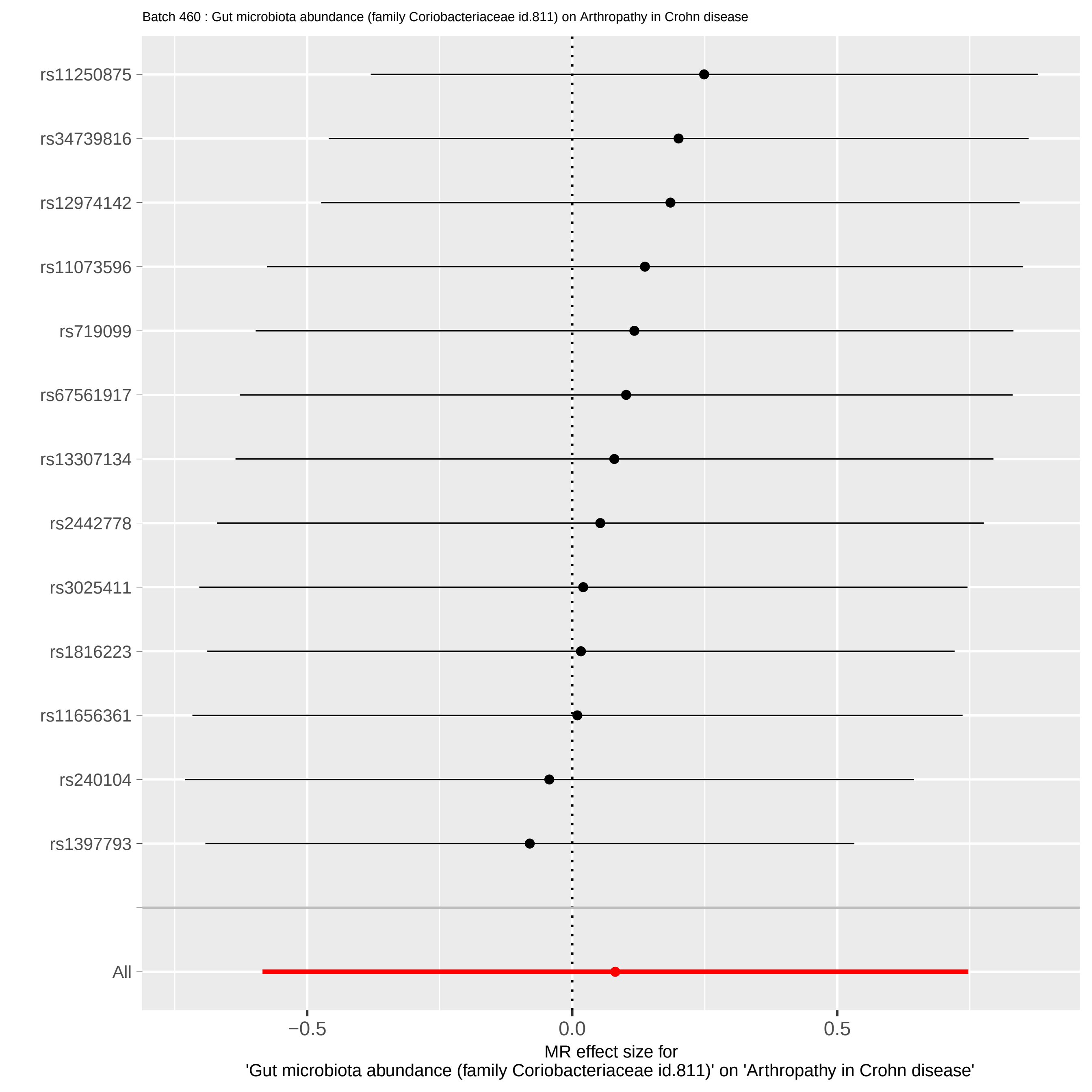
Batch 460 : Gut microbiota abundance (family Coriobacteriaceae id.811) on Arthropathy in Crohn disease
rs11250875
rs34739816
rs12974142
rs11073596
rs719099
rs67561917
rs13307134
rs2442778
rs3025411
rs1816223
rs11656361
rs240104
rs1397793
All
−0.5
0.0
0.5
MR effect size for
'Gut microbiota abundance (family Coriobacteriaceae id.811)' on 'Arthropathy in Crohn disease'

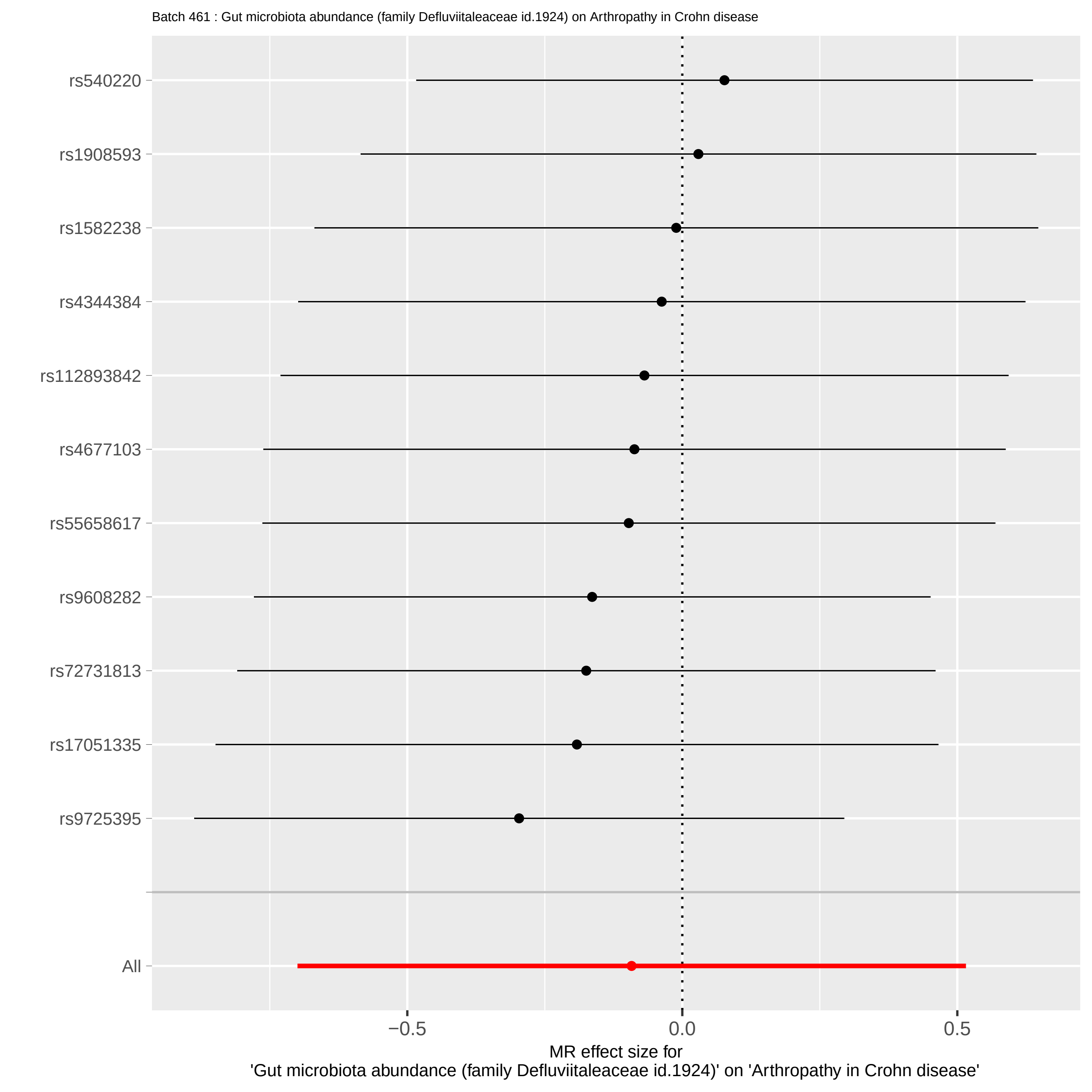

Batch 461 : Gut microbiota abundance (family Defluviitaleaceae id.1924) on Arthropathy in Crohn disease
rs540220
rs1908593
rs1582238
rs4344384
rs112893842
rs4677103
rs55658617
rs9608282
rs72731813
rs17051335
rs9725395
All
−0.5
0.0
0.5
MR effect size for
'Gut microbiota abundance (family Defluviitaleaceae id.1924)' on 'Arthropathy in Crohn disease'

Batch 462 : Gut microbiota abundance (family Desulfovibrionaceae id.3169) on Arthropathy in Crohn disease

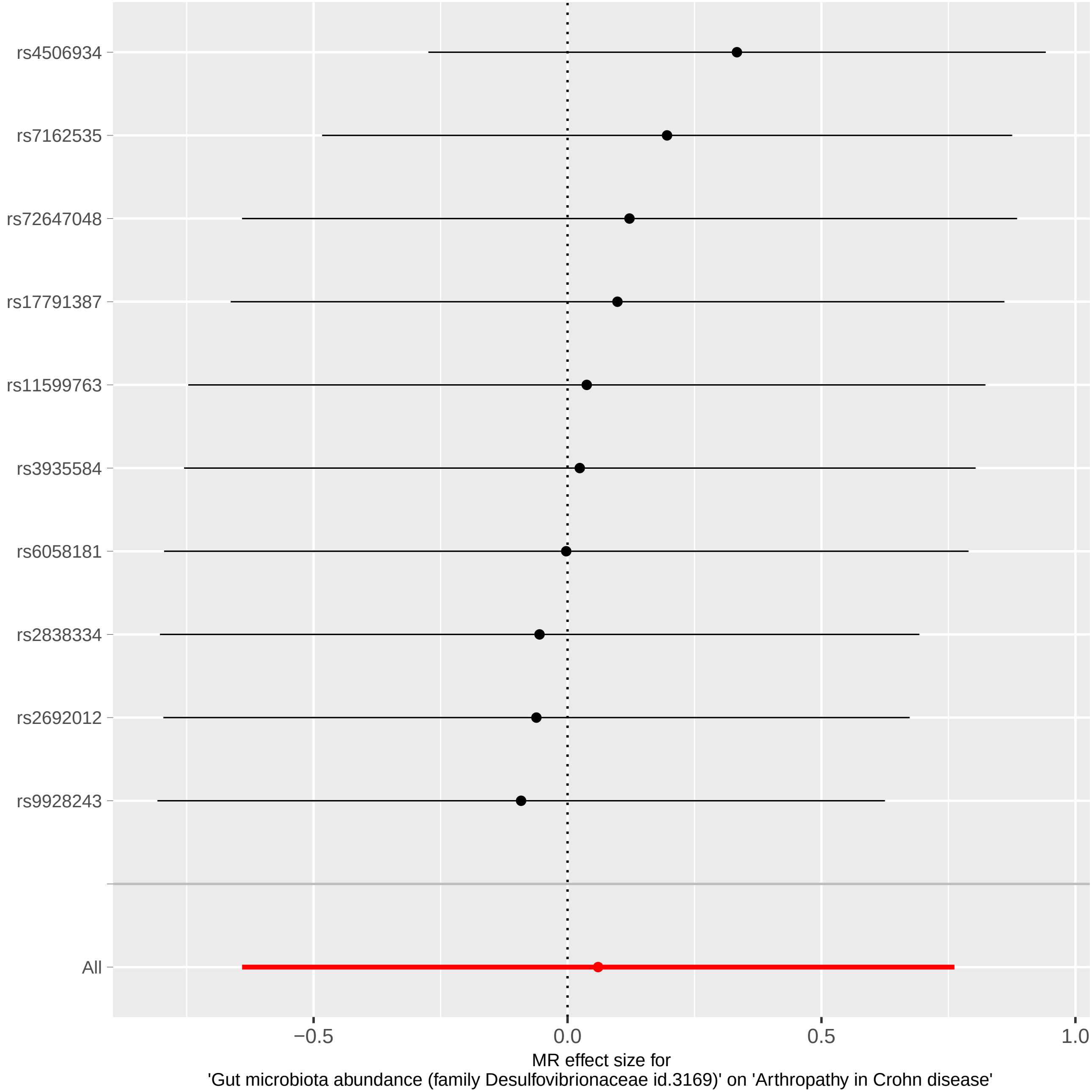

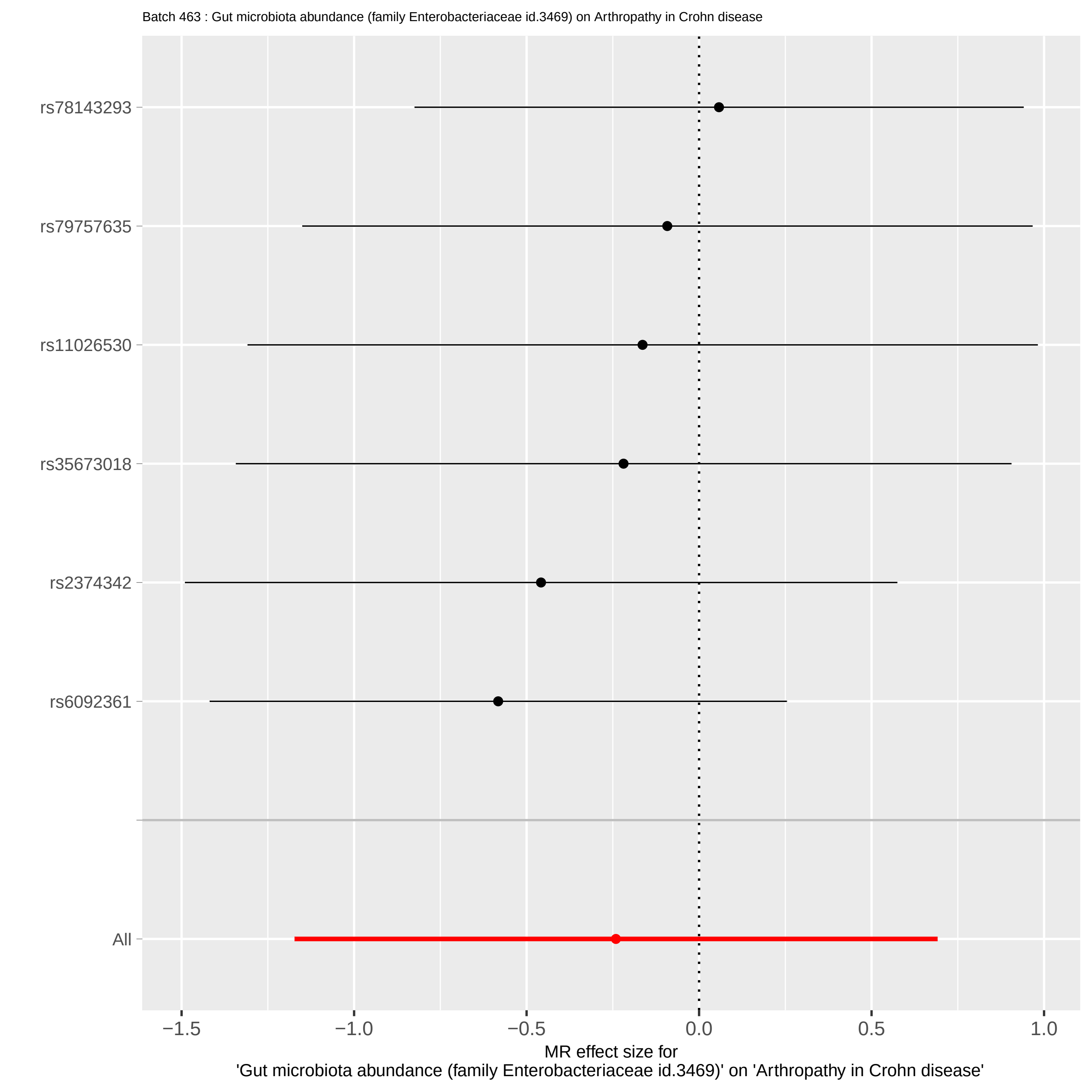

Batch 463 : Gut microbiota abundance (family Enterobacteriaceae id.3469) on Arthropathy in Crohn disease
rs78143293
rs79757635
rs11026530
rs35673018
rs2374342
rs6092361
All
−1.5
−1.0
−0.5
0.0
0.5
1.0
MR effect size for
'Gut microbiota abundance (family Enterobacteriaceae id.3469)' on 'Arthropathy in Crohn disease'

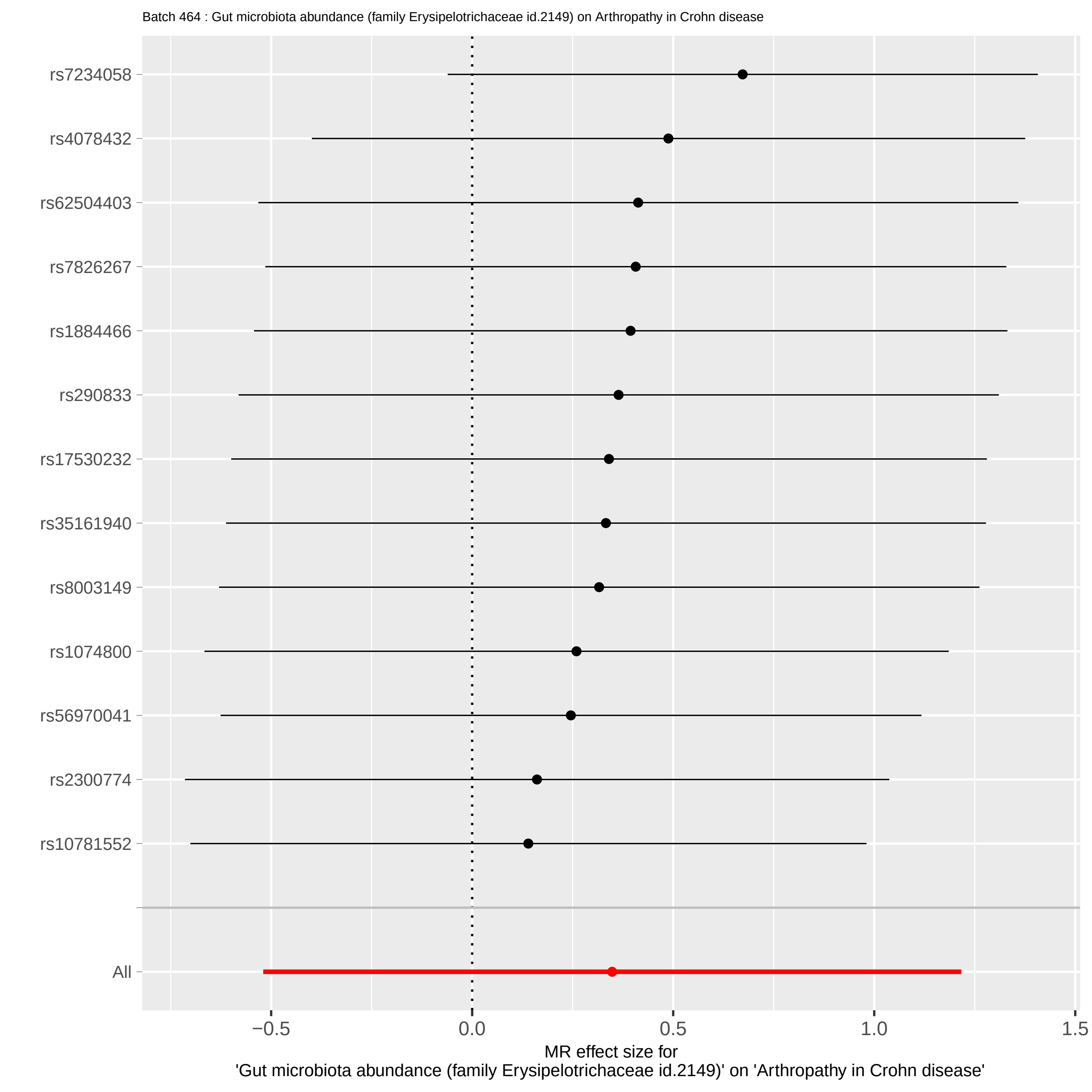

Batch 464 : Gut microbiota abundance (family Erysipelotrichaceae id.2149) on Arthropathy in Crohn disease
rs7234058
rs4078432
rs62504403
rs7826267
rs1884466
rs290833
rs17530232
rs35161940
rs8003149
rs1074800
rs56970041
rs2300774
rs10781552
All
−0.5
0.0
0.5
1.0
1.5
MR effect size for
'Gut microbiota abundance (family Erysipelotrichaceae id.2149)' on 'Arthropathy in Crohn disease'

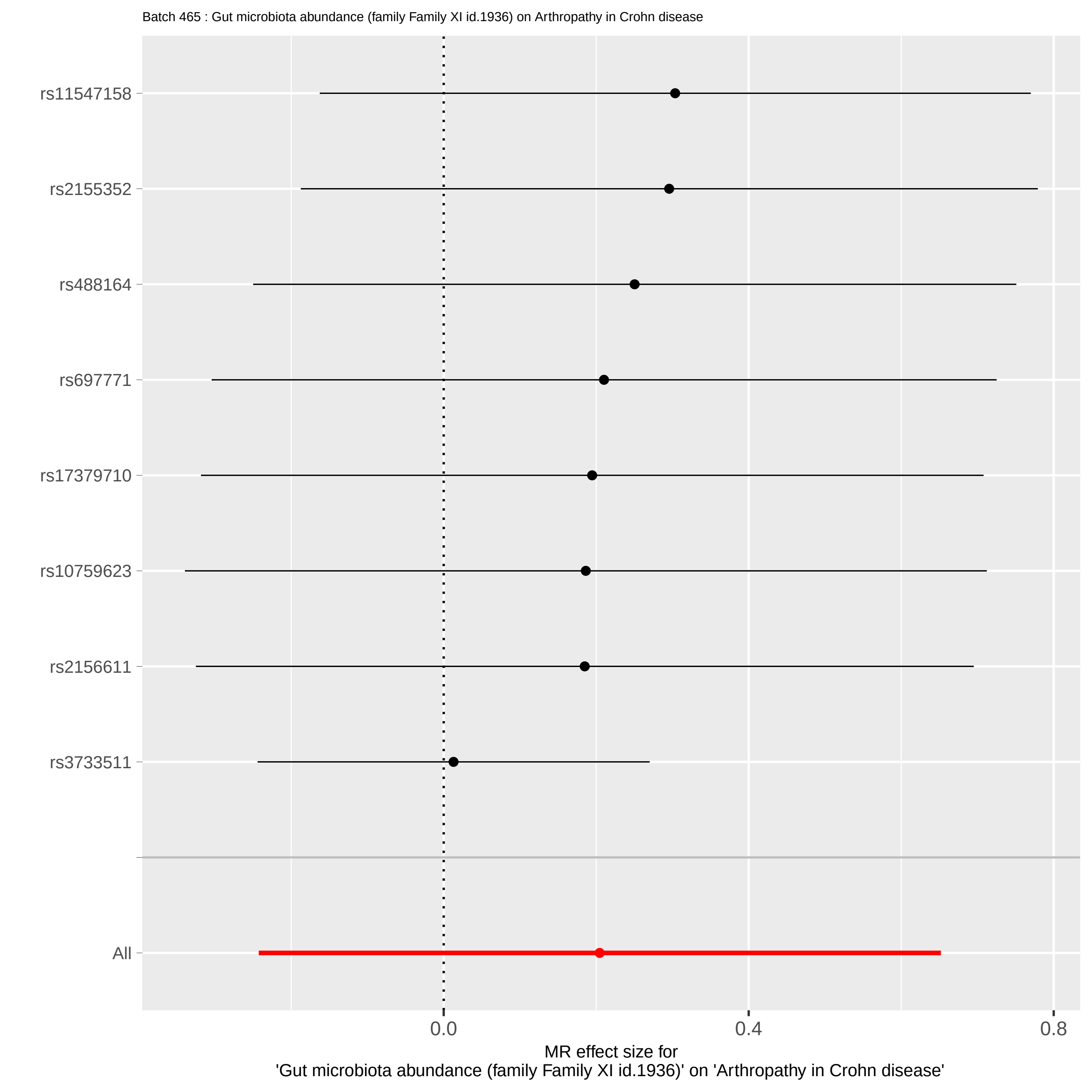
Batch 465 : Gut microbiota abundance (family Family XI id.1936) on Arthropathy in Crohn disease
rs11547158
rs2155352
rs488164
rs697771
rs17379710
rs10759623
rs2156611
rs3733511
All
0.0
0.4
0.8
MR effect size for
'Gut microbiota abundance (family Family XI id.1936)' on 'Arthropathy in Crohn disease'

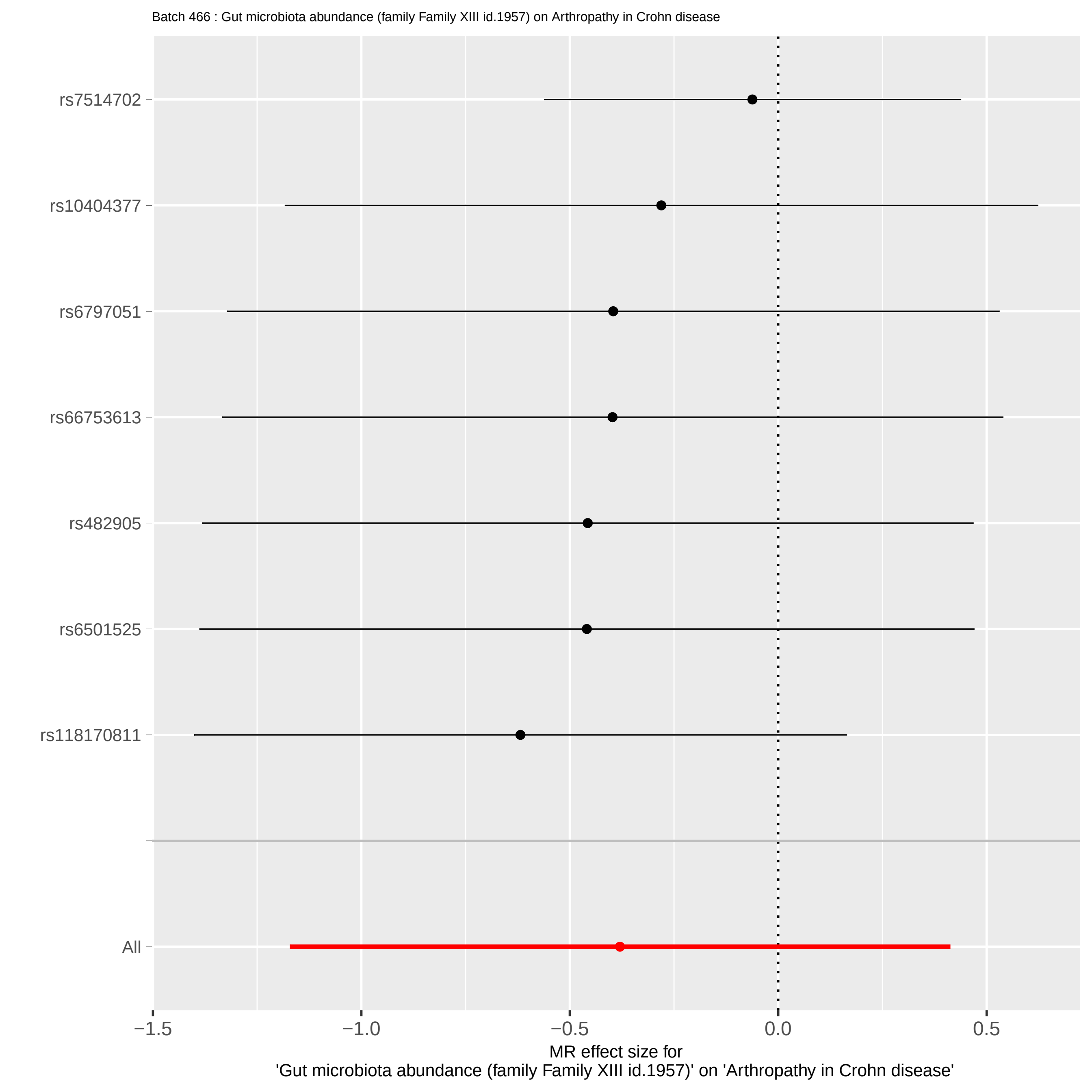
Batch 466 : Gut microbiota abundance (family Family XIII id.1957) on Arthropathy in Crohn disease
rs7514702
rs10404377
rs6797051
rs66753613
rs482905
rs6501525
rs118170811
All
−1.5
−1.0
−0.5
0.0
0.5
MR effect size for
'Gut microbiota abundance (family Family XIII id.1957)' on 'Arthropathy in Crohn disease'

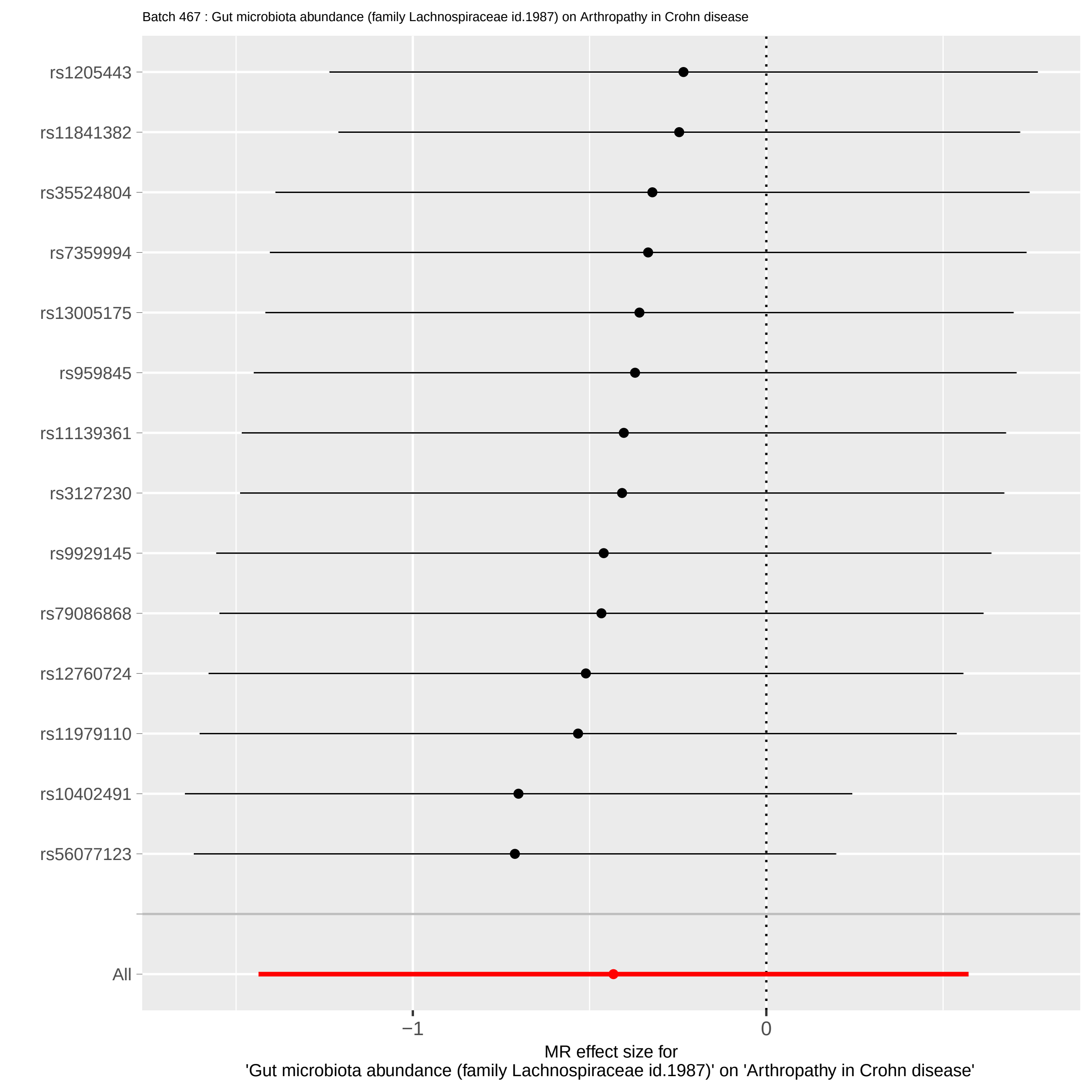
Batch 467 : Gut microbiota abundance (family Lachnospiraceae id.1987) on Arthropathy in Crohn disease
rs1205443
rs11841382
rs35524804
rs7359994
rs13005175
rs959845
rs11139361
rs3127230
rs9929145
rs79086868
rs12760724
rs11979110
rs10402491
rs56077123
All
−1
0
MR effect size for
'Gut microbiota abundance (family Lachnospiraceae id.1987)' on 'Arthropathy in Crohn disease'

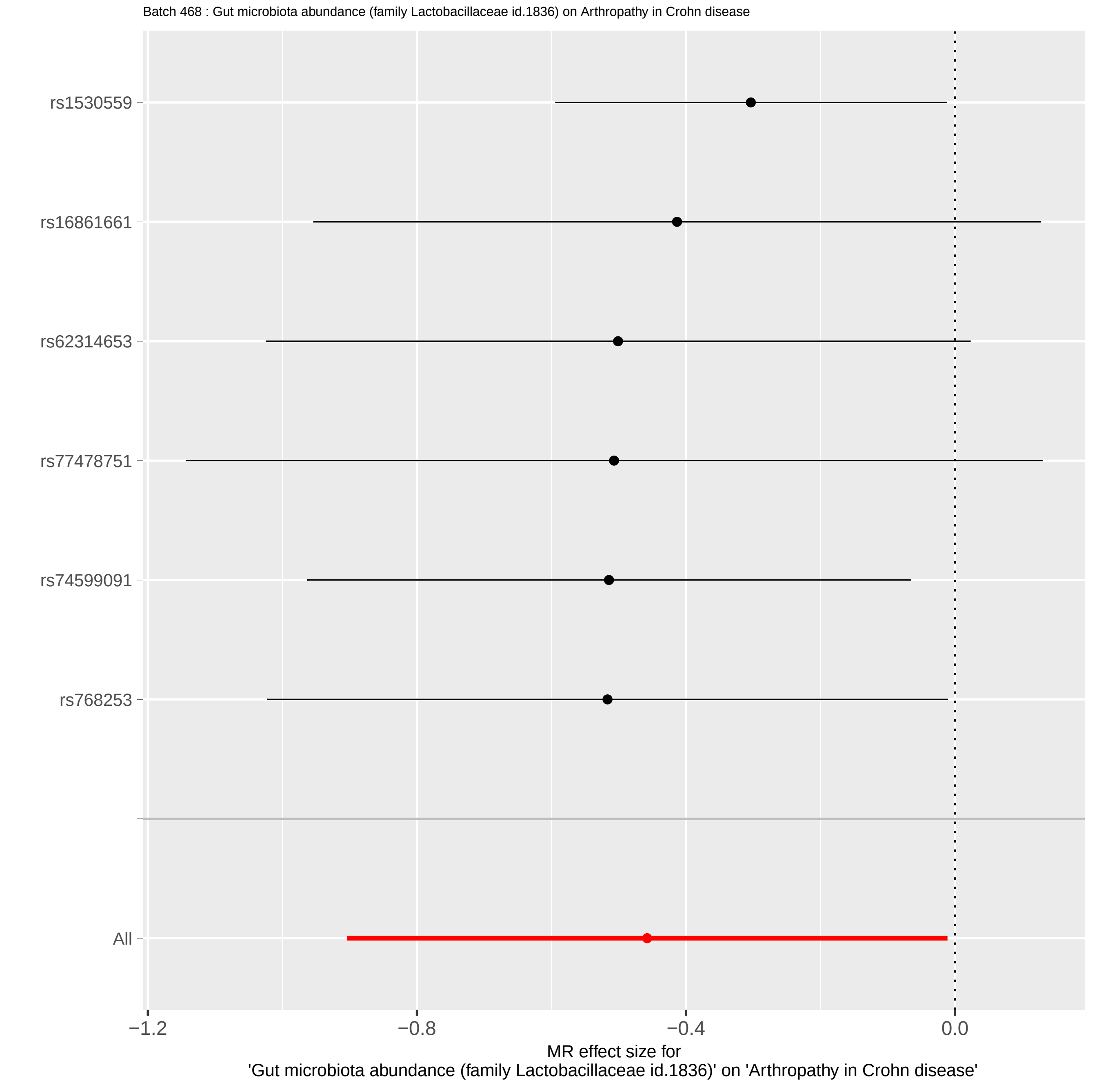
Batch 468 : Gut microbiota abundance (family Lactobacillaceae id.1836) on Arthropathy in Crohn disease
rs1530559
rs16861661
rs62314653
rs77478751
rs74599091
rs768253
All
−1.2
−0.8
−0.4
0.0
MR effect size for
'Gut microbiota abundance (family Lactobacillaceae id.1836)' on 'Arthropathy in Crohn disease'

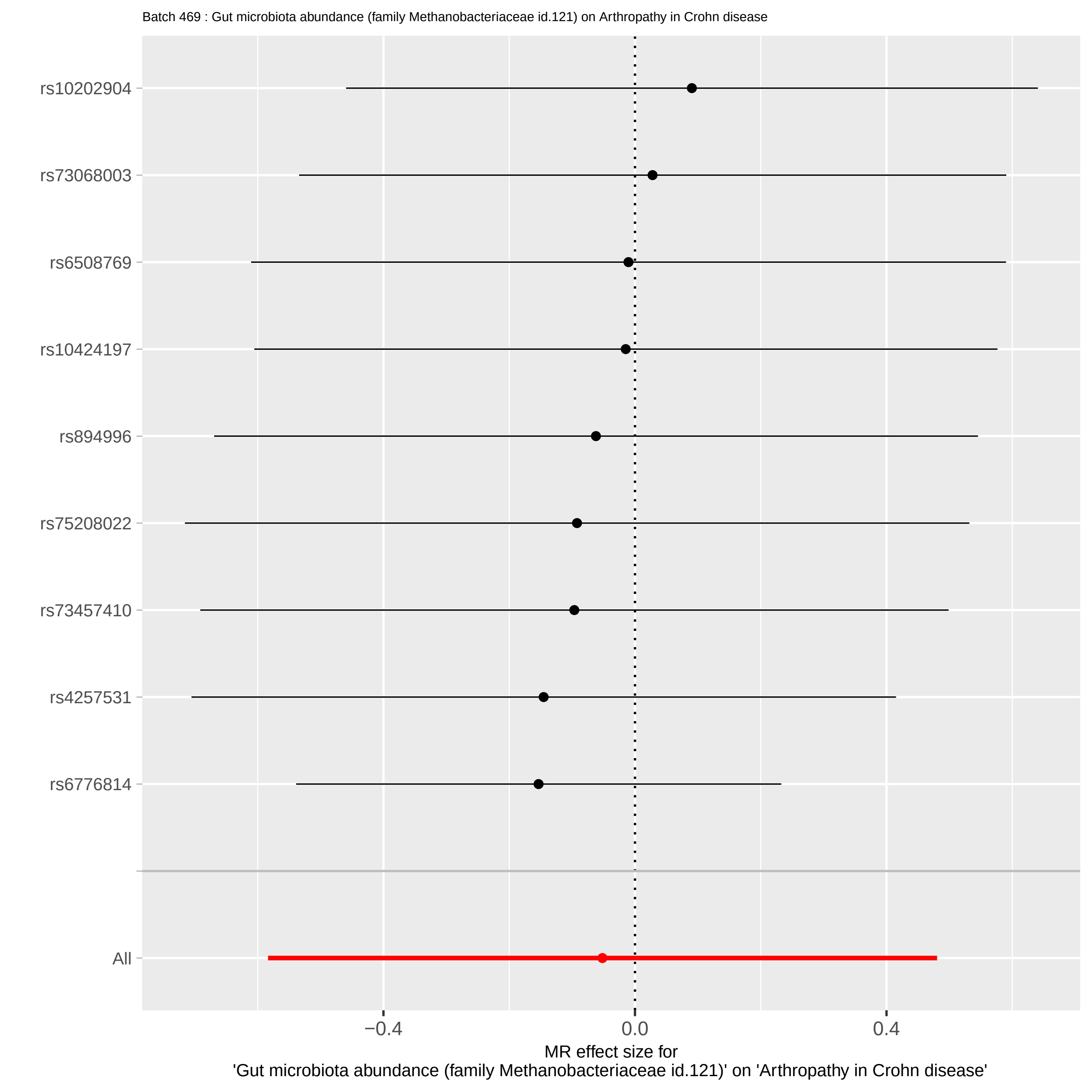
Batch 469 : Gut microbiota abundance (family Methanobacteriaceae id.121) on Arthropathy in Crohn disease
rs10202904
rs73068003
rs6508769
rs10424197
rs894996
rs75208022
rs73457410
rs4257531
rs6776814
All
−0.4
0.0
0.4
MR effect size for
'Gut microbiota abundance (family Methanobacteriaceae id.121)' on 'Arthropathy in Crohn disease'

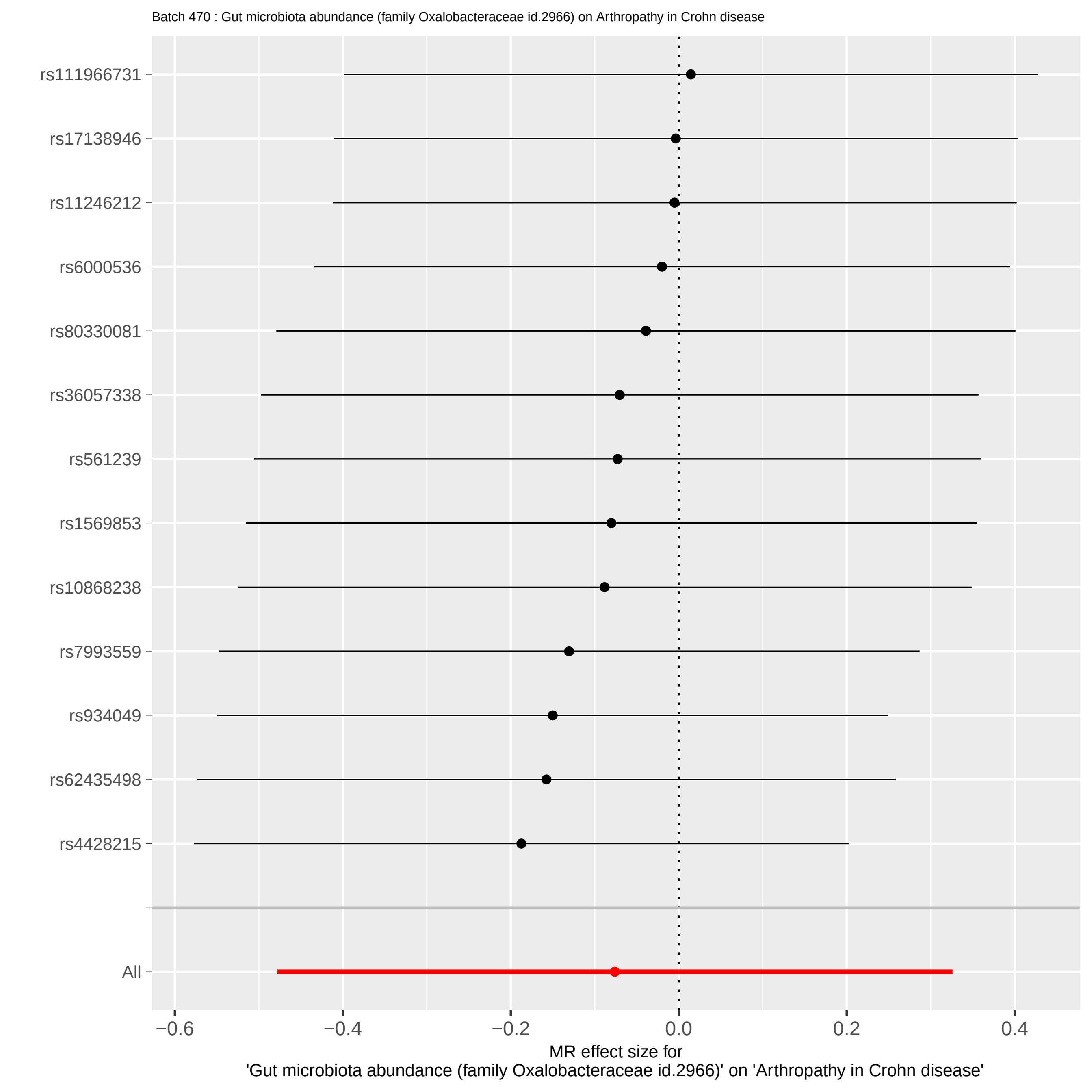

Batch 470 : Gut microbiota abundance (family Oxalobacteraceae id.2966) on Arthropathy in Crohn disease
rs111966731
rs17138946
rs11246212
rs6000536
rs80330081
rs36057338
rs561239
rs1569853
rs10868238
rs7993559
rs934049
rs62435498
rs4428215
All
−0.6
−0.4
−0.2
0.0
0.2
0.4
MR effect size for
'Gut microbiota abundance (family Oxalobacteraceae id.2966)' on 'Arthropathy in Crohn disease'

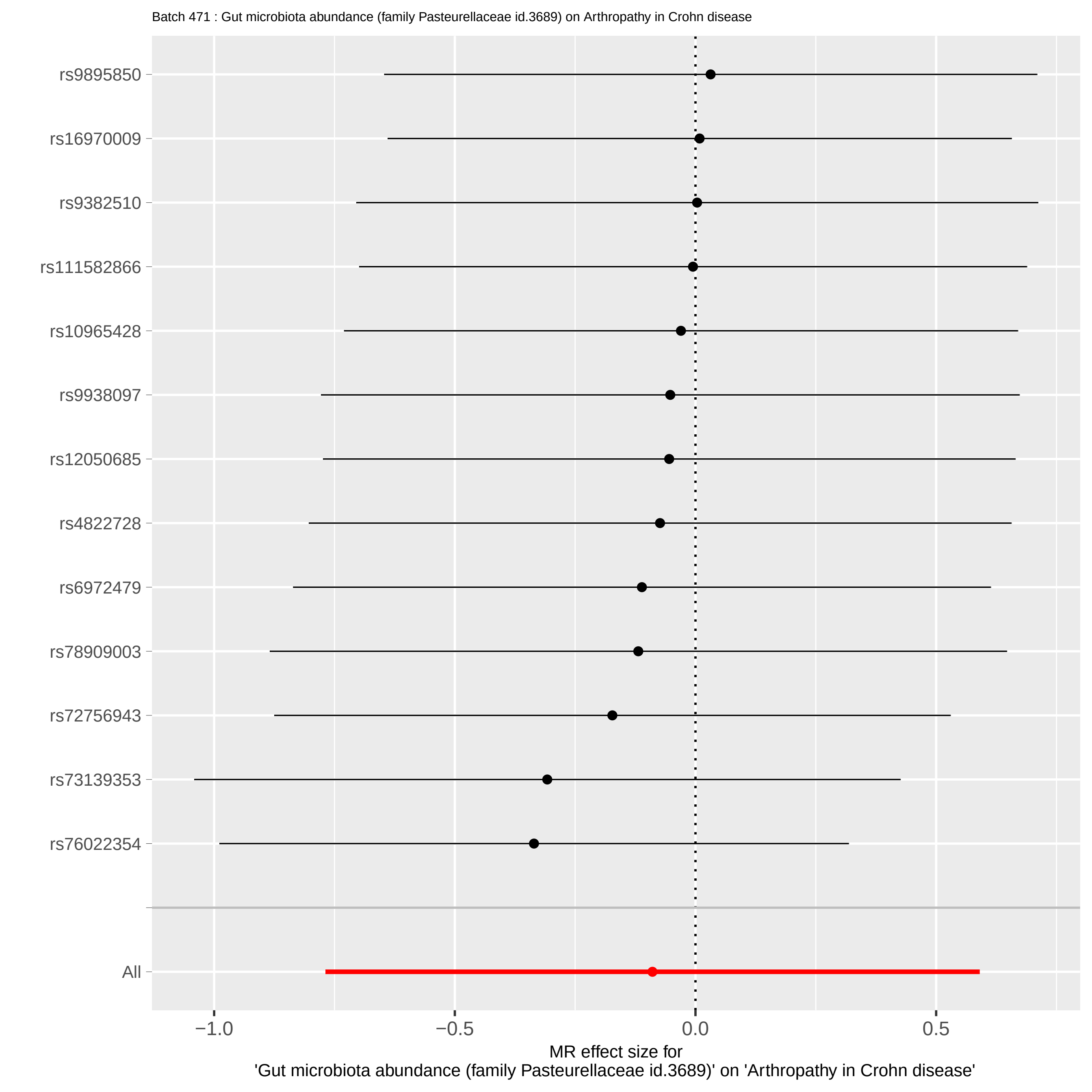

Batch 471 : Gut microbiota abundance (family Pasteurellaceae id.3689) on Arthropathy in Crohn disease
rs9895850
rs16970009
rs9382510
rs111582866
rs10965428
rs9938097
rs12050685
rs4822728
rs6972479
rs78909003
rs72756943
rs73139353
rs76022354
All
−1.0
−0.5
0.0
0.5
MR effect size for
'Gut microbiota abundance (family Pasteurellaceae id.3689)' on 'Arthropathy in Crohn disease'

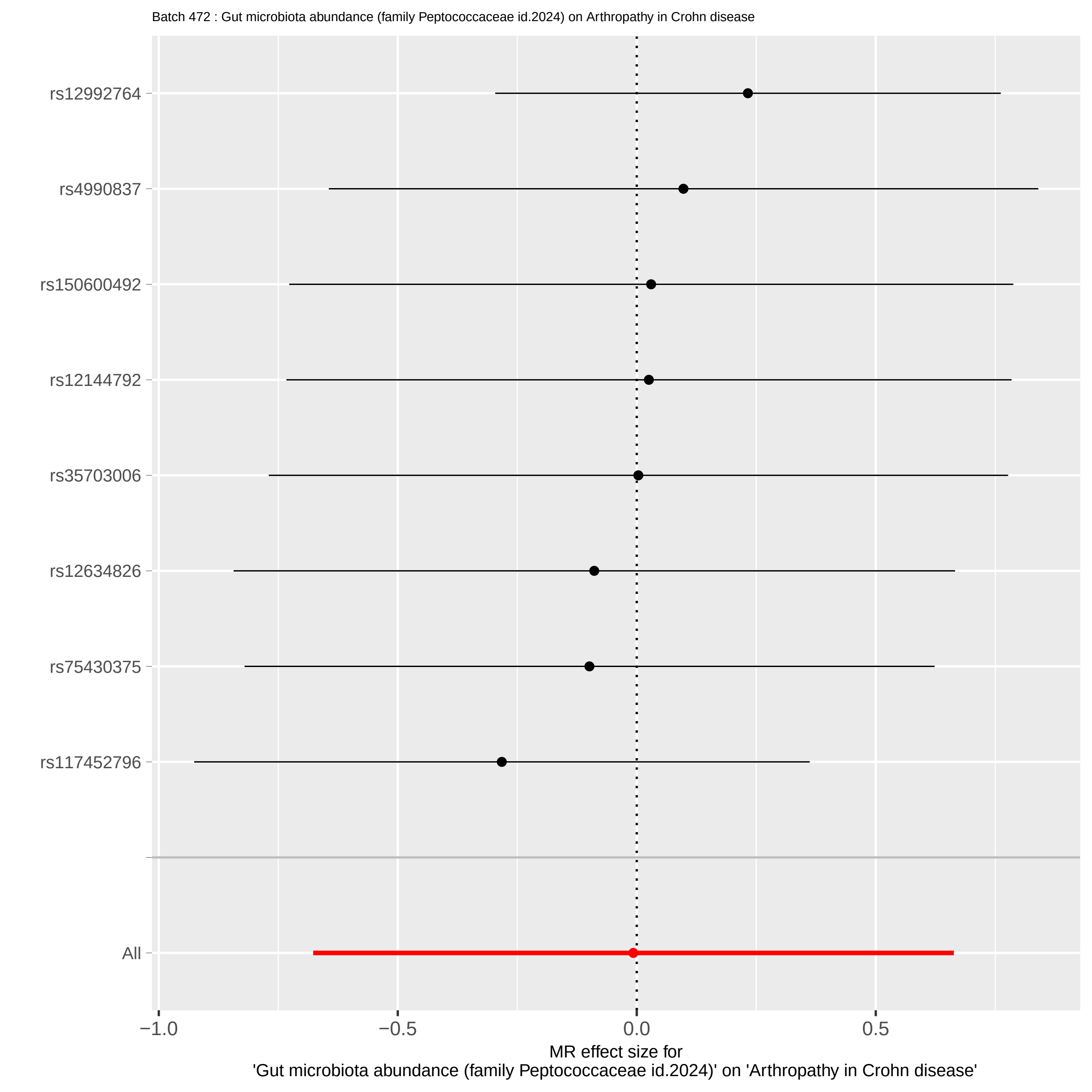
Batch 472 : Gut microbiota abundance (family Peptococcaceae id.2024) on Arthropathy in Crohn disease
rs12992764
rs4990837
rs150600492
rs12144792
rs35703006
rs12634826
rs75430375
rs117452796
All
−1.0
−0.5
0.0
0.5
MR effect size for
'Gut microbiota abundance (family Peptococcaceae id.2024)' on 'Arthropathy in Crohn disease'

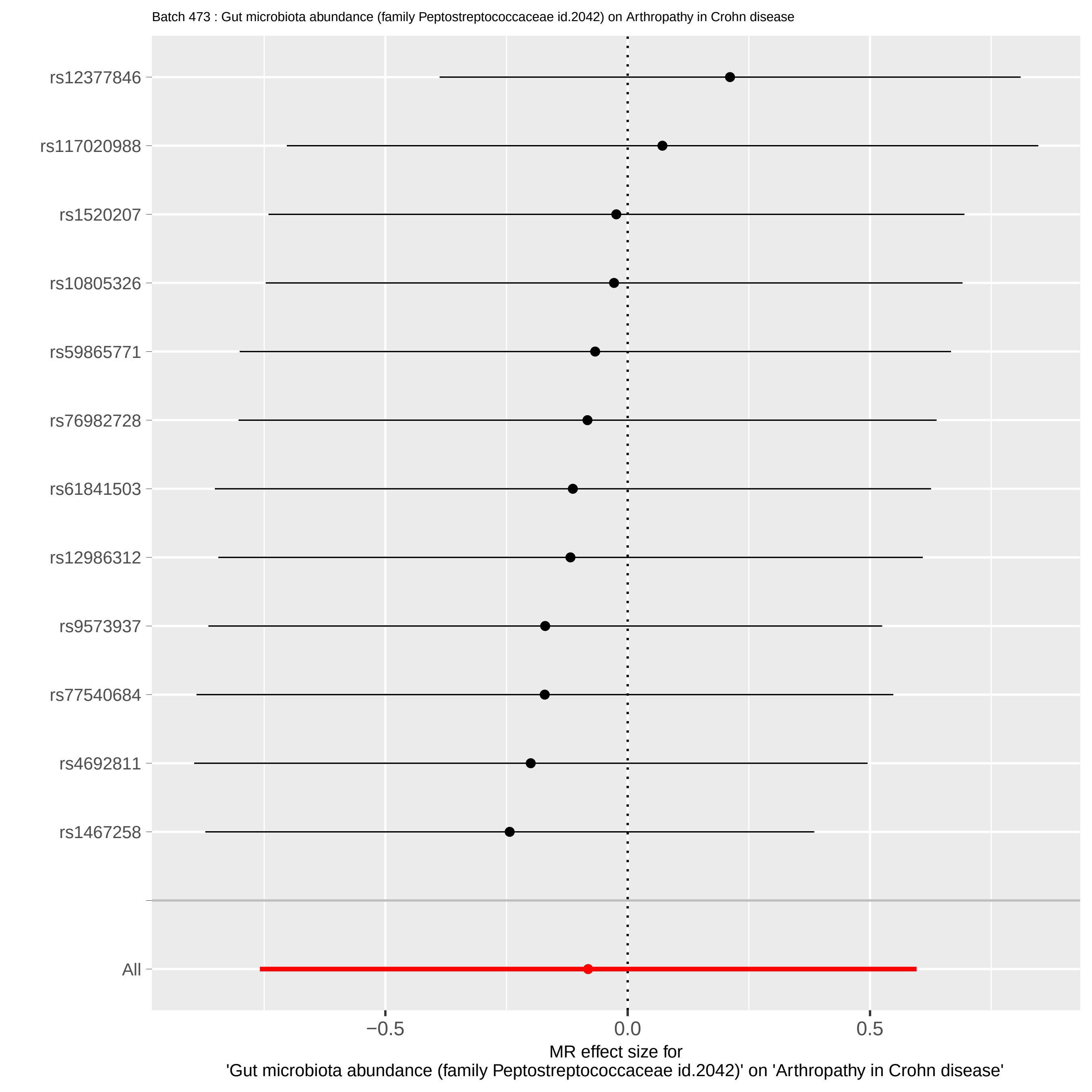
Batch 473 : Gut microbiota abundance (family Peptostreptococcaceae id.2042) on Arthropathy in Crohn disease
rs12377846
rs117020988
rs1520207
rs10805326
rs59865771
rs76982728
rs61841503
rs12986312
rs9573937
rs77540684
rs4692811
rs1467258
All
−0.5
0.0
0.5
MR effect size for
'Gut microbiota abundance (family Peptostreptococcaceae id.2042)' on 'Arthropathy in Crohn disease'

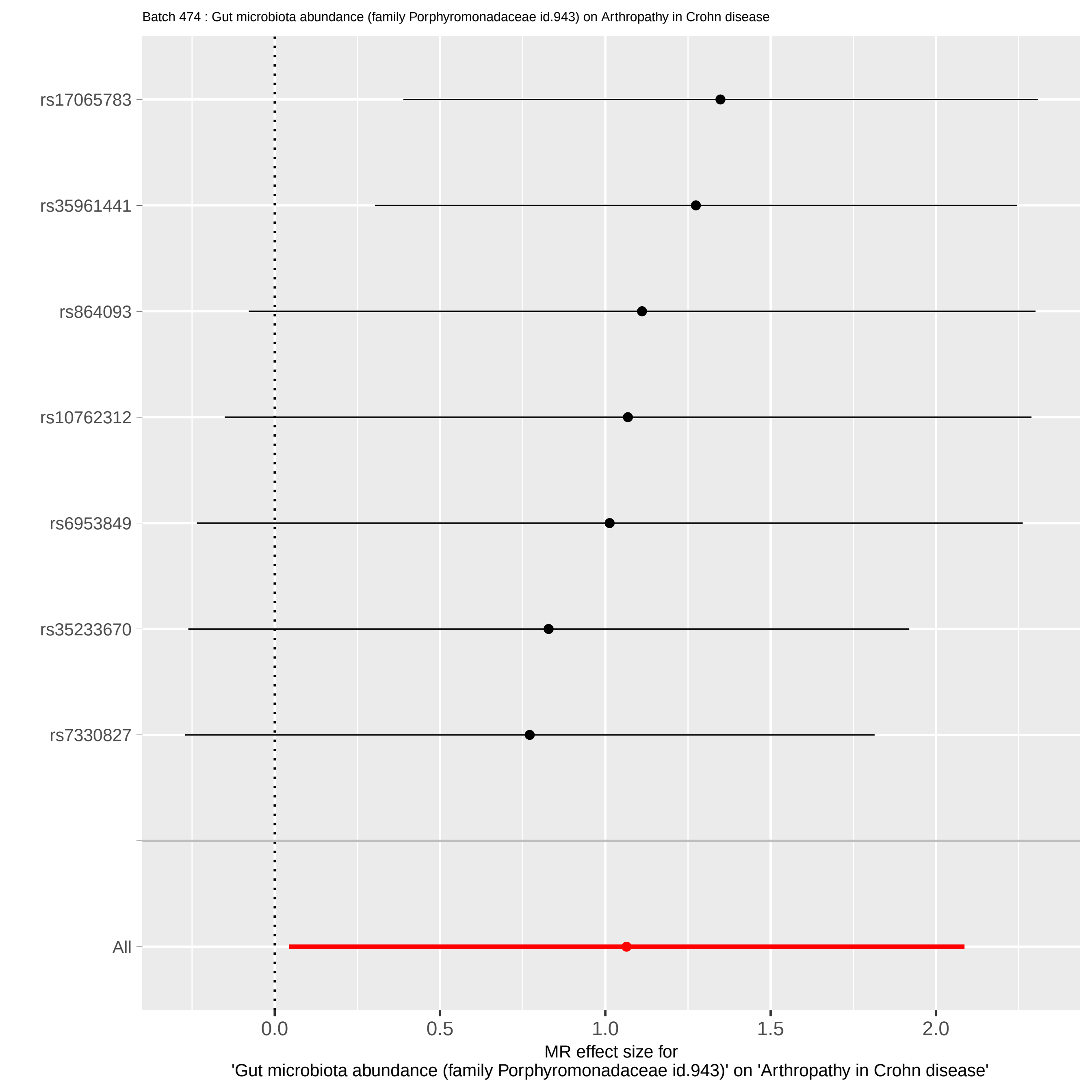

Batch 474 : Gut microbiota abundance (family Porphyromonadaceae id.943) on Arthropathy in Crohn disease
rs17065783
rs35961441
rs864093
rs10762312
rs6953849
rs35233670
rs7330827
All
0.0
0.5
1.0
1.5
2.0
MR effect size for
'Gut microbiota abundance (family Porphyromonadaceae id.943)' on 'Arthropathy in Crohn disease'

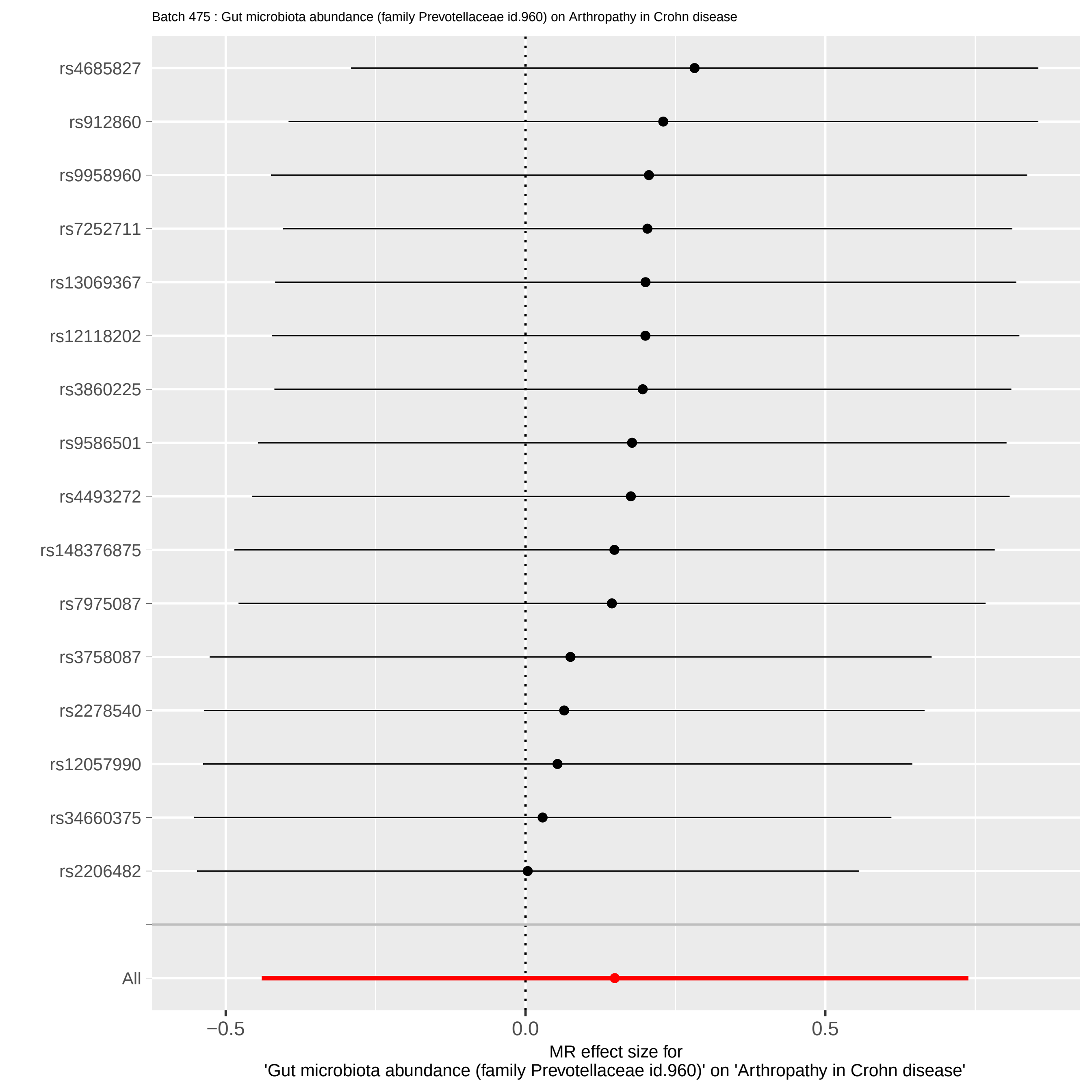
Batch 475 : Gut microbiota abundance (family Prevotellaceae id.960) on Arthropathy in Crohn disease
rs4685827
rs912860
rs9958960
rs7252711
rs13069367
rs12118202
rs3860225
rs9586501
rs4493272
rs148376875
rs7975087
rs3758087
rs2278540
rs12057990
rs34660375
rs2206482
All
−0.5
0.0
0.5
MR effect size for
'Gut microbiota abundance (family Prevotellaceae id.960)' on 'Arthropathy in Crohn disease'

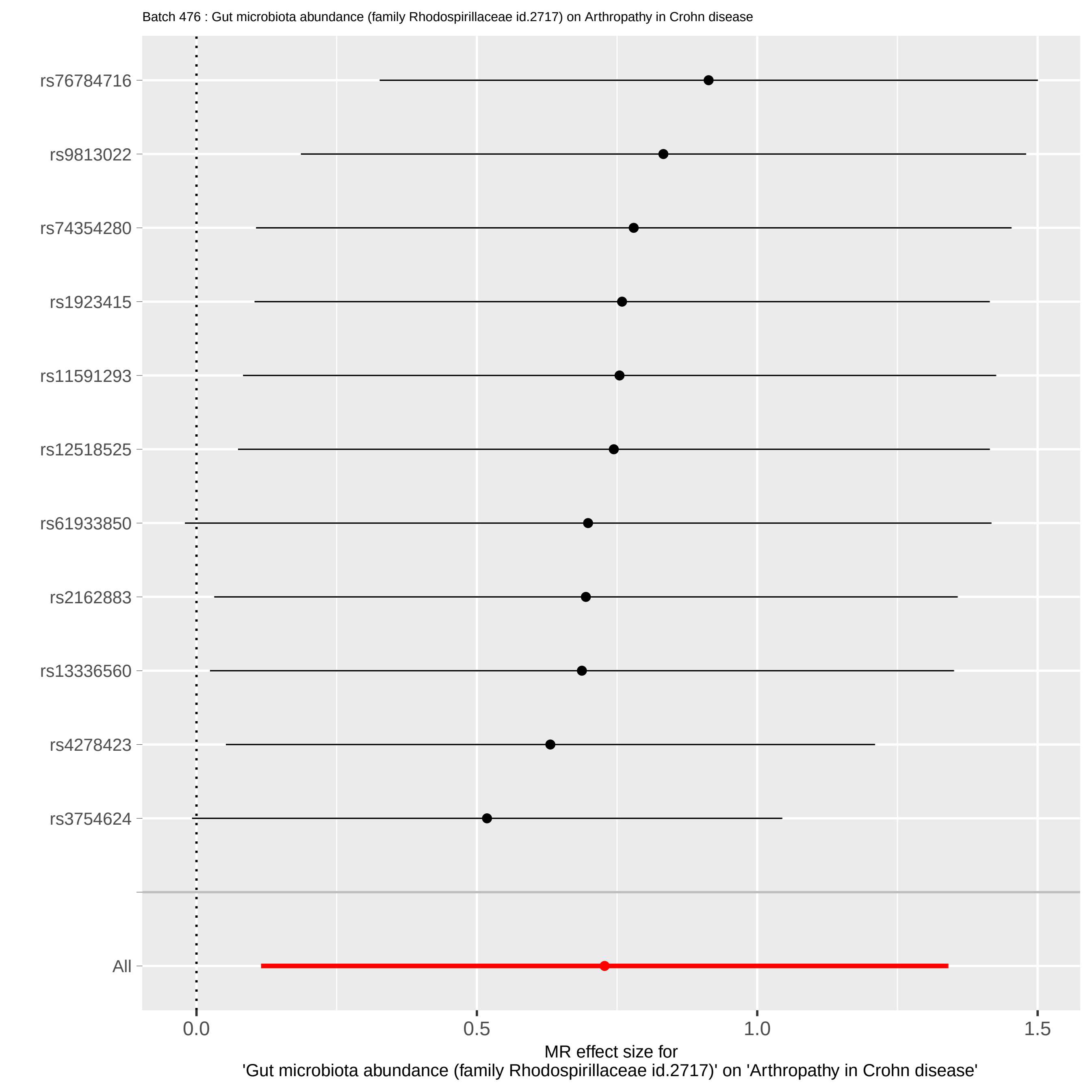

Batch 476 : Gut microbiota abundance (family Rhodospirillaceae id.2717) on Arthropathy in Crohn disease
rs76784716
rs9813022
rs74354280
rs1923415
rs11591293
rs12518525
rs61933850
rs2162883
rs13336560
rs4278423
rs3754624
All
0.0
0.5
1.0
1.5
MR effect size for
'Gut microbiota abundance (family Rhodospirillaceae id.2717)' on 'Arthropathy in Crohn disease'

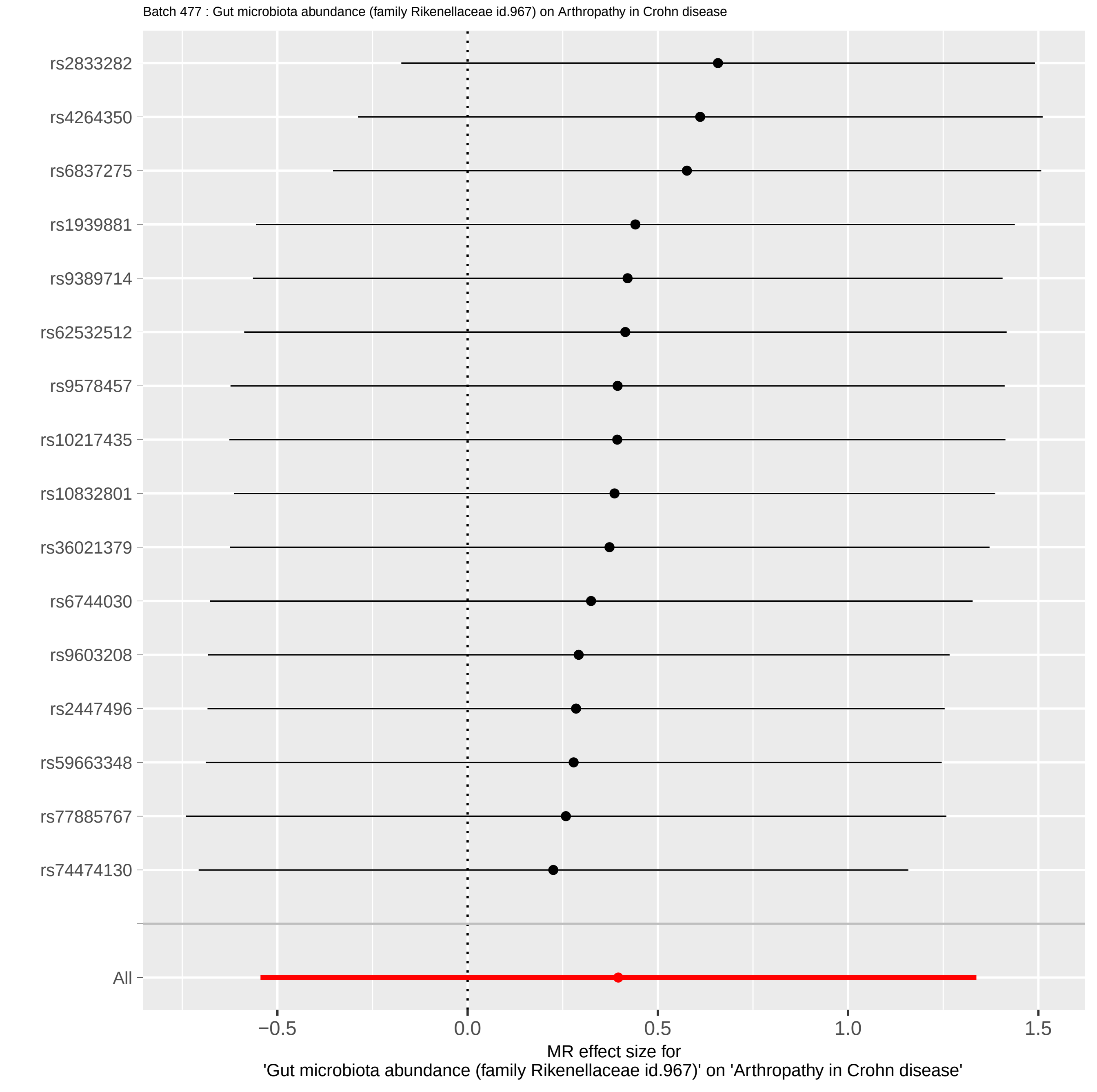

Batch 477 : Gut microbiota abundance (family Rikenellaceae id.967) on Arthropathy in Crohn disease
rs2833282
rs4264350
rs6837275
rs1939881
rs9389714
rs62532512
rs9578457
rs10217435
rs10832801
rs36021379
rs6744030
rs9603208
rs2447496
rs59663348
rs77885767
rs74474130
All
−0.5
0.0
0.5
1.0
1.5
MR effect size for
'Gut microbiota abundance (family Rikenellaceae id.967)' on 'Arthropathy in Crohn disease'

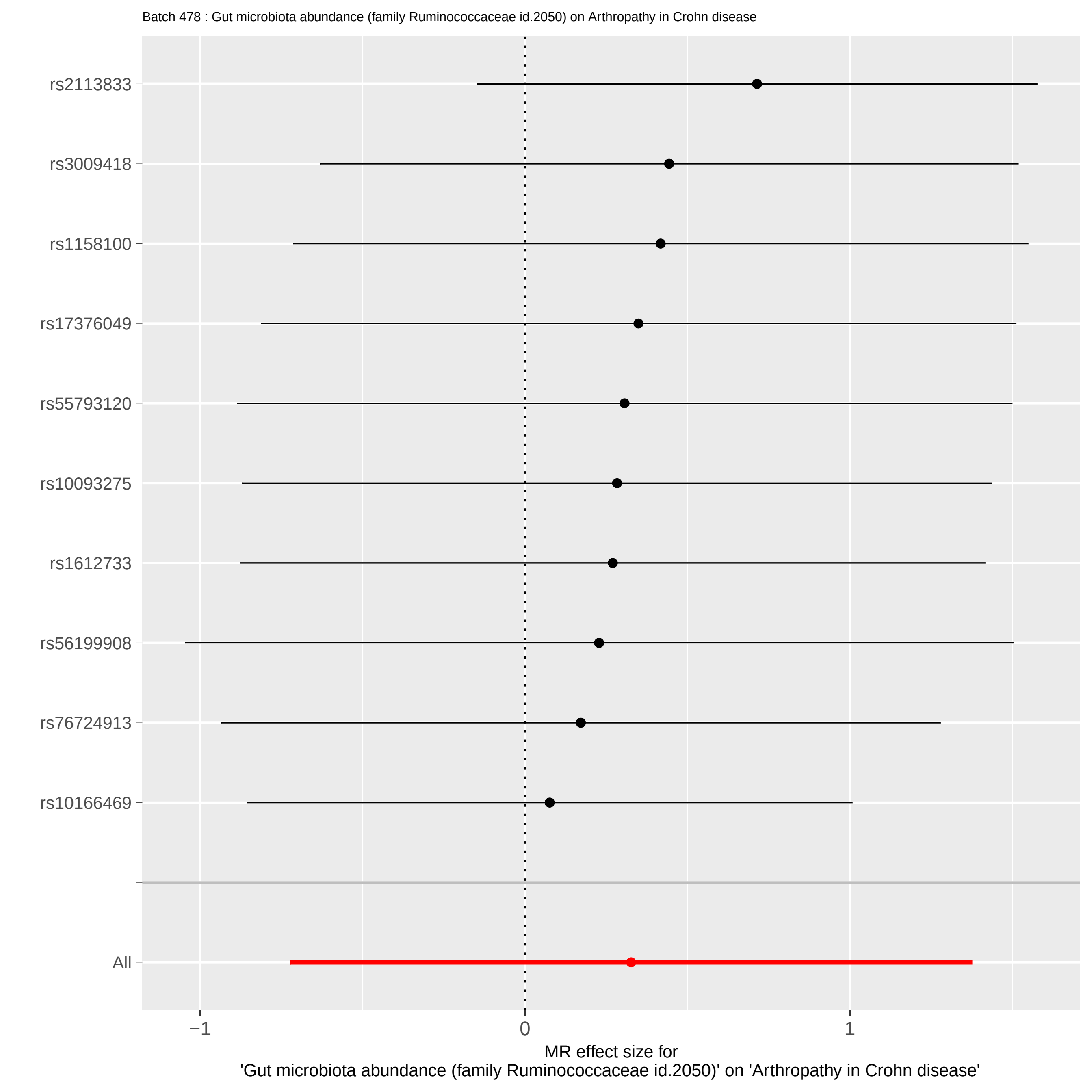
Batch 478 : Gut microbiota abundance (family Ruminococcaceae id.2050) on Arthropathy in Crohn disease
rs2113833
rs3009418
rs1158100
rs17376049
rs55793120
rs10093275
rs1612733
rs56199908
rs76724913
rs10166469
All
−1
0
1
MR effect size for
'Gut microbiota abundance (family Ruminococcaceae id.2050)' on 'Arthropathy in Crohn disease'

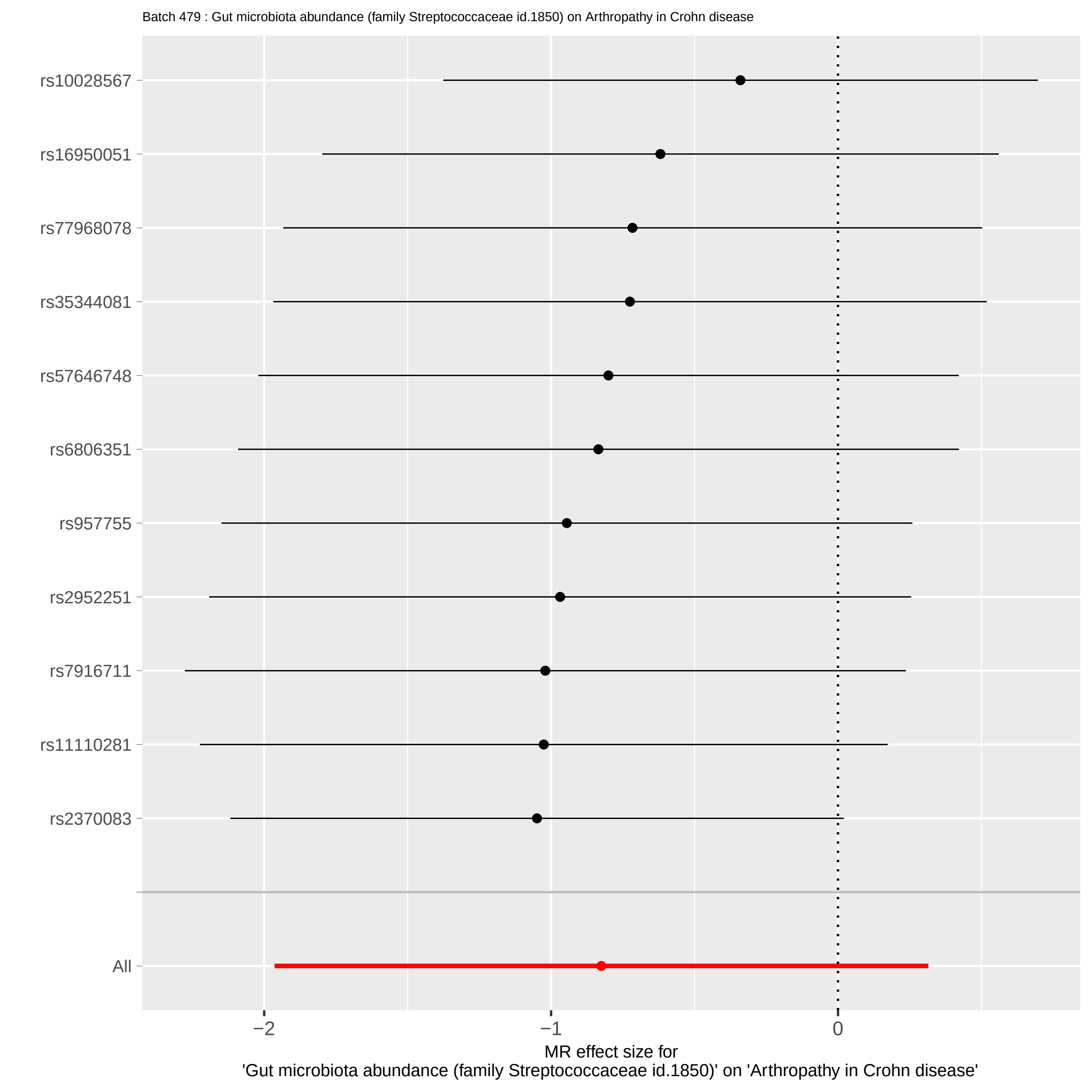

Batch 479 : Gut microbiota abundance (family Streptococcaceae id.1850) on Arthropathy in Crohn disease
rs10028567
rs16950051
rs77968078
rs35344081
rs57646748
rs6806351
rs957755
rs2952251
rs7916711
rs11110281
rs2370083
All
−2
−1
0
MR effect size for
'Gut microbiota abundance (family Streptococcaceae id.1850)' on 'Arthropathy in Crohn disease'

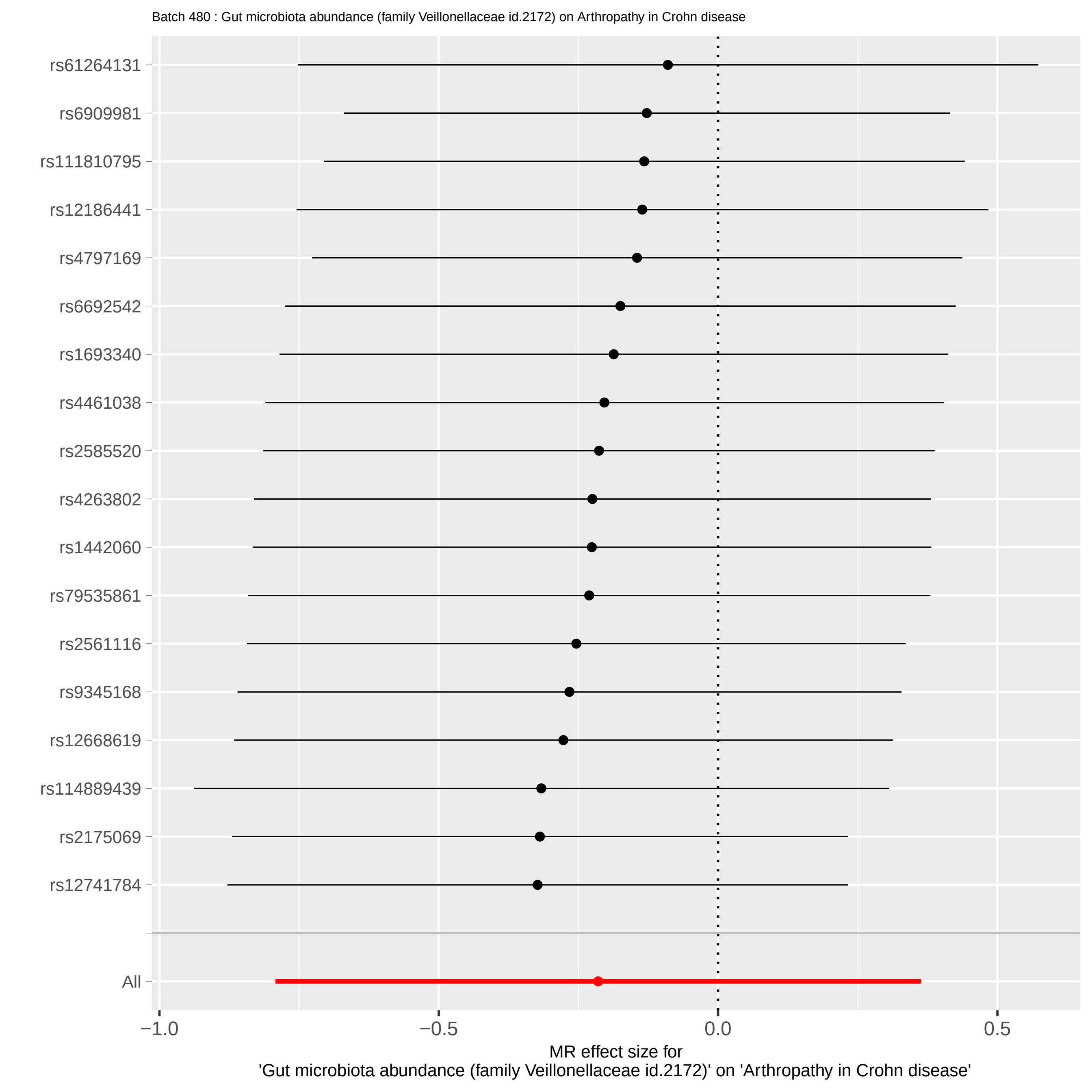

Batch 480 : Gut microbiota abundance (family Veillonellaceae id.2172) on Arthropathy in Crohn disease
rs61264131
rs6909981
rs111810795
rs12186441
rs4797169
rs6692542
rs1693340
rs4461038
rs2585520
rs4263802
rs1442060
rs79535861
rs2561116
rs9345168
rs12668619
rs114889439
rs2175069
rs12741784
All
−1.0
−0.5
0.0
0.5
MR effect size for
'Gut microbiota abundance (family Veillonellaceae id.2172)' on 'Arthropathy in Crohn disease'

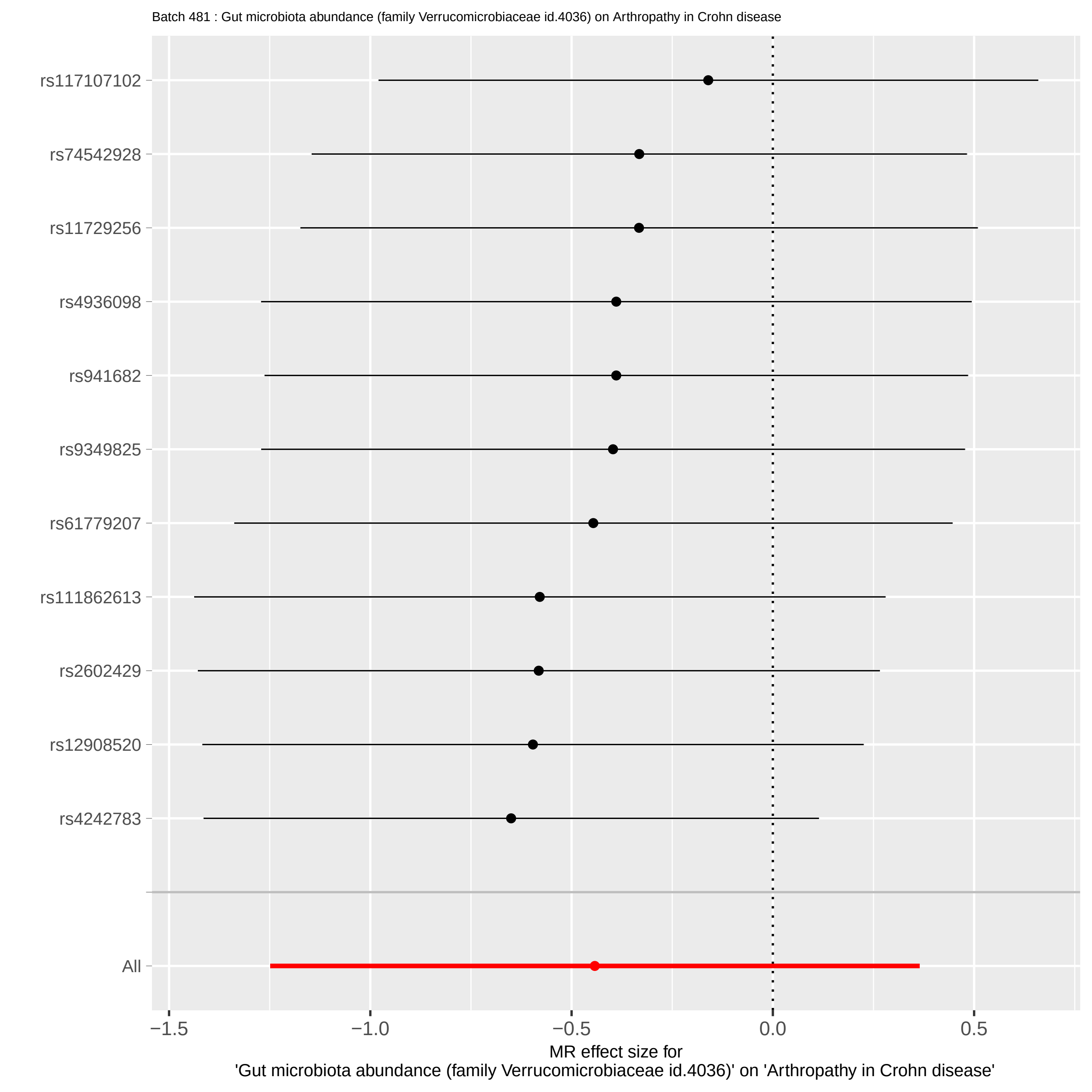

Batch 481 : Gut microbiota abundance (family Verrucomicrobiaceae id.4036) on Arthropathy in Crohn disease
rs117107102
rs74542928
rs11729256
rs4936098
rs941682
rs9349825
rs61779207
rs111862613
rs2602429
rs12908520
rs4242783
All
−1.5
−1.0
−0.5
0.0
0.5
MR effect size for
'Gut microbiota abundance (family Verrucomicrobiaceae id.4036)' on 'Arthropathy in Crohn disease'

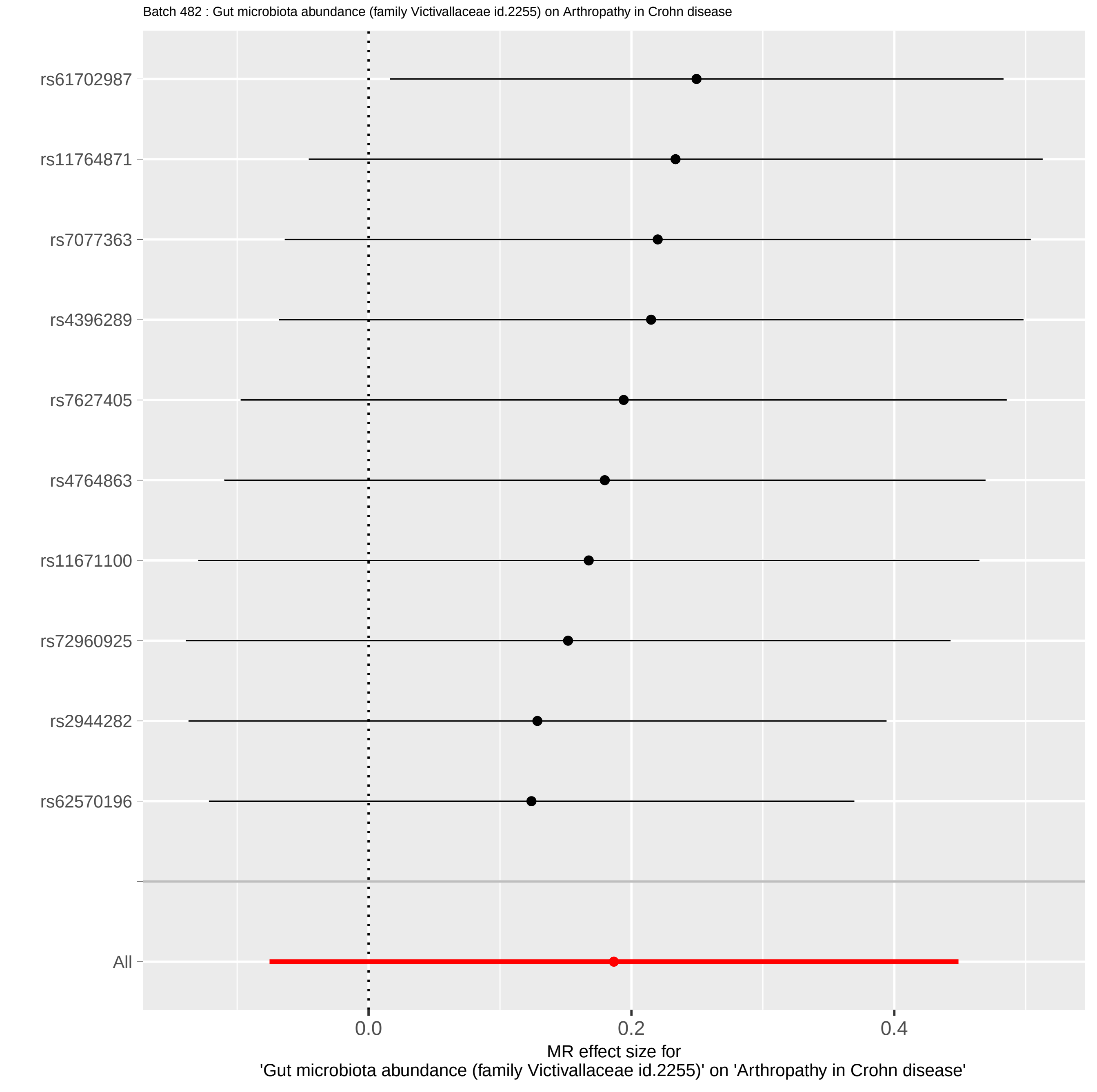
Batch 482 : Gut microbiota abundance (family Victivallaceae id.2255) on Arthropathy in Crohn disease
rs61702987
rs11764871
rs7077363
rs4396289
rs7627405
rs4764863
rs11671100
rs72960925
rs2944282
rs62570196
All
0.0
0.2
0.4
MR effect size for
'Gut microbiota abundance (family Victivallaceae id.2255)' on 'Arthropathy in Crohn disease'

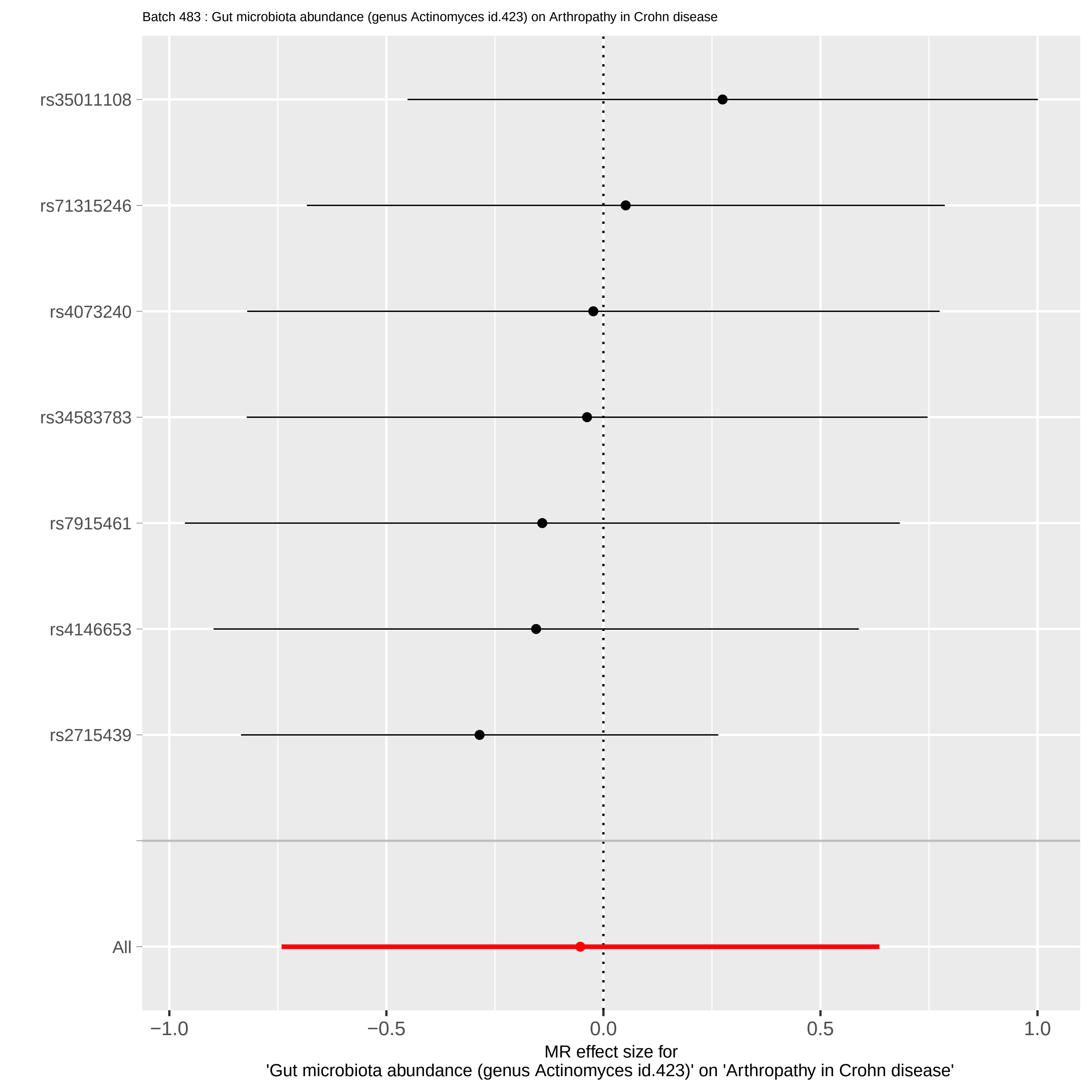

Batch 483 : Gut microbiota abundance (genus Actinomyces id.423) on Arthropathy in Crohn disease
rs35011108
rs71315246
rs4073240
rs34583783
rs7915461
rs4146653
rs2715439
All
−1.0
−0.5
0.0
0.5
1.0
MR effect size for
'Gut microbiota abundance (genus Actinomyces id.423)' on 'Arthropathy in Crohn disease'

Batch 484 : Gut microbiota abundance (genus Adlercreutzia id.812) on Arthropathy in Crohn disease

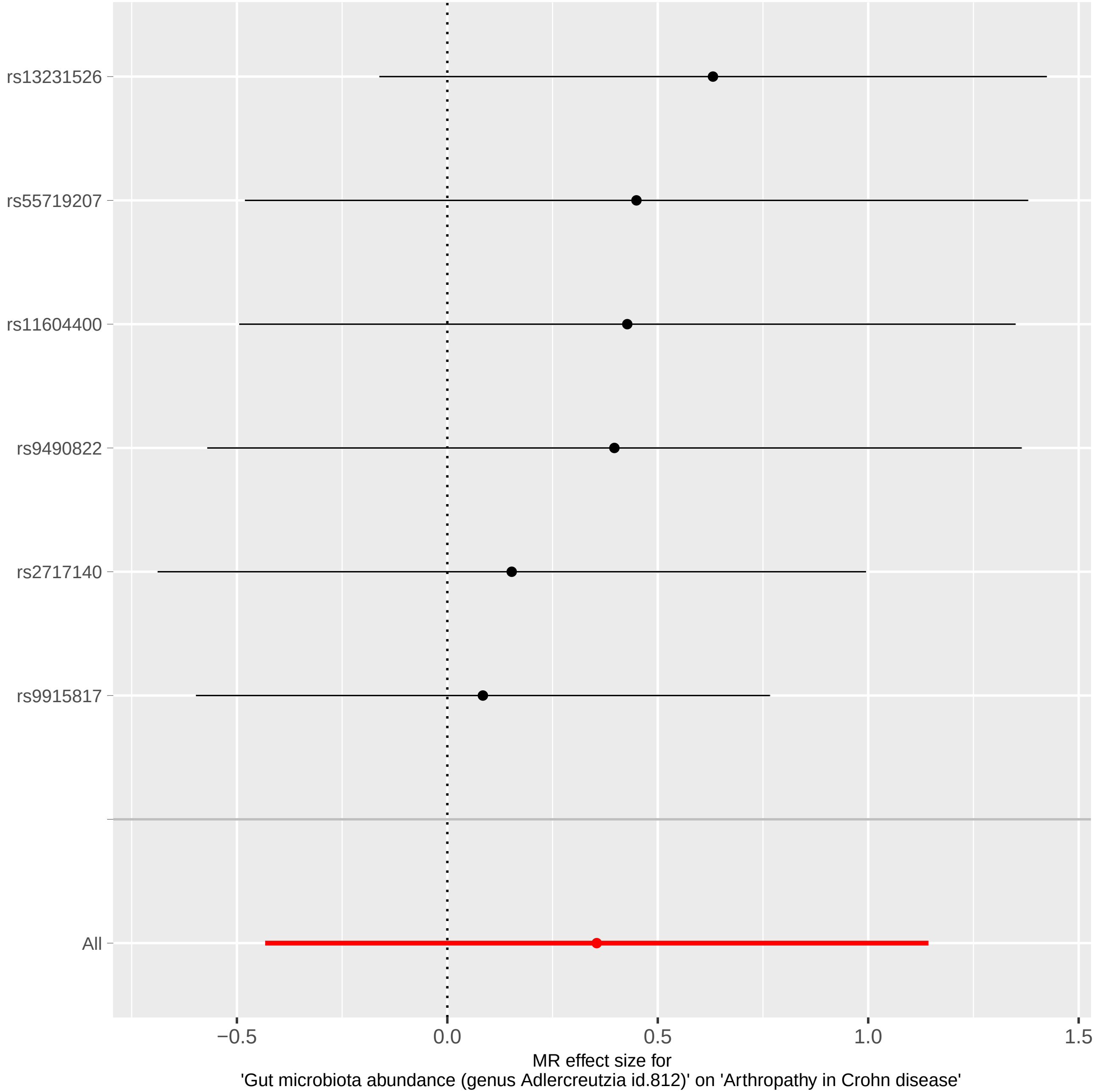

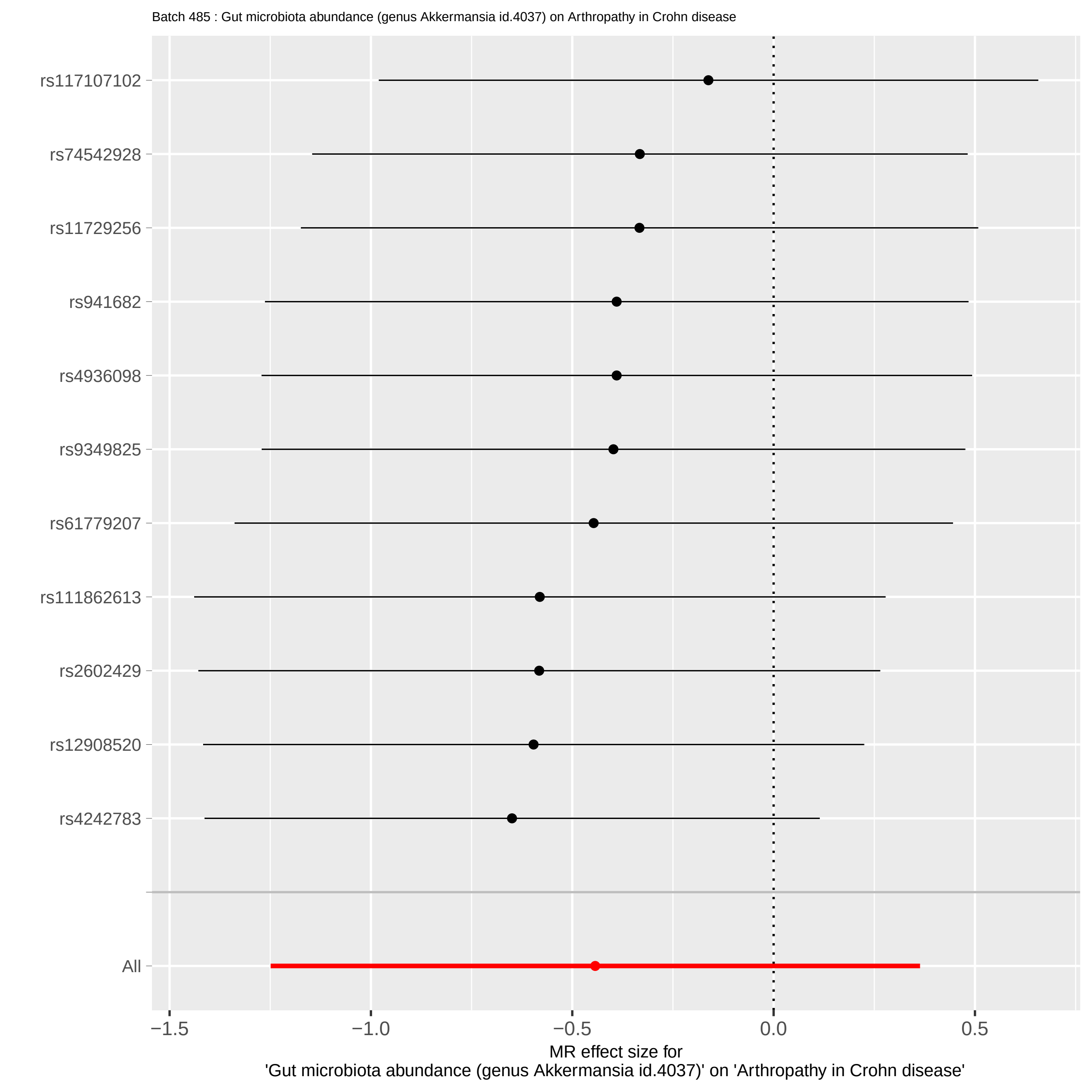
Batch 485 : Gut microbiota abundance (genus Akkermansia id.4037) on Arthropathy in Crohn disease
rs117107102
rs74542928
rs11729256
rs941682
rs4936098
rs9349825
rs61779207
rs111862613
rs2602429
rs12908520
rs4242783
All
−1.5
−1.0
−0.5
0.0
0.5
MR effect size for
'Gut microbiota abundance (genus Akkermansia id.4037)' on 'Arthropathy in Crohn disease'

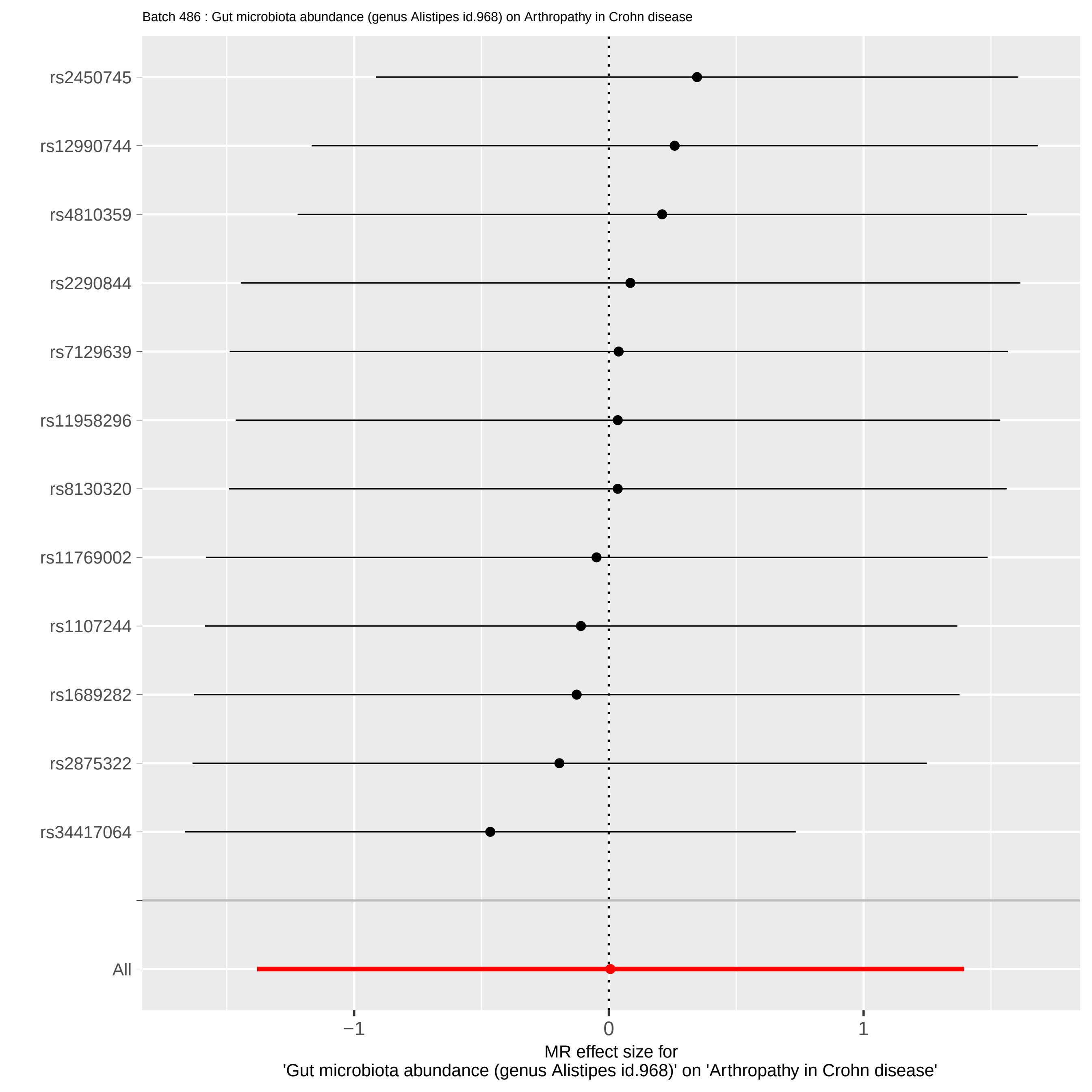
Batch 486 : Gut microbiota abundance (genus Alistipes id.968) on Arthropathy in Crohn disease
rs2450745
rs12990744
rs4810359
rs2290844
rs7129639
rs11958296
rs8130320
rs11769002
rs1107244
rs1689282
rs2875322
rs34417064
All
−1
0
1
MR effect size for
'Gut microbiota abundance (genus Alistipes id.968)' on 'Arthropathy in Crohn disease'

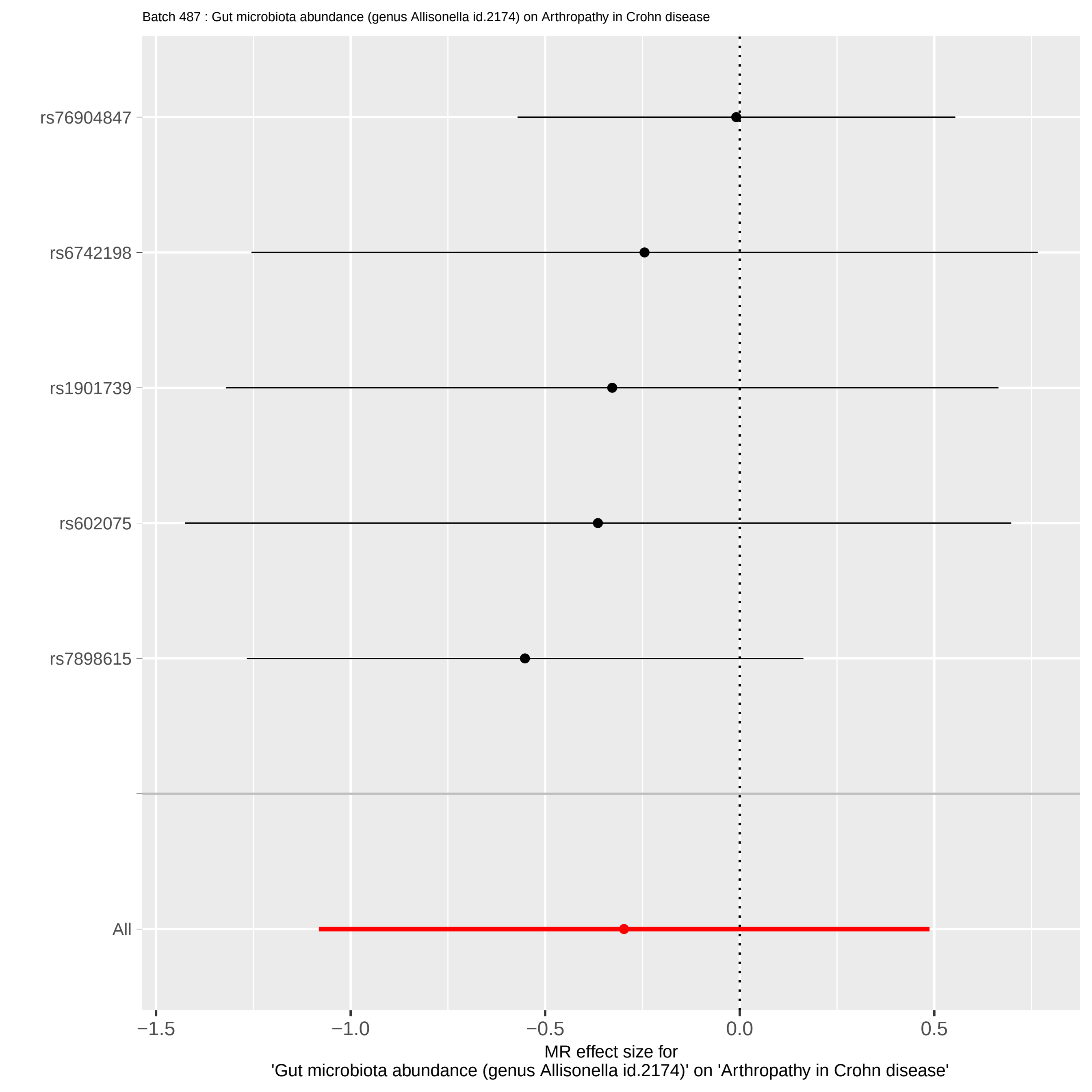

Batch 487 : Gut microbiota abundance (genus Allisonella id.2174) on Arthropathy in Crohn disease
rs76904847
rs6742198
rs1901739
rs602075
rs7898615
All
−1.5
−1.0
−0.5
0.0
0.5
MR effect size for
'Gut microbiota abundance (genus Allisonella id.2174)' on 'Arthropathy in Crohn disease'

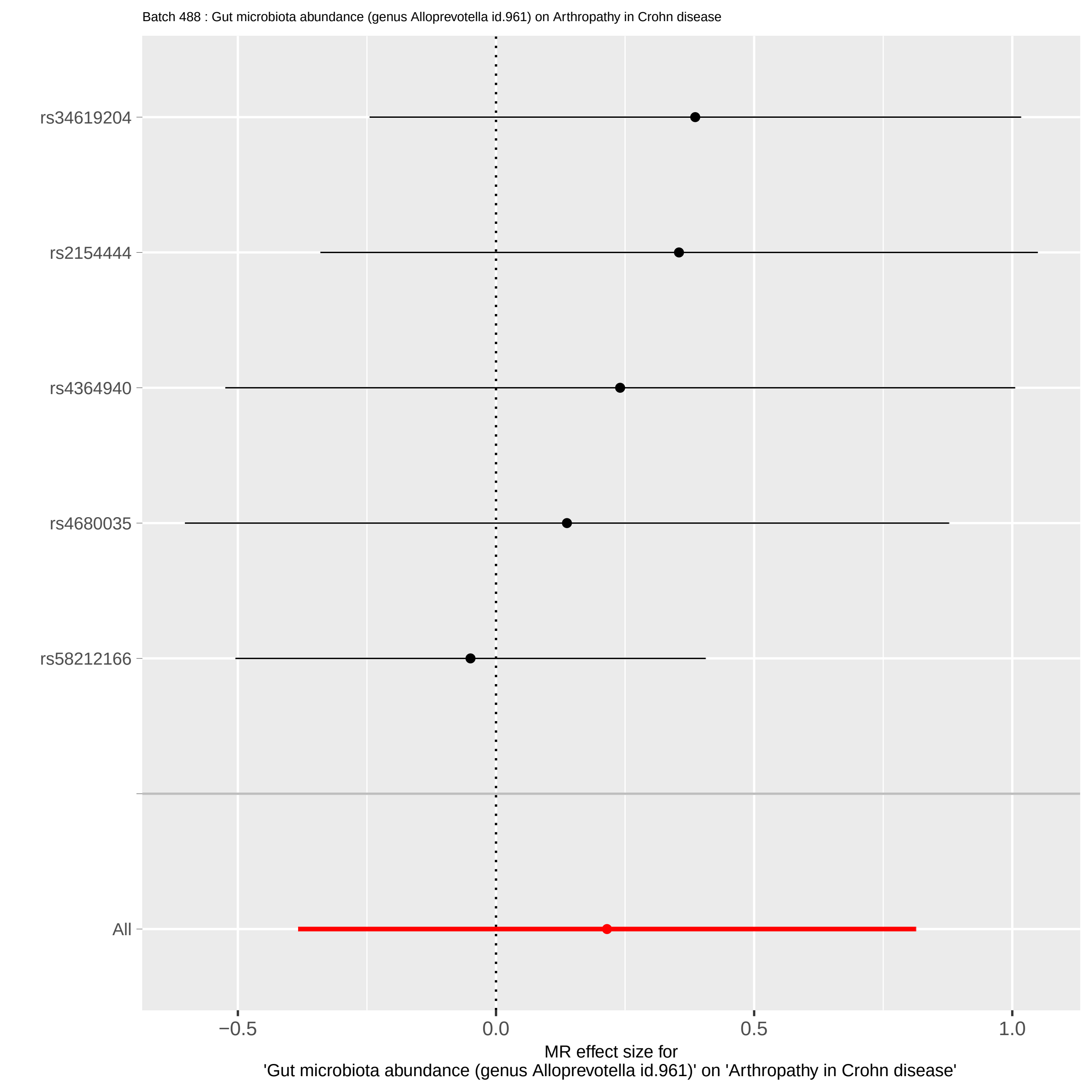

Batch 488 : Gut microbiota abundance (genus Alloprevotella id.961) on Arthropathy in Crohn disease
rs34619204
rs2154444
rs4364940
rs4680035
rs58212166
All
−0.5
0.0
0.5
1.0
MR effect size for
'Gut microbiota abundance (genus Alloprevotella id.961)' on 'Arthropathy in Crohn disease'

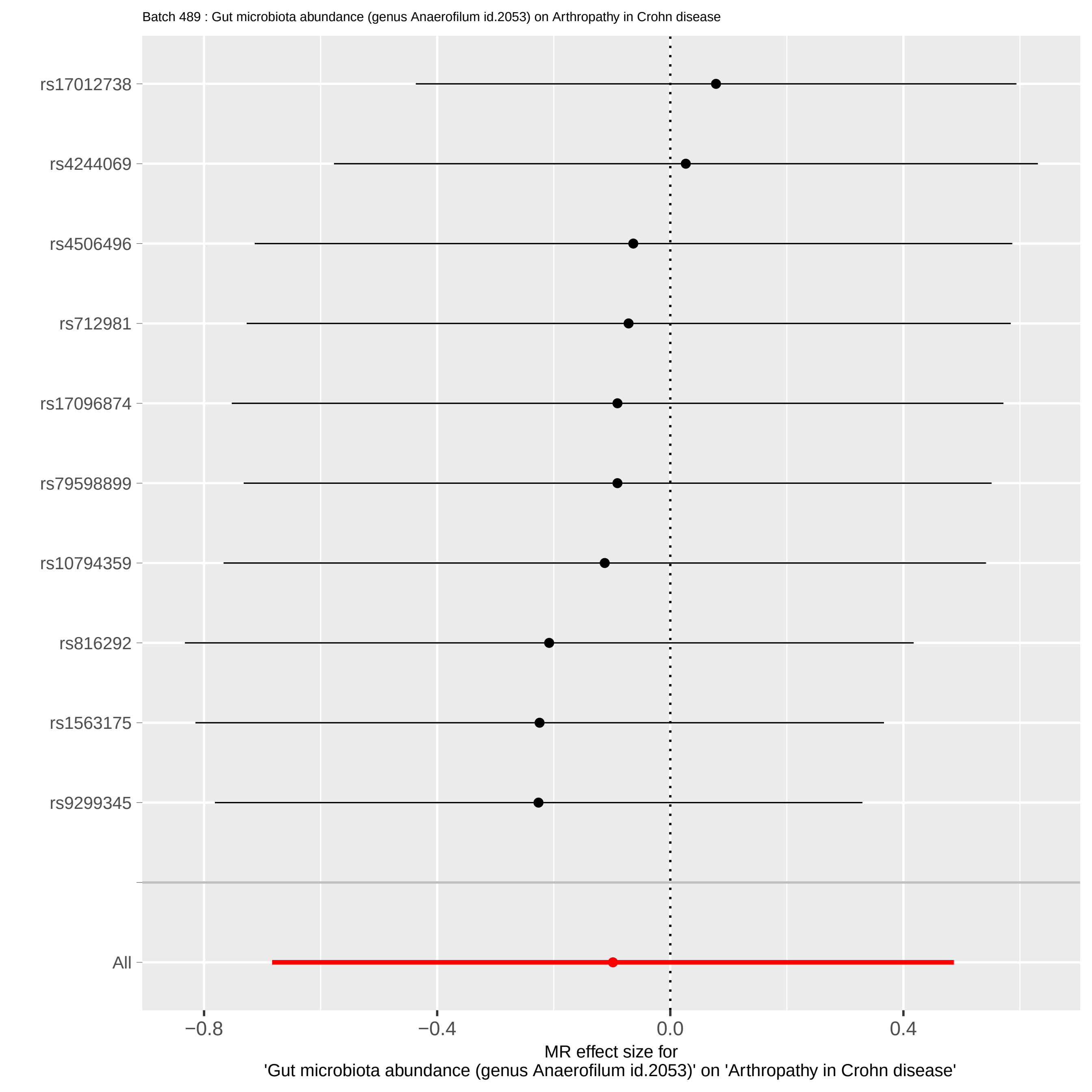

Batch 489 : Gut microbiota abundance (genus Anaerofilum id.2053) on Arthropathy in Crohn disease
rs17012738
rs4244069
rs4506496
rs712981
rs17096874
rs79598899
rs10794359
rs816292
rs1563175
rs9299345
All
−0.8
−0.4
0.0
0.4
MR effect size for
'Gut microbiota abundance (genus Anaerofilum id.2053)' on 'Arthropathy in Crohn disease'

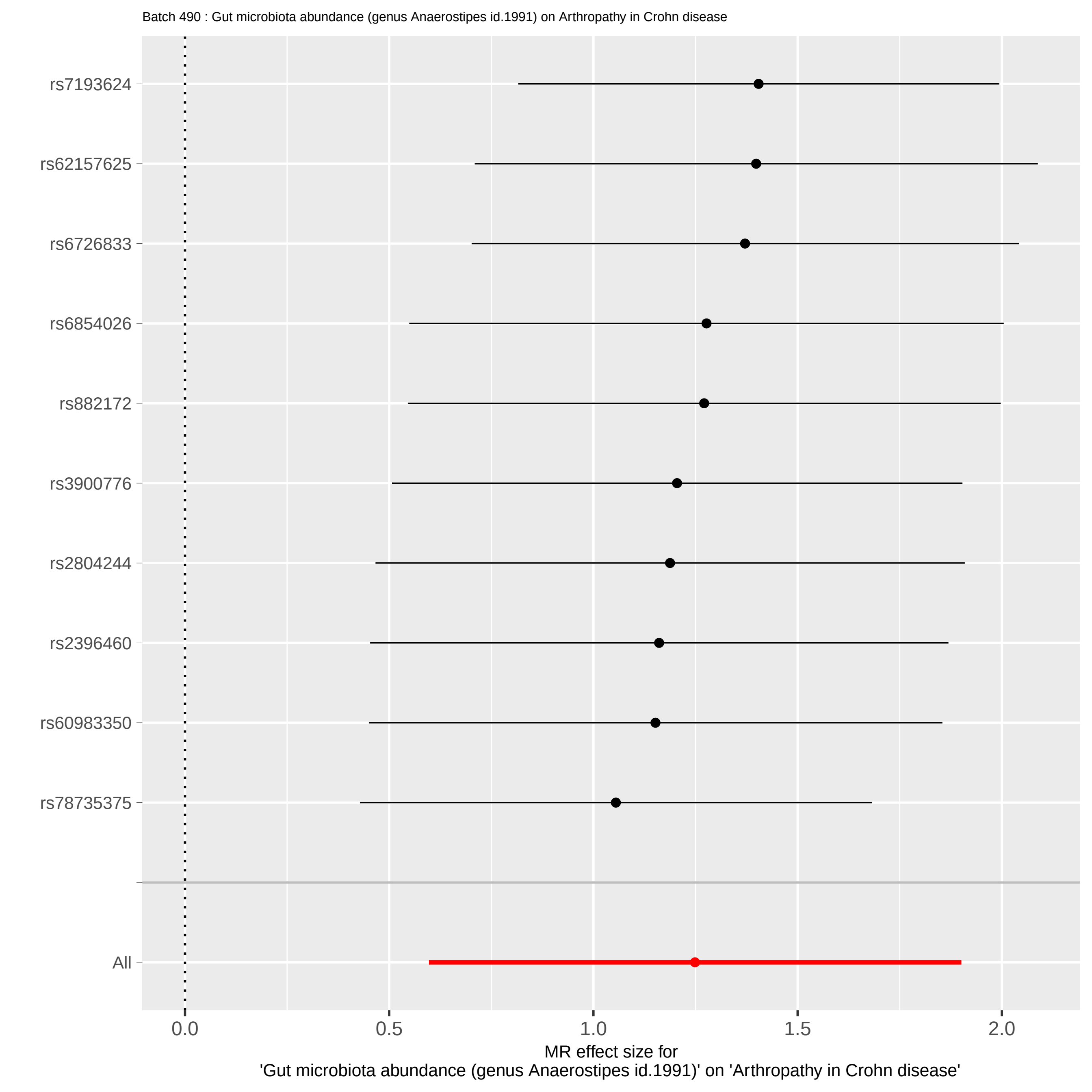
Batch 490 : Gut microbiota abundance (genus Anaerostipes id.1991) on Arthropathy in Crohn disease
rs7193624
rs62157625
rs6726833
rs6854026
rs882172
rs3900776
rs2804244
rs2396460
rs60983350
rs78735375
All
0.0
0.5
1.0
1.5
2.0
MR effect size for
'Gut microbiota abundance (genus Anaerostipes id.1991)' on 'Arthropathy in Crohn disease'

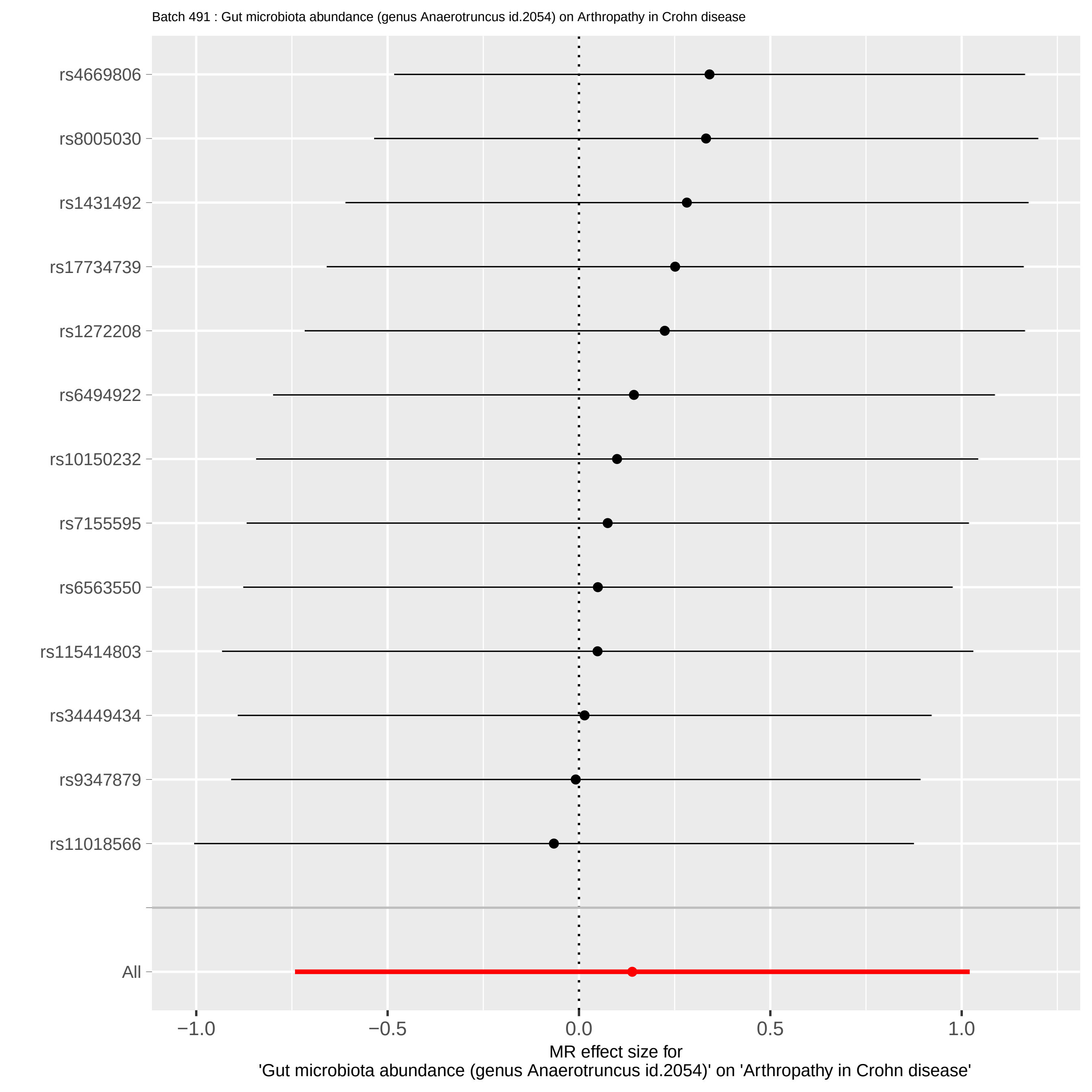
Batch 491 : Gut microbiota abundance (genus Anaerotruncus id.2054) on Arthropathy in Crohn disease
rs4669806
rs8005030
rs1431492
rs17734739
rs1272208
rs6494922
rs10150232
rs7155595
rs6563550
rs115414803
rs34449434
rs9347879
rs11018566
All
−1.0
−0.5
0.0
0.5
1.0
MR effect size for
'Gut microbiota abundance (genus Anaerotruncus id.2054)' on 'Arthropathy in Crohn disease'

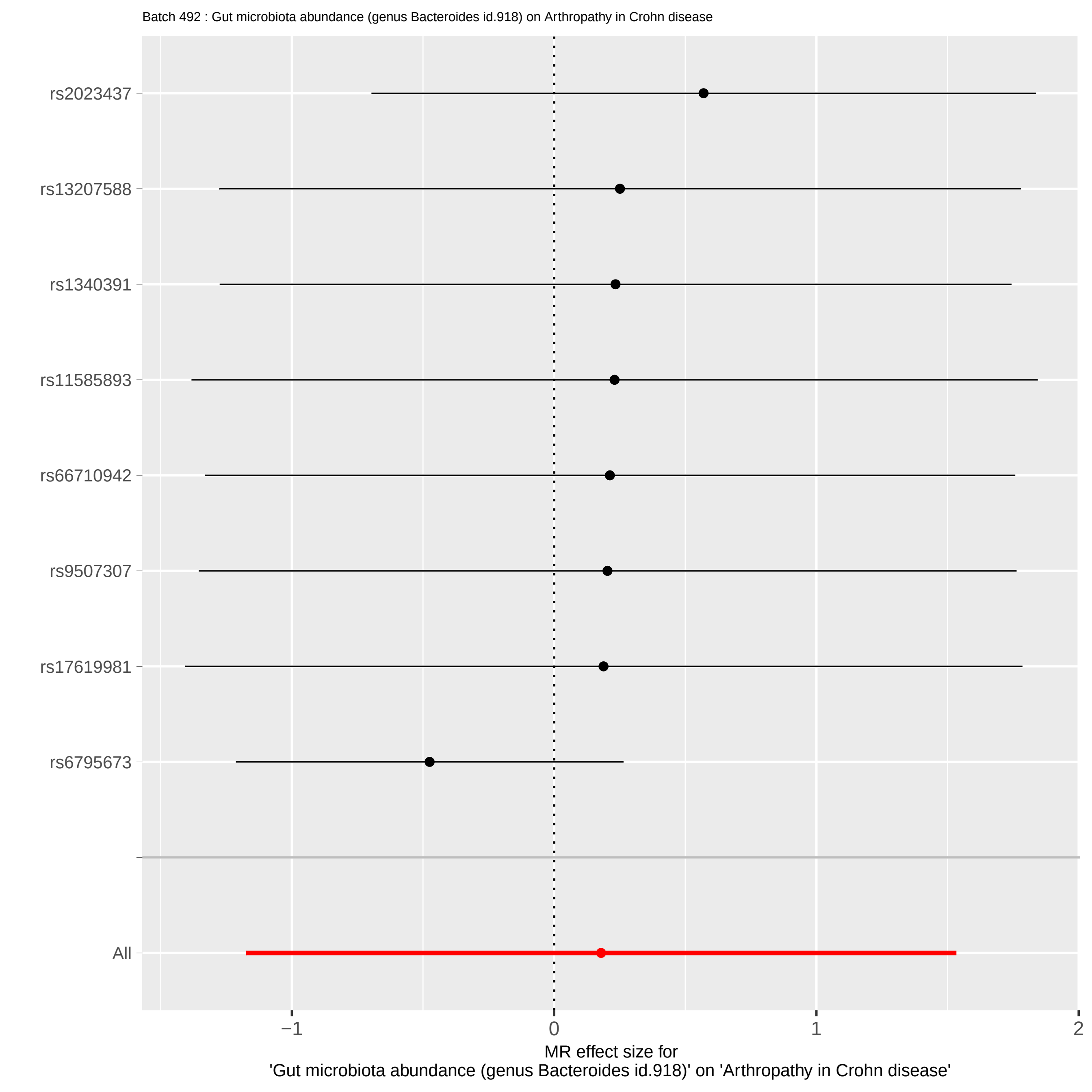
Batch 492 : Gut microbiota abundance (genus Bacteroides id.918) on Arthropathy in Crohn disease
rs2023437
rs13207588
rs1340391
rs11585893
rs66710942
rs9507307
rs17619981
rs6795673
All
−1
0
1
2
MR effect size for
'Gut microbiota abundance (genus Bacteroides id.918)' on 'Arthropathy in Crohn disease'

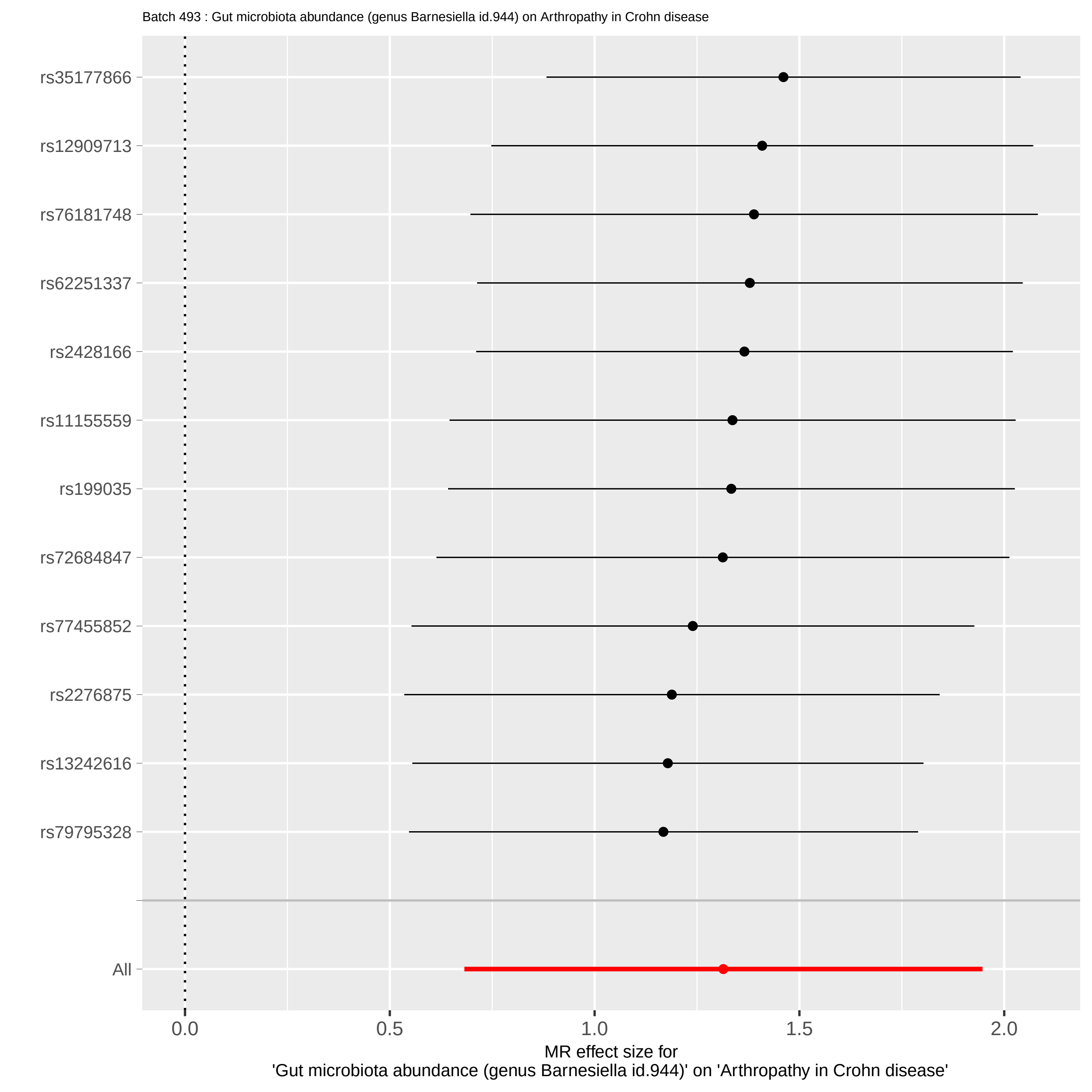

Batch 493 : Gut microbiota abundance (genus Barnesiella id.944) on Arthropathy in Crohn disease
rs35177866
rs12909713
rs76181748
rs62251337
rs2428166
rs11155559
rs199035
rs72684847
rs77455852
rs2276875
rs13242616
rs79795328
All
0.0
0.5
1.0
1.5
2.0
MR effect size for
'Gut microbiota abundance (genus Barnesiella id.944)' on 'Arthropathy in Crohn disease'

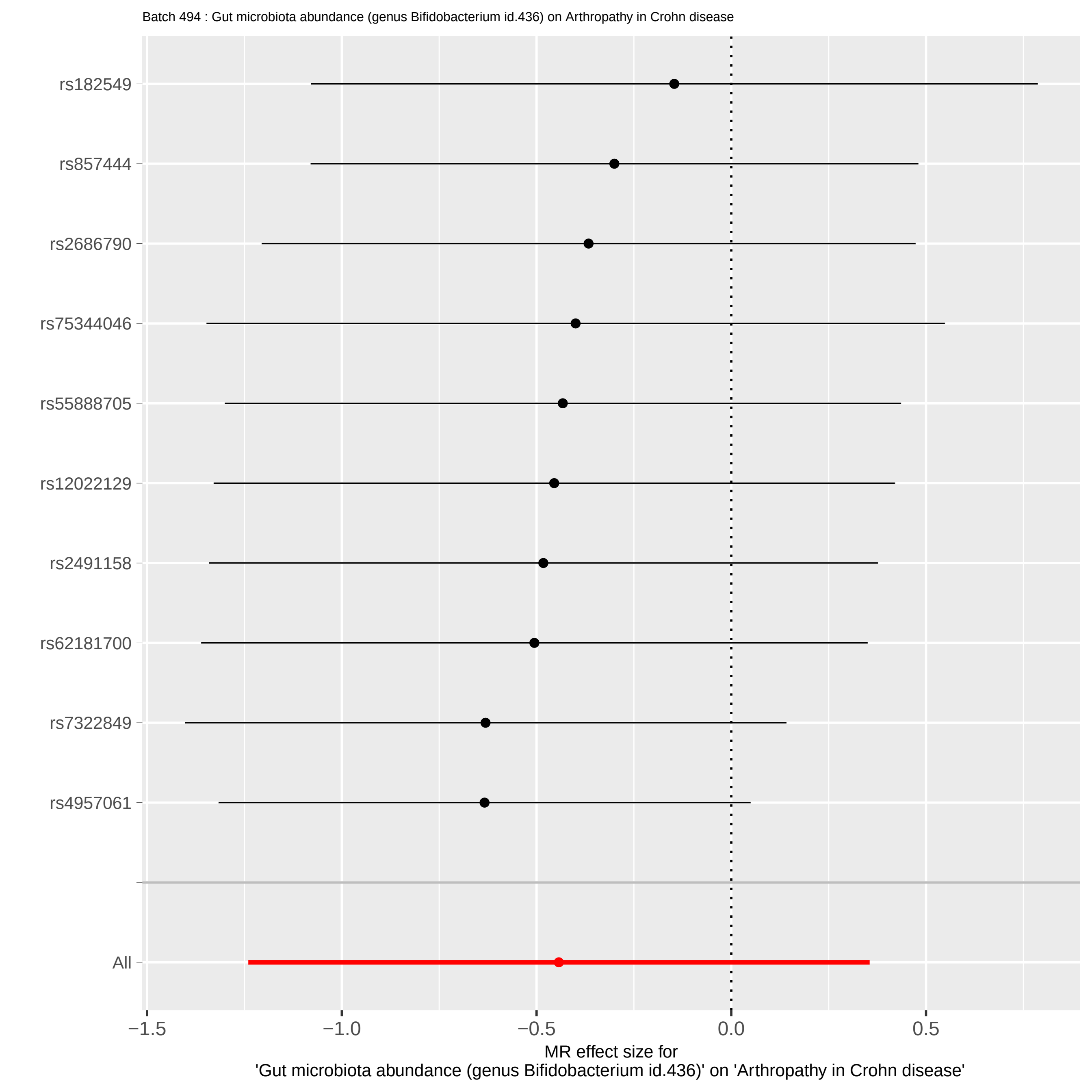
Batch 494 : Gut microbiota abundance (genus Bifidobacterium id.436) on Arthropathy in Crohn disease
rs182549
rs857444
rs2686790
rs75344046
rs55888705
rs12022129
rs2491158
rs62181700
rs7322849
rs4957061
All
−1.5
−1.0
−0.5
0.0
0.5
MR effect size for
'Gut microbiota abundance (genus Bifidobacterium id.436)' on 'Arthropathy in Crohn disease'

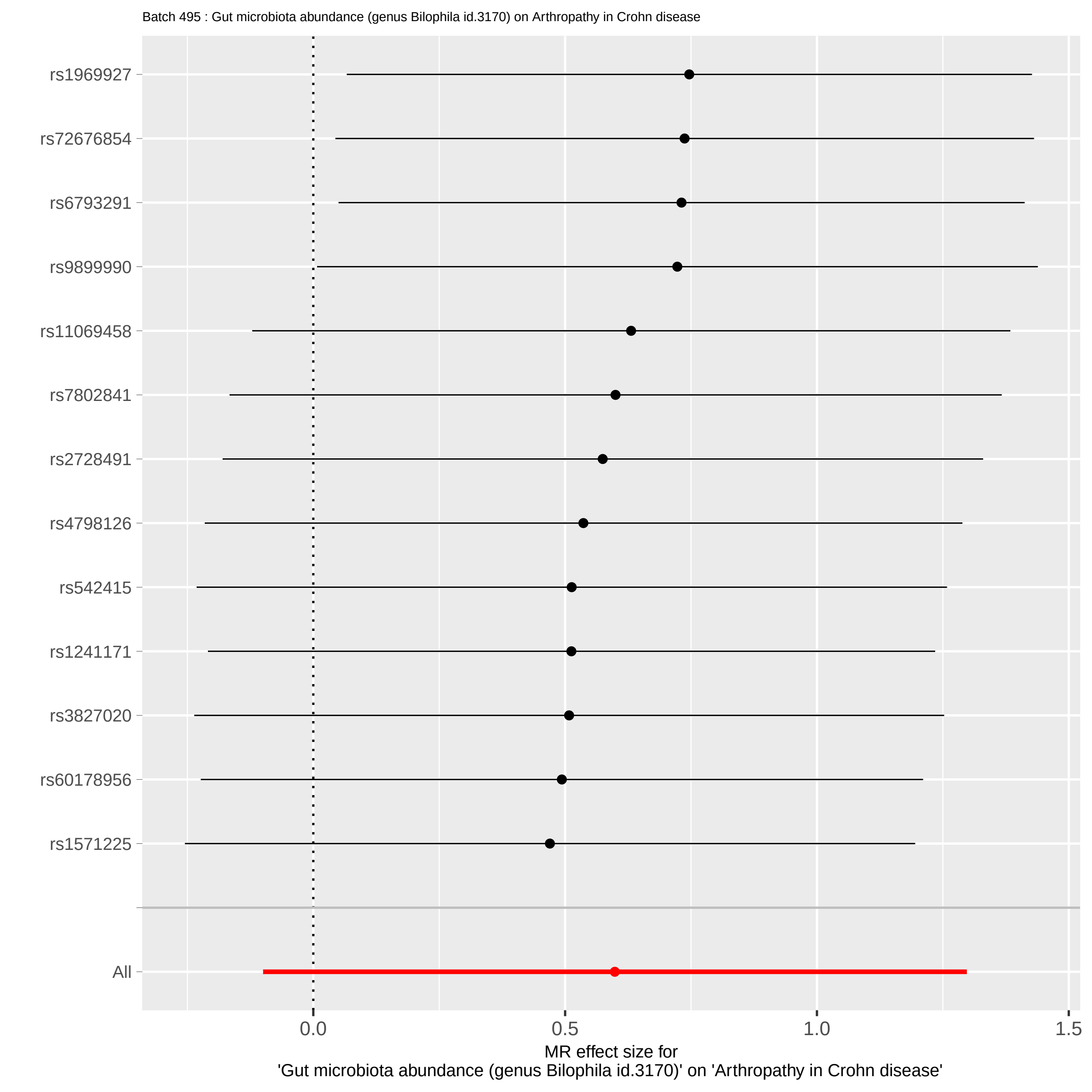

Batch 495 : Gut microbiota abundance (genus Bilophila id.3170) on Arthropathy in Crohn disease
rs1969927
rs72676854
rs6793291
rs9899990
rs11069458
rs7802841
rs2728491
rs4798126
rs542415
rs1241171
rs3827020
rs60178956
rs1571225
All
0.0
0.5
1.0
1.5
MR effect size for
'Gut microbiota abundance (genus Bilophila id.3170)' on 'Arthropathy in Crohn disease'

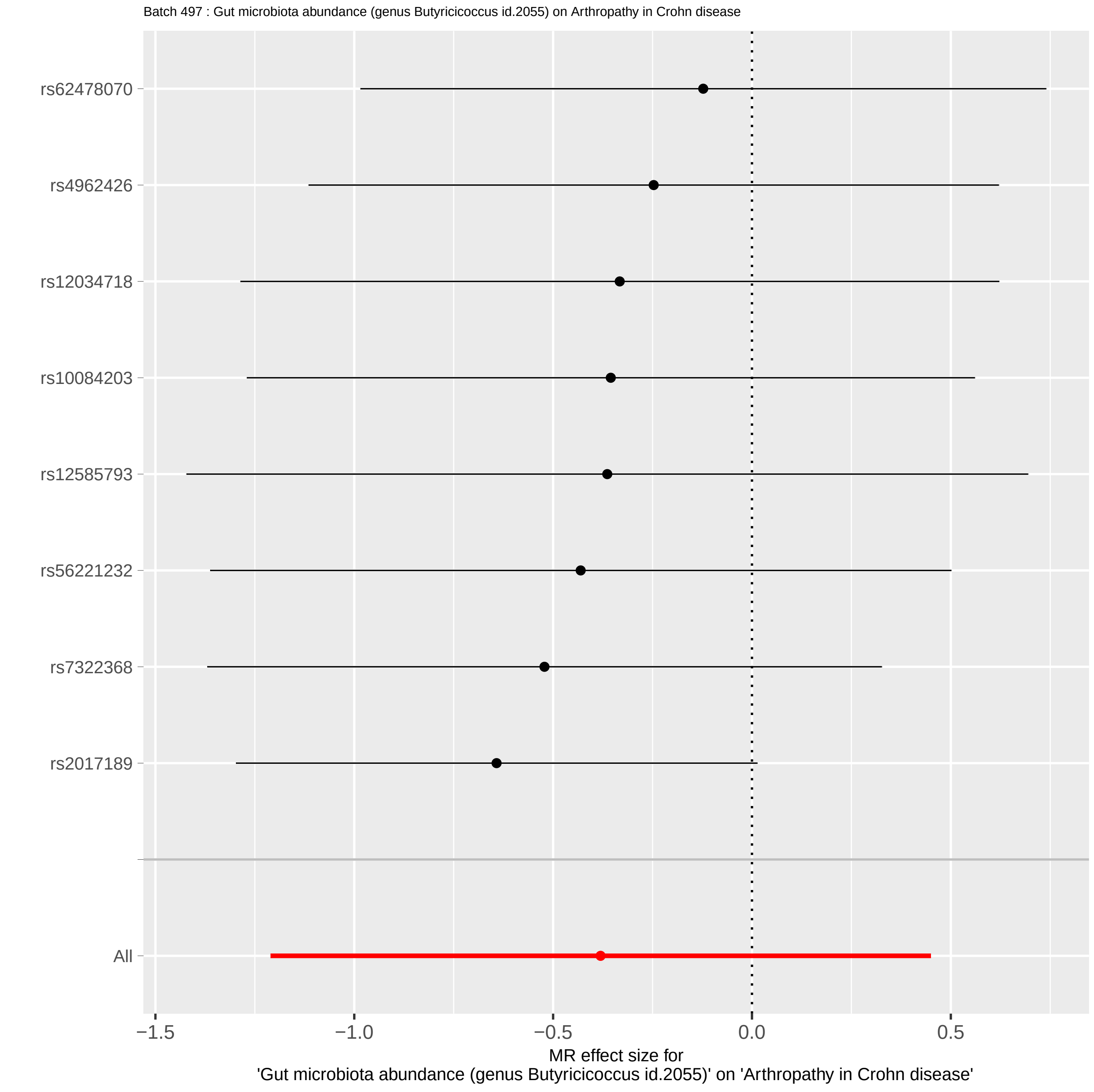
Batch 497 : Gut microbiota abundance (genus Butyricicoccus id.2055) on Arthropathy in Crohn disease
rs62478070
rs4962426
rs12034718
rs10084203
rs12585793
rs56221232
rs7322368
rs2017189
All
−1.5
−1.0
−0.5
0.0
0.5
MR effect size for
'Gut microbiota abundance (genus Butyricicoccus id.2055)' on 'Arthropathy in Crohn disease'

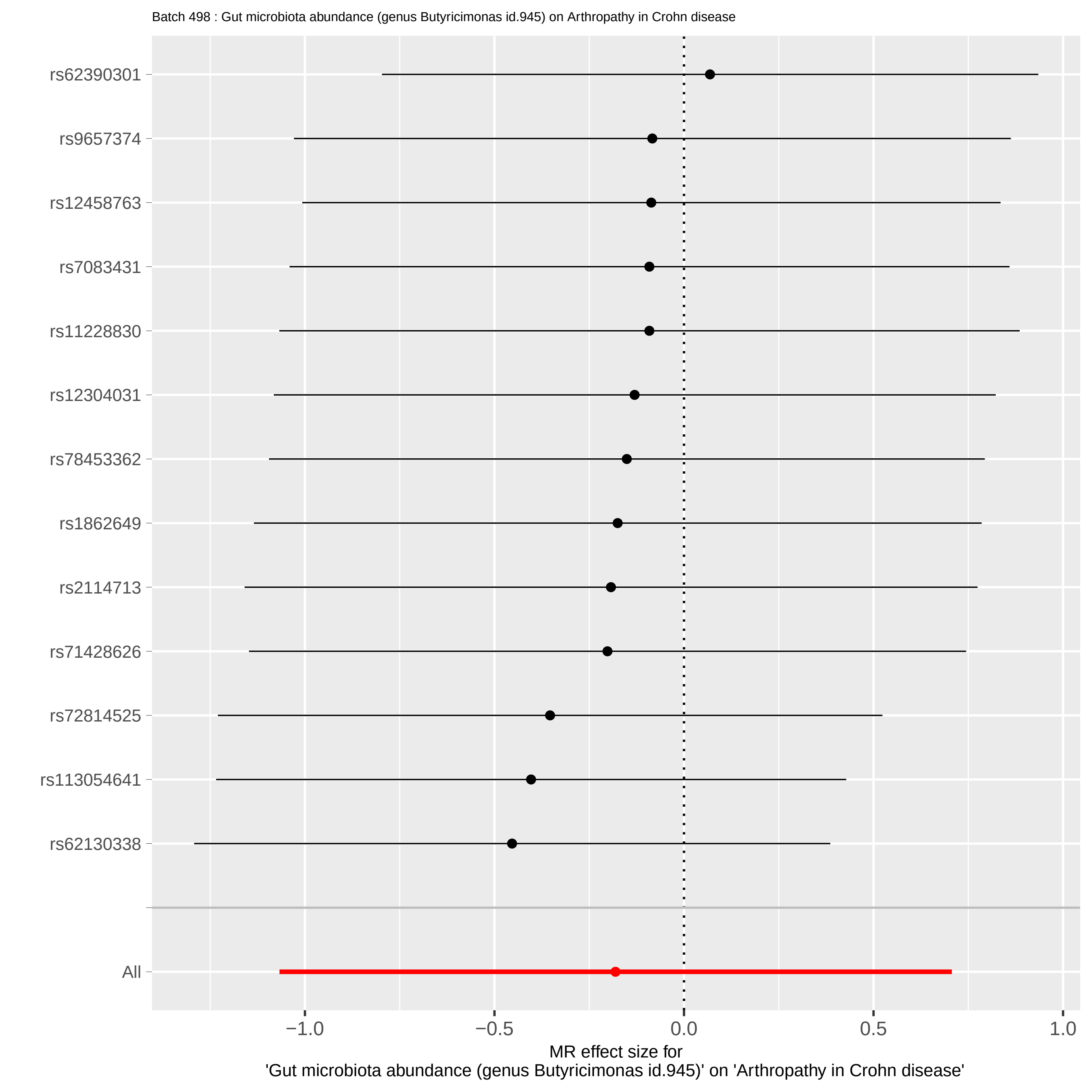

Batch 498 : Gut microbiota abundance (genus Butyricimonas id.945) on Arthropathy in Crohn disease
rs62390301
rs9657374
rs12458763
rs7083431
rs11228830
rs12304031
rs78453362
rs1862649
rs2114713
rs71428626
rs72814525
rs113054641
rs62130338
All
−1.0
−0.5
0.0
0.5
1.0
MR effect size for
'Gut microbiota abundance (genus Butyricimonas id.945)' on 'Arthropathy in Crohn disease'

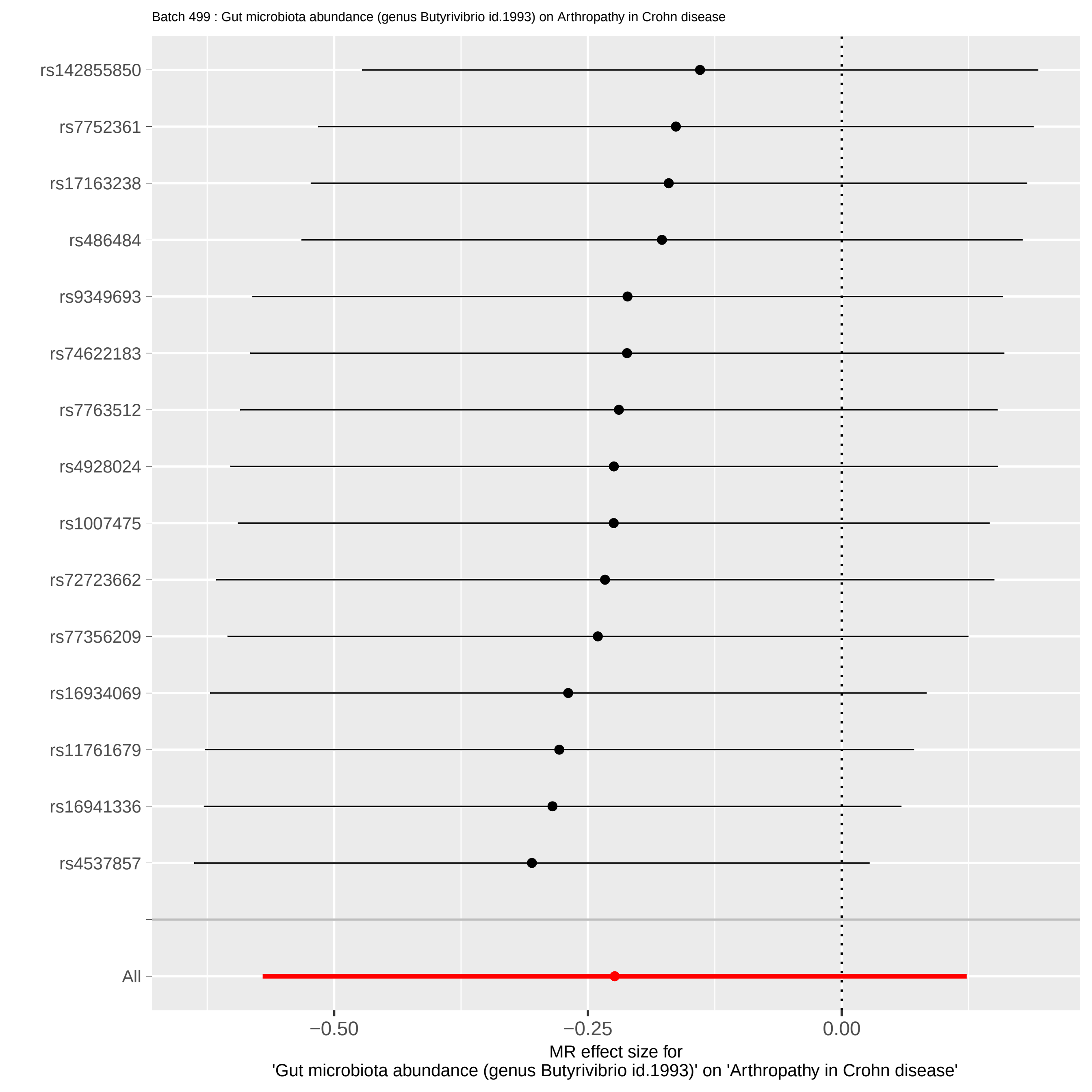
Batch 499 : Gut microbiota abundance (genus Butyrivibrio id.1993) on Arthropathy in Crohn disease
rs142855850
rs7752361
rs17163238
rs486484
rs9349693
rs74622183
rs7763512
rs4928024
rs1007475
rs72723662
rs77356209
rs16934069
rs11761679
rs16941336
rs4537857
All
−0.50
−0.25
0.00
MR effect size for
'Gut microbiota abundance (genus Butyrivibrio id.1993)' on 'Arthropathy in Crohn disease'

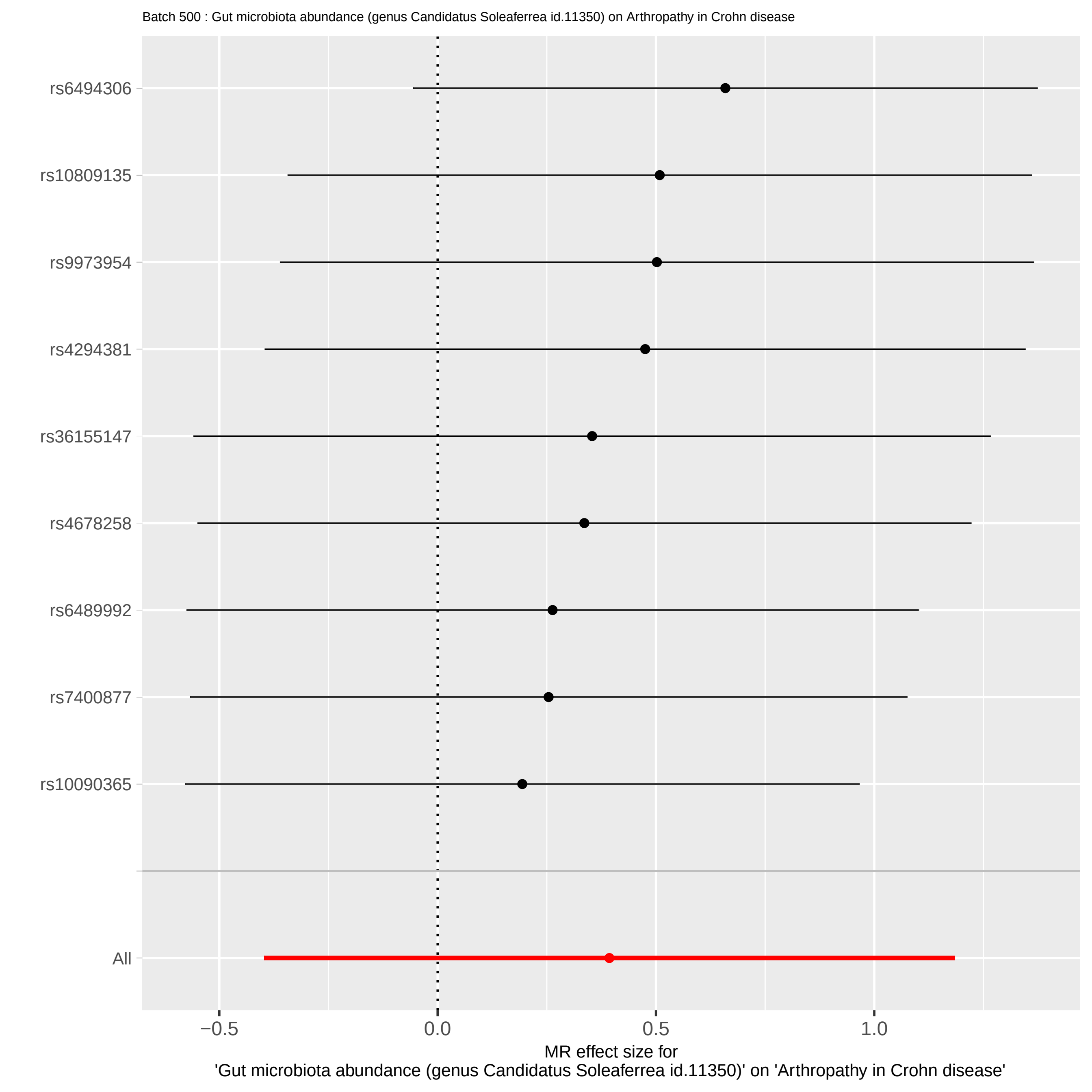

Batch 500 : Gut microbiota abundance (genus Candidatus Soleaferrea id.11350) on Arthropathy in Crohn disease
rs6494306
rs10809135
rs9973954
rs4294381
rs36155147
rs4678258
rs6489992
rs7400877
rs10090365
All
−0.5
0.0
0.5
1.0
MR effect size for
'Gut microbiota abundance (genus Candidatus Soleaferrea id.11350)' on 'Arthropathy in Crohn disease'

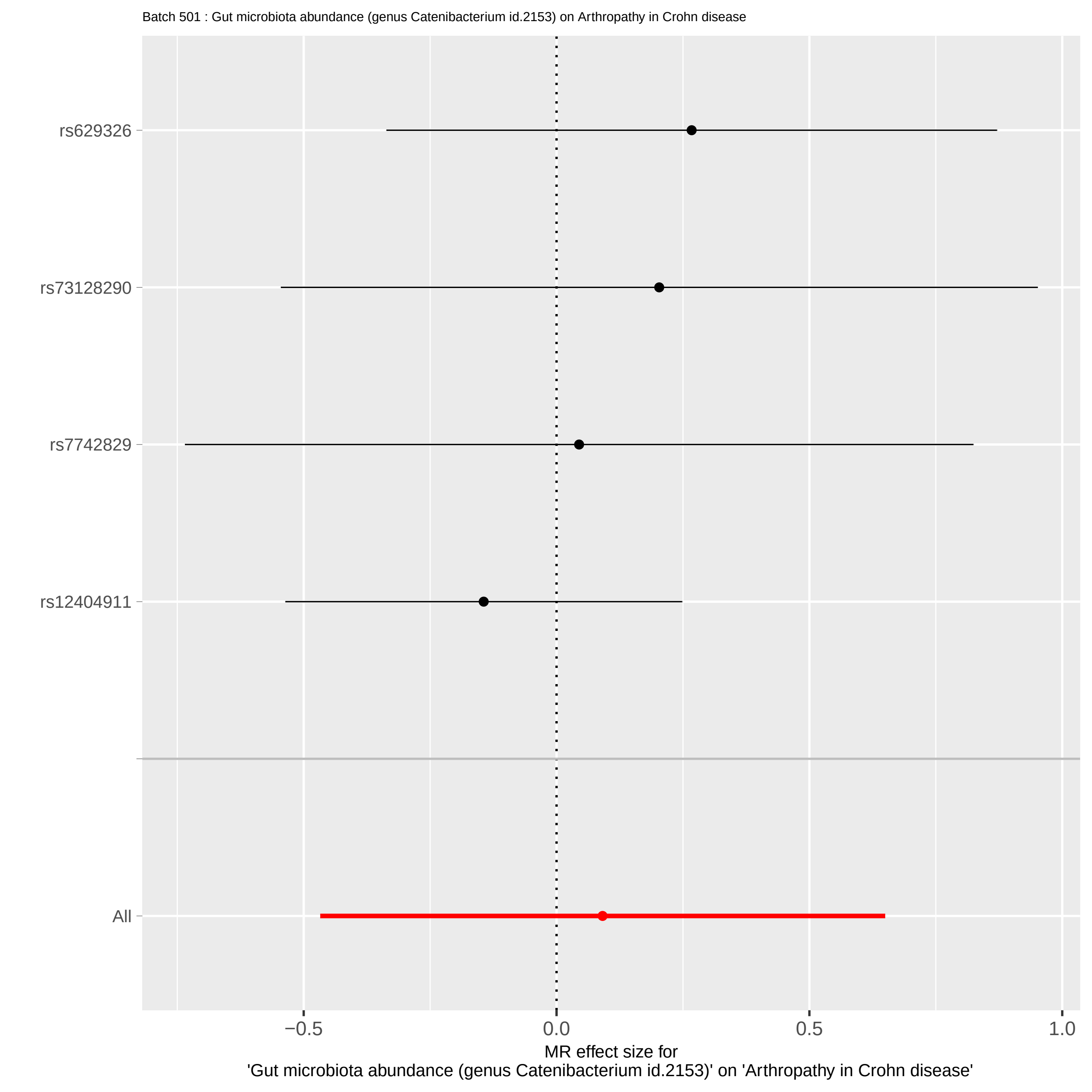
Batch 501 : Gut microbiota abundance (genus Catenibacterium id.2153) on Arthropathy in Crohn disease
rs629326
rs73128290
rs7742829
rs12404911
All
−0.5
0.0
0.5
1.0
MR effect size for
'Gut microbiota abundance (genus Catenibacterium id.2153)' on 'Arthropathy in Crohn disease'

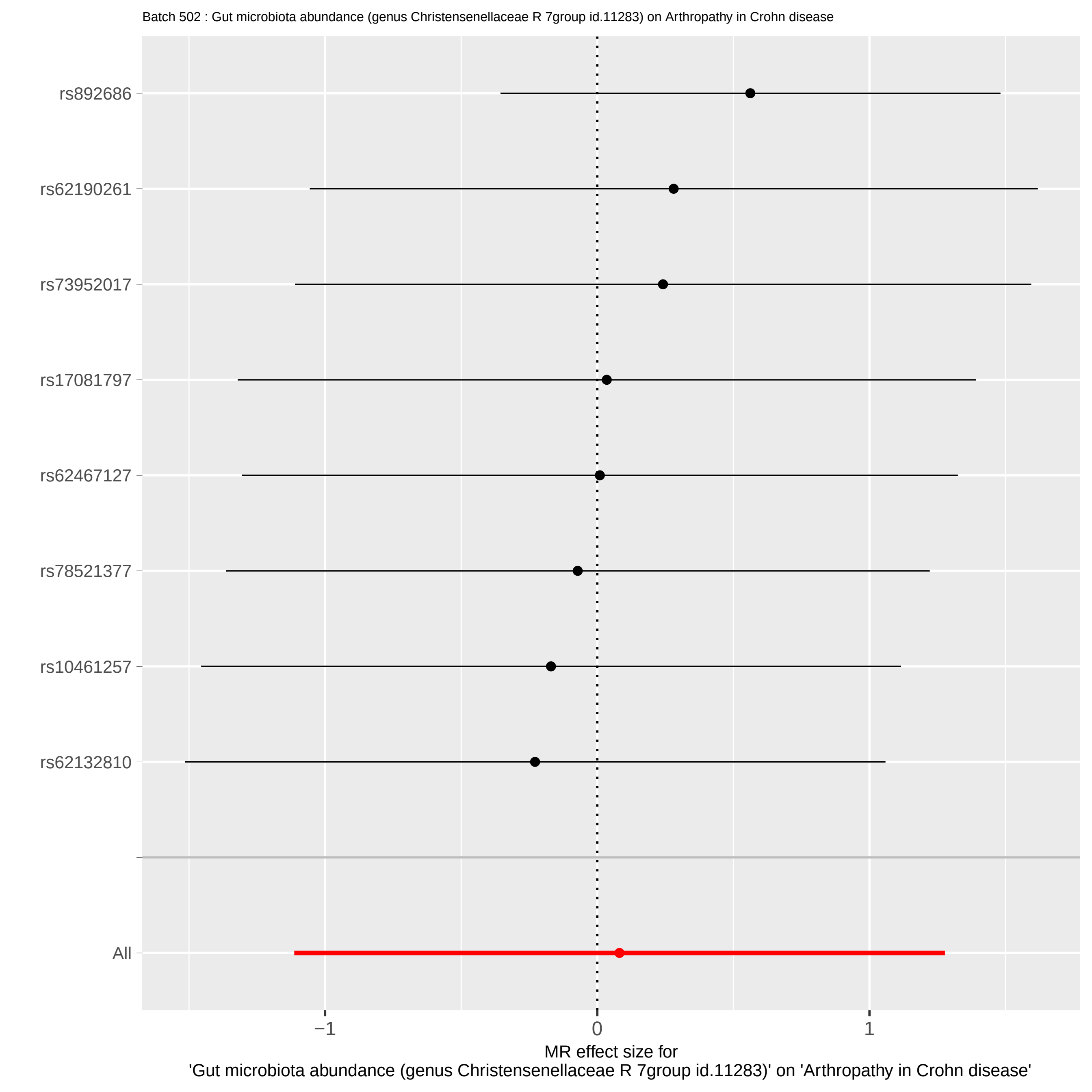

Batch 502 : Gut microbiota abundance (genus Christensenellaceae R 7group id.11283) on Arthropathy in Crohn disease
rs892686
rs62190261
rs73952017
rs17081797
rs62467127
rs78521377
rs10461257
rs62132810
All
−1
0
1
MR effect size for
'Gut microbiota abundance (genus Christensenellaceae R 7group id.11283)' on 'Arthropathy in Crohn disease'

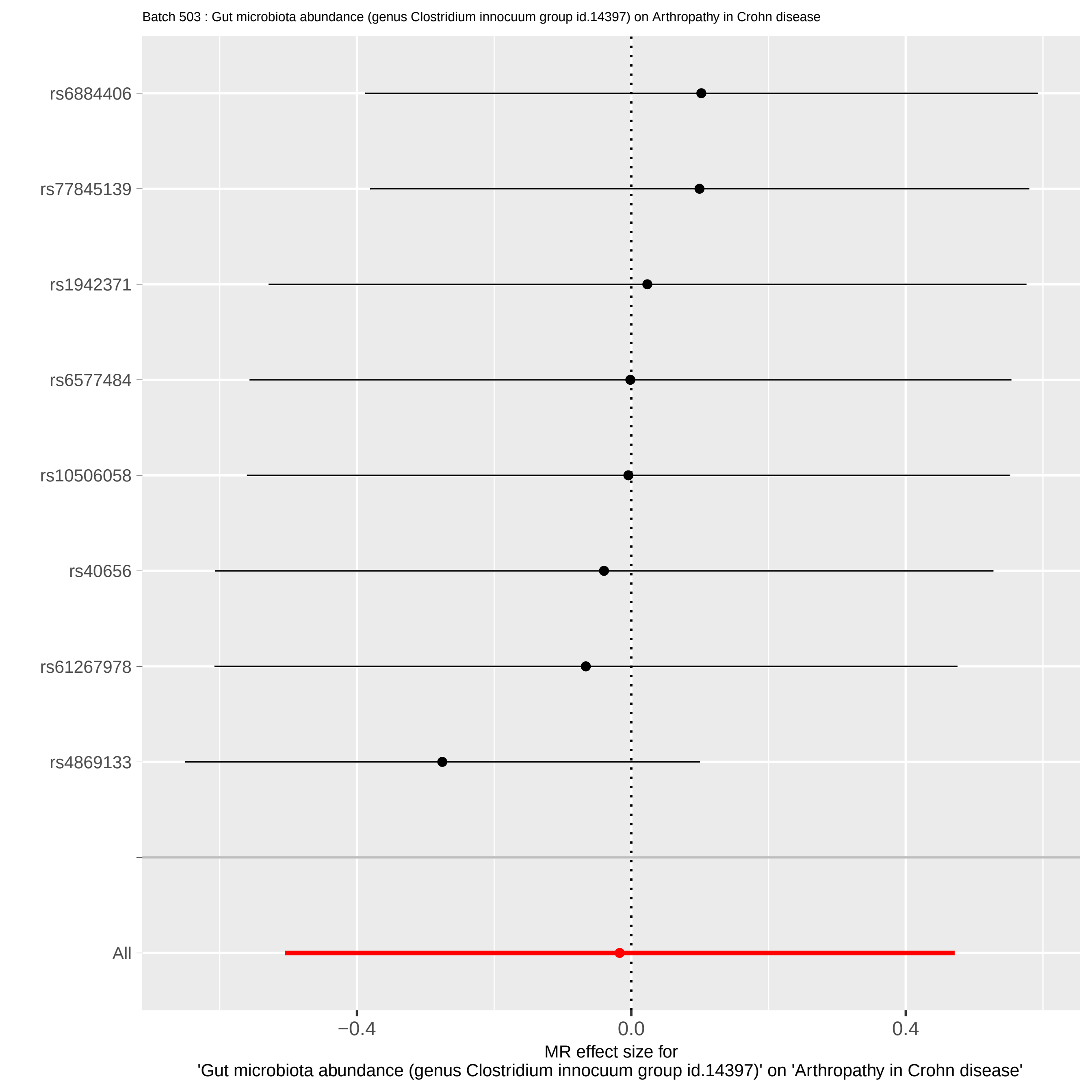

Batch 503 : Gut microbiota abundance (genus Clostridium innocuum group id.14397) on Arthropathy in Crohn disease
rs6884406
rs77845139
rs1942371
rs6577484
rs10506058
rs40656
rs61267978
rs4869133
All
−0.4
0.0
0.4
MR effect size for
'Gut microbiota abundance (genus Clostridium innocuum group id.14397)' on 'Arthropathy in Crohn disease'

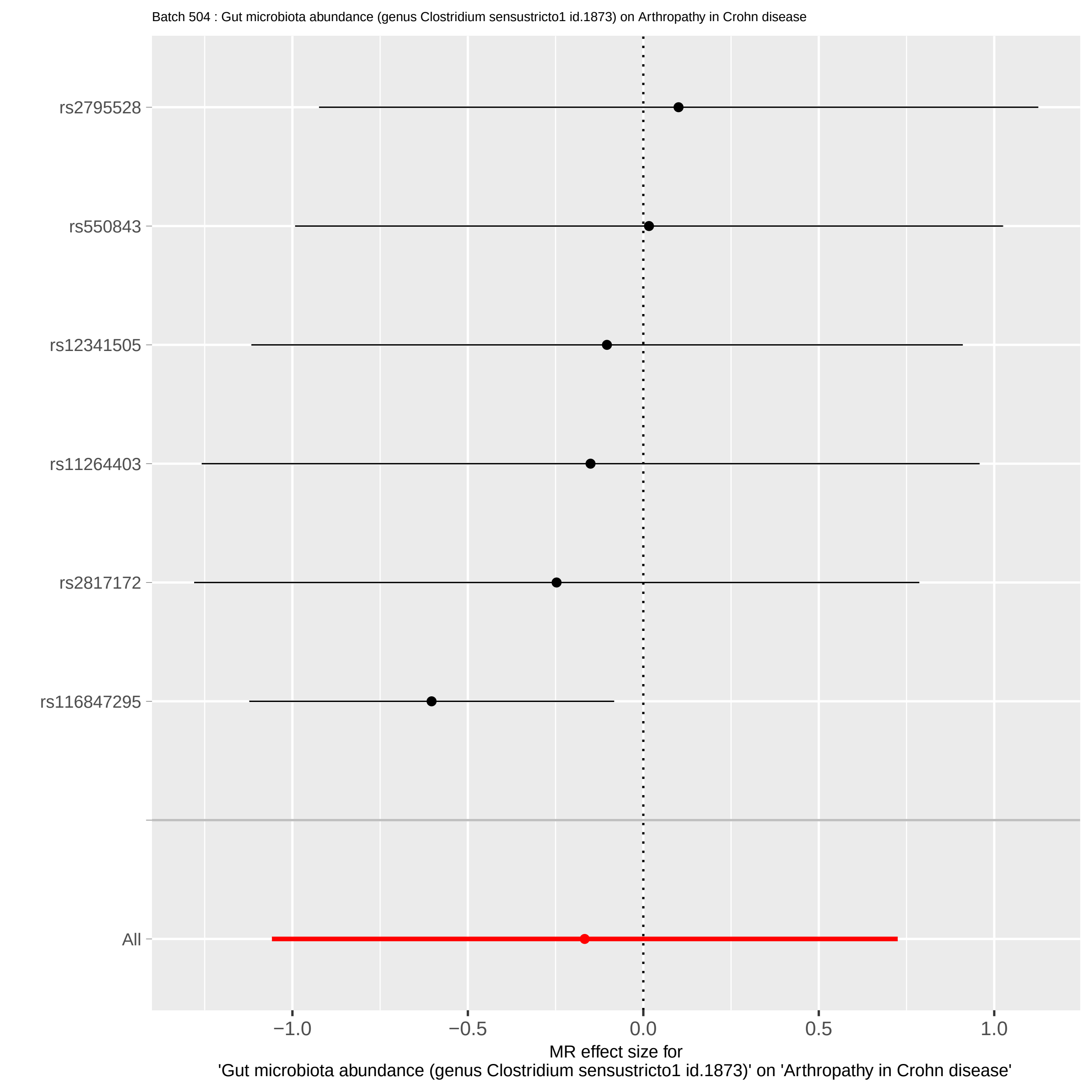

Batch 504 : Gut microbiota abundance (genus Clostridium sensustricto1 id.1873) on Arthropathy in Crohn disease
rs2795528
rs550843
rs12341505
rs11264403
rs2817172
rs116847295
All
−1.0
−0.5
0.0
0.5
1.0
MR effect size for
'Gut microbiota abundance (genus Clostridium sensustricto1 id.1873)' on 'Arthropathy in Crohn disease'

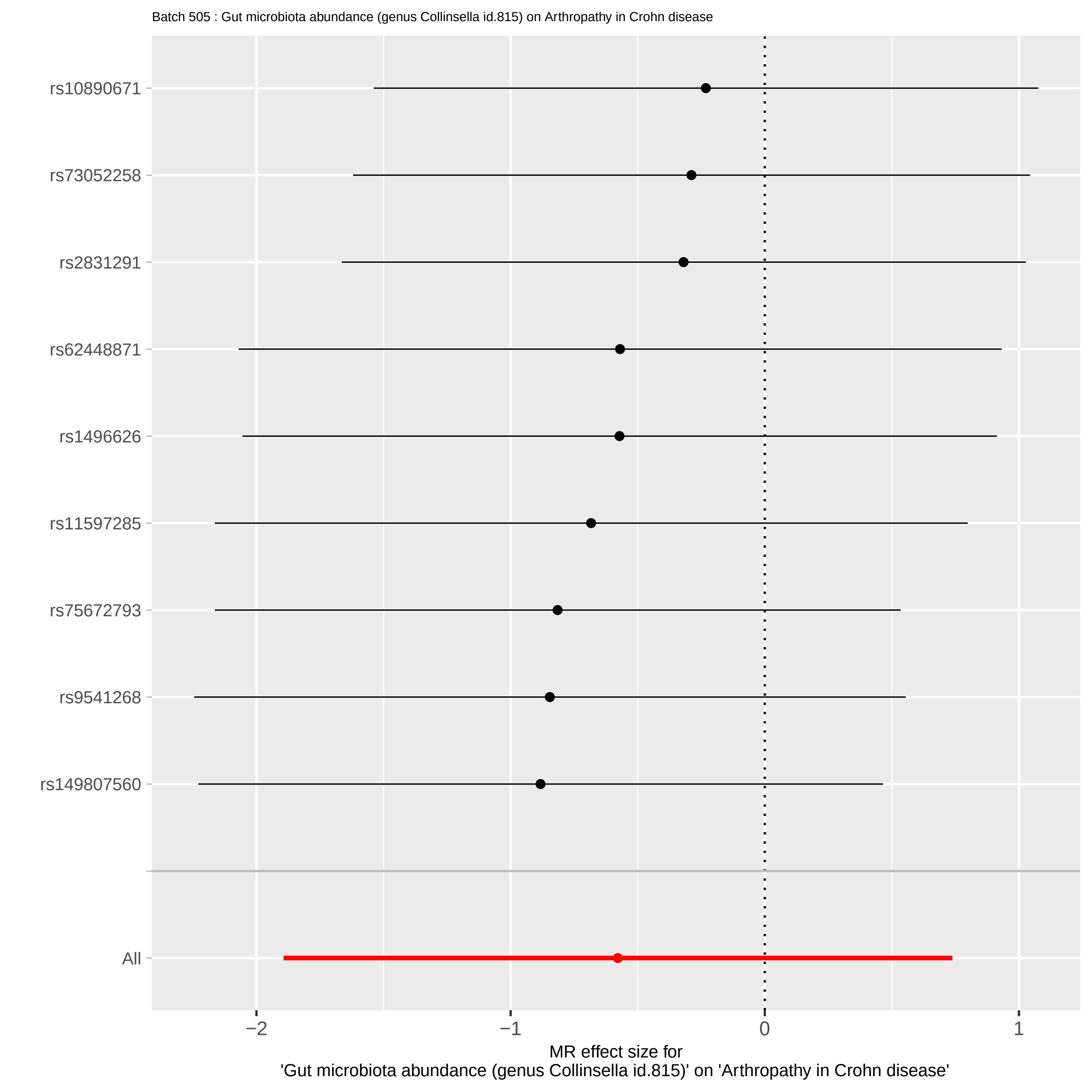

Batch 505 : Gut microbiota abundance (genus Collinsella id.815) on Arthropathy in Crohn disease
rs10890671
rs73052258
rs2831291
rs62448871
rs1496626
rs11597285
rs75672793
rs9541268
rs149807560
All
−2
−1
0
1
MR effect size for
'Gut microbiota abundance (genus Collinsella id.815)' on 'Arthropathy in Crohn disease'

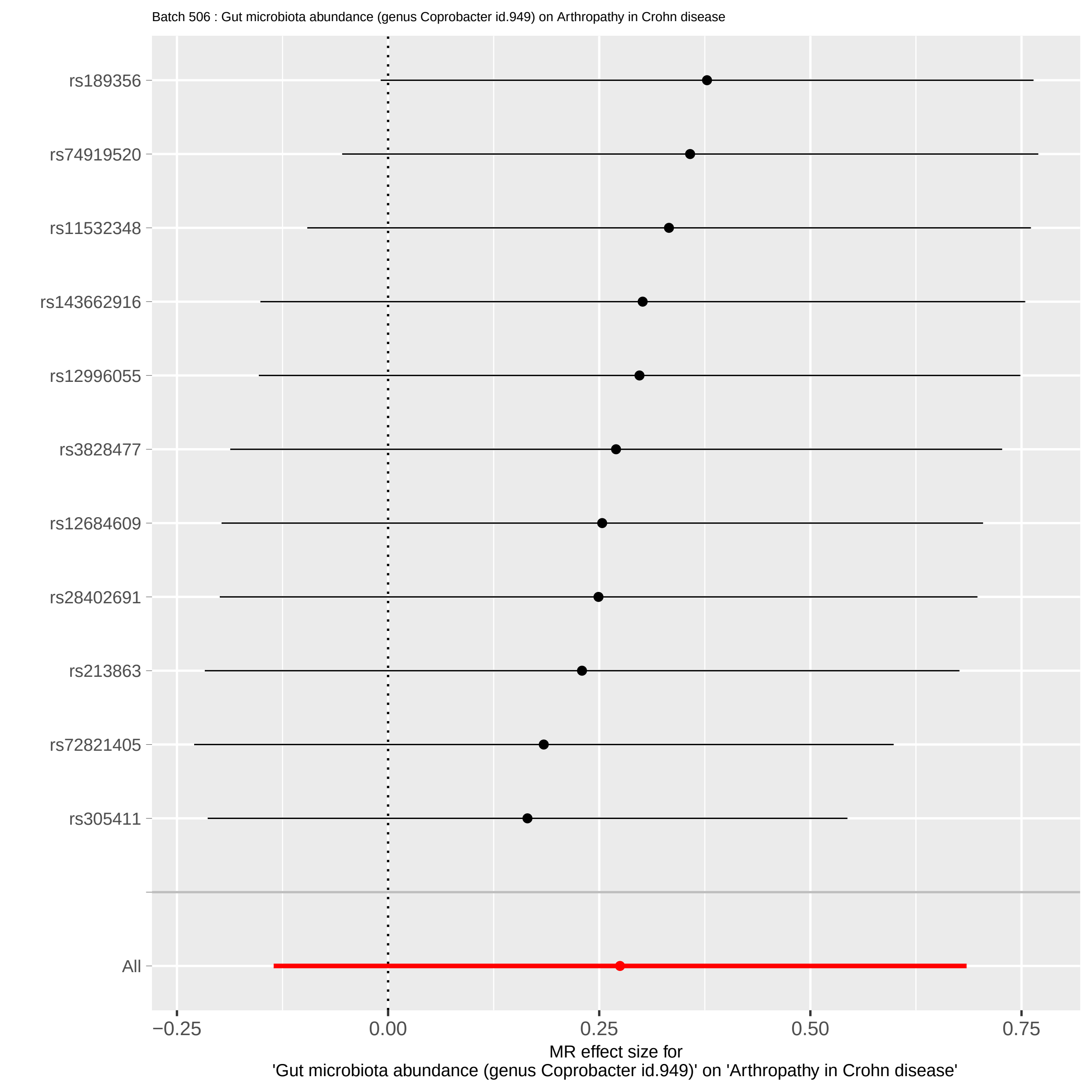

Batch 506 : Gut microbiota abundance (genus Coprobacter id.949) on Arthropathy in Crohn disease
rs189356
rs74919520
rs11532348
rs143662916
rs12996055
rs3828477
rs12684609
rs28402691
rs213863
rs72821405
rs305411
All
−0.25
0.00
0.25
0.50
0.75
MR effect size for
'Gut microbiota abundance (genus Coprobacter id.949)' on 'Arthropathy in Crohn disease'

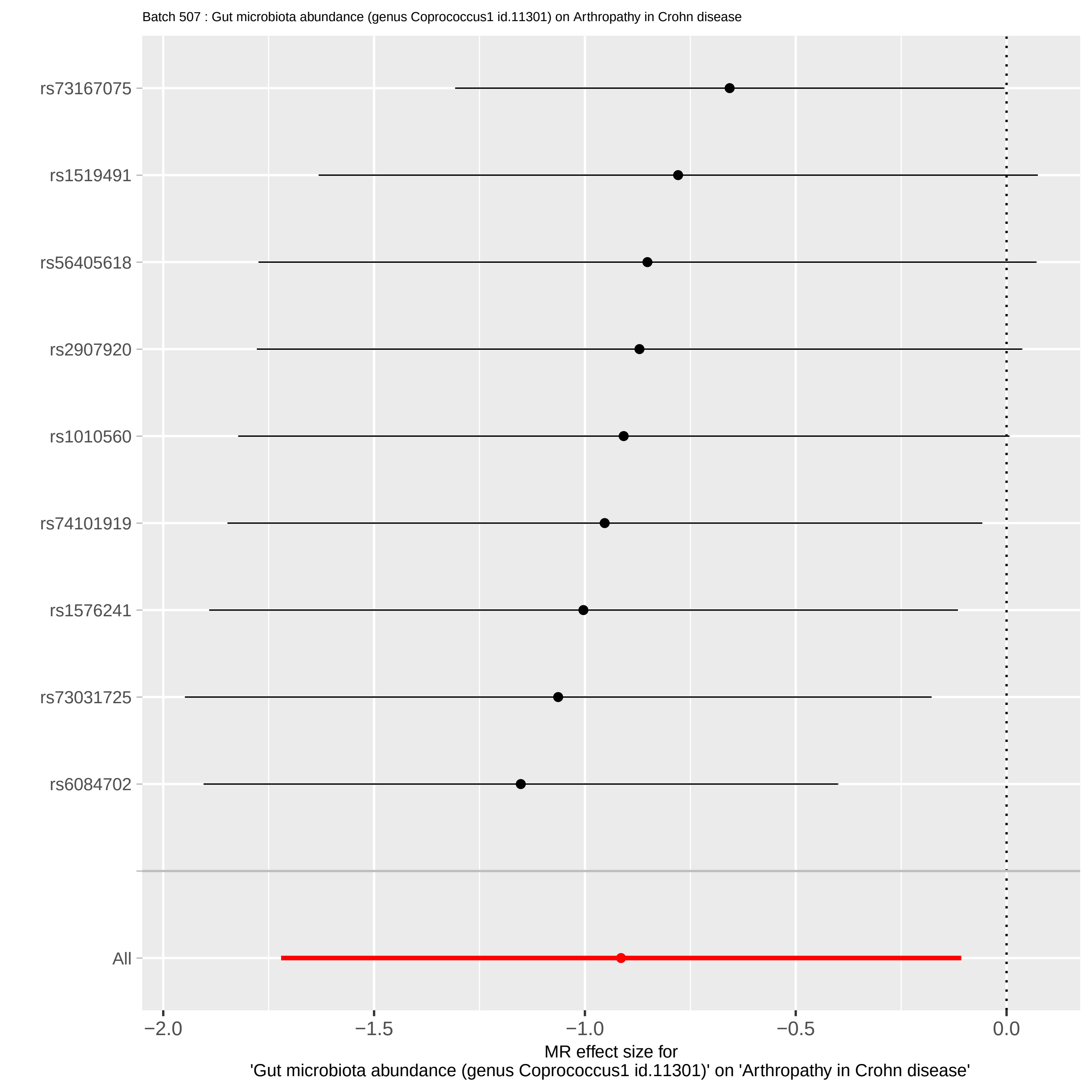

Batch 507 : Gut microbiota abundance (genus Coprococcus1 id.11301) on Arthropathy in Crohn disease
rs73167075
rs1519491
rs56405618
rs2907920
rs1010560
rs74101919
rs1576241
rs73031725
rs6084702
All
−2.0
−1.5
−1.0
−0.5
0.0
MR effect size for
'Gut microbiota abundance (genus Coprococcus1 id.11301)' on 'Arthropathy in Crohn disease'

Batch 508 : Gut microbiota abundance (genus Coprococcus2 id.11302) on Arthropathy in Crohn disease

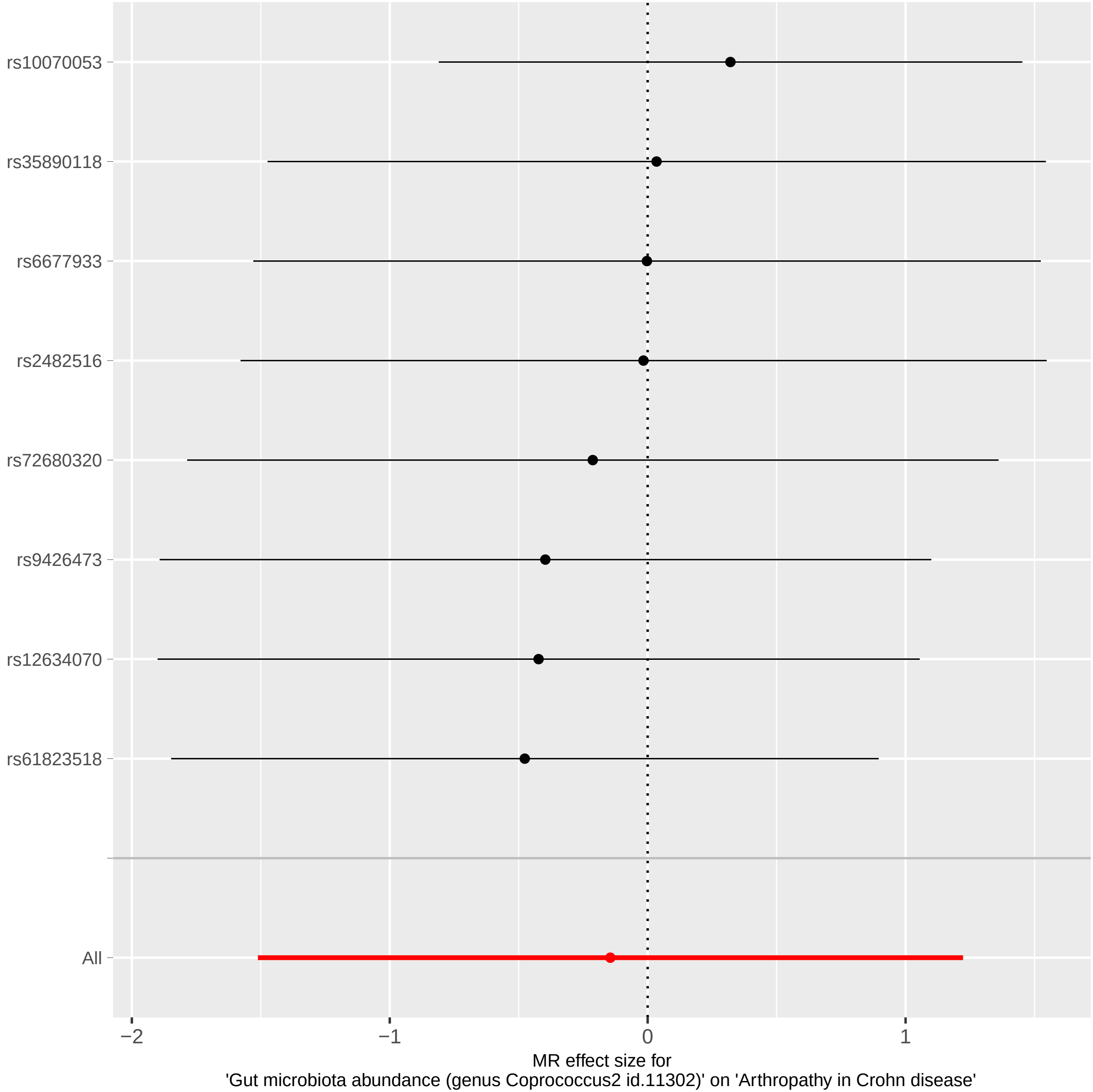

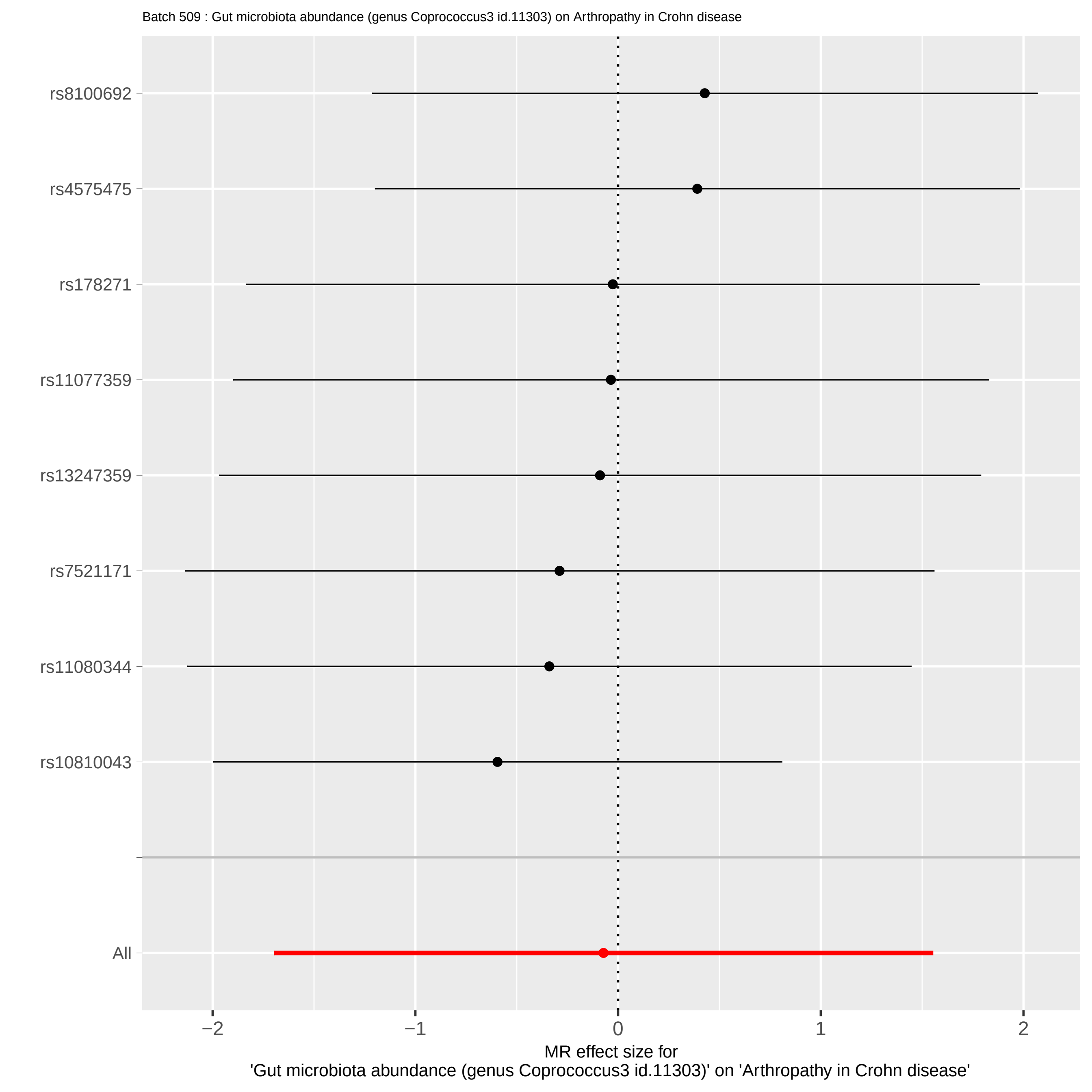
Batch 509 : Gut microbiota abundance (genus Coprococcus3 id.11303) on Arthropathy in Crohn disease
rs8100692
rs4575475
rs178271
rs11077359
rs13247359
rs7521171
rs11080344
rs10810043
All
−2
−1
0
1
2
MR effect size for
'Gut microbiota abundance (genus Coprococcus3 id.11303)' on 'Arthropathy in Crohn disease'

Batch 510 : Gut microbiota abundance (genus Defluviitaleaceae UCG011 id.11287) on Arthropathy in Crohn disease

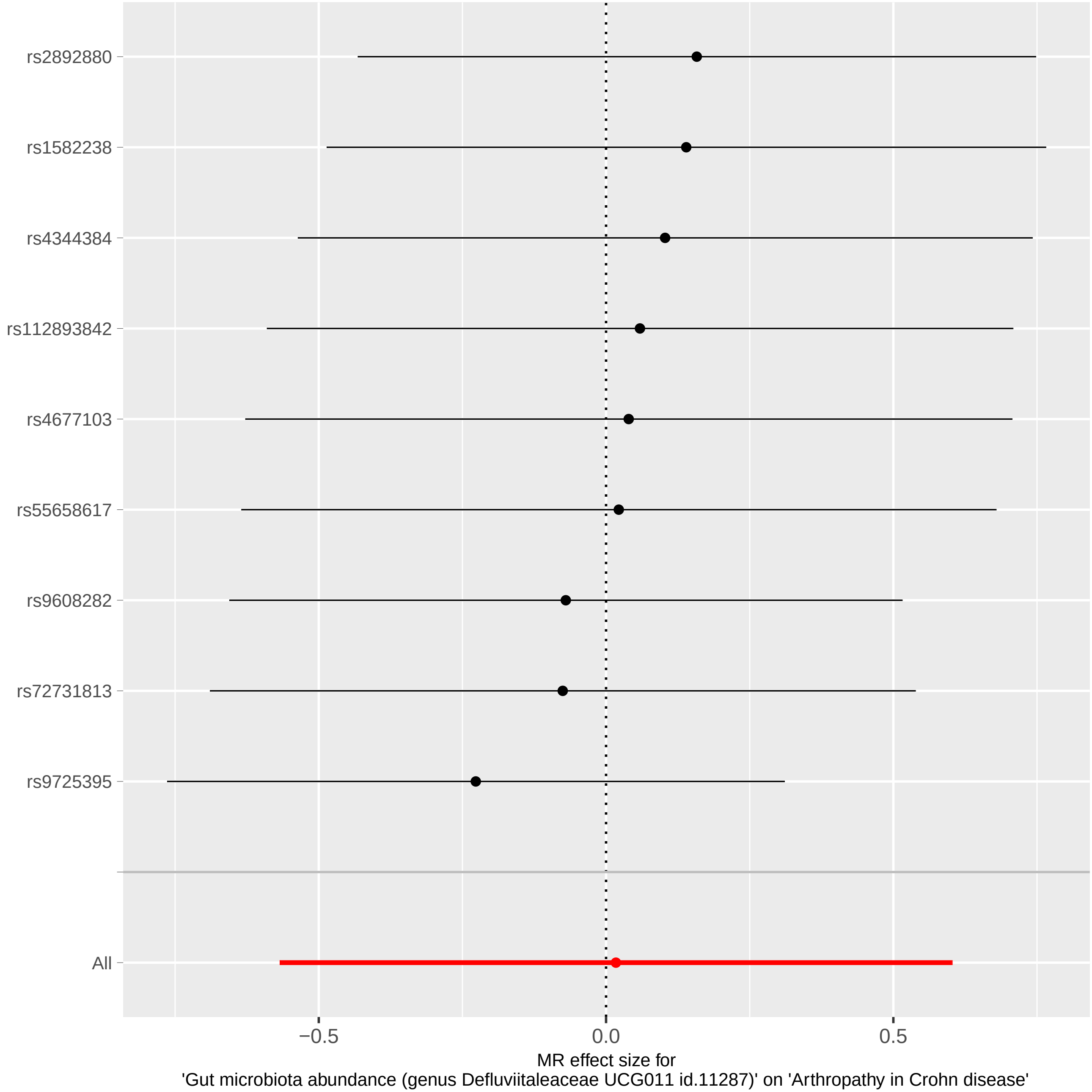

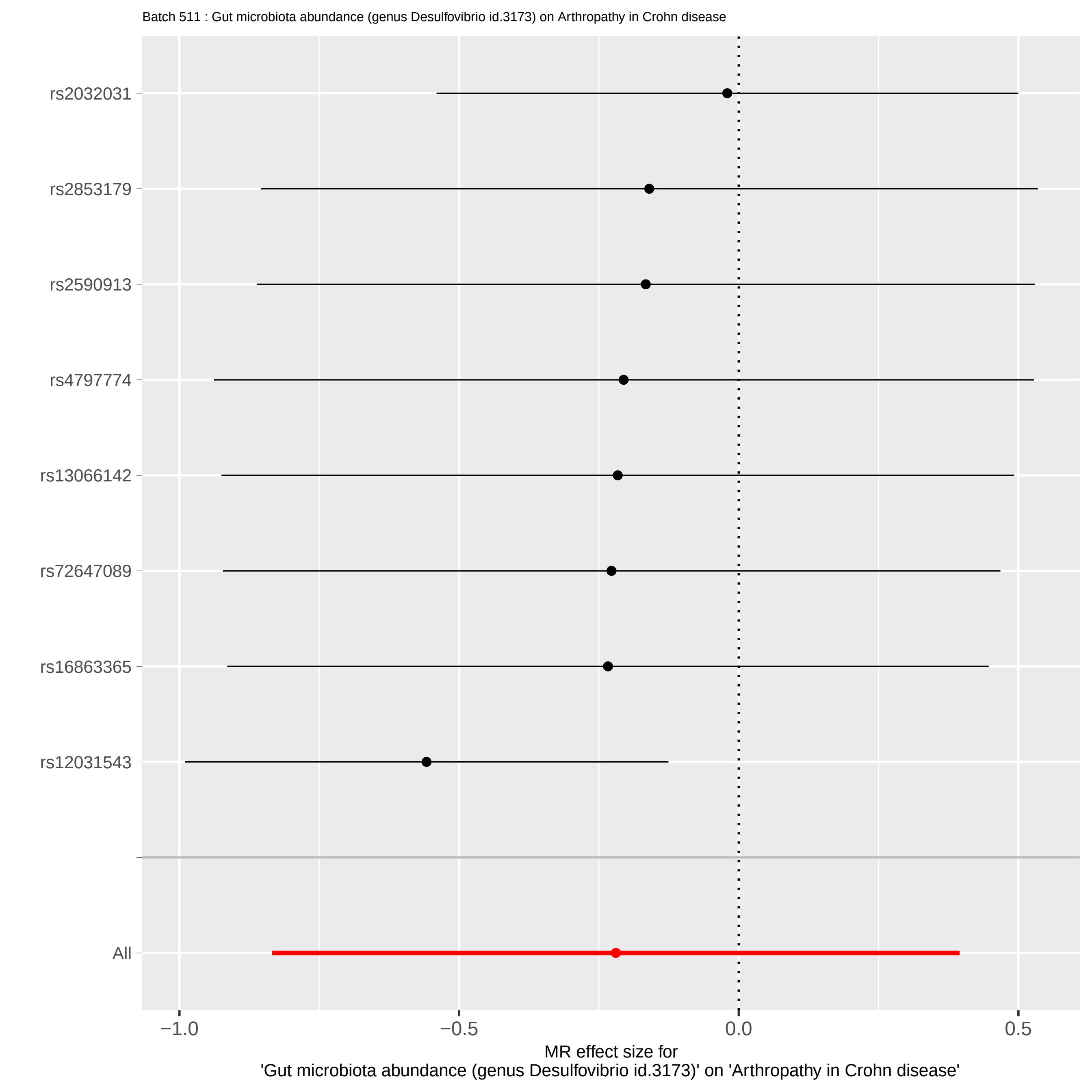

Batch 511 : Gut microbiota abundance (genus Desulfovibrio id.3173) on Arthropathy in Crohn disease
rs2032031
rs2853179
rs2590913
rs4797774
rs13066142
rs72647089
rs16863365
rs12031543
All
−1.0
−0.5
0.0
0.5
MR effect size for
'Gut microbiota abundance (genus Desulfovibrio id.3173)' on 'Arthropathy in Crohn disease'

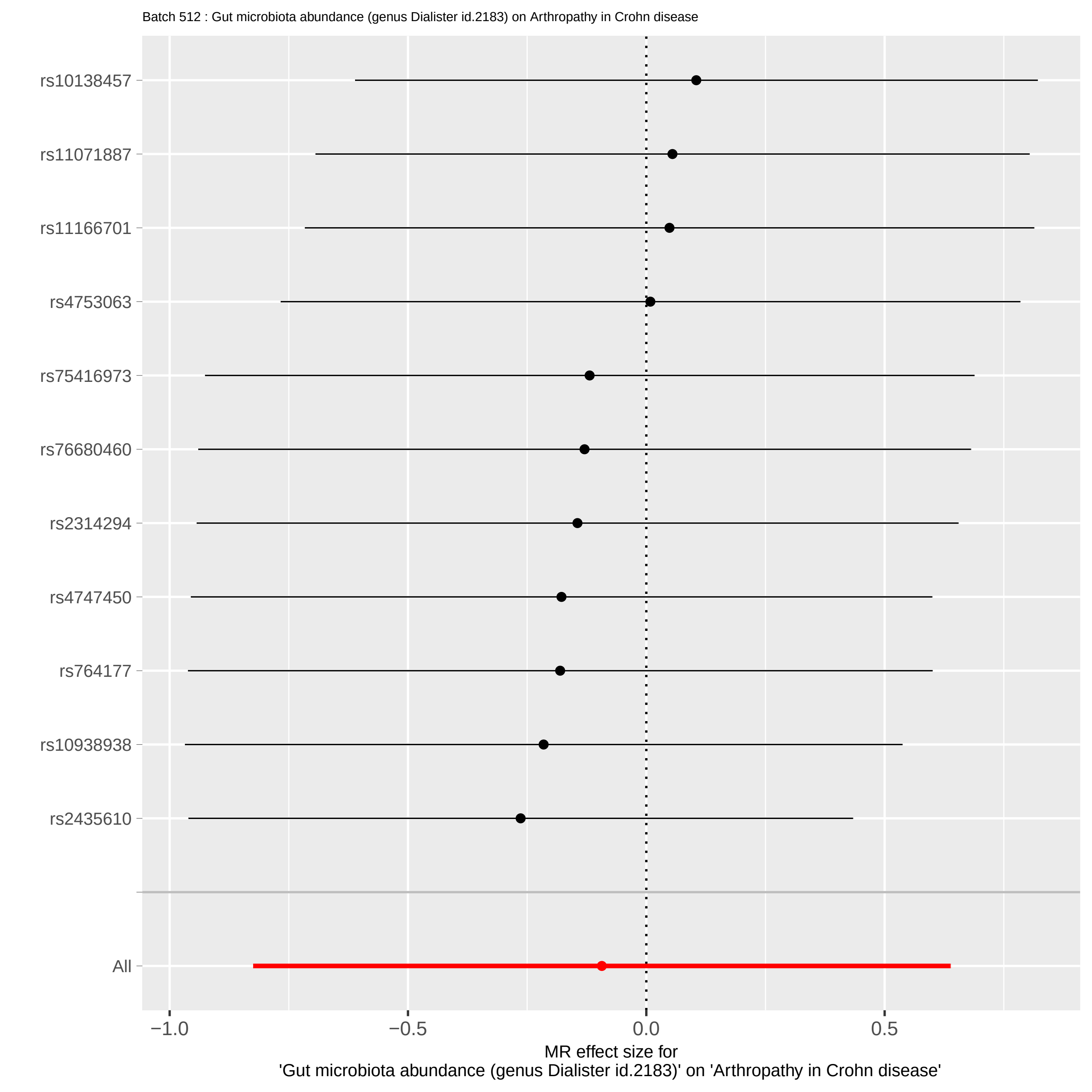

Batch 512 : Gut microbiota abundance (genus Dialister id.2183) on Arthropathy in Crohn disease
rs10138457
rs11071887
rs11166701
rs4753063
rs75416973
rs76680460
rs2314294
rs4747450
rs764177
rs10938938
rs2435610
All
−1.0
−0.5
0.0
0.5
MR effect size for
'Gut microbiota abundance (genus Dialister id.2183)' on 'Arthropathy in Crohn disease'

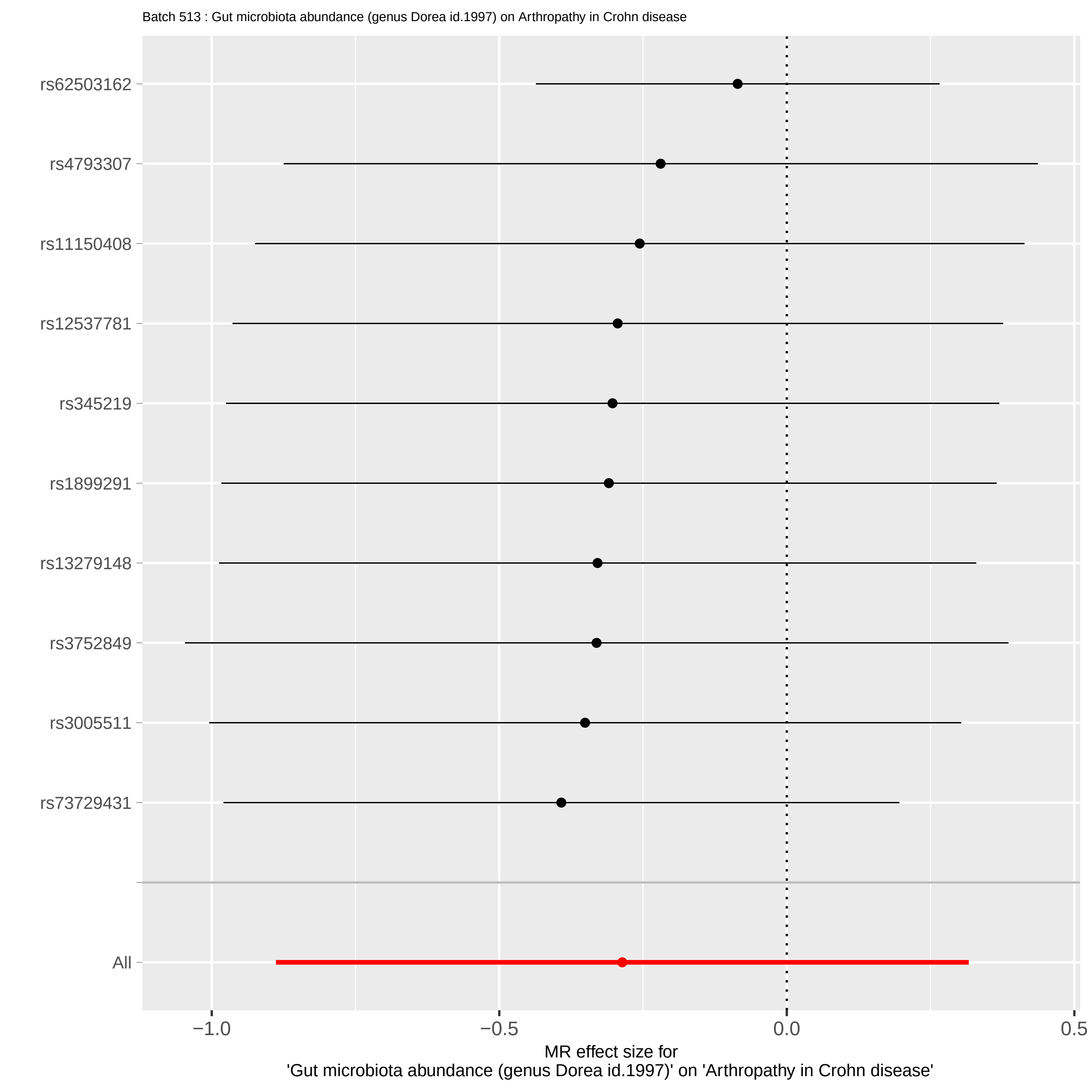
Batch 513 : Gut microbiota abundance (genus Dorea id.1997) on Arthropathy in Crohn disease
rs62503162
rs4793307
rs11150408
rs12537781
rs345219
rs1899291
rs13279148
rs3752849
rs3005511
rs73729431
All
−1.0
−0.5
0.0
0.5
MR effect size for
'Gut microbiota abundance (genus Dorea id.1997)' on 'Arthropathy in Crohn disease'

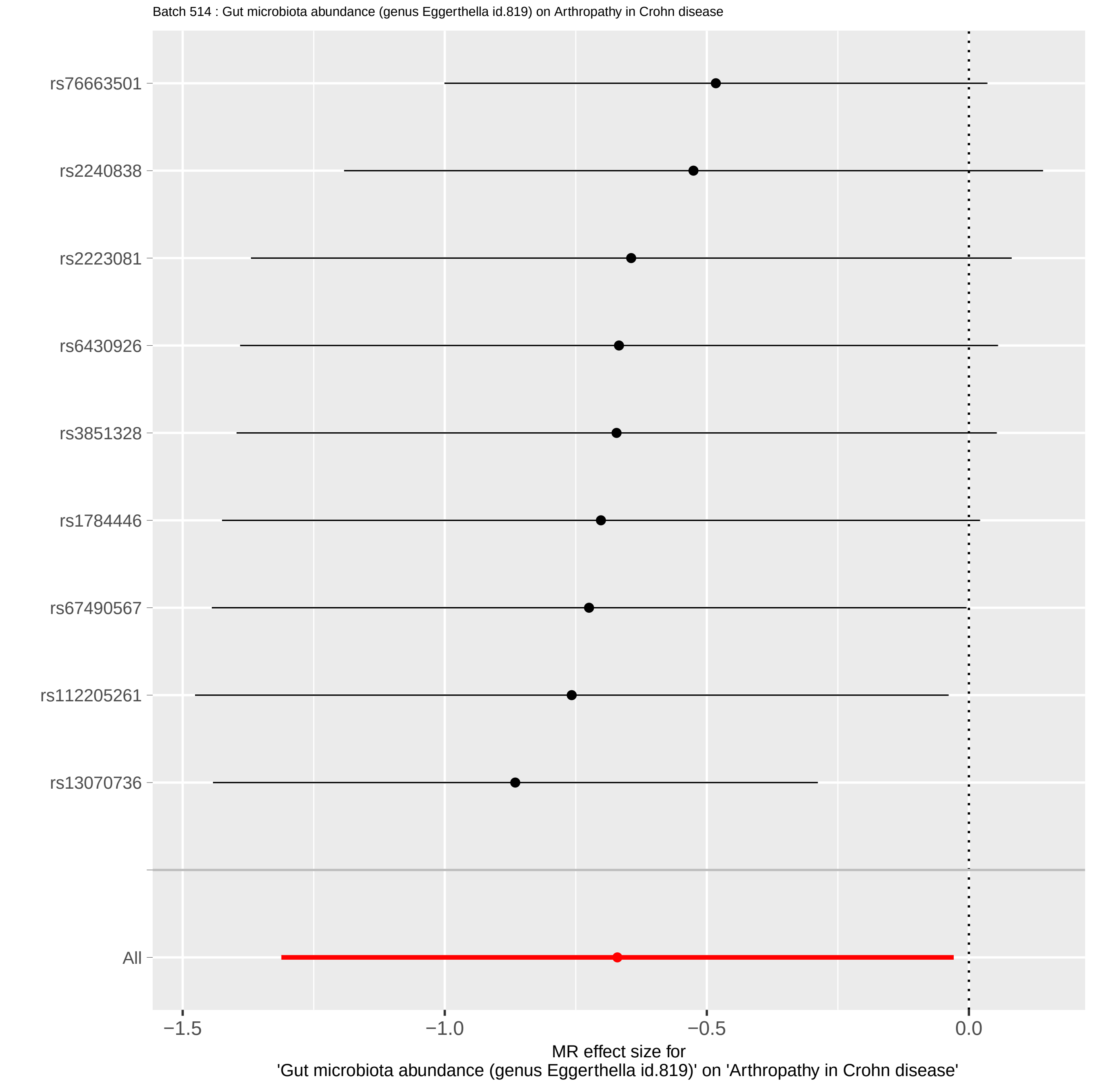

Batch 514 : Gut microbiota abundance (genus Eggerthella id.819) on Arthropathy in Crohn disease
rs76663501
rs2240838
rs2223081
rs6430926
rs3851328
rs1784446
rs67490567
rs112205261
rs13070736
All
−1.5
−1.0
−0.5
0.0
MR effect size for
'Gut microbiota abundance (genus Eggerthella id.819)' on 'Arthropathy in Crohn disease'

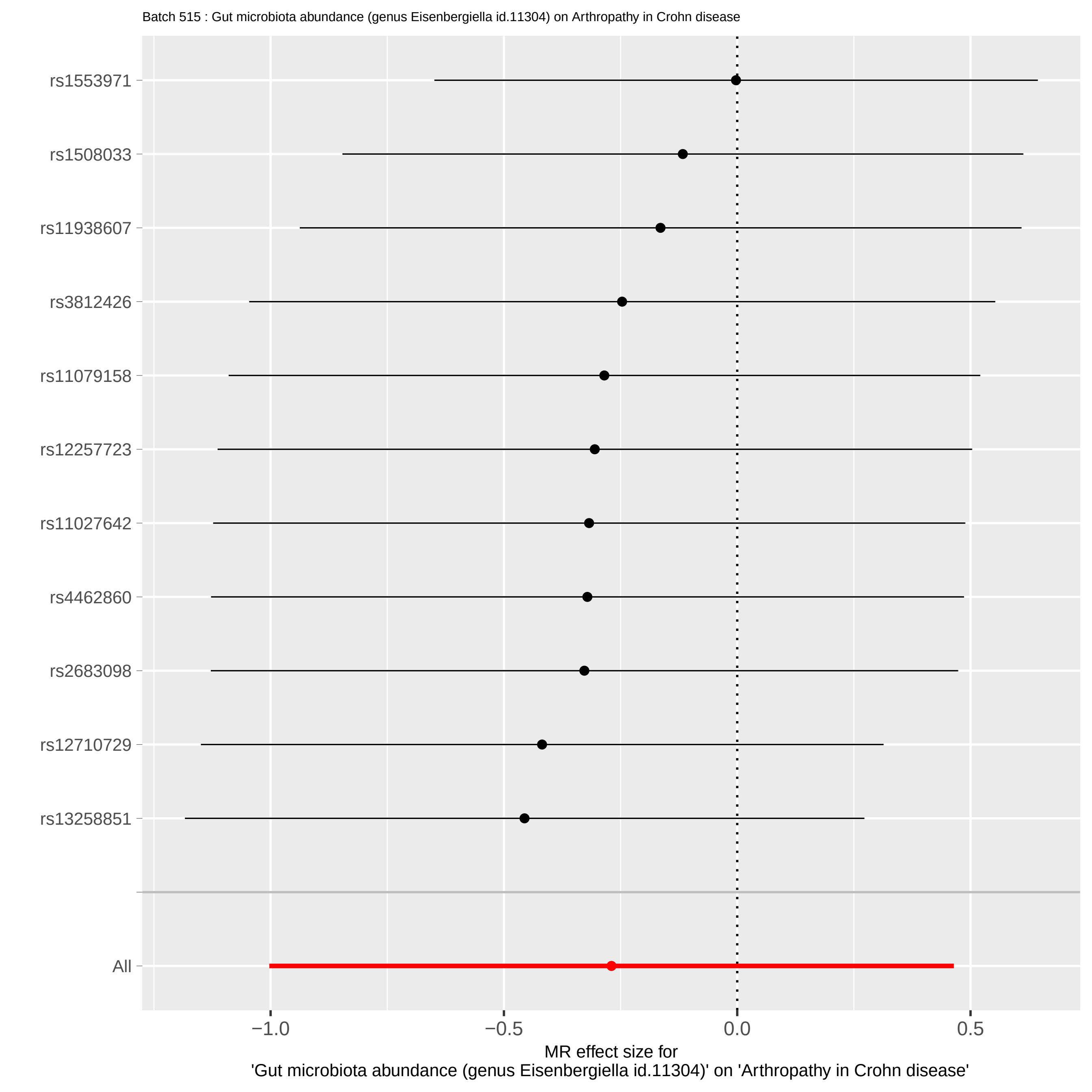
Batch 515 : Gut microbiota abundance (genus Eisenbergiella id.11304) on Arthropathy in Crohn disease
rs1553971
rs1508033
rs11938607
rs3812426
rs11079158
rs12257723
rs11027642
rs4462860
rs2683098
rs12710729
rs13258851
All
−1.0
−0.5
0.0
0.5
MR effect size for
'Gut microbiota abundance (genus Eisenbergiella id.11304)' on 'Arthropathy in Crohn disease'

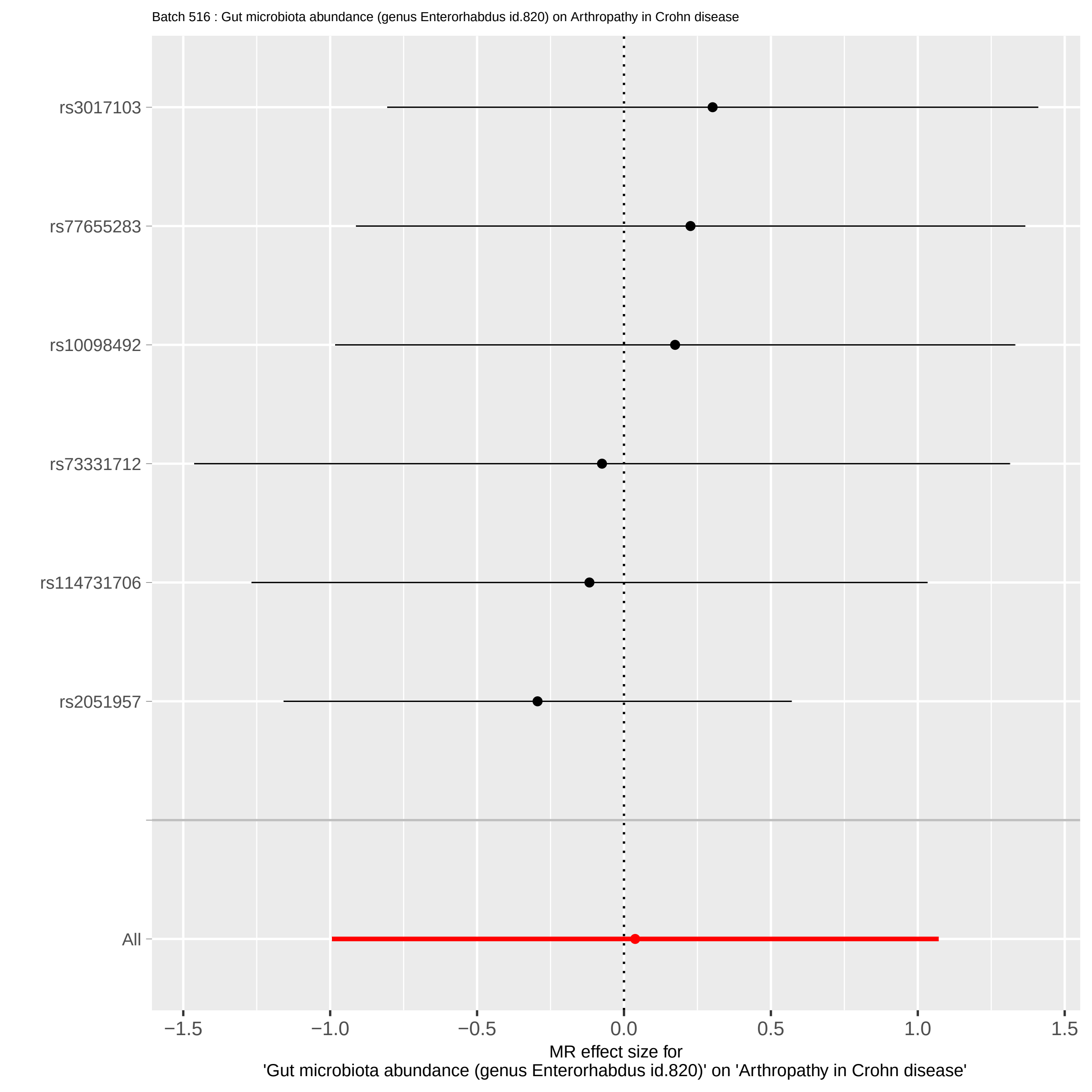
Batch 516 : Gut microbiota abundance (genus Enterorhabdus id.820) on Arthropathy in Crohn disease
rs3017103
rs77655283
rs10098492
rs73331712
rs114731706
rs2051957
All
−1.5
−1.0
−0.5
0.0
0.5
1.0
1.5
MR effect size for
'Gut microbiota abundance (genus Enterorhabdus id.820)' on 'Arthropathy in Crohn disease'

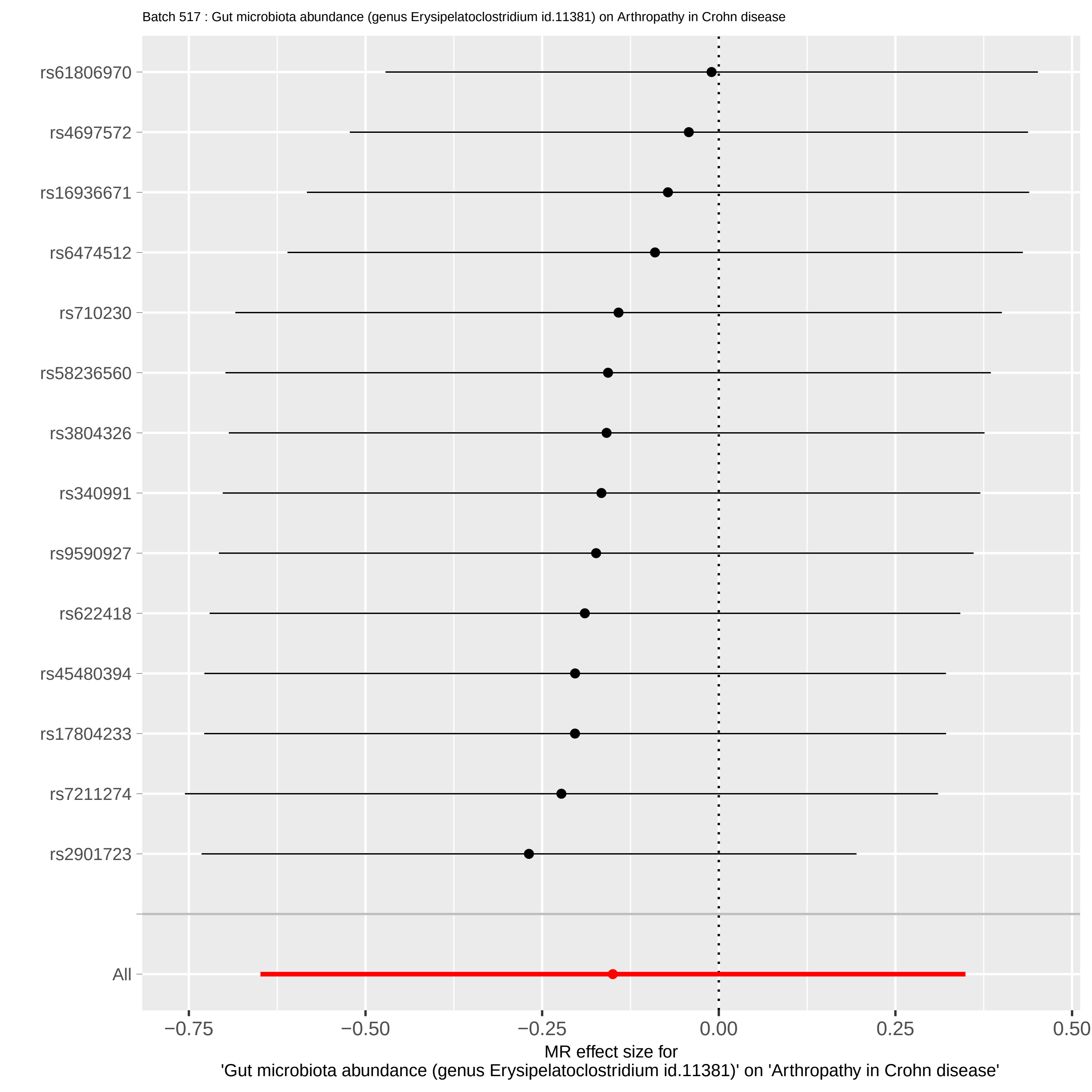

Batch 517 : Gut microbiota abundance (genus Erysipelatoclostridium id.11381) on Arthropathy in Crohn disease
rs61806970
rs4697572
rs16936671
rs6474512
rs710230
rs58236560
rs3804326
rs340991
rs9590927
rs622418
rs45480394
rs17804233
rs7211274
rs2901723
All
−0.75
−0.50
−0.25
0.00
0.25
0.50
MR effect size for
'Gut microbiota abundance (genus Erysipelatoclostridium id.11381)' on 'Arthropathy in Crohn disease'

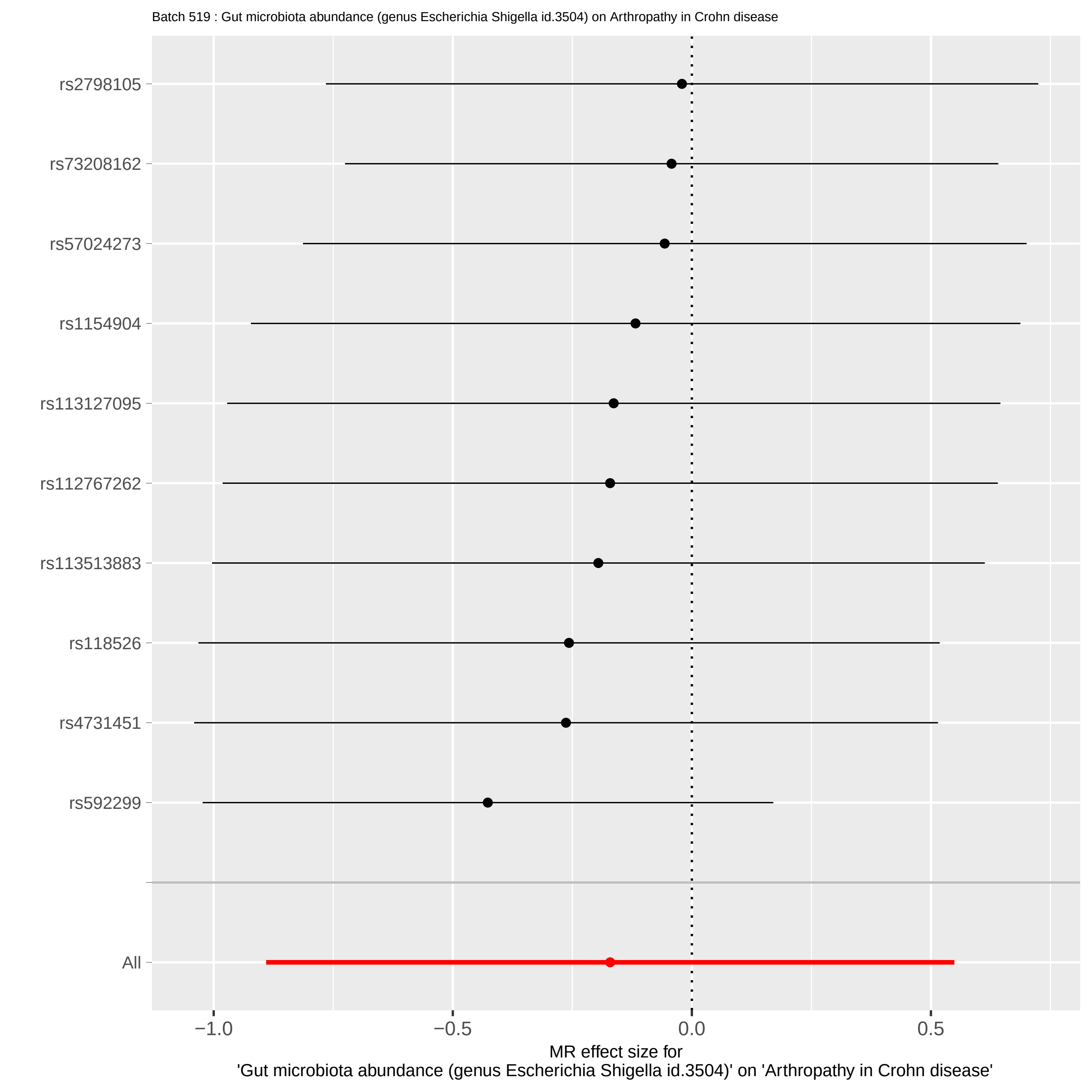

Batch 519 : Gut microbiota abundance (genus Escherichia Shigella id.3504) on Arthropathy in Crohn disease
rs2798105
rs73208162
rs57024273
rs1154904
rs113127095
rs112767262
rs113513883
rs118526
rs4731451
rs592299
All
−1.0
−0.5
0.0
0.5
MR effect size for
'Gut microbiota abundance (genus Escherichia Shigella id.3504)' on 'Arthropathy in Crohn disease'

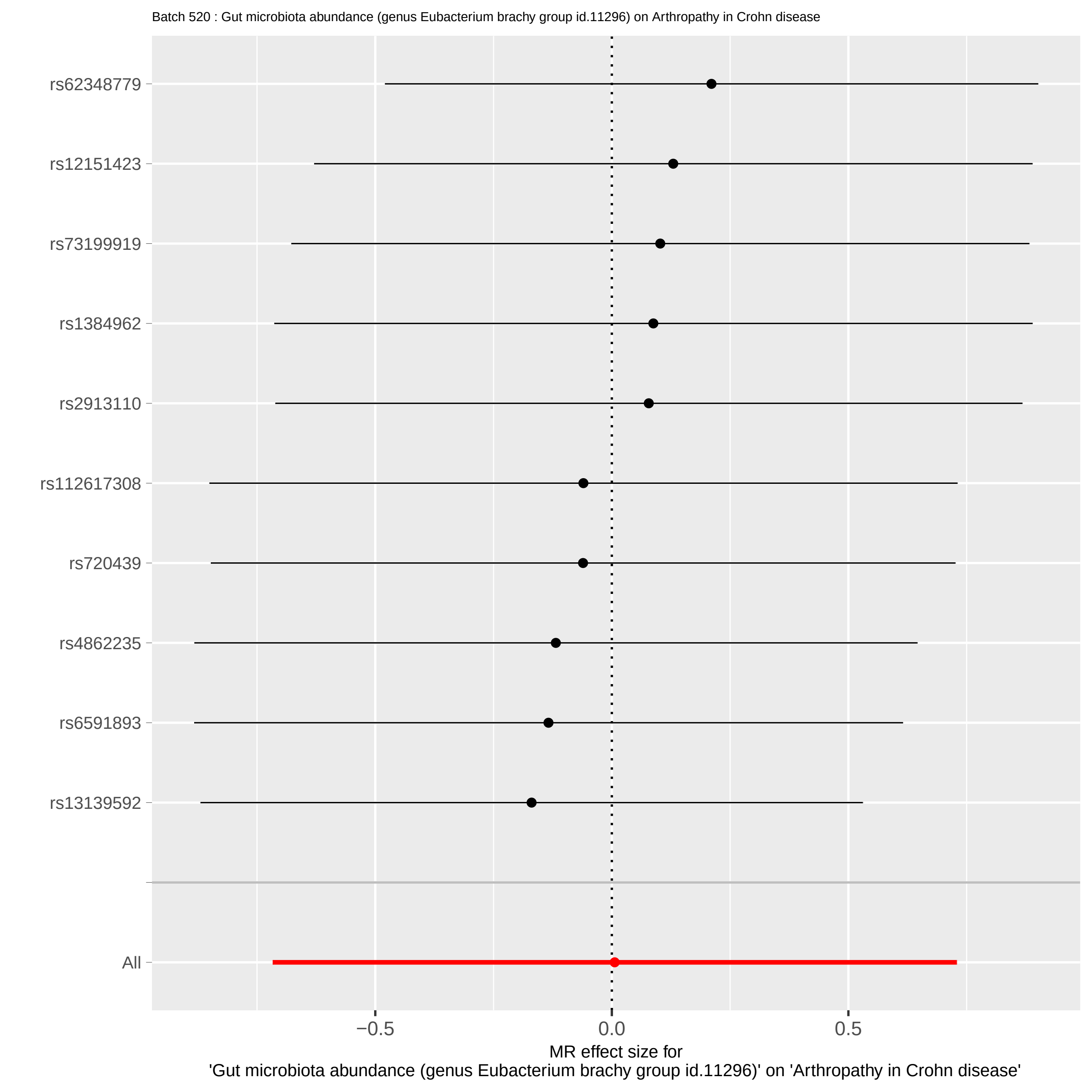

Batch 520 : Gut microbiota abundance (genus Eubacterium brachy group id.11296) on Arthropathy in Crohn disease
rs62348779
rs12151423
rs73199919
rs1384962
rs2913110
rs112617308
rs720439
rs4862235
rs6591893
rs13139592
All
−0.5
0.0
0.5
MR effect size for
'Gut microbiota abundance (genus Eubacterium brachy group id.11296)' on 'Arthropathy in Crohn disease'

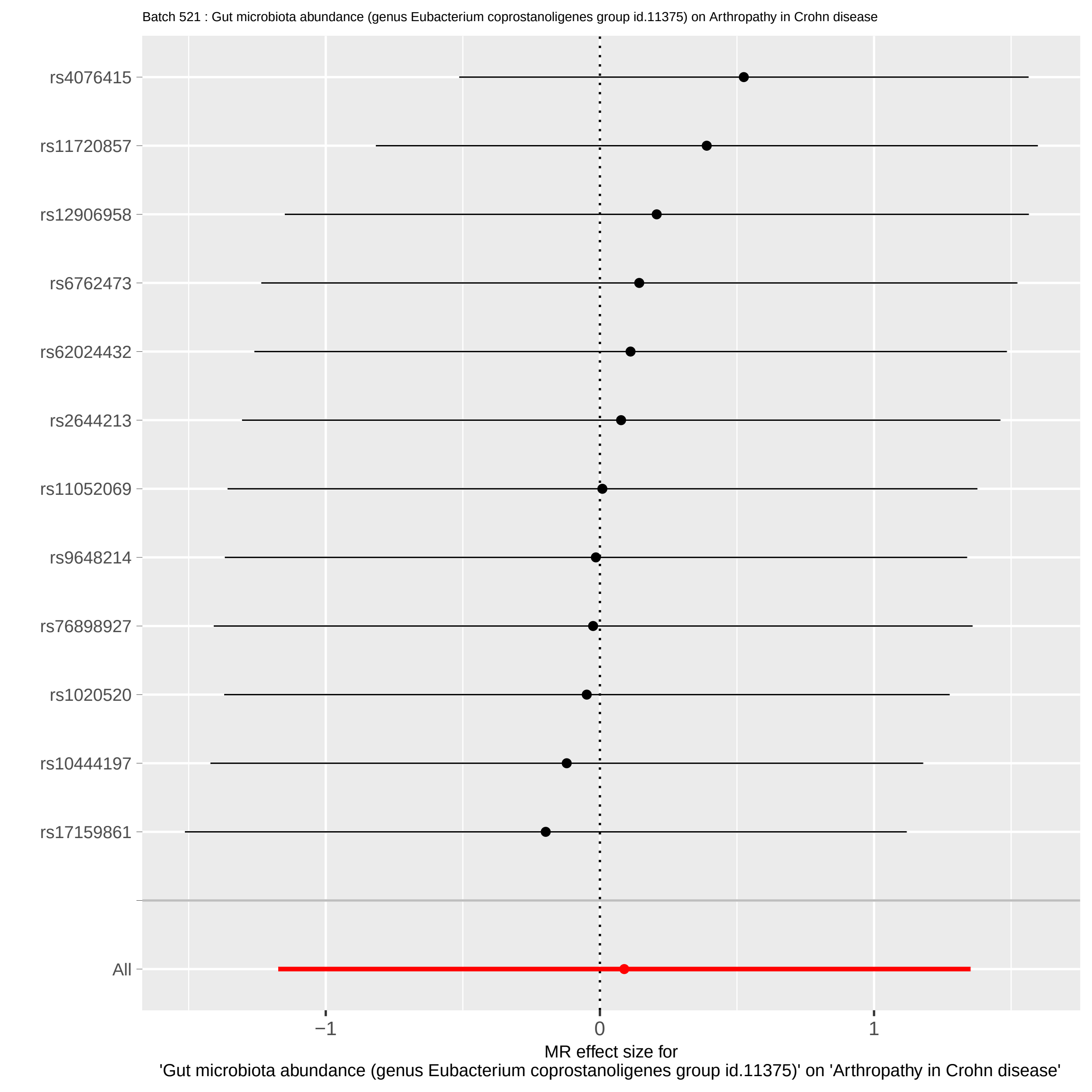
Batch 521 : Gut microbiota abundance (genus Eubacterium coprostanoligenes group id.11375) on Arthropathy in Crohn disease
rs4076415
rs11720857
rs12906958
rs6762473
rs62024432
rs2644213
rs11052069
rs9648214
rs76898927
rs1020520
rs10444197
rs17159861
All
−1
0
1
MR effect size for
'Gut microbiota abundance (genus Eubacterium coprostanoligenes group id.11375)' on 'Arthropathy in Crohn disease'

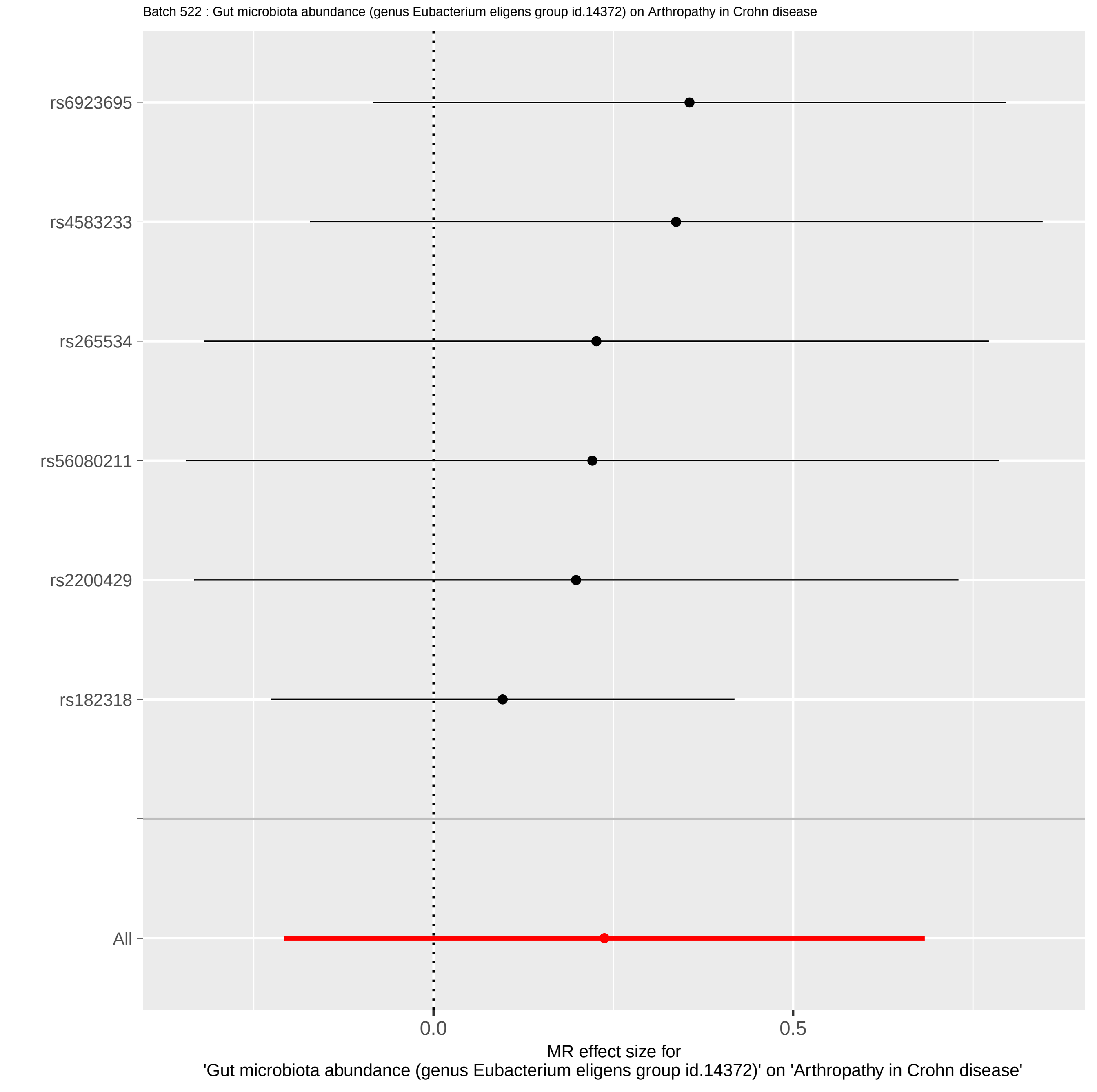
Batch 522 : Gut microbiota abundance (genus Eubacterium eligens group id.14372) on Arthropathy in Crohn disease
rs6923695
rs4583233
rs265534
rs56080211
rs2200429
rs182318
All
0.0
0.5
MR effect size for
'Gut microbiota abundance (genus Eubacterium eligens group id.14372)' on 'Arthropathy in Crohn disease'

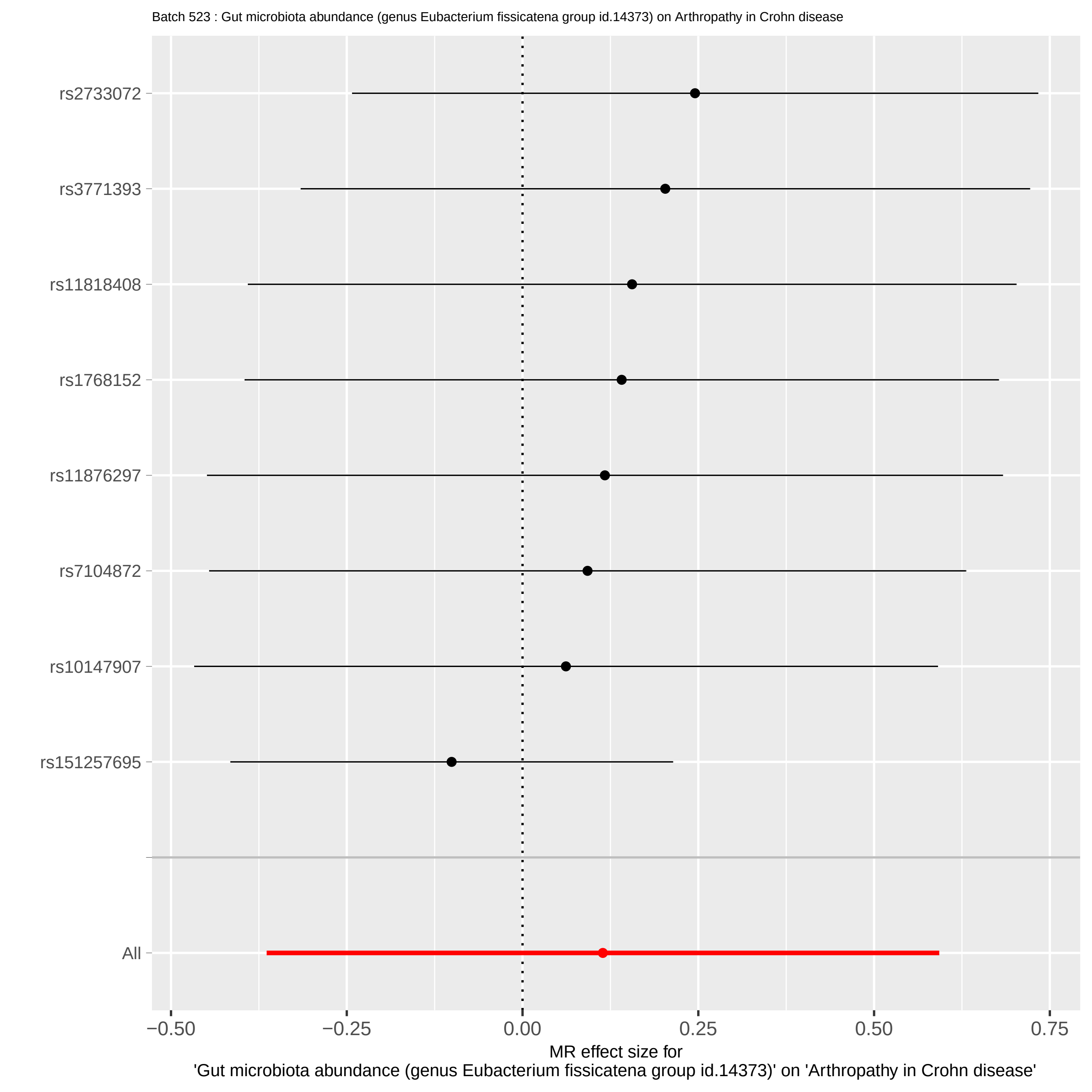
Batch 523 : Gut microbiota abundance (genus Eubacterium fissicatena group id.14373) on Arthropathy in Crohn disease
rs2733072
rs3771393
rs11818408
rs1768152
rs11876297
rs7104872
rs10147907
rs151257695
All
−0.50
−0.25
0.00
0.25
0.50
0.75
MR effect size for
'Gut microbiota abundance (genus Eubacterium fissicatena group id.14373)' on 'Arthropathy in Crohn disease'

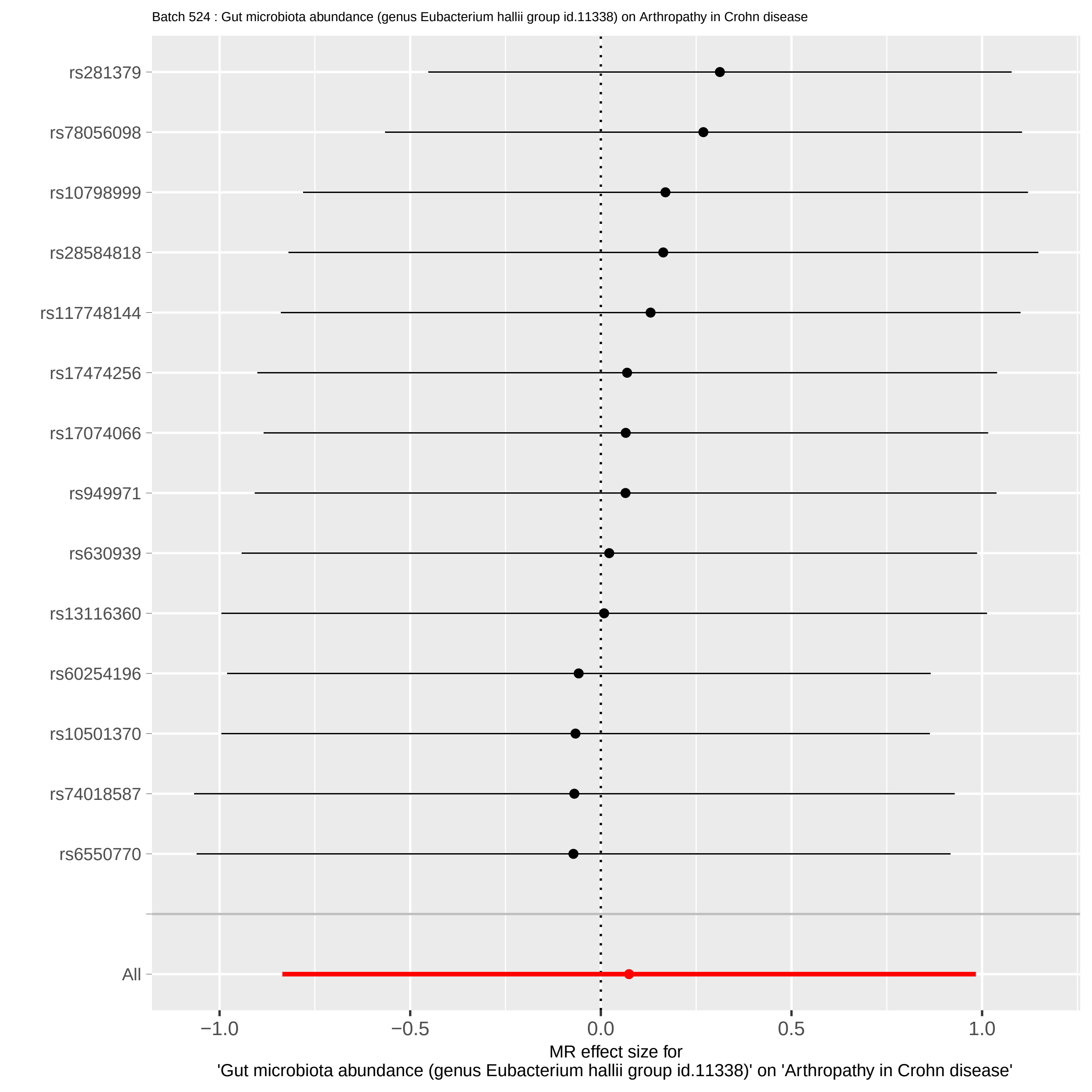

Batch 524 : Gut microbiota abundance (genus Eubacterium hallii group id.11338) on Arthropathy in Crohn disease
rs281379
rs78056098
rs10798999
rs28584818
rs117748144
rs17474256
rs17074066
rs949971
rs630939
rs13116360
rs60254196
rs10501370
rs74018587
rs6550770
All
−1.0
−0.5
0.0
0.5
1.0
MR effect size for
'Gut microbiota abundance (genus Eubacterium hallii group id.11338)' on 'Arthropathy in Crohn disease'

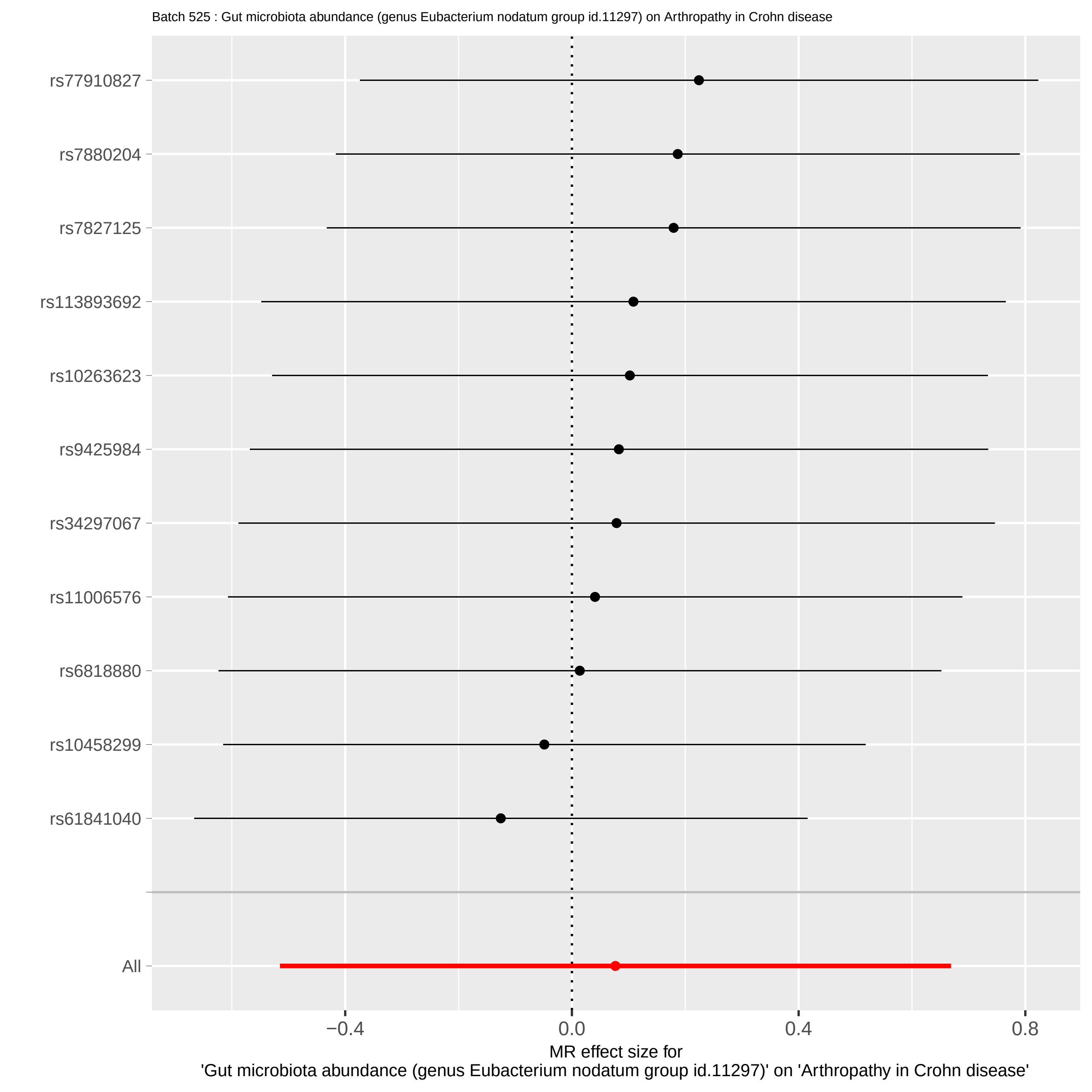

Batch 525 : Gut microbiota abundance (genus Eubacterium nodatum group id.11297) on Arthropathy in Crohn disease
rs77910827
rs7880204
rs7827125
rs113893692
rs10263623
rs9425984
rs34297067
rs11006576
rs6818880
rs10458299
rs61841040
All
−0.4
0.0
0.4
0.8
MR effect size for
'Gut microbiota abundance (genus Eubacterium nodatum group id.11297)' on 'Arthropathy in Crohn disease'

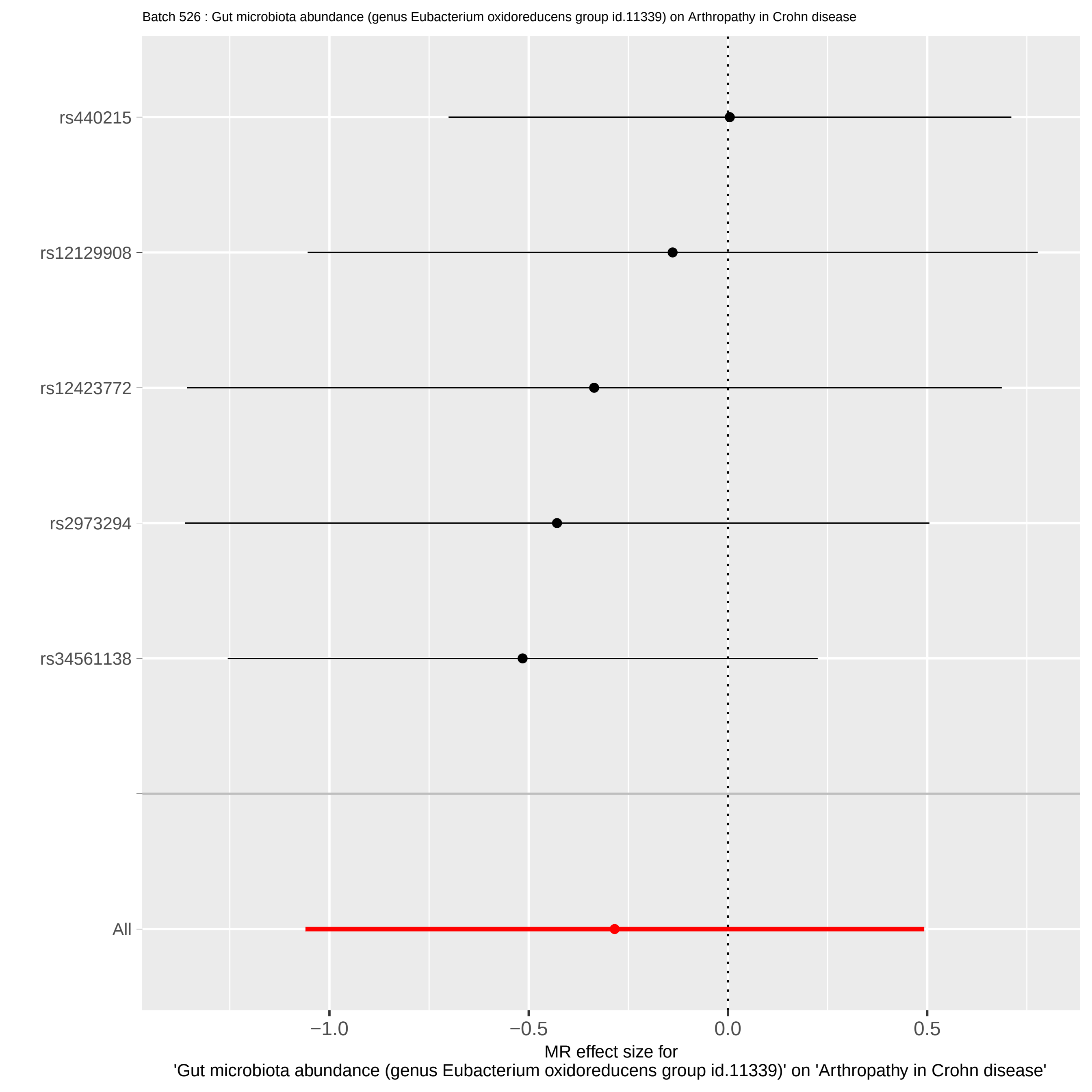

Batch 526 : Gut microbiota abundance (genus Eubacterium oxidoreducens group id.11339) on Arthropathy in Crohn disease
rs440215
rs12129908
rs12423772
rs2973294
rs34561138
All
−1.0
−0.5
0.0
0.5
MR effect size for
'Gut microbiota abundance (genus Eubacterium oxidoreducens group id.11339)' on 'Arthropathy in Crohn disease'

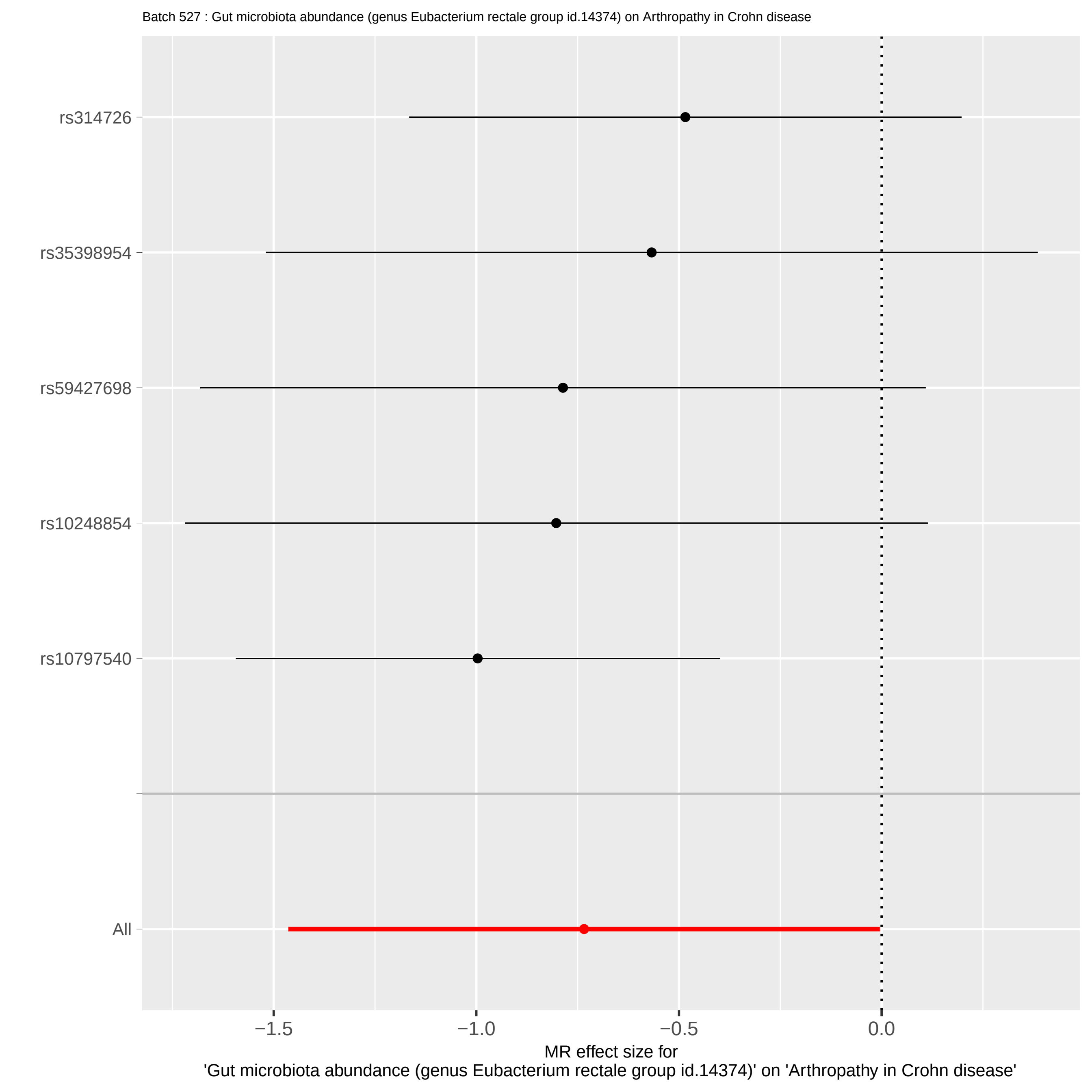
Batch 527 : Gut microbiota abundance (genus Eubacterium rectale group id.14374) on Arthropathy in Crohn disease
rs314726
rs35398954
rs59427698
rs10248854
rs10797540
All
−1.5
−1.0
−0.5
0.0
MR effect size for
'Gut microbiota abundance (genus Eubacterium rectale group id.14374)' on 'Arthropathy in Crohn disease'

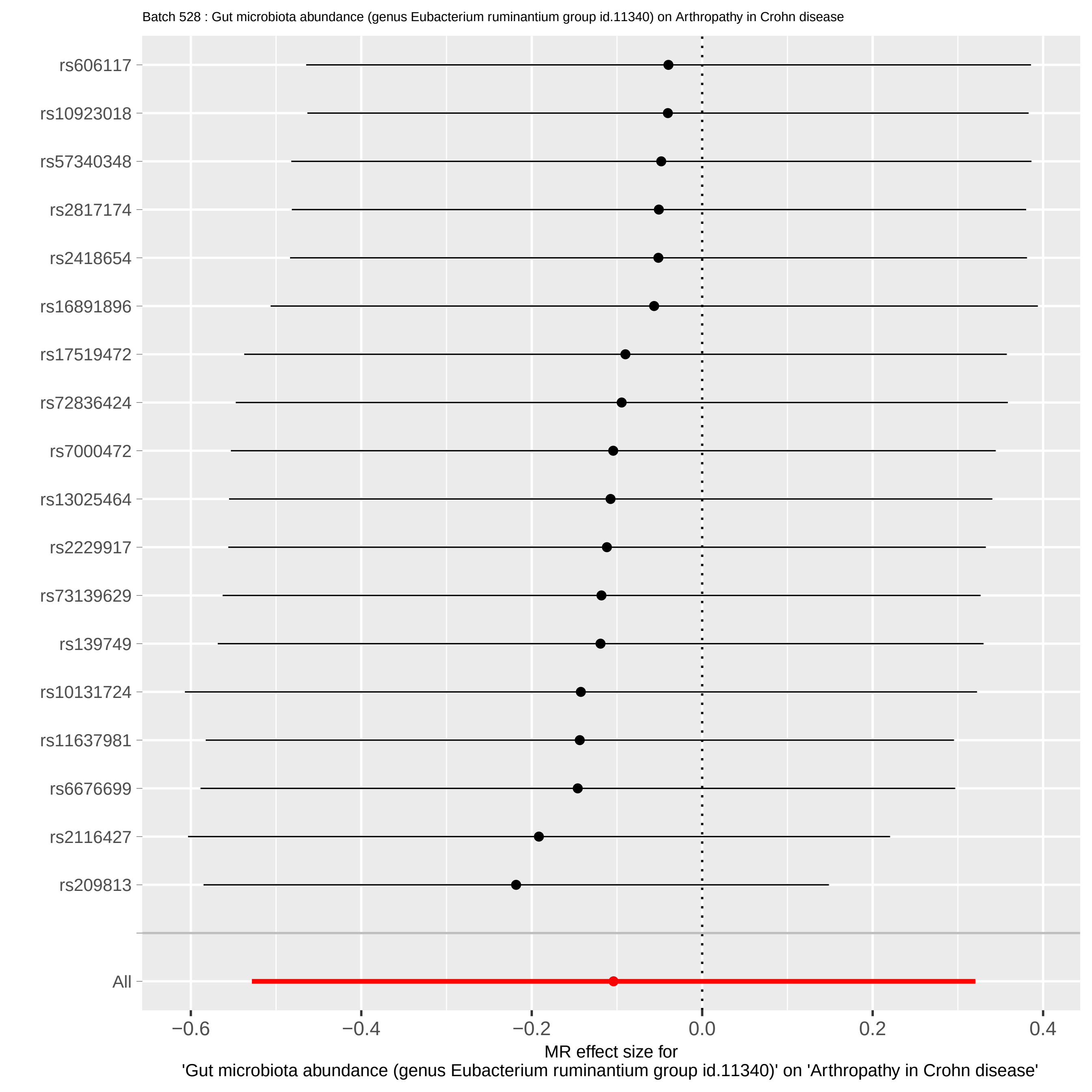

Batch 528 : Gut microbiota abundance (genus Eubacterium ruminantium group id.11340) on Arthropathy in Crohn disease
rs606117
rs10923018
rs57340348
rs2817174
rs2418654
rs16891896
rs17519472
rs72836424
rs7000472
rs13025464
rs2229917
rs73139629
rs139749
rs10131724
rs11637981
rs6676699
rs2116427
rs209813
All
−0.6
−0.4
−0.2
0.0
0.2
0.4
MR effect size for
'Gut microbiota abundance (genus Eubacterium ruminantium group id.11340)' on 'Arthropathy in Crohn disease'

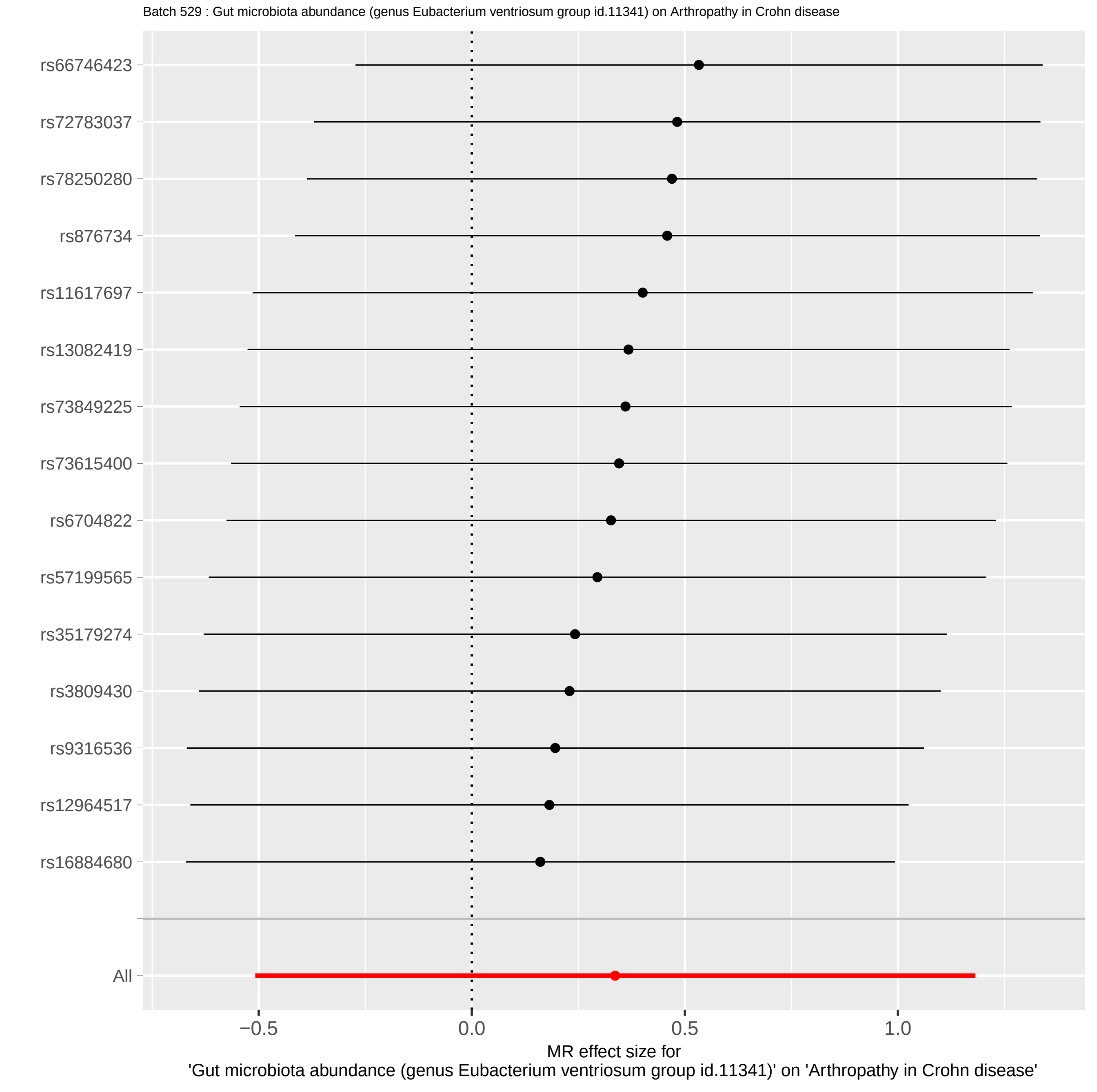

Batch 529 : Gut microbiota abundance (genus Eubacterium ventriosum group id.11341) on Arthropathy in Crohn disease
rs66746423
rs72783037
rs78250280
rs876734
rs11617697
rs13082419
rs73849225
rs73615400
rs6704822
rs57199565
rs35179274
rs3809430
rs9316536
rs12964517
rs16884680
All
−0.5
0.0
0.5
1.0
MR effect size for
'Gut microbiota abundance (genus Eubacterium ventriosum group id.11341)' on 'Arthropathy in Crohn disease'

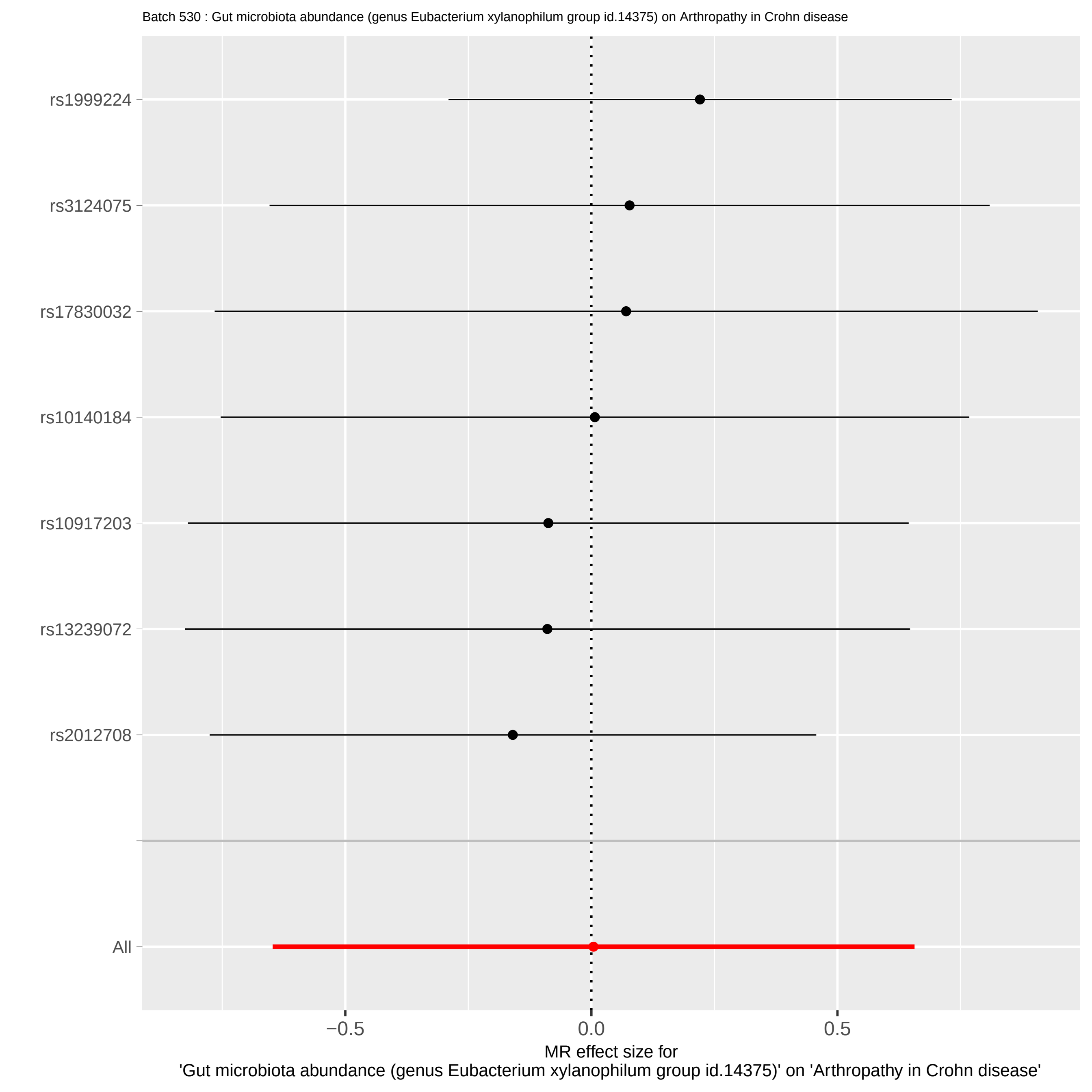

Batch 530 : Gut microbiota abundance (genus Eubacterium xylanophilum group id.14375) on Arthropathy in Crohn disease
rs1999224
rs3124075
rs17830032
rs10140184
rs10917203
rs13239072
rs2012708
All
−0.5
0.0
0.5
MR effect size for
'Gut microbiota abundance (genus Eubacterium xylanophilum group id.14375)' on 'Arthropathy in Crohn disease'

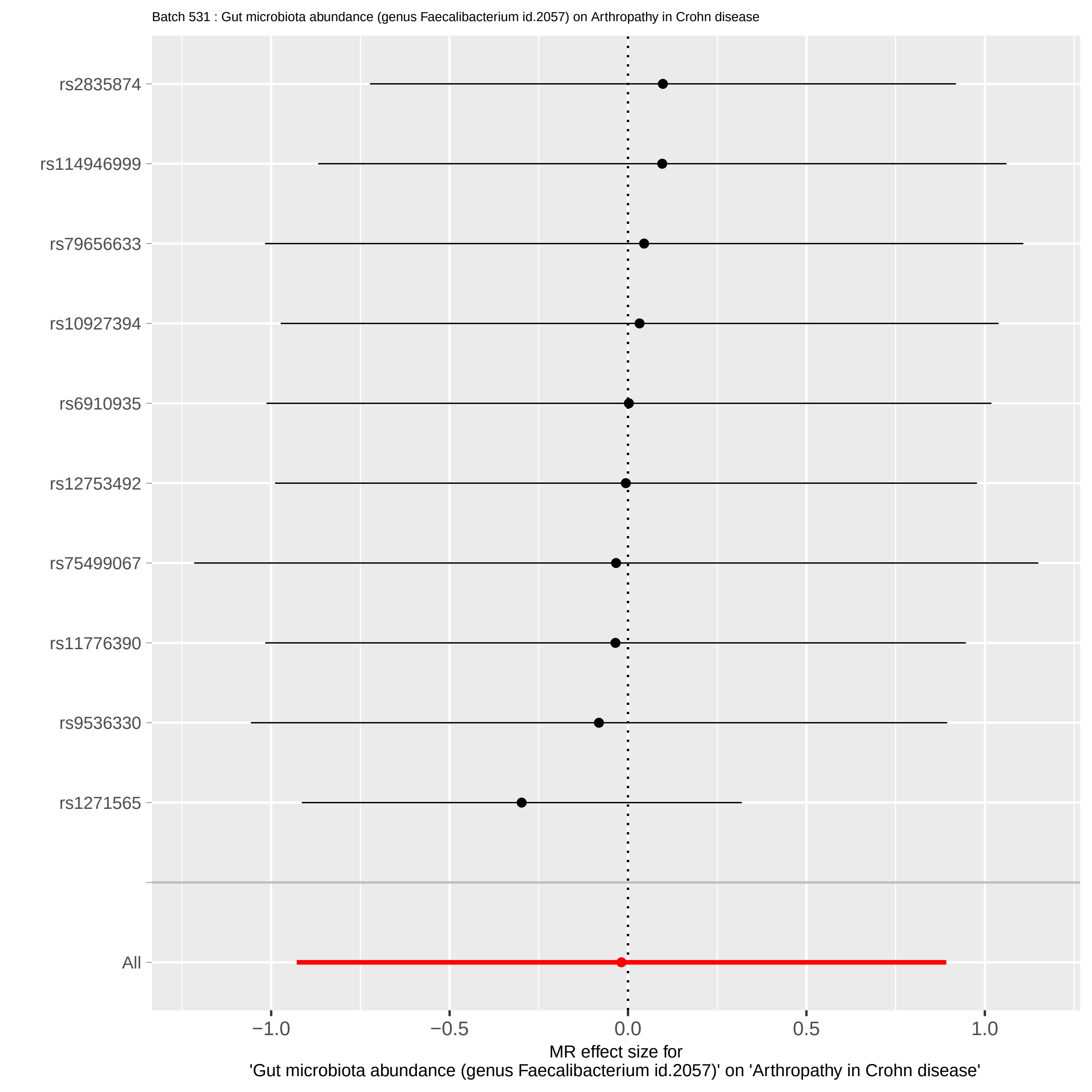

Batch 531 : Gut microbiota abundance (genus Faecalibacterium id.2057) on Arthropathy in Crohn disease
rs2835874
rs114946999
rs79656633
rs10927394
rs6910935
rs12753492
rs75499067
rs11776390
rs9536330
rs1271565
All
−1.0
−0.5
0.0
0.5
1.0
MR effect size for
'Gut microbiota abundance (genus Faecalibacterium id.2057)' on 'Arthropathy in Crohn disease'

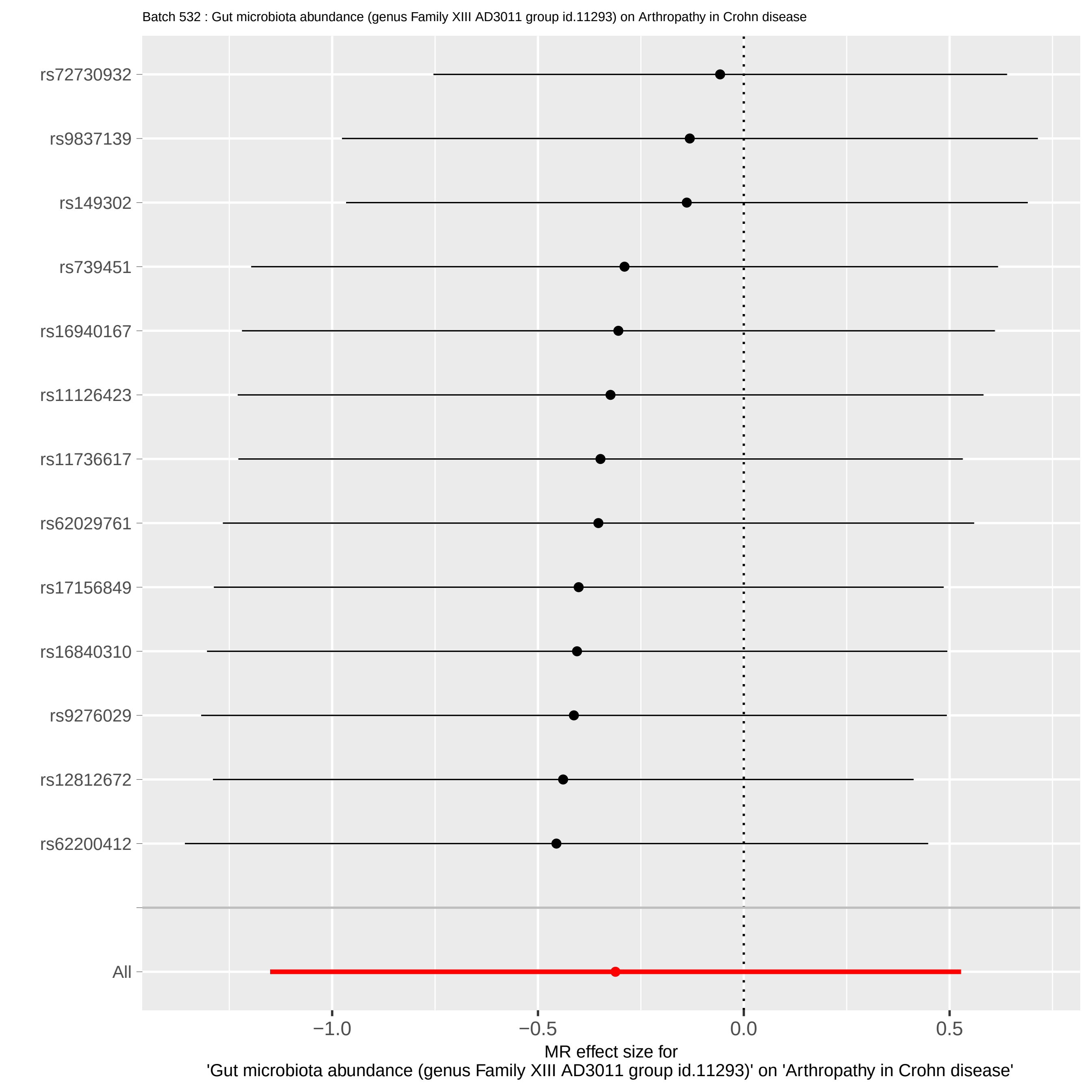
Batch 532 : Gut microbiota abundance (genus Family XIII AD3011 group id.11293) on Arthropathy in Crohn disease
rs72730932
rs9837139
rs149302
rs739451
rs16940167
rs11126423
rs11736617
rs62029761
rs17156849
rs16840310
rs9276029
rs12812672
rs62200412
All
−1.0
−0.5
0.0
0.5
MR effect size for
'Gut microbiota abundance (genus Family XIII AD3011 group id.11293)' on 'Arthropathy in Crohn disease'

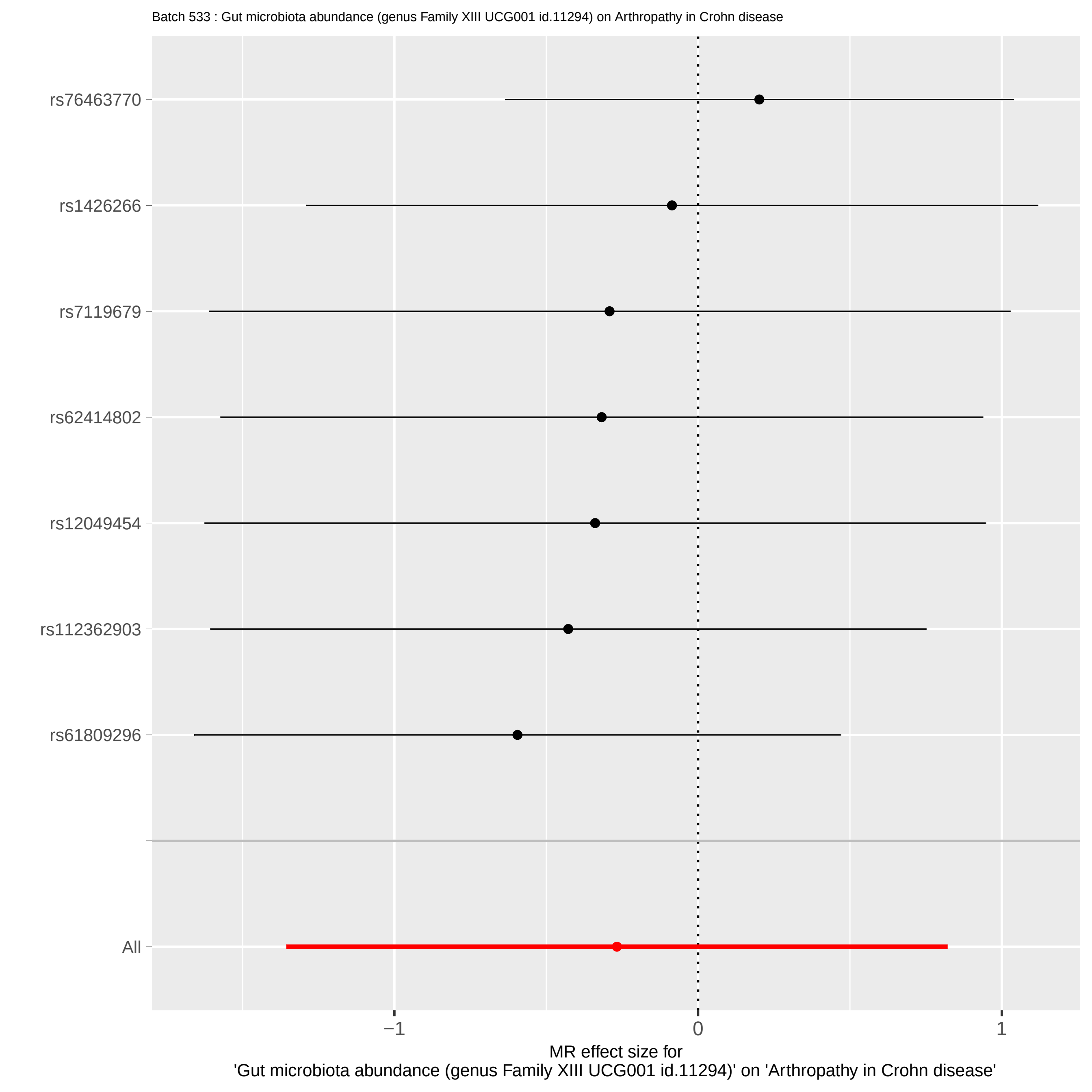

Batch 533 : Gut microbiota abundance (genus Family XIII UCG001 id.11294) on Arthropathy in Crohn disease
rs76463770
rs1426266
rs7119679
rs62414802
rs12049454
rs112362903
rs61809296
All
−1
0
1
MR effect size for
'Gut microbiota abundance (genus Family XIII UCG001 id.11294)' on 'Arthropathy in Crohn disease'

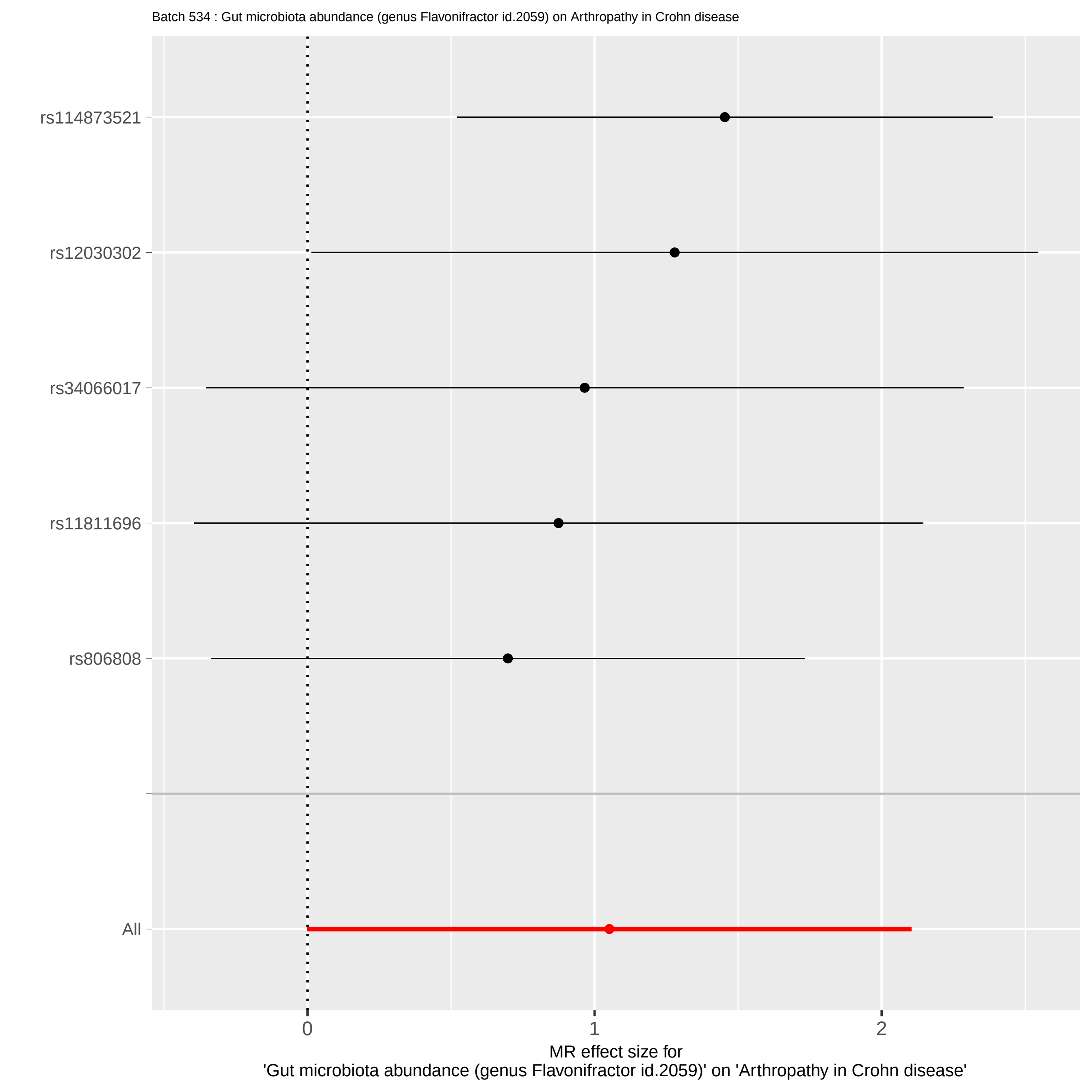
Batch 534 : Gut microbiota abundance (genus Flavonifractor id.2059) on Arthropathy in Crohn disease
rs114873521
rs12030302
rs34066017
rs11811696
rs806808
All
0
1
2
MR effect size for
'Gut microbiota abundance (genus Flavonifractor id.2059)' on 'Arthropathy in Crohn disease'

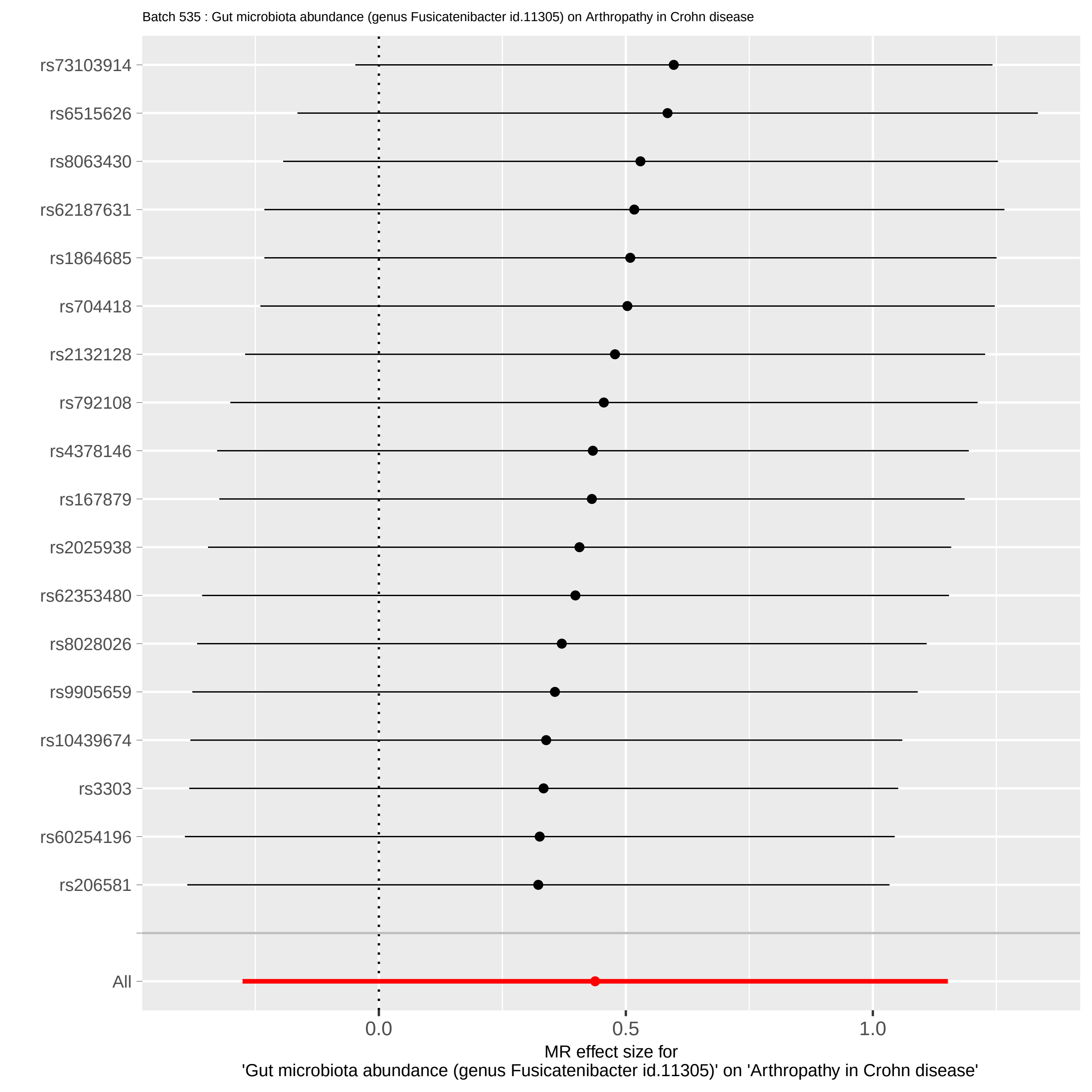

Batch 535 : Gut microbiota abundance (genus Fusicatenibacter id.11305) on Arthropathy in Crohn disease
rs73103914
rs6515626
rs8063430
rs62187631
rs1864685
rs704418
rs2132128
rs792108
rs4378146
rs167879
rs2025938
rs62353480
rs8028026
rs9905659
rs10439674
rs3303
rs60254196
rs206581
All
0.0
0.5
1.0
MR effect size for
'Gut microbiota abundance (genus Fusicatenibacter id.11305)' on 'Arthropathy in Crohn disease'

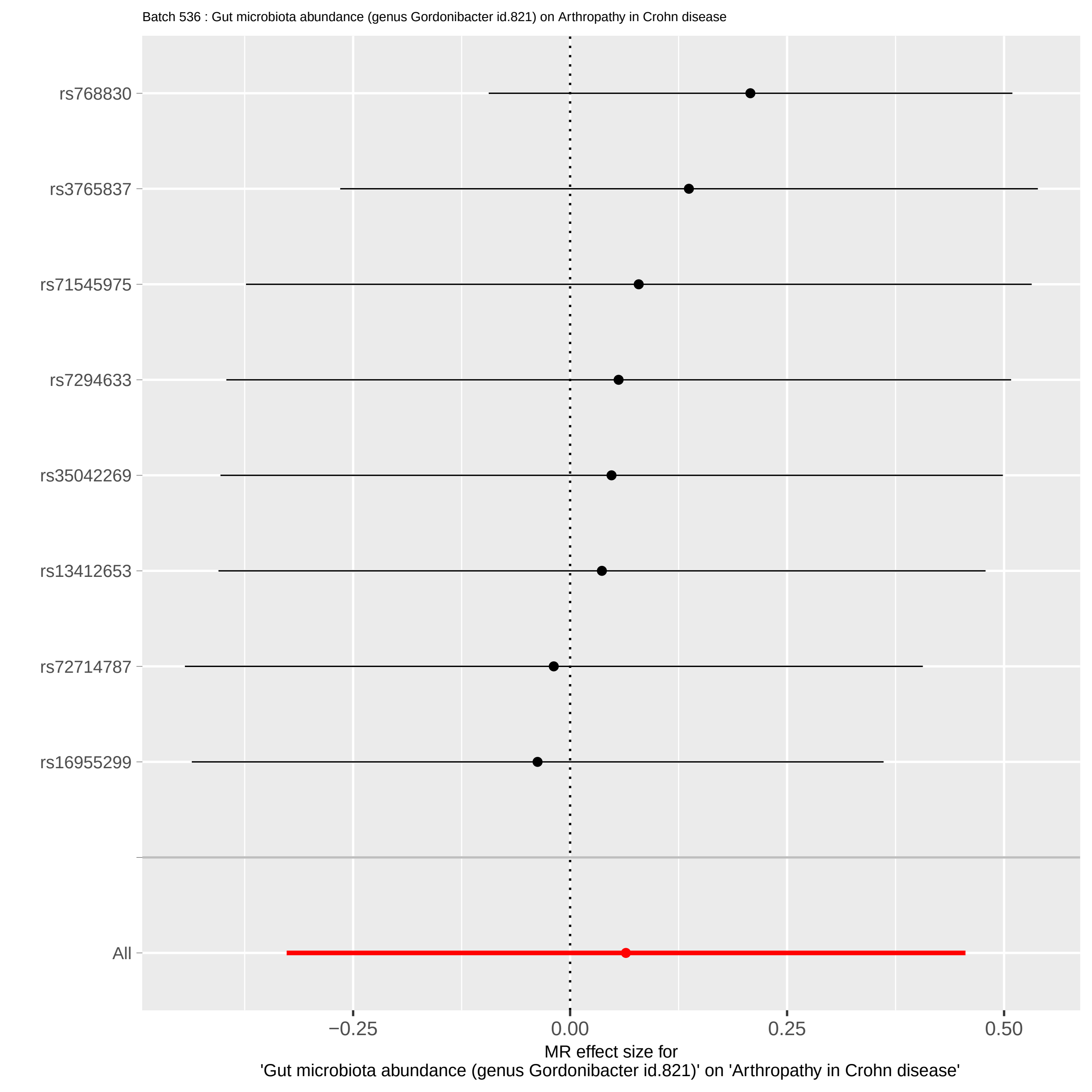
Batch 536 : Gut microbiota abundance (genus Gordonibacter id.821) on Arthropathy in Crohn disease
rs768830
rs3765837
rs71545975
rs7294633
rs35042269
rs13412653
rs72714787
rs16955299
All
−0.25
0.00
0.25
0.50
MR effect size for
'Gut microbiota abundance (genus Gordonibacter id.821)' on 'Arthropathy in Crohn disease'

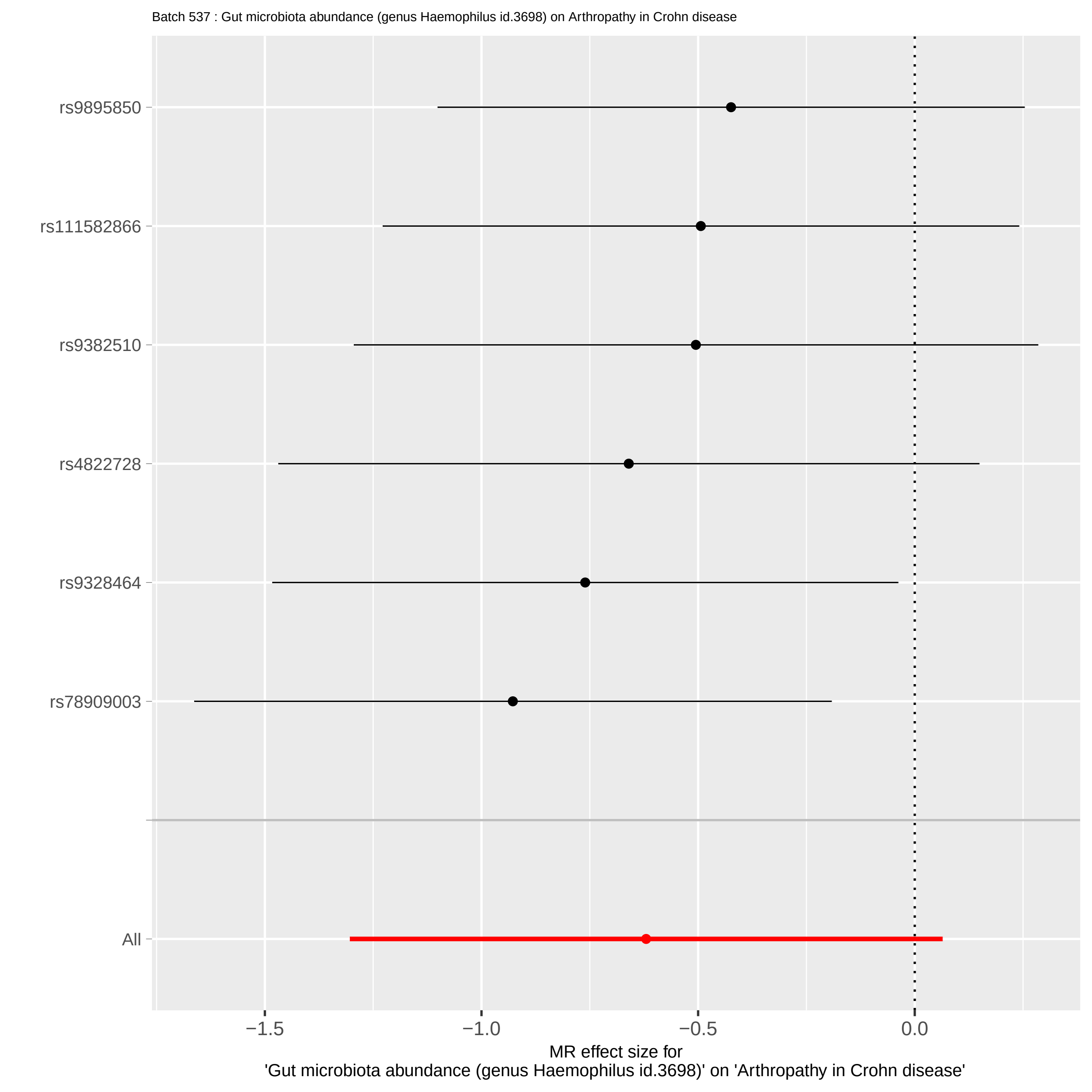

Batch 537 : Gut microbiota abundance (genus Haemophilus id.3698) on Arthropathy in Crohn disease
rs9895850
rs111582866
rs9382510
rs4822728
rs9328464
rs78909003
All
−1.5
−1.0
−0.5
0.0
MR effect size for
'Gut microbiota abundance (genus Haemophilus id.3698)' on 'Arthropathy in Crohn disease'

Batch 538 : Gut microbiota abundance (genus Holdemanella id.11393) on Arthropathy in Crohn disease

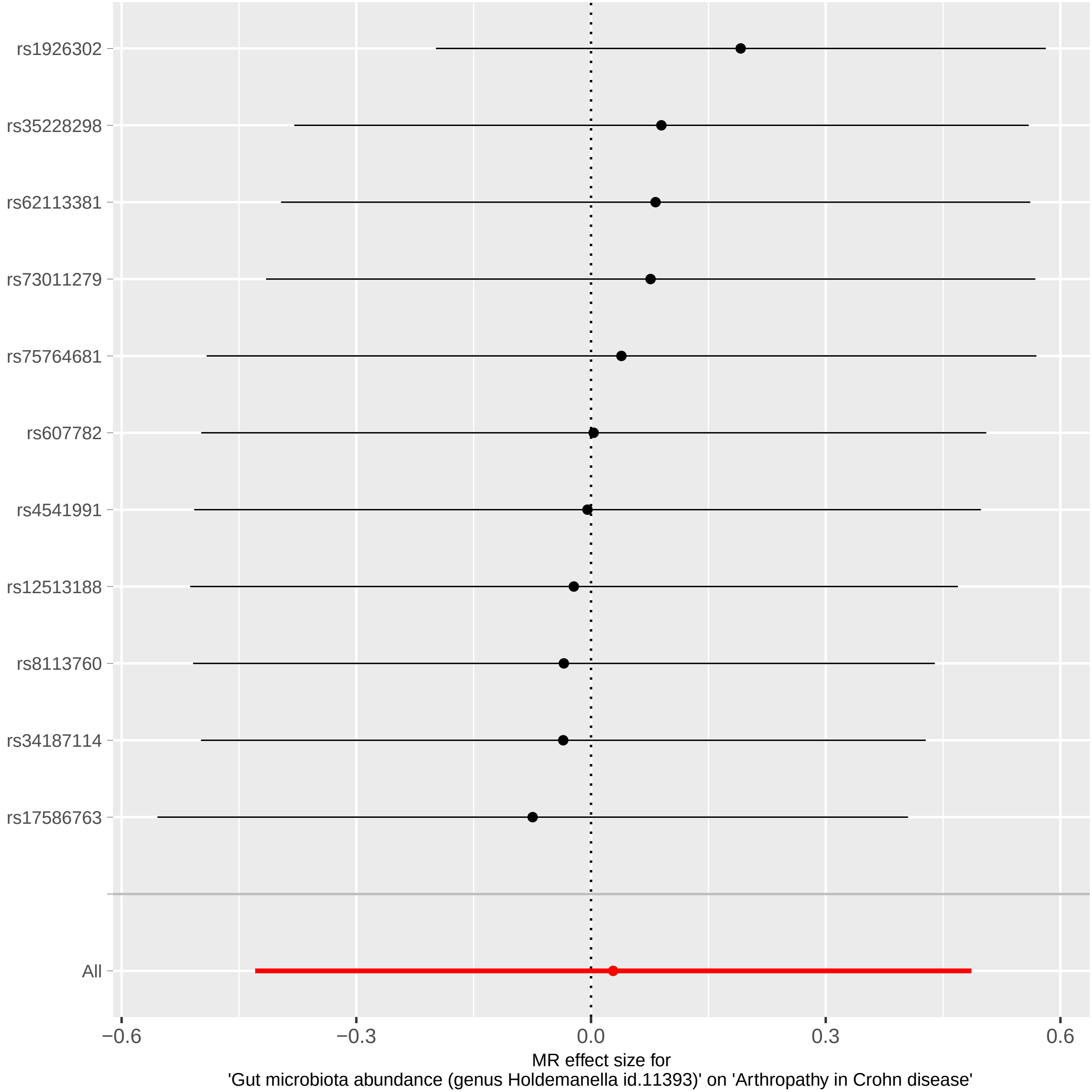

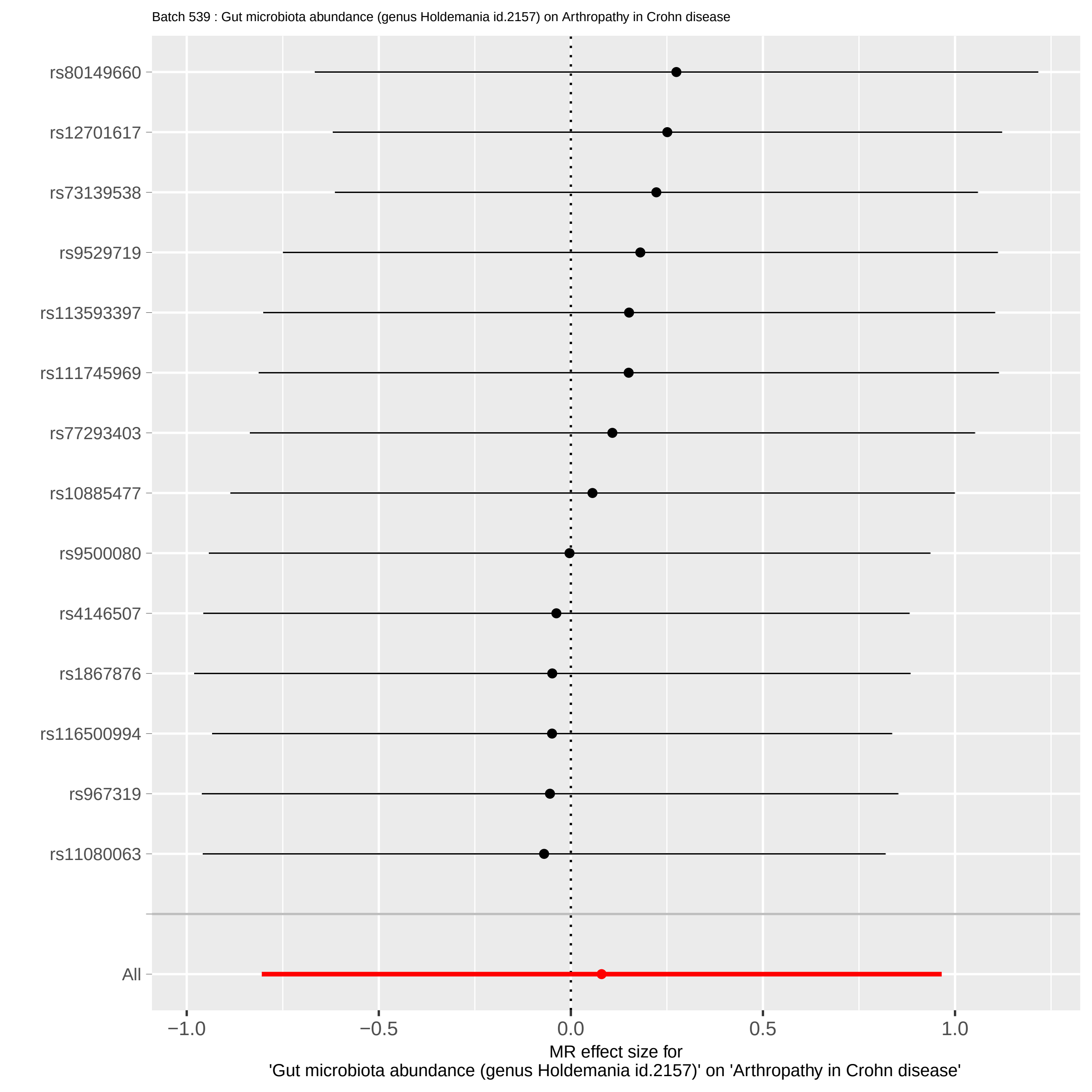
Batch 539 : Gut microbiota abundance (genus Holdemania id.2157) on Arthropathy in Crohn disease
rs80149660
rs12701617
rs73139538
rs9529719
rs113593397
rs111745969
rs77293403
rs10885477
rs9500080
rs4146507
rs1867876
rs116500994
rs967319
rs11080063
All
−1.0
−0.5
0.0
0.5
1.0
MR effect size for
'Gut microbiota abundance (genus Holdemania id.2157)' on 'Arthropathy in Crohn disease'

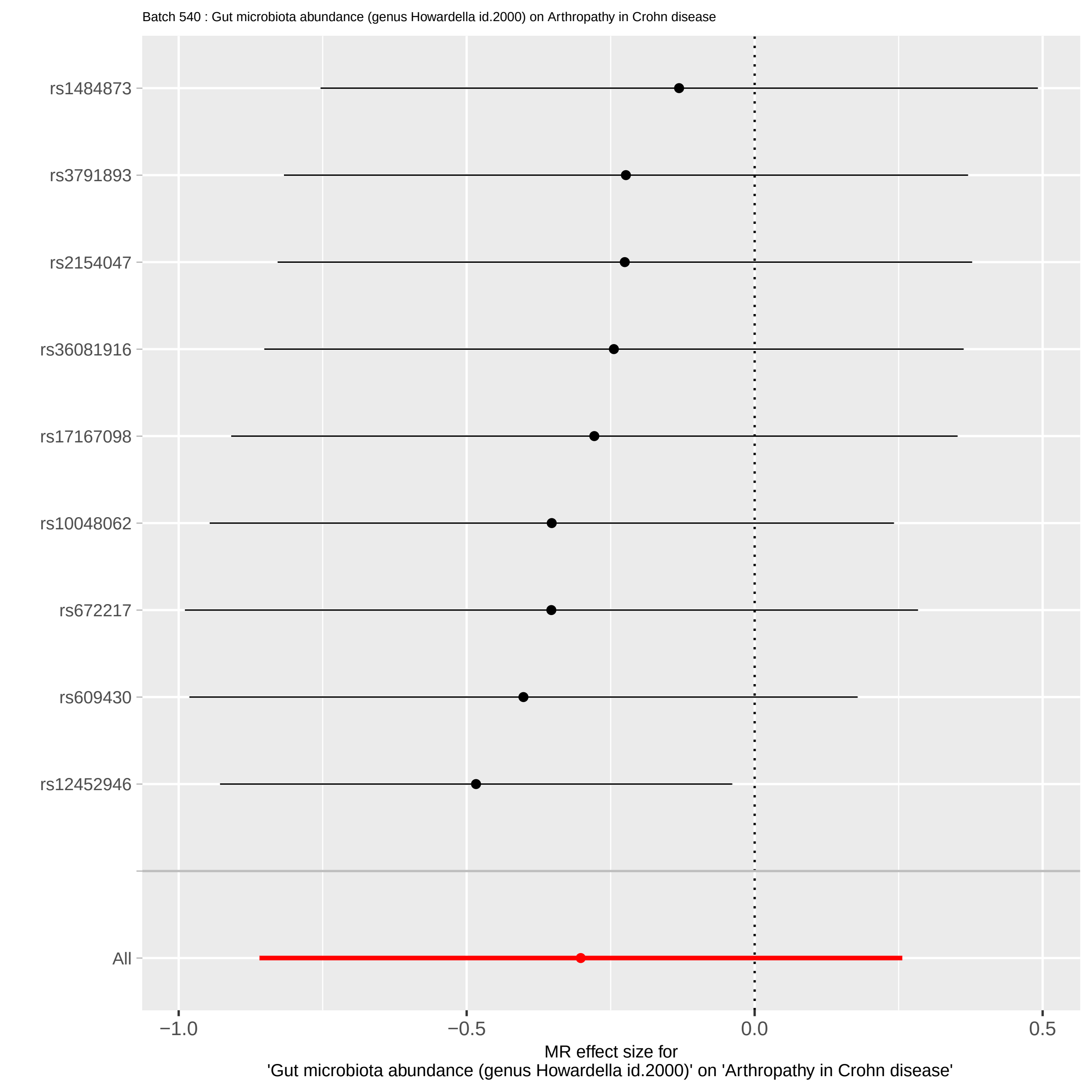
Batch 540 : Gut microbiota abundance (genus Howardella id.2000) on Arthropathy in Crohn disease
rs1484873
rs3791893
rs2154047
rs36081916
rs17167098
rs10048062
rs672217
rs609430
rs12452946
All
−1.0
−0.5
0.0
0.5
MR effect size for
'Gut microbiota abundance (genus Howardella id.2000)' on 'Arthropathy in Crohn disease'

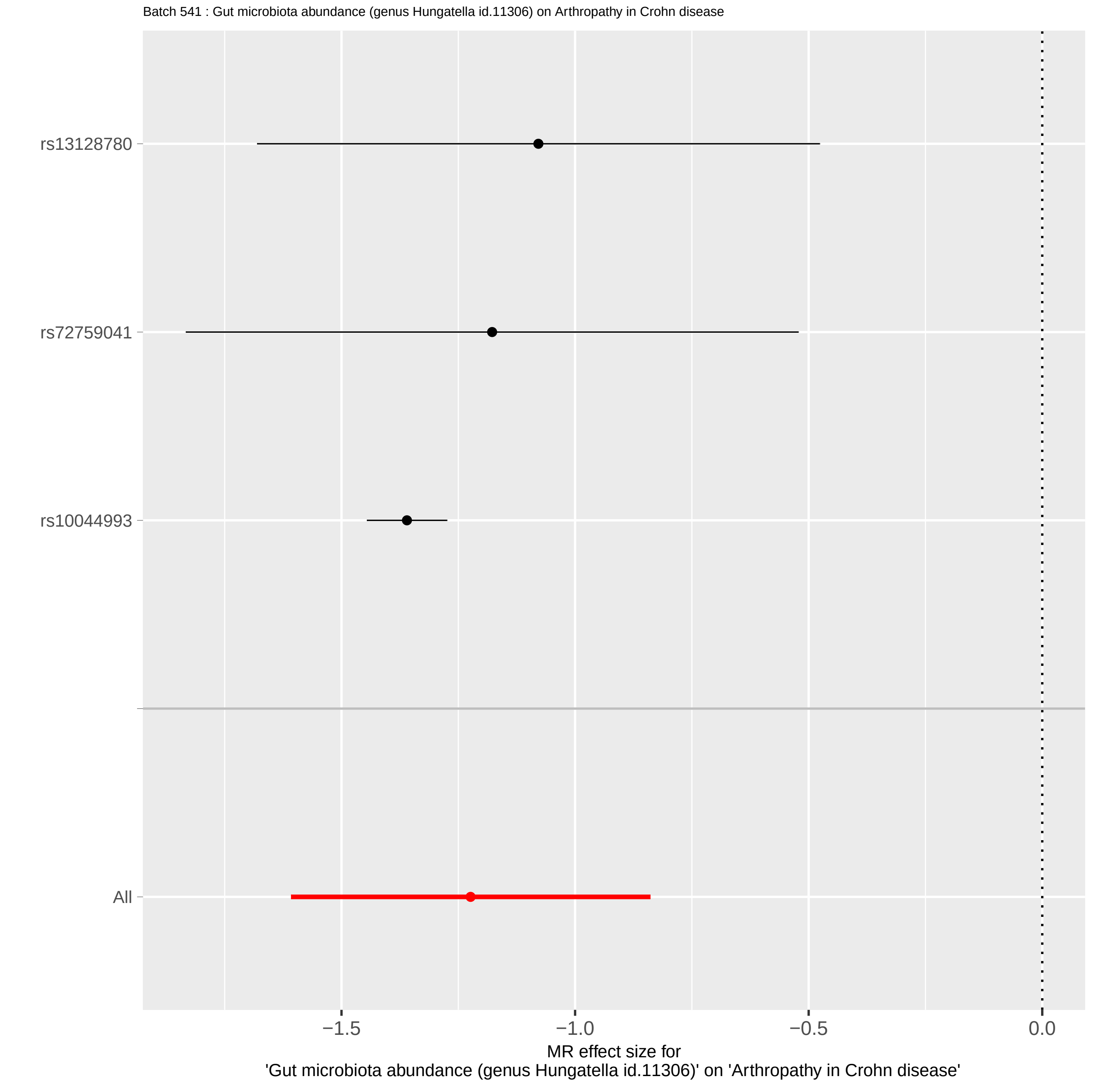
Batch 541 : Gut microbiota abundance (genus Hungatella id.11306) on Arthropathy in Crohn disease
rs13128780
rs72759041
rs10044993
All
−1.5
−1.0
−0.5
0.0
MR effect size for
'Gut microbiota abundance (genus Hungatella id.11306)' on 'Arthropathy in Crohn disease'

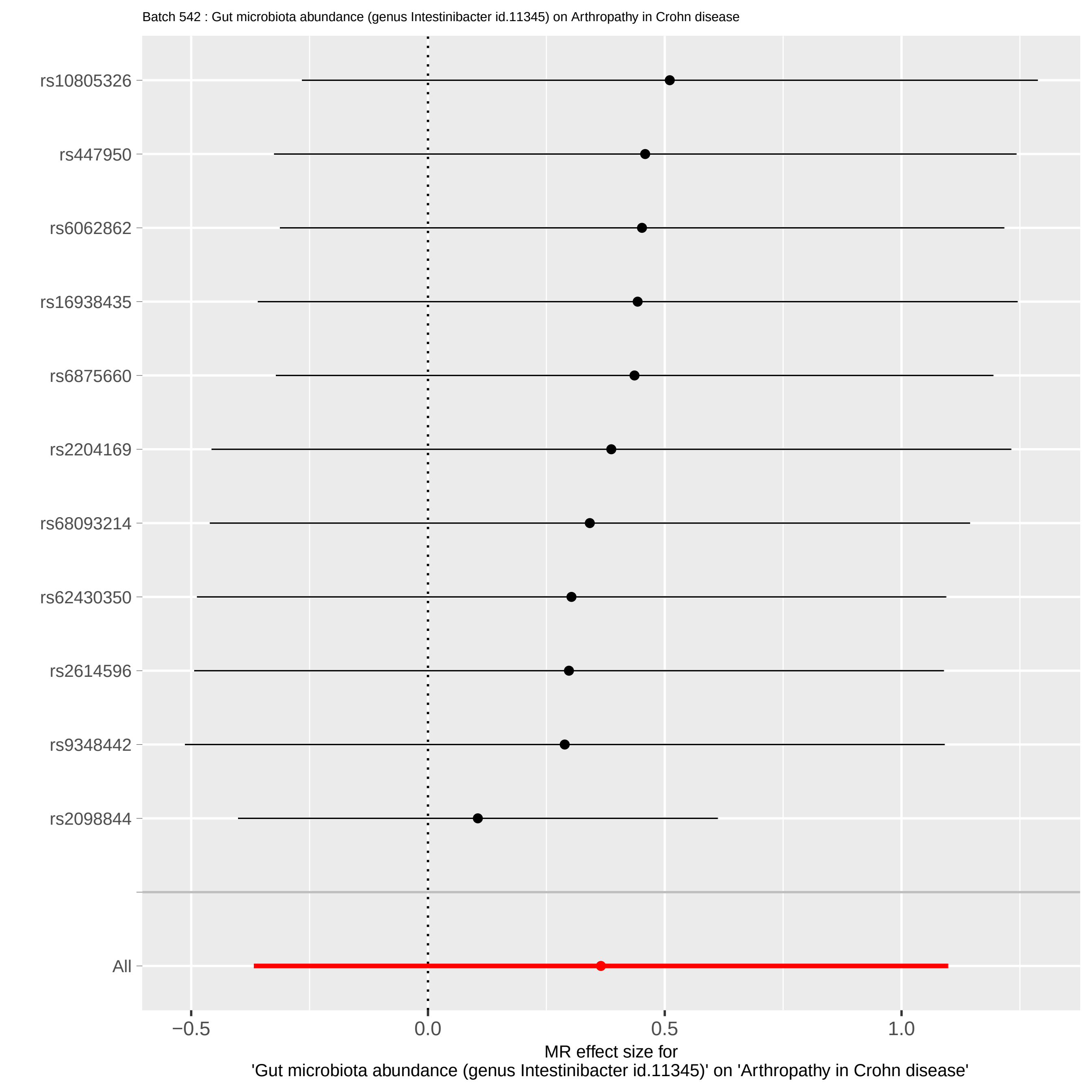

Batch 542 : Gut microbiota abundance (genus Intestinibacter id.11345) on Arthropathy in Crohn disease
rs10805326
rs447950
rs6062862
rs16938435
rs6875660
rs2204169
rs68093214
rs62430350
rs2614596
rs9348442
rs2098844
All
−0.5
0.0
0.5
1.0
MR effect size for
'Gut microbiota abundance (genus Intestinibacter id.11345)' on 'Arthropathy in Crohn disease'

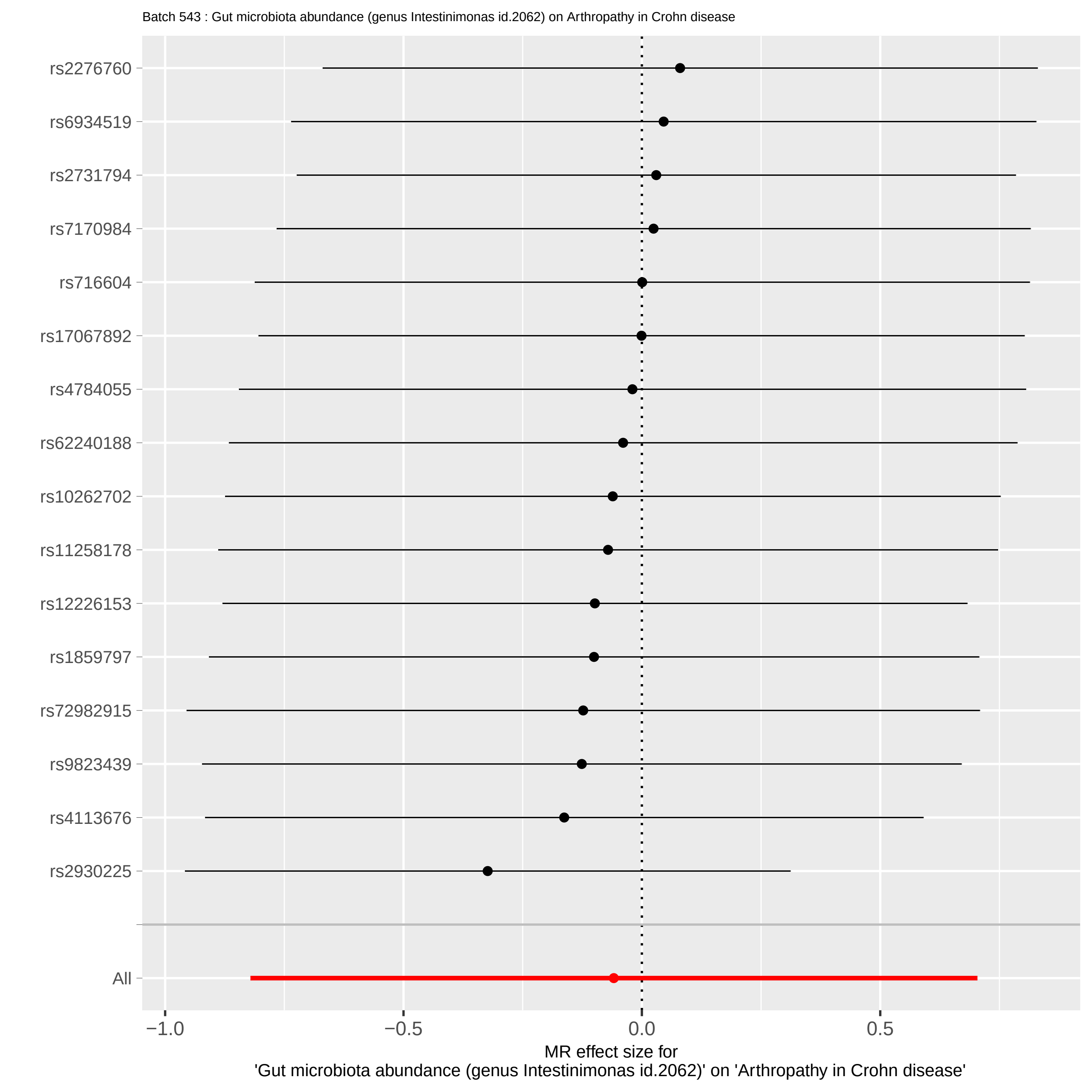

Batch 543 : Gut microbiota abundance (genus Intestinimonas id.2062) on Arthropathy in Crohn disease
rs2276760
rs6934519
rs2731794
rs7170984
rs716604
rs17067892
rs4784055
rs62240188
rs10262702
rs11258178
rs12226153
rs1859797
rs72982915
rs9823439
rs4113676
rs2930225
All
−1.0
−0.5
0.0
0.5
MR effect size for
'Gut microbiota abundance (genus Intestinimonas id.2062)' on 'Arthropathy in Crohn disease'

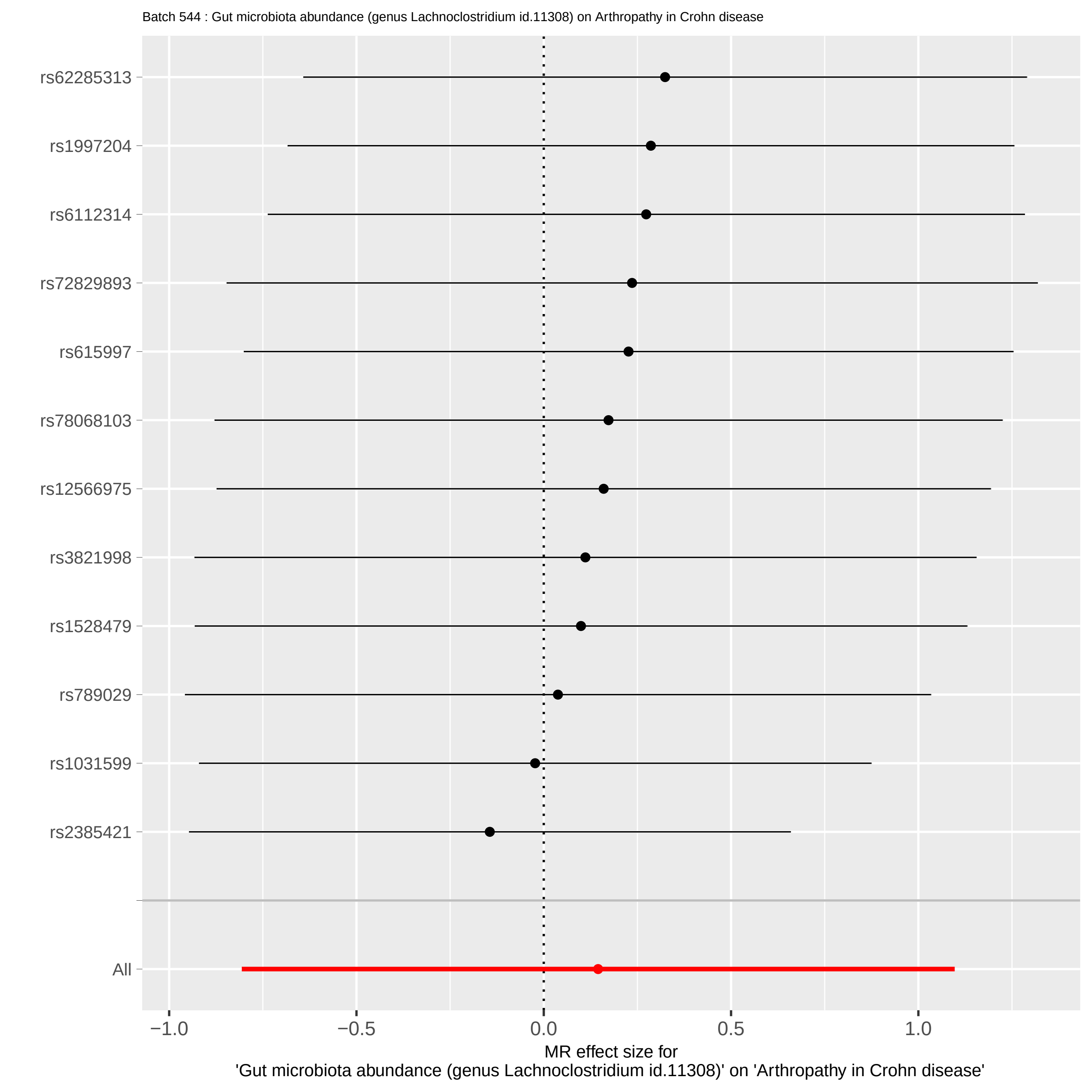
Batch 544 : Gut microbiota abundance (genus Lachnoclostridium id.11308) on Arthropathy in Crohn disease
rs62285313
rs1997204
rs6112314
rs72829893
rs615997
rs78068103
rs12566975
rs3821998
rs1528479
rs789029
rs1031599
rs2385421
All
−1.0
−0.5
0.0
0.5
1.0
MR effect size for
'Gut microbiota abundance (genus Lachnoclostridium id.11308)' on 'Arthropathy in Crohn disease'

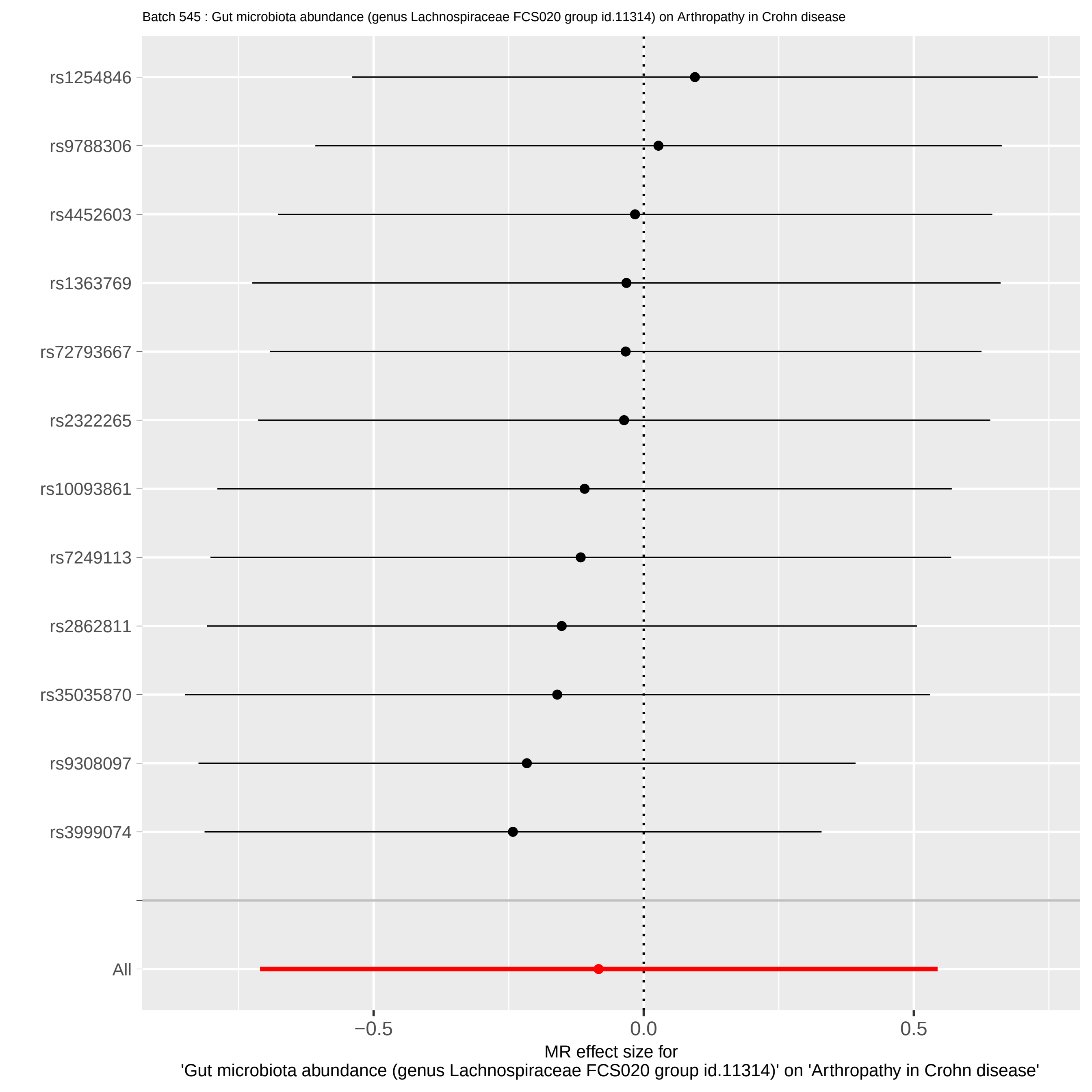

Batch 545 : Gut microbiota abundance (genus Lachnospiraceae FCS020 group id.11314) on Arthropathy in Crohn disease
rs1254846
rs9788306
rs4452603
rs1363769
rs72793667
rs2322265
rs10093861
rs7249113
rs2862811
rs35035870
rs9308097
rs3999074
All
−0.5
0.0
0.5
MR effect size for
'Gut microbiota abundance (genus Lachnospiraceae FCS020 group id.11314)' on 'Arthropathy in Crohn disease'

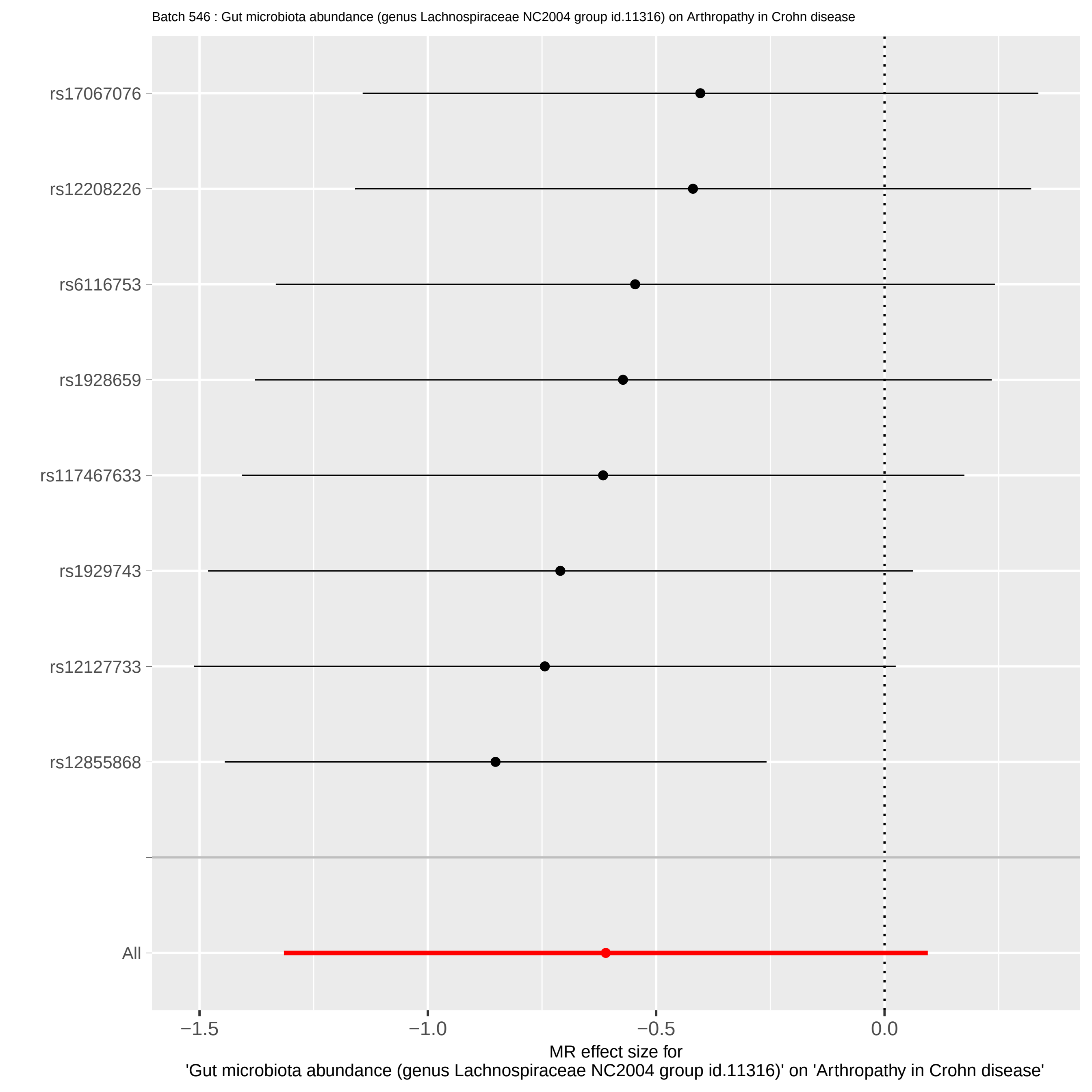

Batch 546 : Gut microbiota abundance (genus Lachnospiraceae NC2004 group id.11316) on Arthropathy in Crohn disease
rs17067076
rs12208226
rs6116753
rs1928659
rs117467633
rs1929743
rs12127733
rs12855868
All
−1.5
−1.0
−0.5
0.0
MR effect size for
'Gut microbiota abundance (genus Lachnospiraceae NC2004 group id.11316)' on 'Arthropathy in Crohn disease'

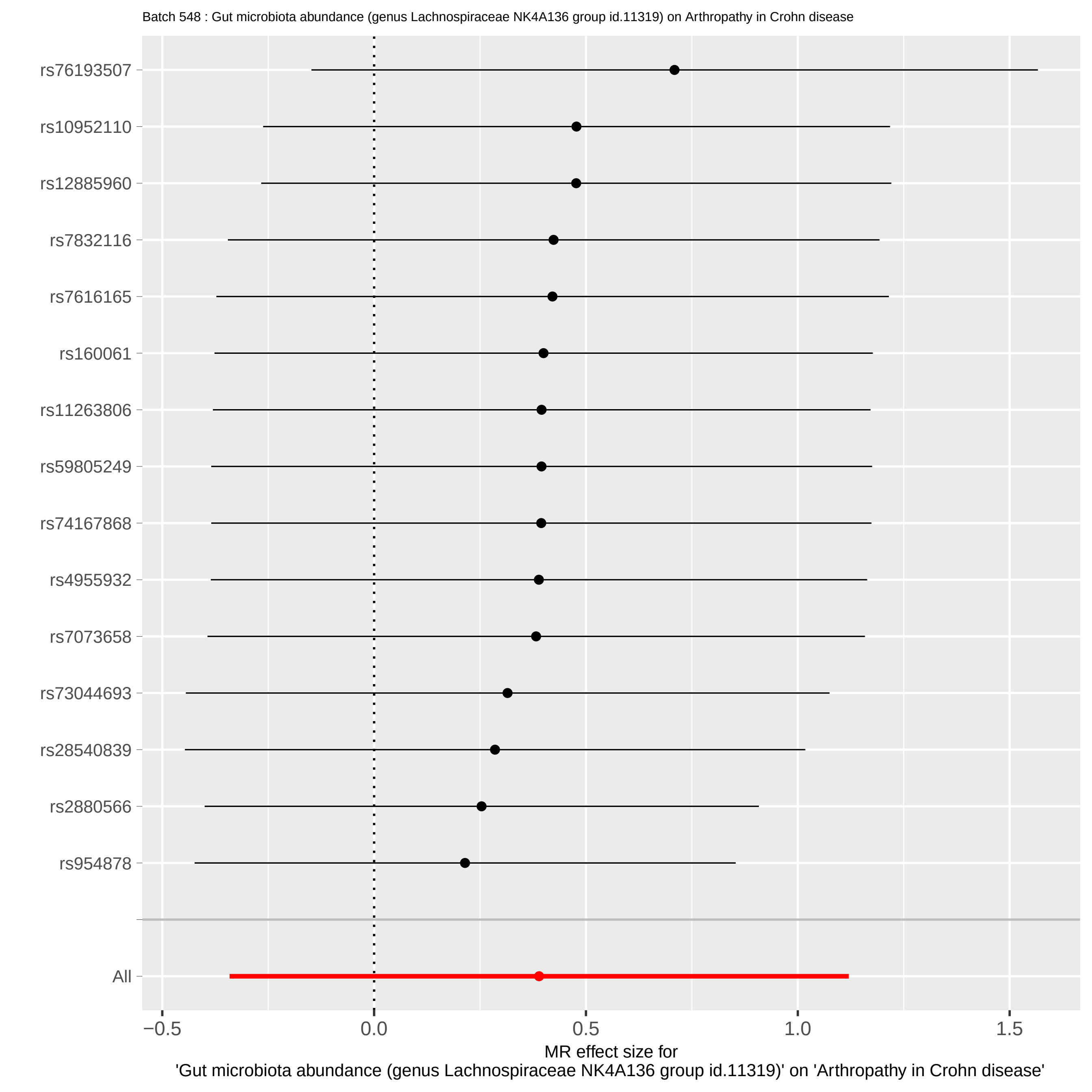

Batch 548 : Gut microbiota abundance (genus Lachnospiraceae NK4A136 group id.11319) on Arthropathy in Crohn disease
rs76193507
rs10952110
rs12885960
rs7832116
rs7616165
rs160061
rs11263806
rs59805249
rs74167868
rs4955932
rs7073658
rs73044693
rs28540839
rs2880566
rs954878
All
−0.5
0.0
0.5
1.0
1.5
MR effect size for
'Gut microbiota abundance (genus Lachnospiraceae NK4A136 group id.11319)' on 'Arthropathy in Crohn disease'

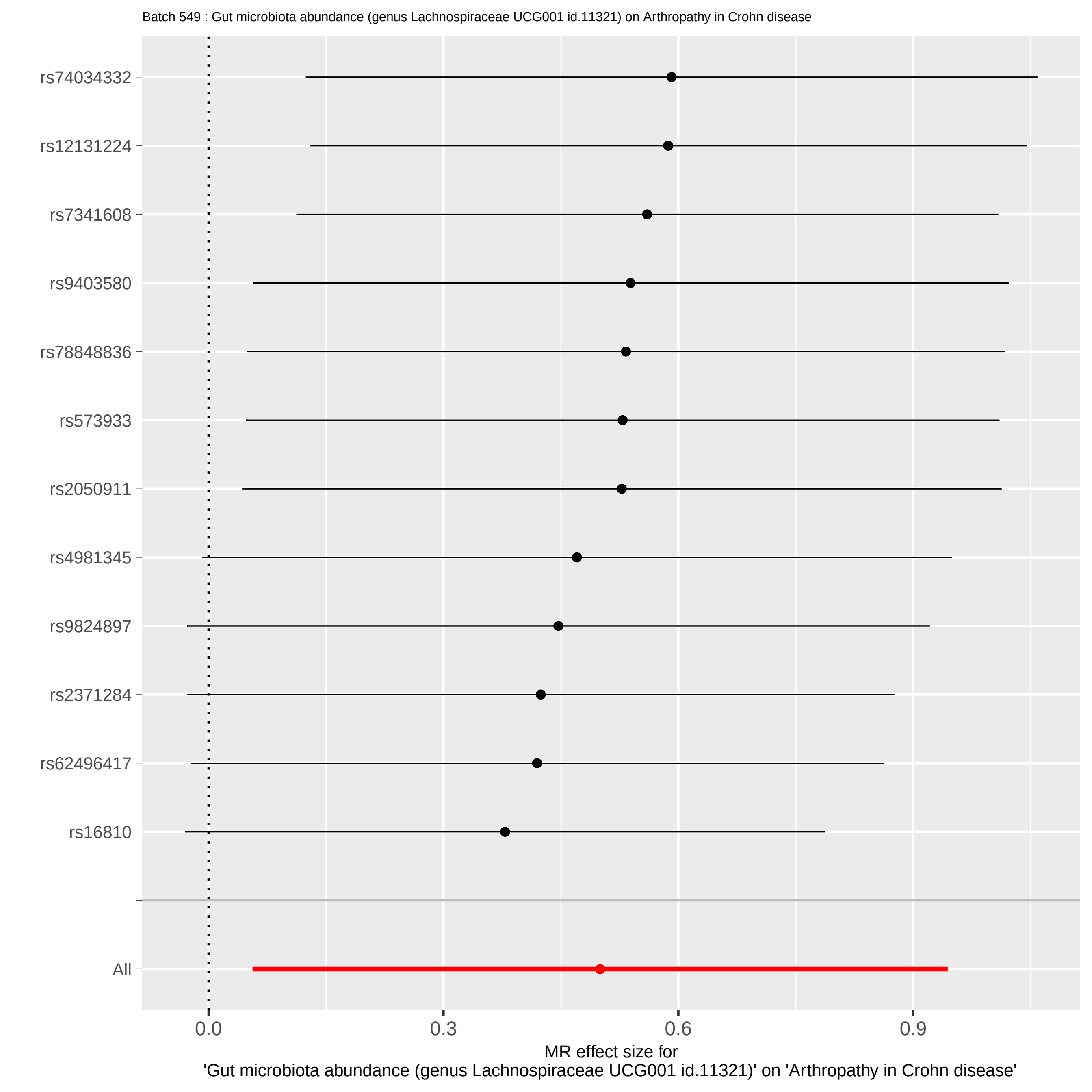

Batch 549 : Gut microbiota abundance (genus Lachnospiraceae UCG001 id.11321) on Arthropathy in Crohn disease
rs74034332
rs12131224
rs7341608
rs9403580
rs78848836
rs573933
rs2050911
rs4981345
rs9824897
rs2371284
rs62496417
rs16810
All
0.0
0.3
0.6
0.9
MR effect size for
'Gut microbiota abundance (genus Lachnospiraceae UCG001 id.11321)' on 'Arthropathy in Crohn disease'

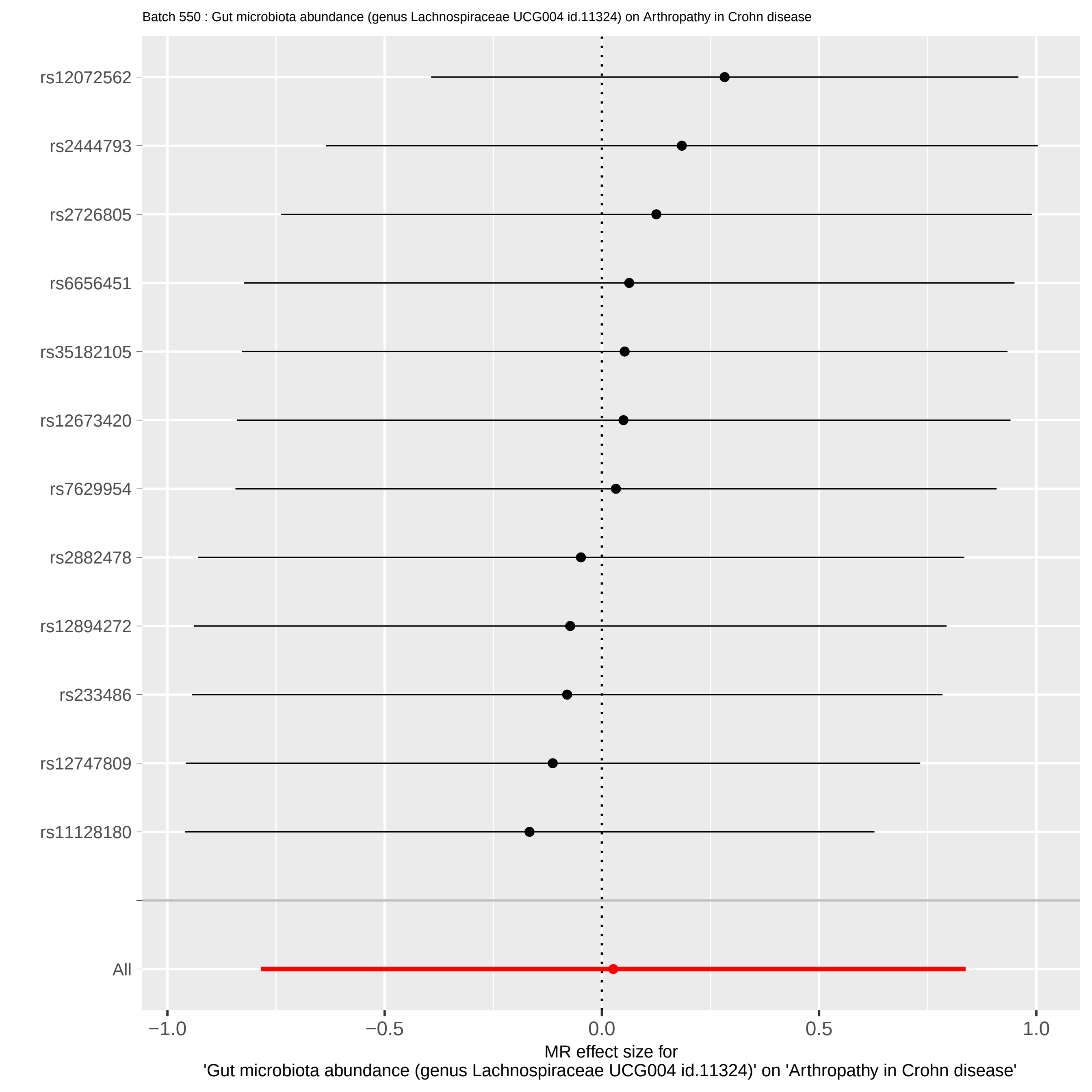
Batch 550 : Gut microbiota abundance (genus Lachnospiraceae UCG004 id.11324) on Arthropathy in Crohn disease
rs12072562
rs2444793
rs2726805
rs6656451
rs35182105
rs12673420
rs7629954
rs2882478
rs12894272
rs233486
rs12747809
rs11128180
All
−1.0
−0.5
0.0
0.5
1.0
MR effect size for
'Gut microbiota abundance (genus Lachnospiraceae UCG004 id.11324)' on 'Arthropathy in Crohn disease'

Batch 551 : Gut microbiota abundance (genus Lachnospiraceae UCG008 id.11328) on Arthropathy in Crohn disease

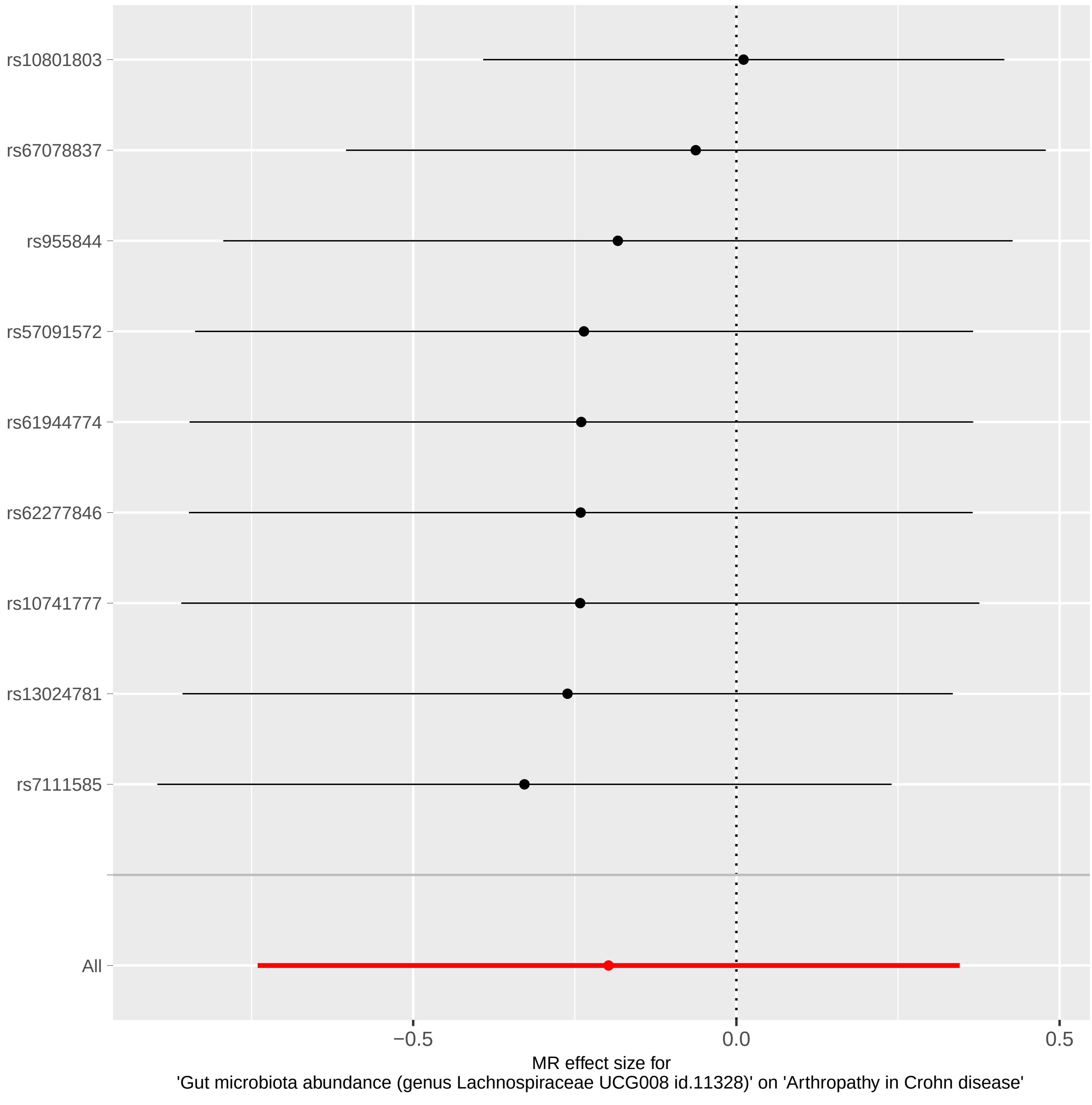

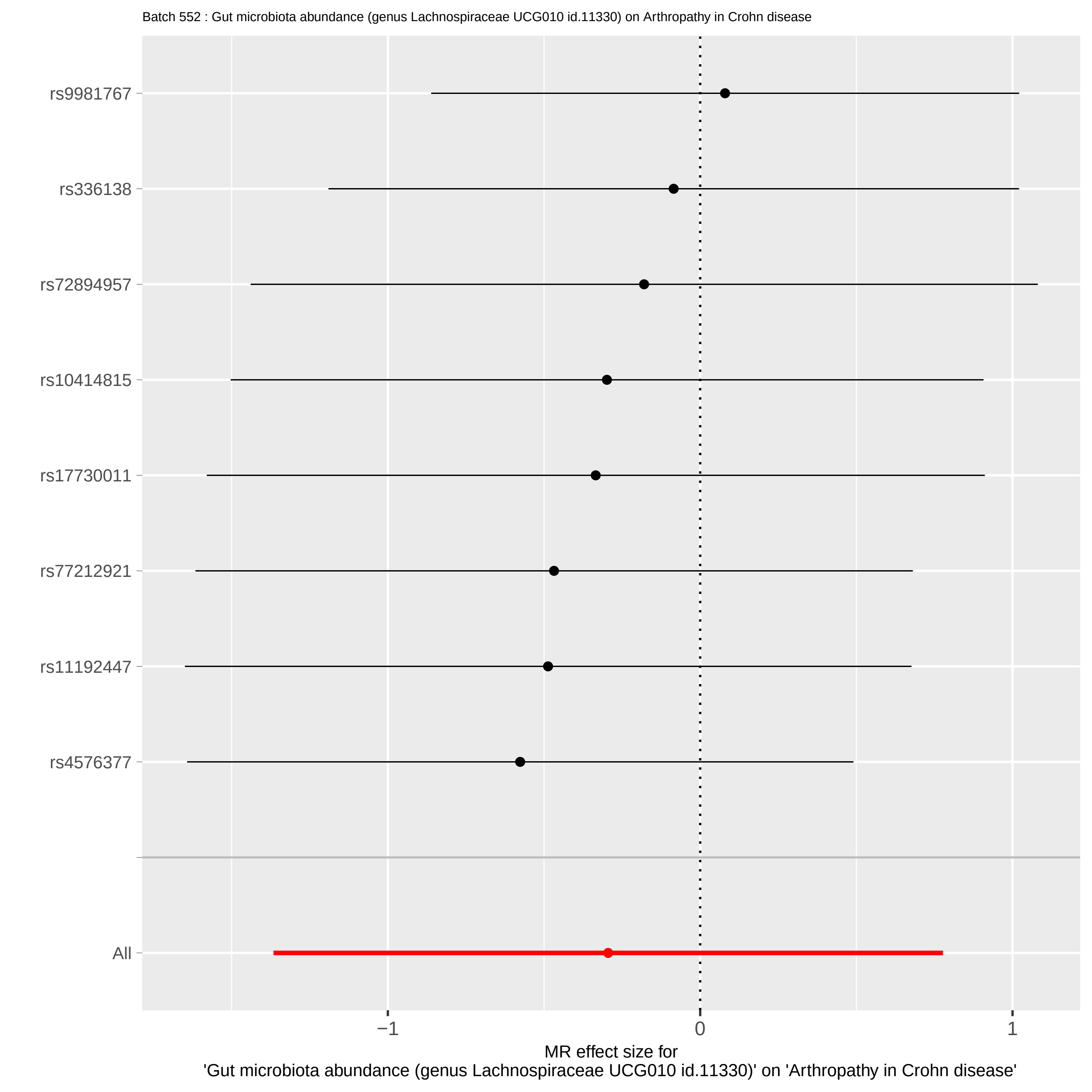

Batch 552 : Gut microbiota abundance (genus Lachnospiraceae UCG010 id.11330) on Arthropathy in Crohn disease
rs9981767
rs336138
rs72894957
rs10414815
rs17730011
rs77212921
rs11192447
rs4576377
All
−1
0
1
MR effect size for
'Gut microbiota abundance (genus Lachnospiraceae UCG010 id.11330)' on 'Arthropathy in Crohn disease'

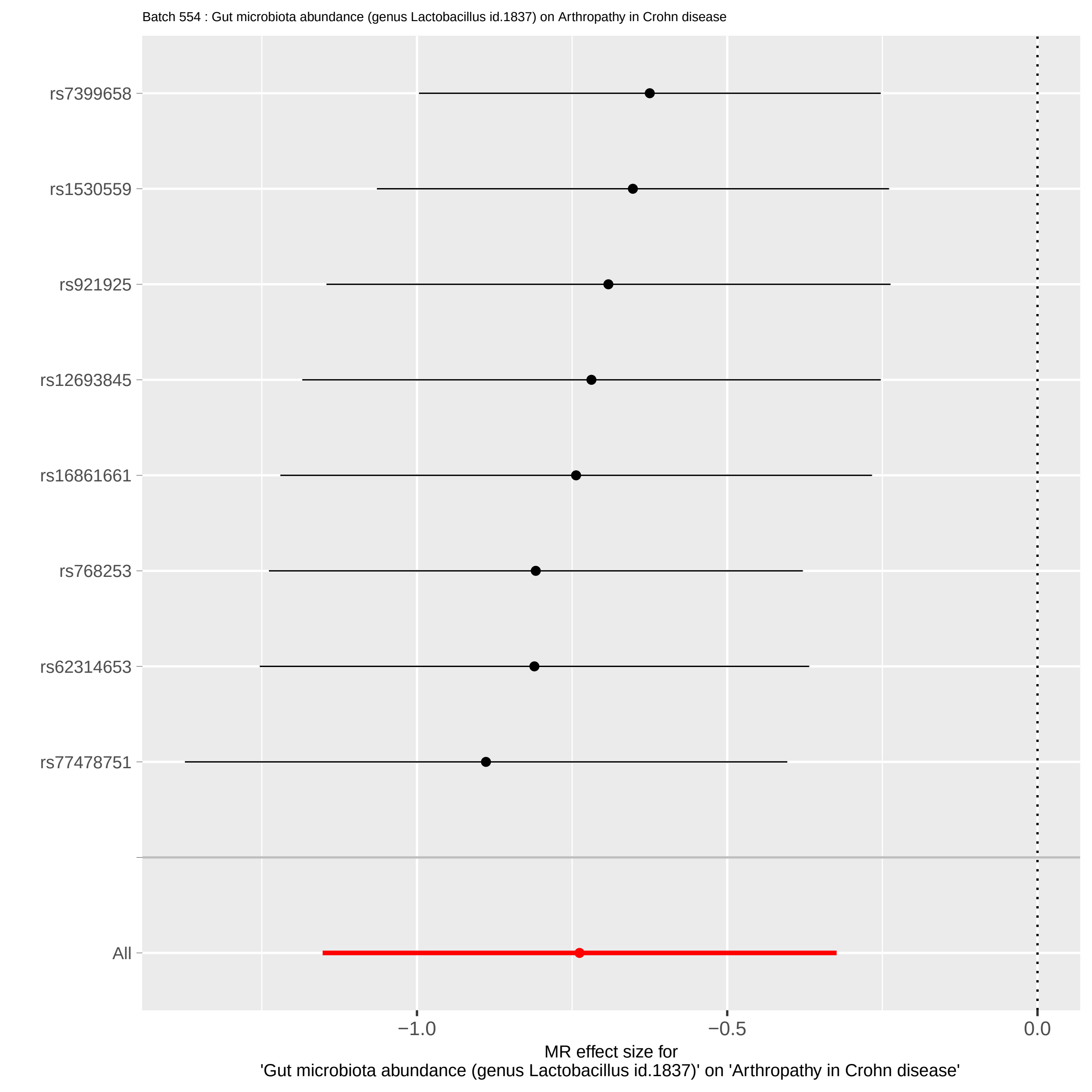
Batch 554 : Gut microbiota abundance (genus Lactobacillus id.1837) on Arthropathy in Crohn disease
rs7399658
rs1530559
rs921925
rs12693845
rs16861661
rs768253
rs62314653
rs77478751
All
−1.0
−0.5
0.0
MR effect size for
'Gut microbiota abundance (genus Lactobacillus id.1837)' on 'Arthropathy in Crohn disease'

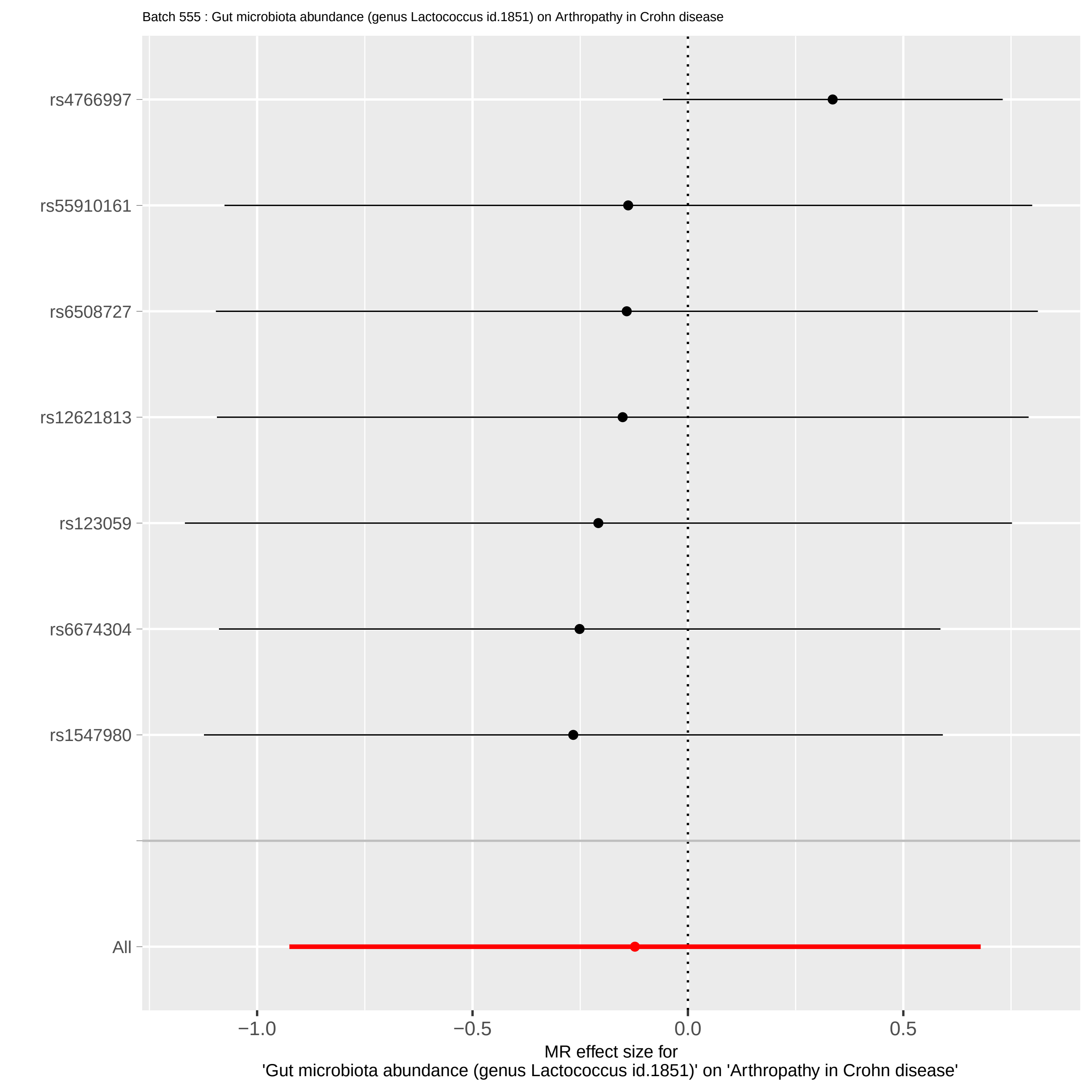
Batch 555 : Gut microbiota abundance (genus Lactococcus id.1851) on Arthropathy in Crohn disease
rs4766997
rs55910161
rs6508727
rs12621813
rs123059
rs6674304
rs1547980
All
−1.0
−0.5
0.0
0.5
MR effect size for
'Gut microbiota abundance (genus Lactococcus id.1851)' on 'Arthropathy in Crohn disease'

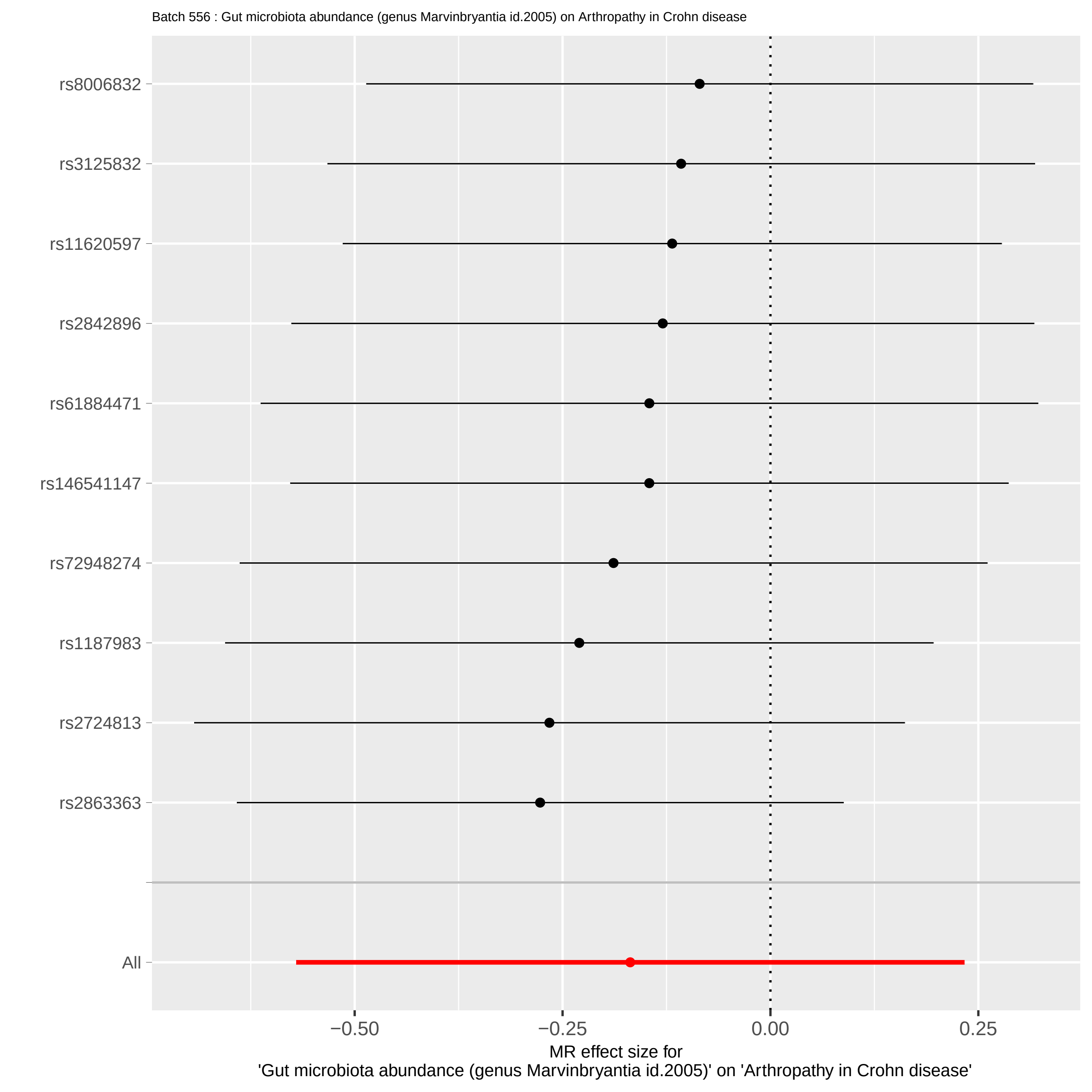

Batch 556 : Gut microbiota abundance (genus Marvinbryantia id.2005) on Arthropathy in Crohn disease
rs8006832
rs3125832
rs11620597
rs2842896
rs61884471
rs146541147
rs72948274
rs1187983
rs2724813
rs2863363
All
−0.50
−0.25
0.00
0.25
MR effect size for
'Gut microbiota abundance (genus Marvinbryantia id.2005)' on 'Arthropathy in Crohn disease'

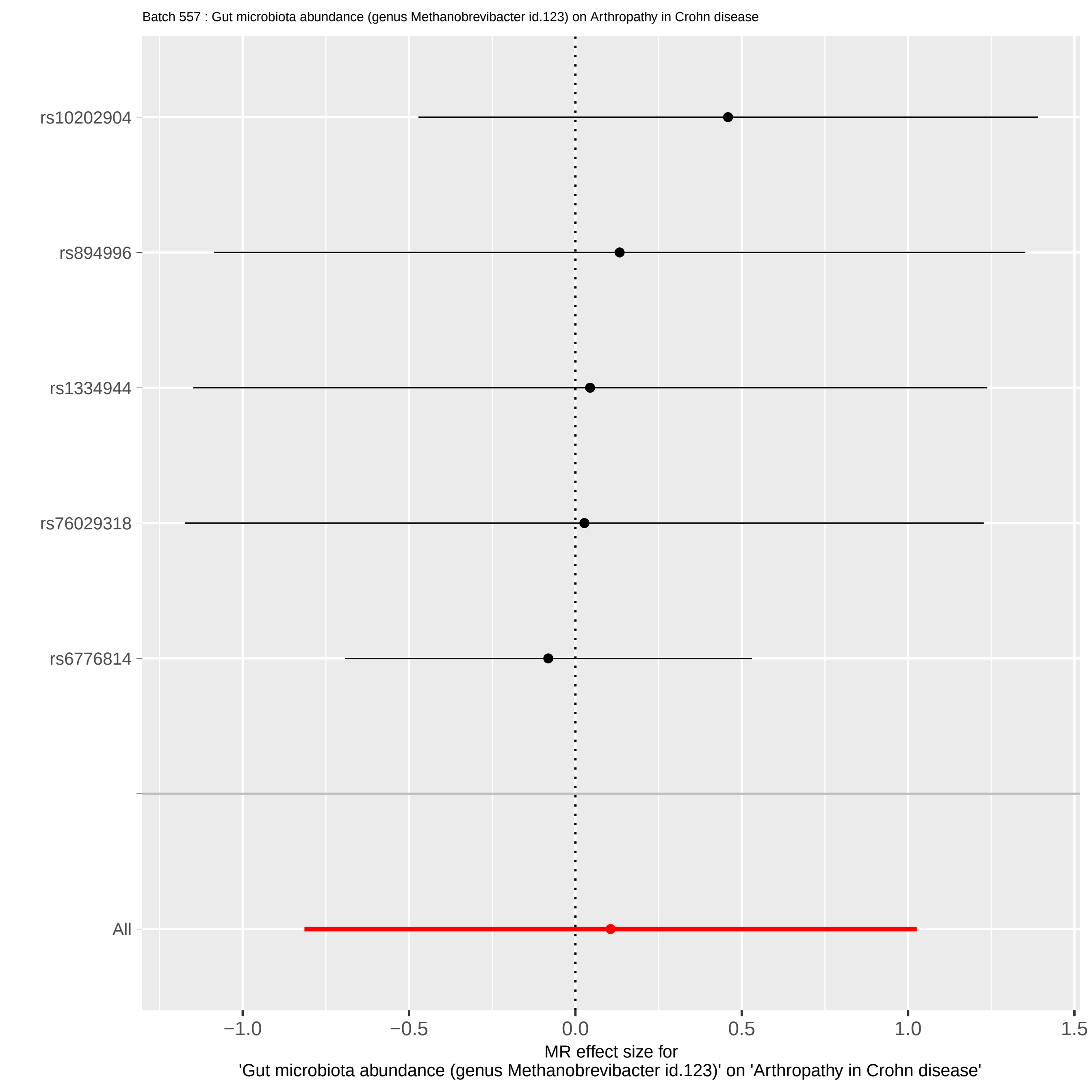
Batch 557 : Gut microbiota abundance (genus Methanobrevibacter id.123) on Arthropathy in Crohn disease
rs10202904
rs894996
rs1334944
rs76029318
rs6776814
All
−1.0
−0.5
0.0
0.5
1.0
1.5
MR effect size for
'Gut microbiota abundance (genus Methanobrevibacter id.123)' on 'Arthropathy in Crohn disease'

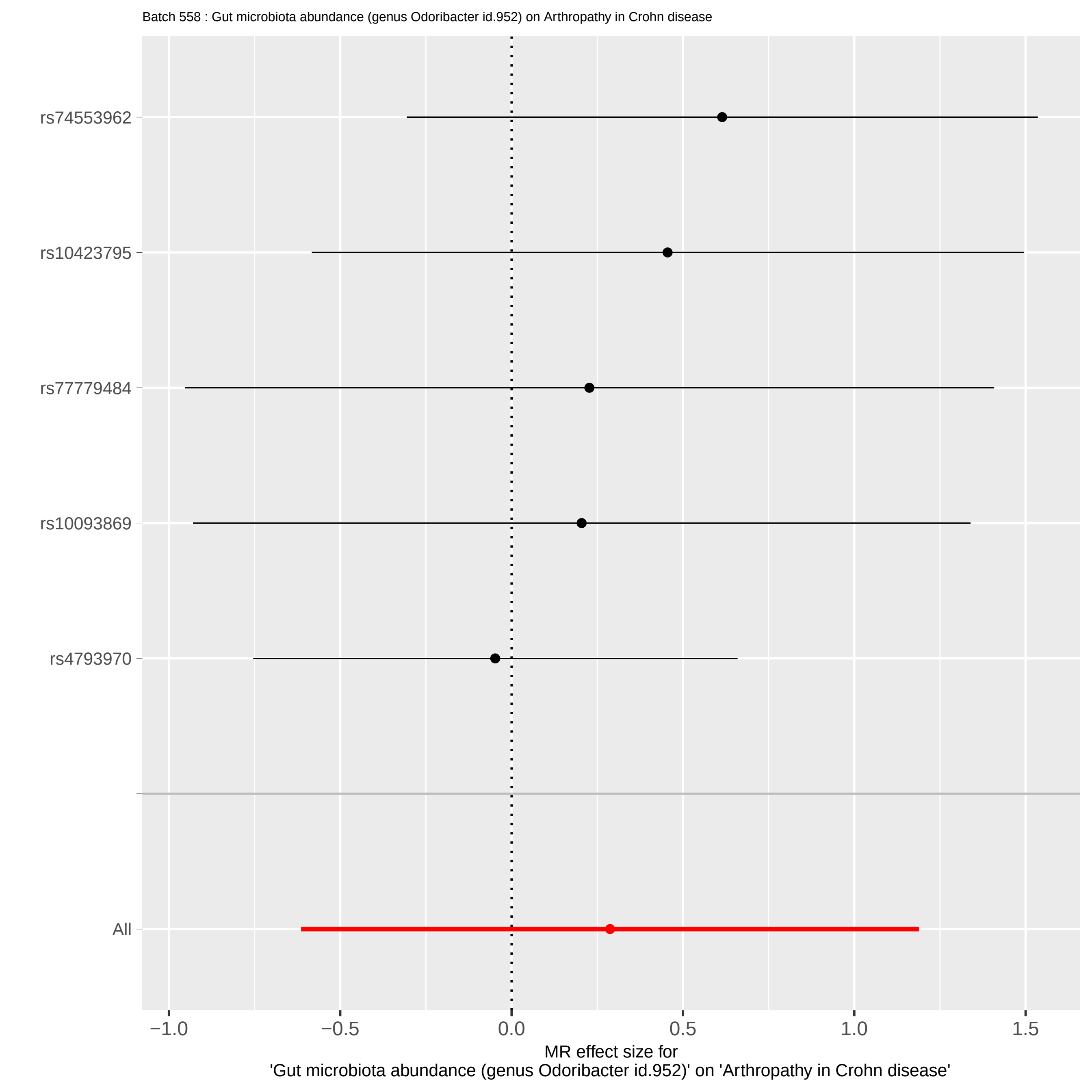

Batch 558 : Gut microbiota abundance (genus Odoribacter id.952) on Arthropathy in Crohn disease
rs74553962
rs10423795
rs77779484
rs10093869
rs4793970
All
−1.0
−0.5
0.0
0.5
1.0
1.5
MR effect size for
'Gut microbiota abundance (genus Odoribacter id.952)' on 'Arthropathy in Crohn disease'

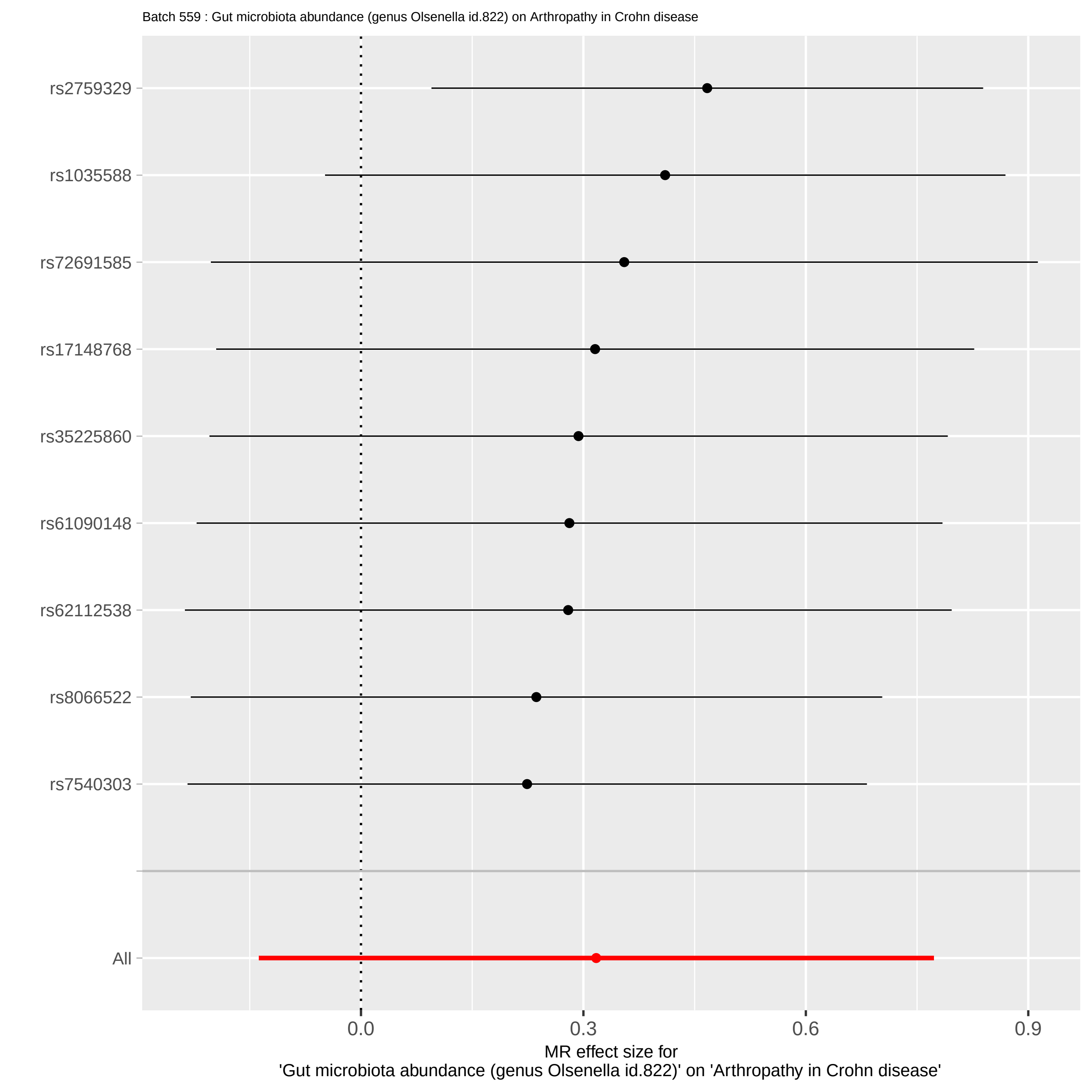

Batch 559 : Gut microbiota abundance (genus Olsenella id.822) on Arthropathy in Crohn disease
rs2759329
rs1035588
rs72691585
rs17148768
rs35225860
rs61090148
rs62112538
rs8066522
rs7540303
All
0.0
0.3
0.6
0.9
MR effect size for
'Gut microbiota abundance (genus Olsenella id.822)' on 'Arthropathy in Crohn disease'

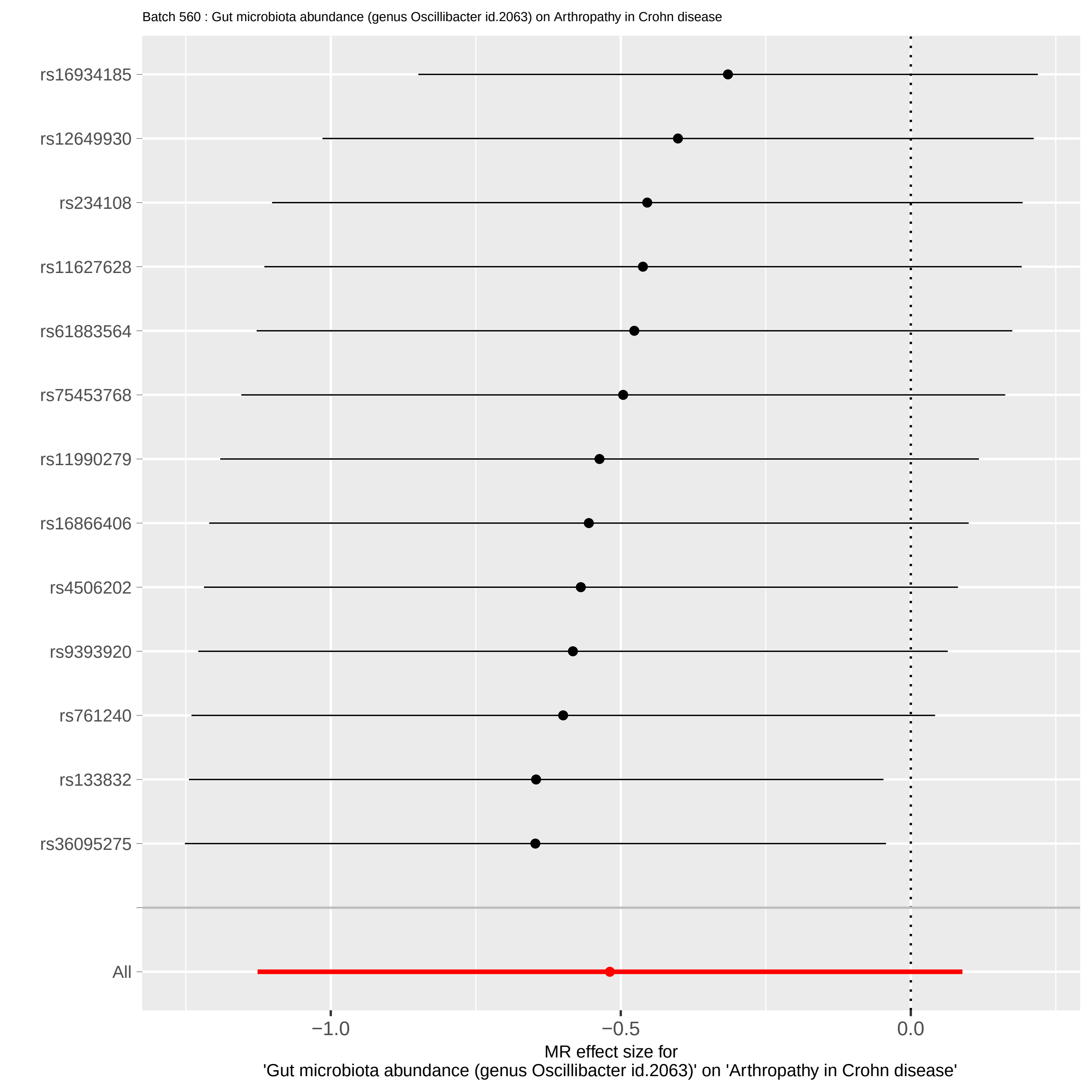

Batch 560 : Gut microbiota abundance (genus Oscillibacter id.2063) on Arthropathy in Crohn disease
rs16934185
rs12649930
rs234108
rs11627628
rs61883564
rs75453768
rs11990279
rs16866406
rs4506202
rs9393920
rs761240
rs133832
rs36095275
All
−1.0
−0.5
0.0
MR effect size for
'Gut microbiota abundance (genus Oscillibacter id.2063)' on 'Arthropathy in Crohn disease'

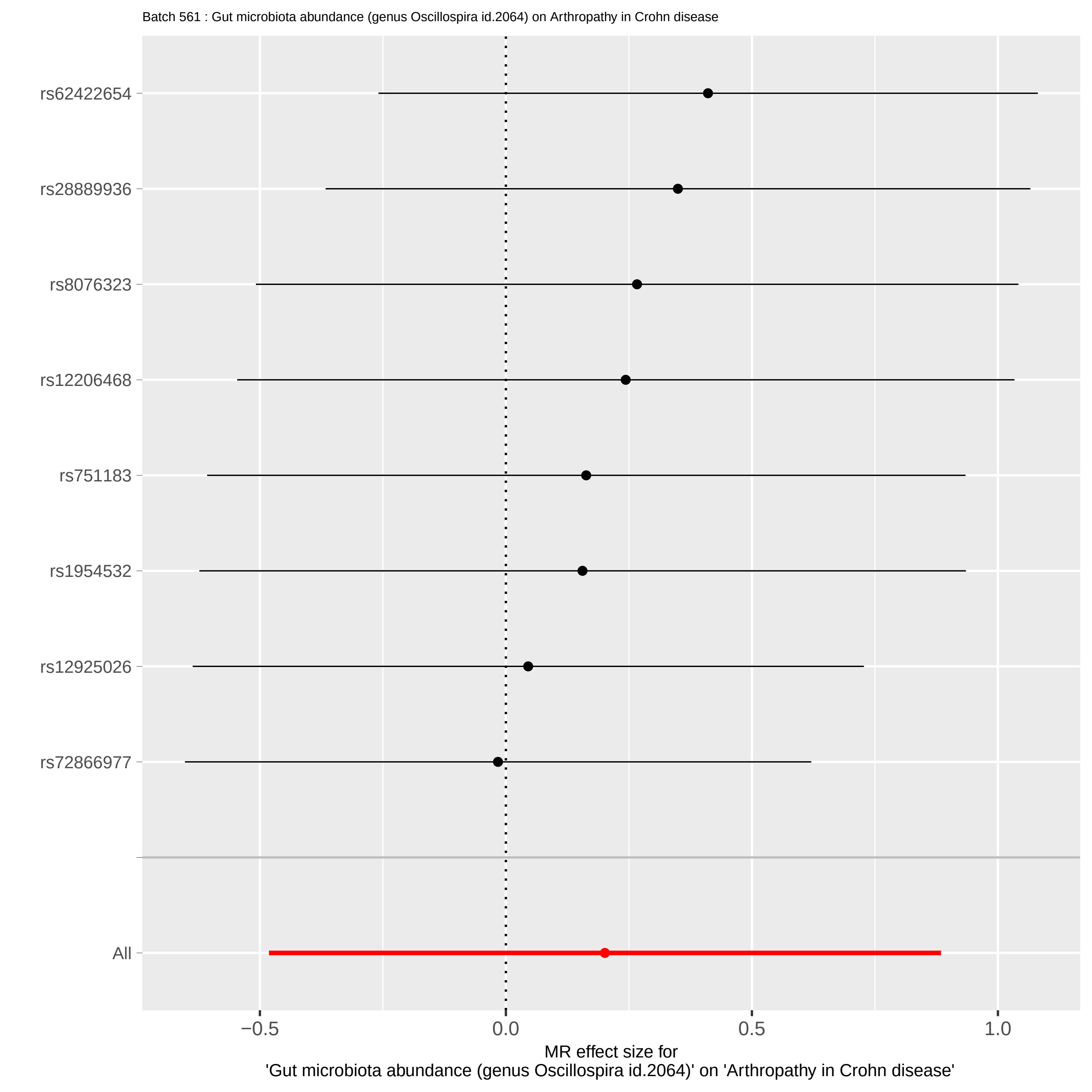
Batch 561 : Gut microbiota abundance (genus Oscillospira id.2064) on Arthropathy in Crohn disease
rs62422654
rs28889936
rs8076323
rs12206468
rs751183
rs1954532
rs12925026
rs72866977
All
−0.5
0.0
0.5
1.0
MR effect size for
'Gut microbiota abundance (genus Oscillospira id.2064)' on 'Arthropathy in Crohn disease'

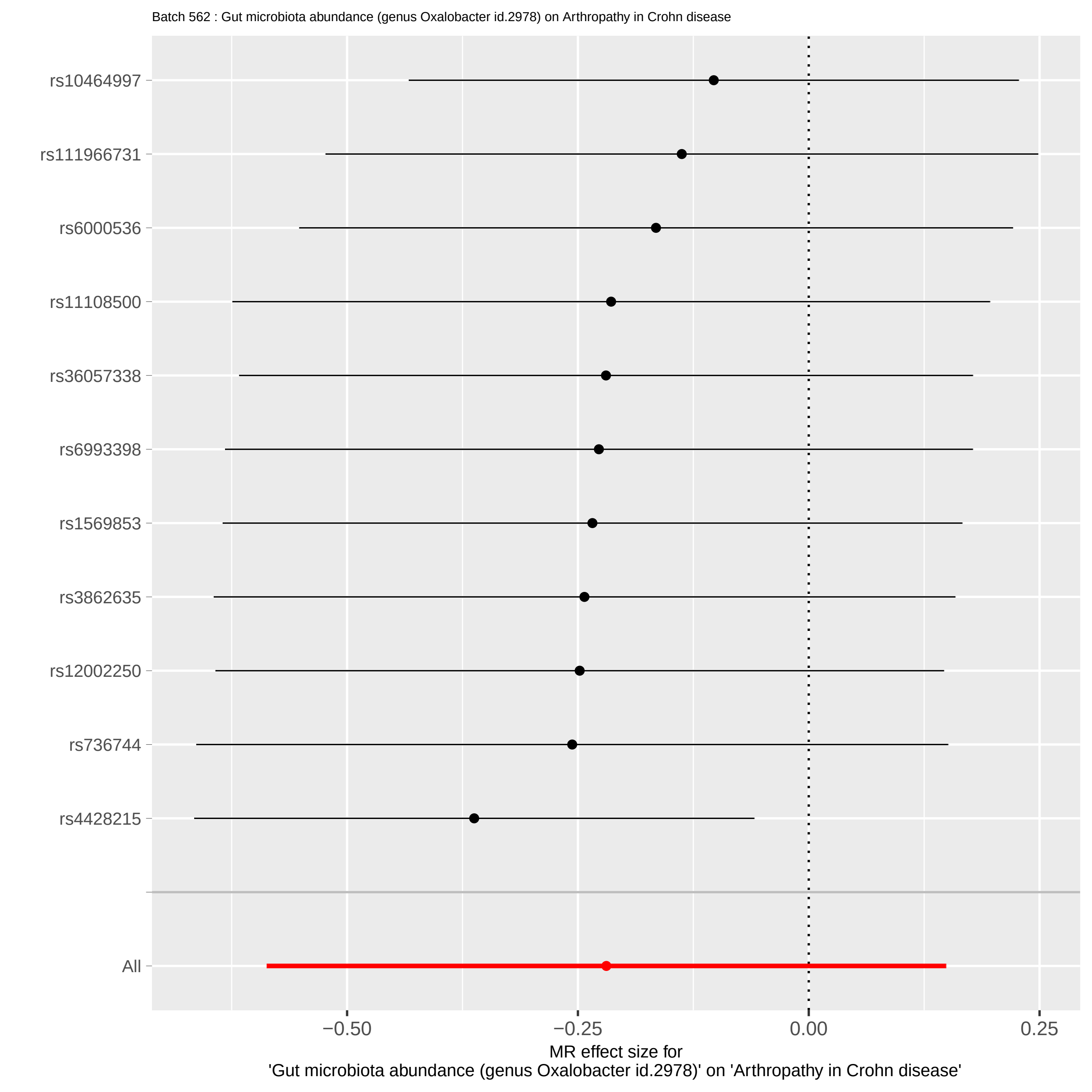

Batch 562 : Gut microbiota abundance (genus Oxalobacter id.2978) on Arthropathy in Crohn disease
rs10464997
rs111966731
rs6000536
rs11108500
rs36057338
rs6993398
rs1569853
rs3862635
rs12002250
rs736744
rs4428215
All
−0.50
−0.25
0.00
0.25
MR effect size for
'Gut microbiota abundance (genus Oxalobacter id.2978)' on 'Arthropathy in Crohn disease'

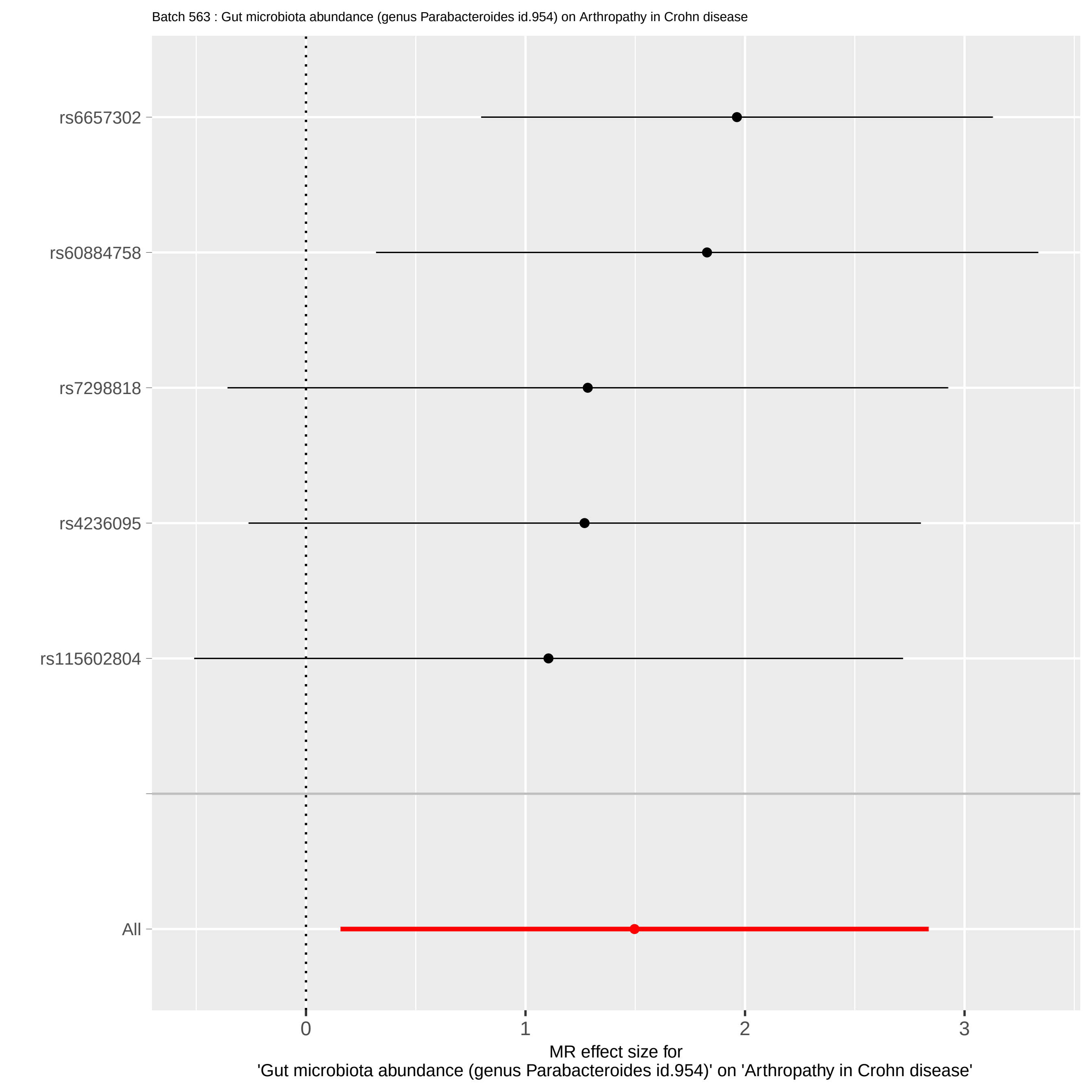

Batch 563 : Gut microbiota abundance (genus Parabacteroides id.954) on Arthropathy in Crohn disease
rs6657302
rs60884758
rs7298818
rs4236095
rs115602804
All
0
1
2
3
MR effect size for
'Gut microbiota abundance (genus Parabacteroides id.954)' on 'Arthropathy in Crohn disease'

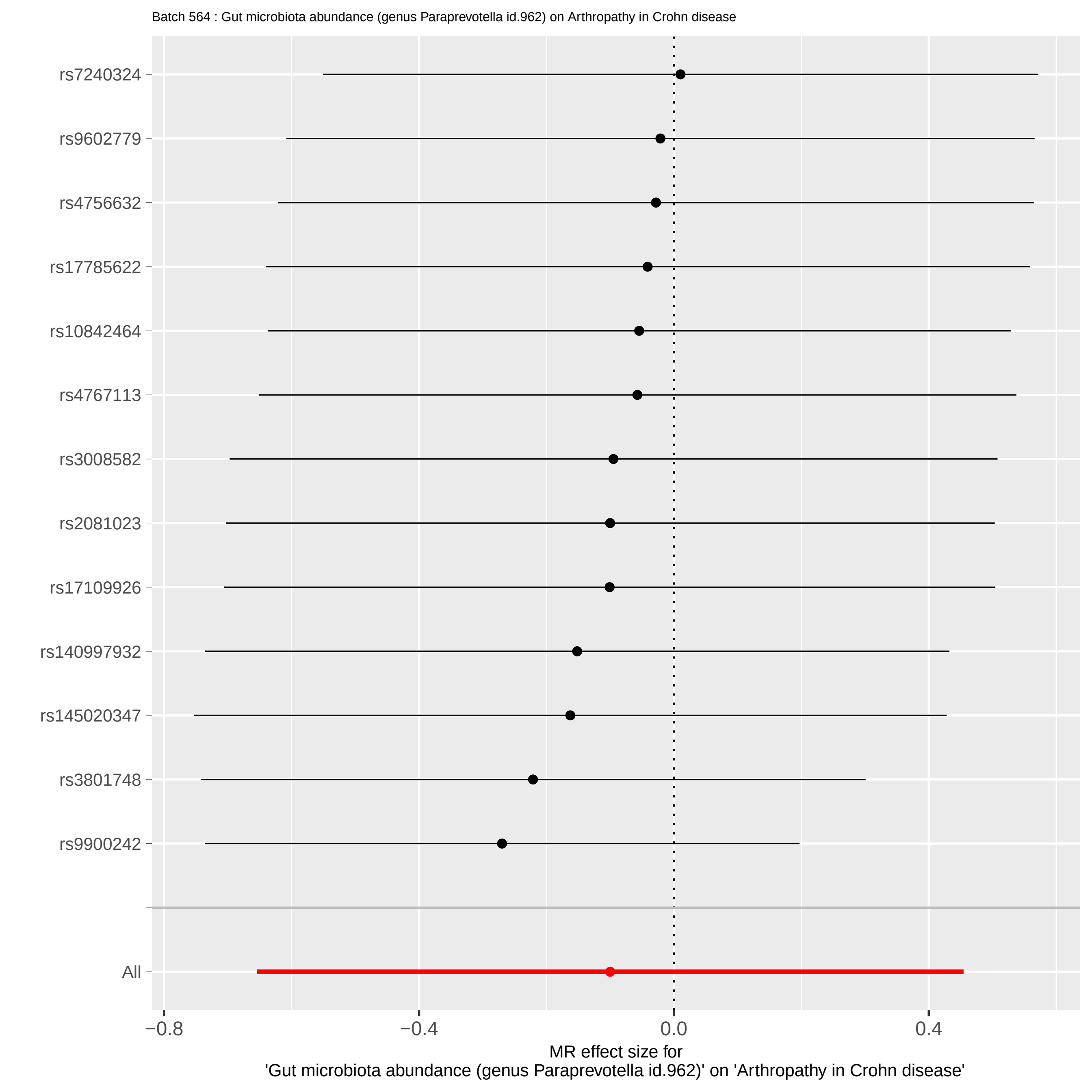

Batch 564 : Gut microbiota abundance (genus Paraprevotella id.962) on Arthropathy in Crohn disease
rs7240324
rs9602779
rs4756632
rs17785622
rs10842464
rs4767113
rs3008582
rs2081023
rs17109926
rs140997932
rs145020347
rs3801748
rs9900242
All
−0.8
−0.4
0.0
0.4
MR effect size for
'Gut microbiota abundance (genus Paraprevotella id.962)' on 'Arthropathy in Crohn disease'

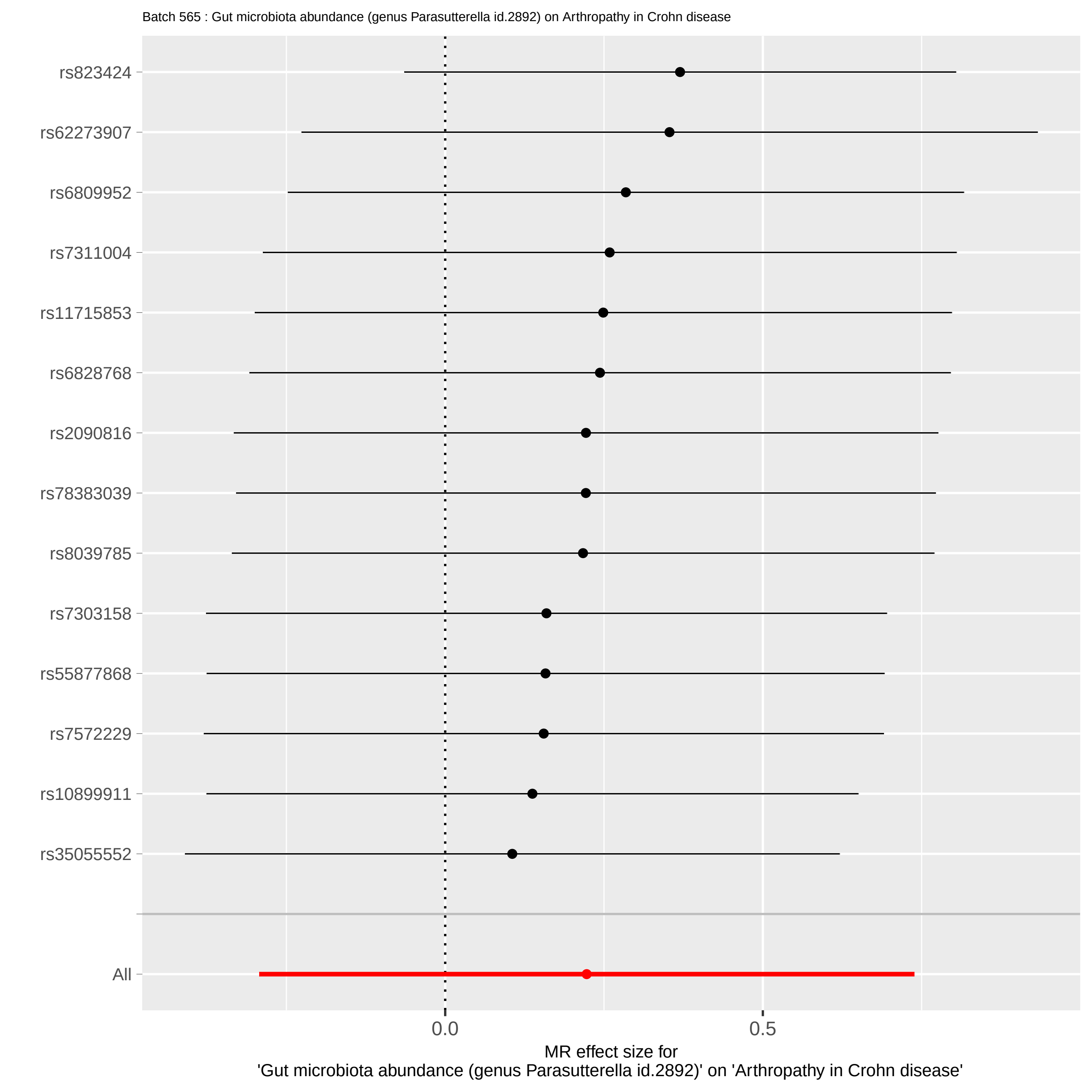

Batch 565 : Gut microbiota abundance (genus Parasutterella id.2892) on Arthropathy in Crohn disease
rs823424
rs62273907
rs6809952
rs7311004
rs11715853
rs6828768
rs2090816
rs78383039
rs8039785
rs7303158
rs55877868
rs7572229
rs10899911
rs35055552
All
0.0
0.5
MR effect size for
'Gut microbiota abundance (genus Parasutterella id.2892)' on 'Arthropathy in Crohn disease'

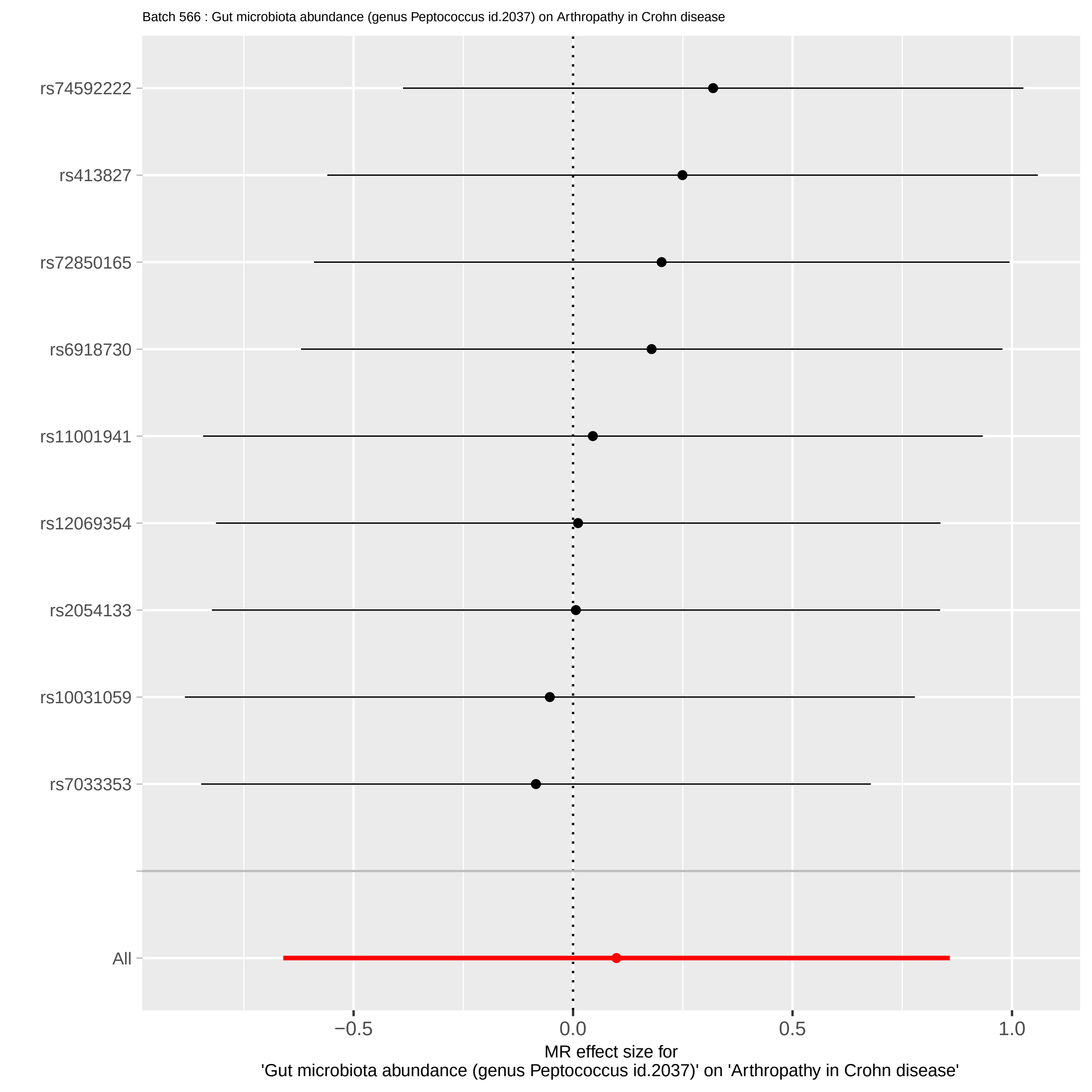

Batch 566 : Gut microbiota abundance (genus Peptococcus id.2037) on Arthropathy in Crohn disease
rs74592222
rs413827
rs72850165
rs6918730
rs11001941
rs12069354
rs2054133
rs10031059
rs7033353
All
−0.5
0.0
0.5
1.0
MR effect size for
'Gut microbiota abundance (genus Peptococcus id.2037)' on 'Arthropathy in Crohn disease'

Batch 567 : Gut microbiota abundance (genus Phascolarctobacterium id.2168) on Arthropathy in Crohn disease

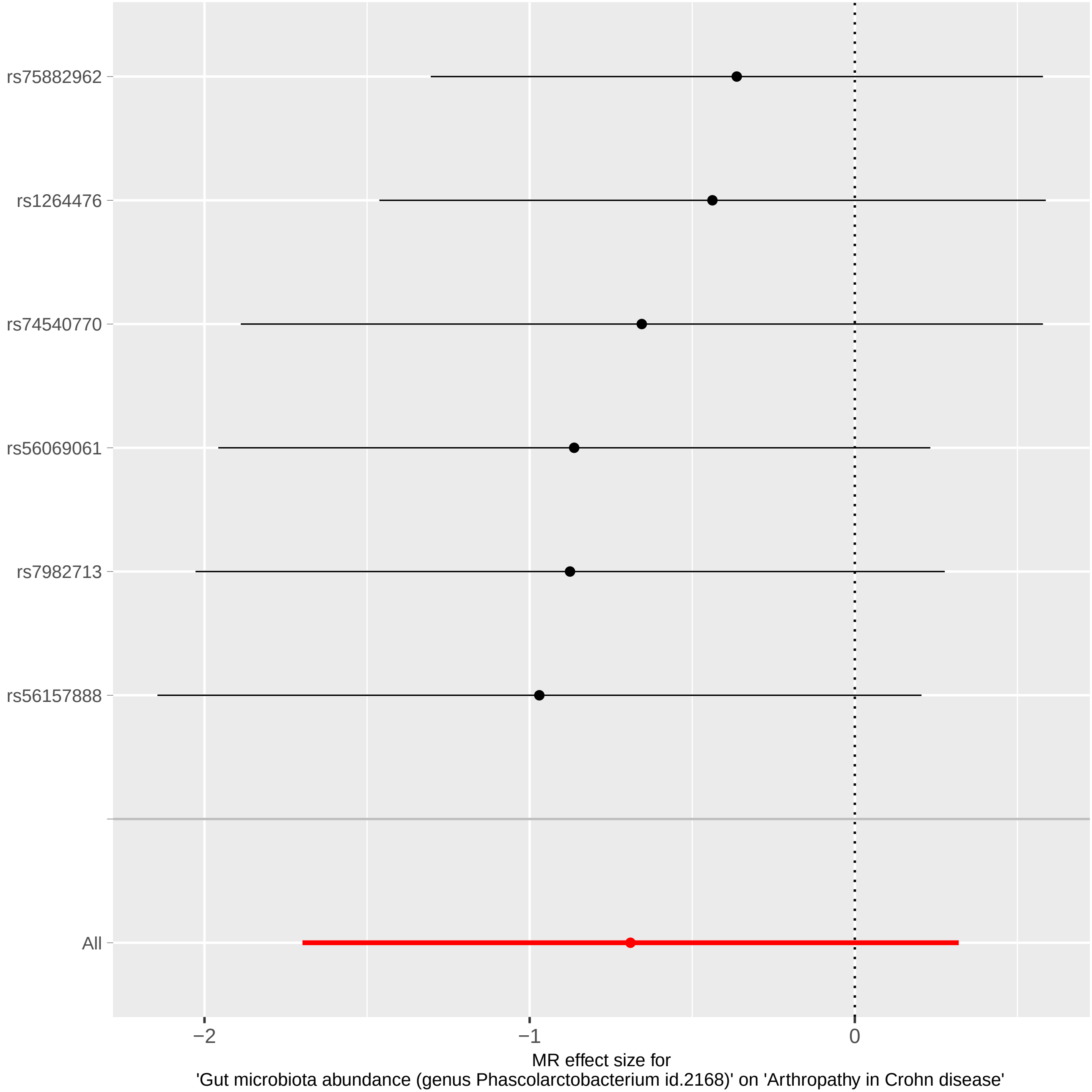

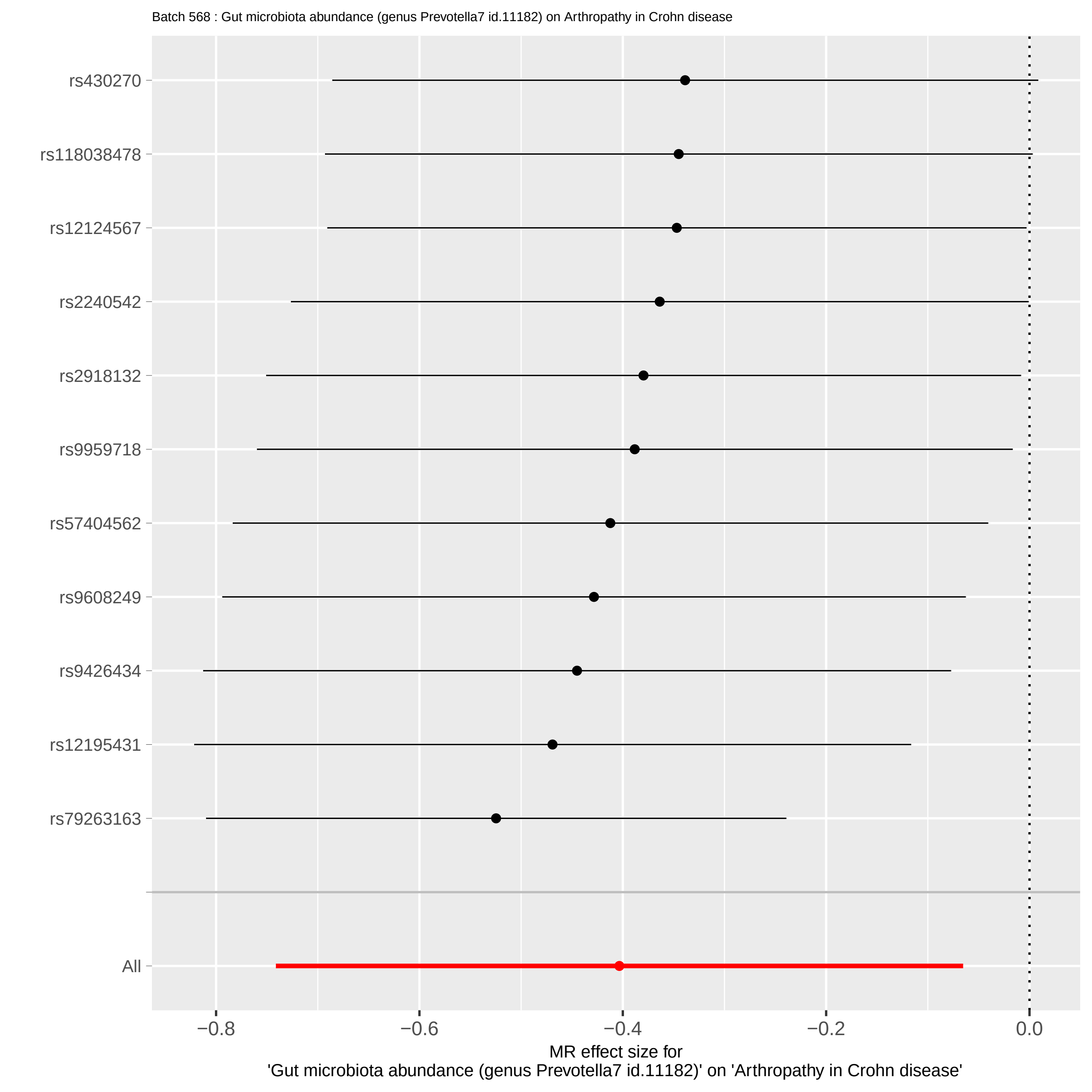

Batch 568 : Gut microbiota abundance (genus Prevotella7 id.11182) on Arthropathy in Crohn disease
rs430270
rs118038478
rs12124567
rs2240542
rs2918132
rs9959718
rs57404562
rs9608249
rs9426434
rs12195431
rs79263163
All
−0.8
−0.6
−0.4
−0.2
0.0
MR effect size for
'Gut microbiota abundance (genus Prevotella7 id.11182)' on 'Arthropathy in Crohn disease'

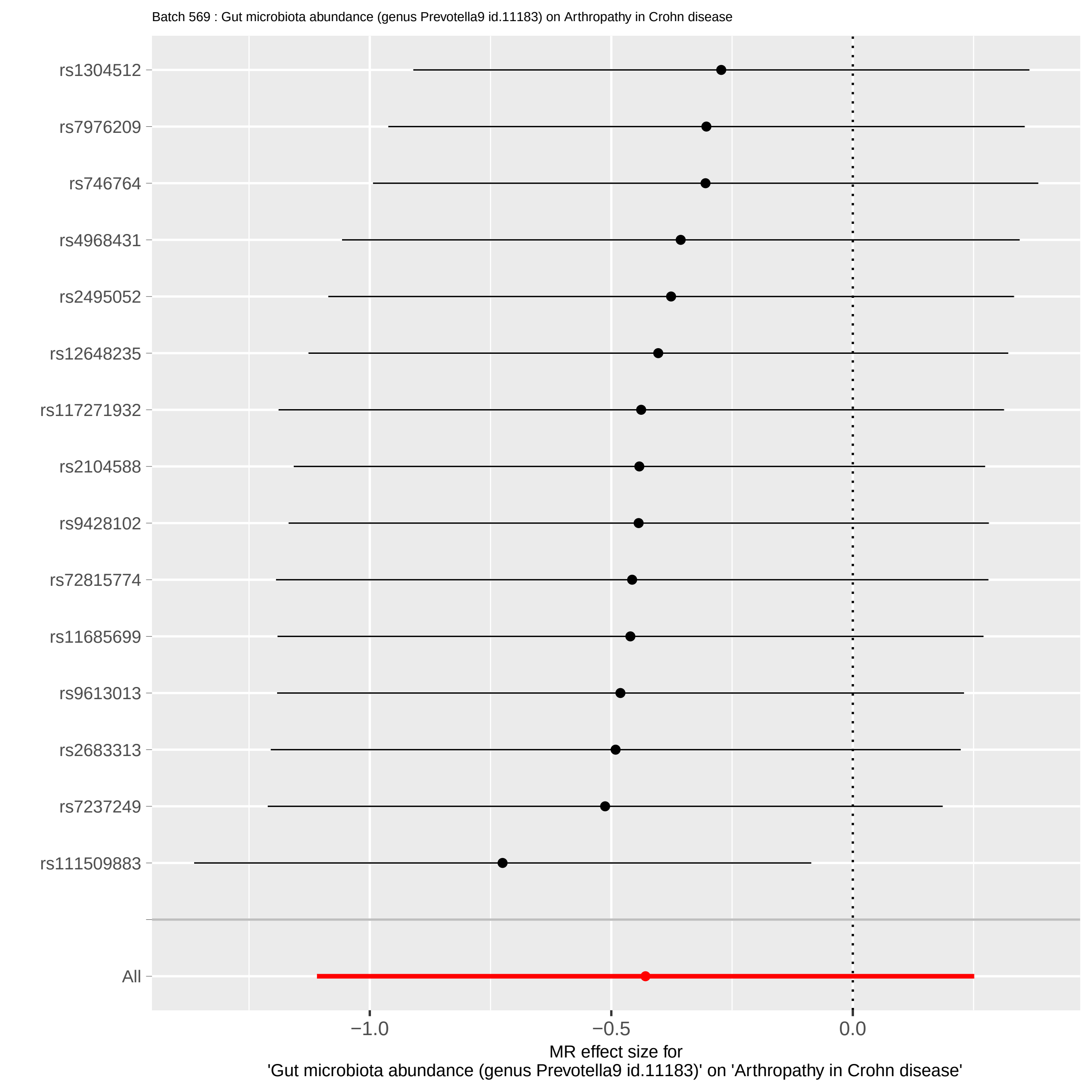

Batch 569 : Gut microbiota abundance (genus Prevotella9 id.11183) on Arthropathy in Crohn disease
rs1304512
rs7976209
rs746764
rs4968431
rs2495052
rs12648235
rs117271932
rs2104588
rs9428102
rs72815774
rs11685699
rs9613013
rs2683313
rs7237249
rs111509883
All
−1.0
−0.5
0.0
MR effect size for
'Gut microbiota abundance (genus Prevotella9 id.11183)' on 'Arthropathy in Crohn disease'

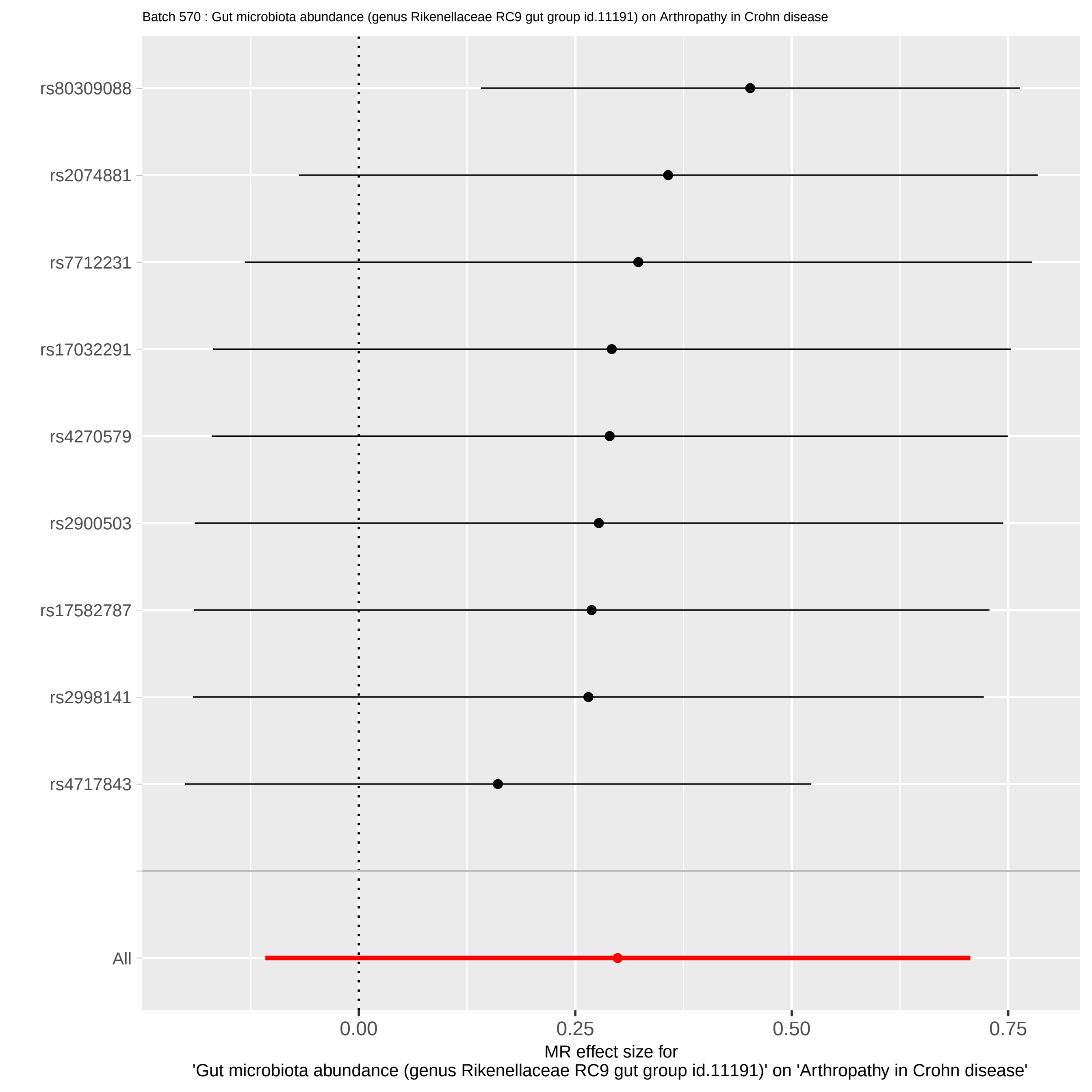

Batch 570 : Gut microbiota abundance (genus Rikenellaceae RC9 gut group id.11191) on Arthropathy in Crohn disease
rs80309088
rs2074881
rs7712231
rs17032291
rs4270579
rs2900503
rs17582787
rs2998141
rs4717843
All
0.00
0.25
0.50
0.75
MR effect size for
'Gut microbiota abundance (genus Rikenellaceae RC9 gut group id.11191)' on 'Arthropathy in Crohn disease'

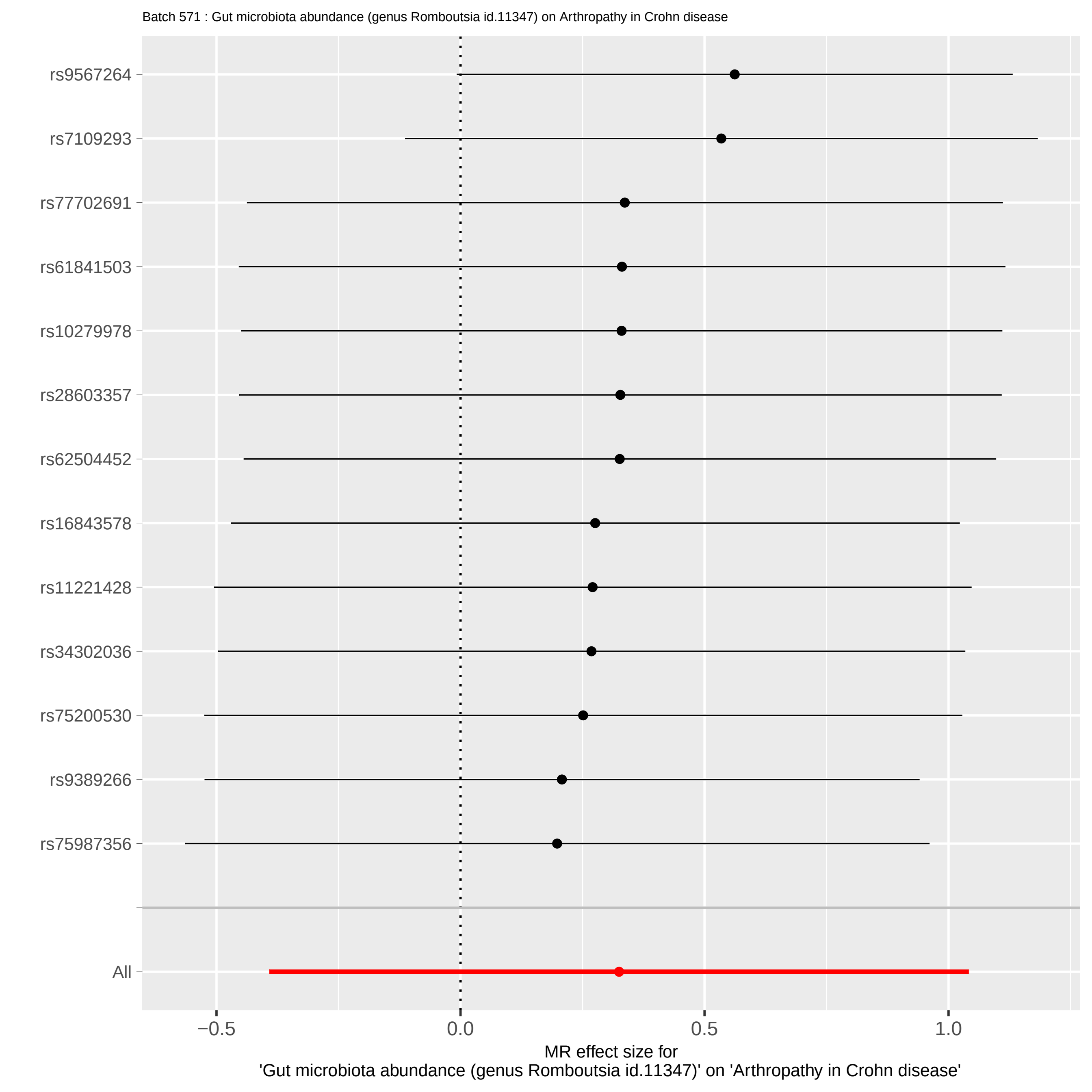
Batch 571 : Gut microbiota abundance (genus Romboutsia id.11347) on Arthropathy in Crohn disease
rs9567264
rs7109293
rs77702691
rs61841503
rs10279978
rs28603357
rs62504452
rs16843578
rs11221428
rs34302036
rs75200530
rs9389266
rs75987356
All
−0.5
0.0
0.5
1.0
MR effect size for
'Gut microbiota abundance (genus Romboutsia id.11347)' on 'Arthropathy in Crohn disease'

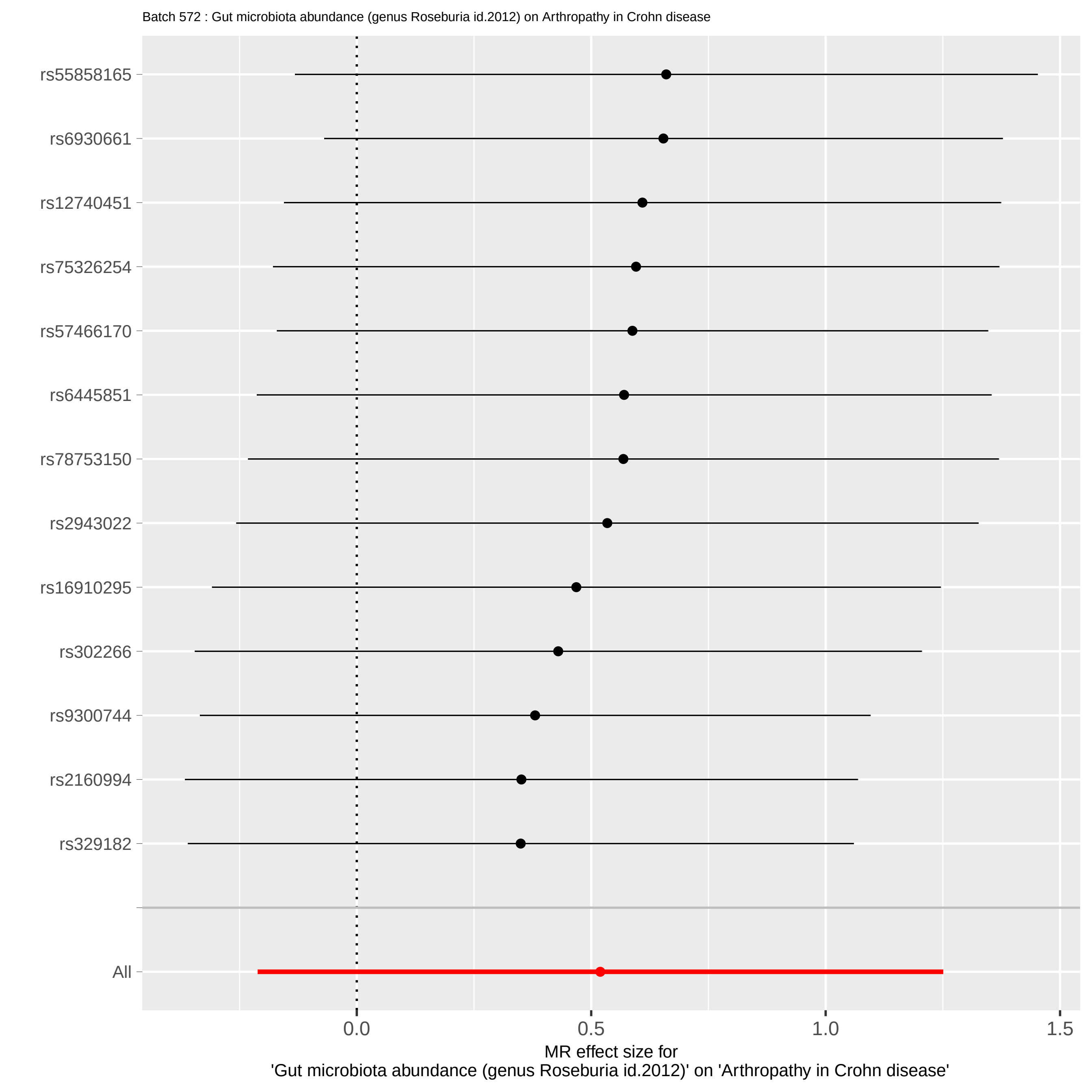
Batch 572 : Gut microbiota abundance (genus Roseburia id.2012) on Arthropathy in Crohn disease
rs55858165
rs6930661
rs12740451
rs75326254
rs57466170
rs6445851
rs78753150
rs2943022
rs16910295
rs302266
rs9300744
rs2160994
rs329182
All
0.0
0.5
1.0
1.5
MR effect size for
'Gut microbiota abundance (genus Roseburia id.2012)' on 'Arthropathy in Crohn disease'

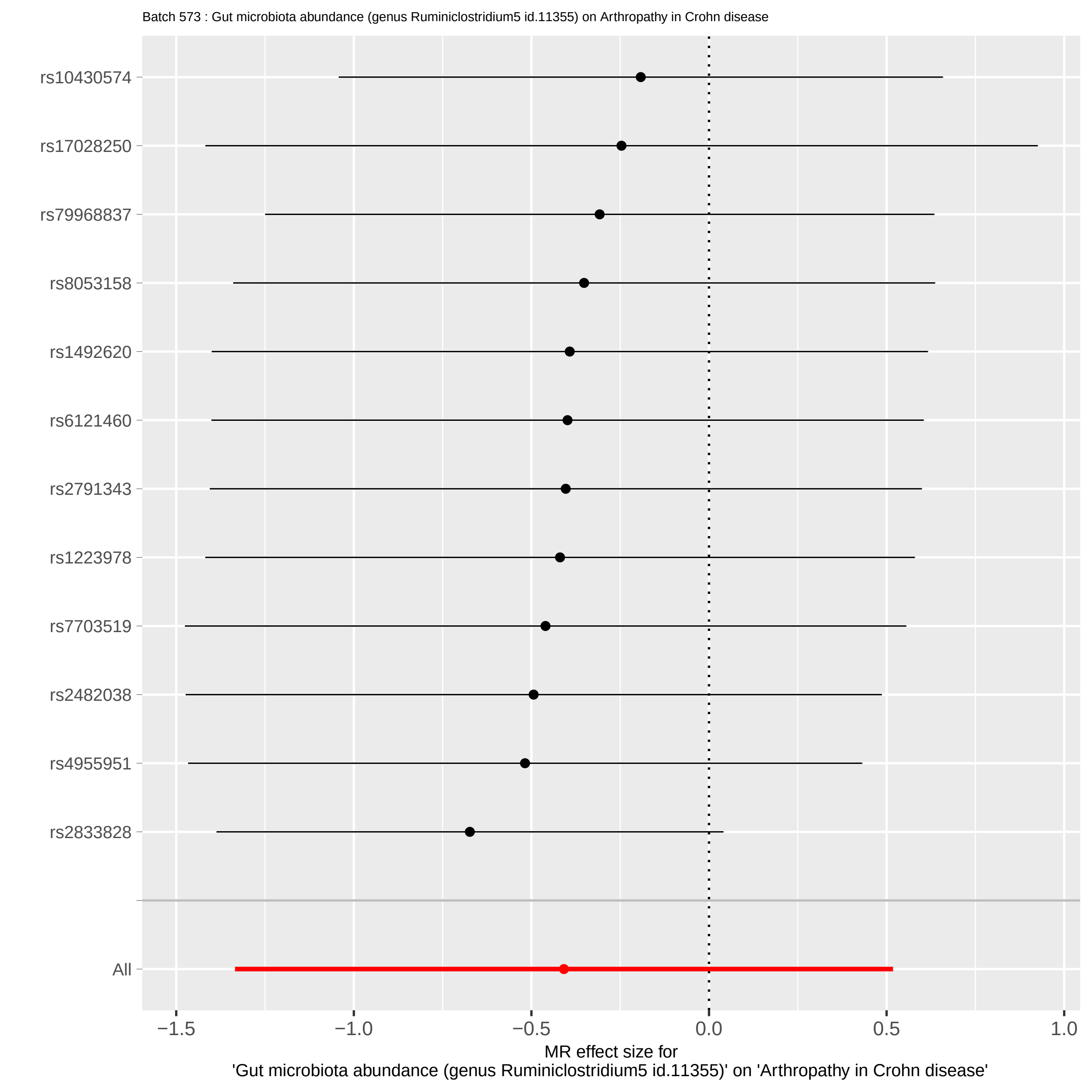
Batch 573 : Gut microbiota abundance (genus Ruminiclostridium5 id.11355) on Arthropathy in Crohn disease
rs10430574
rs17028250
rs79968837
rs8053158
rs1492620
rs6121460
rs2791343
rs1223978
rs7703519
rs2482038
rs4955951
rs2833828
All
−1.5
−1.0
−0.5
0.0
0.5
1.0
MR effect size for
'Gut microbiota abundance (genus Ruminiclostridium5 id.11355)' on 'Arthropathy in Crohn disease'

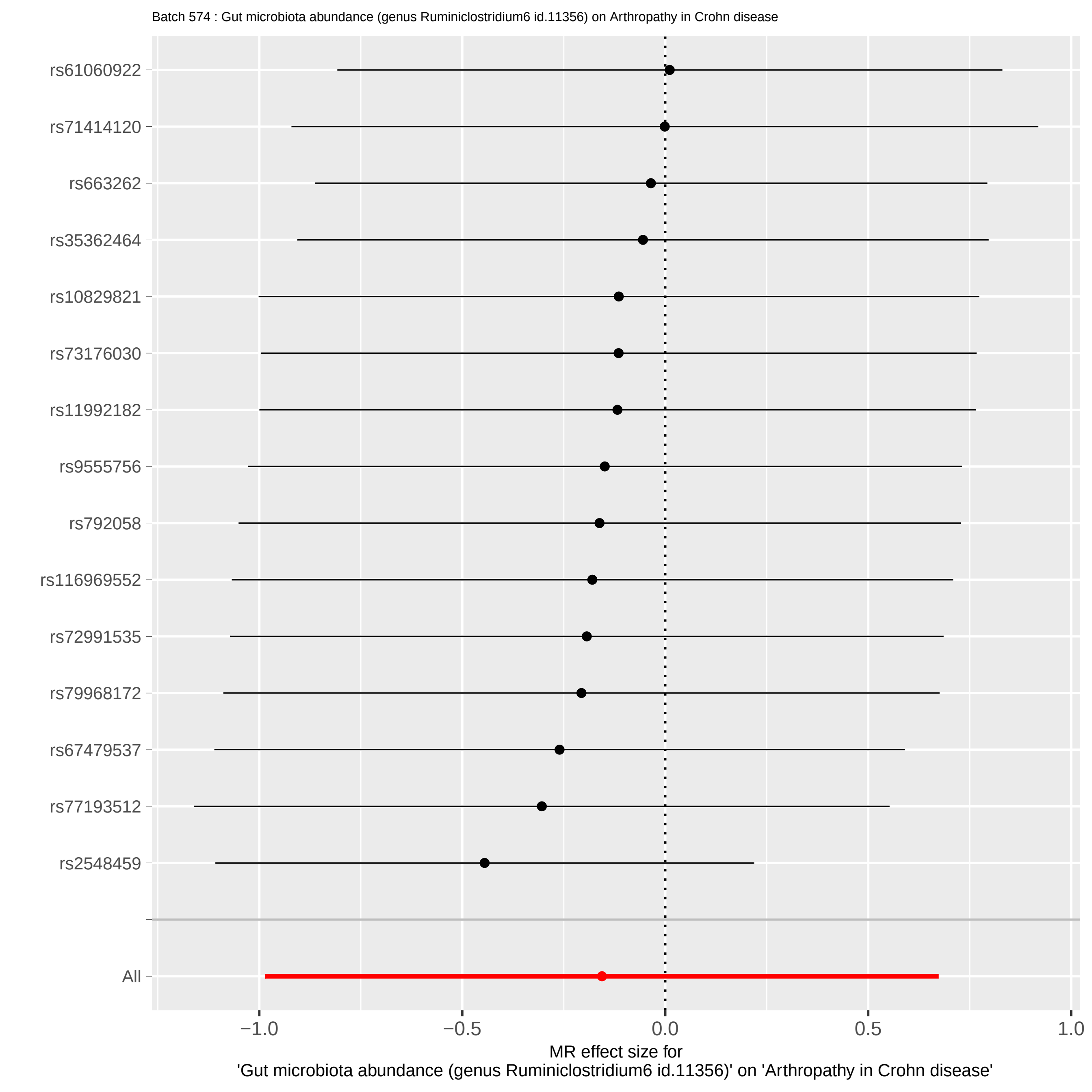
Batch 574 : Gut microbiota abundance (genus Ruminiclostridium6 id.11356) on Arthropathy in Crohn disease
rs61060922
rs71414120
rs663262
rs35362464
rs10829821
rs73176030
rs11992182
rs9555756
rs792058
rs116969552
rs72991535
rs79968172
rs67479537
rs77193512
rs2548459
All
−1.0
−0.5
0.0
0.5
1.0
MR effect size for
'Gut microbiota abundance (genus Ruminiclostridium6 id.11356)' on 'Arthropathy in Crohn disease'

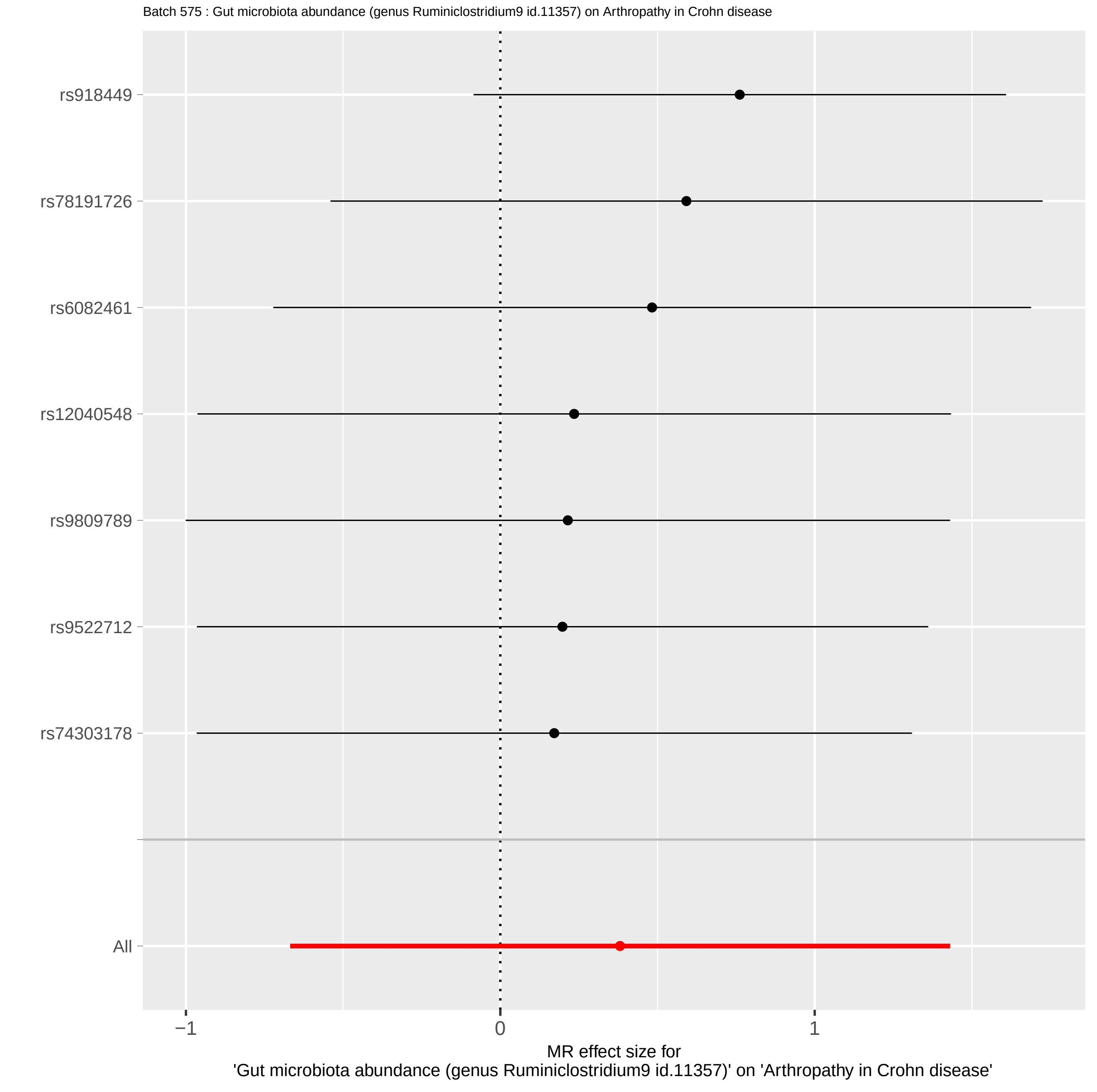

Batch 575 : Gut microbiota abundance (genus Ruminiclostridium9 id.11357) on Arthropathy in Crohn disease
rs918449
rs78191726
rs6082461
rs12040548
rs9809789
rs9522712
rs74303178
All
−1
0
1
MR effect size for
'Gut microbiota abundance (genus Ruminiclostridium9 id.11357)' on 'Arthropathy in Crohn disease'

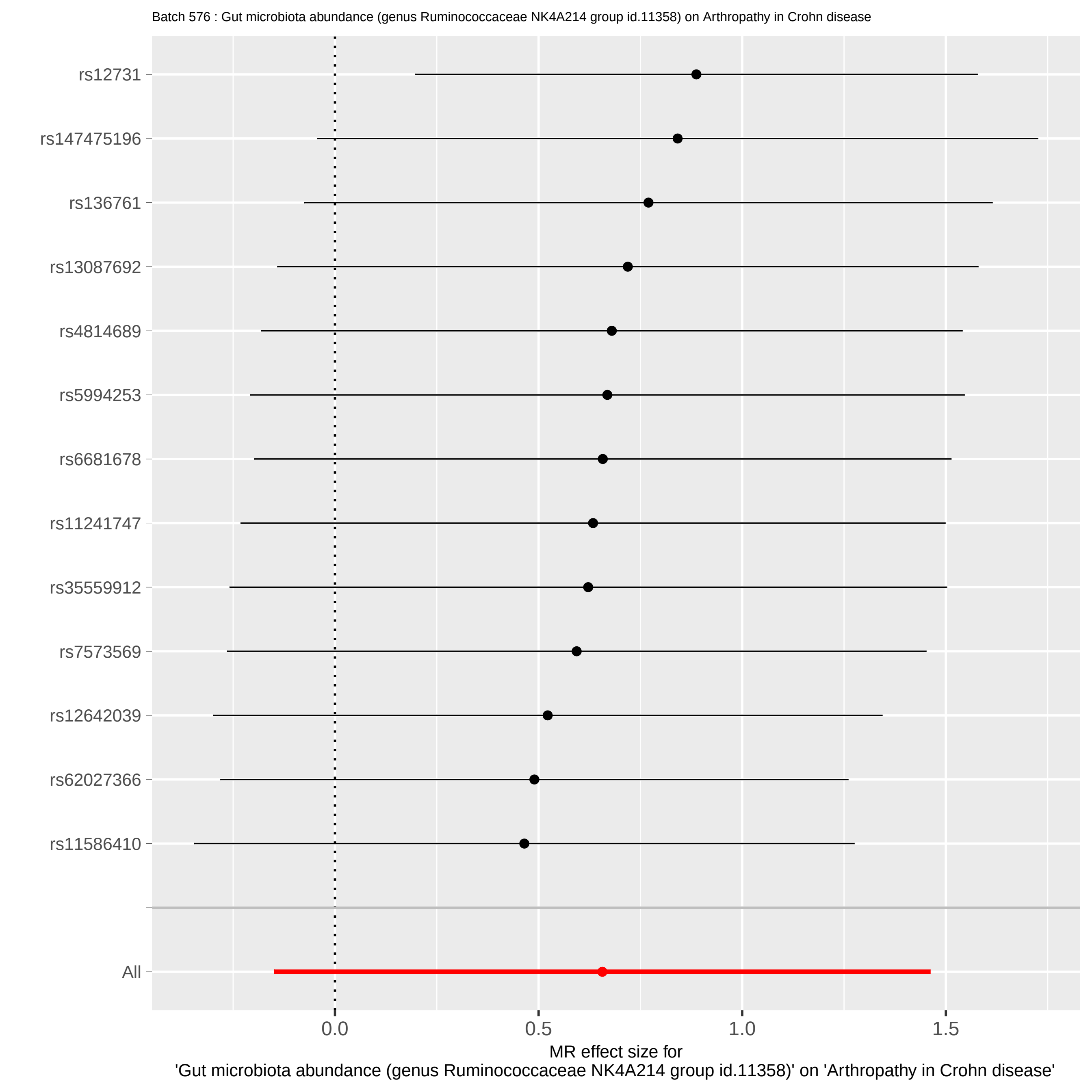
Batch 576 : Gut microbiota abundance (genus Ruminococcaceae NK4A214 group id.11358) on Arthropathy in Crohn disease
rs12731
rs147475196
rs136761
rs13087692
rs4814689
rs5994253
rs6681678
rs11241747
rs35559912
rs7573569
rs12642039
rs62027366
rs11586410
All
0.0
0.5
1.0
1.5
MR effect size for
'Gut microbiota abundance (genus Ruminococcaceae NK4A214 group id.11358)' on 'Arthropathy in Crohn disease'

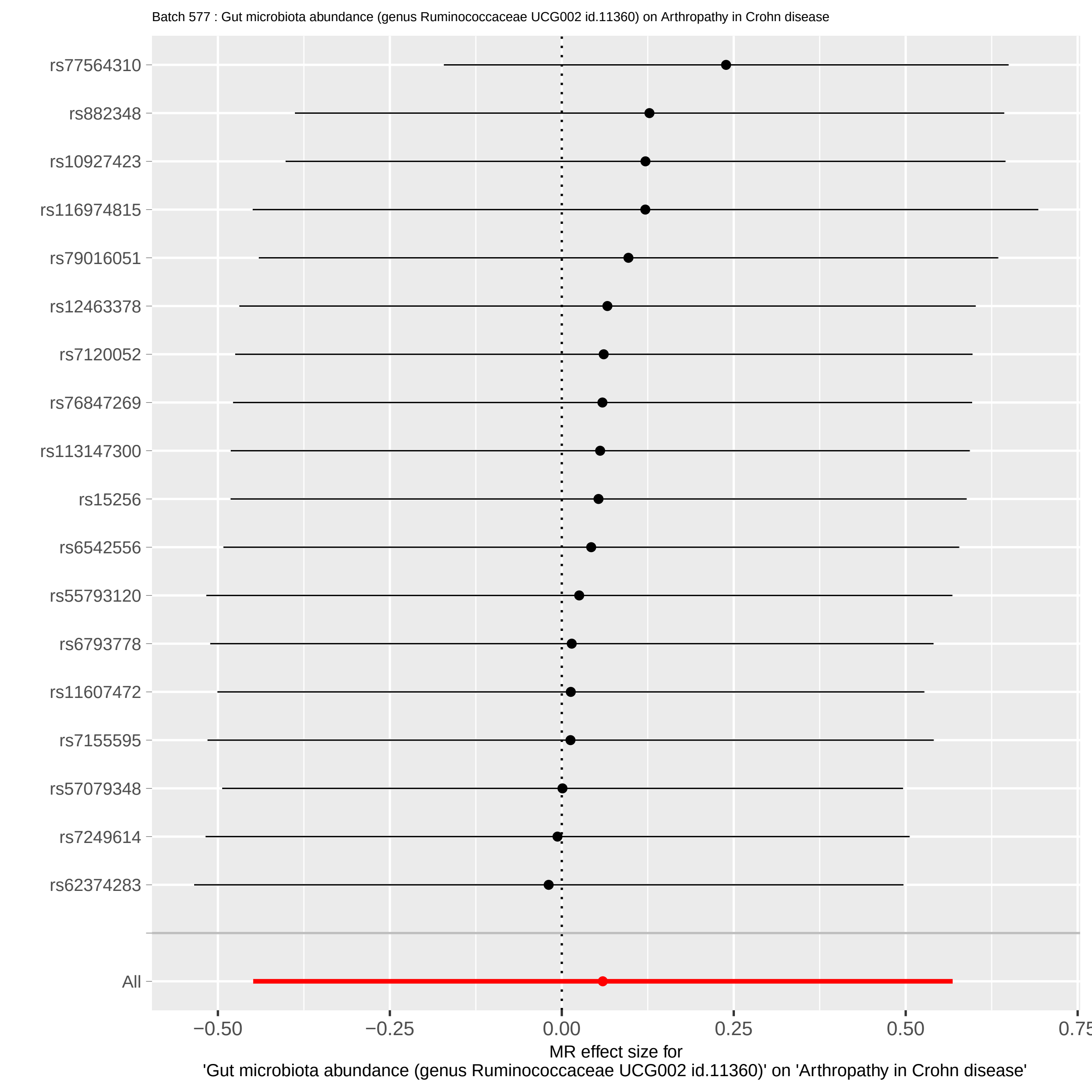
Batch 577 : Gut microbiota abundance (genus Ruminococcaceae UCG002 id.11360) on Arthropathy in Crohn disease
rs77564310
rs882348
rs10927423
rs116974815
rs79016051
rs12463378
rs7120052
rs76847269
rs113147300
rs15256
rs6542556
rs55793120
rs6793778
rs11607472
rs7155595
rs57079348
rs7249614
rs62374283
All
−0.50
−0.25
0.00
0.25
0.50
MR effect size for
'Gut microbiota abundance (genus Ruminococcaceae UCG002 id.11360)' on 'Arthropathy in Crohn disease'

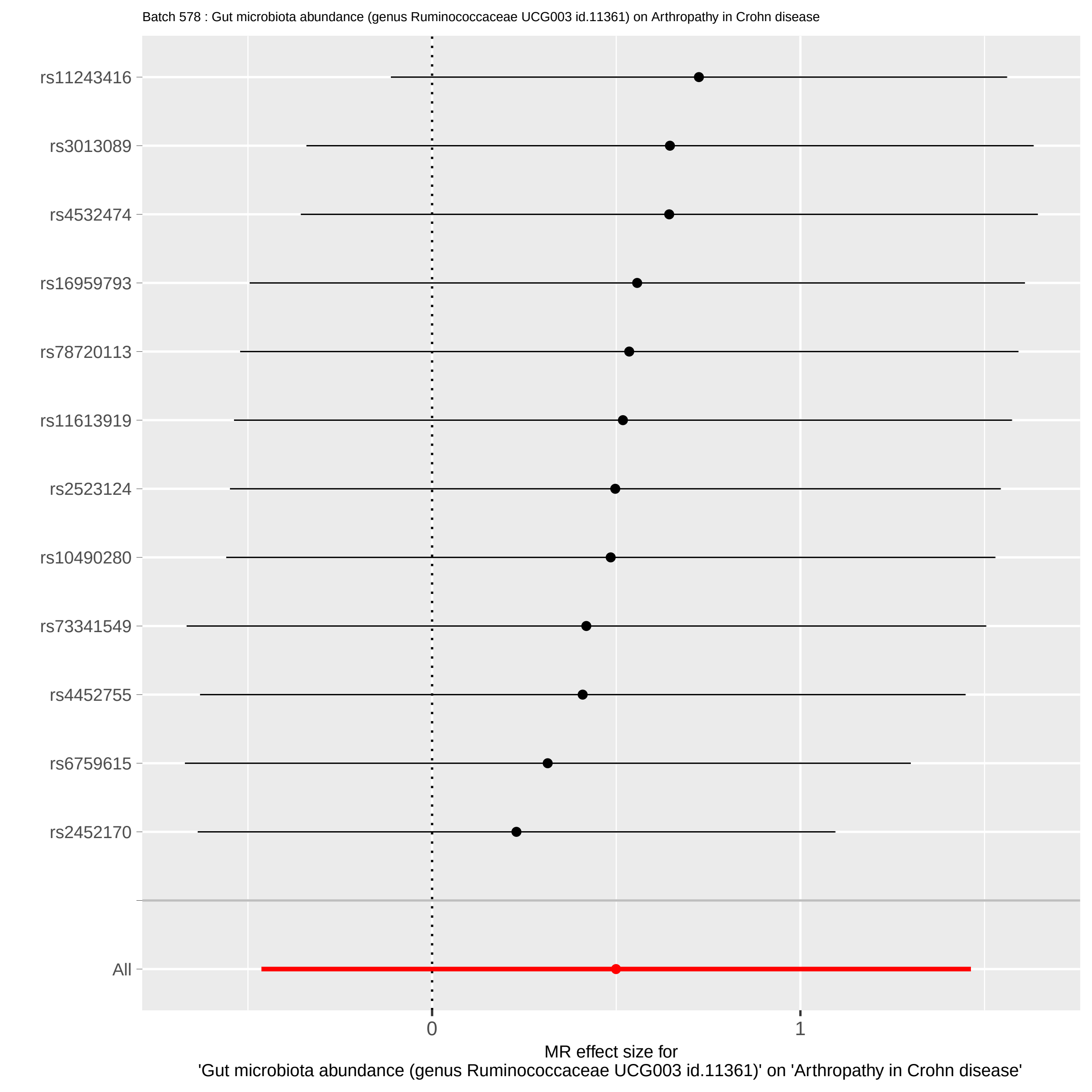
Batch 578 : Gut microbiota abundance (genus Ruminococcaceae UCG003 id.11361) on Arthropathy in Crohn disease
rs11243416
rs3013089
rs4532474
rs16959793
rs78720113
rs11613919
rs2523124
rs10490280
rs73341549
rs4452755
rs6759615
rs2452170
All
0
1
MR effect size for
'Gut microbiota abundance (genus Ruminococcaceae UCG003 id.11361)' on 'Arthropathy in Crohn disease'

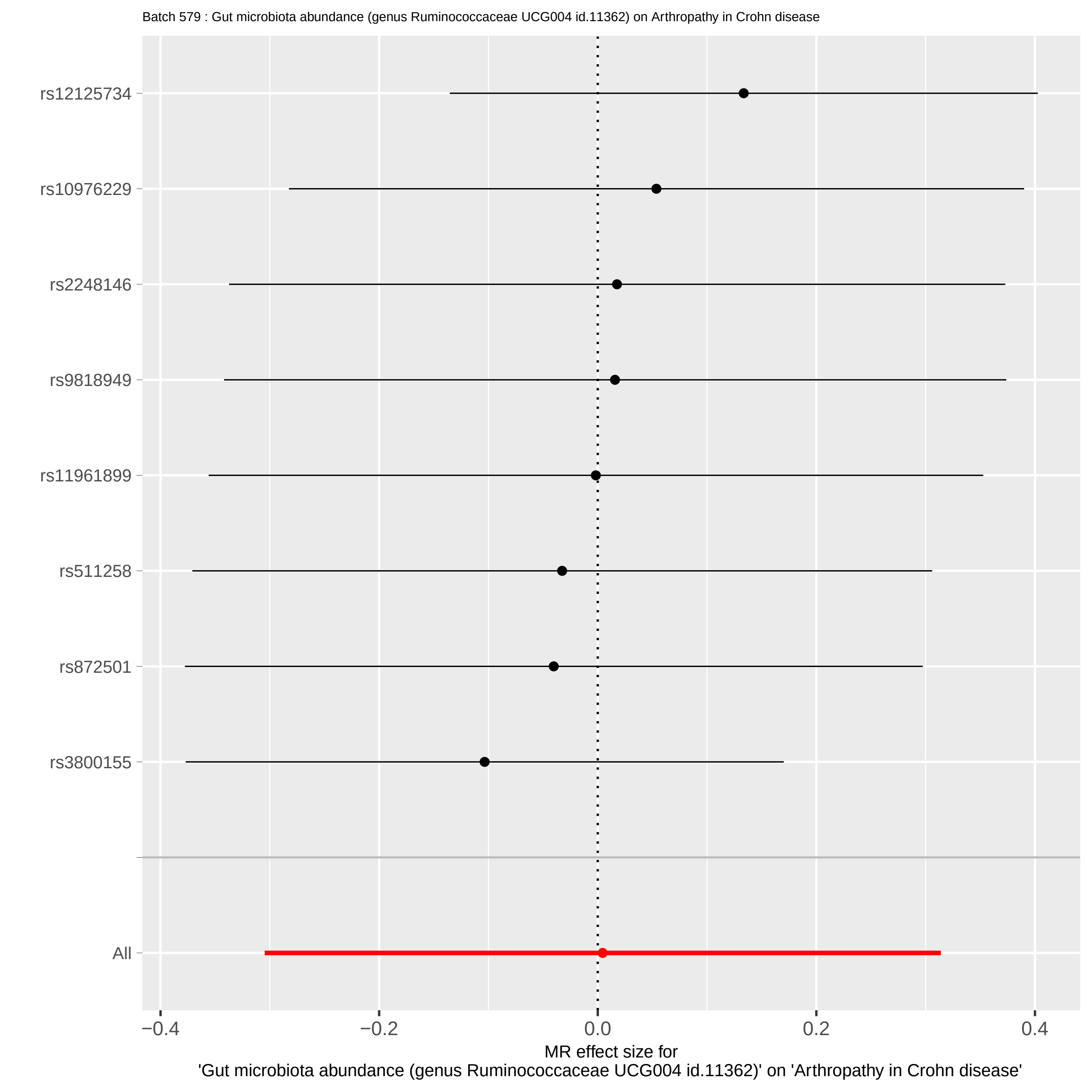

Batch 579 : Gut microbiota abundance (genus Ruminococcaceae UCG004 id.11362) on Arthropathy in Crohn disease
rs12125734
rs10976229
rs2248146
rs9818949
rs11961899
rs511258
rs872501
rs3800155
All
−0.4
−0.2
0.0
0.2
0.4
MR effect size for
'Gut microbiota abundance (genus Ruminococcaceae UCG004 id.11362)' on 'Arthropathy in Crohn disease'

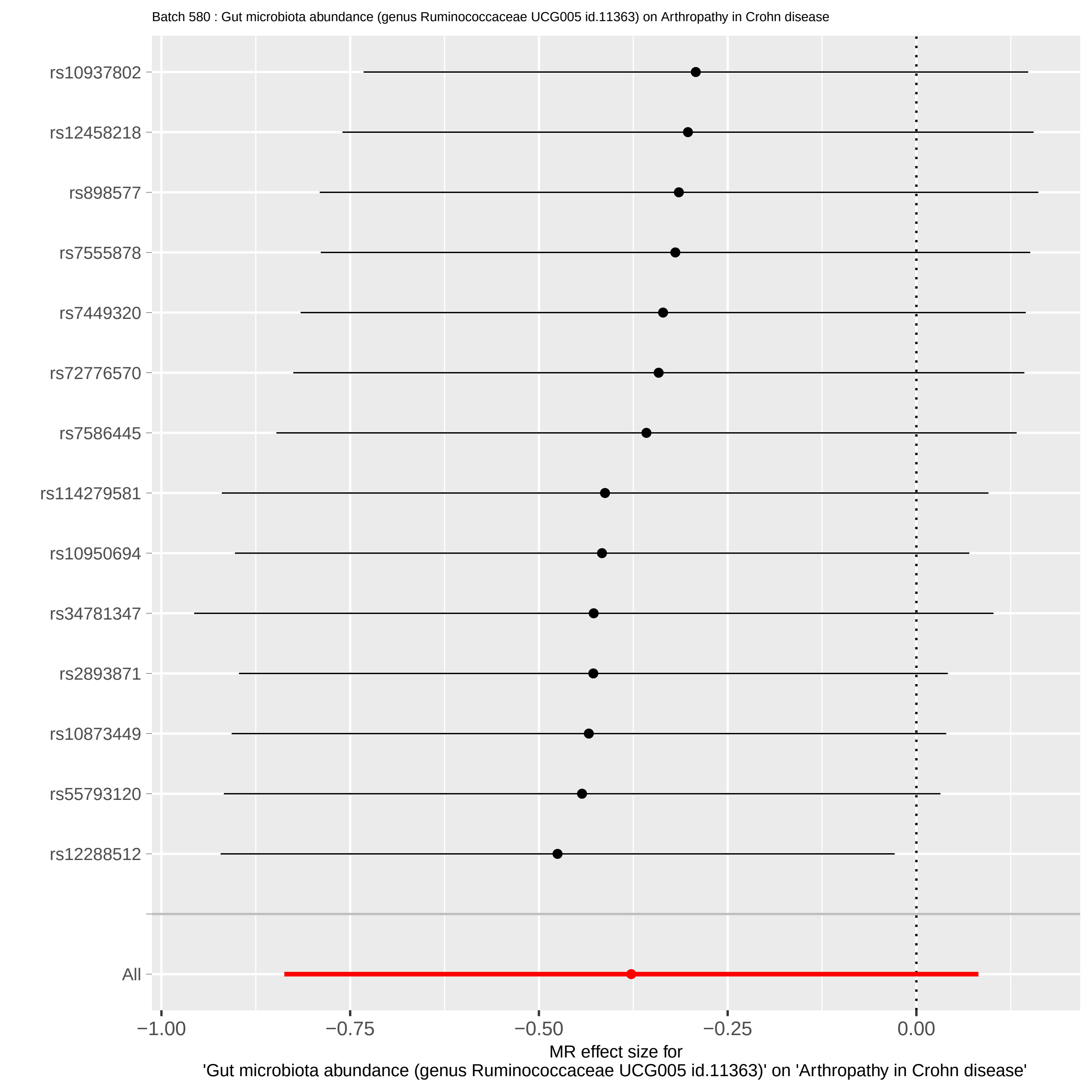

Batch 580 : Gut microbiota abundance (genus Ruminococcaceae UCG005 id.11363) on Arthropathy in Crohn disease
rs10937802
rs12458218
rs898577
rs7555878
rs7449320
rs72776570
rs7586445
rs114279581
rs10950694
rs34781347
rs2893871
rs10873449
rs55793120
rs12288512
All
−1.00
−0.75
−0.50
−0.25
0.00
MR effect size for
'Gut microbiota abundance (genus Ruminococcaceae UCG005 id.11363)' on 'Arthropathy in Crohn disease'

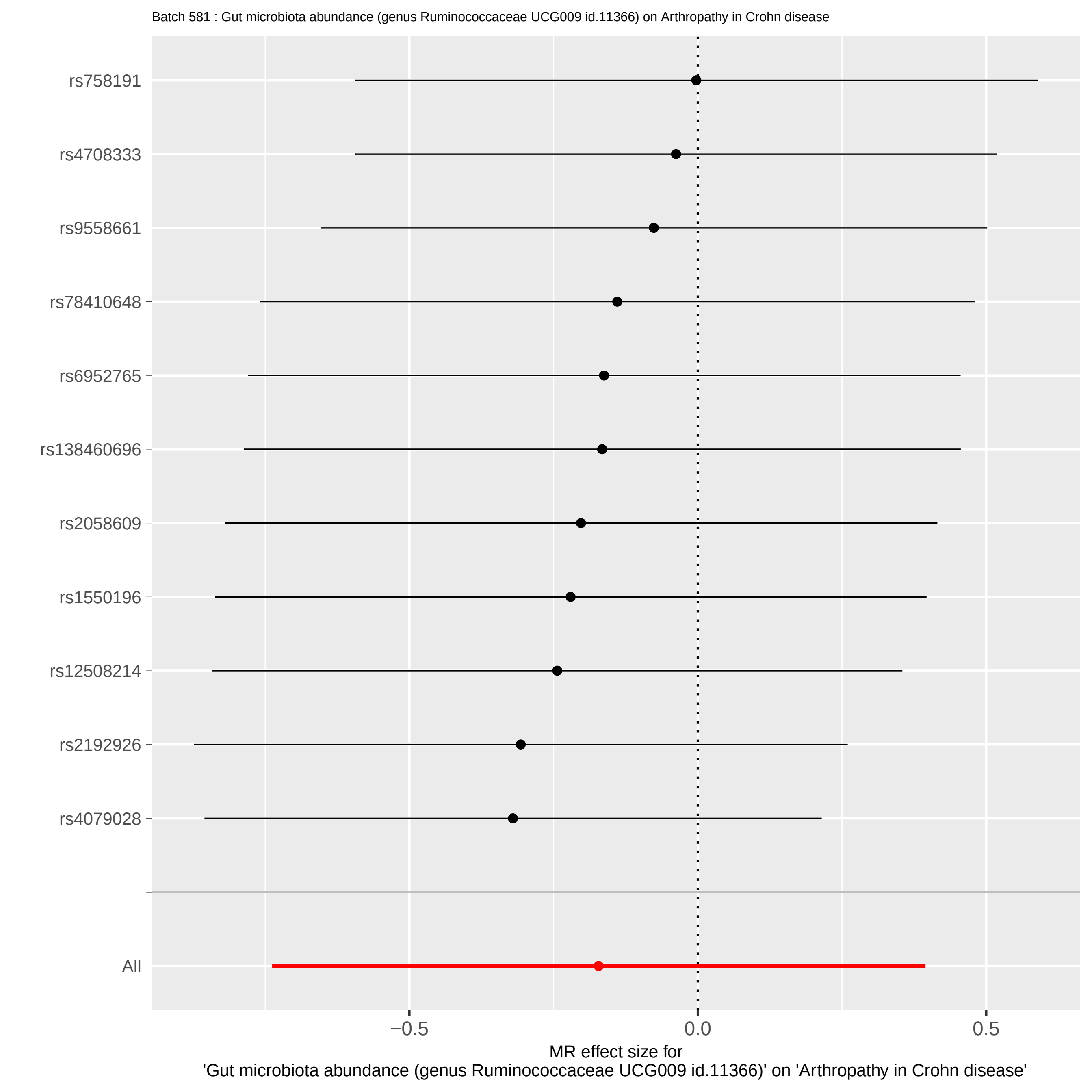

Batch 581 : Gut microbiota abundance (genus Ruminococcaceae UCG009 id.11366) on Arthropathy in Crohn disease
rs758191
rs4708333
rs9558661
rs78410648
rs6952765
rs138460696
rs2058609
rs1550196
rs12508214
rs2192926
rs4079028
All
−0.5
0.0
0.5
MR effect size for
'Gut microbiota abundance (genus Ruminococcaceae UCG009 id.11366)' on 'Arthropathy in Crohn disease'

Batch 582 : Gut microbiota abundance (genus Ruminococcaceae UCG010 id.11367) on Arthropathy in Crohn disease

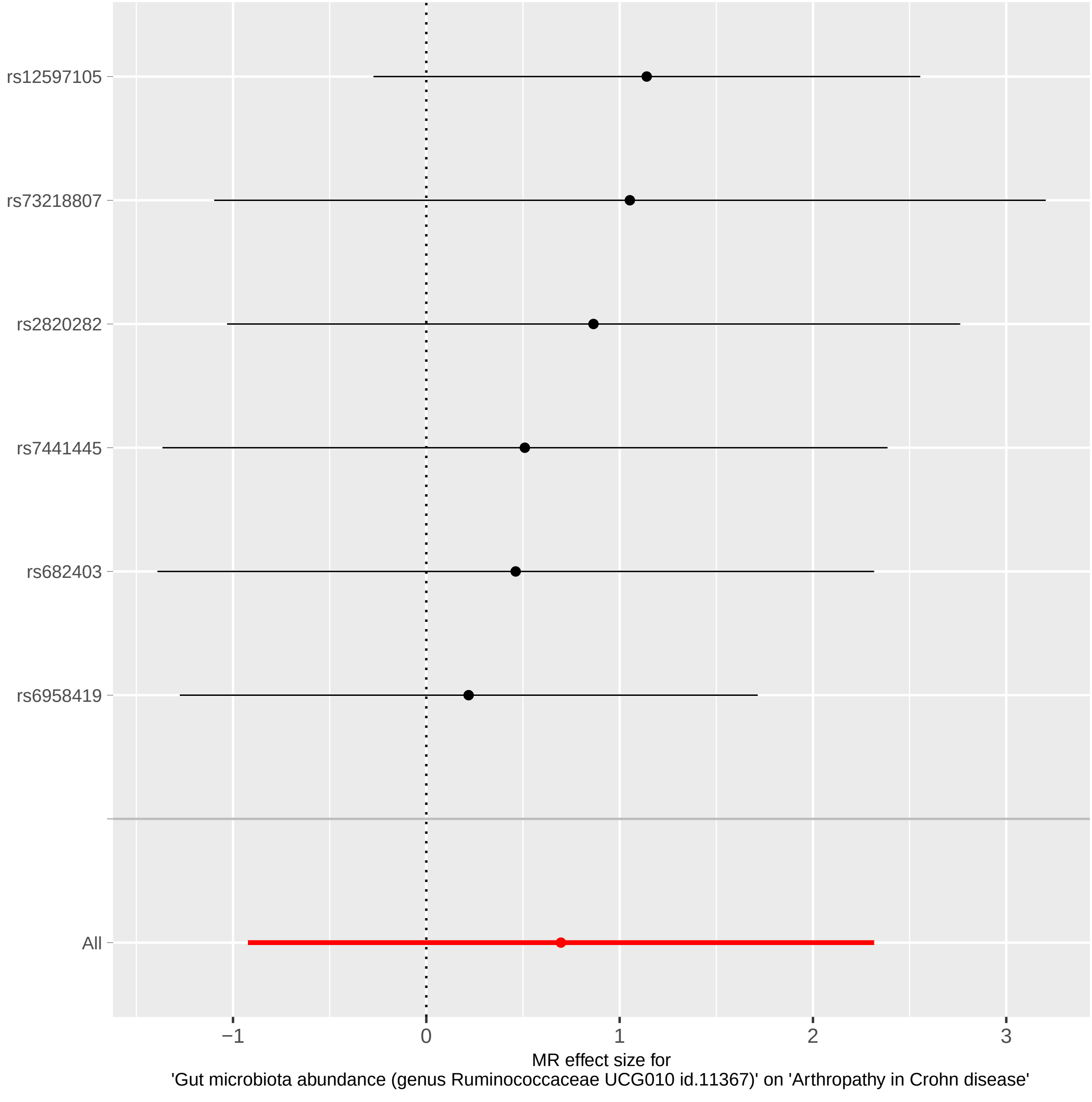

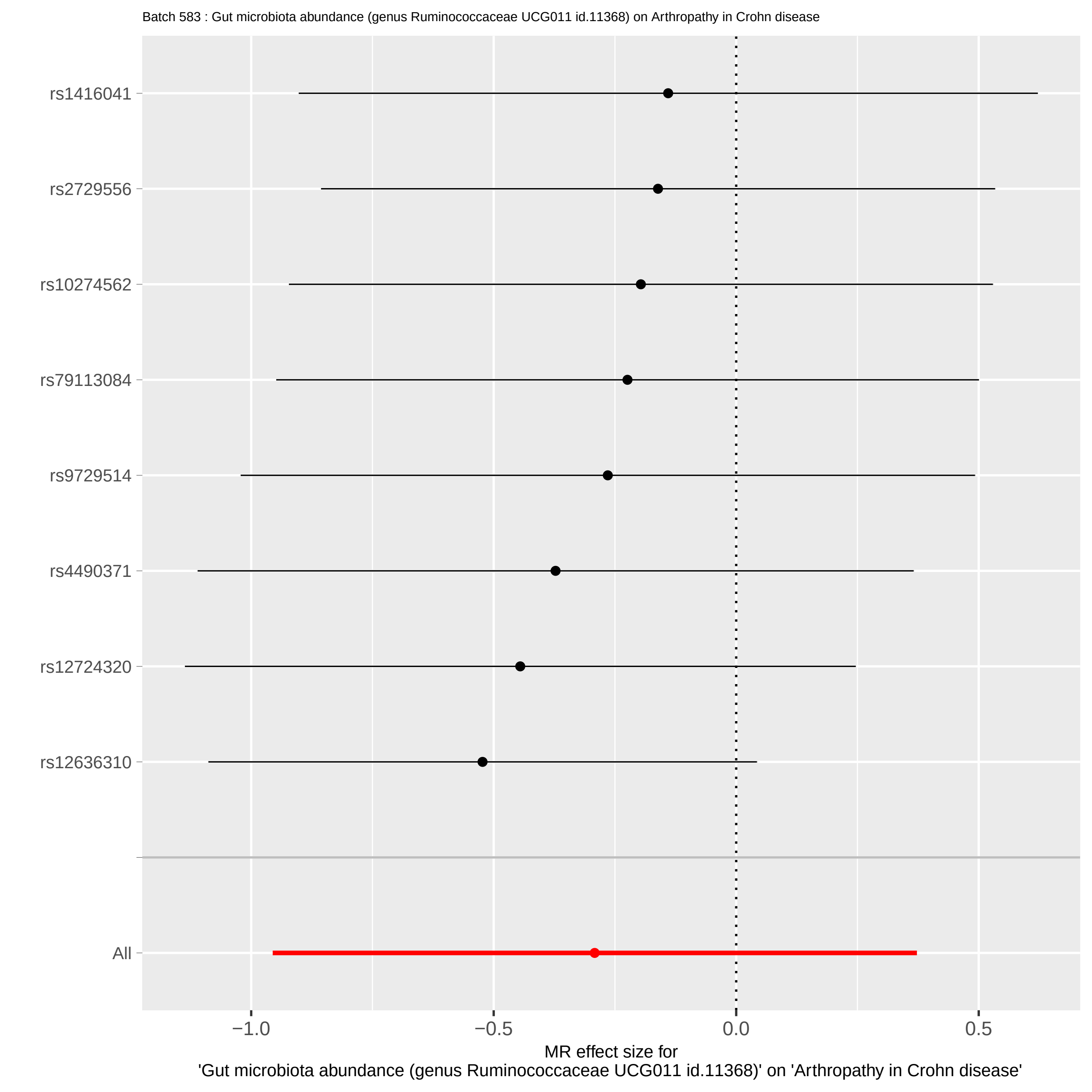
Batch 583 : Gut microbiota abundance (genus Ruminococcaceae UCG011 id.11368) on Arthropathy in Crohn disease
rs1416041
rs2729556
rs10274562
rs79113084
rs9729514
rs4490371
rs12724320
rs12636310
All
−1.0
−0.5
0.0
0.5
MR effect size for
'Gut microbiota abundance (genus Ruminococcaceae UCG011 id.11368)' on 'Arthropathy in Crohn disease'

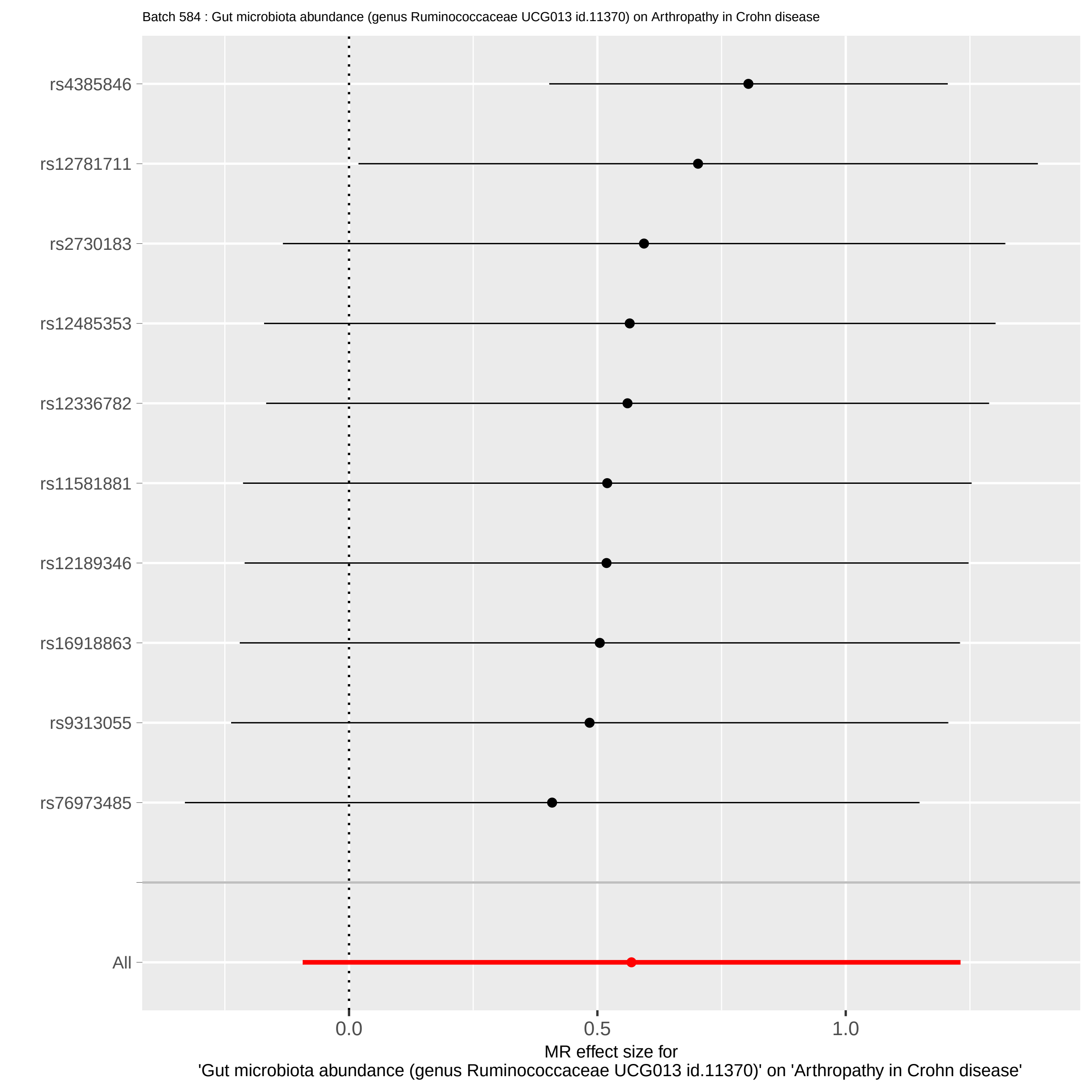

Batch 584 : Gut microbiota abundance (genus Ruminococcaceae UCG013 id.11370) on Arthropathy in Crohn disease
rs4385846
rs12781711
rs2730183
rs12485353
rs12336782
rs11581881
rs12189346
rs16918863
rs9313055
rs76973485
All
0.0
0.5
1.0
MR effect size for
'Gut microbiota abundance (genus Ruminococcaceae UCG013 id.11370)' on 'Arthropathy in Crohn disease'

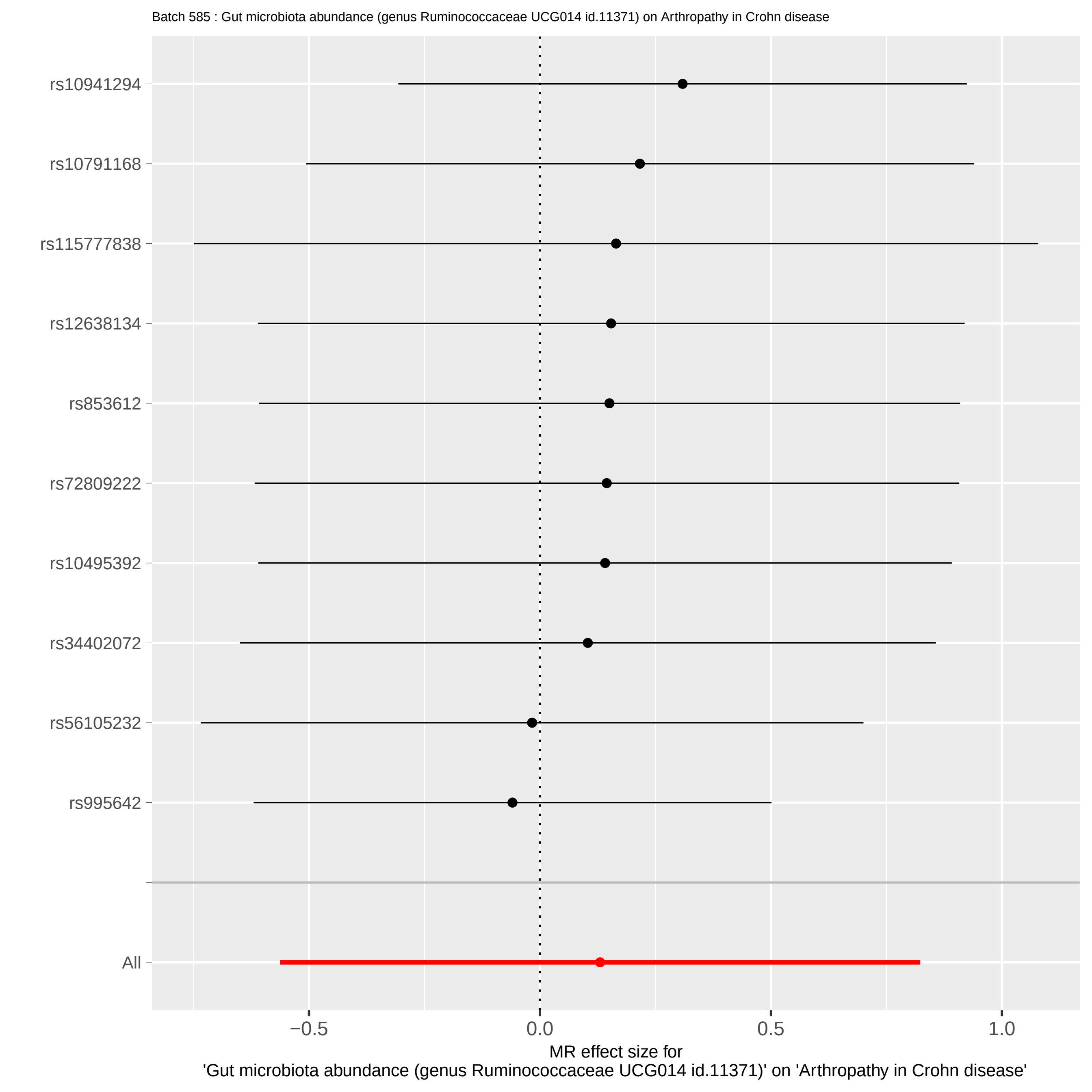

Batch 585 : Gut microbiota abundance (genus Ruminococcaceae UCG014 id.11371) on Arthropathy in Crohn disease
rs10941294
rs10791168
rs115777838
rs12638134
rs853612
rs72809222
rs10495392
rs34402072
rs56105232
rs995642
All
−0.5
0.0
0.5
1.0
MR effect size for
'Gut microbiota abundance (genus Ruminococcaceae UCG014 id.11371)' on 'Arthropathy in Crohn disease'

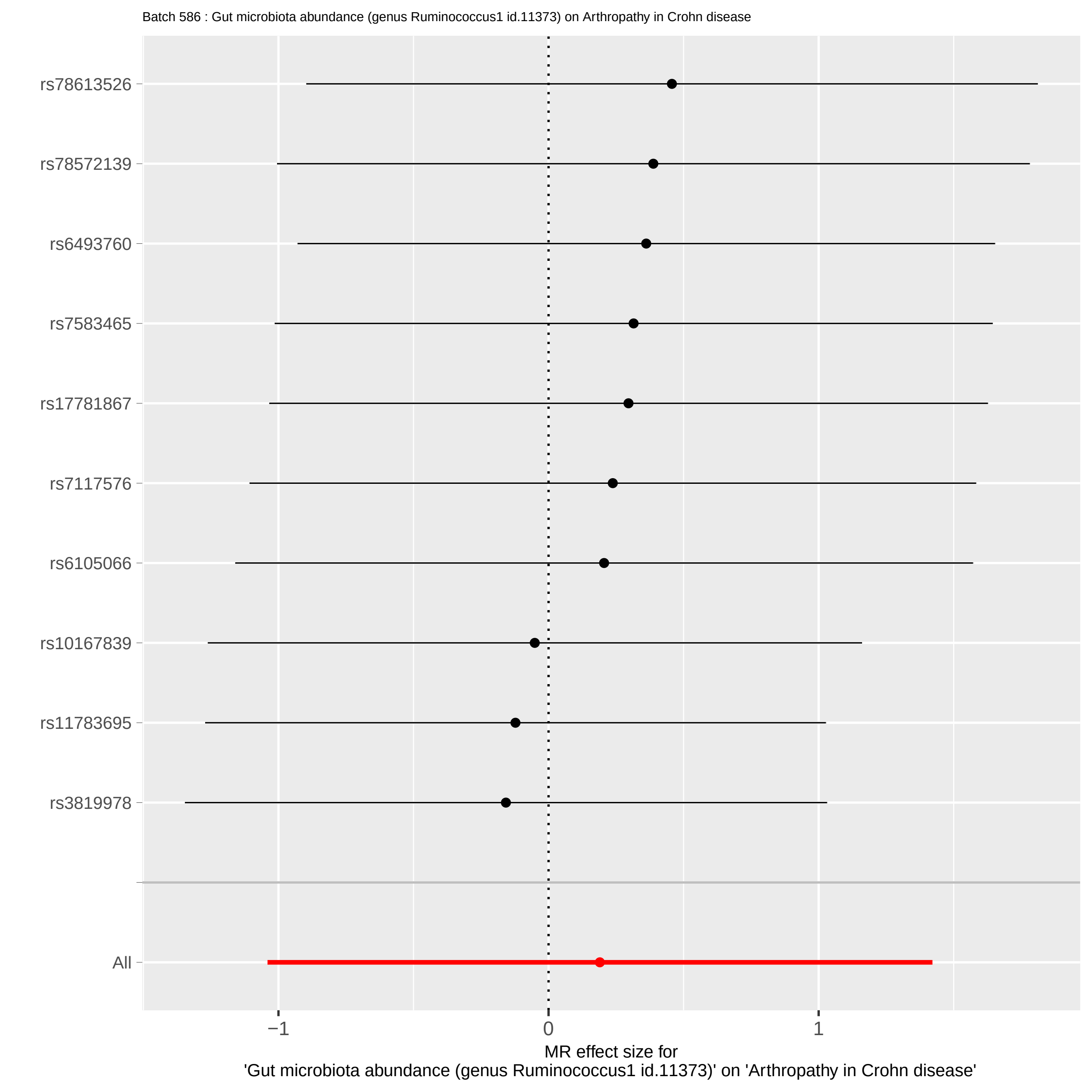

Batch 586 : Gut microbiota abundance (genus Ruminococcus1 id.11373) on Arthropathy in Crohn disease
rs78613526
rs78572139
rs6493760
rs7583465
rs17781867
rs7117576
rs6105066
rs10167839
rs11783695
rs3819978
All
−1
0
1
MR effect size for
'Gut microbiota abundance (genus Ruminococcus1 id.11373)' on 'Arthropathy in Crohn disease'

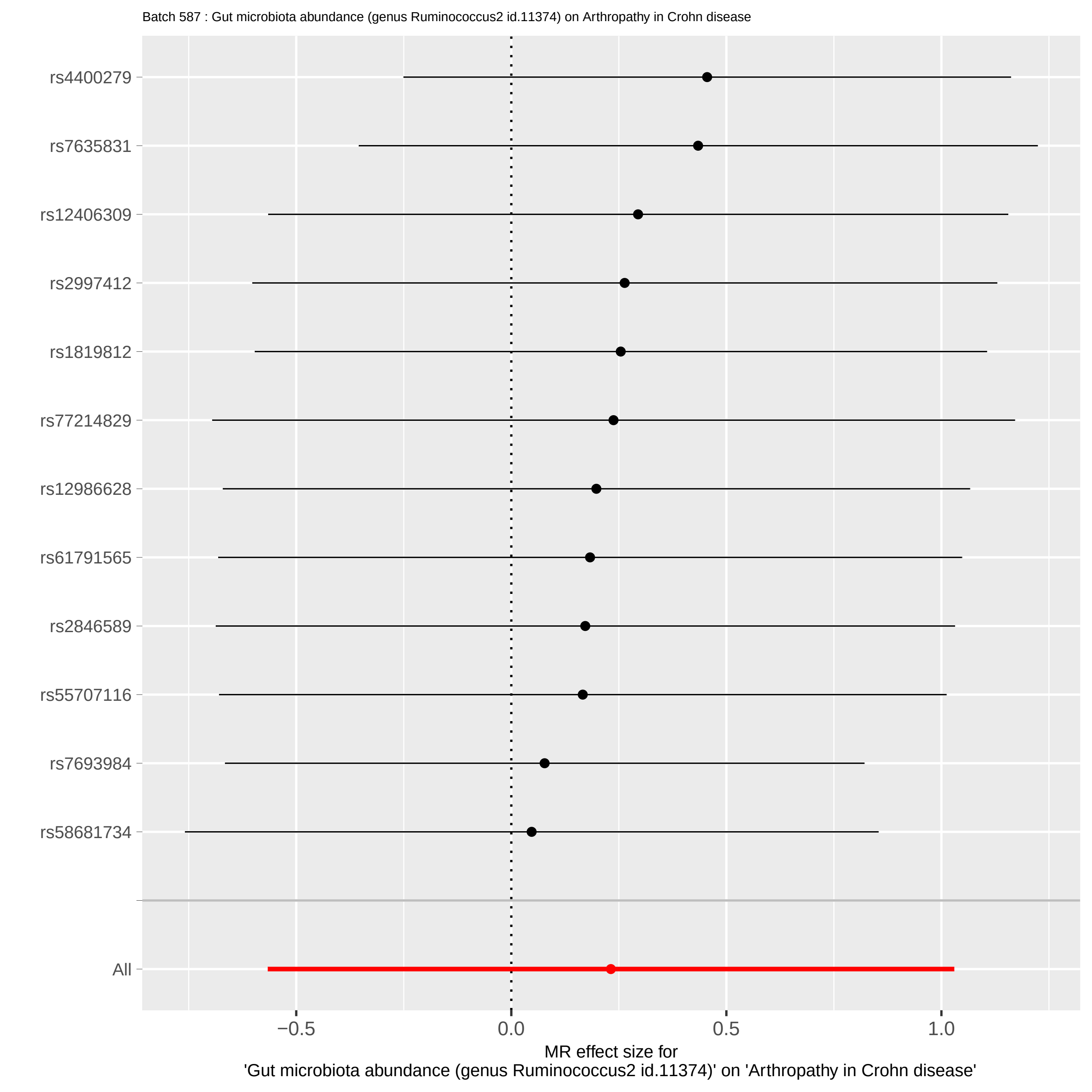
Batch 587 : Gut microbiota abundance (genus Ruminococcus2 id.11374) on Arthropathy in Crohn disease
rs4400279
rs7635831
rs12406309
rs2997412
rs1819812
rs77214829
rs12986628
rs61791565
rs2846589
rs55707116
rs7693984
rs58681734
All
−0.5
0.0
0.5
1.0
MR effect size for
'Gut microbiota abundance (genus Ruminococcus2 id.11374)' on 'Arthropathy in Crohn disease'

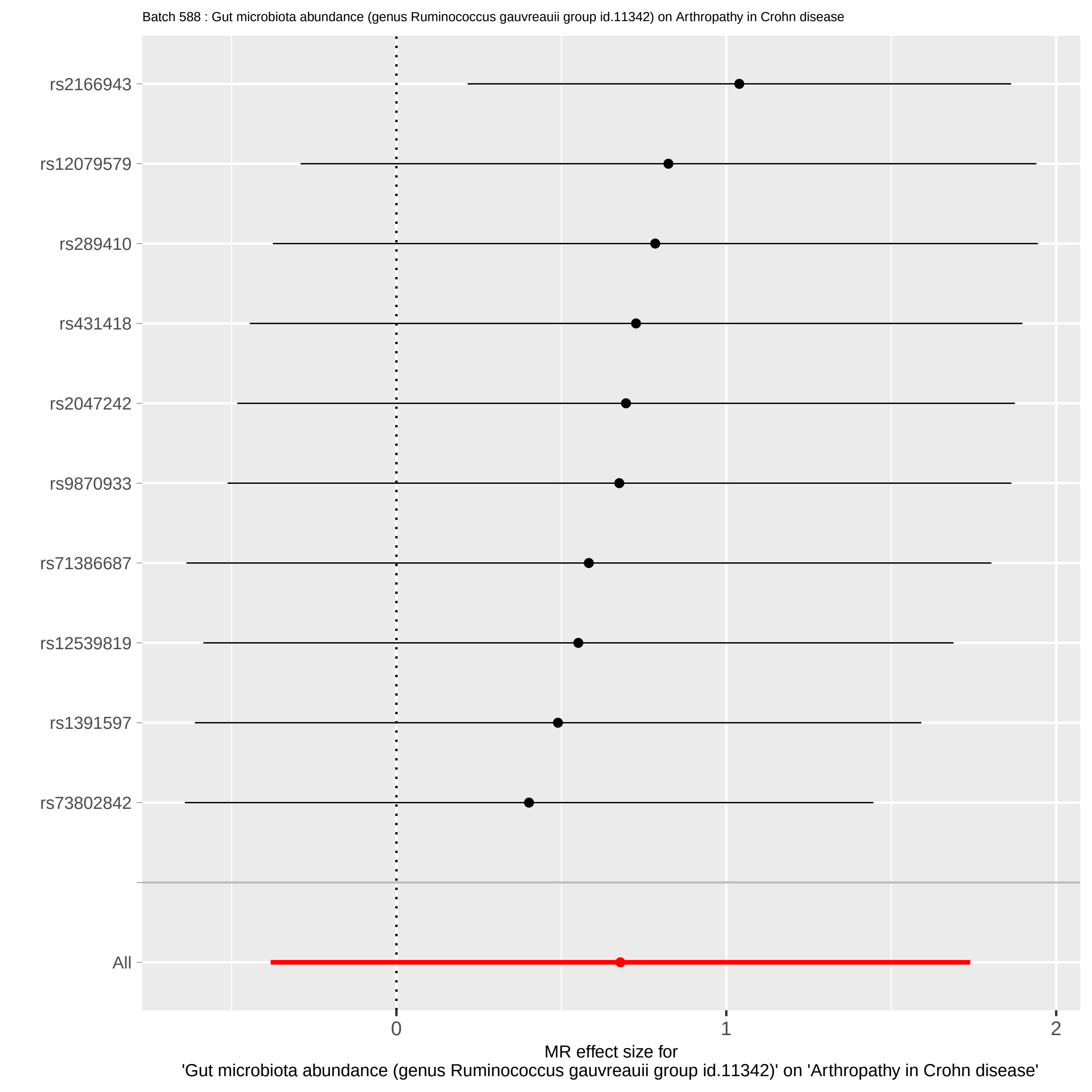
Batch 588 : Gut microbiota abundance (genus Ruminococcus gauvreauii group id.11342) on Arthropathy in Crohn disease
rs2166943
rs12079579
rs289410
rs431418
rs2047242
rs9870933
rs71386687
rs12539819
rs1391597
rs73802842
All
0
1
2
MR effect size for
'Gut microbiota abundance (genus Ruminococcus gauvreauii group id.11342)' on 'Arthropathy in Crohn disease'

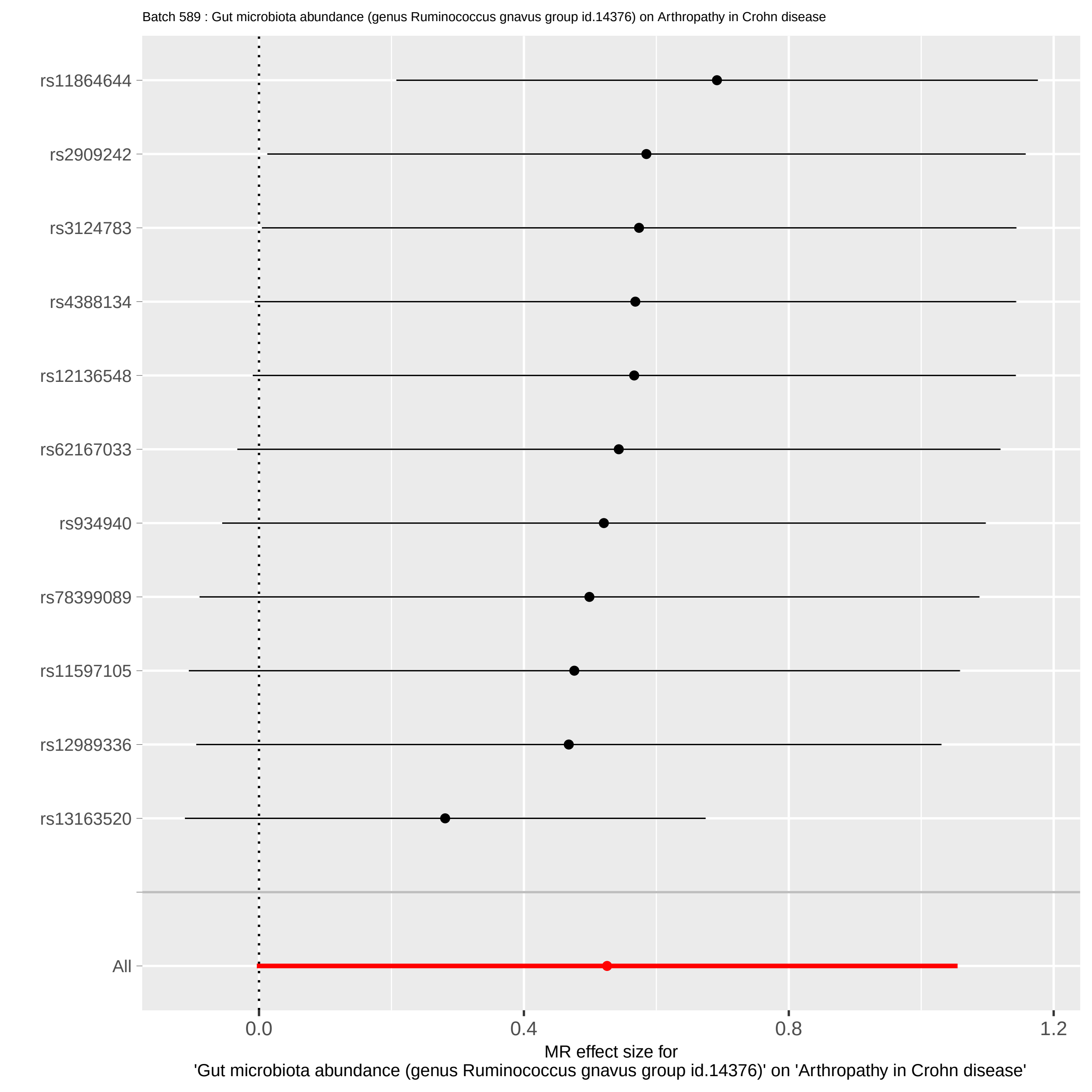

Batch 589 : Gut microbiota abundance (genus Ruminococcus gnavus group id.14376) on Arthropathy in Crohn disease
rs11864644
rs2909242
rs3124783
rs4388134
rs12136548
rs62167033
rs934940
rs78399089
rs11597105
rs12989336
rs13163520
All
0.0
0.4
0.8
1.2
MR effect size for
'Gut microbiota abundance (genus Ruminococcus gnavus group id.14376)' on 'Arthropathy in Crohn disease'

Batch 590 : Gut microbiota abundance (genus Ruminococcus torques group id.14377) on Arthropathy in Crohn disease

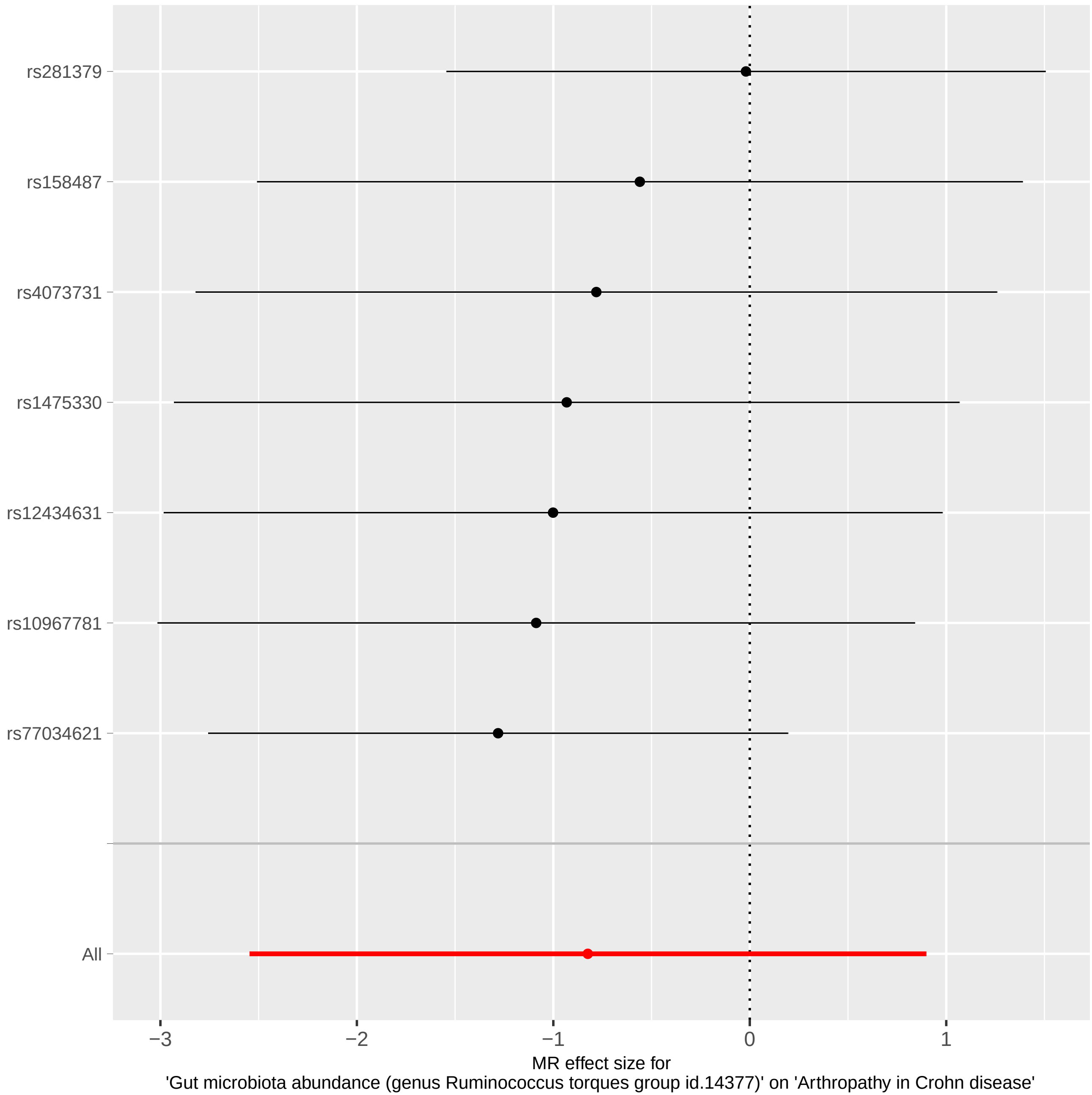

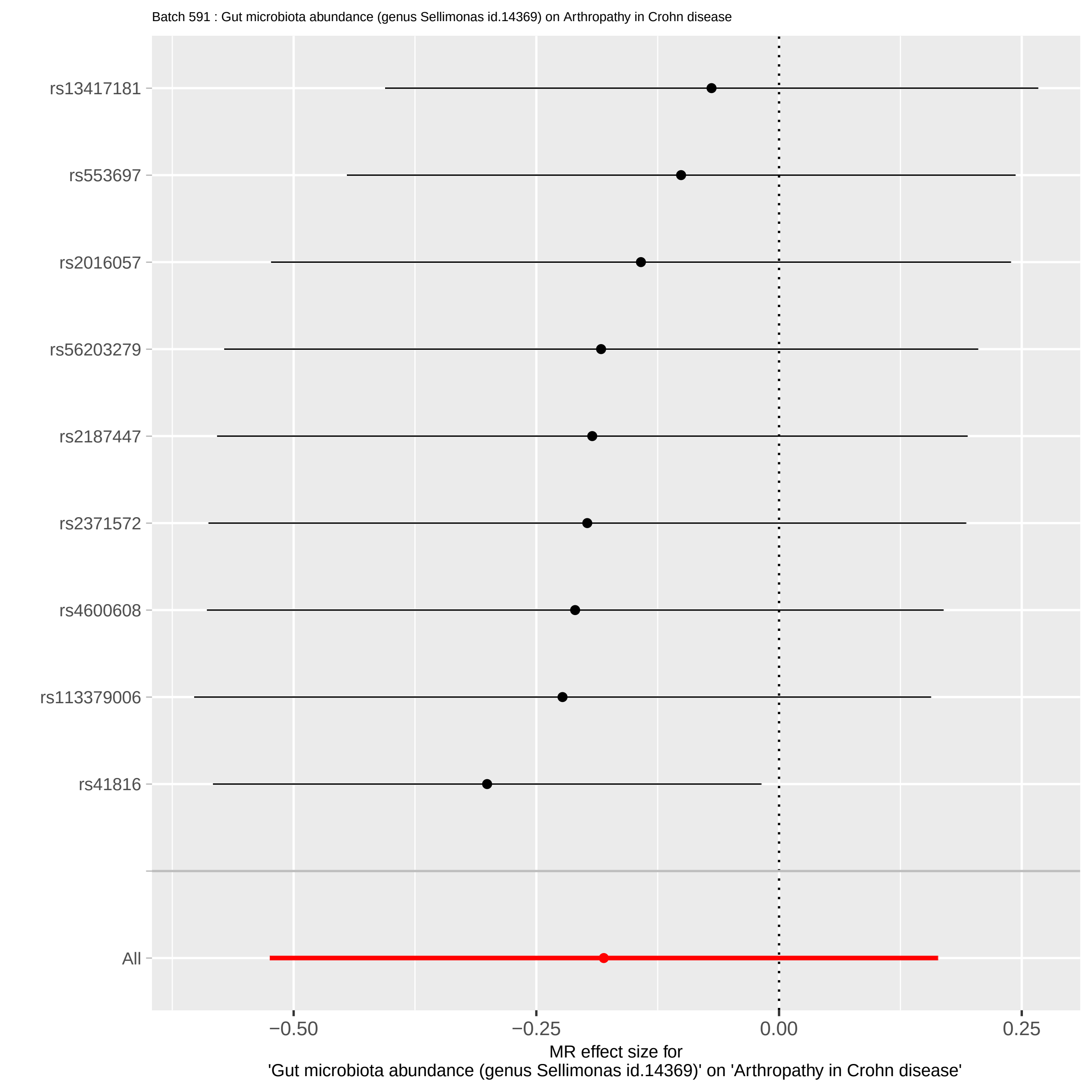

Batch 591 : Gut microbiota abundance (genus Sellimonas id.14369) on Arthropathy in Crohn disease
rs13417181
rs553697
rs2016057
rs56203279
rs2187447
rs2371572
rs4600608
rs113379006
rs41816
All
−0.50
−0.25
0.00
0.25
MR effect size for
'Gut microbiota abundance (genus Sellimonas id.14369)' on 'Arthropathy in Crohn disease'

Batch 592 : Gut microbiota abundance (genus Senegalimassilia id.11160) on Arthropathy in Crohn disease

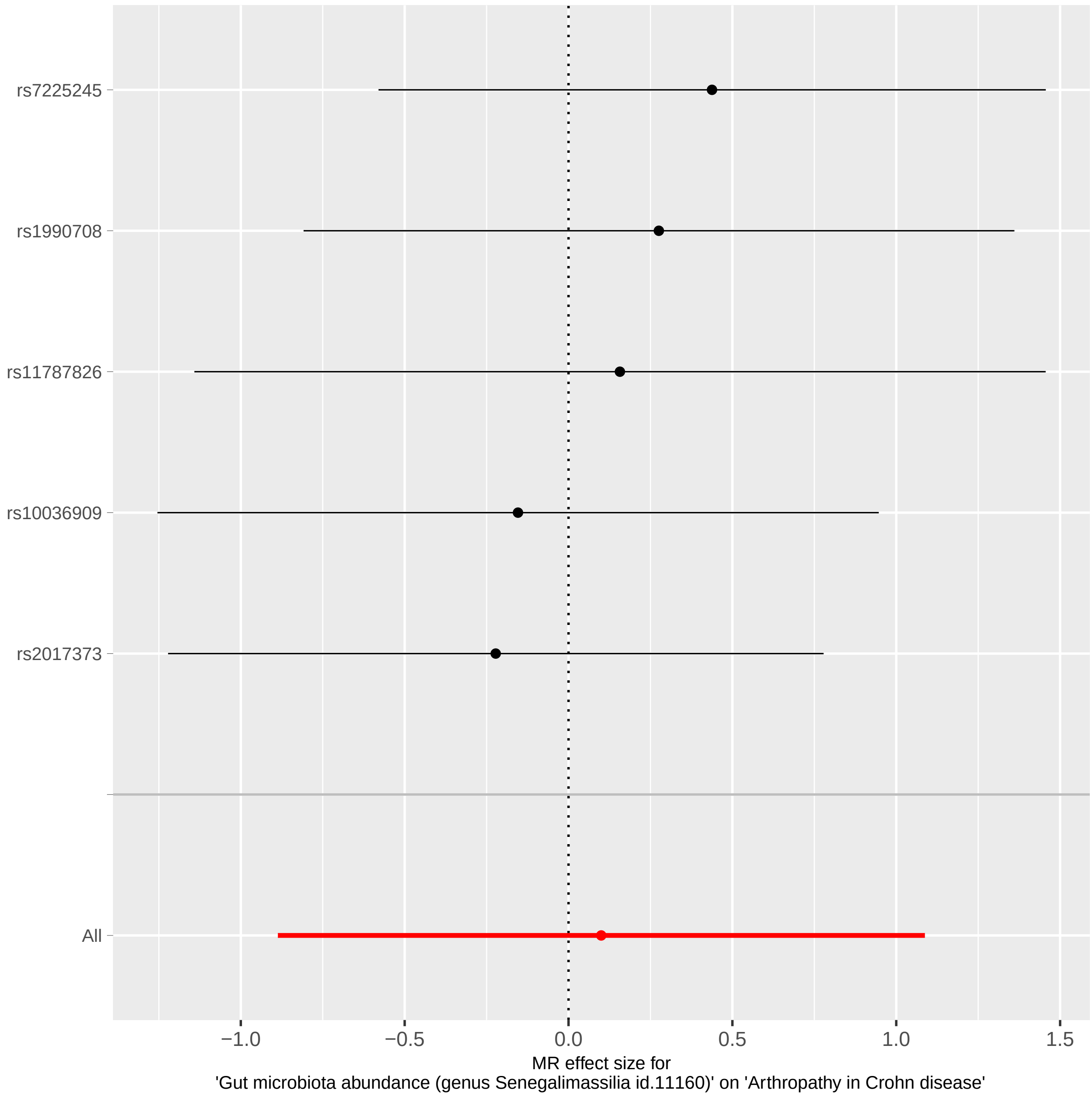

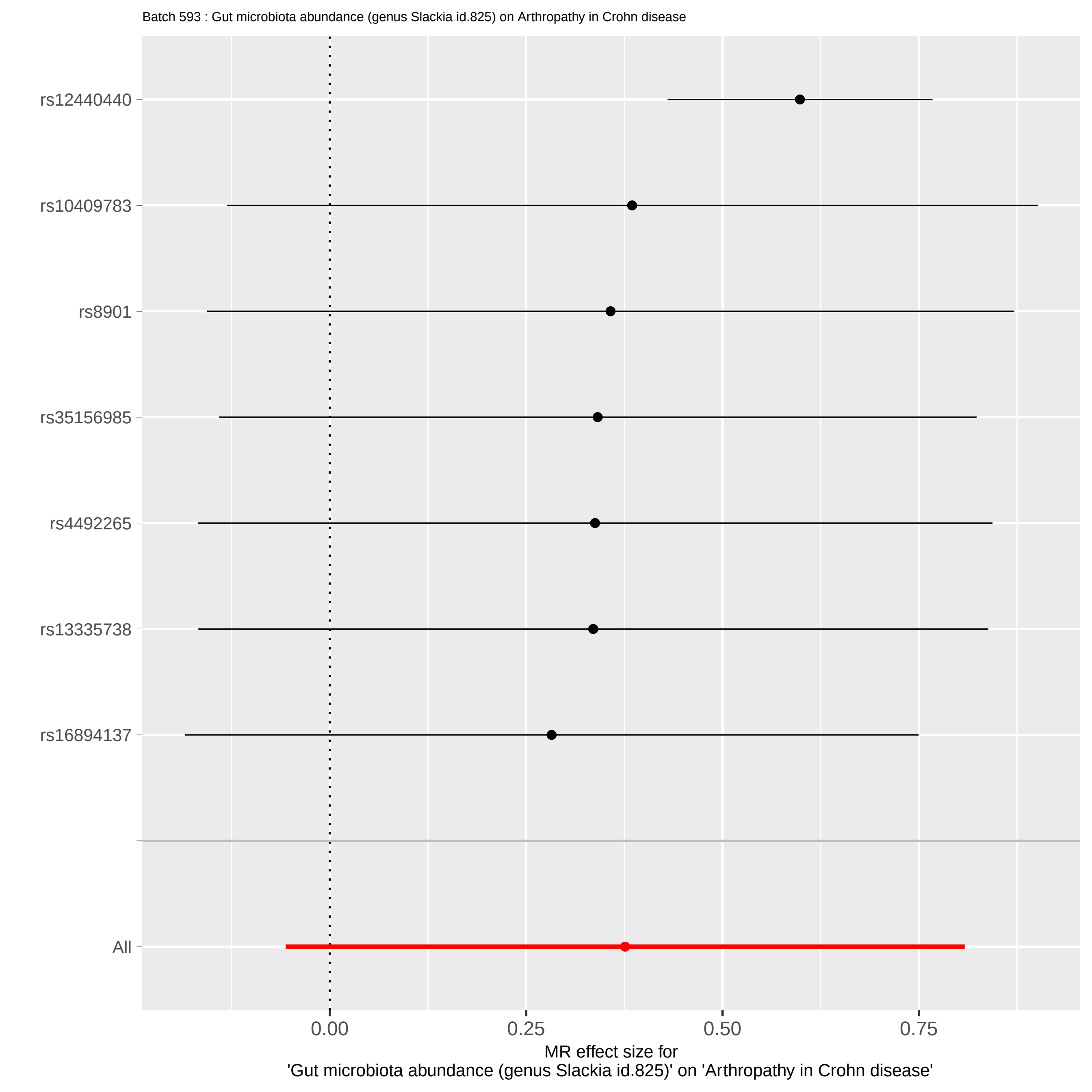
Batch 593 : Gut microbiota abundance (genus Slackia id.825) on Arthropathy in Crohn disease
rs12440440
rs10409783
rs8901
rs35156985
rs4492265
rs13335738
rs16894137
All
0.00
0.25
0.50
0.75
MR effect size for
'Gut microbiota abundance (genus Slackia id.825)' on 'Arthropathy in Crohn disease'

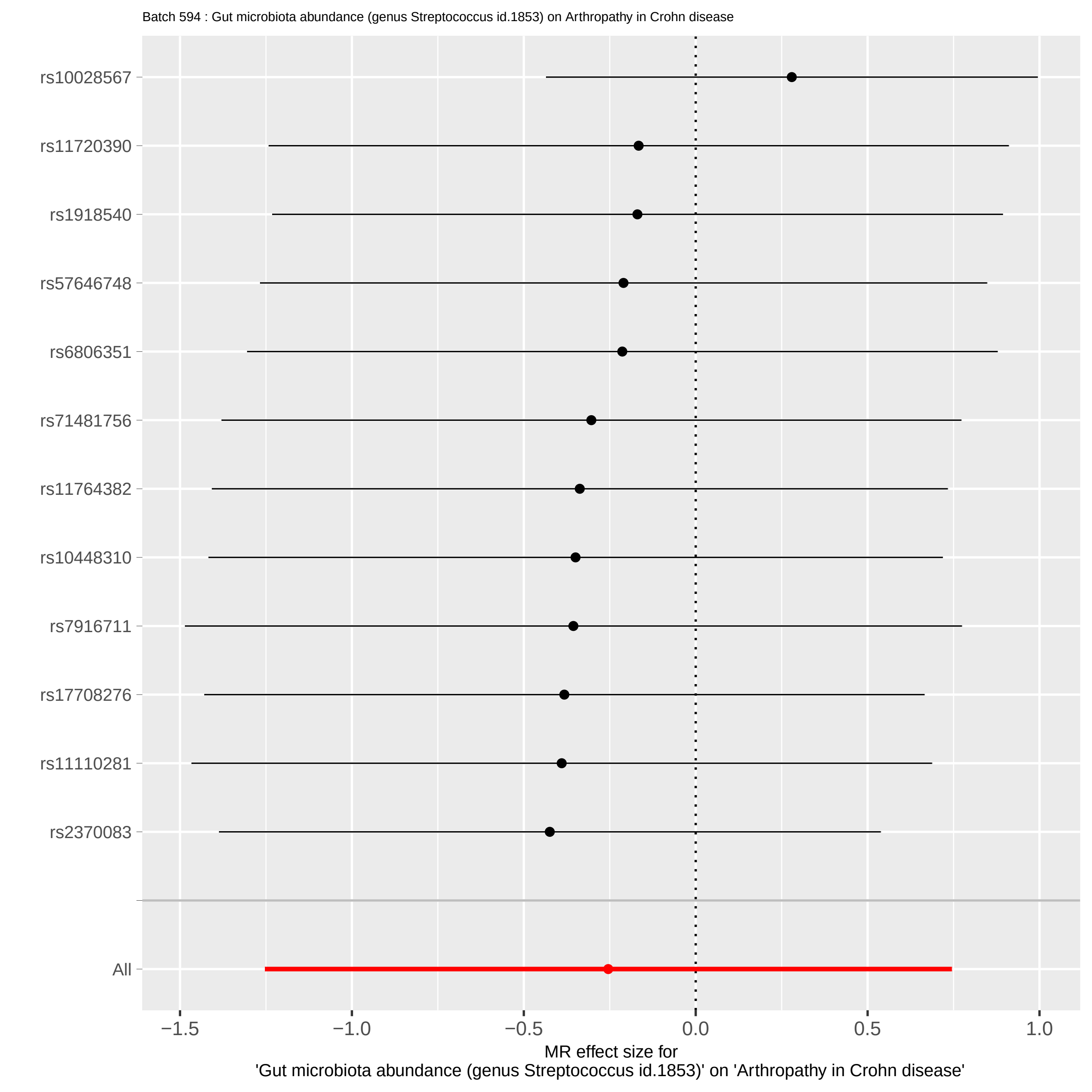
Batch 594 : Gut microbiota abundance (genus Streptococcus id.1853) on Arthropathy in Crohn disease
rs10028567
rs11720390
rs1918540
rs57646748
rs6806351
rs71481756
rs11764382
rs10448310
rs7916711
rs17708276
rs11110281
rs2370083
All
−1.5
−1.0
−0.5
0.0
0.5
1.0
MR effect size for
'Gut microbiota abundance (genus Streptococcus id.1853)' on 'Arthropathy in Crohn disease'

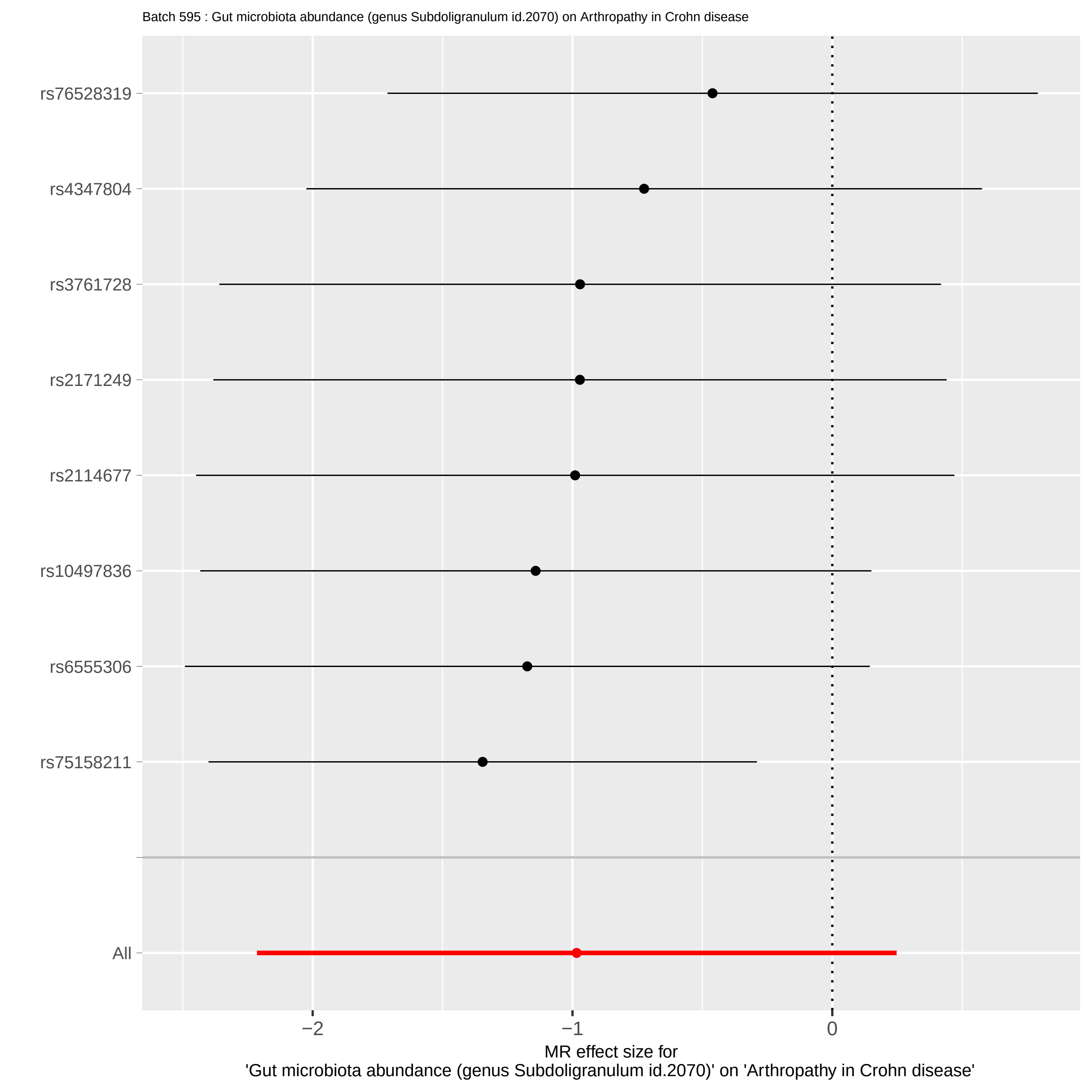
Batch 595 : Gut microbiota abundance (genus Subdoligranulum id.2070) on Arthropathy in Crohn disease
rs76528319
rs4347804
rs3761728
rs2171249
rs2114677
rs10497836
rs6555306
rs75158211
All
−2
−1
0
MR effect size for
'Gut microbiota abundance (genus Subdoligranulum id.2070)' on 'Arthropathy in Crohn disease'

Batch 596 : Gut microbiota abundance (genus Sutterella id.2896) on Arthropathy in Crohn disease

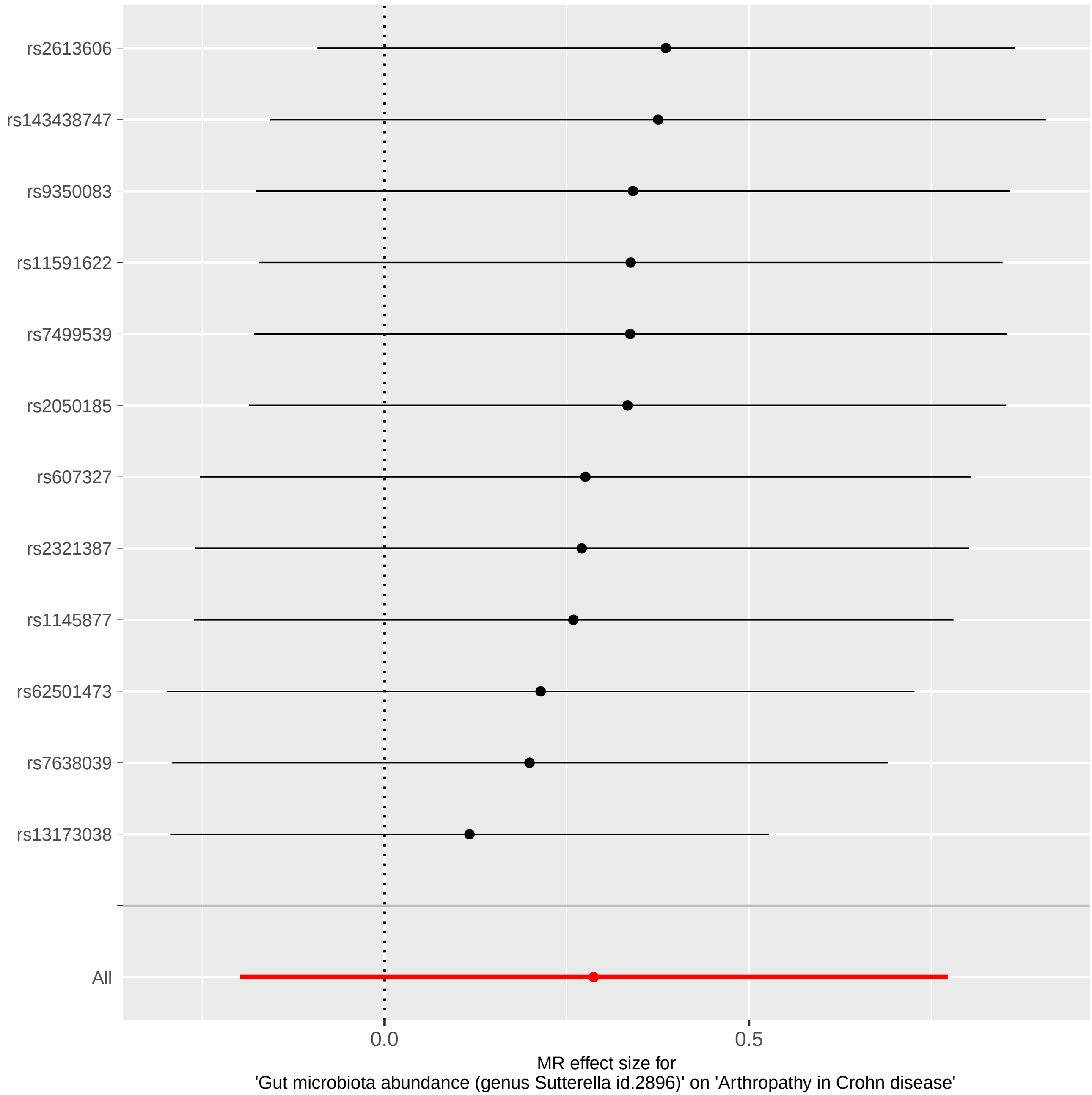

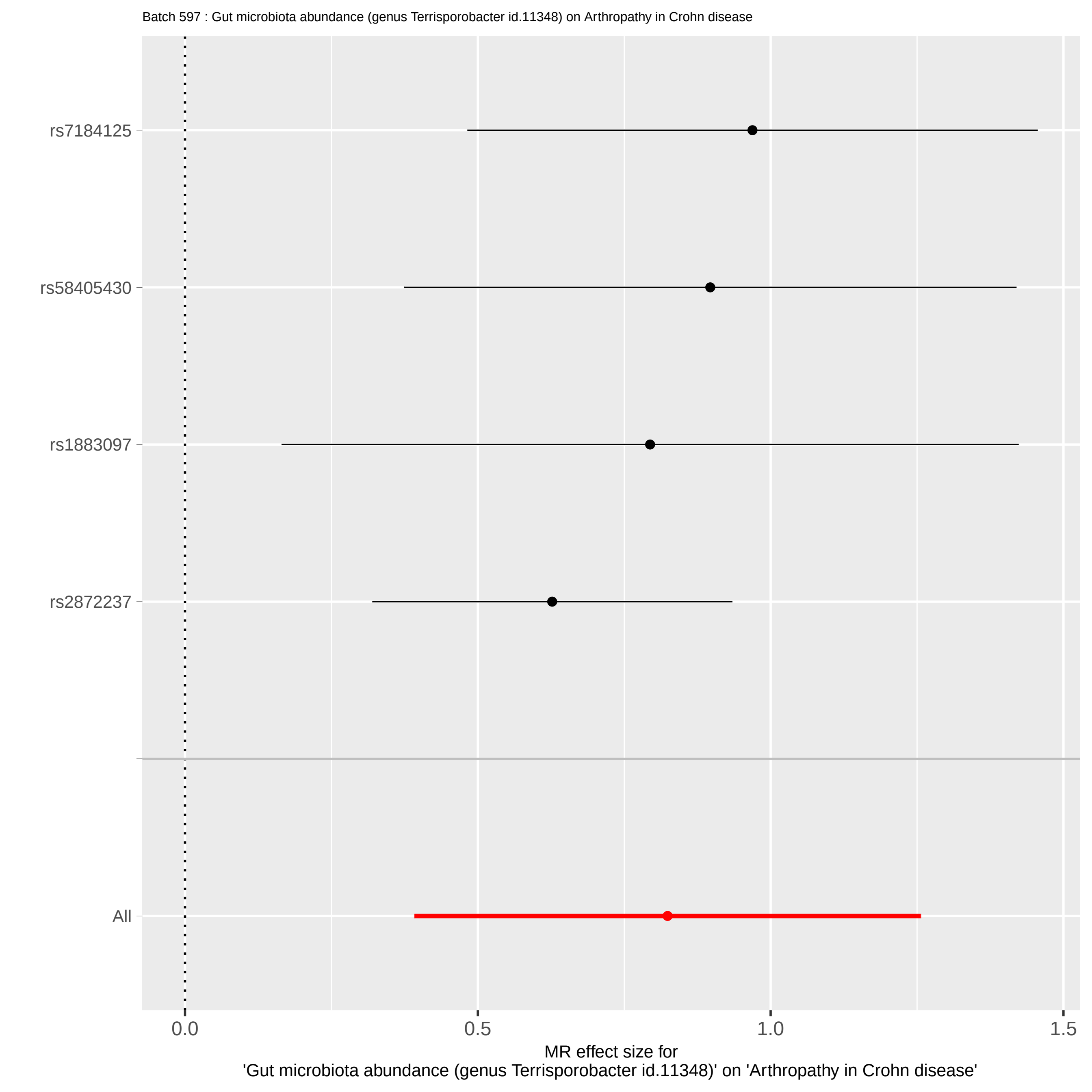

Batch 597 : Gut microbiota abundance (genus Terrisporobacter id.11348) on Arthropathy in Crohn disease
rs7184125
rs58405430
rs1883097
rs2872237
All
0.0
0.5
1.0
1.5
MR effect size for
'Gut microbiota abundance (genus Terrisporobacter id.11348)' on 'Arthropathy in Crohn disease'

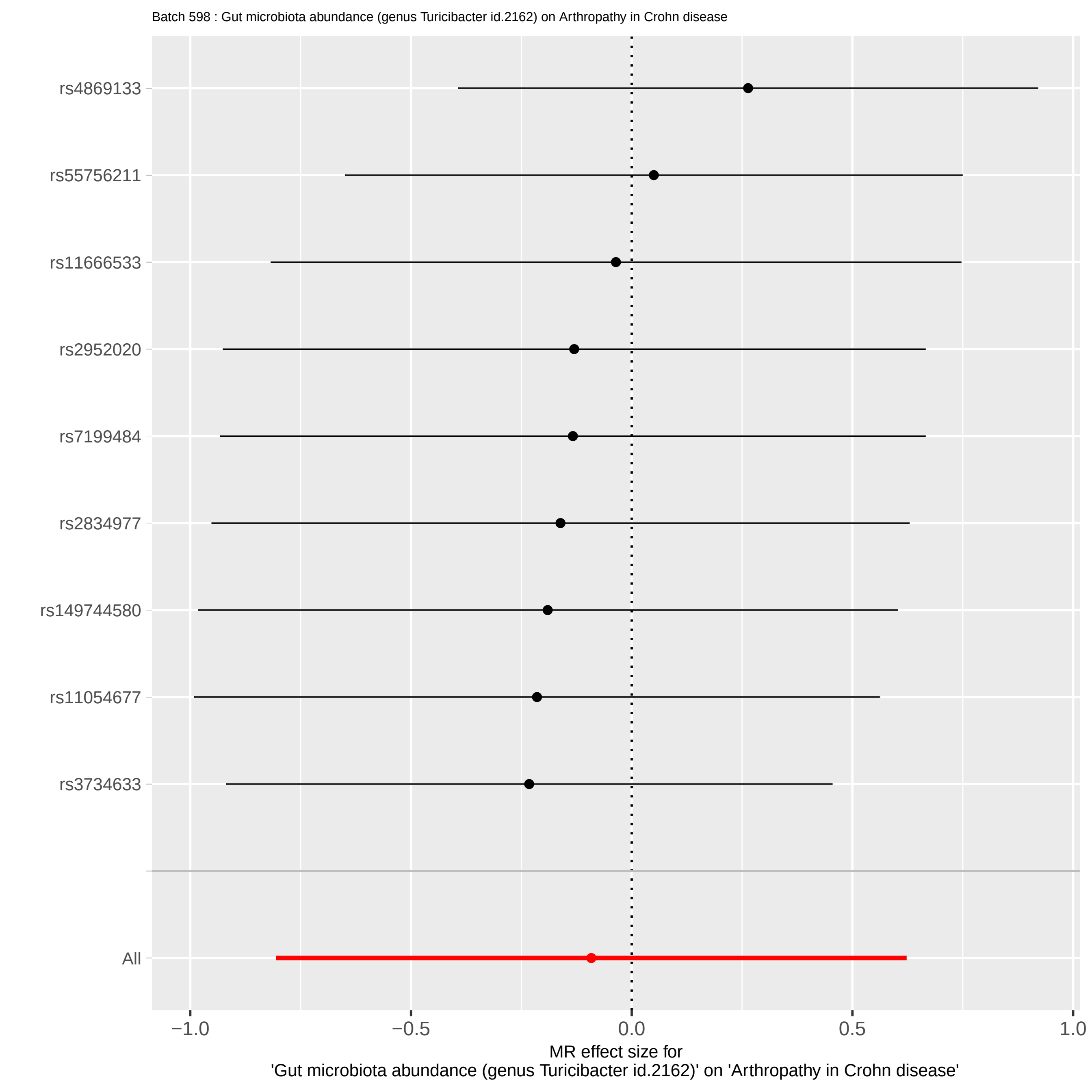
Batch 598 : Gut microbiota abundance (genus Turicibacter id.2162) on Arthropathy in Crohn disease
rs4869133
rs55756211
rs11666533
rs2952020
rs7199484
rs2834977
rs149744580
rs11054677
rs3734633
All
−1.0
−0.5
0.0
0.5
1.0
MR effect size for
'Gut microbiota abundance (genus Turicibacter id.2162)' on 'Arthropathy in Crohn disease'

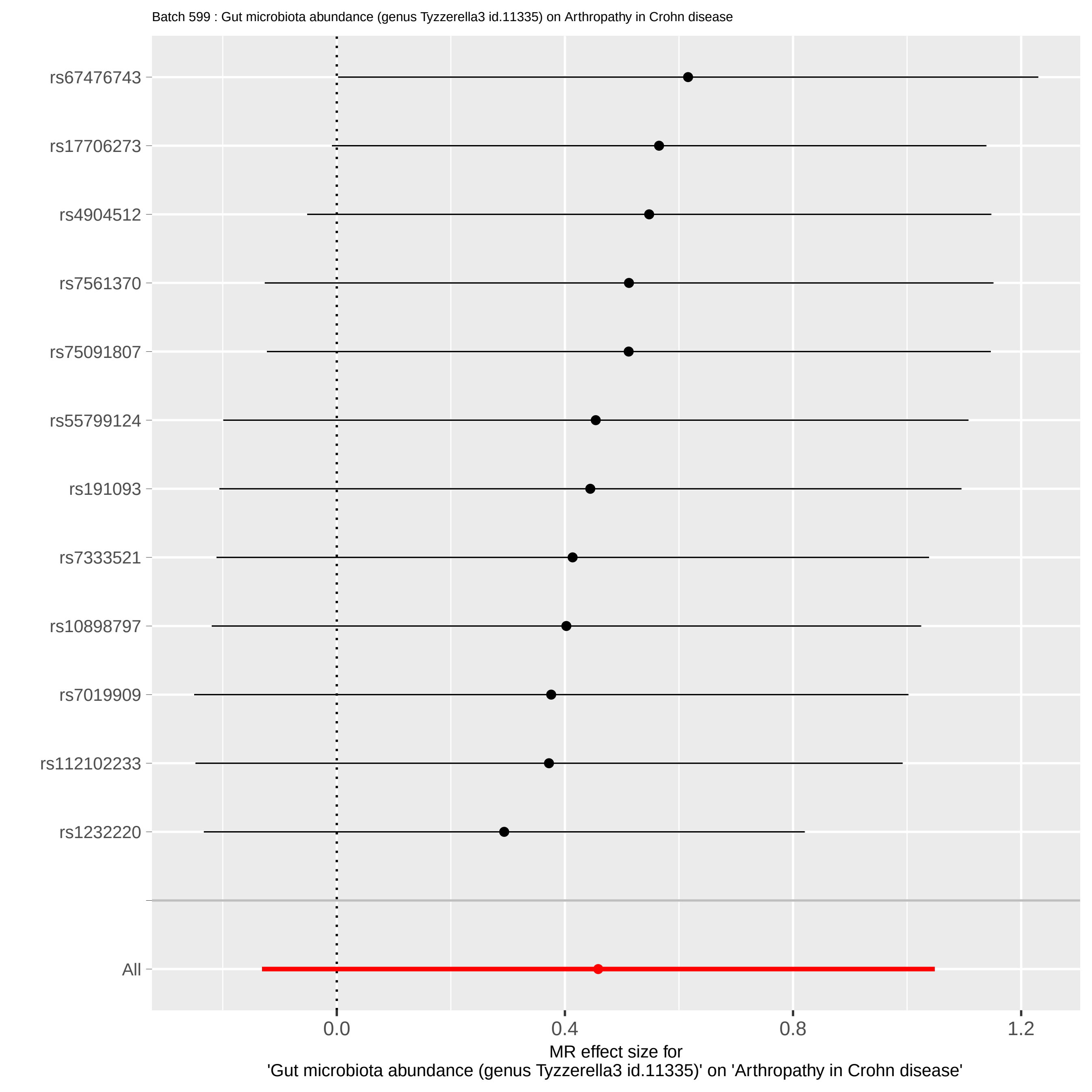

Batch 599 : Gut microbiota abundance (genus Tyzzerella3 id.11335) on Arthropathy in Crohn disease
rs67476743
rs17706273
rs4904512
rs7561370
rs75091807
rs55799124
rs191093
rs7333521
rs10898797
rs7019909
rs112102233
rs1232220
All
0.0
0.4
0.8
1.2
MR effect size for
'Gut microbiota abundance (genus Tyzzerella3 id.11335)' on 'Arthropathy in Crohn disease'

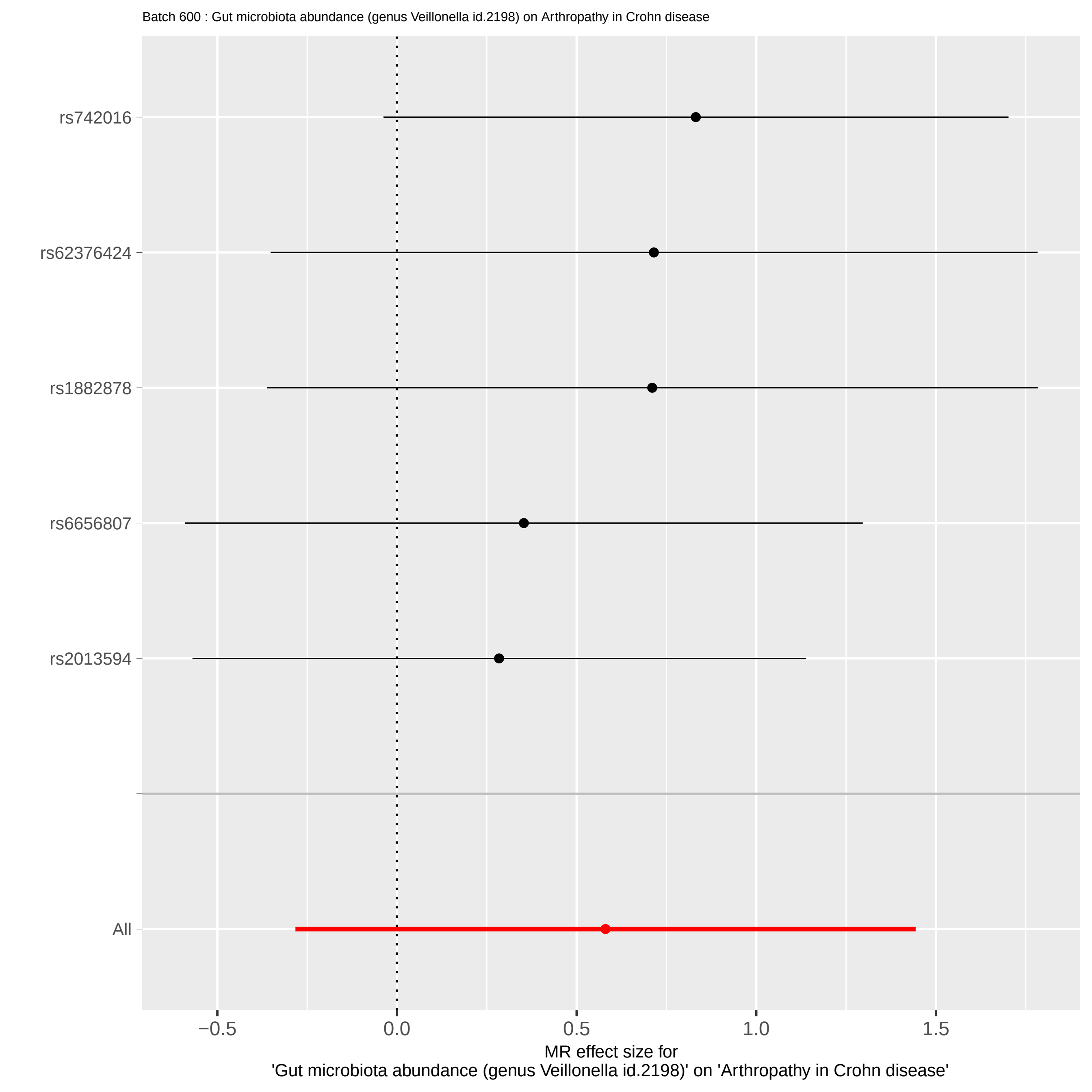
Batch 600 : Gut microbiota abundance (genus Veillonella id.2198) on Arthropathy in Crohn disease
rs742016
rs62376424
rs1882878
rs6656807
rs2013594
All
−0.5
0.0
0.5
1.0
1.5
MR effect size for
'Gut microbiota abundance (genus Veillonella id.2198)' on 'Arthropathy in Crohn disease'

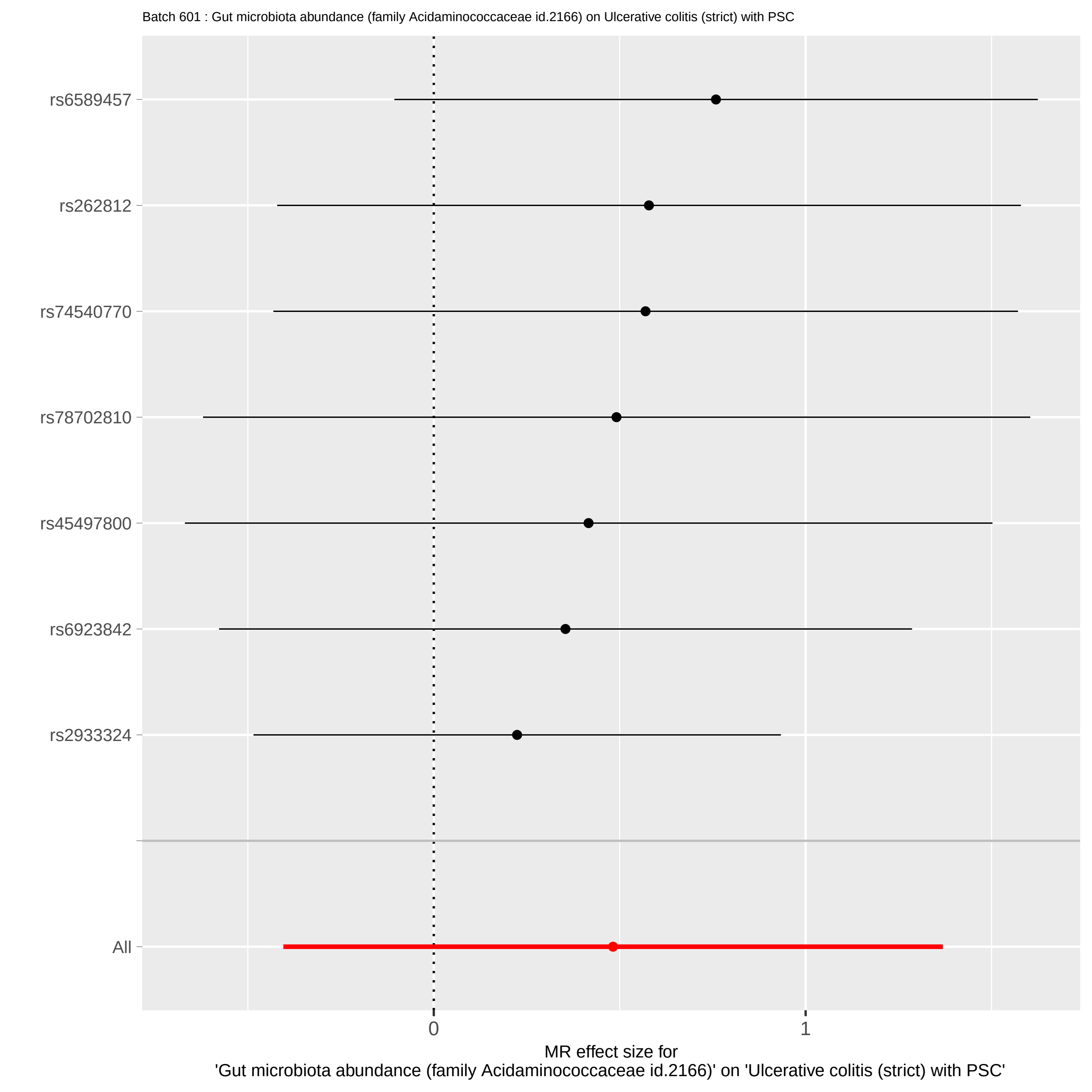
Batch 601 : Gut microbiota abundance (family Acidaminococcaceae id.2166) on Ulcerative colitis (strict) with PSC
rs6589457
rs262812
rs74540770
rs78702810
rs45497800
rs6923842
rs2933324
All
0
1
MR effect size for
'Gut microbiota abundance (family Acidaminococcaceae id.2166)' on 'Ulcerative colitis (strict) with PSC'

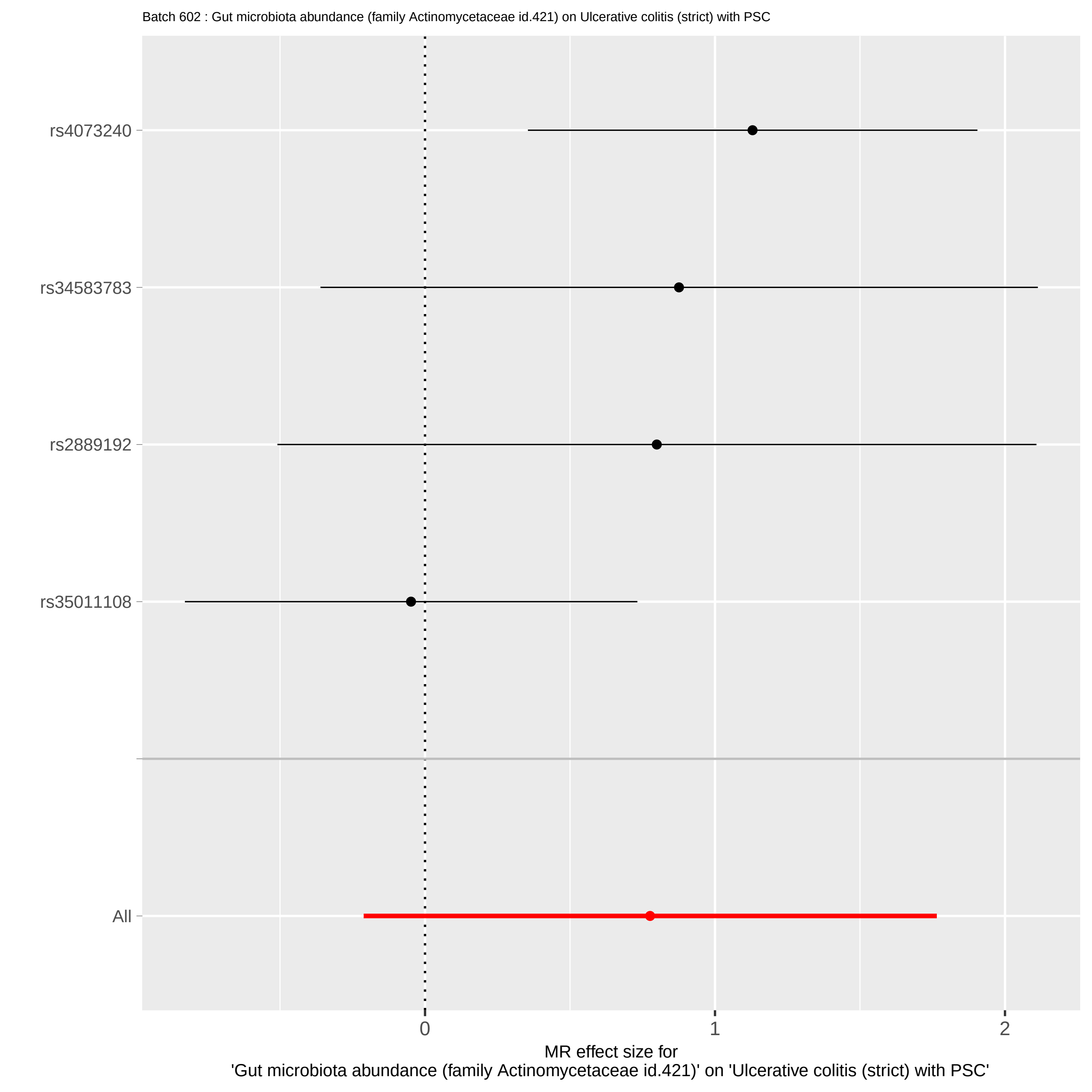

Batch 602 : Gut microbiota abundance (family Actinomycetaceae id.421) on Ulcerative colitis (strict) with PSC
rs4073240
rs34583783
rs2889192
rs35011108
All
0
1
2
MR effect size for
'Gut microbiota abundance (family Actinomycetaceae id.421)' on 'Ulcerative colitis (strict) with PSC'

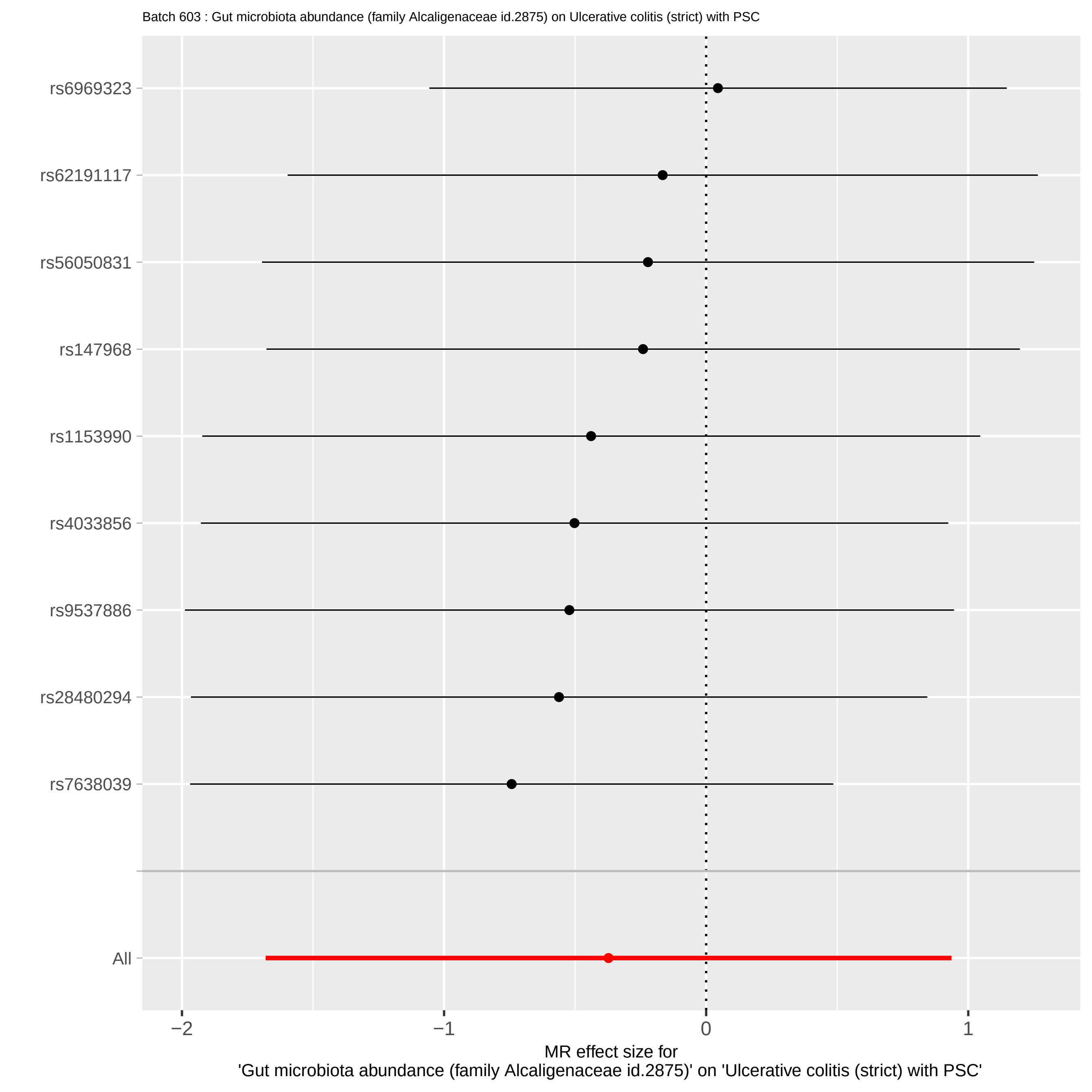

Batch 603 : Gut microbiota abundance (family Alcaligenaceae id.2875) on Ulcerative colitis (strict) with PSC
rs6969323
rs62191117
rs56050831
rs147968
rs1153990
rs4033856
rs9537886
rs28480294
rs7638039
All
−2
−1
0
1
MR effect size for
'Gut microbiota abundance (family Alcaligenaceae id.2875)' on 'Ulcerative colitis (strict) with PSC'

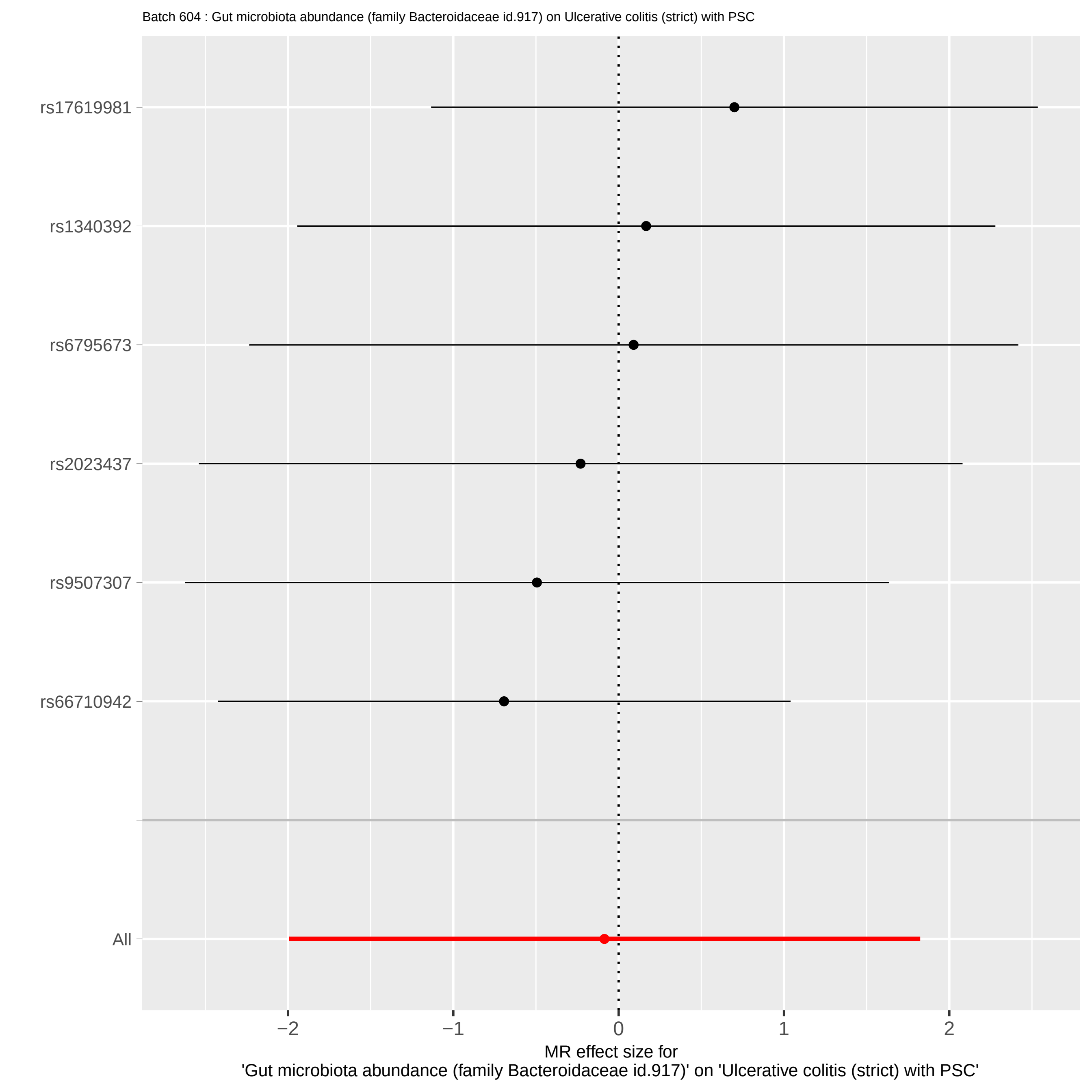
Batch 604 : Gut microbiota abundance (family Bacteroidaceae id.917) on Ulcerative colitis (strict) with PSC
rs17619981
rs1340392
rs6795673
rs2023437
rs9507307
rs66710942
All
−2
−1
0
1
2
MR effect size for
'Gut microbiota abundance (family Bacteroidaceae id.917)' on 'Ulcerative colitis (strict) with PSC'

Batch 605 : Gut microbiota abundance (family Bacteroidales S24 7group id.11173) on Ulcerative colitis (strict) with PSC

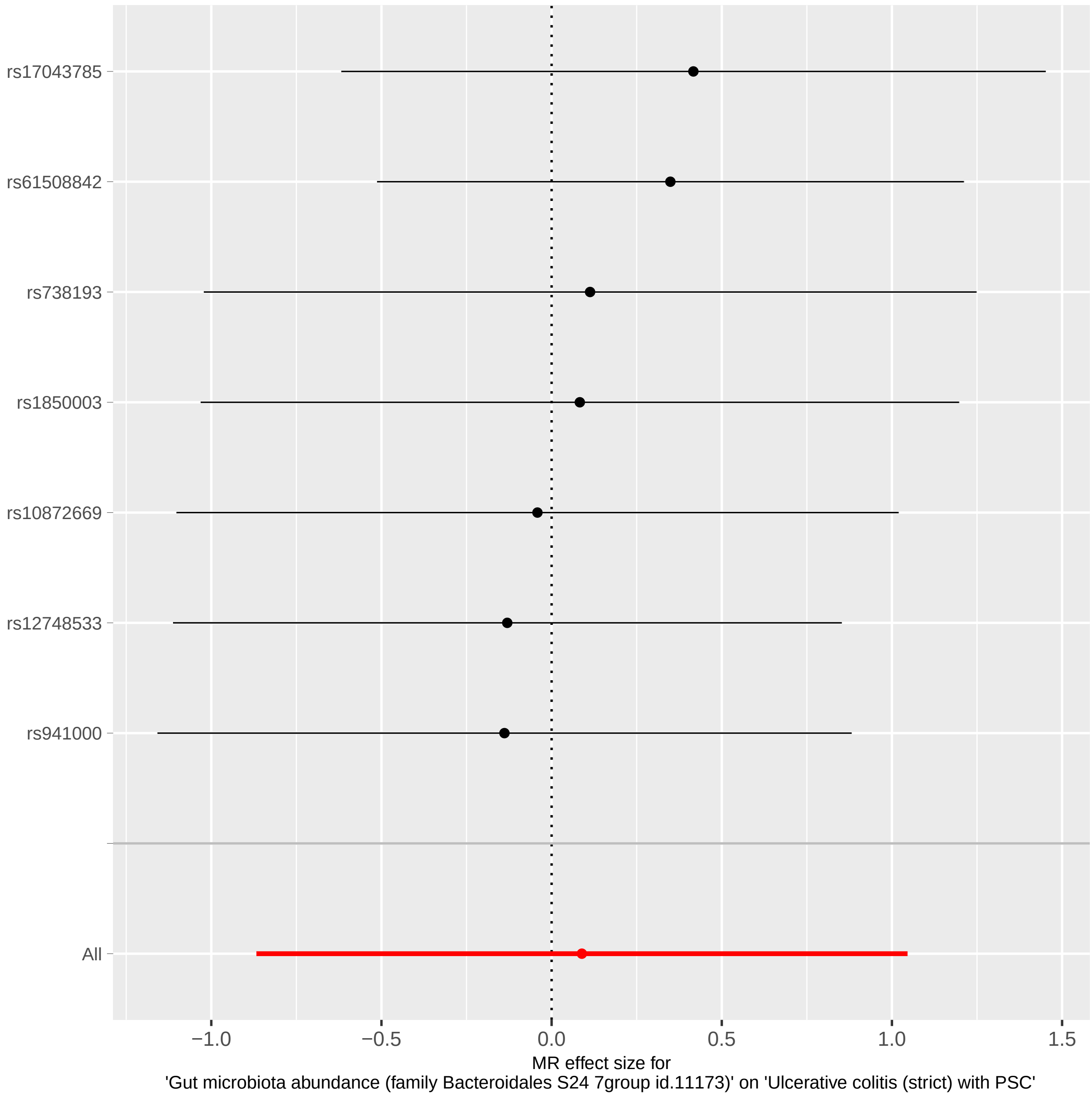

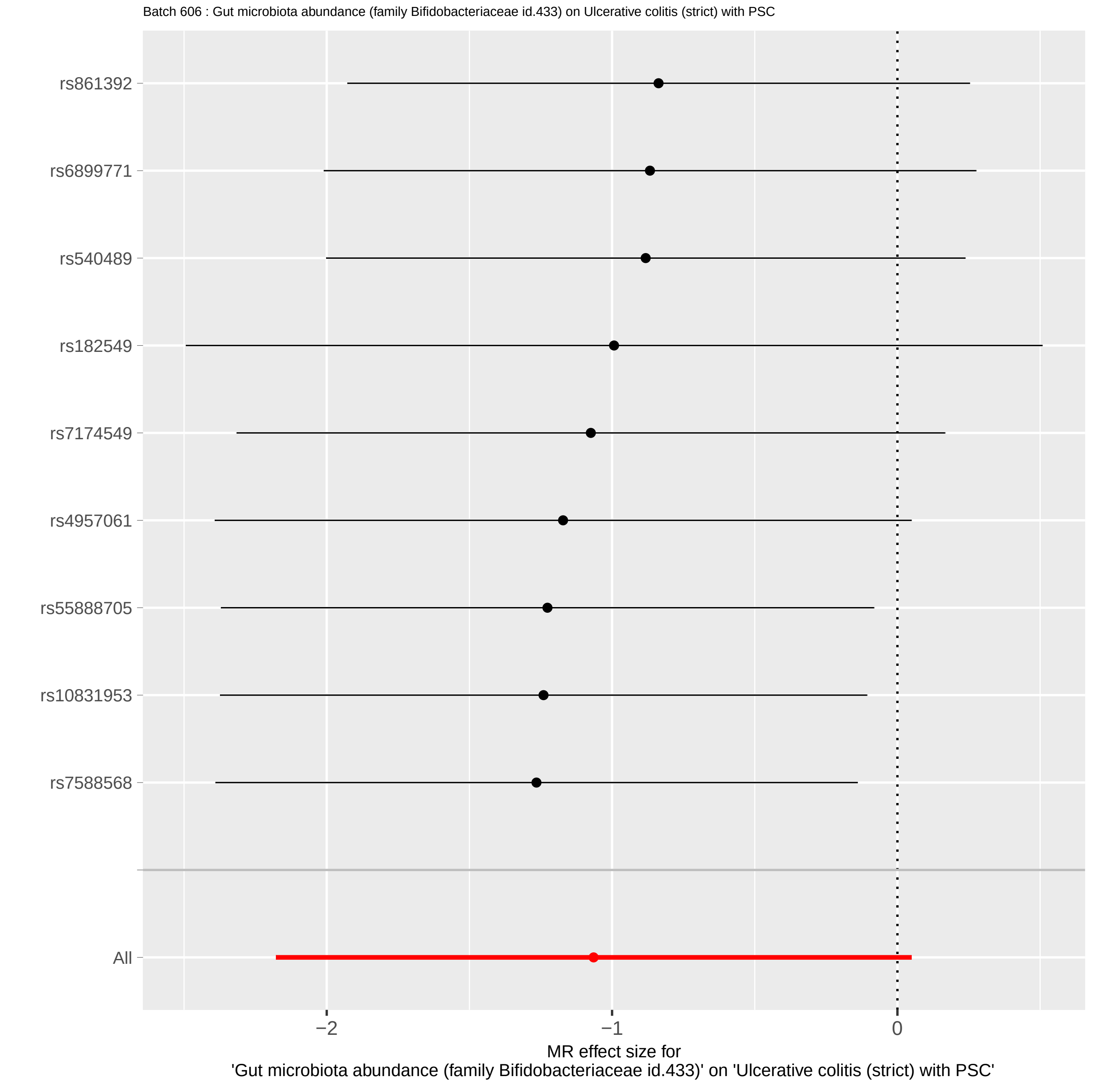

Batch 606 : Gut microbiota abundance (family Bifidobacteriaceae id.433) on Ulcerative colitis (strict) with PSC
rs861392
rs6899771
rs540489
rs182549
rs7174549
rs4957061
rs55888705
rs10831953
rs7588568
All
−2
−1
0
MR effect size for
'Gut microbiota abundance (family Bifidobacteriaceae id.433)' on 'Ulcerative colitis (strict) with PSC'

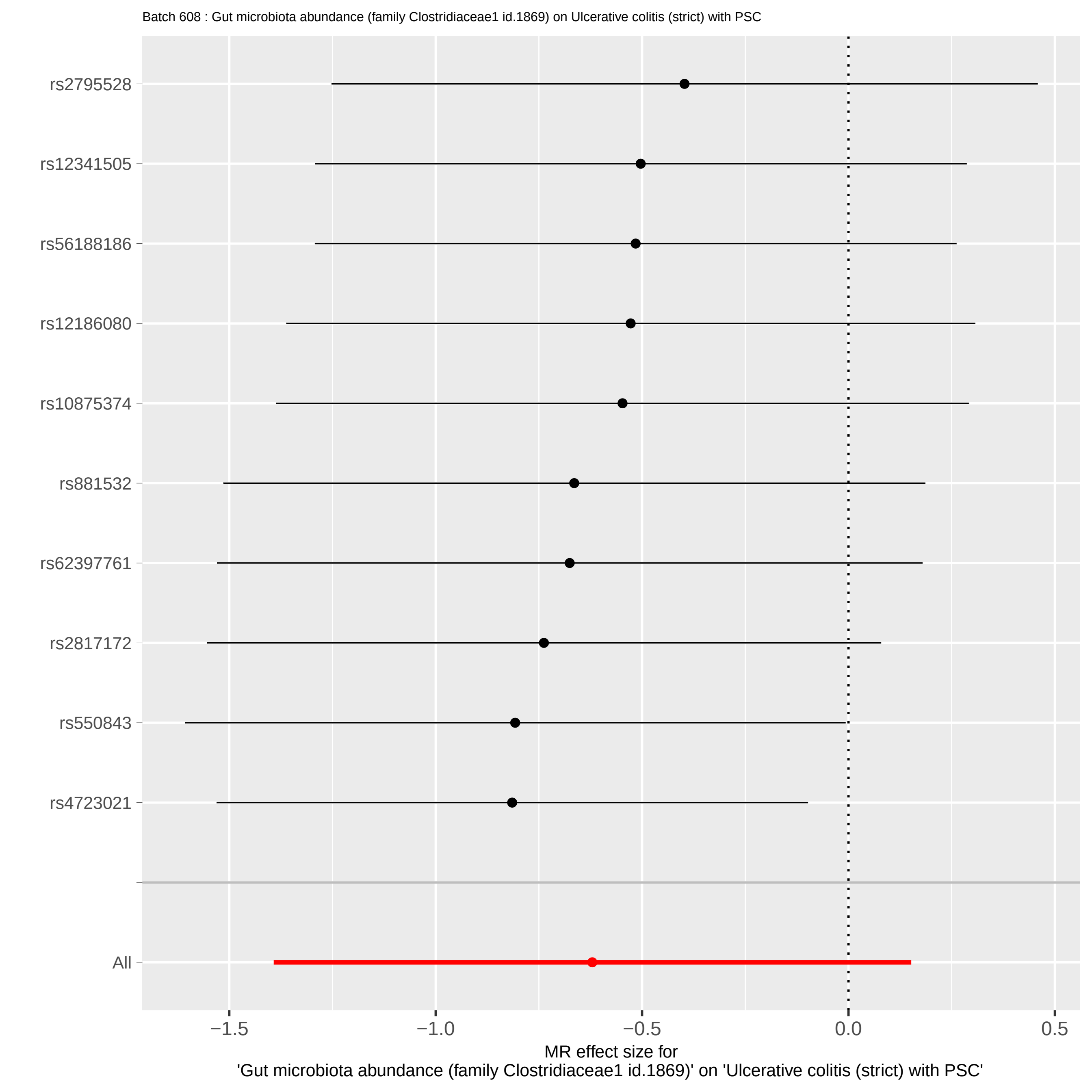

Batch 608 : Gut microbiota abundance (family Clostridiaceae1 id.1869) on Ulcerative colitis (strict) with PSC
rs2795528
rs12341505
rs56188186
rs12186080
rs10875374
rs881532
rs62397761
rs2817172
rs550843
rs4723021
All
−1.5
−1.0
−0.5
0.0
0.5
MR effect size for
'Gut microbiota abundance (family Clostridiaceae1 id.1869)' on 'Ulcerative colitis (strict) with PSC'

Batch 609 : Gut microbiota abundance (family Clostridiales vadin BB60 group id.11286) on Ulcerative colitis (strict) with PSC

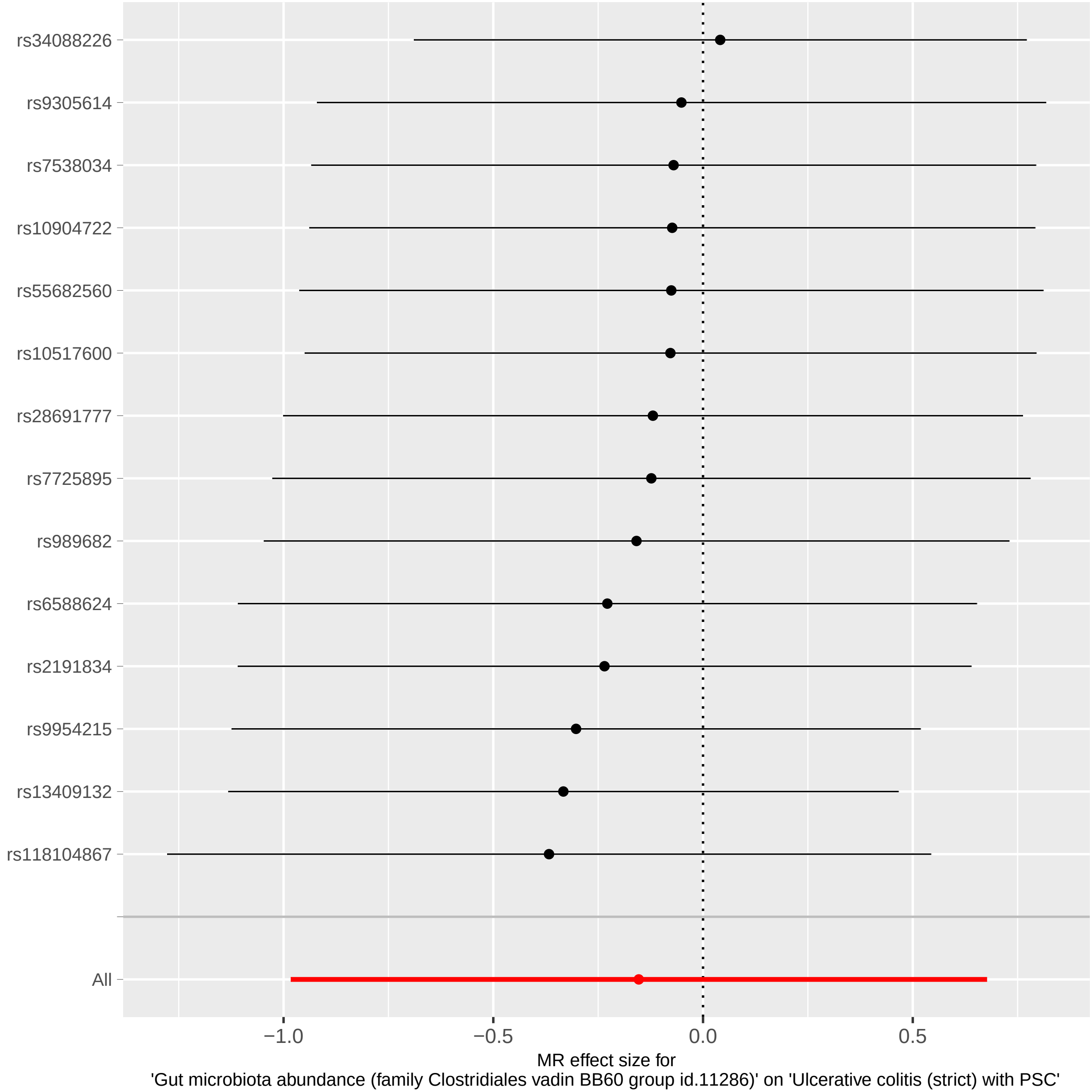

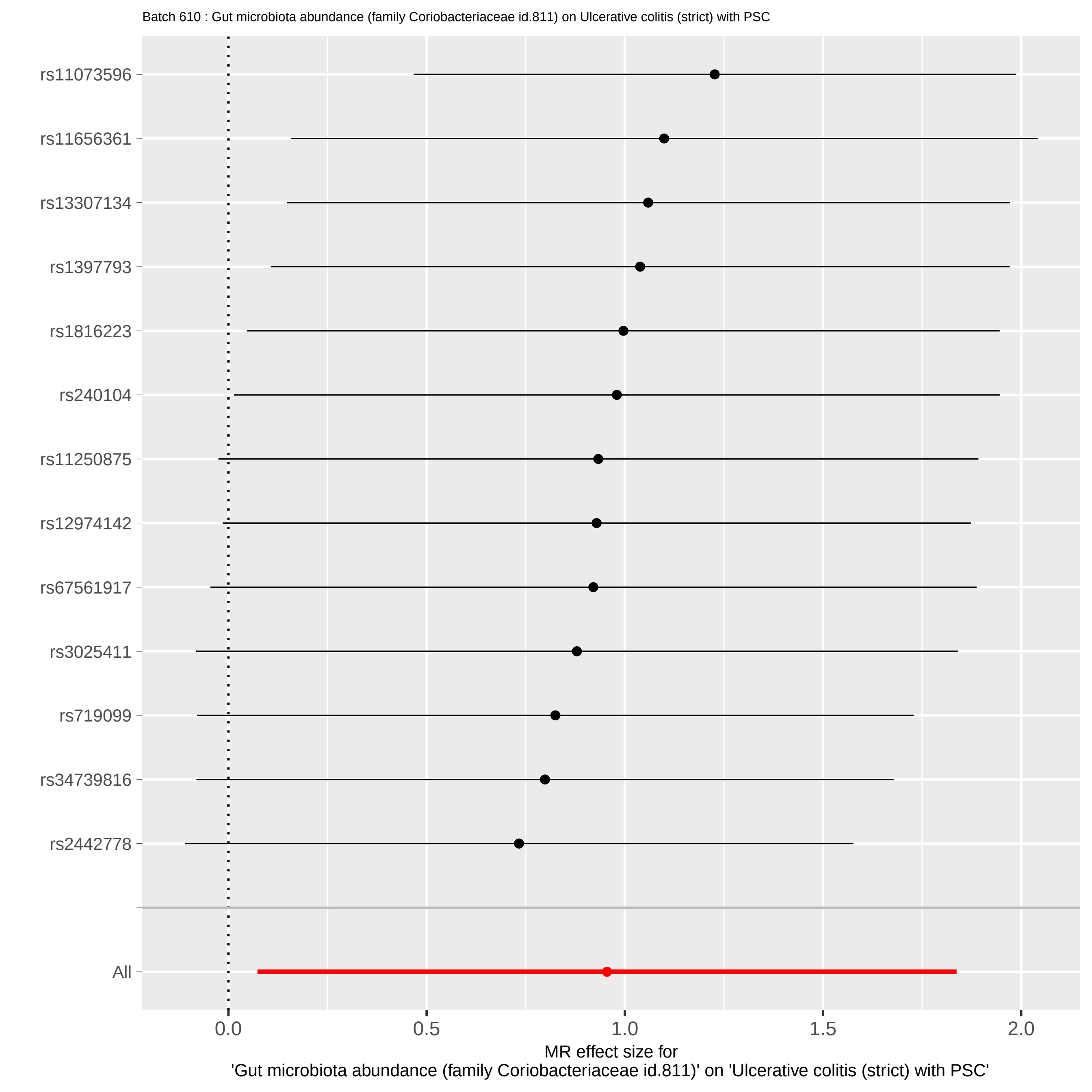

Batch 610 : Gut microbiota abundance (family Coriobacteriaceae id.811) on Ulcerative colitis (strict) with PSC
rs11073596
rs11656361
rs13307134
rs1397793
rs1816223
rs240104
rs11250875
rs12974142
rs67561917
rs3025411
rs719099
rs34739816
rs2442778
All
0.0
0.5
1.0
1.5
2.0
MR effect size for
'Gut microbiota abundance (family Coriobacteriaceae id.811)' on 'Ulcerative colitis (strict) with PSC'

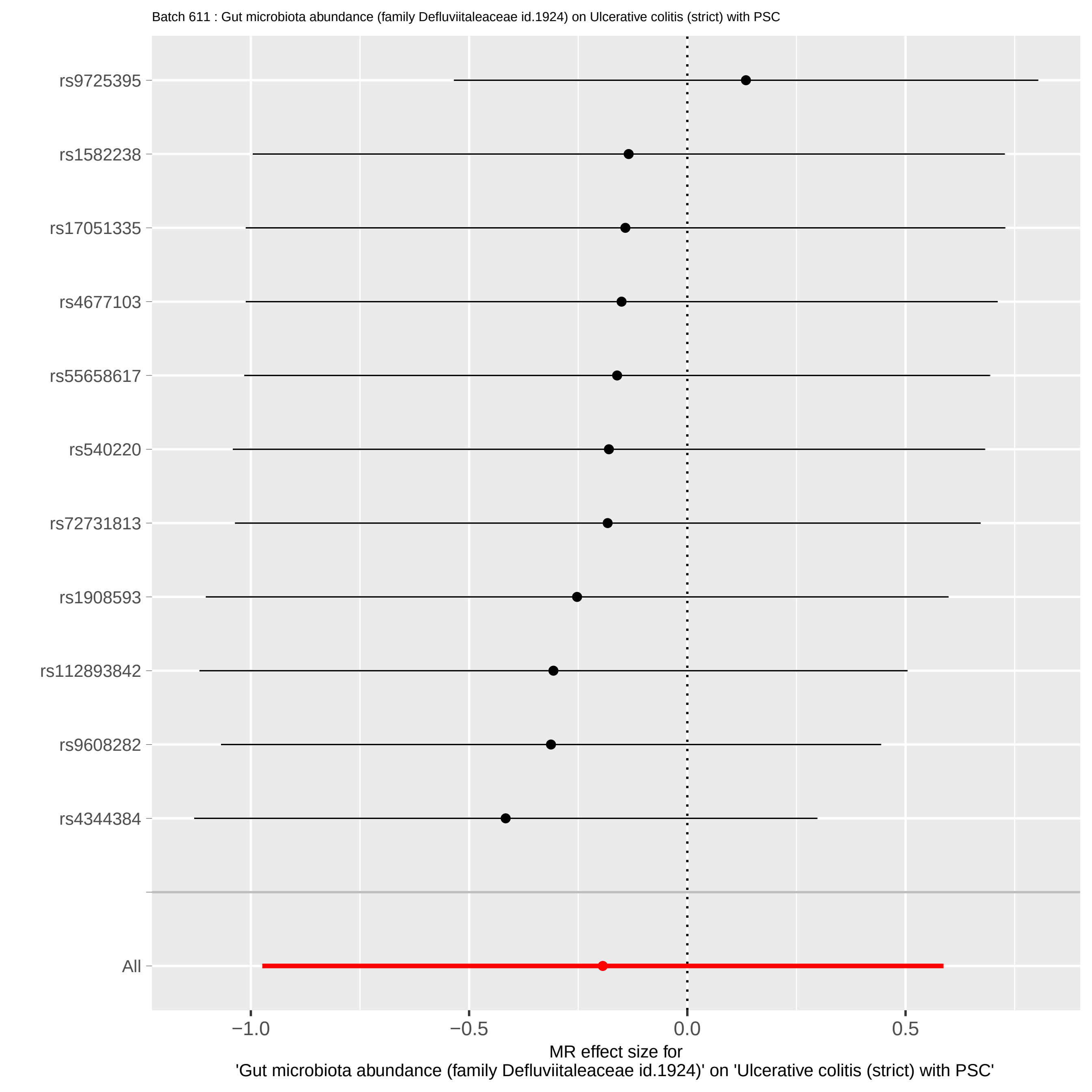

Batch 611 : Gut microbiota abundance (family Defluviitaleaceae id.1924) on Ulcerative colitis (strict) with PSC
rs9725395
rs1582238
rs17051335
rs4677103
rs55658617
rs540220
rs72731813
rs1908593
rs112893842
rs9608282
rs4344384
All
−1.0
−0.5
0.0
0.5
MR effect size for
'Gut microbiota abundance (family Defluviitaleaceae id.1924)' on 'Ulcerative colitis (strict) with PSC'

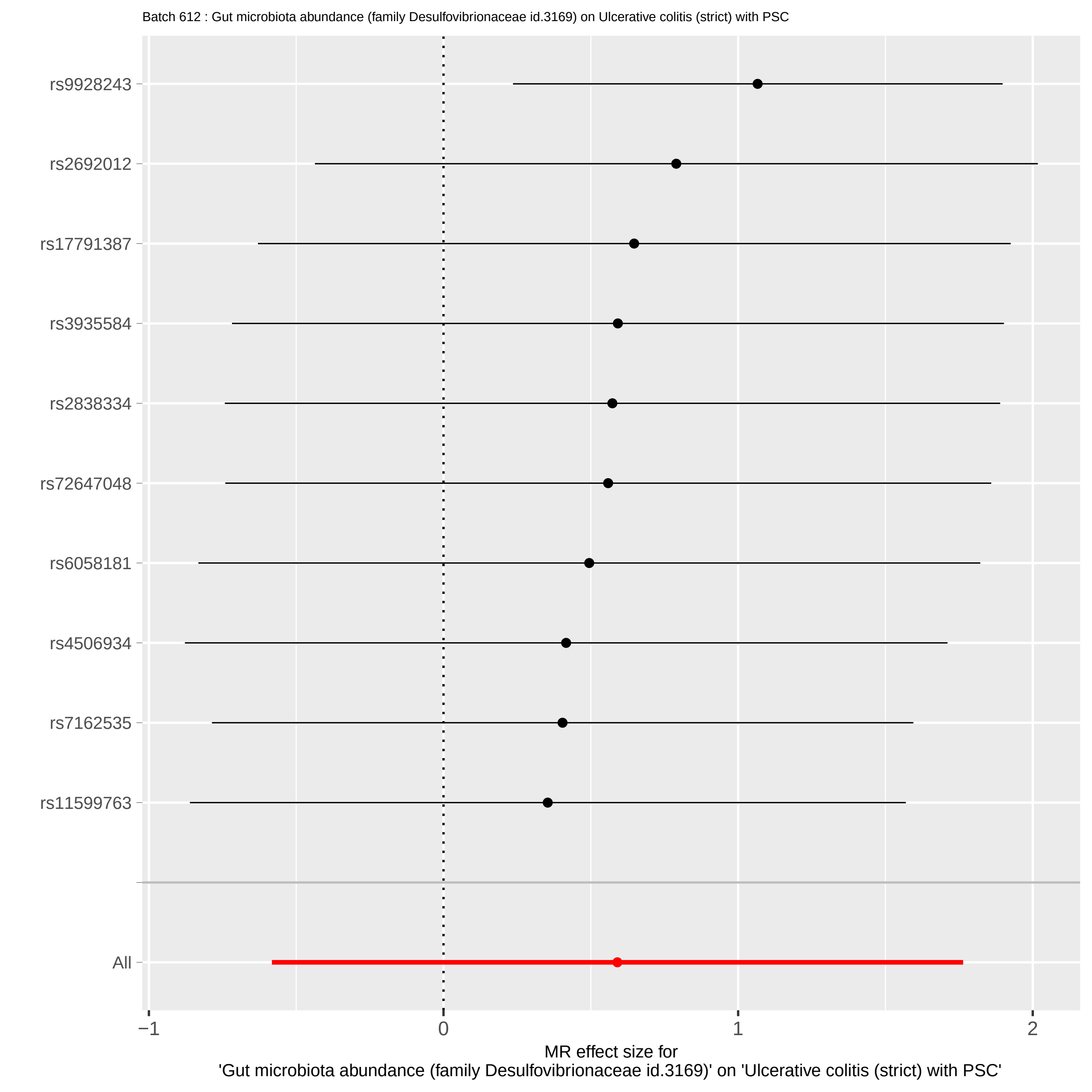

Batch 612 : Gut microbiota abundance (family Desulfovibrionaceae id.3169) on Ulcerative colitis (strict) with PSC
rs9928243
rs2692012
rs17791387
rs3935584
rs2838334
rs72647048
rs6058181
rs4506934
rs7162535
rs11599763
All
−1
0
1
2
MR effect size for
'Gut microbiota abundance (family Desulfovibrionaceae id.3169)' on 'Ulcerative colitis (strict) with PSC'

Batch 613 : Gut microbiota abundance (family Enterobacteriaceae id.3469) on Ulcerative colitis (strict) with PSC

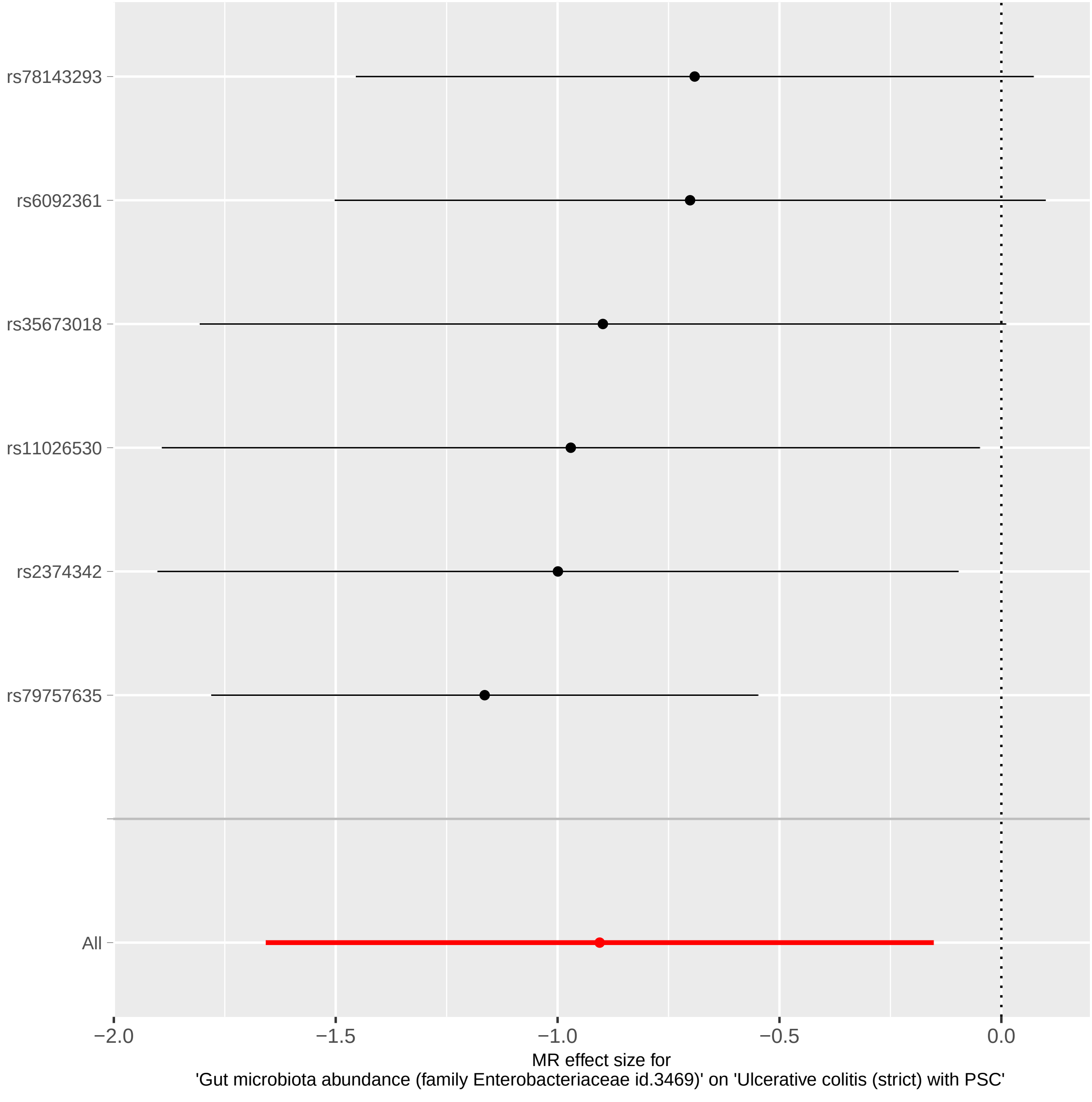

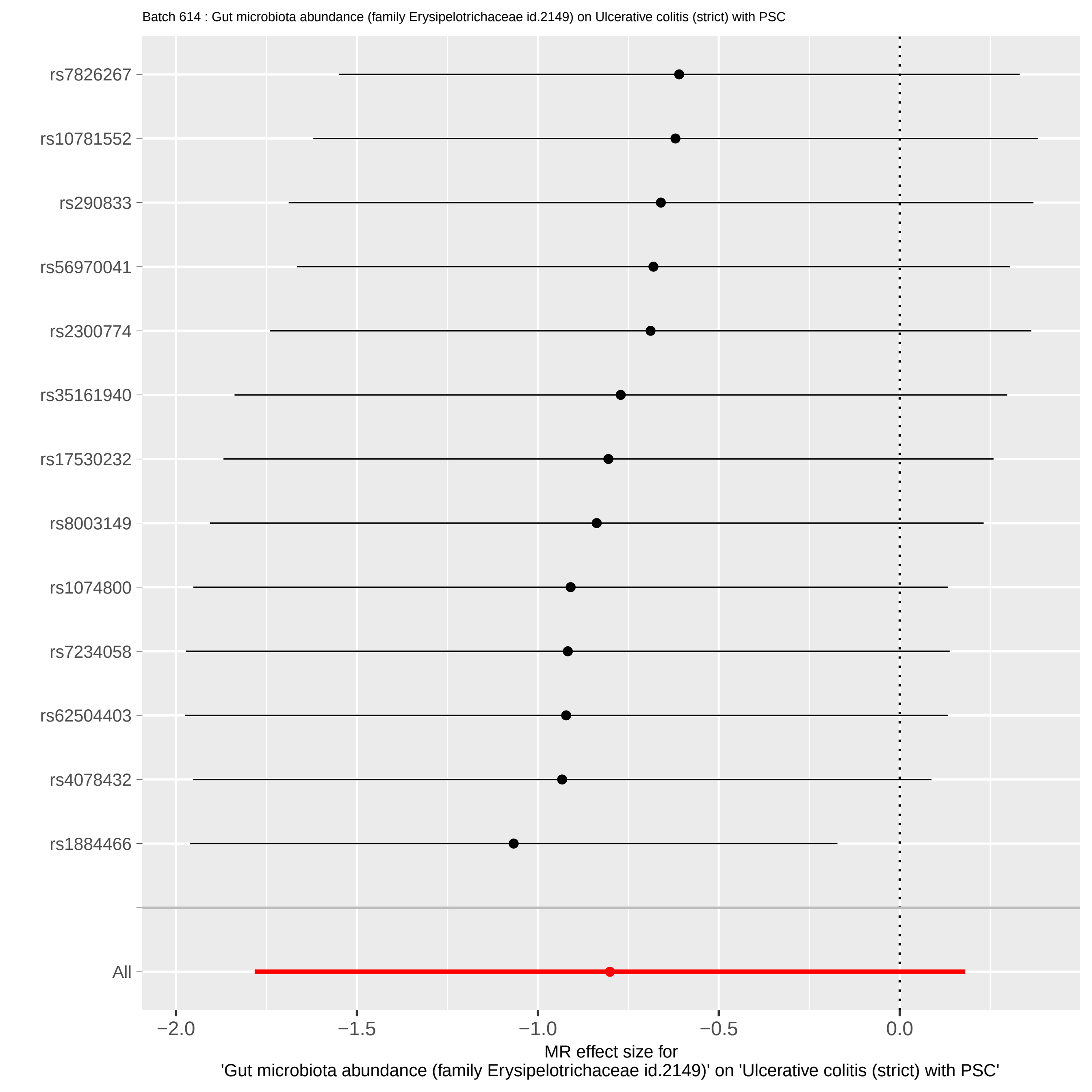

Batch 614 : Gut microbiota abundance (family Erysipelotrichaceae id.2149) on Ulcerative colitis (strict) with PSC
rs7826267
rs10781552
rs290833
rs56970041
rs2300774
rs35161940
rs17530232
rs8003149
rs1074800
rs7234058
rs62504403
rs4078432
rs1884466
All
−2.0
−1.5
−1.0
−0.5
0.0
MR effect size for
'Gut microbiota abundance (family Erysipelotrichaceae id.2149)' on 'Ulcerative colitis (strict) with PSC'

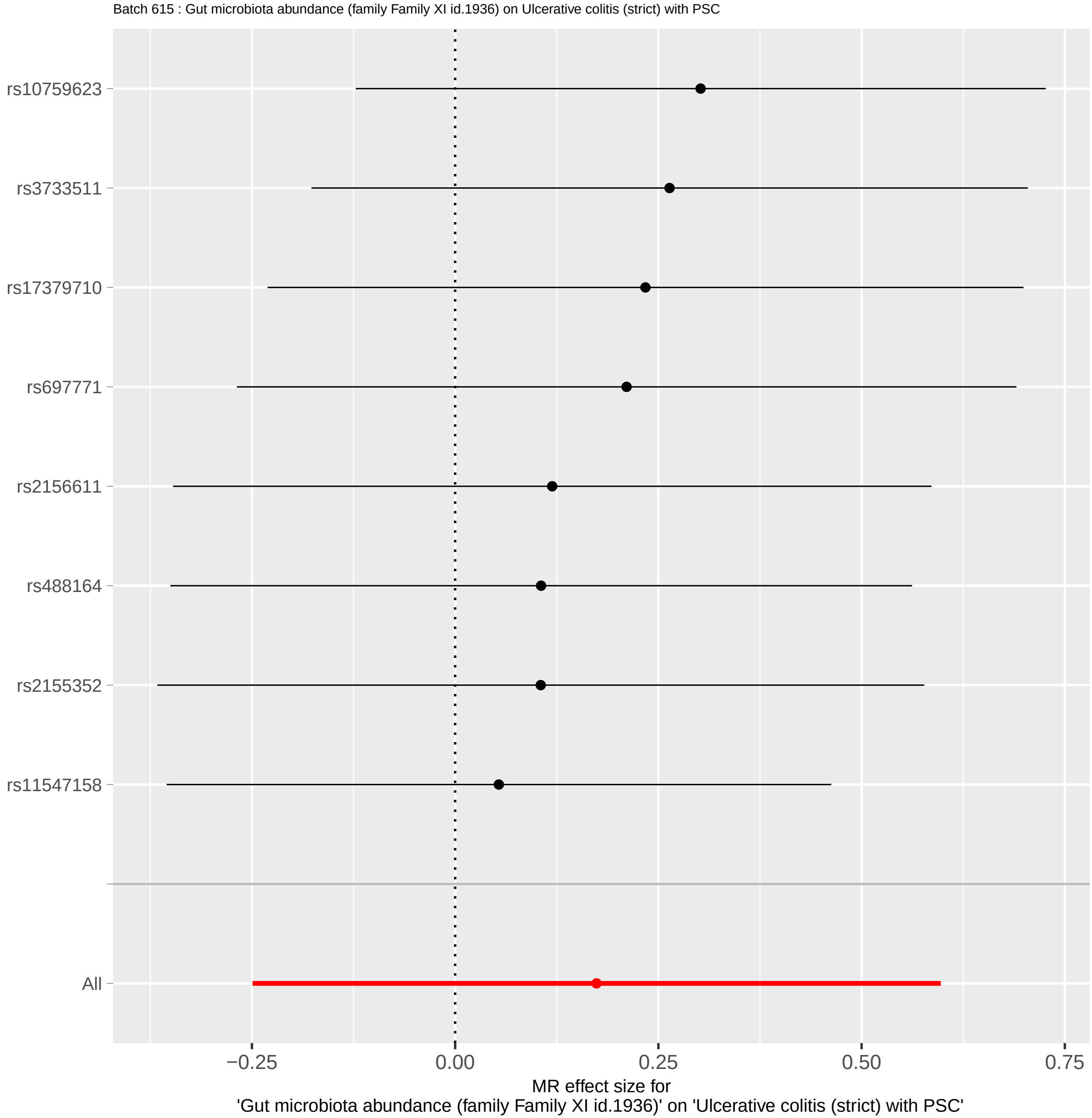

Batch 615 : Gut microbiota abundance (family Family XI id.1936) on Ulcerative colitis (strict) with PSC
rs10759623
rs3733511
rs17379710
rs697771
rs2156611
rs488164
rs2155352
rs11547158
All
−0.25
0.00
0.25
0.50
0.75
MR effect size for
'Gut microbiota abundance (family Family XI id.1936)' on 'Ulcerative colitis (strict) with PSC'

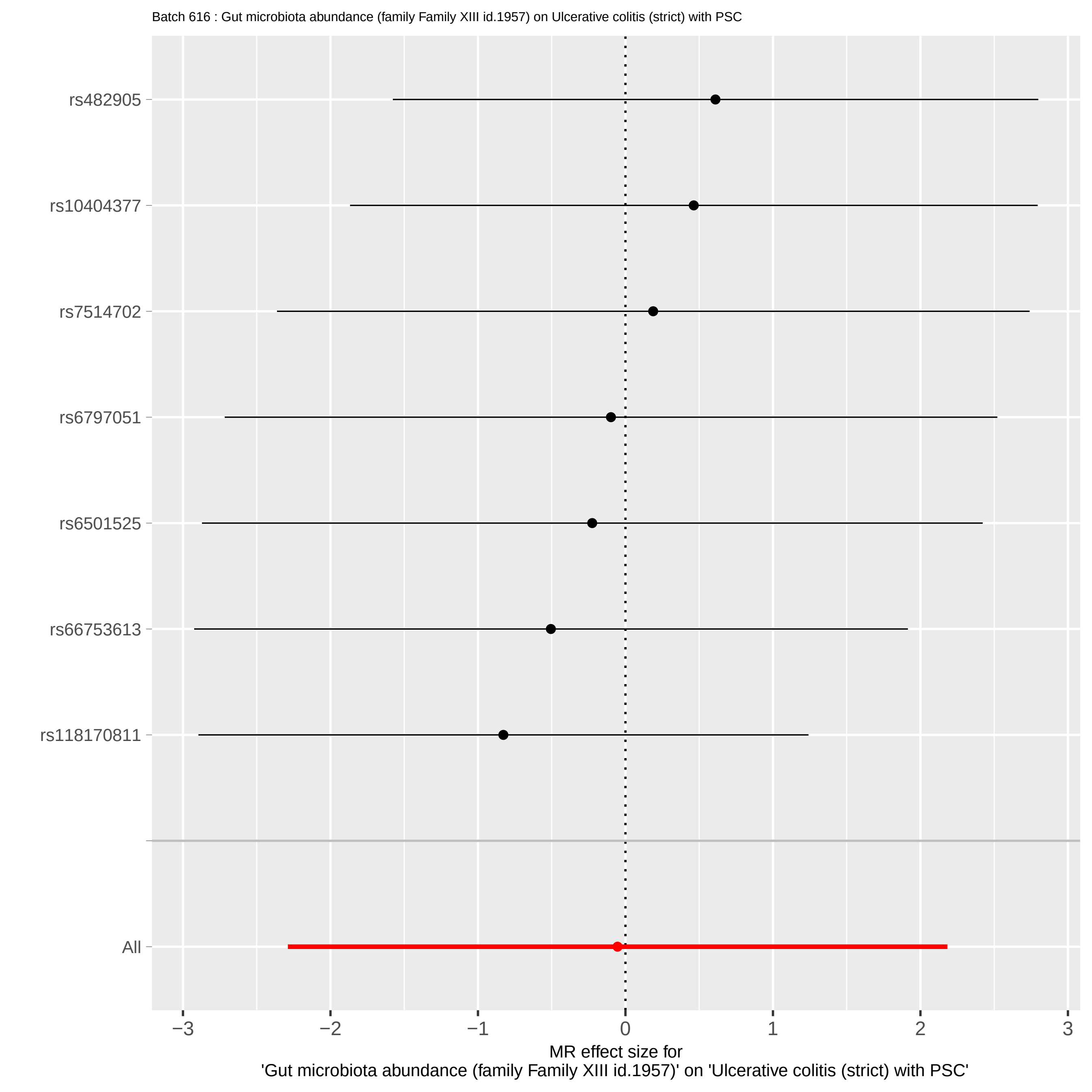
Batch 616 : Gut microbiota abundance (family Family XIII id.1957) on Ulcerative colitis (strict) with PSC
rs482905
rs10404377
rs7514702
rs6797051
rs6501525
rs66753613
rs118170811
All
−3
−2
−1
0
1
2
3
MR effect size for
'Gut microbiota abundance (family Family XIII id.1957)' on 'Ulcerative colitis (strict) with PSC'

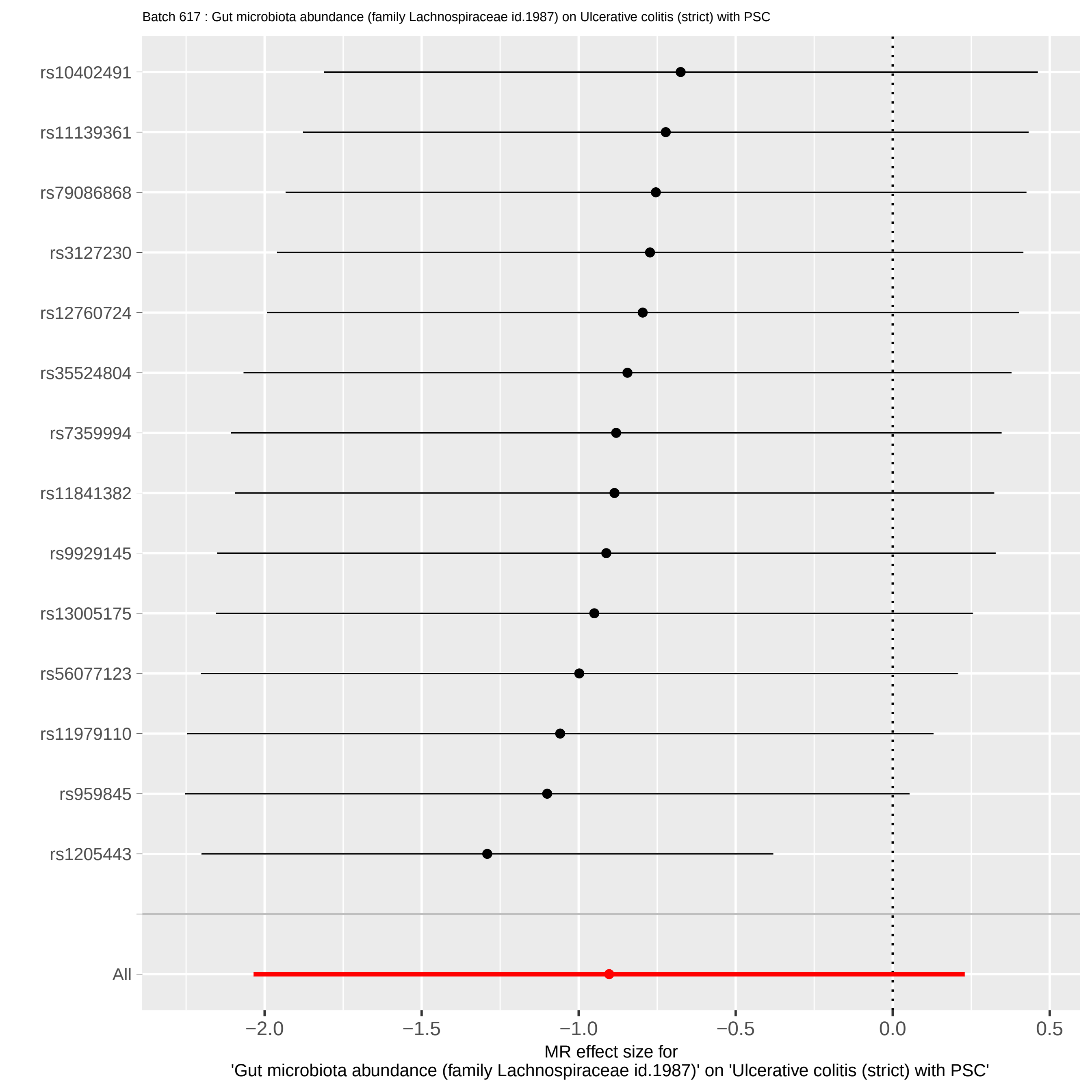
Batch 617 : Gut microbiota abundance (family Lachnospiraceae id.1987) on Ulcerative colitis (strict) with PSC
rs10402491
rs11139361
rs79086868
rs3127230
rs12760724
rs35524804
rs7359994
rs11841382
rs9929145
rs13005175
rs56077123
rs11979110
rs959845
rs1205443
All
−2.0
−1.5
−1.0
−0.5
0.0
0.5
MR effect size for
'Gut microbiota abundance (family Lachnospiraceae id.1987)' on 'Ulcerative colitis (strict) with PSC'

Batch 618 : Gut microbiota abundance (family Lactobacillaceae id.1836) on Ulcerative colitis (strict) with PSC

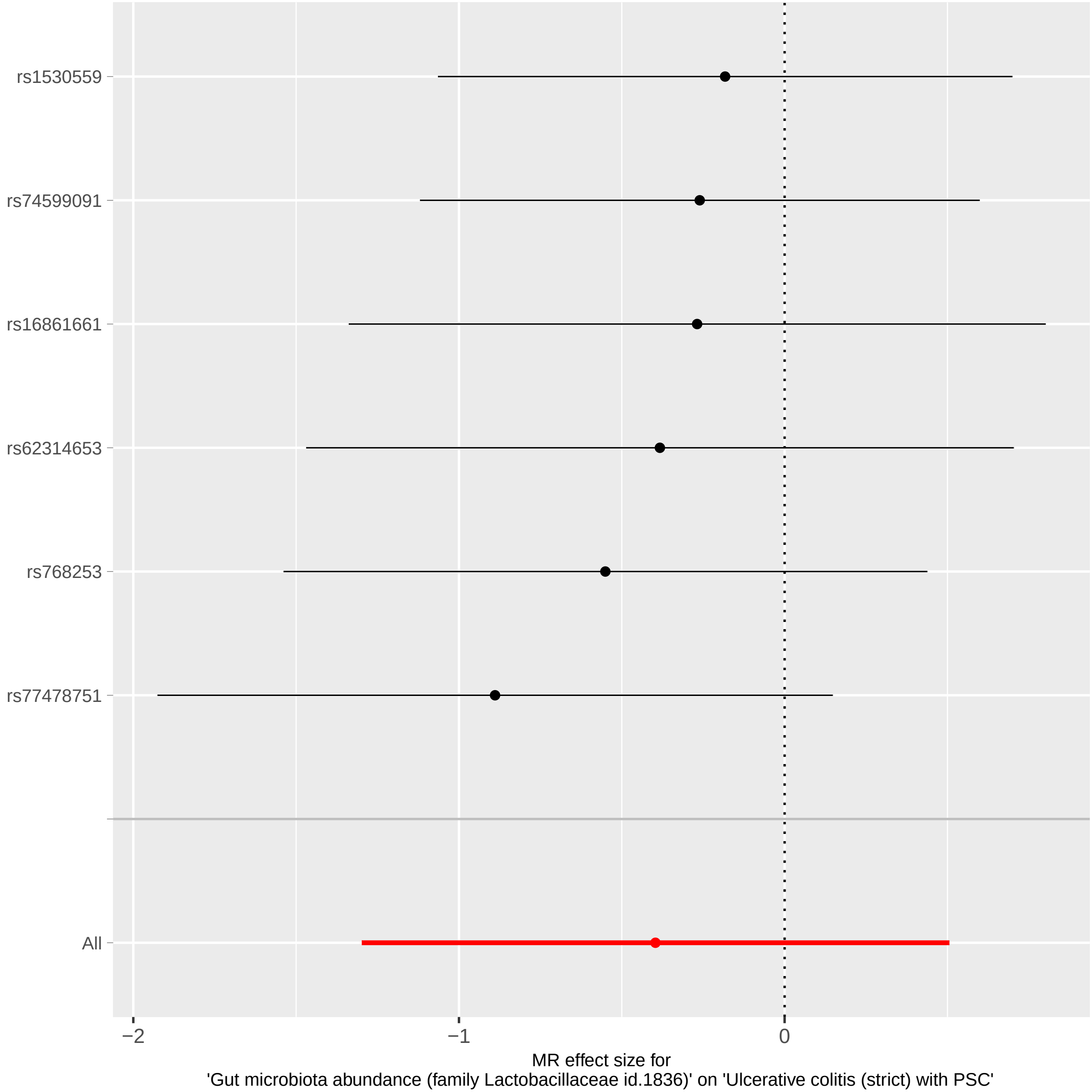

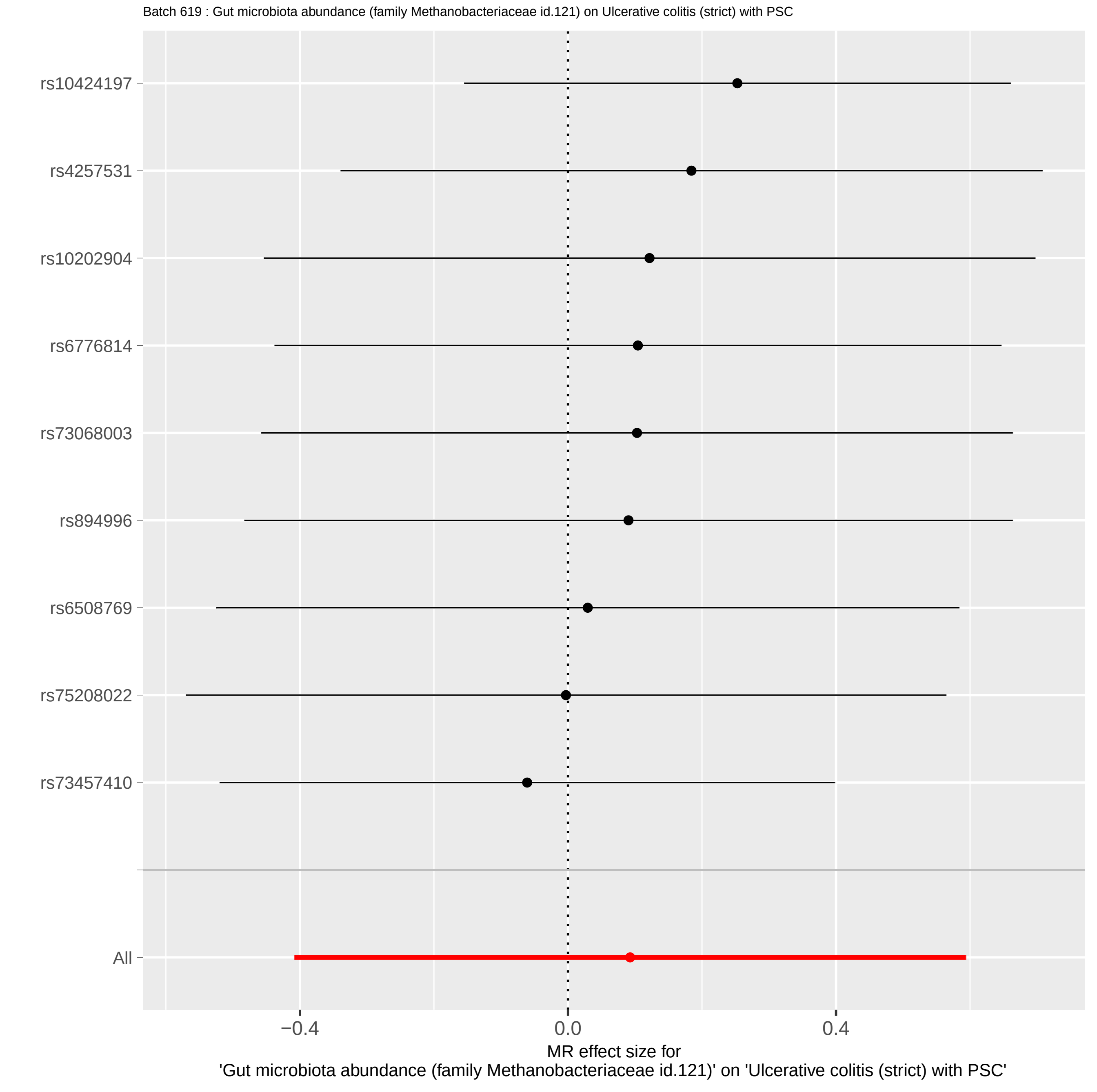
Batch 619 : Gut microbiota abundance (family Methanobacteriaceae id.121) on Ulcerative colitis (strict) with PSC
rs10424197
rs4257531
rs10202904
rs6776814
rs73068003
rs894996
rs6508769
rs75208022
rs73457410
All
−0.4
0.0
0.4
MR effect size for
'Gut microbiota abundance (family Methanobacteriaceae id.121)' on 'Ulcerative colitis (strict) with PSC'

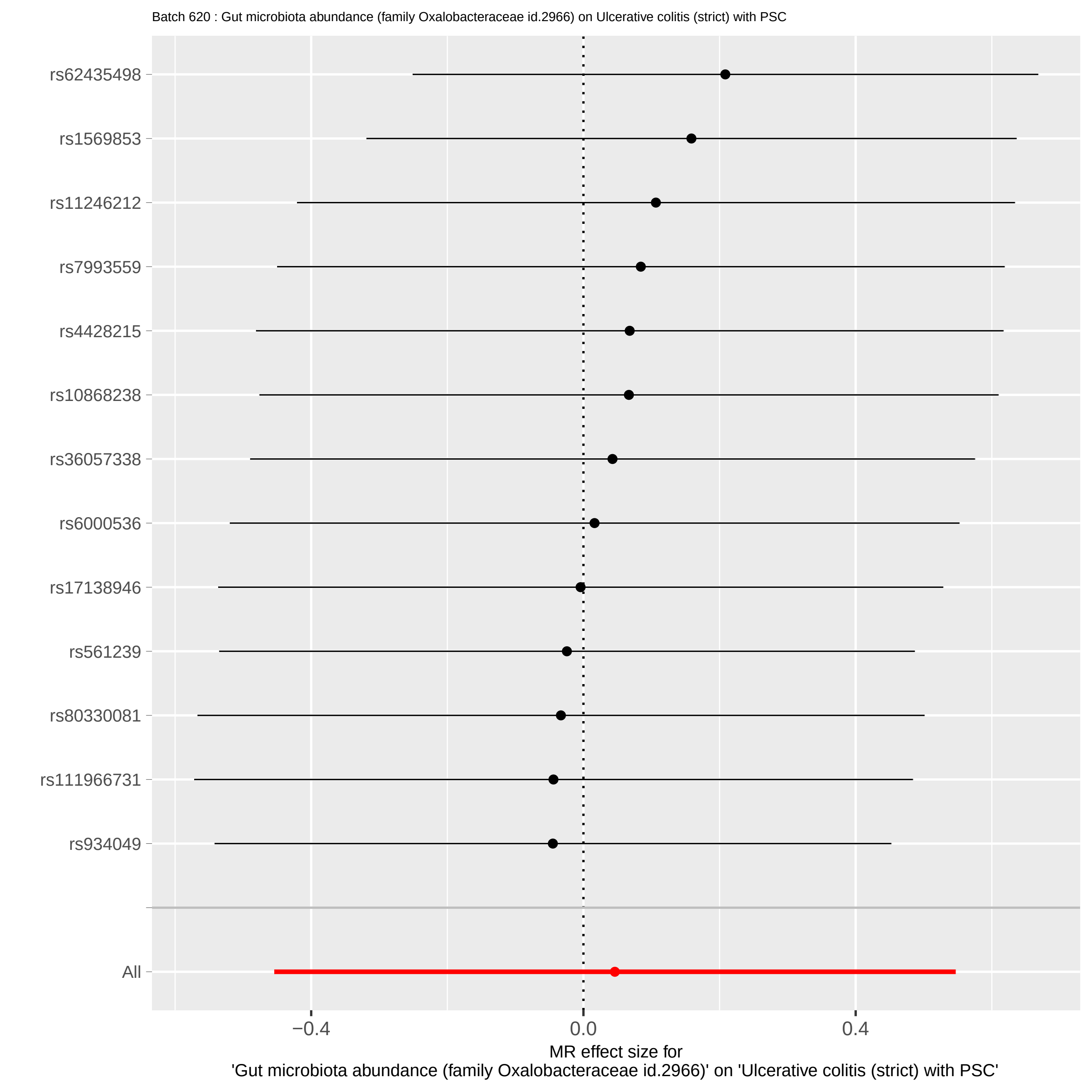
Batch 620 : Gut microbiota abundance (family Oxalobacteraceae id.2966) on Ulcerative colitis (strict) with PSC
rs62435498
rs1569853
rs11246212
rs7993559
rs4428215
rs10868238
rs36057338
rs6000536
rs17138946
rs561239
rs80330081
rs111966731
rs934049
All
−0.4
0.0
0.4
MR effect size for
'Gut microbiota abundance (family Oxalobacteraceae id.2966)' on 'Ulcerative colitis (strict) with PSC'

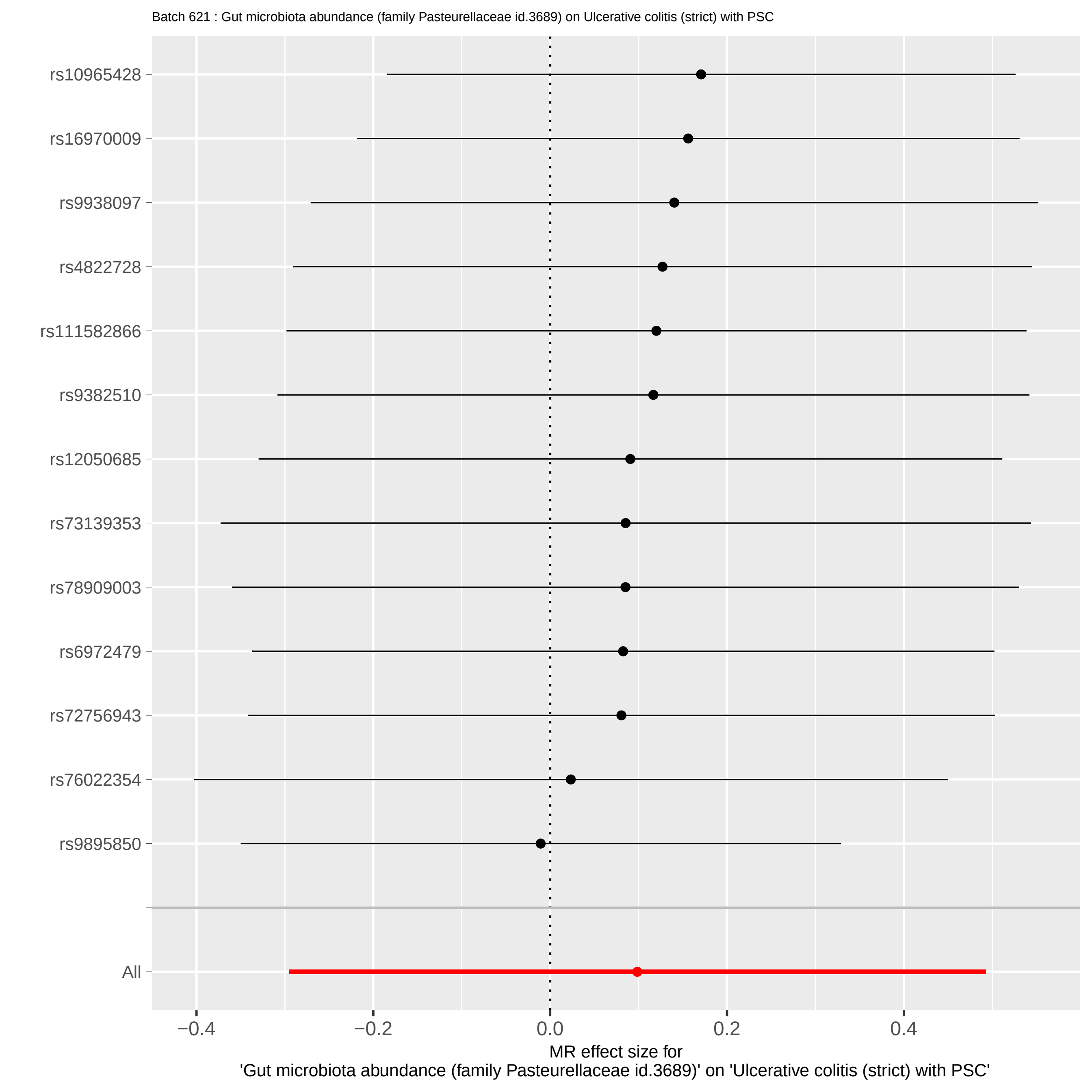

Batch 621 : Gut microbiota abundance (family Pasteurellaceae id.3689) on Ulcerative colitis (strict) with PSC
rs10965428
rs16970009
rs9938097
rs4822728
rs111582866
rs9382510
rs12050685
rs73139353
rs78909003
rs6972479
rs72756943
rs76022354
rs9895850
All
−0.4
−0.2
0.0
0.2
0.4
MR effect size for
'Gut microbiota abundance (family Pasteurellaceae id.3689)' on 'Ulcerative colitis (strict) with PSC'

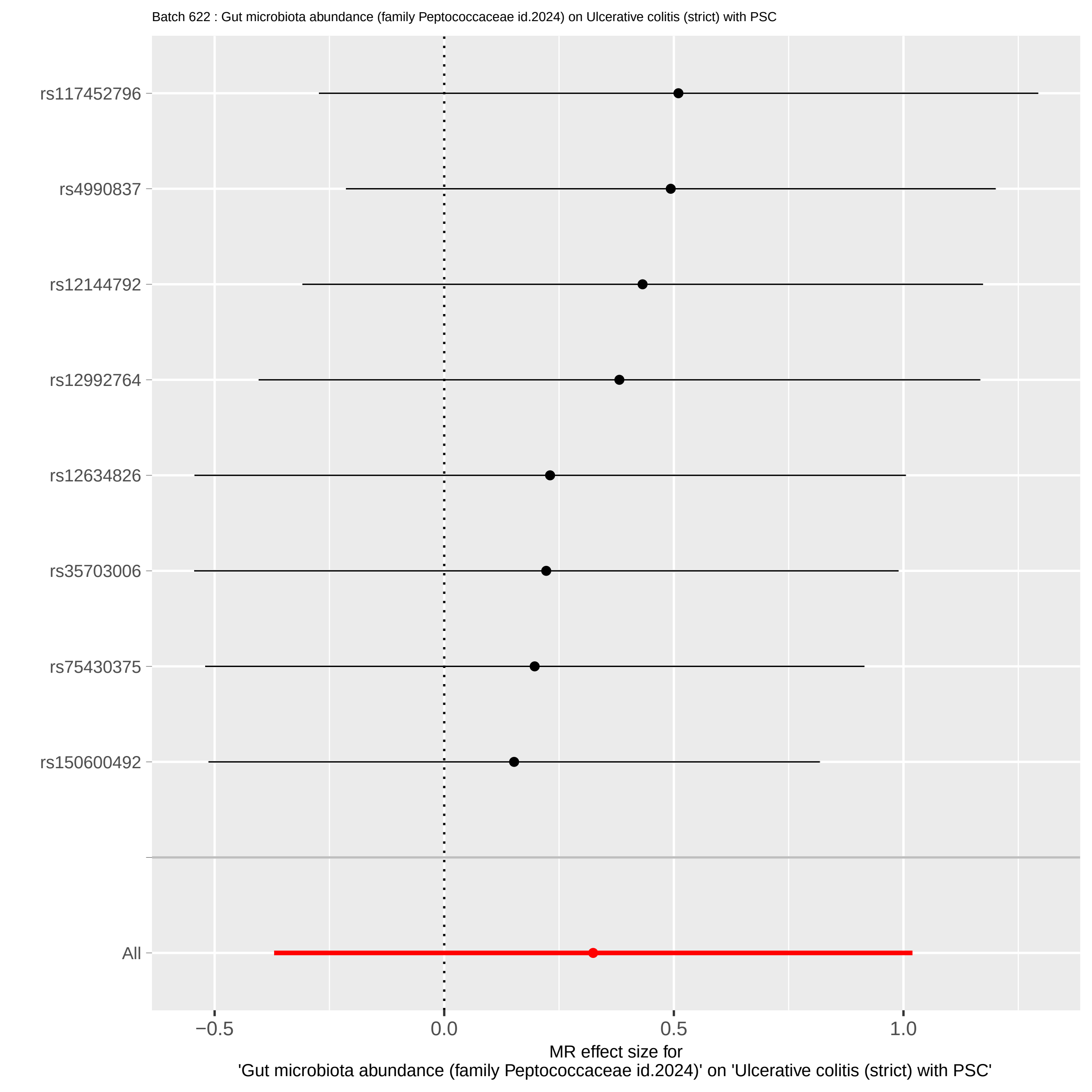

Batch 622 : Gut microbiota abundance (family Peptococcaceae id.2024) on Ulcerative colitis (strict) with PSC
rs117452796
rs4990837
rs12144792
rs12992764
rs12634826
rs35703006
rs75430375
rs150600492
All
−0.5
0.0
0.5
1.0
MR effect size for
'Gut microbiota abundance (family Peptococcaceae id.2024)' on 'Ulcerative colitis (strict) with PSC'

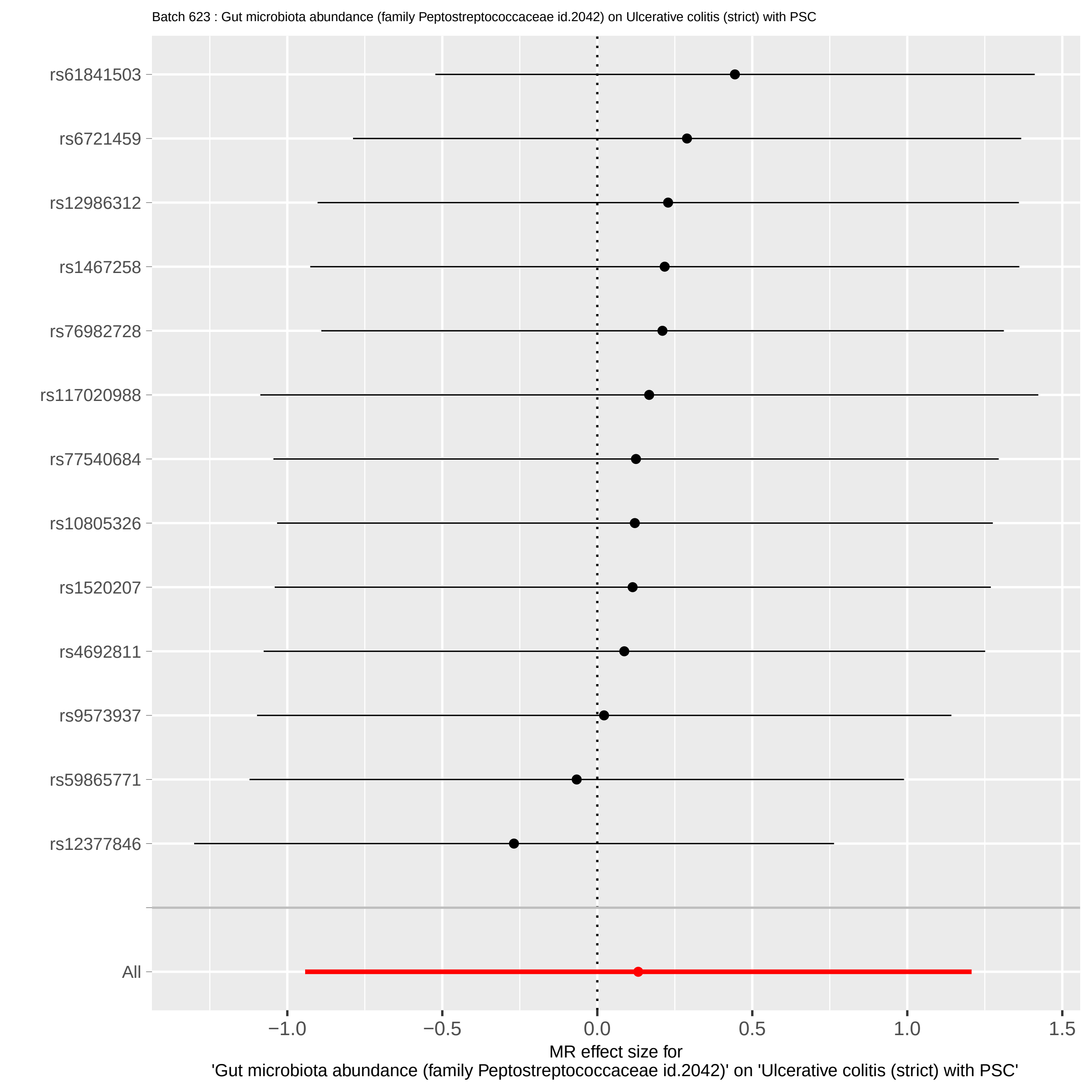
Batch 623 : Gut microbiota abundance (family Peptostreptococcaceae id.2042) on Ulcerative colitis (strict) with PSC
rs61841503
rs6721459
rs12986312
rs1467258
rs76982728
rs117020988
rs77540684
rs10805326
rs1520207
rs4692811
rs9573937
rs59865771
rs12377846
All
−1.0
−0.5
0.0
0.5
1.0
1.5
MR effect size for
'Gut microbiota abundance (family Peptostreptococcaceae id.2042)' on 'Ulcerative colitis (strict) with PSC'

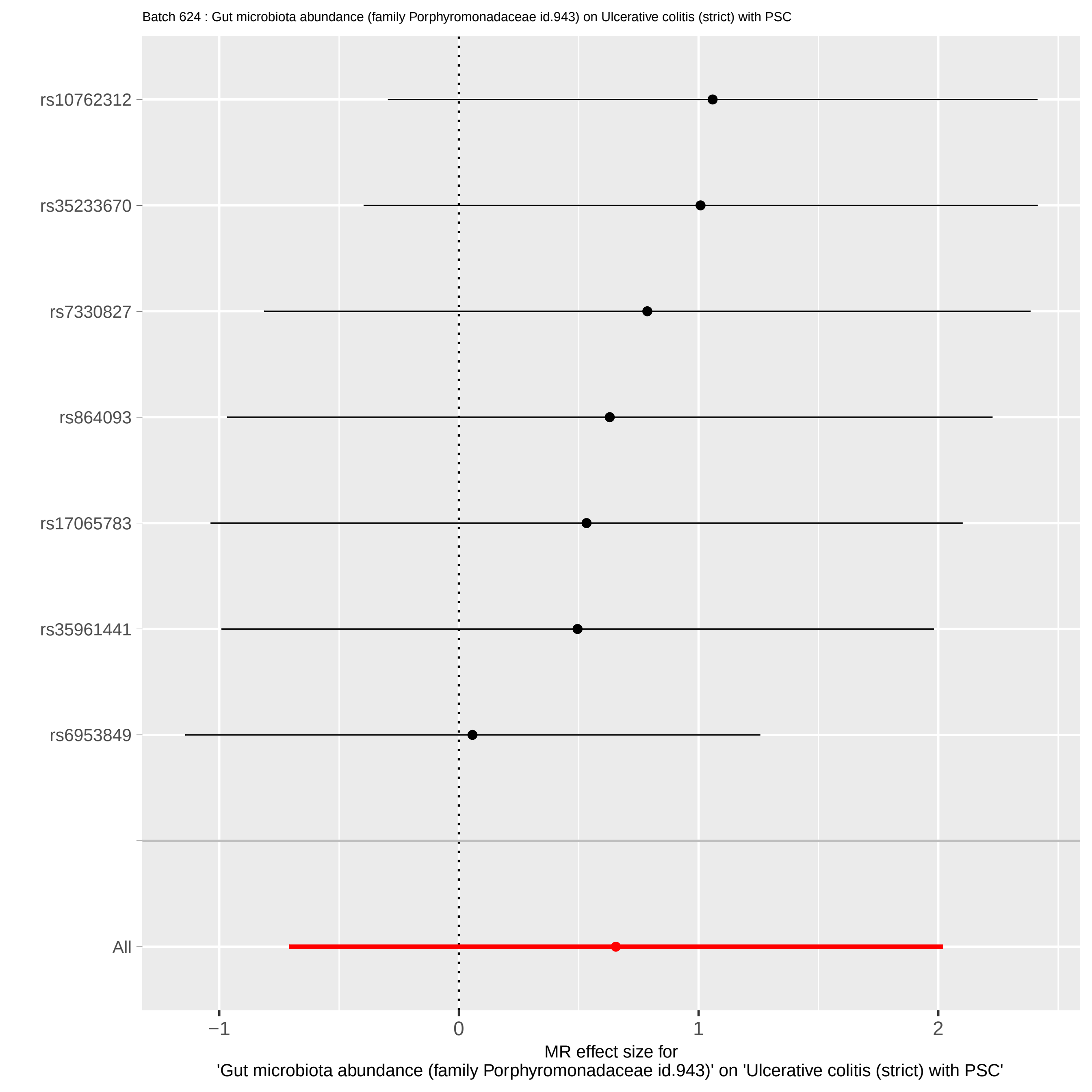
Batch 624 : Gut microbiota abundance (family Porphyromonadaceae id.943) on Ulcerative colitis (strict) with PSC
rs10762312
rs35233670
rs7330827
rs864093
rs17065783
rs35961441
rs6953849
All
−1
0
1
2
MR effect size for
'Gut microbiota abundance (family Porphyromonadaceae id.943)' on 'Ulcerative colitis (strict) with PSC'

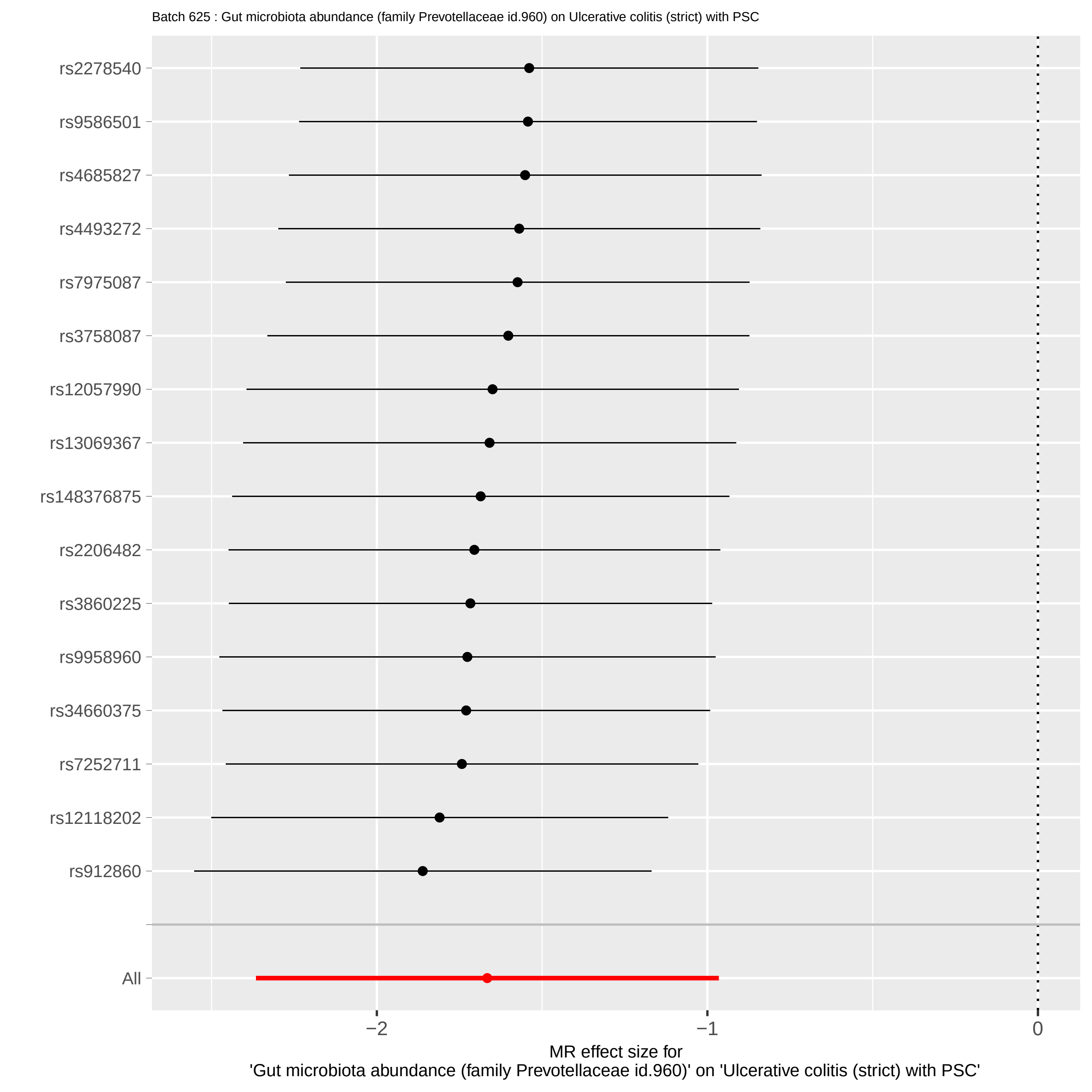

Batch 625 : Gut microbiota abundance (family Prevotellaceae id.960) on Ulcerative colitis (strict) with PSC
rs2278540
rs9586501
rs4685827
rs4493272
rs7975087
rs3758087
rs12057990
rs13069367
rs148376875
rs2206482
rs3860225
rs9958960
rs34660375
rs7252711
rs12118202
rs912860
All
−2
−1
0
MR effect size for
'Gut microbiota abundance (family Prevotellaceae id.960)' on 'Ulcerative colitis (strict) with PSC'

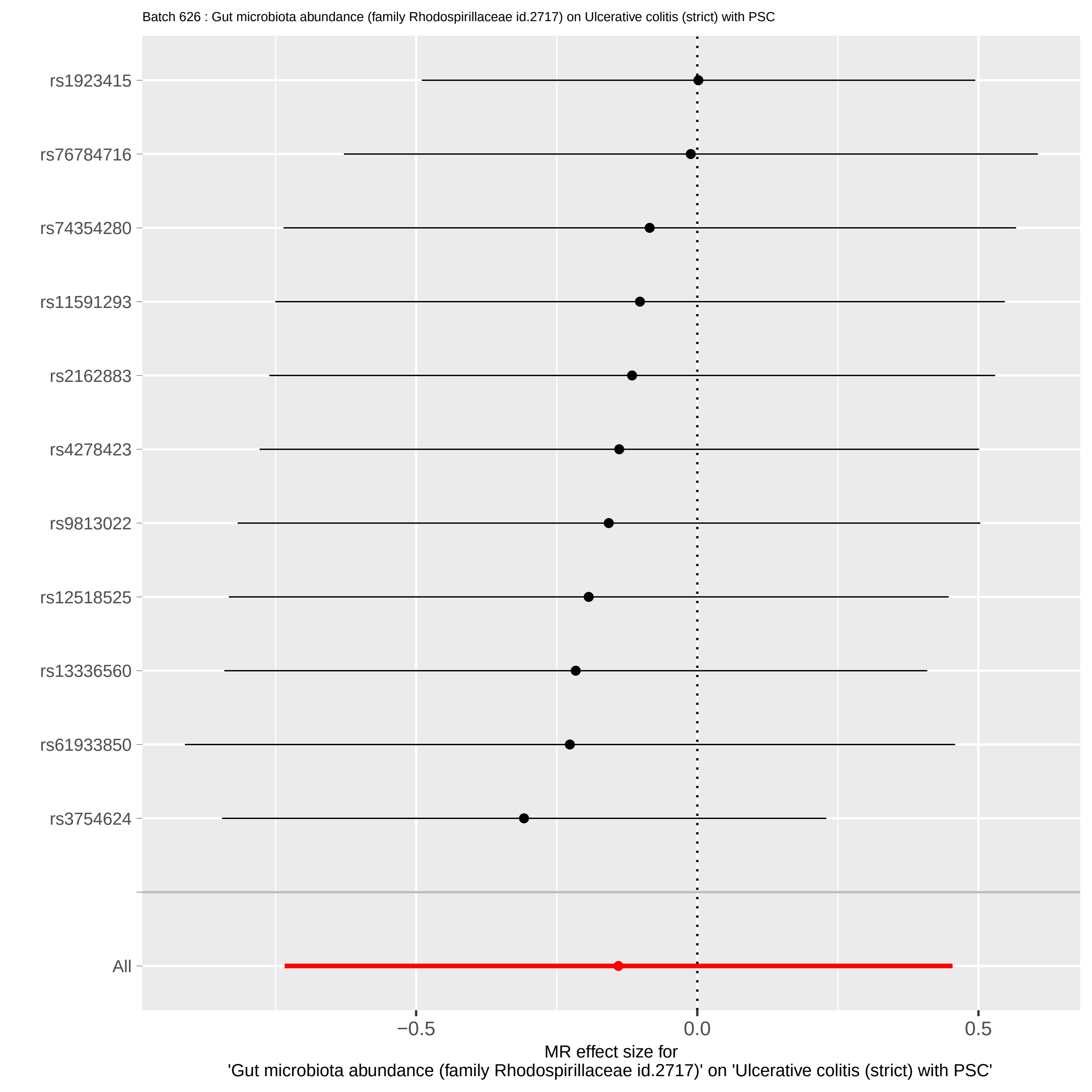
Batch 626 : Gut microbiota abundance (family Rhodospirillaceae id.2717) on Ulcerative colitis (strict) with PSC
rs1923415
rs76784716
rs74354280
rs11591293
rs2162883
rs4278423
rs9813022
rs12518525
rs13336560
rs61933850
rs3754624
All
−0.5
0.0
0.5
MR effect size for
'Gut microbiota abundance (family Rhodospirillaceae id.2717)' on 'Ulcerative colitis (strict) with PSC'

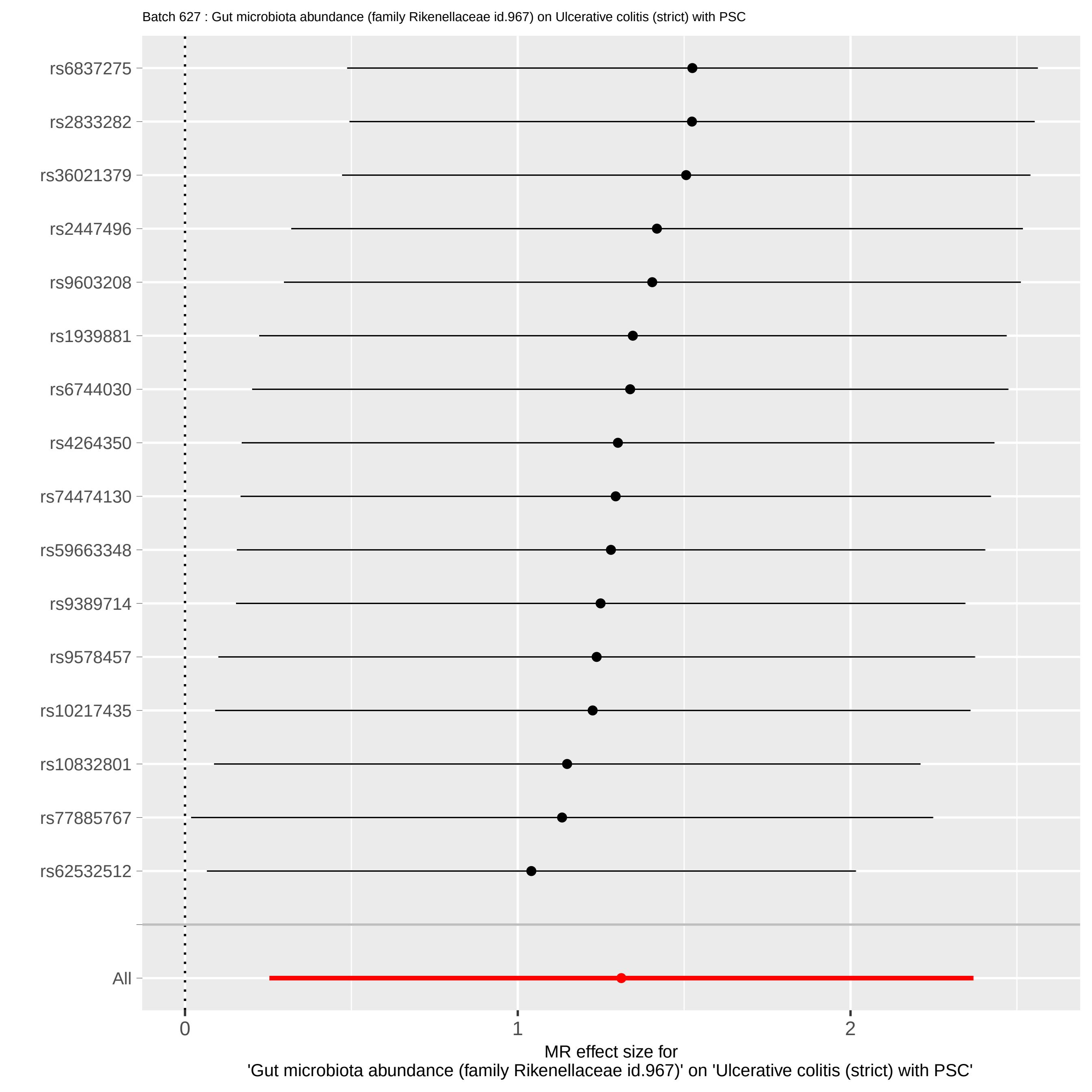
Batch 627 : Gut microbiota abundance (family Rikenellaceae id.967) on Ulcerative colitis (strict) with PSC
rs6837275
rs2833282
rs36021379
rs2447496
rs9603208
rs1939881
rs6744030
rs4264350
rs74474130
rs59663348
rs9389714
rs9578457
rs10217435
rs10832801
rs77885767
rs62532512
All
0
1
2
MR effect size for
'Gut microbiota abundance (family Rikenellaceae id.967)' on 'Ulcerative colitis (strict) with PSC'

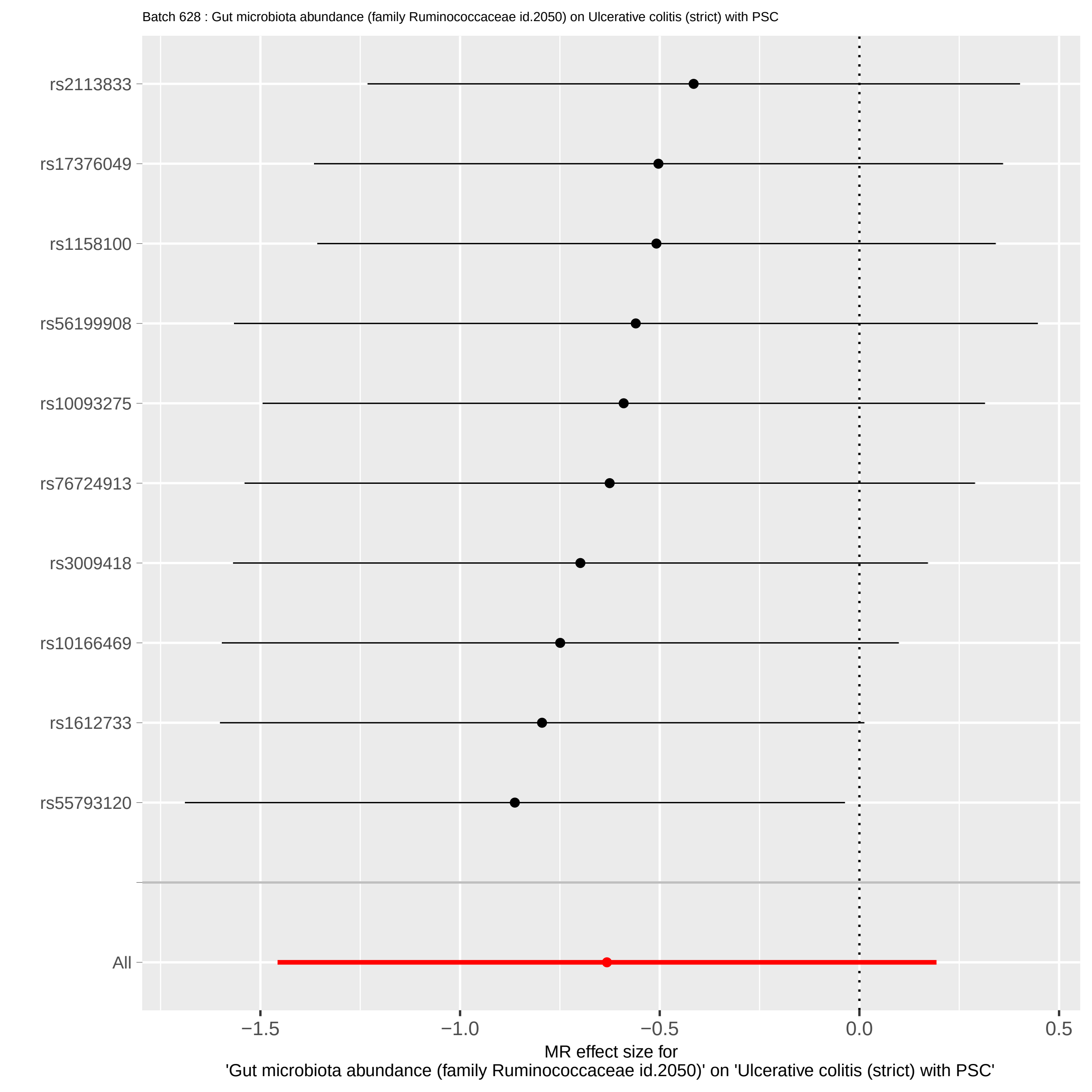
Batch 628 : Gut microbiota abundance (family Ruminococcaceae id.2050) on Ulcerative colitis (strict) with PSC
rs2113833
rs17376049
rs1158100
rs56199908
rs10093275
rs76724913
rs3009418
rs10166469
rs1612733
rs55793120
All
−1.5
−1.0
−0.5
0.0
0.5
MR effect size for
'Gut microbiota abundance (family Ruminococcaceae id.2050)' on 'Ulcerative colitis (strict) with PSC'

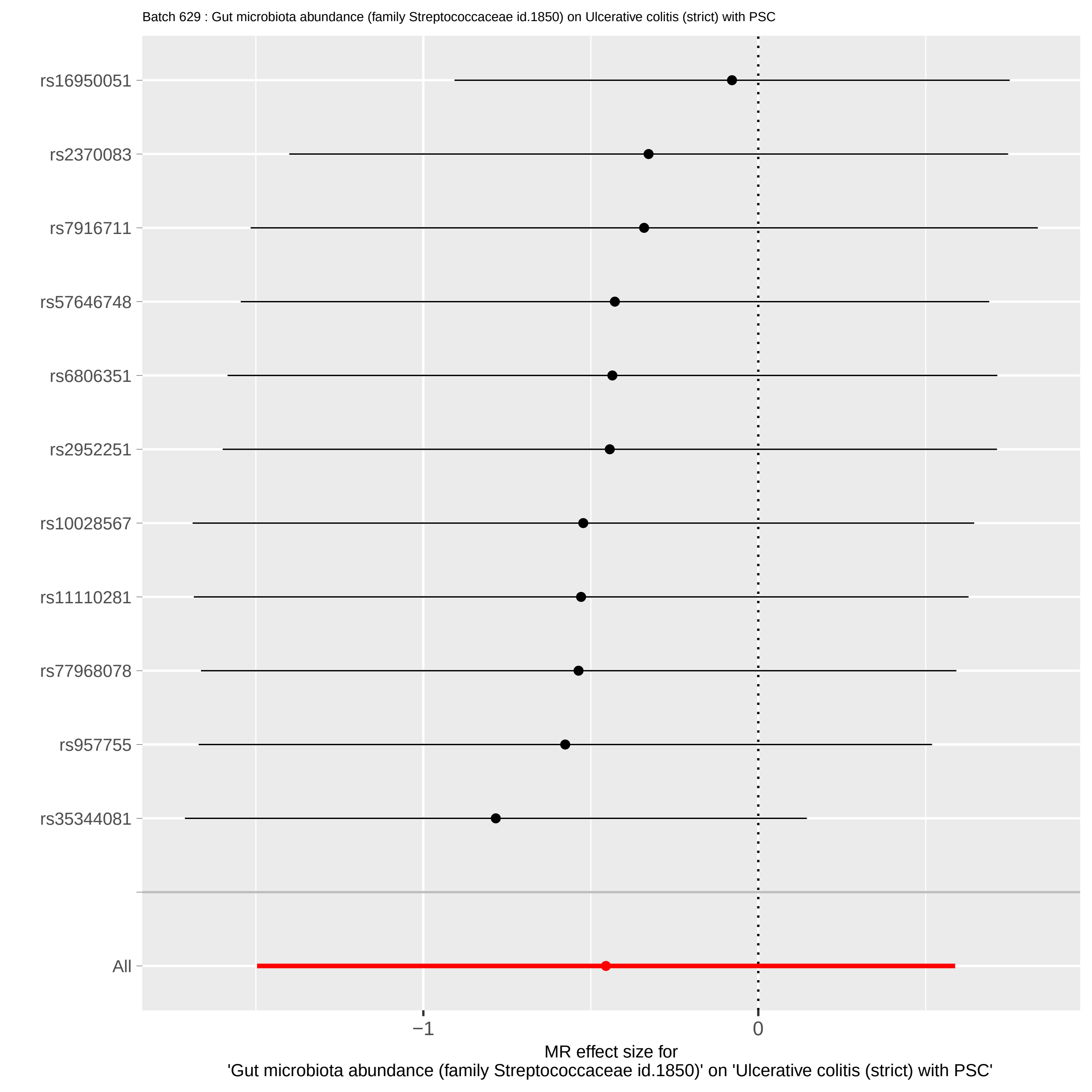

Batch 629 : Gut microbiota abundance (family Streptococcaceae id.1850) on Ulcerative colitis (strict) with PSC
rs16950051
rs2370083
rs7916711
rs57646748
rs6806351
rs2952251
rs10028567
rs11110281
rs77968078
rs957755
rs35344081
All
−1
0
MR effect size for
'Gut microbiota abundance (family Streptococcaceae id.1850)' on 'Ulcerative colitis (strict) with PSC'

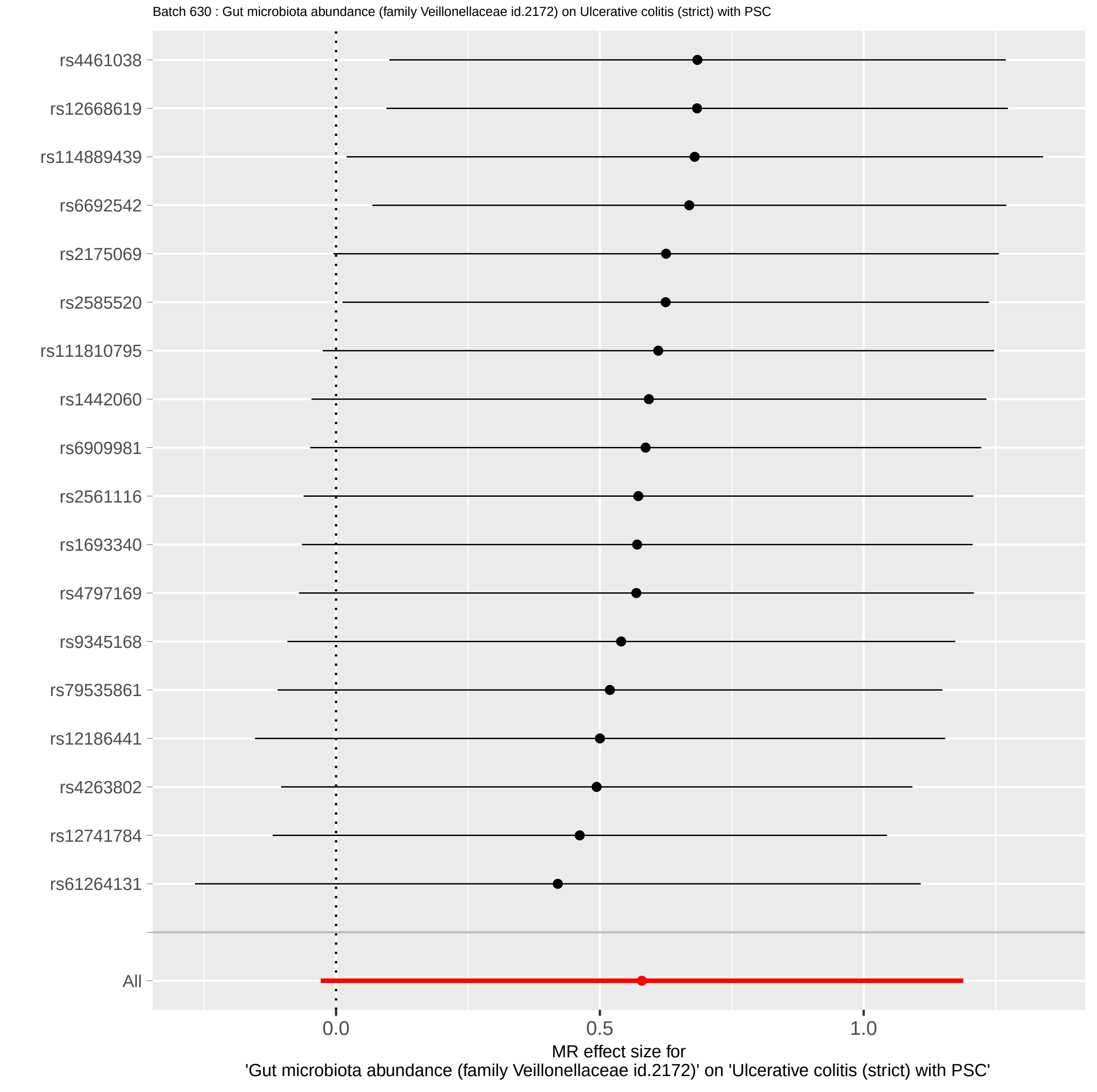

Batch 630 : Gut microbiota abundance (family Veillonellaceae id.2172) on Ulcerative colitis (strict) with PSC
rs4461038
rs12668619
rs114889439
rs6692542
rs2175069
rs2585520
rs111810795
rs1442060
rs6909981
rs2561116
rs1693340
rs4797169
rs9345168
rs79535861
rs12186441
rs4263802
rs12741784
rs61264131
All
0.0
0.5
1.0
MR effect size for
'Gut microbiota abundance (family Veillonellaceae id.2172)' on 'Ulcerative colitis (strict) with PSC'

Batch 631 : Gut microbiota abundance (family Verrucomicrobiaceae id.4036) on Ulcerative colitis (strict) with PSC

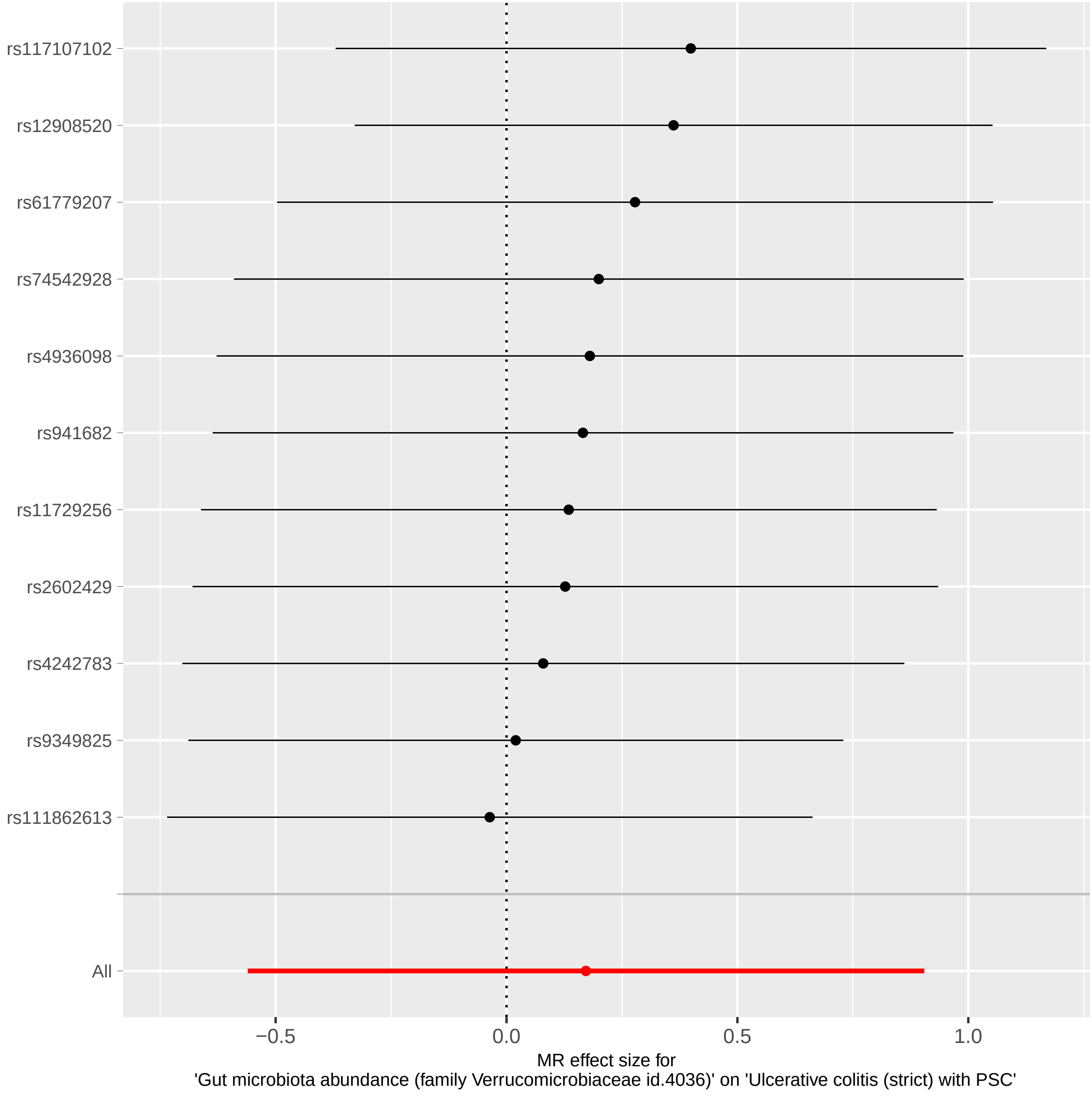

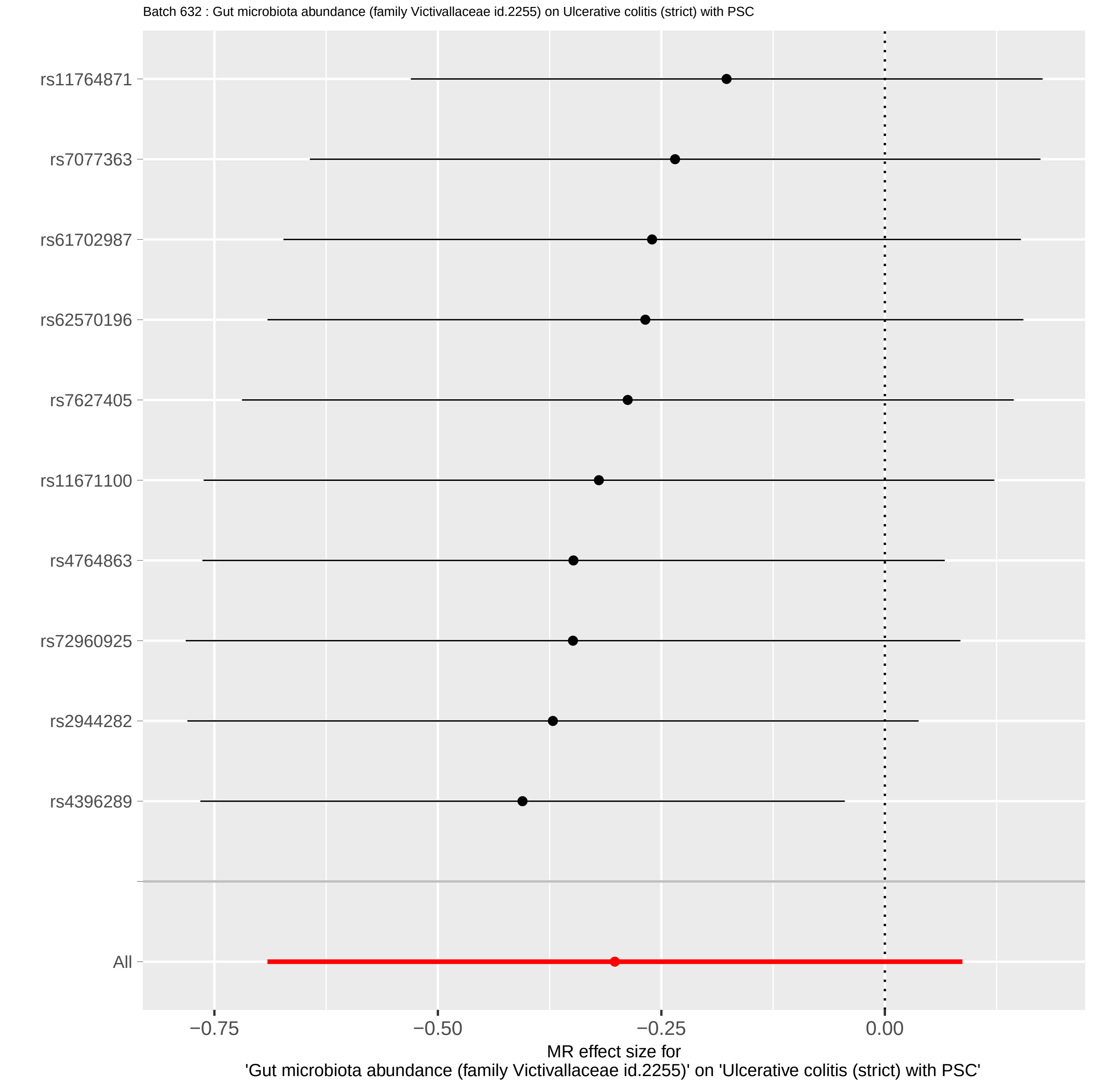
Batch 632 : Gut microbiota abundance (family Victivallaceae id.2255) on Ulcerative colitis (strict) with PSC
rs11764871
rs7077363
rs61702987
rs62570196
rs7627405
rs11671100
rs4764863
rs72960925
rs2944282
rs4396289
All
−0.75
−0.50
−0.25
0.00
MR effect size for
'Gut microbiota abundance (family Victivallaceae id.2255)' on 'Ulcerative colitis (strict) with PSC'

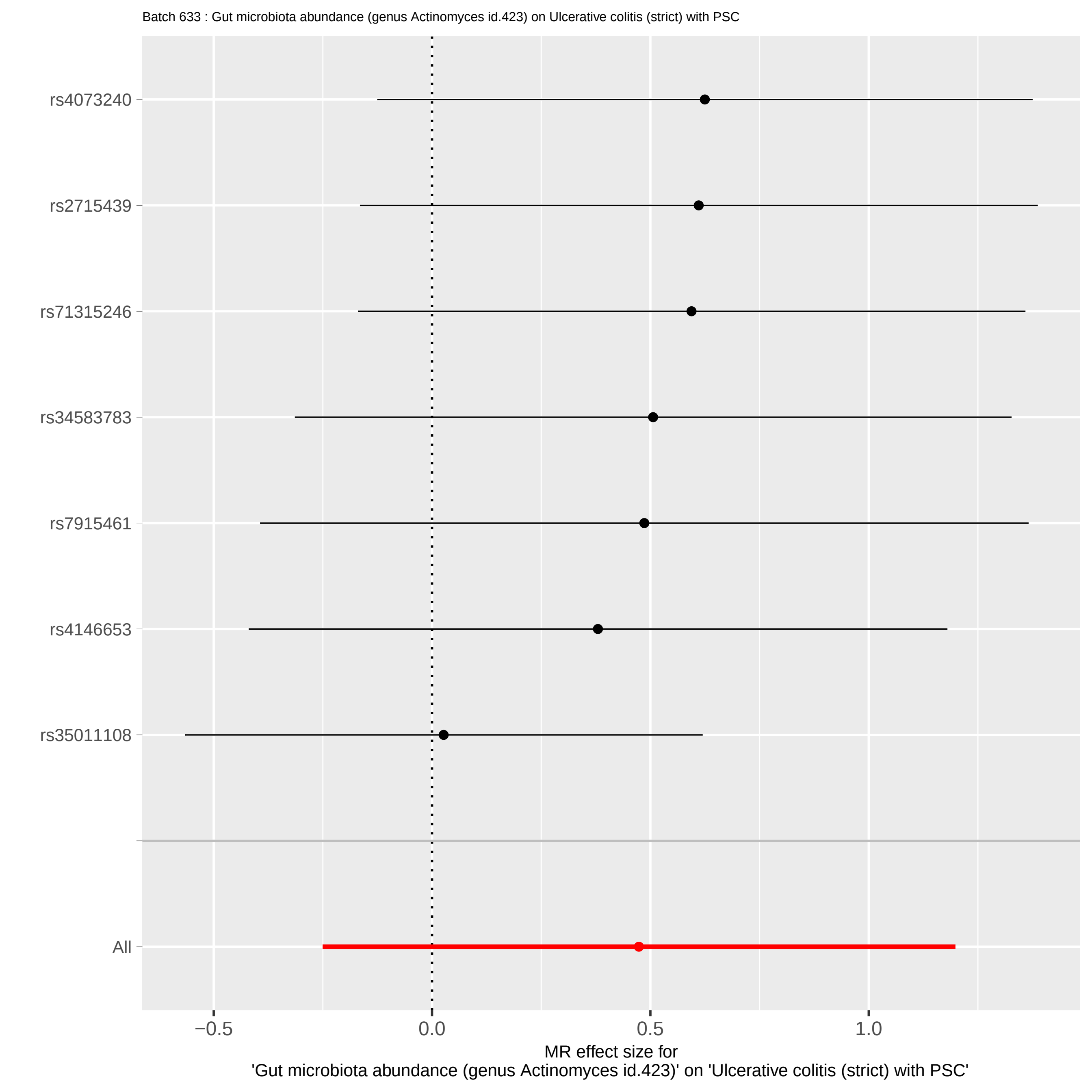

Batch 633 : Gut microbiota abundance (genus Actinomyces id.423) on Ulcerative colitis (strict) with PSC
rs4073240
rs2715439
rs71315246
rs34583783
rs7915461
rs4146653
rs35011108
All
−0.5
0.0
0.5
1.0
MR effect size for
'Gut microbiota abundance (genus Actinomyces id.423)' on 'Ulcerative colitis (strict) with PSC'

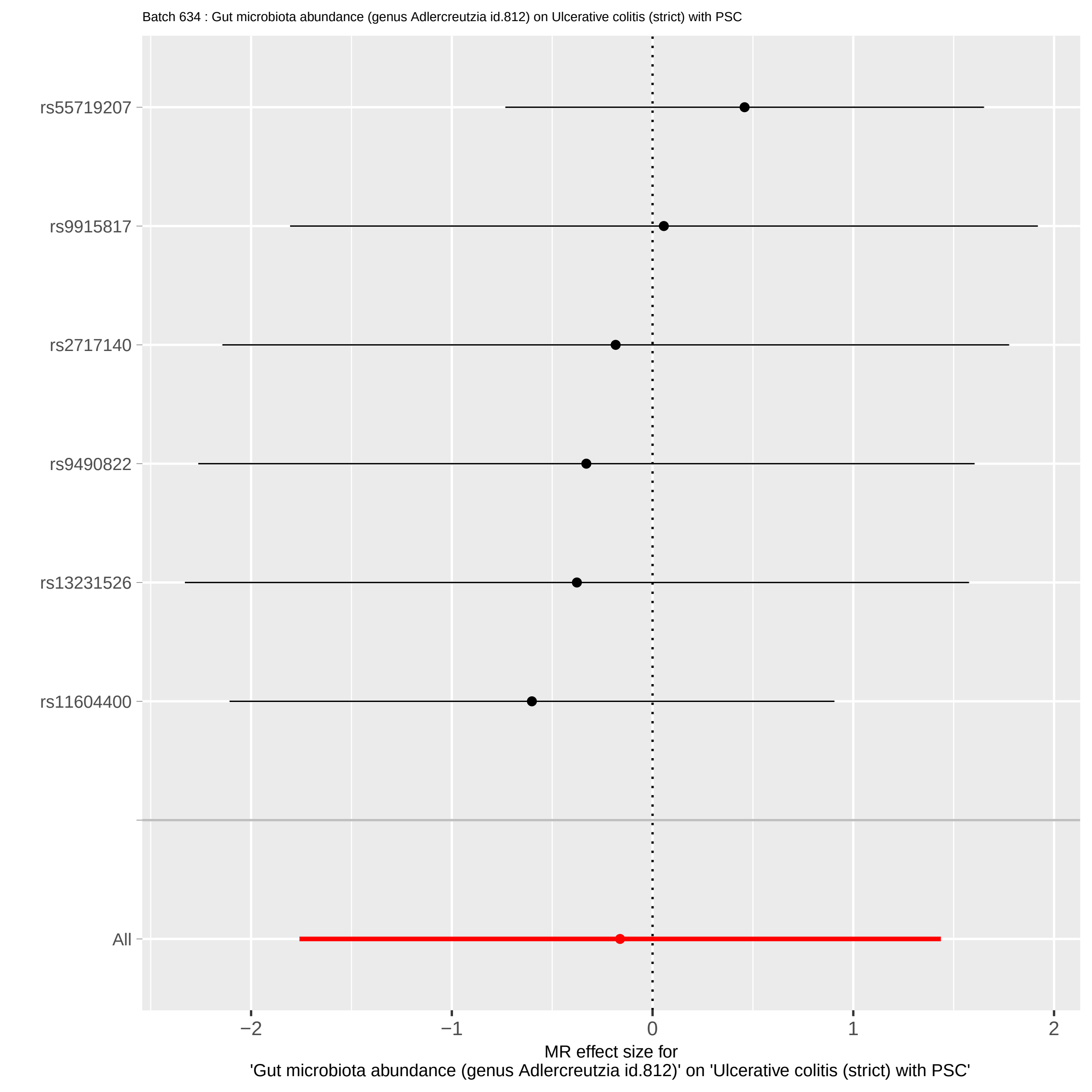

Batch 634 : Gut microbiota abundance (genus Adlercreutzia id.812) on Ulcerative colitis (strict) with PSC
rs55719207
rs9915817
rs2717140
rs9490822
rs13231526
rs11604400
All
−2
−1
0
1
2
MR effect size for
'Gut microbiota abundance (genus Adlercreutzia id.812)' on 'Ulcerative colitis (strict) with PSC'

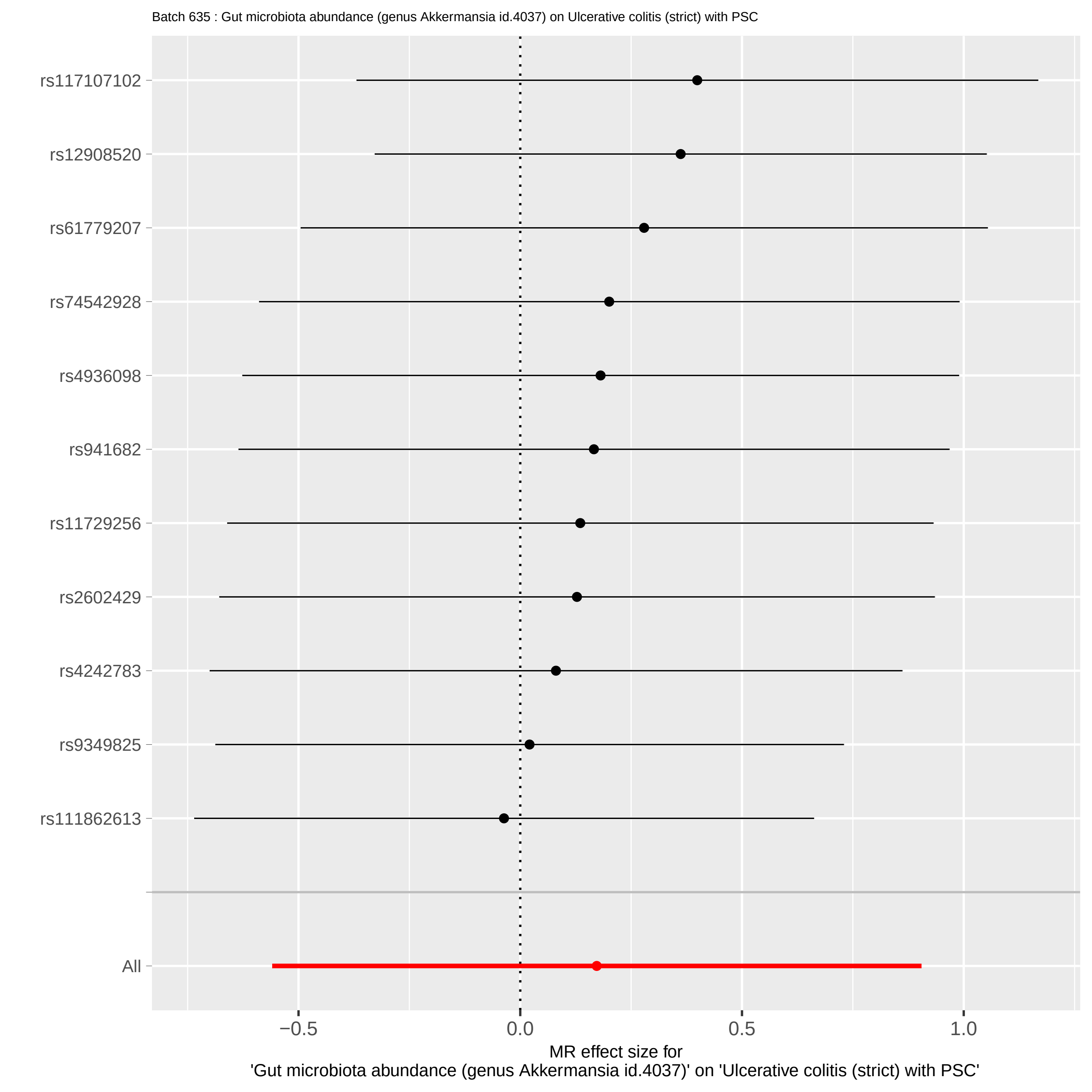

Batch 635 : Gut microbiota abundance (genus Akkermansia id.4037) on Ulcerative colitis (strict) with PSC
rs117107102
rs12908520
rs61779207
rs74542928
rs4936098
rs941682
rs11729256
rs2602429
rs4242783
rs9349825
rs111862613
All
−0.5
0.0
0.5
1.0
MR effect size for
'Gut microbiota abundance (genus Akkermansia id.4037)' on 'Ulcerative colitis (strict) with PSC'

Batch 636 : Gut microbiota abundance (genus Alistipes id.968) on Ulcerative colitis (strict) with PSC

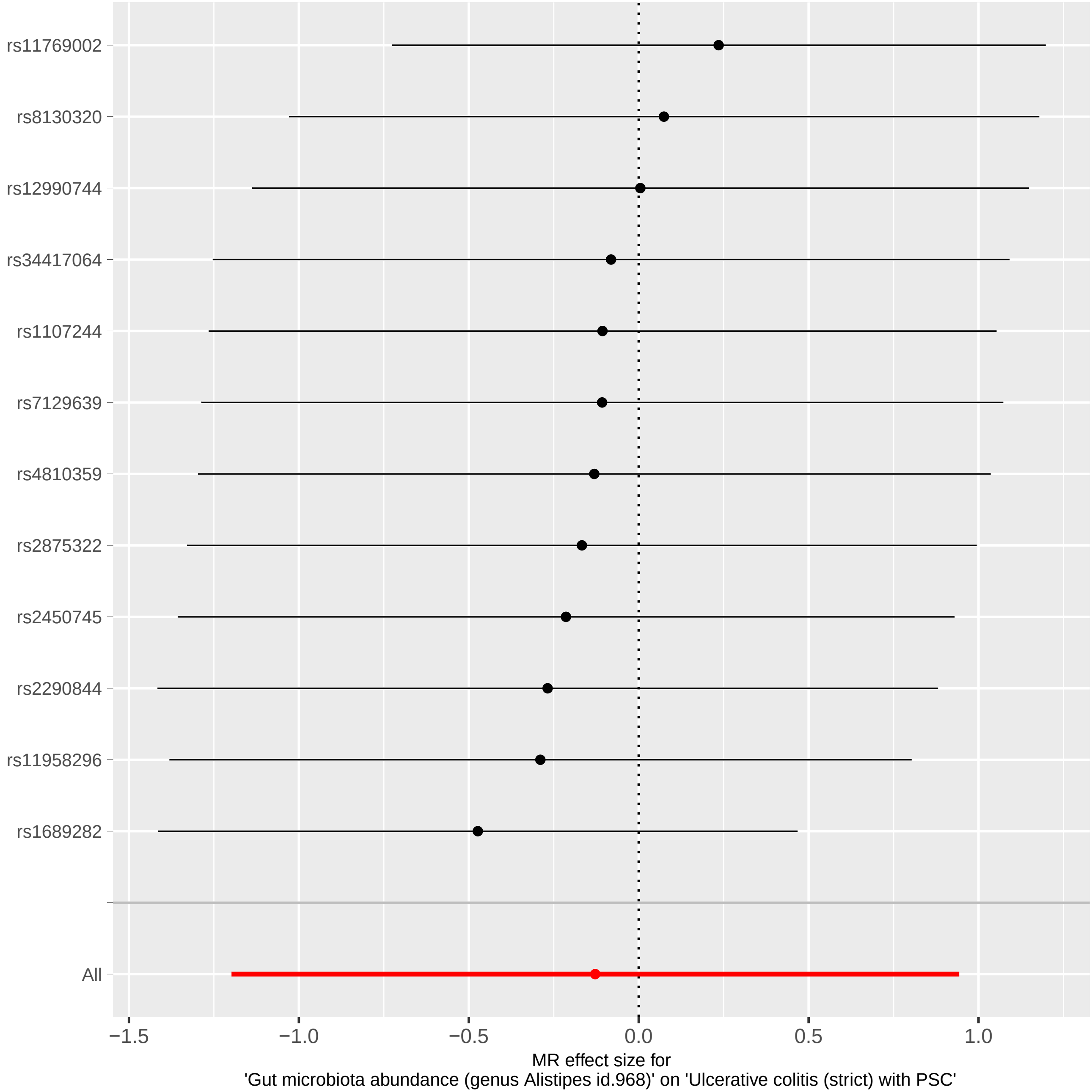

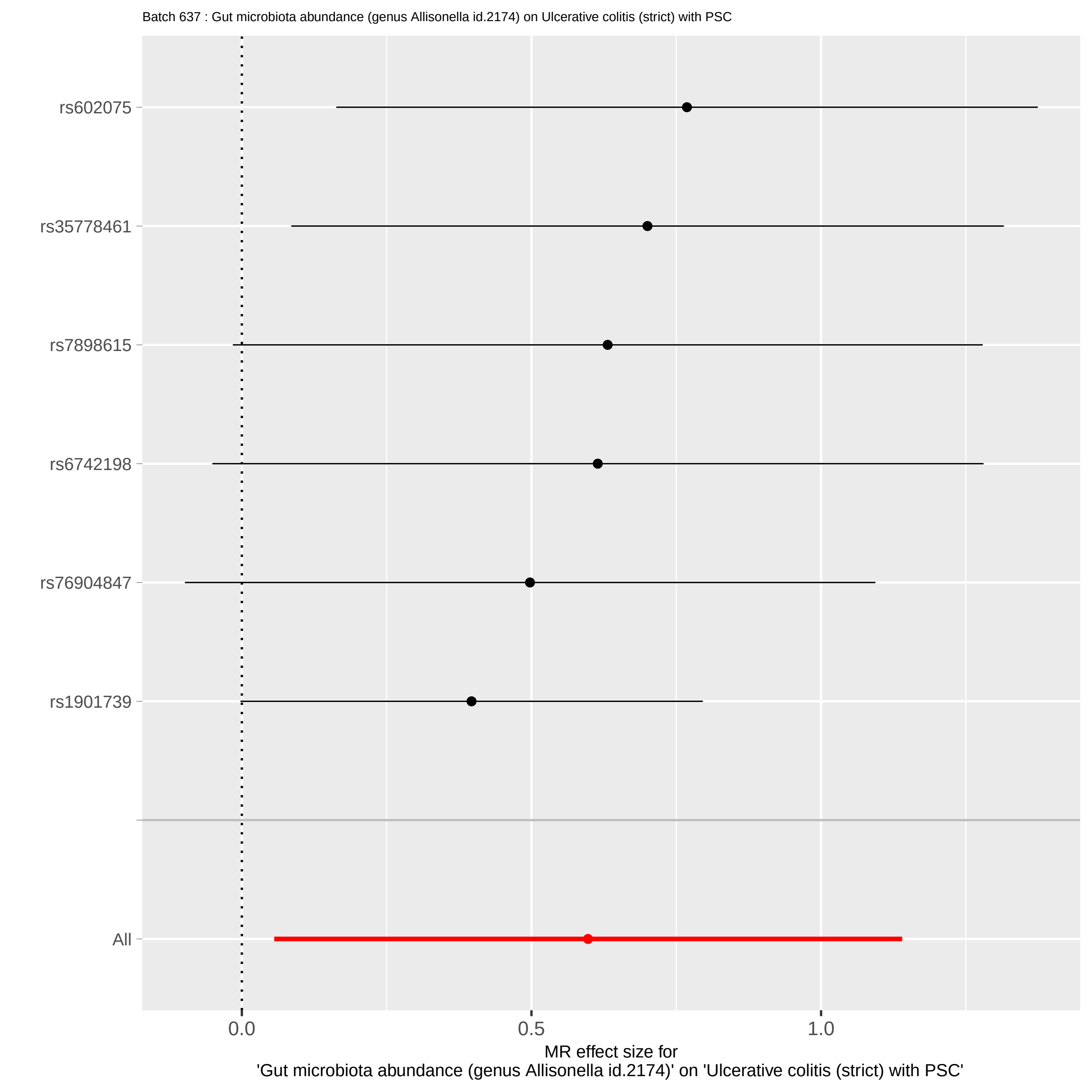

Batch 637 : Gut microbiota abundance (genus Allisonella id.2174) on Ulcerative colitis (strict) with PSC
rs602075
rs35778461
rs7898615
rs6742198
rs76904847
rs1901739
All
0.0
0.5
1.0
MR effect size for
'Gut microbiota abundance (genus Allisonella id.2174)' on 'Ulcerative colitis (strict) with PSC'

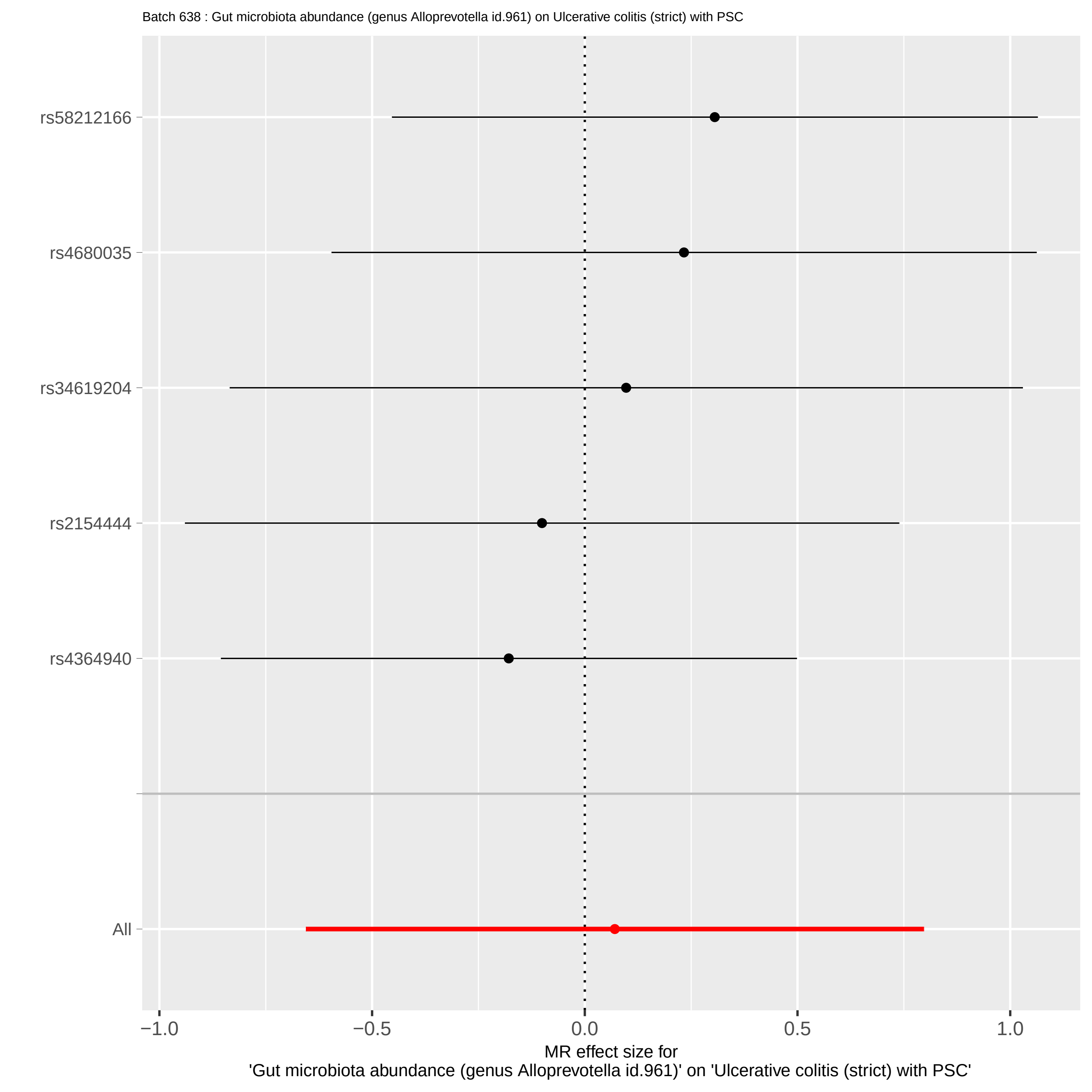

Batch 638 : Gut microbiota abundance (genus Alloprevotella id.961) on Ulcerative colitis (strict) with PSC
rs58212166
rs4680035
rs34619204
rs2154444
rs4364940
All
−1.0
−0.5
0.0
0.5
1.0
MR effect size for
'Gut microbiota abundance (genus Alloprevotella id.961)' on 'Ulcerative colitis (strict) with PSC'

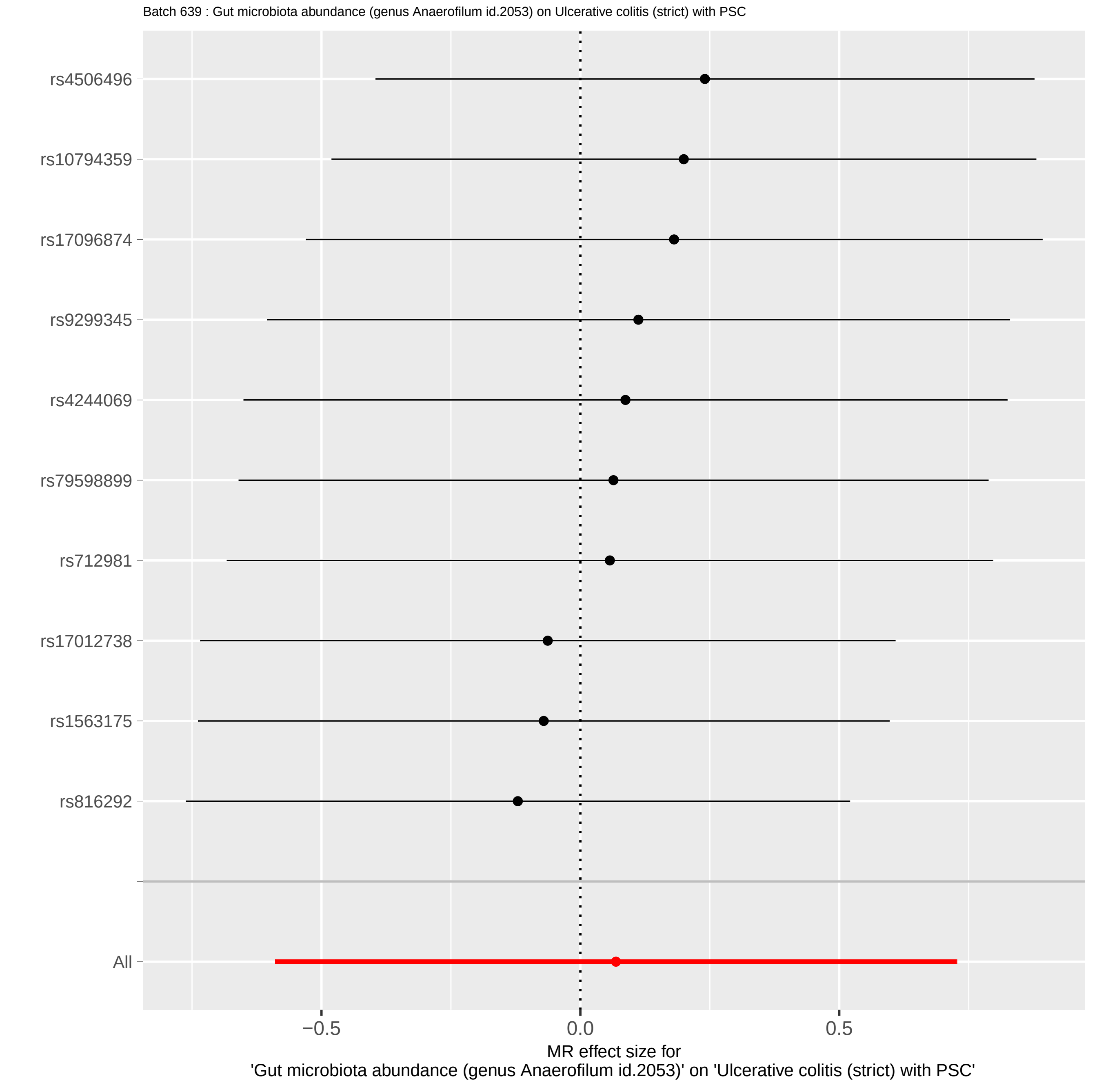
Batch 639 : Gut microbiota abundance (genus Anaerofilum id.2053) on Ulcerative colitis (strict) with PSC
rs4506496
rs10794359
rs17096874
rs9299345
rs4244069
rs79598899
rs712981
rs17012738
rs1563175
rs816292
All
−0.5
0.0
0.5
MR effect size for
'Gut microbiota abundance (genus Anaerofilum id.2053)' on 'Ulcerative colitis (strict) with PSC'

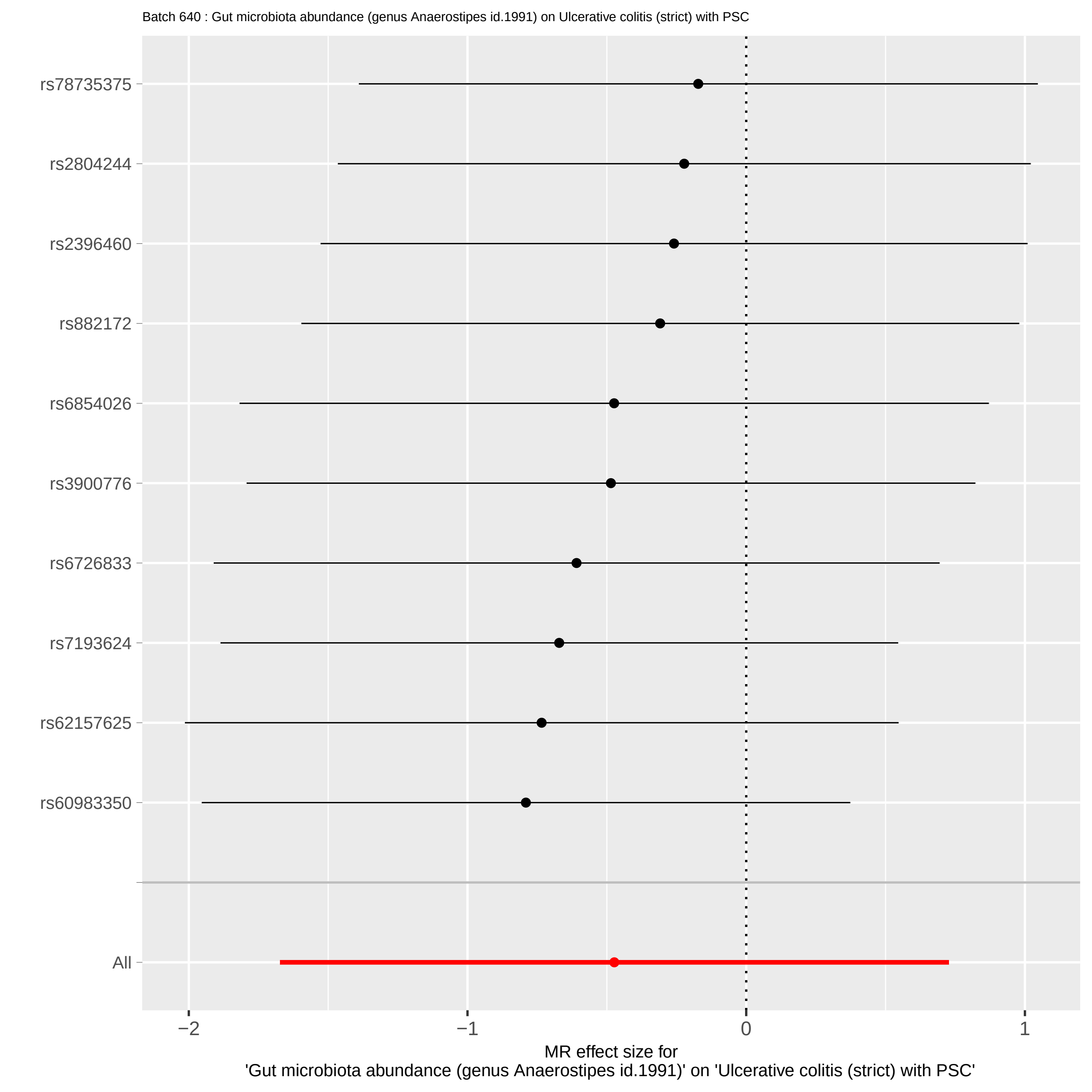

Batch 640 : Gut microbiota abundance (genus Anaerostipes id.1991) on Ulcerative colitis (strict) with PSC
rs78735375
rs2804244
rs2396460
rs882172
rs6854026
rs3900776
rs6726833
rs7193624
rs62157625
rs60983350
All
−2
−1
0
1
MR effect size for
'Gut microbiota abundance (genus Anaerostipes id.1991)' on 'Ulcerative colitis (strict) with PSC'

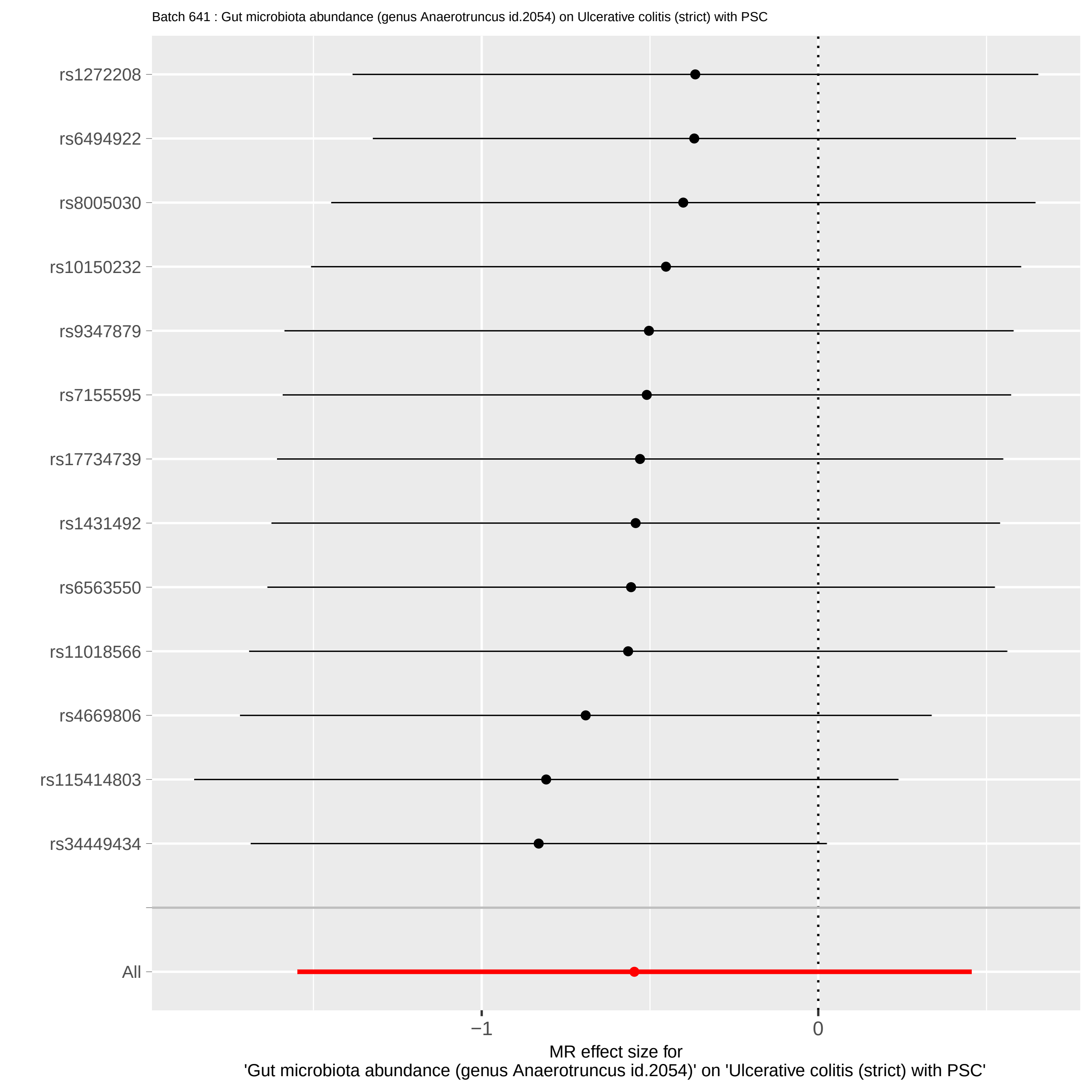
Batch 641 : Gut microbiota abundance (genus Anaerotruncus id.2054) on Ulcerative colitis (strict) with PSC
rs1272208
rs6494922
rs8005030
rs10150232
rs9347879
rs7155595
rs17734739
rs1431492
rs6563550
rs11018566
rs4669806
rs115414803
rs34449434
All
−1
0
MR effect size for
'Gut microbiota abundance (genus Anaerotruncus id.2054)' on 'Ulcerative colitis (strict) with PSC'

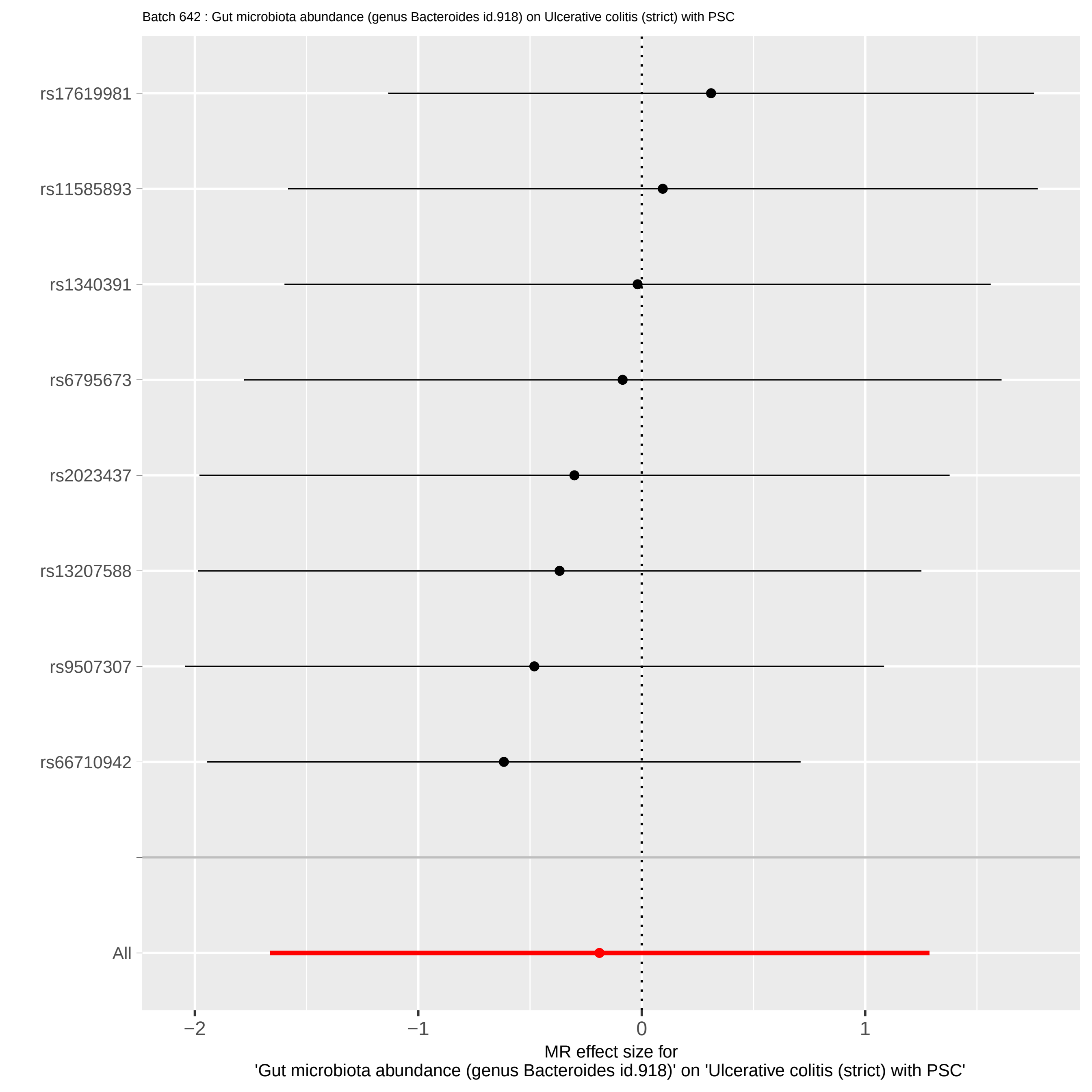

Batch 642 : Gut microbiota abundance (genus Bacteroides id.918) on Ulcerative colitis (strict) with PSC
rs17619981
rs11585893
rs1340391
rs6795673
rs2023437
rs13207588
rs9507307
rs66710942
All
−2
−1
0
1
MR effect size for
'Gut microbiota abundance (genus Bacteroides id.918)' on 'Ulcerative colitis (strict) with PSC'

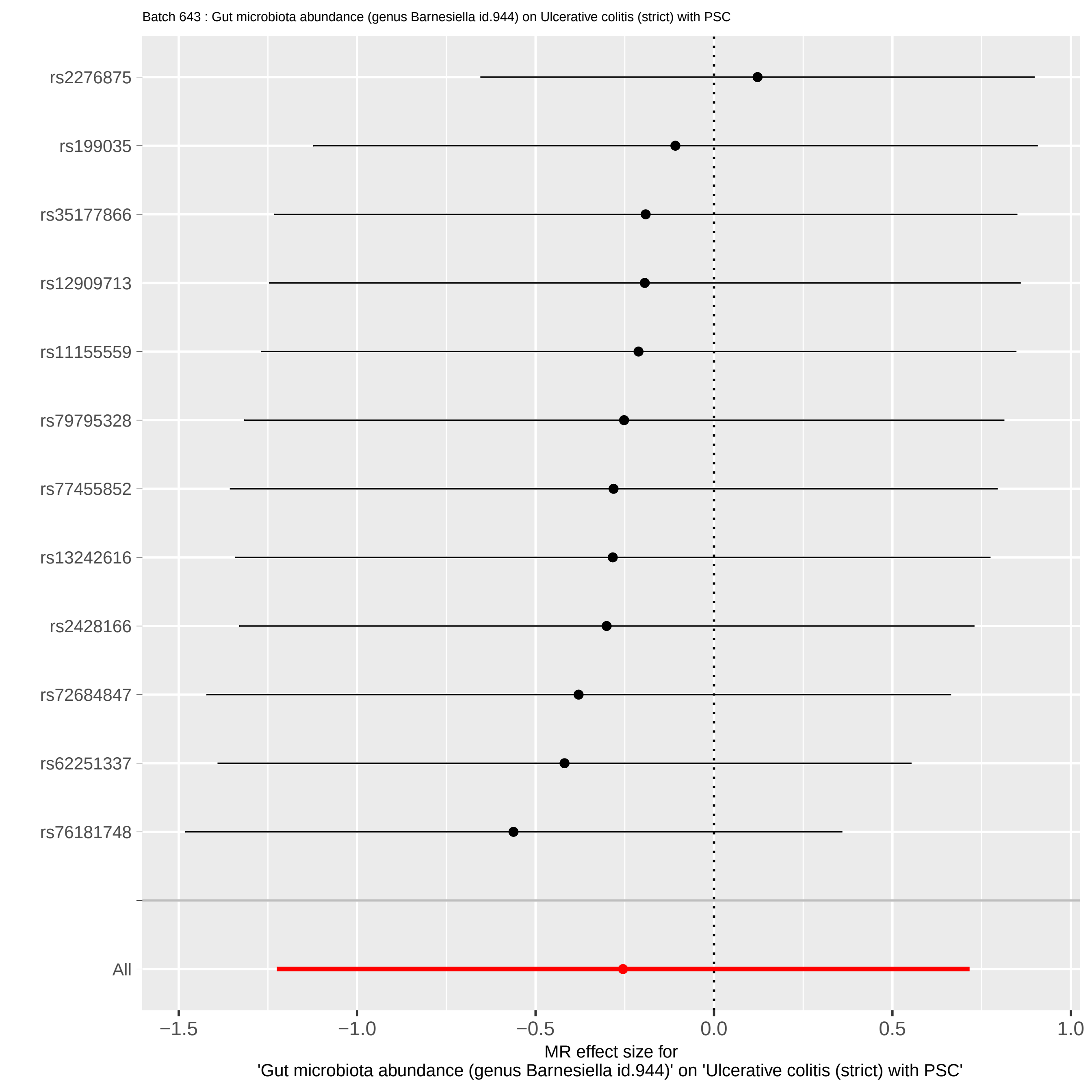
Batch 643 : Gut microbiota abundance (genus Barnesiella id.944) on Ulcerative colitis (strict) with PSC
rs2276875
rs199035
rs35177866
rs12909713
rs11155559
rs79795328
rs77455852
rs13242616
rs2428166
rs72684847
rs62251337
rs76181748
All
−1.5
−1.0
−0.5
0.0
0.5
1.0
MR effect size for
'Gut microbiota abundance (genus Barnesiella id.944)' on 'Ulcerative colitis (strict) with PSC'

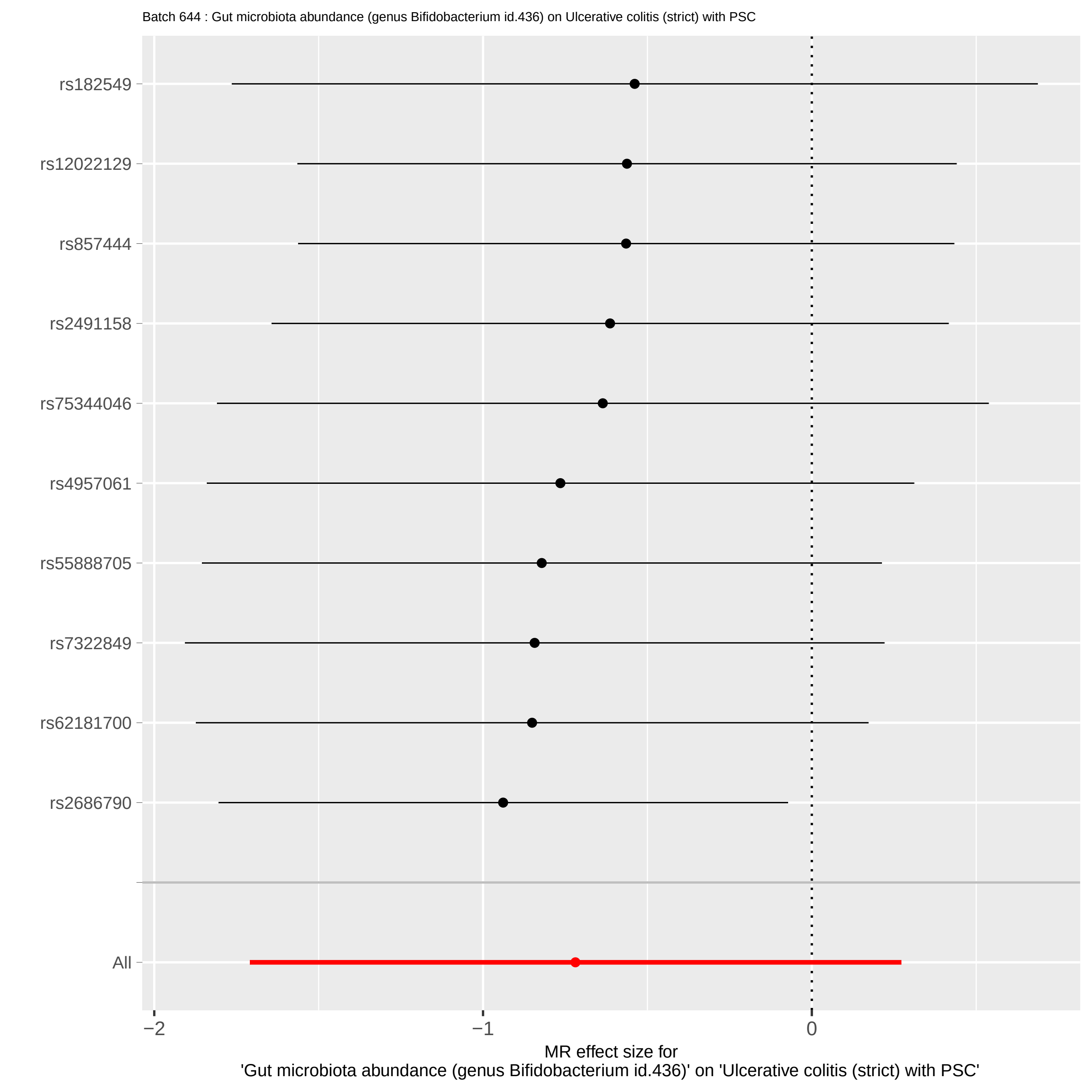

Batch 644 : Gut microbiota abundance (genus Bifidobacterium id.436) on Ulcerative colitis (strict) with PSC
rs182549
rs12022129
rs857444
rs2491158
rs75344046
rs4957061
rs55888705
rs7322849
rs62181700
rs2686790
All
−2
−1
0
MR effect size for
'Gut microbiota abundance (genus Bifidobacterium id.436)' on 'Ulcerative colitis (strict) with PSC'

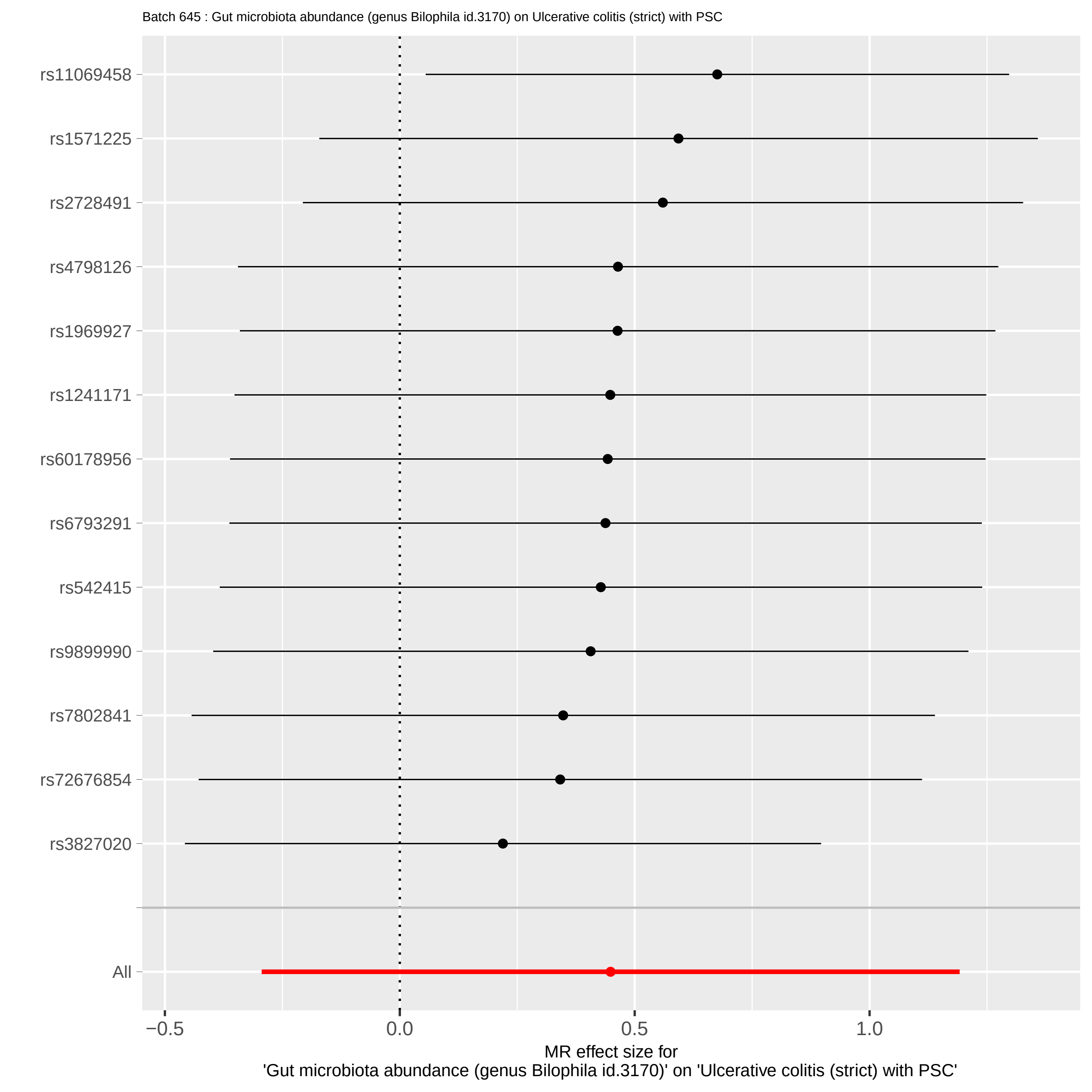
Batch 645 : Gut microbiota abundance (genus Bilophila id.3170) on Ulcerative colitis (strict) with PSC
rs11069458
rs1571225
rs2728491
rs4798126
rs1969927
rs1241171
rs60178956
rs6793291
rs542415
rs9899990
rs7802841
rs72676854
rs3827020
All
−0.5
0.0
0.5
1.0
MR effect size for
'Gut microbiota abundance (genus Bilophila id.3170)' on 'Ulcerative colitis (strict) with PSC'

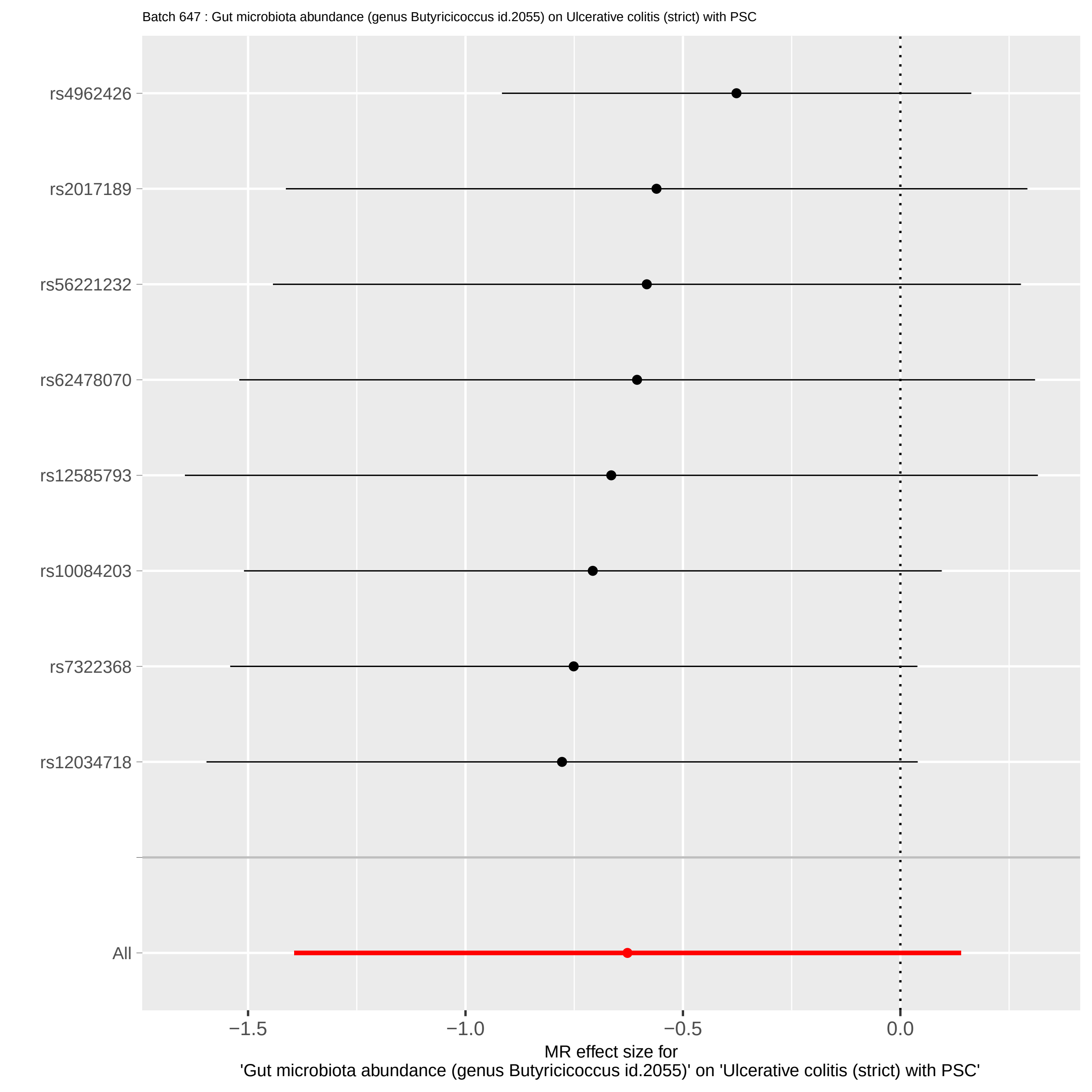
Batch 647 : Gut microbiota abundance (genus Butyricicoccus id.2055) on Ulcerative colitis (strict) with PSC
rs4962426
rs2017189
rs56221232
rs62478070
rs12585793
rs10084203
rs7322368
rs12034718
All
−1.5
−1.0
−0.5
0.0
MR effect size for
'Gut microbiota abundance (genus Butyricicoccus id.2055)' on 'Ulcerative colitis (strict) with PSC'

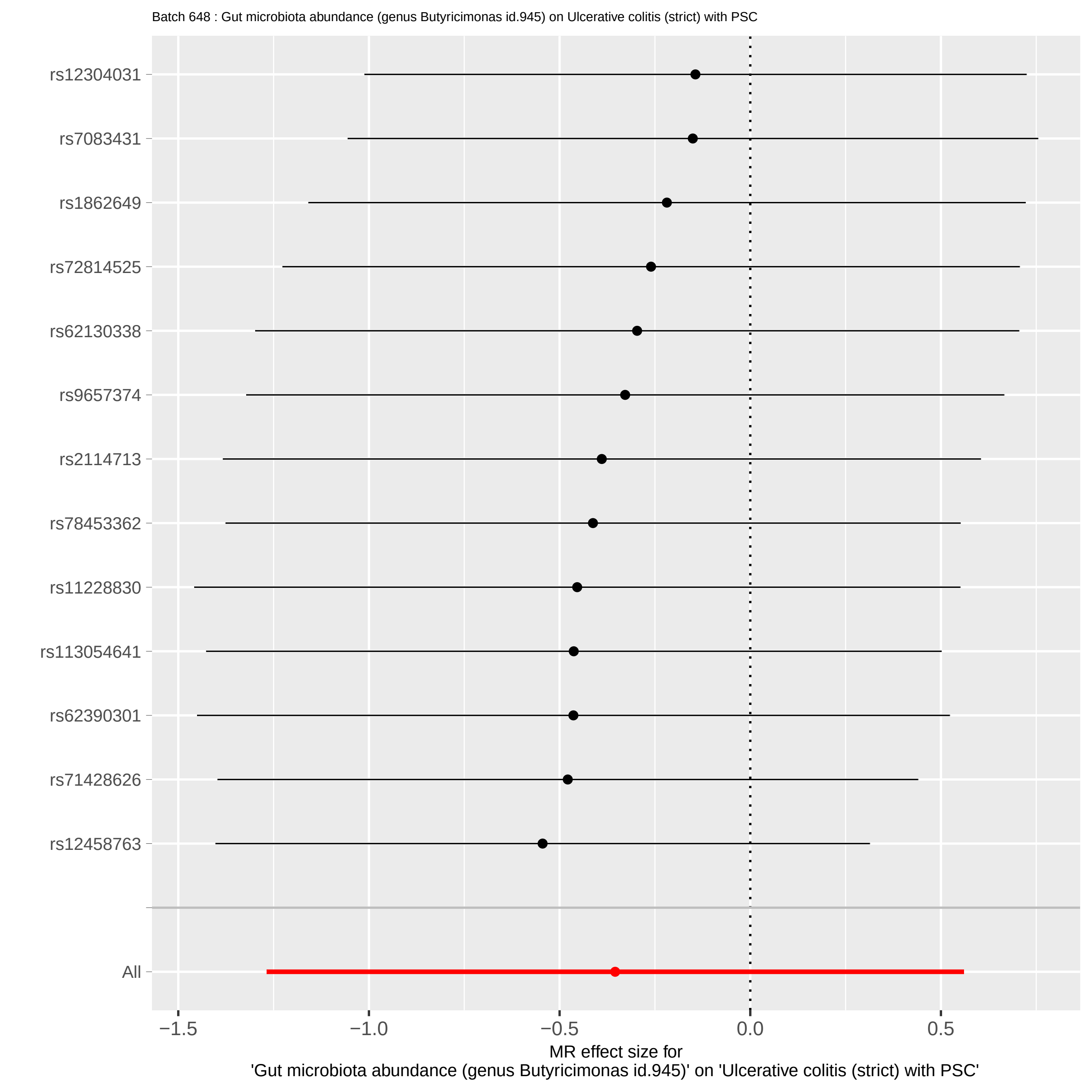
Batch 648 : Gut microbiota abundance (genus Butyricimonas id.945) on Ulcerative colitis (strict) with PSC
rs12304031
rs7083431
rs1862649
rs72814525
rs62130338
rs9657374
rs2114713
rs78453362
rs11228830
rs113054641
rs62390301
rs71428626
rs12458763
All
−1.5
−1.0
−0.5
0.0
0.5
MR effect size for
'Gut microbiota abundance (genus Butyricimonas id.945)' on 'Ulcerative colitis (strict) with PSC'

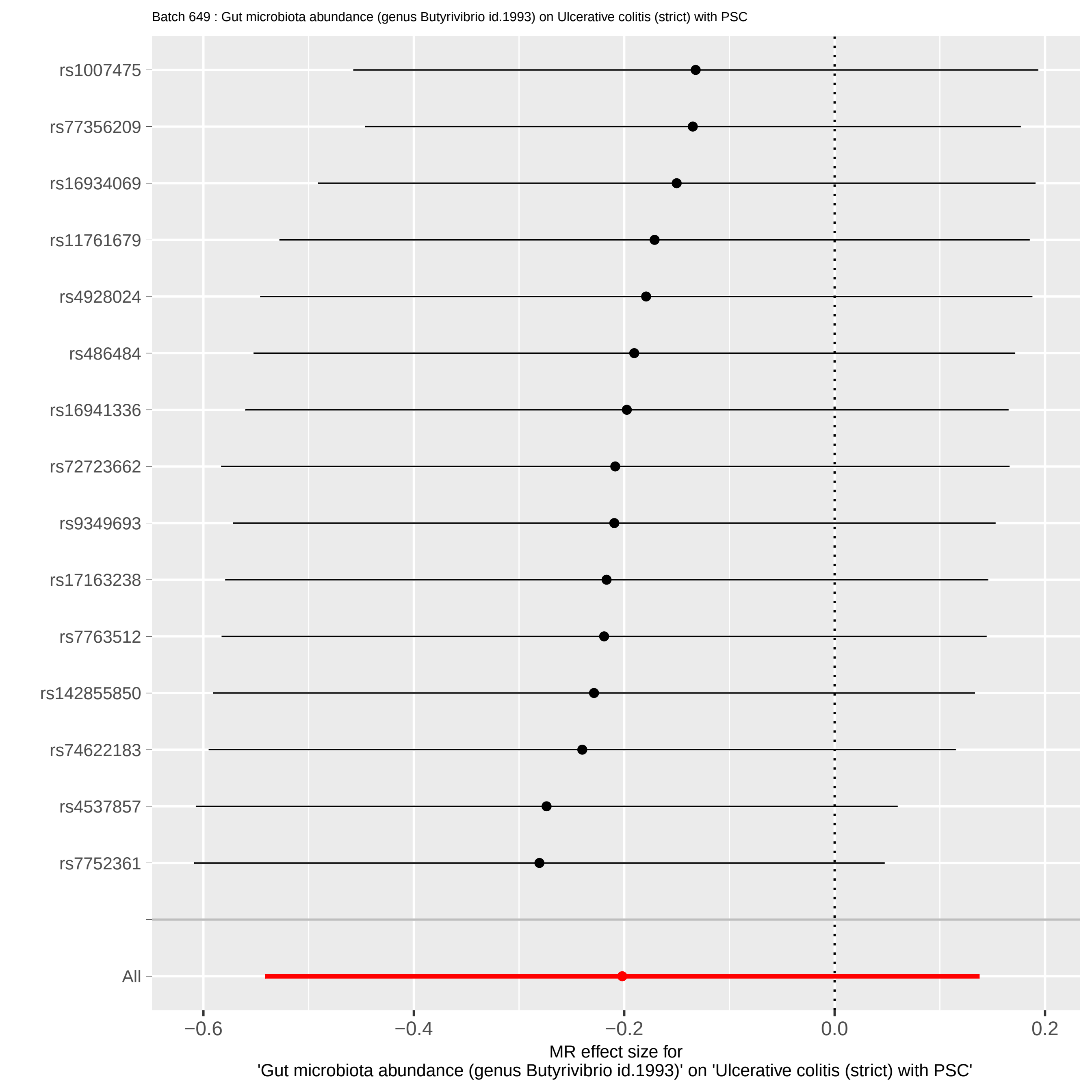

Batch 649 : Gut microbiota abundance (genus Butyrivibrio id.1993) on Ulcerative colitis (strict) with PSC
rs1007475
rs77356209
rs16934069
rs11761679
rs4928024
rs486484
rs16941336
rs72723662
rs9349693
rs17163238
rs7763512
rs142855850
rs74622183
rs4537857
rs7752361
All
−0.6
−0.4
−0.2
0.0
0.2
MR effect size for
'Gut microbiota abundance (genus Butyrivibrio id.1993)' on 'Ulcerative colitis (strict) with PSC'

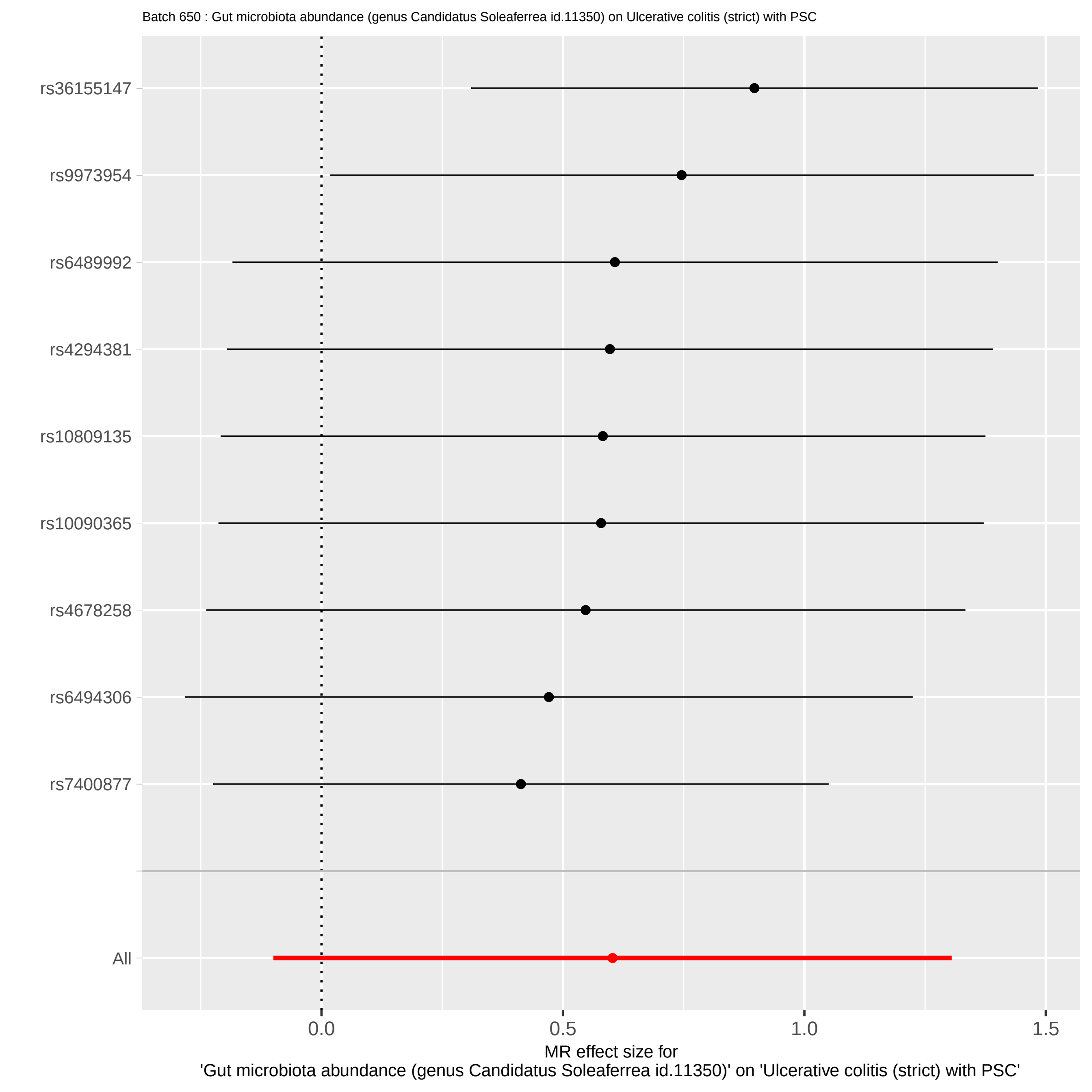

Batch 650 : Gut microbiota abundance (genus Candidatus Soleaferrea id.11350) on Ulcerative colitis (strict) with PSC
rs36155147
rs9973954
rs6489992
rs4294381
rs10809135
rs10090365
rs4678258
rs6494306
rs7400877
All
0.0
0.5
1.0
1.5
MR effect size for
'Gut microbiota abundance (genus Candidatus Soleaferrea id.11350)' on 'Ulcerative colitis (strict) with PSC'

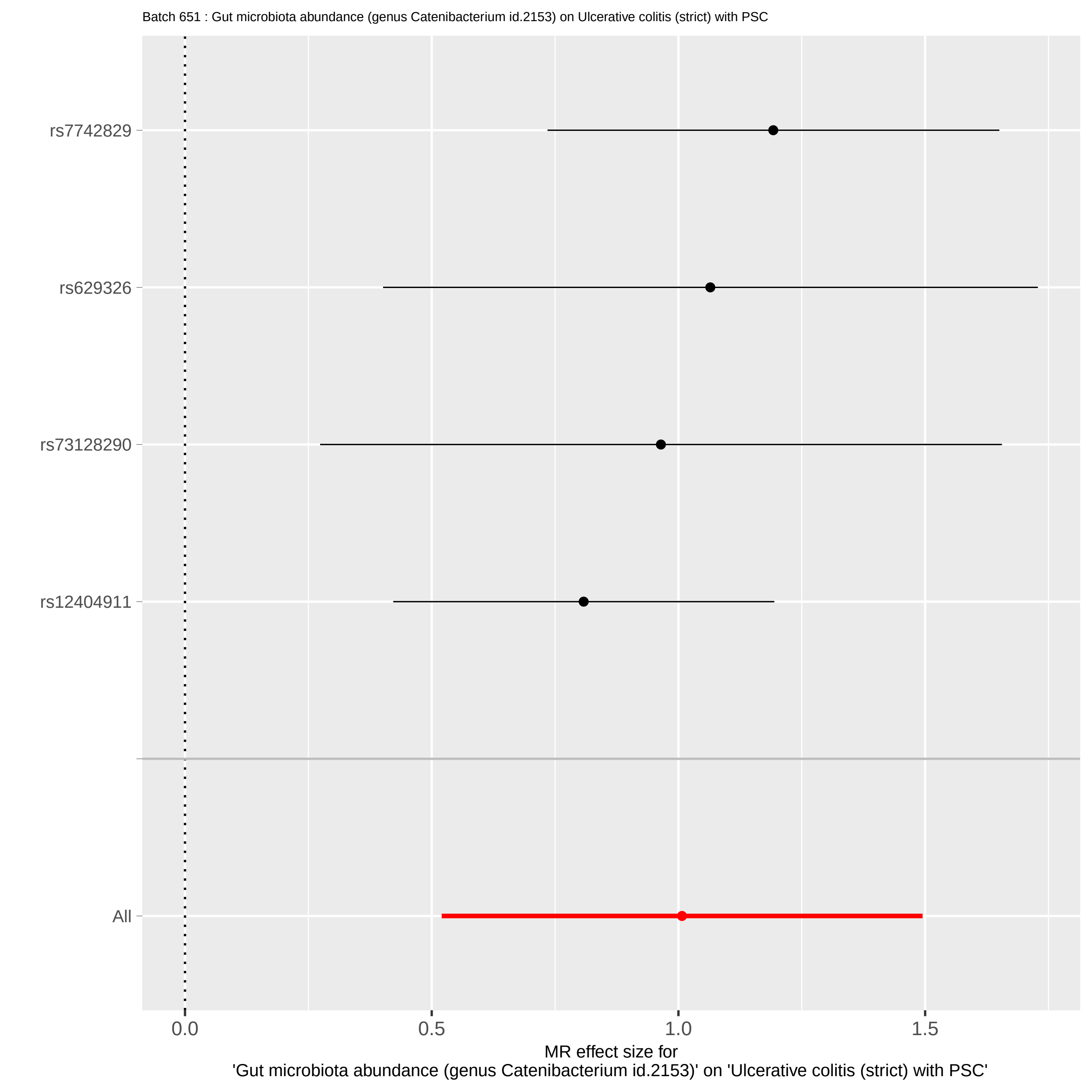

Batch 651 : Gut microbiota abundance (genus Catenibacterium id.2153) on Ulcerative colitis (strict) with PSC
rs7742829
rs629326
rs73128290
rs12404911
All
0.0
0.5
1.0
1.5
MR effect size for
'Gut microbiota abundance (genus Catenibacterium id.2153)' on 'Ulcerative colitis (strict) with PSC'

Batch 652 : Gut microbiota abundance (genus Christensenellaceae R 7group id.11283) on Ulcerative colitis (strict) with PSC

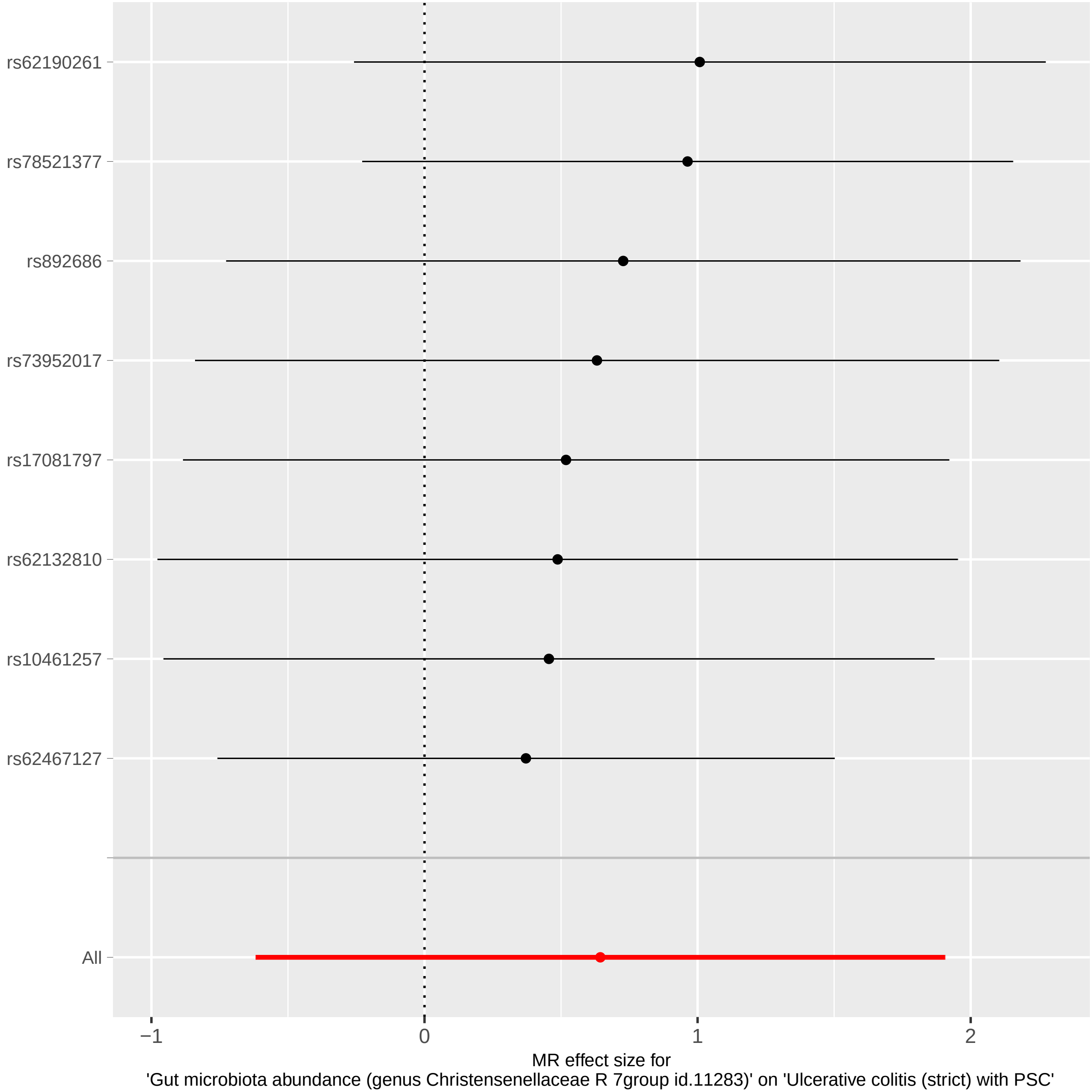

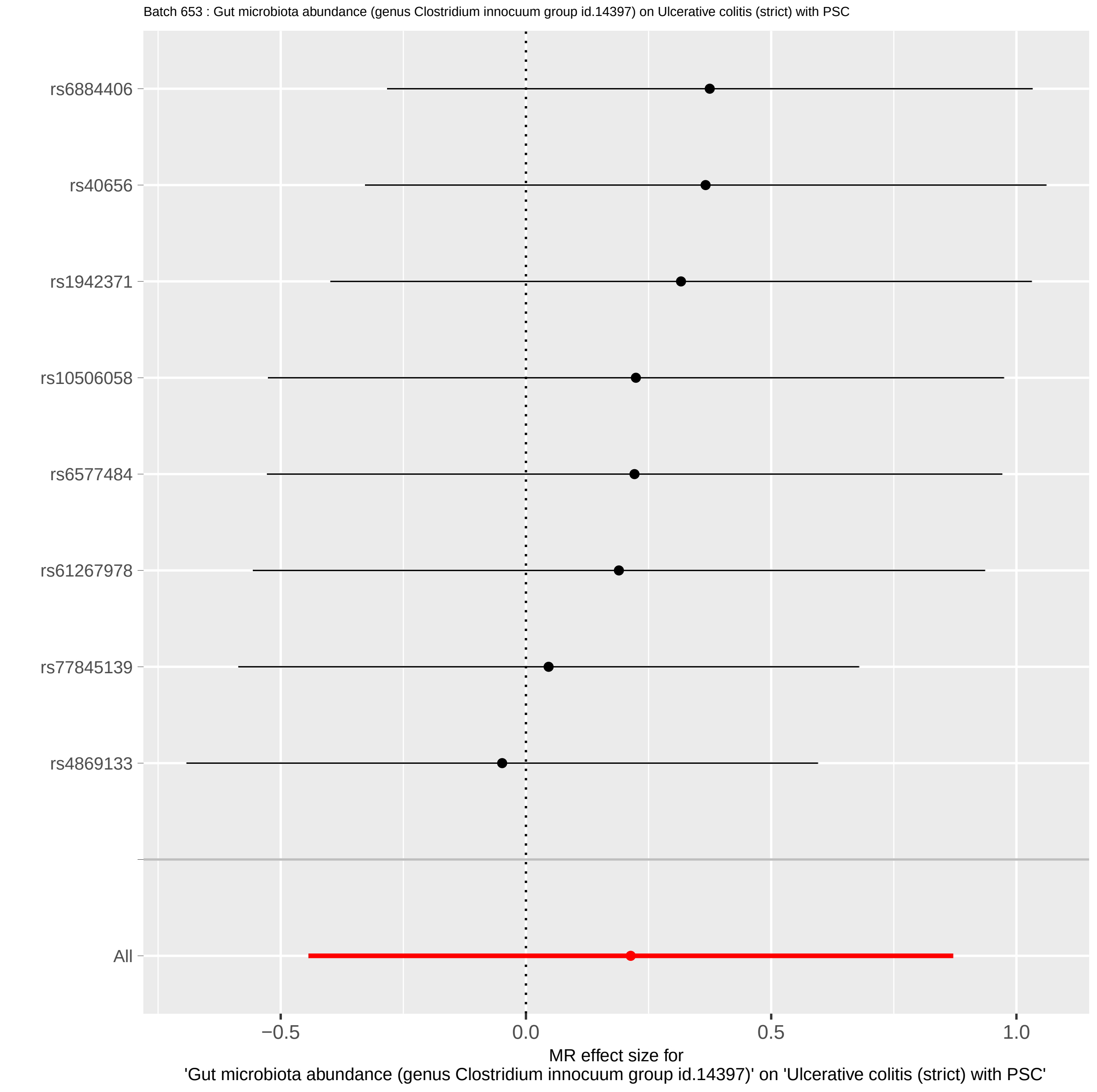
Batch 653 : Gut microbiota abundance (genus Clostridium innocuum group id.14397) on Ulcerative colitis (strict) with PSC
rs6884406
rs40656
rs1942371
rs10506058
rs6577484
rs61267978
rs77845139
rs4869133
All
−0.5
0.0
0.5
1.0
MR effect size for
'Gut microbiota abundance (genus Clostridium innocuum group id.14397)' on 'Ulcerative colitis (strict) with PSC'

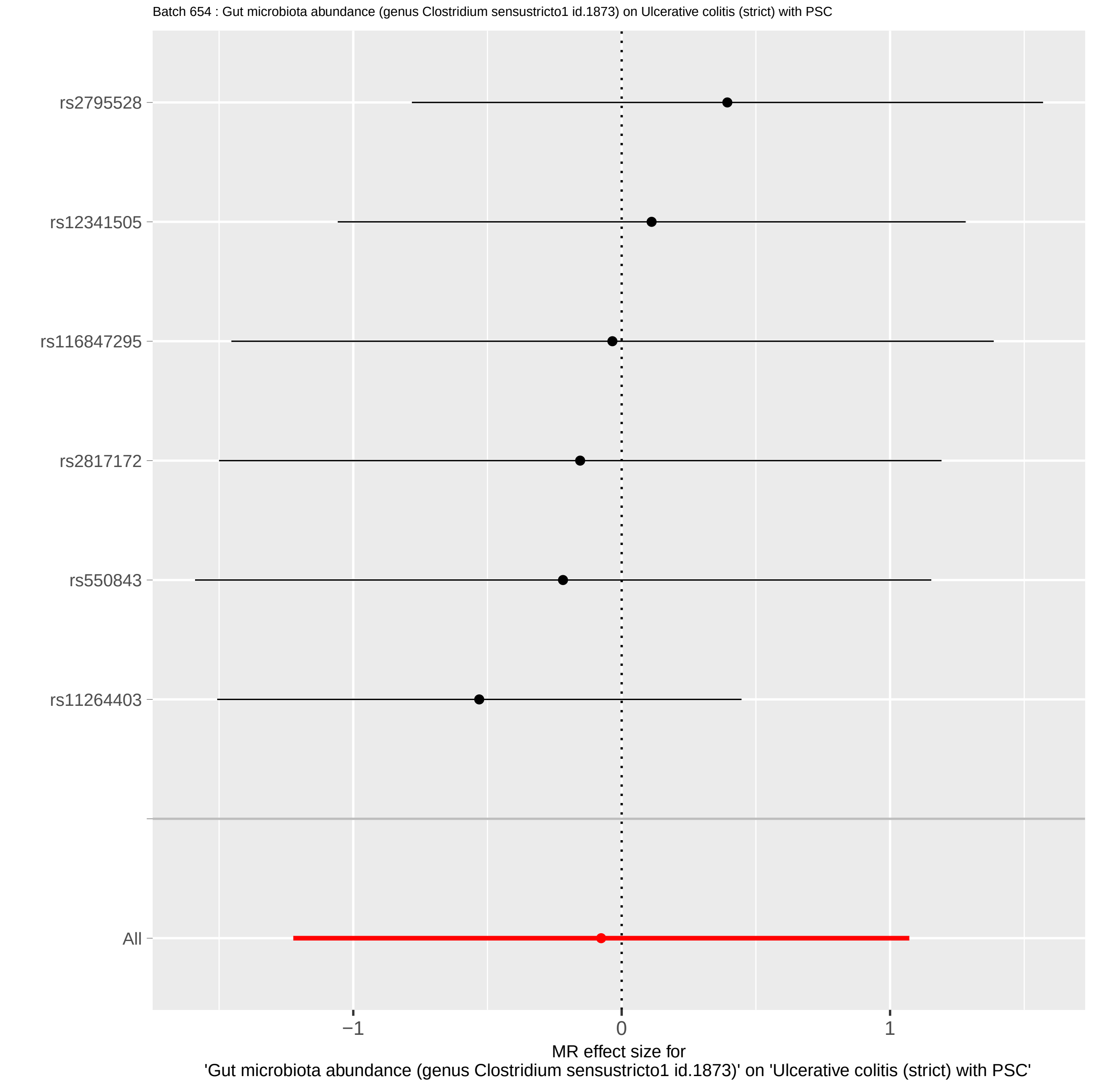

Batch 654 : Gut microbiota abundance (genus Clostridium sensustricto1 id.1873) on Ulcerative colitis (strict) with PSC
rs2795528
rs12341505
rs116847295
rs2817172
rs550843
rs11264403
All
−1
0
1
MR effect size for
'Gut microbiota abundance (genus Clostridium sensustricto1 id.1873)' on 'Ulcerative colitis (strict) with PSC'

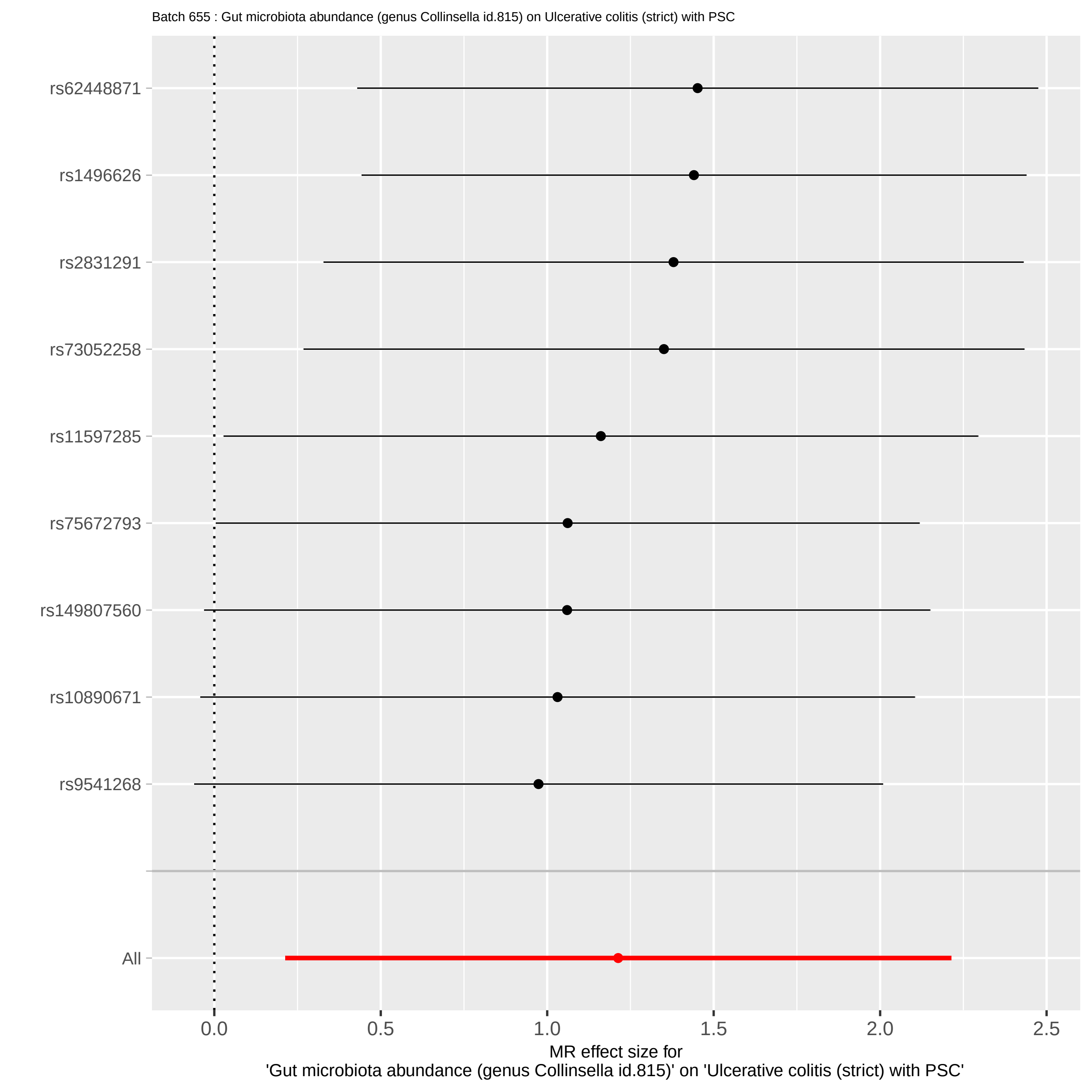
Batch 655 : Gut microbiota abundance (genus Collinsella id.815) on Ulcerative colitis (strict) with PSC
rs62448871
rs1496626
rs2831291
rs73052258
rs11597285
rs75672793
rs149807560
rs10890671
rs9541268
All
0.0
0.5
1.0
1.5
2.0
2.5
MR effect size for
'Gut microbiota abundance (genus Collinsella id.815)' on 'Ulcerative colitis (strict) with PSC'

Batch 656 : Gut microbiota abundance (genus Coprobacter id.949) on Ulcerative colitis (strict) with PSC

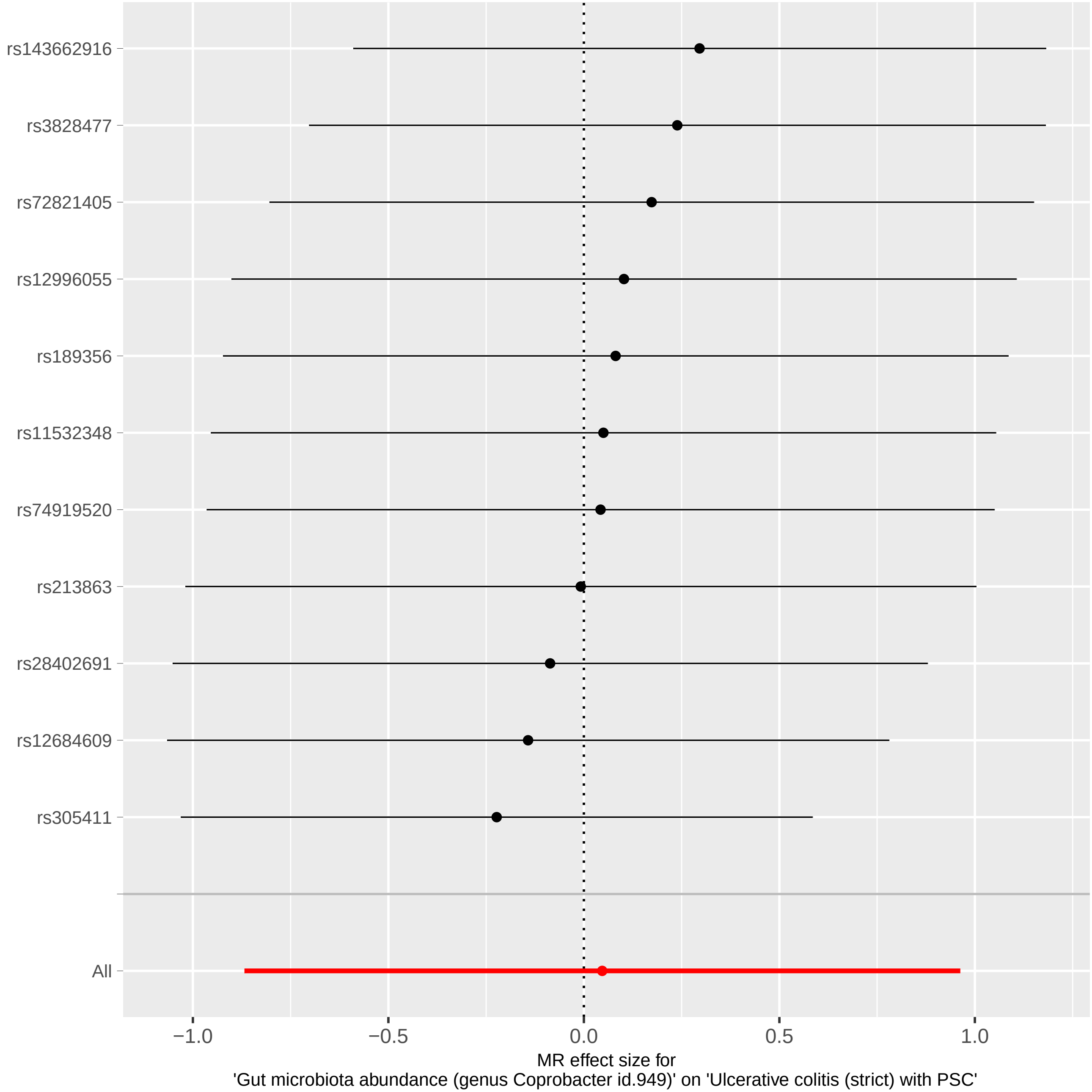

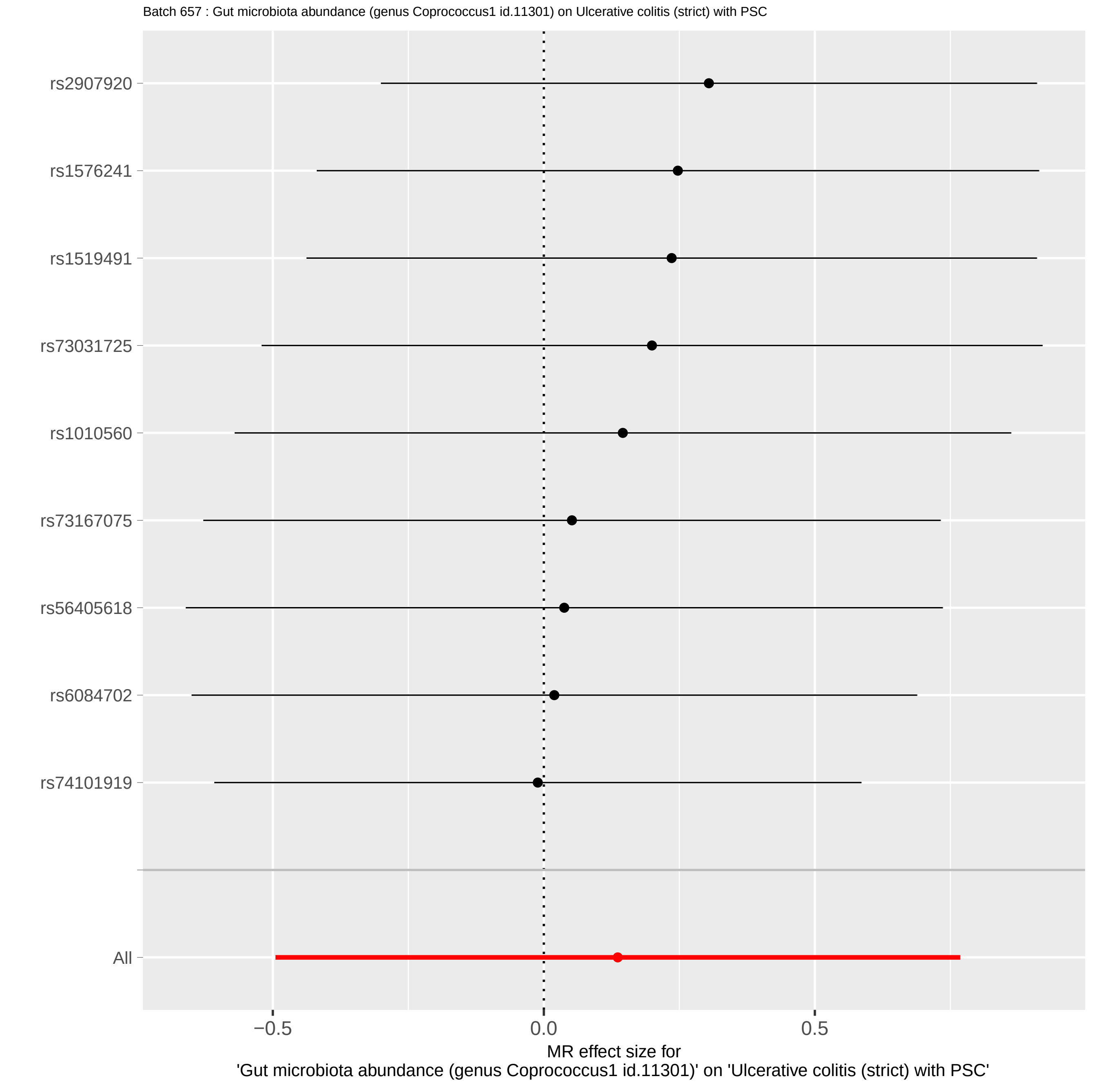
Batch 657 : Gut microbiota abundance (genus Coprococcus1 id.11301) on Ulcerative colitis (strict) with PSC
rs2907920
rs1576241
rs1519491
rs73031725
rs1010560
rs73167075
rs56405618
rs6084702
rs74101919
All
−0.5
0.0
0.5
MR effect size for
'Gut microbiota abundance (genus Coprococcus1 id.11301)' on 'Ulcerative colitis (strict) with PSC'

Batch 658 : Gut microbiota abundance (genus Coprococcus2 id.11302) on Ulcerative colitis (strict) with PSC

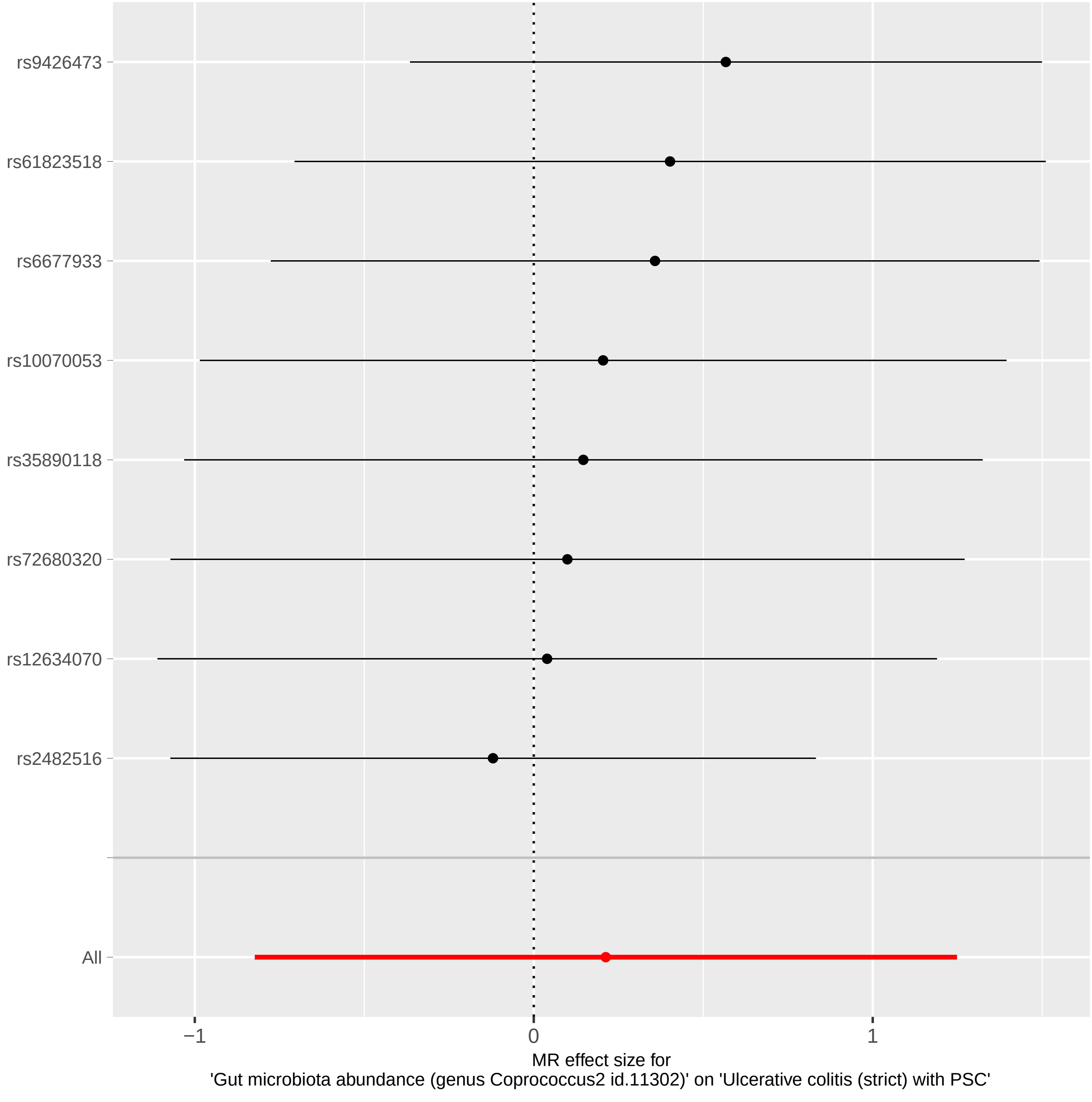

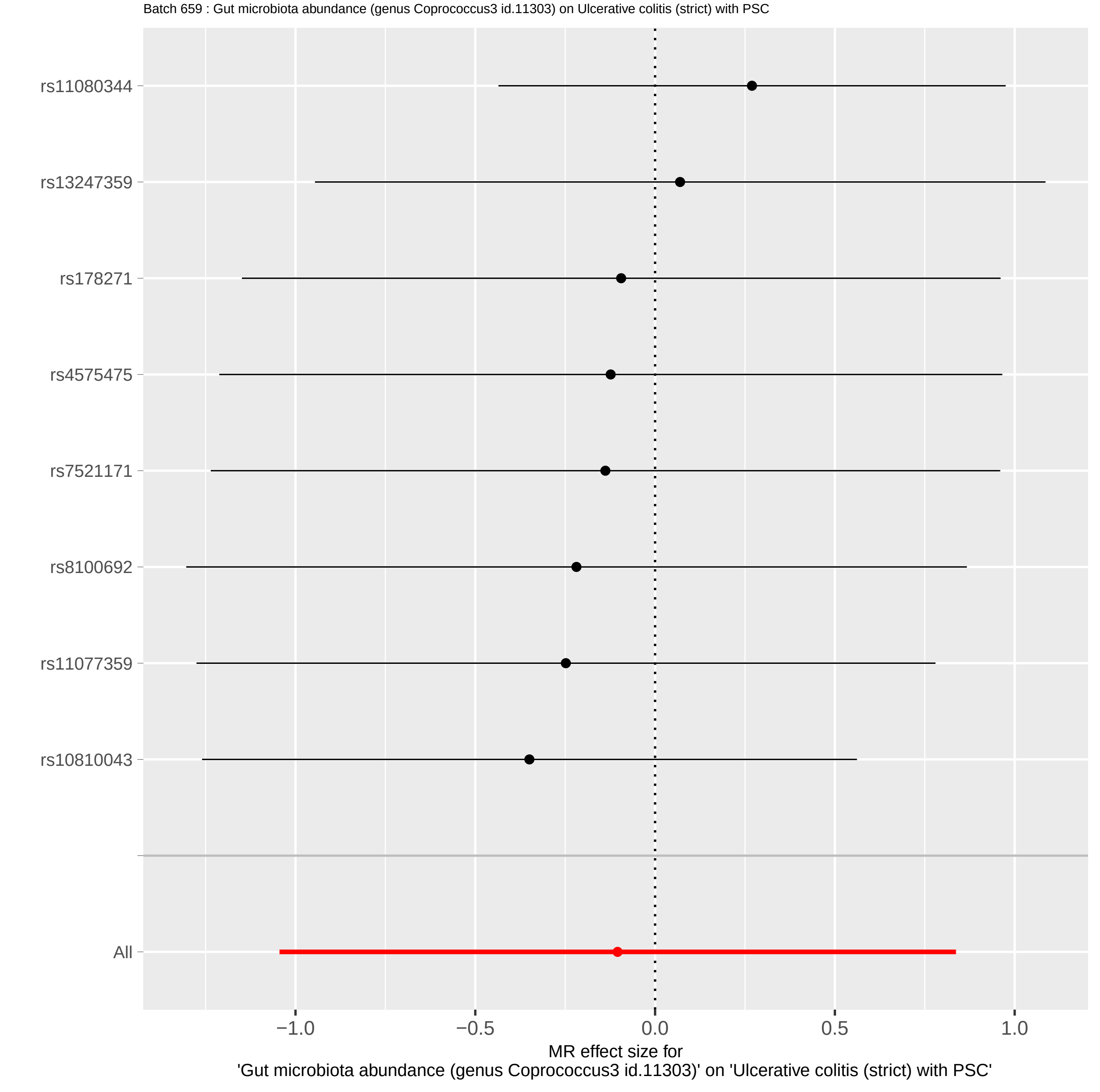

Batch 659 : Gut microbiota abundance (genus Coprococcus3 id.11303) on Ulcerative colitis (strict) with PSC
rs11080344
rs13247359
rs178271
rs4575475
rs7521171
rs8100692
rs11077359
rs10810043
All
−1.0
−0.5
0.0
0.5
1.0
MR effect size for
'Gut microbiota abundance (genus Coprococcus3 id.11303)' on 'Ulcerative colitis (strict) with PSC'

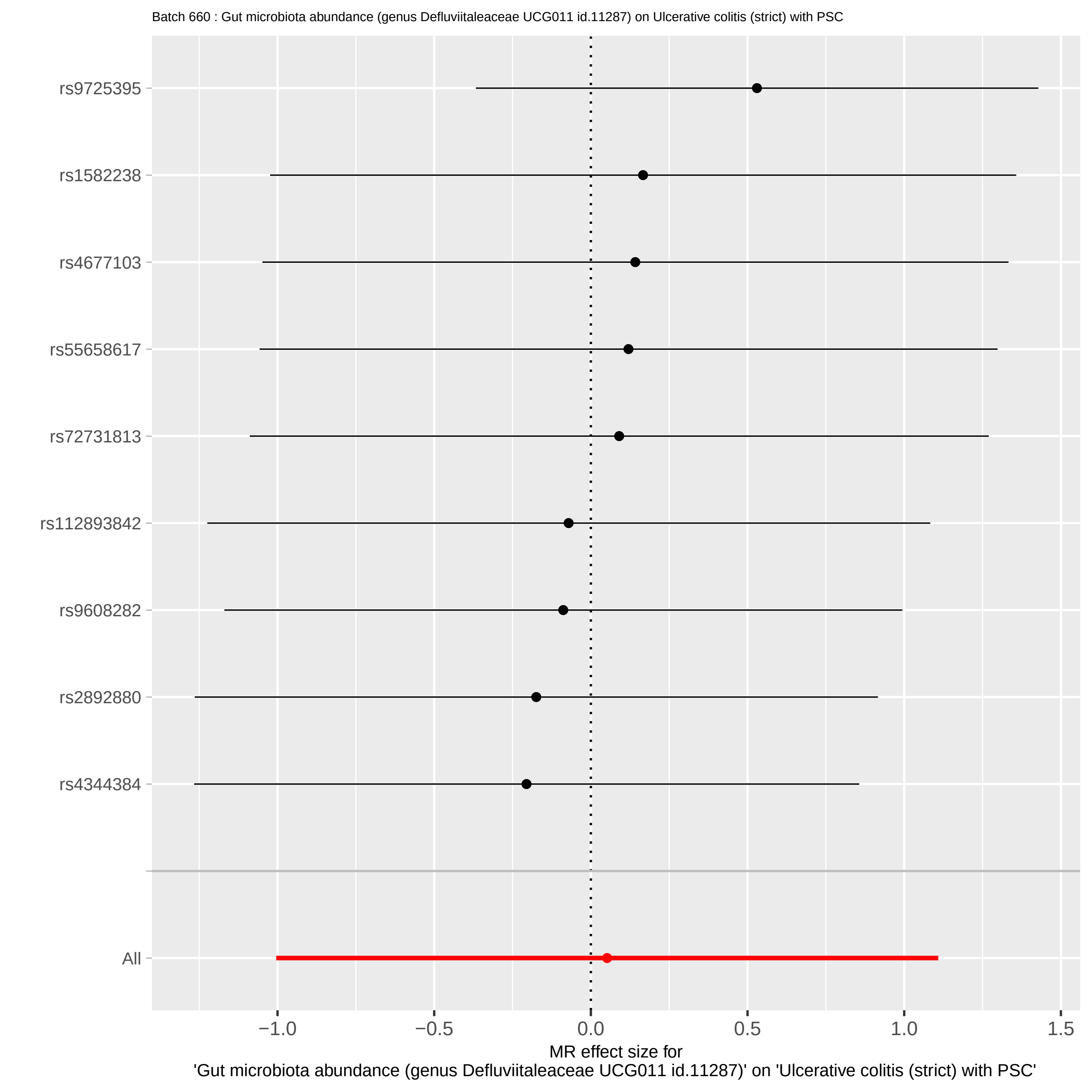
Batch 660 : Gut microbiota abundance (genus Defluviitaleaceae UCG011 id.11287) on Ulcerative colitis (strict) with PSC
rs9725395
rs1582238
rs4677103
rs55658617
rs72731813
rs112893842
rs9608282
rs2892880
rs4344384
All
−1.0
−0.5
0.0
0.5
1.0
1.5
MR effect size for
'Gut microbiota abundance (genus Defluviitaleaceae UCG011 id.11287)' on 'Ulcerative colitis (strict) with PSC'

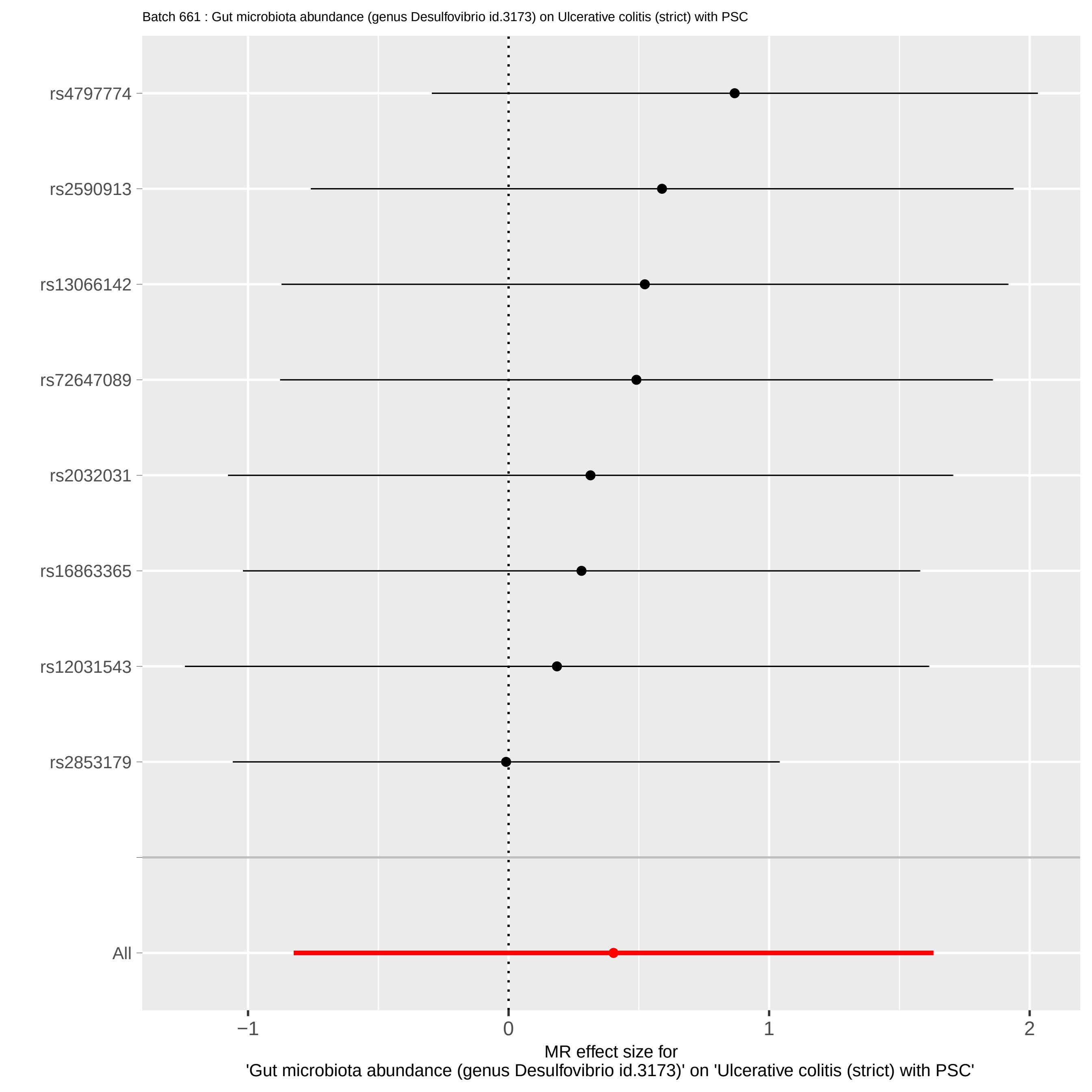

Batch 661 : Gut microbiota abundance (genus Desulfovibrio id.3173) on Ulcerative colitis (strict) with PSC
rs4797774
rs2590913
rs13066142
rs72647089
rs2032031
rs16863365
rs12031543
rs2853179
All
−1
0
1
2
MR effect size for
'Gut microbiota abundance (genus Desulfovibrio id.3173)' on 'Ulcerative colitis (strict) with PSC'

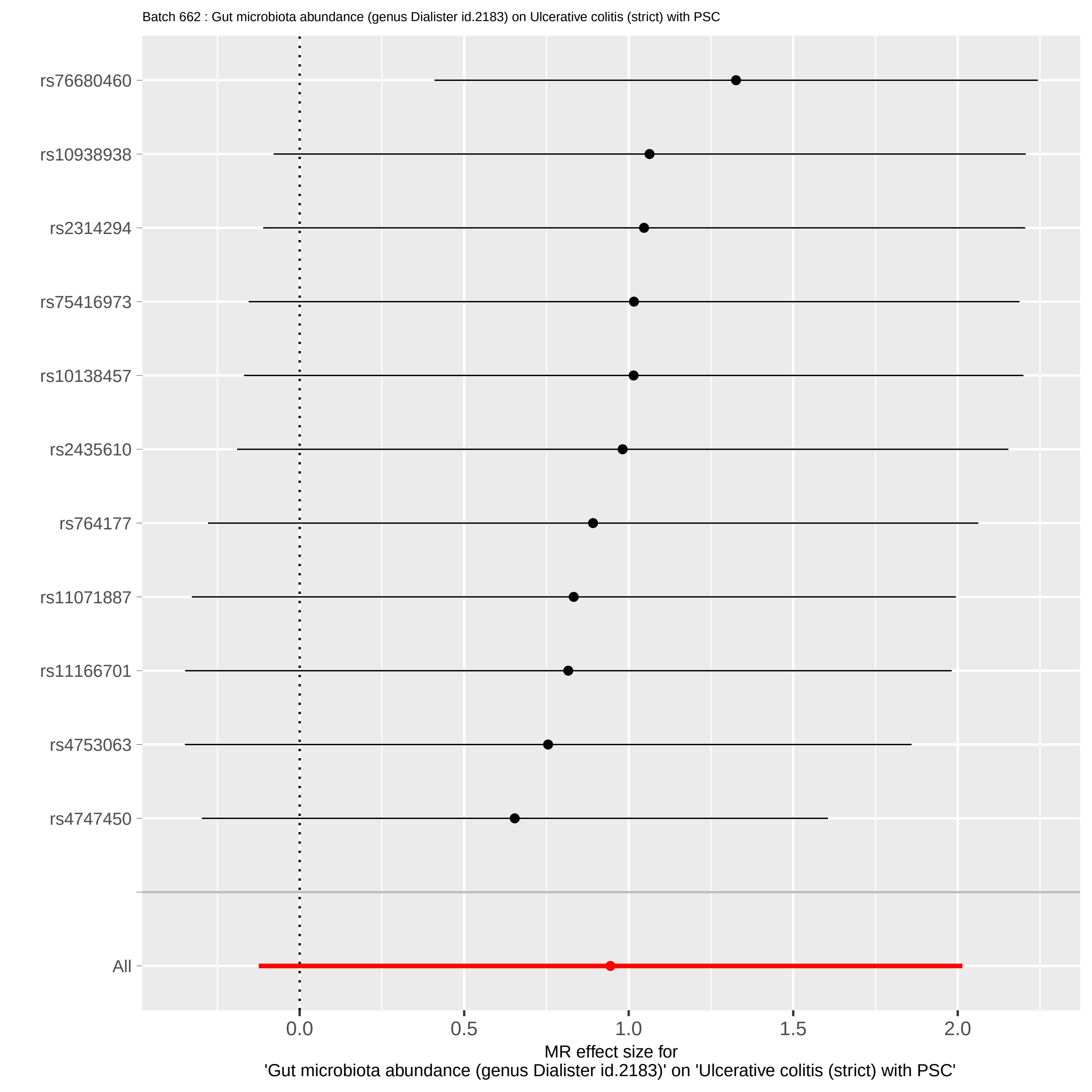
Batch 662 : Gut microbiota abundance (genus Dialister id.2183) on Ulcerative colitis (strict) with PSC
rs76680460
rs10938938
rs2314294
rs75416973
rs10138457
rs2435610
rs764177
rs11071887
rs11166701
rs4753063
rs4747450
All
0.0
0.5
1.0
1.5
2.0
MR effect size for
'Gut microbiota abundance (genus Dialister id.2183)' on 'Ulcerative colitis (strict) with PSC'

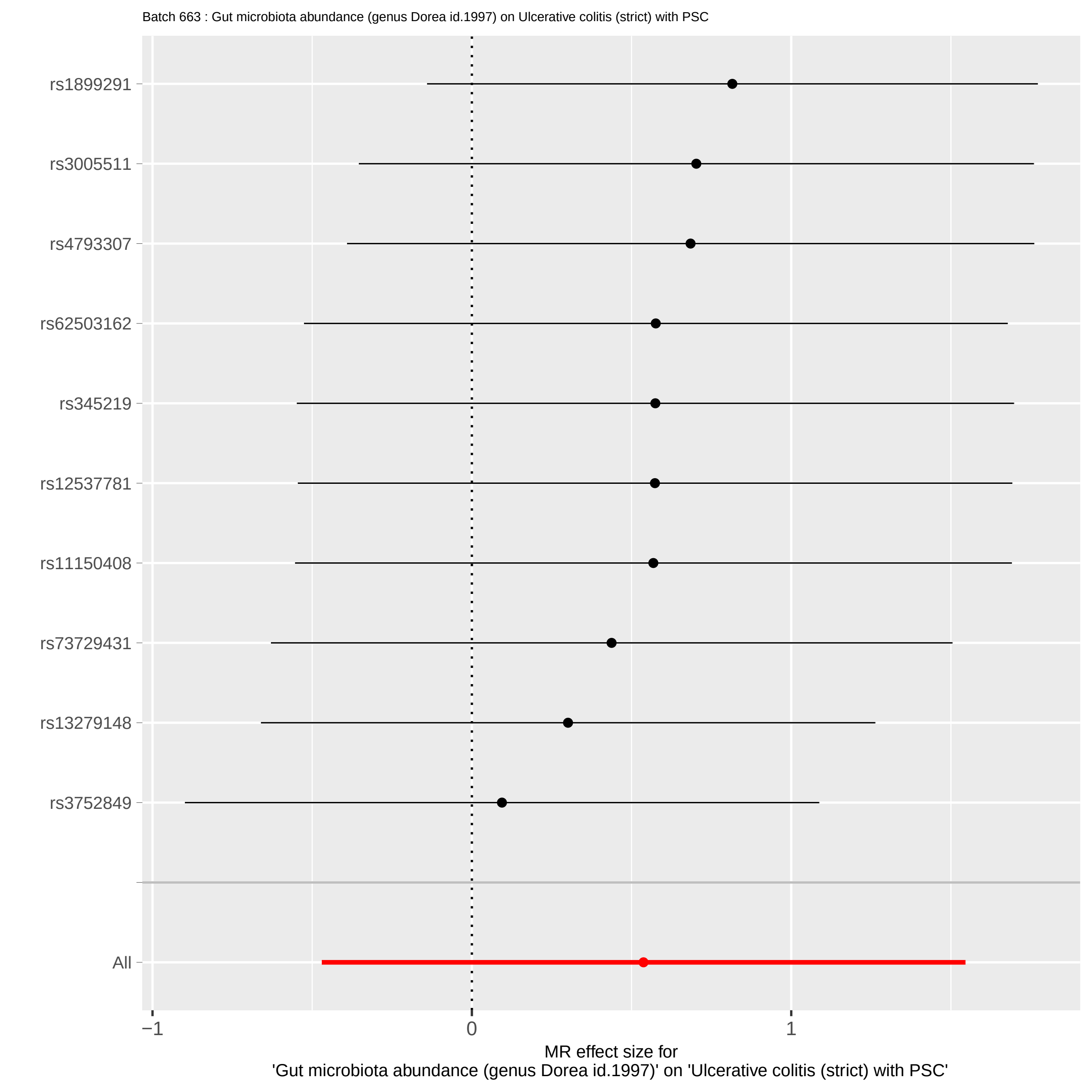
Batch 663 : Gut microbiota abundance (genus Dorea id.1997) on Ulcerative colitis (strict) with PSC
rs1899291
rs3005511
rs4793307
rs62503162
rs345219
rs12537781
rs11150408
rs73729431
rs13279148
rs3752849
All
−1
0
1
MR effect size for
'Gut microbiota abundance (genus Dorea id.1997)' on 'Ulcerative colitis (strict) with PSC'

Batch 664 : Gut microbiota abundance (genus Eggerthella id.819) on Ulcerative colitis (strict) with PSC

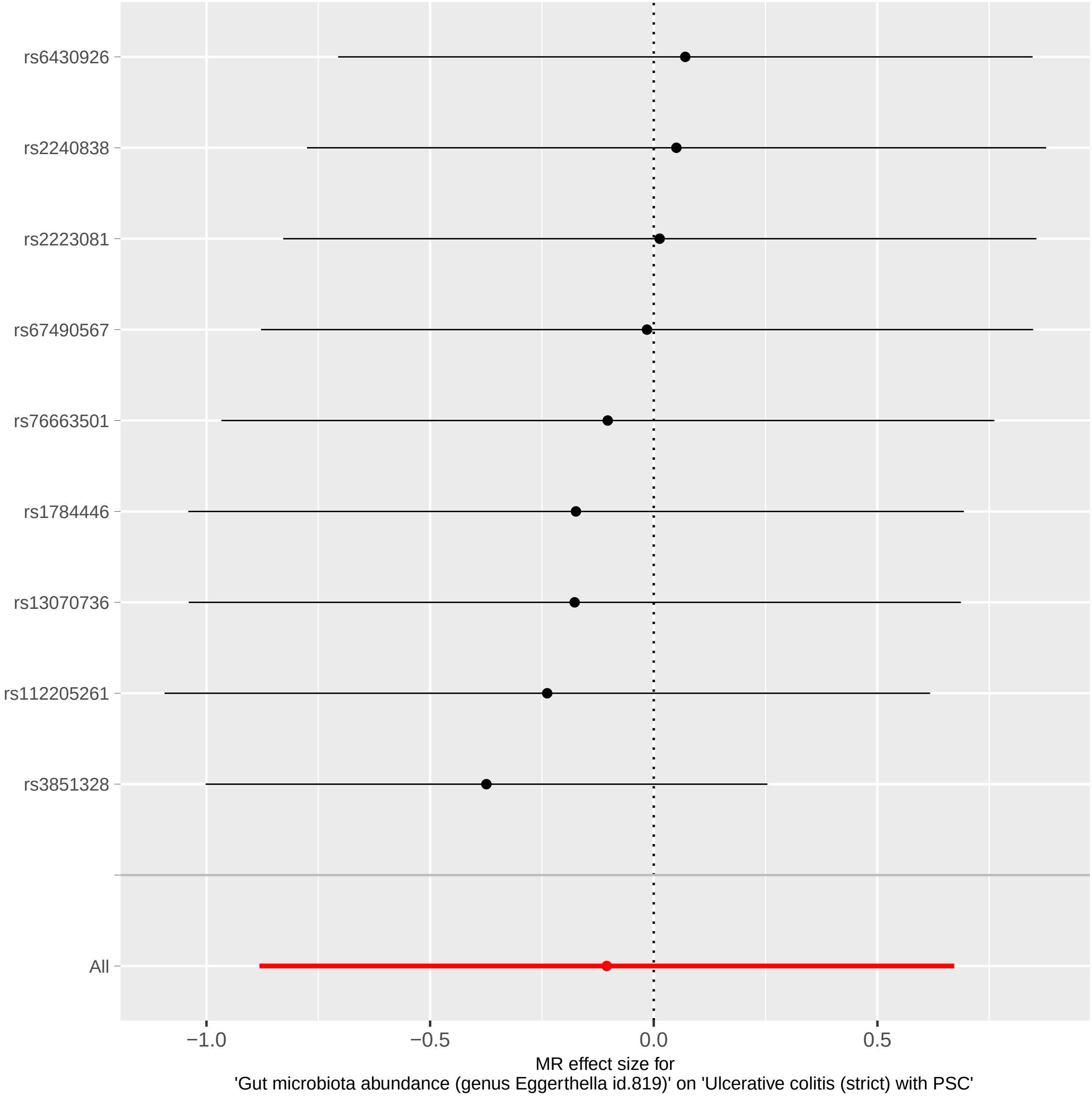

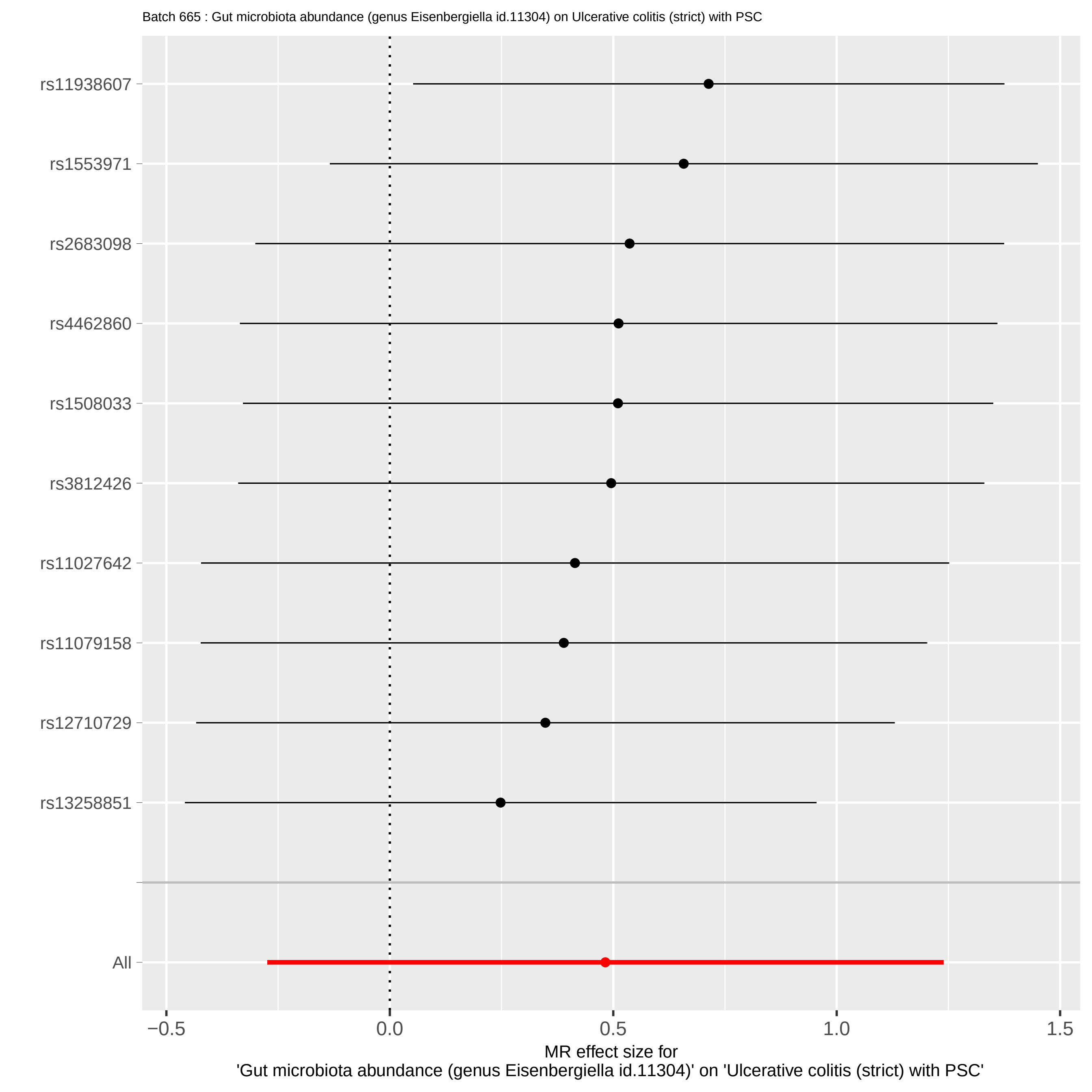

Batch 665 : Gut microbiota abundance (genus Eisenbergiella id.11304) on Ulcerative colitis (strict) with PSC
rs11938607
rs1553971
rs2683098
rs4462860
rs1508033
rs3812426
rs11027642
rs11079158
rs12710729
rs13258851
All
−0.5
0.0
0.5
1.0
1.5
MR effect size for
'Gut microbiota abundance (genus Eisenbergiella id.11304)' on 'Ulcerative colitis (strict) with PSC'

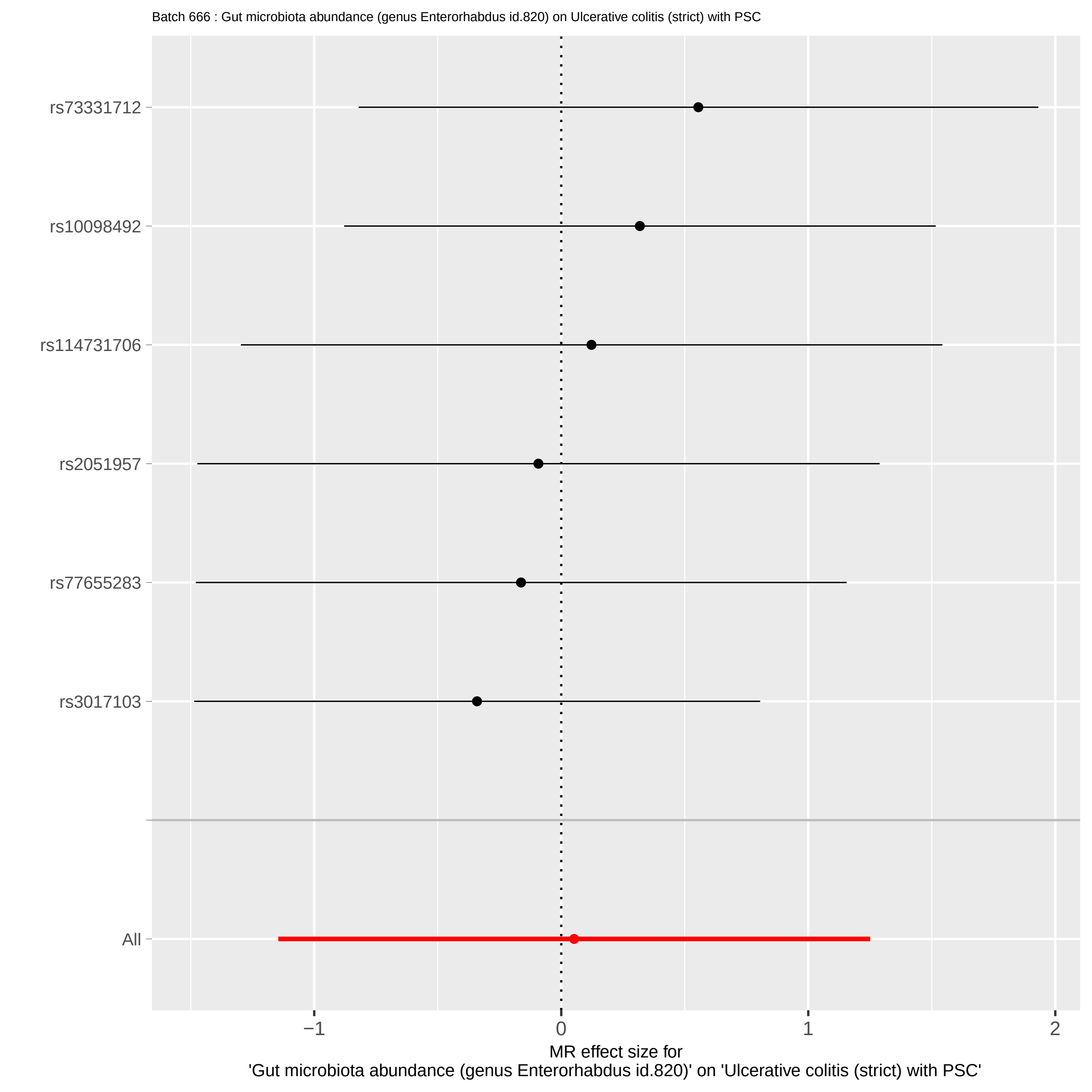
Batch 666 : Gut microbiota abundance (genus Enterorhabdus id.820) on Ulcerative colitis (strict) with PSC
rs73331712
rs10098492
rs114731706
rs2051957
rs77655283
rs3017103
All
−1
0
1
2
MR effect size for
'Gut microbiota abundance (genus Enterorhabdus id.820)' on 'Ulcerative colitis (strict) with PSC'

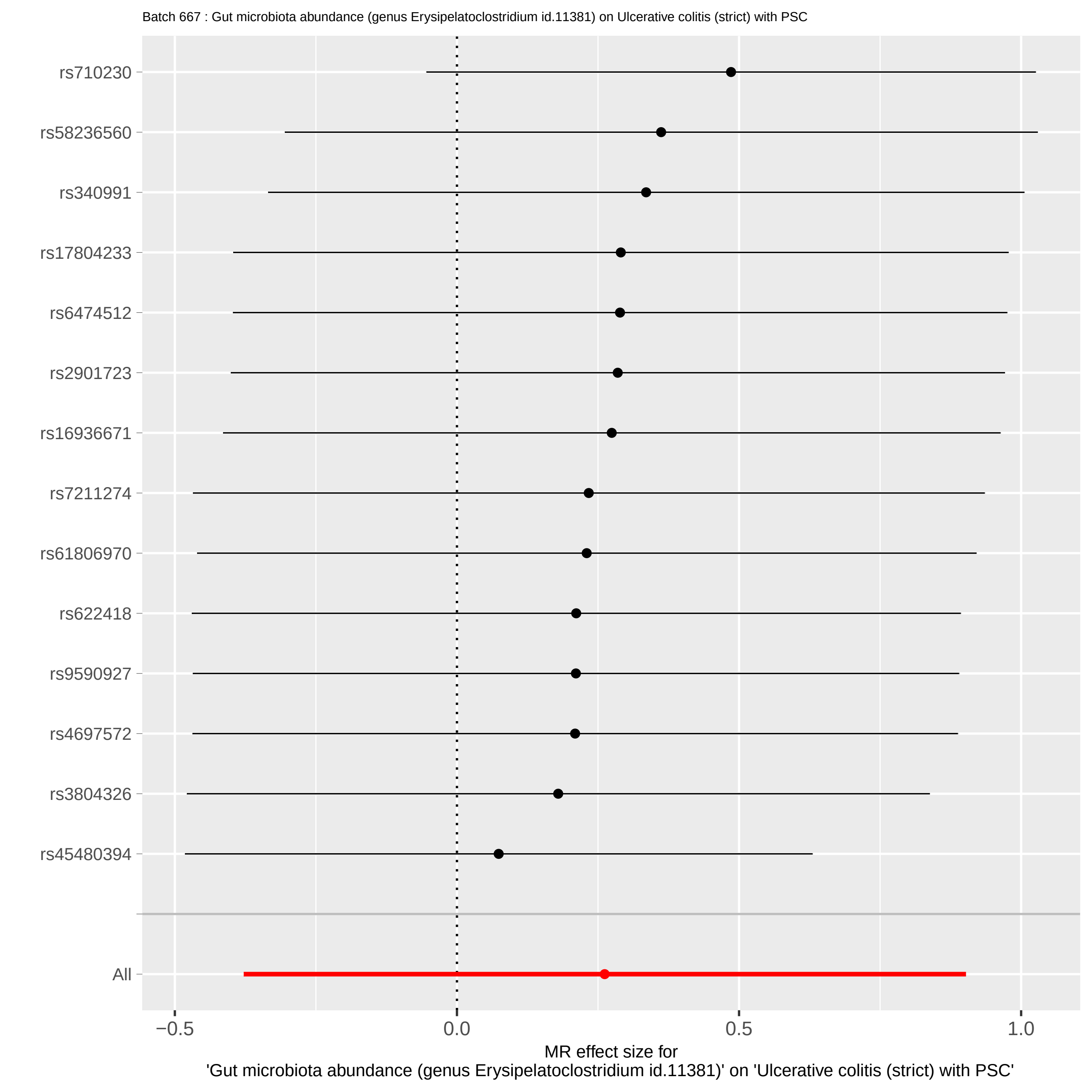

Batch 667 : Gut microbiota abundance (genus Erysipelatoclostridium id.11381) on Ulcerative colitis (strict) with PSC
rs710230
rs58236560
rs340991
rs17804233
rs6474512
rs2901723
rs16936671
rs7211274
rs61806970
rs622418
rs9590927
rs4697572
rs3804326
rs45480394
All
−0.5
0.0
0.5
1.0
MR effect size for
'Gut microbiota abundance (genus Erysipelatoclostridium id.11381)' on 'Ulcerative colitis (strict) with PSC'

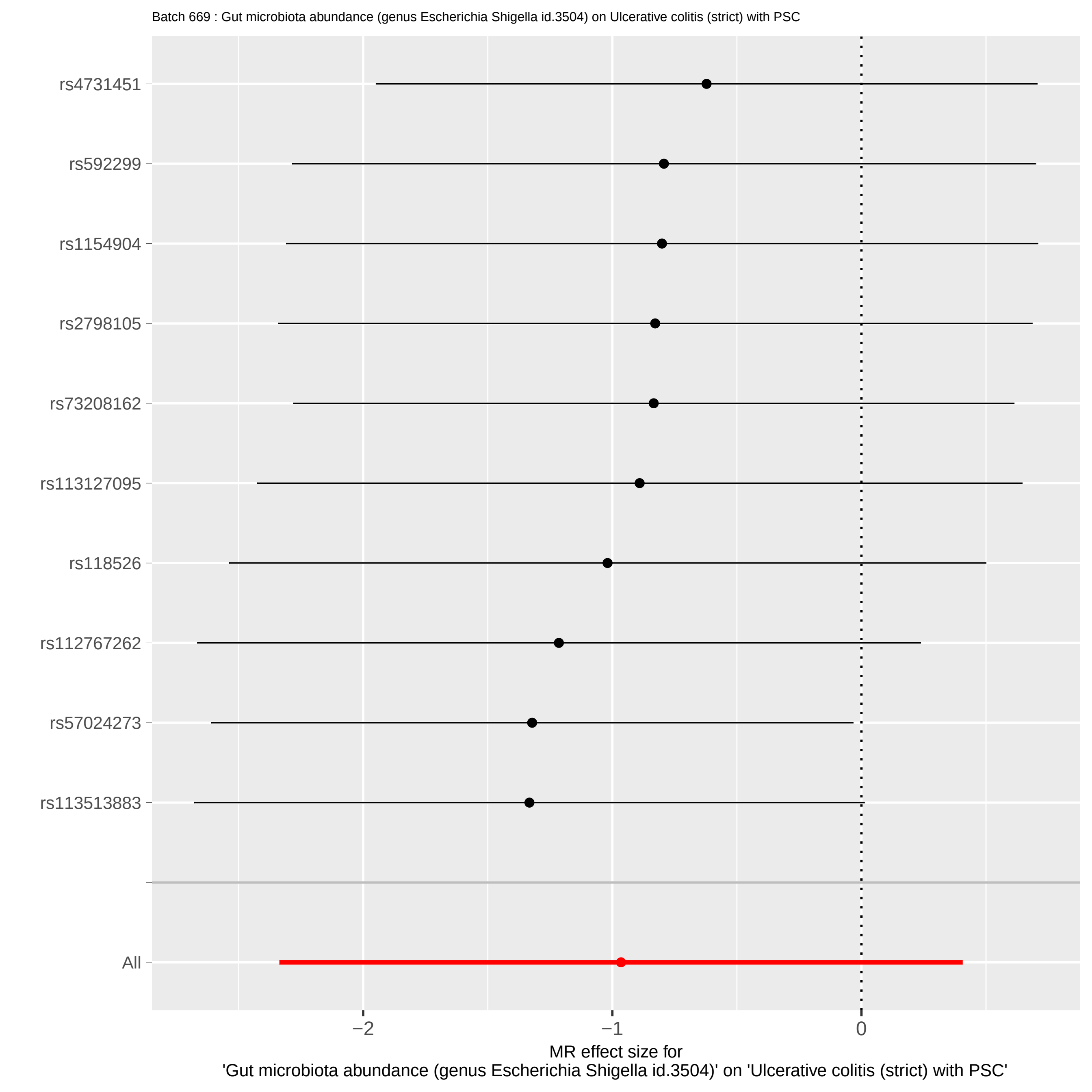

Batch 669 : Gut microbiota abundance (genus Escherichia Shigella id.3504) on Ulcerative colitis (strict) with PSC
rs4731451
rs592299
rs1154904
rs2798105
rs73208162
rs113127095
rs118526
rs112767262
rs57024273
rs113513883
All
−2
−1
0
MR effect size for
'Gut microbiota abundance (genus Escherichia Shigella id.3504)' on 'Ulcerative colitis (strict) with PSC'

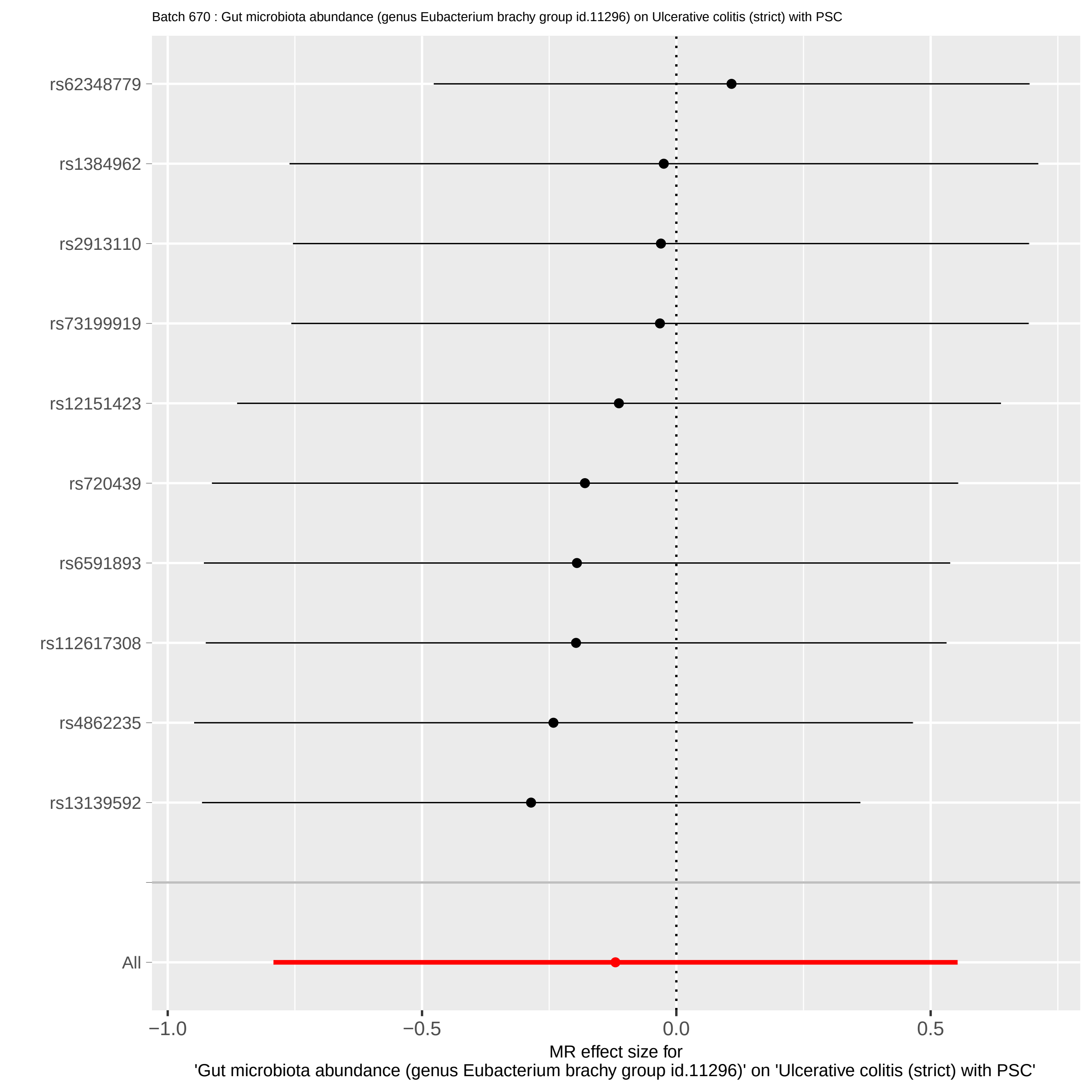
Batch 670 : Gut microbiota abundance (genus Eubacterium brachy group id.11296) on Ulcerative colitis (strict) with PSC
rs62348779
rs1384962
rs2913110
rs73199919
rs12151423
rs720439
rs6591893
rs112617308
rs4862235
rs13139592
All
−1.0
−0.5
0.0
0.5
MR effect size for
'Gut microbiota abundance (genus Eubacterium brachy group id.11296)' on 'Ulcerative colitis (strict) with PSC'

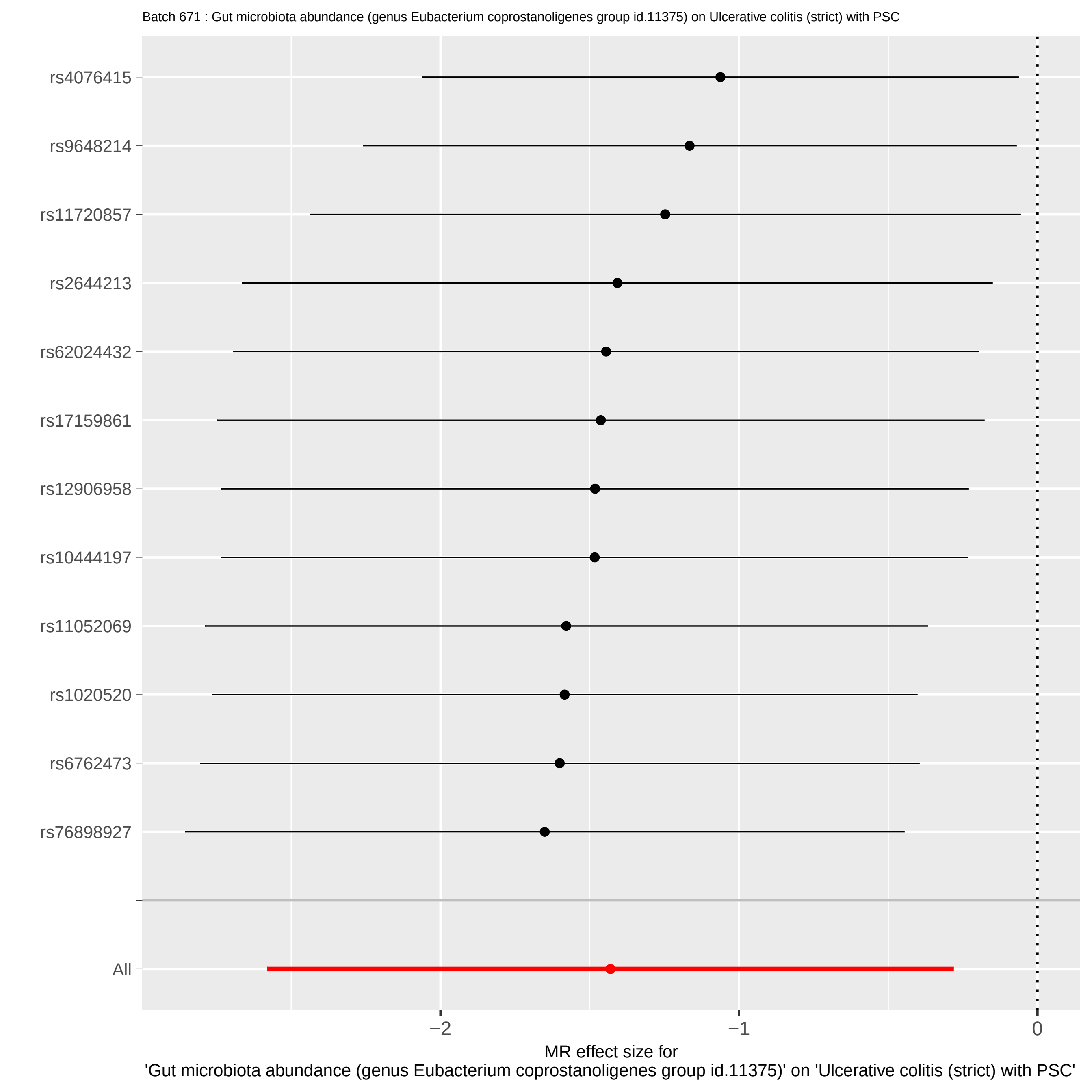

Batch 671 : Gut microbiota abundance (genus Eubacterium coprostanoligenes group id.11375) on Ulcerative colitis (strict) with PSC
rs4076415
rs9648214
rs11720857
rs2644213
rs62024432
rs17159861
rs12906958
rs10444197
rs11052069
rs1020520
rs6762473
rs76898927
All
−2
−1
0
MR effect size for
'Gut microbiota abundance (genus Eubacterium coprostanoligenes group id.11375)' on 'Ulcerative colitis (strict) with PSC'

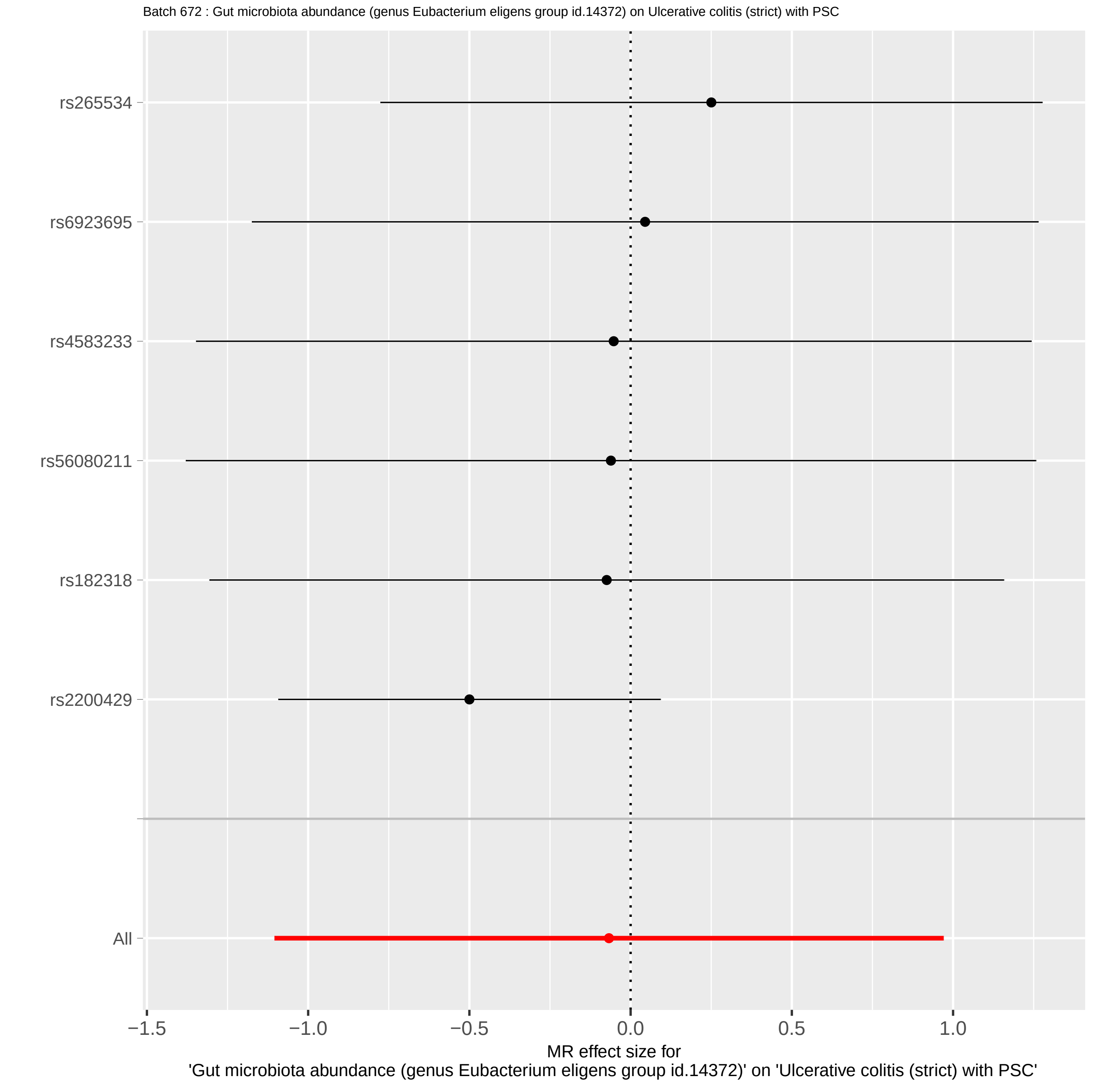
Batch 672 : Gut microbiota abundance (genus Eubacterium eligens group id.14372) on Ulcerative colitis (strict) with PSC
rs265534
rs6923695
rs4583233
rs56080211
rs182318
rs2200429
All
−1.5
−1.0
−0.5
0.0
0.5
1.0
MR effect size for
'Gut microbiota abundance (genus Eubacterium eligens group id.14372)' on 'Ulcerative colitis (strict) with PSC'

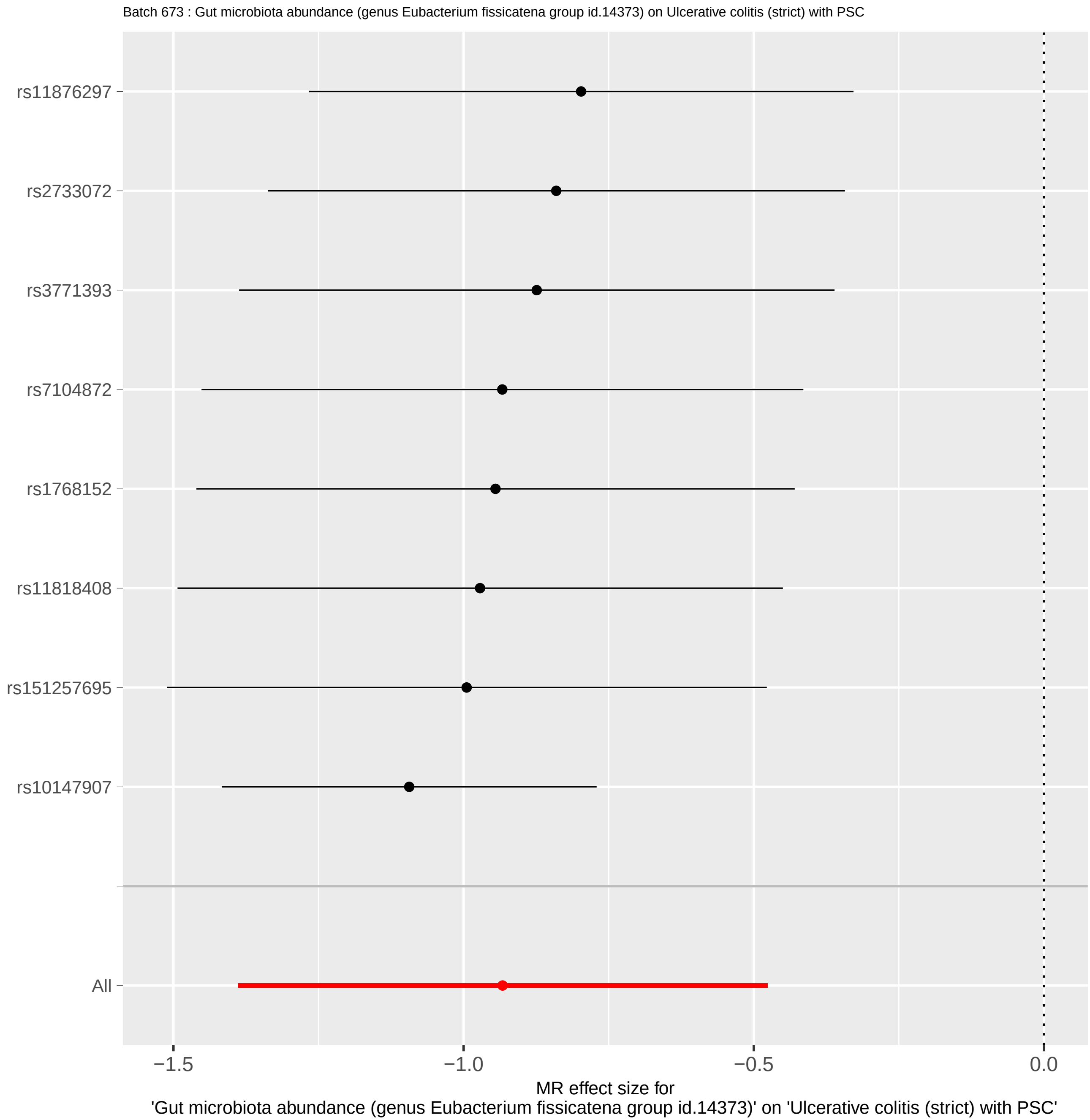

Batch 673 : Gut microbiota abundance (genus Eubacterium fissicatena group id.14373) on Ulcerative colitis (strict) with PSC
rs11876297
rs2733072
rs3771393
rs7104872
rs1768152
rs11818408
rs151257695
rs10147907
All
−1.5
−1.0
−0.5
0.0
MR effect size for
'Gut microbiota abundance (genus Eubacterium fissicatena group id.14373)' on 'Ulcerative colitis (strict) with PSC'

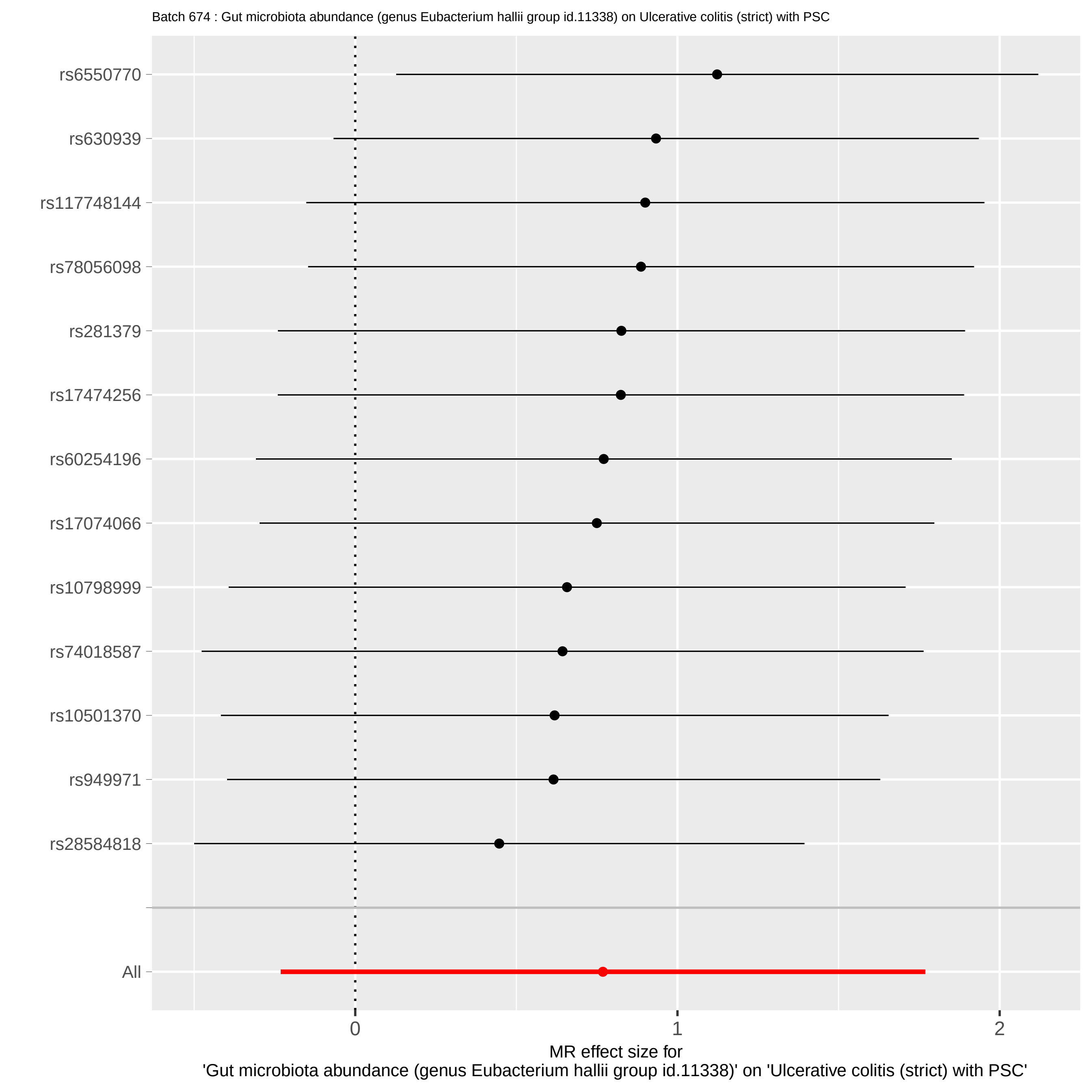

Batch 674 : Gut microbiota abundance (genus Eubacterium hallii group id.11338) on Ulcerative colitis (strict) with PSC
rs6550770
rs630939
rs117748144
rs78056098
rs281379
rs17474256
rs60254196
rs17074066
rs10798999
rs74018587
rs10501370
rs949971
rs28584818
All
0
1
2
MR effect size for
'Gut microbiota abundance (genus Eubacterium hallii group id.11338)' on 'Ulcerative colitis (strict) with PSC'

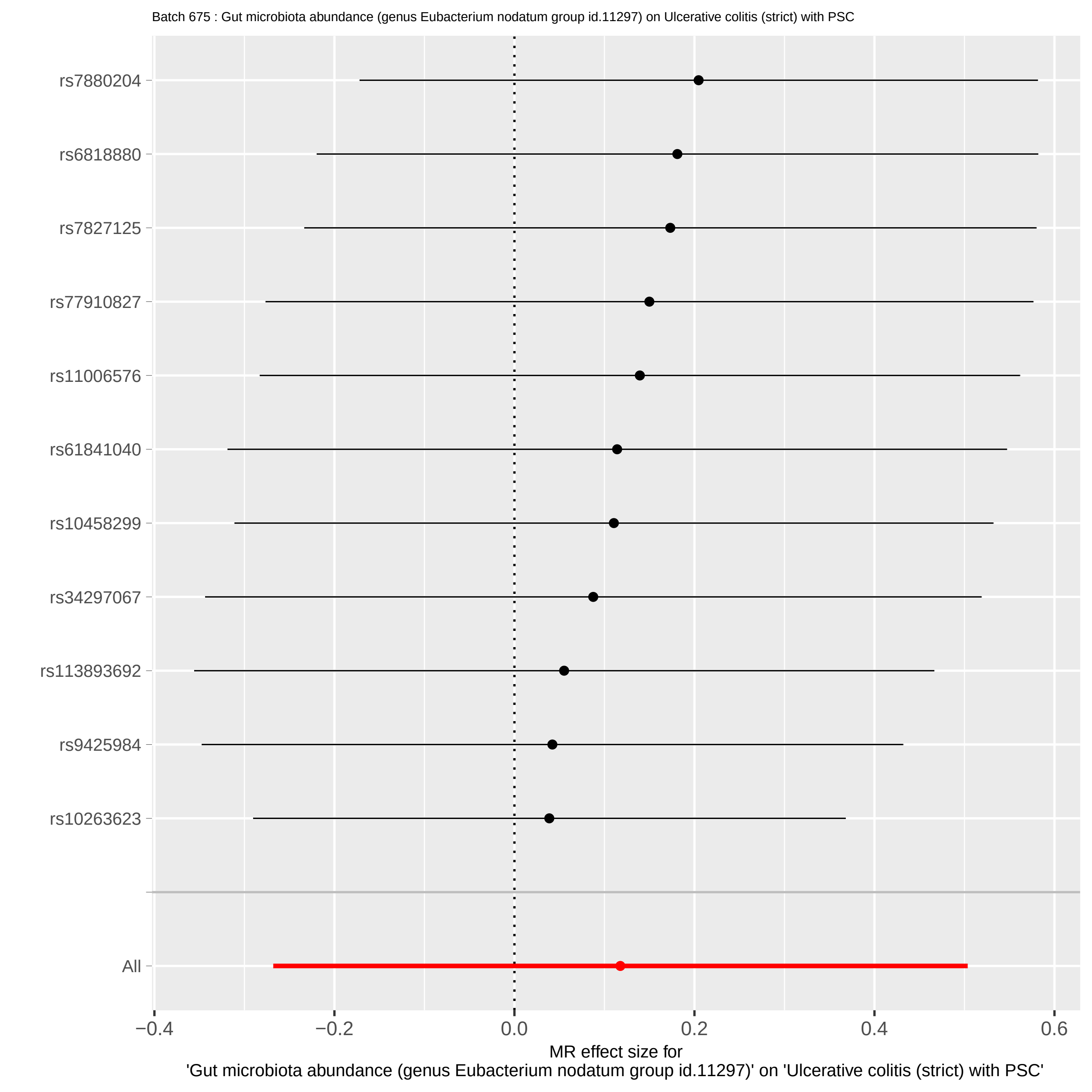
Batch 675 : Gut microbiota abundance (genus Eubacterium nodatum group id.11297) on Ulcerative colitis (strict) with PSC
rs7880204
rs6818880
rs7827125
rs77910827
rs11006576
rs61841040
rs10458299
rs34297067
rs113893692
rs9425984
rs10263623
All
−0.4
−0.2
0.0
0.2
0.4
0.6
MR effect size for
'Gut microbiota abundance (genus Eubacterium nodatum group id.11297)' on 'Ulcerative colitis (strict) with PSC'

Batch 676 : Gut microbiota abundance (genus Eubacterium oxidoreducens group id.11339) on Ulcerative colitis (strict) with PSC

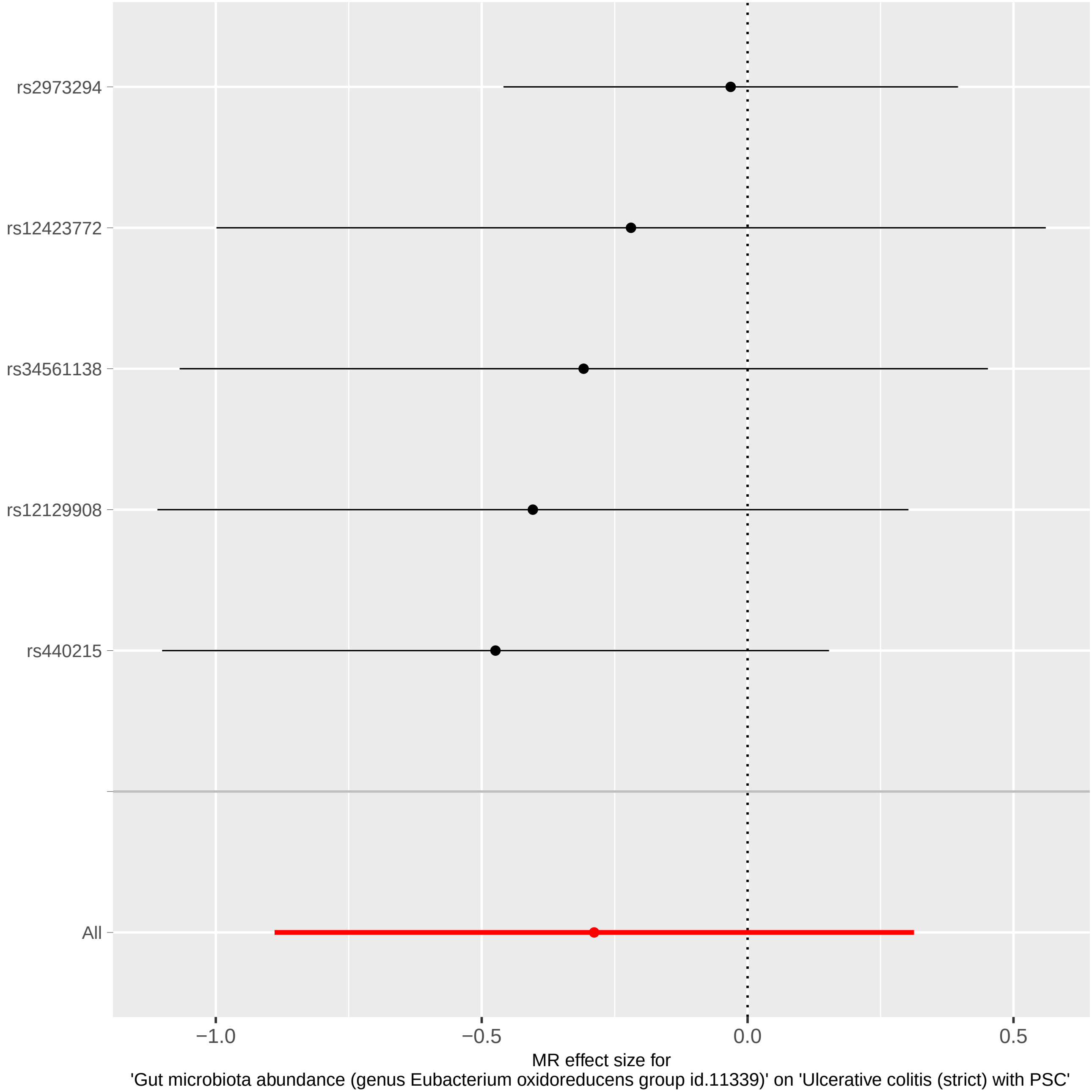

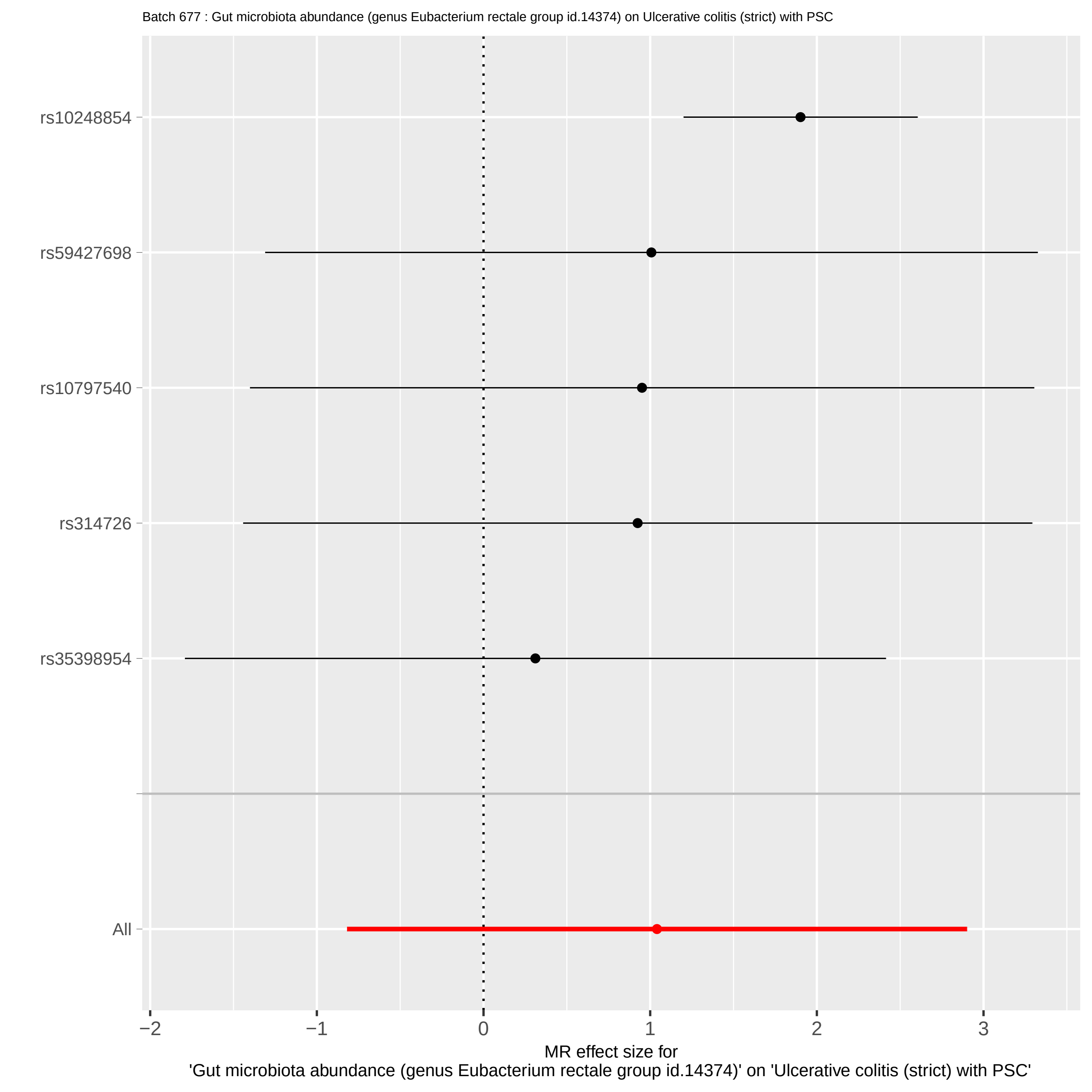
Batch 677 : Gut microbiota abundance (genus Eubacterium rectale group id.14374) on Ulcerative colitis (strict) with PSC
rs10248854
rs59427698
rs10797540
rs314726
rs35398954
All
−2
−1
0
1
2
3
MR effect size for
'Gut microbiota abundance (genus Eubacterium rectale group id.14374)' on 'Ulcerative colitis (strict) with PSC'

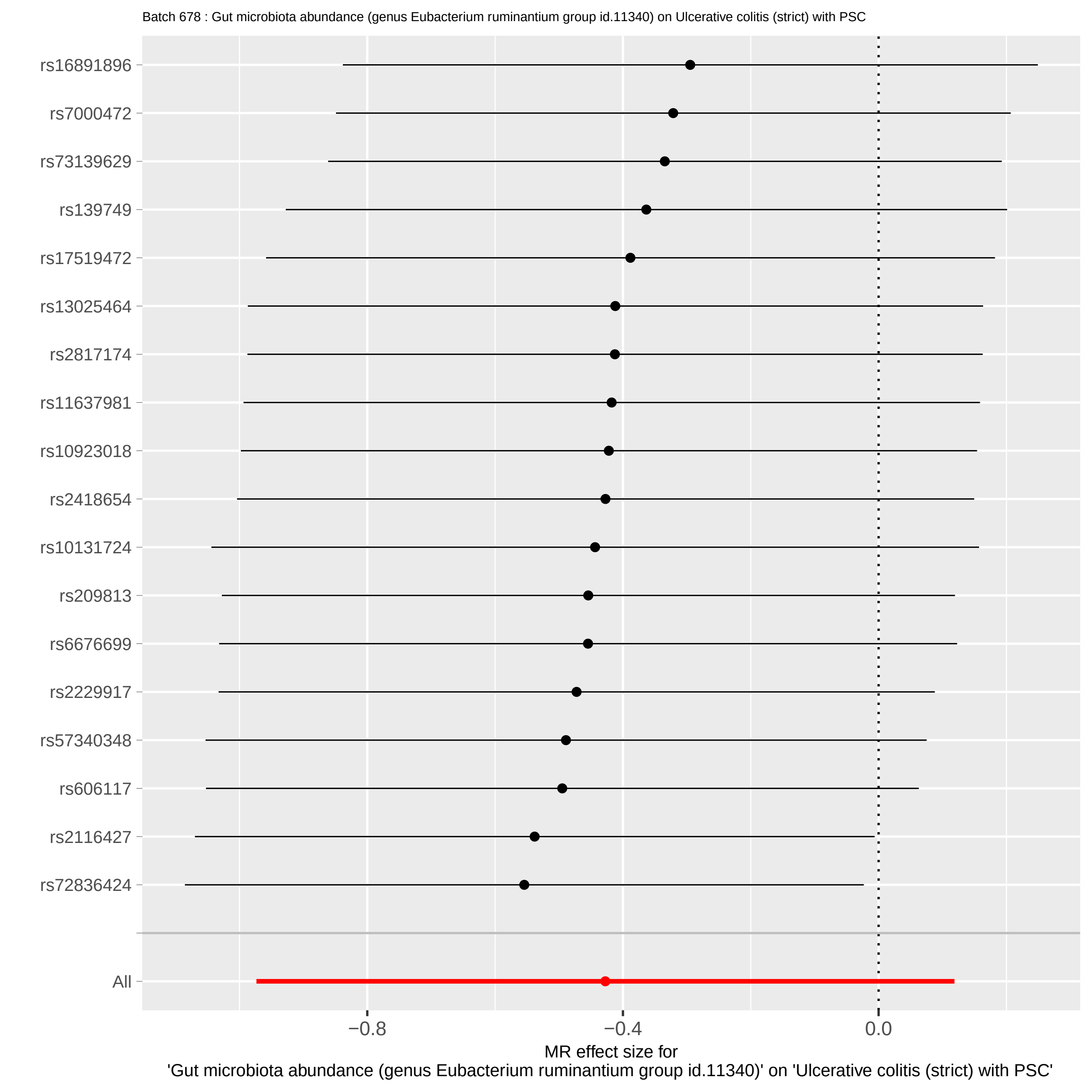

Batch 678 : Gut microbiota abundance (genus Eubacterium ruminantium group id.11340) on Ulcerative colitis (strict) with PSC
rs16891896
rs7000472
rs73139629
rs139749
rs17519472
rs13025464
rs2817174
rs11637981
rs10923018
rs2418654
rs10131724
rs209813
rs6676699
rs2229917
rs57340348
rs606117
rs2116427
rs72836424
All
−0.8
−0.4
0.0
MR effect size for
'Gut microbiota abundance (genus Eubacterium ruminantium group id.11340)' on 'Ulcerative colitis (strict) with PSC'

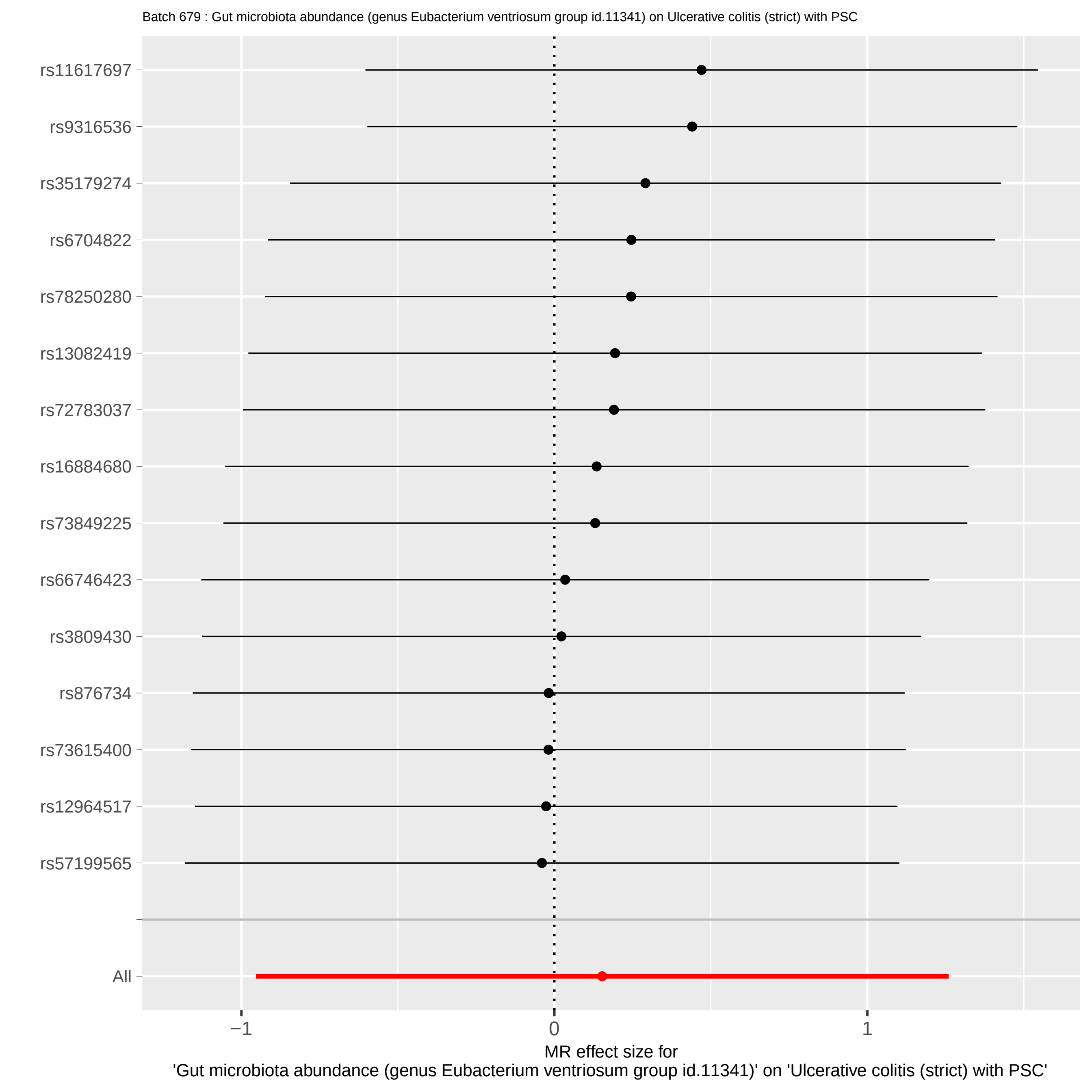
Batch 679 : Gut microbiota abundance (genus Eubacterium ventriosum group id.11341) on Ulcerative colitis (strict) with PSC
rs11617697
rs9316536
rs35179274
rs6704822
rs78250280
rs13082419
rs72783037
rs16884680
rs73849225
rs66746423
rs3809430
rs876734
rs73615400
rs12964517
rs57199565
All
−1
0
1
MR effect size for
'Gut microbiota abundance (genus Eubacterium ventriosum group id.11341)' on 'Ulcerative colitis (strict) with PSC'

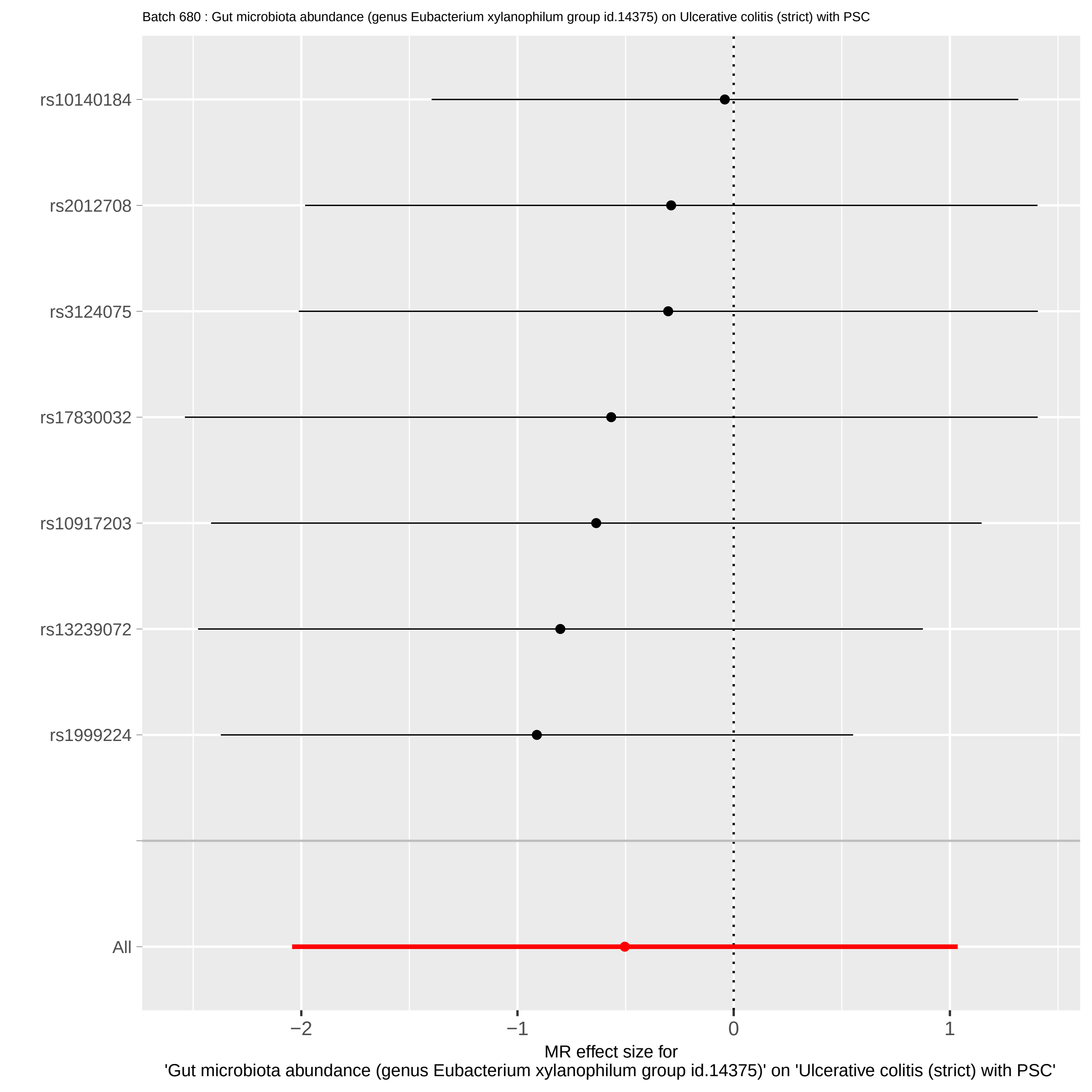

Batch 680 : Gut microbiota abundance (genus Eubacterium xylanophilum group id.14375) on Ulcerative colitis (strict) with PSC
rs10140184
rs2012708
rs3124075
rs17830032
rs10917203
rs13239072
rs1999224
All
−2
−1
0
1
MR effect size for
'Gut microbiota abundance (genus Eubacterium xylanophilum group id.14375)' on 'Ulcerative colitis (strict) with PSC'

Batch 681 : Gut microbiota abundance (genus Faecalibacterium id.2057) on Ulcerative colitis (strict) with PSC

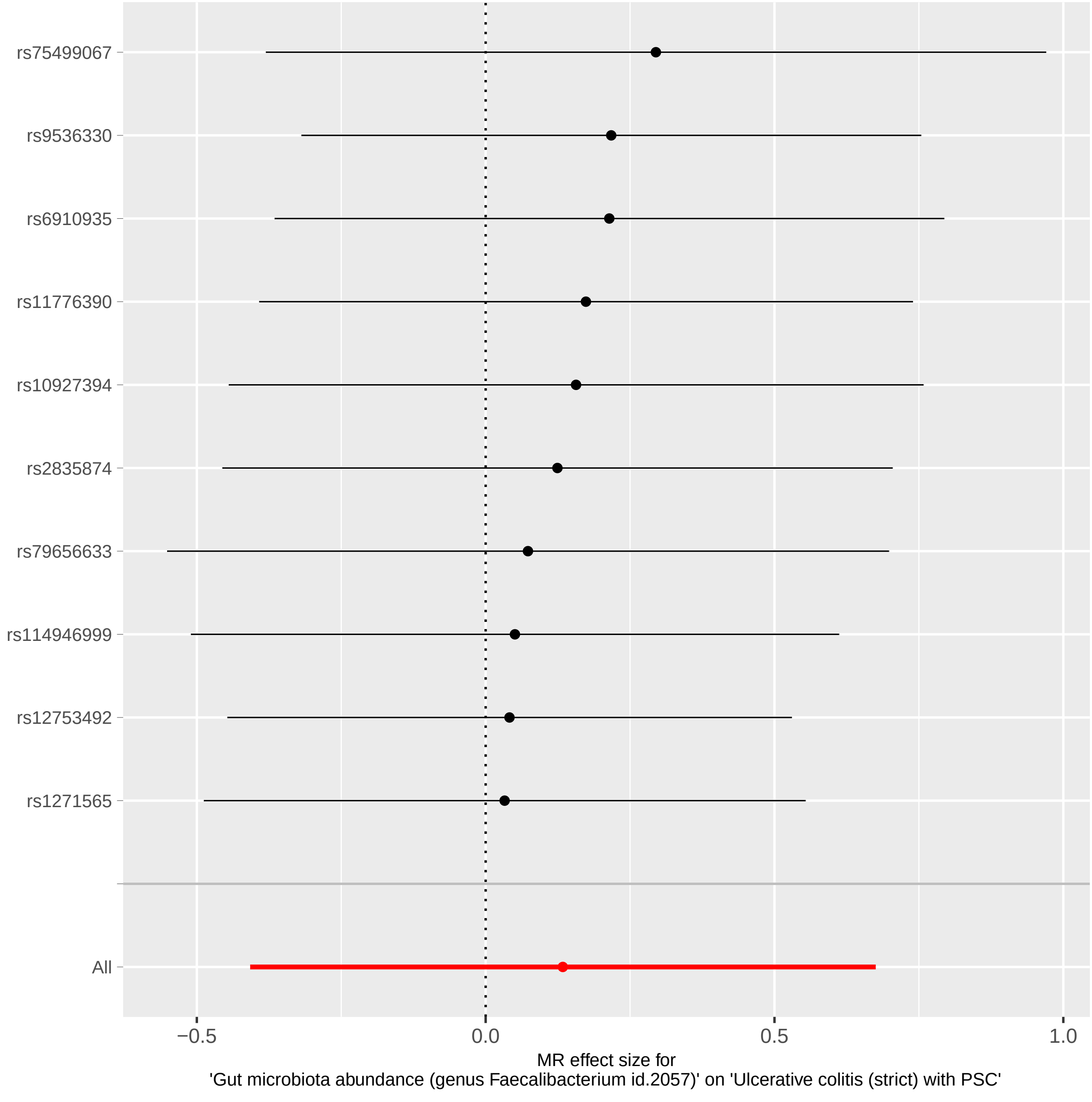

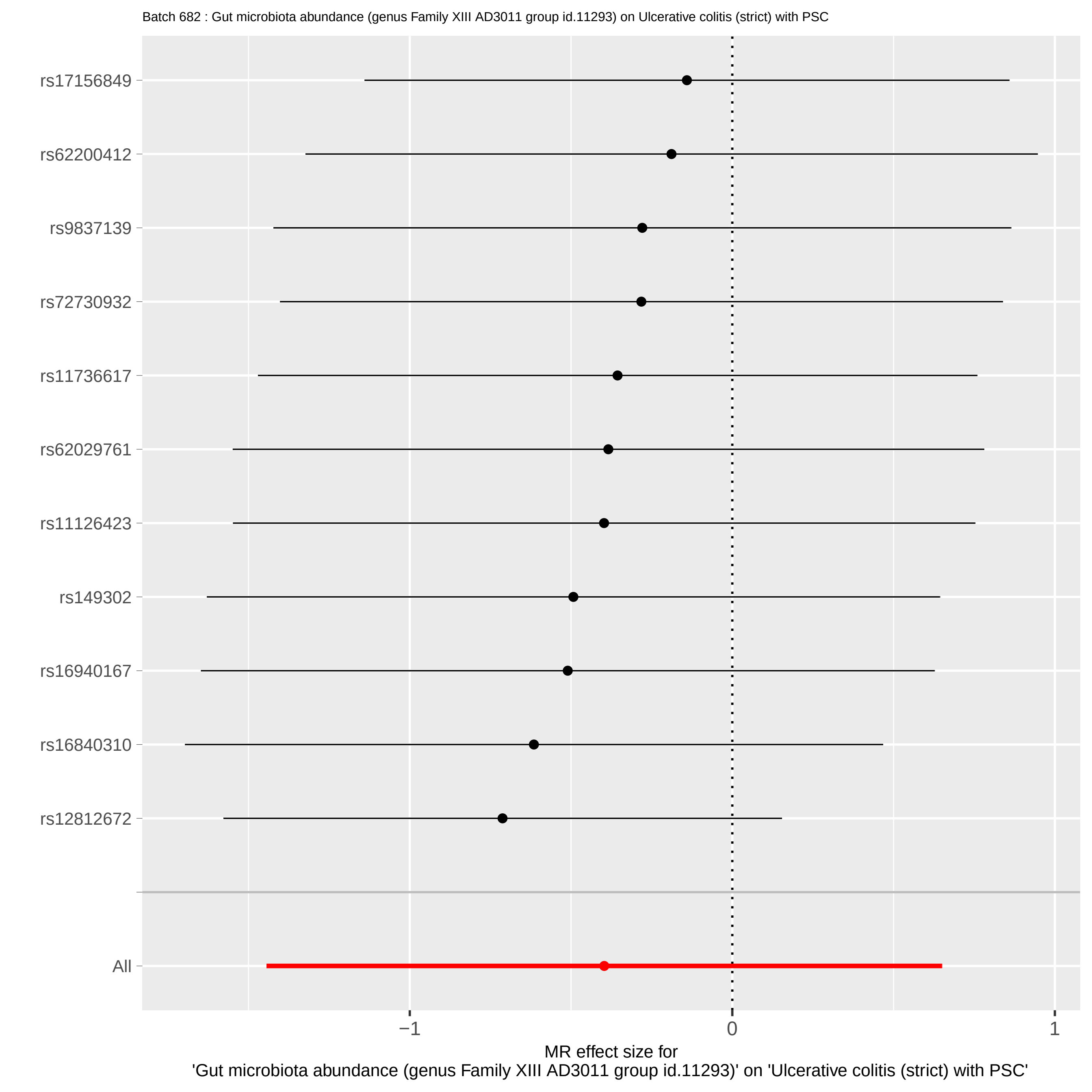
Batch 682 : Gut microbiota abundance (genus Family XIII AD3011 group id.11293) on Ulcerative colitis (strict) with PSC
rs17156849
rs62200412
rs9837139
rs72730932
rs11736617
rs62029761
rs11126423
rs149302
rs16940167
rs16840310
rs12812672
All
−1
0
1
MR effect size for
'Gut microbiota abundance (genus Family XIII AD3011 group id.11293)' on 'Ulcerative colitis (strict) with PSC'

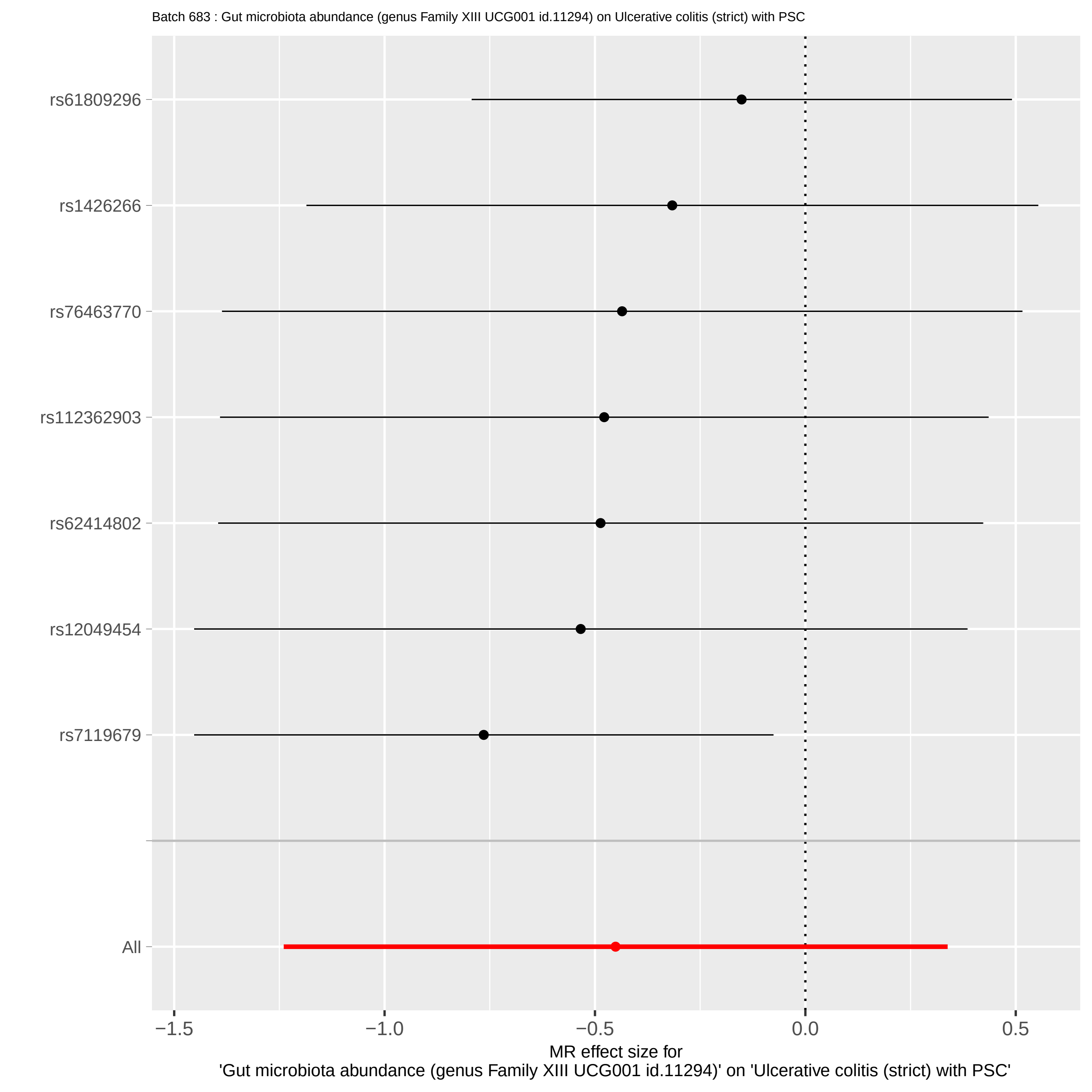
Batch 683 : Gut microbiota abundance (genus Family XIII UCG001 id.11294) on Ulcerative colitis (strict) with PSC
rs61809296
rs1426266
rs76463770
rs112362903
rs62414802
rs12049454
rs7119679
All
−1.5
−1.0
−0.5
0.0
0.5
MR effect size for
'Gut microbiota abundance (genus Family XIII UCG001 id.11294)' on 'Ulcerative colitis (strict) with PSC'

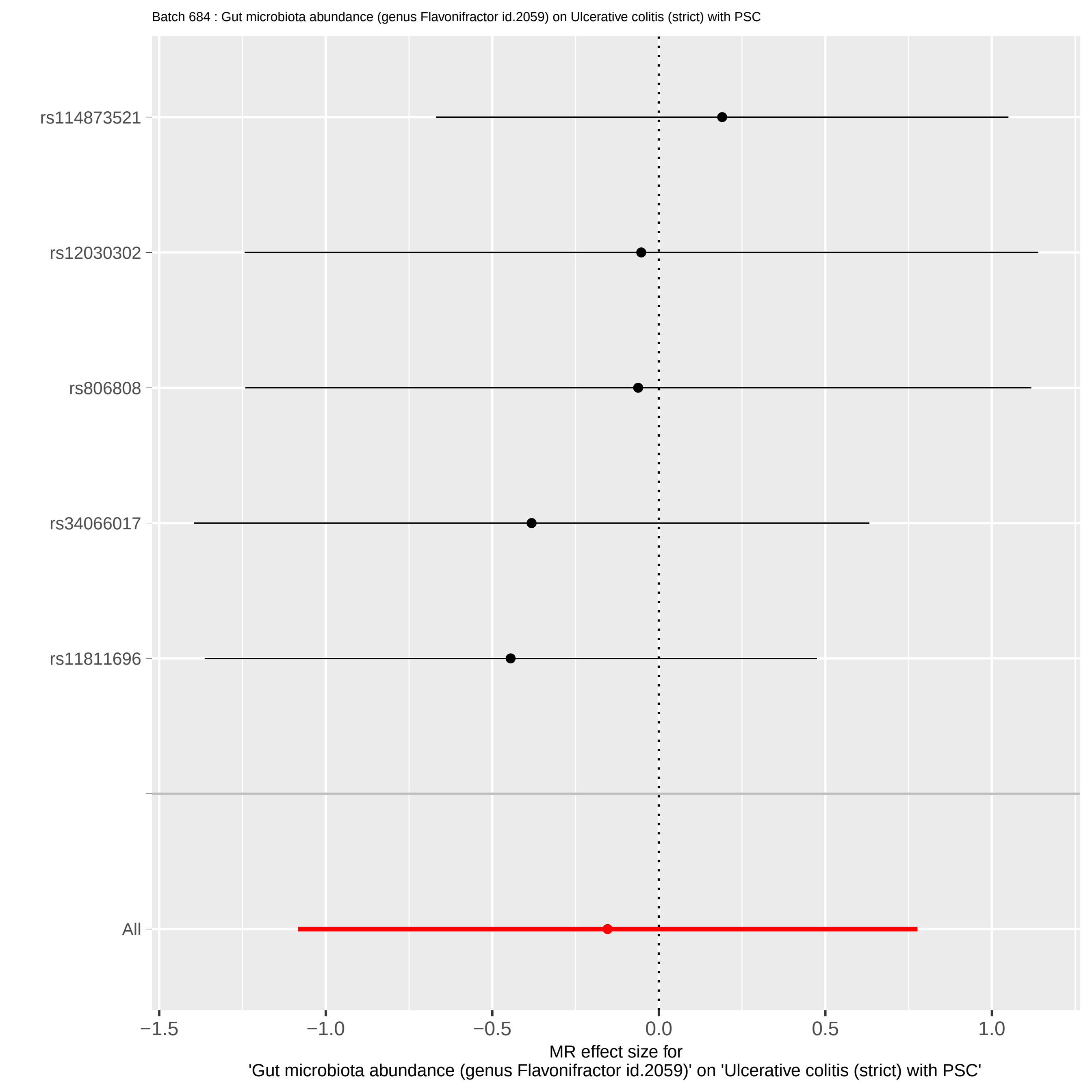

Batch 684 : Gut microbiota abundance (genus Flavonifractor id.2059) on Ulcerative colitis (strict) with PSC
rs114873521
rs12030302
rs806808
rs34066017
rs11811696
All
−1.5
−1.0
−0.5
0.0
0.5
1.0
MR effect size for
'Gut microbiota abundance (genus Flavonifractor id.2059)' on 'Ulcerative colitis (strict) with PSC'

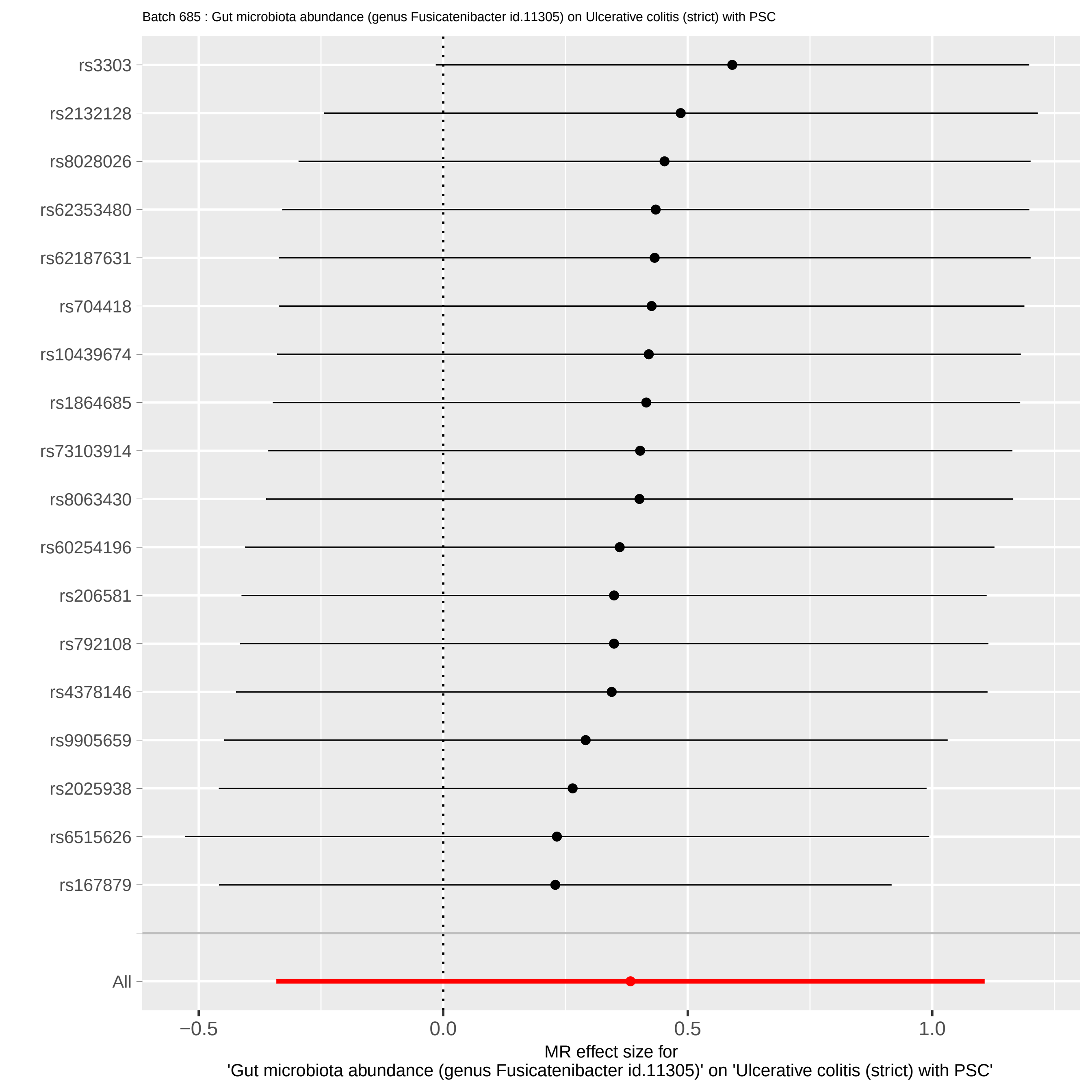

Batch 685 : Gut microbiota abundance (genus Fusicatenibacter id.11305) on Ulcerative colitis (strict) with PSC
rs3303
rs2132128
rs8028026
rs62353480
rs62187631
rs704418
rs10439674
rs1864685
rs73103914
rs8063430
rs60254196
rs206581
rs792108
rs4378146
rs9905659
rs2025938
rs6515626
rs167879
All
−0.5
0.0
0.5
1.0
MR effect size for
'Gut microbiota abundance (genus Fusicatenibacter id.11305)' on 'Ulcerative colitis (strict) with PSC'

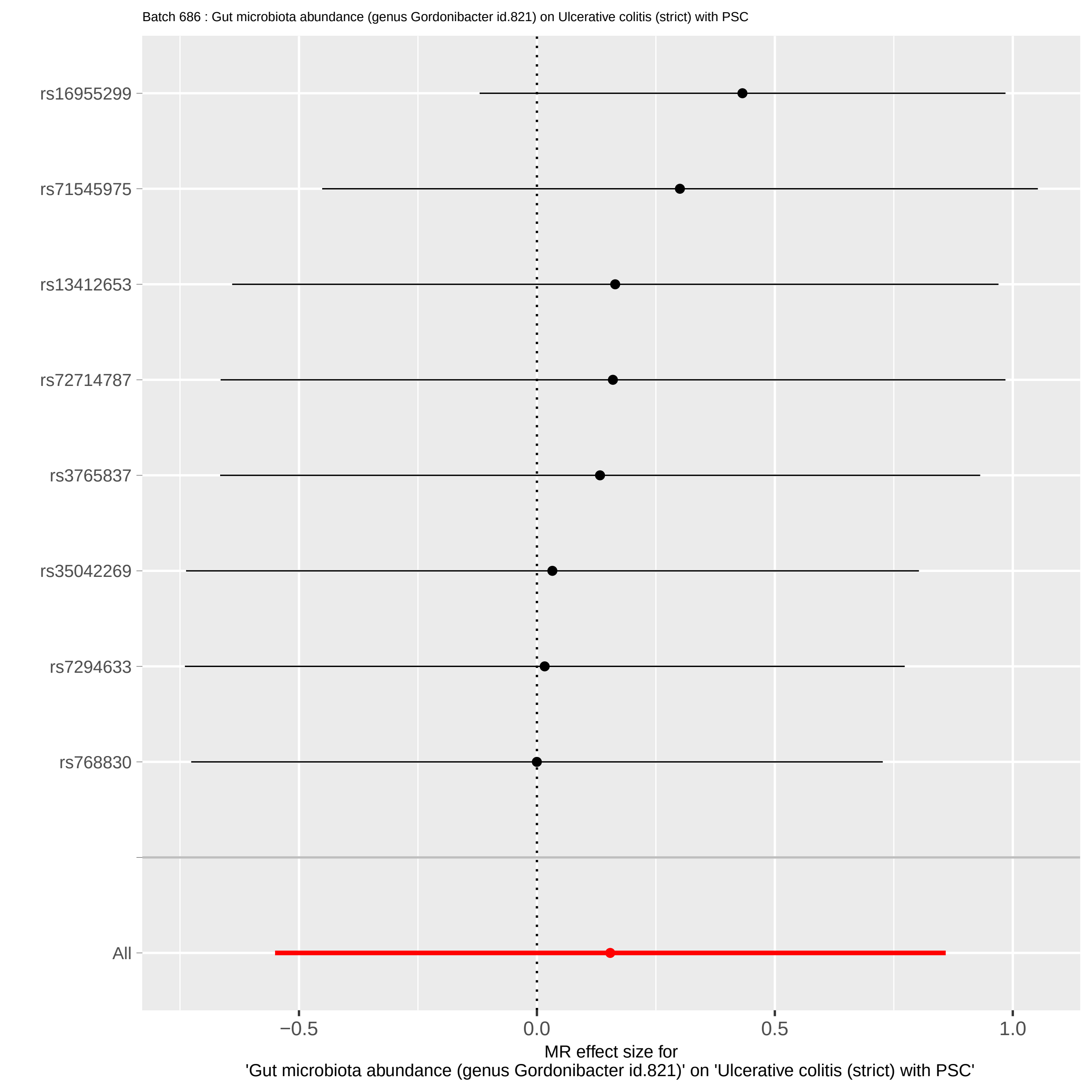
Batch 686 : Gut microbiota abundance (genus Gordonibacter id.821) on Ulcerative colitis (strict) with PSC
rs16955299
rs71545975
rs13412653
rs72714787
rs3765837
rs35042269
rs7294633
rs768830
All
−0.5
0.0
0.5
1.0
MR effect size for
'Gut microbiota abundance (genus Gordonibacter id.821)' on 'Ulcerative colitis (strict) with PSC'

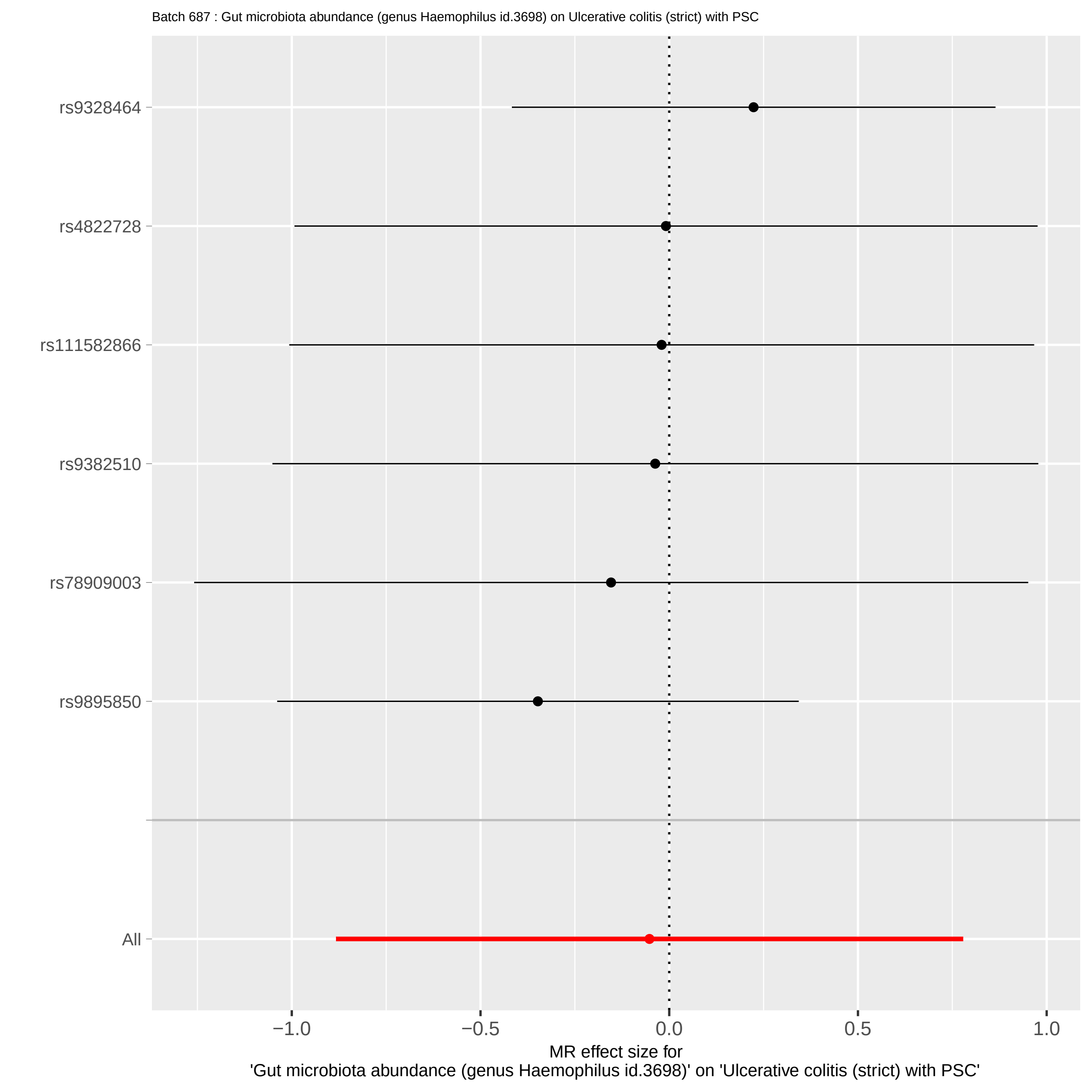

Batch 687 : Gut microbiota abundance (genus Haemophilus id.3698) on Ulcerative colitis (strict) with PSC
rs9328464
rs4822728
rs111582866
rs9382510
rs78909003
rs9895850
All
−1.0
−0.5
0.0
0.5
1.0
MR effect size for
'Gut microbiota abundance (genus Haemophilus id.3698)' on 'Ulcerative colitis (strict) with PSC'

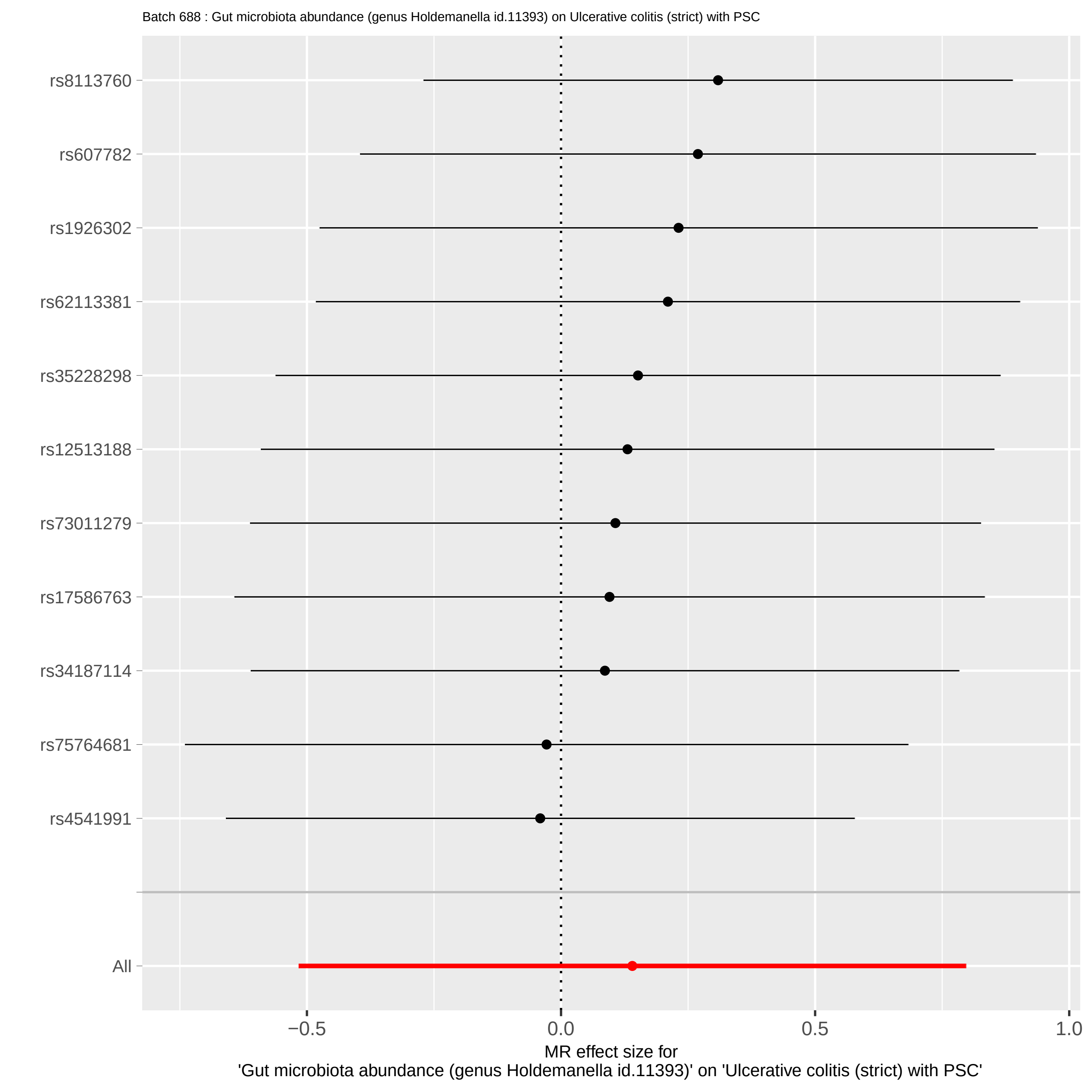

Batch 688 : Gut microbiota abundance (genus Holdemanella id.11393) on Ulcerative colitis (strict) with PSC
rs8113760
rs607782
rs1926302
rs62113381
rs35228298
rs12513188
rs73011279
rs17586763
rs34187114
rs75764681
rs4541991
All
−0.5
0.0
0.5
1.0
MR effect size for
'Gut microbiota abundance (genus Holdemanella id.11393)' on 'Ulcerative colitis (strict) with PSC'

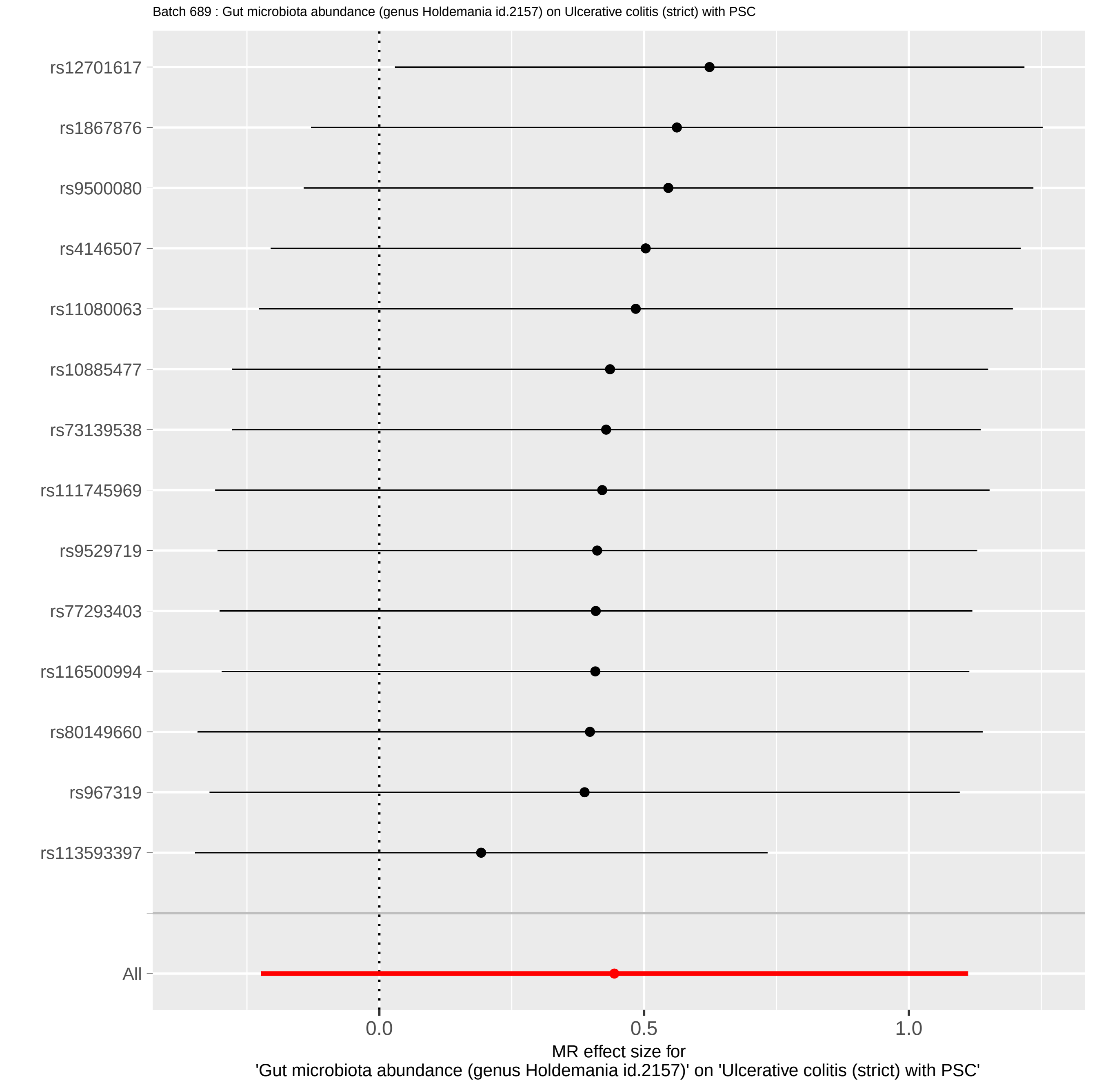

Batch 689 : Gut microbiota abundance (genus Holdemania id.2157) on Ulcerative colitis (strict) with PSC
rs12701617
rs1867876
rs9500080
rs4146507
rs11080063
rs10885477
rs73139538
rs111745969
rs9529719
rs77293403
rs116500994
rs80149660
rs967319
rs113593397
All
0.0
0.5
1.0
MR effect size for
'Gut microbiota abundance (genus Holdemania id.2157)' on 'Ulcerative colitis (strict) with PSC'

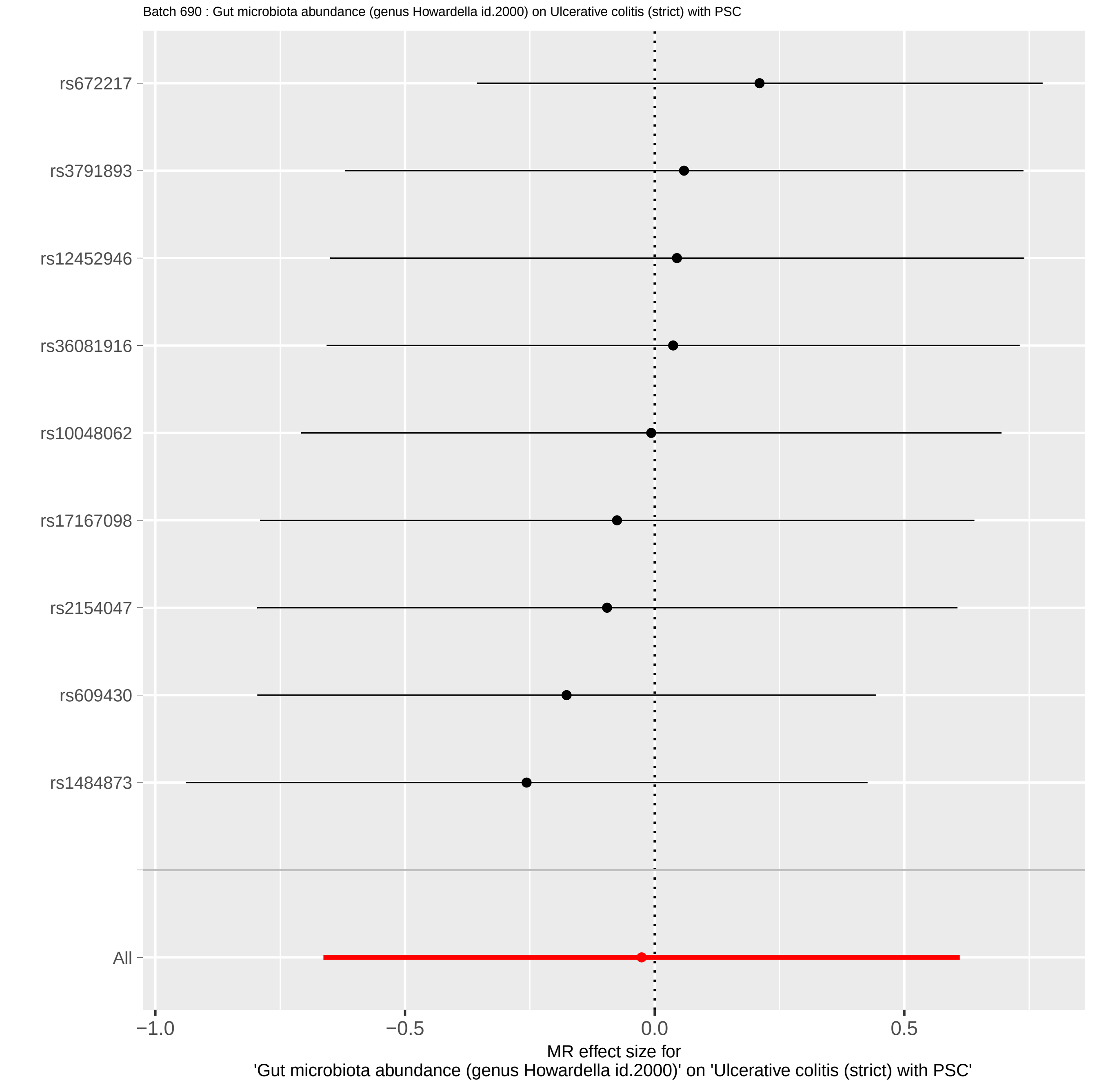
Batch 690 : Gut microbiota abundance (genus Howardella id.2000) on Ulcerative colitis (strict) with PSC
rs672217
rs3791893
rs12452946
rs36081916
rs10048062
rs17167098
rs2154047
rs609430
rs1484873
All
−1.0
−0.5
0.0
0.5
MR effect size for
'Gut microbiota abundance (genus Howardella id.2000)' on 'Ulcerative colitis (strict) with PSC'

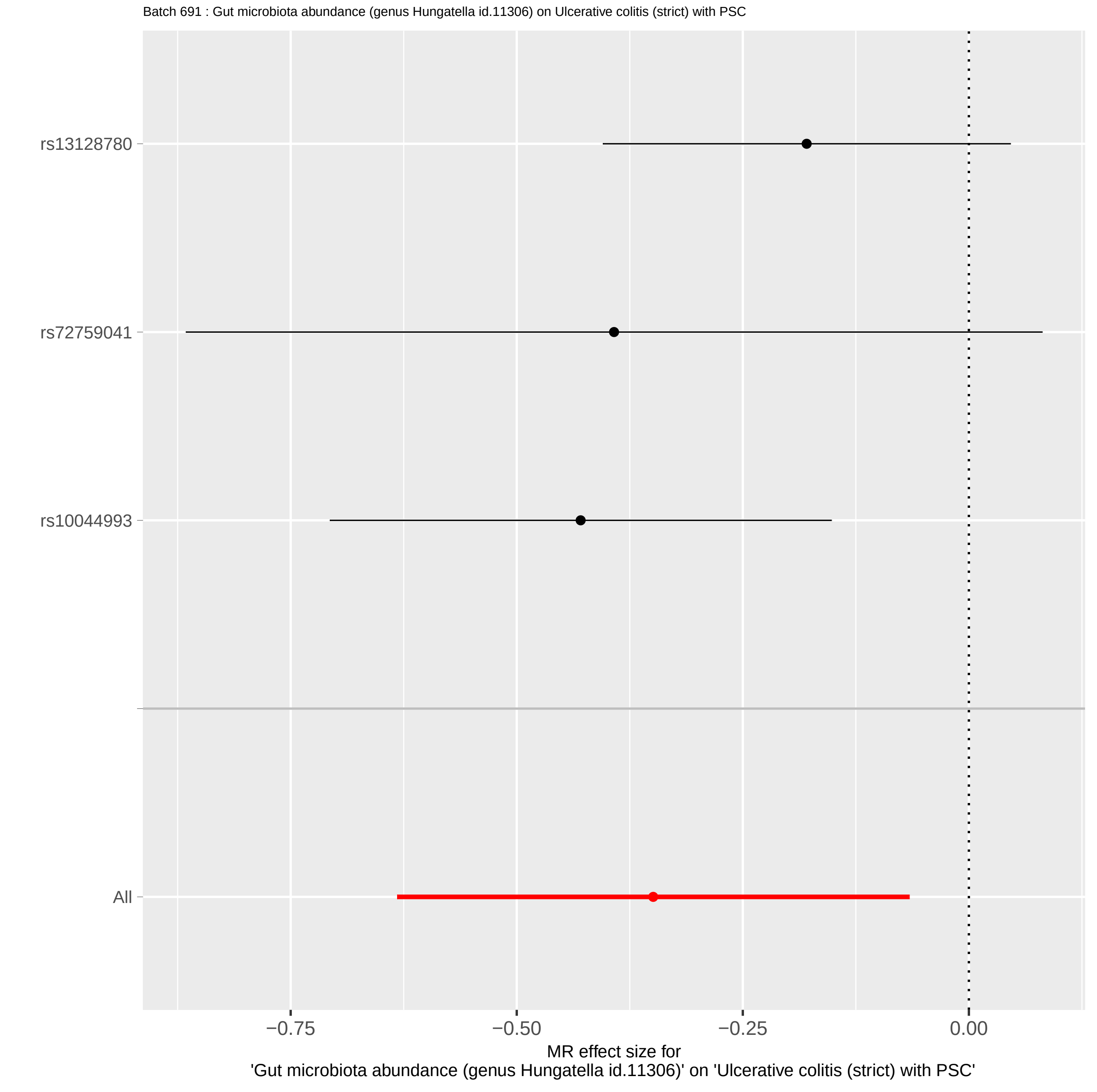

Batch 691 : Gut microbiota abundance (genus Hungatella id.11306) on Ulcerative colitis (strict) with PSC
rs13128780
rs72759041
rs10044993
All
−0.75
−0.50
−0.25
0.00
MR effect size for
'Gut microbiota abundance (genus Hungatella id.11306)' on 'Ulcerative colitis (strict) with PSC'

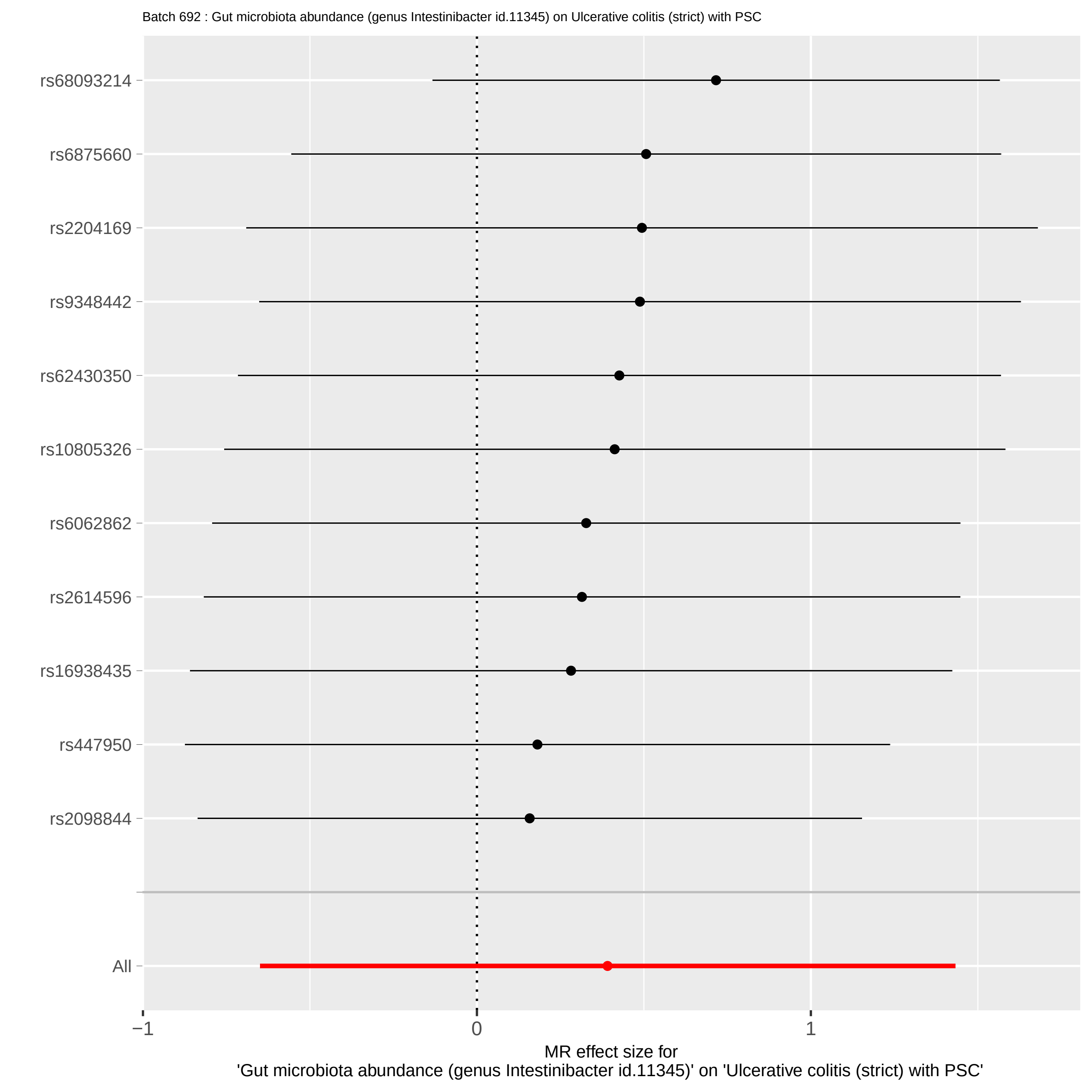
Batch 692 : Gut microbiota abundance (genus Intestinibacter id.11345) on Ulcerative colitis (strict) with PSC
rs68093214
rs6875660
rs2204169
rs9348442
rs62430350
rs10805326
rs6062862
rs2614596
rs16938435
rs447950
rs2098844
All
−1
0
1
MR effect size for
'Gut microbiota abundance (genus Intestinibacter id.11345)' on 'Ulcerative colitis (strict) with PSC'

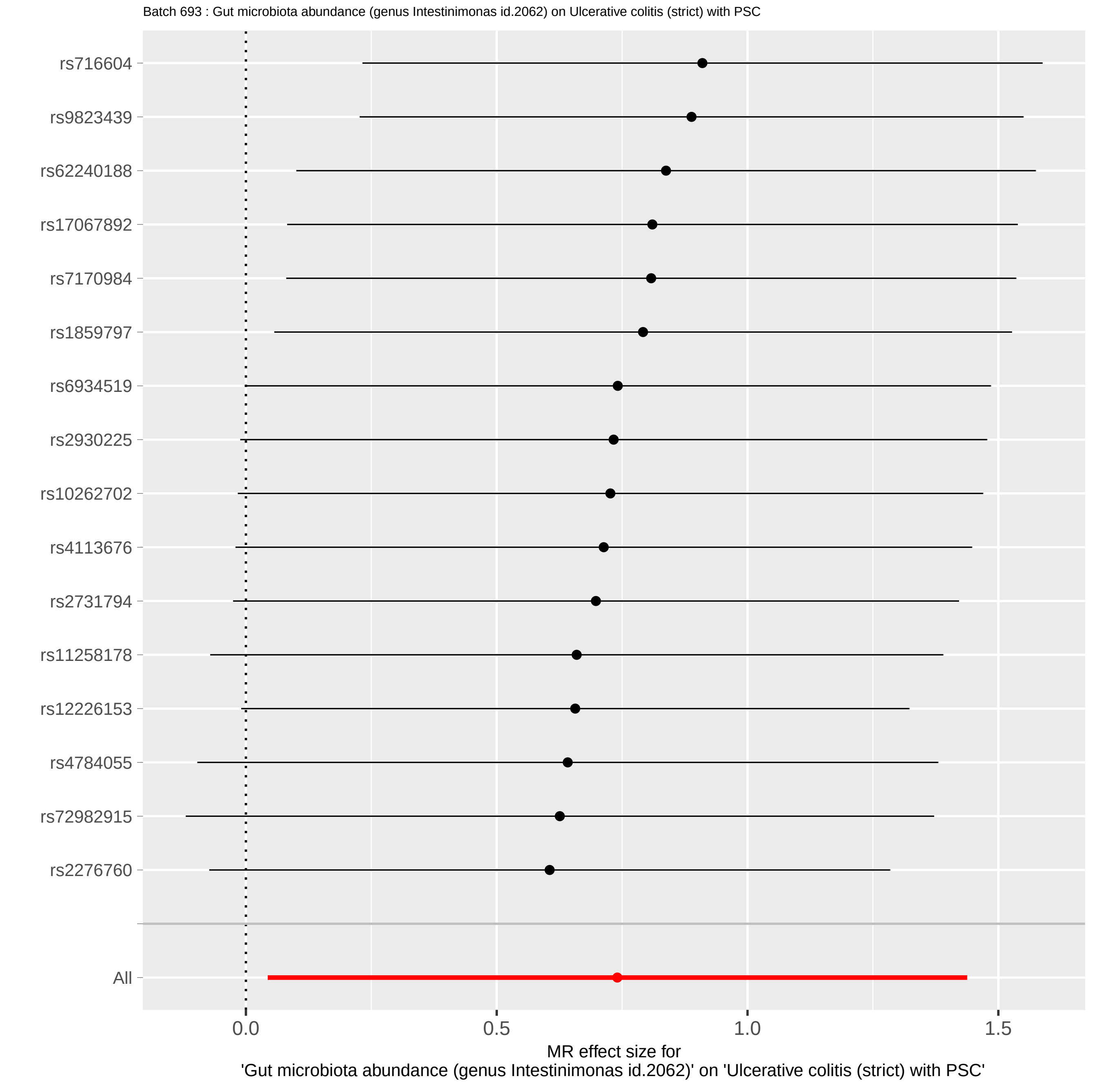
Batch 693 : Gut microbiota abundance (genus Intestinimonas id.2062) on Ulcerative colitis (strict) with PSC
rs716604
rs9823439
rs62240188
rs17067892
rs7170984
rs1859797
rs6934519
rs2930225
rs10262702
rs4113676
rs2731794
rs11258178
rs12226153
rs4784055
rs72982915
rs2276760
All
0.0
0.5
1.0
1.5
MR effect size for
'Gut microbiota abundance (genus Intestinimonas id.2062)' on 'Ulcerative colitis (strict) with PSC'

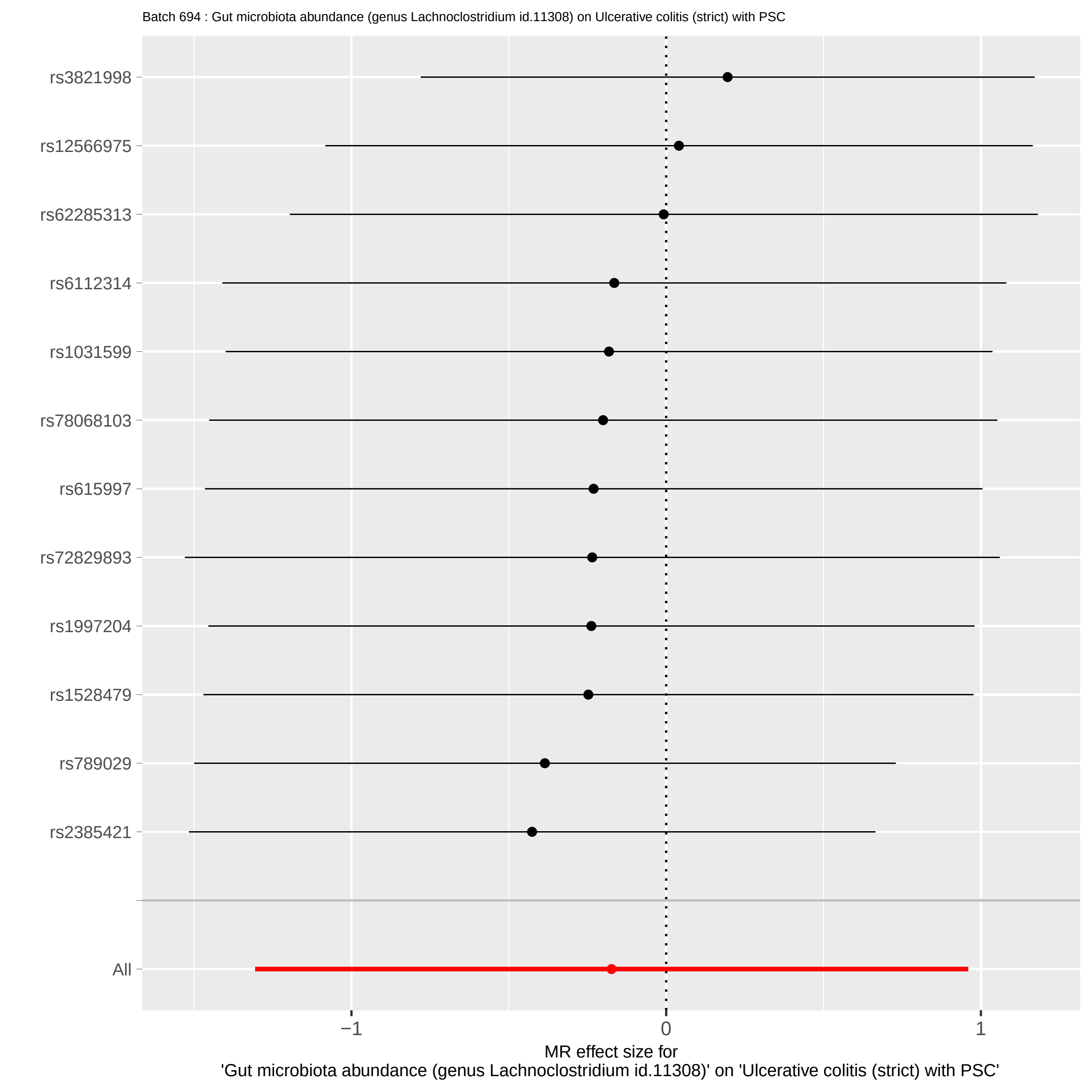

Batch 694 : Gut microbiota abundance (genus Lachnoclostridium id.11308) on Ulcerative colitis (strict) with PSC
rs3821998
rs12566975
rs62285313
rs6112314
rs1031599
rs78068103
rs615997
rs72829893
rs1997204
rs1528479
rs789029
rs2385421
All
−1
0
1
MR effect size for
'Gut microbiota abundance (genus Lachnoclostridium id.11308)' on 'Ulcerative colitis (strict) with PSC'

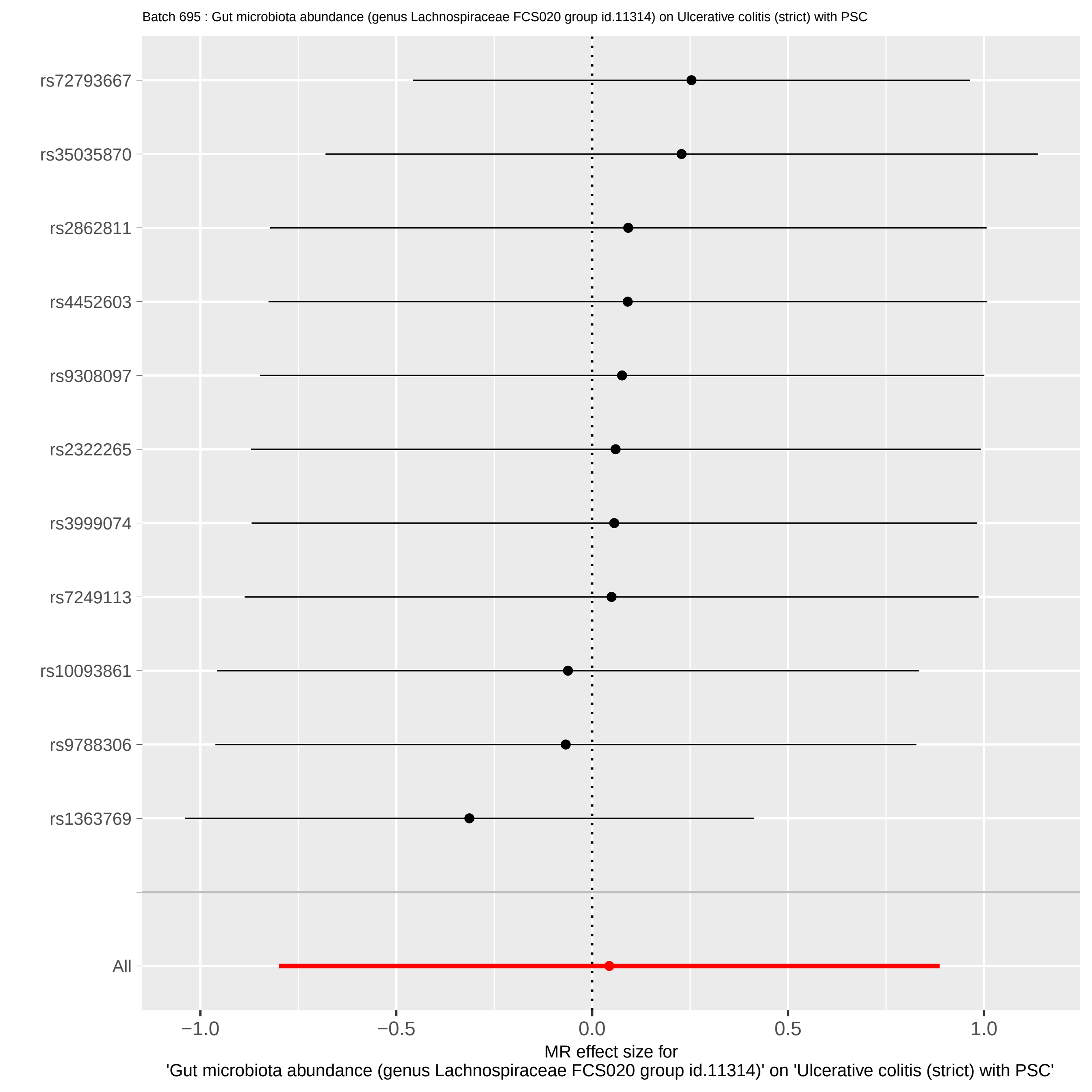
Batch 695 : Gut microbiota abundance (genus Lachnospiraceae FCS020 group id.11314) on Ulcerative colitis (strict) with PSC
rs72793667
rs35035870
rs2862811
rs4452603
rs9308097
rs2322265
rs3999074
rs7249113
rs10093861
rs9788306
rs1363769
All
−1.0
−0.5
0.0
0.5
1.0
MR effect size for
'Gut microbiota abundance (genus Lachnospiraceae FCS020 group id.11314)' on 'Ulcerative colitis (strict) with PSC'

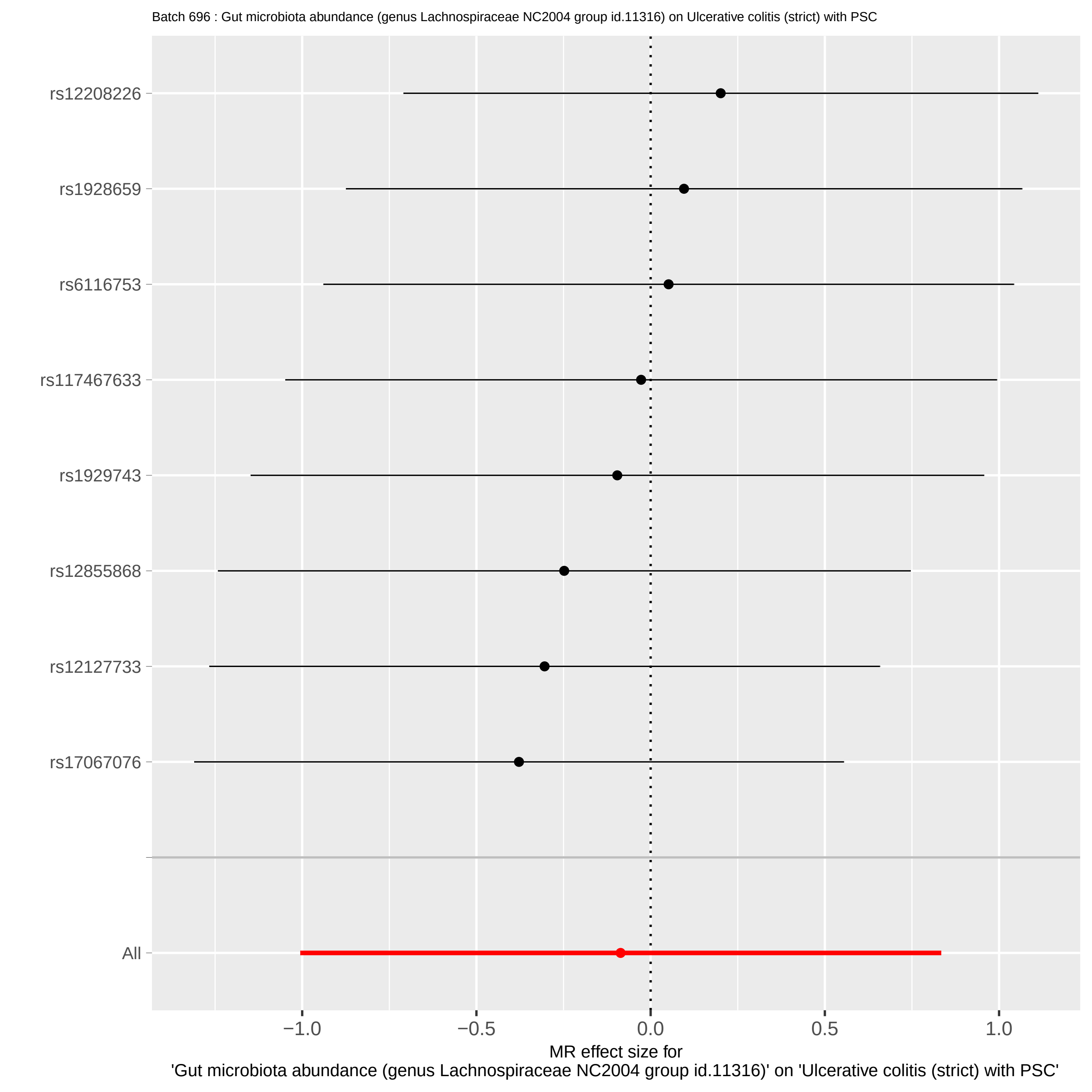
Batch 696 : Gut microbiota abundance (genus Lachnospiraceae NC2004 group id.11316) on Ulcerative colitis (strict) with PSC
rs12208226
rs1928659
rs6116753
rs117467633
rs1929743
rs12855868
rs12127733
rs17067076
All
−1.0
−0.5
0.0
0.5
1.0
MR effect size for
'Gut microbiota abundance (genus Lachnospiraceae NC2004 group id.11316)' on 'Ulcerative colitis (strict) with PSC'

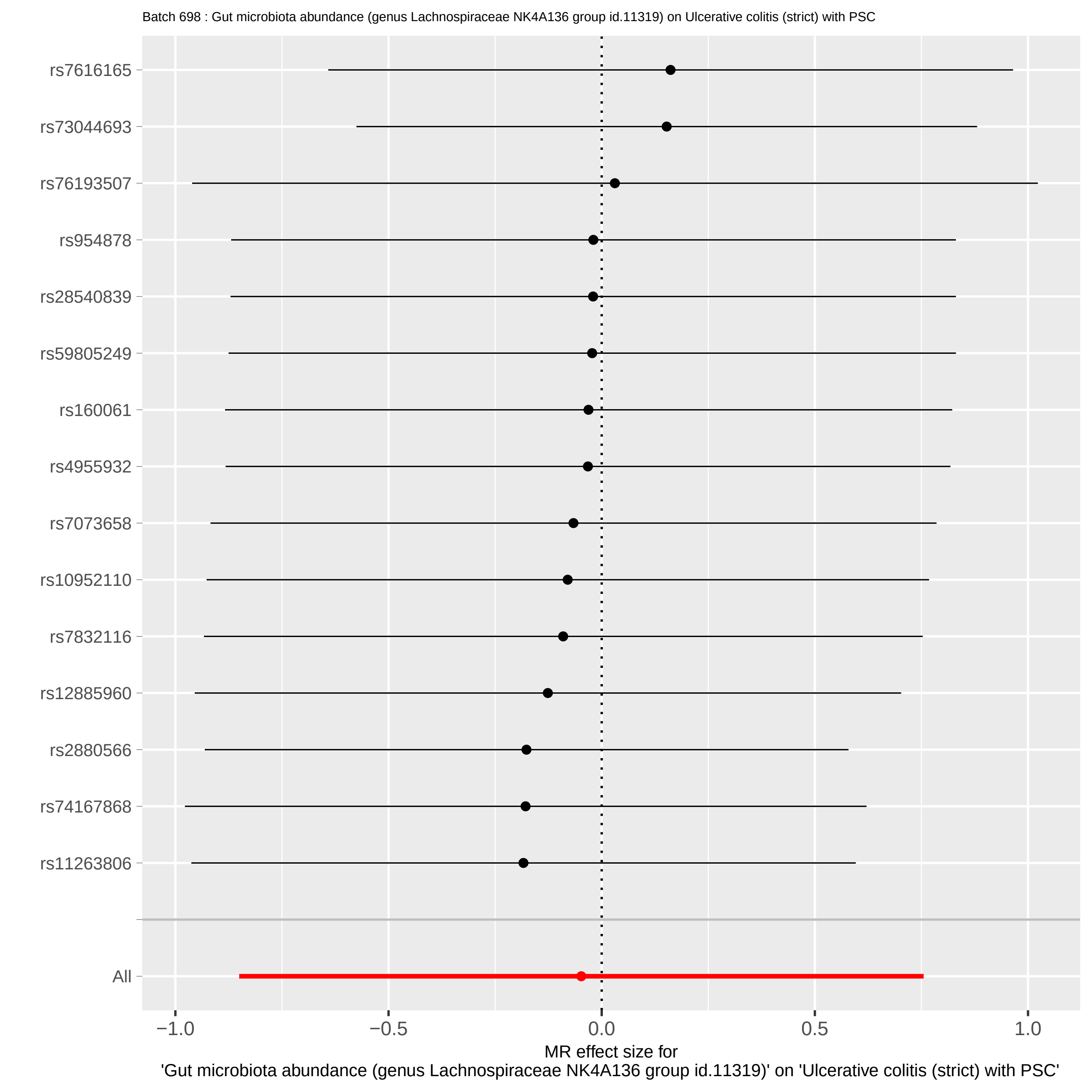
Batch 698 : Gut microbiota abundance (genus Lachnospiraceae NK4A136 group id.11319) on Ulcerative colitis (strict) with PSC
rs7616165
rs73044693
rs76193507
rs954878
rs28540839
rs59805249
rs160061
rs4955932
rs7073658
rs10952110
rs7832116
rs12885960
rs2880566
rs74167868
rs11263806
All
−1.0
−0.5
0.0
0.5
1.0
MR effect size for
'Gut microbiota abundance (genus Lachnospiraceae NK4A136 group id.11319)' on 'Ulcerative colitis (strict) with PSC'

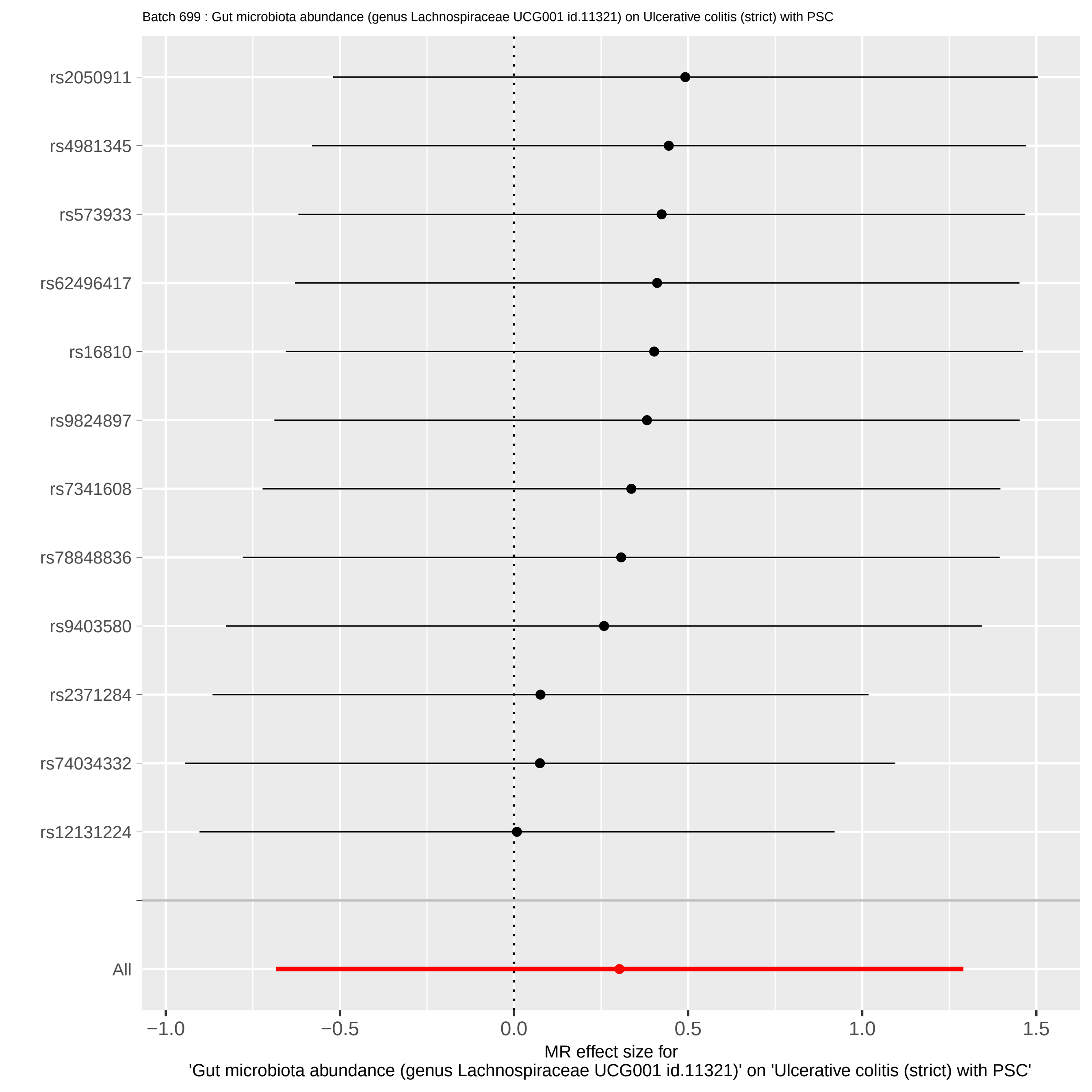

Batch 699 : Gut microbiota abundance (genus Lachnospiraceae UCG001 id.11321) on Ulcerative colitis (strict) with PSC
rs2050911
rs4981345
rs573933
rs62496417
rs16810
rs9824897
rs7341608
rs78848836
rs9403580
rs2371284
rs74034332
rs12131224
All
−1.0
−0.5
0.0
0.5
1.0
1.5
MR effect size for
'Gut microbiota abundance (genus Lachnospiraceae UCG001 id.11321)' on 'Ulcerative colitis (strict) with PSC'

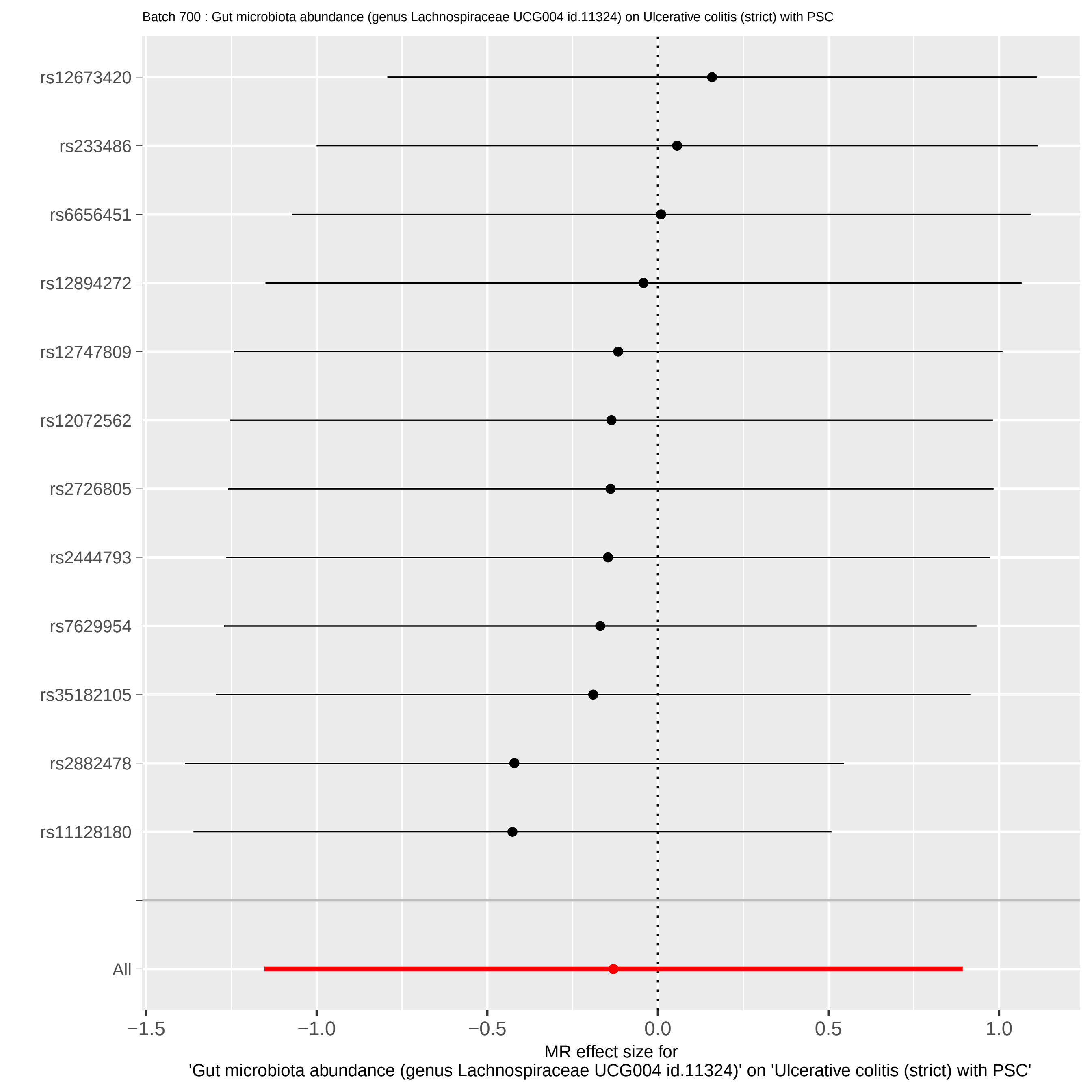
Batch 700 : Gut microbiota abundance (genus Lachnospiraceae UCG004 id.11324) on Ulcerative colitis (strict) with PSC
rs12673420
rs233486
rs6656451
rs12894272
rs12747809
rs12072562
rs2726805
rs2444793
rs7629954
rs35182105
rs2882478
rs11128180
All
−1.5
−1.0
−0.5
0.0
0.5
1.0
MR effect size for
'Gut microbiota abundance (genus Lachnospiraceae UCG004 id.11324)' on 'Ulcerative colitis (strict) with PSC'

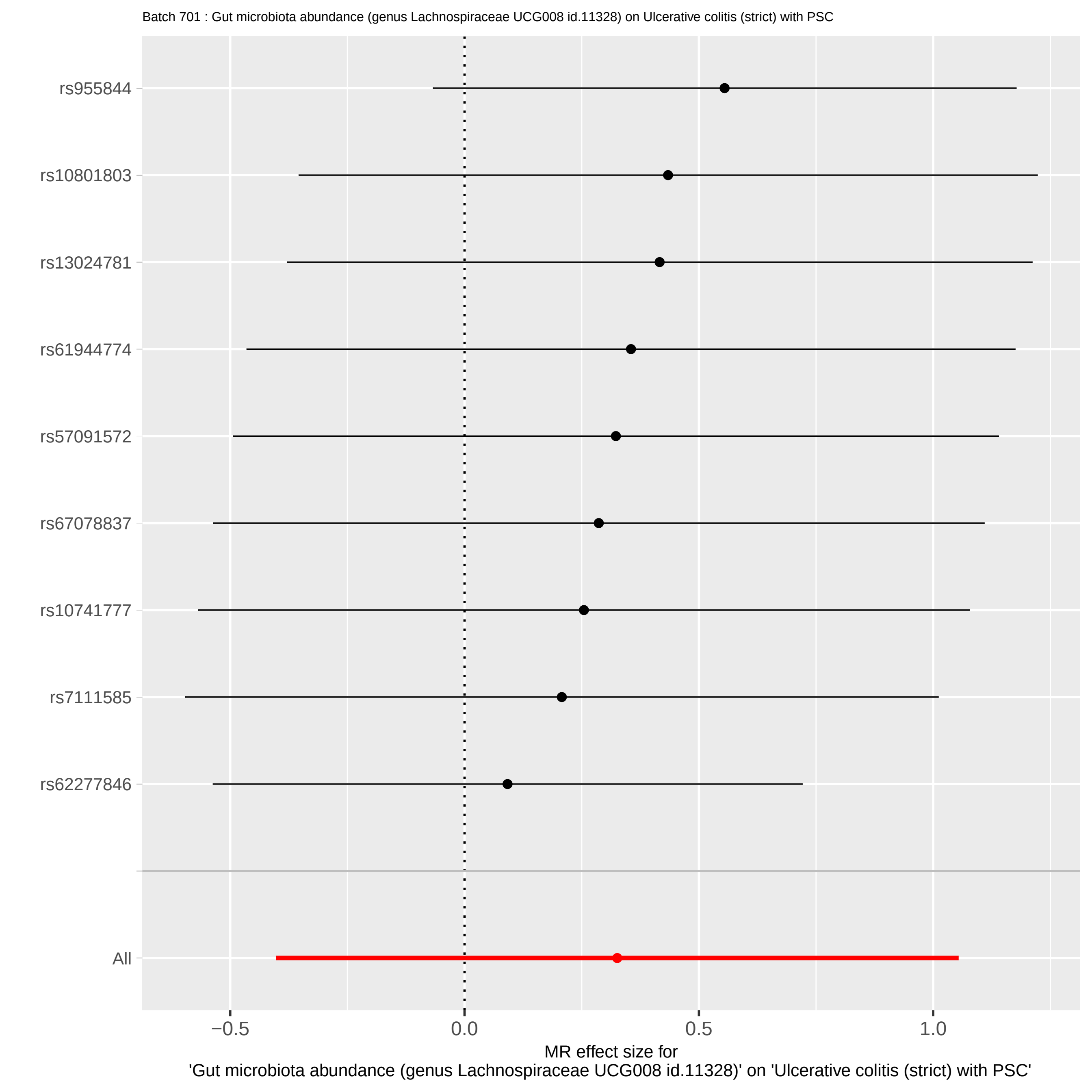
Batch 701 : Gut microbiota abundance (genus Lachnospiraceae UCG008 id.11328) on Ulcerative colitis (strict) with PSC
rs955844
rs10801803
rs13024781
rs61944774
rs57091572
rs67078837
rs10741777
rs7111585
rs62277846
All
−0.5
0.0
0.5
1.0
MR effect size for
'Gut microbiota abundance (genus Lachnospiraceae UCG008 id.11328)' on 'Ulcerative colitis (strict) with PSC'

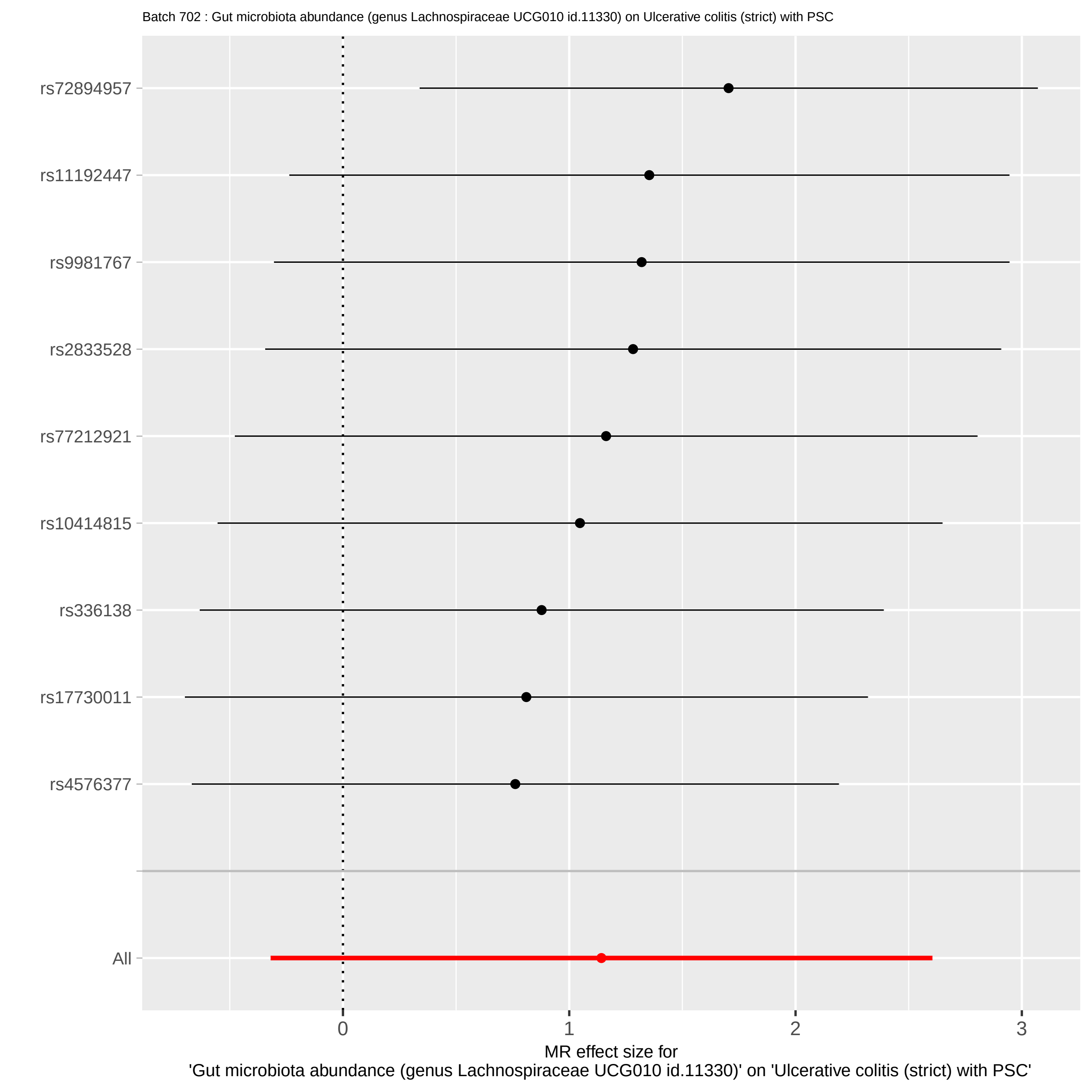

Batch 702 : Gut microbiota abundance (genus Lachnospiraceae UCG010 id.11330) on Ulcerative colitis (strict) with PSC
rs72894957
rs11192447
rs9981767
rs2833528
rs77212921
rs10414815
rs336138
rs17730011
rs4576377
All
0
1
2
3
MR effect size for
'Gut microbiota abundance (genus Lachnospiraceae UCG010 id.11330)' on 'Ulcerative colitis (strict) with PSC'

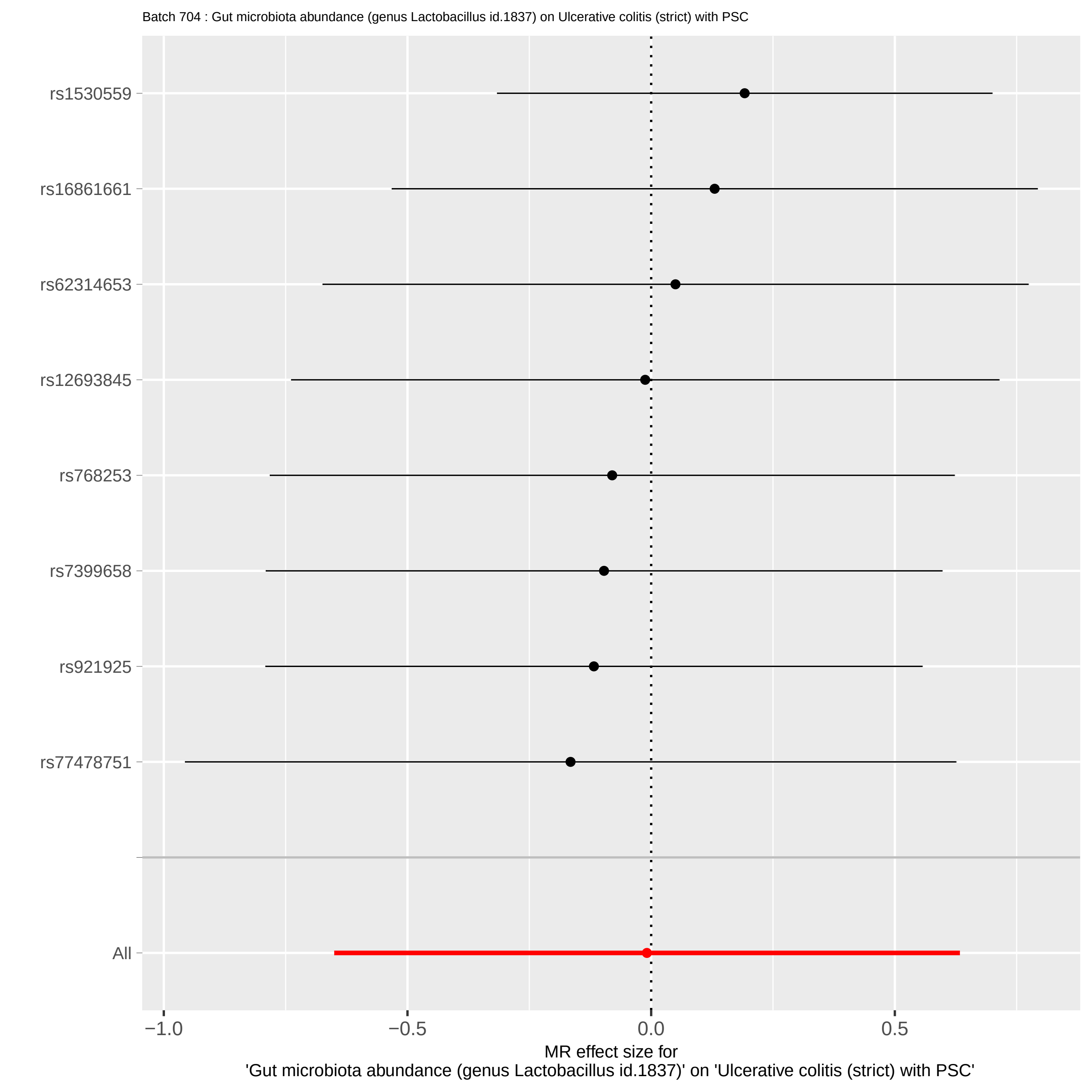

Batch 704 : Gut microbiota abundance (genus Lactobacillus id.1837) on Ulcerative colitis (strict) with PSC
rs1530559
rs16861661
rs62314653
rs12693845
rs768253
rs7399658
rs921925
rs77478751
All
−1.0
−0.5
0.0
0.5
MR effect size for
'Gut microbiota abundance (genus Lactobacillus id.1837)' on 'Ulcerative colitis (strict) with PSC'

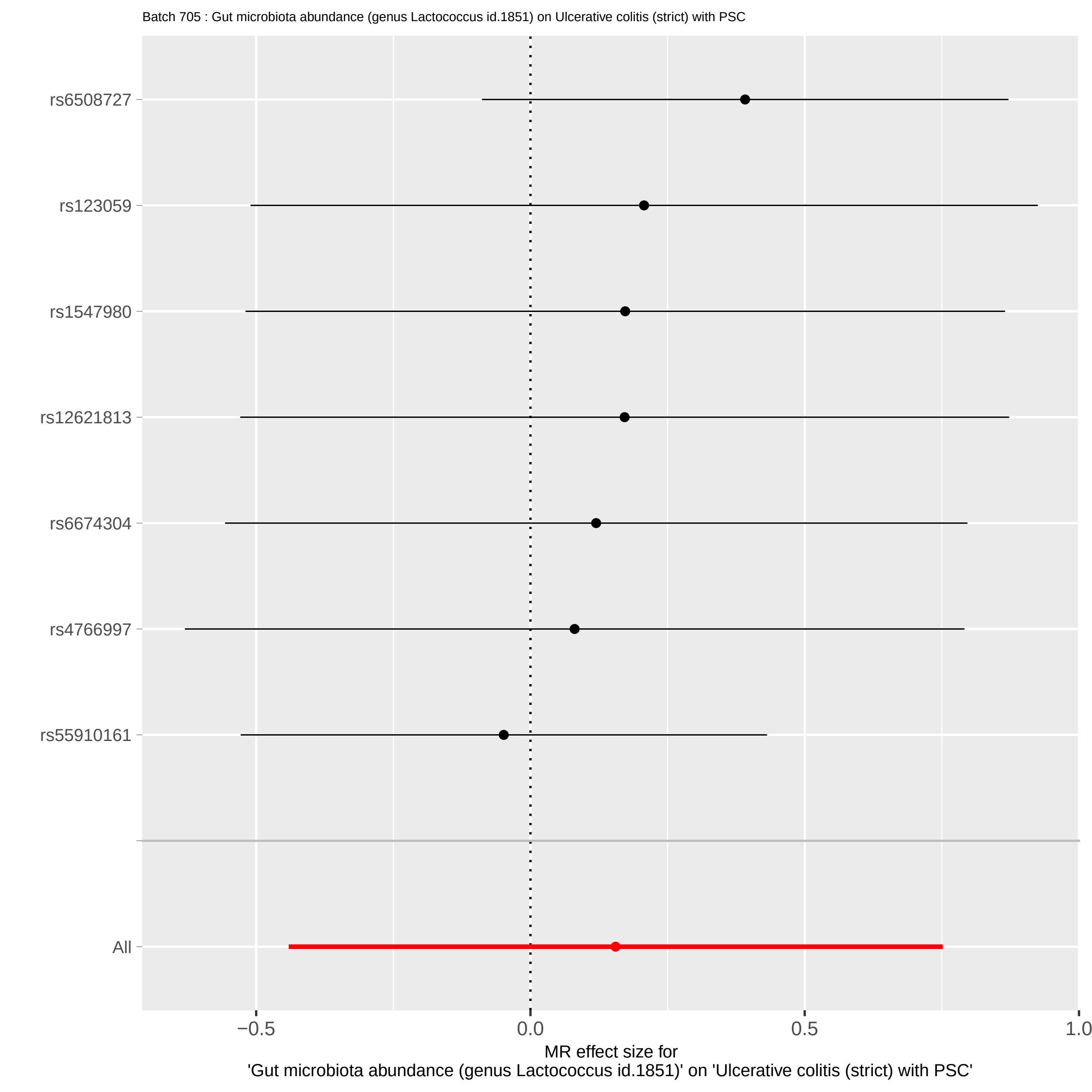
Batch 705 : Gut microbiota abundance (genus Lactococcus id.1851) on Ulcerative colitis (strict) with PSC
rs6508727
rs123059
rs1547980
rs12621813
rs6674304
rs4766997
rs55910161
All
−0.5
0.0
0.5
1.0
MR effect size for
'Gut microbiota abundance (genus Lactococcus id.1851)' on 'Ulcerative colitis (strict) with PSC'

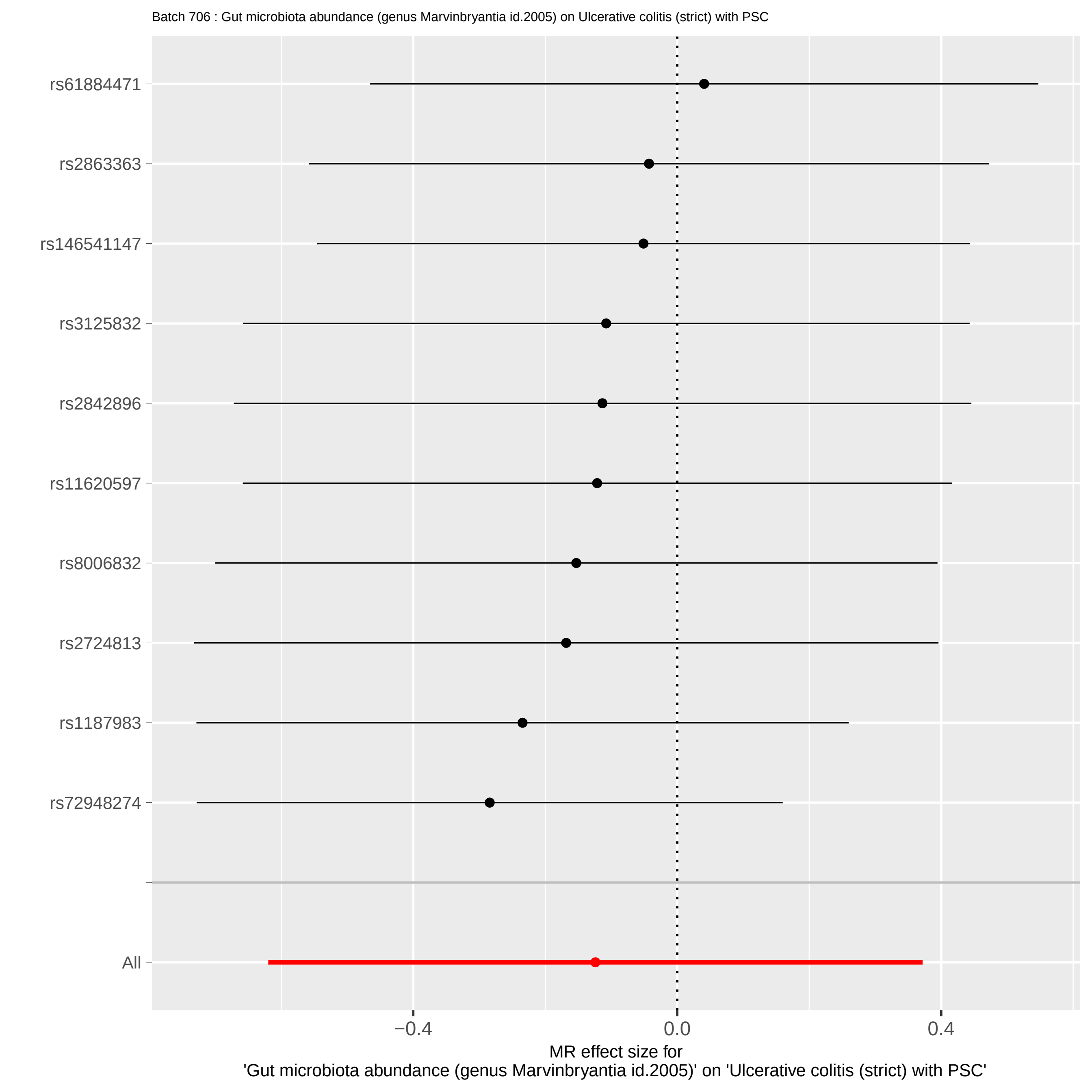

Batch 706 : Gut microbiota abundance (genus Marvinbryantia id.2005) on Ulcerative colitis (strict) with PSC
rs61884471
rs2863363
rs146541147
rs3125832
rs2842896
rs11620597
rs8006832
rs2724813
rs1187983
rs72948274
All
−0.4
0.0
0.4
MR effect size for
'Gut microbiota abundance (genus Marvinbryantia id.2005)' on 'Ulcerative colitis (strict) with PSC'

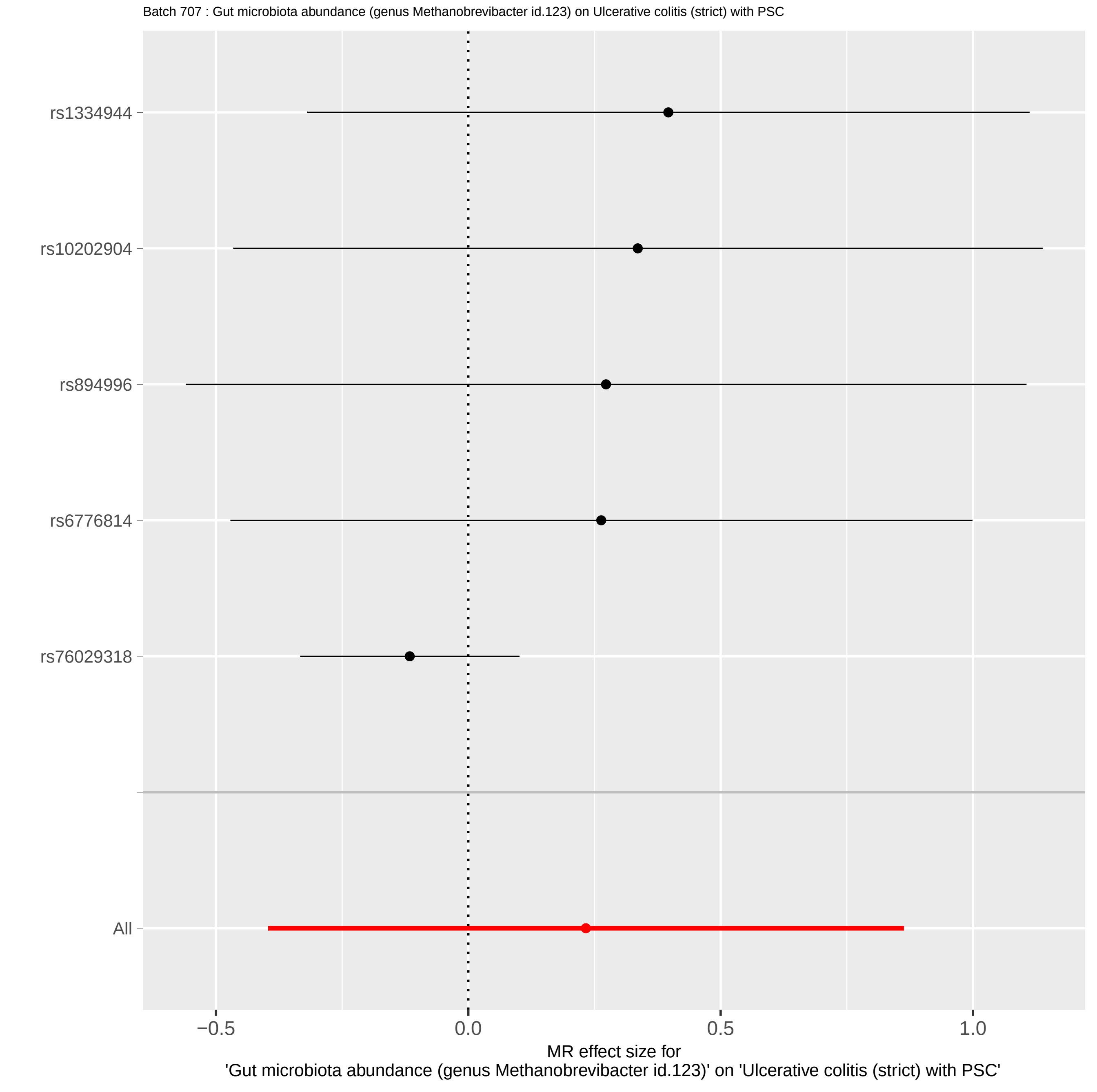
Batch 707 : Gut microbiota abundance (genus Methanobrevibacter id.123) on Ulcerative colitis (strict) with PSC
rs1334944
rs10202904
rs894996
rs6776814
rs76029318
All
−0.5
0.0
0.5
1.0
MR effect size for
'Gut microbiota abundance (genus Methanobrevibacter id.123)' on 'Ulcerative colitis (strict) with PSC'

Batch 708 : Gut microbiota abundance (genus Odoribacter id.952) on Ulcerative colitis (strict) with PSC

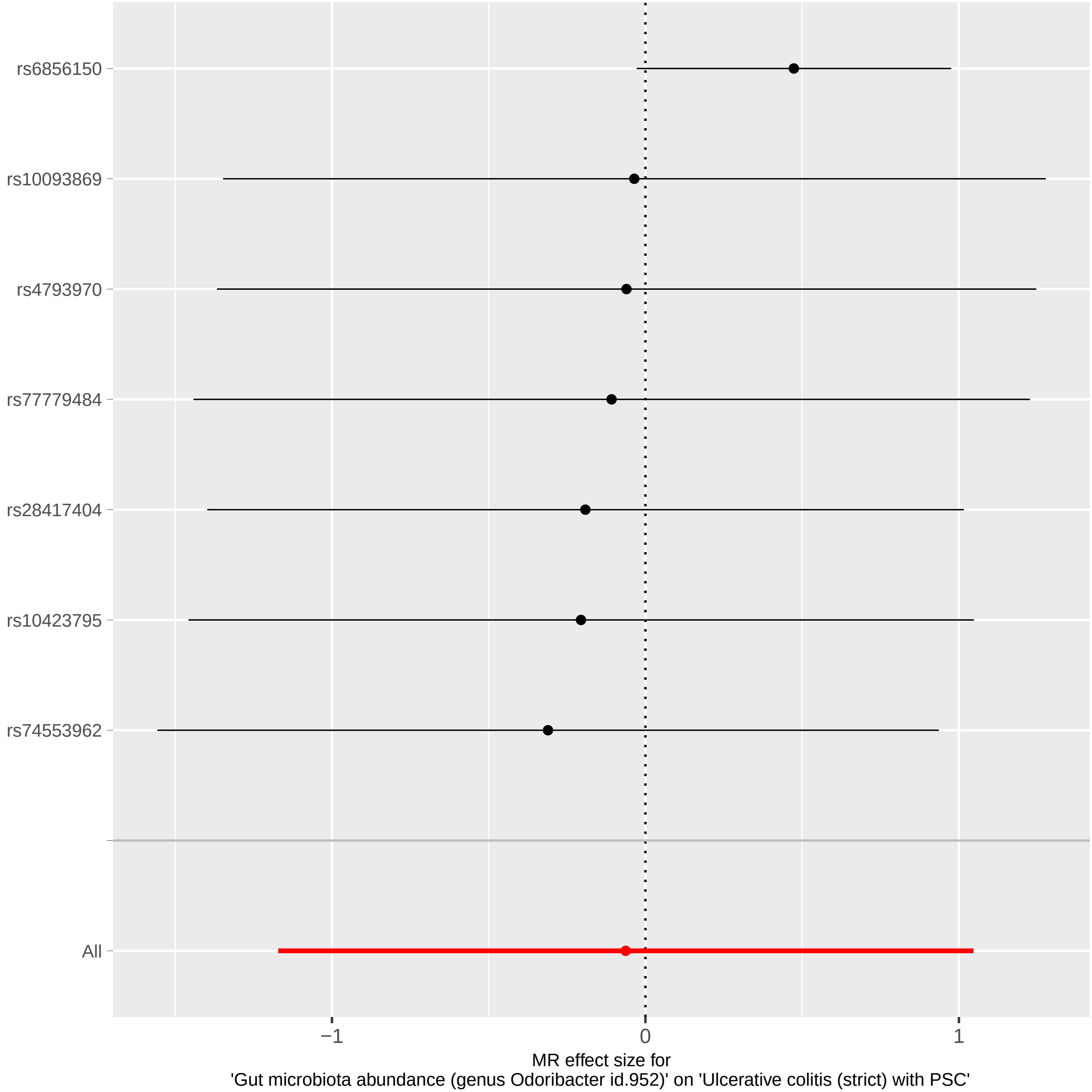

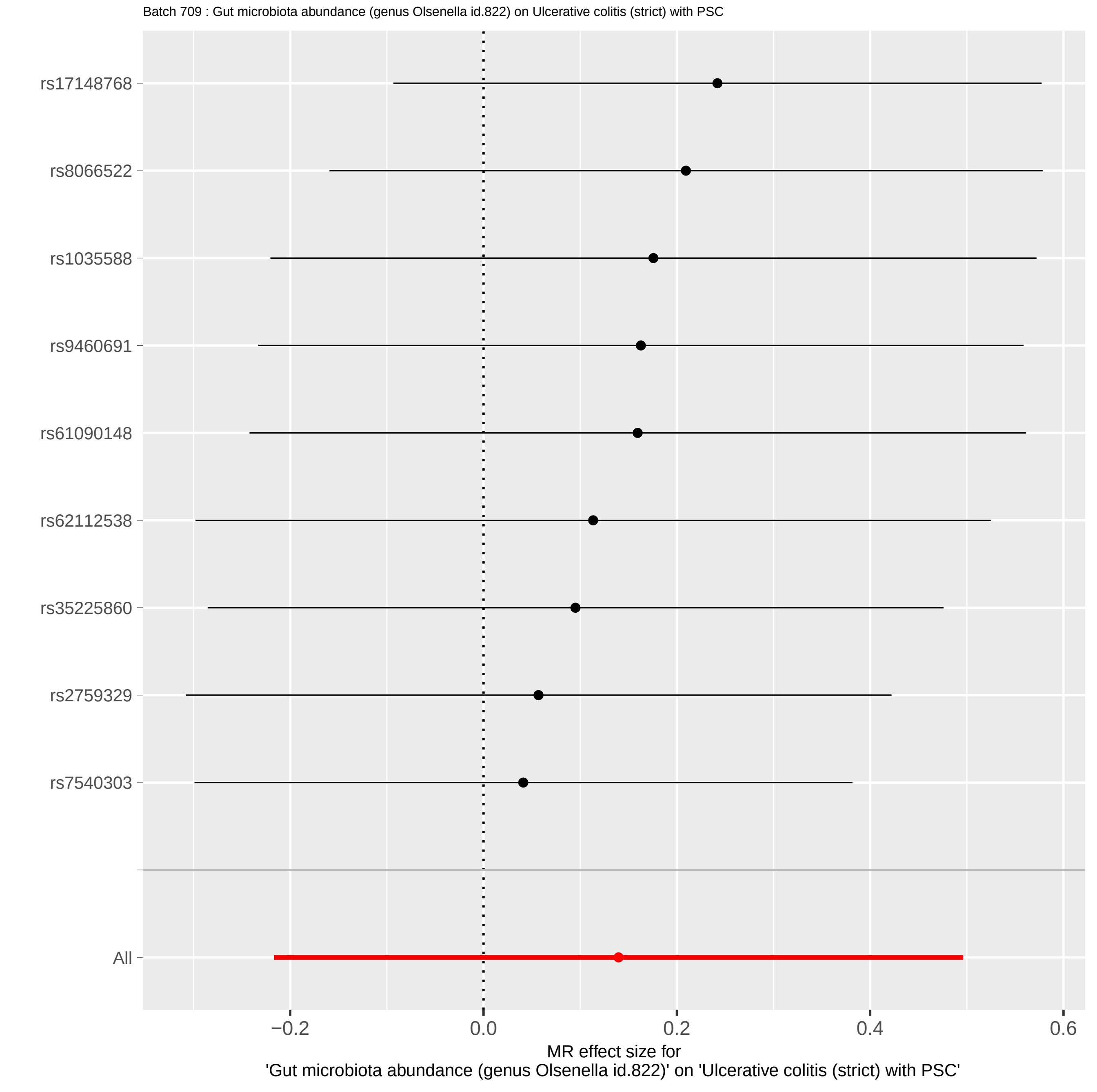

Batch 709 : Gut microbiota abundance (genus Olsenella id.822) on Ulcerative colitis (strict) with PSC
rs17148768
rs8066522
rs1035588
rs9460691
rs61090148
rs62112538
rs35225860
rs2759329
rs7540303
All
−0.2
0.0
0.2
0.4
0.6
MR effect size for
'Gut microbiota abundance (genus Olsenella id.822)' on 'Ulcerative colitis (strict) with PSC'

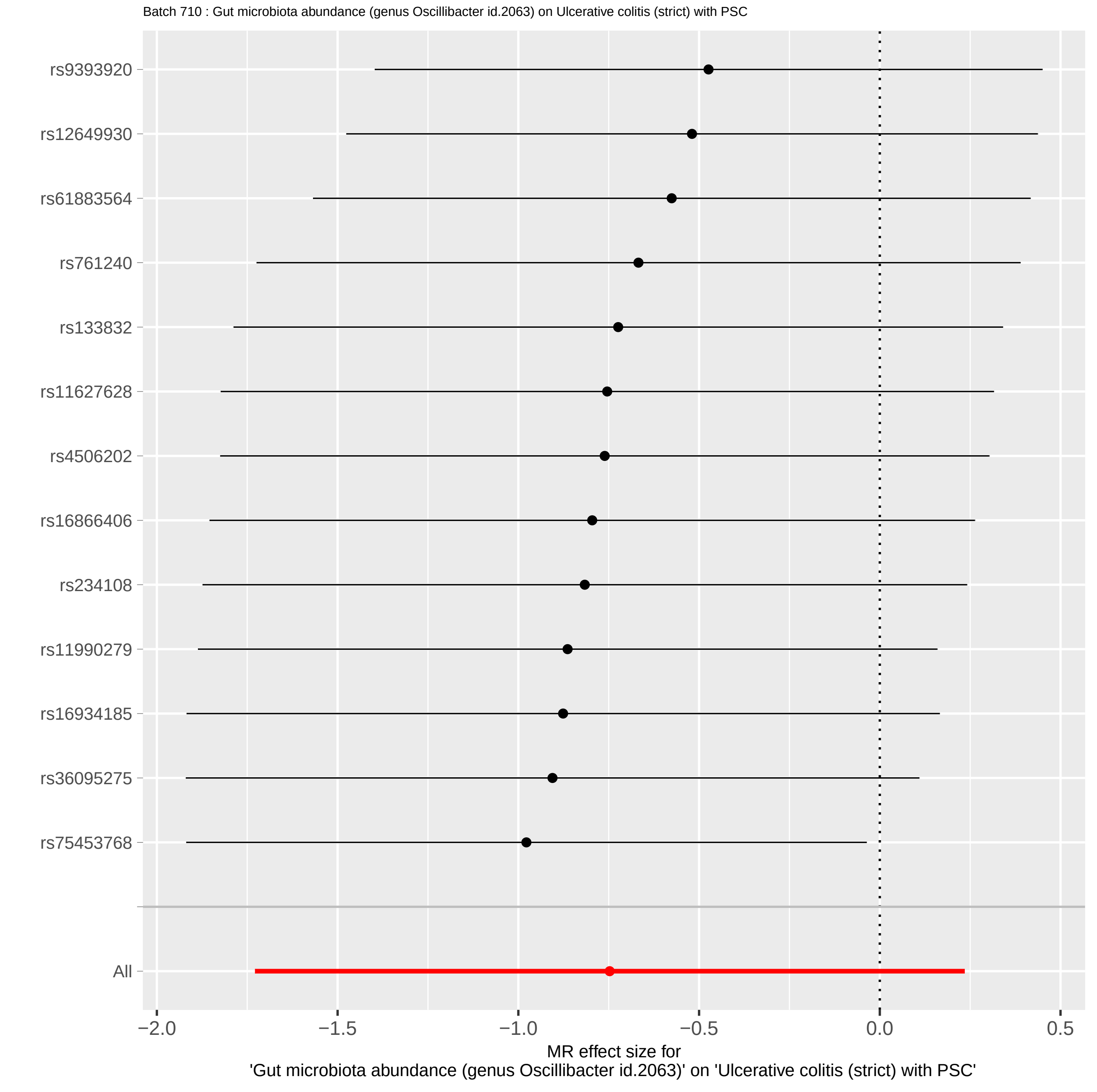

Batch 710 : Gut microbiota abundance (genus Oscillibacter id.2063) on Ulcerative colitis (strict) with PSC
rs9393920
rs12649930
rs61883564
rs761240
rs133832
rs11627628
rs4506202
rs16866406
rs234108
rs11990279
rs16934185
rs36095275
rs75453768
All
−2.0
−1.5
−1.0
−0.5
0.0
0.5
MR effect size for
'Gut microbiota abundance (genus Oscillibacter id.2063)' on 'Ulcerative colitis (strict) with PSC'

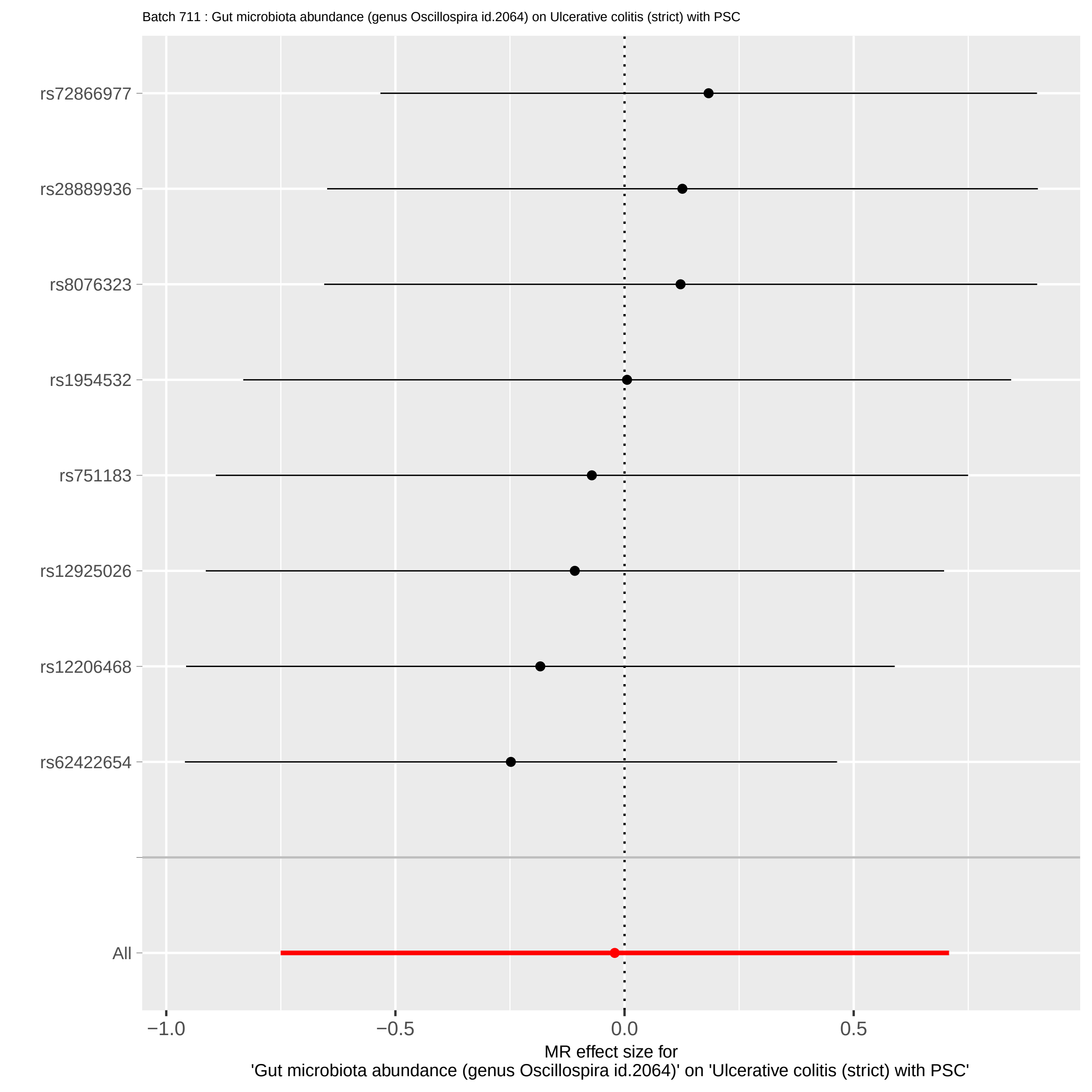

Batch 711 : Gut microbiota abundance (genus Oscillospira id.2064) on Ulcerative colitis (strict) with PSC
rs72866977
rs28889936
rs8076323
rs1954532
rs751183
rs12925026
rs12206468
rs62422654
All
−1.0
−0.5
0.0
0.5
MR effect size for
'Gut microbiota abundance (genus Oscillospira id.2064)' on 'Ulcerative colitis (strict) with PSC'

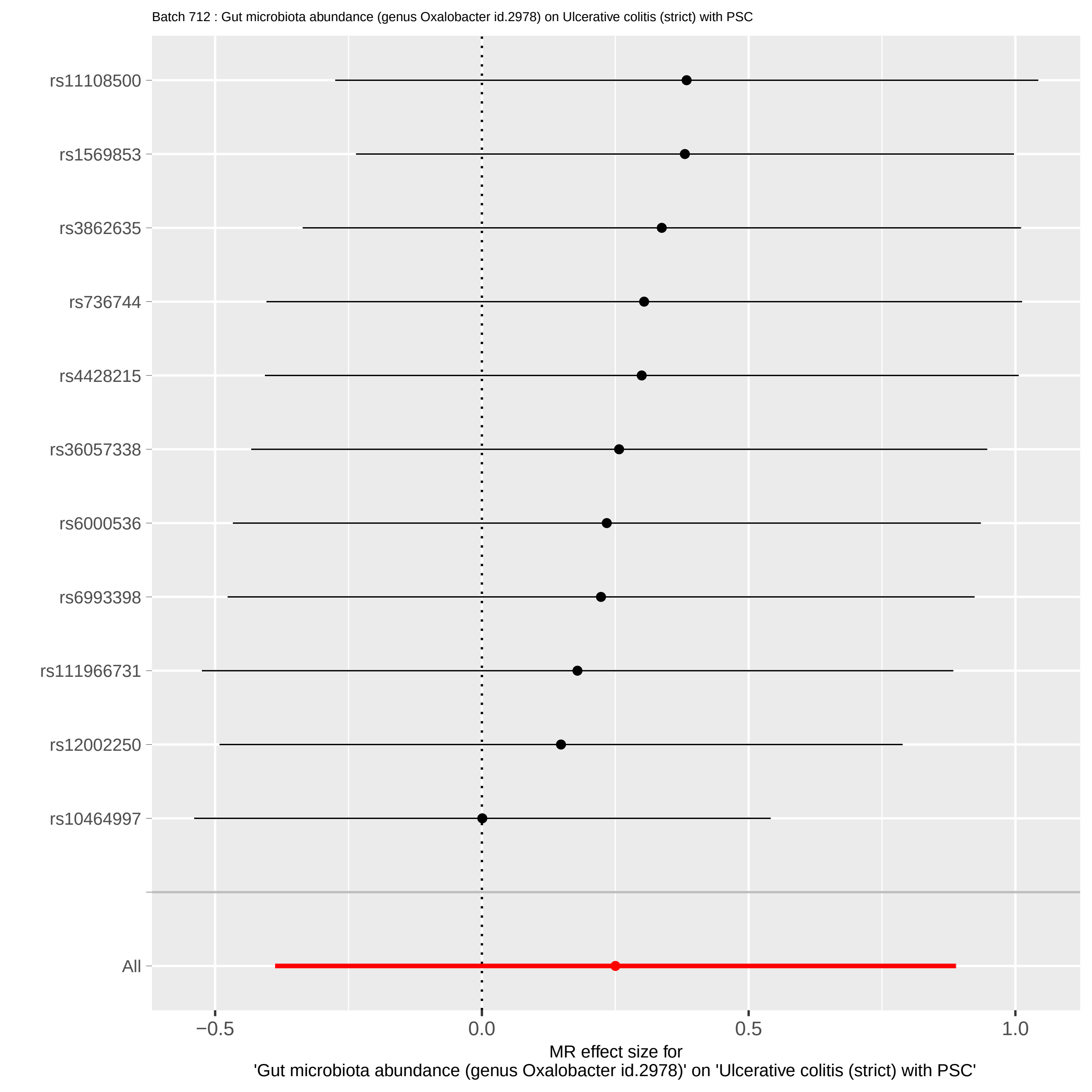

Batch 712 : Gut microbiota abundance (genus Oxalobacter id.2978) on Ulcerative colitis (strict) with PSC
rs11108500
rs1569853
rs3862635
rs736744
rs4428215
rs36057338
rs6000536
rs6993398
rs111966731
rs12002250
rs10464997
All
−0.5
0.0
0.5
1.0
MR effect size for
'Gut microbiota abundance (genus Oxalobacter id.2978)' on 'Ulcerative colitis (strict) with PSC'

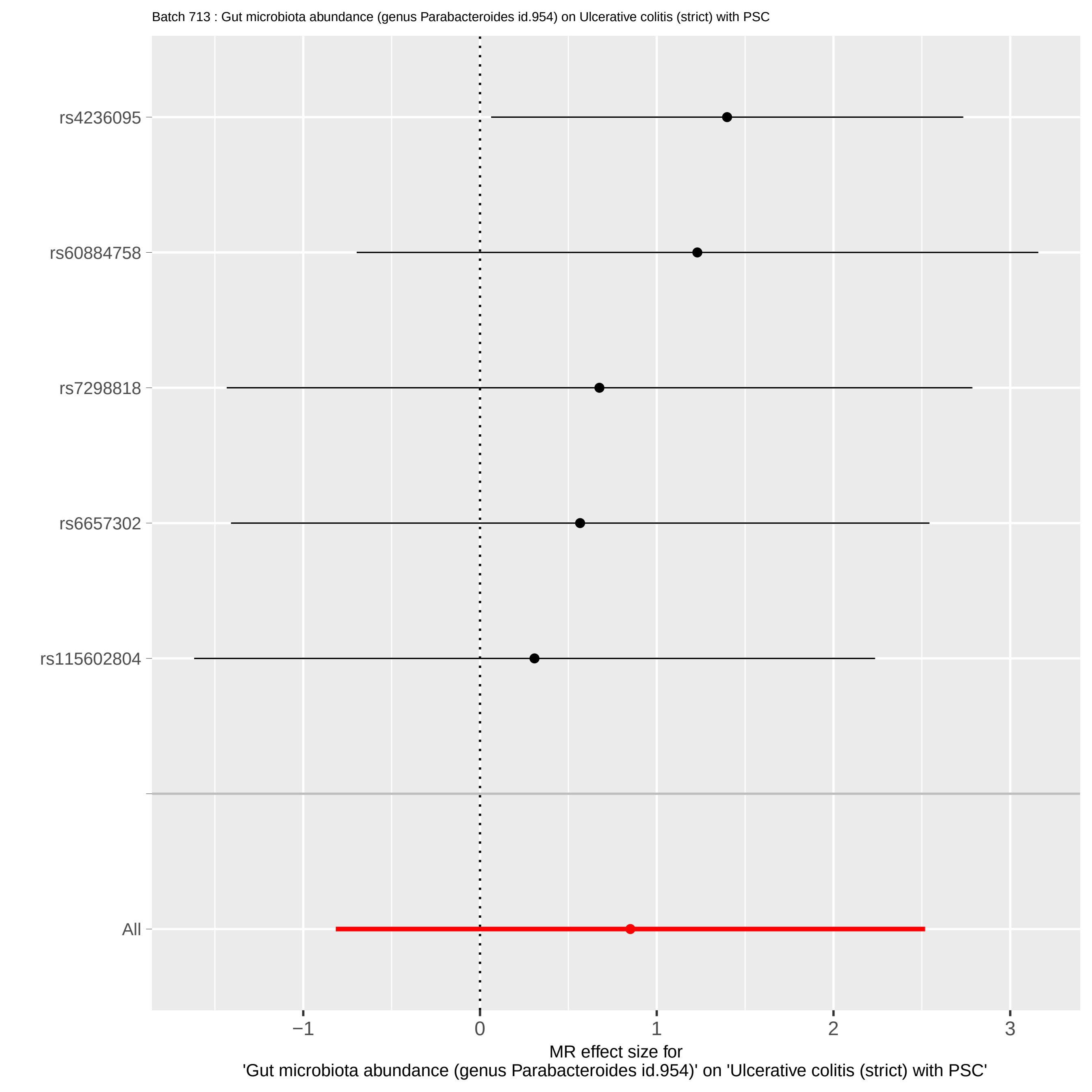

Batch 713 : Gut microbiota abundance (genus Parabacteroides id.954) on Ulcerative colitis (strict) with PSC
rs4236095
rs60884758
rs7298818
rs6657302
rs115602804
All
−1
0
1
2
3
MR effect size for
'Gut microbiota abundance (genus Parabacteroides id.954)' on 'Ulcerative colitis (strict) with PSC'

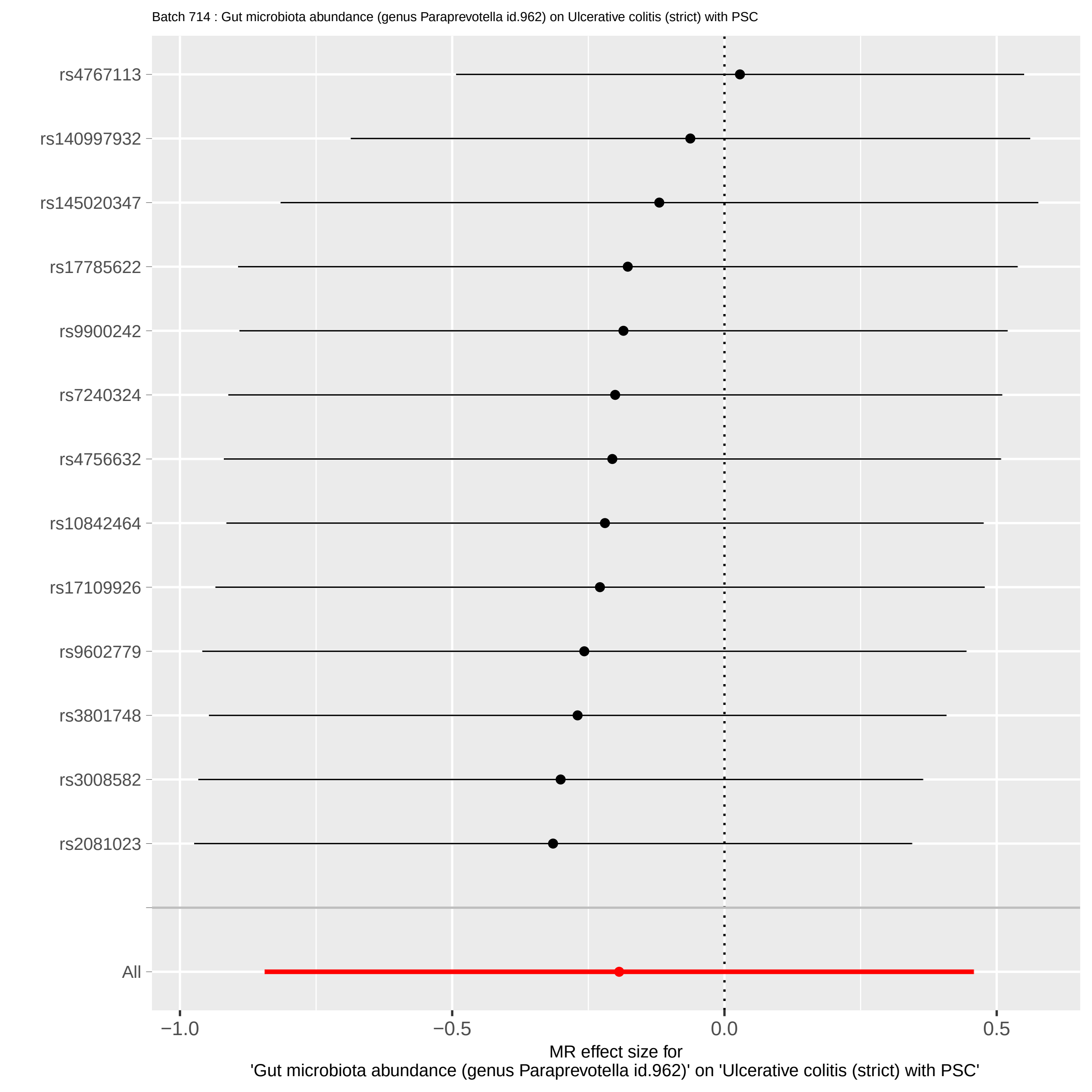

Batch 714 : Gut microbiota abundance (genus Paraprevotella id.962) on Ulcerative colitis (strict) with PSC
rs4767113
rs140997932
rs145020347
rs17785622
rs9900242
rs7240324
rs4756632
rs10842464
rs17109926
rs9602779
rs3801748
rs3008582
rs2081023
All
−1.0
−0.5
0.0
0.5
MR effect size for
'Gut microbiota abundance (genus Paraprevotella id.962)' on 'Ulcerative colitis (strict) with PSC'

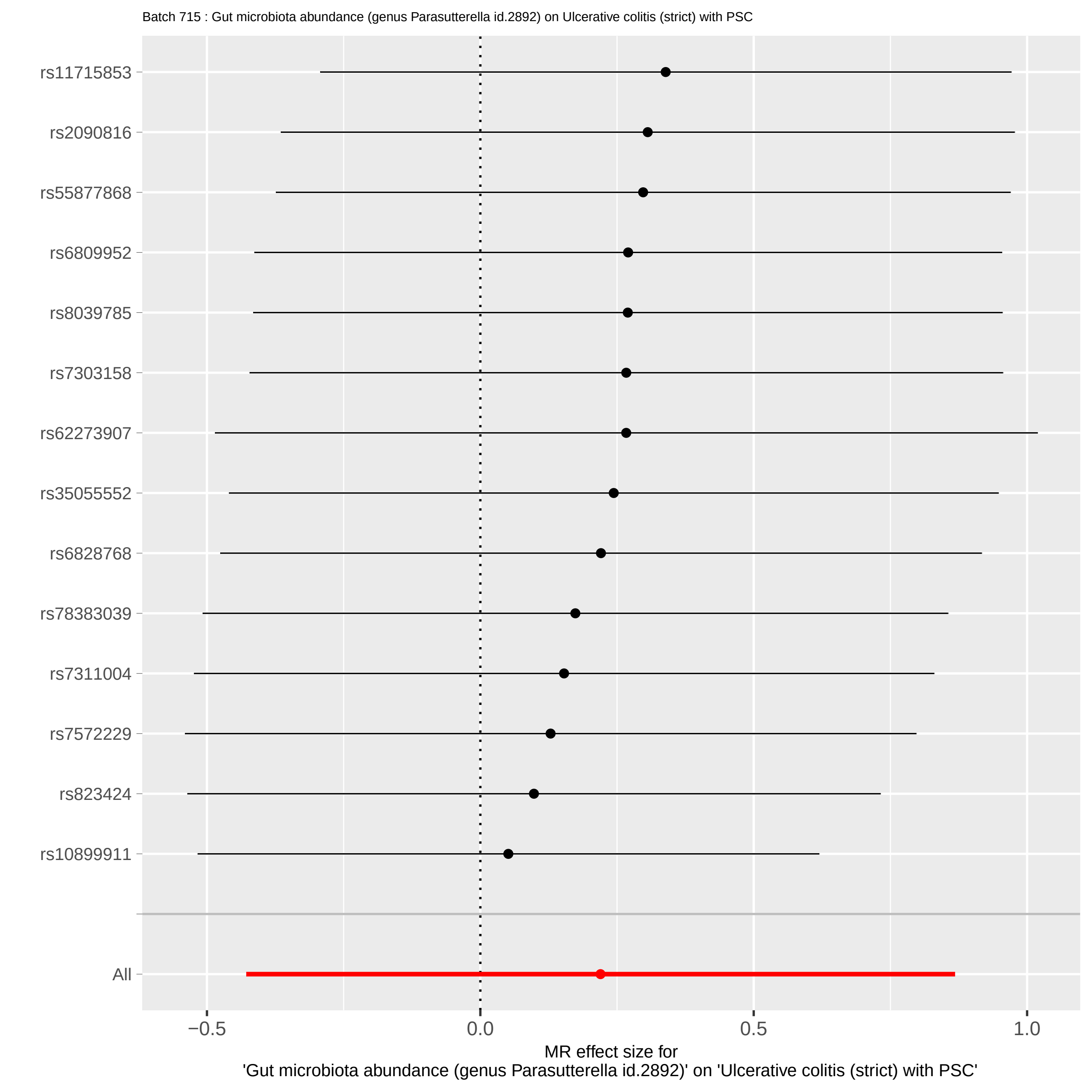

Batch 715 : Gut microbiota abundance (genus Parasutterella id.2892) on Ulcerative colitis (strict) with PSC
rs11715853
rs2090816
rs55877868
rs6809952
rs8039785
rs7303158
rs62273907
rs35055552
rs6828768
rs78383039
rs7311004
rs7572229
rs823424
rs10899911
All
−0.5
0.0
0.5
1.0
MR effect size for
'Gut microbiota abundance (genus Parasutterella id.2892)' on 'Ulcerative colitis (strict) with PSC'

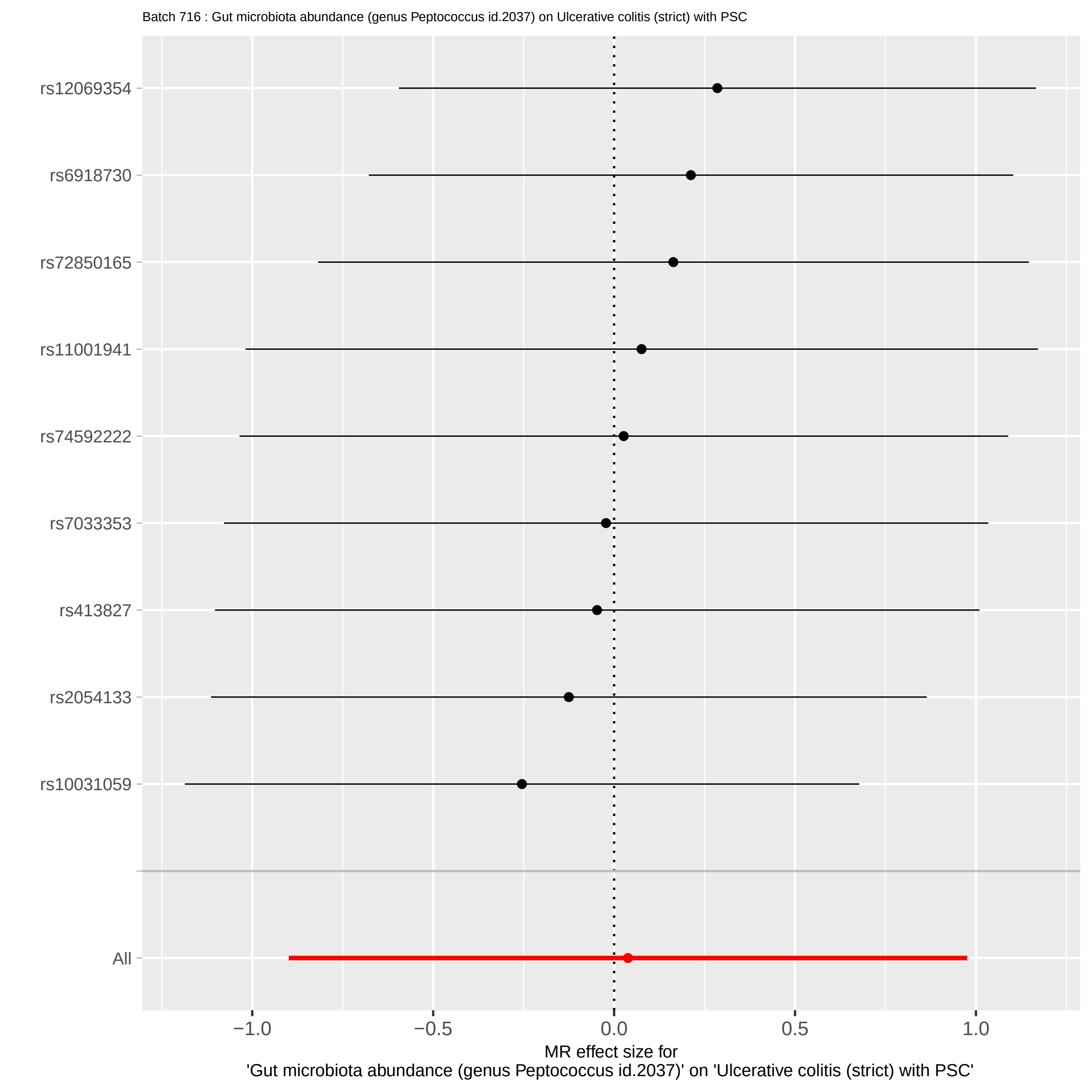

Batch 716 : Gut microbiota abundance (genus Peptococcus id.2037) on Ulcerative colitis (strict) with PSC
rs12069354
rs6918730
rs72850165
rs11001941
rs74592222
rs7033353
rs413827
rs2054133
rs10031059
All
−1.0
−0.5
0.0
0.5
1.0
MR effect size for
'Gut microbiota abundance (genus Peptococcus id.2037)' on 'Ulcerative colitis (strict) with PSC'

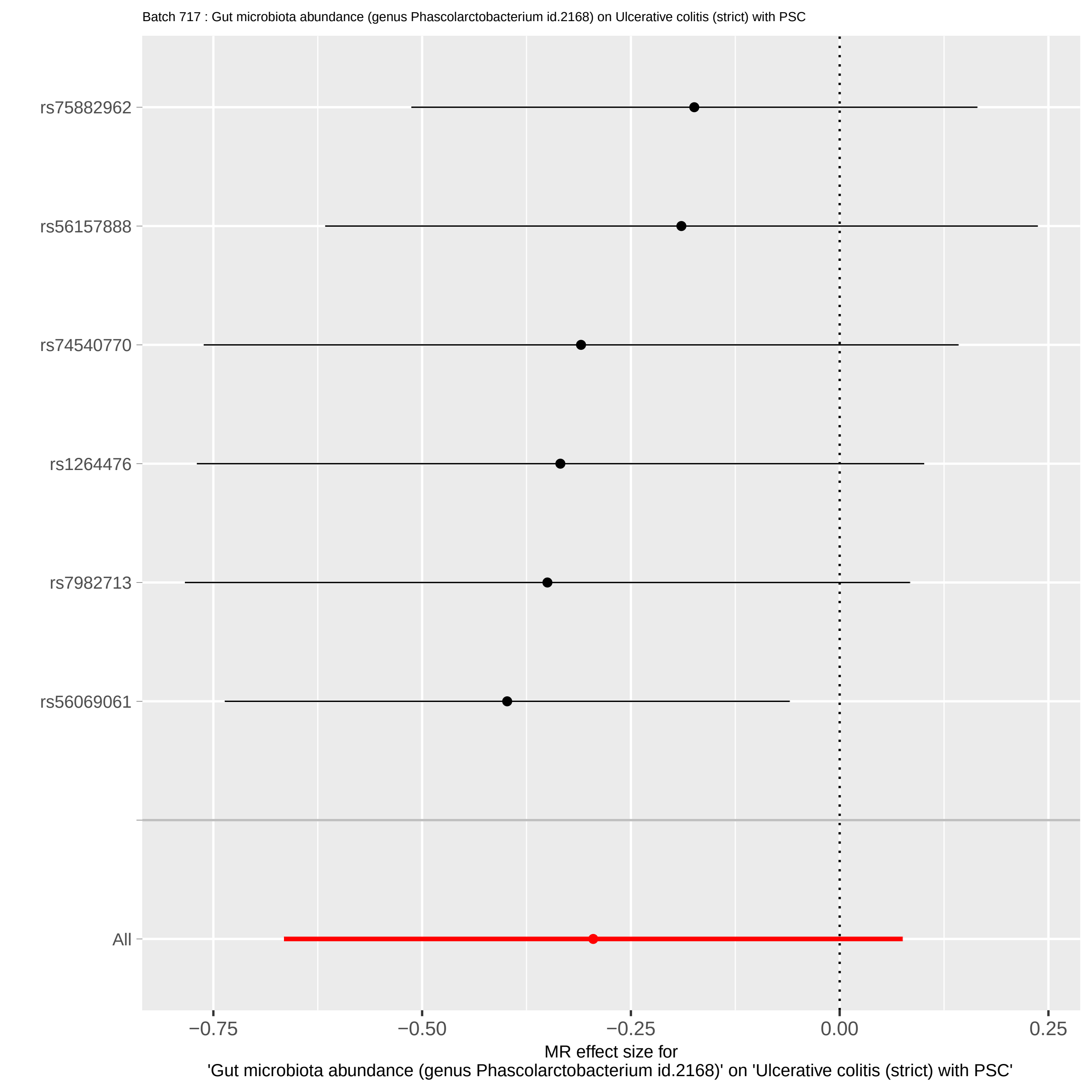
Batch 717 : Gut microbiota abundance (genus Phascolarctobacterium id.2168) on Ulcerative colitis (strict) with PSC
rs75882962
rs56157888
rs74540770
rs1264476
rs7982713
rs56069061
All
−0.75
−0.50
−0.25
0.00
0.25
MR effect size for
'Gut microbiota abundance (genus Phascolarctobacterium id.2168)' on 'Ulcerative colitis (strict) with PSC'

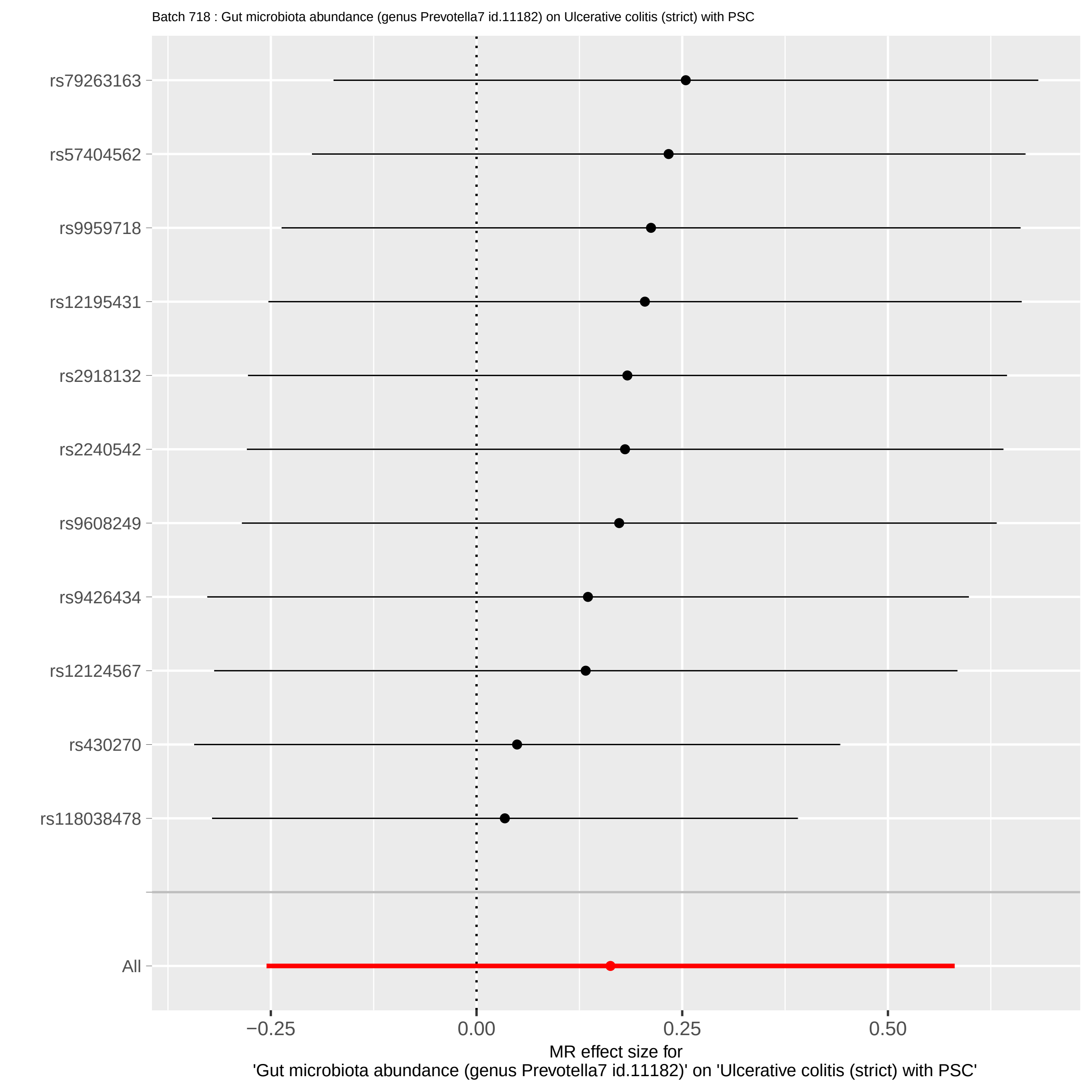

Batch 718 : Gut microbiota abundance (genus Prevotella7 id.11182) on Ulcerative colitis (strict) with PSC
rs79263163
rs57404562
rs9959718
rs12195431
rs2918132
rs2240542
rs9608249
rs9426434
rs12124567
rs430270
rs118038478
All
−0.25
0.00
0.25
0.50
MR effect size for
'Gut microbiota abundance (genus Prevotella7 id.11182)' on 'Ulcerative colitis (strict) with PSC'

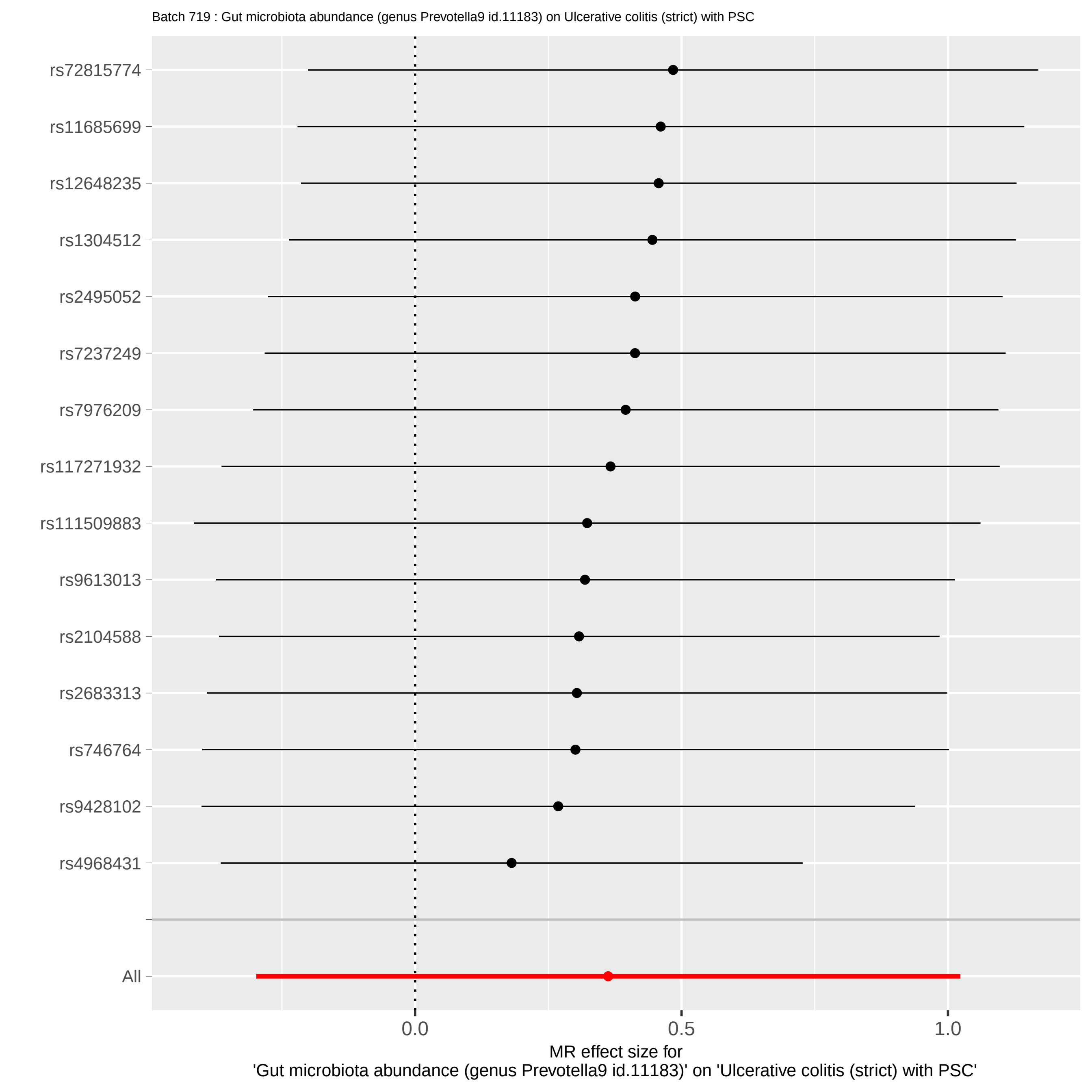

Batch 719 : Gut microbiota abundance (genus Prevotella9 id.11183) on Ulcerative colitis (strict) with PSC
rs72815774
rs11685699
rs12648235
rs1304512
rs2495052
rs7237249
rs7976209
rs117271932
rs111509883
rs9613013
rs2104588
rs2683313
rs746764
rs9428102
rs4968431
All
0.0
0.5
1.0
MR effect size for
'Gut microbiota abundance (genus Prevotella9 id.11183)' on 'Ulcerative colitis (strict) with PSC'

Batch 720 : Gut microbiota abundance (genus Rikenellaceae RC9 gut group id.11191) on Ulcerative colitis (strict) with PSC

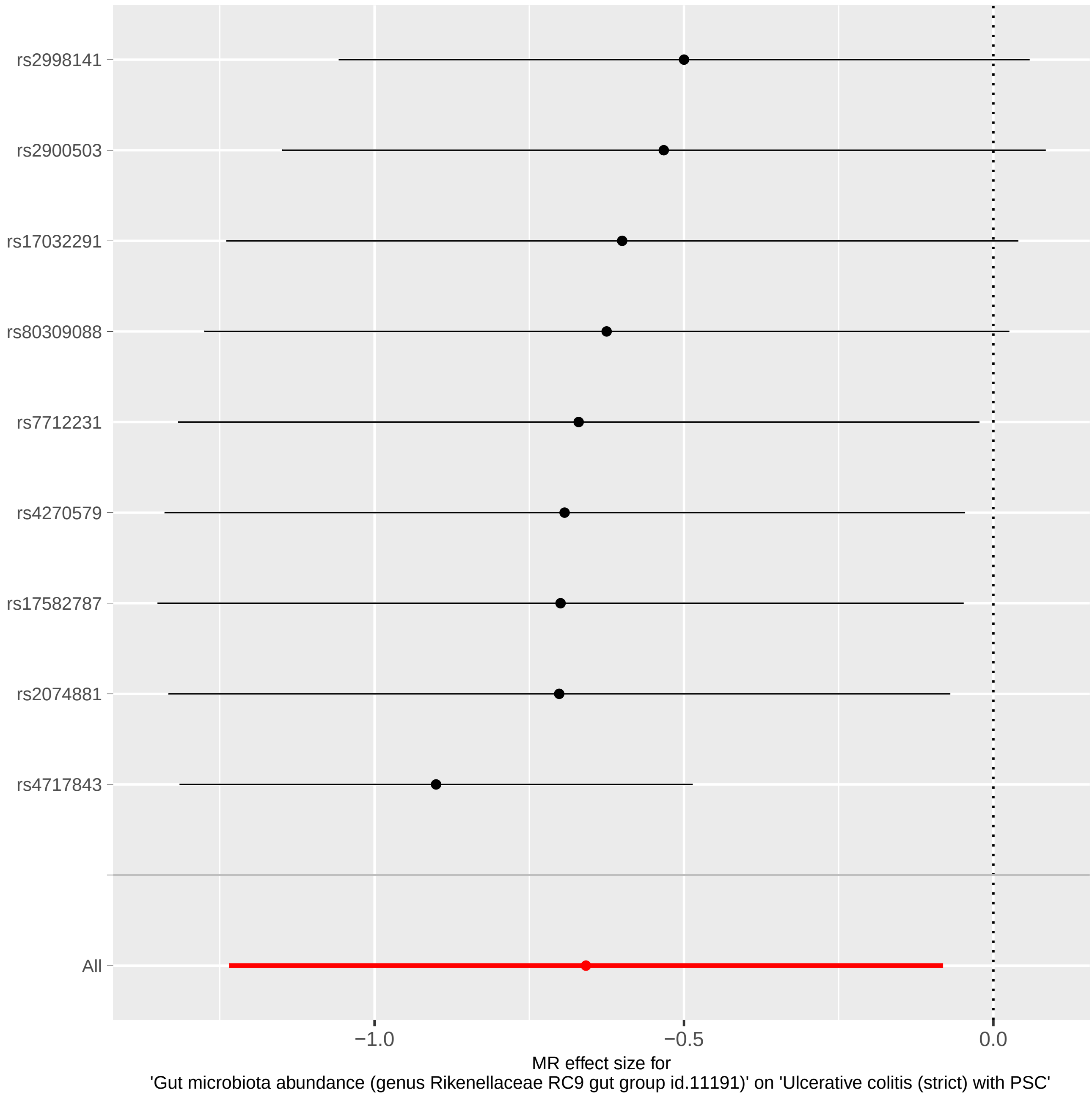

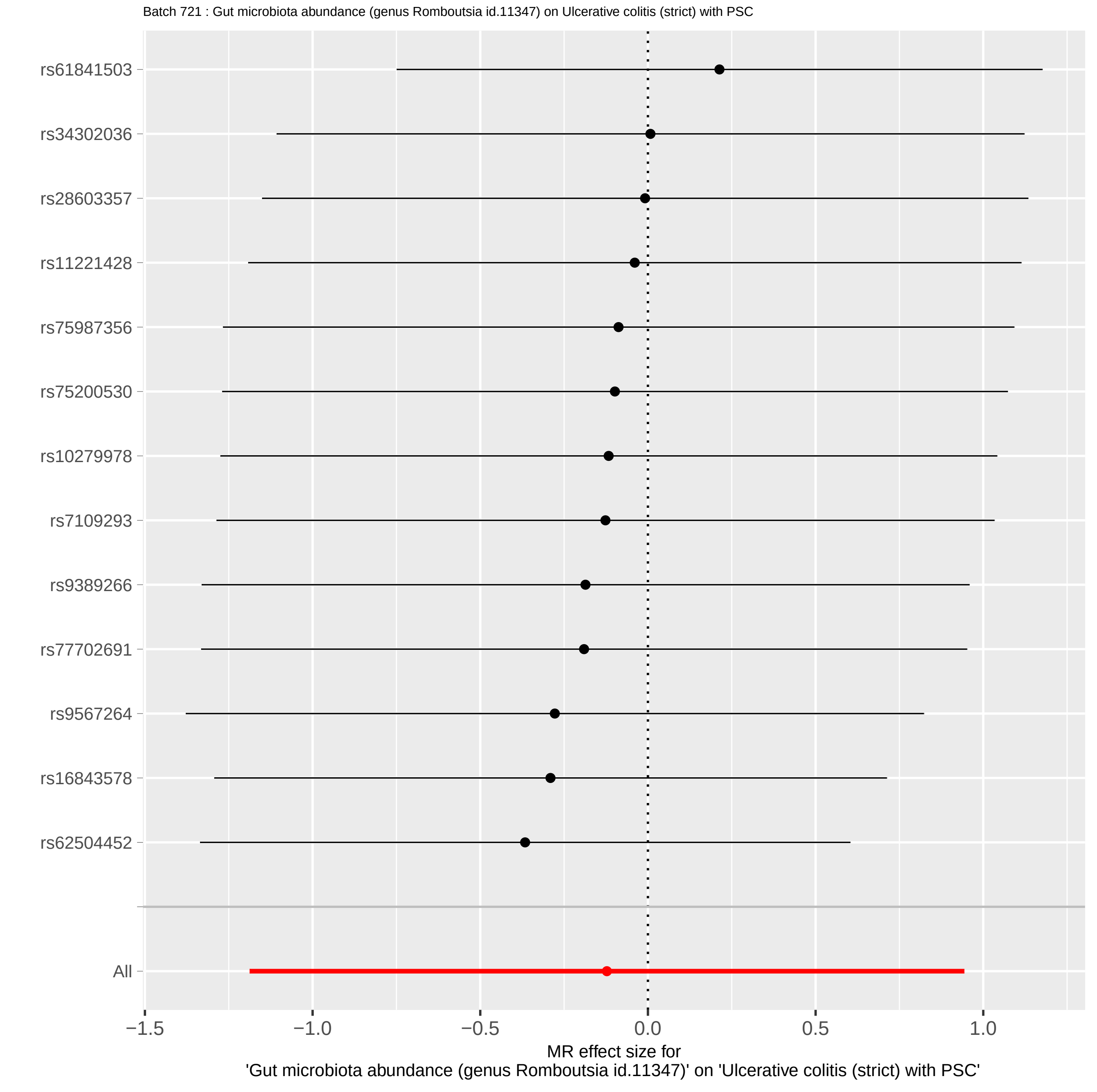
Batch 721 : Gut microbiota abundance (genus Romboutsia id.11347) on Ulcerative colitis (strict) with PSC
rs61841503
rs34302036
rs28603357
rs11221428
rs75987356
rs75200530
rs10279978
rs7109293
rs9389266
rs77702691
rs9567264
rs16843578
rs62504452
All
−1.5
−1.0
−0.5
0.0
0.5
1.0
MR effect size for
'Gut microbiota abundance (genus Romboutsia id.11347)' on 'Ulcerative colitis (strict) with PSC'

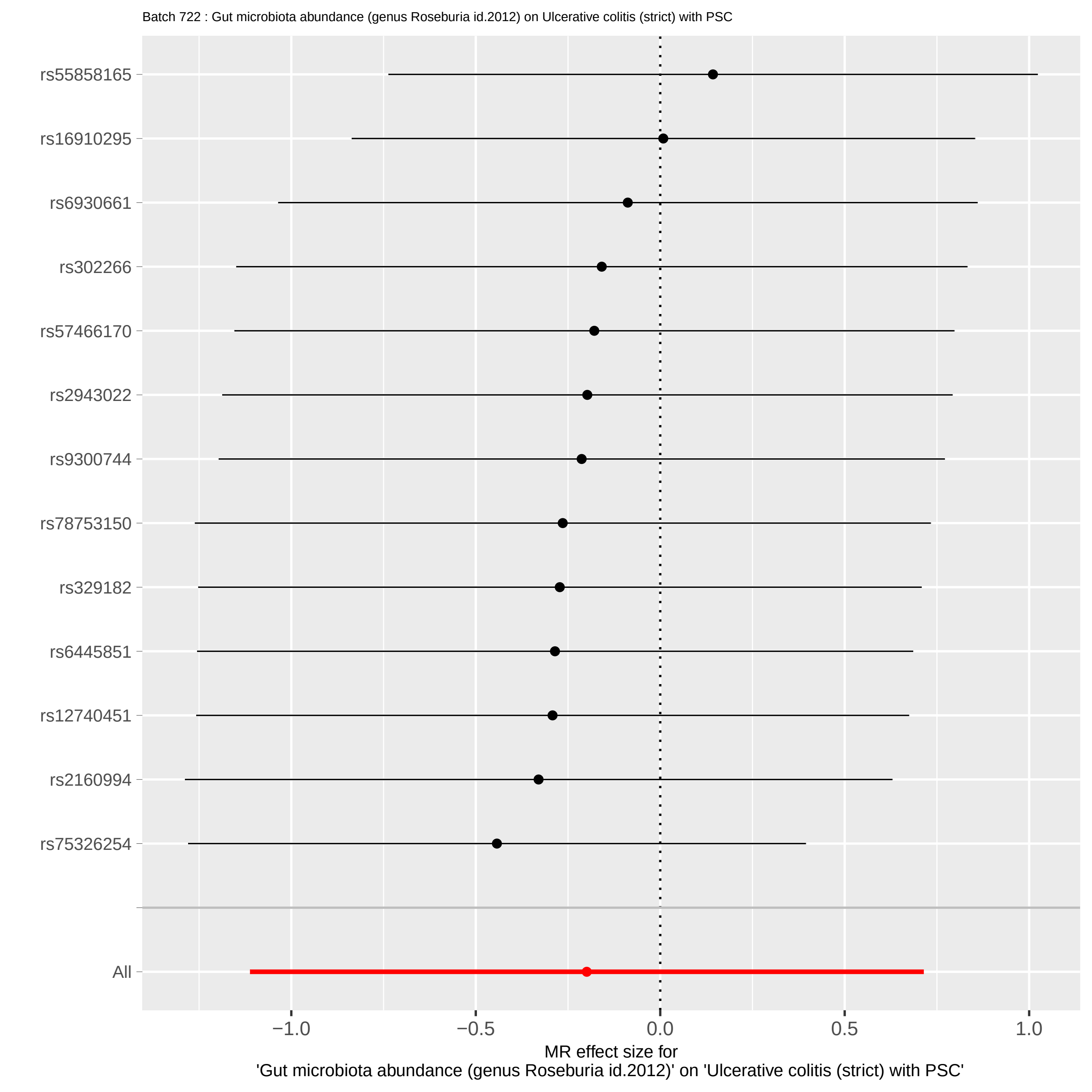

Batch 722 : Gut microbiota abundance (genus Roseburia id.2012) on Ulcerative colitis (strict) with PSC
rs55858165
rs16910295
rs6930661
rs302266
rs57466170
rs2943022
rs9300744
rs78753150
rs329182
rs6445851
rs12740451
rs2160994
rs75326254
All
−1.0
−0.5
0.0
0.5
1.0
MR effect size for
'Gut microbiota abundance (genus Roseburia id.2012)' on 'Ulcerative colitis (strict) with PSC'

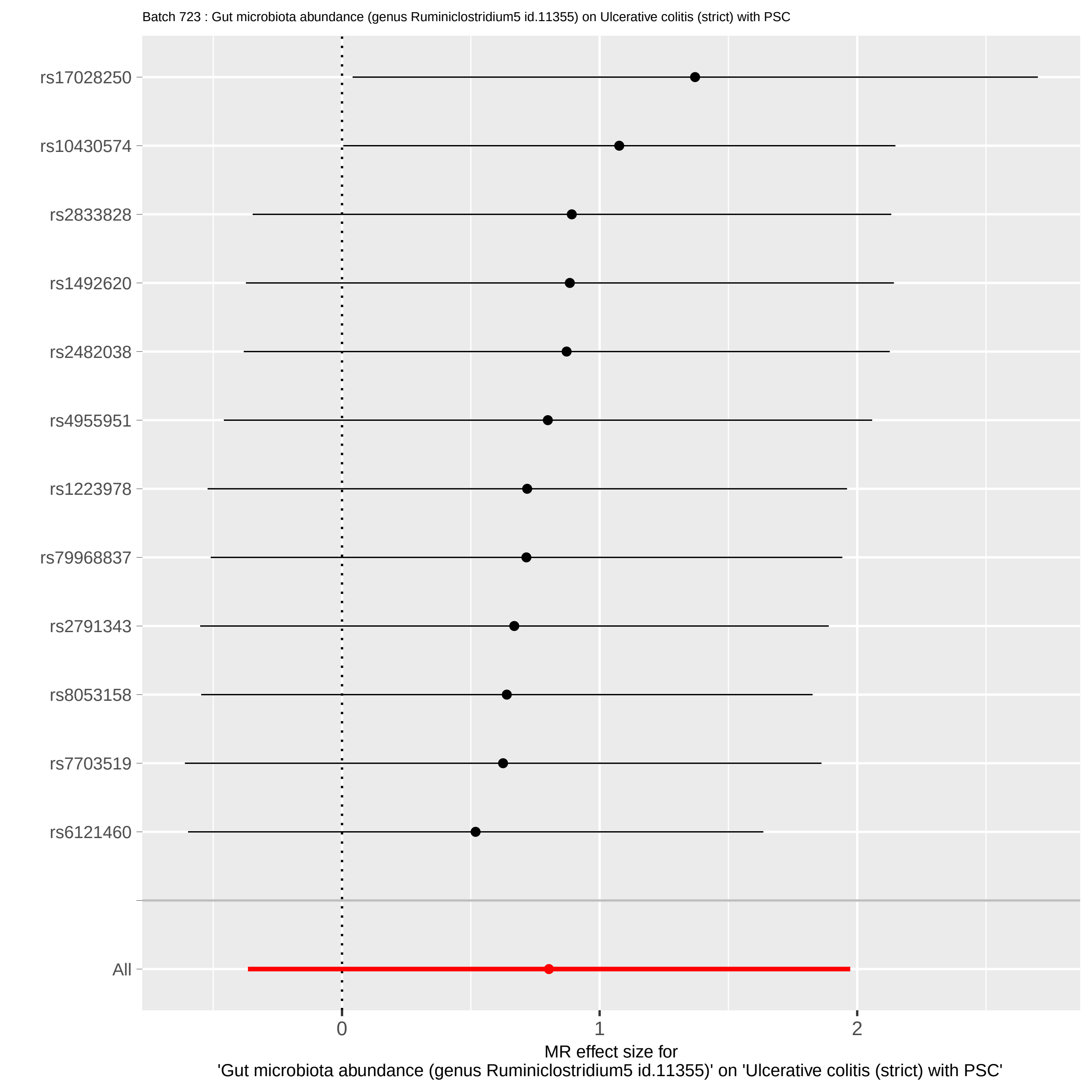
Batch 723 : Gut microbiota abundance (genus Ruminiclostridium5 id.11355) on Ulcerative colitis (strict) with PSC
rs17028250
rs10430574
rs2833828
rs1492620
rs2482038
rs4955951
rs1223978
rs79968837
rs2791343
rs8053158
rs7703519
rs6121460
All
0
1
2
MR effect size for
'Gut microbiota abundance (genus Ruminiclostridium5 id.11355)' on 'Ulcerative colitis (strict) with PSC'

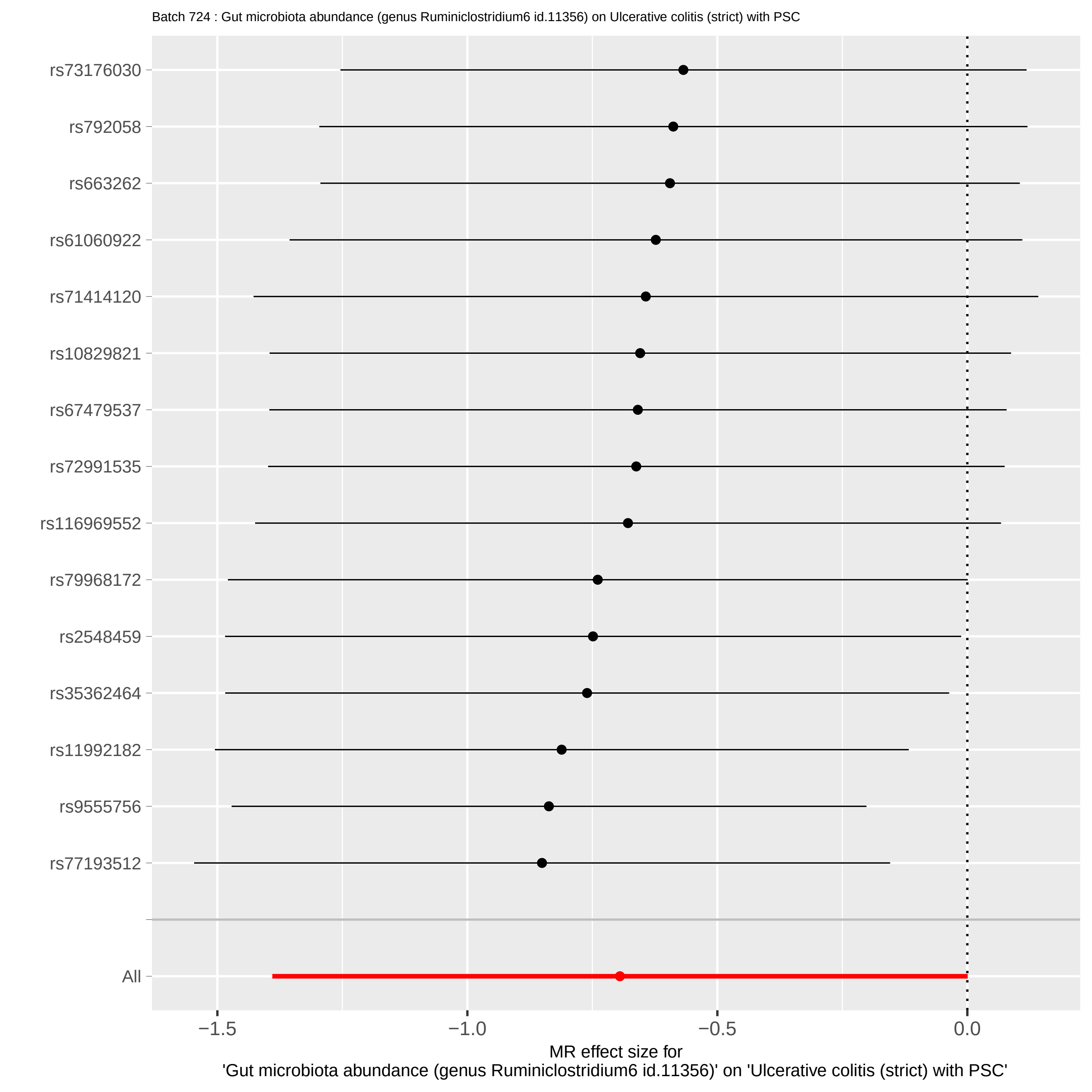
Batch 724 : Gut microbiota abundance (genus Ruminiclostridium6 id.11356) on Ulcerative colitis (strict) with PSC
rs73176030
rs792058
rs663262
rs61060922
rs71414120
rs10829821
rs67479537
rs72991535
rs116969552
rs79968172
rs2548459
rs35362464
rs11992182
rs9555756
rs77193512
All
−1.5
−1.0
−0.5
0.0
MR effect size for
'Gut microbiota abundance (genus Ruminiclostridium6 id.11356)' on 'Ulcerative colitis (strict) with PSC'

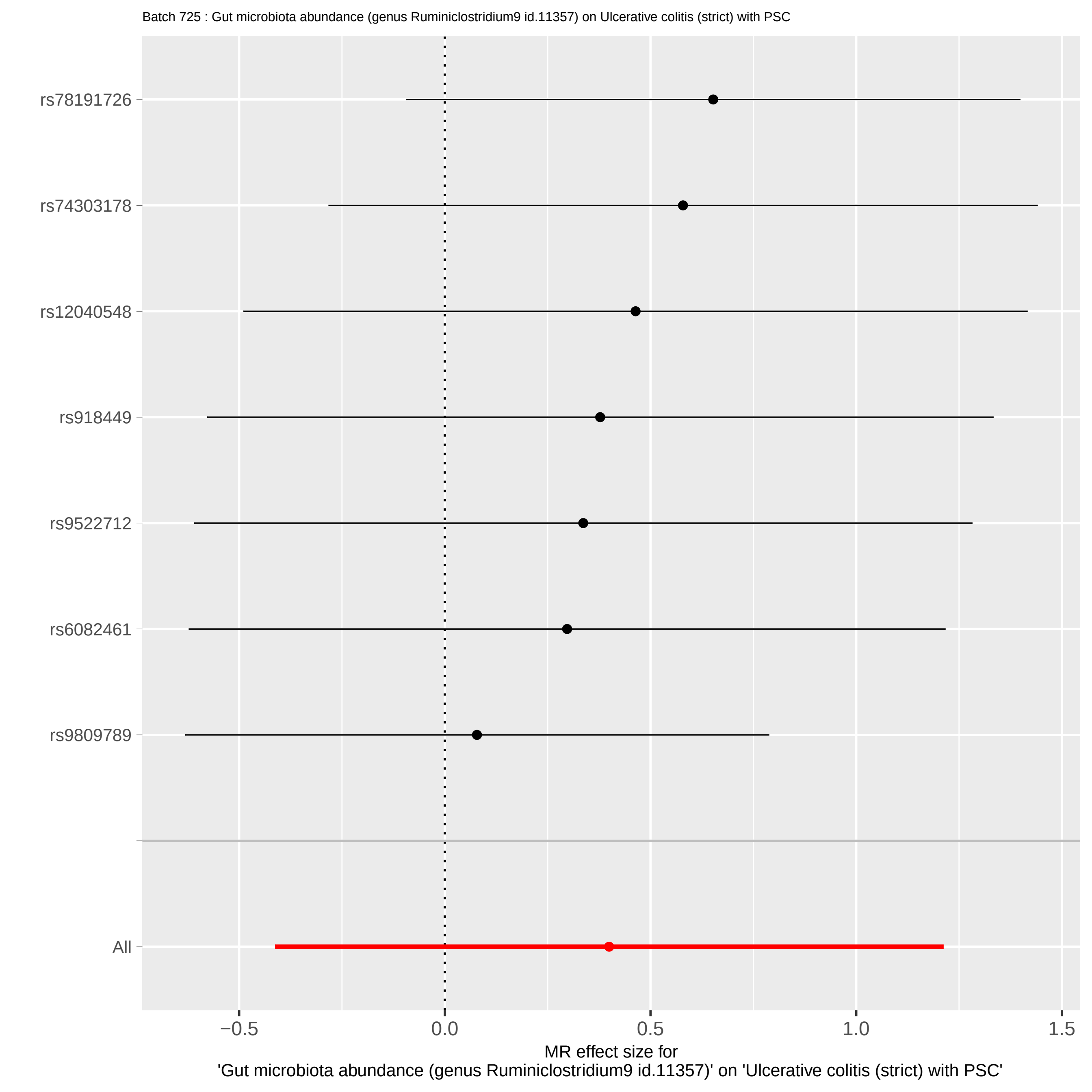
Batch 725 : Gut microbiota abundance (genus Ruminiclostridium9 id.11357) on Ulcerative colitis (strict) with PSC
rs78191726
rs74303178
rs12040548
rs918449
rs9522712
rs6082461
rs9809789
All
−0.5
0.0
0.5
1.0
1.5
MR effect size for
'Gut microbiota abundance (genus Ruminiclostridium9 id.11357)' on 'Ulcerative colitis (strict) with PSC'

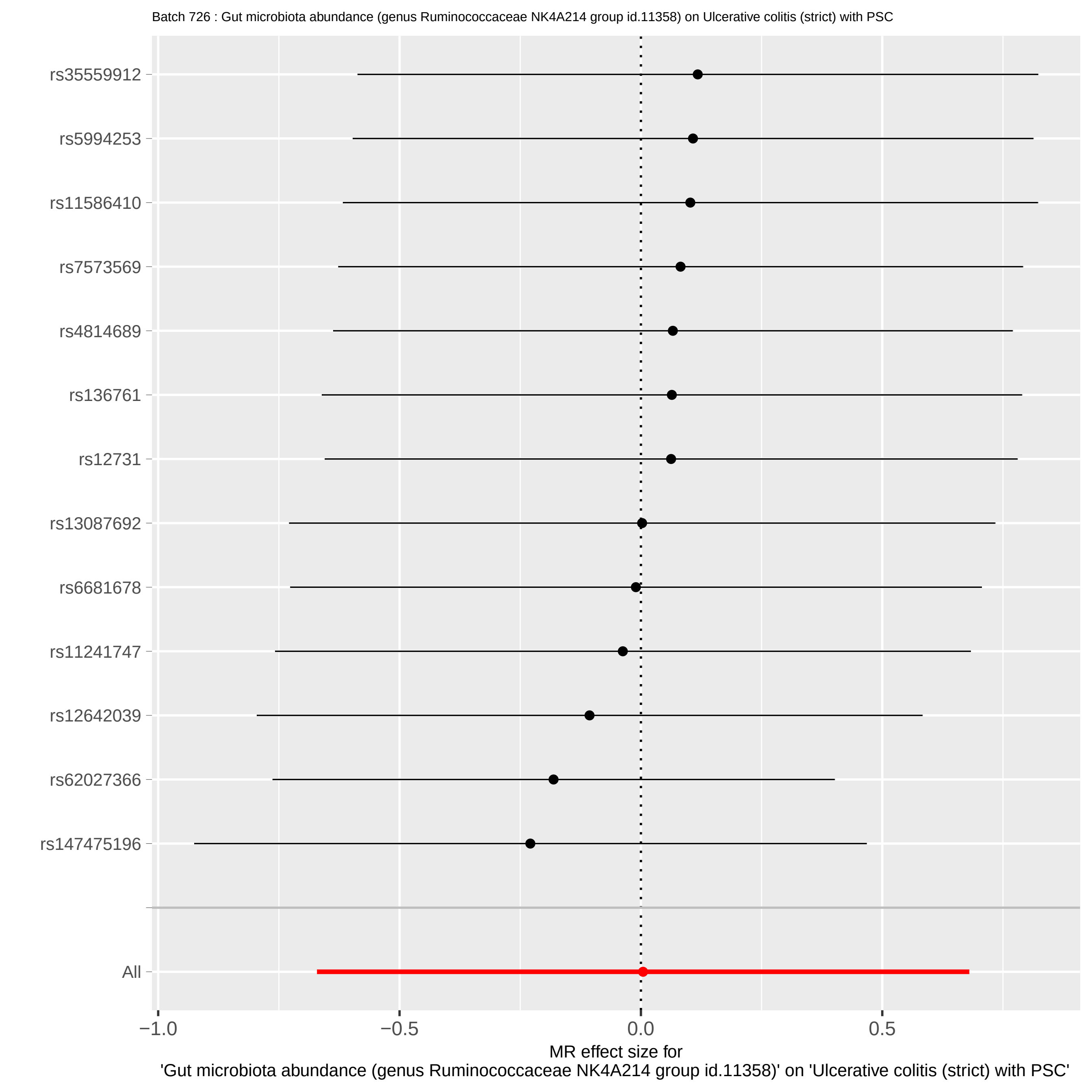

Batch 726 : Gut microbiota abundance (genus Ruminococcaceae NK4A214 group id.11358) on Ulcerative colitis (strict) with PSC
rs35559912
rs5994253
rs11586410
rs7573569
rs4814689
rs136761
rs12731
rs13087692
rs6681678
rs11241747
rs12642039
rs62027366
rs147475196
All
−1.0
−0.5
0.0
0.5
MR effect size for
'Gut microbiota abundance (genus Ruminococcaceae NK4A214 group id.11358)' on 'Ulcerative colitis (strict) with PSC'

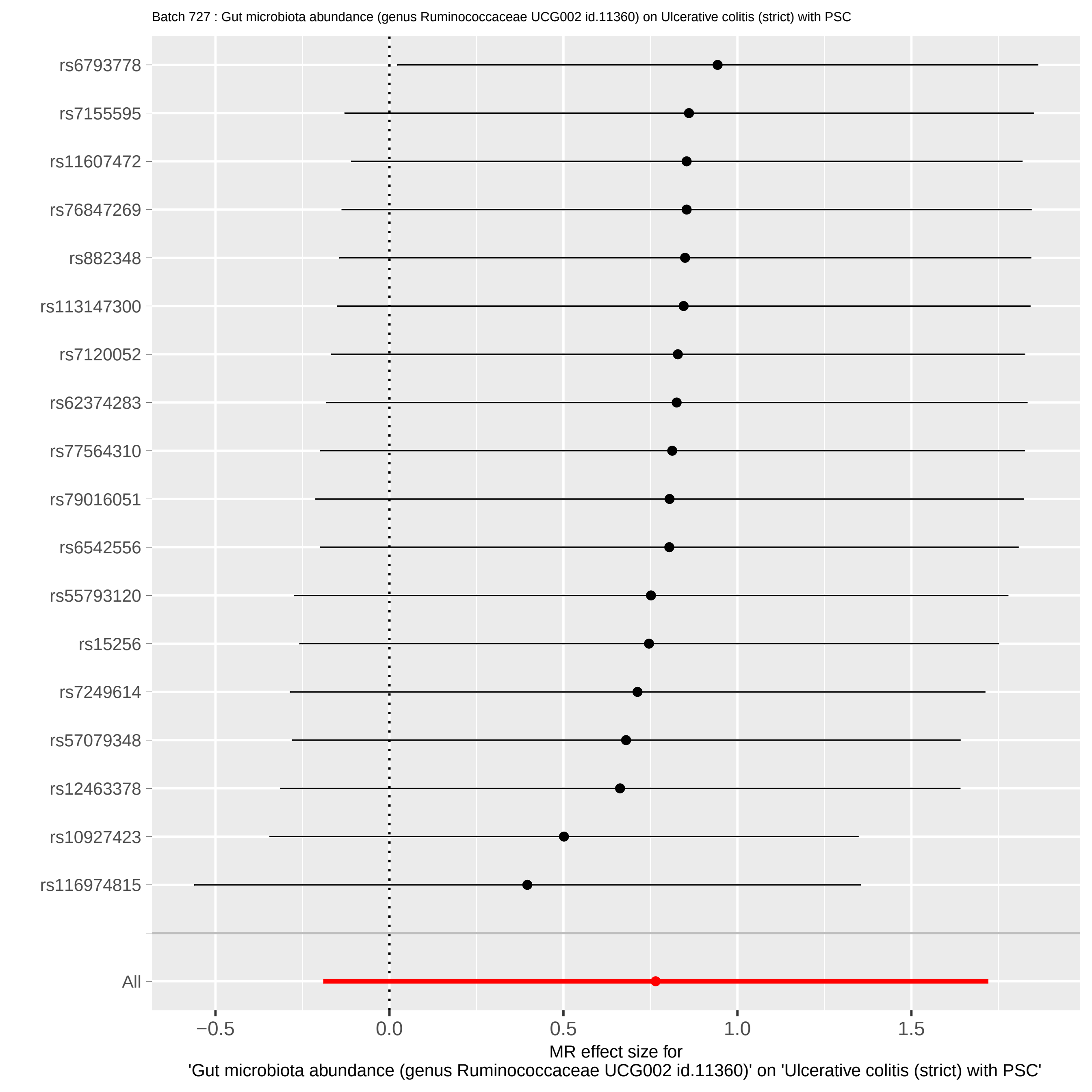
Batch 727 : Gut microbiota abundance (genus Ruminococcaceae UCG002 id.11360) on Ulcerative colitis (strict) with PSC
rs6793778
rs7155595
rs11607472
rs76847269
rs882348
rs113147300
rs7120052
rs62374283
rs77564310
rs79016051
rs6542556
rs55793120
rs15256
rs7249614
rs57079348
rs12463378
rs10927423
rs116974815
All
−0.5
0.0
0.5
1.0
1.5
MR effect size for
'Gut microbiota abundance (genus Ruminococcaceae UCG002 id.11360)' on 'Ulcerative colitis (strict) with PSC'

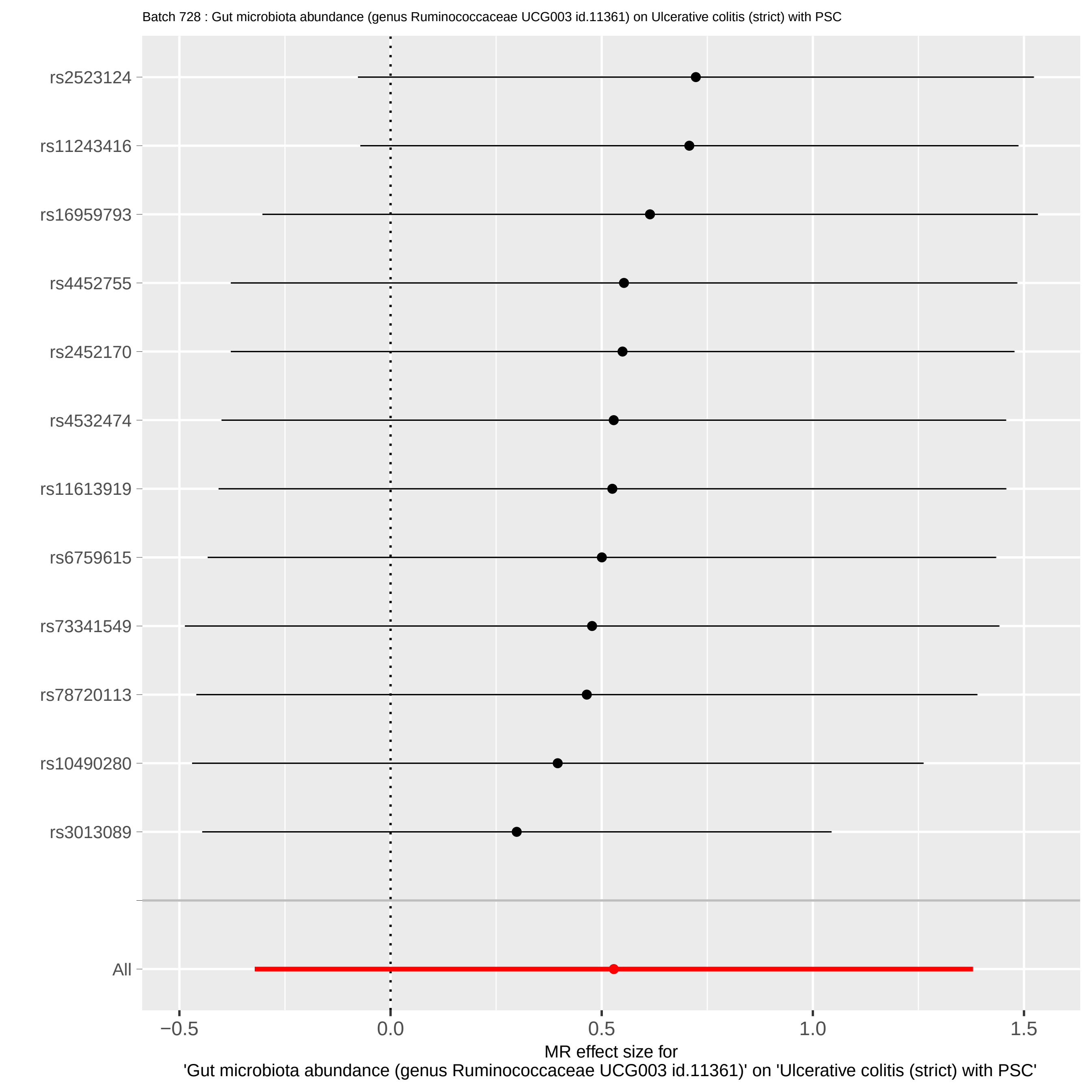

Batch 728 : Gut microbiota abundance (genus Ruminococcaceae UCG003 id.11361) on Ulcerative colitis (strict) with PSC
rs2523124
rs11243416
rs16959793
rs4452755
rs2452170
rs4532474
rs11613919
rs6759615
rs73341549
rs78720113
rs10490280
rs3013089
All
−0.5
0.0
0.5
1.0
1.5
MR effect size for
'Gut microbiota abundance (genus Ruminococcaceae UCG003 id.11361)' on 'Ulcerative colitis (strict) with PSC'

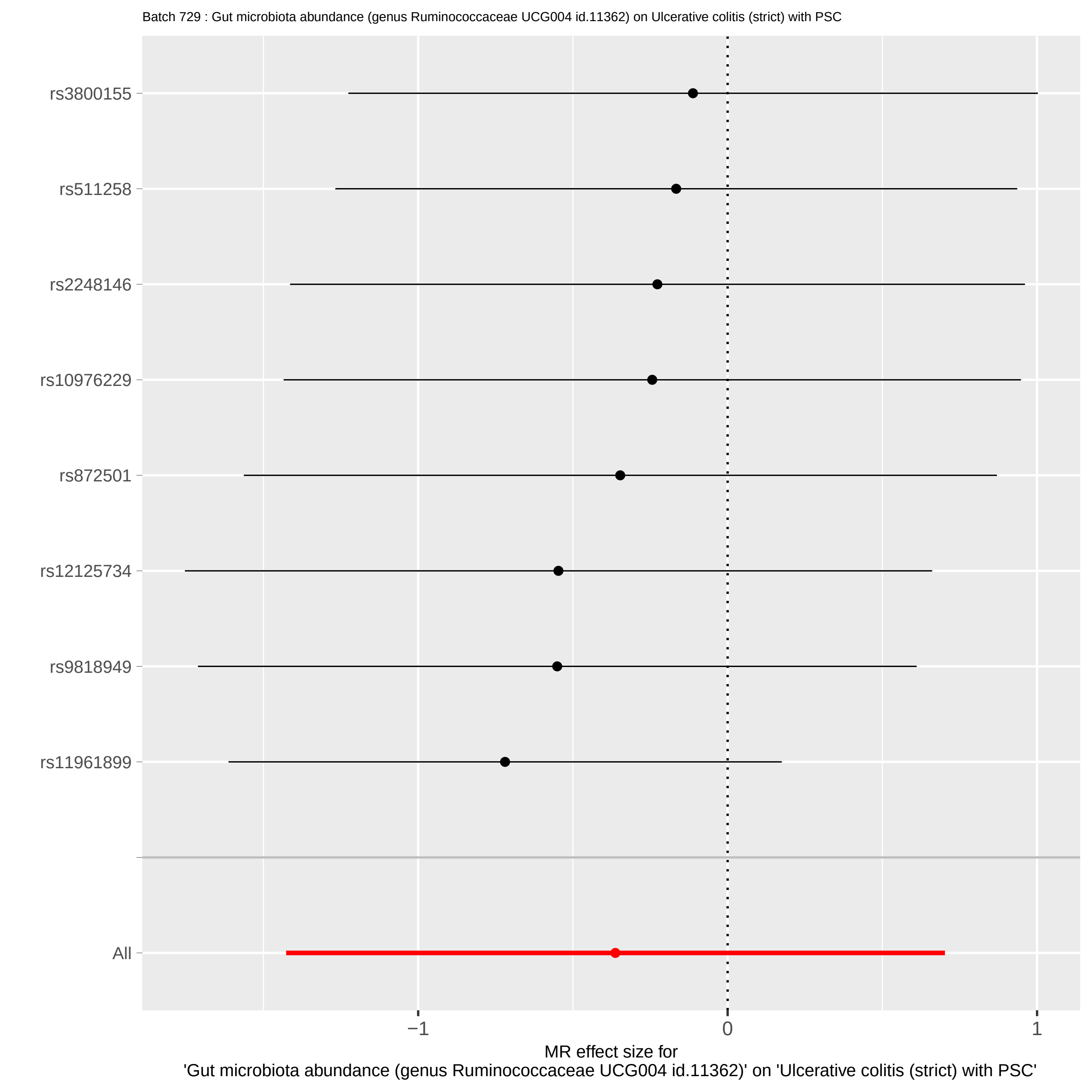

Batch 729 : Gut microbiota abundance (genus Ruminococcaceae UCG004 id.11362) on Ulcerative colitis (strict) with PSC
rs3800155
rs511258
rs2248146
rs10976229
rs872501
rs12125734
rs9818949
rs11961899
All
−1
0
1
MR effect size for
'Gut microbiota abundance (genus Ruminococcaceae UCG004 id.11362)' on 'Ulcerative colitis (strict) with PSC'

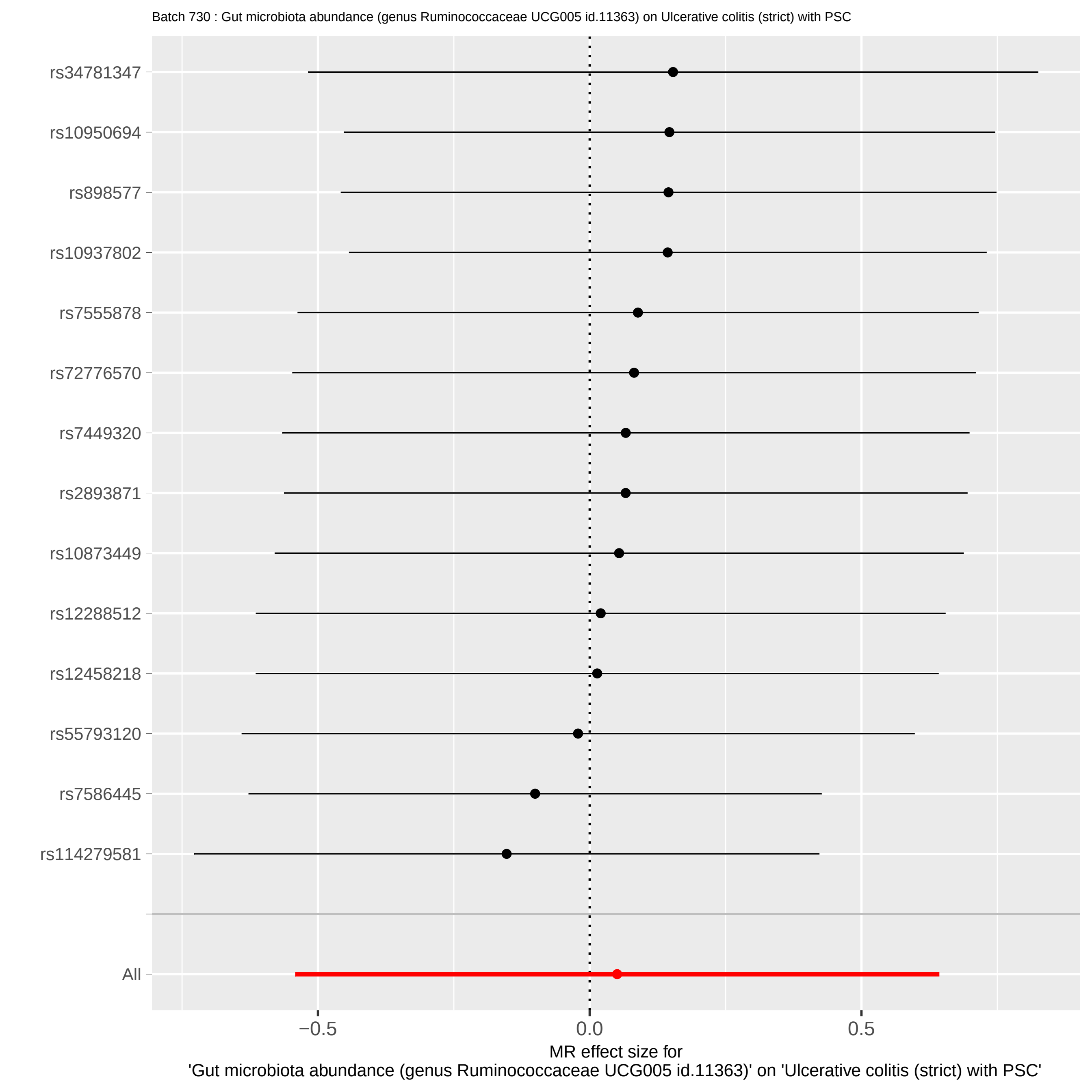

Batch 730 : Gut microbiota abundance (genus Ruminococcaceae UCG005 id.11363) on Ulcerative colitis (strict) with PSC
rs34781347
rs10950694
rs898577
rs10937802
rs7555878
rs72776570
rs7449320
rs2893871
rs10873449
rs12288512
rs12458218
rs55793120
rs7586445
rs114279581
All
−0.5
0.0
0.5
MR effect size for
'Gut microbiota abundance (genus Ruminococcaceae UCG005 id.11363)' on 'Ulcerative colitis (strict) with PSC'

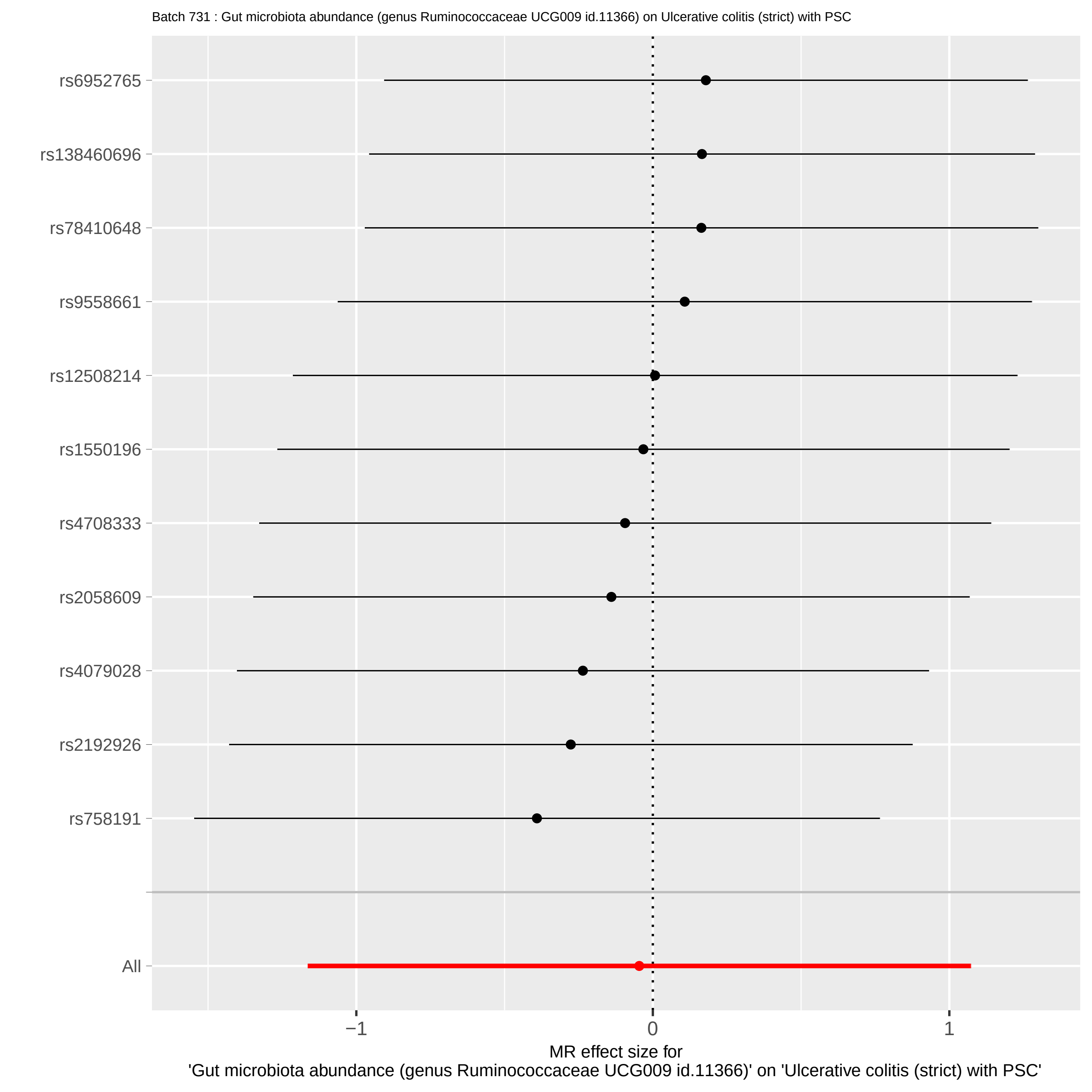

Batch 731 : Gut microbiota abundance (genus Ruminococcaceae UCG009 id.11366) on Ulcerative colitis (strict) with PSC
rs6952765
rs138460696
rs78410648
rs9558661
rs12508214
rs1550196
rs4708333
rs2058609
rs4079028
rs2192926
rs758191
All
−1
0
1
MR effect size for
'Gut microbiota abundance (genus Ruminococcaceae UCG009 id.11366)' on 'Ulcerative colitis (strict) with PSC'

Batch 732 : Gut microbiota abundance (genus Ruminococcaceae UCG010 id.11367) on Ulcerative colitis (strict) with PSC

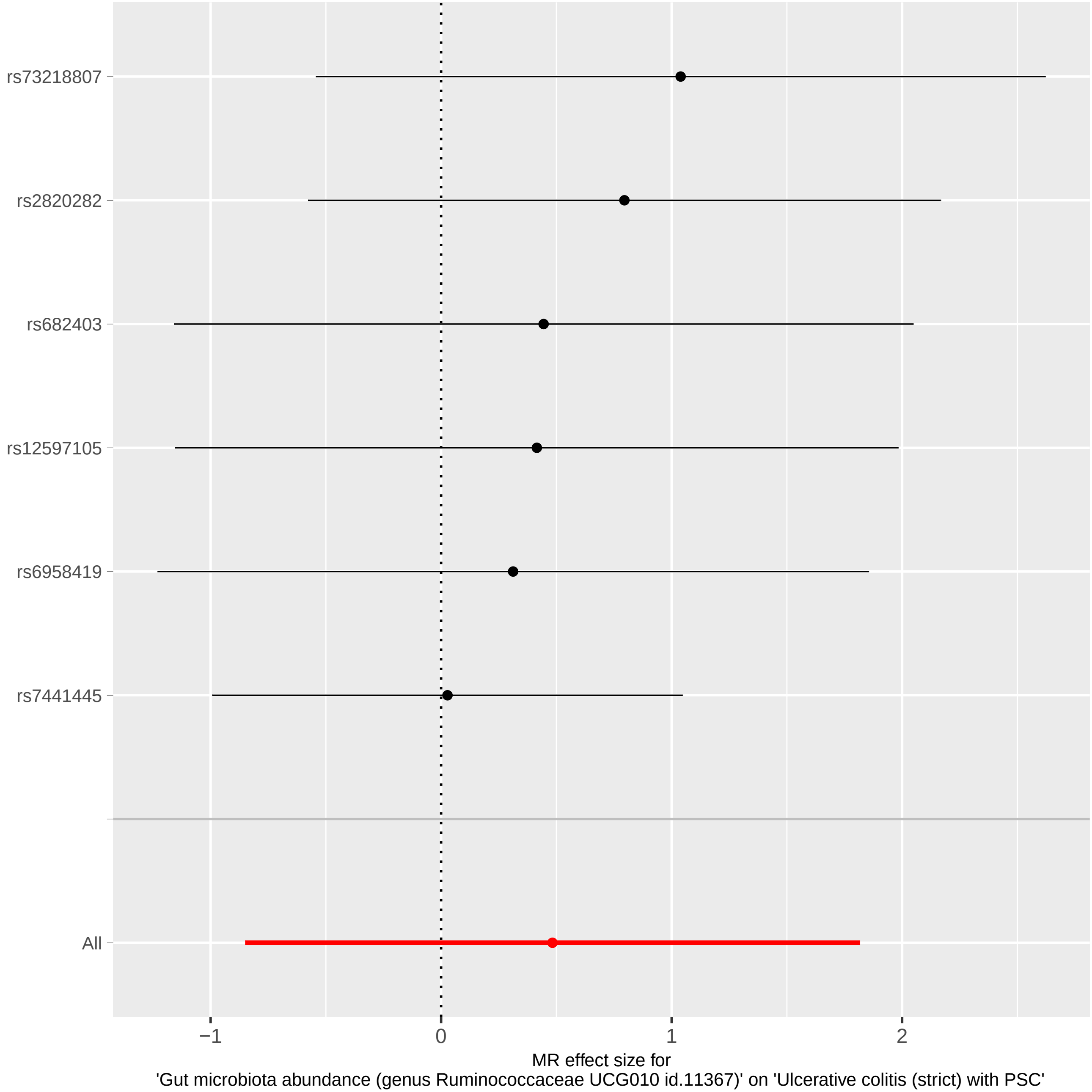

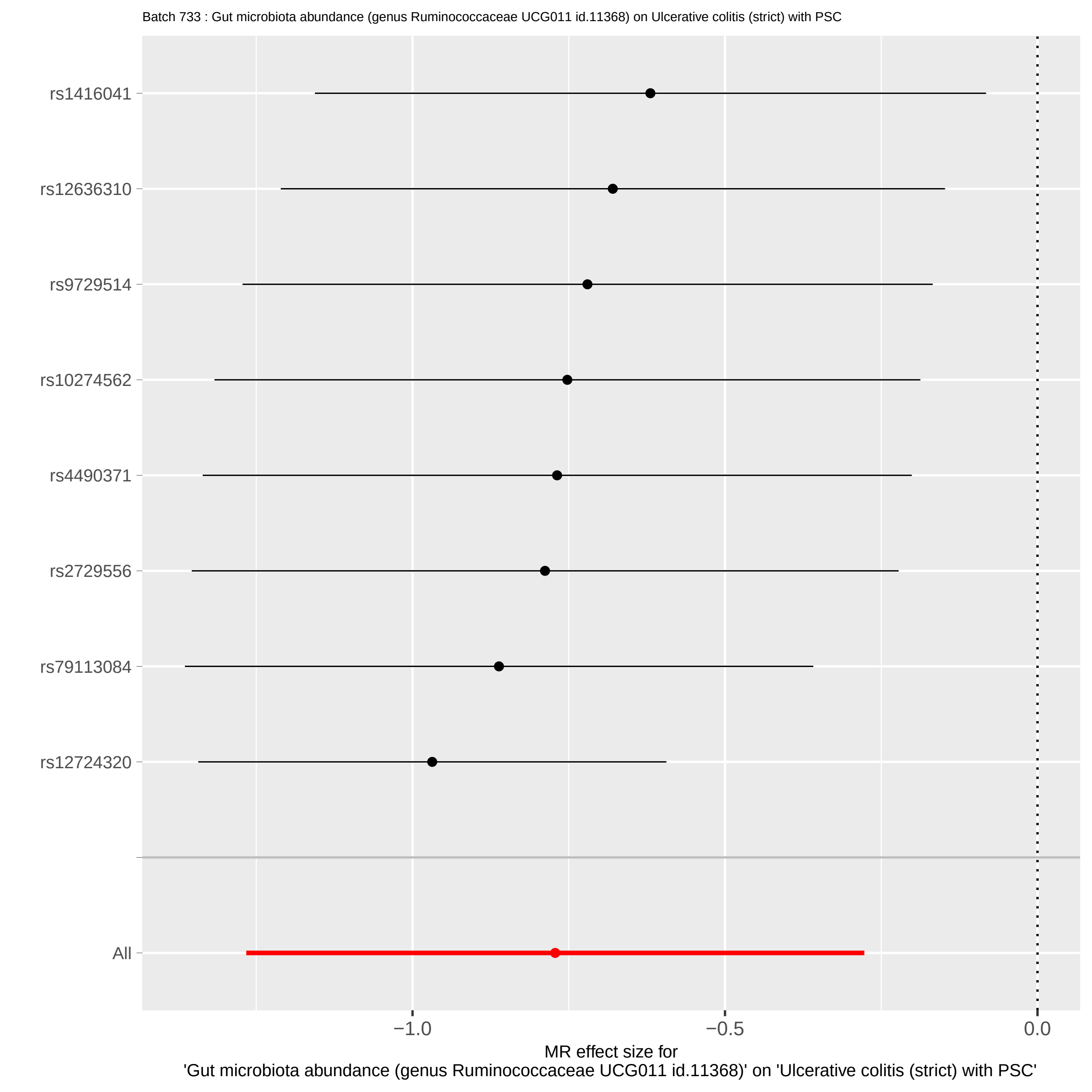

Batch 733 : Gut microbiota abundance (genus Ruminococcaceae UCG011 id.11368) on Ulcerative colitis (strict) with PSC
rs1416041
rs12636310
rs9729514
rs10274562
rs4490371
rs2729556
rs79113084
rs12724320
All
−1.0
−0.5
0.0
MR effect size for
'Gut microbiota abundance (genus Ruminococcaceae UCG011 id.11368)' on 'Ulcerative colitis (strict) with PSC'

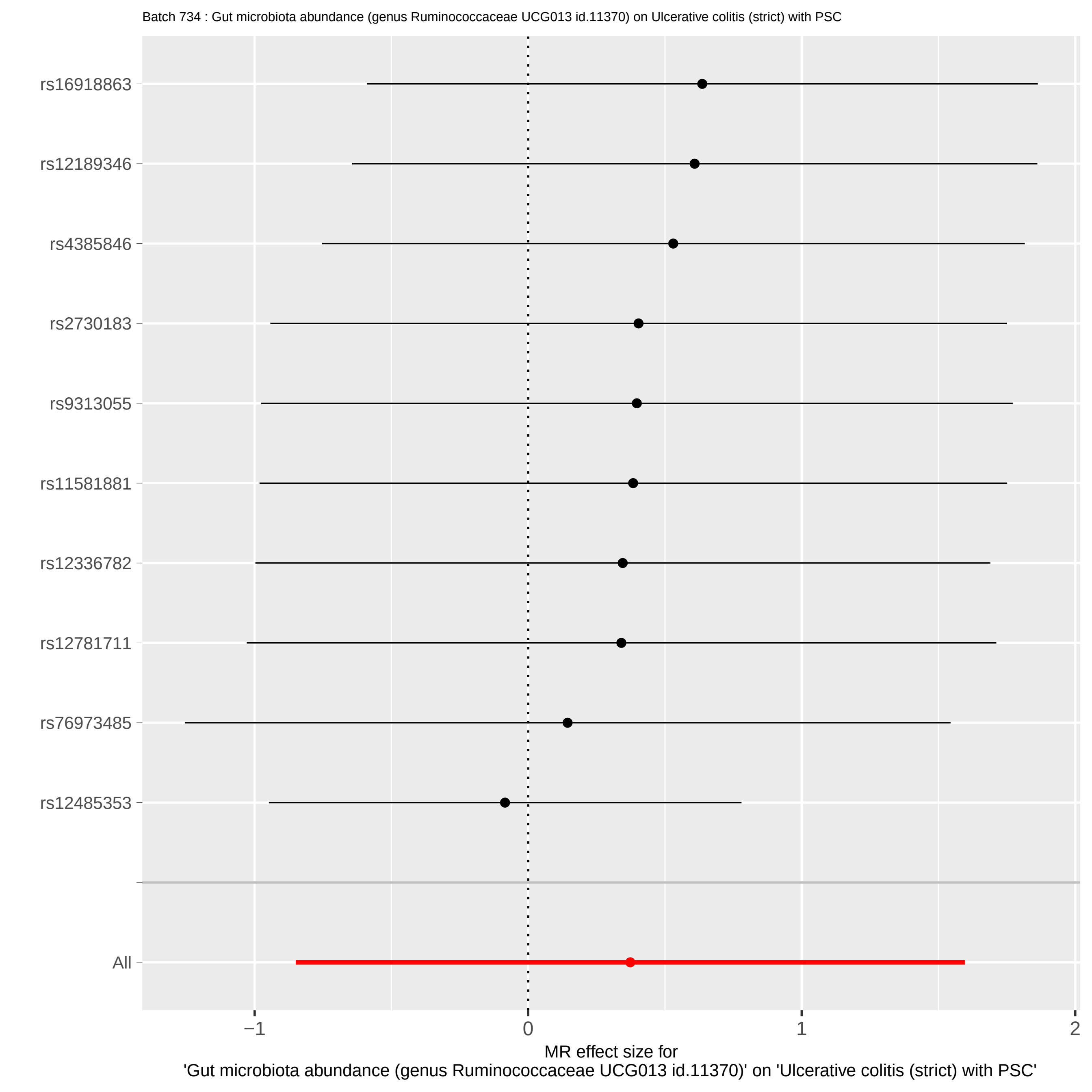
Batch 734 : Gut microbiota abundance (genus Ruminococcaceae UCG013 id.11370) on Ulcerative colitis (strict) with PSC
rs16918863
rs12189346
rs4385846
rs2730183
rs9313055
rs11581881
rs12336782
rs12781711
rs76973485
rs12485353
All
−1
0
1
2
MR effect size for
'Gut microbiota abundance (genus Ruminococcaceae UCG013 id.11370)' on 'Ulcerative colitis (strict) with PSC'

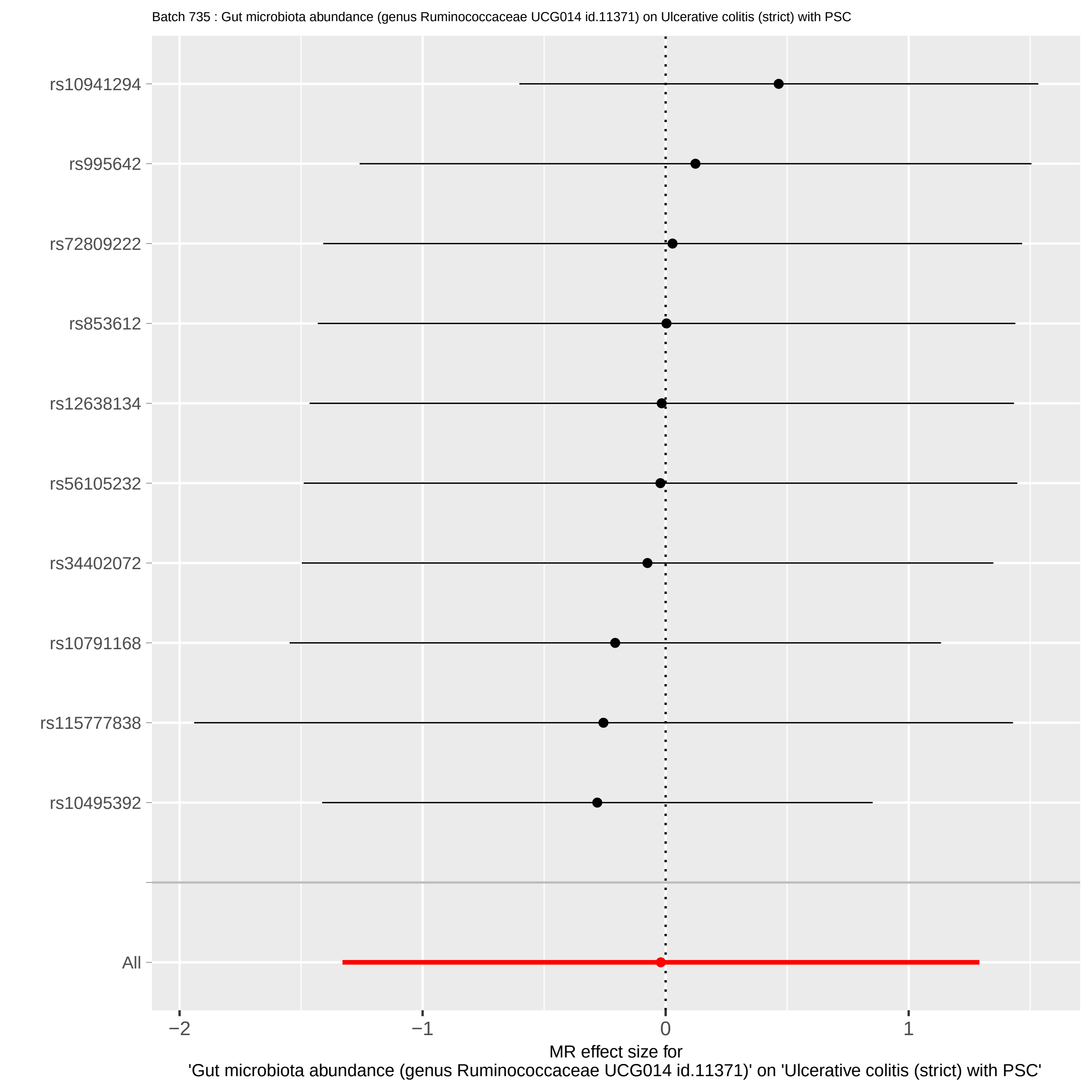

Batch 735 : Gut microbiota abundance (genus Ruminococcaceae UCG014 id.11371) on Ulcerative colitis (strict) with PSC
rs10941294
rs995642
rs72809222
rs853612
rs12638134
rs56105232
rs34402072
rs10791168
rs115777838
rs10495392
All
−2
−1
0
1
MR effect size for
'Gut microbiota abundance (genus Ruminococcaceae UCG014 id.11371)' on 'Ulcerative colitis (strict) with PSC'

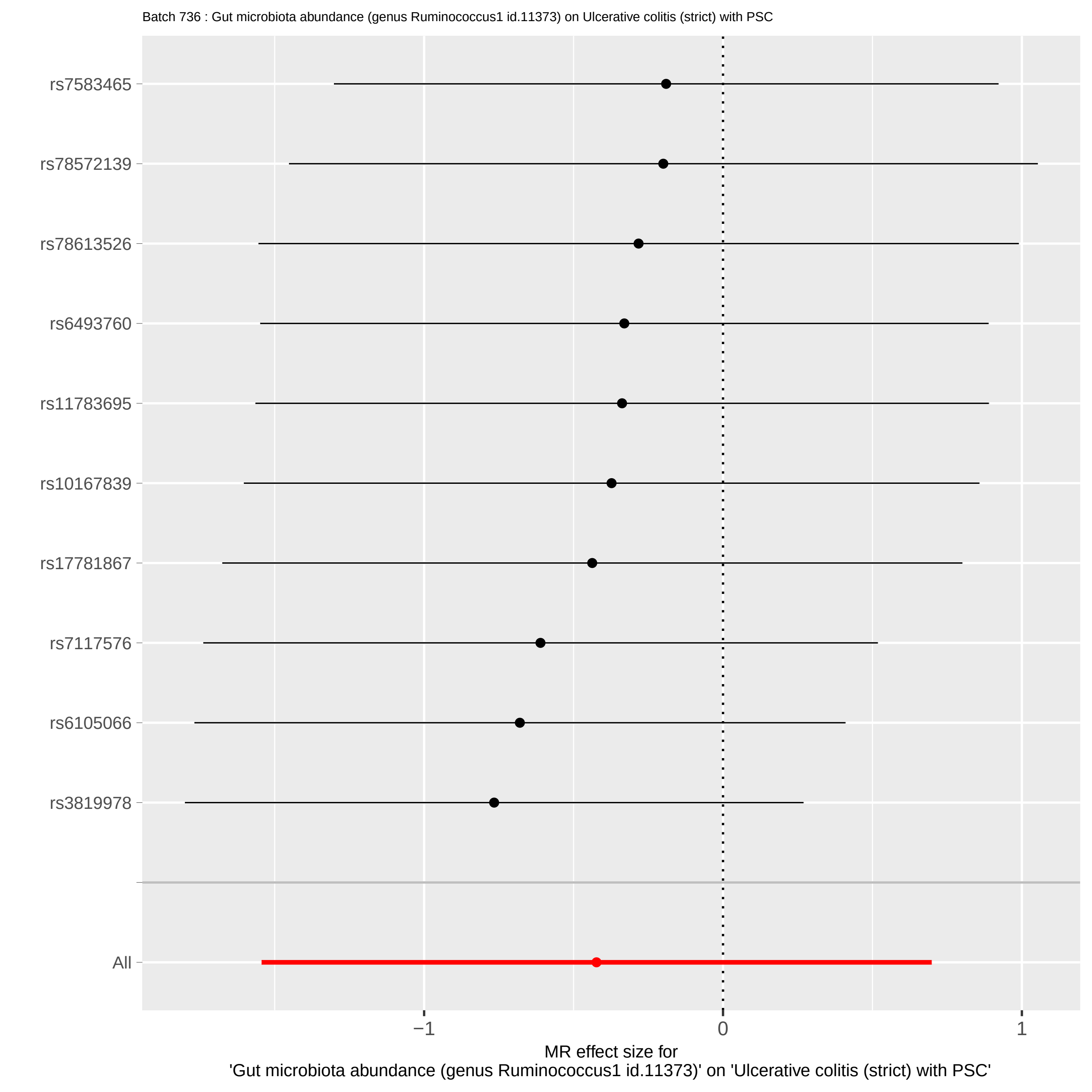
Batch 736 : Gut microbiota abundance (genus Ruminococcus1 id.11373) on Ulcerative colitis (strict) with PSC
rs7583465
rs78572139
rs78613526
rs6493760
rs11783695
rs10167839
rs17781867
rs7117576
rs6105066
rs3819978
All
−1
0
1
MR effect size for
'Gut microbiota abundance (genus Ruminococcus1 id.11373)' on 'Ulcerative colitis (strict) with PSC'

Batch 737 : Gut microbiota abundance (genus Ruminococcus2 id.11374) on Ulcerative colitis (strict) with PSC

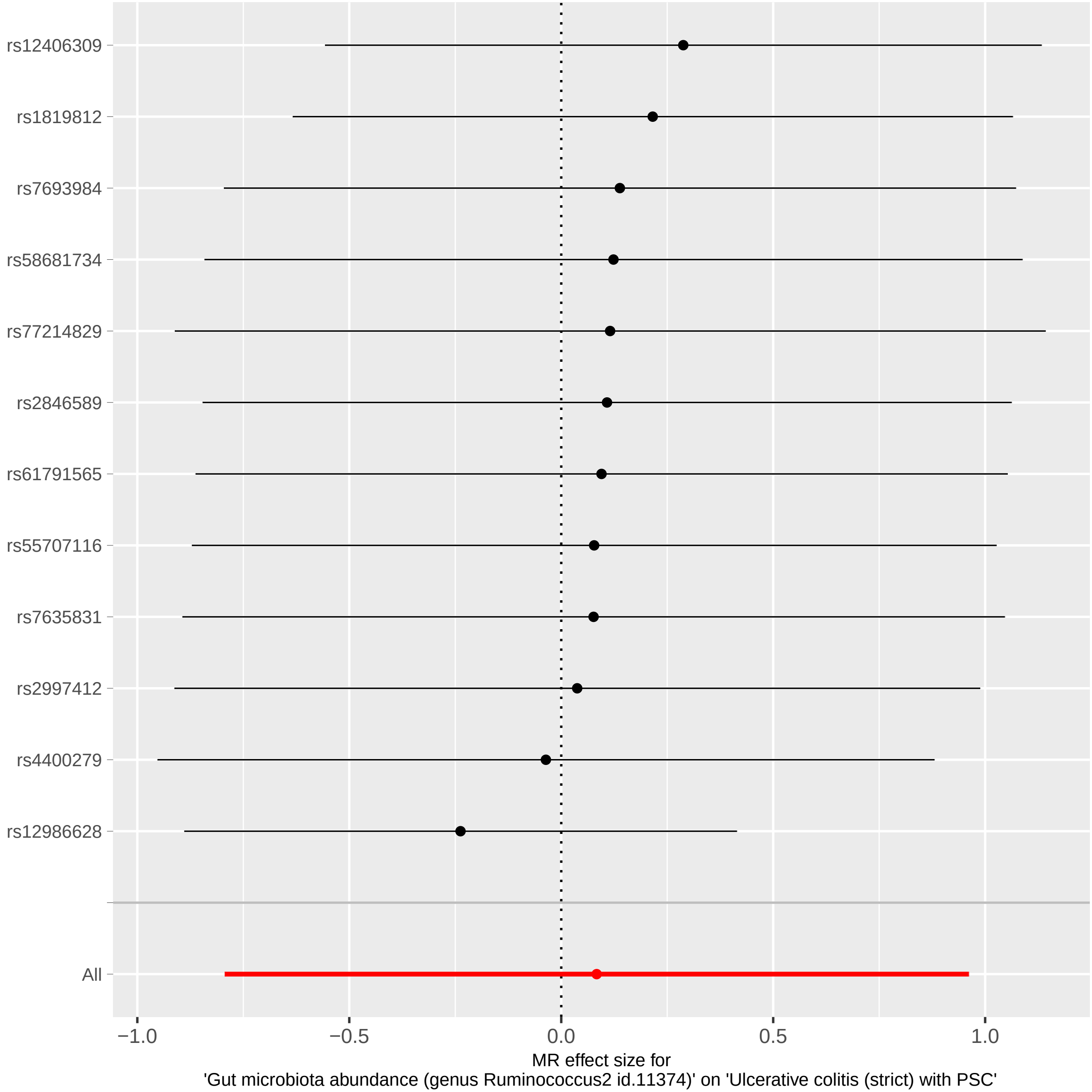

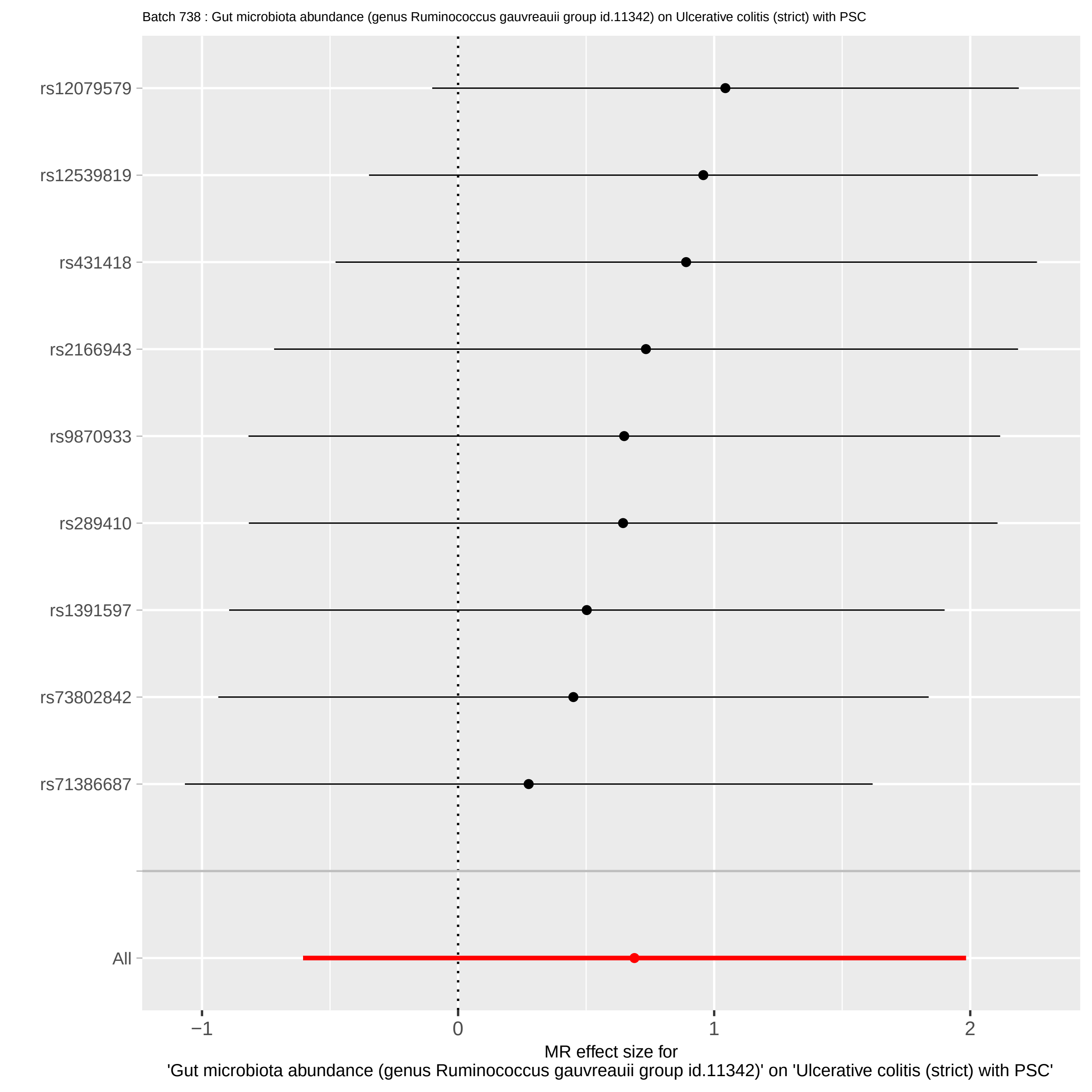

Batch 738 : Gut microbiota abundance (genus Ruminococcus gauvreauii group id.11342) on Ulcerative colitis (strict) with PSC
rs12079579
rs12539819
rs431418
rs2166943
rs9870933
rs289410
rs1391597
rs73802842
rs71386687
All
−1
0
1
2
MR effect size for
'Gut microbiota abundance (genus Ruminococcus gauvreauii group id.11342)' on 'Ulcerative colitis (strict) with PSC'

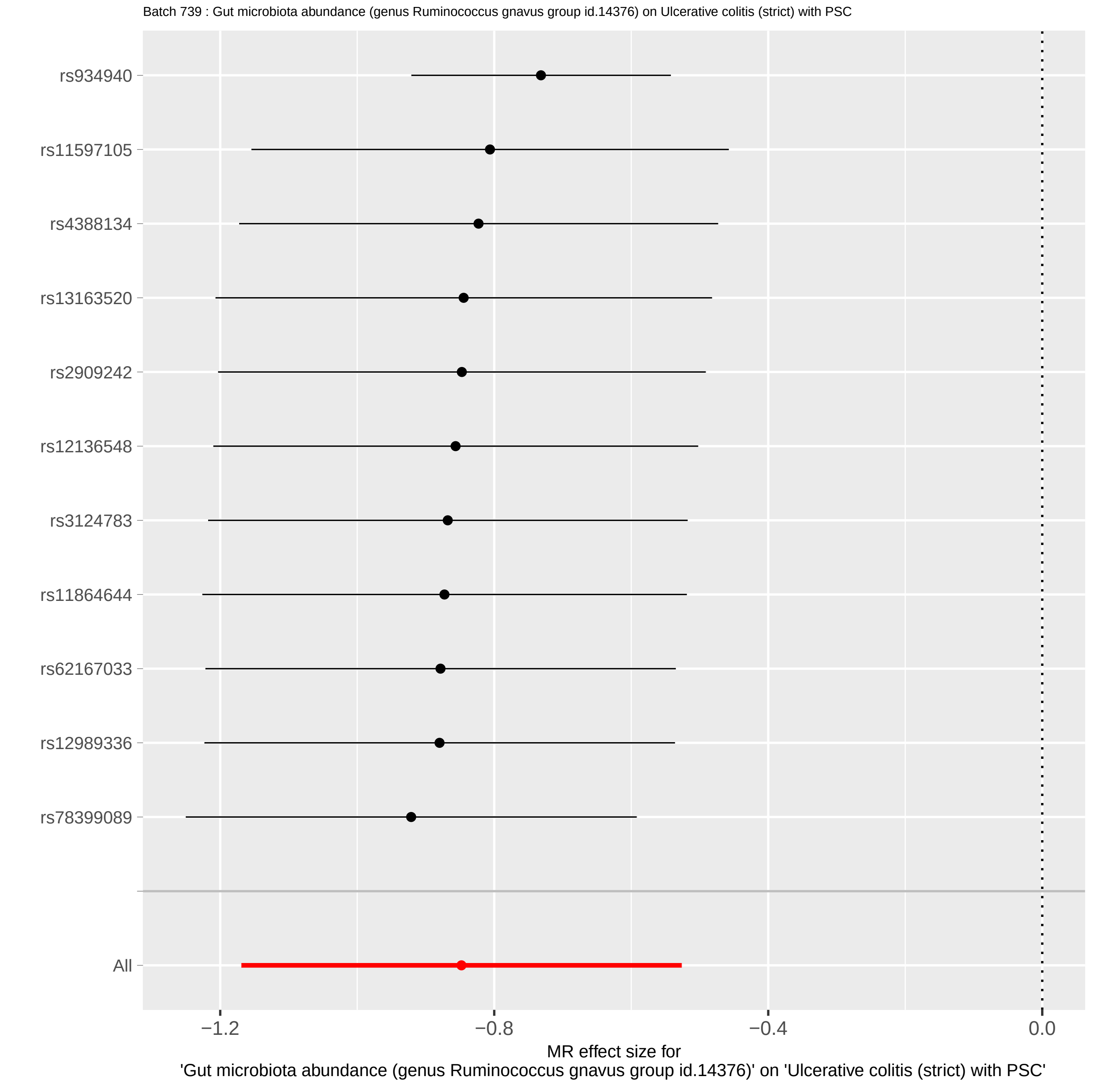

Batch 739 : Gut microbiota abundance (genus Ruminococcus gnavus group id.14376) on Ulcerative colitis (strict) with PSC
rs934940
rs11597105
rs4388134
rs13163520
rs2909242
rs12136548
rs3124783
rs11864644
rs62167033
rs12989336
rs78399089
All
−1.2
−0.8
−0.4
0.0
MR effect size for
'Gut microbiota abundance (genus Ruminococcus gnavus group id.14376)' on 'Ulcerative colitis (strict) with PSC'

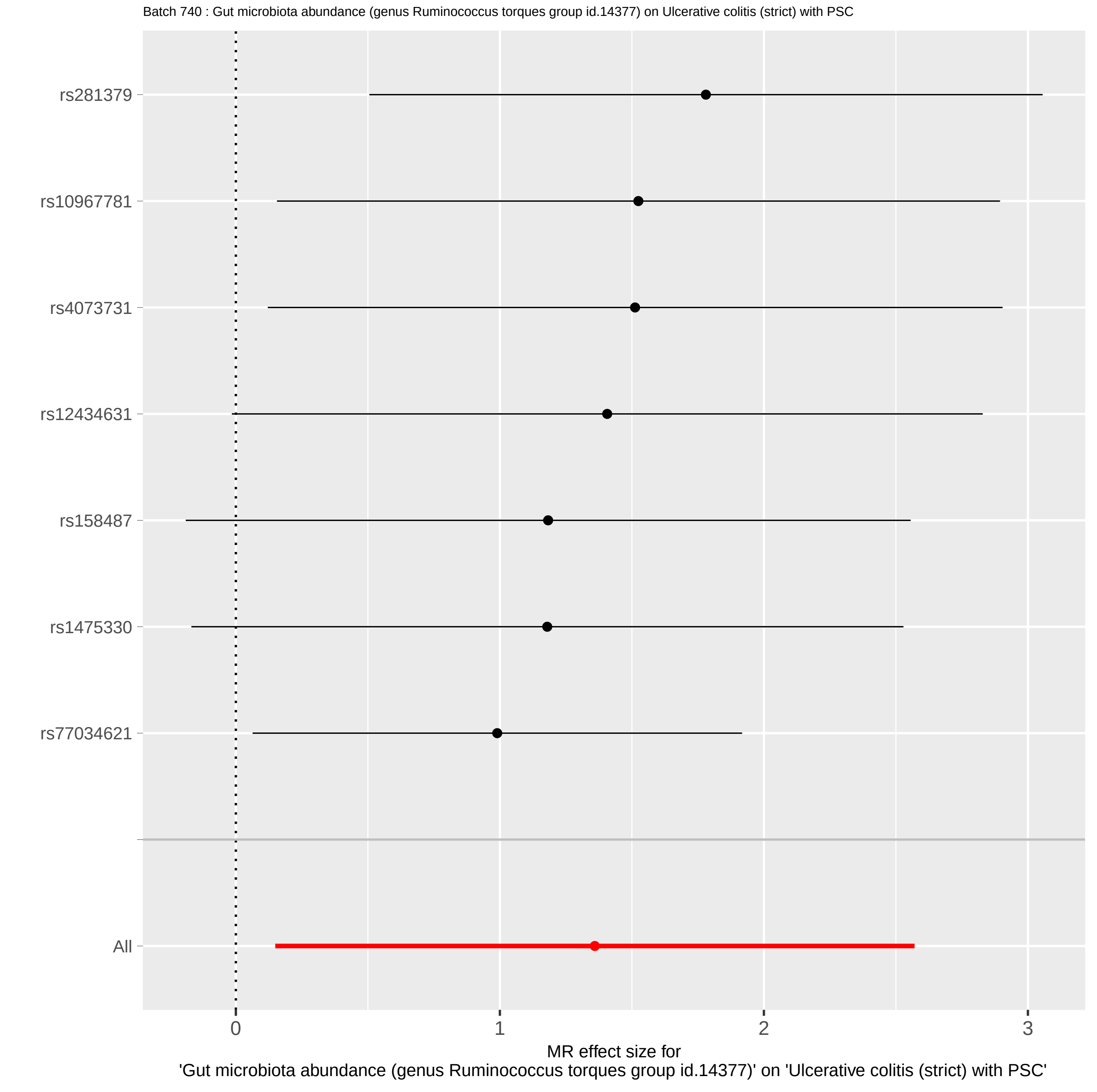

Batch 740 : Gut microbiota abundance (genus Ruminococcus torques group id.14377) on Ulcerative colitis (strict) with PSC
rs281379
rs10967781
rs4073731
rs12434631
rs158487
rs1475330
rs77034621
All
0
1
2
3
MR effect size for
'Gut microbiota abundance (genus Ruminococcus torques group id.14377)' on 'Ulcerative colitis (strict) with PSC'

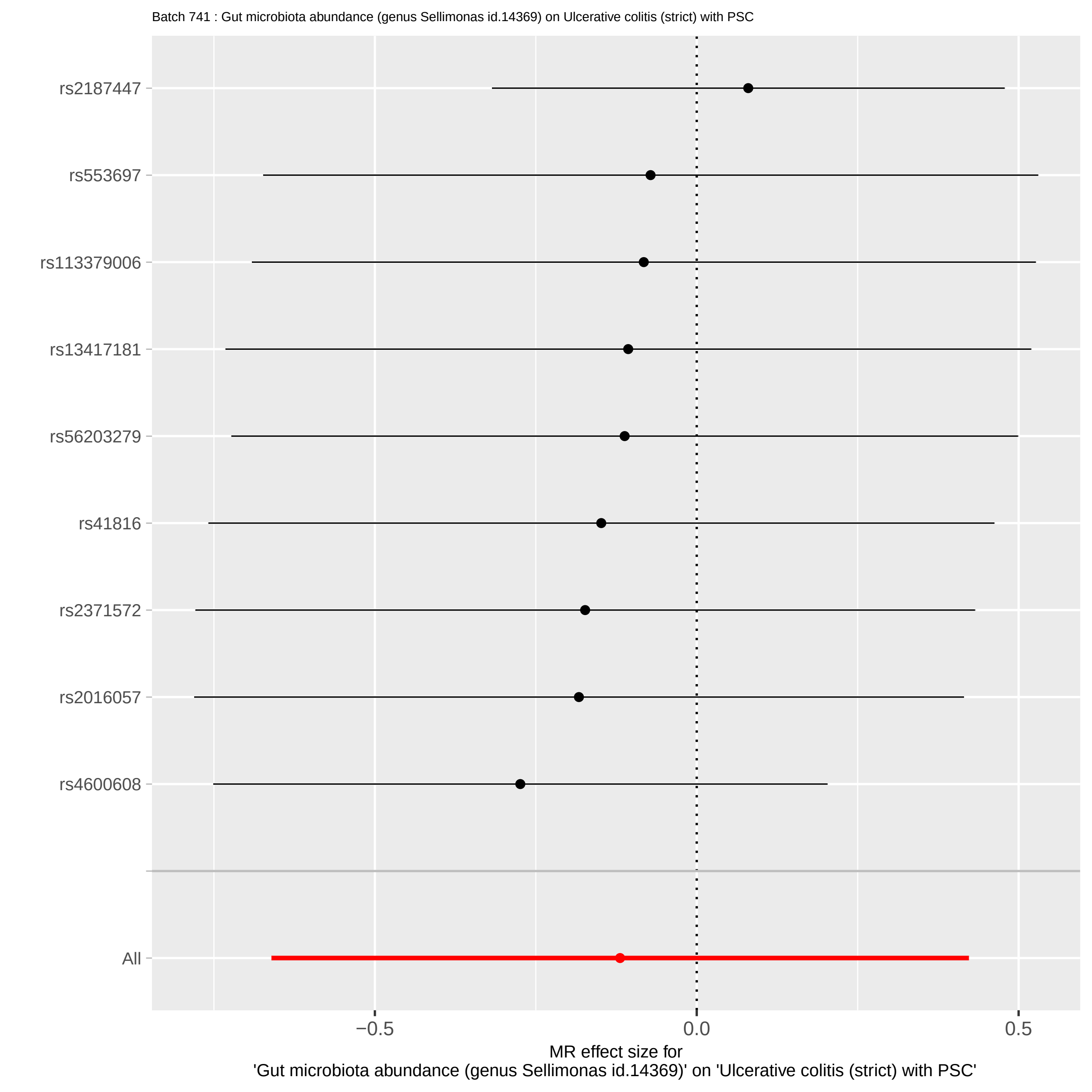

Batch 741 : Gut microbiota abundance (genus Sellimonas id.14369) on Ulcerative colitis (strict) with PSC
rs2187447
rs553697
rs113379006
rs13417181
rs56203279
rs41816
rs2371572
rs2016057
rs4600608
All
−0.5
0.0
0.5
MR effect size for
'Gut microbiota abundance (genus Sellimonas id.14369)' on 'Ulcerative colitis (strict) with PSC'

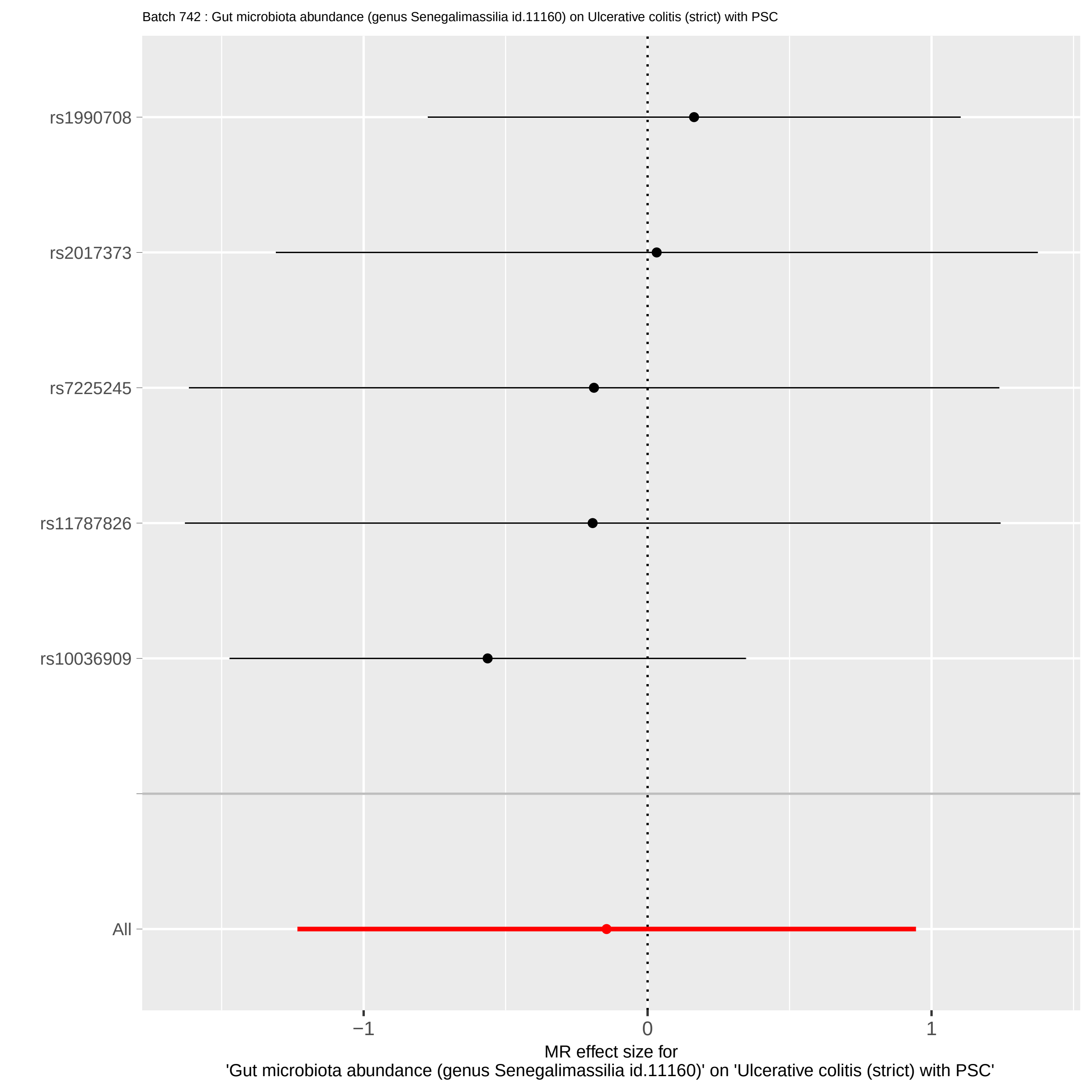

Batch 742 : Gut microbiota abundance (genus Senegalimassilia id.11160) on Ulcerative colitis (strict) with PSC
rs1990708
rs2017373
rs7225245
rs11787826
rs10036909
All
−1
0
1
MR effect size for
'Gut microbiota abundance (genus Senegalimassilia id.11160)' on 'Ulcerative colitis (strict) with PSC'

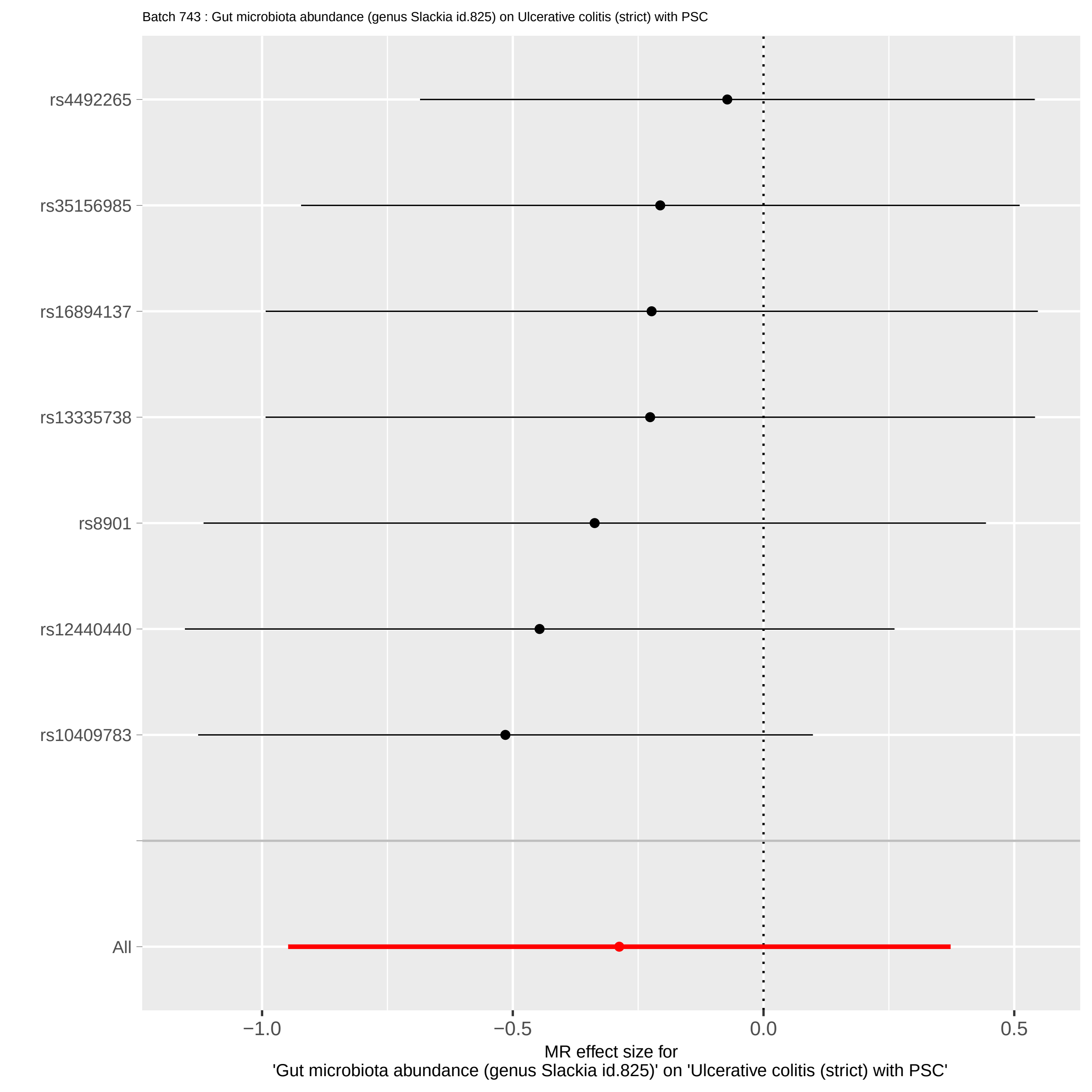
Batch 743 : Gut microbiota abundance (genus Slackia id.825) on Ulcerative colitis (strict) with PSC
rs4492265
rs35156985
rs16894137
rs13335738
rs8901
rs12440440
rs10409783
All
−1.0
−0.5
0.0
0.5
MR effect size for
'Gut microbiota abundance (genus Slackia id.825)' on 'Ulcerative colitis (strict) with PSC'

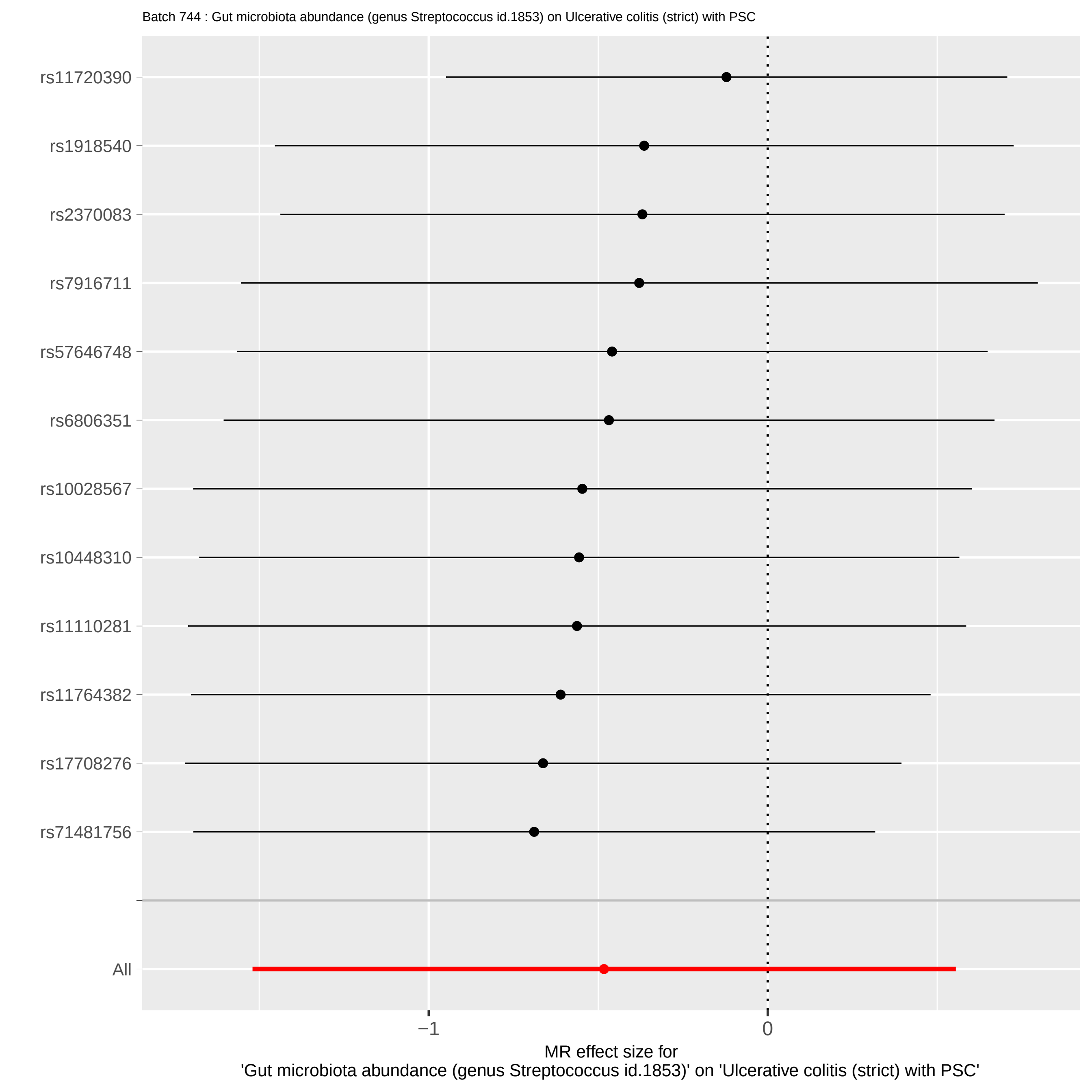

Batch 744 : Gut microbiota abundance (genus Streptococcus id.1853) on Ulcerative colitis (strict) with PSC
rs11720390
rs1918540
rs2370083
rs7916711
rs57646748
rs6806351
rs10028567
rs10448310
rs11110281
rs11764382
rs17708276
rs71481756
All
−1
0
MR effect size for
'Gut microbiota abundance (genus Streptococcus id.1853)' on 'Ulcerative colitis (strict) with PSC'

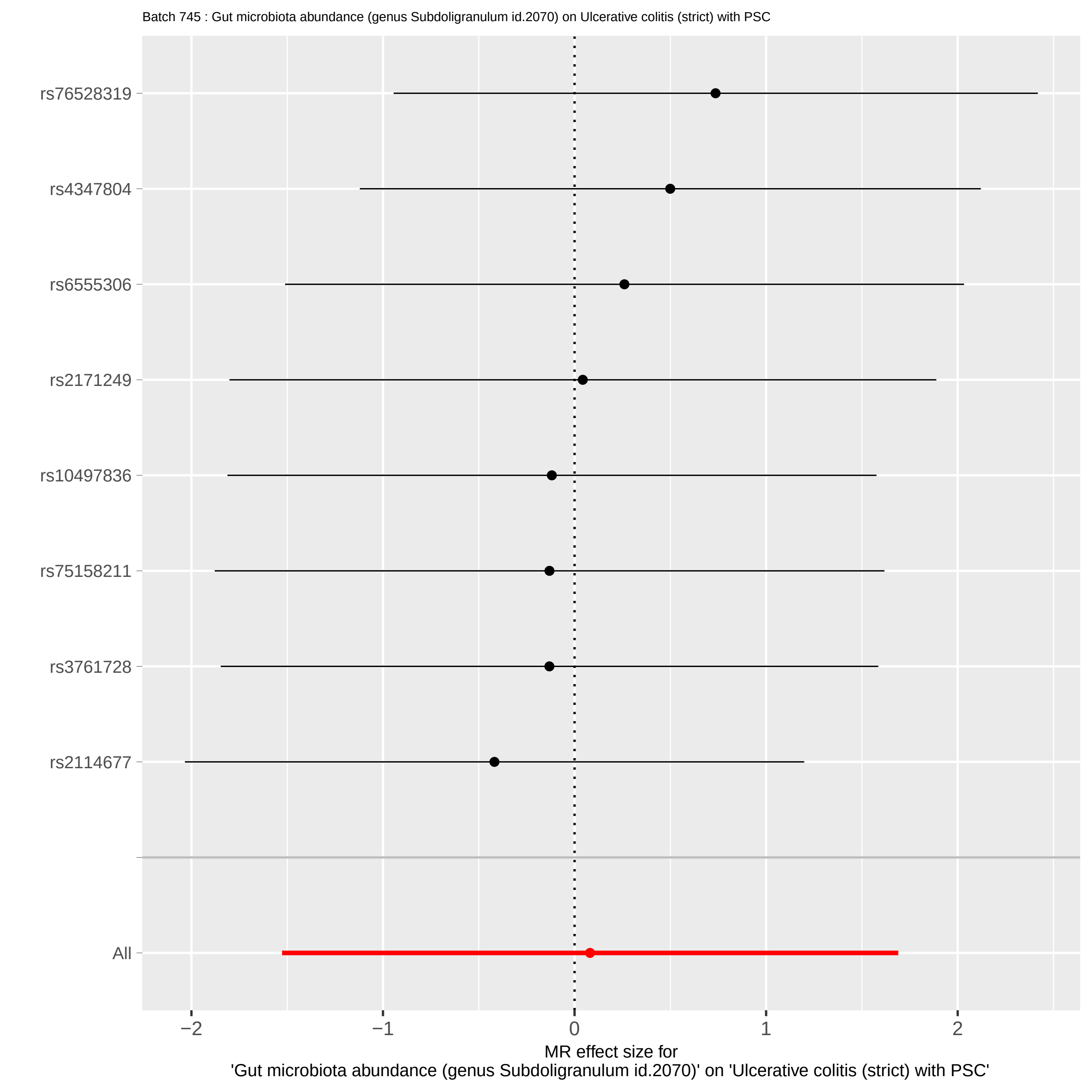
Batch 745 : Gut microbiota abundance (genus Subdoligranulum id.2070) on Ulcerative colitis (strict) with PSC
rs76528319
rs4347804
rs6555306
rs2171249
rs10497836
rs75158211
rs3761728
rs2114677
All
−2
−1
0
1
2
MR effect size for
'Gut microbiota abundance (genus Subdoligranulum id.2070)' on 'Ulcerative colitis (strict) with PSC'

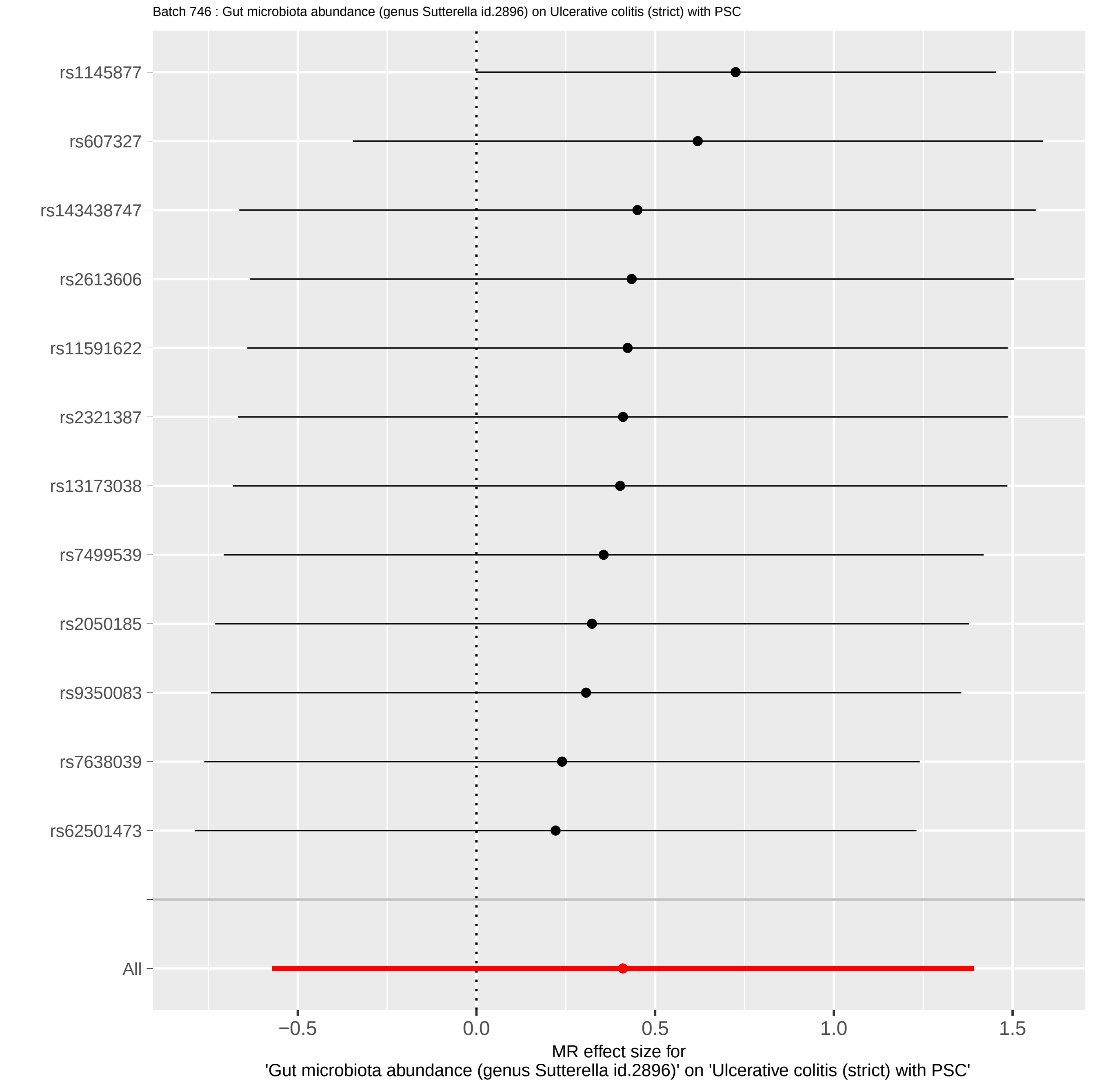

Batch 746 : Gut microbiota abundance (genus Sutterella id.2896) on Ulcerative colitis (strict) with PSC
rs1145877
rs607327
rs143438747
rs2613606
rs11591622
rs2321387
rs13173038
rs7499539
rs2050185
rs9350083
rs7638039
rs62501473
All
−0.5
0.0
0.5
1.0
1.5
MR effect size for
'Gut microbiota abundance (genus Sutterella id.2896)' on 'Ulcerative colitis (strict) with PSC'

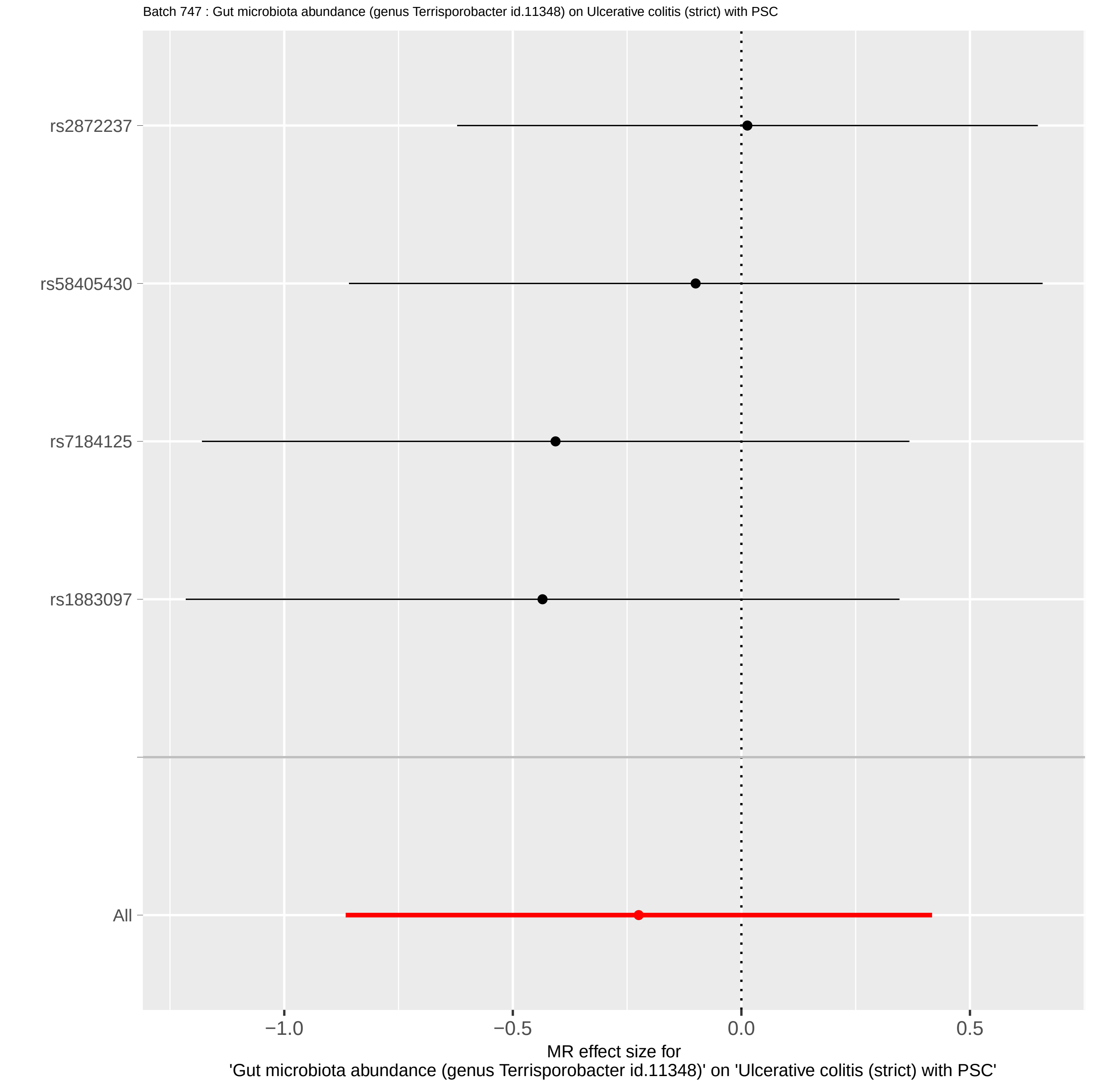

Batch 747 : Gut microbiota abundance (genus Terrisporobacter id.11348) on Ulcerative colitis (strict) with PSC
rs2872237
rs58405430
rs7184125
rs1883097
All
−1.0
−0.5
0.0
0.5
MR effect size for
'Gut microbiota abundance (genus Terrisporobacter id.11348)' on 'Ulcerative colitis (strict) with PSC'

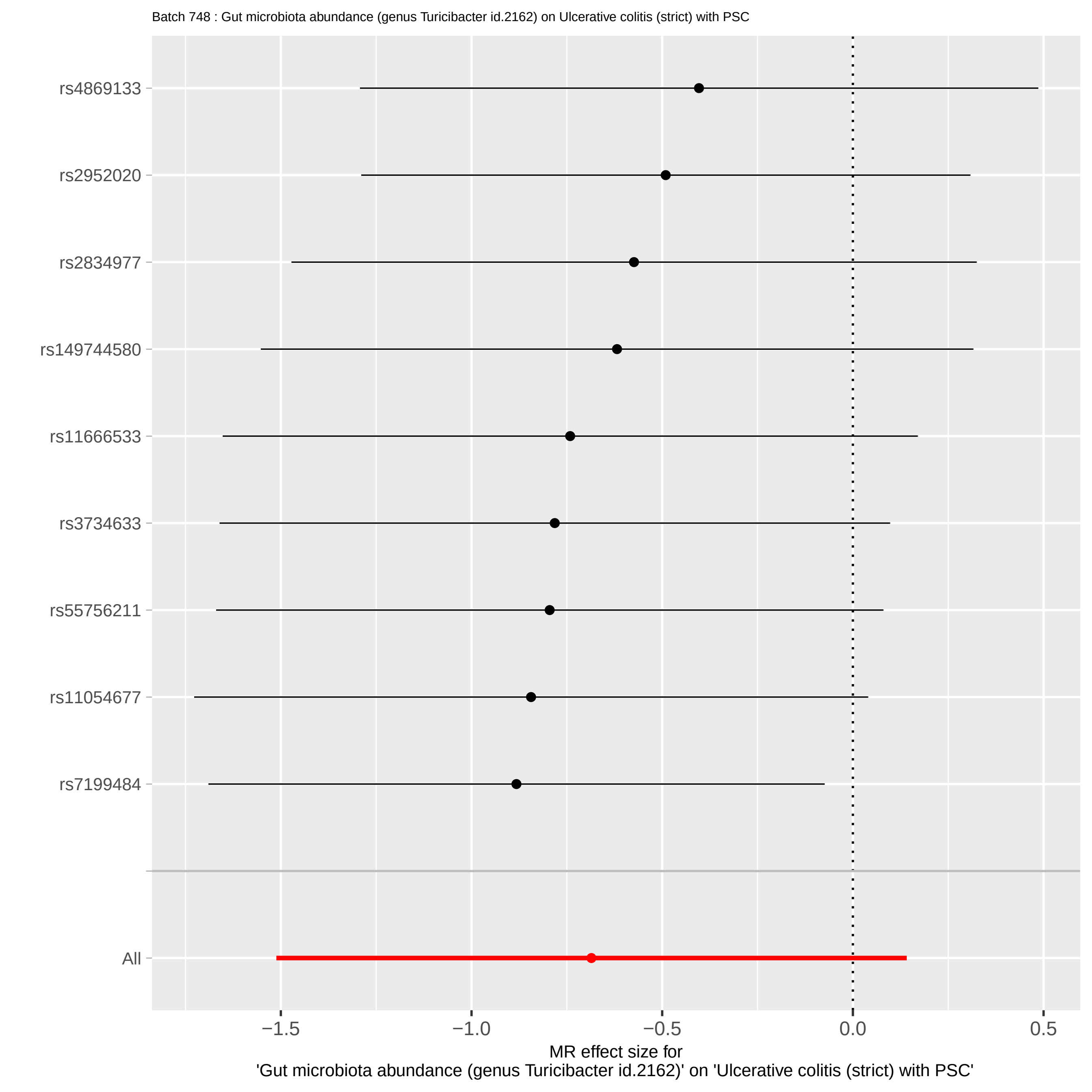

Batch 748 : Gut microbiota abundance (genus Turicibacter id.2162) on Ulcerative colitis (strict) with PSC
rs4869133
rs2952020
rs2834977
rs149744580
rs11666533
rs3734633
rs55756211
rs11054677
rs7199484
All
−1.5
−1.0
−0.5
0.0
0.5
MR effect size for
'Gut microbiota abundance (genus Turicibacter id.2162)' on 'Ulcerative colitis (strict) with PSC'

Batch 749 : Gut microbiota abundance (genus Tyzzerella3 id.11335) on Ulcerative colitis (strict) with PSC

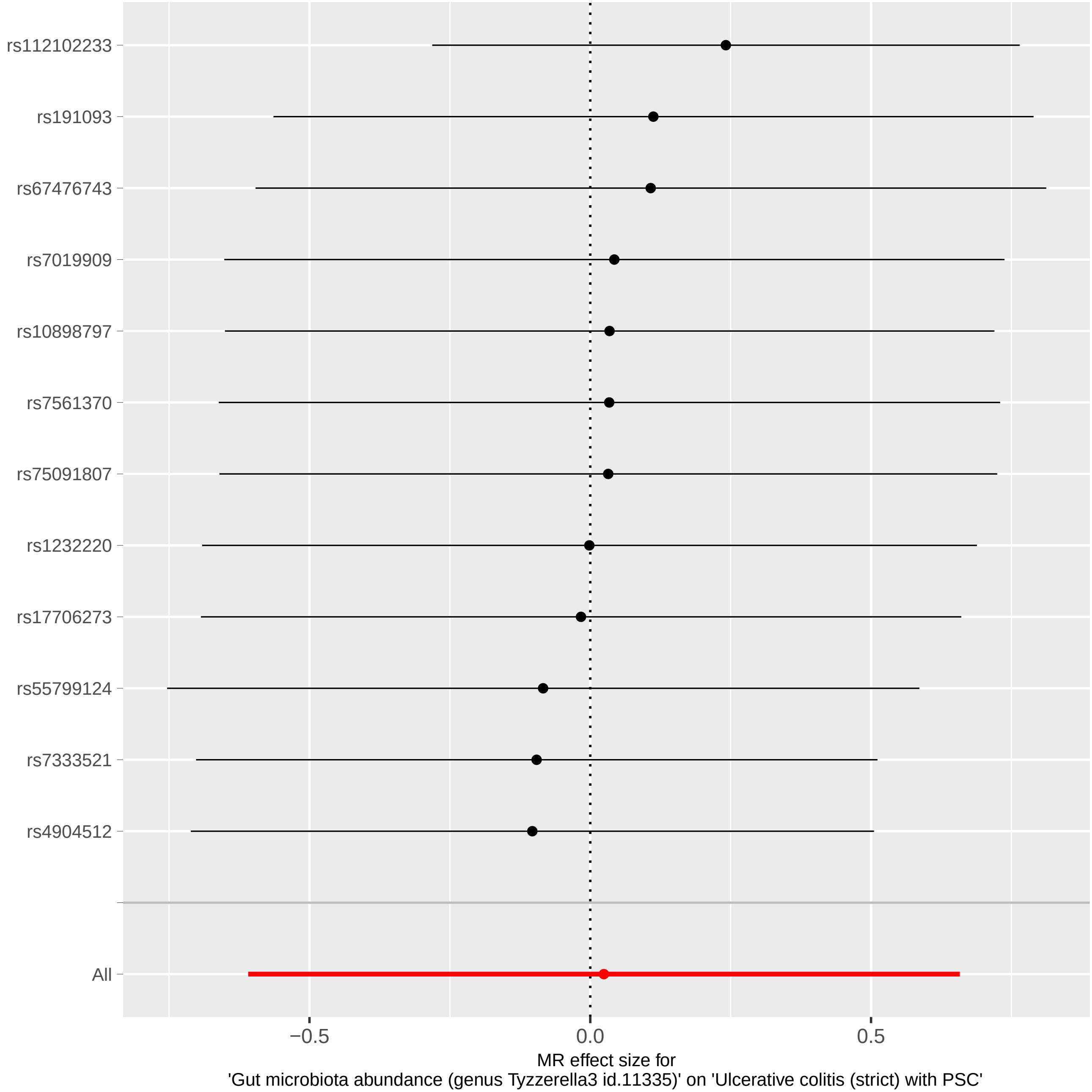

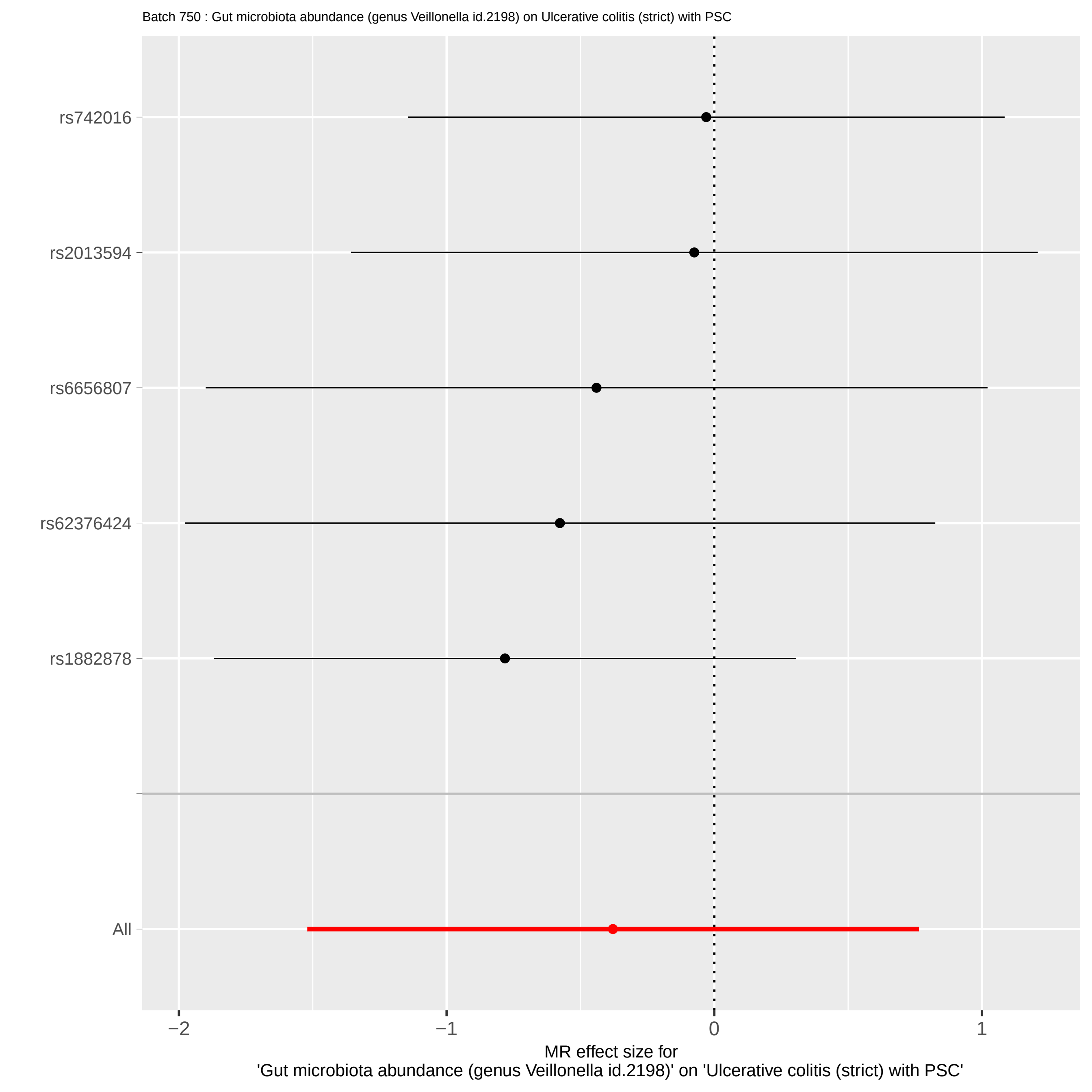
Batch 750 : Gut microbiota abundance (genus Veillonella id.2198) on Ulcerative colitis (strict) with PSC
rs742016
rs2013594
rs6656807
rs62376424
rs1882878
All
−2
−1
0
1
MR effect size for
'Gut microbiota abundance (genus Veillonella id.2198)' on 'Ulcerative colitis (strict) with PSC'

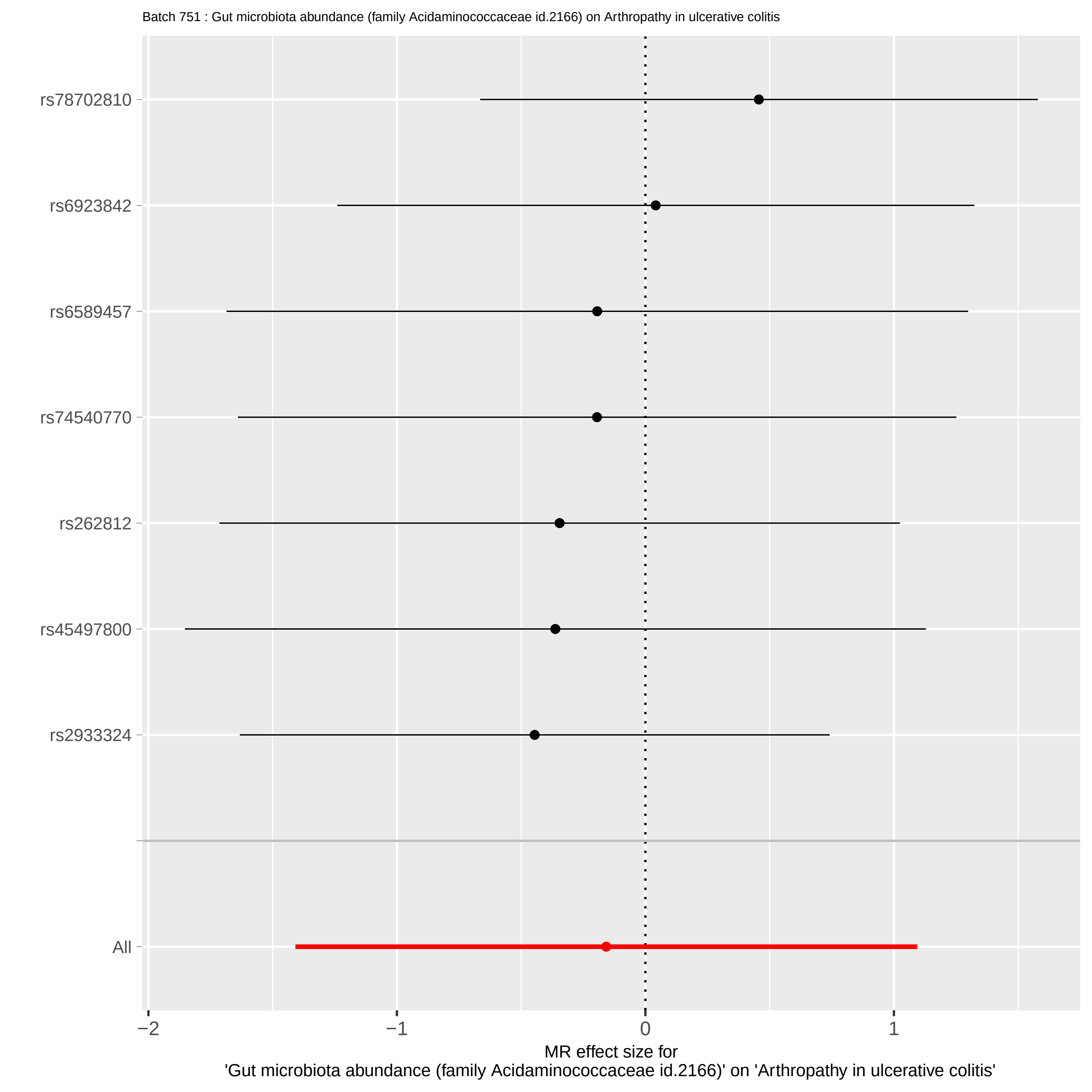
Batch 751 : Gut microbiota abundance (family Acidaminococcaceae id.2166) on Arthropathy in ulcerative colitis
rs78702810
rs6923842
rs6589457
rs74540770
rs262812
rs45497800
rs2933324
All
−2
−1
0
1
MR effect size for
'Gut microbiota abundance (family Acidaminococcaceae id.2166)' on 'Arthropathy in ulcerative colitis'

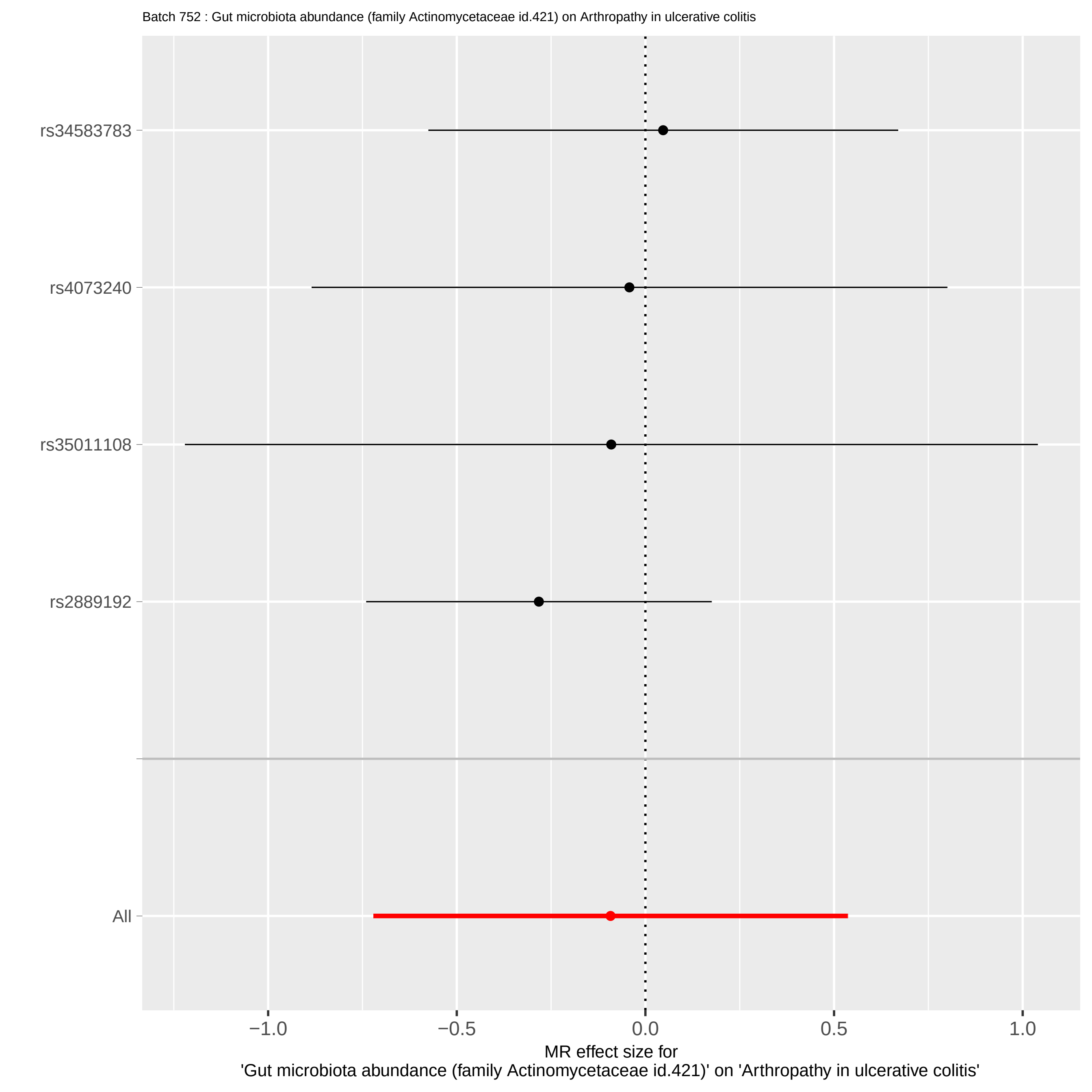
Batch 752 : Gut microbiota abundance (family Actinomycetaceae id.421) on Arthropathy in ulcerative colitis
rs34583783
rs4073240
rs35011108
rs2889192
All
−1.0
−0.5
0.0
0.5
1.0
MR effect size for
'Gut microbiota abundance (family Actinomycetaceae id.421)' on 'Arthropathy in ulcerative colitis'

Batch 753 : Gut microbiota abundance (family Alcaligenaceae id.2875) on Arthropathy in ulcerative colitis

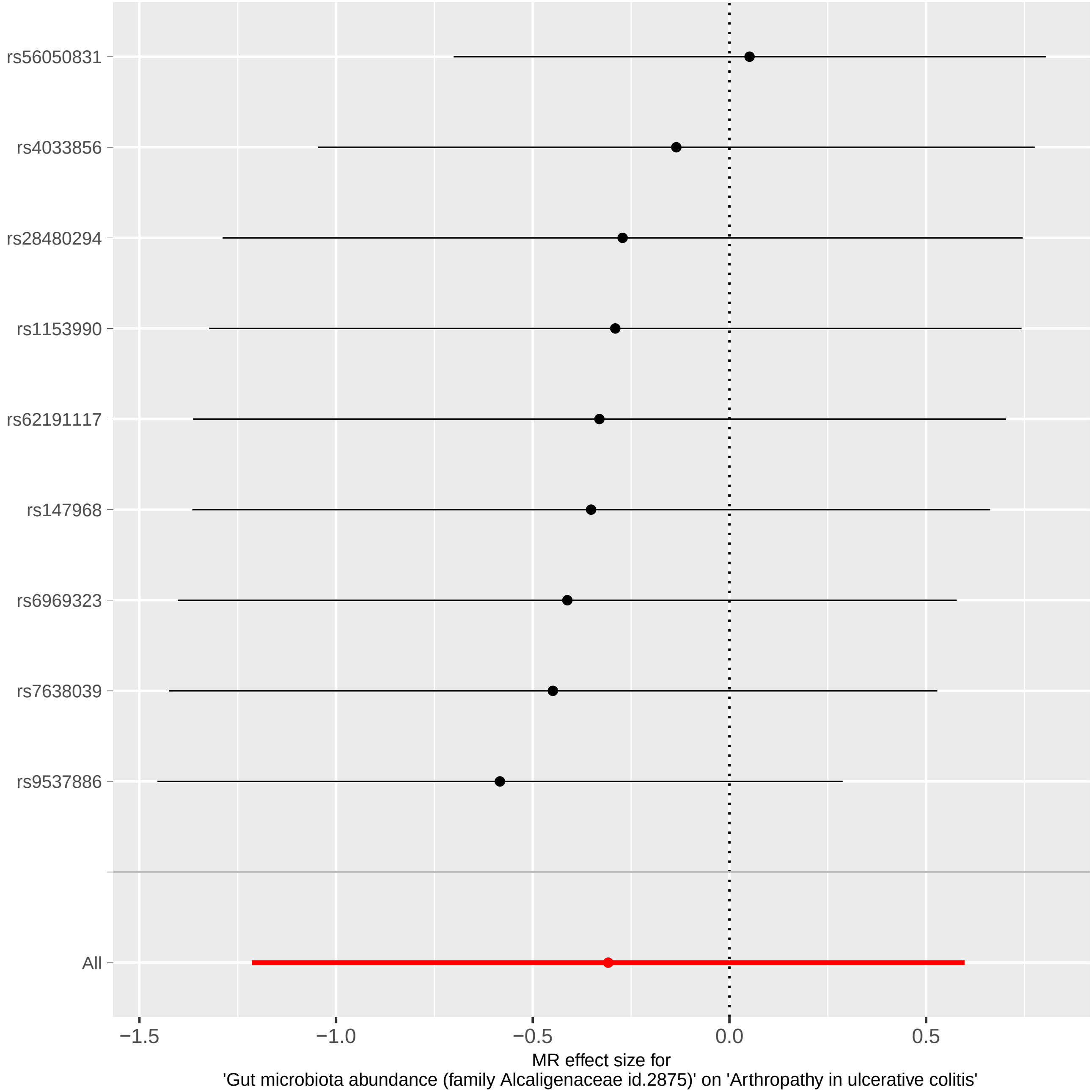

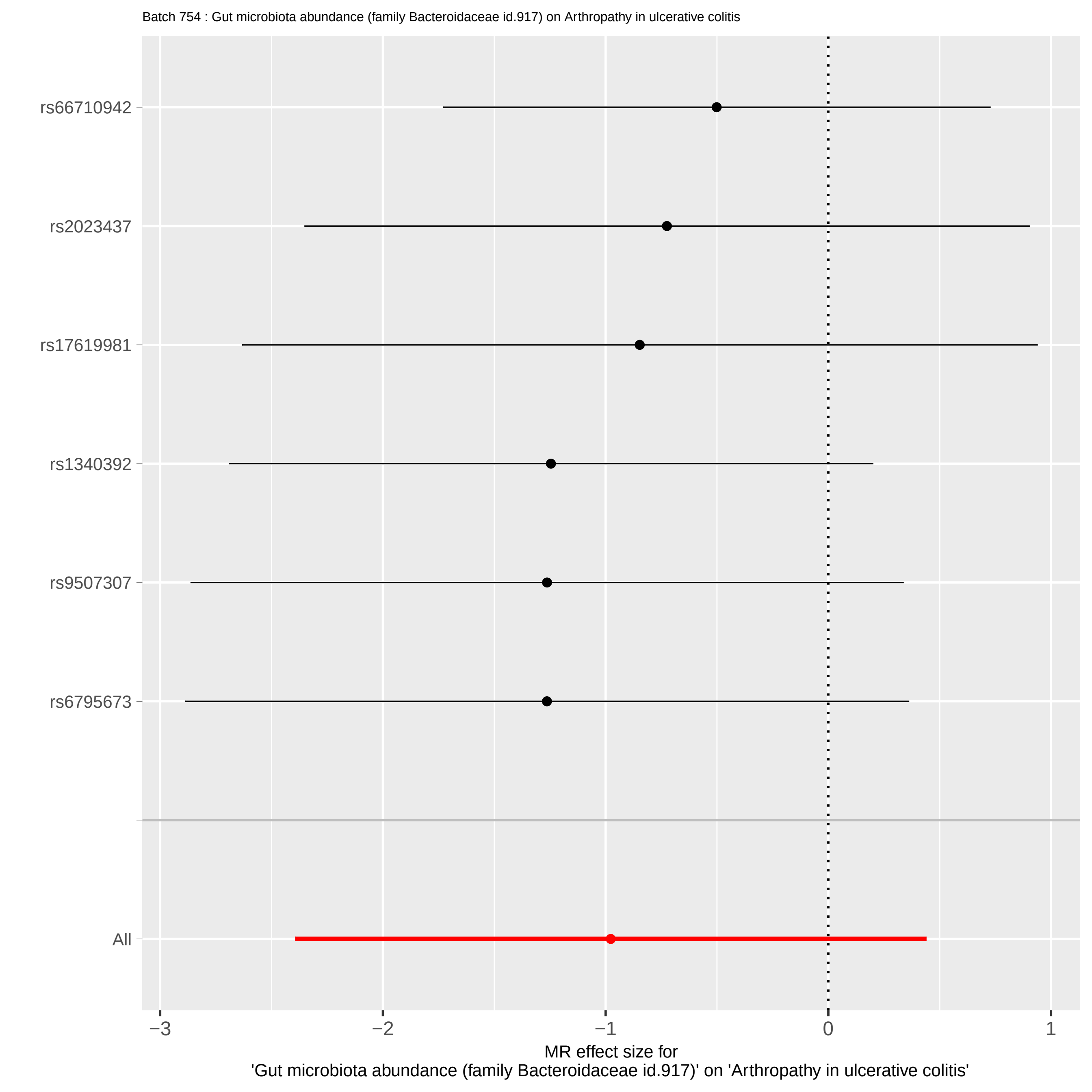
Batch 754 : Gut microbiota abundance (family Bacteroidaceae id.917) on Arthropathy in ulcerative colitis
rs66710942
rs2023437
rs17619981
rs1340392
rs9507307
rs6795673
All
−3
−2
−1
0
1
MR effect size for
'Gut microbiota abundance (family Bacteroidaceae id.917)' on 'Arthropathy in ulcerative colitis'

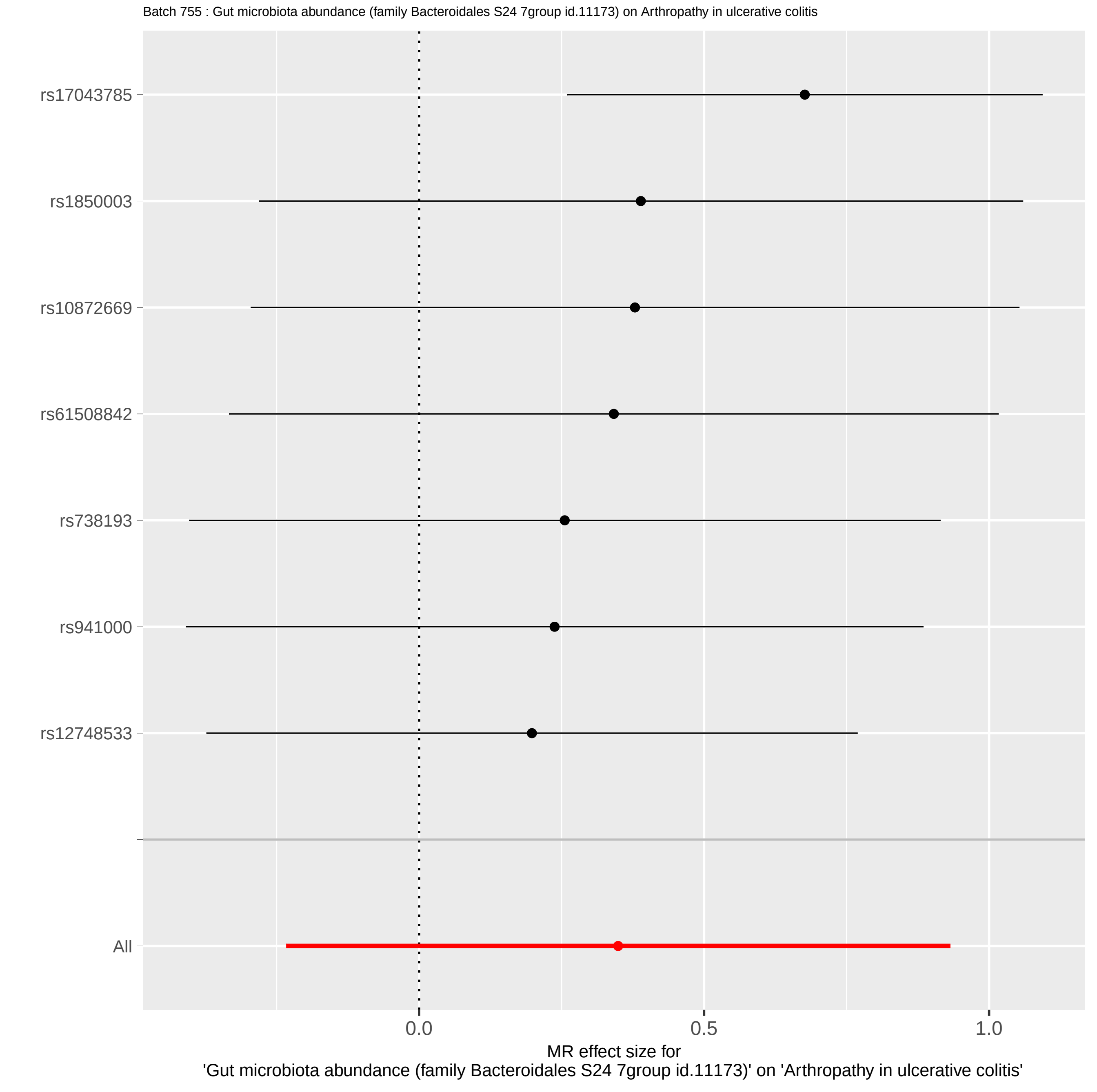

Batch 755 : Gut microbiota abundance (family Bacteroidales S24 7group id.11173) on Arthropathy in ulcerative colitis
rs17043785
rs1850003
rs10872669
rs61508842
rs738193
rs941000
rs12748533
All
0.0
0.5
1.0
MR effect size for
'Gut microbiota abundance (family Bacteroidales S24 7group id.11173)' on 'Arthropathy in ulcerative colitis'

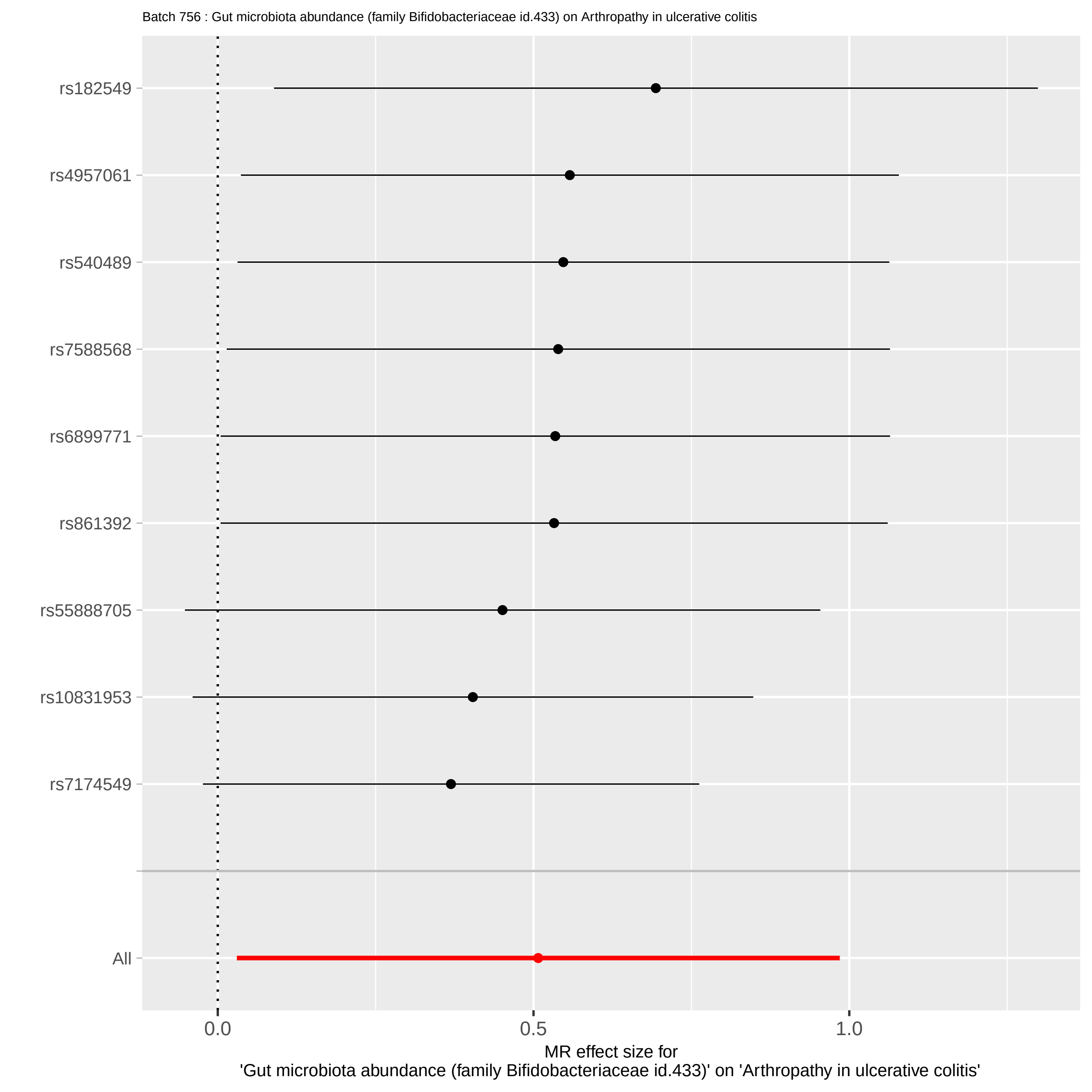
Batch 756 : Gut microbiota abundance (family Bifidobacteriaceae id.433) on Arthropathy in ulcerative colitis
rs182549
rs4957061
rs540489
rs7588568
rs6899771
rs861392
rs55888705
rs10831953
rs7174549
All
0.0
0.5
1.0
MR effect size for
'Gut microbiota abundance (family Bifidobacteriaceae id.433)' on 'Arthropathy in ulcerative colitis'

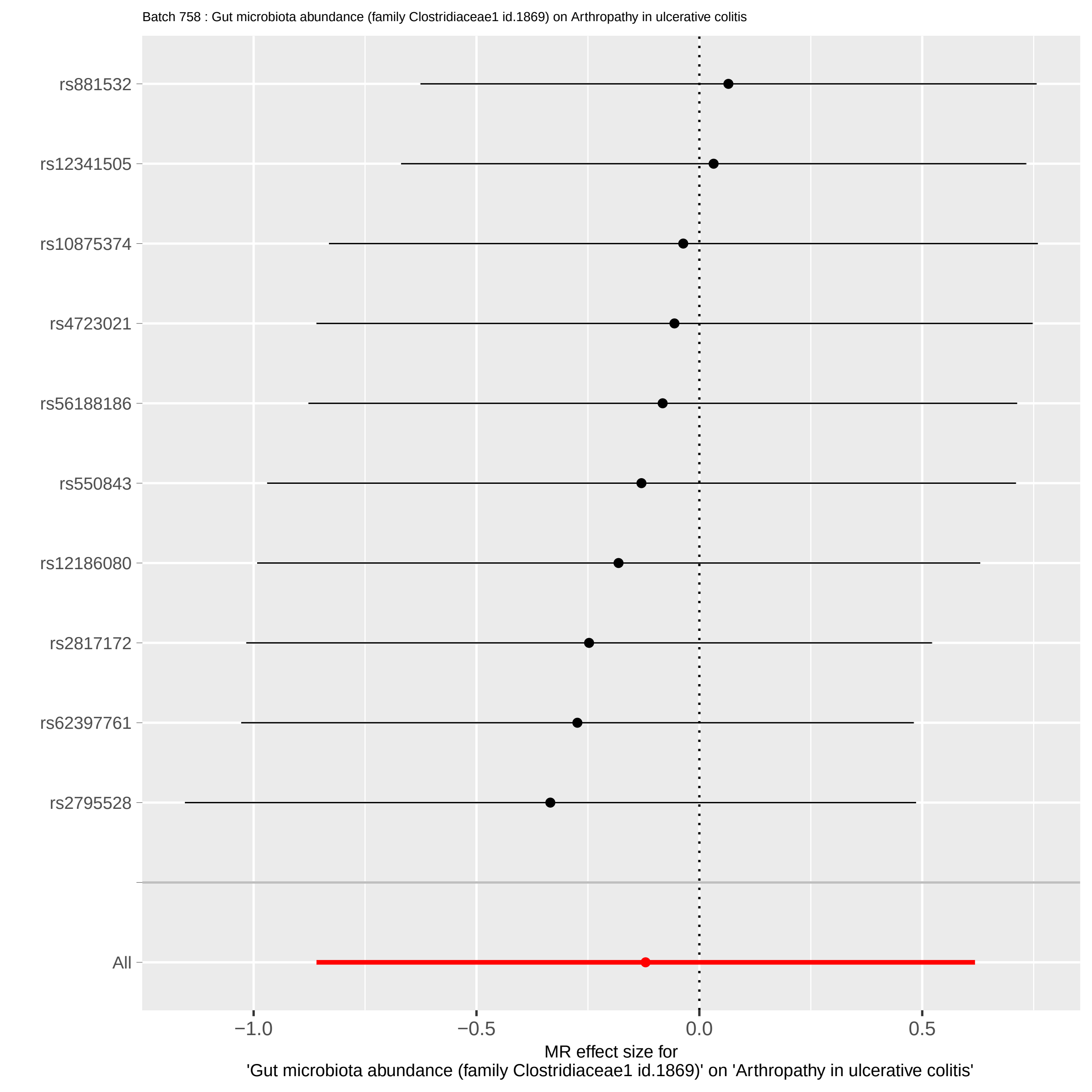

Batch 758 : Gut microbiota abundance (family Clostridiaceae1 id.1869) on Arthropathy in ulcerative colitis
rs881532
rs12341505
rs10875374
rs4723021
rs56188186
rs550843
rs12186080
rs2817172
rs62397761
rs2795528
All
−1.0
−0.5
0.0
0.5
MR effect size for
'Gut microbiota abundance (family Clostridiaceae1 id.1869)' on 'Arthropathy in ulcerative colitis'

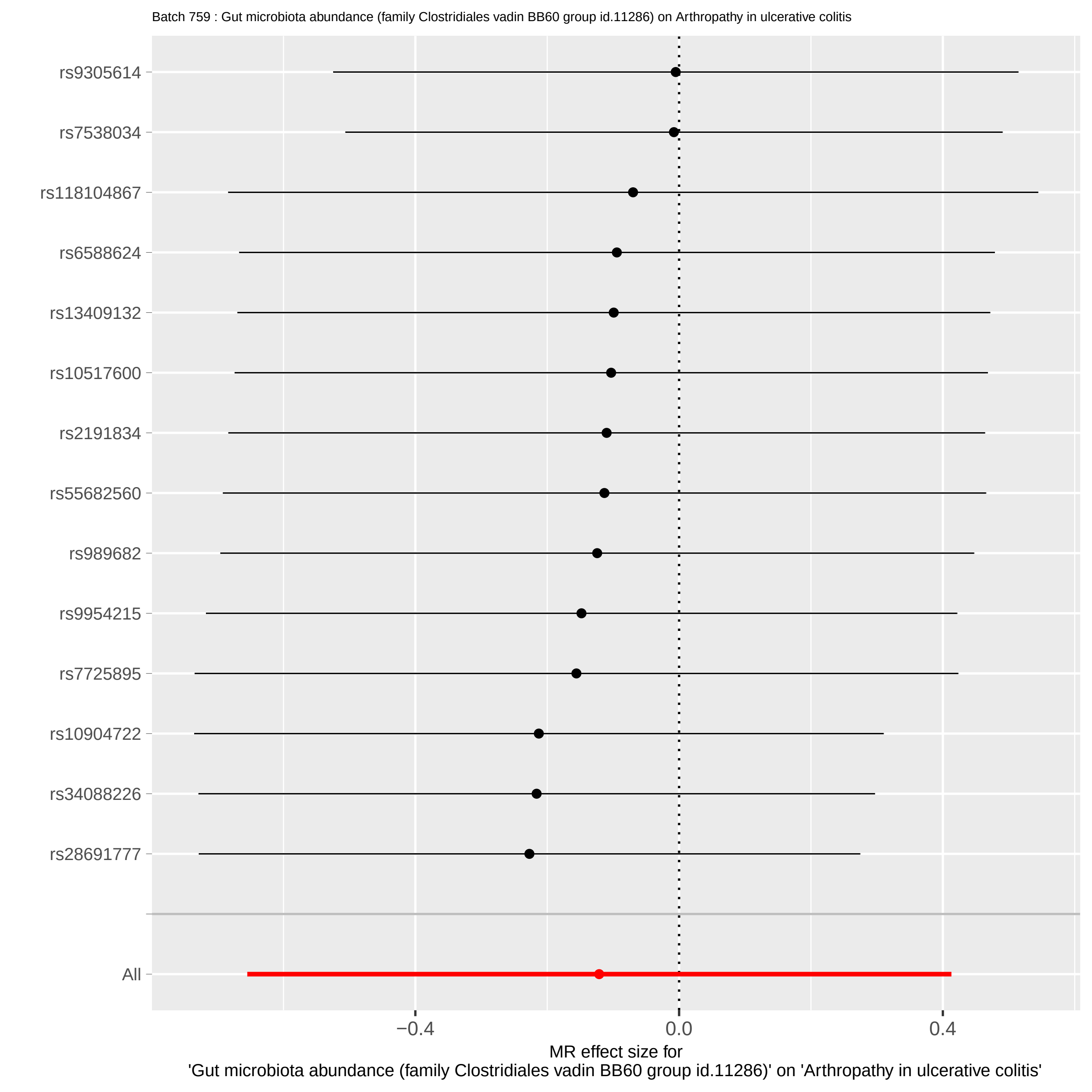

Batch 759 : Gut microbiota abundance (family Clostridiales vadin BB60 group id.11286) on Arthropathy in ulcerative colitis
rs9305614
rs7538034
rs118104867
rs6588624
rs13409132
rs10517600
rs2191834
rs55682560
rs989682
rs9954215
rs7725895
rs10904722
rs34088226
rs28691777
All
−0.4
0.0
0.4
MR effect size for
'Gut microbiota abundance (family Clostridiales vadin BB60 group id.11286)' on 'Arthropathy in ulcerative colitis'

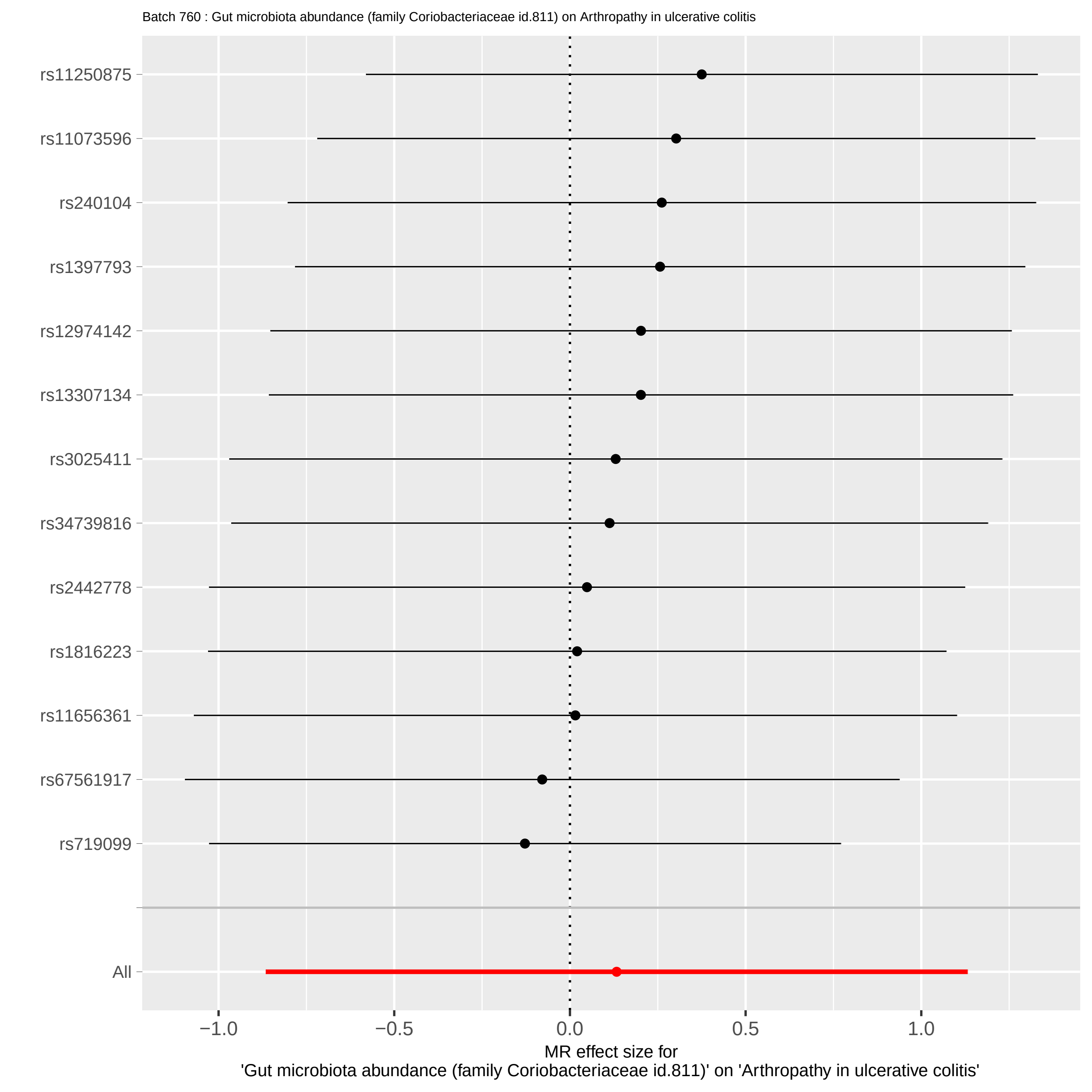
Batch 760 : Gut microbiota abundance (family Coriobacteriaceae id.811) on Arthropathy in ulcerative colitis
rs11250875
rs11073596
rs240104
rs1397793
rs12974142
rs13307134
rs3025411
rs34739816
rs2442778
rs1816223
rs11656361
rs67561917
rs719099
All
−1.0
−0.5
0.0
0.5
1.0
MR effect size for
'Gut microbiota abundance (family Coriobacteriaceae id.811)' on 'Arthropathy in ulcerative colitis'

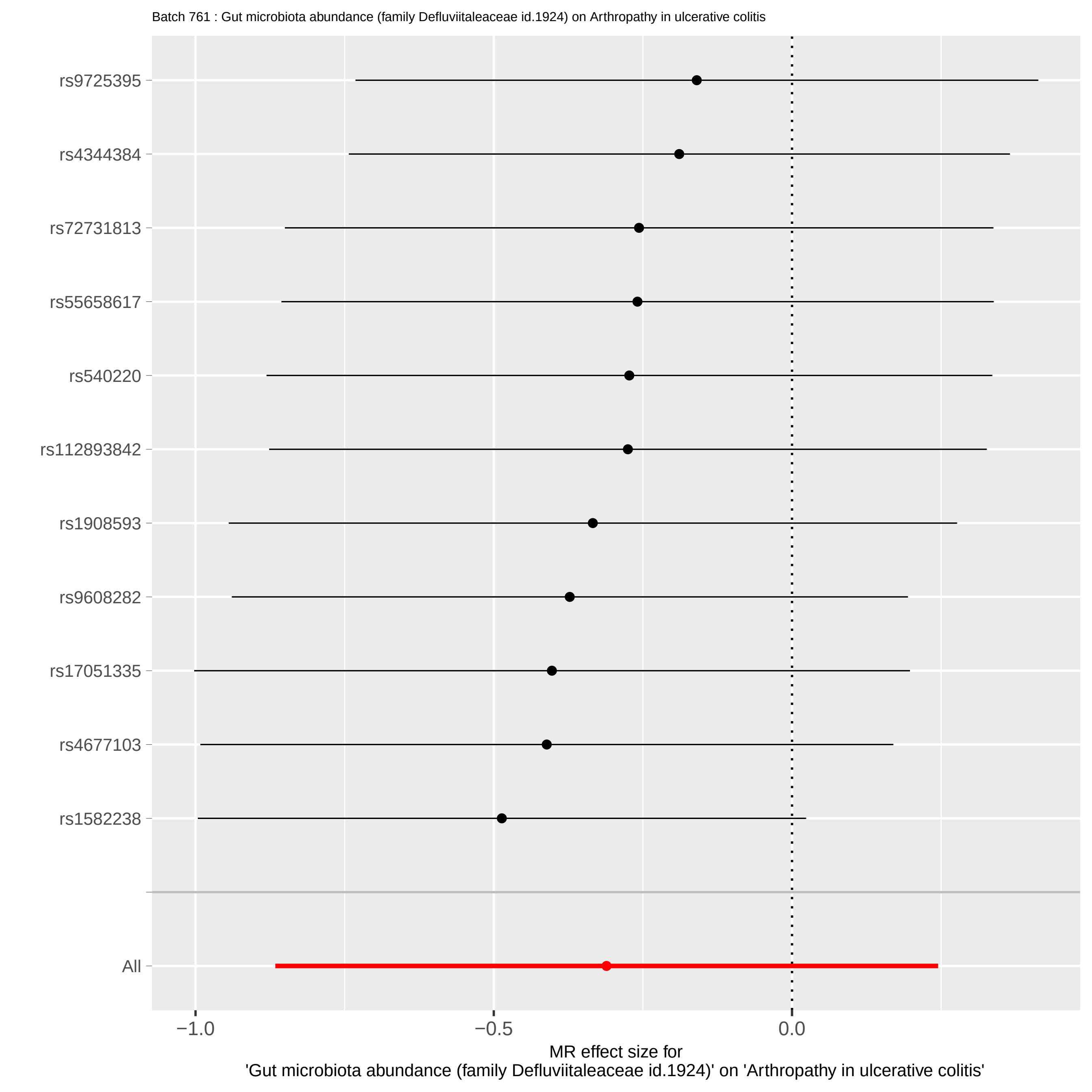
Batch 761 : Gut microbiota abundance (family Defluviitaleaceae id.1924) on Arthropathy in ulcerative colitis
rs9725395
rs4344384
rs72731813
rs55658617
rs540220
rs112893842
rs1908593
rs9608282
rs17051335
rs4677103
rs1582238
All
−1.0
−0.5
0.0
MR effect size for
'Gut microbiota abundance (family Defluviitaleaceae id.1924)' on 'Arthropathy in ulcerative colitis'

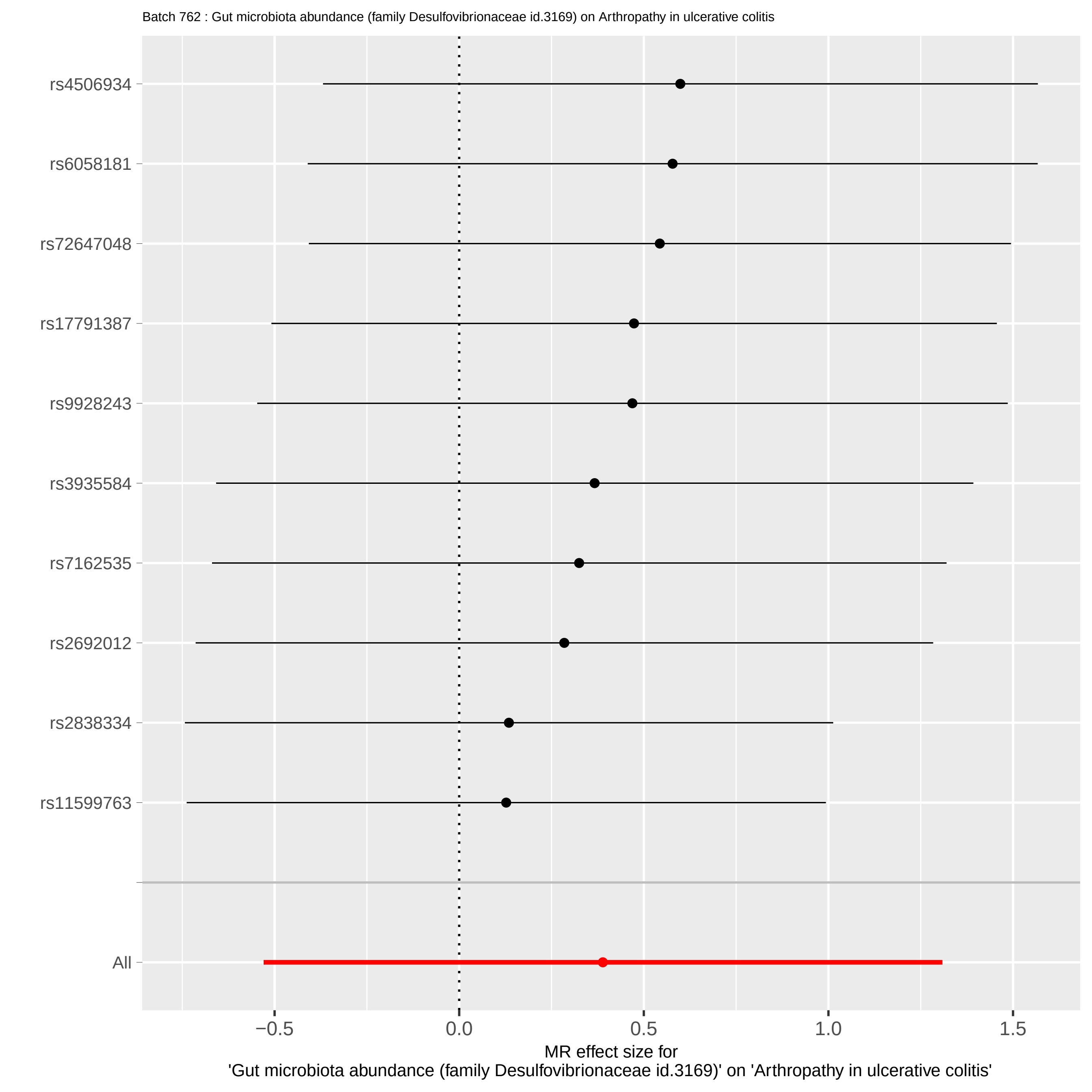

Batch 762 : Gut microbiota abundance (family Desulfovibrionaceae id.3169) on Arthropathy in ulcerative colitis
rs4506934
rs6058181
rs72647048
rs17791387
rs9928243
rs3935584
rs7162535
rs2692012
rs2838334
rs11599763
All
−0.5
0.0
0.5
1.0
1.5
MR effect size for
'Gut microbiota abundance (family Desulfovibrionaceae id.3169)' on 'Arthropathy in ulcerative colitis'

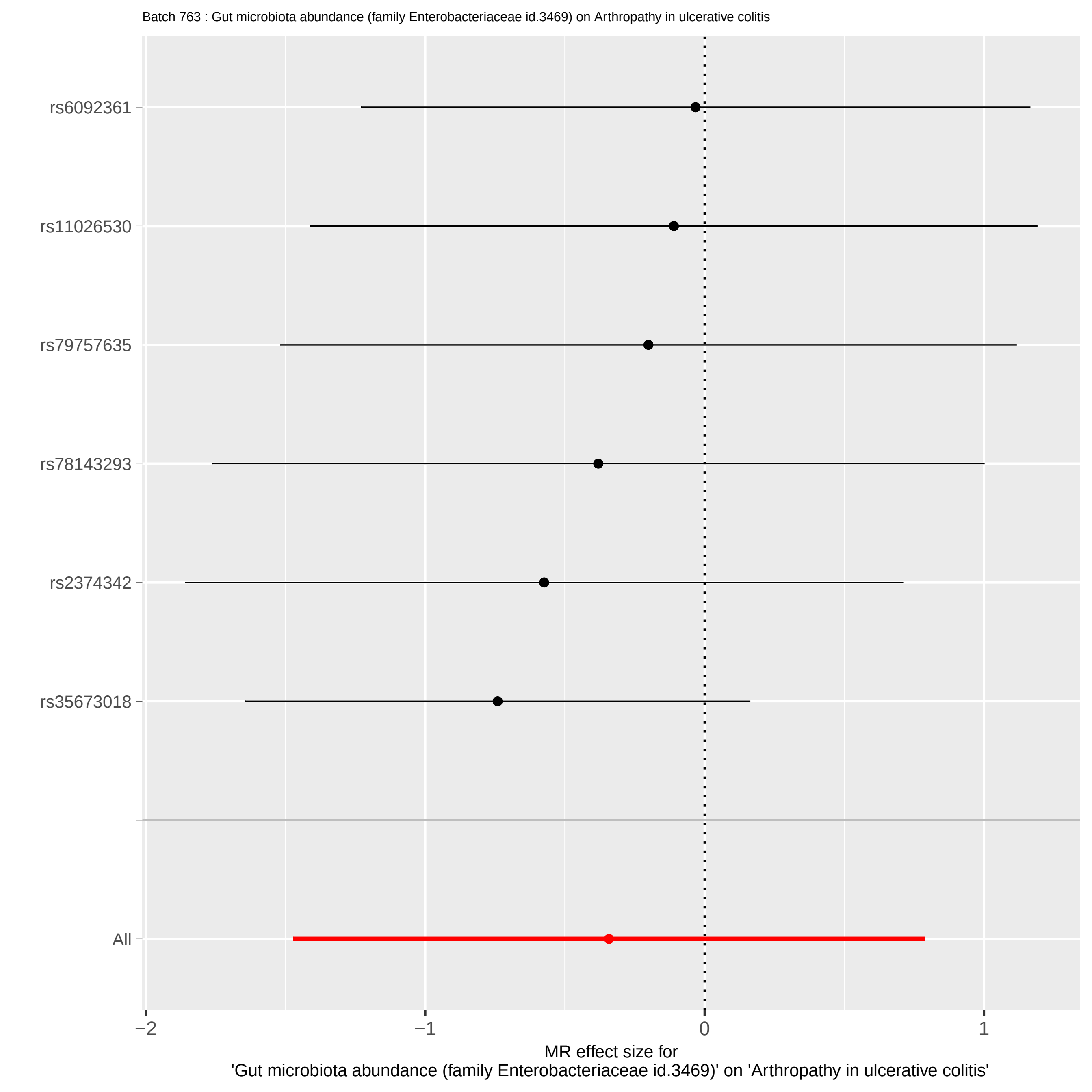
Batch 763 : Gut microbiota abundance (family Enterobacteriaceae id.3469) on Arthropathy in ulcerative colitis
rs6092361
rs11026530
rs79757635
rs78143293
rs2374342
rs35673018
All
−2
−1
0
1
MR effect size for
'Gut microbiota abundance (family Enterobacteriaceae id.3469)' on 'Arthropathy in ulcerative colitis'

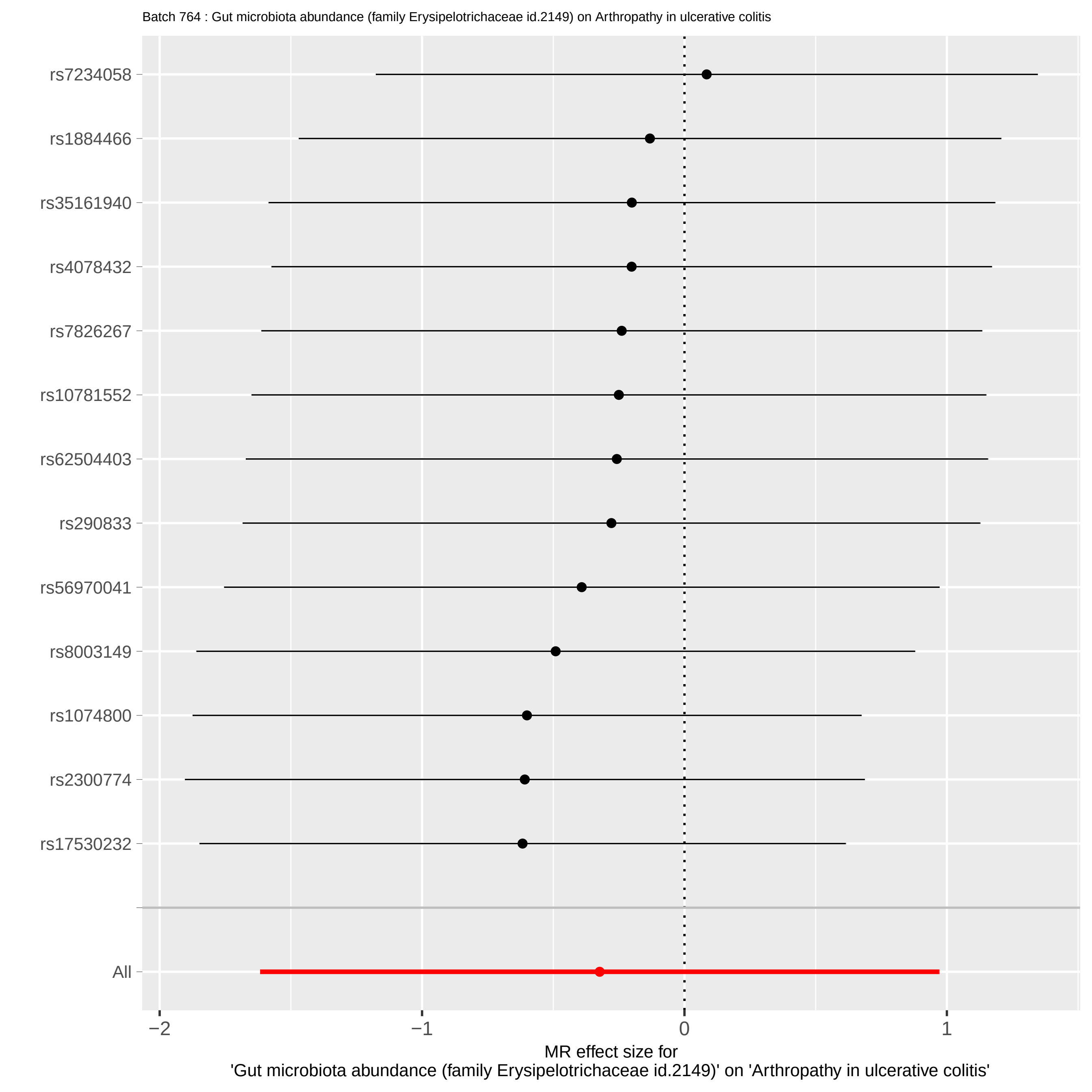

Batch 764 : Gut microbiota abundance (family Erysipelotrichaceae id.2149) on Arthropathy in ulcerative colitis
rs7234058
rs1884466
rs35161940
rs4078432
rs7826267
rs10781552
rs62504403
rs290833
rs56970041
rs8003149
rs1074800
rs2300774
rs17530232
All
−2
−1
0
1
MR effect size for
'Gut microbiota abundance (family Erysipelotrichaceae id.2149)' on 'Arthropathy in ulcerative colitis'

Batch 765 : Gut microbiota abundance (family Family XI id.1936) on Arthropathy in ulcerative colitis

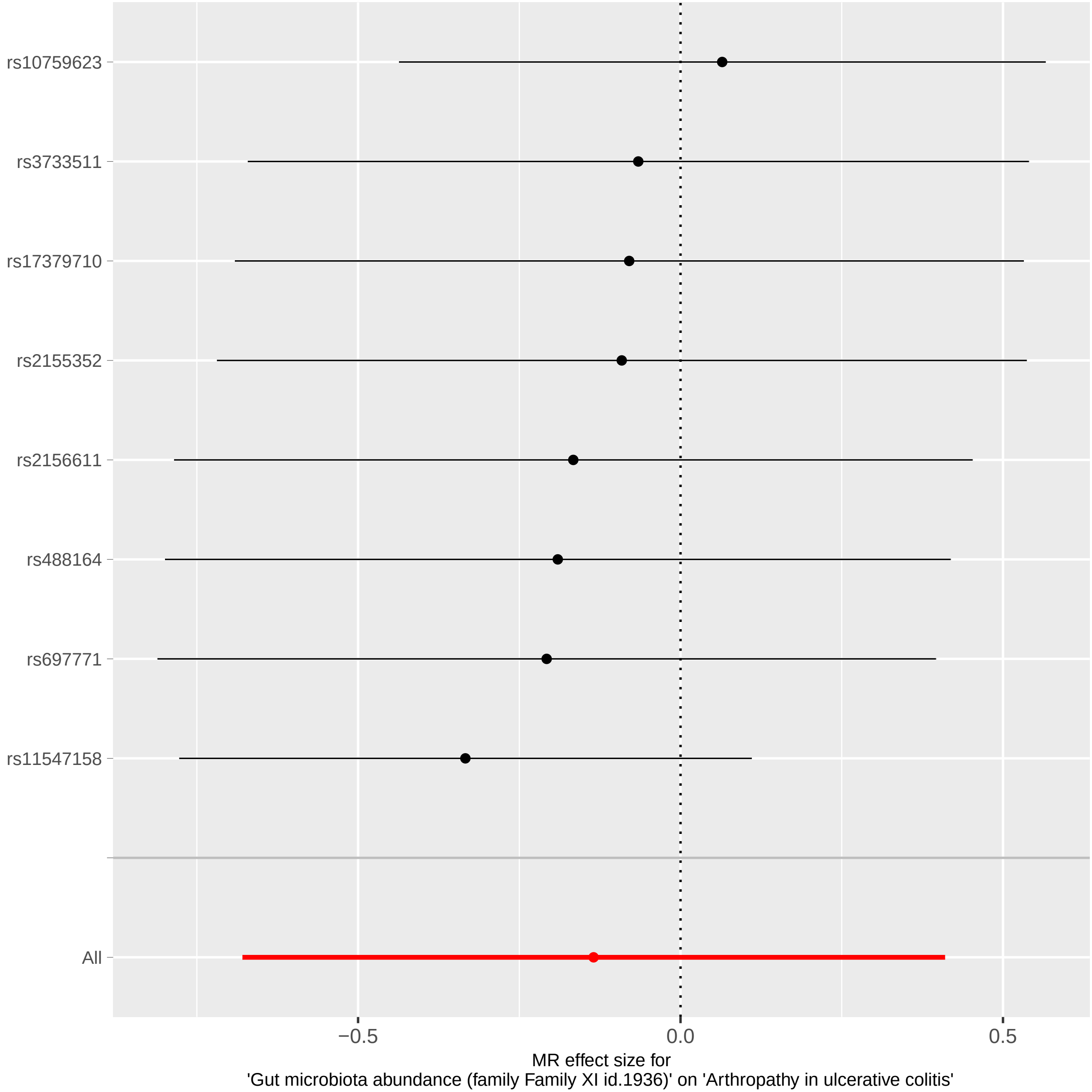

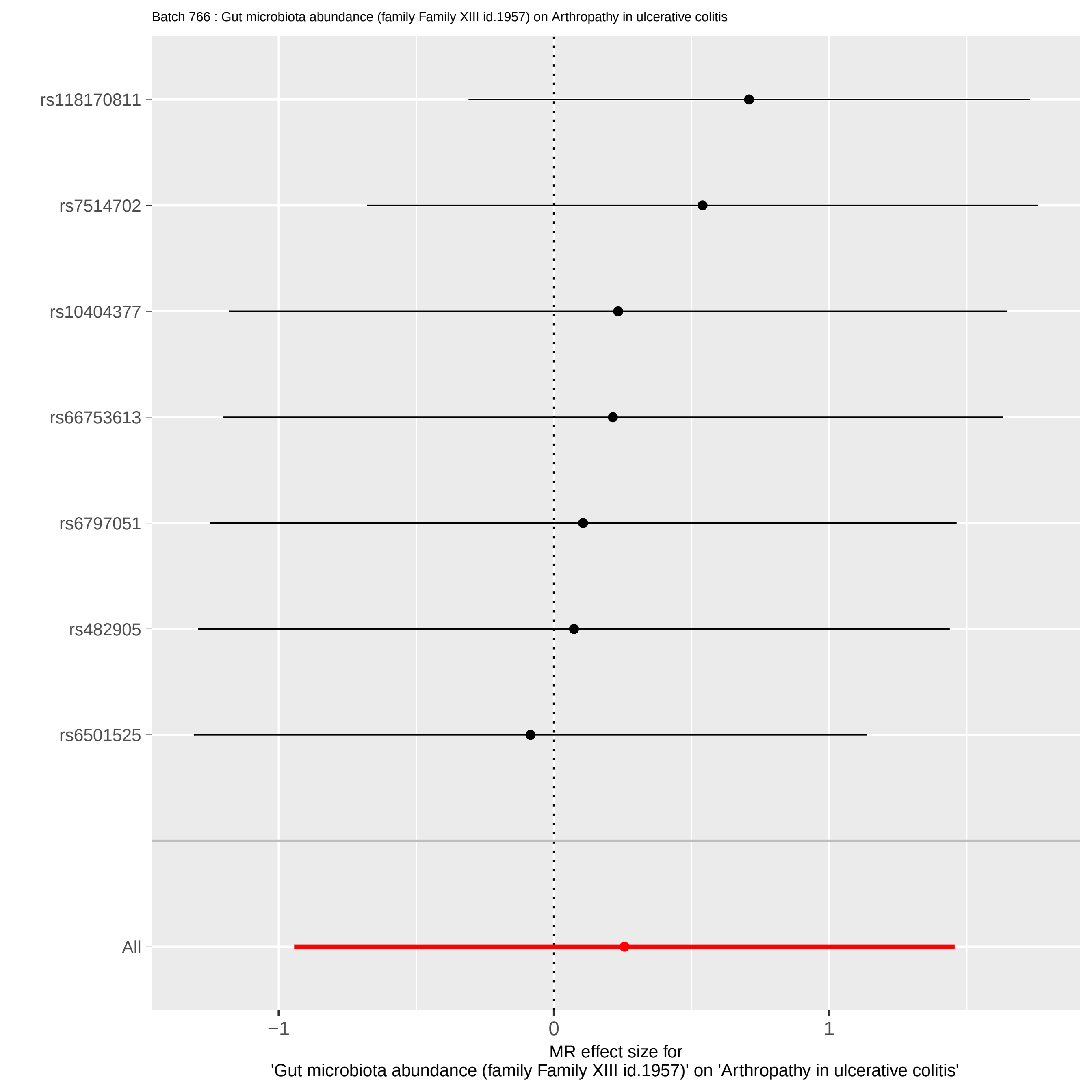
Batch 766 : Gut microbiota abundance (family Family XIII id.1957) on Arthropathy in ulcerative colitis
rs118170811
rs7514702
rs10404377
rs66753613
rs6797051
rs482905
rs6501525
All
−1
0
1
MR effect size for
'Gut microbiota abundance (family Family XIII id.1957)' on 'Arthropathy in ulcerative colitis'

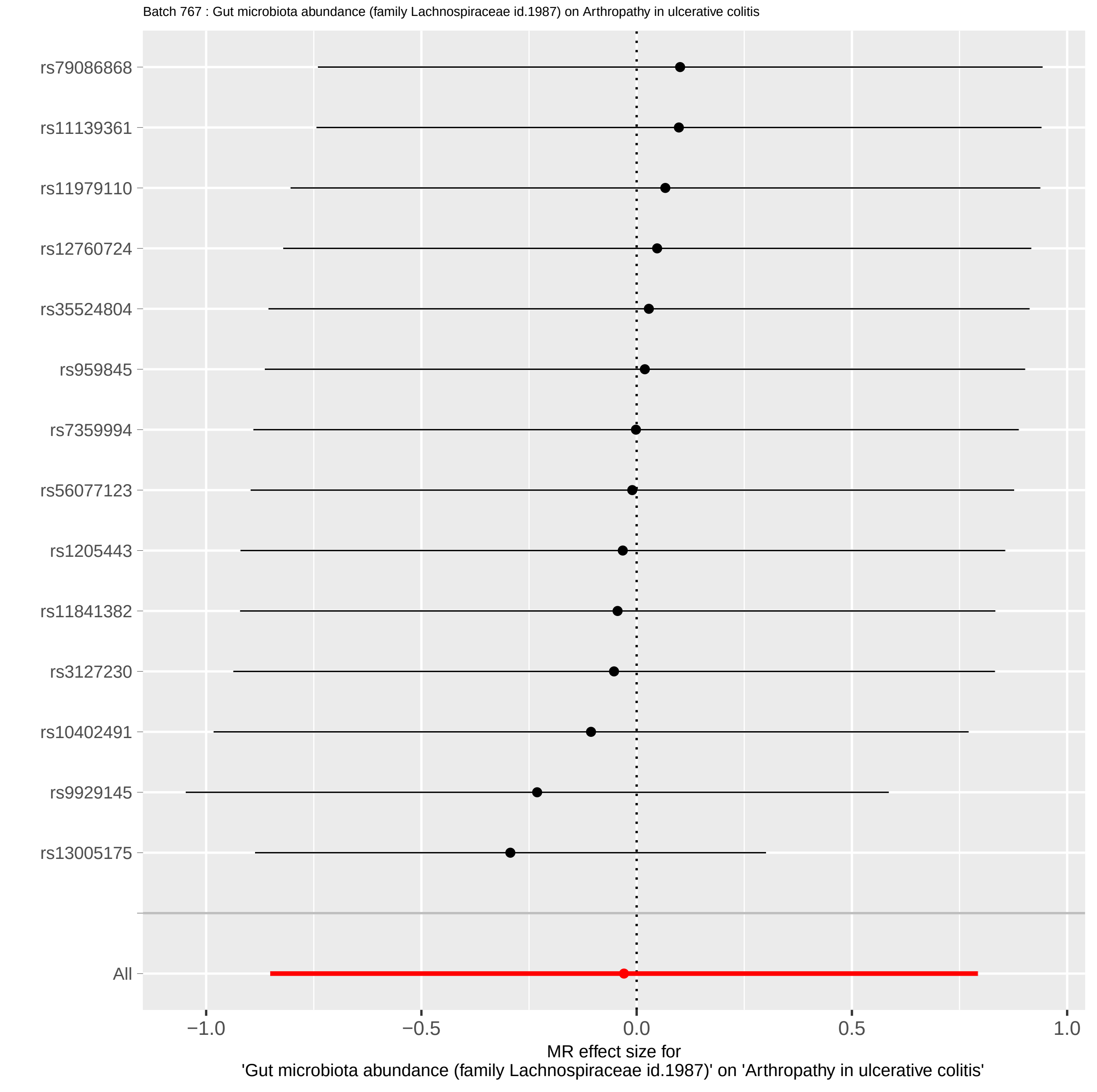

Batch 767 : Gut microbiota abundance (family Lachnospiraceae id.1987) on Arthropathy in ulcerative colitis
rs79086868
rs11139361
rs11979110
rs12760724
rs35524804
rs959845
rs7359994
rs56077123
rs1205443
rs11841382
rs3127230
rs10402491
rs9929145
rs13005175
All
−1.0
−0.5
0.0
0.5
1.0
MR effect size for
'Gut microbiota abundance (family Lachnospiraceae id.1987)' on 'Arthropathy in ulcerative colitis'

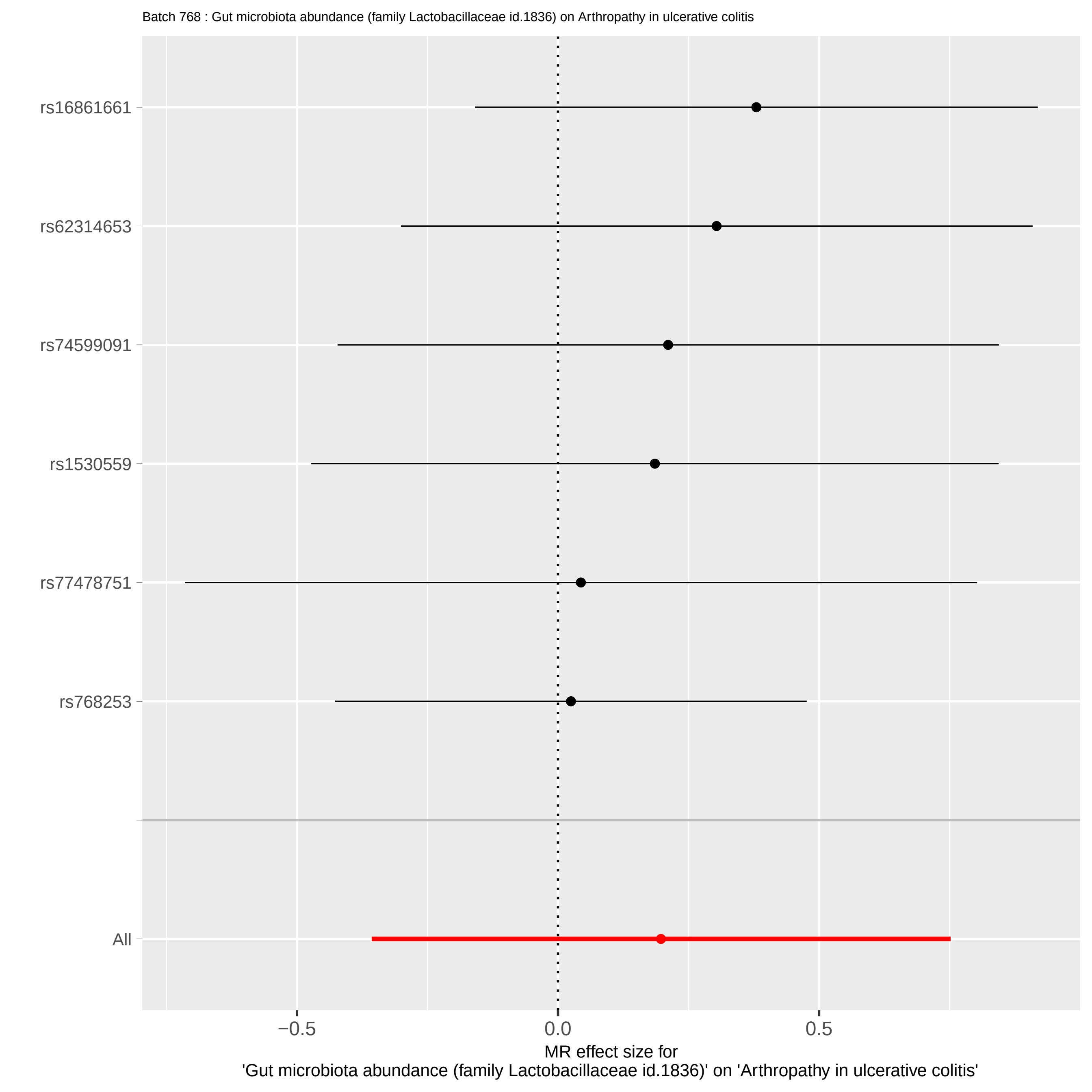
Batch 768 : Gut microbiota abundance (family Lactobacillaceae id.1836) on Arthropathy in ulcerative colitis
rs16861661
rs62314653
rs74599091
rs1530559
rs77478751
rs768253
All
−0.5
0.0
0.5
MR effect size for
'Gut microbiota abundance (family Lactobacillaceae id.1836)' on 'Arthropathy in ulcerative colitis'

Batch 769 : Gut microbiota abundance (family Methanobacteriaceae id.121) on Arthropathy in ulcerative colitis

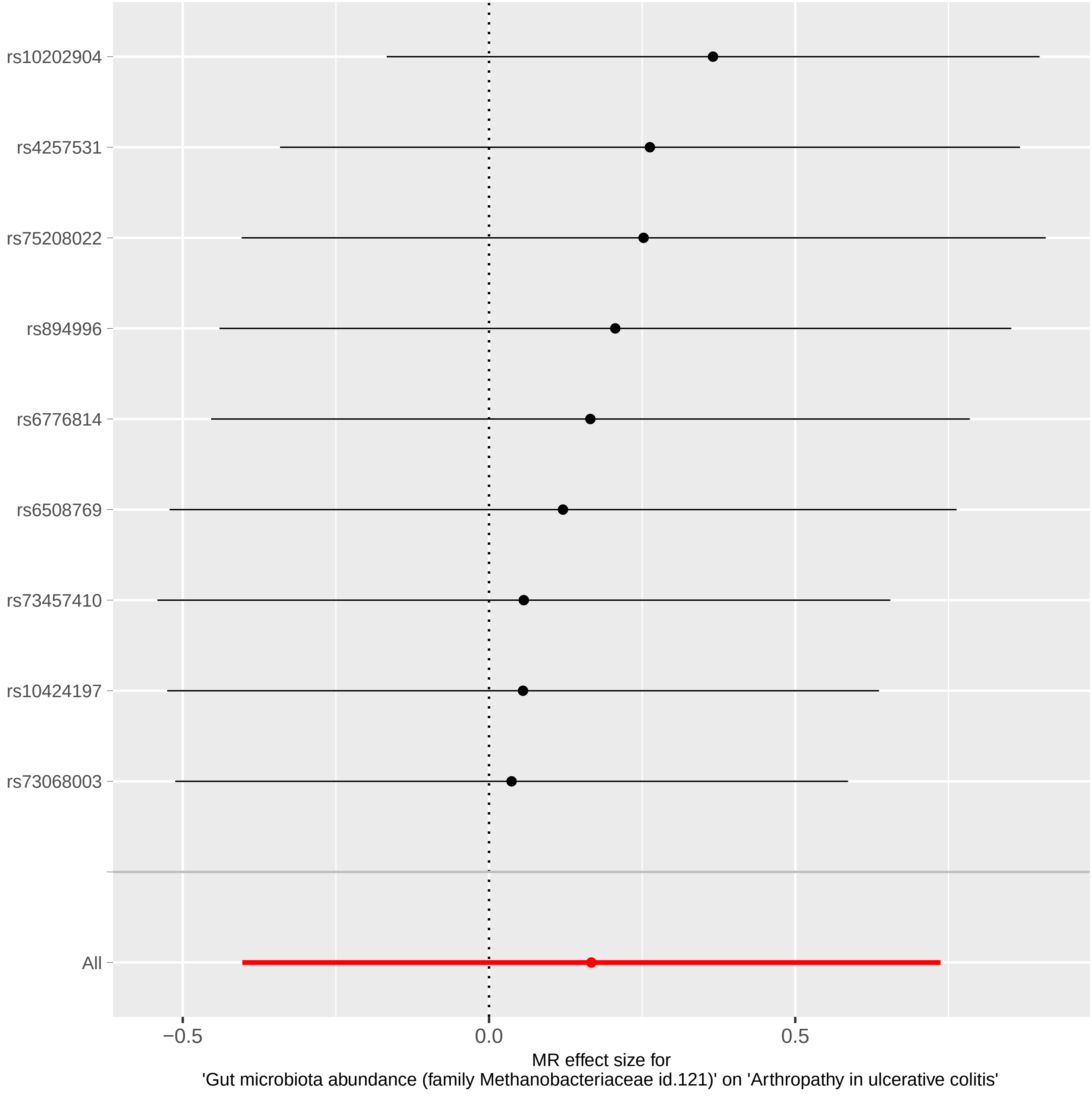

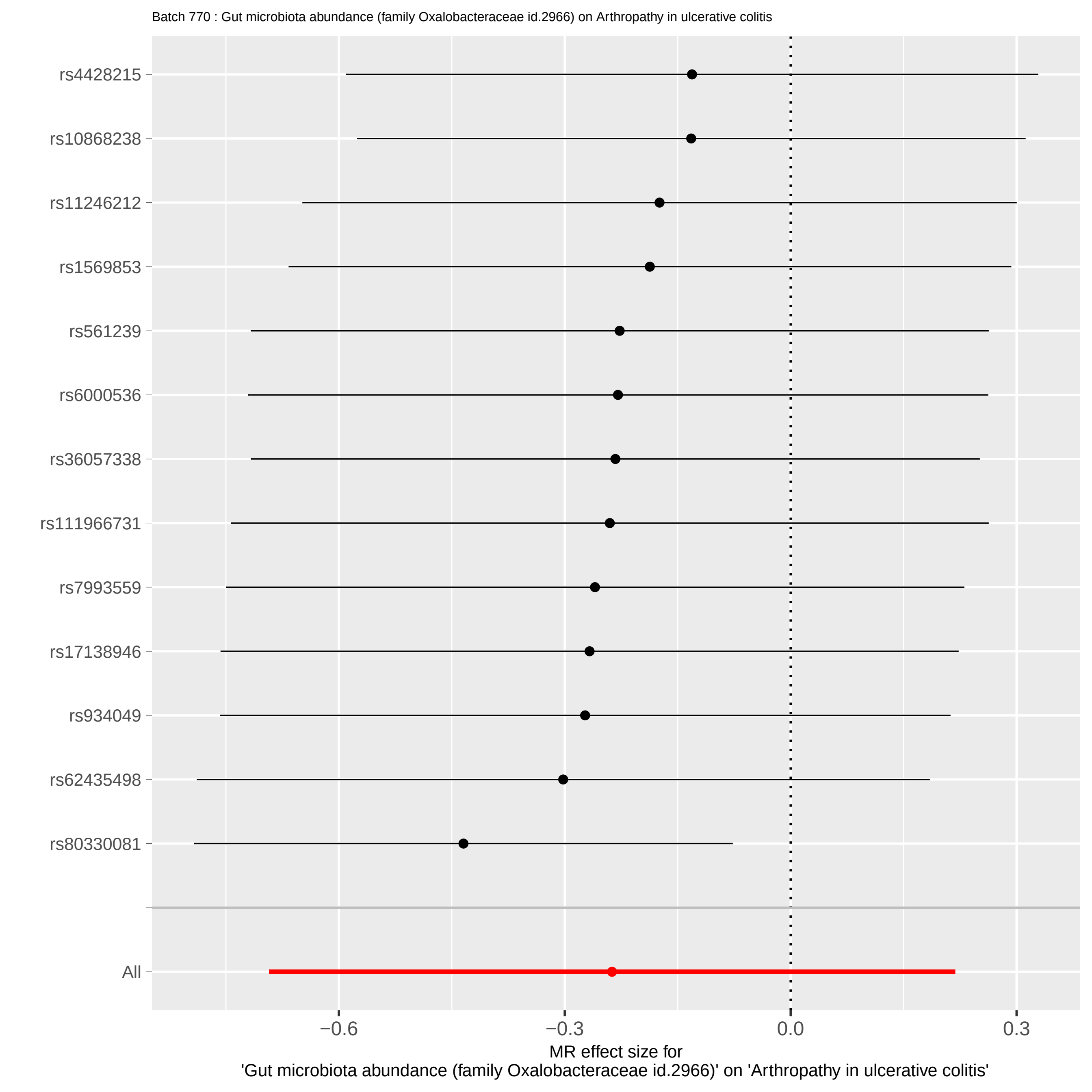

Batch 770 : Gut microbiota abundance (family Oxalobacteraceae id.2966) on Arthropathy in ulcerative colitis
rs4428215
rs10868238
rs11246212
rs1569853
rs561239
rs6000536
rs36057338
rs111966731
rs7993559
rs17138946
rs934049
rs62435498
rs80330081
All
−0.6
−0.3
0.0
0.3
MR effect size for
'Gut microbiota abundance (family Oxalobacteraceae id.2966)' on 'Arthropathy in ulcerative colitis'

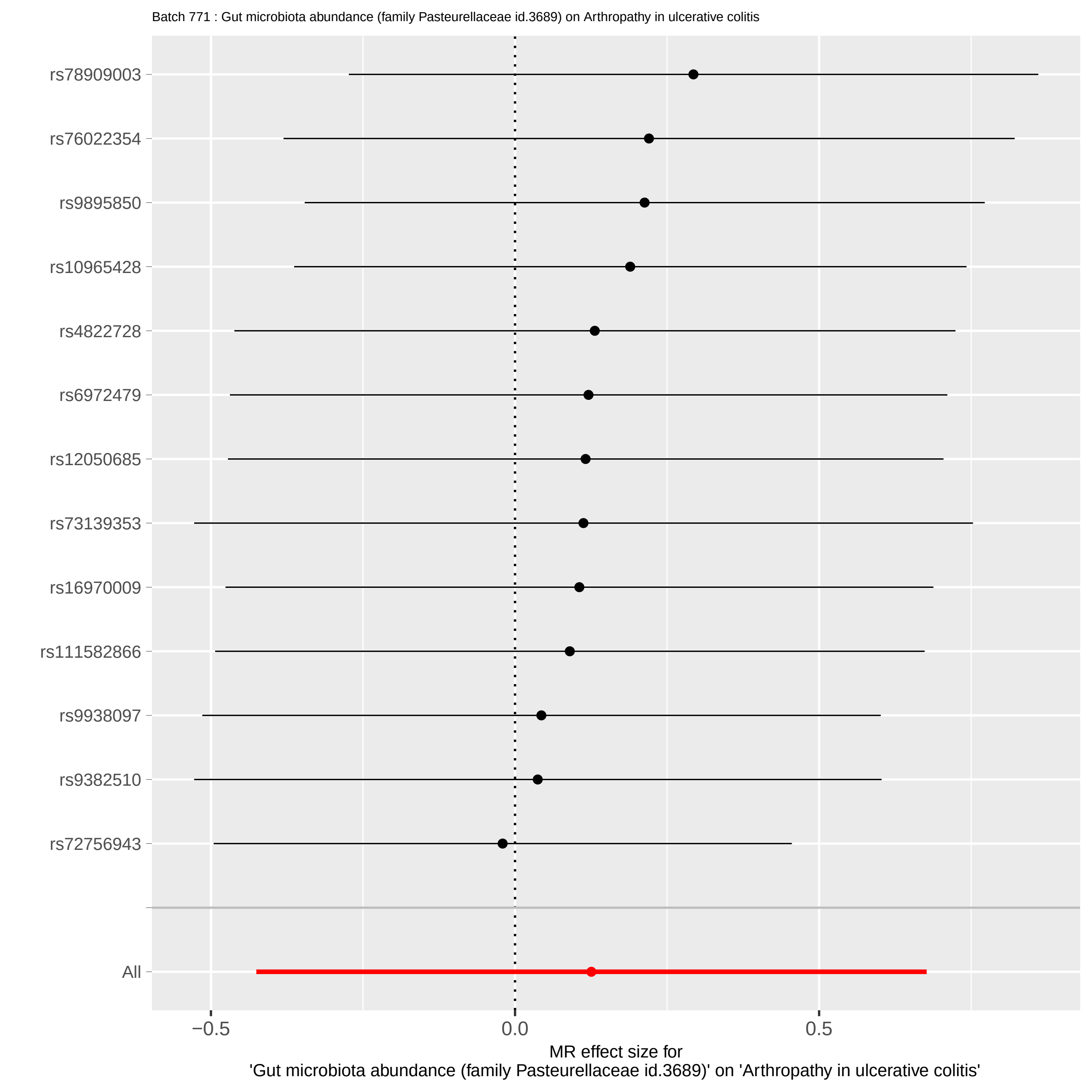

Batch 771 : Gut microbiota abundance (family Pasteurellaceae id.3689) on Arthropathy in ulcerative colitis
rs78909003
rs76022354
rs9895850
rs10965428
rs4822728
rs6972479
rs12050685
rs73139353
rs16970009
rs111582866
rs9938097
rs9382510
rs72756943
All
−0.5
0.0
0.5
MR effect size for
'Gut microbiota abundance (family Pasteurellaceae id.3689)' on 'Arthropathy in ulcerative colitis'

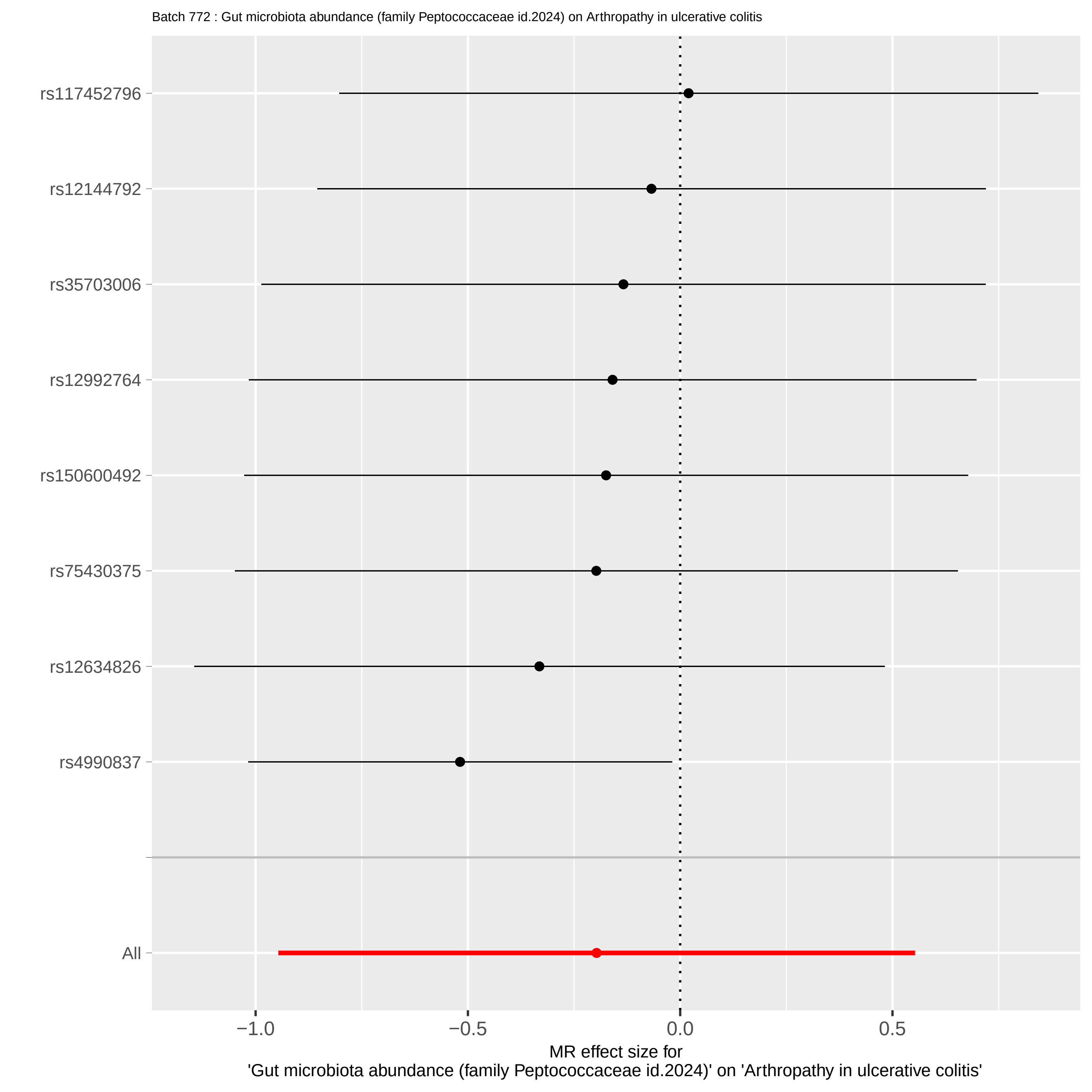

Batch 772 : Gut microbiota abundance (family Peptococcaceae id.2024) on Arthropathy in ulcerative colitis
rs117452796
rs12144792
rs35703006
rs12992764
rs150600492
rs75430375
rs12634826
rs4990837
All
−1.0
−0.5
0.0
0.5
MR effect size for
'Gut microbiota abundance (family Peptococcaceae id.2024)' on 'Arthropathy in ulcerative colitis'

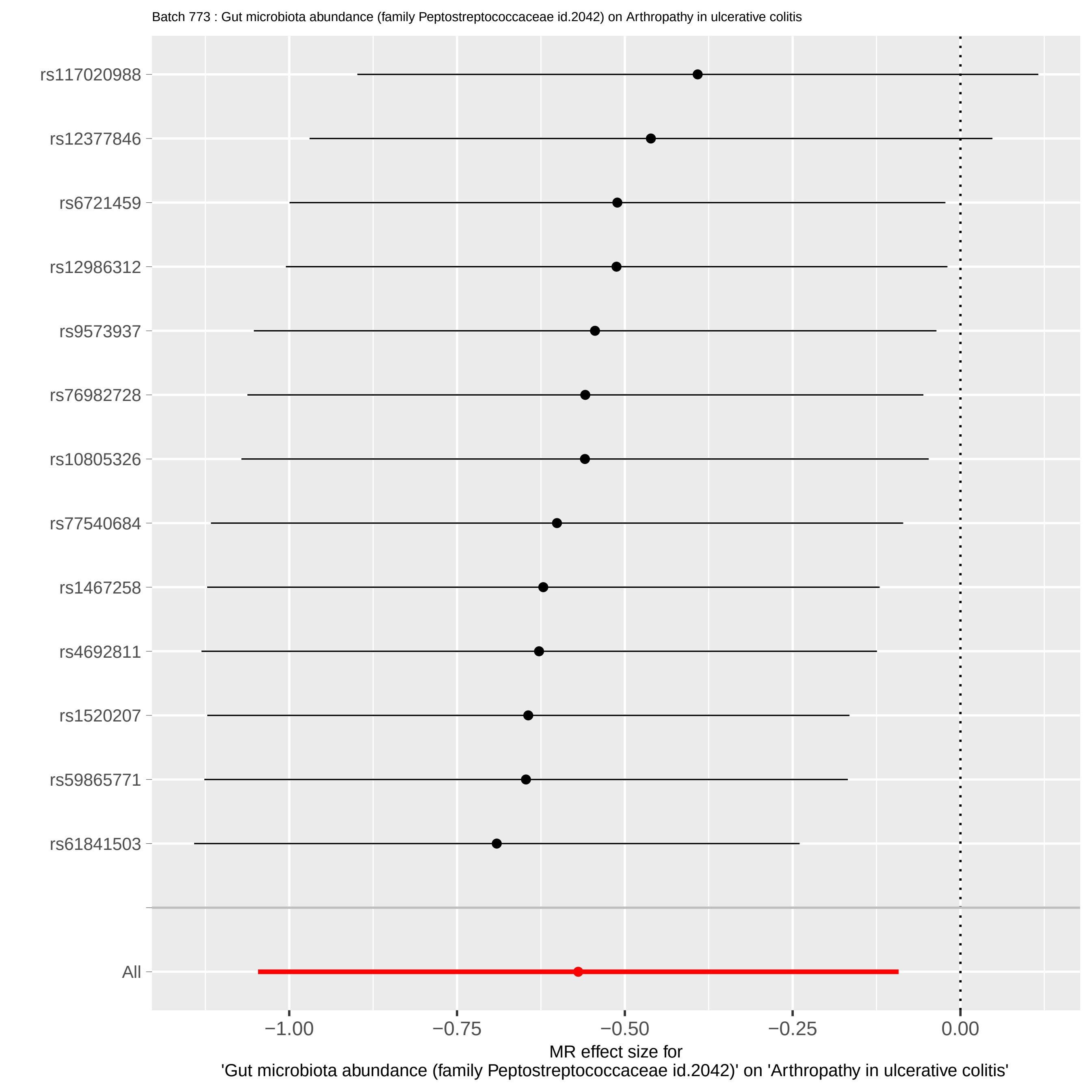

Batch 773 : Gut microbiota abundance (family Peptostreptococcaceae id.2042) on Arthropathy in ulcerative colitis
rs117020988
rs12377846
rs6721459
rs12986312
rs9573937
rs76982728
rs10805326
rs77540684
rs1467258
rs4692811
rs1520207
rs59865771
rs61841503
All
−1.00
−0.75
−0.50
−0.25
0.00
MR effect size for
'Gut microbiota abundance (family Peptostreptococcaceae id.2042)' on 'Arthropathy in ulcerative colitis'

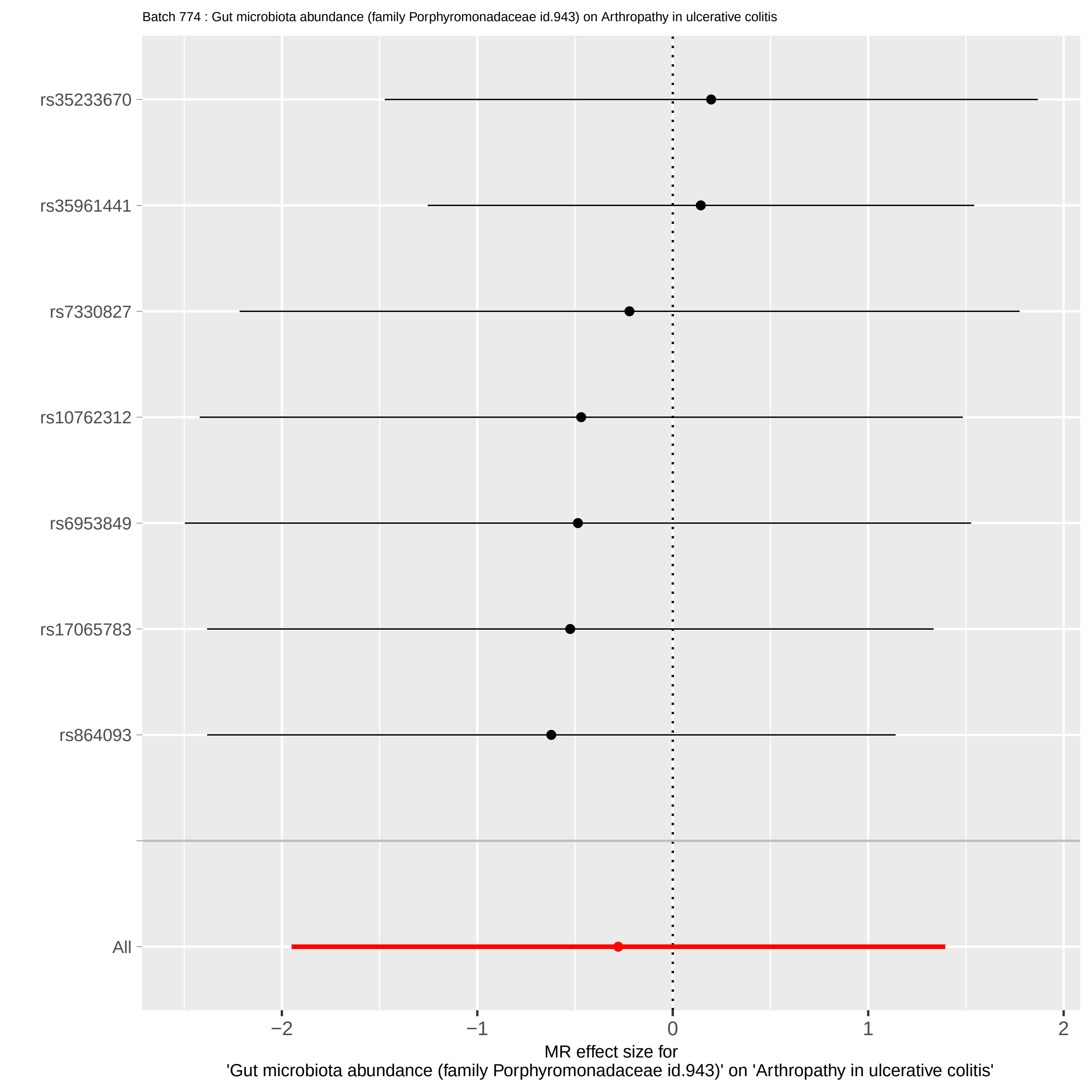

Batch 774 : Gut microbiota abundance (family Porphyromonadaceae id.943) on Arthropathy in ulcerative colitis
rs35233670
rs35961441
rs7330827
rs10762312
rs6953849
rs17065783
rs864093
All
−2
−1
0
1
2
MR effect size for
'Gut microbiota abundance (family Porphyromonadaceae id.943)' on 'Arthropathy in ulcerative colitis'

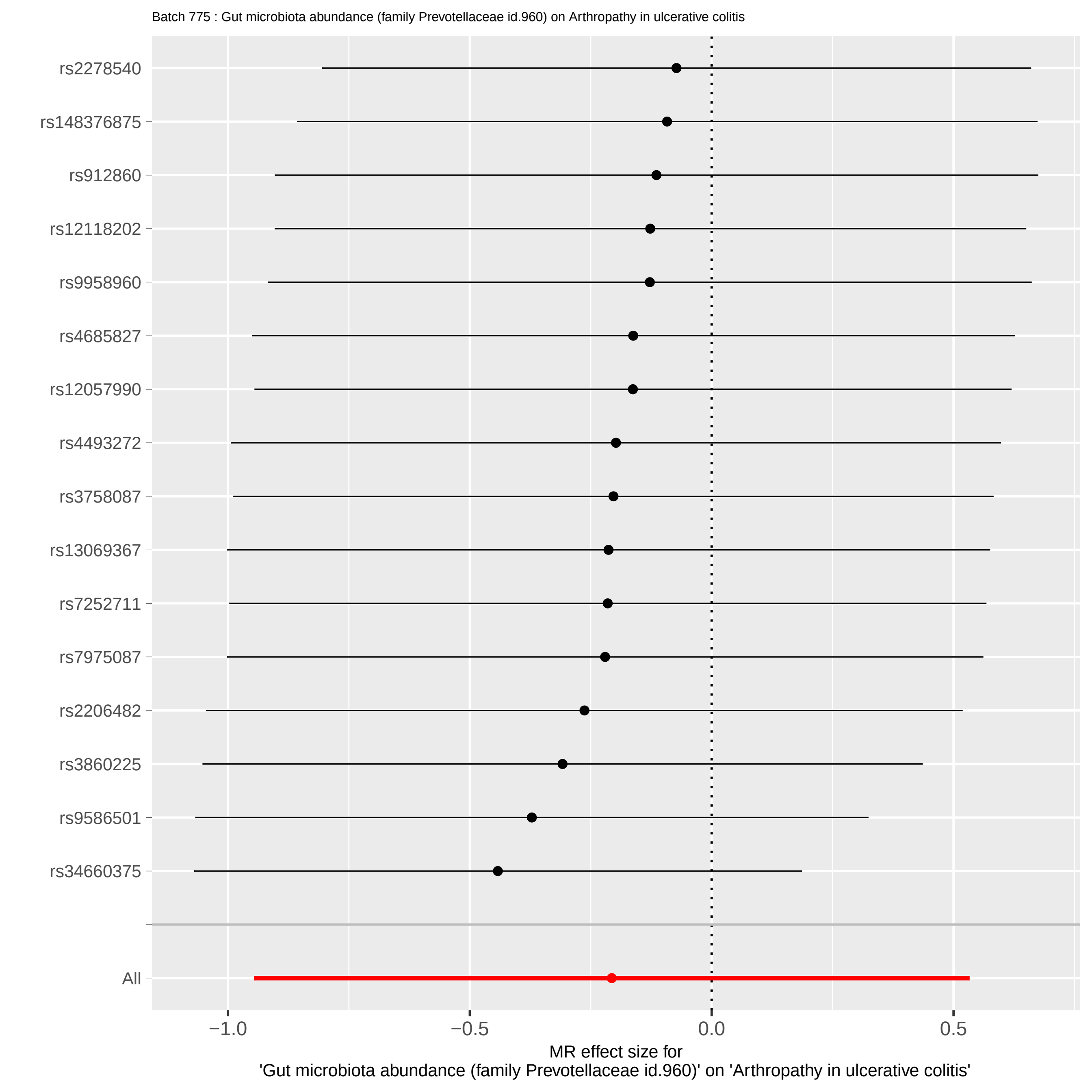

Batch 775 : Gut microbiota abundance (family Prevotellaceae id.960) on Arthropathy in ulcerative colitis
rs2278540
rs148376875
rs912860
rs12118202
rs9958960
rs4685827
rs12057990
rs4493272
rs3758087
rs13069367
rs7252711
rs7975087
rs2206482
rs3860225
rs9586501
rs34660375
All
−1.0
−0.5
0.0
0.5
MR effect size for
'Gut microbiota abundance (family Prevotellaceae id.960)' on 'Arthropathy in ulcerative colitis'

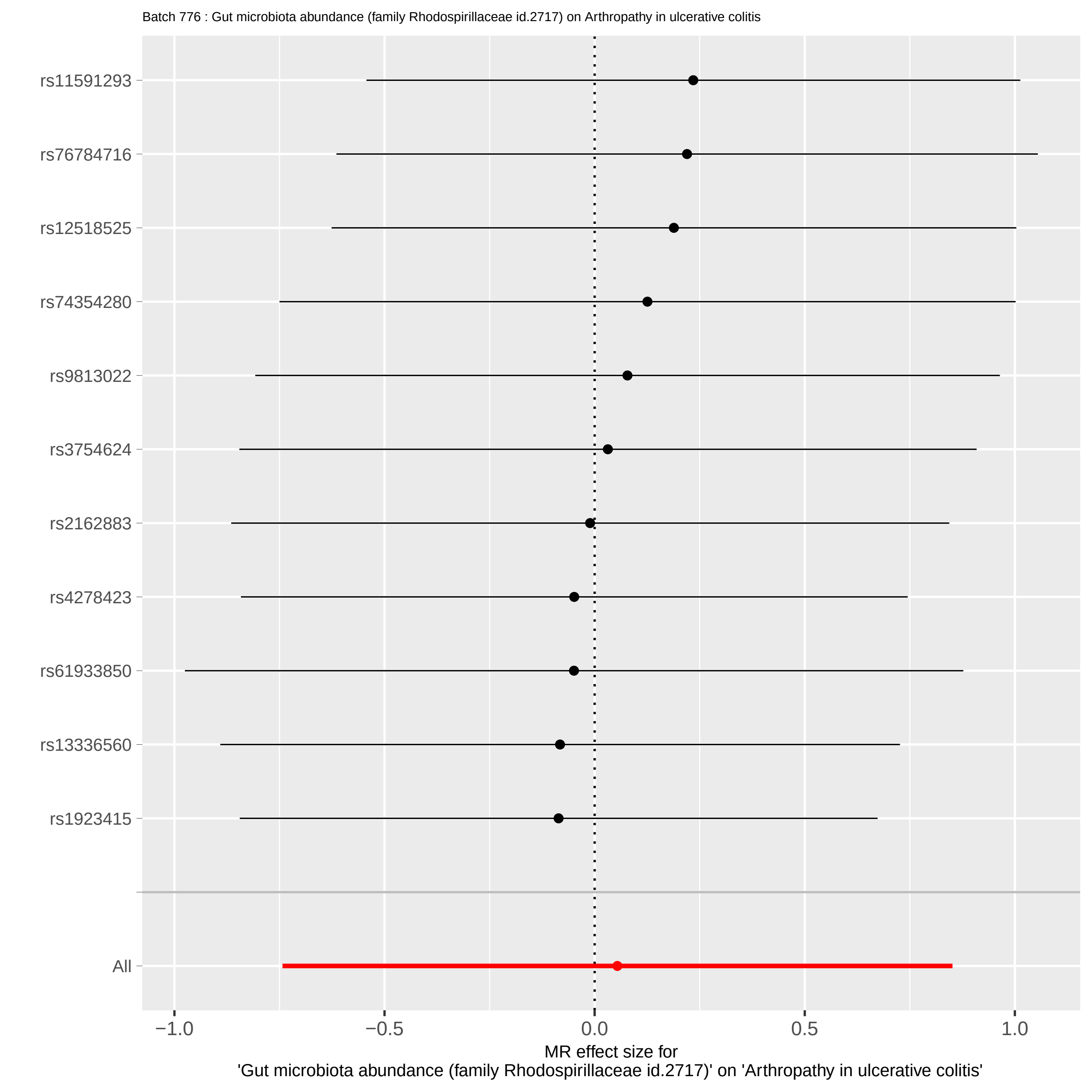
Batch 776 : Gut microbiota abundance (family Rhodospirillaceae id.2717) on Arthropathy in ulcerative colitis
rs11591293
rs76784716
rs12518525
rs74354280
rs9813022
rs3754624
rs2162883
rs4278423
rs61933850
rs13336560
rs1923415
All
−1.0
−0.5
0.0
0.5
1.0
MR effect size for
'Gut microbiota abundance (family Rhodospirillaceae id.2717)' on 'Arthropathy in ulcerative colitis'

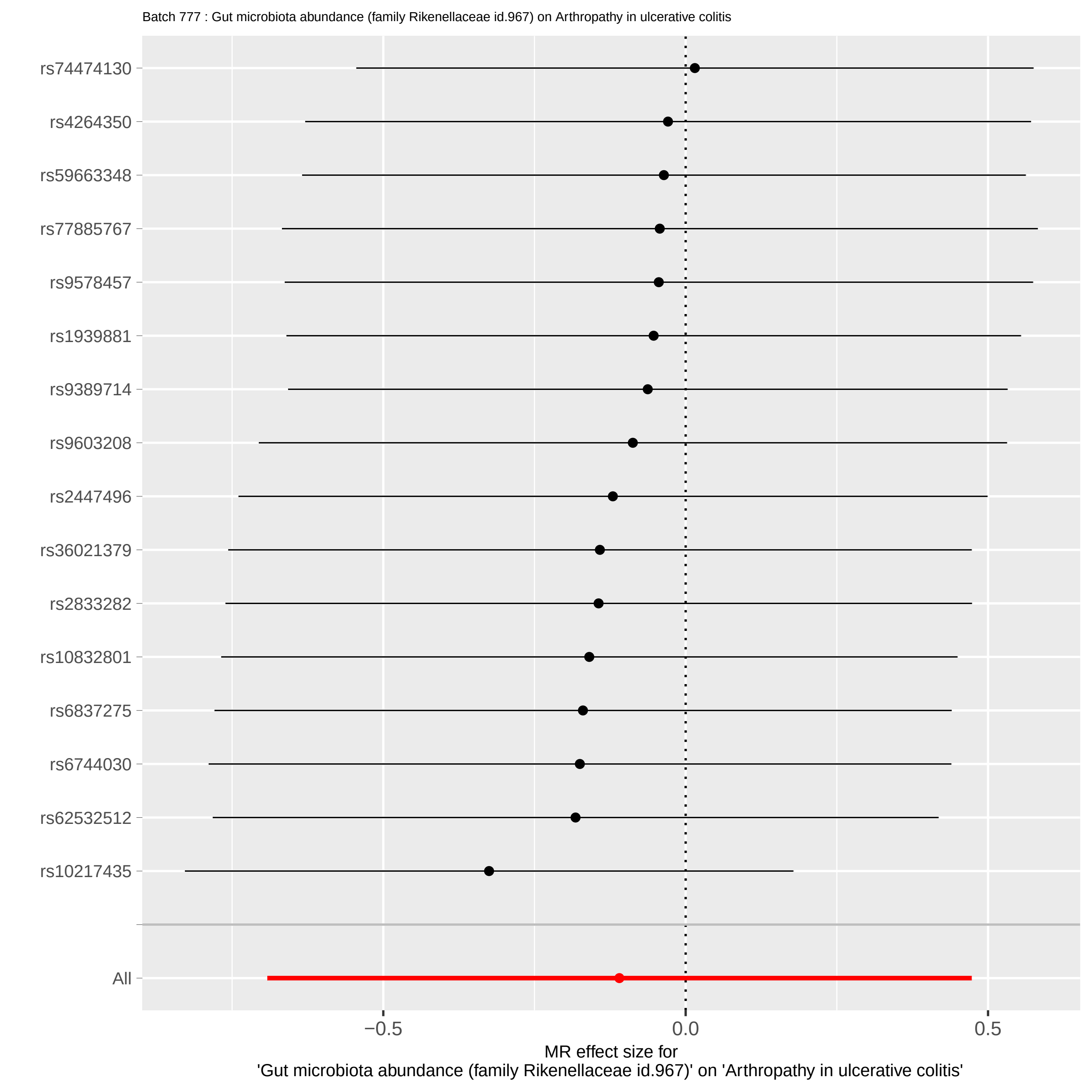

Batch 777 : Gut microbiota abundance (family Rikenellaceae id.967) on Arthropathy in ulcerative colitis
rs74474130
rs4264350
rs59663348
rs77885767
rs9578457
rs1939881
rs9389714
rs9603208
rs2447496
rs36021379
rs2833282
rs10832801
rs6837275
rs6744030
rs62532512
rs10217435
All
−0.5
0.0
0.5
MR effect size for
'Gut microbiota abundance (family Rikenellaceae id.967)' on 'Arthropathy in ulcerative colitis'

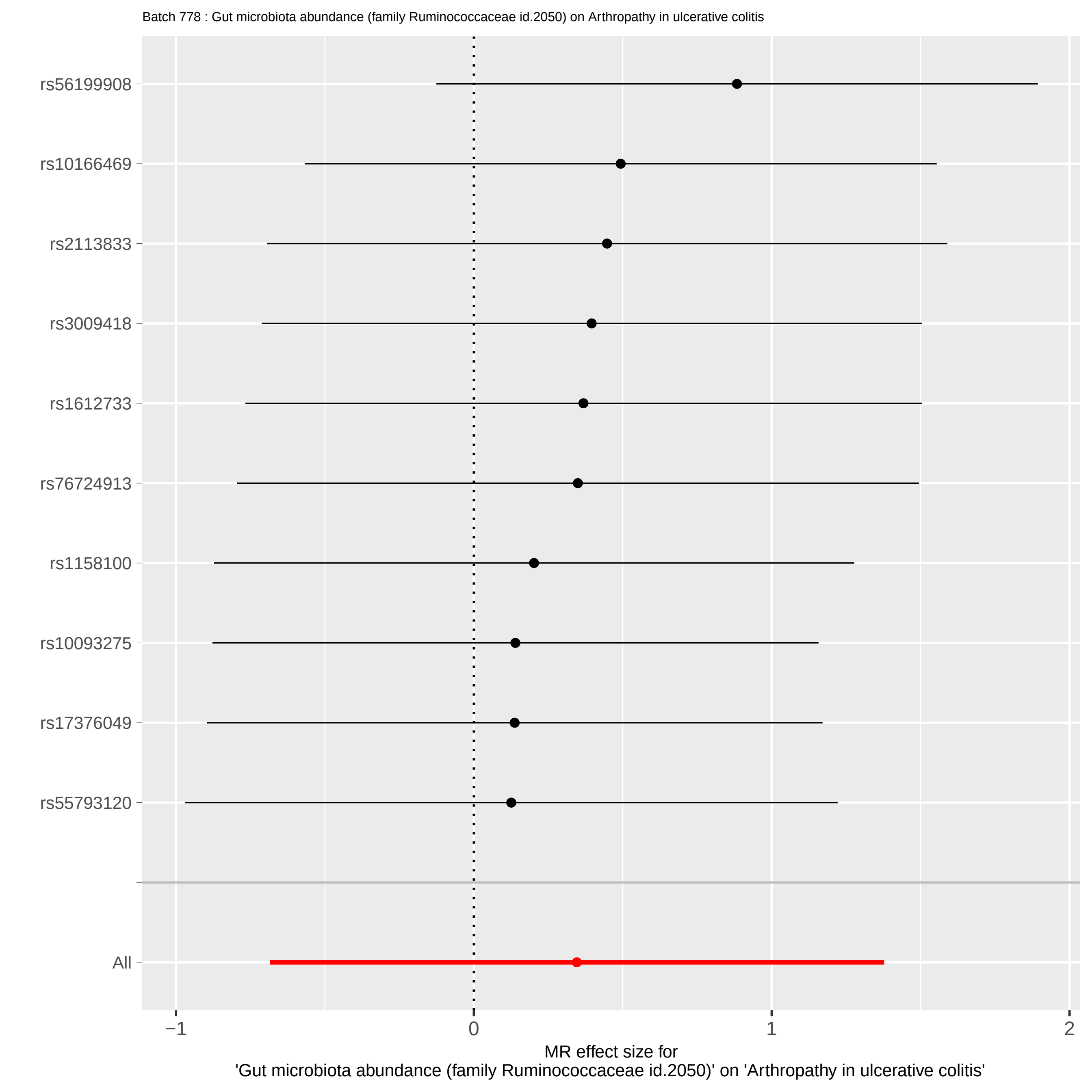
Batch 778 : Gut microbiota abundance (family Ruminococcaceae id.2050) on Arthropathy in ulcerative colitis
rs56199908
rs10166469
rs2113833
rs3009418
rs1612733
rs76724913
rs1158100
rs10093275
rs17376049
rs55793120
All
−1
0
1
2
MR effect size for
'Gut microbiota abundance (family Ruminococcaceae id.2050)' on 'Arthropathy in ulcerative colitis'

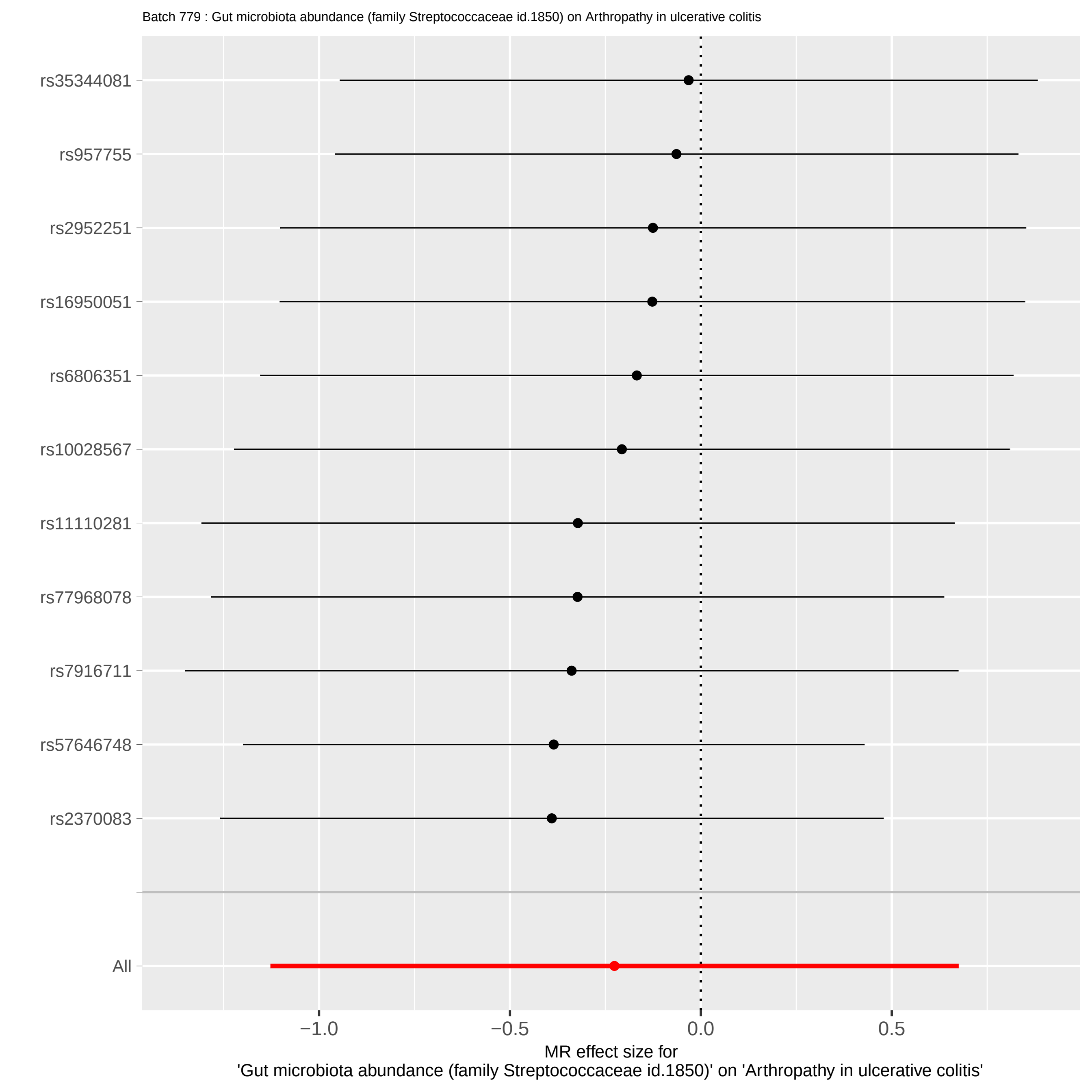

Batch 779 : Gut microbiota abundance (family Streptococcaceae id.1850) on Arthropathy in ulcerative colitis
rs35344081
rs957755
rs2952251
rs16950051
rs6806351
rs10028567
rs11110281
rs77968078
rs7916711
rs57646748
rs2370083
All
−1.0
−0.5
0.0
0.5
MR effect size for
'Gut microbiota abundance (family Streptococcaceae id.1850)' on 'Arthropathy in ulcerative colitis'

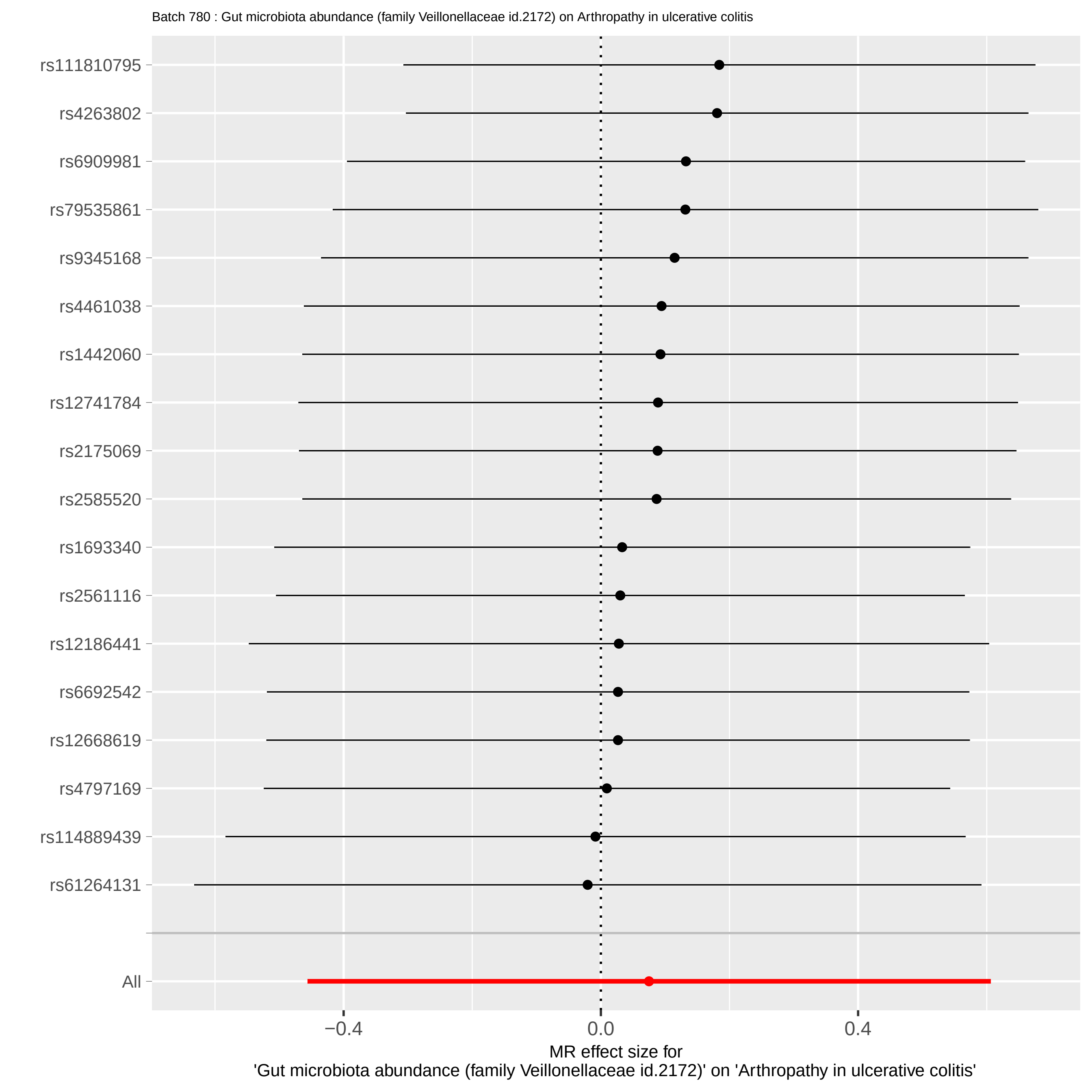
Batch 780 : Gut microbiota abundance (family Veillonellaceae id.2172) on Arthropathy in ulcerative colitis
rs111810795
rs4263802
rs6909981
rs79535861
rs9345168
rs4461038
rs1442060
rs12741784
rs2175069
rs2585520
rs1693340
rs2561116
rs12186441
rs6692542
rs12668619
rs4797169
rs114889439
rs61264131
All
−0.4
0.0
0.4
MR effect size for
'Gut microbiota abundance (family Veillonellaceae id.2172)' on 'Arthropathy in ulcerative colitis'

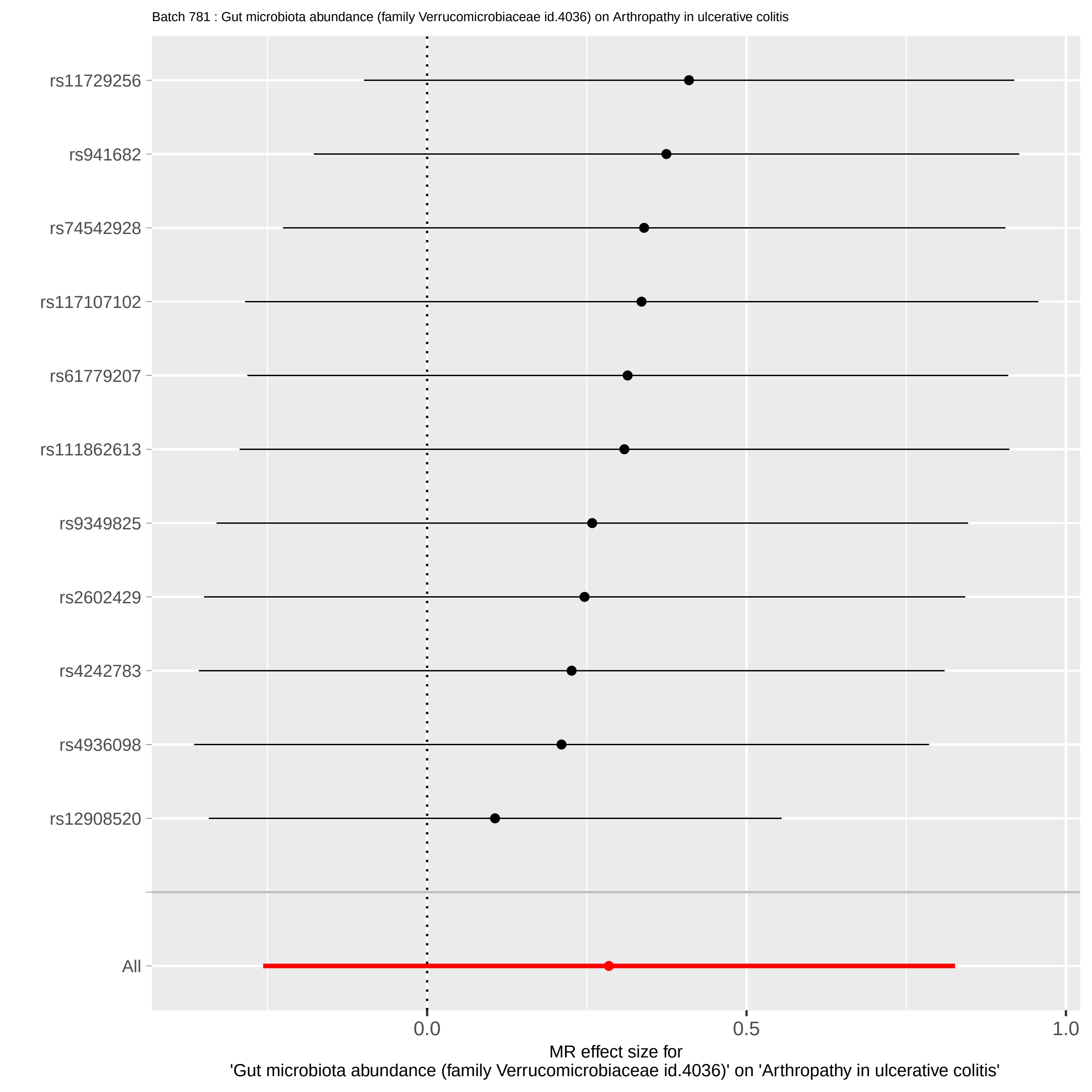

Batch 781 : Gut microbiota abundance (family Verrucomicrobiaceae id.4036) on Arthropathy in ulcerative colitis
rs11729256
rs941682
rs74542928
rs117107102
rs61779207
rs111862613
rs9349825
rs2602429
rs4242783
rs4936098
rs12908520
All
0.0
0.5
1.0
MR effect size for
'Gut microbiota abundance (family Verrucomicrobiaceae id.4036)' on 'Arthropathy in ulcerative colitis'

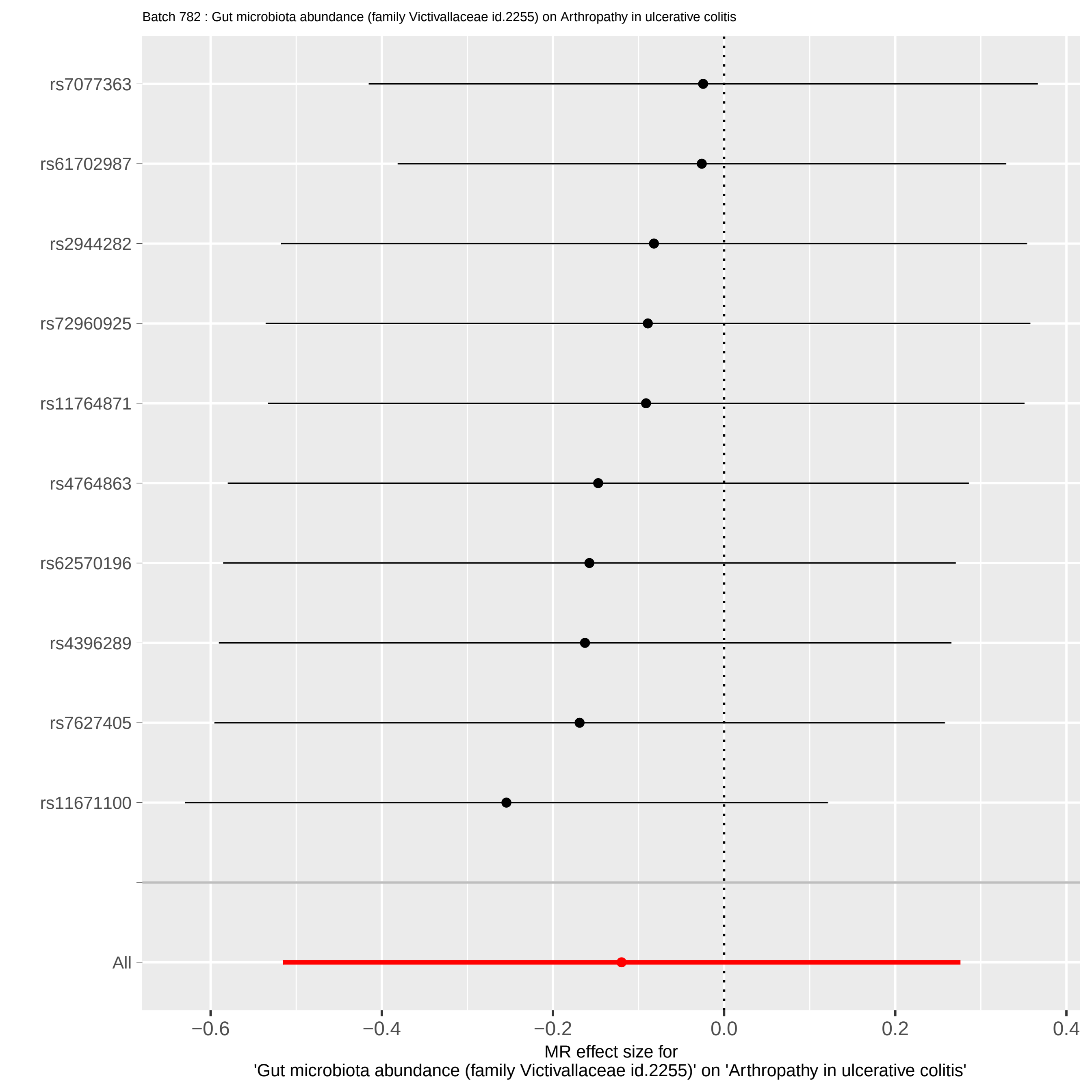
Batch 782 : Gut microbiota abundance (family Victivallaceae id.2255) on Arthropathy in ulcerative colitis
rs7077363
rs61702987
rs2944282
rs72960925
rs11764871
rs4764863
rs62570196
rs4396289
rs7627405
rs11671100
All
−0.6
−0.4
−0.2
0.0
0.2
0.4
MR effect size for
'Gut microbiota abundance (family Victivallaceae id.2255)' on 'Arthropathy in ulcerative colitis'

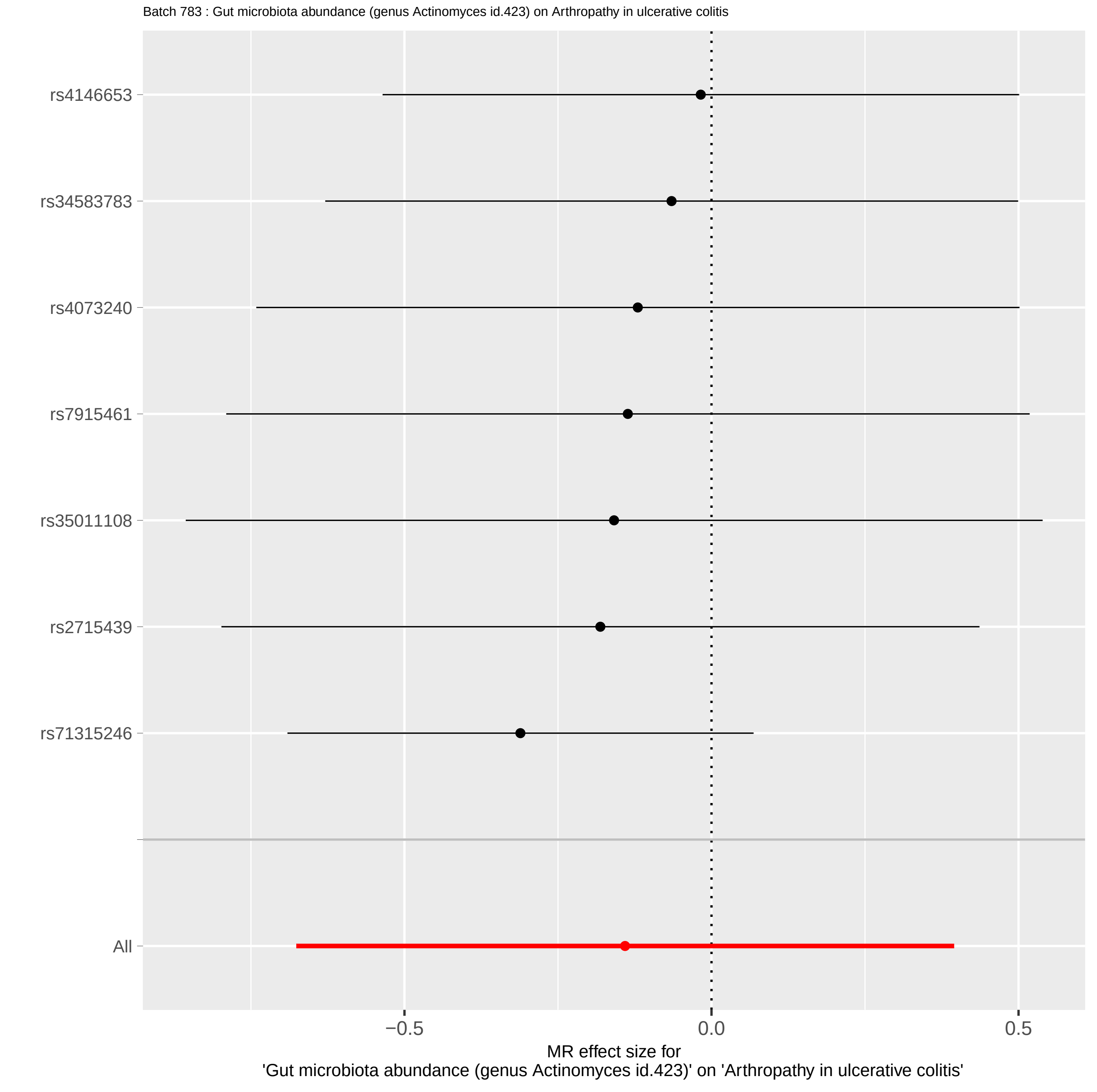
Batch 783 : Gut microbiota abundance (genus Actinomyces id.423) on Arthropathy in ulcerative colitis
rs4146653
rs34583783
rs4073240
rs7915461
rs35011108
rs2715439
rs71315246
All
−0.5
0.0
0.5
MR effect size for
'Gut microbiota abundance (genus Actinomyces id.423)' on 'Arthropathy in ulcerative colitis'

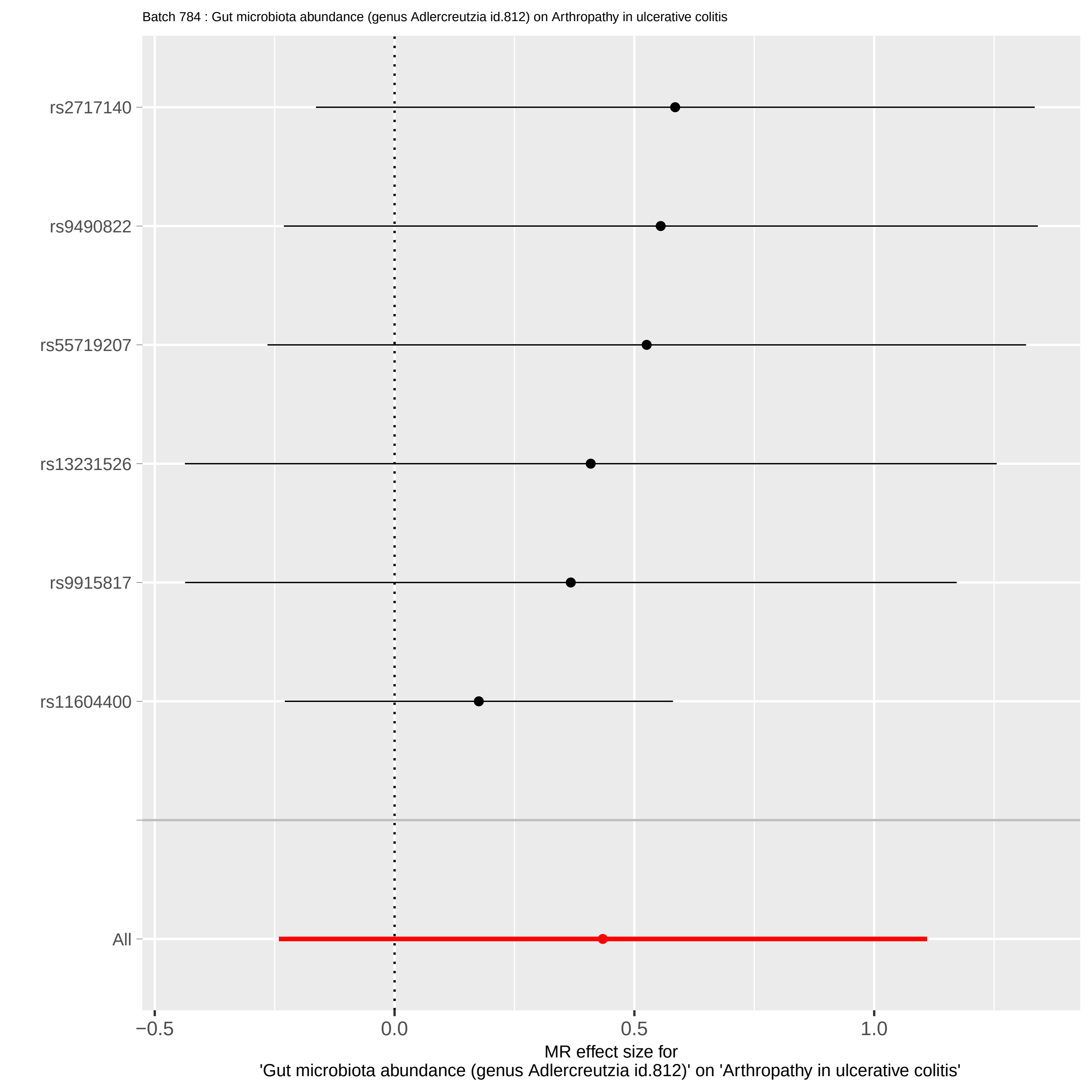
Batch 784 : Gut microbiota abundance (genus Adlercreutzia id.812) on Arthropathy in ulcerative colitis
rs2717140
rs9490822
rs55719207
rs13231526
rs9915817
rs11604400
All
−0.5
0.0
0.5
1.0
MR effect size for
'Gut microbiota abundance (genus Adlercreutzia id.812)' on 'Arthropathy in ulcerative colitis'

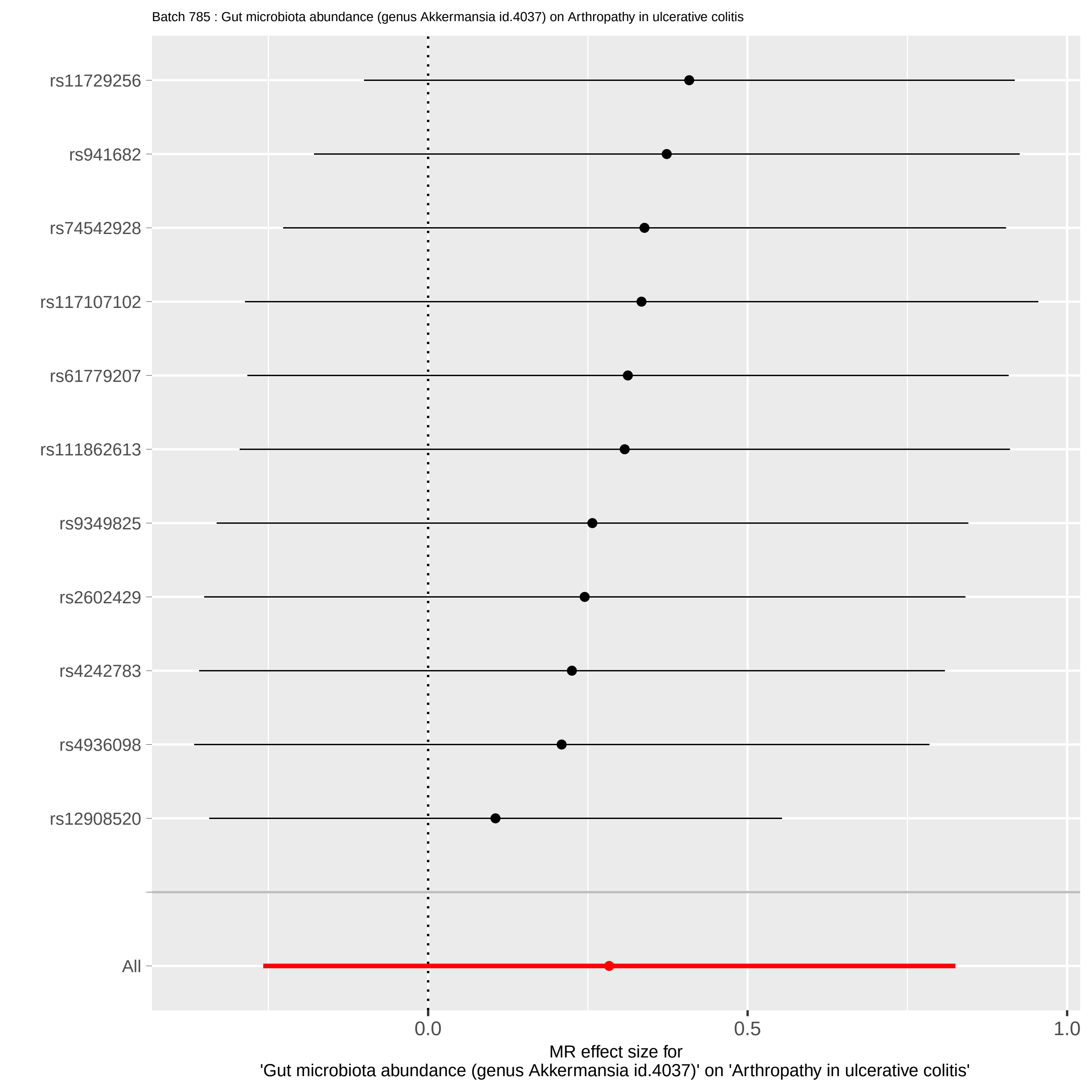
Batch 785 : Gut microbiota abundance (genus Akkermansia id.4037) on Arthropathy in ulcerative colitis
rs11729256
rs941682
rs74542928
rs117107102
rs61779207
rs111862613
rs9349825
rs2602429
rs4242783
rs4936098
rs12908520
All
0.0
0.5
1.0
MR effect size for
'Gut microbiota abundance (genus Akkermansia id.4037)' on 'Arthropathy in ulcerative colitis'

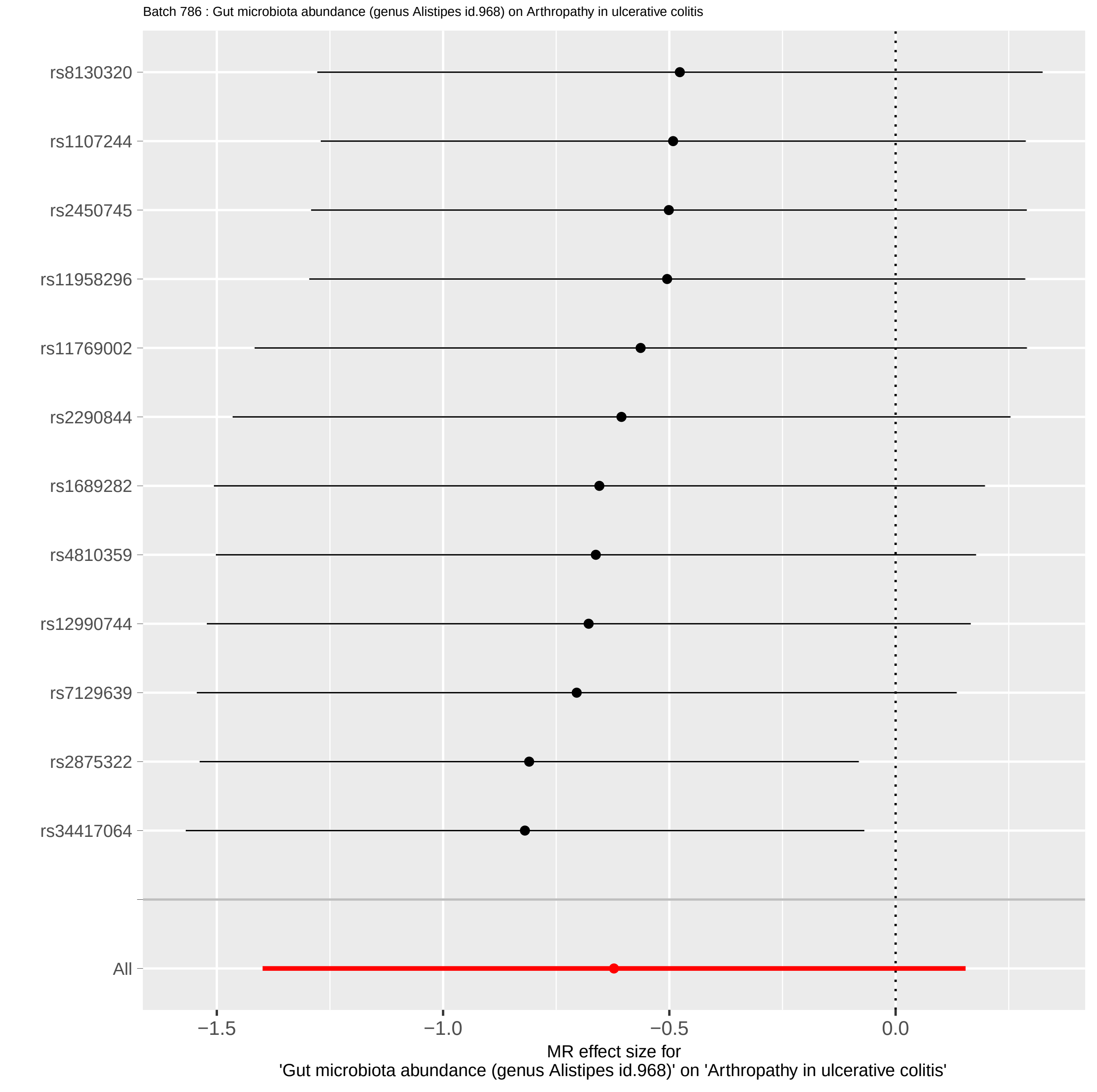

Batch 786 : Gut microbiota abundance (genus Alistipes id.968) on Arthropathy in ulcerative colitis
rs8130320
rs1107244
rs2450745
rs11958296
rs11769002
rs2290844
rs1689282
rs4810359
rs12990744
rs7129639
rs2875322
rs34417064
All
−1.5
−1.0
−0.5
0.0
MR effect size for
'Gut microbiota abundance (genus Alistipes id.968)' on 'Arthropathy in ulcerative colitis'

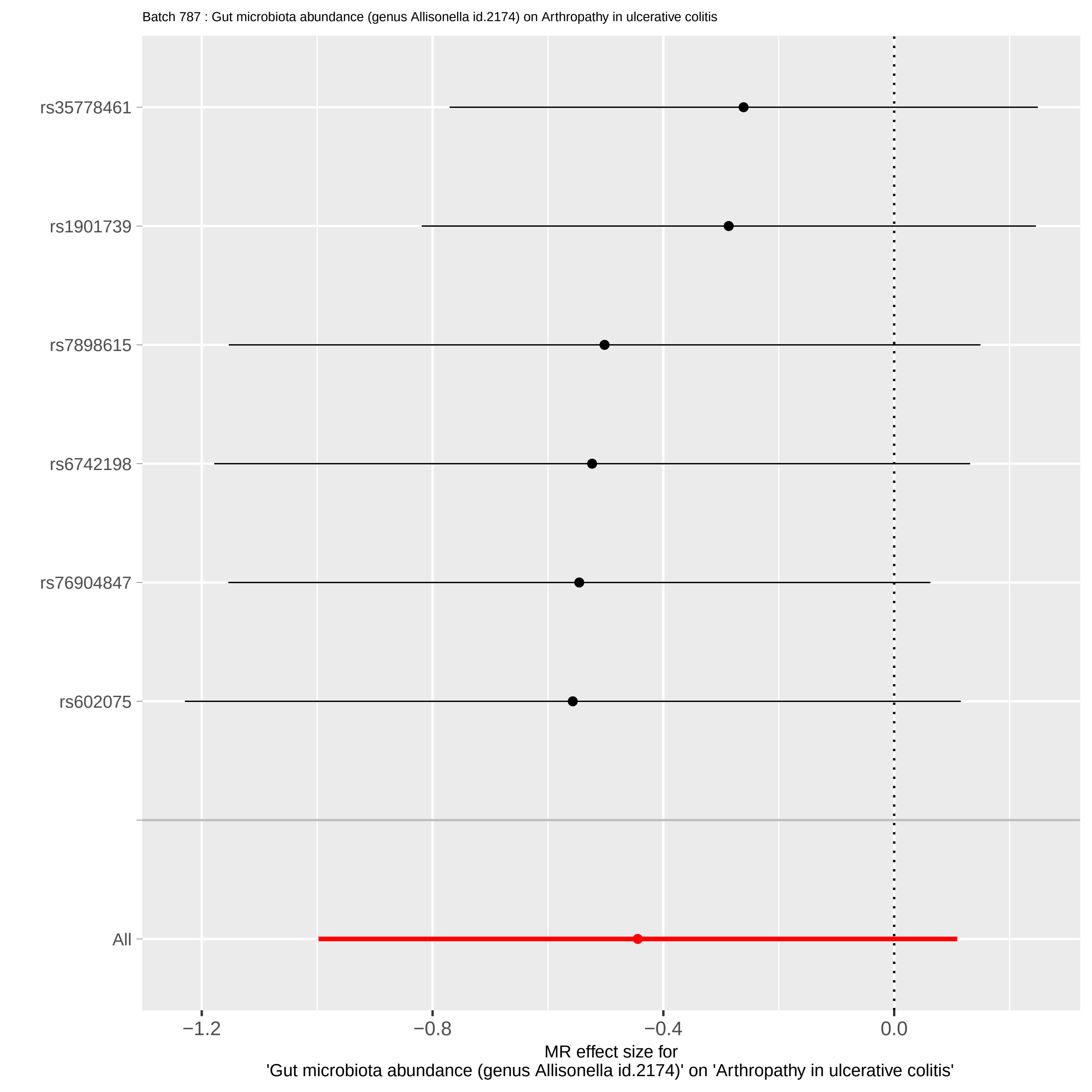
Batch 787 : Gut microbiota abundance (genus Allisonella id.2174) on Arthropathy in ulcerative colitis
rs35778461
rs1901739
rs7898615
rs6742198
rs76904847
rs602075
All
−1.2
−0.8
−0.4
0.0
MR effect size for
'Gut microbiota abundance (genus Allisonella id.2174)' on 'Arthropathy in ulcerative colitis'

Batch 788 : Gut microbiota abundance (genus Alloprevotella id.961) on Arthropathy in ulcerative colitis

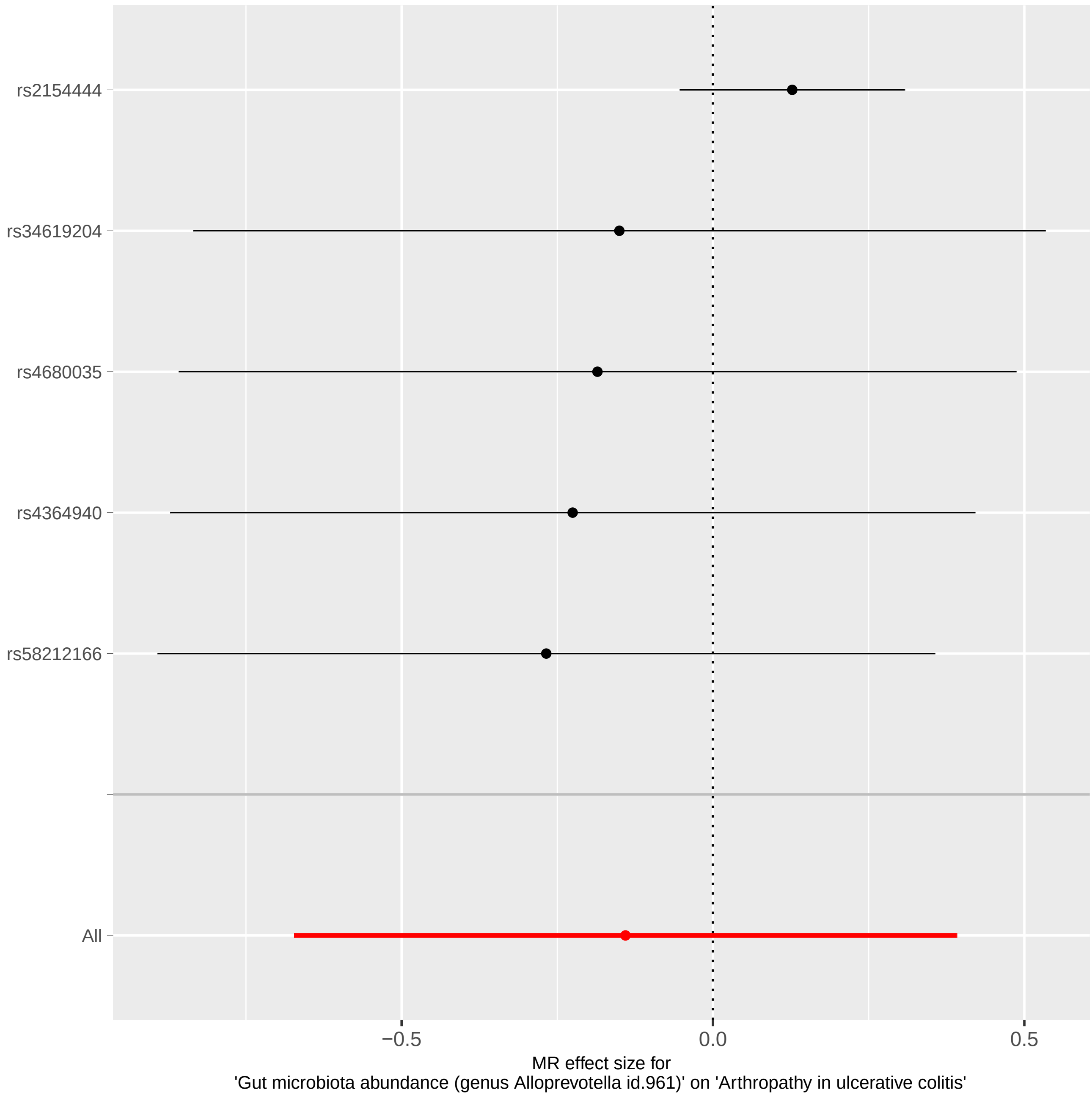

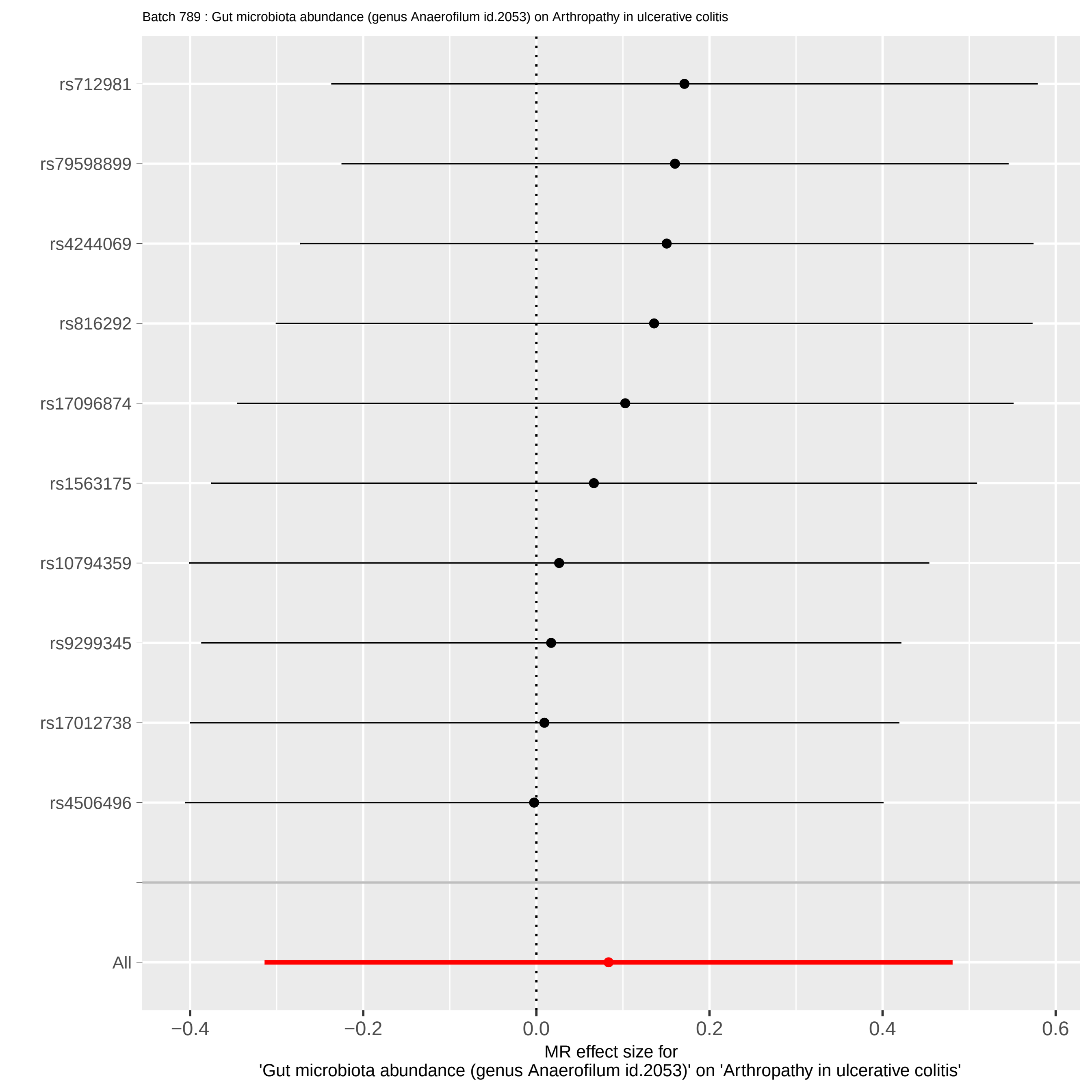

Batch 789 : Gut microbiota abundance (genus Anaerofilum id.2053) on Arthropathy in ulcerative colitis
rs712981
rs79598899
rs4244069
rs816292
rs17096874
rs1563175
rs10794359
rs9299345
rs17012738
rs4506496
All
−0.4
−0.2
0.0
0.2
0.4
0.6
MR effect size for
'Gut microbiota abundance (genus Anaerofilum id.2053)' on 'Arthropathy in ulcerative colitis'

Batch 790 : Gut microbiota abundance (genus Anaerostipes id.1991) on Arthropathy in ulcerative colitis

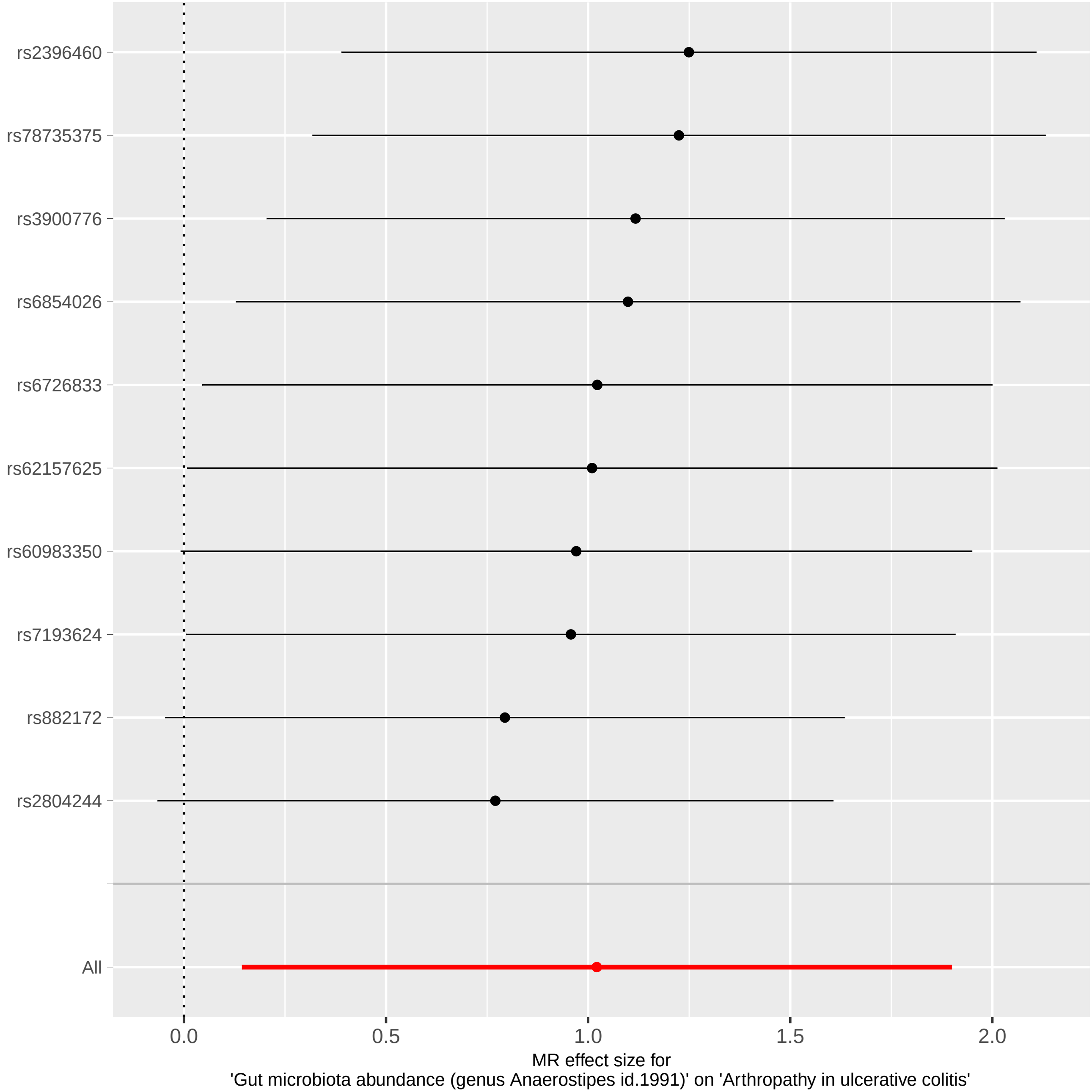

Batch 791 : Gut microbiota abundance (genus Anaerotruncus id.2054) on Arthropathy in ulcerative colitis

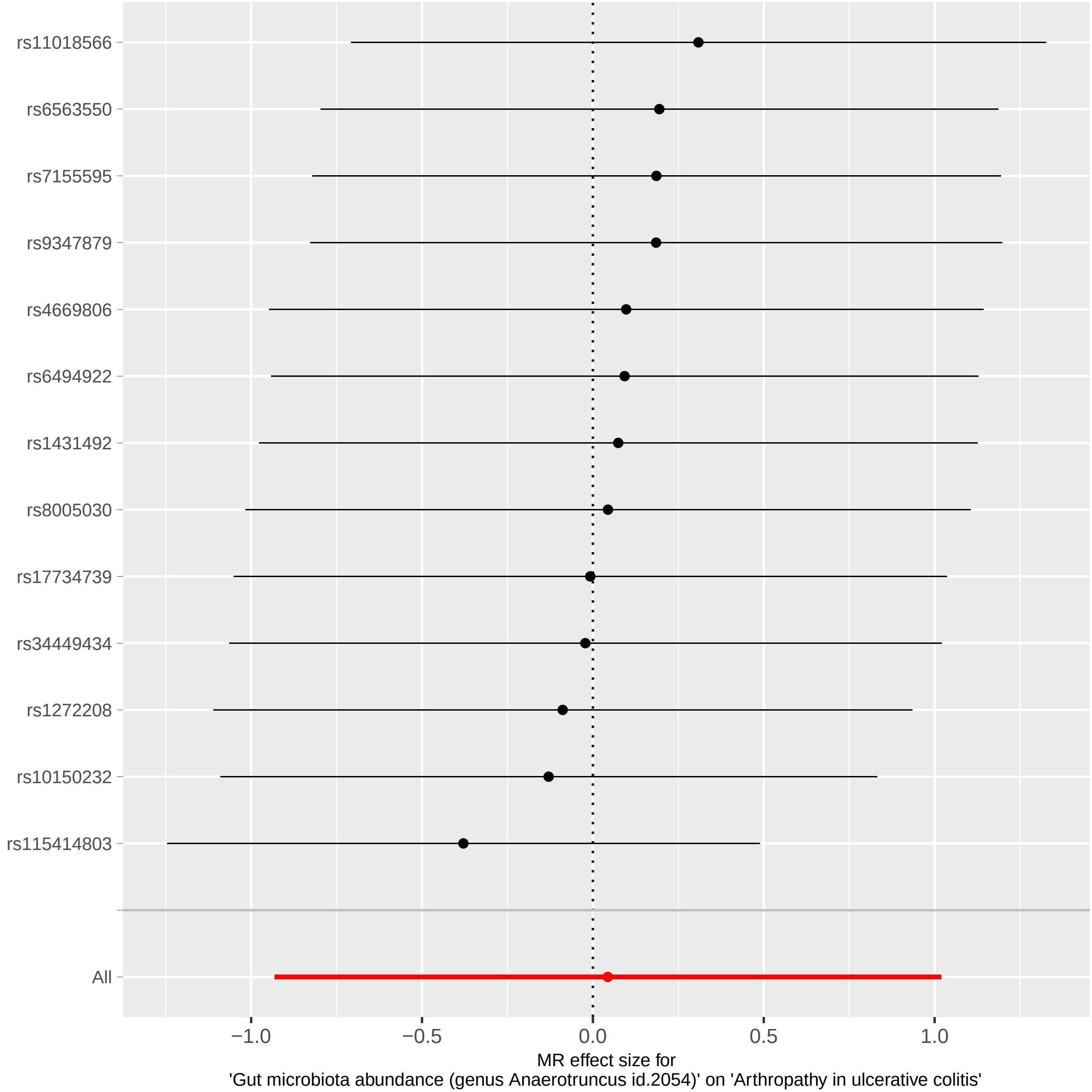

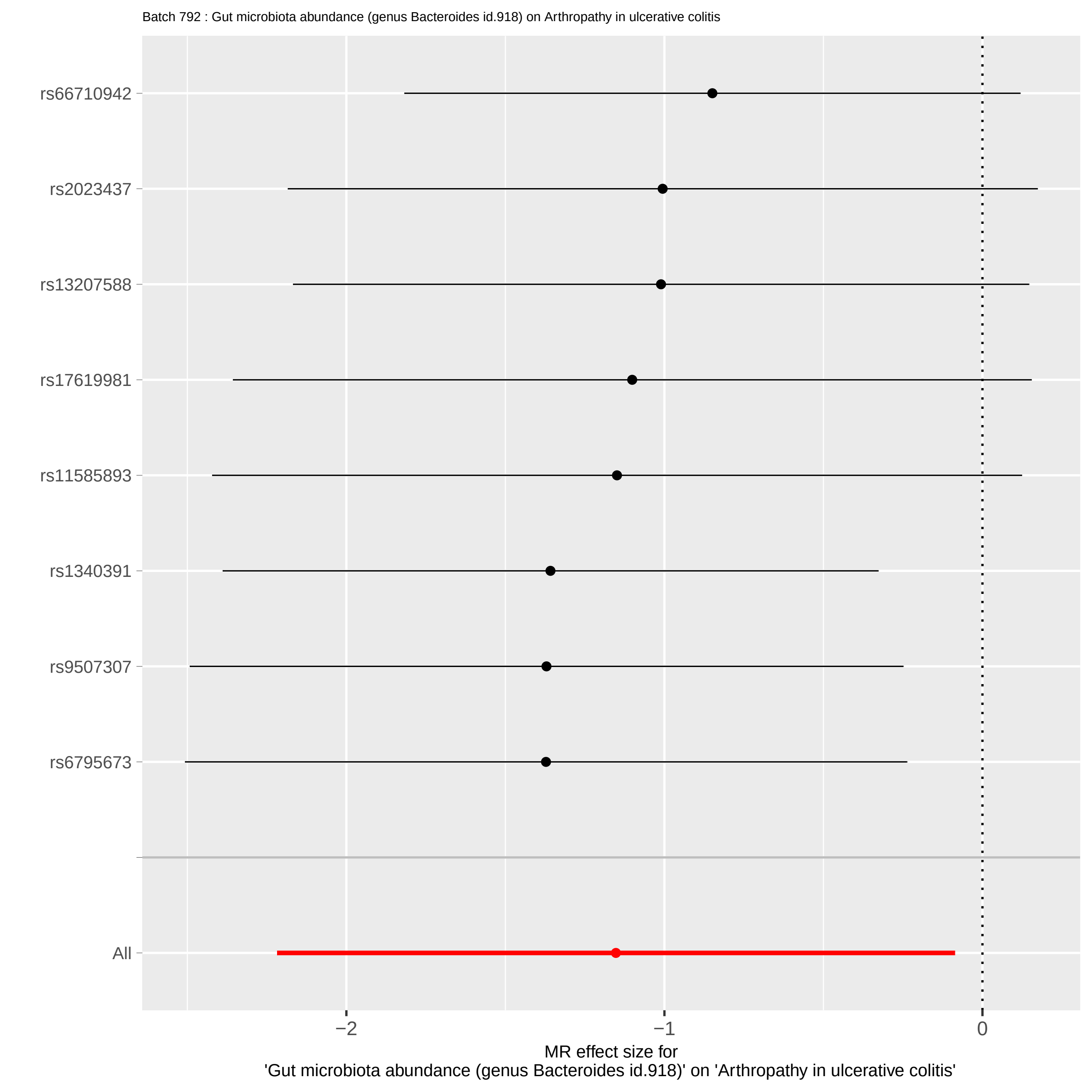

Batch 792 : Gut microbiota abundance (genus Bacteroides id.918) on Arthropathy in ulcerative colitis
rs66710942
rs2023437
rs13207588
rs17619981
rs11585893
rs1340391
rs9507307
rs6795673
All
−2
−1
0
MR effect size for
'Gut microbiota abundance (genus Bacteroides id.918)' on 'Arthropathy in ulcerative colitis'

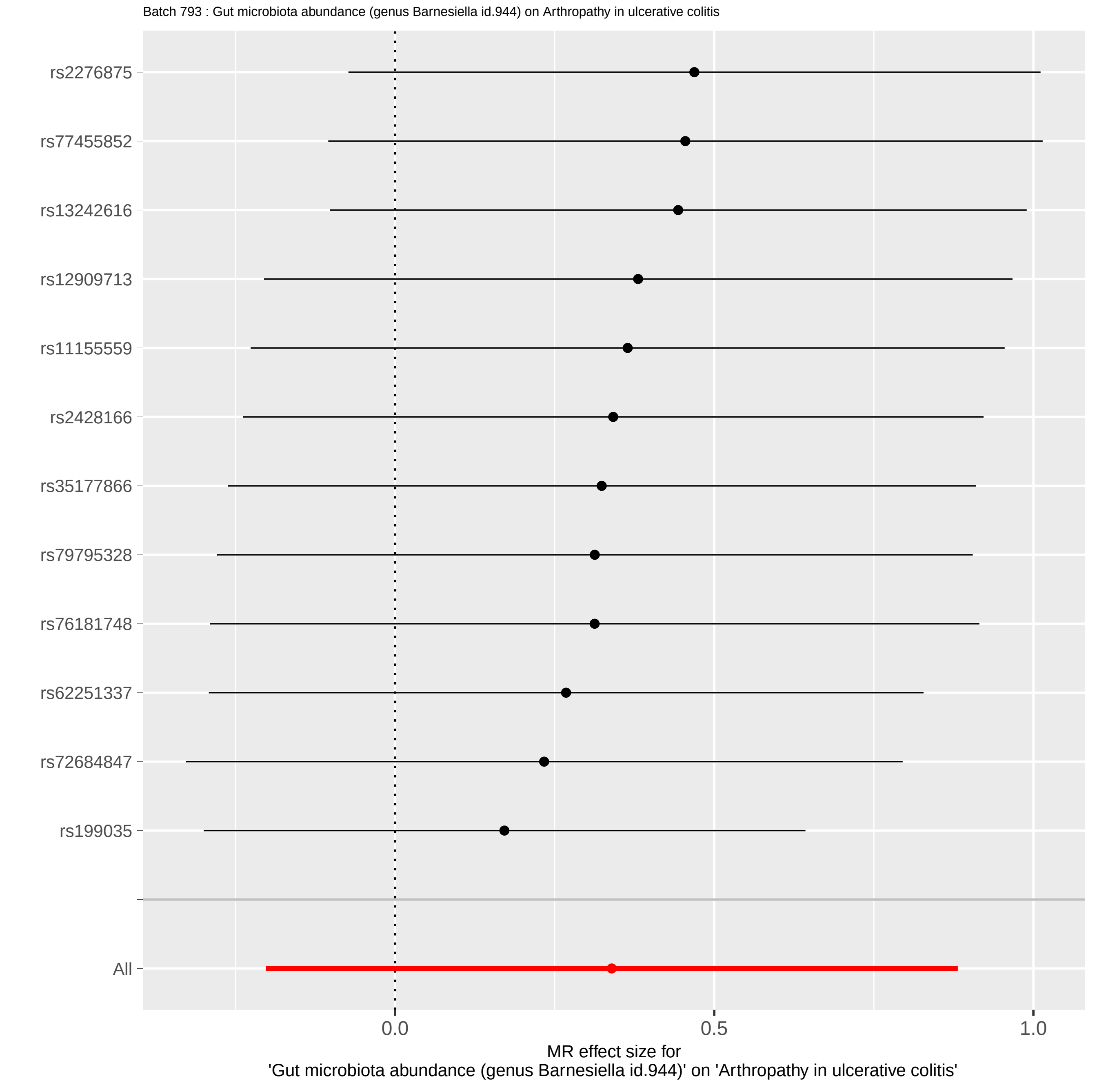

Batch 793 : Gut microbiota abundance (genus Barnesiella id.944) on Arthropathy in ulcerative colitis
rs2276875
rs77455852
rs13242616
rs12909713
rs11155559
rs2428166
rs35177866
rs79795328
rs76181748
rs62251337
rs72684847
rs199035
All
0.0
0.5
1.0
MR effect size for
'Gut microbiota abundance (genus Barnesiella id.944)' on 'Arthropathy in ulcerative colitis'

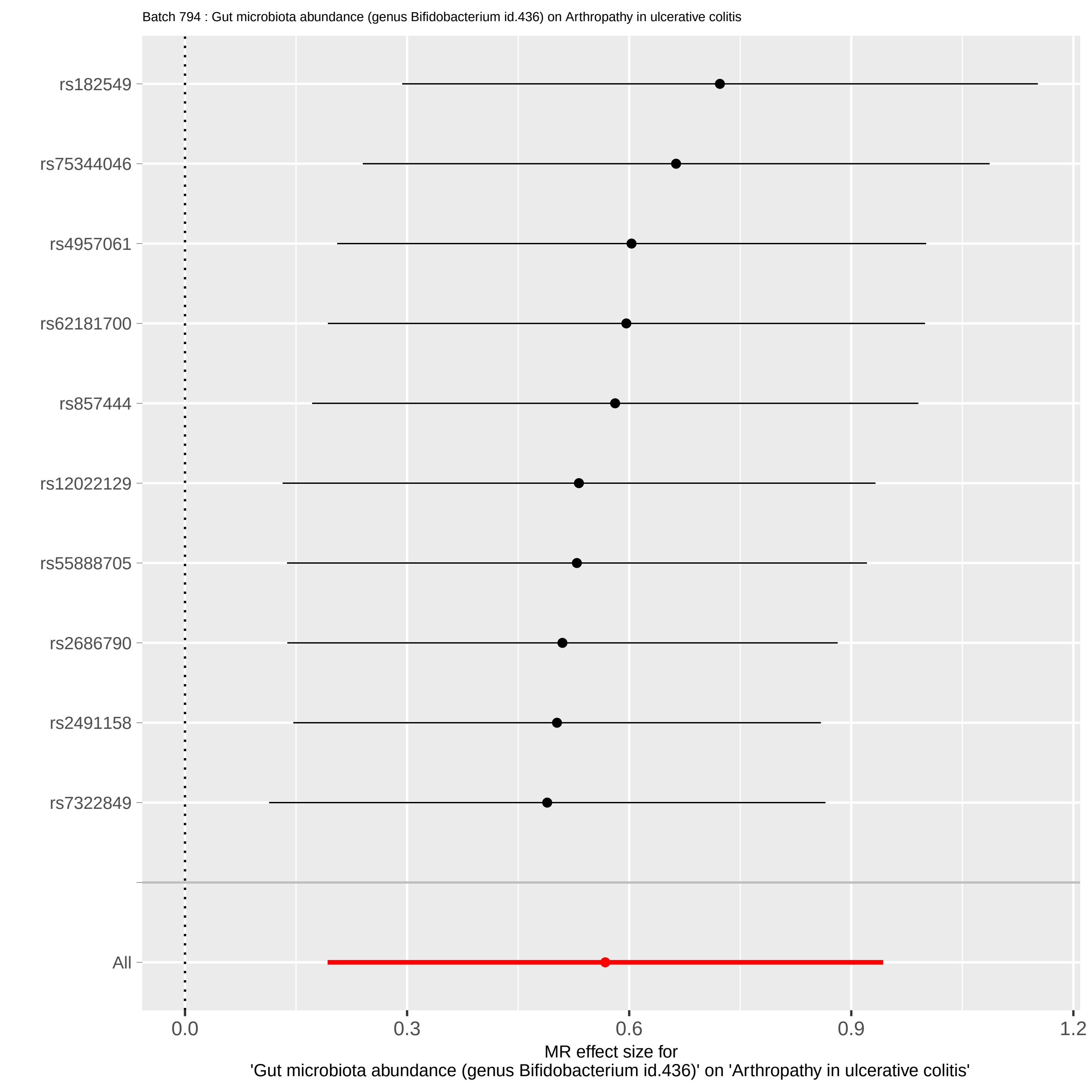

Batch 794 : Gut microbiota abundance (genus Bifidobacterium id.436) on Arthropathy in ulcerative colitis
rs182549
rs75344046
rs4957061
rs62181700
rs857444
rs12022129
rs55888705
rs2686790
rs2491158
rs7322849
All
0.0
0.3
0.6
0.9
1.2
MR effect size for
'Gut microbiota abundance (genus Bifidobacterium id.436)' on 'Arthropathy in ulcerative colitis'

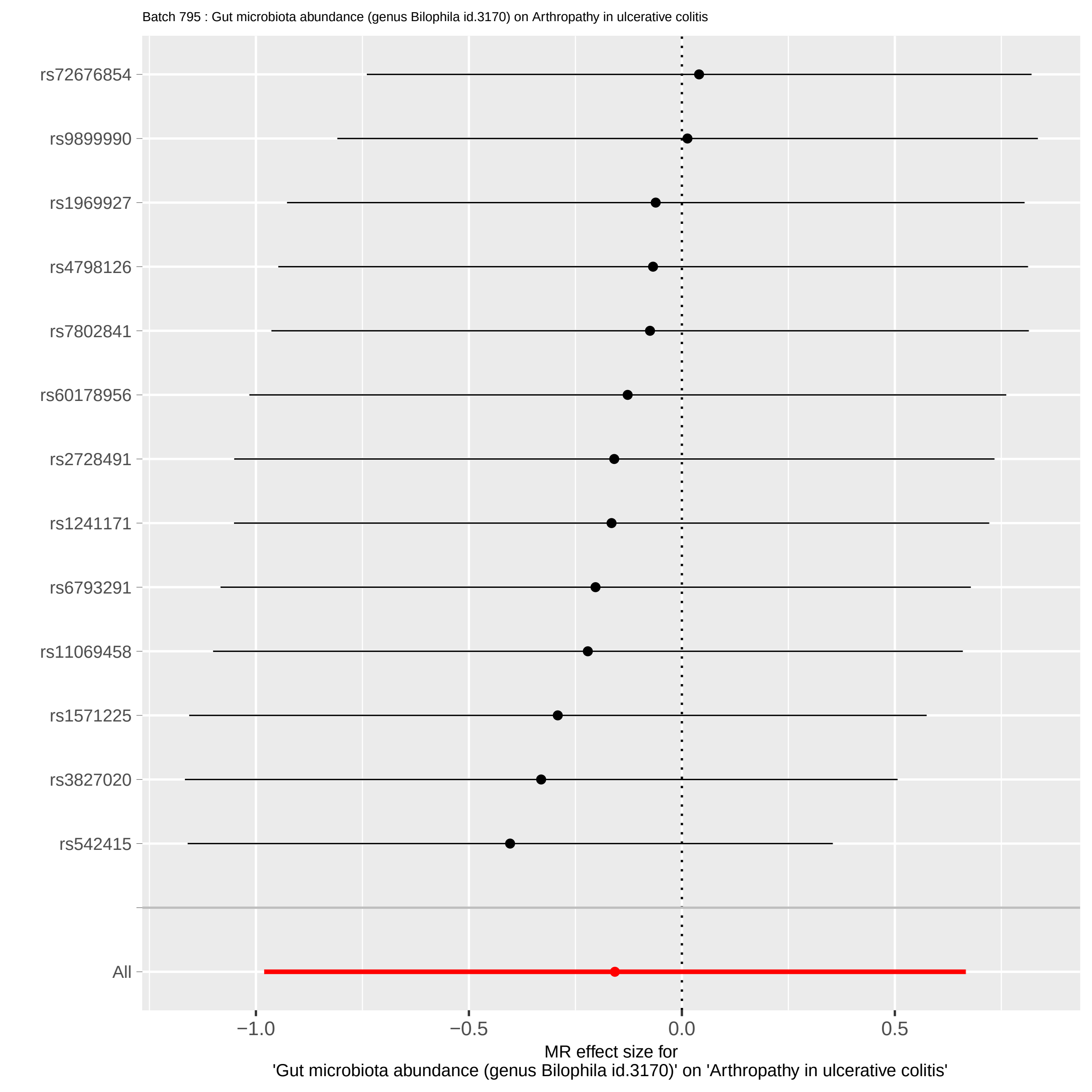

Batch 795 : Gut microbiota abundance (genus Bilophila id.3170) on Arthropathy in ulcerative colitis
rs72676854
rs9899990
rs1969927
rs4798126
rs7802841
rs60178956
rs2728491
rs1241171
rs6793291
rs11069458
rs1571225
rs3827020
rs542415
All
−1.0
−0.5
0.0
0.5
MR effect size for
'Gut microbiota abundance (genus Bilophila id.3170)' on 'Arthropathy in ulcerative colitis'

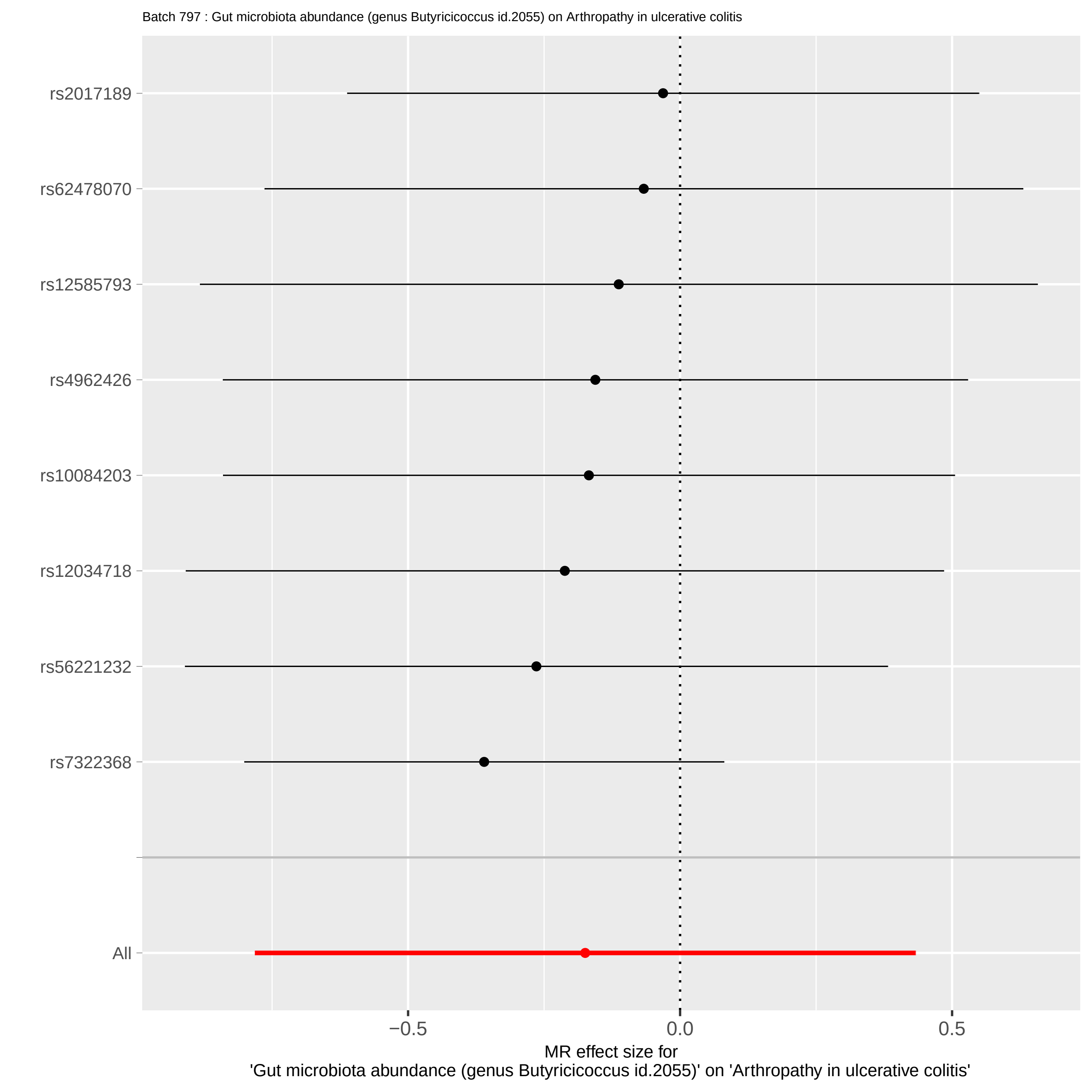

Batch 797 : Gut microbiota abundance (genus Butyricicoccus id.2055) on Arthropathy in ulcerative colitis
rs2017189
rs62478070
rs12585793
rs4962426
rs10084203
rs12034718
rs56221232
rs7322368
All
−0.5
0.0
0.5
MR effect size for
'Gut microbiota abundance (genus Butyricicoccus id.2055)' on 'Arthropathy in ulcerative colitis'

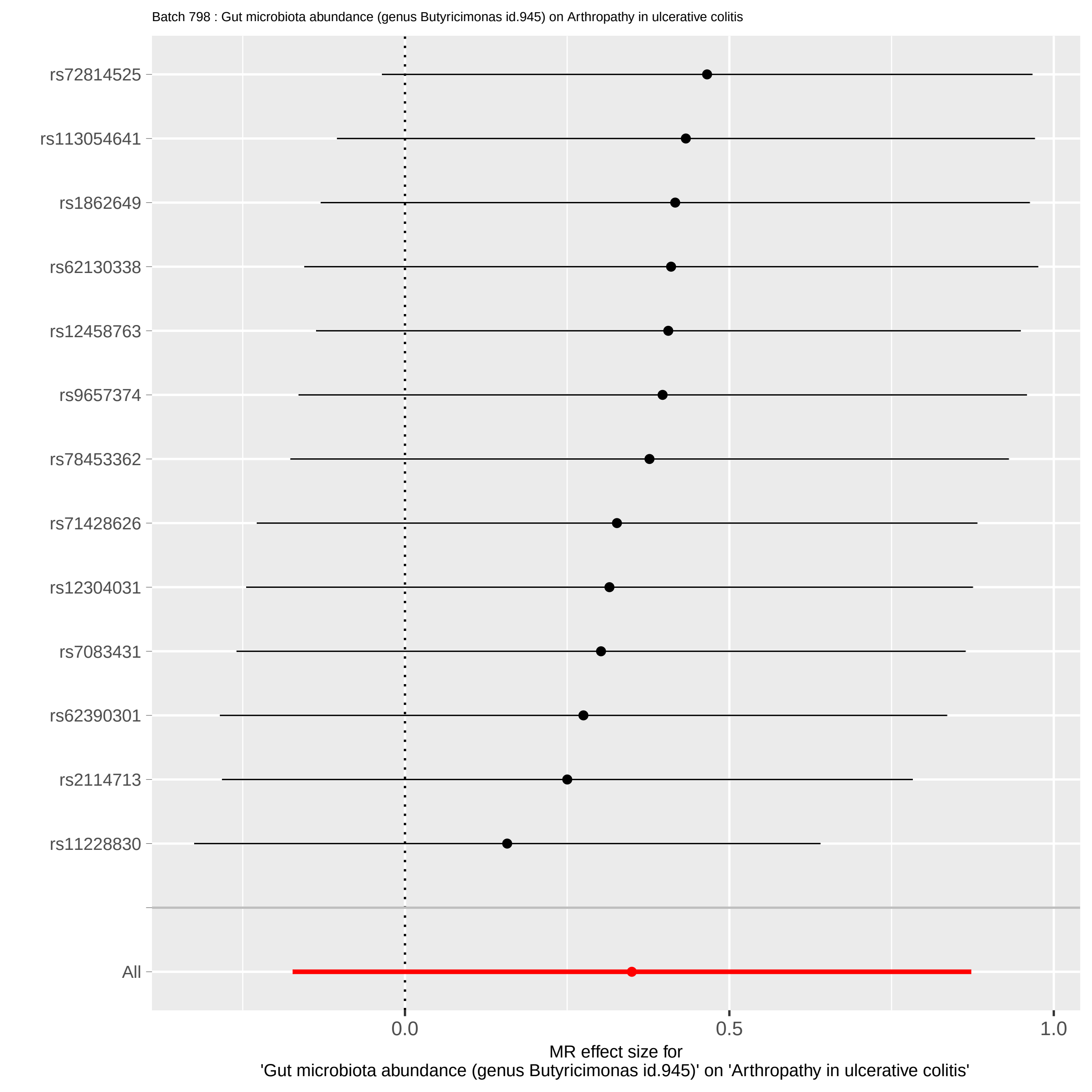

Batch 798 : Gut microbiota abundance (genus Butyricimonas id.945) on Arthropathy in ulcerative colitis
rs72814525
rs113054641
rs1862649
rs62130338
rs12458763
rs9657374
rs78453362
rs71428626
rs12304031
rs7083431
rs62390301
rs2114713
rs11228830
All
0.0
0.5
1.0
MR effect size for
'Gut microbiota abundance (genus Butyricimonas id.945)' on 'Arthropathy in ulcerative colitis'

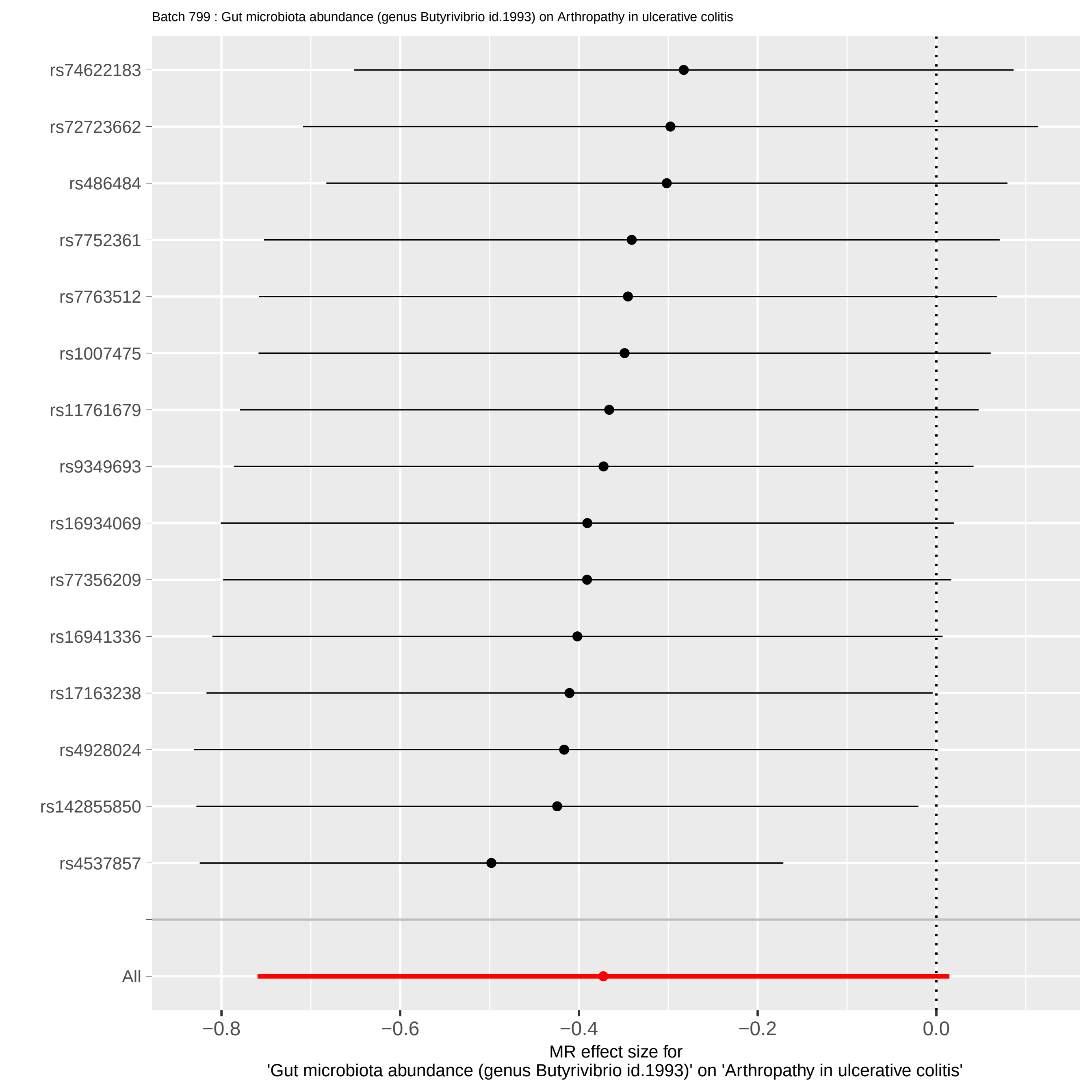
Batch 799 : Gut microbiota abundance (genus Butyrivibrio id.1993) on Arthropathy in ulcerative colitis
rs74622183
rs72723662
rs486484
rs7752361
rs7763512
rs1007475
rs11761679
rs9349693
rs16934069
rs77356209
rs16941336
rs17163238
rs4928024
rs142855850
rs4537857
All
−0.8
−0.6
−0.4
−0.2
0.0
MR effect size for
'Gut microbiota abundance (genus Butyrivibrio id.1993)' on 'Arthropathy in ulcerative colitis'

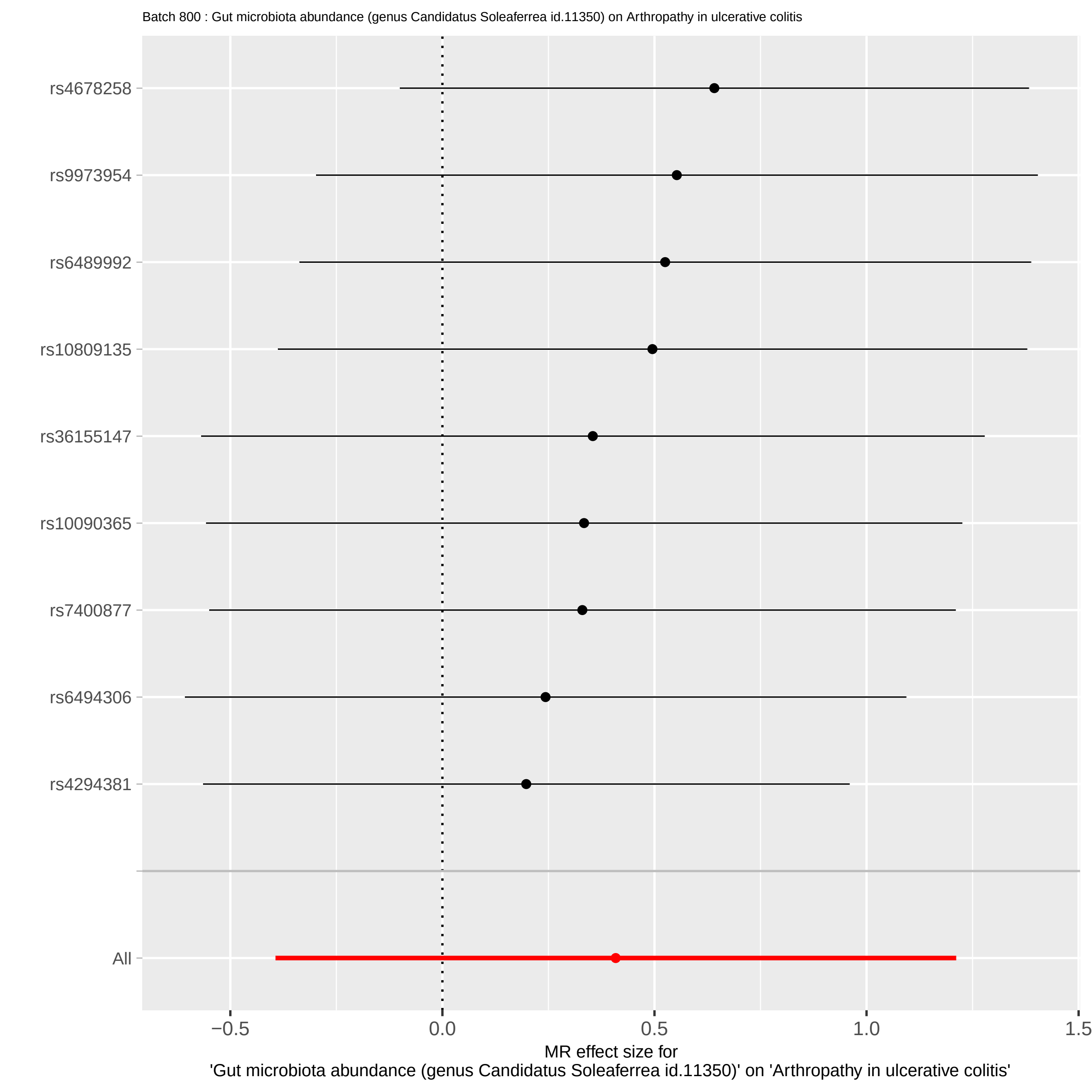

Batch 800 : Gut microbiota abundance (genus Candidatus Soleaferrea id.11350) on Arthropathy in ulcerative colitis
rs4678258
rs9973954
rs6489992
rs10809135
rs36155147
rs10090365
rs7400877
rs6494306
rs4294381
All
−0.5
0.0
0.5
1.0
1.5
MR effect size for
'Gut microbiota abundance (genus Candidatus Soleaferrea id.11350)' on 'Arthropathy in ulcerative colitis'

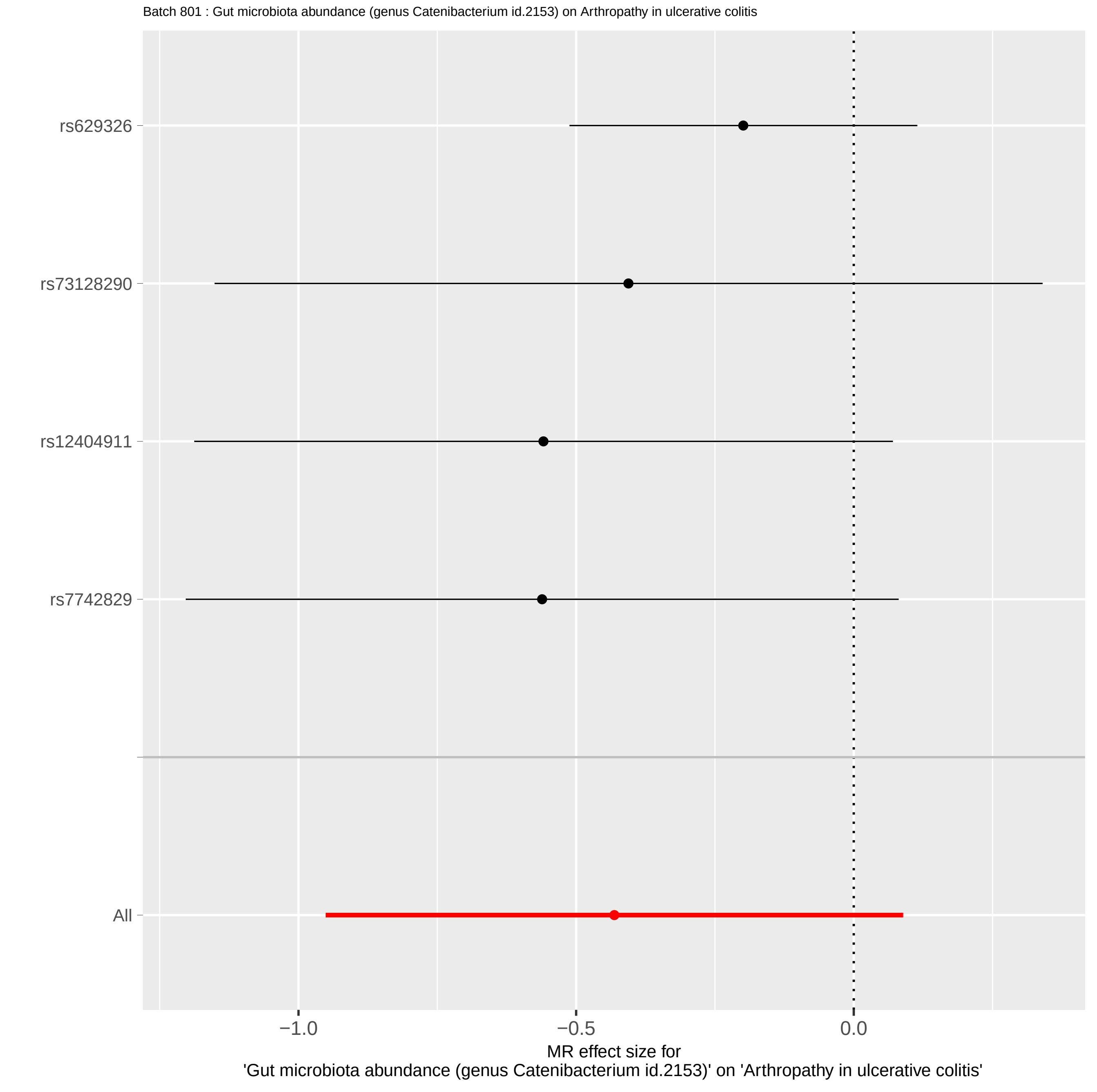
Batch 801 : Gut microbiota abundance (genus Catenibacterium id.2153) on Arthropathy in ulcerative colitis
rs629326
rs73128290
rs12404911
rs7742829
All
−1.0
−0.5
0.0
MR effect size for
'Gut microbiota abundance (genus Catenibacterium id.2153)' on 'Arthropathy in ulcerative colitis'

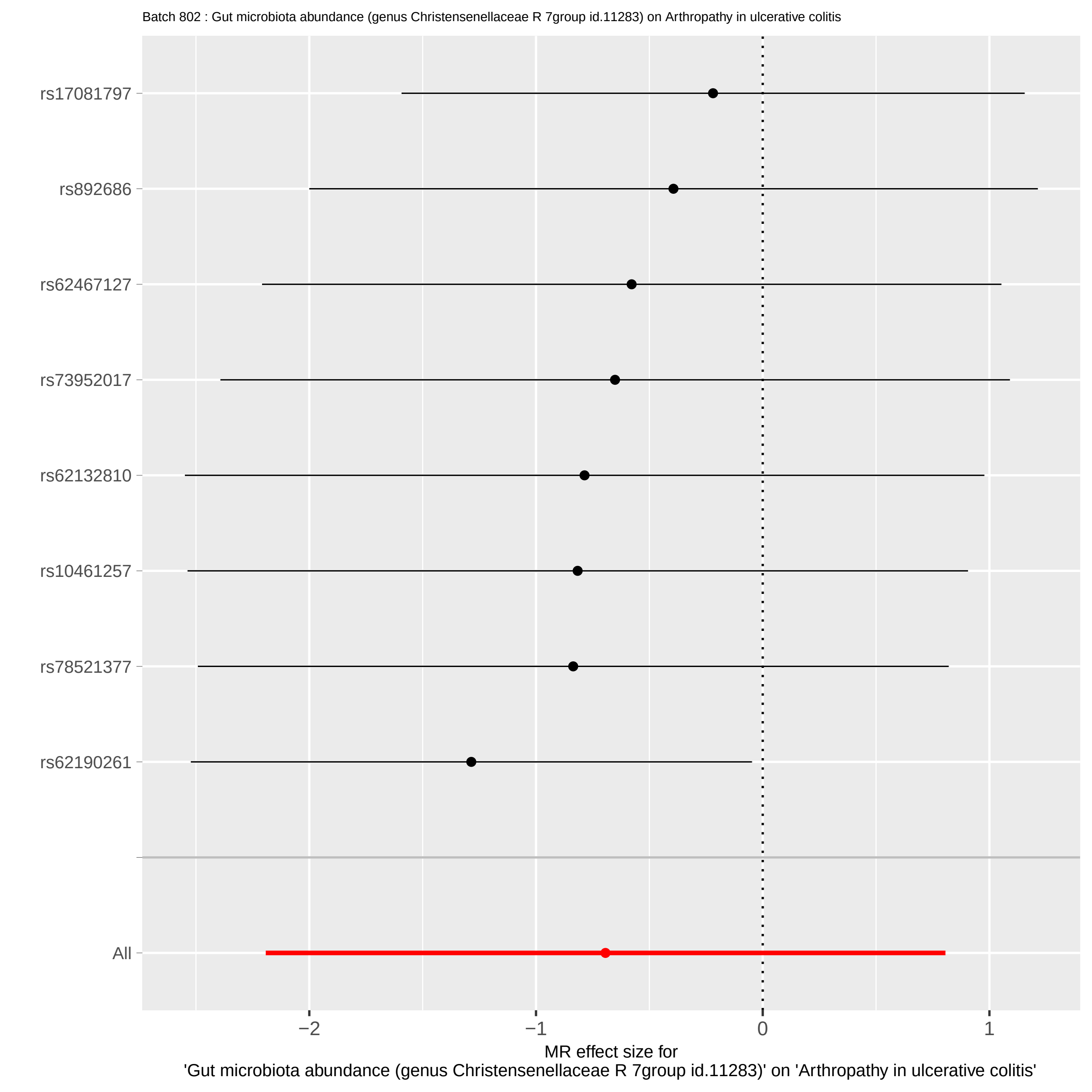
Batch 802 : Gut microbiota abundance (genus Christensenellaceae R 7group id.11283) on Arthropathy in ulcerative colitis
rs17081797
rs892686
rs62467127
rs73952017
rs62132810
rs10461257
rs78521377
rs62190261
All
−2
−1
0
1
MR effect size for
'Gut microbiota abundance (genus Christensenellaceae R 7group id.11283)' on 'Arthropathy in ulcerative colitis'

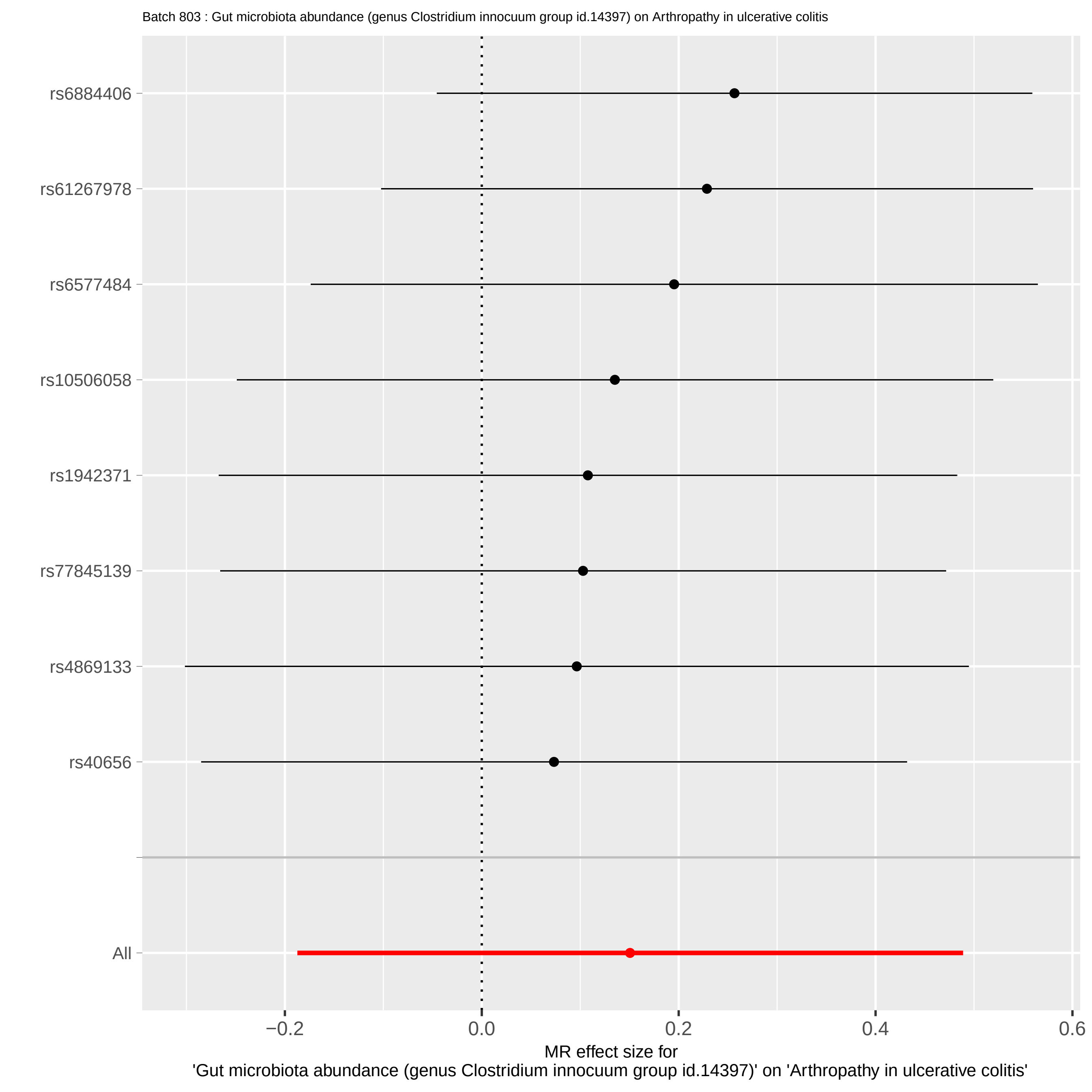

Batch 803 : Gut microbiota abundance (genus Clostridium innocuum group id.14397) on Arthropathy in ulcerative colitis
rs6884406
rs61267978
rs6577484
rs10506058
rs1942371
rs77845139
rs4869133
rs40656
All
−0.2
0.0
0.2
0.4
0.6
MR effect size for
'Gut microbiota abundance (genus Clostridium innocuum group id.14397)' on 'Arthropathy in ulcerative colitis'

Batch 804 : Gut microbiota abundance (genus Clostridium sensustricto1 id.1873) on Arthropathy in ulcerative colitis

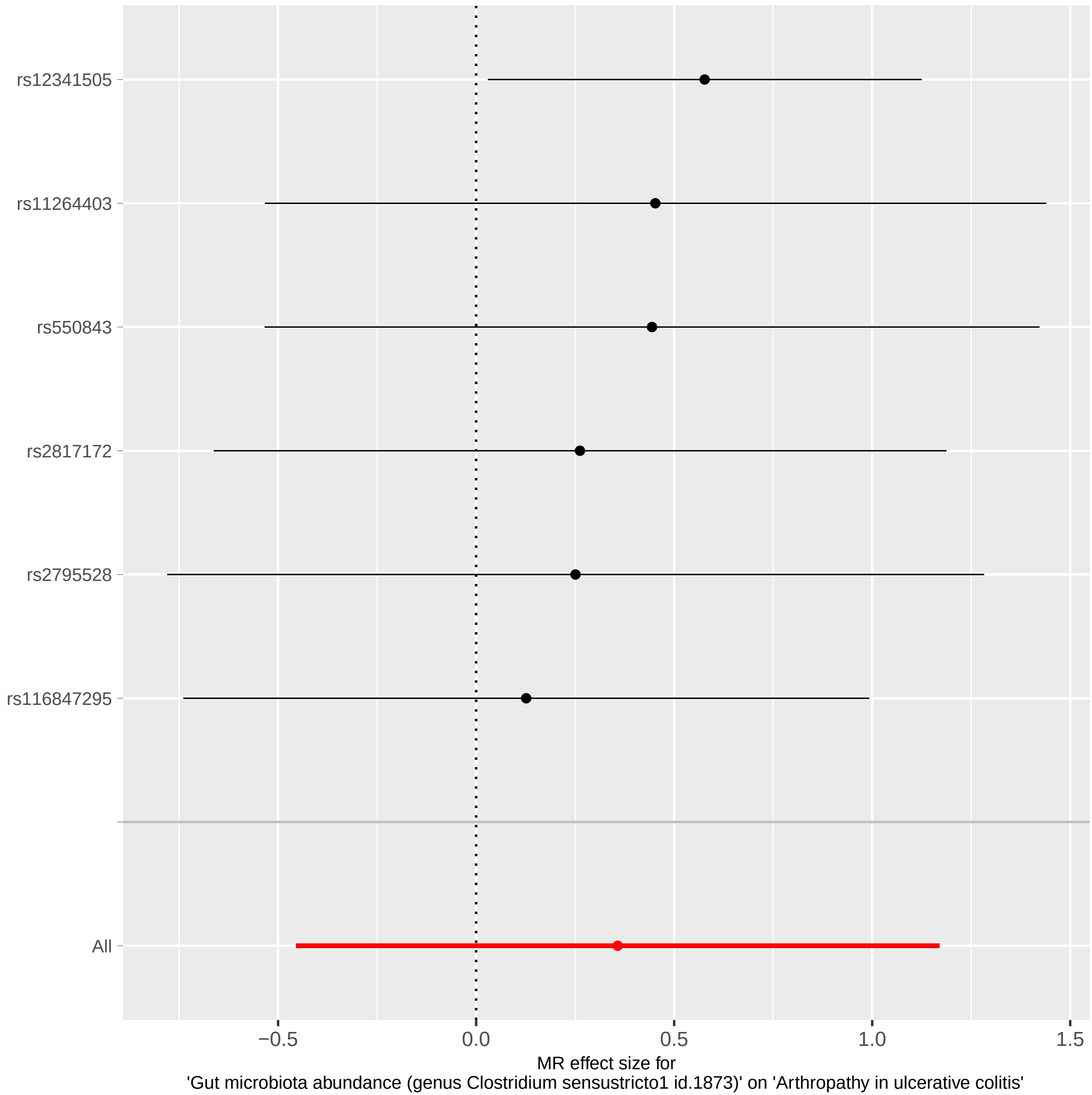

Batch 805 : Gut microbiota abundance (genus Collinsella id.815) on Arthropathy in ulcerative colitis

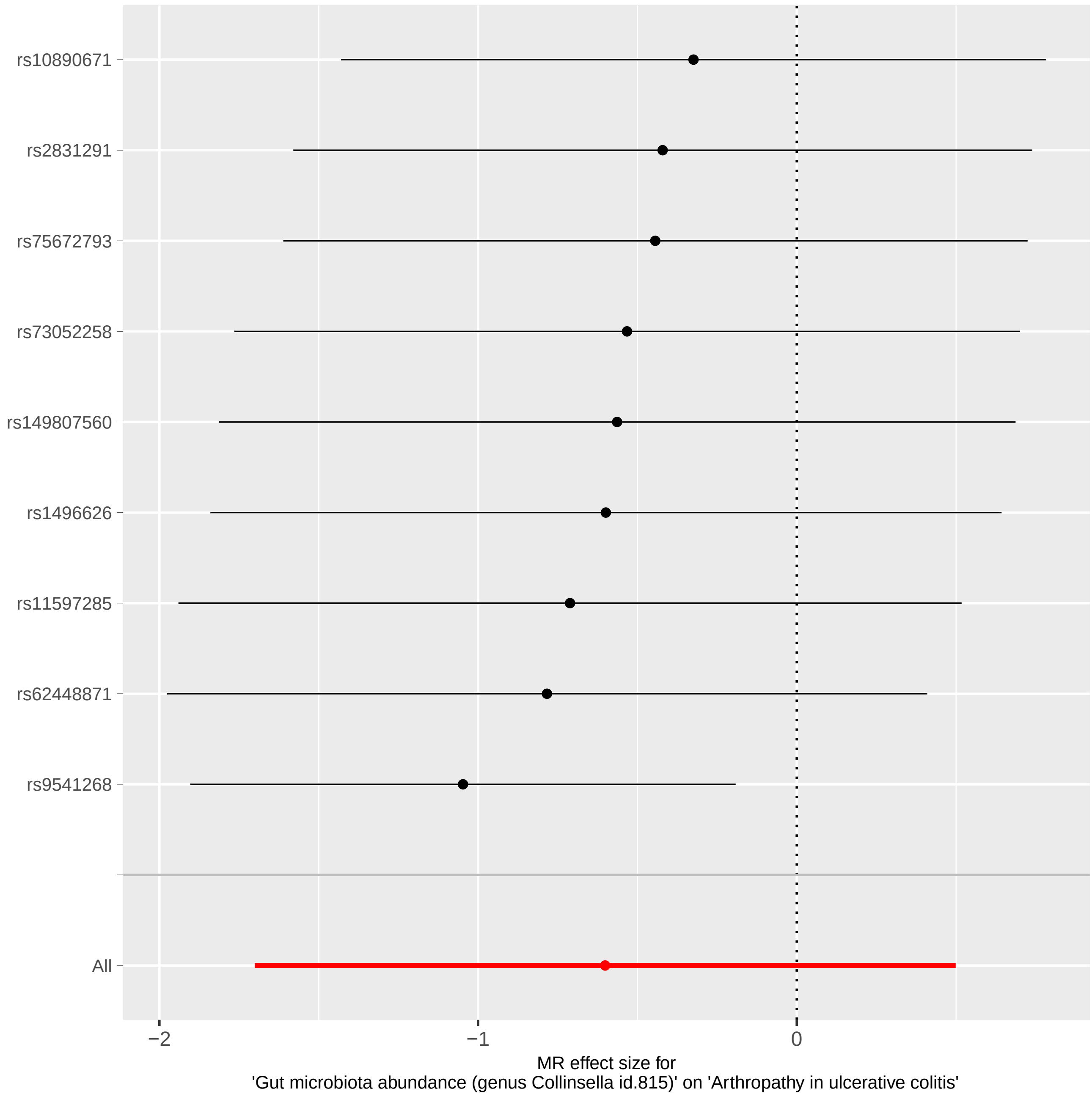

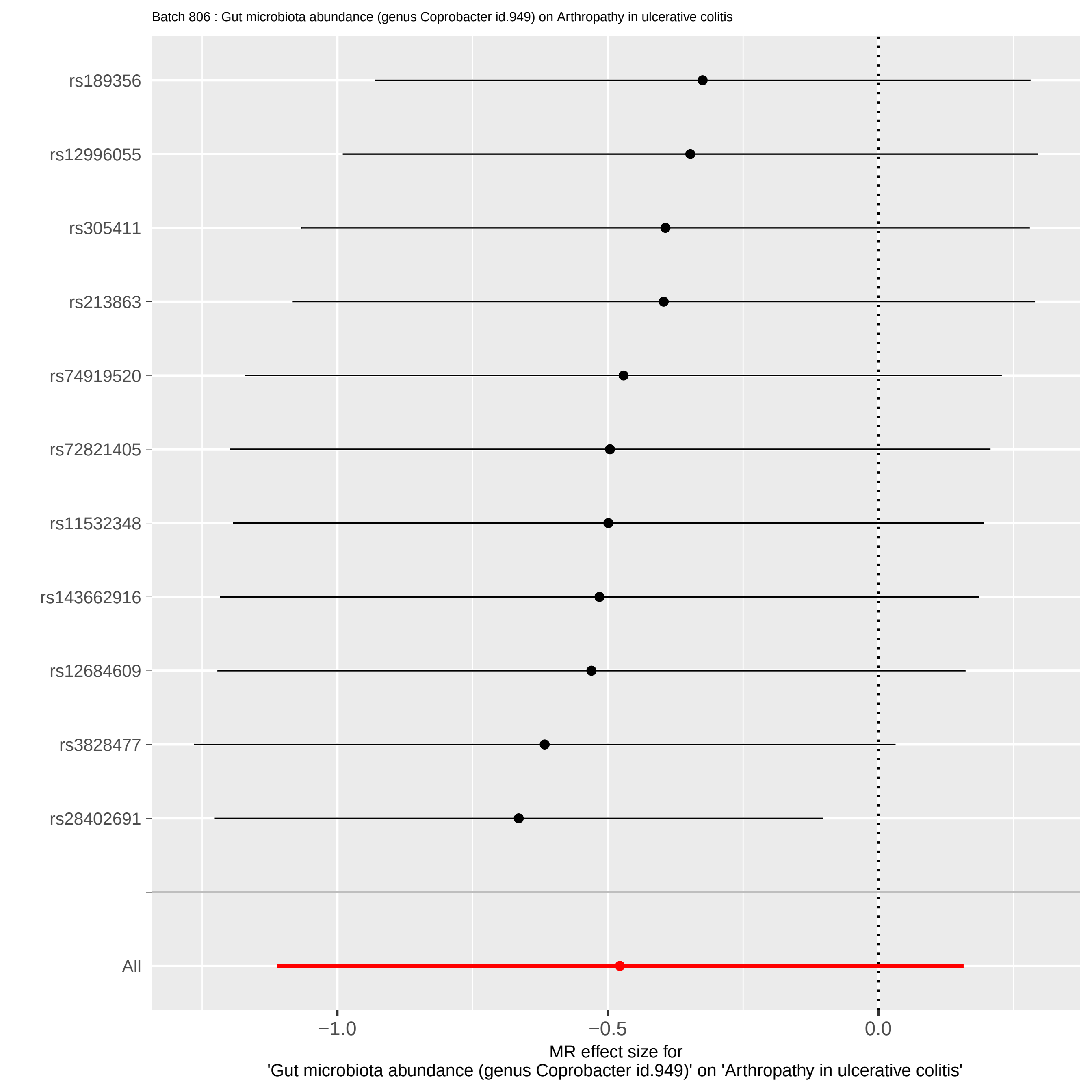

Batch 806 : Gut microbiota abundance (genus Coprobacter id.949) on Arthropathy in ulcerative colitis
rs189356
rs12996055
rs305411
rs213863
rs74919520
rs72821405
rs11532348
rs143662916
rs12684609
rs3828477
rs28402691
All
−1.0
−0.5
0.0
MR effect size for
'Gut microbiota abundance (genus Coprobacter id.949)' on 'Arthropathy in ulcerative colitis'

Batch 807 : Gut microbiota abundance (genus Coprococcus1 id.11301) on Arthropathy in ulcerative colitis

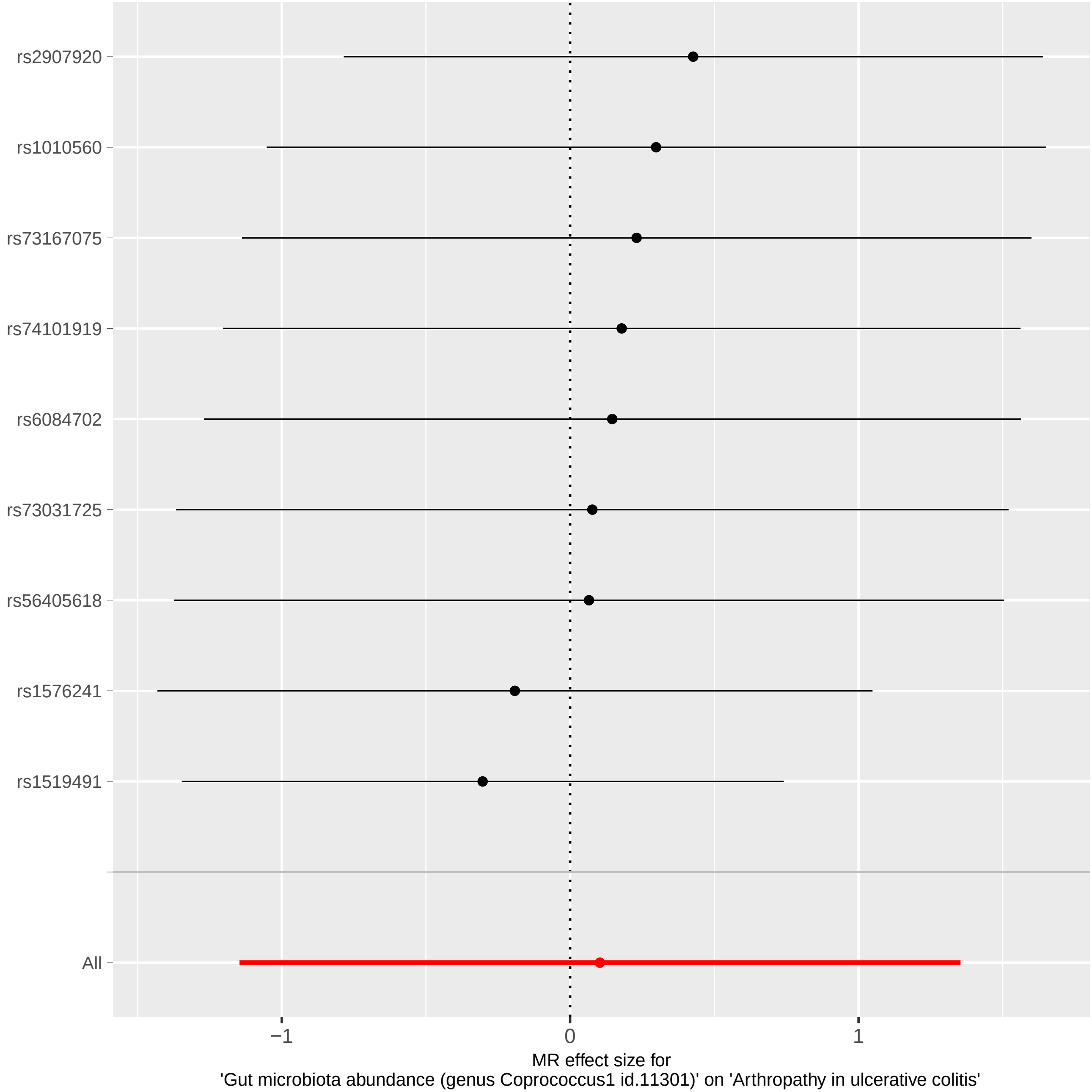

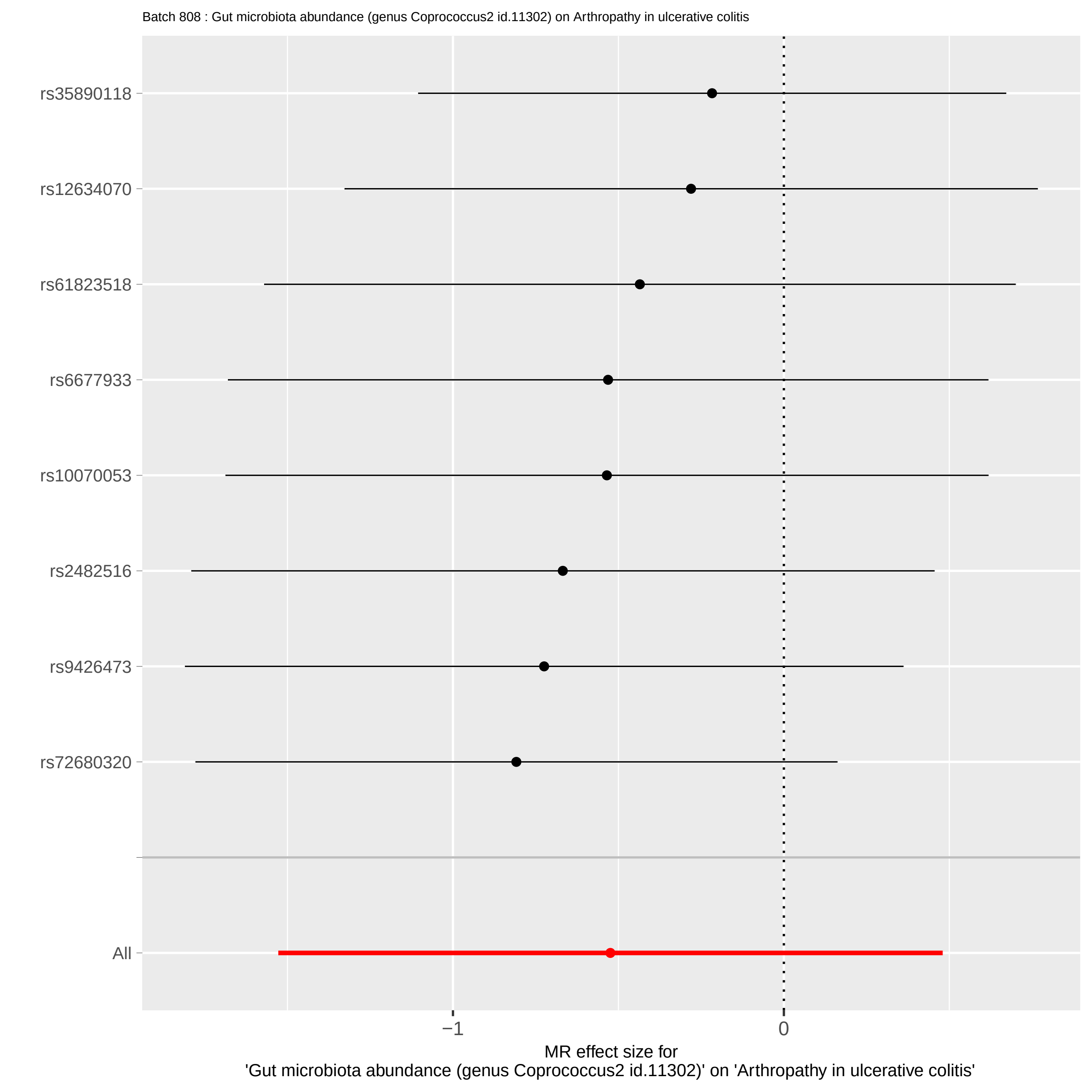
Batch 808 : Gut microbiota abundance (genus Coprococcus2 id.11302) on Arthropathy in ulcerative colitis
rs35890118
rs12634070
rs61823518
rs6677933
rs10070053
rs2482516
rs9426473
rs72680320
All
−1
0
MR effect size for
'Gut microbiota abundance (genus Coprococcus2 id.11302)' on 'Arthropathy in ulcerative colitis'

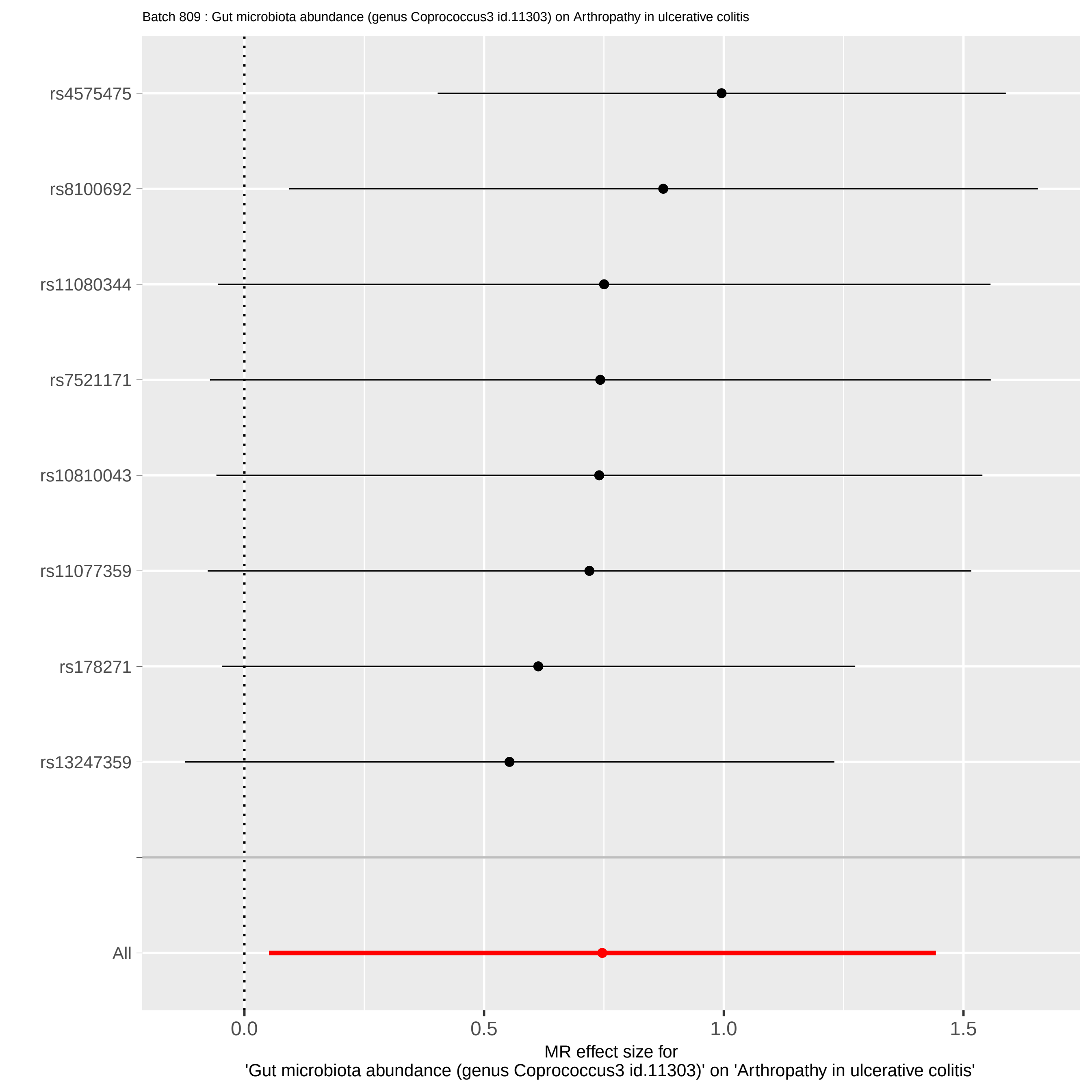
Batch 809 : Gut microbiota abundance (genus Coprococcus3 id.11303) on Arthropathy in ulcerative colitis
rs4575475
rs8100692
rs11080344
rs7521171
rs10810043
rs11077359
rs178271
rs13247359
All
0.0
0.5
1.0
1.5
MR effect size for
'Gut microbiota abundance (genus Coprococcus3 id.11303)' on 'Arthropathy in ulcerative colitis'

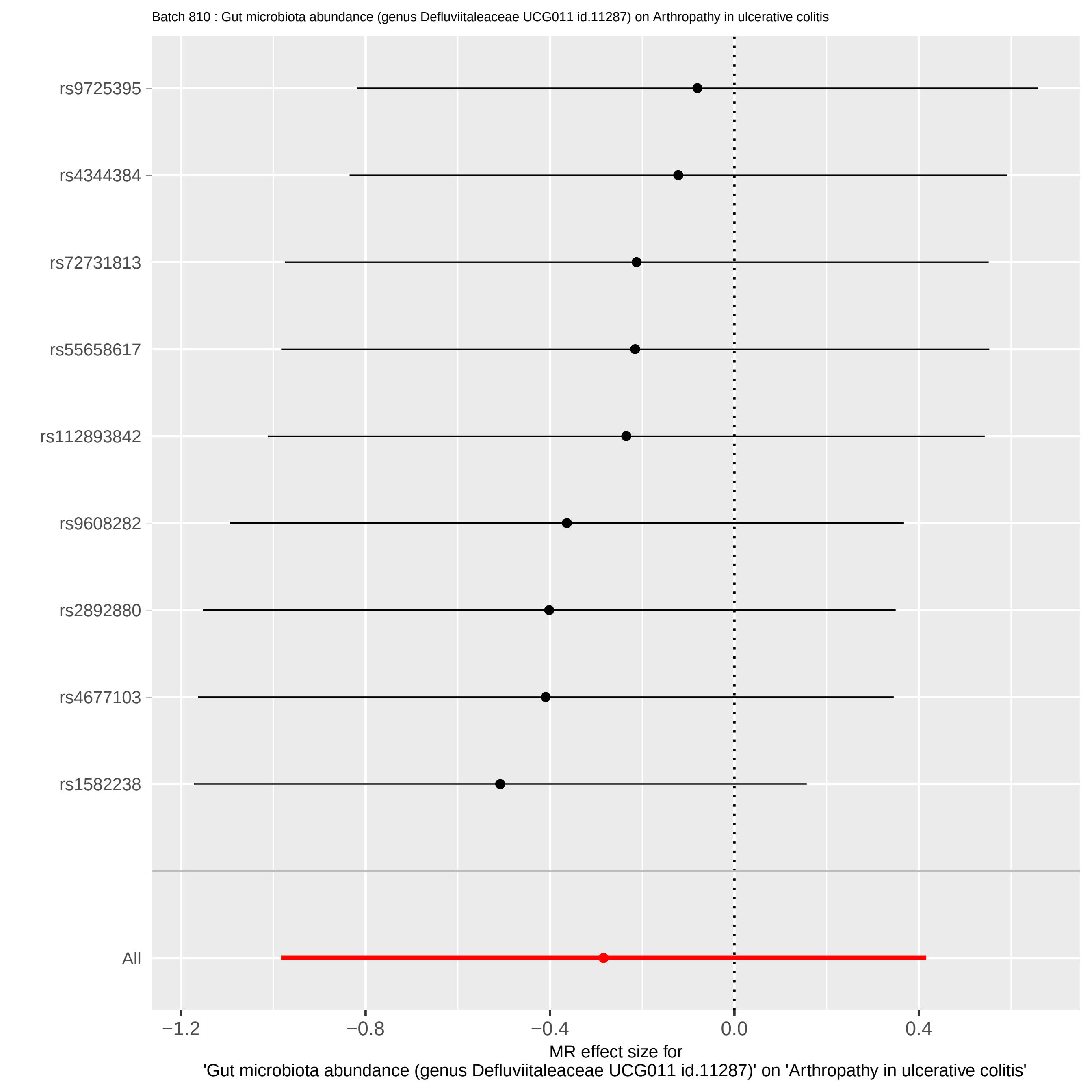
Batch 810 : Gut microbiota abundance (genus Defluviitaleaceae UCG011 id.11287) on Arthropathy in ulcerative colitis
rs9725395
rs4344384
rs72731813
rs55658617
rs112893842
rs9608282
rs2892880
rs4677103
rs1582238
All
−1.2
−0.8
−0.4
0.0
0.4
MR effect size for
'Gut microbiota abundance (genus Defluviitaleaceae UCG011 id.11287)' on 'Arthropathy in ulcerative colitis'

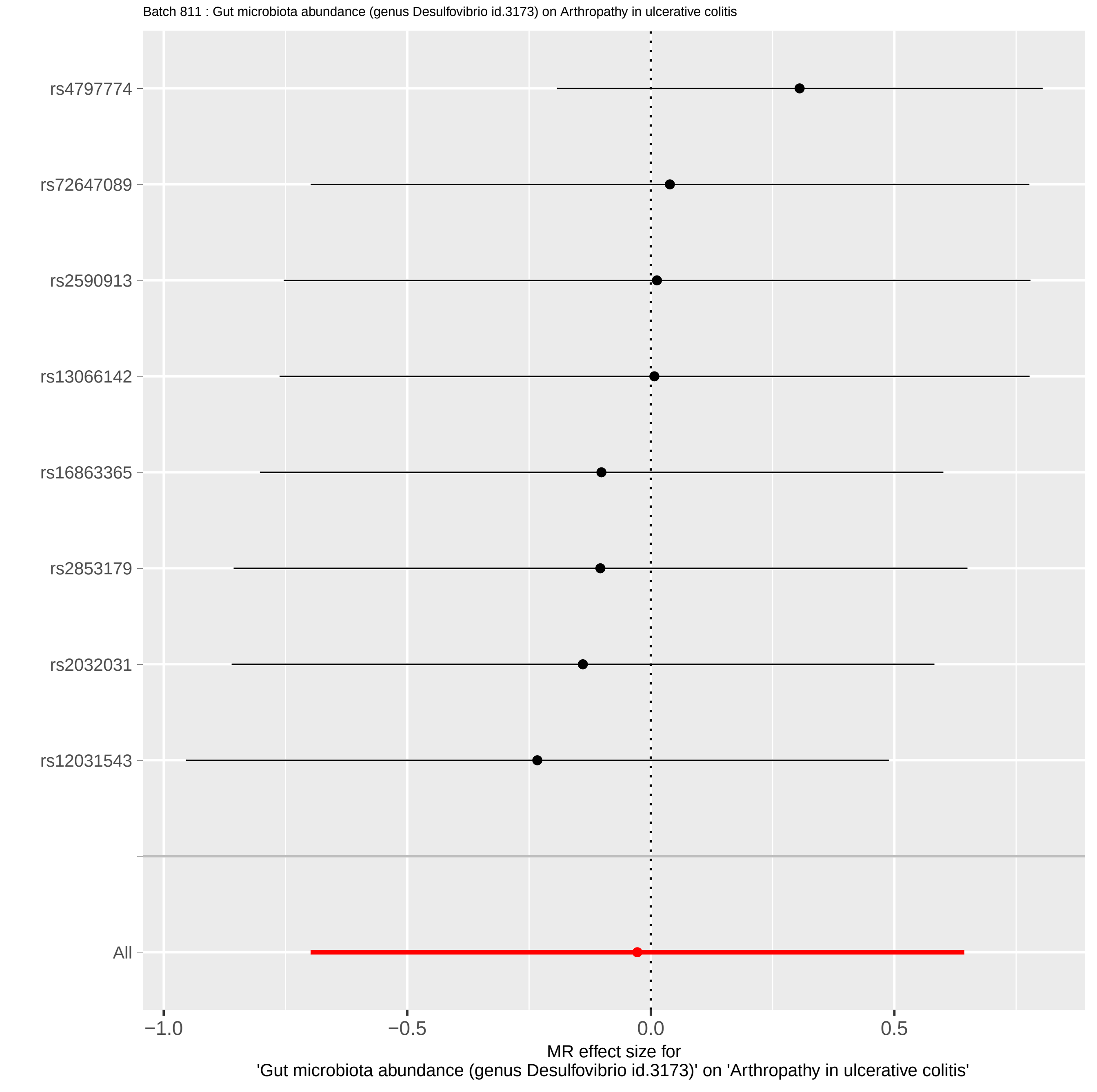

Batch 811 : Gut microbiota abundance (genus Desulfovibrio id.3173) on Arthropathy in ulcerative colitis
rs4797774
rs72647089
rs2590913
rs13066142
rs16863365
rs2853179
rs2032031
rs12031543
All
−1.0
−0.5
0.0
0.5
MR effect size for
'Gut microbiota abundance (genus Desulfovibrio id.3173)' on 'Arthropathy in ulcerative colitis'

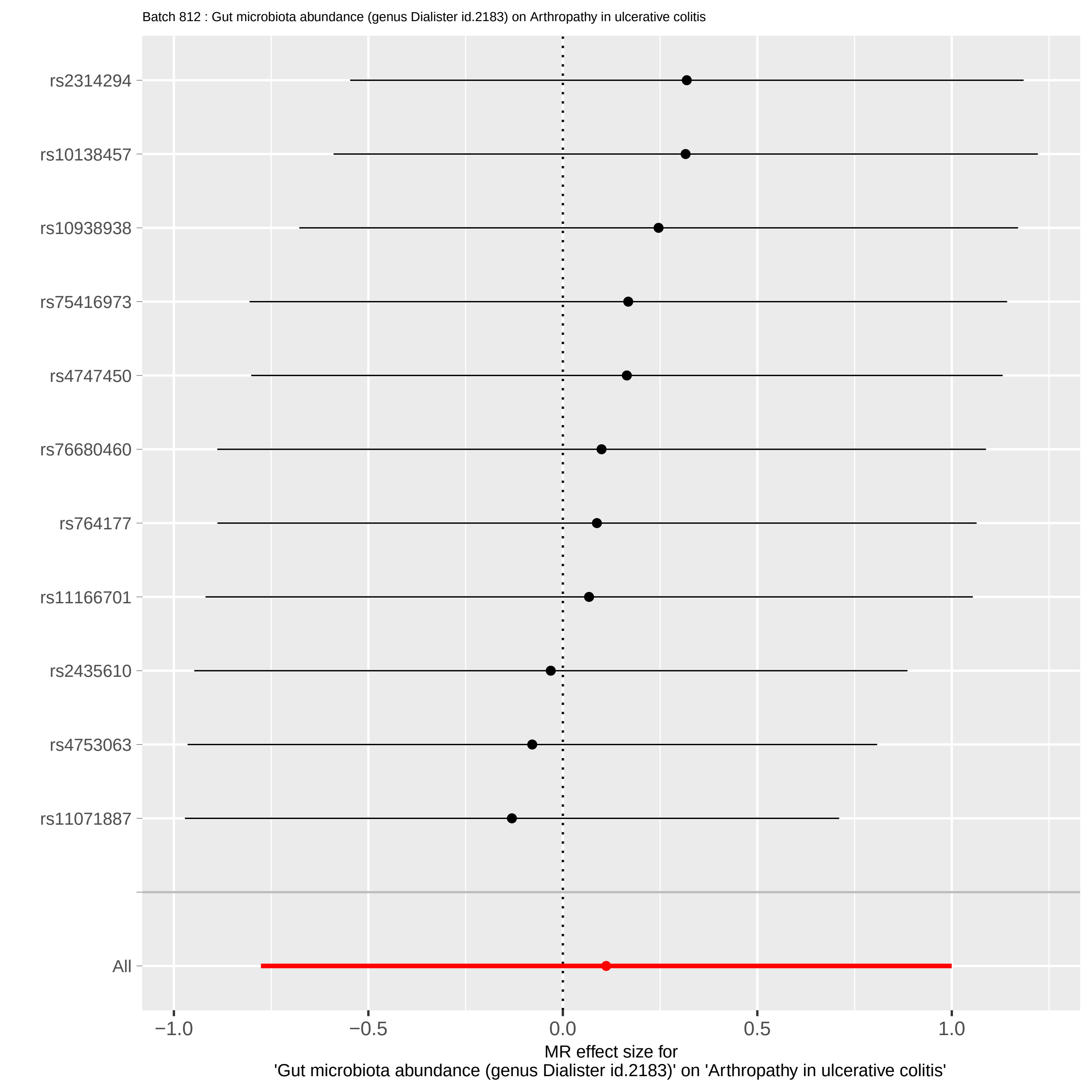
Batch 812 : Gut microbiota abundance (genus Dialister id.2183) on Arthropathy in ulcerative colitis
rs2314294
rs10138457
rs10938938
rs75416973
rs4747450
rs76680460
rs764177
rs11166701
rs2435610
rs4753063
rs11071887
All
−1.0
−0.5
0.0
0.5
1.0
MR effect size for
'Gut microbiota abundance (genus Dialister id.2183)' on 'Arthropathy in ulcerative colitis'

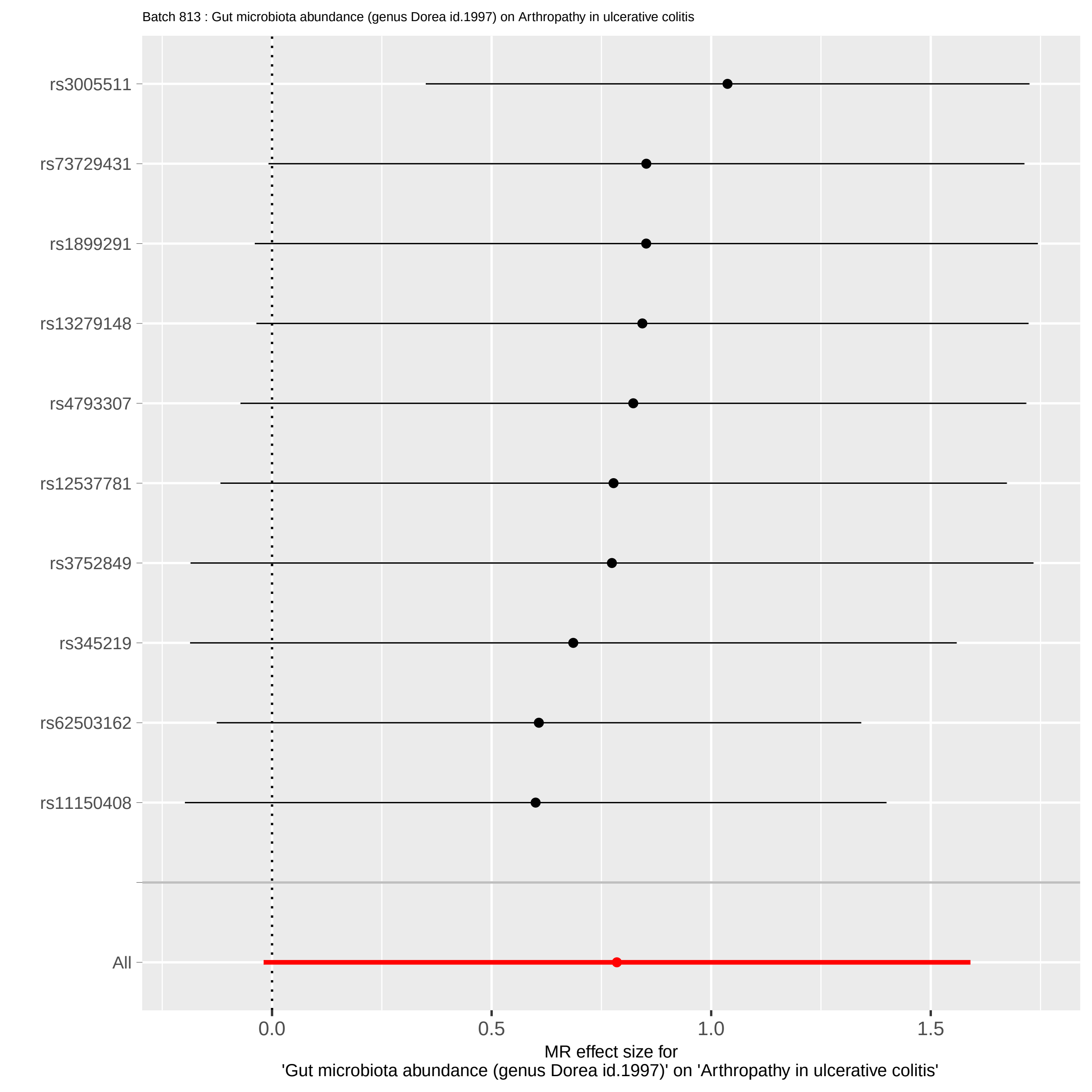

Batch 813 : Gut microbiota abundance (genus Dorea id.1997) on Arthropathy in ulcerative colitis
rs3005511
rs73729431
rs1899291
rs13279148
rs4793307
rs12537781
rs3752849
rs345219
rs62503162
rs11150408
All
0.0
0.5
1.0
1.5
MR effect size for
'Gut microbiota abundance (genus Dorea id.1997)' on 'Arthropathy in ulcerative colitis'

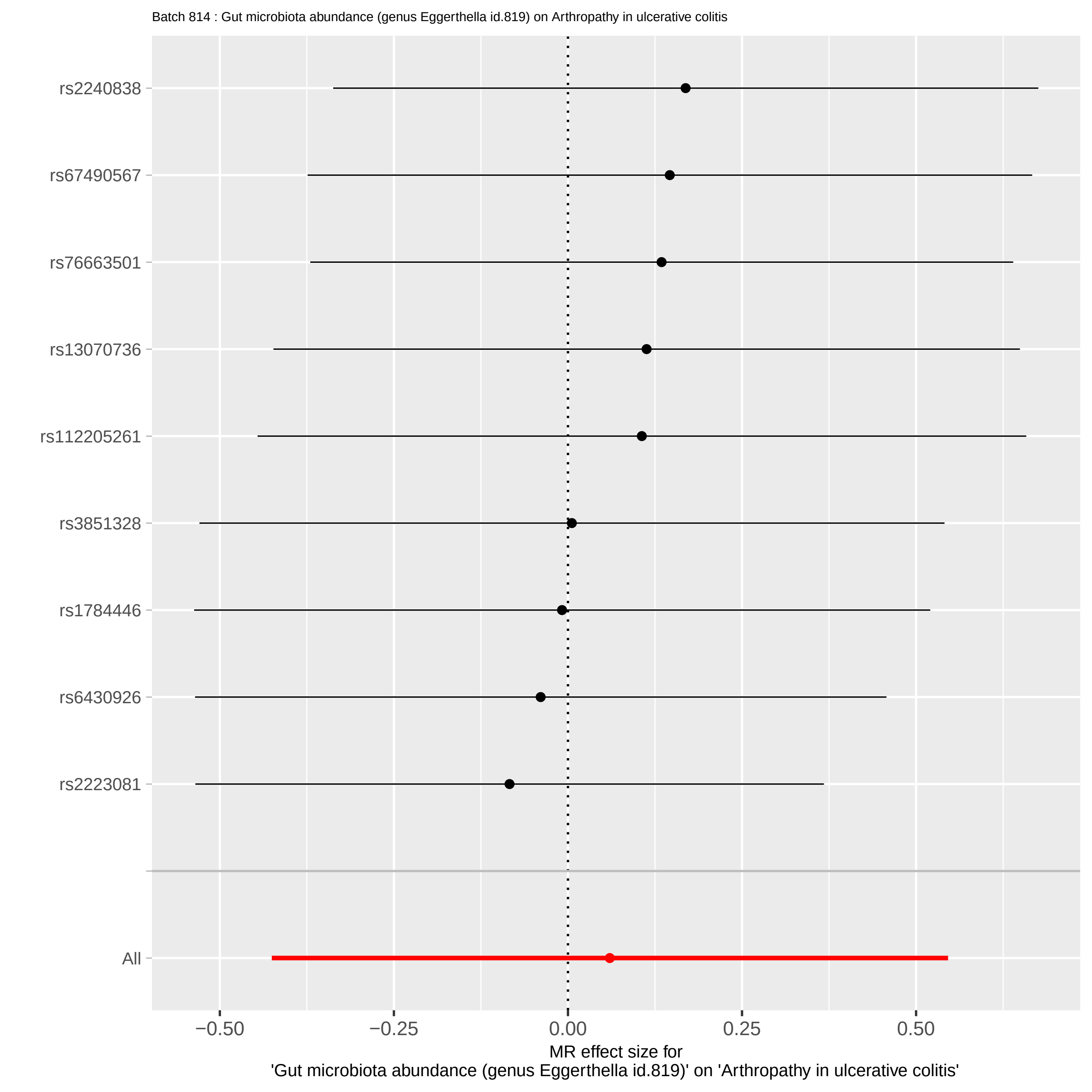

Batch 814 : Gut microbiota abundance (genus Eggerthella id.819) on Arthropathy in ulcerative colitis
rs2240838
rs67490567
rs76663501
rs13070736
rs112205261
rs3851328
rs1784446
rs6430926
rs2223081
All
−0.50
−0.25
0.00
0.25
0.50
MR effect size for
'Gut microbiota abundance (genus Eggerthella id.819)' on 'Arthropathy in ulcerative colitis'

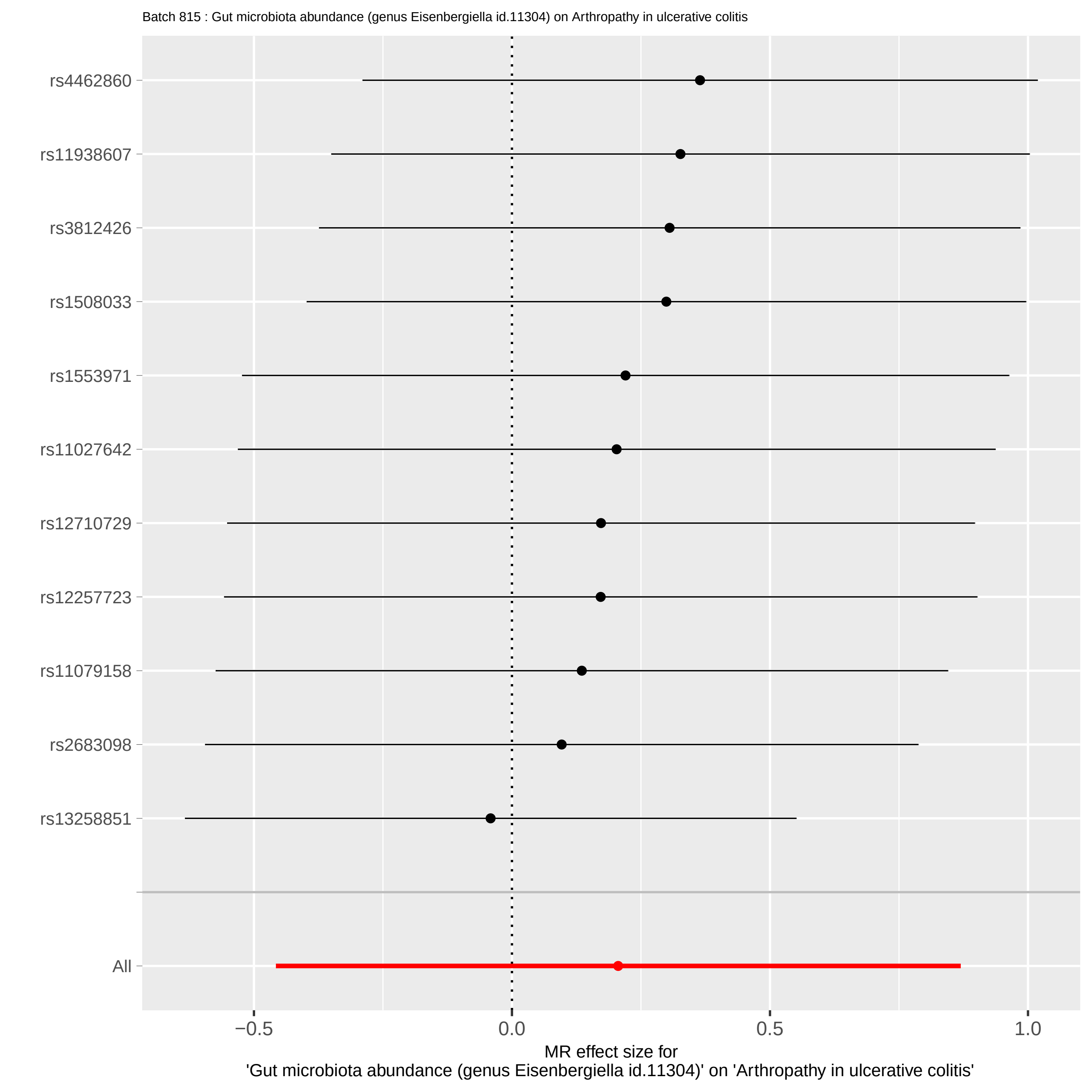

Batch 815 : Gut microbiota abundance (genus Eisenbergiella id.11304) on Arthropathy in ulcerative colitis
rs4462860
rs11938607
rs3812426
rs1508033
rs1553971
rs11027642
rs12710729
rs12257723
rs11079158
rs2683098
rs13258851
All
−0.5
0.0
0.5
1.0
MR effect size for
'Gut microbiota abundance (genus Eisenbergiella id.11304)' on 'Arthropathy in ulcerative colitis'

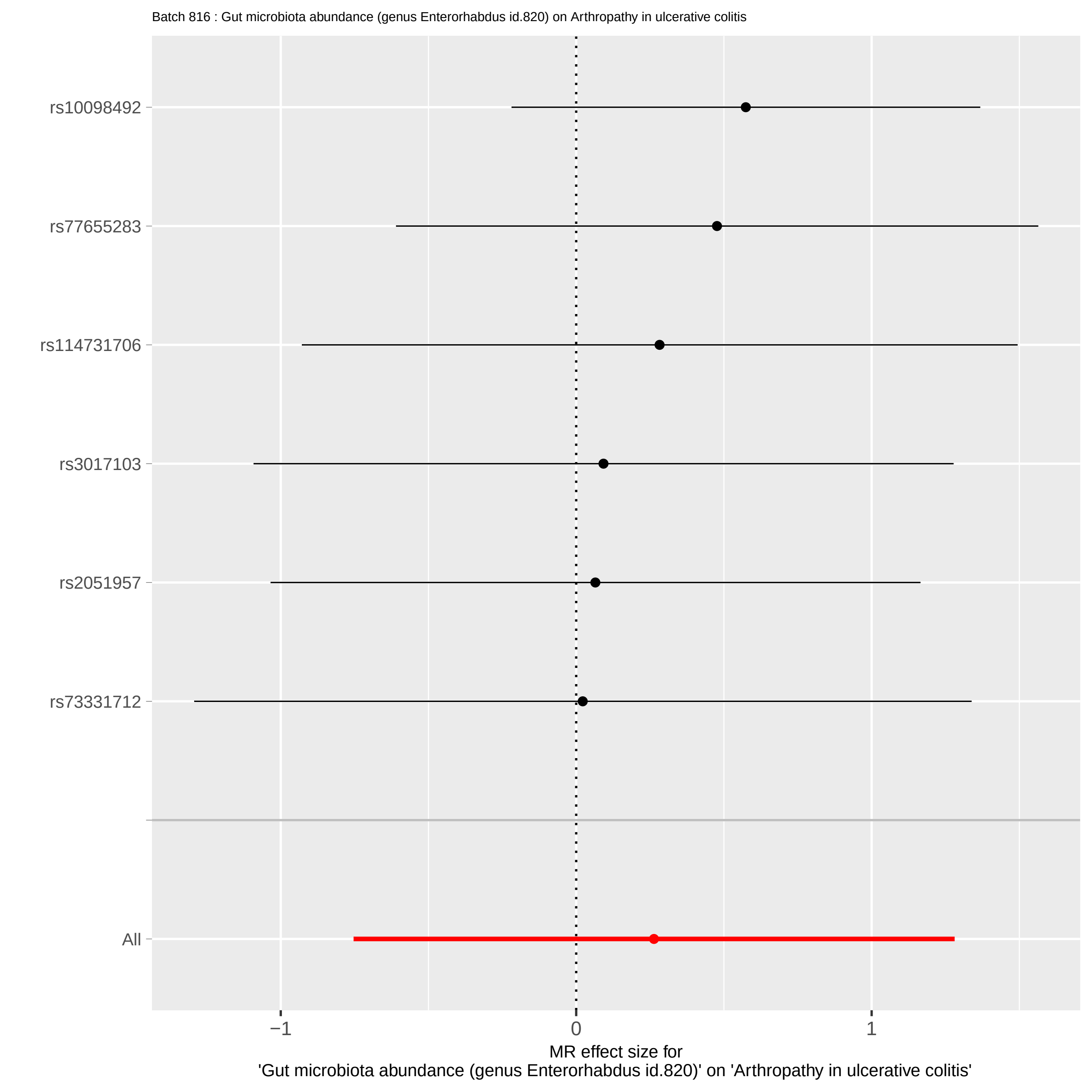
Batch 816 : Gut microbiota abundance (genus Enterorhabdus id.820) on Arthropathy in ulcerative colitis
rs10098492
rs77655283
rs114731706
rs3017103
rs2051957
rs73331712
All
−1
0
1
MR effect size for
'Gut microbiota abundance (genus Enterorhabdus id.820)' on 'Arthropathy in ulcerative colitis'

Batch 817 : Gut microbiota abundance (genus Erysipelatoclostridium id.11381) on Arthropathy in ulcerative colitis

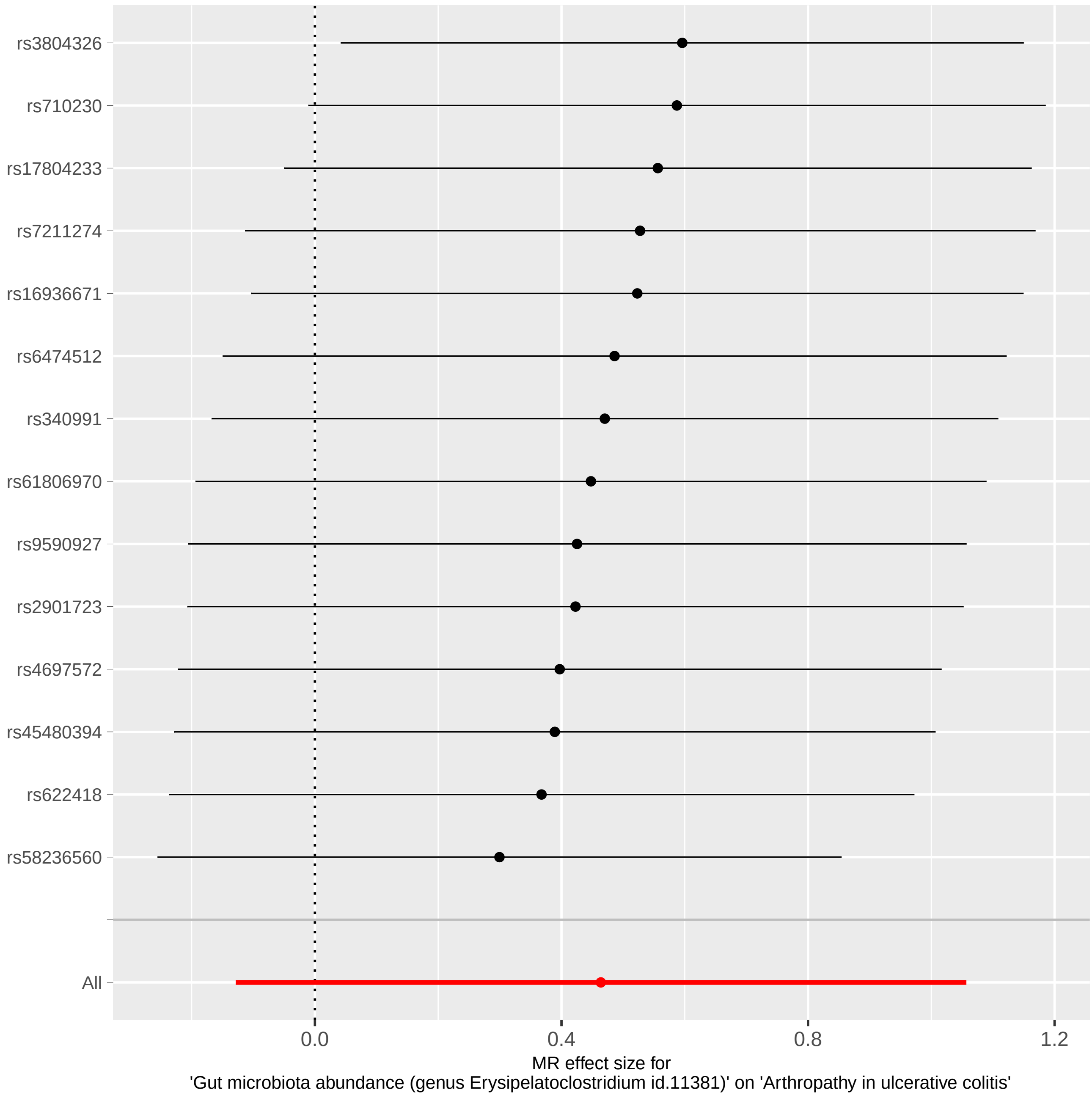

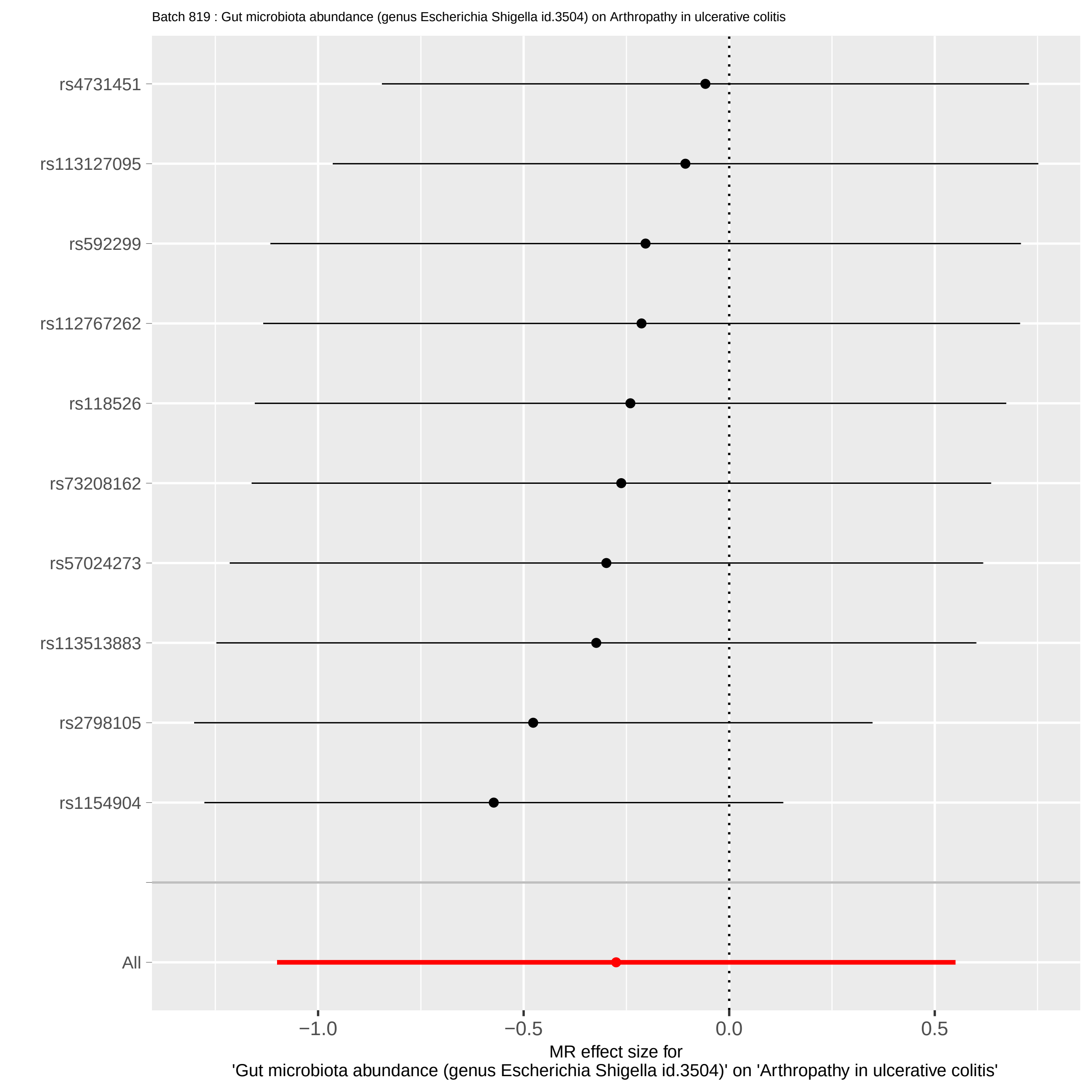

Batch 819 : Gut microbiota abundance (genus Escherichia Shigella id.3504) on Arthropathy in ulcerative colitis
rs4731451
rs113127095
rs592299
rs112767262
rs118526
rs73208162
rs57024273
rs113513883
rs2798105
rs1154904
All
−1.0
−0.5
0.0
0.5
MR effect size for
'Gut microbiota abundance (genus Escherichia Shigella id.3504)' on 'Arthropathy in ulcerative colitis'

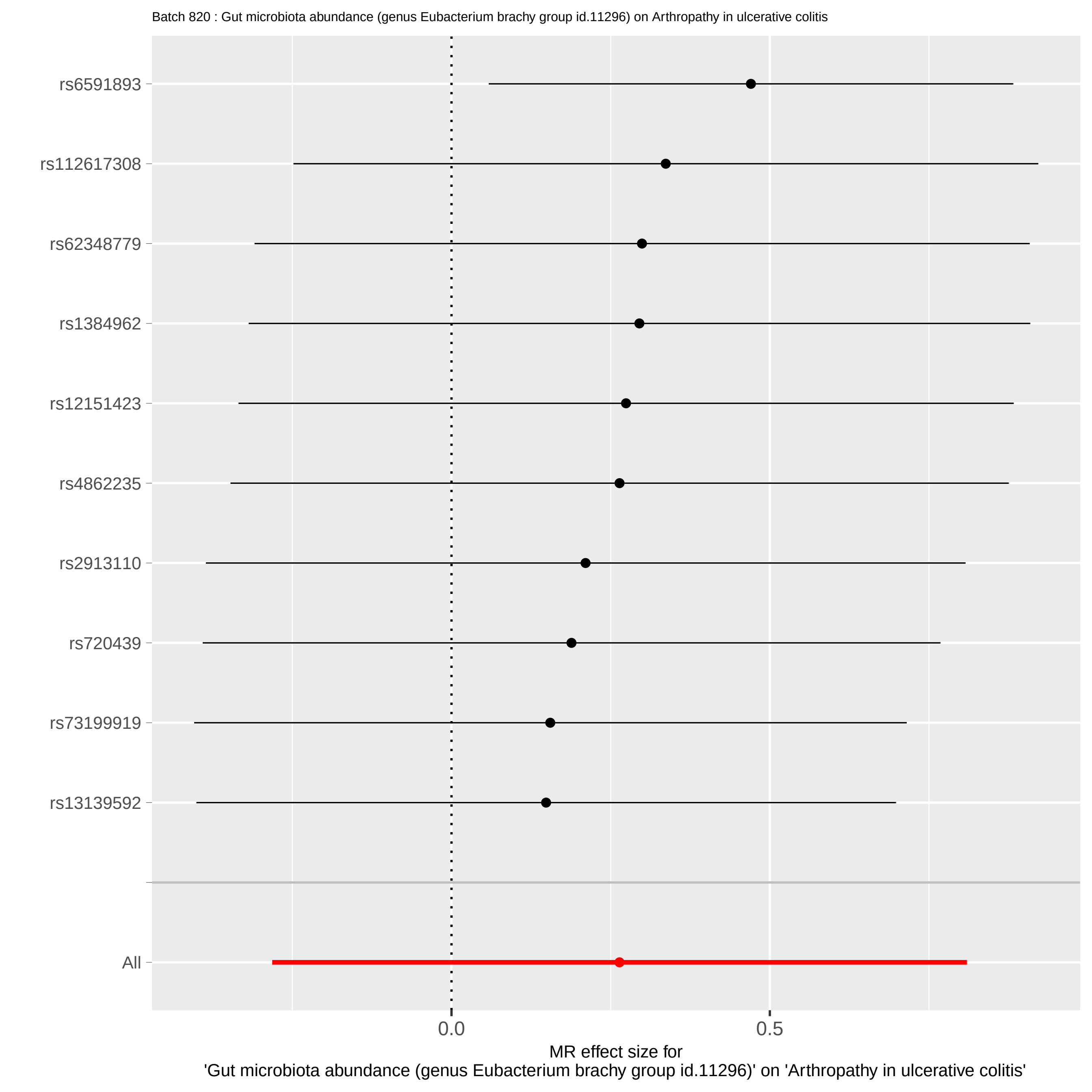

Batch 820 : Gut microbiota abundance (genus Eubacterium brachy group id.11296) on Arthropathy in ulcerative colitis
rs6591893
rs112617308
rs62348779
rs1384962
rs12151423
rs4862235
rs2913110
rs720439
rs73199919
rs13139592
All
0.0
0.5
MR effect size for
'Gut microbiota abundance (genus Eubacterium brachy group id.11296)' on 'Arthropathy in ulcerative colitis'

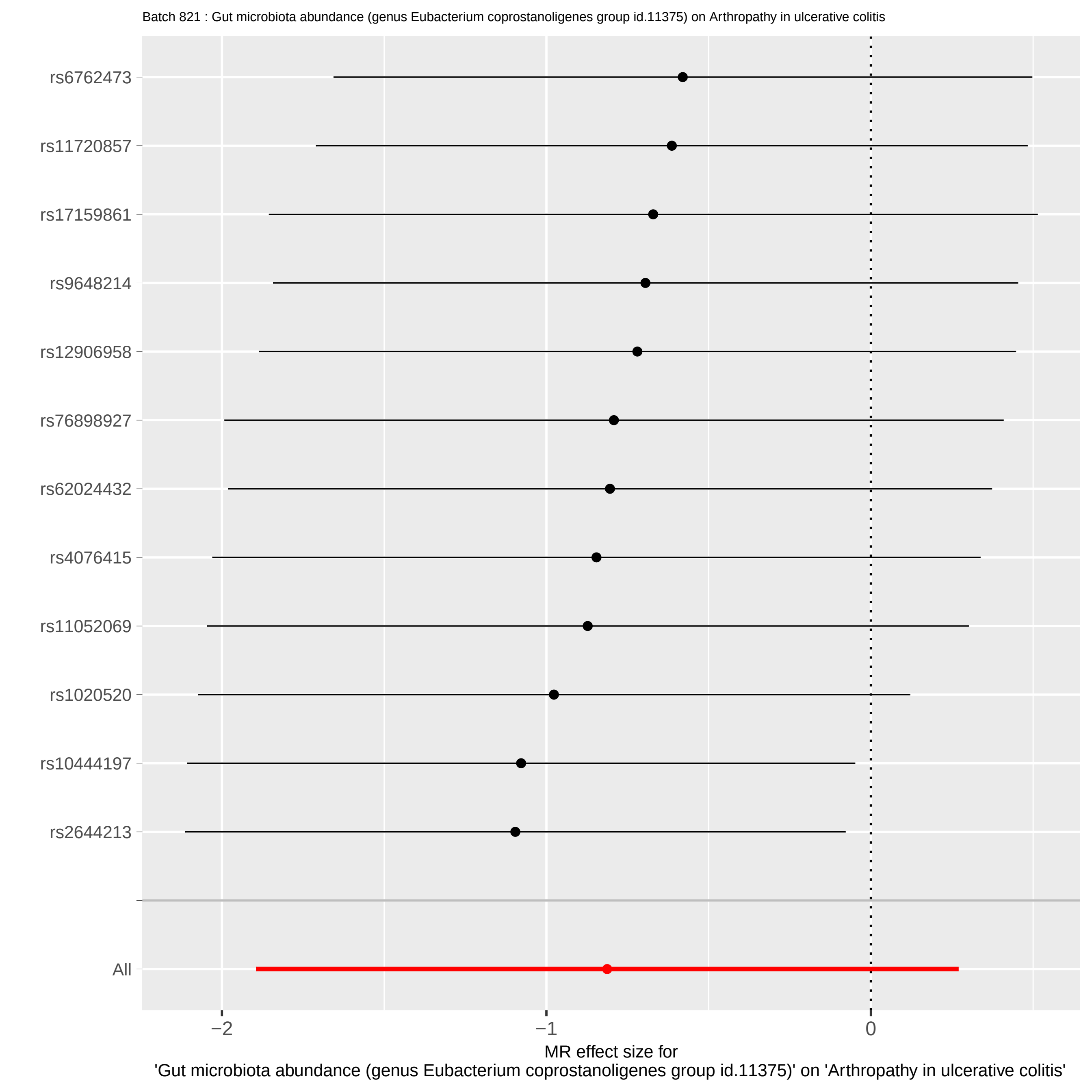

Batch 821 : Gut microbiota abundance (genus Eubacterium coprostanoligenes group id.11375) on Arthropathy in ulcerative colitis
rs6762473
rs11720857
rs17159861
rs9648214
rs12906958
rs76898927
rs62024432
rs4076415
rs11052069
rs1020520
rs10444197
rs2644213
All
−2
−1
0
MR effect size for
'Gut microbiota abundance (genus Eubacterium coprostanoligenes group id.11375)' on 'Arthropathy in ulcerative colitis'

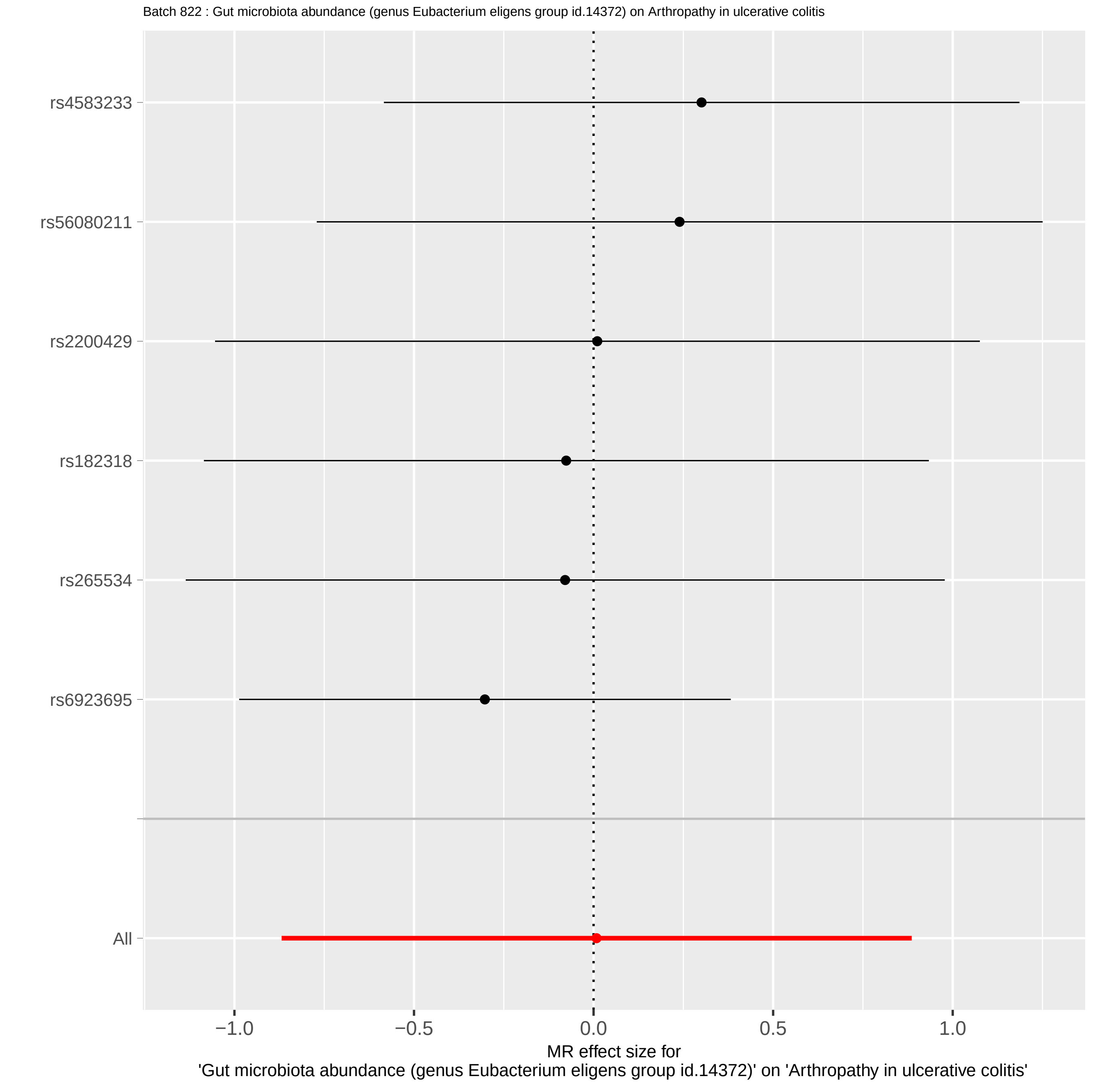

Batch 822 : Gut microbiota abundance (genus Eubacterium eligens group id.14372) on Arthropathy in ulcerative colitis
rs4583233
rs56080211
rs2200429
rs182318
rs265534
rs6923695
All
−1.0
−0.5
0.0
0.5
1.0
MR effect size for
'Gut microbiota abundance (genus Eubacterium eligens group id.14372)' on 'Arthropathy in ulcerative colitis'

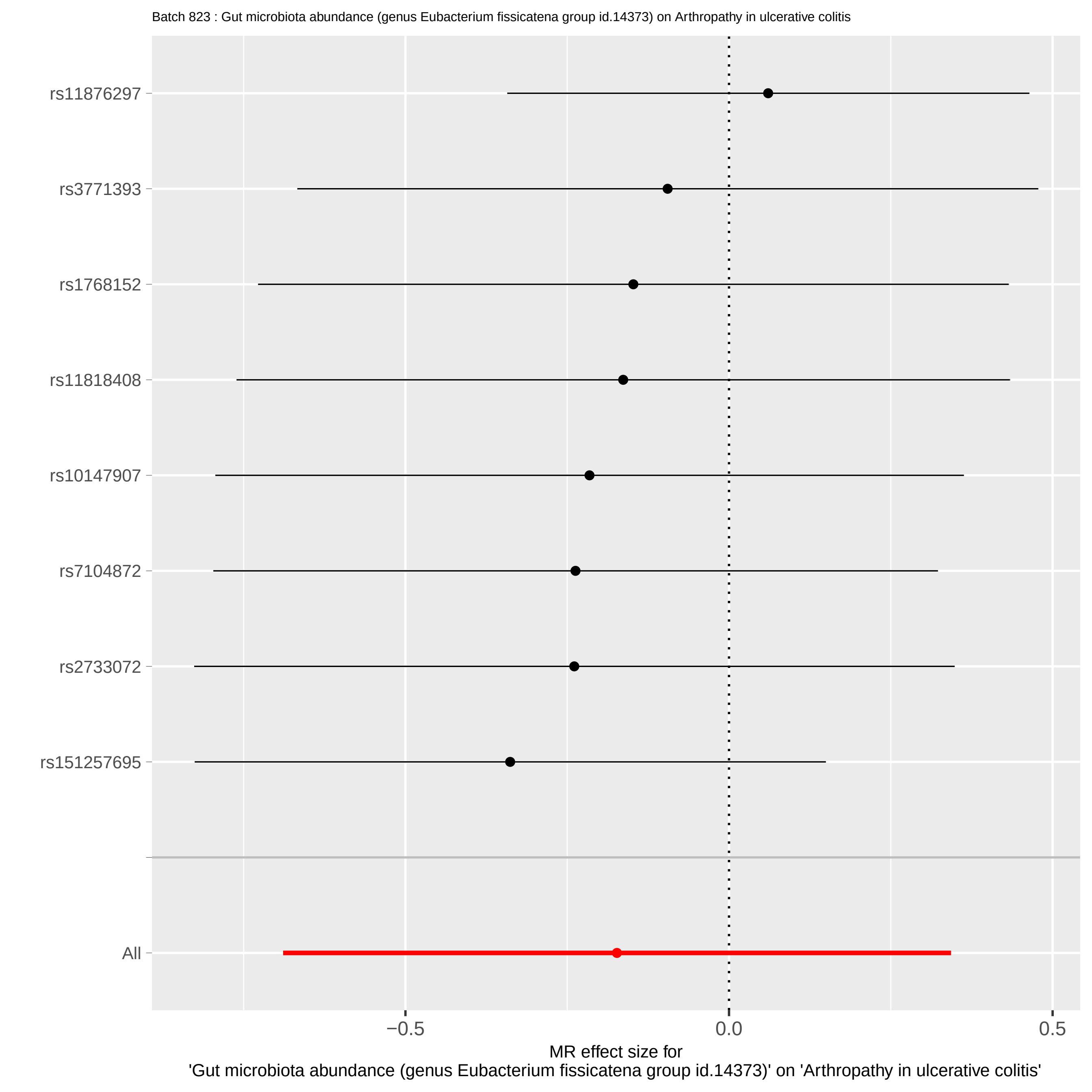

Batch 823 : Gut microbiota abundance (genus Eubacterium fissicatena group id.14373) on Arthropathy in ulcerative colitis
rs11876297
rs3771393
rs1768152
rs11818408
rs10147907
rs7104872
rs2733072
rs151257695
All
−0.5
0.0
0.5
MR effect size for
'Gut microbiota abundance (genus Eubacterium fissicatena group id.14373)' on 'Arthropathy in ulcerative colitis'

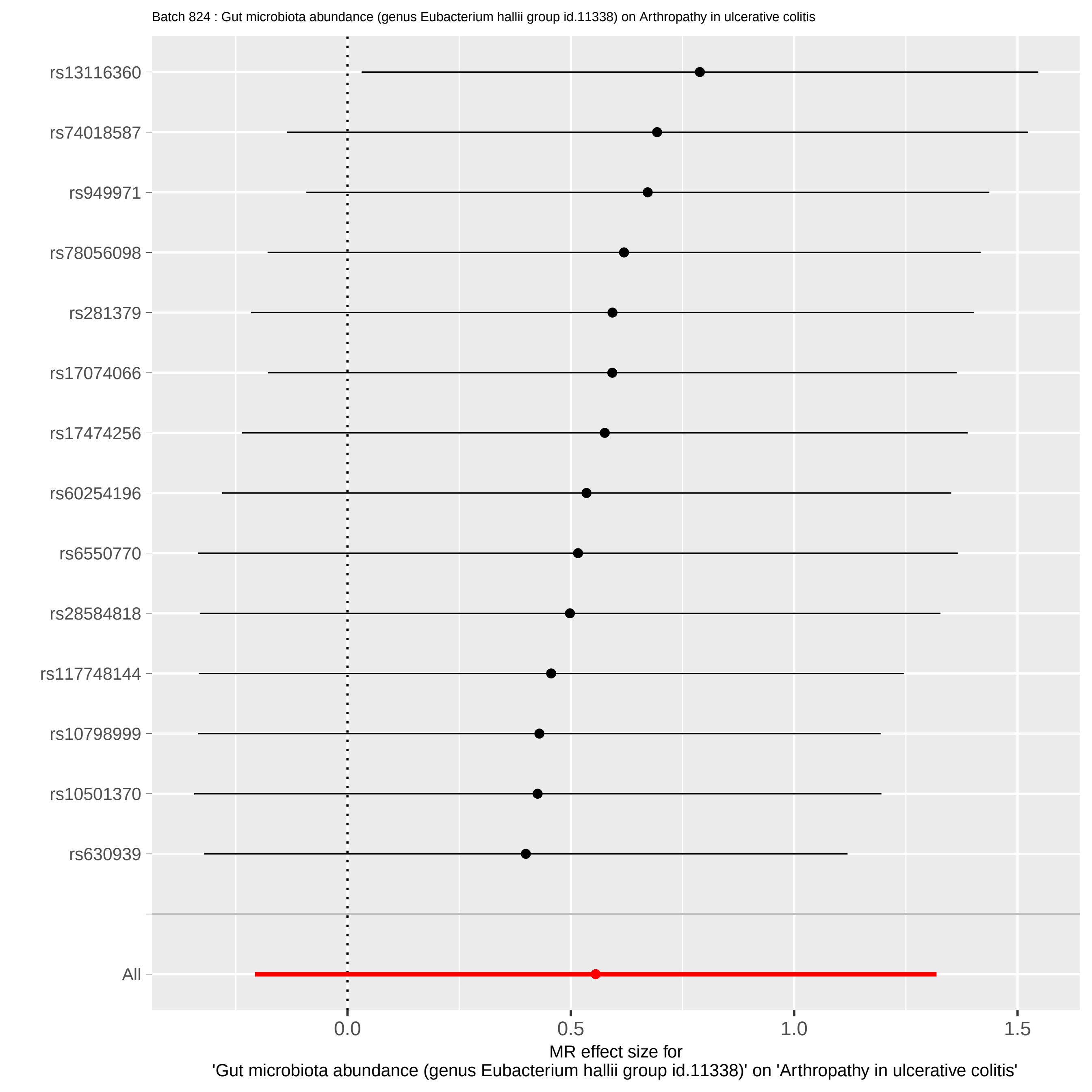

Batch 824 : Gut microbiota abundance (genus Eubacterium hallii group id.11338) on Arthropathy in ulcerative colitis
rs13116360
rs74018587
rs949971
rs78056098
rs281379
rs17074066
rs17474256
rs60254196
rs6550770
rs28584818
rs117748144
rs10798999
rs10501370
rs630939
All
0.0
0.5
1.0
1.5
MR effect size for
'Gut microbiota abundance (genus Eubacterium hallii group id.11338)' on 'Arthropathy in ulcerative colitis'

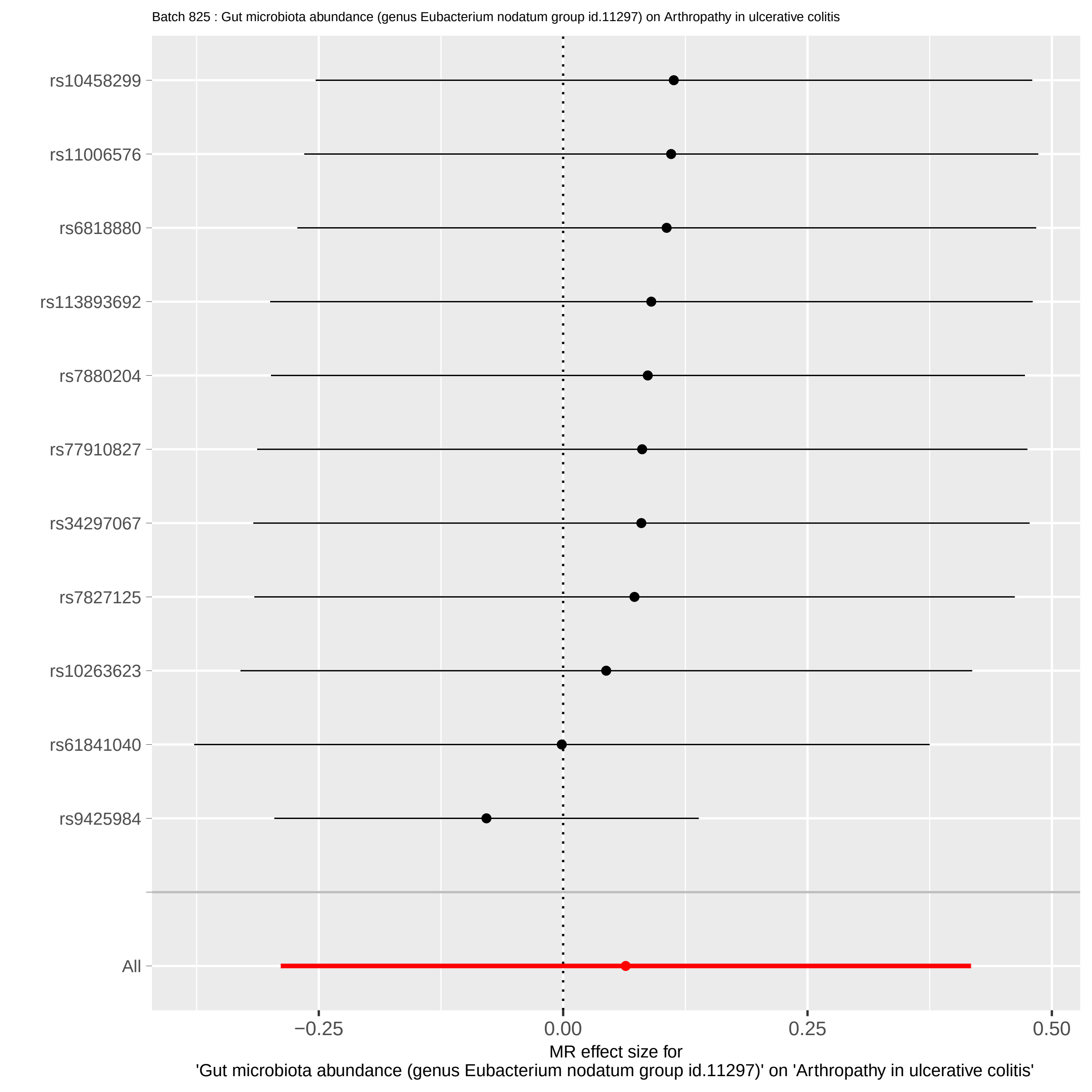

Batch 825 : Gut microbiota abundance (genus Eubacterium nodatum group id.11297) on Arthropathy in ulcerative colitis
rs10458299
rs11006576
rs6818880
rs113893692
rs7880204
rs77910827
rs34297067
rs7827125
rs10263623
rs61841040
rs9425984
All
−0.25
0.00
0.25
0.50
MR effect size for
'Gut microbiota abundance (genus Eubacterium nodatum group id.11297)' on 'Arthropathy in ulcerative colitis'

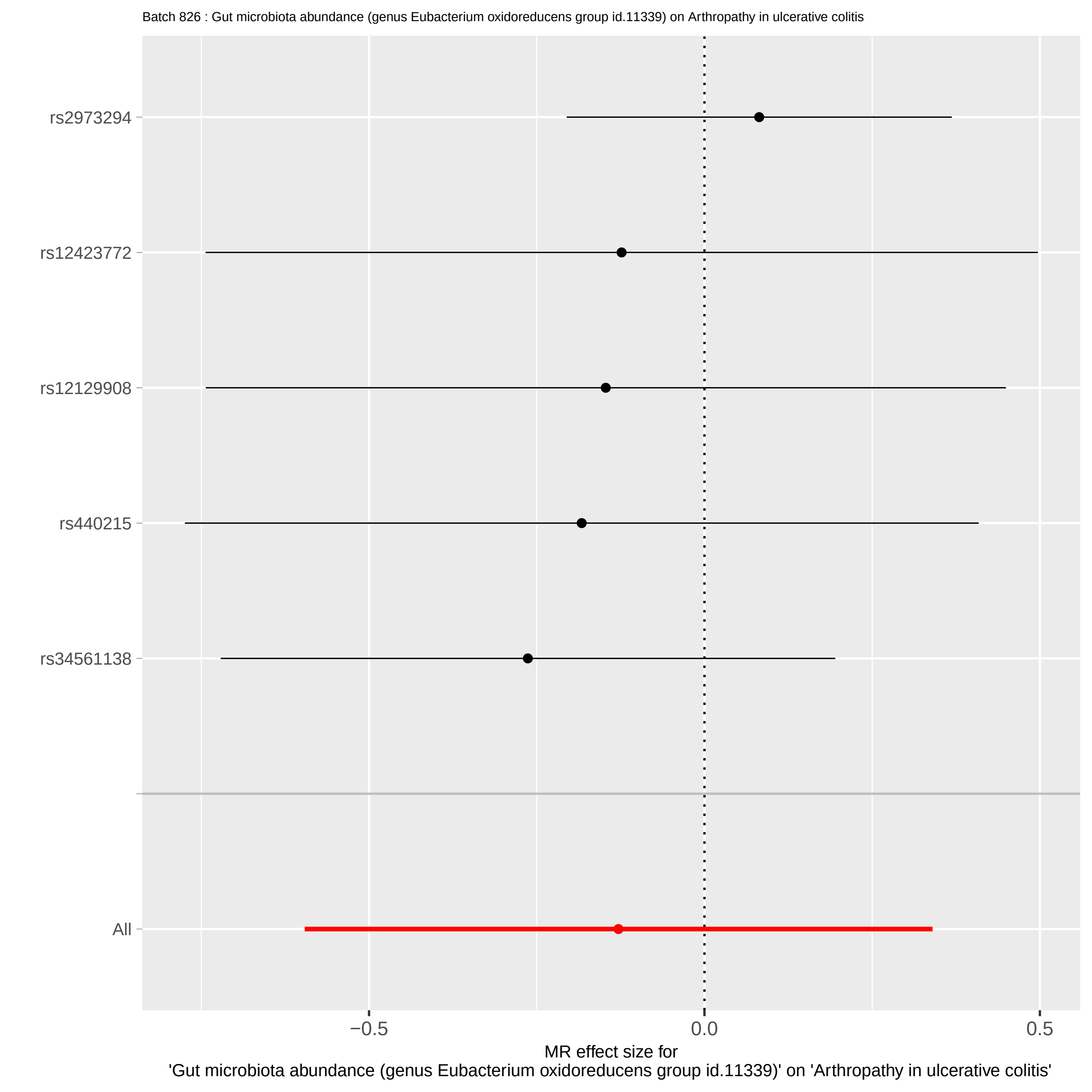
Batch 826 : Gut microbiota abundance (genus Eubacterium oxidoreducens group id.11339) on Arthropathy in ulcerative colitis
rs2973294
rs12423772
rs12129908
rs440215
rs34561138
All
−0.5
0.0
0.5
MR effect size for
'Gut microbiota abundance (genus Eubacterium oxidoreducens group id.11339)' on 'Arthropathy in ulcerative colitis'

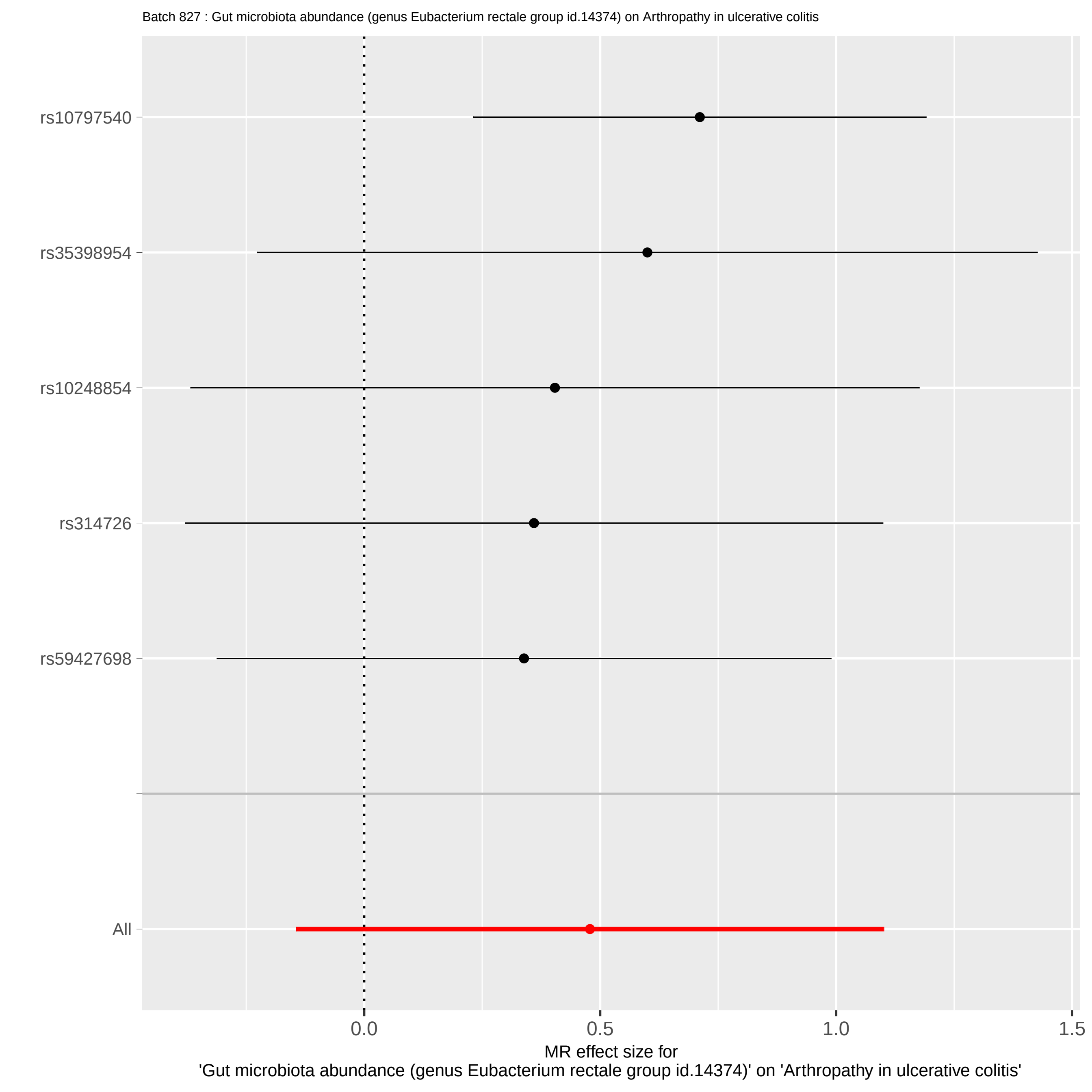

Batch 827 : Gut microbiota abundance (genus Eubacterium rectale group id.14374) on Arthropathy in ulcerative colitis
rs10797540
rs35398954
rs10248854
rs314726
rs59427698
All
0.0
0.5
1.0
1.5
MR effect size for
'Gut microbiota abundance (genus Eubacterium rectale group id.14374)' on 'Arthropathy in ulcerative colitis'

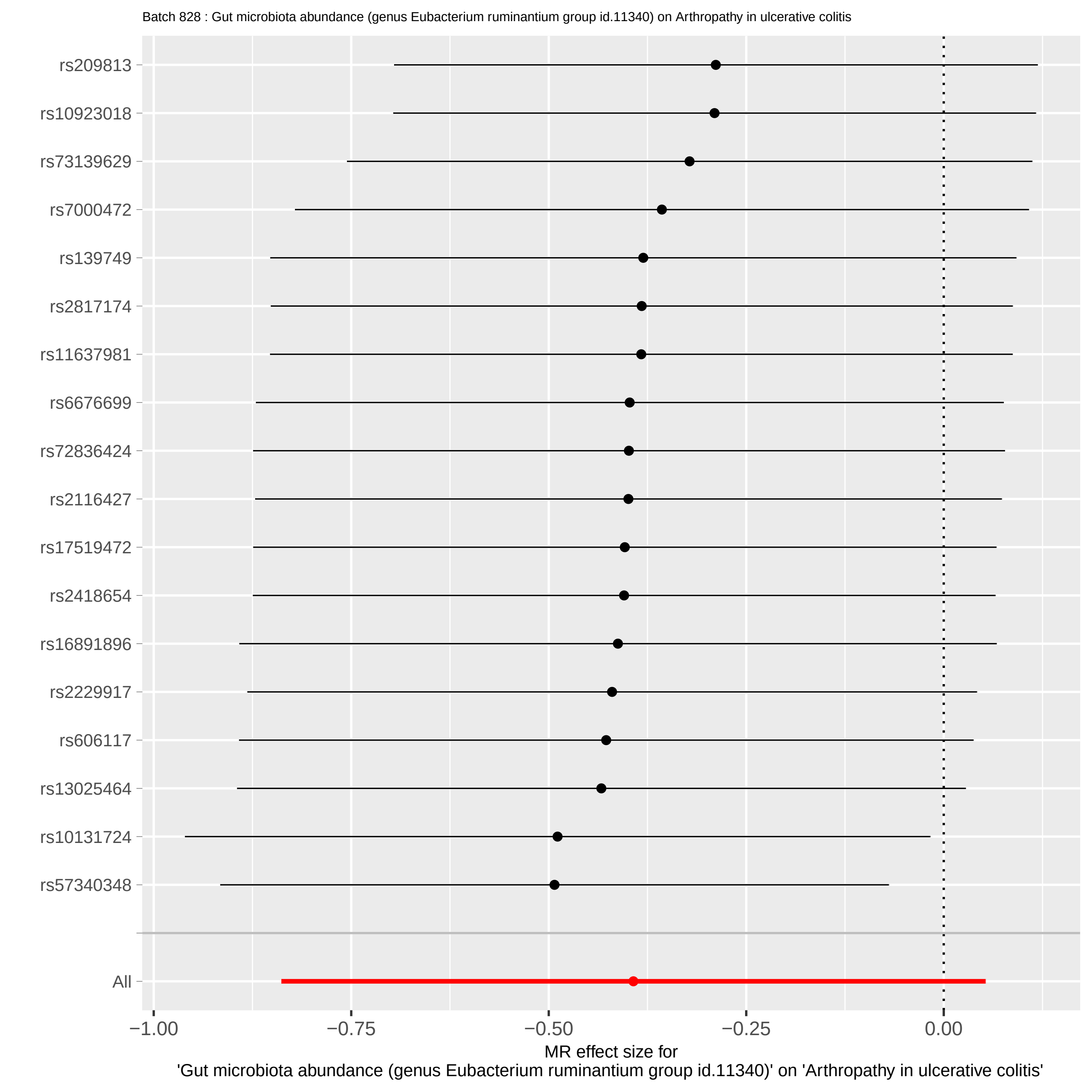
Batch 828 : Gut microbiota abundance (genus Eubacterium ruminantium group id.11340) on Arthropathy in ulcerative colitis
rs209813
rs10923018
rs73139629
rs7000472
rs139749
rs2817174
rs11637981
rs6676699
rs72836424
rs2116427
rs17519472
rs2418654
rs16891896
rs2229917
rs606117
rs13025464
rs10131724
rs57340348
All
−1.00
−0.75
−0.50
−0.25
0.00
MR effect size for
'Gut microbiota abundance (genus Eubacterium ruminantium group id.11340)' on 'Arthropathy in ulcerative colitis'

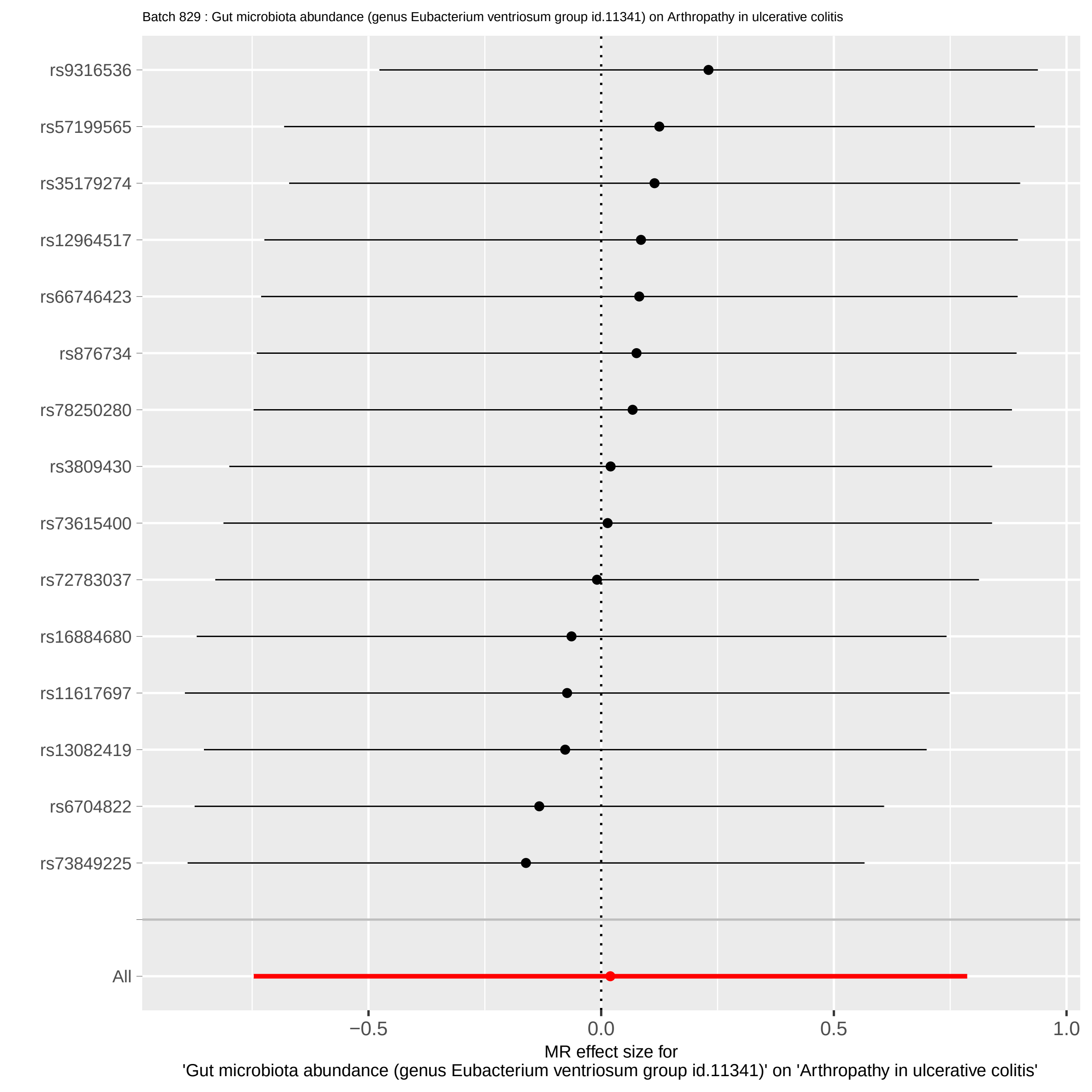

Batch 829 : Gut microbiota abundance (genus Eubacterium ventriosum group id.11341) on Arthropathy in ulcerative colitis
rs9316536
rs57199565
rs35179274
rs12964517
rs66746423
rs876734
rs78250280
rs3809430
rs73615400
rs72783037
rs16884680
rs11617697
rs13082419
rs6704822
rs73849225
All
−0.5
0.0
0.5
1.0
MR effect size for
'Gut microbiota abundance (genus Eubacterium ventriosum group id.11341)' on 'Arthropathy in ulcerative colitis'

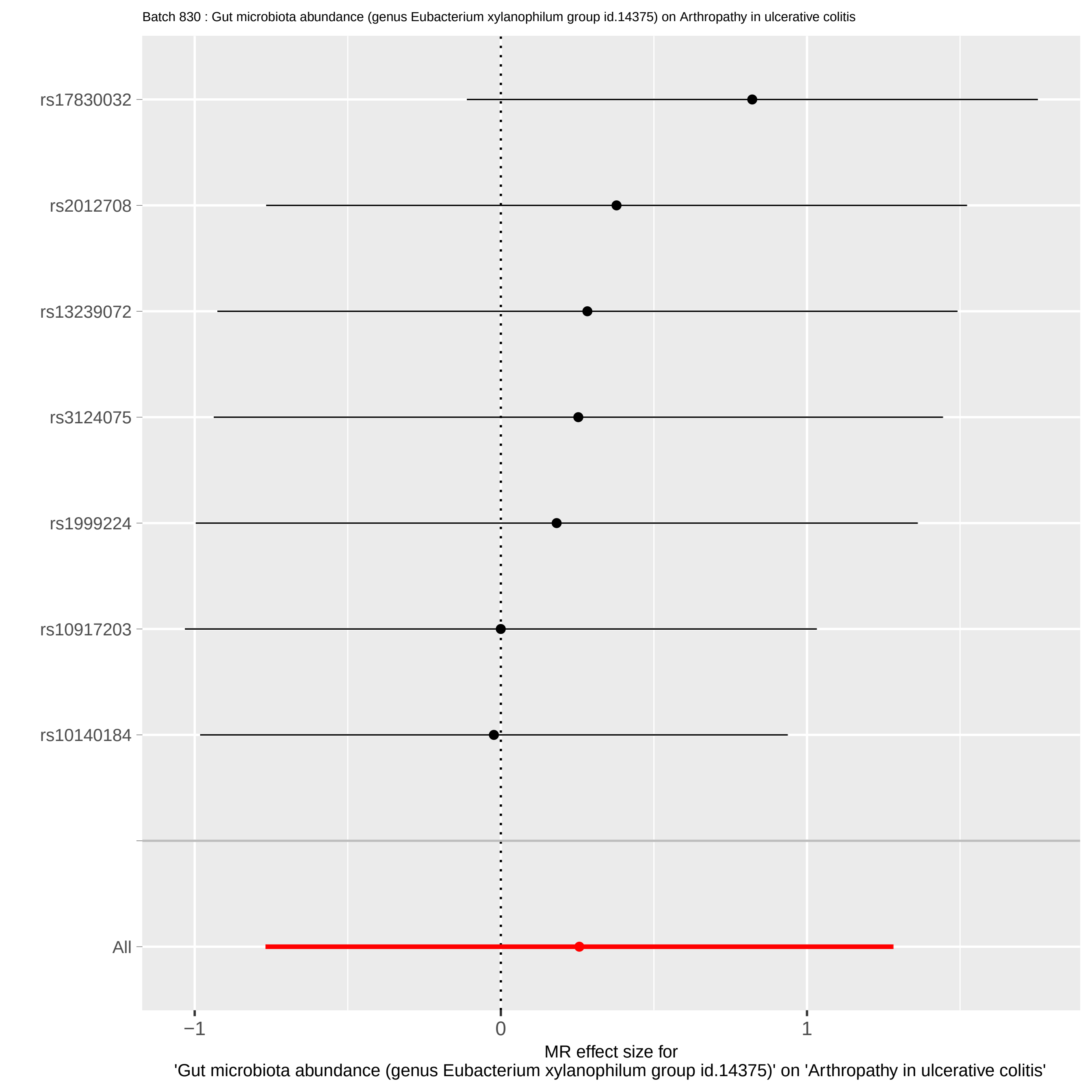
Batch 830 : Gut microbiota abundance (genus Eubacterium xylanophilum group id.14375) on Arthropathy in ulcerative colitis
rs17830032
rs2012708
rs13239072
rs3124075
rs1999224
rs10917203
rs10140184
All
−1
0
1
MR effect size for
'Gut microbiota abundance (genus Eubacterium xylanophilum group id.14375)' on 'Arthropathy in ulcerative colitis'

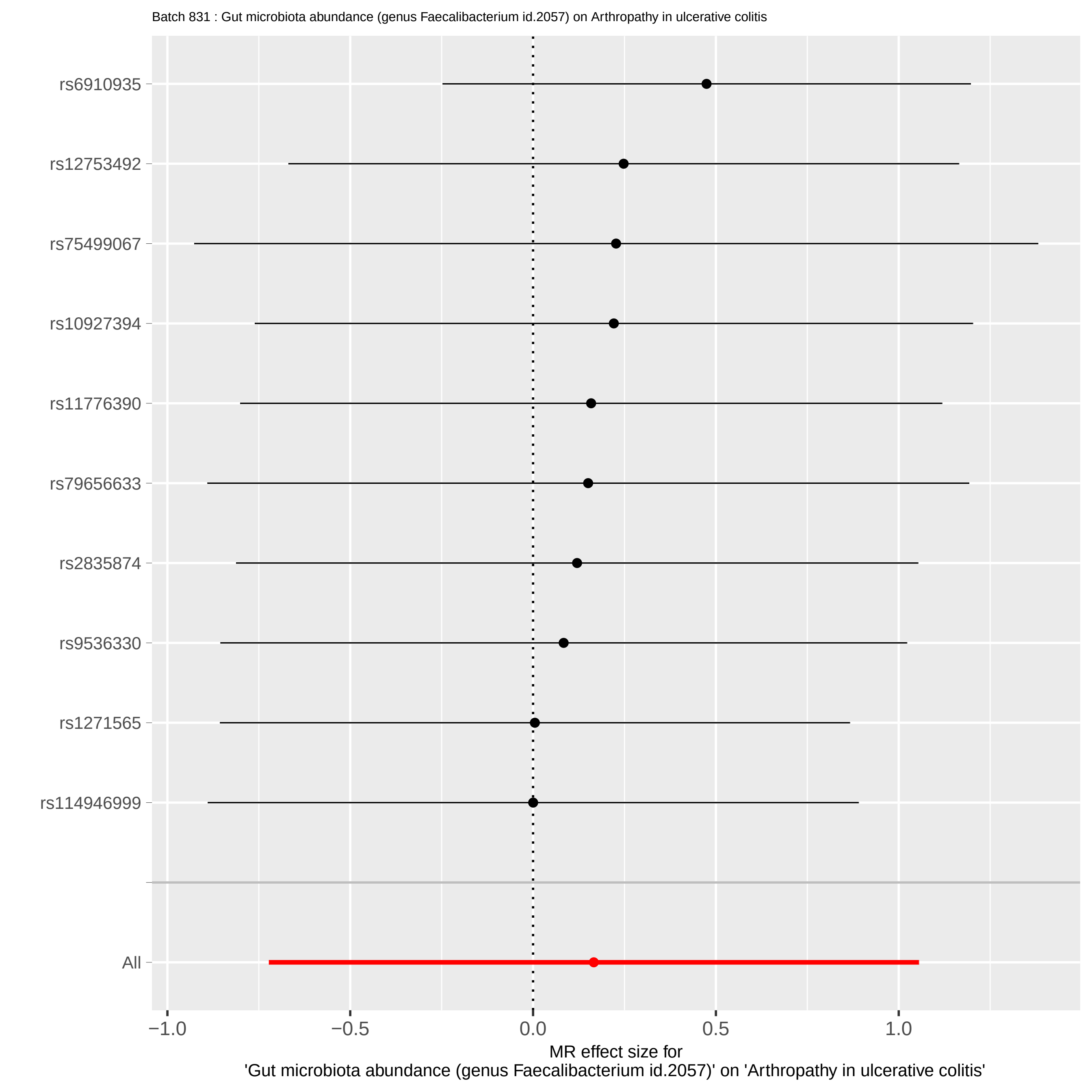

Batch 831 : Gut microbiota abundance (genus Faecalibacterium id.2057) on Arthropathy in ulcerative colitis
rs6910935
rs12753492
rs75499067
rs10927394
rs11776390
rs79656633
rs2835874
rs9536330
rs1271565
rs114946999
All
−1.0
−0.5
0.0
0.5
1.0
MR effect size for
'Gut microbiota abundance (genus Faecalibacterium id.2057)' on 'Arthropathy in ulcerative colitis'

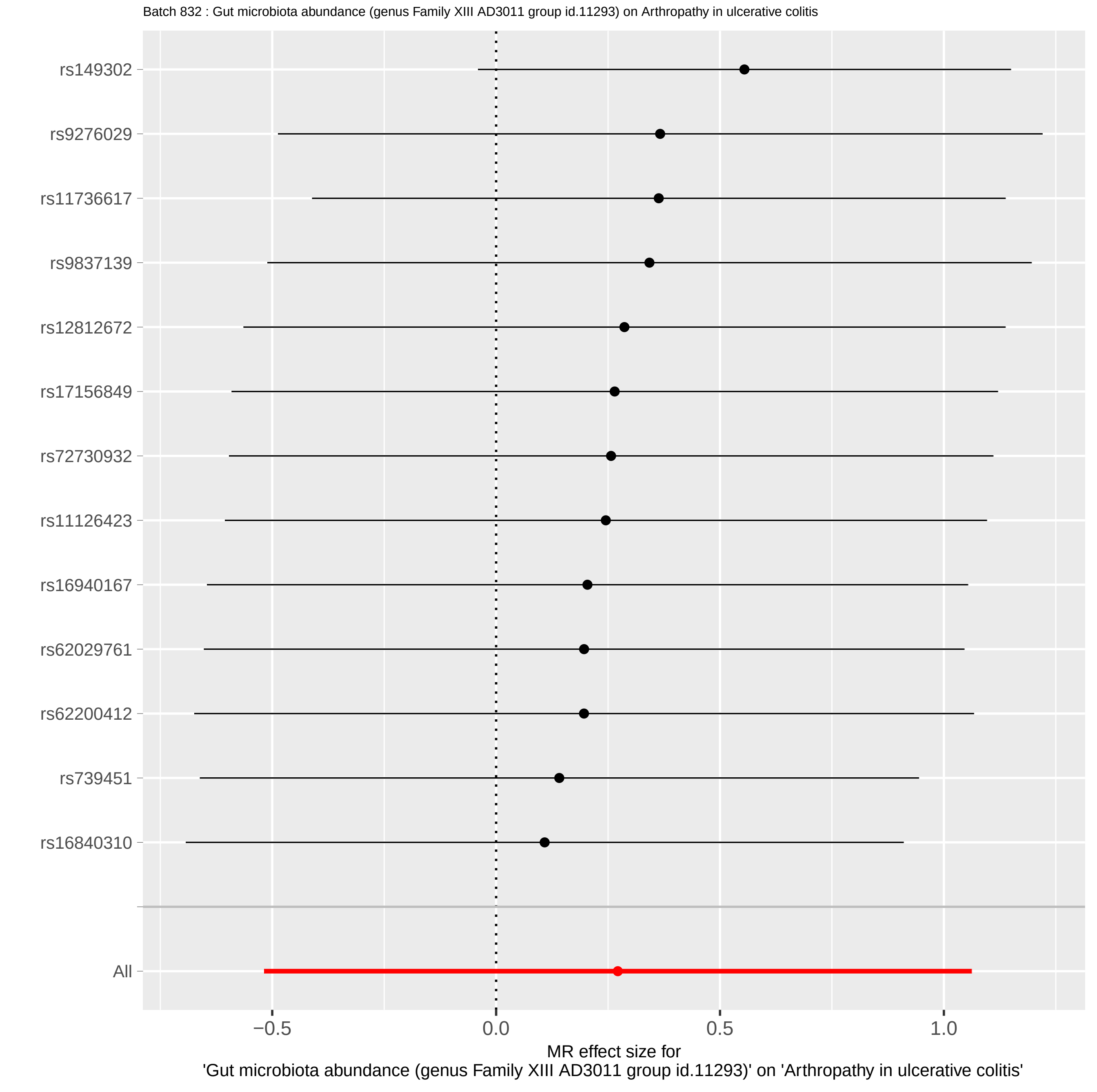
Batch 832 : Gut microbiota abundance (genus Family XIII AD3011 group id.11293) on Arthropathy in ulcerative colitis
rs149302
rs9276029
rs11736617
rs9837139
rs12812672
rs17156849
rs72730932
rs11126423
rs16940167
rs62029761
rs62200412
rs739451
rs16840310
All
−0.5
0.0
0.5
1.0
MR effect size for
'Gut microbiota abundance (genus Family XIII AD3011 group id.11293)' on 'Arthropathy in ulcerative colitis'

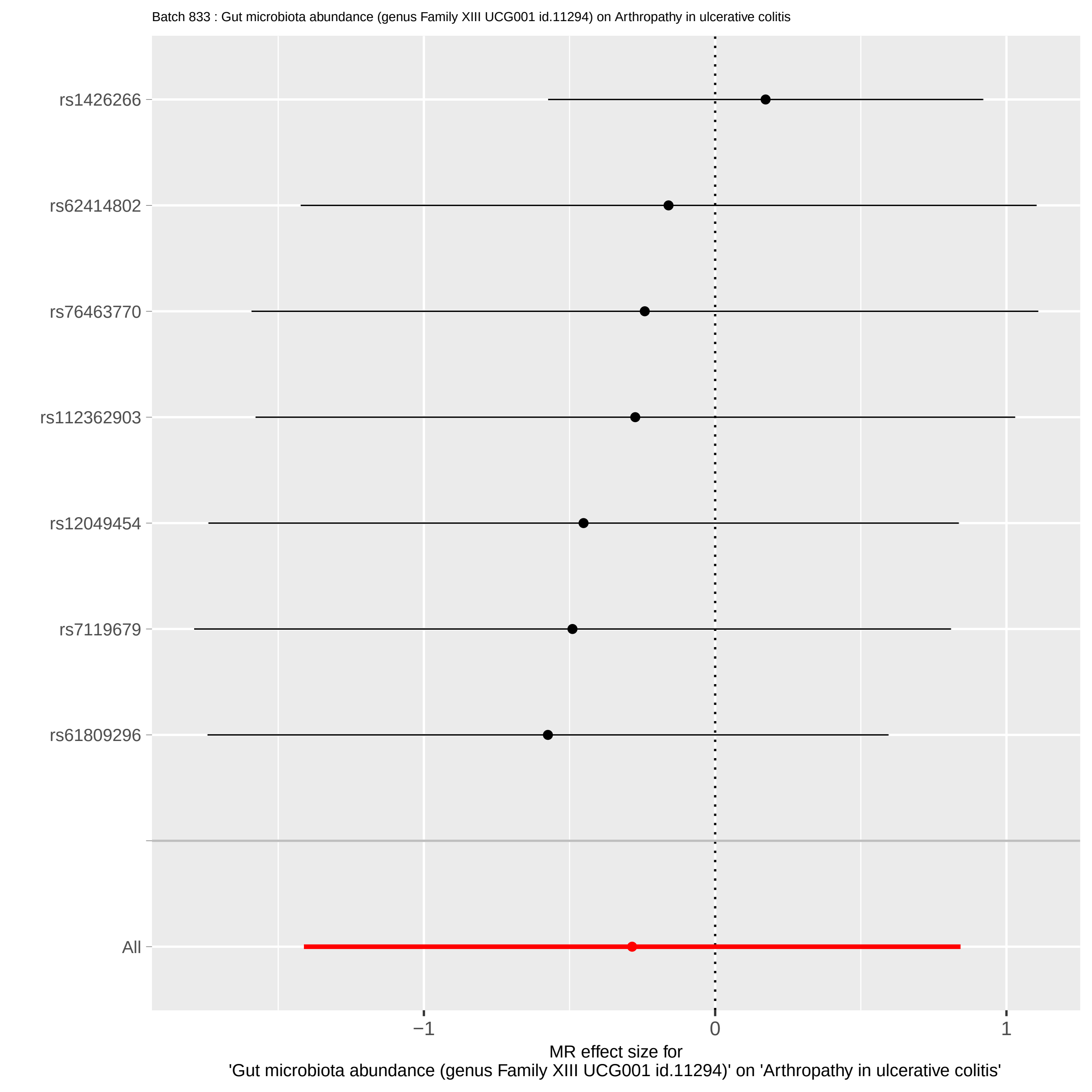

Batch 833 : Gut microbiota abundance (genus Family XIII UCG001 id.11294) on Arthropathy in ulcerative colitis
rs1426266
rs62414802
rs76463770
rs112362903
rs12049454
rs7119679
rs61809296
All
−1
0
1
MR effect size for
'Gut microbiota abundance (genus Family XIII UCG001 id.11294)' on 'Arthropathy in ulcerative colitis'

Batch 834 : Gut microbiota abundance (genus Flavonifractor id.2059) on Arthropathy in ulcerative colitis

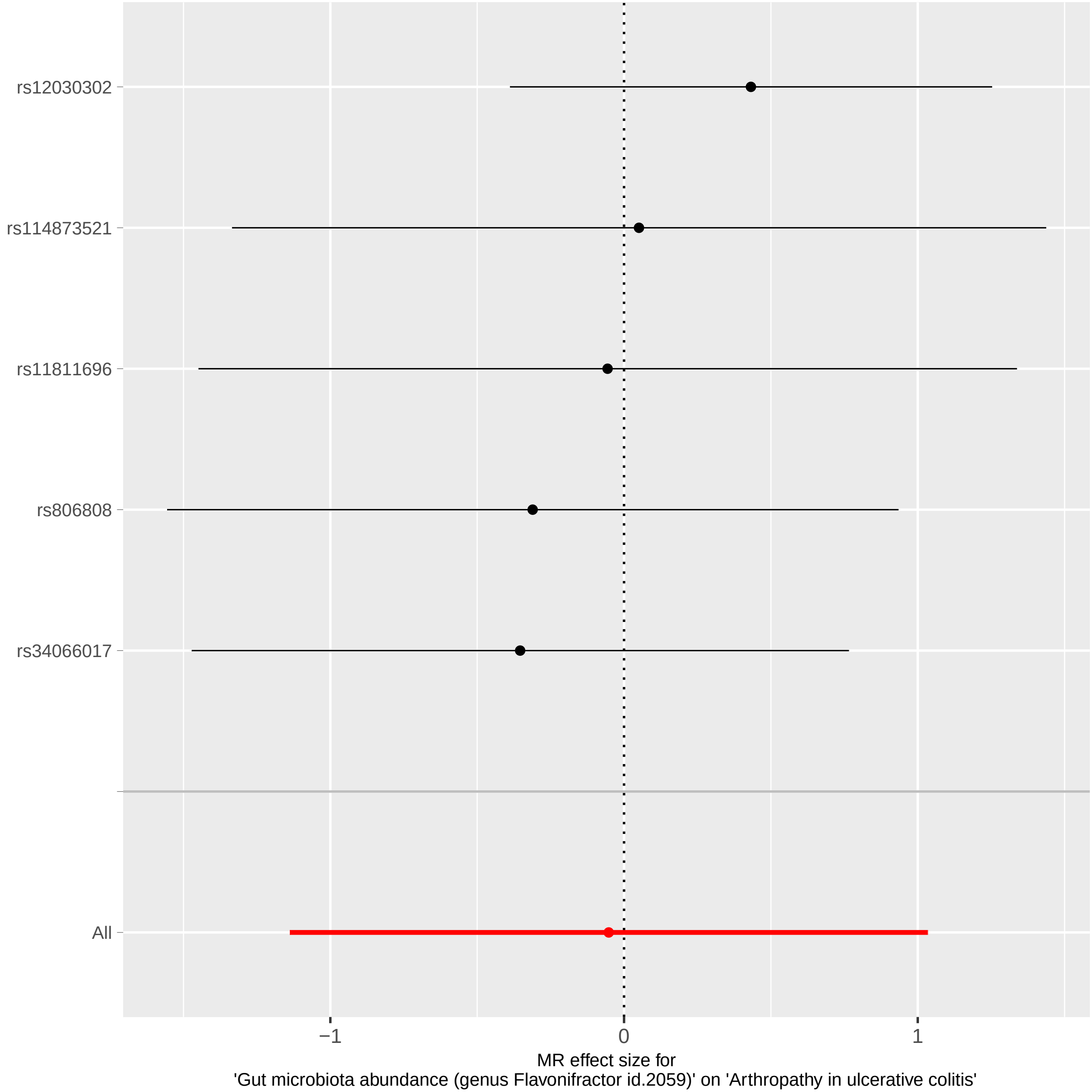

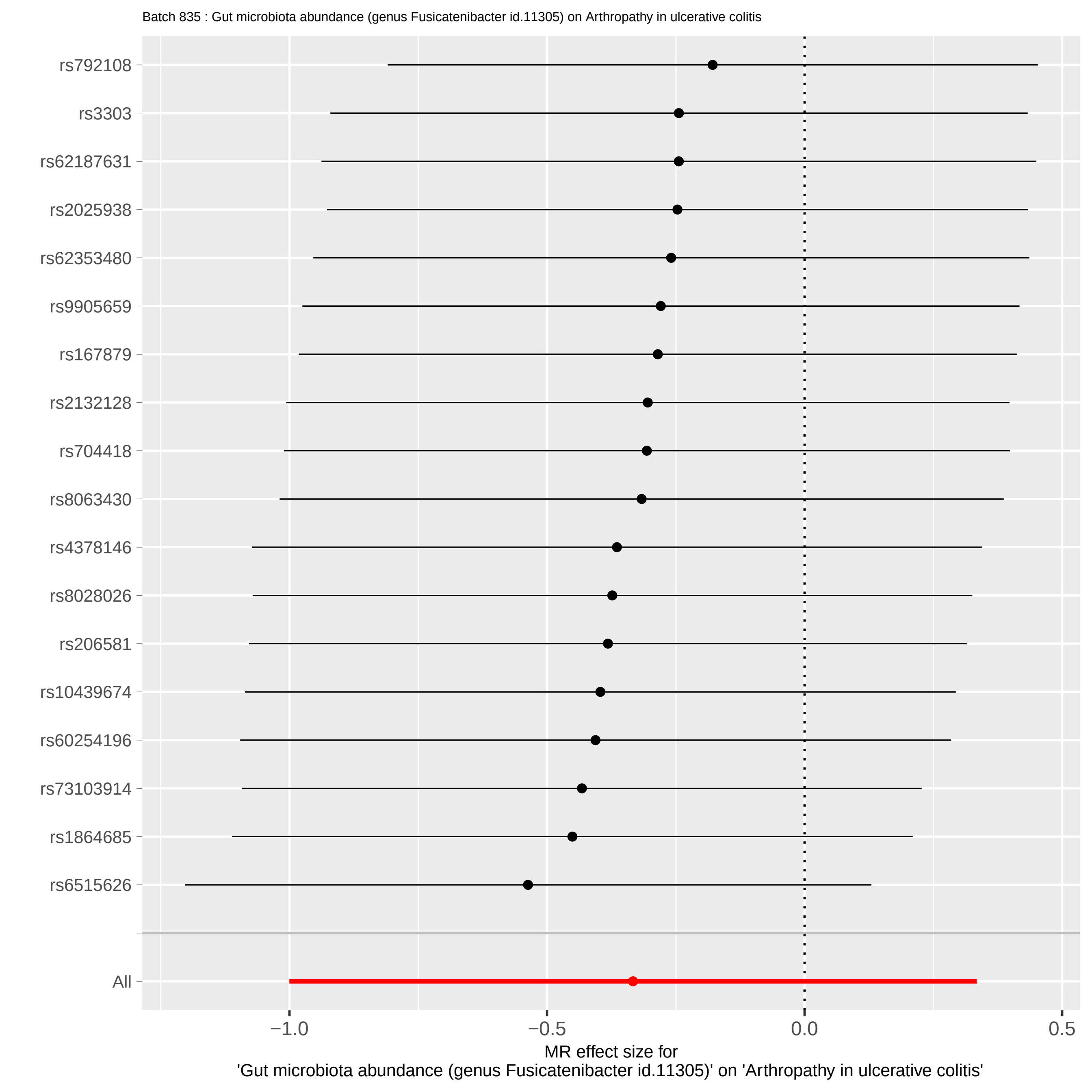
Batch 835 : Gut microbiota abundance (genus Fusicatenibacter id.11305) on Arthropathy in ulcerative colitis
rs792108
rs3303
rs62187631
rs2025938
rs62353480
rs9905659
rs167879
rs2132128
rs704418
rs8063430
rs4378146
rs8028026
rs206581
rs10439674
rs60254196
rs73103914
rs1864685
rs6515626
All
−1.0
−0.5
0.0
0.5
MR effect size for
'Gut microbiota abundance (genus Fusicatenibacter id.11305)' on 'Arthropathy in ulcerative colitis'

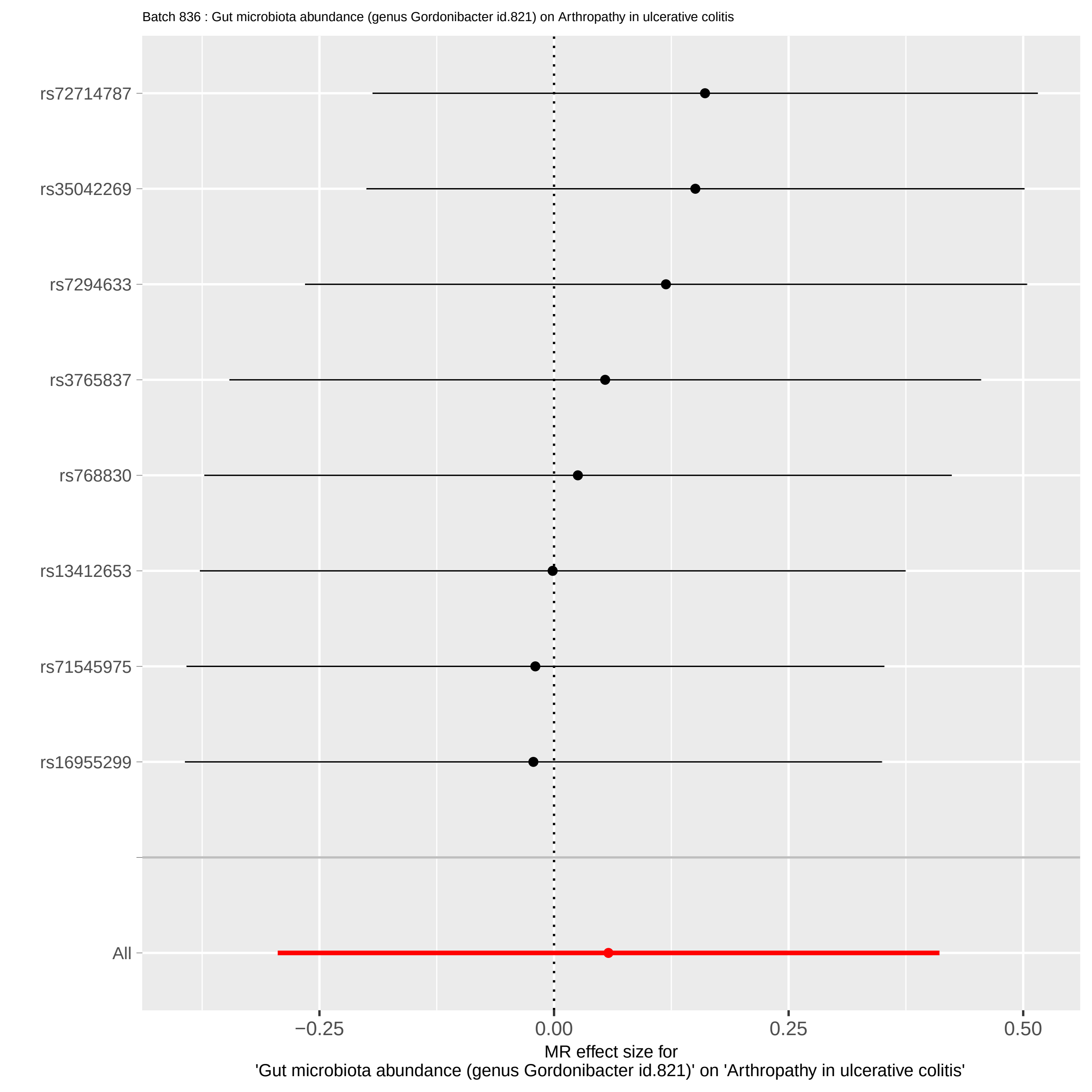
Batch 836 : Gut microbiota abundance (genus Gordonibacter id.821) on Arthropathy in ulcerative colitis
rs72714787
rs35042269
rs7294633
rs3765837
rs768830
rs13412653
rs71545975
rs16955299
All
−0.25
0.00
0.25
0.50
MR effect size for
'Gut microbiota abundance (genus Gordonibacter id.821)' on 'Arthropathy in ulcerative colitis'

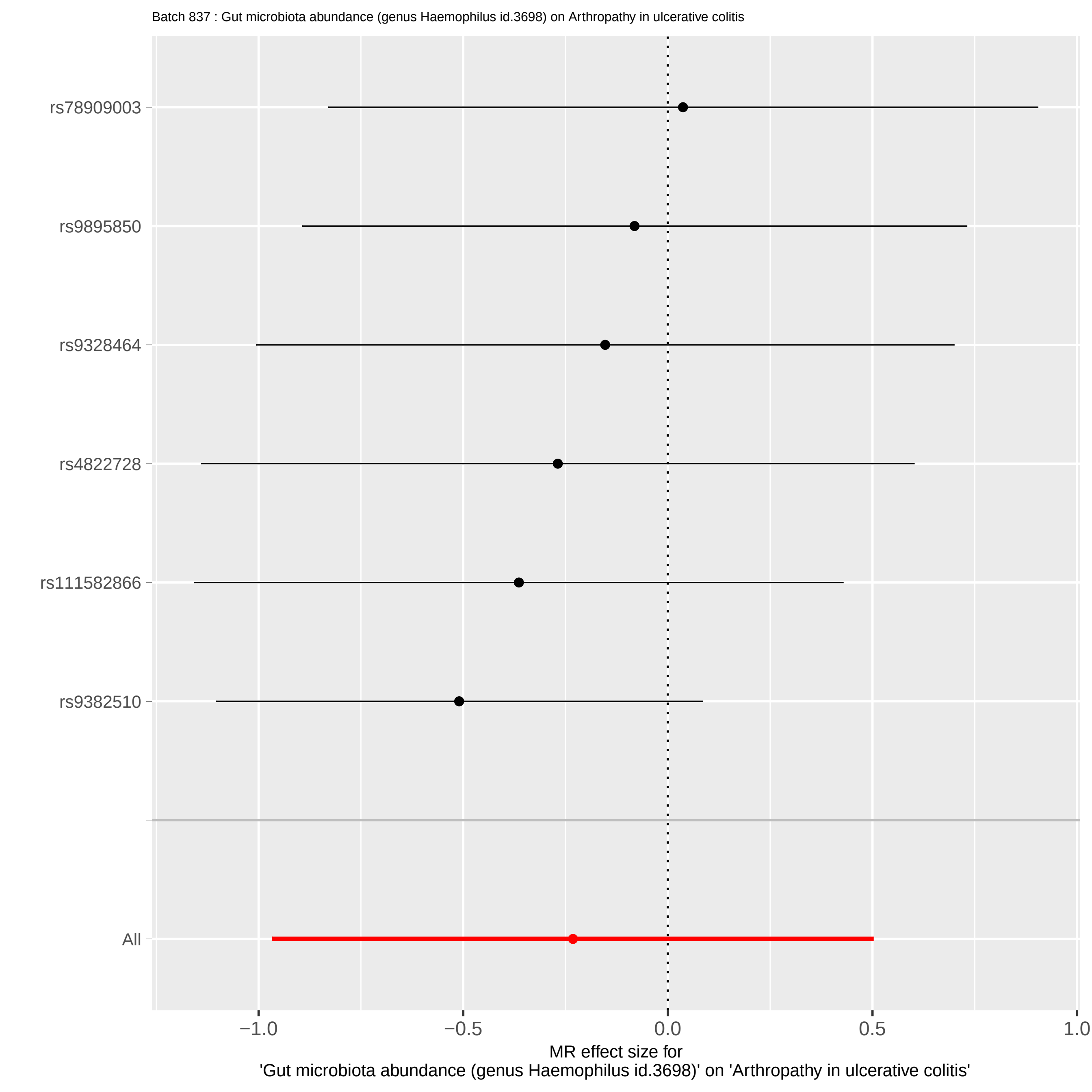

Batch 837 : Gut microbiota abundance (genus Haemophilus id.3698) on Arthropathy in ulcerative colitis
rs78909003
rs9895850
rs9328464
rs4822728
rs111582866
rs9382510
All
−1.0
−0.5
0.0
0.5
1.0
MR effect size for
'Gut microbiota abundance (genus Haemophilus id.3698)' on 'Arthropathy in ulcerative colitis'

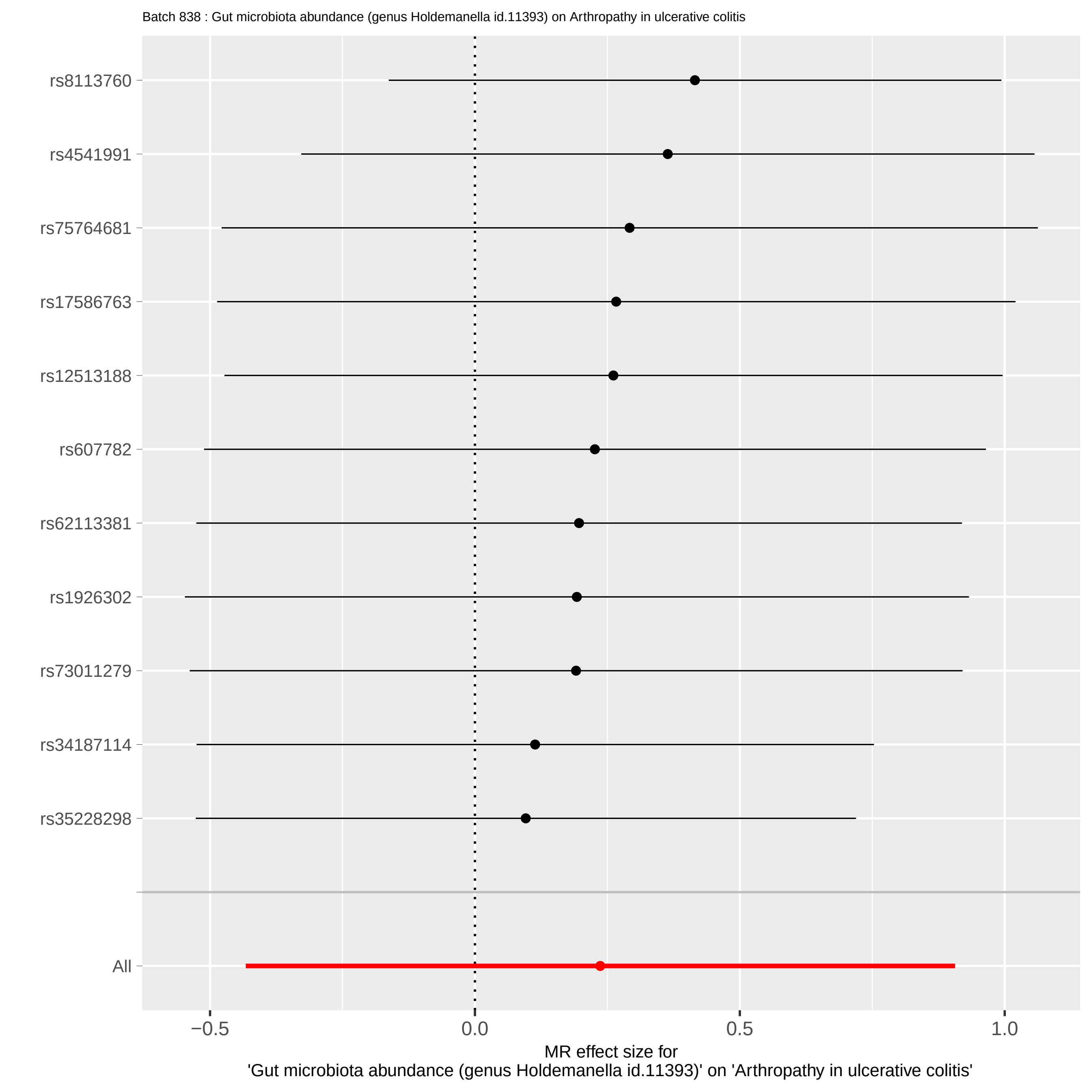

Batch 838 : Gut microbiota abundance (genus Holdemanella id.11393) on Arthropathy in ulcerative colitis
rs8113760
rs4541991
rs75764681
rs17586763
rs12513188
rs607782
rs62113381
rs1926302
rs73011279
rs34187114
rs35228298
All
−0.5
0.0
0.5
1.0
MR effect size for
'Gut microbiota abundance (genus Holdemanella id.11393)' on 'Arthropathy in ulcerative colitis'

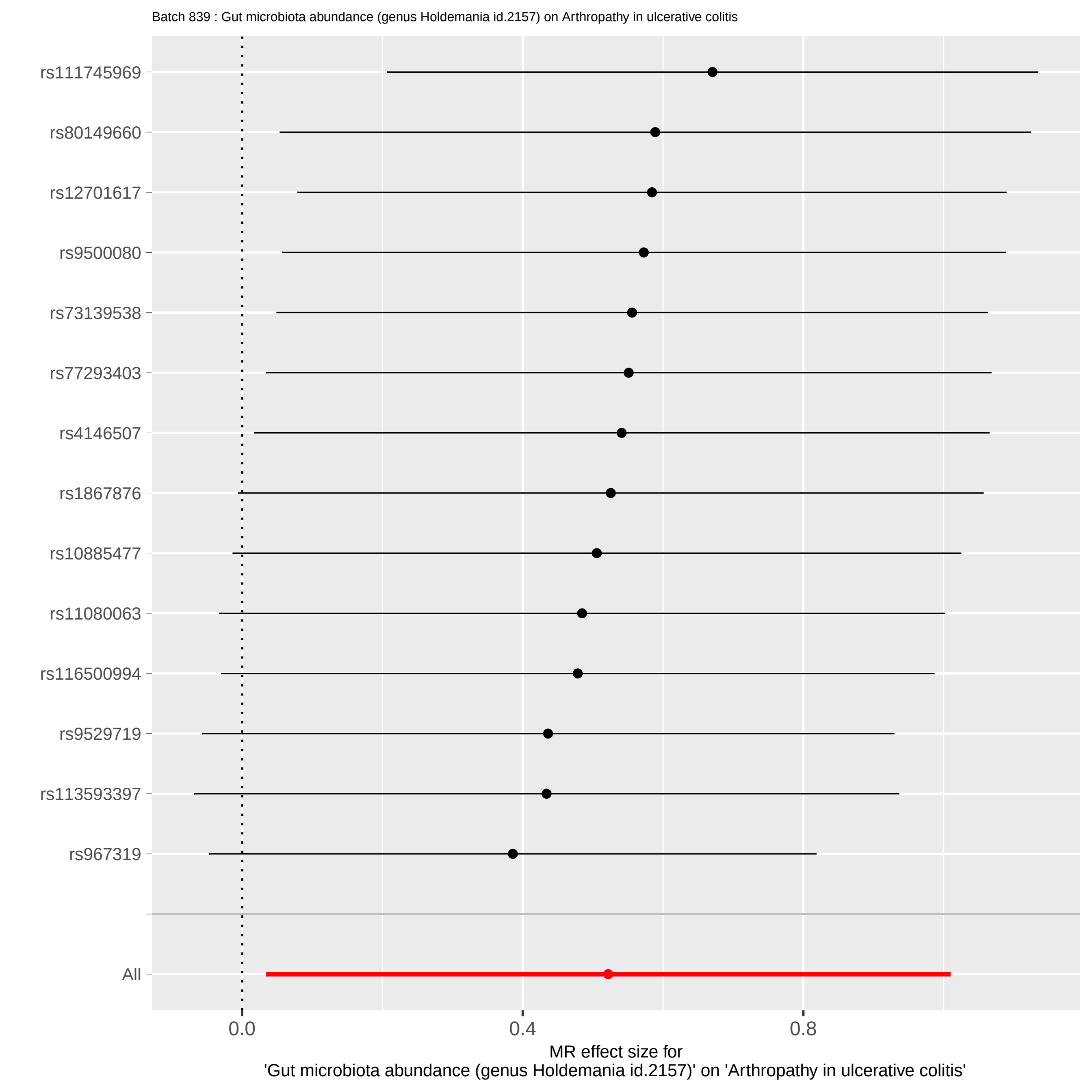
Batch 839 : Gut microbiota abundance (genus Holdemania id.2157) on Arthropathy in ulcerative colitis
rs111745969
rs80149660
rs12701617
rs9500080
rs73139538
rs77293403
rs4146507
rs1867876
rs10885477
rs11080063
rs116500994
rs9529719
rs113593397
rs967319
All
0.0
0.4
0.8
MR effect size for
'Gut microbiota abundance (genus Holdemania id.2157)' on 'Arthropathy in ulcerative colitis'

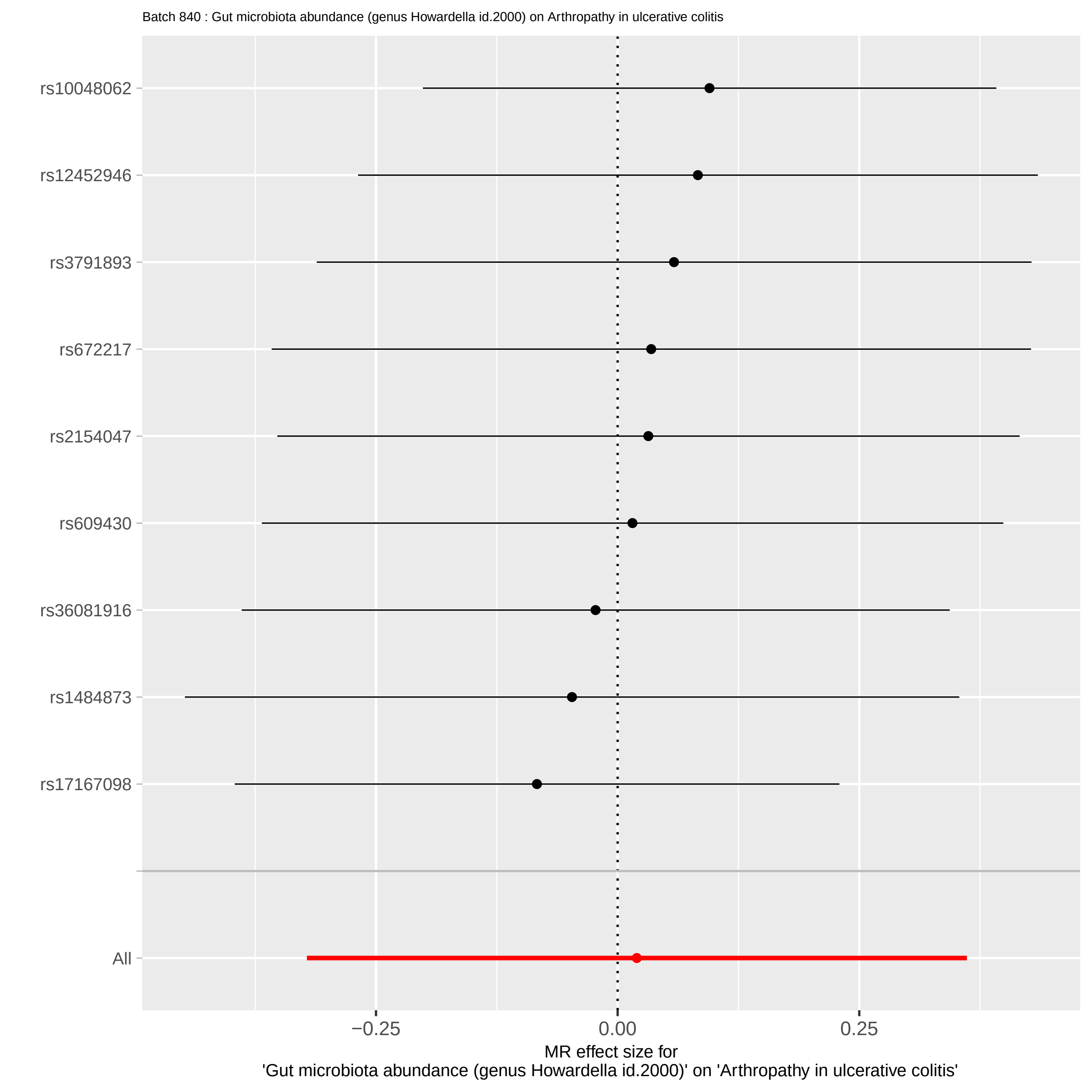
Batch 840 : Gut microbiota abundance (genus Howardella id.2000) on Arthropathy in ulcerative colitis
rs10048062
rs12452946
rs3791893
rs672217
rs2154047
rs609430
rs36081916
rs1484873
rs17167098
All
−0.25
0.00
0.25
MR effect size for
'Gut microbiota abundance (genus Howardella id.2000)' on 'Arthropathy in ulcerative colitis'

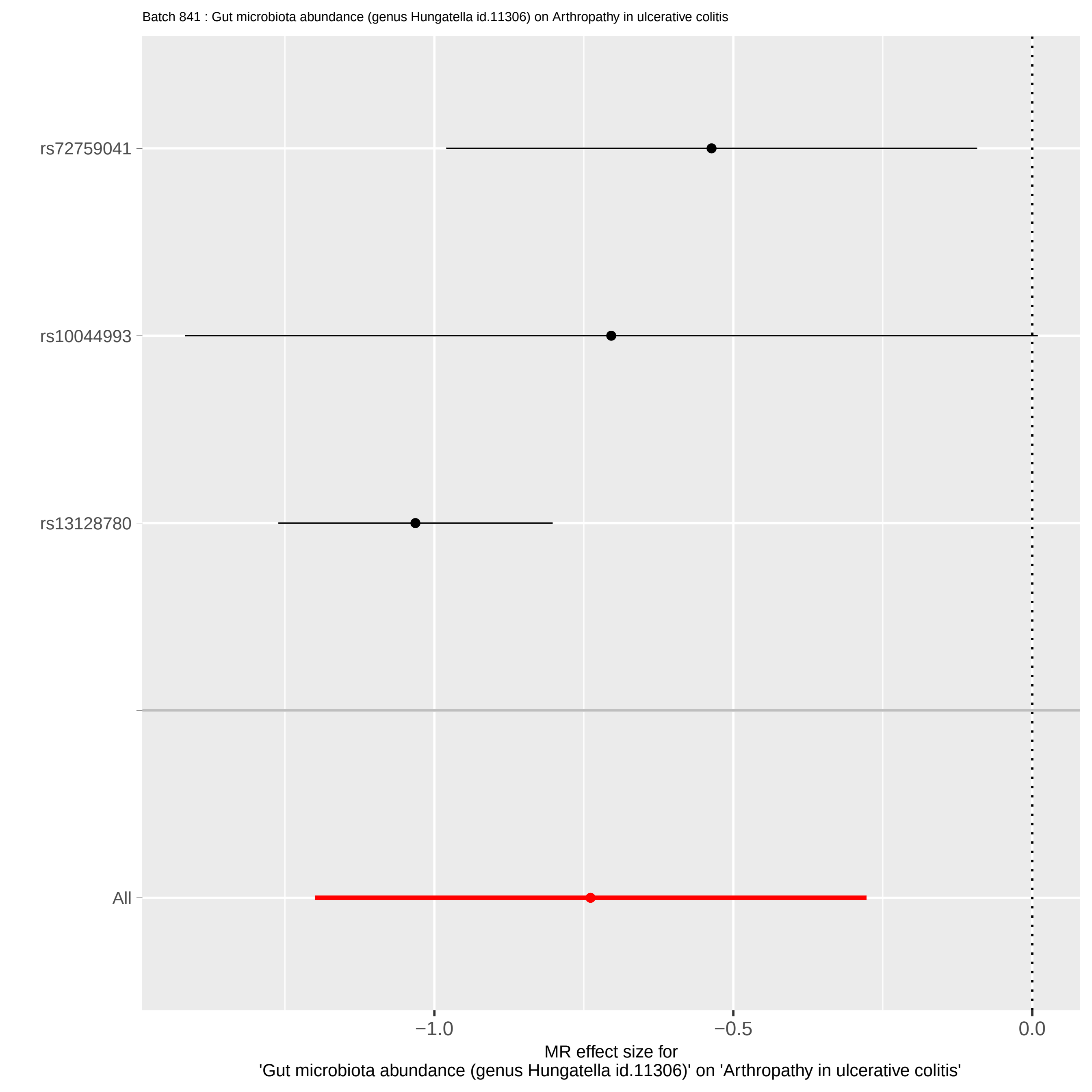

Batch 841 : Gut microbiota abundance (genus Hungatella id.11306) on Arthropathy in ulcerative colitis
rs72759041
rs10044993
rs13128780
All
−1.0
−0.5
0.0
MR effect size for
'Gut microbiota abundance (genus Hungatella id.11306)' on 'Arthropathy in ulcerative colitis'

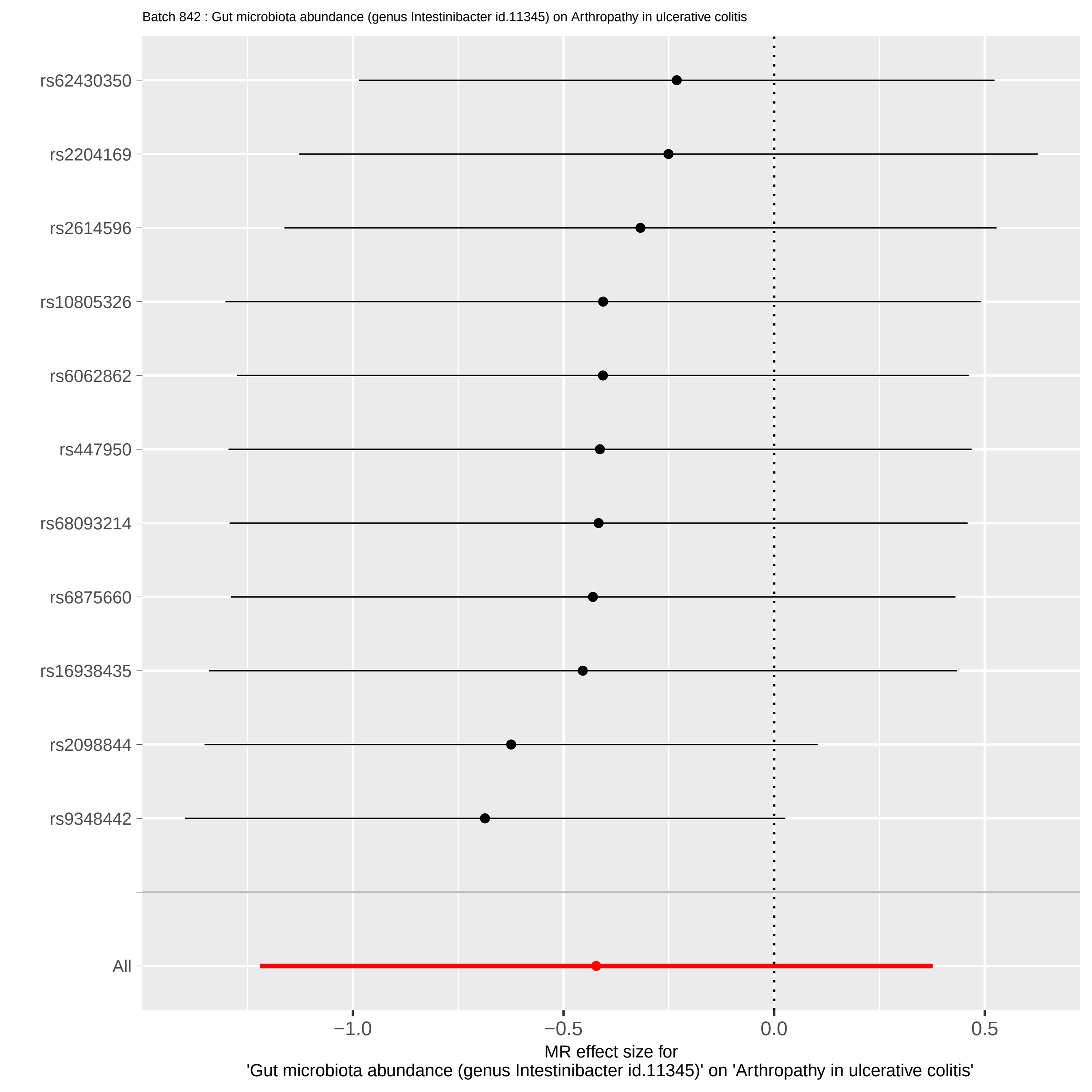

Batch 842 : Gut microbiota abundance (genus Intestinibacter id.11345) on Arthropathy in ulcerative colitis
rs62430350
rs2204169
rs2614596
rs10805326
rs6062862
rs447950
rs68093214
rs6875660
rs16938435
rs2098844
rs9348442
All
−1.0
−0.5
0.0
0.5
MR effect size for
'Gut microbiota abundance (genus Intestinibacter id.11345)' on 'Arthropathy in ulcerative colitis'

Batch 843 : Gut microbiota abundance (genus Intestinimonas id.2062) on Arthropathy in ulcerative colitis

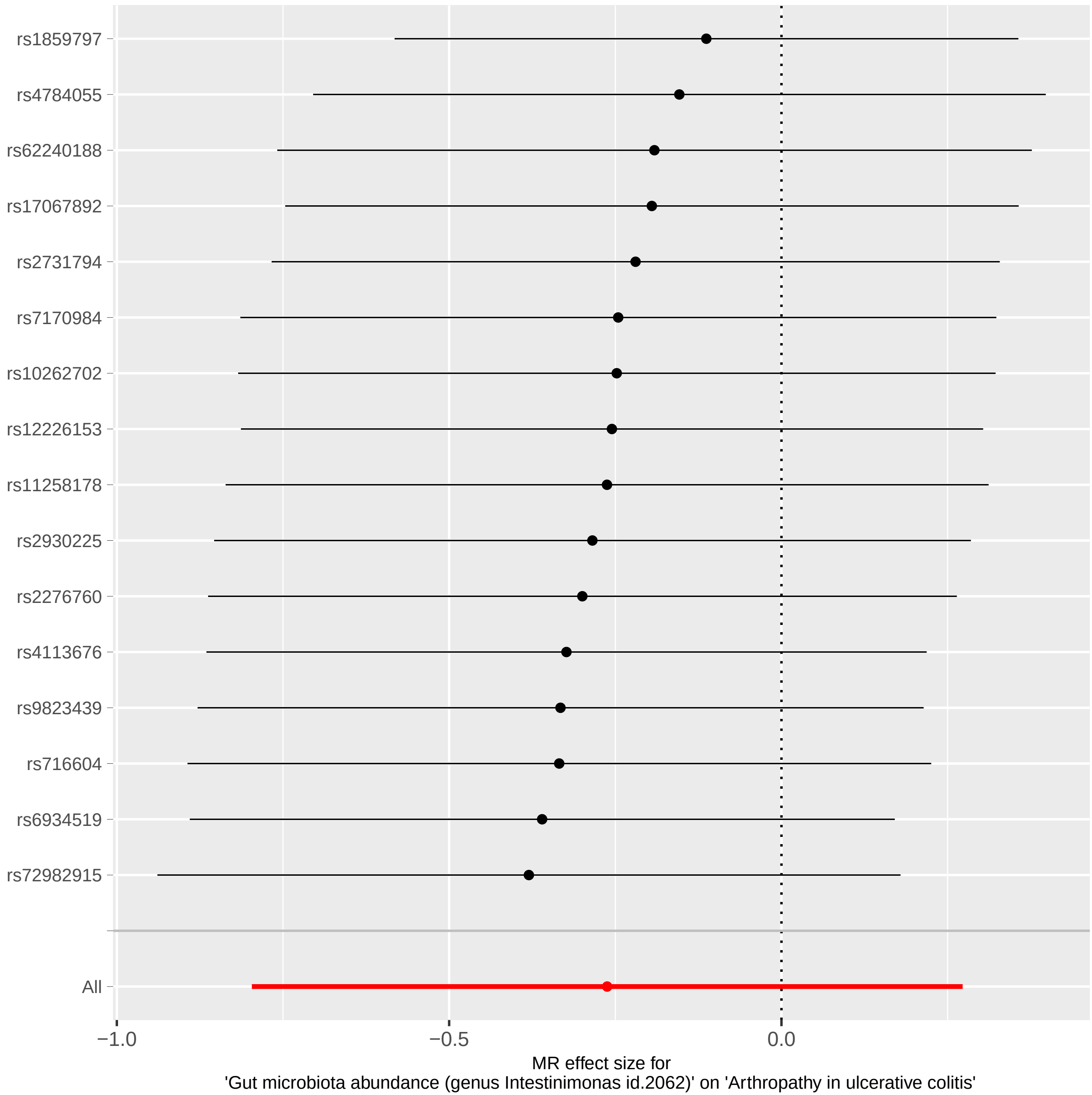

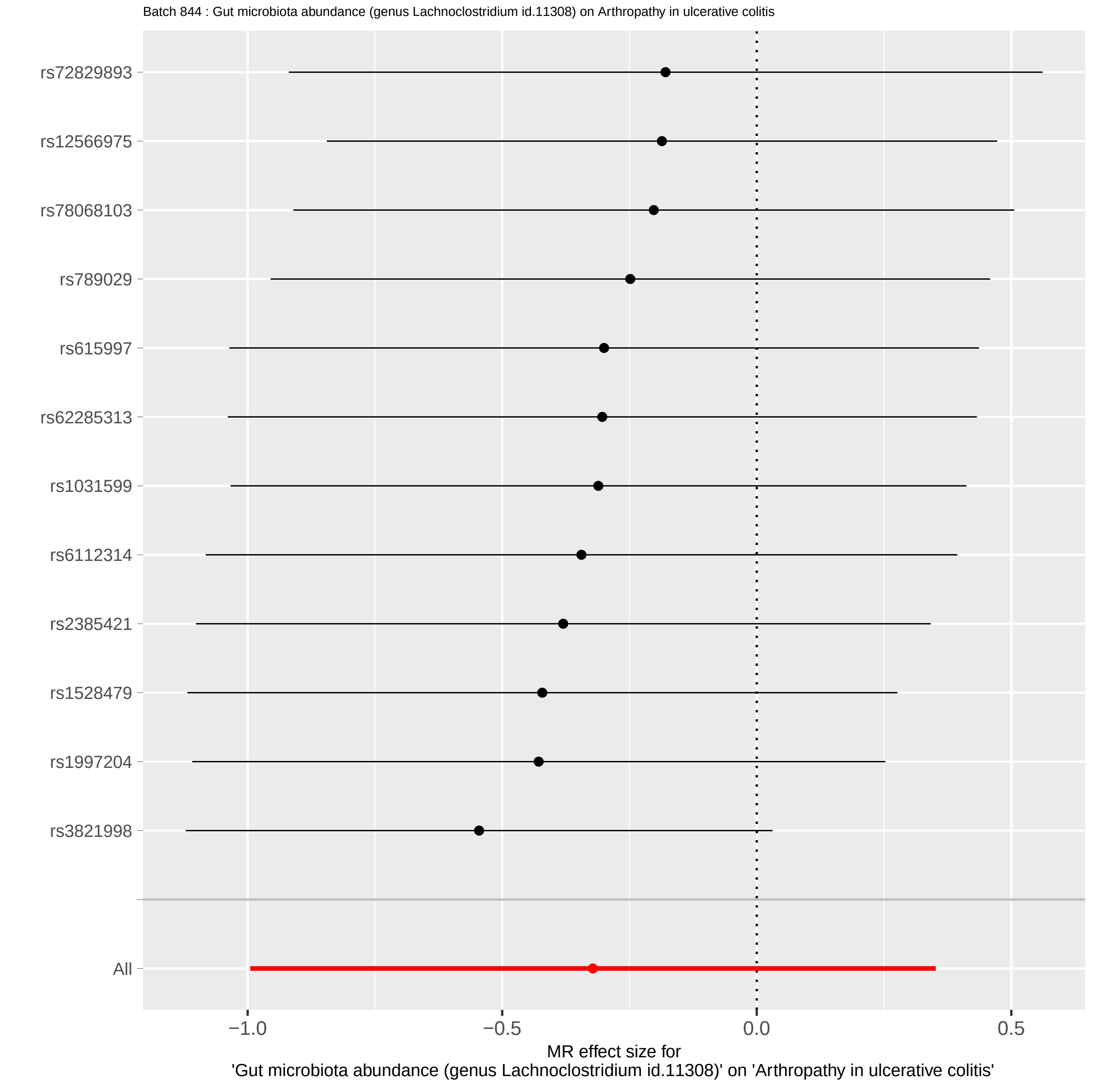
Batch 844 : Gut microbiota abundance (genus Lachnoclostridium id.11308) on Arthropathy in ulcerative colitis
rs72829893
rs12566975
rs78068103
rs789029
rs615997
rs62285313
rs1031599
rs6112314
rs2385421
rs1528479
rs1997204
rs3821998
All
−1.0
−0.5
0.0
0.5
MR effect size for
'Gut microbiota abundance (genus Lachnoclostridium id.11308)' on 'Arthropathy in ulcerative colitis'

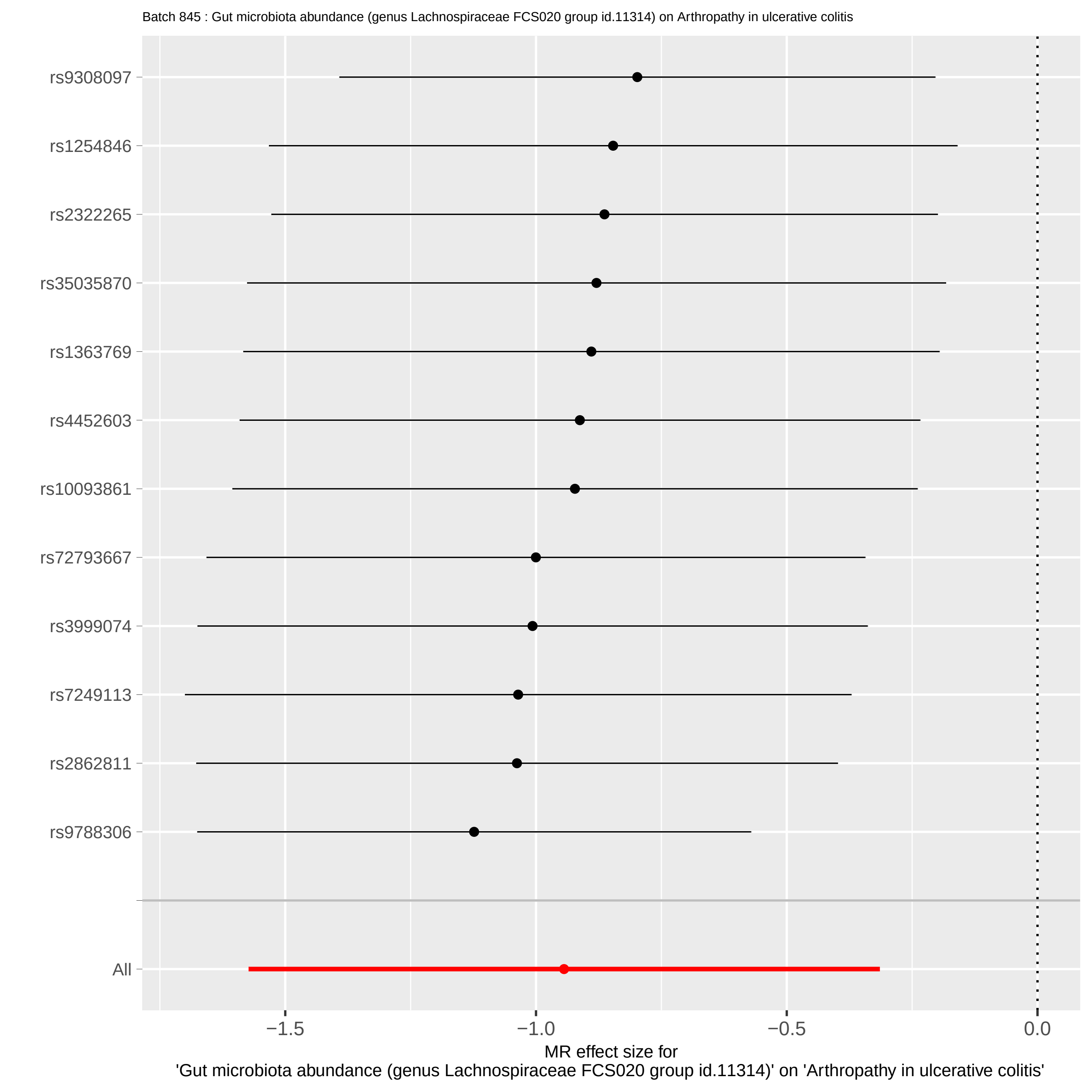

Batch 845 : Gut microbiota abundance (genus Lachnospiraceae FCS020 group id.11314) on Arthropathy in ulcerative colitis
rs9308097
rs1254846
rs2322265
rs35035870
rs1363769
rs4452603
rs10093861
rs72793667
rs3999074
rs7249113
rs2862811
rs9788306
All
−1.5
−1.0
−0.5
0.0
MR effect size for
'Gut microbiota abundance (genus Lachnospiraceae FCS020 group id.11314)' on 'Arthropathy in ulcerative colitis'

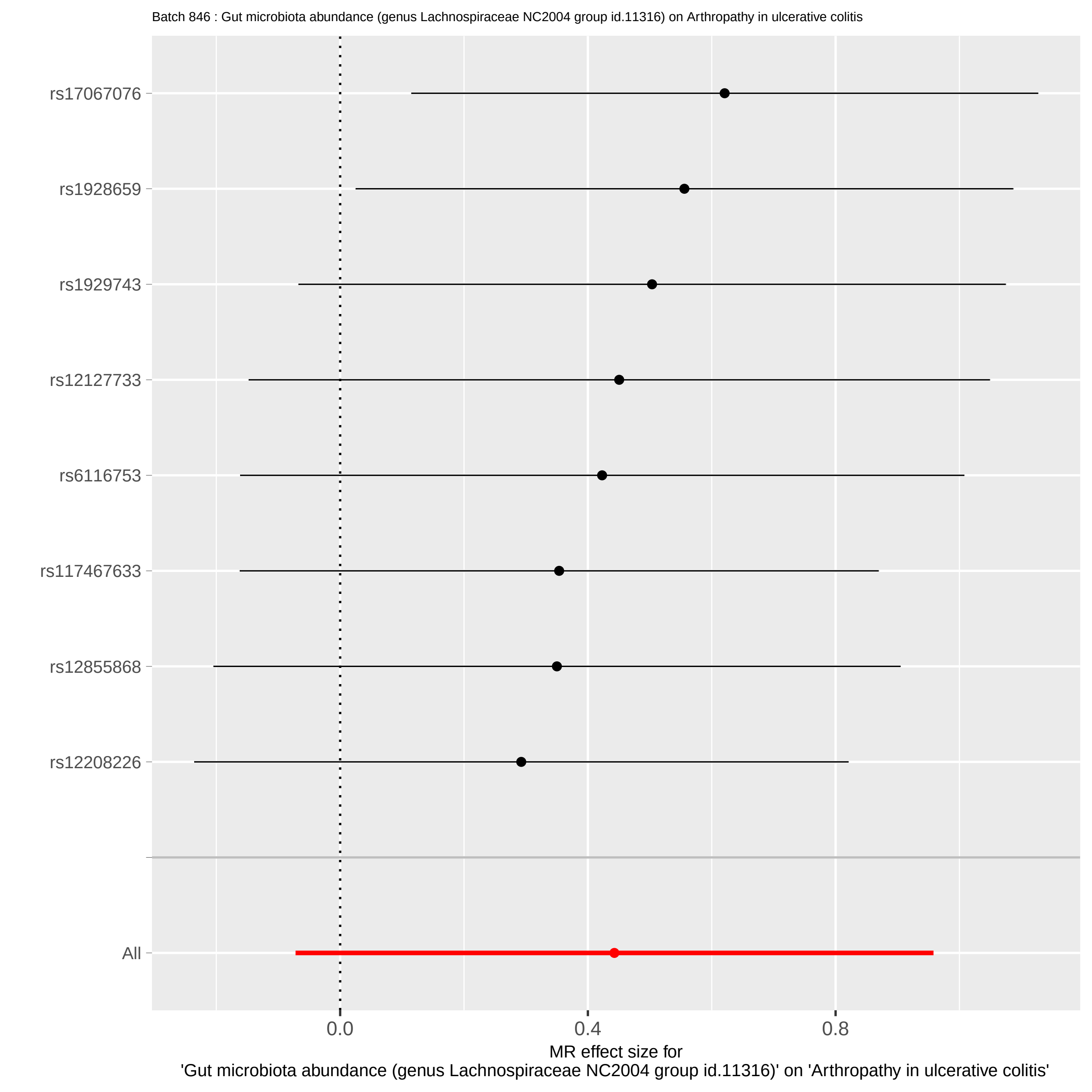

Batch 846 : Gut microbiota abundance (genus Lachnospiraceae NC2004 group id.11316) on Arthropathy in ulcerative colitis
rs17067076
rs1928659
rs1929743
rs12127733
rs6116753
rs117467633
rs12855868
rs12208226
All
0.0
0.4
0.8
MR effect size for
'Gut microbiota abundance (genus Lachnospiraceae NC2004 group id.11316)' on 'Arthropathy in ulcerative colitis'

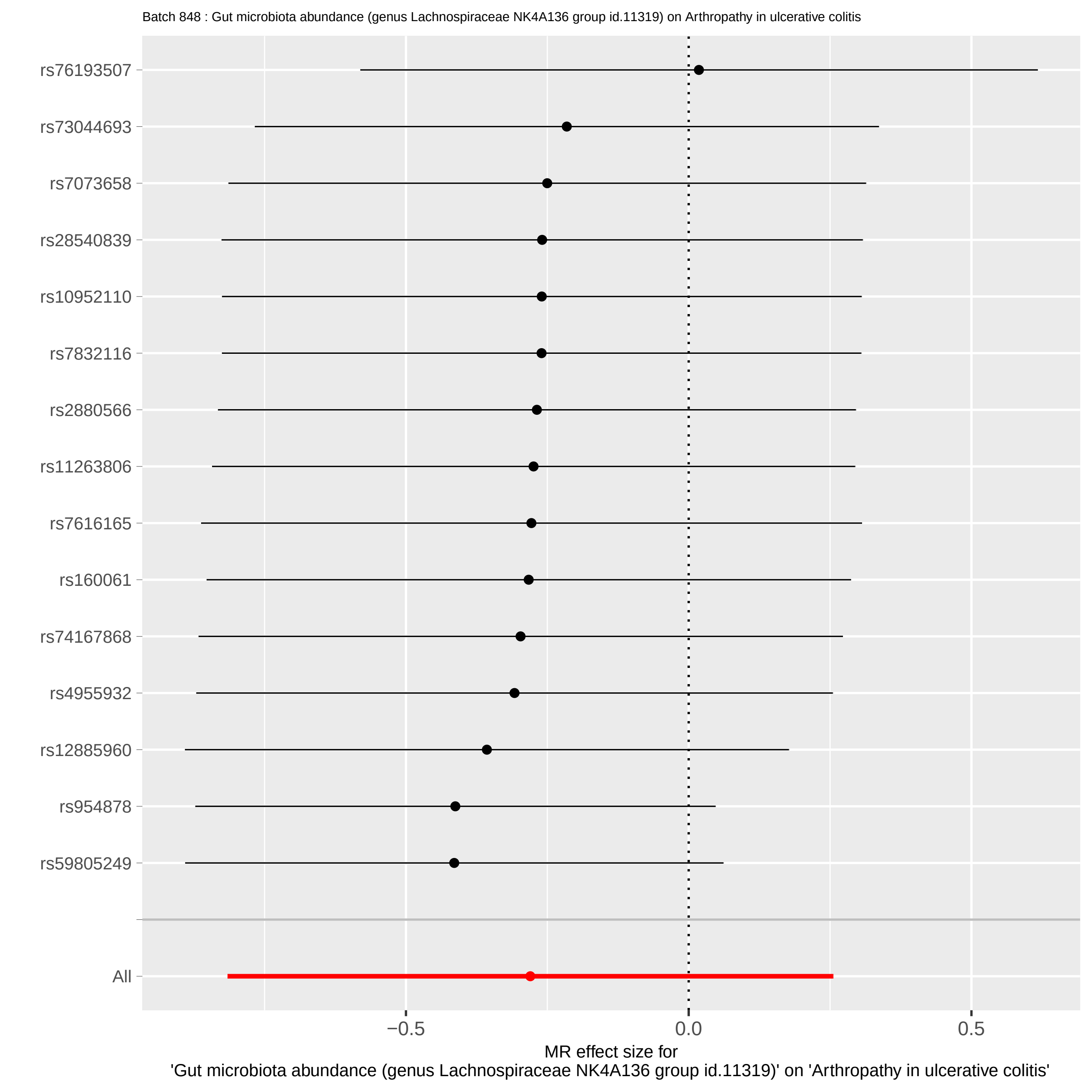

Batch 848 : Gut microbiota abundance (genus Lachnospiraceae NK4A136 group id.11319) on Arthropathy in ulcerative colitis
rs76193507
rs73044693
rs7073658
rs28540839
rs10952110
rs7832116
rs2880566
rs11263806
rs7616165
rs160061
rs74167868
rs4955932
rs12885960
rs954878
rs59805249
All
−0.5
0.0
0.5
MR effect size for
'Gut microbiota abundance (genus Lachnospiraceae NK4A136 group id.11319)' on 'Arthropathy in ulcerative colitis'

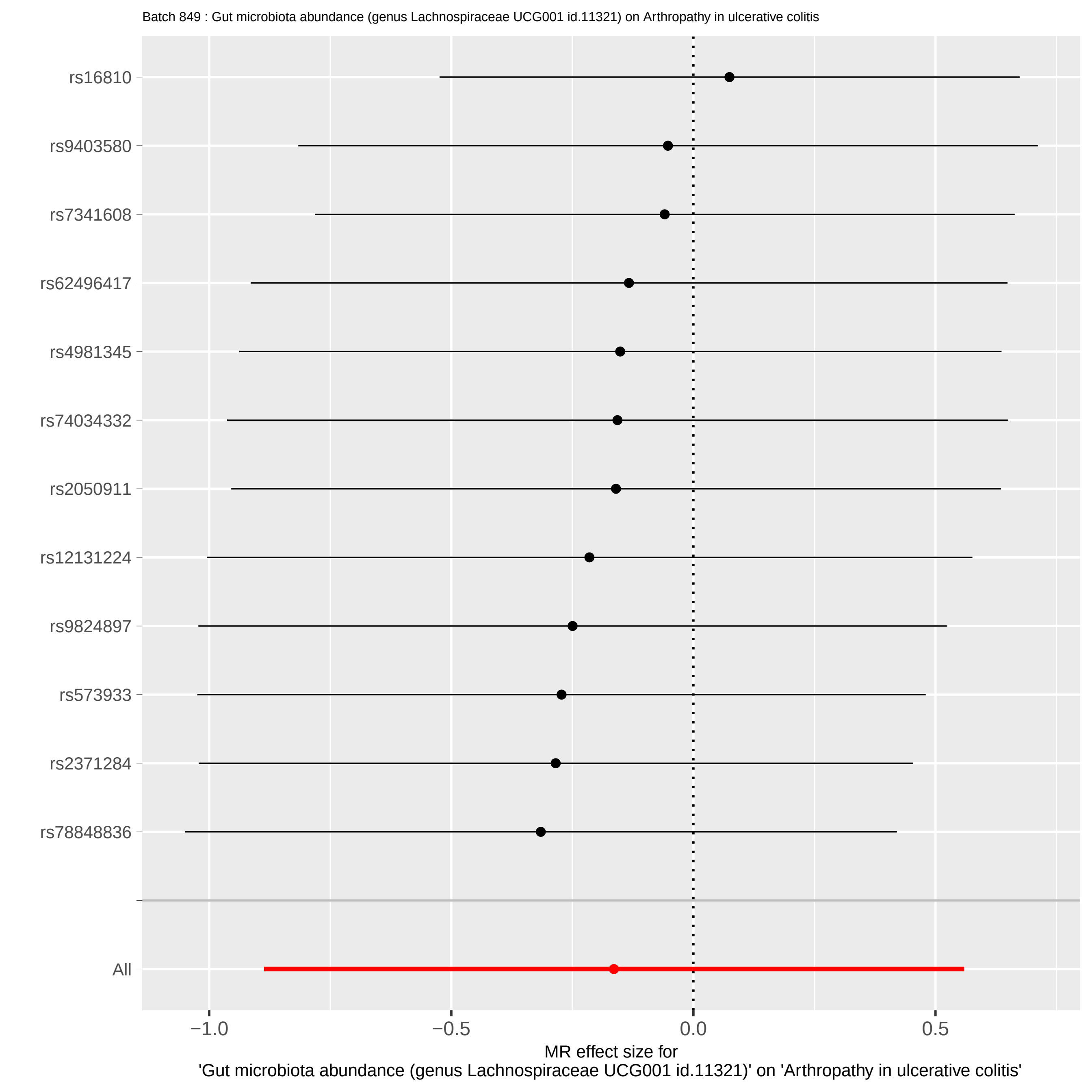

Batch 849 : Gut microbiota abundance (genus Lachnospiraceae UCG001 id.11321) on Arthropathy in ulcerative colitis
rs16810
rs9403580
rs7341608
rs62496417
rs4981345
rs74034332
rs2050911
rs12131224
rs9824897
rs573933
rs2371284
rs78848836
All
−1.0
−0.5
0.0
0.5
MR effect size for
'Gut microbiota abundance (genus Lachnospiraceae UCG001 id.11321)' on 'Arthropathy in ulcerative colitis'

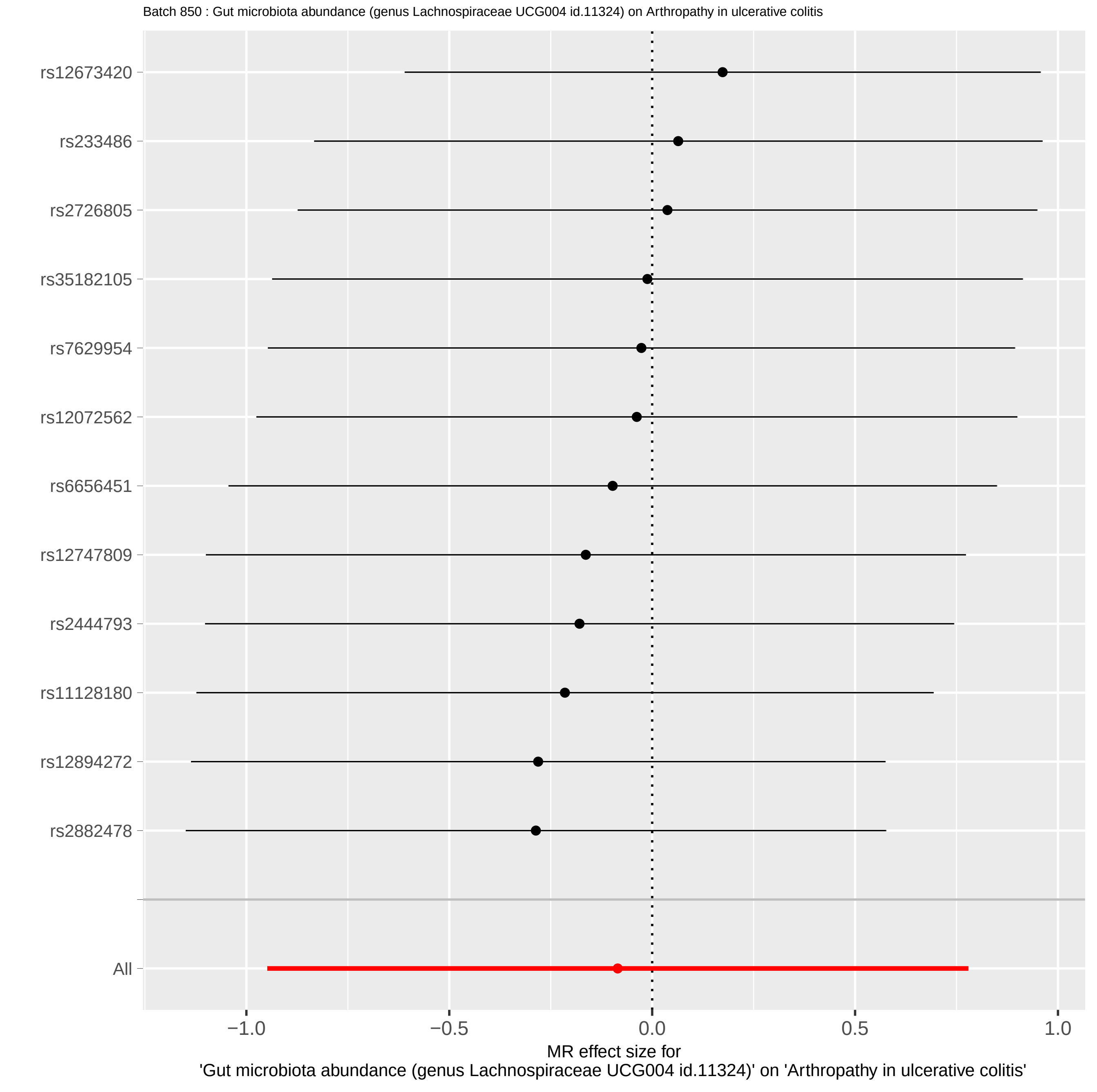

Batch 850 : Gut microbiota abundance (genus Lachnospiraceae UCG004 id.11324) on Arthropathy in ulcerative colitis
rs12673420
rs233486
rs2726805
rs35182105
rs7629954
rs12072562
rs6656451
rs12747809
rs2444793
rs11128180
rs12894272
rs2882478
All
−1.0
−0.5
0.0
0.5
1.0
MR effect size for
'Gut microbiota abundance (genus Lachnospiraceae UCG004 id.11324)' on 'Arthropathy in ulcerative colitis'

Batch 851 : Gut microbiota abundance (genus Lachnospiraceae UCG008 id.11328) on Arthropathy in ulcerative colitis

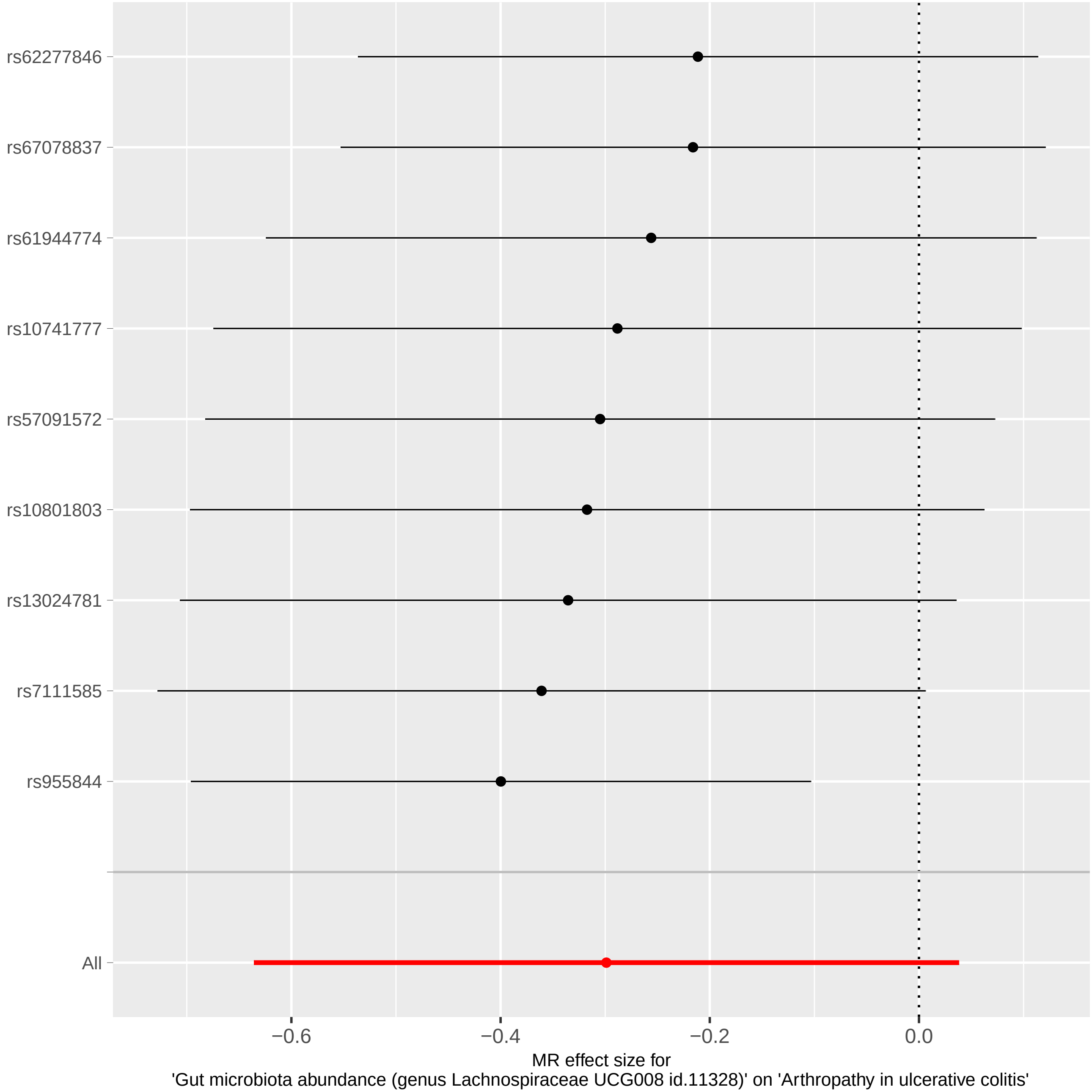

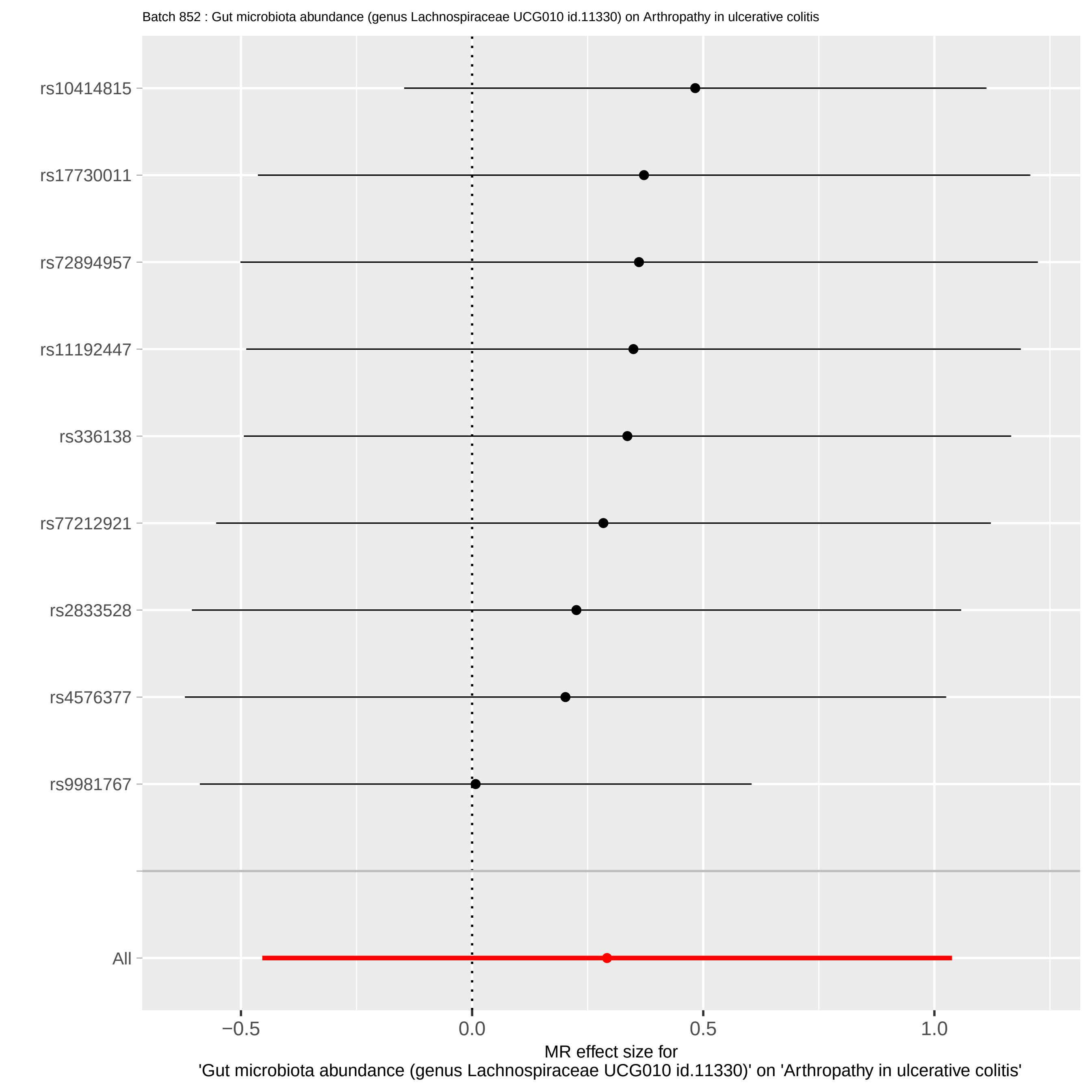
Batch 852 : Gut microbiota abundance (genus Lachnospiraceae UCG010 id.11330) on Arthropathy in ulcerative colitis
rs10414815
rs17730011
rs72894957
rs11192447
rs336138
rs77212921
rs2833528
rs4576377
rs9981767
All
−0.5
0.0
0.5
1.0
MR effect size for
'Gut microbiota abundance (genus Lachnospiraceae UCG010 id.11330)' on 'Arthropathy in ulcerative colitis'

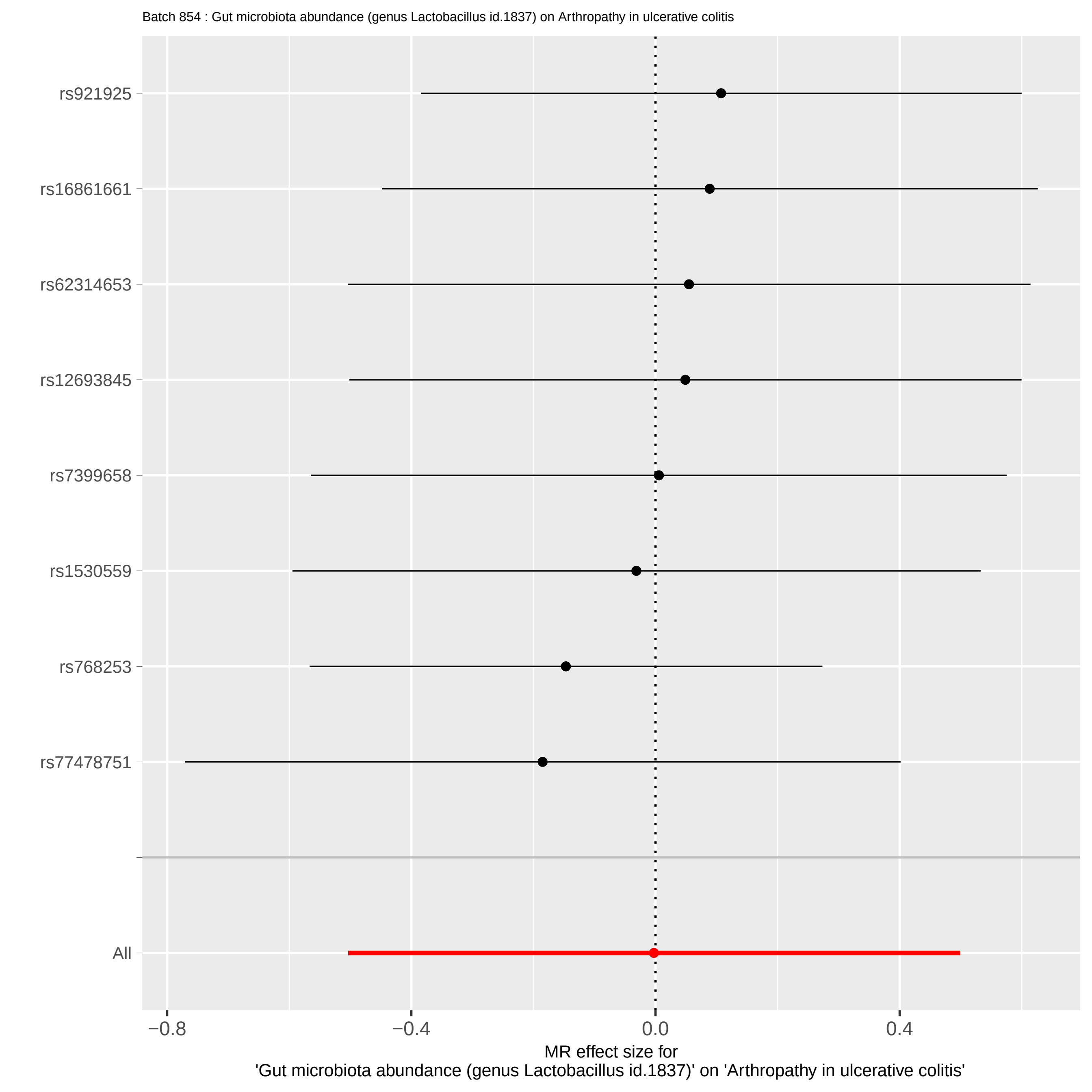
Batch 854 : Gut microbiota abundance (genus Lactobacillus id.1837) on Arthropathy in ulcerative colitis
rs921925
rs16861661
rs62314653
rs12693845
rs7399658
rs1530559
rs768253
rs77478751
All
−0.8
−0.4
0.0
0.4
MR effect size for
'Gut microbiota abundance (genus Lactobacillus id.1837)' on 'Arthropathy in ulcerative colitis'

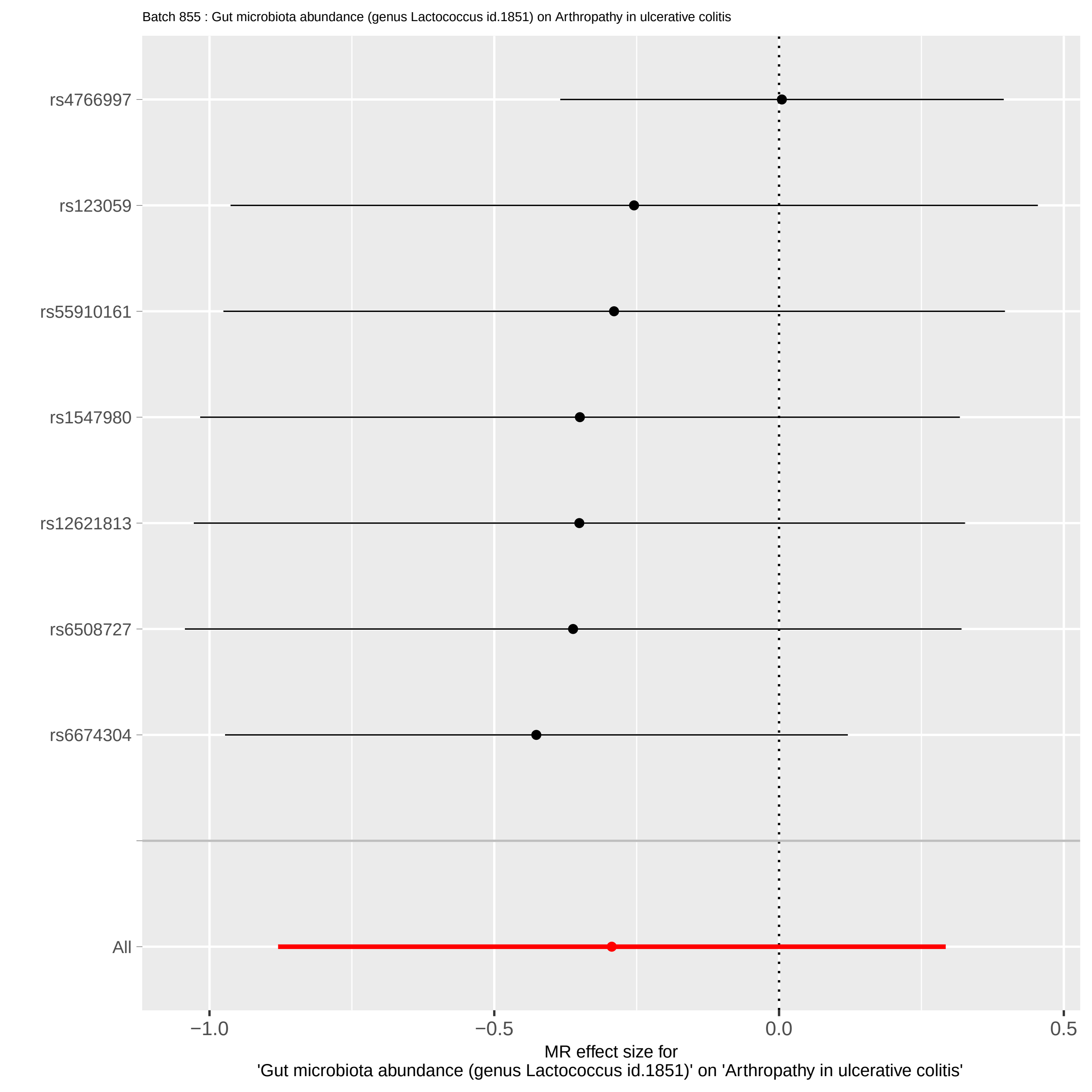

Batch 855 : Gut microbiota abundance (genus Lactococcus id.1851) on Arthropathy in ulcerative colitis
rs4766997
rs123059
rs55910161
rs1547980
rs12621813
rs6508727
rs6674304
All
−1.0
−0.5
0.0
0.5
MR effect size for
'Gut microbiota abundance (genus Lactococcus id.1851)' on 'Arthropathy in ulcerative colitis'

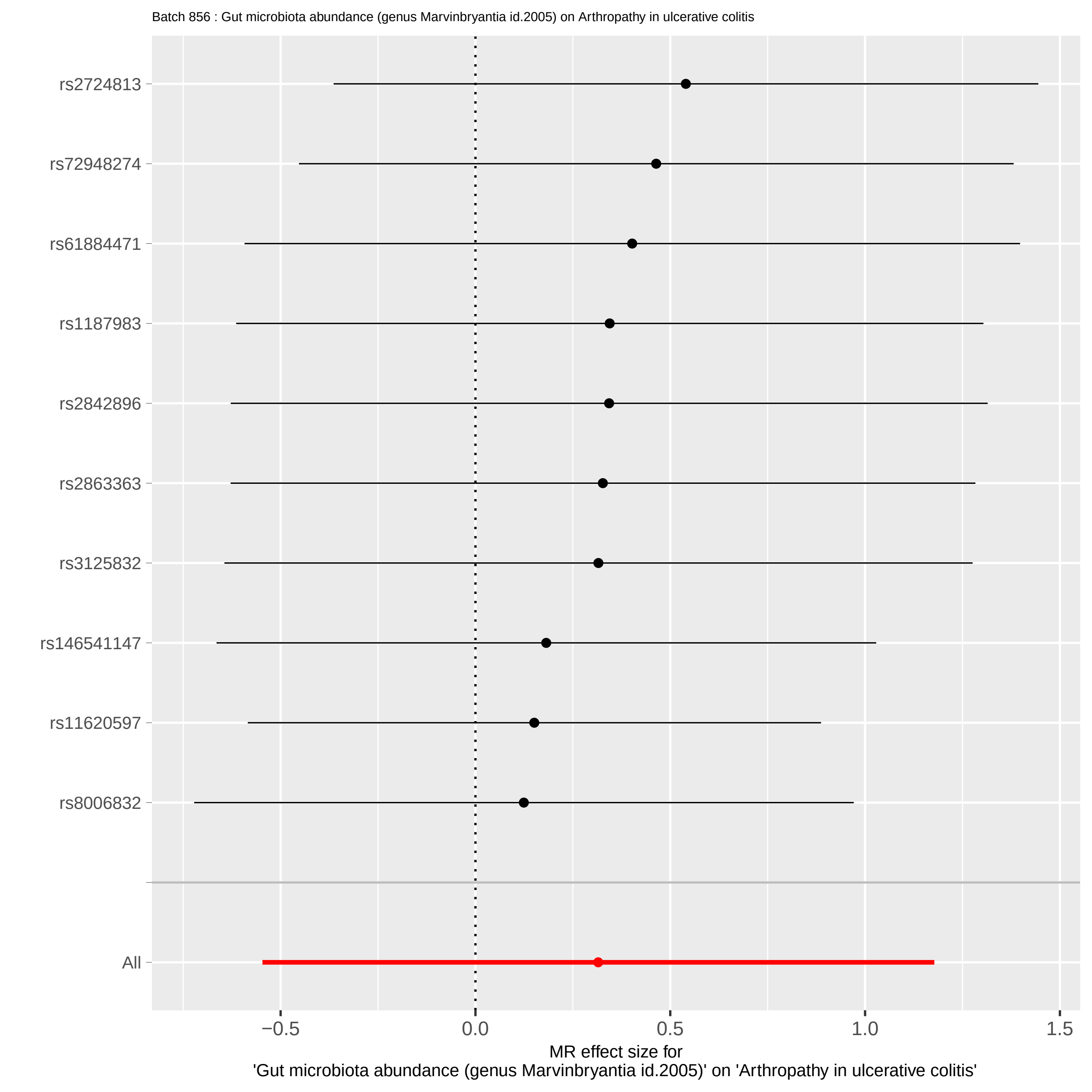
Batch 856 : Gut microbiota abundance (genus Marvinbryantia id.2005) on Arthropathy in ulcerative colitis
rs2724813
rs72948274
rs61884471
rs1187983
rs2842896
rs2863363
rs3125832
rs146541147
rs11620597
rs8006832
All
−0.5
0.0
0.5
1.0
1.5
MR effect size for
'Gut microbiota abundance (genus Marvinbryantia id.2005)' on 'Arthropathy in ulcerative colitis'

Batch 857 : Gut microbiota abundance (genus Methanobrevibacter id.123) on Arthropathy in ulcerative colitis

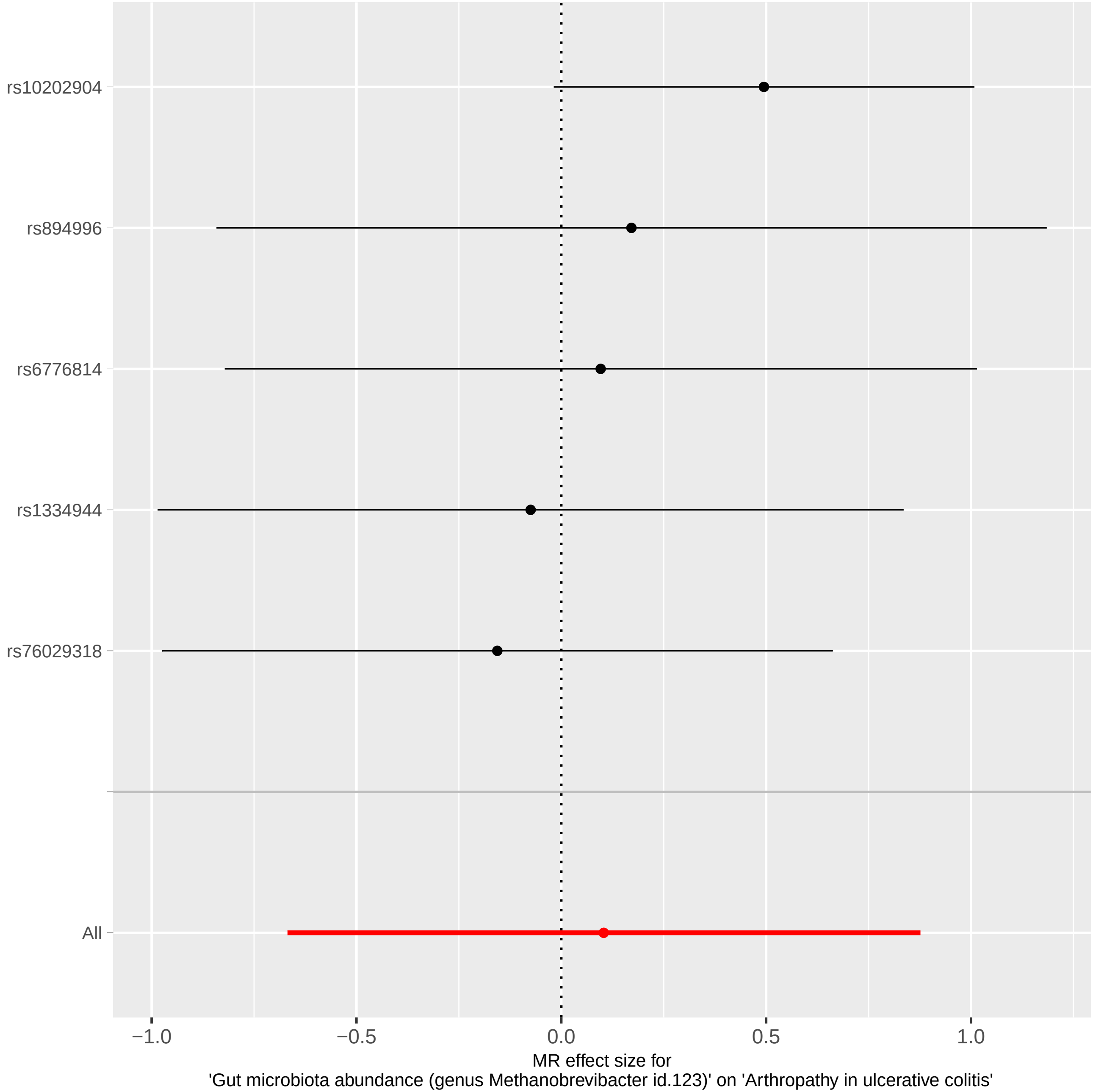

Batch 858 : Gut microbiota abundance (genus Odoribacter id.952) on Arthropathy in ulcerative colitis

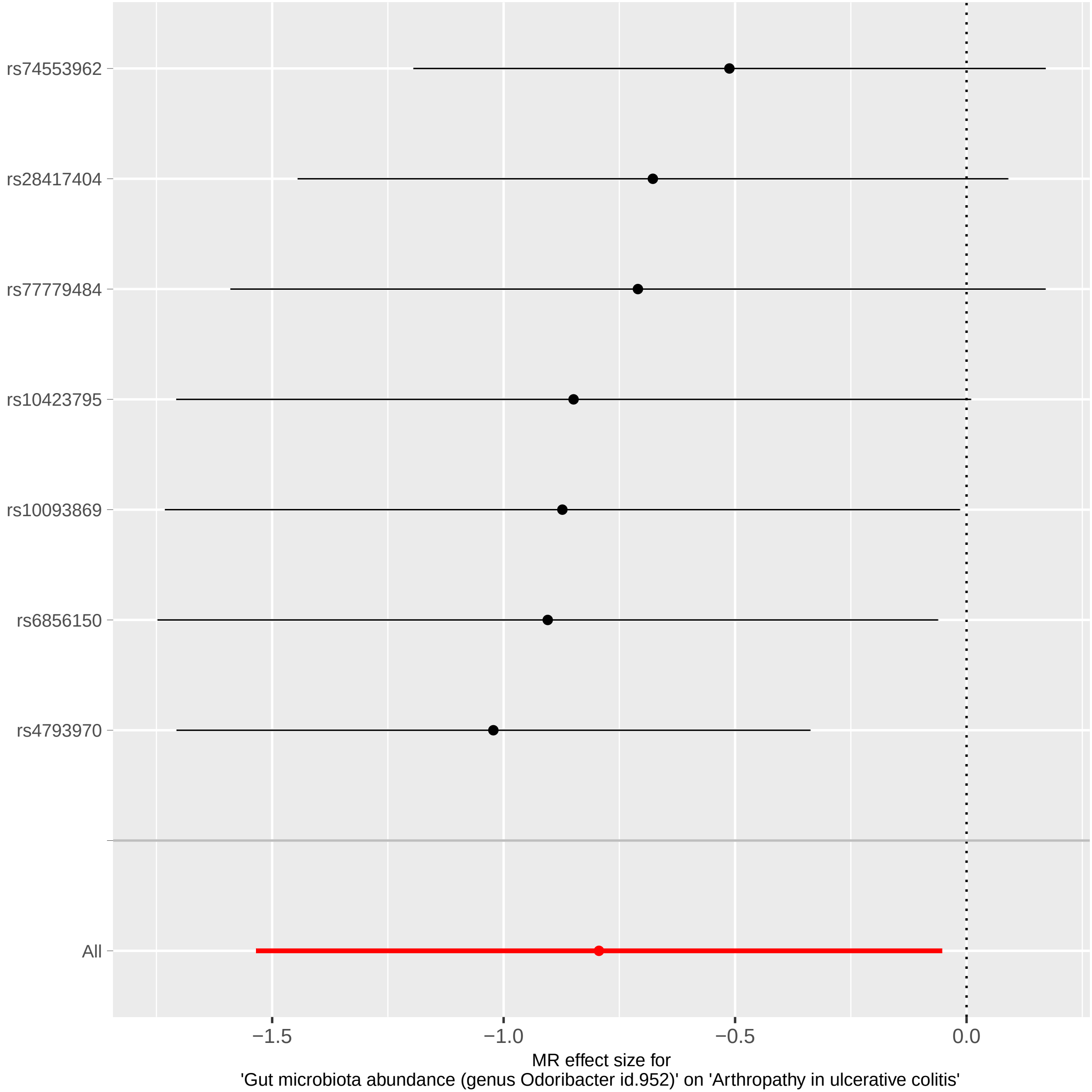

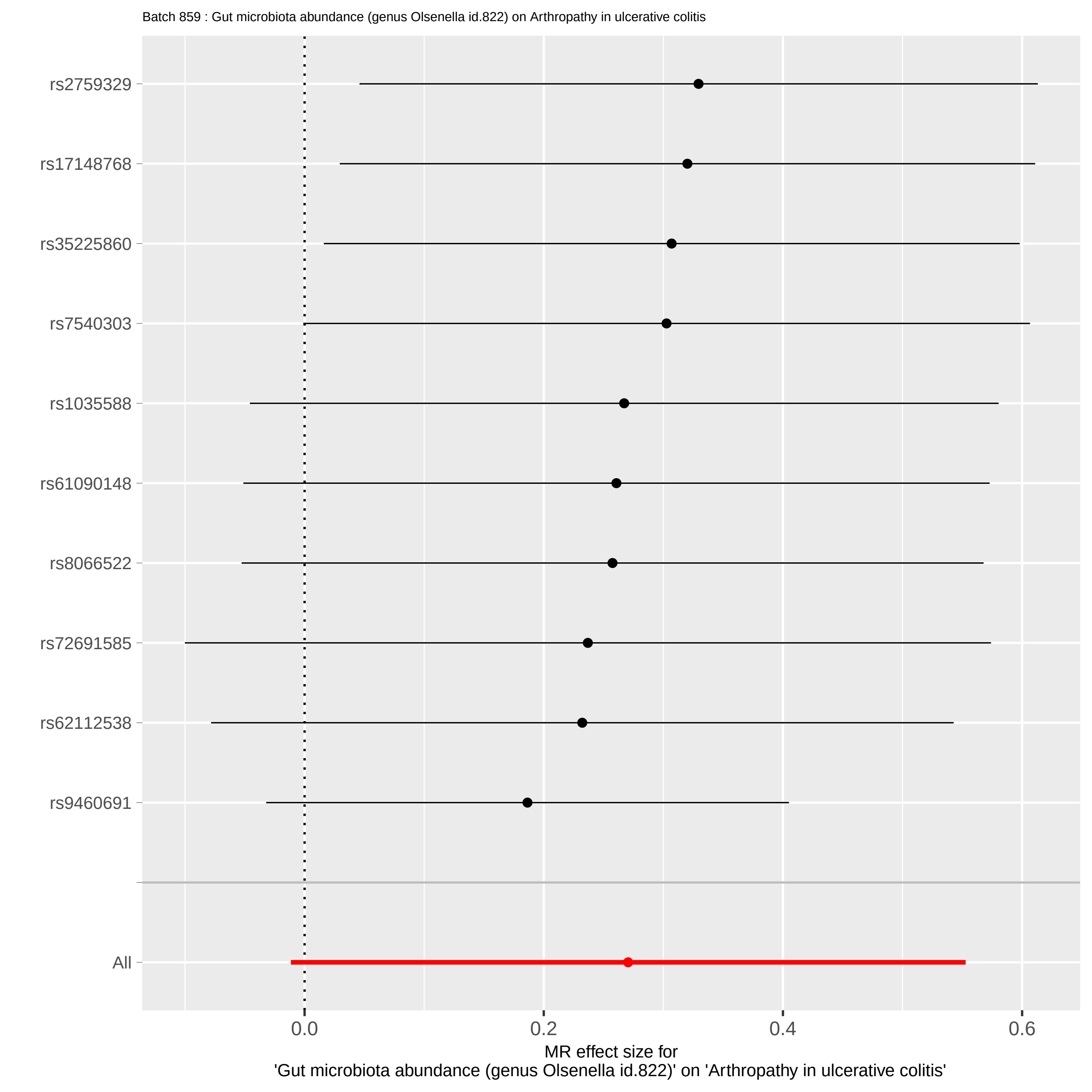

Batch 859 : Gut microbiota abundance (genus Olsenella id.822) on Arthropathy in ulcerative colitis
rs2759329
rs17148768
rs35225860
rs7540303
rs1035588
rs61090148
rs8066522
rs72691585
rs62112538
rs9460691
All
0.0
0.2
0.4
0.6
MR effect size for
'Gut microbiota abundance (genus Olsenella id.822)' on 'Arthropathy in ulcerative colitis'

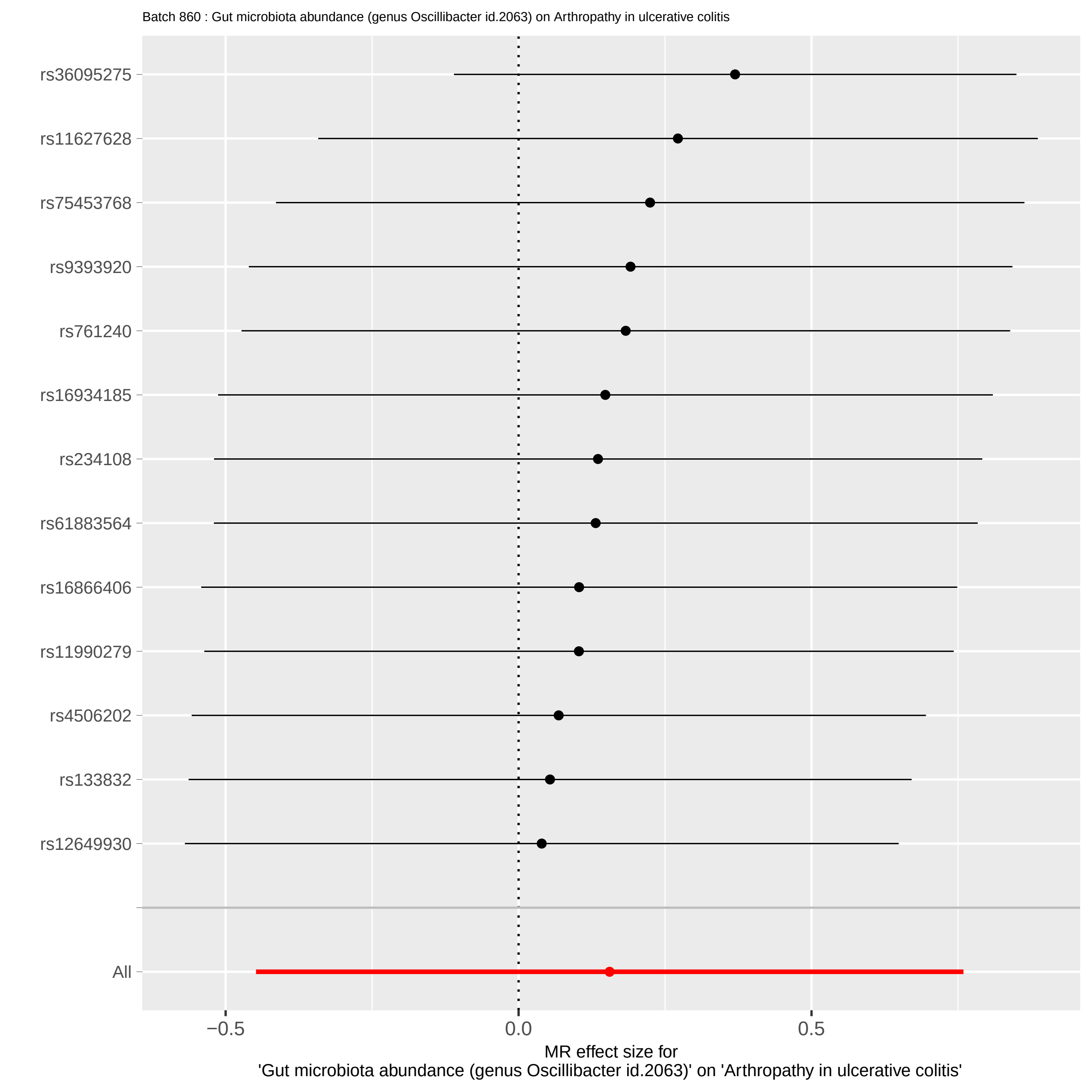
Batch 860 : Gut microbiota abundance (genus Oscillibacter id.2063) on Arthropathy in ulcerative colitis
rs36095275
rs11627628
rs75453768
rs9393920
rs761240
rs16934185
rs234108
rs61883564
rs16866406
rs11990279
rs4506202
rs133832
rs12649930
All
−0.5
0.0
0.5
MR effect size for
'Gut microbiota abundance (genus Oscillibacter id.2063)' on 'Arthropathy in ulcerative colitis'

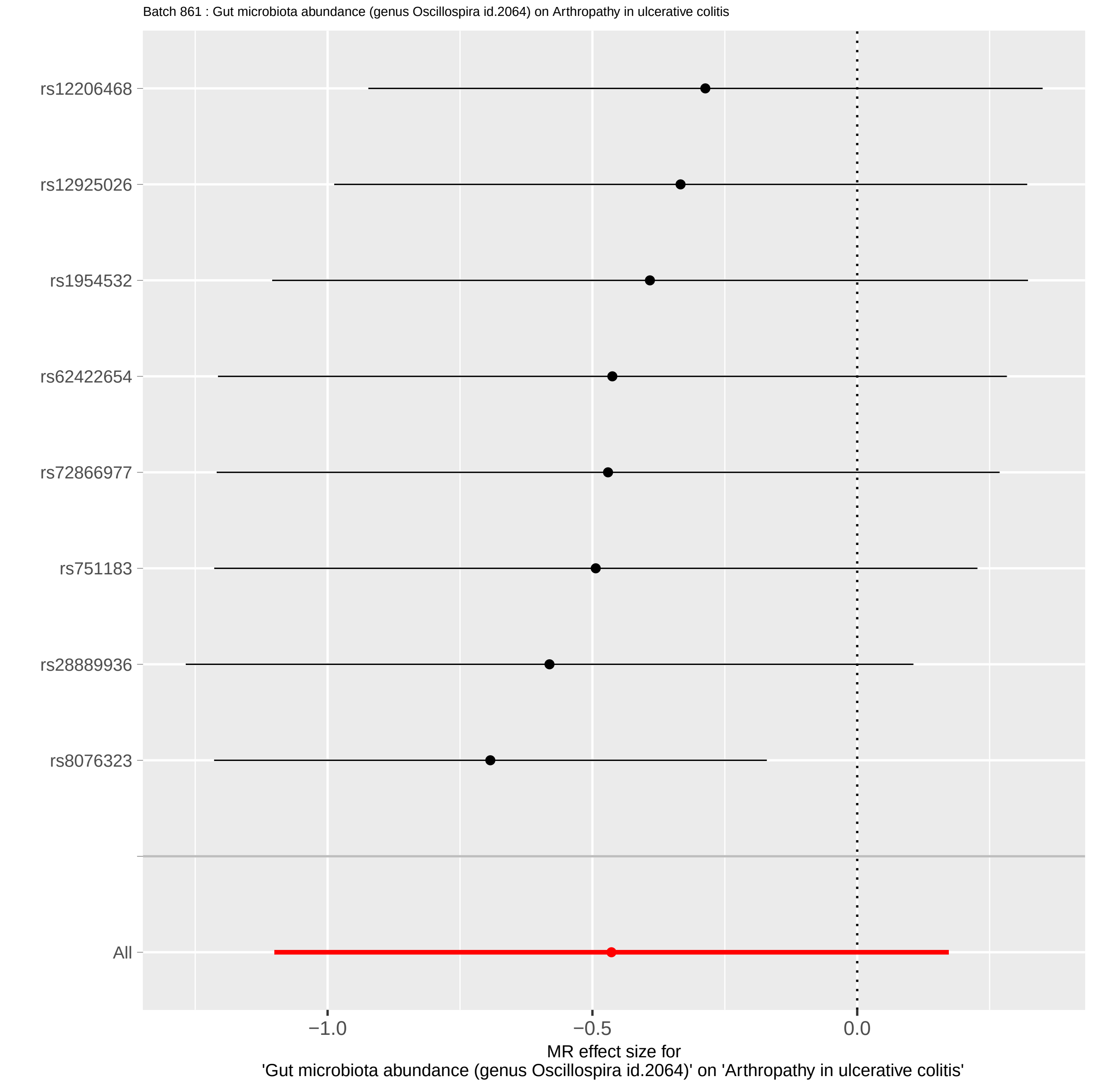

Batch 861 : Gut microbiota abundance (genus Oscillospira id.2064) on Arthropathy in ulcerative colitis
rs12206468
rs12925026
rs1954532
rs62422654
rs72866977
rs751183
rs28889936
rs8076323
All
−1.0
−0.5
0.0
MR effect size for
'Gut microbiota abundance (genus Oscillospira id.2064)' on 'Arthropathy in ulcerative colitis'

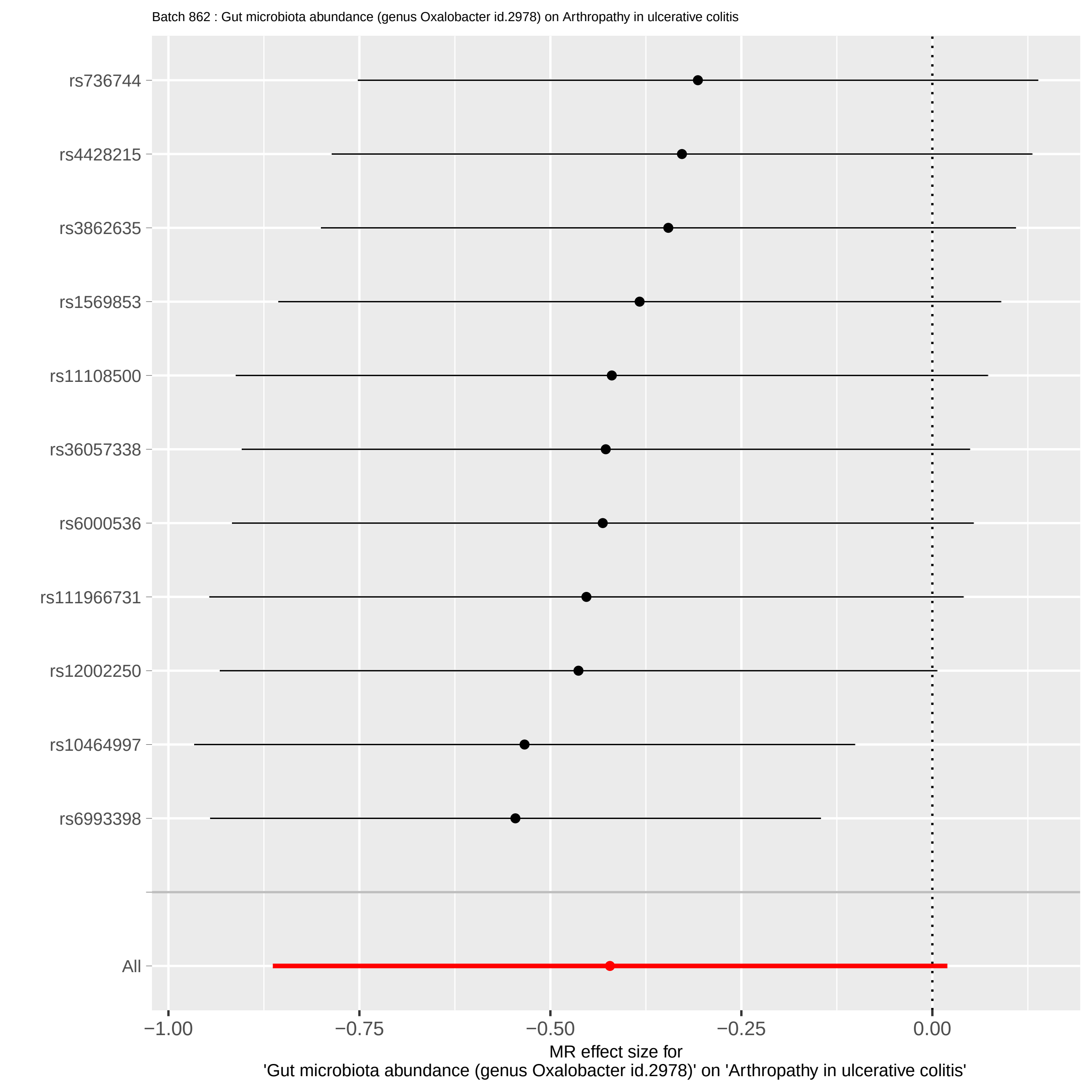
Batch 862 : Gut microbiota abundance (genus Oxalobacter id.2978) on Arthropathy in ulcerative colitis
rs736744
rs4428215
rs3862635
rs1569853
rs11108500
rs36057338
rs6000536
rs111966731
rs12002250
rs10464997
rs6993398
All
−1.00
−0.75
−0.50
−0.25
0.00
MR effect size for
'Gut microbiota abundance (genus Oxalobacter id.2978)' on 'Arthropathy in ulcerative colitis'

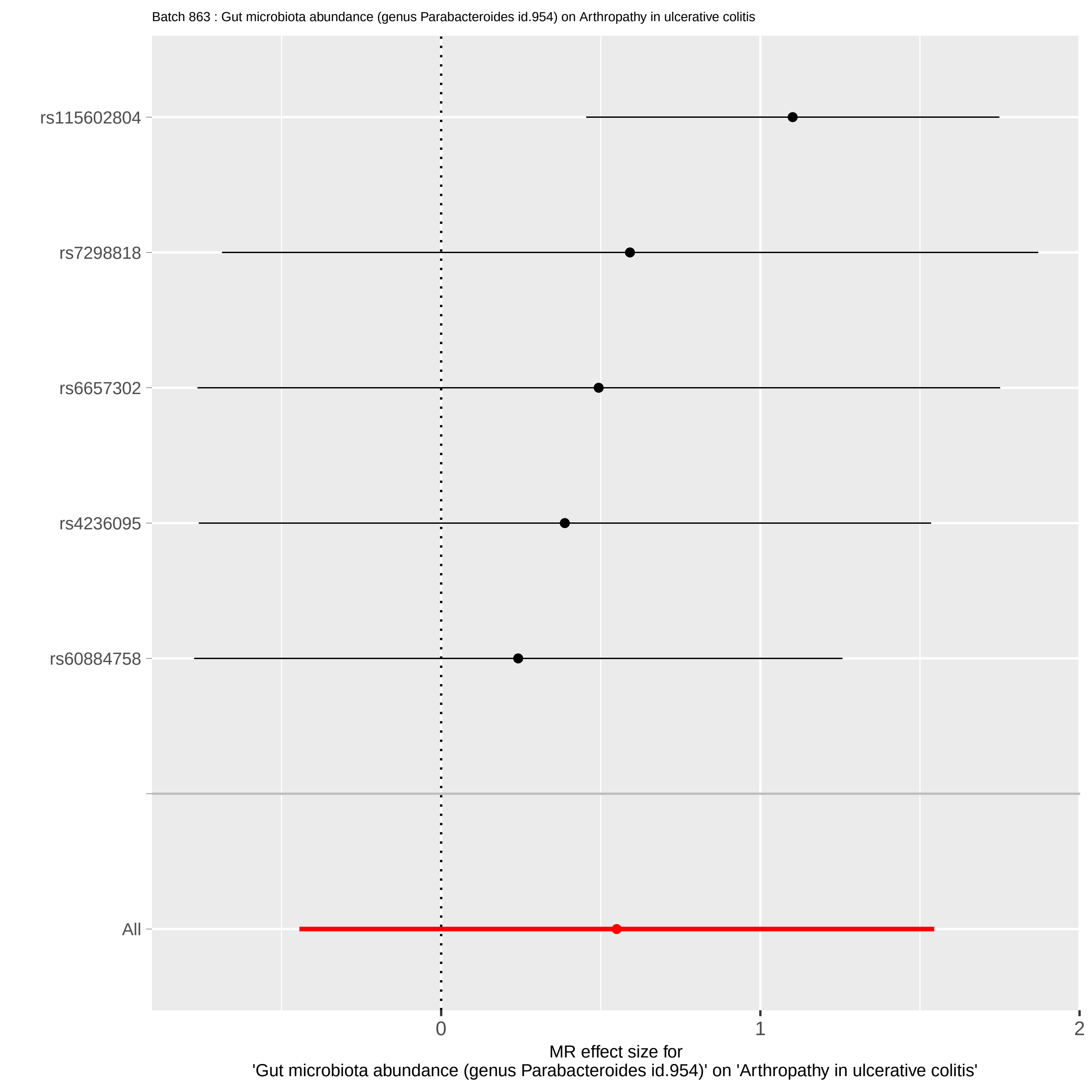
Batch 863 : Gut microbiota abundance (genus Parabacteroides id.954) on Arthropathy in ulcerative colitis
rs115602804
rs7298818
rs6657302
rs4236095
rs60884758
All
0
1
2
MR effect size for
'Gut microbiota abundance (genus Parabacteroides id.954)' on 'Arthropathy in ulcerative colitis'

Batch 864 : Gut microbiota abundance (genus Paraprevotella id.962) on Arthropathy in ulcerative colitis

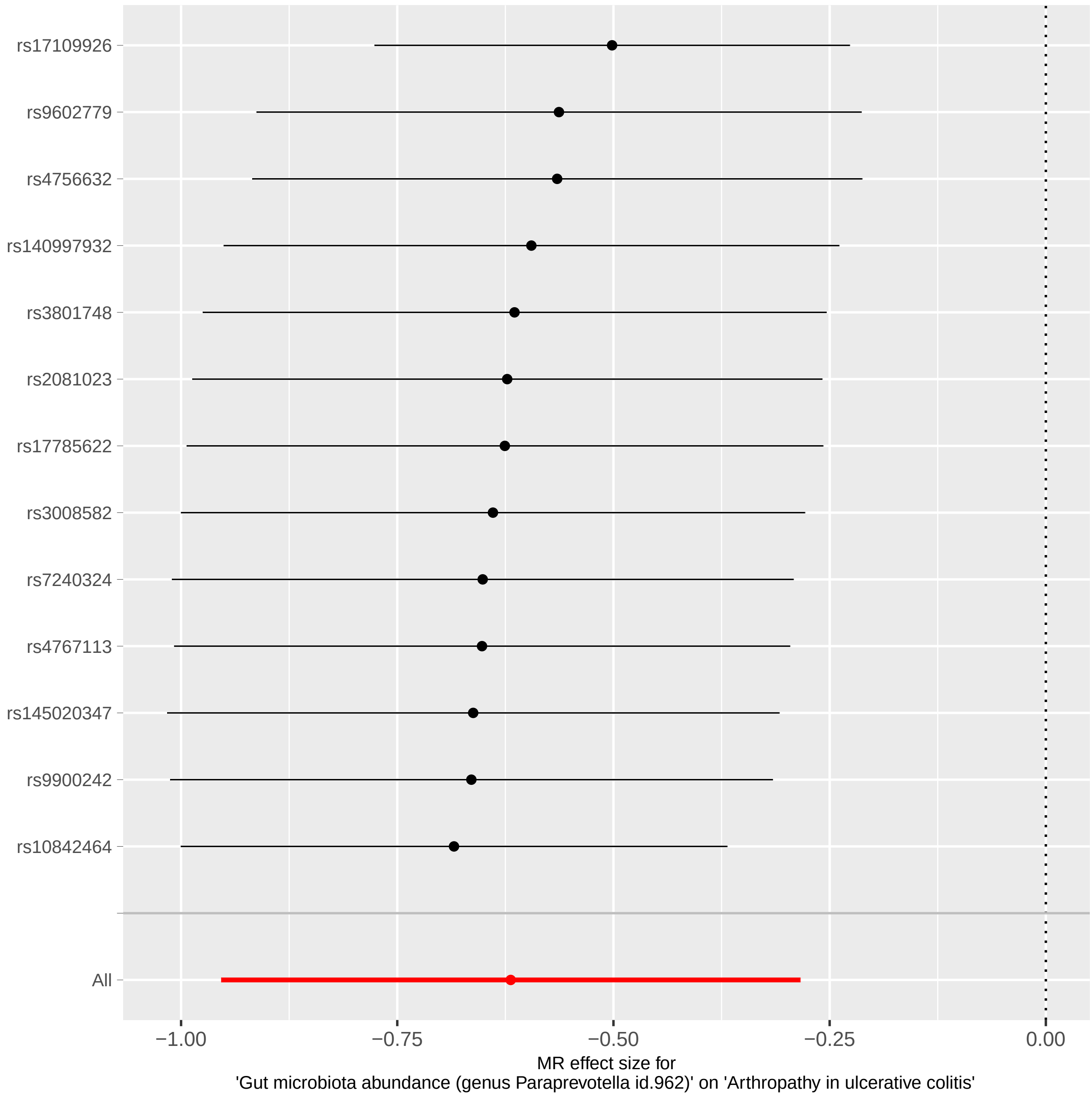

Batch 865 : Gut microbiota abundance (genus Parasutterella id.2892) on Arthropathy in ulcerative colitis

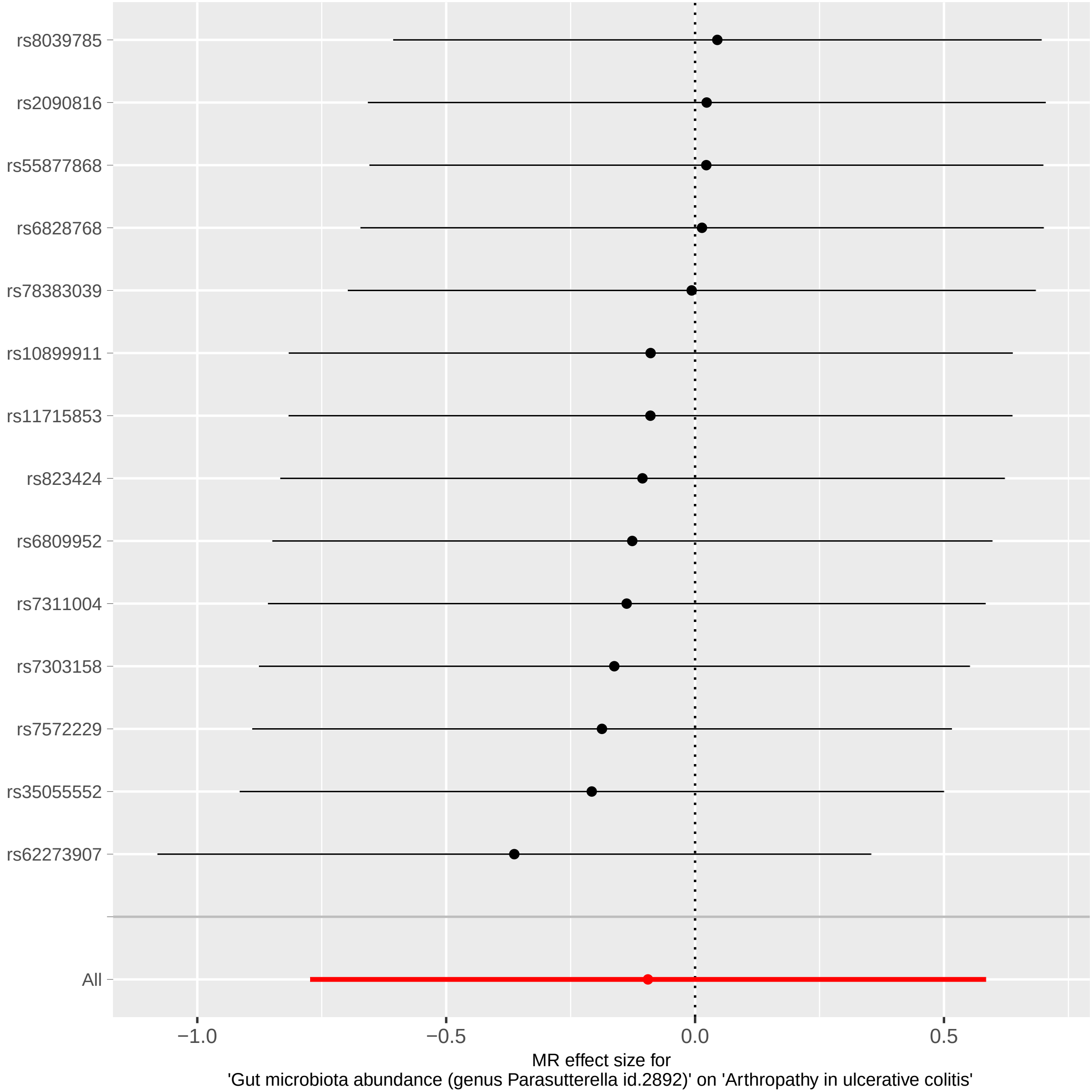

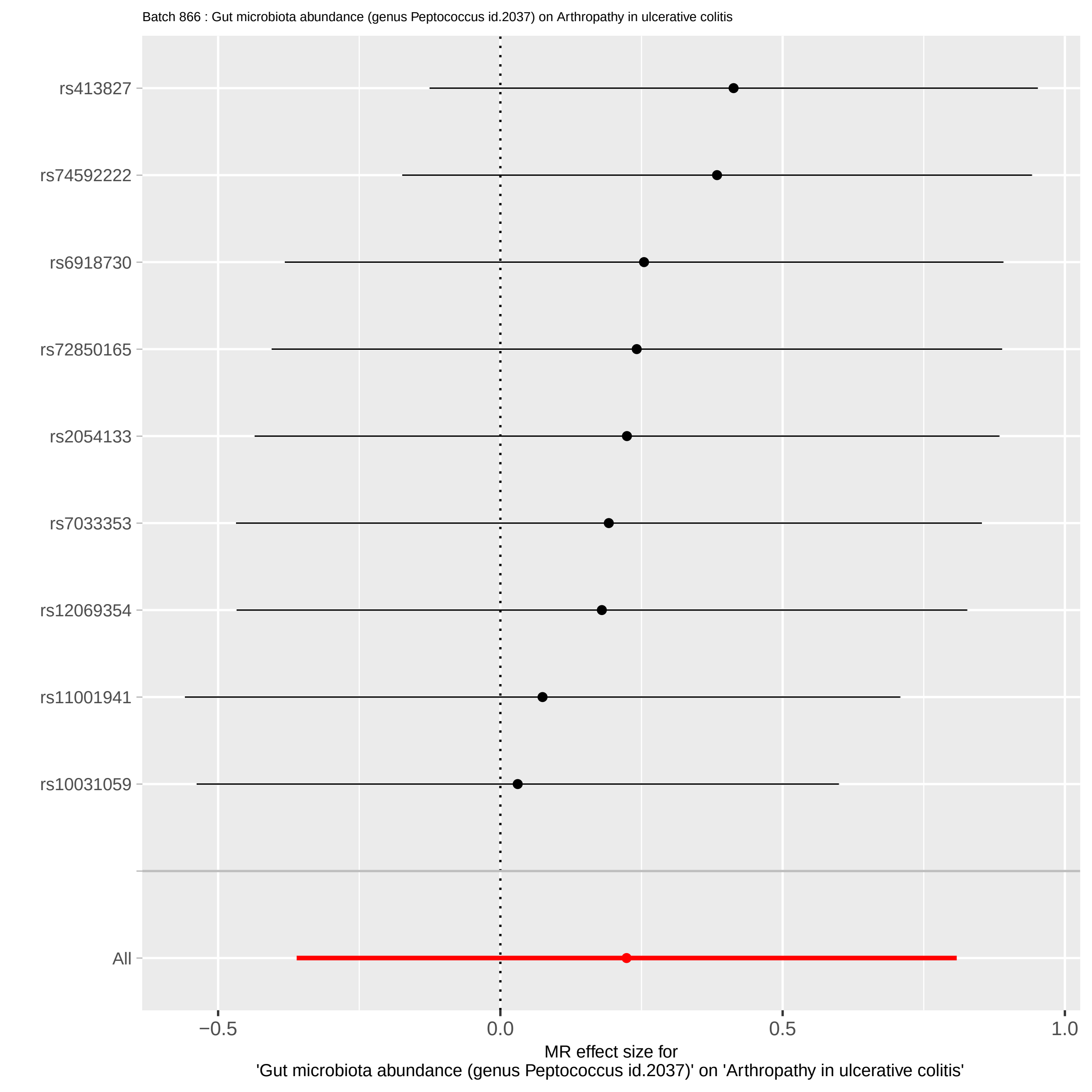

Batch 866 : Gut microbiota abundance (genus Peptococcus id.2037) on Arthropathy in ulcerative colitis
rs413827
rs74592222
rs6918730
rs72850165
rs2054133
rs7033353
rs12069354
rs11001941
rs10031059
All
−0.5
0.0
0.5
1.0
MR effect size for
'Gut microbiota abundance (genus Peptococcus id.2037)' on 'Arthropathy in ulcerative colitis'

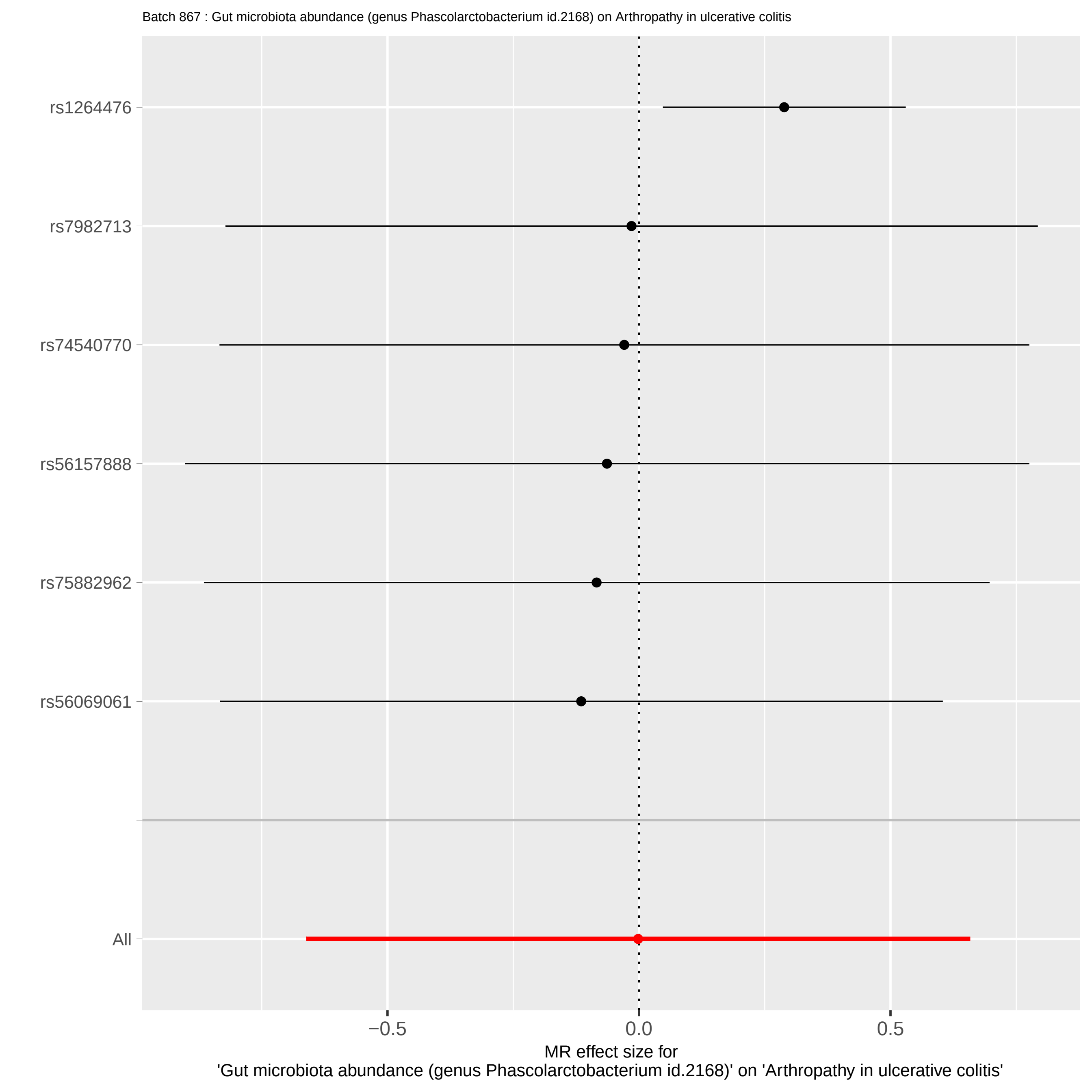

Batch 867 : Gut microbiota abundance (genus Phascolarctobacterium id.2168) on Arthropathy in ulcerative colitis
rs1264476
rs7982713
rs74540770
rs56157888
rs75882962
rs56069061
All
−0.5
0.0
0.5
MR effect size for
'Gut microbiota abundance (genus Phascolarctobacterium id.2168)' on 'Arthropathy in ulcerative colitis'

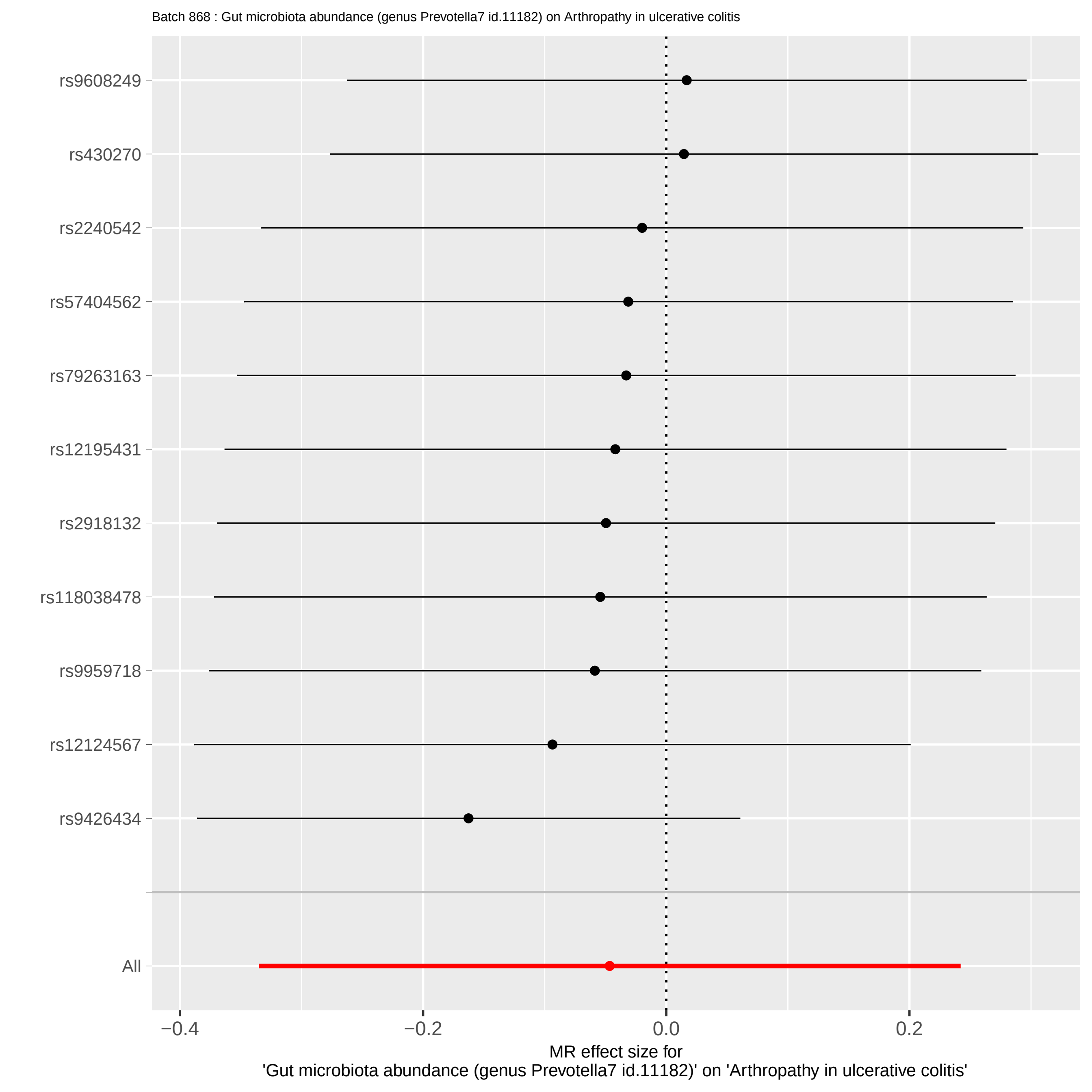

Batch 868 : Gut microbiota abundance (genus Prevotella7 id.11182) on Arthropathy in ulcerative colitis
rs9608249
rs430270
rs2240542
rs57404562
rs79263163
rs12195431
rs2918132
rs118038478
rs9959718
rs12124567
rs9426434
All
−0.4
−0.2
0.0
0.2
MR effect size for
'Gut microbiota abundance (genus Prevotella7 id.11182)' on 'Arthropathy in ulcerative colitis'

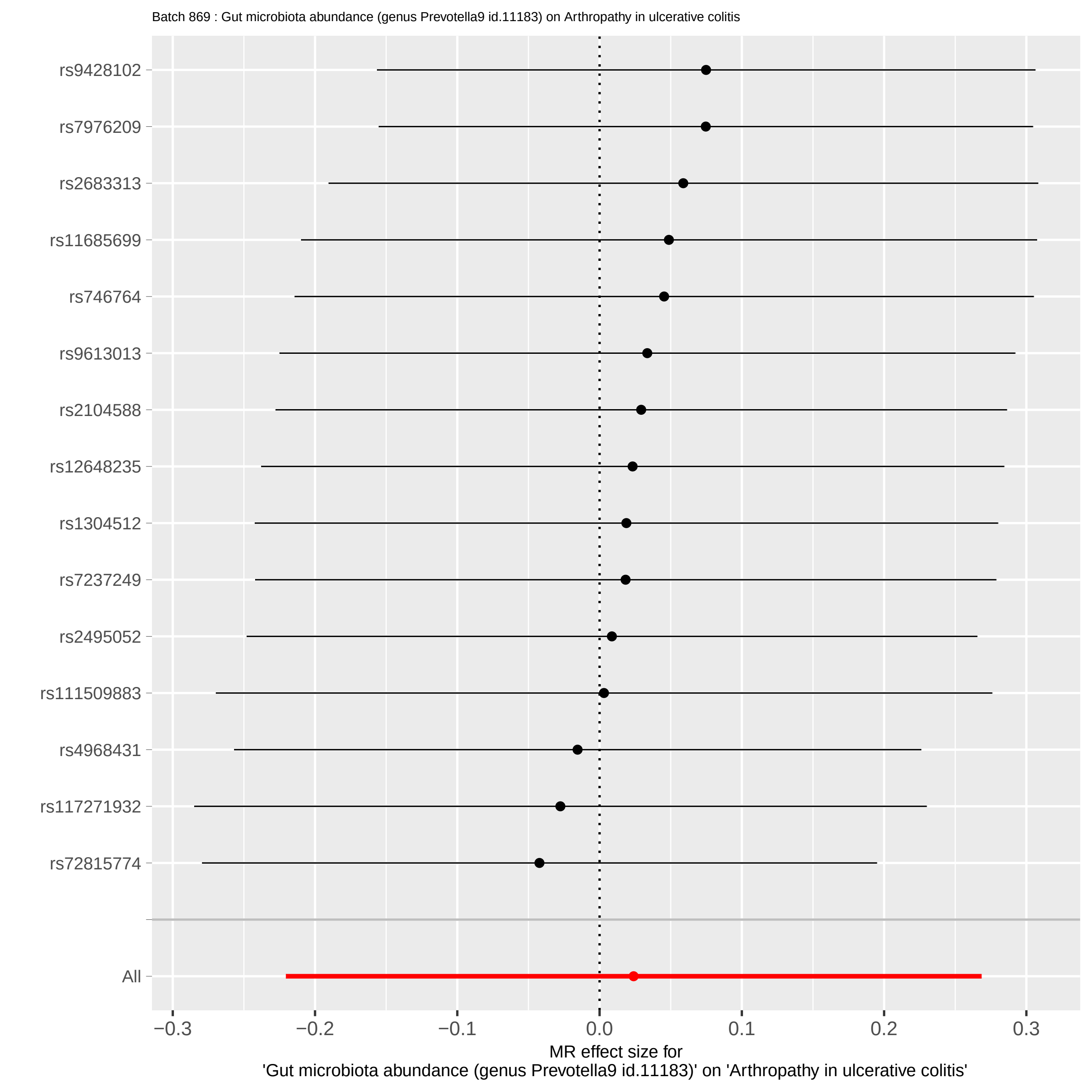

Batch 869 : Gut microbiota abundance (genus Prevotella9 id.11183) on Arthropathy in ulcerative colitis
rs9428102
rs7976209
rs2683313
rs11685699
rs746764
rs9613013
rs2104588
rs12648235
rs1304512
rs7237249
rs2495052
rs111509883
rs4968431
rs117271932
rs72815774
All
−0.3
−0.2
−0.1
0.0
0.1
0.2
0.3
MR effect size for
'Gut microbiota abundance (genus Prevotella9 id.11183)' on 'Arthropathy in ulcerative colitis'

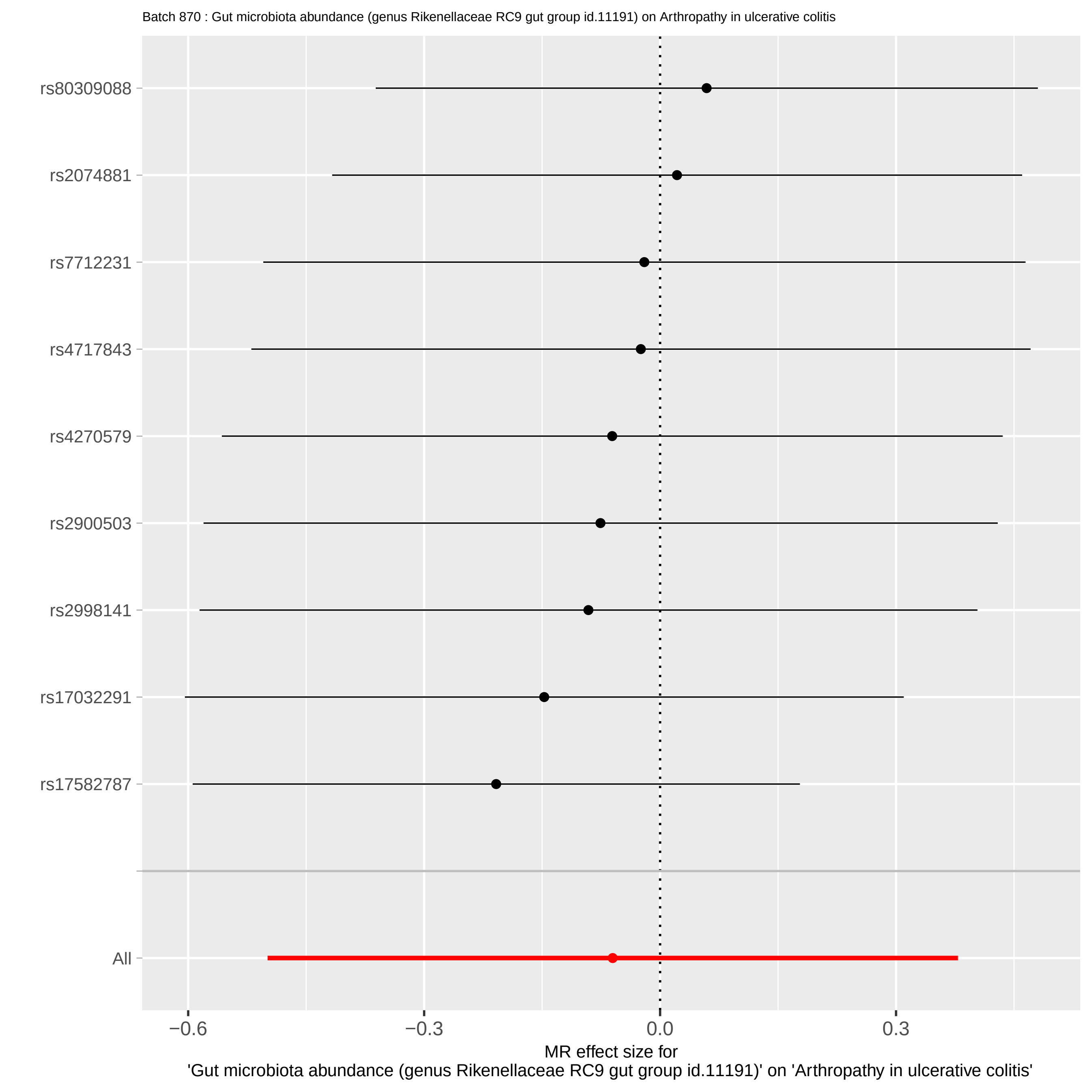

Batch 870 : Gut microbiota abundance (genus Rikenellaceae RC9 gut group id.11191) on Arthropathy in ulcerative colitis
rs80309088
rs2074881
rs7712231
rs4717843
rs4270579
rs2900503
rs2998141
rs17032291
rs17582787
All
−0.6
−0.3
0.0
0.3
MR effect size for
'Gut microbiota abundance (genus Rikenellaceae RC9 gut group id.11191)' on 'Arthropathy in ulcerative colitis'

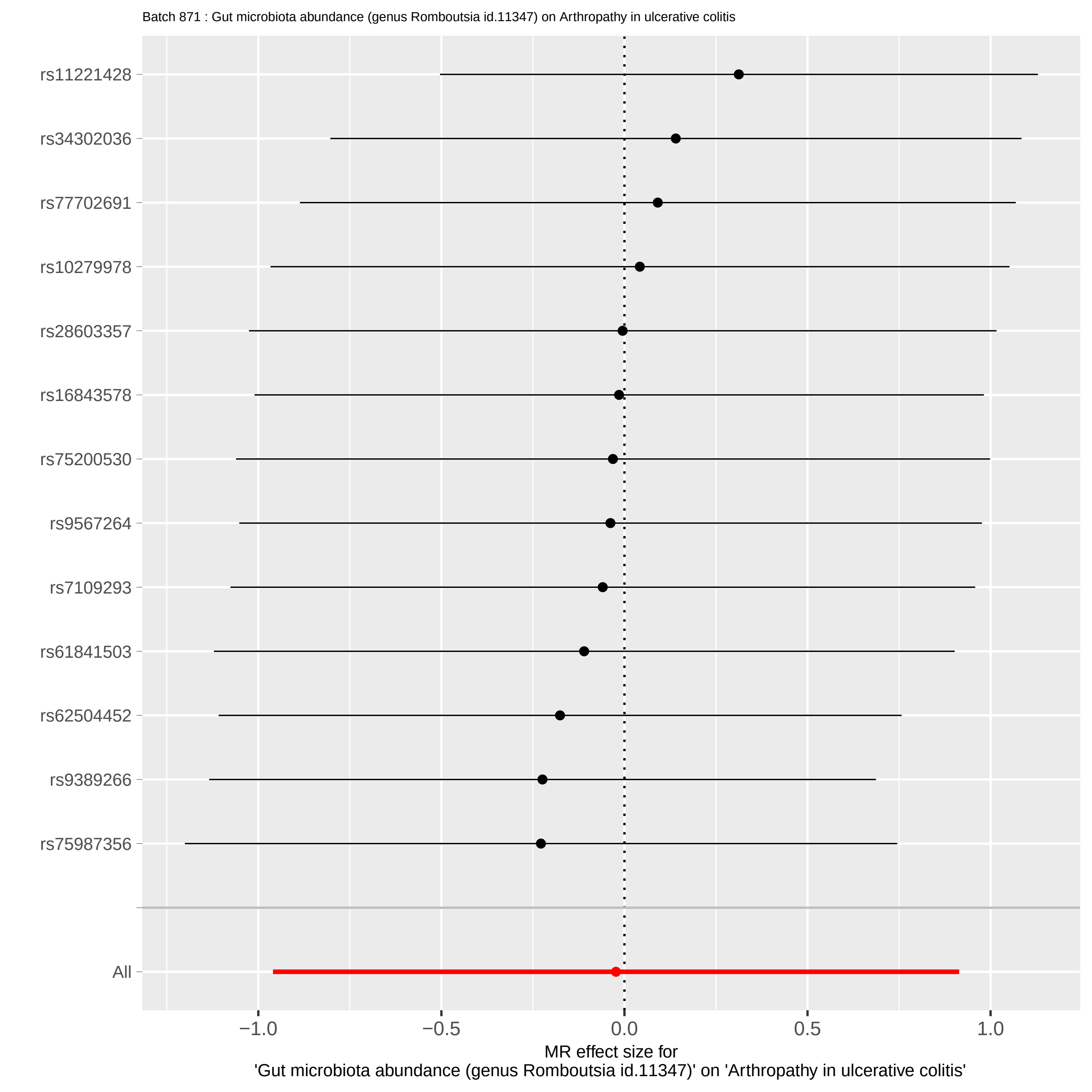

Batch 871 : Gut microbiota abundance (genus Romboutsia id.11347) on Arthropathy in ulcerative colitis
rs11221428
rs34302036
rs77702691
rs10279978
rs28603357
rs16843578
rs75200530
rs9567264
rs7109293
rs61841503
rs62504452
rs9389266
rs75987356
All
−1.0
−0.5
0.0
0.5
1.0
MR effect size for
'Gut microbiota abundance (genus Romboutsia id.11347)' on 'Arthropathy in ulcerative colitis'

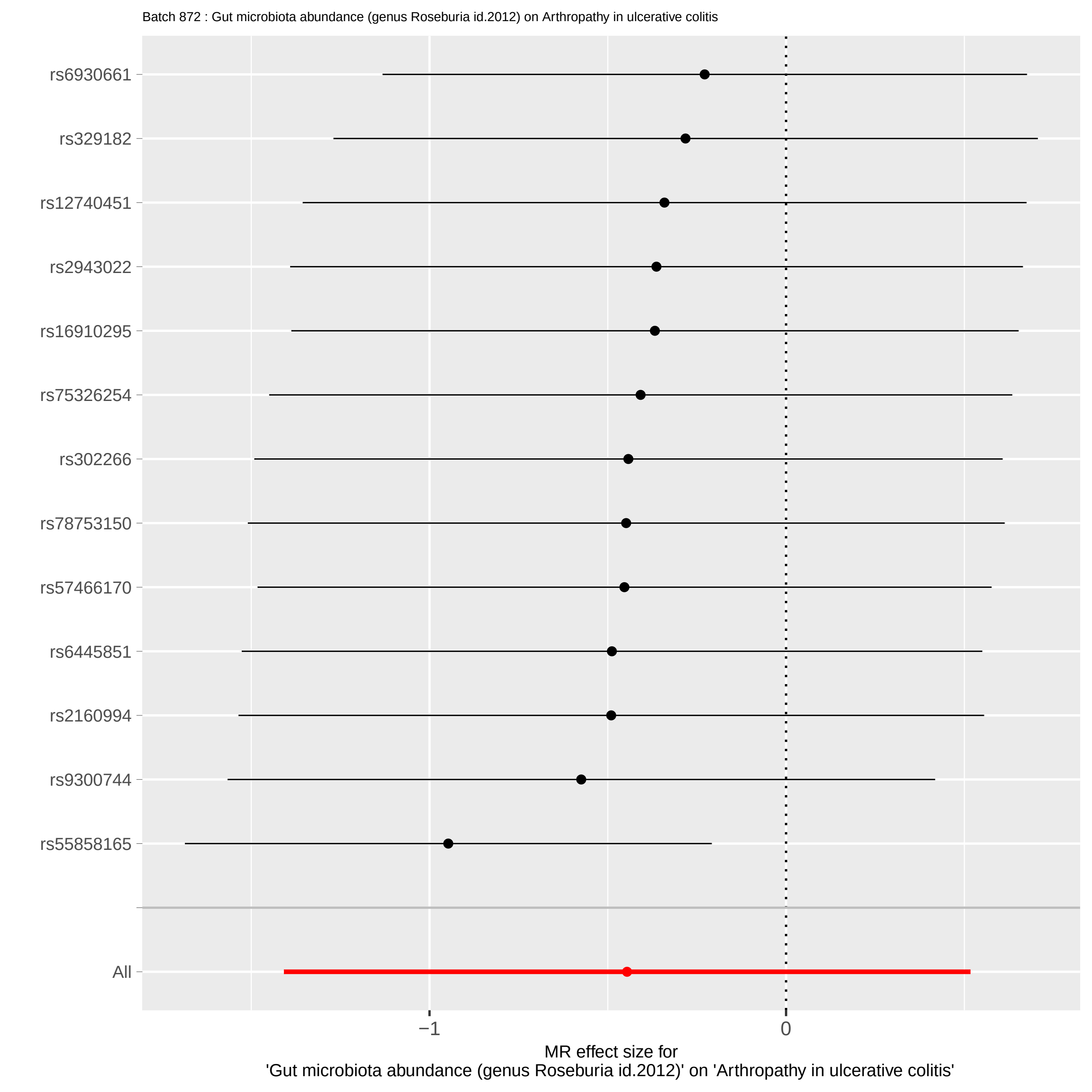

Batch 872 : Gut microbiota abundance (genus Roseburia id.2012) on Arthropathy in ulcerative colitis
rs6930661
rs329182
rs12740451
rs2943022
rs16910295
rs75326254
rs302266
rs78753150
rs57466170
rs6445851
rs2160994
rs9300744
rs55858165
All
−1
0
MR effect size for
'Gut microbiota abundance (genus Roseburia id.2012)' on 'Arthropathy in ulcerative colitis'

Batch 873 : Gut microbiota abundance (genus Ruminiclostridium5 id.11355) on Arthropathy in ulcerative colitis

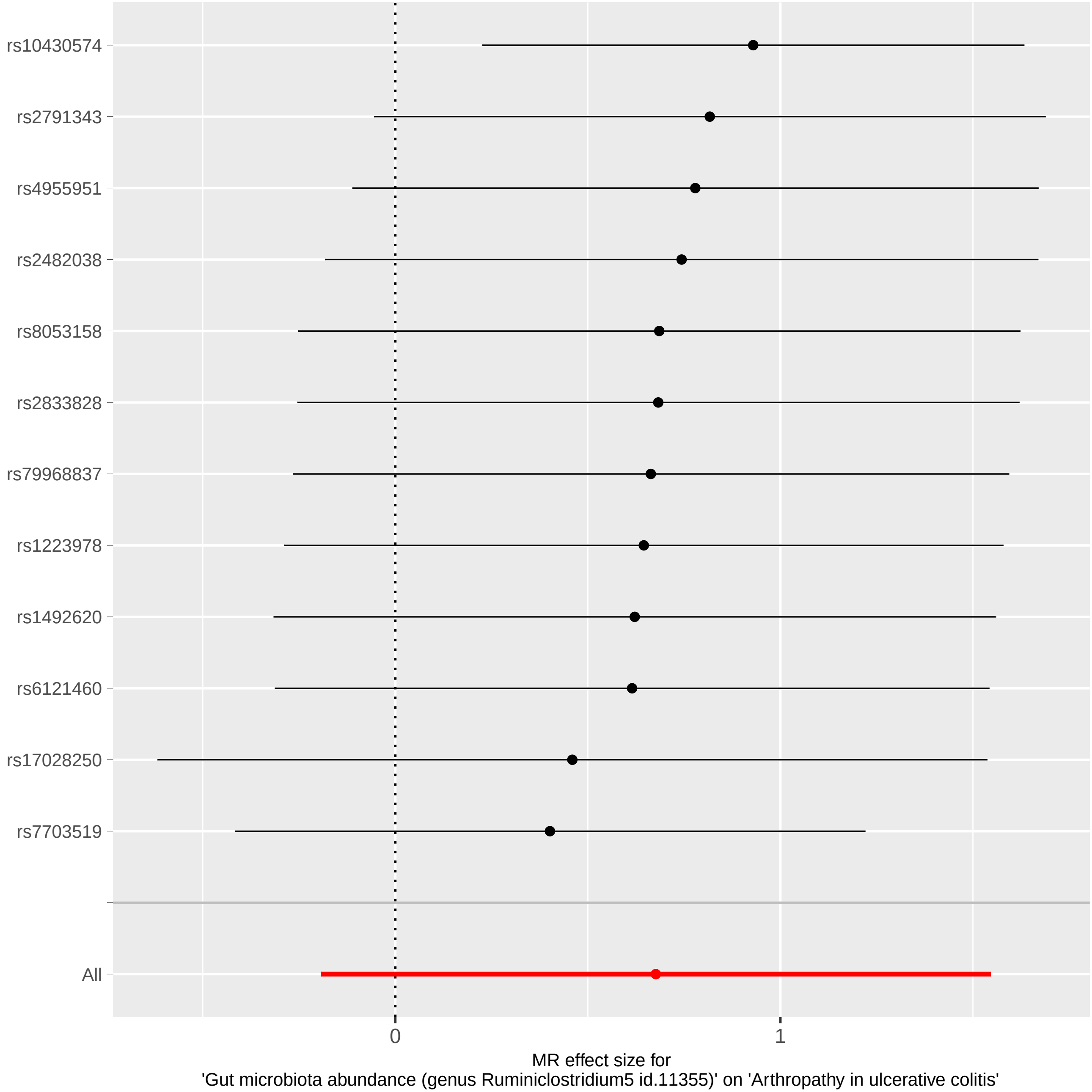

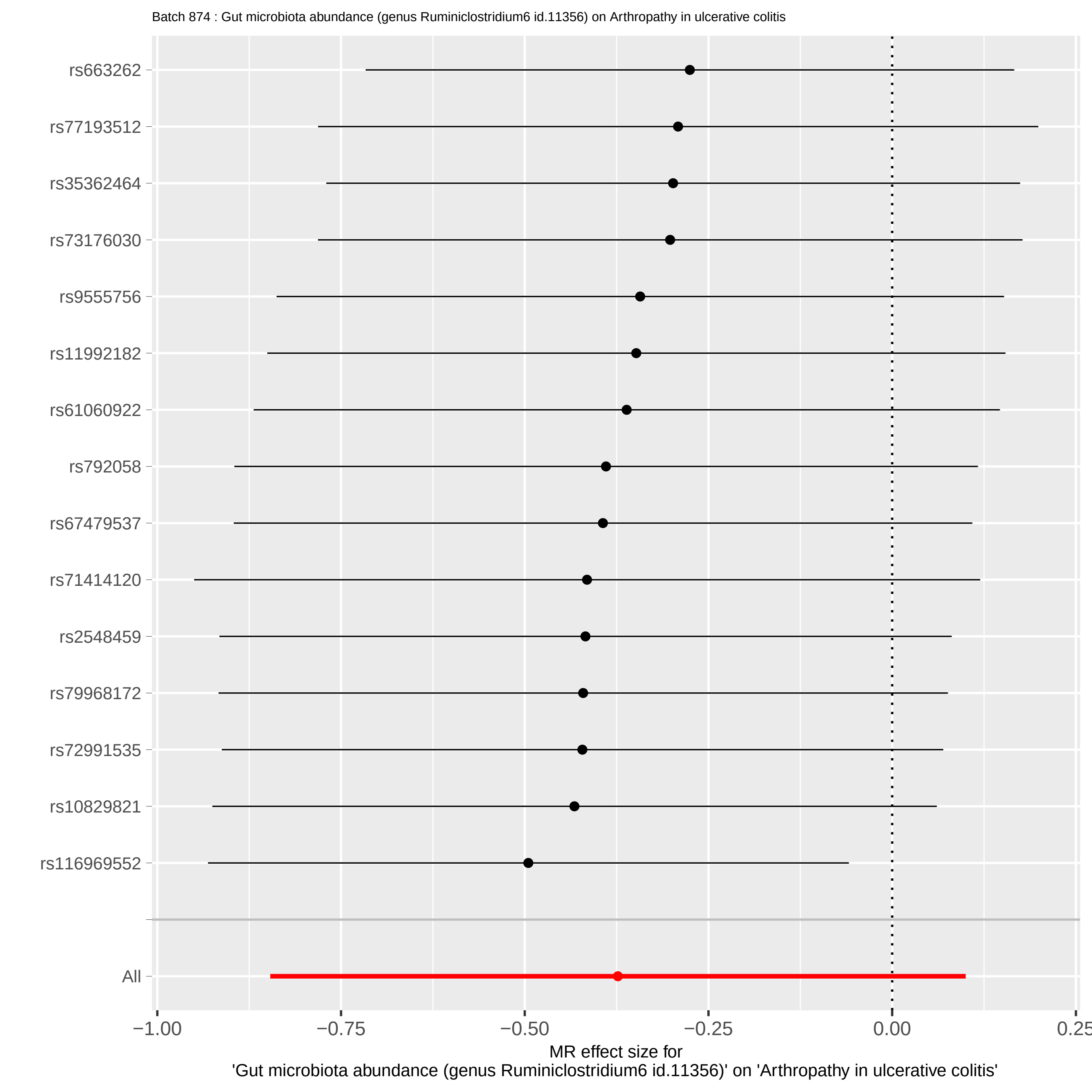
Batch 874 : Gut microbiota abundance (genus Ruminiclostridium6 id.11356) on Arthropathy in ulcerative colitis
rs663262
rs77193512
rs35362464
rs73176030
rs9555756
rs11992182
rs61060922
rs792058
rs67479537
rs71414120
rs2548459
rs79968172
rs72991535
rs10829821
rs116969552
All
−1.00
−0.75
−0.50
−0.25
0.00
0.25
MR effect size for
'Gut microbiota abundance (genus Ruminiclostridium6 id.11356)' on 'Arthropathy in ulcerative colitis'

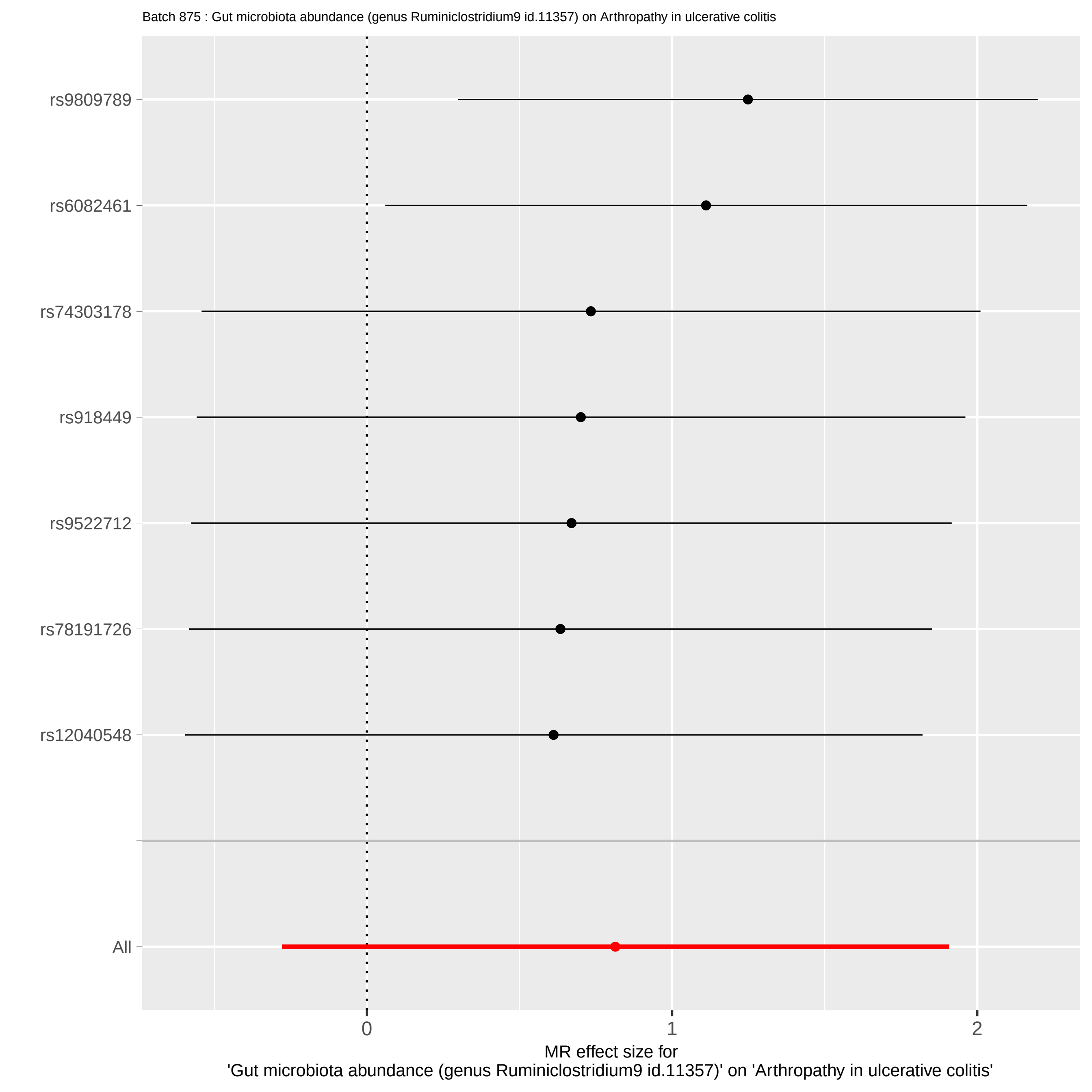
Batch 875 : Gut microbiota abundance (genus Ruminiclostridium9 id.11357) on Arthropathy in ulcerative colitis
rs9809789
rs6082461
rs74303178
rs918449
rs9522712
rs78191726
rs12040548
All
0
1
2
MR effect size for
'Gut microbiota abundance (genus Ruminiclostridium9 id.11357)' on 'Arthropathy in ulcerative colitis'

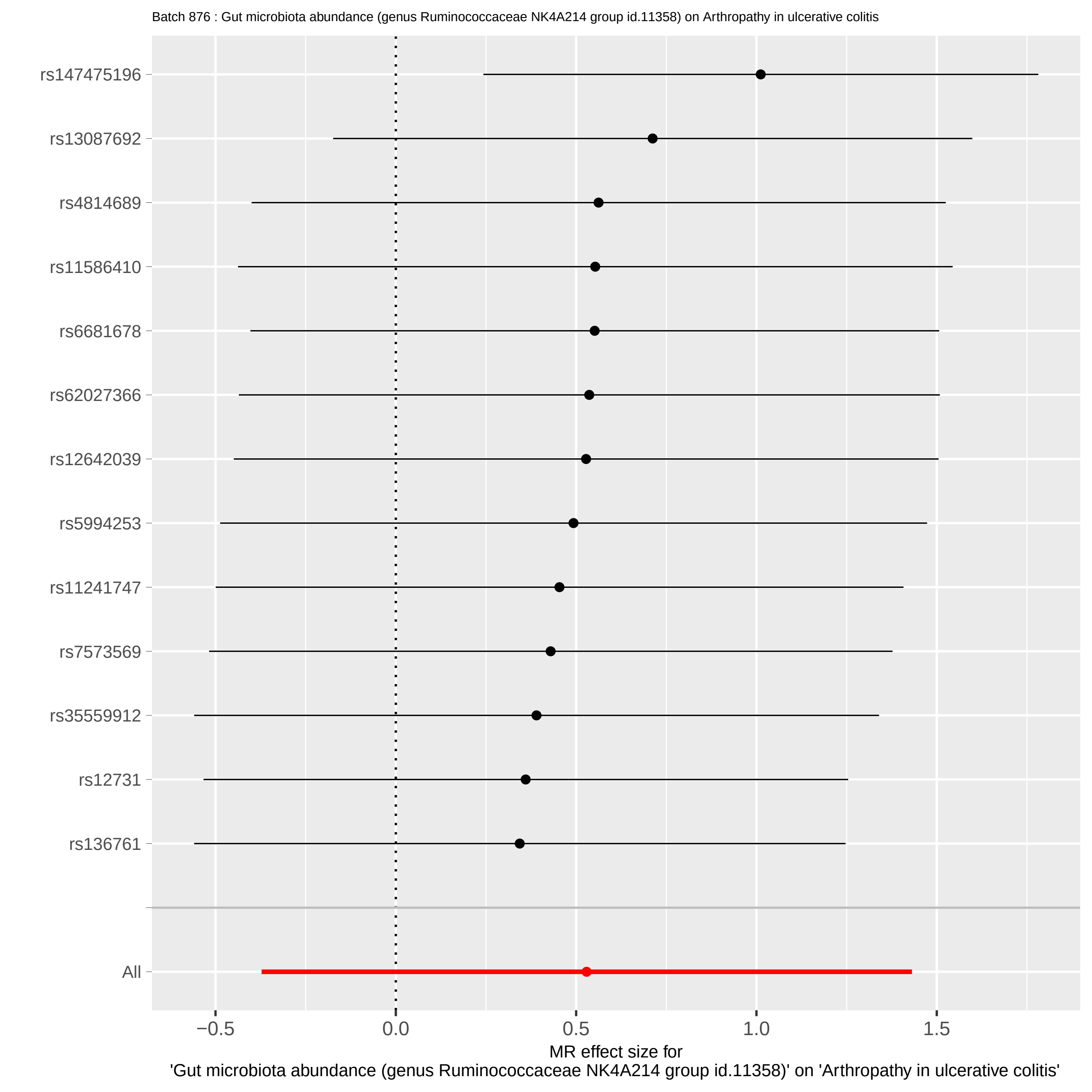
Batch 876 : Gut microbiota abundance (genus Ruminococcaceae NK4A214 group id.11358) on Arthropathy in ulcerative colitis
rs147475196
rs13087692
rs4814689
rs11586410
rs6681678
rs62027366
rs12642039
rs5994253
rs11241747
rs7573569
rs35559912
rs12731
rs136761
All
−0.5
0.0
0.5
1.0
1.5
MR effect size for
'Gut microbiota abundance (genus Ruminococcaceae NK4A214 group id.11358)' on 'Arthropathy in ulcerative colitis'

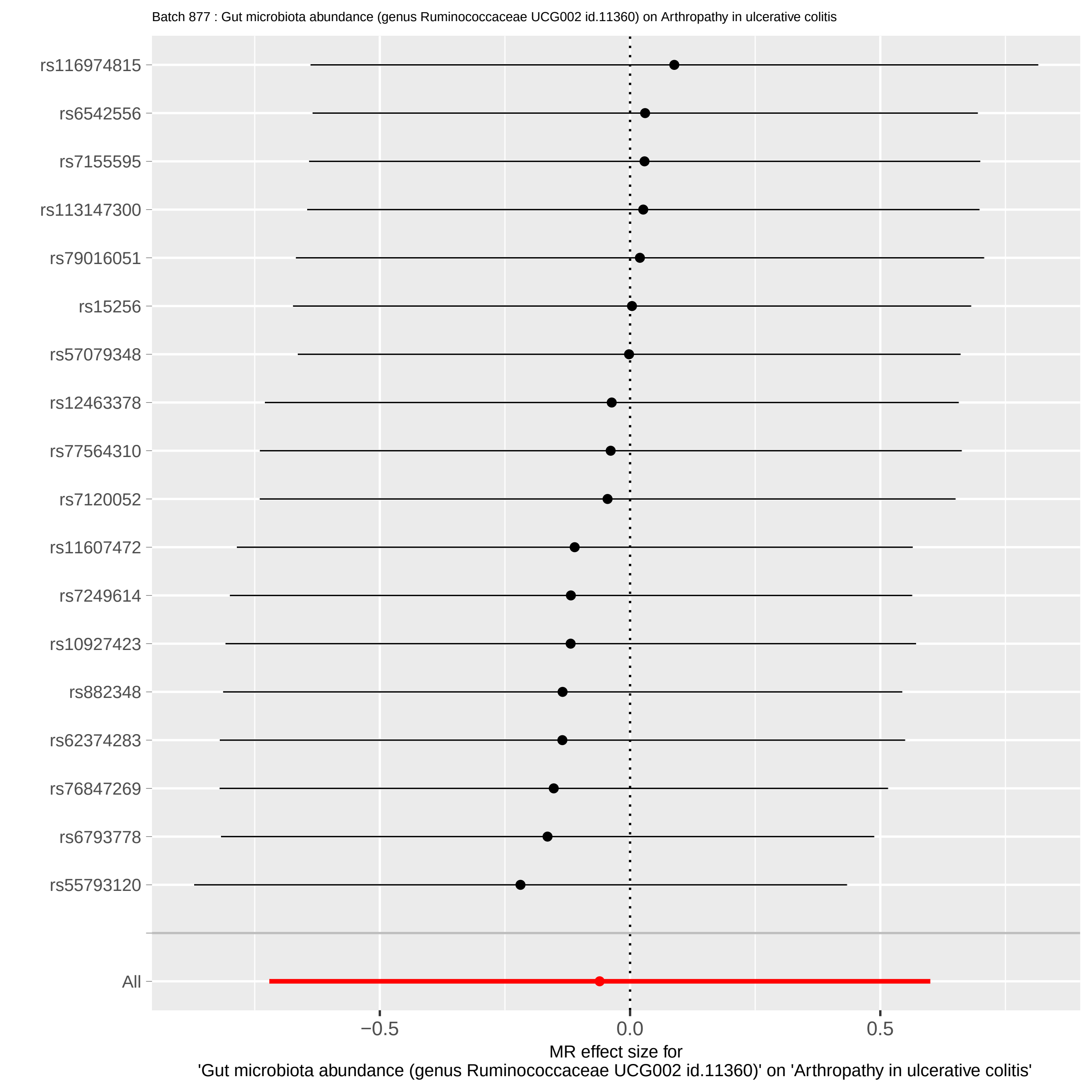

Batch 877 : Gut microbiota abundance (genus Ruminococcaceae UCG002 id.11360) on Arthropathy in ulcerative colitis
rs116974815
rs6542556
rs7155595
rs113147300
rs79016051
rs15256
rs57079348
rs12463378
rs77564310
rs7120052
rs11607472
rs7249614
rs10927423
rs882348
rs62374283
rs76847269
rs6793778
rs55793120
All
−0.5
0.0
0.5
MR effect size for
'Gut microbiota abundance (genus Ruminococcaceae UCG002 id.11360)' on 'Arthropathy in ulcerative colitis'

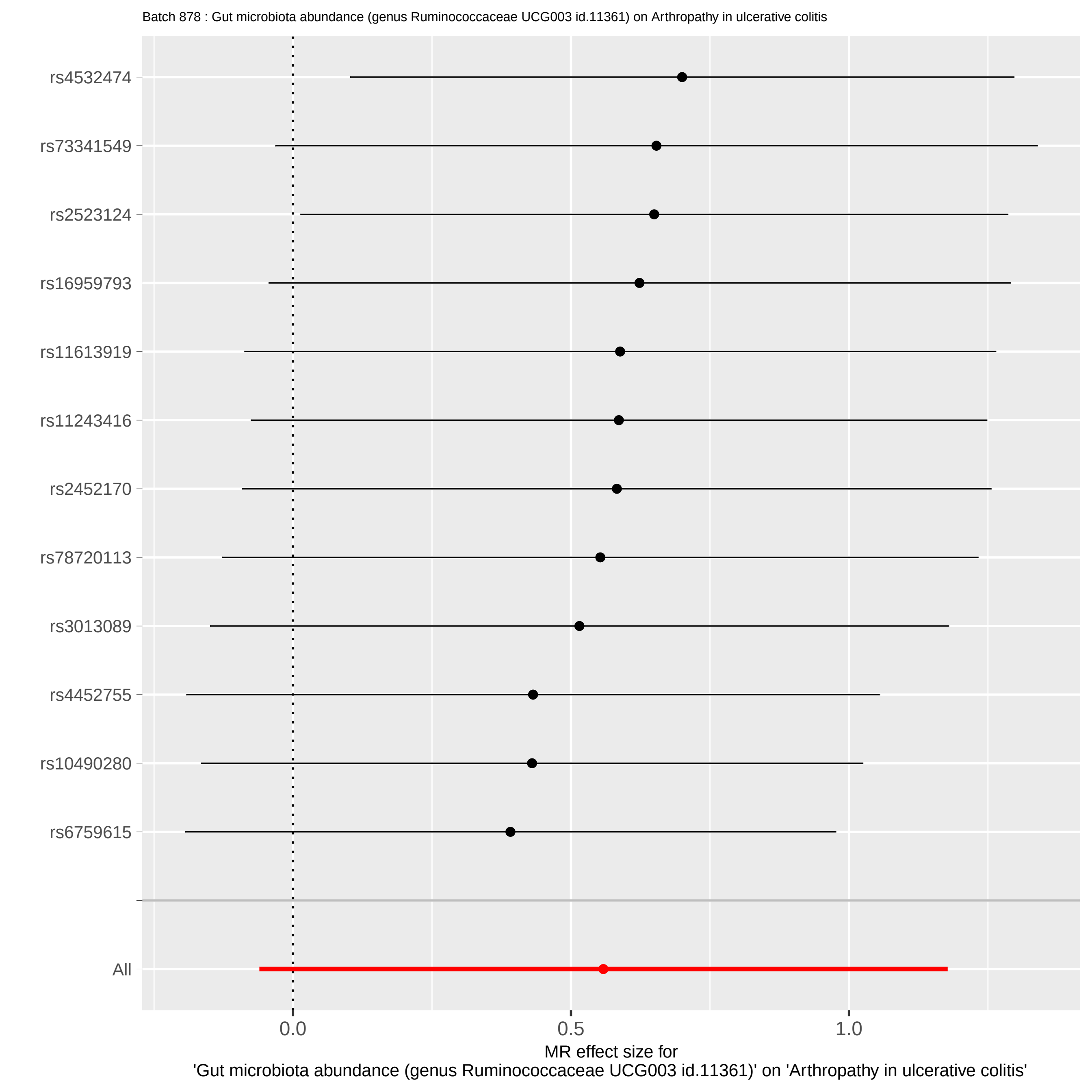
Batch 878 : Gut microbiota abundance (genus Ruminococcaceae UCG003 id.11361) on Arthropathy in ulcerative colitis
rs4532474
rs73341549
rs2523124
rs16959793
rs11613919
rs11243416
rs2452170
rs78720113
rs3013089
rs4452755
rs10490280
rs6759615
All
0.0
0.5
1.0
MR effect size for
'Gut microbiota abundance (genus Ruminococcaceae UCG003 id.11361)' on 'Arthropathy in ulcerative colitis'

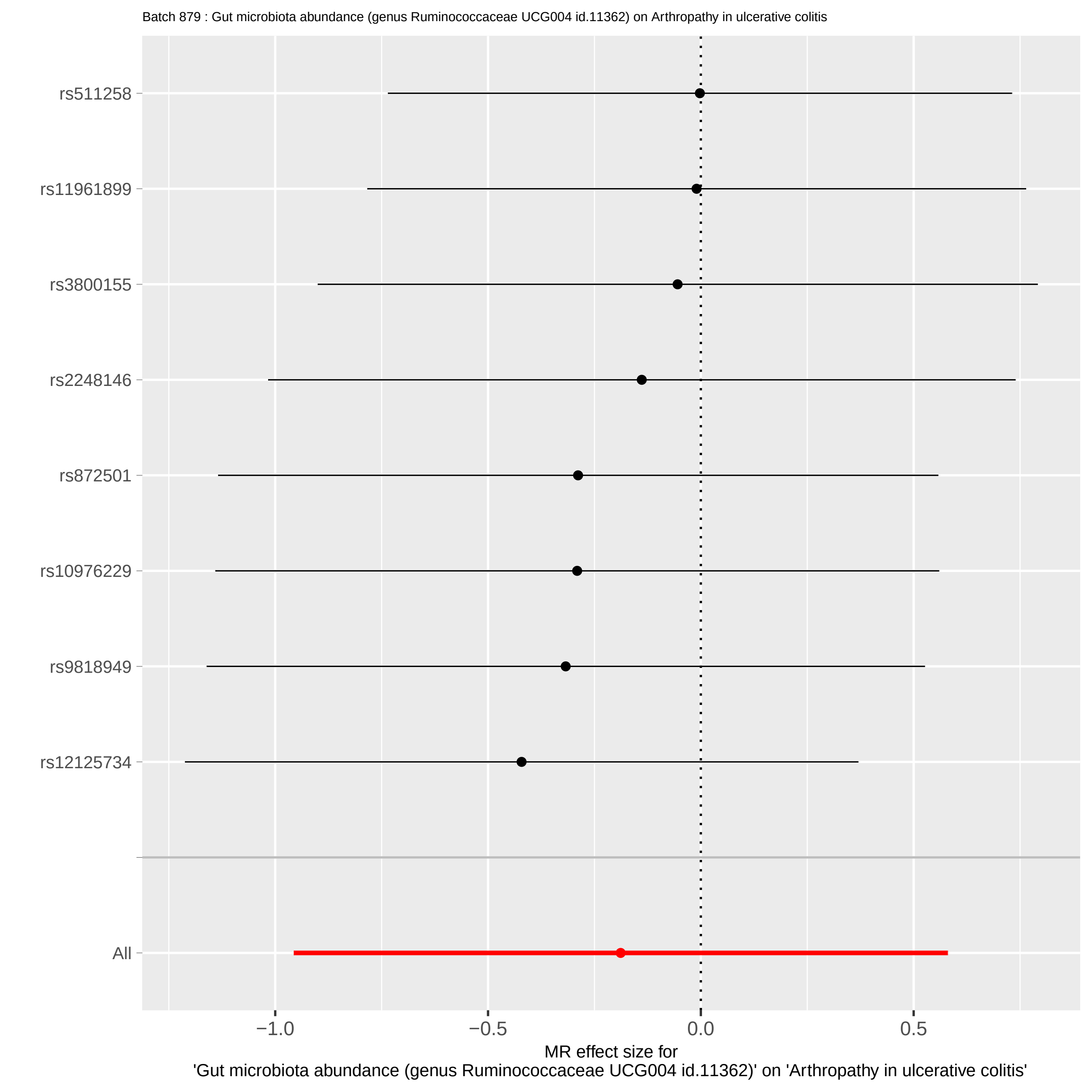
Batch 879 : Gut microbiota abundance (genus Ruminococcaceae UCG004 id.11362) on Arthropathy in ulcerative colitis
rs511258
rs11961899
rs3800155
rs2248146
rs872501
rs10976229
rs9818949
rs12125734
All
−1.0
−0.5
0.0
0.5
MR effect size for
'Gut microbiota abundance (genus Ruminococcaceae UCG004 id.11362)' on 'Arthropathy in ulcerative colitis'

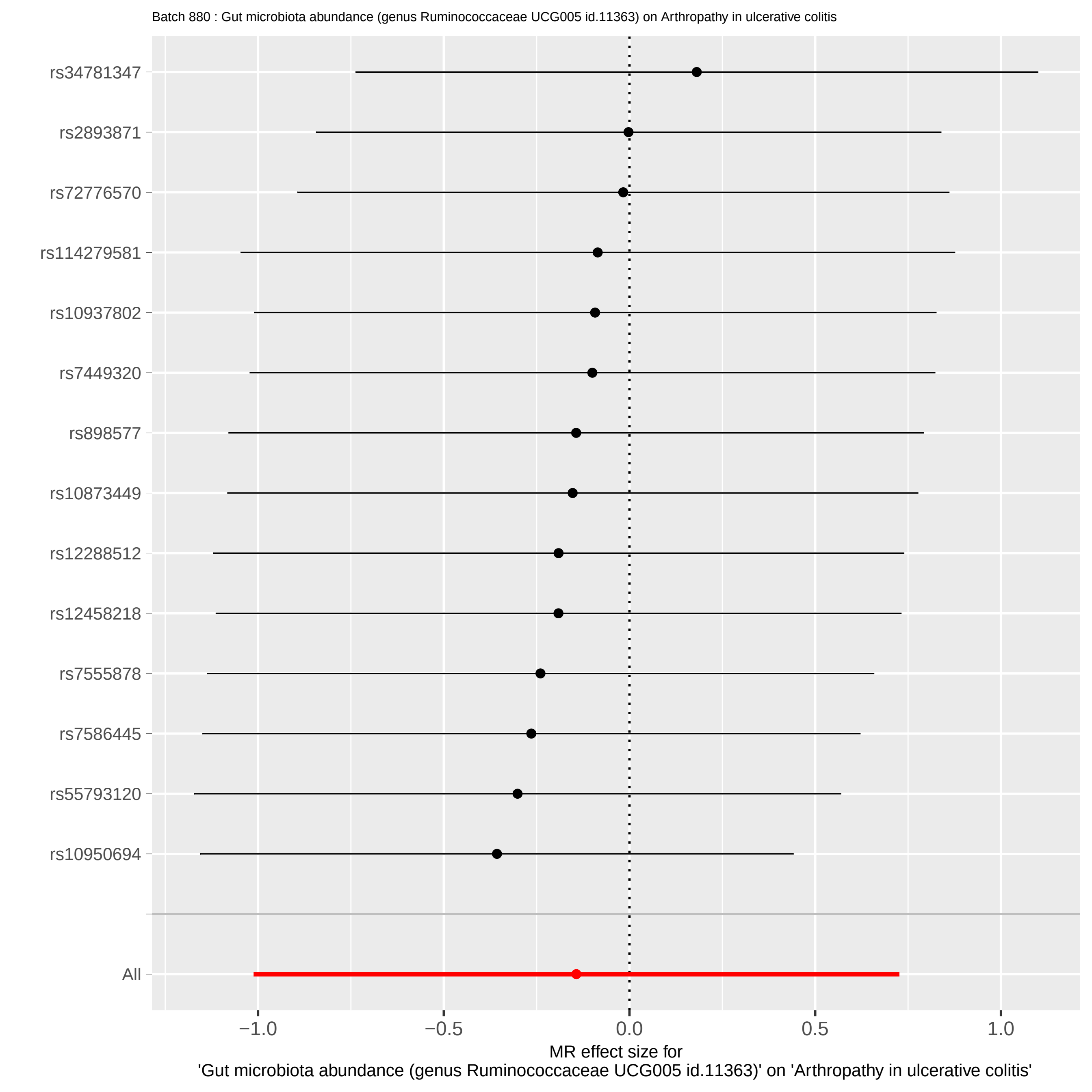
Batch 880 : Gut microbiota abundance (genus Ruminococcaceae UCG005 id.11363) on Arthropathy in ulcerative colitis
rs34781347
rs2893871
rs72776570
rs114279581
rs10937802
rs7449320
rs898577
rs10873449
rs12288512
rs12458218
rs7555878
rs7586445
rs55793120
rs10950694
All
−1.0
−0.5
0.0
0.5
1.0
MR effect size for
'Gut microbiota abundance (genus Ruminococcaceae UCG005 id.11363)' on 'Arthropathy in ulcerative colitis'

Batch 881 : Gut microbiota abundance (genus Ruminococcaceae UCG009 id.11366) on Arthropathy in ulcerative colitis

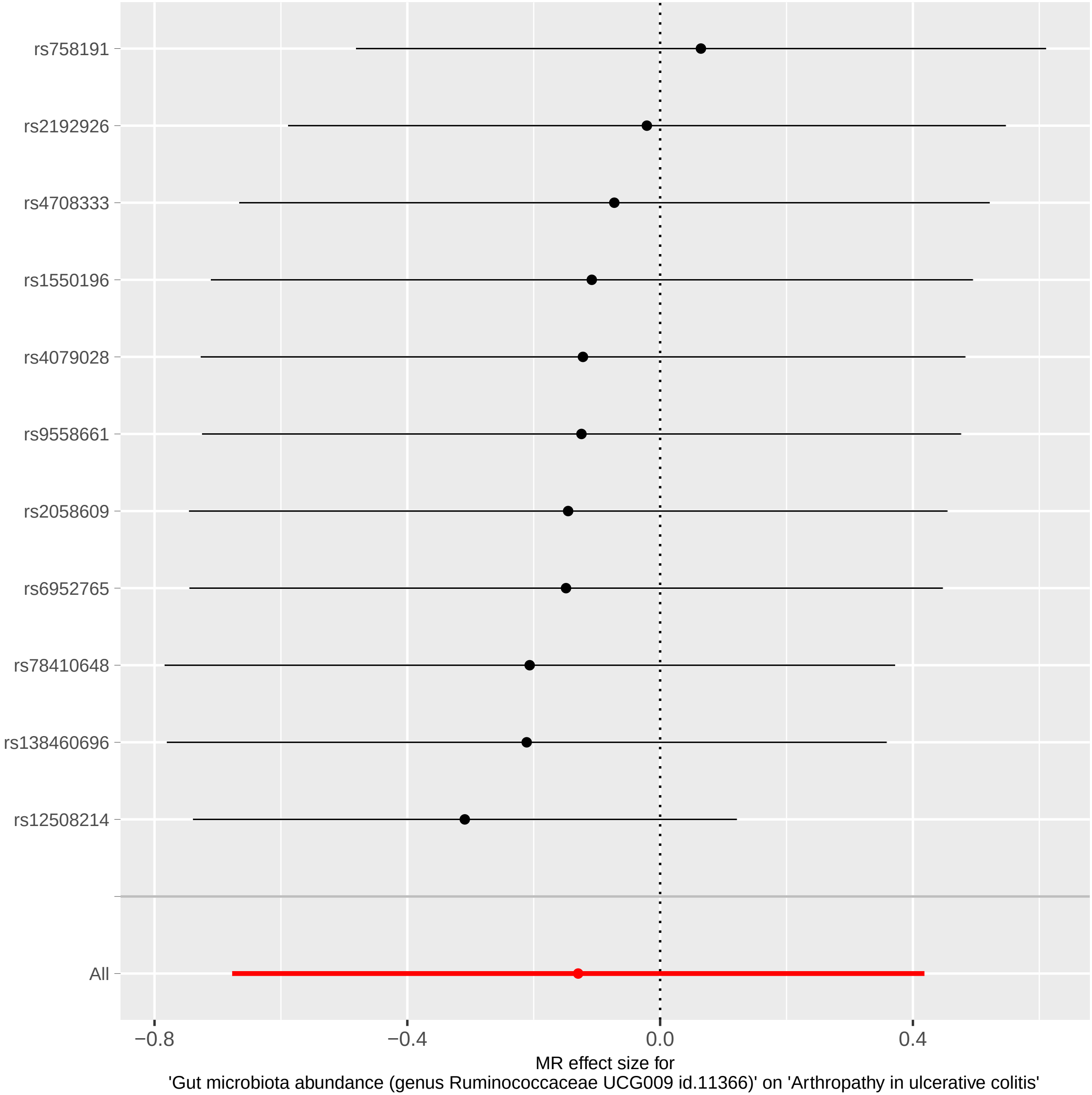

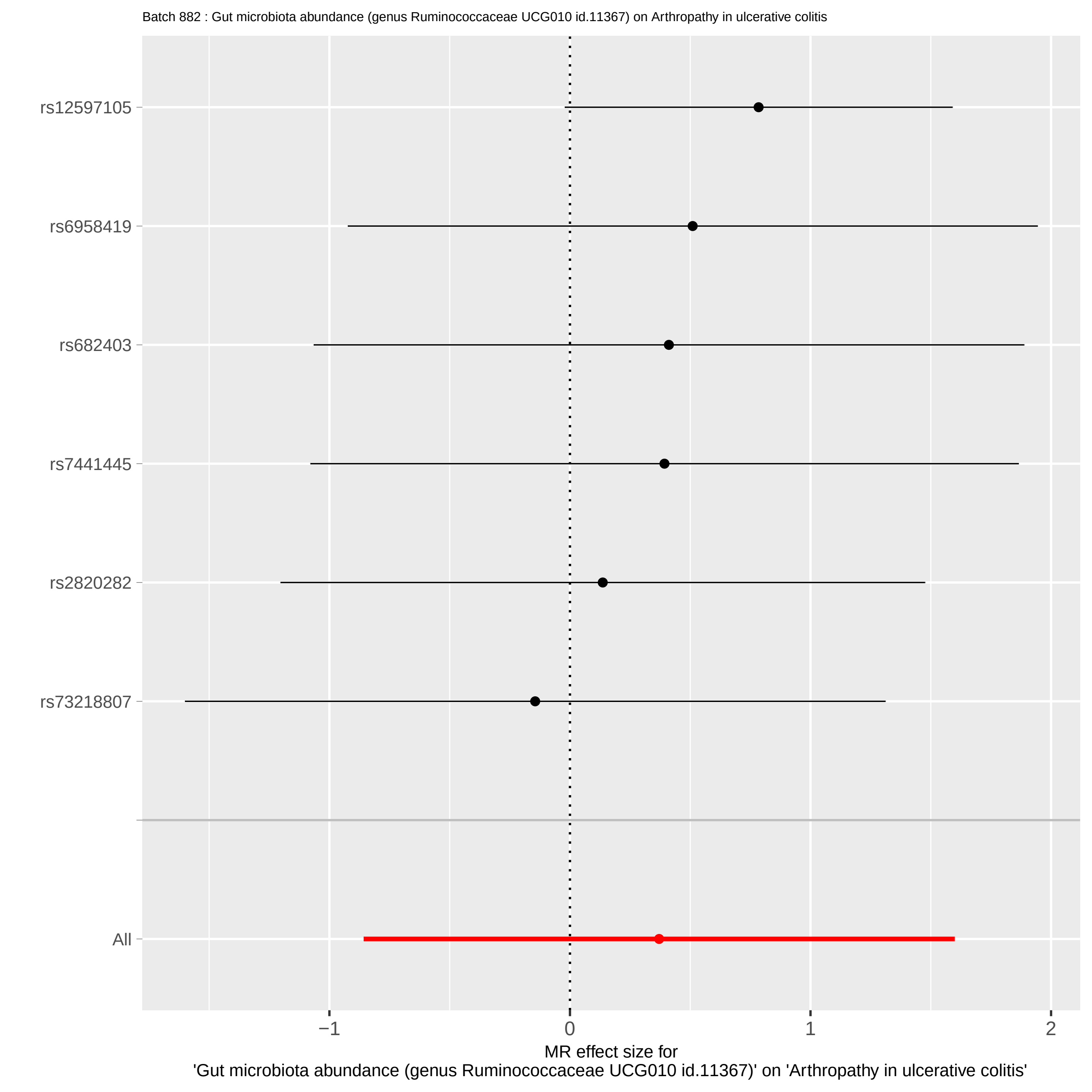

Batch 882 : Gut microbiota abundance (genus Ruminococcaceae UCG010 id.11367) on Arthropathy in ulcerative colitis
rs12597105
rs6958419
rs682403
rs7441445
rs2820282
rs73218807
All
−1
0
1
2
MR effect size for
'Gut microbiota abundance (genus Ruminococcaceae UCG010 id.11367)' on 'Arthropathy in ulcerative colitis'

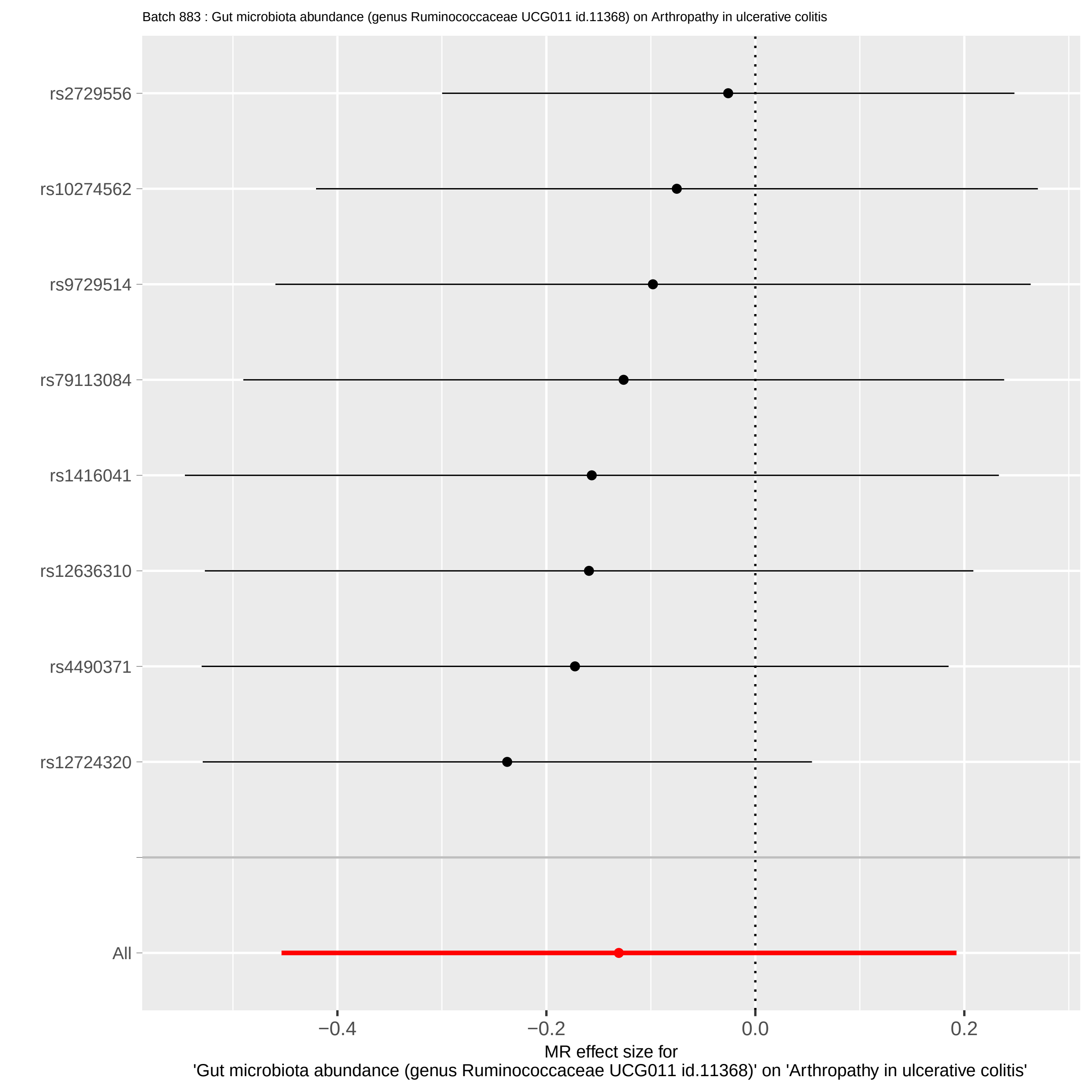
Batch 883 : Gut microbiota abundance (genus Ruminococcaceae UCG011 id.11368) on Arthropathy in ulcerative colitis
rs2729556
rs10274562
rs9729514
rs79113084
rs1416041
rs12636310
rs4490371
rs12724320
All
−0.4
−0.2
0.0
0.2
MR effect size for
'Gut microbiota abundance (genus Ruminococcaceae UCG011 id.11368)' on 'Arthropathy in ulcerative colitis'

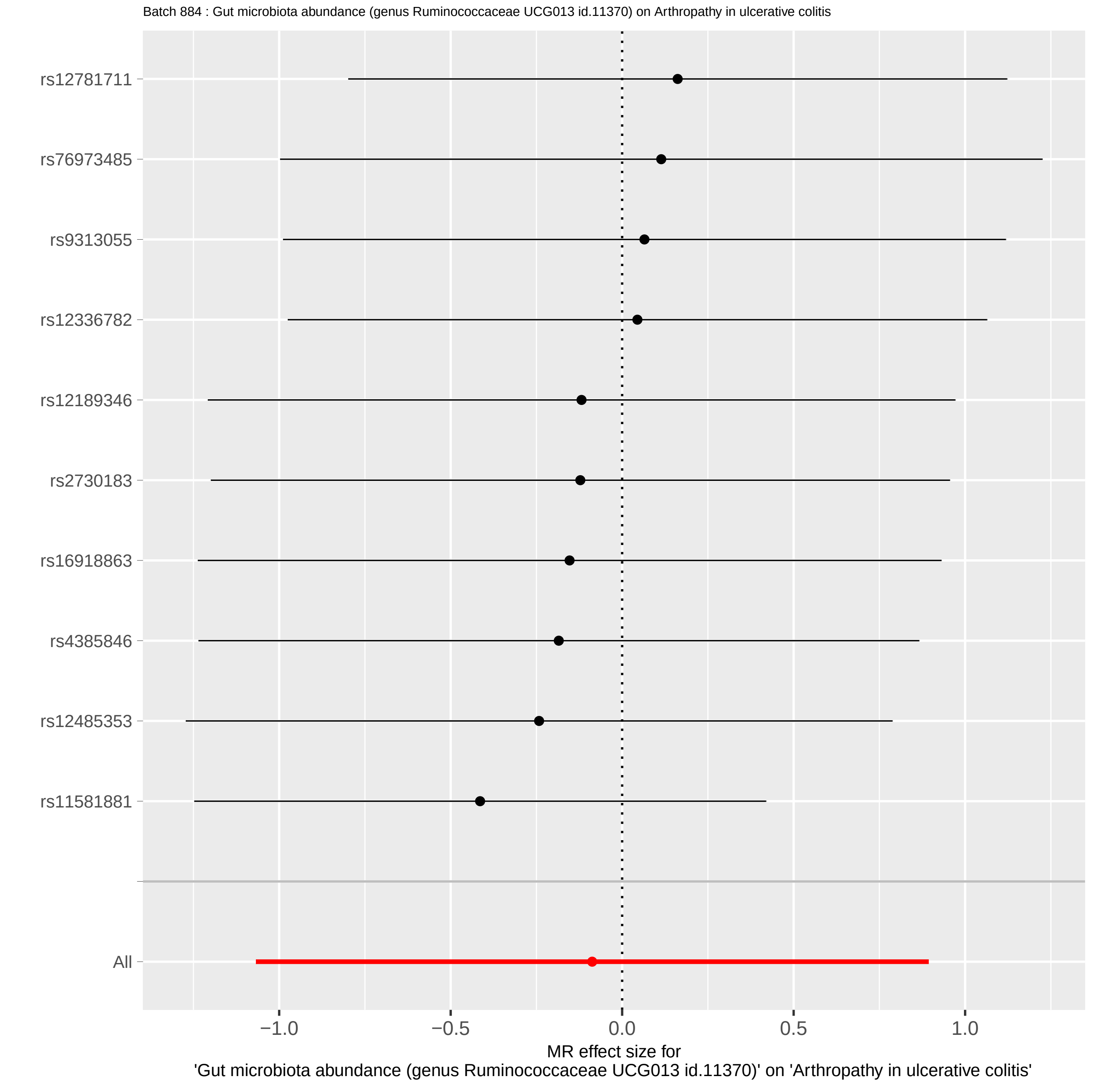

Batch 884 : Gut microbiota abundance (genus Ruminococcaceae UCG013 id.11370) on Arthropathy in ulcerative colitis
rs12781711
rs76973485
rs9313055
rs12336782
rs12189346
rs2730183
rs16918863
rs4385846
rs12485353
rs11581881
All
−1.0
−0.5
0.0
0.5
1.0
MR effect size for
'Gut microbiota abundance (genus Ruminococcaceae UCG013 id.11370)' on 'Arthropathy in ulcerative colitis'

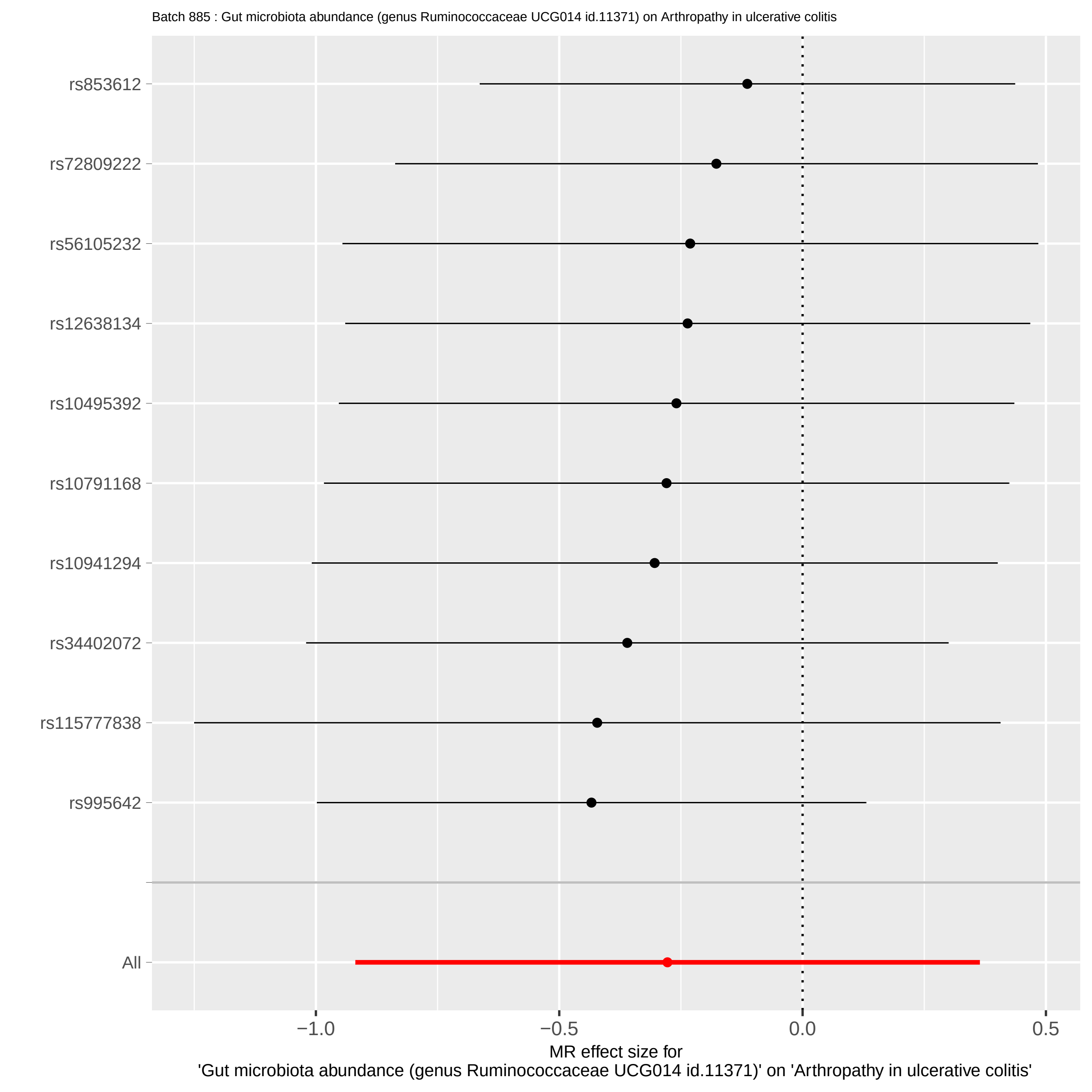
Batch 885 : Gut microbiota abundance (genus Ruminococcaceae UCG014 id.11371) on Arthropathy in ulcerative colitis
rs853612
rs72809222
rs56105232
rs12638134
rs10495392
rs10791168
rs10941294
rs34402072
rs115777838
rs995642
All
−1.0
−0.5
0.0
0.5
MR effect size for
'Gut microbiota abundance (genus Ruminococcaceae UCG014 id.11371)' on 'Arthropathy in ulcerative colitis'

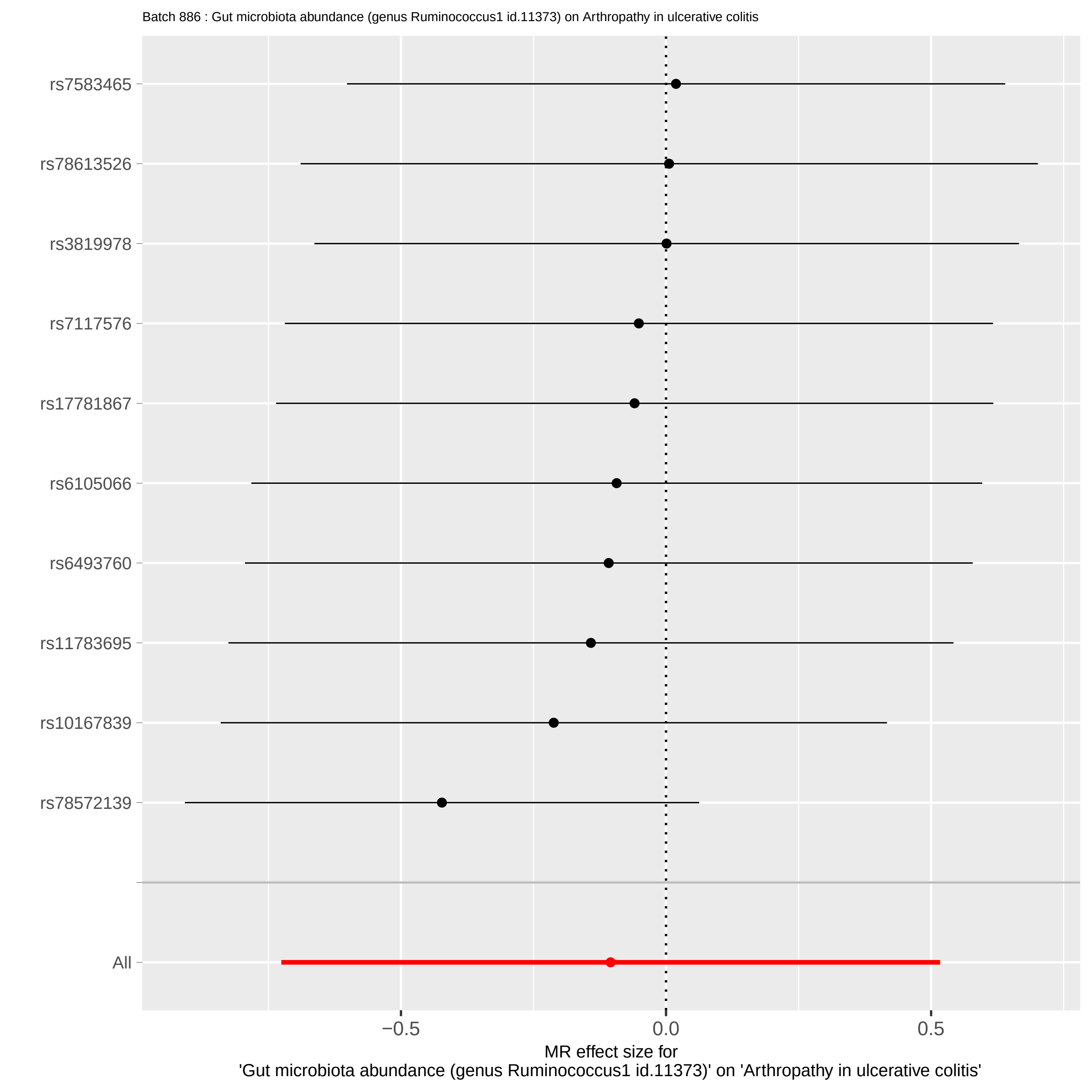
Batch 886 : Gut microbiota abundance (genus Ruminococcus1 id.11373) on Arthropathy in ulcerative colitis
rs7583465
rs78613526
rs3819978
rs7117576
rs17781867
rs6105066
rs6493760
rs11783695
rs10167839
rs78572139
All
−0.5
0.0
0.5
MR effect size for
'Gut microbiota abundance (genus Ruminococcus1 id.11373)' on 'Arthropathy in ulcerative colitis'

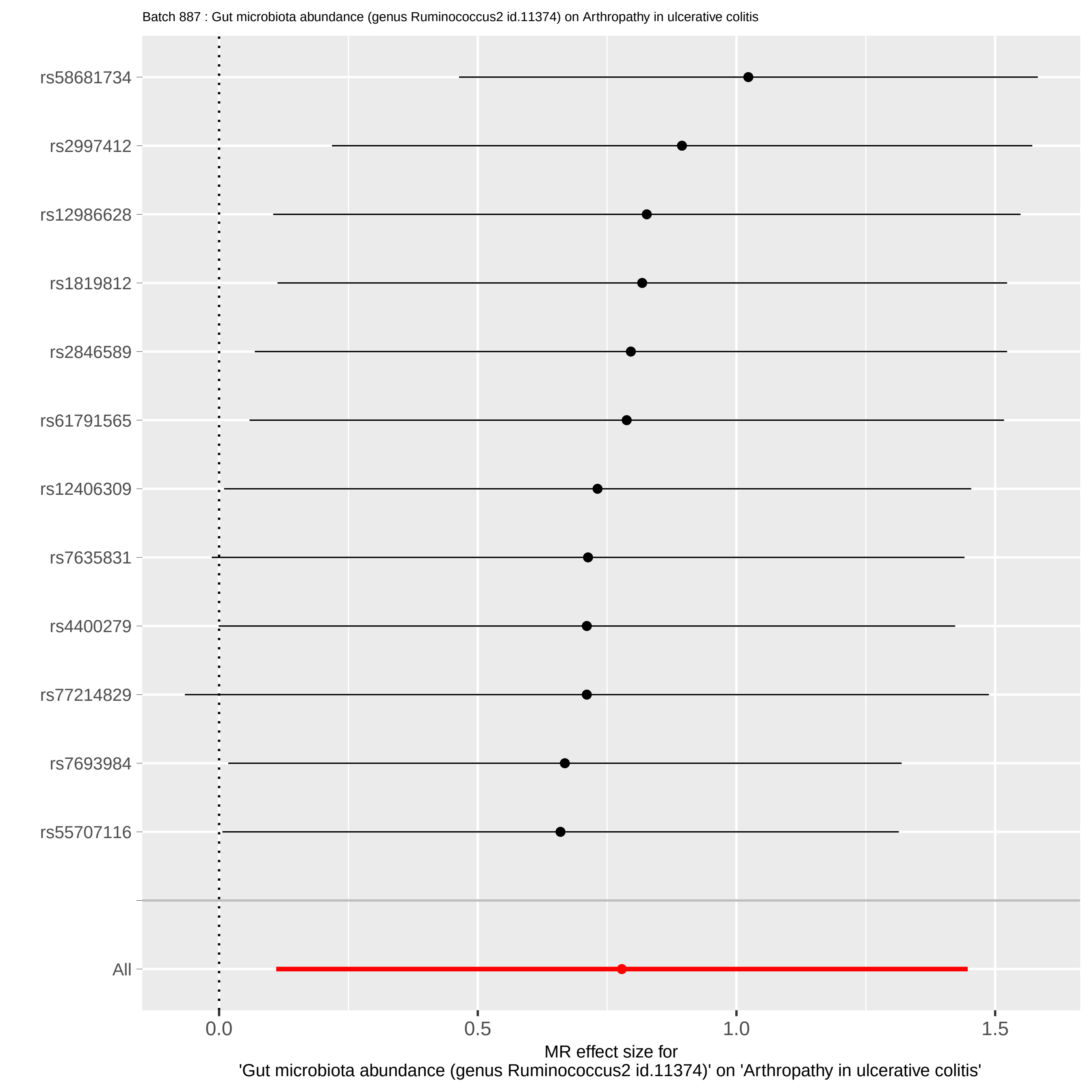

Batch 887 : Gut microbiota abundance (genus Ruminococcus2 id.11374) on Arthropathy in ulcerative colitis
rs58681734
rs2997412
rs12986628
rs1819812
rs2846589
rs61791565
rs12406309
rs7635831
rs4400279
rs77214829
rs7693984
rs55707116
All
0.0
0.5
1.0
1.5
MR effect size for
'Gut microbiota abundance (genus Ruminococcus2 id.11374)' on 'Arthropathy in ulcerative colitis'

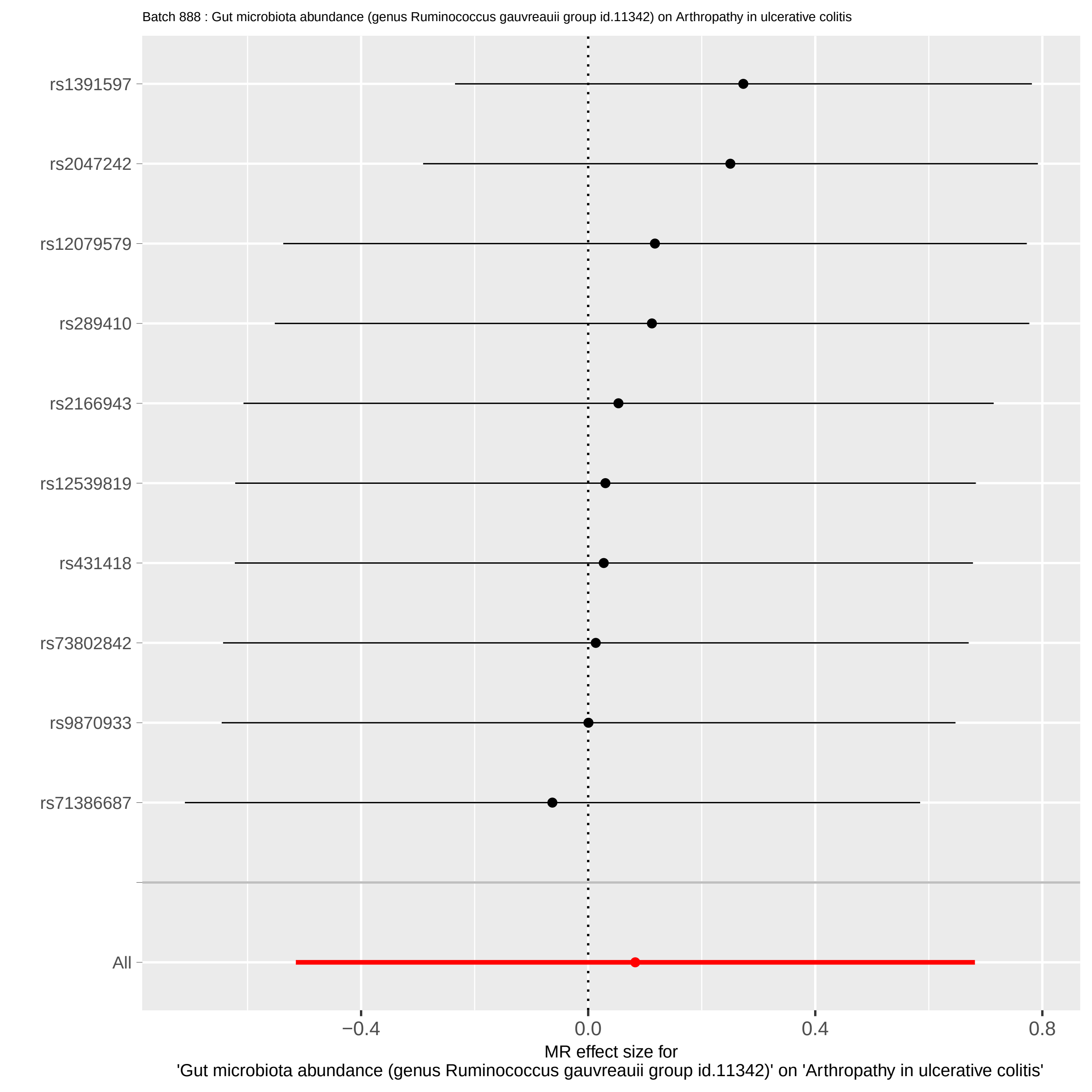
Batch 888 : Gut microbiota abundance (genus Ruminococcus gauvreauii group id.11342) on Arthropathy in ulcerative colitis
rs1391597
rs2047242
rs12079579
rs289410
rs2166943
rs12539819
rs431418
rs73802842
rs9870933
rs71386687
All
−0.4
0.0
0.4
0.8
MR effect size for
'Gut microbiota abundance (genus Ruminococcus gauvreauii group id.11342)' on 'Arthropathy in ulcerative colitis'

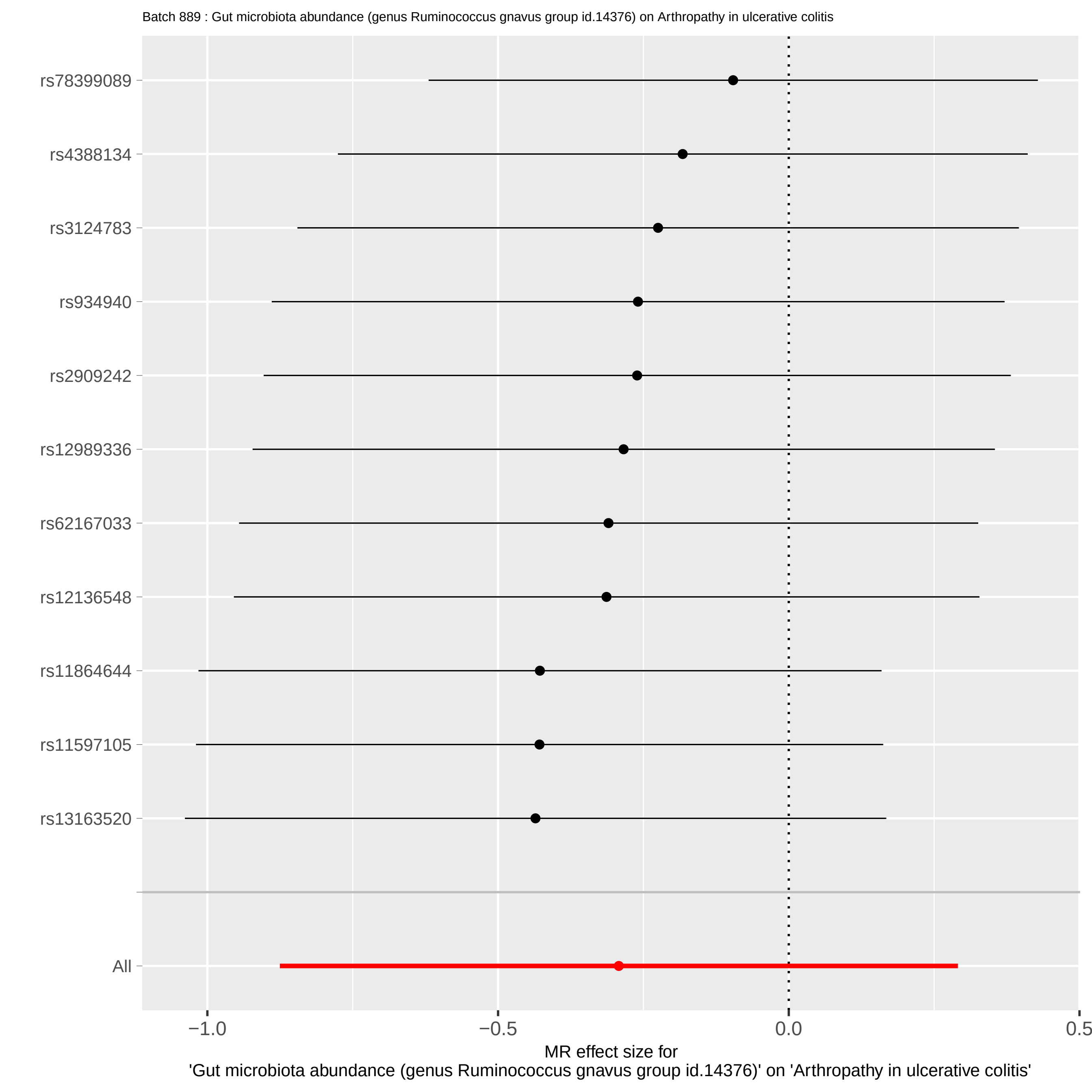
Batch 889 : Gut microbiota abundance (genus Ruminococcus gnavus group id.14376) on Arthropathy in ulcerative colitis
rs78399089
rs4388134
rs3124783
rs934940
rs2909242
rs12989336
rs62167033
rs12136548
rs11864644
rs11597105
rs13163520
All
−1.0
−0.5
0.0
0.5
MR effect size for
'Gut microbiota abundance (genus Ruminococcus gnavus group id.14376)' on 'Arthropathy in ulcerative colitis'

Batch 890 : Gut microbiota abundance (genus Ruminococcus torques group id.14377) on Arthropathy in ulcerative colitis

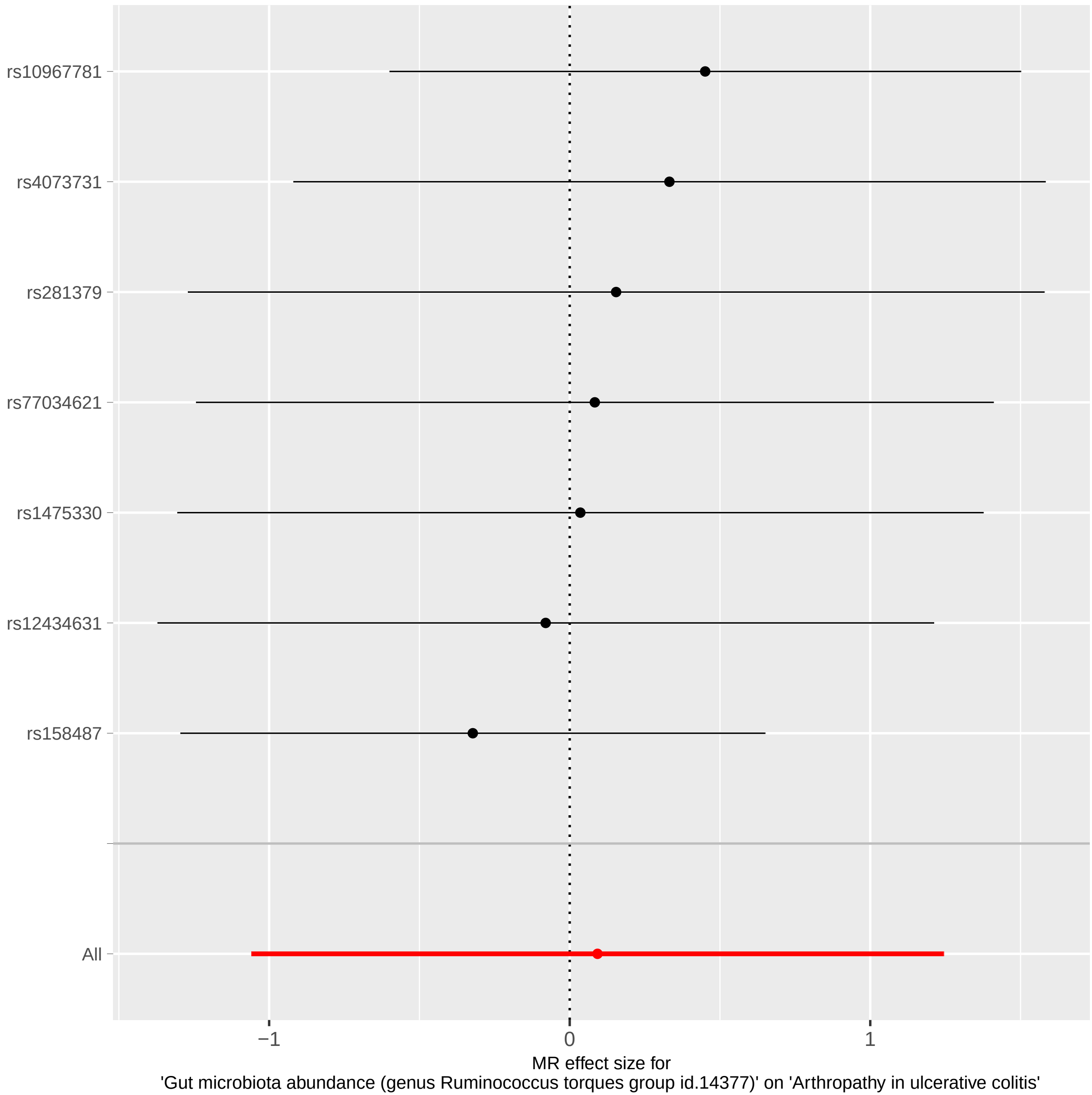

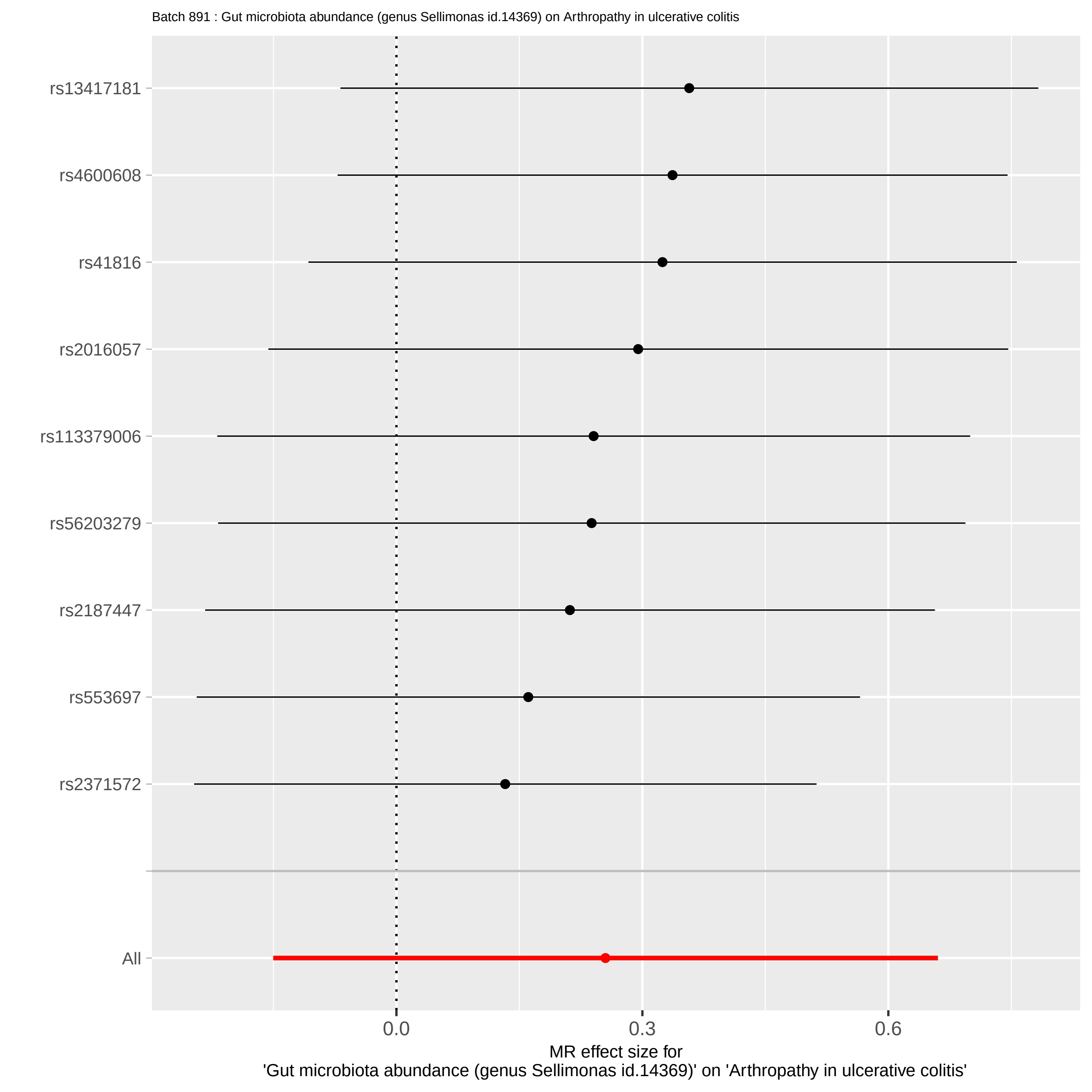
Batch 891 : Gut microbiota abundance (genus Sellimonas id.14369) on Arthropathy in ulcerative colitis
rs13417181
rs4600608
rs41816
rs2016057
rs113379006
rs56203279
rs2187447
rs553697
rs2371572
All
0.0
0.3
0.6
MR effect size for
'Gut microbiota abundance (genus Sellimonas id.14369)' on 'Arthropathy in ulcerative colitis'

Batch 892 : Gut microbiota abundance (genus Senegalimassilia id.11160) on Arthropathy in ulcerative colitis

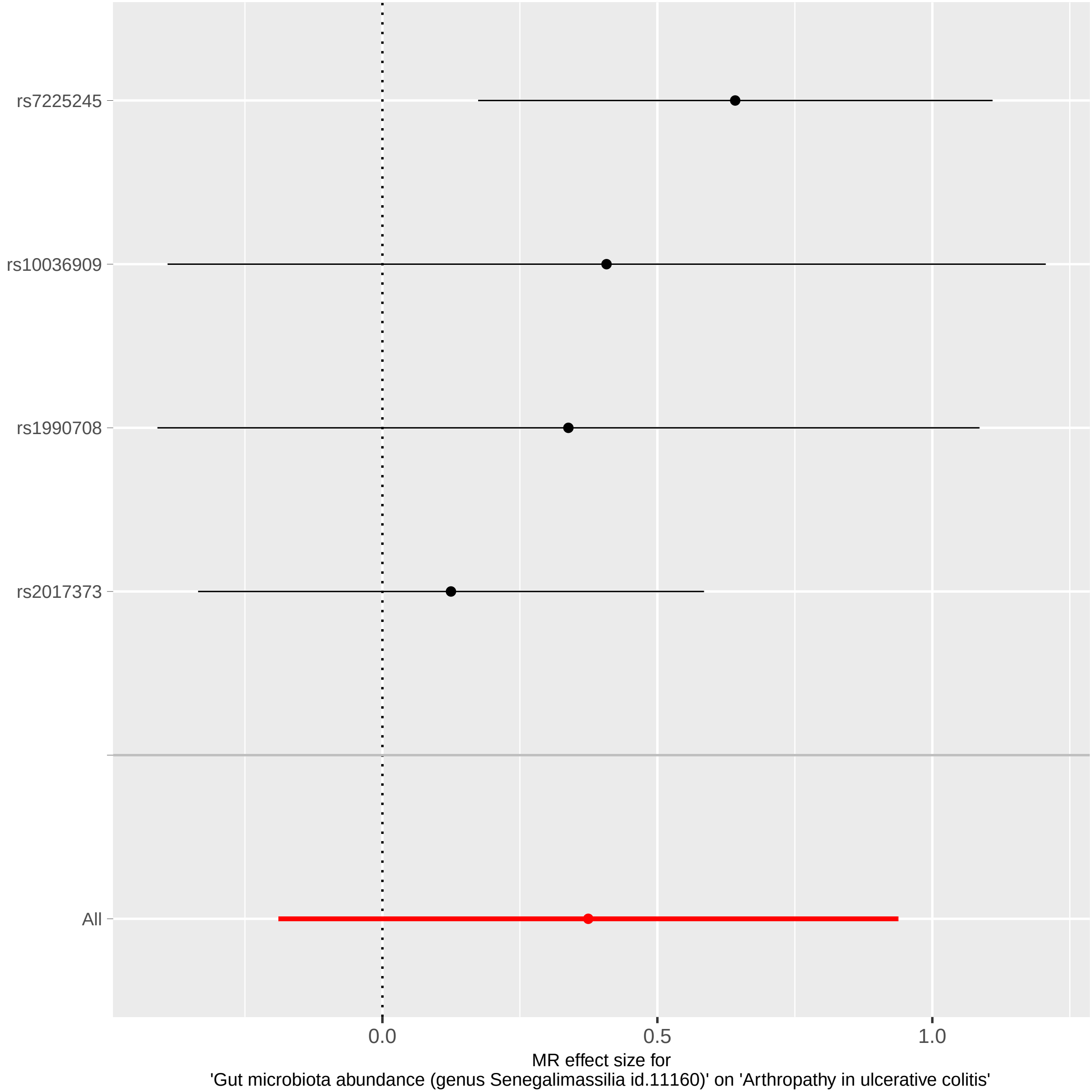

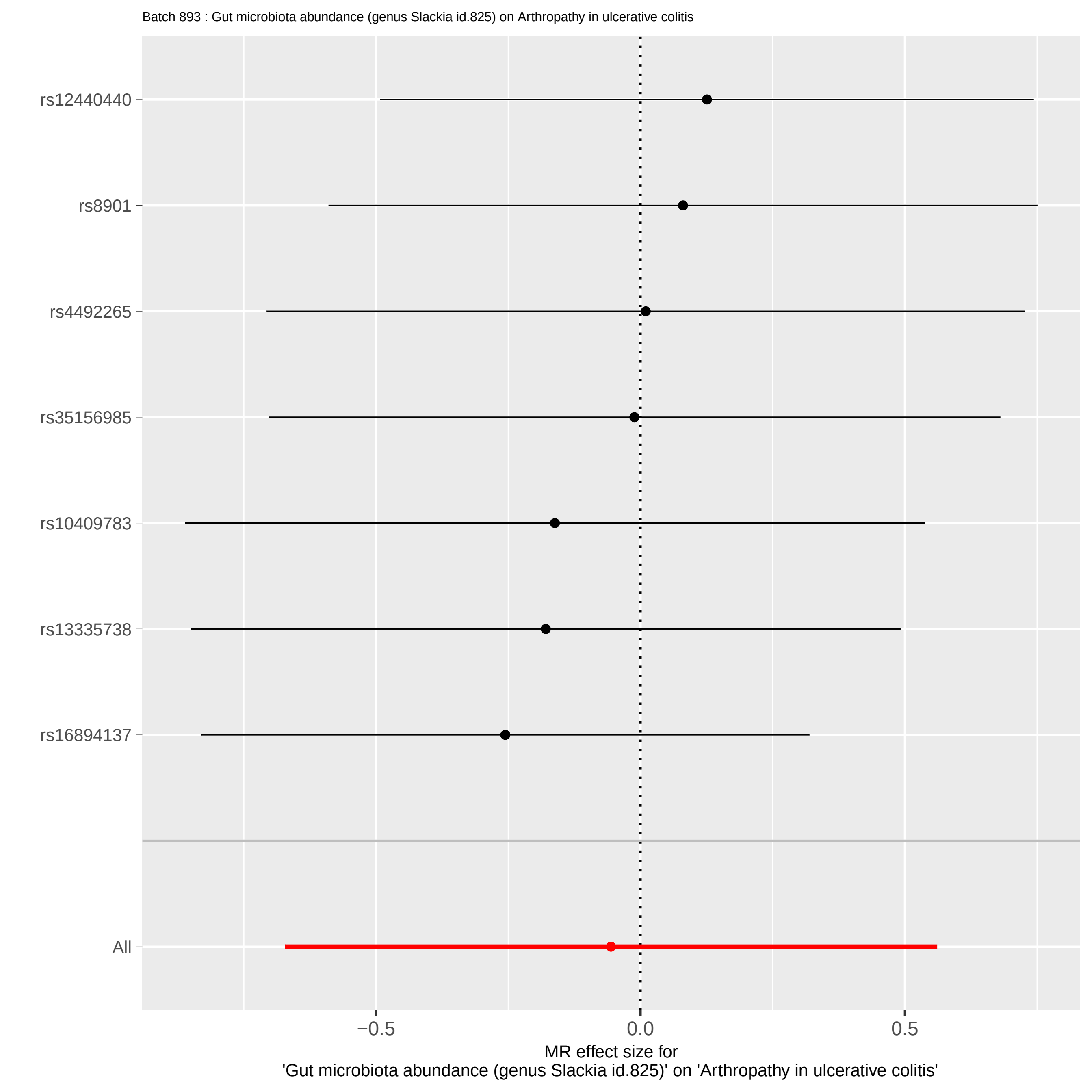
Batch 893 : Gut microbiota abundance (genus Slackia id.825) on Arthropathy in ulcerative colitis
rs12440440
rs8901
rs4492265
rs35156985
rs10409783
rs13335738
rs16894137
All
−0.5
0.0
0.5
MR effect size for
'Gut microbiota abundance (genus Slackia id.825)' on 'Arthropathy in ulcerative colitis'

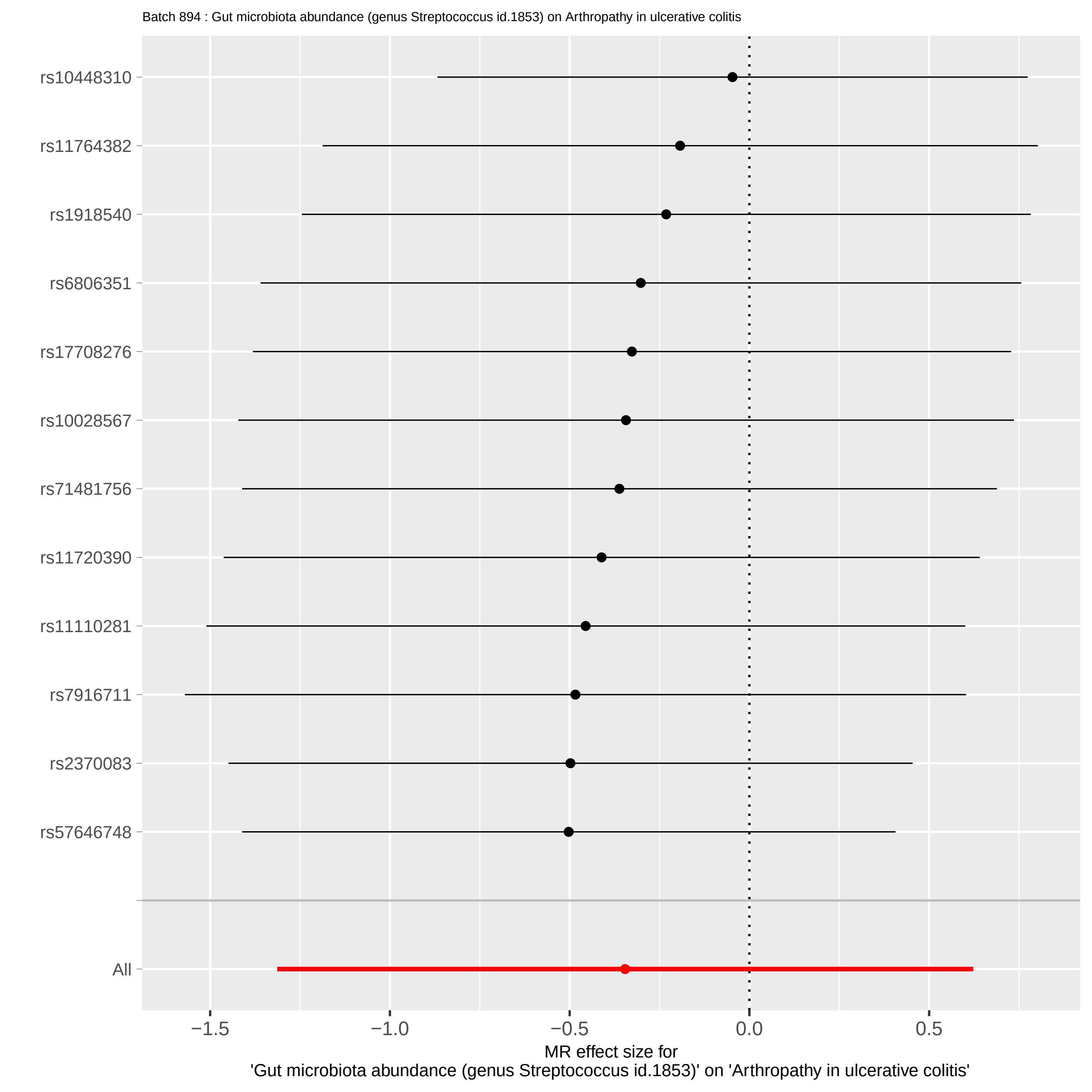
Batch 894 : Gut microbiota abundance (genus Streptococcus id.1853) on Arthropathy in ulcerative colitis
rs10448310
rs11764382
rs1918540
rs6806351
rs17708276
rs10028567
rs71481756
rs11720390
rs11110281
rs7916711
rs2370083
rs57646748
All
−1.5
−1.0
−0.5
0.0
0.5
MR effect size for
'Gut microbiota abundance (genus Streptococcus id.1853)' on 'Arthropathy in ulcerative colitis'

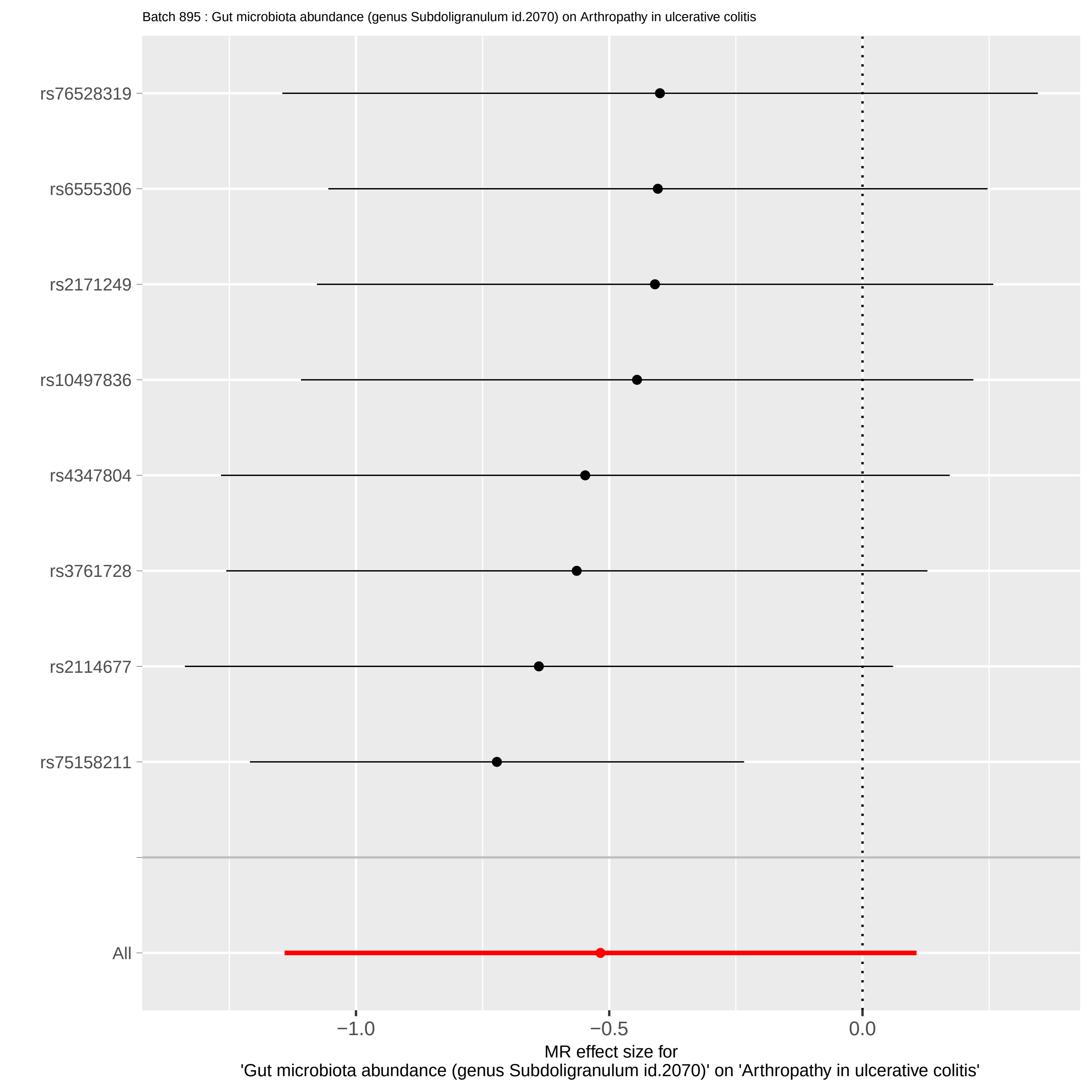

Batch 895 : Gut microbiota abundance (genus Subdoligranulum id.2070) on Arthropathy in ulcerative colitis
rs76528319
rs6555306
rs2171249
rs10497836
rs4347804
rs3761728
rs2114677
rs75158211
All
−1.0
−0.5
0.0
MR effect size for
'Gut microbiota abundance (genus Subdoligranulum id.2070)' on 'Arthropathy in ulcerative colitis'

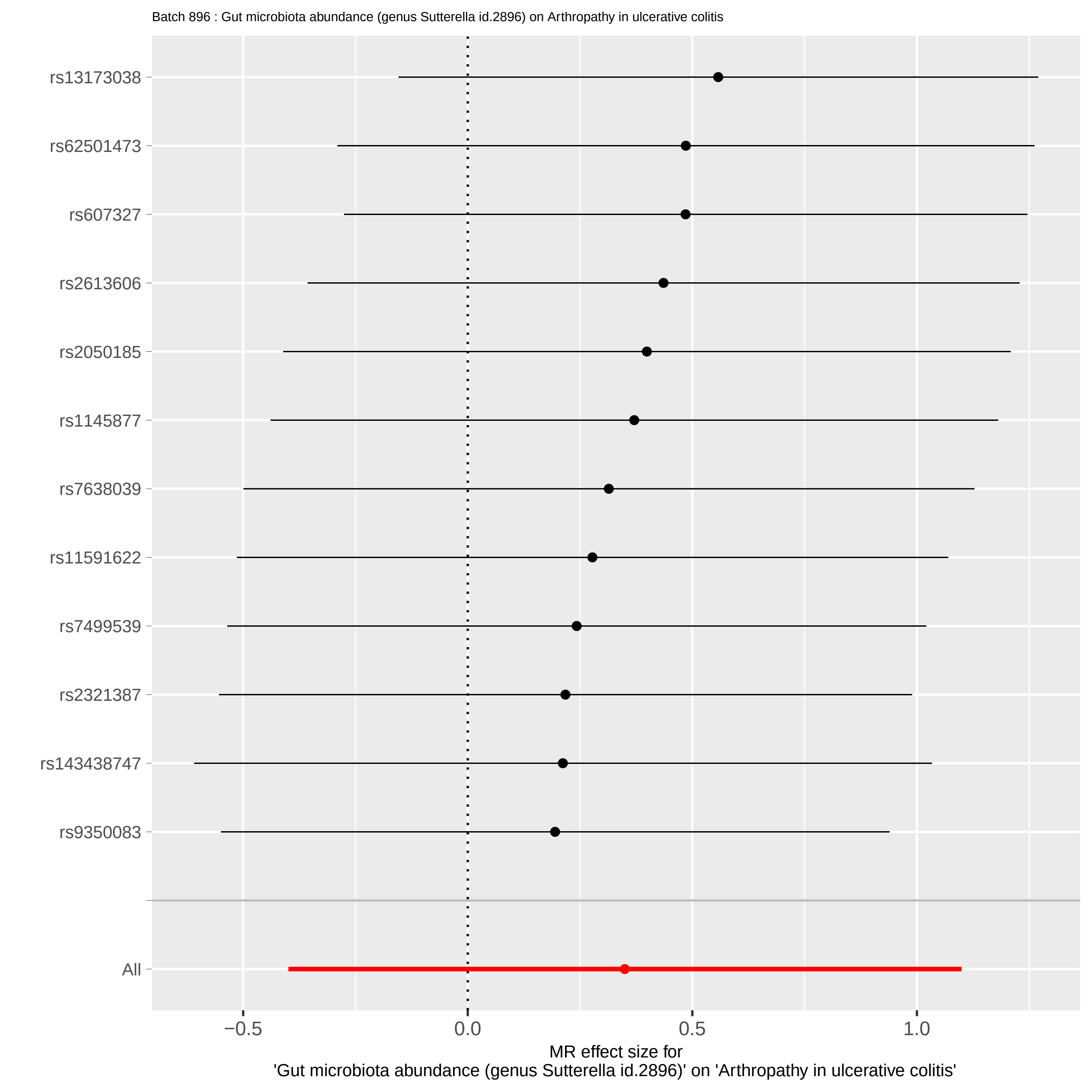

Batch 896 : Gut microbiota abundance (genus Sutterella id.2896) on Arthropathy in ulcerative colitis
rs13173038
rs62501473
rs607327
rs2613606
rs2050185
rs1145877
rs7638039
rs11591622
rs7499539
rs2321387
rs143438747
rs9350083
All
−0.5
0.0
0.5
1.0
MR effect size for
'Gut microbiota abundance (genus Sutterella id.2896)' on 'Arthropathy in ulcerative colitis'

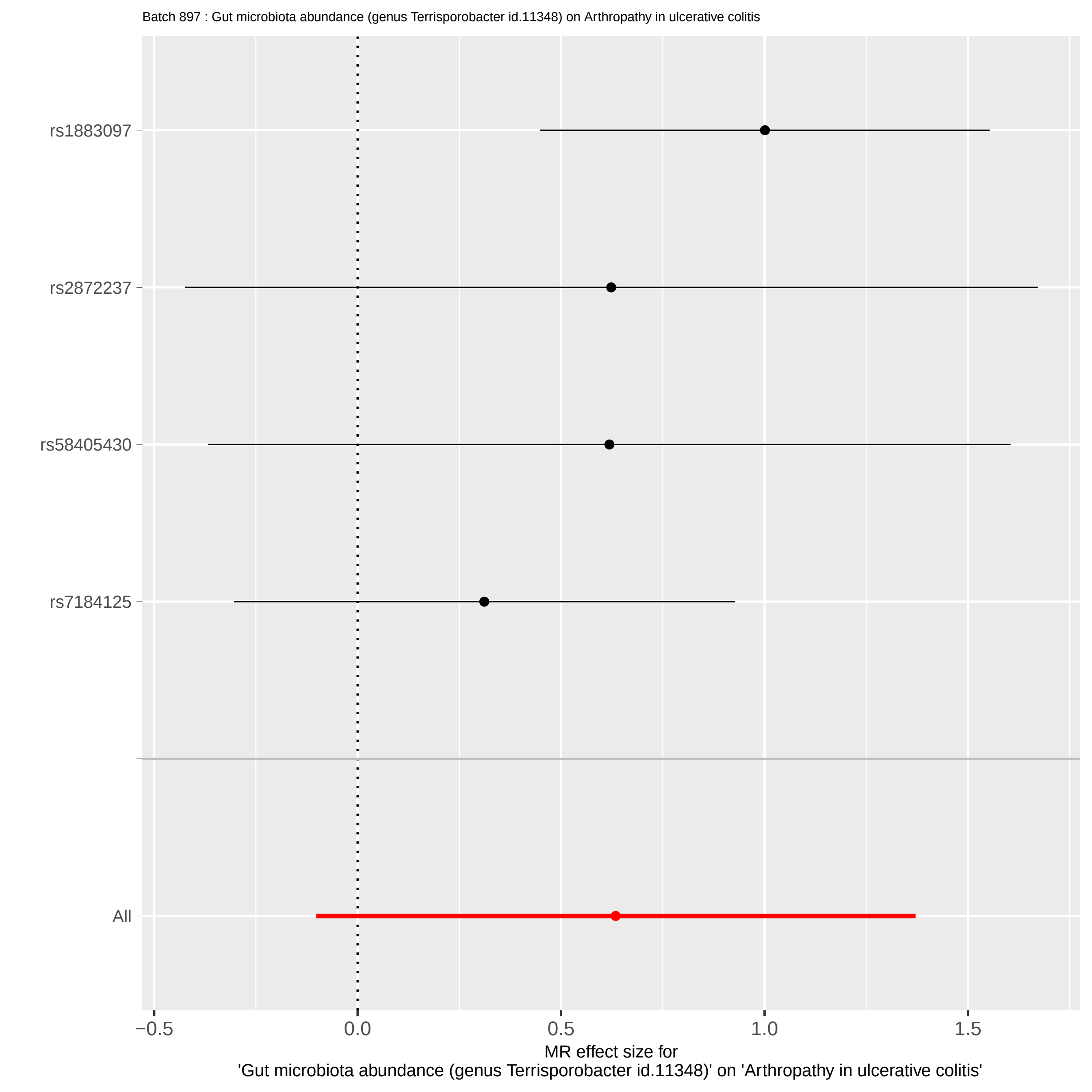

Batch 897 : Gut microbiota abundance (genus Terrisporobacter id.11348) on Arthropathy in ulcerative colitis
rs1883097
rs2872237
rs58405430
rs7184125
All
−0.5
0.0
0.5
1.0
1.5
MR effect size for
'Gut microbiota abundance (genus Terrisporobacter id.11348)' on 'Arthropathy in ulcerative colitis'

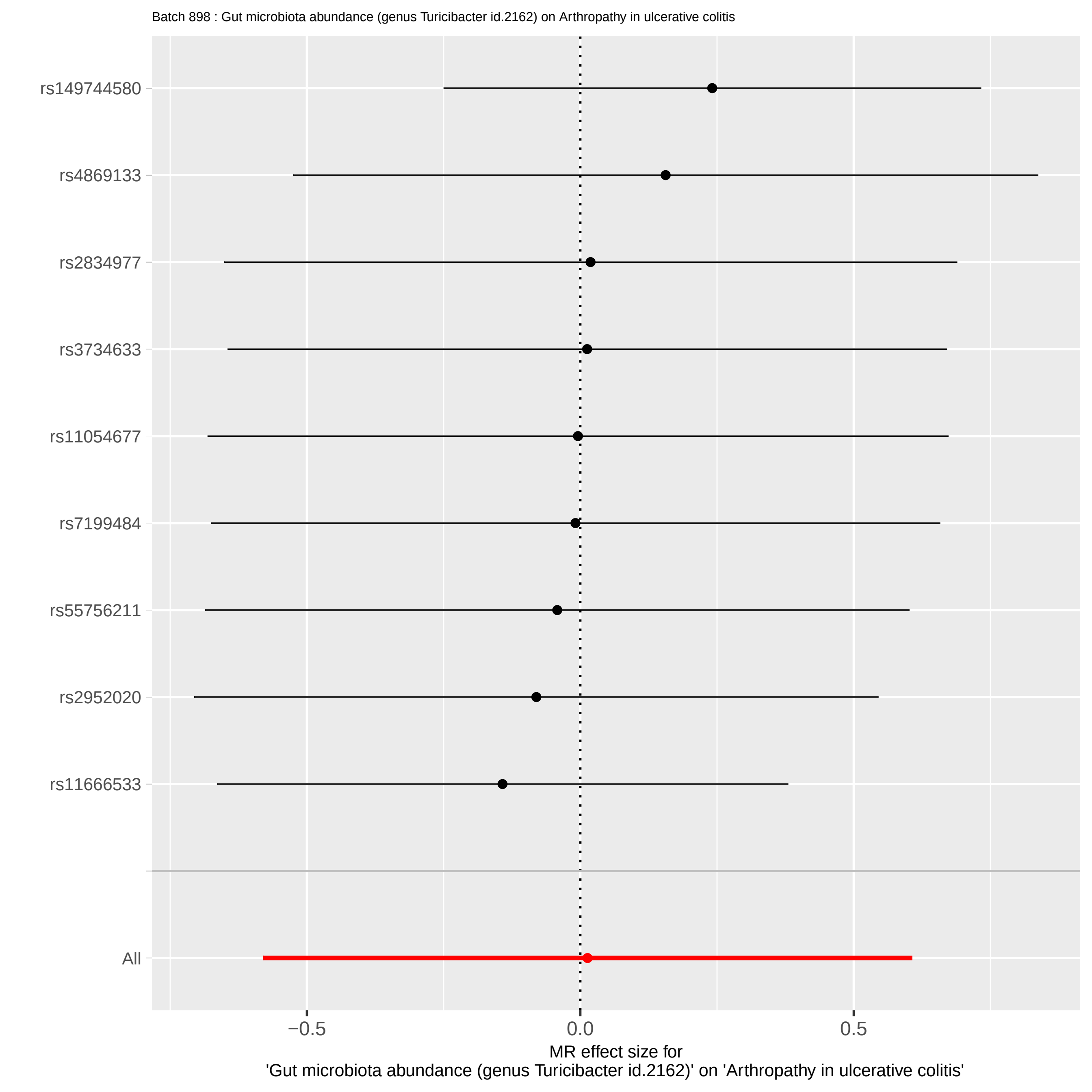

Batch 898 : Gut microbiota abundance (genus Turicibacter id.2162) on Arthropathy in ulcerative colitis
rs149744580
rs4869133
rs2834977
rs3734633
rs11054677
rs7199484
rs55756211
rs2952020
rs11666533
All
−0.5
0.0
0.5
MR effect size for
'Gut microbiota abundance (genus Turicibacter id.2162)' on 'Arthropathy in ulcerative colitis'

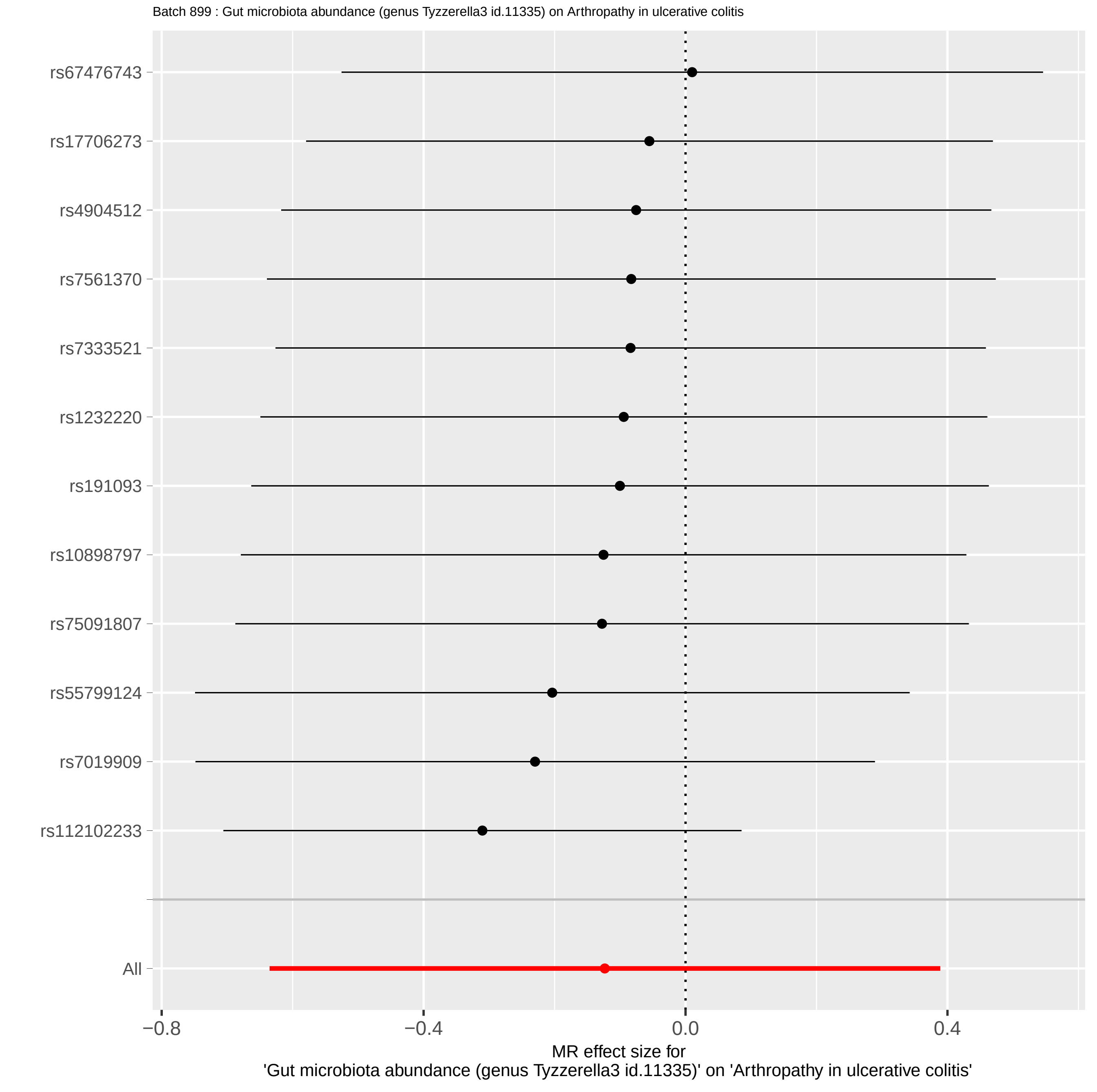

Batch 899 : Gut microbiota abundance (genus Tyzzerella3 id.11335) on Arthropathy in ulcerative colitis
rs67476743
rs17706273
rs4904512
rs7561370
rs7333521
rs1232220
rs191093
rs10898797
rs75091807
rs55799124
rs7019909
rs112102233
All
−0.8
−0.4
0.0
0.4
MR effect size for
'Gut microbiota abundance (genus Tyzzerella3 id.11335)' on 'Arthropathy in ulcerative colitis'

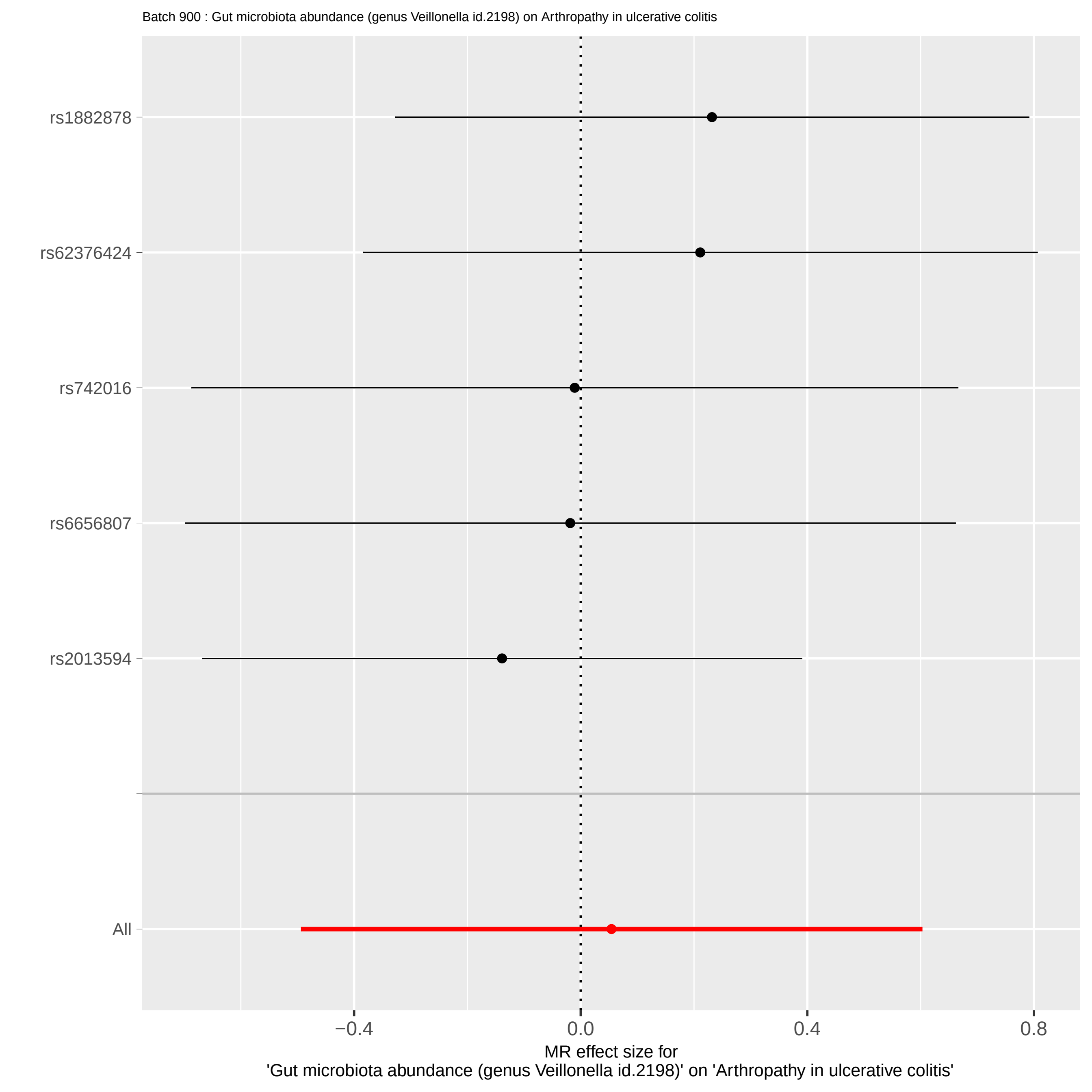
Batch 900 : Gut microbiota abundance (genus Veillonella id.2198) on Arthropathy in ulcerative colitis
rs1882878
rs62376424
rs742016
rs6656807
rs2013594
All
−0.4
0.0
0.4
0.8
MR effect size for
'Gut microbiota abundance (genus Veillonella id.2198)' on 'Arthropathy in ulcerative colitis'

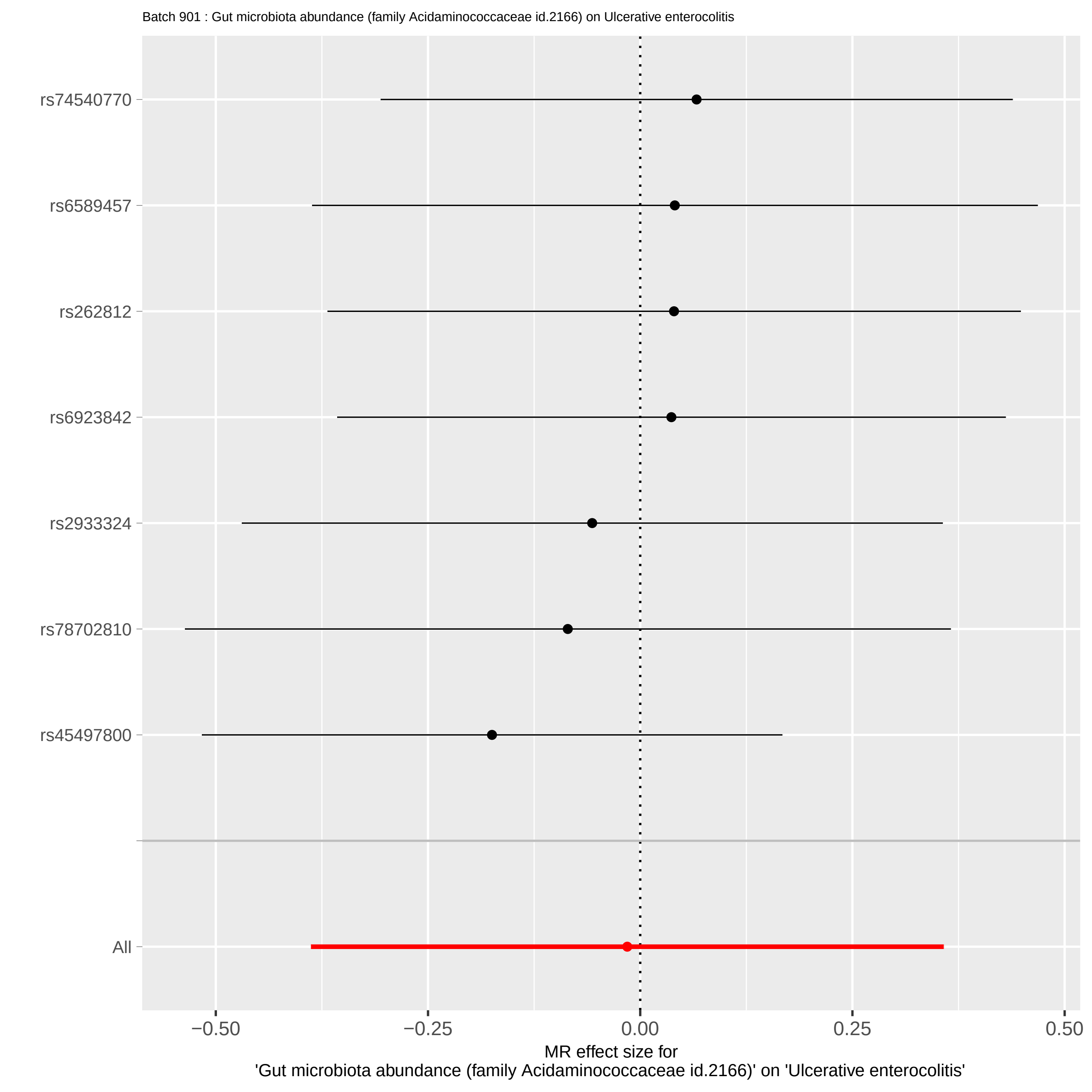
Batch 901 : Gut microbiota abundance (family Acidaminococcaceae id.2166) on Ulcerative enterocolitis
rs74540770
rs6589457
rs262812
rs6923842
rs2933324
rs78702810
rs45497800
All
−0.50
−0.25
0.00
0.25
0.50
MR effect size for
'Gut microbiota abundance (family Acidaminococcaceae id.2166)' on 'Ulcerative enterocolitis'

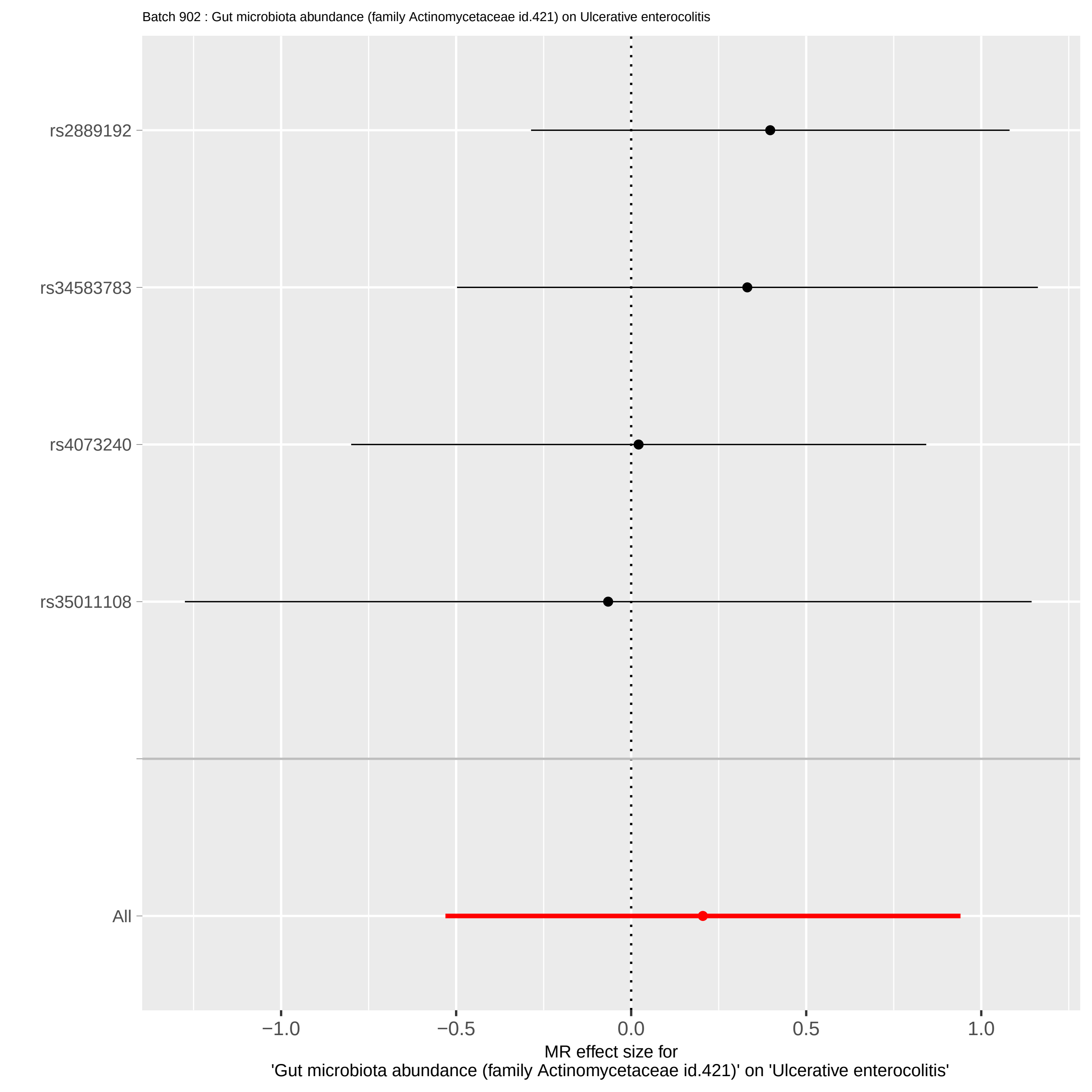
Batch 902 : Gut microbiota abundance (family Actinomycetaceae id.421) on Ulcerative enterocolitis
rs2889192
rs34583783
rs4073240
rs35011108
All
−1.0
−0.5
0.0
0.5
1.0
MR effect size for
'Gut microbiota abundance (family Actinomycetaceae id.421)' on 'Ulcerative enterocolitis'

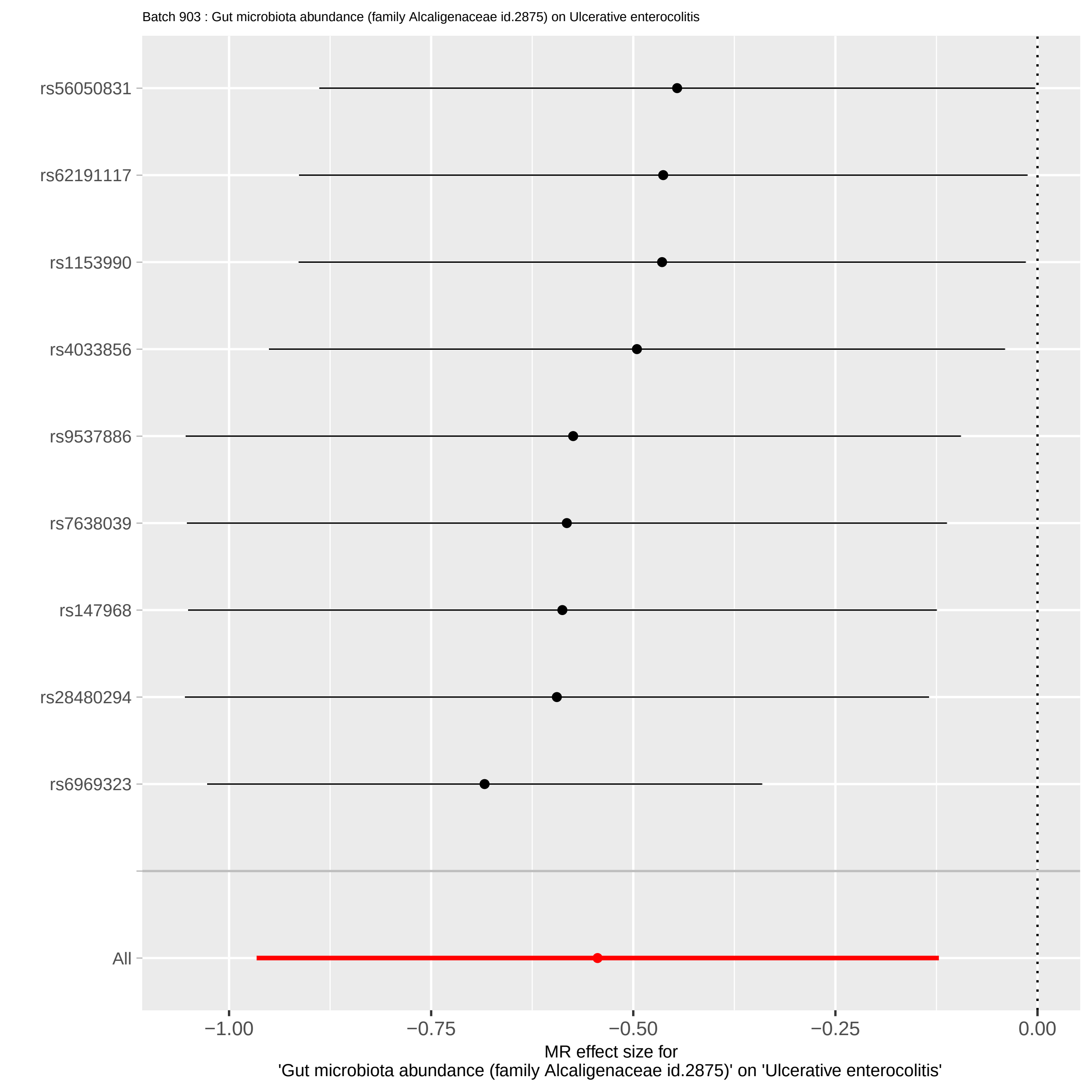
Batch 903 : Gut microbiota abundance (family Alcaligenaceae id.2875) on Ulcerative enterocolitis
rs56050831
rs62191117
rs1153990
rs4033856
rs9537886
rs7638039
rs147968
rs28480294
rs6969323
All
−1.00
−0.75
−0.50
−0.25
0.00
MR effect size for
'Gut microbiota abundance (family Alcaligenaceae id.2875)' on 'Ulcerative enterocolitis'

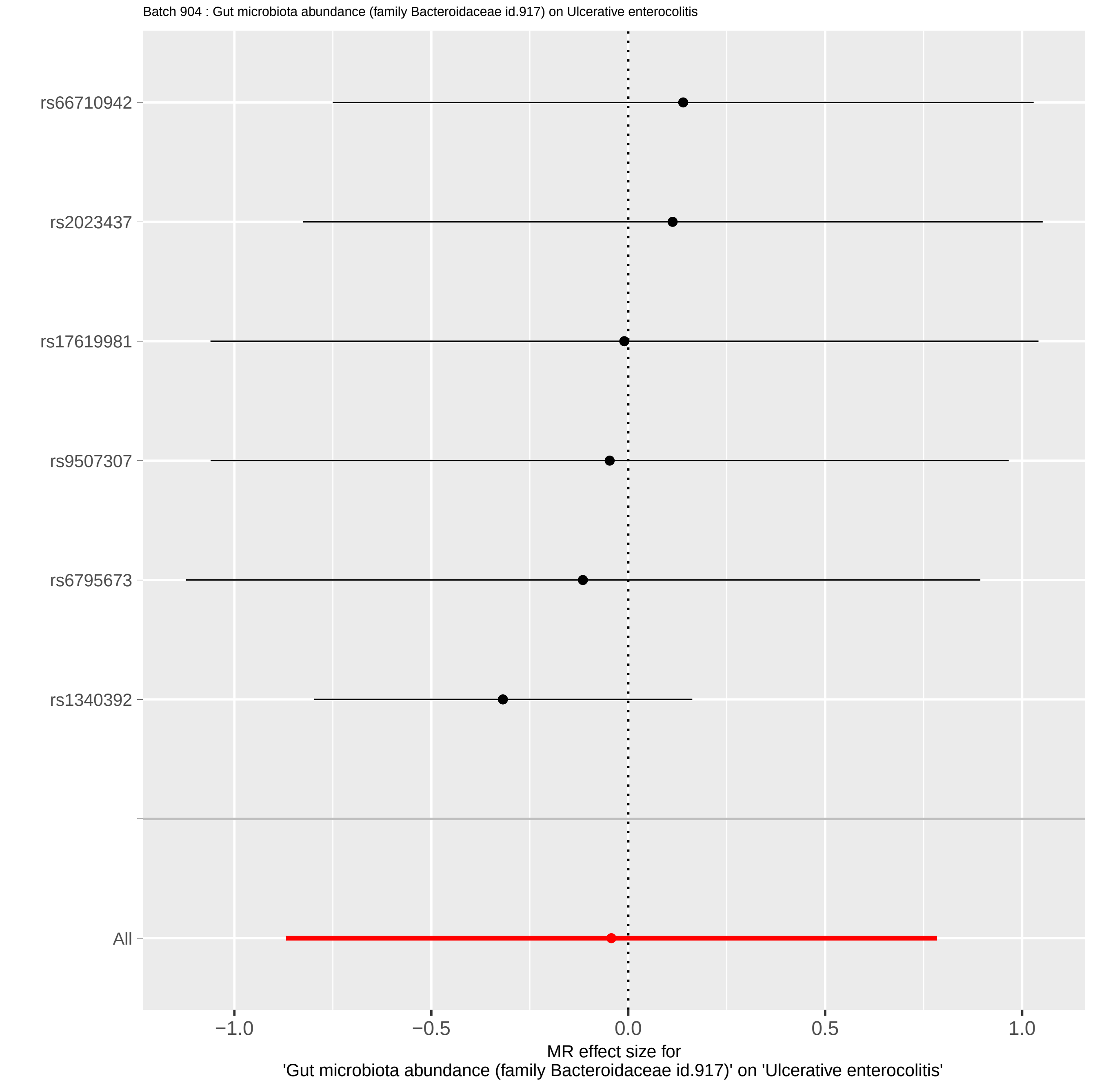

Batch 904 : Gut microbiota abundance (family Bacteroidaceae id.917) on Ulcerative enterocolitis
rs66710942
rs2023437
rs17619981
rs9507307
rs6795673
rs1340392
All
−1.0
−0.5
0.0
0.5
1.0
MR effect size for
'Gut microbiota abundance (family Bacteroidaceae id.917)' on 'Ulcerative enterocolitis'

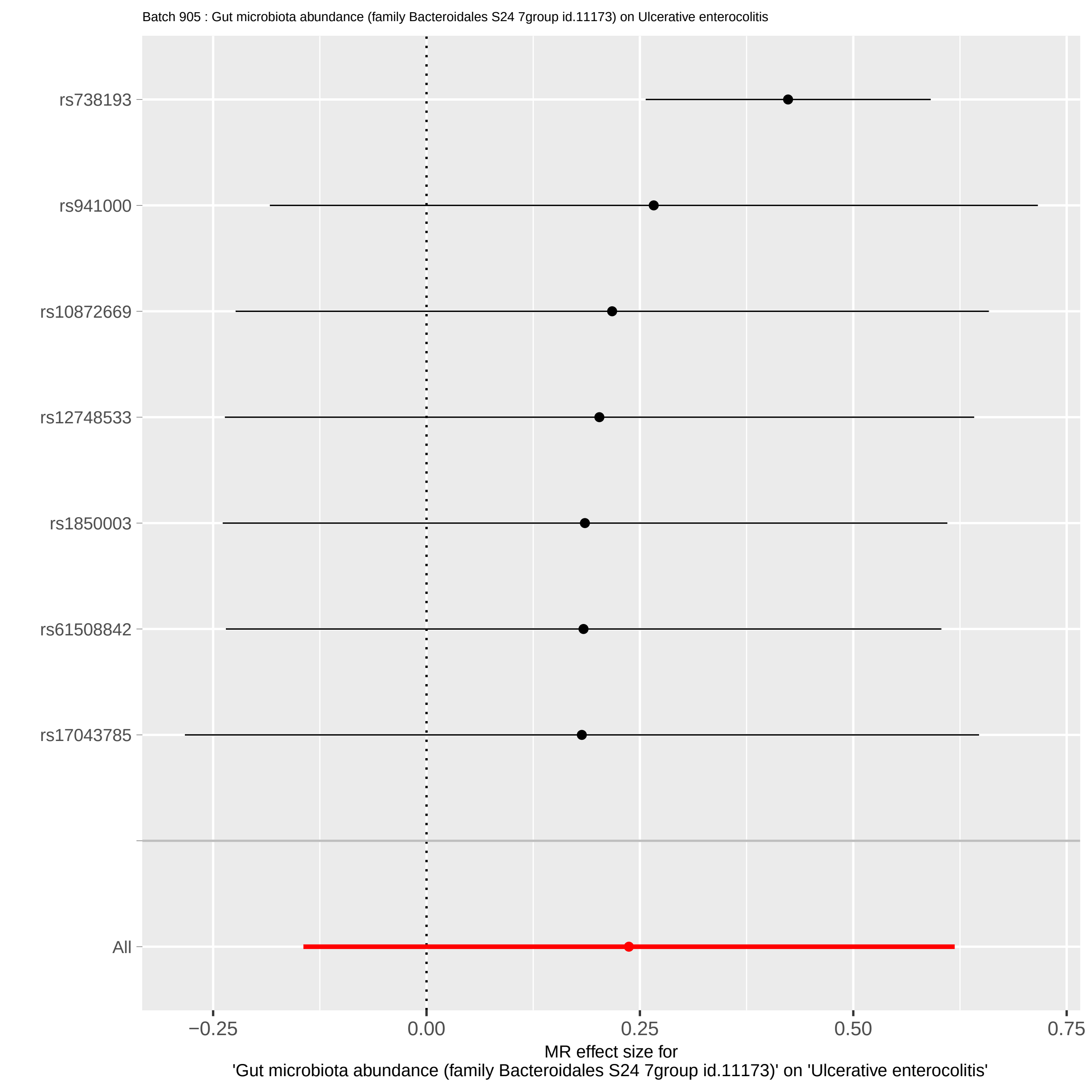
Batch 905 : Gut microbiota abundance (family Bacteroidales S24 7group id.11173) on Ulcerative enterocolitis
rs738193
rs941000
rs10872669
rs12748533
rs1850003
rs61508842
rs17043785
All
−0.25
0.00
0.25
0.50
0.75
MR effect size for
'Gut microbiota abundance (family Bacteroidales S24 7group id.11173)' on 'Ulcerative enterocolitis'

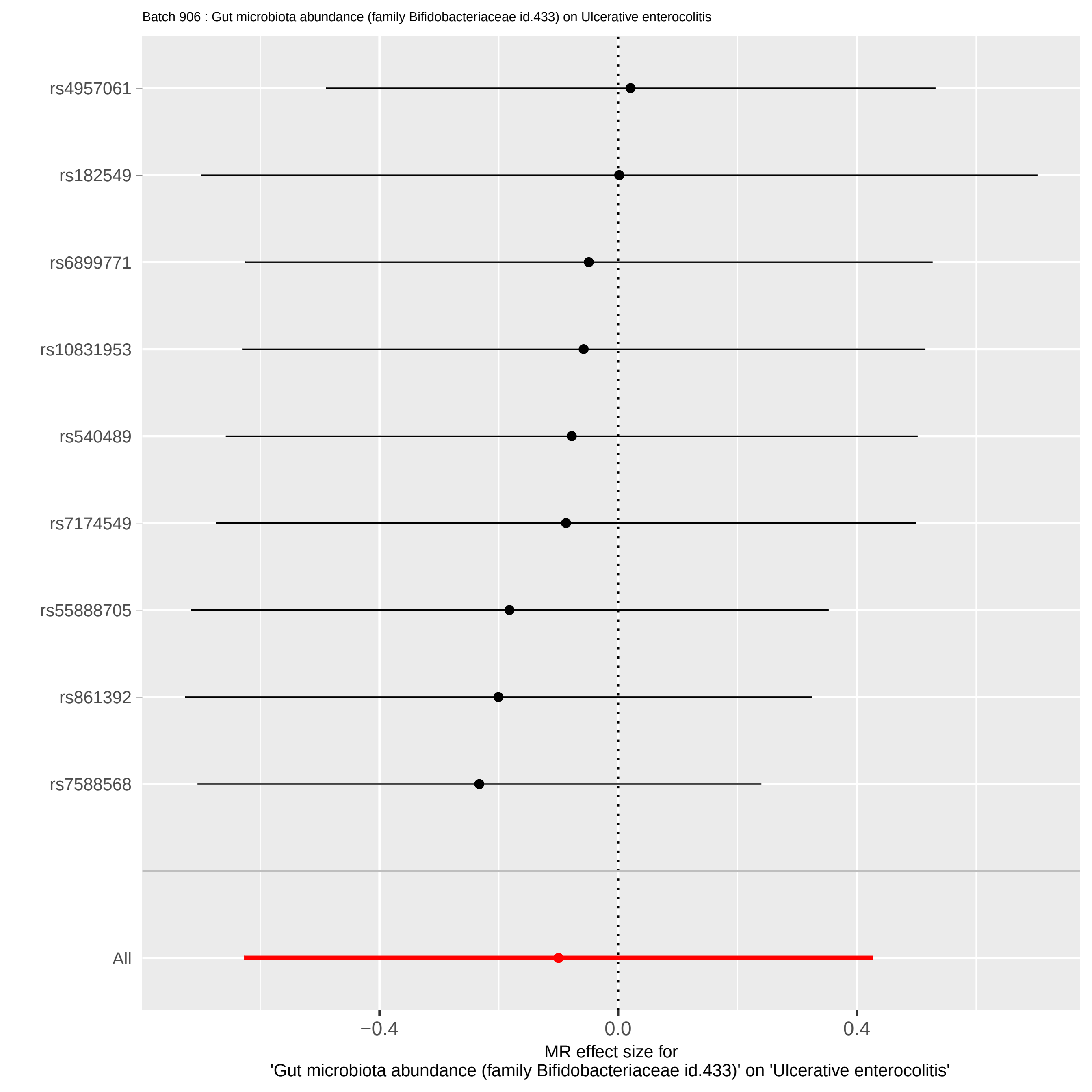

Batch 906 : Gut microbiota abundance (family Bifidobacteriaceae id.433) on Ulcerative enterocolitis
rs4957061
rs182549
rs6899771
rs10831953
rs540489
rs7174549
rs55888705
rs861392
rs7588568
All
−0.4
0.0
0.4
MR effect size for
'Gut microbiota abundance (family Bifidobacteriaceae id.433)' on 'Ulcerative enterocolitis'

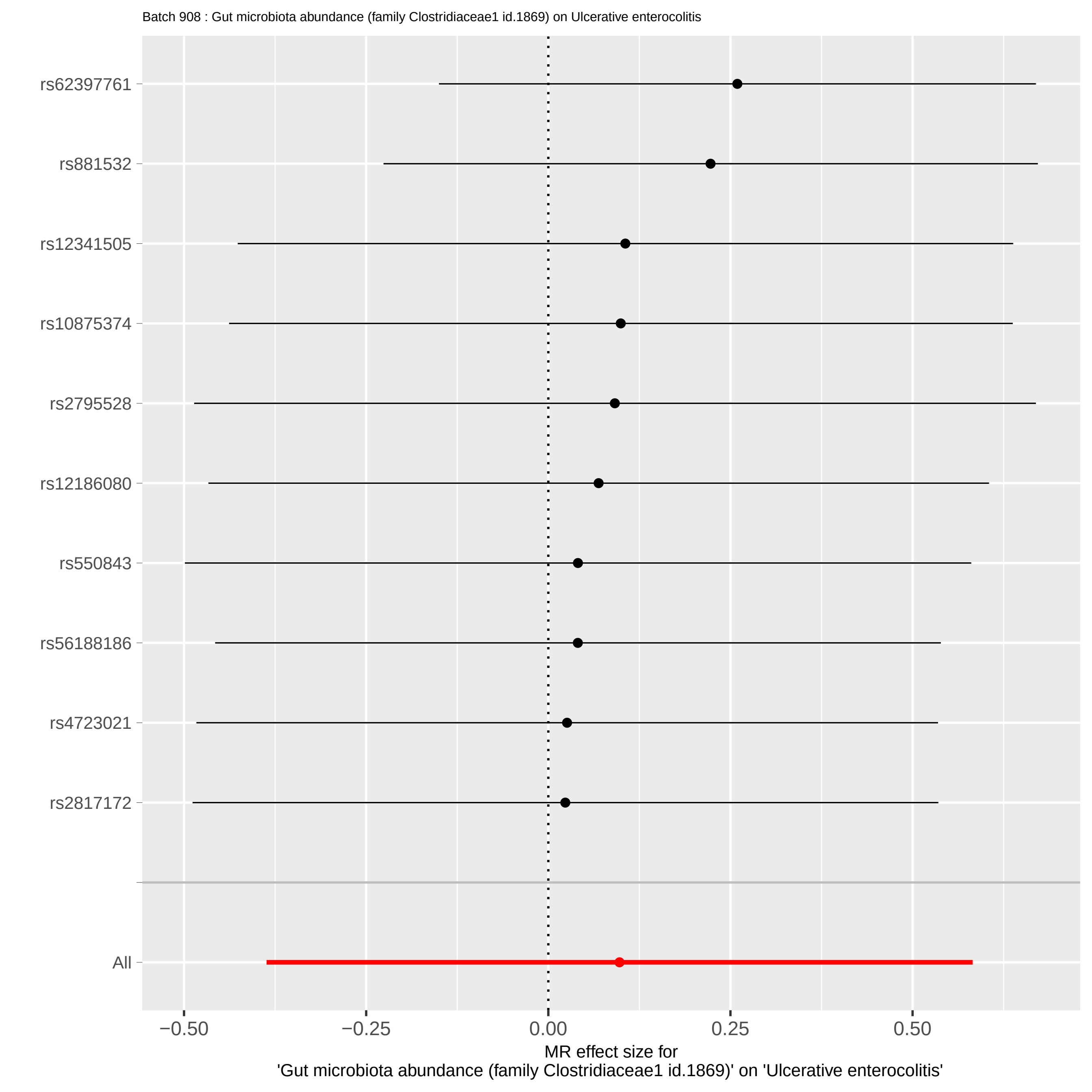

Batch 908 : Gut microbiota abundance (family Clostridiaceae1 id.1869) on Ulcerative enterocolitis
rs62397761
rs881532
rs12341505
rs10875374
rs2795528
rs12186080
rs550843
rs56188186
rs4723021
rs2817172
All
−0.50
−0.25
0.00
0.25
0.50
MR effect size for
'Gut microbiota abundance (family Clostridiaceae1 id.1869)' on 'Ulcerative enterocolitis'

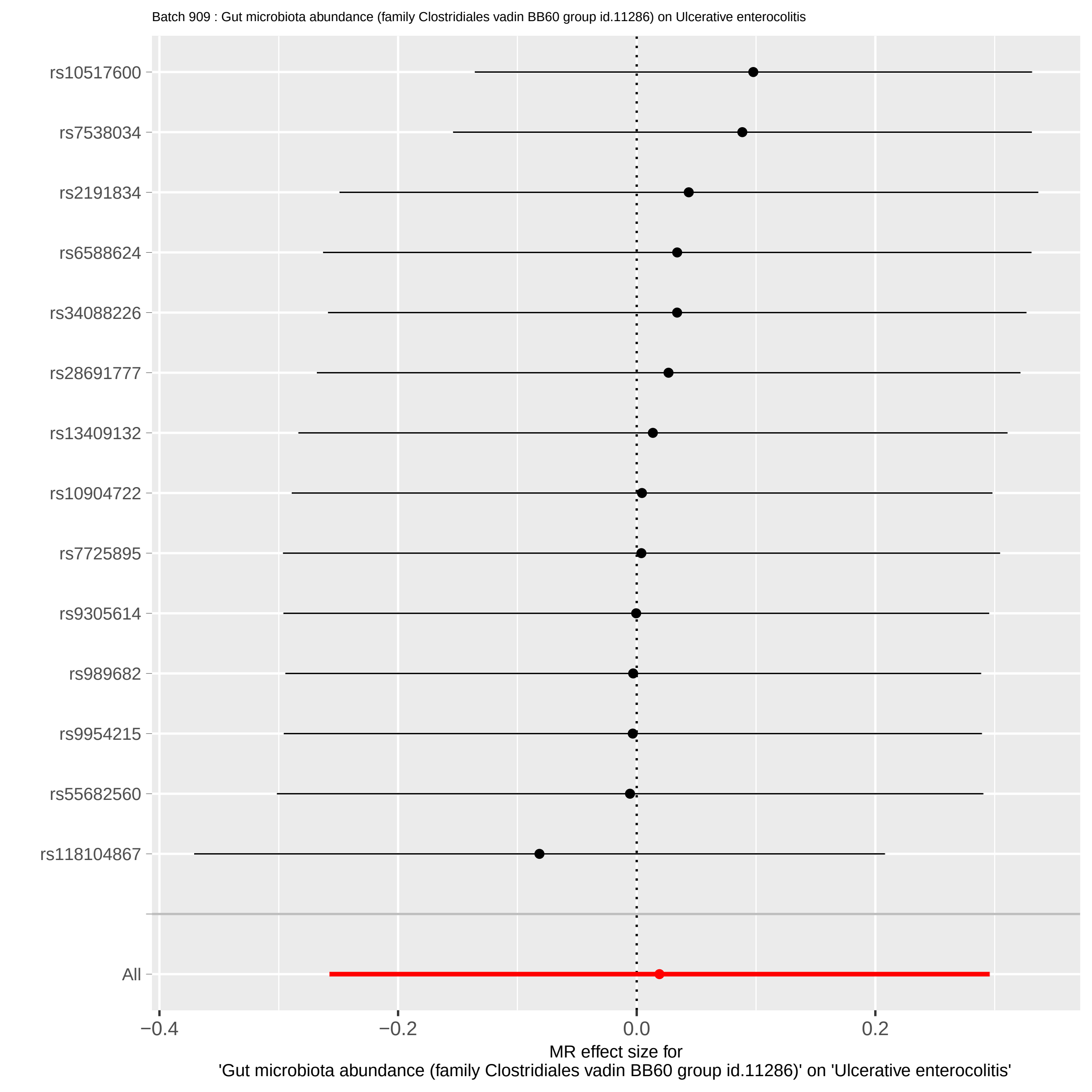
Batch 909 : Gut microbiota abundance (family Clostridiales vadin BB60 group id.11286) on Ulcerative enterocolitis
rs10517600
rs7538034
rs2191834
rs6588624
rs34088226
rs28691777
rs13409132
rs10904722
rs7725895
rs9305614
rs989682
rs9954215
rs55682560
rs118104867
All
−0.4
−0.2
0.0
0.2
MR effect size for
'Gut microbiota abundance (family Clostridiales vadin BB60 group id.11286)' on 'Ulcerative enterocolitis'

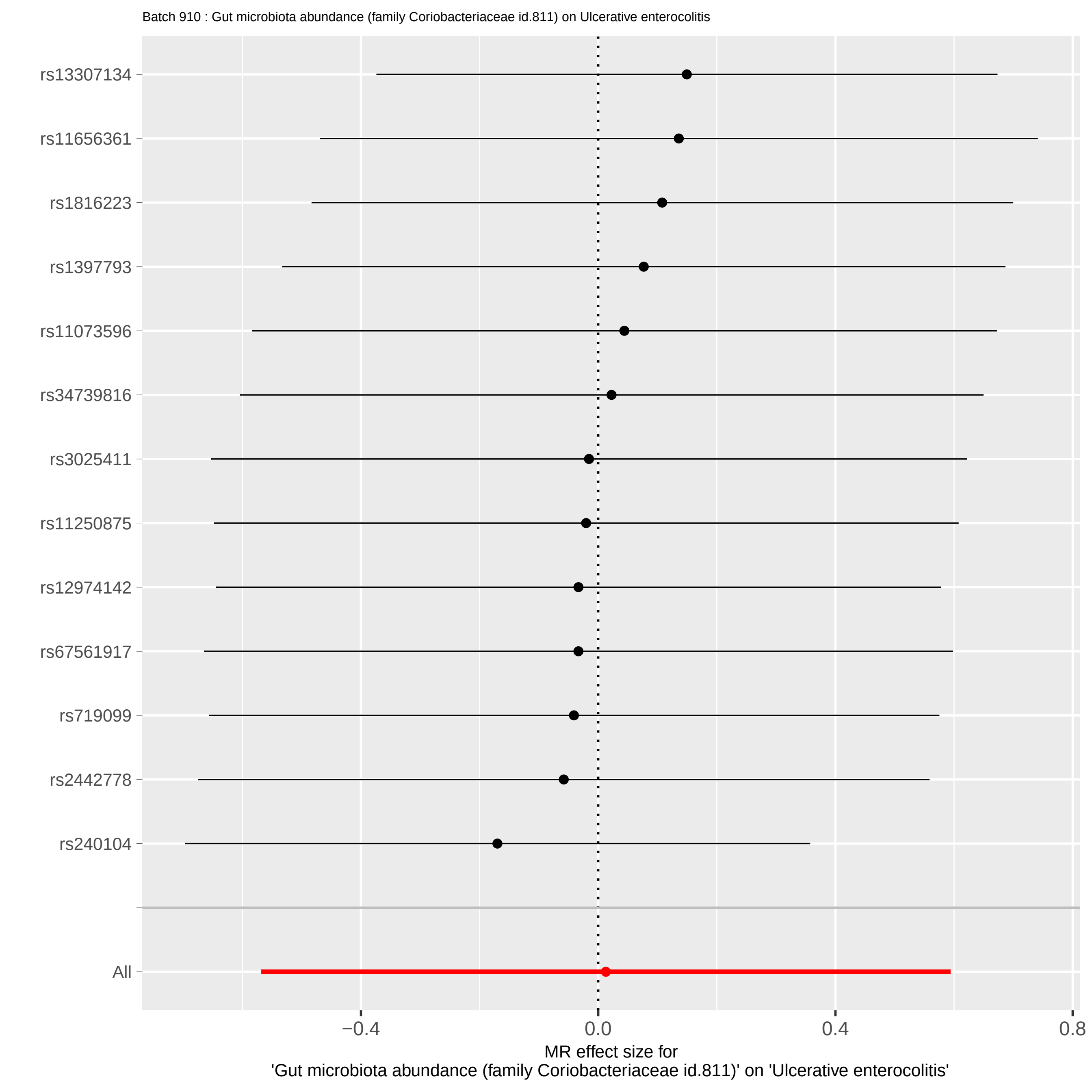
Batch 910 : Gut microbiota abundance (family Coriobacteriaceae id.811) on Ulcerative enterocolitis
rs13307134
rs11656361
rs1816223
rs1397793
rs11073596
rs34739816
rs3025411
rs11250875
rs12974142
rs67561917
rs719099
rs2442778
rs240104
All
−0.4
0.0
0.4
0.8
MR effect size for
'Gut microbiota abundance (family Coriobacteriaceae id.811)' on 'Ulcerative enterocolitis'

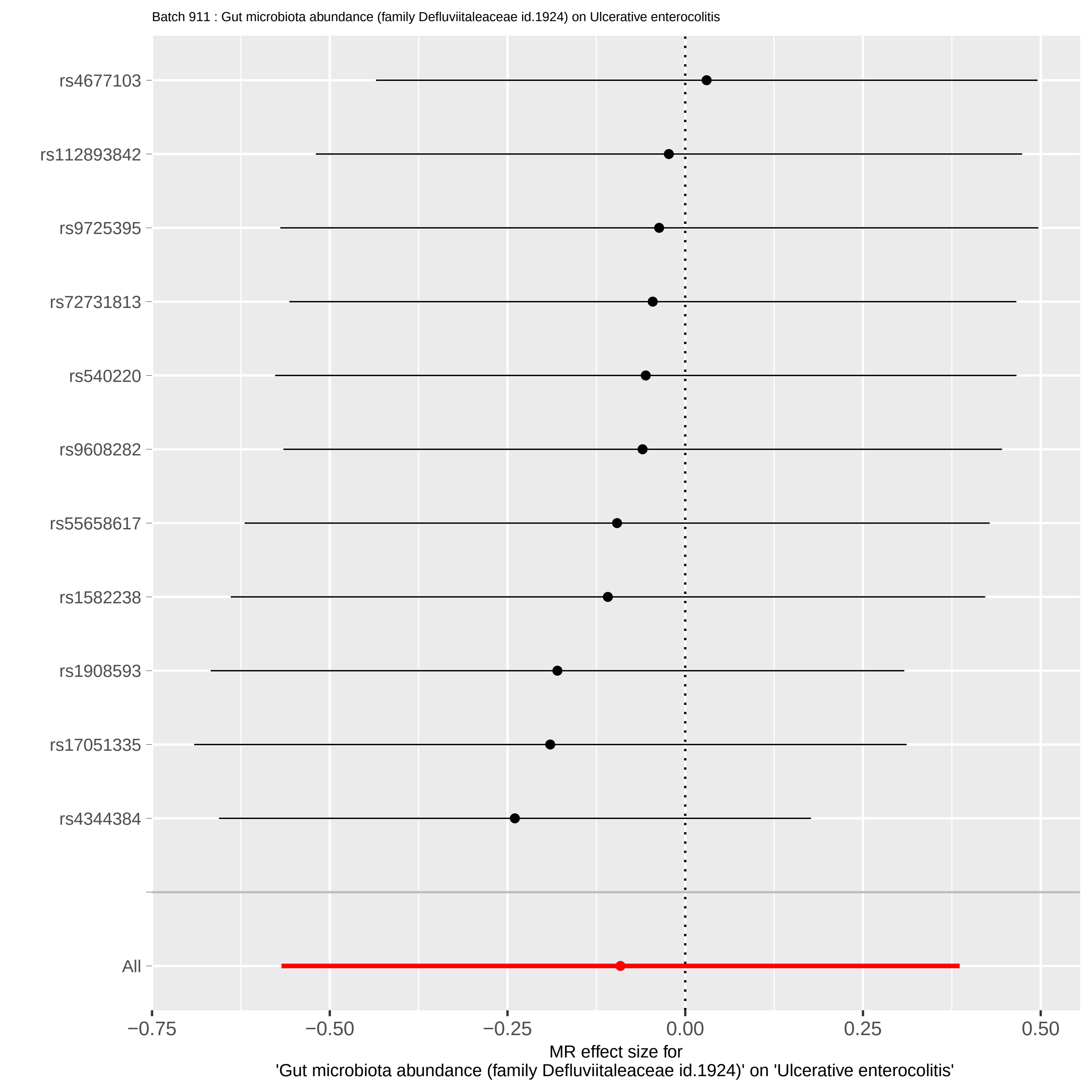
Batch 911 : Gut microbiota abundance (family Defluviitaleaceae id.1924) on Ulcerative enterocolitis
rs4677103
rs112893842
rs9725395
rs72731813
rs540220
rs9608282
rs55658617
rs1582238
rs1908593
rs17051335
rs4344384
All
−0.75
−0.50
−0.25
0.00
0.25
0.50
MR effect size for
'Gut microbiota abundance (family Defluviitaleaceae id.1924)' on 'Ulcerative enterocolitis'

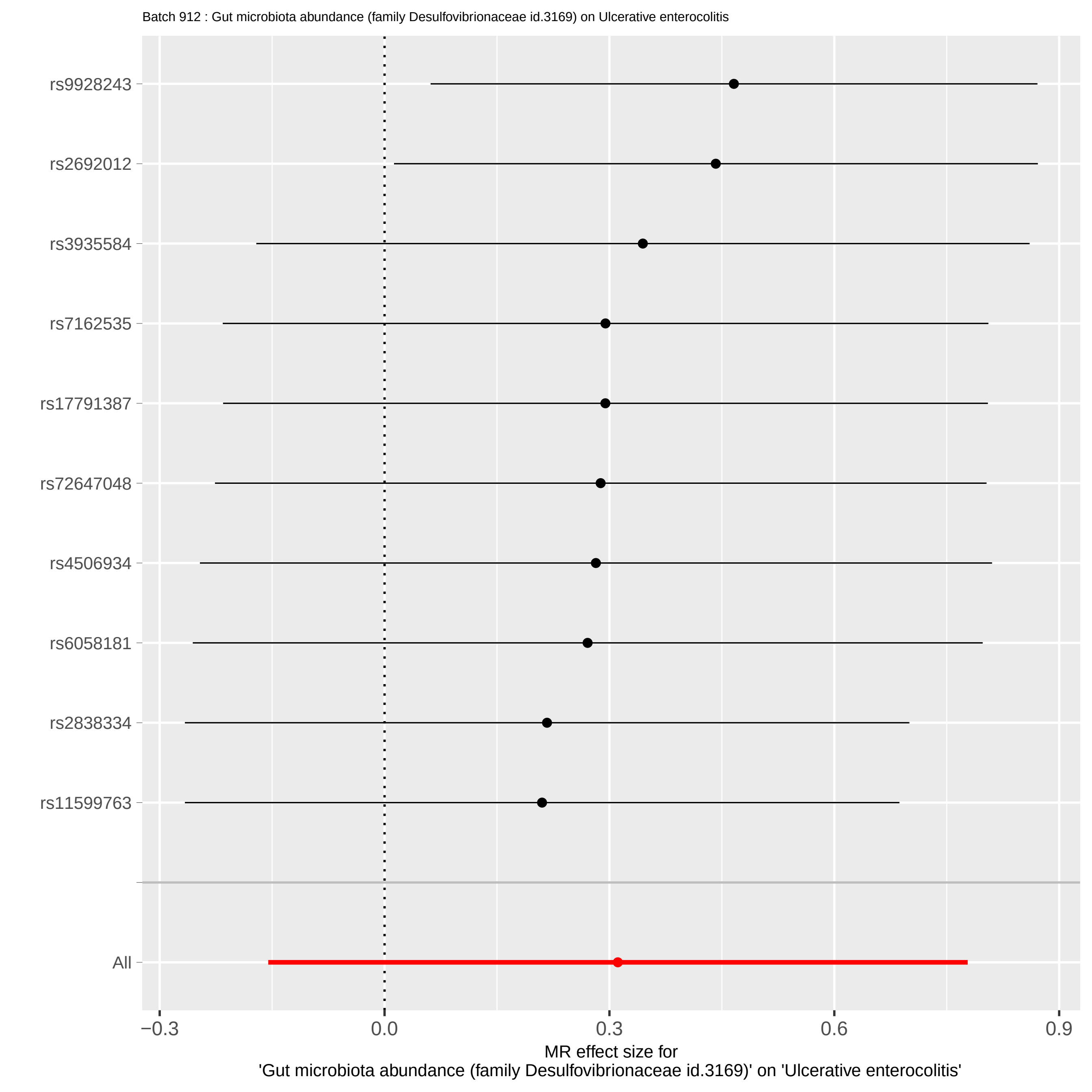
Batch 912 : Gut microbiota abundance (family Desulfovibrionaceae id.3169) on Ulcerative enterocolitis
rs9928243
rs2692012
rs3935584
rs7162535
rs17791387
rs72647048
rs4506934
rs6058181
rs2838334
rs11599763
All
−0.3
0.0
0.3
0.6
0.9
MR effect size for
'Gut microbiota abundance (family Desulfovibrionaceae id.3169)' on 'Ulcerative enterocolitis'

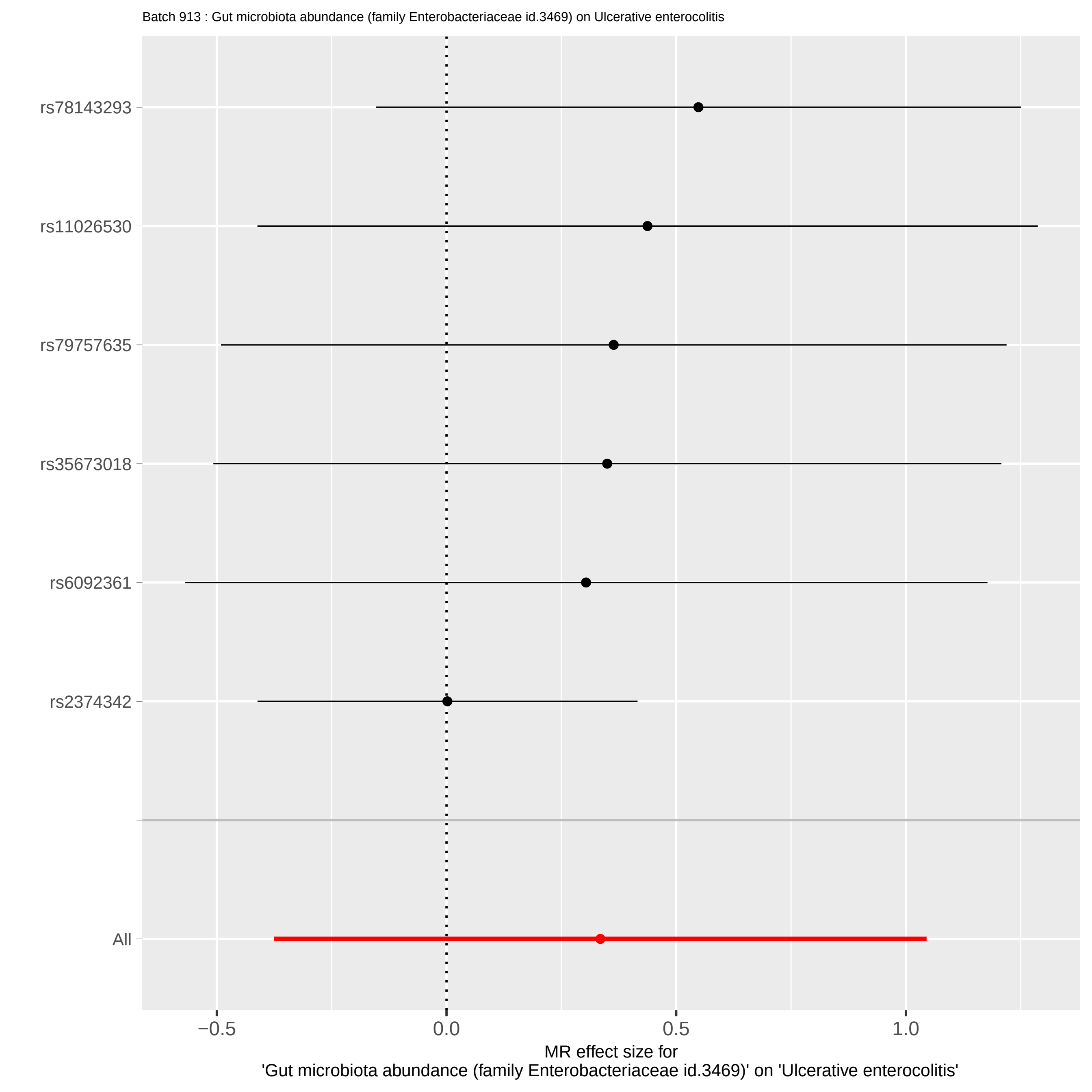

Batch 913 : Gut microbiota abundance (family Enterobacteriaceae id.3469) on Ulcerative enterocolitis
rs78143293
rs11026530
rs79757635
rs35673018
rs6092361
rs2374342
All
−0.5
0.0
0.5
1.0
MR effect size for
'Gut microbiota abundance (family Enterobacteriaceae id.3469)' on 'Ulcerative enterocolitis'

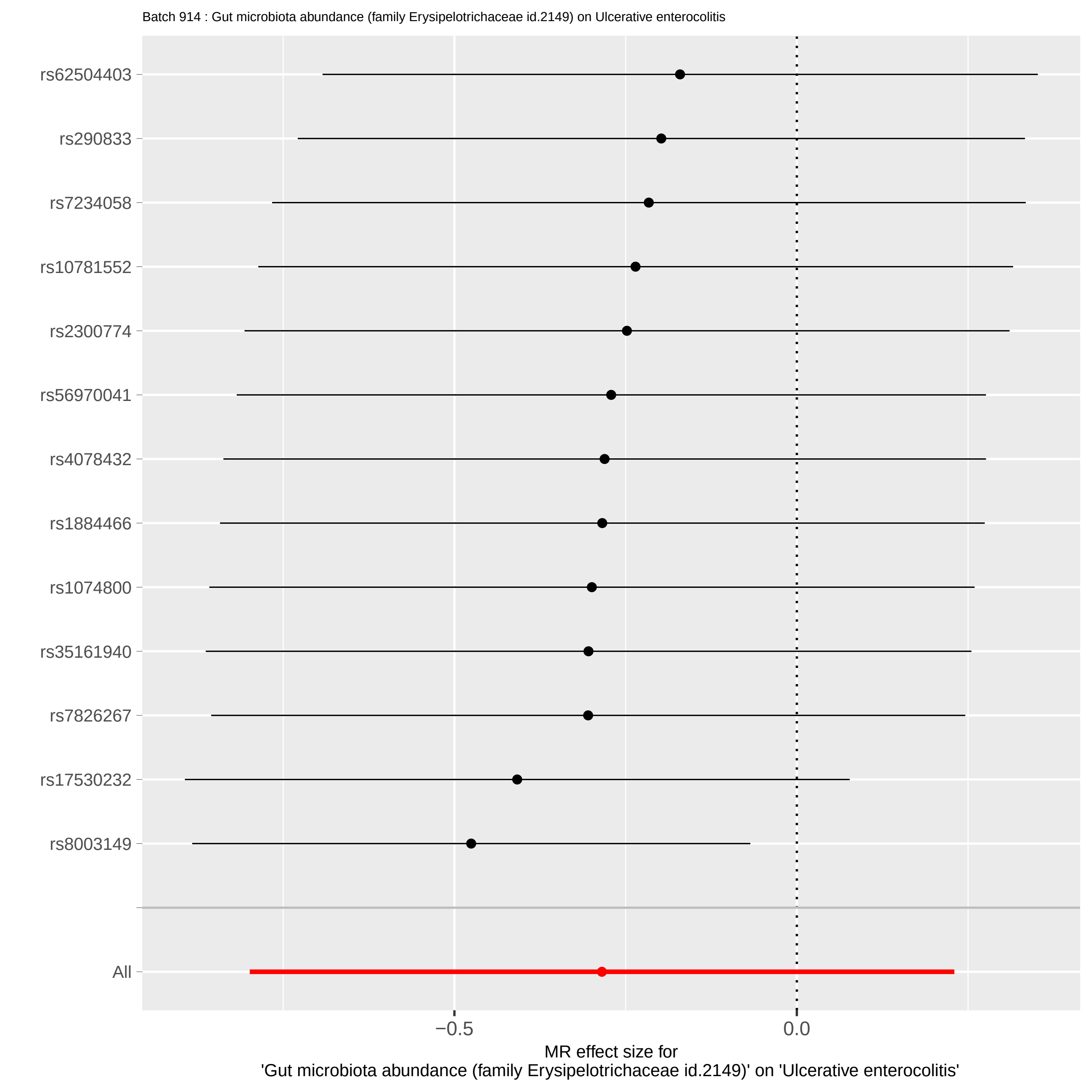

Batch 914 : Gut microbiota abundance (family Erysipelotrichaceae id.2149) on Ulcerative enterocolitis
rs62504403
rs290833
rs7234058
rs10781552
rs2300774
rs56970041
rs4078432
rs1884466
rs1074800
rs35161940
rs7826267
rs17530232
rs8003149
All
−0.5
0.0
MR effect size for
'Gut microbiota abundance (family Erysipelotrichaceae id.2149)' on 'Ulcerative enterocolitis'

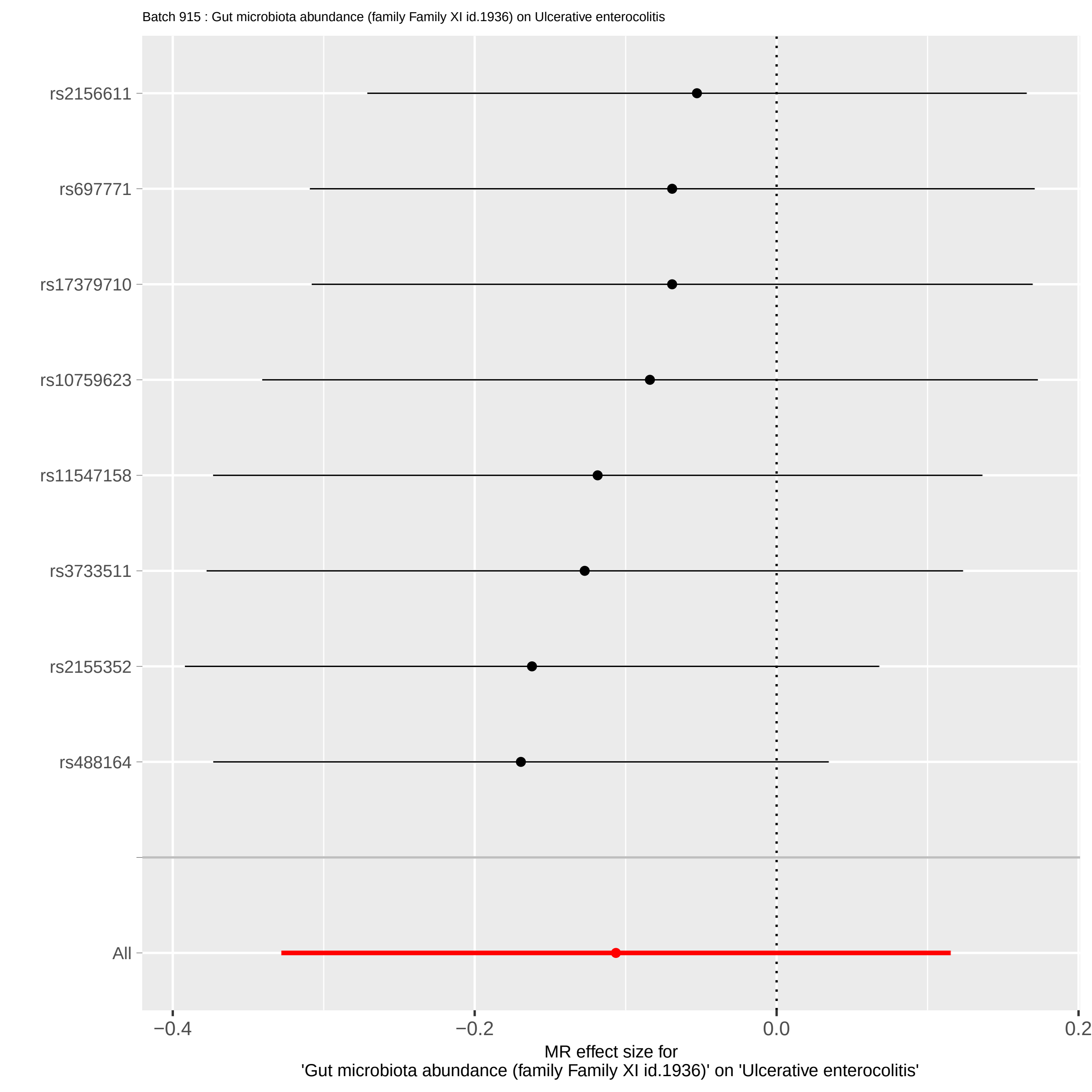
Batch 915 : Gut microbiota abundance (family Family XI id.1936) on Ulcerative enterocolitis
rs2156611
rs697771
rs17379710
rs10759623
rs11547158
rs3733511
rs2155352
rs488164
All
−0.4
−0.2
0.0
0.2
MR effect size for
'Gut microbiota abundance (family Family XI id.1936)' on 'Ulcerative enterocolitis'

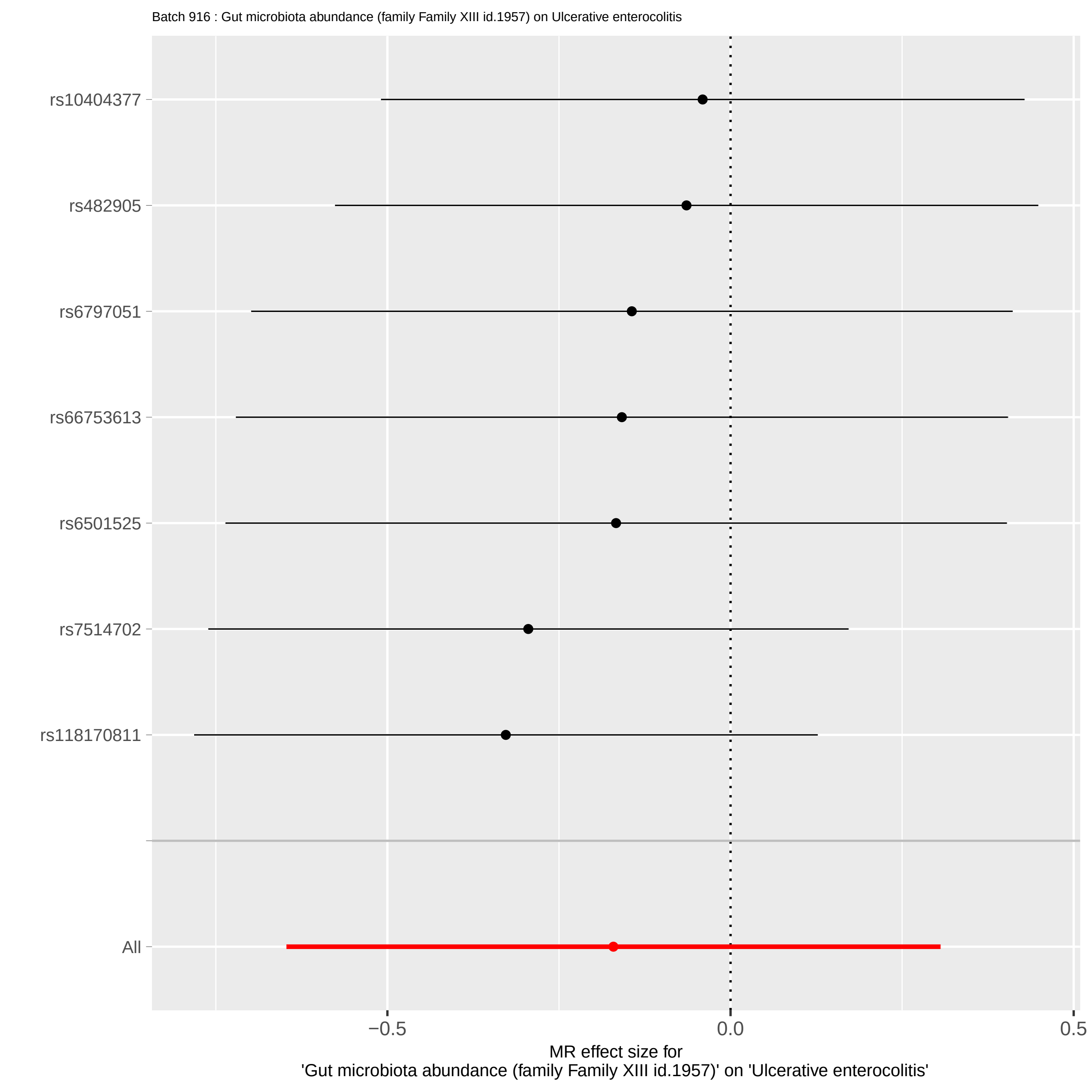
Batch 916 : Gut microbiota abundance (family Family XIII id.1957) on Ulcerative enterocolitis
rs10404377
rs482905
rs6797051
rs66753613
rs6501525
rs7514702
rs118170811
All
−0.5
0.0
0.5
MR effect size for
'Gut microbiota abundance (family Family XIII id.1957)' on 'Ulcerative enterocolitis'

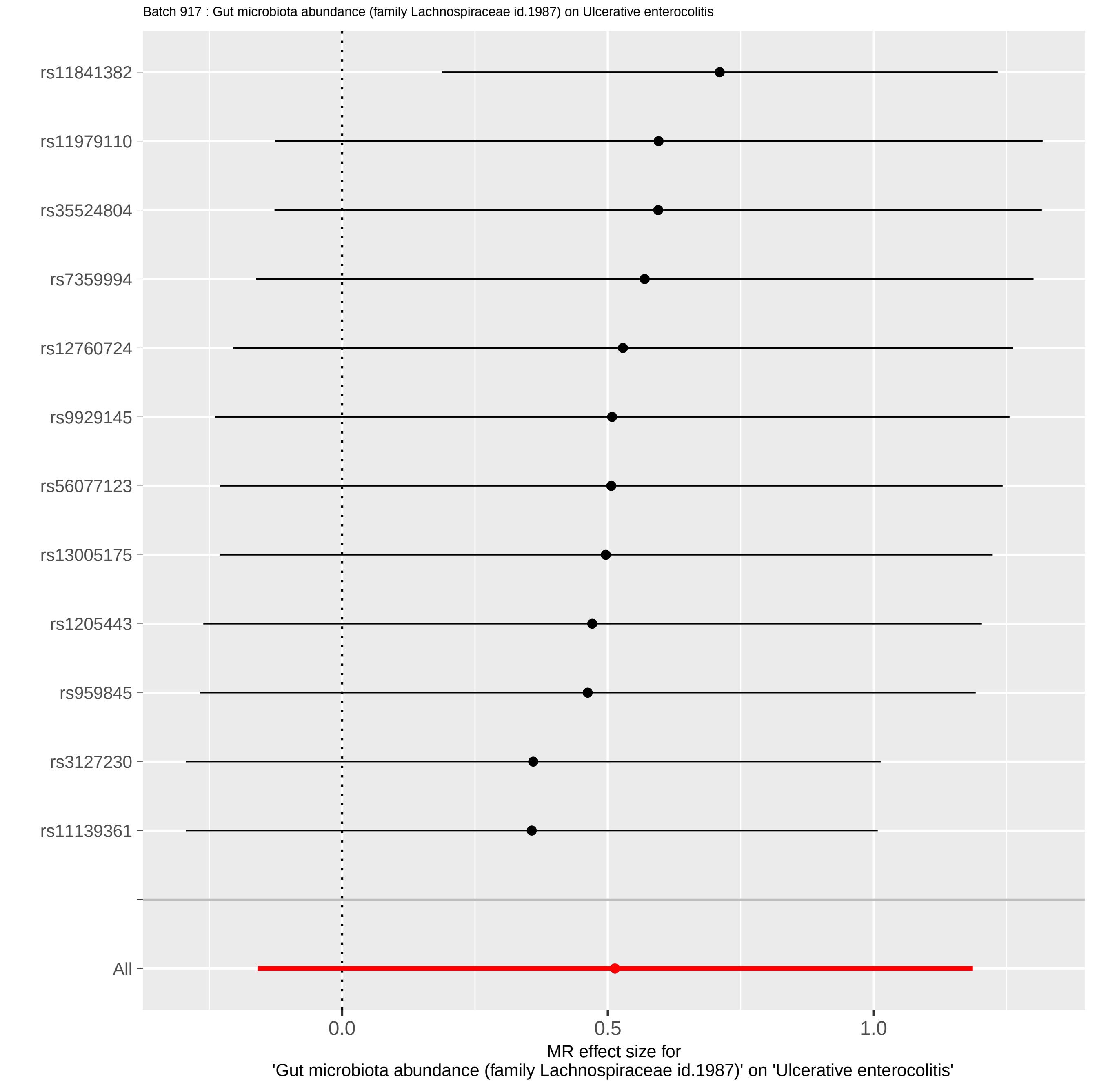

Batch 917 : Gut microbiota abundance (family Lachnospiraceae id.1987) on Ulcerative enterocolitis
rs11841382
rs11979110
rs35524804
rs7359994
rs12760724
rs9929145
rs56077123
rs13005175
rs1205443
rs959845
rs3127230
rs11139361
All
0.0
0.5
1.0
MR effect size for
'Gut microbiota abundance (family Lachnospiraceae id.1987)' on 'Ulcerative enterocolitis'

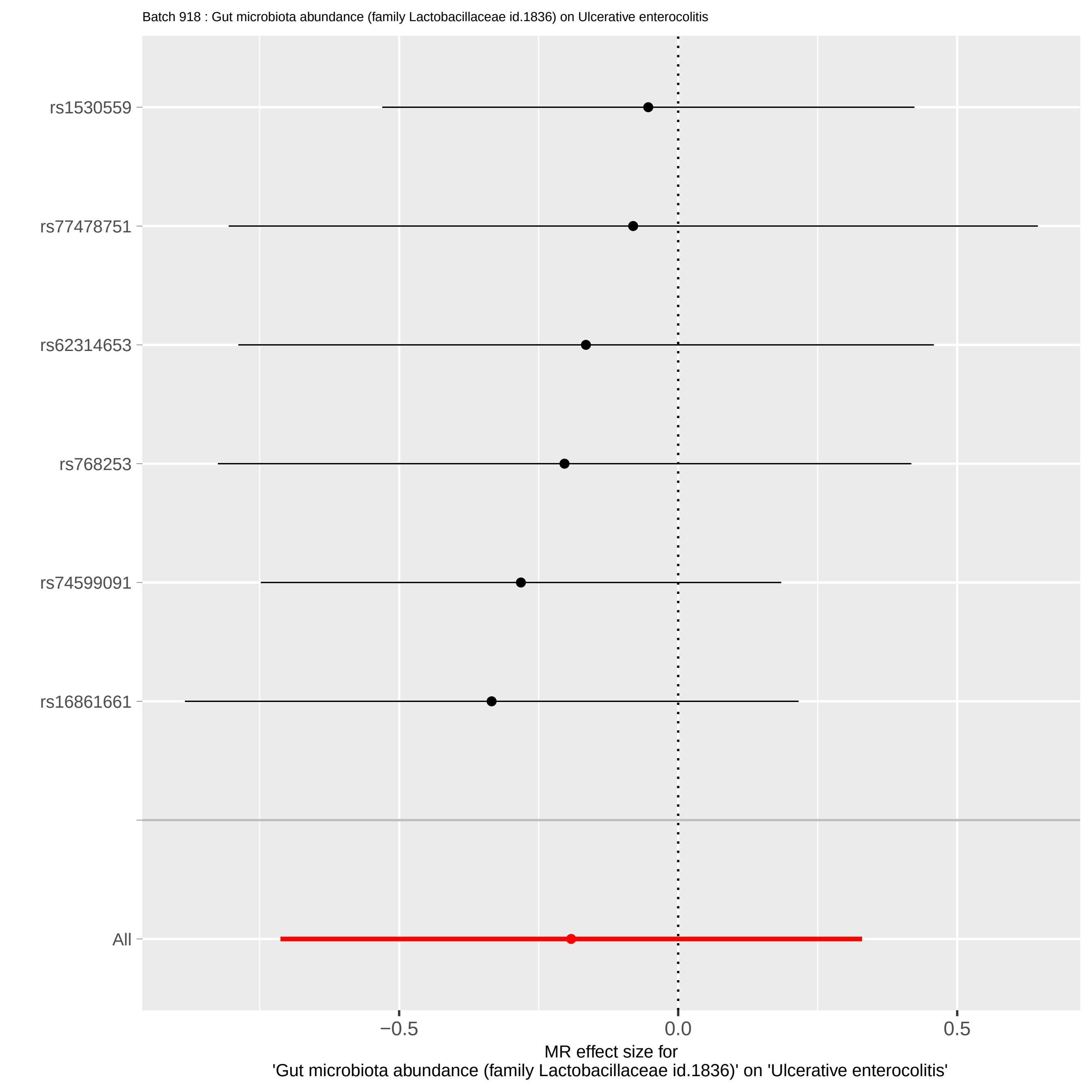
Batch 918 : Gut microbiota abundance (family Lactobacillaceae id.1836) on Ulcerative enterocolitis
rs1530559
rs77478751
rs62314653
rs768253
rs74599091
rs16861661
All
−0.5
0.0
0.5
MR effect size for
'Gut microbiota abundance (family Lactobacillaceae id.1836)' on 'Ulcerative enterocolitis'

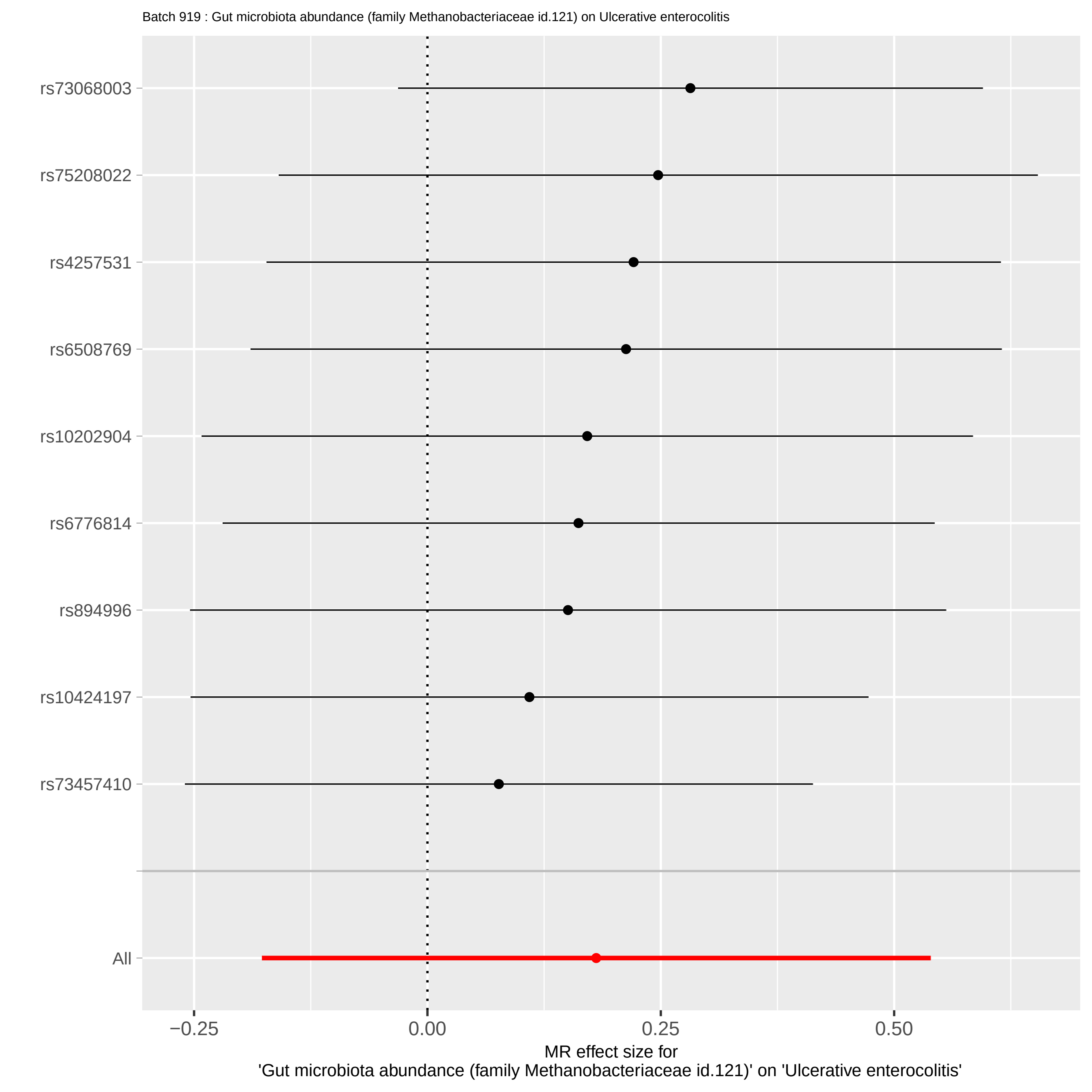
Batch 919 : Gut microbiota abundance (family Methanobacteriaceae id.121) on Ulcerative enterocolitis
rs73068003
rs75208022
rs4257531
rs6508769
rs10202904
rs6776814
rs894996
rs10424197
rs73457410
All
−0.25
0.00
0.25
0.50
MR effect size for
'Gut microbiota abundance (family Methanobacteriaceae id.121)' on 'Ulcerative enterocolitis'

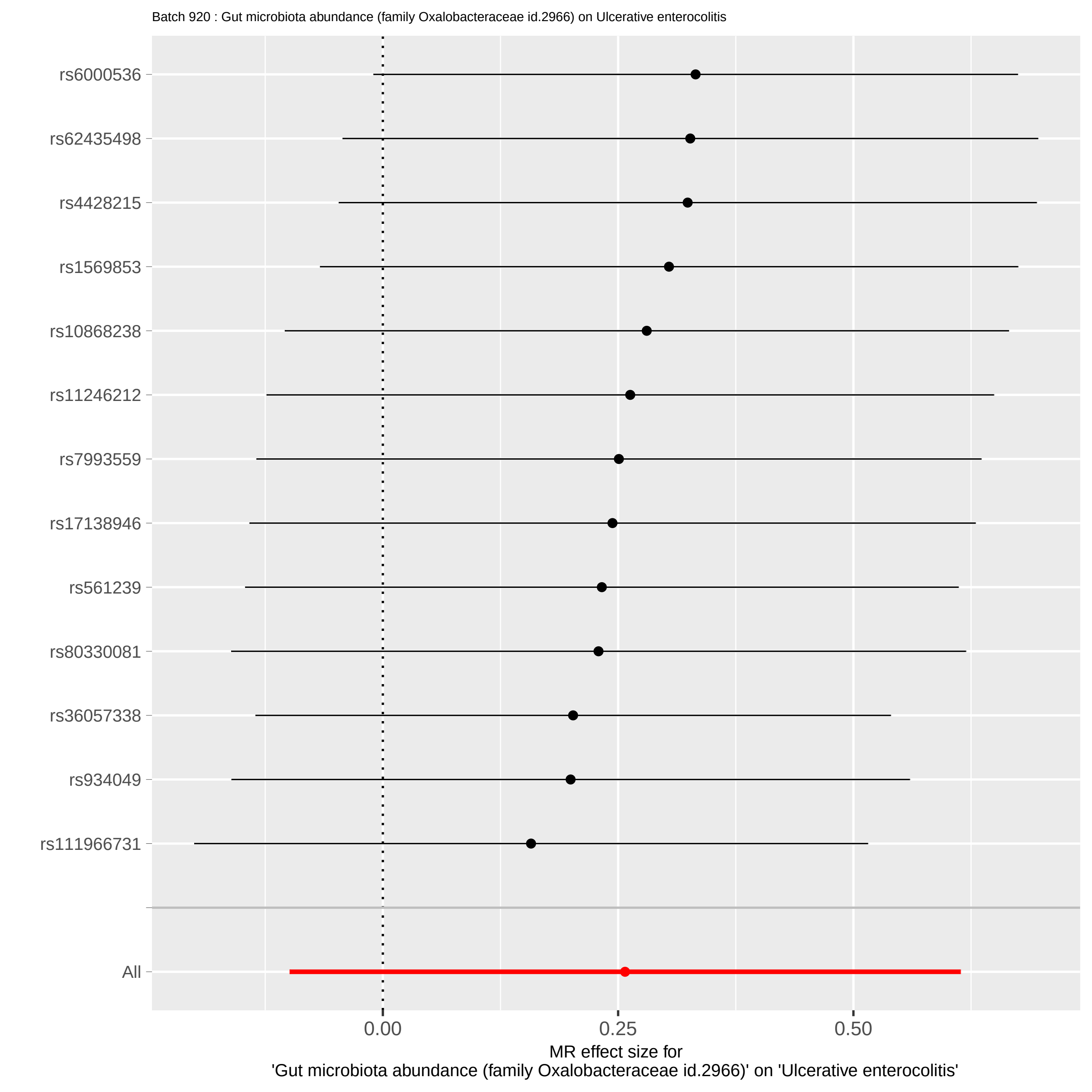

Batch 920 : Gut microbiota abundance (family Oxalobacteraceae id.2966) on Ulcerative enterocolitis
rs6000536
rs62435498
rs4428215
rs1569853
rs10868238
rs11246212
rs7993559
rs17138946
rs561239
rs80330081
rs36057338
rs934049
rs111966731
All
0.00
0.25
0.50
MR effect size for
'Gut microbiota abundance (family Oxalobacteraceae id.2966)' on 'Ulcerative enterocolitis'

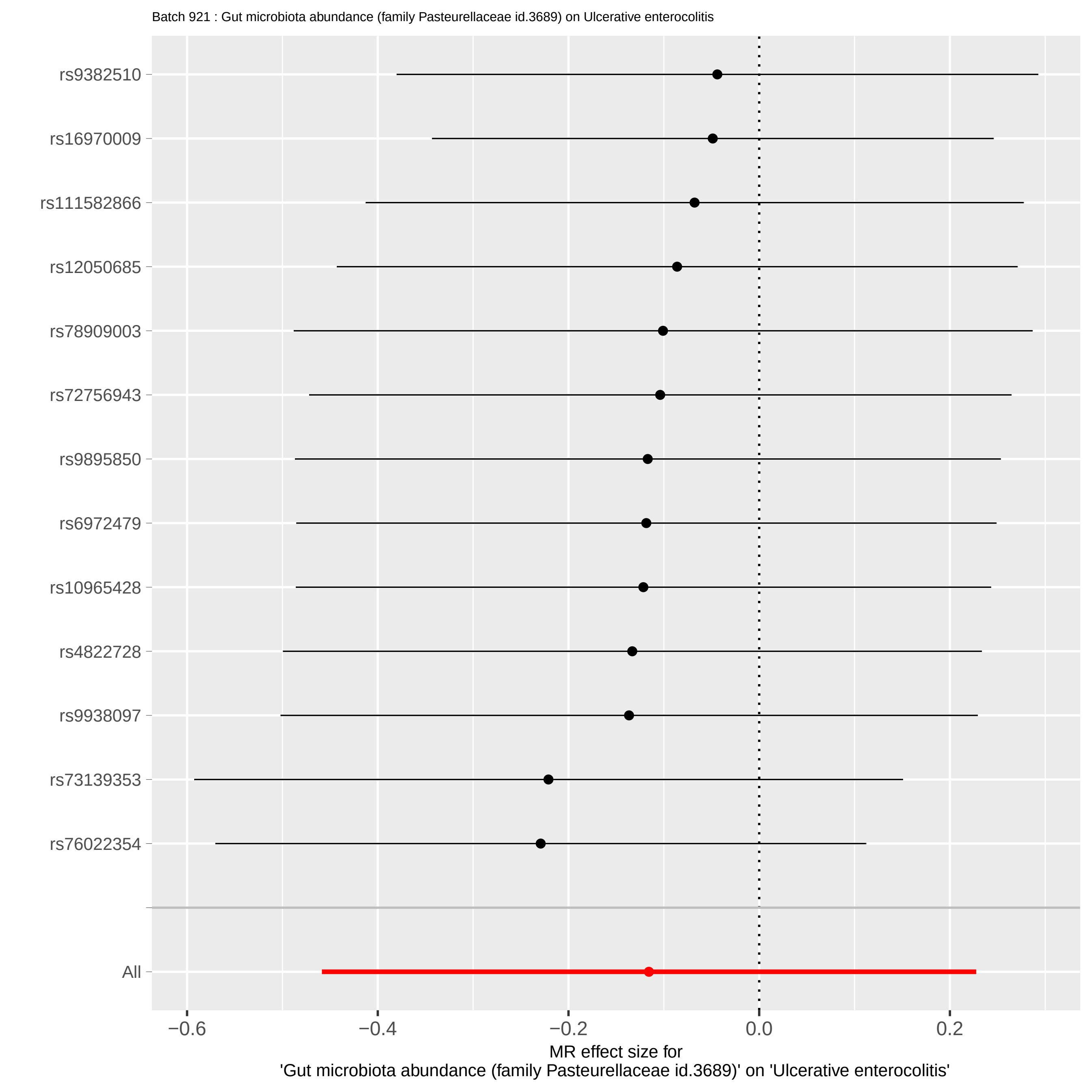

Batch 921 : Gut microbiota abundance (family Pasteurellaceae id.3689) on Ulcerative enterocolitis
rs9382510
rs16970009
rs111582866
rs12050685
rs78909003
rs72756943
rs9895850
rs6972479
rs10965428
rs4822728
rs9938097
rs73139353
rs76022354
All
−0.6
−0.4
−0.2
0.0
0.2
MR effect size for
'Gut microbiota abundance (family Pasteurellaceae id.3689)' on 'Ulcerative enterocolitis'

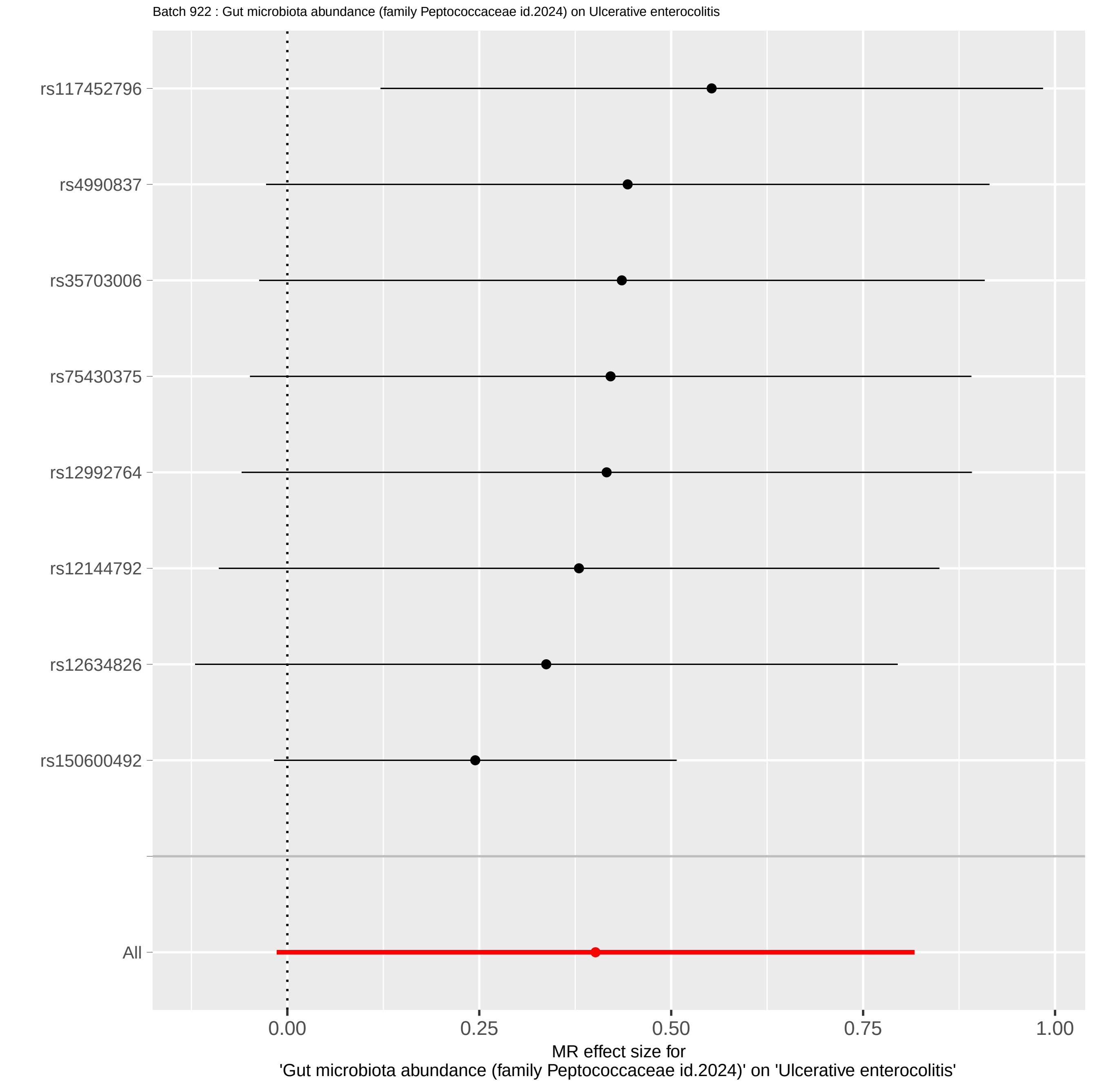
Batch 922 : Gut microbiota abundance (family Peptococcaceae id.2024) on Ulcerative enterocolitis
rs117452796
rs4990837
rs35703006
rs75430375
rs12992764
rs12144792
rs12634826
rs150600492
All
0.00
0.25
0.50
0.75
1.00
MR effect size for
'Gut microbiota abundance (family Peptococcaceae id.2024)' on 'Ulcerative enterocolitis'

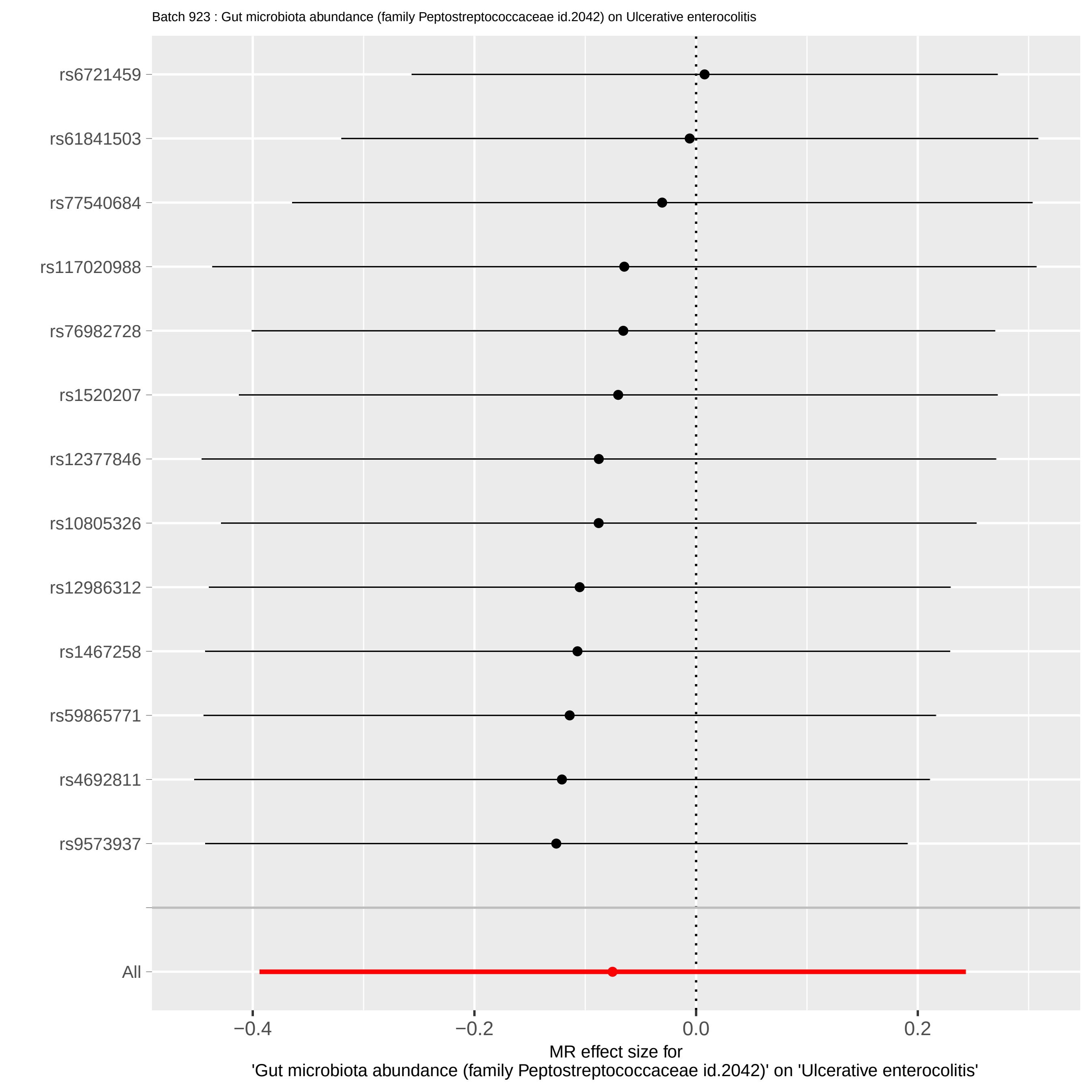
Batch 923 : Gut microbiota abundance (family Peptostreptococcaceae id.2042) on Ulcerative enterocolitis
rs6721459
rs61841503
rs77540684
rs117020988
rs76982728
rs1520207
rs12377846
rs10805326
rs12986312
rs1467258
rs59865771
rs4692811
rs9573937
All
−0.4
−0.2
0.0
0.2
MR effect size for
'Gut microbiota abundance (family Peptostreptococcaceae id.2042)' on 'Ulcerative enterocolitis'

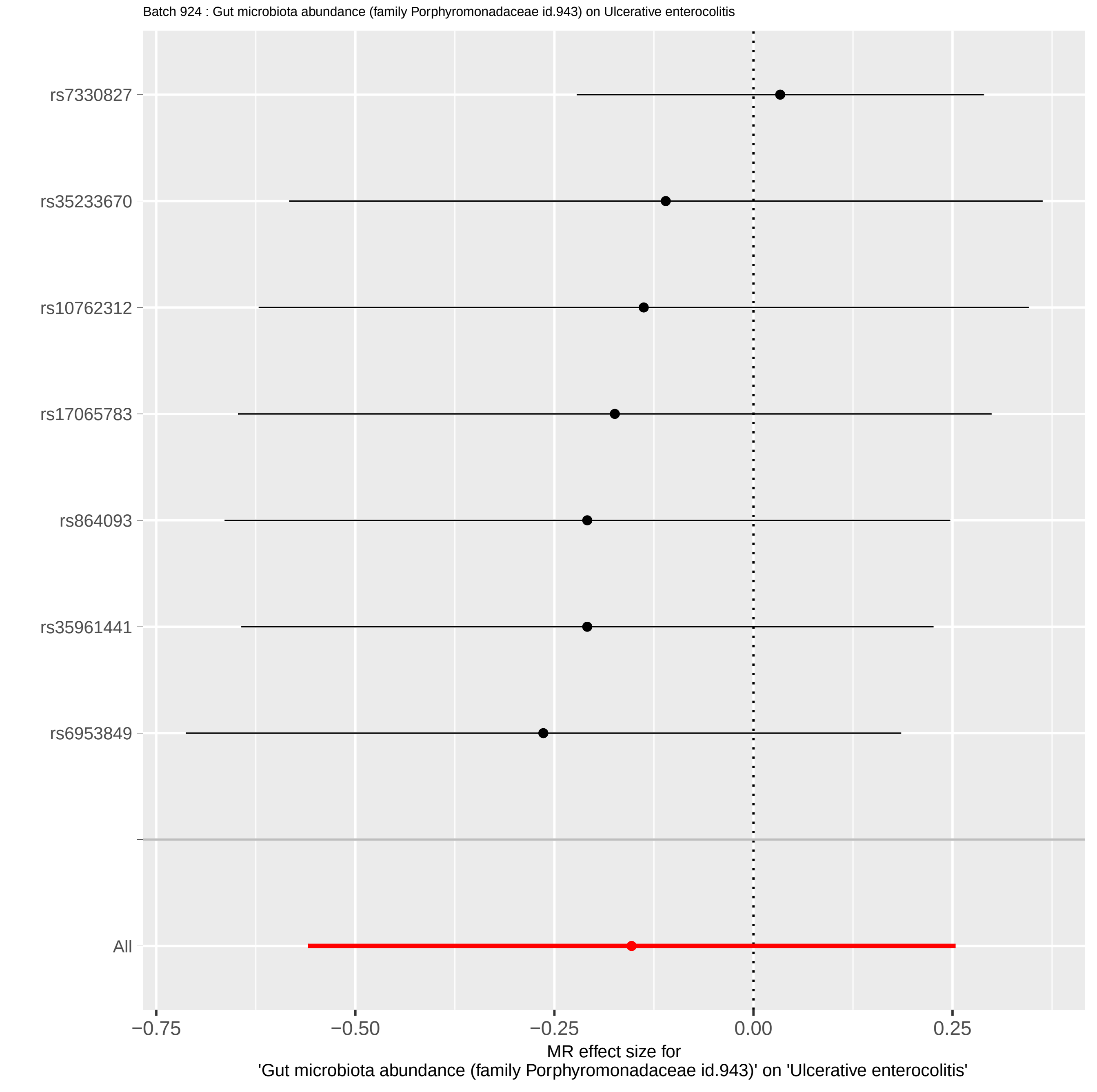
Batch 924 : Gut microbiota abundance (family Porphyromonadaceae id.943) on Ulcerative enterocolitis
rs7330827
rs35233670
rs10762312
rs17065783
rs864093
rs35961441
rs6953849
All
−0.75
−0.50
−0.25
0.00
0.25
MR effect size for
'Gut microbiota abundance (family Porphyromonadaceae id.943)' on 'Ulcerative enterocolitis'

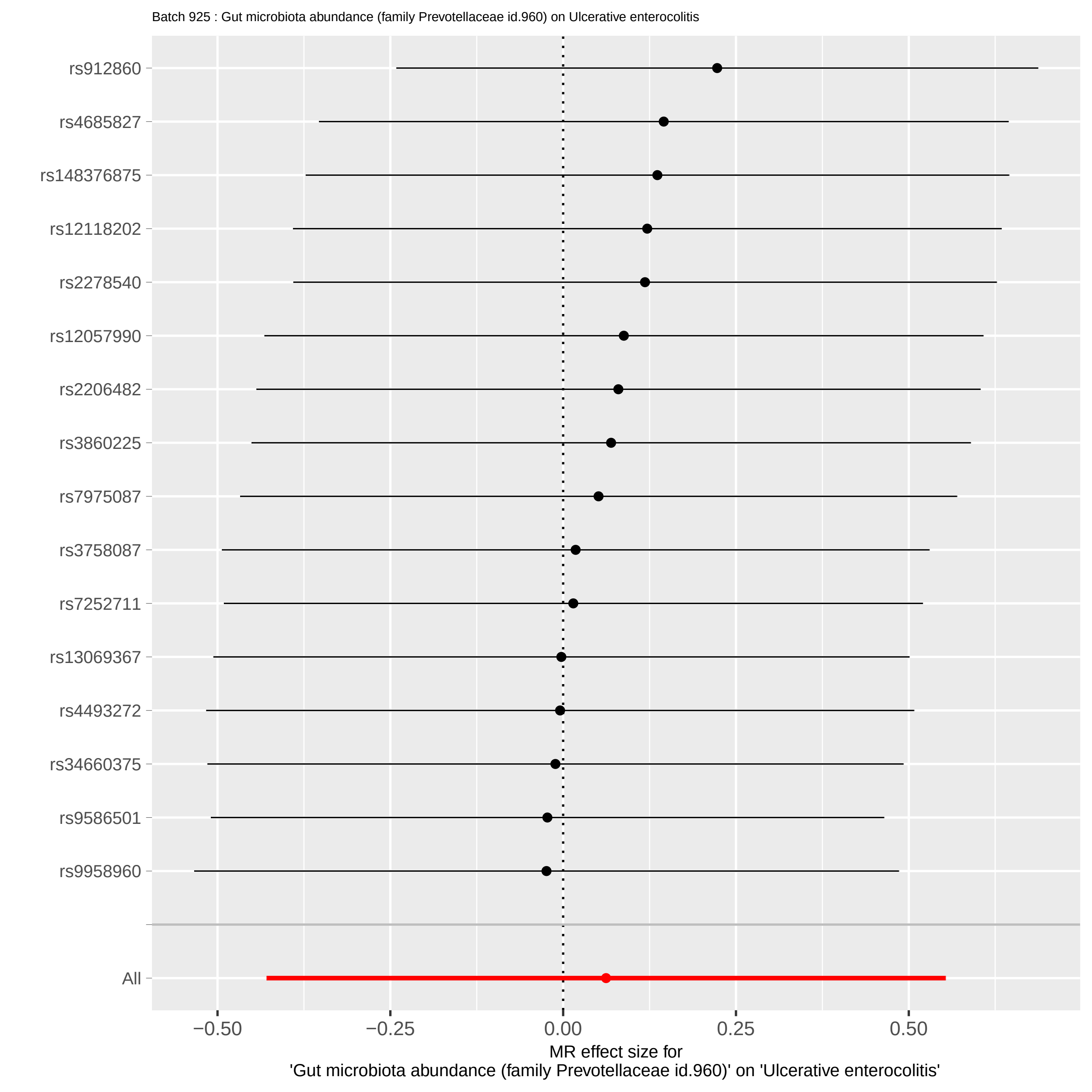
Batch 925 : Gut microbiota abundance (family Prevotellaceae id.960) on Ulcerative enterocolitis
rs912860
rs4685827
rs148376875
rs12118202
rs2278540
rs12057990
rs2206482
rs3860225
rs7975087
rs3758087
rs7252711
rs13069367
rs4493272
rs34660375
rs9586501
rs9958960
All
−0.50
−0.25
0.00
0.25
0.50
MR effect size for
'Gut microbiota abundance (family Prevotellaceae id.960)' on 'Ulcerative enterocolitis'

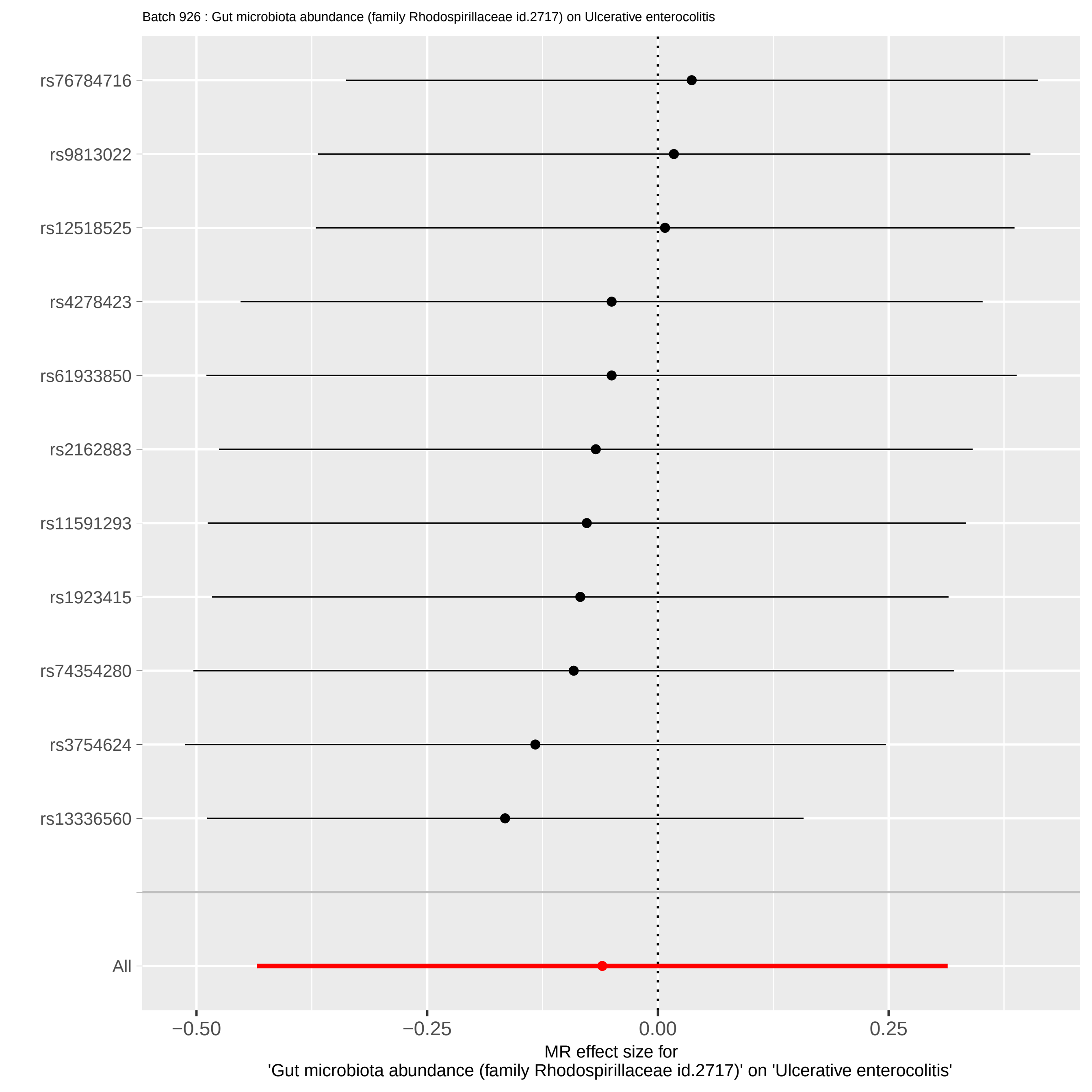
Batch 926 : Gut microbiota abundance (family Rhodospirillaceae id.2717) on Ulcerative enterocolitis
rs76784716
rs9813022
rs12518525
rs4278423
rs61933850
rs2162883
rs11591293
rs1923415
rs74354280
rs3754624
rs13336560
All
−0.50
−0.25
0.00
0.25
MR effect size for
'Gut microbiota abundance (family Rhodospirillaceae id.2717)' on 'Ulcerative enterocolitis'

Batch 927 : Gut microbiota abundance (family Rikenellaceae id.967) on Ulcerative enterocolitis

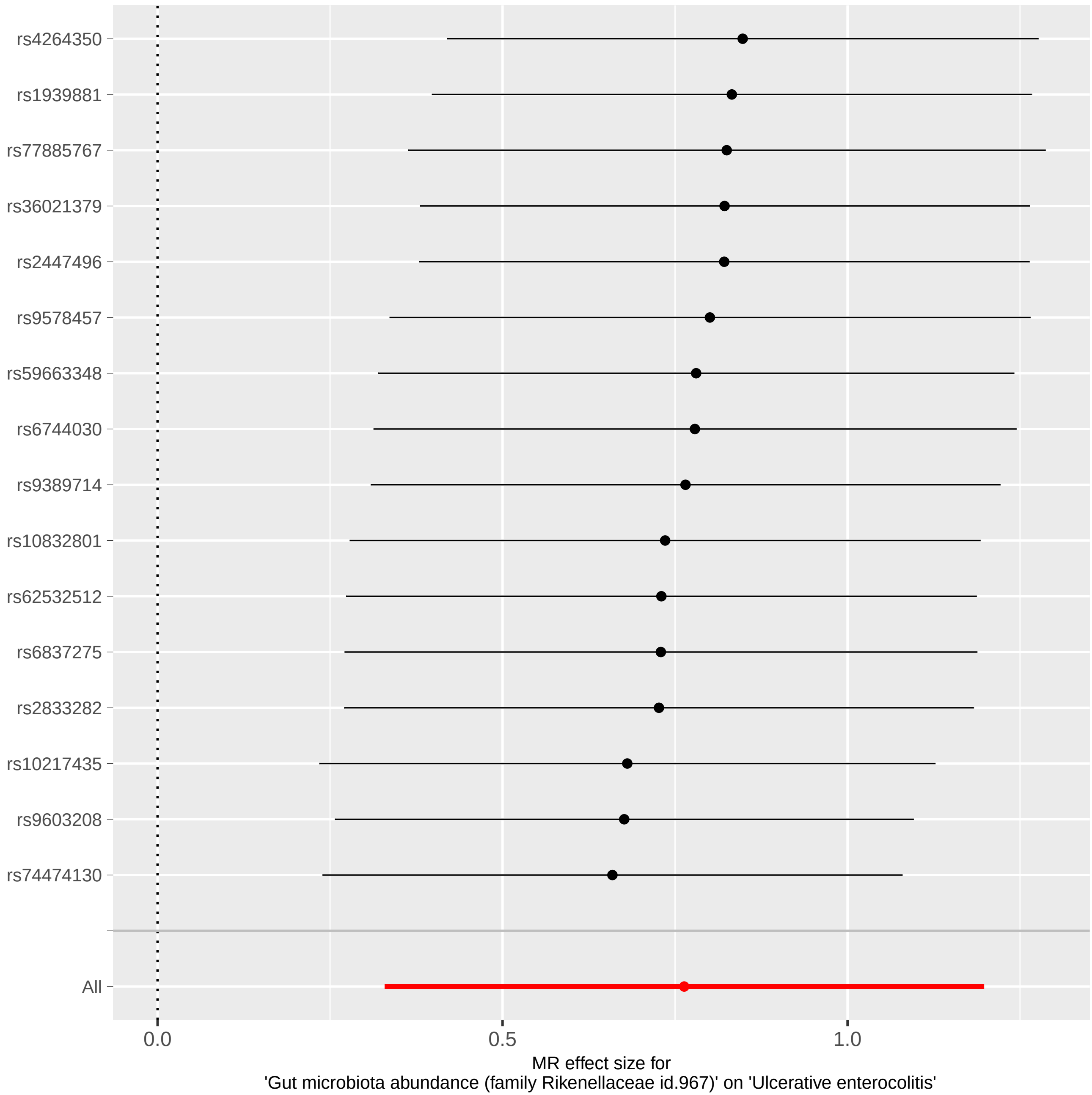

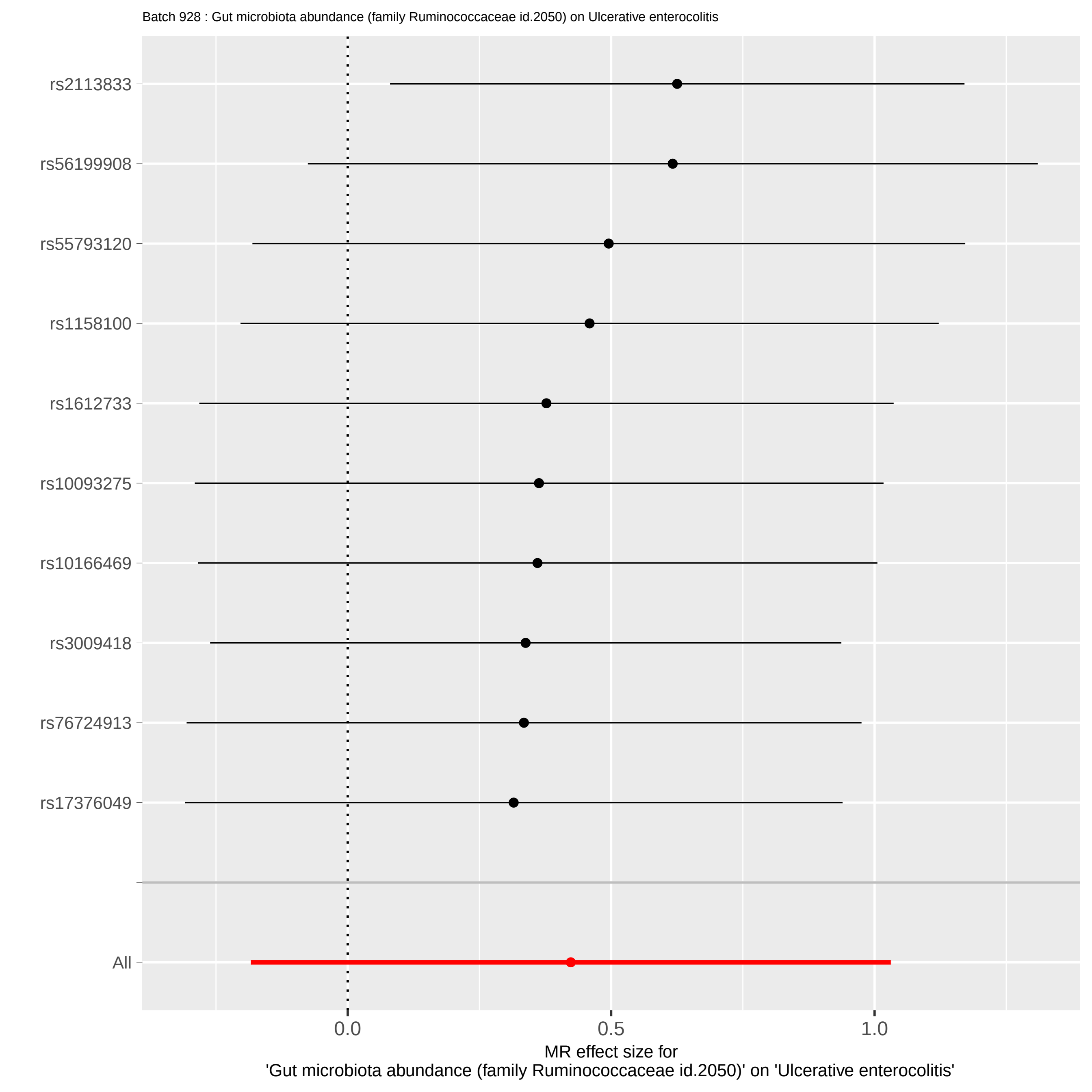
Batch 928 : Gut microbiota abundance (family Ruminococcaceae id.2050) on Ulcerative enterocolitis
rs2113833
rs56199908
rs55793120
rs1158100
rs1612733
rs10093275
rs10166469
rs3009418
rs76724913
rs17376049
All
0.0
0.5
1.0
MR effect size for
'Gut microbiota abundance (family Ruminococcaceae id.2050)' on 'Ulcerative enterocolitis'

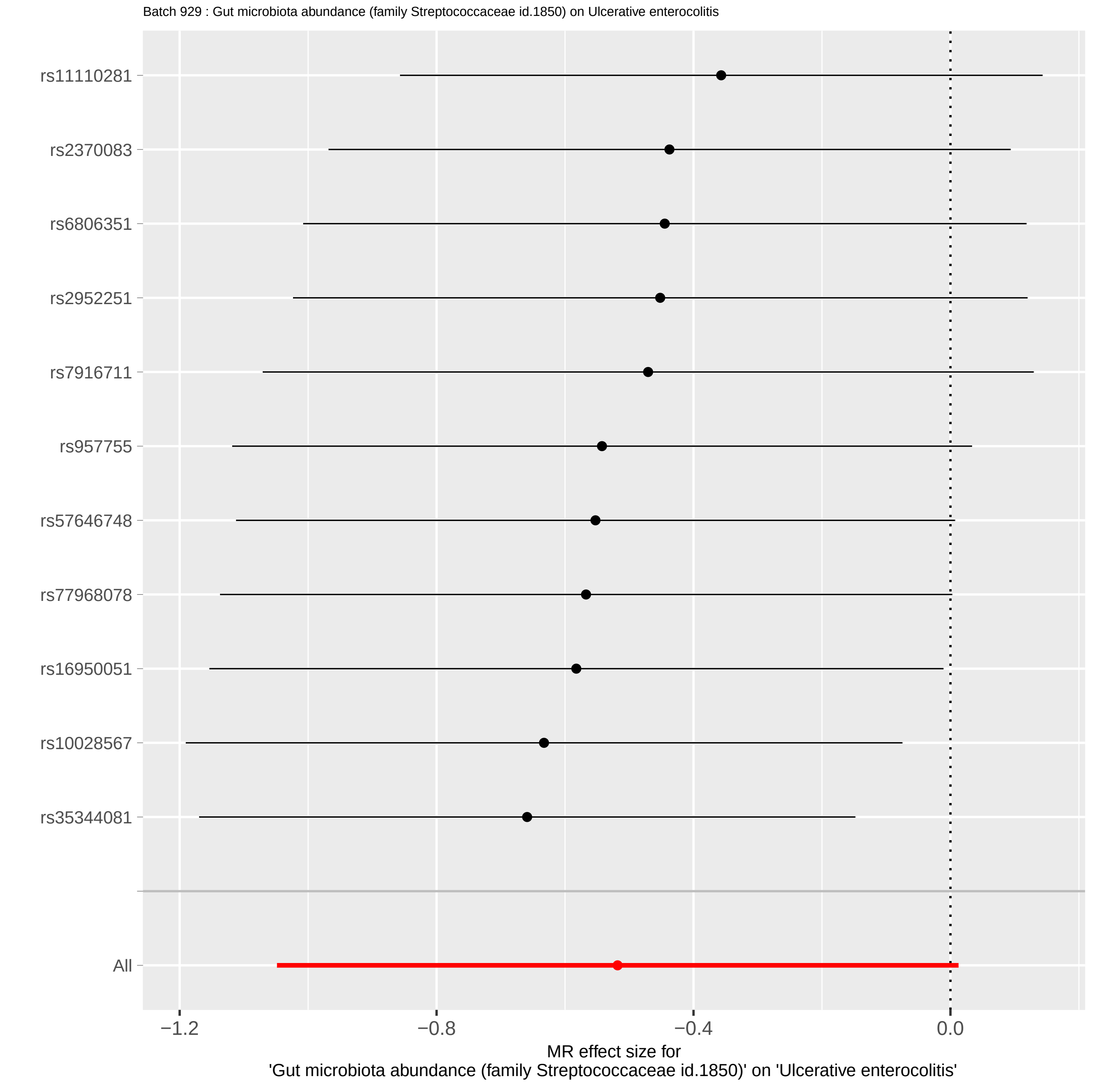

Batch 929 : Gut microbiota abundance (family Streptococcaceae id.1850) on Ulcerative enterocolitis
rs11110281
rs2370083
rs6806351
rs2952251
rs7916711
rs957755
rs57646748
rs77968078
rs16950051
rs10028567
rs35344081
All
−1.2
−0.8
−0.4
0.0
MR effect size for
'Gut microbiota abundance (family Streptococcaceae id.1850)' on 'Ulcerative enterocolitis'

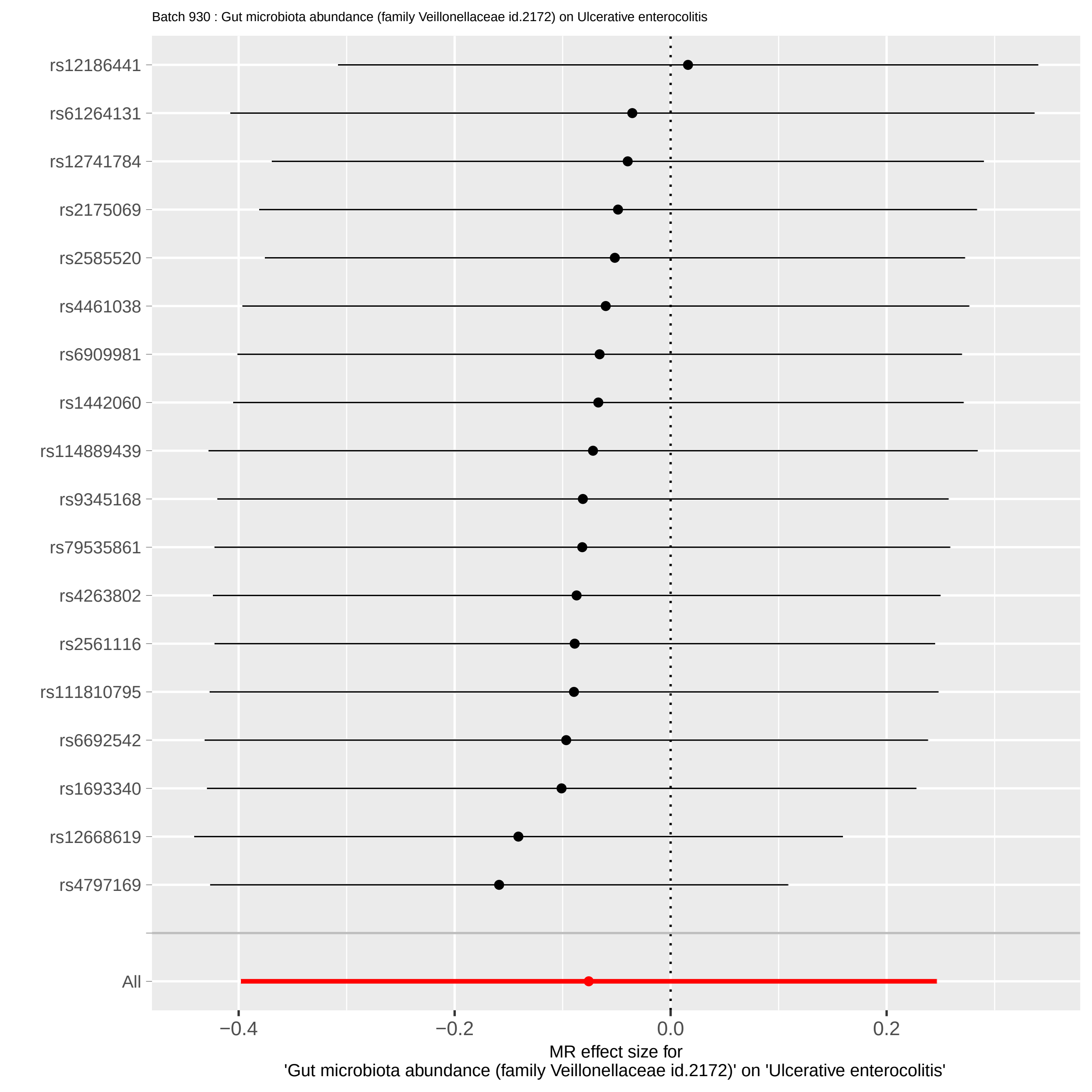

Batch 930 : Gut microbiota abundance (family Veillonellaceae id.2172) on Ulcerative enterocolitis
rs12186441
rs61264131
rs12741784
rs2175069
rs2585520
rs4461038
rs6909981
rs1442060
rs114889439
rs9345168
rs79535861
rs4263802
rs2561116
rs111810795
rs6692542
rs1693340
rs12668619
rs4797169
All
−0.4
−0.2
0.0
0.2
MR effect size for
'Gut microbiota abundance (family Veillonellaceae id.2172)' on 'Ulcerative enterocolitis'

Batch 931 : Gut microbiota abundance (family Verrucomicrobiaceae id.4036) on Ulcerative enterocolitis

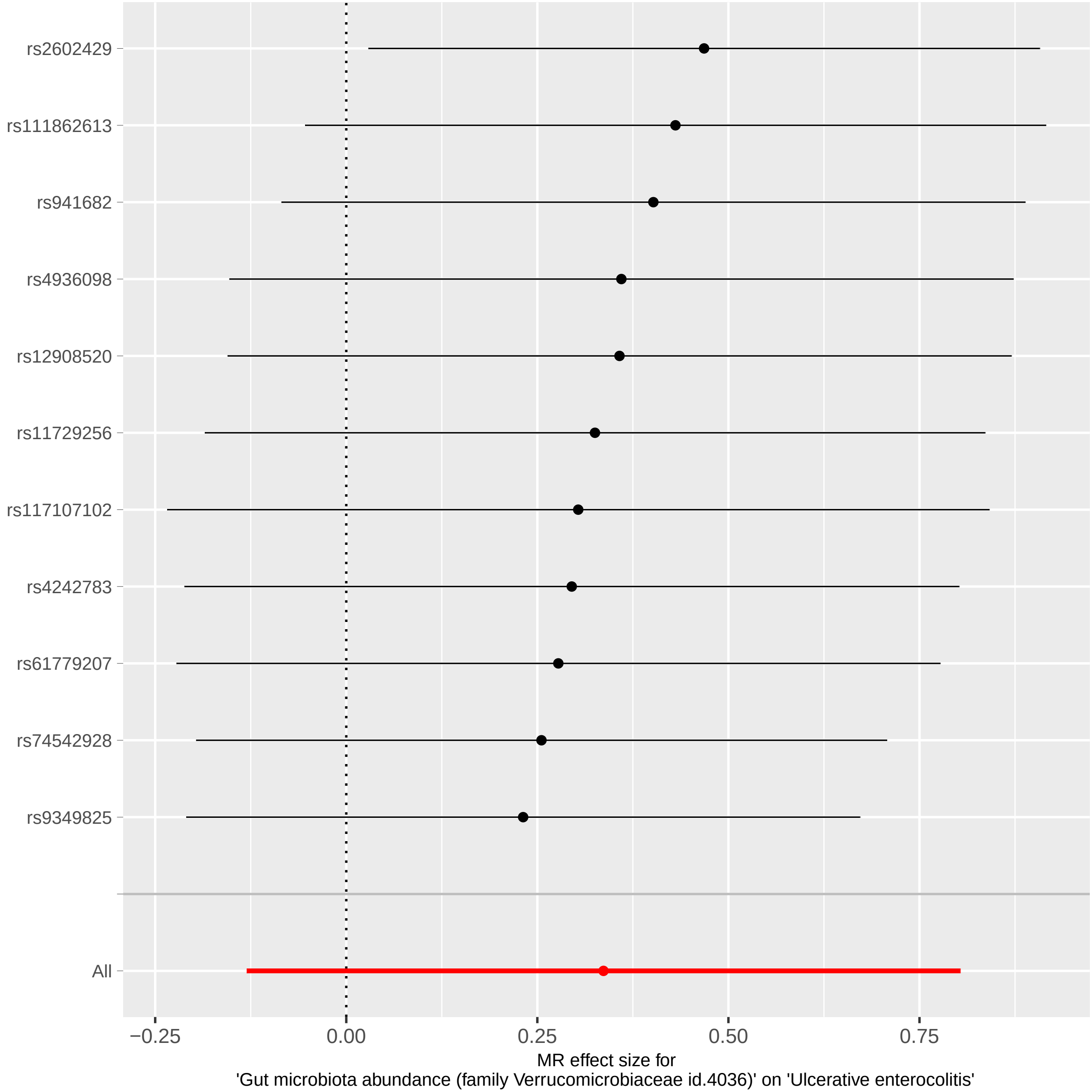

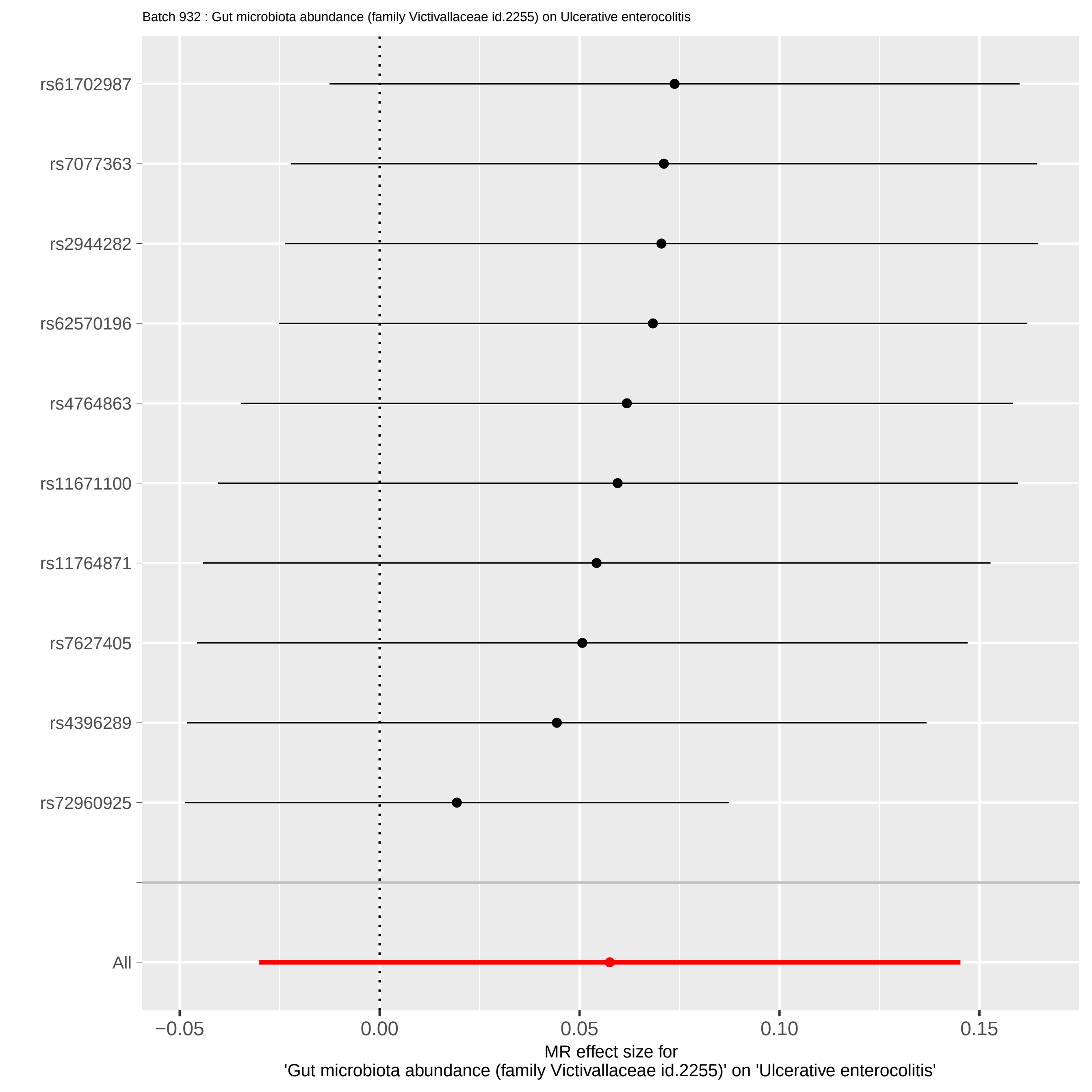
Batch 932 : Gut microbiota abundance (family Victivallaceae id.2255) on Ulcerative enterocolitis
rs61702987
rs7077363
rs2944282
rs62570196
rs4764863
rs11671100
rs11764871
rs7627405
rs4396289
rs72960925
All
−0.05
0.00
0.05
0.10
0.15
MR effect size for
'Gut microbiota abundance (family Victivallaceae id.2255)' on 'Ulcerative enterocolitis'

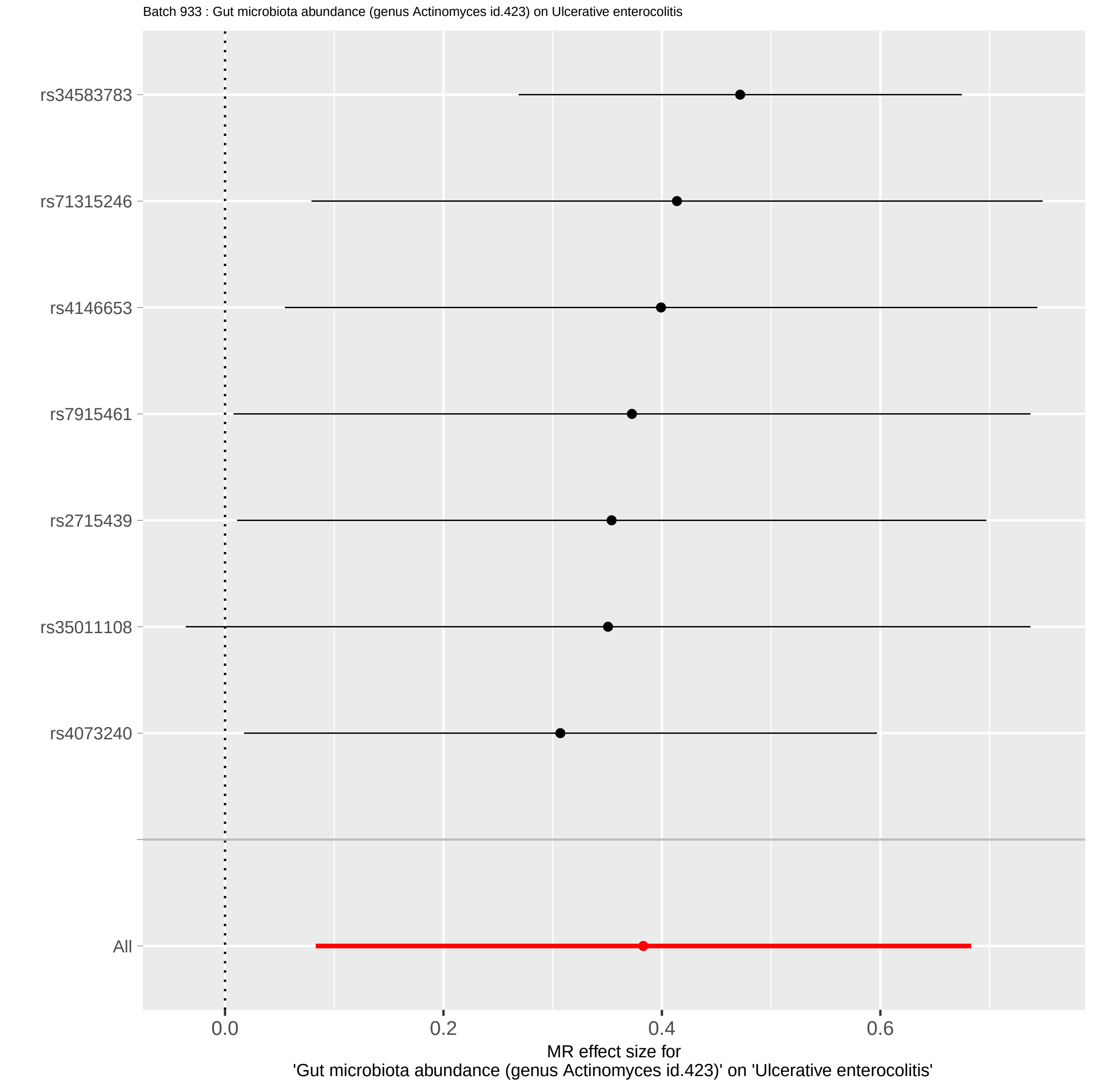

Batch 933 : Gut microbiota abundance (genus Actinomyces id.423) on Ulcerative enterocolitis
rs34583783
rs71315246
rs4146653
rs7915461
rs2715439
rs35011108
rs4073240
All
0.0
0.2
0.4
0.6
MR effect size for
'Gut microbiota abundance (genus Actinomyces id.423)' on 'Ulcerative enterocolitis'

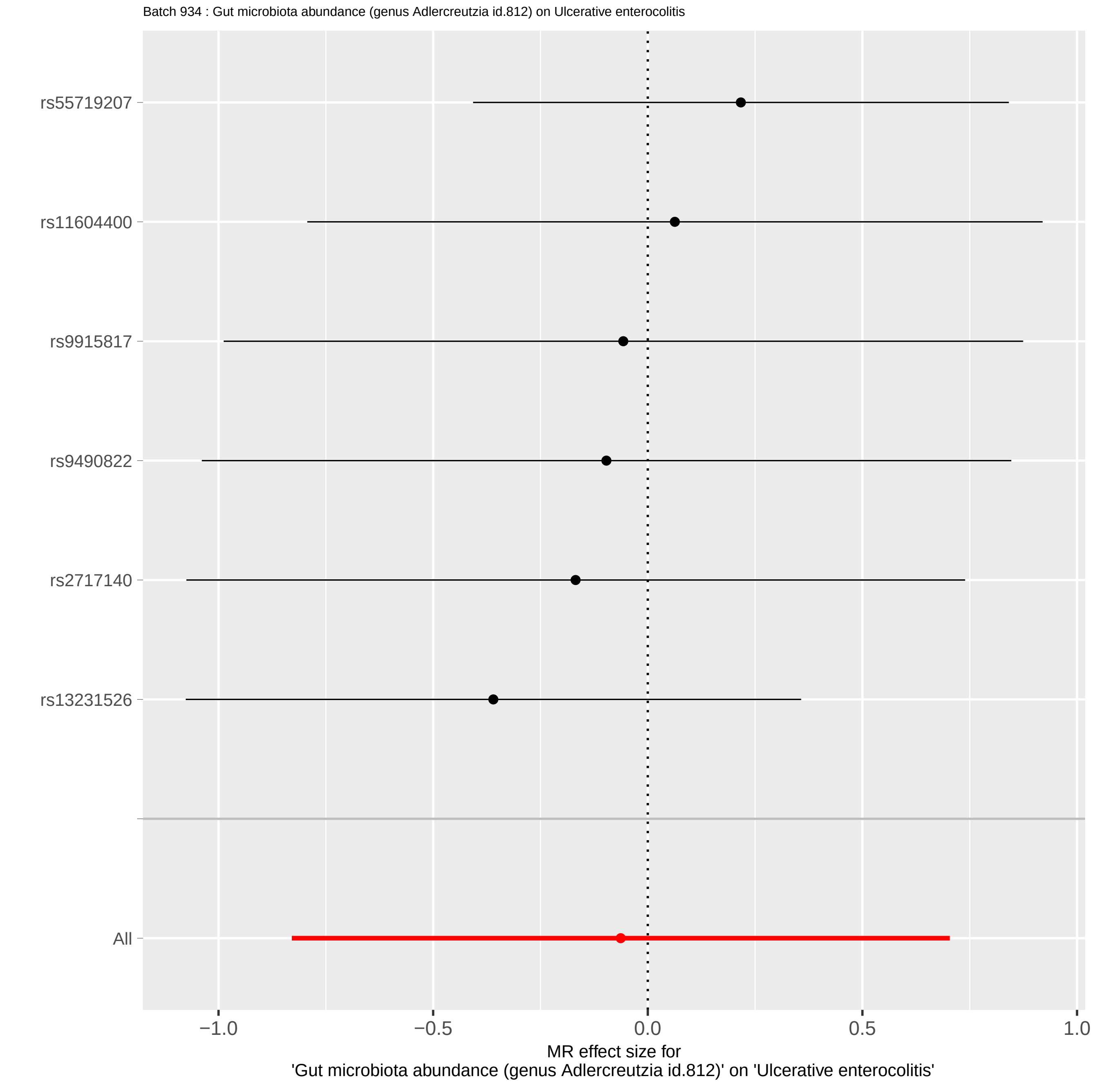
Batch 934 : Gut microbiota abundance (genus Adlercreutzia id.812) on Ulcerative enterocolitis
rs55719207
rs11604400
rs9915817
rs9490822
rs2717140
rs13231526
All
−1.0
−0.5
0.0
0.5
1.0
MR effect size for
'Gut microbiota abundance (genus Adlercreutzia id.812)' on 'Ulcerative enterocolitis'

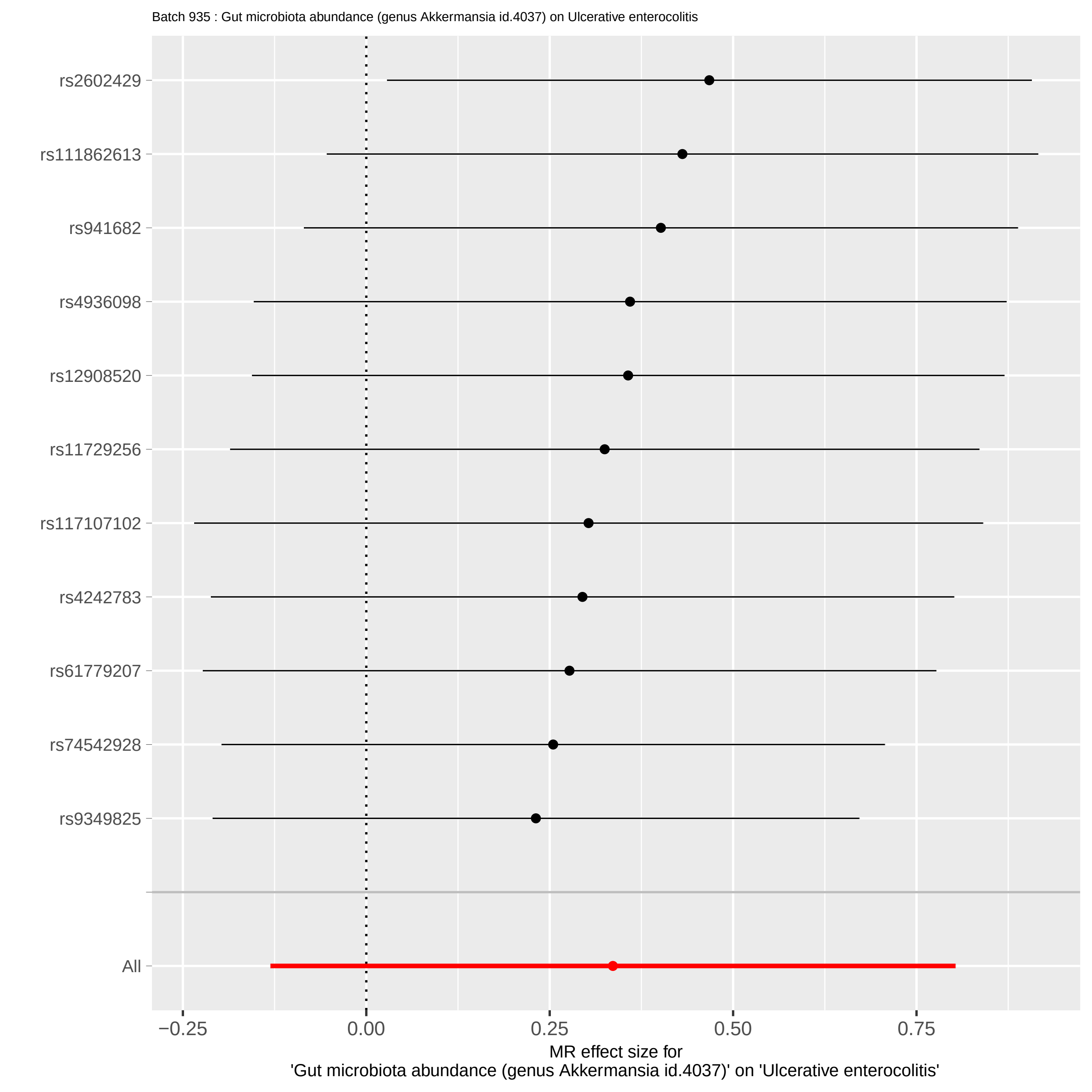

Batch 935 : Gut microbiota abundance (genus Akkermansia id.4037) on Ulcerative enterocolitis
rs2602429
rs111862613
rs941682
rs4936098
rs12908520
rs11729256
rs117107102
rs4242783
rs61779207
rs74542928
rs9349825
All
−0.25
0.00
0.25
0.50
0.75
MR effect size for
'Gut microbiota abundance (genus Akkermansia id.4037)' on 'Ulcerative enterocolitis'

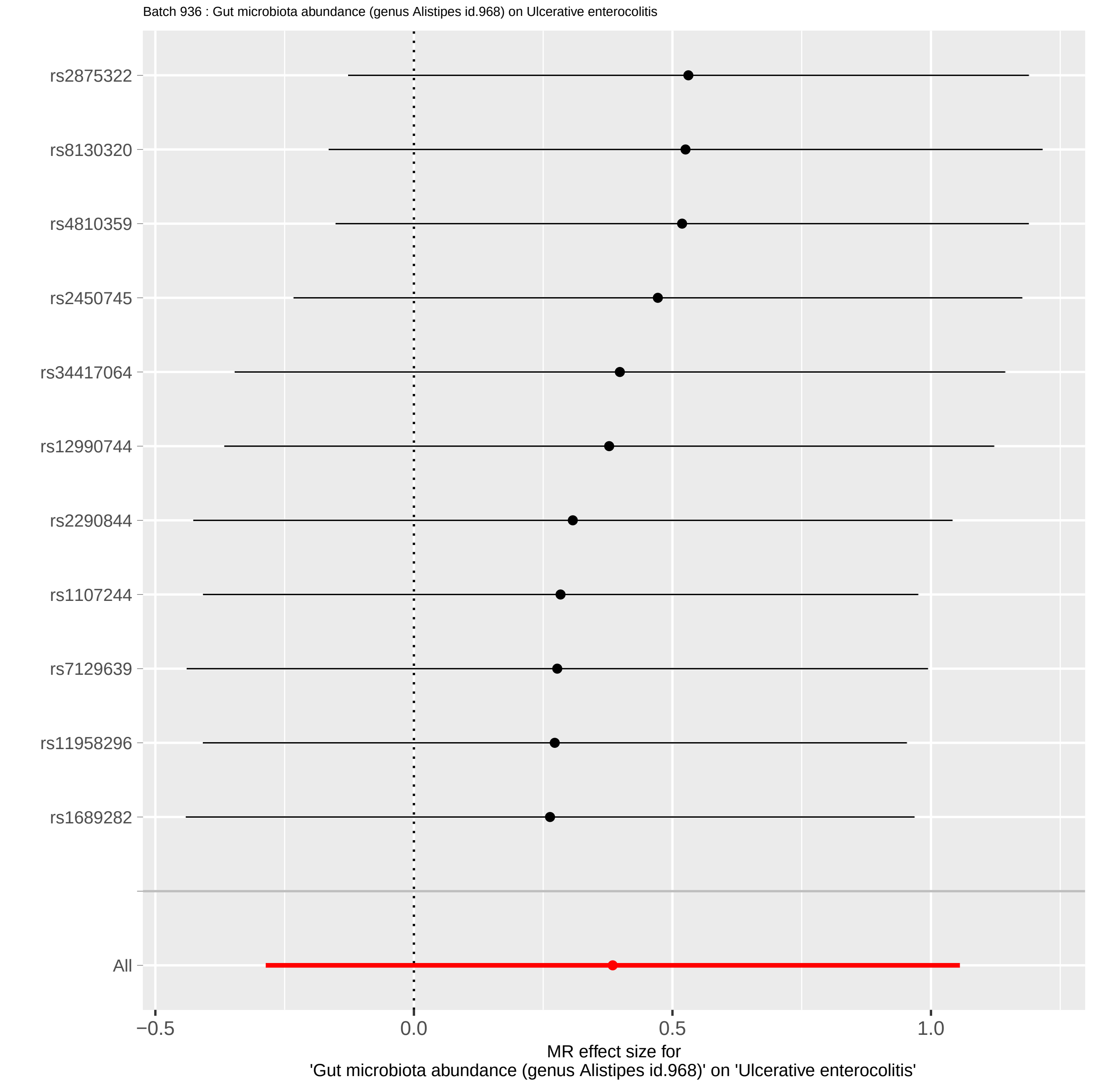
Batch 936 : Gut microbiota abundance (genus Alistipes id.968) on Ulcerative enterocolitis
rs2875322
rs8130320
rs4810359
rs2450745
rs34417064
rs12990744
rs2290844
rs1107244
rs7129639
rs11958296
rs1689282
All
−0.5
0.0
0.5
1.0
MR effect size for
'Gut microbiota abundance (genus Alistipes id.968)' on 'Ulcerative enterocolitis'

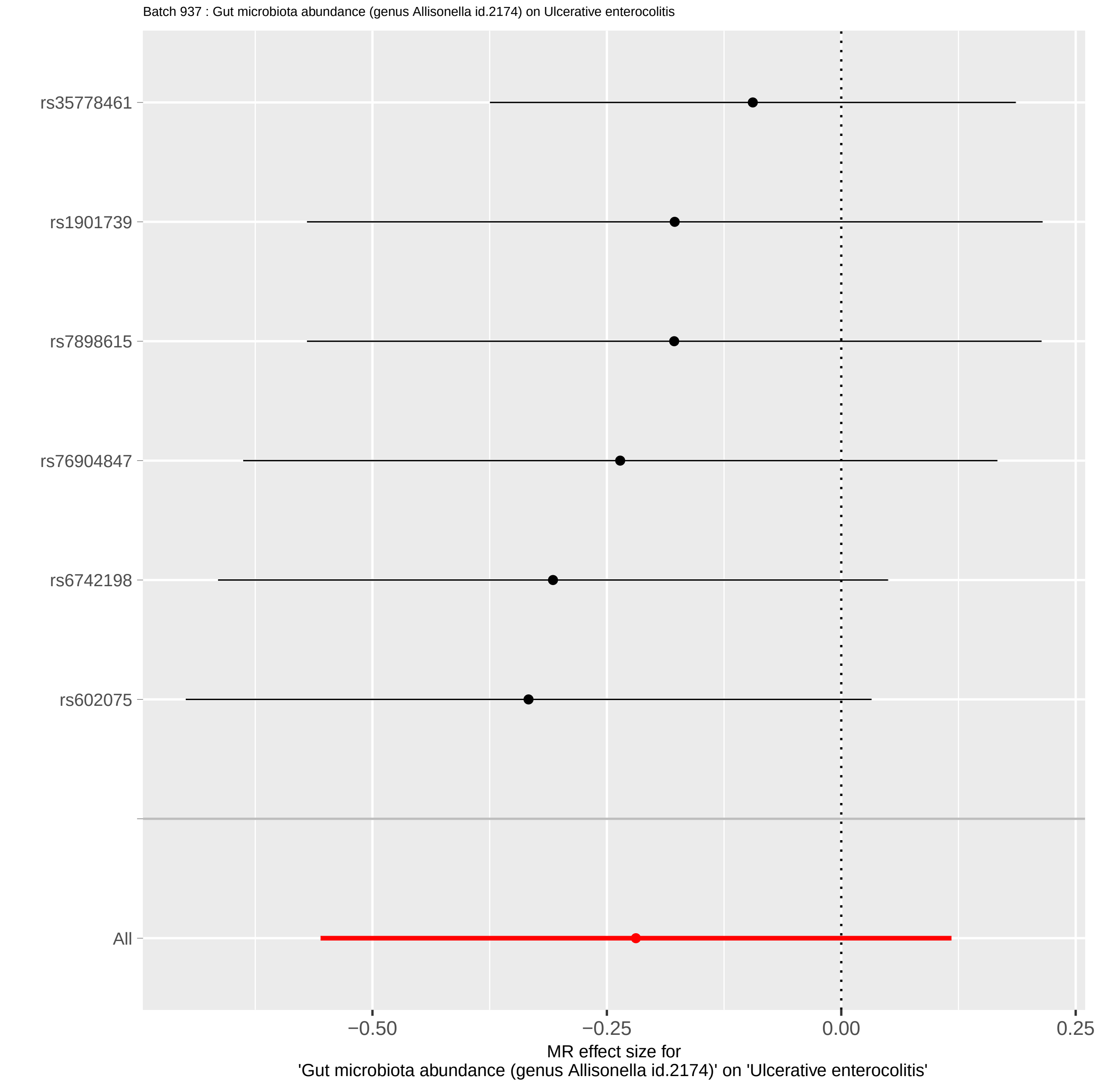
Batch 937 : Gut microbiota abundance (genus Allisonella id.2174) on Ulcerative enterocolitis
rs35778461
rs1901739
rs7898615
rs76904847
rs6742198
rs602075
All
−0.50
−0.25
0.00
0.25
MR effect size for
'Gut microbiota abundance (genus Allisonella id.2174)' on 'Ulcerative enterocolitis'

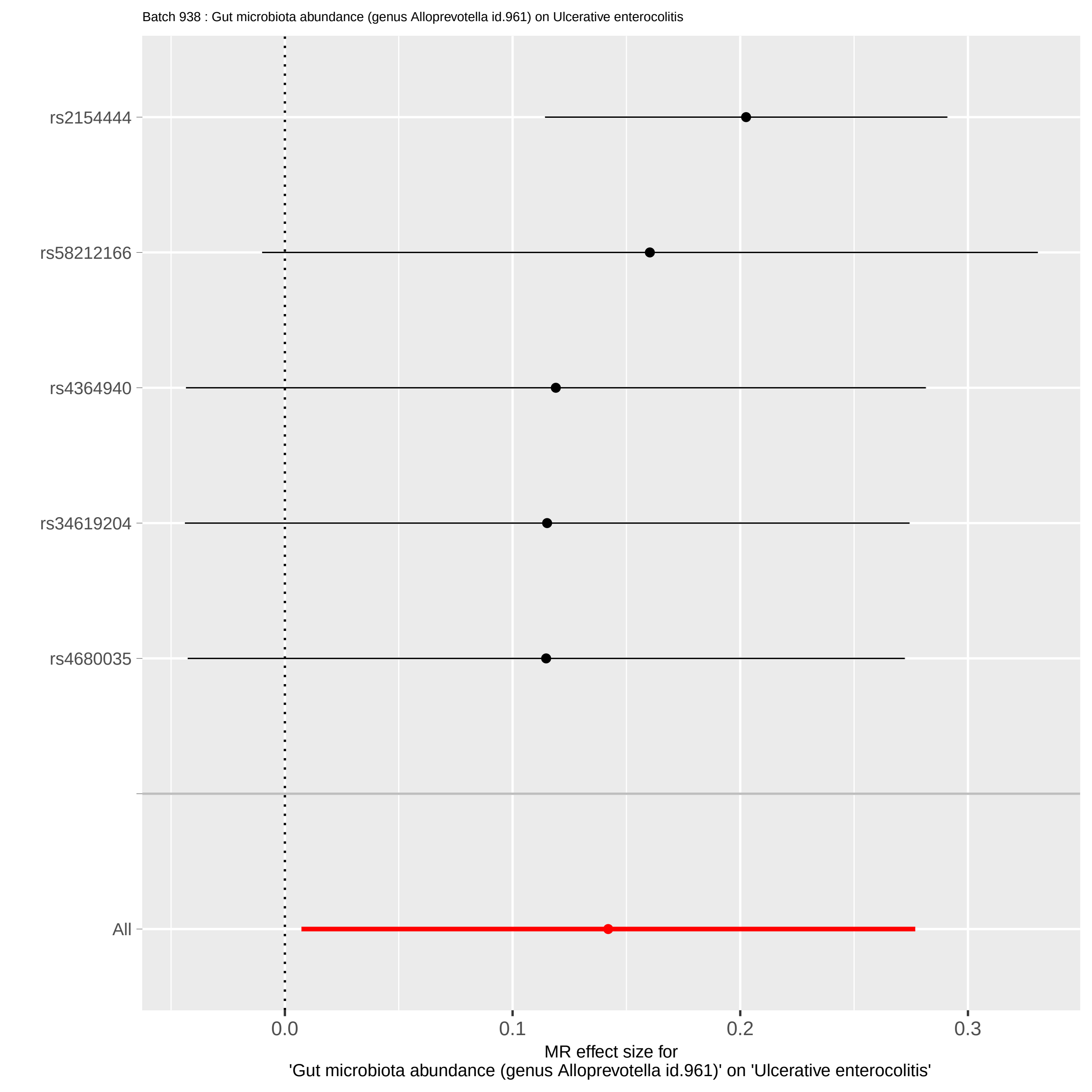
Batch 938 : Gut microbiota abundance (genus Alloprevotella id.961) on Ulcerative enterocolitis
rs2154444
rs58212166
rs4364940
rs34619204
rs4680035
All
0.0
0.1
0.2
0.3
MR effect size for
'Gut microbiota abundance (genus Alloprevotella id.961)' on 'Ulcerative enterocolitis'

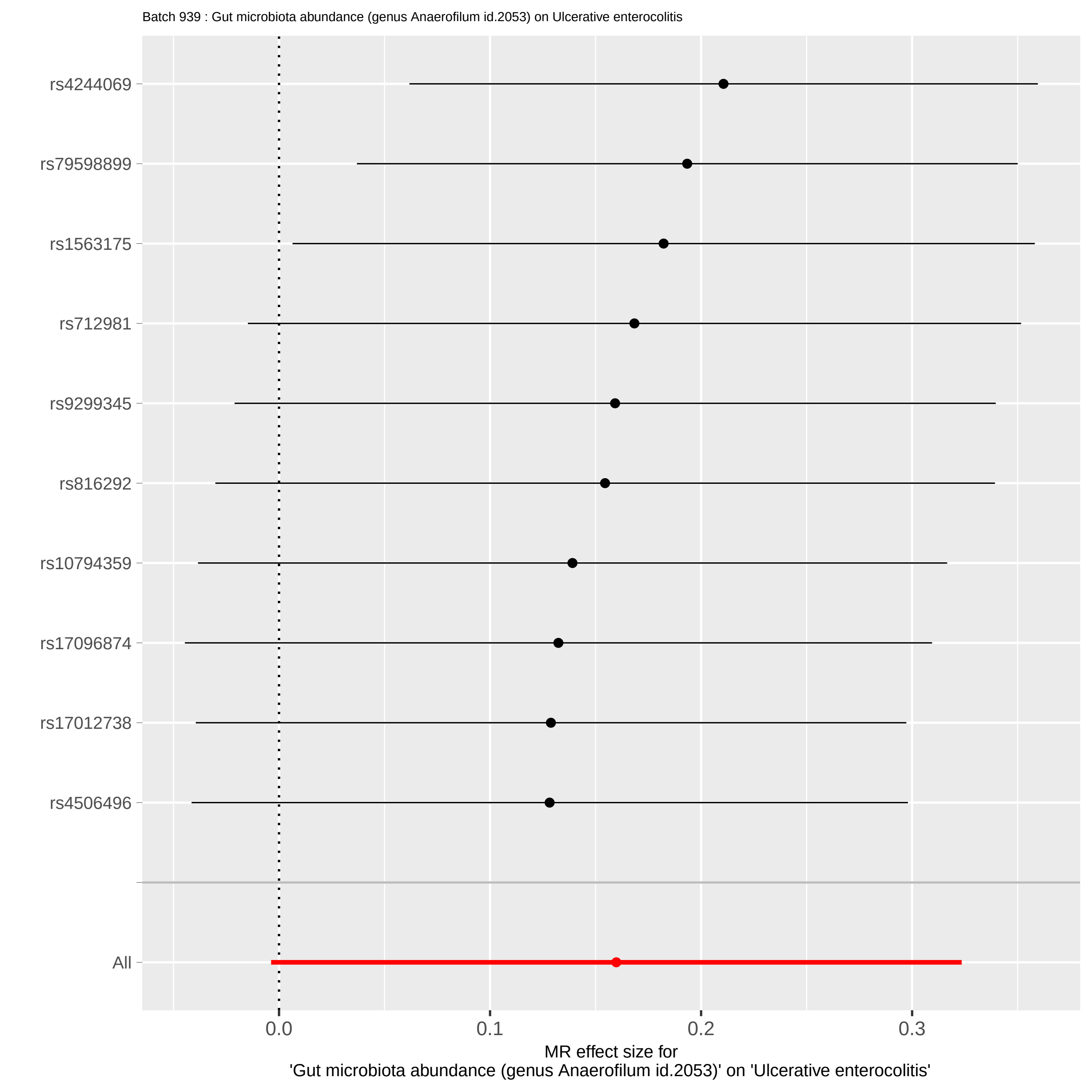

Batch 939 : Gut microbiota abundance (genus Anaerofilum id.2053) on Ulcerative enterocolitis
rs4244069
rs79598899
rs1563175
rs712981
rs9299345
rs816292
rs10794359
rs17096874
rs17012738
rs4506496
All
0.0
0.1
0.2
0.3
MR effect size for
'Gut microbiota abundance (genus Anaerofilum id.2053)' on 'Ulcerative enterocolitis'

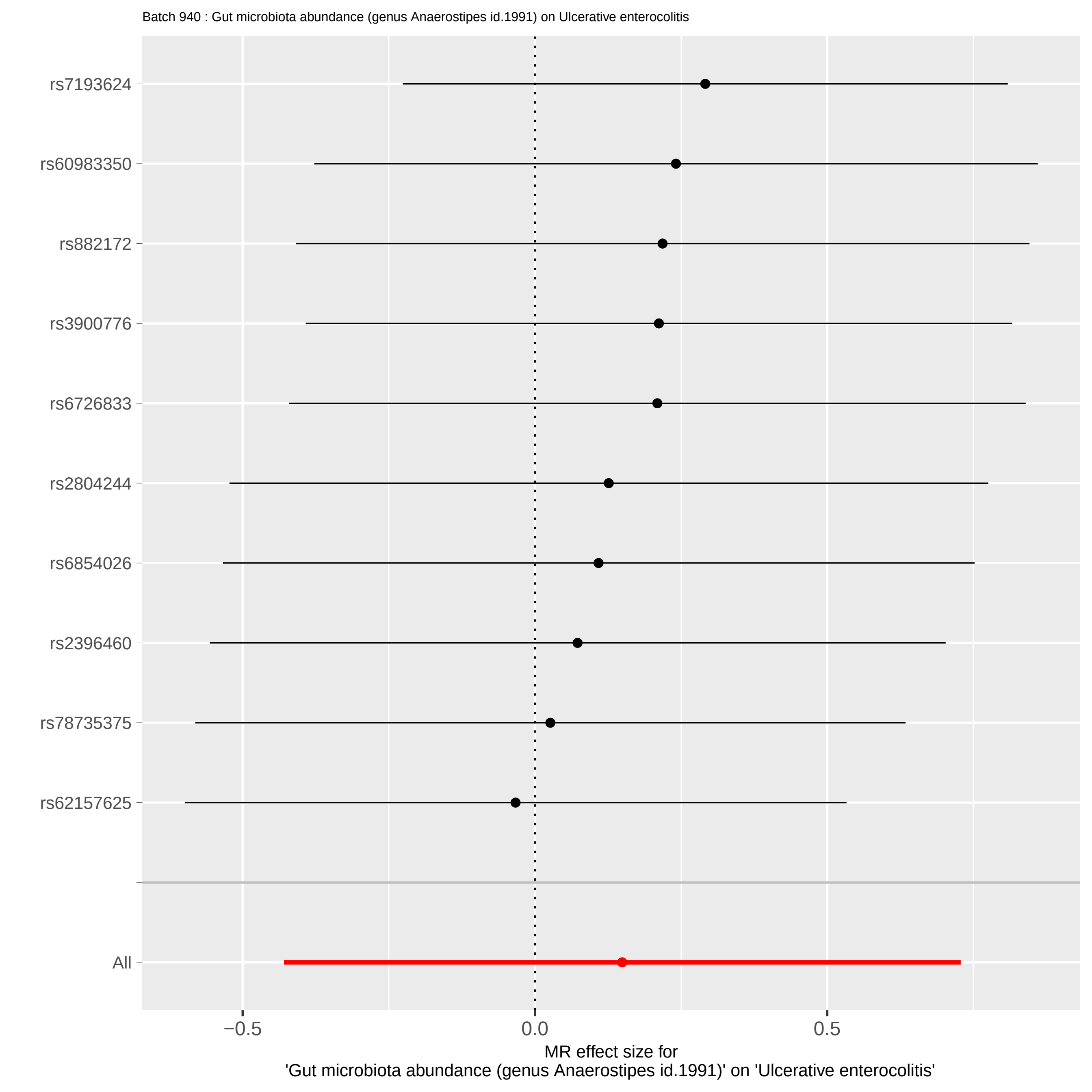

Batch 940 : Gut microbiota abundance (genus Anaerostipes id.1991) on Ulcerative enterocolitis
rs7193624
rs60983350
rs882172
rs3900776
rs6726833
rs2804244
rs6854026
rs2396460
rs78735375
rs62157625
All
−0.5
0.0
0.5
MR effect size for
'Gut microbiota abundance (genus Anaerostipes id.1991)' on 'Ulcerative enterocolitis'

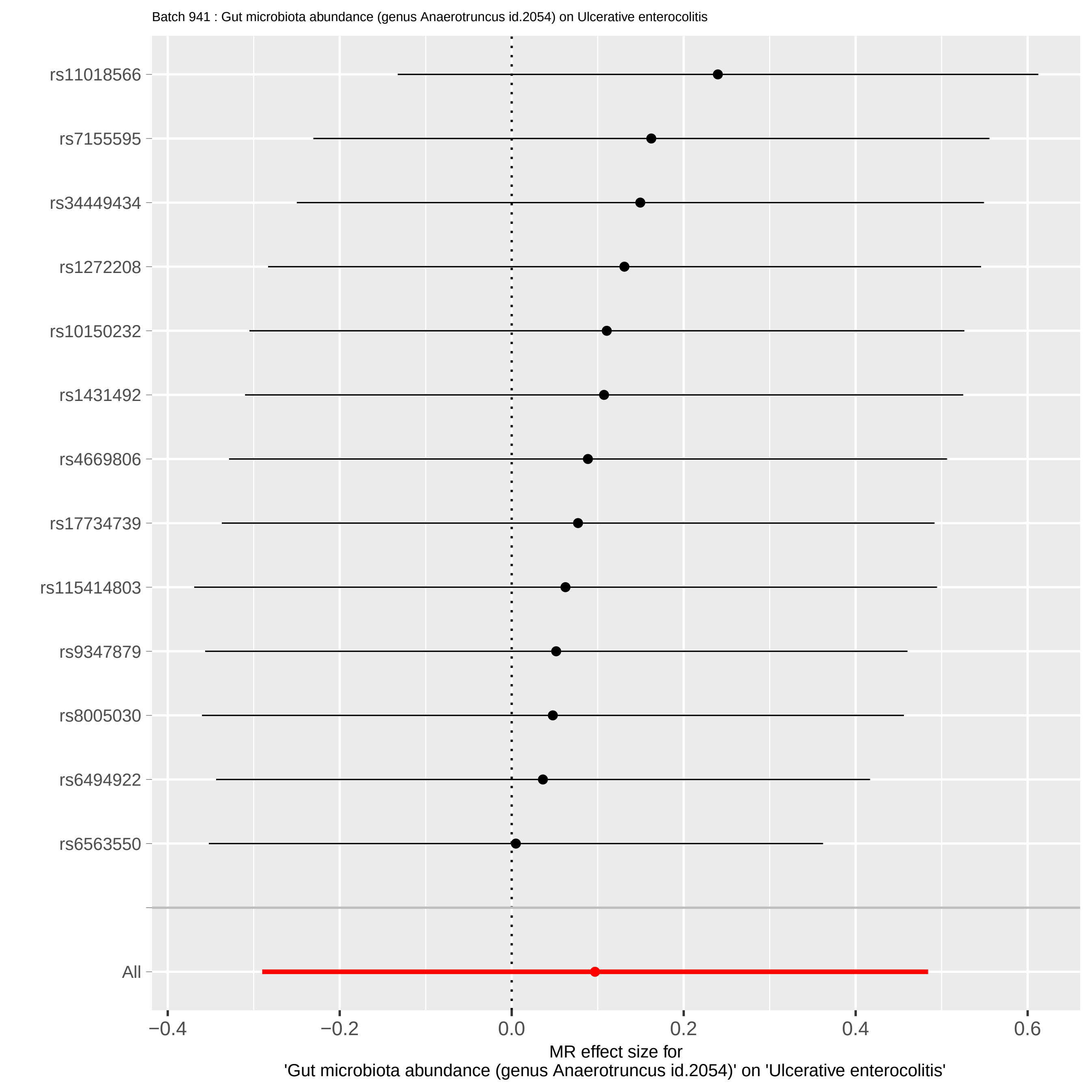

Batch 941 : Gut microbiota abundance (genus Anaerotruncus id.2054) on Ulcerative enterocolitis
rs11018566
rs7155595
rs34449434
rs1272208
rs10150232
rs1431492
rs4669806
rs17734739
rs115414803
rs9347879
rs8005030
rs6494922
rs6563550
All
−0.4
−0.2
0.0
0.2
0.4
0.6
MR effect size for
'Gut microbiota abundance (genus Anaerotruncus id.2054)' on 'Ulcerative enterocolitis'

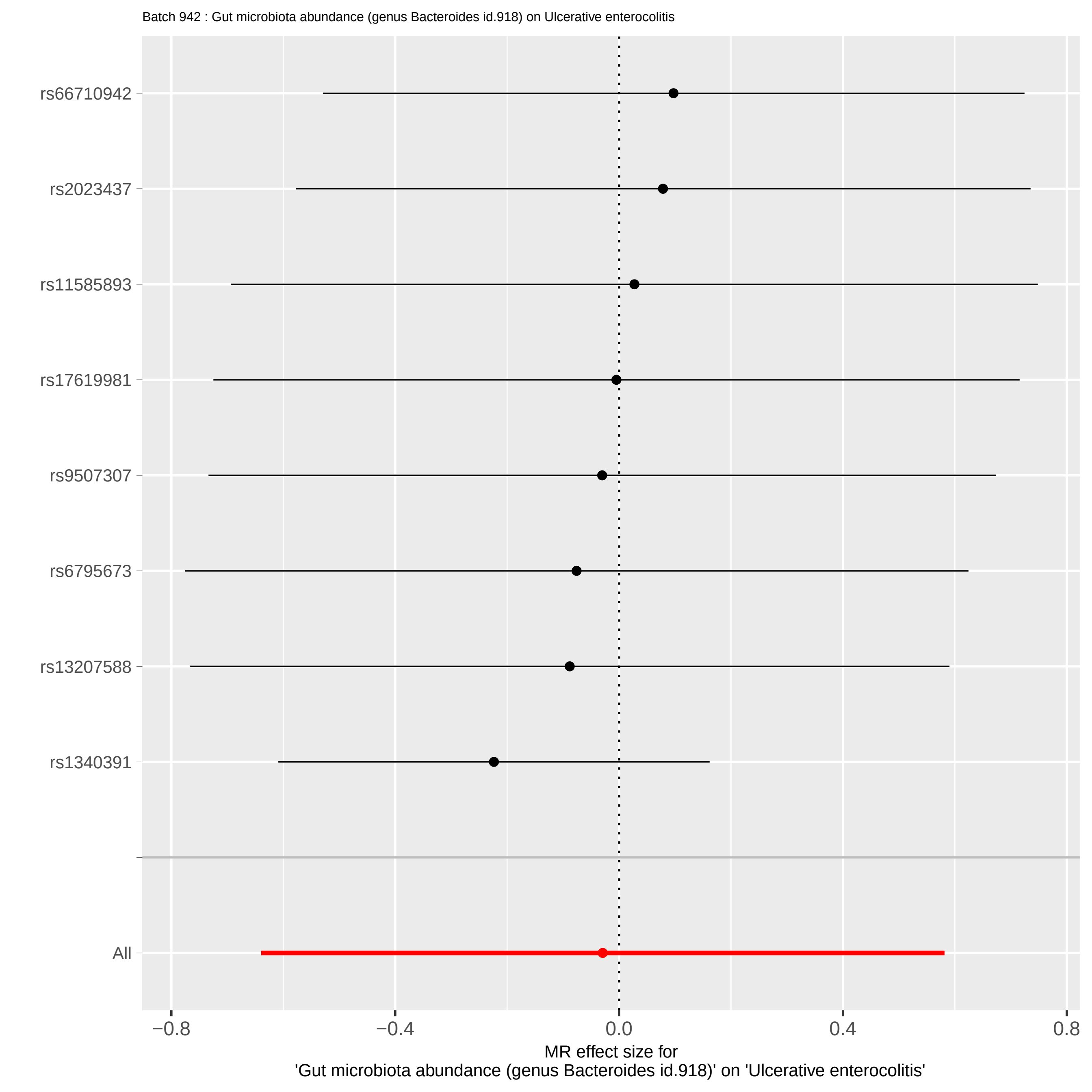
Batch 942 : Gut microbiota abundance (genus Bacteroides id.918) on Ulcerative enterocolitis
rs66710942
rs2023437
rs11585893
rs17619981
rs9507307
rs6795673
rs13207588
rs1340391
All
−0.8
−0.4
0.0
0.4
0.8
MR effect size for
'Gut microbiota abundance (genus Bacteroides id.918)' on 'Ulcerative enterocolitis'

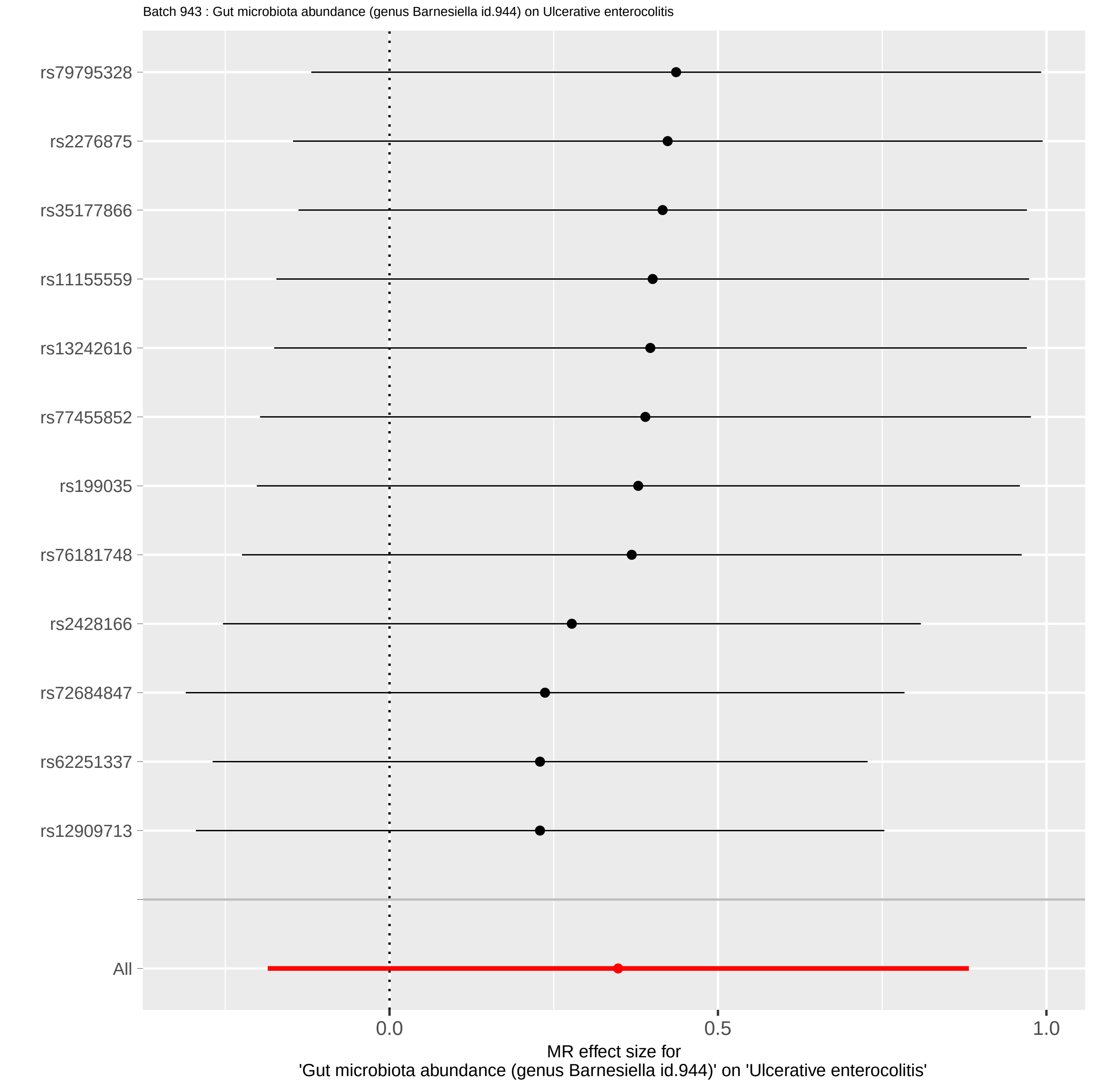

Batch 943 : Gut microbiota abundance (genus Barnesiella id.944) on Ulcerative enterocolitis
rs79795328
rs2276875
rs35177866
rs11155559
rs13242616
rs77455852
rs199035
rs76181748
rs2428166
rs72684847
rs62251337
rs12909713
All
0.0
0.5
1.0
MR effect size for
'Gut microbiota abundance (genus Barnesiella id.944)' on 'Ulcerative enterocolitis'

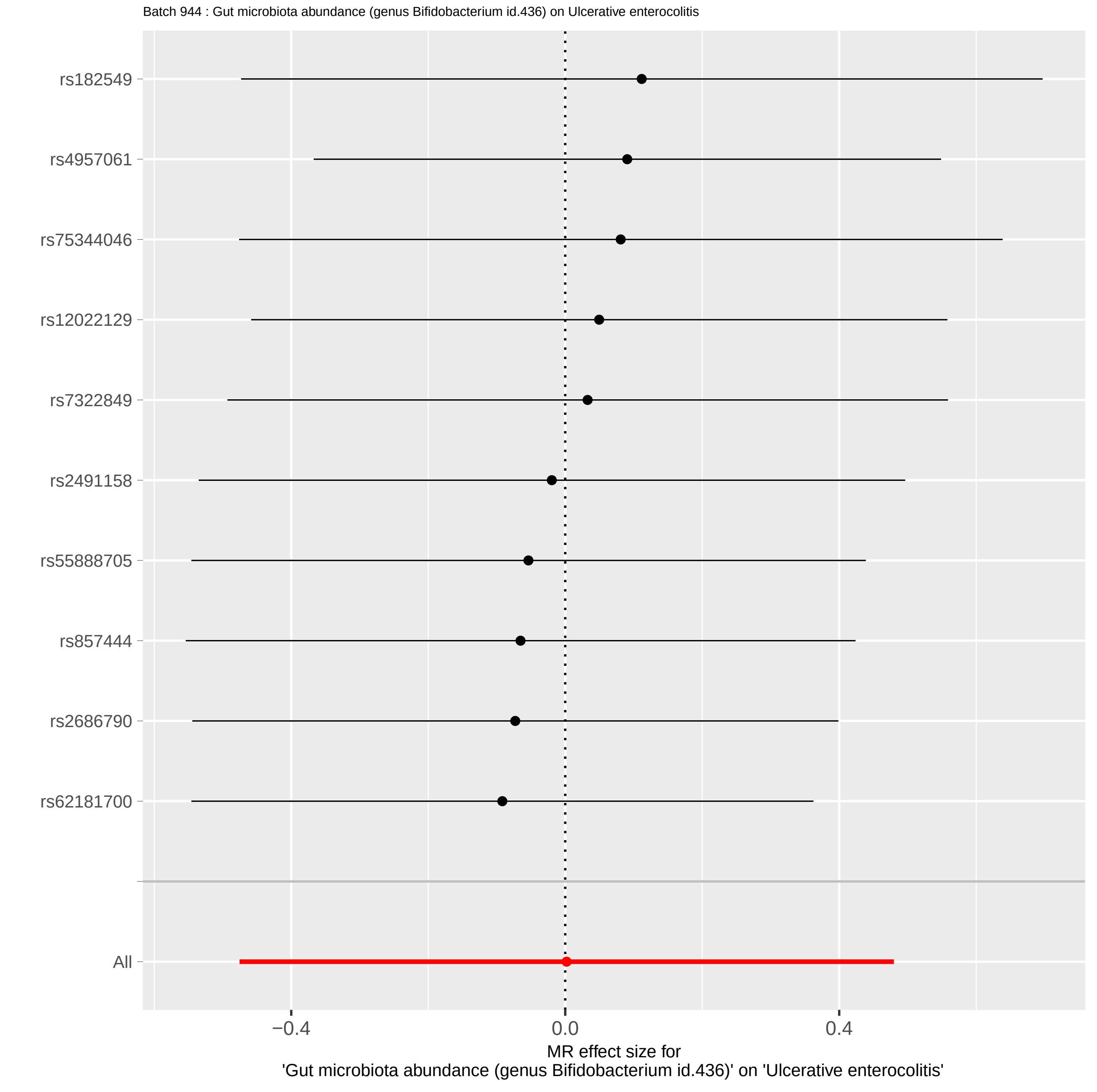

Batch 944 : Gut microbiota abundance (genus Bifidobacterium id.436) on Ulcerative enterocolitis
rs182549
rs4957061
rs75344046
rs12022129
rs7322849
rs2491158
rs55888705
rs857444
rs2686790
rs62181700
All
−0.4
0.0
0.4
MR effect size for
'Gut microbiota abundance (genus Bifidobacterium id.436)' on 'Ulcerative enterocolitis'

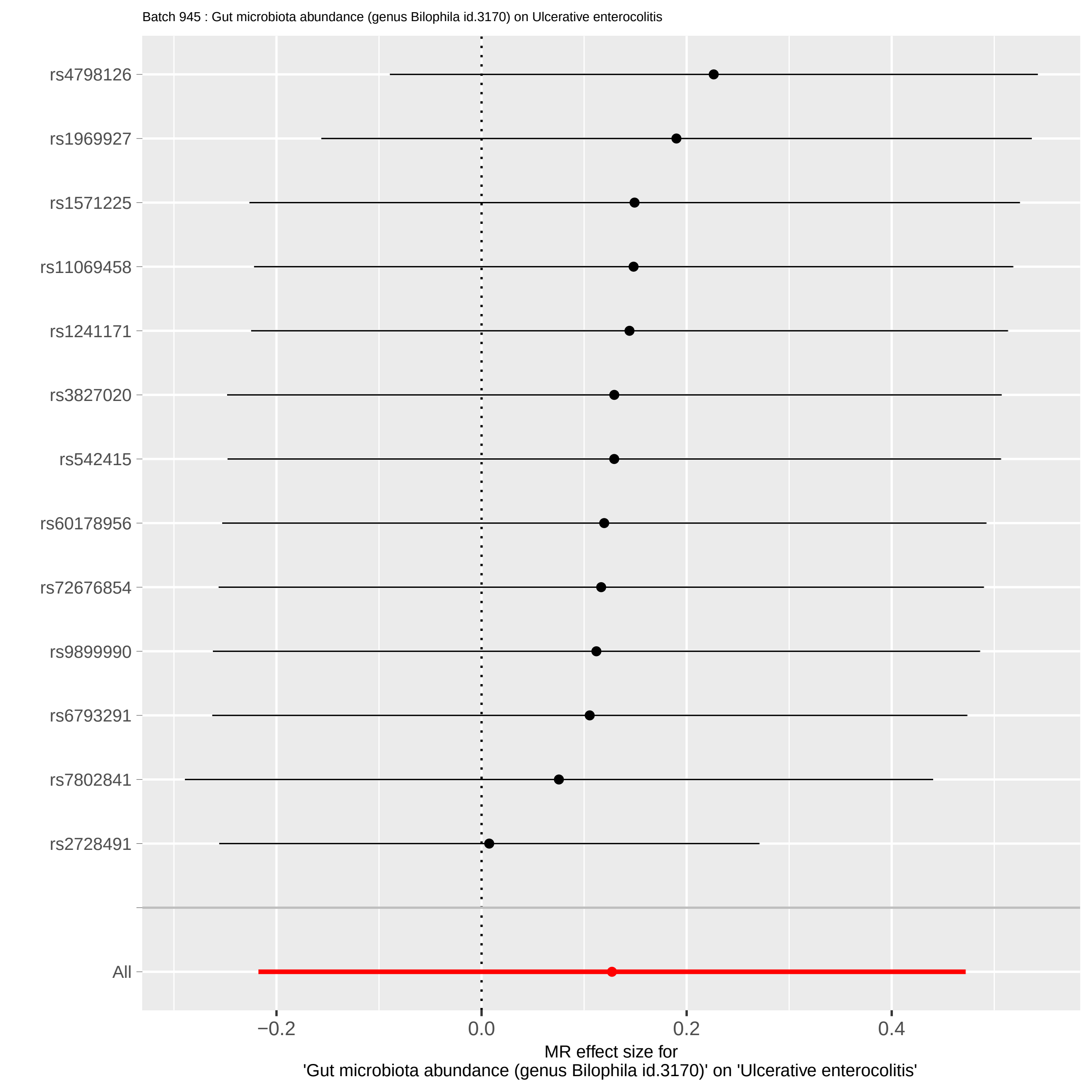
Batch 945 : Gut microbiota abundance (genus Bilophila id.3170) on Ulcerative enterocolitis
rs4798126
rs1969927
rs1571225
rs11069458
rs1241171
rs3827020
rs542415
rs60178956
rs72676854
rs9899990
rs6793291
rs7802841
rs2728491
All
−0.2
0.0
0.2
0.4
MR effect size for
'Gut microbiota abundance (genus Bilophila id.3170)' on 'Ulcerative enterocolitis'

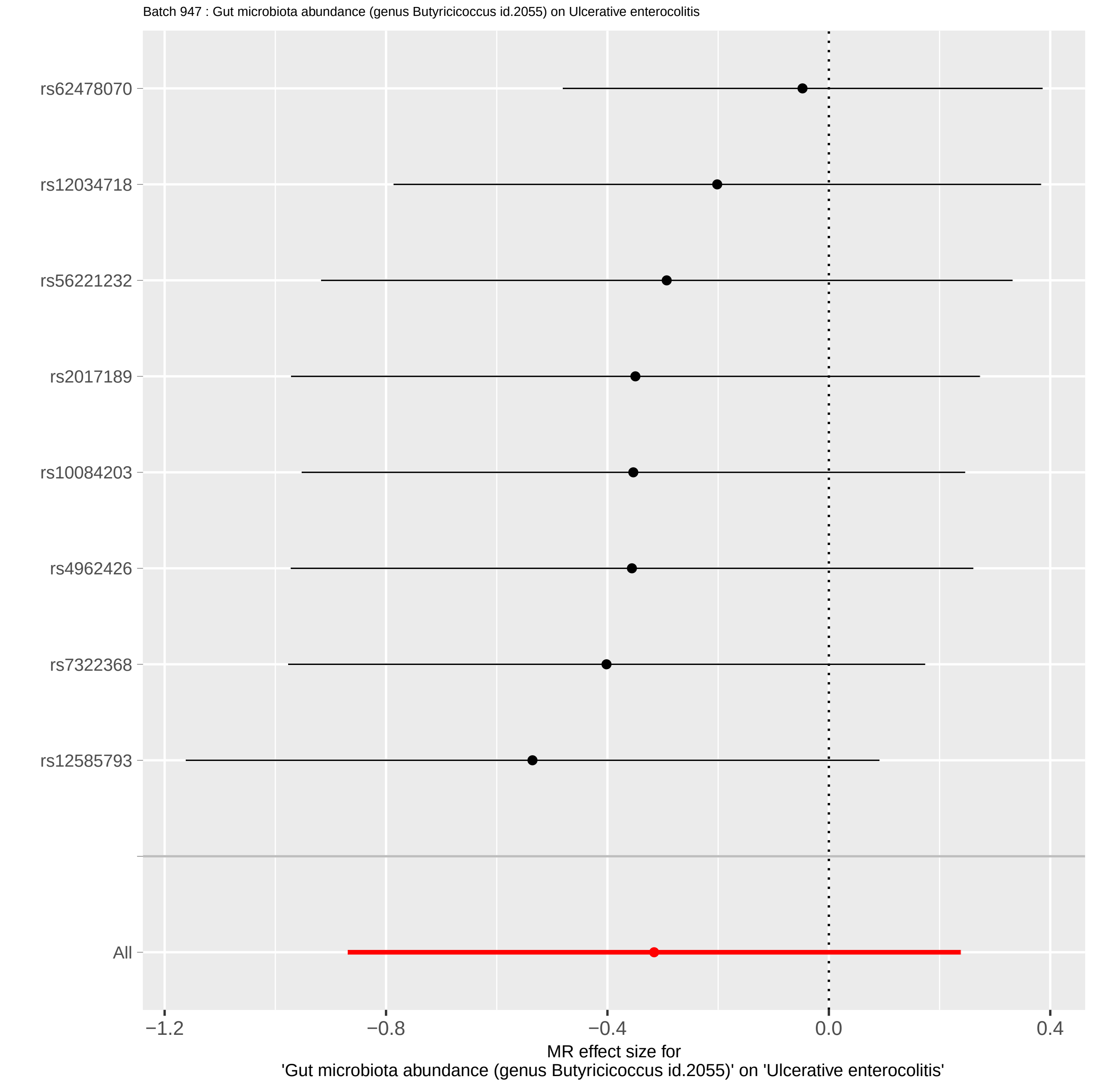
Batch 947 : Gut microbiota abundance (genus Butyricicoccus id.2055) on Ulcerative enterocolitis
rs62478070
rs12034718
rs56221232
rs2017189
rs10084203
rs4962426
rs7322368
rs12585793
All
−1.2
−0.8
−0.4
0.0
0.4
MR effect size for
'Gut microbiota abundance (genus Butyricicoccus id.2055)' on 'Ulcerative enterocolitis'

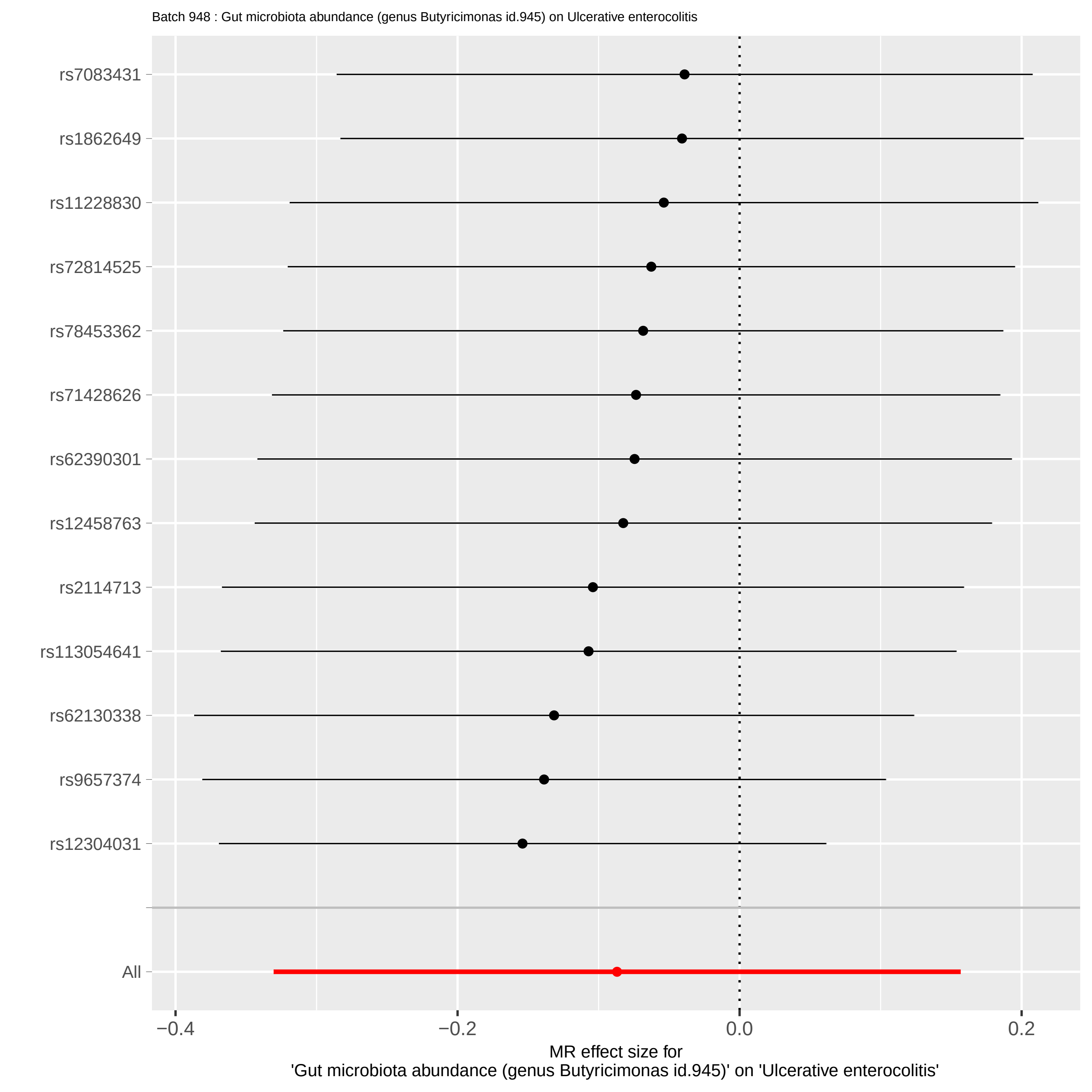

Batch 948 : Gut microbiota abundance (genus Butyricimonas id.945) on Ulcerative enterocolitis
rs7083431
rs1862649
rs11228830
rs72814525
rs78453362
rs71428626
rs62390301
rs12458763
rs2114713
rs113054641
rs62130338
rs9657374
rs12304031
All
−0.4
−0.2
0.0
0.2
MR effect size for
'Gut microbiota abundance (genus Butyricimonas id.945)' on 'Ulcerative enterocolitis'

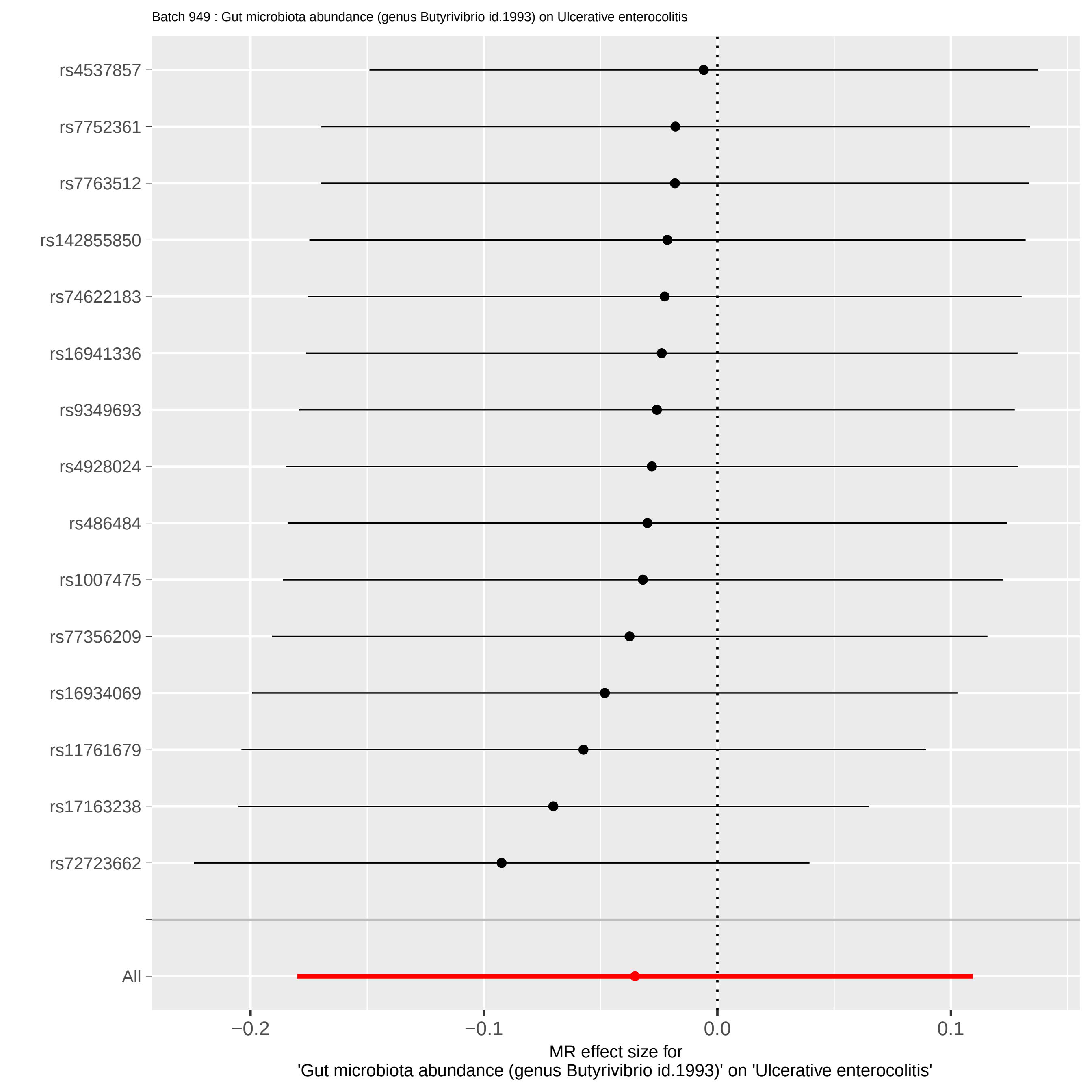

Batch 949 : Gut microbiota abundance (genus Butyrivibrio id.1993) on Ulcerative enterocolitis
rs4537857
rs7752361
rs7763512
rs142855850
rs74622183
rs16941336
rs9349693
rs4928024
rs486484
rs1007475
rs77356209
rs16934069
rs11761679
rs17163238
rs72723662
All
−0.2
−0.1
0.0
0.1
MR effect size for
'Gut microbiota abundance (genus Butyrivibrio id.1993)' on 'Ulcerative enterocolitis'

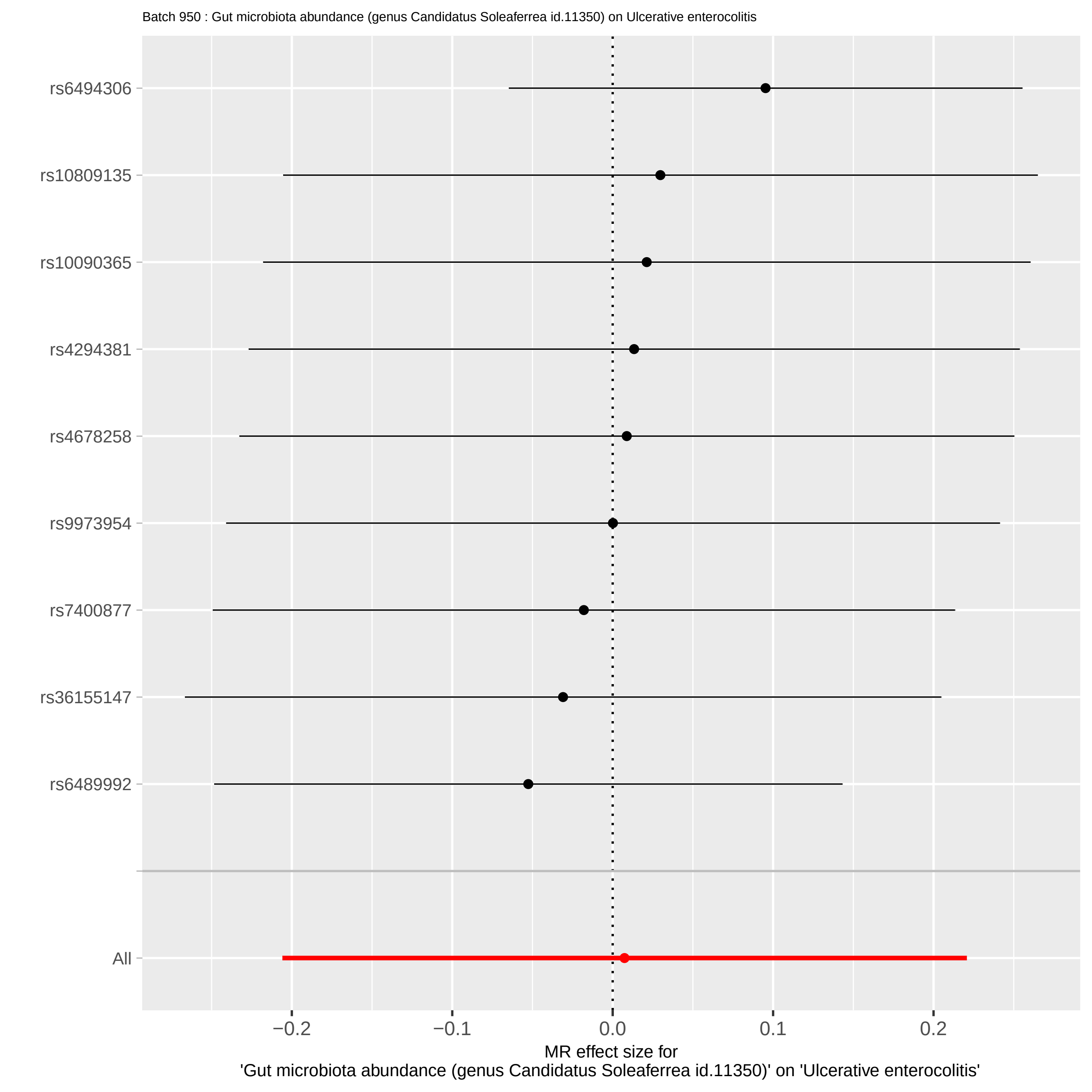

Batch 950 : Gut microbiota abundance (genus Candidatus Soleaferrea id.11350) on Ulcerative enterocolitis
rs6494306
rs10809135
rs10090365
rs4294381
rs4678258
rs9973954
rs7400877
rs36155147
rs6489992
All
−0.2
−0.1
0.0
0.1
0.2
MR effect size for
'Gut microbiota abundance (genus Candidatus Soleaferrea id.11350)' on 'Ulcerative enterocolitis'

Batch 951 : Gut microbiota abundance (genus Catenibacterium id.2153) on Ulcerative enterocolitis

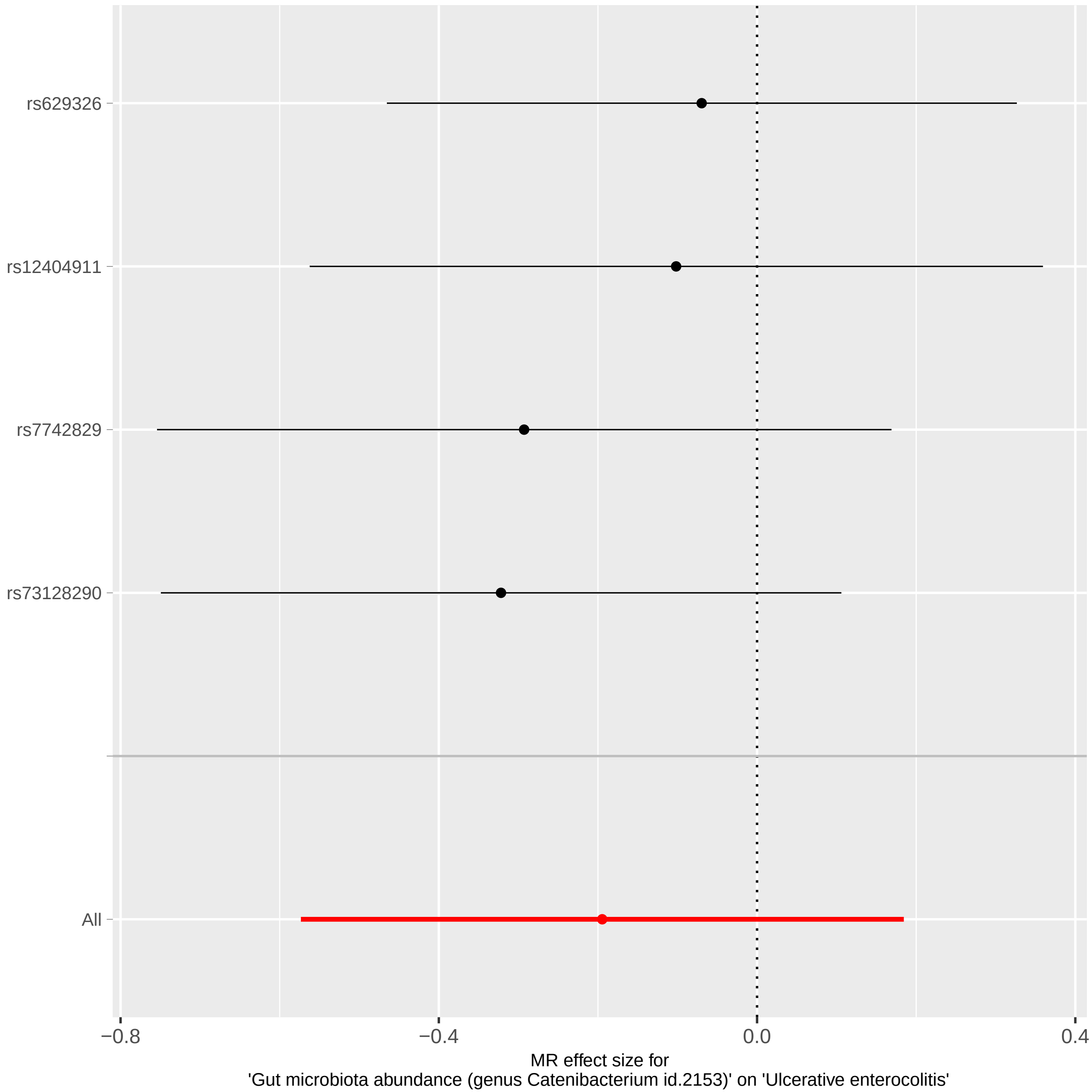

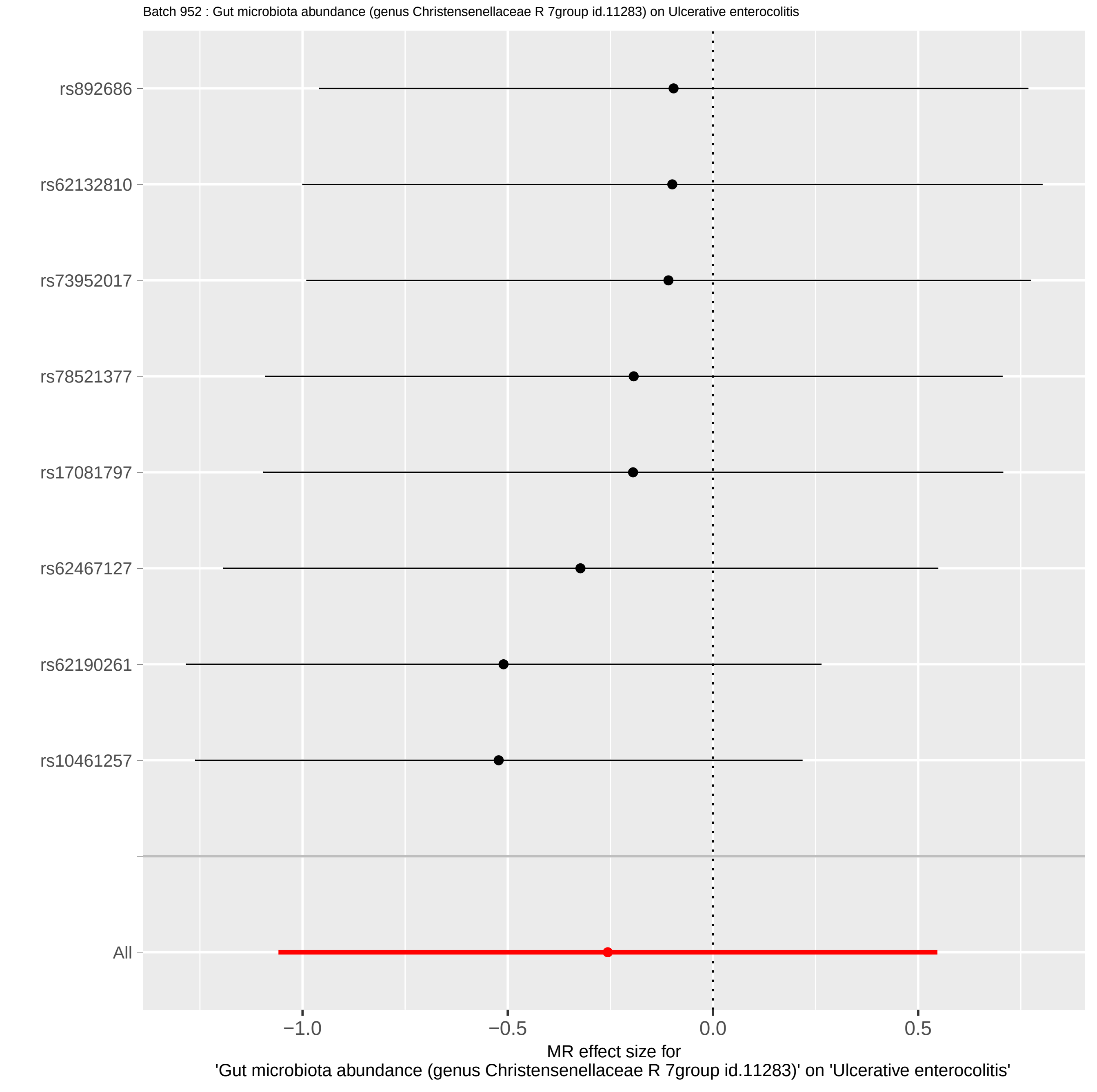
Batch 952 : Gut microbiota abundance (genus Christensenellaceae R 7group id.11283) on Ulcerative enterocolitis
rs892686
rs62132810
rs73952017
rs78521377
rs17081797
rs62467127
rs62190261
rs10461257
All
−1.0
−0.5
0.0
0.5
MR effect size for
'Gut microbiota abundance (genus Christensenellaceae R 7group id.11283)' on 'Ulcerative enterocolitis'

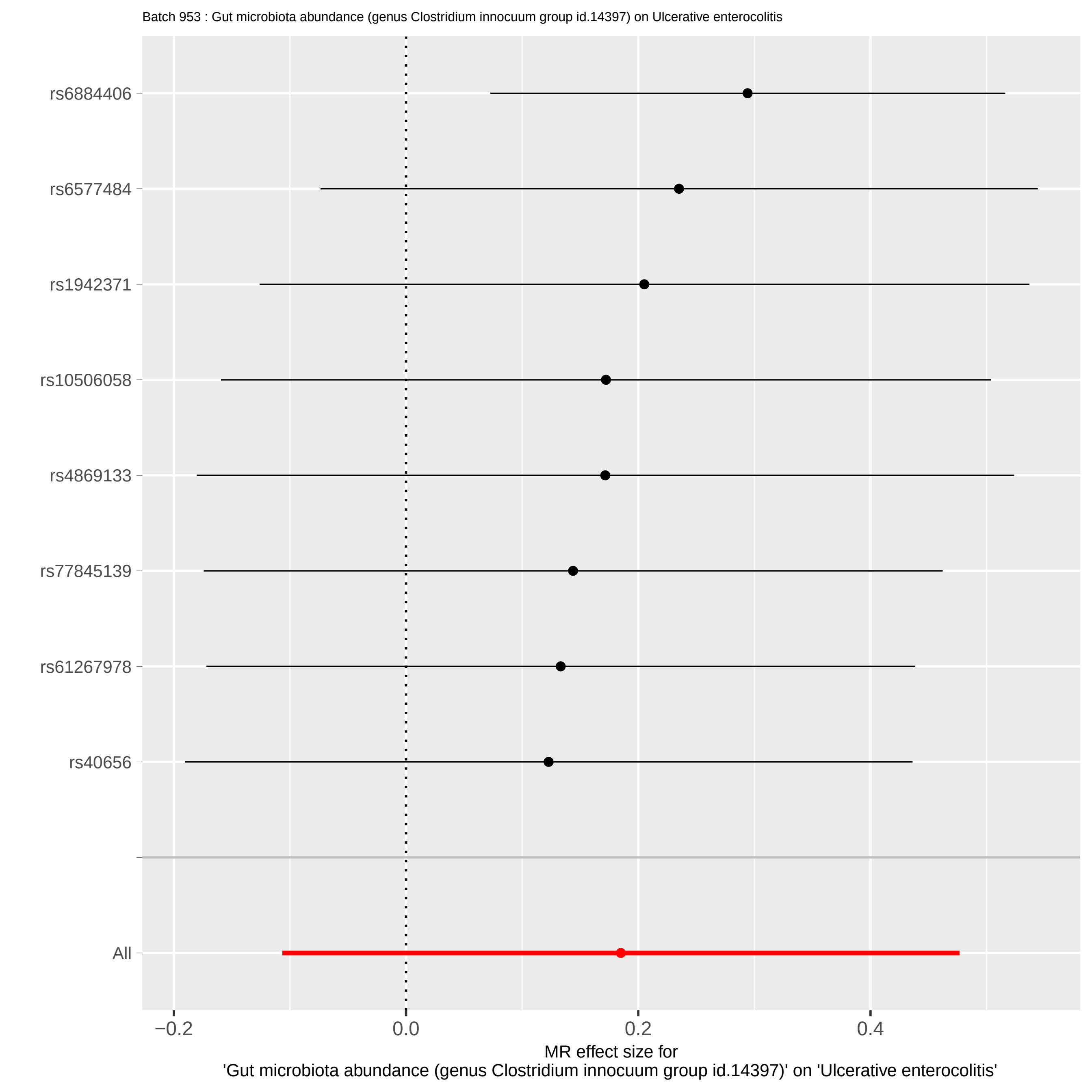
Batch 953 : Gut microbiota abundance (genus Clostridium innocuum group id.14397) on Ulcerative enterocolitis
rs6884406
rs6577484
rs1942371
rs10506058
rs4869133
rs77845139
rs61267978
rs40656
All
−0.2
0.0
0.2
0.4
MR effect size for
'Gut microbiota abundance (genus Clostridium innocuum group id.14397)' on 'Ulcerative enterocolitis'

Batch 954 : Gut microbiota abundance (genus Clostridium sensustricto1 id.1873) on Ulcerative enterocolitis

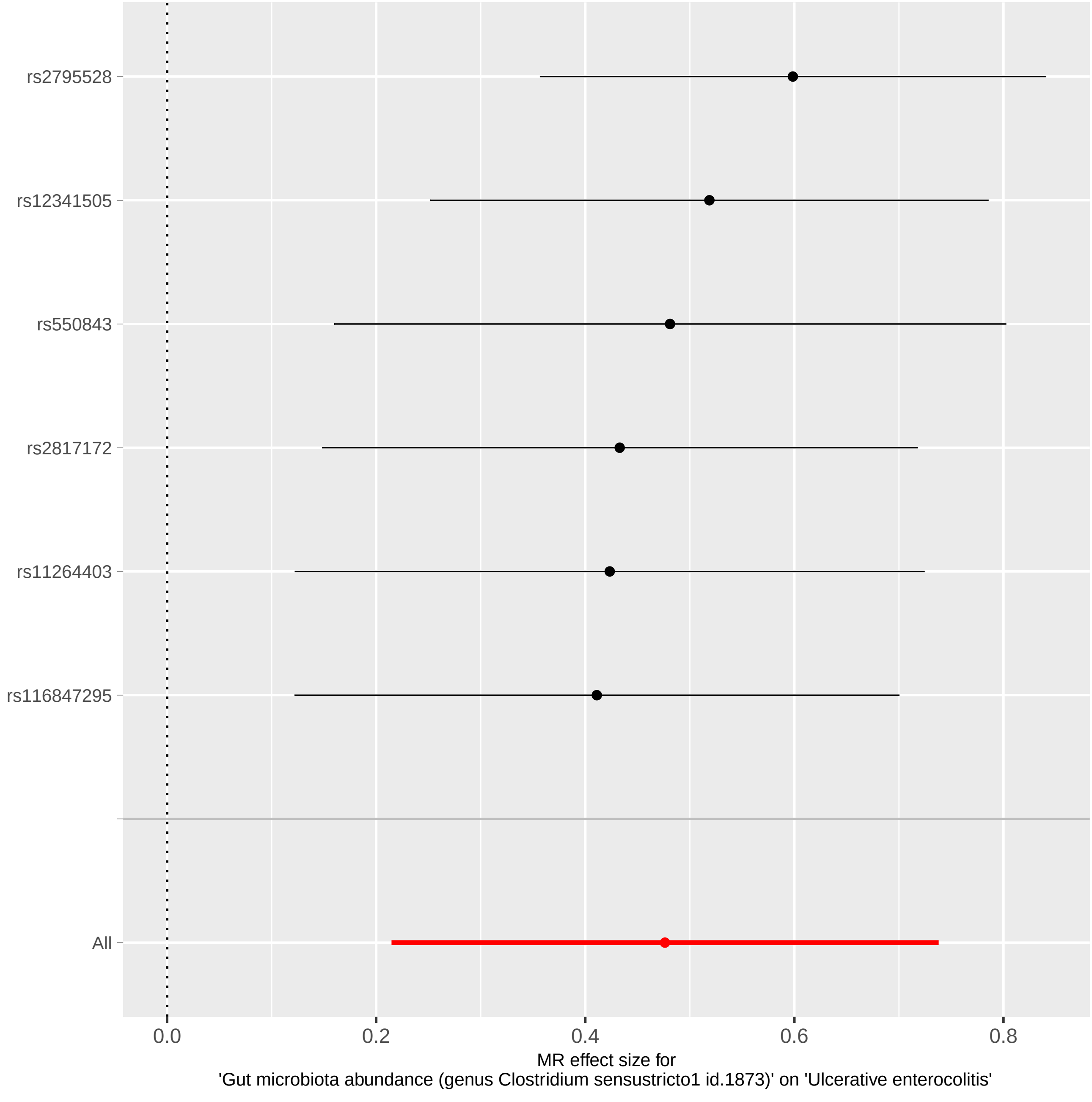

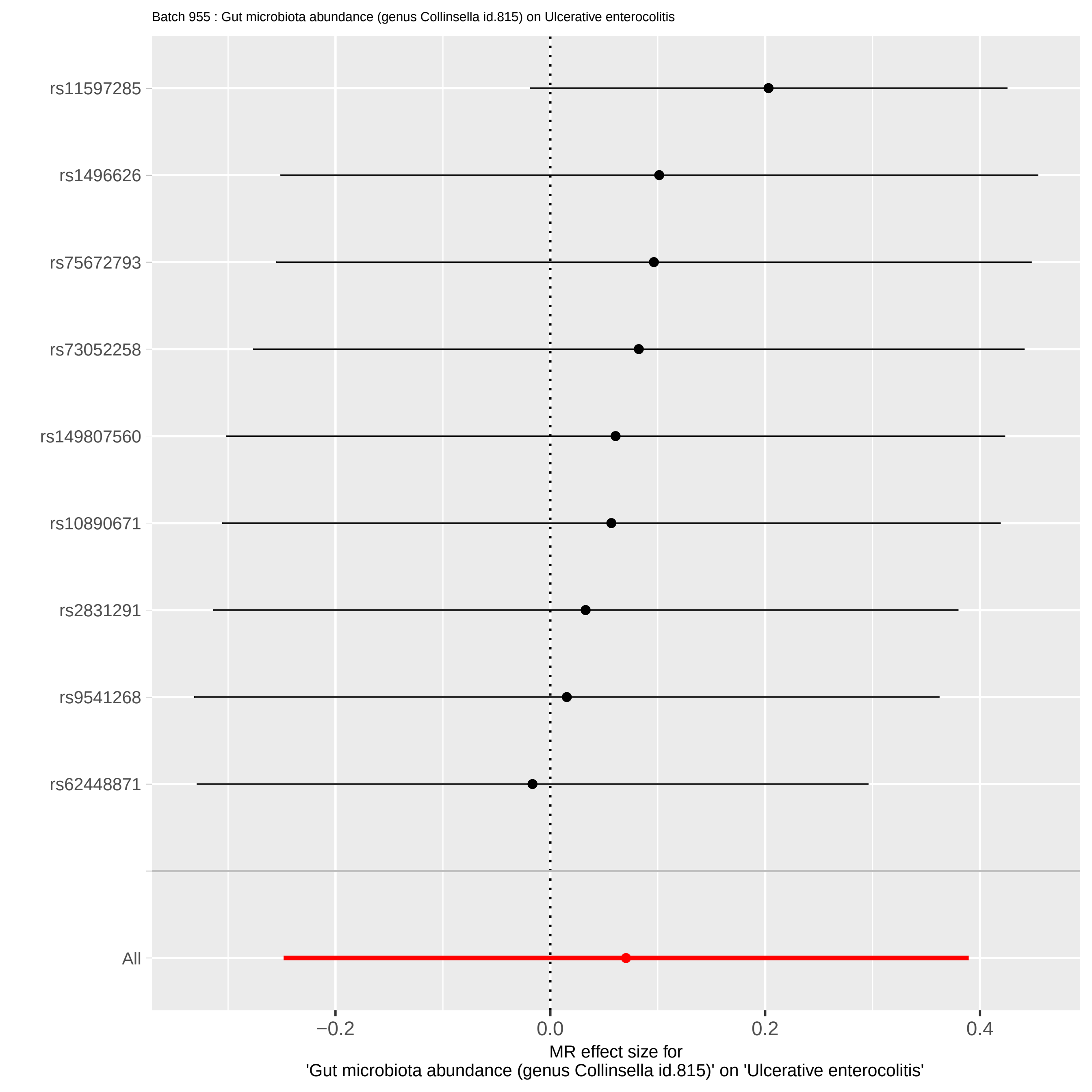
Batch 955 : Gut microbiota abundance (genus Collinsella id.815) on Ulcerative enterocolitis
rs11597285
rs1496626
rs75672793
rs73052258
rs149807560
rs10890671
rs2831291
rs9541268
rs62448871
All
−0.2
0.0
0.2
0.4
MR effect size for
'Gut microbiota abundance (genus Collinsella id.815)' on 'Ulcerative enterocolitis'

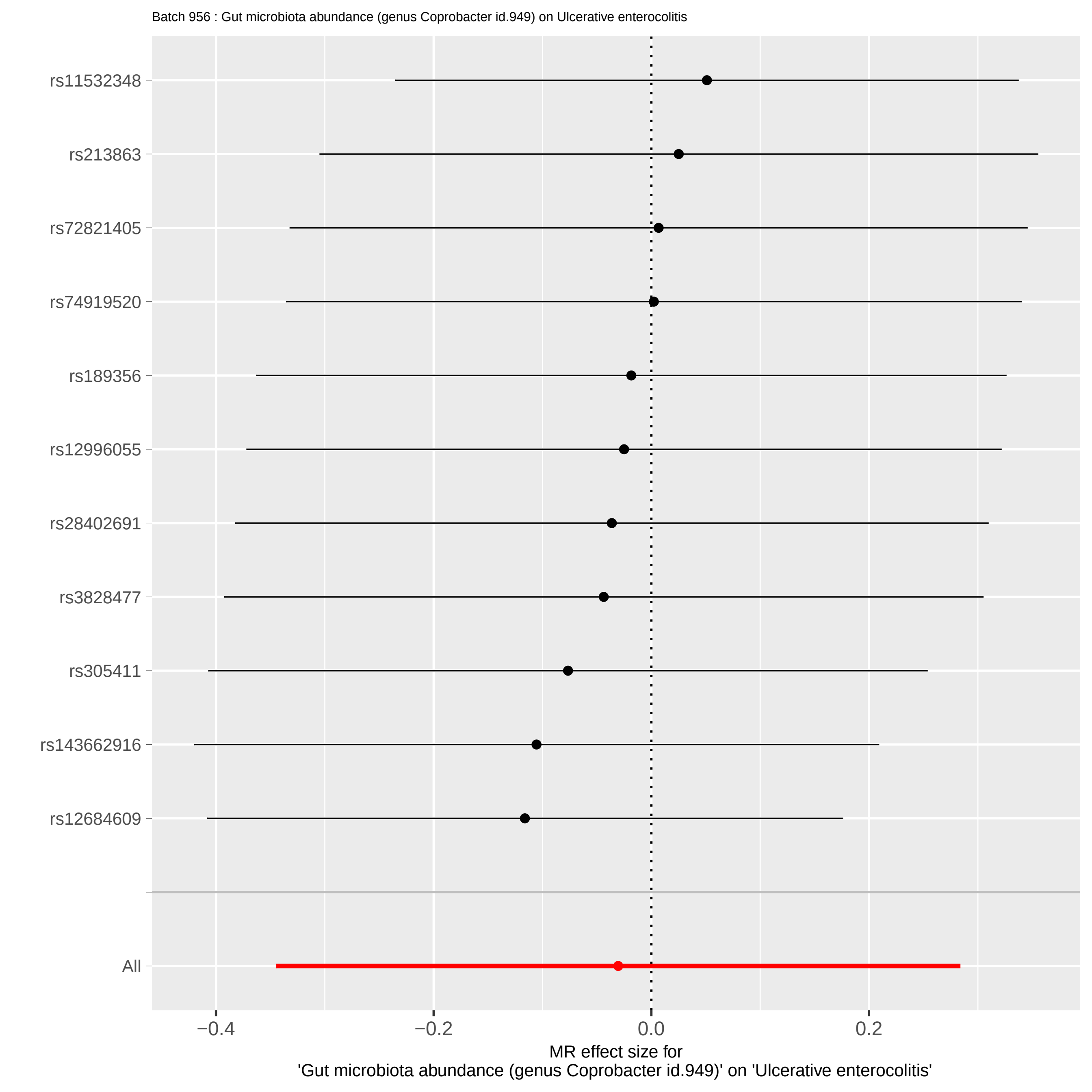
Batch 956 : Gut microbiota abundance (genus Coprobacter id.949) on Ulcerative enterocolitis
rs11532348
rs213863
rs72821405
rs74919520
rs189356
rs12996055
rs28402691
rs3828477
rs305411
rs143662916
rs12684609
All
−0.4
−0.2
0.0
0.2
MR effect size for
'Gut microbiota abundance (genus Coprobacter id.949)' on 'Ulcerative enterocolitis'

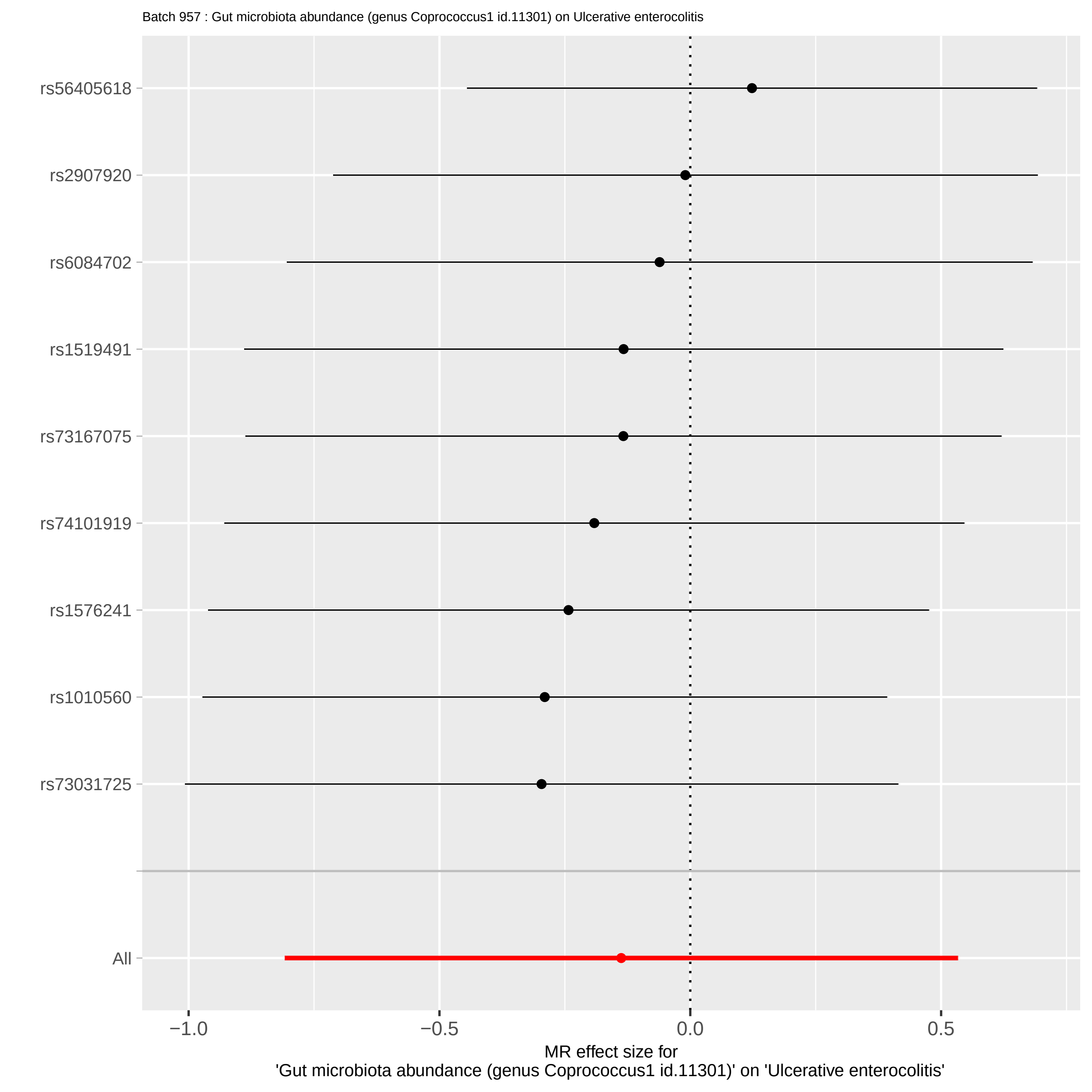

Batch 957 : Gut microbiota abundance (genus Coprococcus1 id.11301) on Ulcerative enterocolitis
rs56405618
rs2907920
rs6084702
rs1519491
rs73167075
rs74101919
rs1576241
rs1010560
rs73031725
All
−1.0
−0.5
0.0
0.5
MR effect size for
'Gut microbiota abundance (genus Coprococcus1 id.11301)' on 'Ulcerative enterocolitis'

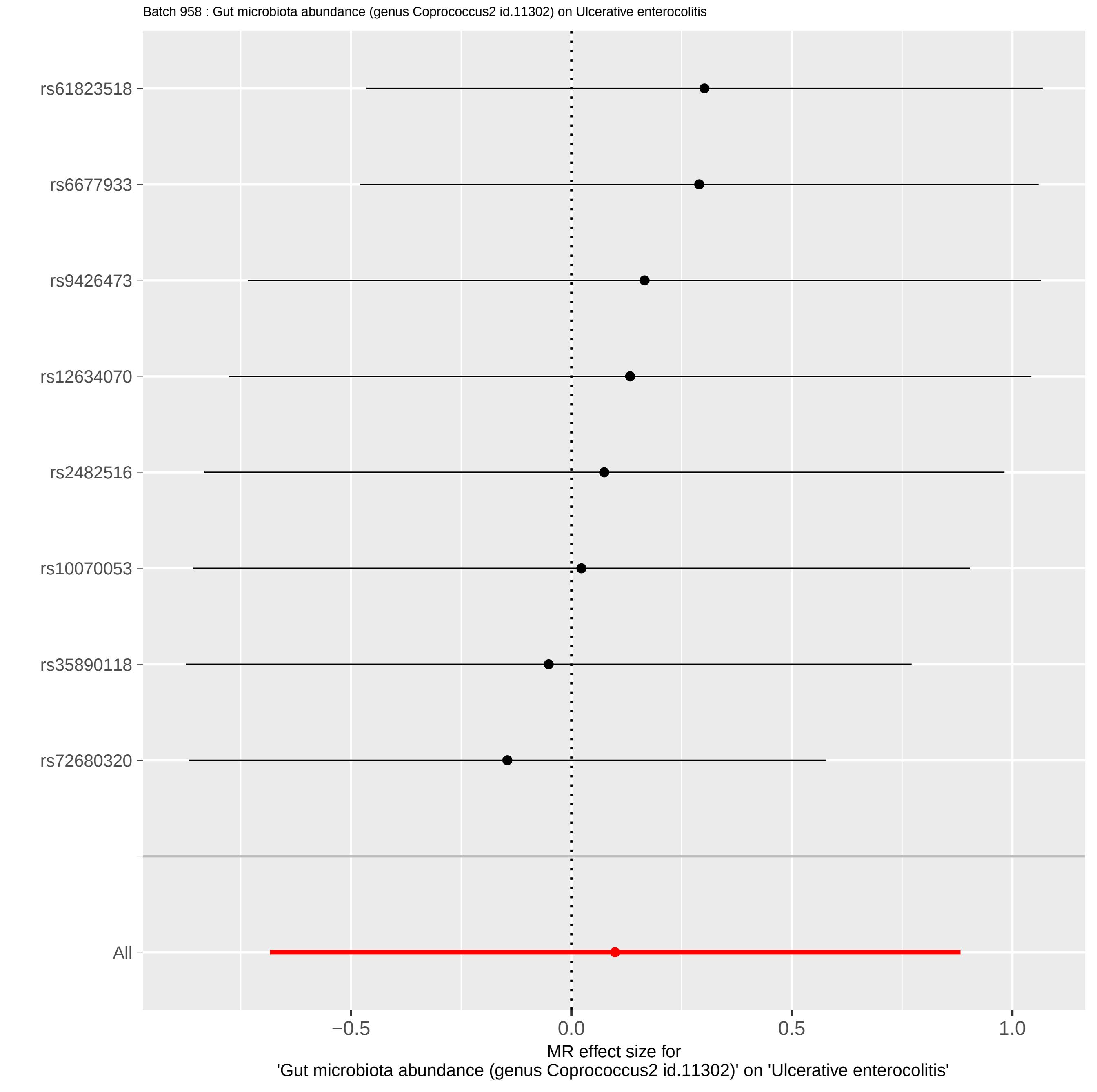

Batch 958 : Gut microbiota abundance (genus Coprococcus2 id.11302) on Ulcerative enterocolitis
rs61823518
rs6677933
rs9426473
rs12634070
rs2482516
rs10070053
rs35890118
rs72680320
All
−0.5
0.0
0.5
1.0
MR effect size for
'Gut microbiota abundance (genus Coprococcus2 id.11302)' on 'Ulcerative enterocolitis'

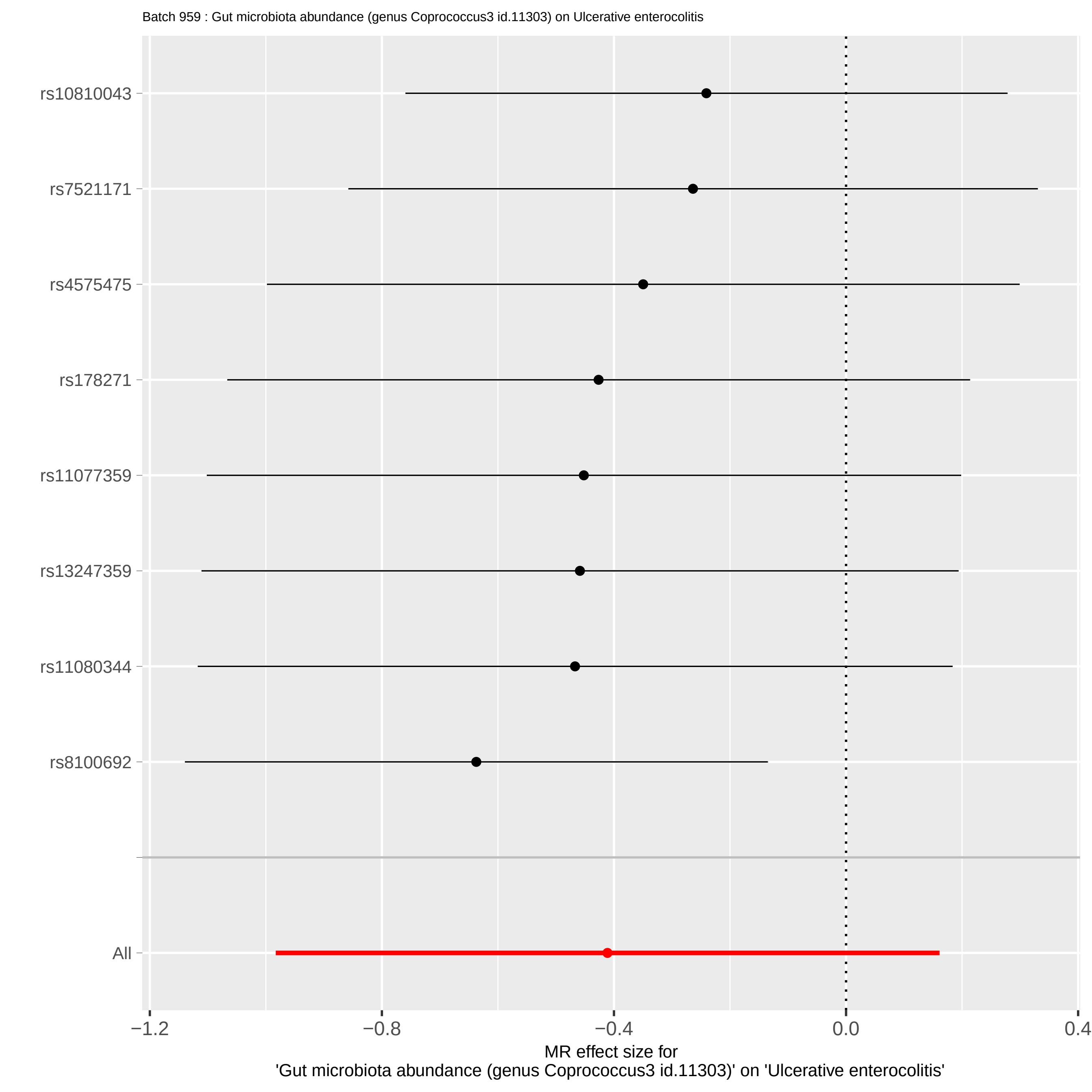
Batch 959 : Gut microbiota abundance (genus Coprococcus3 id.11303) on Ulcerative enterocolitis
rs10810043
rs7521171
rs4575475
rs178271
rs11077359
rs13247359
rs11080344
rs8100692
All
−1.2
−0.8
−0.4
0.0
0.4
MR effect size for
'Gut microbiota abundance (genus Coprococcus3 id.11303)' on 'Ulcerative enterocolitis'

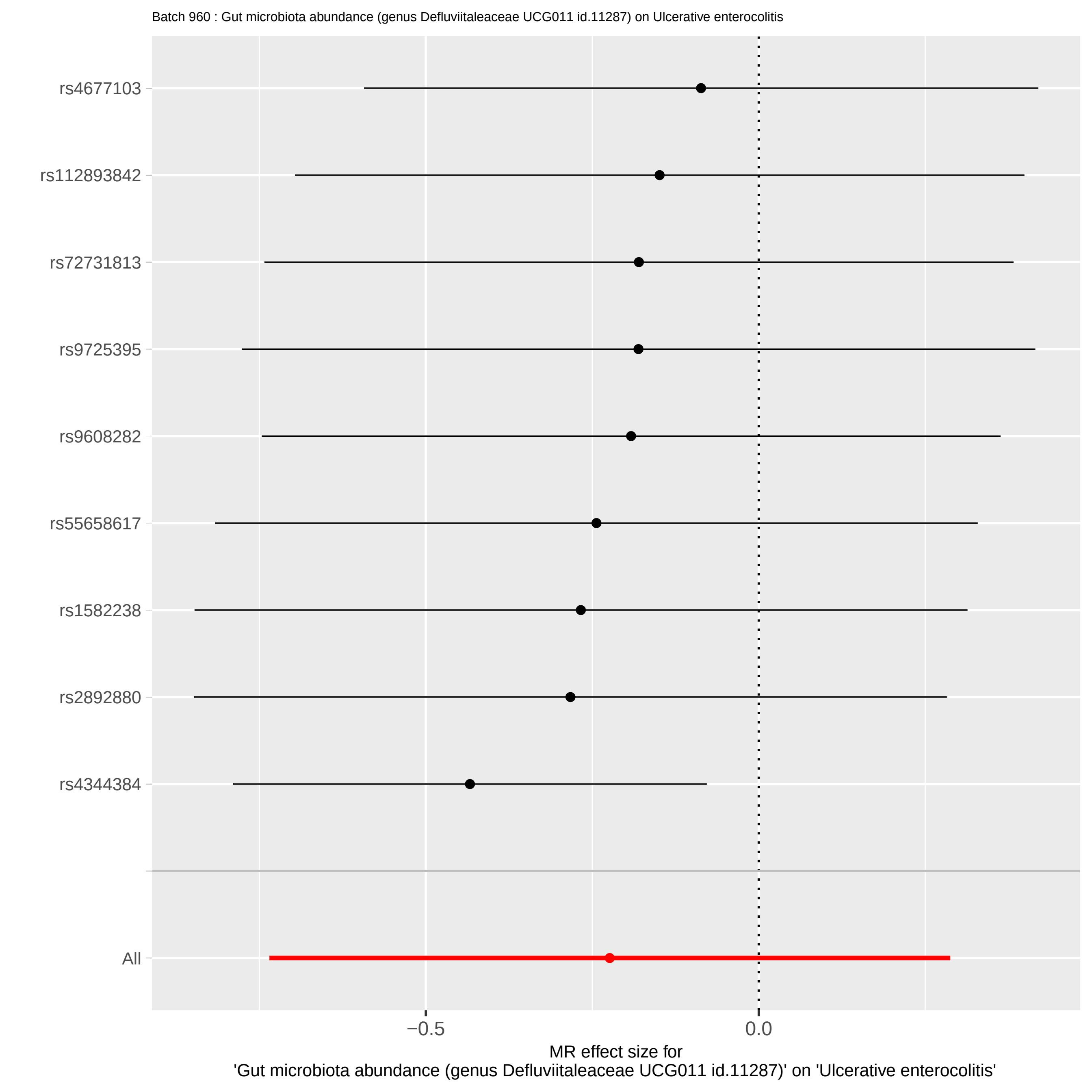
Batch 960 : Gut microbiota abundance (genus Defluviitaleaceae UCG011 id.11287) on Ulcerative enterocolitis
rs4677103
rs112893842
rs72731813
rs9725395
rs9608282
rs55658617
rs1582238
rs2892880
rs4344384
All
−0.5
0.0
MR effect size for
'Gut microbiota abundance (genus Defluviitaleaceae UCG011 id.11287)' on 'Ulcerative enterocolitis'

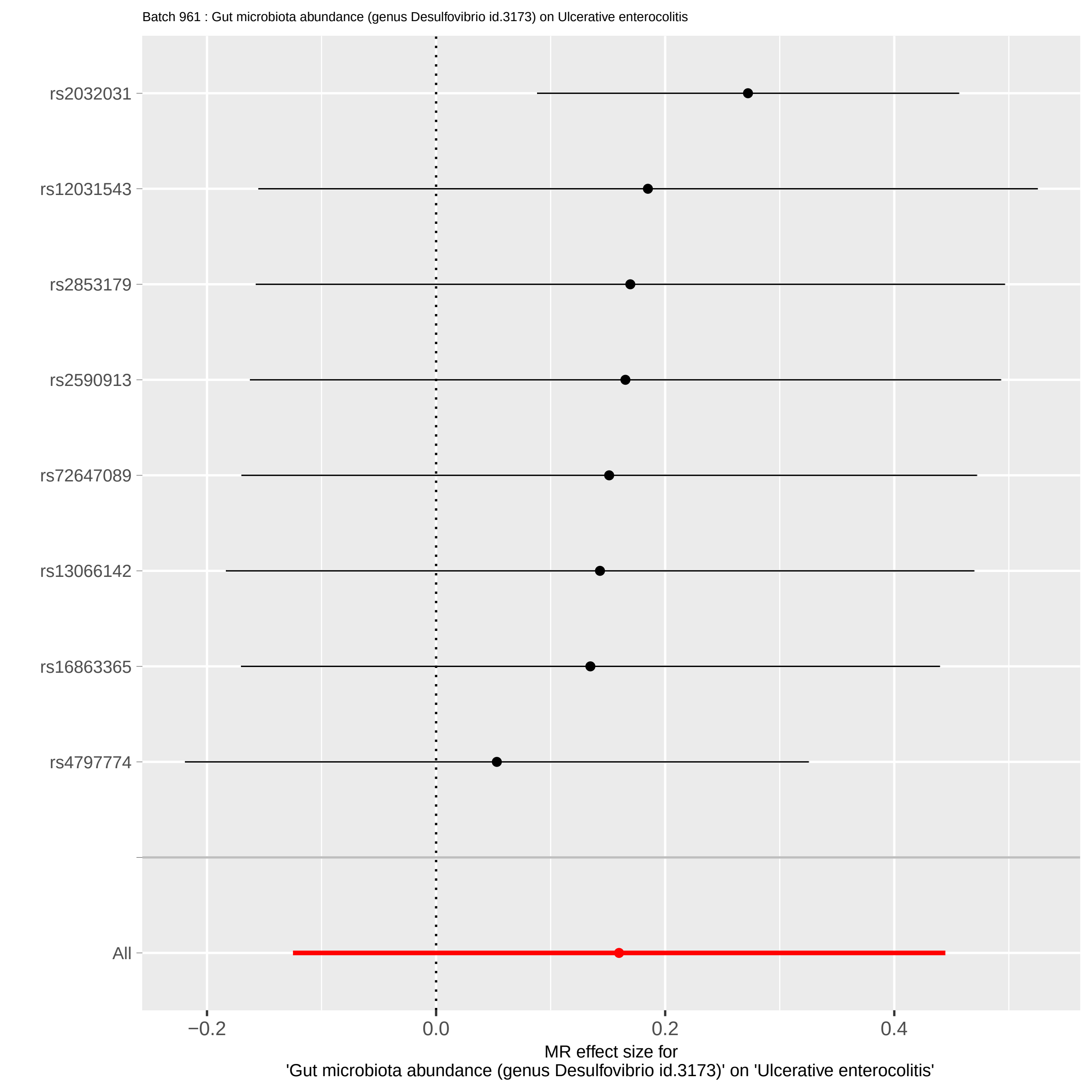

Batch 961 : Gut microbiota abundance (genus Desulfovibrio id.3173) on Ulcerative enterocolitis
rs2032031
rs12031543
rs2853179
rs2590913
rs72647089
rs13066142
rs16863365
rs4797774
All
−0.2
0.0
0.2
0.4
MR effect size for
'Gut microbiota abundance (genus Desulfovibrio id.3173)' on 'Ulcerative enterocolitis'

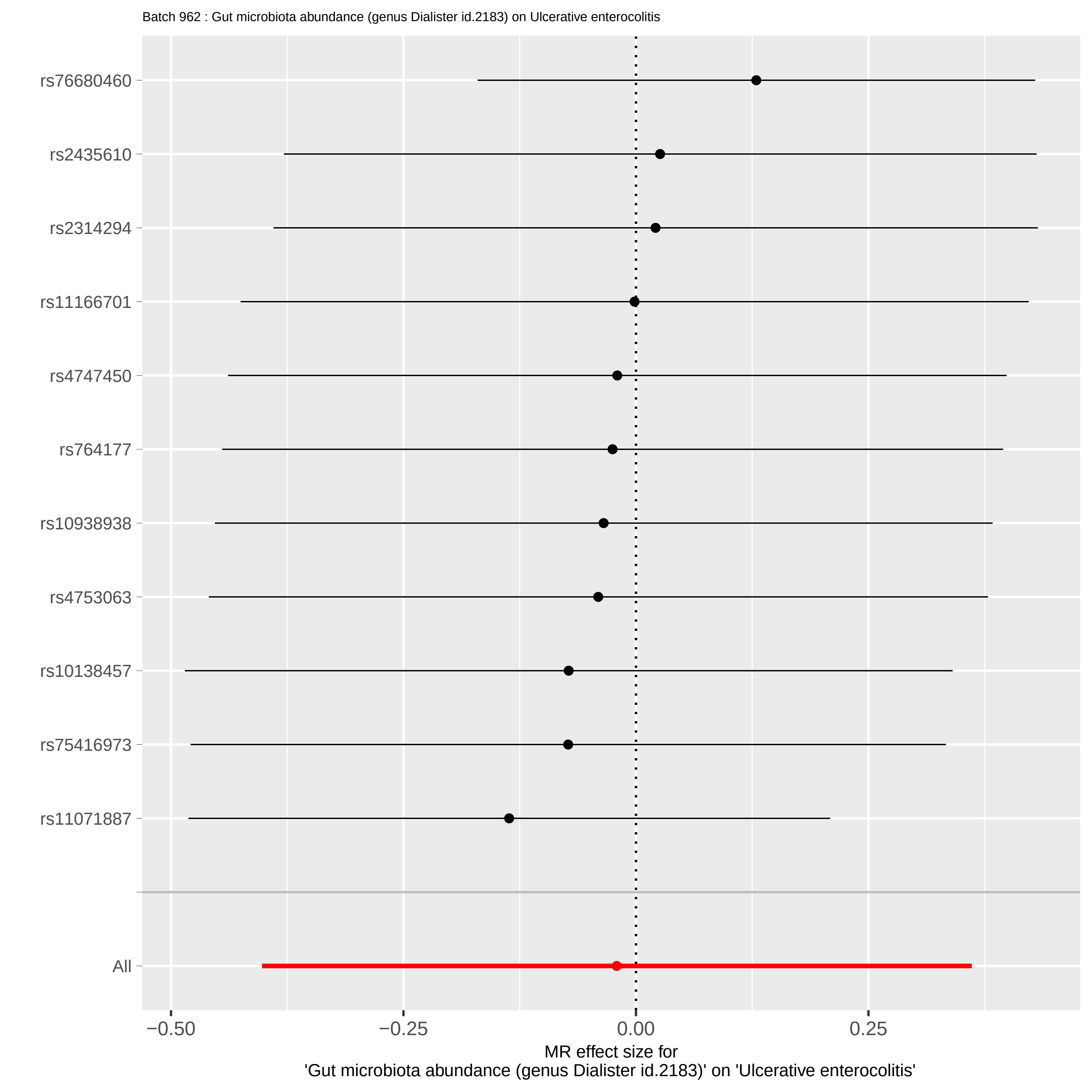
Batch 962 : Gut microbiota abundance (genus Dialister id.2183) on Ulcerative enterocolitis
rs76680460
rs2435610
rs2314294
rs11166701
rs4747450
rs764177
rs10938938
rs4753063
rs10138457
rs75416973
rs11071887
All
−0.50
−0.25
0.00
0.25
MR effect size for
'Gut microbiota abundance (genus Dialister id.2183)' on 'Ulcerative enterocolitis'

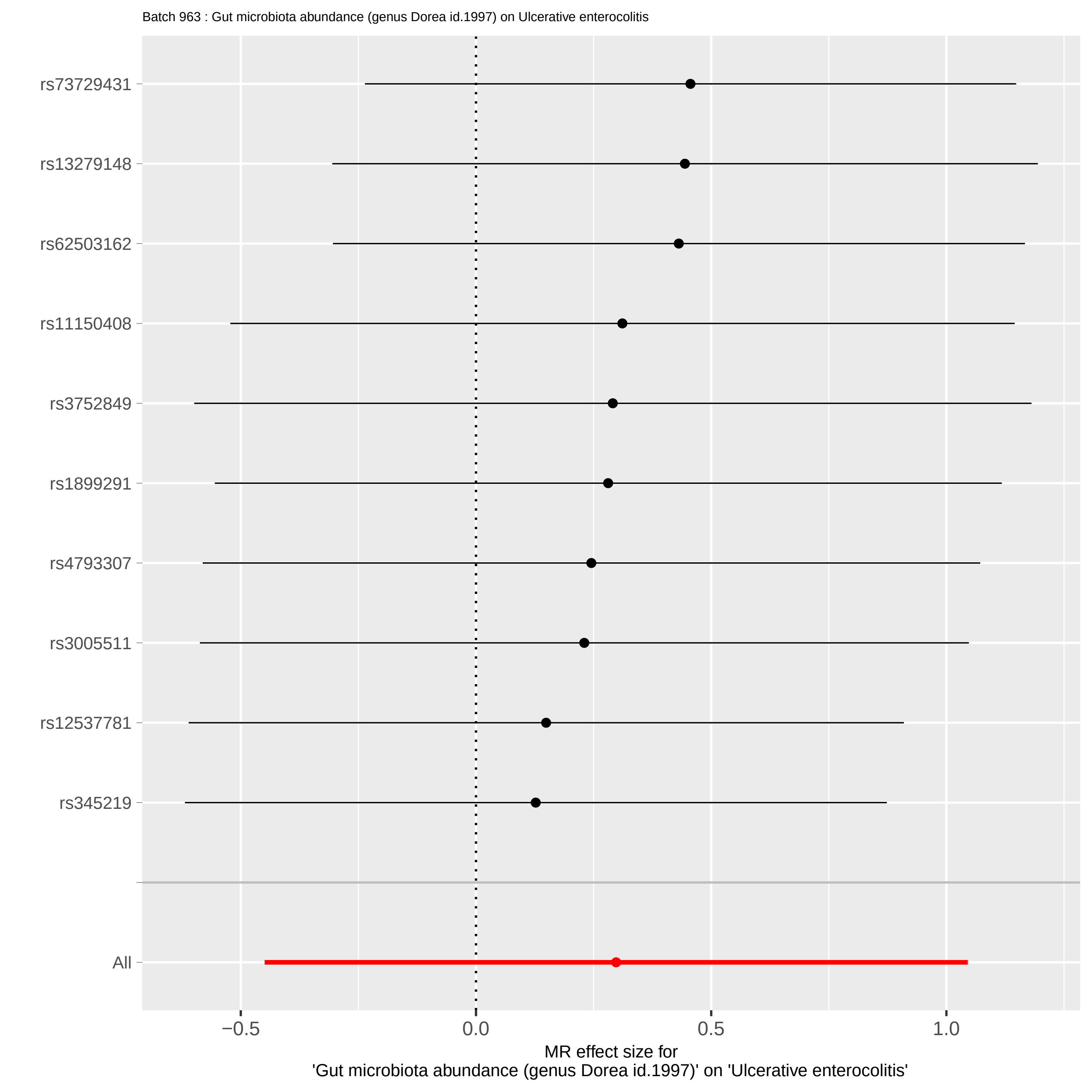
Batch 963 : Gut microbiota abundance (genus Dorea id.1997) on Ulcerative enterocolitis
rs73729431
rs13279148
rs62503162
rs11150408
rs3752849
rs1899291
rs4793307
rs3005511
rs12537781
rs345219
All
−0.5
0.0
0.5
1.0
MR effect size for
'Gut microbiota abundance (genus Dorea id.1997)' on 'Ulcerative enterocolitis'

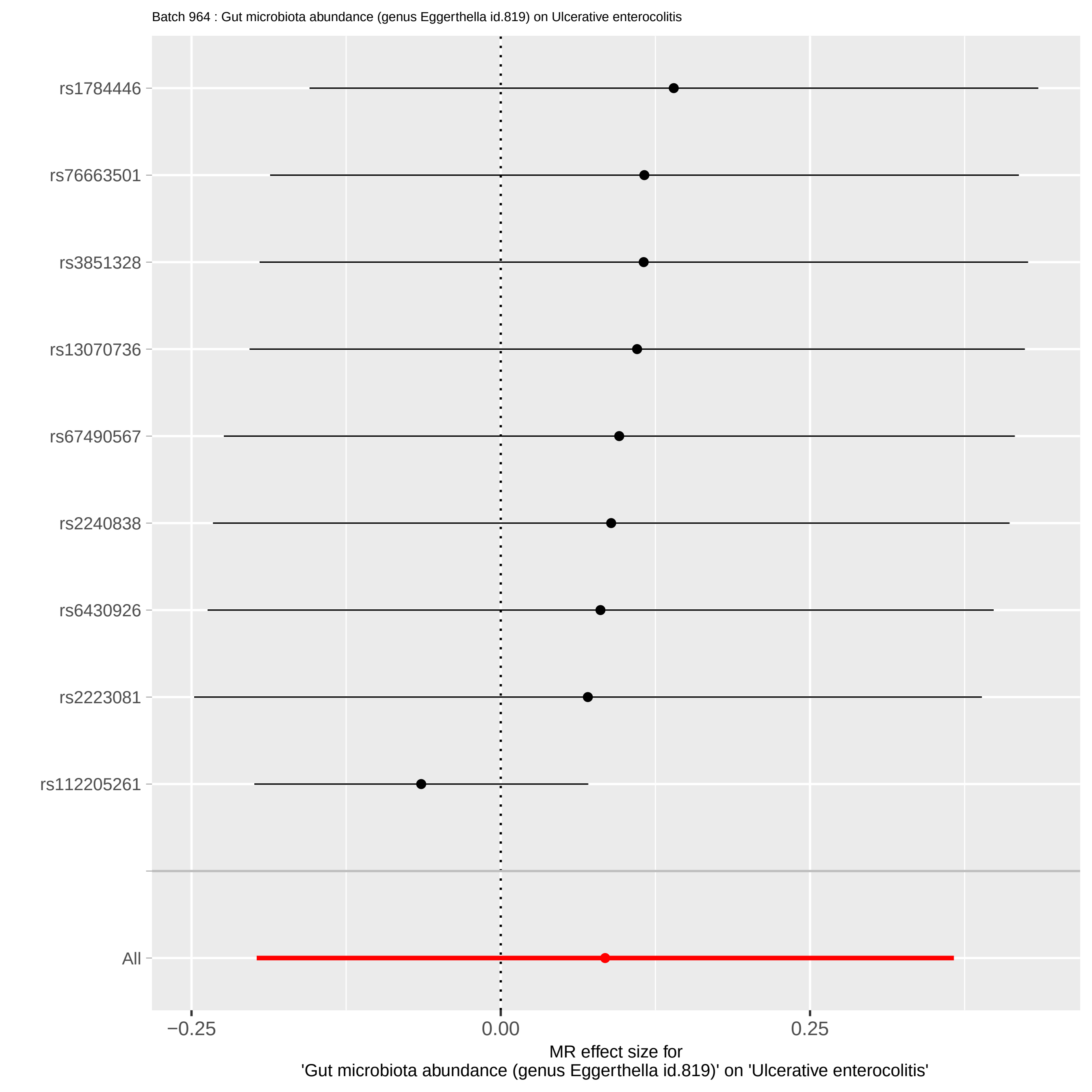
Batch 964 : Gut microbiota abundance (genus Eggerthella id.819) on Ulcerative enterocolitis
rs1784446
rs76663501
rs3851328
rs13070736
rs67490567
rs2240838
rs6430926
rs2223081
rs112205261
All
−0.25
0.00
0.25
MR effect size for
'Gut microbiota abundance (genus Eggerthella id.819)' on 'Ulcerative enterocolitis'

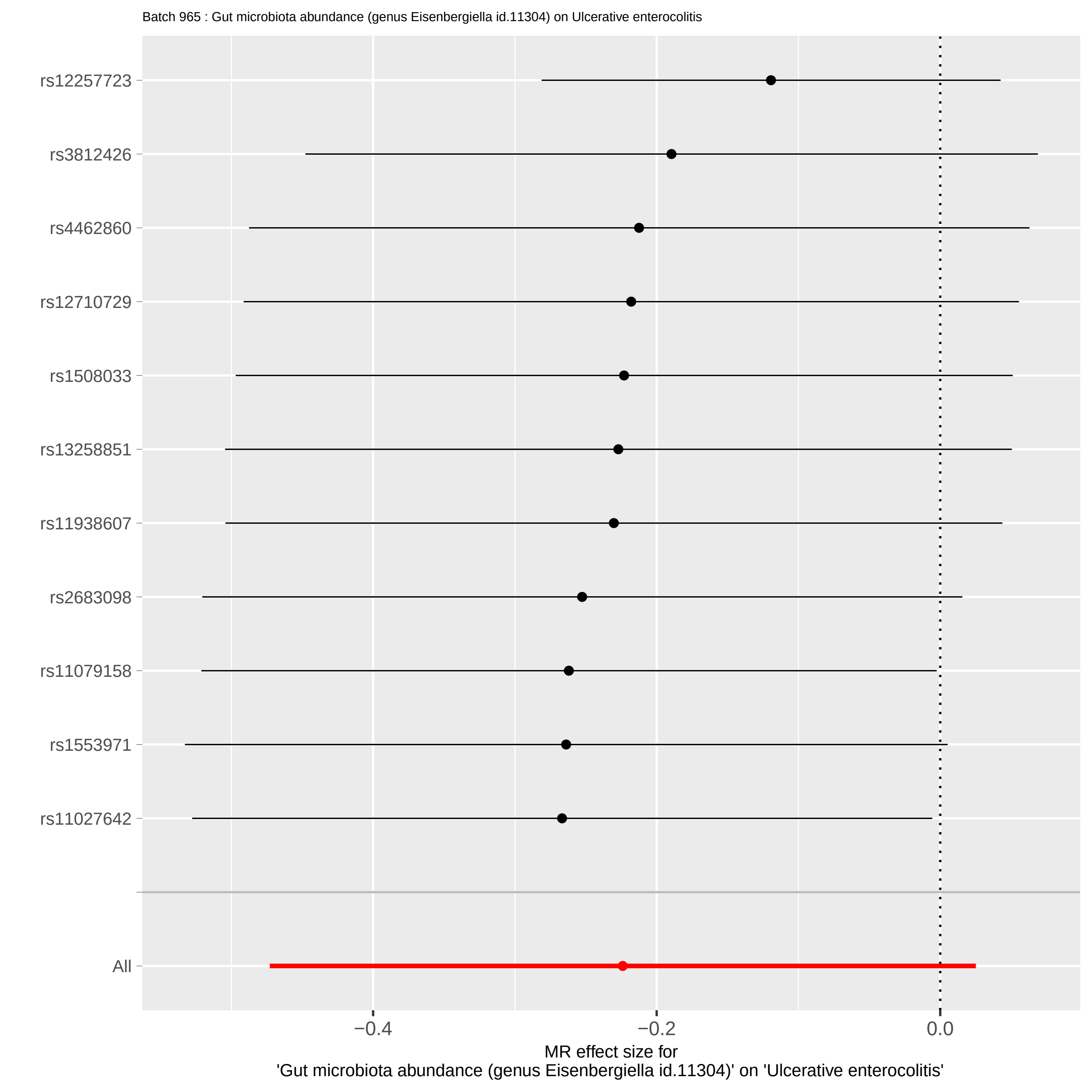

Batch 965 : Gut microbiota abundance (genus Eisenbergiella id.11304) on Ulcerative enterocolitis
rs12257723
rs3812426
rs4462860
rs12710729
rs1508033
rs13258851
rs11938607
rs2683098
rs11079158
rs1553971
rs11027642
All
−0.4
−0.2
0.0
MR effect size for
'Gut microbiota abundance (genus Eisenbergiella id.11304)' on 'Ulcerative enterocolitis'

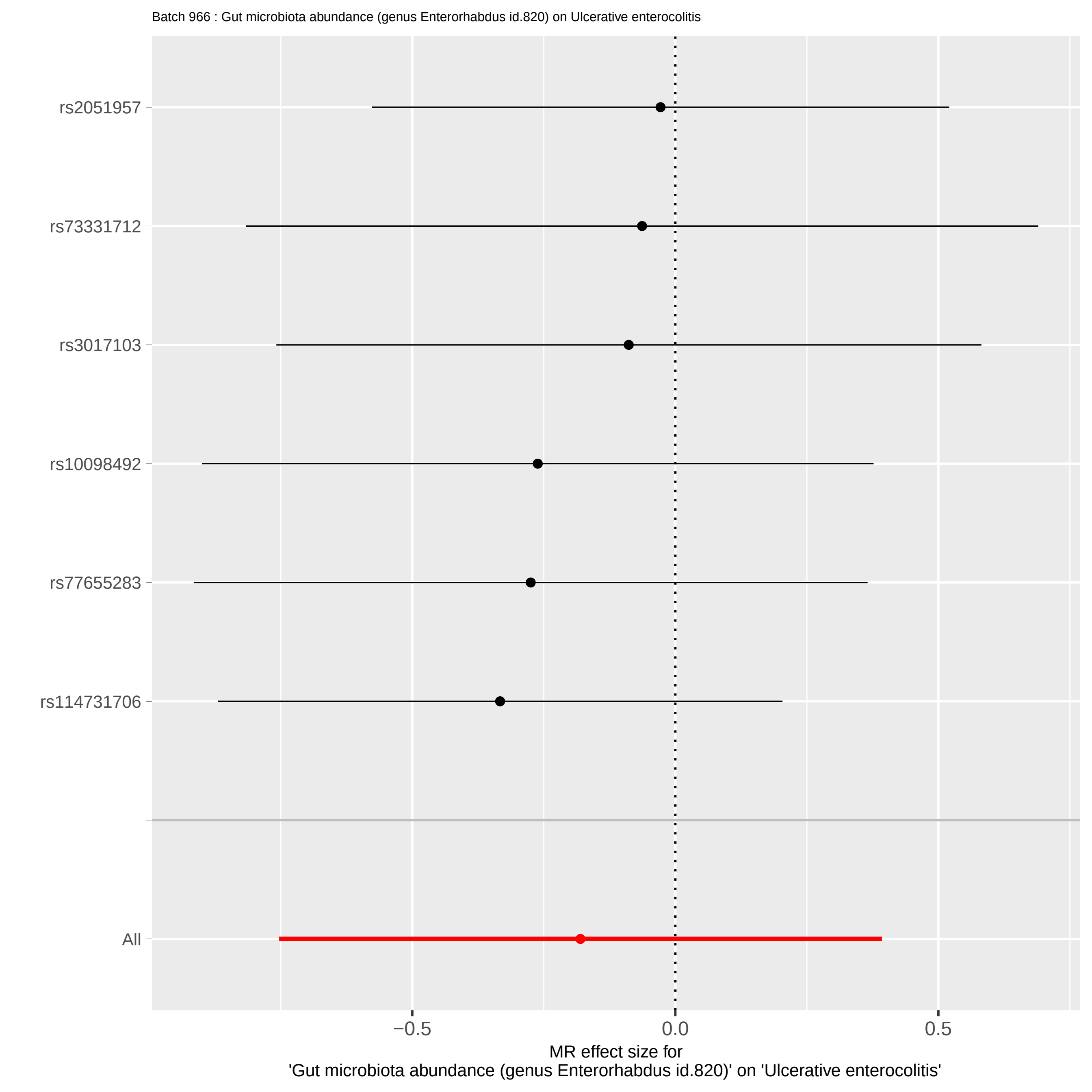
Batch 966 : Gut microbiota abundance (genus Enterorhabdus id.820) on Ulcerative enterocolitis
rs2051957
rs73331712
rs3017103
rs10098492
rs77655283
rs114731706
All
−0.5
0.0
0.5
MR effect size for
'Gut microbiota abundance (genus Enterorhabdus id.820)' on 'Ulcerative enterocolitis'

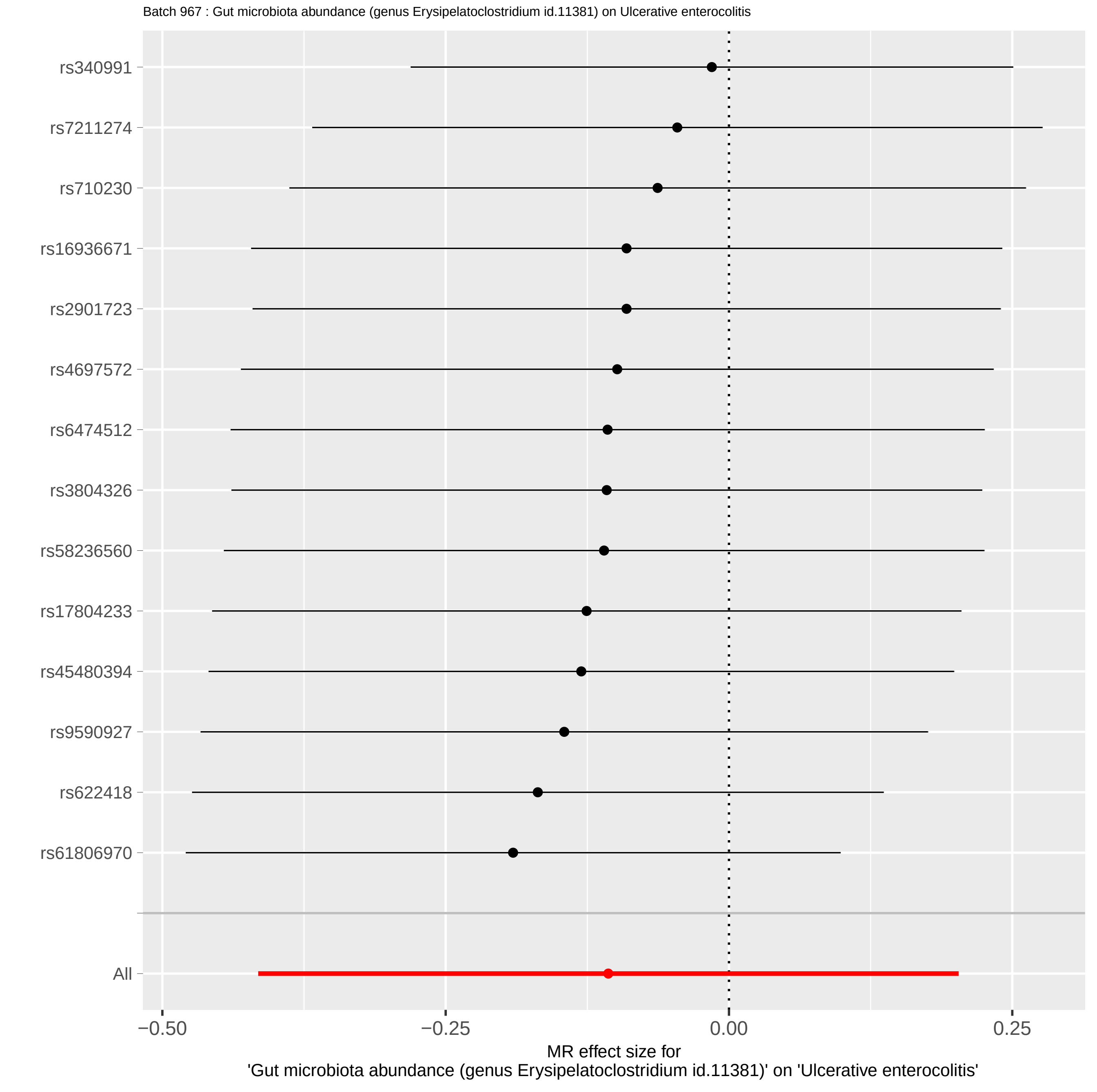

Batch 967 : Gut microbiota abundance (genus Erysipelatoclostridium id.11381) on Ulcerative enterocolitis
rs340991
rs7211274
rs710230
rs16936671
rs2901723
rs4697572
rs6474512
rs3804326
rs58236560
rs17804233
rs45480394
rs9590927
rs622418
rs61806970
All
−0.50
−0.25
0.00
0.25
MR effect size for
'Gut microbiota abundance (genus Erysipelatoclostridium id.11381)' on 'Ulcerative enterocolitis'

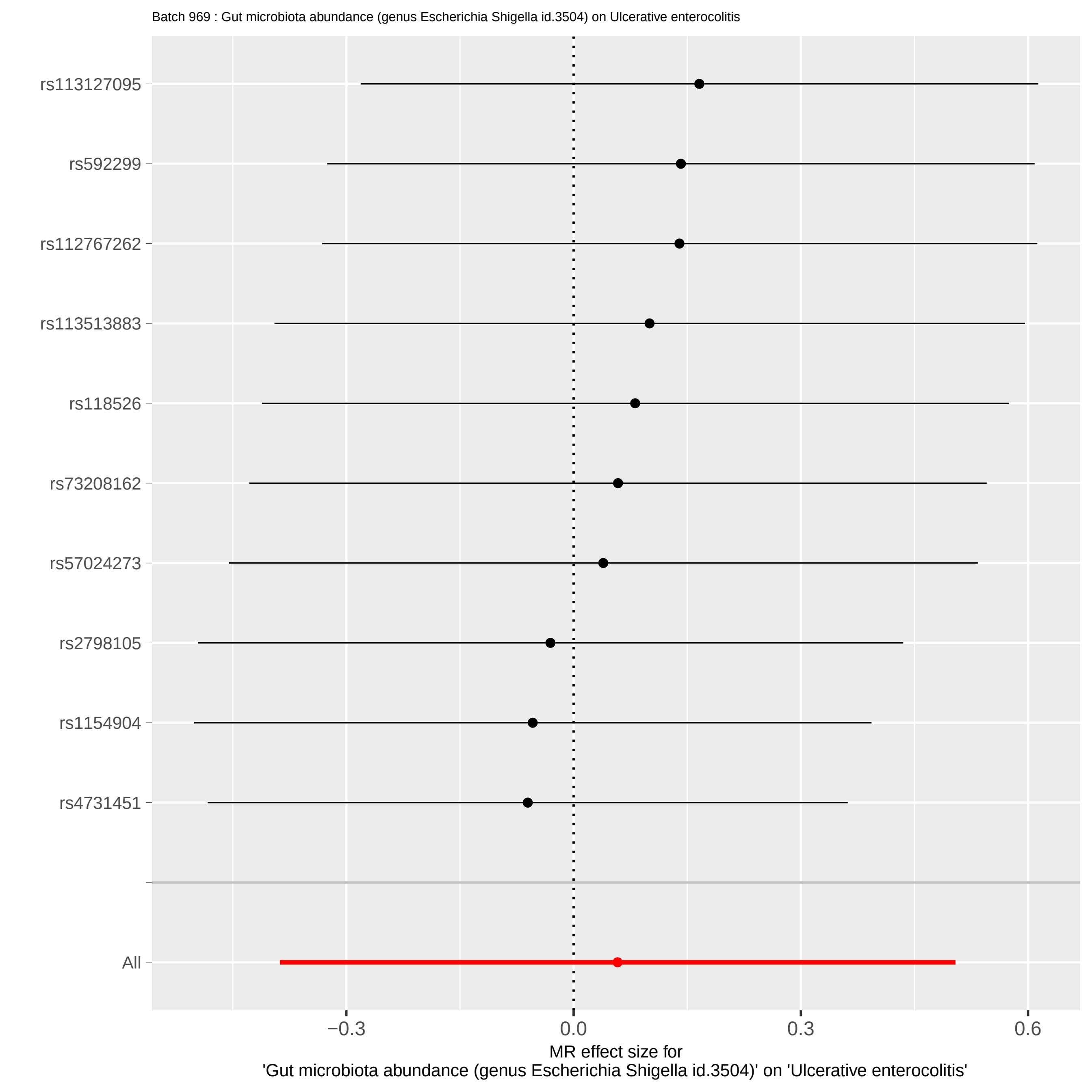

Batch 969 : Gut microbiota abundance (genus Escherichia Shigella id.3504) on Ulcerative enterocolitis
rs113127095
rs592299
rs112767262
rs113513883
rs118526
rs73208162
rs57024273
rs2798105
rs1154904
rs4731451
All
−0.3
0.0
0.3
0.6
MR effect size for
'Gut microbiota abundance (genus Escherichia Shigella id.3504)' on 'Ulcerative enterocolitis'

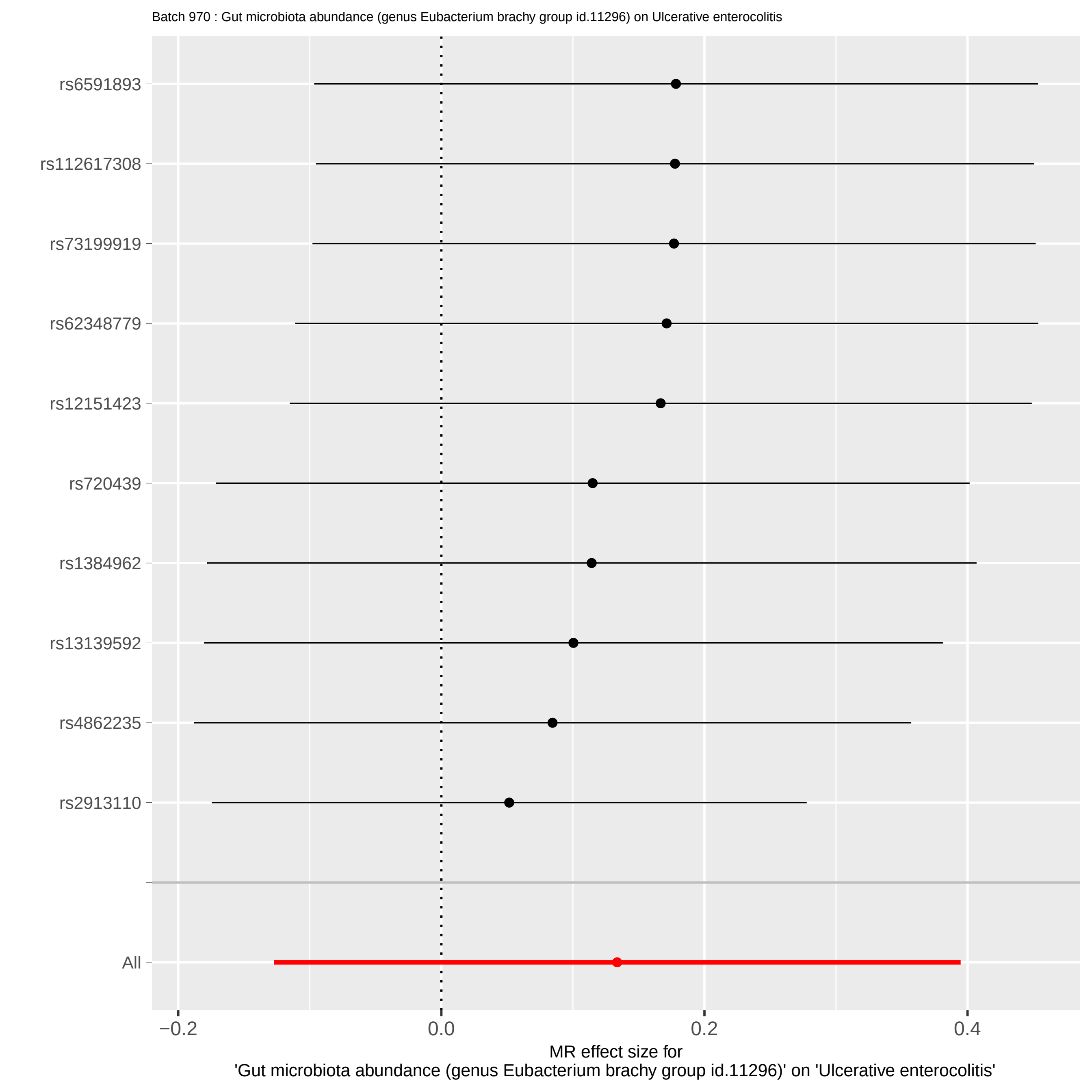

Batch 970 : Gut microbiota abundance (genus Eubacterium brachy group id.11296) on Ulcerative enterocolitis
rs6591893
rs112617308
rs73199919
rs62348779
rs12151423
rs720439
rs1384962
rs13139592
rs4862235
rs2913110
All
−0.2
0.0
0.2
0.4
MR effect size for
'Gut microbiota abundance (genus Eubacterium brachy group id.11296)' on 'Ulcerative enterocolitis'

Batch 971 : Gut microbiota abundance (genus Eubacterium coprostanoligenes group id.11375) on Ulcerative enterocolitis

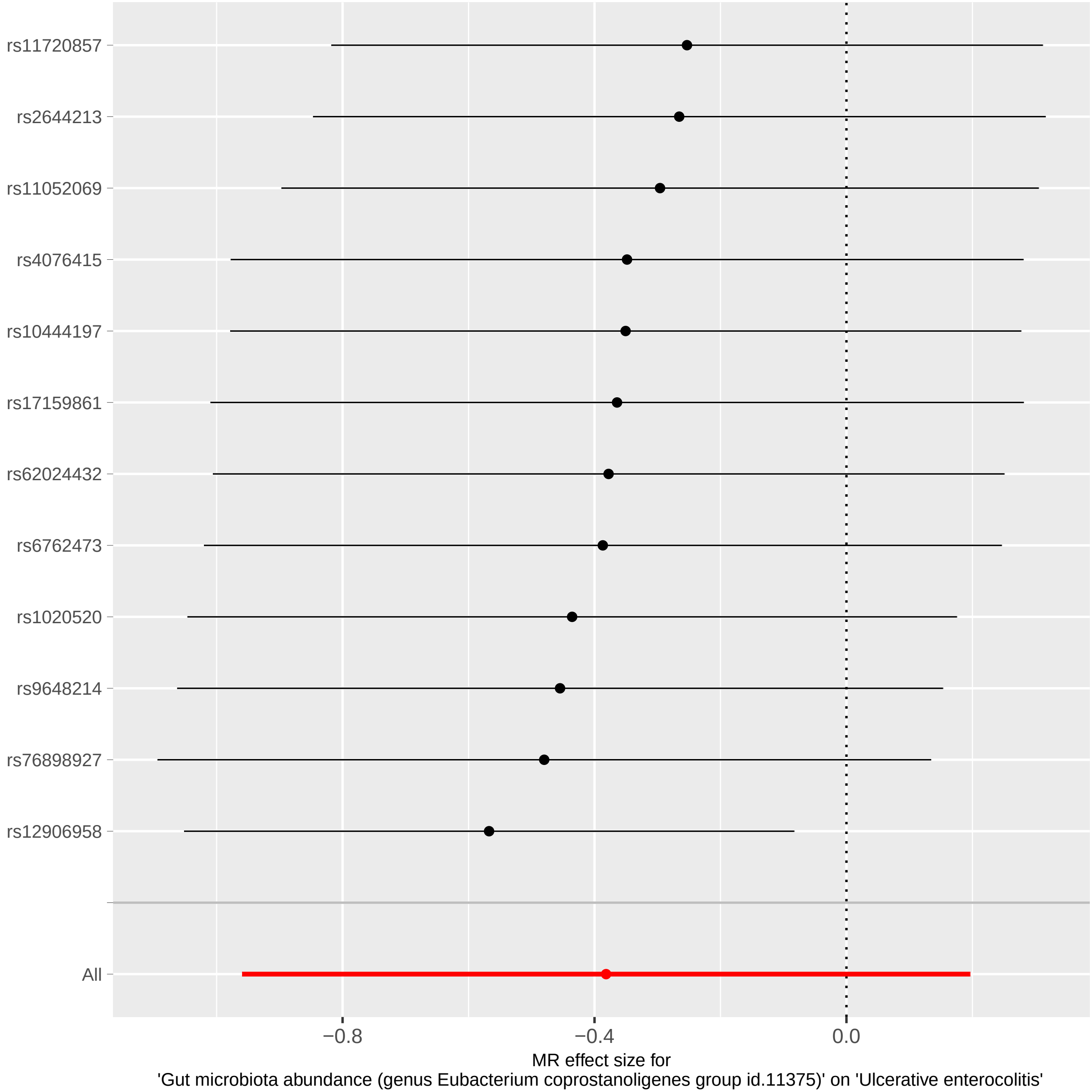

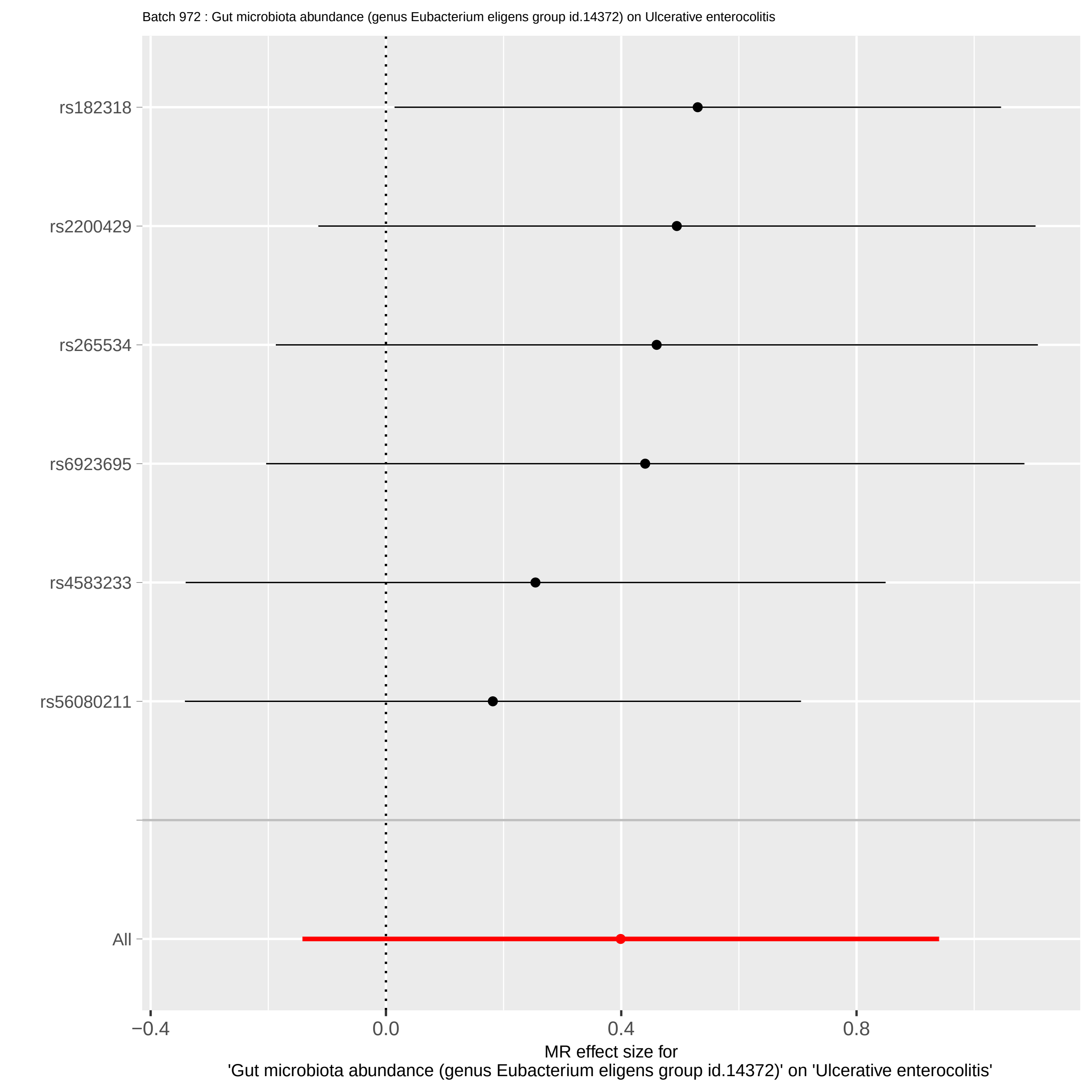

Batch 972 : Gut microbiota abundance (genus Eubacterium eligens group id.14372) on Ulcerative enterocolitis
rs182318
rs2200429
rs265534
rs6923695
rs4583233
rs56080211
All
−0.4
0.0
0.4
0.8
MR effect size for
'Gut microbiota abundance (genus Eubacterium eligens group id.14372)' on 'Ulcerative enterocolitis'

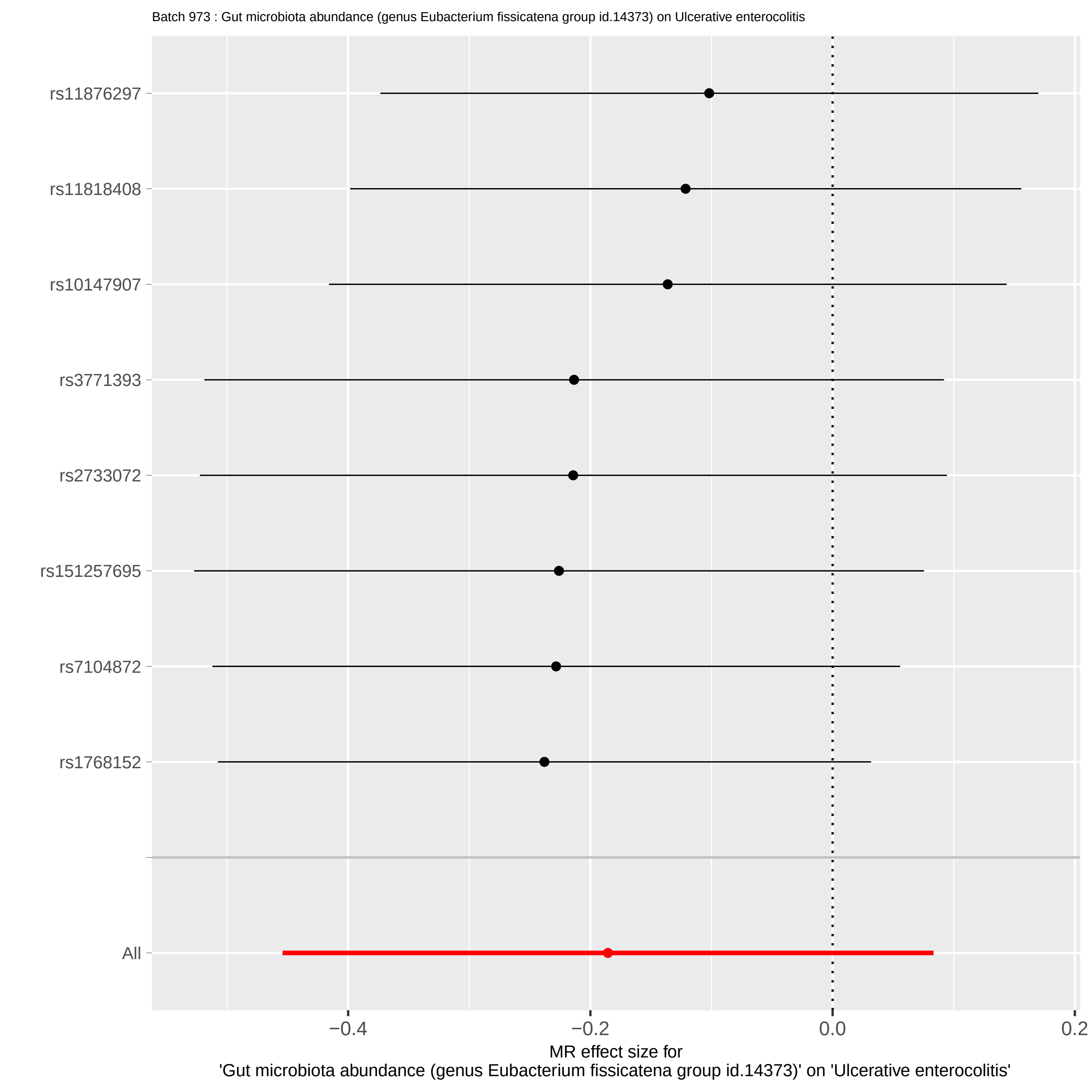
Batch 973 : Gut microbiota abundance (genus Eubacterium fissicatena group id.14373) on Ulcerative enterocolitis
rs11876297
rs11818408
rs10147907
rs3771393
rs2733072
rs151257695
rs7104872
rs1768152
All
−0.4
−0.2
0.0
0.2
MR effect size for
'Gut microbiota abundance (genus Eubacterium fissicatena group id.14373)' on 'Ulcerative enterocolitis'

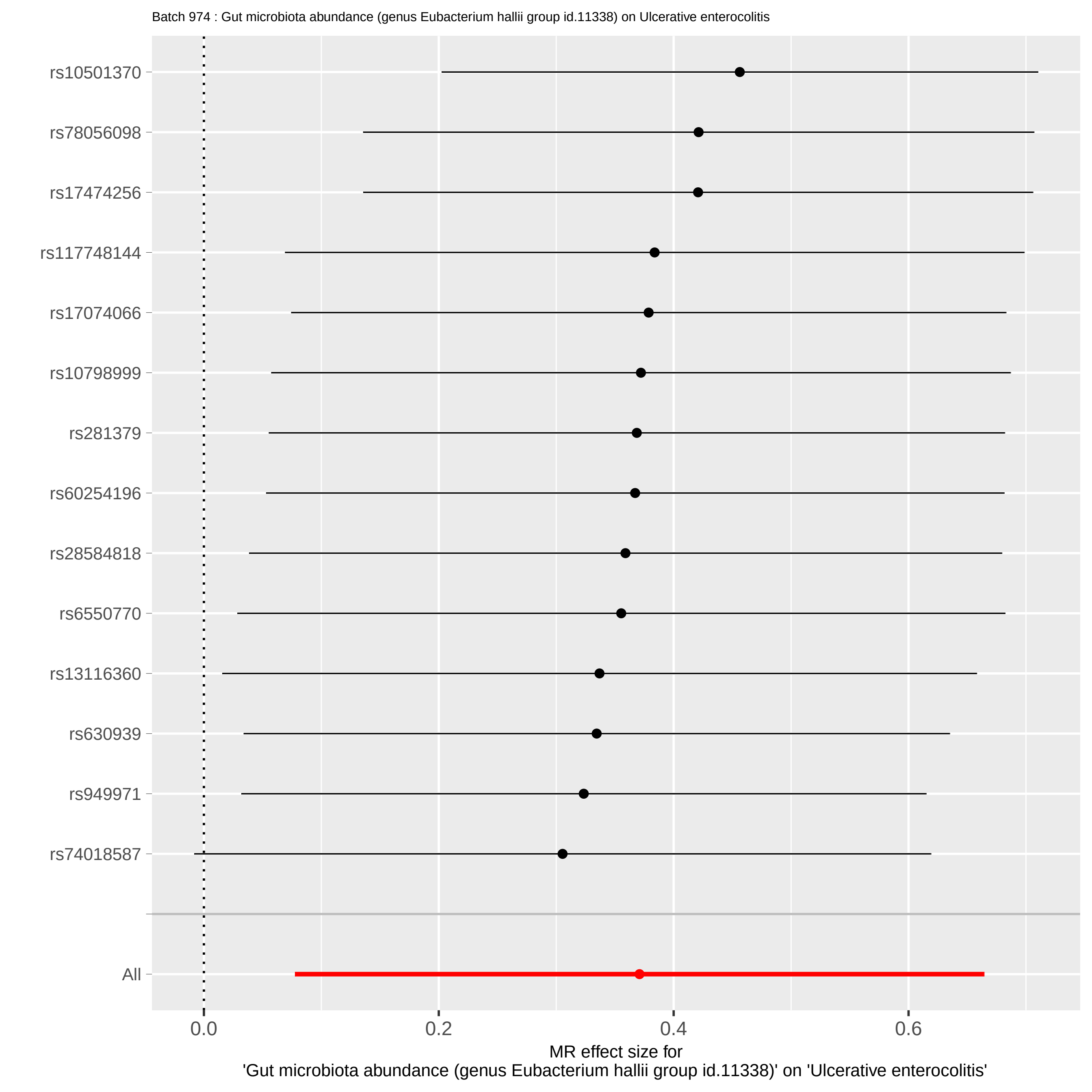
Batch 974 : Gut microbiota abundance (genus Eubacterium hallii group id.11338) on Ulcerative enterocolitis
rs10501370
rs78056098
rs17474256
rs117748144
rs17074066
rs10798999
rs281379
rs60254196
rs28584818
rs6550770
rs13116360
rs630939
rs949971
rs74018587
All
0.0
0.2
0.4
0.6
MR effect size for
'Gut microbiota abundance (genus Eubacterium hallii group id.11338)' on 'Ulcerative enterocolitis'

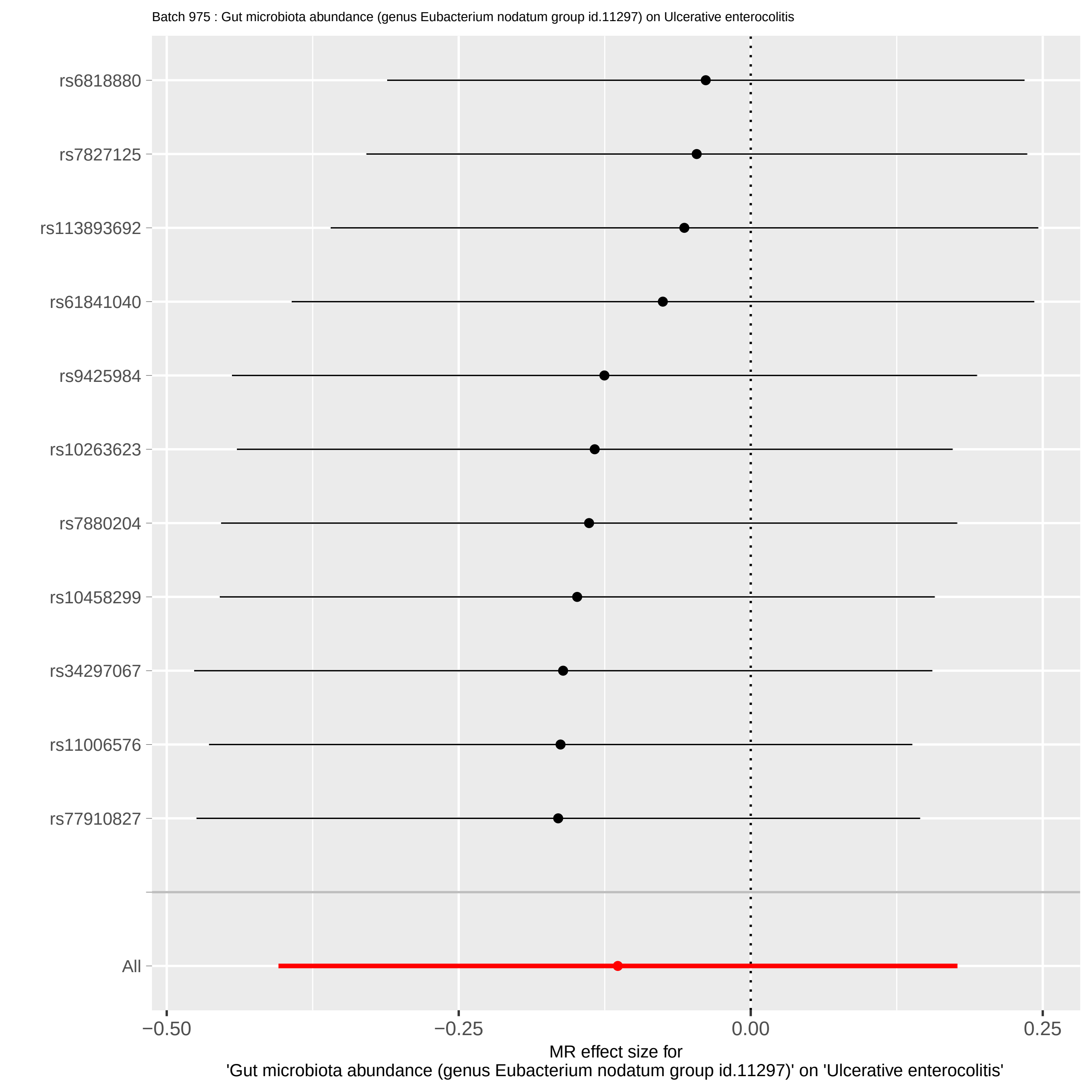

Batch 975 : Gut microbiota abundance (genus Eubacterium nodatum group id.11297) on Ulcerative enterocolitis
rs6818880
rs7827125
rs113893692
rs61841040
rs9425984
rs10263623
rs7880204
rs10458299
rs34297067
rs11006576
rs77910827
All
−0.50
−0.25
0.00
0.25
MR effect size for
'Gut microbiota abundance (genus Eubacterium nodatum group id.11297)' on 'Ulcerative enterocolitis'

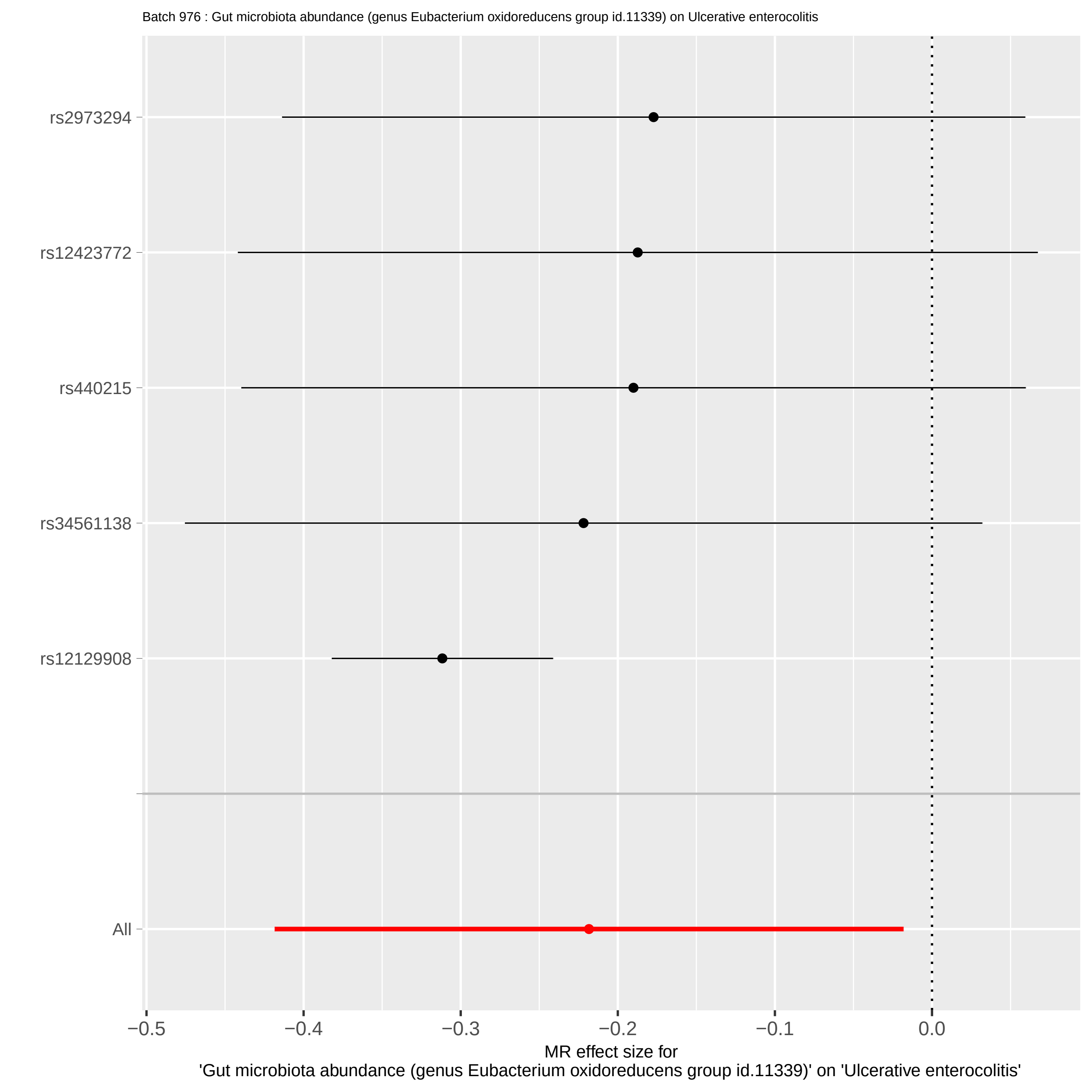

Batch 976 : Gut microbiota abundance (genus Eubacterium oxidoreducens group id.11339) on Ulcerative enterocolitis
rs2973294
rs12423772
rs440215
rs34561138
rs12129908
All
−0.5
−0.4
−0.3
−0.2
−0.1
0.0
MR effect size for
'Gut microbiota abundance (genus Eubacterium oxidoreducens group id.11339)' on 'Ulcerative enterocolitis'

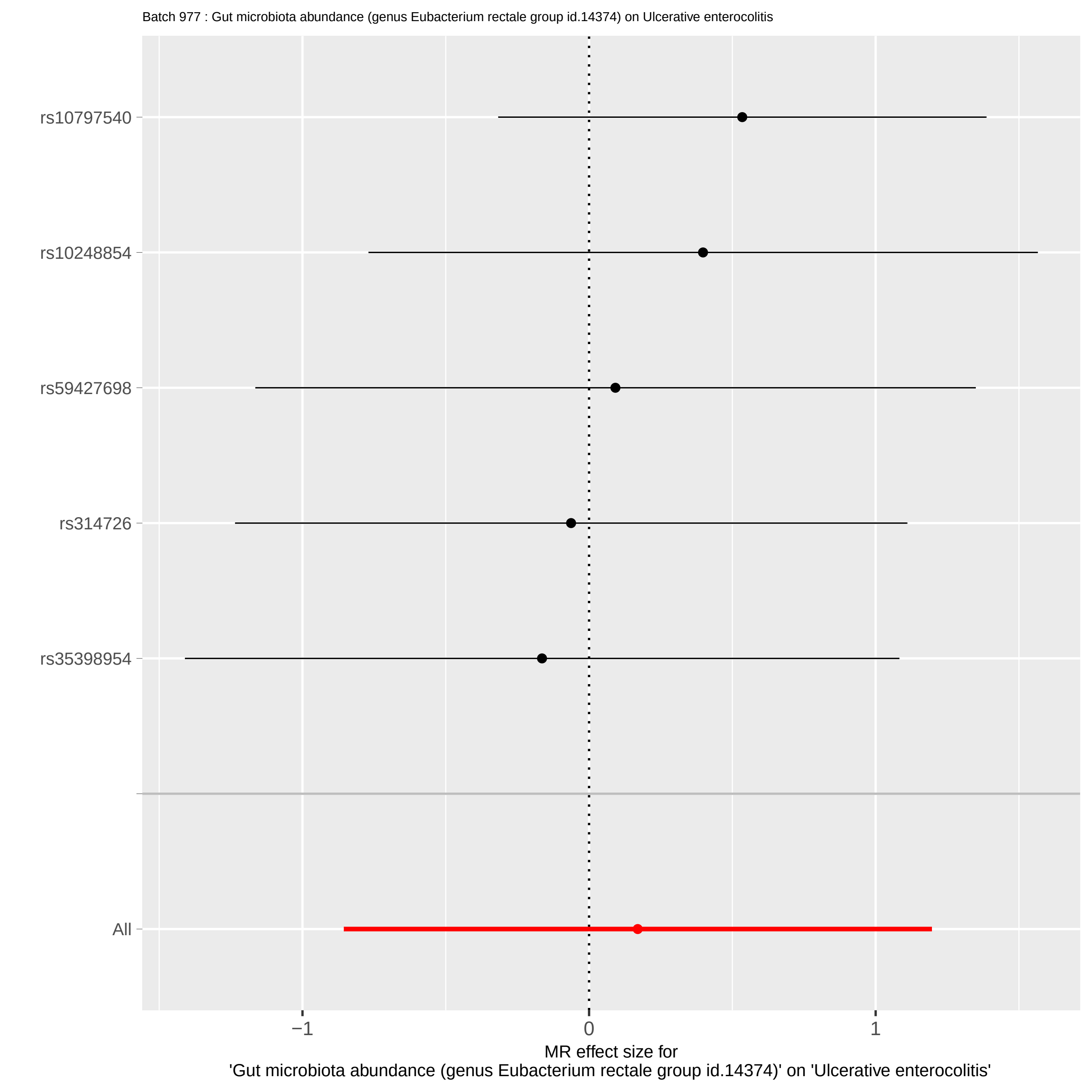

Batch 977 : Gut microbiota abundance (genus Eubacterium rectale group id.14374) on Ulcerative enterocolitis
rs10797540
rs10248854
rs59427698
rs314726
rs35398954
All
−1
0
1
MR effect size for
'Gut microbiota abundance (genus Eubacterium rectale group id.14374)' on 'Ulcerative enterocolitis'

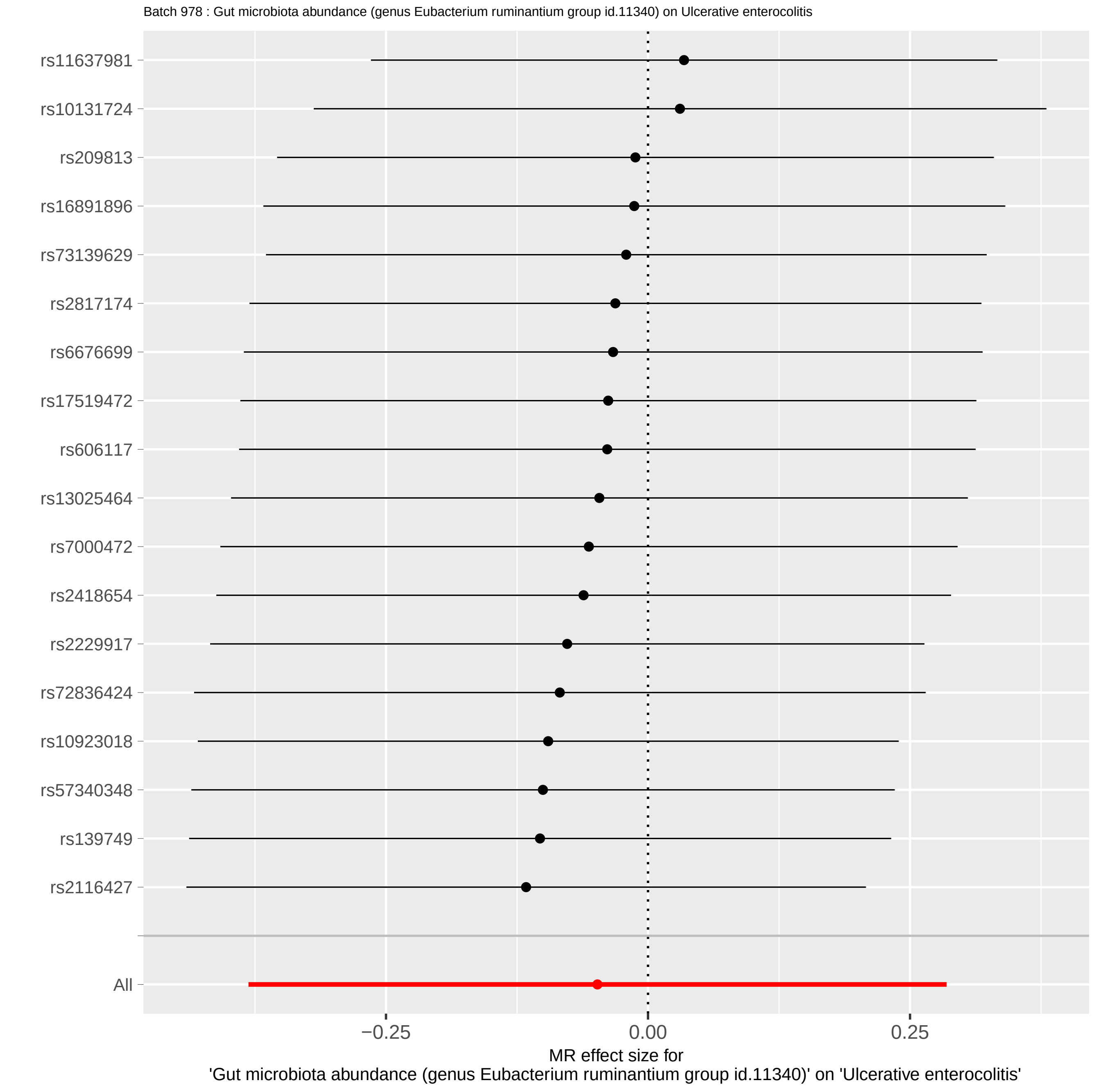

Batch 978 : Gut microbiota abundance (genus Eubacterium ruminantium group id.11340) on Ulcerative enterocolitis
rs11637981
rs10131724
rs209813
rs16891896
rs73139629
rs2817174
rs6676699
rs17519472
rs606117
rs13025464
rs7000472
rs2418654
rs2229917
rs72836424
rs10923018
rs57340348
rs139749
rs2116427
All
−0.25
0.00
0.25
MR effect size for
'Gut microbiota abundance (genus Eubacterium ruminantium group id.11340)' on 'Ulcerative enterocolitis'

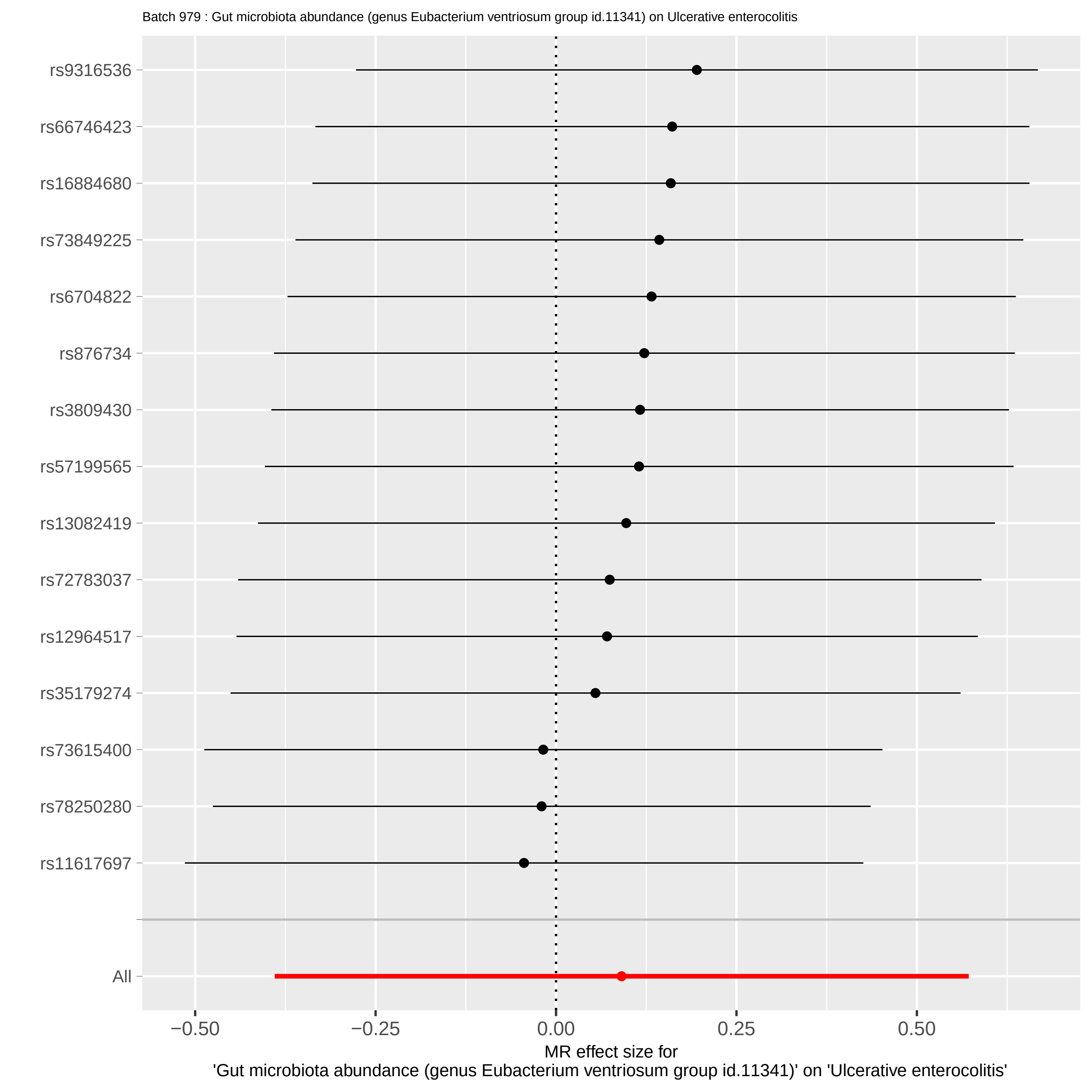
Batch 979 : Gut microbiota abundance (genus Eubacterium ventriosum group id.11341) on Ulcerative enterocolitis
rs9316536
rs66746423
rs16884680
rs73849225
rs6704822
rs876734
rs3809430
rs57199565
rs13082419
rs72783037
rs12964517
rs35179274
rs73615400
rs78250280
rs11617697
All
−0.50
−0.25
0.00
0.25
0.50
MR effect size for
'Gut microbiota abundance (genus Eubacterium ventriosum group id.11341)' on 'Ulcerative enterocolitis'

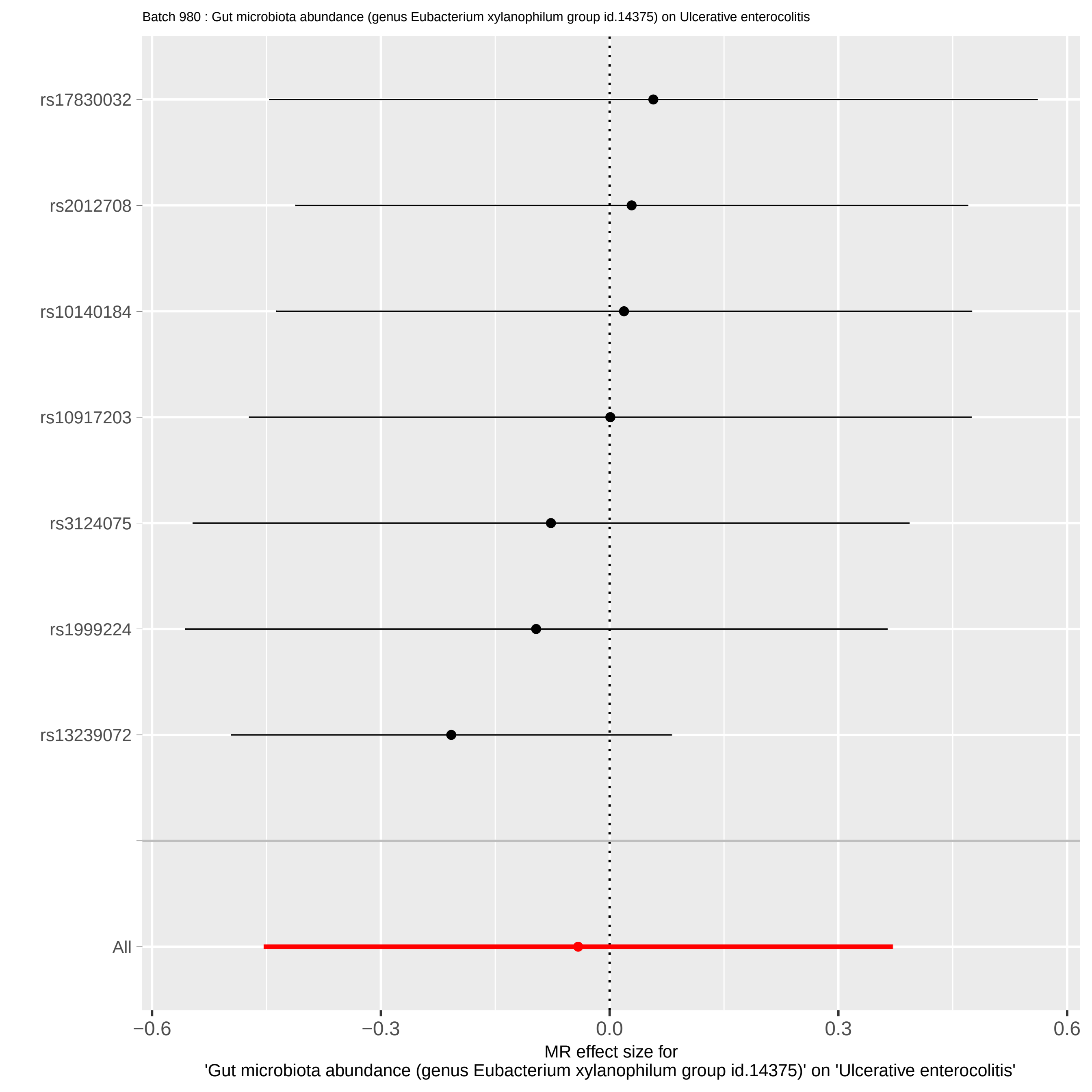
Batch 980 : Gut microbiota abundance (genus Eubacterium xylanophilum group id.14375) on Ulcerative enterocolitis
rs17830032
rs2012708
rs10140184
rs10917203
rs3124075
rs1999224
rs13239072
All
−0.6
−0.3
0.0
0.3
0.6
MR effect size for
'Gut microbiota abundance (genus Eubacterium xylanophilum group id.14375)' on 'Ulcerative enterocolitis'

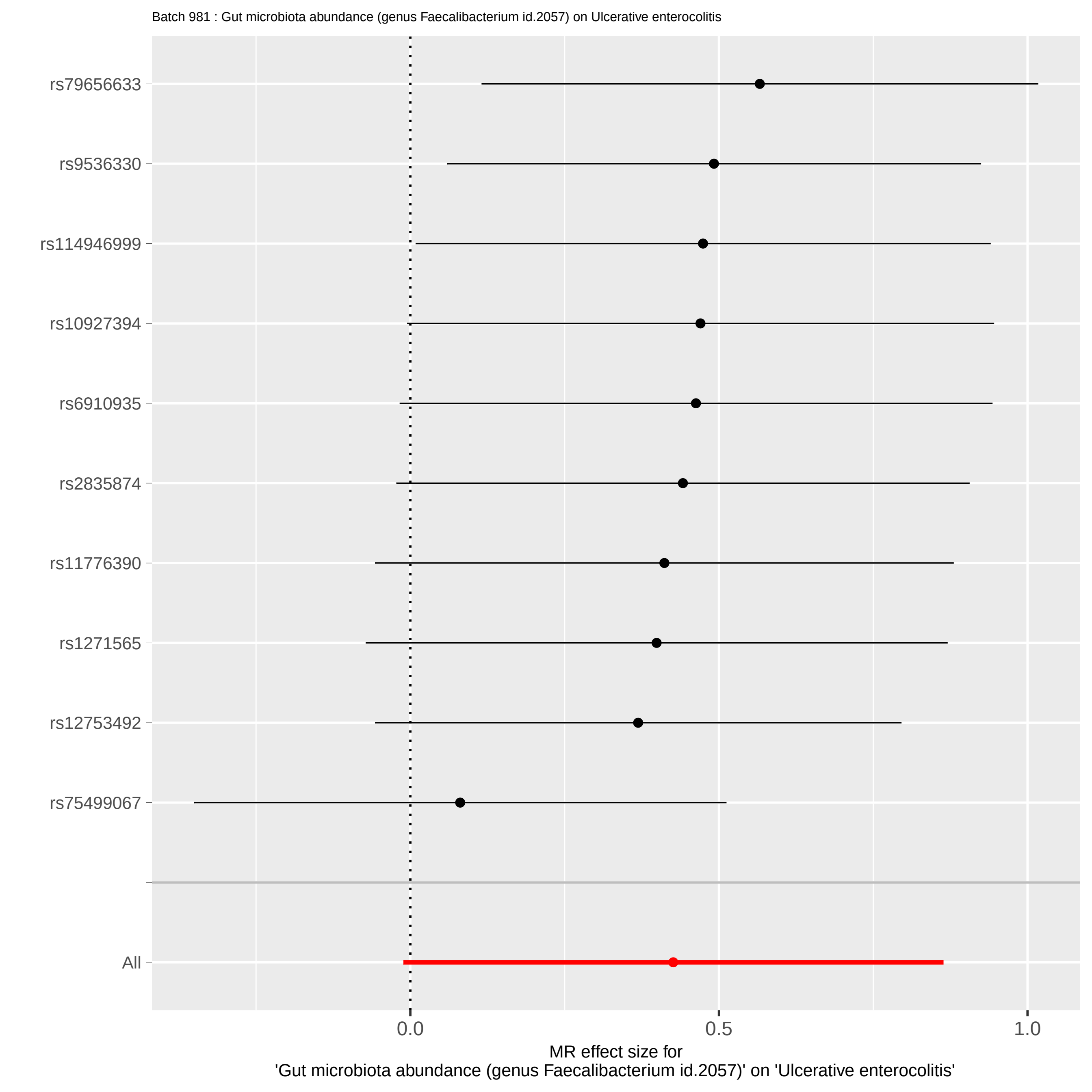
Batch 981 : Gut microbiota abundance (genus Faecalibacterium id.2057) on Ulcerative enterocolitis
rs79656633
rs9536330
rs114946999
rs10927394
rs6910935
rs2835874
rs11776390
rs1271565
rs12753492
rs75499067
All
0.0
0.5
1.0
MR effect size for
'Gut microbiota abundance (genus Faecalibacterium id.2057)' on 'Ulcerative enterocolitis'

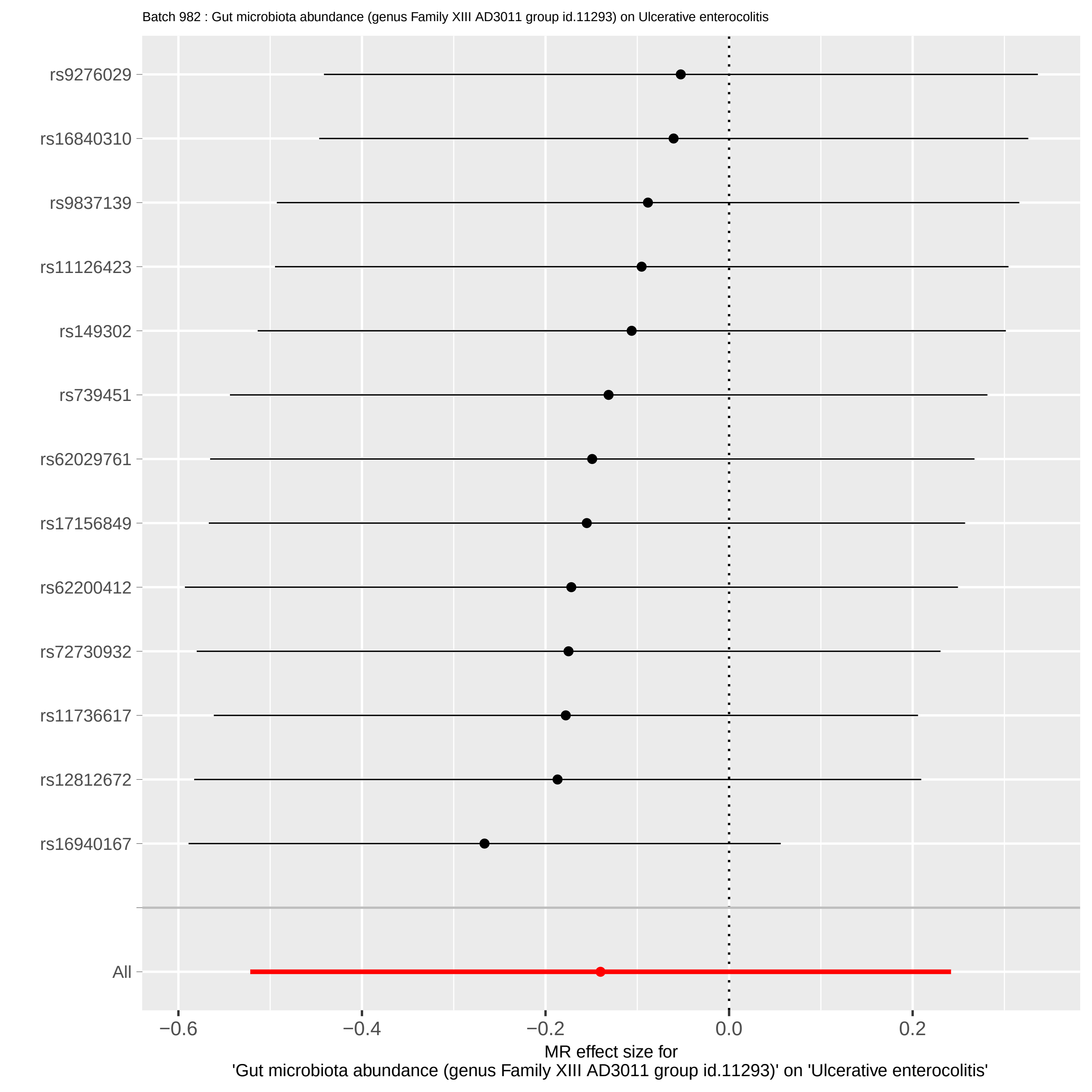
Batch 982 : Gut microbiota abundance (genus Family XIII AD3011 group id.11293) on Ulcerative enterocolitis
rs9276029
rs16840310
rs9837139
rs11126423
rs149302
rs739451
rs62029761
rs17156849
rs62200412
rs72730932
rs11736617
rs12812672
rs16940167
All
−0.6
−0.4
−0.2
0.0
0.2
MR effect size for
'Gut microbiota abundance (genus Family XIII AD3011 group id.11293)' on 'Ulcerative enterocolitis'

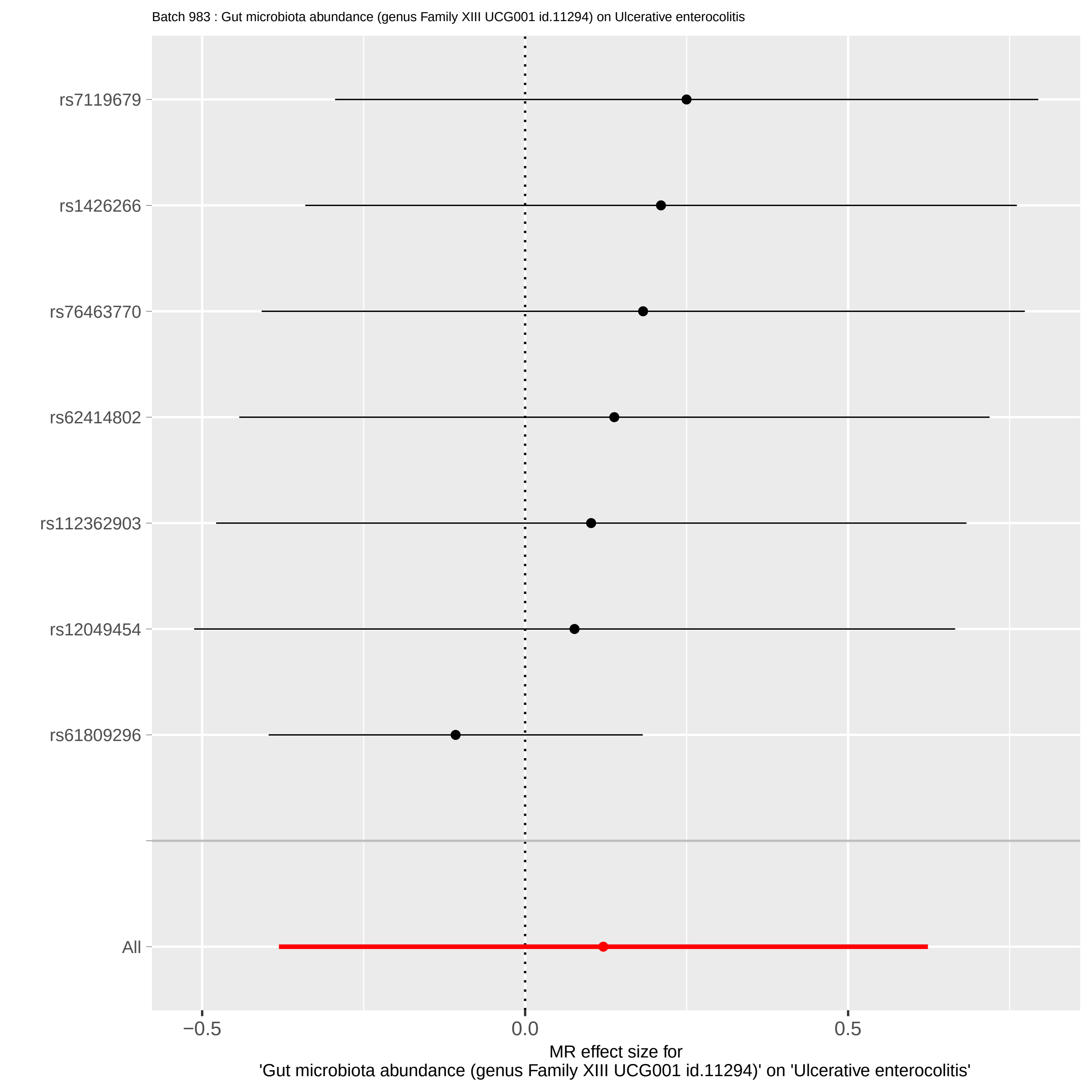
Batch 983 : Gut microbiota abundance (genus Family XIII UCG001 id.11294) on Ulcerative enterocolitis
rs7119679
rs1426266
rs76463770
rs62414802
rs112362903
rs12049454
rs61809296
All
−0.5
0.0
0.5
MR effect size for
'Gut microbiota abundance (genus Family XIII UCG001 id.11294)' on 'Ulcerative enterocolitis'

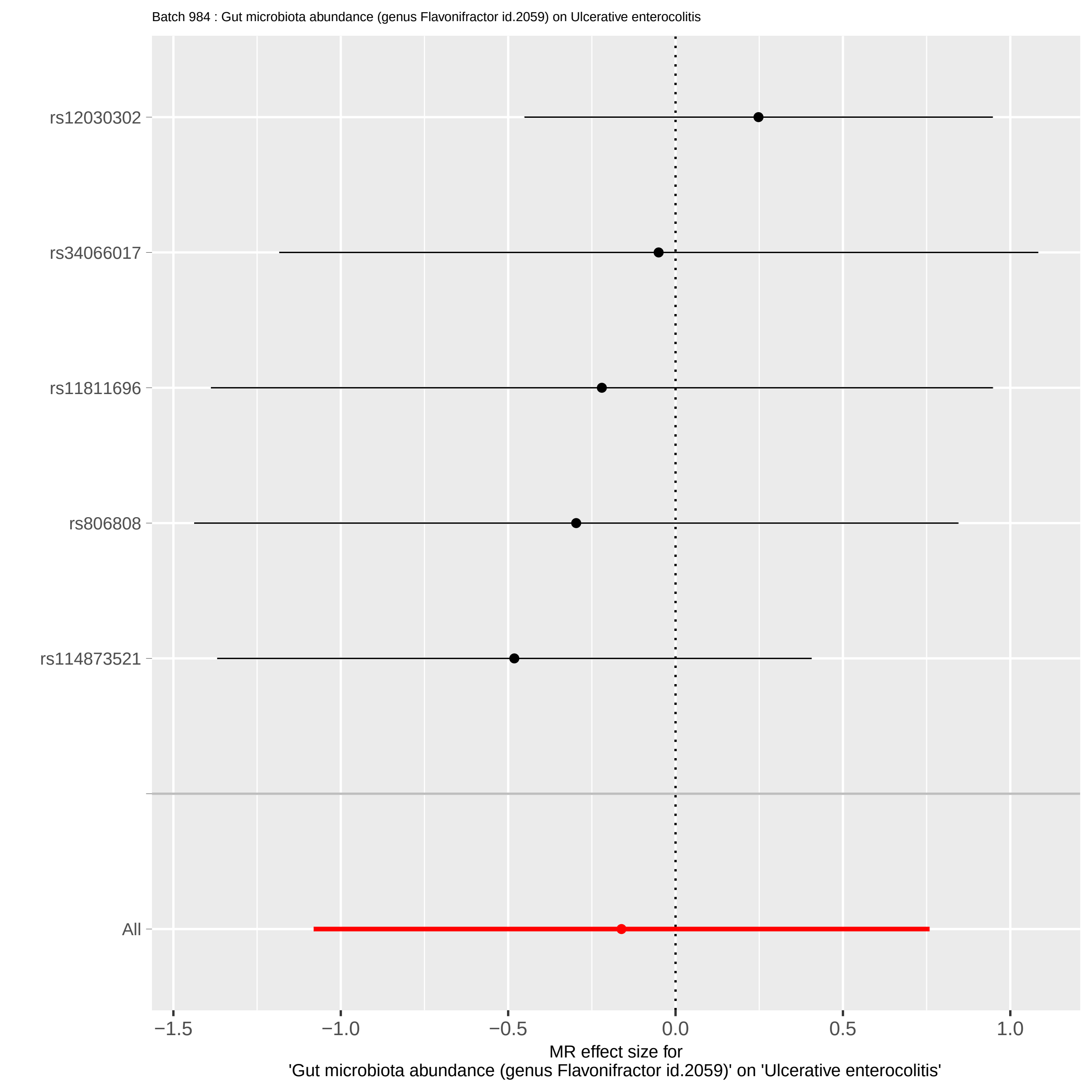

Batch 984 : Gut microbiota abundance (genus Flavonifractor id.2059) on Ulcerative enterocolitis
rs12030302
rs34066017
rs11811696
rs806808
rs114873521
All
−1.5
−1.0
−0.5
0.0
0.5
1.0
MR effect size for
'Gut microbiota abundance (genus Flavonifractor id.2059)' on 'Ulcerative enterocolitis'

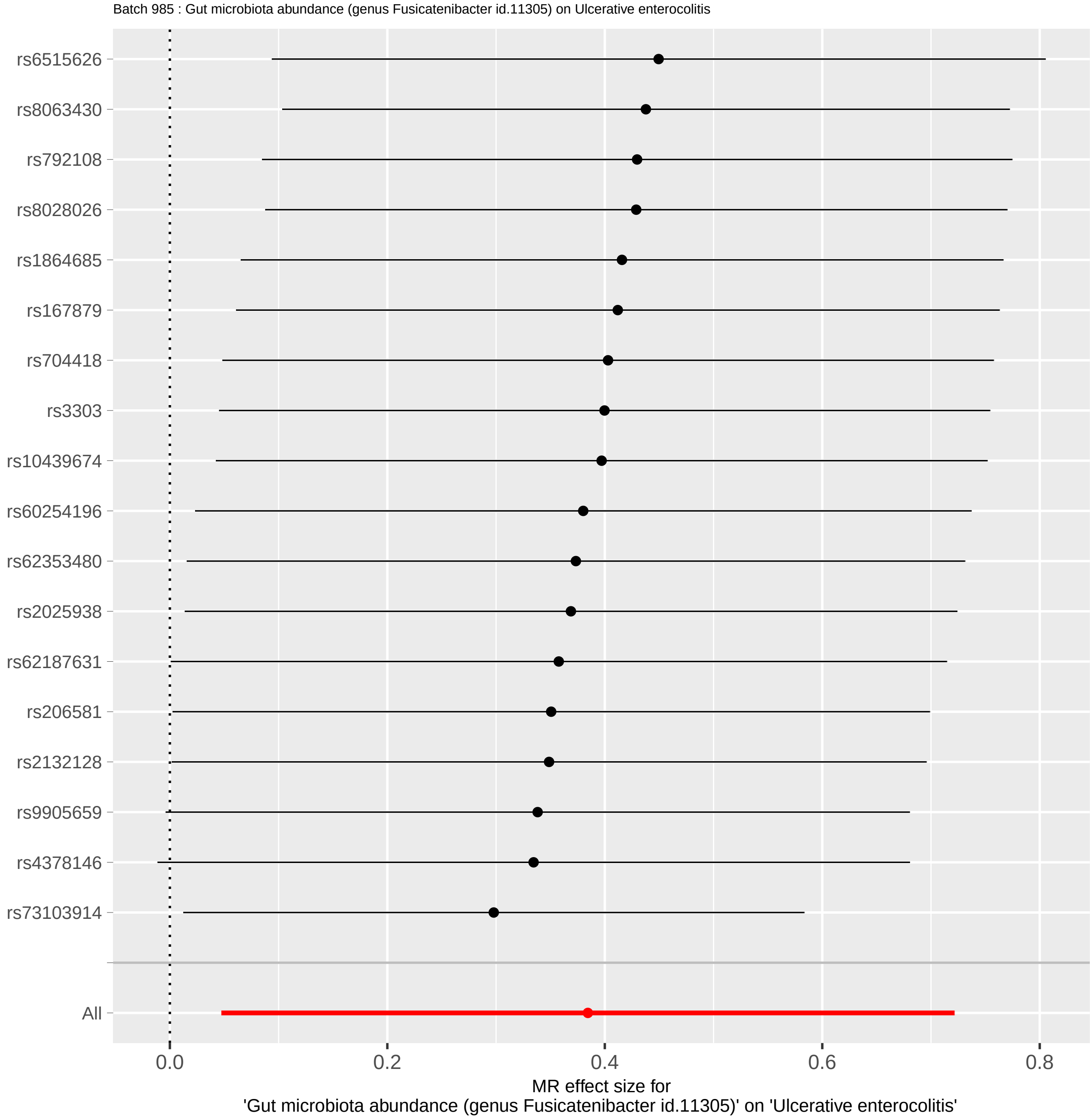
Batch 985 : Gut microbiota abundance (genus Fusicatenibacter id.11305) on Ulcerative enterocolitis
rs6515626
rs8063430
rs792108
rs8028026
rs1864685
rs167879
rs704418
rs3303
rs10439674
rs60254196
rs62353480
rs2025938
rs62187631
rs206581
rs2132128
rs9905659
rs4378146
rs73103914
All
0.0
0.2
0.4
0.6
0.8
MR effect size for
'Gut microbiota abundance (genus Fusicatenibacter id.11305)' on 'Ulcerative enterocolitis'

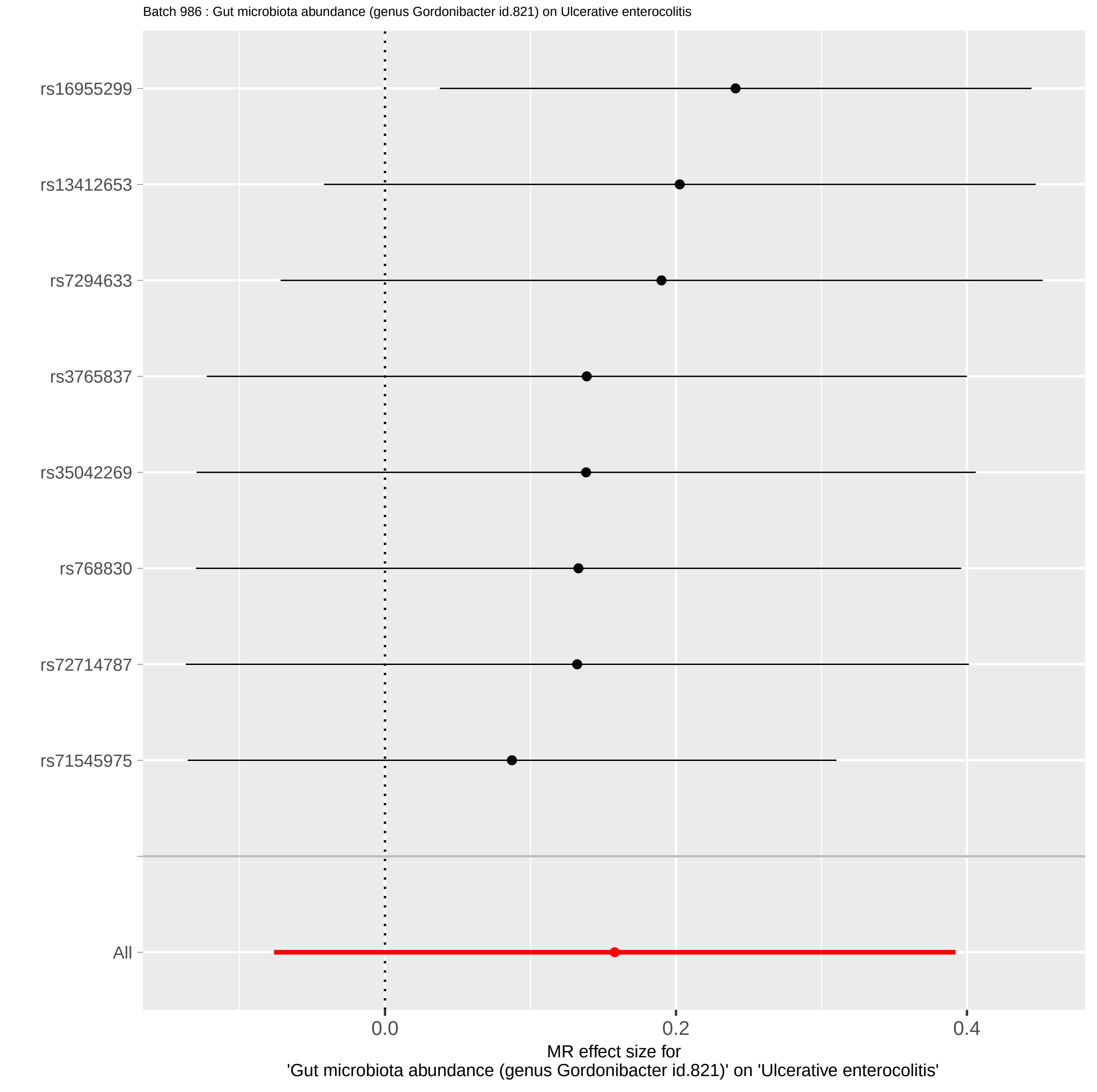

Batch 986 : Gut microbiota abundance (genus Gordonibacter id.821) on Ulcerative enterocolitis
rs16955299
rs13412653
rs7294633
rs3765837
rs35042269
rs768830
rs72714787
rs71545975
All
0.0
0.2
0.4
MR effect size for
'Gut microbiota abundance (genus Gordonibacter id.821)' on 'Ulcerative enterocolitis'

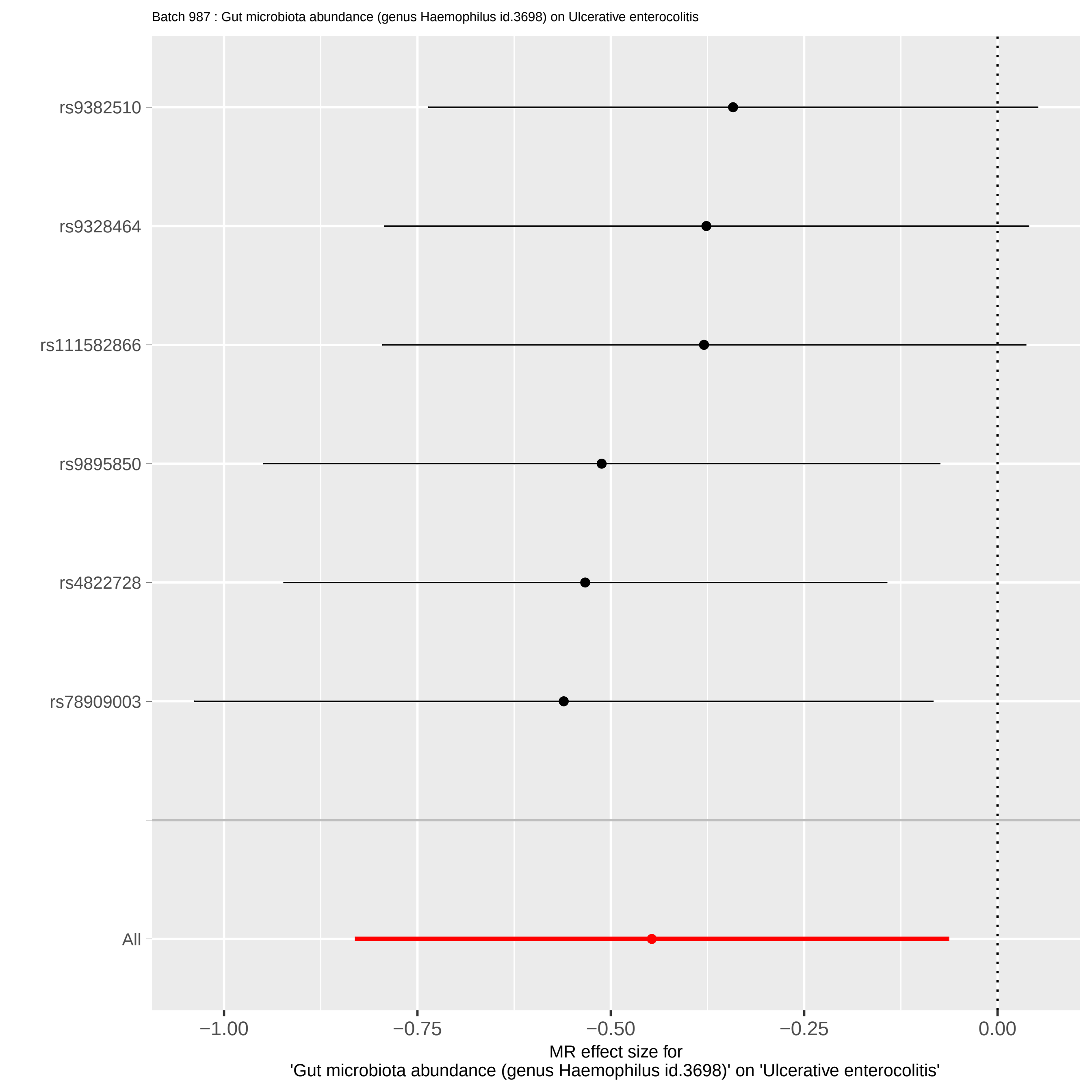
Batch 987 : Gut microbiota abundance (genus Haemophilus id.3698) on Ulcerative enterocolitis
rs9382510
rs9328464
rs111582866
rs9895850
rs4822728
rs78909003
All
−1.00
−0.75
−0.50
−0.25
0.00
MR effect size for
'Gut microbiota abundance (genus Haemophilus id.3698)' on 'Ulcerative enterocolitis'

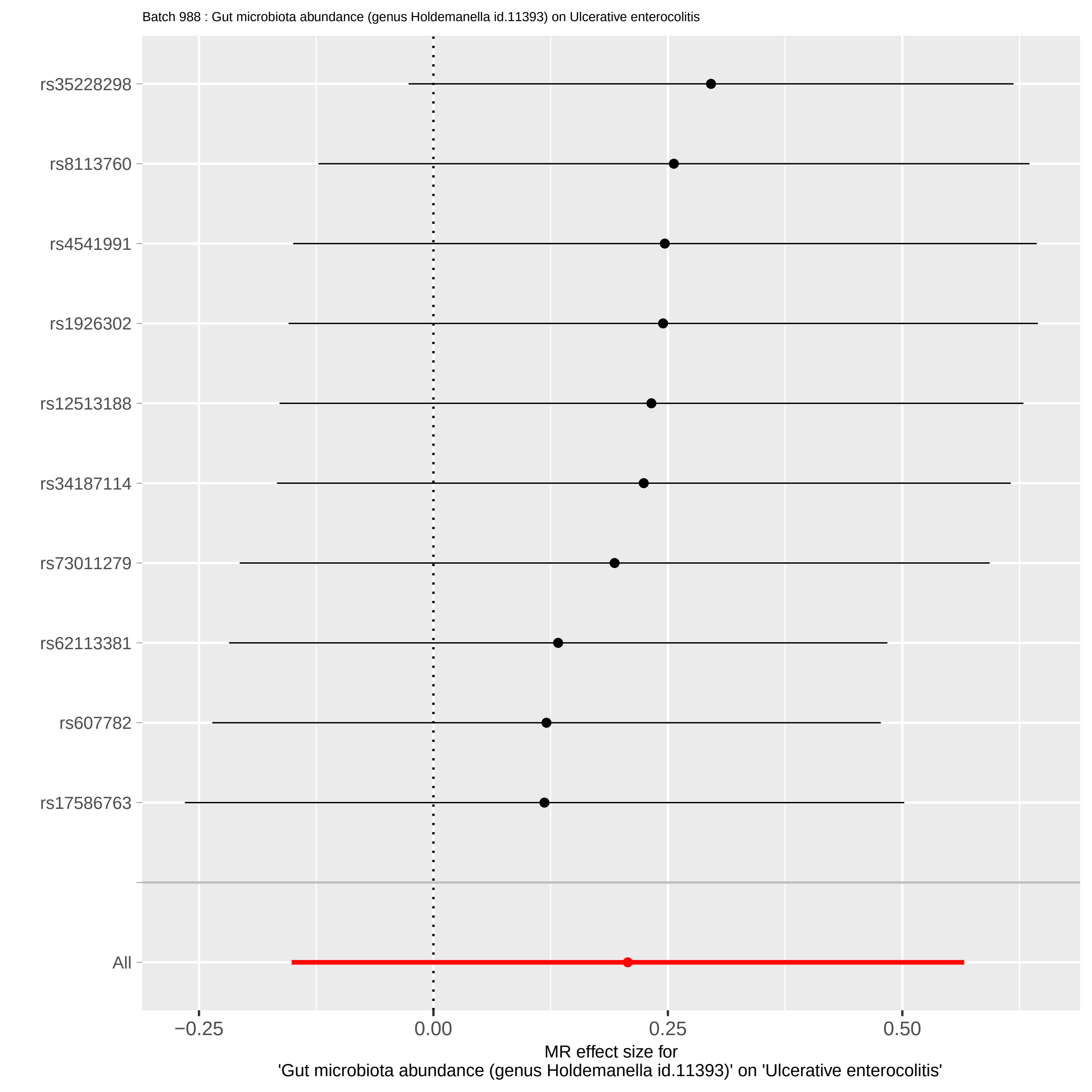

Batch 988 : Gut microbiota abundance (genus Holdemanella id.11393) on Ulcerative enterocolitis
rs35228298
rs8113760
rs4541991
rs1926302
rs12513188
rs34187114
rs73011279
rs62113381
rs607782
rs17586763
All
−0.25
0.00
0.25
0.50
MR effect size for
'Gut microbiota abundance (genus Holdemanella id.11393)' on 'Ulcerative enterocolitis'

Batch 989 : Gut microbiota abundance (genus Holdemania id.2157) on Ulcerative enterocolitis

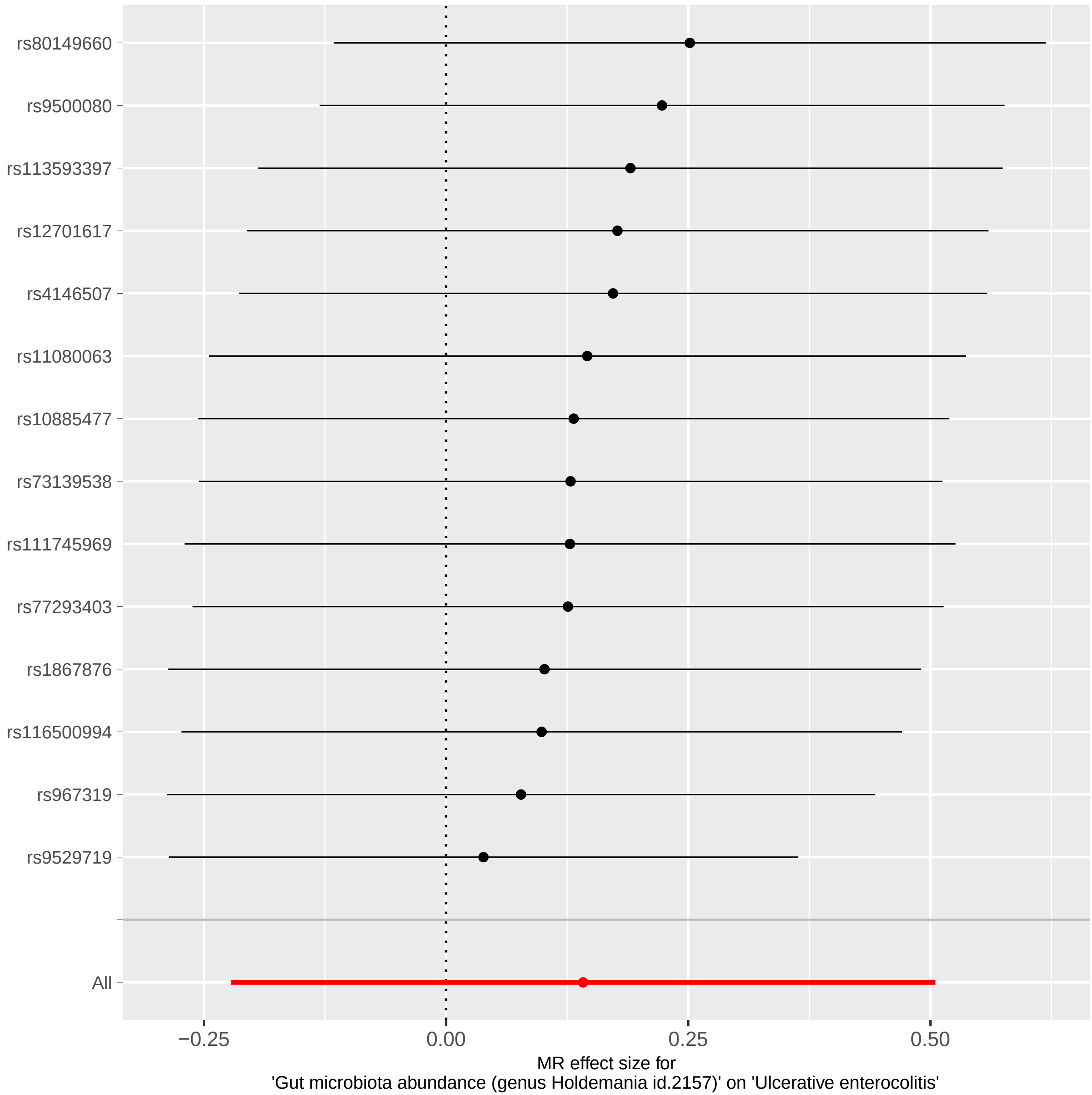

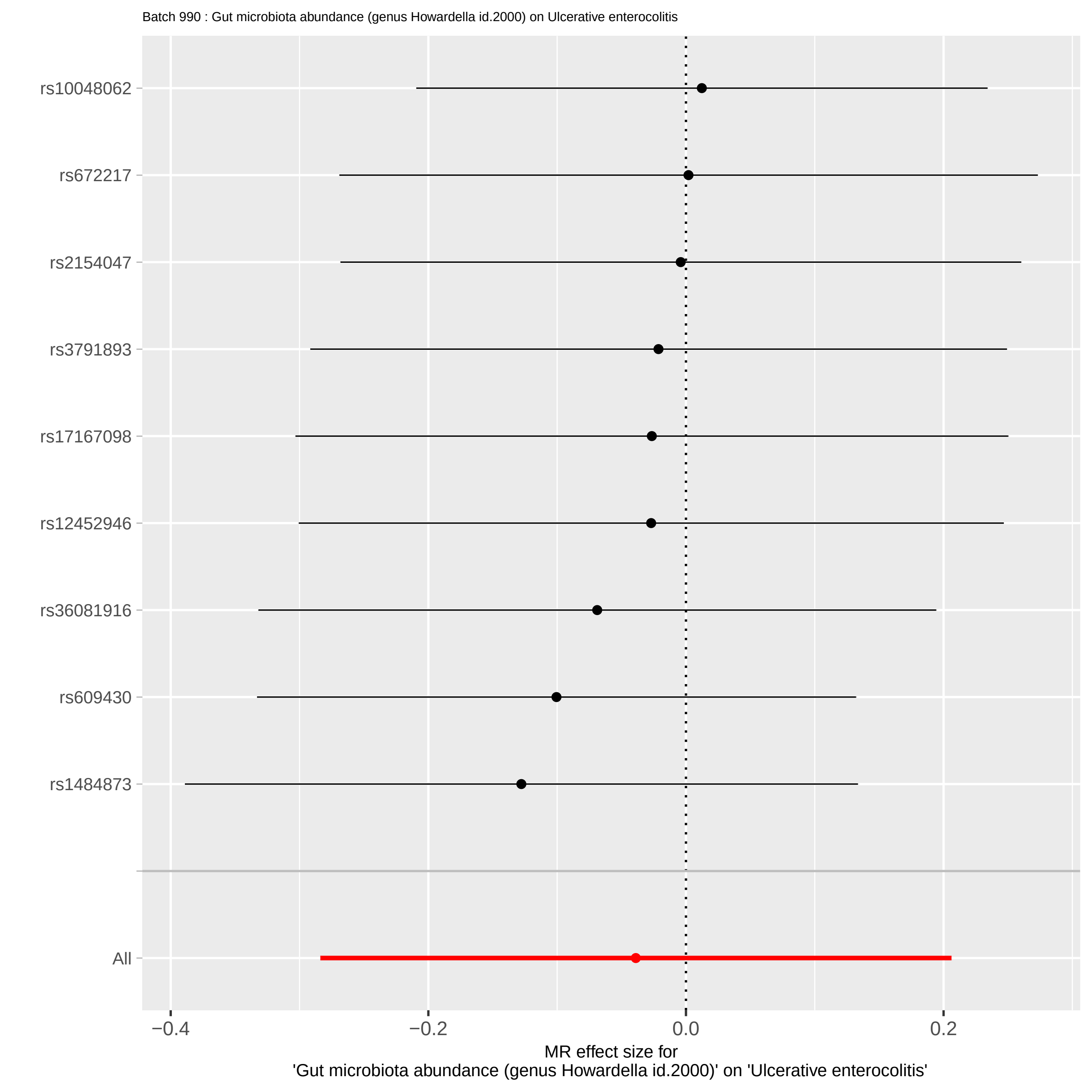
Batch 990 : Gut microbiota abundance (genus Howardella id.2000) on Ulcerative enterocolitis
rs10048062
rs672217
rs2154047
rs3791893
rs17167098
rs12452946
rs36081916
rs609430
rs1484873
All
−0.4
−0.2
0.0
0.2
MR effect size for
'Gut microbiota abundance (genus Howardella id.2000)' on 'Ulcerative enterocolitis'

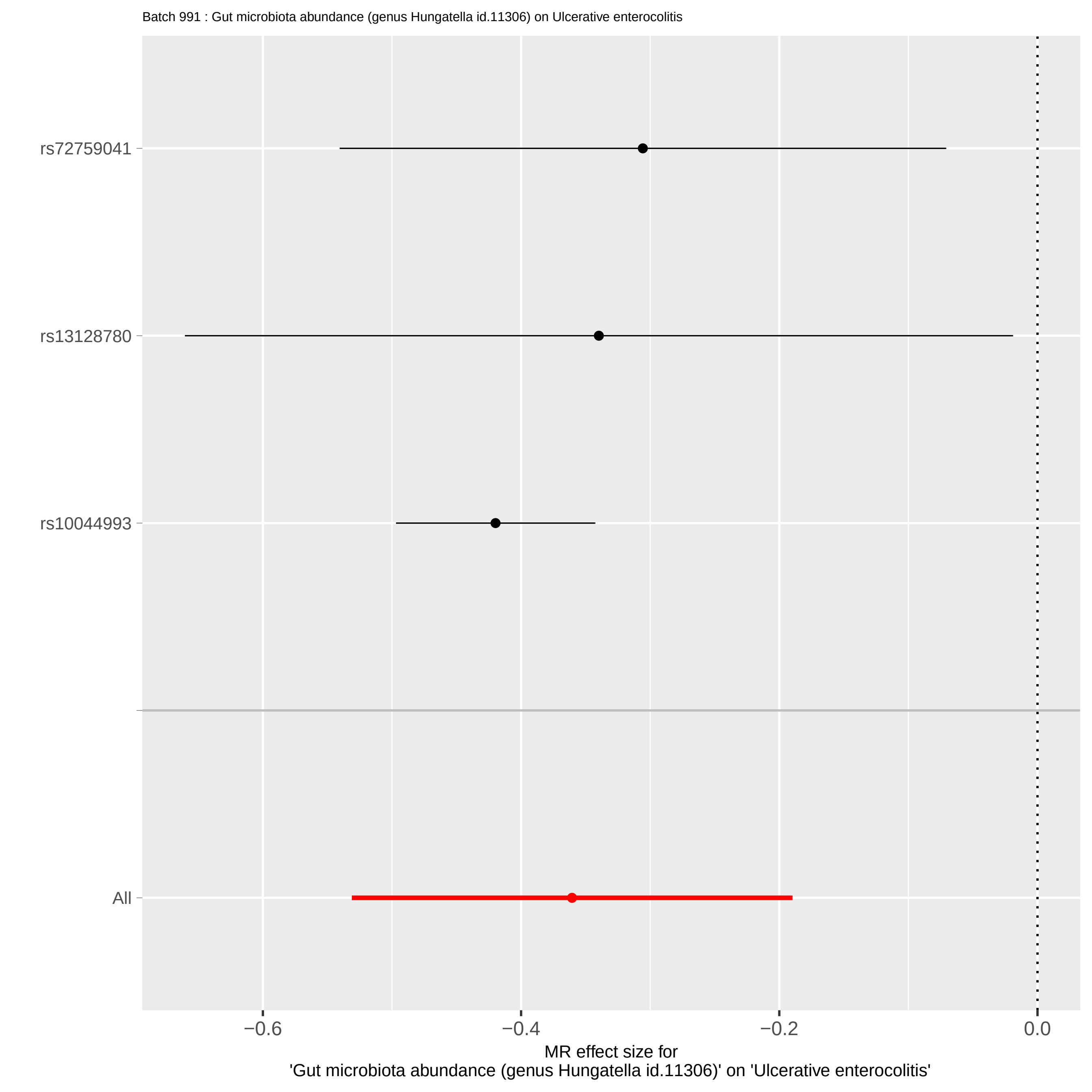

Batch 991 : Gut microbiota abundance (genus Hungatella id.11306) on Ulcerative enterocolitis
rs72759041
rs13128780
rs10044993
All
−0.6
−0.4
−0.2
0.0
MR effect size for
'Gut microbiota abundance (genus Hungatella id.11306)' on 'Ulcerative enterocolitis'

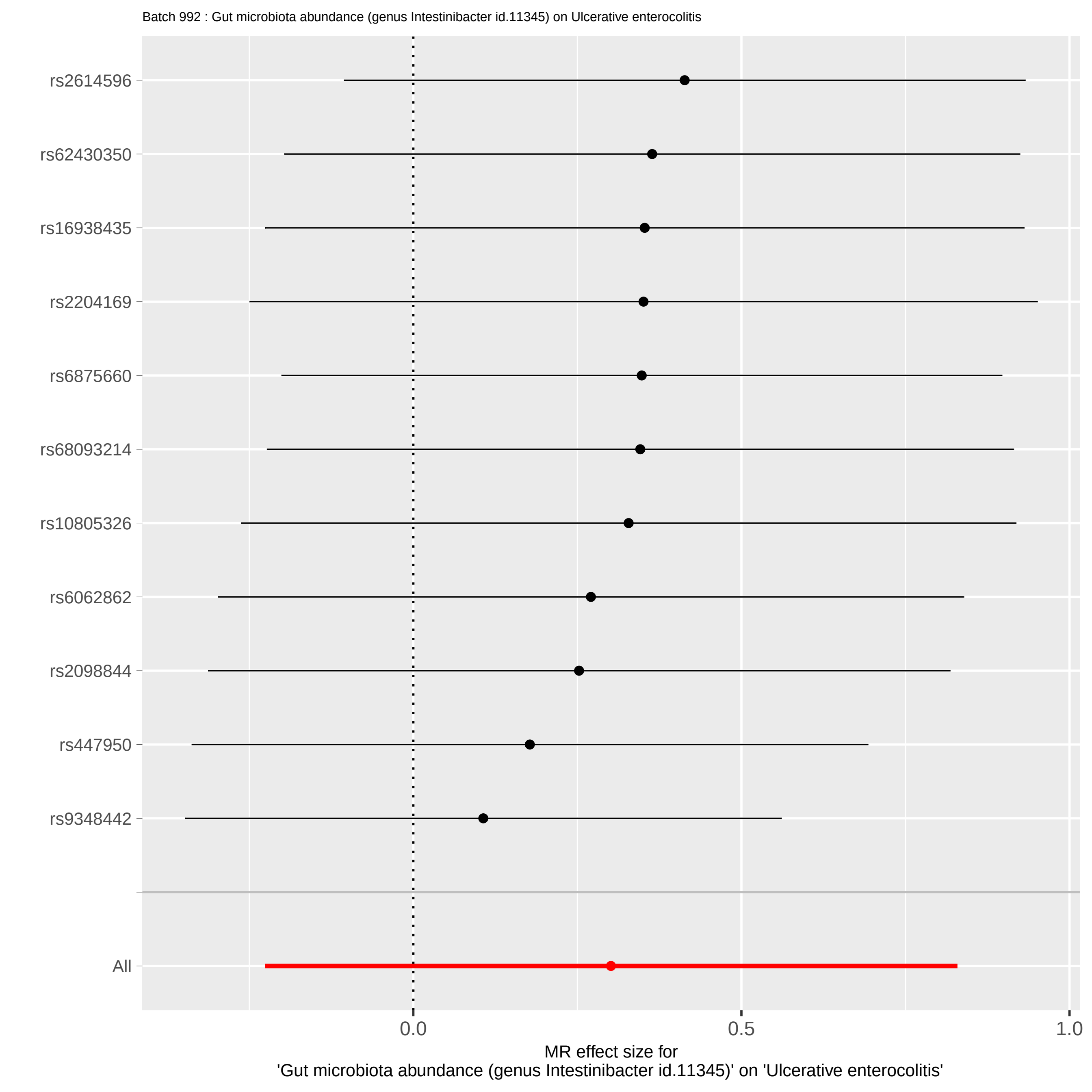
Batch 992 : Gut microbiota abundance (genus Intestinibacter id.11345) on Ulcerative enterocolitis
rs2614596
rs62430350
rs16938435
rs2204169
rs6875660
rs68093214
rs10805326
rs6062862
rs2098844
rs447950
rs9348442
All
0.0
0.5
1.0
MR effect size for
'Gut microbiota abundance (genus Intestinibacter id.11345)' on 'Ulcerative enterocolitis'

Batch 993 : Gut microbiota abundance (genus Intestinimonas id.2062) on Ulcerative enterocolitis

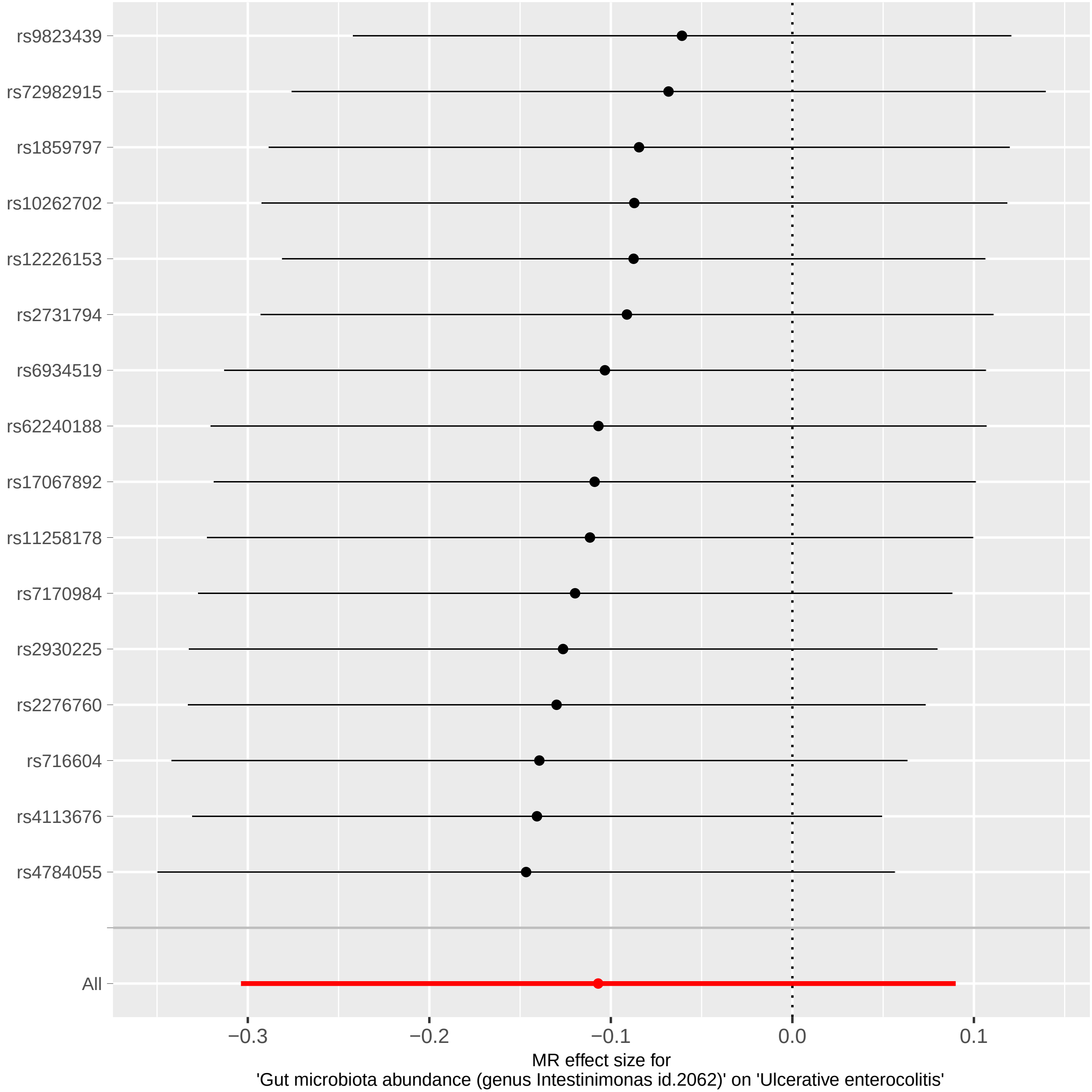

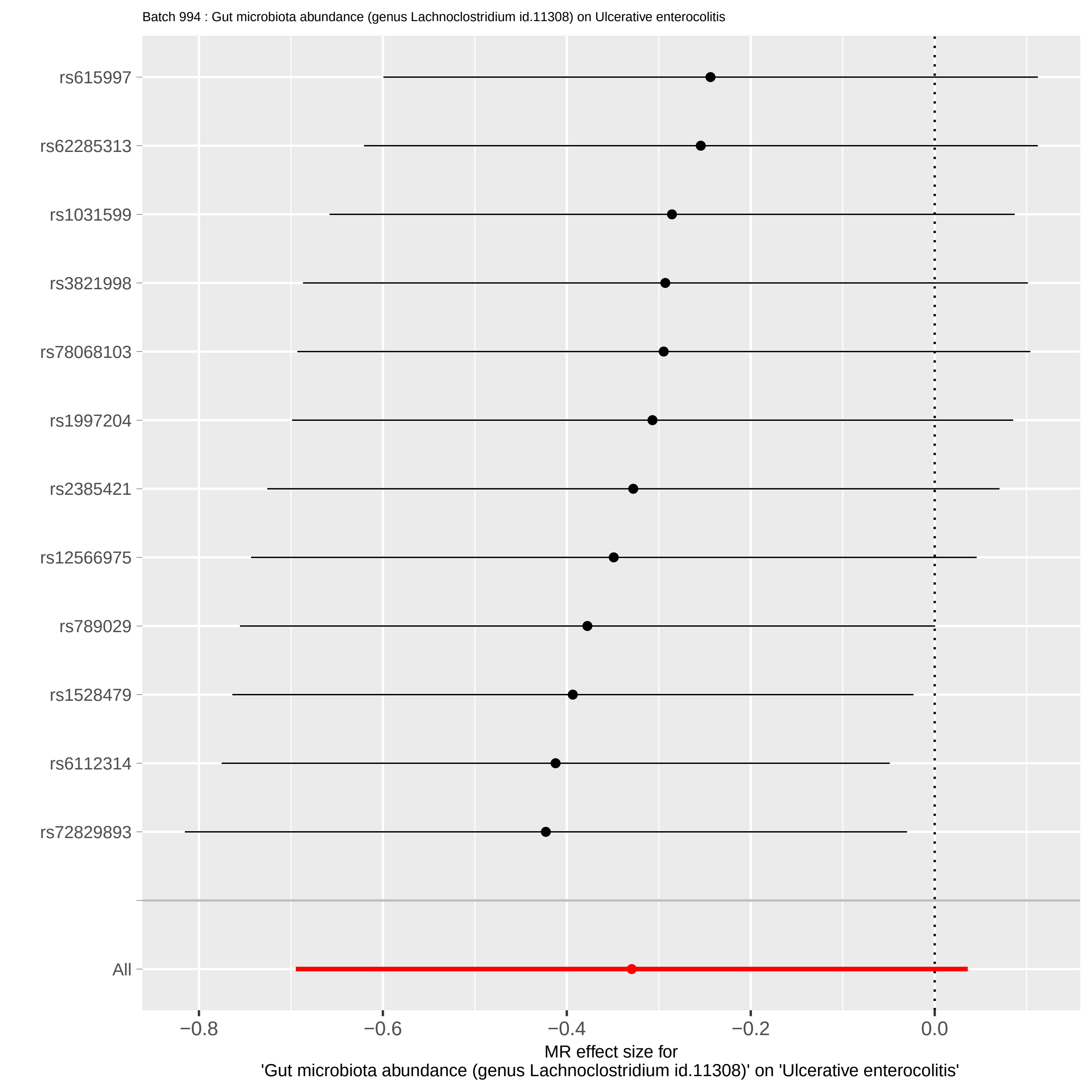
Batch 994 : Gut microbiota abundance (genus Lachnoclostridium id.11308) on Ulcerative enterocolitis
rs615997
rs62285313
rs1031599
rs3821998
rs78068103
rs1997204
rs2385421
rs12566975
rs789029
rs1528479
rs6112314
rs72829893
All
−0.8
−0.6
−0.4
−0.2
0.0
MR effect size for
'Gut microbiota abundance (genus Lachnoclostridium id.11308)' on 'Ulcerative enterocolitis'

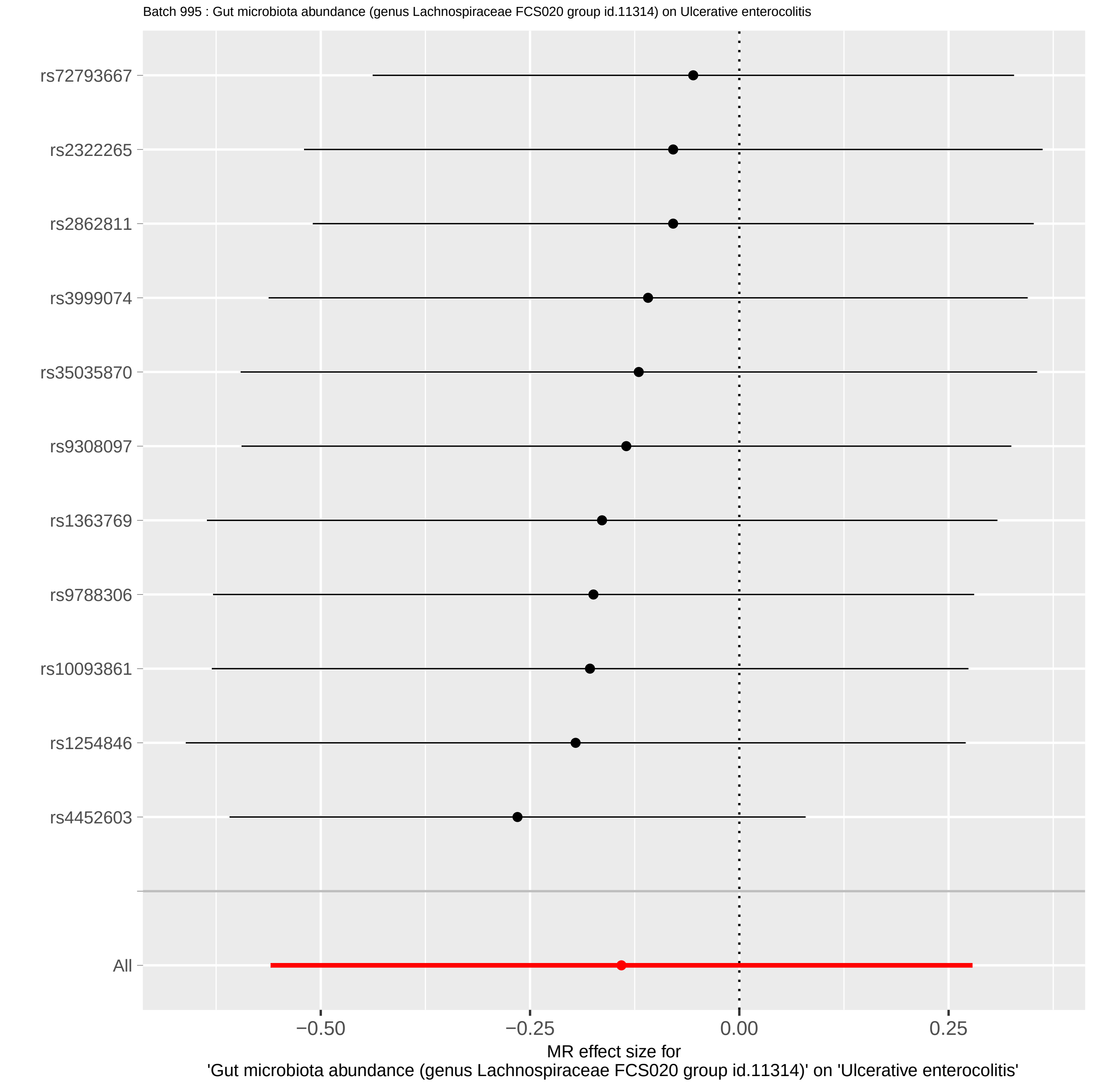

Batch 995 : Gut microbiota abundance (genus Lachnospiraceae FCS020 group id.11314) on Ulcerative enterocolitis
rs72793667
rs2322265
rs2862811
rs3999074
rs35035870
rs9308097
rs1363769
rs9788306
rs10093861
rs1254846
rs4452603
All
−0.50
−0.25
0.00
0.25
MR effect size for
'Gut microbiota abundance (genus Lachnospiraceae FCS020 group id.11314)' on 'Ulcerative enterocolitis'

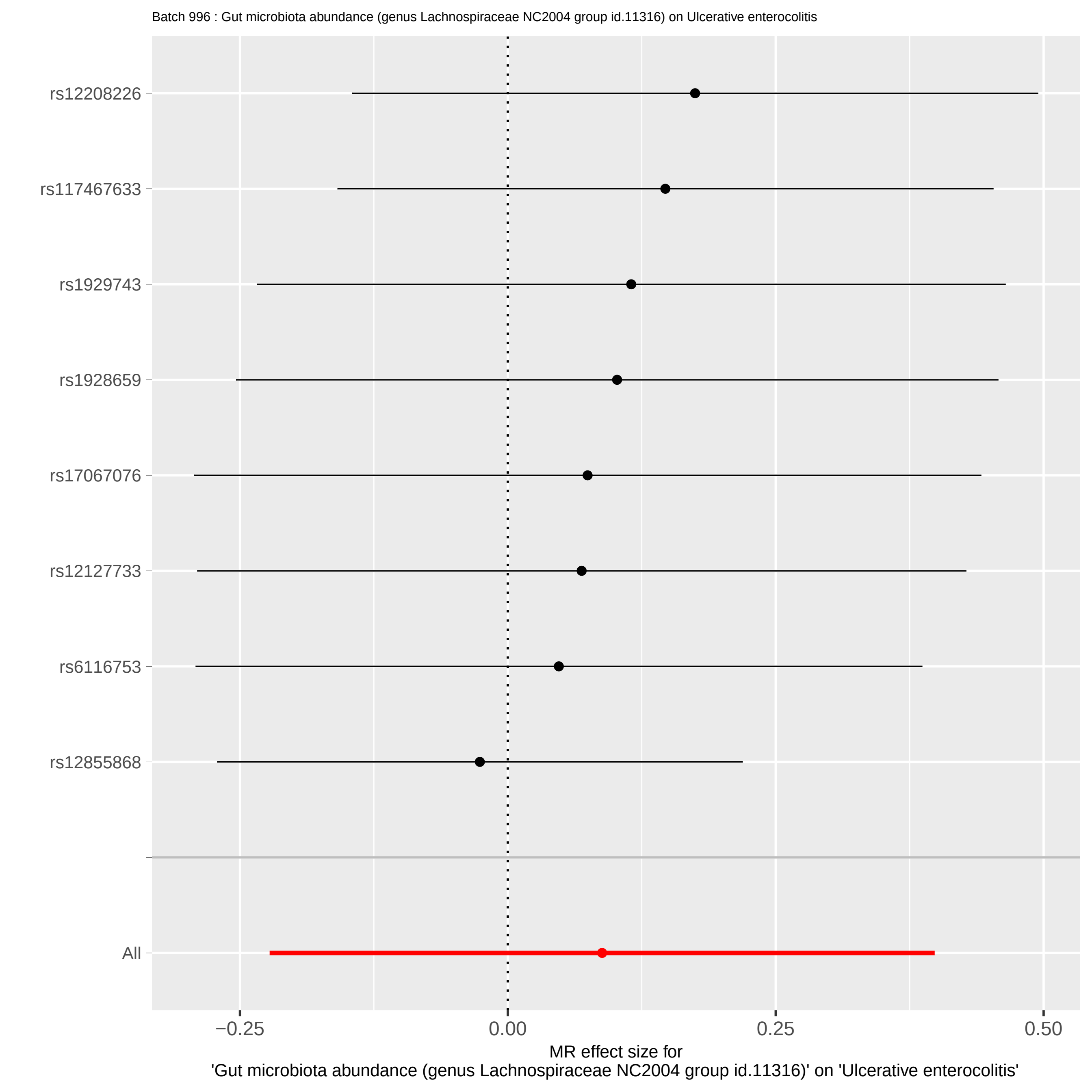

Batch 996 : Gut microbiota abundance (genus Lachnospiraceae NC2004 group id.11316) on Ulcerative enterocolitis
rs12208226
rs117467633
rs1929743
rs1928659
rs17067076
rs12127733
rs6116753
rs12855868
All
−0.25
0.00
0.25
0.50
MR effect size for
'Gut microbiota abundance (genus Lachnospiraceae NC2004 group id.11316)' on 'Ulcerative enterocolitis'

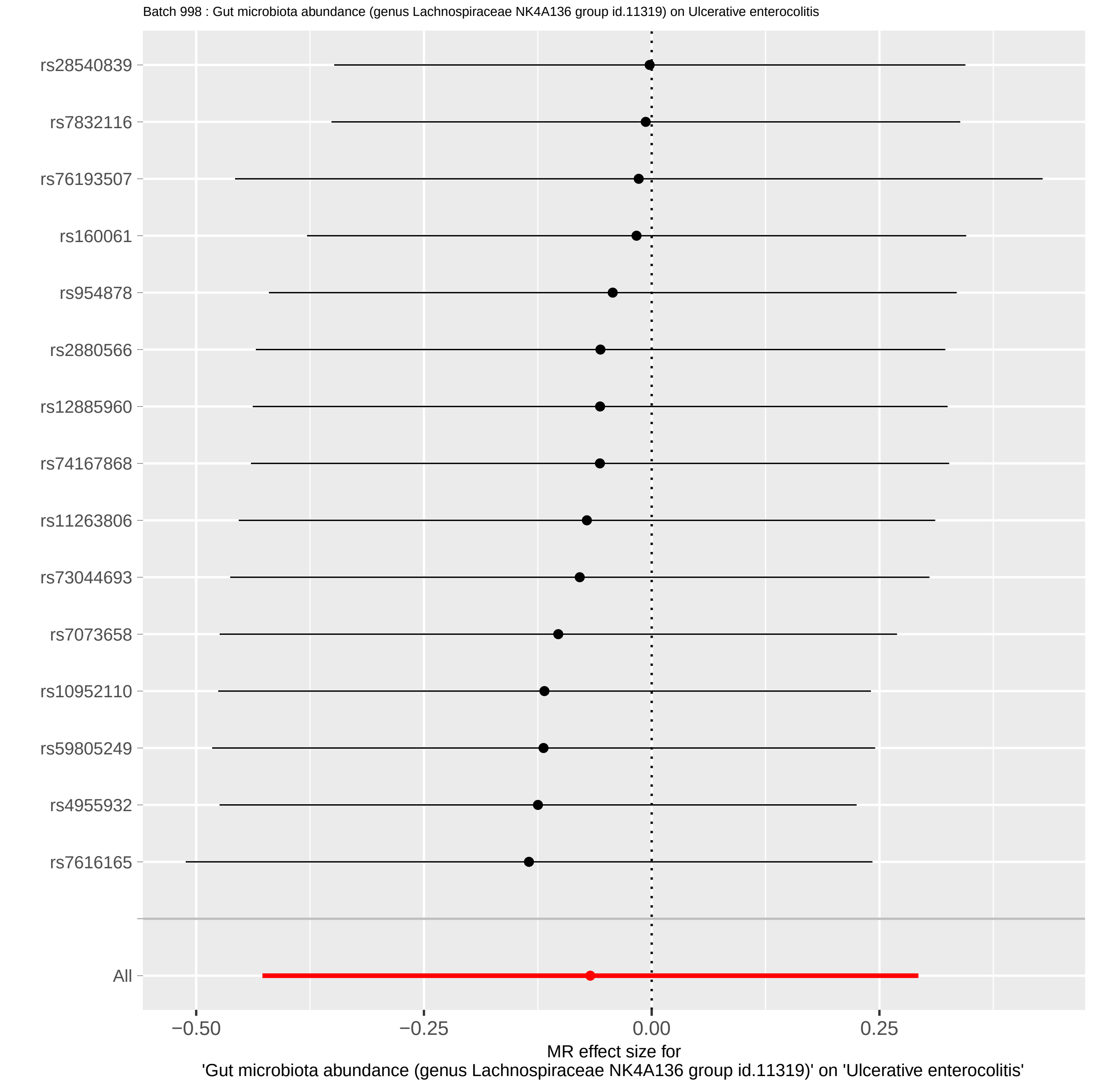

Batch 998 : Gut microbiota abundance (genus Lachnospiraceae NK4A136 group id.11319) on Ulcerative enterocolitis
rs28540839
rs7832116
rs76193507
rs160061
rs954878
rs2880566
rs12885960
rs74167868
rs11263806
rs73044693
rs7073658
rs10952110
rs59805249
rs4955932
rs7616165
All
−0.50
−0.25
0.00
0.25
MR effect size for
'Gut microbiota abundance (genus Lachnospiraceae NK4A136 group id.11319)' on 'Ulcerative enterocolitis'

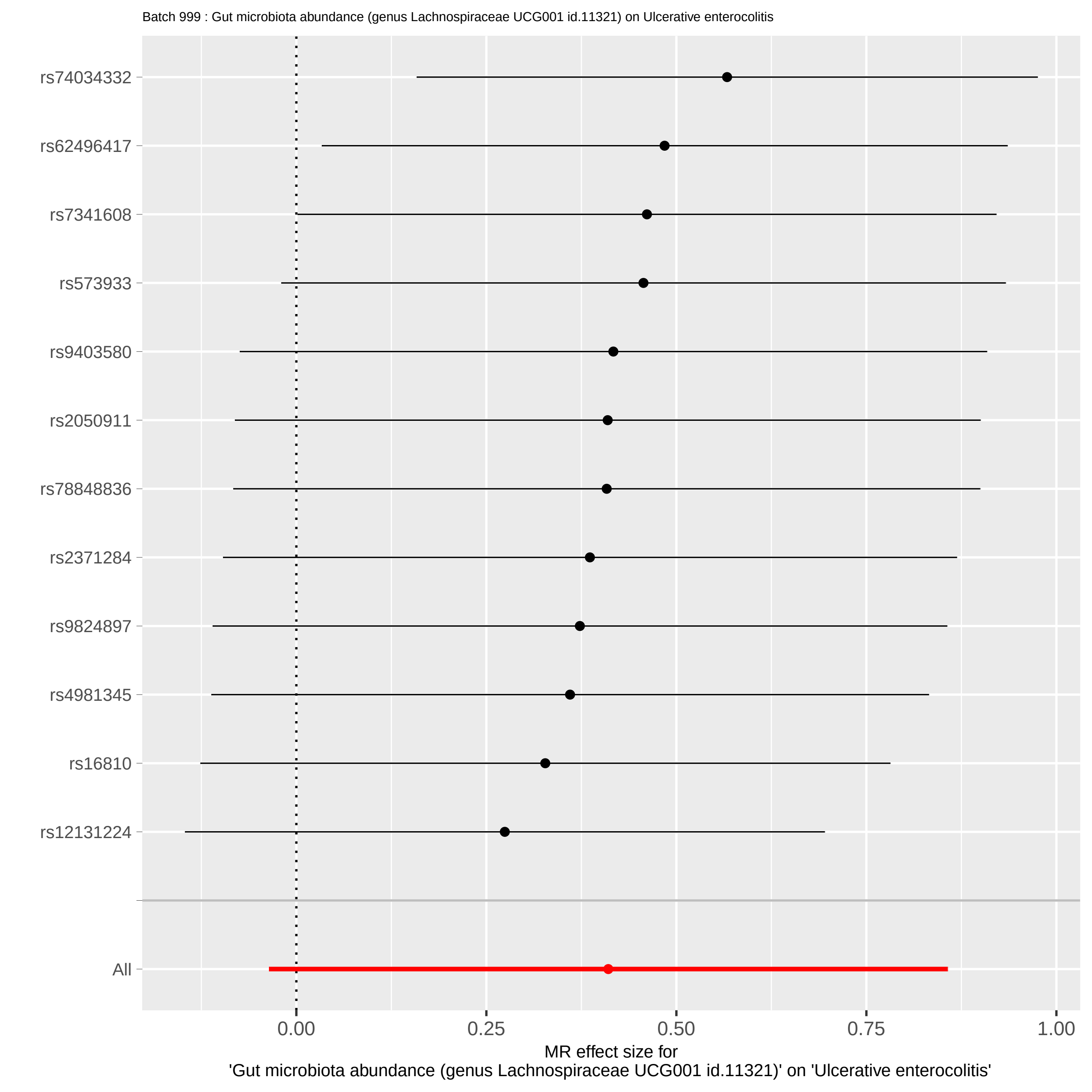
Batch 999 : Gut microbiota abundance (genus Lachnospiraceae UCG001 id.11321) on Ulcerative enterocolitis
rs74034332
rs62496417
rs7341608
rs573933
rs9403580
rs2050911
rs78848836
rs2371284
rs9824897
rs4981345
rs16810
rs12131224
All
0.00
0.25
0.50
0.75
1.00
MR effect size for
'Gut microbiota abundance (genus Lachnospiraceae UCG001 id.11321)' on 'Ulcerative enterocolitis'

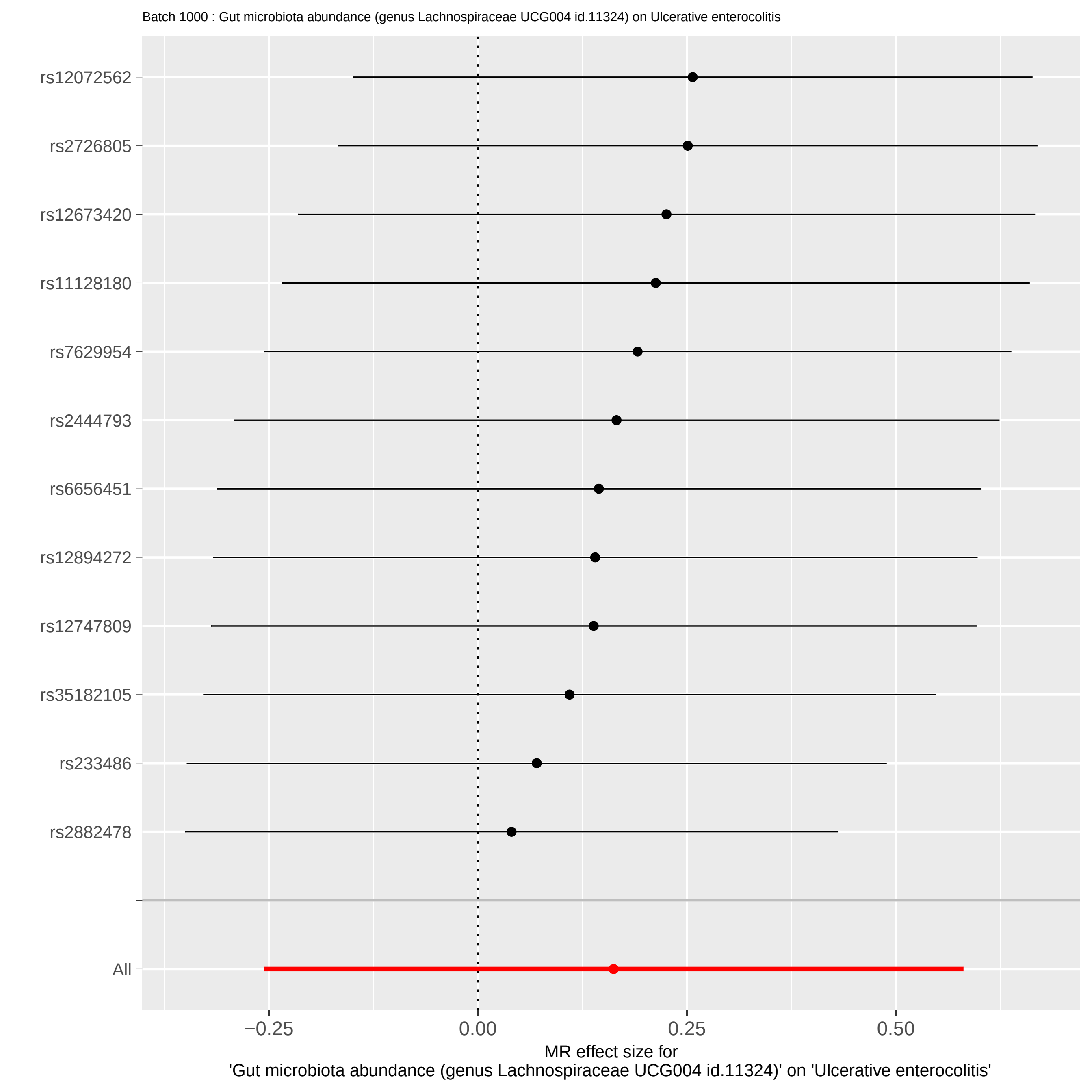

Batch 1000 : Gut microbiota abundance (genus Lachnospiraceae UCG004 id.11324) on Ulcerative enterocolitis
rs12072562
rs2726805
rs12673420
rs11128180
rs7629954
rs2444793
rs6656451
rs12894272
rs12747809
rs35182105
rs233486
rs2882478
All
−0.25
0.00
0.25
0.50
MR effect size for
'Gut microbiota abundance (genus Lachnospiraceae UCG004 id.11324)' on 'Ulcerative enterocolitis'

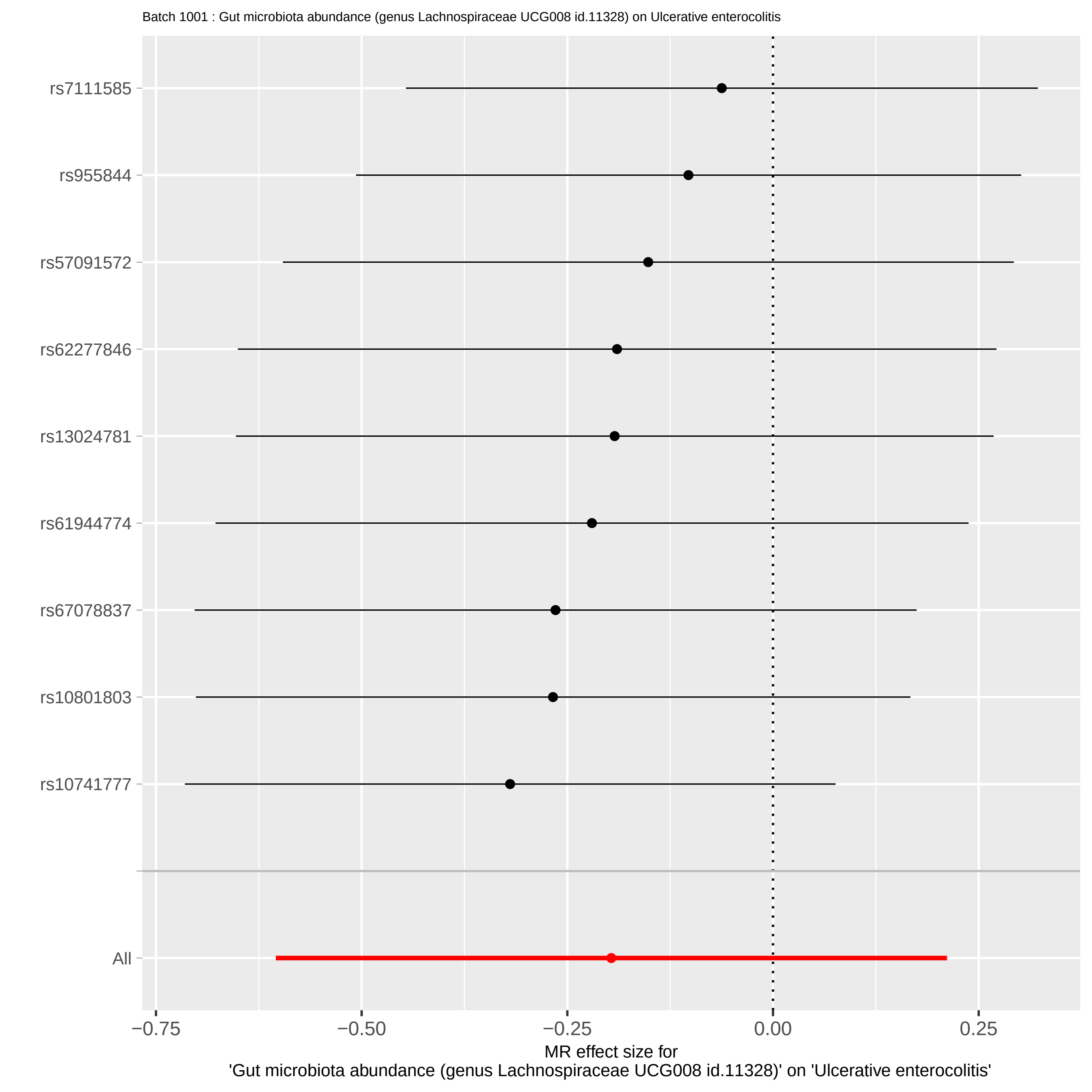
Batch 1001 : Gut microbiota abundance (genus Lachnospiraceae UCG008 id.11328) on Ulcerative enterocolitis
rs7111585
rs955844
rs57091572
rs62277846
rs13024781
rs61944774
rs67078837
rs10801803
rs10741777
All
−0.75
−0.50
−0.25
0.00
0.25
MR effect size for
'Gut microbiota abundance (genus Lachnospiraceae UCG008 id.11328)' on 'Ulcerative enterocolitis'

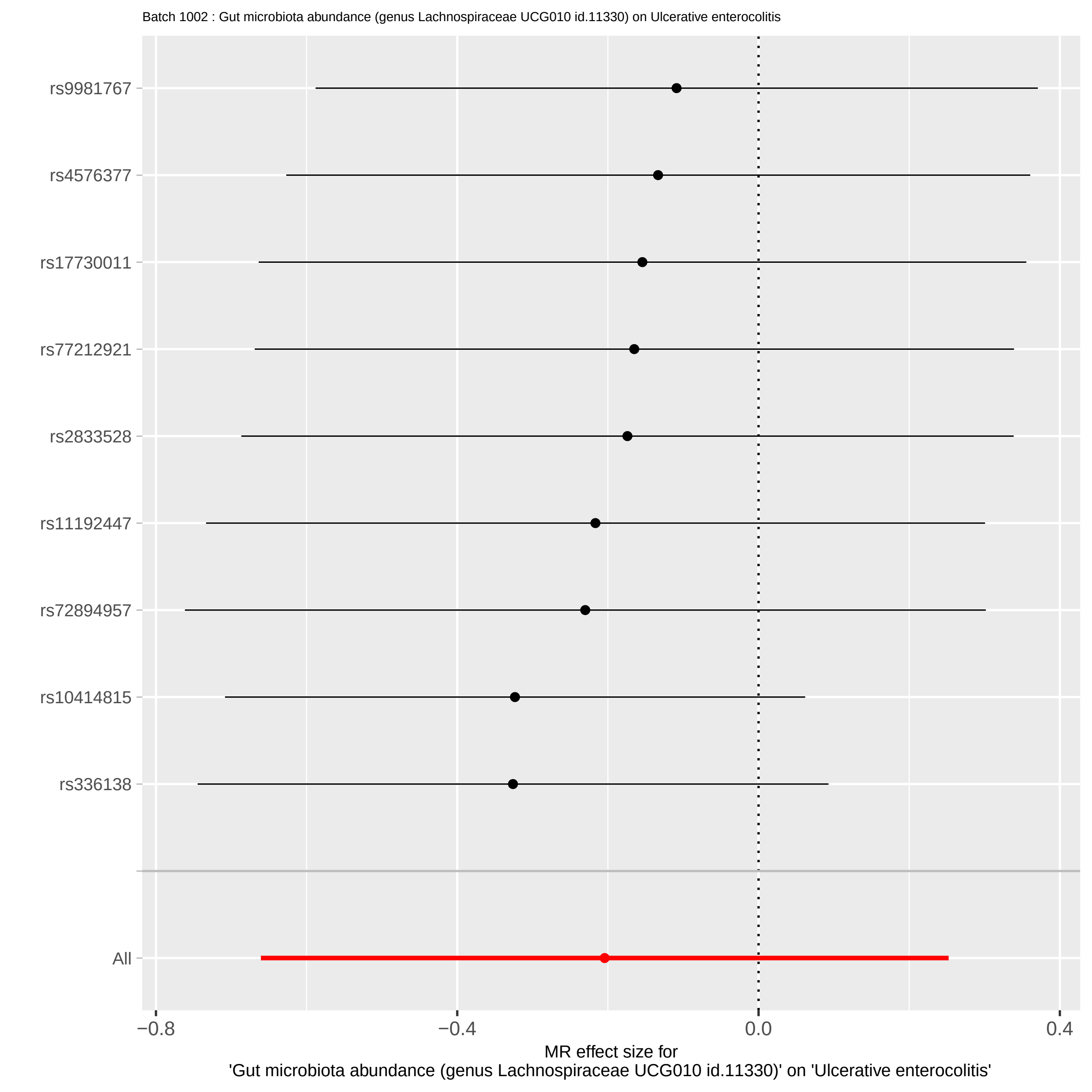

Batch 1002 : Gut microbiota abundance (genus Lachnospiraceae UCG010 id.11330) on Ulcerative enterocolitis
rs9981767
rs4576377
rs17730011
rs77212921
rs2833528
rs11192447
rs72894957
rs10414815
rs336138
All
−0.8
−0.4
0.0
0.4
MR effect size for
'Gut microbiota abundance (genus Lachnospiraceae UCG010 id.11330)' on 'Ulcerative enterocolitis'

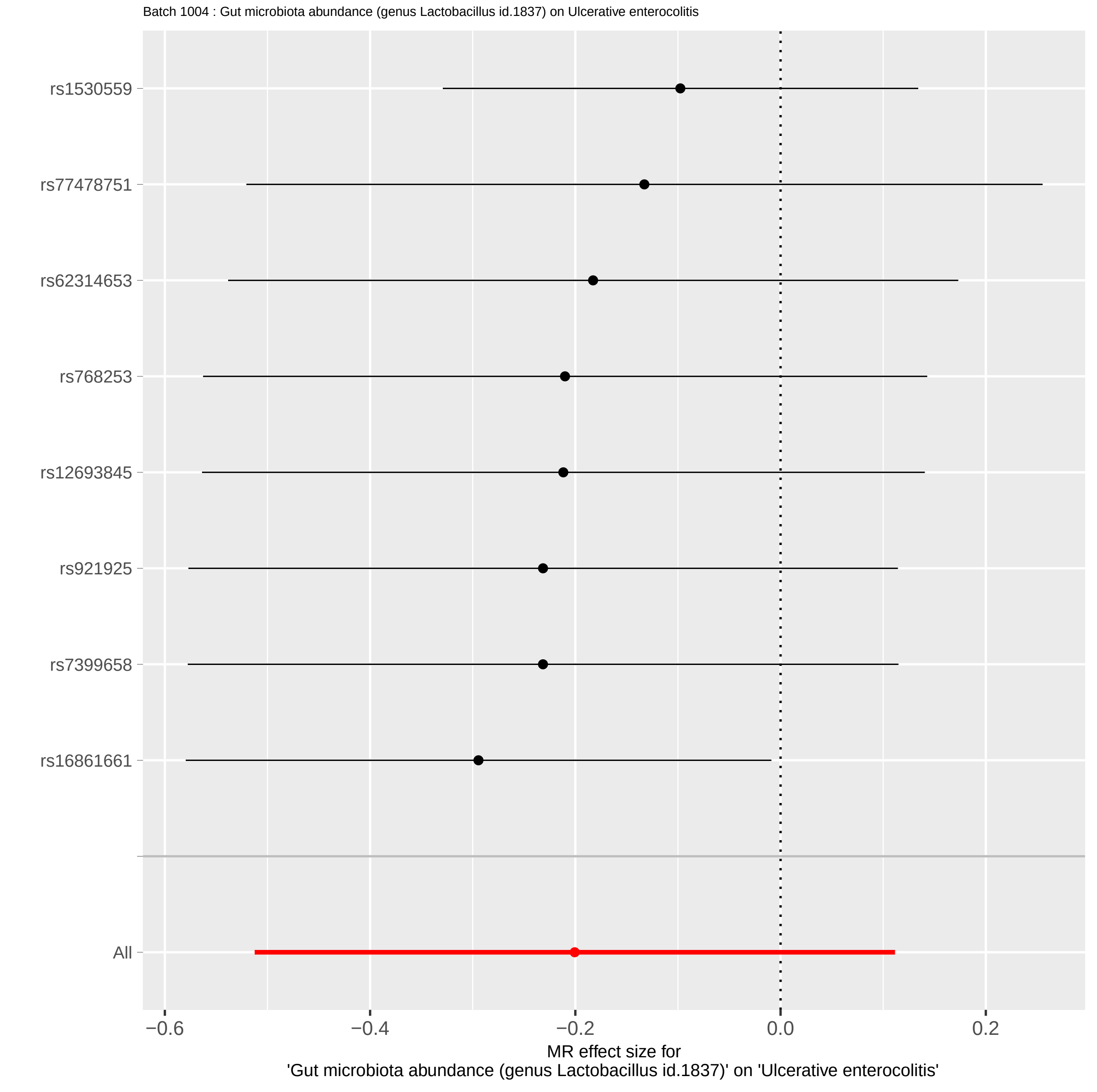

Batch 1004 : Gut microbiota abundance (genus Lactobacillus id.1837) on Ulcerative enterocolitis
rs1530559
rs77478751
rs62314653
rs768253
rs12693845
rs921925
rs7399658
rs16861661
All
−0.6
−0.4
−0.2
0.0
0.2
MR effect size for
'Gut microbiota abundance (genus Lactobacillus id.1837)' on 'Ulcerative enterocolitis'

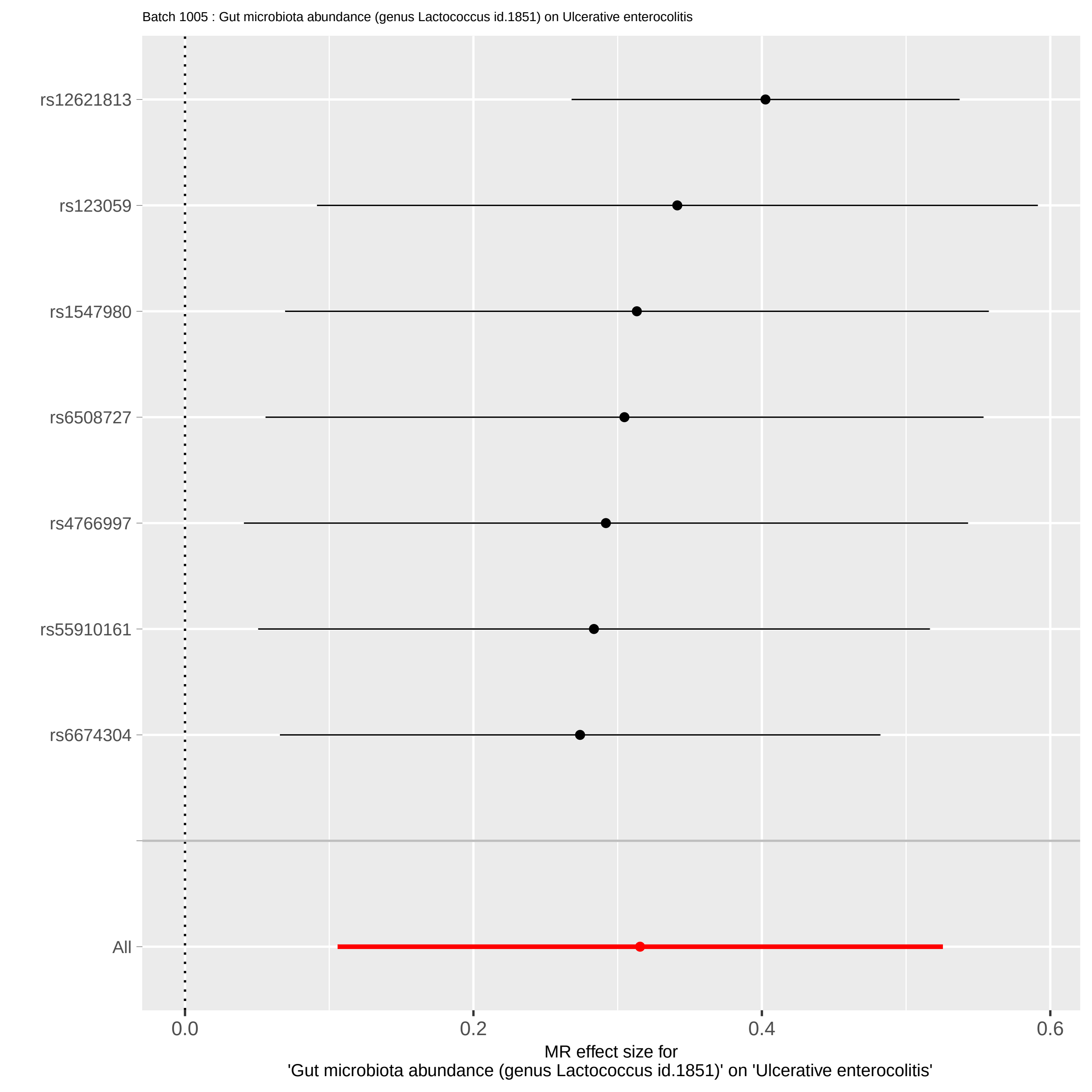
Batch 1005 : Gut microbiota abundance (genus Lactococcus id.1851) on Ulcerative enterocolitis
rs12621813
rs123059
rs1547980
rs6508727
rs4766997
rs55910161
rs6674304
All
0.0
0.2
0.4
0.6
MR effect size for
'Gut microbiota abundance (genus Lactococcus id.1851)' on 'Ulcerative enterocolitis'

Batch 1006 : Gut microbiota abundance (genus Marvinbryantia id.2005) on Ulcerative enterocolitis

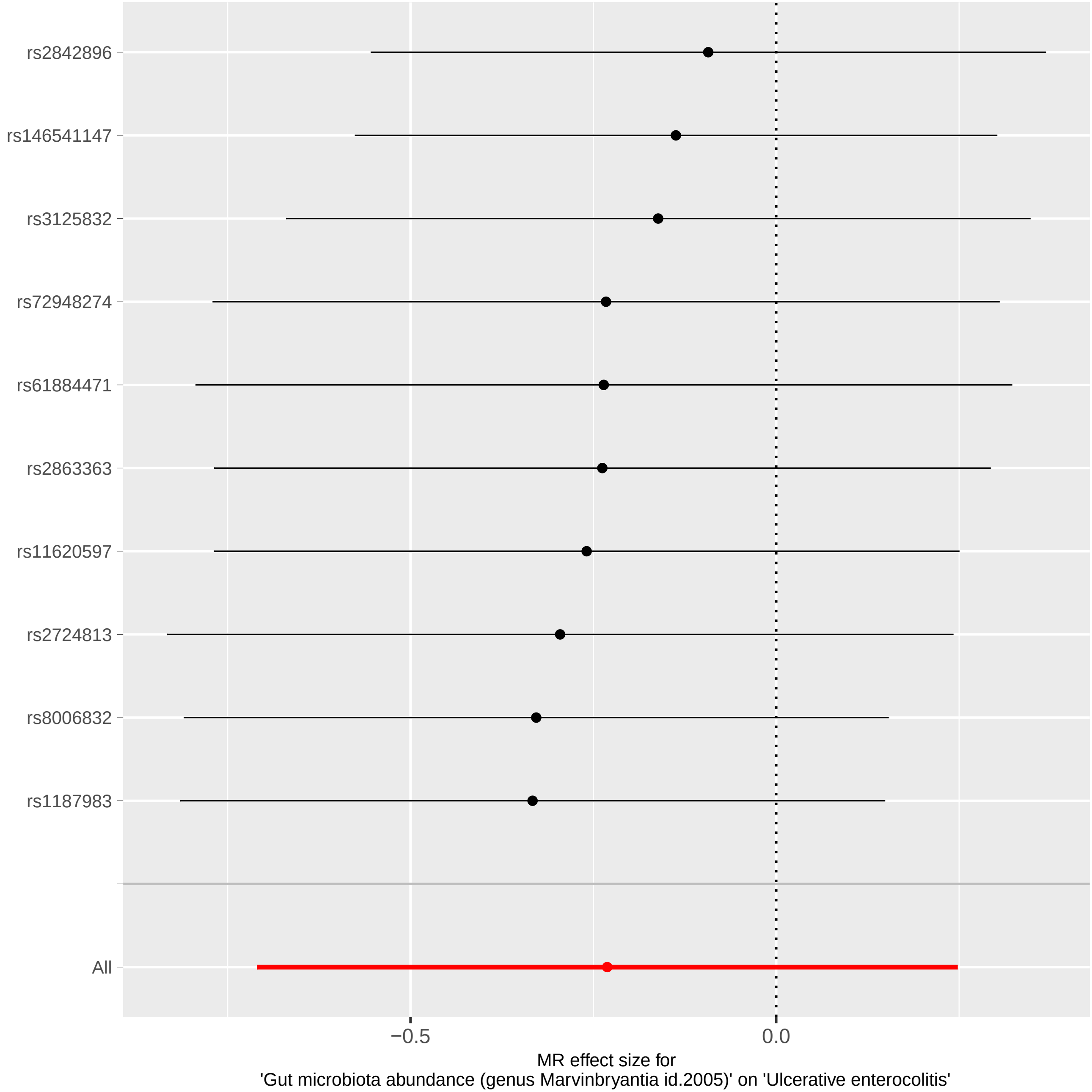

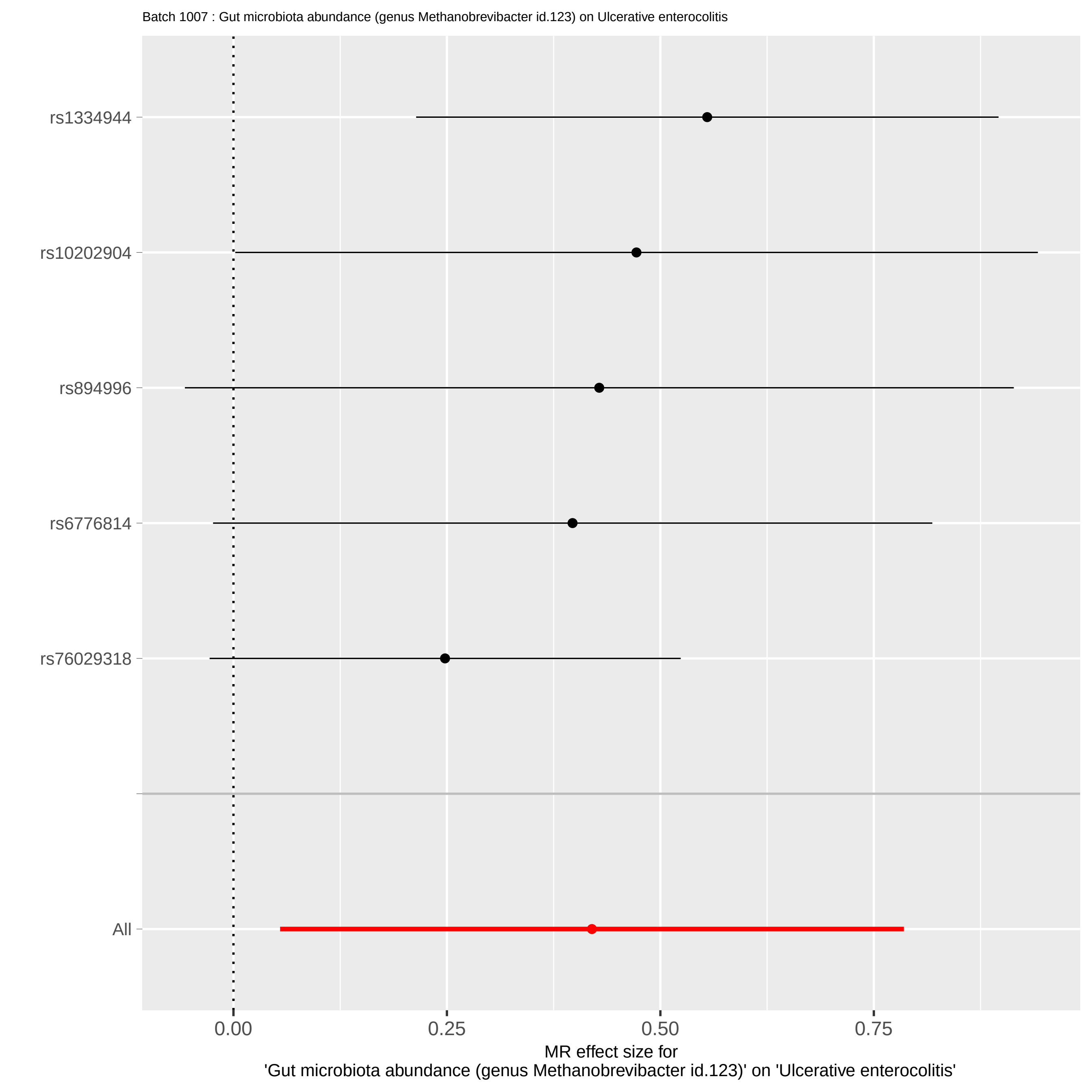

Batch 1007 : Gut microbiota abundance (genus Methanobrevibacter id.123) on Ulcerative enterocolitis
rs1334944
rs10202904
rs894996
rs6776814
rs76029318
All
0.00
0.25
0.50
0.75
MR effect size for
'Gut microbiota abundance (genus Methanobrevibacter id.123)' on 'Ulcerative enterocolitis'

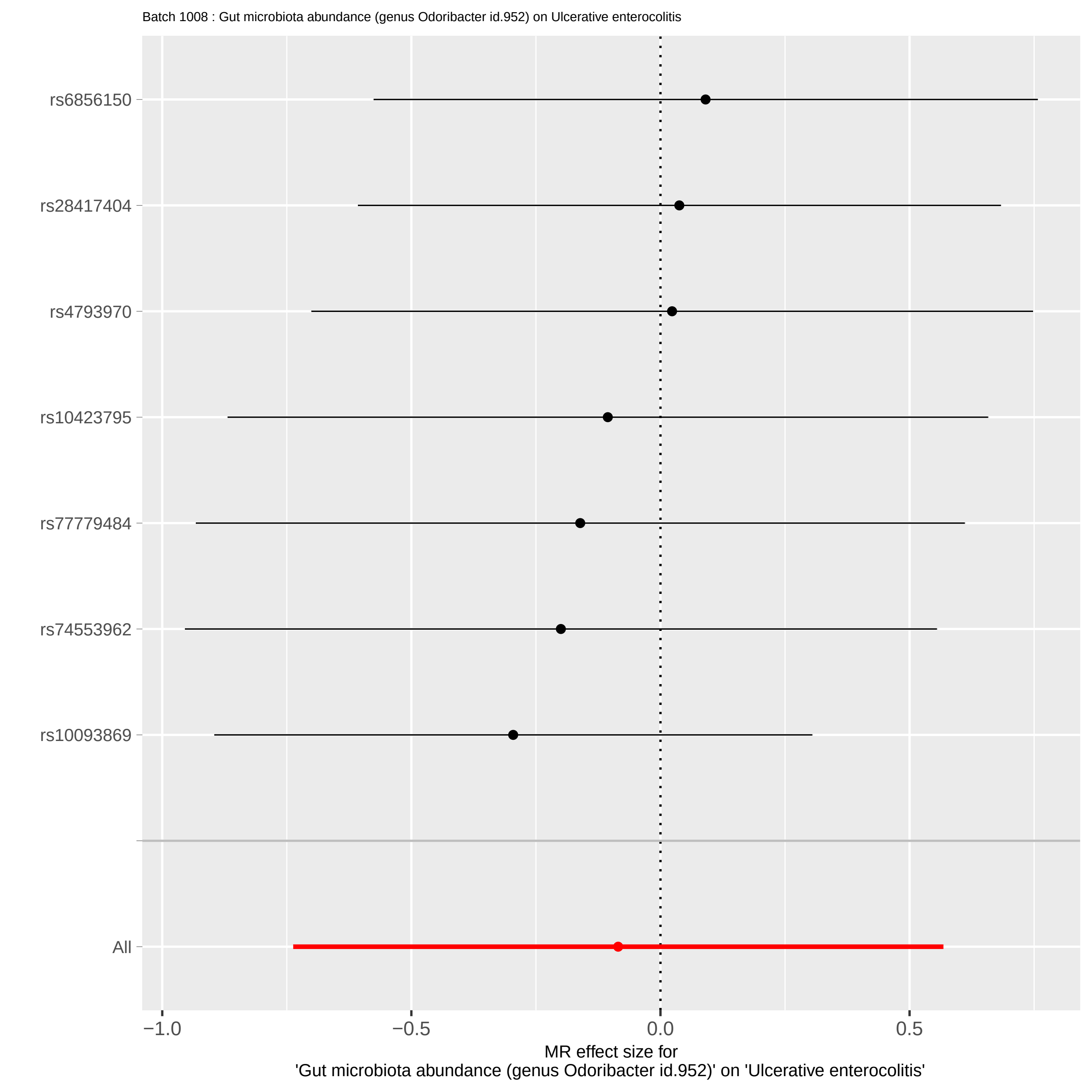

Batch 1008 : Gut microbiota abundance (genus Odoribacter id.952) on Ulcerative enterocolitis
rs6856150
rs28417404
rs4793970
rs10423795
rs77779484
rs74553962
rs10093869
All
−1.0
−0.5
0.0
0.5
MR effect size for
'Gut microbiota abundance (genus Odoribacter id.952)' on 'Ulcerative enterocolitis'

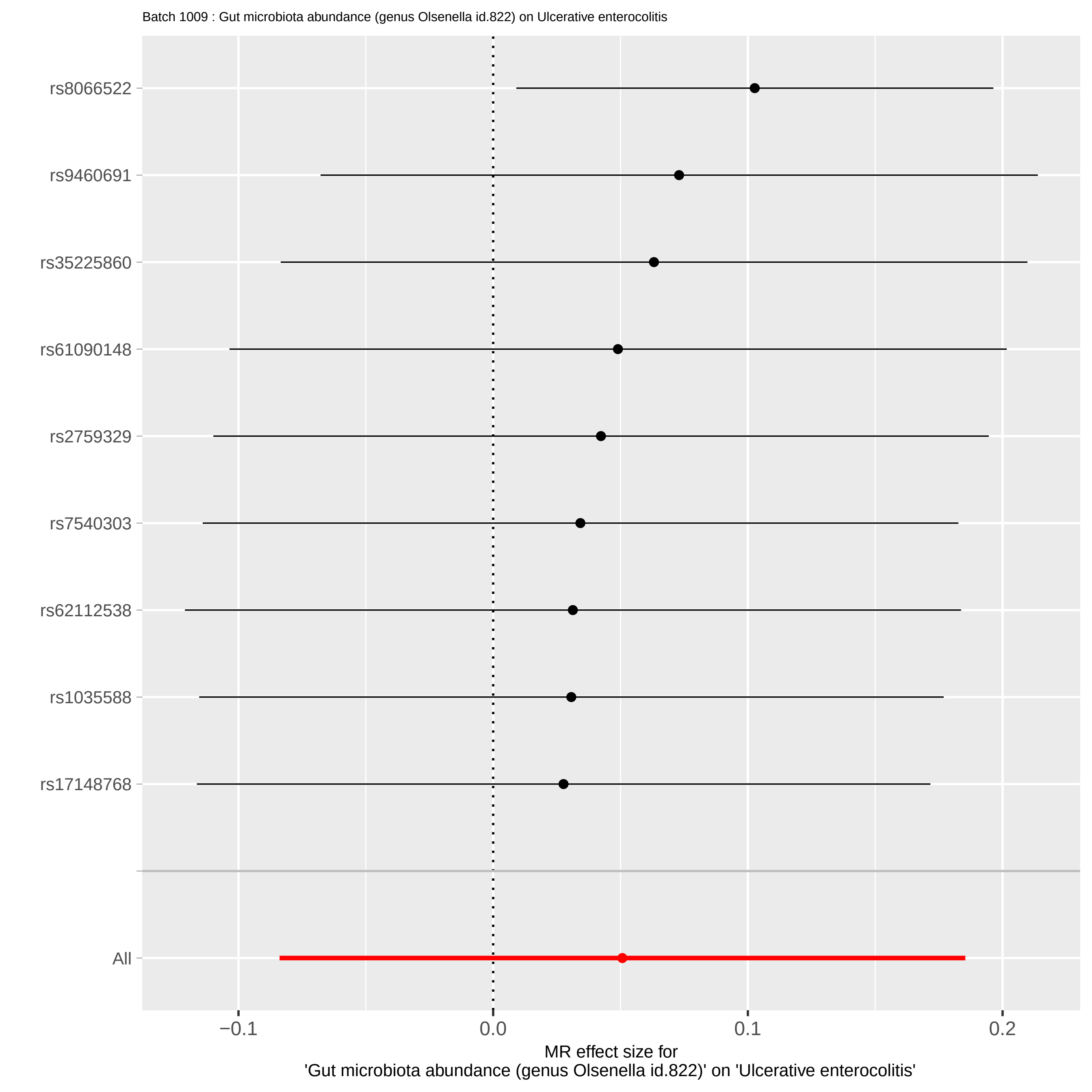

Batch 1009 : Gut microbiota abundance (genus Olsenella id.822) on Ulcerative enterocolitis
rs8066522
rs9460691
rs35225860
rs61090148
rs2759329
rs7540303
rs62112538
rs1035588
rs17148768
All
−0.1
0.0
0.1
0.2
MR effect size for
'Gut microbiota abundance (genus Olsenella id.822)' on 'Ulcerative enterocolitis'

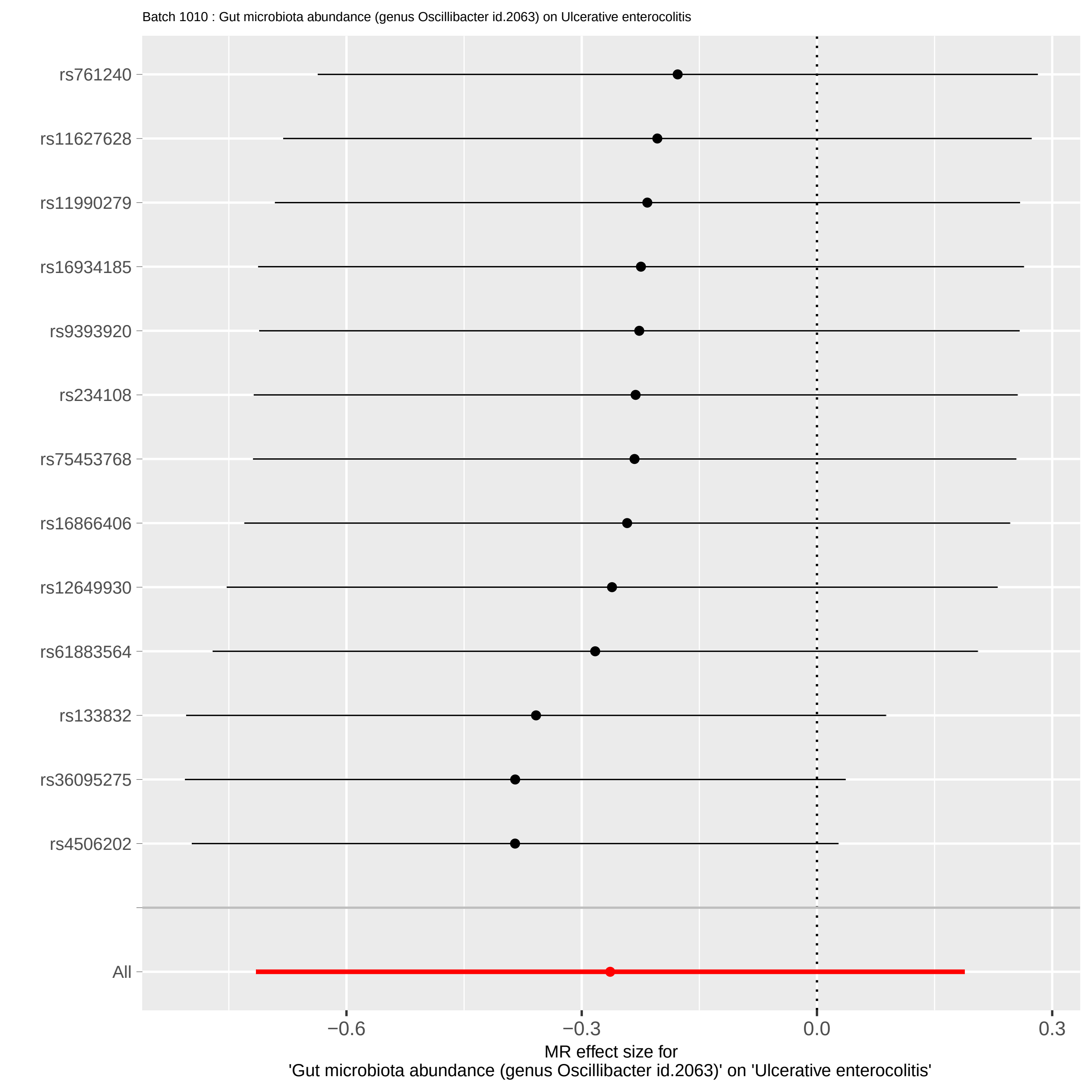

Batch 1010 : Gut microbiota abundance (genus Oscillibacter id.2063) on Ulcerative enterocolitis
rs761240
rs11627628
rs11990279
rs16934185
rs9393920
rs234108
rs75453768
rs16866406
rs12649930
rs61883564
rs133832
rs36095275
rs4506202
All
−0.6
−0.3
0.0
0.3
MR effect size for
'Gut microbiota abundance (genus Oscillibacter id.2063)' on 'Ulcerative enterocolitis'

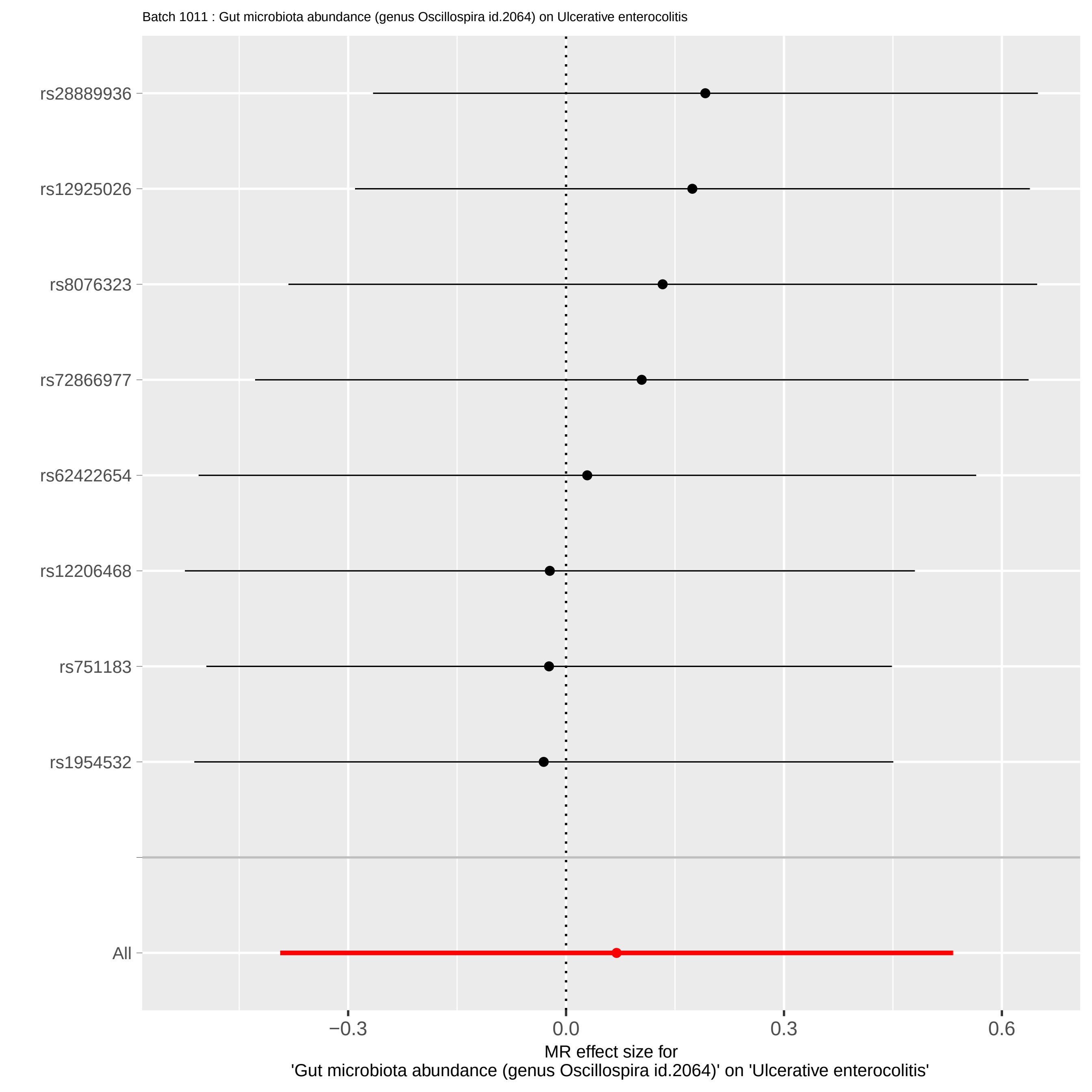

Batch 1011 : Gut microbiota abundance (genus Oscillospira id.2064) on Ulcerative enterocolitis
rs28889936
rs12925026
rs8076323
rs72866977
rs62422654
rs12206468
rs751183
rs1954532
All
−0.3
0.0
0.3
0.6
MR effect size for
'Gut microbiota abundance (genus Oscillospira id.2064)' on 'Ulcerative enterocolitis'

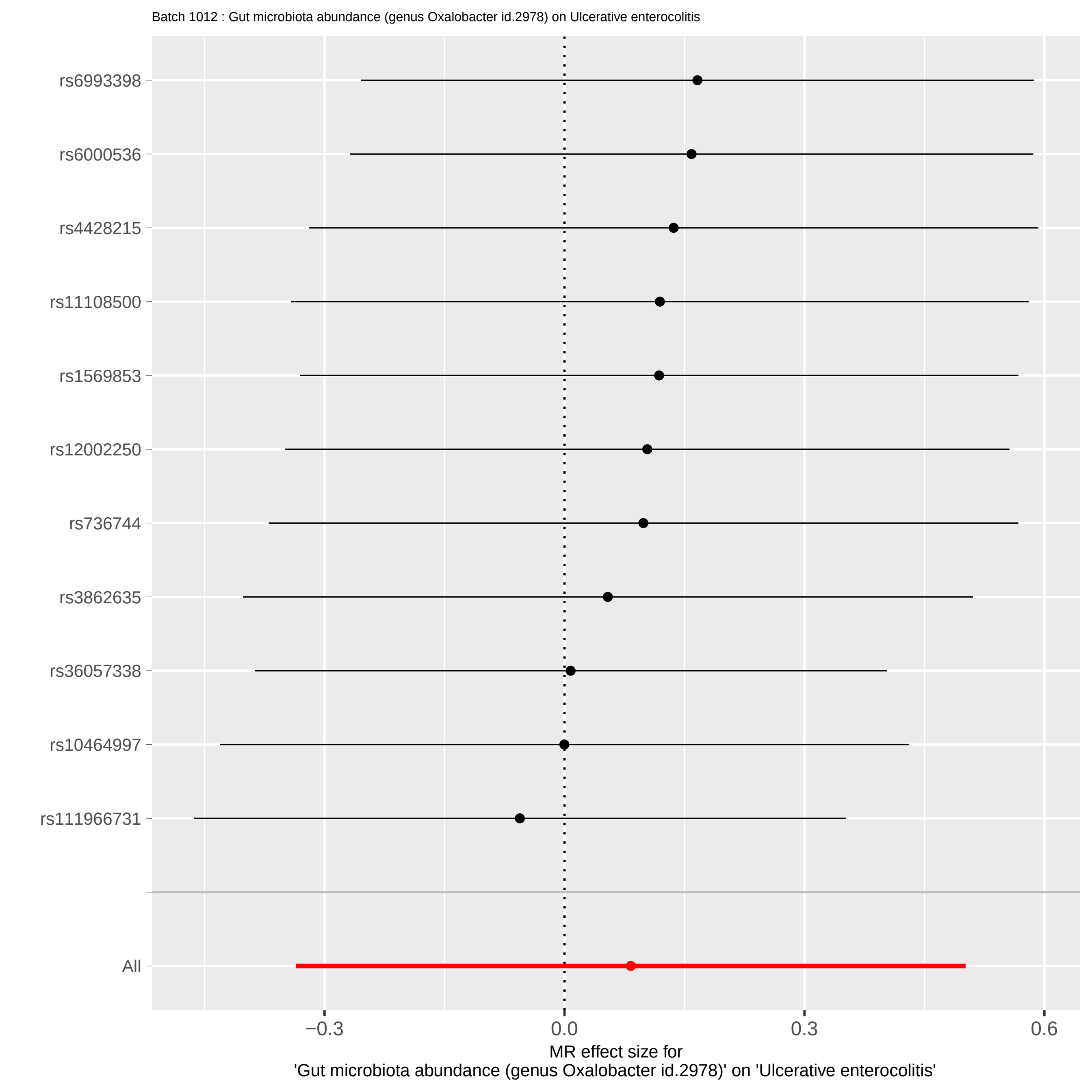

Batch 1012 : Gut microbiota abundance (genus Oxalobacter id.2978) on Ulcerative enterocolitis
rs6993398
rs6000536
rs4428215
rs11108500
rs1569853
rs12002250
rs736744
rs3862635
rs36057338
rs10464997
rs111966731
All
−0.3
0.0
0.3
0.6
MR effect size for
'Gut microbiota abundance (genus Oxalobacter id.2978)' on 'Ulcerative enterocolitis'

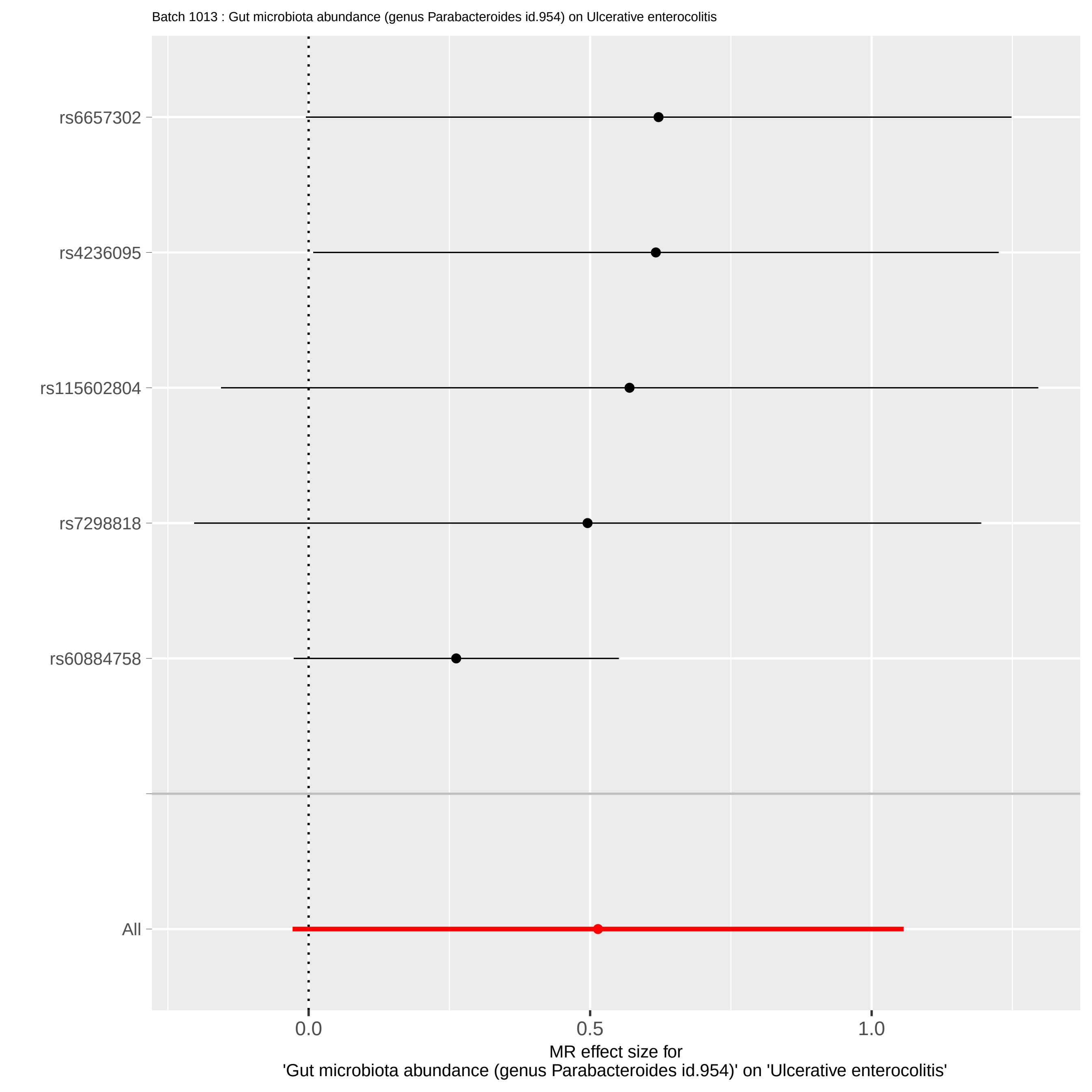

Batch 1013 : Gut microbiota abundance (genus Parabacteroides id.954) on Ulcerative enterocolitis
rs6657302
rs4236095
rs115602804
rs7298818
rs60884758
All
0.0
0.5
1.0
MR effect size for
'Gut microbiota abundance (genus Parabacteroides id.954)' on 'Ulcerative enterocolitis'

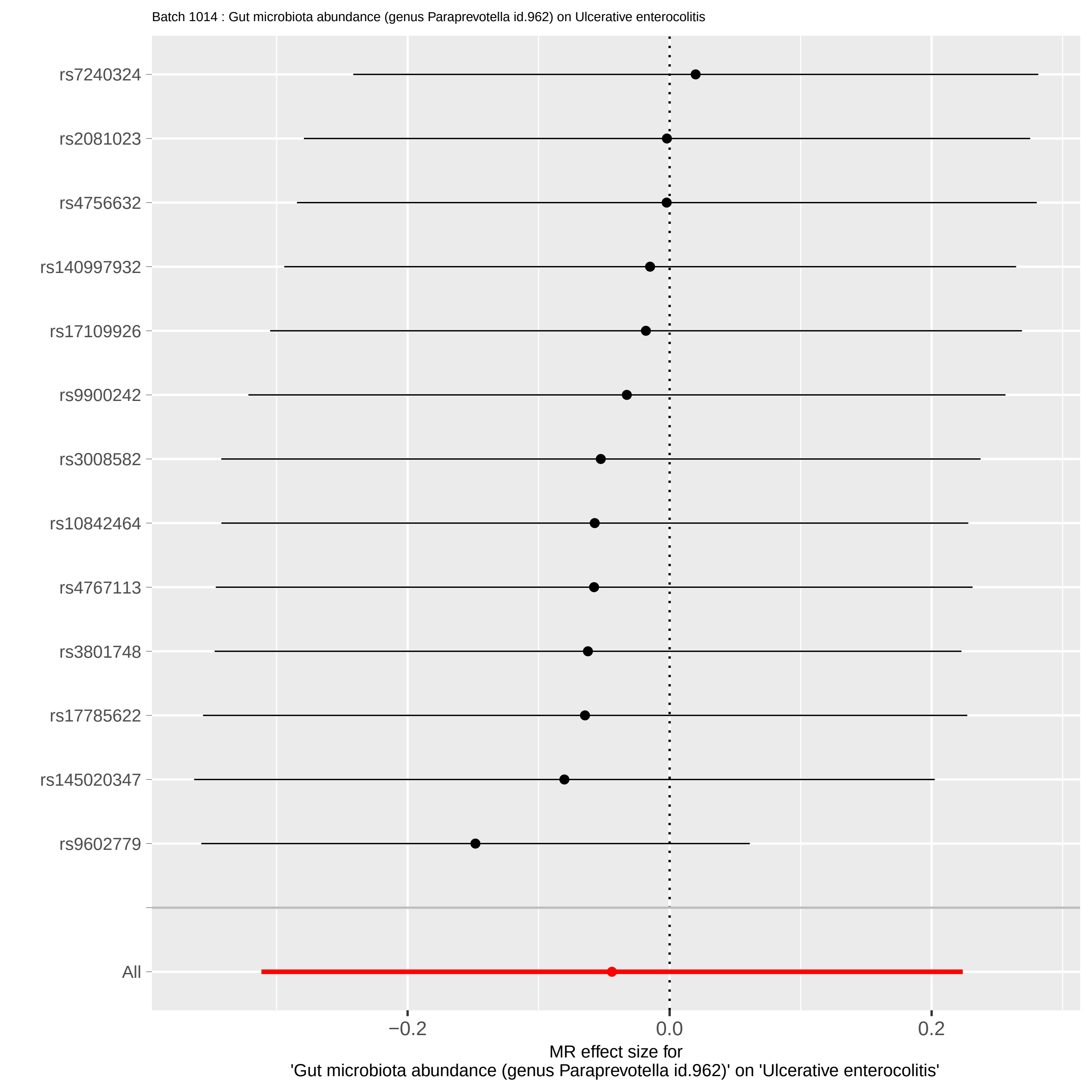
Batch 1014 : Gut microbiota abundance (genus Paraprevotella id.962) on Ulcerative enterocolitis
rs7240324
rs2081023
rs4756632
rs140997932
rs17109926
rs9900242
rs3008582
rs10842464
rs4767113
rs3801748
rs17785622
rs145020347
rs9602779
All
−0.2
0.0
0.2
MR effect size for
'Gut microbiota abundance (genus Paraprevotella id.962)' on 'Ulcerative enterocolitis'

Batch 1015 : Gut microbiota abundance (genus Parasutterella id.2892) on Ulcerative enterocolitis

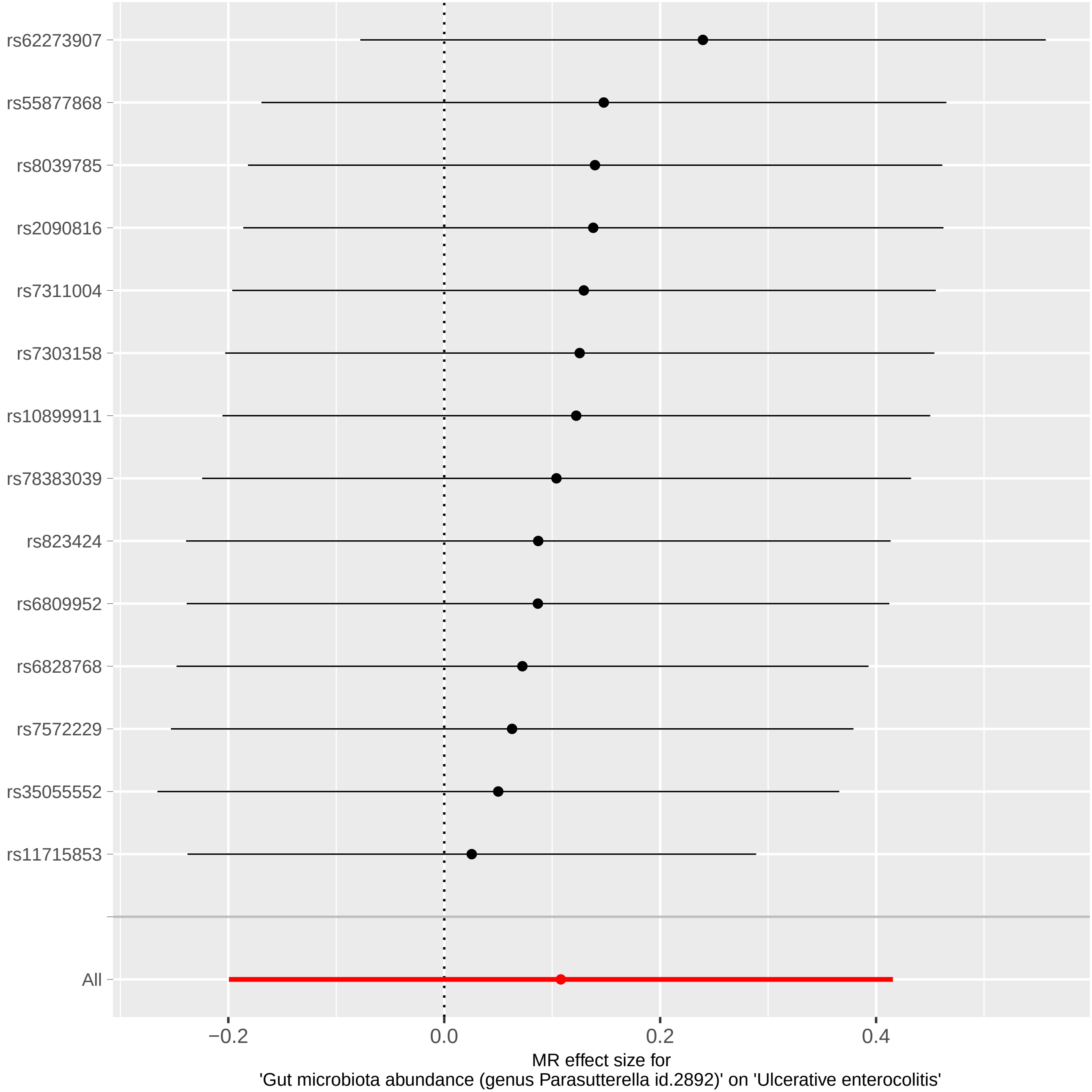

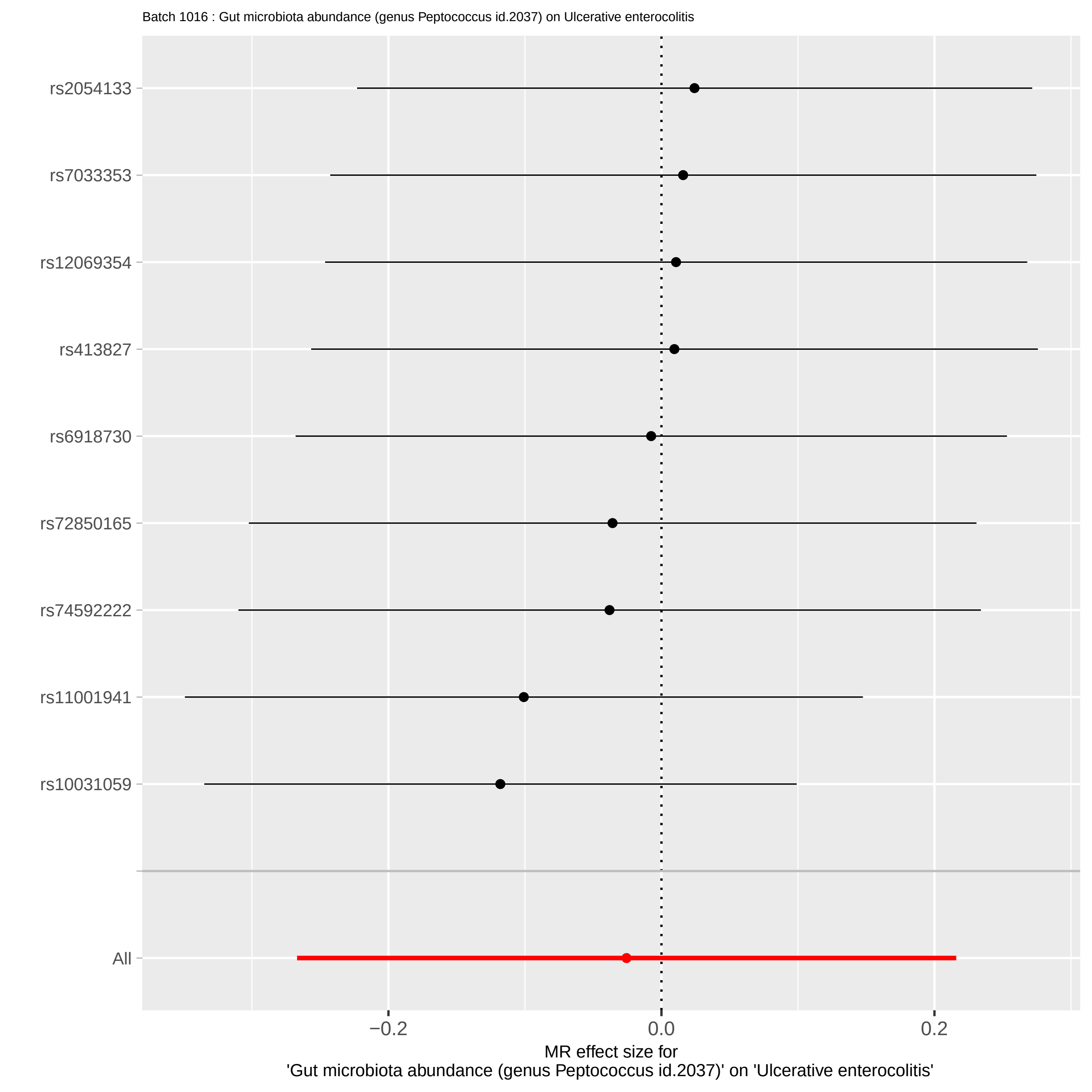
Batch 1016 : Gut microbiota abundance (genus Peptococcus id.2037) on Ulcerative enterocolitis
rs2054133
rs7033353
rs12069354
rs413827
rs6918730
rs72850165
rs74592222
rs11001941
rs10031059
All
−0.2
0.0
0.2
MR effect size for
'Gut microbiota abundance (genus Peptococcus id.2037)' on 'Ulcerative enterocolitis'

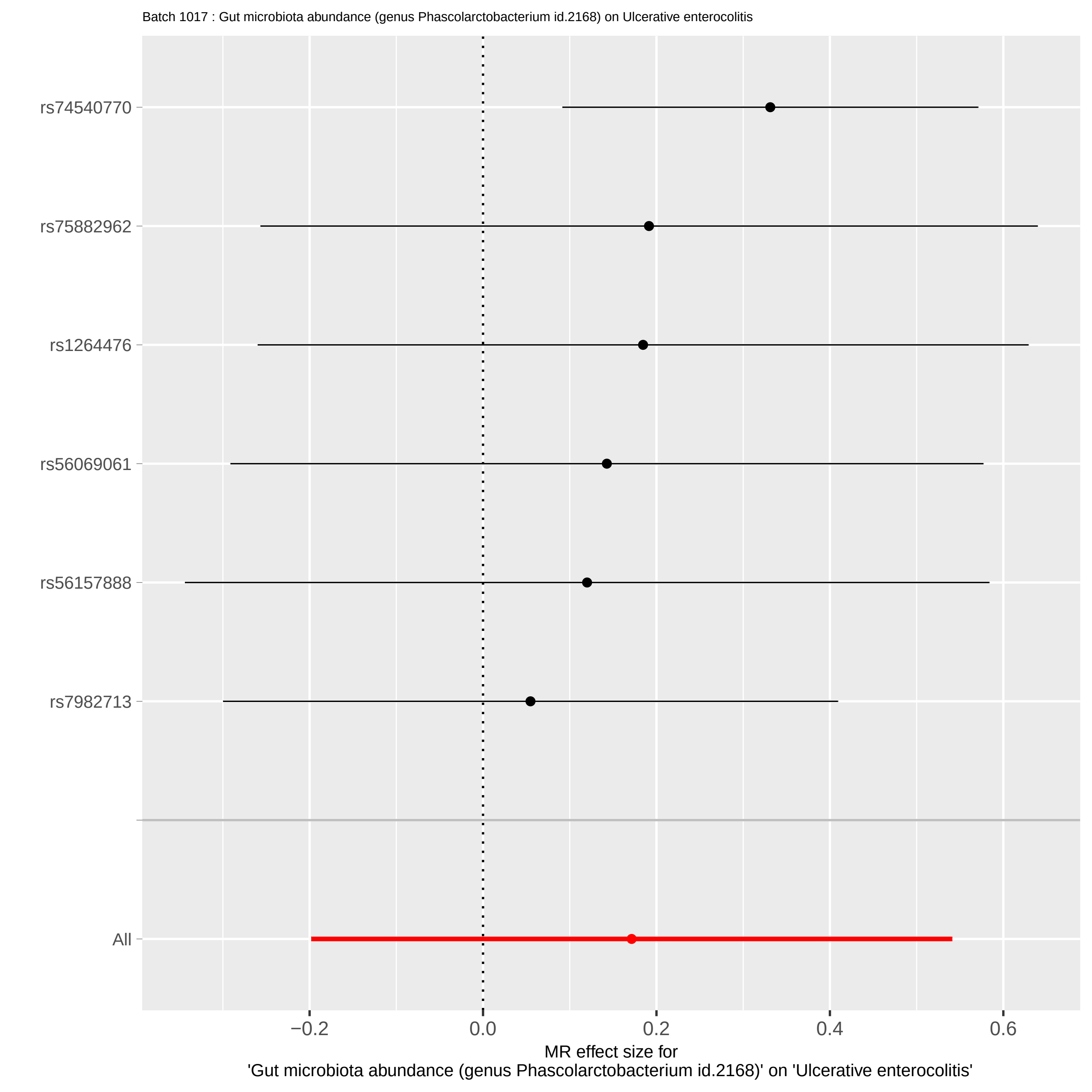
Batch 1017 : Gut microbiota abundance (genus Phascolarctobacterium id.2168) on Ulcerative enterocolitis
rs74540770
rs75882962
rs1264476
rs56069061
rs56157888
rs7982713
All
−0.2
0.0
0.2
0.4
0.6
MR effect size for
'Gut microbiota abundance (genus Phascolarctobacterium id.2168)' on 'Ulcerative enterocolitis'

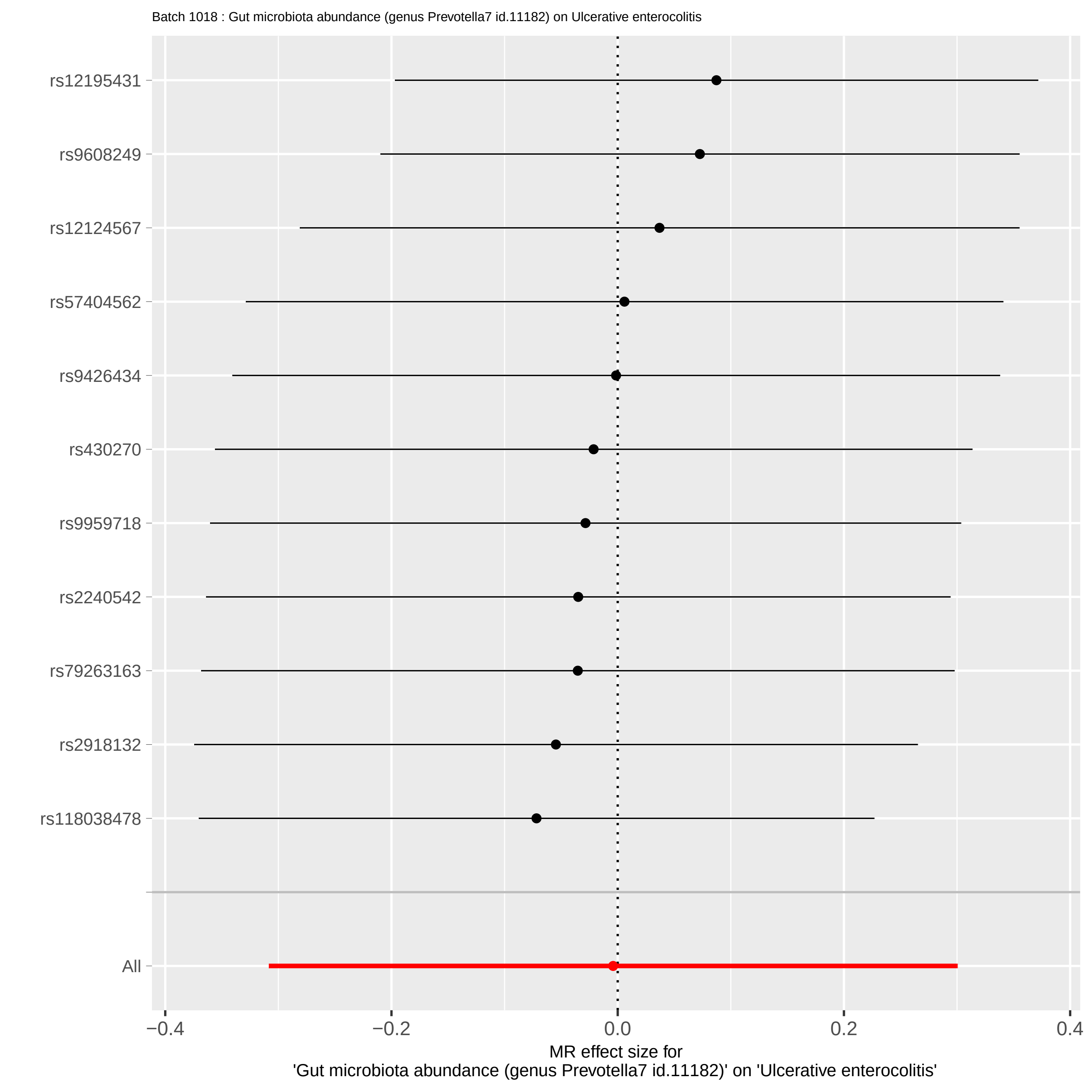

Batch 1018 : Gut microbiota abundance (genus Prevotella7 id.11182) on Ulcerative enterocolitis
rs12195431
rs9608249
rs12124567
rs57404562
rs9426434
rs430270
rs9959718
rs2240542
rs79263163
rs2918132
rs118038478
All
−0.4
−0.2
0.0
0.2
0.4
MR effect size for
'Gut microbiota abundance (genus Prevotella7 id.11182)' on 'Ulcerative enterocolitis'

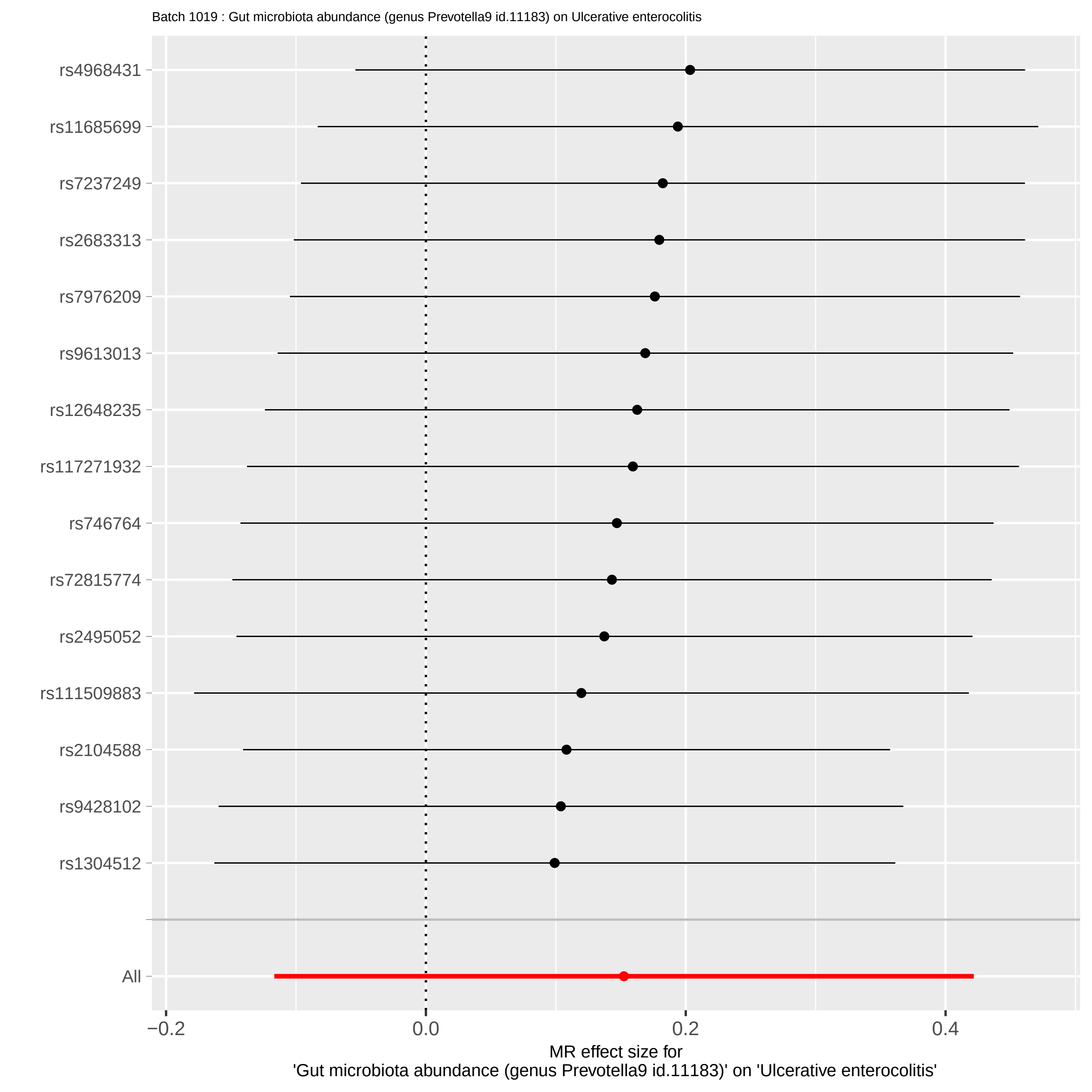
Batch 1019 : Gut microbiota abundance (genus Prevotella9 id.11183) on Ulcerative enterocolitis
rs4968431
rs11685699
rs7237249
rs2683313
rs7976209
rs9613013
rs12648235
rs117271932
rs746764
rs72815774
rs2495052
rs111509883
rs2104588
rs9428102
rs1304512
All
−0.2
0.0
0.2
0.4
MR effect size for
'Gut microbiota abundance (genus Prevotella9 id.11183)' on 'Ulcerative enterocolitis'

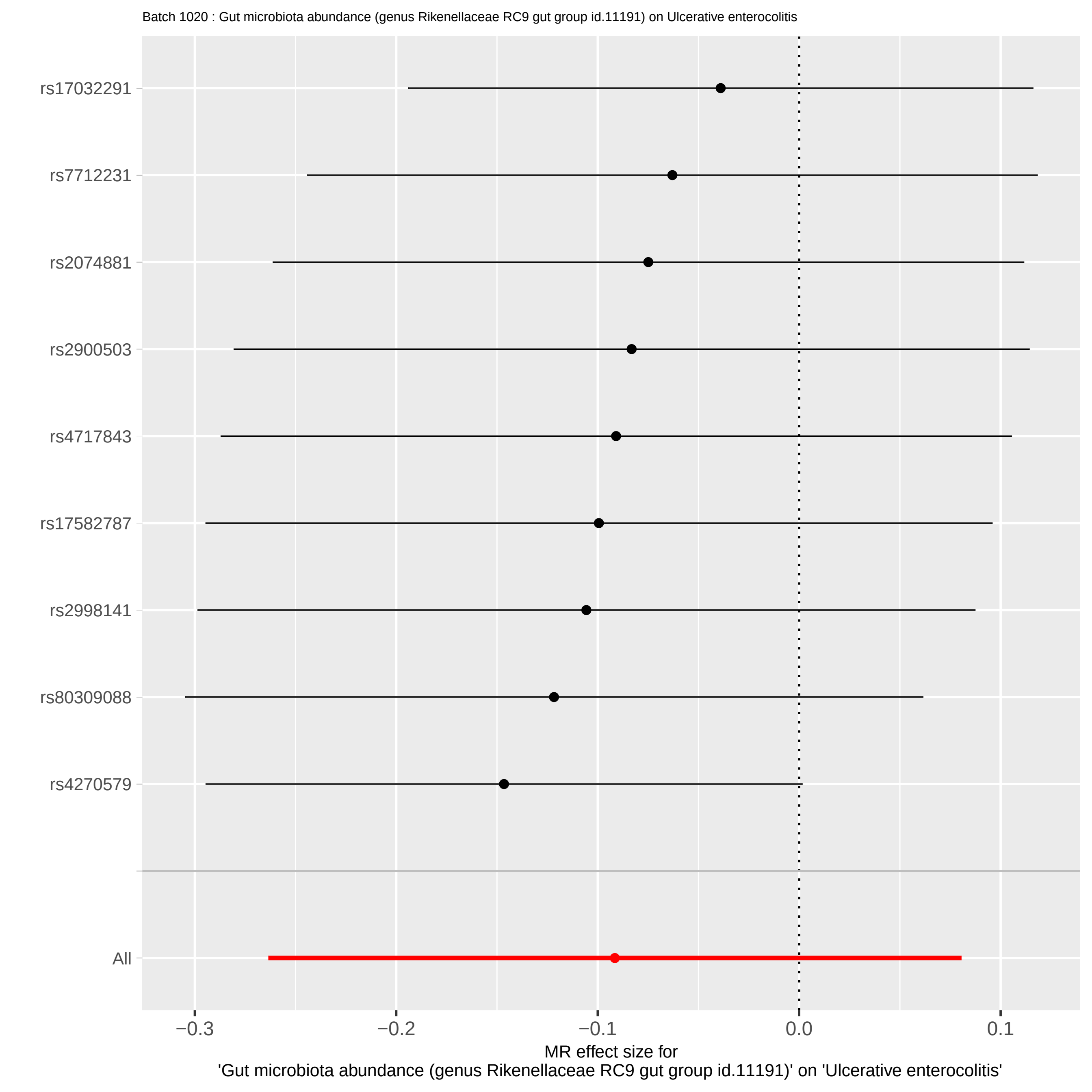
Batch 1020 : Gut microbiota abundance (genus Rikenellaceae RC9 gut group id.11191) on Ulcerative enterocolitis
rs17032291
rs7712231
rs2074881
rs2900503
rs4717843
rs17582787
rs2998141
rs80309088
rs4270579
All
−0.3
−0.2
−0.1
0.0
0.1
MR effect size for
'Gut microbiota abundance (genus Rikenellaceae RC9 gut group id.11191)' on 'Ulcerative enterocolitis'

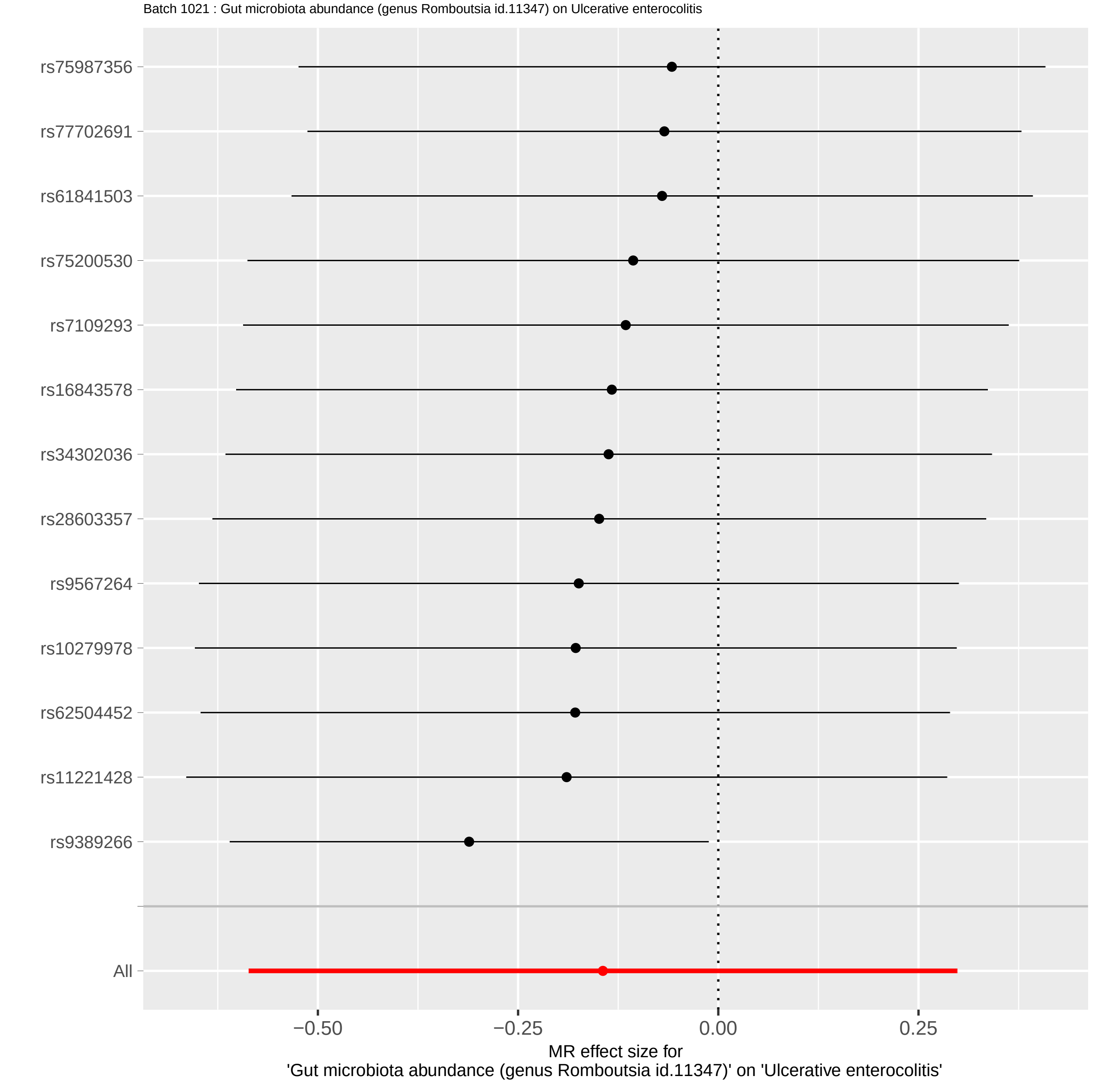

Batch 1021 : Gut microbiota abundance (genus Romboutsia id.11347) on Ulcerative enterocolitis
rs75987356
rs77702691
rs61841503
rs75200530
rs7109293
rs16843578
rs34302036
rs28603357
rs9567264
rs10279978
rs62504452
rs11221428
rs9389266
All
−0.50
−0.25
0.00
0.25
MR effect size for
'Gut microbiota abundance (genus Romboutsia id.11347)' on 'Ulcerative enterocolitis'

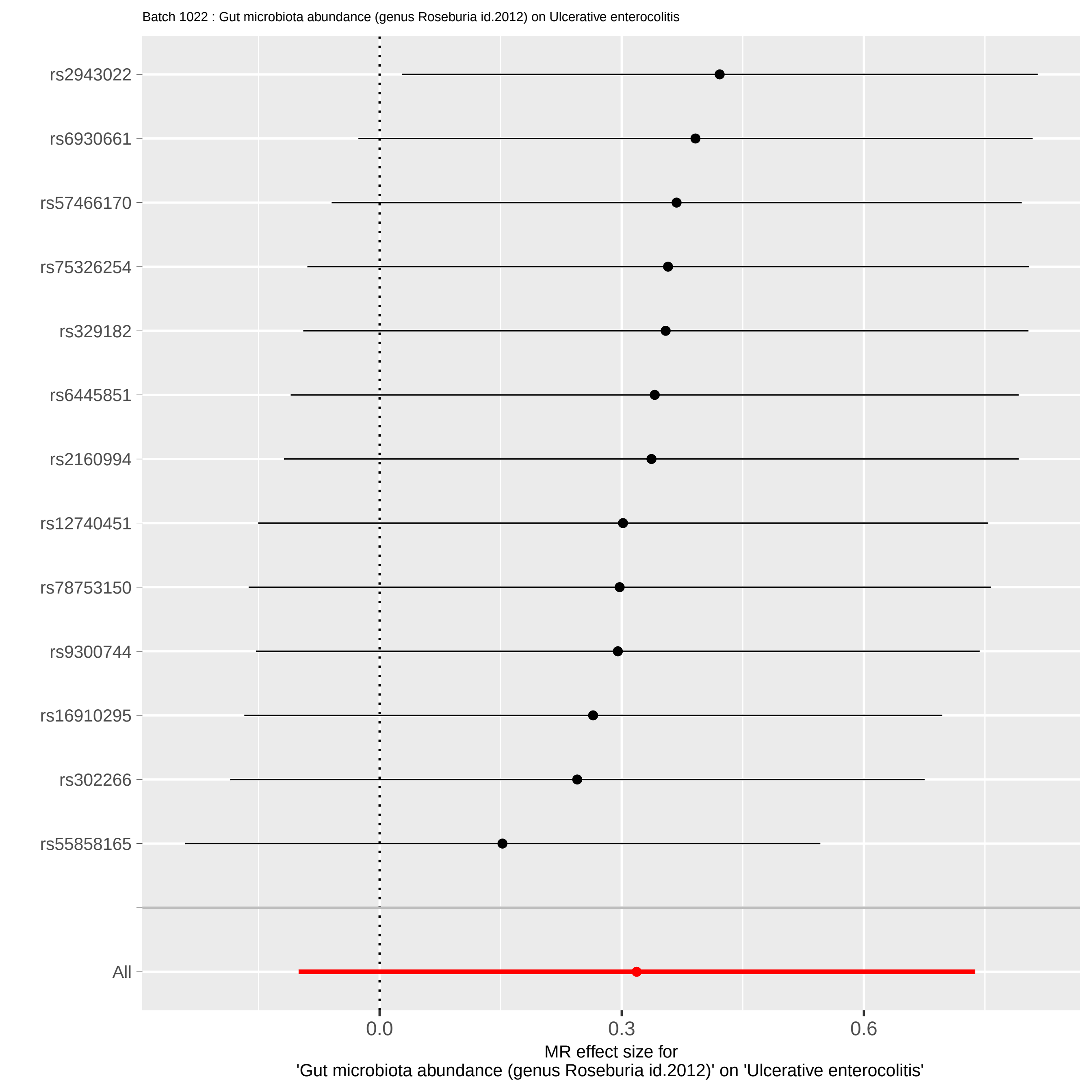
Batch 1022 : Gut microbiota abundance (genus Roseburia id.2012) on Ulcerative enterocolitis
rs2943022
rs6930661
rs57466170
rs75326254
rs329182
rs6445851
rs2160994
rs12740451
rs78753150
rs9300744
rs16910295
rs302266
rs55858165
All
0.0
0.3
0.6
MR effect size for
'Gut microbiota abundance (genus Roseburia id.2012)' on 'Ulcerative enterocolitis'

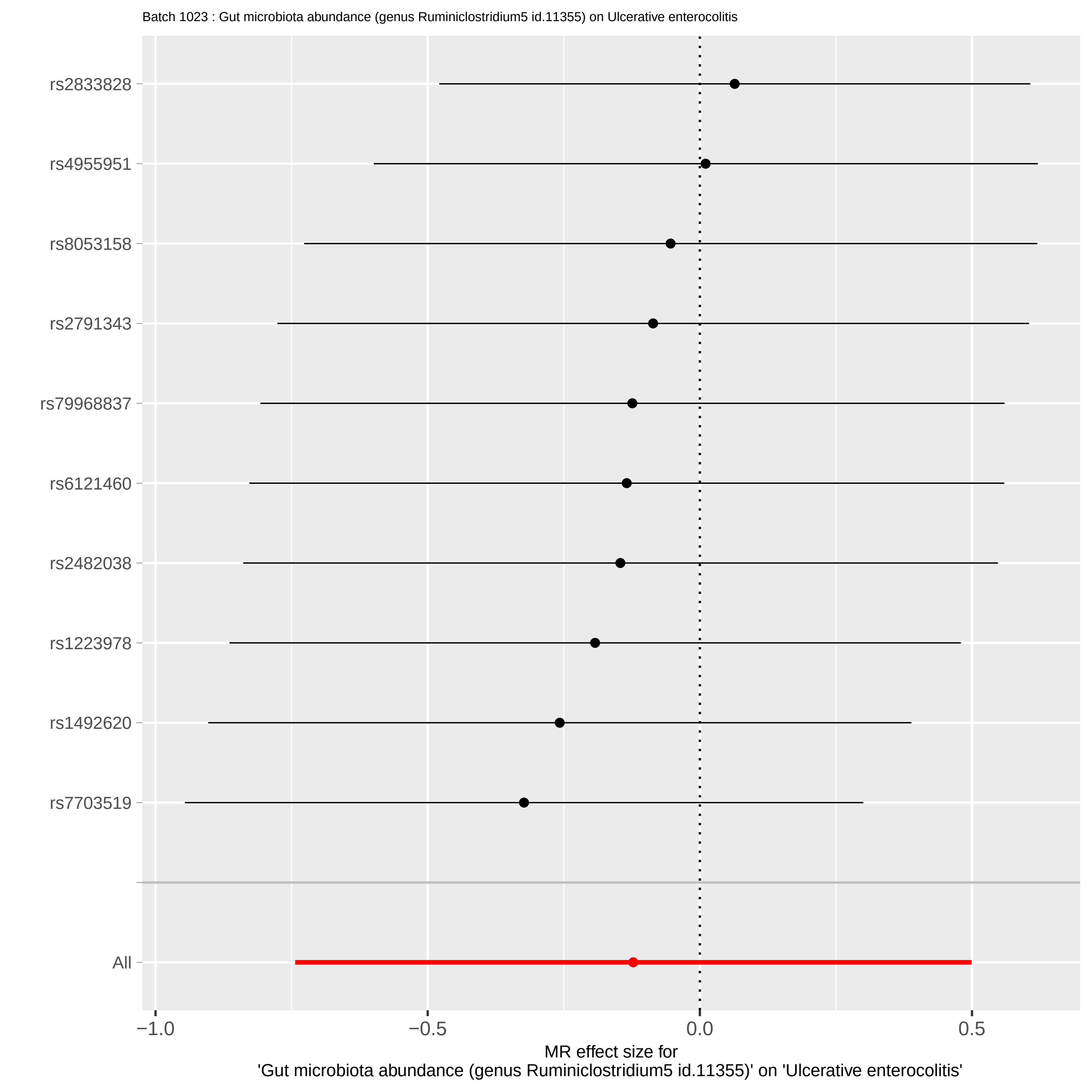

Batch 1023 : Gut microbiota abundance (genus Ruminiclostridium5 id.11355) on Ulcerative enterocolitis
rs2833828
rs4955951
rs8053158
rs2791343
rs79968837
rs6121460
rs2482038
rs1223978
rs1492620
rs7703519
All
−1.0
−0.5
0.0
0.5
MR effect size for
'Gut microbiota abundance (genus Ruminiclostridium5 id.11355)' on 'Ulcerative enterocolitis'

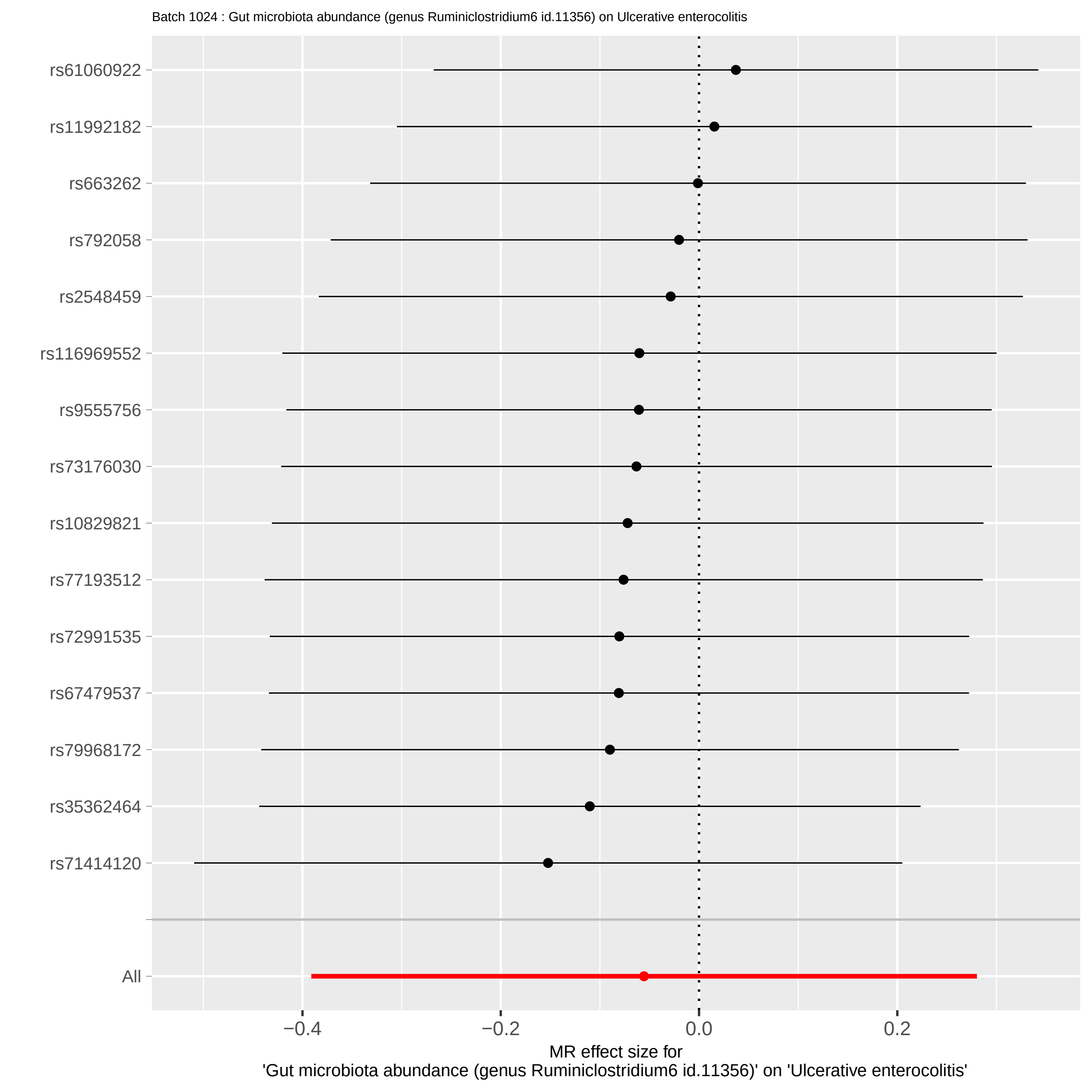

Batch 1024 : Gut microbiota abundance (genus Ruminiclostridium6 id.11356) on Ulcerative enterocolitis
rs61060922
rs11992182
rs663262
rs792058
rs2548459
rs116969552
rs9555756
rs73176030
rs10829821
rs77193512
rs72991535
rs67479537
rs79968172
rs35362464
rs71414120
All
−0.4
−0.2
0.0
0.2
MR effect size for
'Gut microbiota abundance (genus Ruminiclostridium6 id.11356)' on 'Ulcerative enterocolitis'

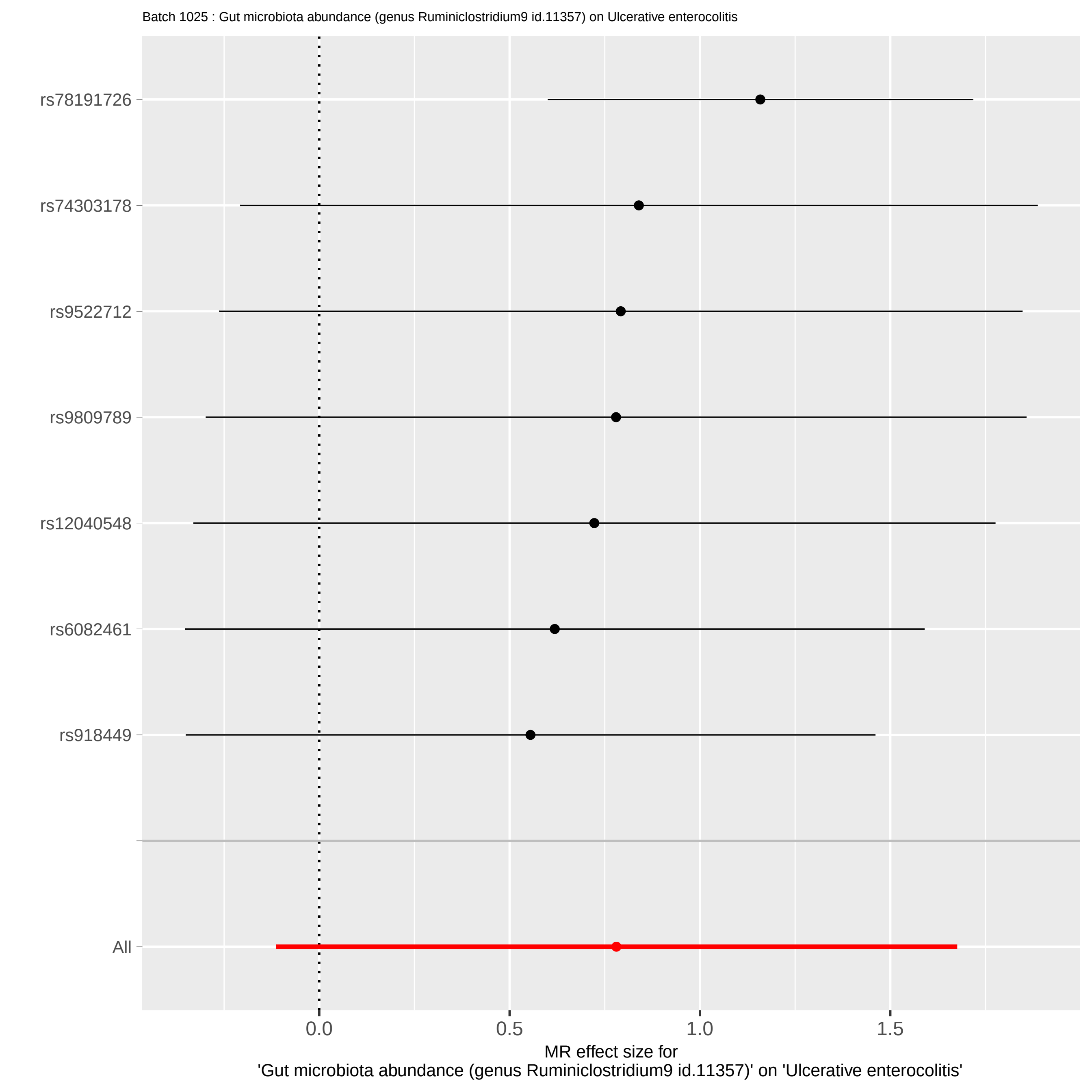

Batch 1025 : Gut microbiota abundance (genus Ruminiclostridium9 id.11357) on Ulcerative enterocolitis
rs78191726
rs74303178
rs9522712
rs9809789
rs12040548
rs6082461
rs918449
All
0.0
0.5
1.0
1.5
MR effect size for
'Gut microbiota abundance (genus Ruminiclostridium9 id.11357)' on 'Ulcerative enterocolitis'

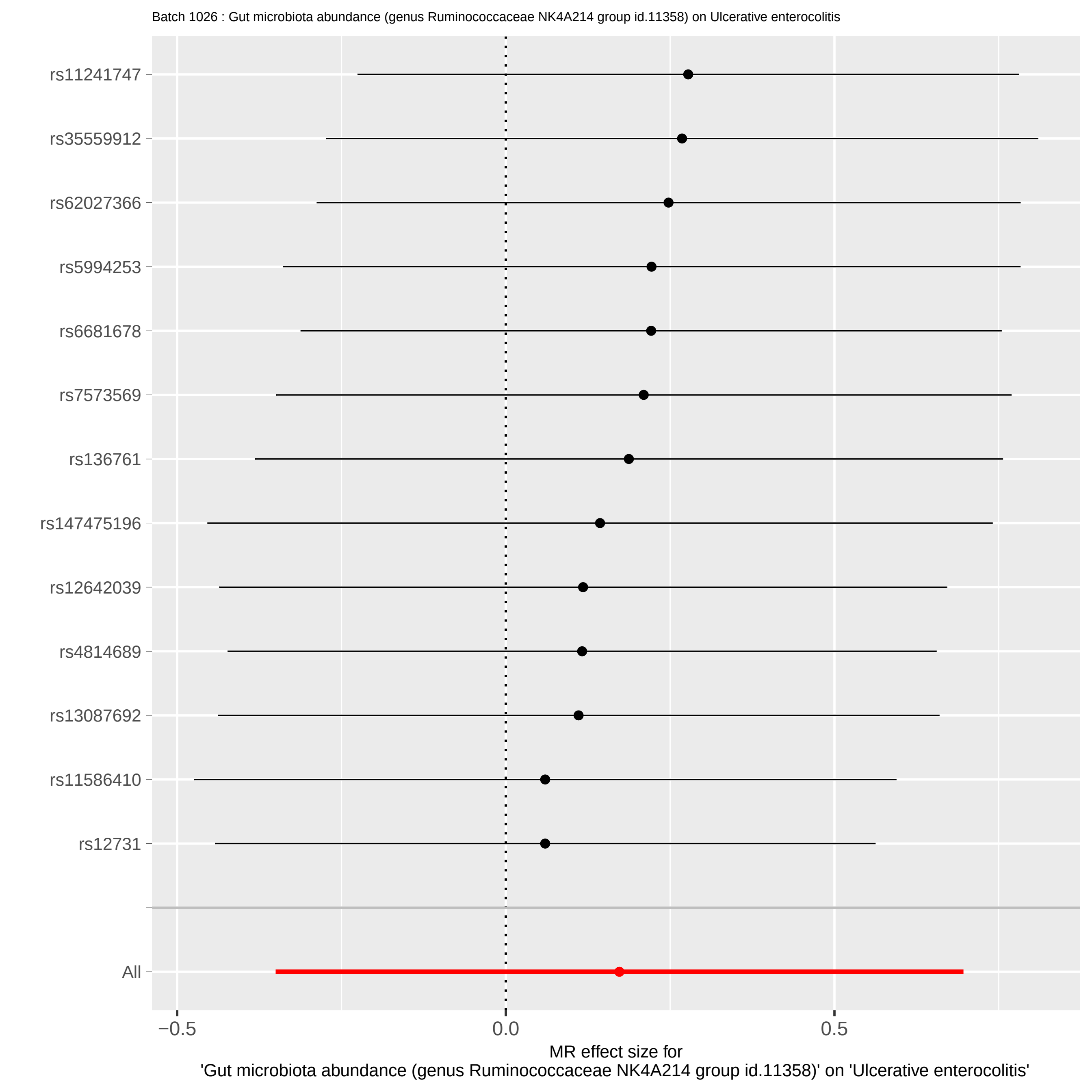

Batch 1026 : Gut microbiota abundance (genus Ruminococcaceae NK4A214 group id.11358) on Ulcerative enterocolitis
rs11241747
rs35559912
rs62027366
rs5994253
rs6681678
rs7573569
rs136761
rs147475196
rs12642039
rs4814689
rs13087692
rs11586410
rs12731
All
−0.5
0.0
0.5
MR effect size for
'Gut microbiota abundance (genus Ruminococcaceae NK4A214 group id.11358)' on 'Ulcerative enterocolitis'

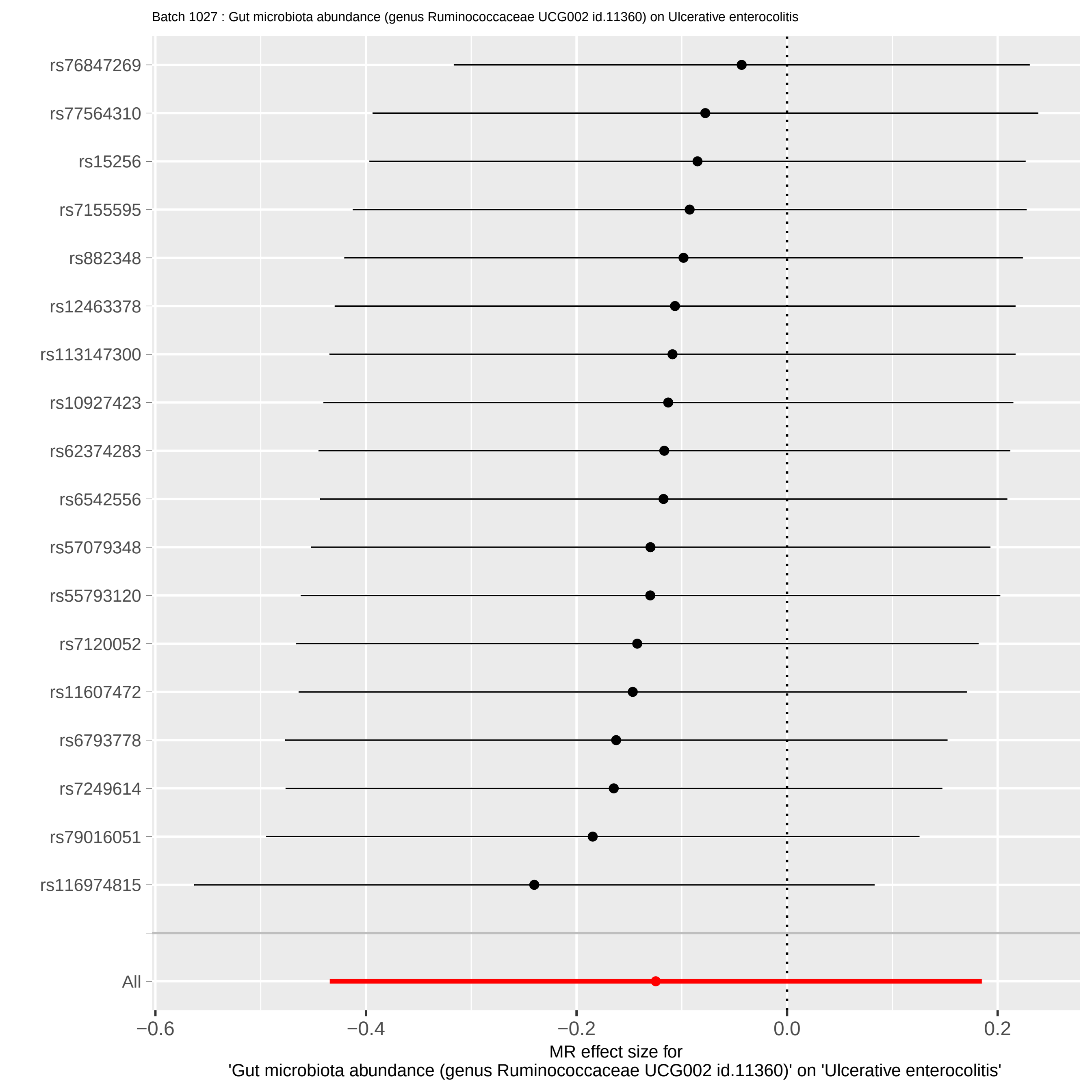

Batch 1027 : Gut microbiota abundance (genus Ruminococcaceae UCG002 id.11360) on Ulcerative enterocolitis
rs76847269
rs77564310
rs15256
rs7155595
rs882348
rs12463378
rs113147300
rs10927423
rs62374283
rs6542556
rs57079348
rs55793120
rs7120052
rs11607472
rs6793778
rs7249614
rs79016051
rs116974815
All
−0.6
−0.4
−0.2
0.0
0.2
MR effect size for
'Gut microbiota abundance (genus Ruminococcaceae UCG002 id.11360)' on 'Ulcerative enterocolitis'

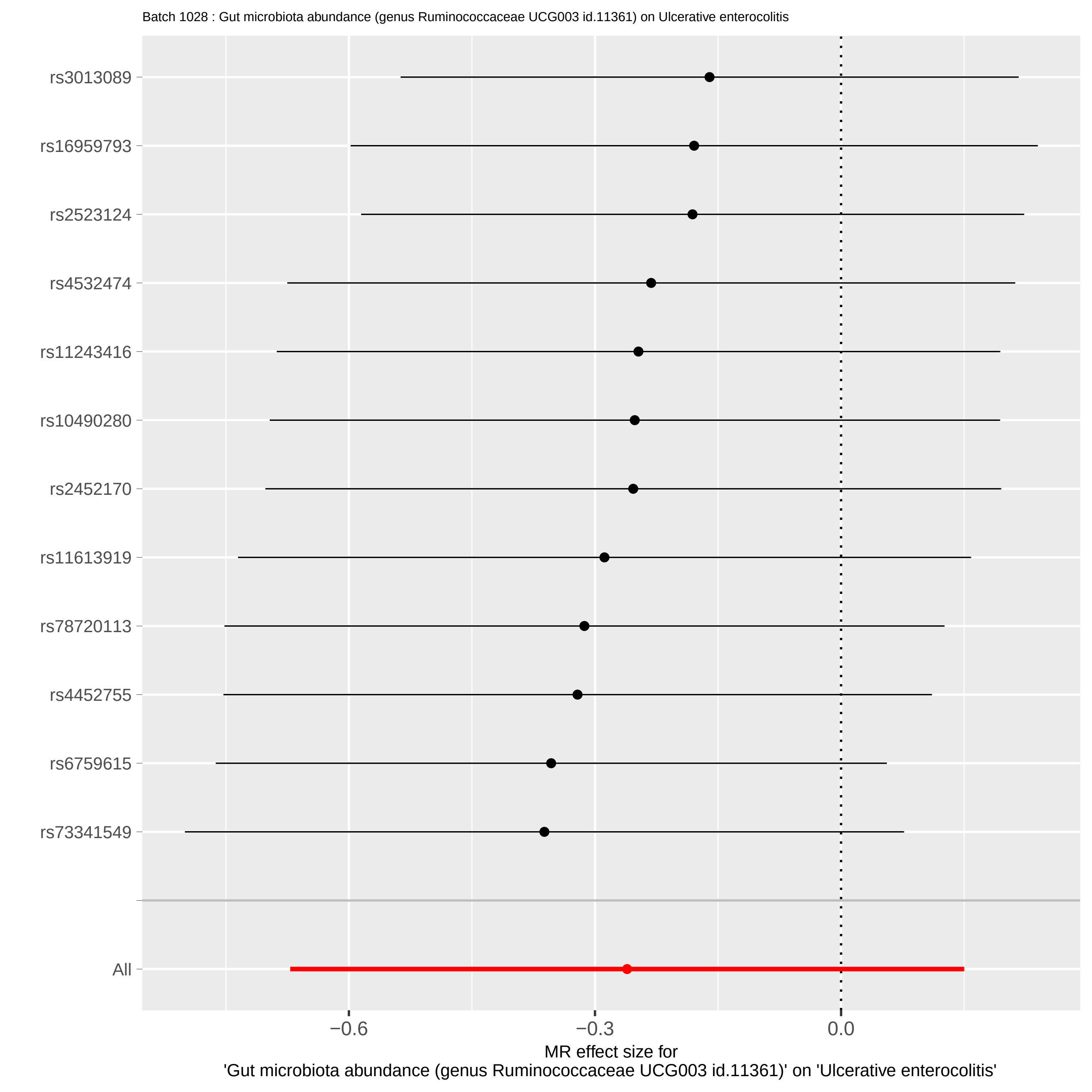

Batch 1028 : Gut microbiota abundance (genus Ruminococcaceae UCG003 id.11361) on Ulcerative enterocolitis
rs3013089
rs16959793
rs2523124
rs4532474
rs11243416
rs10490280
rs2452170
rs11613919
rs78720113
rs4452755
rs6759615
rs73341549
All
−0.6
−0.3
0.0
MR effect size for
'Gut microbiota abundance (genus Ruminococcaceae UCG003 id.11361)' on 'Ulcerative enterocolitis'

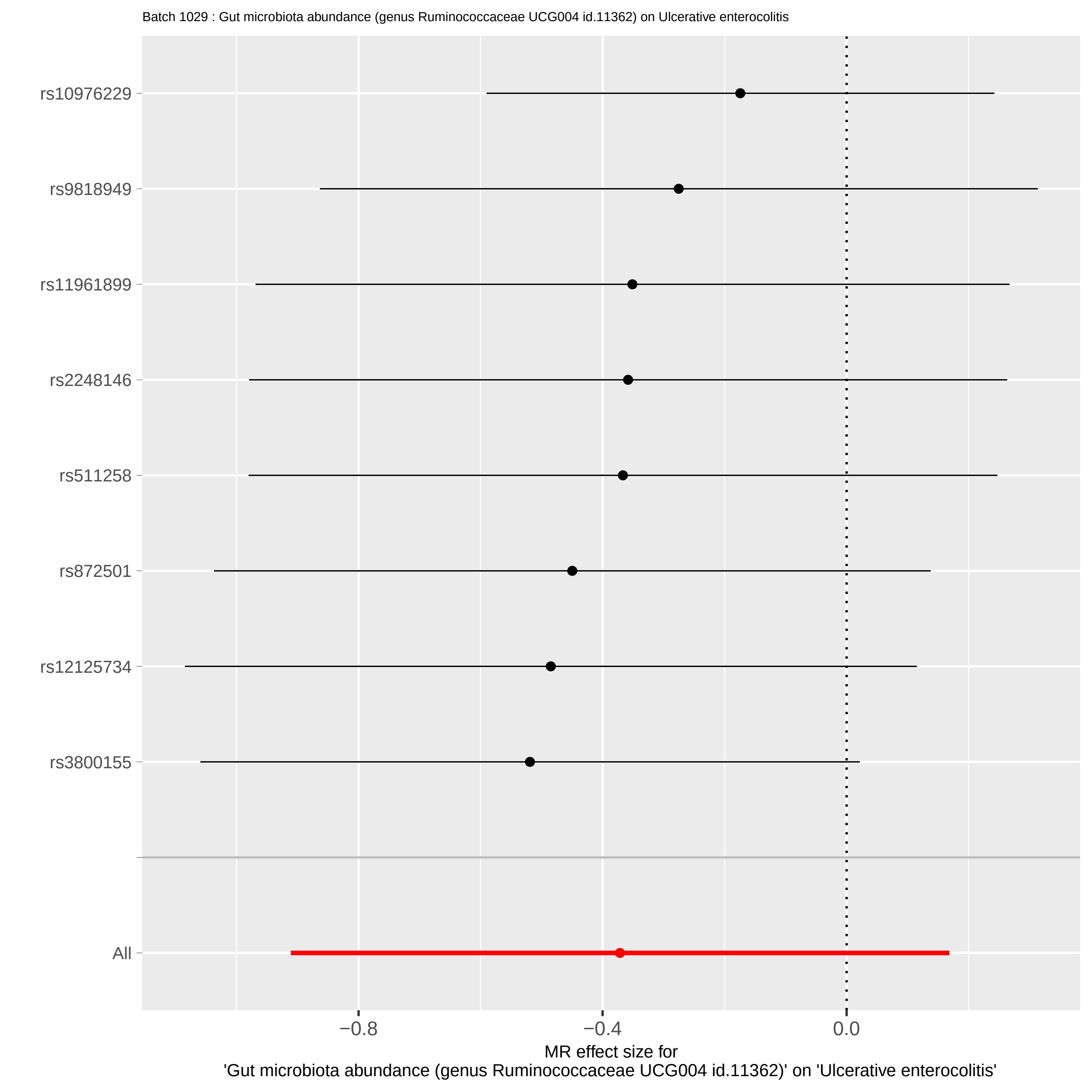

Batch 1029 : Gut microbiota abundance (genus Ruminococcaceae UCG004 id.11362) on Ulcerative enterocolitis
rs10976229
rs9818949
rs11961899
rs2248146
rs511258
rs872501
rs12125734
rs3800155
All
−0.8
−0.4
0.0
MR effect size for
'Gut microbiota abundance (genus Ruminococcaceae UCG004 id.11362)' on 'Ulcerative enterocolitis'

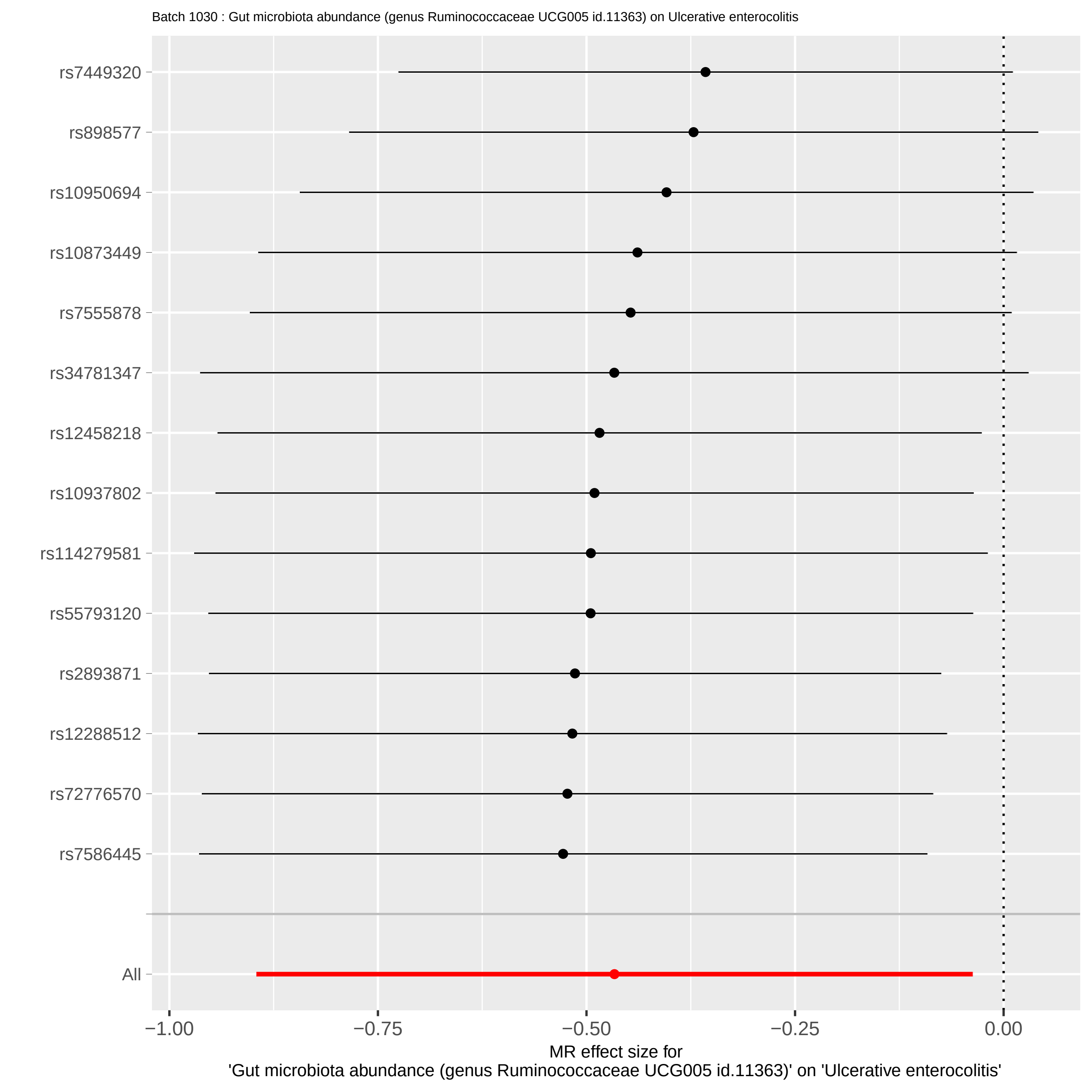
Batch 1030 : Gut microbiota abundance (genus Ruminococcaceae UCG005 id.11363) on Ulcerative enterocolitis
rs7449320
rs898577
rs10950694
rs10873449
rs7555878
rs34781347
rs12458218
rs10937802
rs114279581
rs55793120
rs2893871
rs12288512
rs72776570
rs7586445
All
−1.00
−0.75
−0.50
−0.25
0.00
MR effect size for
'Gut microbiota abundance (genus Ruminococcaceae UCG005 id.11363)' on 'Ulcerative enterocolitis'

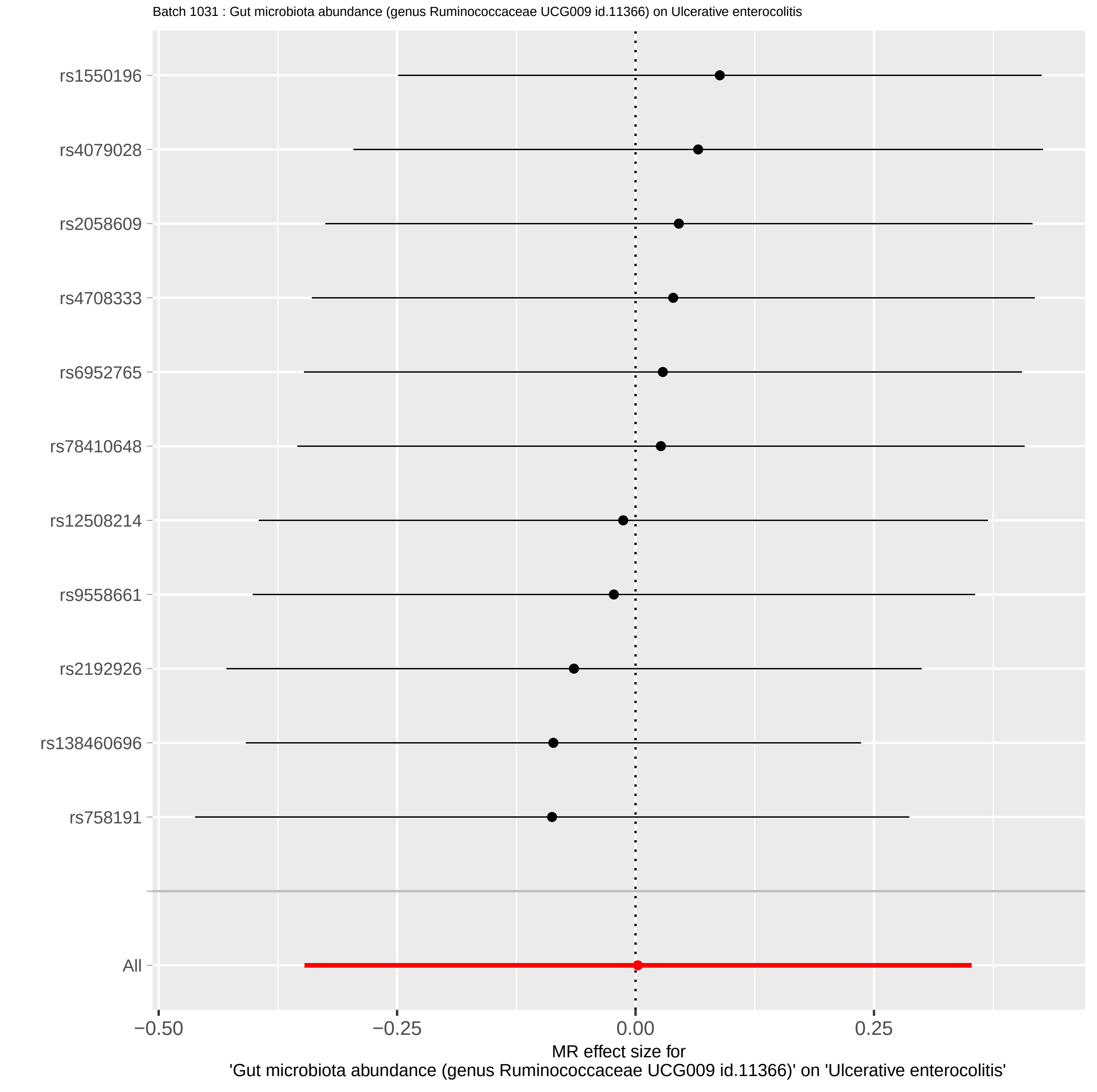
Batch 1031 : Gut microbiota abundance (genus Ruminococcaceae UCG009 id.11366) on Ulcerative enterocolitis
rs1550196
rs4079028
rs2058609
rs4708333
rs6952765
rs78410648
rs12508214
rs9558661
rs2192926
rs138460696
rs758191
All
−0.50
−0.25
0.00
0.25
MR effect size for
'Gut microbiota abundance (genus Ruminococcaceae UCG009 id.11366)' on 'Ulcerative enterocolitis'

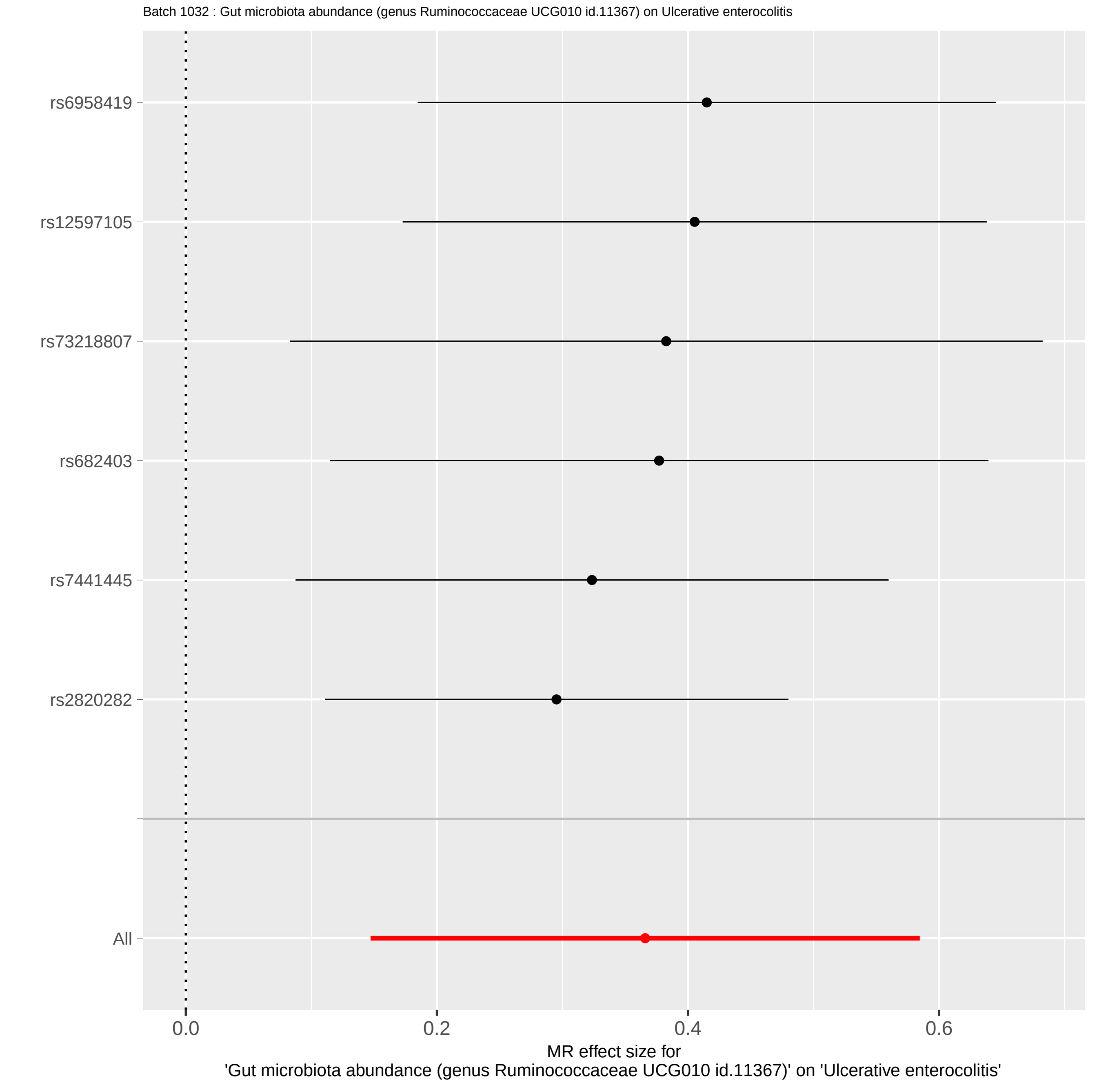
Batch 1032 : Gut microbiota abundance (genus Ruminococcaceae UCG010 id.11367) on Ulcerative enterocolitis
rs6958419
rs12597105
rs73218807
rs682403
rs7441445
rs2820282
All
0.0
0.2
0.4
0.6
MR effect size for
'Gut microbiota abundance (genus Ruminococcaceae UCG010 id.11367)' on 'Ulcerative enterocolitis'

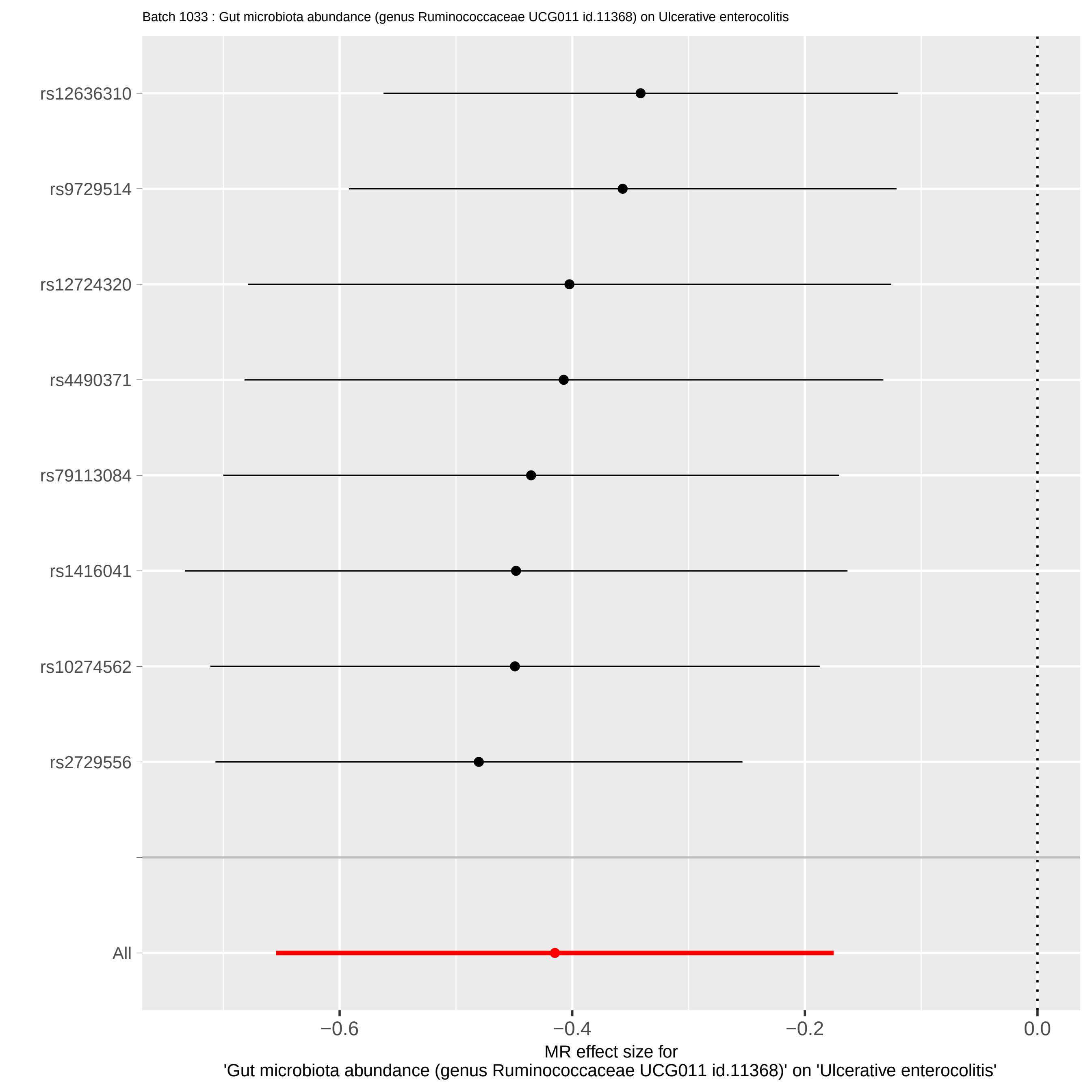

Batch 1033 : Gut microbiota abundance (genus Ruminococcaceae UCG011 id.11368) on Ulcerative enterocolitis
rs12636310
rs9729514
rs12724320
rs4490371
rs79113084
rs1416041
rs10274562
rs2729556
All
−0.6
−0.4
−0.2
0.0
MR effect size for
'Gut microbiota abundance (genus Ruminococcaceae UCG011 id.11368)' on 'Ulcerative enterocolitis'

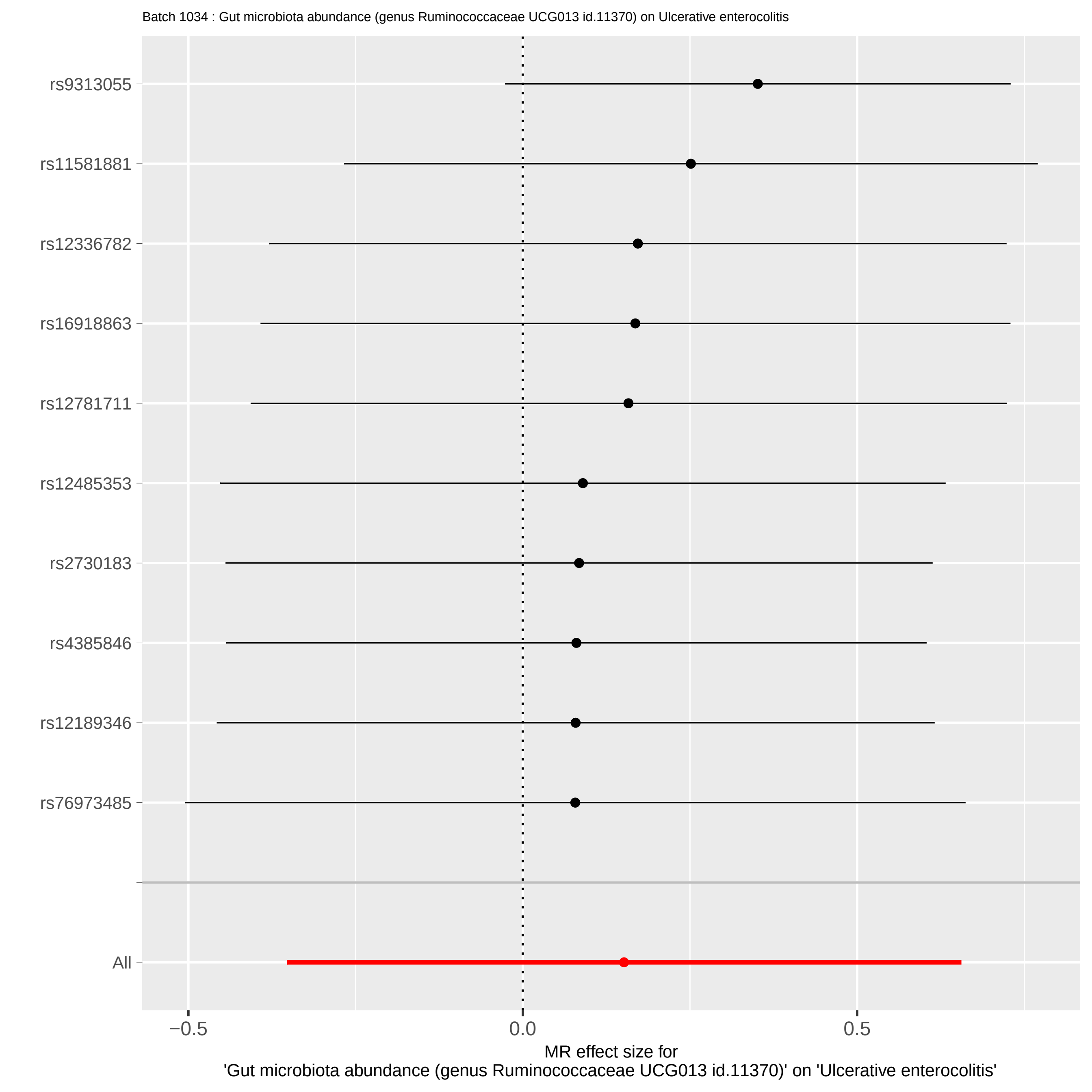
Batch 1034 : Gut microbiota abundance (genus Ruminococcaceae UCG013 id.11370) on Ulcerative enterocolitis
rs9313055
rs11581881
rs12336782
rs16918863
rs12781711
rs12485353
rs2730183
rs4385846
rs12189346
rs76973485
All
−0.5
0.0
0.5
MR effect size for
'Gut microbiota abundance (genus Ruminococcaceae UCG013 id.11370)' on 'Ulcerative enterocolitis'

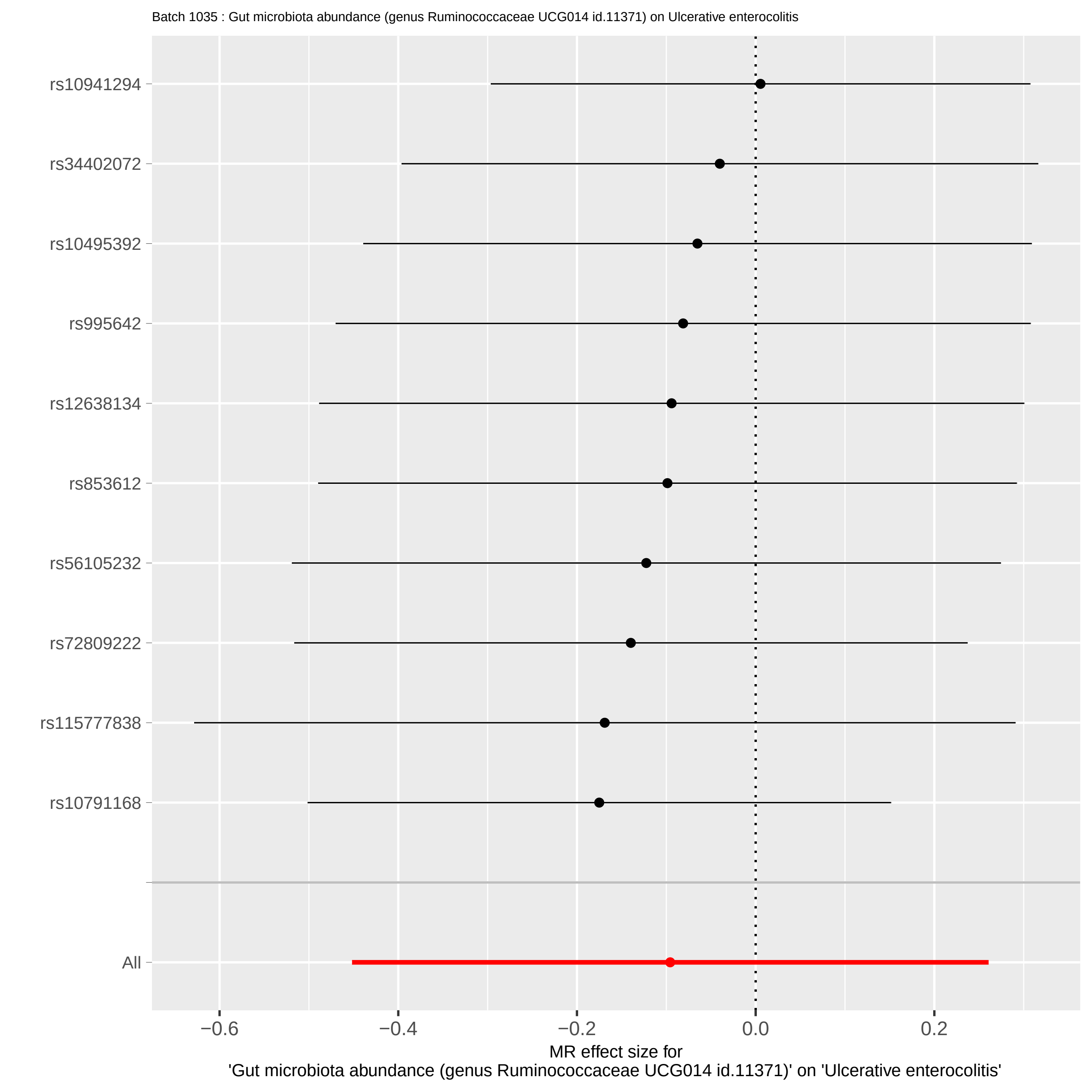

Batch 1035 : Gut microbiota abundance (genus Ruminococcaceae UCG014 id.11371) on Ulcerative enterocolitis
rs10941294
rs34402072
rs10495392
rs995642
rs12638134
rs853612
rs56105232
rs72809222
rs115777838
rs10791168
All
−0.6
−0.4
−0.2
0.0
0.2
MR effect size for
'Gut microbiota abundance (genus Ruminococcaceae UCG014 id.11371)' on 'Ulcerative enterocolitis'

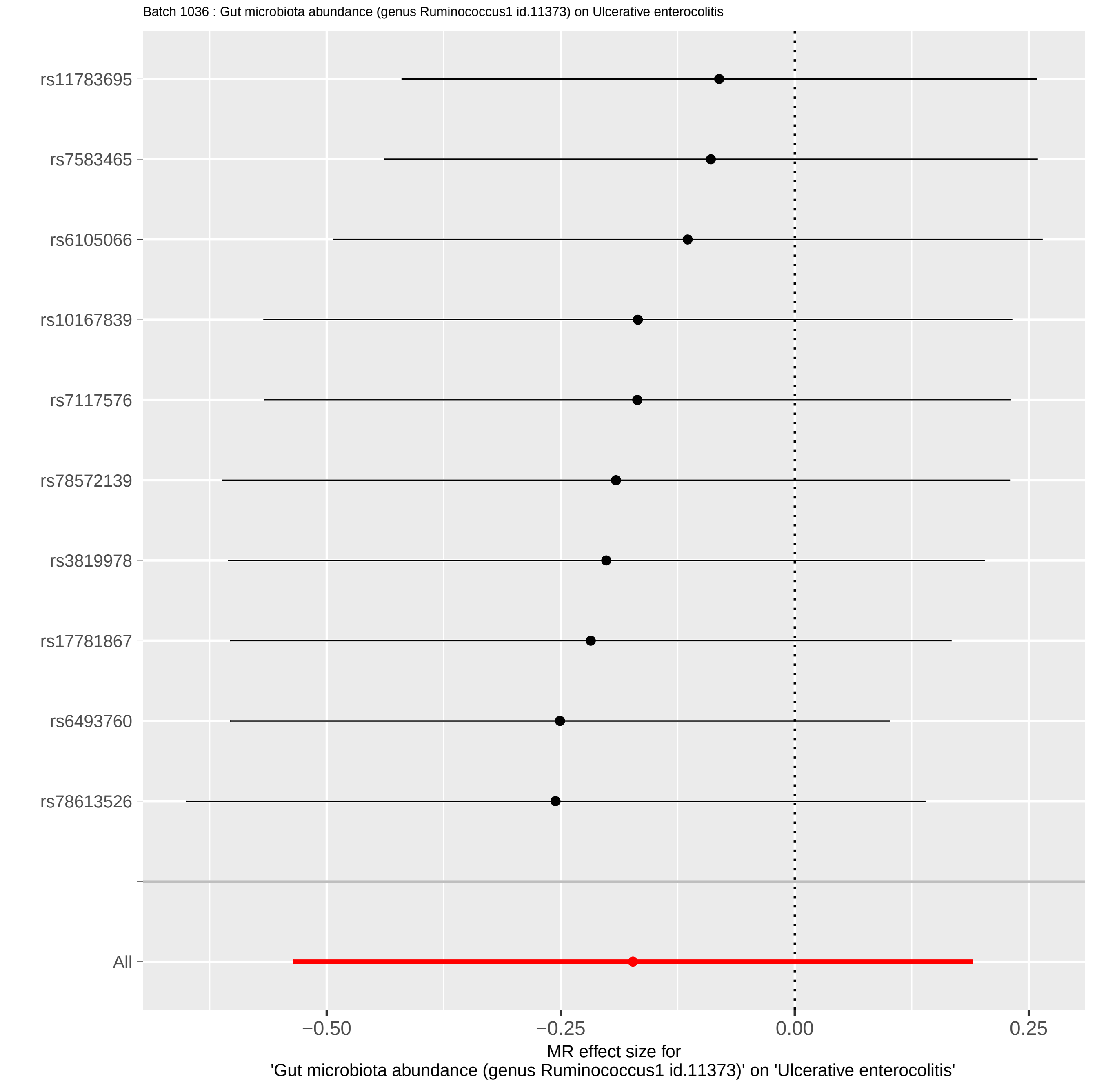

Batch 1036 : Gut microbiota abundance (genus Ruminococcus1 id.11373) on Ulcerative enterocolitis
rs11783695
rs7583465
rs6105066
rs10167839
rs7117576
rs78572139
rs3819978
rs17781867
rs6493760
rs78613526
All
−0.50
−0.25
0.00
0.25
MR effect size for
'Gut microbiota abundance (genus Ruminococcus1 id.11373)' on 'Ulcerative enterocolitis'

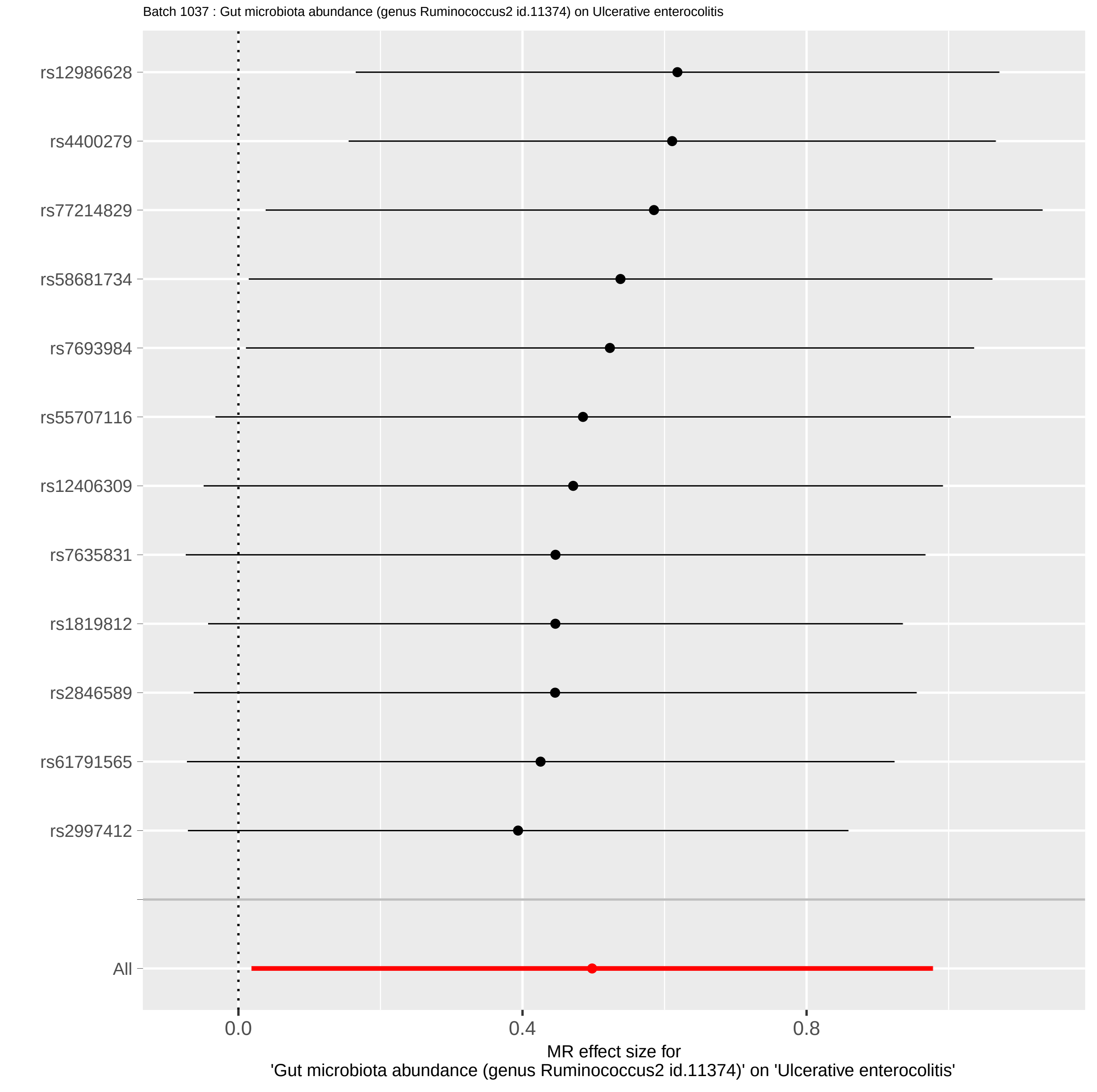

Batch 1037 : Gut microbiota abundance (genus Ruminococcus2 id.11374) on Ulcerative enterocolitis
rs12986628
rs4400279
rs77214829
rs58681734
rs7693984
rs55707116
rs12406309
rs7635831
rs1819812
rs2846589
rs61791565
rs2997412
All
0.0
0.4
0.8
MR effect size for
'Gut microbiota abundance (genus Ruminococcus2 id.11374)' on 'Ulcerative enterocolitis'

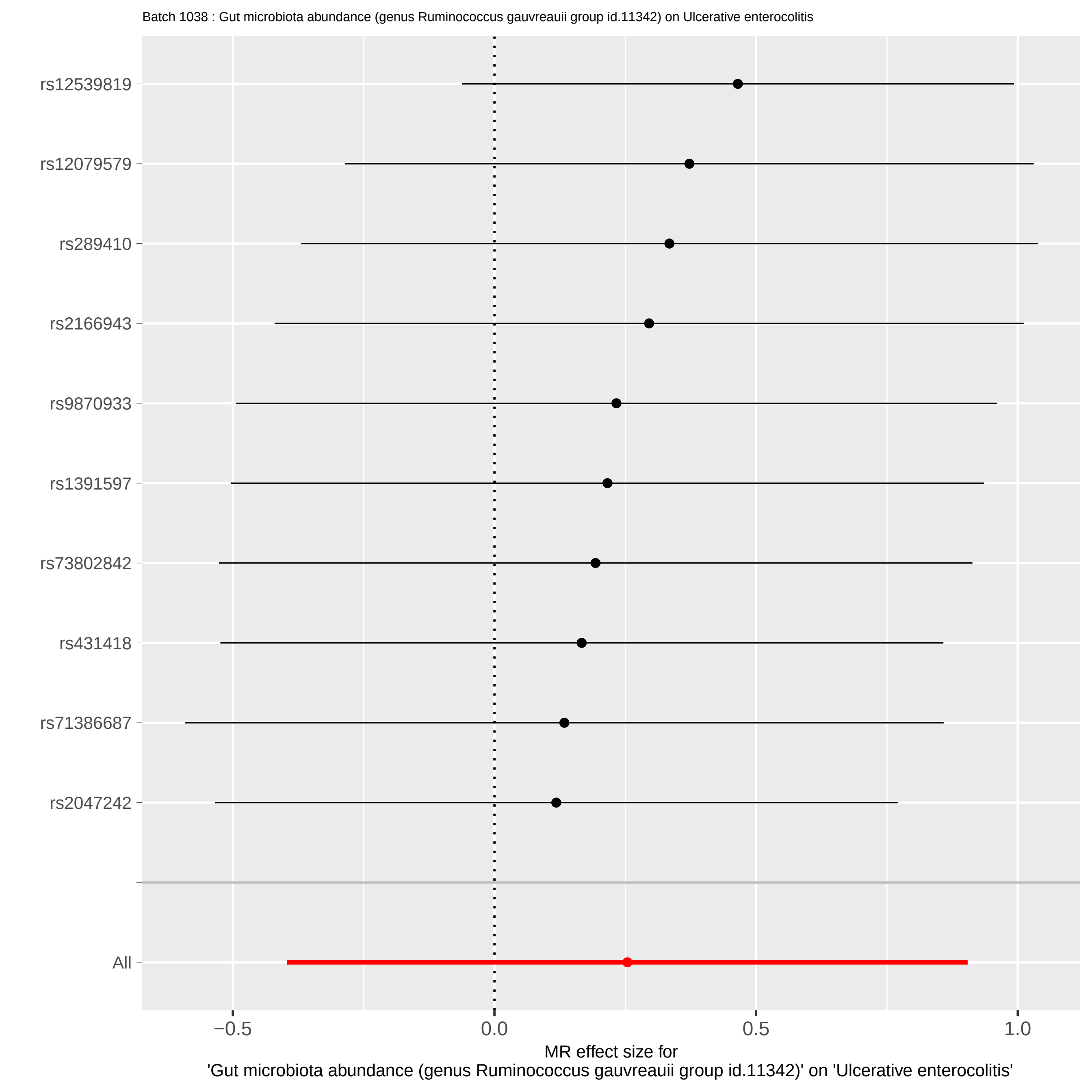

Batch 1038 : Gut microbiota abundance (genus Ruminococcus gauvreauii group id.11342) on Ulcerative enterocolitis
rs12539819
rs12079579
rs289410
rs2166943
rs9870933
rs1391597
rs73802842
rs431418
rs71386687
rs2047242
All
−0.5
0.0
0.5
1.0
MR effect size for
'Gut microbiota abundance (genus Ruminococcus gauvreauii group id.11342)' on 'Ulcerative enterocolitis'

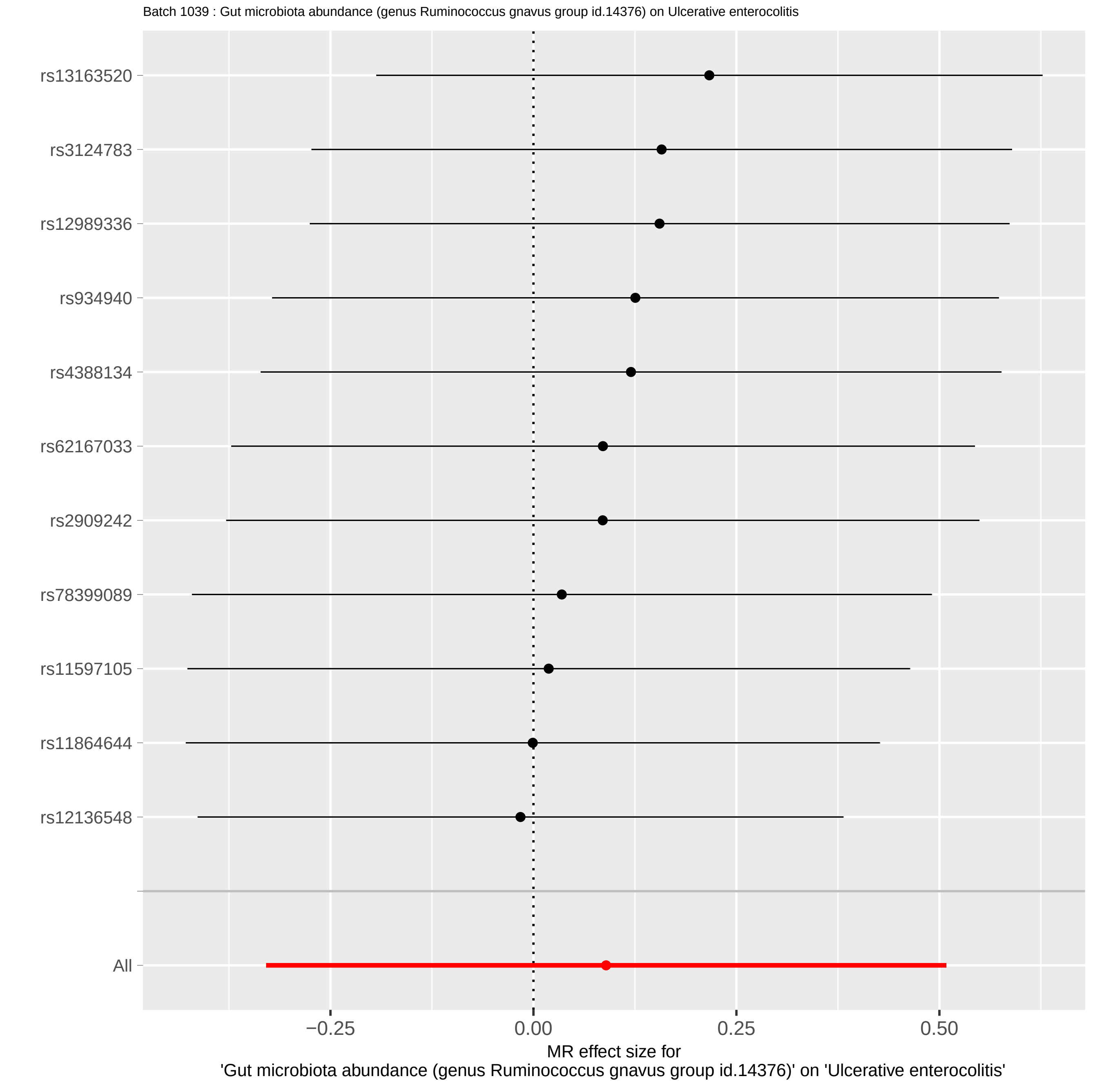

Batch 1039 : Gut microbiota abundance (genus Ruminococcus gnavus group id.14376) on Ulcerative enterocolitis
rs13163520
rs3124783
rs12989336
rs934940
rs4388134
rs62167033
rs2909242
rs78399089
rs11597105
rs11864644
rs12136548
All
−0.25
0.00
0.25
0.50
MR effect size for
'Gut microbiota abundance (genus Ruminococcus gnavus group id.14376)' on 'Ulcerative enterocolitis'

Batch 1040 : Gut microbiota abundance (genus Ruminococcus torques group id.14377) on Ulcerative enterocolitis

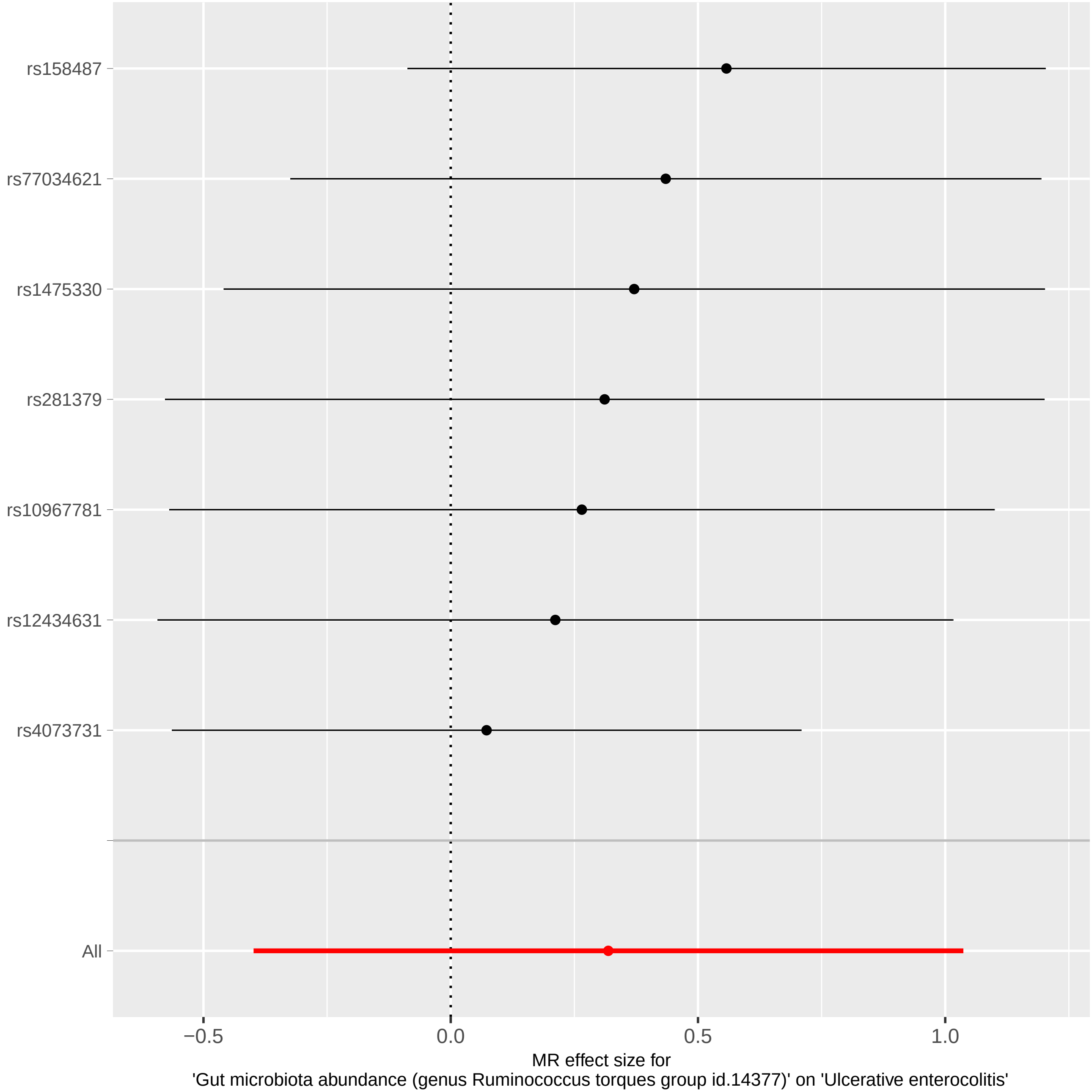

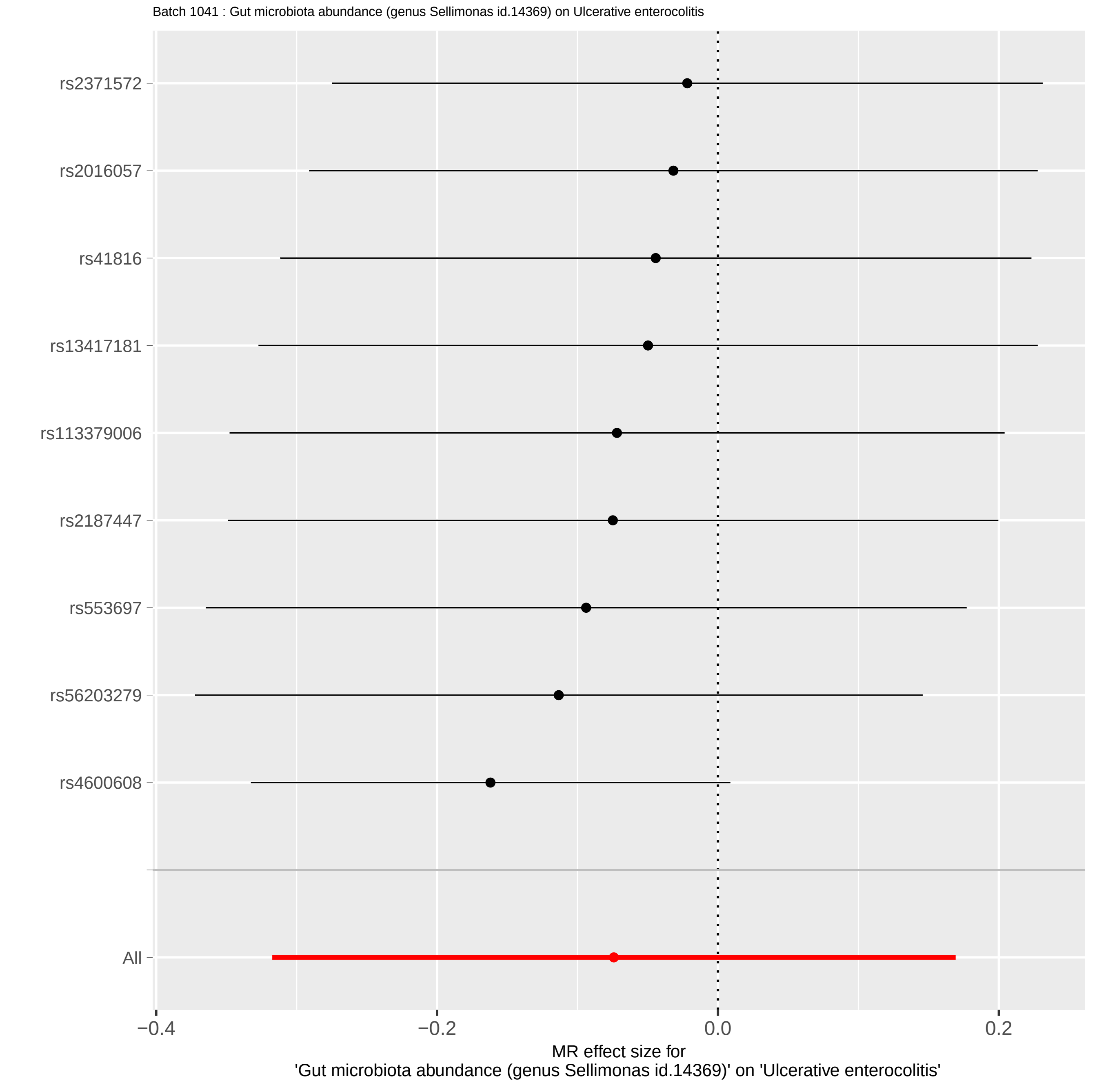

Batch 1041 : Gut microbiota abundance (genus Sellimonas id.14369) on Ulcerative enterocolitis
rs2371572
rs2016057
rs41816
rs13417181
rs113379006
rs2187447
rs553697
rs56203279
rs4600608
All
−0.4
−0.2
0.0
0.2
MR effect size for
'Gut microbiota abundance (genus Sellimonas id.14369)' on 'Ulcerative enterocolitis'

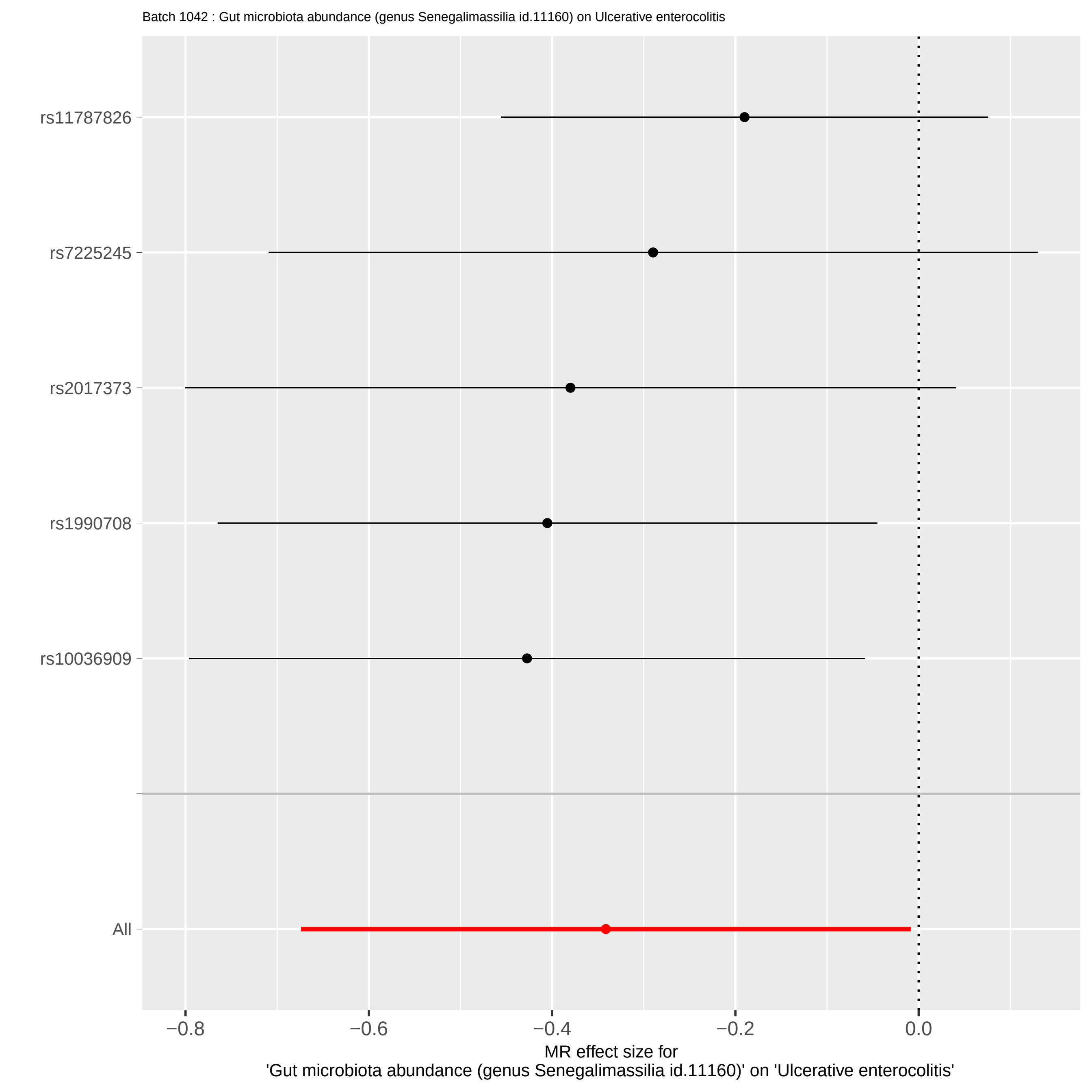

Batch 1042 : Gut microbiota abundance (genus Senegalimassilia id.11160) on Ulcerative enterocolitis
rs11787826
rs7225245
rs2017373
rs1990708
rs10036909
All
−0.8
−0.6
−0.4
−0.2
0.0
MR effect size for
'Gut microbiota abundance (genus Senegalimassilia id.11160)' on 'Ulcerative enterocolitis'

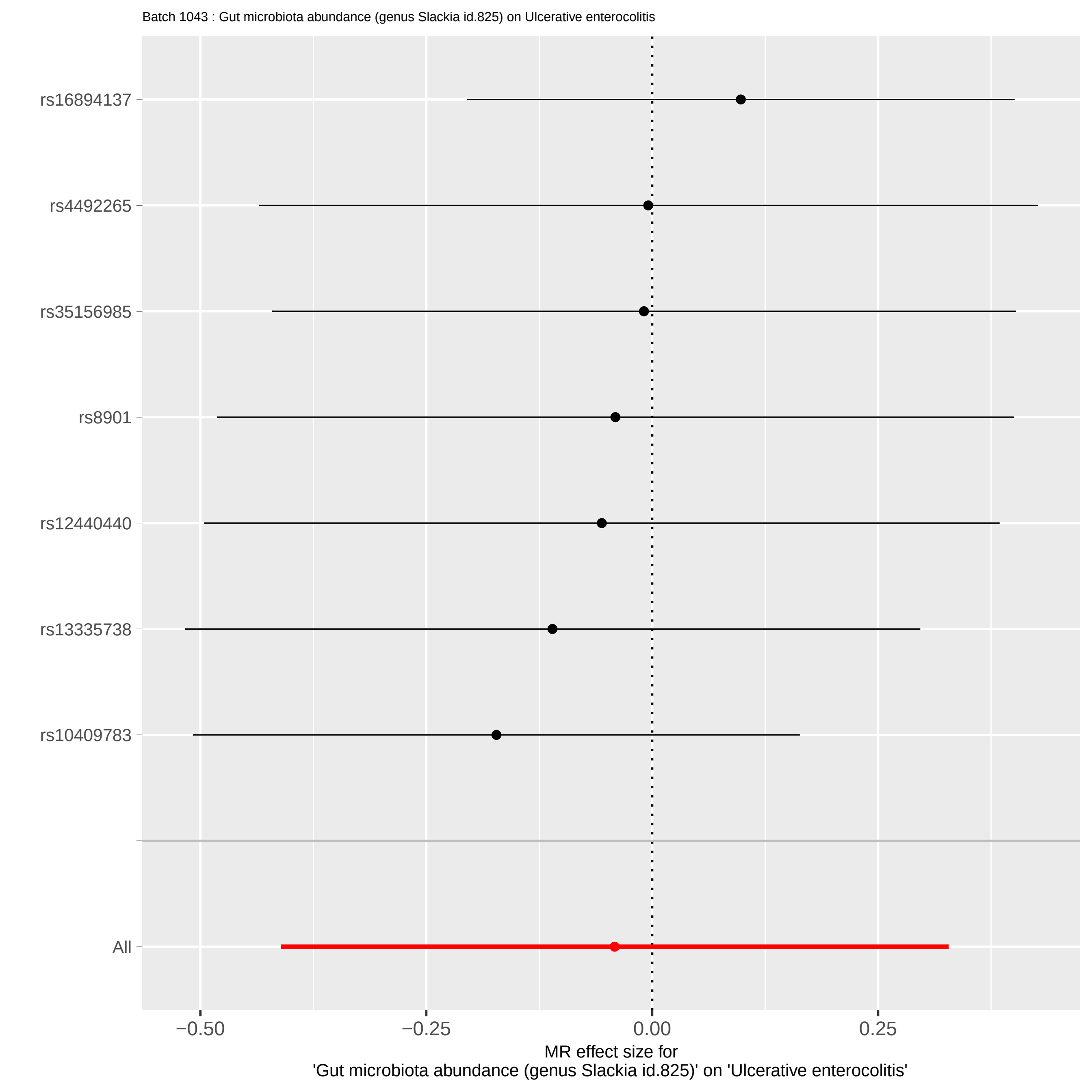
Batch 1043 : Gut microbiota abundance (genus Slackia id.825) on Ulcerative enterocolitis
rs16894137
rs4492265
rs35156985
rs8901
rs12440440
rs13335738
rs10409783
All
−0.50
−0.25
0.00
0.25
MR effect size for
'Gut microbiota abundance (genus Slackia id.825)' on 'Ulcerative enterocolitis'

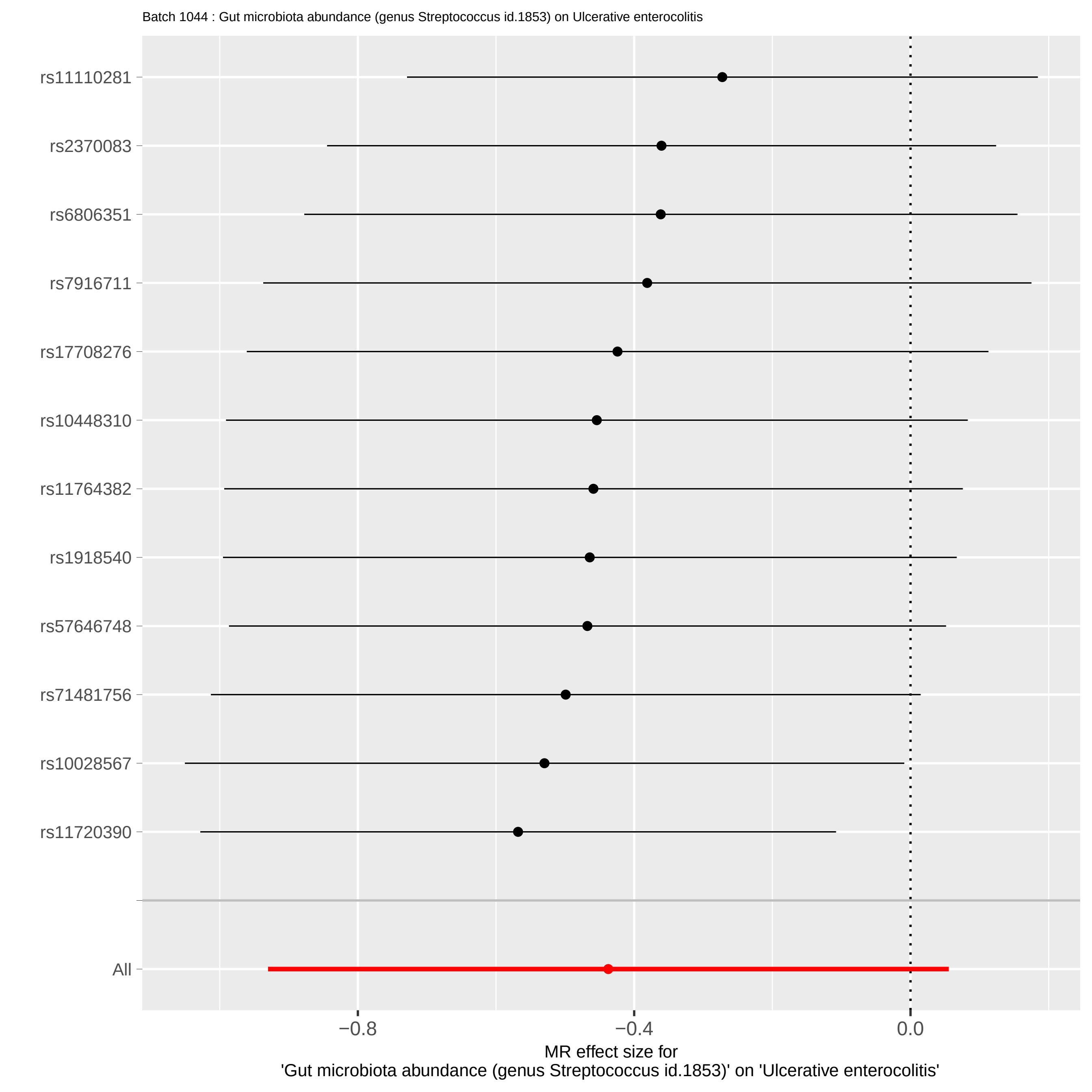

Batch 1044 : Gut microbiota abundance (genus Streptococcus id.1853) on Ulcerative enterocolitis
rs11110281
rs2370083
rs6806351
rs7916711
rs17708276
rs10448310
rs11764382
rs1918540
rs57646748
rs71481756
rs10028567
rs11720390
All
−0.8
−0.4
0.0
MR effect size for
'Gut microbiota abundance (genus Streptococcus id.1853)' on 'Ulcerative enterocolitis'

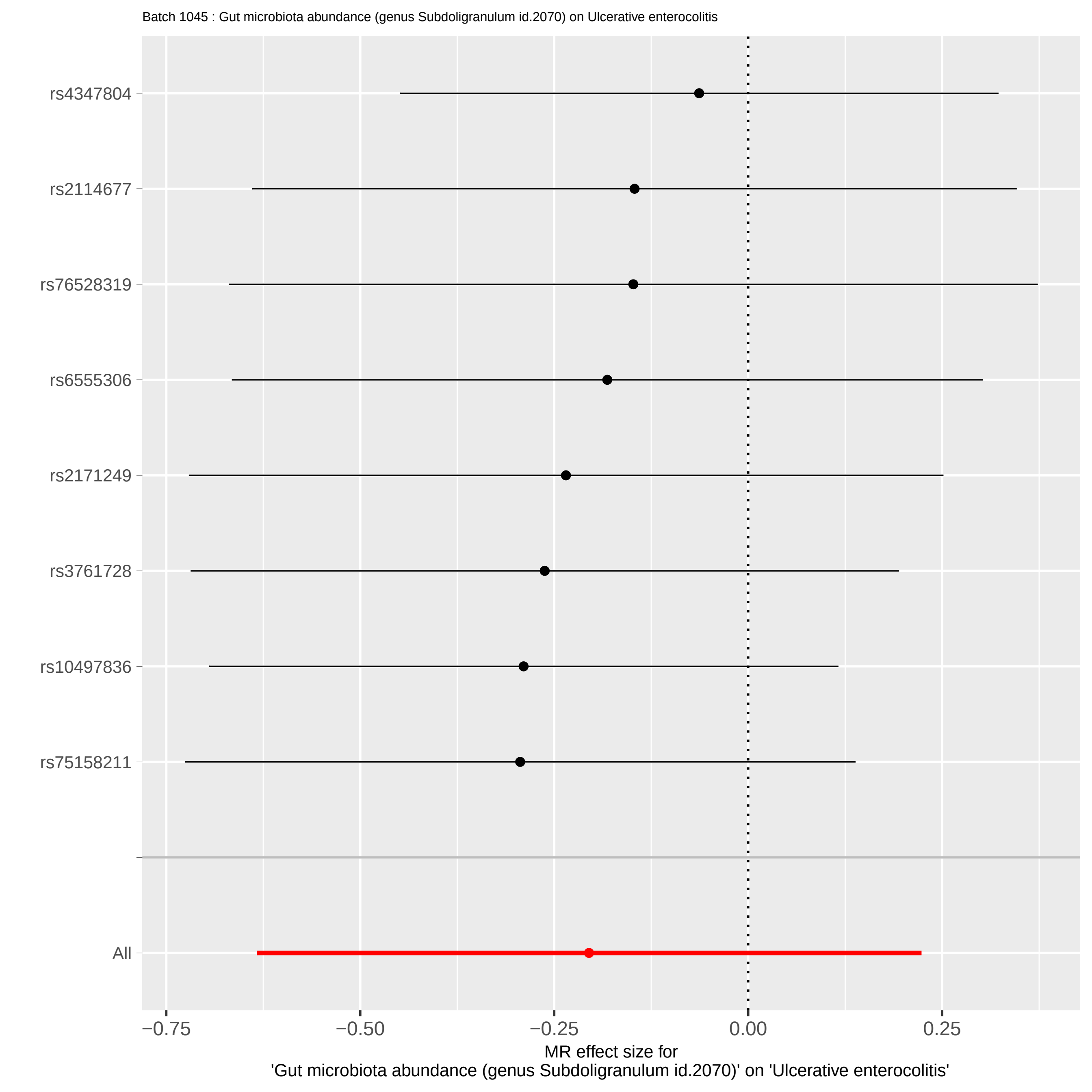
Batch 1045 : Gut microbiota abundance (genus Subdoligranulum id.2070) on Ulcerative enterocolitis
rs4347804
rs2114677
rs76528319
rs6555306
rs2171249
rs3761728
rs10497836
rs75158211
All
−0.75
−0.50
−0.25
0.00
0.25
MR effect size for
'Gut microbiota abundance (genus Subdoligranulum id.2070)' on 'Ulcerative enterocolitis'

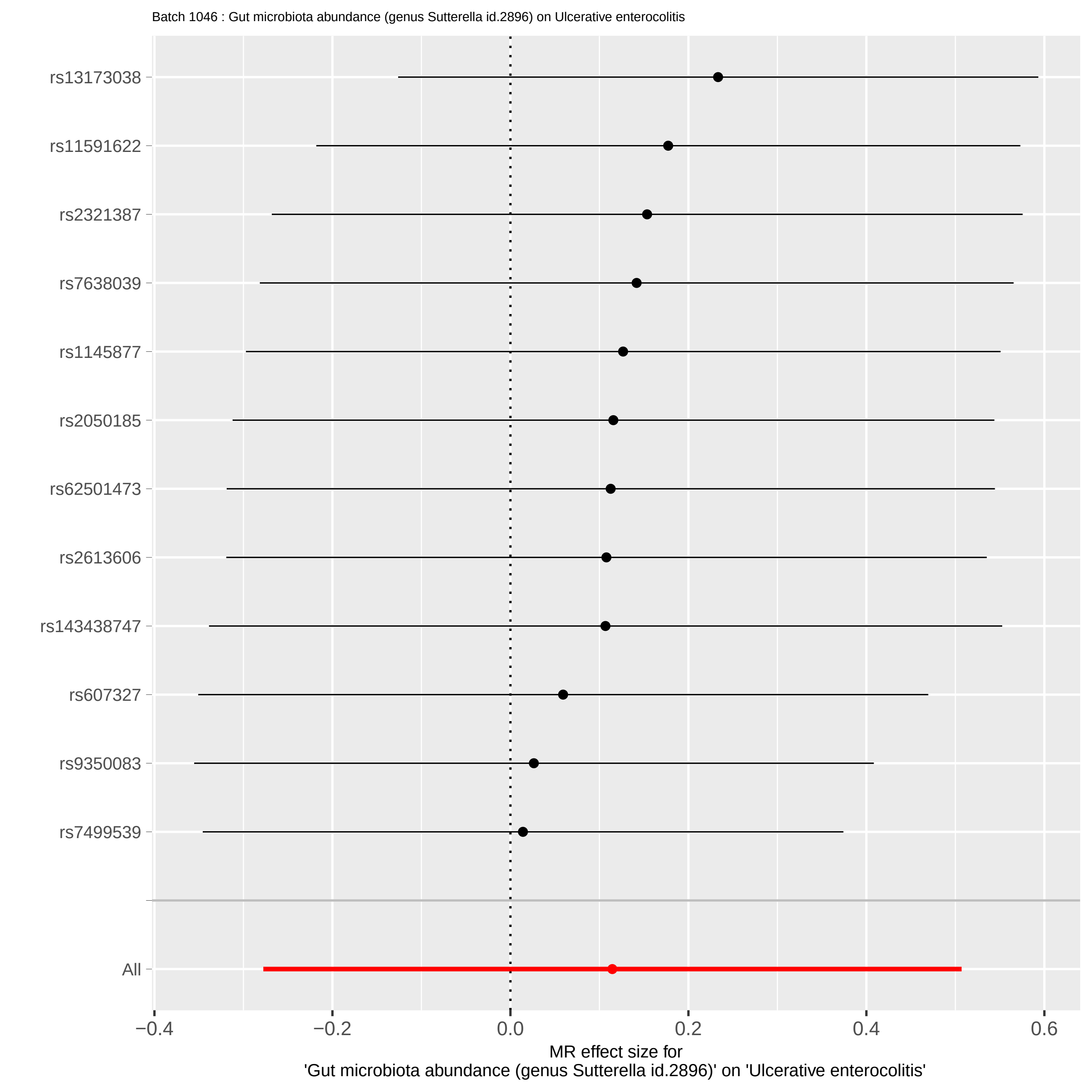

Batch 1046 : Gut microbiota abundance (genus Sutterella id.2896) on Ulcerative enterocolitis
rs13173038
rs11591622
rs2321387
rs7638039
rs1145877
rs2050185
rs62501473
rs2613606
rs143438747
rs607327
rs9350083
rs7499539
All
−0.4
−0.2
0.0
0.2
0.4
0.6
MR effect size for
'Gut microbiota abundance (genus Sutterella id.2896)' on 'Ulcerative enterocolitis'

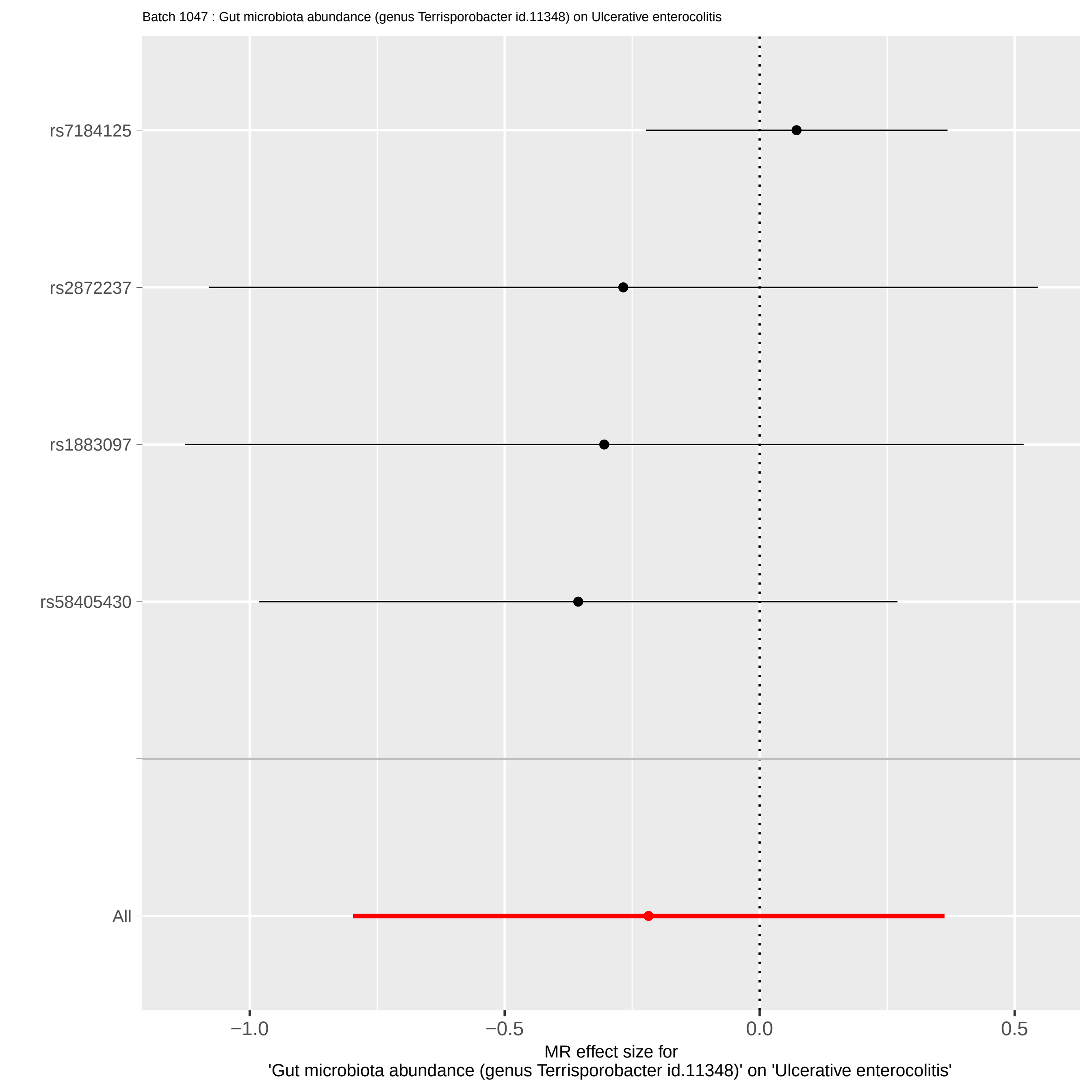
Batch 1047 : Gut microbiota abundance (genus Terrisporobacter id.11348) on Ulcerative enterocolitis
rs7184125
rs2872237
rs1883097
rs58405430
All
−1.0
−0.5
0.0
0.5
MR effect size for
'Gut microbiota abundance (genus Terrisporobacter id.11348)' on 'Ulcerative enterocolitis'

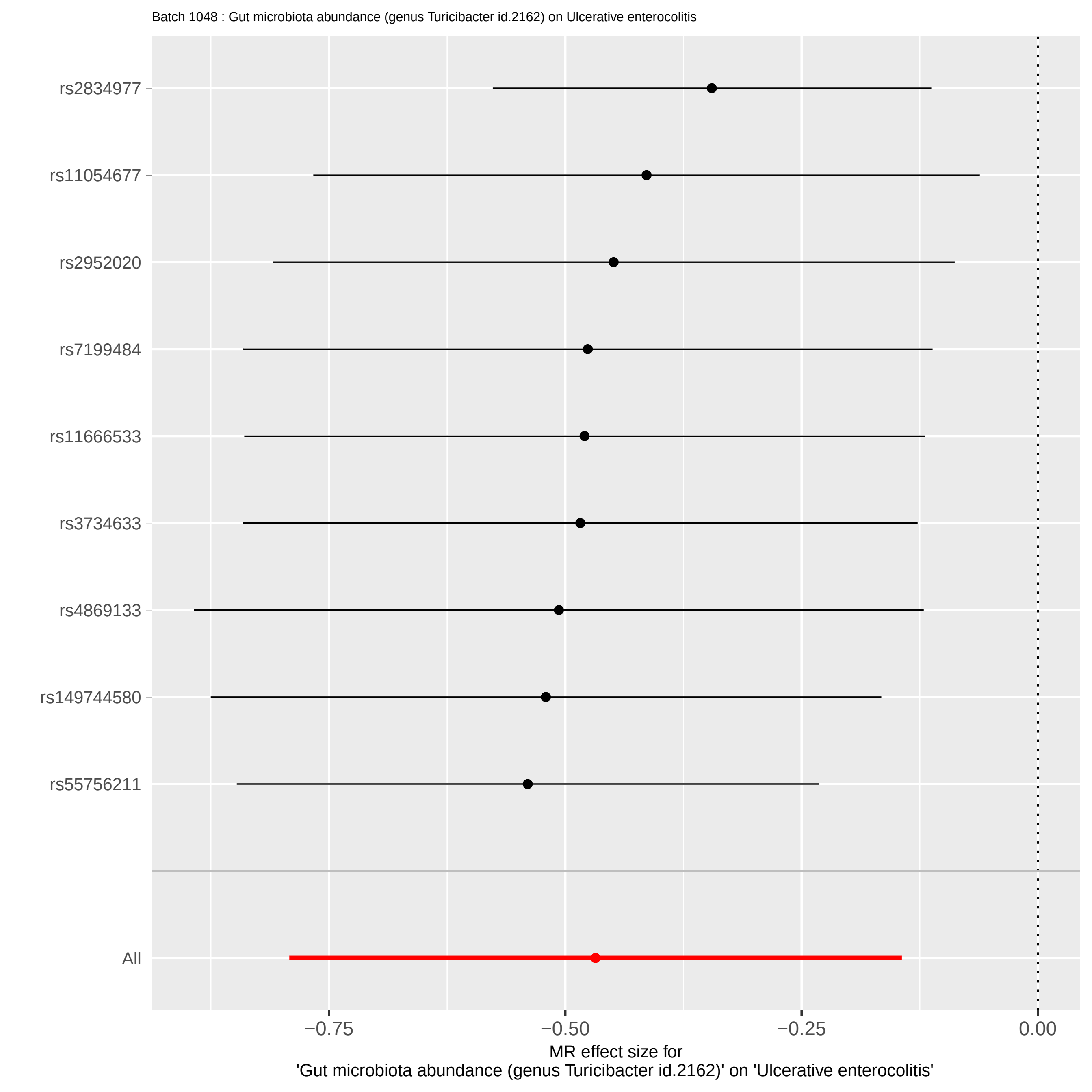

Batch 1048 : Gut microbiota abundance (genus Turicibacter id.2162) on Ulcerative enterocolitis
rs2834977
rs11054677
rs2952020
rs7199484
rs11666533
rs3734633
rs4869133
rs149744580
rs55756211
All
−0.75
−0.50
−0.25
0.00
MR effect size for
'Gut microbiota abundance (genus Turicibacter id.2162)' on 'Ulcerative enterocolitis'

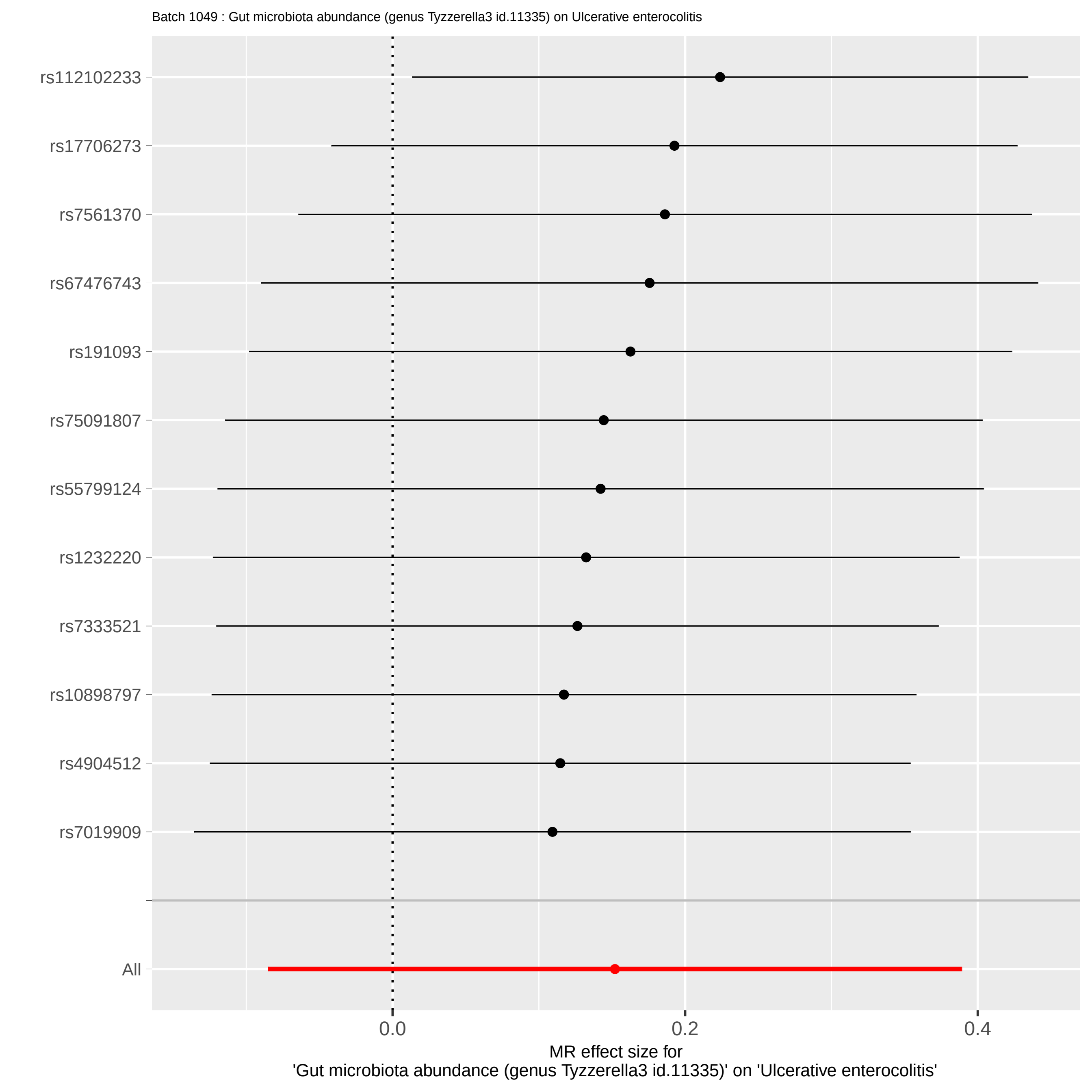

Batch 1049 : Gut microbiota abundance (genus Tyzzerella3 id.11335) on Ulcerative enterocolitis
rs112102233
rs17706273
rs7561370
rs67476743
rs191093
rs75091807
rs55799124
rs1232220
rs7333521
rs10898797
rs4904512
rs7019909
All
0.0
0.2
0.4
MR effect size for
'Gut microbiota abundance (genus Tyzzerella3 id.11335)' on 'Ulcerative enterocolitis'

Batch 1050 : Gut microbiota abundance (genus Veillonella id.2198) on Ulcerative enterocolitis

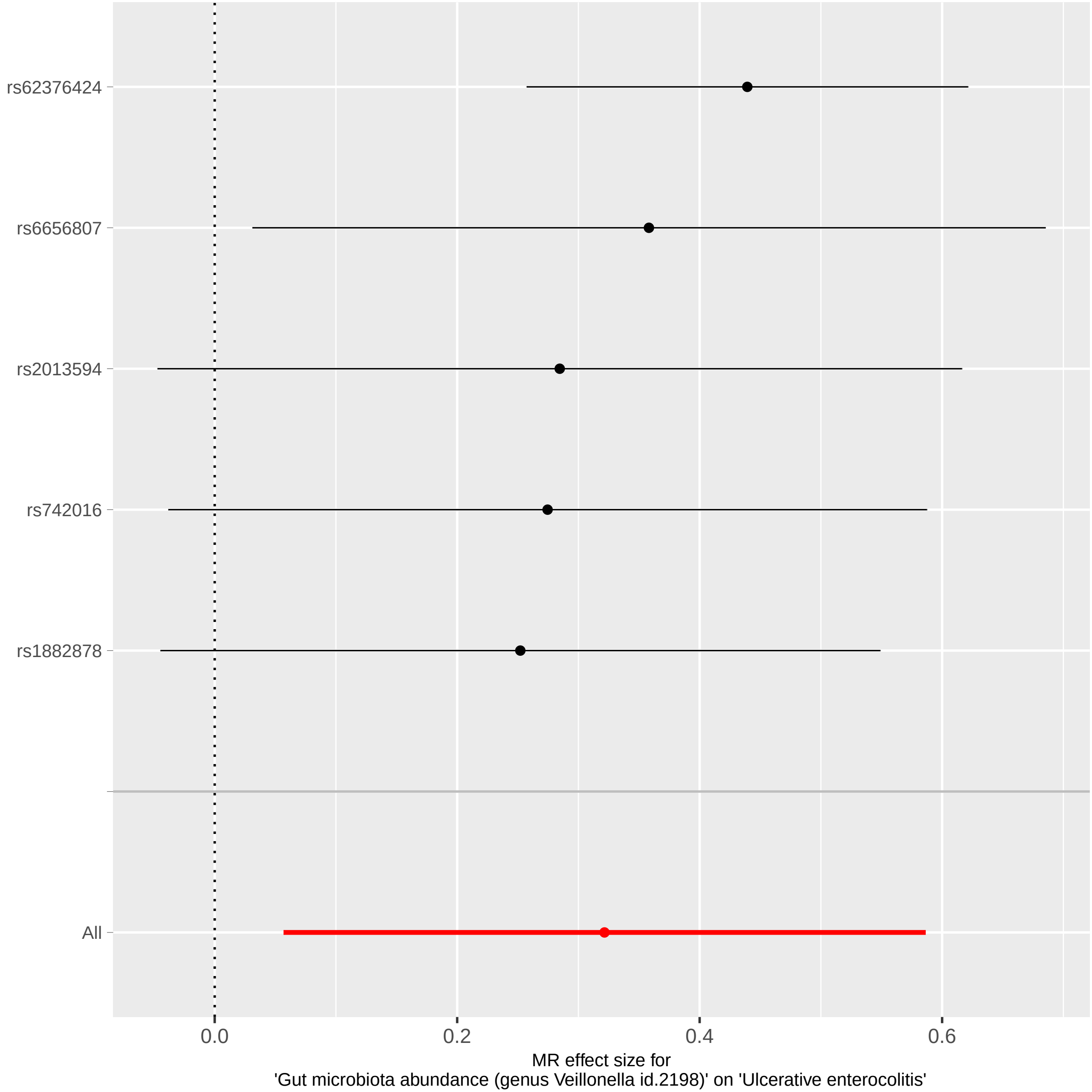

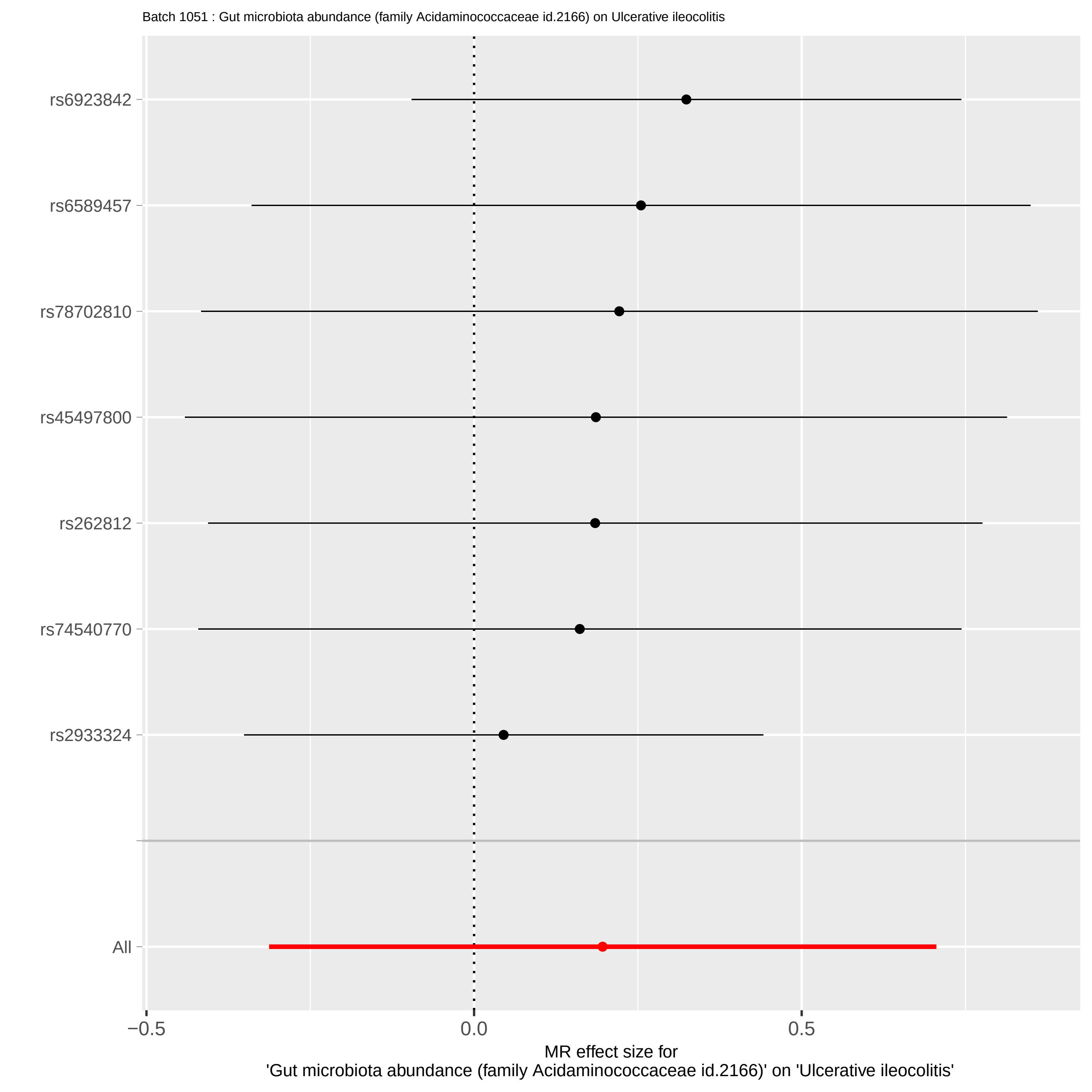

Batch 1051 : Gut microbiota abundance (family Acidaminococcaceae id.2166) on Ulcerative ileocolitis
rs6923842
rs6589457
rs78702810
rs45497800
rs262812
rs74540770
rs2933324
All
−0.5
0.0
0.5
MR effect size for
'Gut microbiota abundance (family Acidaminococcaceae id.2166)' on 'Ulcerative ileocolitis'

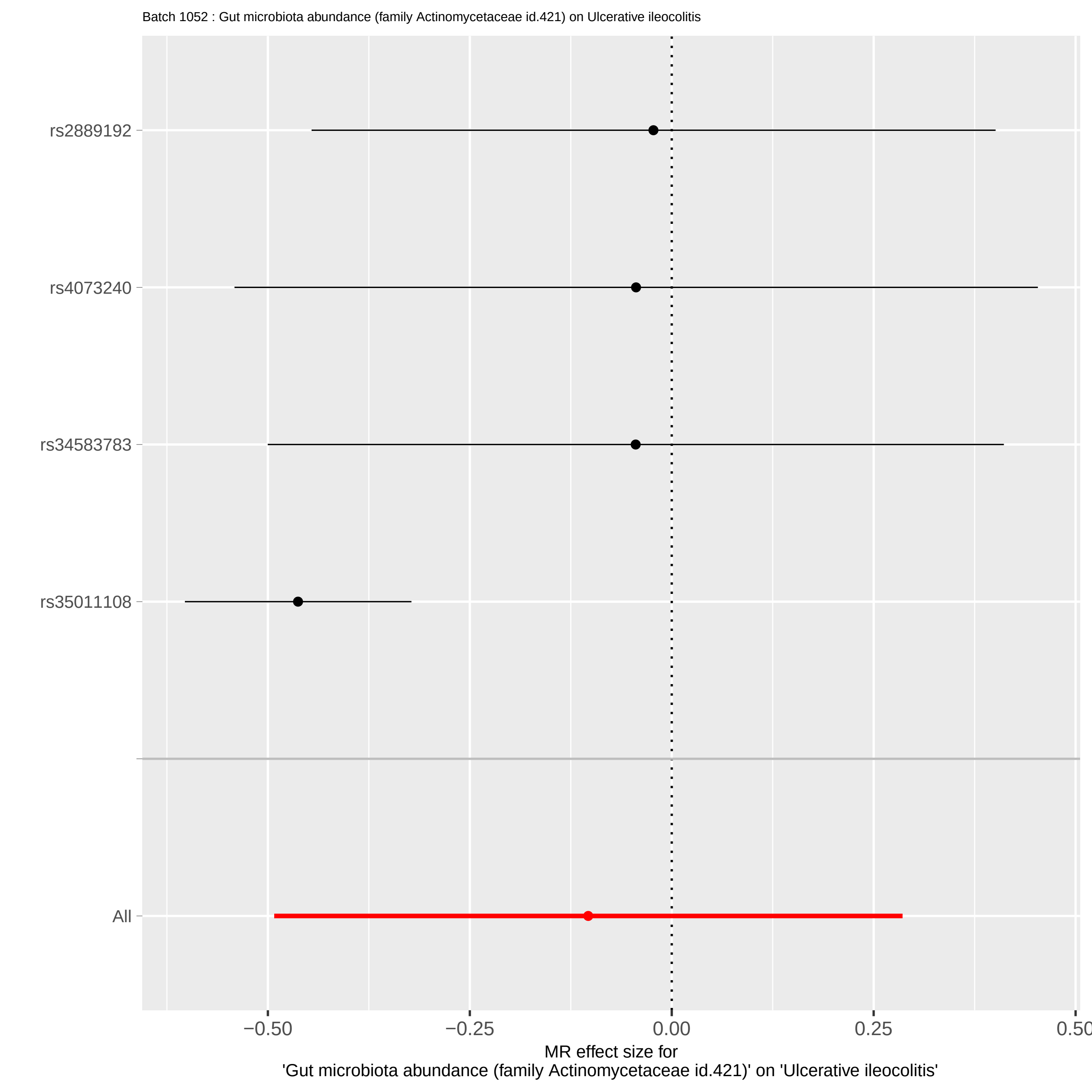
Batch 1052 : Gut microbiota abundance (family Actinomycetaceae id.421) on Ulcerative ileocolitis
rs2889192
rs4073240
rs34583783
rs35011108
All
−0.50
−0.25
0.00
0.25
0.50
MR effect size for
'Gut microbiota abundance (family Actinomycetaceae id.421)' on 'Ulcerative ileocolitis'

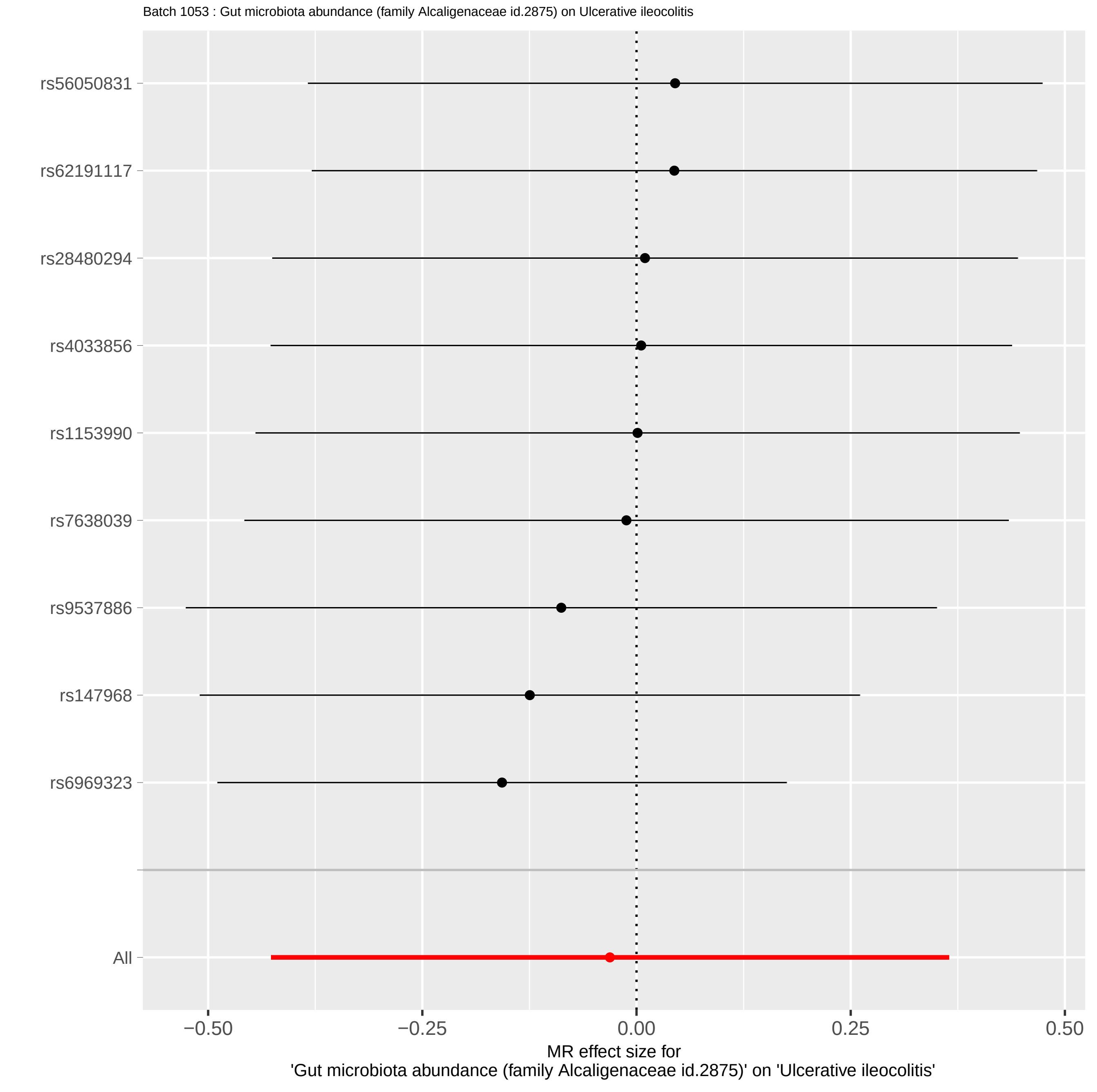

Batch 1053 : Gut microbiota abundance (family Alcaligenaceae id.2875) on Ulcerative ileocolitis
rs56050831
rs62191117
rs28480294
rs4033856
rs1153990
rs7638039
rs9537886
rs147968
rs6969323
All
−0.50
−0.25
0.00
0.25
0.50
MR effect size for
'Gut microbiota abundance (family Alcaligenaceae id.2875)' on 'Ulcerative ileocolitis'

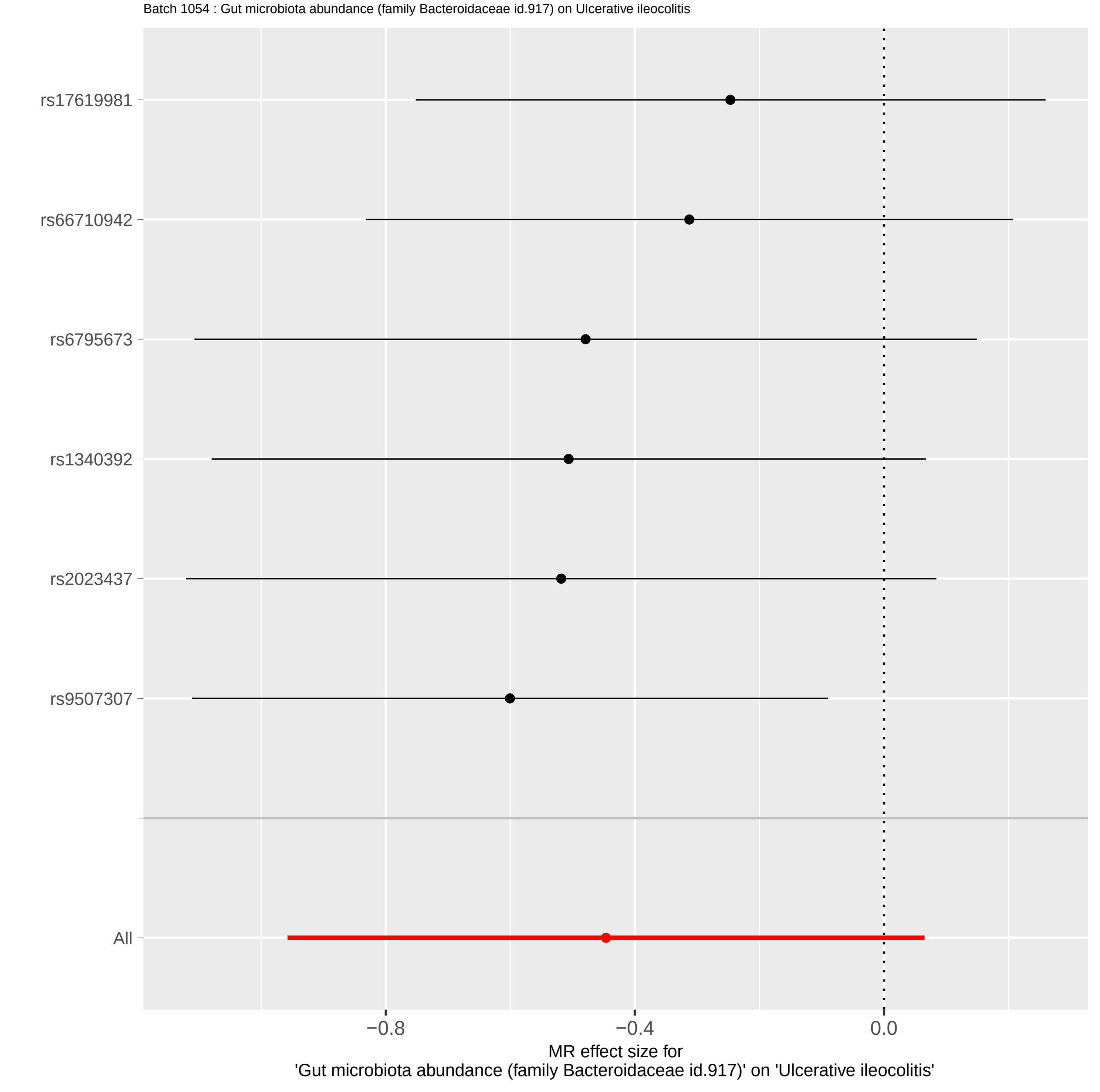
Batch 1054 : Gut microbiota abundance (family Bacteroidaceae id.917) on Ulcerative ileocolitis
rs17619981
rs66710942
rs6795673
rs1340392
rs2023437
rs9507307
All
−0.8
−0.4
0.0
MR effect size for
'Gut microbiota abundance (family Bacteroidaceae id.917)' on 'Ulcerative ileocolitis'

Batch 1055 : Gut microbiota abundance (family Bacteroidales S24 7group id.11173) on Ulcerative ileocolitis

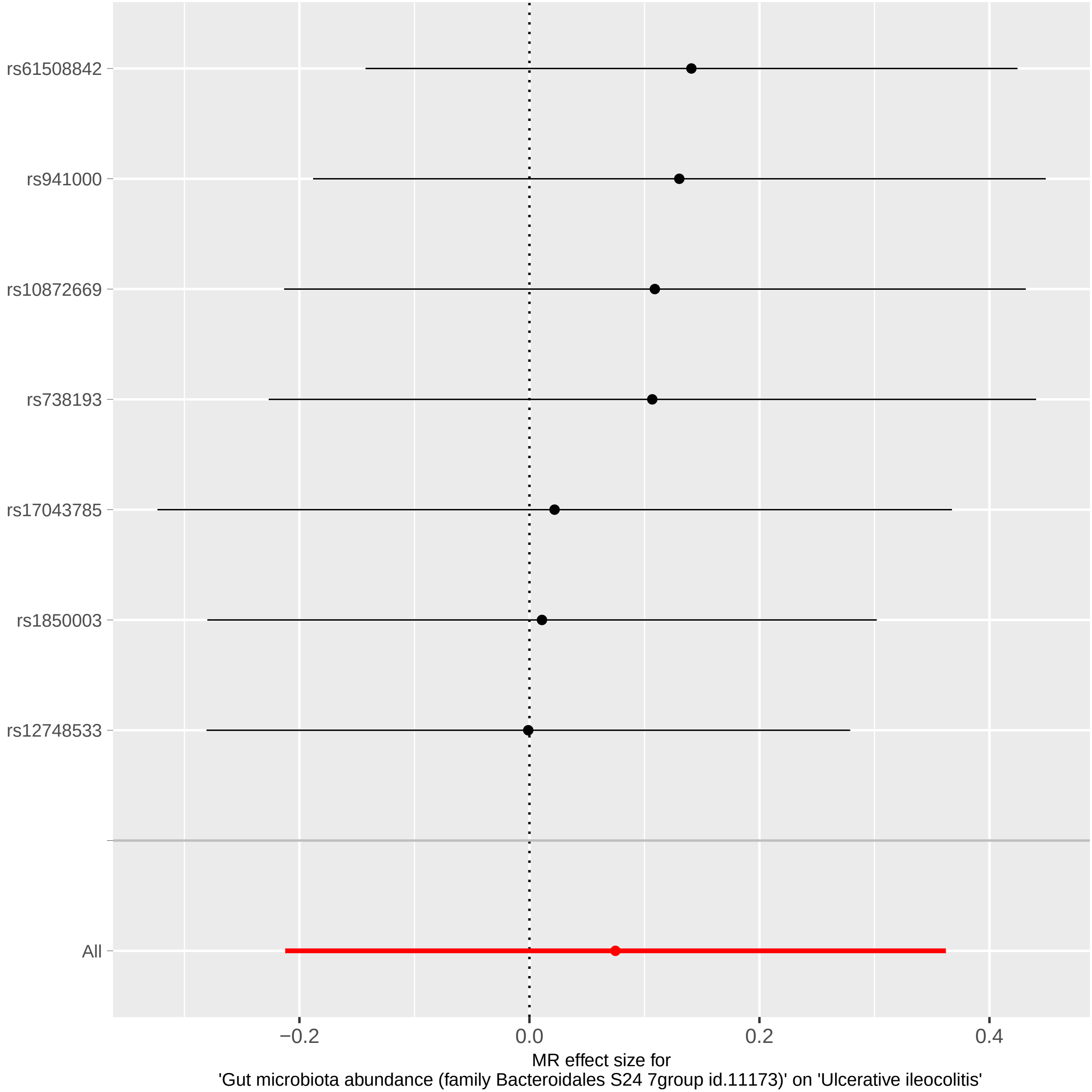

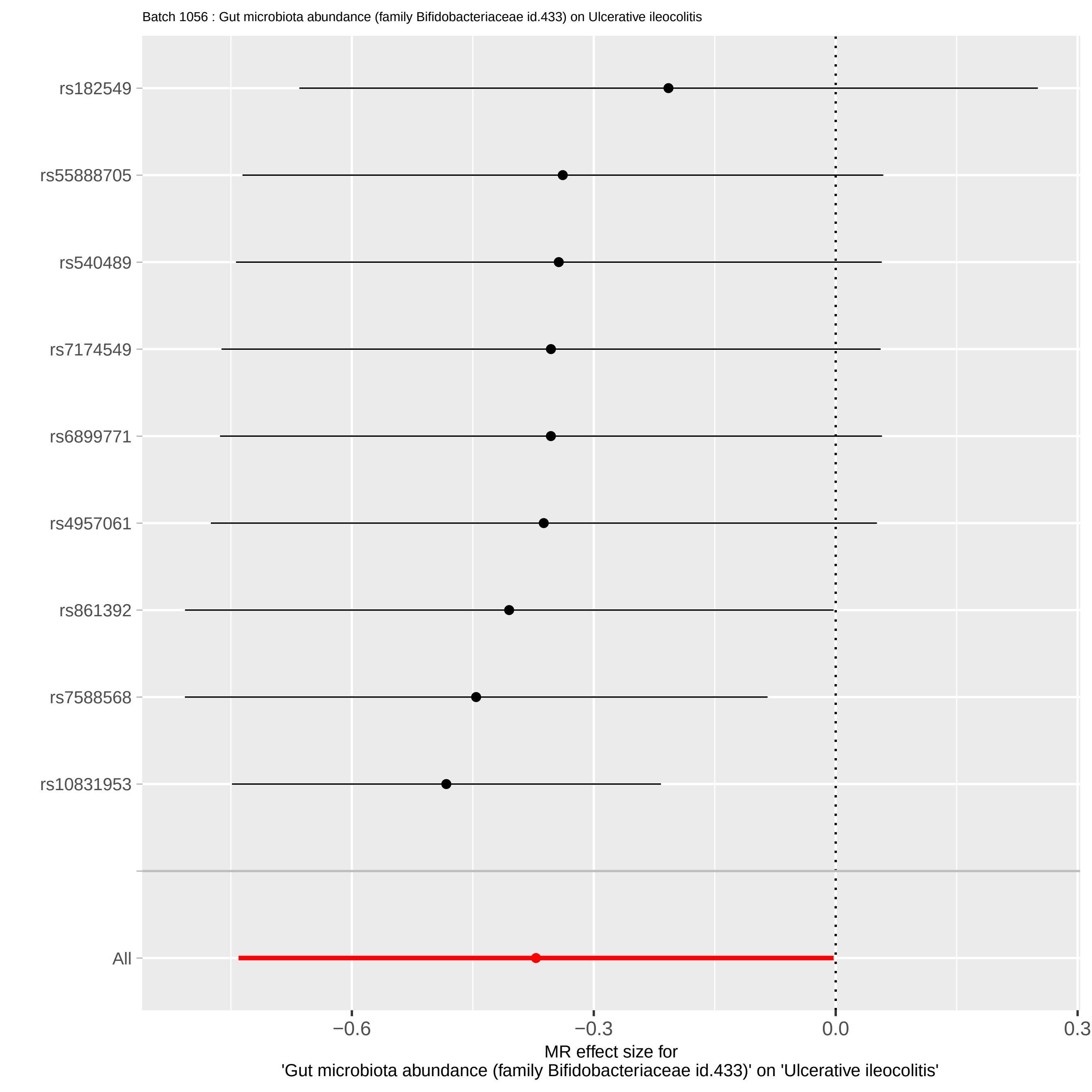

Batch 1056 : Gut microbiota abundance (family Bifidobacteriaceae id.433) on Ulcerative ileocolitis
rs182549
rs55888705
rs540489
rs7174549
rs6899771
rs4957061
rs861392
rs7588568
rs10831953
All
−0.6
−0.3
0.0
0.3
MR effect size for
'Gut microbiota abundance (family Bifidobacteriaceae id.433)' on 'Ulcerative ileocolitis'

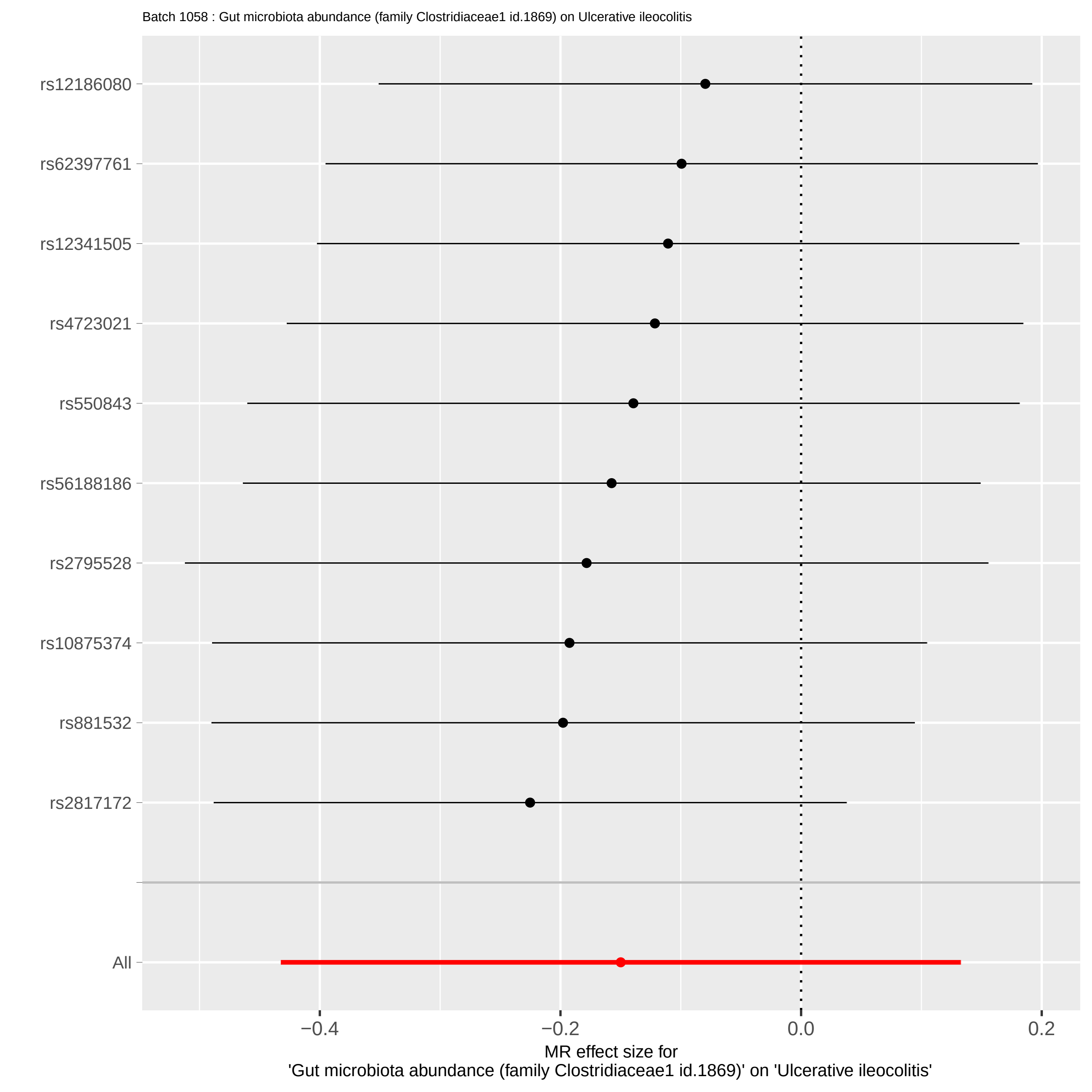

Batch 1058 : Gut microbiota abundance (family Clostridiaceae1 id.1869) on Ulcerative ileocolitis
rs12186080
rs62397761
rs12341505
rs4723021
rs550843
rs56188186
rs2795528
rs10875374
rs881532
rs2817172
All
−0.4
−0.2
0.0
0.2
MR effect size for
'Gut microbiota abundance (family Clostridiaceae1 id.1869)' on 'Ulcerative ileocolitis'

Batch 1059 : Gut microbiota abundance (family Clostridiales vadin BB60 group id.11286) on Ulcerative ileocolitis

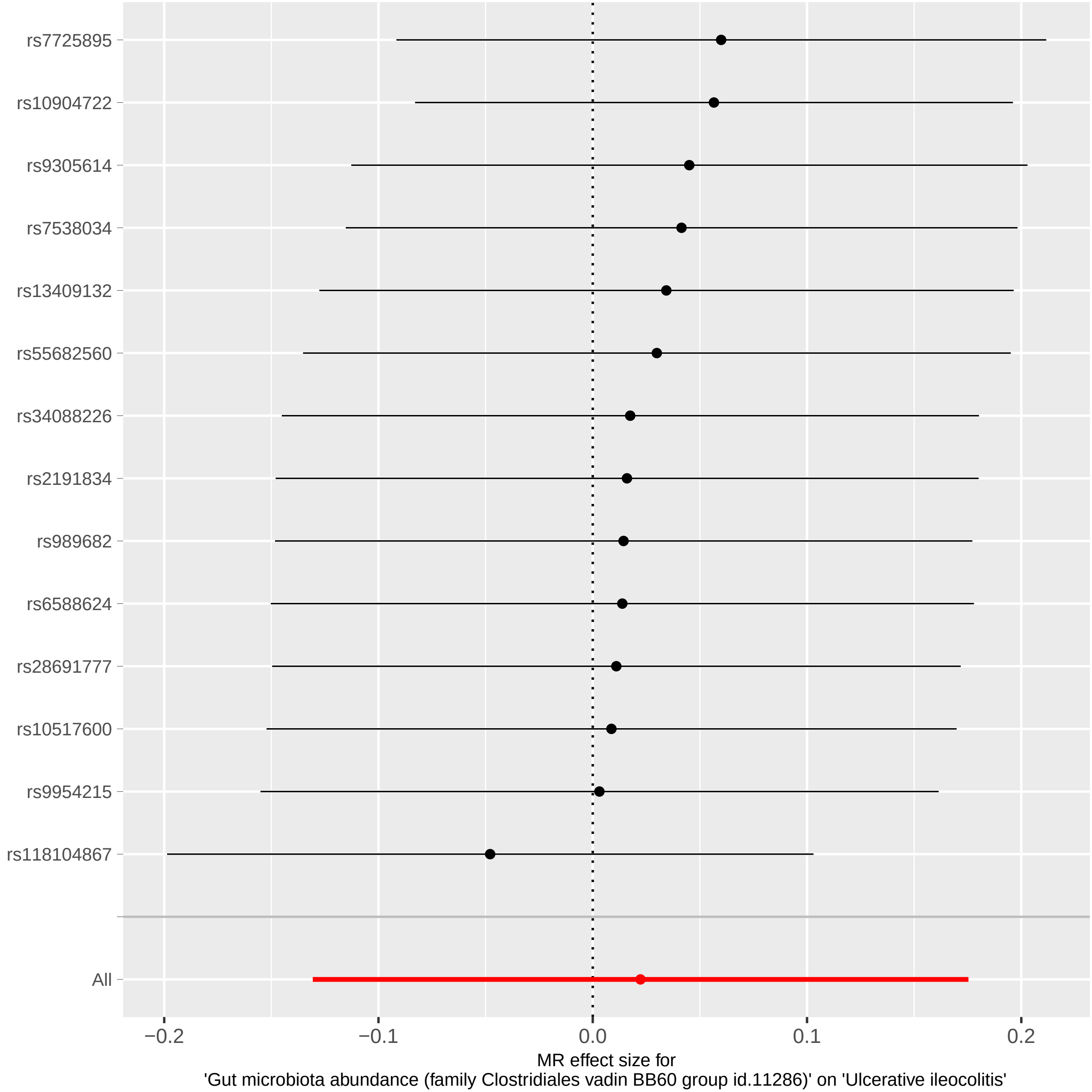

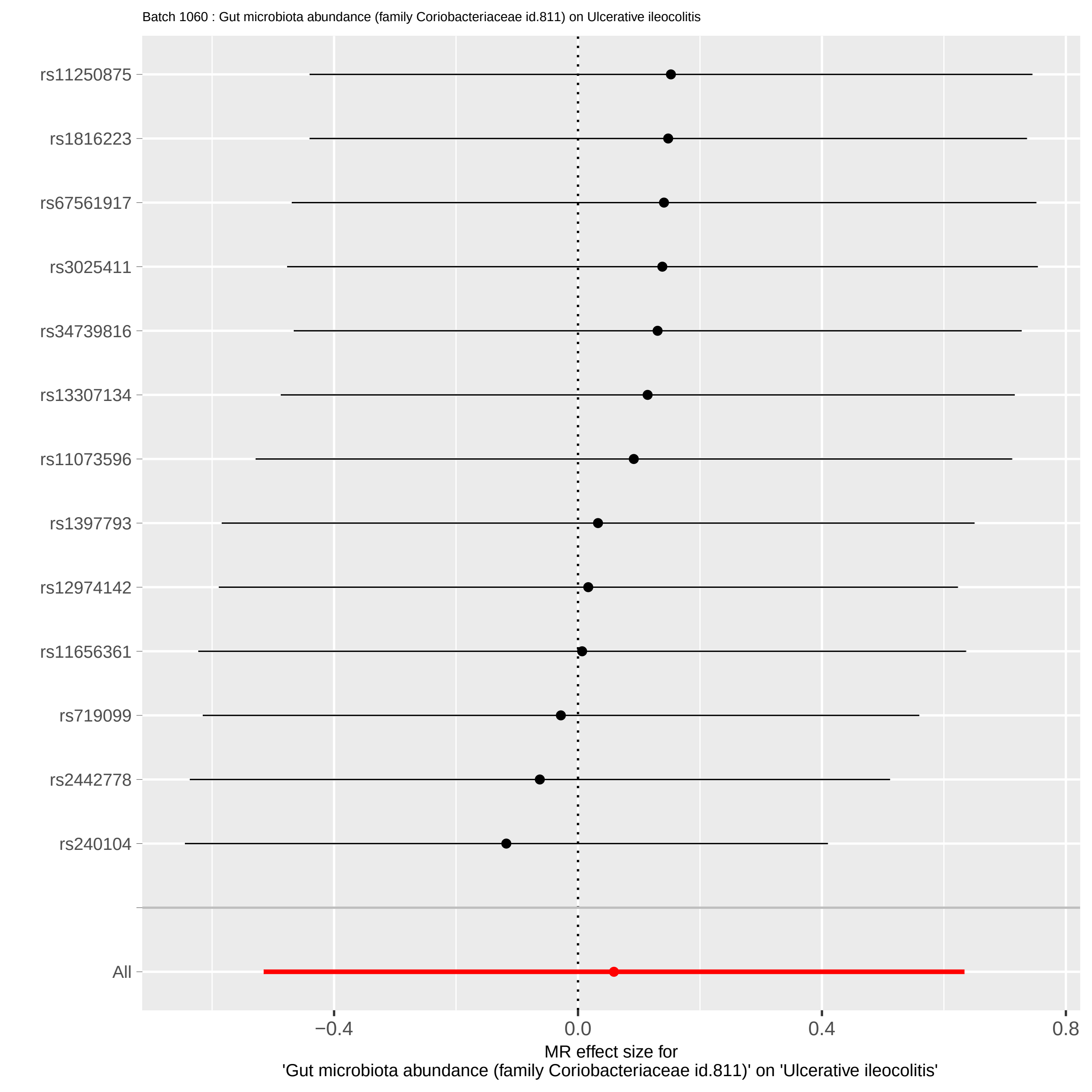

Batch 1060 : Gut microbiota abundance (family Coriobacteriaceae id.811) on Ulcerative ileocolitis
rs11250875
rs1816223
rs67561917
rs3025411
rs34739816
rs13307134
rs11073596
rs1397793
rs12974142
rs11656361
rs719099
rs2442778
rs240104
All
−0.4
0.0
0.4
0.8
MR effect size for
'Gut microbiota abundance (family Coriobacteriaceae id.811)' on 'Ulcerative ileocolitis'

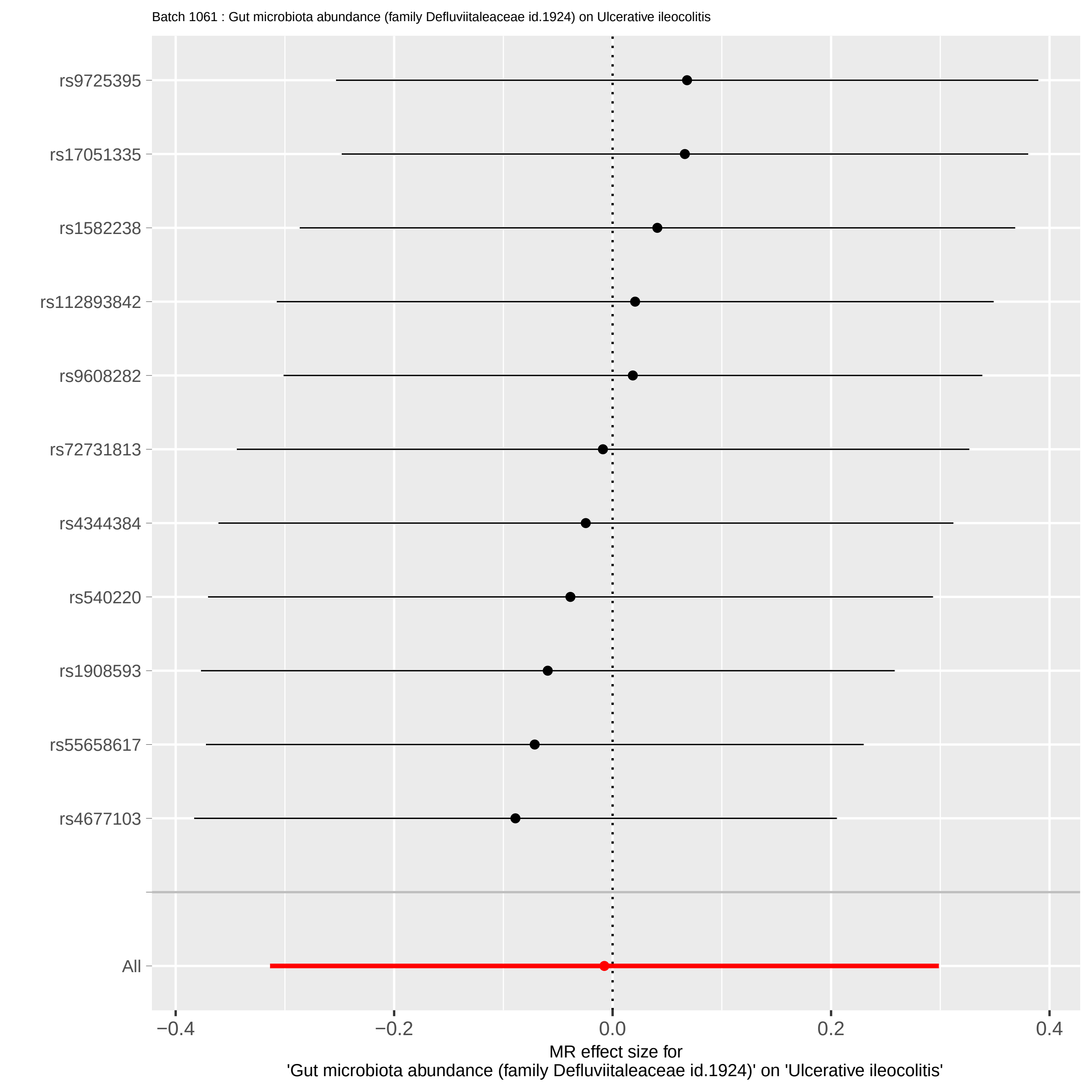

Batch 1061 : Gut microbiota abundance (family Defluviitaleaceae id.1924) on Ulcerative ileocolitis
rs9725395
rs17051335
rs1582238
rs112893842
rs9608282
rs72731813
rs4344384
rs540220
rs1908593
rs55658617
rs4677103
All
−0.4
−0.2
0.0
0.2
0.4
MR effect size for
'Gut microbiota abundance (family Defluviitaleaceae id.1924)' on 'Ulcerative ileocolitis'

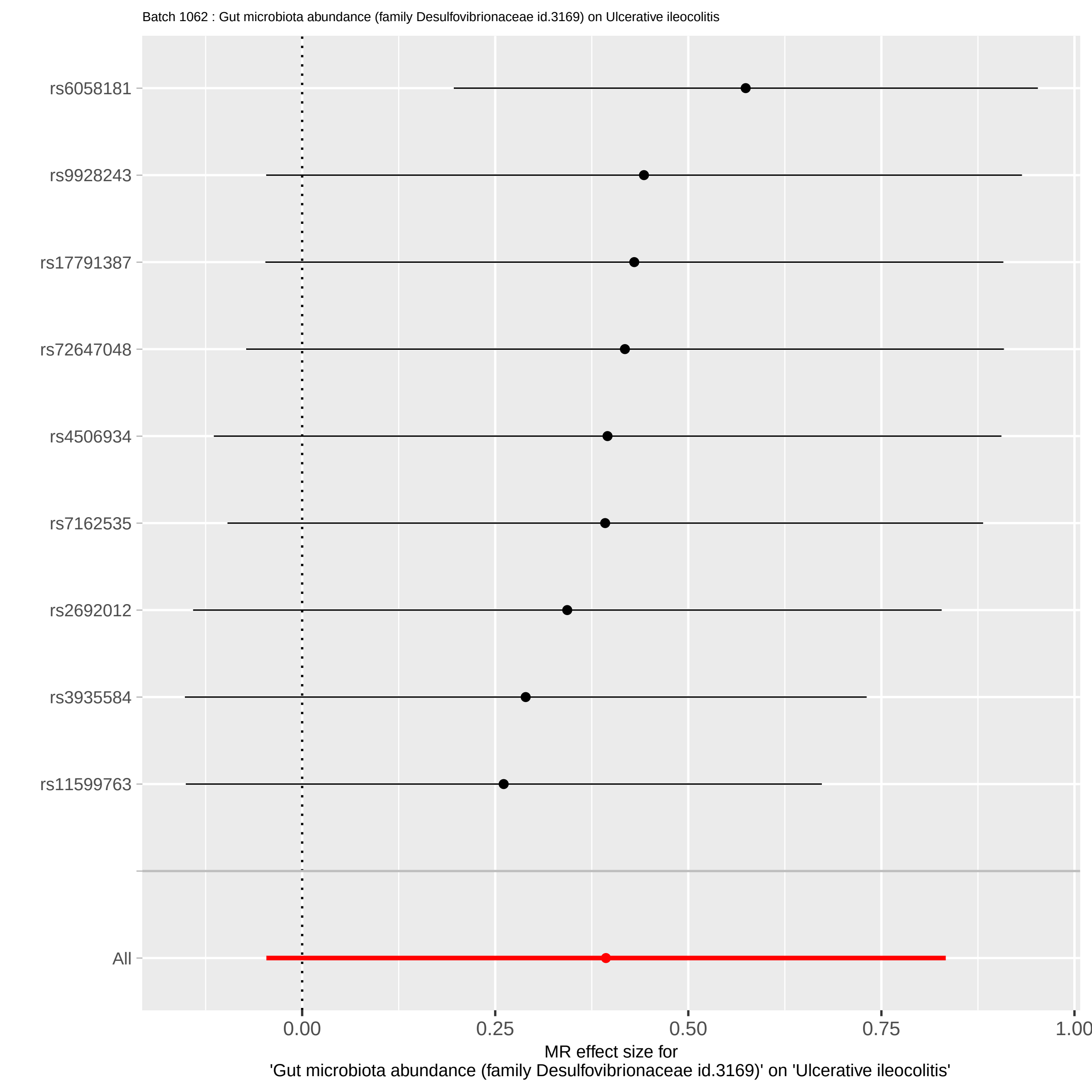

Batch 1062 : Gut microbiota abundance (family Desulfovibrionaceae id.3169) on Ulcerative ileocolitis
rs6058181
rs9928243
rs17791387
rs72647048
rs4506934
rs7162535
rs2692012
rs3935584
rs11599763
All
0.00
0.25
0.50
0.75
1.00
MR effect size for
'Gut microbiota abundance (family Desulfovibrionaceae id.3169)' on 'Ulcerative ileocolitis'

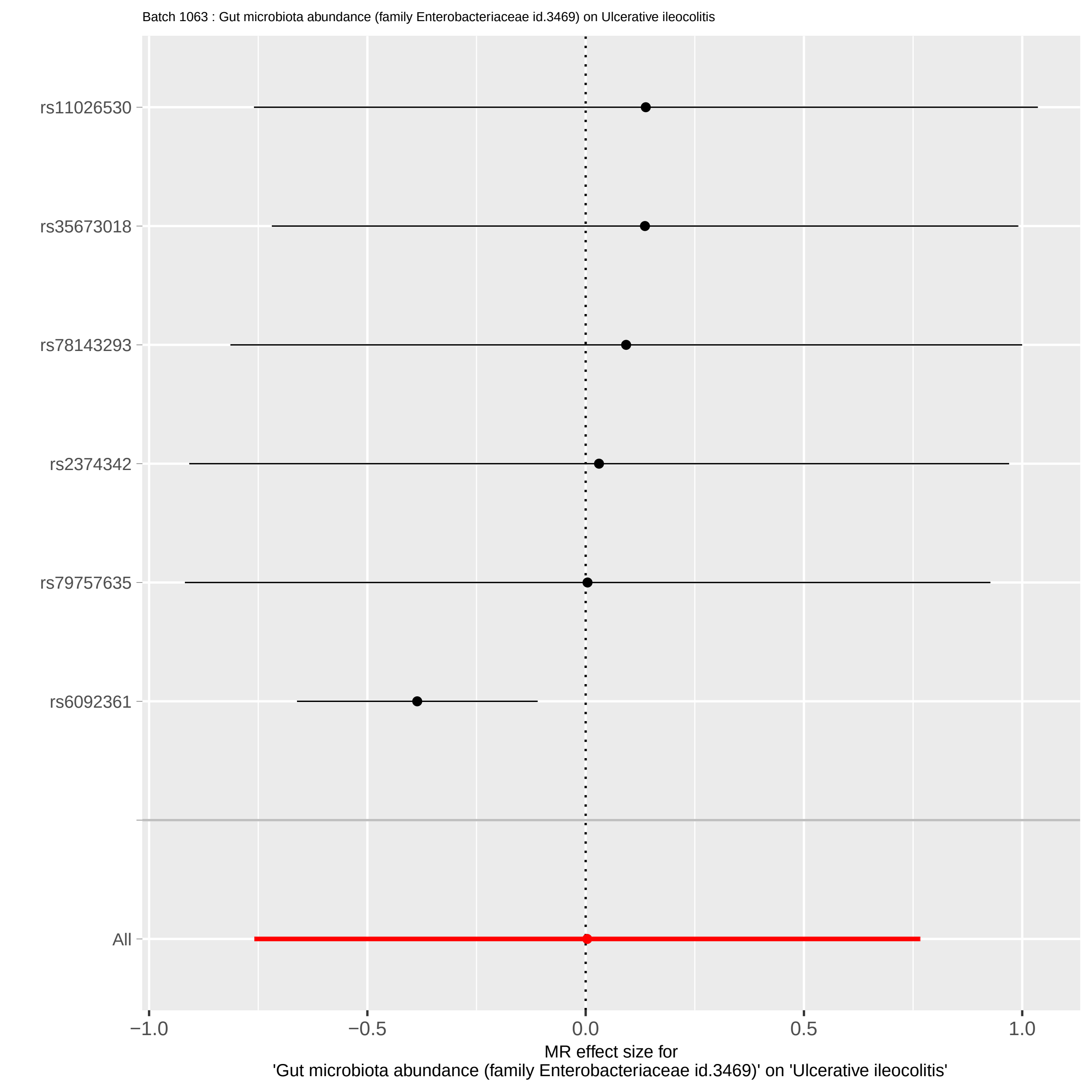
Batch 1063 : Gut microbiota abundance (family Enterobacteriaceae id.3469) on Ulcerative ileocolitis
rs11026530
rs35673018
rs78143293
rs2374342
rs79757635
rs6092361
All
−1.0
−0.5
0.0
0.5
1.0
MR effect size for
'Gut microbiota abundance (family Enterobacteriaceae id.3469)' on 'Ulcerative ileocolitis'

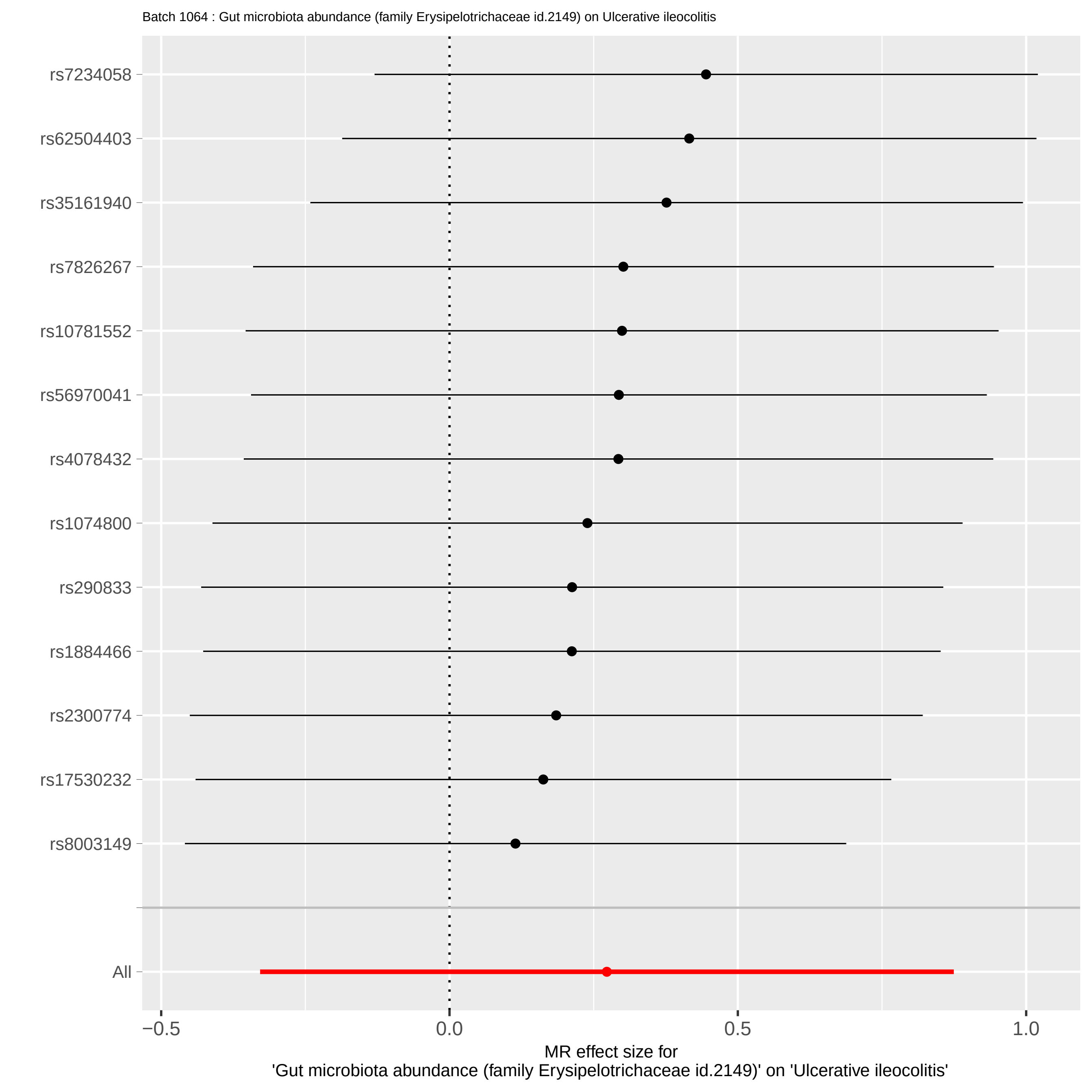
Batch 1064 : Gut microbiota abundance (family Erysipelotrichaceae id.2149) on Ulcerative ileocolitis
rs7234058
rs62504403
rs35161940
rs7826267
rs10781552
rs56970041
rs4078432
rs1074800
rs290833
rs1884466
rs2300774
rs17530232
rs8003149
All
−0.5
0.0
0.5
1.0
MR effect size for
'Gut microbiota abundance (family Erysipelotrichaceae id.2149)' on 'Ulcerative ileocolitis'

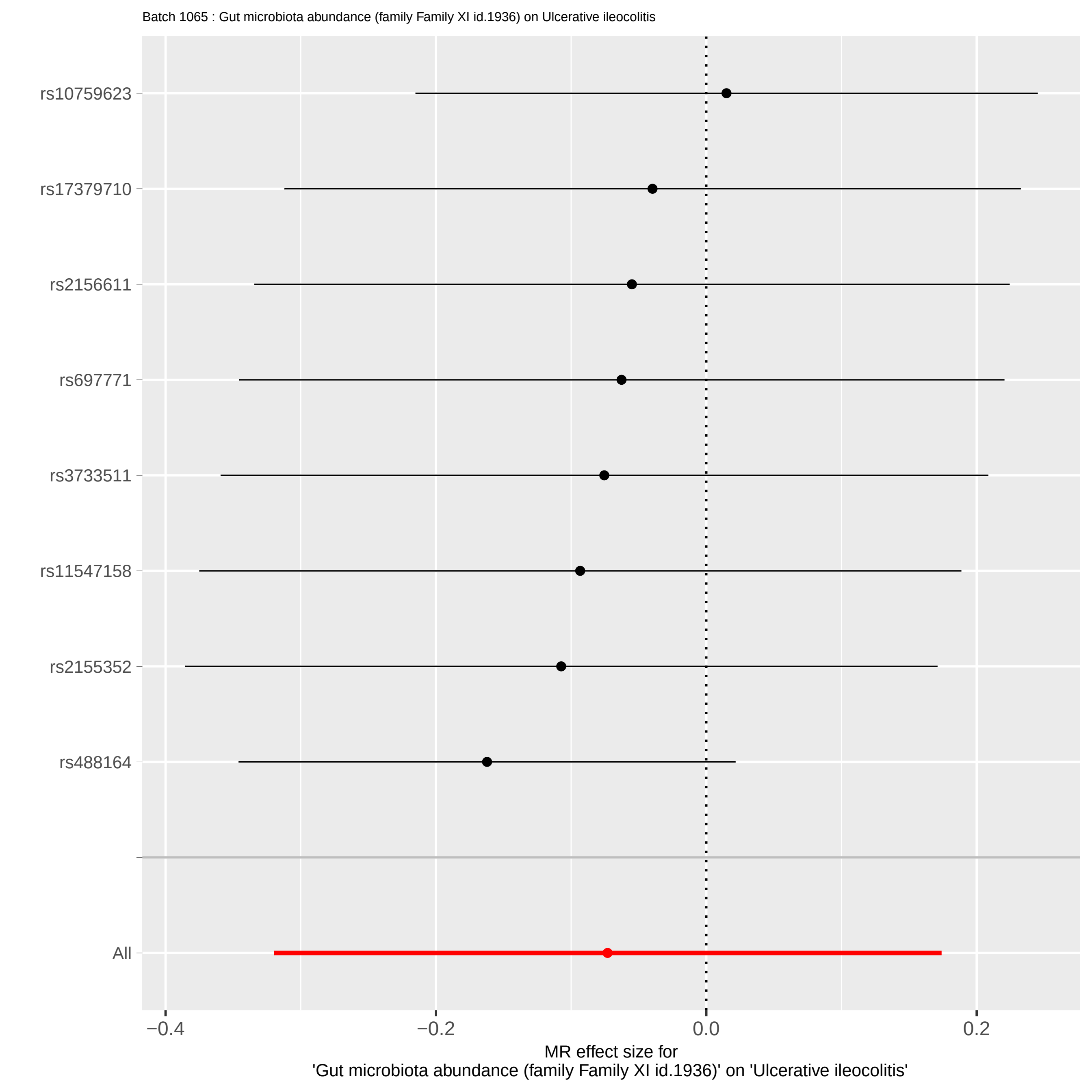

Batch 1065 : Gut microbiota abundance (family Family XI id.1936) on Ulcerative ileocolitis
rs10759623
rs17379710
rs2156611
rs697771
rs3733511
rs11547158
rs2155352
rs488164
All
−0.4
−0.2
0.0
0.2
MR effect size for
'Gut microbiota abundance (family Family XI id.1936)' on 'Ulcerative ileocolitis'

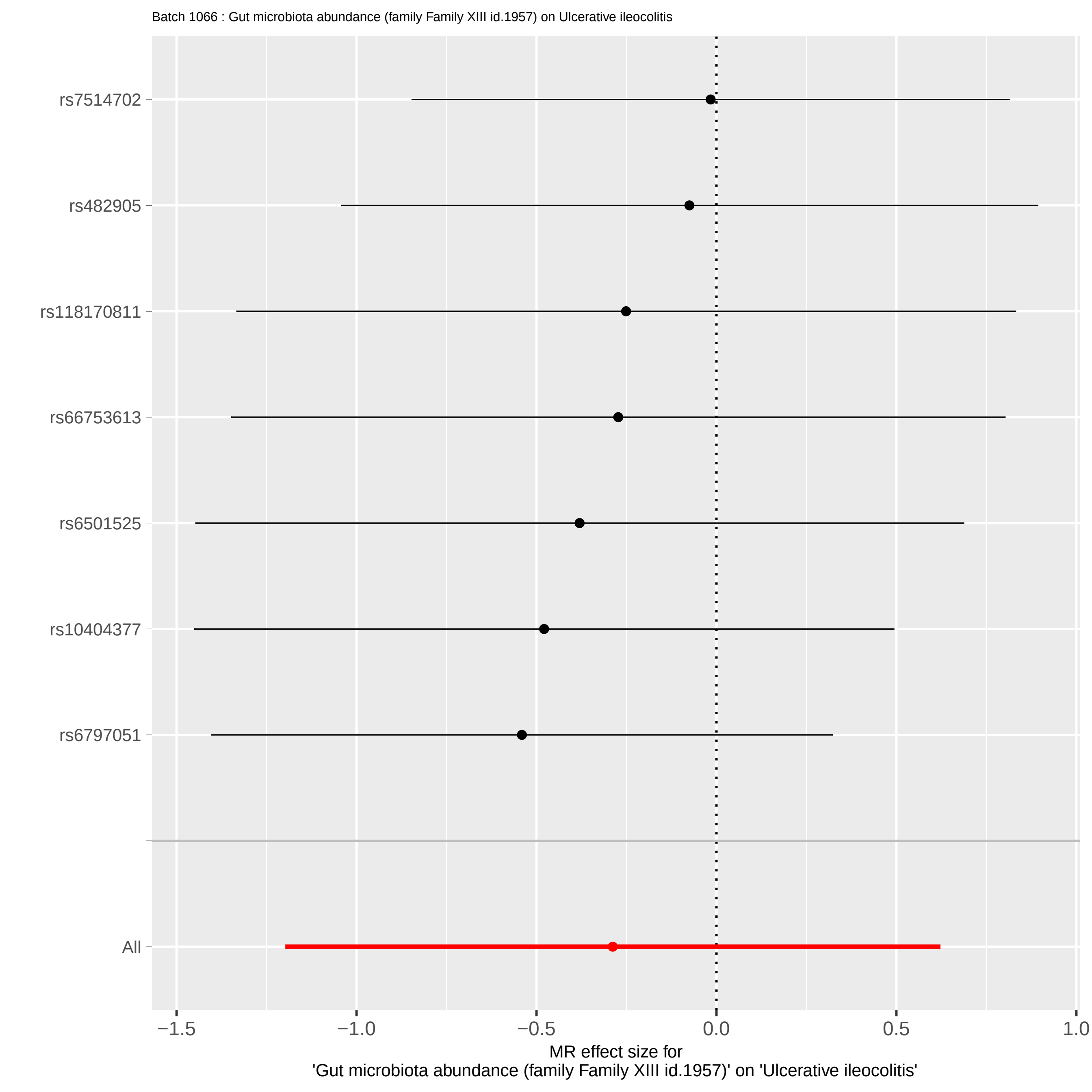

Batch 1066 : Gut microbiota abundance (family Family XIII id.1957) on Ulcerative ileocolitis
rs7514702
rs482905
rs118170811
rs66753613
rs6501525
rs10404377
rs6797051
All
−1.5
−1.0
−0.5
0.0
0.5
1.0
MR effect size for
'Gut microbiota abundance (family Family XIII id.1957)' on 'Ulcerative ileocolitis'

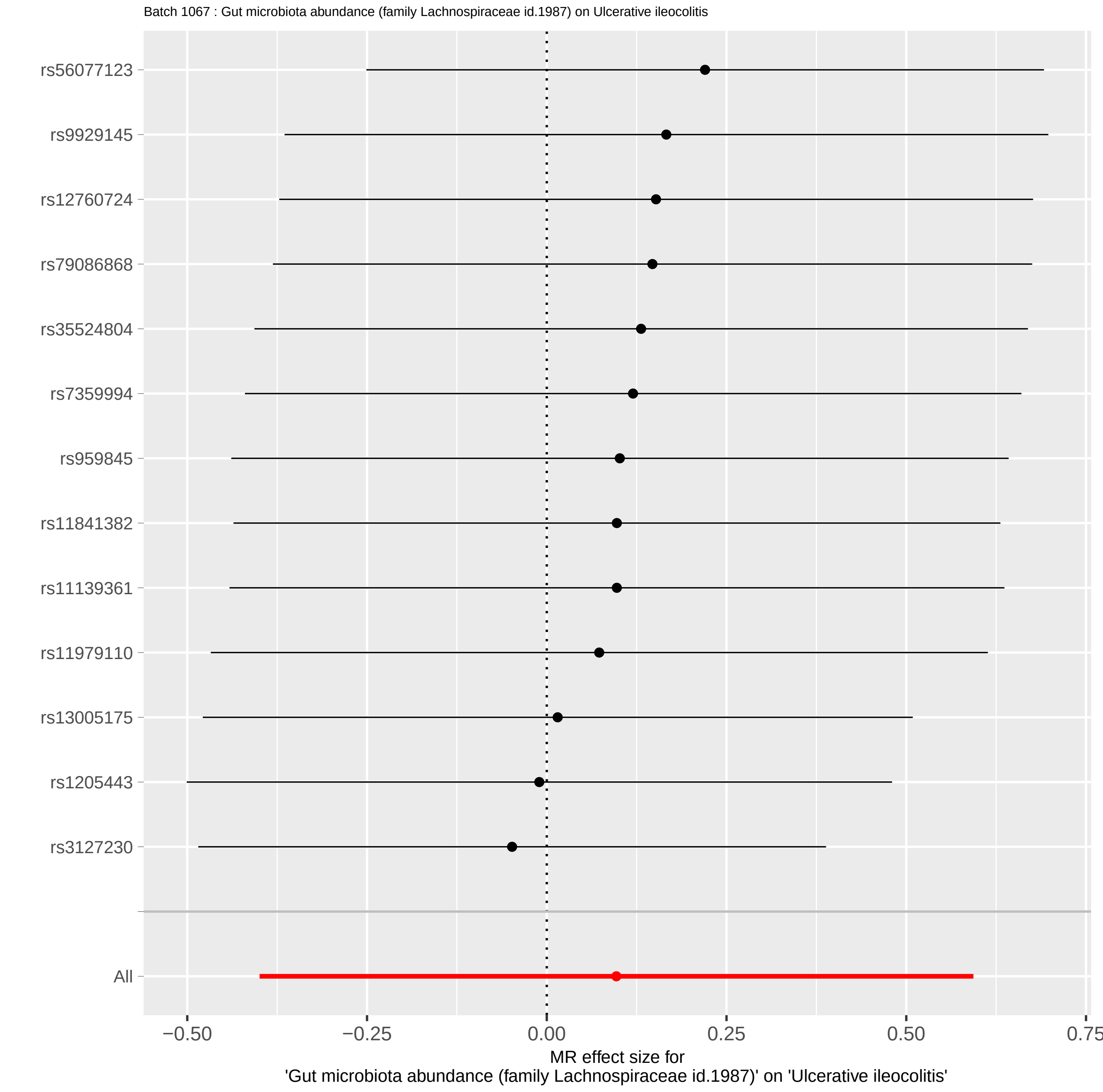

Batch 1067 : Gut microbiota abundance (family Lachnospiraceae id.1987) on Ulcerative ileocolitis
rs56077123
rs9929145
rs12760724
rs79086868
rs35524804
rs7359994
rs959845
rs11841382
rs11139361
rs11979110
rs13005175
rs1205443
rs3127230
All
−0.50
−0.25
0.00
0.25
0.50
0.75
MR effect size for
'Gut microbiota abundance (family Lachnospiraceae id.1987)' on 'Ulcerative ileocolitis'

Batch 1068 : Gut microbiota abundance (family Lactobacillaceae id.1836) on Ulcerative ileocolitis

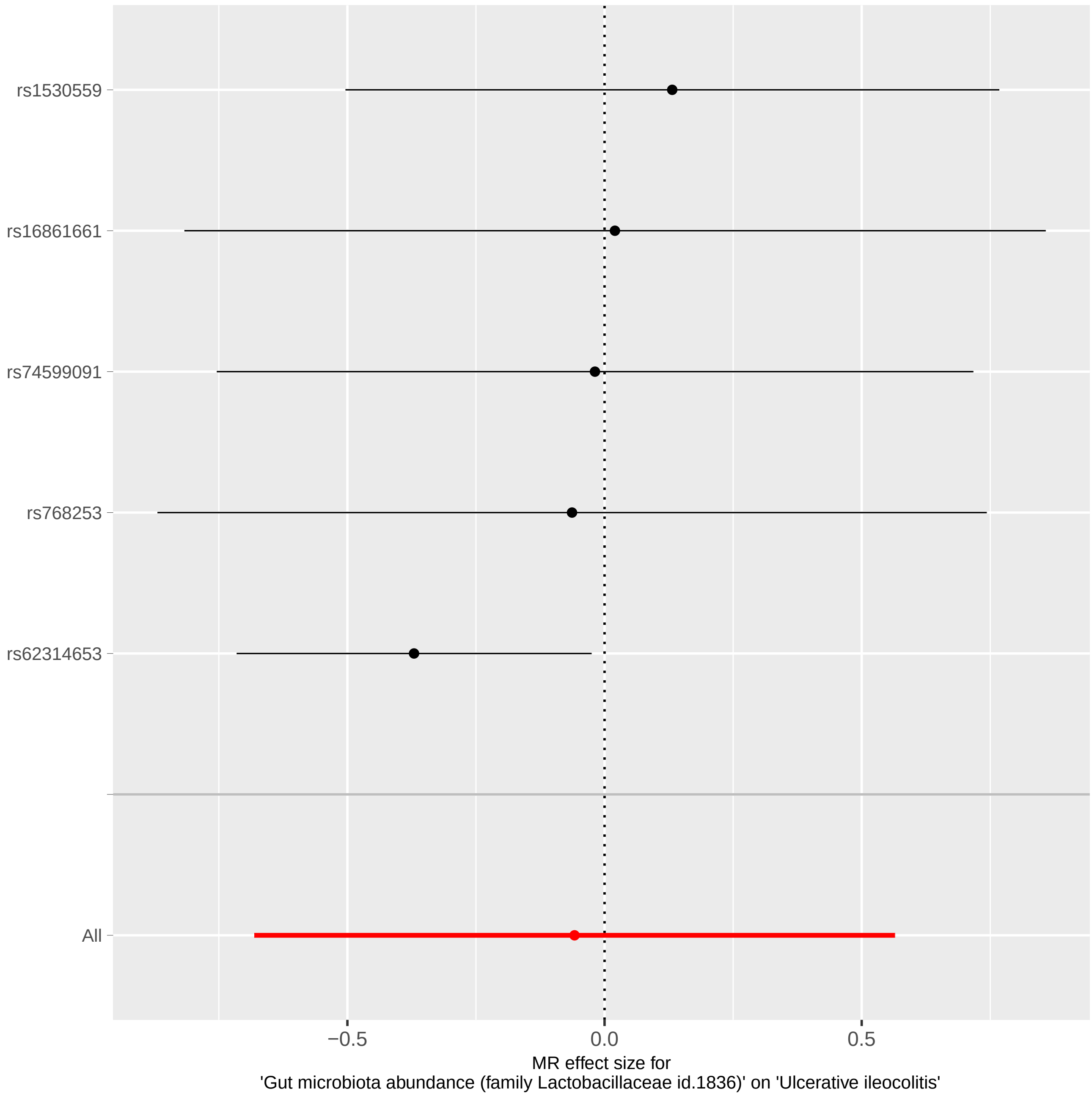

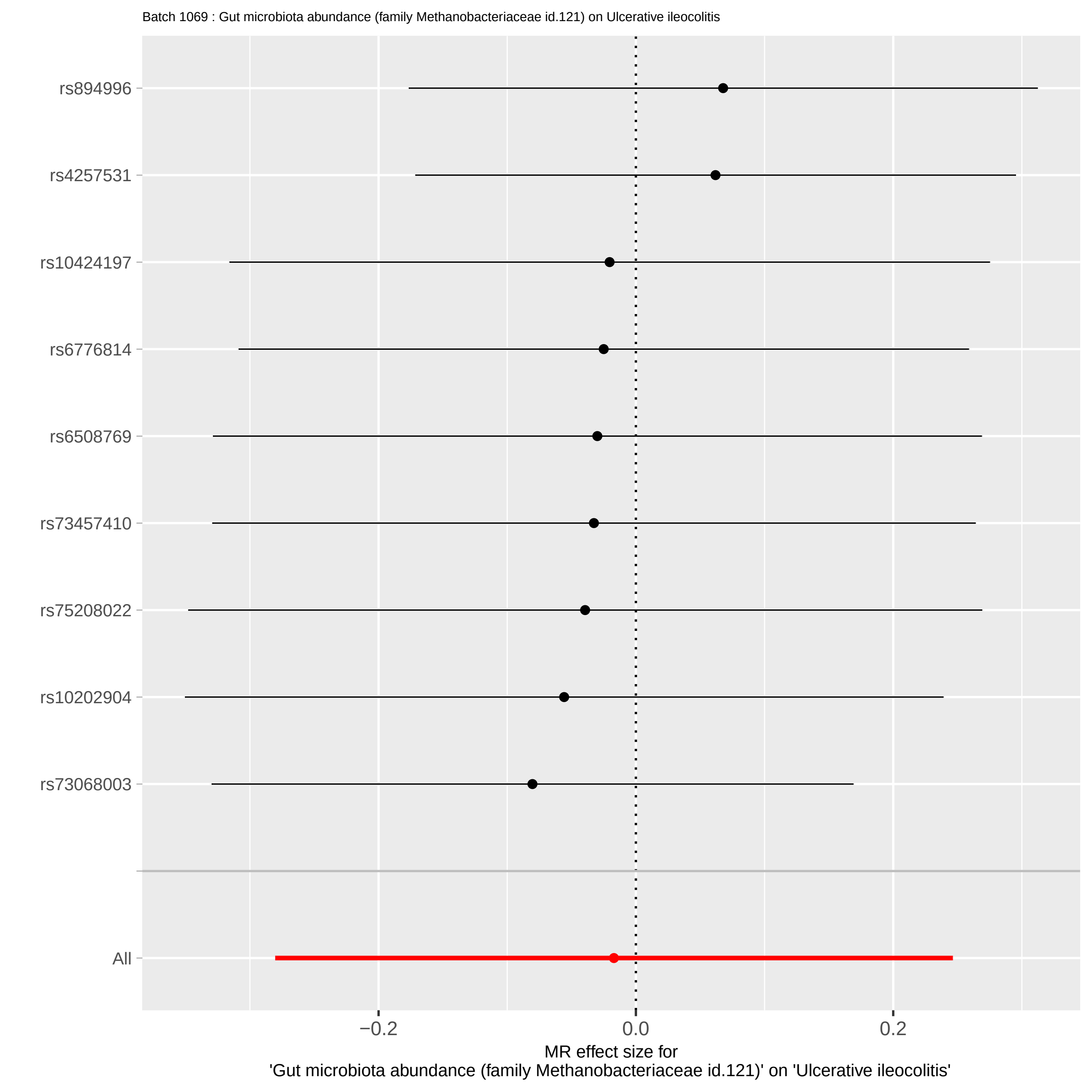

Batch 1069 : Gut microbiota abundance (family Methanobacteriaceae id.121) on Ulcerative ileocolitis
rs894996
rs4257531
rs10424197
rs6776814
rs6508769
rs73457410
rs75208022
rs10202904
rs73068003
All
−0.2
0.0
0.2
MR effect size for
'Gut microbiota abundance (family Methanobacteriaceae id.121)' on 'Ulcerative ileocolitis'

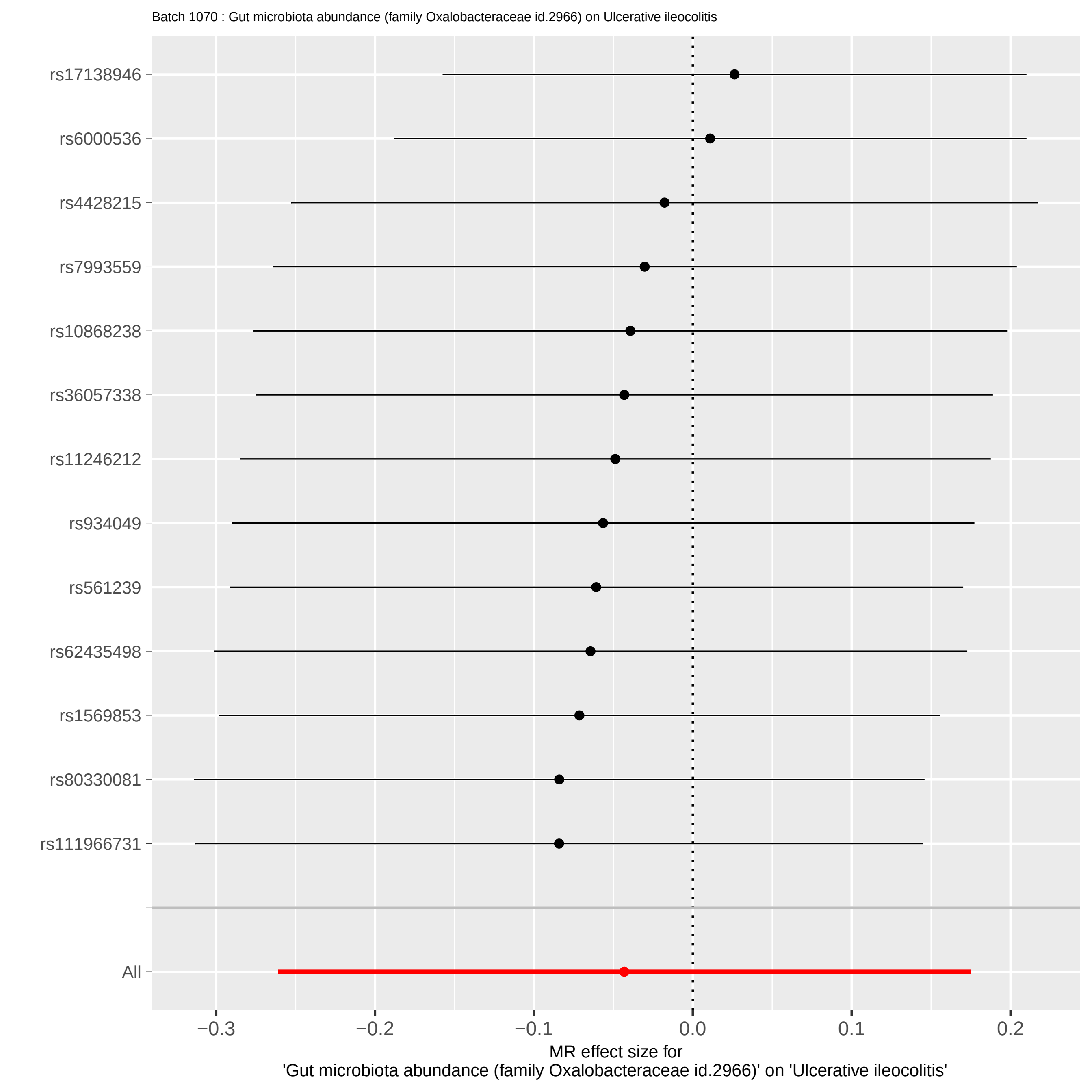

Batch 1070 : Gut microbiota abundance (family Oxalobacteraceae id.2966) on Ulcerative ileocolitis
rs17138946
rs6000536
rs4428215
rs7993559
rs10868238
rs36057338
rs11246212
rs934049
rs561239
rs62435498
rs1569853
rs80330081
rs111966731
All
−0.3
−0.2
−0.1
0.0
0.1
0.2
MR effect size for
'Gut microbiota abundance (family Oxalobacteraceae id.2966)' on 'Ulcerative ileocolitis'

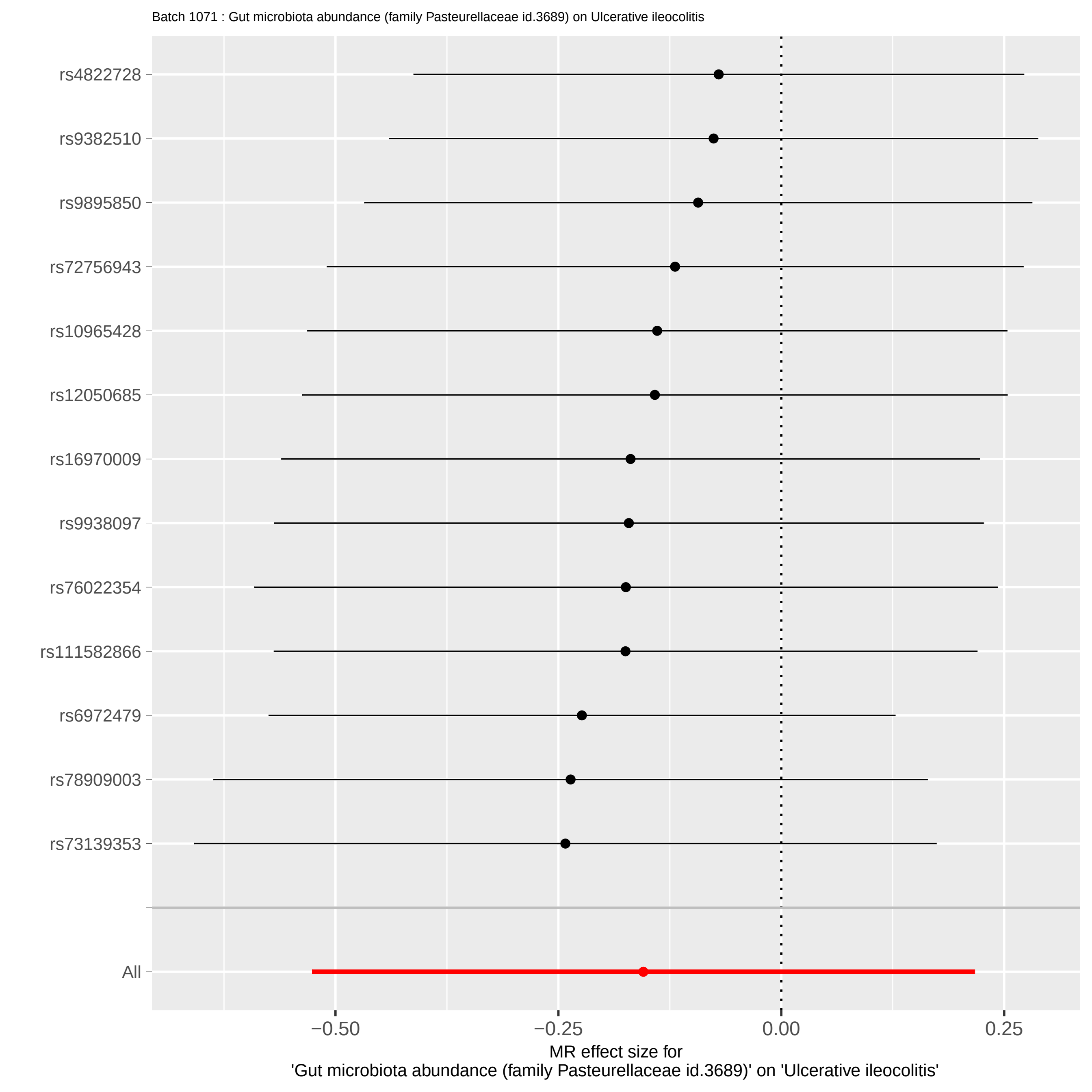
Batch 1071 : Gut microbiota abundance (family Pasteurellaceae id.3689) on Ulcerative ileocolitis
rs4822728
rs9382510
rs9895850
rs72756943
rs10965428
rs12050685
rs16970009
rs9938097
rs76022354
rs111582866
rs6972479
rs78909003
rs73139353
All
−0.50
−0.25
0.00
0.25
MR effect size for
'Gut microbiota abundance (family Pasteurellaceae id.3689)' on 'Ulcerative ileocolitis'

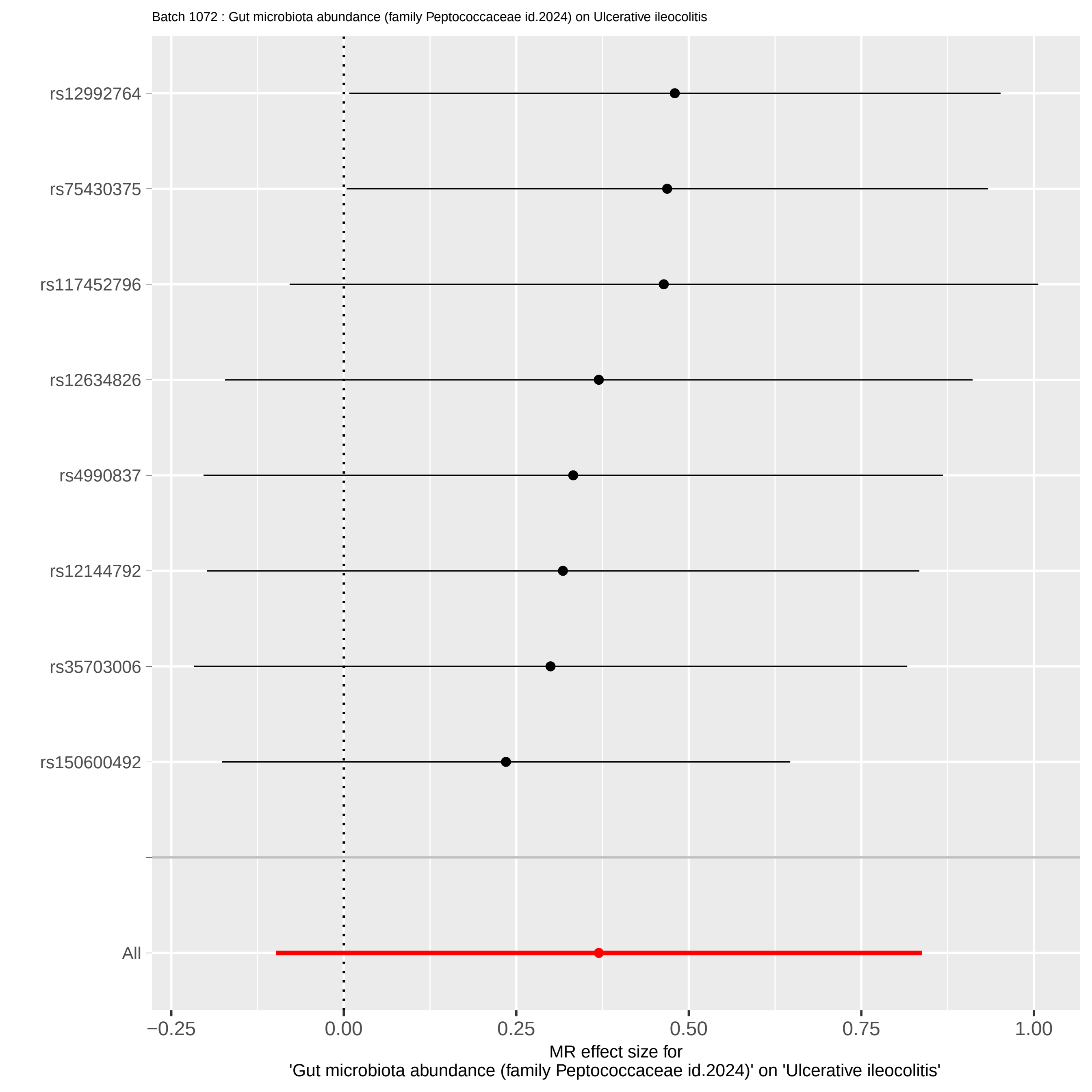

Batch 1072 : Gut microbiota abundance (family Peptococcaceae id.2024) on Ulcerative ileocolitis
rs12992764
rs75430375
rs117452796
rs12634826
rs4990837
rs12144792
rs35703006
rs150600492
All
−0.25
0.00
0.25
0.50
0.75
1.00
MR effect size for
'Gut microbiota abundance (family Peptococcaceae id.2024)' on 'Ulcerative ileocolitis'

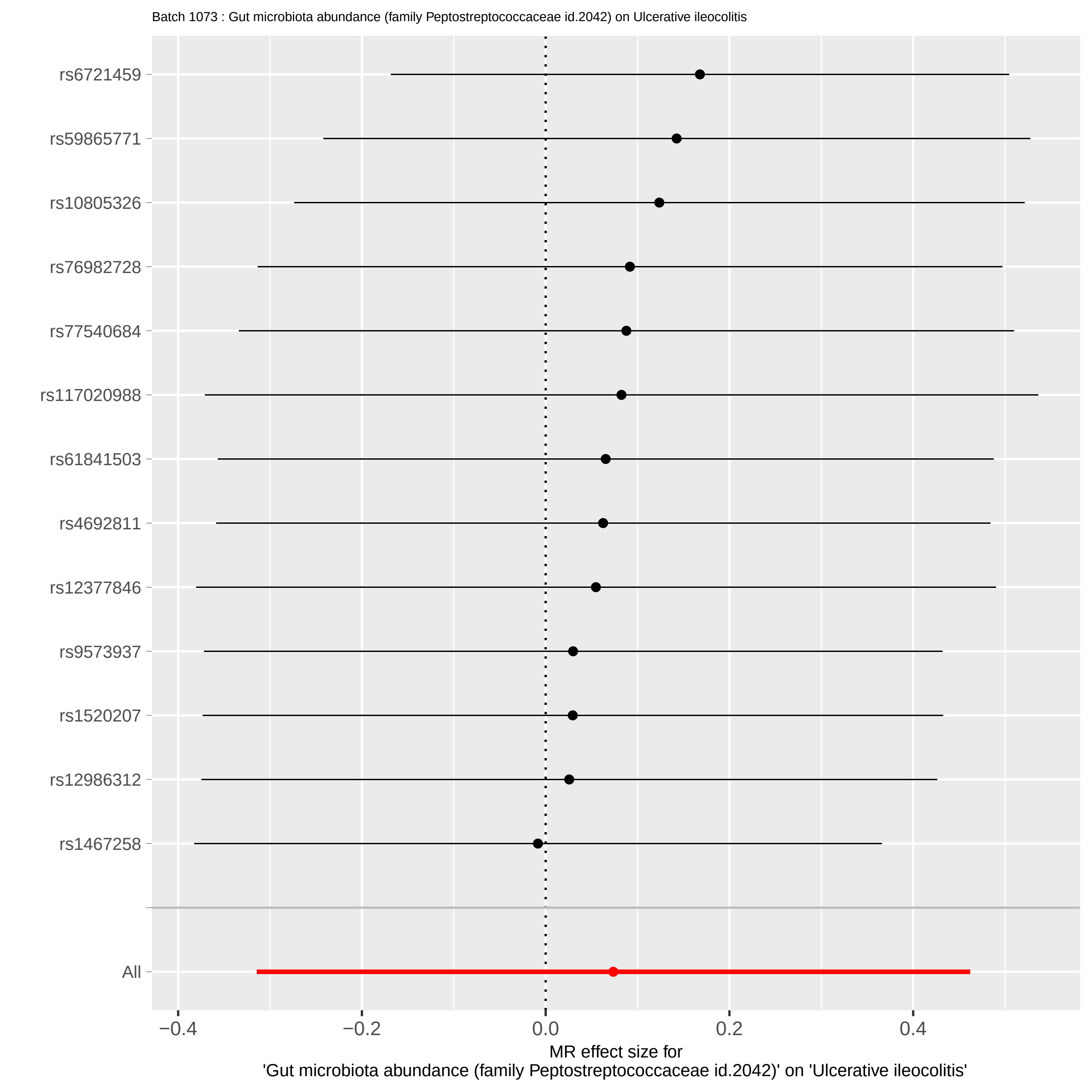

Batch 1073 : Gut microbiota abundance (family Peptostreptococcaceae id.2042) on Ulcerative ileocolitis
rs6721459
rs59865771
rs10805326
rs76982728
rs77540684
rs117020988
rs61841503
rs4692811
rs12377846
rs9573937
rs1520207
rs12986312
rs1467258
All
−0.4
−0.2
0.0
0.2
0.4
MR effect size for
'Gut microbiota abundance (family Peptostreptococcaceae id.2042)' on 'Ulcerative ileocolitis'

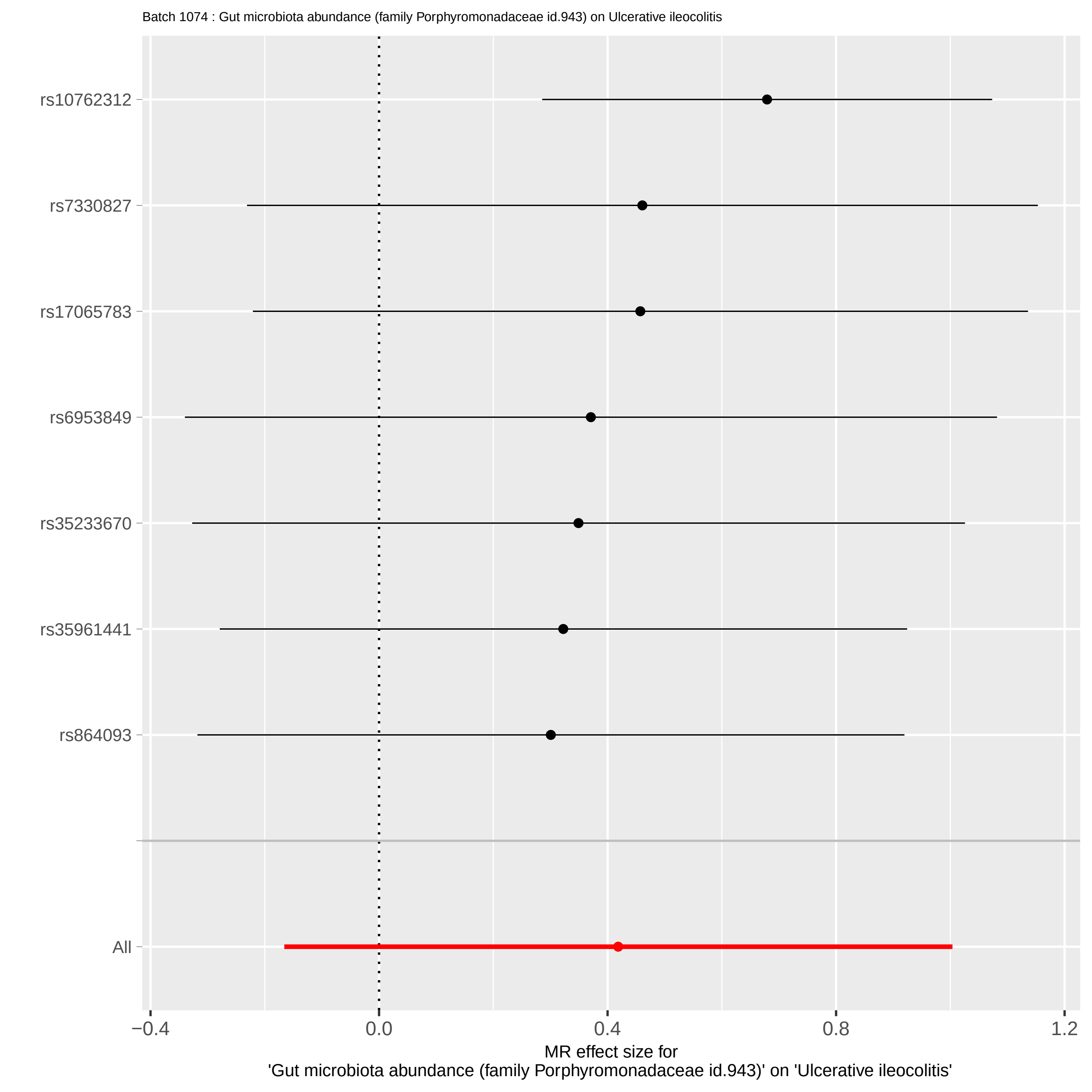
Batch 1074 : Gut microbiota abundance (family Porphyromonadaceae id.943) on Ulcerative ileocolitis
rs10762312
rs7330827
rs17065783
rs6953849
rs35233670
rs35961441
rs864093
All
−0.4
0.0
0.4
0.8
1.2
MR effect size for
'Gut microbiota abundance (family Porphyromonadaceae id.943)' on 'Ulcerative ileocolitis'

Batch 1075 : Gut microbiota abundance (family Prevotellaceae id.960) on Ulcerative ileocolitis

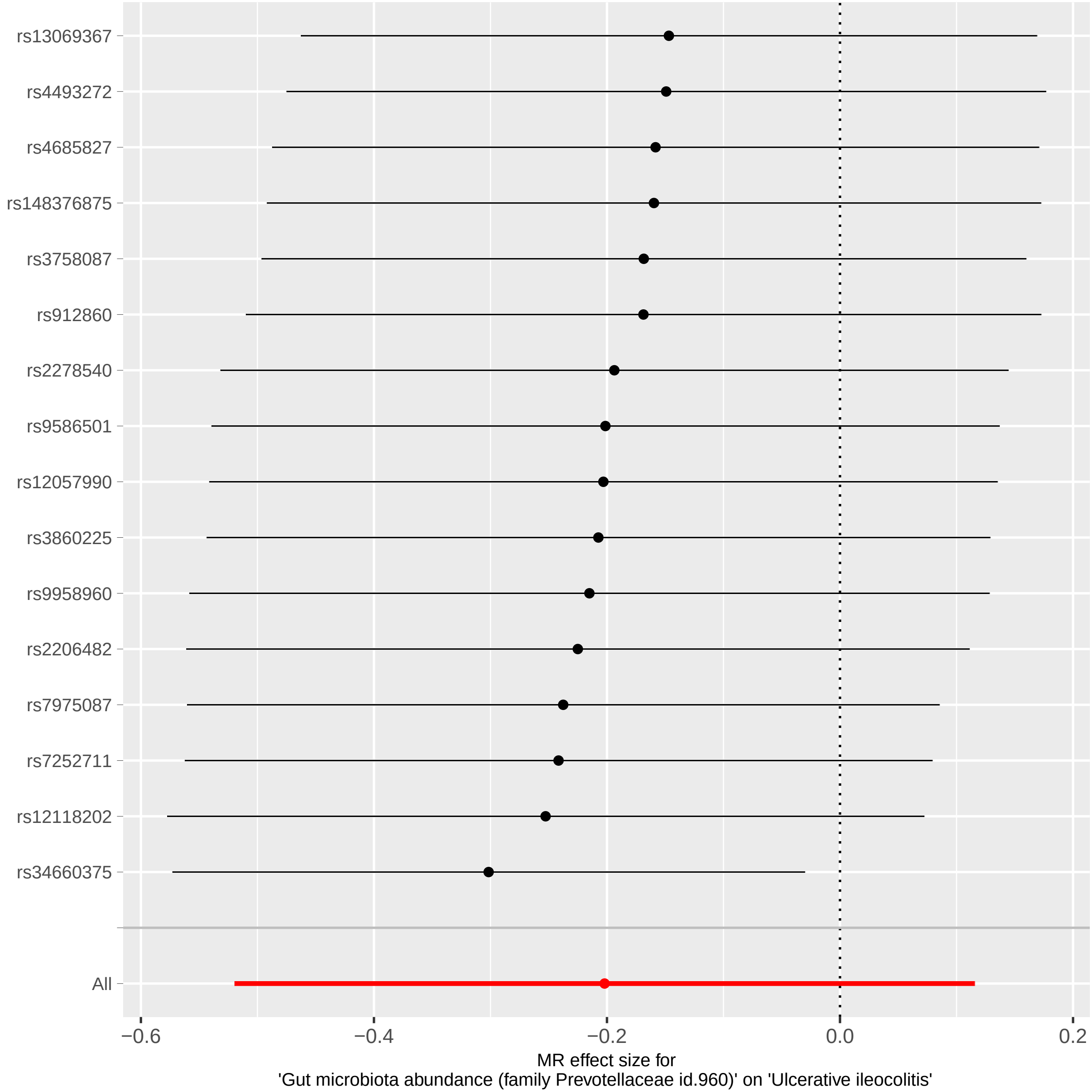

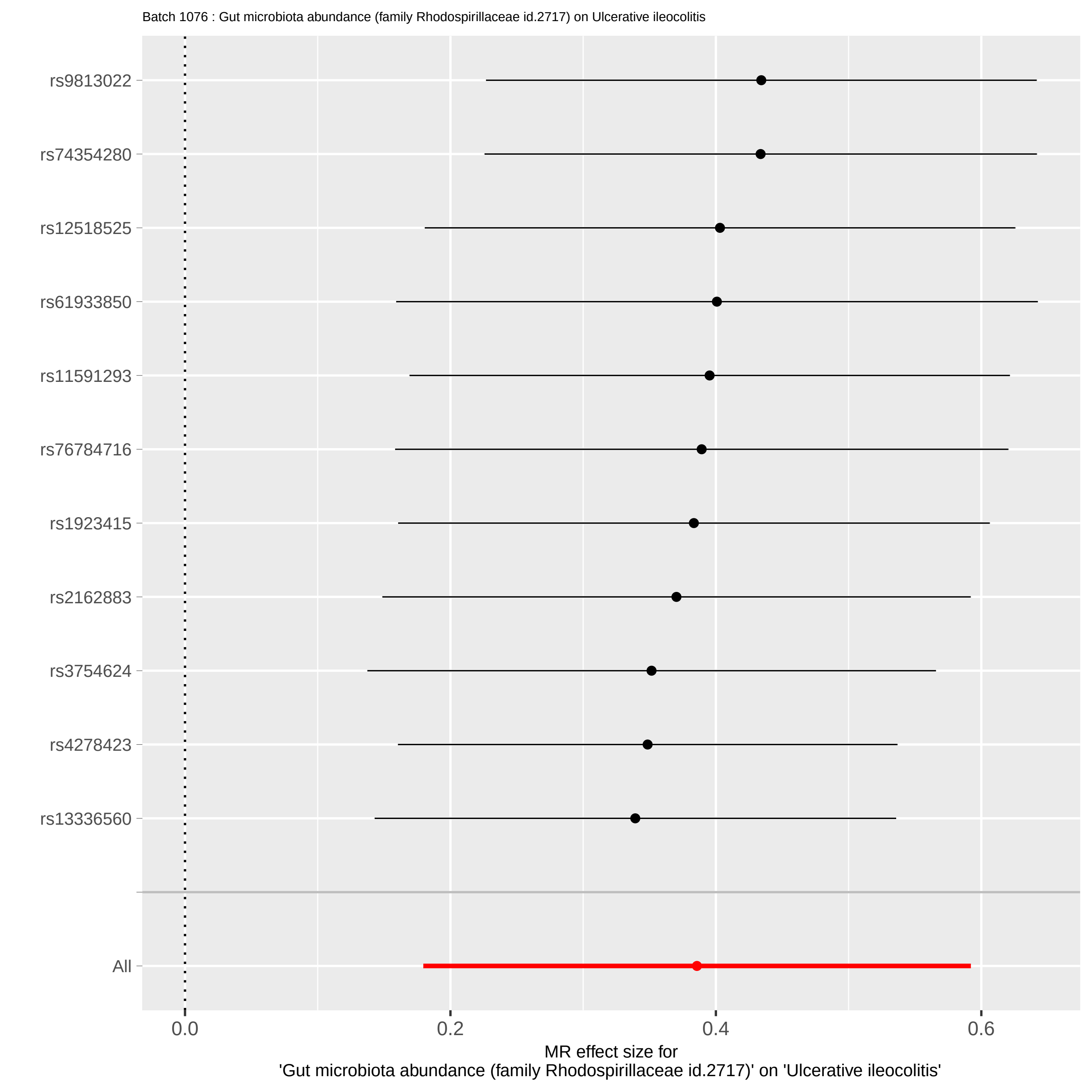

Batch 1076 : Gut microbiota abundance (family Rhodospirillaceae id.2717) on Ulcerative ileocolitis
rs9813022
rs74354280
rs12518525
rs61933850
rs11591293
rs76784716
rs1923415
rs2162883
rs3754624
rs4278423
rs13336560
All
0.0
0.2
0.4
0.6
MR effect size for
'Gut microbiota abundance (family Rhodospirillaceae id.2717)' on 'Ulcerative ileocolitis'

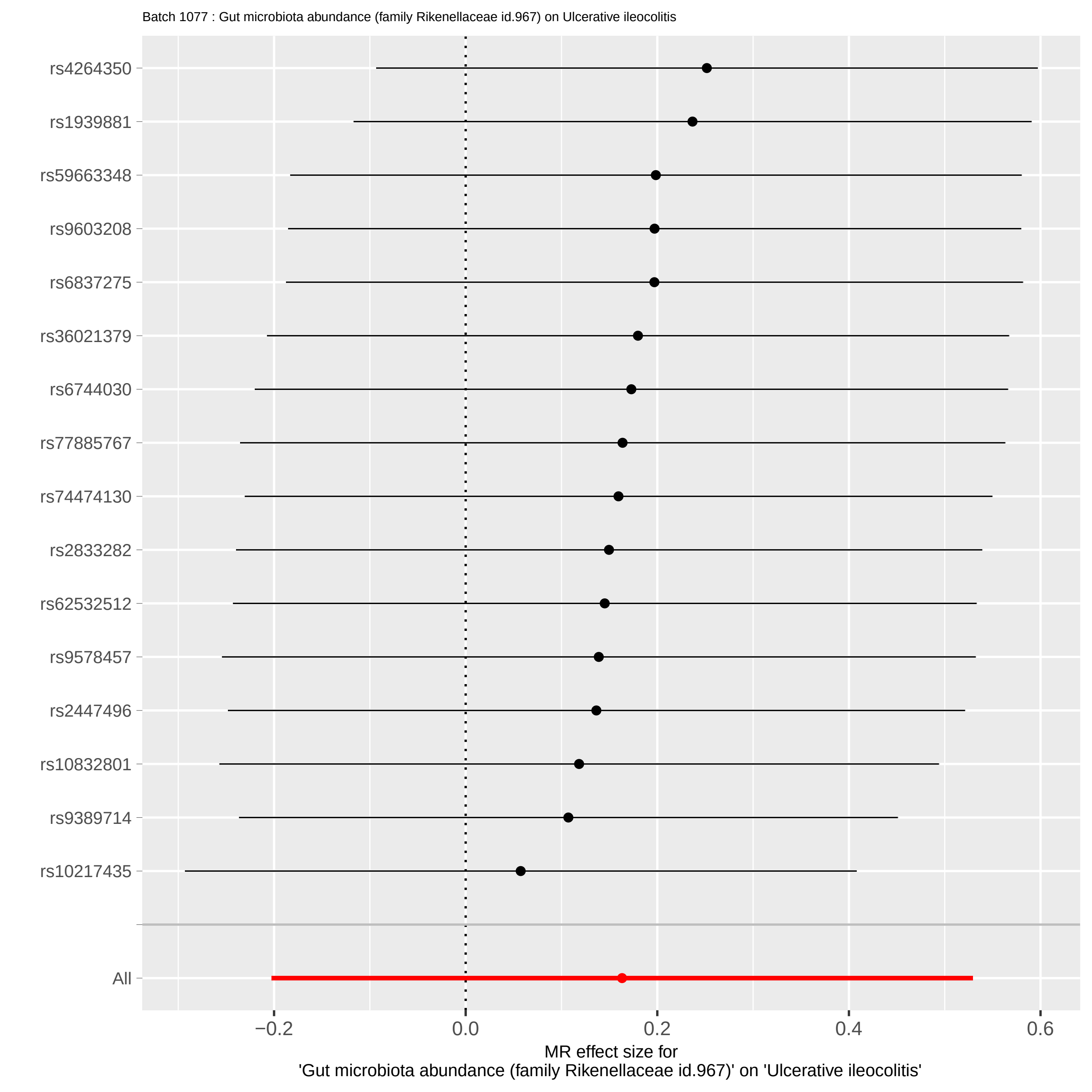

Batch 1077 : Gut microbiota abundance (family Rikenellaceae id.967) on Ulcerative ileocolitis
rs4264350
rs1939881
rs59663348
rs9603208
rs6837275
rs36021379
rs6744030
rs77885767
rs74474130
rs2833282
rs62532512
rs9578457
rs2447496
rs10832801
rs9389714
rs10217435
All
−0.2
0.0
0.2
0.4
0.6
MR effect size for
'Gut microbiota abundance (family Rikenellaceae id.967)' on 'Ulcerative ileocolitis'

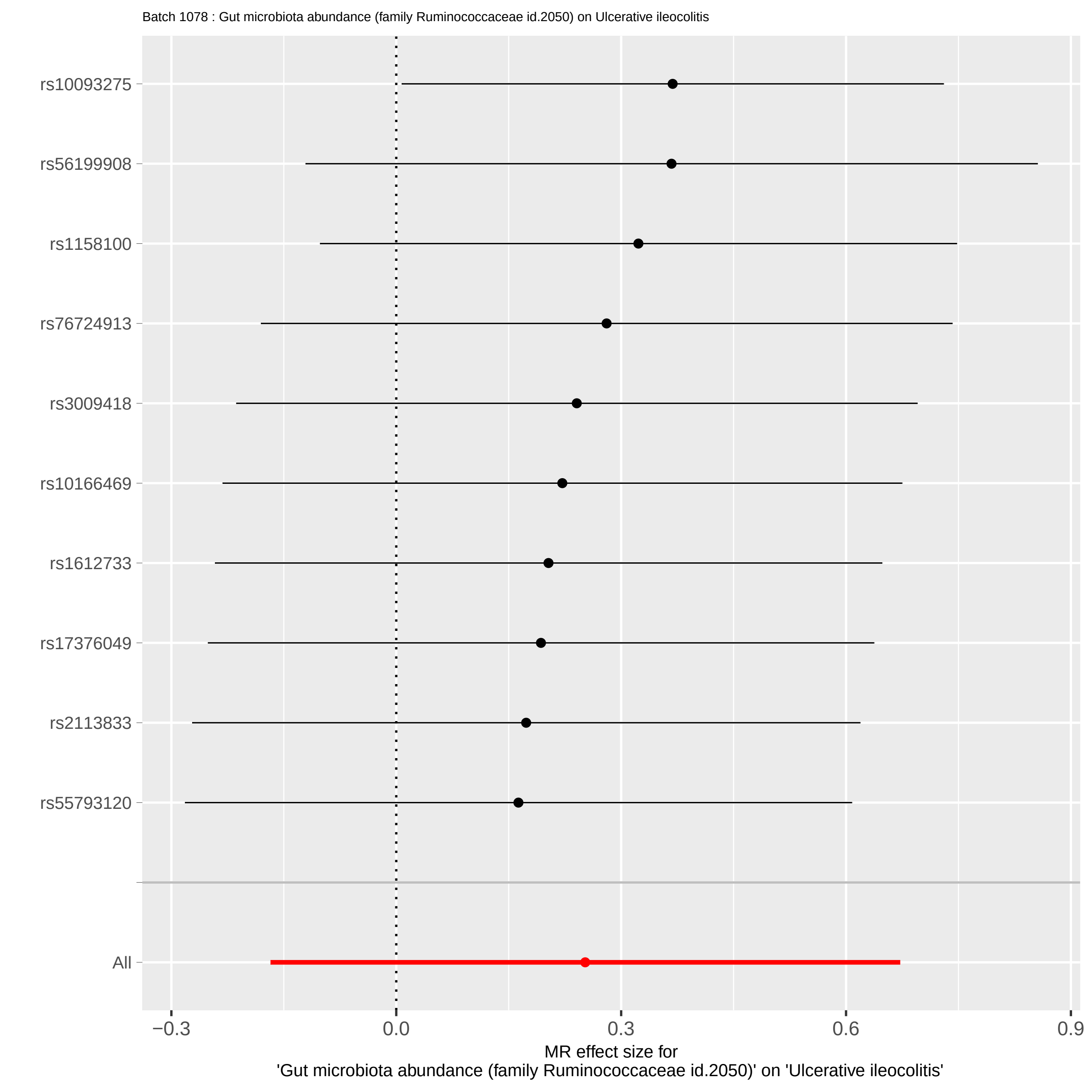

Batch 1078 : Gut microbiota abundance (family Ruminococcaceae id.2050) on Ulcerative ileocolitis
rs10093275
rs56199908
rs1158100
rs76724913
rs3009418
rs10166469
rs1612733
rs17376049
rs2113833
rs55793120
All
−0.3
0.0
0.3
0.6
0.9
MR effect size for
'Gut microbiota abundance (family Ruminococcaceae id.2050)' on 'Ulcerative ileocolitis'

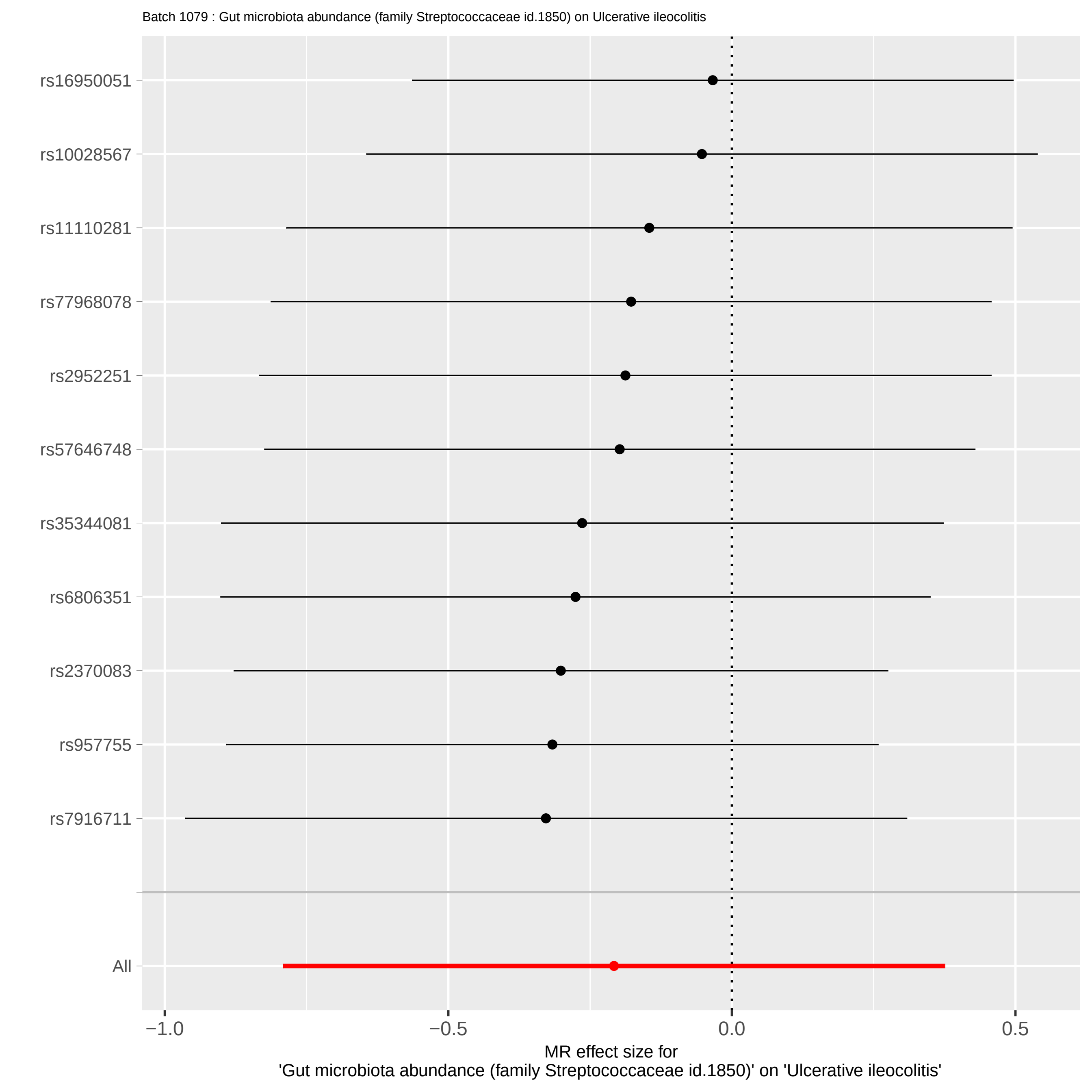

Batch 1079 : Gut microbiota abundance (family Streptococcaceae id.1850) on Ulcerative ileocolitis
rs16950051
rs10028567
rs11110281
rs77968078
rs2952251
rs57646748
rs35344081
rs6806351
rs2370083
rs957755
rs7916711
All
−1.0
−0.5
0.0
0.5
MR effect size for
'Gut microbiota abundance (family Streptococcaceae id.1850)' on 'Ulcerative ileocolitis'

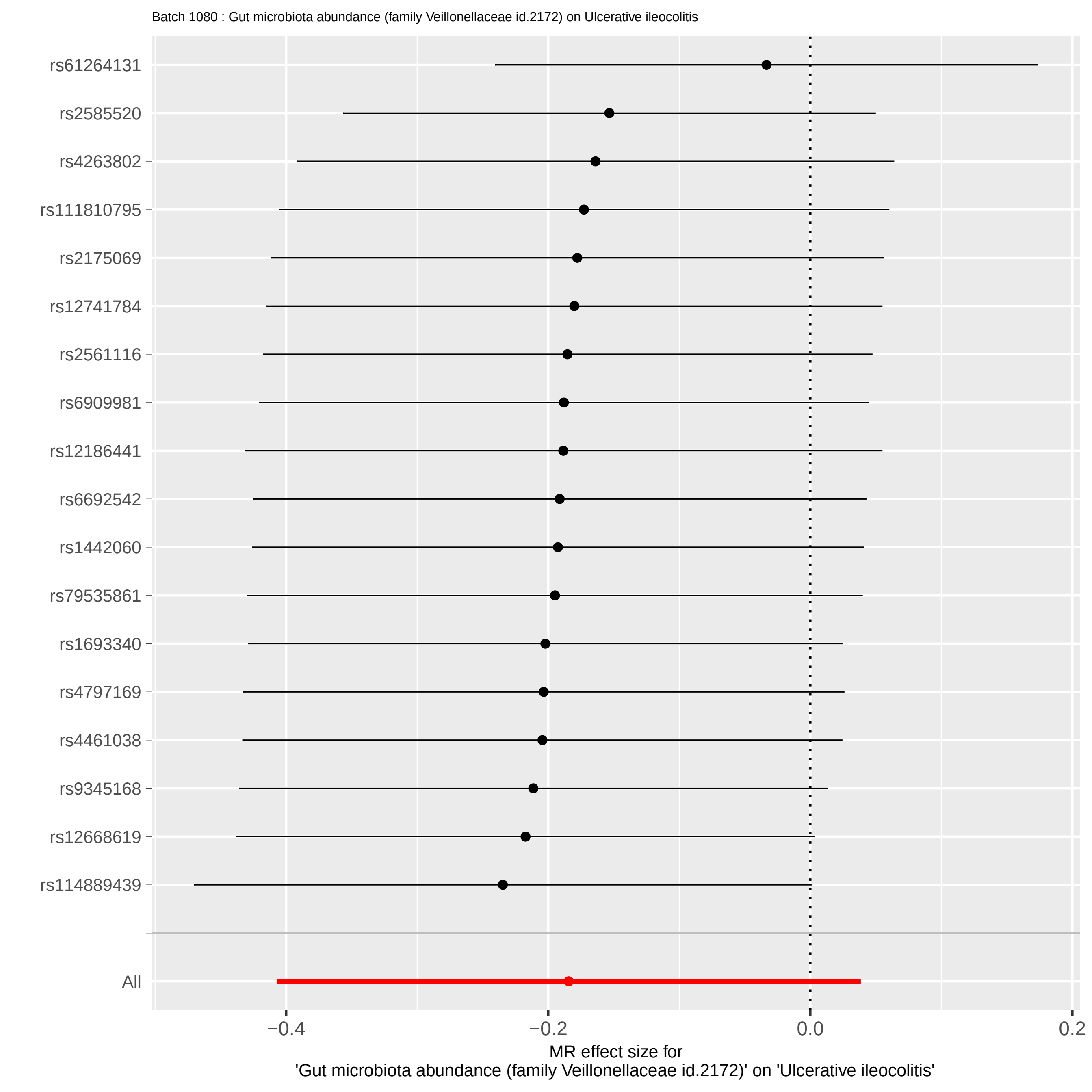

Batch 1080 : Gut microbiota abundance (family Veillonellaceae id.2172) on Ulcerative ileocolitis
rs61264131
rs2585520
rs4263802
rs111810795
rs2175069
rs12741784
rs2561116
rs6909981
rs12186441
rs6692542
rs1442060
rs79535861
rs1693340
rs4797169
rs4461038
rs9345168
rs12668619
rs114889439
All
−0.4
−0.2
0.0
0.2
MR effect size for
'Gut microbiota abundance (family Veillonellaceae id.2172)' on 'Ulcerative ileocolitis'

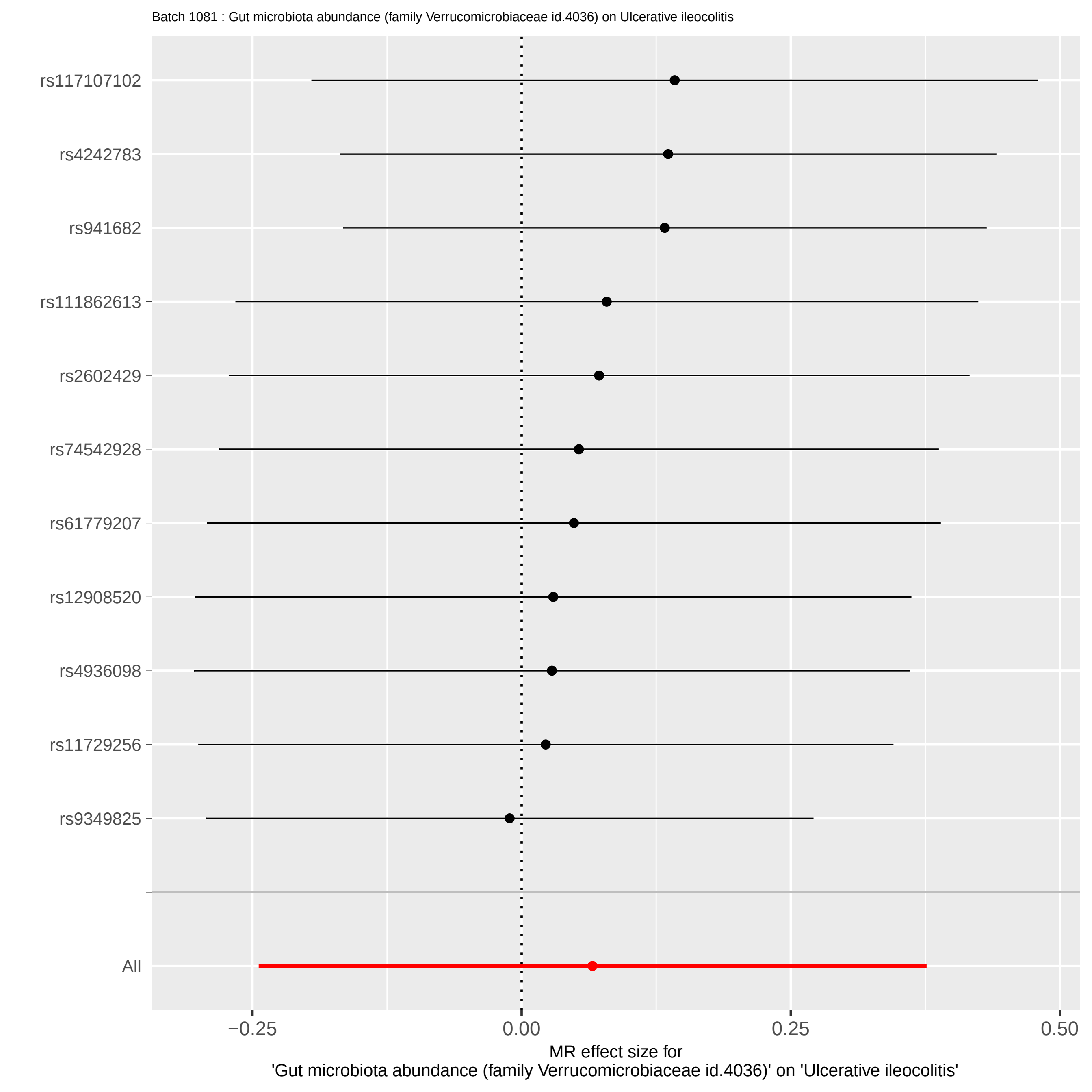

Batch 1081 : Gut microbiota abundance (family Verrucomicrobiaceae id.4036) on Ulcerative ileocolitis
rs117107102
rs4242783
rs941682
rs111862613
rs2602429
rs74542928
rs61779207
rs12908520
rs4936098
rs11729256
rs9349825
All
−0.25
0.00
0.25
0.50
MR effect size for
'Gut microbiota abundance (family Verrucomicrobiaceae id.4036)' on 'Ulcerative ileocolitis'

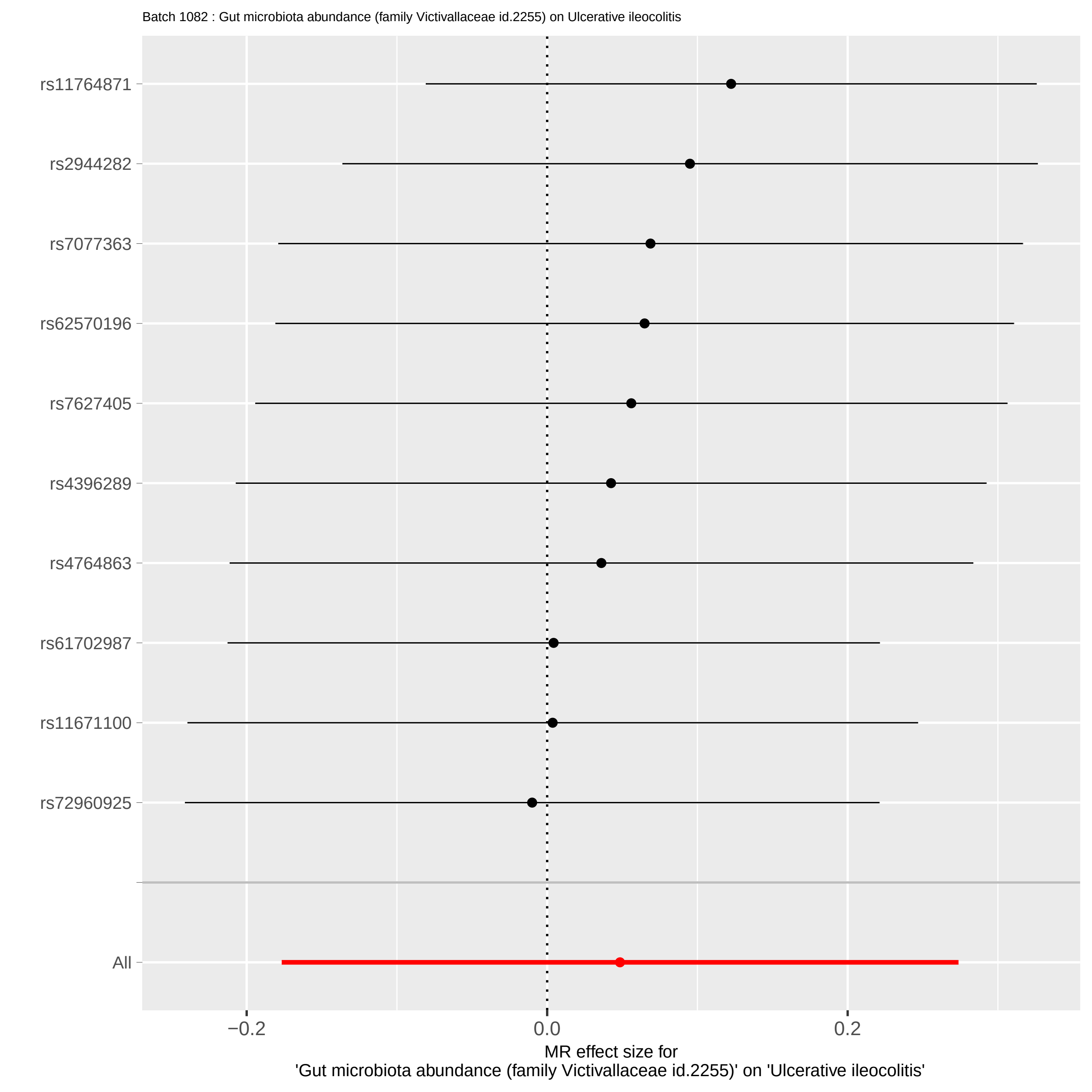

Batch 1082 : Gut microbiota abundance (family Victivallaceae id.2255) on Ulcerative ileocolitis
rs11764871
rs2944282
rs7077363
rs62570196
rs7627405
rs4396289
rs4764863
rs61702987
rs11671100
rs72960925
All
−0.2
0.0
0.2
MR effect size for
'Gut microbiota abundance (family Victivallaceae id.2255)' on 'Ulcerative ileocolitis'

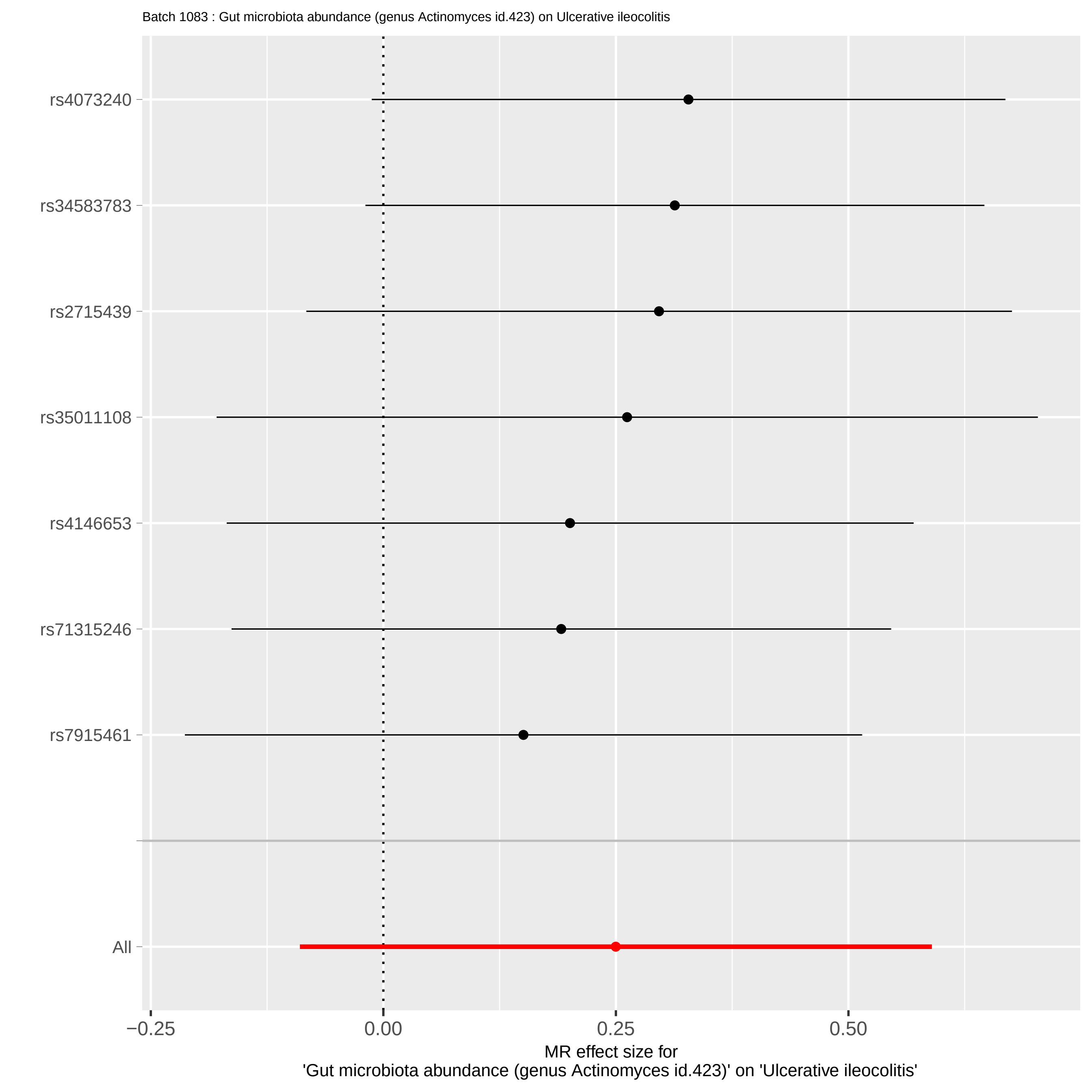
Batch 1083 : Gut microbiota abundance (genus Actinomyces id.423) on Ulcerative ileocolitis
rs4073240
rs34583783
rs2715439
rs35011108
rs4146653
rs71315246
rs7915461
All
−0.25
0.00
0.25
0.50
MR effect size for
'Gut microbiota abundance (genus Actinomyces id.423)' on 'Ulcerative ileocolitis'

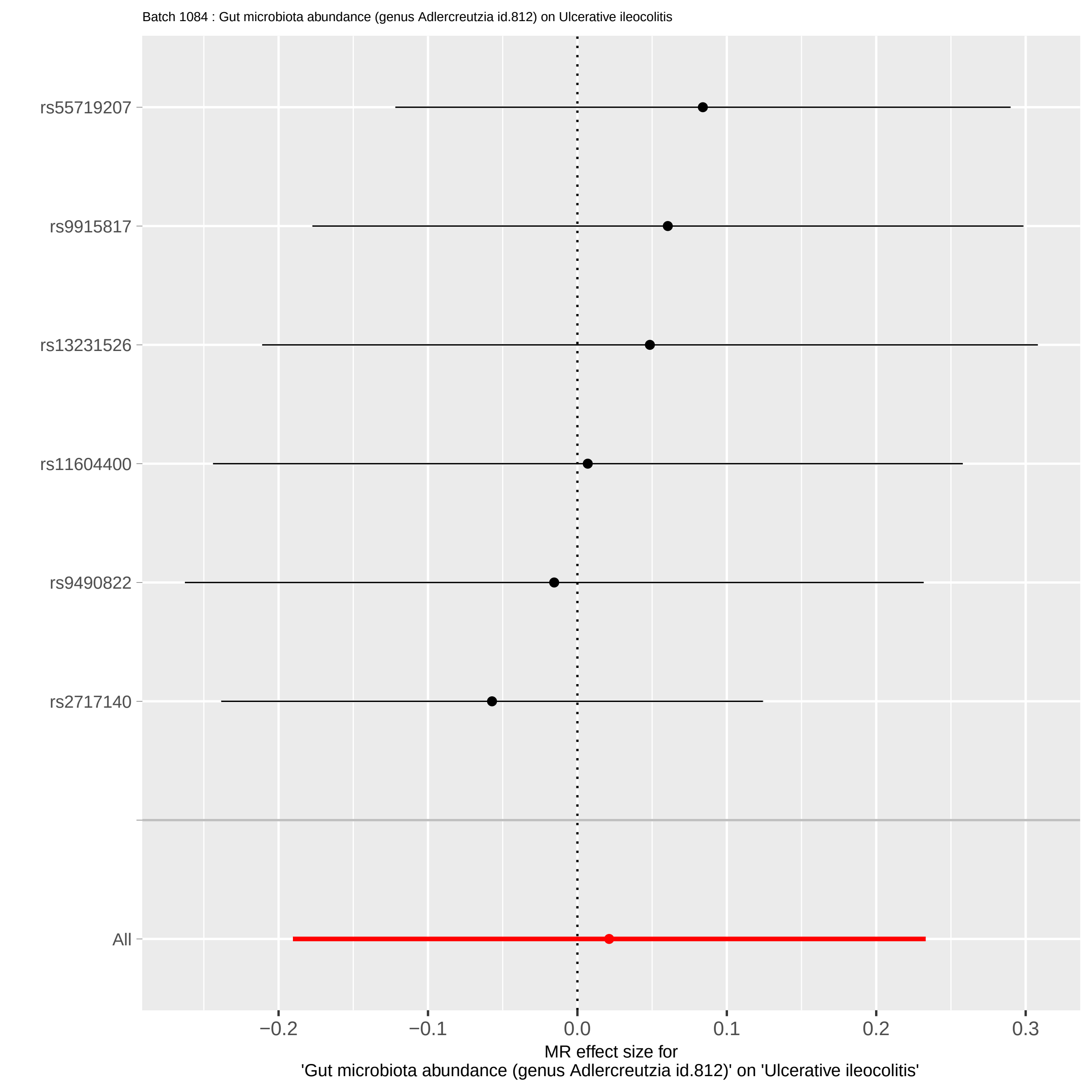

Batch 1084 : Gut microbiota abundance (genus Adlercreutzia id.812) on Ulcerative ileocolitis
rs55719207
rs9915817
rs13231526
rs11604400
rs9490822
rs2717140
All
−0.2
−0.1
0.0
0.1
0.2
0.3
MR effect size for
'Gut microbiota abundance (genus Adlercreutzia id.812)' on 'Ulcerative ileocolitis'

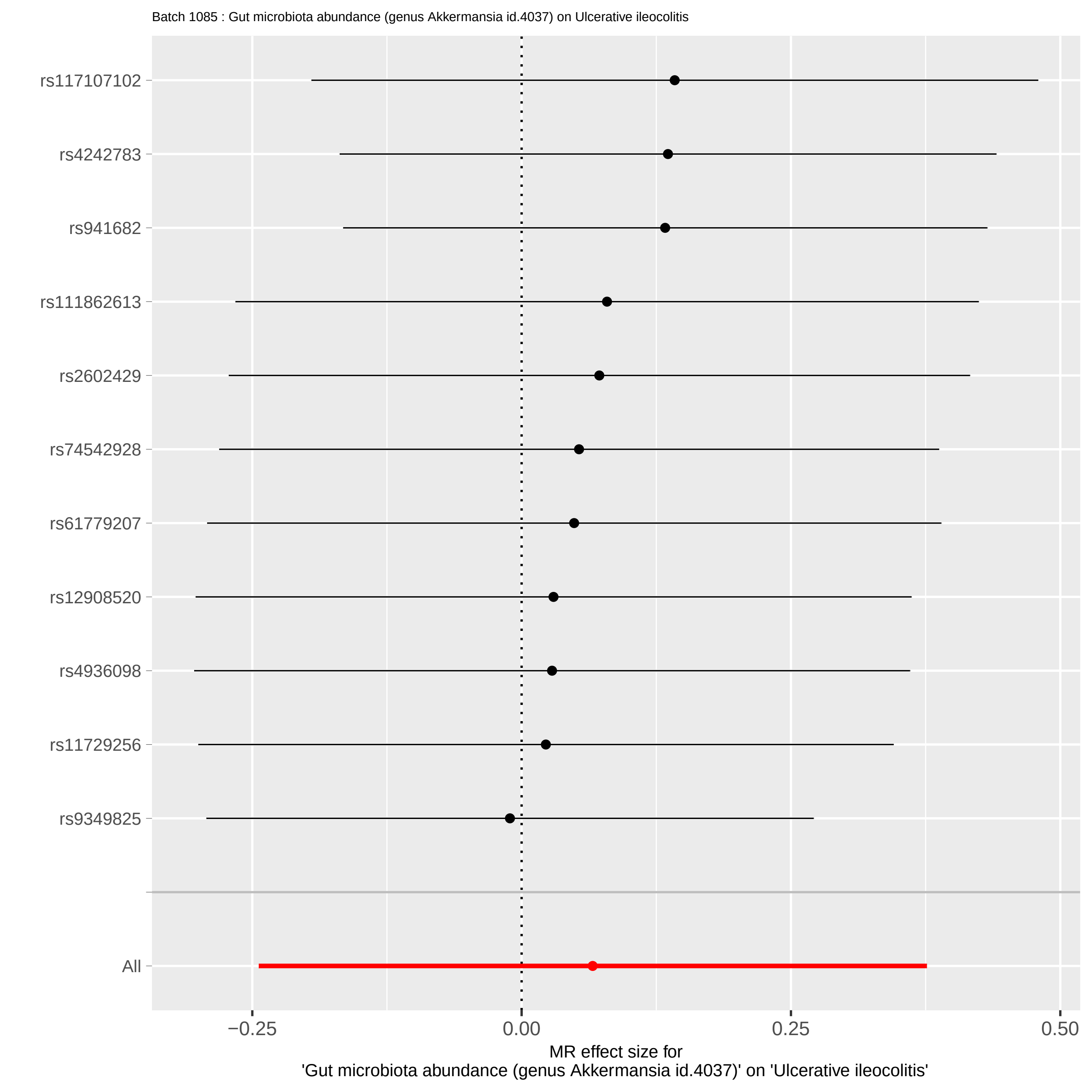
Batch 1085 : Gut microbiota abundance (genus Akkermansia id.4037) on Ulcerative ileocolitis
rs117107102
rs4242783
rs941682
rs111862613
rs2602429
rs74542928
rs61779207
rs12908520
rs4936098
rs11729256
rs9349825
All
−0.25
0.00
0.25
0.50
MR effect size for
'Gut microbiota abundance (genus Akkermansia id.4037)' on 'Ulcerative ileocolitis'

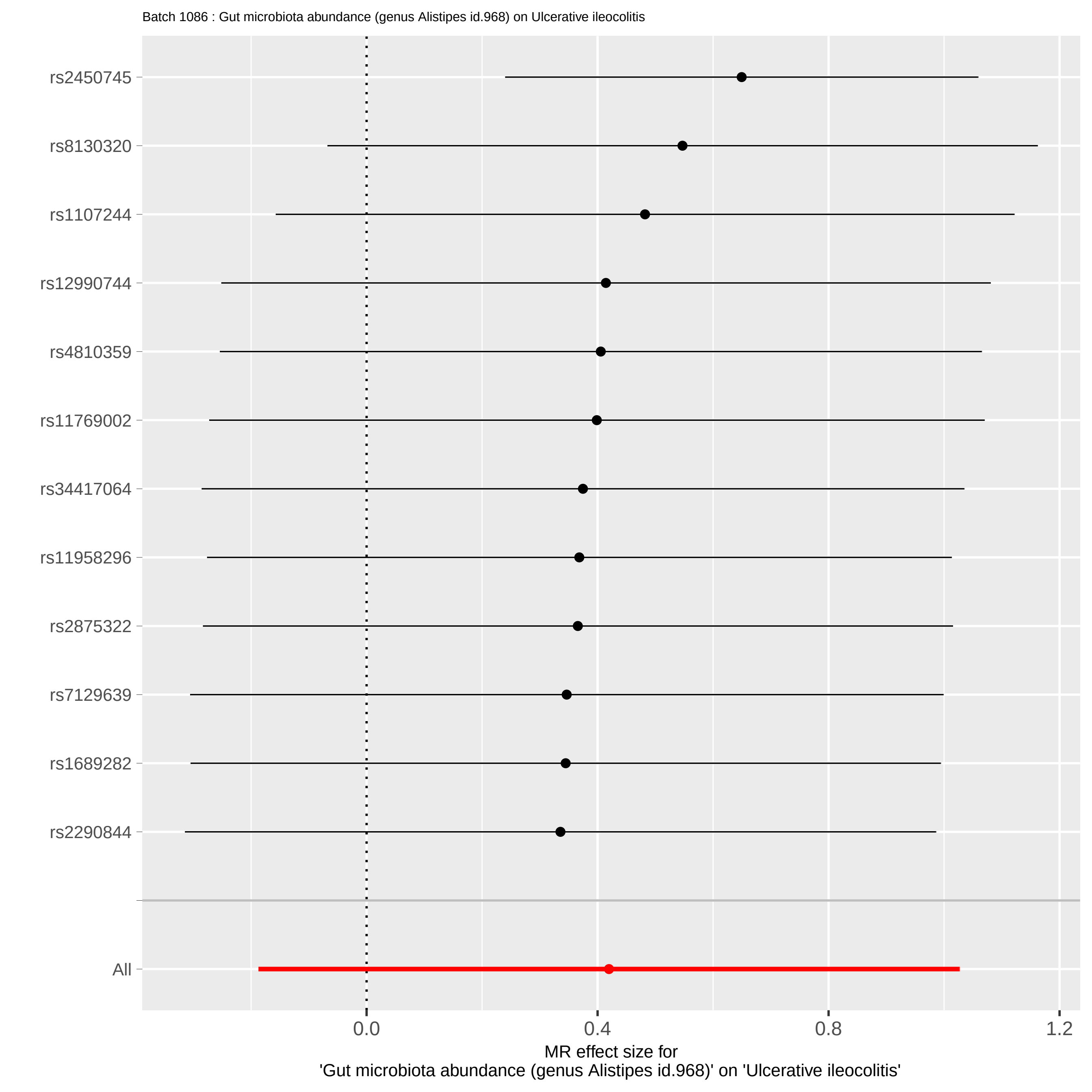

Batch 1086 : Gut microbiota abundance (genus Alistipes id.968) on Ulcerative ileocolitis
rs2450745
rs8130320
rs1107244
rs12990744
rs4810359
rs11769002
rs34417064
rs11958296
rs2875322
rs7129639
rs1689282
rs2290844
All
0.0
0.4
0.8
1.2
MR effect size for
'Gut microbiota abundance (genus Alistipes id.968)' on 'Ulcerative ileocolitis'

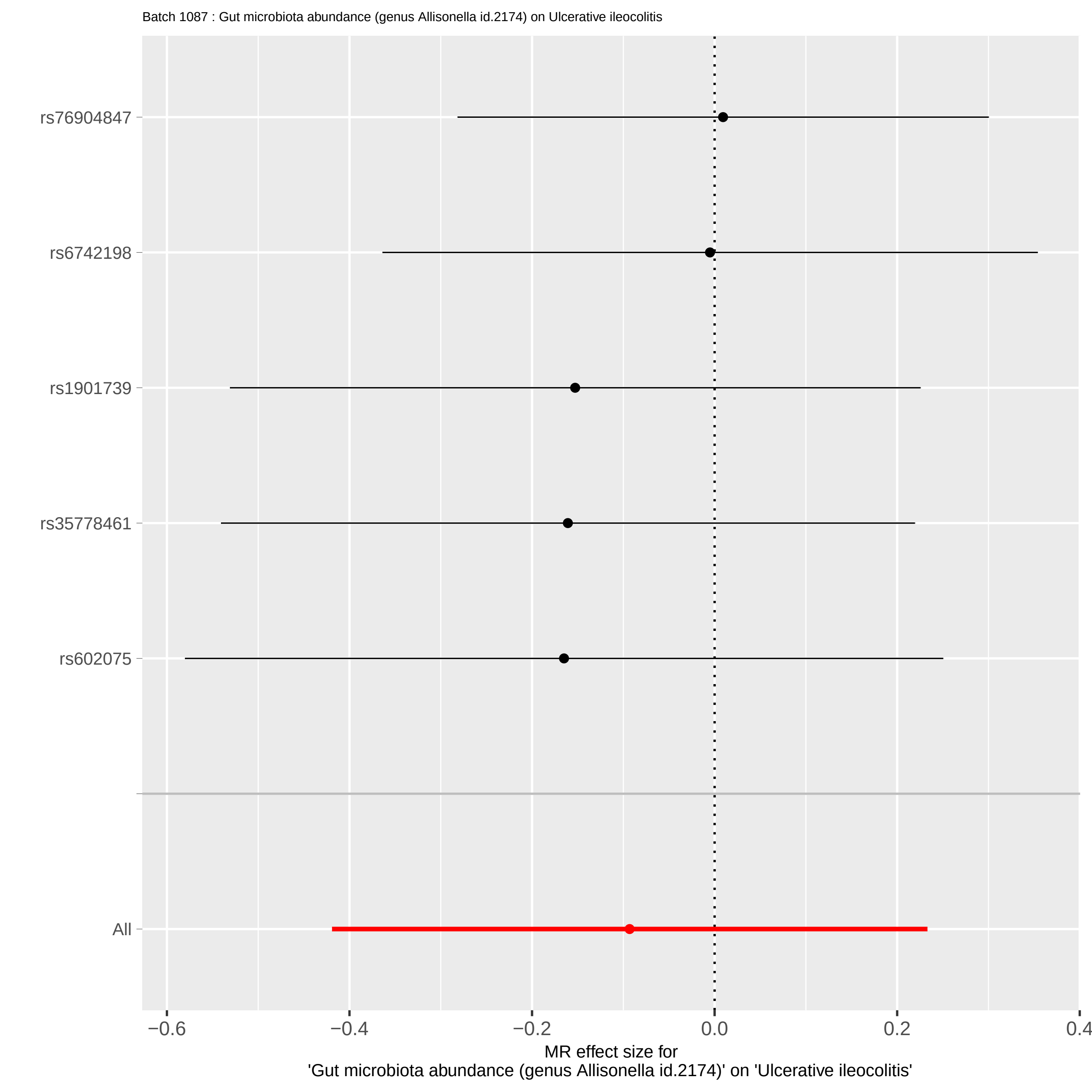
Batch 1087 : Gut microbiota abundance (genus Allisonella id.2174) on Ulcerative ileocolitis
rs76904847
rs6742198
rs1901739
rs35778461
rs602075
All
−0.6
−0.4
−0.2
0.0
0.2
0.4
MR effect size for
'Gut microbiota abundance (genus Allisonella id.2174)' on 'Ulcerative ileocolitis'

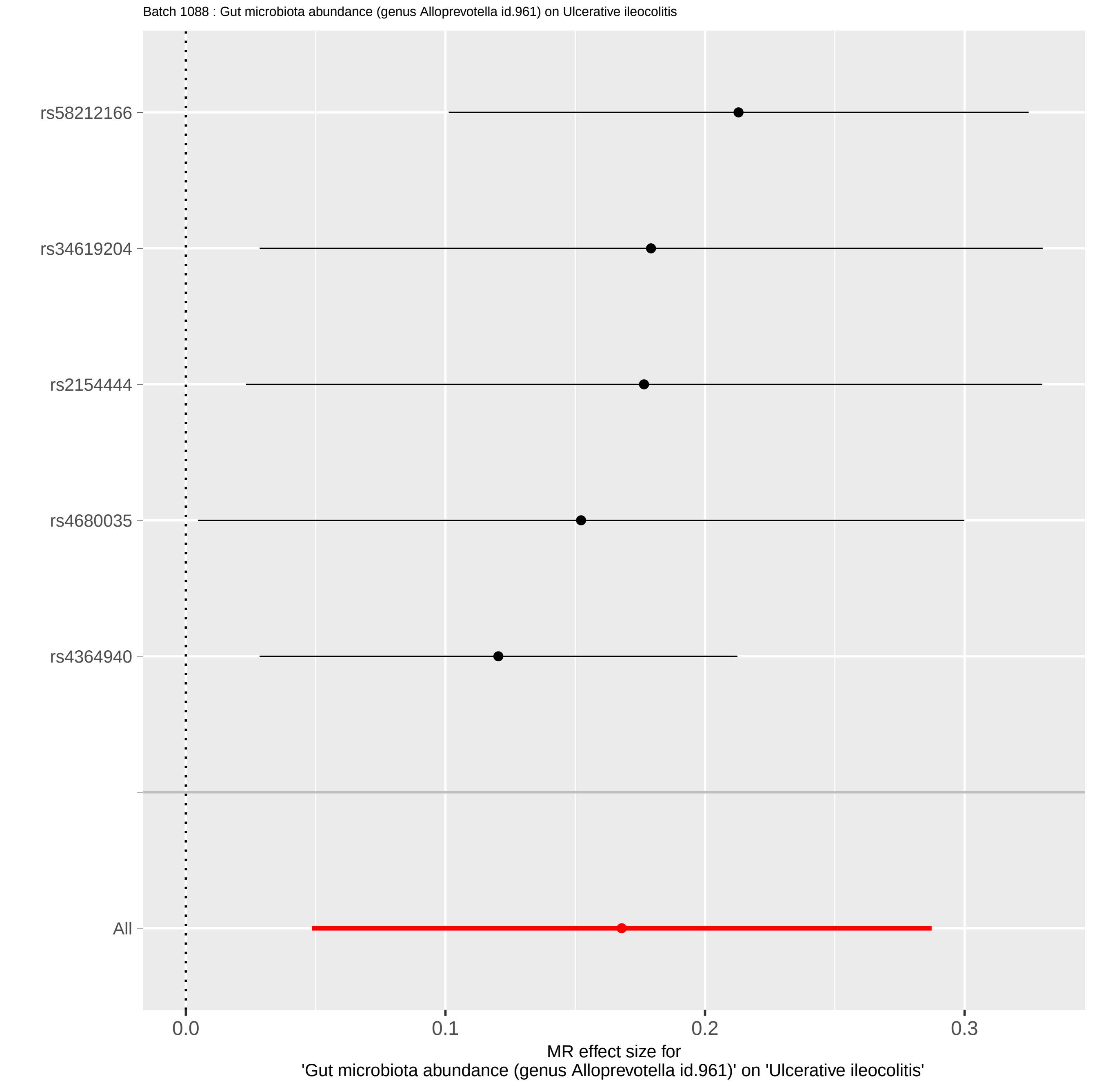

Batch 1088 : Gut microbiota abundance (genus Alloprevotella id.961) on Ulcerative ileocolitis
rs58212166
rs34619204
rs2154444
rs4680035
rs4364940
All
0.0
0.1
0.2
0.3
MR effect size for
'Gut microbiota abundance (genus Alloprevotella id.961)' on 'Ulcerative ileocolitis'

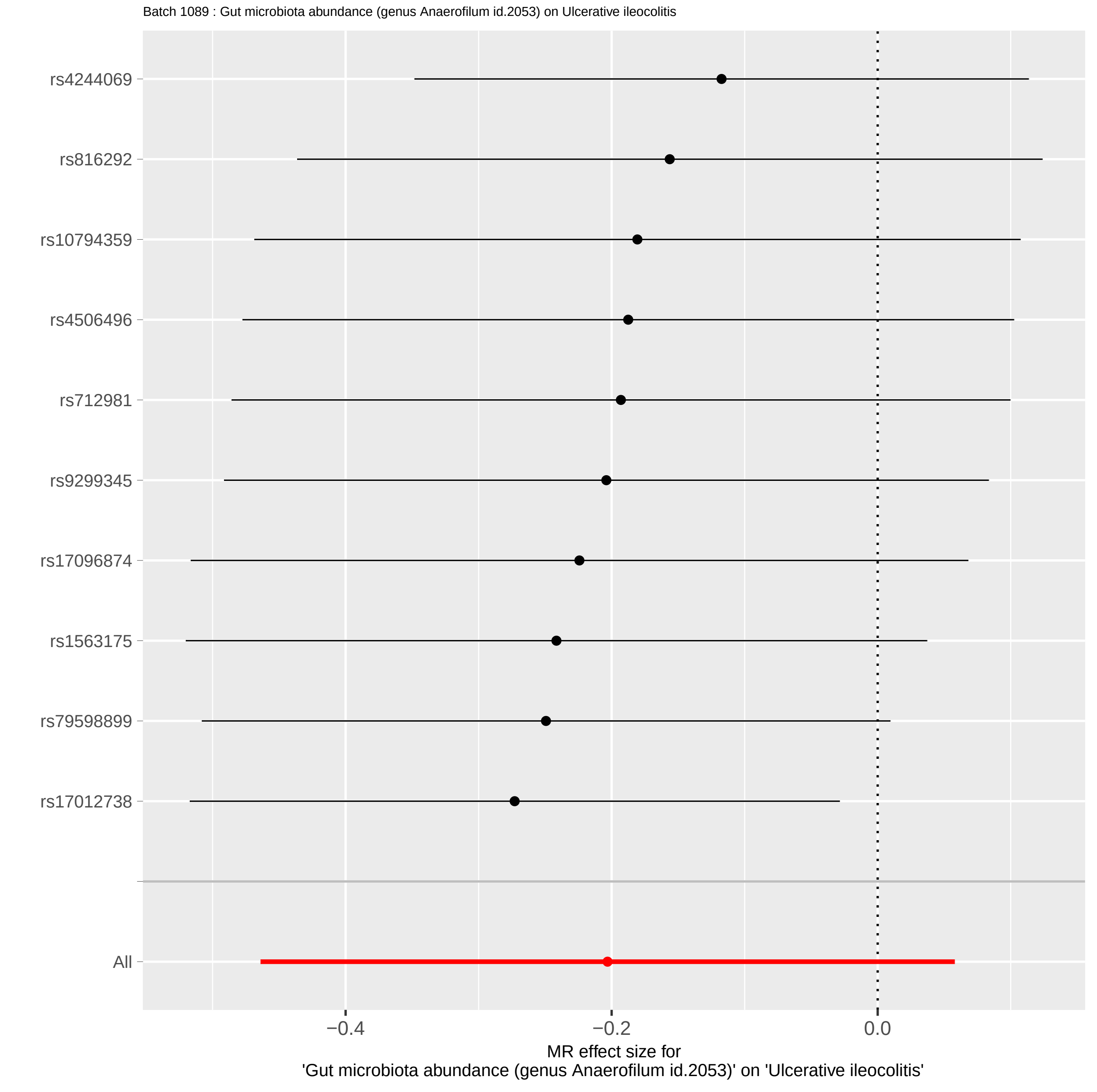

Batch 1089 : Gut microbiota abundance (genus Anaerofilum id.2053) on Ulcerative ileocolitis
rs4244069
rs816292
rs10794359
rs4506496
rs712981
rs9299345
rs17096874
rs1563175
rs79598899
rs17012738
All
−0.4
−0.2
0.0
MR effect size for
'Gut microbiota abundance (genus Anaerofilum id.2053)' on 'Ulcerative ileocolitis'

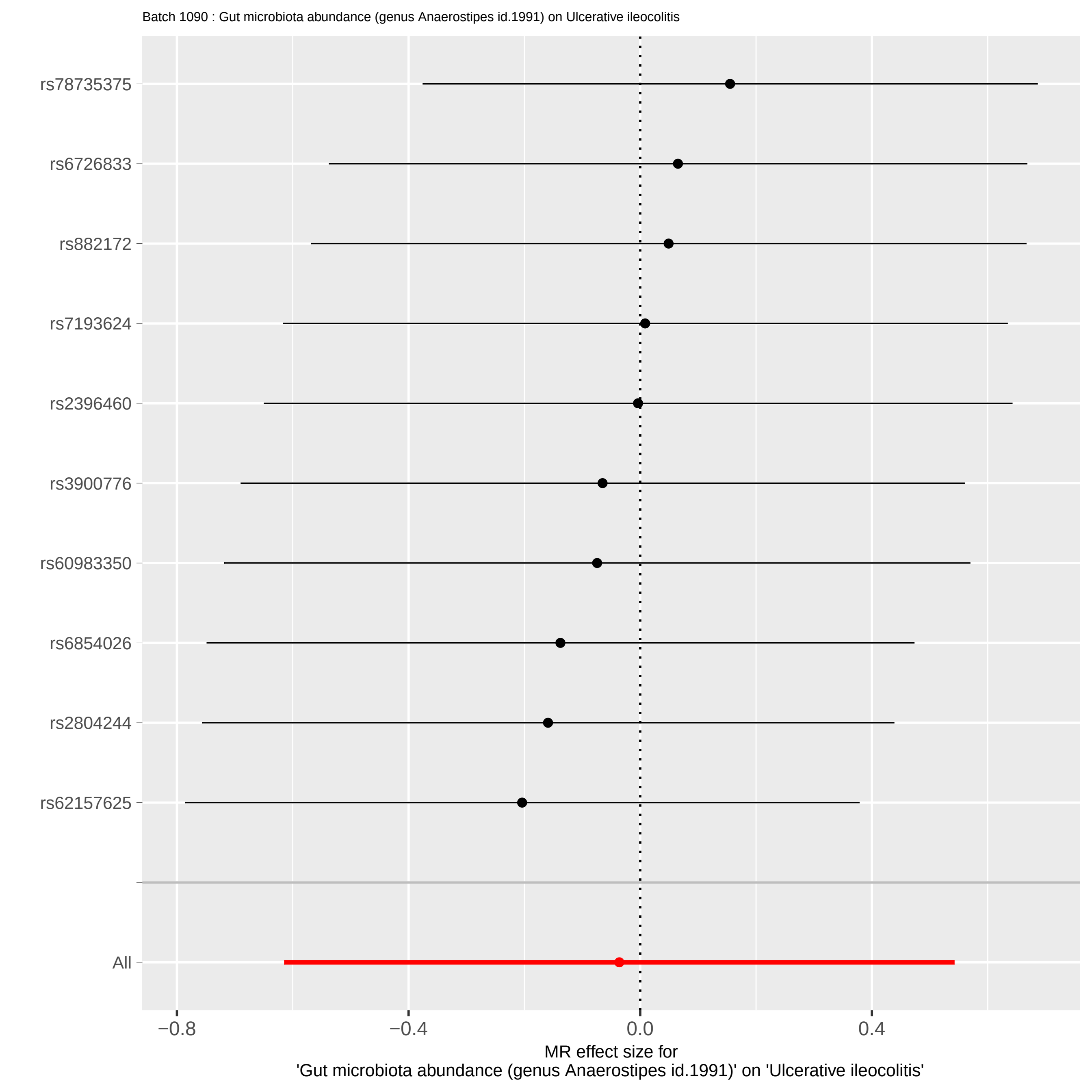

Batch 1090 : Gut microbiota abundance (genus Anaerostipes id.1991) on Ulcerative ileocolitis
rs78735375
rs6726833
rs882172
rs7193624
rs2396460
rs3900776
rs60983350
rs6854026
rs2804244
rs62157625
All
−0.8
−0.4
0.0
0.4
MR effect size for
'Gut microbiota abundance (genus Anaerostipes id.1991)' on 'Ulcerative ileocolitis'

Batch 1091 : Gut microbiota abundance (genus Anaerotruncus id.2054) on Ulcerative ileocolitis

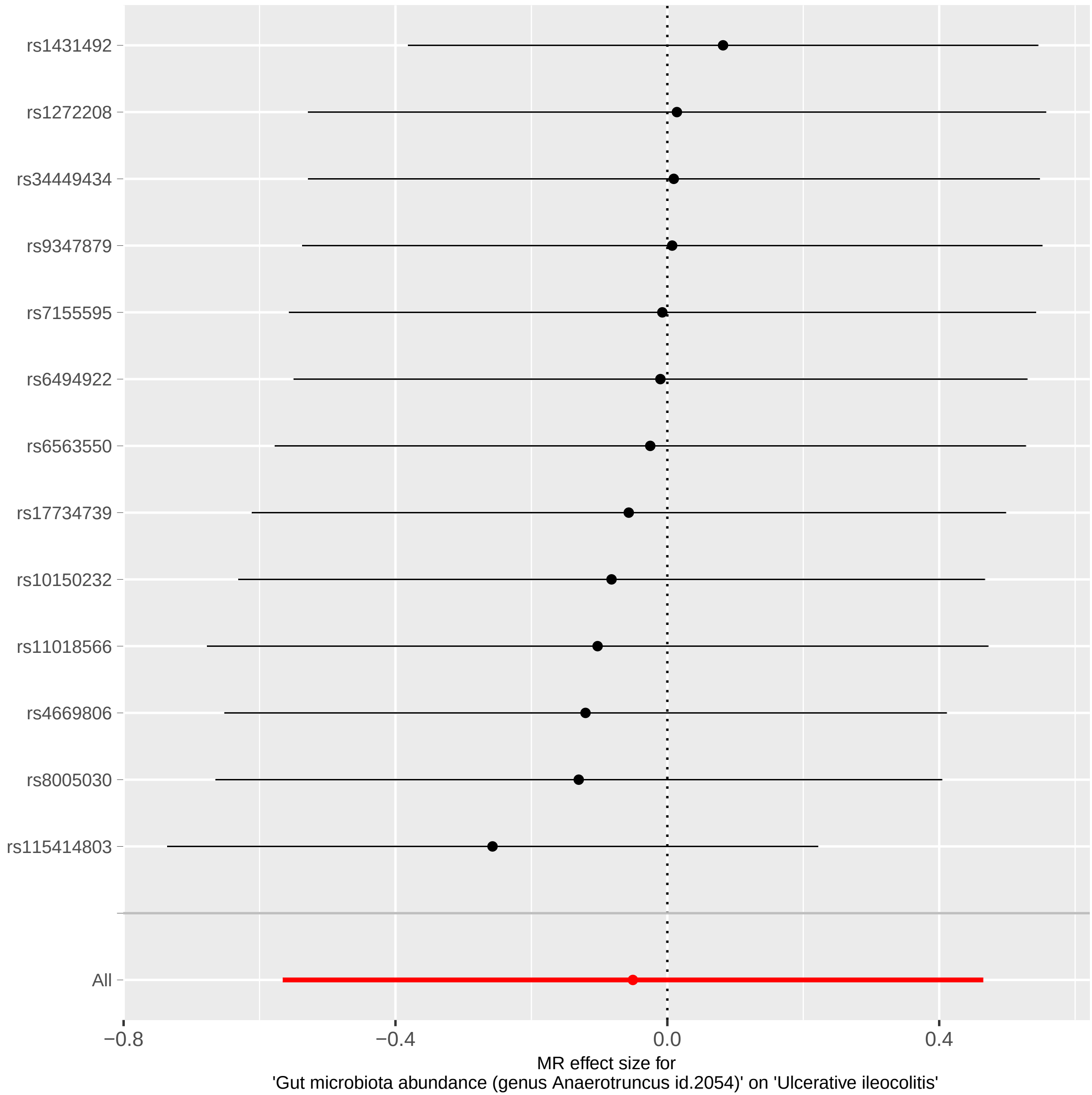

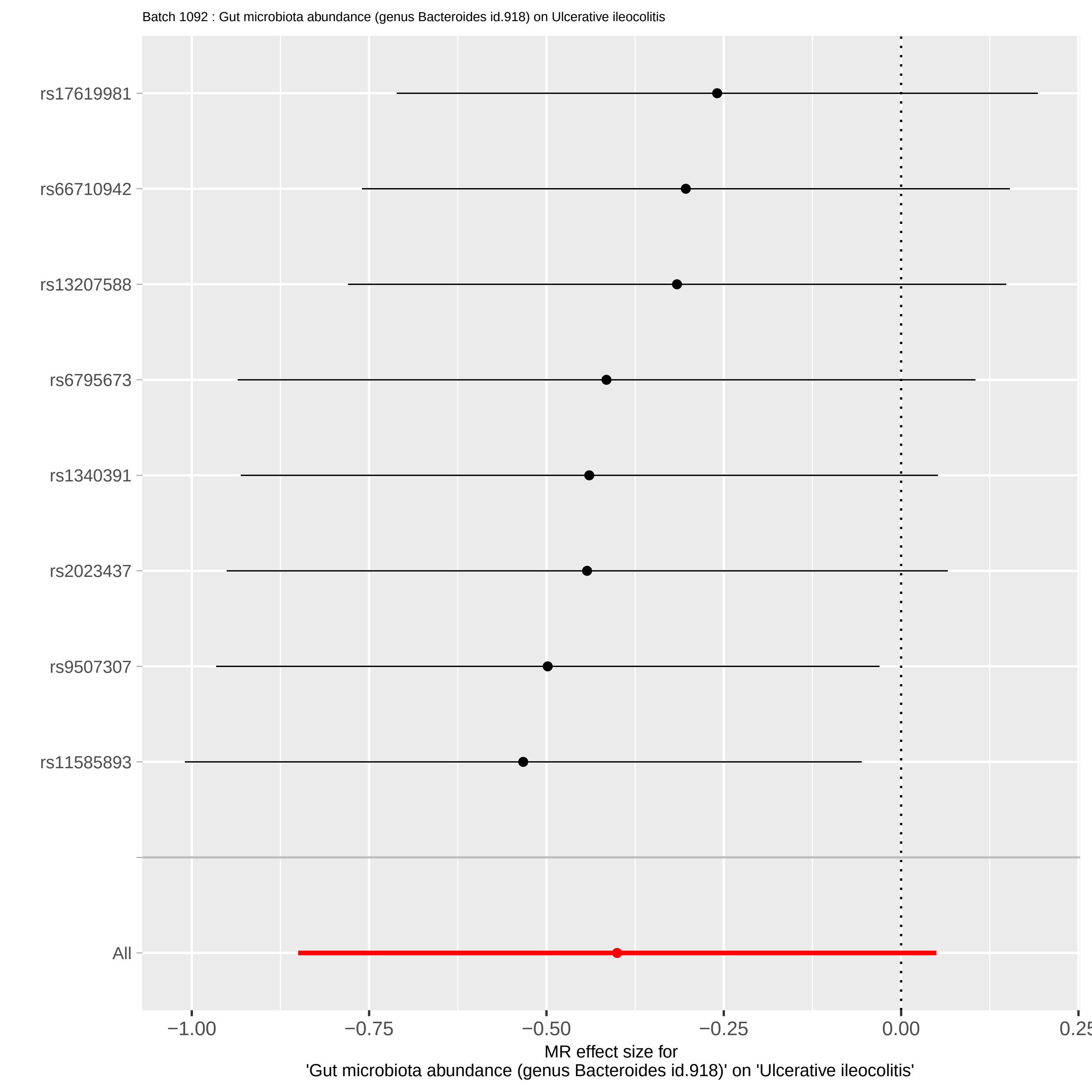

Batch 1092 : Gut microbiota abundance (genus Bacteroides id.918) on Ulcerative ileocolitis
rs17619981
rs66710942
rs13207588
rs6795673
rs1340391
rs2023437
rs9507307
rs11585893
All
−1.00
−0.75
−0.50
−0.25
0.00
0.25
MR effect size for
'Gut microbiota abundance (genus Bacteroides id.918)' on 'Ulcerative ileocolitis'

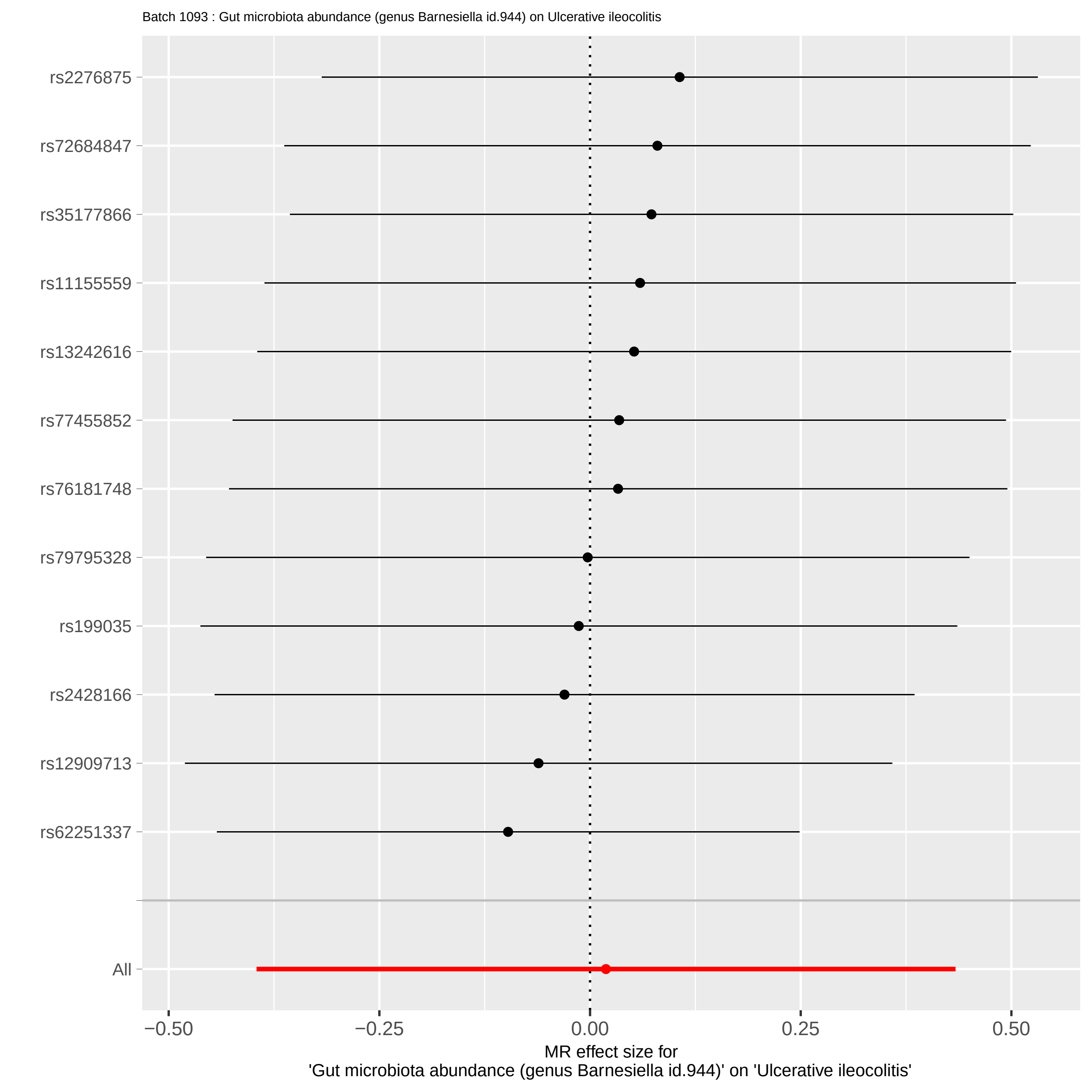

Batch 1093 : Gut microbiota abundance (genus Barnesiella id.944) on Ulcerative ileocolitis
rs2276875
rs72684847
rs35177866
rs11155559
rs13242616
rs77455852
rs76181748
rs79795328
rs199035
rs2428166
rs12909713
rs62251337
All
−0.50
−0.25
0.00
0.25
0.50
MR effect size for
'Gut microbiota abundance (genus Barnesiella id.944)' on 'Ulcerative ileocolitis'

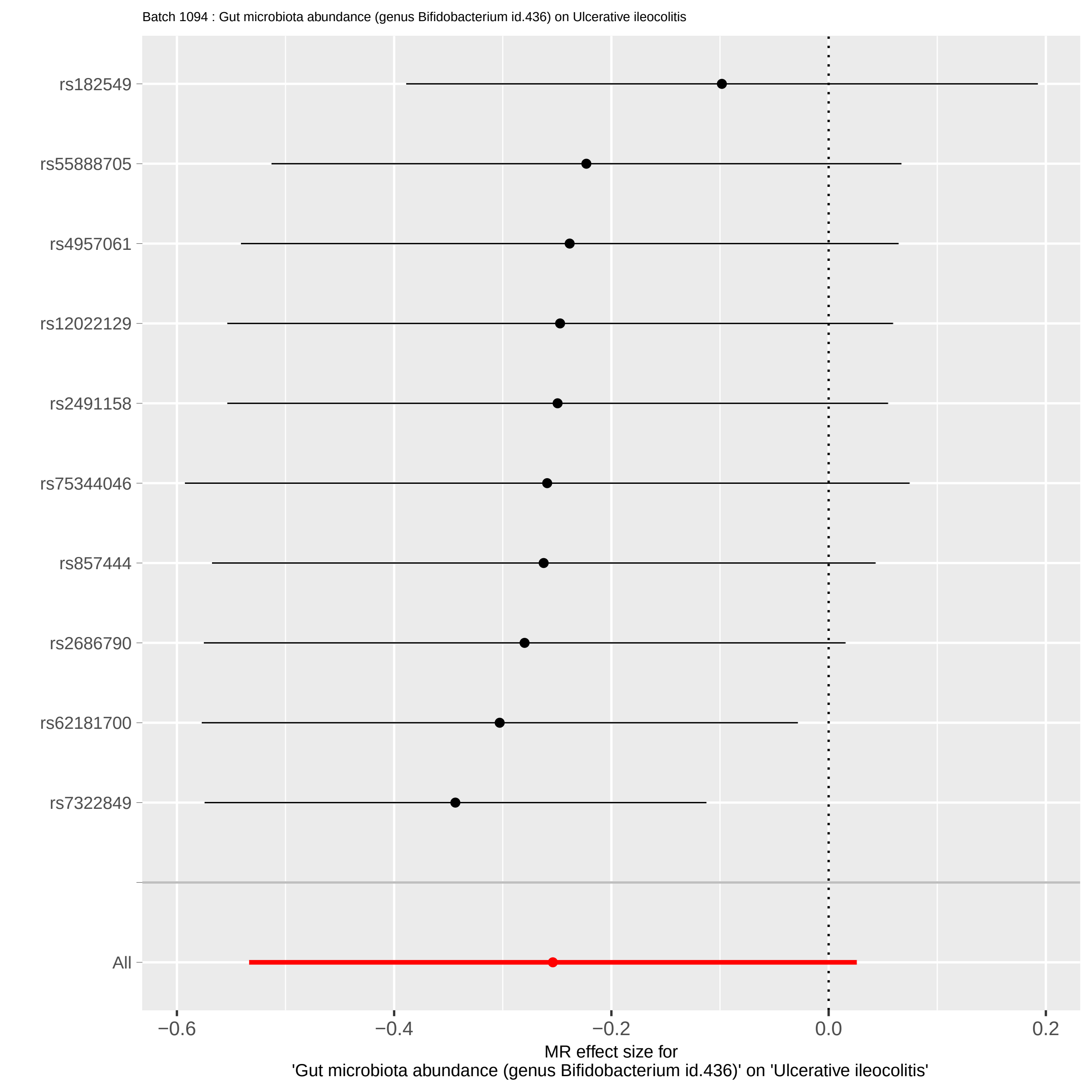

Batch 1094 : Gut microbiota abundance (genus Bifidobacterium id.436) on Ulcerative ileocolitis
rs182549
rs55888705
rs4957061
rs12022129
rs2491158
rs75344046
rs857444
rs2686790
rs62181700
rs7322849
All
−0.6
−0.4
−0.2
0.0
0.2
MR effect size for
'Gut microbiota abundance (genus Bifidobacterium id.436)' on 'Ulcerative ileocolitis'

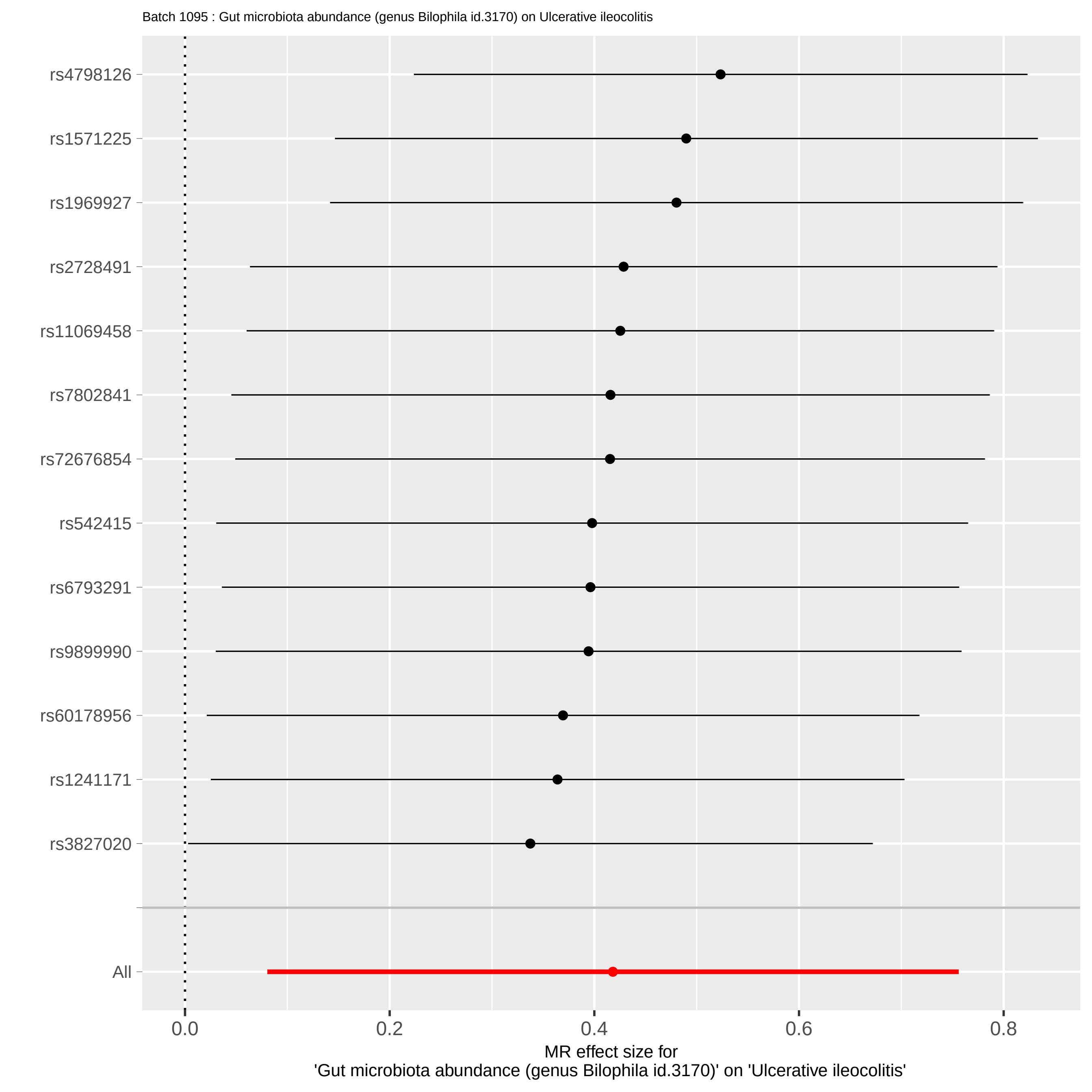
Batch 1095 : Gut microbiota abundance (genus Bilophila id.3170) on Ulcerative ileocolitis
rs4798126
rs1571225
rs1969927
rs2728491
rs11069458
rs7802841
rs72676854
rs542415
rs6793291
rs9899990
rs60178956
rs1241171
rs3827020
All
0.0
0.2
0.4
0.6
0.8
MR effect size for
'Gut microbiota abundance (genus Bilophila id.3170)' on 'Ulcerative ileocolitis'

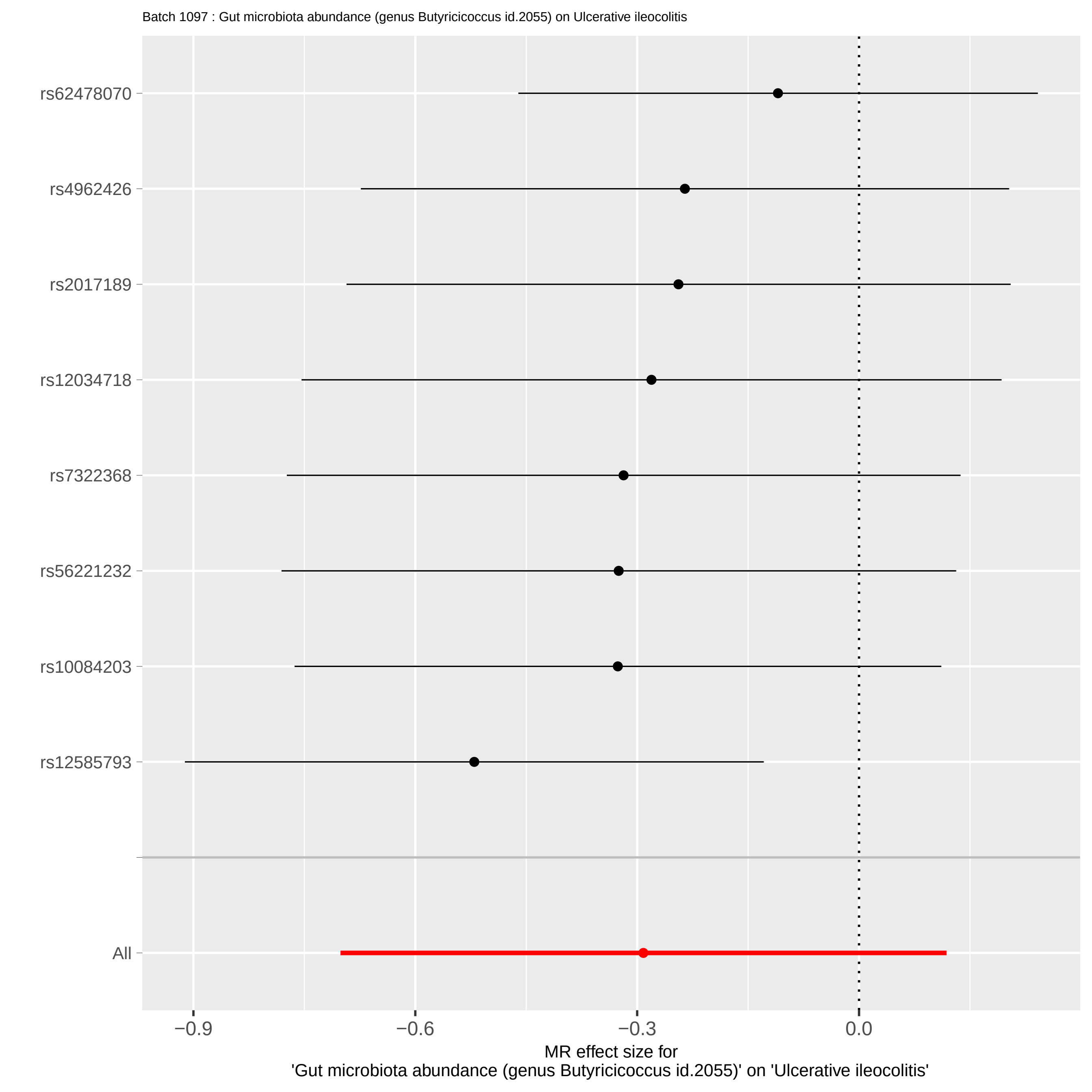

Batch 1097 : Gut microbiota abundance (genus Butyricicoccus id.2055) on Ulcerative ileocolitis
rs62478070
rs4962426
rs2017189
rs12034718
rs7322368
rs56221232
rs10084203
rs12585793
All
−0.9
−0.6
−0.3
0.0
MR effect size for
'Gut microbiota abundance (genus Butyricicoccus id.2055)' on 'Ulcerative ileocolitis'

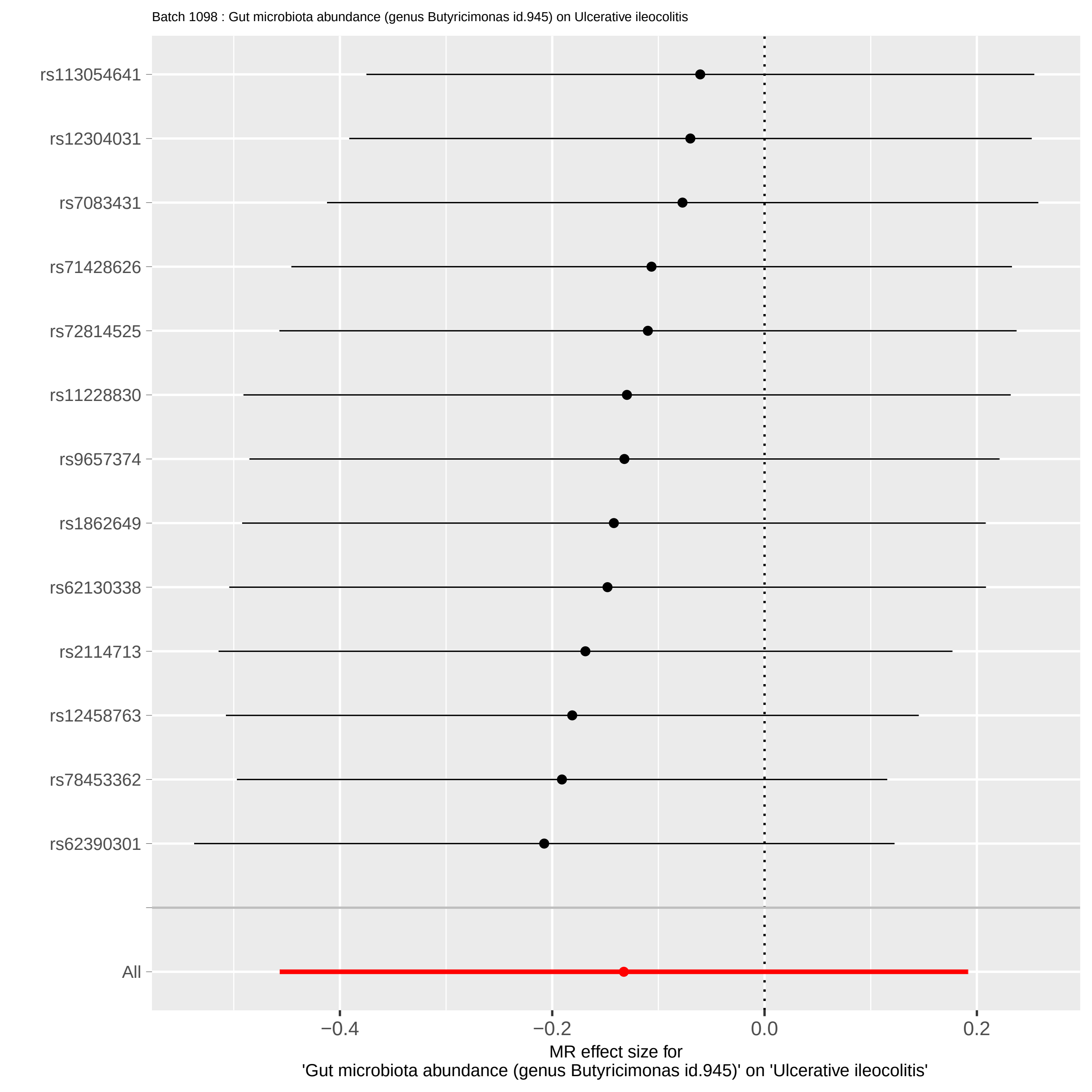

Batch 1098 : Gut microbiota abundance (genus Butyricimonas id.945) on Ulcerative ileocolitis
rs113054641
rs12304031
rs7083431
rs71428626
rs72814525
rs11228830
rs9657374
rs1862649
rs62130338
rs2114713
rs12458763
rs78453362
rs62390301
All
−0.4
−0.2
0.0
0.2
MR effect size for
'Gut microbiota abundance (genus Butyricimonas id.945)' on 'Ulcerative ileocolitis'

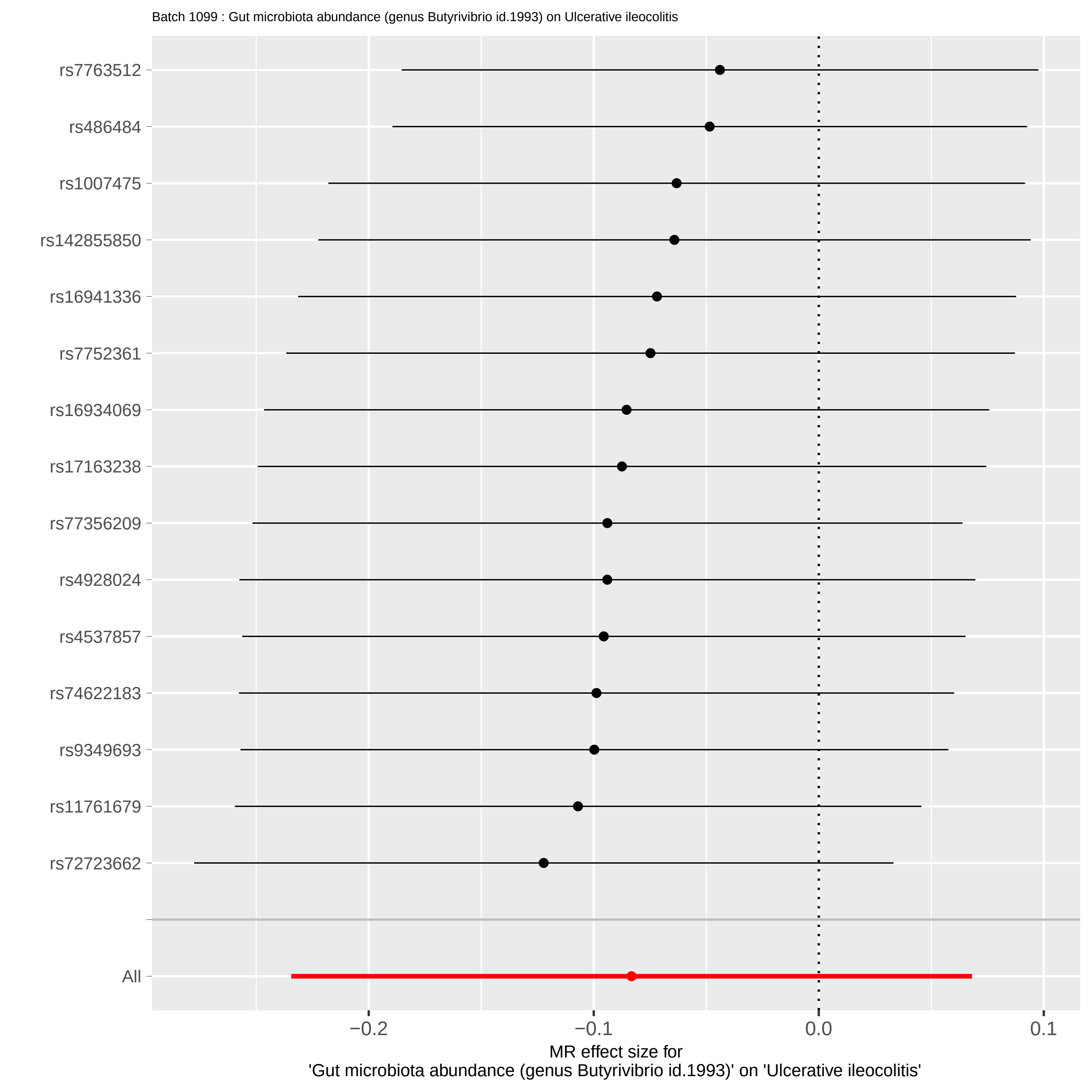
Batch 1099 : Gut microbiota abundance (genus Butyrivibrio id.1993) on Ulcerative ileocolitis
rs7763512
rs486484
rs1007475
rs142855850
rs16941336
rs7752361
rs16934069
rs17163238
rs77356209
rs4928024
rs4537857
rs74622183
rs9349693
rs11761679
rs72723662
All
−0.2
−0.1
0.0
0.1
MR effect size for
'Gut microbiota abundance (genus Butyrivibrio id.1993)' on 'Ulcerative ileocolitis'

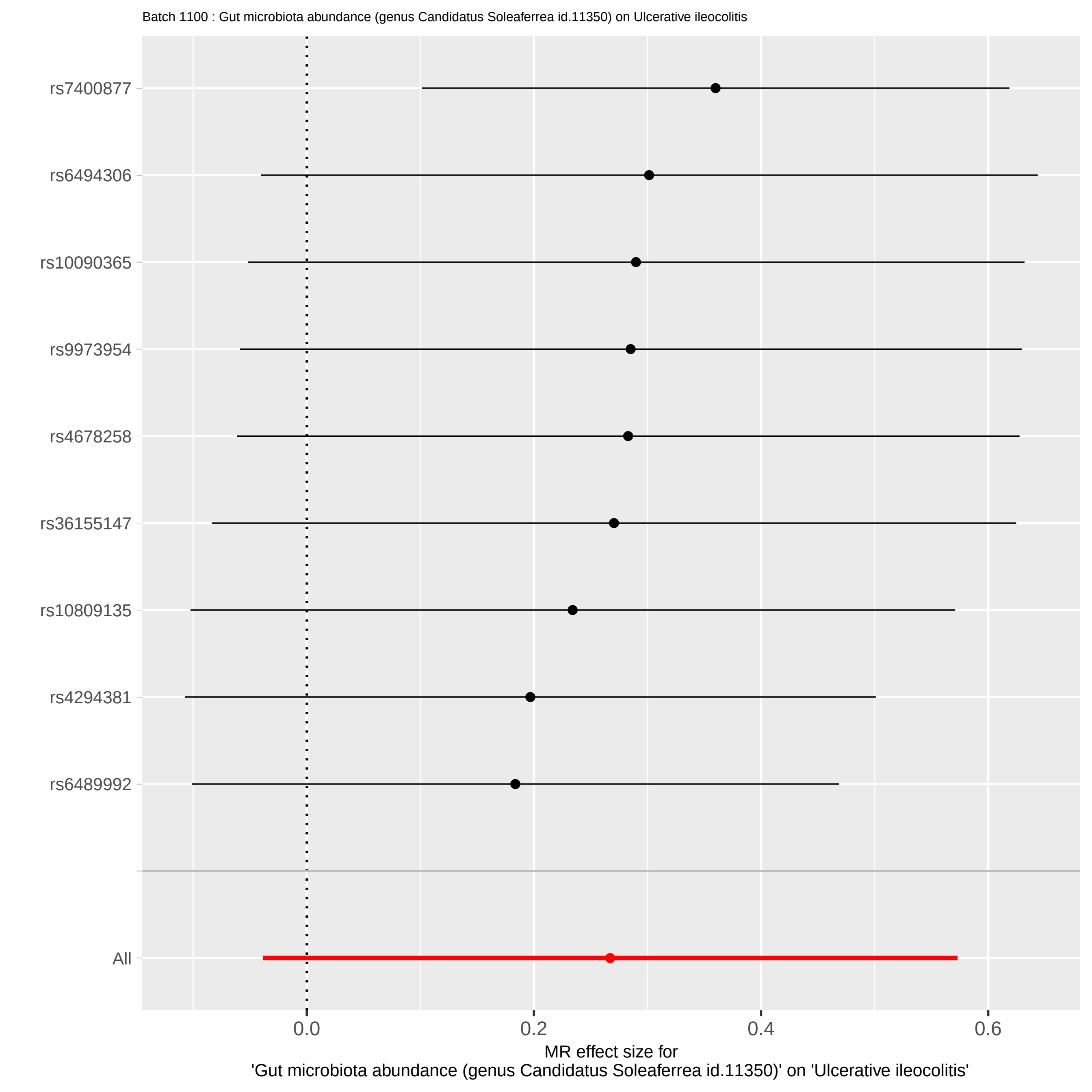
Batch 1100 : Gut microbiota abundance (genus Candidatus Soleaferrea id.11350) on Ulcerative ileocolitis
rs7400877
rs6494306
rs10090365
rs9973954
rs4678258
rs36155147
rs10809135
rs4294381
rs6489992
All
0.0
0.2
0.4
0.6
MR effect size for
'Gut microbiota abundance (genus Candidatus Soleaferrea id.11350)' on 'Ulcerative ileocolitis'

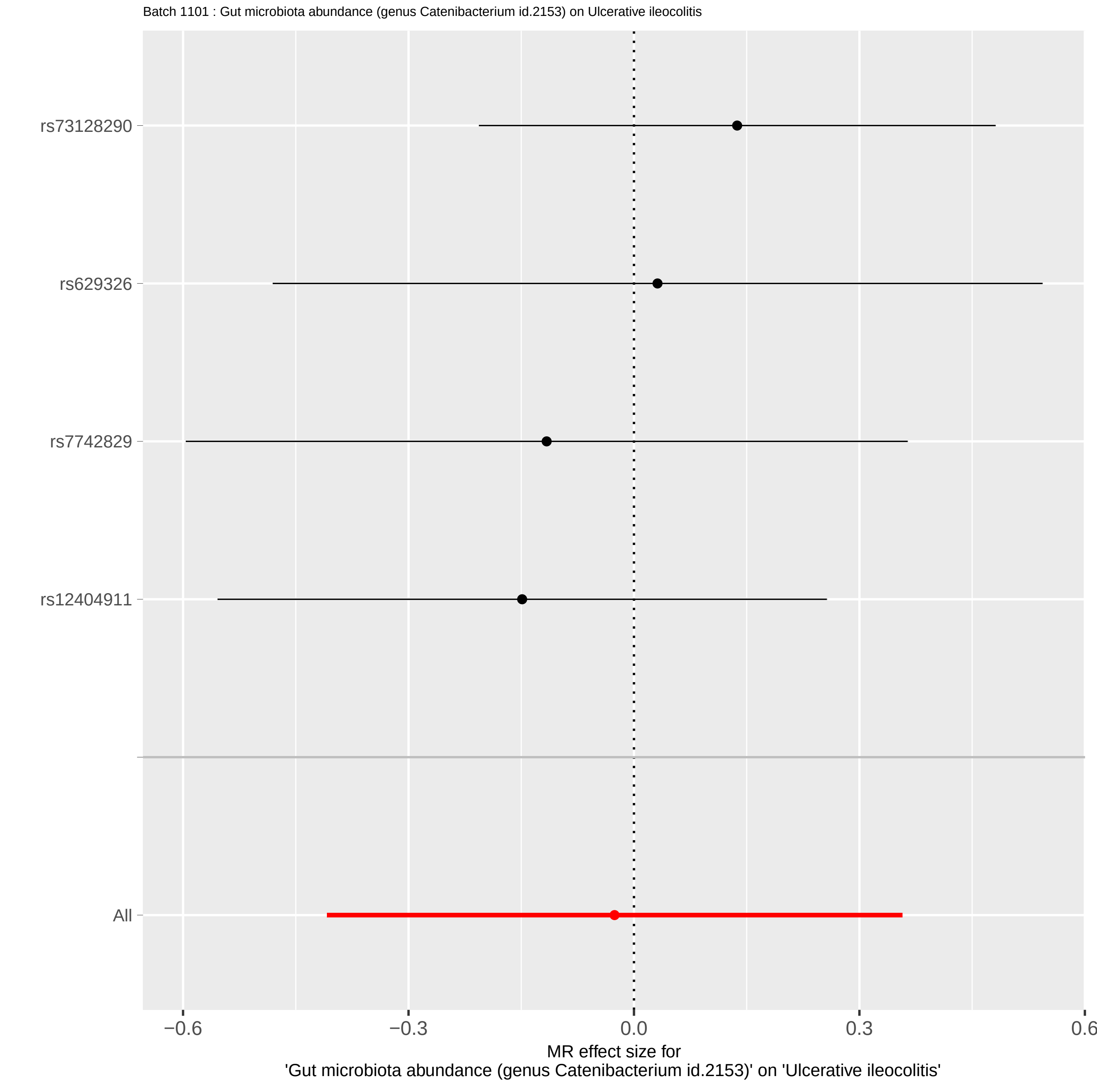

Batch 1101 : Gut microbiota abundance (genus Catenibacterium id.2153) on Ulcerative ileocolitis
rs73128290
rs629326
rs7742829
rs12404911
All
−0.6
−0.3
0.0
0.3
0.6
MR effect size for
'Gut microbiota abundance (genus Catenibacterium id.2153)' on 'Ulcerative ileocolitis'

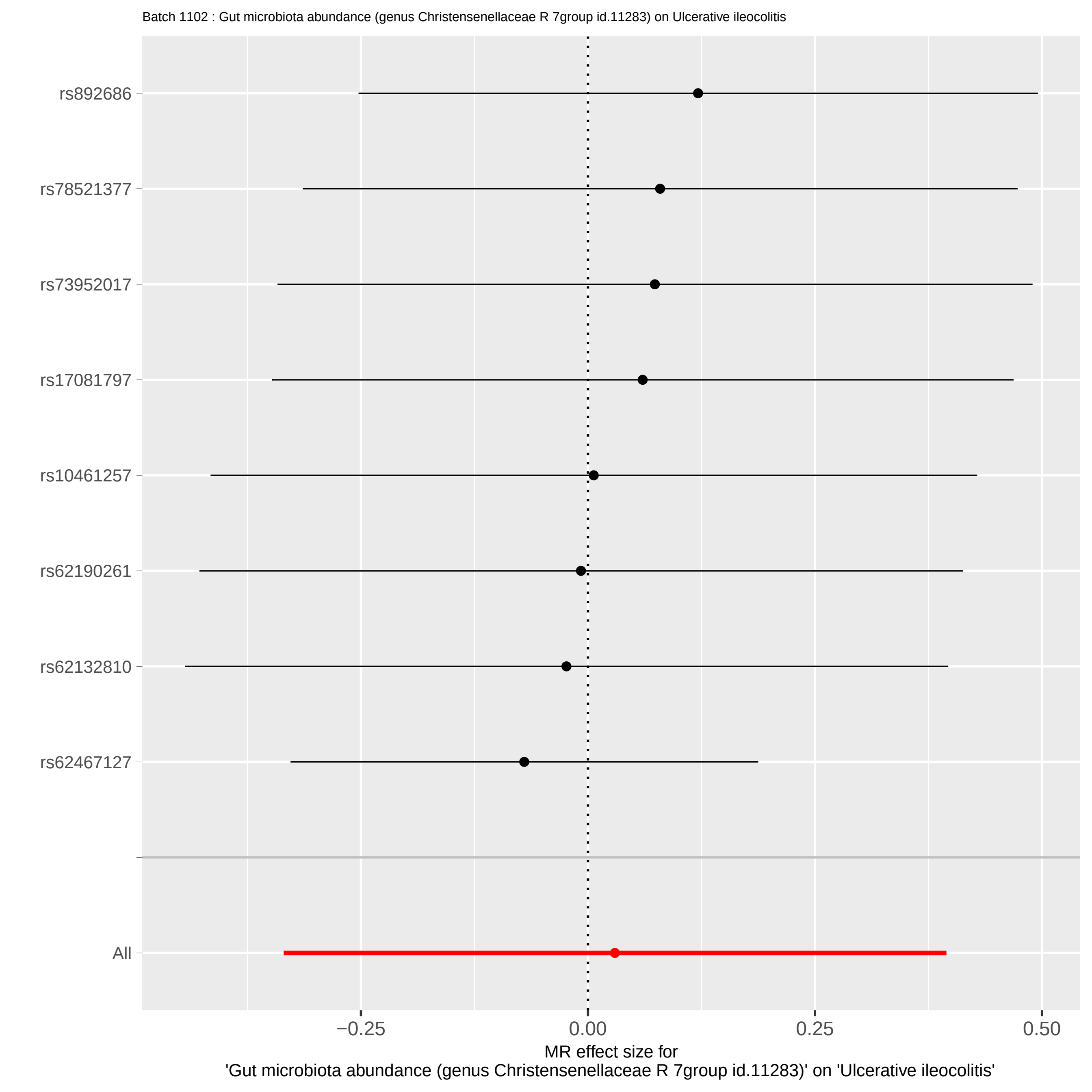

Batch 1102 : Gut microbiota abundance (genus Christensenellaceae R 7group id.11283) on Ulcerative ileocolitis
rs892686
rs78521377
rs73952017
rs17081797
rs10461257
rs62190261
rs62132810
rs62467127
All
−0.25
0.00
0.25
0.50
MR effect size for
'Gut microbiota abundance (genus Christensenellaceae R 7group id.11283)' on 'Ulcerative ileocolitis'

Batch 1103 : Gut microbiota abundance (genus Clostridium innocuum group id.14397) on Ulcerative ileocolitis

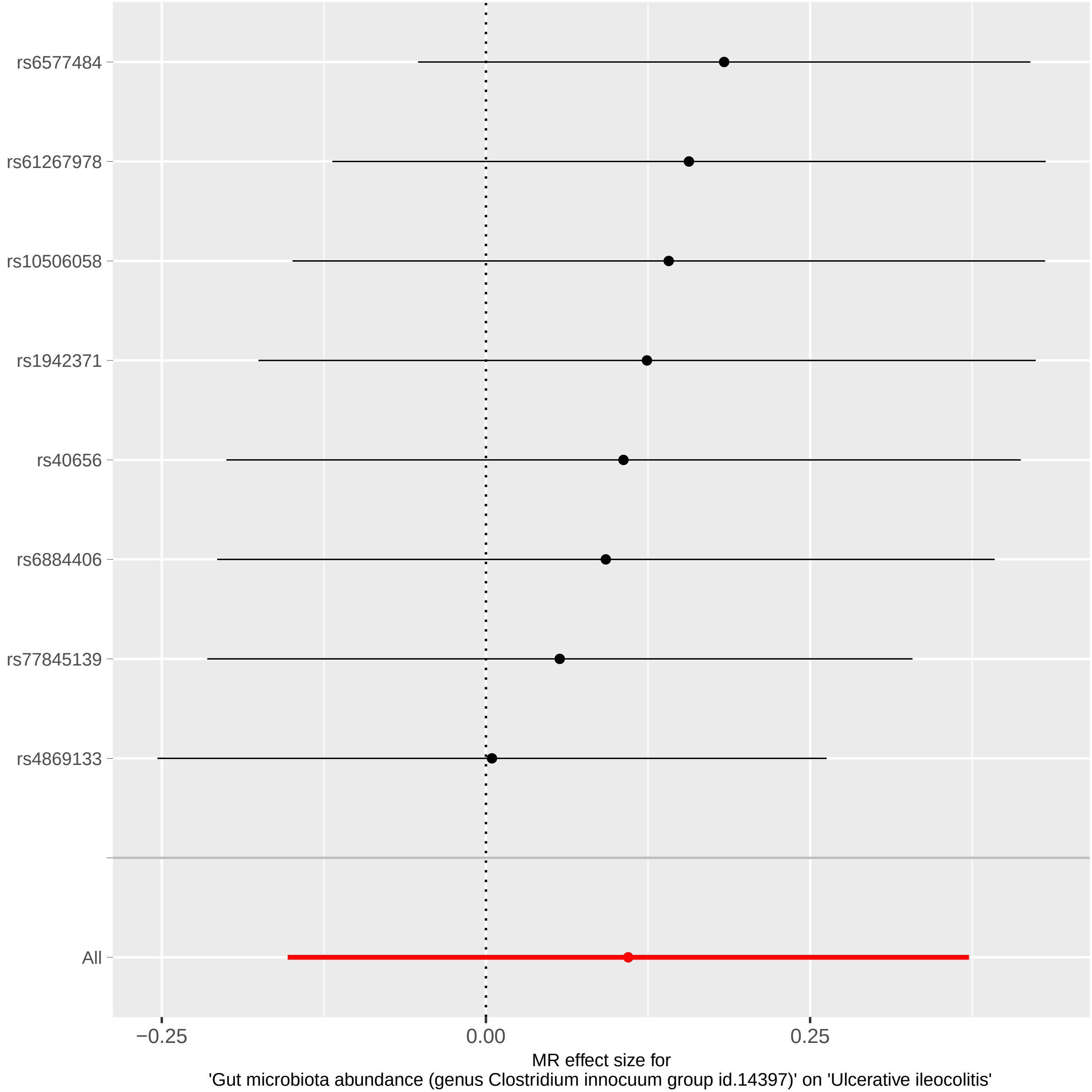

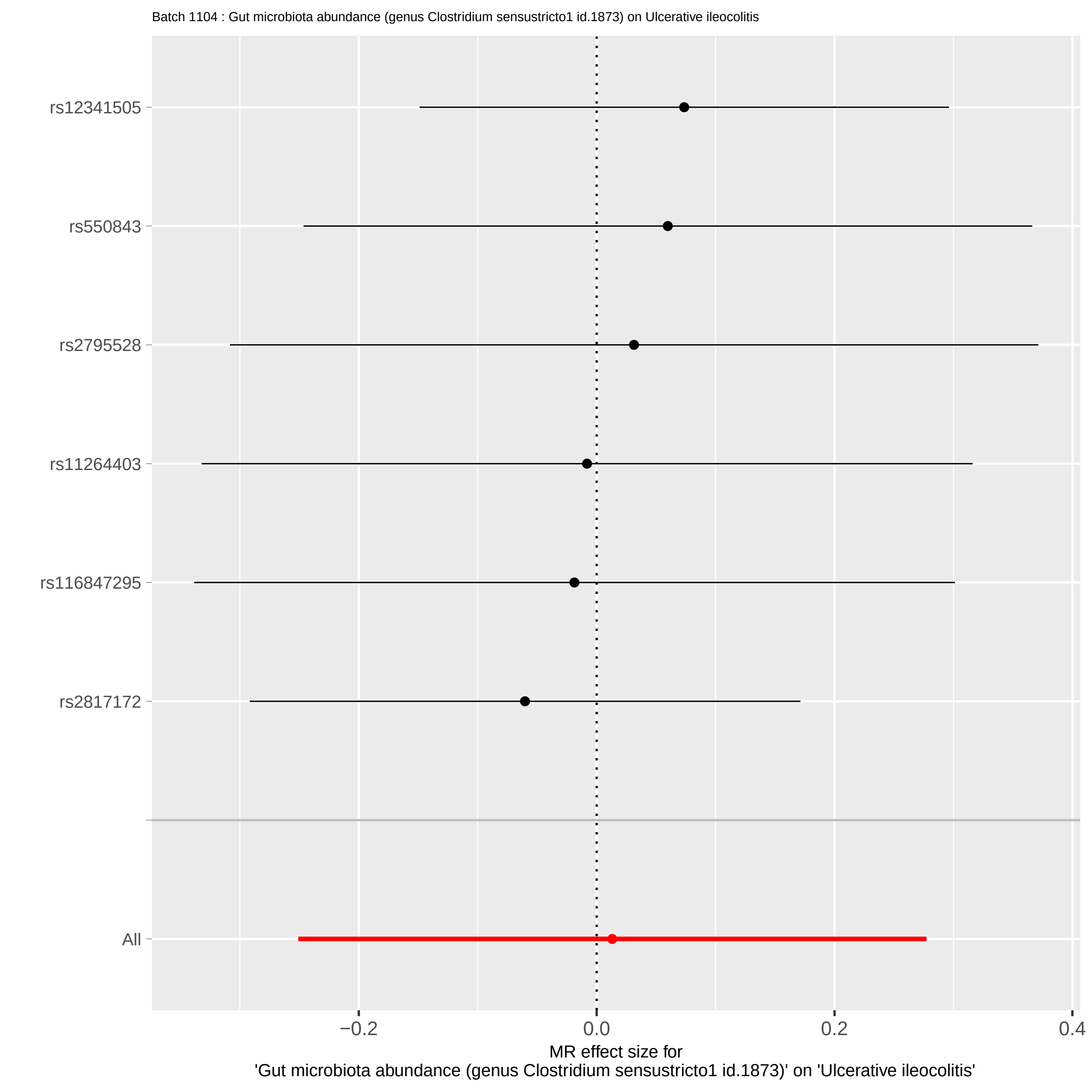

Batch 1104 : Gut microbiota abundance (genus Clostridium sensustricto1 id.1873) on Ulcerative ileocolitis
rs12341505
rs550843
rs2795528
rs11264403
rs116847295
rs2817172
All
−0.2
0.0
0.2
0.4
MR effect size for
'Gut microbiota abundance (genus Clostridium sensustricto1 id.1873)' on 'Ulcerative ileocolitis'

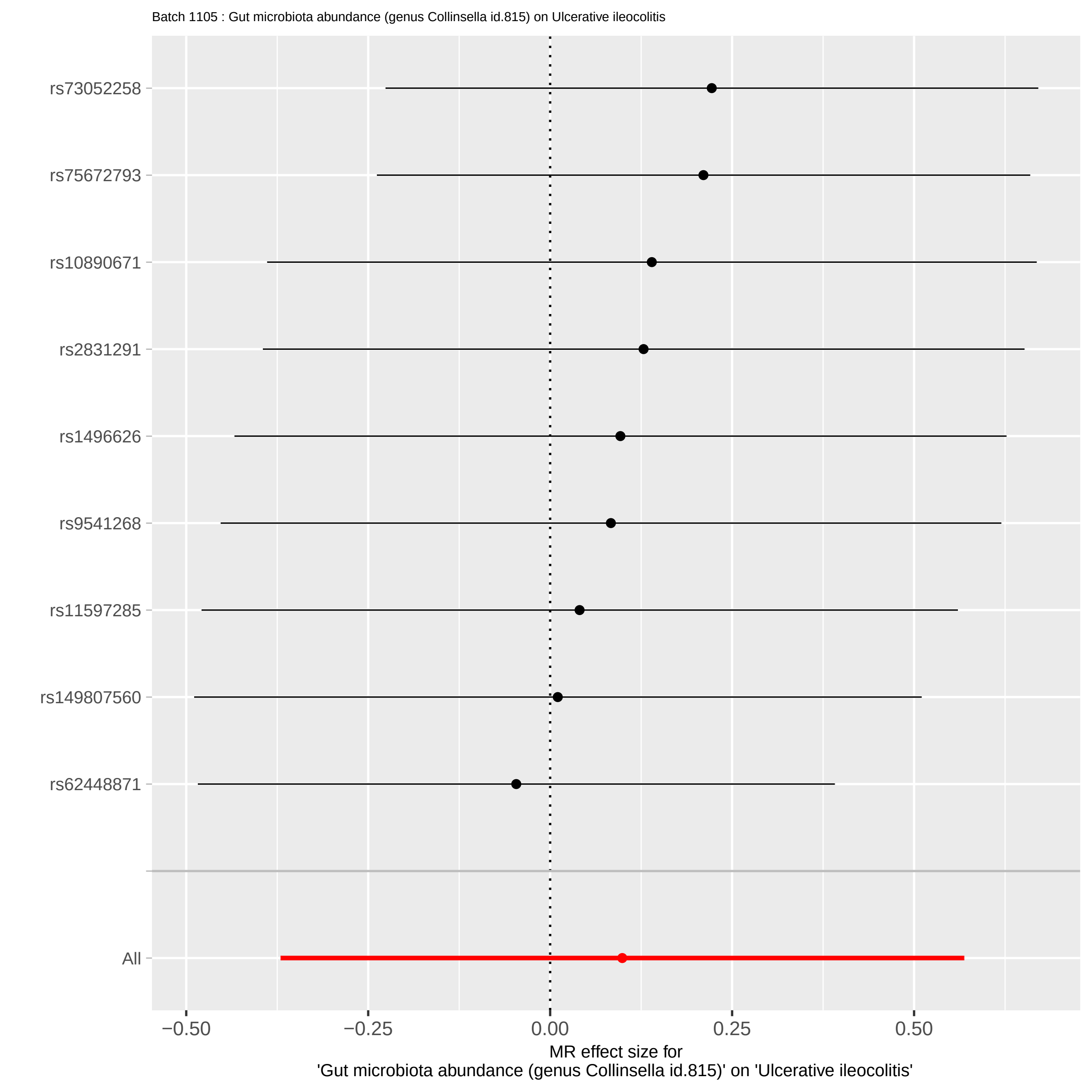

Batch 1105 : Gut microbiota abundance (genus Collinsella id.815) on Ulcerative ileocolitis
rs73052258
rs75672793
rs10890671
rs2831291
rs1496626
rs9541268
rs11597285
rs149807560
rs62448871
All
−0.50
−0.25
0.00
0.25
0.50
MR effect size for
'Gut microbiota abundance (genus Collinsella id.815)' on 'Ulcerative ileocolitis'

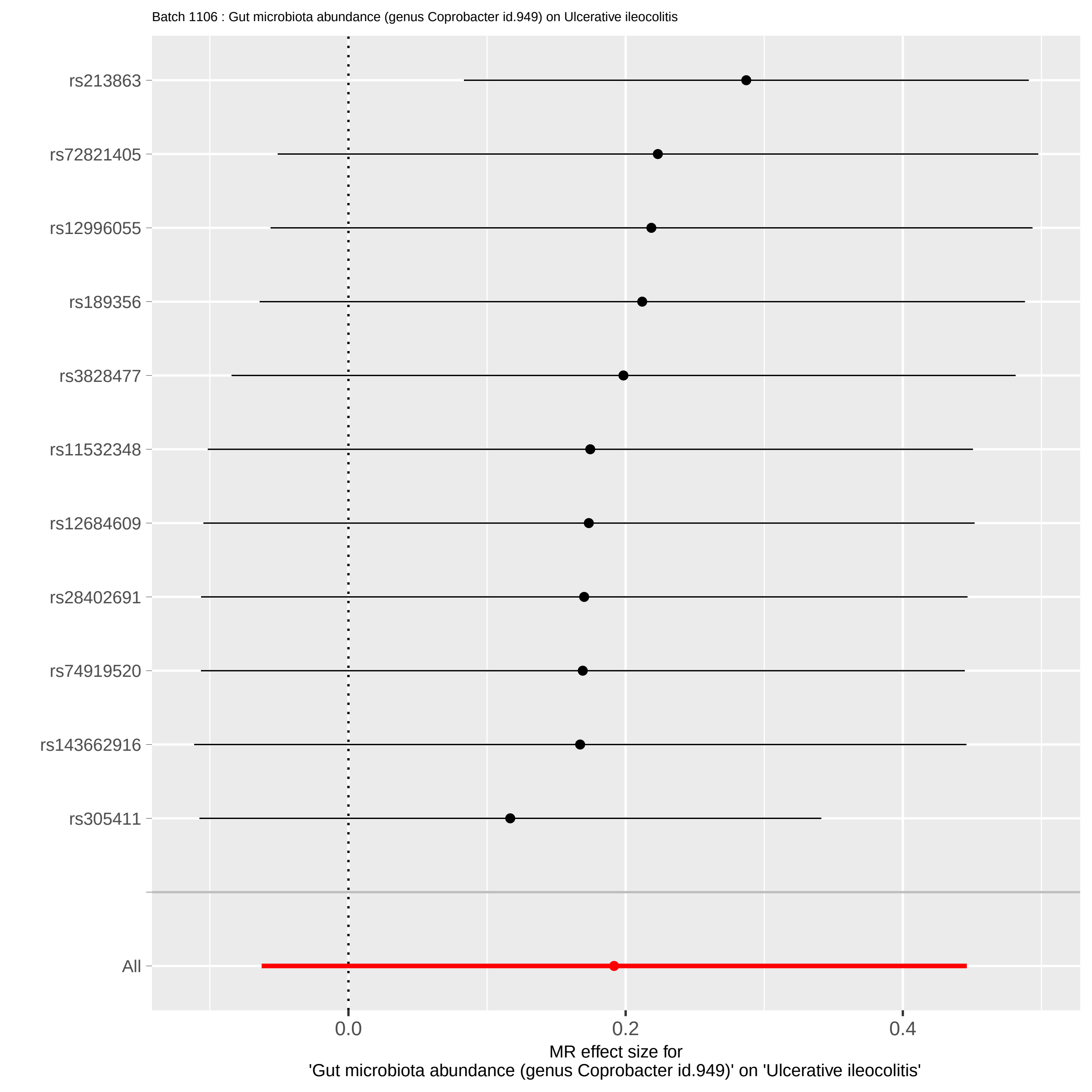

Batch 1106 : Gut microbiota abundance (genus Coprobacter id.949) on Ulcerative ileocolitis
rs213863
rs72821405
rs12996055
rs189356
rs3828477
rs11532348
rs12684609
rs28402691
rs74919520
rs143662916
rs305411
All
0.0
0.2
0.4
MR effect size for
'Gut microbiota abundance (genus Coprobacter id.949)' on 'Ulcerative ileocolitis'

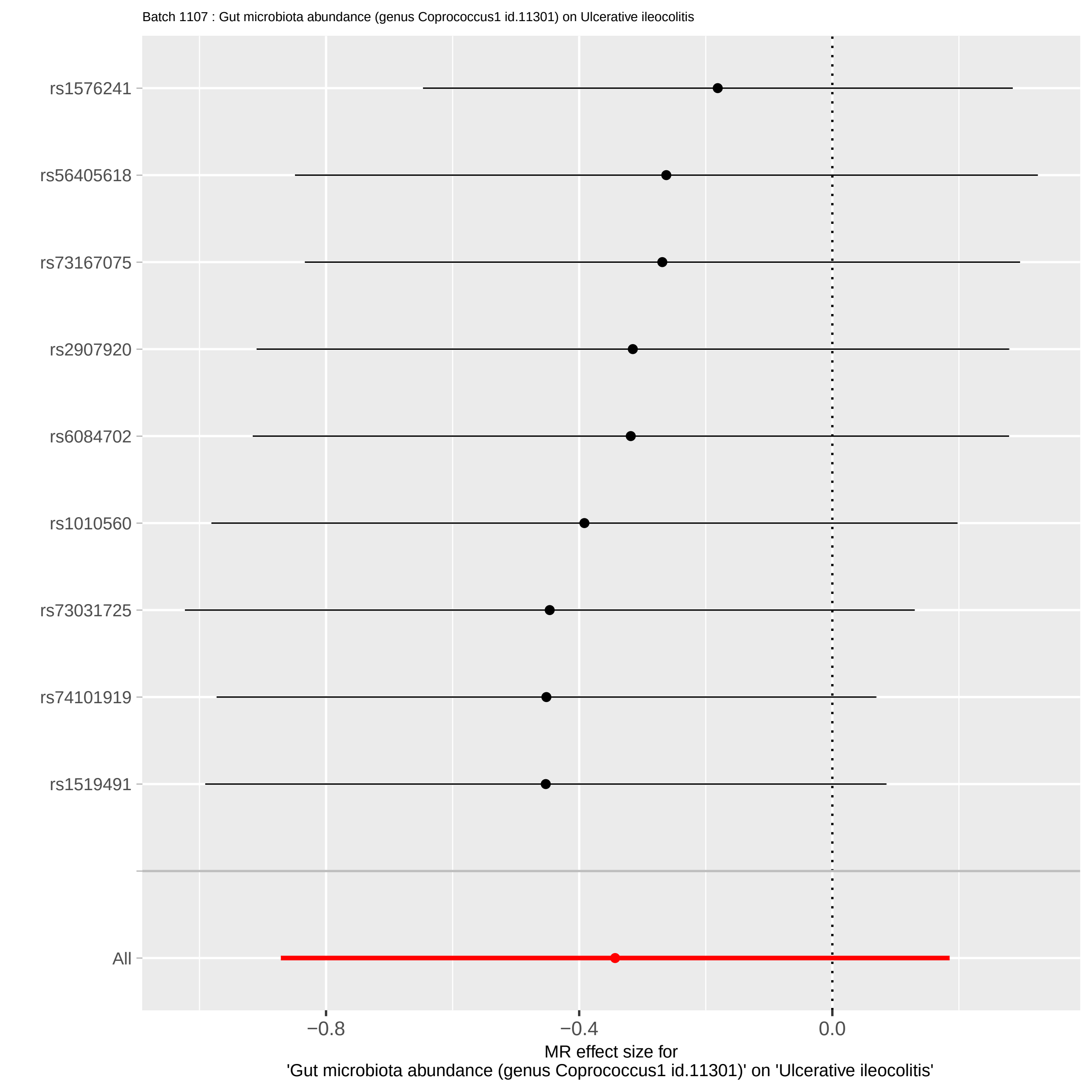
Batch 1107 : Gut microbiota abundance (genus Coprococcus1 id.11301) on Ulcerative ileocolitis
rs1576241
rs56405618
rs73167075
rs2907920
rs6084702
rs1010560
rs73031725
rs74101919
rs1519491
All
−0.8
−0.4
0.0
MR effect size for
'Gut microbiota abundance (genus Coprococcus1 id.11301)' on 'Ulcerative ileocolitis'

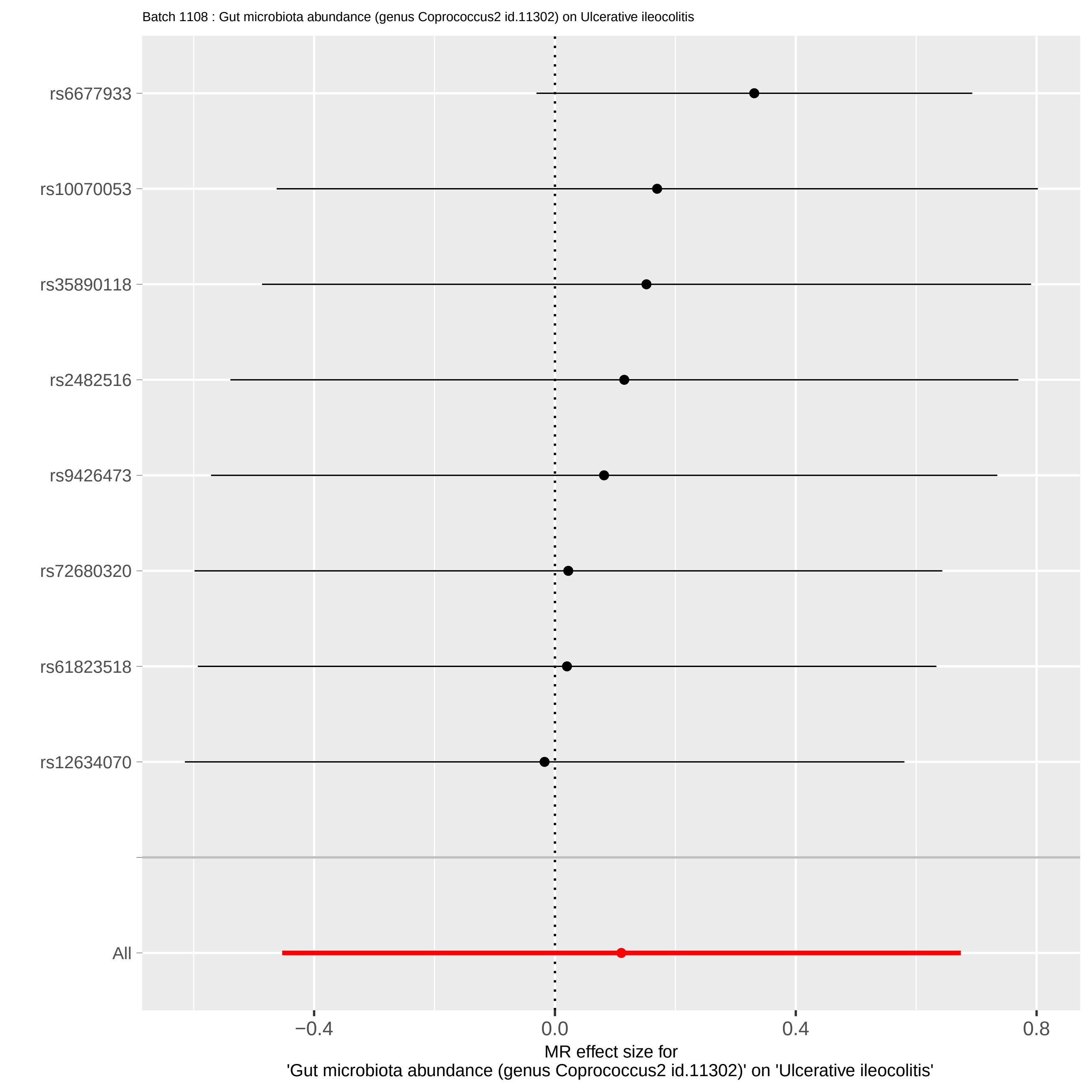

Batch 1108 : Gut microbiota abundance (genus Coprococcus2 id.11302) on Ulcerative ileocolitis
rs6677933
rs10070053
rs35890118
rs2482516
rs9426473
rs72680320
rs61823518
rs12634070
All
−0.4
0.0
0.4
0.8
MR effect size for
'Gut microbiota abundance (genus Coprococcus2 id.11302)' on 'Ulcerative ileocolitis'

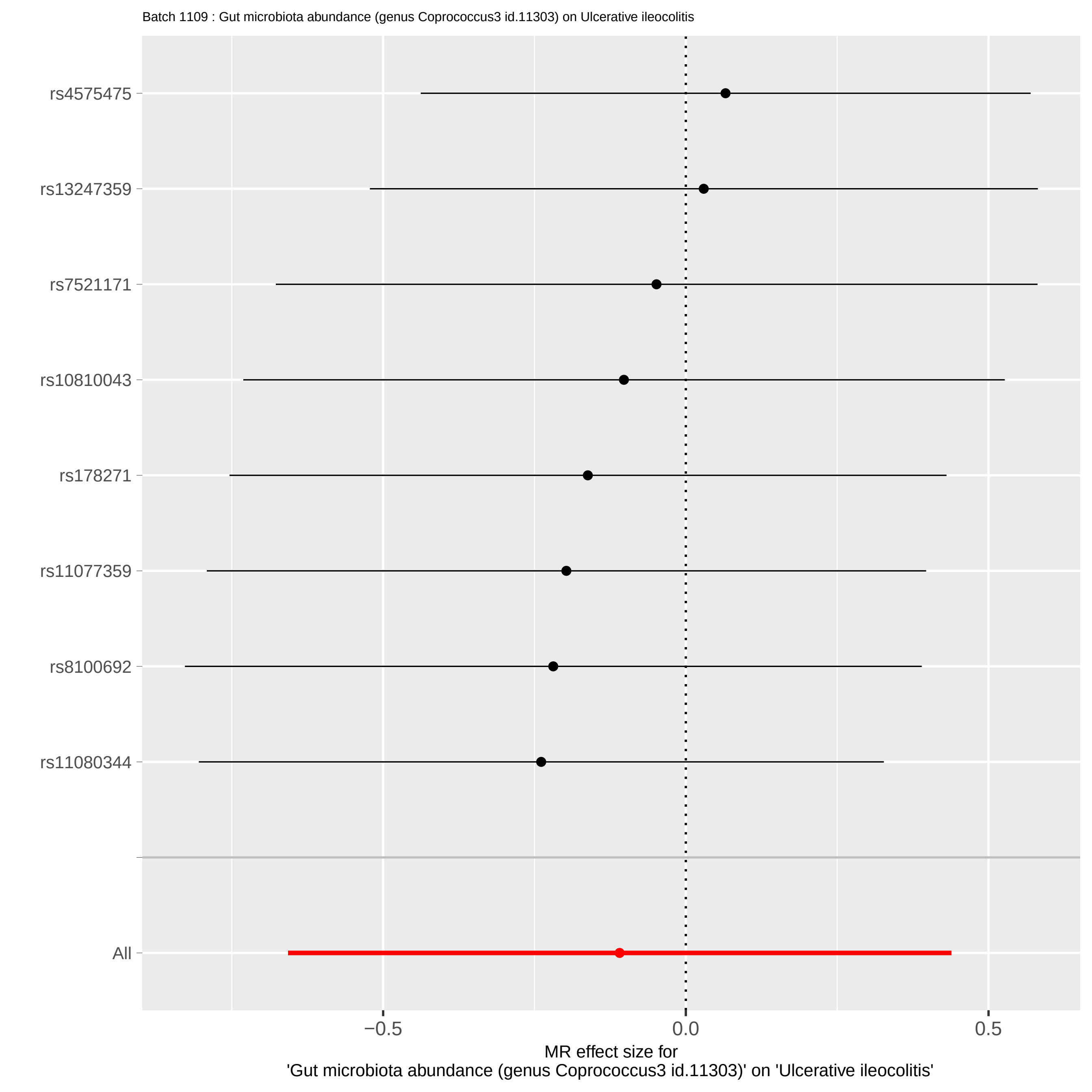

Batch 1109 : Gut microbiota abundance (genus Coprococcus3 id.11303) on Ulcerative ileocolitis
rs4575475
rs13247359
rs7521171
rs10810043
rs178271
rs11077359
rs8100692
rs11080344
All
−0.5
0.0
0.5
MR effect size for
'Gut microbiota abundance (genus Coprococcus3 id.11303)' on 'Ulcerative ileocolitis'

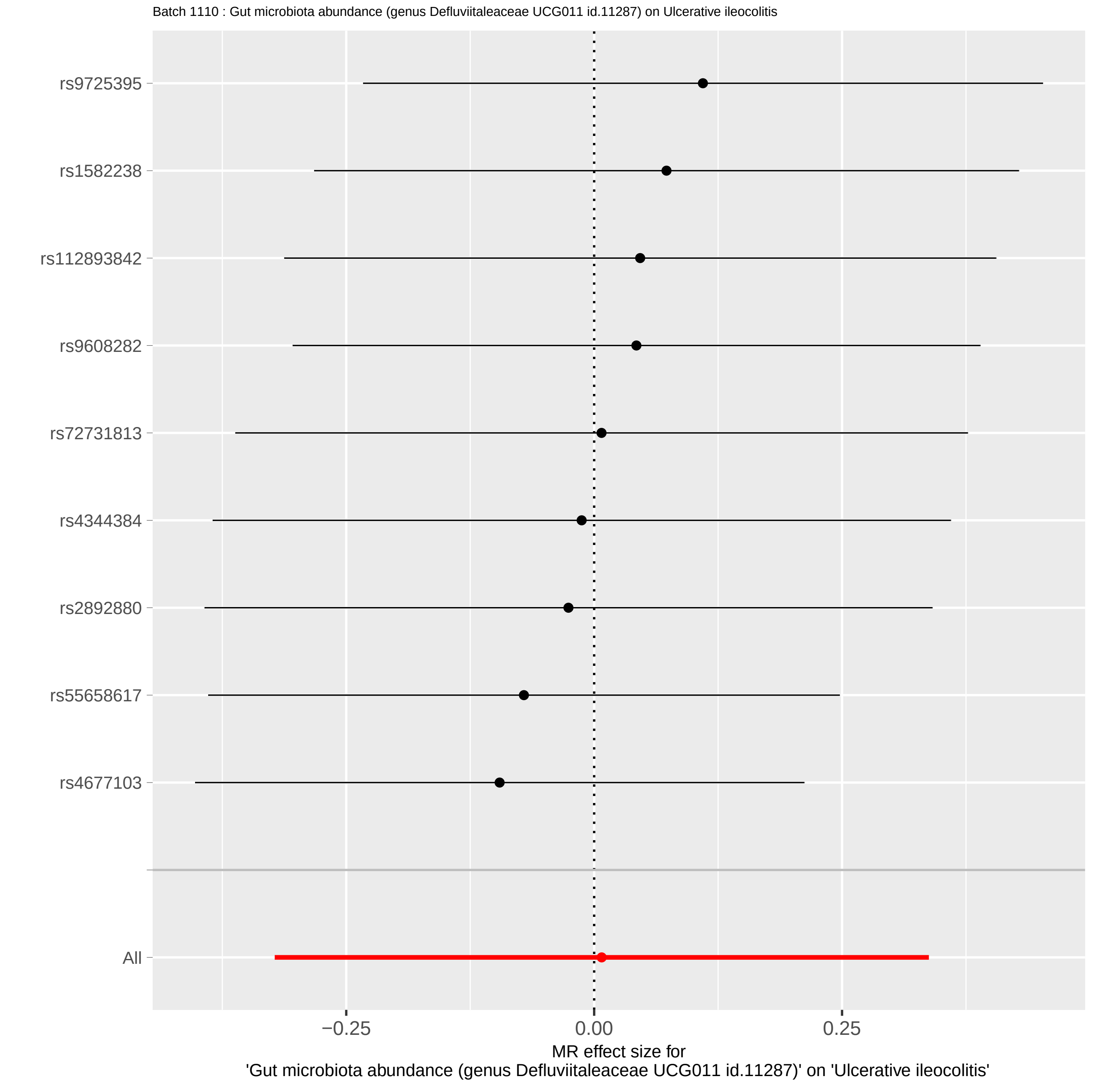

Batch 1110 : Gut microbiota abundance (genus Defluviitaleaceae UCG011 id.11287) on Ulcerative ileocolitis
rs9725395
rs1582238
rs112893842
rs9608282
rs72731813
rs4344384
rs2892880
rs55658617
rs4677103
All
−0.25
0.00
0.25
MR effect size for
'Gut microbiota abundance (genus Defluviitaleaceae UCG011 id.11287)' on 'Ulcerative ileocolitis'

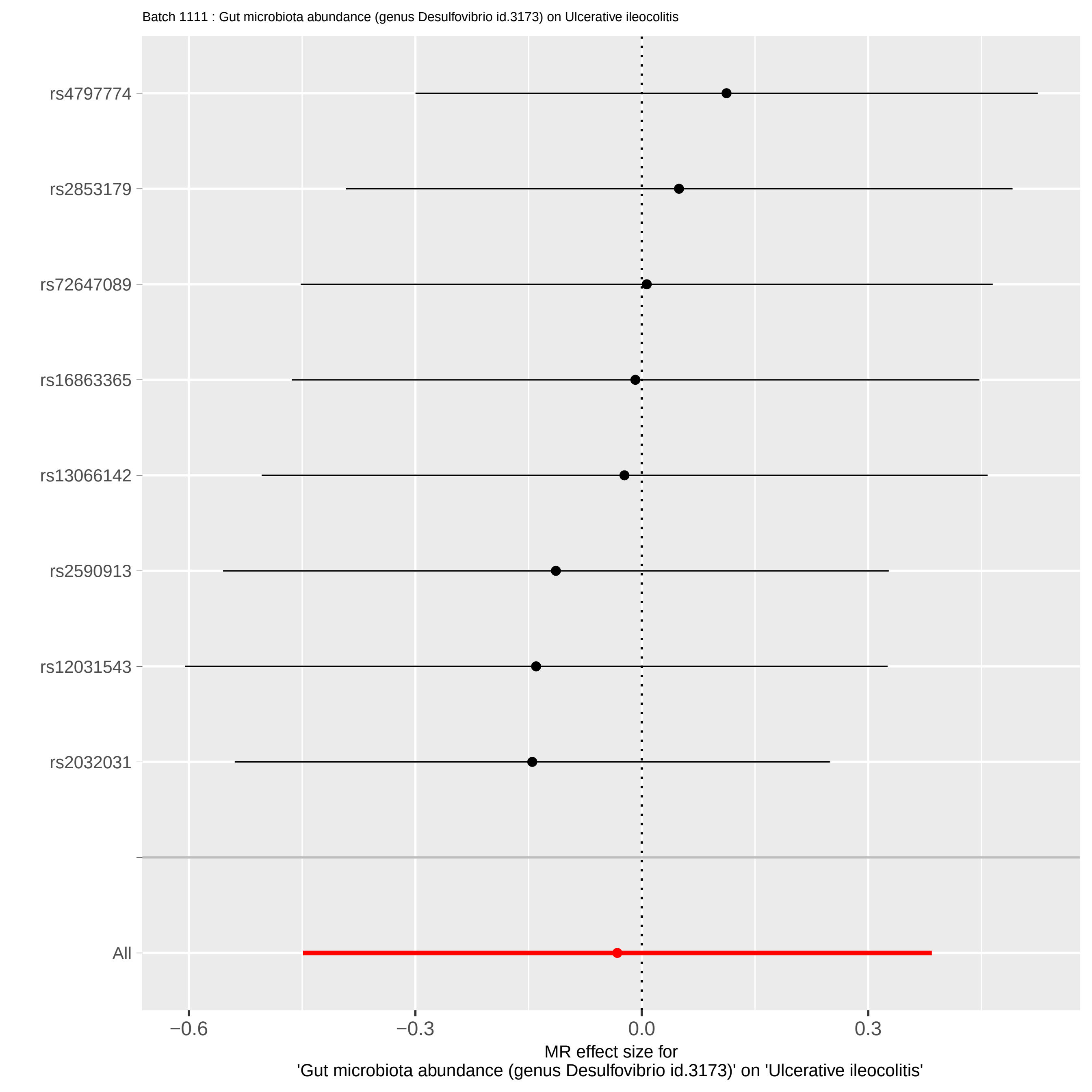

Batch 1111 : Gut microbiota abundance (genus Desulfovibrio id.3173) on Ulcerative ileocolitis
rs4797774
rs2853179
rs72647089
rs16863365
rs13066142
rs2590913
rs12031543
rs2032031
All
−0.6
−0.3
0.0
0.3
MR effect size for
'Gut microbiota abundance (genus Desulfovibrio id.3173)' on 'Ulcerative ileocolitis'

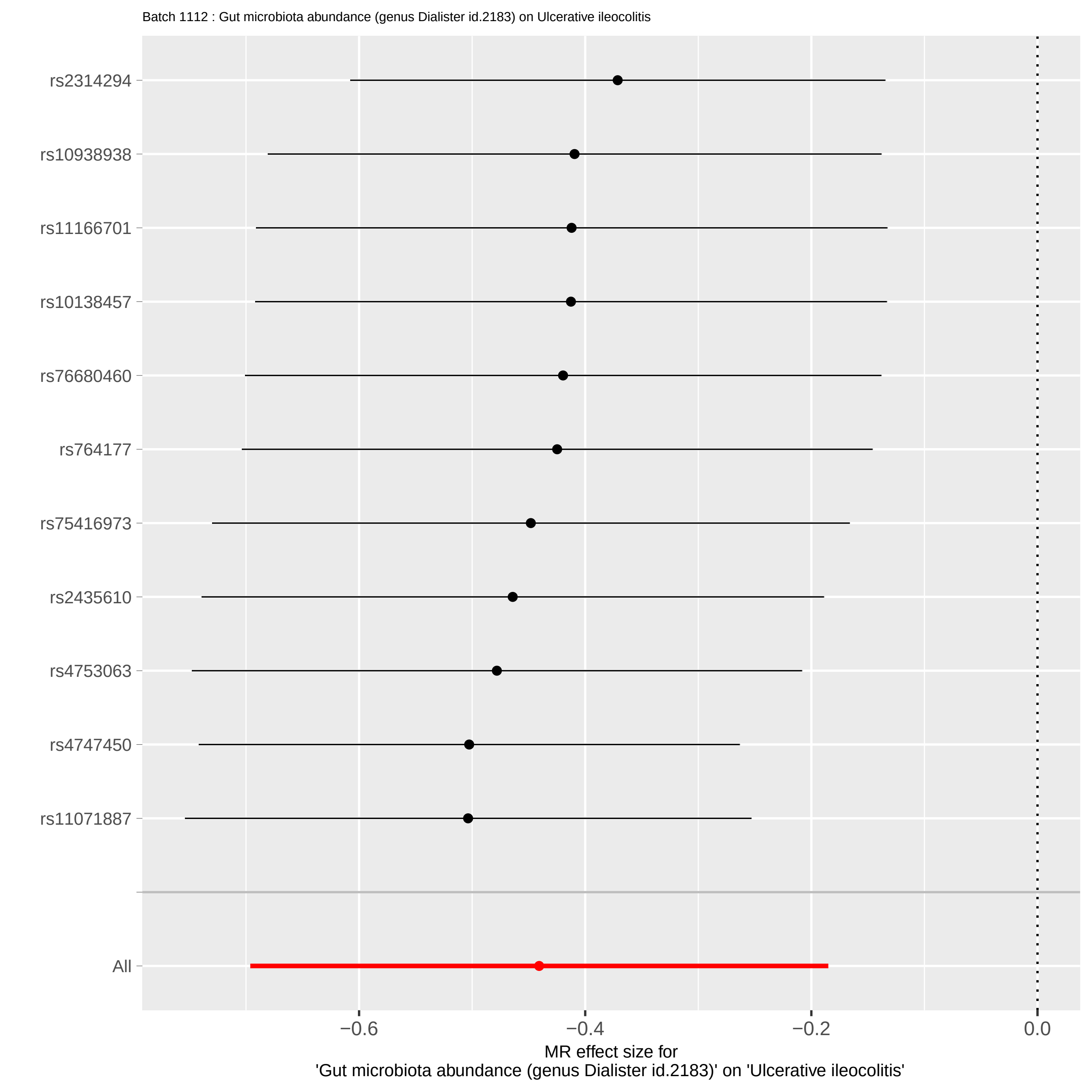
Batch 1112 : Gut microbiota abundance (genus Dialister id.2183) on Ulcerative ileocolitis
rs2314294
rs10938938
rs11166701
rs10138457
rs76680460
rs764177
rs75416973
rs2435610
rs4753063
rs4747450
rs11071887
All
−0.6
−0.4
−0.2
0.0
MR effect size for
'Gut microbiota abundance (genus Dialister id.2183)' on 'Ulcerative ileocolitis'

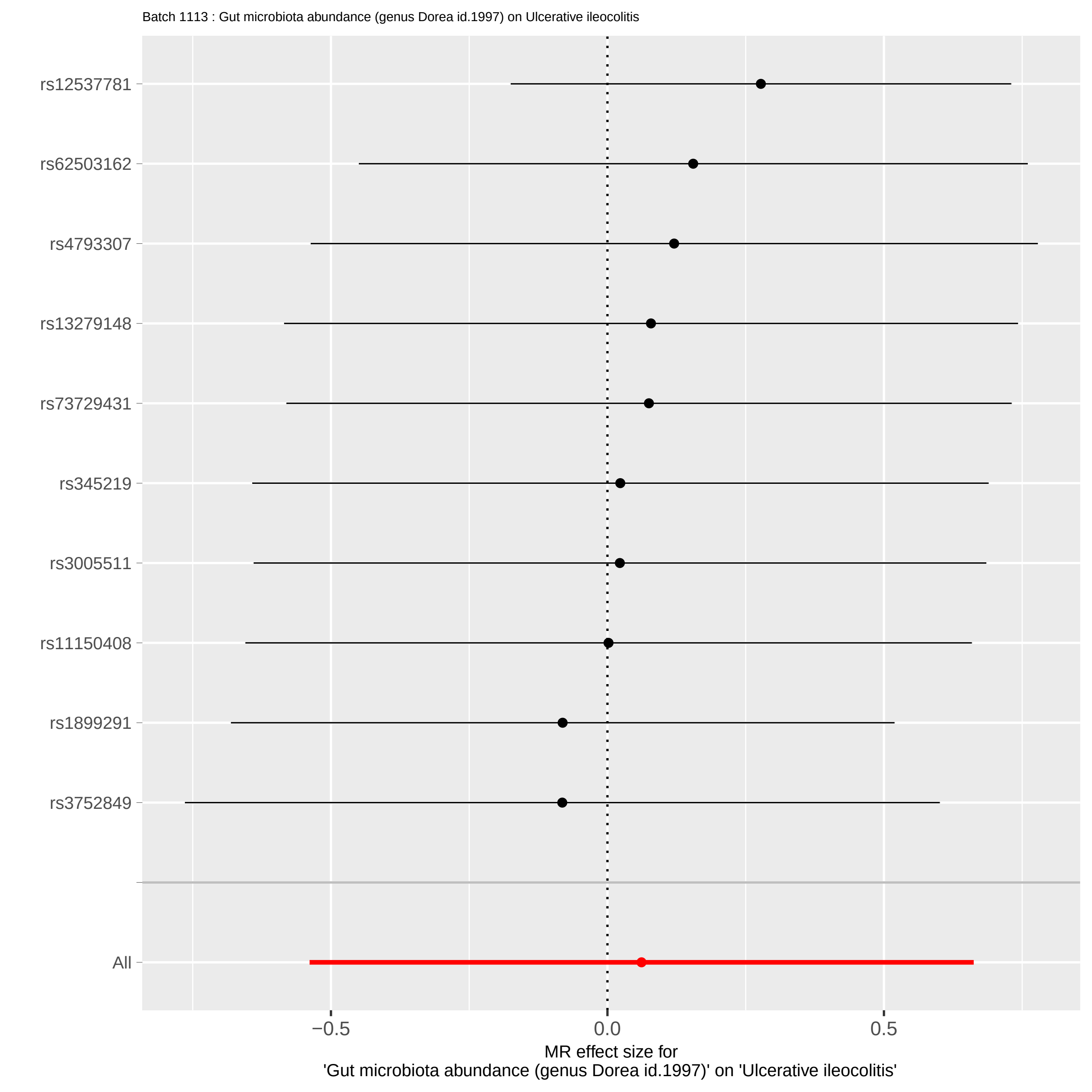

Batch 1113 : Gut microbiota abundance (genus Dorea id.1997) on Ulcerative ileocolitis
rs12537781
rs62503162
rs4793307
rs13279148
rs73729431
rs345219
rs3005511
rs11150408
rs1899291
rs3752849
All
−0.5
0.0
0.5
MR effect size for
'Gut microbiota abundance (genus Dorea id.1997)' on 'Ulcerative ileocolitis'

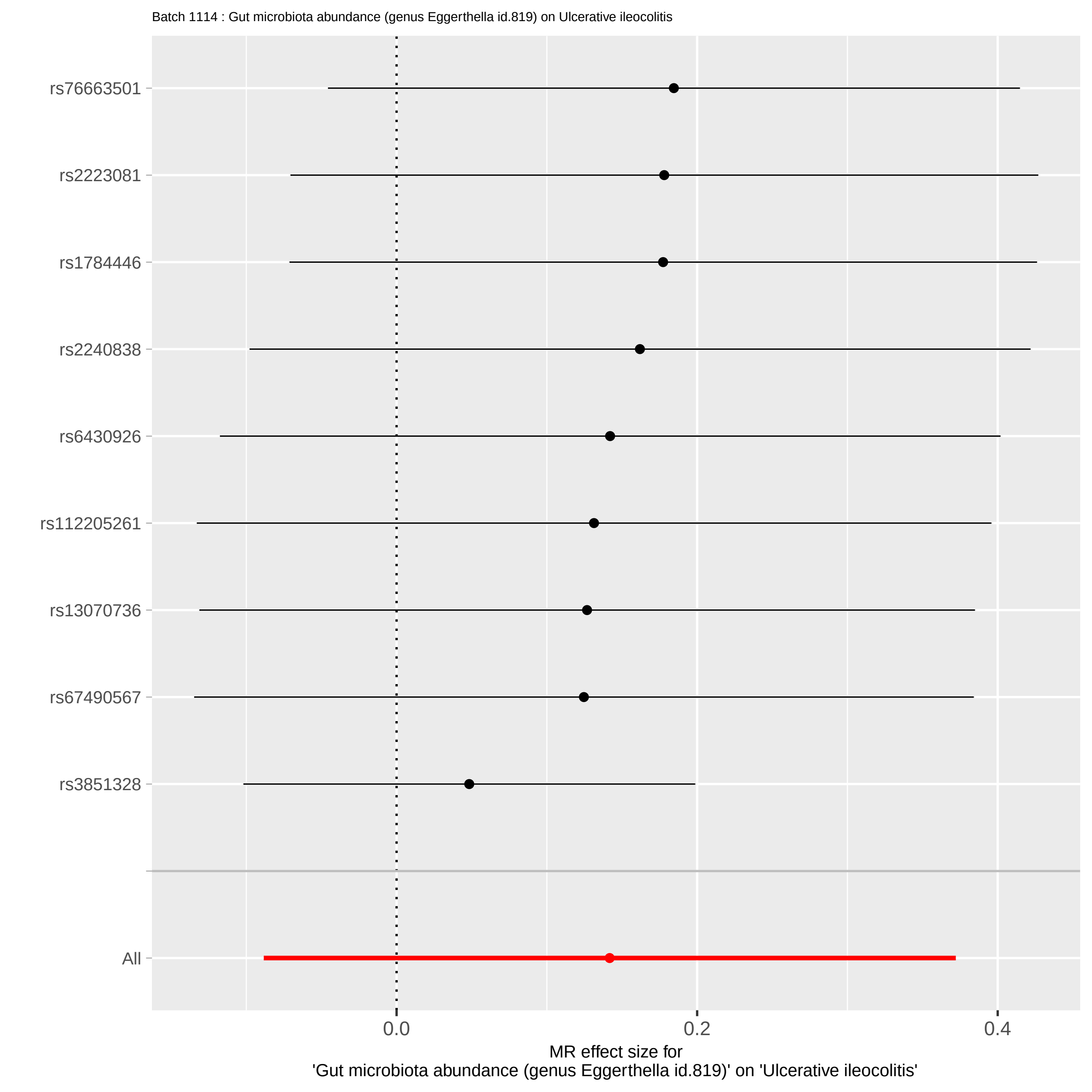
Batch 1114 : Gut microbiota abundance (genus Eggerthella id.819) on Ulcerative ileocolitis
rs76663501
rs2223081
rs1784446
rs2240838
rs6430926
rs112205261
rs13070736
rs67490567
rs3851328
All
0.0
0.2
0.4
MR effect size for
'Gut microbiota abundance (genus Eggerthella id.819)' on 'Ulcerative ileocolitis'

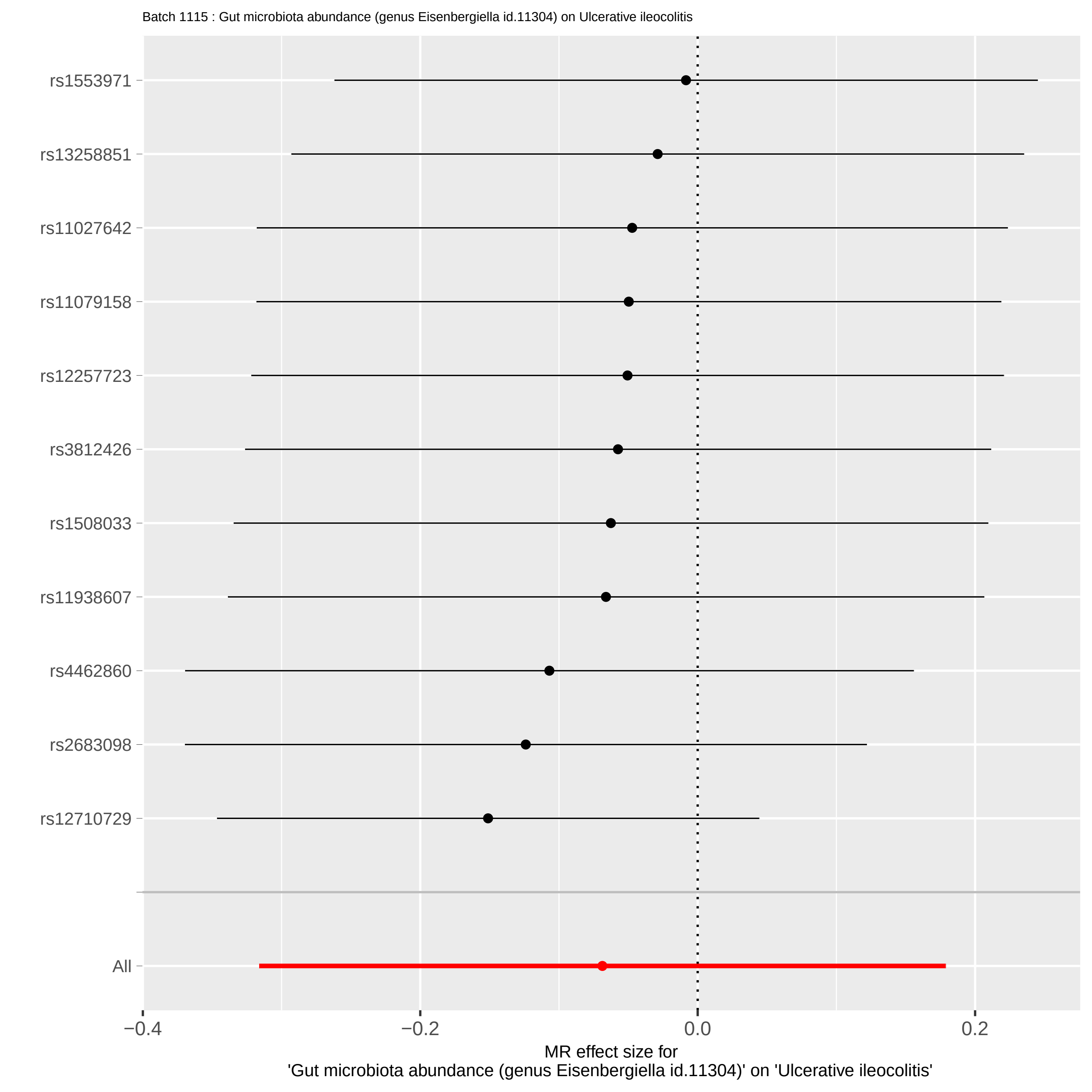

Batch 1115 : Gut microbiota abundance (genus Eisenbergiella id.11304) on Ulcerative ileocolitis
rs1553971
rs13258851
rs11027642
rs11079158
rs12257723
rs3812426
rs1508033
rs11938607
rs4462860
rs2683098
rs12710729
All
−0.4
−0.2
0.0
0.2
MR effect size for
'Gut microbiota abundance (genus Eisenbergiella id.11304)' on 'Ulcerative ileocolitis'

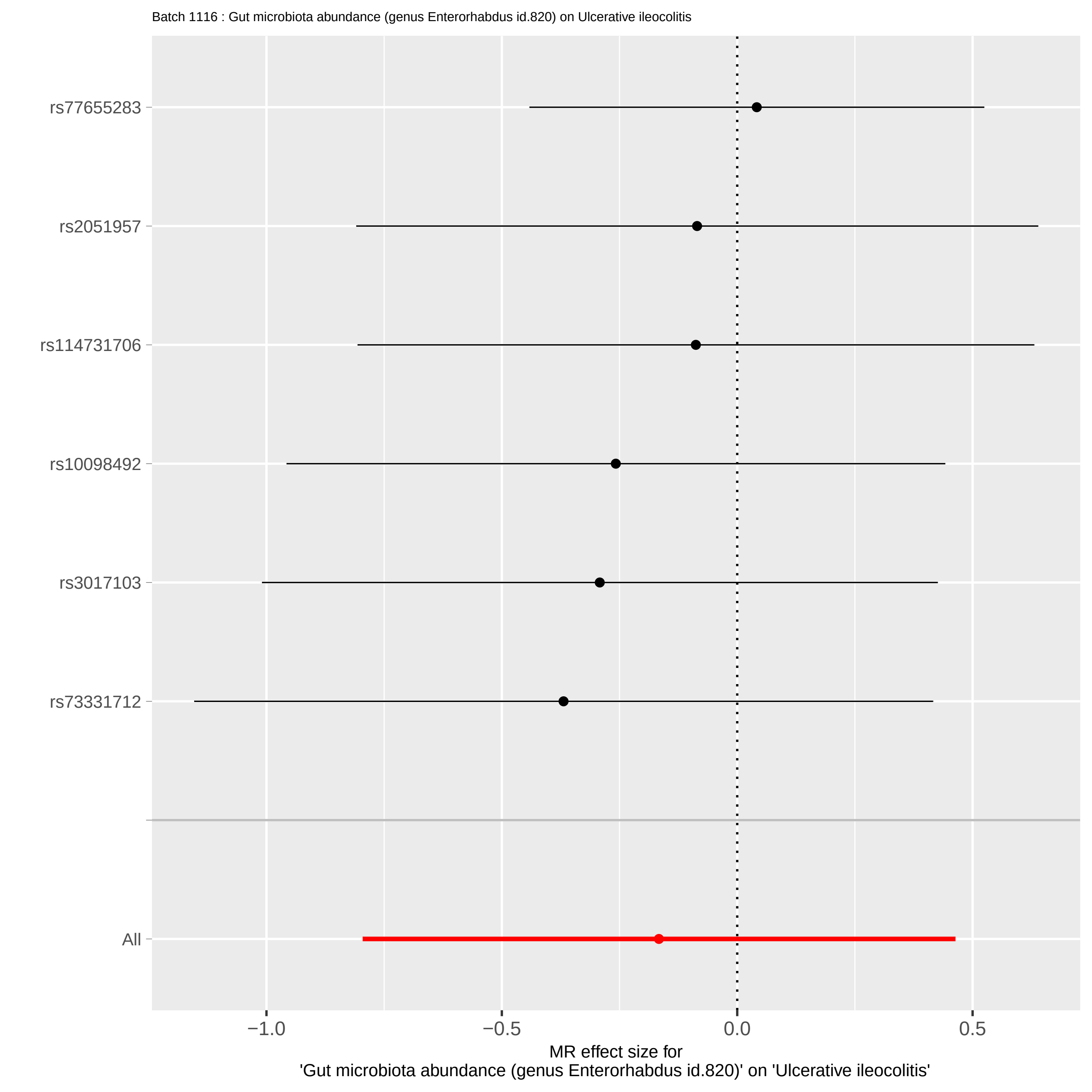
Batch 1116 : Gut microbiota abundance (genus Enterorhabdus id.820) on Ulcerative ileocolitis
rs77655283
rs2051957
rs114731706
rs10098492
rs3017103
rs73331712
All
−1.0
−0.5
0.0
0.5
MR effect size for
'Gut microbiota abundance (genus Enterorhabdus id.820)' on 'Ulcerative ileocolitis'

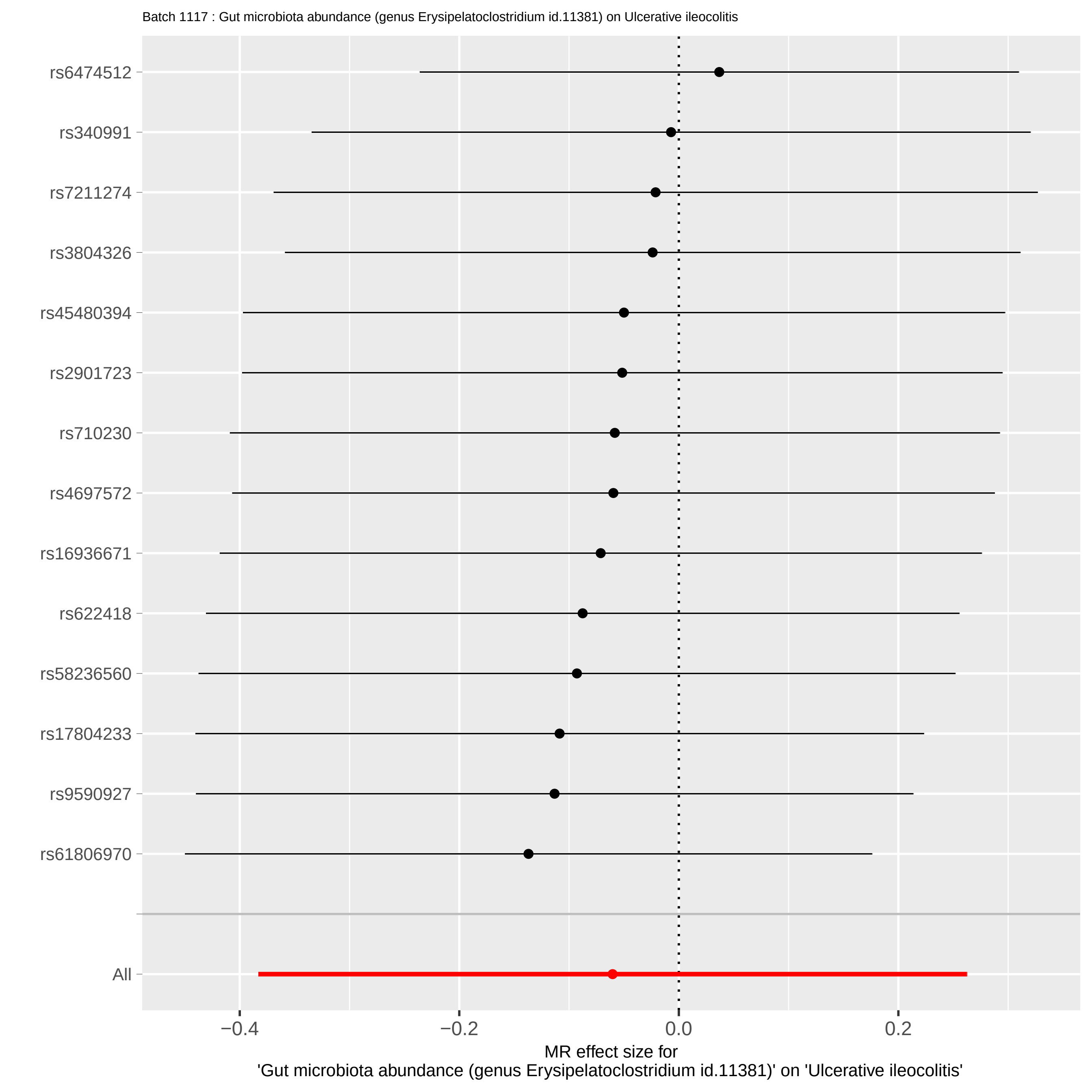

Batch 1117 : Gut microbiota abundance (genus Erysipelatoclostridium id.11381) on Ulcerative ileocolitis
rs6474512
rs340991
rs7211274
rs3804326
rs45480394
rs2901723
rs710230
rs4697572
rs16936671
rs622418
rs58236560
rs17804233
rs9590927
rs61806970
All
−0.4
−0.2
0.0
0.2
MR effect size for
'Gut microbiota abundance (genus Erysipelatoclostridium id.11381)' on 'Ulcerative ileocolitis'

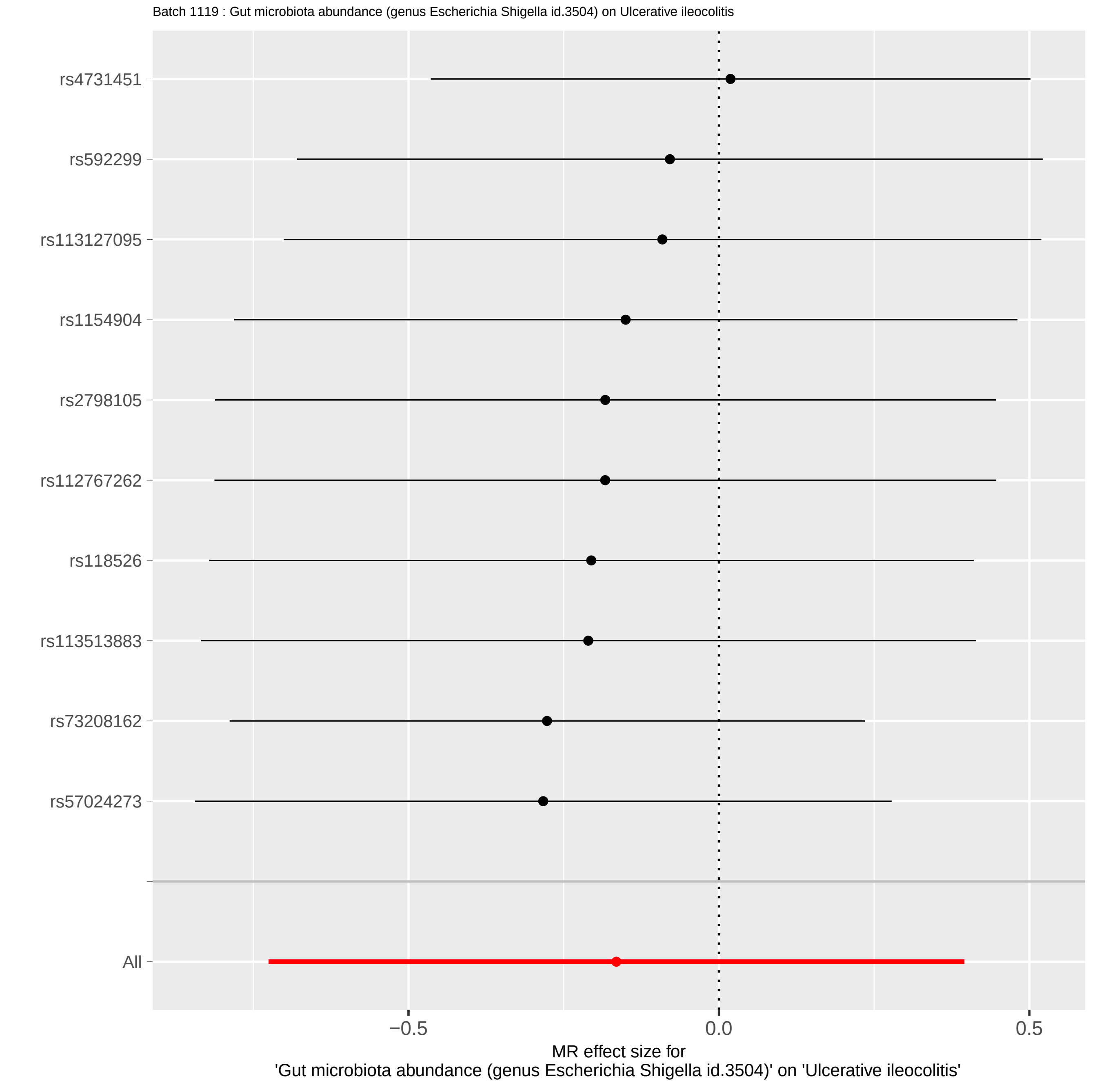

Batch 1119 : Gut microbiota abundance (genus Escherichia Shigella id.3504) on Ulcerative ileocolitis
rs4731451
rs592299
rs113127095
rs1154904
rs2798105
rs112767262
rs118526
rs113513883
rs73208162
rs57024273
All
−0.5
0.0
0.5
MR effect size for
'Gut microbiota abundance (genus Escherichia Shigella id.3504)' on 'Ulcerative ileocolitis'

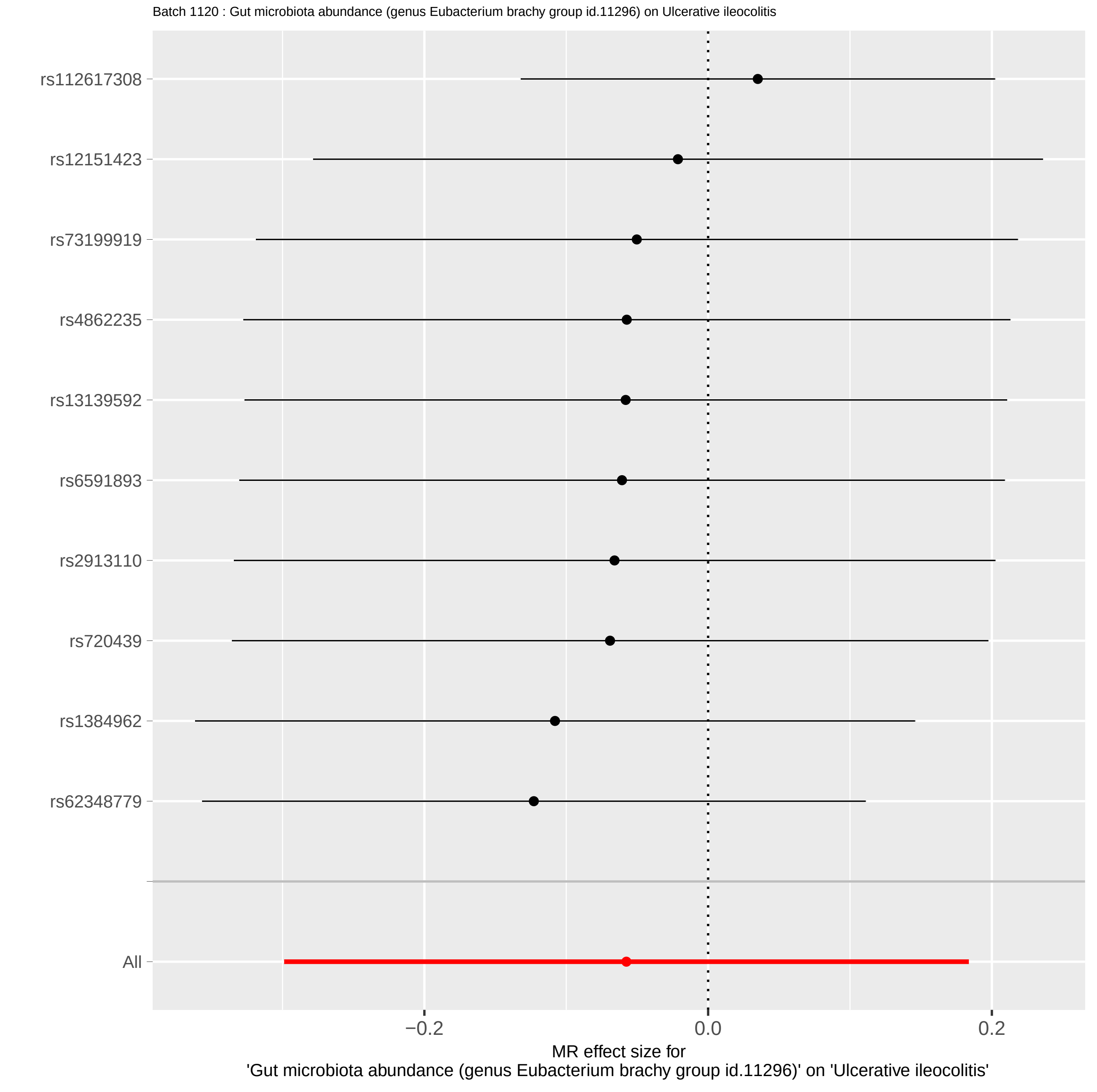
Batch 1120 : Gut microbiota abundance (genus Eubacterium brachy group id.11296) on Ulcerative ileocolitis
rs112617308
rs12151423
rs73199919
rs4862235
rs13139592
rs6591893
rs2913110
rs720439
rs1384962
rs62348779
All
−0.2
0.0
0.2
MR effect size for
'Gut microbiota abundance (genus Eubacterium brachy group id.11296)' on 'Ulcerative ileocolitis'

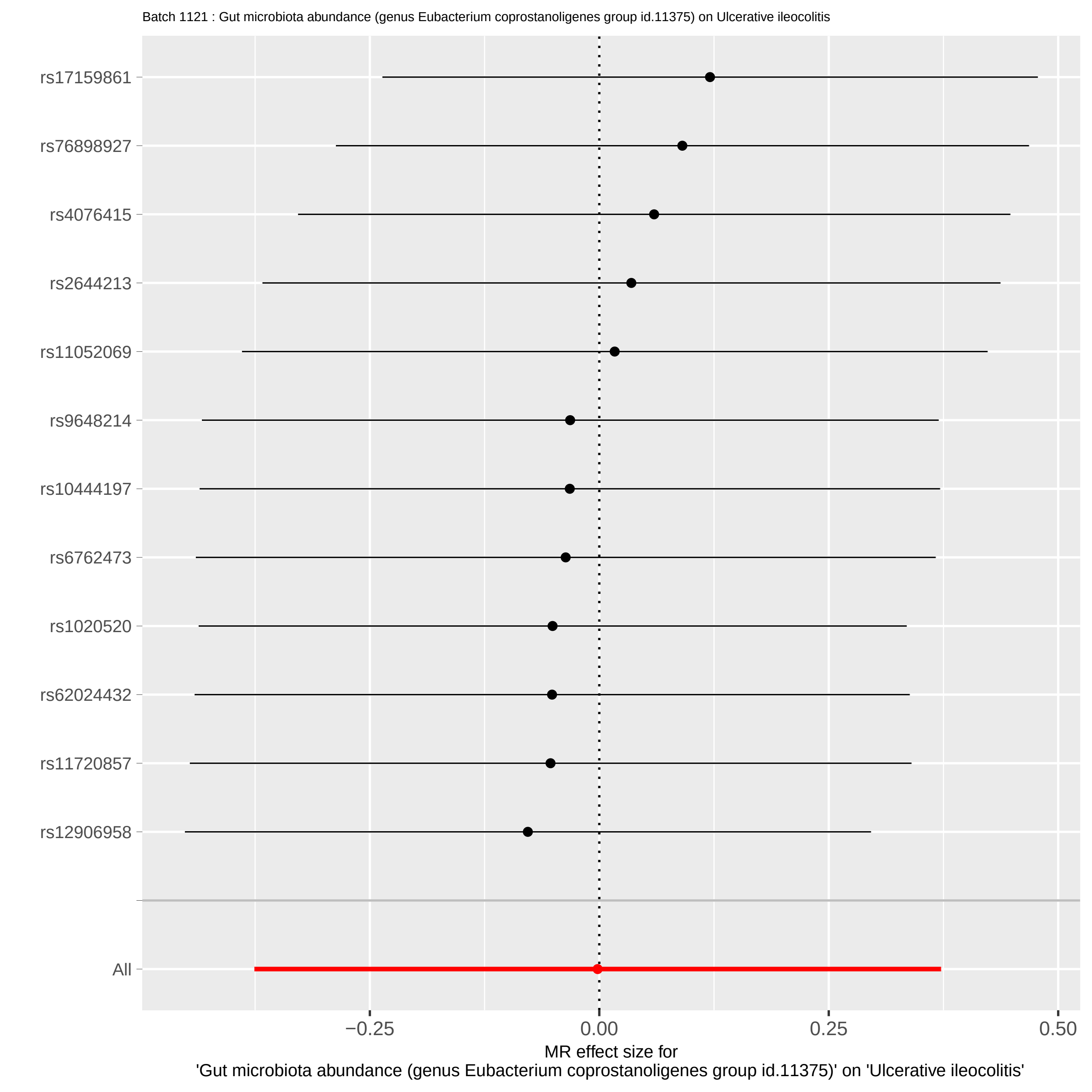

Batch 1121 : Gut microbiota abundance (genus Eubacterium coprostanoligenes group id.11375) on Ulcerative ileocolitis
rs17159861
rs76898927
rs4076415
rs2644213
rs11052069
rs9648214
rs10444197
rs6762473
rs1020520
rs62024432
rs11720857
rs12906958
All
−0.25
0.00
0.25
0.50
MR effect size for
'Gut microbiota abundance (genus Eubacterium coprostanoligenes group id.11375)' on 'Ulcerative ileocolitis'

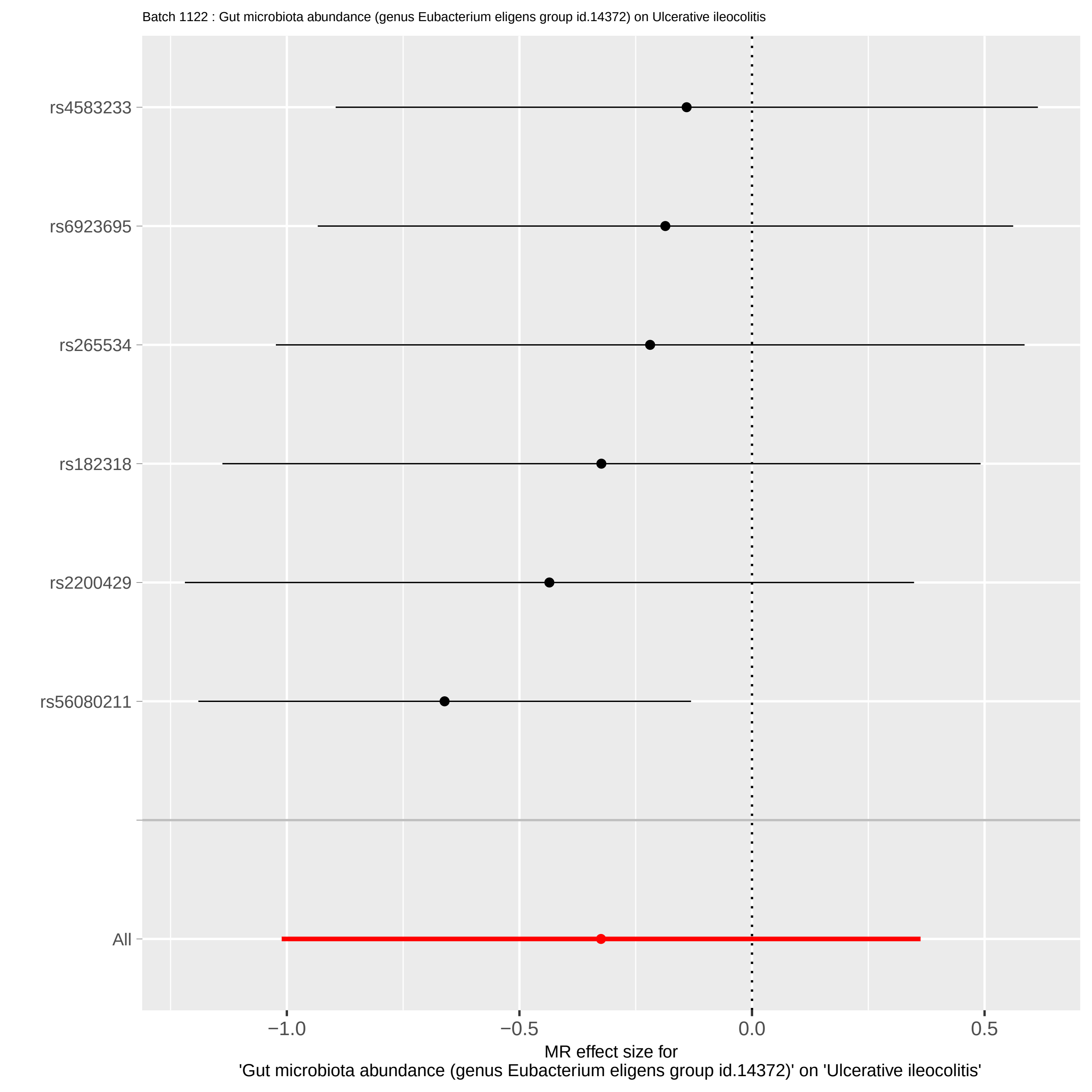
Batch 1122 : Gut microbiota abundance (genus Eubacterium eligens group id.14372) on Ulcerative ileocolitis
rs4583233
rs6923695
rs265534
rs182318
rs2200429
rs56080211
All
−1.0
−0.5
0.0
0.5
MR effect size for
'Gut microbiota abundance (genus Eubacterium eligens group id.14372)' on 'Ulcerative ileocolitis'

Batch 1123 : Gut microbiota abundance (genus Eubacterium fissicatena group id.14373) on Ulcerative ileocolitis

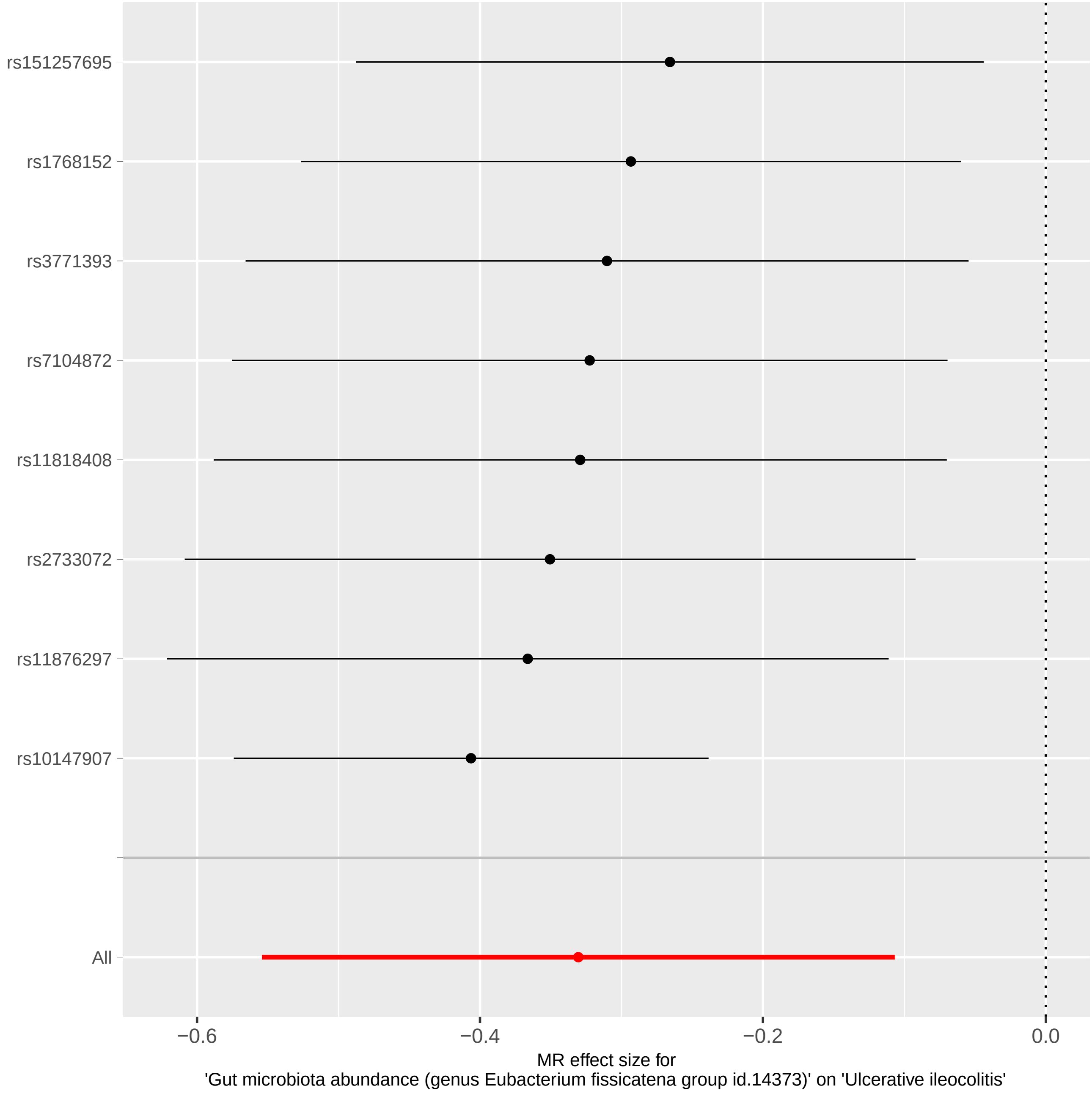

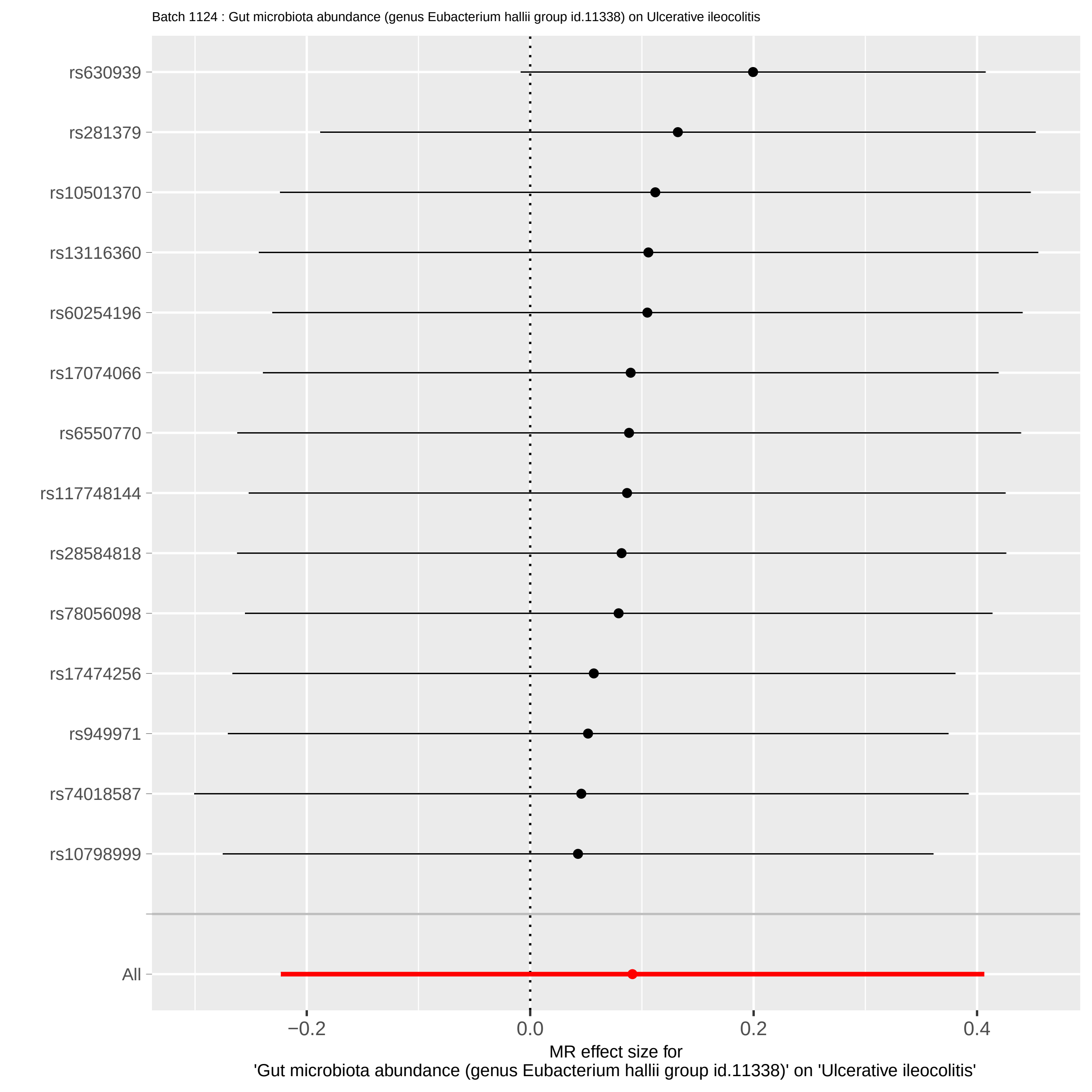
Batch 1124 : Gut microbiota abundance (genus Eubacterium hallii group id.11338) on Ulcerative ileocolitis
rs630939
rs281379
rs10501370
rs13116360
rs60254196
rs17074066
rs6550770
rs117748144
rs28584818
rs78056098
rs17474256
rs949971
rs74018587
rs10798999
All
−0.2
0.0
0.2
0.4
MR effect size for
'Gut microbiota abundance (genus Eubacterium hallii group id.11338)' on 'Ulcerative ileocolitis'

Batch 1125 : Gut microbiota abundance (genus Eubacterium nodatum group id.11297) on Ulcerative ileocolitis

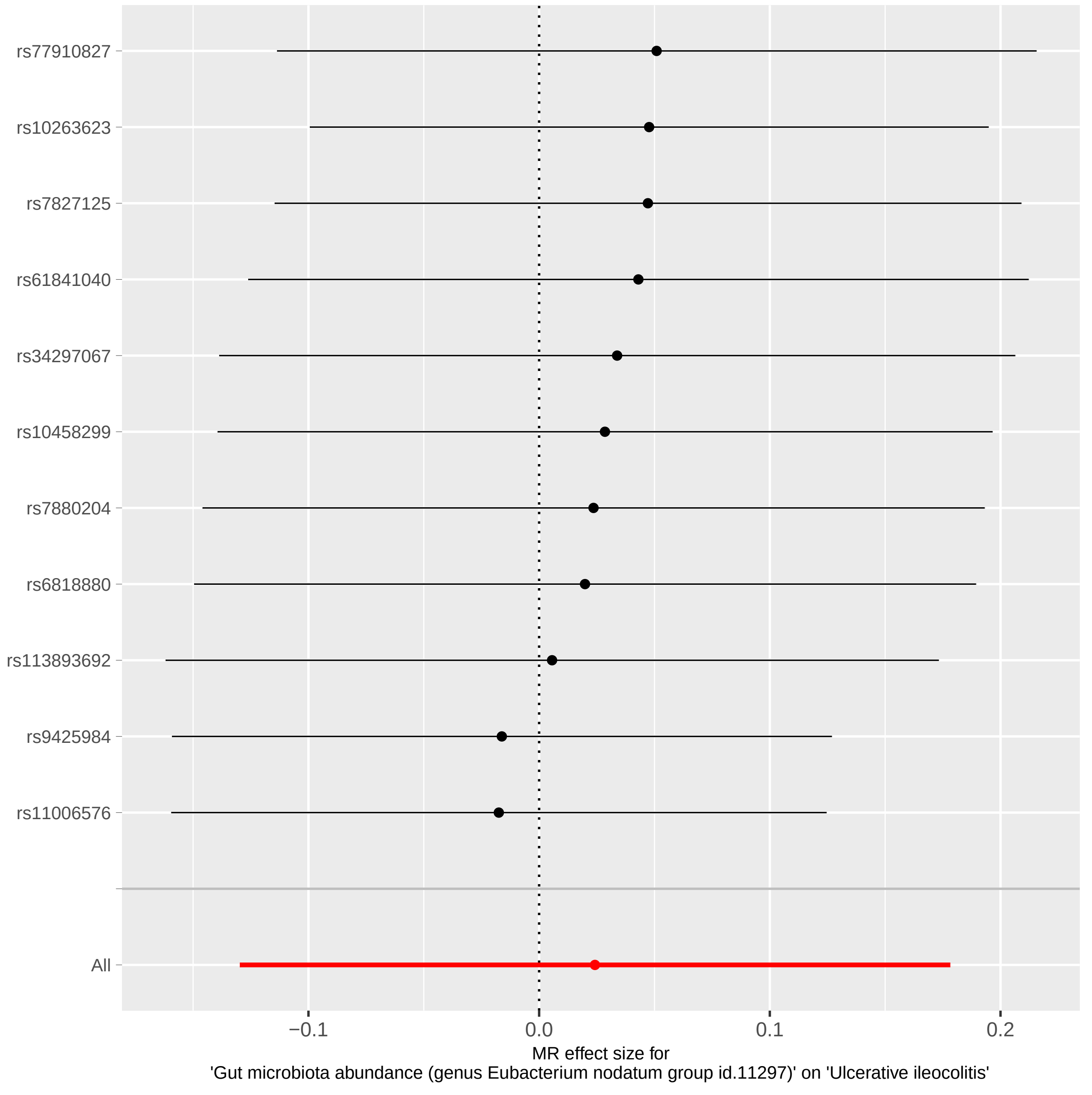

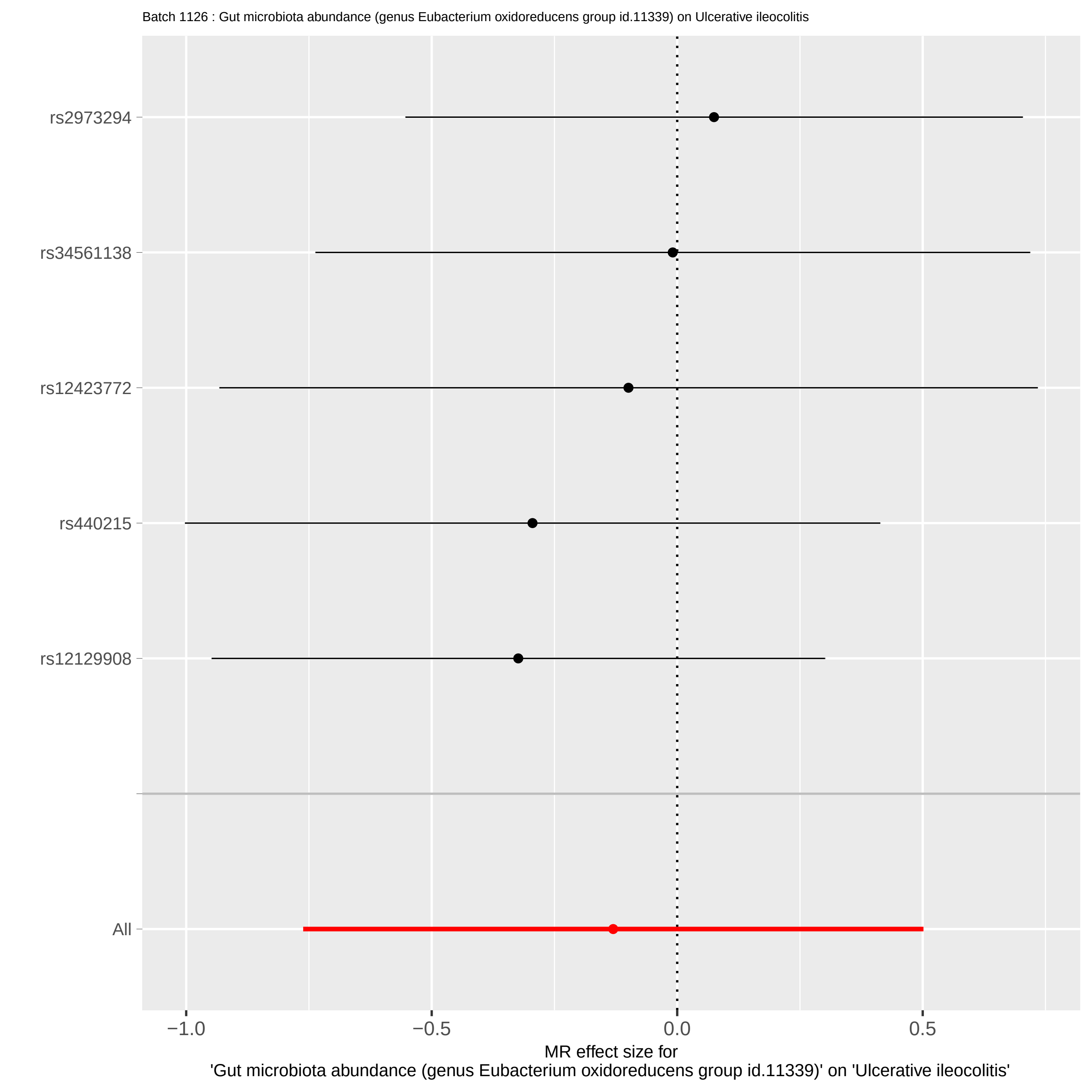

Batch 1126 : Gut microbiota abundance (genus Eubacterium oxidoreducens group id.11339) on Ulcerative ileocolitis
rs2973294
rs34561138
rs12423772
rs440215
rs12129908
All
−1.0
−0.5
0.0
0.5
MR effect size for
'Gut microbiota abundance (genus Eubacterium oxidoreducens group id.11339)' on 'Ulcerative ileocolitis'

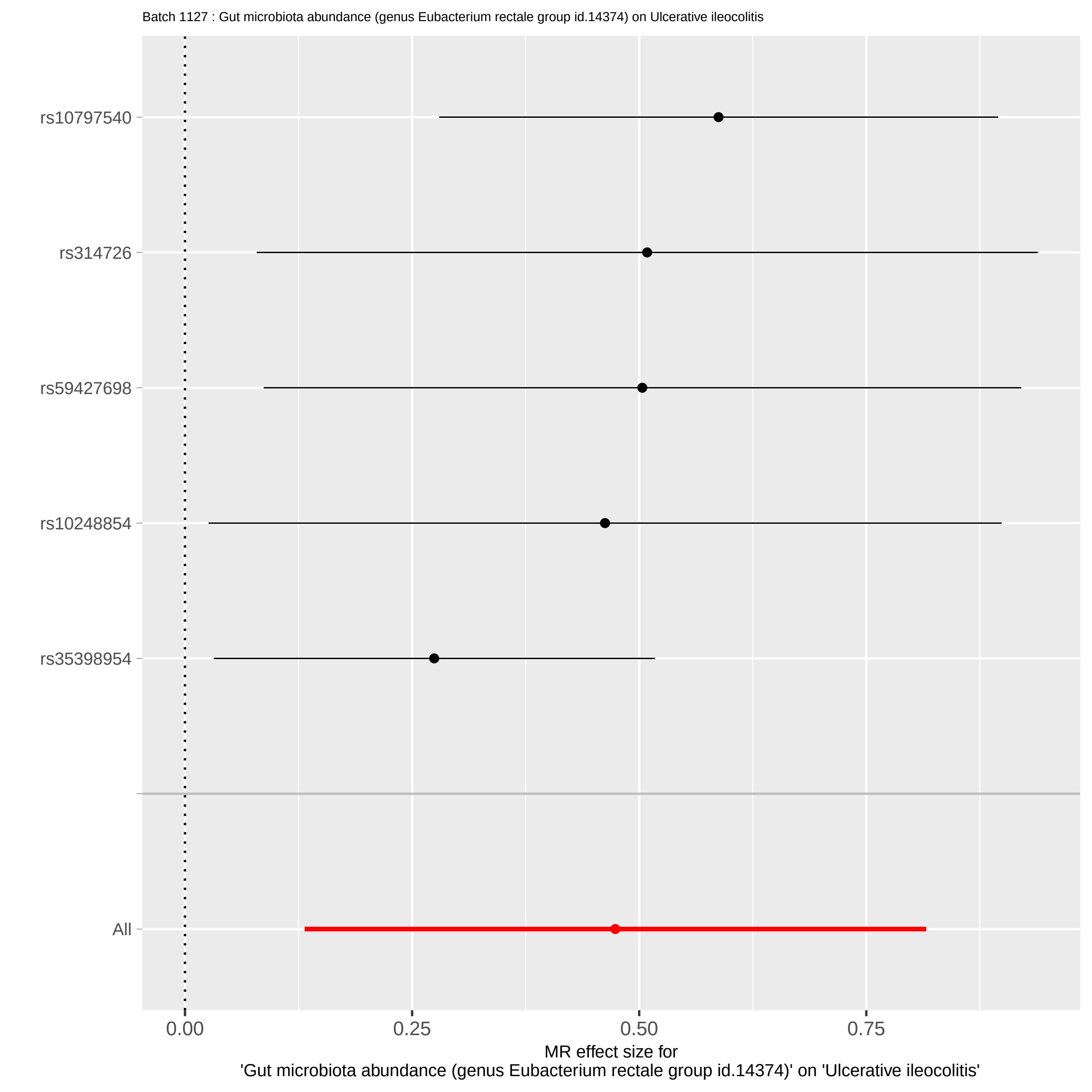

Batch 1127 : Gut microbiota abundance (genus Eubacterium rectale group id.14374) on Ulcerative ileocolitis
rs10797540
rs314726
rs59427698
rs10248854
rs35398954
All
0.00
0.25
0.50
0.75
MR effect size for
'Gut microbiota abundance (genus Eubacterium rectale group id.14374)' on 'Ulcerative ileocolitis'

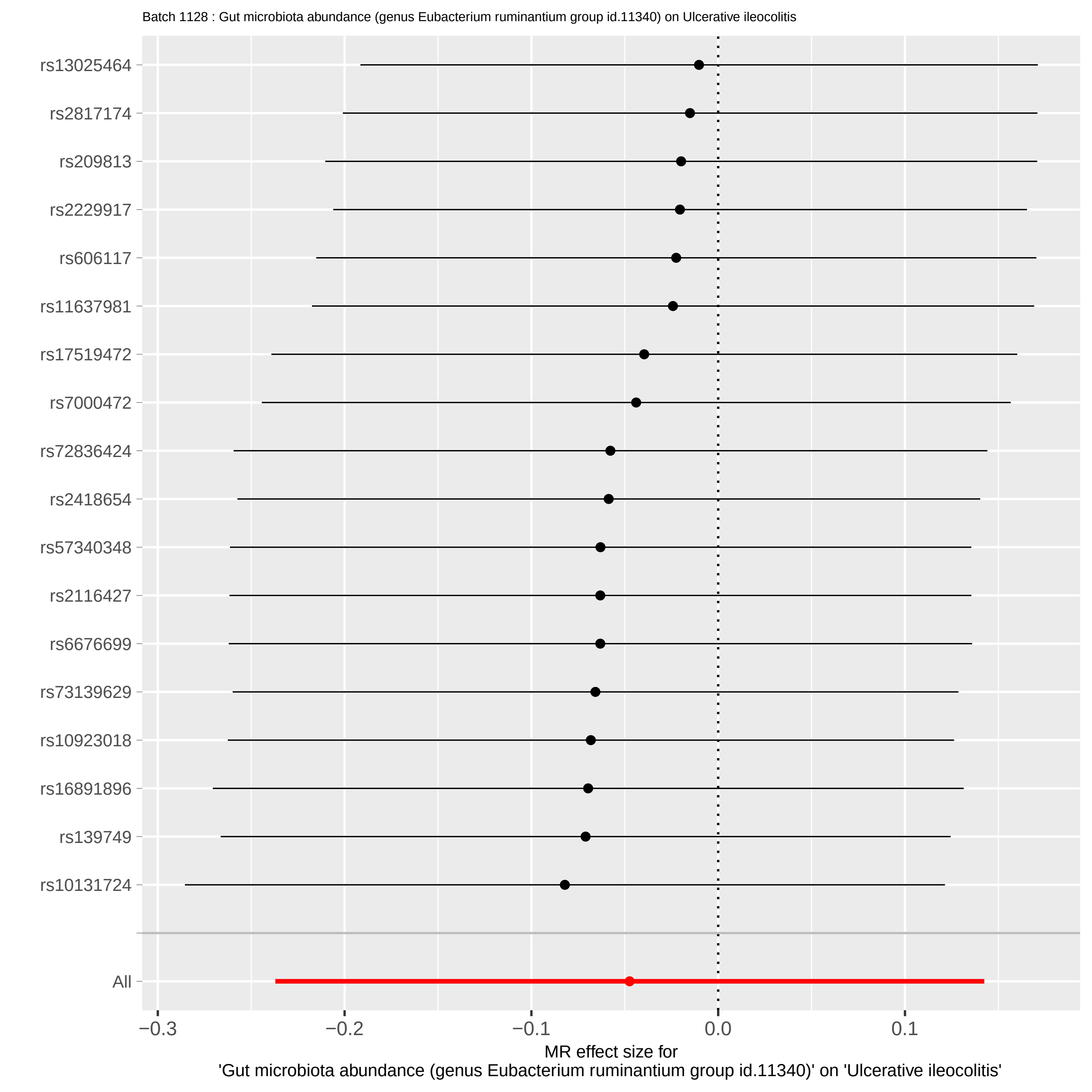
Batch 1128 : Gut microbiota abundance (genus Eubacterium ruminantium group id.11340) on Ulcerative ileocolitis
rs13025464
rs2817174
rs209813
rs2229917
rs606117
rs11637981
rs17519472
rs7000472
rs72836424
rs2418654
rs57340348
rs2116427
rs6676699
rs73139629
rs10923018
rs16891896
rs139749
rs10131724
All
−0.3
−0.2
−0.1
0.0
0.1
MR effect size for
'Gut microbiota abundance (genus Eubacterium ruminantium group id.11340)' on 'Ulcerative ileocolitis'

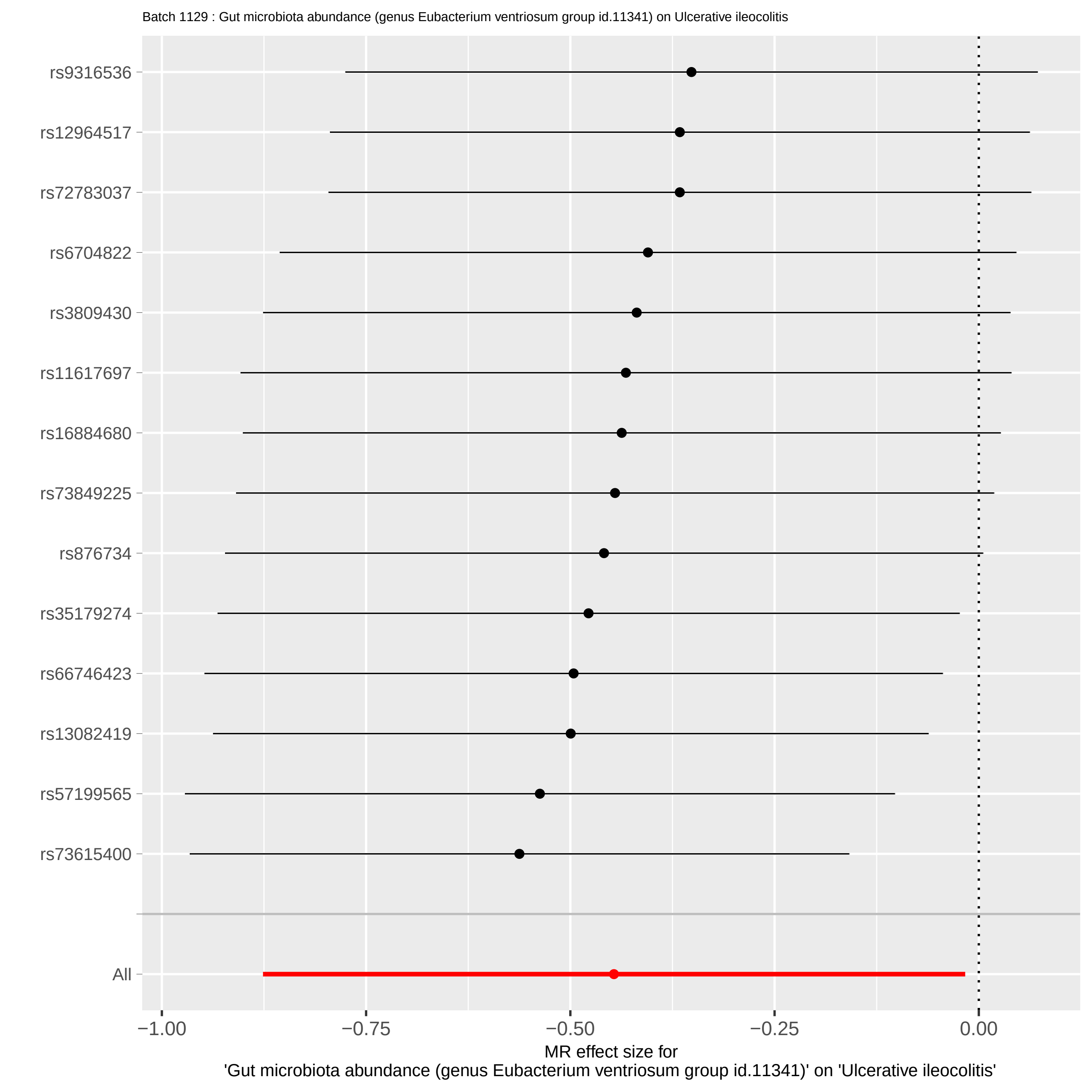

Batch 1129 : Gut microbiota abundance (genus Eubacterium ventriosum group id.11341) on Ulcerative ileocolitis
rs9316536
rs12964517
rs72783037
rs6704822
rs3809430
rs11617697
rs16884680
rs73849225
rs876734
rs35179274
rs66746423
rs13082419
rs57199565
rs73615400
All
−1.00
−0.75
−0.50
−0.25
0.00
MR effect size for
'Gut microbiota abundance (genus Eubacterium ventriosum group id.11341)' on 'Ulcerative ileocolitis'

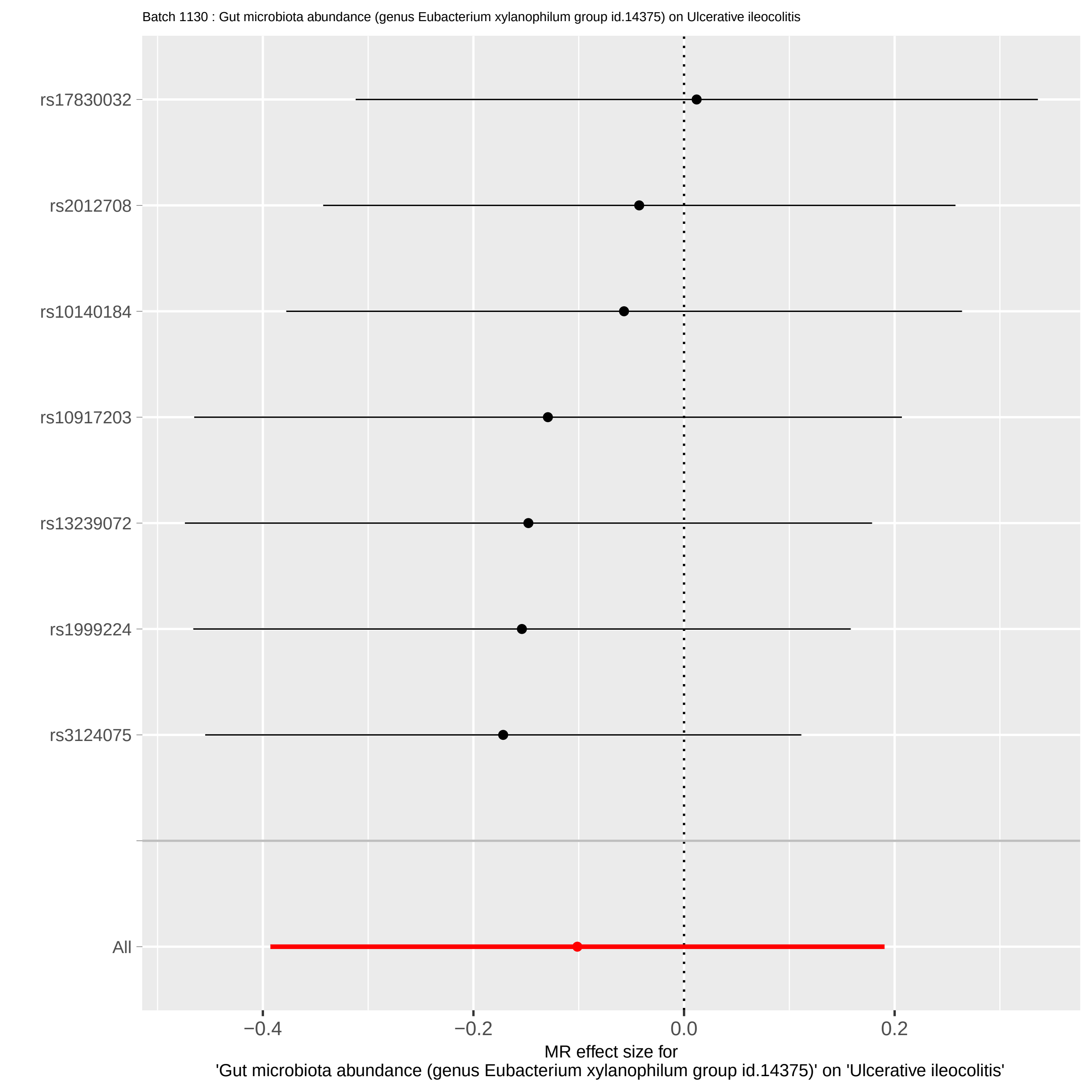
Batch 1130 : Gut microbiota abundance (genus Eubacterium xylanophilum group id.14375) on Ulcerative ileocolitis
rs17830032
rs2012708
rs10140184
rs10917203
rs13239072
rs1999224
rs3124075
All
−0.4
−0.2
0.0
0.2
MR effect size for
'Gut microbiota abundance (genus Eubacterium xylanophilum group id.14375)' on 'Ulcerative ileocolitis'

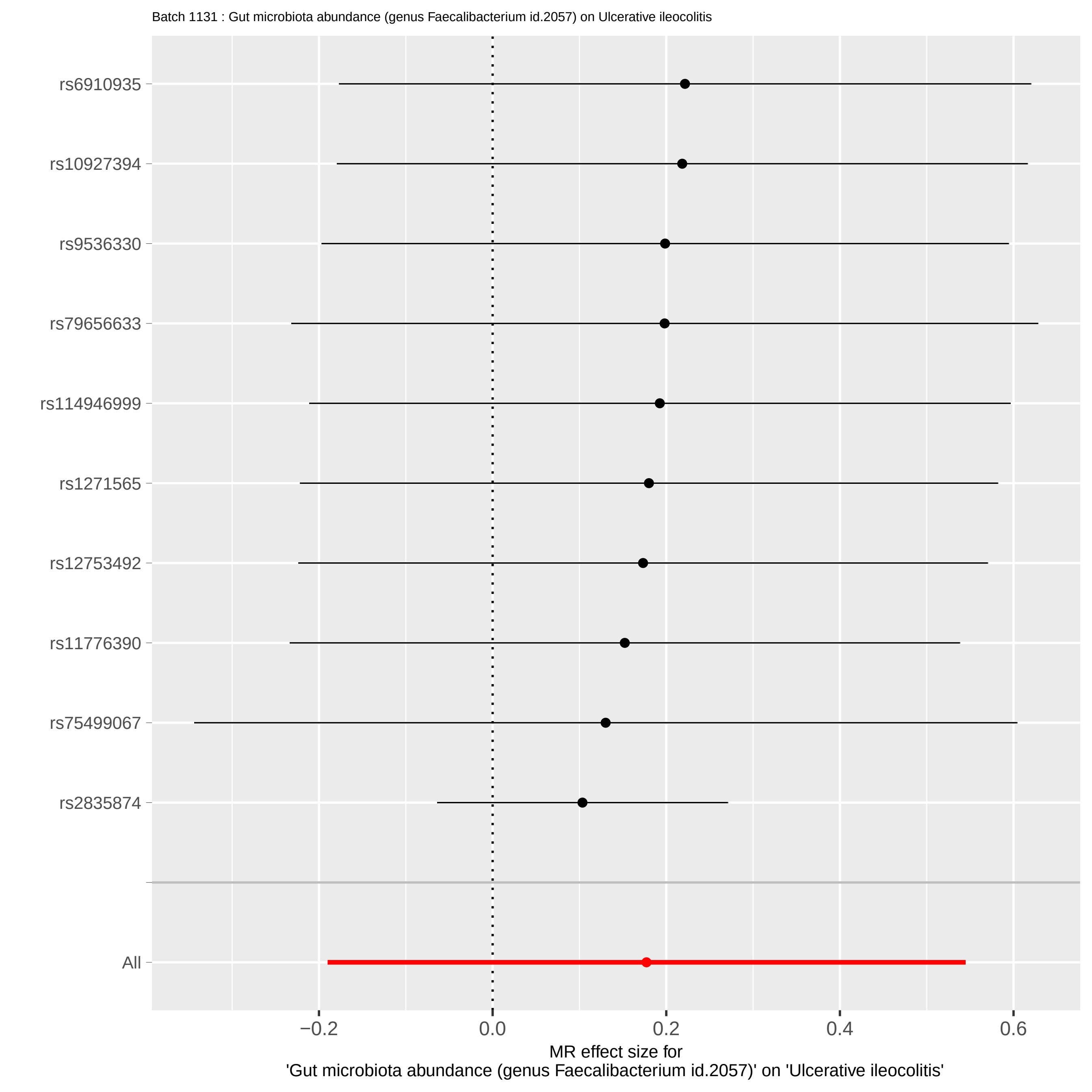
Batch 1131 : Gut microbiota abundance (genus Faecalibacterium id.2057) on Ulcerative ileocolitis
rs6910935
rs10927394
rs9536330
rs79656633
rs114946999
rs1271565
rs12753492
rs11776390
rs75499067
rs2835874
All
−0.2
0.0
0.2
0.4
0.6
MR effect size for
'Gut microbiota abundance (genus Faecalibacterium id.2057)' on 'Ulcerative ileocolitis'

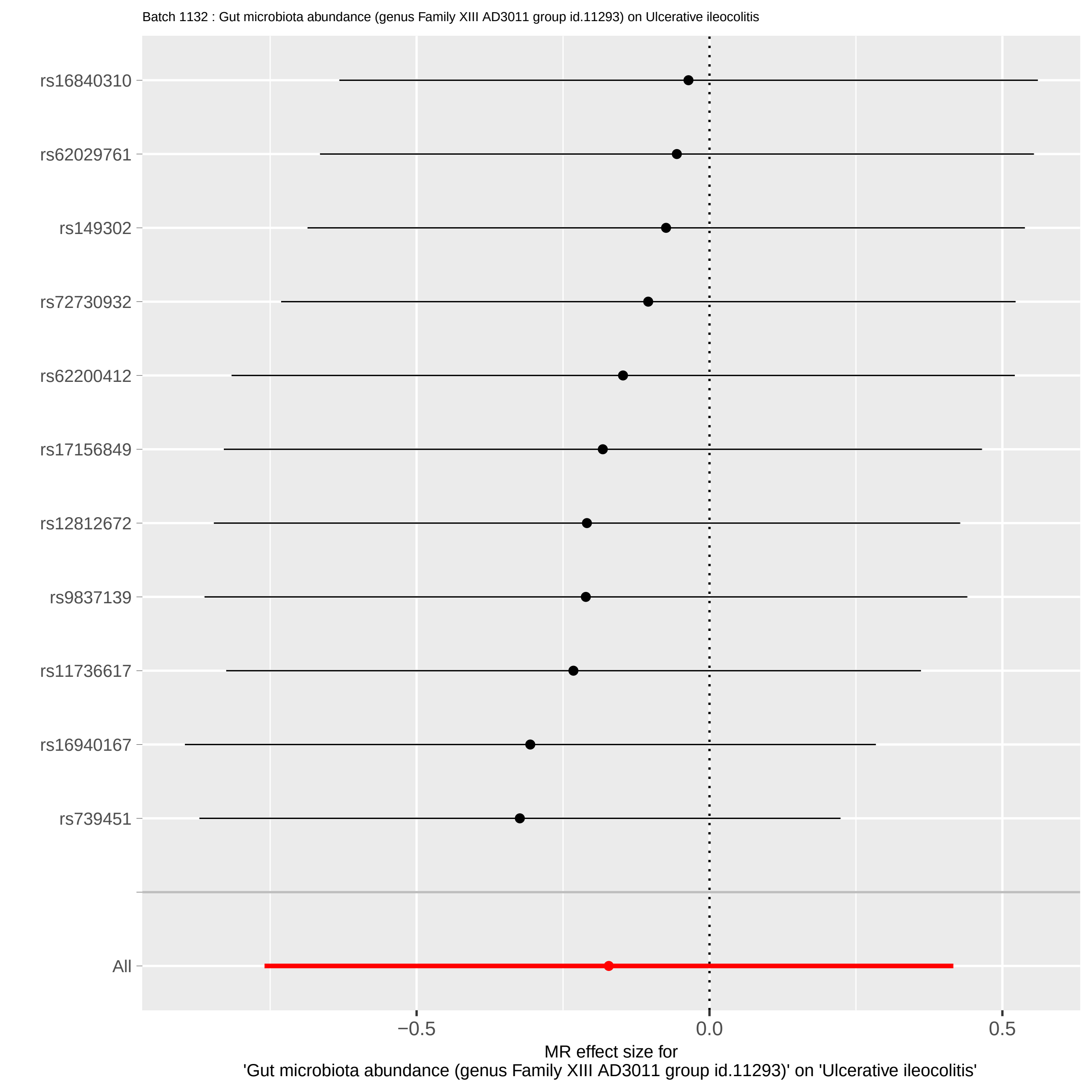

Batch 1132 : Gut microbiota abundance (genus Family XIII AD3011 group id.11293) on Ulcerative ileocolitis
rs16840310
rs62029761
rs149302
rs72730932
rs62200412
rs17156849
rs12812672
rs9837139
rs11736617
rs16940167
rs739451
All
−0.5
0.0
0.5
MR effect size for
'Gut microbiota abundance (genus Family XIII AD3011 group id.11293)' on 'Ulcerative ileocolitis'

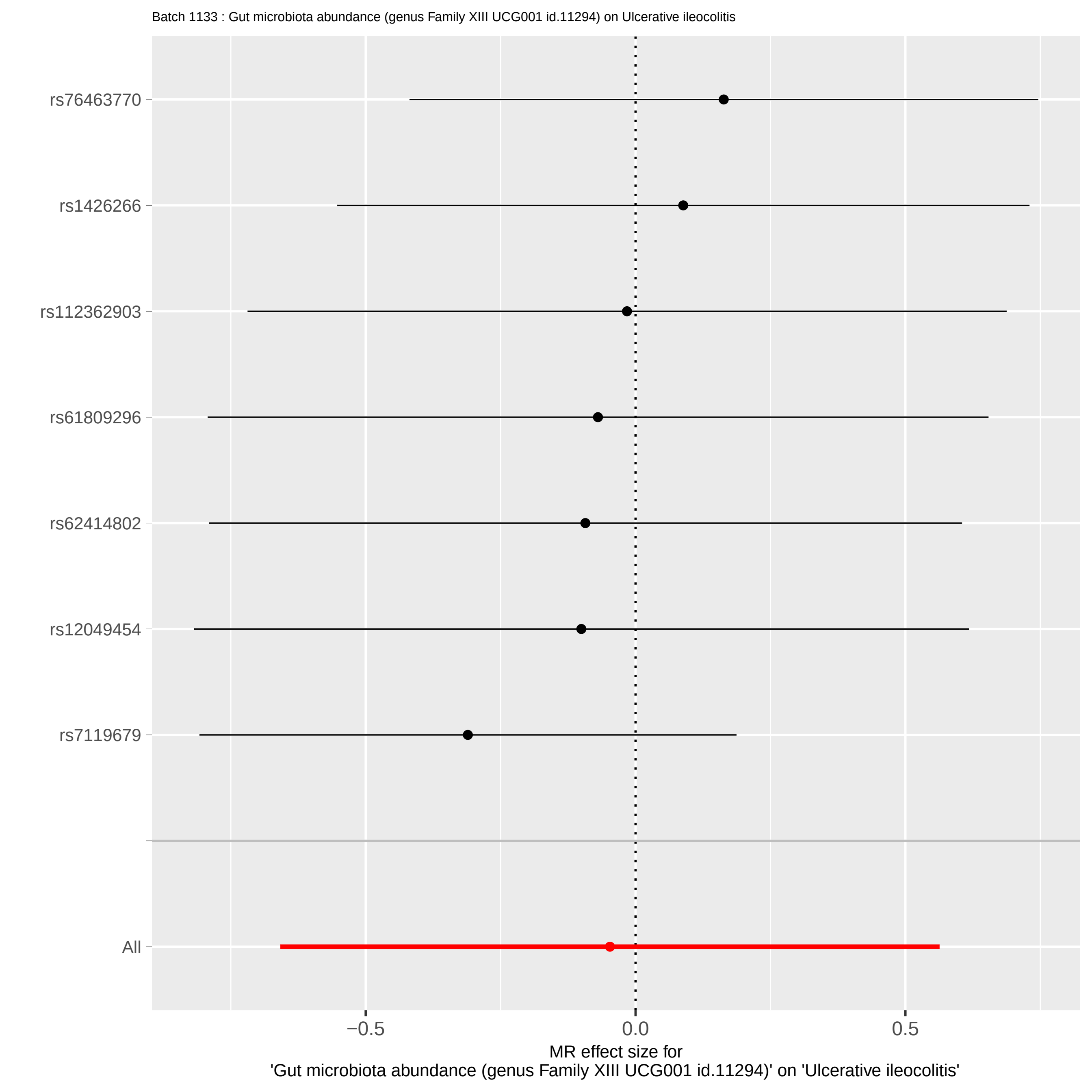

Batch 1133 : Gut microbiota abundance (genus Family XIII UCG001 id.11294) on Ulcerative ileocolitis
rs76463770
rs1426266
rs112362903
rs61809296
rs62414802
rs12049454
rs7119679
All
−0.5
0.0
0.5
MR effect size for
'Gut microbiota abundance (genus Family XIII UCG001 id.11294)' on 'Ulcerative ileocolitis'

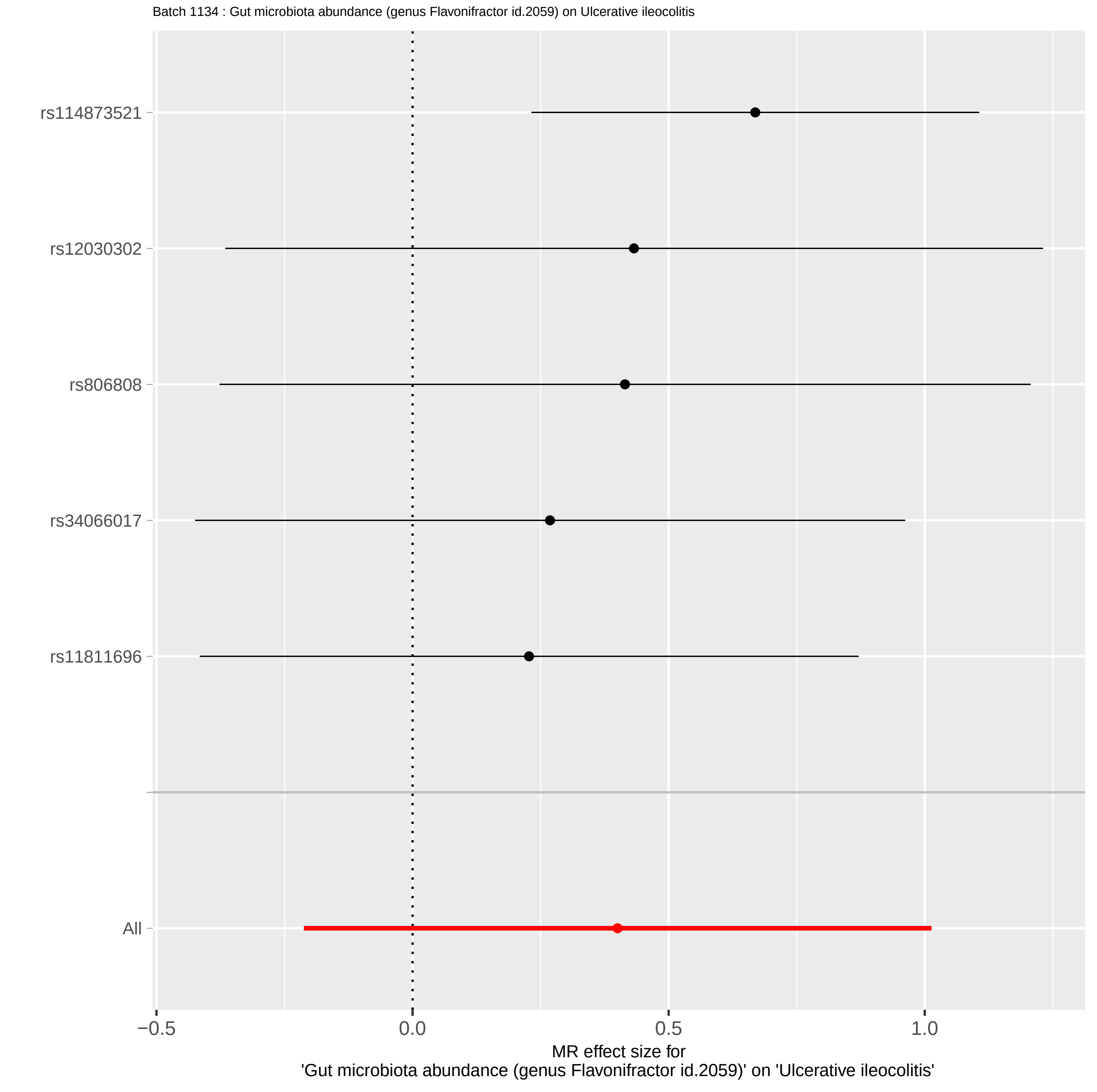

Batch 1134 : Gut microbiota abundance (genus Flavonifractor id.2059) on Ulcerative ileocolitis
rs114873521
rs12030302
rs806808
rs34066017
rs11811696
All
−0.5
0.0
0.5
1.0
MR effect size for
'Gut microbiota abundance (genus Flavonifractor id.2059)' on 'Ulcerative ileocolitis'

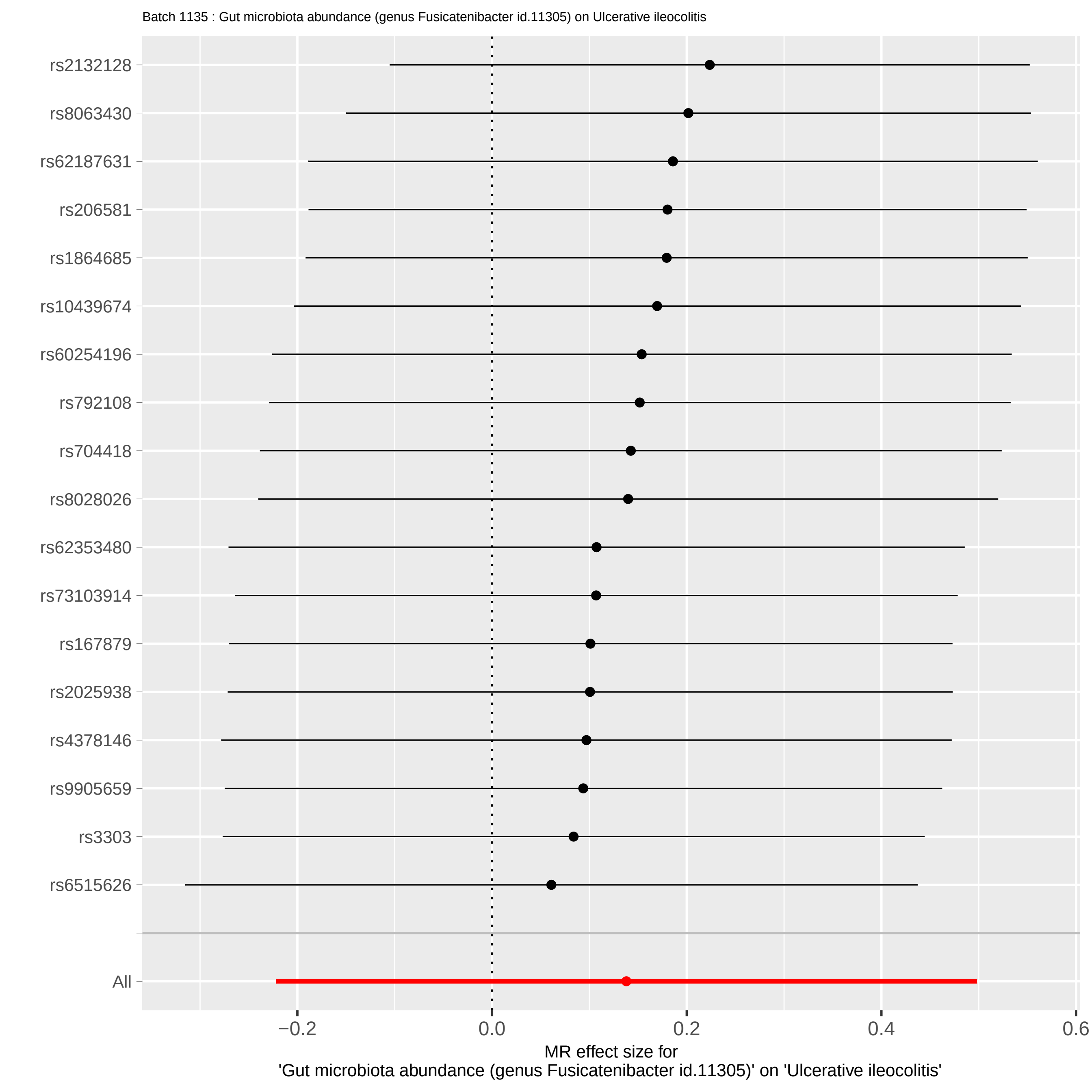

Batch 1135 : Gut microbiota abundance (genus Fusicatenibacter id.11305) on Ulcerative ileocolitis
rs2132128
rs8063430
rs62187631
rs206581
rs1864685
rs10439674
rs60254196
rs792108
rs704418
rs8028026
rs62353480
rs73103914
rs167879
rs2025938
rs4378146
rs9905659
rs3303
rs6515626
All
−0.2
0.0
0.2
0.4
0.6
MR effect size for
'Gut microbiota abundance (genus Fusicatenibacter id.11305)' on 'Ulcerative ileocolitis'

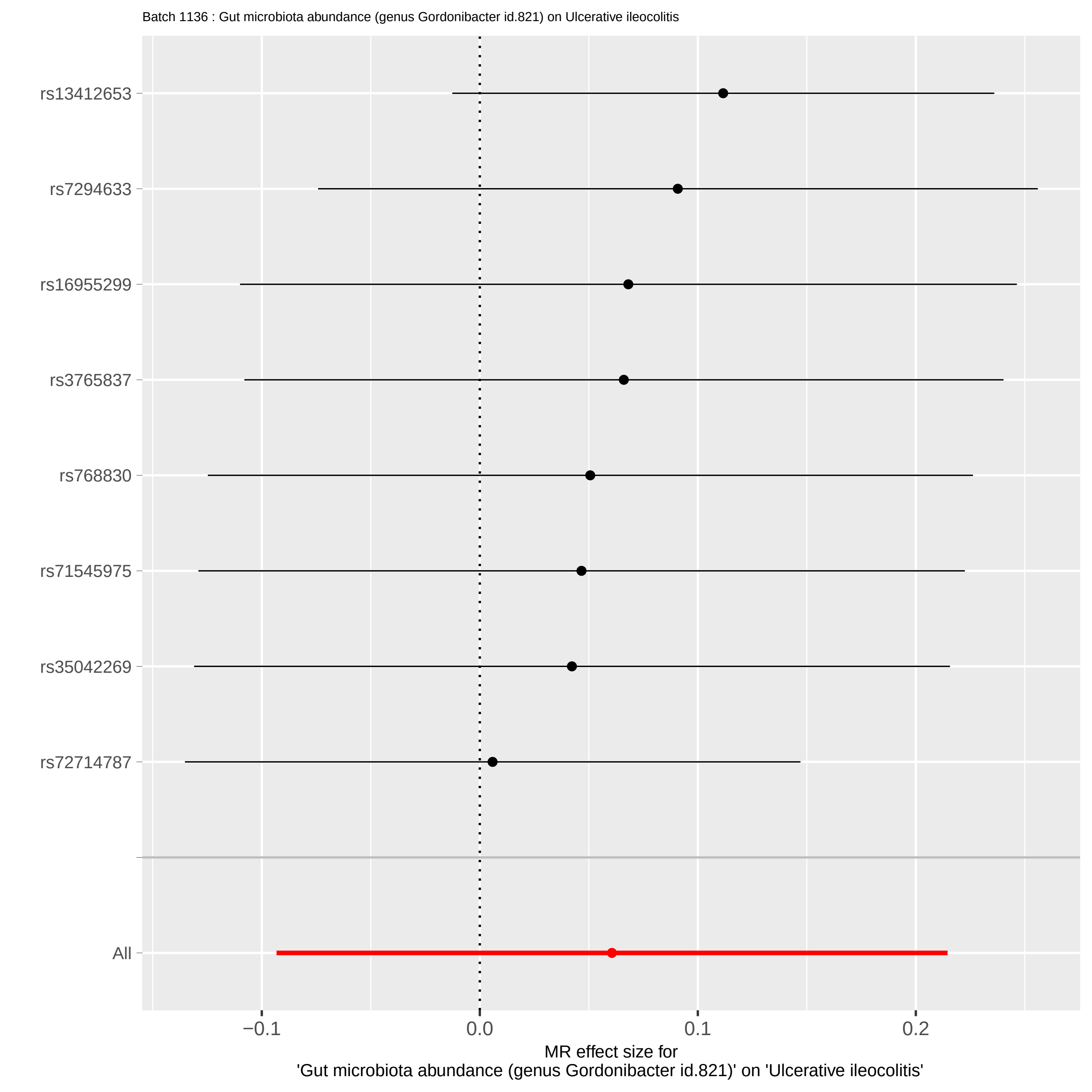

Batch 1136 : Gut microbiota abundance (genus Gordonibacter id.821) on Ulcerative ileocolitis
rs13412653
rs7294633
rs16955299
rs3765837
rs768830
rs71545975
rs35042269
rs72714787
All
−0.1
0.0
0.1
0.2
MR effect size for
'Gut microbiota abundance (genus Gordonibacter id.821)' on 'Ulcerative ileocolitis'

Batch 1137 : Gut microbiota abundance (genus Haemophilus id.3698) on Ulcerative ileocolitis

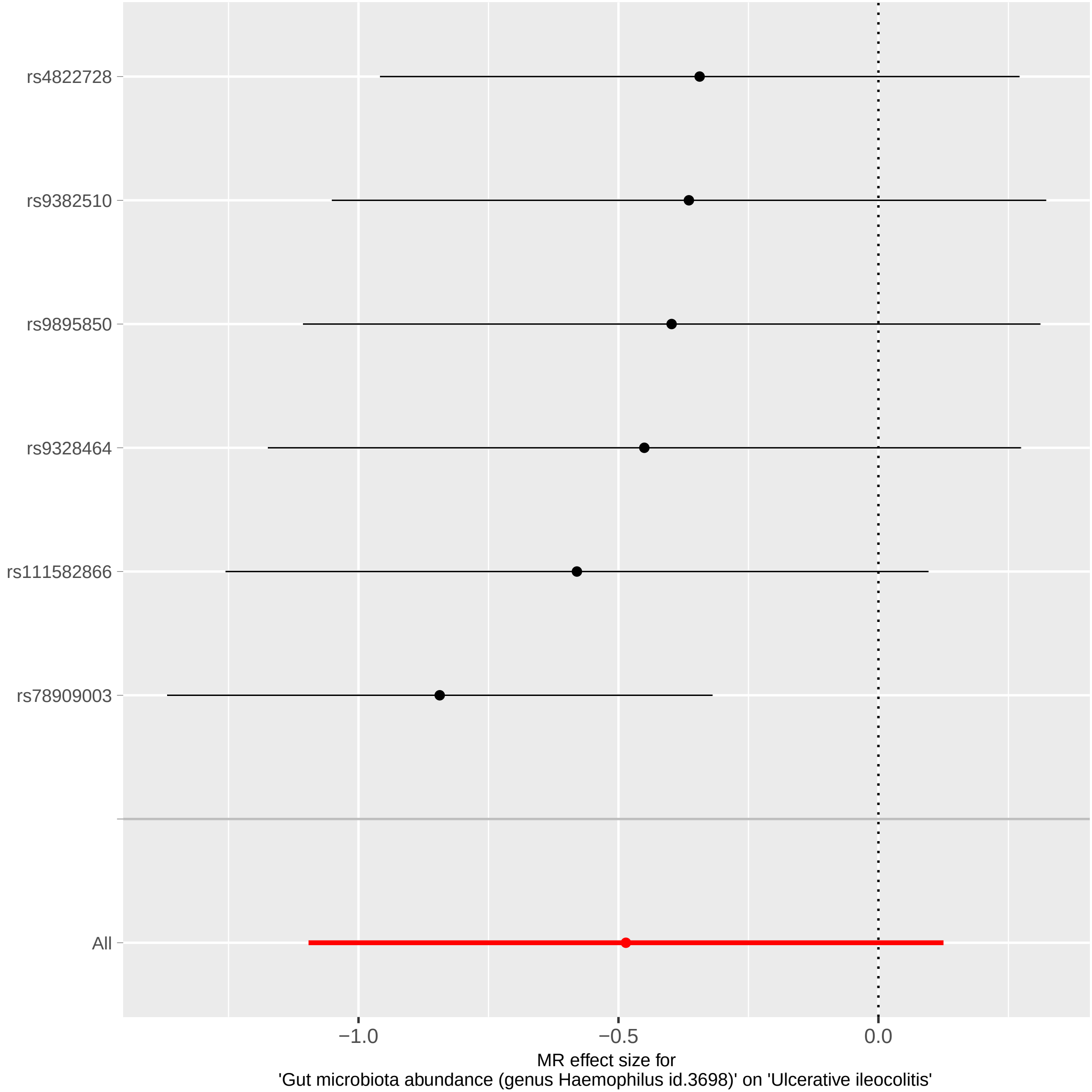

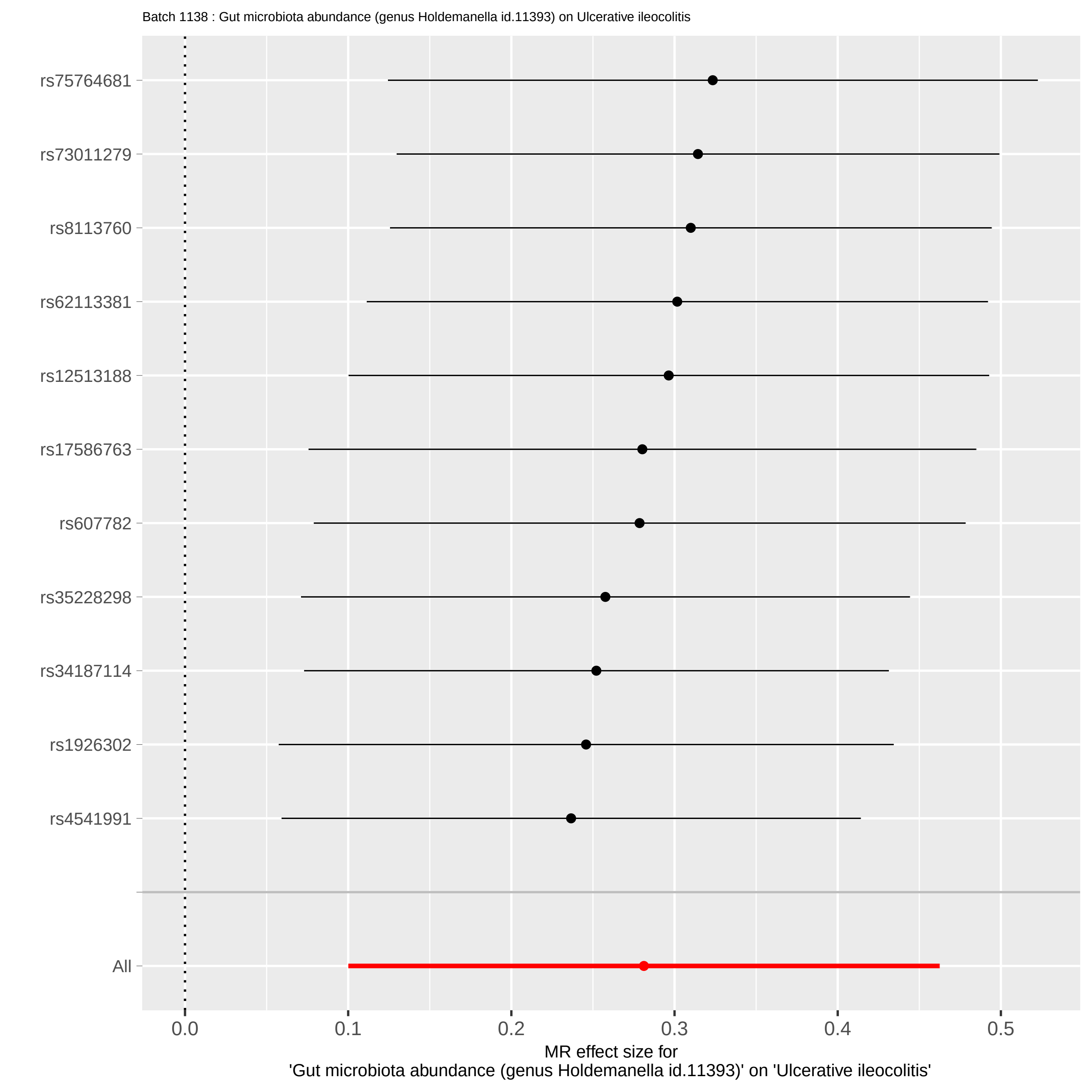

Batch 1138 : Gut microbiota abundance (genus Holdemanella id.11393) on Ulcerative ileocolitis
rs75764681
rs73011279
rs8113760
rs62113381
rs12513188
rs17586763
rs607782
rs35228298
rs34187114
rs1926302
rs4541991
All
0.0
0.1
0.2
0.3
0.4
0.5
MR effect size for
'Gut microbiota abundance (genus Holdemanella id.11393)' on 'Ulcerative ileocolitis'

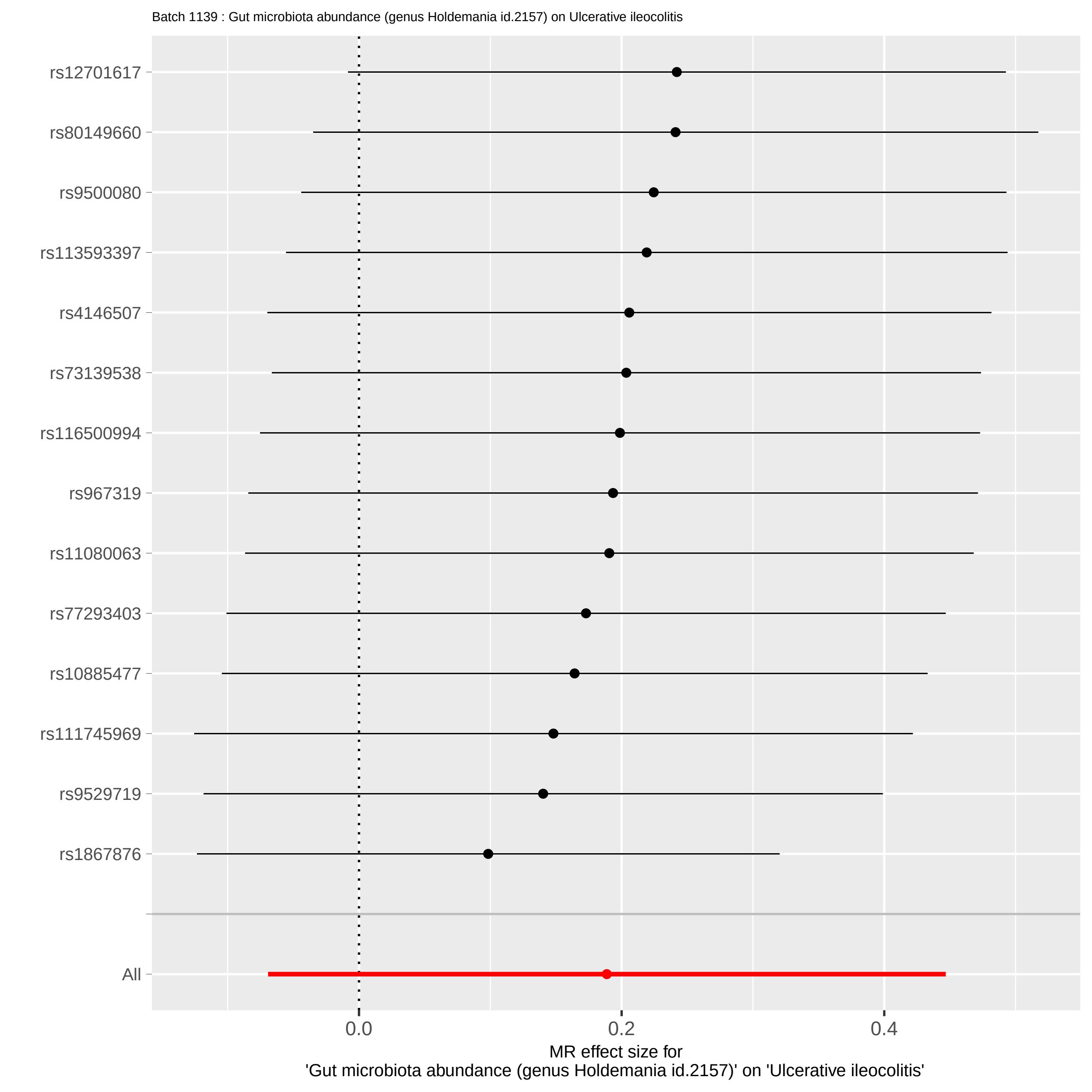

Batch 1139 : Gut microbiota abundance (genus Holdemania id.2157) on Ulcerative ileocolitis
rs12701617
rs80149660
rs9500080
rs113593397
rs4146507
rs73139538
rs116500994
rs967319
rs11080063
rs77293403
rs10885477
rs111745969
rs9529719
rs1867876
All
0.0
0.2
0.4
MR effect size for
'Gut microbiota abundance (genus Holdemania id.2157)' on 'Ulcerative ileocolitis'

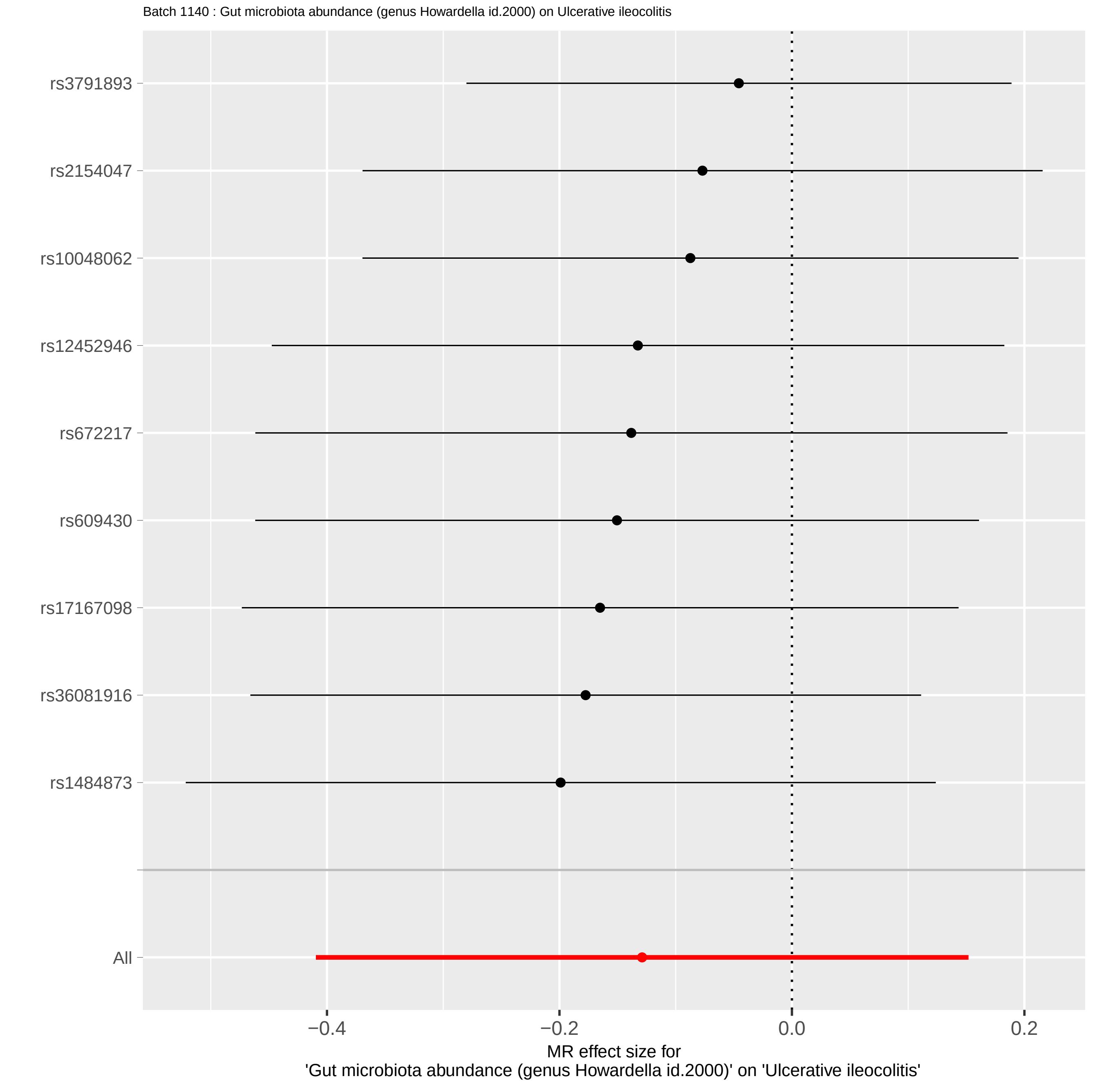

Batch 1140 : Gut microbiota abundance (genus Howardella id.2000) on Ulcerative ileocolitis
rs3791893
rs2154047
rs10048062
rs12452946
rs672217
rs609430
rs17167098
rs36081916
rs1484873
All
−0.4
−0.2
0.0
0.2
MR effect size for
'Gut microbiota abundance (genus Howardella id.2000)' on 'Ulcerative ileocolitis'

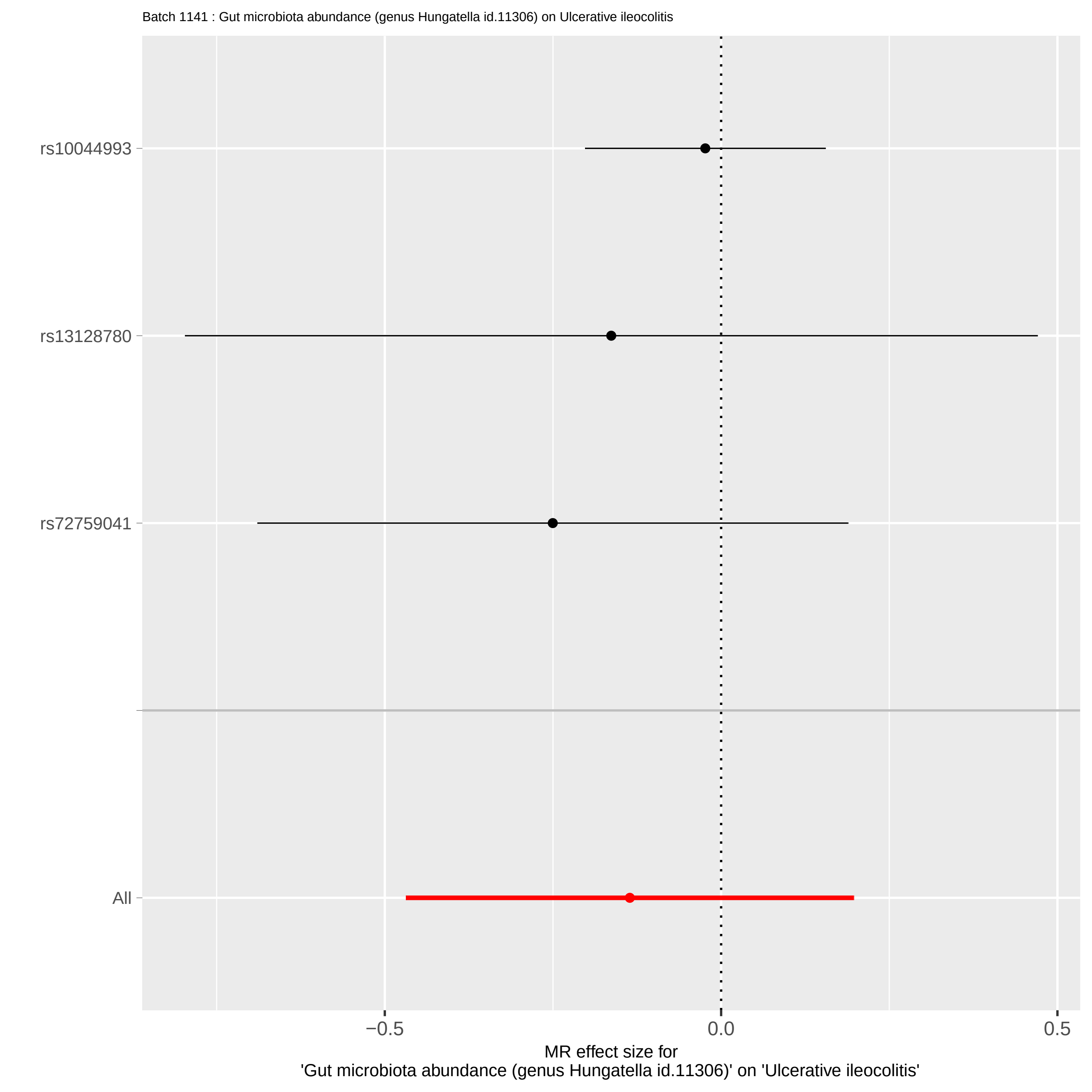
Batch 1141 : Gut microbiota abundance (genus Hungatella id.11306) on Ulcerative ileocolitis
rs10044993
rs13128780
rs72759041
All
−0.5
0.0
0.5
MR effect size for
'Gut microbiota abundance (genus Hungatella id.11306)' on 'Ulcerative ileocolitis'

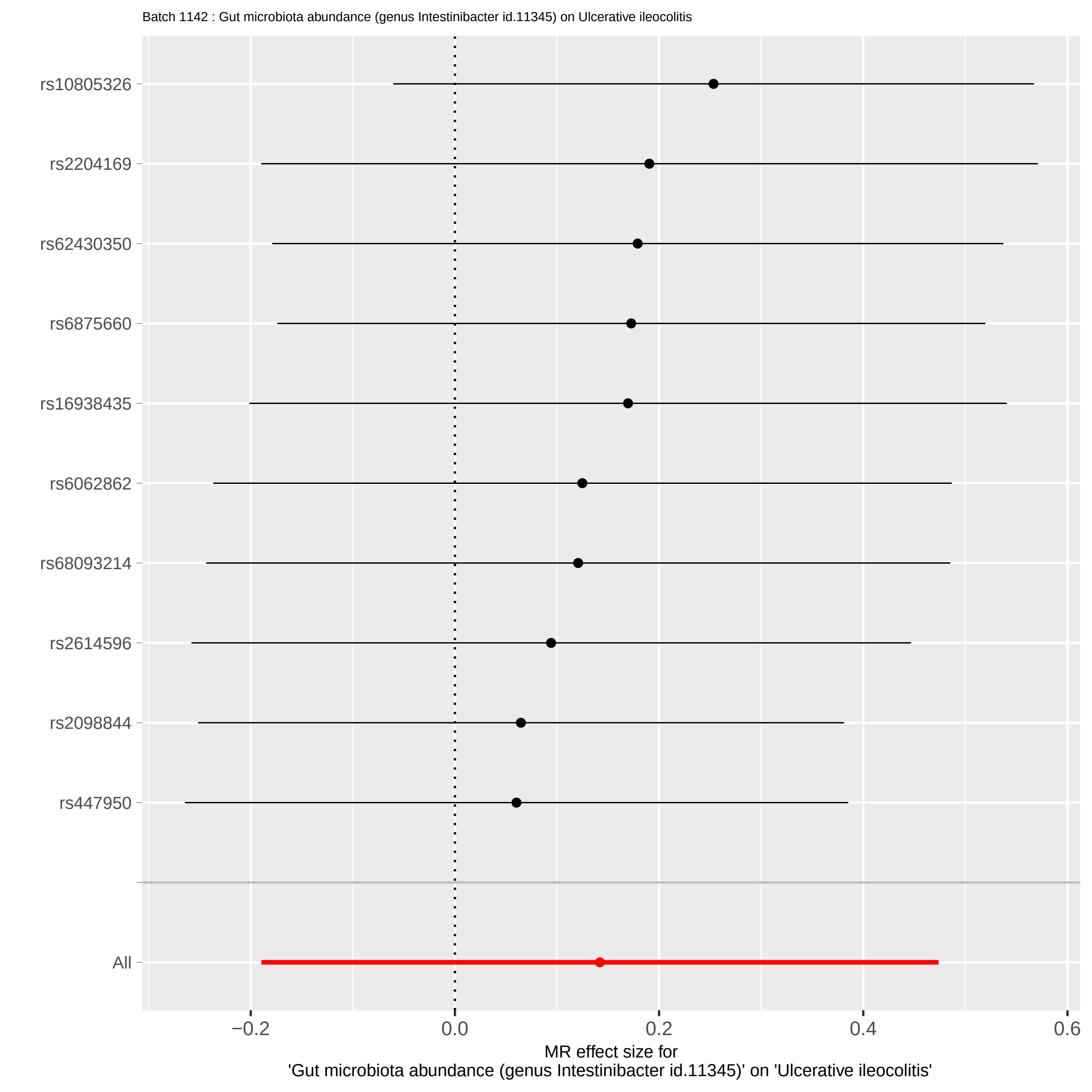

Batch 1142 : Gut microbiota abundance (genus Intestinibacter id.11345) on Ulcerative ileocolitis
rs10805326
rs2204169
rs62430350
rs6875660
rs16938435
rs6062862
rs68093214
rs2614596
rs2098844
rs447950
All
−0.2
0.0
0.2
0.4
0.6
MR effect size for
'Gut microbiota abundance (genus Intestinibacter id.11345)' on 'Ulcerative ileocolitis'

Batch 1143 : Gut microbiota abundance (genus Intestinimonas id.2062) on Ulcerative ileocolitis

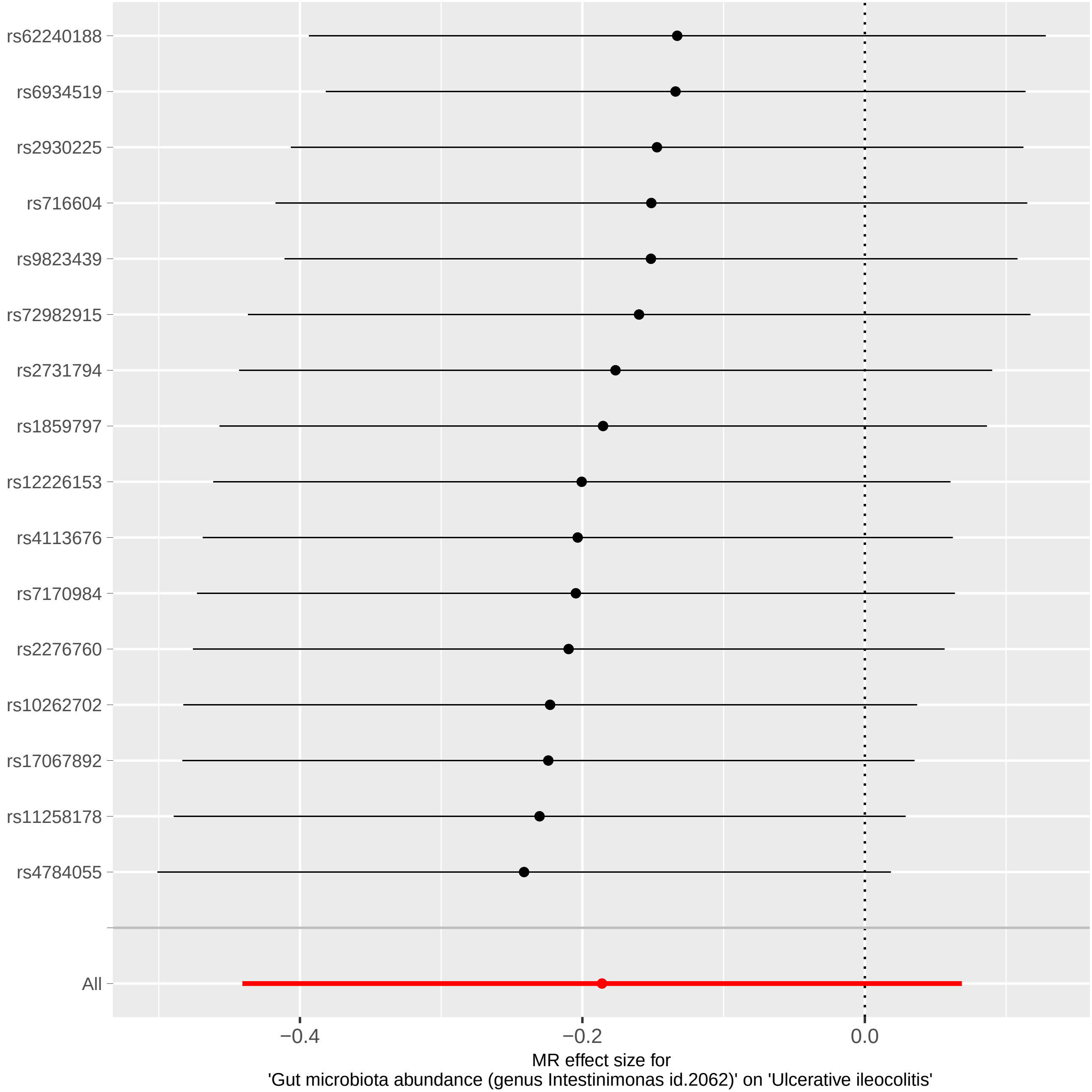

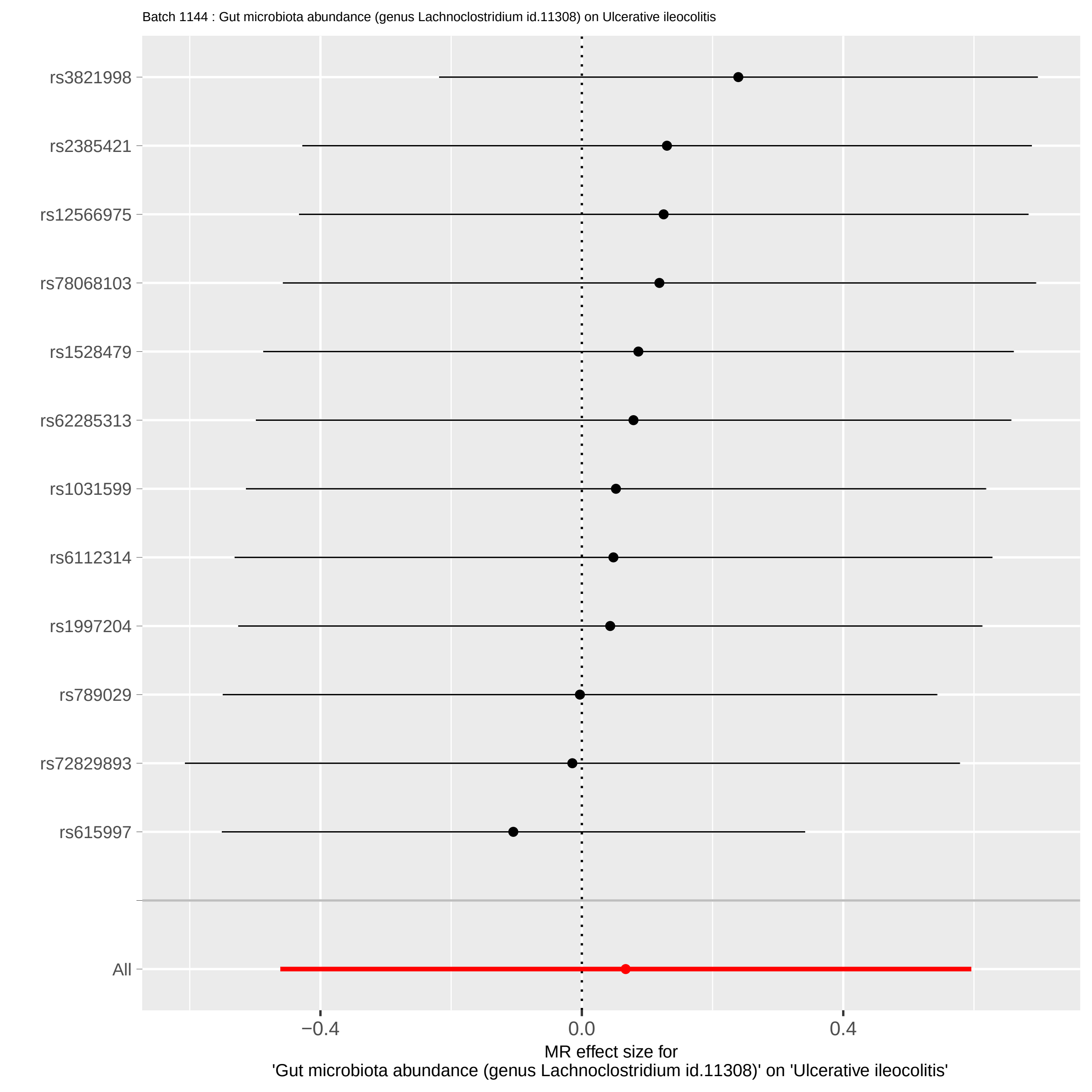
Batch 1144 : Gut microbiota abundance (genus Lachnoclostridium id.11308) on Ulcerative ileocolitis
rs3821998
rs2385421
rs12566975
rs78068103
rs1528479
rs62285313
rs1031599
rs6112314
rs1997204
rs789029
rs72829893
rs615997
All
−0.4
0.0
0.4
MR effect size for
'Gut microbiota abundance (genus Lachnoclostridium id.11308)' on 'Ulcerative ileocolitis'

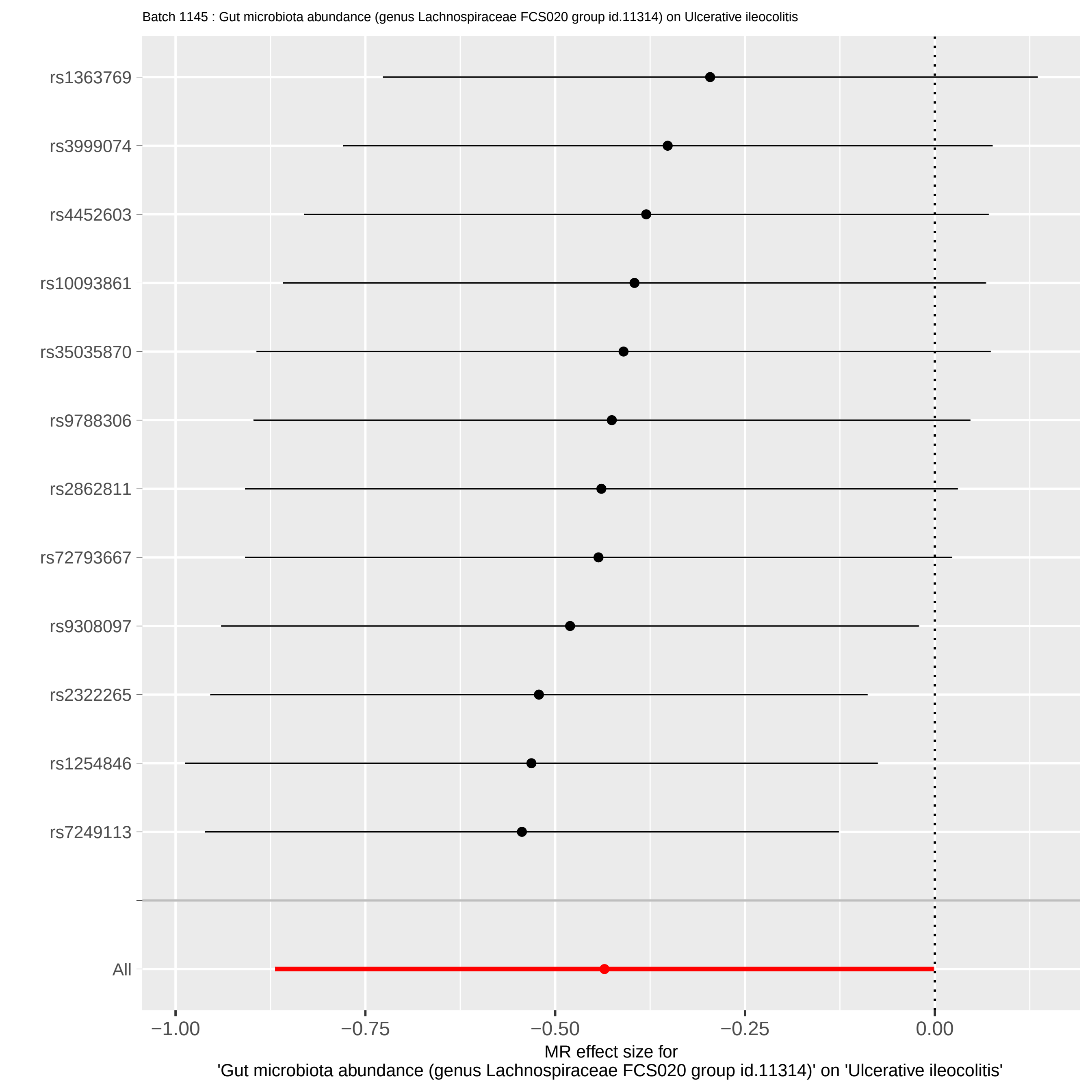

Batch 1145 : Gut microbiota abundance (genus Lachnospiraceae FCS020 group id.11314) on Ulcerative ileocolitis
rs1363769
rs3999074
rs4452603
rs10093861
rs35035870
rs9788306
rs2862811
rs72793667
rs9308097
rs2322265
rs1254846
rs7249113
All
−1.00
−0.75
−0.50
−0.25
0.00
MR effect size for
'Gut microbiota abundance (genus Lachnospiraceae FCS020 group id.11314)' on 'Ulcerative ileocolitis'

Batch 1146 : Gut microbiota abundance (genus Lachnospiraceae NC2004 group id.11316) on Ulcerative ileocolitis

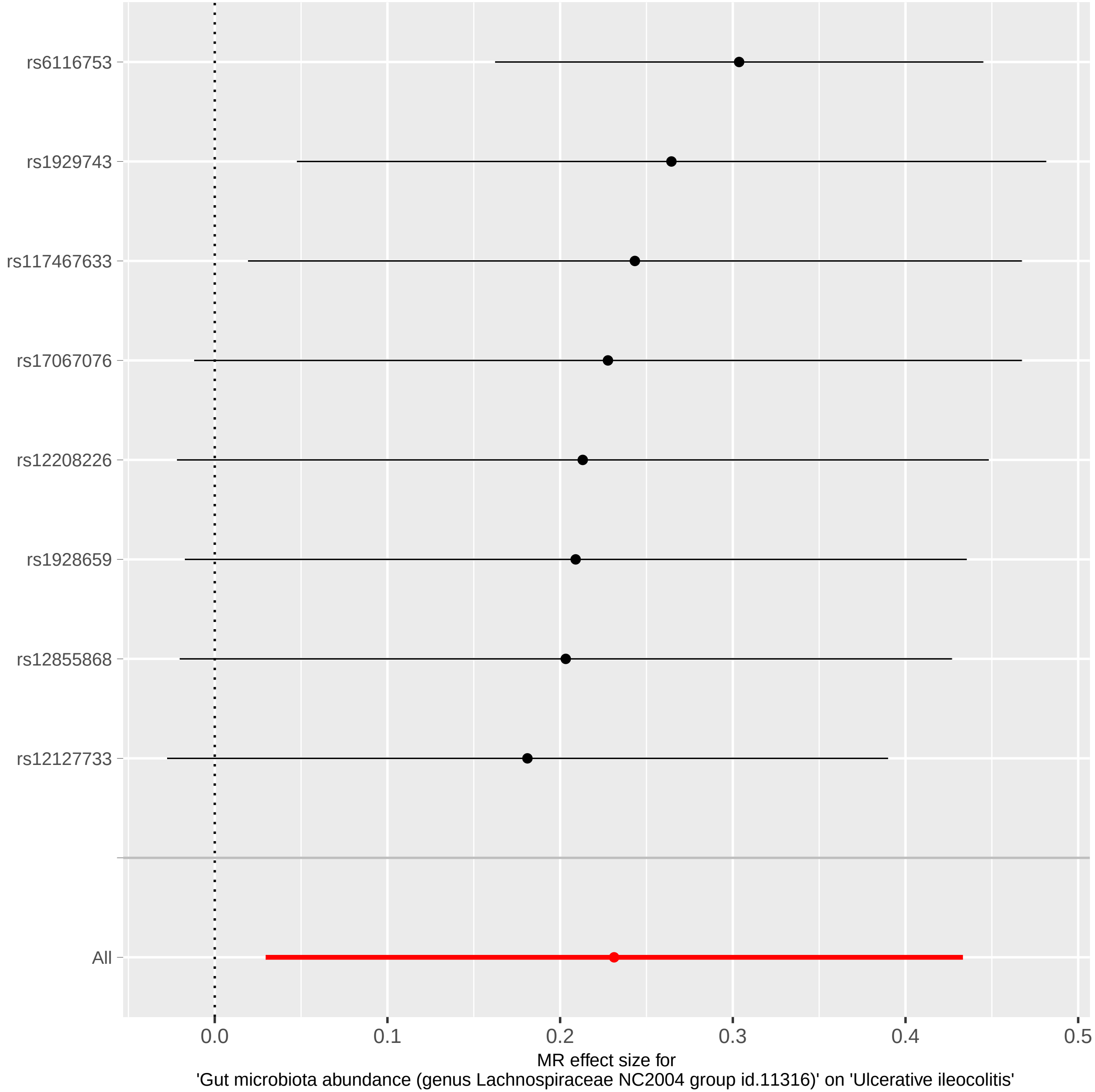

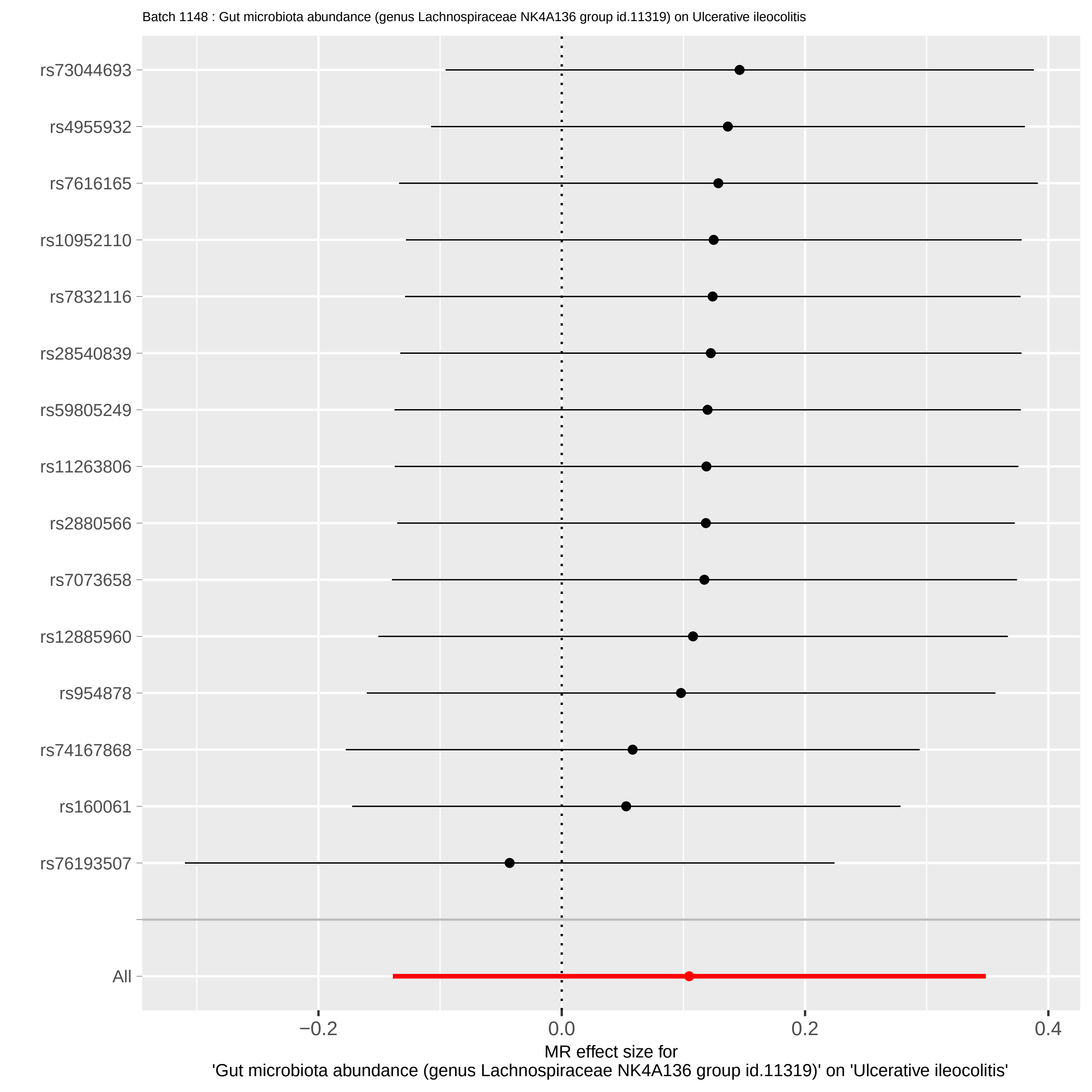

Batch 1148 : Gut microbiota abundance (genus Lachnospiraceae NK4A136 group id.11319) on Ulcerative ileocolitis
rs73044693
rs4955932
rs7616165
rs10952110
rs7832116
rs28540839
rs59805249
rs11263806
rs2880566
rs7073658
rs12885960
rs954878
rs74167868
rs160061
rs76193507
All
−0.2
0.0
0.2
0.4
MR effect size for
'Gut microbiota abundance (genus Lachnospiraceae NK4A136 group id.11319)' on 'Ulcerative ileocolitis'

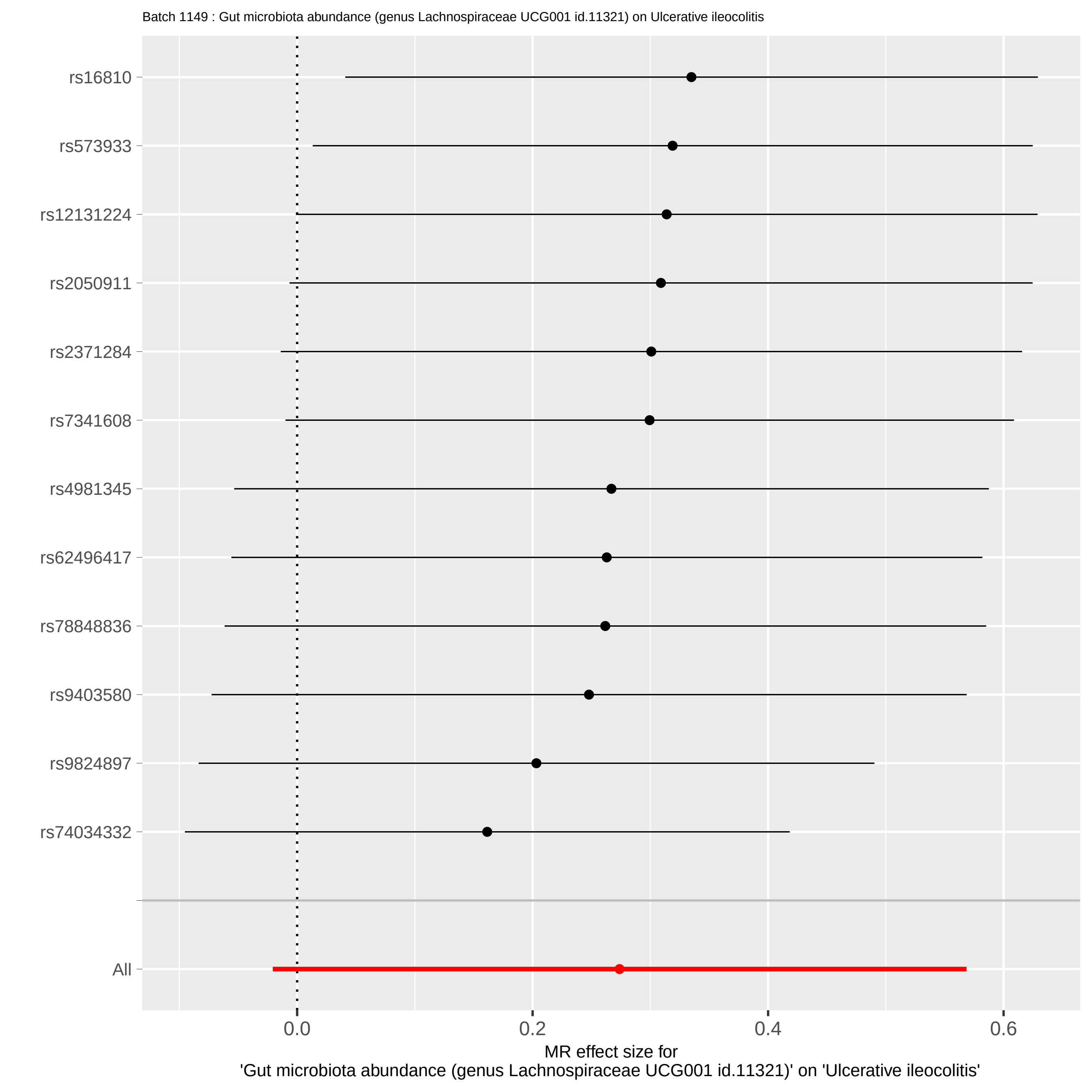

Batch 1149 : Gut microbiota abundance (genus Lachnospiraceae UCG001 id.11321) on Ulcerative ileocolitis
rs16810
rs573933
rs12131224
rs2050911
rs2371284
rs7341608
rs4981345
rs62496417
rs78848836
rs9403580
rs9824897
rs74034332
All
0.0
0.2
0.4
0.6
MR effect size for
'Gut microbiota abundance (genus Lachnospiraceae UCG001 id.11321)' on 'Ulcerative ileocolitis'

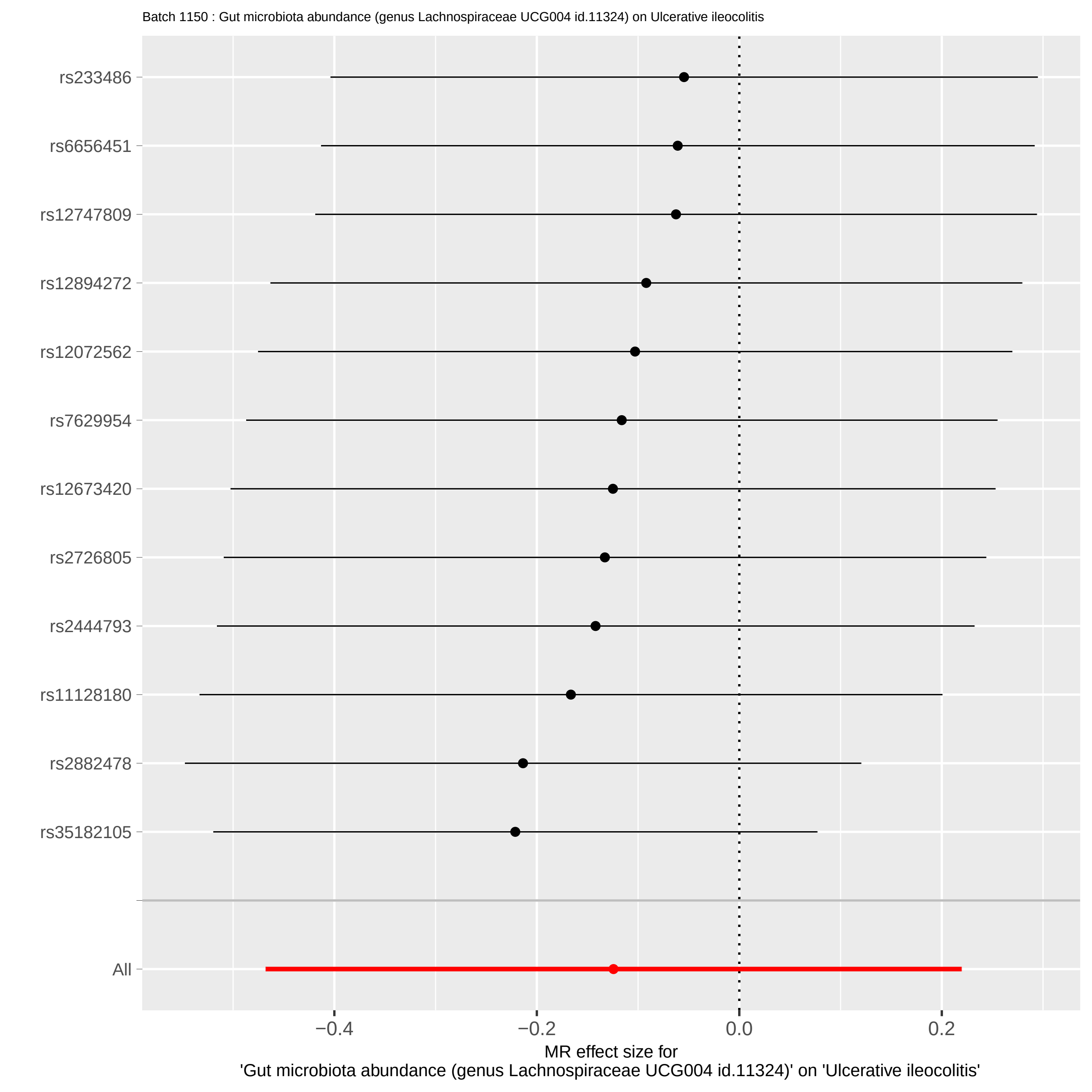
Batch 1150 : Gut microbiota abundance (genus Lachnospiraceae UCG004 id.11324) on Ulcerative ileocolitis
rs233486
rs6656451
rs12747809
rs12894272
rs12072562
rs7629954
rs12673420
rs2726805
rs2444793
rs11128180
rs2882478
rs35182105
All
−0.4
−0.2
0.0
0.2
MR effect size for
'Gut microbiota abundance (genus Lachnospiraceae UCG004 id.11324)' on 'Ulcerative ileocolitis'

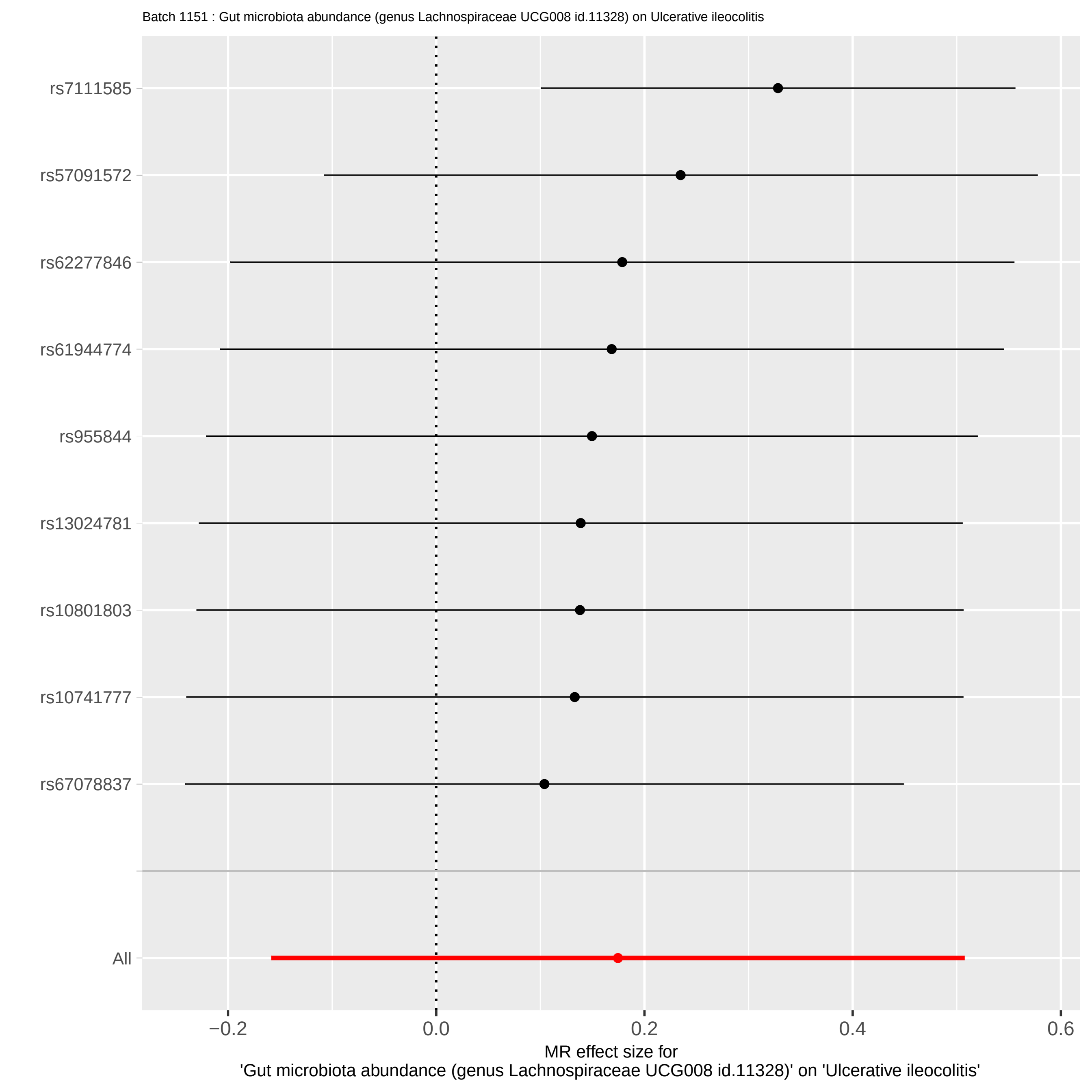

Batch 1151 : Gut microbiota abundance (genus Lachnospiraceae UCG008 id.11328) on Ulcerative ileocolitis
rs7111585
rs57091572
rs62277846
rs61944774
rs955844
rs13024781
rs10801803
rs10741777
rs67078837
All
−0.2
0.0
0.2
0.4
0.6
MR effect size for
'Gut microbiota abundance (genus Lachnospiraceae UCG008 id.11328)' on 'Ulcerative ileocolitis'

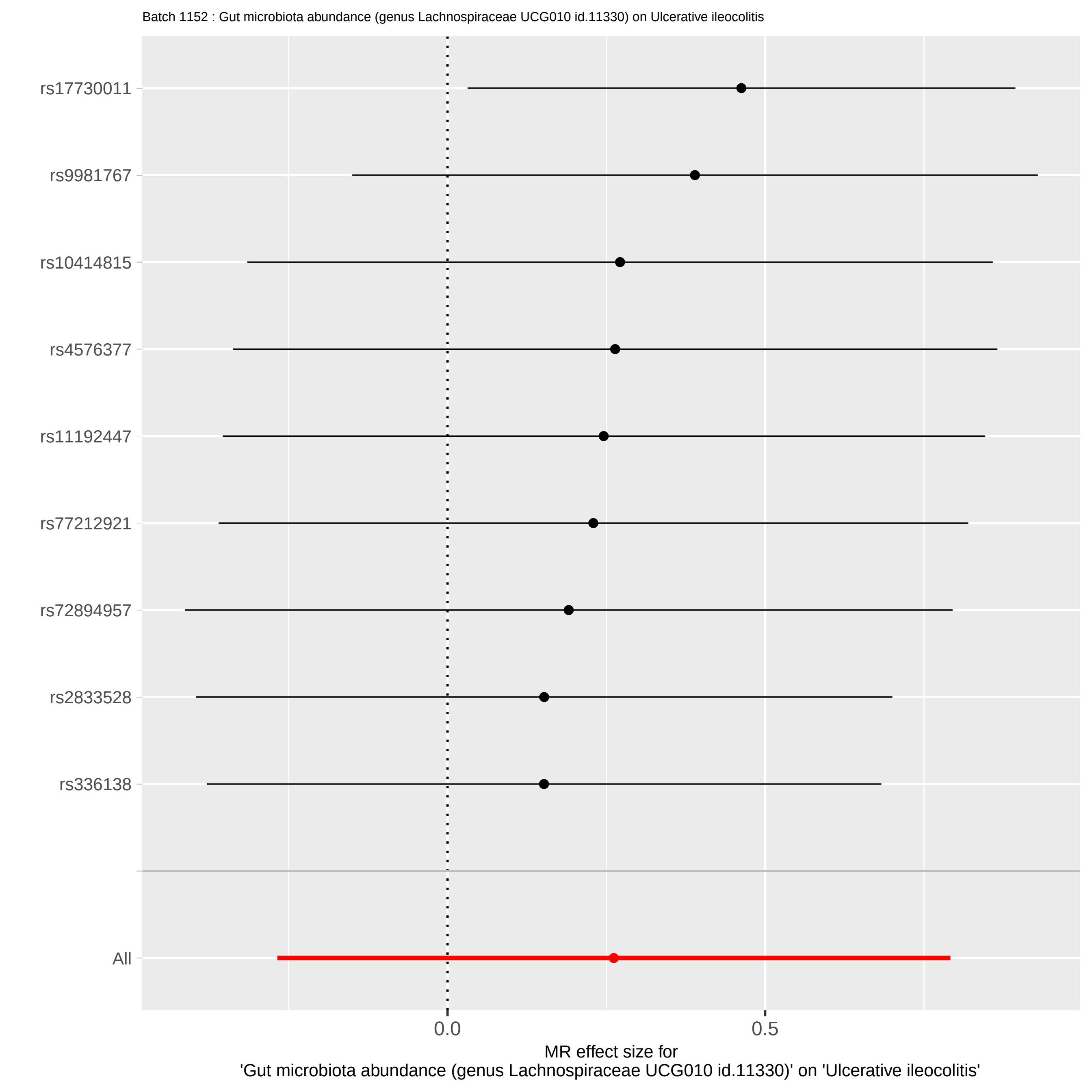

Batch 1152 : Gut microbiota abundance (genus Lachnospiraceae UCG010 id.11330) on Ulcerative ileocolitis
rs17730011
rs9981767
rs10414815
rs4576377
rs11192447
rs77212921
rs72894957
rs2833528
rs336138
All
0.0
0.5
MR effect size for
'Gut microbiota abundance (genus Lachnospiraceae UCG010 id.11330)' on 'Ulcerative ileocolitis'

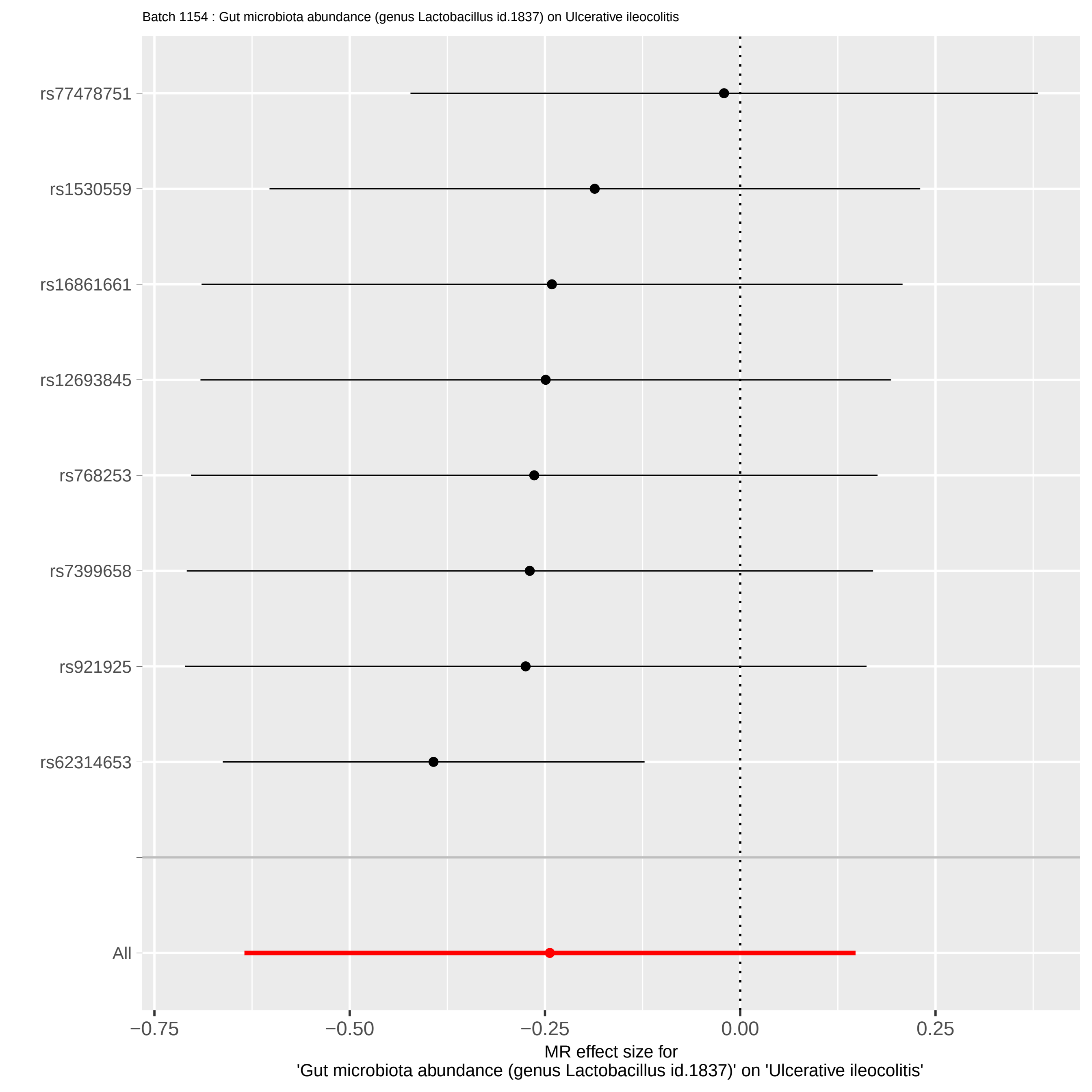

Batch 1154 : Gut microbiota abundance (genus Lactobacillus id.1837) on Ulcerative ileocolitis
rs77478751
rs1530559
rs16861661
rs12693845
rs768253
rs7399658
rs921925
rs62314653
All
−0.75
−0.50
−0.25
0.00
0.25
MR effect size for
'Gut microbiota abundance (genus Lactobacillus id.1837)' on 'Ulcerative ileocolitis'

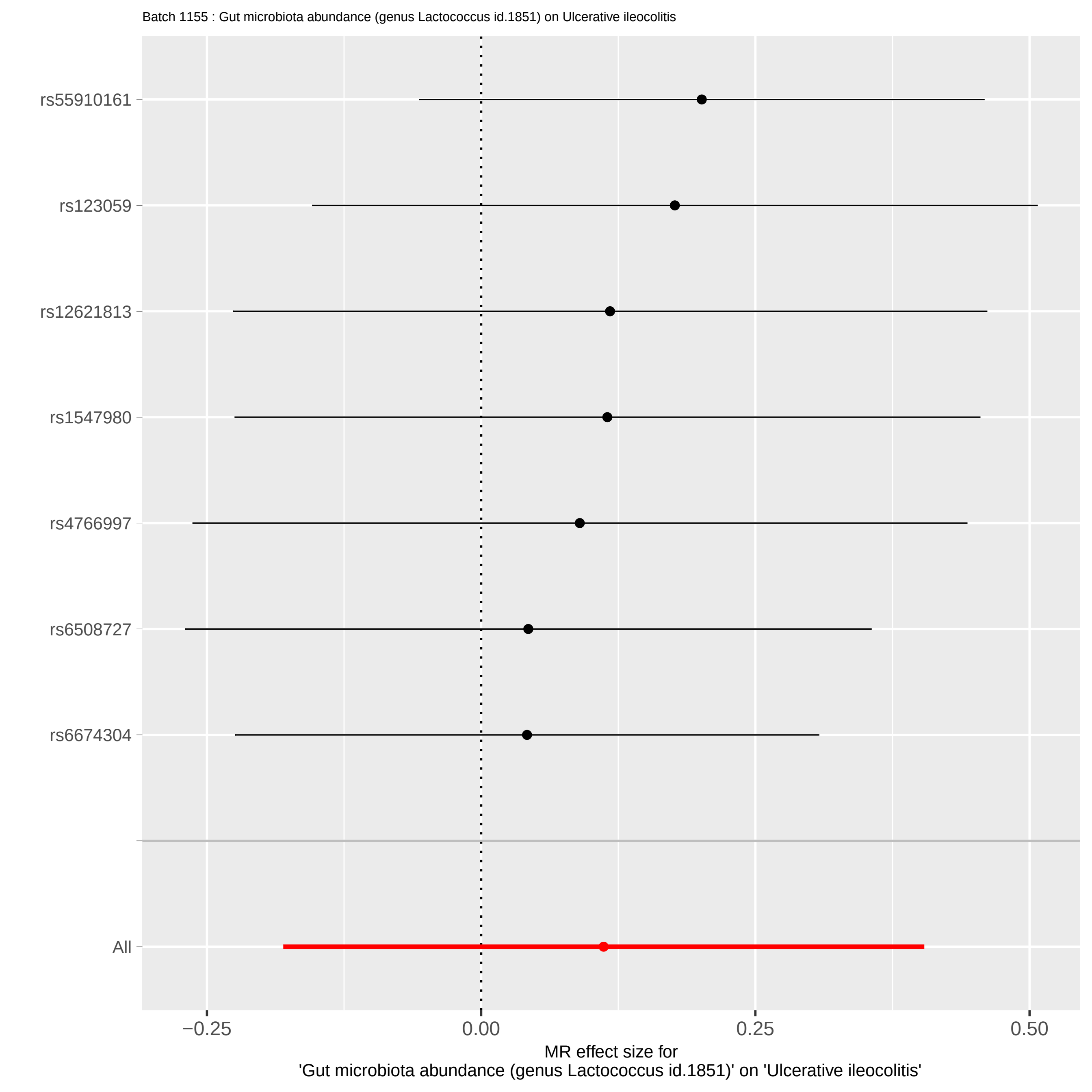

Batch 1155 : Gut microbiota abundance (genus Lactococcus id.1851) on Ulcerative ileocolitis
rs55910161
rs123059
rs12621813
rs1547980
rs4766997
rs6508727
rs6674304
All
−0.25
0.00
0.25
0.50
MR effect size for
'Gut microbiota abundance (genus Lactococcus id.1851)' on 'Ulcerative ileocolitis'

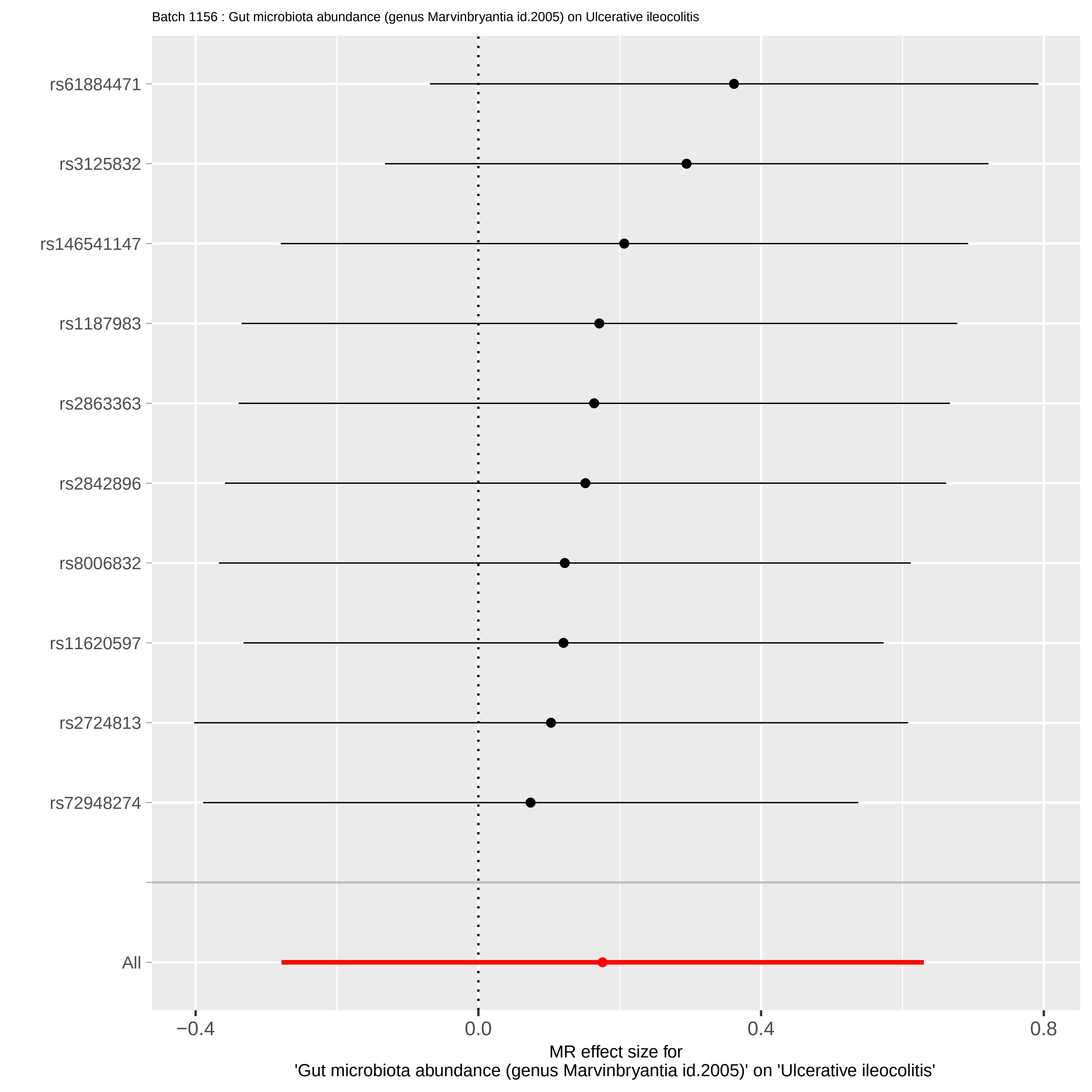

Batch 1156 : Gut microbiota abundance (genus Marvinbryantia id.2005) on Ulcerative ileocolitis
rs61884471
rs3125832
rs146541147
rs1187983
rs2863363
rs2842896
rs8006832
rs11620597
rs2724813
rs72948274
All
−0.4
0.0
0.4
0.8
MR effect size for
'Gut microbiota abundance (genus Marvinbryantia id.2005)' on 'Ulcerative ileocolitis'

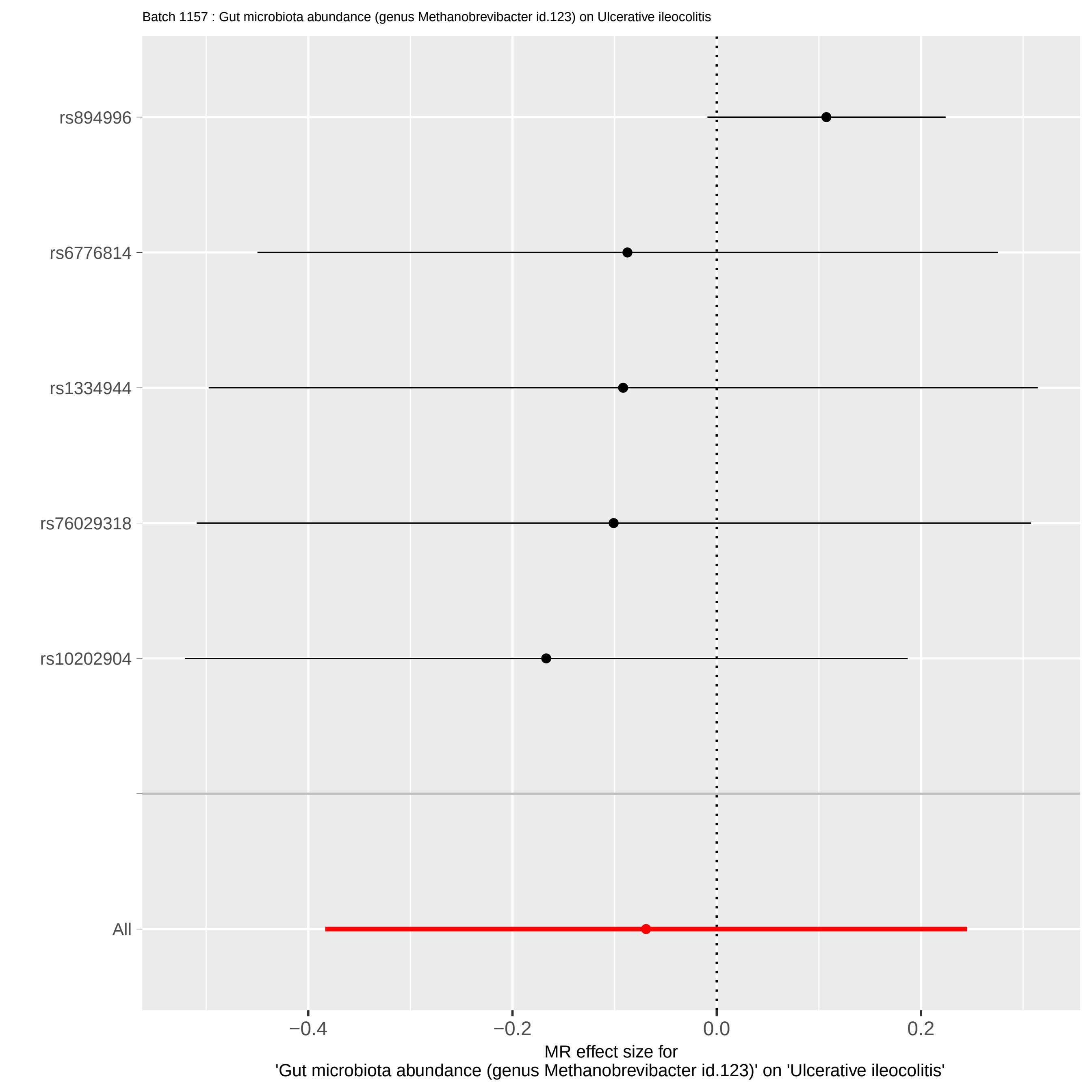
Batch 1157 : Gut microbiota abundance (genus Methanobrevibacter id.123) on Ulcerative ileocolitis
rs894996
rs6776814
rs1334944
rs76029318
rs10202904
All
−0.4
−0.2
0.0
0.2
MR effect size for
'Gut microbiota abundance (genus Methanobrevibacter id.123)' on 'Ulcerative ileocolitis'

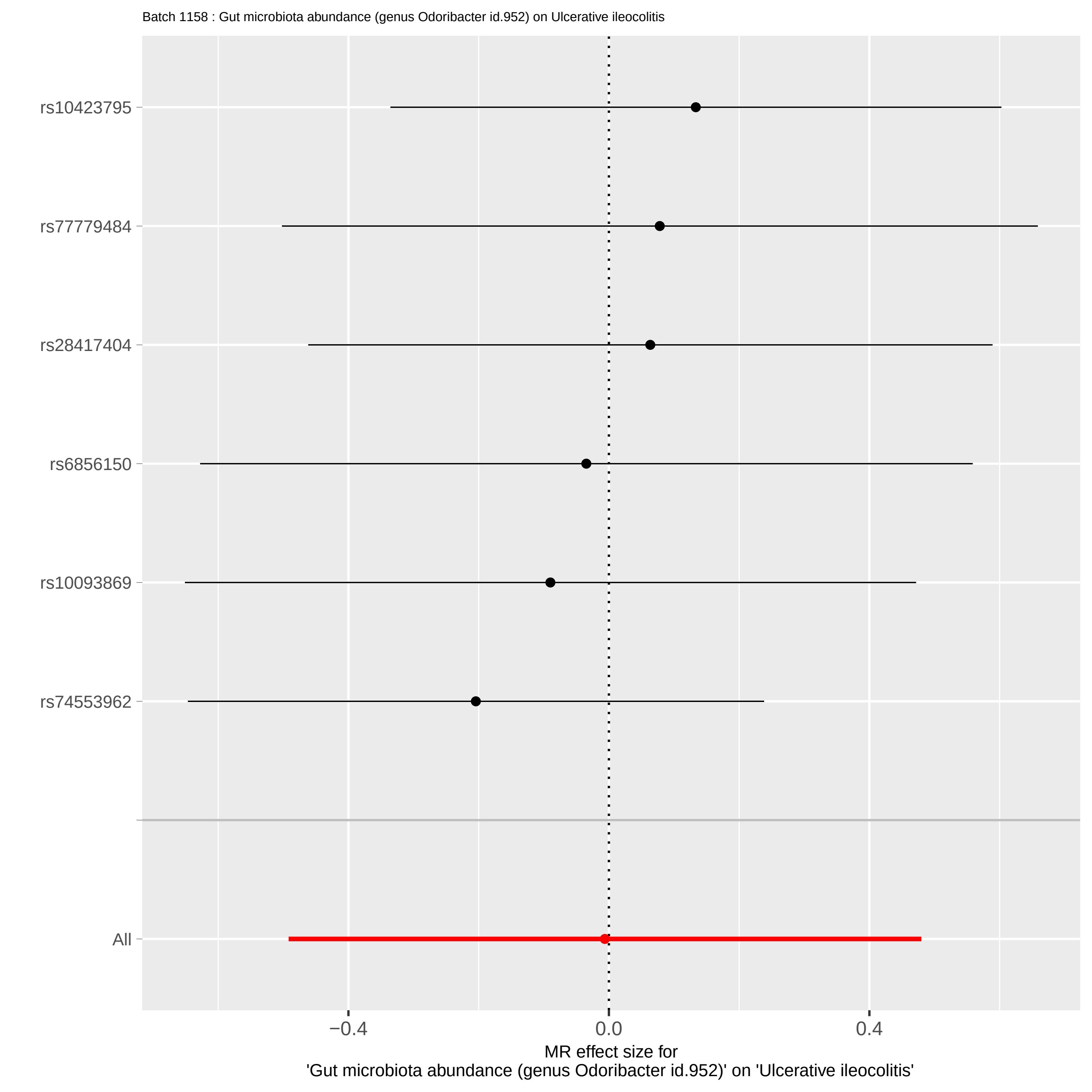

Batch 1158 : Gut microbiota abundance (genus Odoribacter id.952) on Ulcerative ileocolitis
rs10423795
rs77779484
rs28417404
rs6856150
rs10093869
rs74553962
All
−0.4
0.0
0.4
MR effect size for
'Gut microbiota abundance (genus Odoribacter id.952)' on 'Ulcerative ileocolitis'

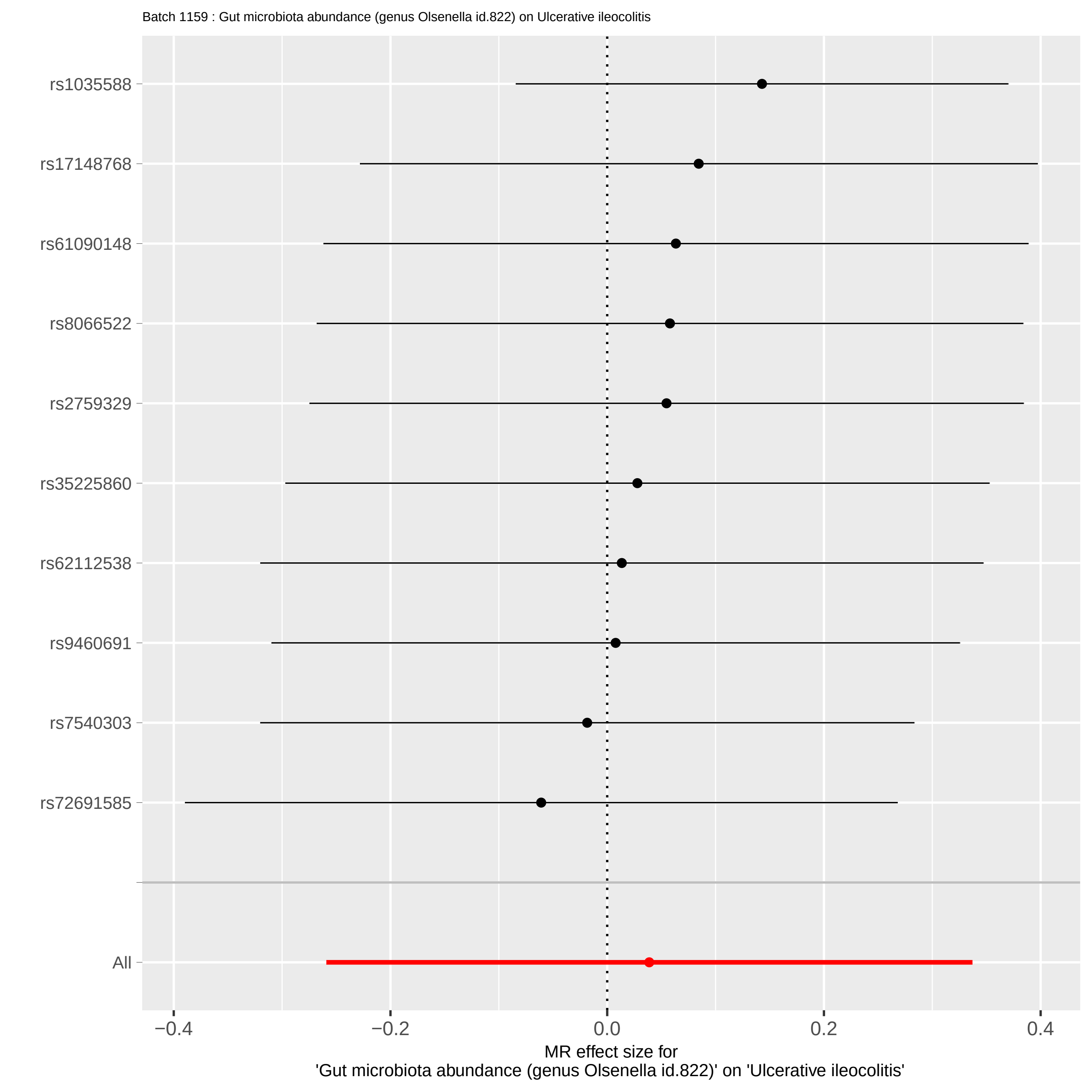
Batch 1159 : Gut microbiota abundance (genus Olsenella id.822) on Ulcerative ileocolitis
rs1035588
rs17148768
rs61090148
rs8066522
rs2759329
rs35225860
rs62112538
rs9460691
rs7540303
rs72691585
All
−0.4
−0.2
0.0
0.2
0.4
MR effect size for
'Gut microbiota abundance (genus Olsenella id.822)' on 'Ulcerative ileocolitis'

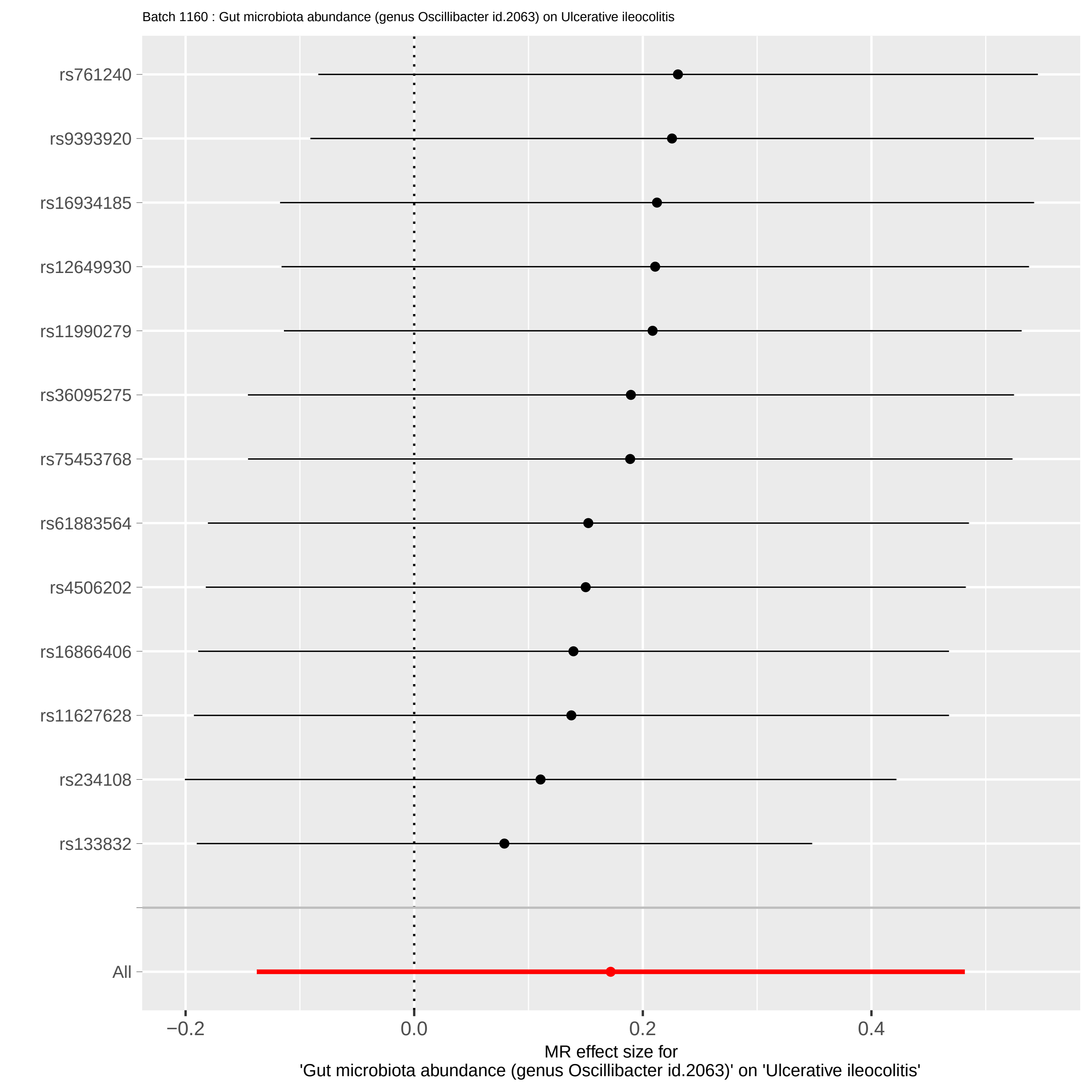
Batch 1160 : Gut microbiota abundance (genus Oscillibacter id.2063) on Ulcerative ileocolitis
rs761240
rs9393920
rs16934185
rs12649930
rs11990279
rs36095275
rs75453768
rs61883564
rs4506202
rs16866406
rs11627628
rs234108
rs133832
All
−0.2
0.0
0.2
0.4
MR effect size for
'Gut microbiota abundance (genus Oscillibacter id.2063)' on 'Ulcerative ileocolitis'

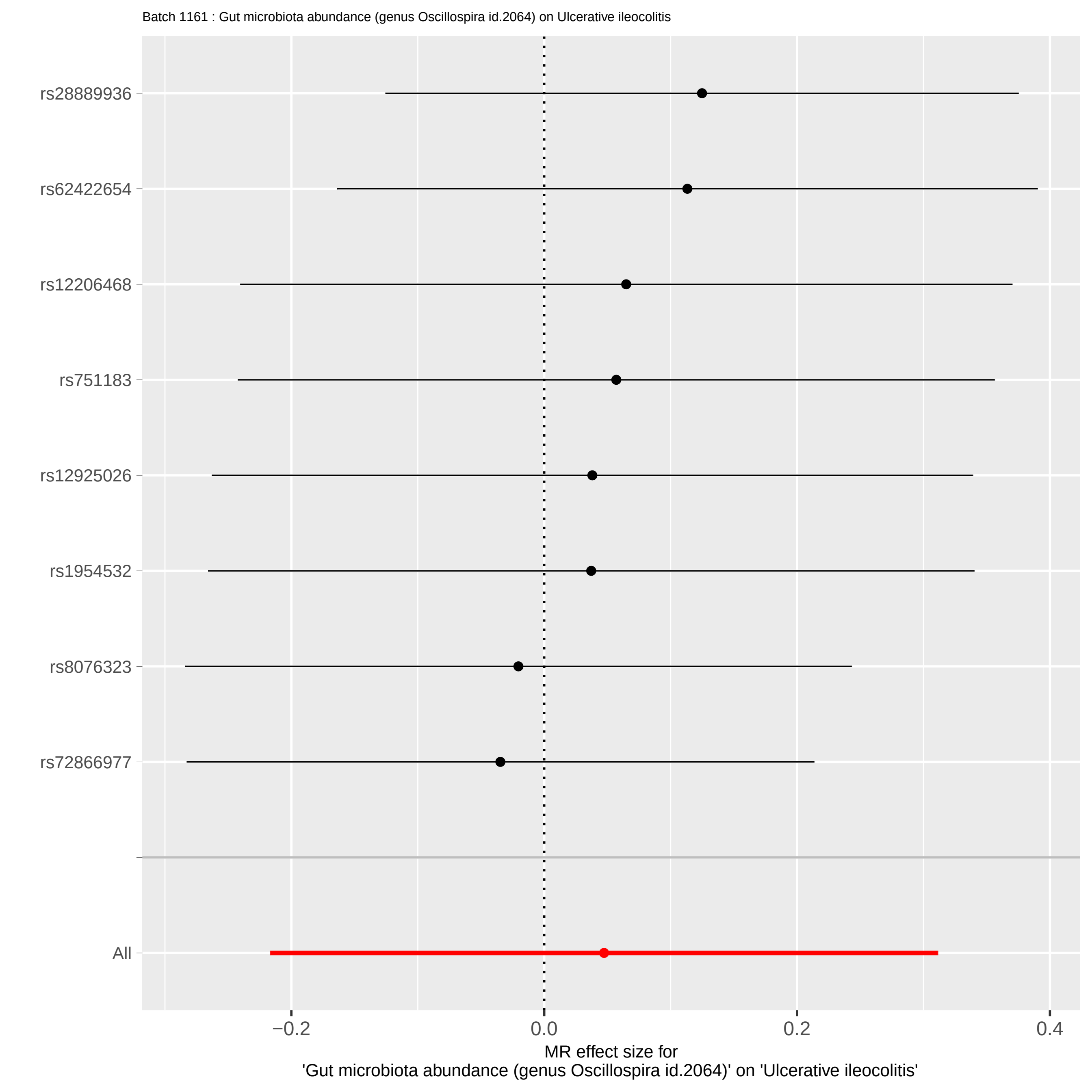

Batch 1161 : Gut microbiota abundance (genus Oscillospira id.2064) on Ulcerative ileocolitis
rs28889936
rs62422654
rs12206468
rs751183
rs12925026
rs1954532
rs8076323
rs72866977
All
−0.2
0.0
0.2
0.4
MR effect size for
'Gut microbiota abundance (genus Oscillospira id.2064)' on 'Ulcerative ileocolitis'

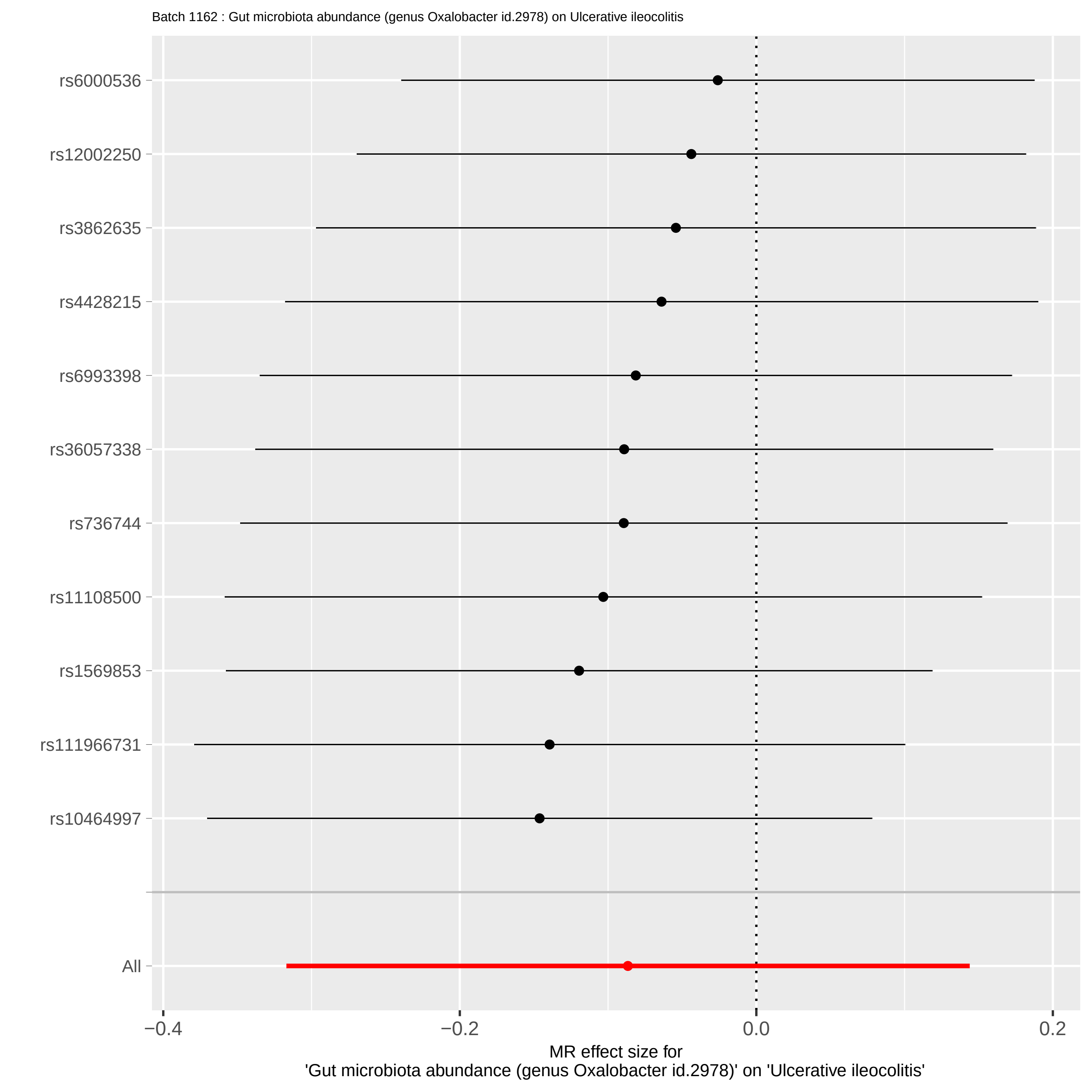

Batch 1162 : Gut microbiota abundance (genus Oxalobacter id.2978) on Ulcerative ileocolitis
rs6000536
rs12002250
rs3862635
rs4428215
rs6993398
rs36057338
rs736744
rs11108500
rs1569853
rs111966731
rs10464997
All
−0.4
−0.2
0.0
0.2
MR effect size for
'Gut microbiota abundance (genus Oxalobacter id.2978)' on 'Ulcerative ileocolitis'

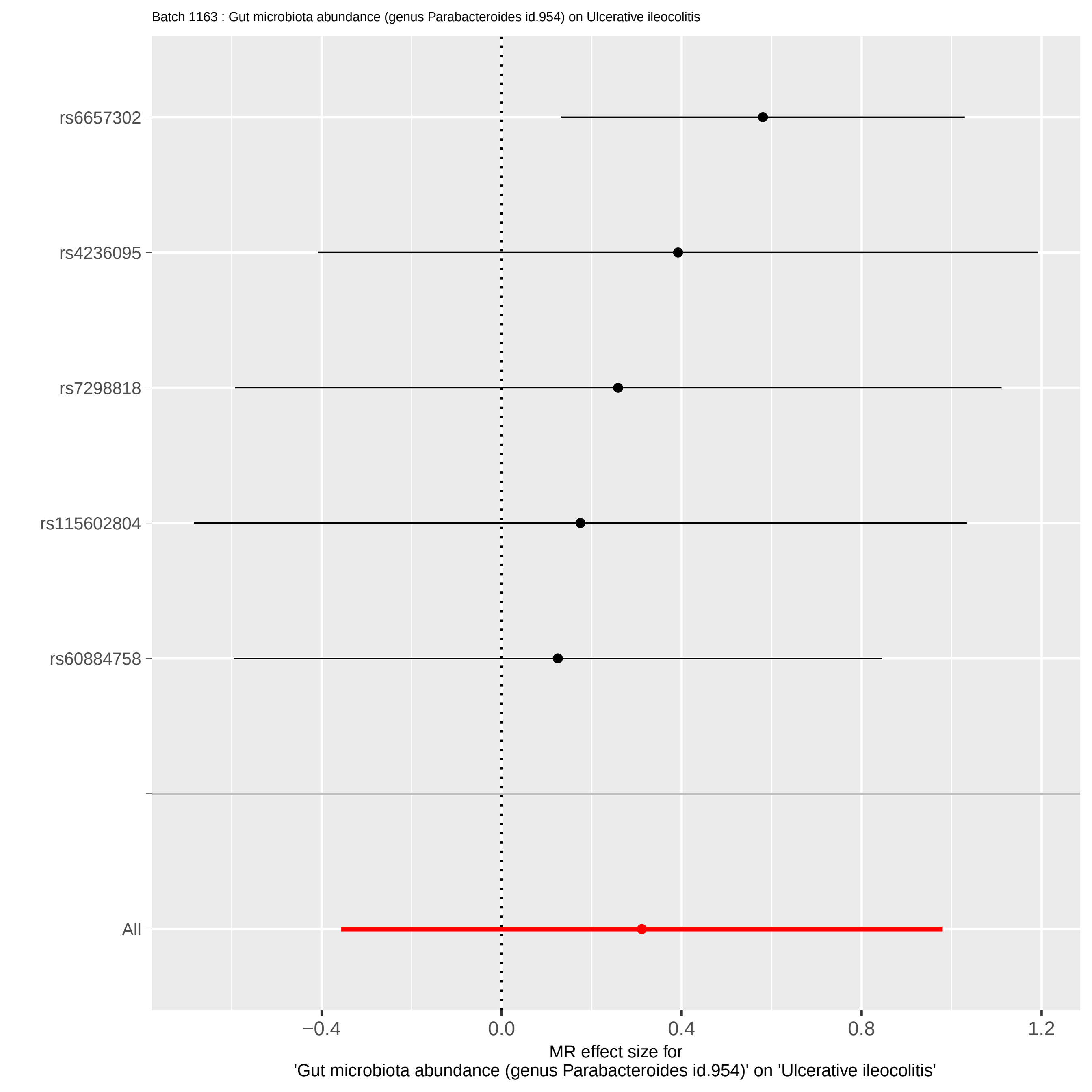

Batch 1163 : Gut microbiota abundance (genus Parabacteroides id.954) on Ulcerative ileocolitis
rs6657302
rs4236095
rs7298818
rs115602804
rs60884758
All
−0.4
0.0
0.4
0.8
1.2
MR effect size for
'Gut microbiota abundance (genus Parabacteroides id.954)' on 'Ulcerative ileocolitis'

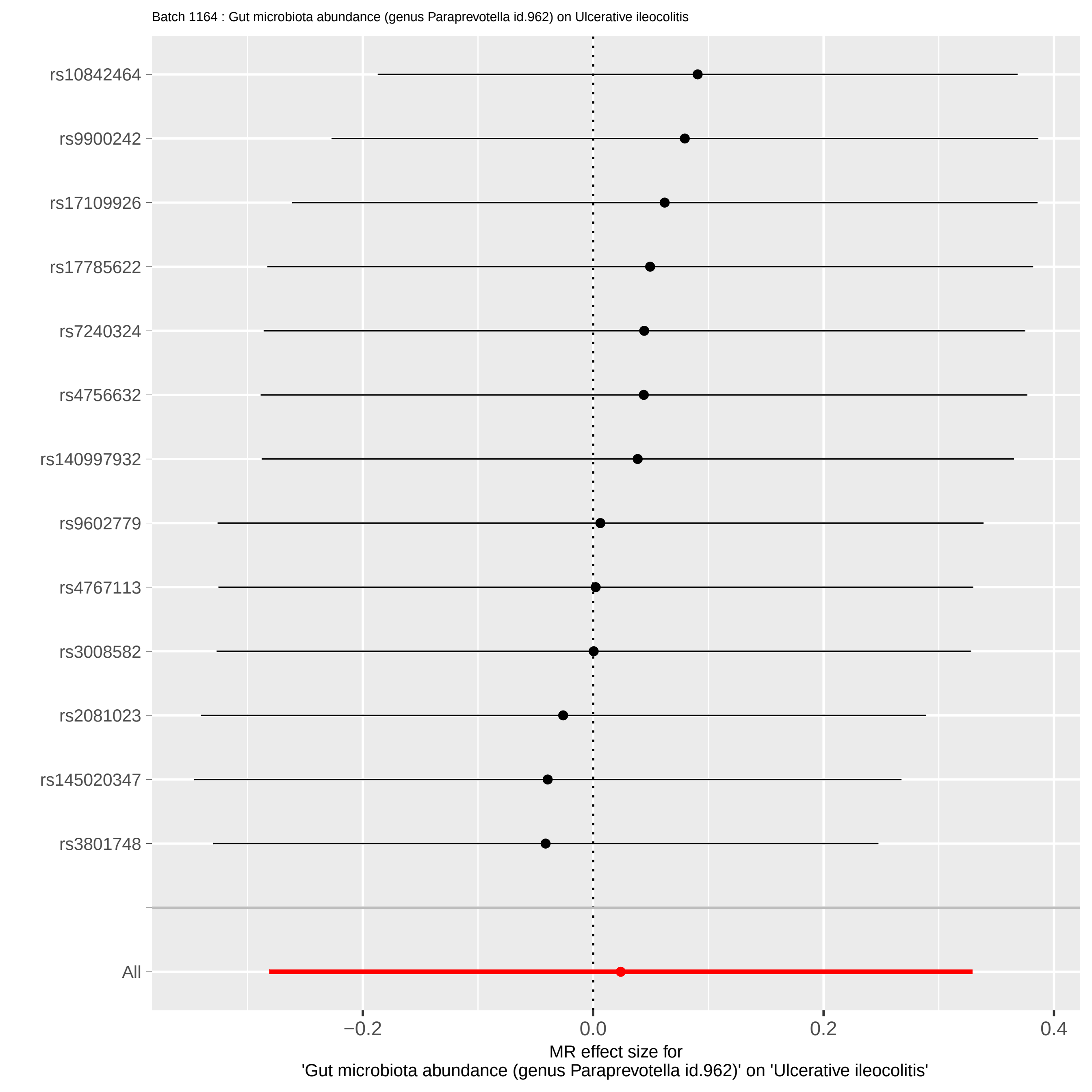
Batch 1164 : Gut microbiota abundance (genus Paraprevotella id.962) on Ulcerative ileocolitis
rs10842464
rs9900242
rs17109926
rs17785622
rs7240324
rs4756632
rs140997932
rs9602779
rs4767113
rs3008582
rs2081023
rs145020347
rs3801748
All
−0.2
0.0
0.2
0.4
MR effect size for
'Gut microbiota abundance (genus Paraprevotella id.962)' on 'Ulcerative ileocolitis'

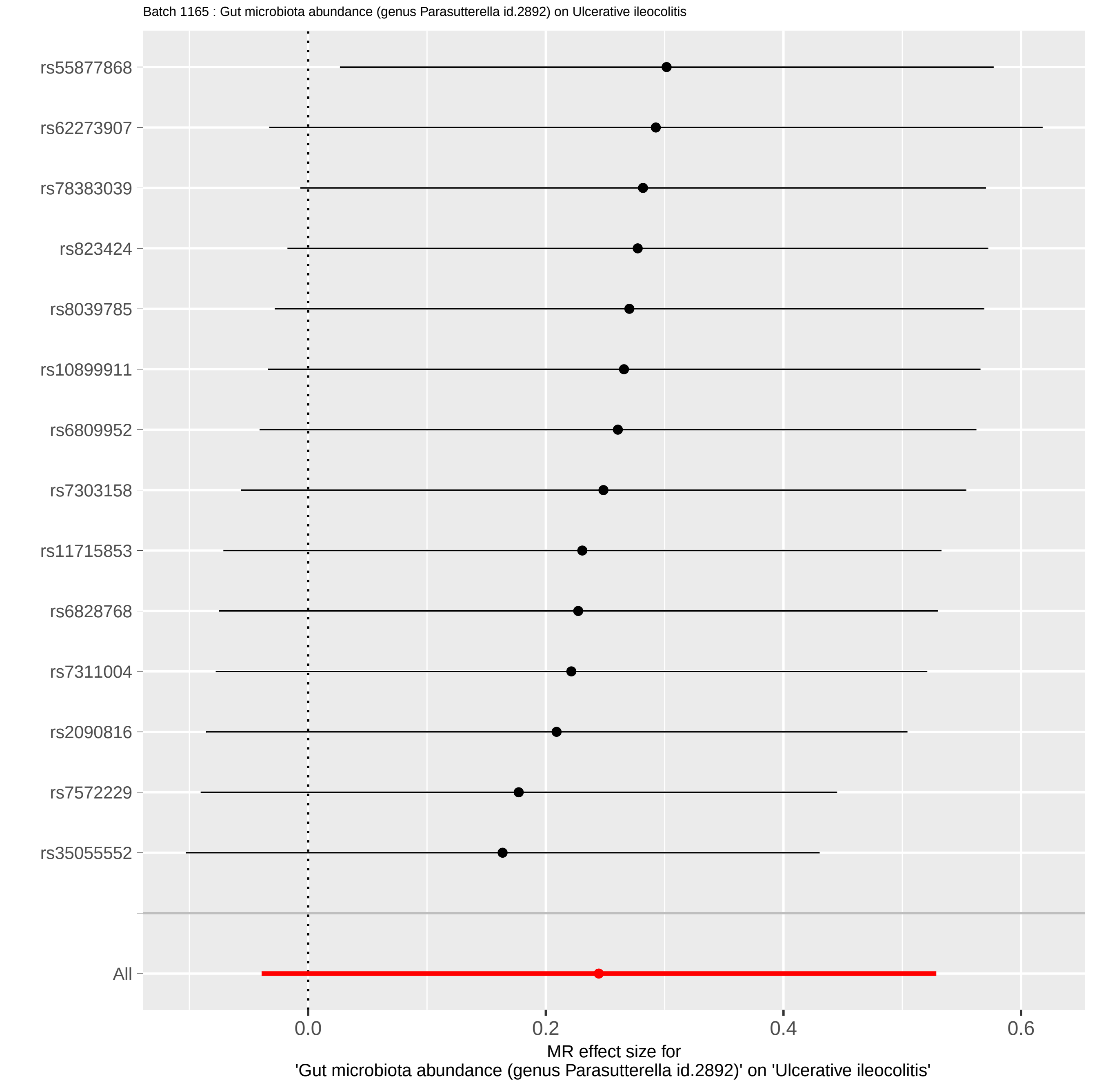

Batch 1165 : Gut microbiota abundance (genus Parasutterella id.2892) on Ulcerative ileocolitis
rs55877868
rs62273907
rs78383039
rs823424
rs8039785
rs10899911
rs6809952
rs7303158
rs11715853
rs6828768
rs7311004
rs2090816
rs7572229
rs35055552
All
0.0
0.2
0.4
0.6
MR effect size for
'Gut microbiota abundance (genus Parasutterella id.2892)' on 'Ulcerative ileocolitis'

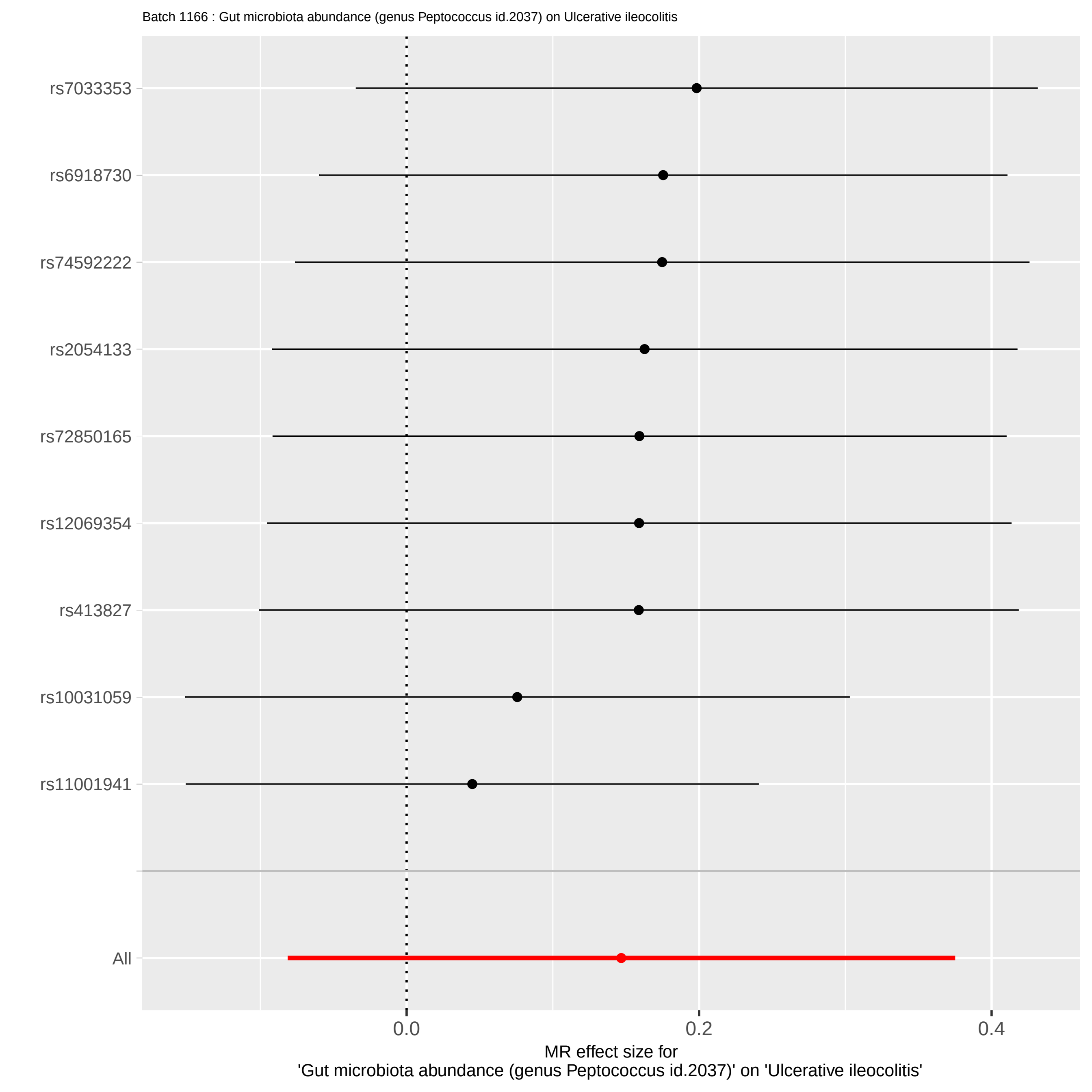

Batch 1166 : Gut microbiota abundance (genus Peptococcus id.2037) on Ulcerative ileocolitis
rs7033353
rs6918730
rs74592222
rs2054133
rs72850165
rs12069354
rs413827
rs10031059
rs11001941
All
0.0
0.2
0.4
MR effect size for
'Gut microbiota abundance (genus Peptococcus id.2037)' on 'Ulcerative ileocolitis'

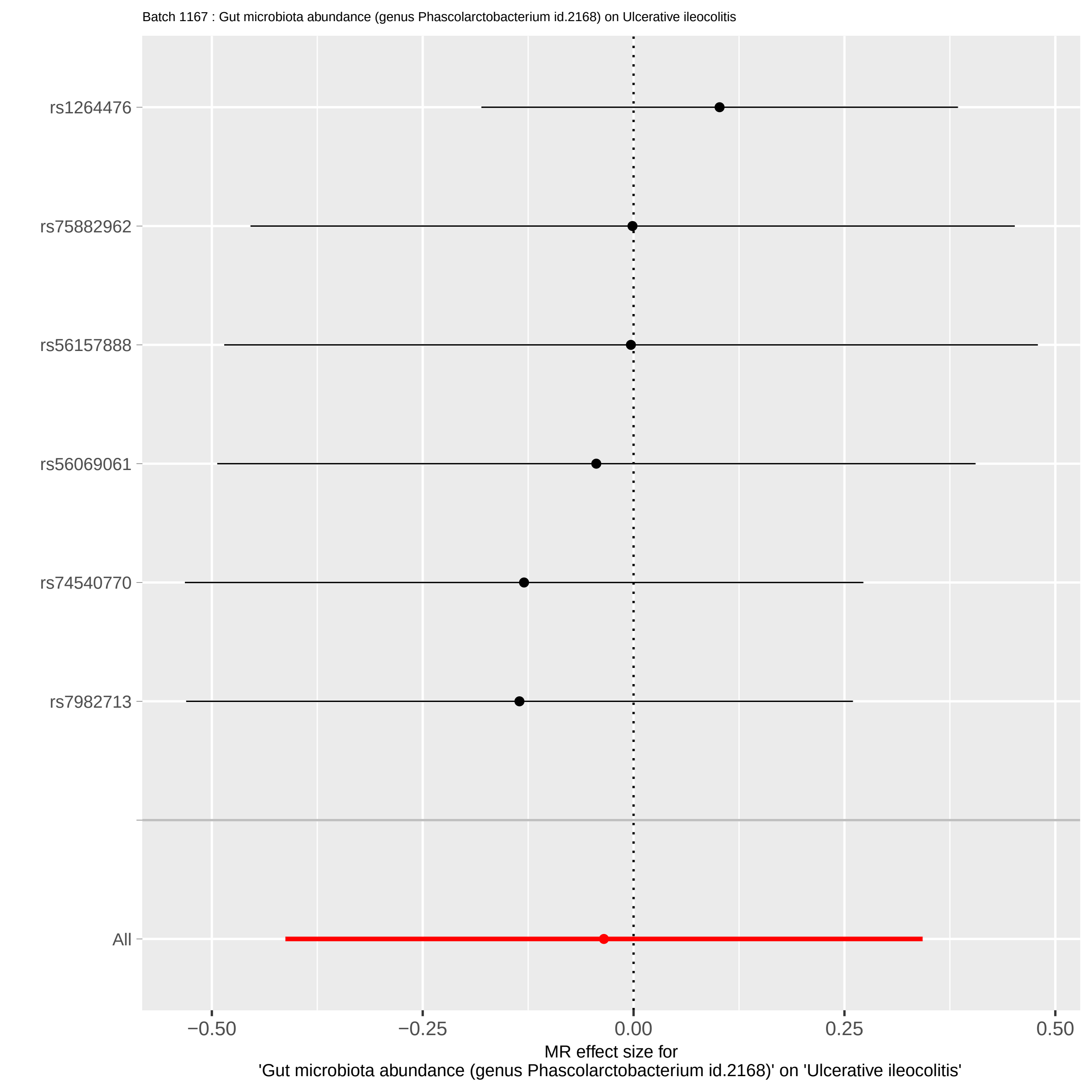

Batch 1167 : Gut microbiota abundance (genus Phascolarctobacterium id.2168) on Ulcerative ileocolitis
rs1264476
rs75882962
rs56157888
rs56069061
rs74540770
rs7982713
All
−0.50
−0.25
0.00
0.25
0.50
MR effect size for
'Gut microbiota abundance (genus Phascolarctobacterium id.2168)' on 'Ulcerative ileocolitis'

Batch 1168 : Gut microbiota abundance (genus Prevotella7 id.11182) on Ulcerative ileocolitis

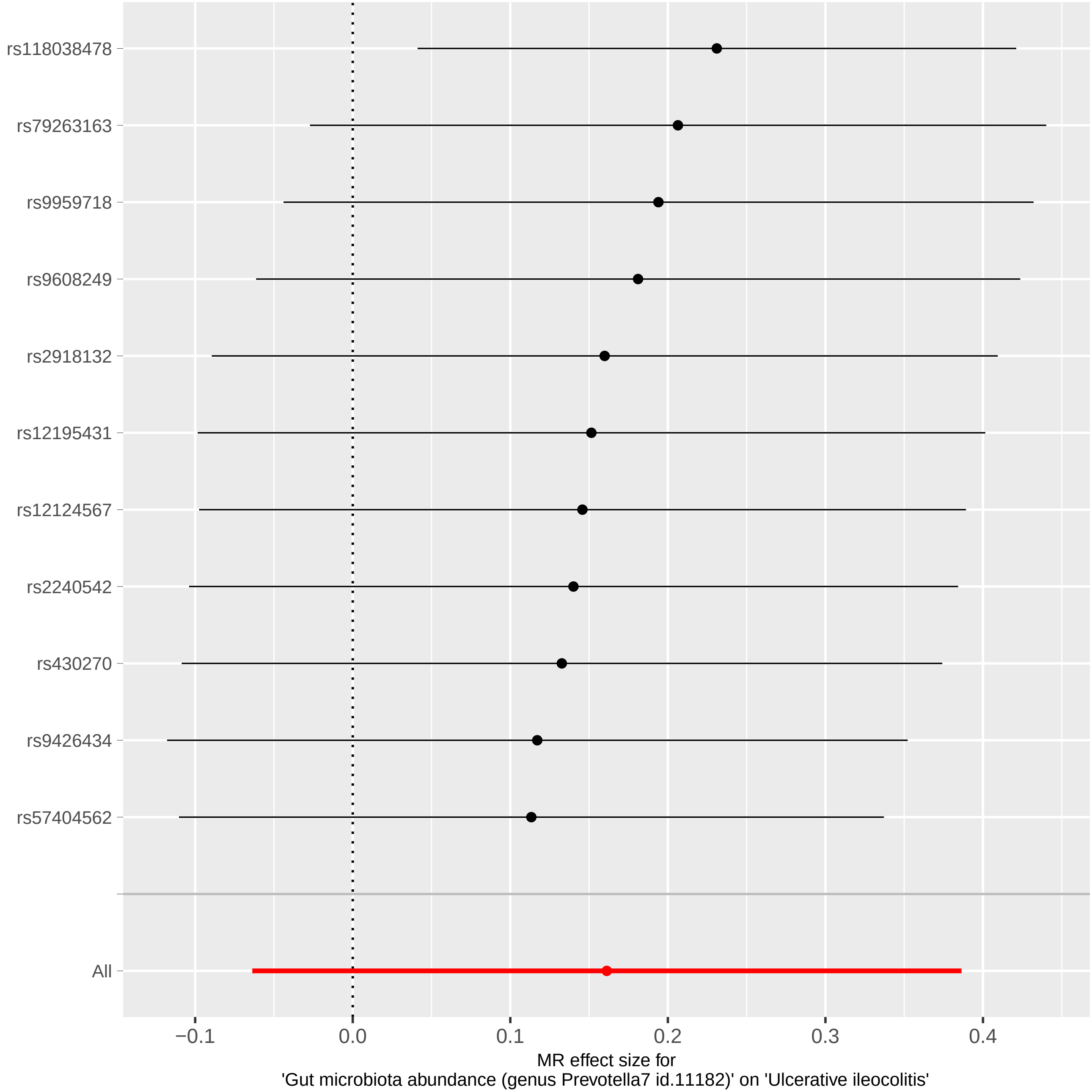

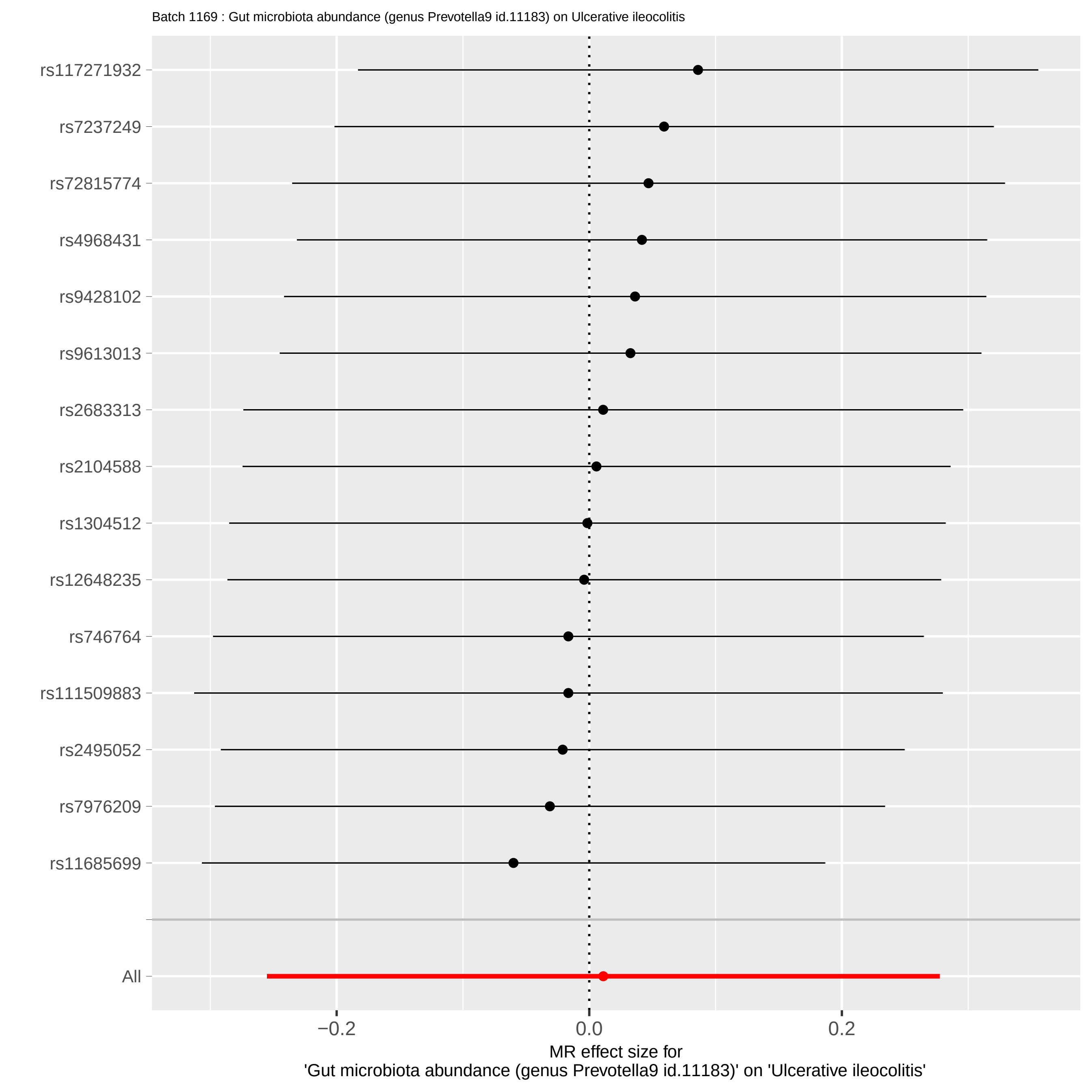
Batch 1169 : Gut microbiota abundance (genus Prevotella9 id.11183) on Ulcerative ileocolitis
rs117271932
rs7237249
rs72815774
rs4968431
rs9428102
rs9613013
rs2683313
rs2104588
rs1304512
rs12648235
rs746764
rs111509883
rs2495052
rs7976209
rs11685699
All
−0.2
0.0
0.2
MR effect size for
'Gut microbiota abundance (genus Prevotella9 id.11183)' on 'Ulcerative ileocolitis'

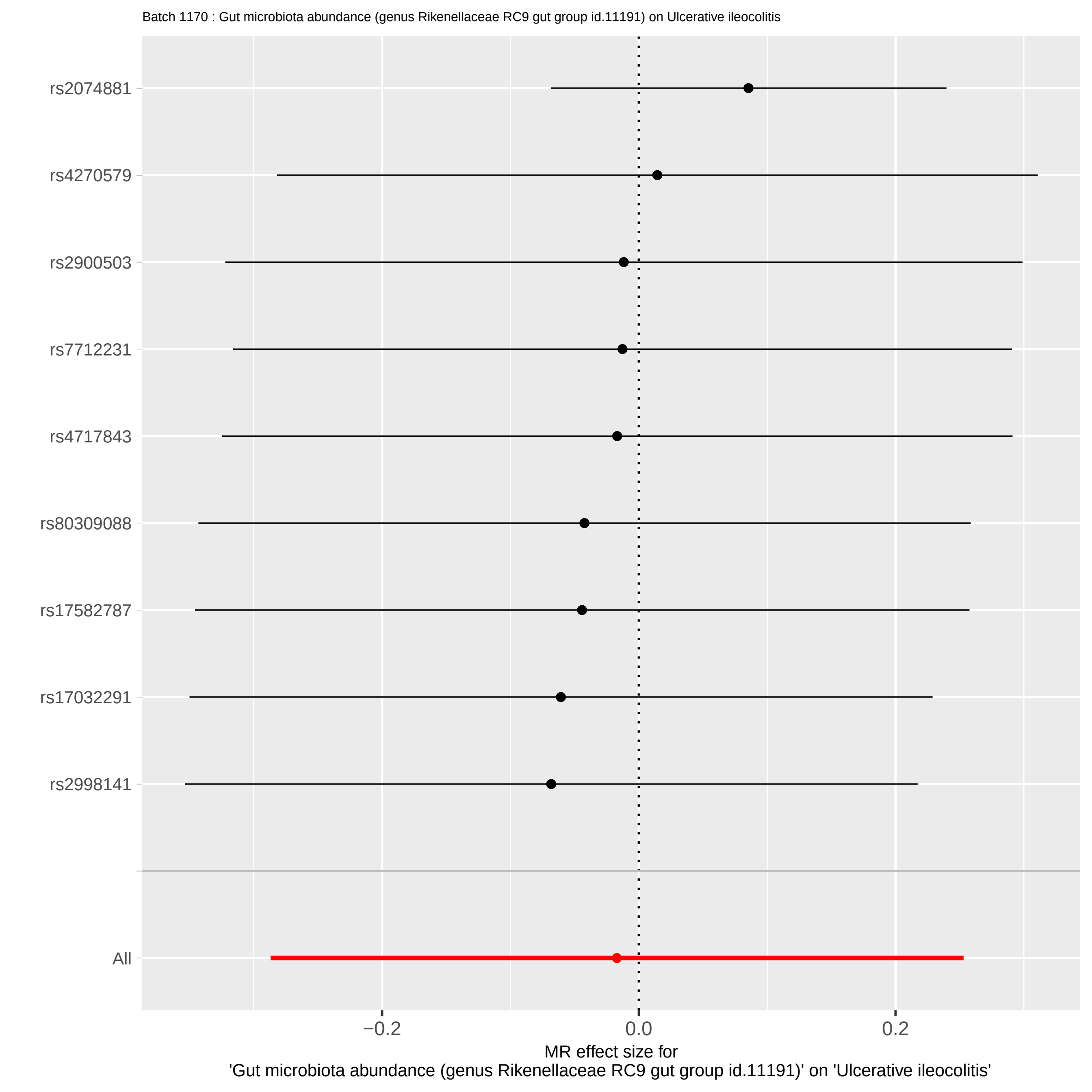

Batch 1170 : Gut microbiota abundance (genus Rikenellaceae RC9 gut group id.11191) on Ulcerative ileocolitis
rs2074881
rs4270579
rs2900503
rs7712231
rs4717843
rs80309088
rs17582787
rs17032291
rs2998141
All
−0.2
0.0
0.2
MR effect size for
'Gut microbiota abundance (genus Rikenellaceae RC9 gut group id.11191)' on 'Ulcerative ileocolitis'

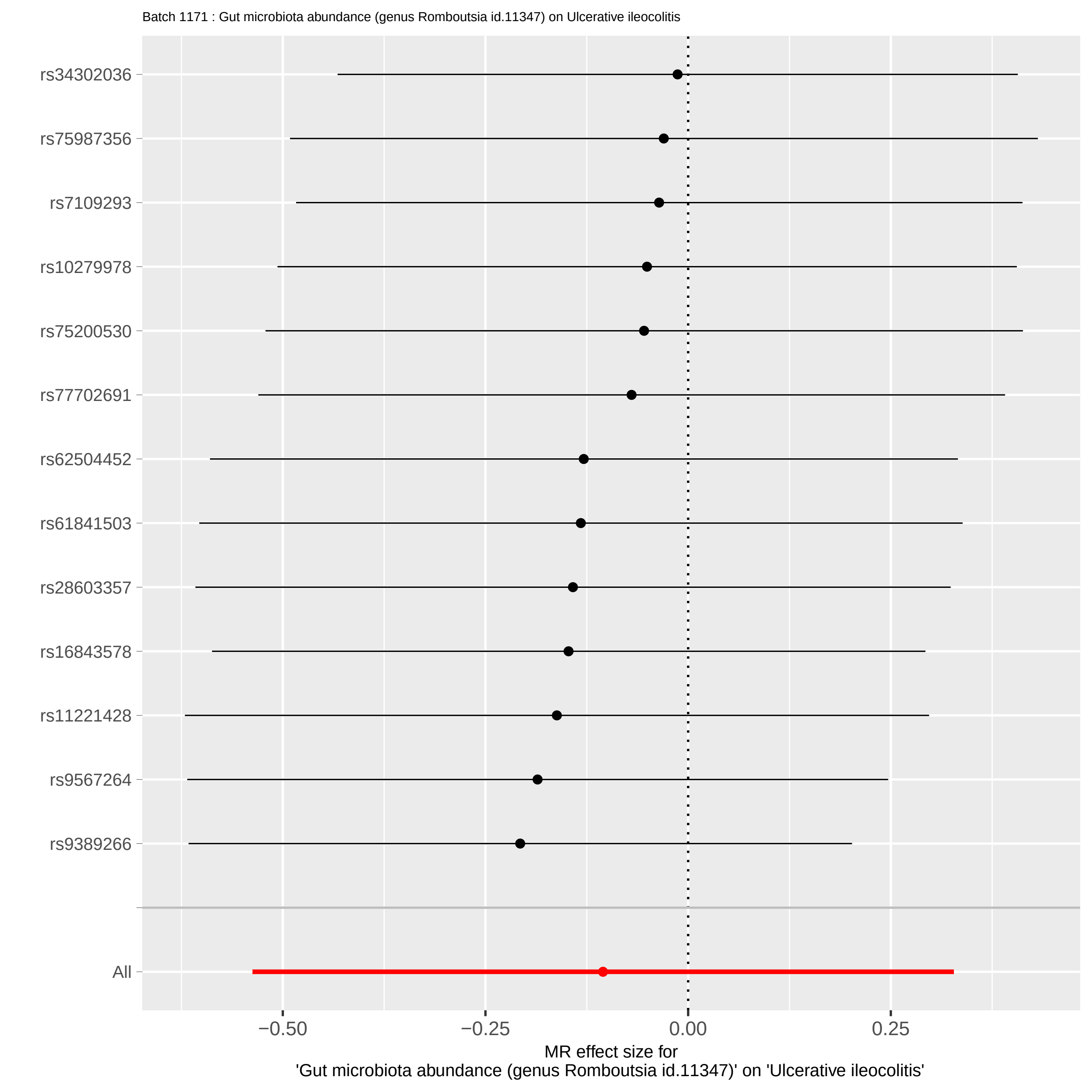

Batch 1171 : Gut microbiota abundance (genus Romboutsia id.11347) on Ulcerative ileocolitis
rs34302036
rs75987356
rs7109293
rs10279978
rs75200530
rs77702691
rs62504452
rs61841503
rs28603357
rs16843578
rs11221428
rs9567264
rs9389266
All
−0.50
−0.25
0.00
0.25
MR effect size for
'Gut microbiota abundance (genus Romboutsia id.11347)' on 'Ulcerative ileocolitis'

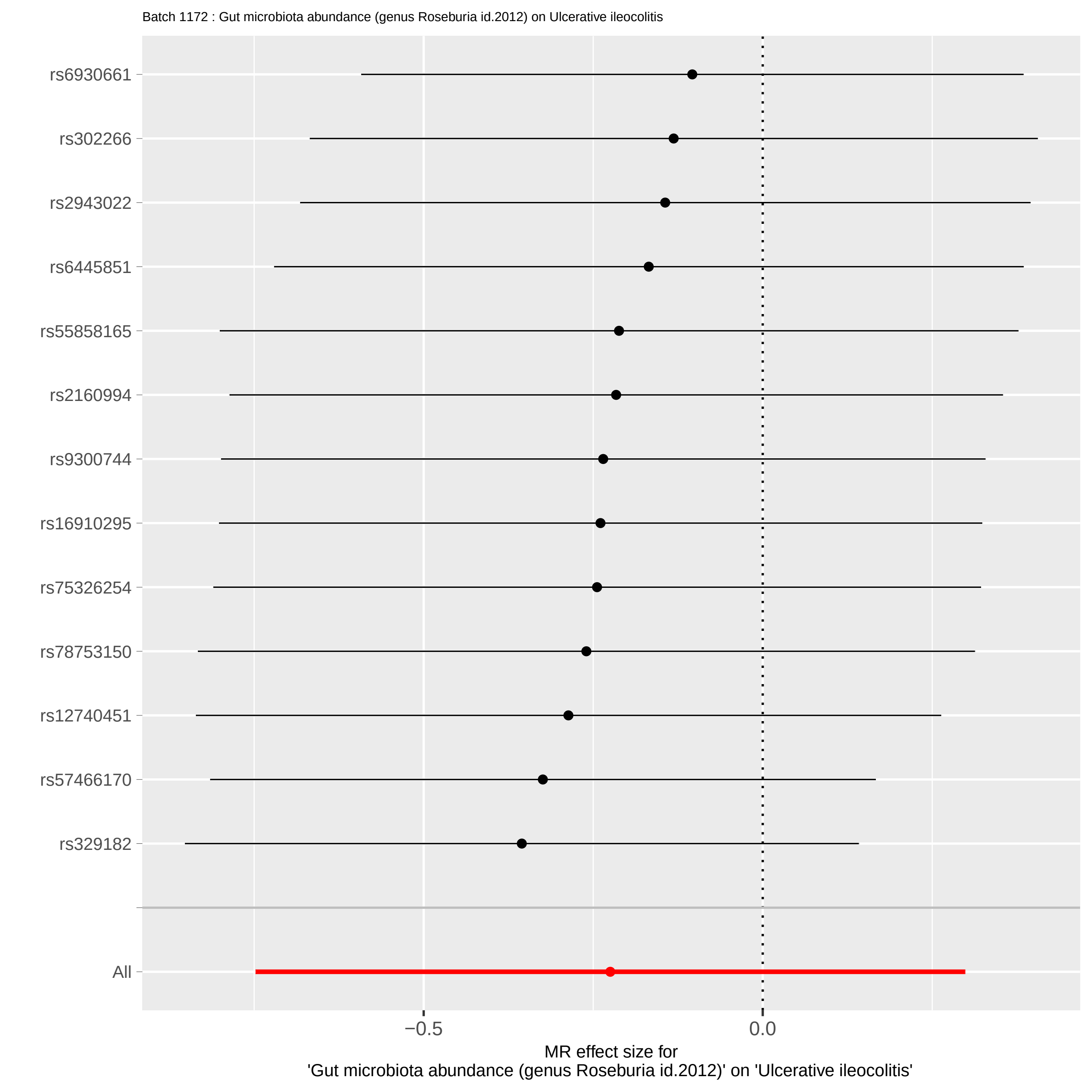

Batch 1172 : Gut microbiota abundance (genus Roseburia id.2012) on Ulcerative ileocolitis
rs6930661
rs302266
rs2943022
rs6445851
rs55858165
rs2160994
rs9300744
rs16910295
rs75326254
rs78753150
rs12740451
rs57466170
rs329182
All
−0.5
0.0
MR effect size for
'Gut microbiota abundance (genus Roseburia id.2012)' on 'Ulcerative ileocolitis'

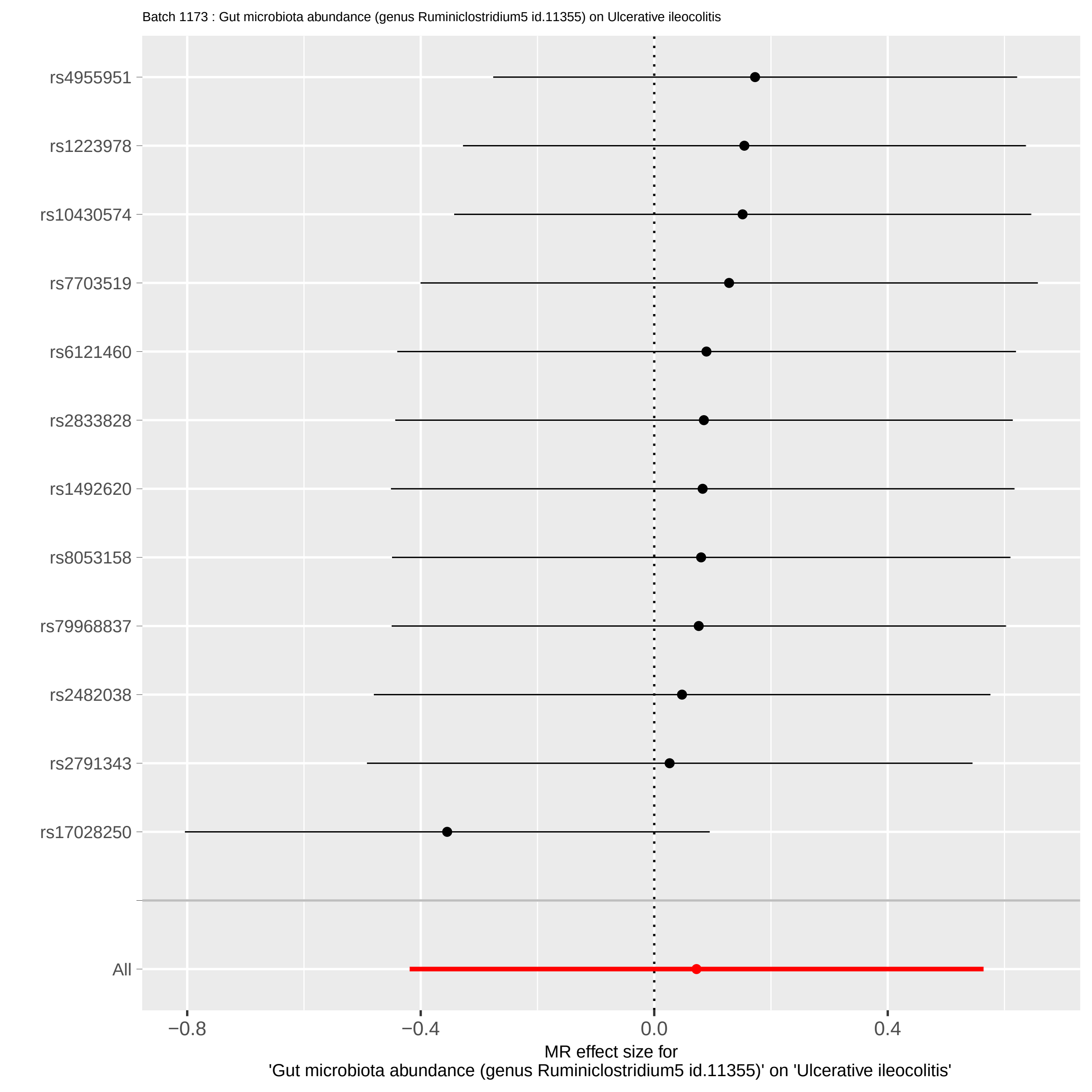

Batch 1173 : Gut microbiota abundance (genus Ruminiclostridium5 id.11355) on Ulcerative ileocolitis
rs4955951
rs1223978
rs10430574
rs7703519
rs6121460
rs2833828
rs1492620
rs8053158
rs79968837
rs2482038
rs2791343
rs17028250
All
−0.8
−0.4
0.0
0.4
MR effect size for
'Gut microbiota abundance (genus Ruminiclostridium5 id.11355)' on 'Ulcerative ileocolitis'

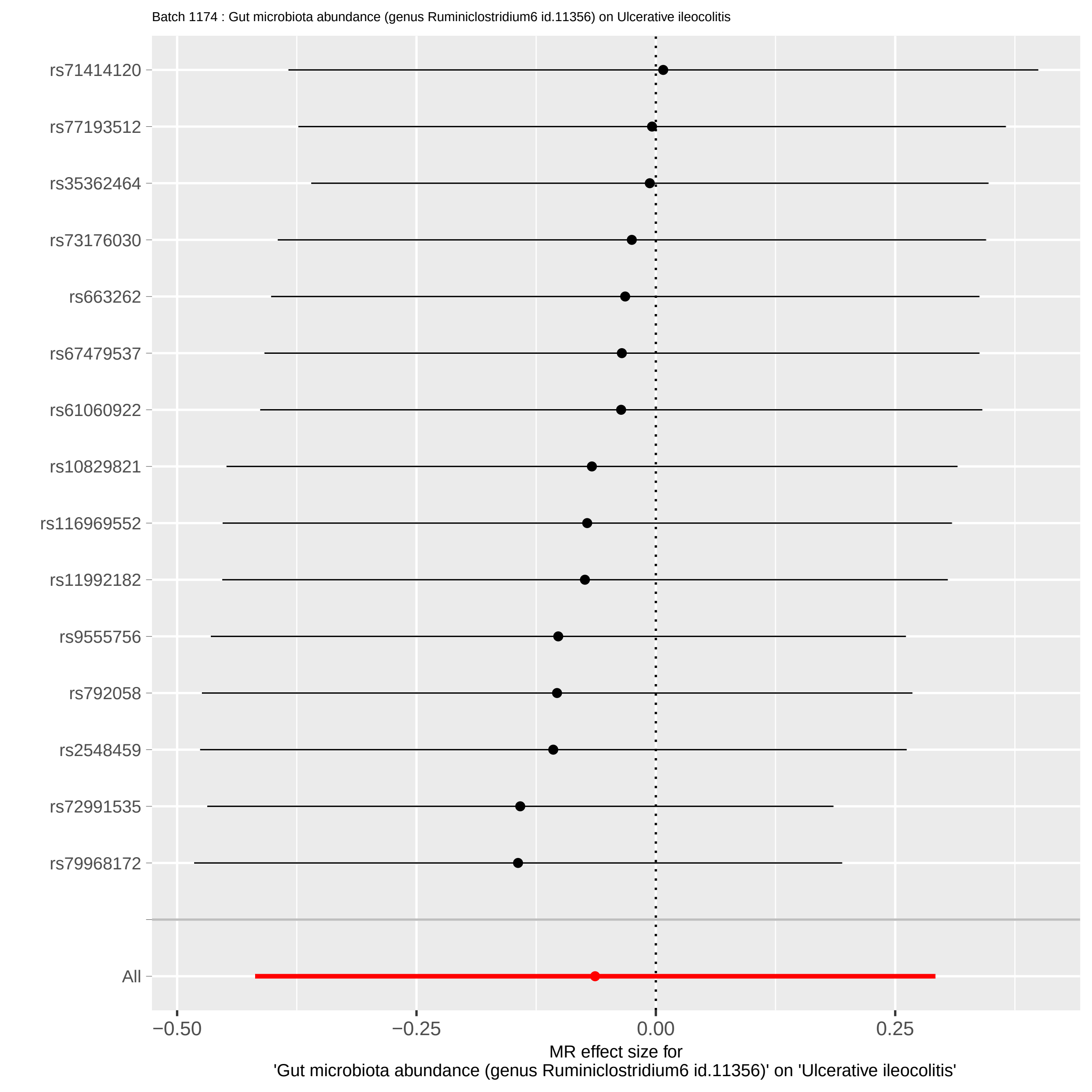
Batch 1174 : Gut microbiota abundance (genus Ruminiclostridium6 id.11356) on Ulcerative ileocolitis
rs71414120
rs77193512
rs35362464
rs73176030
rs663262
rs67479537
rs61060922
rs10829821
rs116969552
rs11992182
rs9555756
rs792058
rs2548459
rs72991535
rs79968172
All
−0.50
−0.25
0.00
0.25
MR effect size for
'Gut microbiota abundance (genus Ruminiclostridium6 id.11356)' on 'Ulcerative ileocolitis'

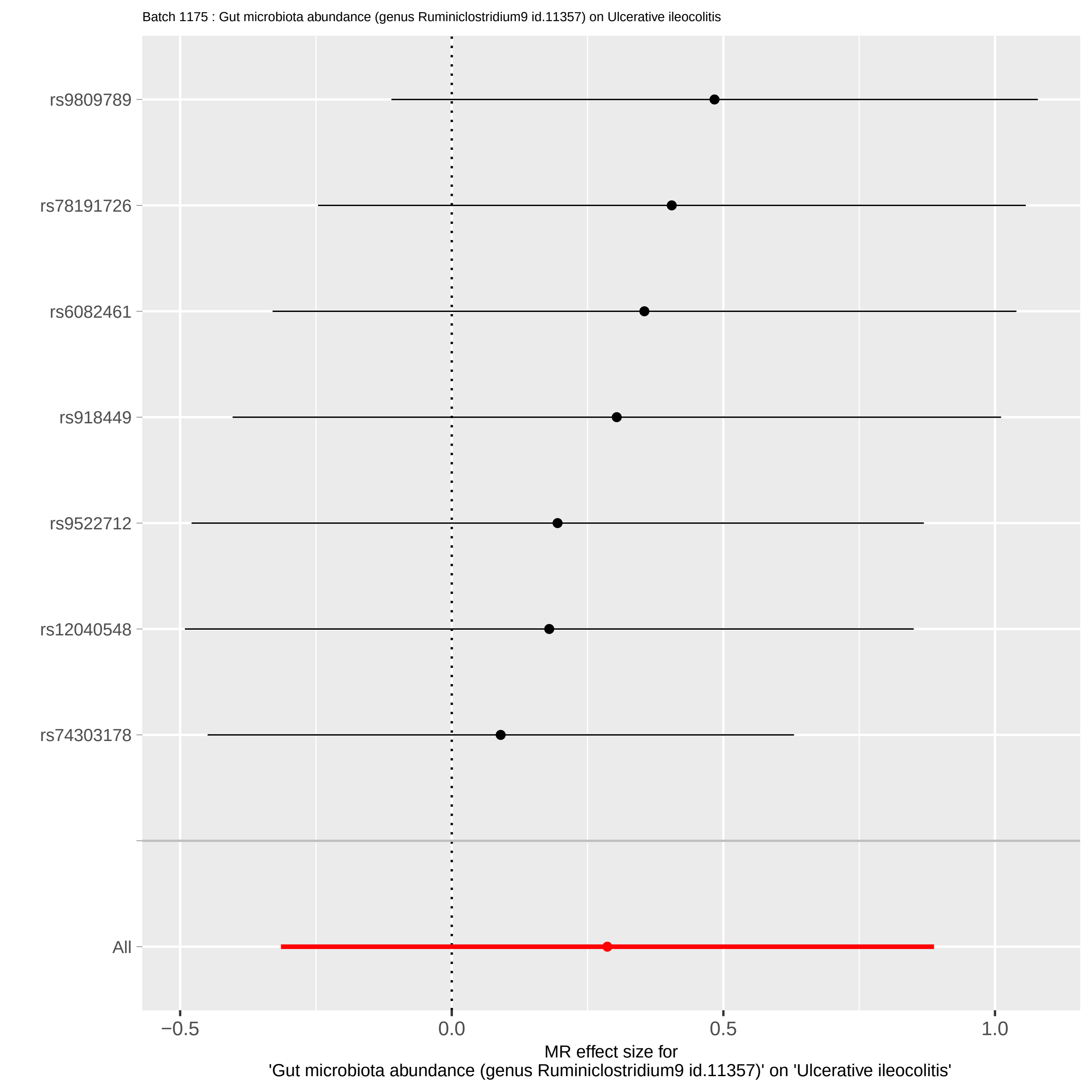
Batch 1175 : Gut microbiota abundance (genus Ruminiclostridium9 id.11357) on Ulcerative ileocolitis
rs9809789
rs78191726
rs6082461
rs918449
rs9522712
rs12040548
rs74303178
All
−0.5
0.0
0.5
1.0
MR effect size for
'Gut microbiota abundance (genus Ruminiclostridium9 id.11357)' on 'Ulcerative ileocolitis'

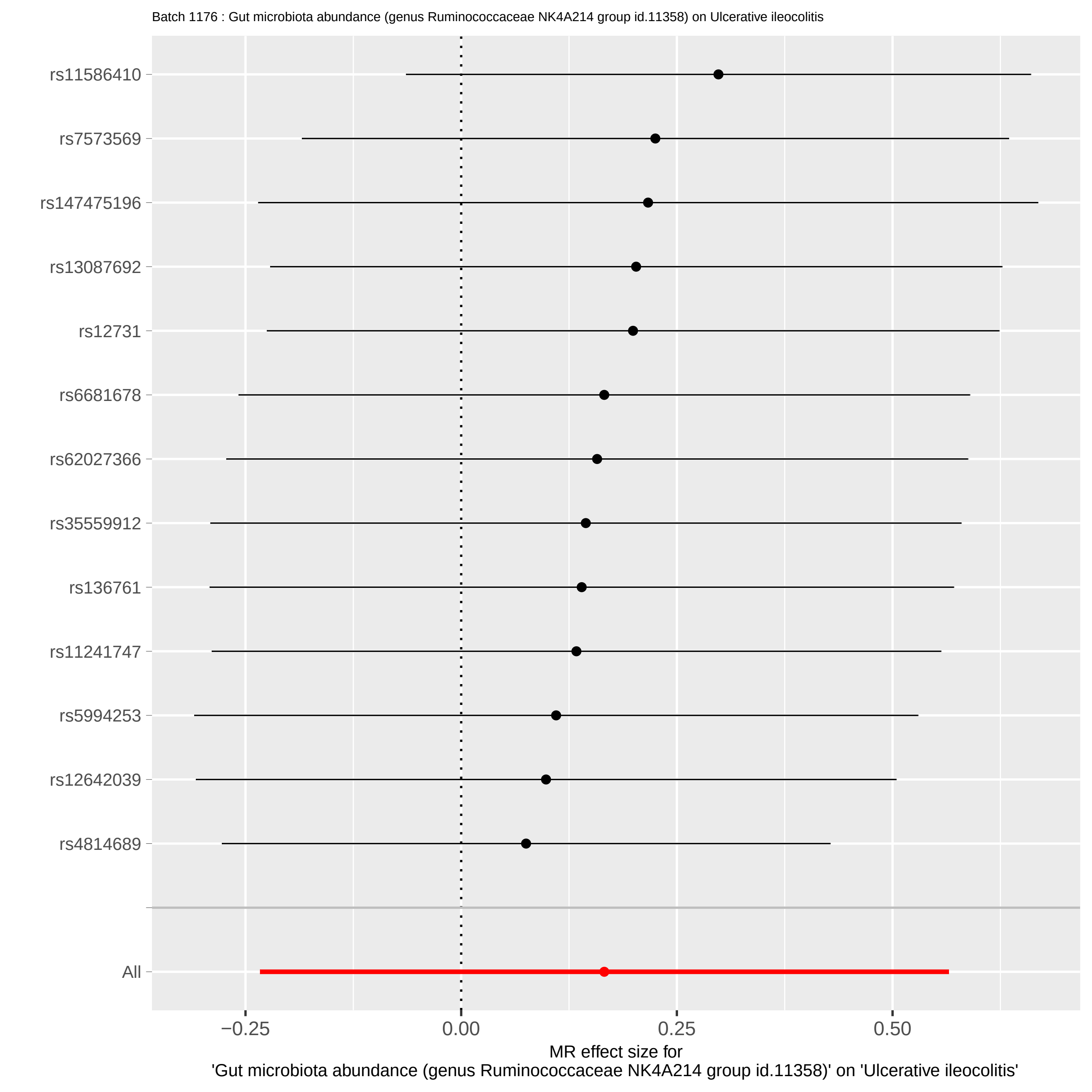
Batch 1176 : Gut microbiota abundance (genus Ruminococcaceae NK4A214 group id.11358) on Ulcerative ileocolitis
rs11586410
rs7573569
rs147475196
rs13087692
rs12731
rs6681678
rs62027366
rs35559912
rs136761
rs11241747
rs5994253
rs12642039
rs4814689
All
−0.25
0.00
0.25
0.50
MR effect size for
'Gut microbiota abundance (genus Ruminococcaceae NK4A214 group id.11358)' on 'Ulcerative ileocolitis'

Batch 1177 : Gut microbiota abundance (genus Ruminococcaceae UCG002 id.11360) on Ulcerative ileocolitis

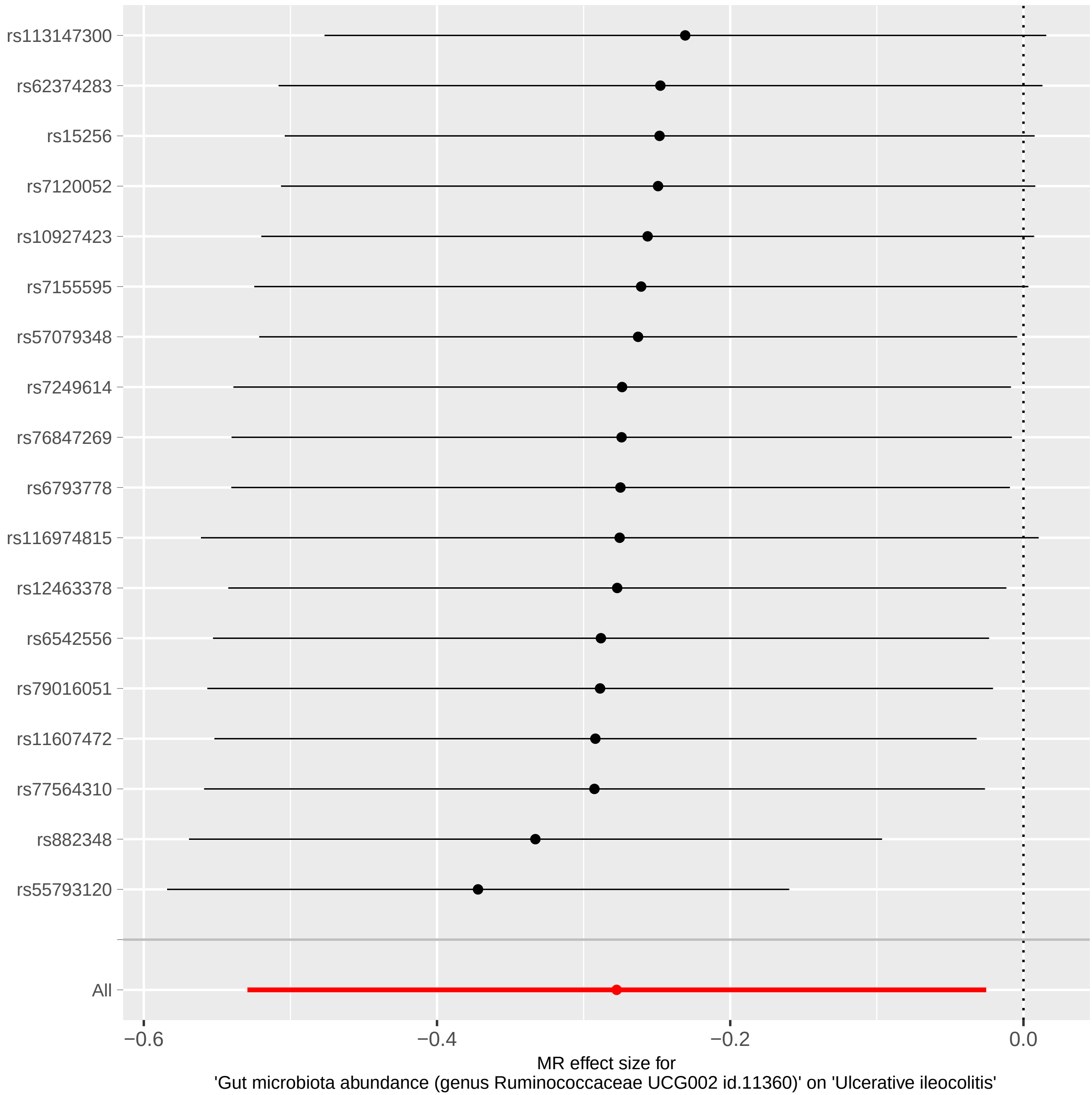

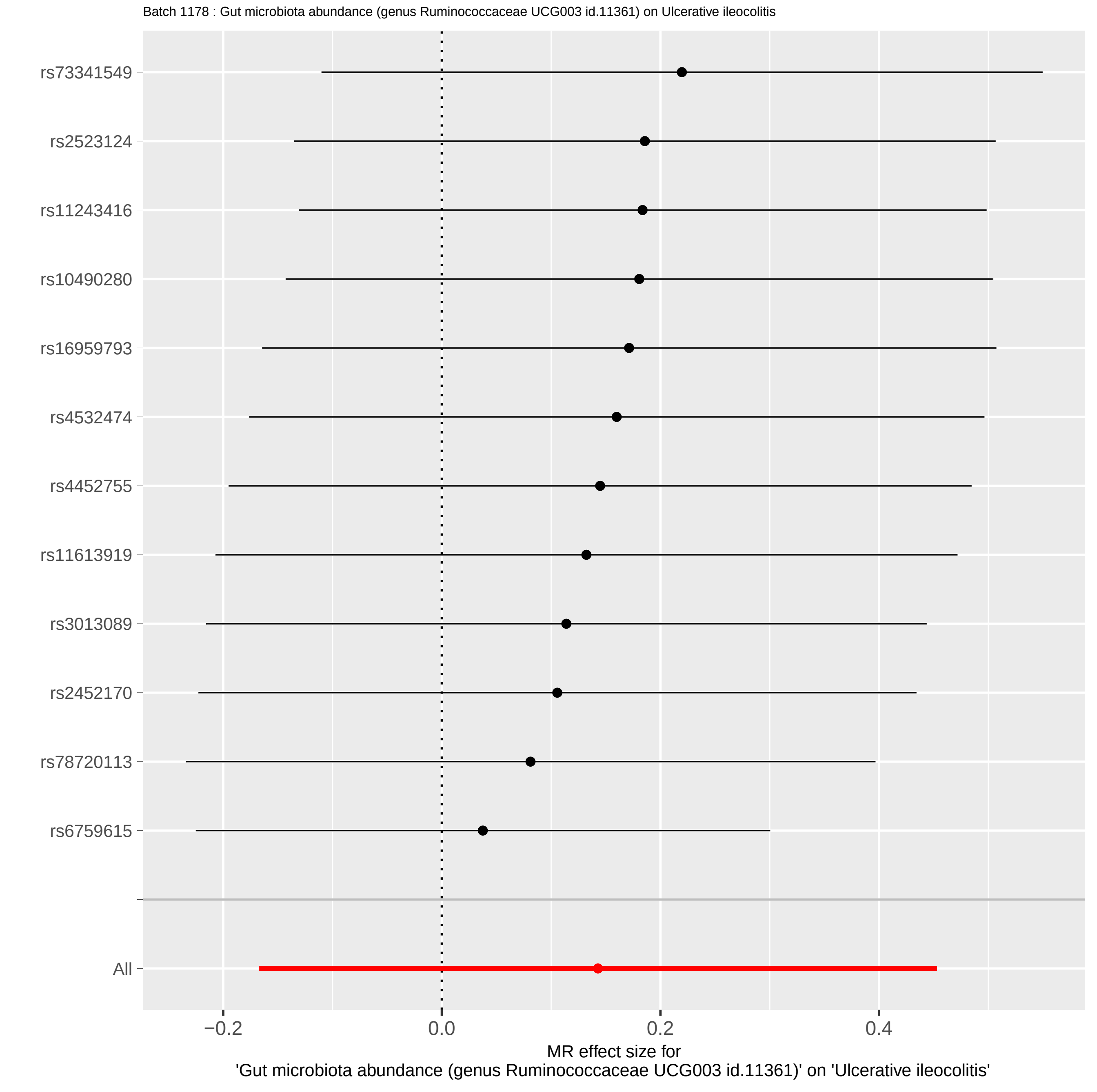

Batch 1178 : Gut microbiota abundance (genus Ruminococcaceae UCG003 id.11361) on Ulcerative ileocolitis
rs73341549
rs2523124
rs11243416
rs10490280
rs16959793
rs4532474
rs4452755
rs11613919
rs3013089
rs2452170
rs78720113
rs6759615
All
−0.2
0.0
0.2
0.4
MR effect size for
'Gut microbiota abundance (genus Ruminococcaceae UCG003 id.11361)' on 'Ulcerative ileocolitis'

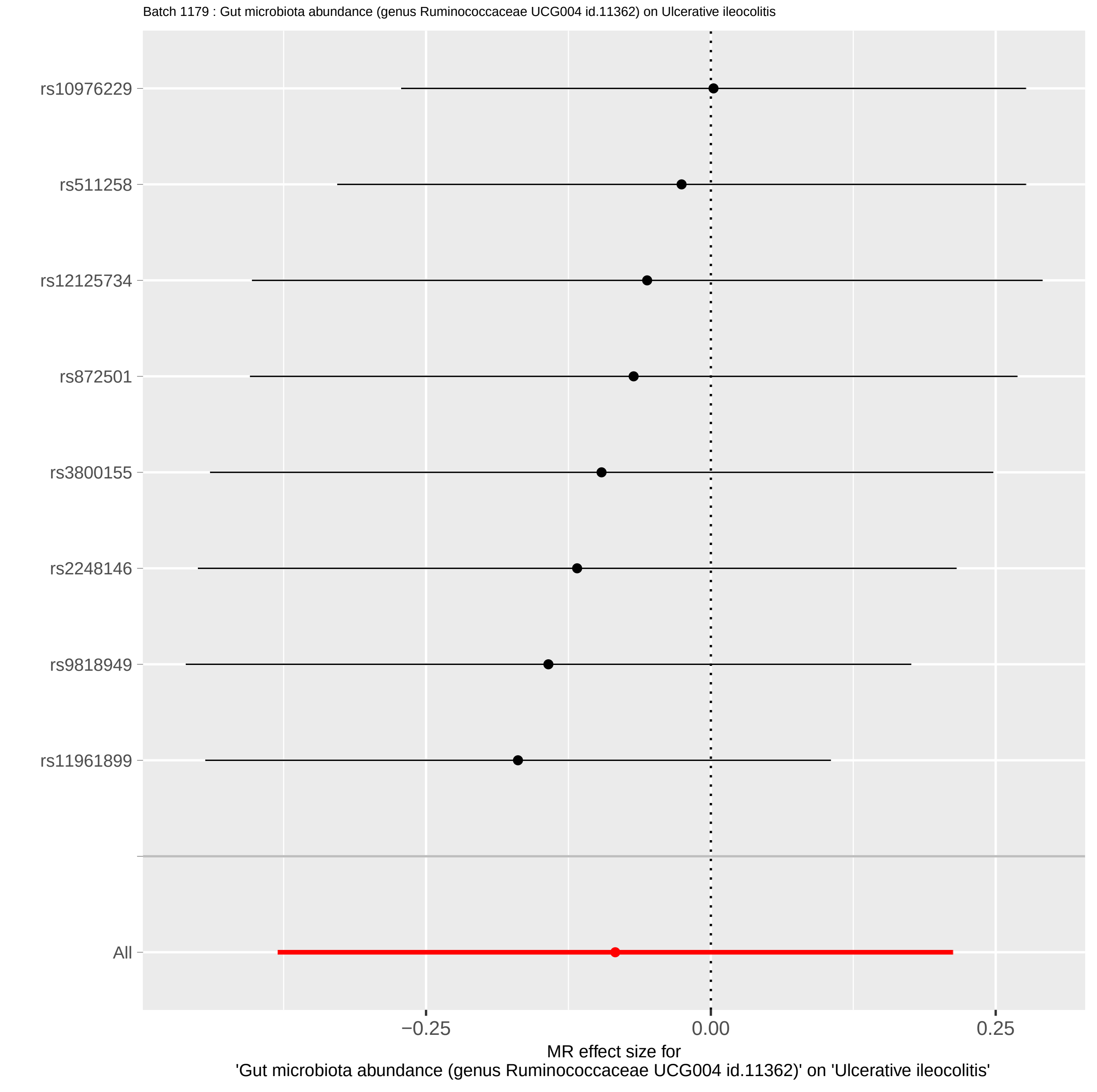

Batch 1179 : Gut microbiota abundance (genus Ruminococcaceae UCG004 id.11362) on Ulcerative ileocolitis
rs10976229
rs511258
rs12125734
rs872501
rs3800155
rs2248146
rs9818949
rs11961899
All
−0.25
0.00
0.25
MR effect size for
'Gut microbiota abundance (genus Ruminococcaceae UCG004 id.11362)' on 'Ulcerative ileocolitis'

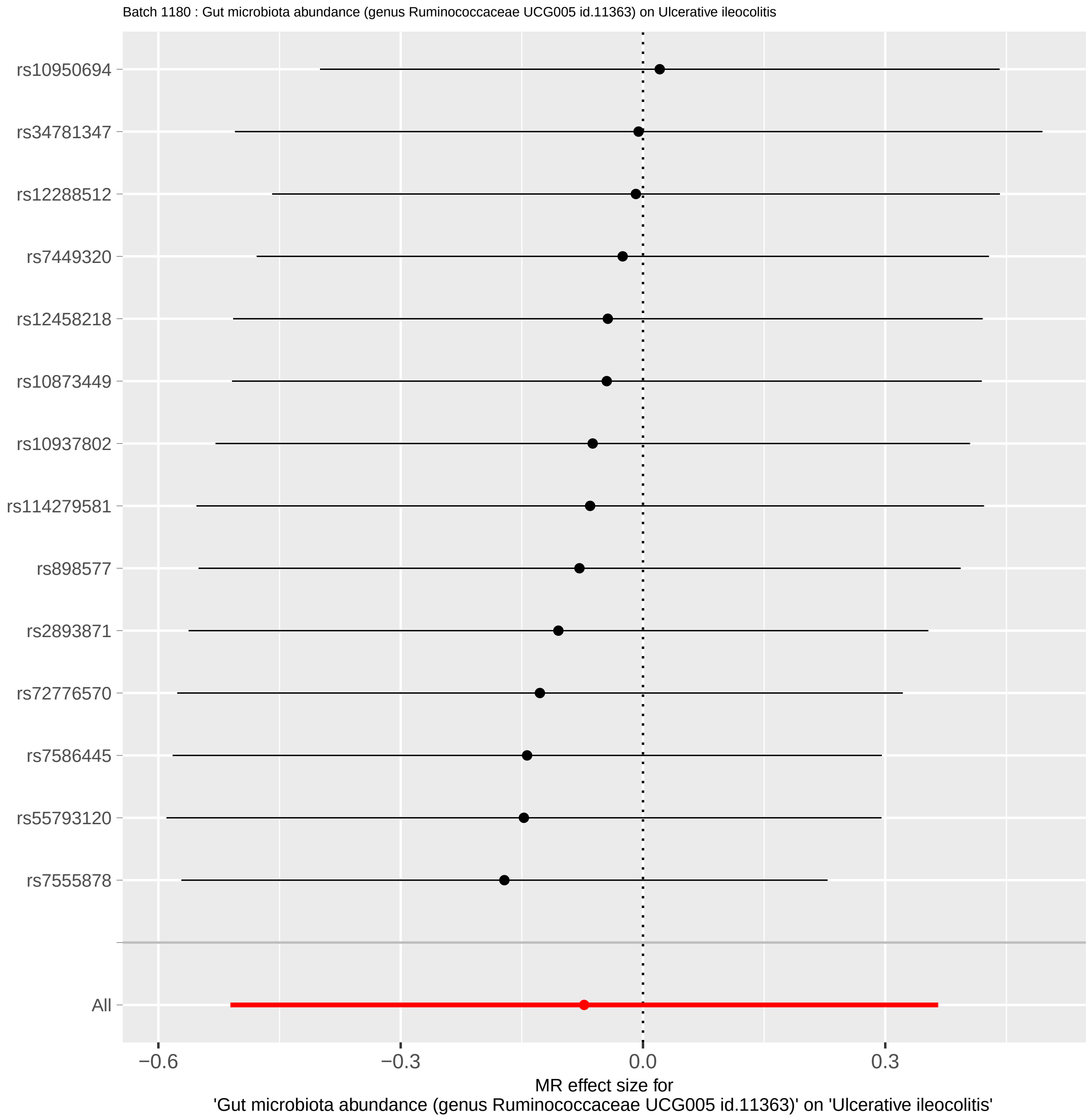

Batch 1180 : Gut microbiota abundance (genus Ruminococcaceae UCG005 id.11363) on Ulcerative ileocolitis
rs10950694
rs34781347
rs12288512
rs7449320
rs12458218
rs10873449
rs10937802
rs114279581
rs898577
rs2893871
rs72776570
rs7586445
rs55793120
rs7555878
All
−0.6
−0.3
0.0
0.3
MR effect size for
'Gut microbiota abundance (genus Ruminococcaceae UCG005 id.11363)' on 'Ulcerative ileocolitis'

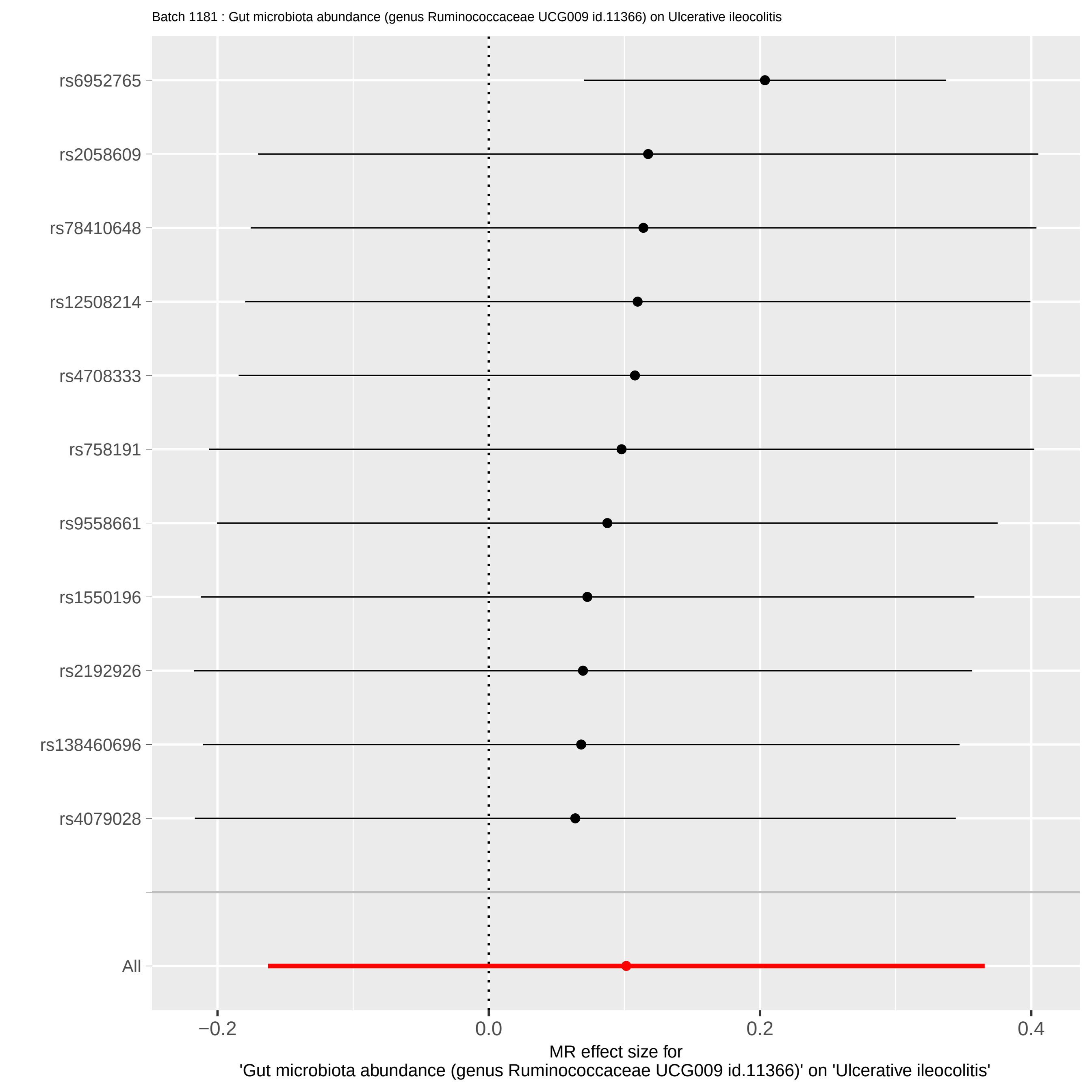

Batch 1181 : Gut microbiota abundance (genus Ruminococcaceae UCG009 id.11366) on Ulcerative ileocolitis
rs6952765
rs2058609
rs78410648
rs12508214
rs4708333
rs758191
rs9558661
rs1550196
rs2192926
rs138460696
rs4079028
All
−0.2
0.0
0.2
0.4
MR effect size for
'Gut microbiota abundance (genus Ruminococcaceae UCG009 id.11366)' on 'Ulcerative ileocolitis'

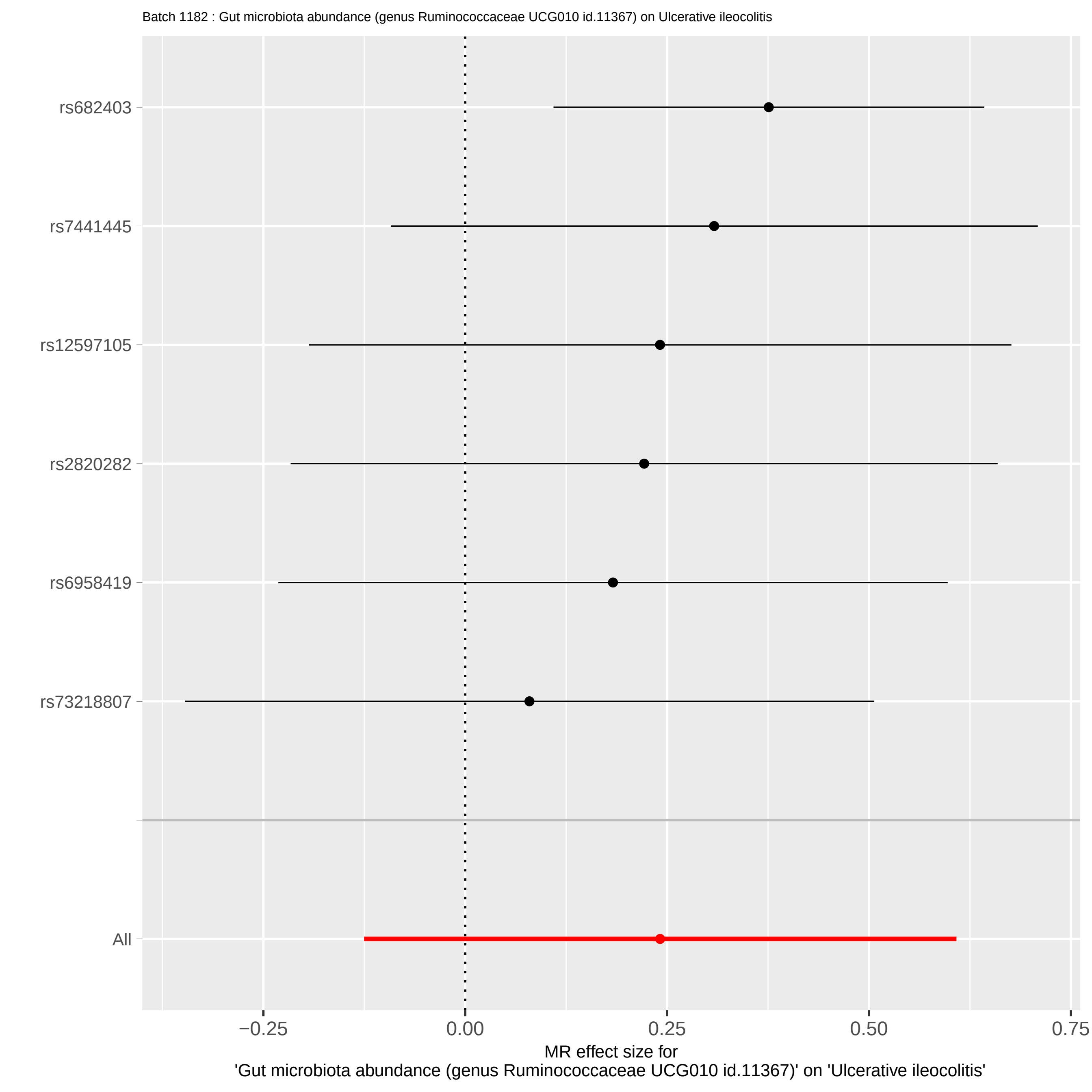

Batch 1182 : Gut microbiota abundance (genus Ruminococcaceae UCG010 id.11367) on Ulcerative ileocolitis
rs682403
rs7441445
rs12597105
rs2820282
rs6958419
rs73218807
All
−0.25
0.00
0.25
0.50
0.75
MR effect size for
'Gut microbiota abundance (genus Ruminococcaceae UCG010 id.11367)' on 'Ulcerative ileocolitis'

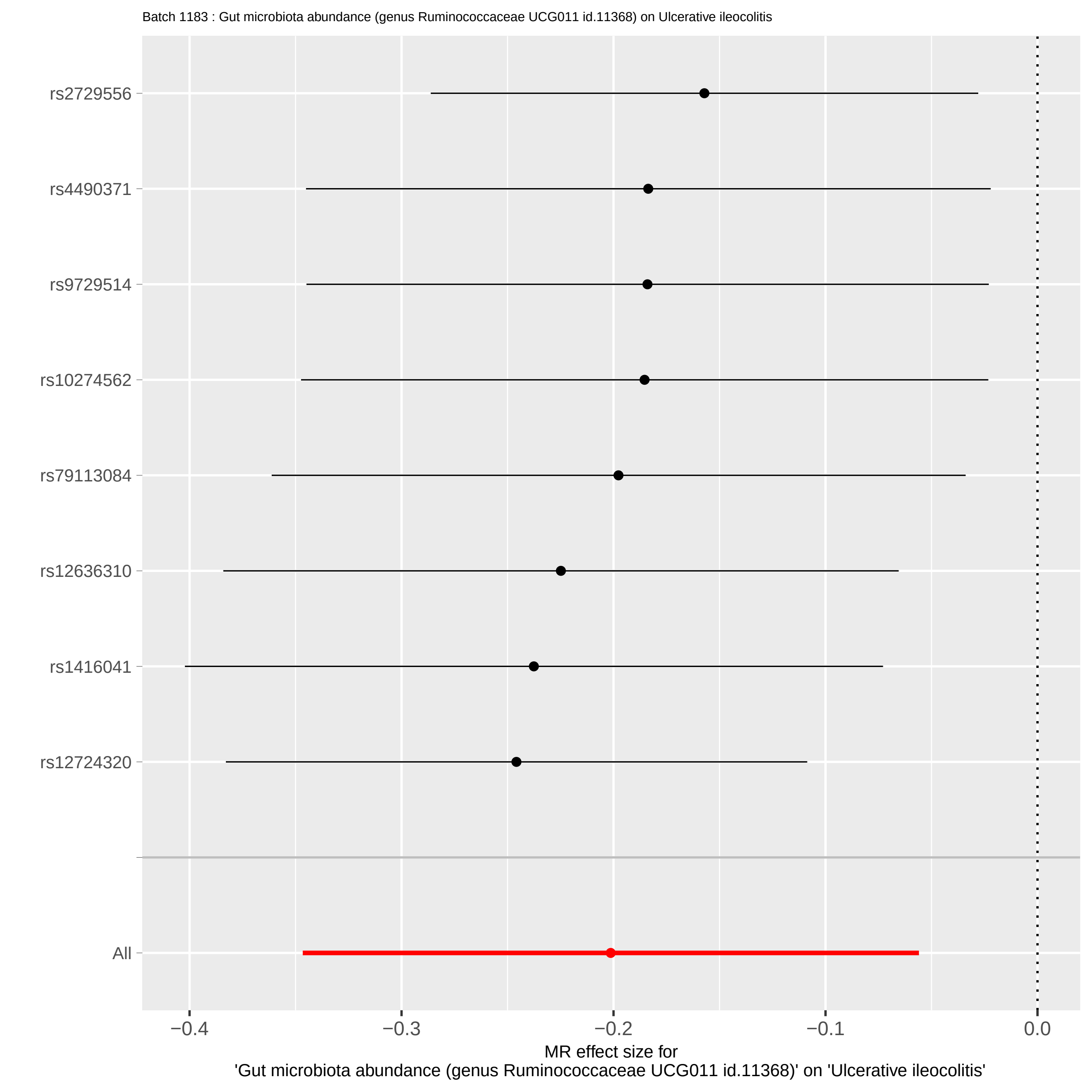

Batch 1183 : Gut microbiota abundance (genus Ruminococcaceae UCG011 id.11368) on Ulcerative ileocolitis
rs2729556
rs4490371
rs9729514
rs10274562
rs79113084
rs12636310
rs1416041
rs12724320
All
−0.4
−0.3
−0.2
−0.1
0.0
MR effect size for
'Gut microbiota abundance (genus Ruminococcaceae UCG011 id.11368)' on 'Ulcerative ileocolitis'

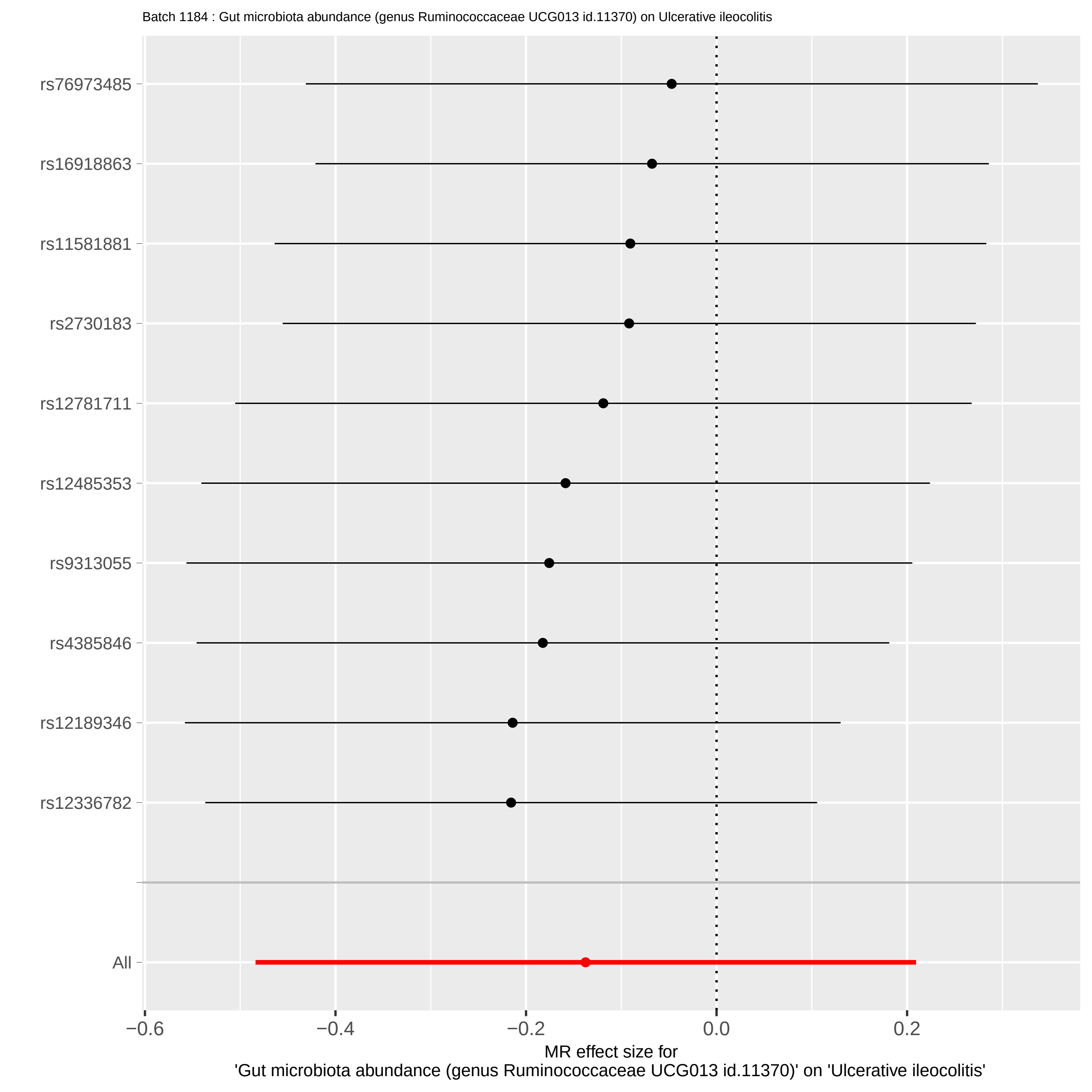

Batch 1184 : Gut microbiota abundance (genus Ruminococcaceae UCG013 id.11370) on Ulcerative ileocolitis
rs76973485
rs16918863
rs11581881
rs2730183
rs12781711
rs12485353
rs9313055
rs4385846
rs12189346
rs12336782
All
−0.6
−0.4
−0.2
0.0
0.2
MR effect size for
'Gut microbiota abundance (genus Ruminococcaceae UCG013 id.11370)' on 'Ulcerative ileocolitis'

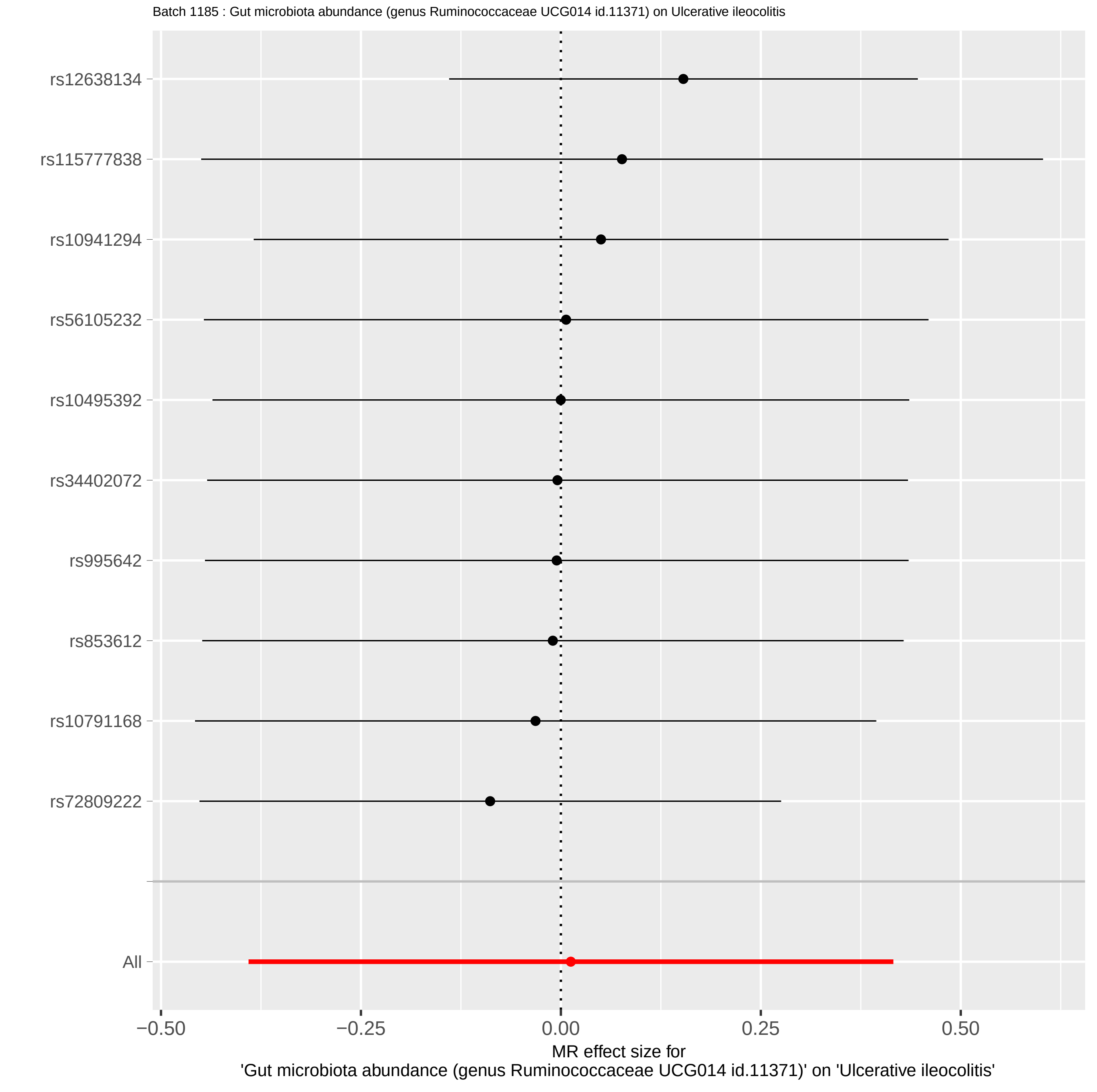

Batch 1185 : Gut microbiota abundance (genus Ruminococcaceae UCG014 id.11371) on Ulcerative ileocolitis
rs12638134
rs115777838
rs10941294
rs56105232
rs10495392
rs34402072
rs995642
rs853612
rs10791168
rs72809222
All
−0.50
−0.25
0.00
0.25
0.50
MR effect size for
'Gut microbiota abundance (genus Ruminococcaceae UCG014 id.11371)' on 'Ulcerative ileocolitis'

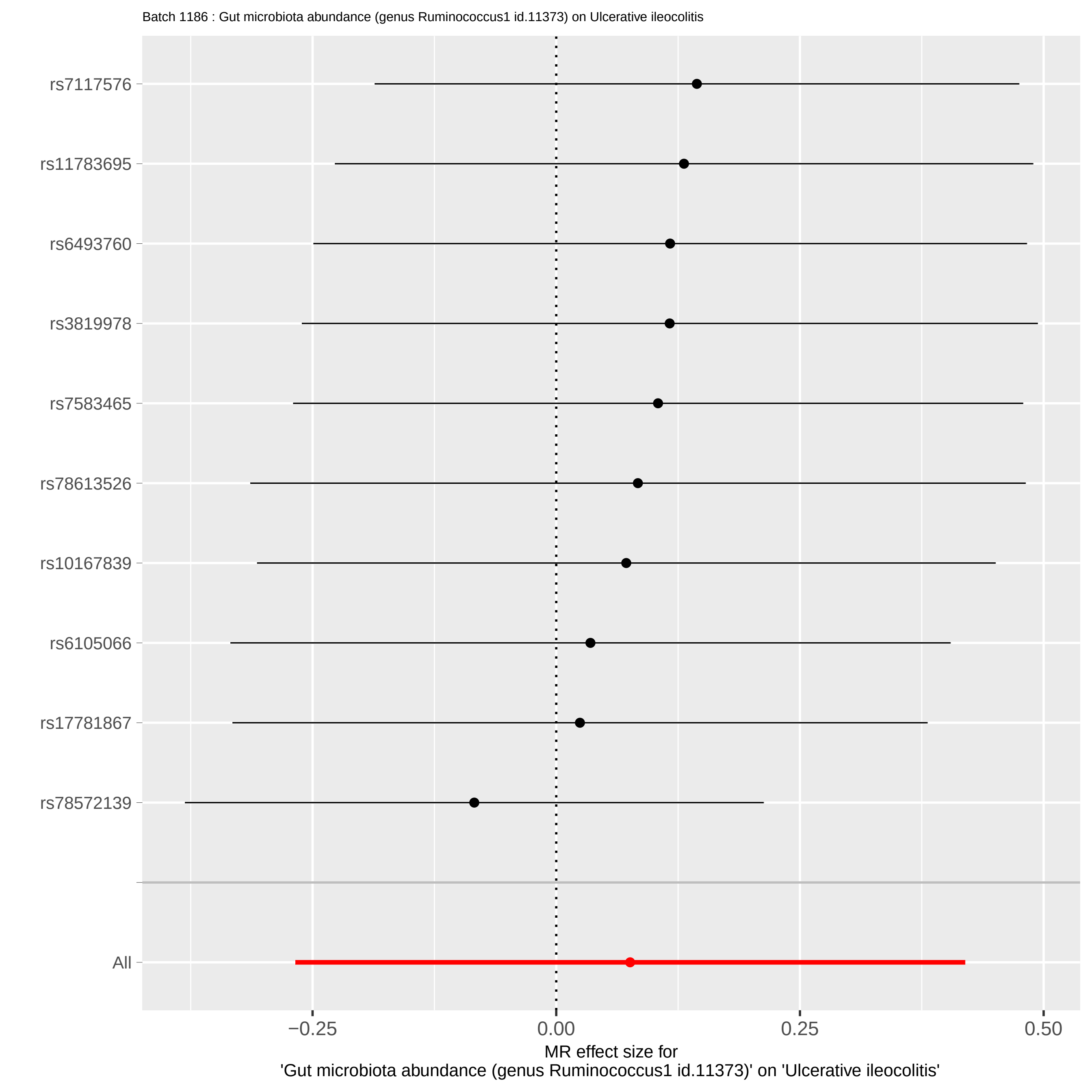

Batch 1186 : Gut microbiota abundance (genus Ruminococcus1 id.11373) on Ulcerative ileocolitis
rs7117576
rs11783695
rs6493760
rs3819978
rs7583465
rs78613526
rs10167839
rs6105066
rs17781867
rs78572139
All
−0.25
0.00
0.25
0.50
MR effect size for
'Gut microbiota abundance (genus Ruminococcus1 id.11373)' on 'Ulcerative ileocolitis'

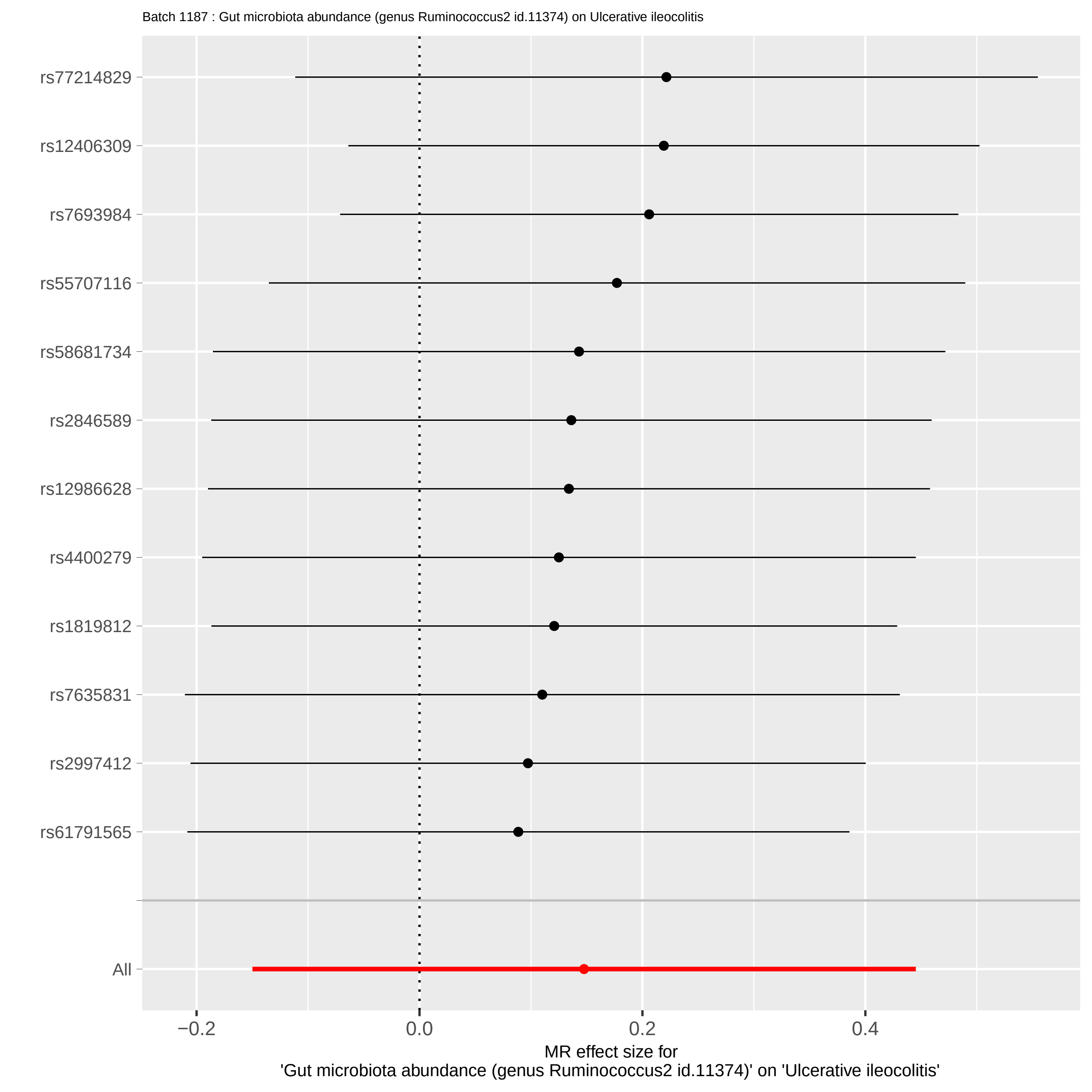

Batch 1187 : Gut microbiota abundance (genus Ruminococcus2 id.11374) on Ulcerative ileocolitis
rs77214829
rs12406309
rs7693984
rs55707116
rs58681734
rs2846589
rs12986628
rs4400279
rs1819812
rs7635831
rs2997412
rs61791565
All
−0.2
0.0
0.2
0.4
MR effect size for
'Gut microbiota abundance (genus Ruminococcus2 id.11374)' on 'Ulcerative ileocolitis'

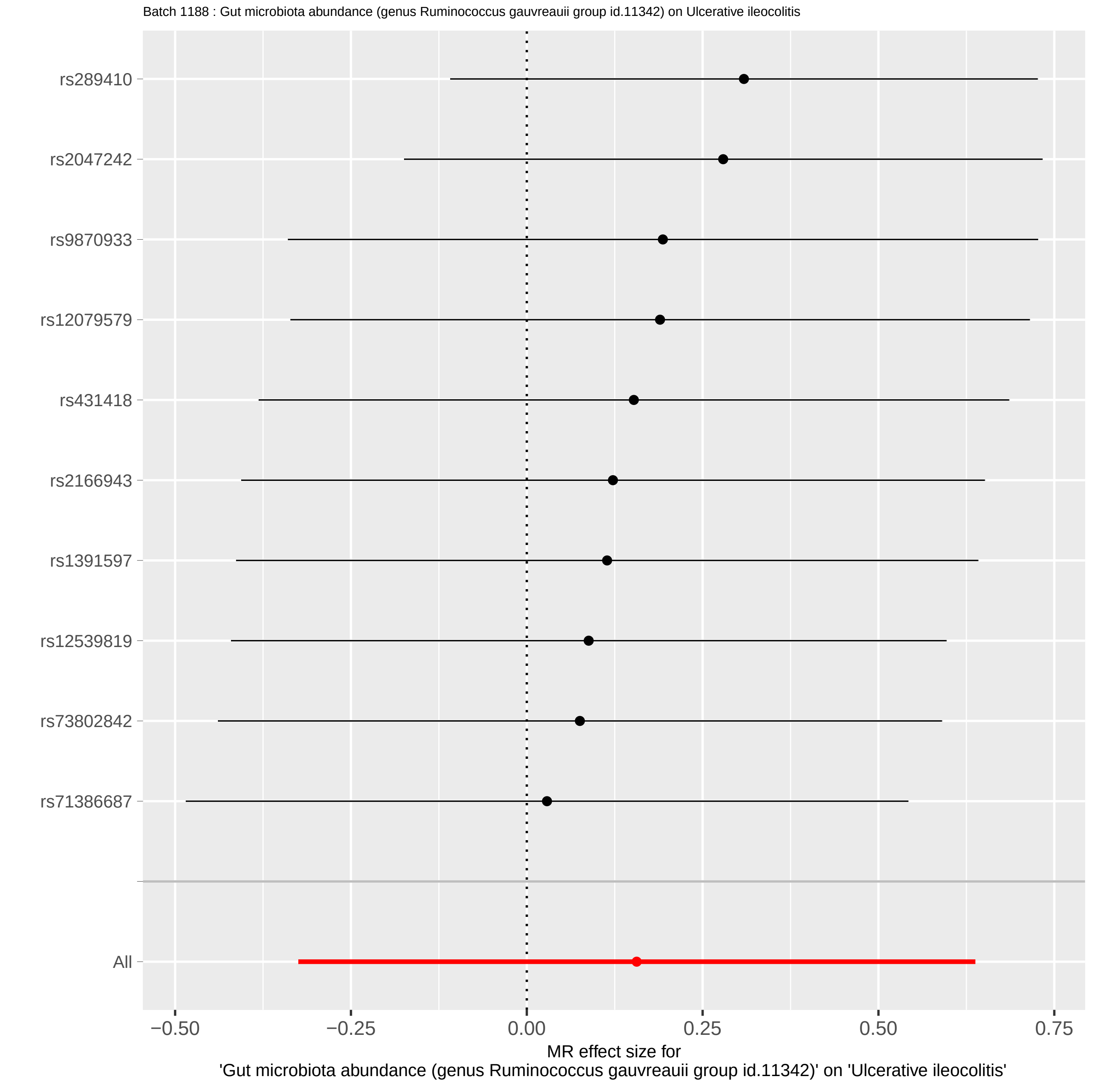

Batch 1188 : Gut microbiota abundance (genus Ruminococcus gauvreauii group id.11342) on Ulcerative ileocolitis
rs289410
rs2047242
rs9870933
rs12079579
rs431418
rs2166943
rs1391597
rs12539819
rs73802842
rs71386687
All
−0.50
−0.25
0.00
0.25
0.50
0.75
MR effect size for
'Gut microbiota abundance (genus Ruminococcus gauvreauii group id.11342)' on 'Ulcerative ileocolitis'

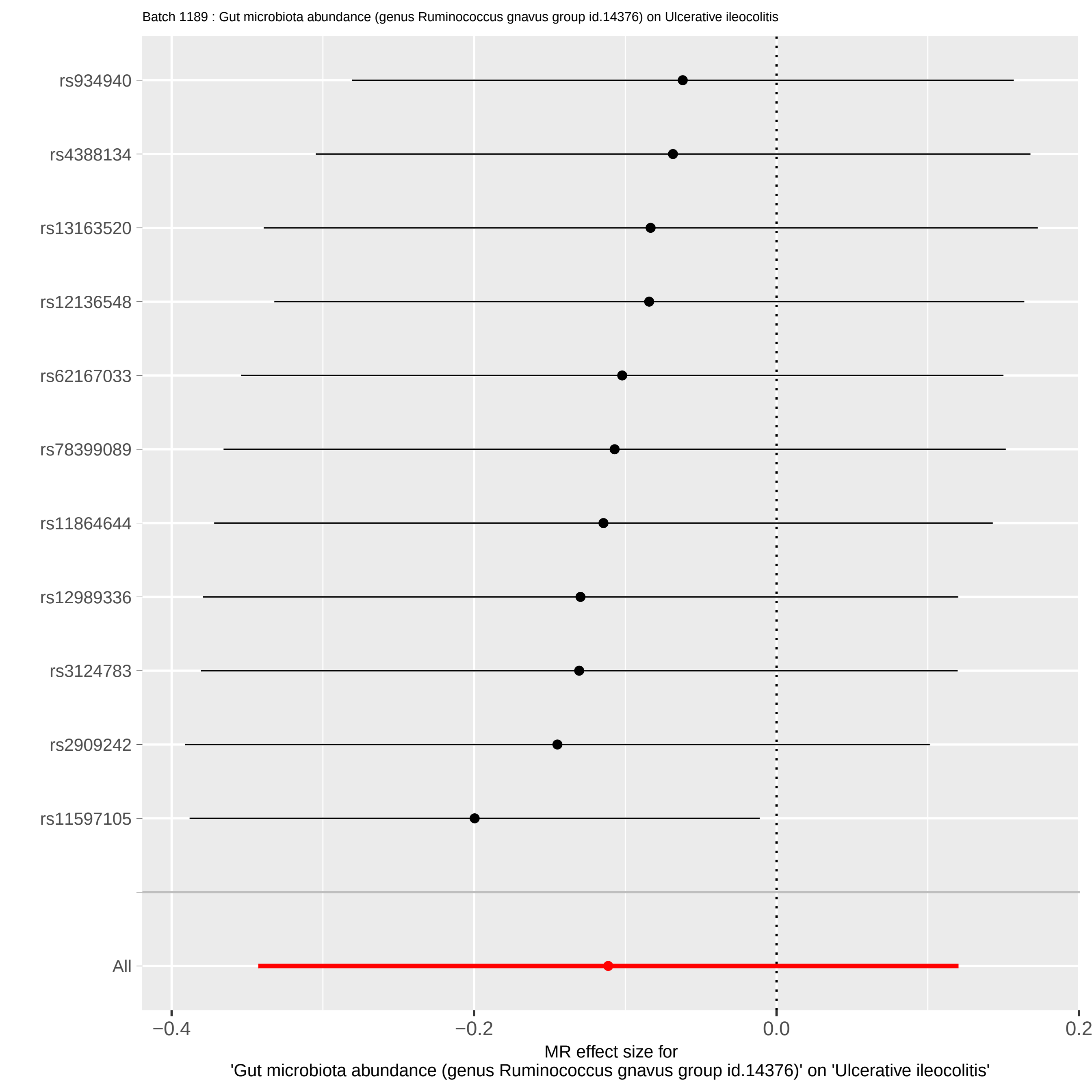
Batch 1189 : Gut microbiota abundance (genus Ruminococcus gnavus group id.14376) on Ulcerative ileocolitis
rs934940
rs4388134
rs13163520
rs12136548
rs62167033
rs78399089
rs11864644
rs12989336
rs3124783
rs2909242
rs11597105
All
−0.4
−0.2
0.0
0.2
MR effect size for
'Gut microbiota abundance (genus Ruminococcus gnavus group id.14376)' on 'Ulcerative ileocolitis'

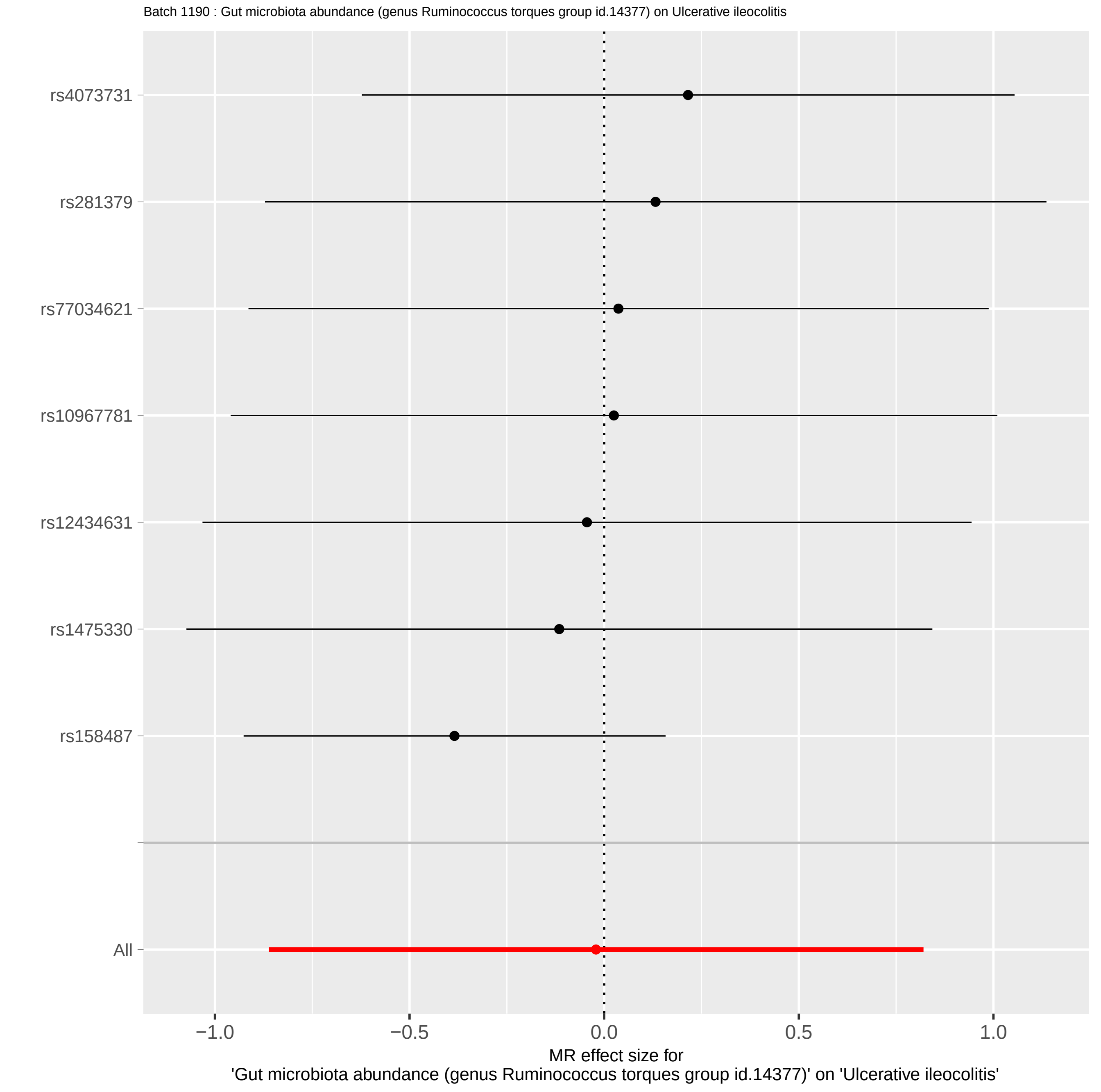

Batch 1190 : Gut microbiota abundance (genus Ruminococcus torques group id.14377) on Ulcerative ileocolitis
rs4073731
rs281379
rs77034621
rs10967781
rs12434631
rs1475330
rs158487
All
−1.0
−0.5
0.0
0.5
1.0
MR effect size for
'Gut microbiota abundance (genus Ruminococcus torques group id.14377)' on 'Ulcerative ileocolitis'

Batch 1191 : Gut microbiota abundance (genus Sellimonas id.14369) on Ulcerative ileocolitis

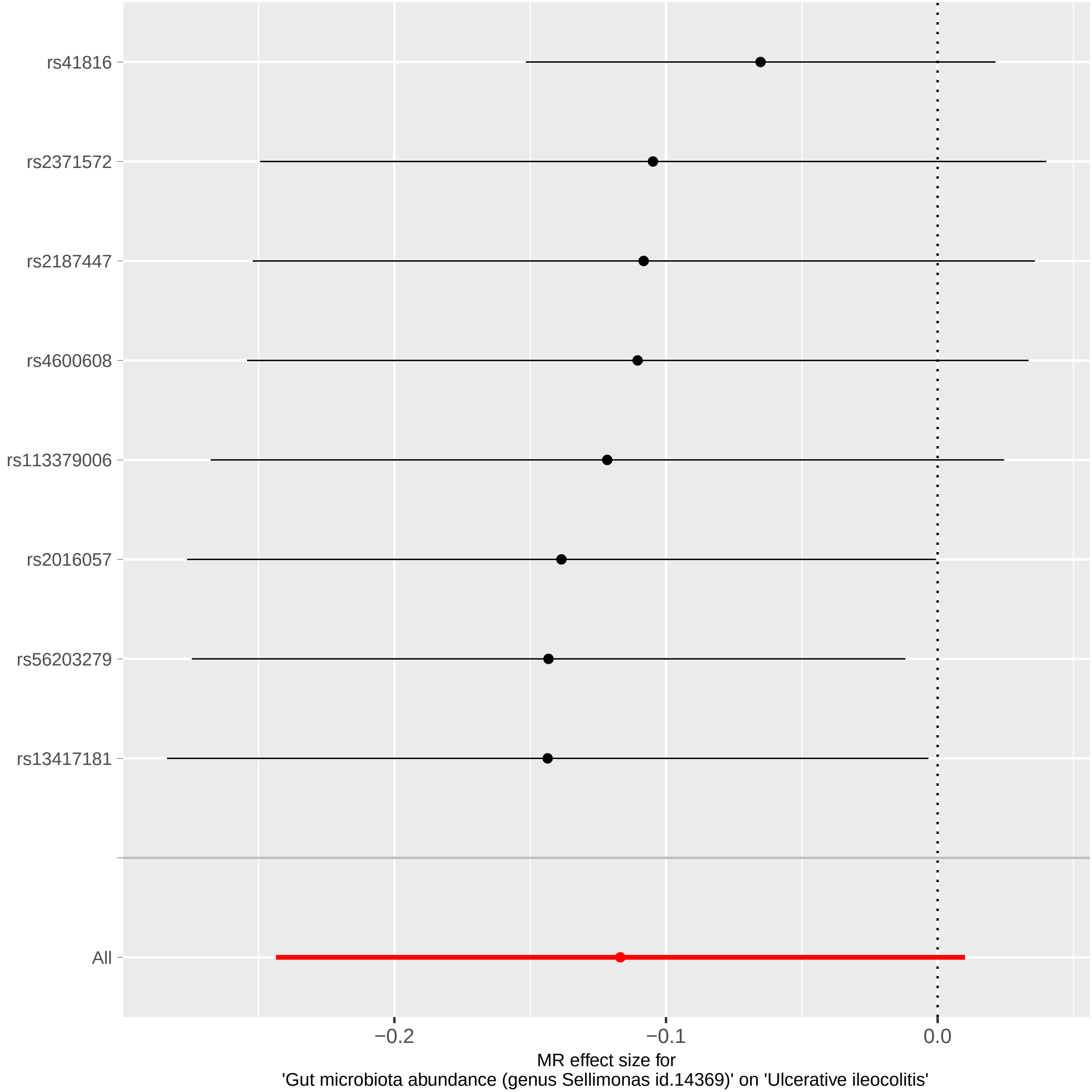

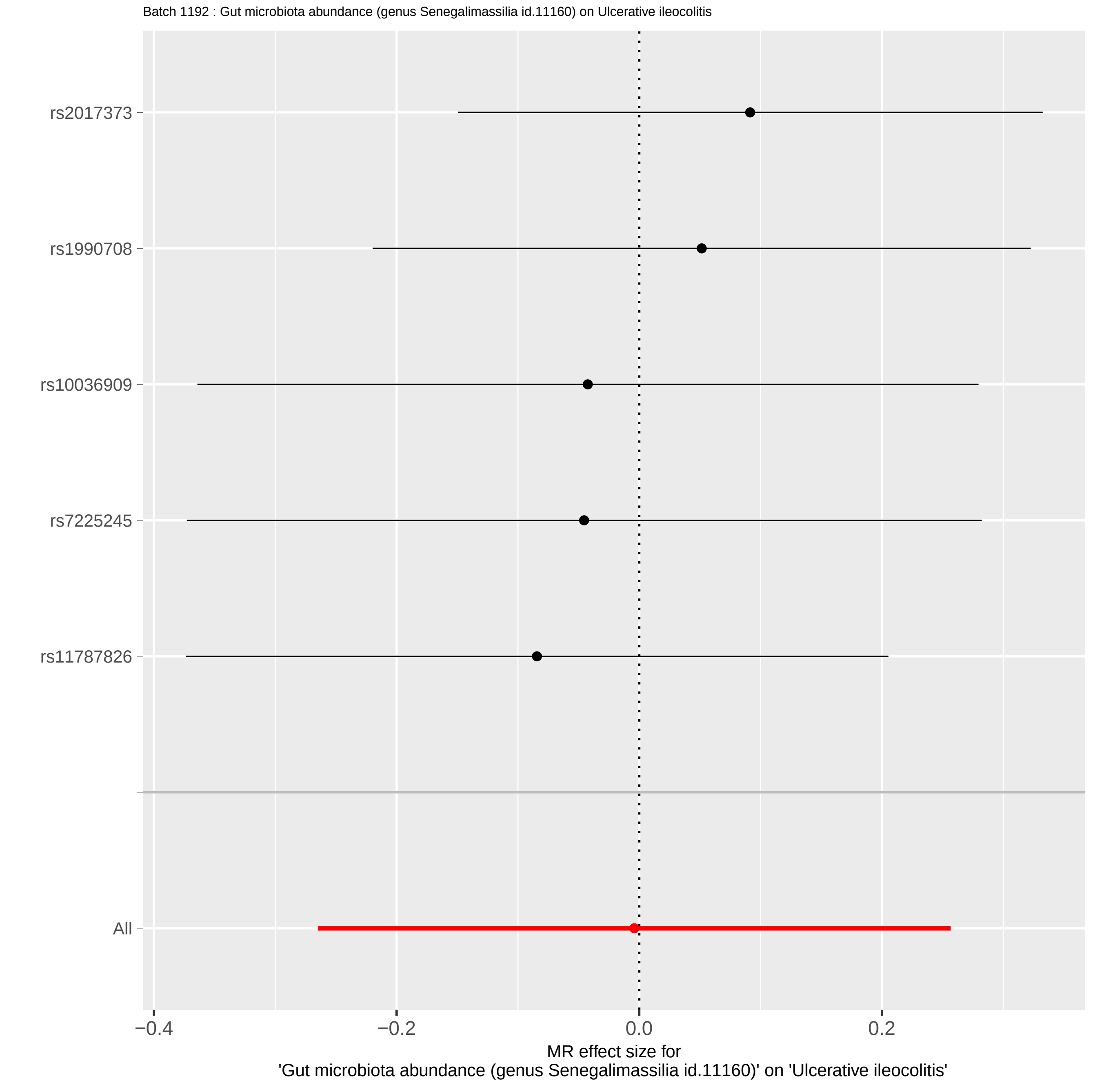

Batch 1192 : Gut microbiota abundance (genus Senegalimassilia id.11160) on Ulcerative ileocolitis
rs2017373
rs1990708
rs10036909
rs7225245
rs11787826
All
−0.4
−0.2
0.0
0.2
MR effect size for
'Gut microbiota abundance (genus Senegalimassilia id.11160)' on 'Ulcerative ileocolitis'

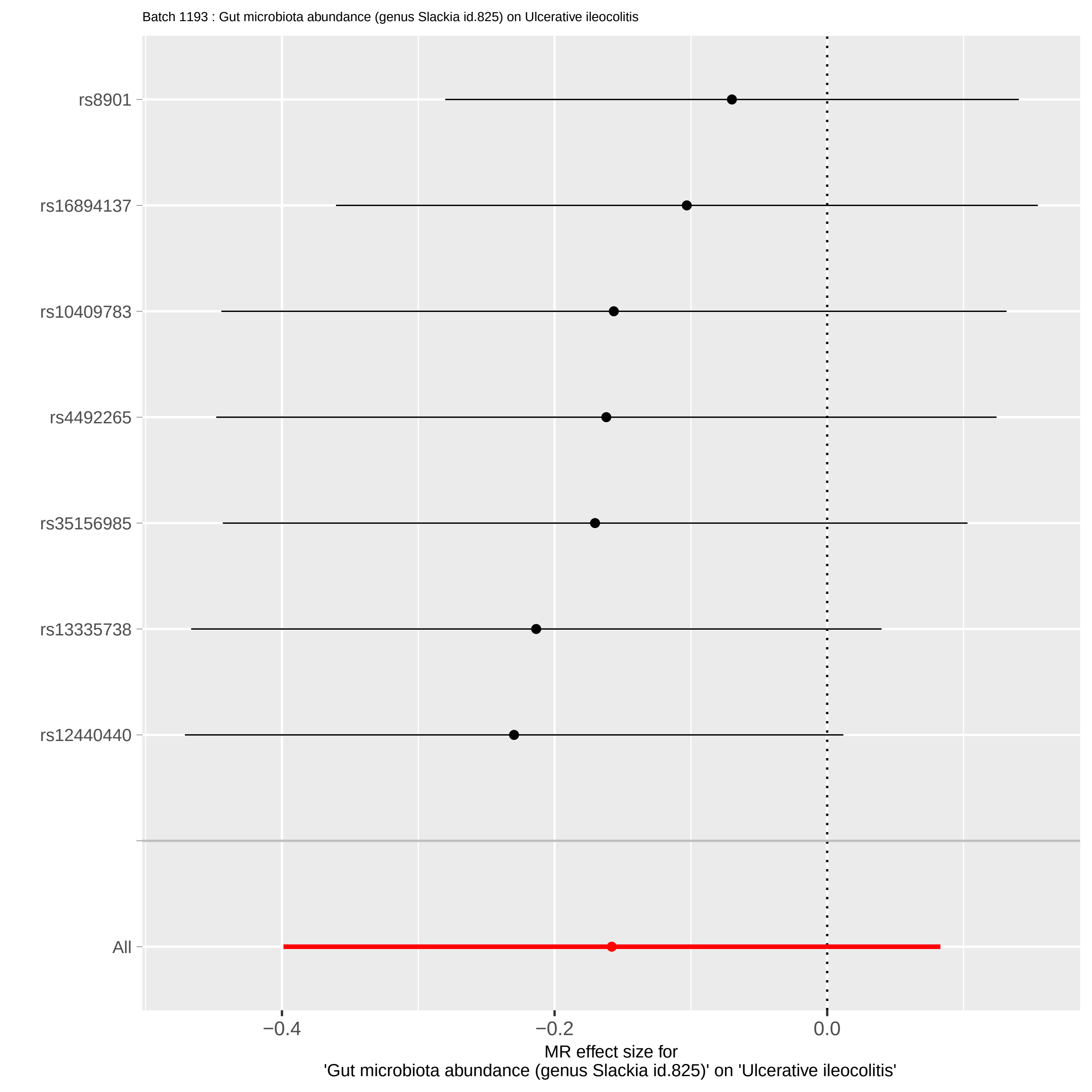

Batch 1193 : Gut microbiota abundance (genus Slackia id.825) on Ulcerative ileocolitis
rs8901
rs16894137
rs10409783
rs4492265
rs35156985
rs13335738
rs12440440
All
−0.4
−0.2
0.0
MR effect size for
'Gut microbiota abundance (genus Slackia id.825)' on 'Ulcerative ileocolitis'

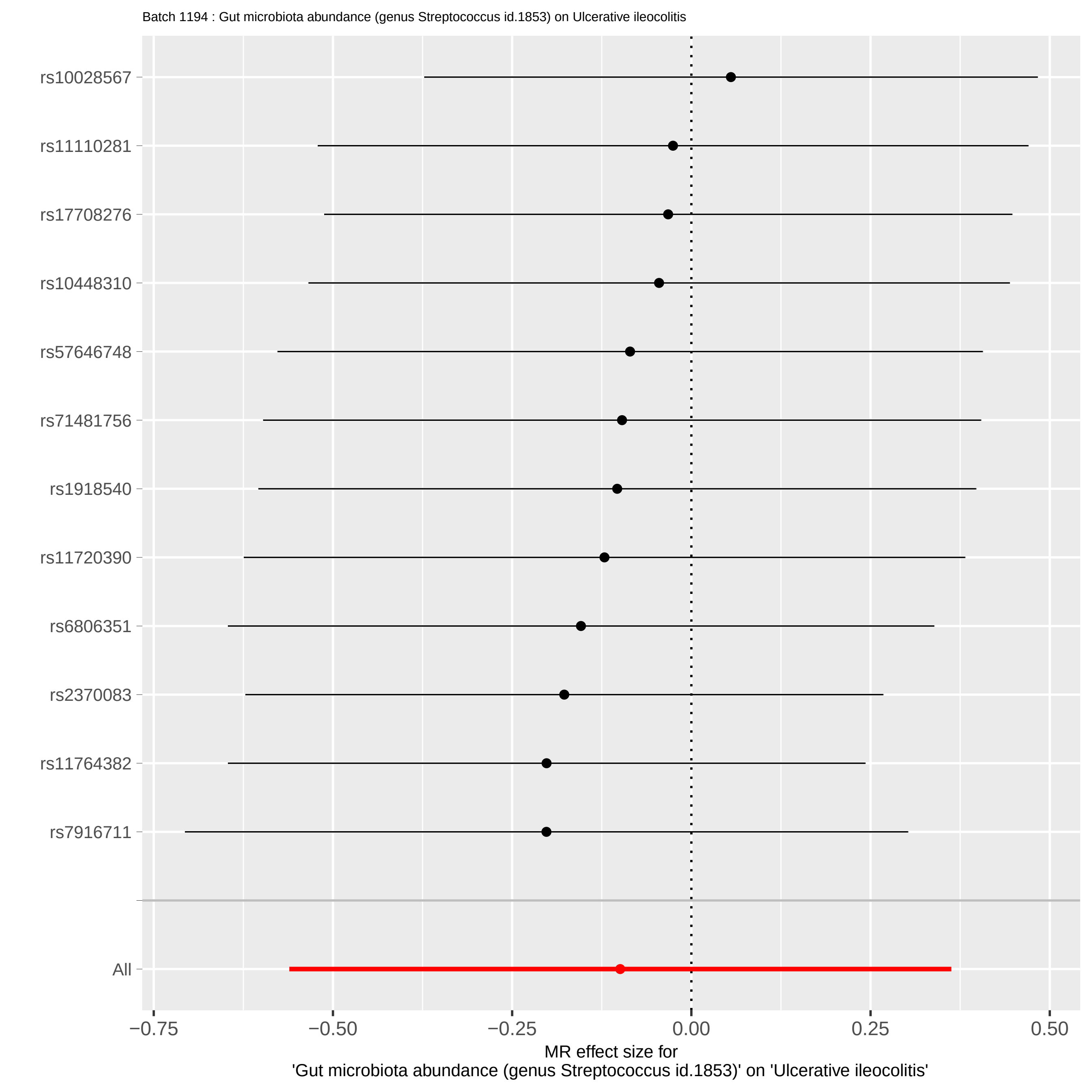

Batch 1194 : Gut microbiota abundance (genus Streptococcus id.1853) on Ulcerative ileocolitis
rs10028567
rs11110281
rs17708276
rs10448310
rs57646748
rs71481756
rs1918540
rs11720390
rs6806351
rs2370083
rs11764382
rs7916711
All
−0.75
−0.50
−0.25
0.00
0.25
0.50
MR effect size for
'Gut microbiota abundance (genus Streptococcus id.1853)' on 'Ulcerative ileocolitis'

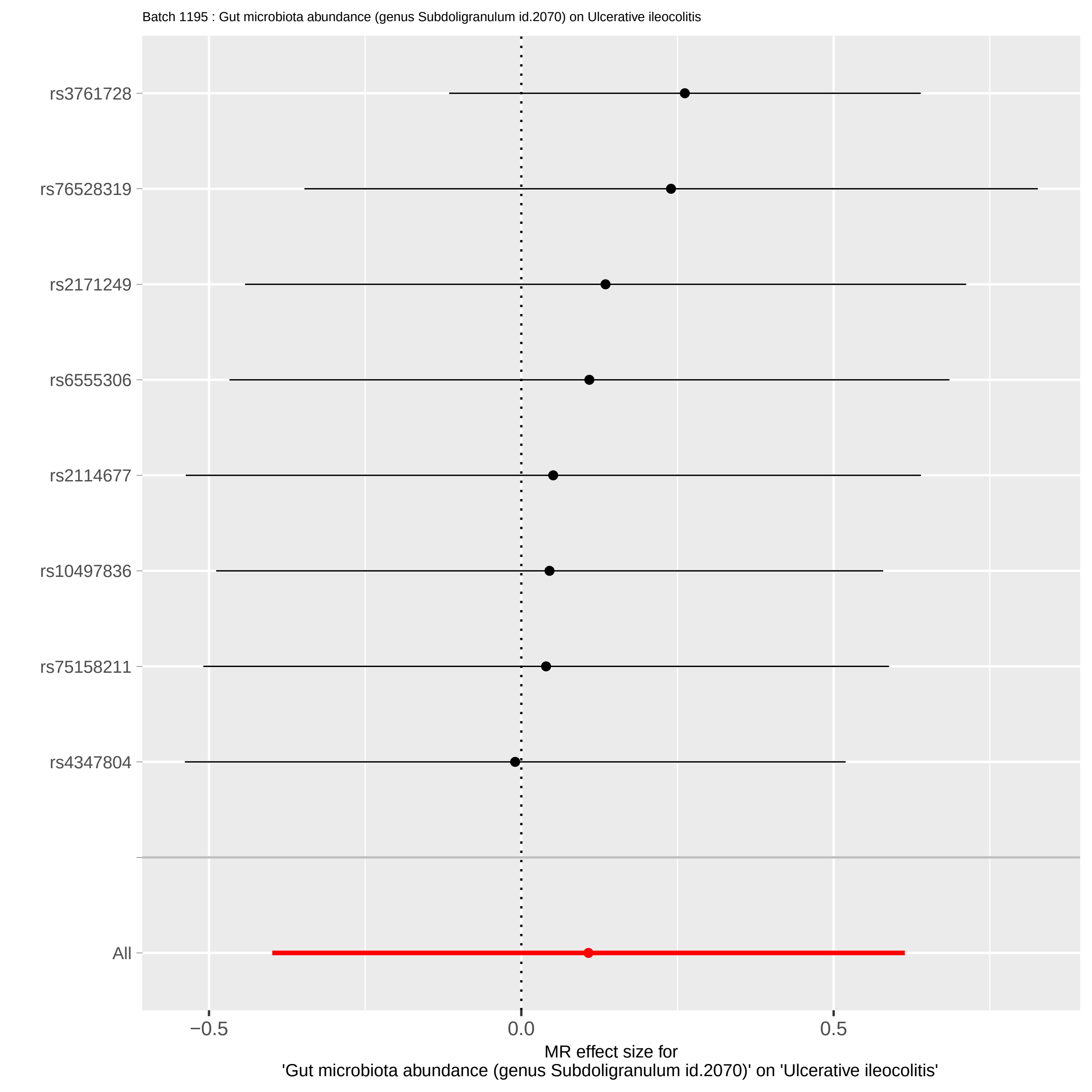

Batch 1195 : Gut microbiota abundance (genus Subdoligranulum id.2070) on Ulcerative ileocolitis
rs3761728
rs76528319
rs2171249
rs6555306
rs2114677
rs10497836
rs75158211
rs4347804
All
−0.5
0.0
0.5
MR effect size for
'Gut microbiota abundance (genus Subdoligranulum id.2070)' on 'Ulcerative ileocolitis'

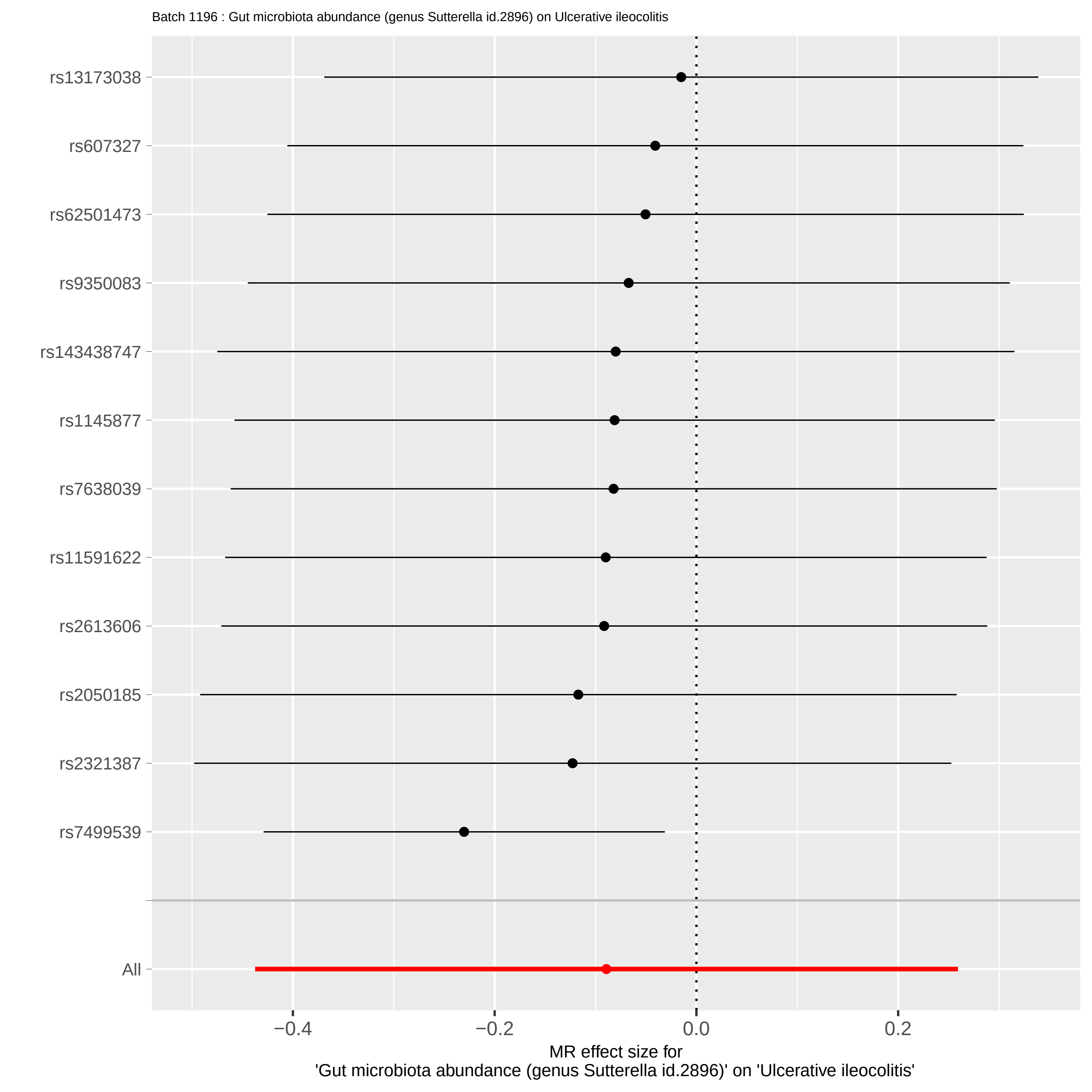
Batch 1196 : Gut microbiota abundance (genus Sutterella id.2896) on Ulcerative ileocolitis
rs13173038
rs607327
rs62501473
rs9350083
rs143438747
rs1145877
rs7638039
rs11591622
rs2613606
rs2050185
rs2321387
rs7499539
All
−0.4
−0.2
0.0
0.2
MR effect size for
'Gut microbiota abundance (genus Sutterella id.2896)' on 'Ulcerative ileocolitis'

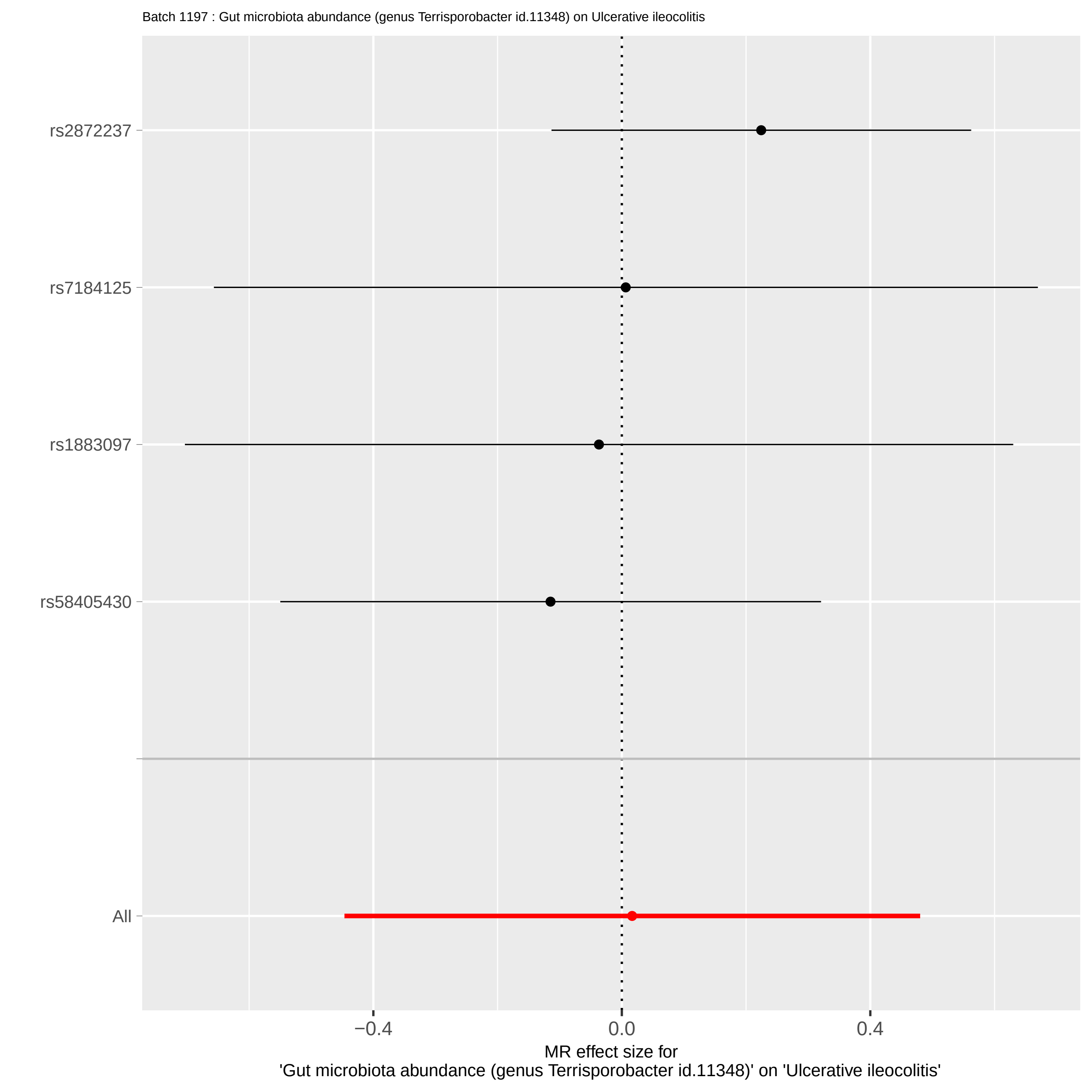
Batch 1197 : Gut microbiota abundance (genus Terrisporobacter id.11348) on Ulcerative ileocolitis
rs2872237
rs7184125
rs1883097
rs58405430
All
−0.4
0.0
0.4
MR effect size for
'Gut microbiota abundance (genus Terrisporobacter id.11348)' on 'Ulcerative ileocolitis'

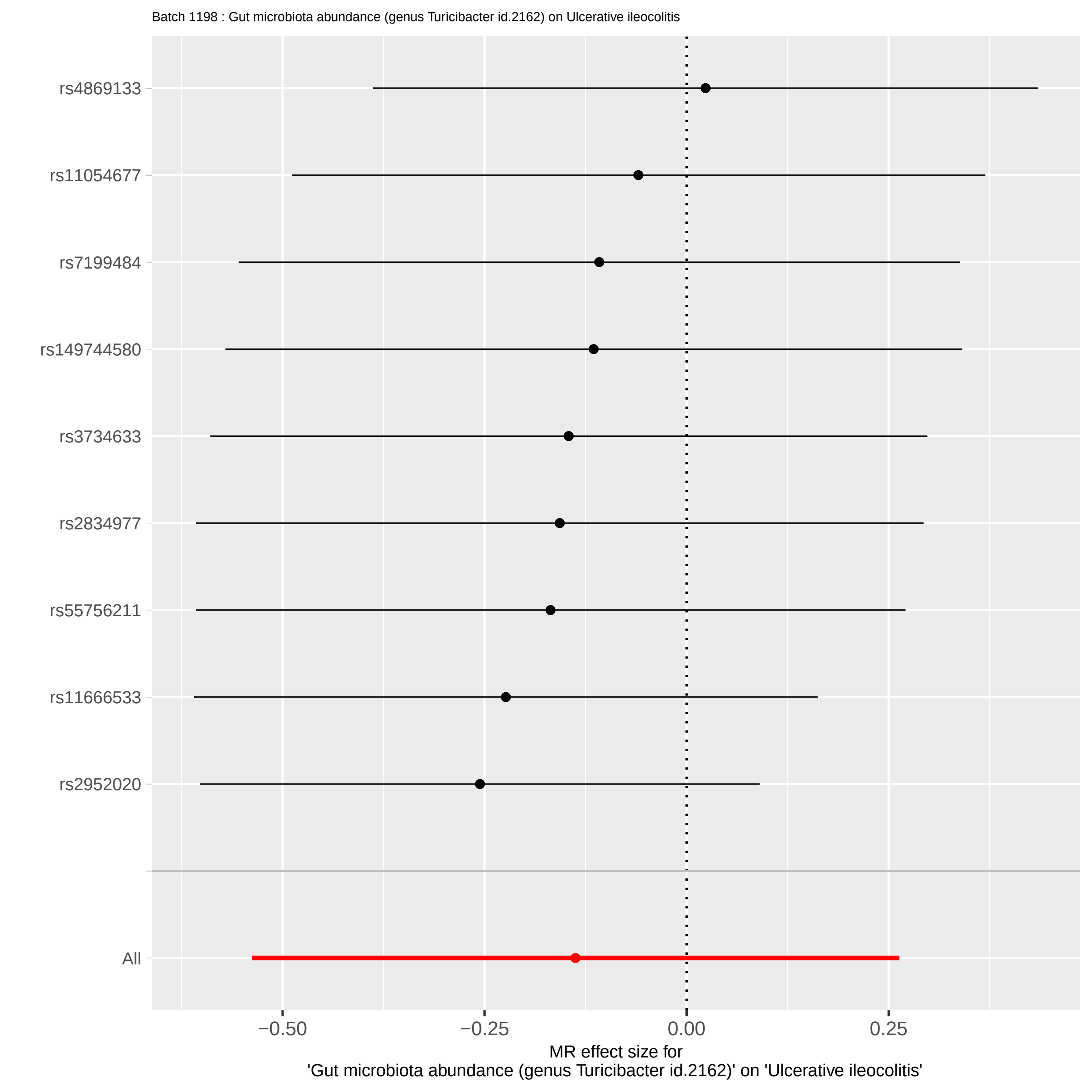
Batch 1198 : Gut microbiota abundance (genus Turicibacter id.2162) on Ulcerative ileocolitis
rs4869133
rs11054677
rs7199484
rs149744580
rs3734633
rs2834977
rs55756211
rs11666533
rs2952020
All
−0.50
−0.25
0.00
0.25
MR effect size for
'Gut microbiota abundance (genus Turicibacter id.2162)' on 'Ulcerative ileocolitis'

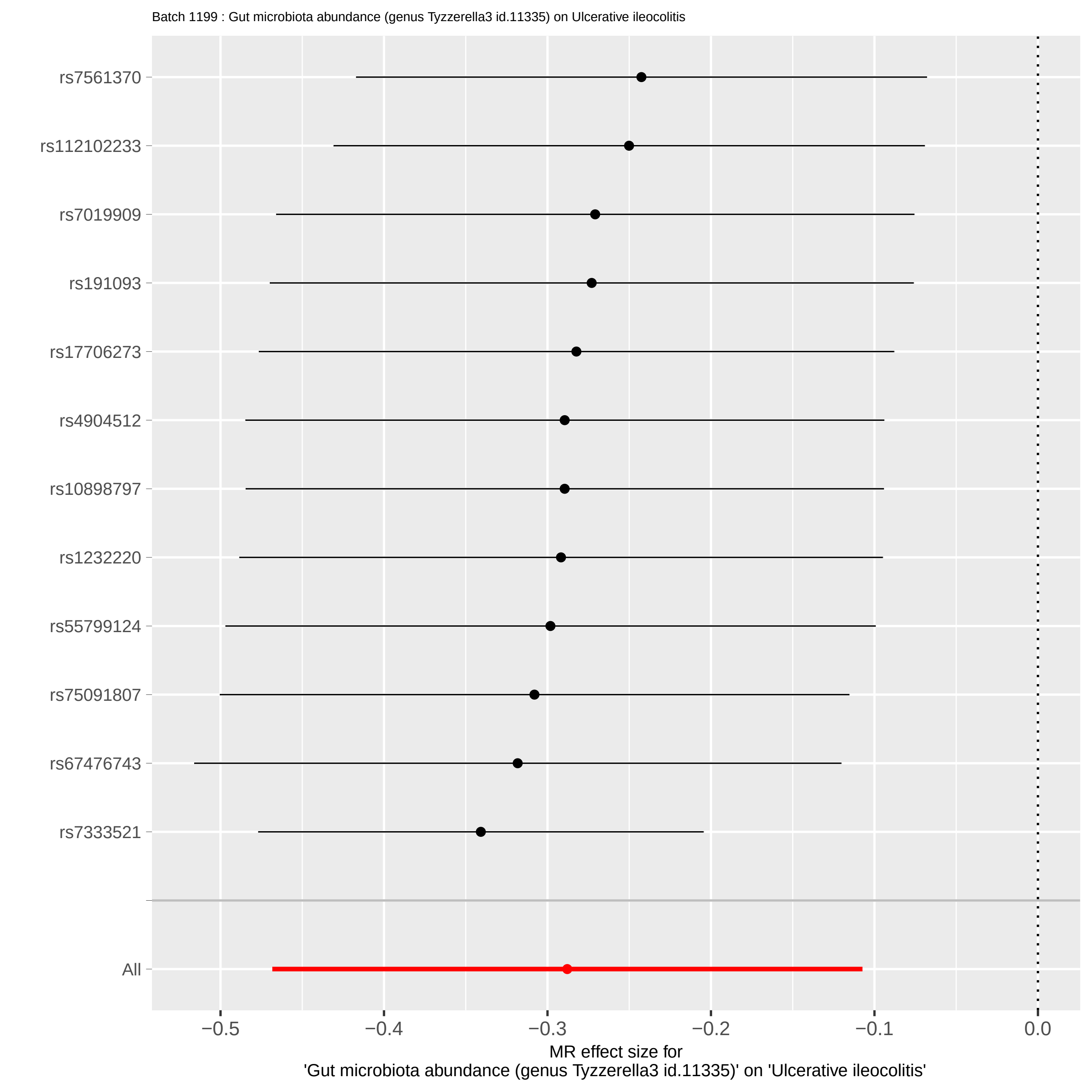

Batch 1199 : Gut microbiota abundance (genus Tyzzerella3 id.11335) on Ulcerative ileocolitis
rs7561370
rs112102233
rs7019909
rs191093
rs17706273
rs4904512
rs10898797
rs1232220
rs55799124
rs75091807
rs67476743
rs7333521
All
−0.5
−0.4
−0.3
−0.2
−0.1
0.0
MR effect size for
'Gut microbiota abundance (genus Tyzzerella3 id.11335)' on 'Ulcerative ileocolitis'

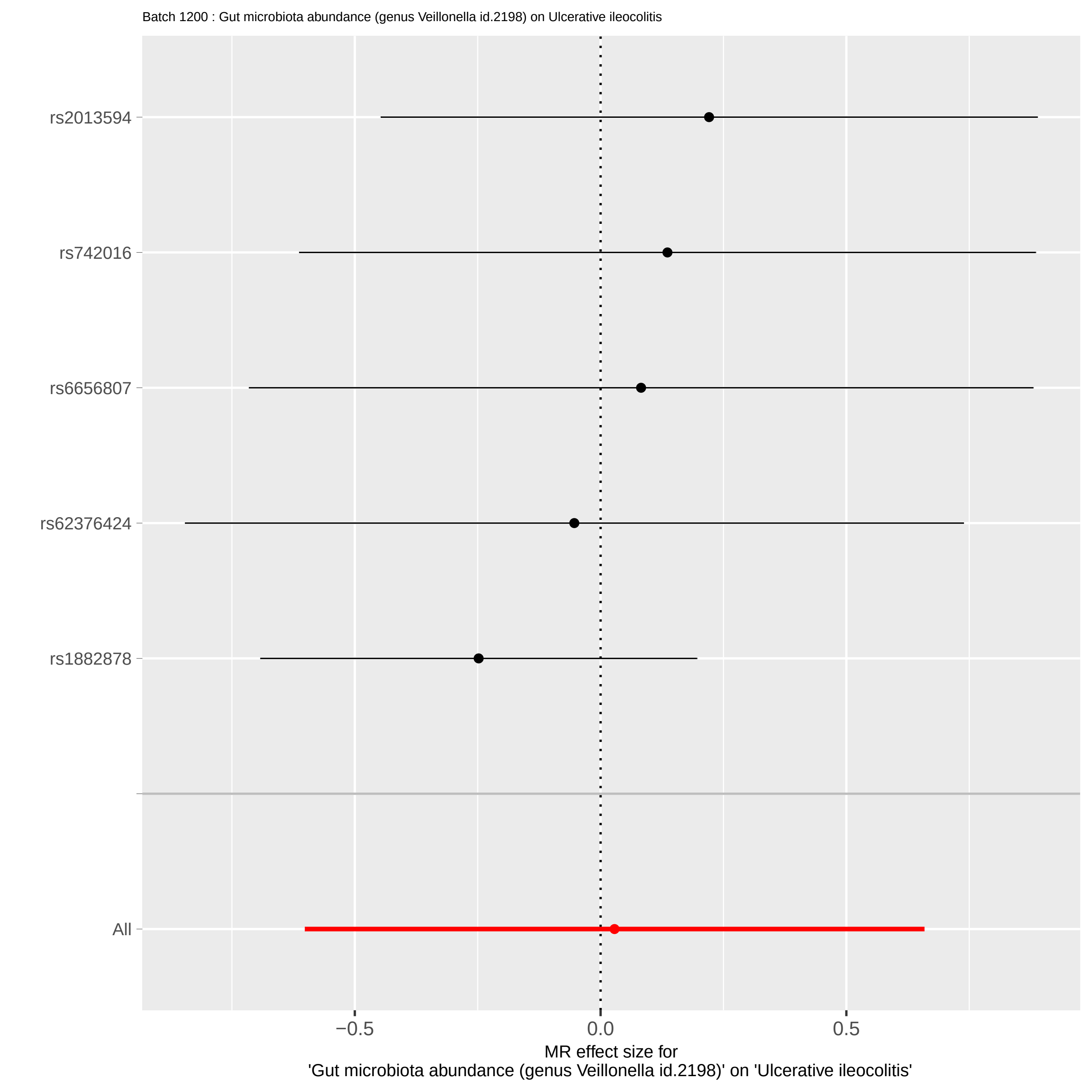
Batch 1200 : Gut microbiota abundance (genus Veillonella id.2198) on Ulcerative ileocolitis
rs2013594
rs742016
rs6656807
rs62376424
rs1882878
All
−0.5
0.0
0.5
MR effect size for
'Gut microbiota abundance (genus Veillonella id.2198)' on 'Ulcerative ileocolitis'

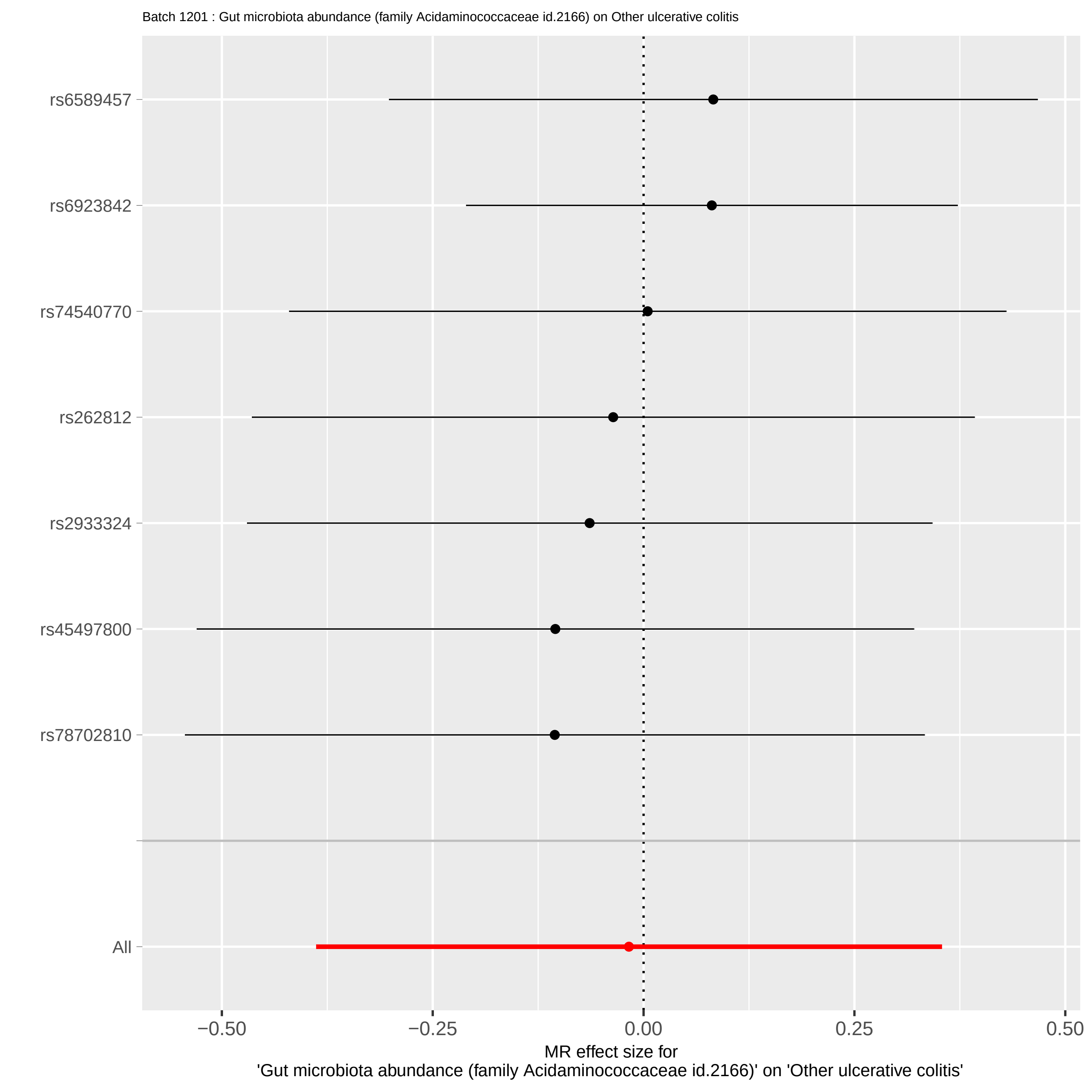
Batch 1201 : Gut microbiota abundance (family Acidaminococcaceae id.2166) on Other ulcerative colitis
rs6589457
rs6923842
rs74540770
rs262812
rs2933324
rs45497800
rs78702810
All
−0.50
−0.25
0.00
0.25
0.50
MR effect size for
'Gut microbiota abundance (family Acidaminococcaceae id.2166)' on 'Other ulcerative colitis'

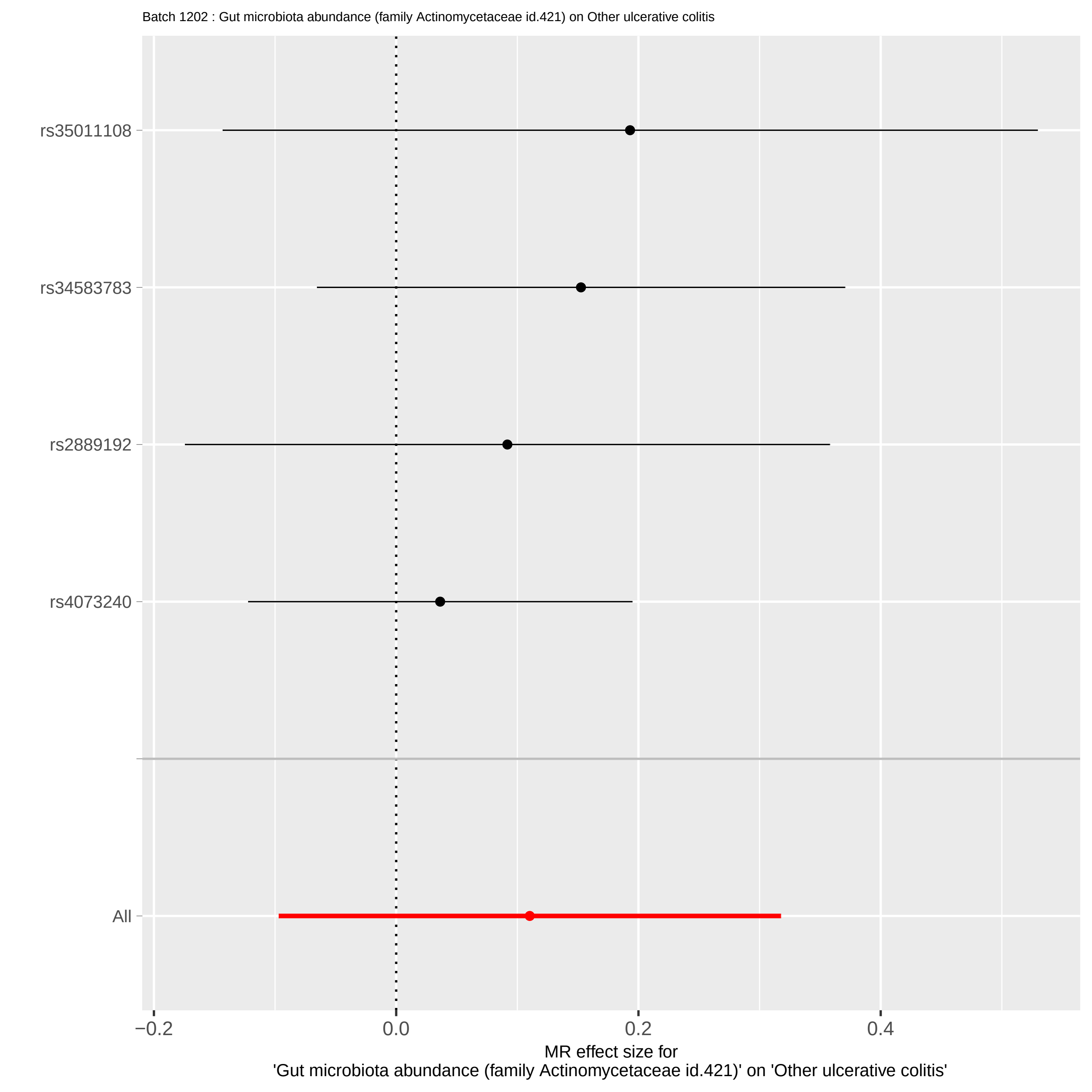

Batch 1202 : Gut microbiota abundance (family Actinomycetaceae id.421) on Other ulcerative colitis
rs35011108
rs34583783
rs2889192
rs4073240
All
−0.2
0.0
0.2
0.4
MR effect size for
'Gut microbiota abundance (family Actinomycetaceae id.421)' on 'Other ulcerative colitis'

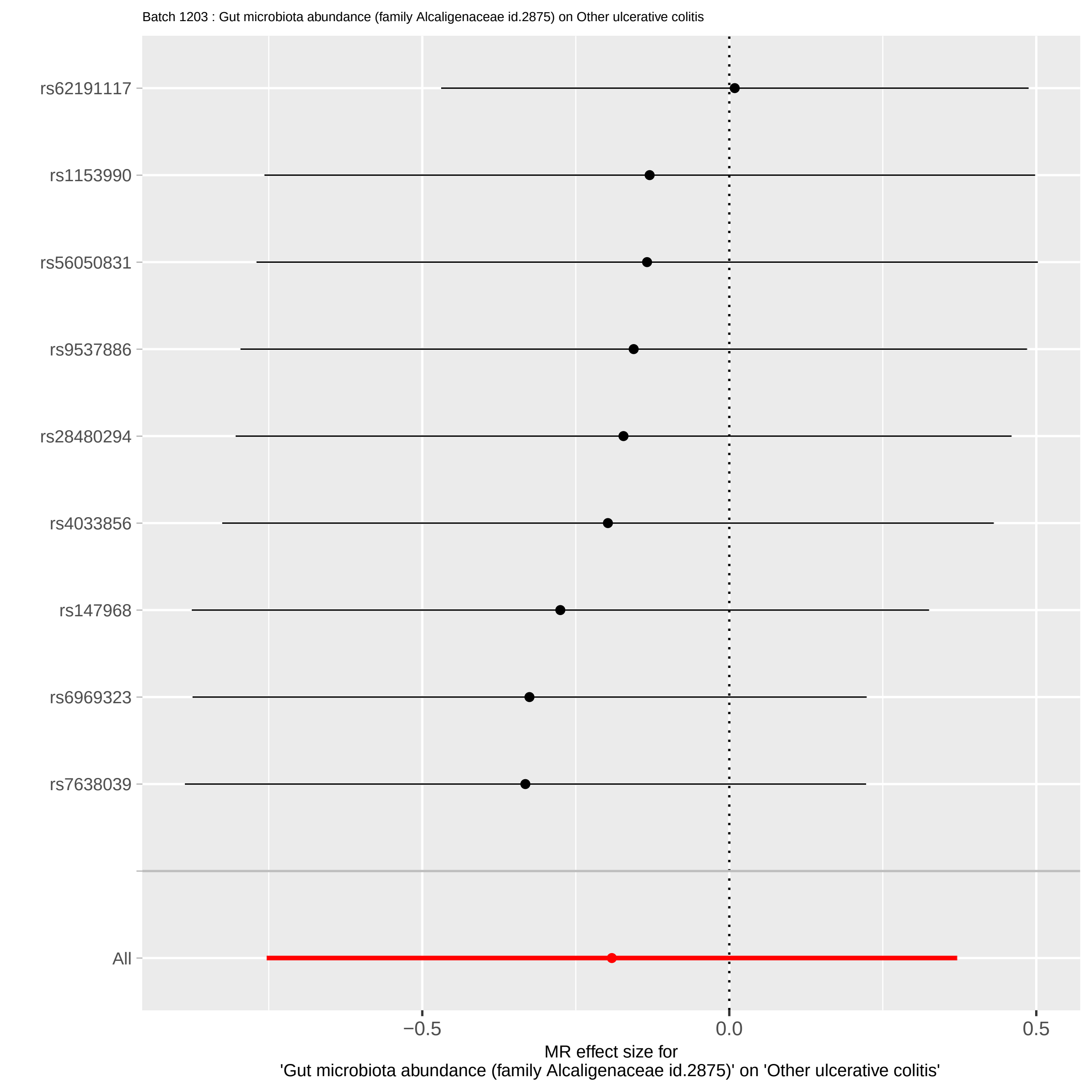
Batch 1203 : Gut microbiota abundance (family Alcaligenaceae id.2875) on Other ulcerative colitis
rs62191117
rs1153990
rs56050831
rs9537886
rs28480294
rs4033856
rs147968
rs6969323
rs7638039
All
−0.5
0.0
0.5
MR effect size for
'Gut microbiota abundance (family Alcaligenaceae id.2875)' on 'Other ulcerative colitis'

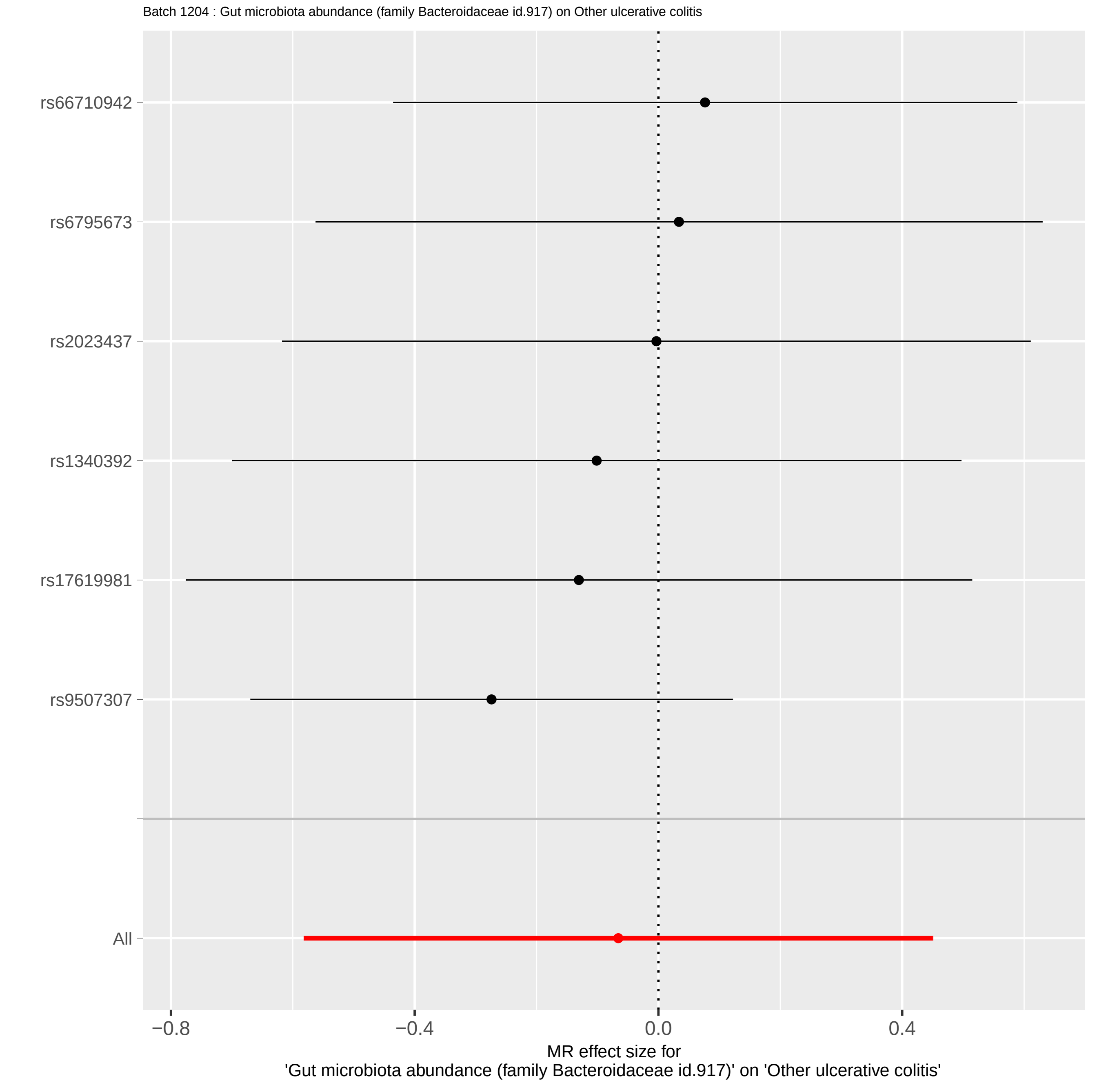

Batch 1204 : Gut microbiota abundance (family Bacteroidaceae id.917) on Other ulcerative colitis
rs66710942
rs6795673
rs2023437
rs1340392
rs17619981
rs9507307
All
−0.8
−0.4
0.0
0.4
MR effect size for
'Gut microbiota abundance (family Bacteroidaceae id.917)' on 'Other ulcerative colitis'

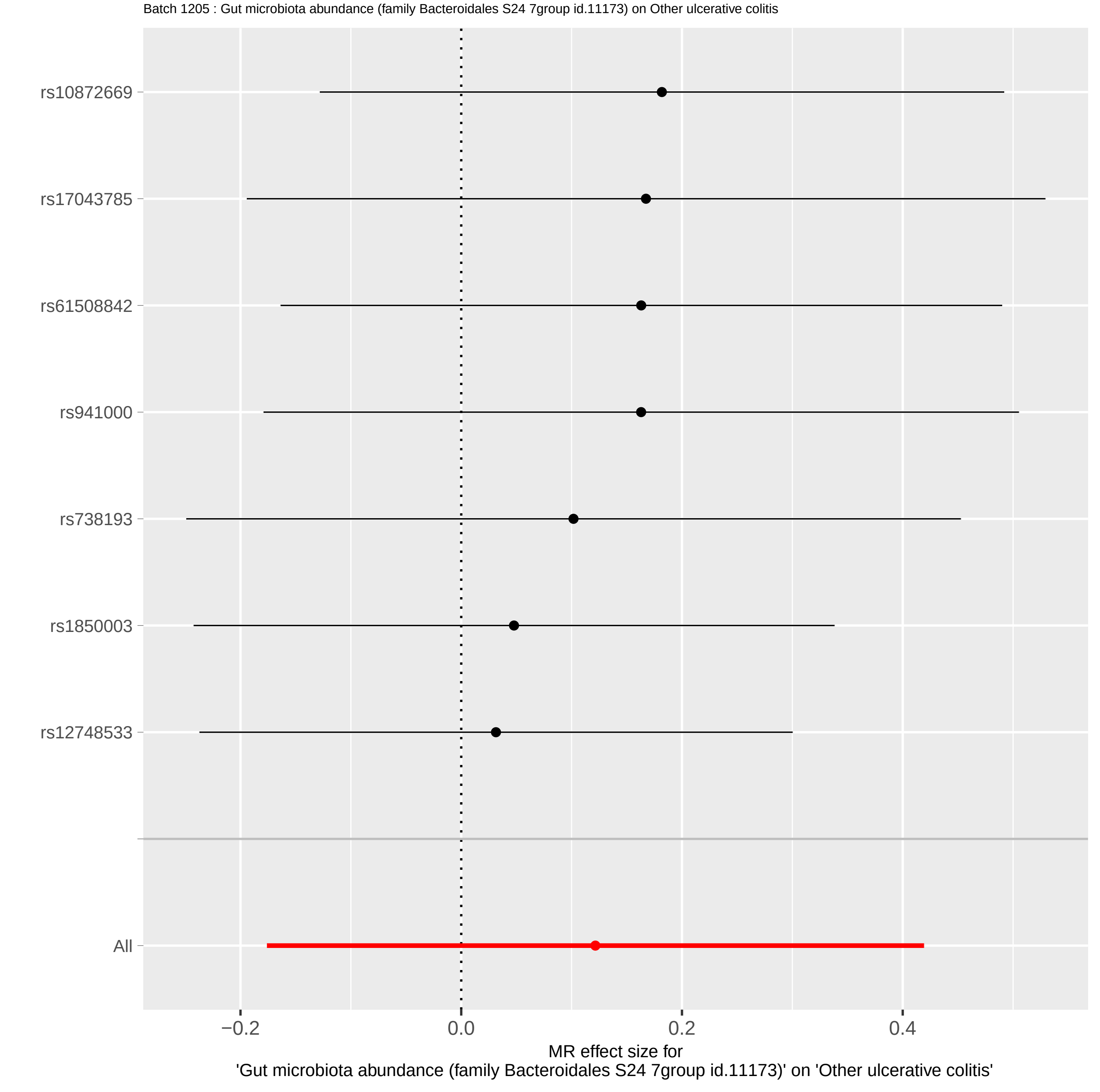

Batch 1205 : Gut microbiota abundance (family Bacteroidales S24 7group id.11173) on Other ulcerative colitis
rs10872669
rs17043785
rs61508842
rs941000
rs738193
rs1850003
rs12748533
All
−0.2
0.0
0.2
0.4
MR effect size for
'Gut microbiota abundance (family Bacteroidales S24 7group id.11173)' on 'Other ulcerative colitis'

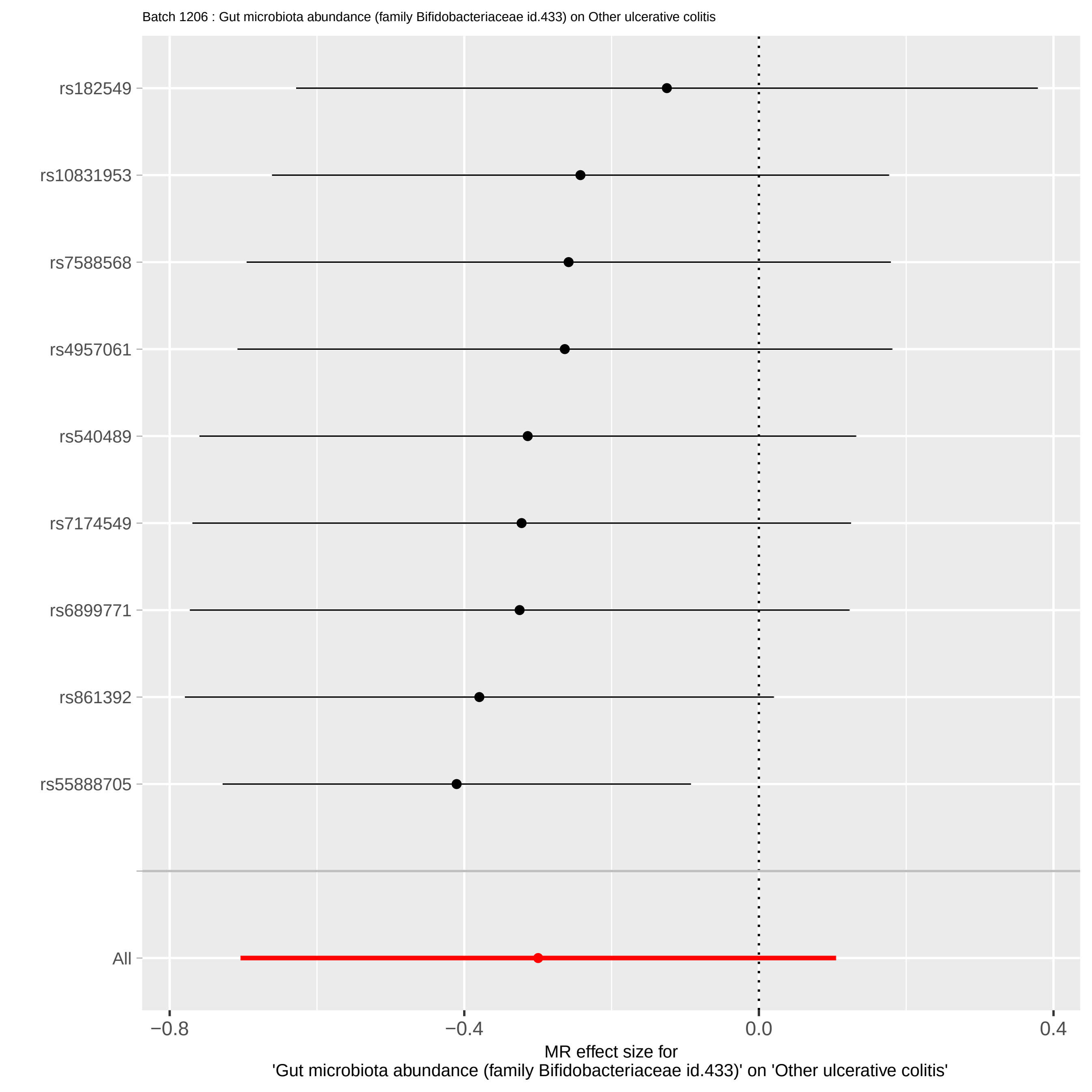

Batch 1206 : Gut microbiota abundance (family Bifidobacteriaceae id.433) on Other ulcerative colitis
rs182549
rs10831953
rs7588568
rs4957061
rs540489
rs7174549
rs6899771
rs861392
rs55888705
All
−0.8
−0.4
0.0
0.4
MR effect size for
'Gut microbiota abundance (family Bifidobacteriaceae id.433)' on 'Other ulcerative colitis'

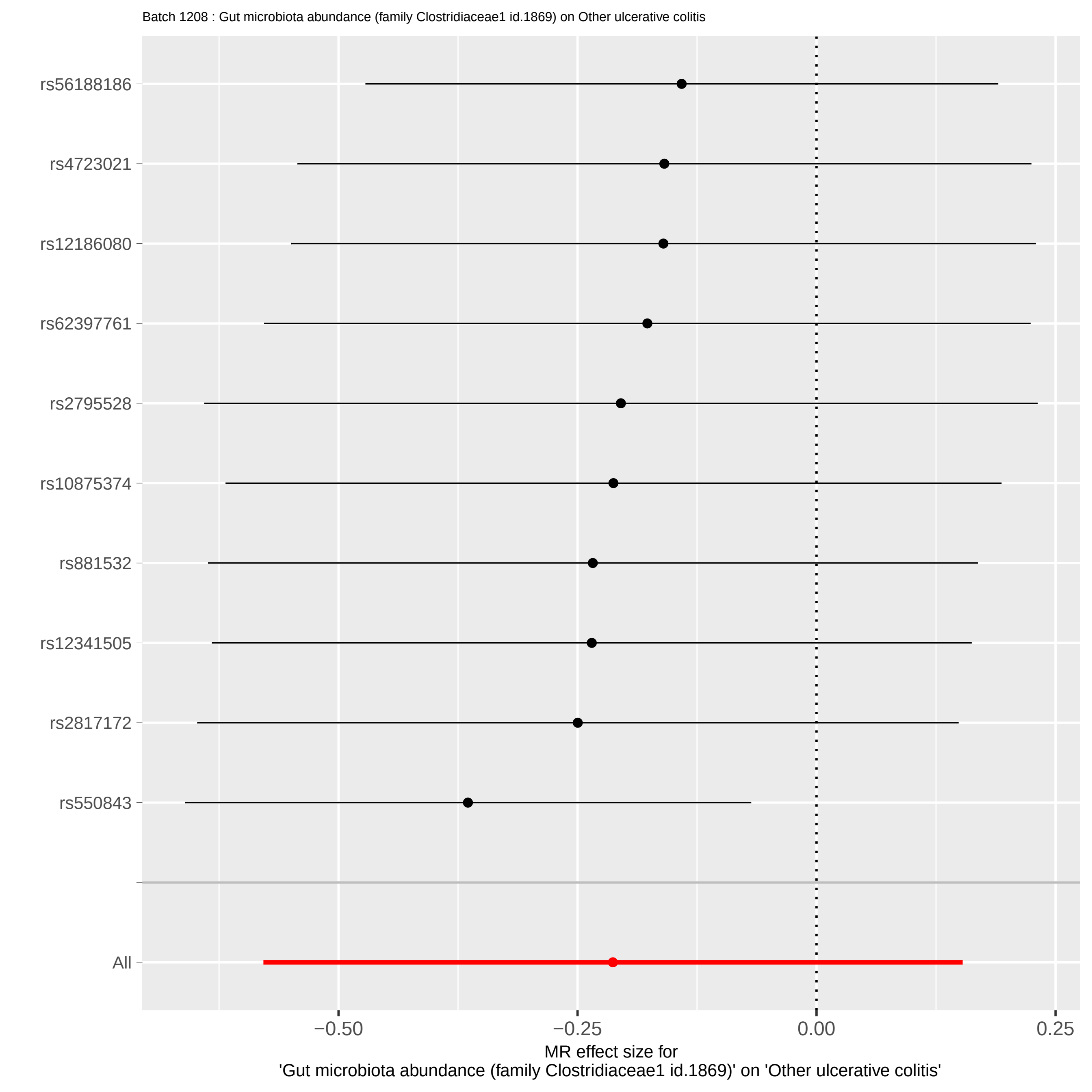

Batch 1208 : Gut microbiota abundance (family Clostridiaceae1 id.1869) on Other ulcerative colitis
rs56188186
rs4723021
rs12186080
rs62397761
rs2795528
rs10875374
rs881532
rs12341505
rs2817172
rs550843
All
−0.50
−0.25
0.00
0.25
MR effect size for
'Gut microbiota abundance (family Clostridiaceae1 id.1869)' on 'Other ulcerative colitis'

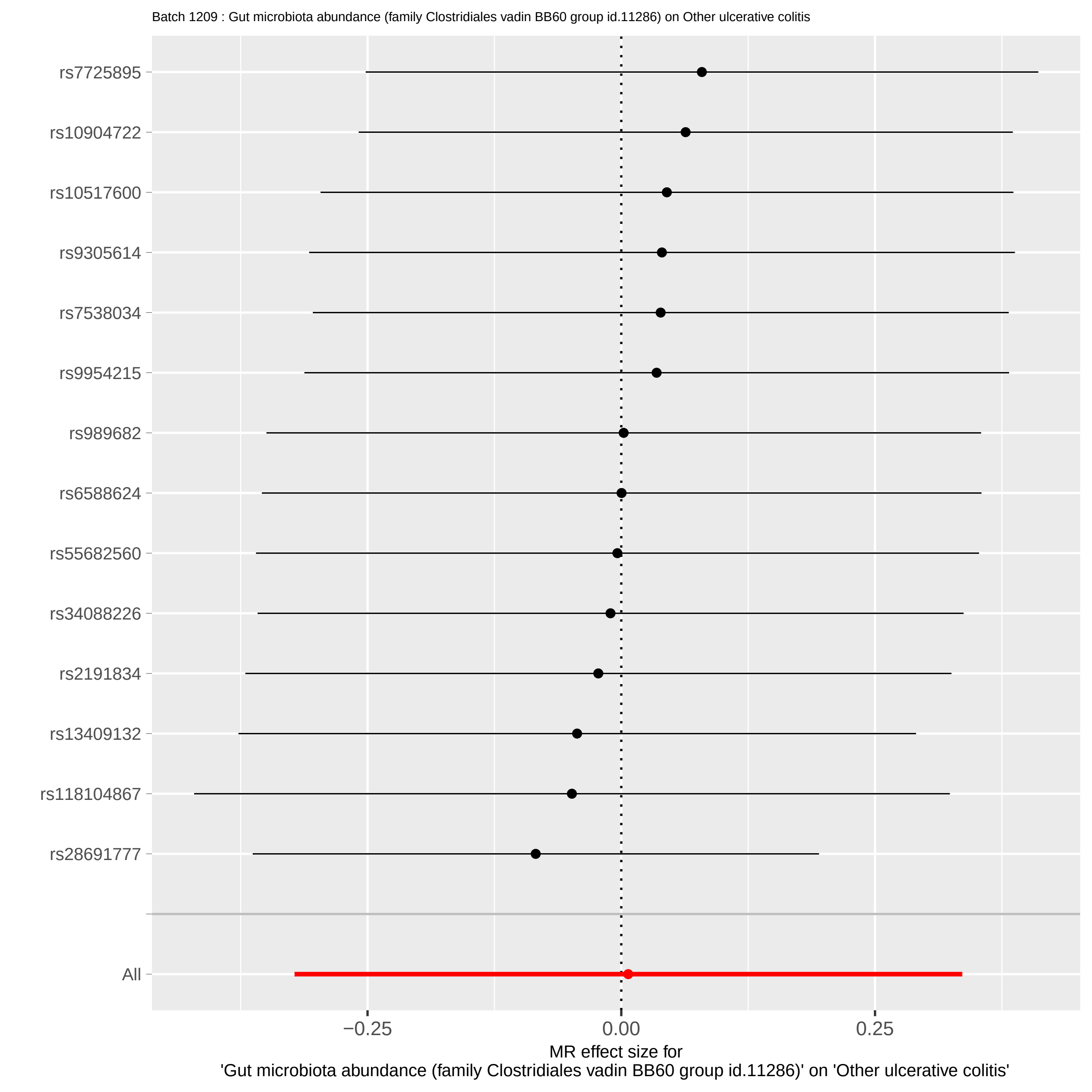
Batch 1209 : Gut microbiota abundance (family Clostridiales vadin BB60 group id.11286) on Other ulcerative colitis
rs7725895
rs10904722
rs10517600
rs9305614
rs7538034
rs9954215
rs989682
rs6588624
rs55682560
rs34088226
rs2191834
rs13409132
rs118104867
rs28691777
All
−0.25
0.00
0.25
MR effect size for
'Gut microbiota abundance (family Clostridiales vadin BB60 group id.11286)' on 'Other ulcerative colitis'

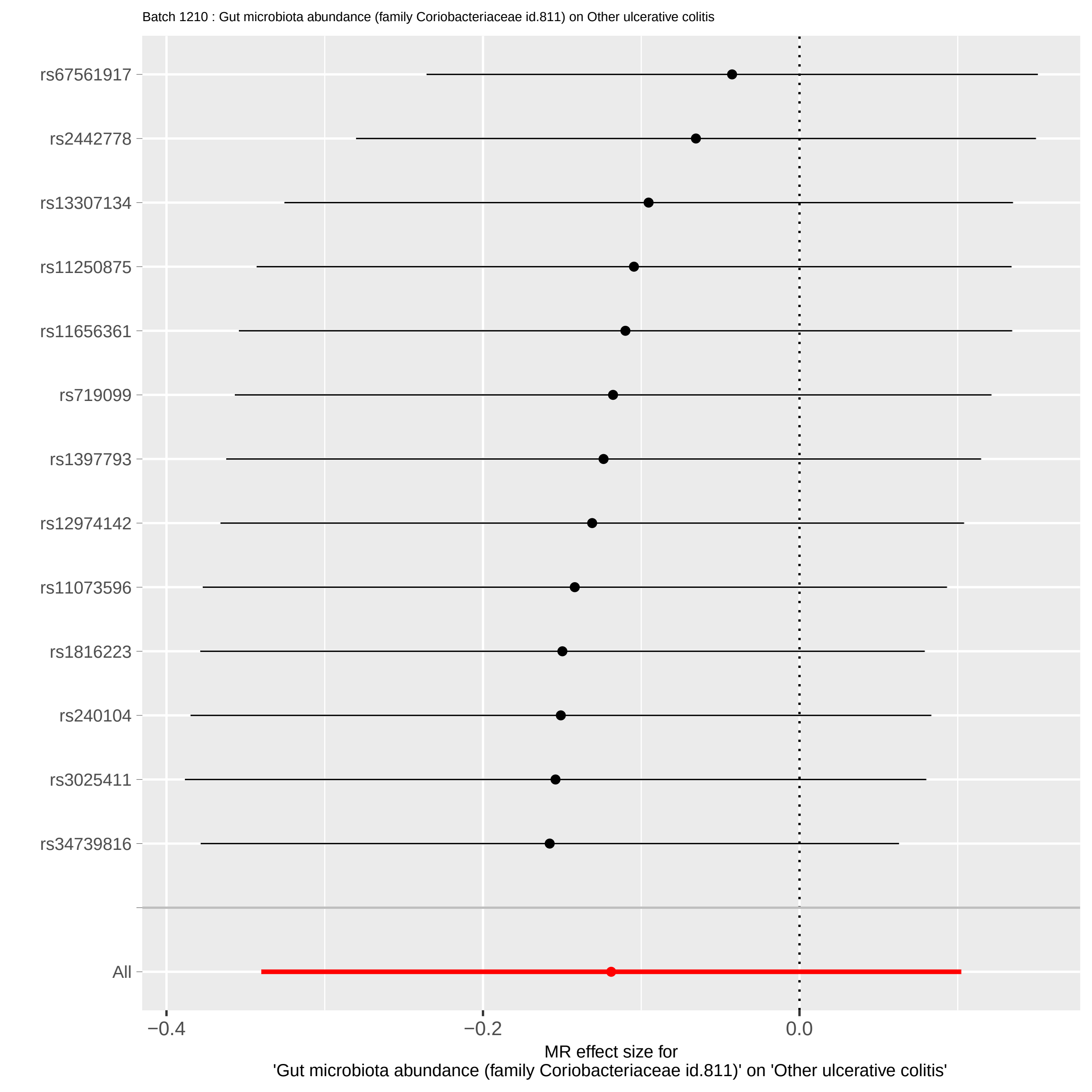
Batch 1210 : Gut microbiota abundance (family Coriobacteriaceae id.811) on Other ulcerative colitis
rs67561917
rs2442778
rs13307134
rs11250875
rs11656361
rs719099
rs1397793
rs12974142
rs11073596
rs1816223
rs240104
rs3025411
rs34739816
All
−0.4
−0.2
0.0
MR effect size for
'Gut microbiota abundance (family Coriobacteriaceae id.811)' on 'Other ulcerative colitis'

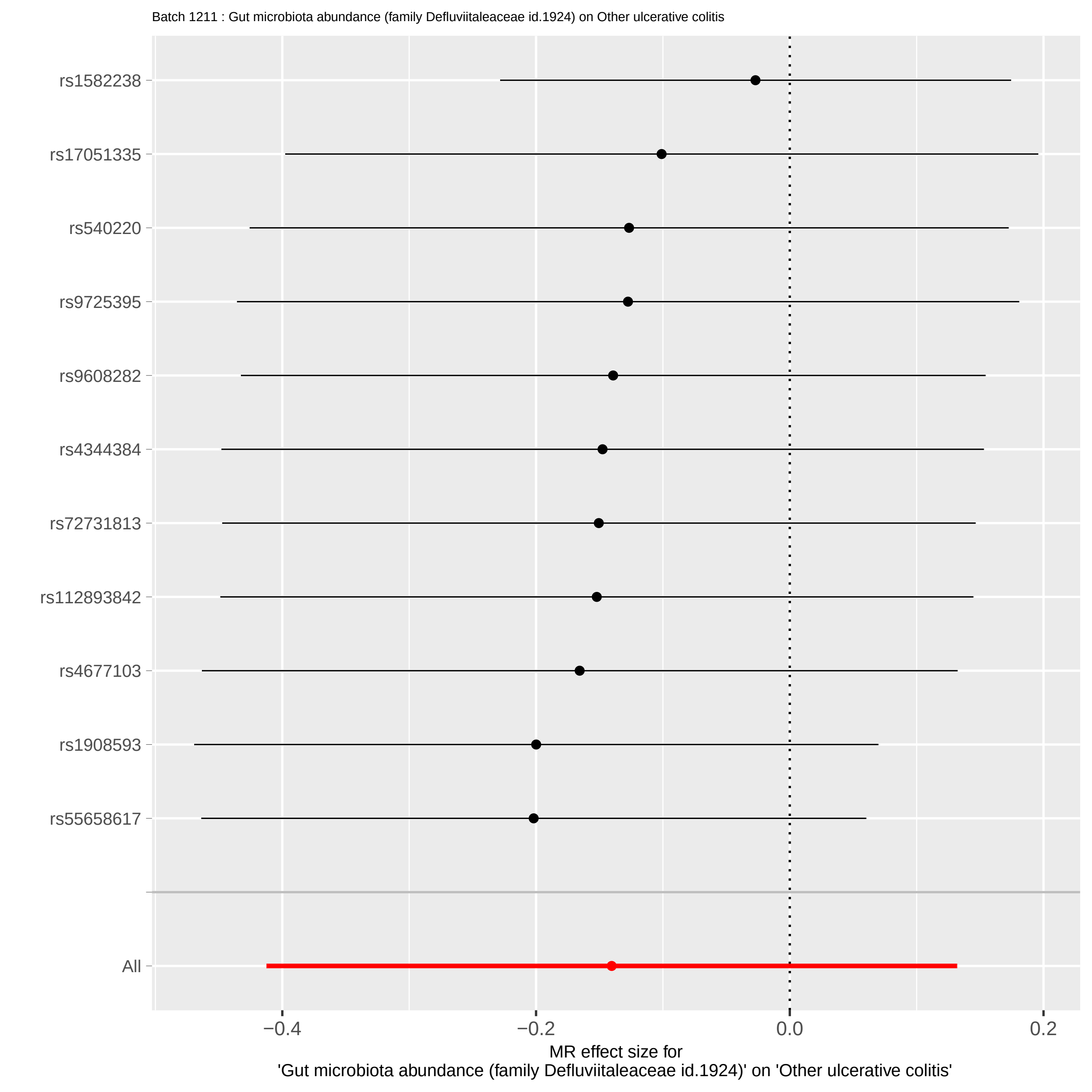

Batch 1211 : Gut microbiota abundance (family Defluviitaleaceae id.1924) on Other ulcerative colitis
rs1582238
rs17051335
rs540220
rs9725395
rs9608282
rs4344384
rs72731813
rs112893842
rs4677103
rs1908593
rs55658617
All
−0.4
−0.2
0.0
0.2
MR effect size for
'Gut microbiota abundance (family Defluviitaleaceae id.1924)' on 'Other ulcerative colitis'

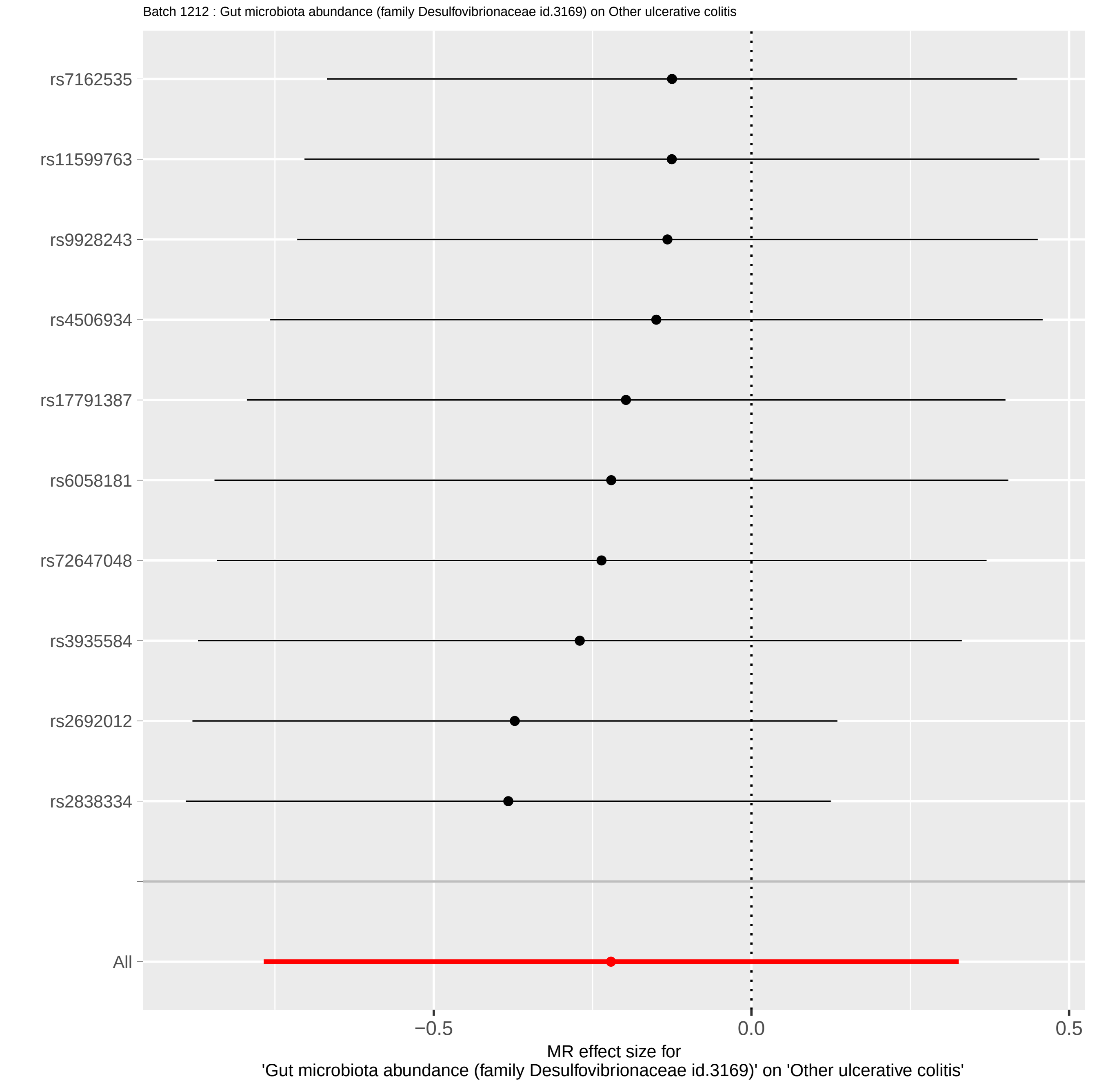

Batch 1212 : Gut microbiota abundance (family Desulfovibrionaceae id.3169) on Other ulcerative colitis
rs7162535
rs11599763
rs9928243
rs4506934
rs17791387
rs6058181
rs72647048
rs3935584
rs2692012
rs2838334
All
−0.5
0.0
0.5
MR effect size for
'Gut microbiota abundance (family Desulfovibrionaceae id.3169)' on 'Other ulcerative colitis'

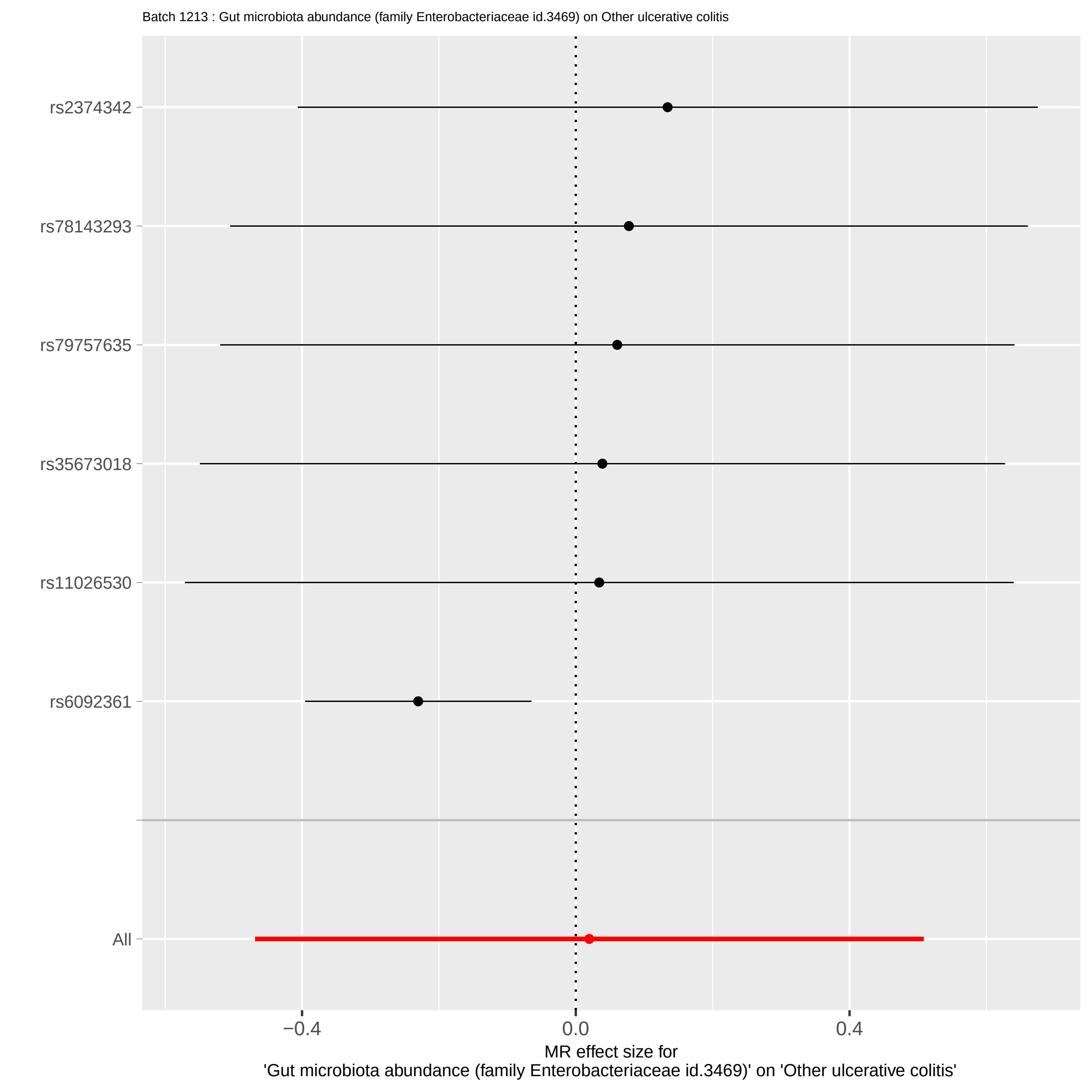

Batch 1213 : Gut microbiota abundance (family Enterobacteriaceae id.3469) on Other ulcerative colitis
rs2374342
rs78143293
rs79757635
rs35673018
rs11026530
rs6092361
All
−0.4
0.0
0.4
MR effect size for
'Gut microbiota abundance (family Enterobacteriaceae id.3469)' on 'Other ulcerative colitis'

Batch 1214 : Gut microbiota abundance (family Erysipelotrichaceae id.2149) on Other ulcerative colitis

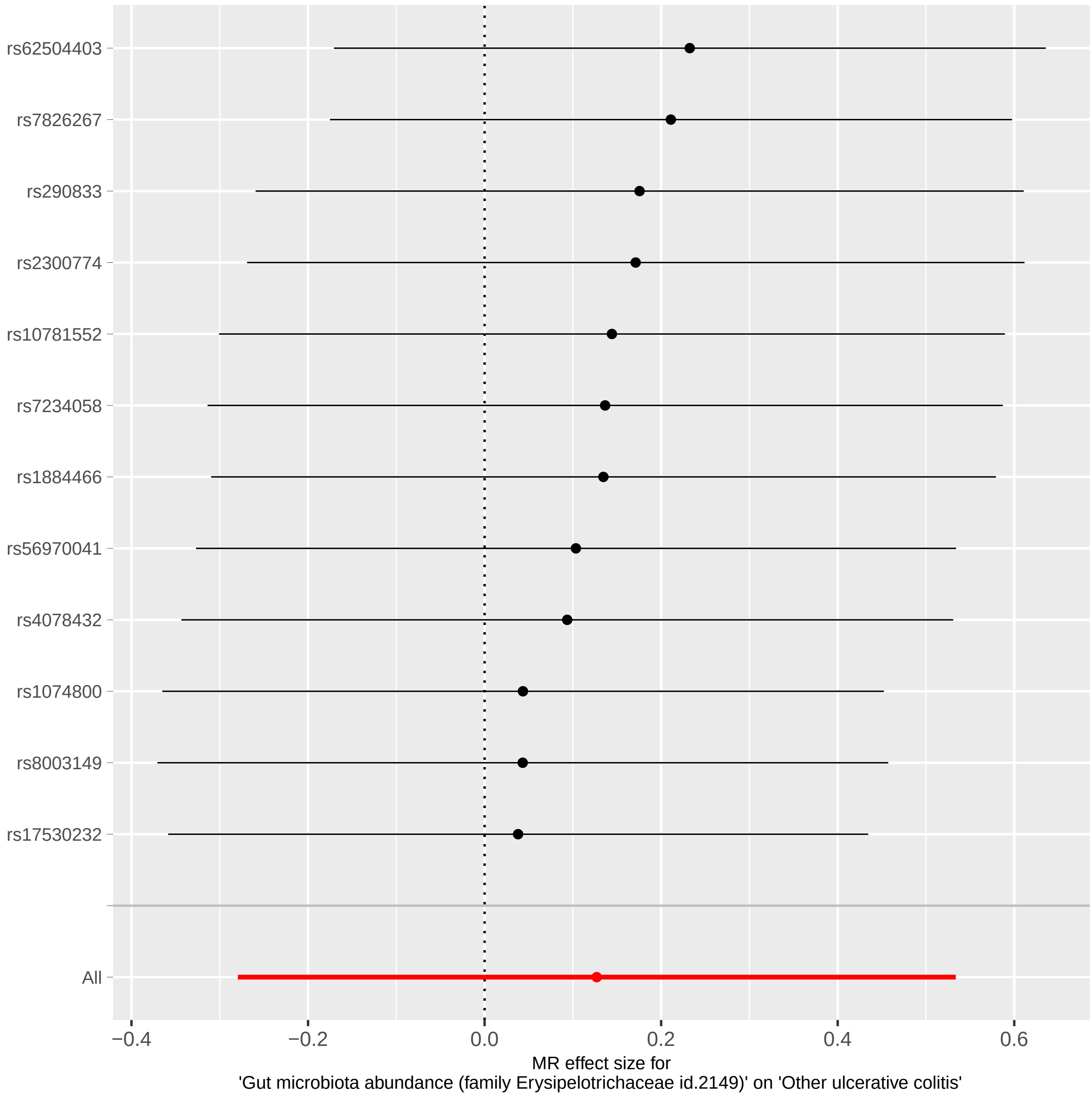

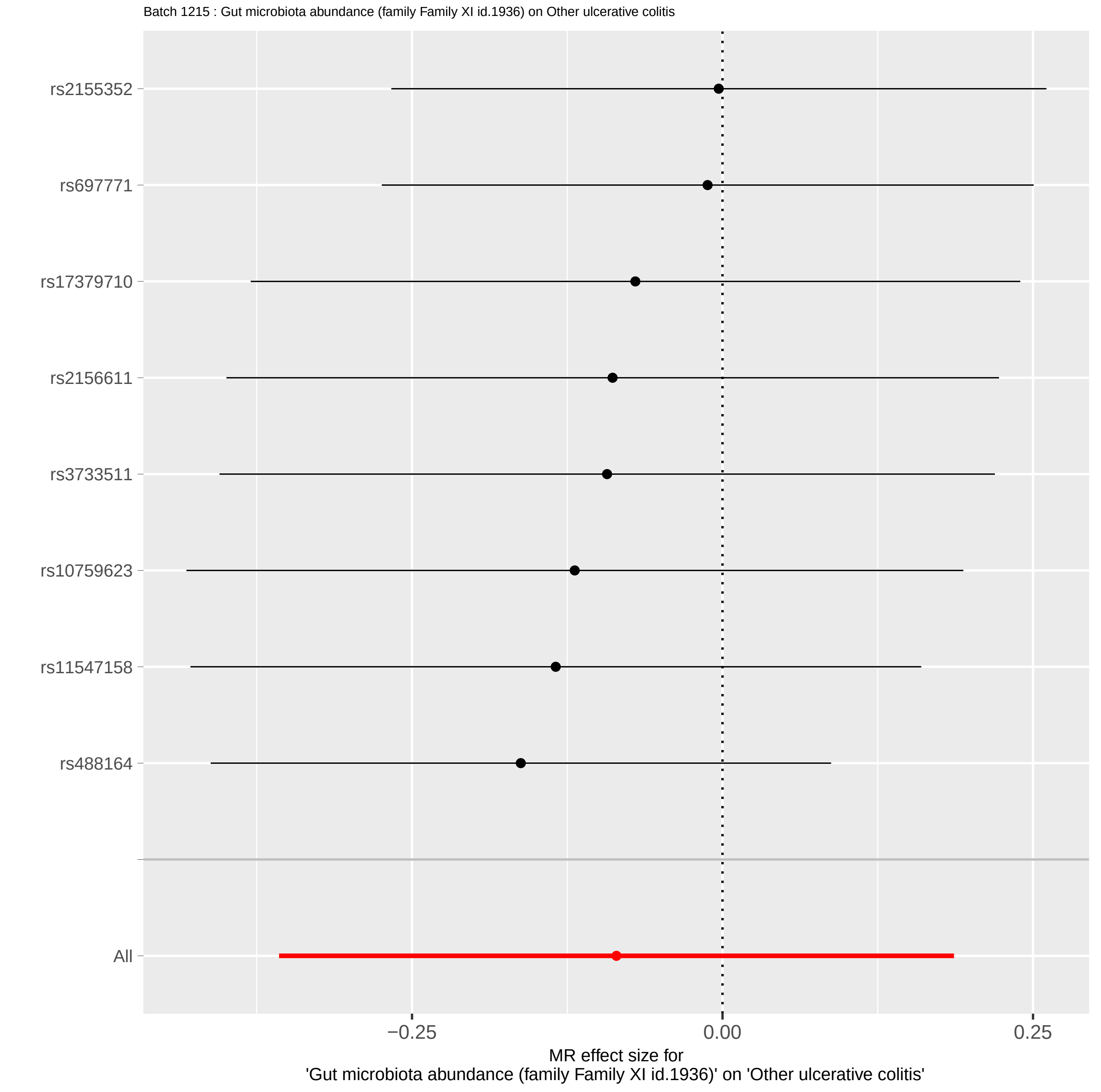

Batch 1215 : Gut microbiota abundance (family Family XI id.1936) on Other ulcerative colitis
rs2155352
rs697771
rs17379710
rs2156611
rs3733511
rs10759623
rs11547158
rs488164
All
−0.25
0.00
0.25
MR effect size for
'Gut microbiota abundance (family Family XI id.1936)' on 'Other ulcerative colitis'

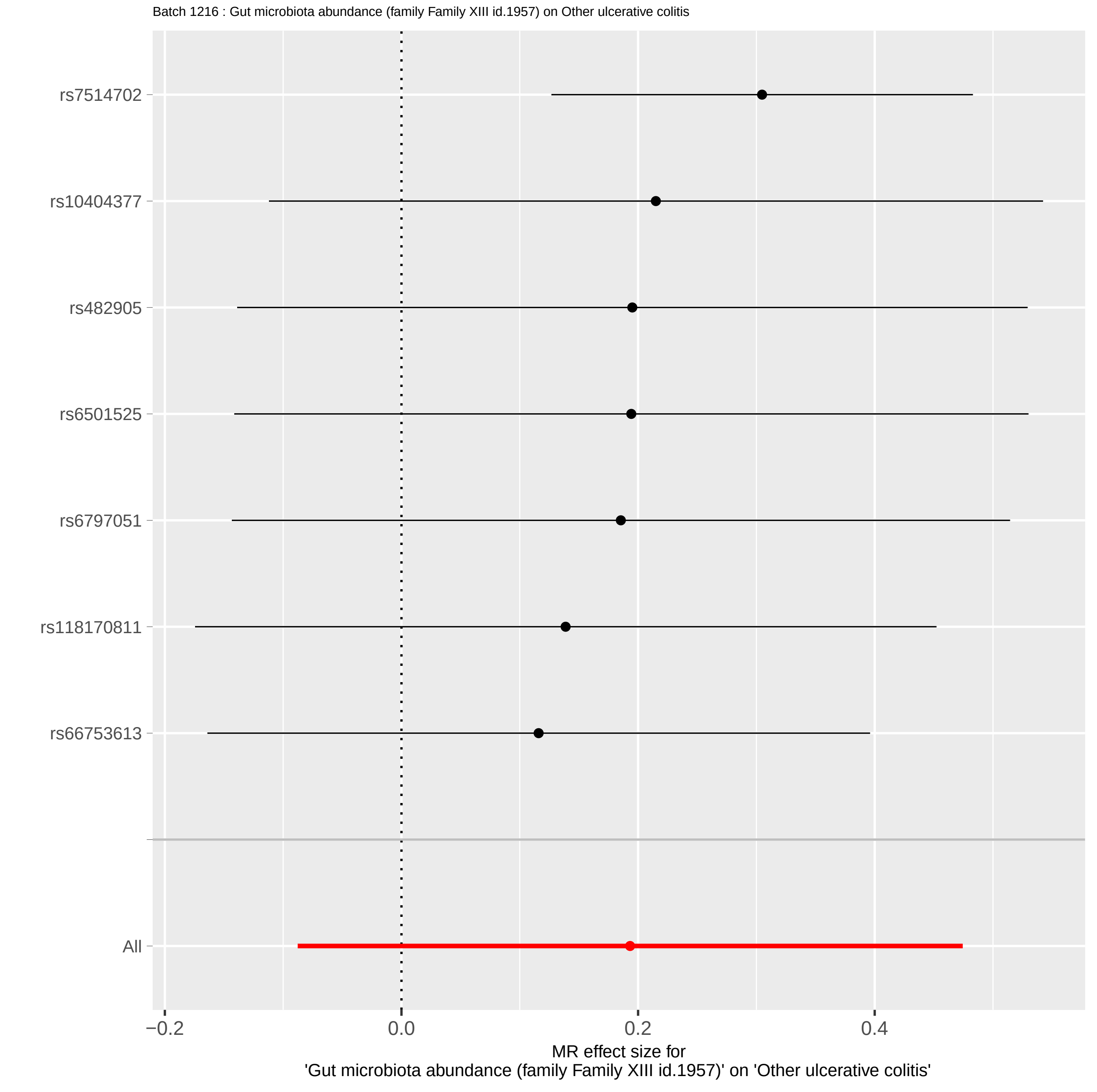

Batch 1216 : Gut microbiota abundance (family Family XIII id.1957) on Other ulcerative colitis
rs7514702
rs10404377
rs482905
rs6501525
rs6797051
rs118170811
rs66753613
All
−0.2
0.0
0.2
0.4
MR effect size for
'Gut microbiota abundance (family Family XIII id.1957)' on 'Other ulcerative colitis'

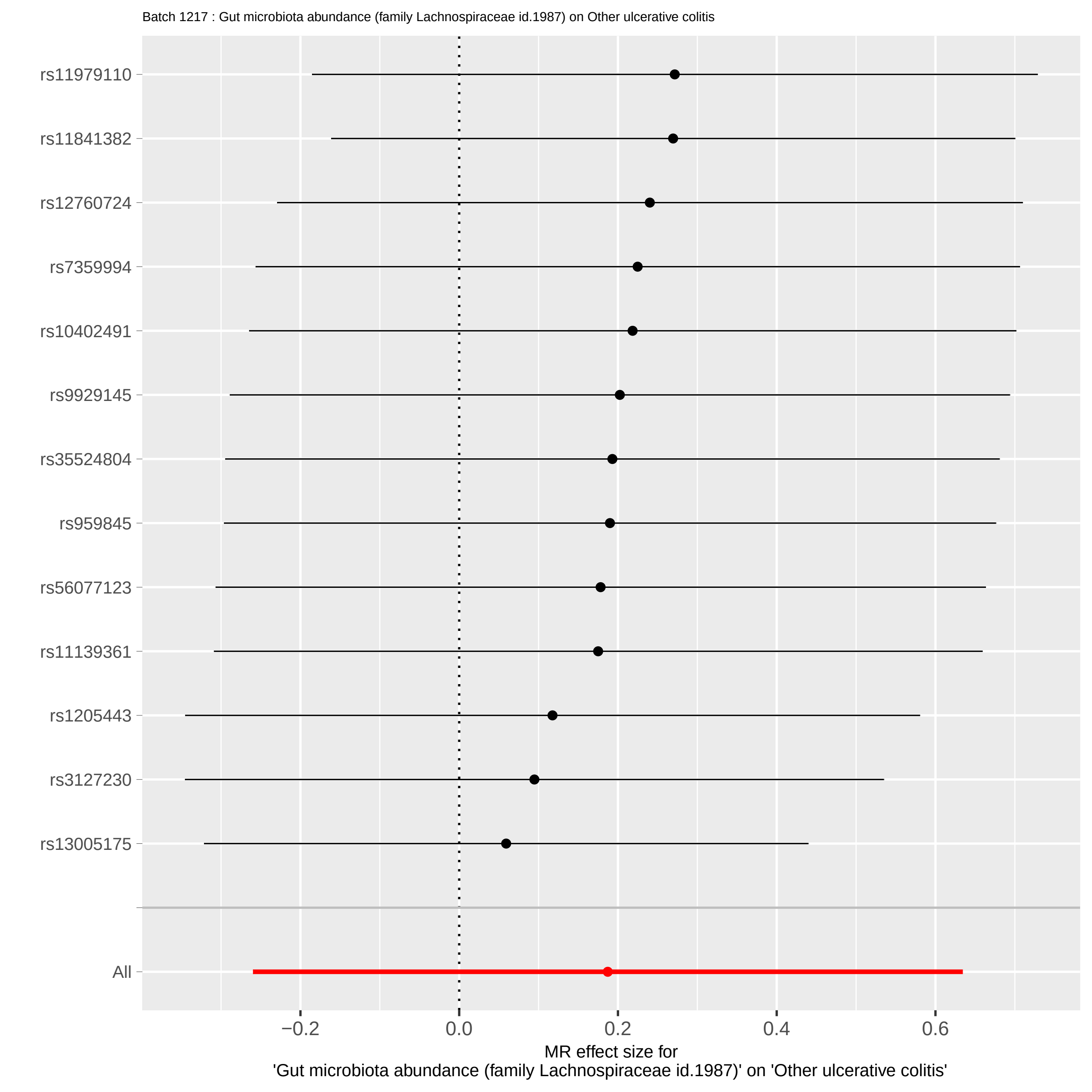

Batch 1217 : Gut microbiota abundance (family Lachnospiraceae id.1987) on Other ulcerative colitis
rs11979110
rs11841382
rs12760724
rs7359994
rs10402491
rs9929145
rs35524804
rs959845
rs56077123
rs11139361
rs1205443
rs3127230
rs13005175
All
−0.2
0.0
0.2
0.4
0.6
MR effect size for
'Gut microbiota abundance (family Lachnospiraceae id.1987)' on 'Other ulcerative colitis'

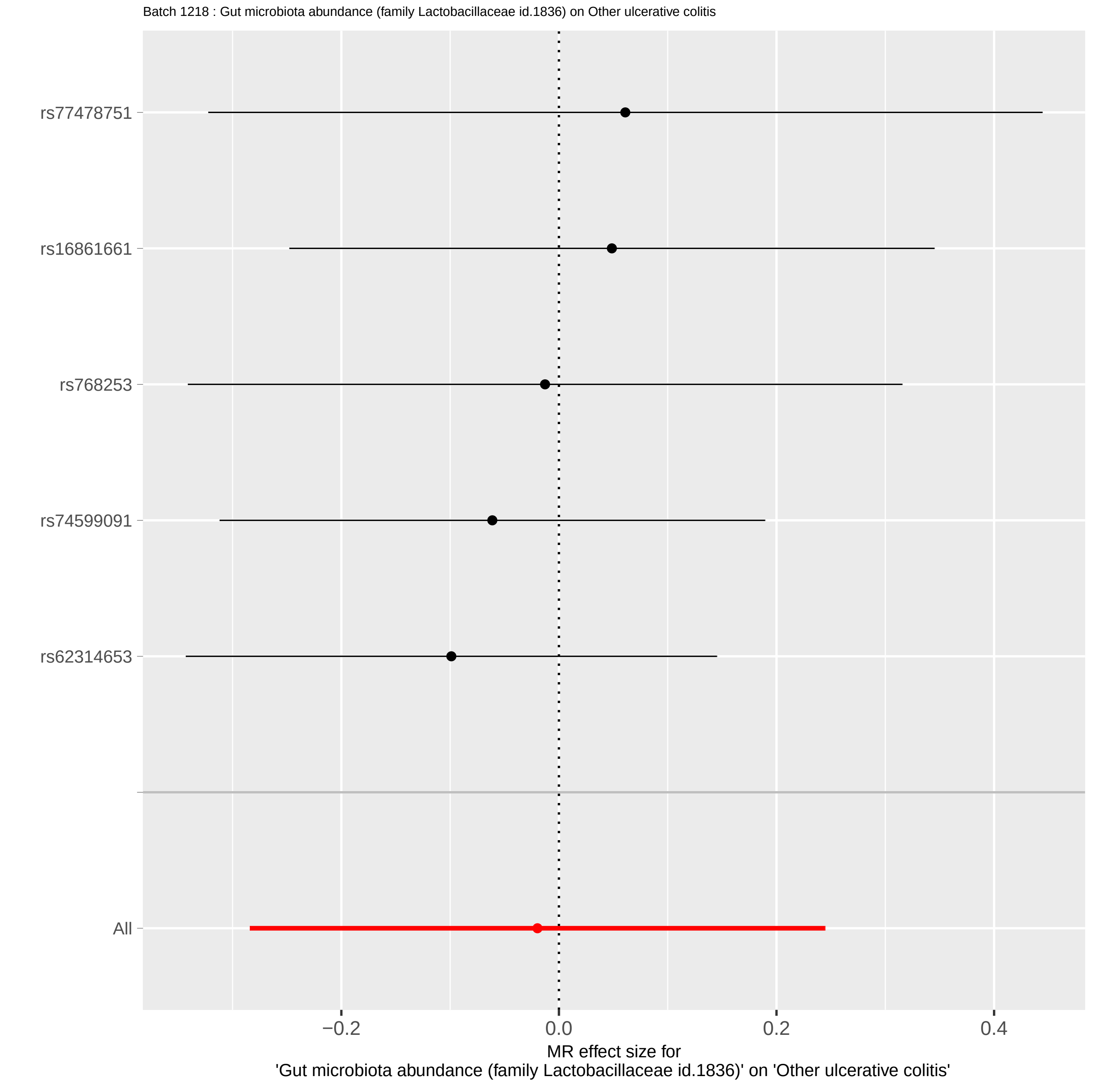
Batch 1218 : Gut microbiota abundance (family Lactobacillaceae id.1836) on Other ulcerative colitis
rs77478751
rs16861661
rs768253
rs74599091
rs62314653
All
−0.2
0.0
0.2
0.4
MR effect size for
'Gut microbiota abundance (family Lactobacillaceae id.1836)' on 'Other ulcerative colitis'

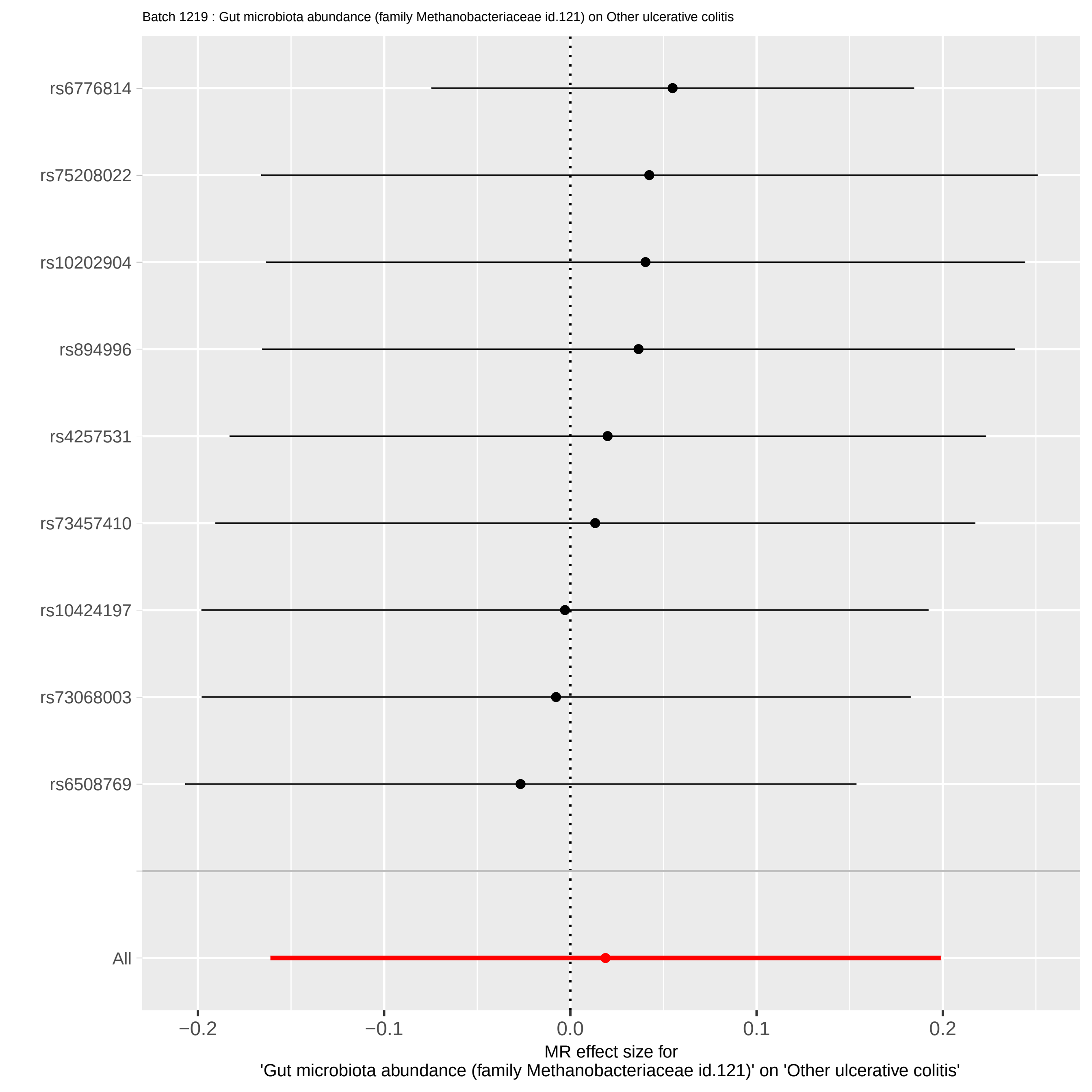

Batch 1219 : Gut microbiota abundance (family Methanobacteriaceae id.121) on Other ulcerative colitis
rs6776814
rs75208022
rs10202904
rs894996
rs4257531
rs73457410
rs10424197
rs73068003
rs6508769
All
−0.2
−0.1
0.0
0.1
0.2
MR effect size for
'Gut microbiota abundance (family Methanobacteriaceae id.121)' on 'Other ulcerative colitis'

Batch 1220 : Gut microbiota abundance (family Oxalobacteraceae id.2966) on Other ulcerative colitis

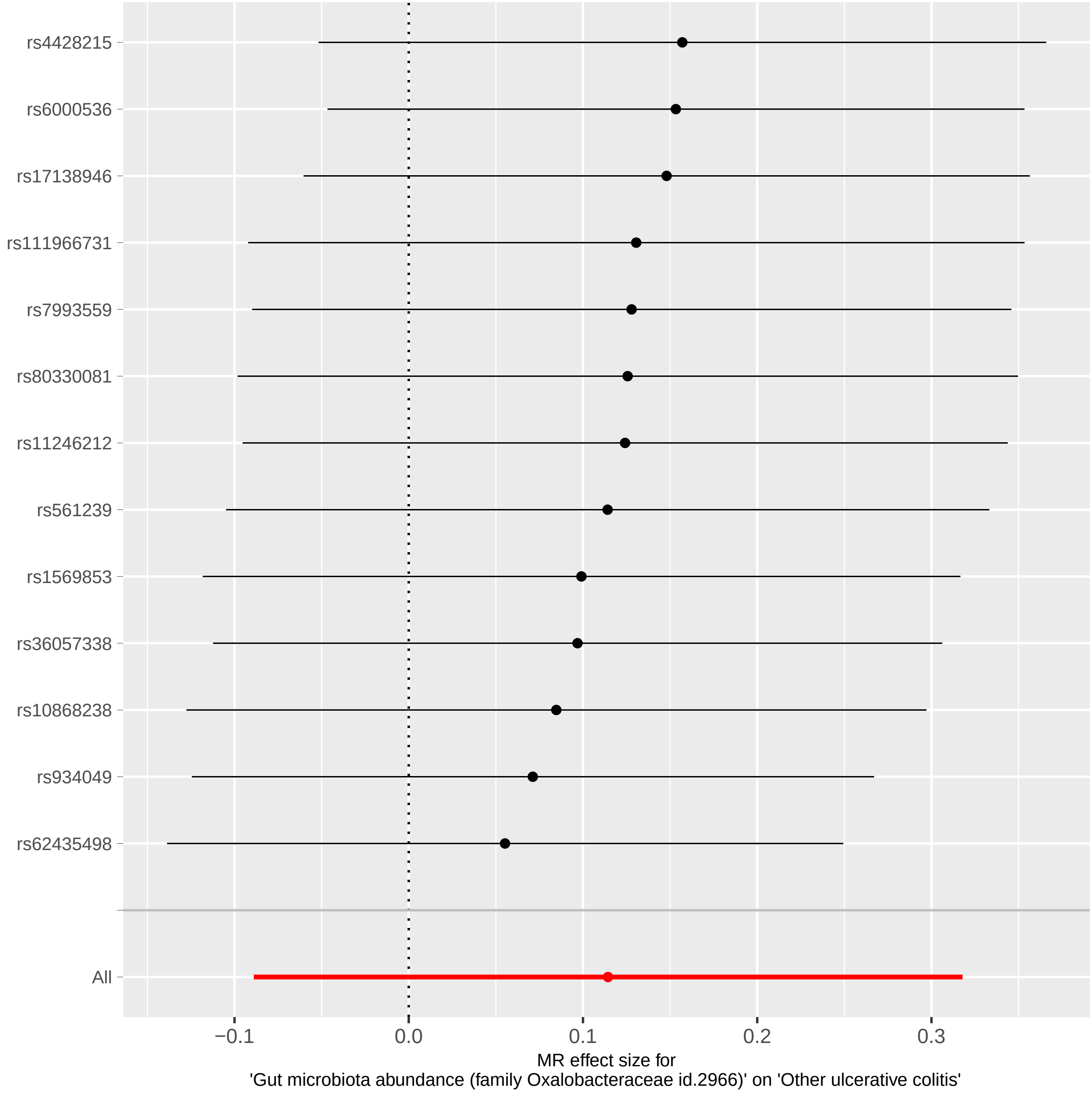

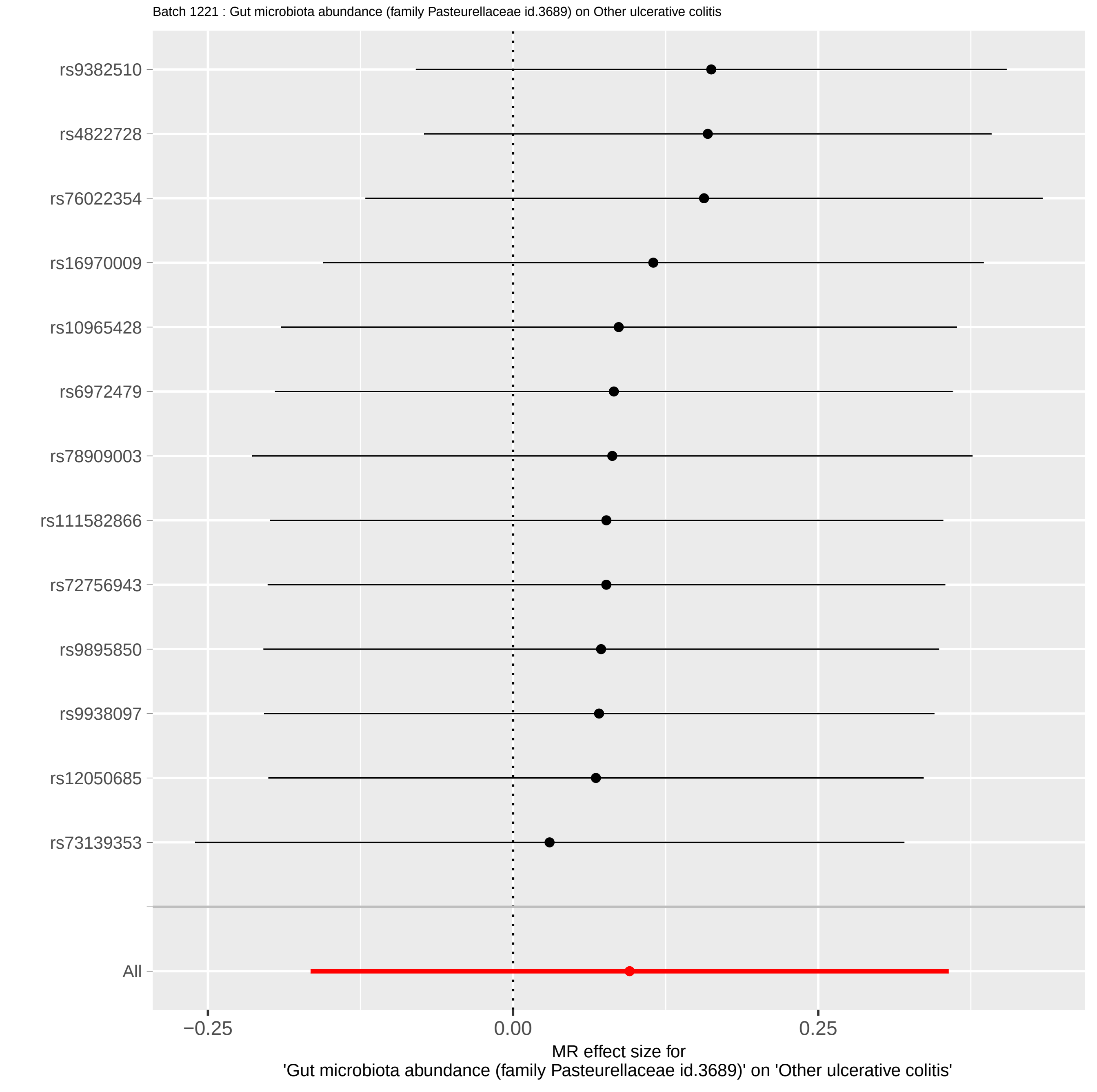
Batch 1221 : Gut microbiota abundance (family Pasteurellaceae id.3689) on Other ulcerative colitis
rs9382510
rs4822728
rs76022354
rs16970009
rs10965428
rs6972479
rs78909003
rs111582866
rs72756943
rs9895850
rs9938097
rs12050685
rs73139353
All
−0.25
0.00
0.25
MR effect size for
'Gut microbiota abundance (family Pasteurellaceae id.3689)' on 'Other ulcerative colitis'

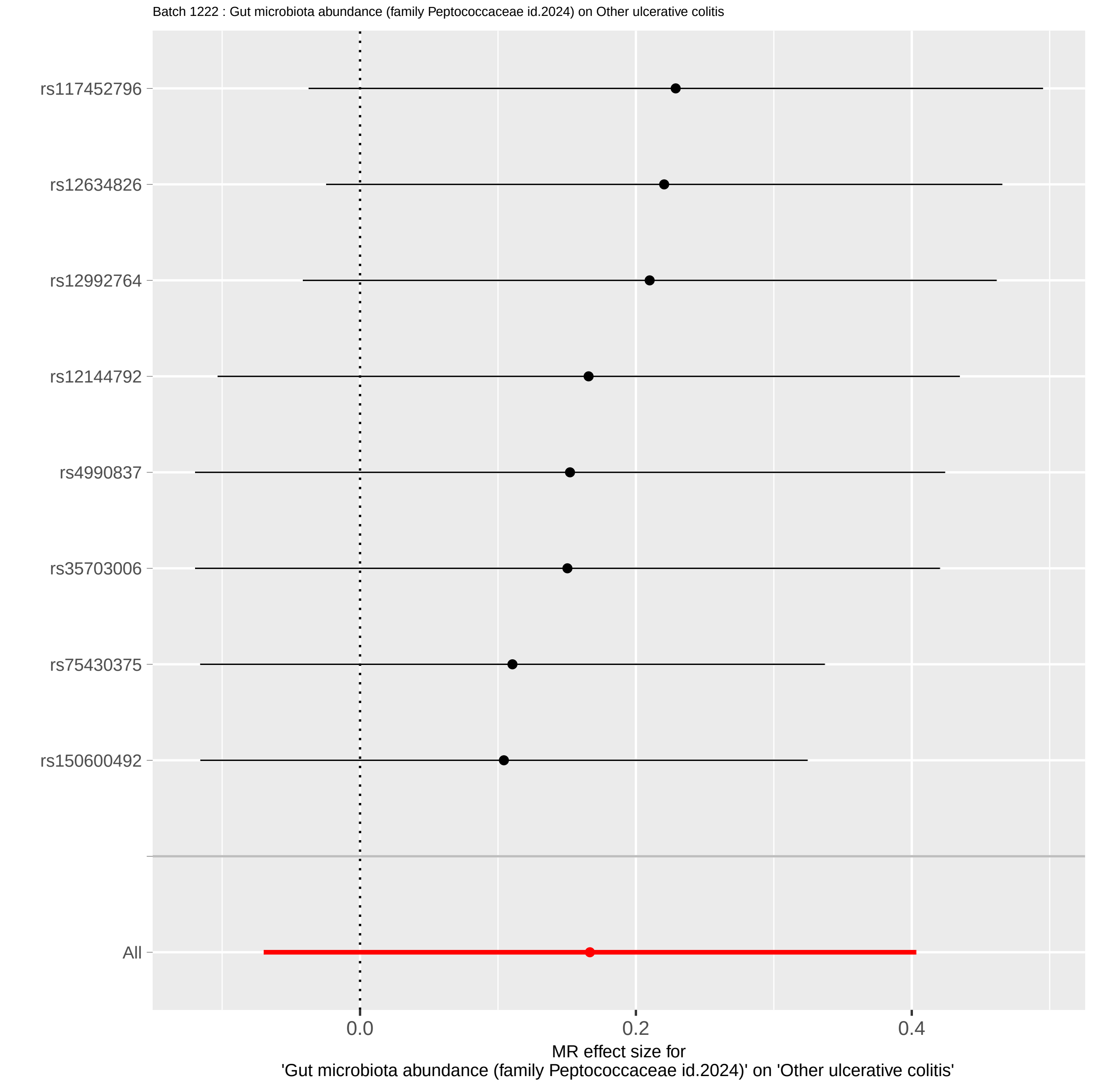

Batch 1222 : Gut microbiota abundance (family Peptococcaceae id.2024) on Other ulcerative colitis
rs117452796
rs12634826
rs12992764
rs12144792
rs4990837
rs35703006
rs75430375
rs150600492
All
0.0
0.2
0.4
MR effect size for
'Gut microbiota abundance (family Peptococcaceae id.2024)' on 'Other ulcerative colitis'

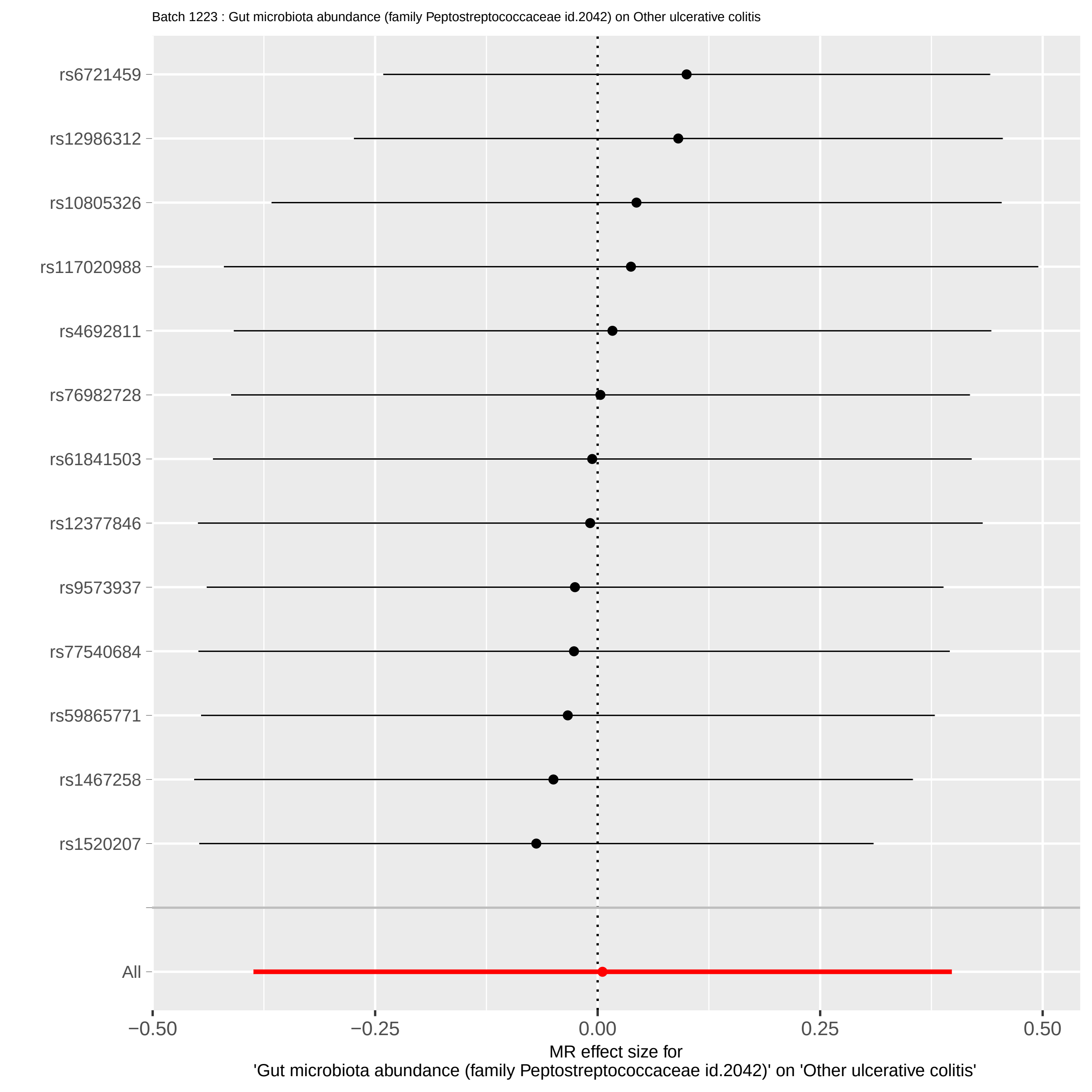
Batch 1223 : Gut microbiota abundance (family Peptostreptococcaceae id.2042) on Other ulcerative colitis
rs6721459
rs12986312
rs10805326
rs117020988
rs4692811
rs76982728
rs61841503
rs12377846
rs9573937
rs77540684
rs59865771
rs1467258
rs1520207
All
−0.50
−0.25
0.00
0.25
0.50
MR effect size for
'Gut microbiota abundance (family Peptostreptococcaceae id.2042)' on 'Other ulcerative colitis'

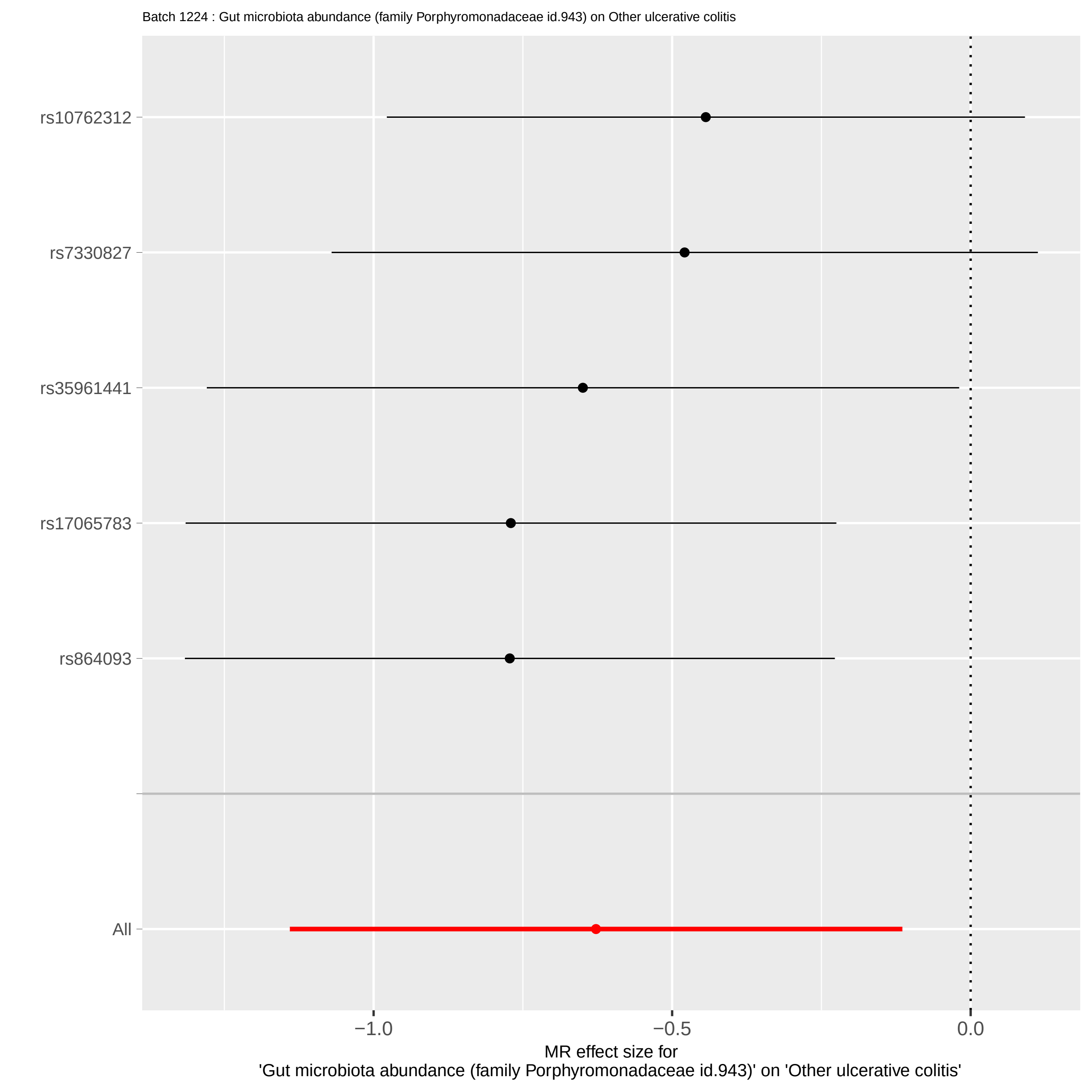

Batch 1224 : Gut microbiota abundance (family Porphyromonadaceae id.943) on Other ulcerative colitis
rs10762312
rs7330827
rs35961441
rs17065783
rs864093
All
−1.0
−0.5
0.0
MR effect size for
'Gut microbiota abundance (family Porphyromonadaceae id.943)' on 'Other ulcerative colitis'

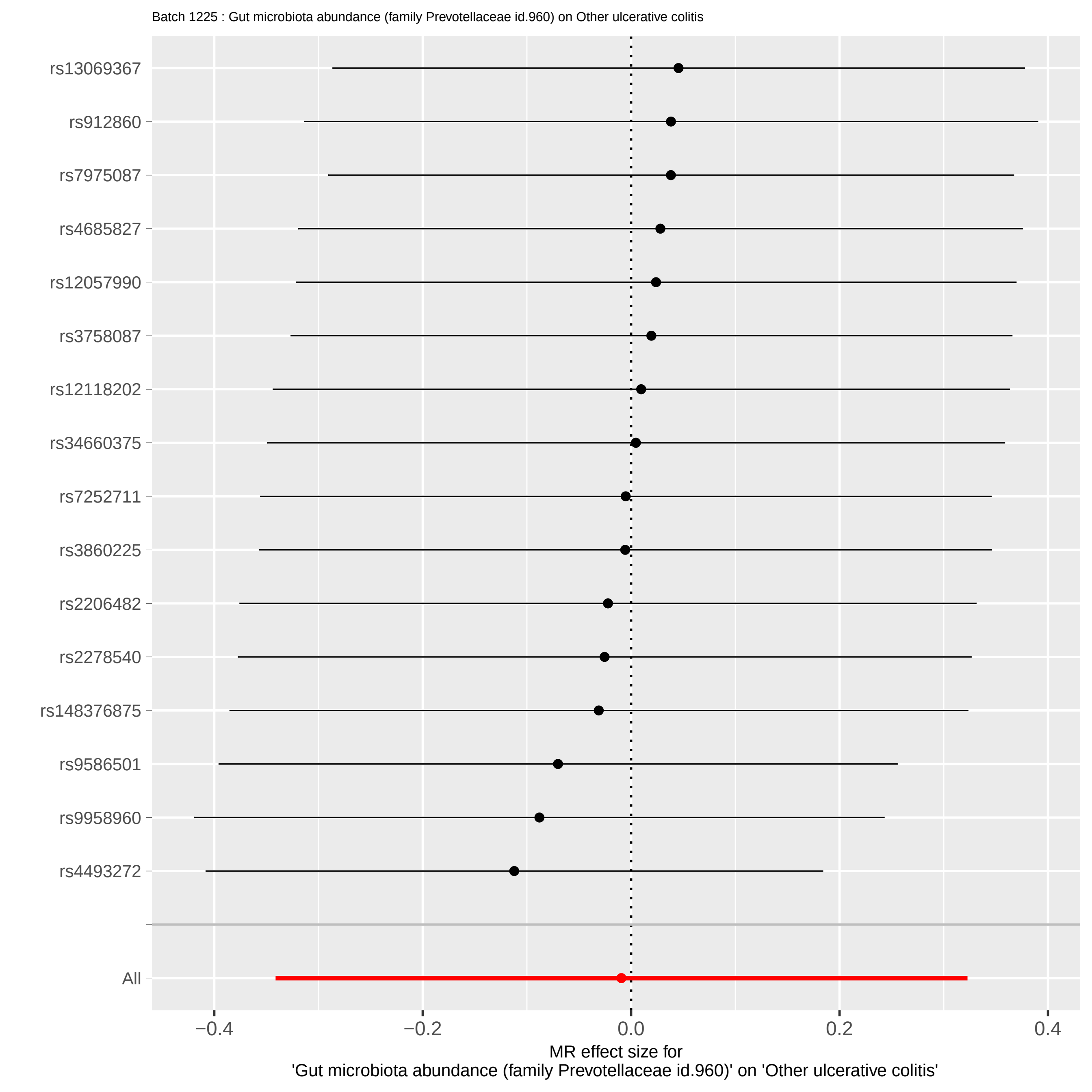

Batch 1225 : Gut microbiota abundance (family Prevotellaceae id.960) on Other ulcerative colitis
rs13069367
rs912860
rs7975087
rs4685827
rs12057990
rs3758087
rs12118202
rs34660375
rs7252711
rs3860225
rs2206482
rs2278540
rs148376875
rs9586501
rs9958960
rs4493272
All
−0.4
−0.2
0.0
0.2
0.4
MR effect size for
'Gut microbiota abundance (family Prevotellaceae id.960)' on 'Other ulcerative colitis'

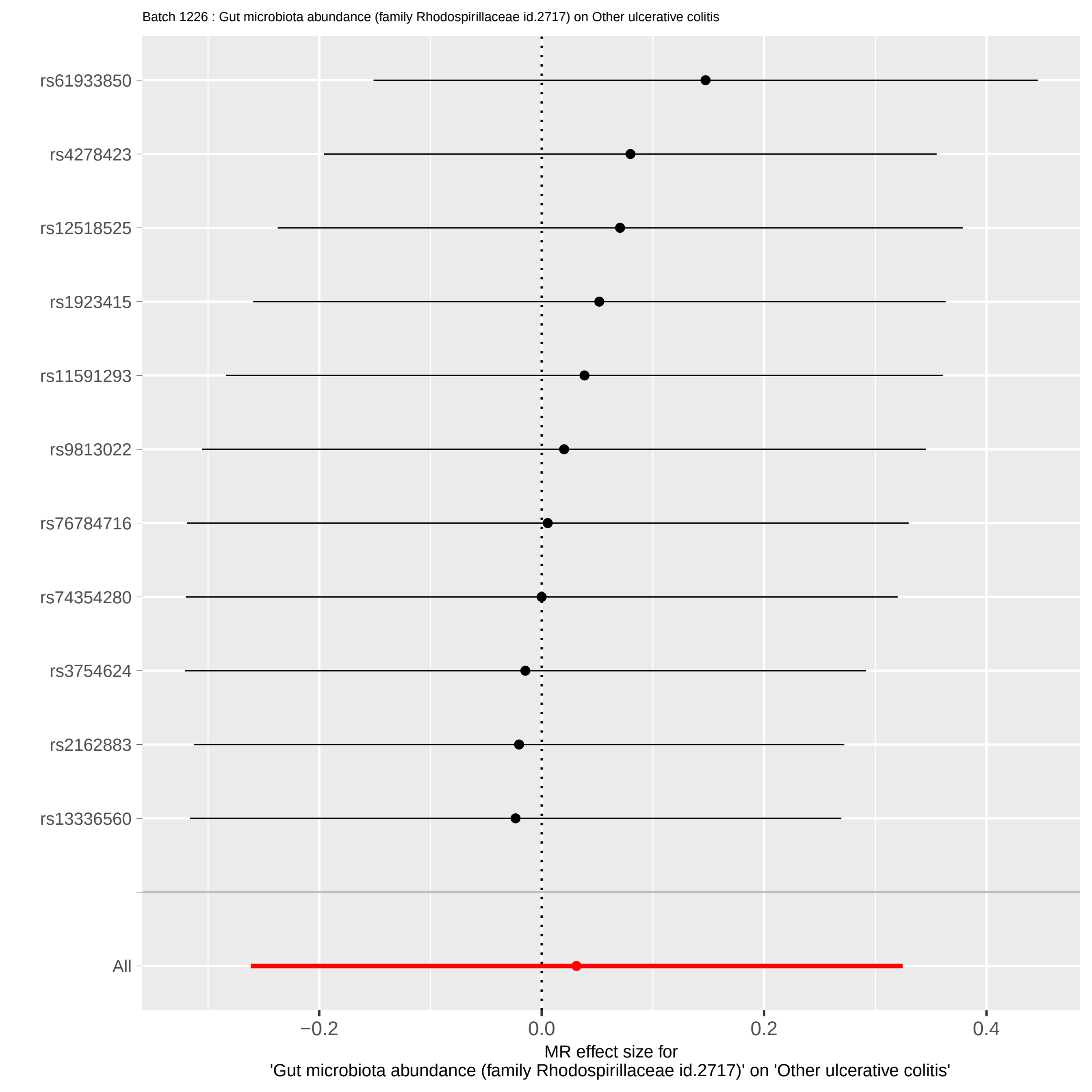
Batch 1226 : Gut microbiota abundance (family Rhodospirillaceae id.2717) on Other ulcerative colitis
rs61933850
rs4278423
rs12518525
rs1923415
rs11591293
rs9813022
rs76784716
rs74354280
rs3754624
rs2162883
rs13336560
All
−0.2
0.0
0.2
0.4
MR effect size for
'Gut microbiota abundance (family Rhodospirillaceae id.2717)' on 'Other ulcerative colitis'

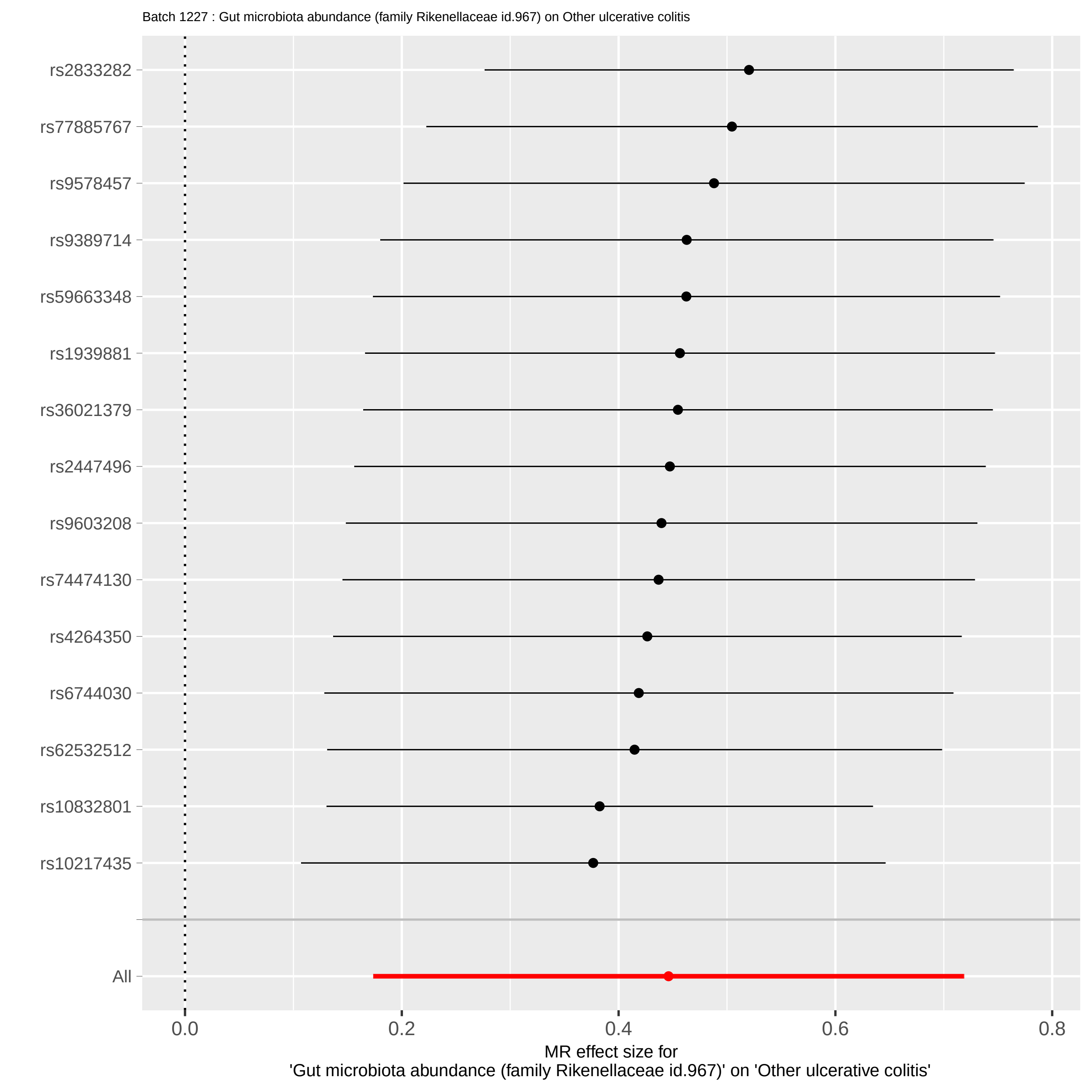

Batch 1227 : Gut microbiota abundance (family Rikenellaceae id.967) on Other ulcerative colitis
rs2833282
rs77885767
rs9578457
rs9389714
rs59663348
rs1939881
rs36021379
rs2447496
rs9603208
rs74474130
rs4264350
rs6744030
rs62532512
rs10832801
rs10217435
All
0.0
0.2
0.4
0.6
0.8
MR effect size for
'Gut microbiota abundance (family Rikenellaceae id.967)' on 'Other ulcerative colitis'

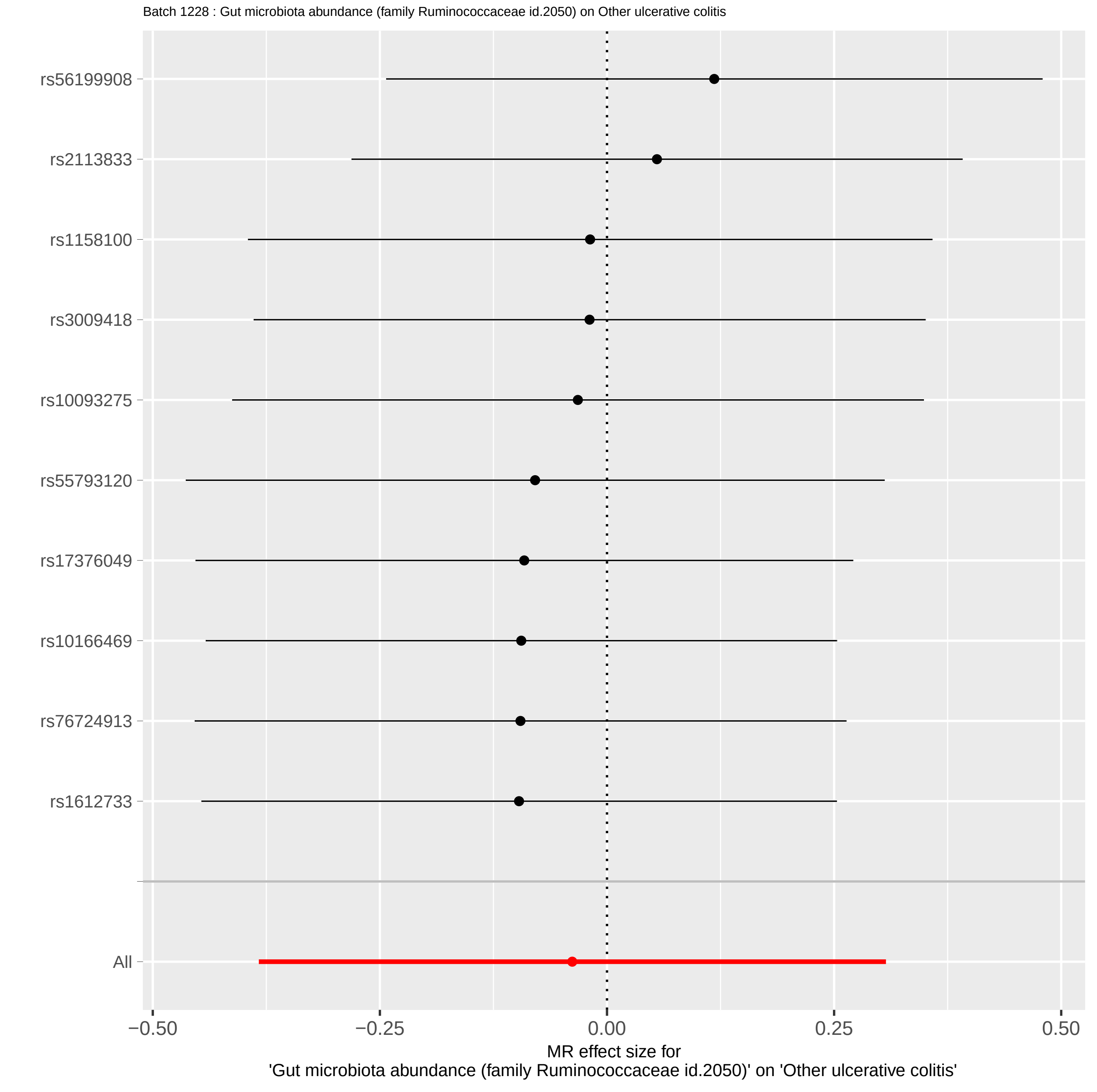
Batch 1228 : Gut microbiota abundance (family Ruminococcaceae id.2050) on Other ulcerative colitis
rs56199908
rs2113833
rs1158100
rs3009418
rs10093275
rs55793120
rs17376049
rs10166469
rs76724913
rs1612733
All
−0.50
−0.25
0.00
0.25
0.50
MR effect size for
'Gut microbiota abundance (family Ruminococcaceae id.2050)' on 'Other ulcerative colitis'

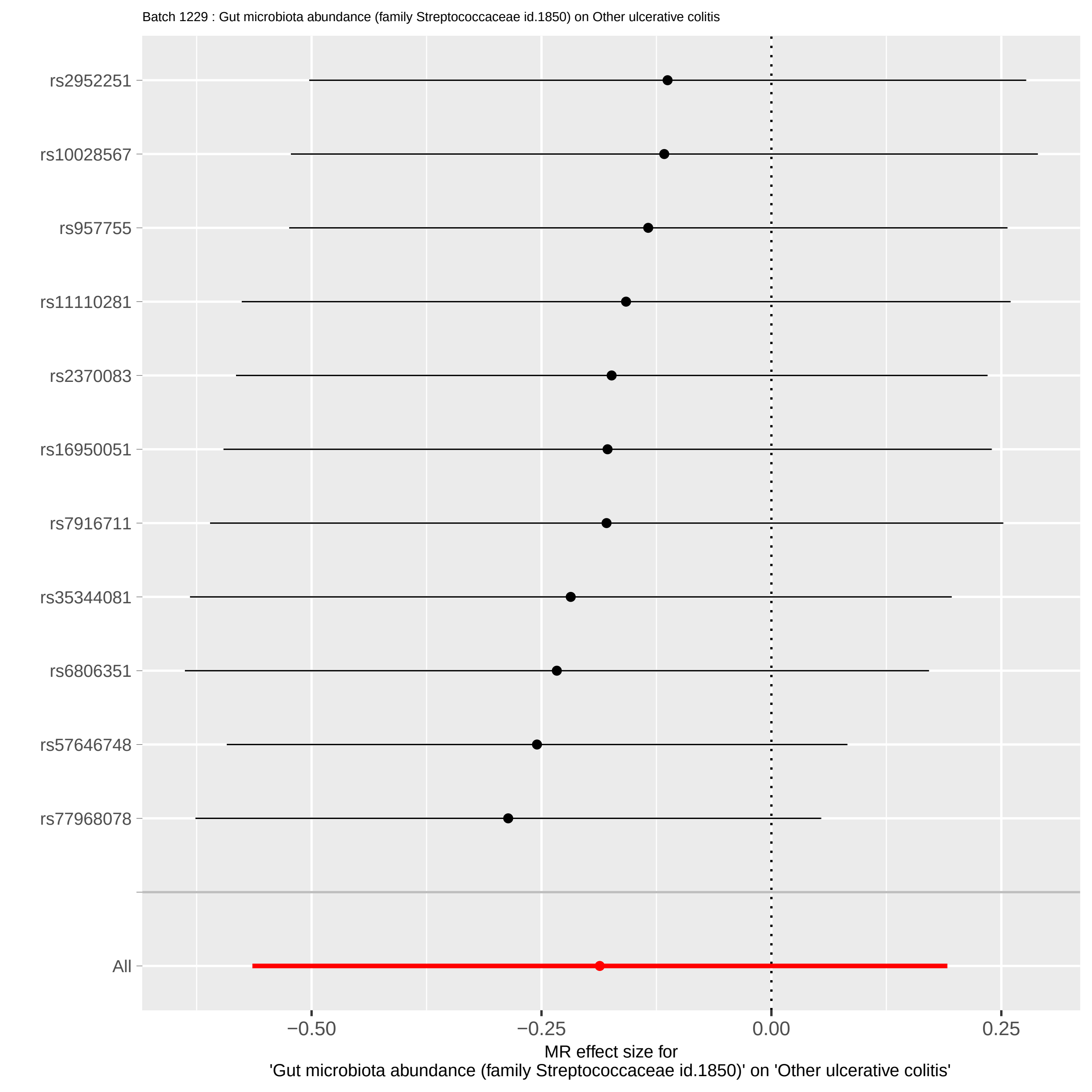

Batch 1229 : Gut microbiota abundance (family Streptococcaceae id.1850) on Other ulcerative colitis
rs2952251
rs10028567
rs957755
rs11110281
rs2370083
rs16950051
rs7916711
rs35344081
rs6806351
rs57646748
rs77968078
All
−0.50
−0.25
0.00
0.25
MR effect size for
'Gut microbiota abundance (family Streptococcaceae id.1850)' on 'Other ulcerative colitis'

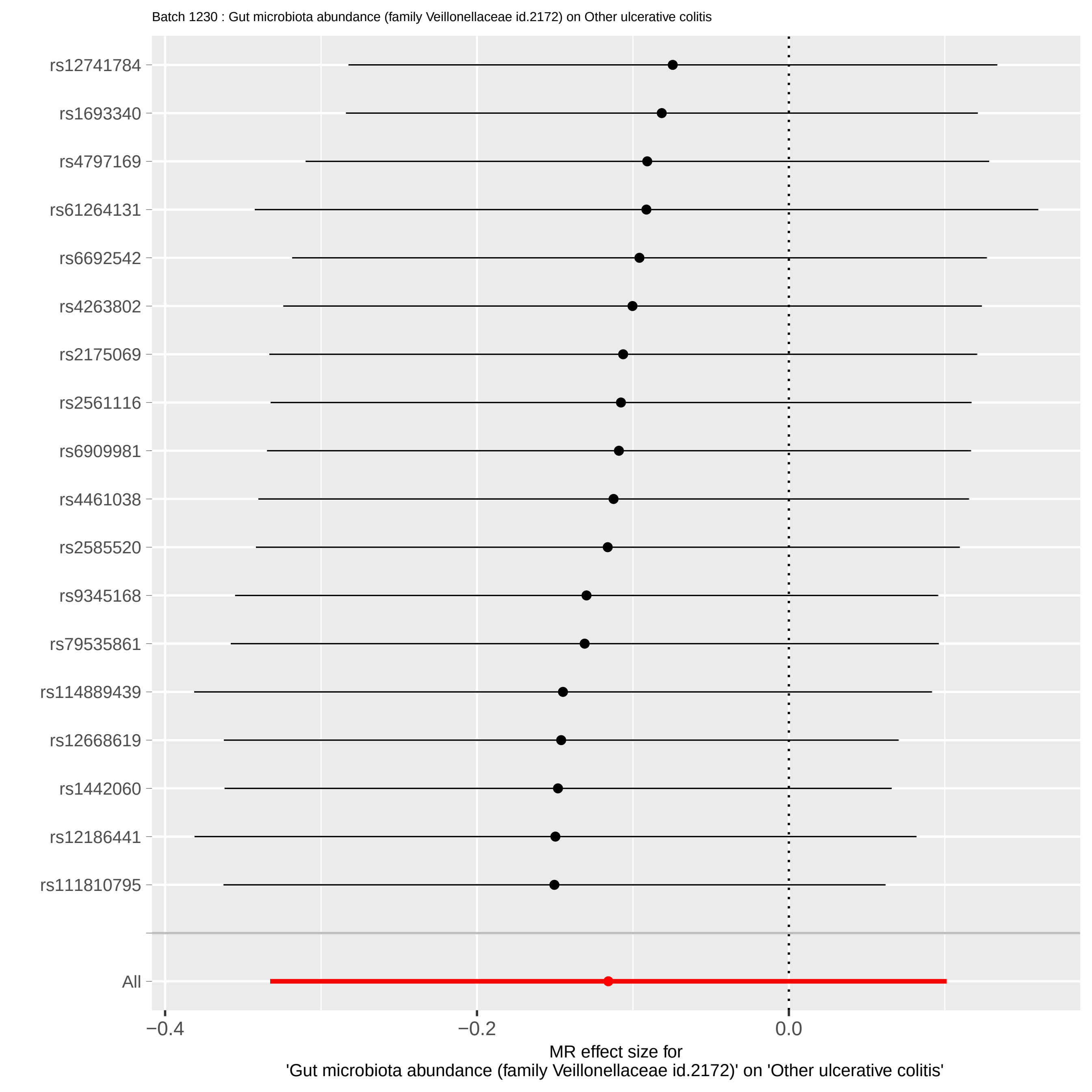

Batch 1230 : Gut microbiota abundance (family Veillonellaceae id.2172) on Other ulcerative colitis
rs12741784
rs1693340
rs4797169
rs61264131
rs6692542
rs4263802
rs2175069
rs2561116
rs6909981
rs4461038
rs2585520
rs9345168
rs79535861
rs114889439
rs12668619
rs1442060
rs12186441
rs111810795
All
−0.4
−0.2
0.0
MR effect size for
'Gut microbiota abundance (family Veillonellaceae id.2172)' on 'Other ulcerative colitis'

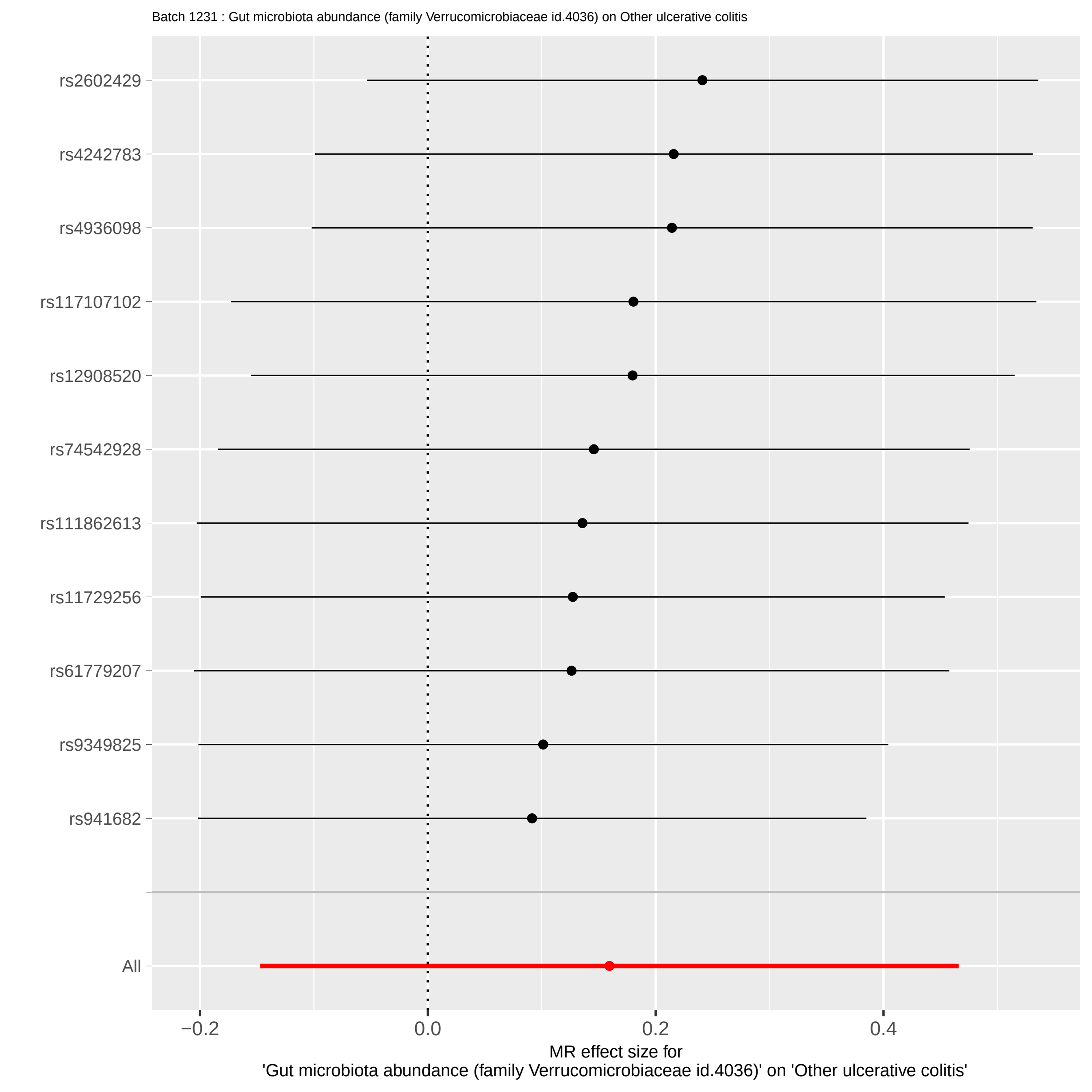

Batch 1231 : Gut microbiota abundance (family Verrucomicrobiaceae id.4036) on Other ulcerative colitis
rs2602429
rs4242783
rs4936098
rs117107102
rs12908520
rs74542928
rs111862613
rs11729256
rs61779207
rs9349825
rs941682
All
−0.2
0.0
0.2
0.4
MR effect size for
'Gut microbiota abundance (family Verrucomicrobiaceae id.4036)' on 'Other ulcerative colitis'

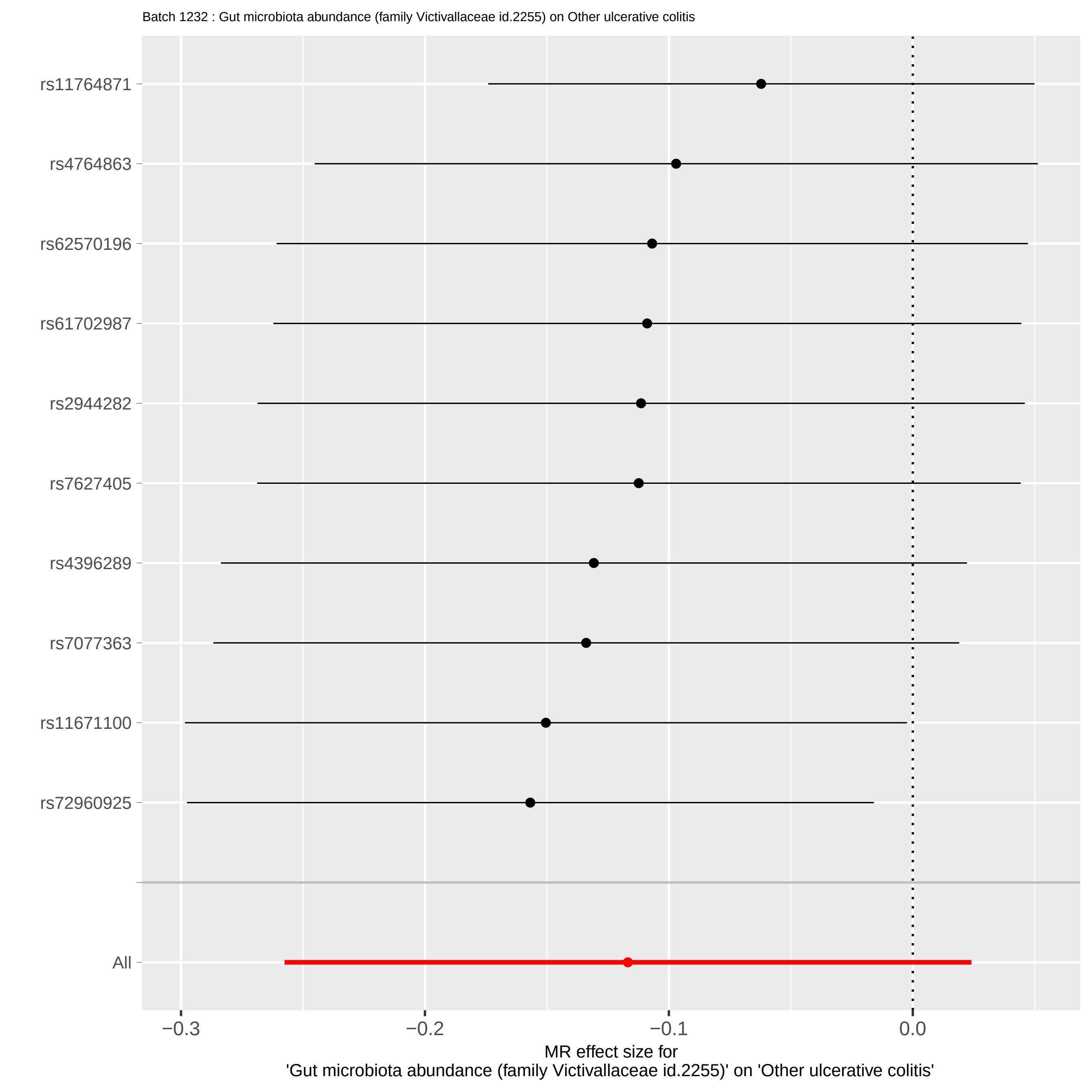
Batch 1232 : Gut microbiota abundance (family Victivallaceae id.2255) on Other ulcerative colitis
rs11764871
rs4764863
rs62570196
rs61702987
rs2944282
rs7627405
rs4396289
rs7077363
rs11671100
rs72960925
All
−0.3
−0.2
−0.1
0.0
MR effect size for
'Gut microbiota abundance (family Victivallaceae id.2255)' on 'Other ulcerative colitis'

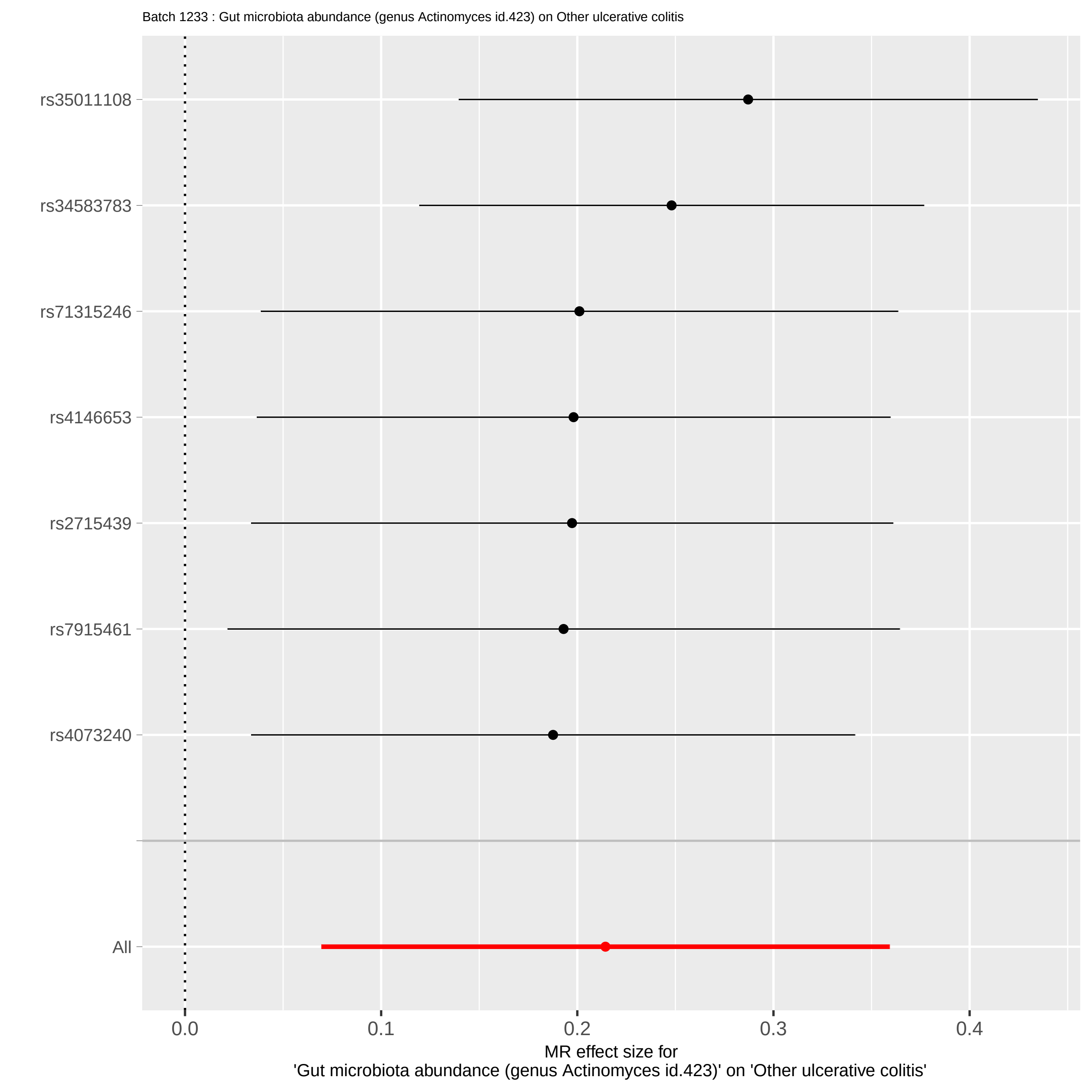

Batch 1233 : Gut microbiota abundance (genus Actinomyces id.423) on Other ulcerative colitis
rs35011108
rs34583783
rs71315246
rs4146653
rs2715439
rs7915461
rs4073240
All
0.0
0.1
0.2
0.3
0.4
MR effect size for
'Gut microbiota abundance (genus Actinomyces id.423)' on 'Other ulcerative colitis'

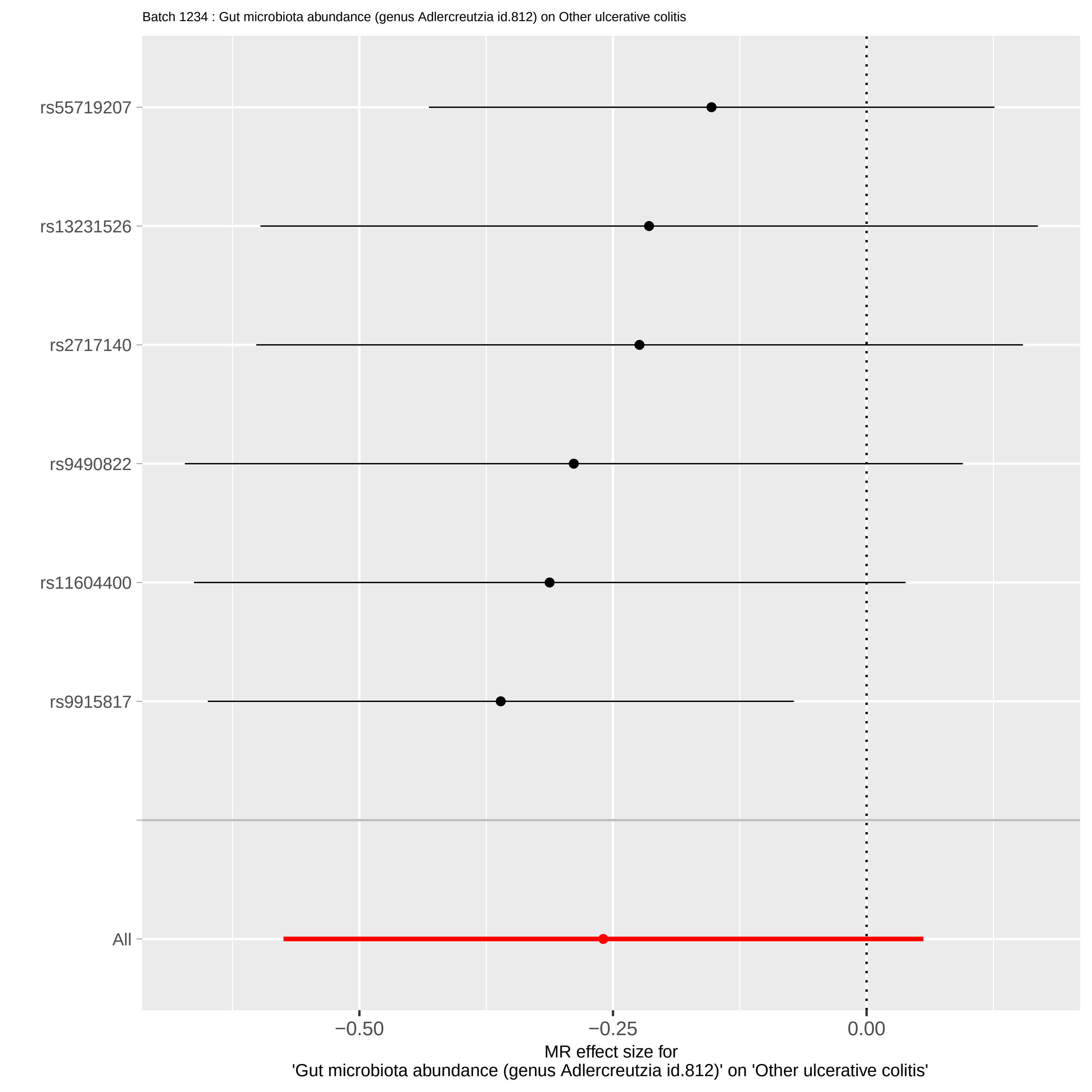
Batch 1234 : Gut microbiota abundance (genus Adlercreutzia id.812) on Other ulcerative colitis
rs55719207
rs13231526
rs2717140
rs9490822
rs11604400
rs9915817
All
−0.50
−0.25
0.00
MR effect size for
'Gut microbiota abundance (genus Adlercreutzia id.812)' on 'Other ulcerative colitis'

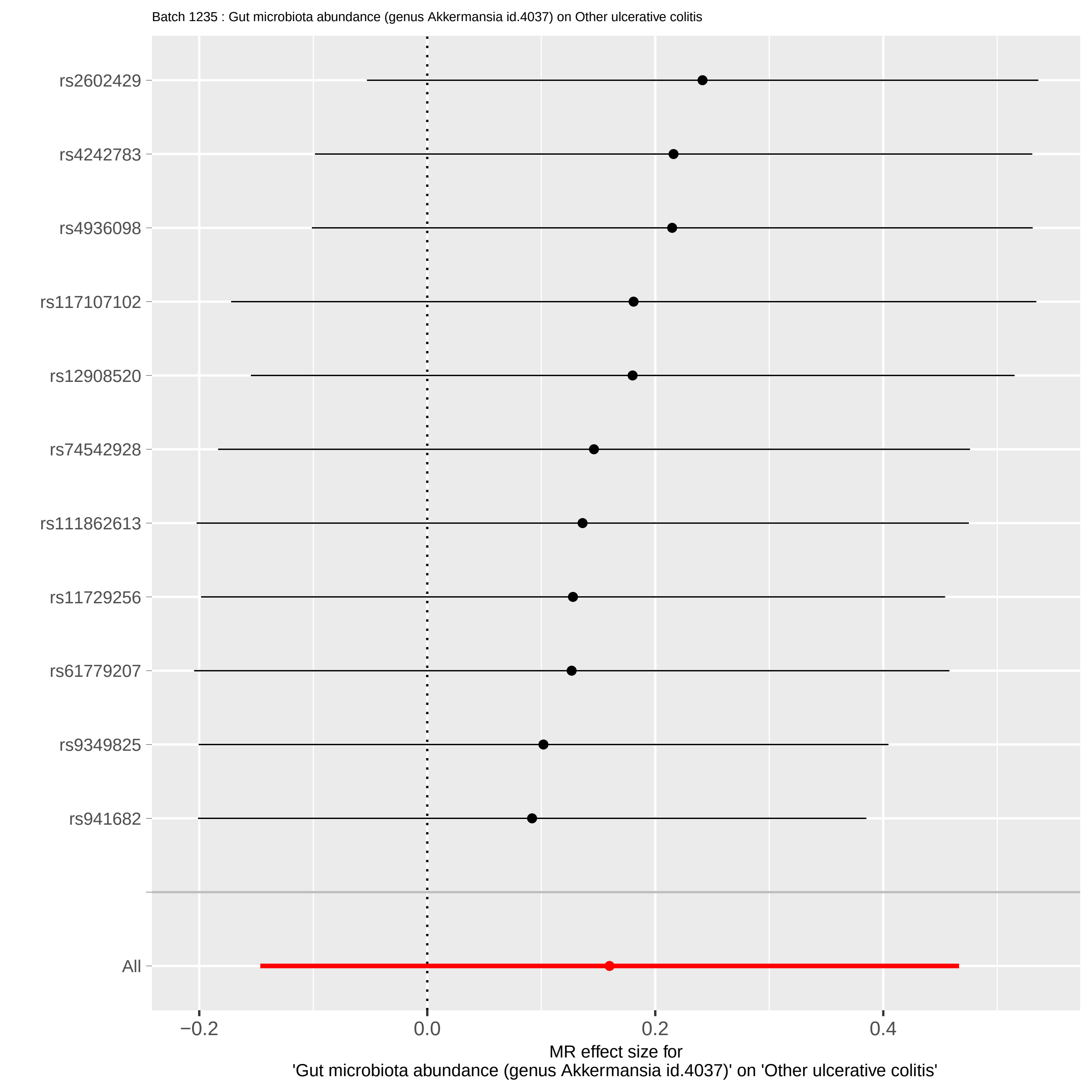

Batch 1235 : Gut microbiota abundance (genus Akkermansia id.4037) on Other ulcerative colitis
rs2602429
rs4242783
rs4936098
rs117107102
rs12908520
rs74542928
rs111862613
rs11729256
rs61779207
rs9349825
rs941682
All
−0.2
0.0
0.2
0.4
MR effect size for
'Gut microbiota abundance (genus Akkermansia id.4037)' on 'Other ulcerative colitis'

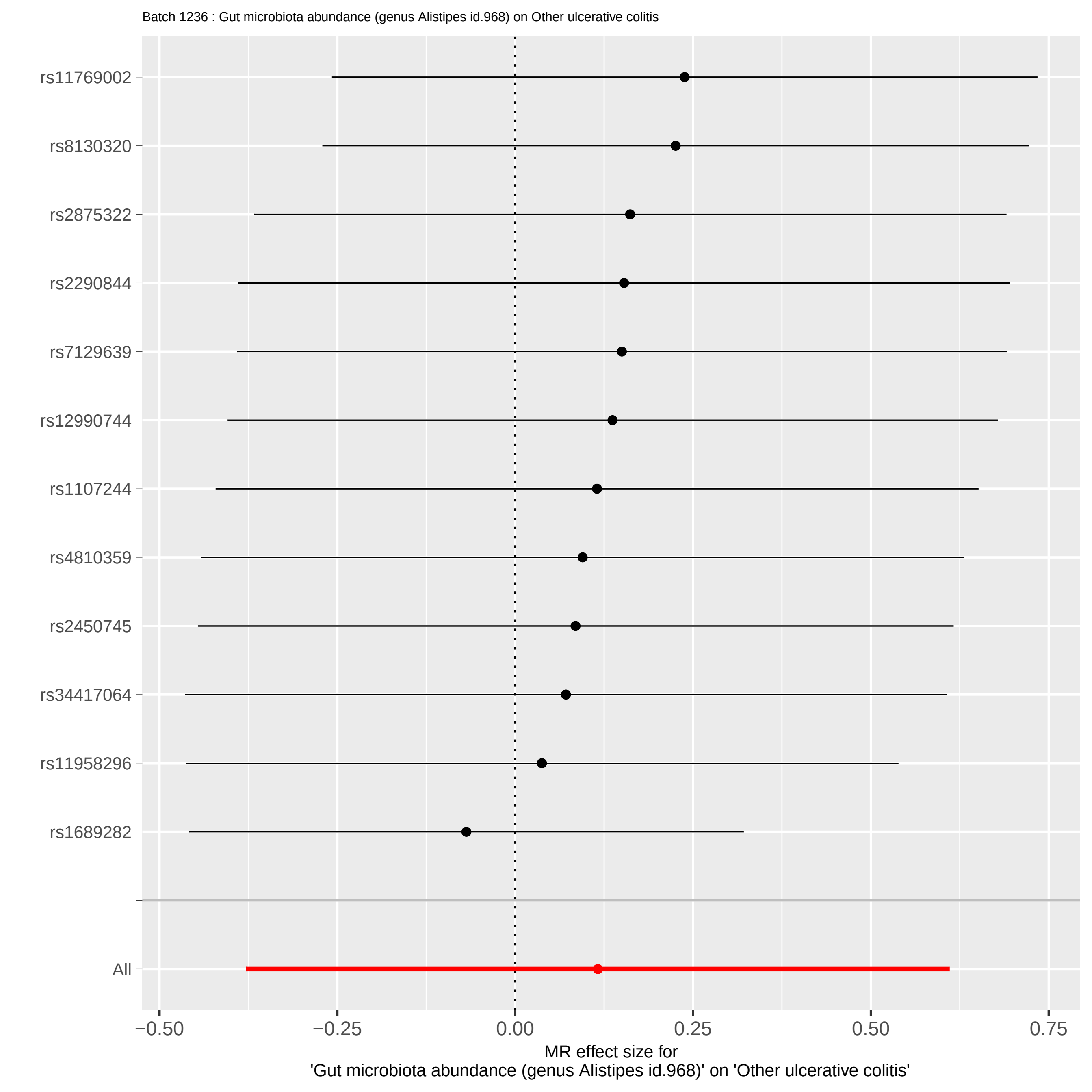

Batch 1236 : Gut microbiota abundance (genus Alistipes id.968) on Other ulcerative colitis
rs11769002
rs8130320
rs2875322
rs2290844
rs7129639
rs12990744
rs1107244
rs4810359
rs2450745
rs34417064
rs11958296
rs1689282
All
−0.50
−0.25
0.00
0.25
0.50
0.75
MR effect size for
'Gut microbiota abundance (genus Alistipes id.968)' on 'Other ulcerative colitis'

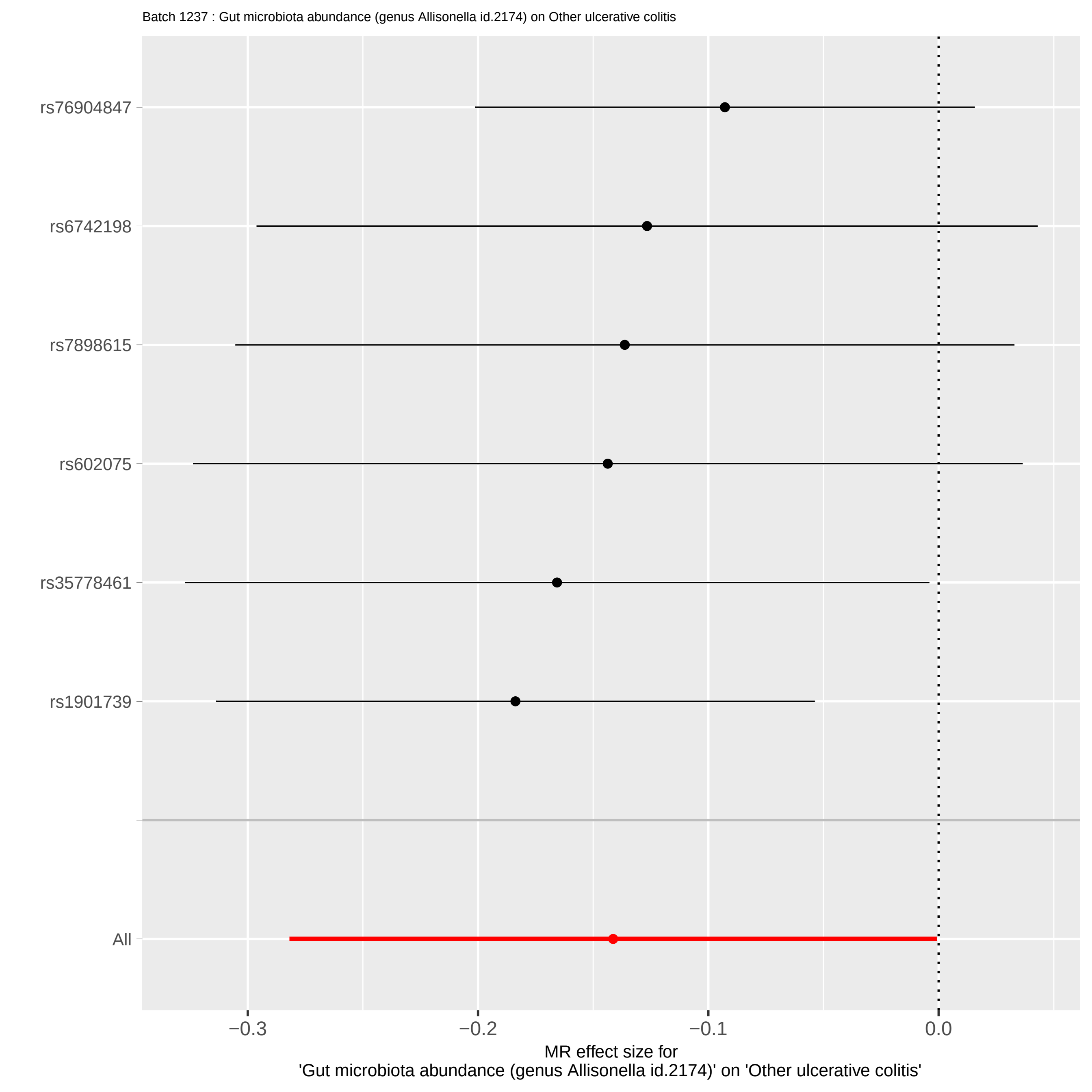

Batch 1237 : Gut microbiota abundance (genus Allisonella id.2174) on Other ulcerative colitis
rs76904847
rs6742198
rs7898615
rs602075
rs35778461
rs1901739
All
−0.3
−0.2
−0.1
0.0
MR effect size for
'Gut microbiota abundance (genus Allisonella id.2174)' on 'Other ulcerative colitis'

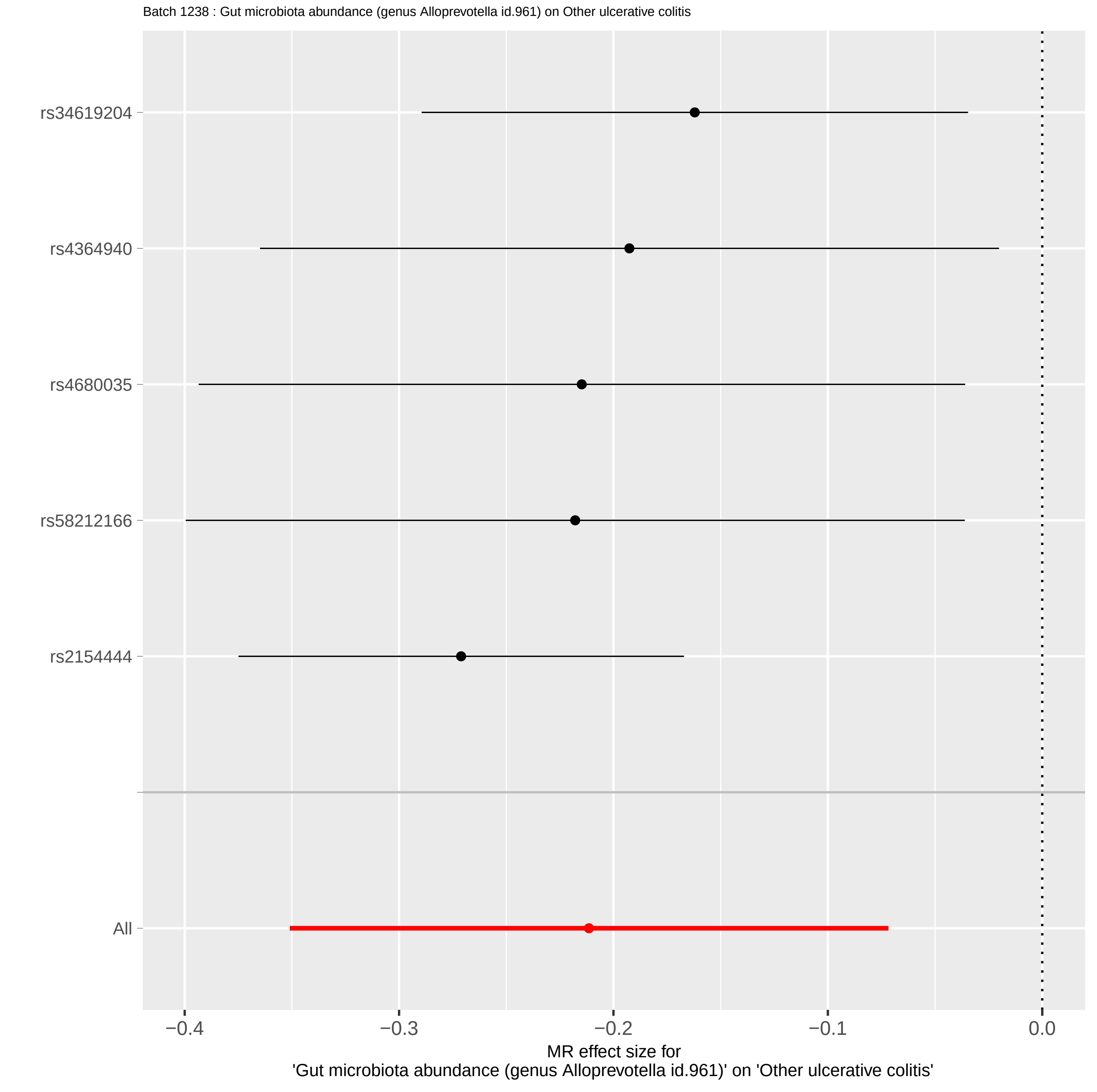
Batch 1238 : Gut microbiota abundance (genus Alloprevotella id.961) on Other ulcerative colitis
rs34619204
rs4364940
rs4680035
rs58212166
rs2154444
All
−0.4
−0.3
−0.2
−0.1
0.0
MR effect size for
'Gut microbiota abundance (genus Alloprevotella id.961)' on 'Other ulcerative colitis'

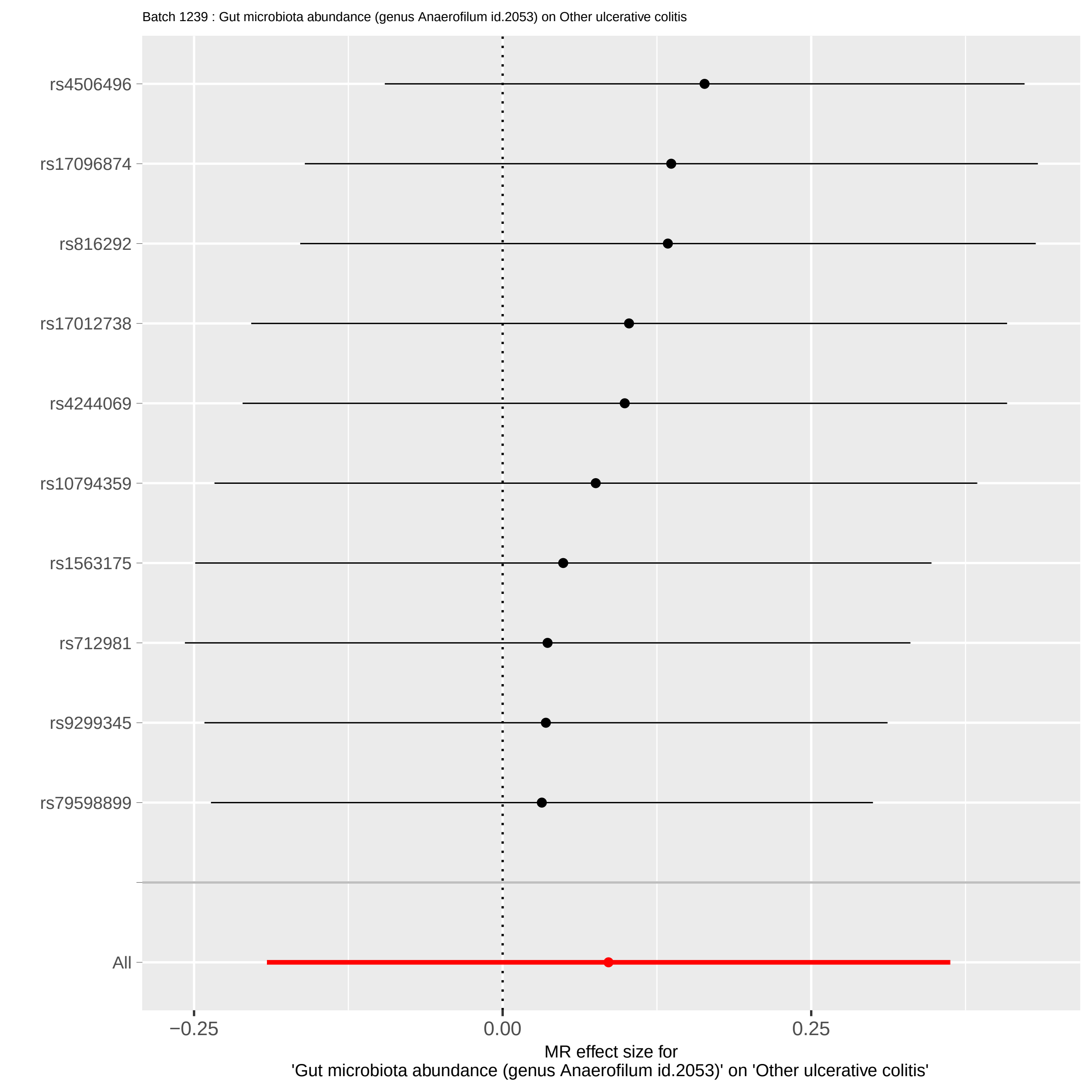

Batch 1239 : Gut microbiota abundance (genus Anaerofilum id.2053) on Other ulcerative colitis
rs4506496
rs17096874
rs816292
rs17012738
rs4244069
rs10794359
rs1563175
rs712981
rs9299345
rs79598899
All
−0.25
0.00
0.25
MR effect size for
'Gut microbiota abundance (genus Anaerofilum id.2053)' on 'Other ulcerative colitis'

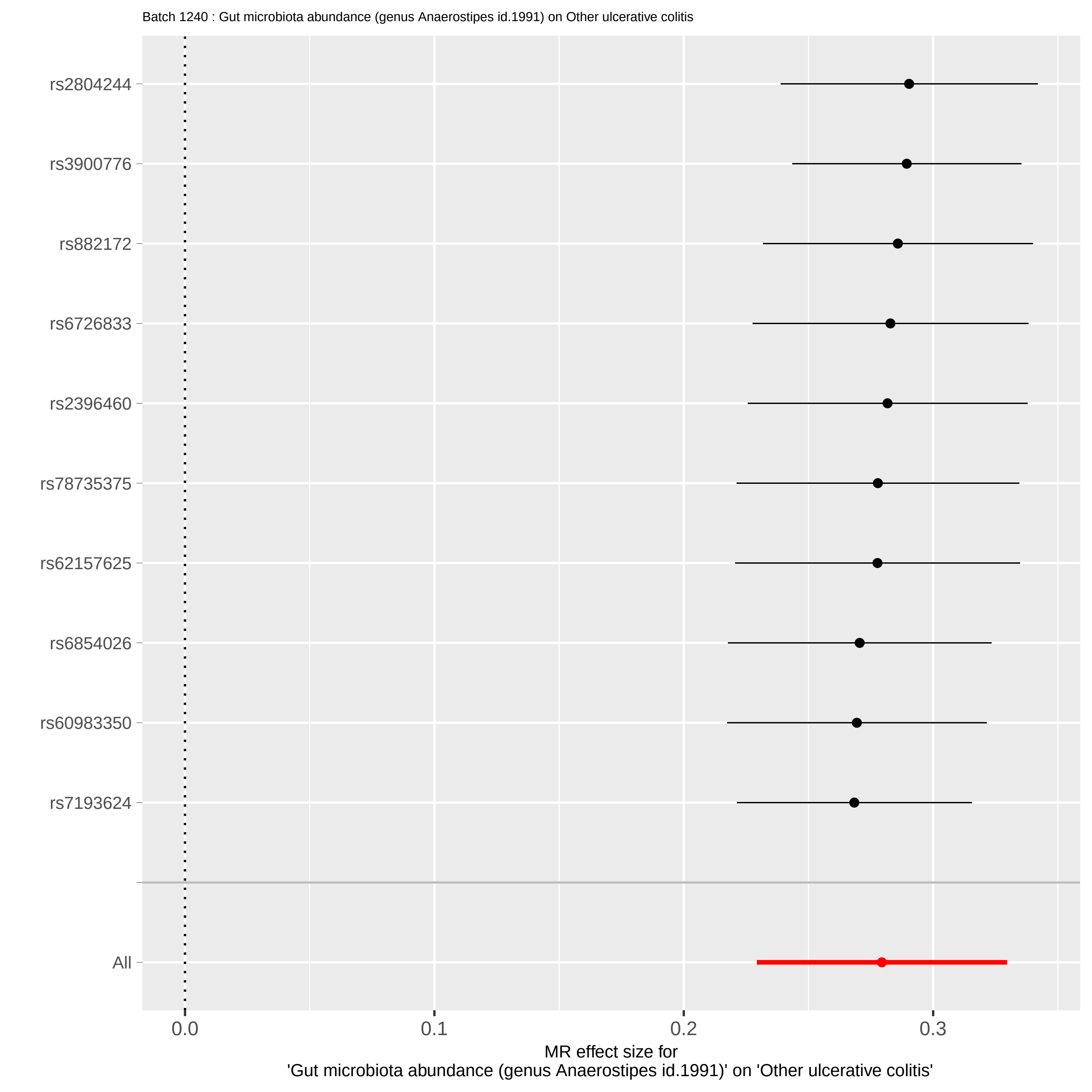
Batch 1240 : Gut microbiota abundance (genus Anaerostipes id.1991) on Other ulcerative colitis
rs2804244
rs3900776
rs882172
rs6726833
rs2396460
rs78735375
rs62157625
rs6854026
rs60983350
rs7193624
All
0.0
0.1
0.2
0.3
MR effect size for
'Gut microbiota abundance (genus Anaerostipes id.1991)' on 'Other ulcerative colitis'

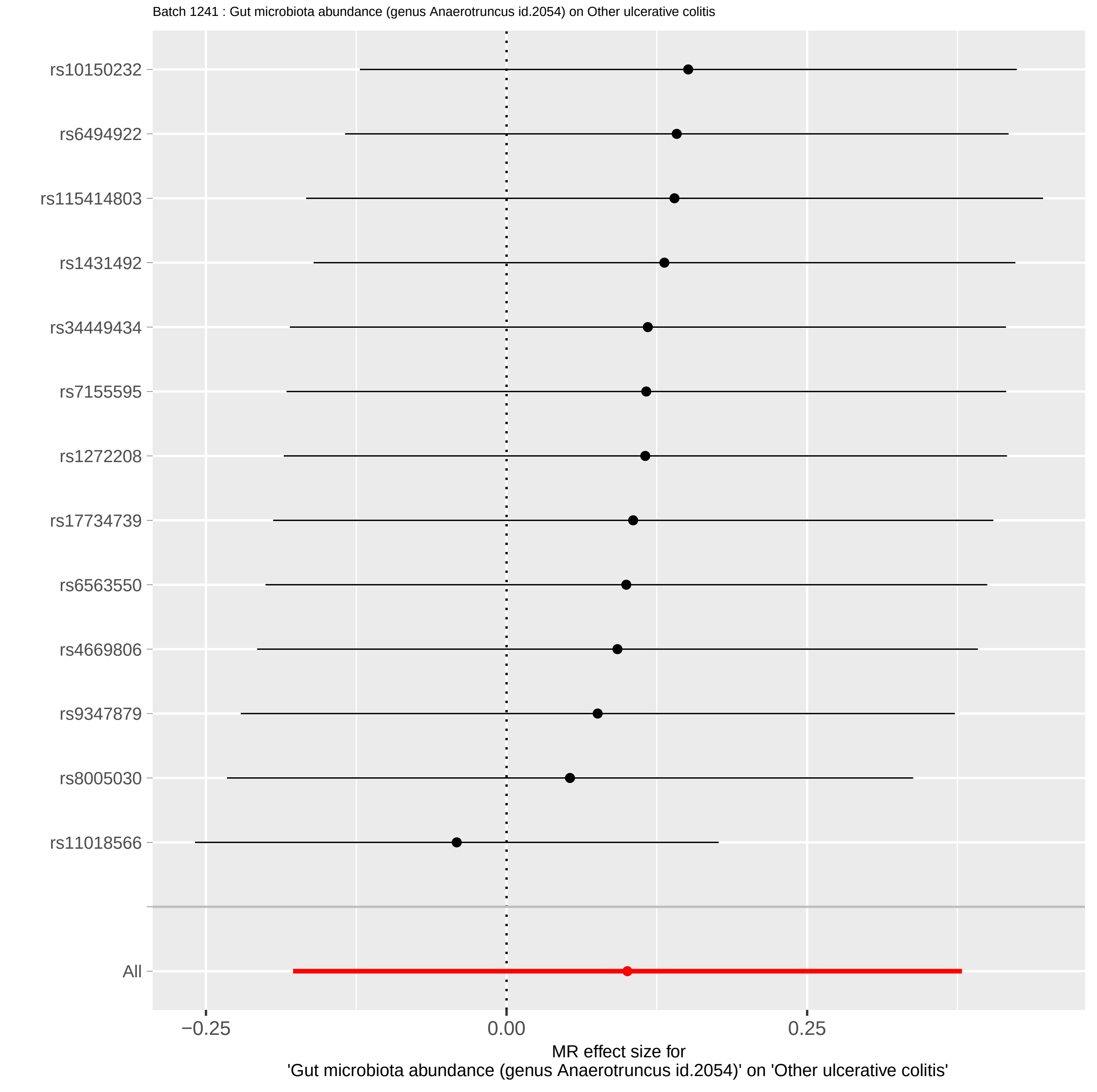

Batch 1241 : Gut microbiota abundance (genus Anaerotruncus id.2054) on Other ulcerative colitis
rs10150232
rs6494922
rs115414803
rs1431492
rs34449434
rs7155595
rs1272208
rs17734739
rs6563550
rs4669806
rs9347879
rs8005030
rs11018566
All
−0.25
0.00
0.25
MR effect size for
'Gut microbiota abundance (genus Anaerotruncus id.2054)' on 'Other ulcerative colitis'

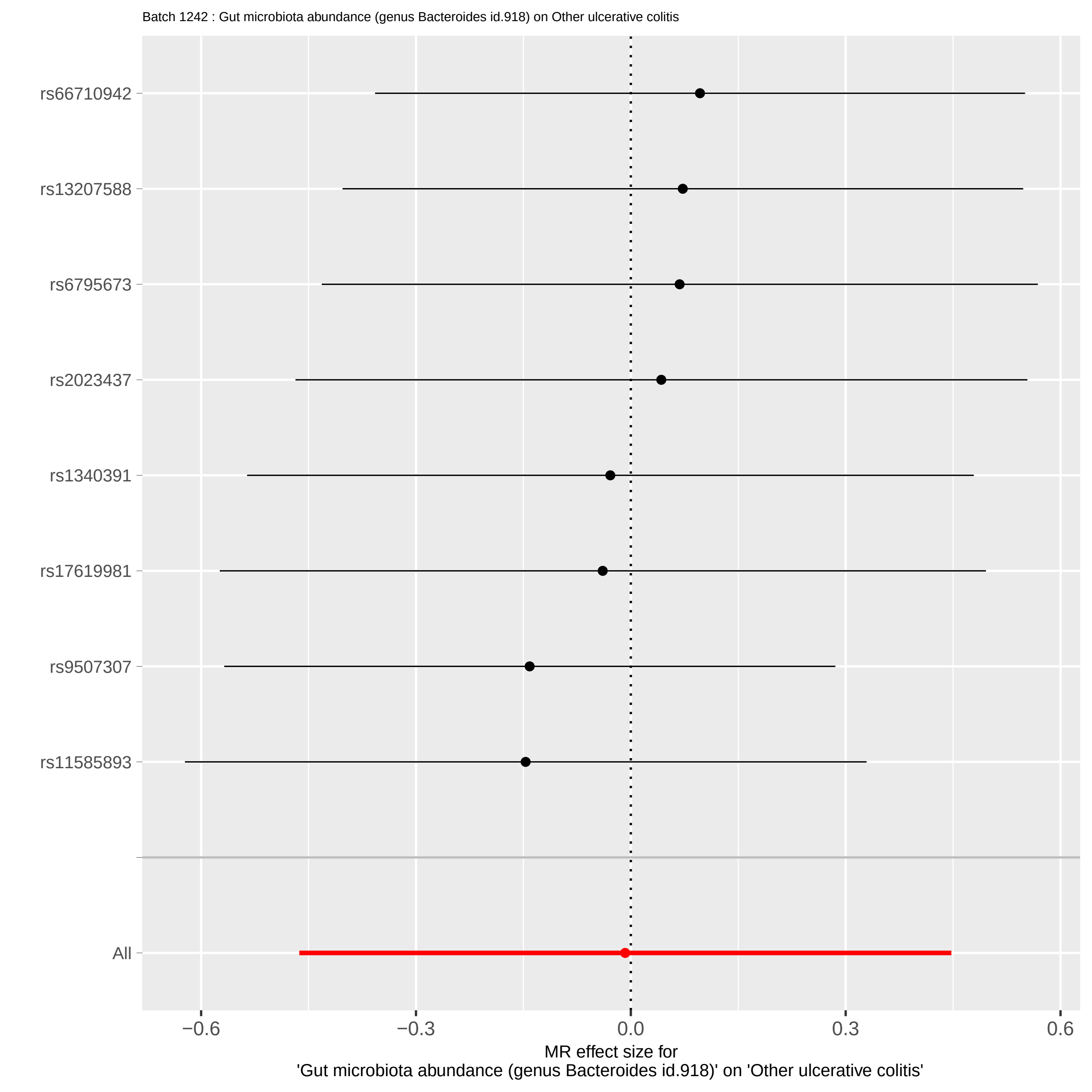

Batch 1242 : Gut microbiota abundance (genus Bacteroides id.918) on Other ulcerative colitis
rs66710942
rs13207588
rs6795673
rs2023437
rs1340391
rs17619981
rs9507307
rs11585893
All
−0.6
−0.3
0.0
0.3
0.6
MR effect size for
'Gut microbiota abundance (genus Bacteroides id.918)' on 'Other ulcerative colitis'

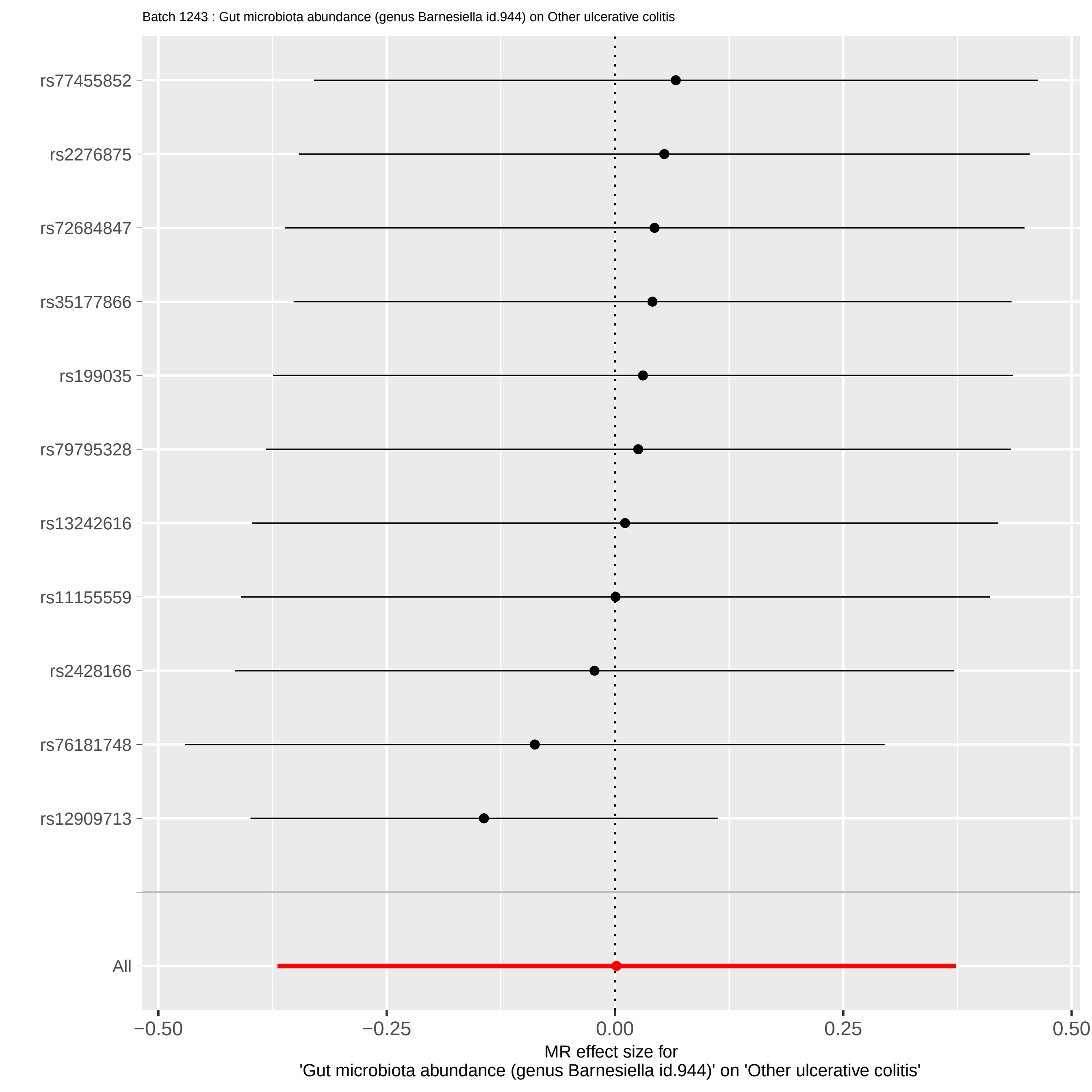

Batch 1243 : Gut microbiota abundance (genus Barnesiella id.944) on Other ulcerative colitis
rs77455852
rs2276875
rs72684847
rs35177866
rs199035
rs79795328
rs13242616
rs11155559
rs2428166
rs76181748
rs12909713
All
−0.50
−0.25
0.00
0.25
0.50
MR effect size for
'Gut microbiota abundance (genus Barnesiella id.944)' on 'Other ulcerative colitis'

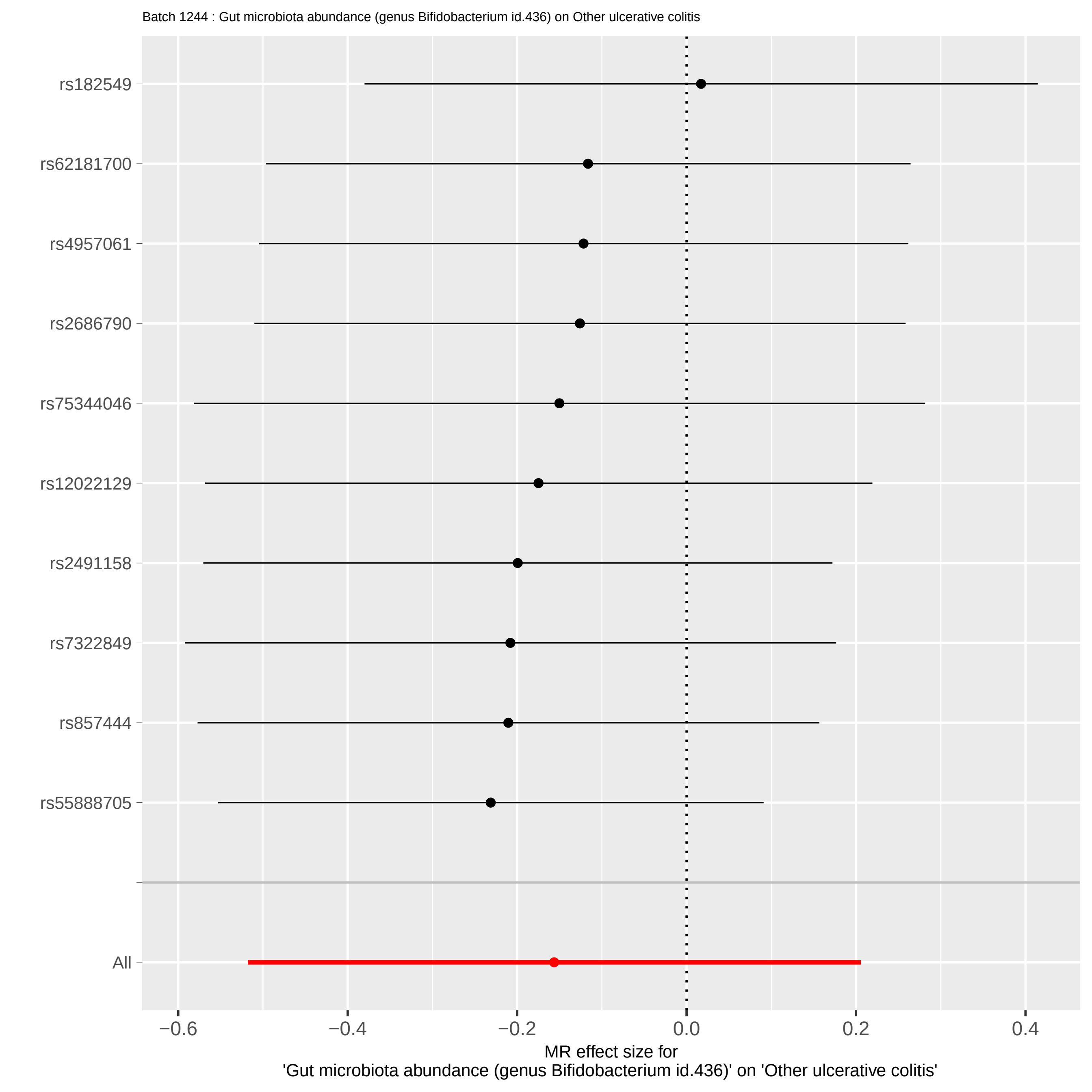

Batch 1244 : Gut microbiota abundance (genus Bifidobacterium id.436) on Other ulcerative colitis
rs182549
rs62181700
rs4957061
rs2686790
rs75344046
rs12022129
rs2491158
rs7322849
rs857444
rs55888705
All
−0.6
−0.4
−0.2
0.0
0.2
0.4
MR effect size for
'Gut microbiota abundance (genus Bifidobacterium id.436)' on 'Other ulcerative colitis'

Batch 1245 : Gut microbiota abundance (genus Bilophila id.3170) on Other ulcerative colitis

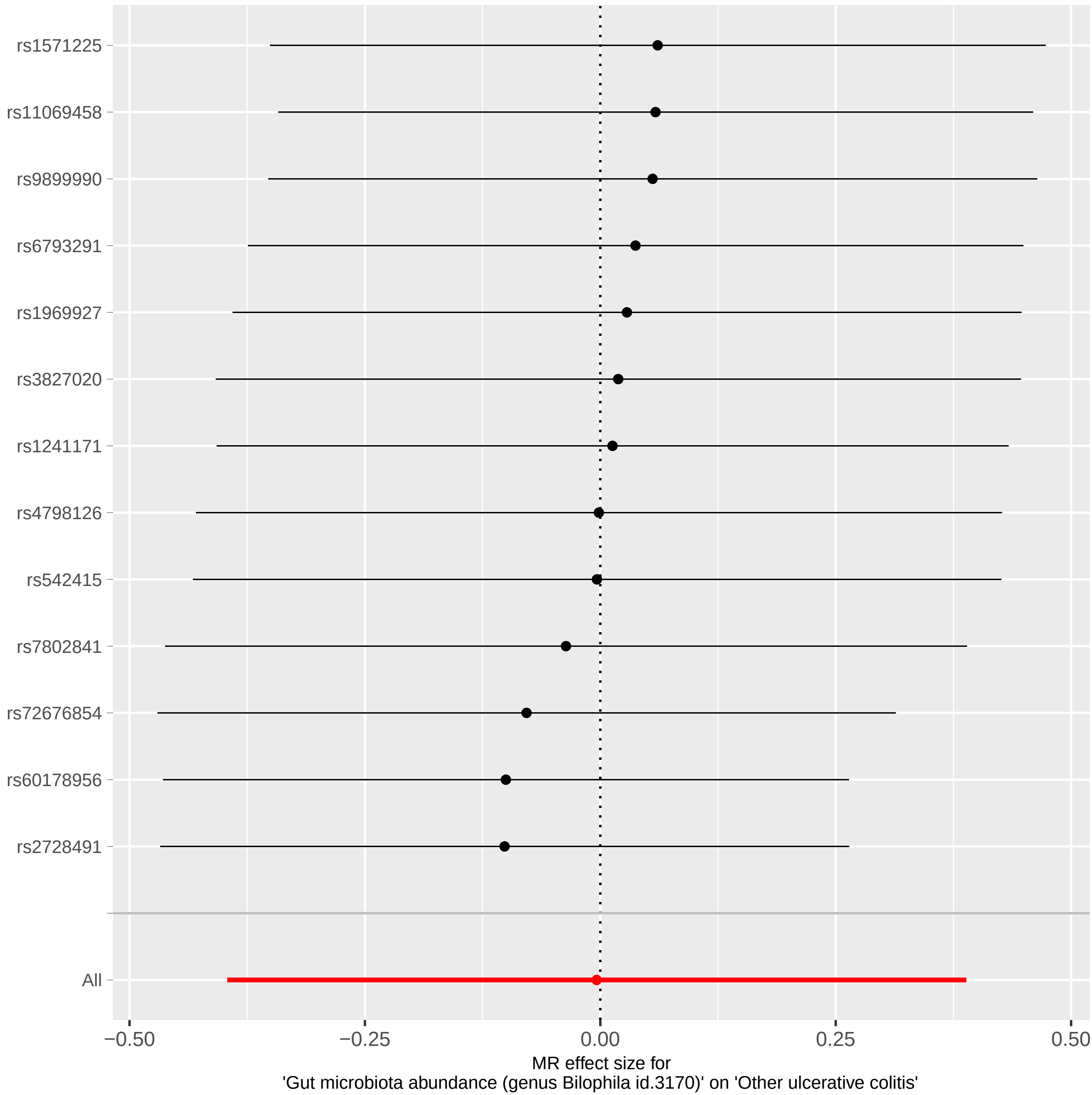

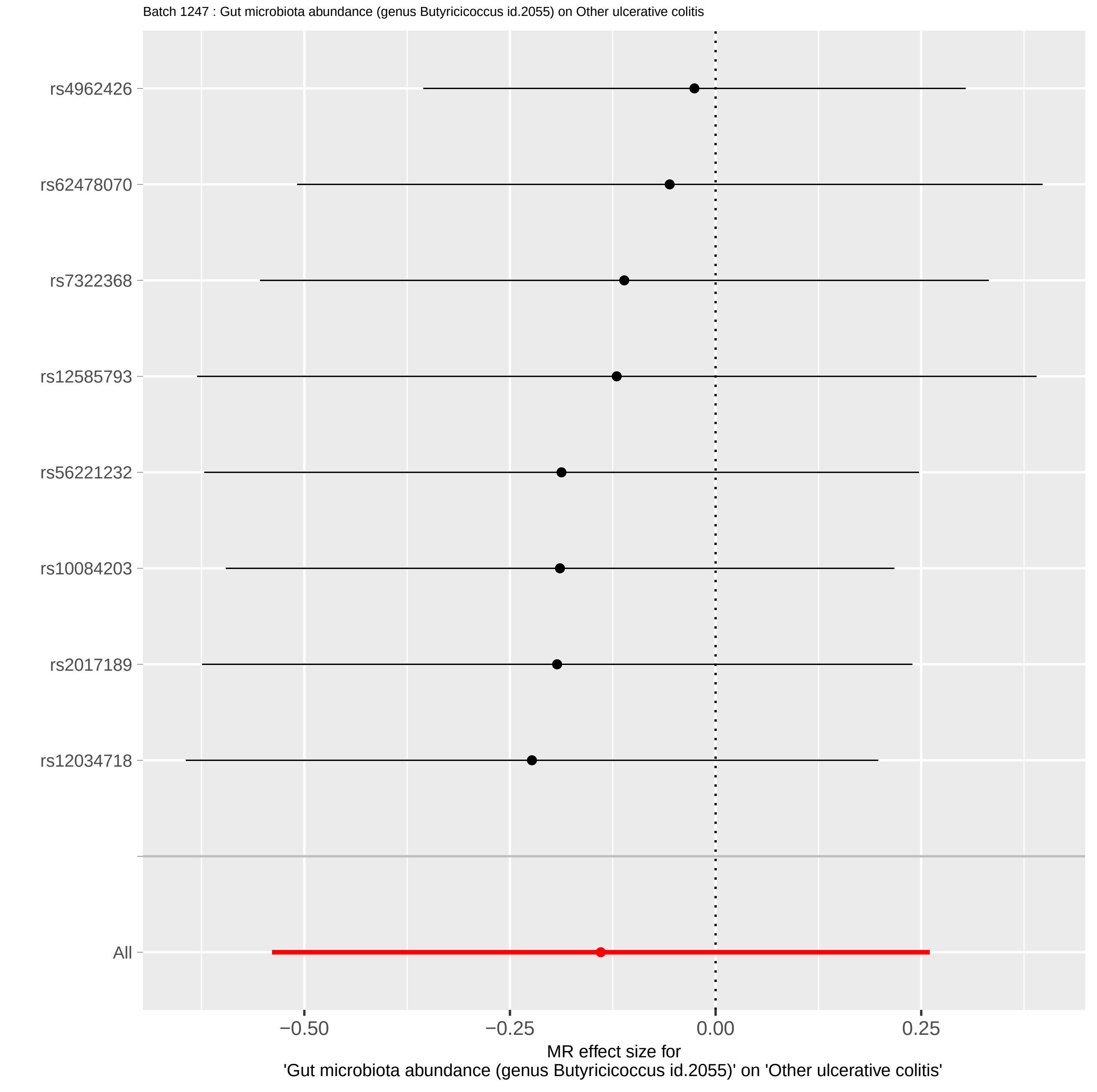

Batch 1247 : Gut microbiota abundance (genus Butyricicoccus id.2055) on Other ulcerative colitis
rs4962426
rs62478070
rs7322368
rs12585793
rs56221232
rs10084203
rs2017189
rs12034718
All
−0.50
−0.25
0.00
0.25
MR effect size for
'Gut microbiota abundance (genus Butyricicoccus id.2055)' on 'Other ulcerative colitis'

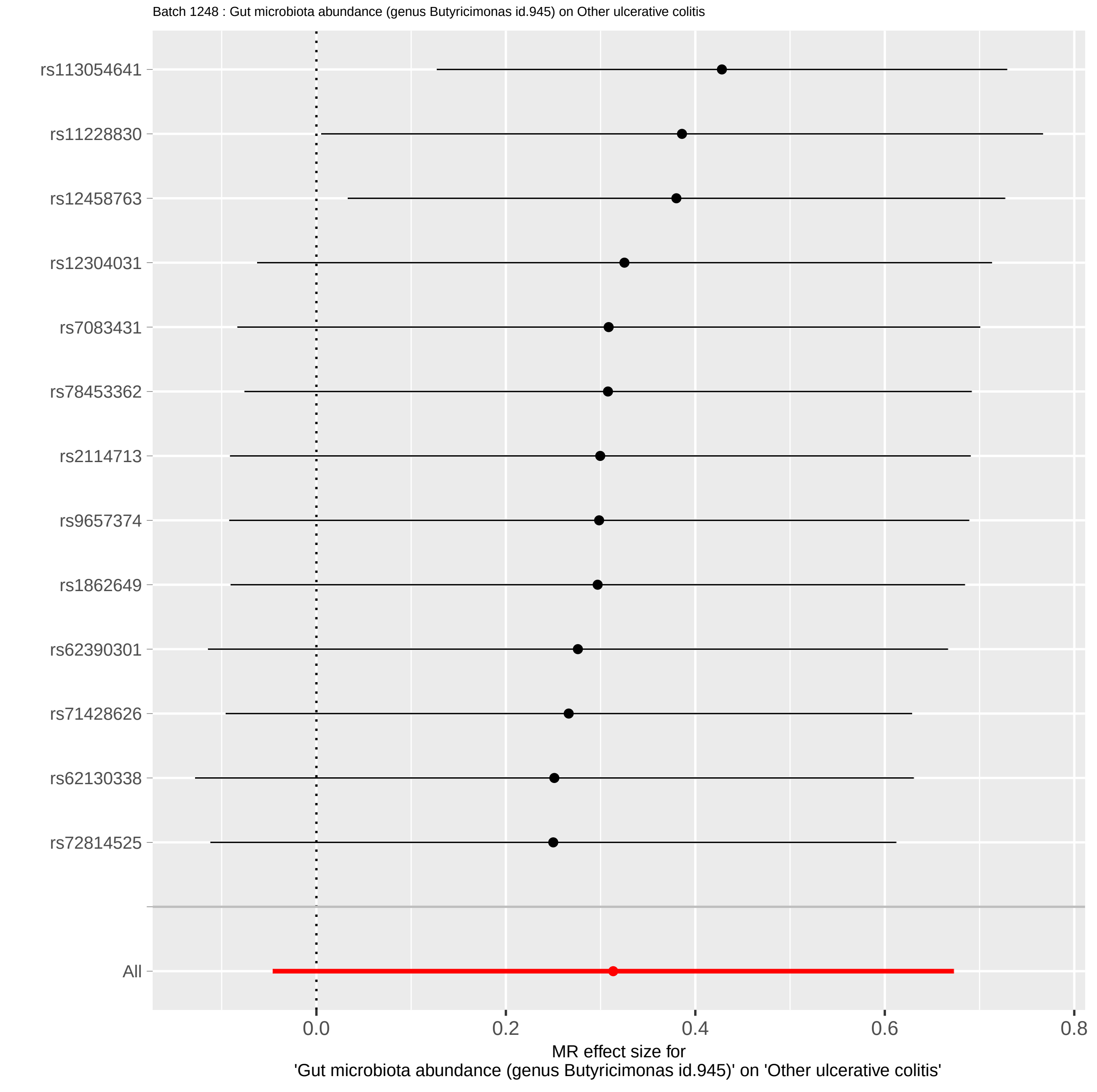
Batch 1248 : Gut microbiota abundance (genus Butyricimonas id.945) on Other ulcerative colitis
rs113054641
rs11228830
rs12458763
rs12304031
rs7083431
rs78453362
rs2114713
rs9657374
rs1862649
rs62390301
rs71428626
rs62130338
rs72814525
All
0.0
0.2
0.4
0.6
0.8
MR effect size for
'Gut microbiota abundance (genus Butyricimonas id.945)' on 'Other ulcerative colitis'

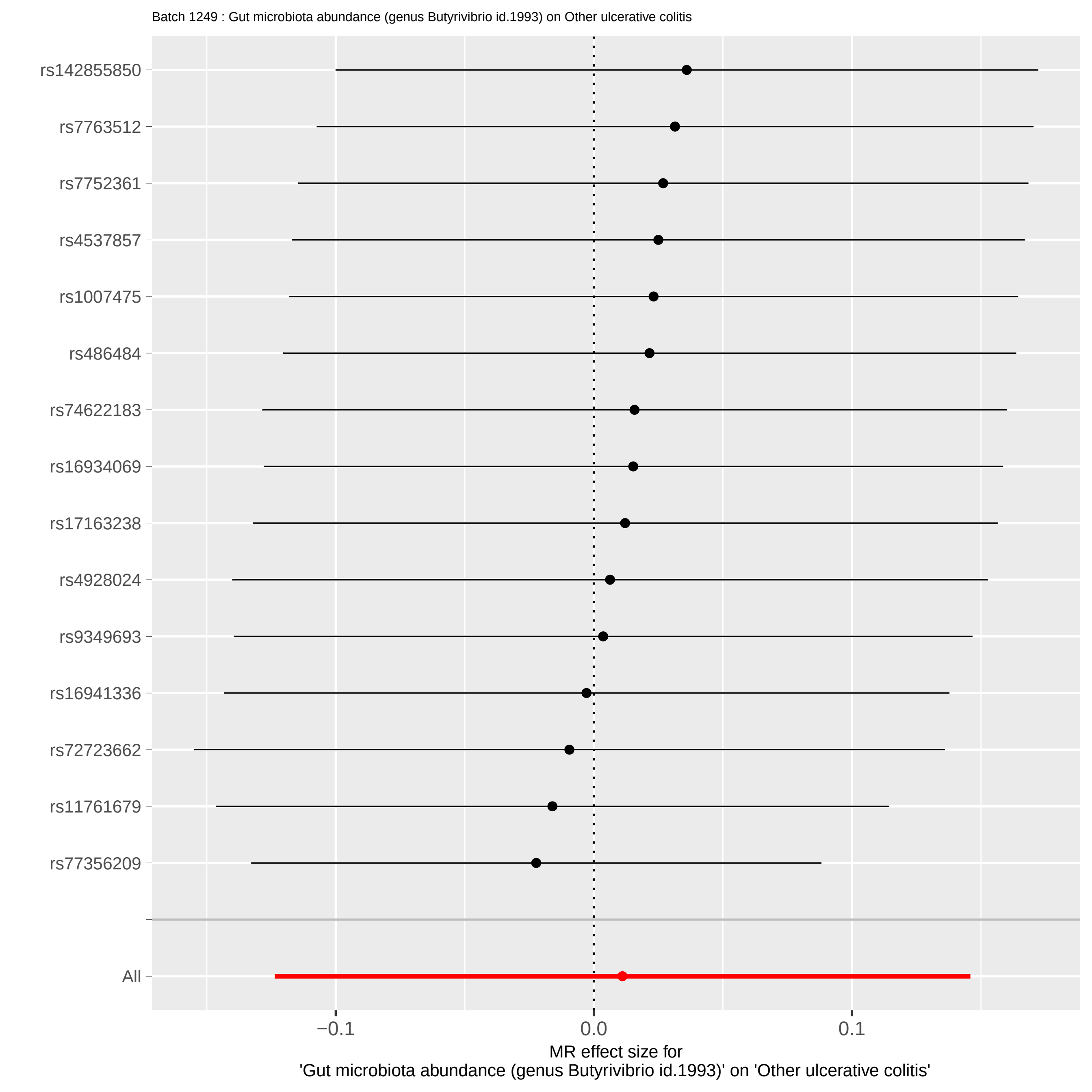
Batch 1249 : Gut microbiota abundance (genus Butyrivibrio id.1993) on Other ulcerative colitis
rs142855850
rs7763512
rs7752361
rs4537857
rs1007475
rs486484
rs74622183
rs16934069
rs17163238
rs4928024
rs9349693
rs16941336
rs72723662
rs11761679
rs77356209
All
−0.1
0.0
0.1
MR effect size for
'Gut microbiota abundance (genus Butyrivibrio id.1993)' on 'Other ulcerative colitis'

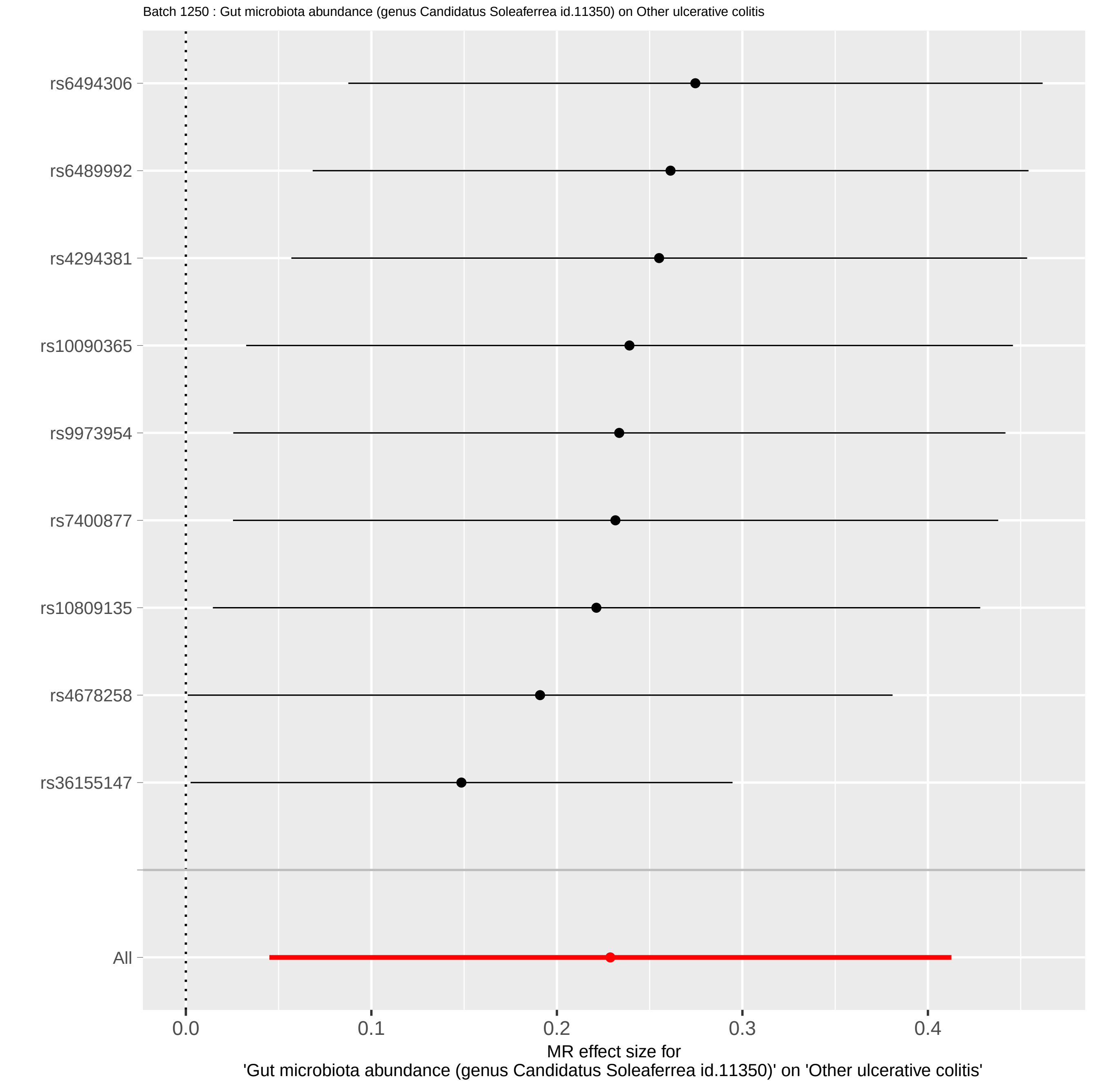
Batch 1250 : Gut microbiota abundance (genus Candidatus Soleaferrea id.11350) on Other ulcerative colitis
rs6494306
rs6489992
rs4294381
rs10090365
rs9973954
rs7400877
rs10809135
rs4678258
rs36155147
All
0.0
0.1
0.2
0.3
0.4
MR effect size for
'Gut microbiota abundance (genus Candidatus Soleaferrea id.11350)' on 'Other ulcerative colitis'

Batch 1251 : Gut microbiota abundance (genus Catenibacterium id.2153) on Other ulcerative colitis

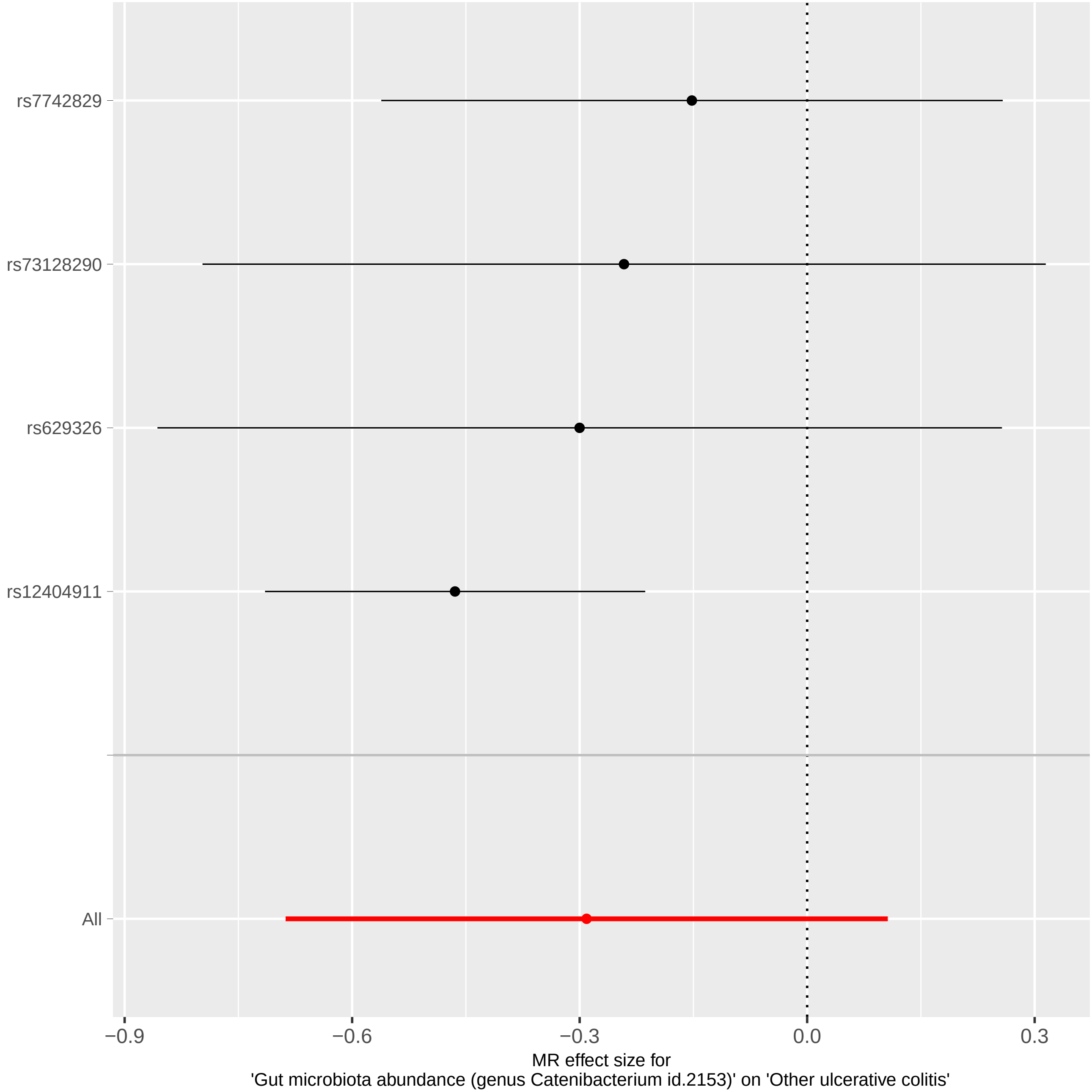

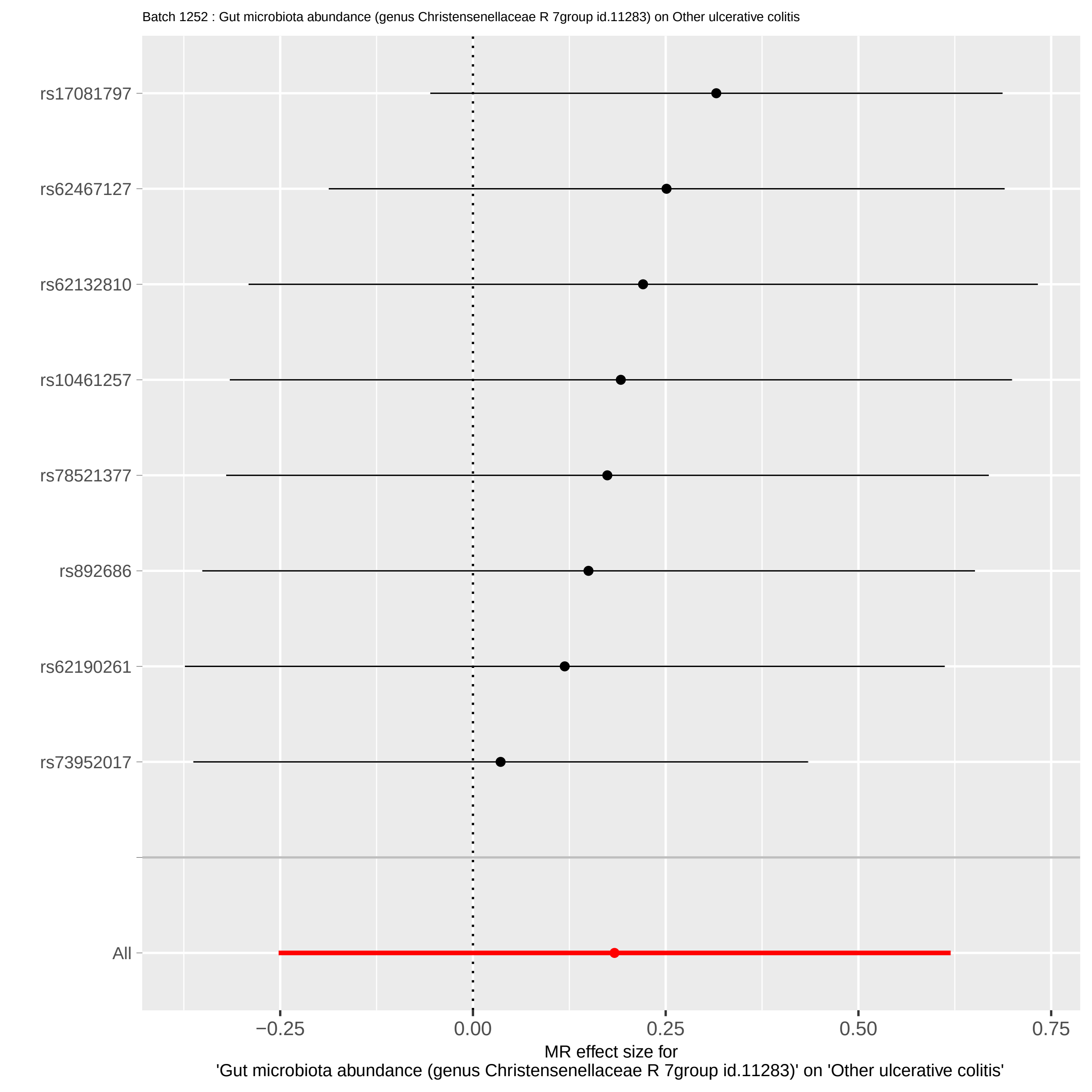

Batch 1252 : Gut microbiota abundance (genus Christensenellaceae R 7group id.11283) on Other ulcerative colitis
rs17081797
rs62467127
rs62132810
rs10461257
rs78521377
rs892686
rs62190261
rs73952017
All
−0.25
0.00
0.25
0.50
0.75
MR effect size for
'Gut microbiota abundance (genus Christensenellaceae R 7group id.11283)' on 'Other ulcerative colitis'

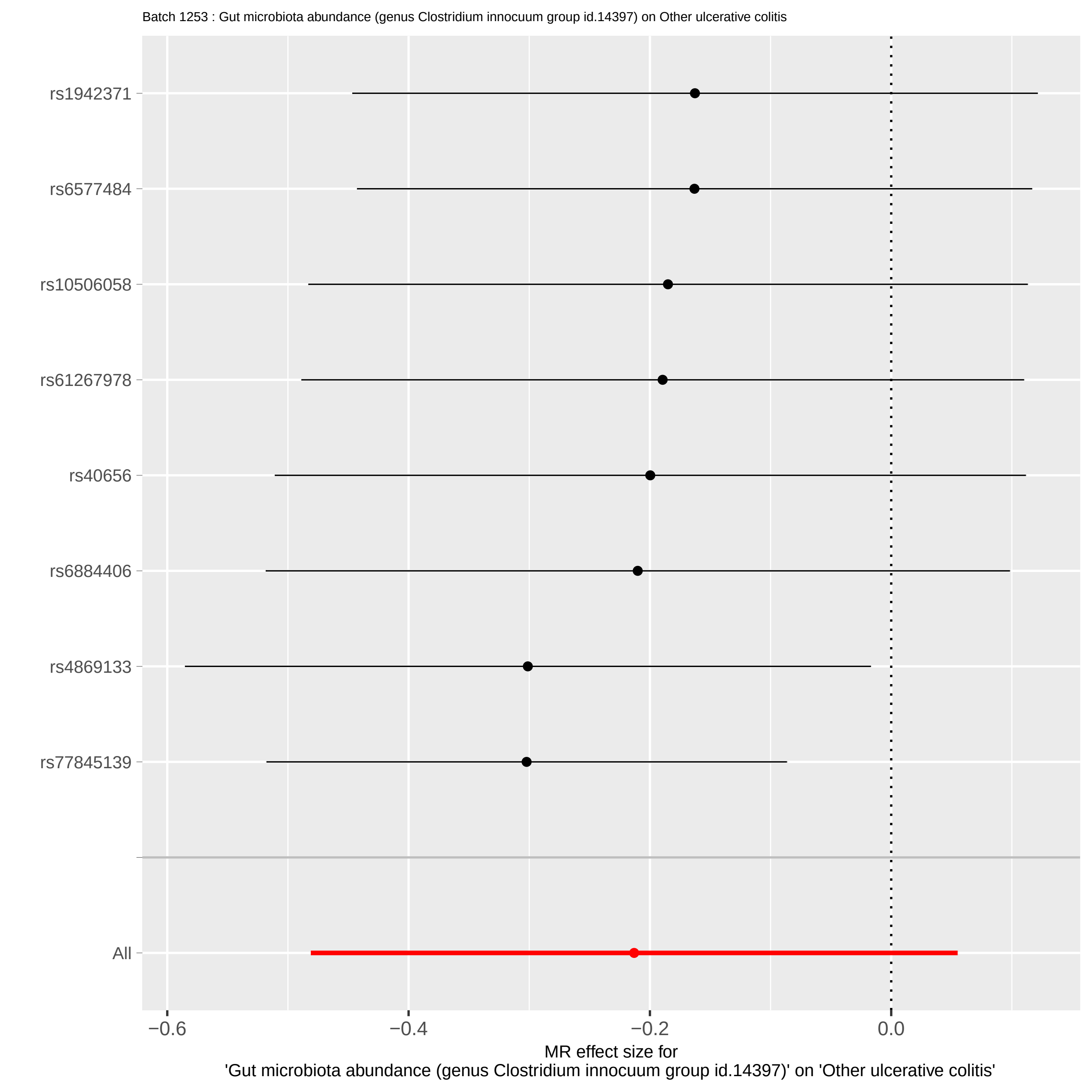

Batch 1253 : Gut microbiota abundance (genus Clostridium innocuum group id.14397) on Other ulcerative colitis
rs1942371
rs6577484
rs10506058
rs61267978
rs40656
rs6884406
rs4869133
rs77845139
All
−0.6
−0.4
−0.2
0.0
MR effect size for
'Gut microbiota abundance (genus Clostridium innocuum group id.14397)' on 'Other ulcerative colitis'

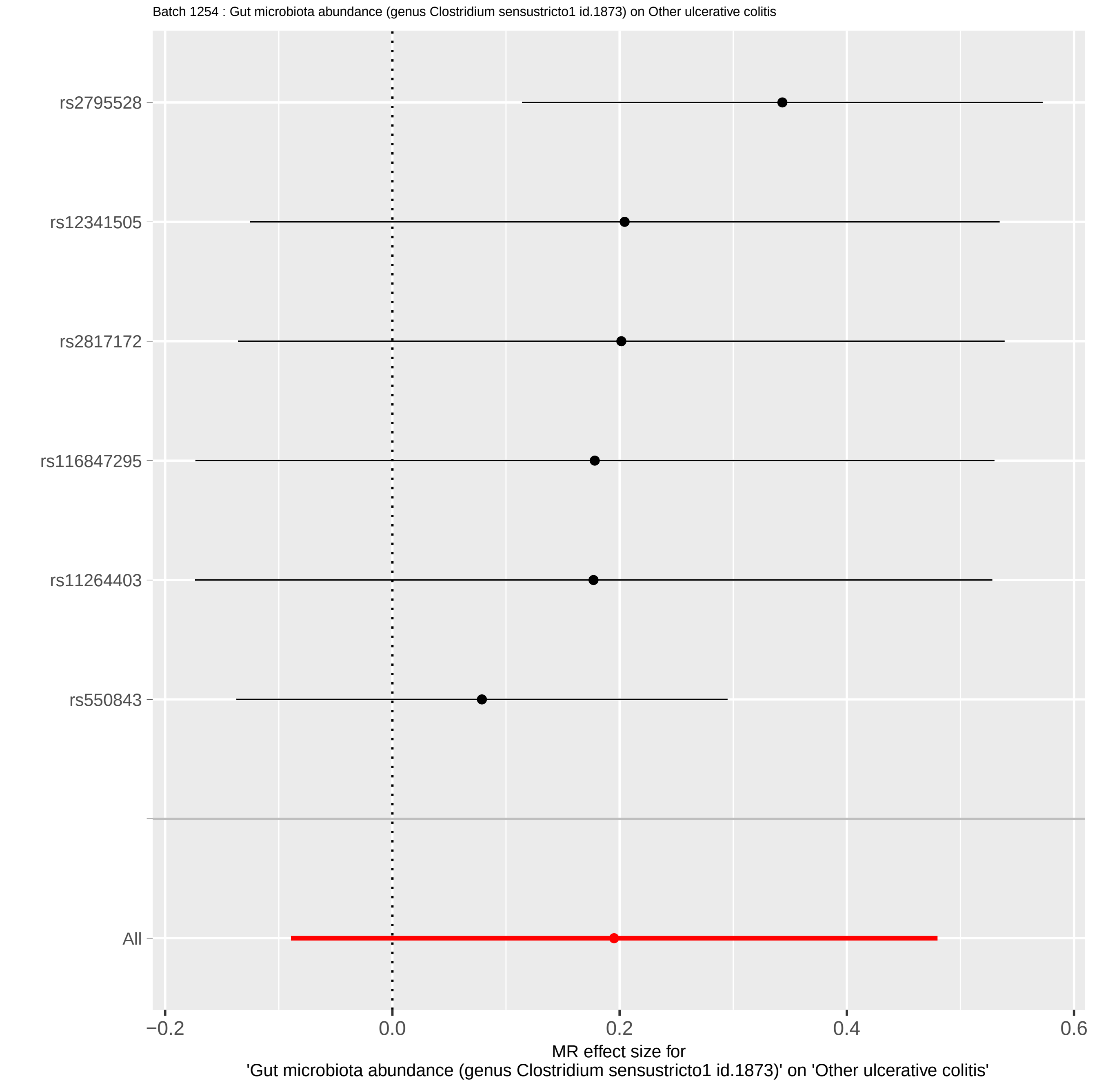

Batch 1254 : Gut microbiota abundance (genus Clostridium sensustricto1 id.1873) on Other ulcerative colitis
rs2795528
rs12341505
rs2817172
rs116847295
rs11264403
rs550843
All
−0.2
0.0
0.2
0.4
0.6
MR effect size for
'Gut microbiota abundance (genus Clostridium sensustricto1 id.1873)' on 'Other ulcerative colitis'

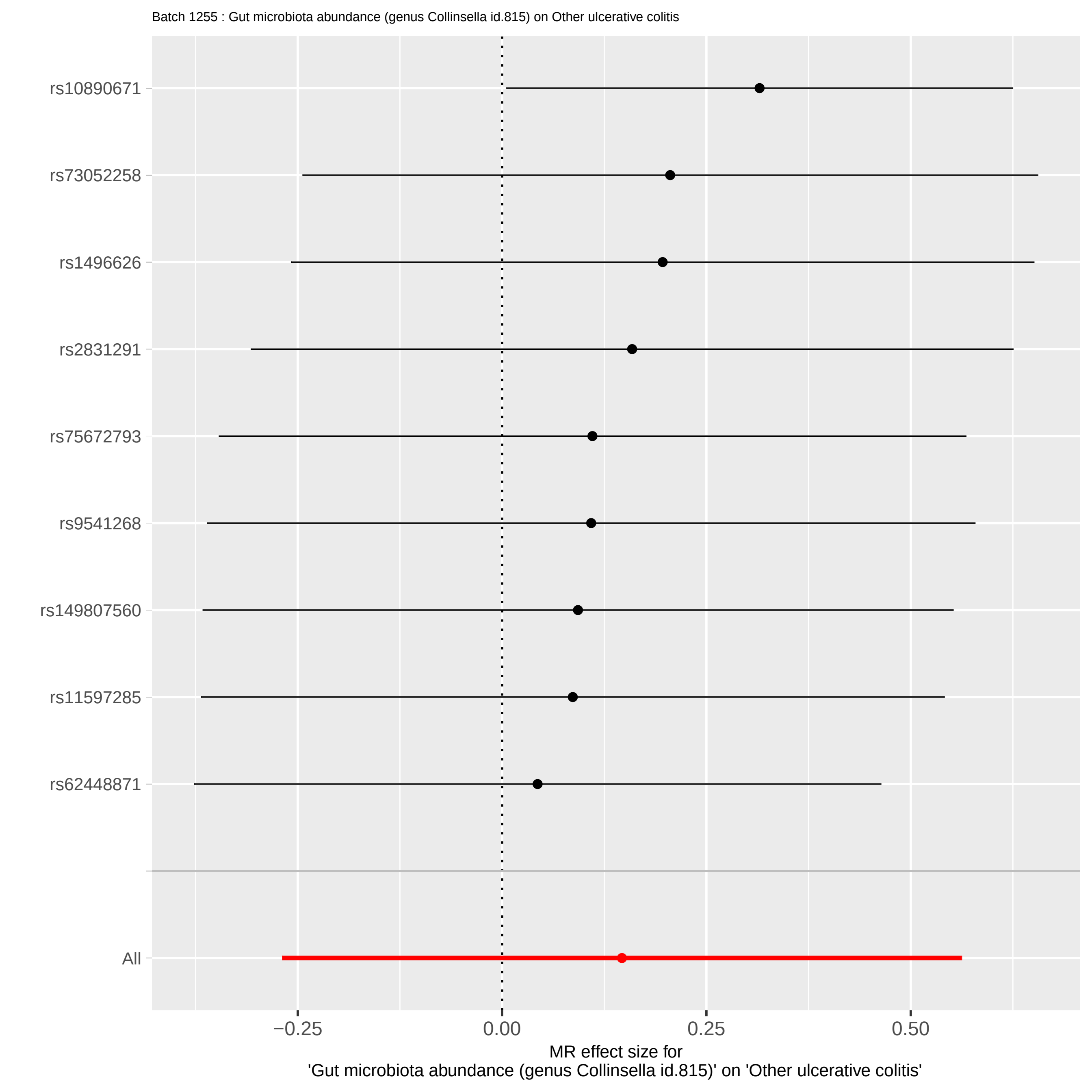

Batch 1255 : Gut microbiota abundance (genus Collinsella id.815) on Other ulcerative colitis
rs10890671
rs73052258
rs1496626
rs2831291
rs75672793
rs9541268
rs149807560
rs11597285
rs62448871
All
−0.25
0.00
0.25
0.50
MR effect size for
'Gut microbiota abundance (genus Collinsella id.815)' on 'Other ulcerative colitis'

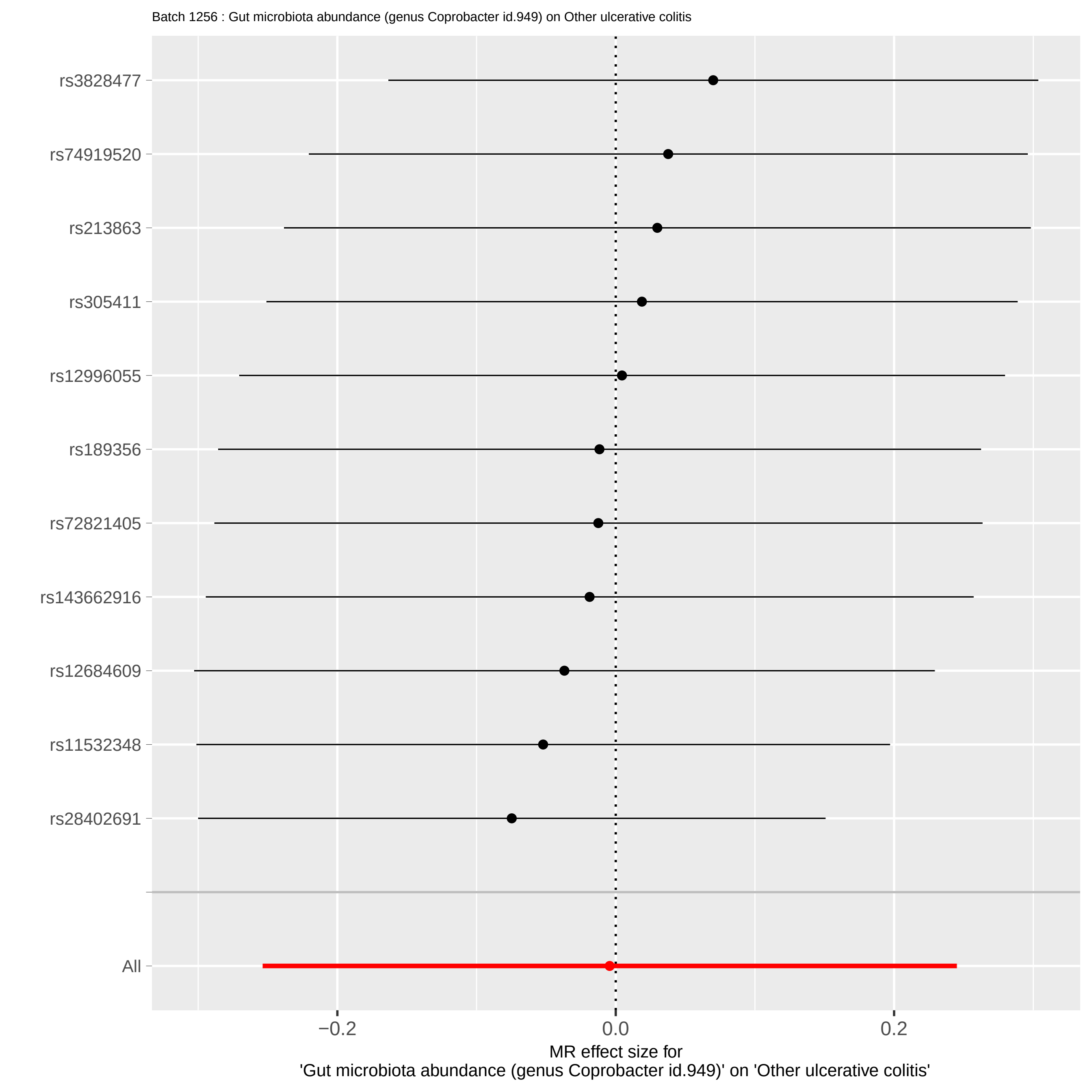
Batch 1256 : Gut microbiota abundance (genus Coprobacter id.949) on Other ulcerative colitis
rs3828477
rs74919520
rs213863
rs305411
rs12996055
rs189356
rs72821405
rs143662916
rs12684609
rs11532348
rs28402691
All
−0.2
0.0
0.2
MR effect size for
'Gut microbiota abundance (genus Coprobacter id.949)' on 'Other ulcerative colitis'

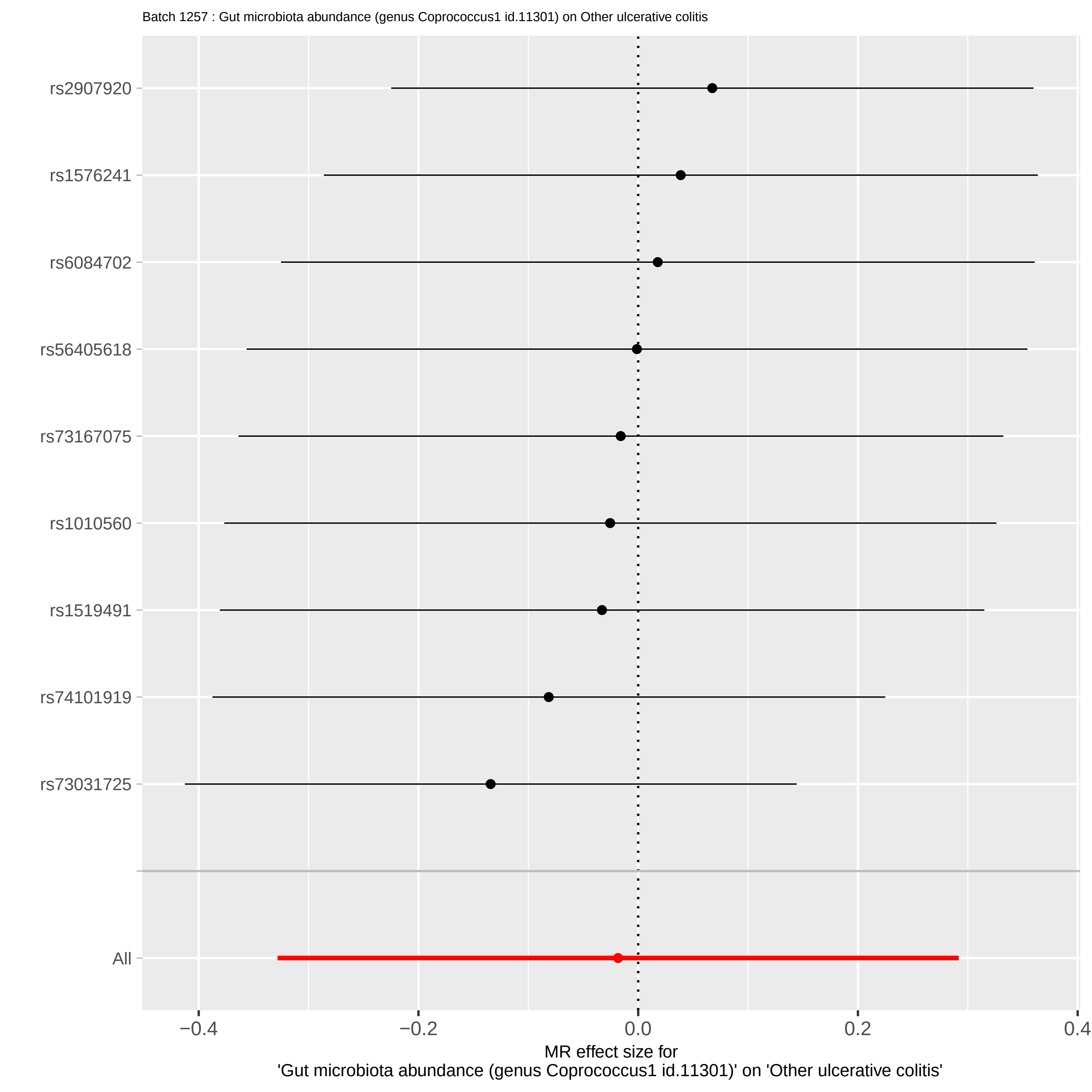

Batch 1257 : Gut microbiota abundance (genus Coprococcus1 id.11301) on Other ulcerative colitis
rs2907920
rs1576241
rs6084702
rs56405618
rs73167075
rs1010560
rs1519491
rs74101919
rs73031725
All
−0.4
−0.2
0.0
0.2
0.4
MR effect size for
'Gut microbiota abundance (genus Coprococcus1 id.11301)' on 'Other ulcerative colitis'

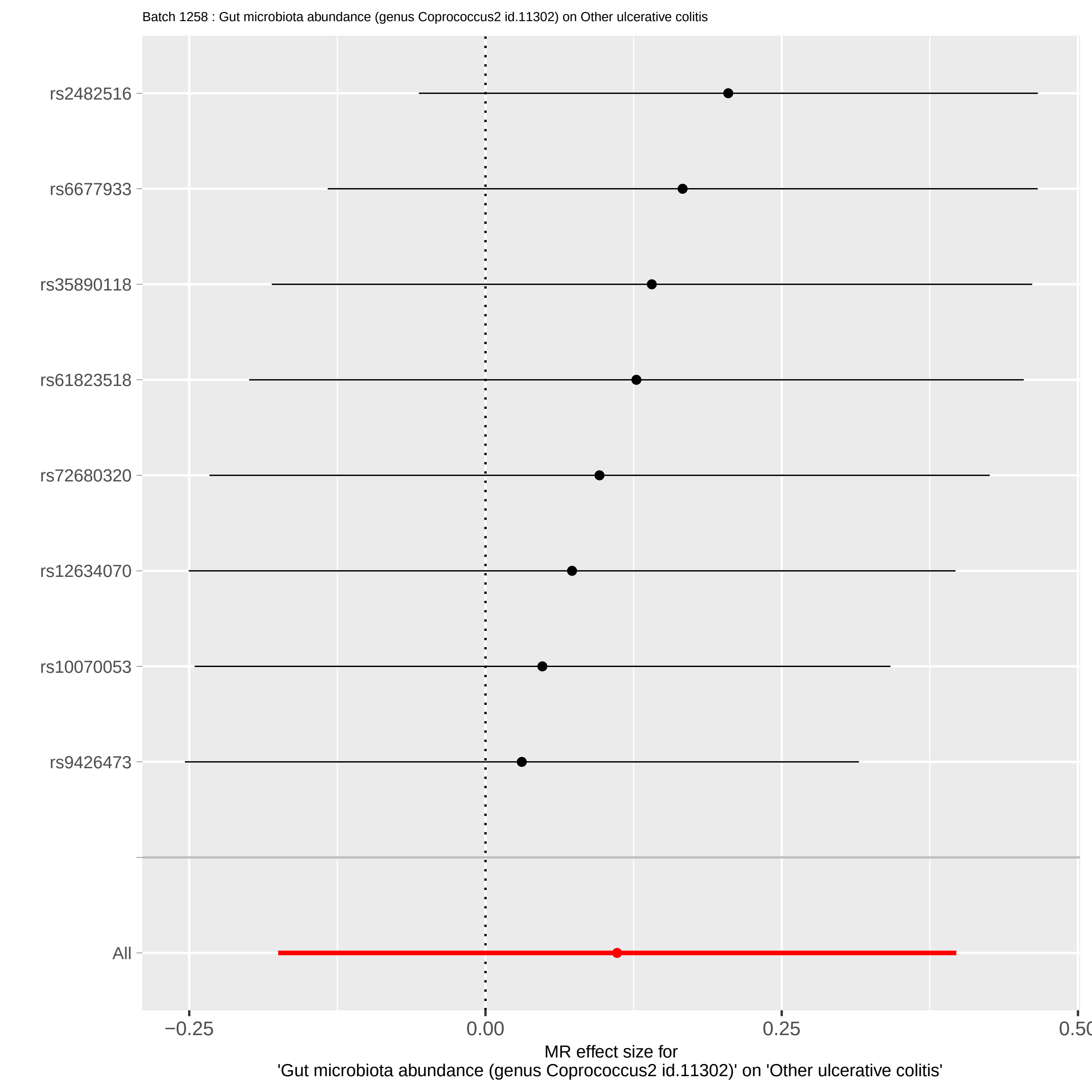
Batch 1258 : Gut microbiota abundance (genus Coprococcus2 id.11302) on Other ulcerative colitis
rs2482516
rs6677933
rs35890118
rs61823518
rs72680320
rs12634070
rs10070053
rs9426473
All
−0.25
0.00
0.25
0.50
MR effect size for
'Gut microbiota abundance (genus Coprococcus2 id.11302)' on 'Other ulcerative colitis'

Batch 1259 : Gut microbiota abundance (genus Coprococcus3 id.11303) on Other ulcerative colitis

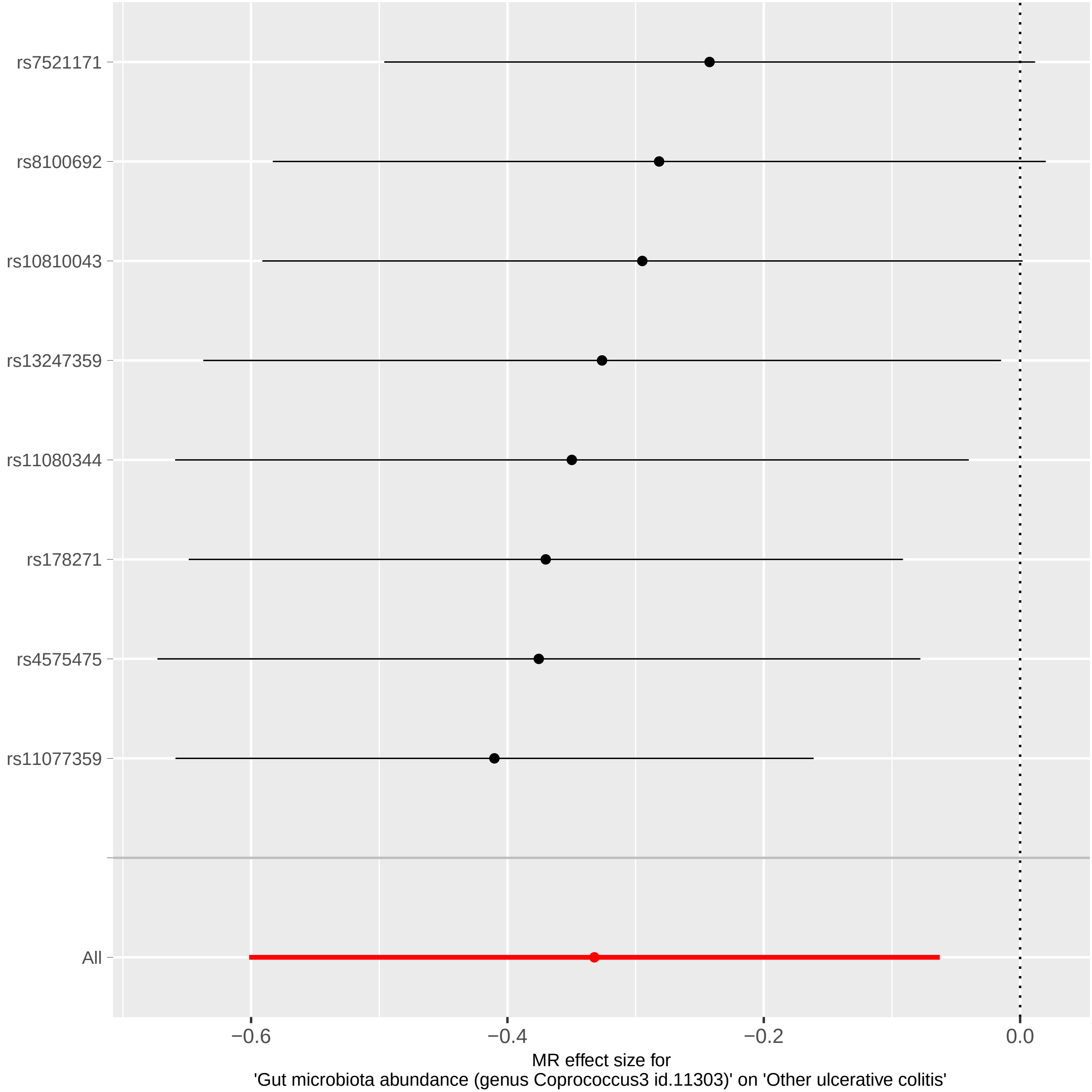

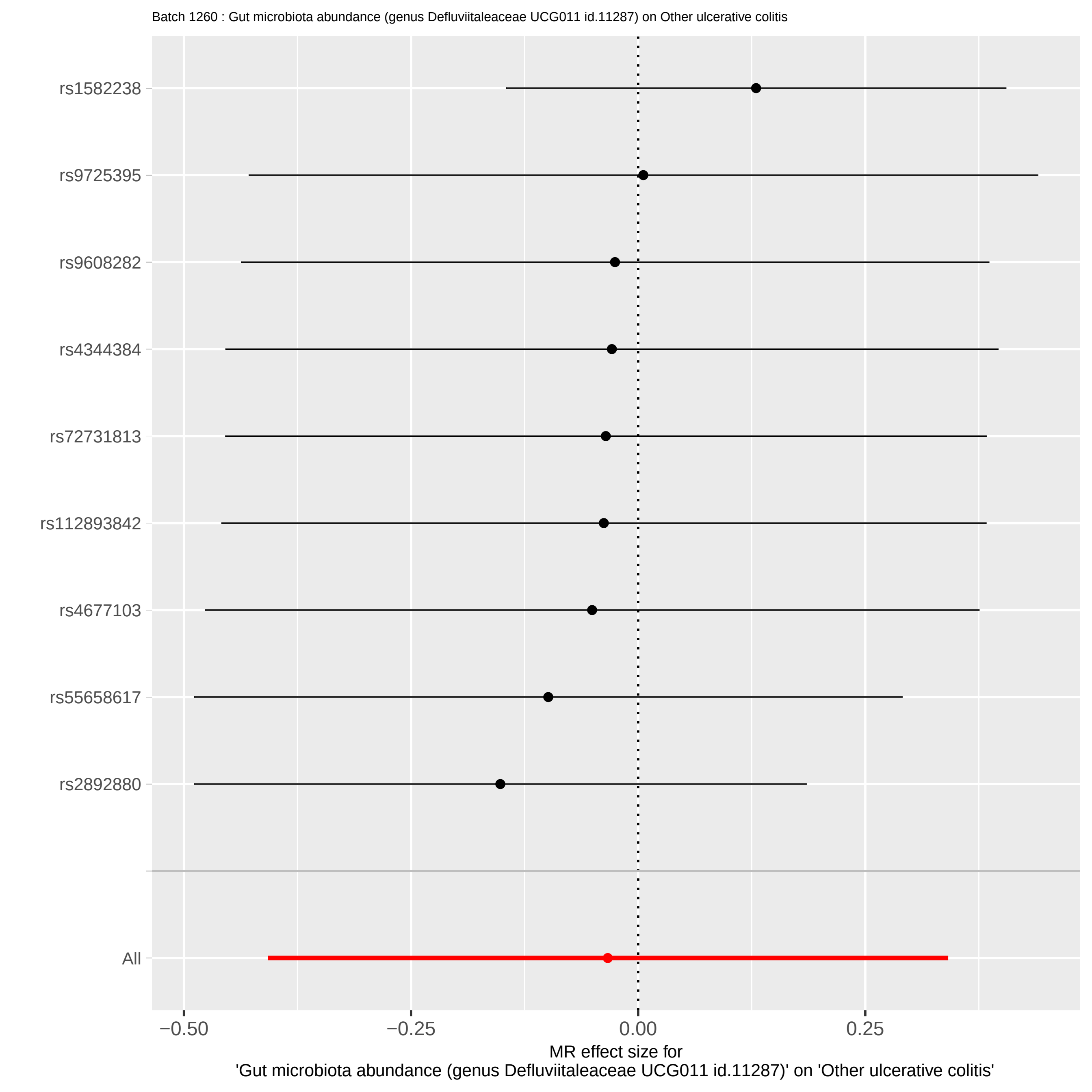
Batch 1260 : Gut microbiota abundance (genus Defluviitaleaceae UCG011 id.11287) on Other ulcerative colitis
rs1582238
rs9725395
rs9608282
rs4344384
rs72731813
rs112893842
rs4677103
rs55658617
rs2892880
All
−0.50
−0.25
0.00
0.25
MR effect size for
'Gut microbiota abundance (genus Defluviitaleaceae UCG011 id.11287)' on 'Other ulcerative colitis'

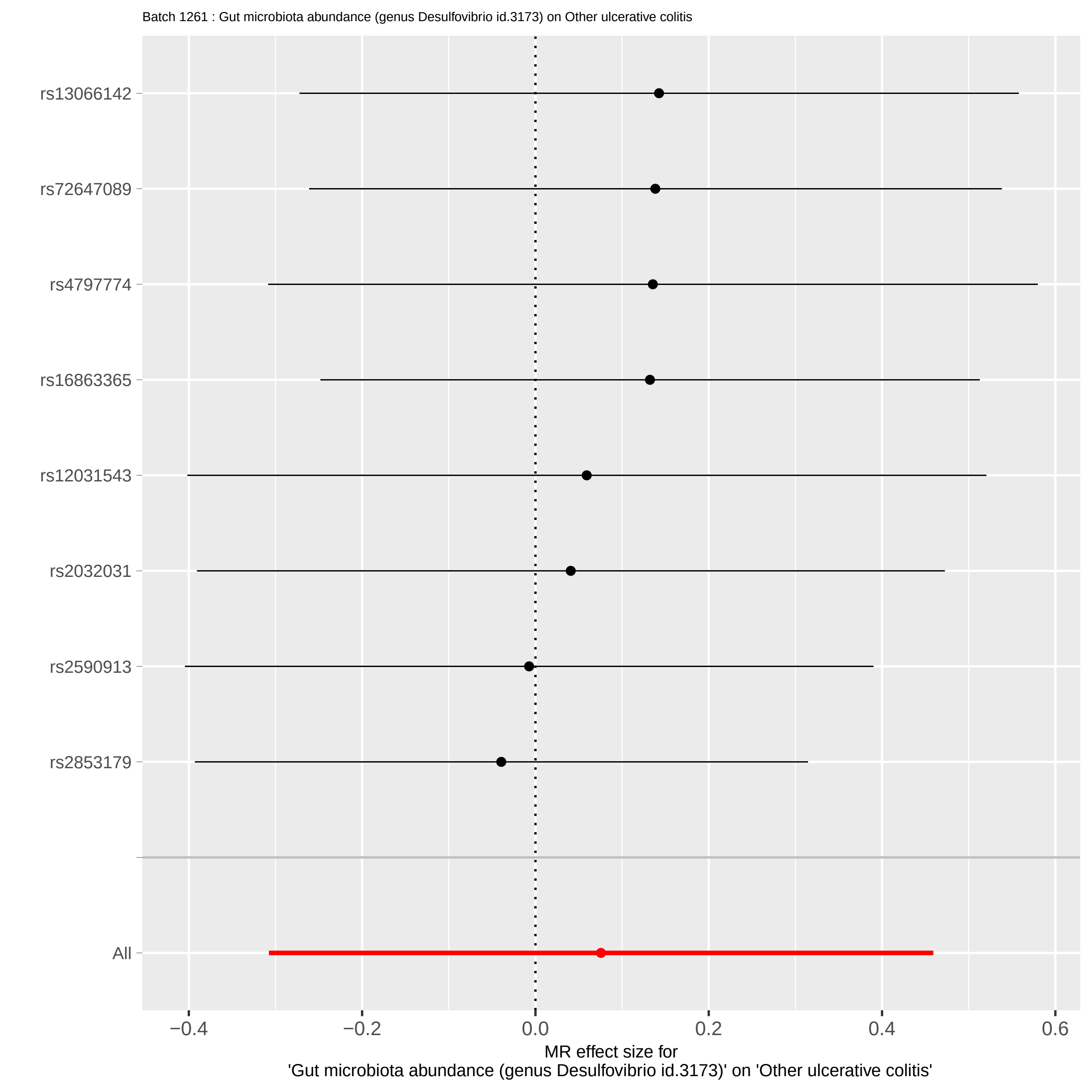

Batch 1261 : Gut microbiota abundance (genus Desulfovibrio id.3173) on Other ulcerative colitis
rs13066142
rs72647089
rs4797774
rs16863365
rs12031543
rs2032031
rs2590913
rs2853179
All
−0.4
−0.2
0.0
0.2
0.4
0.6
MR effect size for
'Gut microbiota abundance (genus Desulfovibrio id.3173)' on 'Other ulcerative colitis'

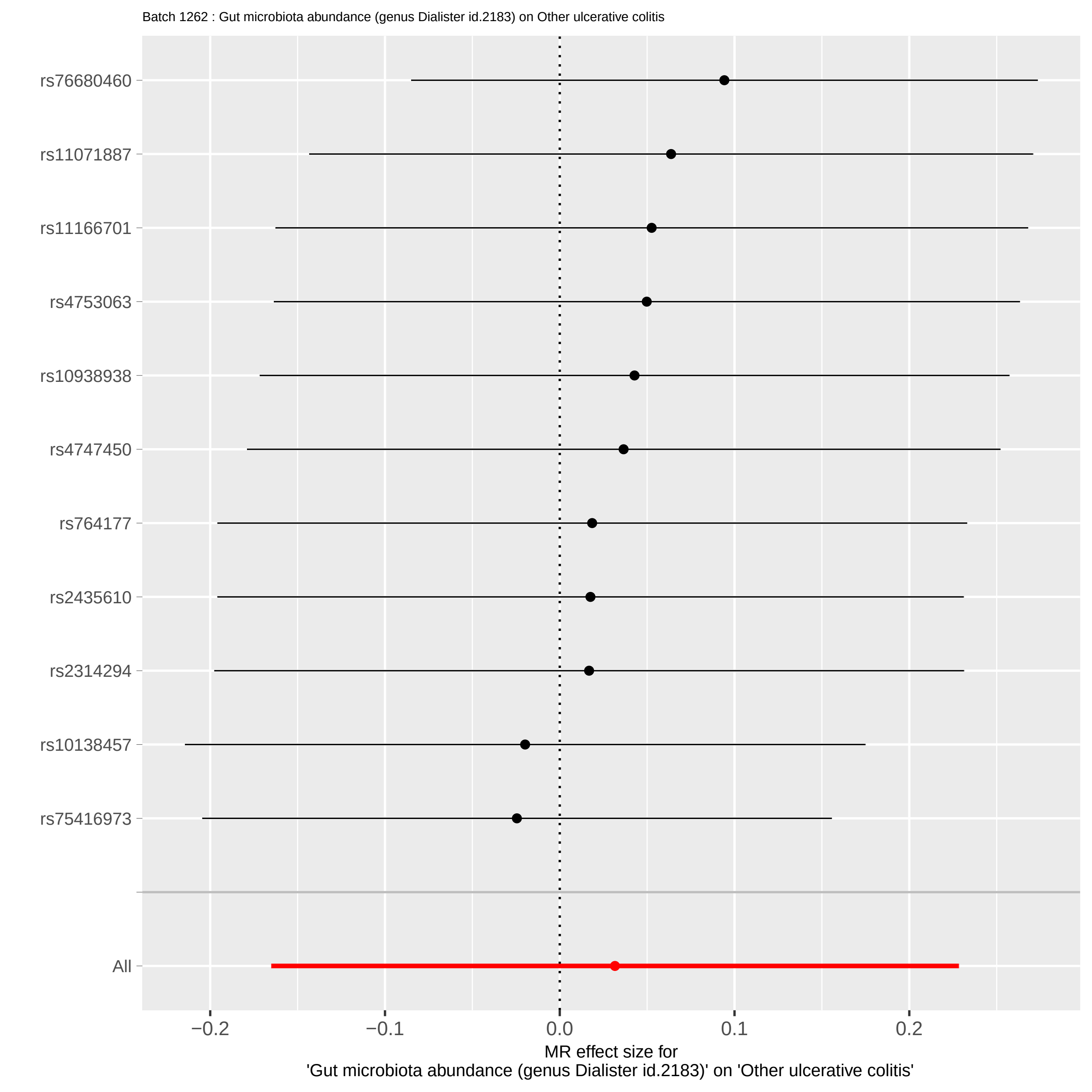

Batch 1262 : Gut microbiota abundance (genus Dialister id.2183) on Other ulcerative colitis
rs76680460
rs11071887
rs11166701
rs4753063
rs10938938
rs4747450
rs764177
rs2435610
rs2314294
rs10138457
rs75416973
All
−0.2
−0.1
0.0
0.1
0.2
MR effect size for
'Gut microbiota abundance (genus Dialister id.2183)' on 'Other ulcerative colitis'

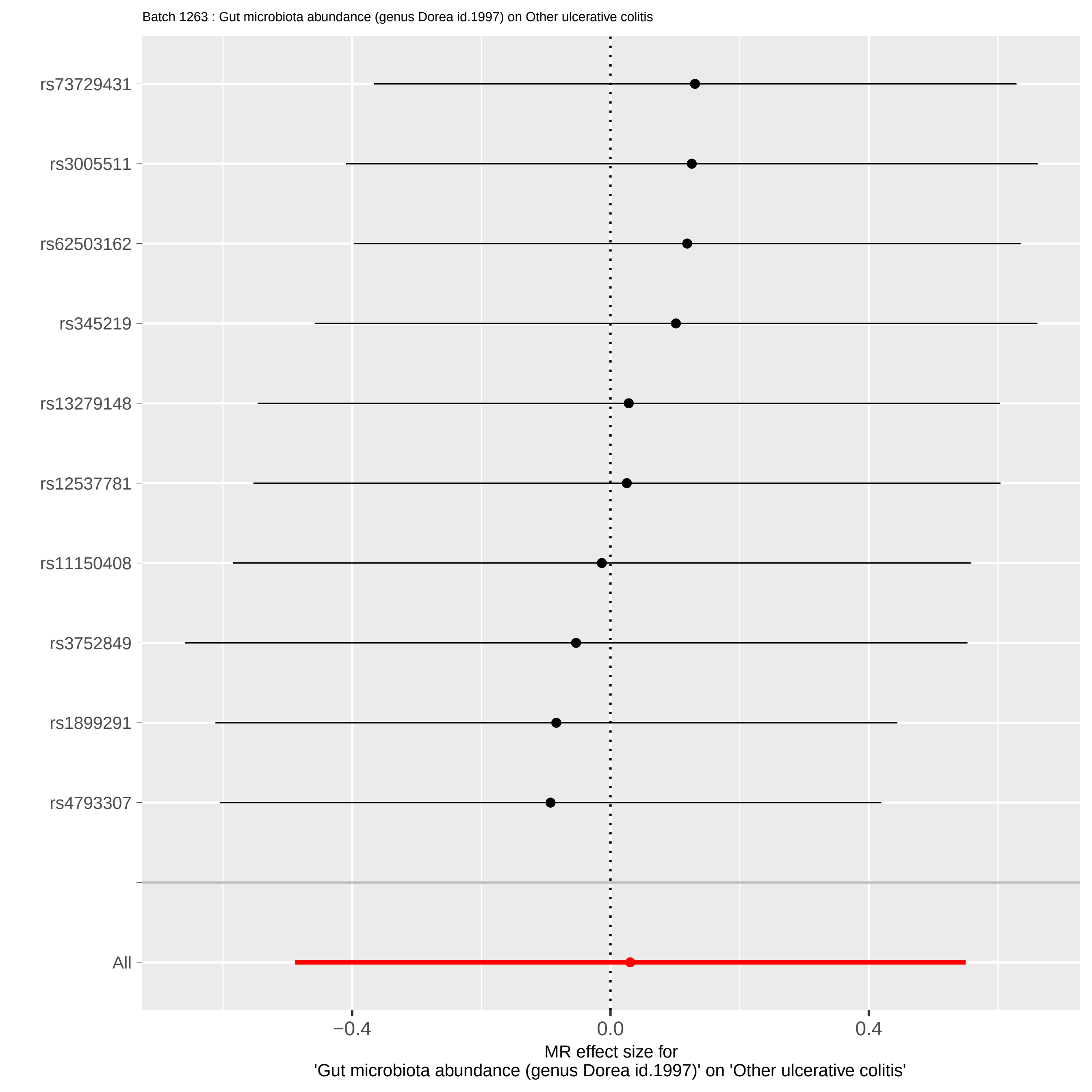

Batch 1263 : Gut microbiota abundance (genus Dorea id.1997) on Other ulcerative colitis
rs73729431
rs3005511
rs62503162
rs345219
rs13279148
rs12537781
rs11150408
rs3752849
rs1899291
rs4793307
All
−0.4
0.0
0.4
MR effect size for
'Gut microbiota abundance (genus Dorea id.1997)' on 'Other ulcerative colitis'

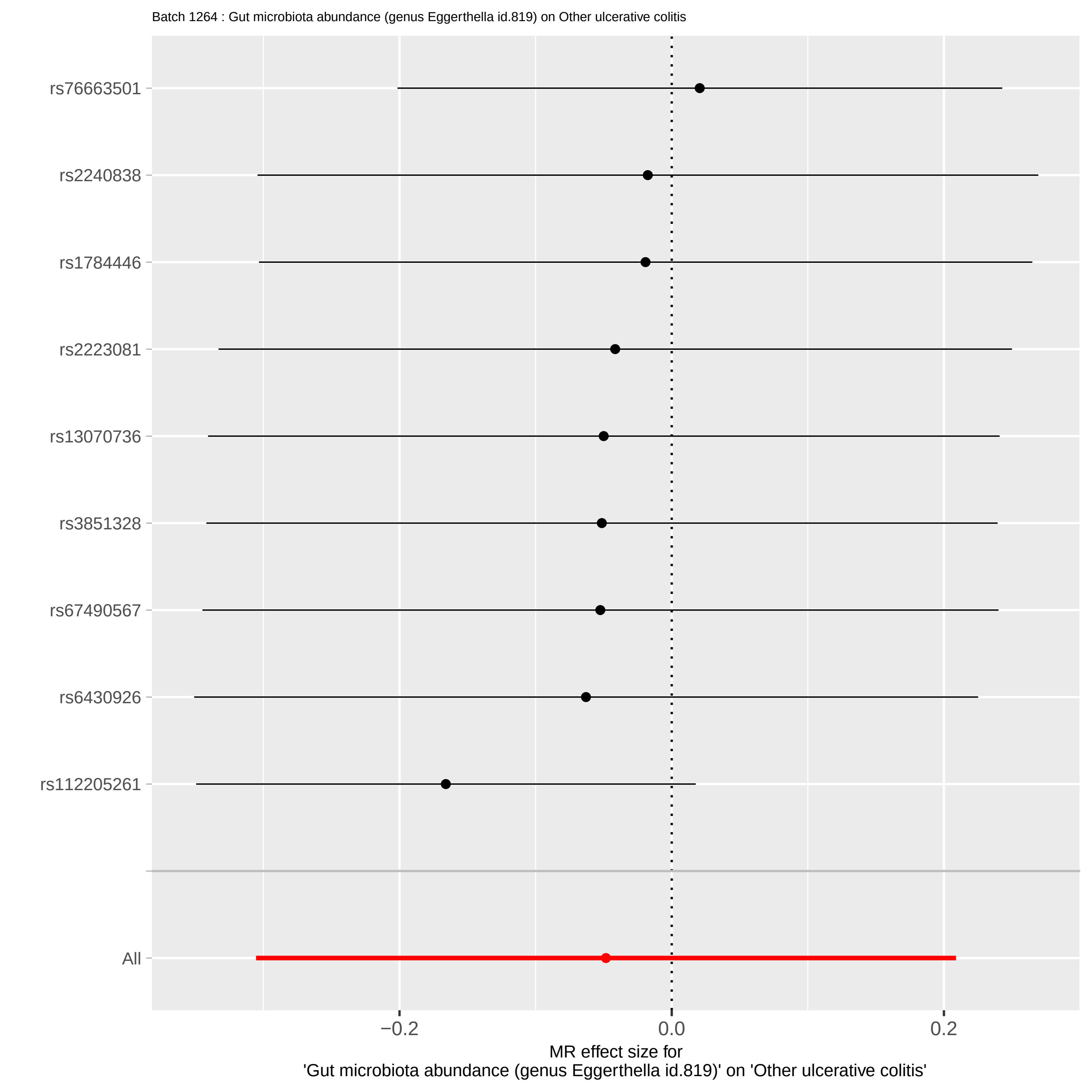

Batch 1264 : Gut microbiota abundance (genus Eggerthella id.819) on Other ulcerative colitis
rs76663501
rs2240838
rs1784446
rs2223081
rs13070736
rs3851328
rs67490567
rs6430926
rs112205261
All
−0.2
0.0
0.2
MR effect size for
'Gut microbiota abundance (genus Eggerthella id.819)' on 'Other ulcerative colitis'

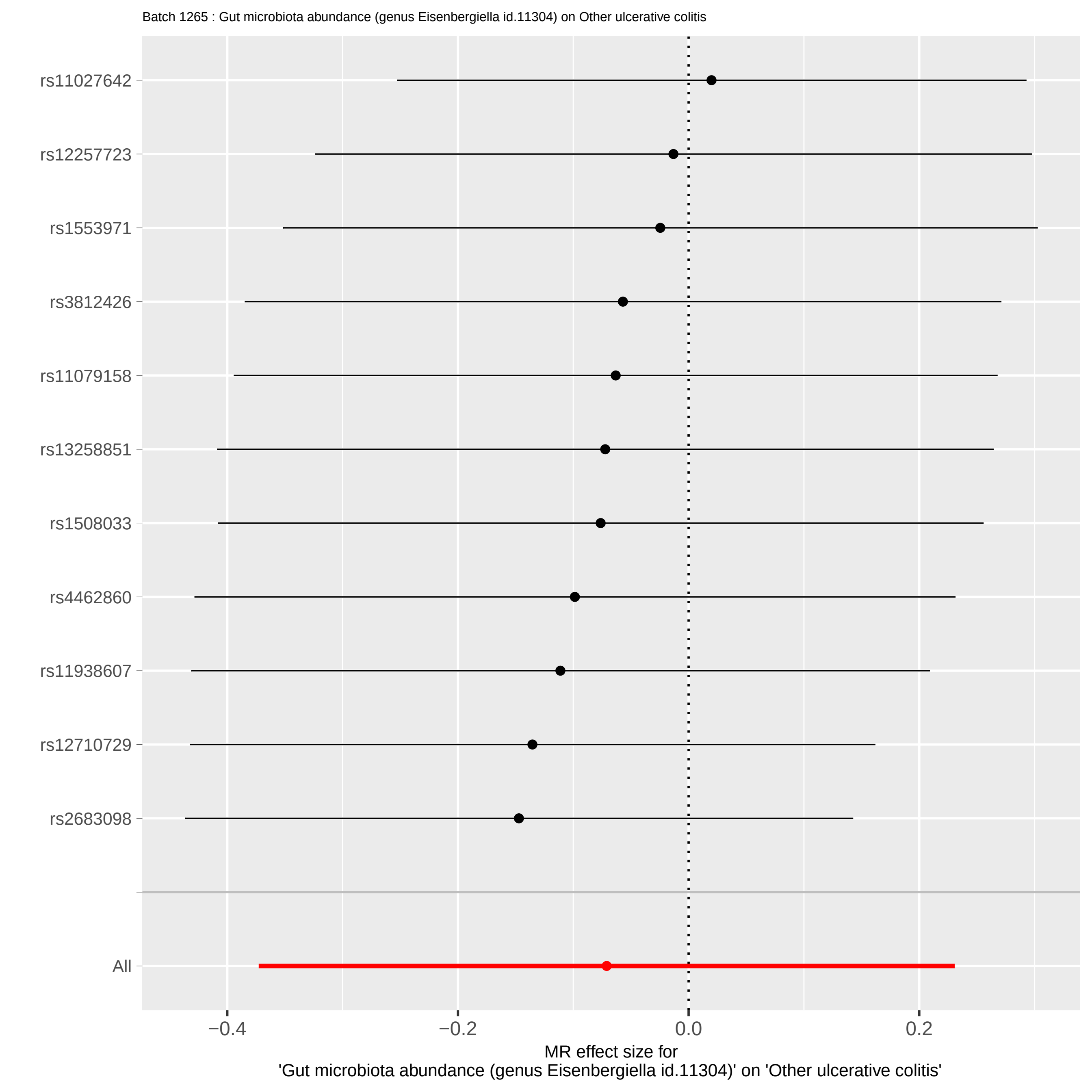
Batch 1265 : Gut microbiota abundance (genus Eisenbergiella id.11304) on Other ulcerative colitis
rs11027642
rs12257723
rs1553971
rs3812426
rs11079158
rs13258851
rs1508033
rs4462860
rs11938607
rs12710729
rs2683098
All
−0.4
−0.2
0.0
0.2
MR effect size for
'Gut microbiota abundance (genus Eisenbergiella id.11304)' on 'Other ulcerative colitis'

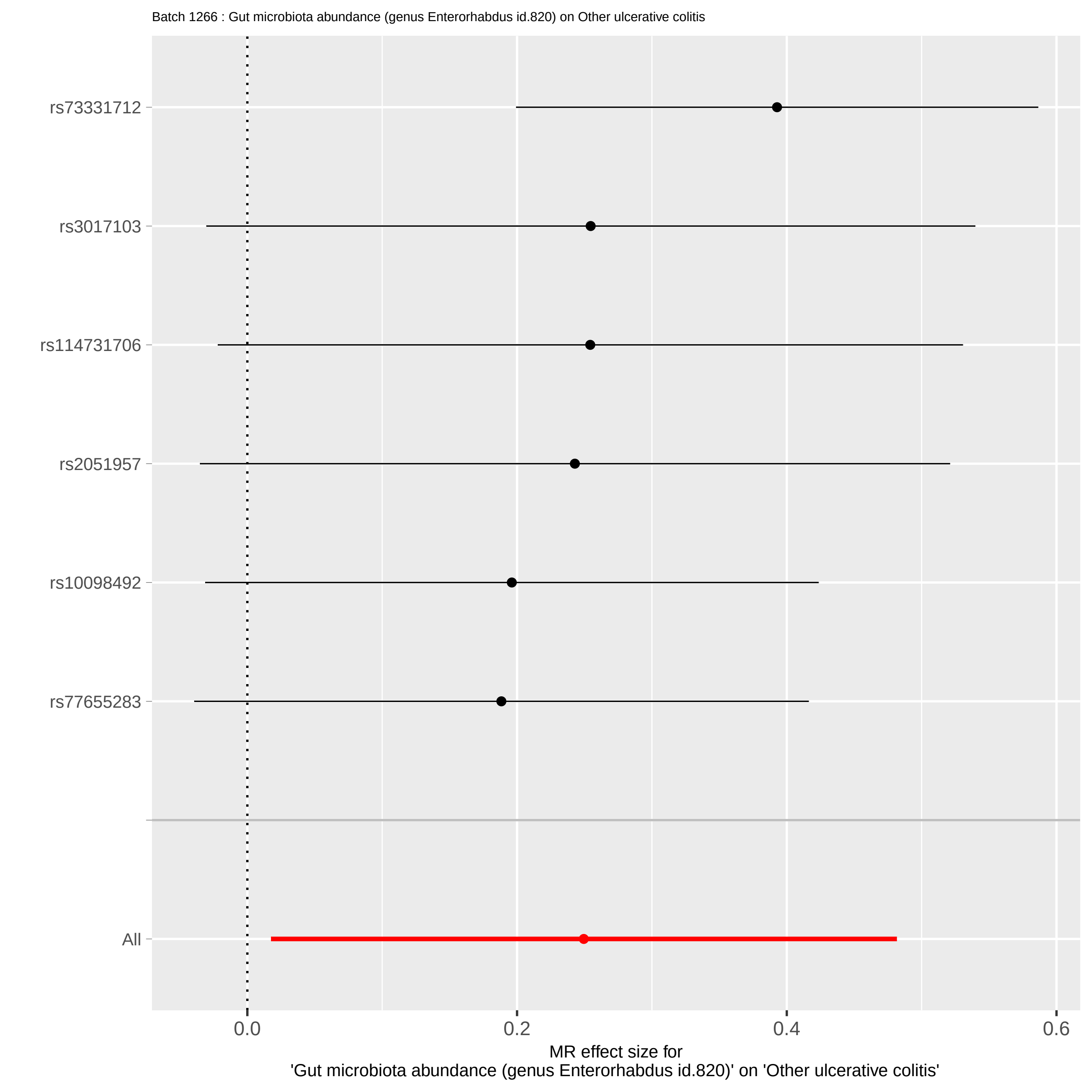

Batch 1266 : Gut microbiota abundance (genus Enterorhabdus id.820) on Other ulcerative colitis
rs73331712
rs3017103
rs114731706
rs2051957
rs10098492
rs77655283
All
0.0
0.2
0.4
0.6
MR effect size for
'Gut microbiota abundance (genus Enterorhabdus id.820)' on 'Other ulcerative colitis'

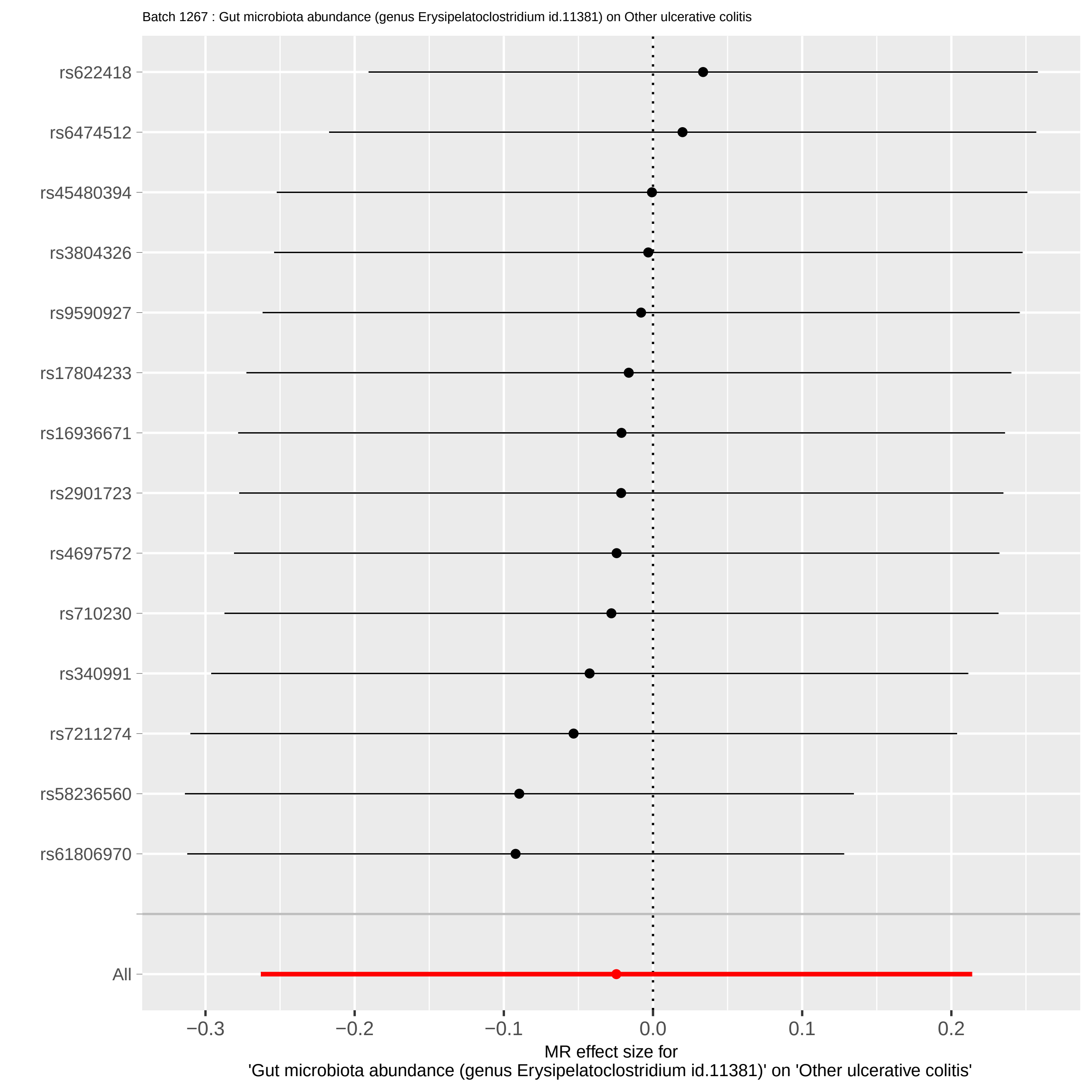

Batch 1267 : Gut microbiota abundance (genus Erysipelatoclostridium id.11381) on Other ulcerative colitis
rs622418
rs6474512
rs45480394
rs3804326
rs9590927
rs17804233
rs16936671
rs2901723
rs4697572
rs710230
rs340991
rs7211274
rs58236560
rs61806970
All
−0.3
−0.2
−0.1
0.0
0.1
0.2
MR effect size for
'Gut microbiota abundance (genus Erysipelatoclostridium id.11381)' on 'Other ulcerative colitis'

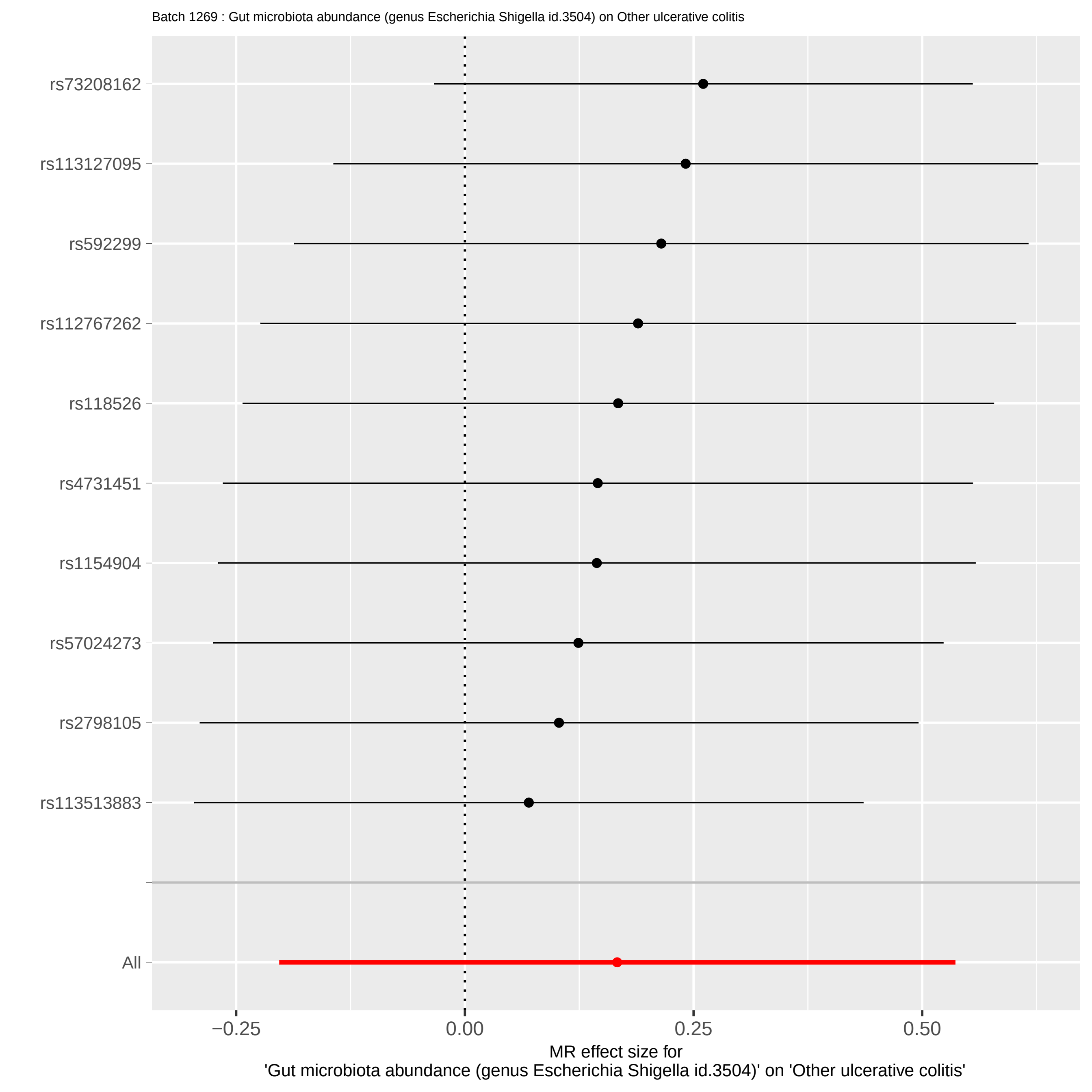

Batch 1269 : Gut microbiota abundance (genus Escherichia Shigella id.3504) on Other ulcerative colitis
rs73208162
rs113127095
rs592299
rs112767262
rs118526
rs4731451
rs1154904
rs57024273
rs2798105
rs113513883
All
−0.25
0.00
0.25
0.50
MR effect size for
'Gut microbiota abundance (genus Escherichia Shigella id.3504)' on 'Other ulcerative colitis'

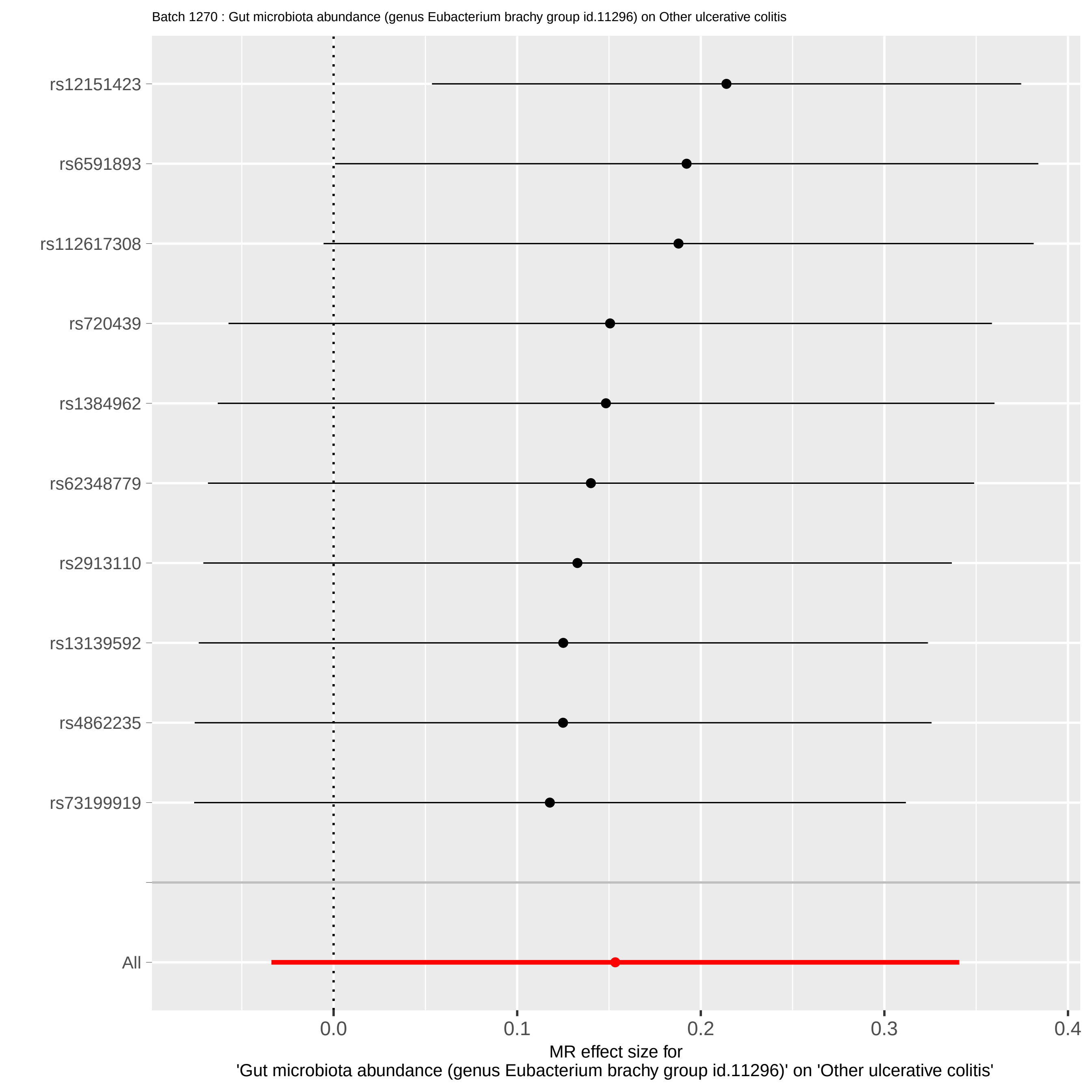

Batch 1270 : Gut microbiota abundance (genus Eubacterium brachy group id.11296) on Other ulcerative colitis
rs12151423
rs6591893
rs112617308
rs720439
rs1384962
rs62348779
rs2913110
rs13139592
rs4862235
rs73199919
All
0.0
0.1
0.2
0.3
0.4
MR effect size for
'Gut microbiota abundance (genus Eubacterium brachy group id.11296)' on 'Other ulcerative colitis'

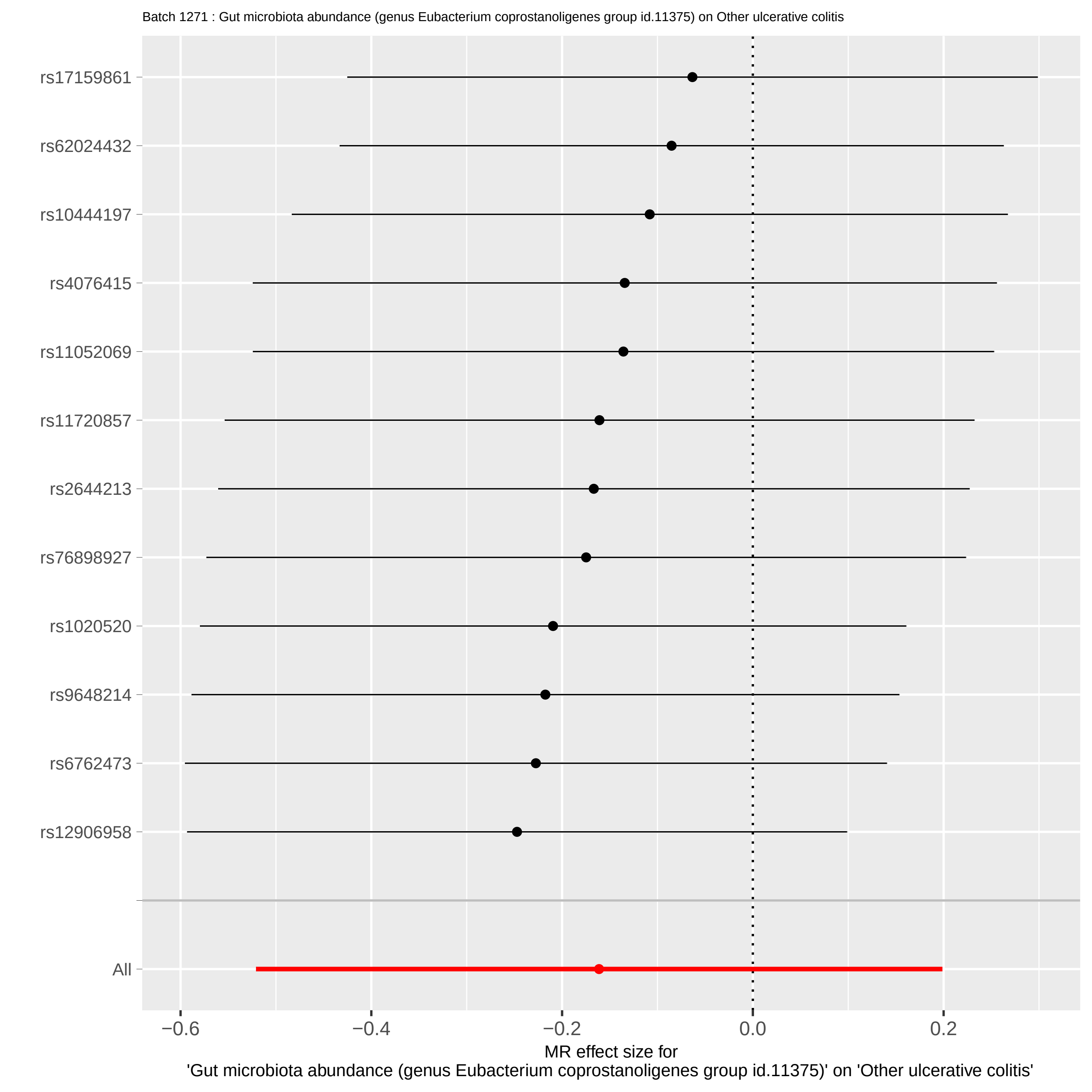

Batch 1271 : Gut microbiota abundance (genus Eubacterium coprostanoligenes group id.11375) on Other ulcerative colitis
rs17159861
rs62024432
rs10444197
rs4076415
rs11052069
rs11720857
rs2644213
rs76898927
rs1020520
rs9648214
rs6762473
rs12906958
All
−0.6
−0.4
−0.2
0.0
0.2
MR effect size for
'Gut microbiota abundance (genus Eubacterium coprostanoligenes group id.11375)' on 'Other ulcerative colitis'

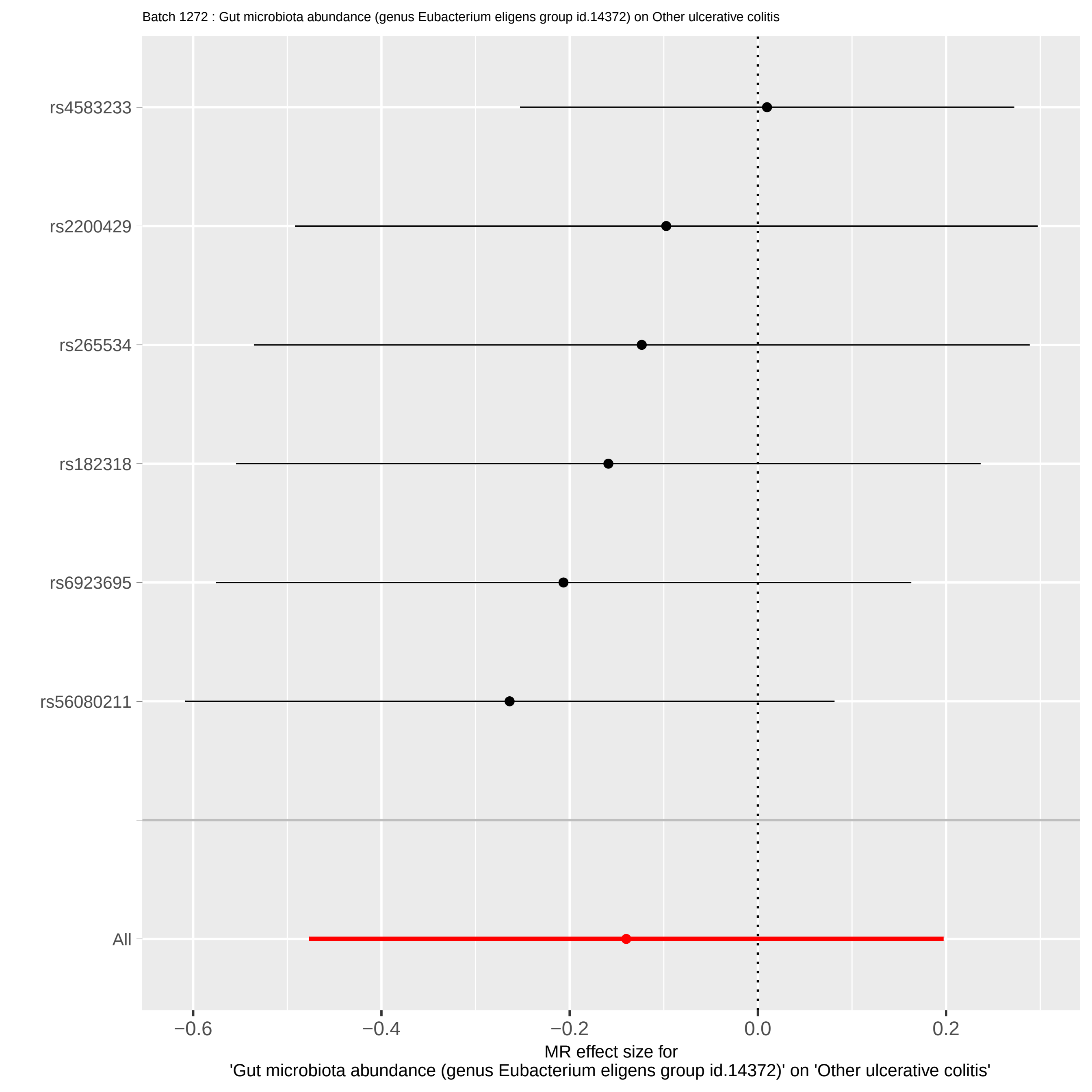

Batch 1272 : Gut microbiota abundance (genus Eubacterium eligens group id.14372) on Other ulcerative colitis
rs4583233
rs2200429
rs265534
rs182318
rs6923695
rs56080211
All
−0.6
−0.4
−0.2
0.0
0.2
MR effect size for
'Gut microbiota abundance (genus Eubacterium eligens group id.14372)' on 'Other ulcerative colitis'

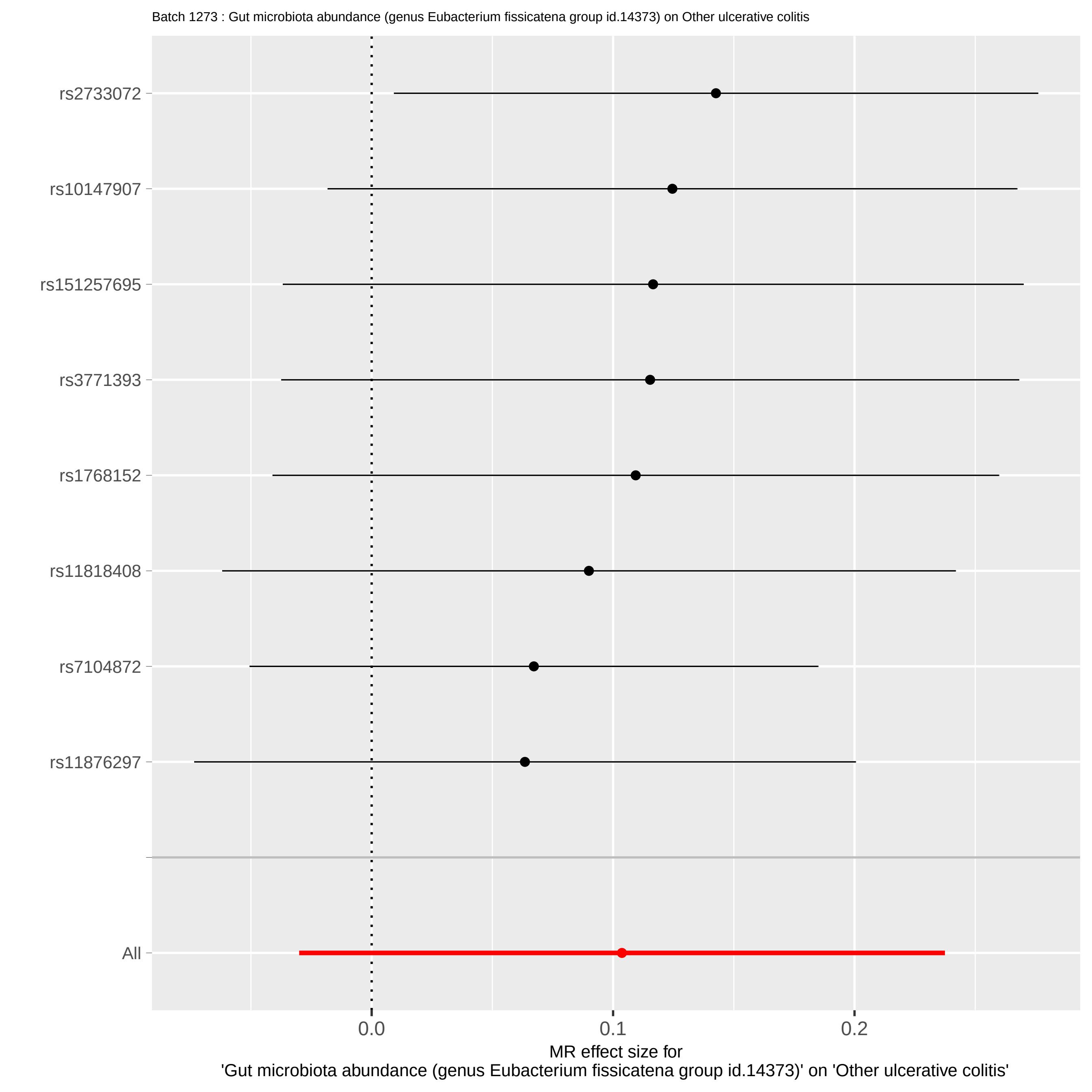

Batch 1273 : Gut microbiota abundance (genus Eubacterium fissicatena group id.14373) on Other ulcerative colitis
rs2733072
rs10147907
rs151257695
rs3771393
rs1768152
rs11818408
rs7104872
rs11876297
All
0.0
0.1
0.2
MR effect size for
'Gut microbiota abundance (genus Eubacterium fissicatena group id.14373)' on 'Other ulcerative colitis'

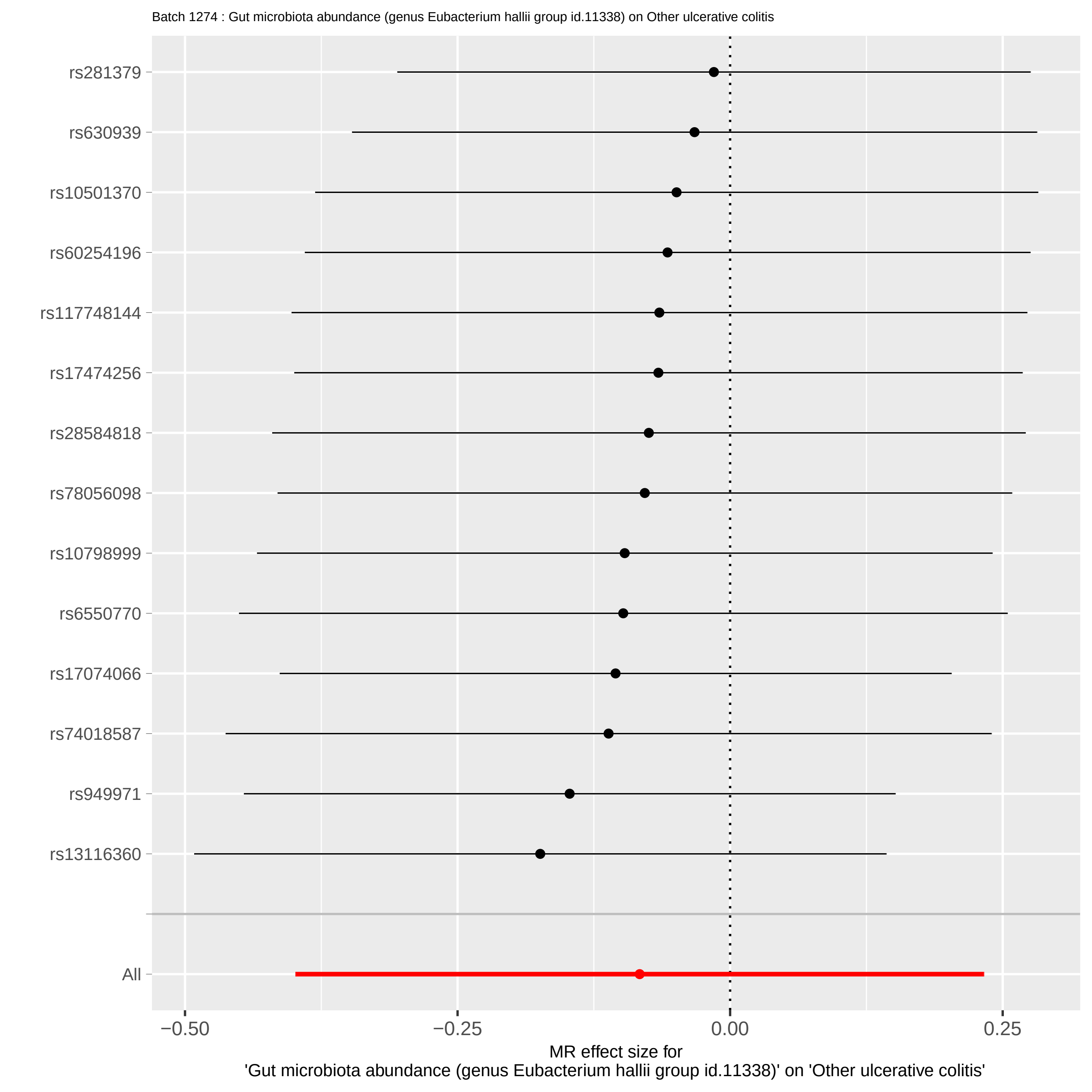

Batch 1274 : Gut microbiota abundance (genus Eubacterium hallii group id.11338) on Other ulcerative colitis
rs281379
rs630939
rs10501370
rs60254196
rs117748144
rs17474256
rs28584818
rs78056098
rs10798999
rs6550770
rs17074066
rs74018587
rs949971
rs13116360
All
−0.50
−0.25
0.00
0.25
MR effect size for
'Gut microbiota abundance (genus Eubacterium hallii group id.11338)' on 'Other ulcerative colitis'

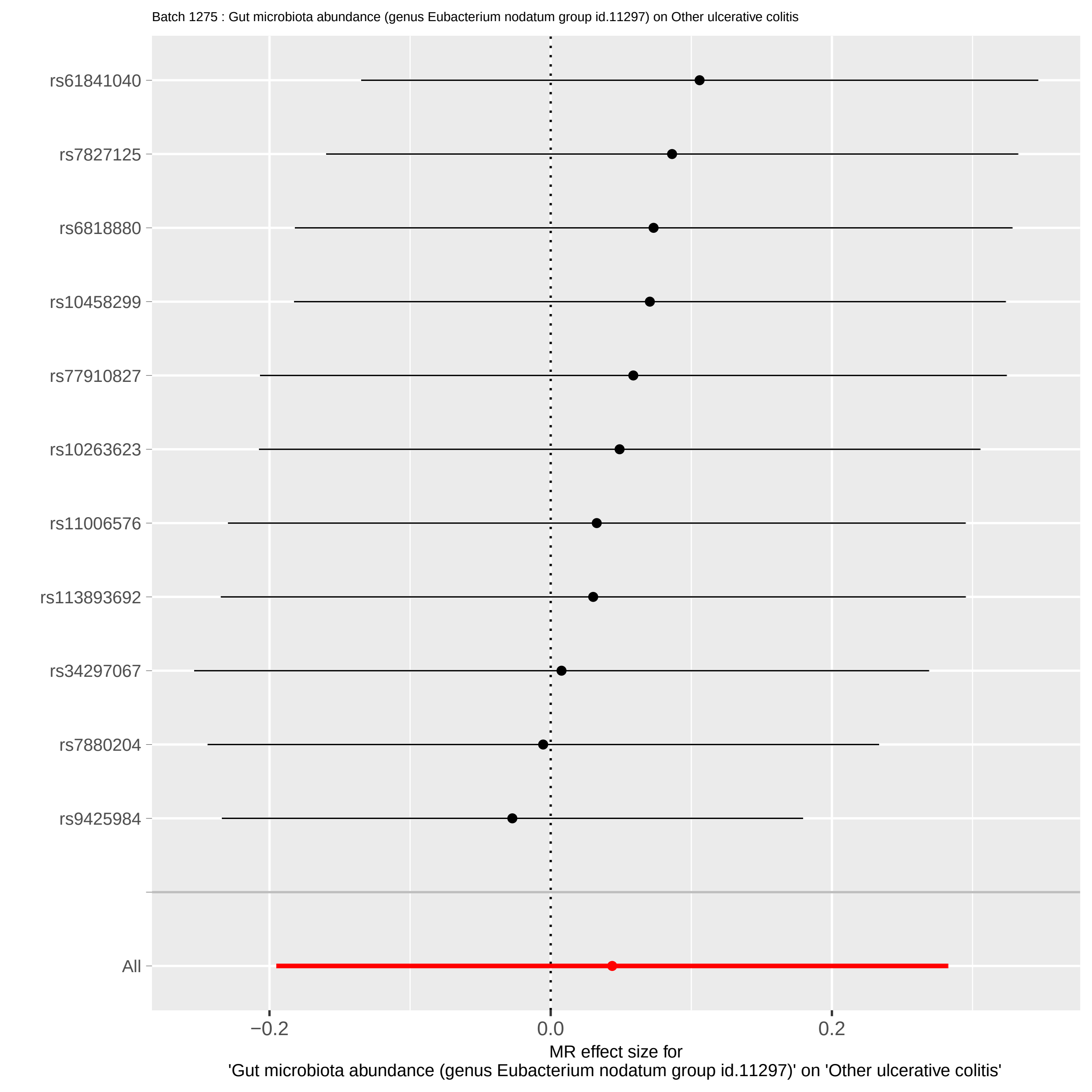

Batch 1275 : Gut microbiota abundance (genus Eubacterium nodatum group id.11297) on Other ulcerative colitis
rs61841040
rs7827125
rs6818880
rs10458299
rs77910827
rs10263623
rs11006576
rs113893692
rs34297067
rs7880204
rs9425984
All
−0.2
0.0
0.2
MR effect size for
'Gut microbiota abundance (genus Eubacterium nodatum group id.11297)' on 'Other ulcerative colitis'

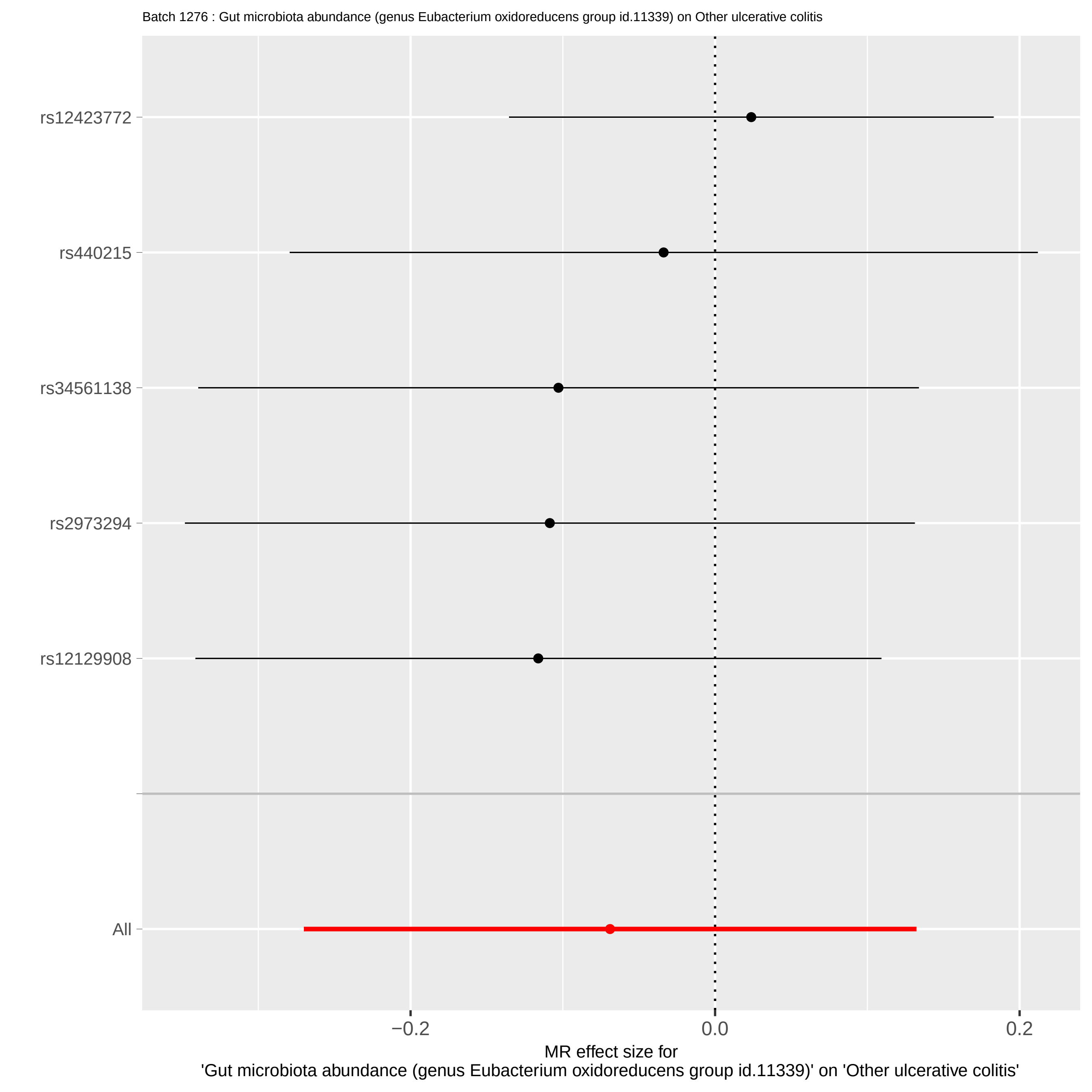

Batch 1276 : Gut microbiota abundance (genus Eubacterium oxidoreducens group id.11339) on Other ulcerative colitis
rs12423772
rs440215
rs34561138
rs2973294
rs12129908
All
−0.2
0.0
0.2
MR effect size for
'Gut microbiota abundance (genus Eubacterium oxidoreducens group id.11339)' on 'Other ulcerative colitis'

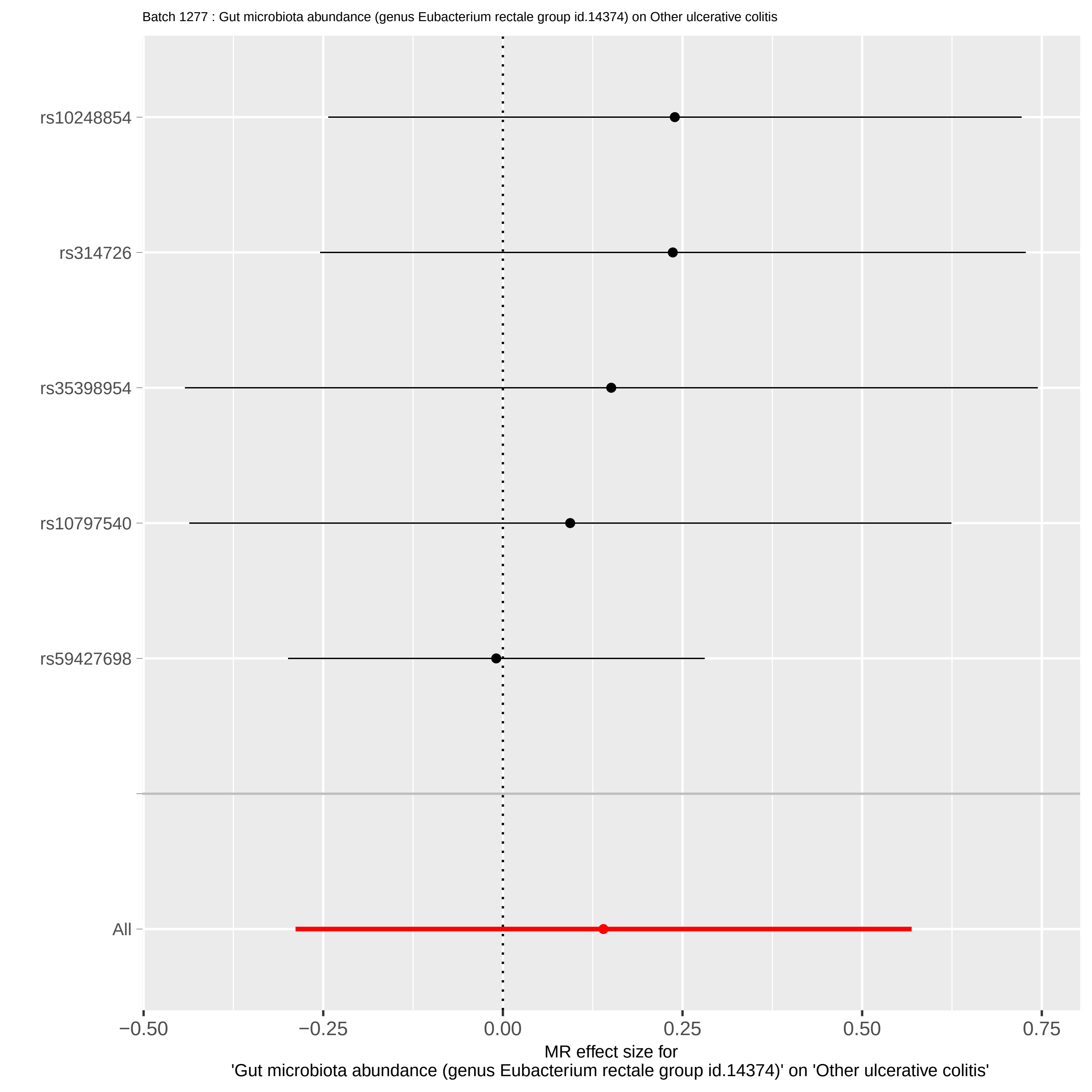

Batch 1277 : Gut microbiota abundance (genus Eubacterium rectale group id.14374) on Other ulcerative colitis
rs10248854
rs314726
rs35398954
rs10797540
rs59427698
All
−0.50
−0.25
0.00
0.25
0.50
0.75
MR effect size for
'Gut microbiota abundance (genus Eubacterium rectale group id.14374)' on 'Other ulcerative colitis'

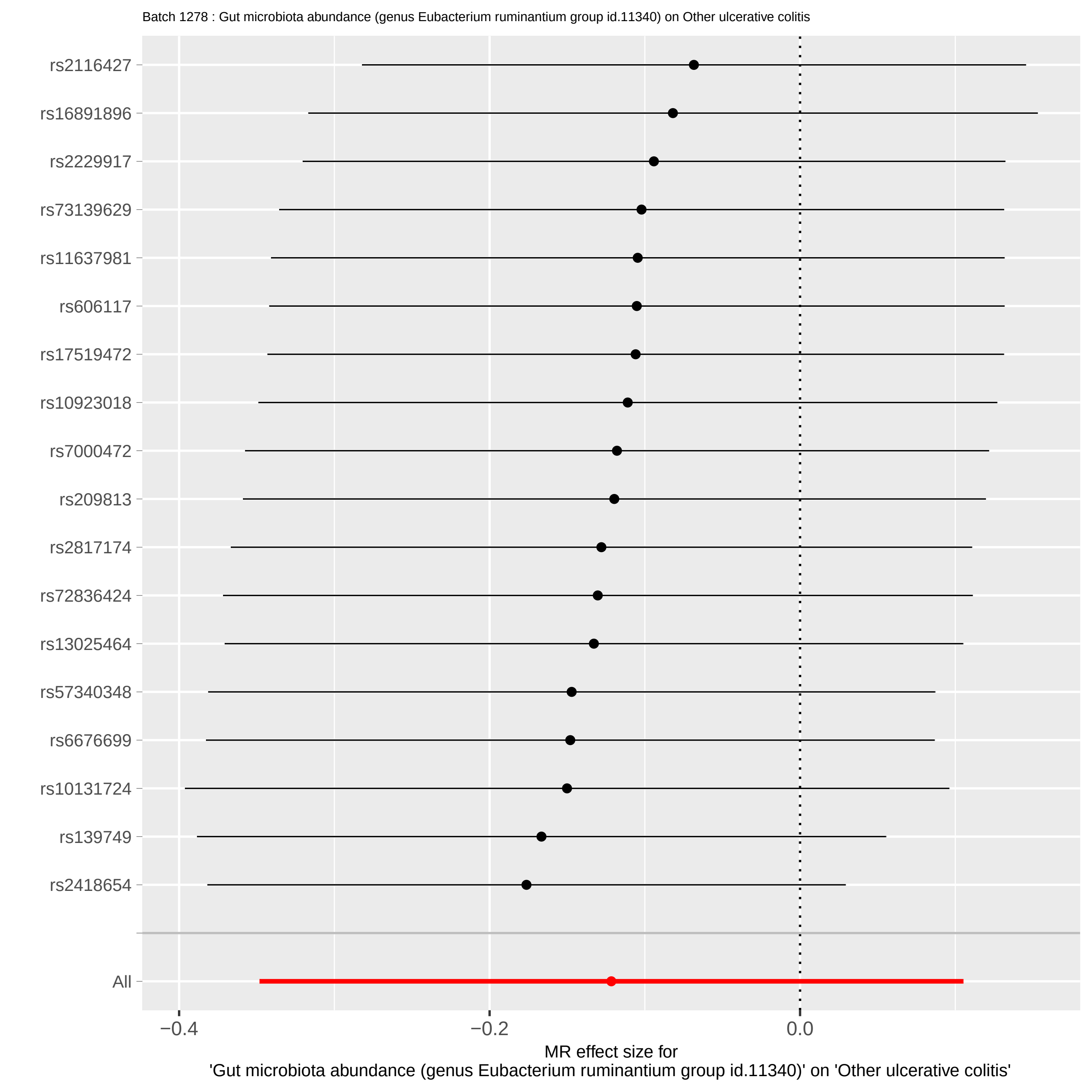
Batch 1278 : Gut microbiota abundance (genus Eubacterium ruminantium group id.11340) on Other ulcerative colitis
rs2116427
rs16891896
rs2229917
rs73139629
rs11637981
rs606117
rs17519472
rs10923018
rs7000472
rs209813
rs2817174
rs72836424
rs13025464
rs57340348
rs6676699
rs10131724
rs139749
rs2418654
All
−0.4
−0.2
0.0
MR effect size for
'Gut microbiota abundance (genus Eubacterium ruminantium group id.11340)' on 'Other ulcerative colitis'

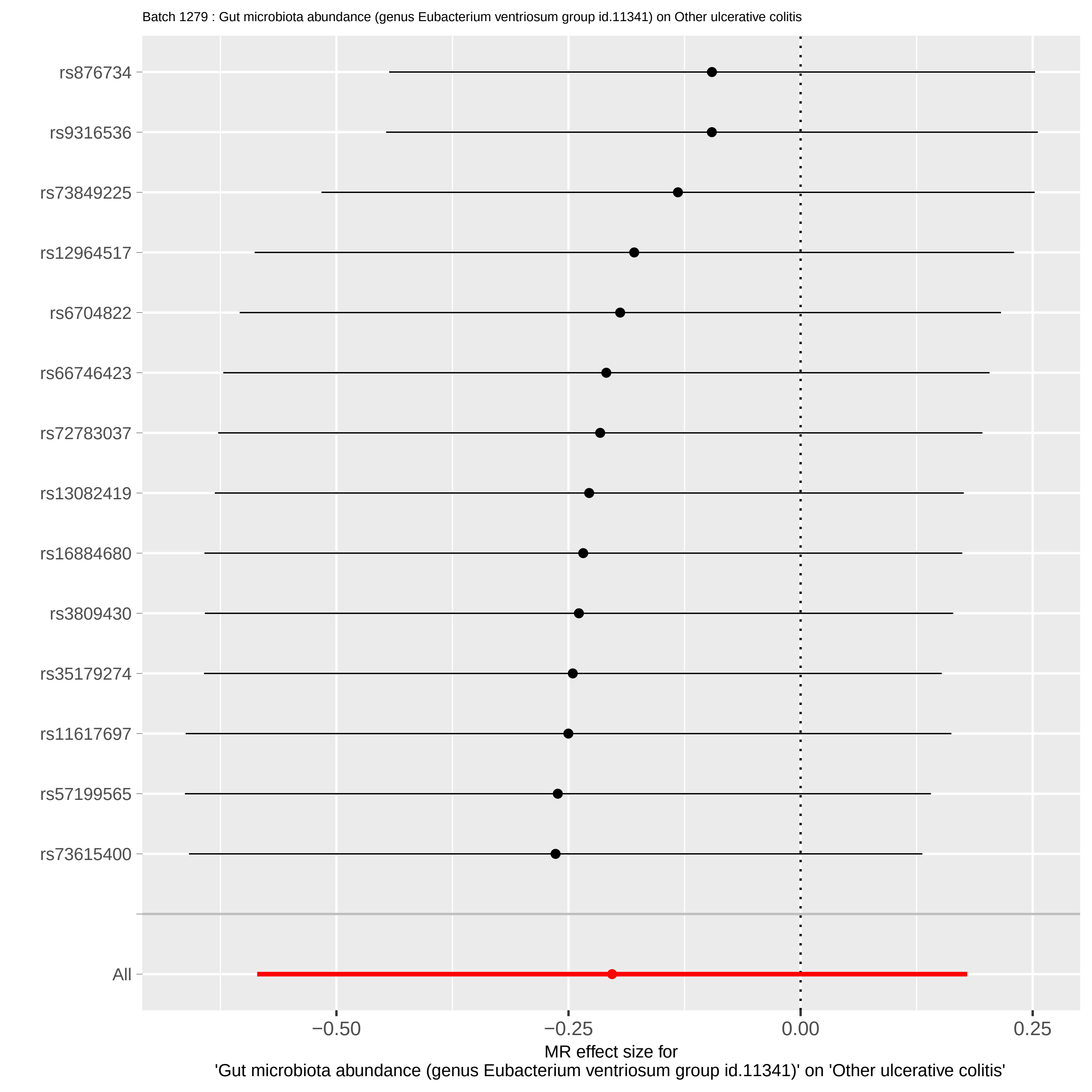

Batch 1279 : Gut microbiota abundance (genus Eubacterium ventriosum group id.11341) on Other ulcerative colitis
rs876734
rs9316536
rs73849225
rs12964517
rs6704822
rs66746423
rs72783037
rs13082419
rs16884680
rs3809430
rs35179274
rs11617697
rs57199565
rs73615400
All
−0.50
−0.25
0.00
0.25
MR effect size for
'Gut microbiota abundance (genus Eubacterium ventriosum group id.11341)' on 'Other ulcerative colitis'

Batch 1280 : Gut microbiota abundance (genus Eubacterium xylanophilum group id.14375) on Other ulcerative colitis

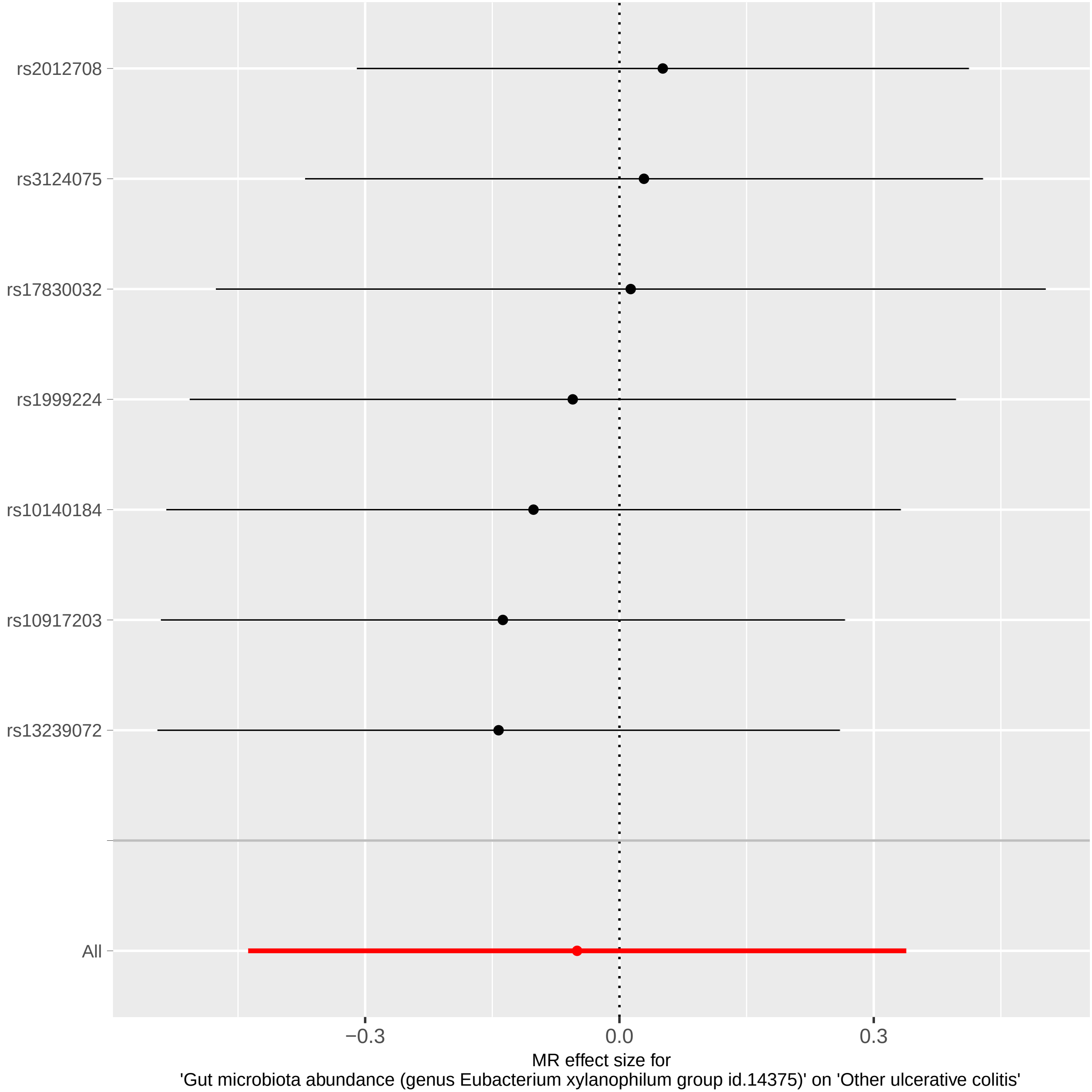

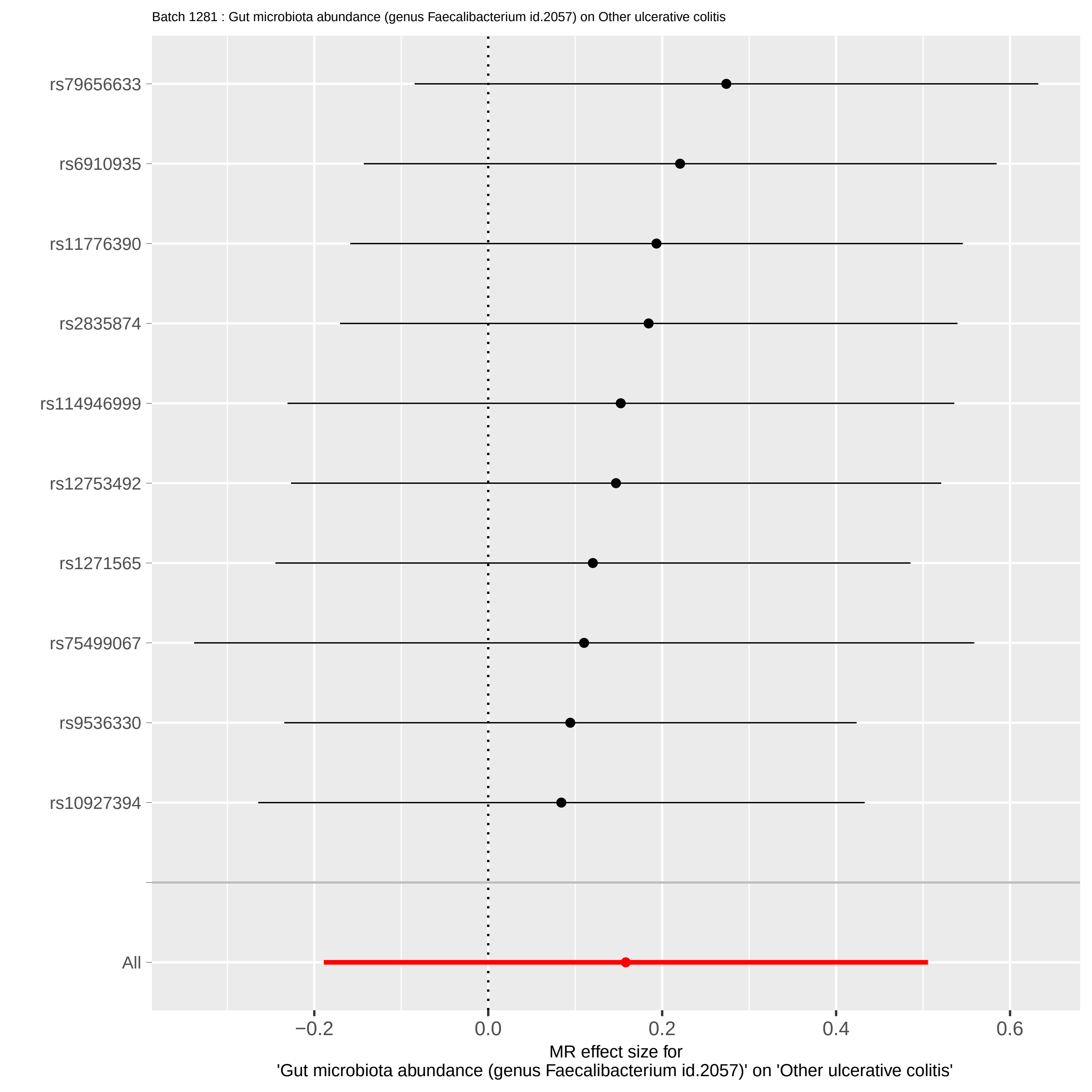
Batch 1281 : Gut microbiota abundance (genus Faecalibacterium id.2057) on Other ulcerative colitis
rs79656633
rs6910935
rs11776390
rs2835874
rs114946999
rs12753492
rs1271565
rs75499067
rs9536330
rs10927394
All
−0.2
0.0
0.2
0.4
0.6
MR effect size for
'Gut microbiota abundance (genus Faecalibacterium id.2057)' on 'Other ulcerative colitis'

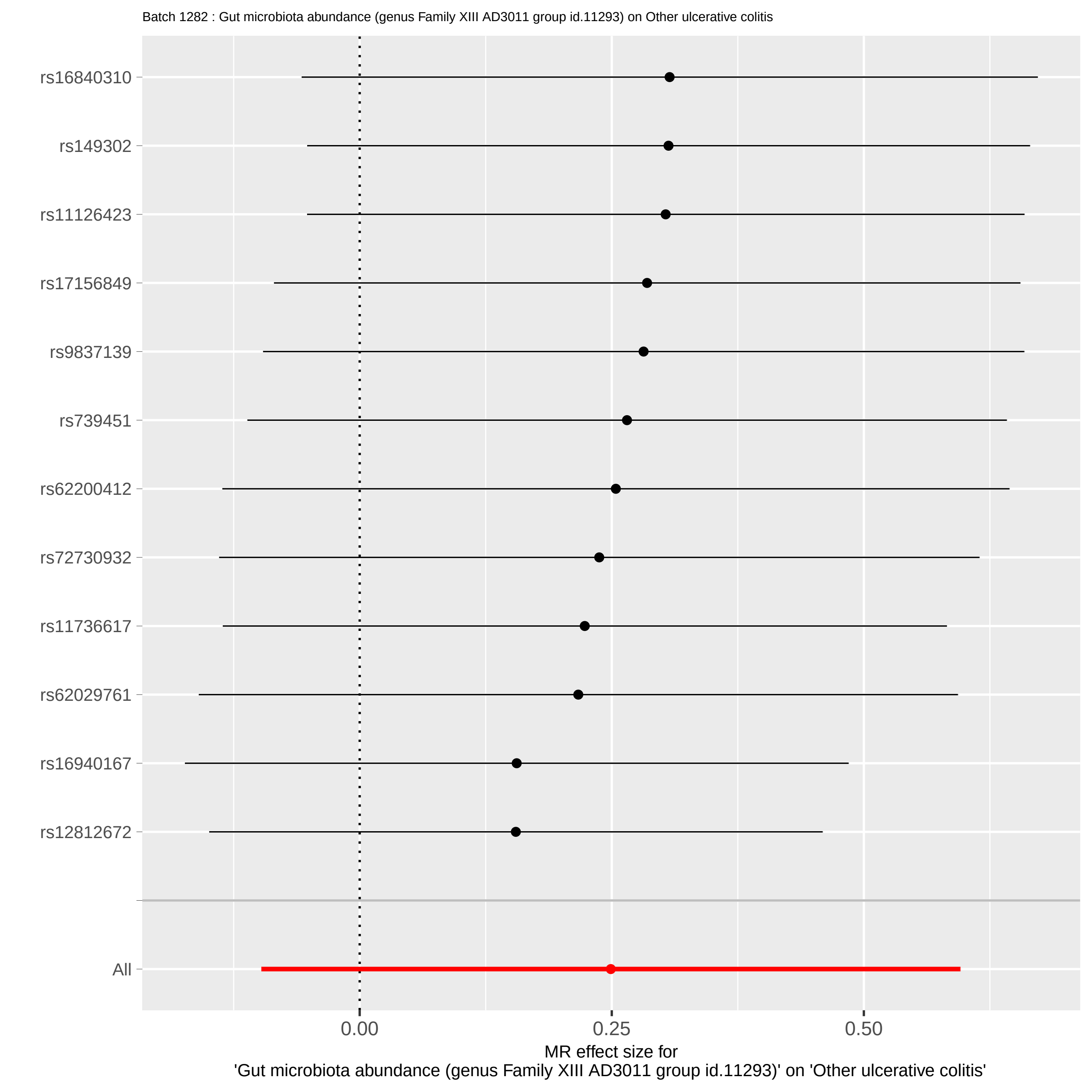

Batch 1282 : Gut microbiota abundance (genus Family XIII AD3011 group id.11293) on Other ulcerative colitis
rs16840310
rs149302
rs11126423
rs17156849
rs9837139
rs739451
rs62200412
rs72730932
rs11736617
rs62029761
rs16940167
rs12812672
All
0.00
0.25
0.50
MR effect size for
'Gut microbiota abundance (genus Family XIII AD3011 group id.11293)' on 'Other ulcerative colitis'

Batch 1283 : Gut microbiota abundance (genus Family XIII UCG001 id.11294) on Other ulcerative colitis

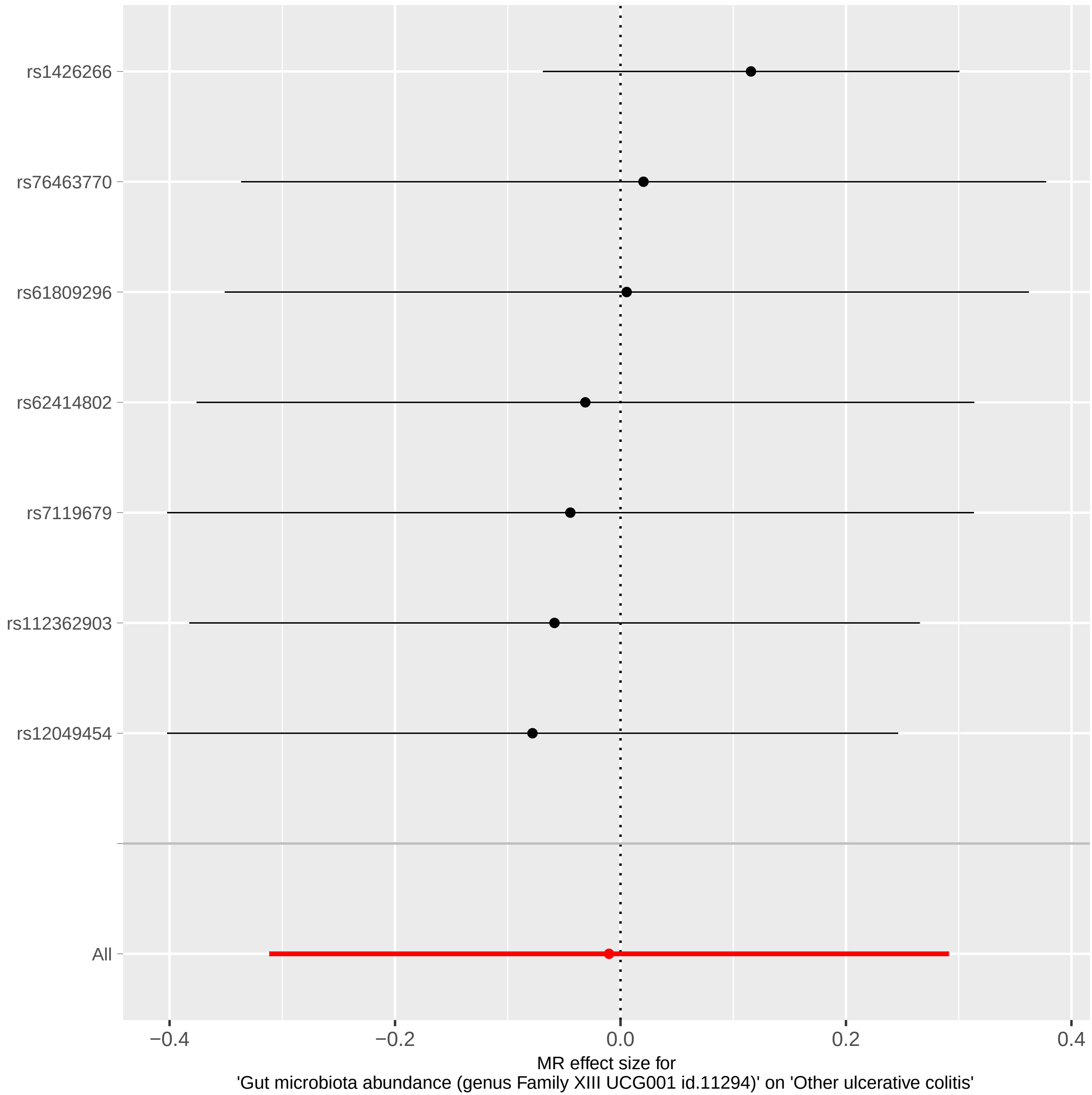

Batch 1284 : Gut microbiota abundance (genus Flavonifractor id.2059) on Other ulcerative colitis

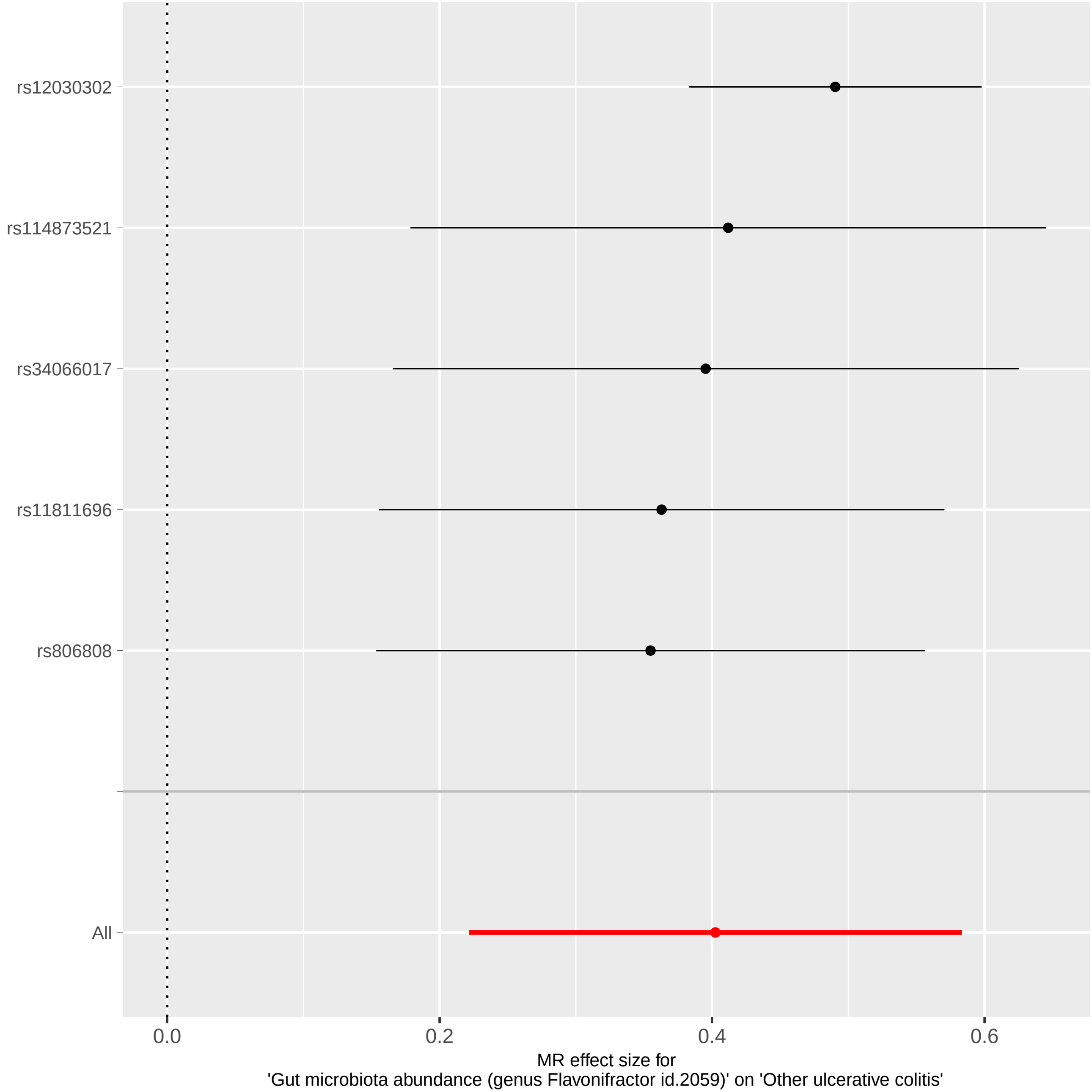

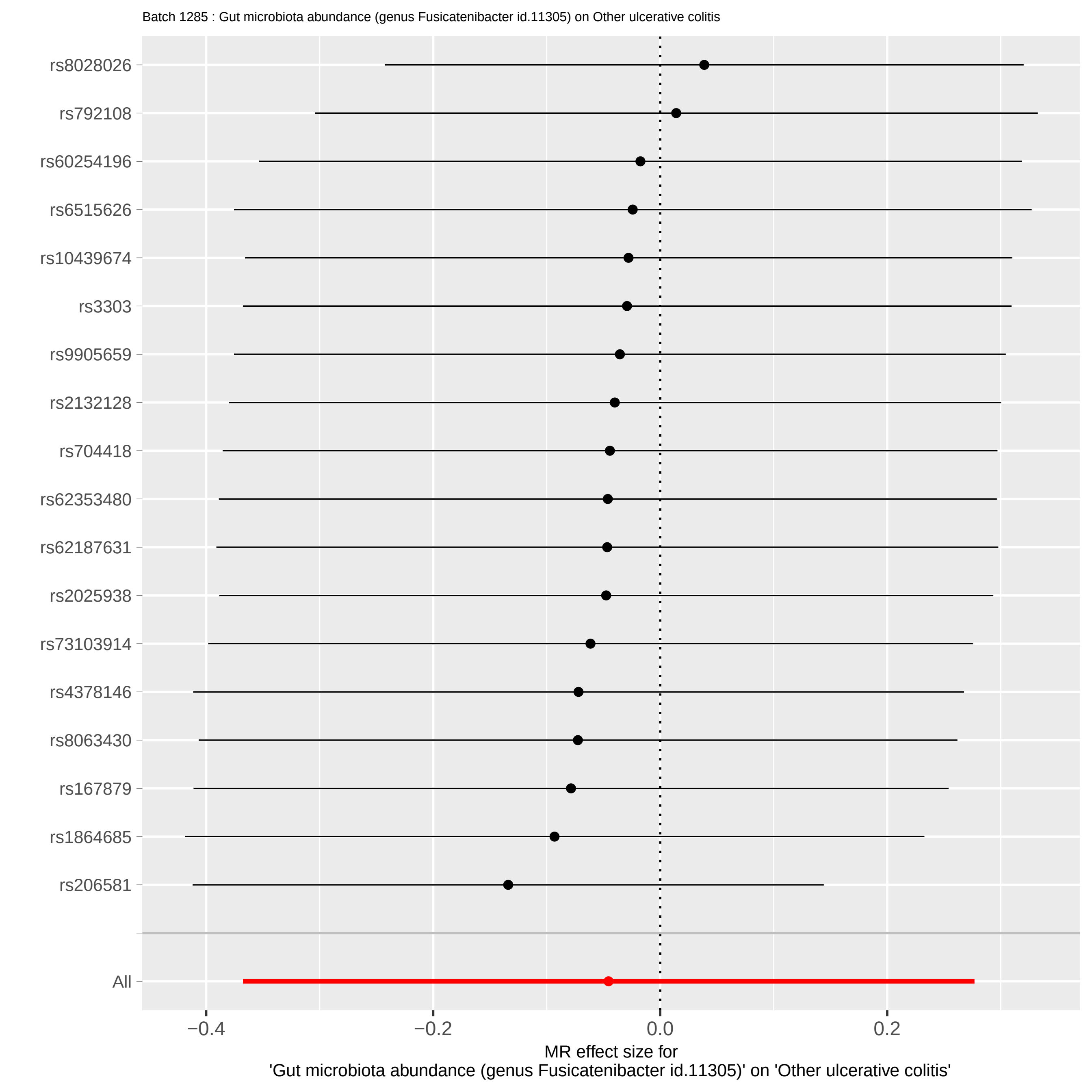
Batch 1285 : Gut microbiota abundance (genus Fusicatenibacter id.11305) on Other ulcerative colitis
rs8028026
rs792108
rs60254196
rs6515626
rs10439674
rs3303
rs9905659
rs2132128
rs704418
rs62353480
rs62187631
rs2025938
rs73103914
rs4378146
rs8063430
rs167879
rs1864685
rs206581
All
−0.4
−0.2
0.0
0.2
MR effect size for
'Gut microbiota abundance (genus Fusicatenibacter id.11305)' on 'Other ulcerative colitis'

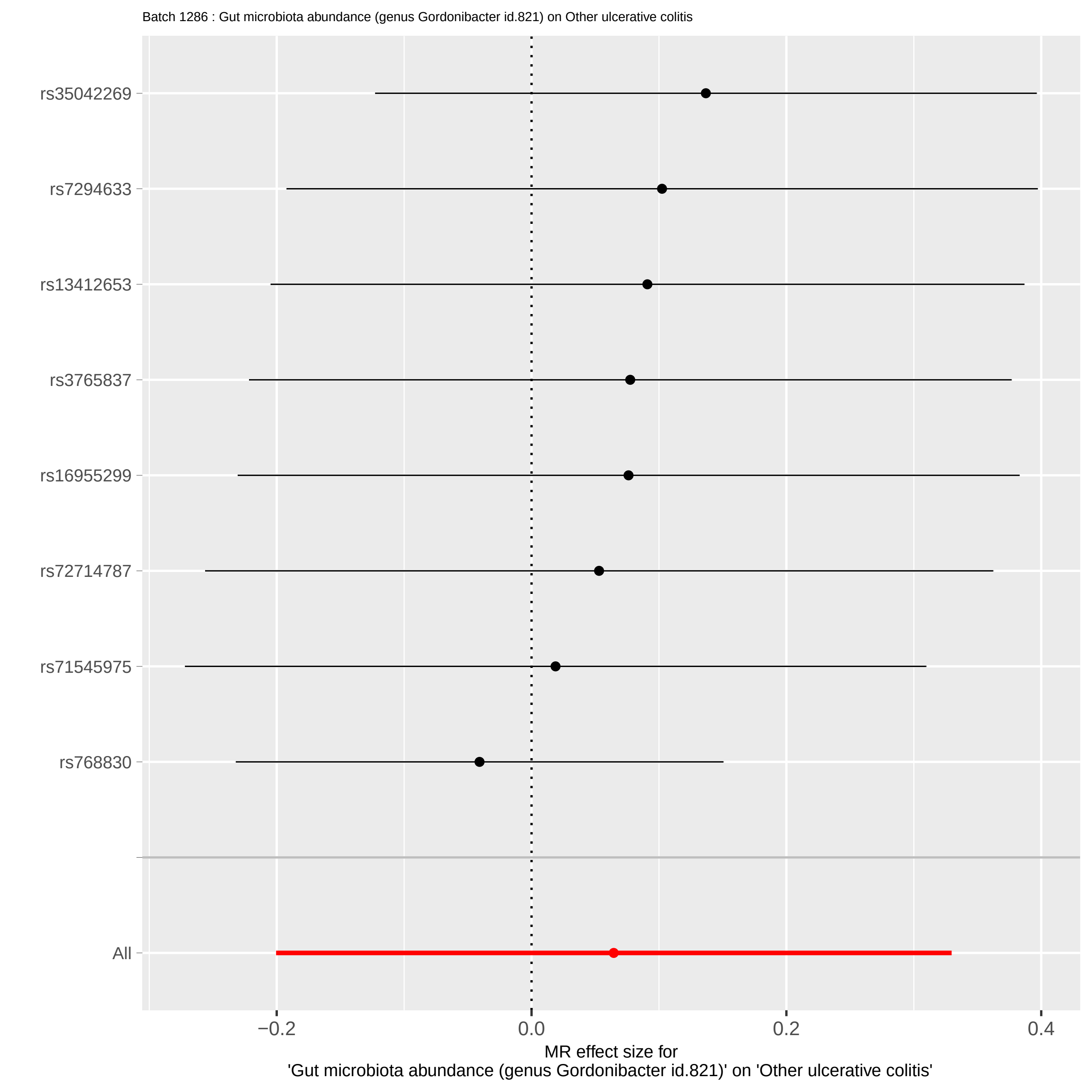

Batch 1286 : Gut microbiota abundance (genus Gordonibacter id.821) on Other ulcerative colitis
rs35042269
rs7294633
rs13412653
rs3765837
rs16955299
rs72714787
rs71545975
rs768830
All
−0.2
0.0
0.2
0.4
MR effect size for
'Gut microbiota abundance (genus Gordonibacter id.821)' on 'Other ulcerative colitis'

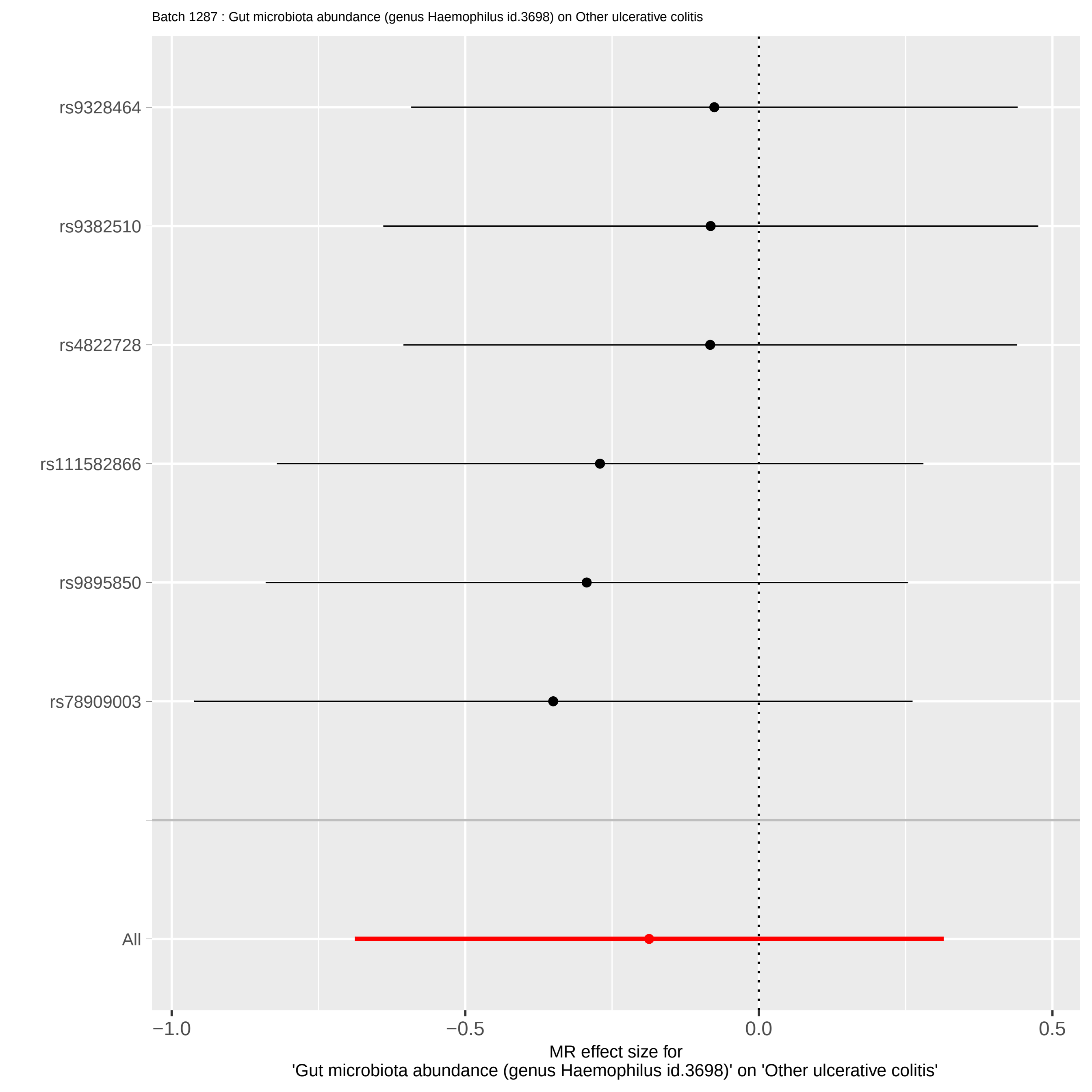
Batch 1287 : Gut microbiota abundance (genus Haemophilus id.3698) on Other ulcerative colitis
rs9328464
rs9382510
rs4822728
rs111582866
rs9895850
rs78909003
All
−1.0
−0.5
0.0
0.5
MR effect size for
'Gut microbiota abundance (genus Haemophilus id.3698)' on 'Other ulcerative colitis'

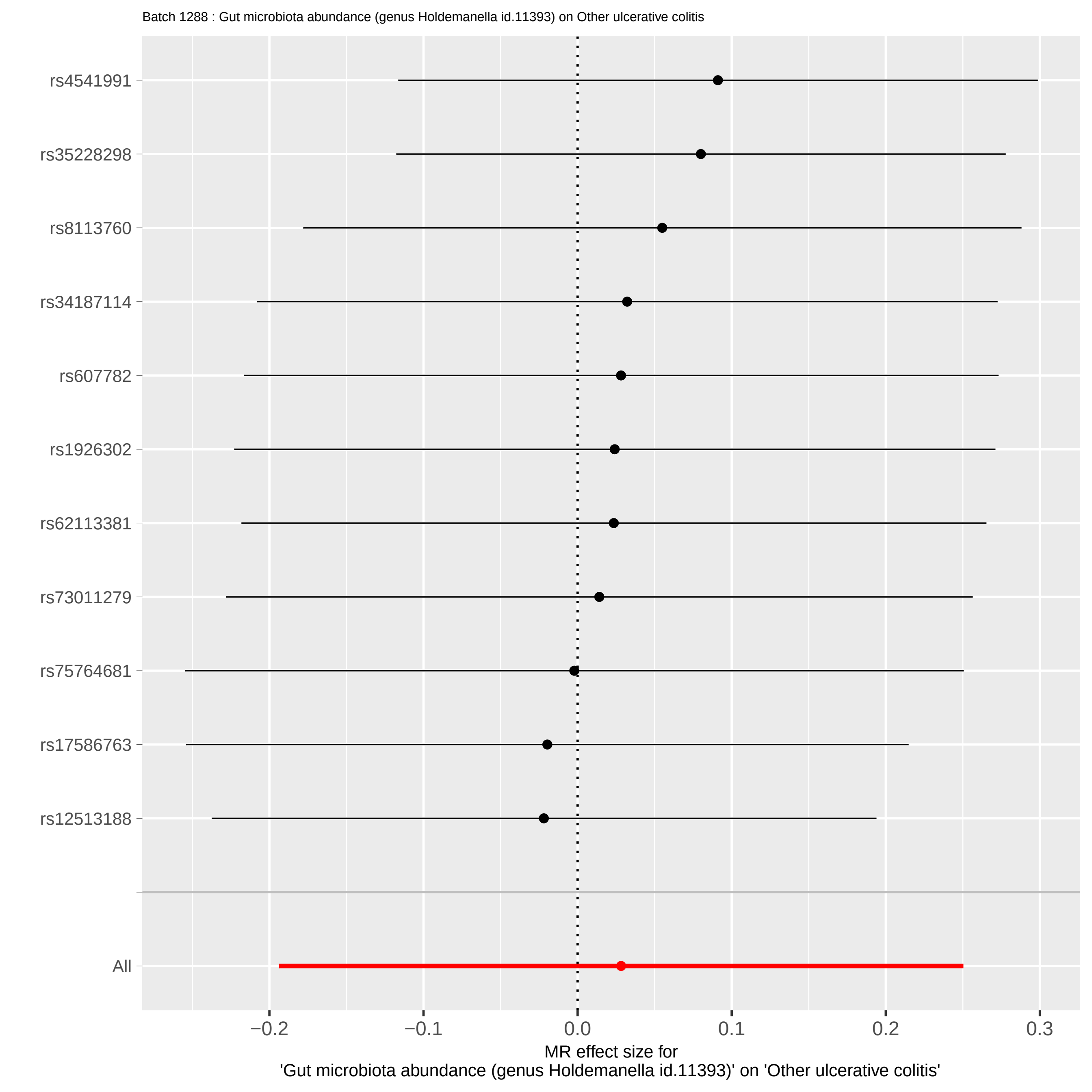
Batch 1288 : Gut microbiota abundance (genus Holdemanella id.11393) on Other ulcerative colitis
rs4541991
rs35228298
rs8113760
rs34187114
rs607782
rs1926302
rs62113381
rs73011279
rs75764681
rs17586763
rs12513188
All
−0.2
−0.1
0.0
0.1
0.2
0.3
MR effect size for
'Gut microbiota abundance (genus Holdemanella id.11393)' on 'Other ulcerative colitis'

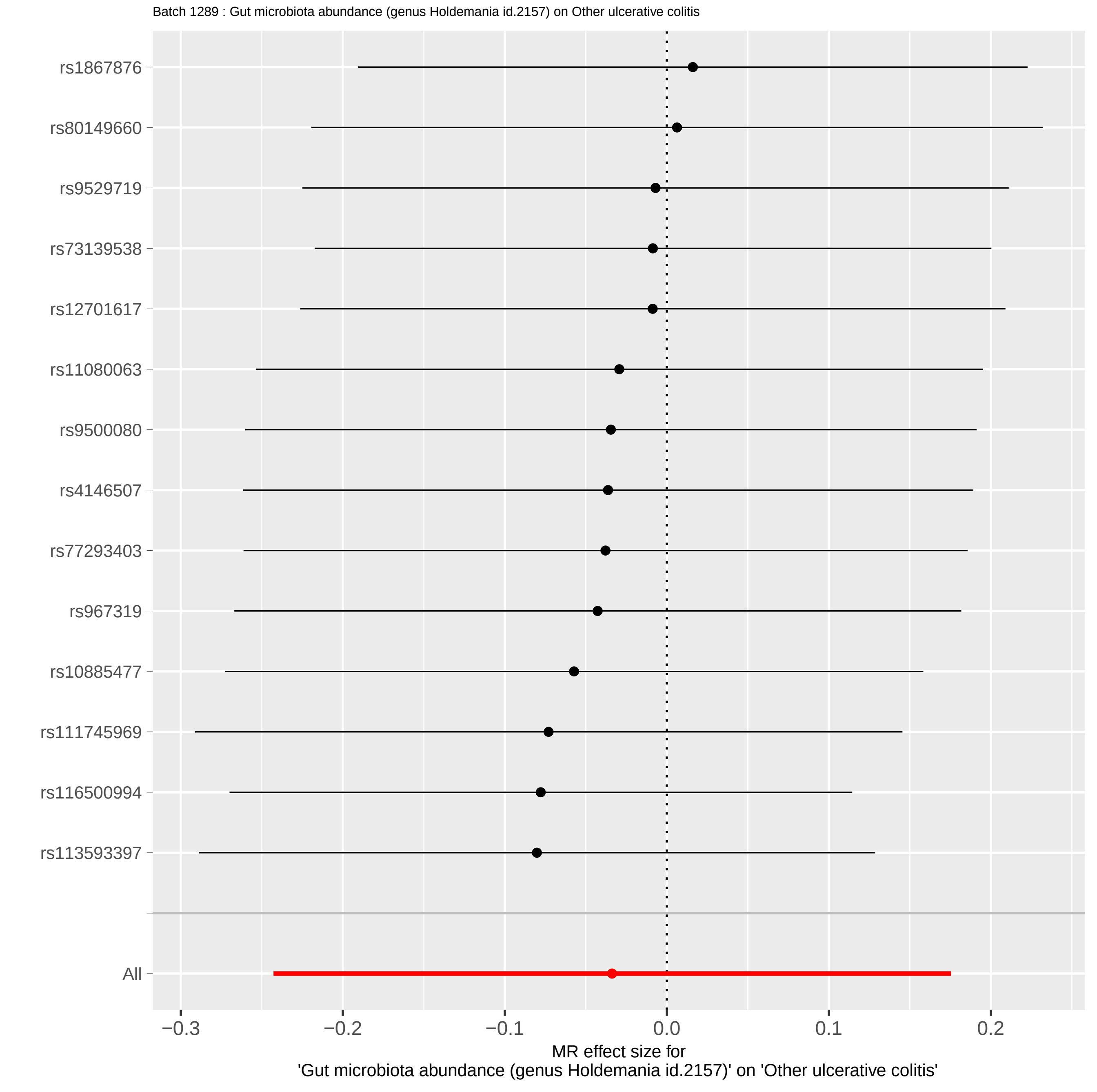
Batch 1289 : Gut microbiota abundance (genus Holdemania id.2157) on Other ulcerative colitis
rs1867876
rs80149660
rs9529719
rs73139538
rs12701617
rs11080063
rs9500080
rs4146507
rs77293403
rs967319
rs10885477
rs111745969
rs116500994
rs113593397
All
−0.3
−0.2
−0.1
0.0
0.1
0.2
MR effect size for
'Gut microbiota abundance (genus Holdemania id.2157)' on 'Other ulcerative colitis'

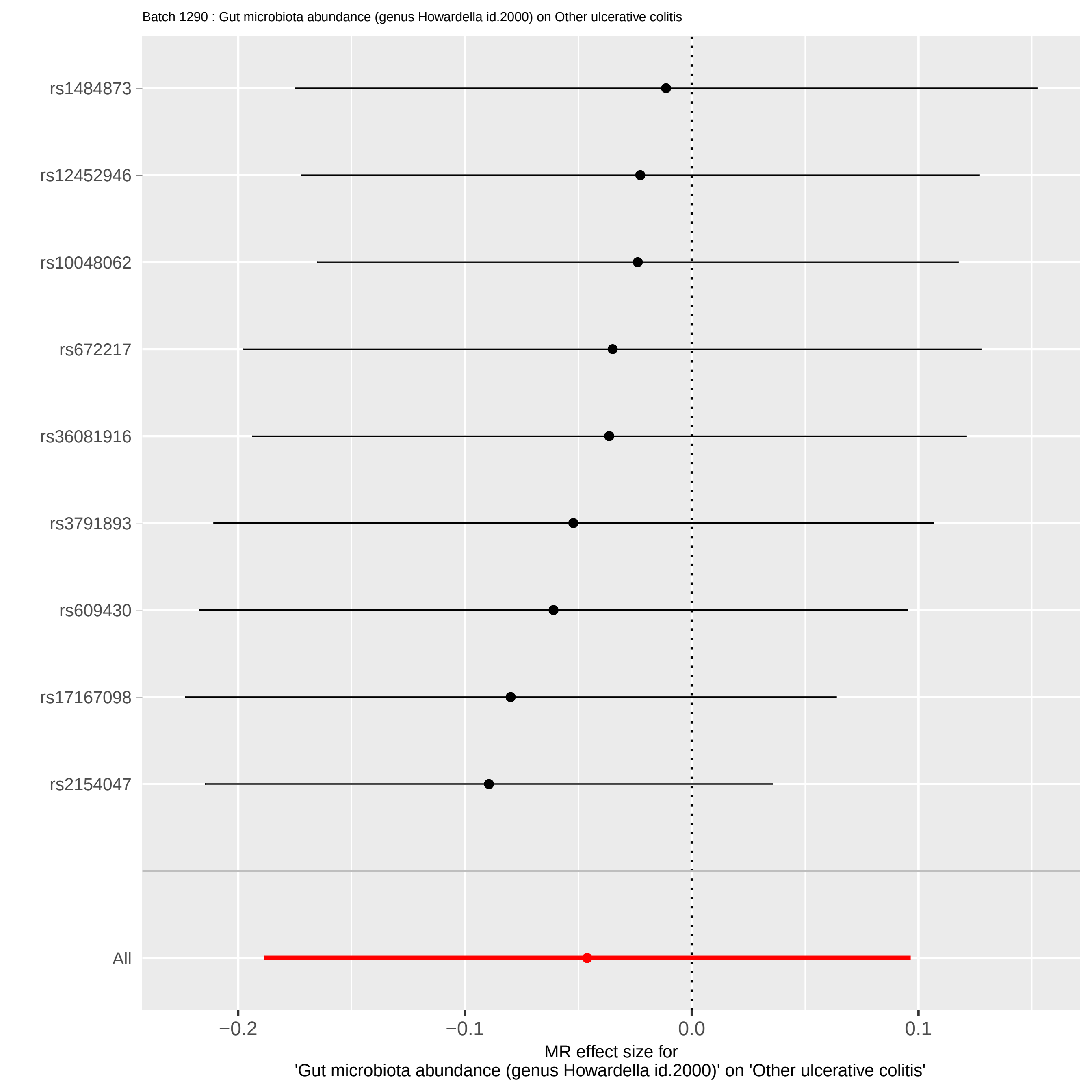
Batch 1290 : Gut microbiota abundance (genus Howardella id.2000) on Other ulcerative colitis
rs1484873
rs12452946
rs10048062
rs672217
rs36081916
rs3791893
rs609430
rs17167098
rs2154047
All
−0.2
−0.1
0.0
0.1
MR effect size for
'Gut microbiota abundance (genus Howardella id.2000)' on 'Other ulcerative colitis'

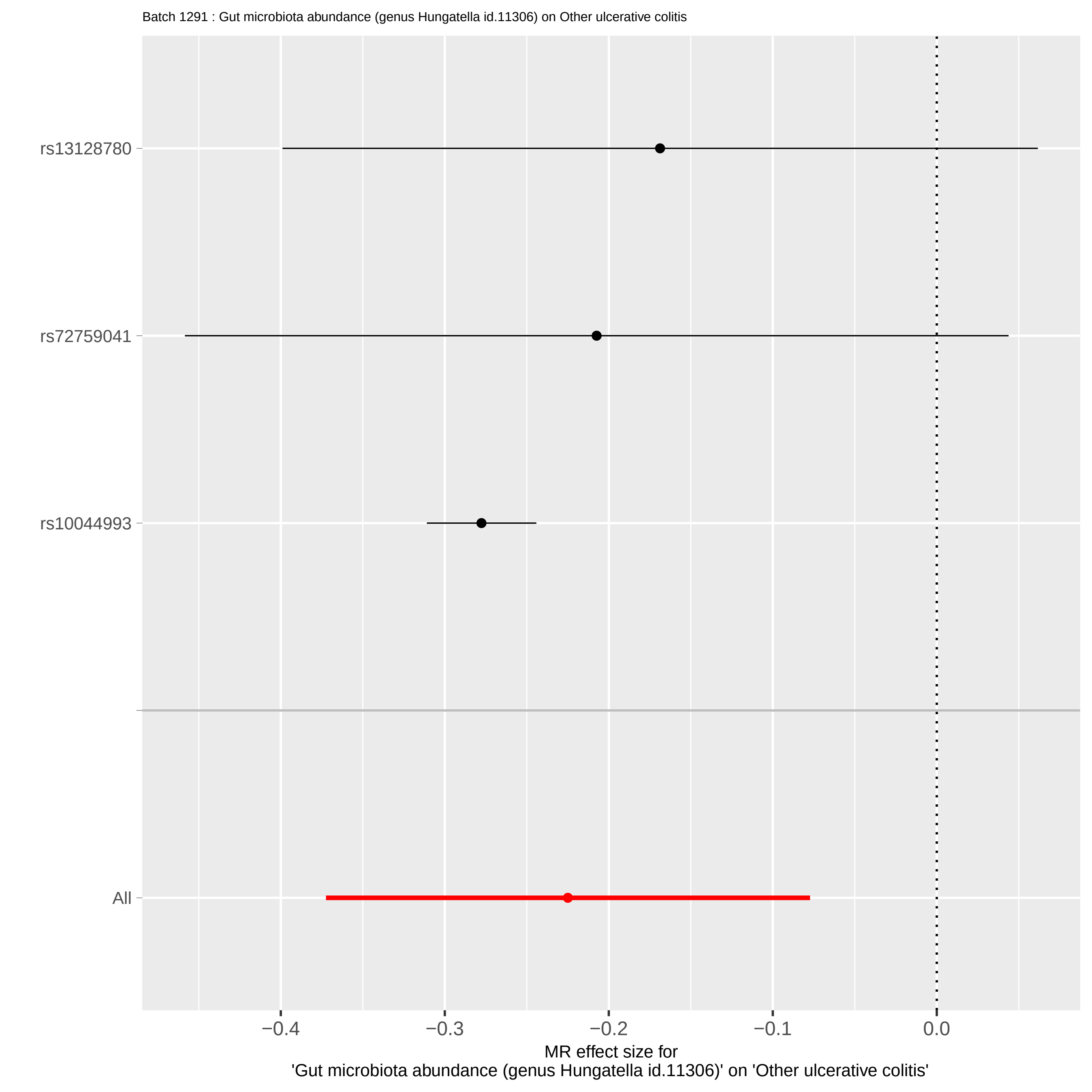
Batch 1291 : Gut microbiota abundance (genus Hungatella id.11306) on Other ulcerative colitis
rs13128780
rs72759041
rs10044993
All
−0.4
−0.3
−0.2
−0.1
0.0
MR effect size for
'Gut microbiota abundance (genus Hungatella id.11306)' on 'Other ulcerative colitis'

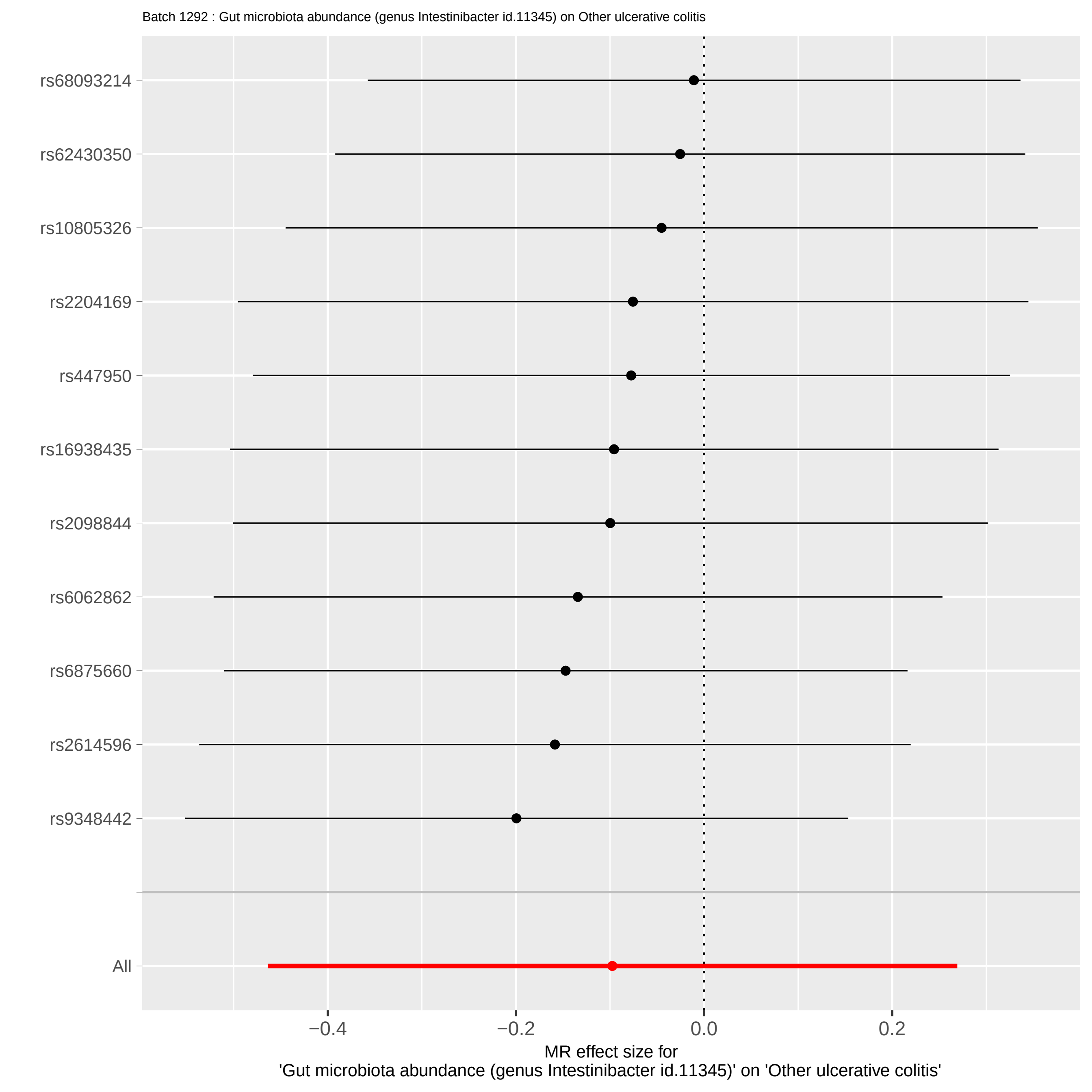

Batch 1292 : Gut microbiota abundance (genus Intestinibacter id.11345) on Other ulcerative colitis
rs68093214
rs62430350
rs10805326
rs2204169
rs447950
rs16938435
rs2098844
rs6062862
rs6875660
rs2614596
rs9348442
All
−0.4
−0.2
0.0
0.2
MR effect size for
'Gut microbiota abundance (genus Intestinibacter id.11345)' on 'Other ulcerative colitis'

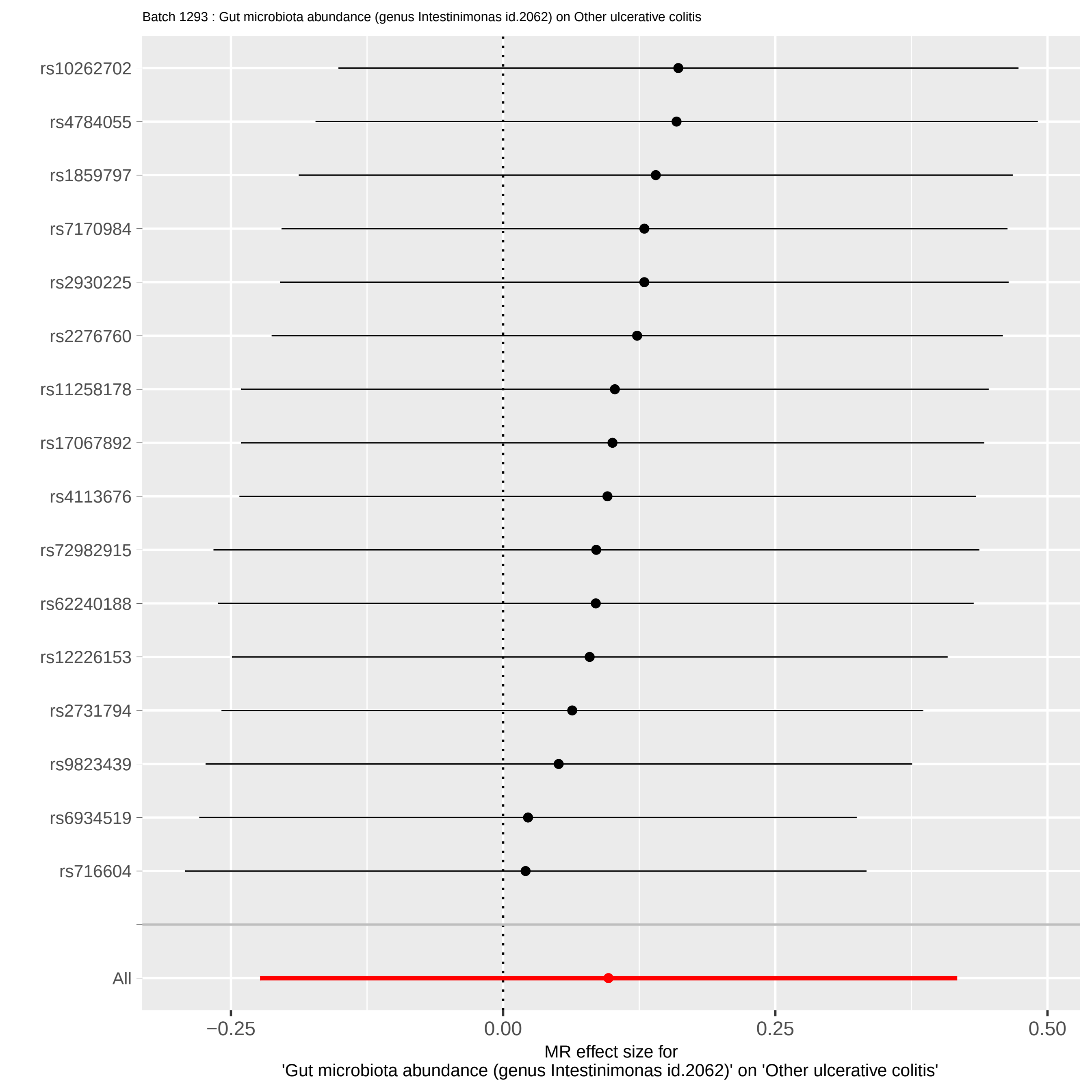

Batch 1293 : Gut microbiota abundance (genus Intestinimonas id.2062) on Other ulcerative colitis
rs10262702
rs4784055
rs1859797
rs7170984
rs2930225
rs2276760
rs11258178
rs17067892
rs4113676
rs72982915
rs62240188
rs12226153
rs2731794
rs9823439
rs6934519
rs716604
All
−0.25
0.00
0.25
0.50
MR effect size for
'Gut microbiota abundance (genus Intestinimonas id.2062)' on 'Other ulcerative colitis'

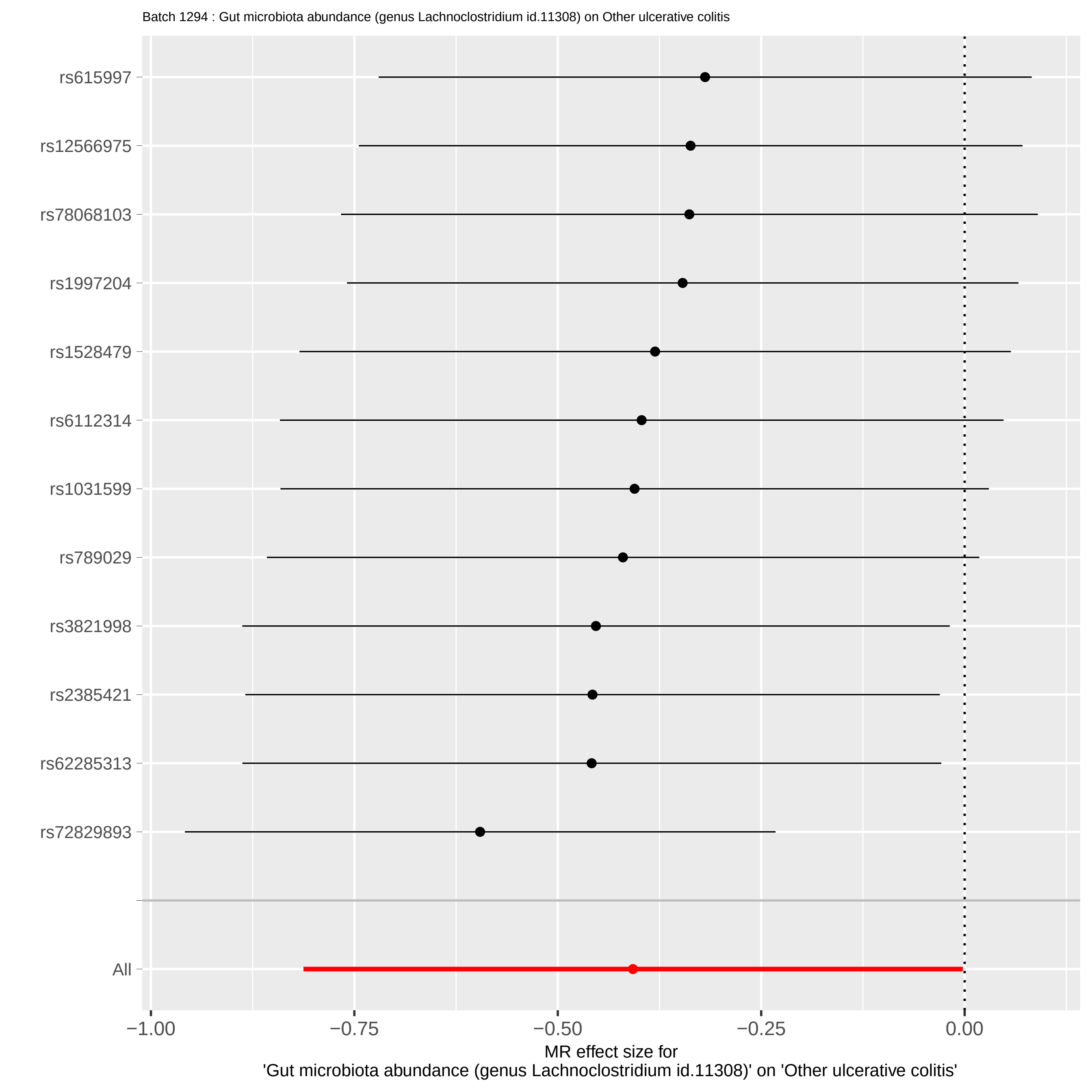

Batch 1294 : Gut microbiota abundance (genus Lachnoclostridium id.11308) on Other ulcerative colitis
rs615997
rs12566975
rs78068103
rs1997204
rs1528479
rs6112314
rs1031599
rs789029
rs3821998
rs2385421
rs62285313
rs72829893
All
−1.00
−0.75
−0.50
−0.25
0.00
MR effect size for
'Gut microbiota abundance (genus Lachnoclostridium id.11308)' on 'Other ulcerative colitis'

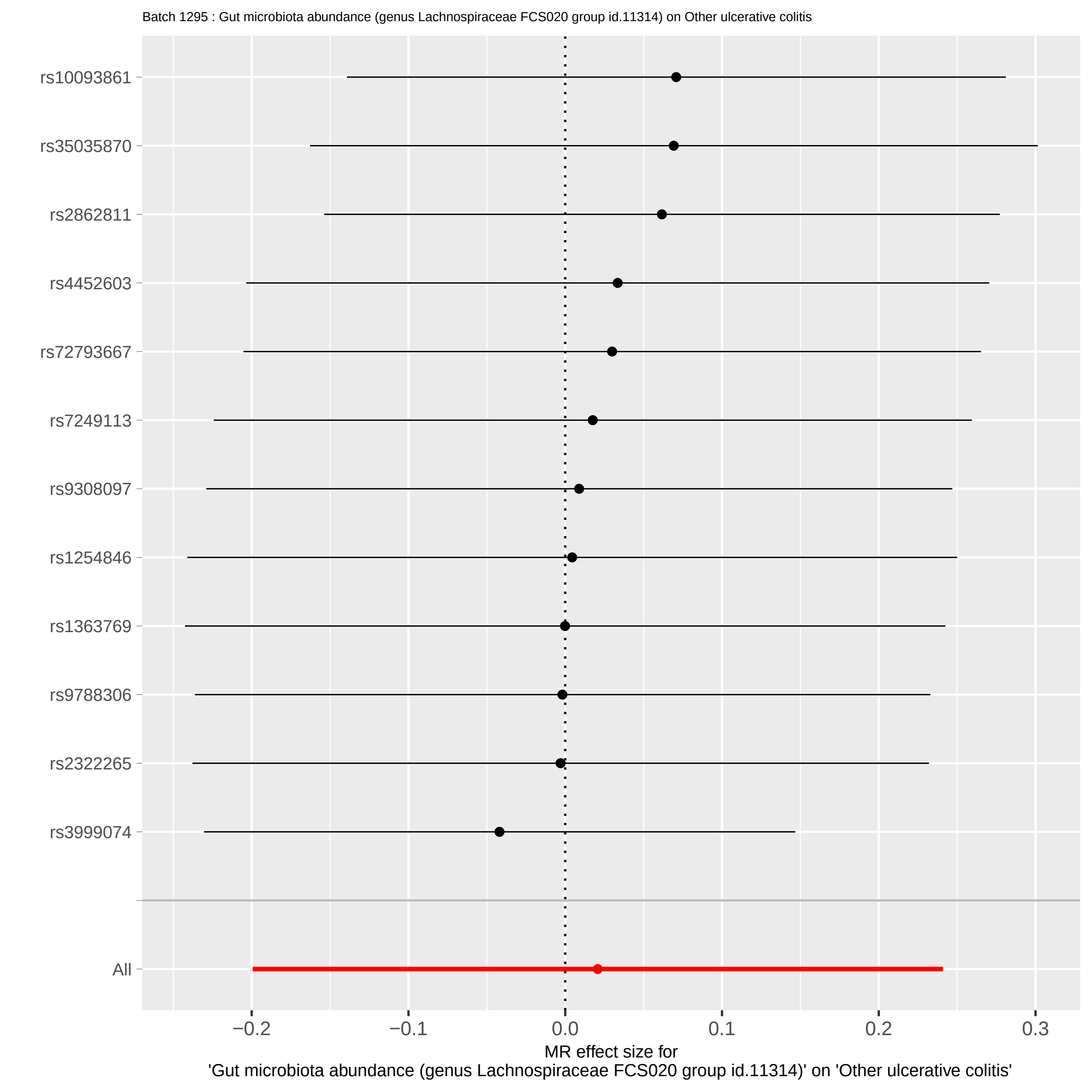

Batch 1295 : Gut microbiota abundance (genus Lachnospiraceae FCS020 group id.11314) on Other ulcerative colitis
rs10093861
rs35035870
rs2862811
rs4452603
rs72793667
rs7249113
rs9308097
rs1254846
rs1363769
rs9788306
rs2322265
rs3999074
All
−0.2
−0.1
0.0
0.1
0.2
0.3
MR effect size for
'Gut microbiota abundance (genus Lachnospiraceae FCS020 group id.11314)' on 'Other ulcerative colitis'

Batch 1296 : Gut microbiota abundance (genus Lachnospiraceae NC2004 group id.11316) on Other ulcerative colitis

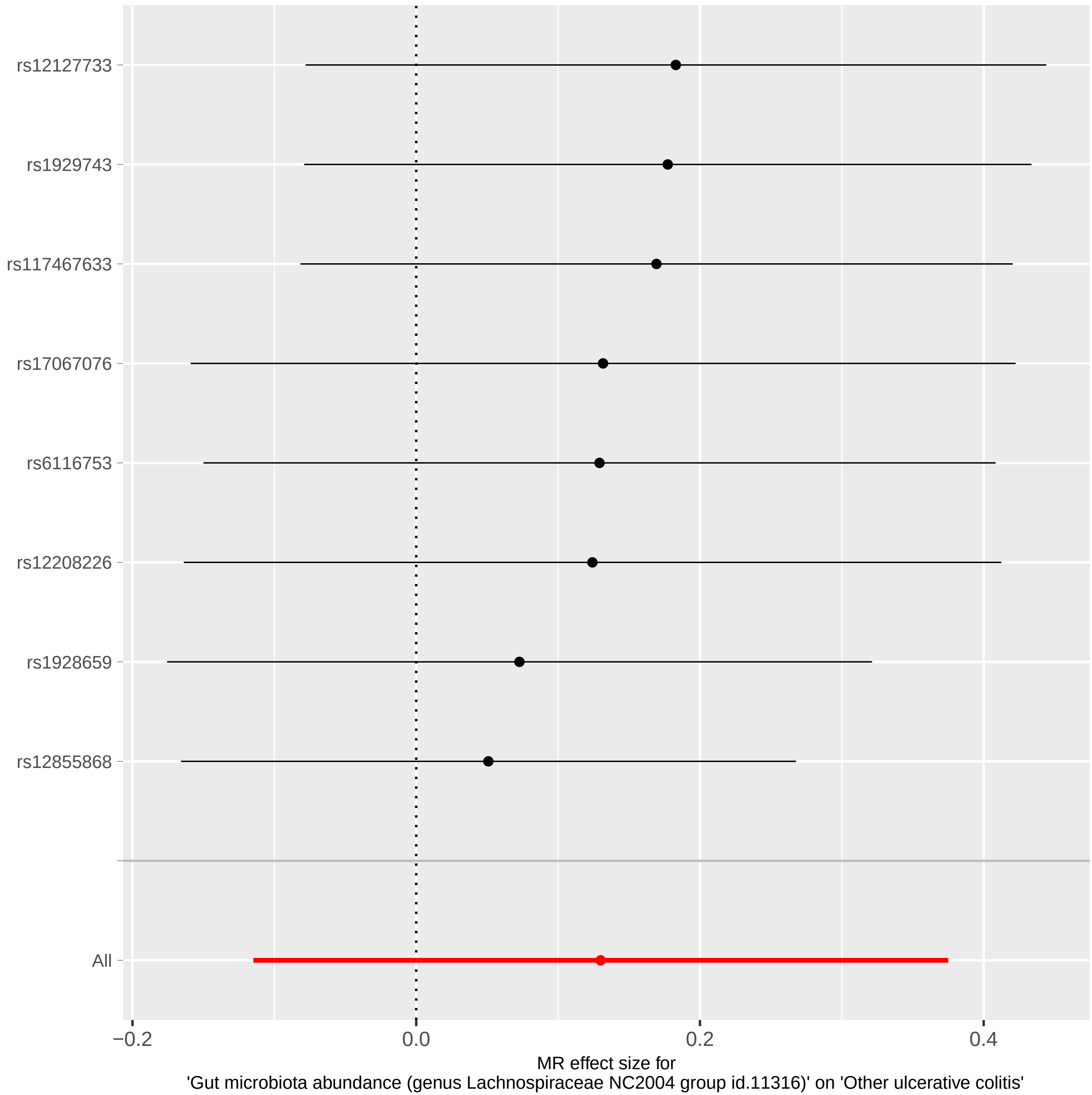

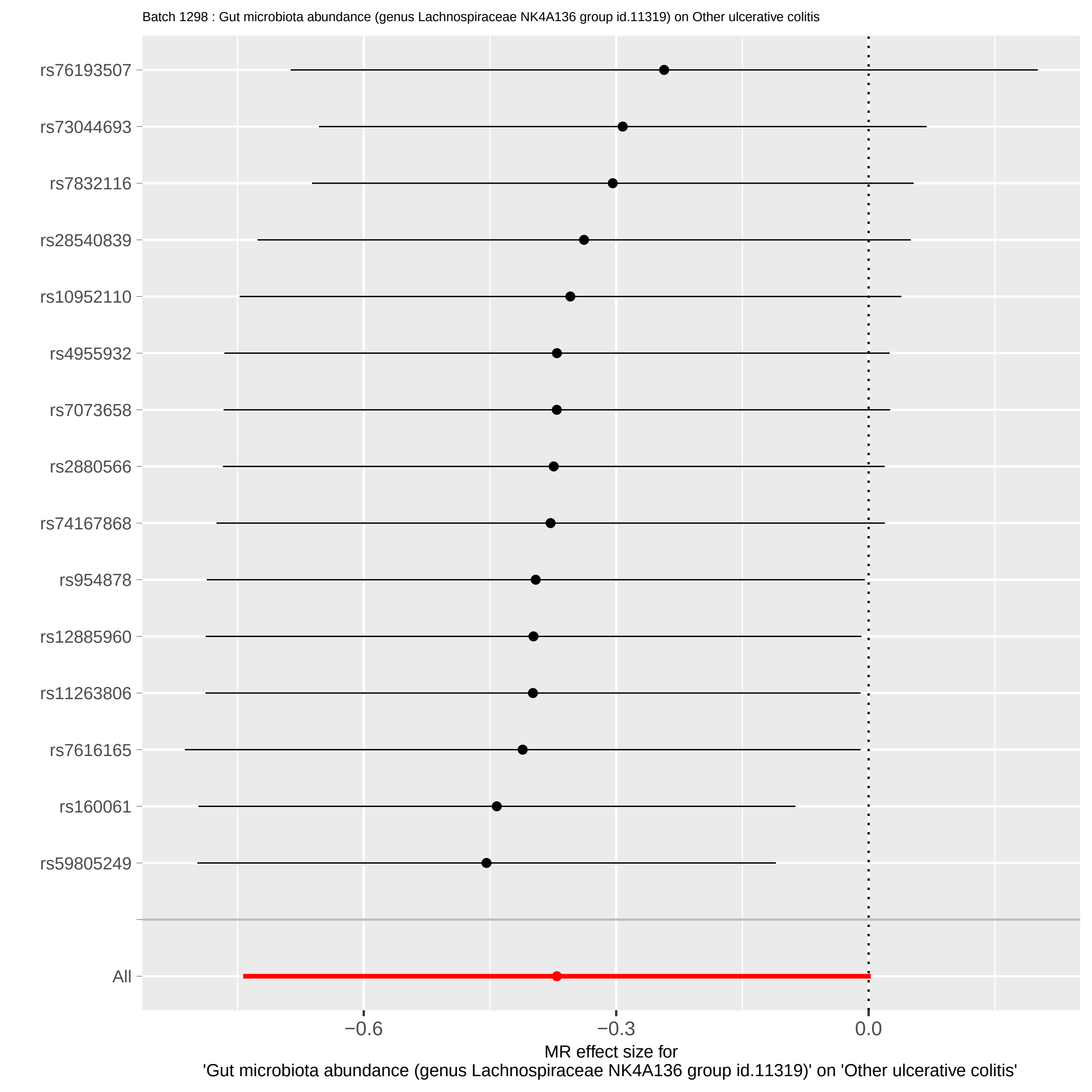

Batch 1298 : Gut microbiota abundance (genus Lachnospiraceae NK4A136 group id.11319) on Other ulcerative colitis
rs76193507
rs73044693
rs7832116
rs28540839
rs10952110
rs4955932
rs7073658
rs2880566
rs74167868
rs954878
rs12885960
rs11263806
rs7616165
rs160061
rs59805249
All
−0.6
−0.3
0.0
MR effect size for
'Gut microbiota abundance (genus Lachnospiraceae NK4A136 group id.11319)' on 'Other ulcerative colitis'

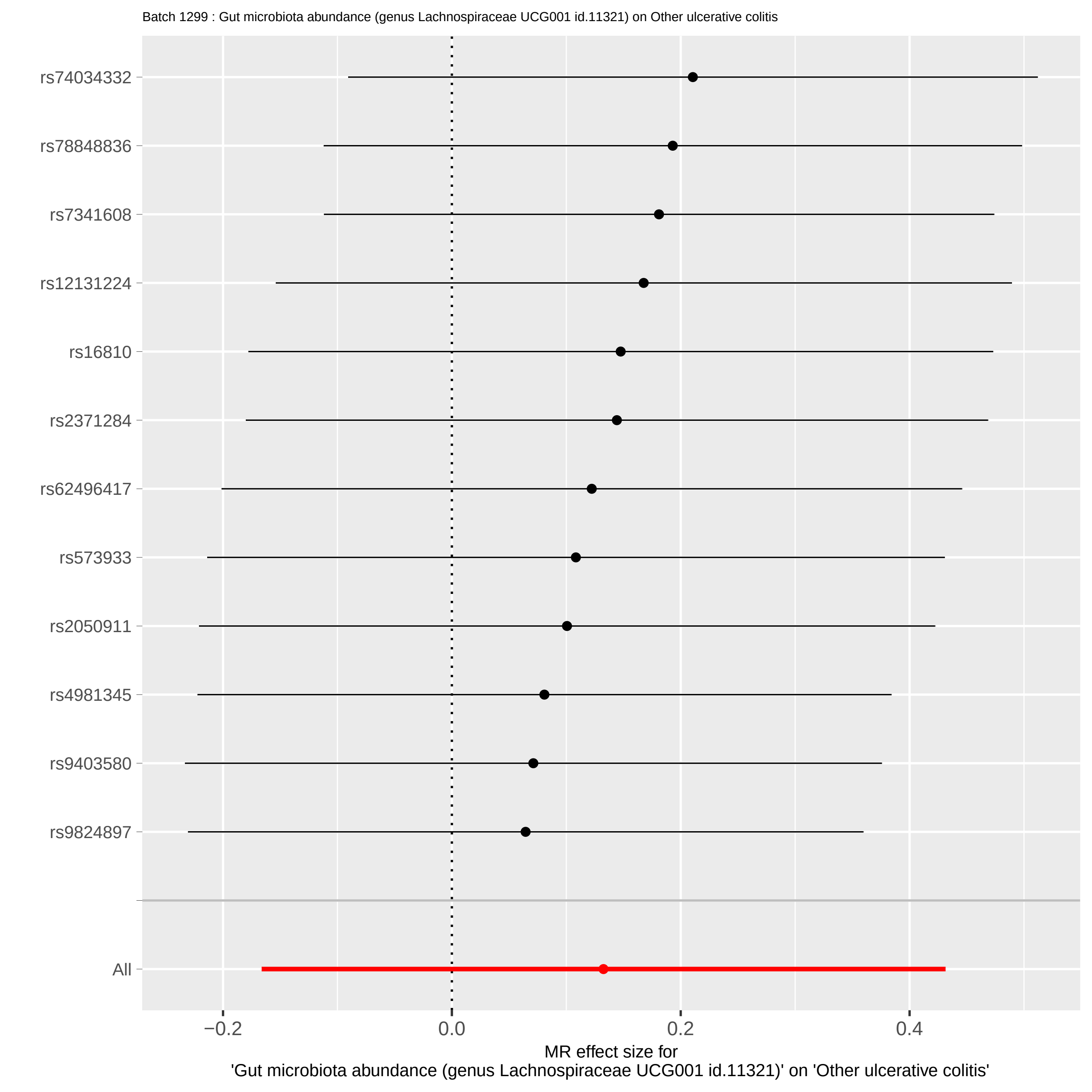

Batch 1299 : Gut microbiota abundance (genus Lachnospiraceae UCG001 id.11321) on Other ulcerative colitis
rs74034332
rs78848836
rs7341608
rs12131224
rs16810
rs2371284
rs62496417
rs573933
rs2050911
rs4981345
rs9403580
rs9824897
All
−0.2
0.0
0.2
0.4
MR effect size for
'Gut microbiota abundance (genus Lachnospiraceae UCG001 id.11321)' on 'Other ulcerative colitis'

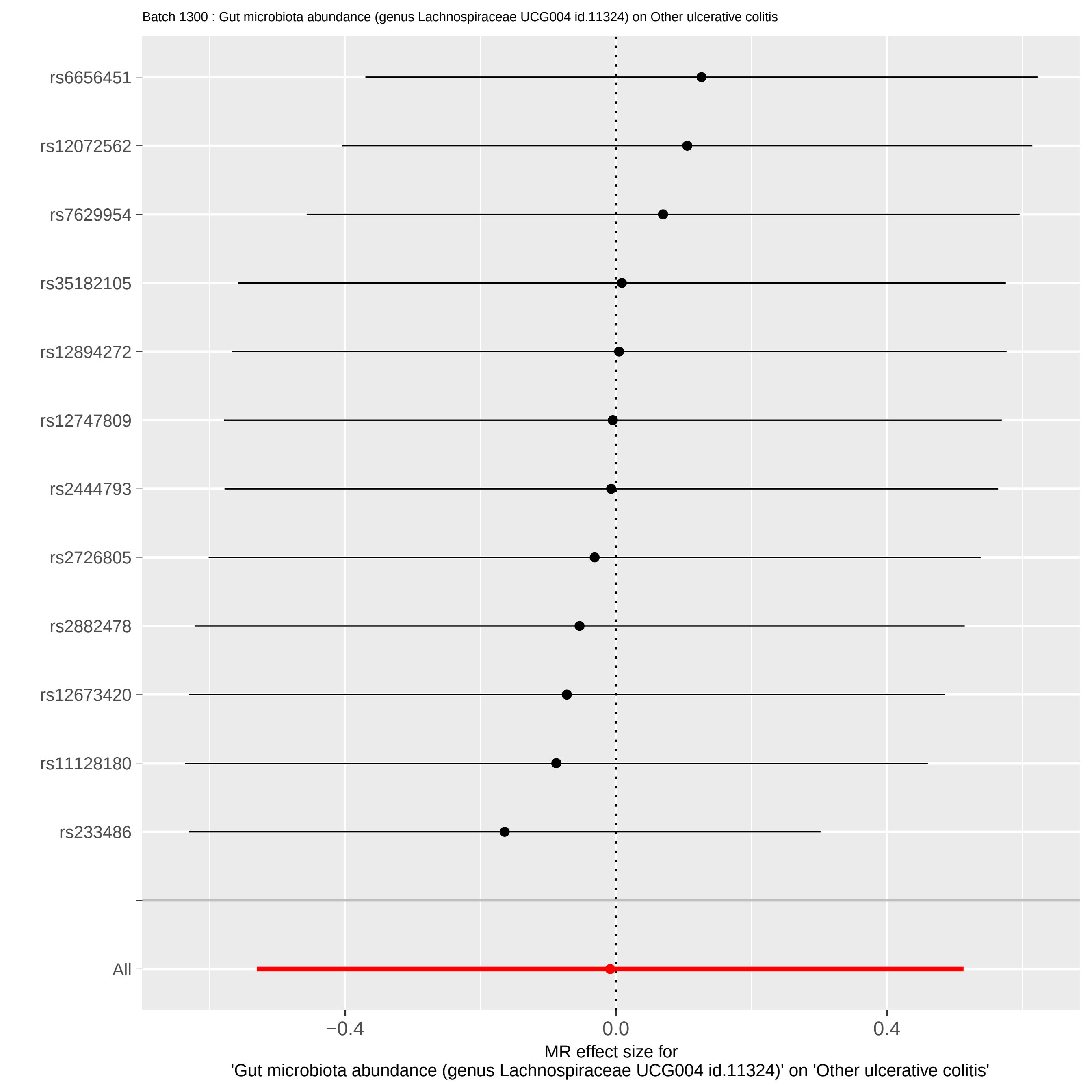
Batch 1300 : Gut microbiota abundance (genus Lachnospiraceae UCG004 id.11324) on Other ulcerative colitis
rs6656451
rs12072562
rs7629954
rs35182105
rs12894272
rs12747809
rs2444793
rs2726805
rs2882478
rs12673420
rs11128180
rs233486
All
−0.4
0.0
0.4
MR effect size for
'Gut microbiota abundance (genus Lachnospiraceae UCG004 id.11324)' on 'Other ulcerative colitis'

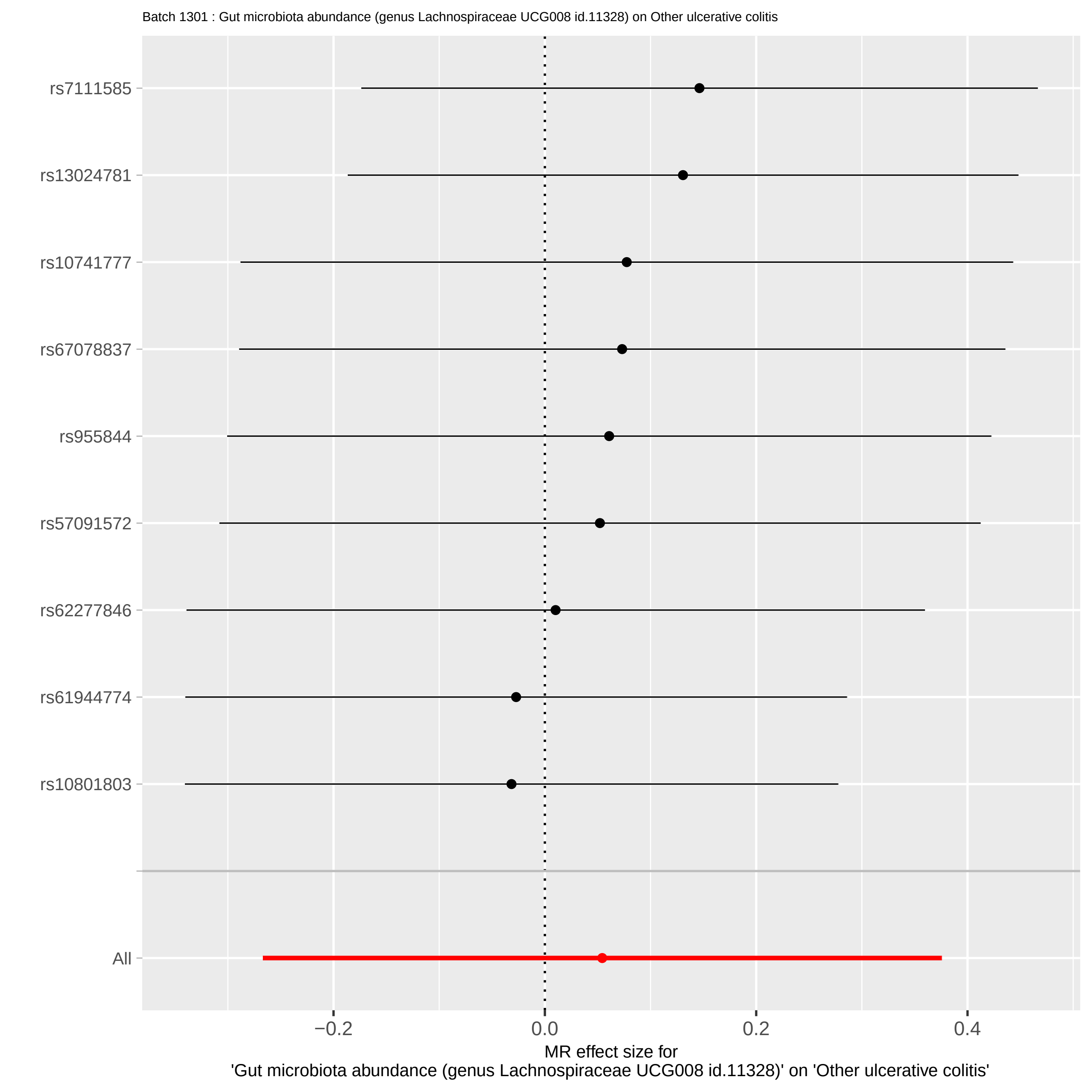

Batch 1301 : Gut microbiota abundance (genus Lachnospiraceae UCG008 id.11328) on Other ulcerative colitis
rs7111585
rs13024781
rs10741777
rs67078837
rs955844
rs57091572
rs62277846
rs61944774
rs10801803
All
−0.2
0.0
0.2
0.4
MR effect size for
'Gut microbiota abundance (genus Lachnospiraceae UCG008 id.11328)' on 'Other ulcerative colitis'

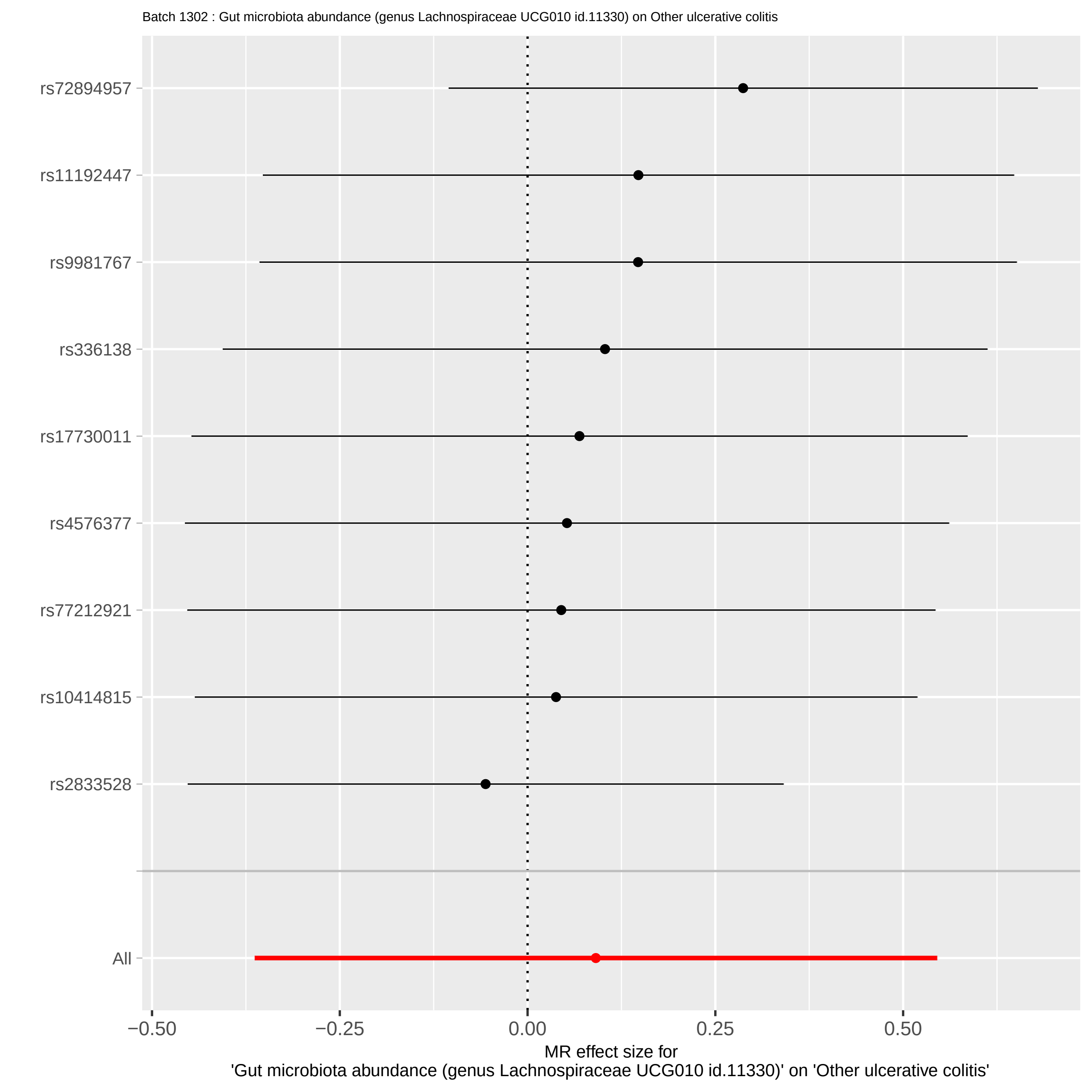

Batch 1302 : Gut microbiota abundance (genus Lachnospiraceae UCG010 id.11330) on Other ulcerative colitis
rs72894957
rs11192447
rs9981767
rs336138
rs17730011
rs4576377
rs77212921
rs10414815
rs2833528
All
−0.50
−0.25
0.00
0.25
0.50
MR effect size for
'Gut microbiota abundance (genus Lachnospiraceae UCG010 id.11330)' on 'Other ulcerative colitis'

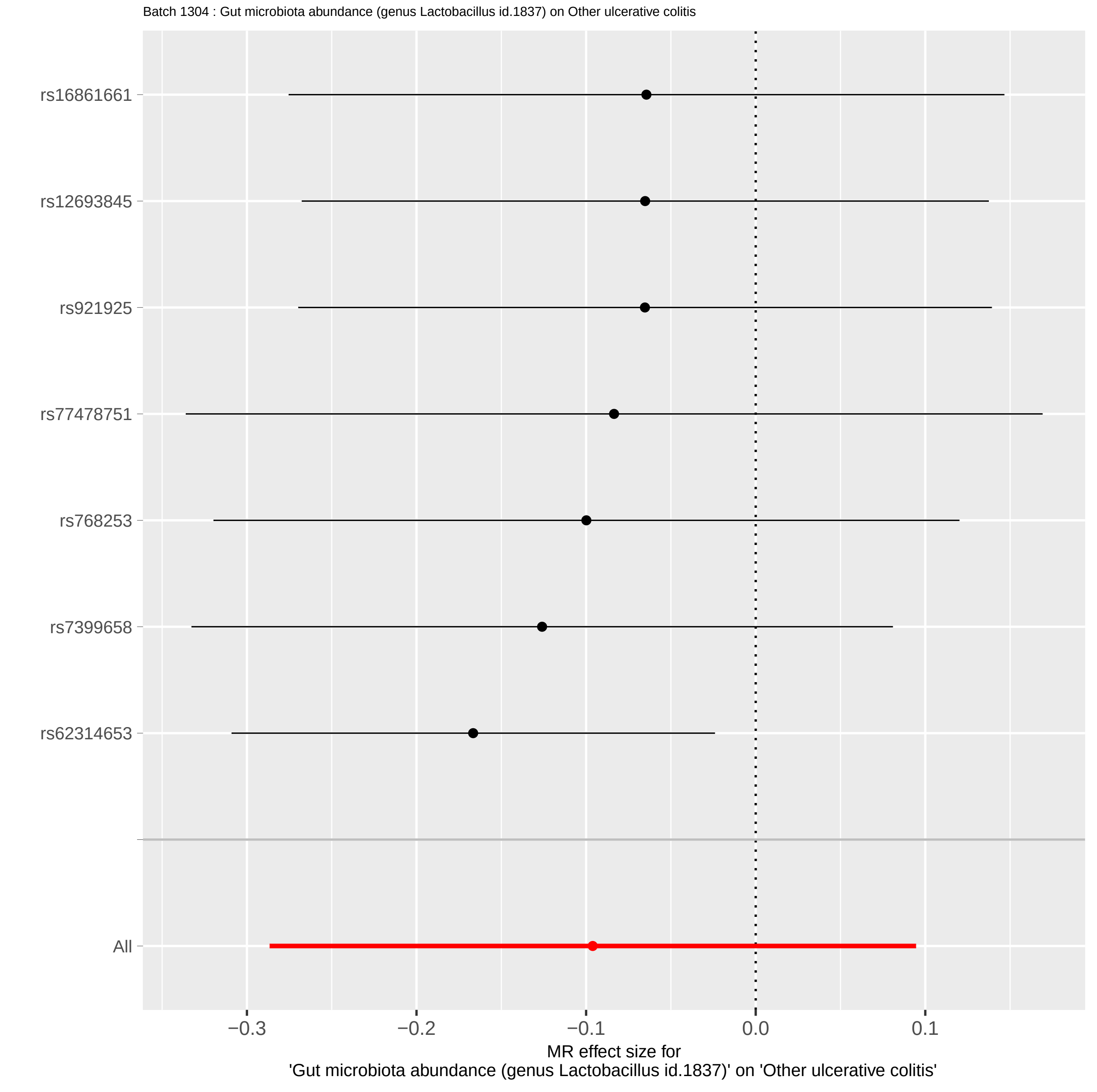

Batch 1304 : Gut microbiota abundance (genus Lactobacillus id.1837) on Other ulcerative colitis
rs16861661
rs12693845
rs921925
rs77478751
rs768253
rs7399658
rs62314653
All
−0.3
−0.2
−0.1
0.0
0.1
MR effect size for
'Gut microbiota abundance (genus Lactobacillus id.1837)' on 'Other ulcerative colitis'

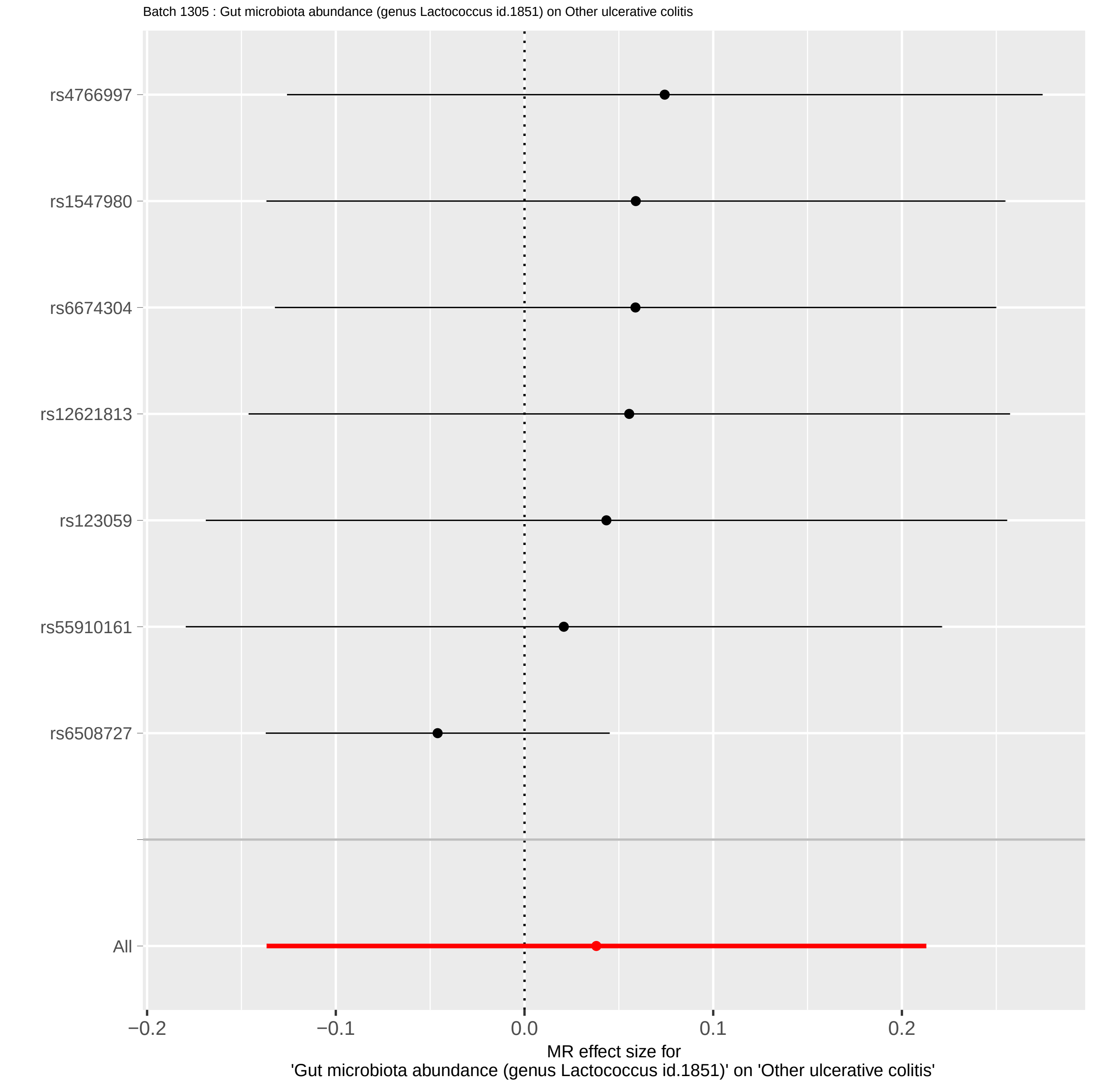
Batch 1305 : Gut microbiota abundance (genus Lactococcus id.1851) on Other ulcerative colitis
rs4766997
rs1547980
rs6674304
rs12621813
rs123059
rs55910161
rs6508727
All
−0.2
−0.1
0.0
0.1
0.2
MR effect size for
'Gut microbiota abundance (genus Lactococcus id.1851)' on 'Other ulcerative colitis'

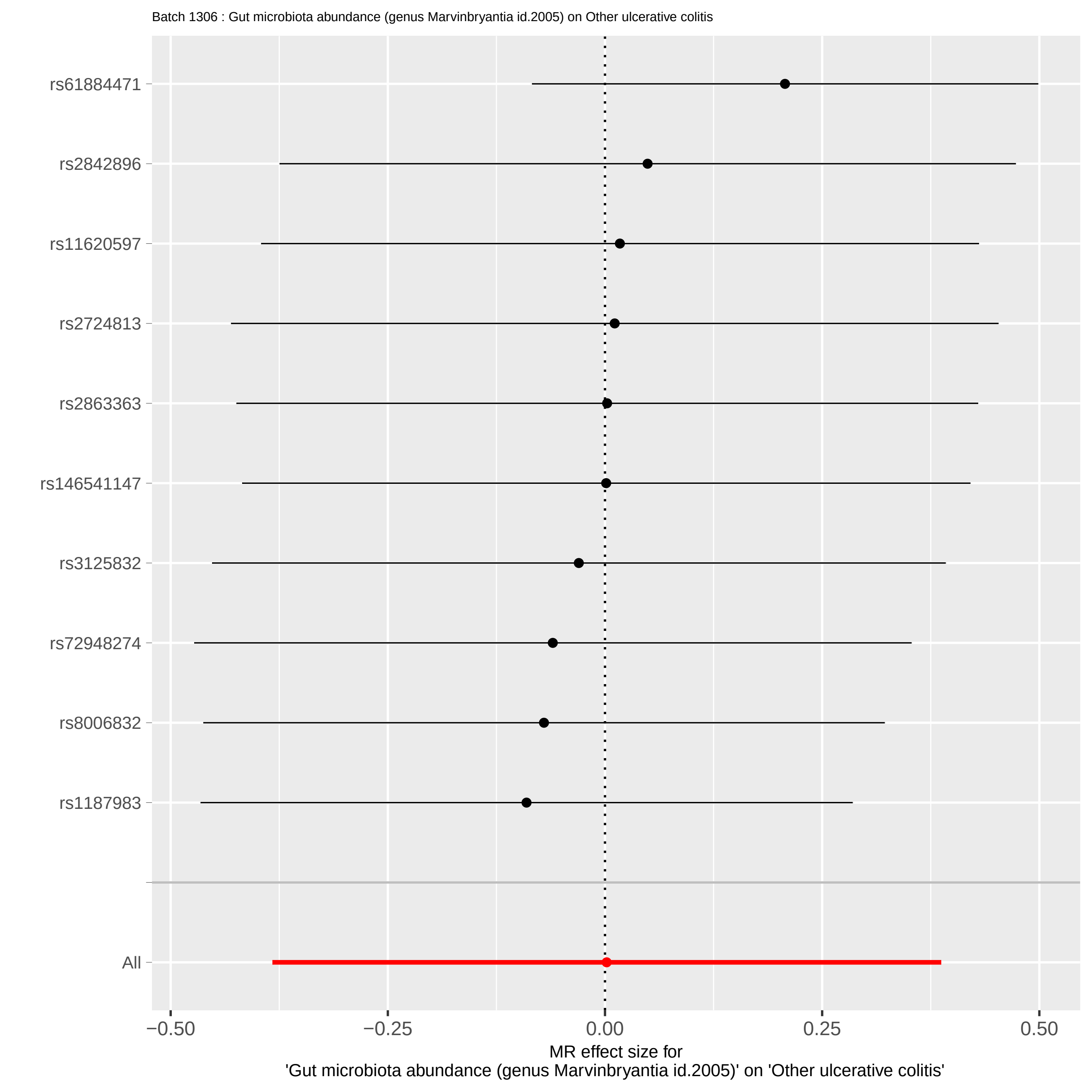

Batch 1306 : Gut microbiota abundance (genus Marvinbryantia id.2005) on Other ulcerative colitis
rs61884471
rs2842896
rs11620597
rs2724813
rs2863363
rs146541147
rs3125832
rs72948274
rs8006832
rs1187983
All
−0.50
−0.25
0.00
0.25
0.50
MR effect size for
'Gut microbiota abundance (genus Marvinbryantia id.2005)' on 'Other ulcerative colitis'

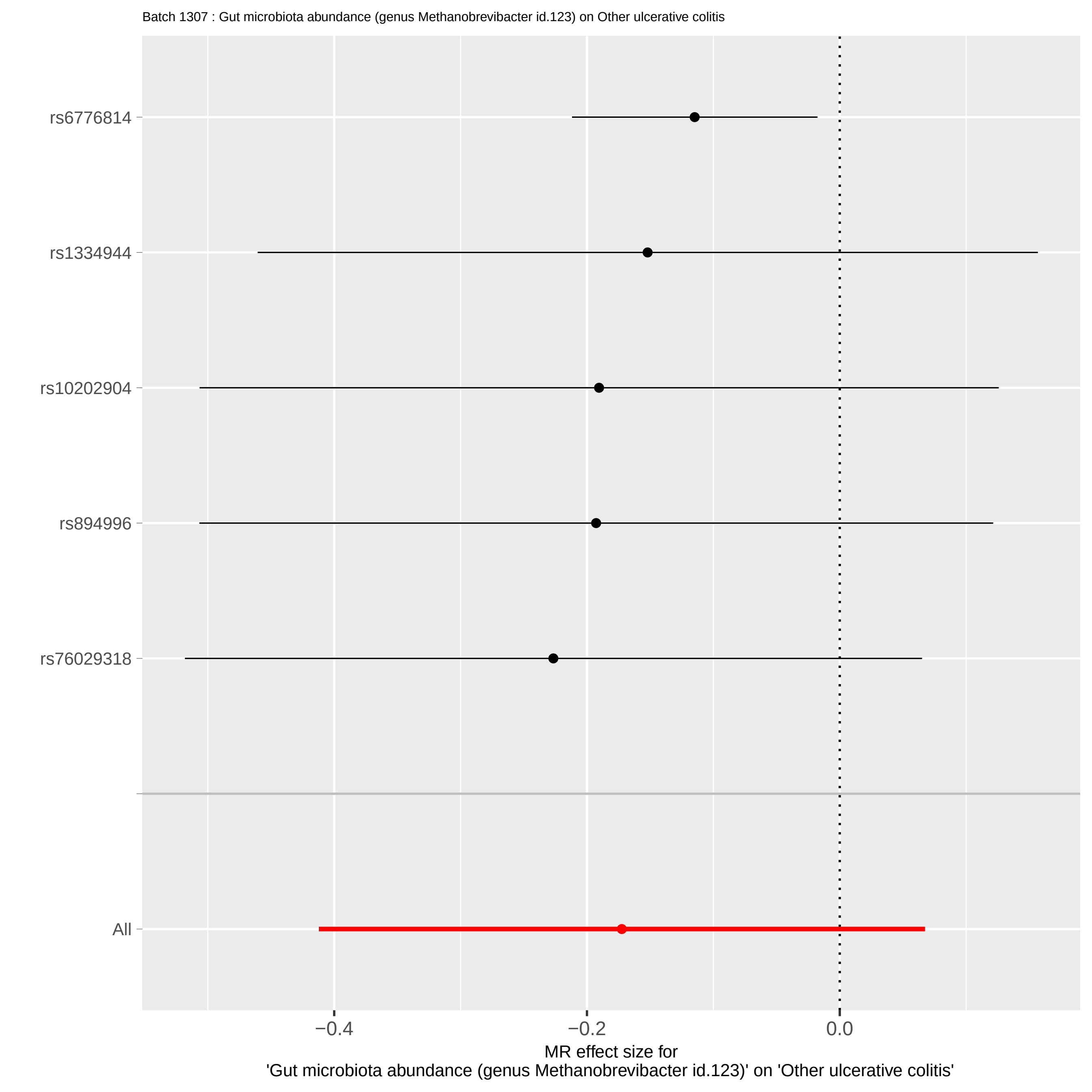
Batch 1307 : Gut microbiota abundance (genus Methanobrevibacter id.123) on Other ulcerative colitis
rs6776814
rs1334944
rs10202904
rs894996
rs76029318
All
−0.4
−0.2
0.0
MR effect size for
'Gut microbiota abundance (genus Methanobrevibacter id.123)' on 'Other ulcerative colitis'

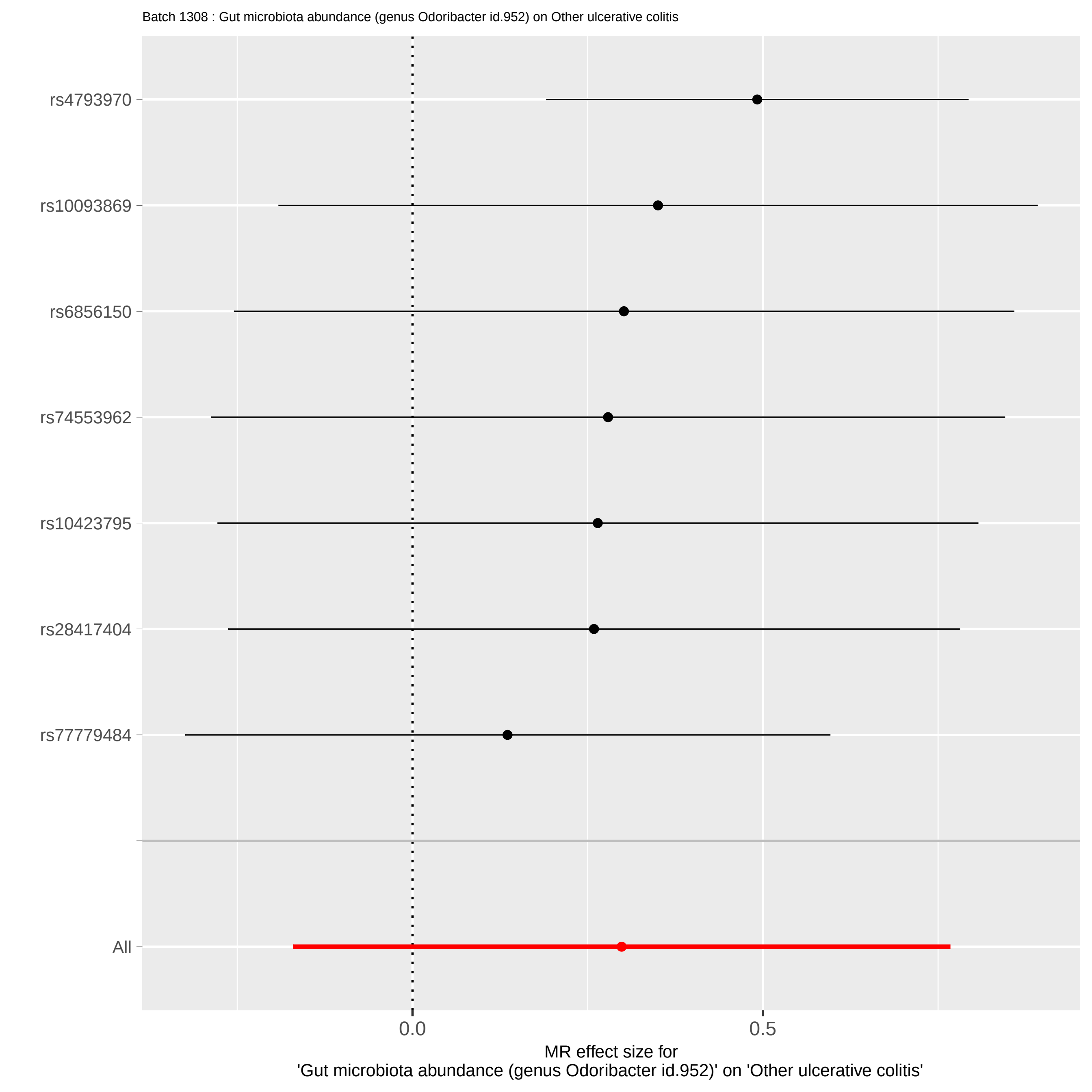

Batch 1308 : Gut microbiota abundance (genus Odoribacter id.952) on Other ulcerative colitis
rs4793970
rs10093869
rs6856150
rs74553962
rs10423795
rs28417404
rs77779484
All
0.0
0.5
MR effect size for
'Gut microbiota abundance (genus Odoribacter id.952)' on 'Other ulcerative colitis'

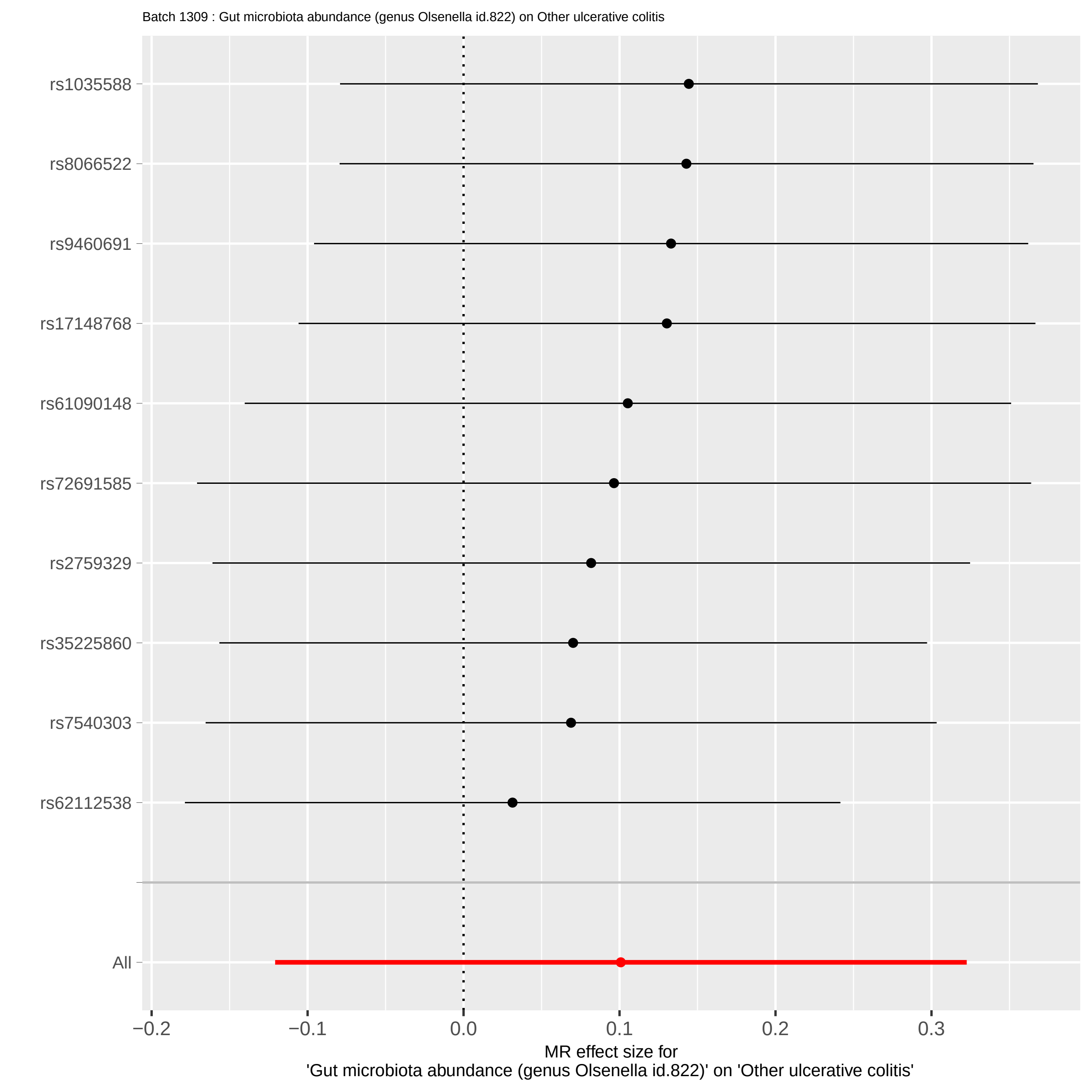

Batch 1309 : Gut microbiota abundance (genus Olsenella id.822) on Other ulcerative colitis
rs1035588
rs8066522
rs9460691
rs17148768
rs61090148
rs72691585
rs2759329
rs35225860
rs7540303
rs62112538
All
−0.2
−0.1
0.0
0.1
0.2
0.3
MR effect size for
'Gut microbiota abundance (genus Olsenella id.822)' on 'Other ulcerative colitis'

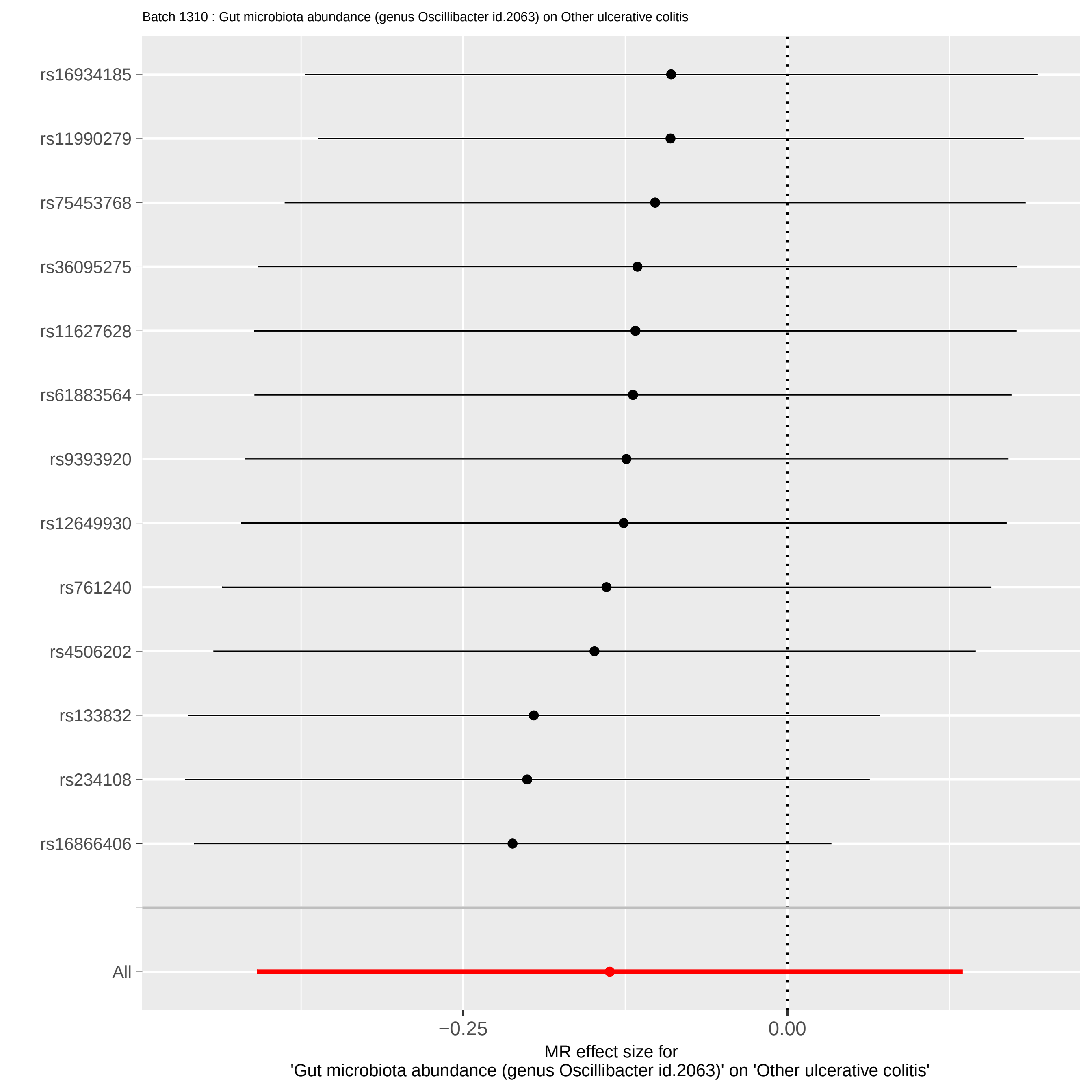
Batch 1310 : Gut microbiota abundance (genus Oscillibacter id.2063) on Other ulcerative colitis
rs16934185
rs11990279
rs75453768
rs36095275
rs11627628
rs61883564
rs9393920
rs12649930
rs761240
rs4506202
rs133832
rs234108
rs16866406
All
−0.25
0.00
MR effect size for
'Gut microbiota abundance (genus Oscillibacter id.2063)' on 'Other ulcerative colitis'

Batch 1311 : Gut microbiota abundance (genus Oscillospira id.2064) on Other ulcerative colitis

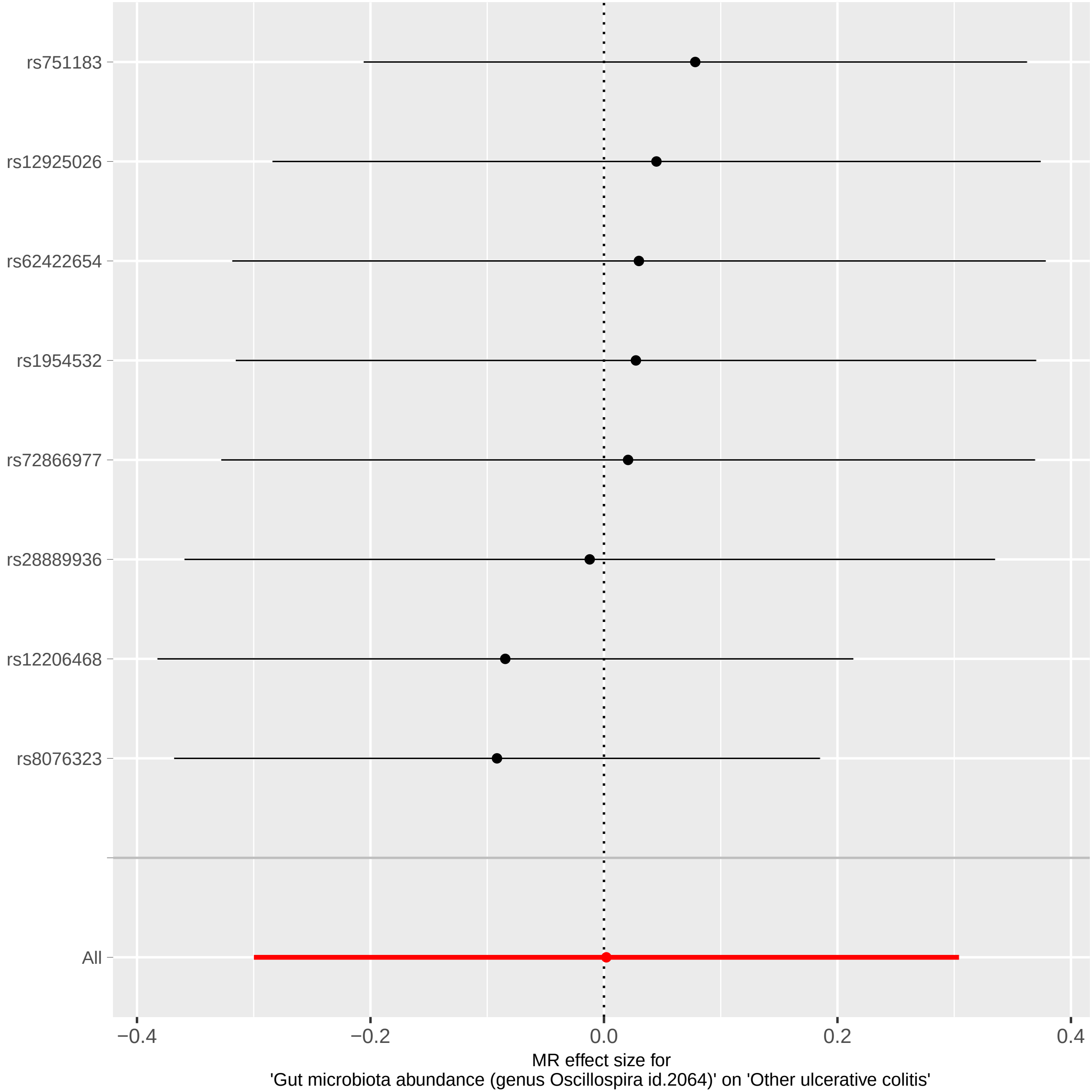

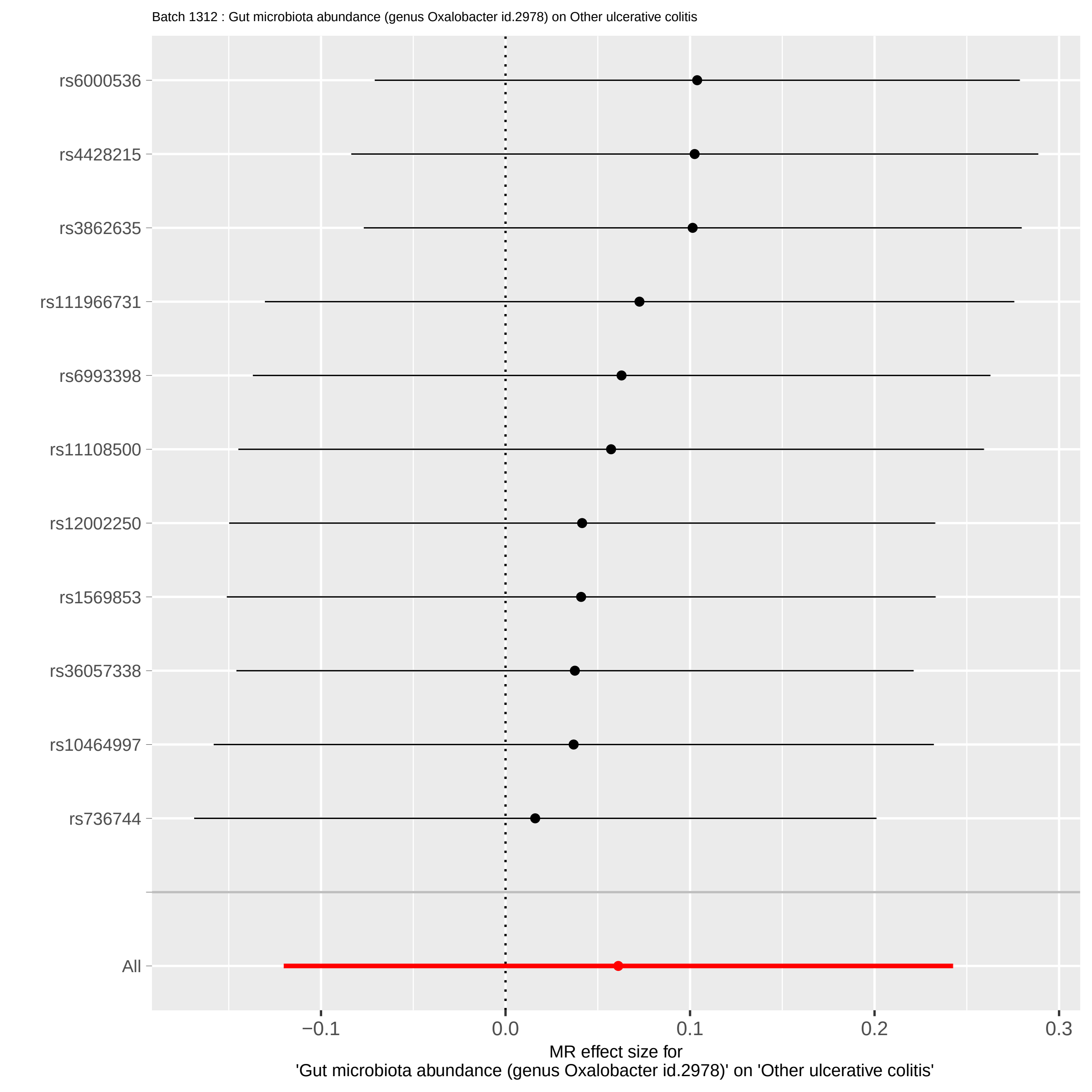
Batch 1312 : Gut microbiota abundance (genus Oxalobacter id.2978) on Other ulcerative colitis
rs6000536
rs4428215
rs3862635
rs111966731
rs6993398
rs11108500
rs12002250
rs1569853
rs36057338
rs10464997
rs736744
All
−0.1
0.0
0.1
0.2
0.3
MR effect size for
'Gut microbiota abundance (genus Oxalobacter id.2978)' on 'Other ulcerative colitis'

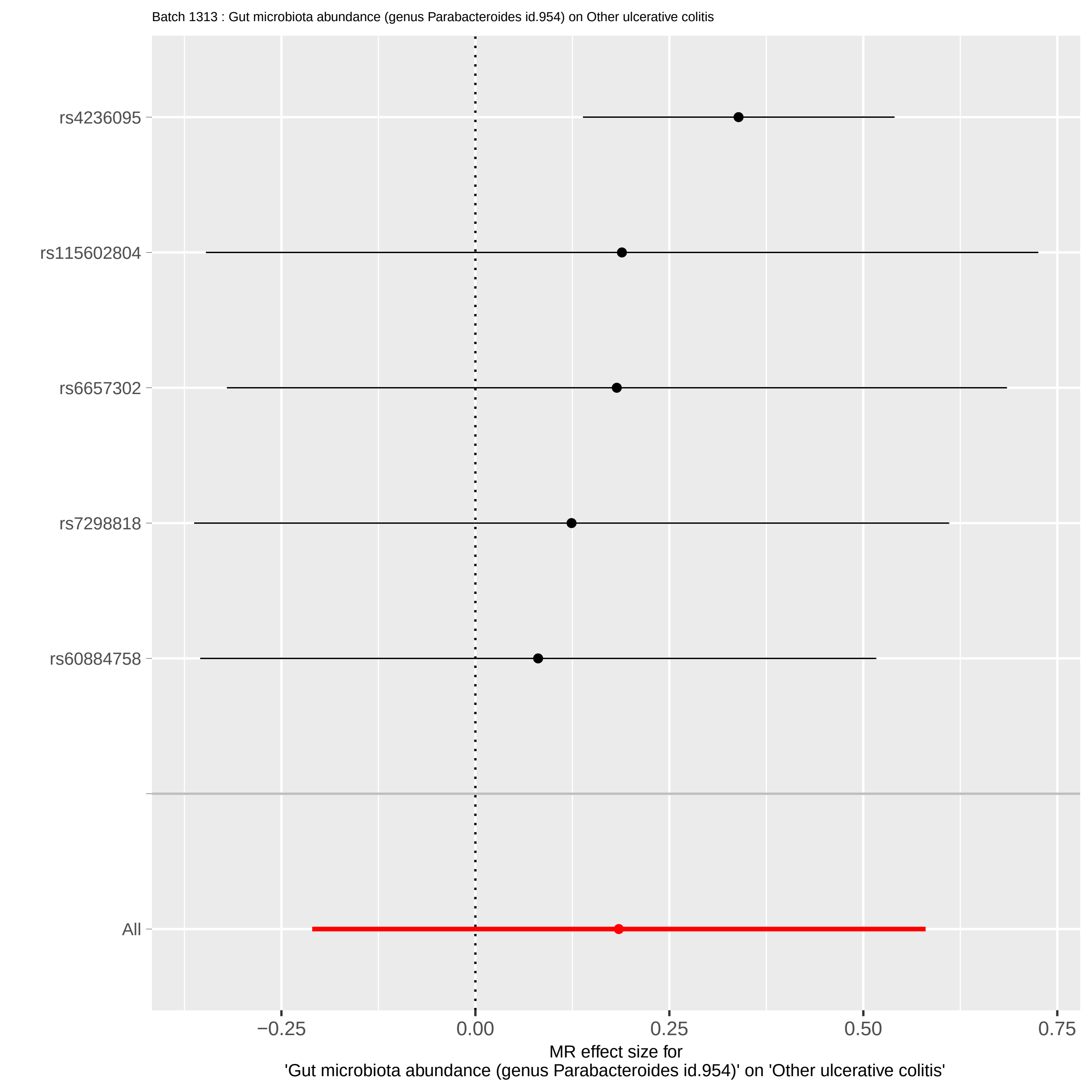
Batch 1313 : Gut microbiota abundance (genus Parabacteroides id.954) on Other ulcerative colitis
rs4236095
rs115602804
rs6657302
rs7298818
rs60884758
All
−0.25
0.00
0.25
0.50
0.75
MR effect size for
'Gut microbiota abundance (genus Parabacteroides id.954)' on 'Other ulcerative colitis'

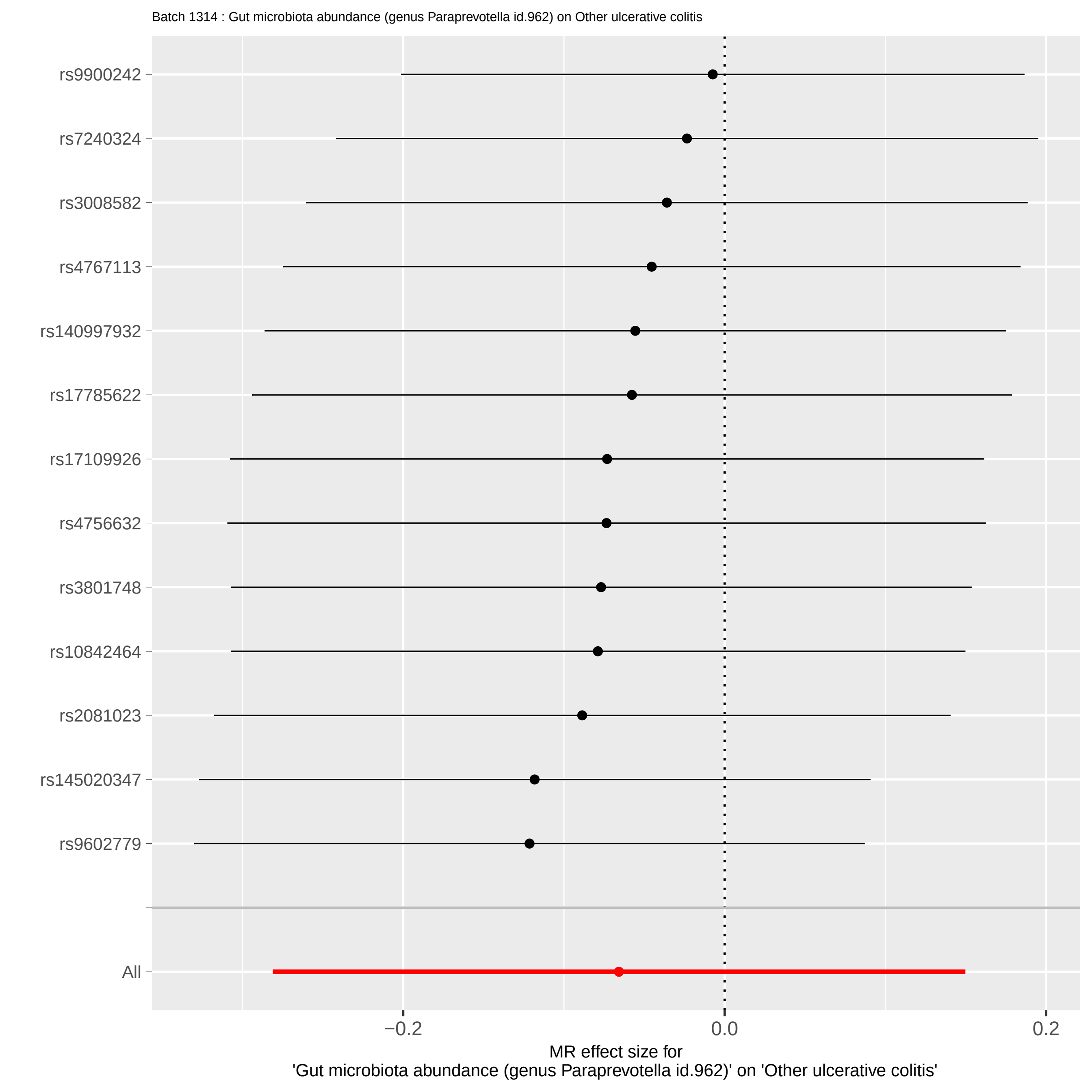

Batch 1314 : Gut microbiota abundance (genus Paraprevotella id.962) on Other ulcerative colitis
rs9900242
rs7240324
rs3008582
rs4767113
rs140997932
rs17785622
rs17109926
rs4756632
rs3801748
rs10842464
rs2081023
rs145020347
rs9602779
All
−0.2
0.0
0.2
MR effect size for
'Gut microbiota abundance (genus Paraprevotella id.962)' on 'Other ulcerative colitis'

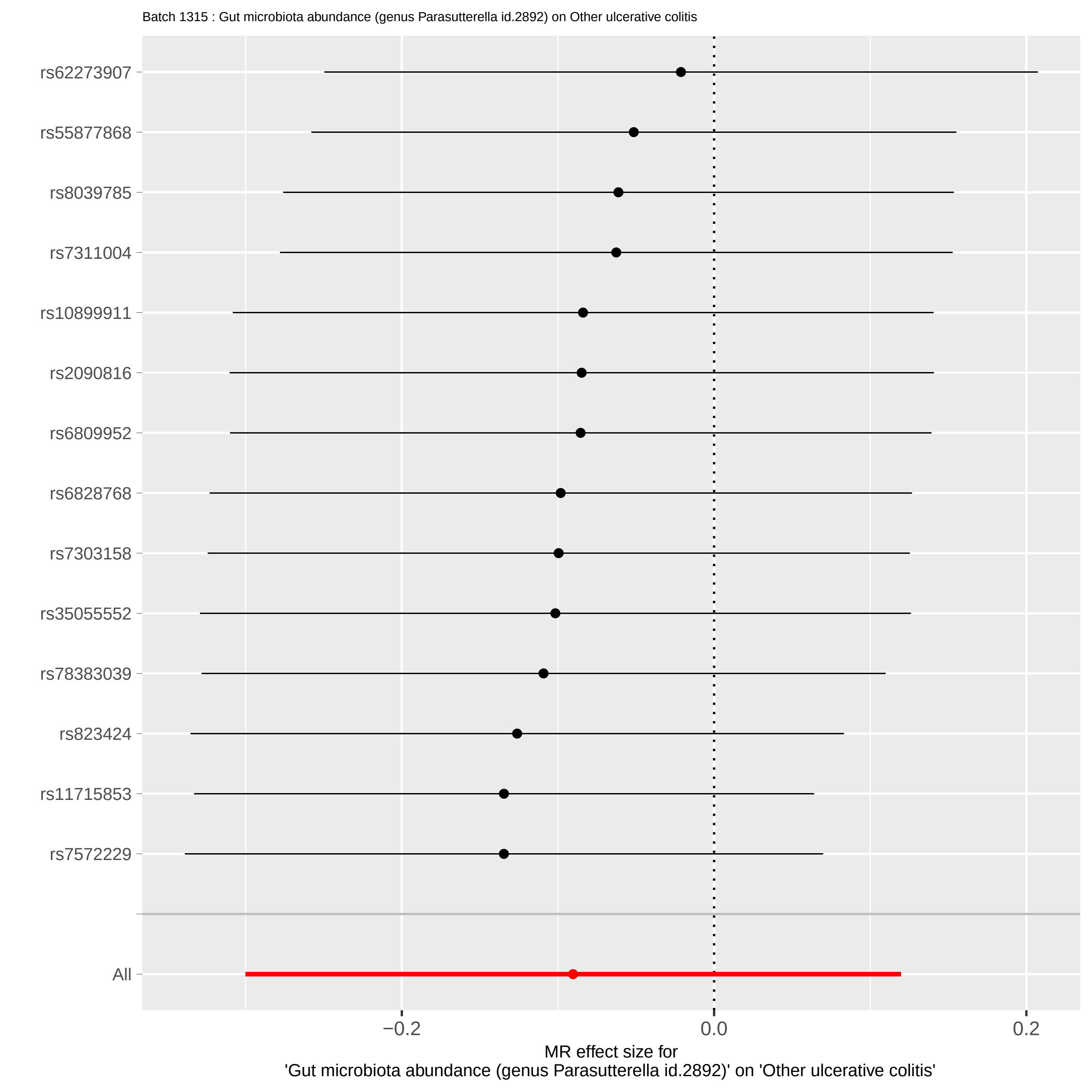

Batch 1315 : Gut microbiota abundance (genus Parasutterella id.2892) on Other ulcerative colitis
rs62273907
rs55877868
rs8039785
rs7311004
rs10899911
rs2090816
rs6809952
rs6828768
rs7303158
rs35055552
rs78383039
rs823424
rs11715853
rs7572229
All
−0.2
0.0
0.2
MR effect size for
'Gut microbiota abundance (genus Parasutterella id.2892)' on 'Other ulcerative colitis'

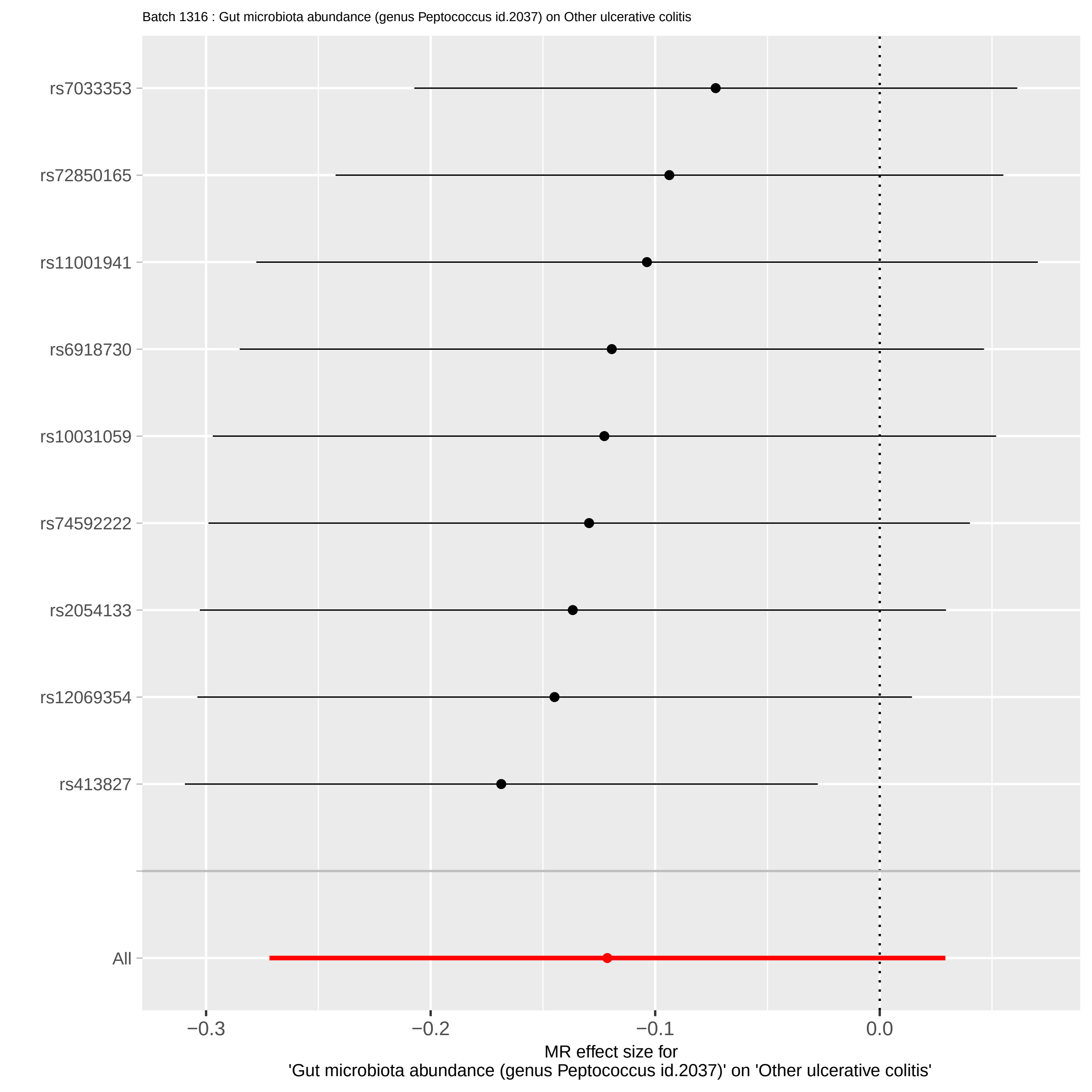

Batch 1316 : Gut microbiota abundance (genus Peptococcus id.2037) on Other ulcerative colitis
rs7033353
rs72850165
rs11001941
rs6918730
rs10031059
rs74592222
rs2054133
rs12069354
rs413827
All
−0.3
−0.2
−0.1
0.0
MR effect size for
'Gut microbiota abundance (genus Peptococcus id.2037)' on 'Other ulcerative colitis'

Batch 1317 : Gut microbiota abundance (genus Phascolarctobacterium id.2168) on Other ulcerative colitis

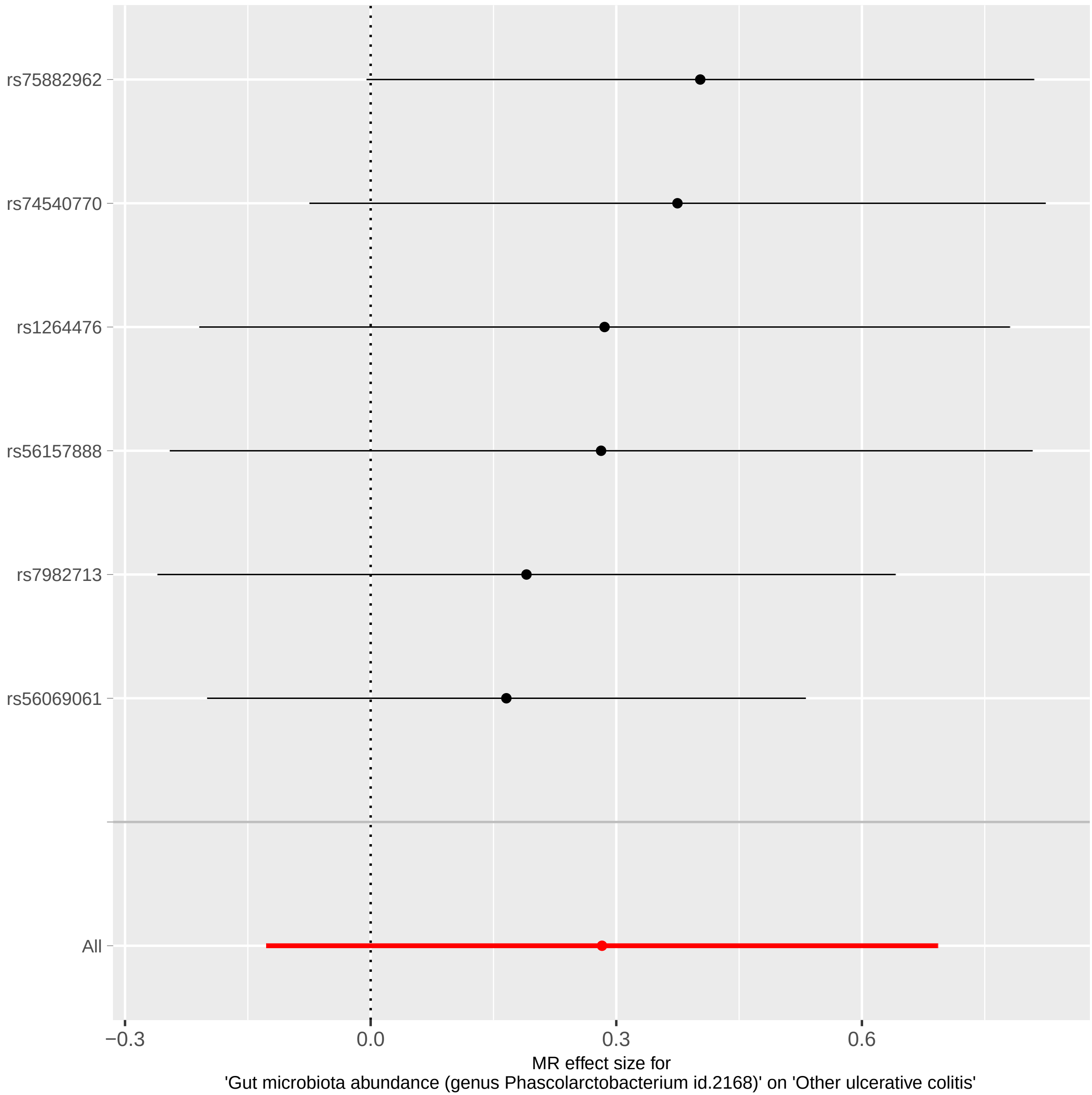

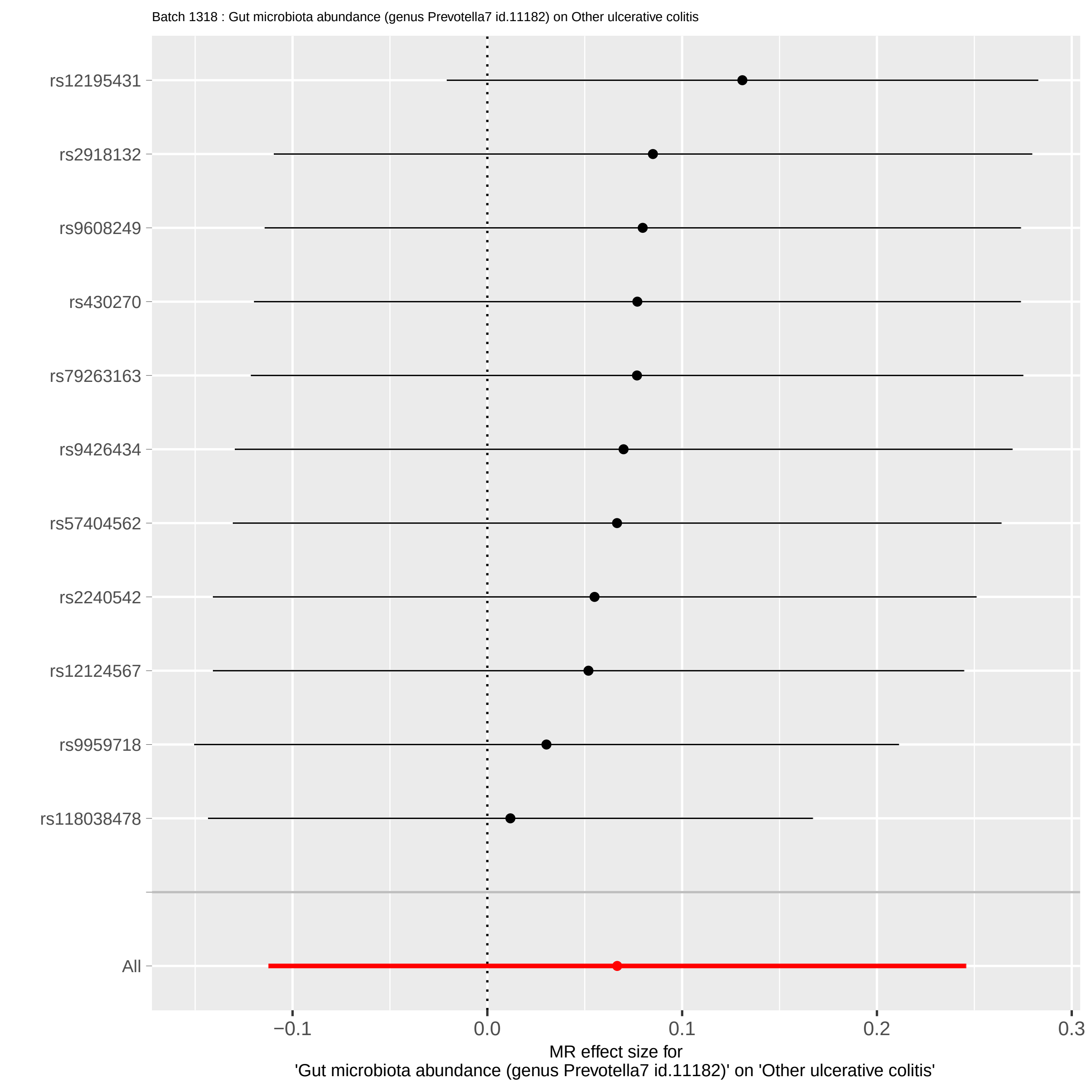

Batch 1318 : Gut microbiota abundance (genus Prevotella7 id.11182) on Other ulcerative colitis
rs12195431
rs2918132
rs9608249
rs430270
rs79263163
rs9426434
rs57404562
rs2240542
rs12124567
rs9959718
rs118038478
All
−0.1
0.0
0.1
0.2
0.3
MR effect size for
'Gut microbiota abundance (genus Prevotella7 id.11182)' on 'Other ulcerative colitis'

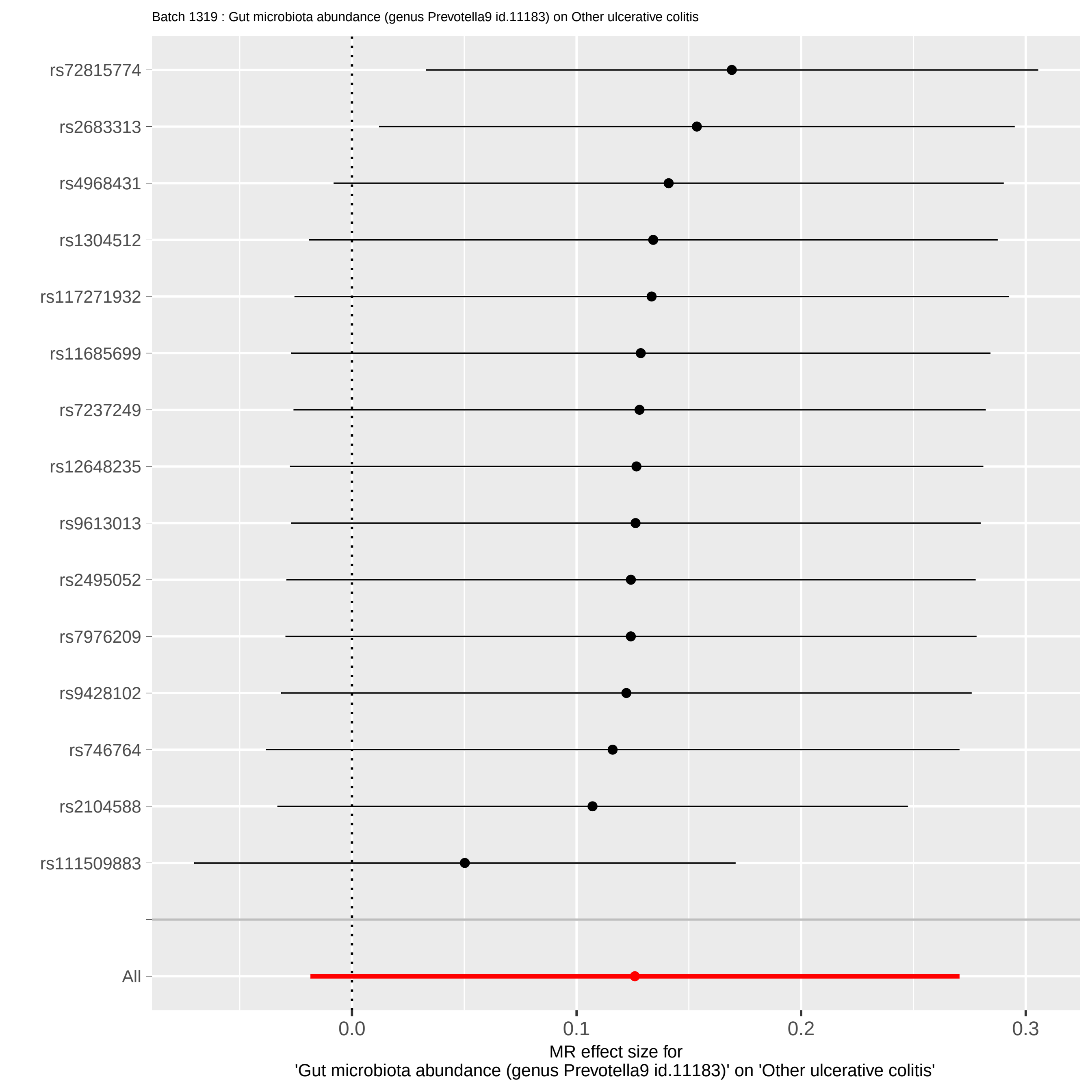

Batch 1319 : Gut microbiota abundance (genus Prevotella9 id.11183) on Other ulcerative colitis
rs72815774
rs2683313
rs4968431
rs1304512
rs117271932
rs11685699
rs7237249
rs12648235
rs9613013
rs2495052
rs7976209
rs9428102
rs746764
rs2104588
rs111509883
All
0.0
0.1
0.2
0.3
MR effect size for
'Gut microbiota abundance (genus Prevotella9 id.11183)' on 'Other ulcerative colitis'

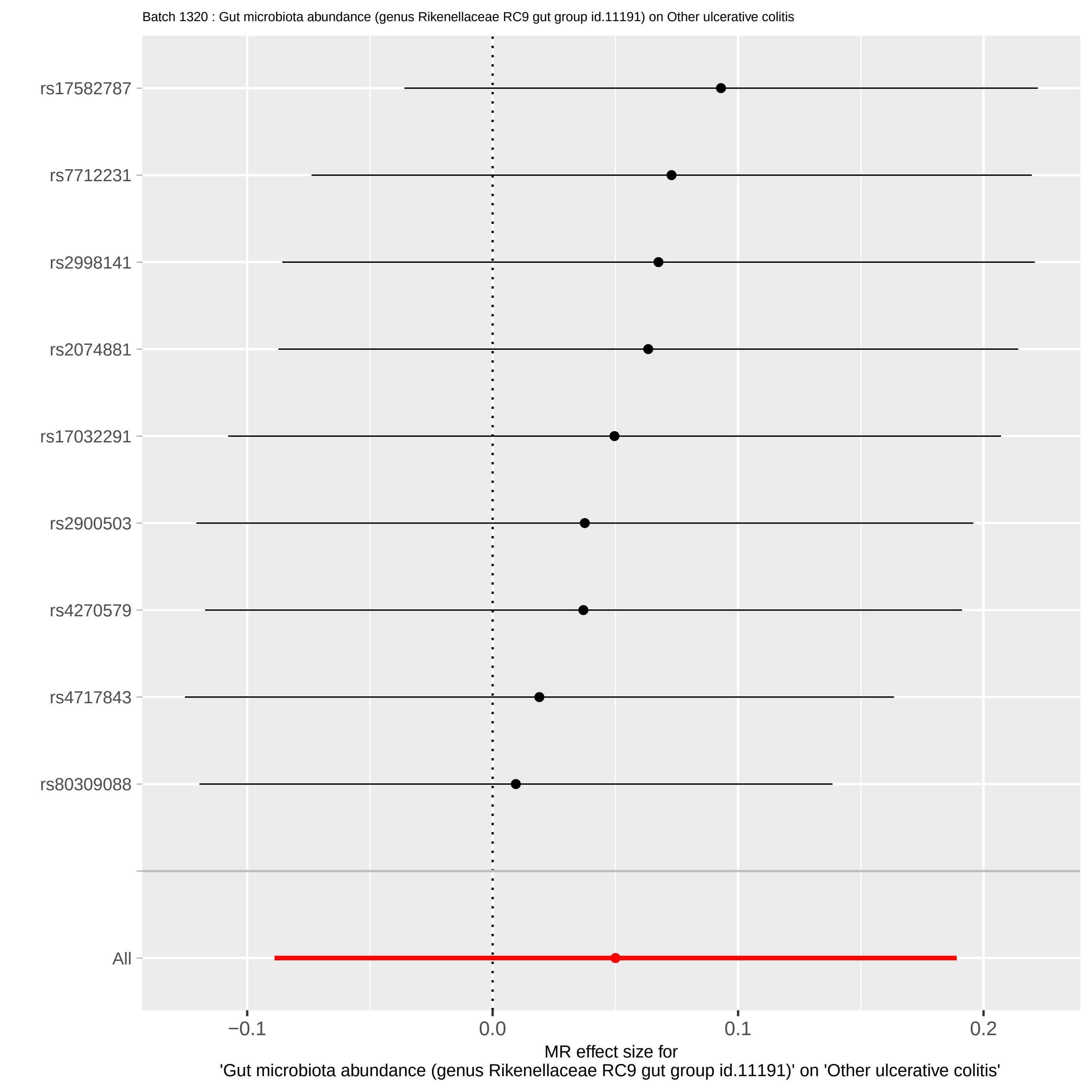

Batch 1320 : Gut microbiota abundance (genus Rikenellaceae RC9 gut group id.11191) on Other ulcerative colitis
rs17582787
rs7712231
rs2998141
rs2074881
rs17032291
rs2900503
rs4270579
rs4717843
rs80309088
All
−0.1
0.0
0.1
0.2
MR effect size for
'Gut microbiota abundance (genus Rikenellaceae RC9 gut group id.11191)' on 'Other ulcerative colitis'

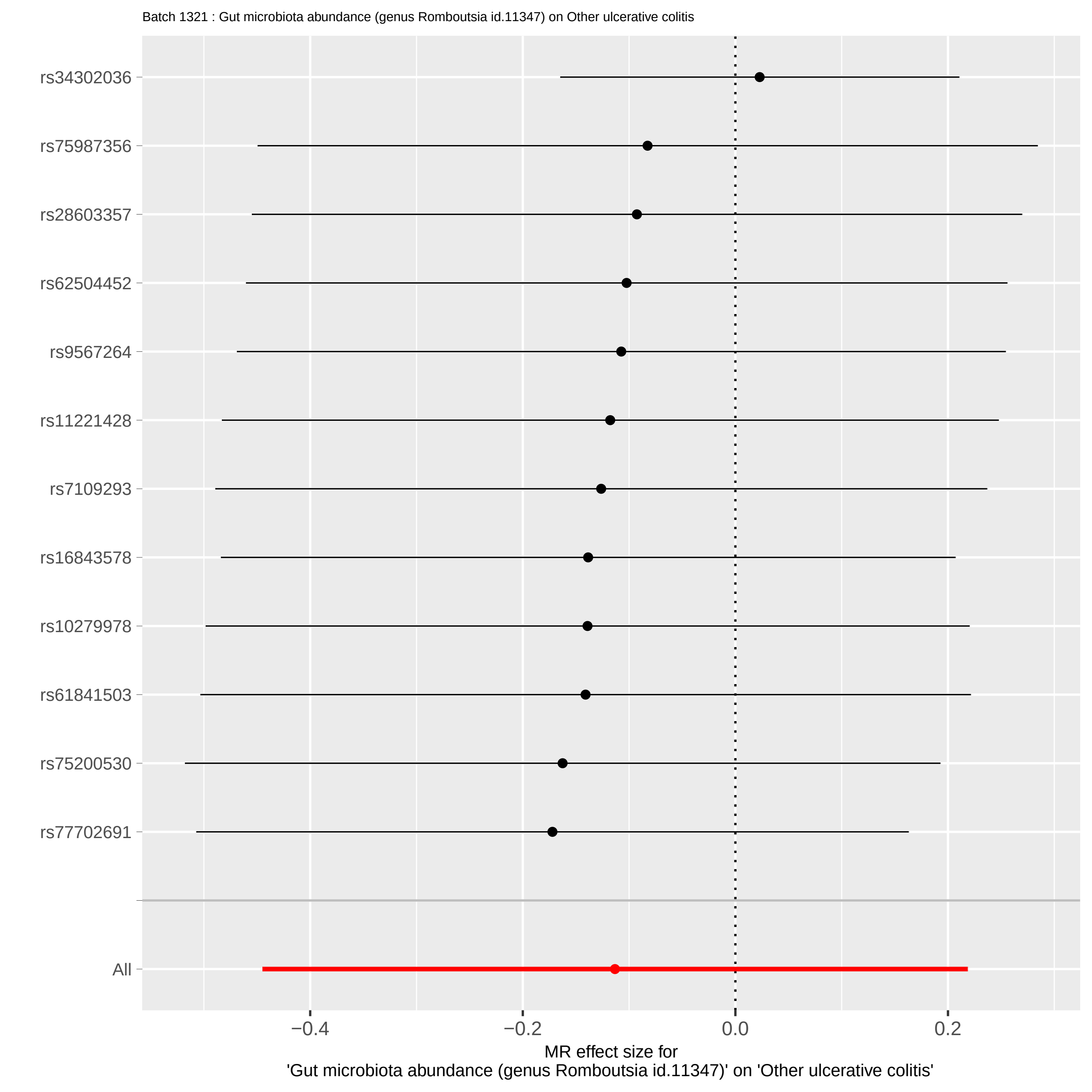
Batch 1321 : Gut microbiota abundance (genus Romboutsia id.11347) on Other ulcerative colitis
rs34302036
rs75987356
rs28603357
rs62504452
rs9567264
rs11221428
rs7109293
rs16843578
rs10279978
rs61841503
rs75200530
rs77702691
All
−0.4
−0.2
0.0
0.2
MR effect size for
'Gut microbiota abundance (genus Romboutsia id.11347)' on 'Other ulcerative colitis'

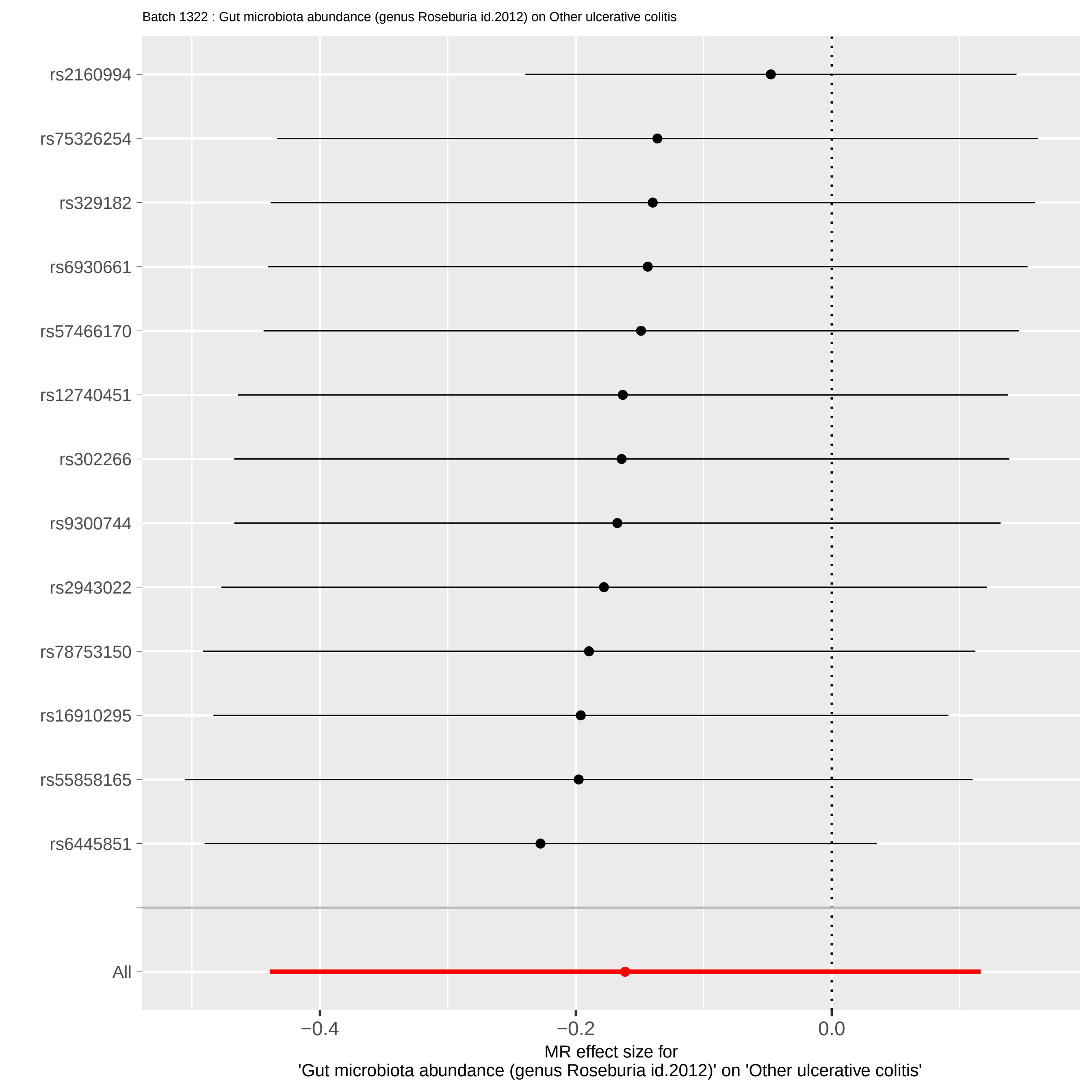

Batch 1322 : Gut microbiota abundance (genus Roseburia id.2012) on Other ulcerative colitis
rs2160994
rs75326254
rs329182
rs6930661
rs57466170
rs12740451
rs302266
rs9300744
rs2943022
rs78753150
rs16910295
rs55858165
rs6445851
All
−0.4
−0.2
0.0
MR effect size for
'Gut microbiota abundance (genus Roseburia id.2012)' on 'Other ulcerative colitis'

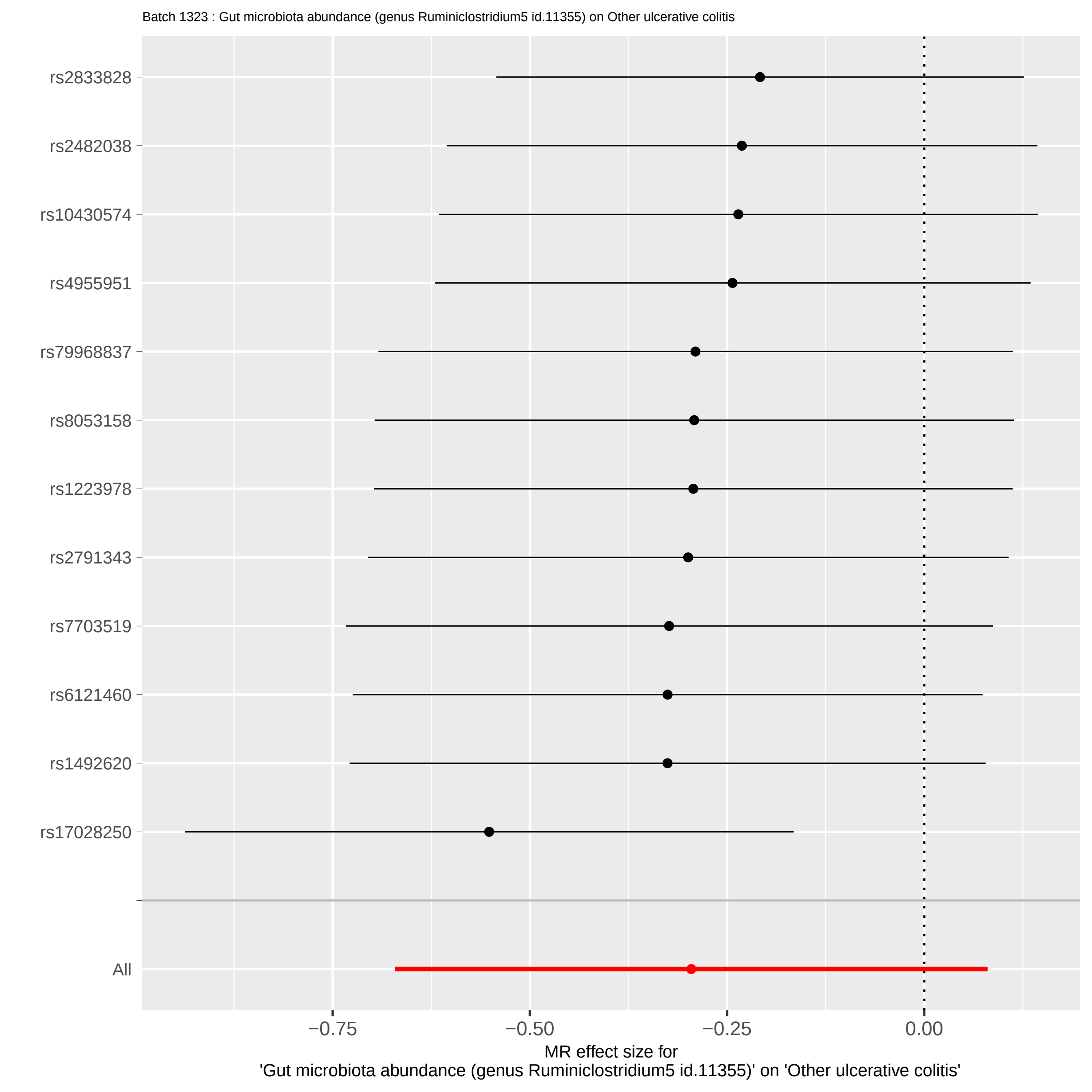

Batch 1323 : Gut microbiota abundance (genus Ruminiclostridium5 id.11355) on Other ulcerative colitis
rs2833828
rs2482038
rs10430574
rs4955951
rs79968837
rs8053158
rs1223978
rs2791343
rs7703519
rs6121460
rs1492620
rs17028250
All
−0.75
−0.50
−0.25
0.00
MR effect size for
'Gut microbiota abundance (genus Ruminiclostridium5 id.11355)' on 'Other ulcerative colitis'

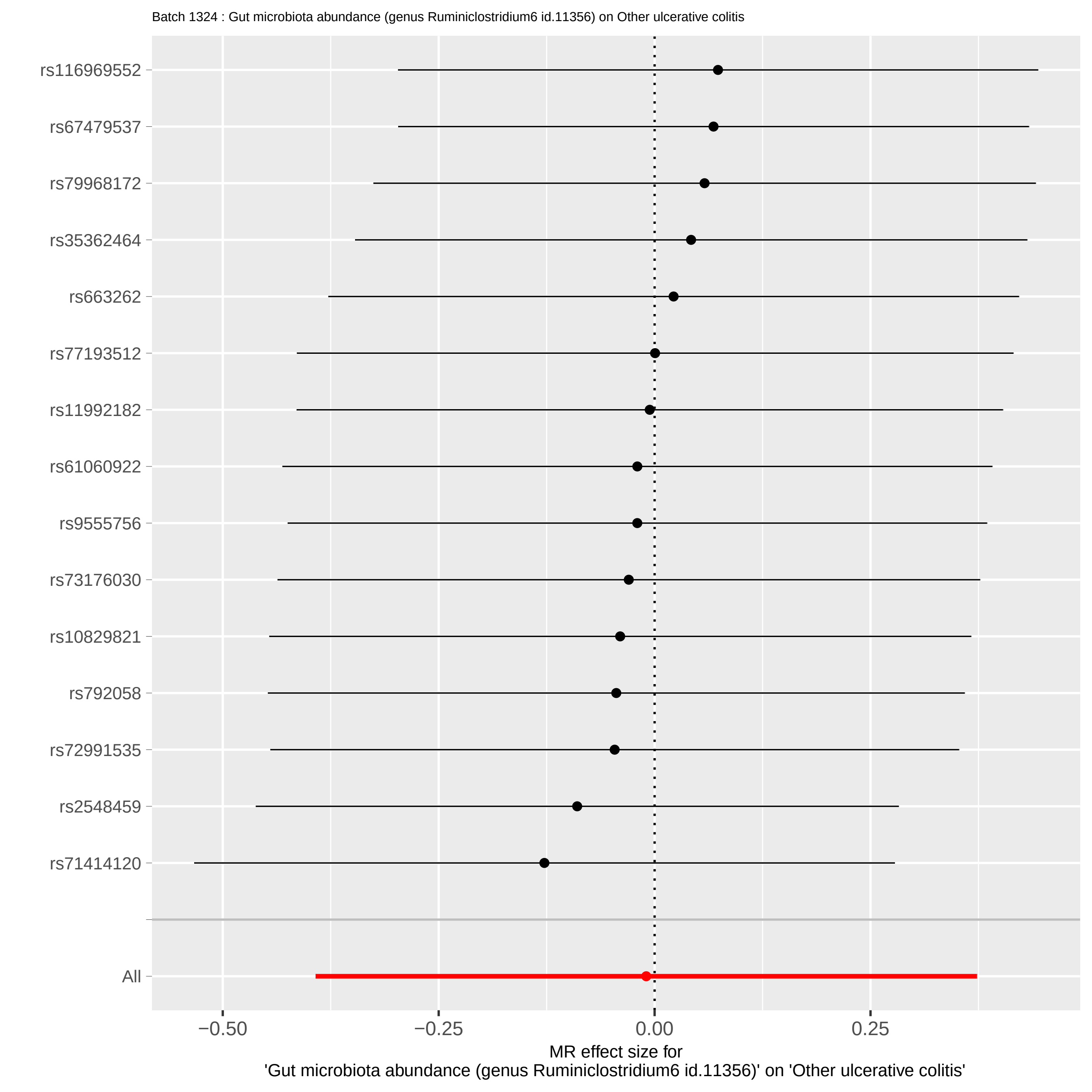
Batch 1324 : Gut microbiota abundance (genus Ruminiclostridium6 id.11356) on Other ulcerative colitis
rs116969552
rs67479537
rs79968172
rs35362464
rs663262
rs77193512
rs11992182
rs61060922
rs9555756
rs73176030
rs10829821
rs792058
rs72991535
rs2548459
rs71414120
All
−0.50
−0.25
0.00
0.25
MR effect size for
'Gut microbiota abundance (genus Ruminiclostridium6 id.11356)' on 'Other ulcerative colitis'

Batch 1325 : Gut microbiota abundance (genus Ruminiclostridium9 id.11357) on Other ulcerative colitis

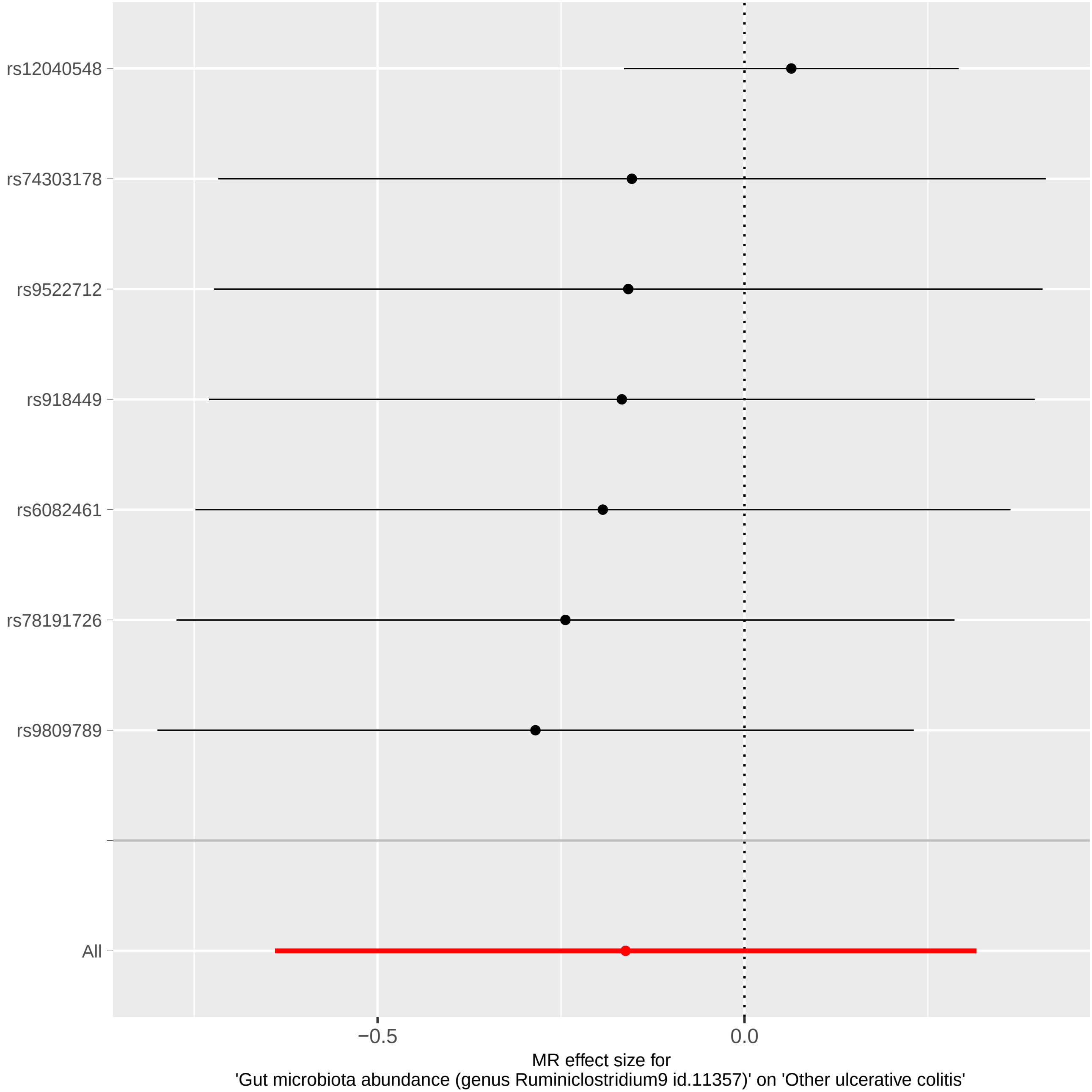

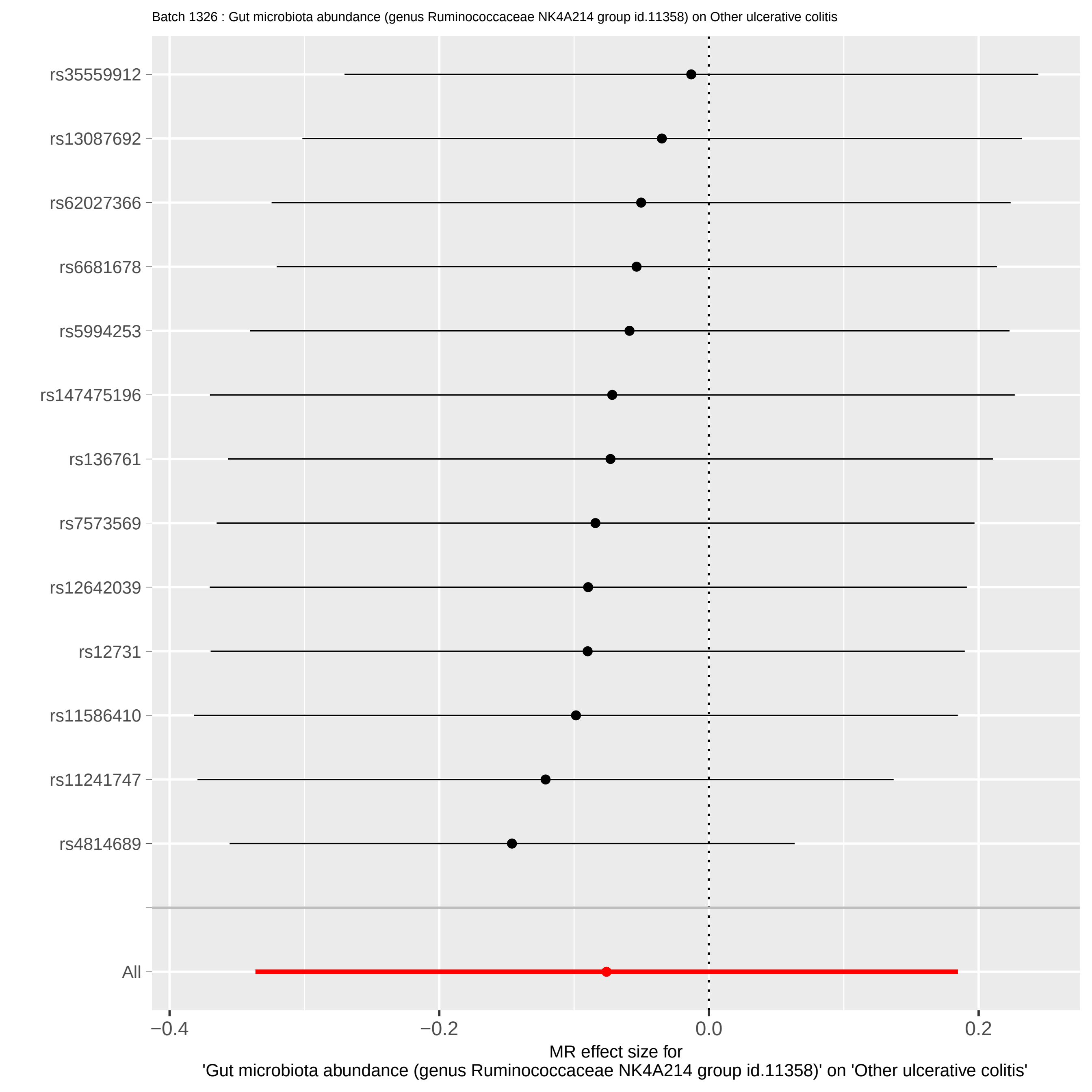

Batch 1326 : Gut microbiota abundance (genus Ruminococcaceae NK4A214 group id.11358) on Other ulcerative colitis
rs35559912
rs13087692
rs62027366
rs6681678
rs5994253
rs147475196
rs136761
rs7573569
rs12642039
rs12731
rs11586410
rs11241747
rs4814689
All
−0.4
−0.2
0.0
0.2
MR effect size for
'Gut microbiota abundance (genus Ruminococcaceae NK4A214 group id.11358)' on 'Other ulcerative colitis'

Batch 1327 : Gut microbiota abundance (genus Ruminococcaceae UCG002 id.11360) on Other ulcerative colitis

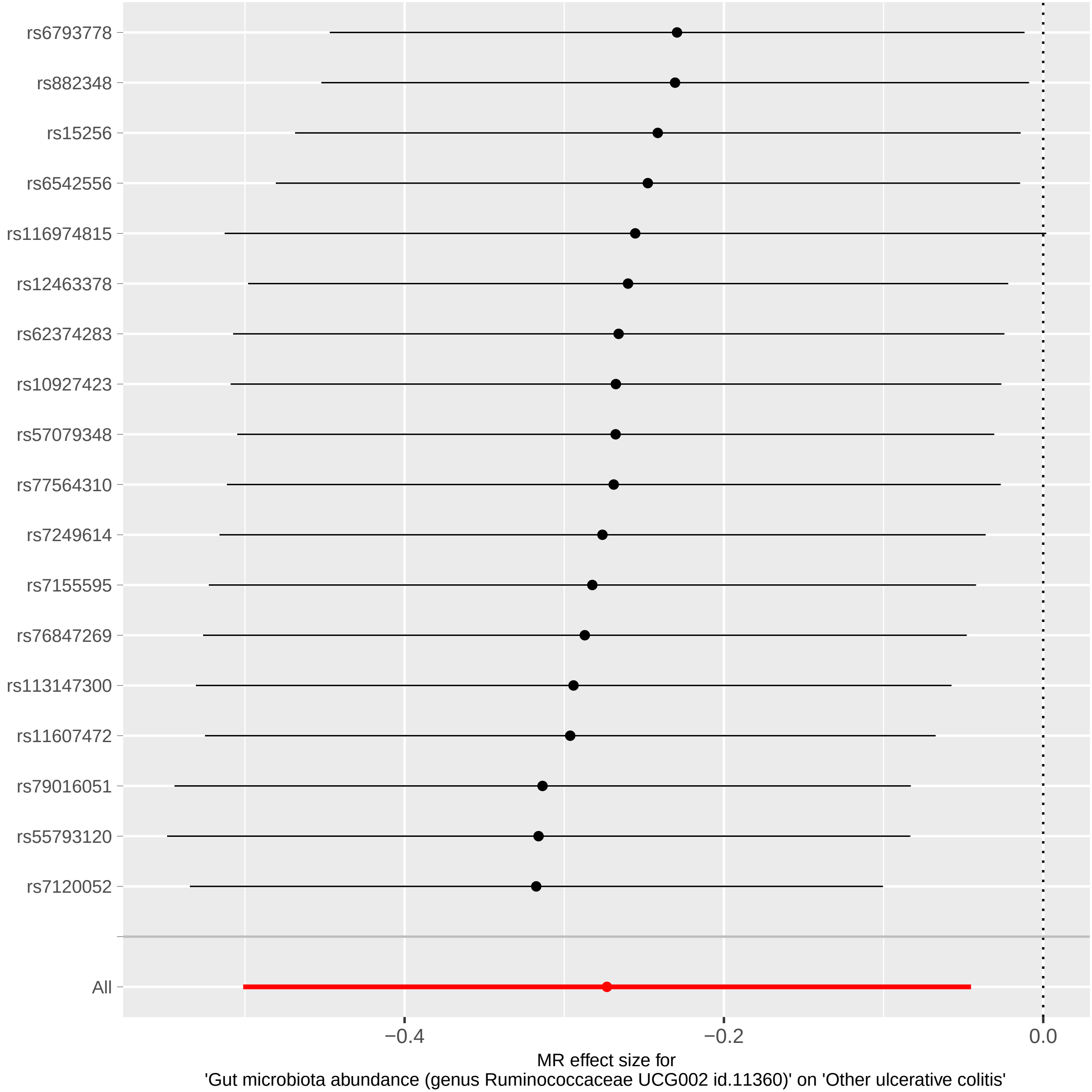

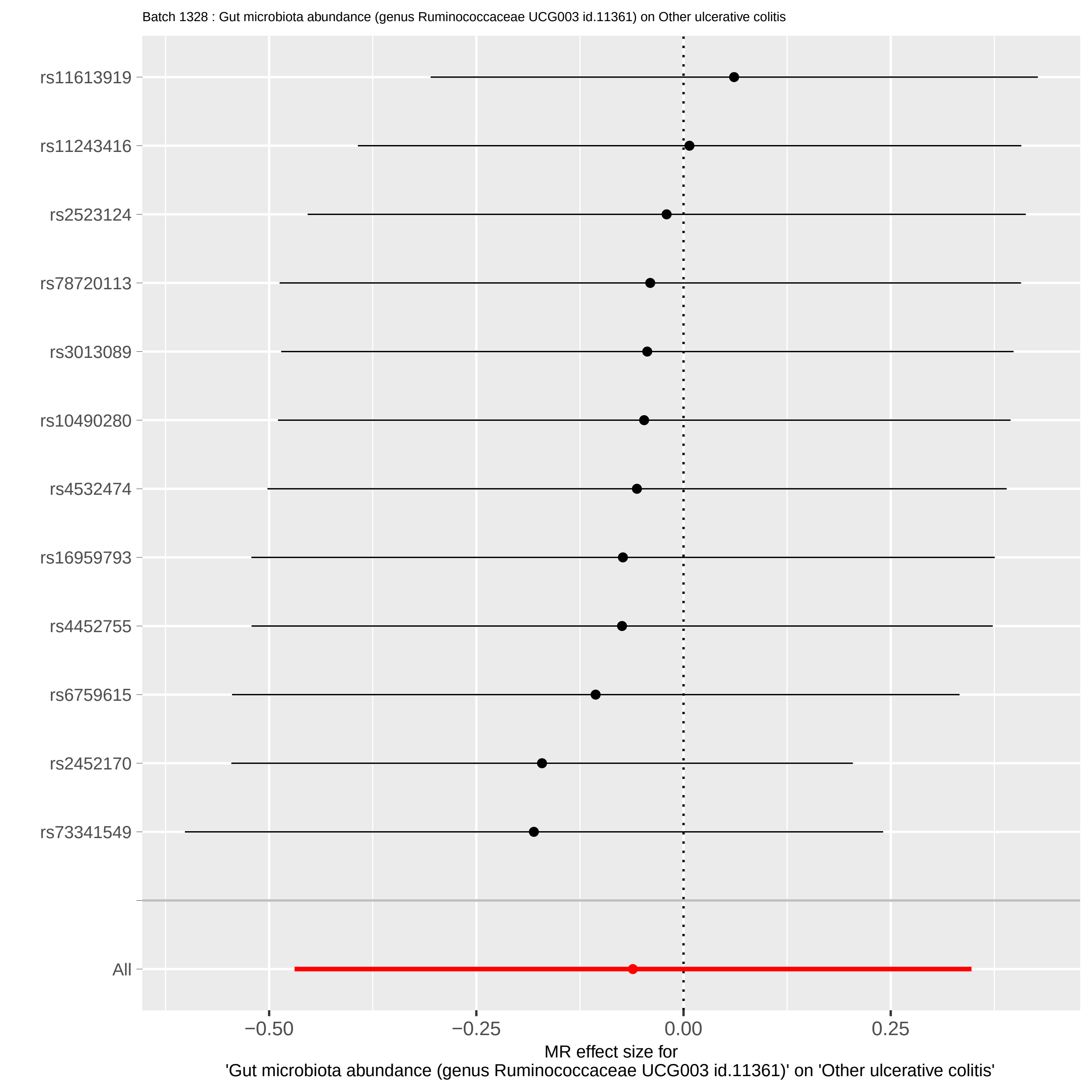

Batch 1328 : Gut microbiota abundance (genus Ruminococcaceae UCG003 id.11361) on Other ulcerative colitis
rs11613919
rs11243416
rs2523124
rs78720113
rs3013089
rs10490280
rs4532474
rs16959793
rs4452755
rs6759615
rs2452170
rs73341549
All
−0.50
−0.25
0.00
0.25
MR effect size for
'Gut microbiota abundance (genus Ruminococcaceae UCG003 id.11361)' on 'Other ulcerative colitis'

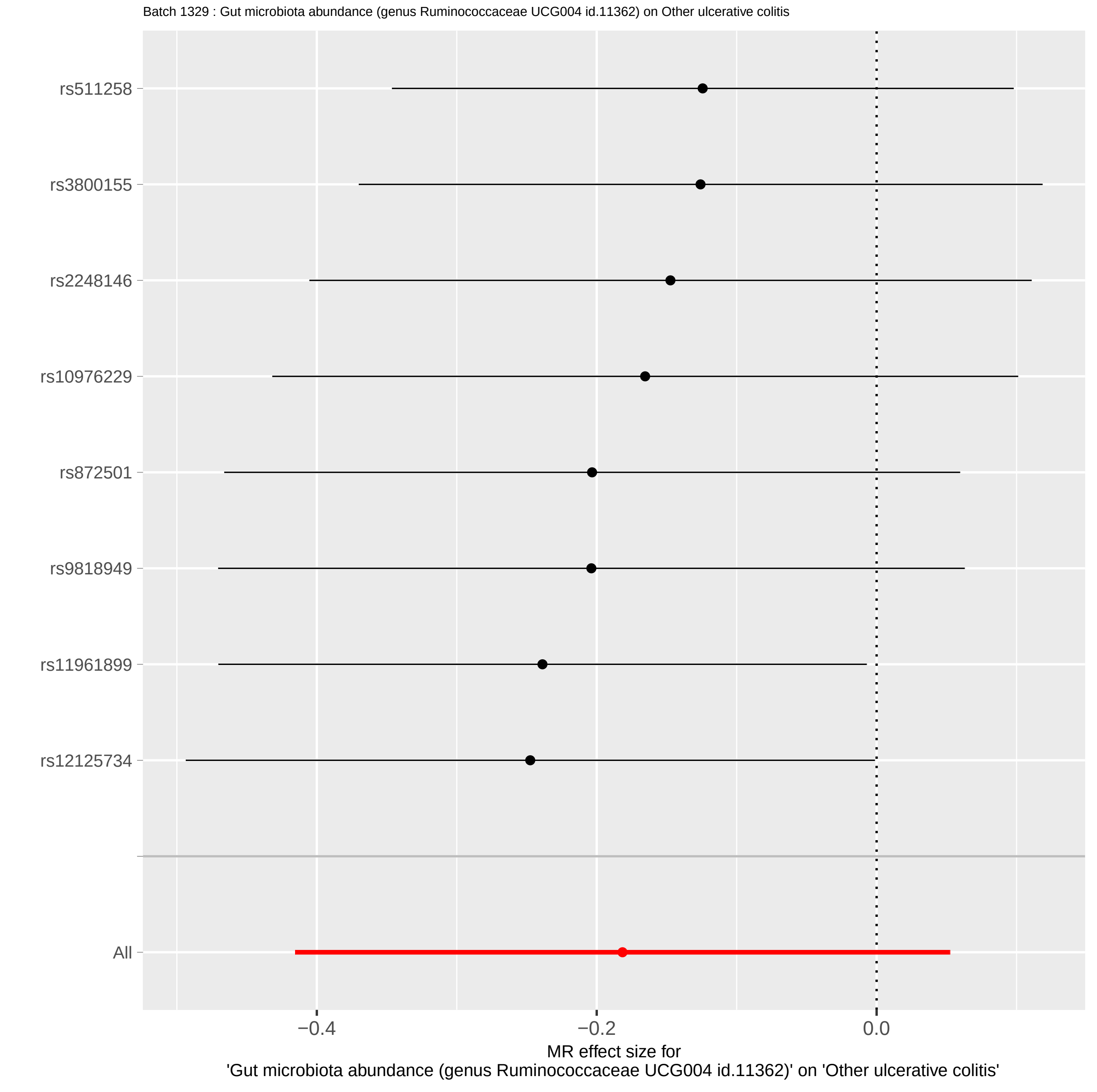

Batch 1329 : Gut microbiota abundance (genus Ruminococcaceae UCG004 id.11362) on Other ulcerative colitis
rs511258
rs3800155
rs2248146
rs10976229
rs872501
rs9818949
rs11961899
rs12125734
All
−0.4
−0.2
0.0
MR effect size for
'Gut microbiota abundance (genus Ruminococcaceae UCG004 id.11362)' on 'Other ulcerative colitis'

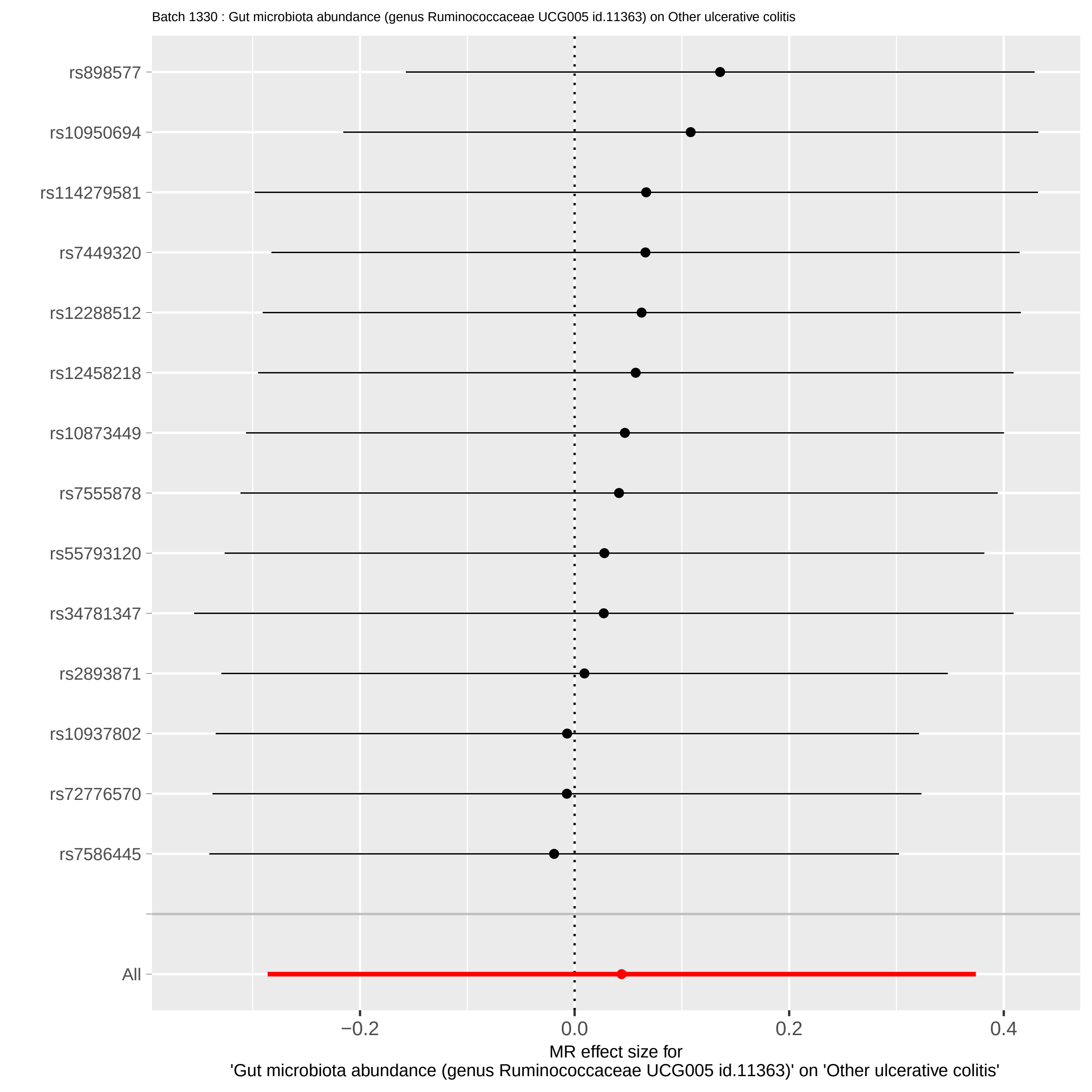

Batch 1330 : Gut microbiota abundance (genus Ruminococcaceae UCG005 id.11363) on Other ulcerative colitis
rs898577
rs10950694
rs114279581
rs7449320
rs12288512
rs12458218
rs10873449
rs7555878
rs55793120
rs34781347
rs2893871
rs10937802
rs72776570
rs7586445
All
−0.2
0.0
0.2
0.4
MR effect size for
'Gut microbiota abundance (genus Ruminococcaceae UCG005 id.11363)' on 'Other ulcerative colitis'

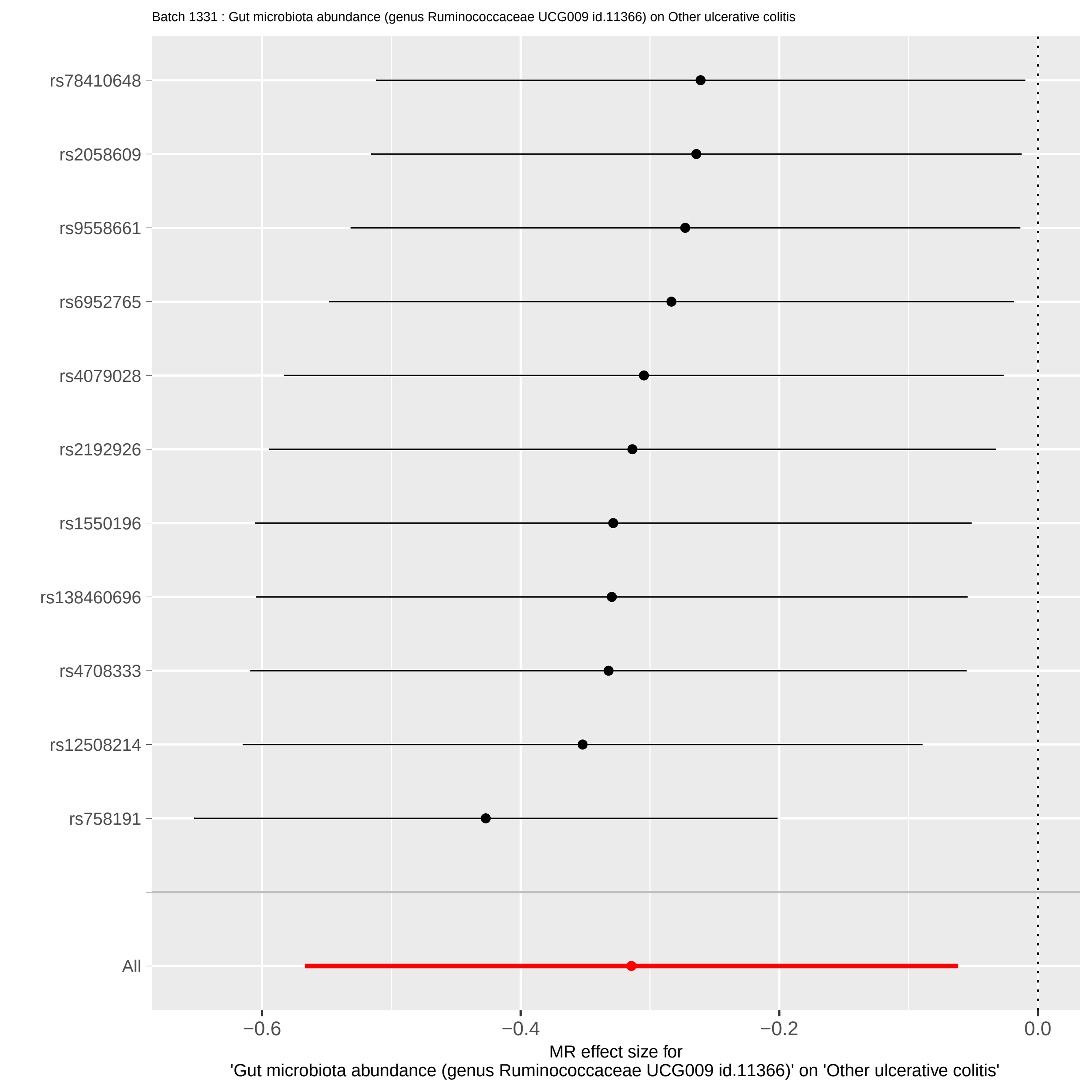
Batch 1331 : Gut microbiota abundance (genus Ruminococcaceae UCG009 id.11366) on Other ulcerative colitis
rs78410648
rs2058609
rs9558661
rs6952765
rs4079028
rs2192926
rs1550196
rs138460696
rs4708333
rs12508214
rs758191
All
−0.6
−0.4
−0.2
0.0
MR effect size for
'Gut microbiota abundance (genus Ruminococcaceae UCG009 id.11366)' on 'Other ulcerative colitis'

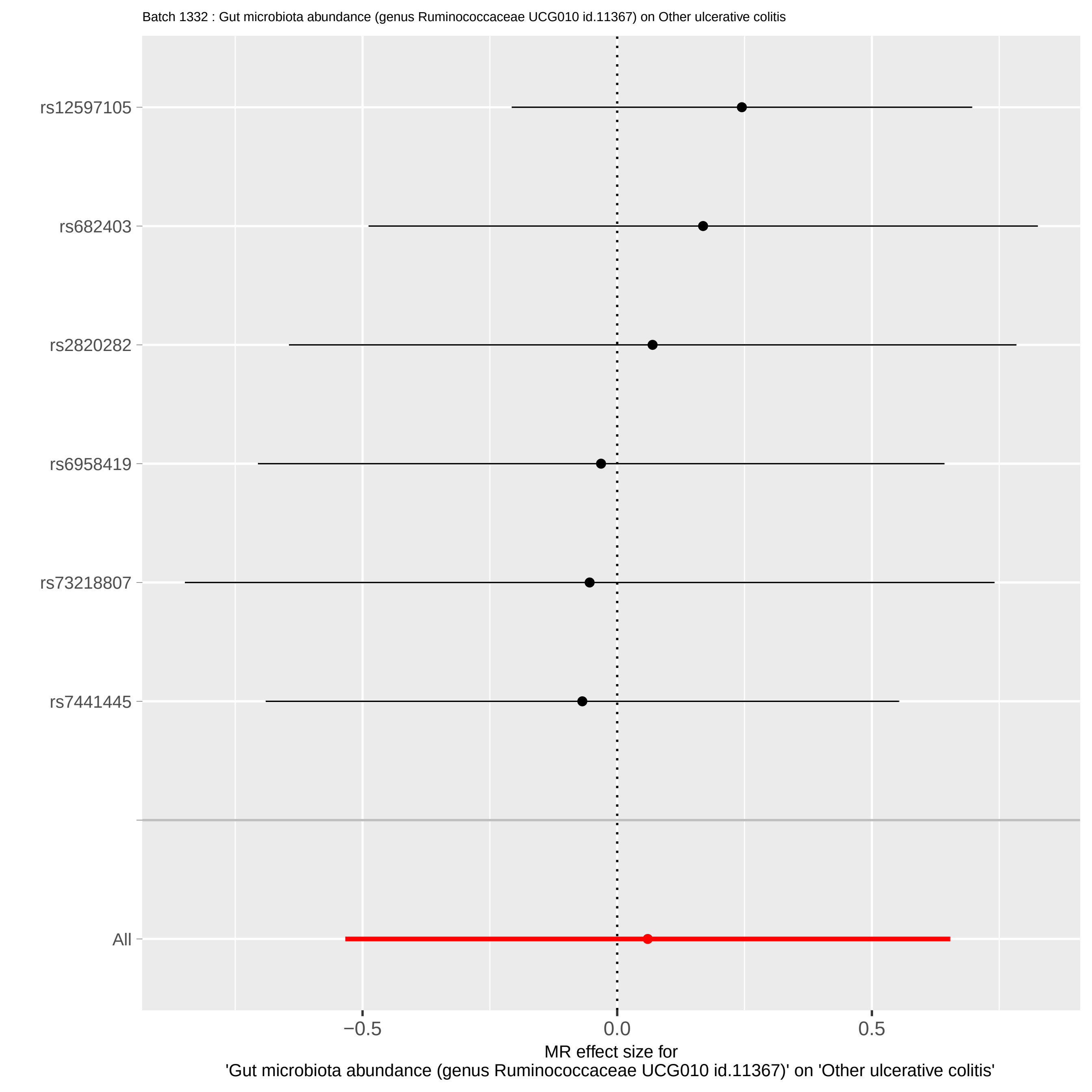

Batch 1332 : Gut microbiota abundance (genus Ruminococcaceae UCG010 id.11367) on Other ulcerative colitis
rs12597105
rs682403
rs2820282
rs6958419
rs73218807
rs7441445
All
−0.5
0.0
0.5
MR effect size for
'Gut microbiota abundance (genus Ruminococcaceae UCG010 id.11367)' on 'Other ulcerative colitis'

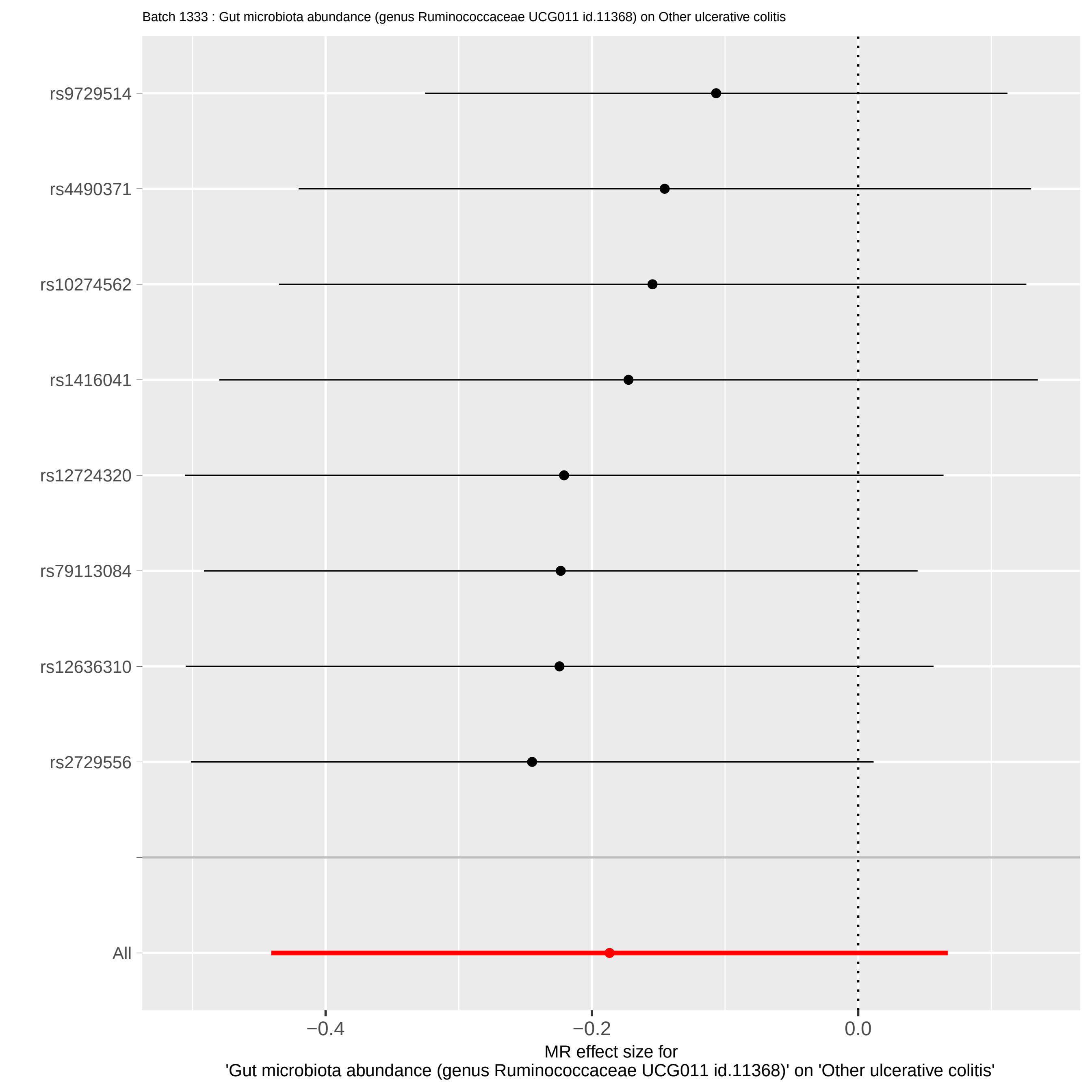

Batch 1333 : Gut microbiota abundance (genus Ruminococcaceae UCG011 id.11368) on Other ulcerative colitis
rs9729514
rs4490371
rs10274562
rs1416041
rs12724320
rs79113084
rs12636310
rs2729556
All
−0.4
−0.2
0.0
MR effect size for
'Gut microbiota abundance (genus Ruminococcaceae UCG011 id.11368)' on 'Other ulcerative colitis'

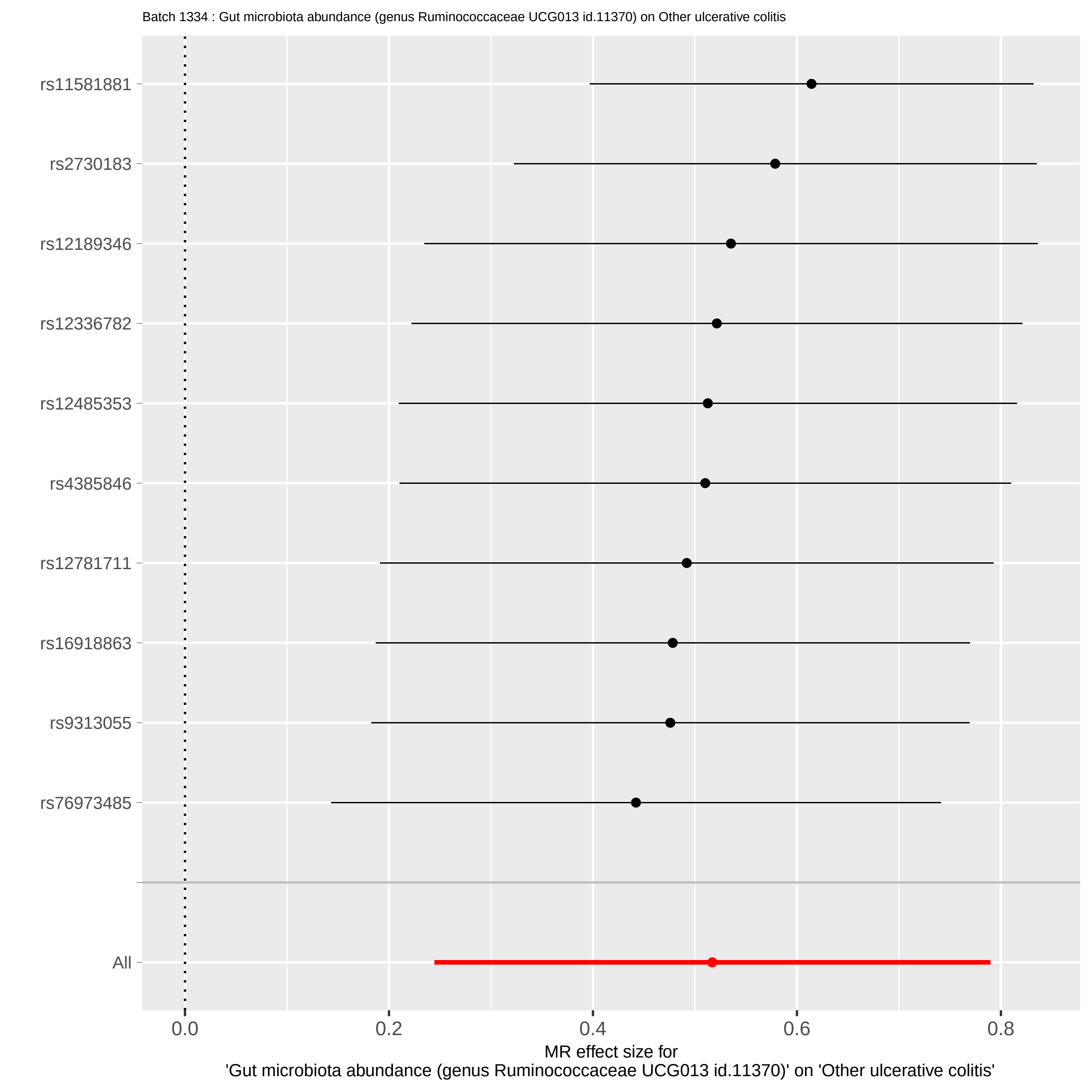
Batch 1334 : Gut microbiota abundance (genus Ruminococcaceae UCG013 id.11370) on Other ulcerative colitis
rs11581881
rs2730183
rs12189346
rs12336782
rs12485353
rs4385846
rs12781711
rs16918863
rs9313055
rs76973485
All
0.0
0.2
0.4
0.6
0.8
MR effect size for
'Gut microbiota abundance (genus Ruminococcaceae UCG013 id.11370)' on 'Other ulcerative colitis'

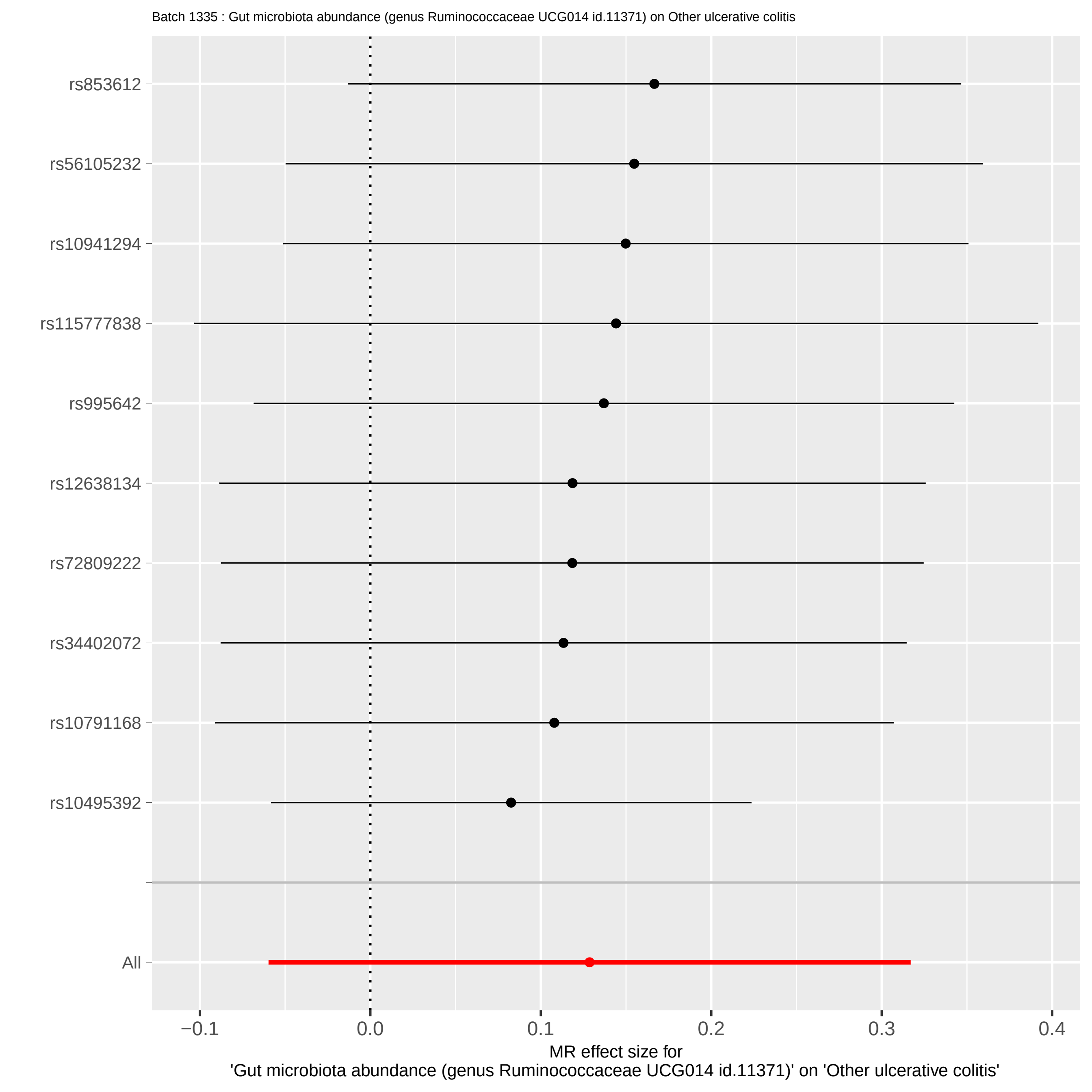
Batch 1335 : Gut microbiota abundance (genus Ruminococcaceae UCG014 id.11371) on Other ulcerative colitis
rs853612
rs56105232
rs10941294
rs115777838
rs995642
rs12638134
rs72809222
rs34402072
rs10791168
rs10495392
All
−0.1
0.0
0.1
0.2
0.3
0.4
MR effect size for
'Gut microbiota abundance (genus Ruminococcaceae UCG014 id.11371)' on 'Other ulcerative colitis'

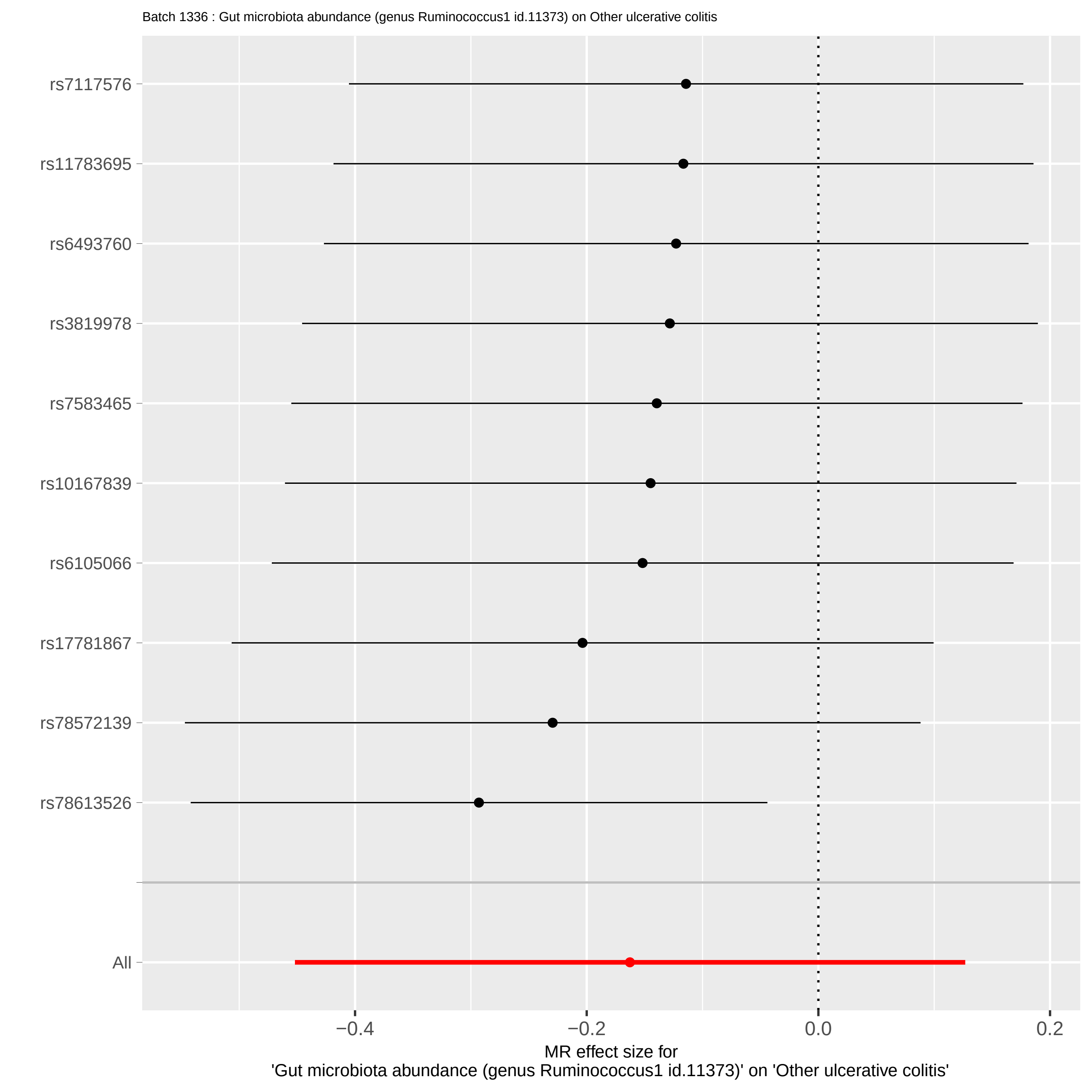

Batch 1336 : Gut microbiota abundance (genus Ruminococcus1 id.11373) on Other ulcerative colitis
rs7117576
rs11783695
rs6493760
rs3819978
rs7583465
rs10167839
rs6105066
rs17781867
rs78572139
rs78613526
All
−0.4
−0.2
0.0
0.2
MR effect size for
'Gut microbiota abundance (genus Ruminococcus1 id.11373)' on 'Other ulcerative colitis'

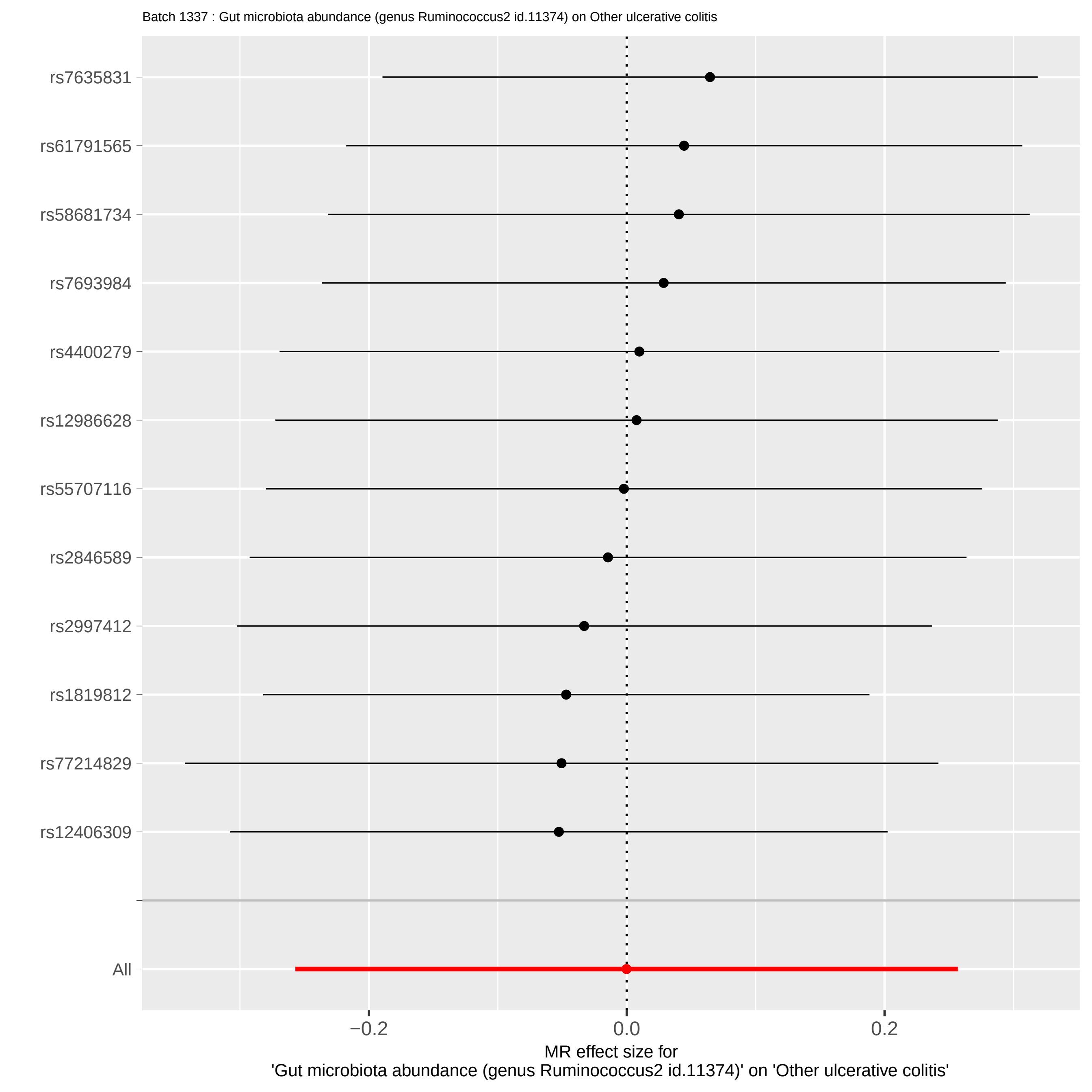

Batch 1337 : Gut microbiota abundance (genus Ruminococcus2 id.11374) on Other ulcerative colitis
rs7635831
rs61791565
rs58681734
rs7693984
rs4400279
rs12986628
rs55707116
rs2846589
rs2997412
rs1819812
rs77214829
rs12406309
All
−0.2
0.0
0.2
MR effect size for
'Gut microbiota abundance (genus Ruminococcus2 id.11374)' on 'Other ulcerative colitis'

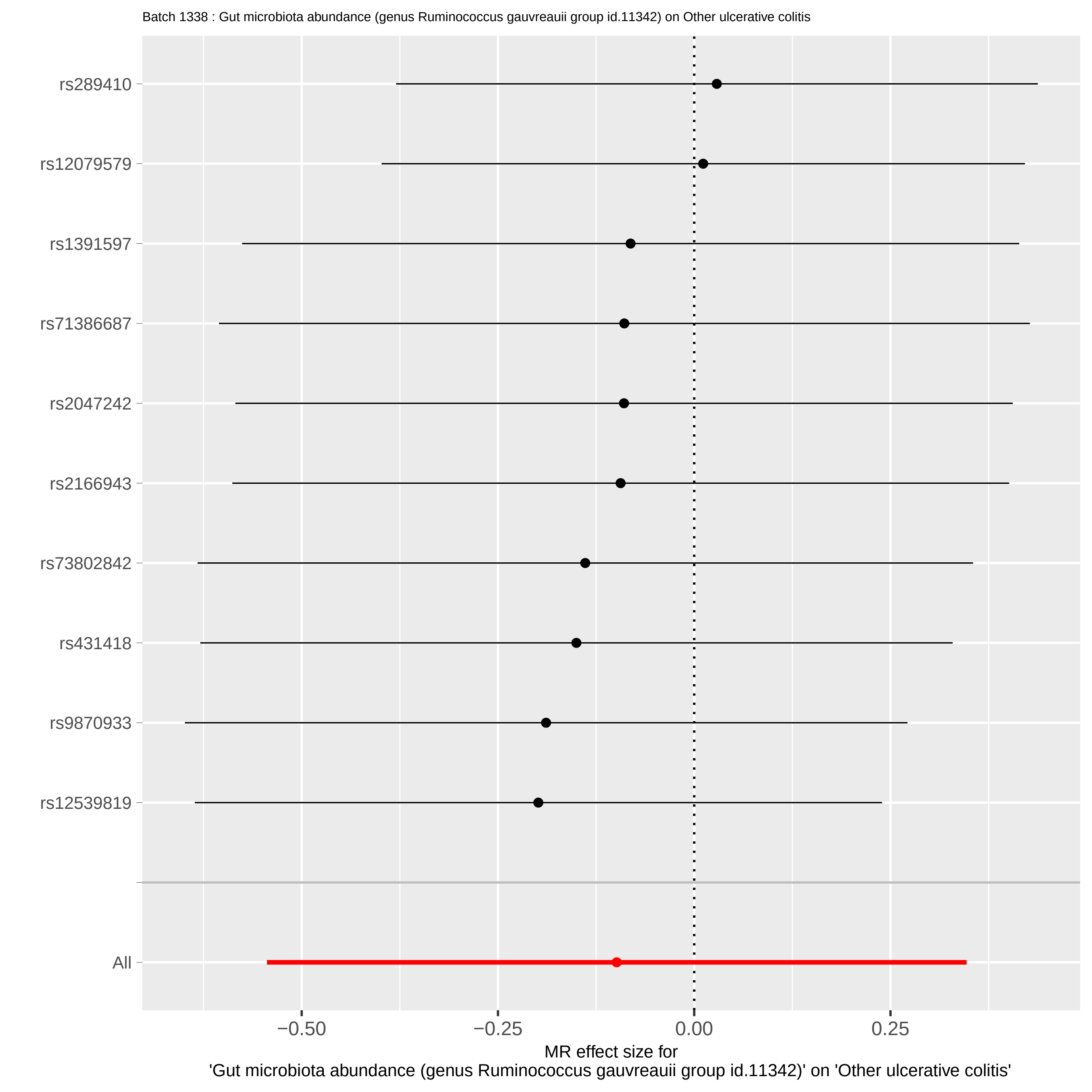

Batch 1338 : Gut microbiota abundance (genus Ruminococcus gauvreauii group id.11342) on Other ulcerative colitis
rs289410
rs12079579
rs1391597
rs71386687
rs2047242
rs2166943
rs73802842
rs431418
rs9870933
rs12539819
All
−0.50
−0.25
0.00
0.25
MR effect size for
'Gut microbiota abundance (genus Ruminococcus gauvreauii group id.11342)' on 'Other ulcerative colitis'

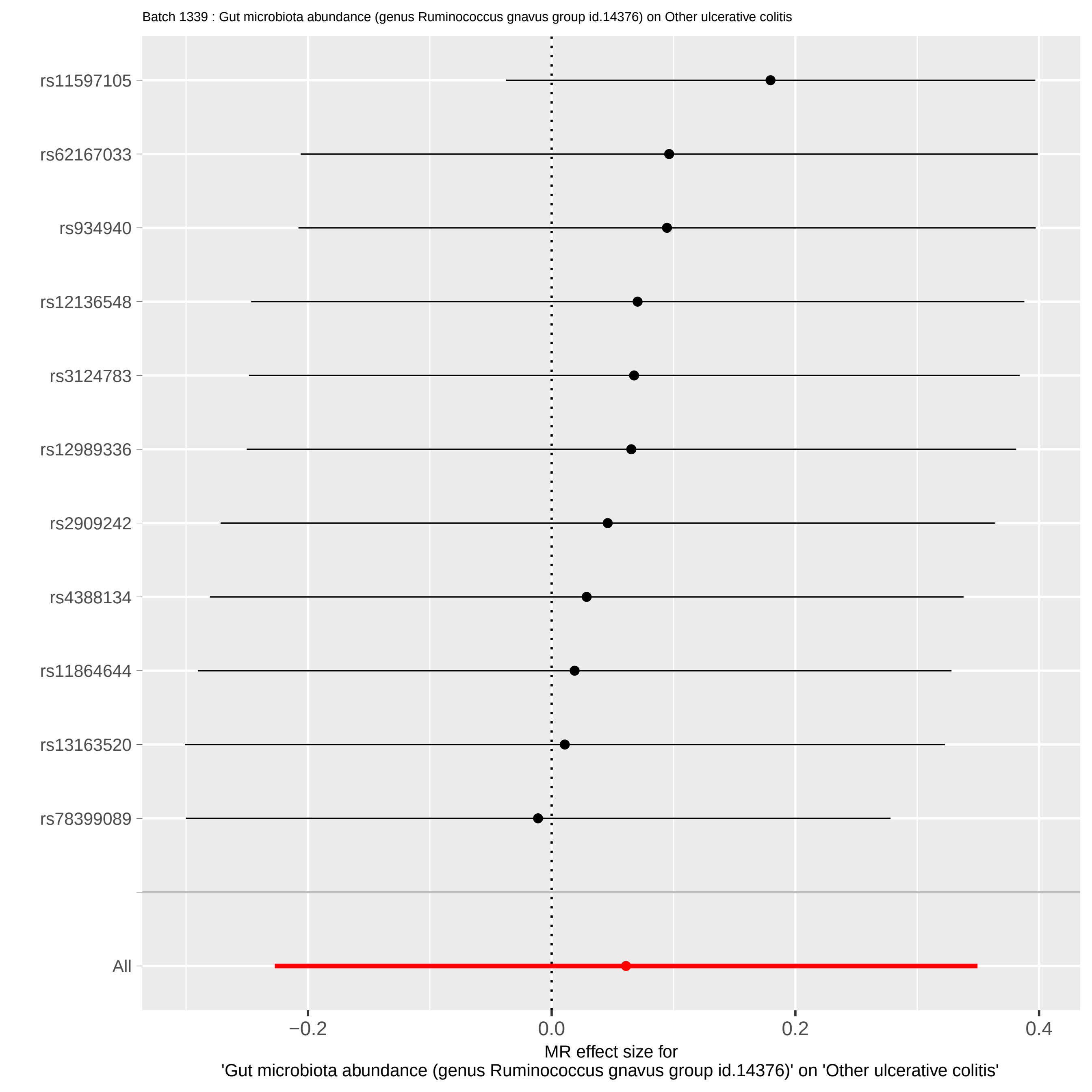

Batch 1339 : Gut microbiota abundance (genus Ruminococcus gnavus group id.14376) on Other ulcerative colitis
rs11597105
rs62167033
rs934940
rs12136548
rs3124783
rs12989336
rs2909242
rs4388134
rs11864644
rs13163520
rs78399089
All
−0.2
0.0
0.2
0.4
MR effect size for
'Gut microbiota abundance (genus Ruminococcus gnavus group id.14376)' on 'Other ulcerative colitis'

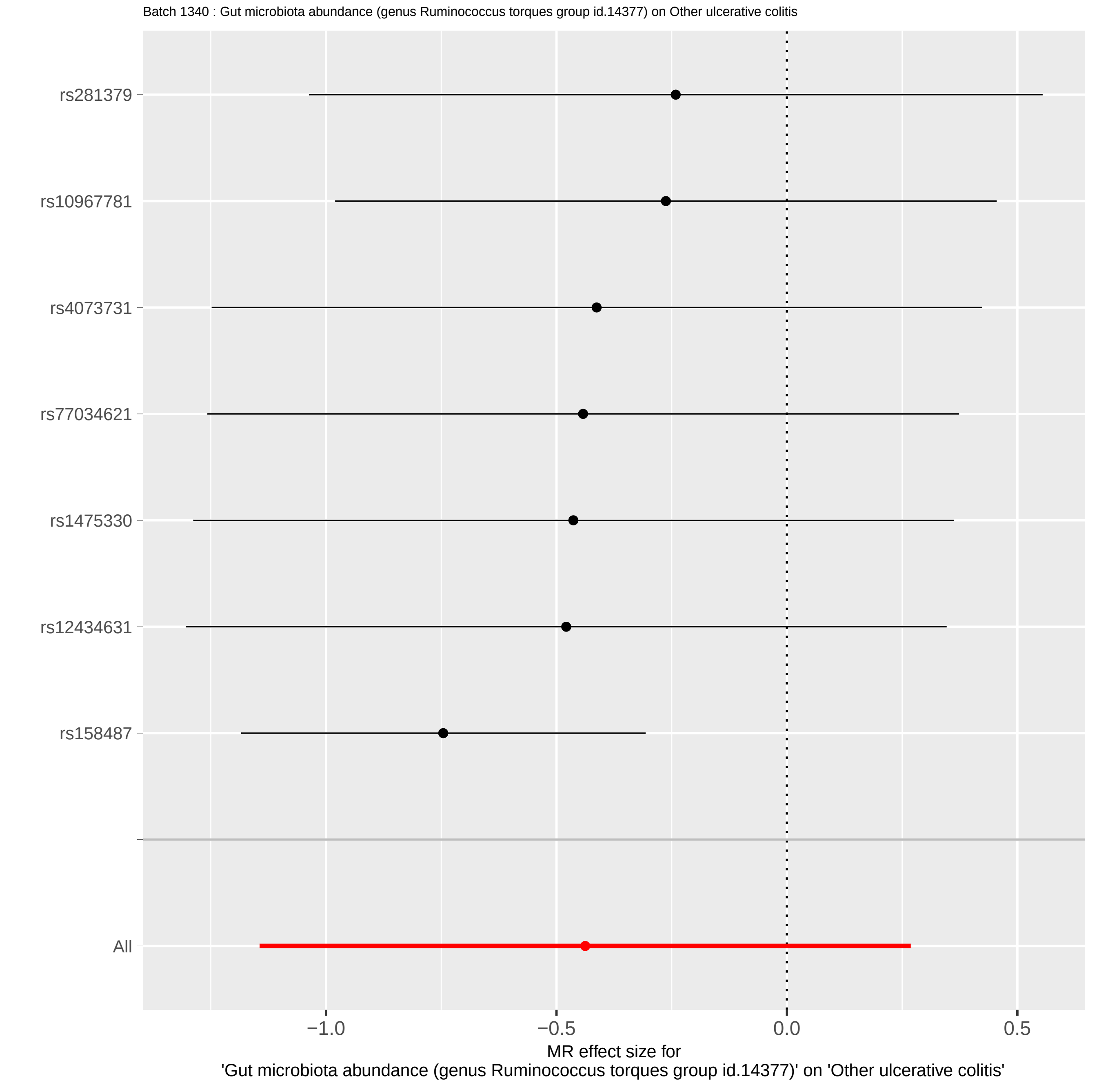

Batch 1340 : Gut microbiota abundance (genus Ruminococcus torques group id.14377) on Other ulcerative colitis
rs281379
rs10967781
rs4073731
rs77034621
rs1475330
rs12434631
rs158487
All
−1.0
−0.5
0.0
0.5
MR effect size for
'Gut microbiota abundance (genus Ruminococcus torques group id.14377)' on 'Other ulcerative colitis'

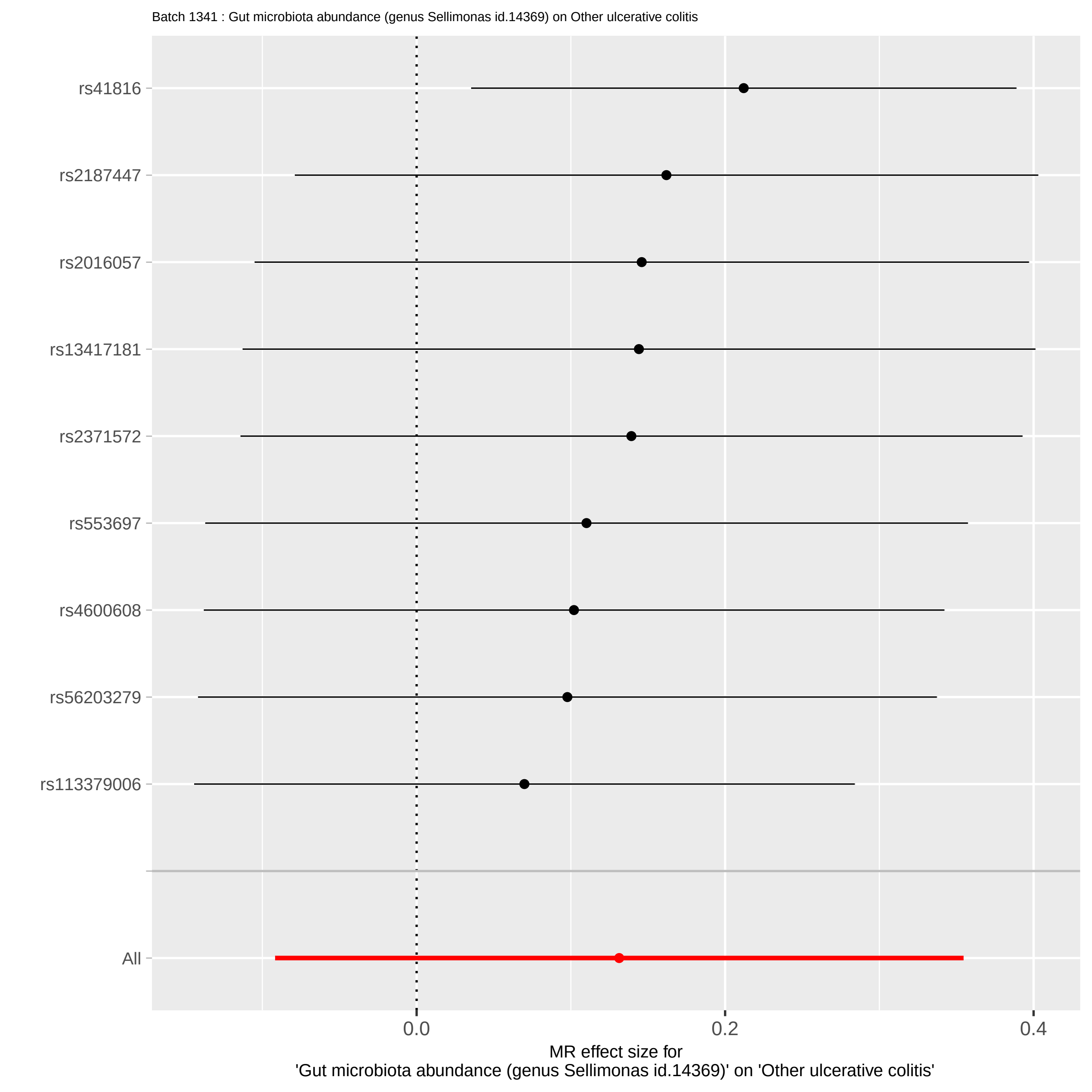

Batch 1341 : Gut microbiota abundance (genus Sellimonas id.14369) on Other ulcerative colitis
rs41816
rs2187447
rs2016057
rs13417181
rs2371572
rs553697
rs4600608
rs56203279
rs113379006
All
0.0
0.2
0.4
MR effect size for
'Gut microbiota abundance (genus Sellimonas id.14369)' on 'Other ulcerative colitis'

Batch 1342 : Gut microbiota abundance (genus Senegalimassilia id.11160) on Other ulcerative colitis

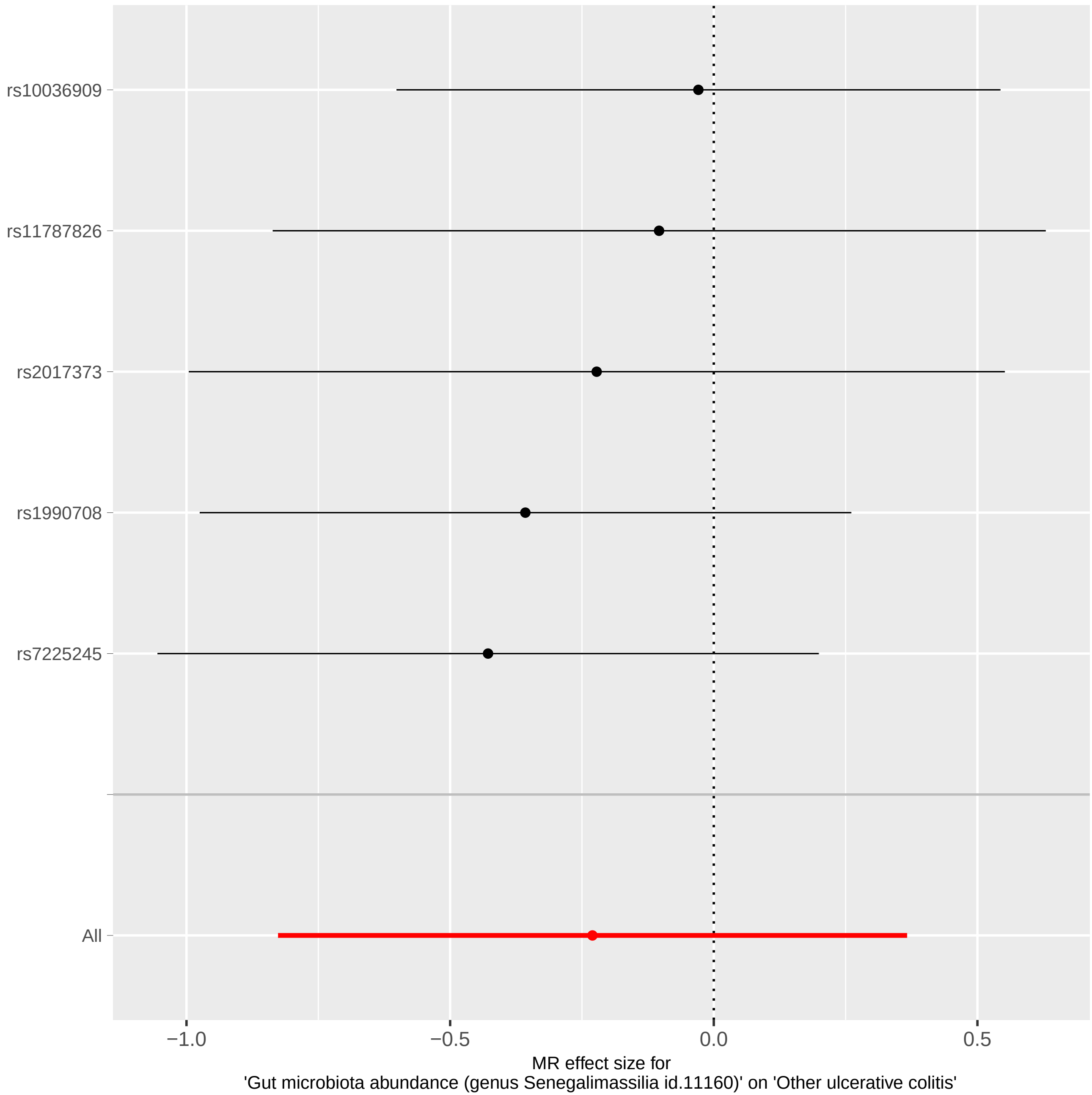

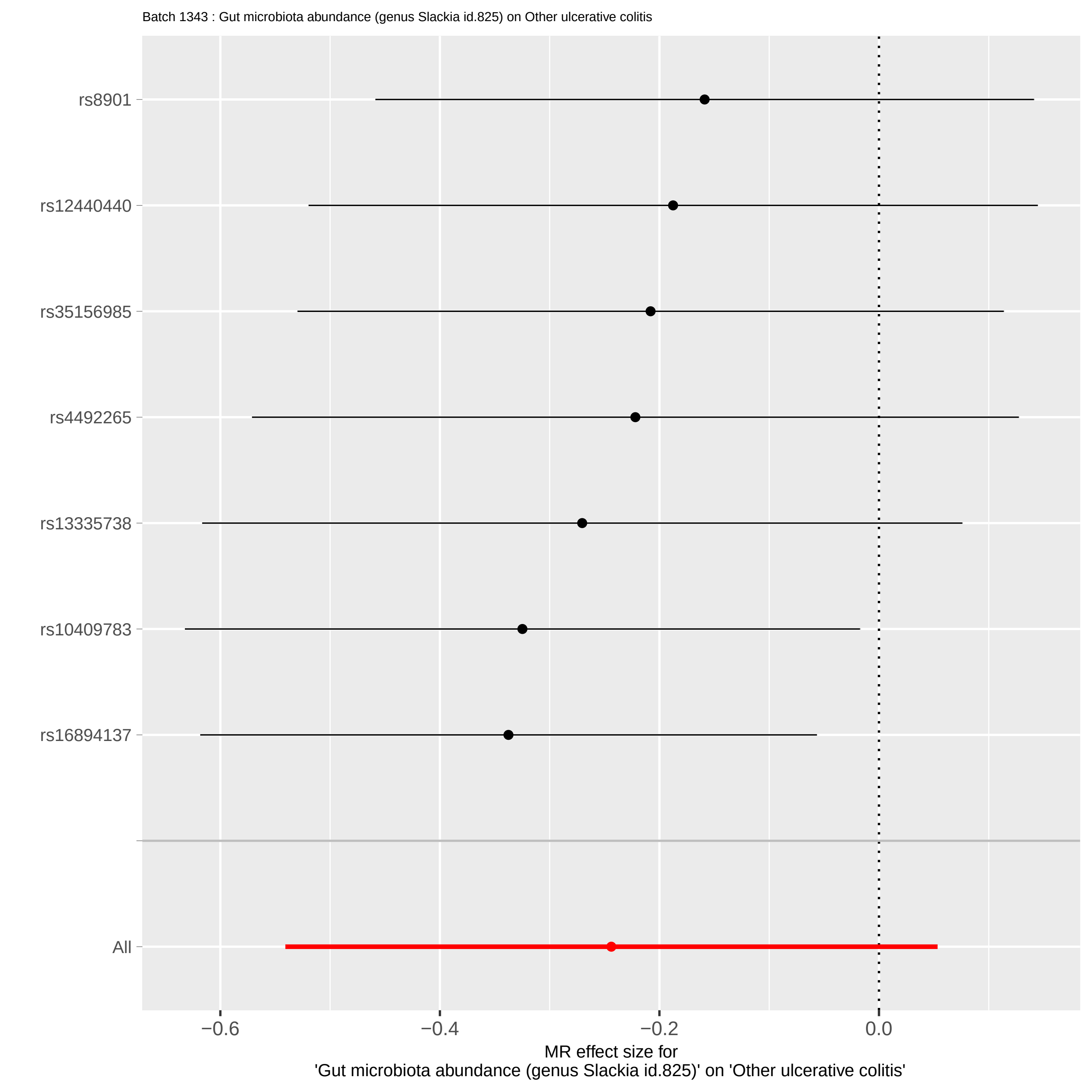

Batch 1343 : Gut microbiota abundance (genus Slackia id.825) on Other ulcerative colitis
rs8901
rs12440440
rs35156985
rs4492265
rs13335738
rs10409783
rs16894137
All
−0.6
−0.4
−0.2
0.0
MR effect size for
'Gut microbiota abundance (genus Slackia id.825)' on 'Other ulcerative colitis'

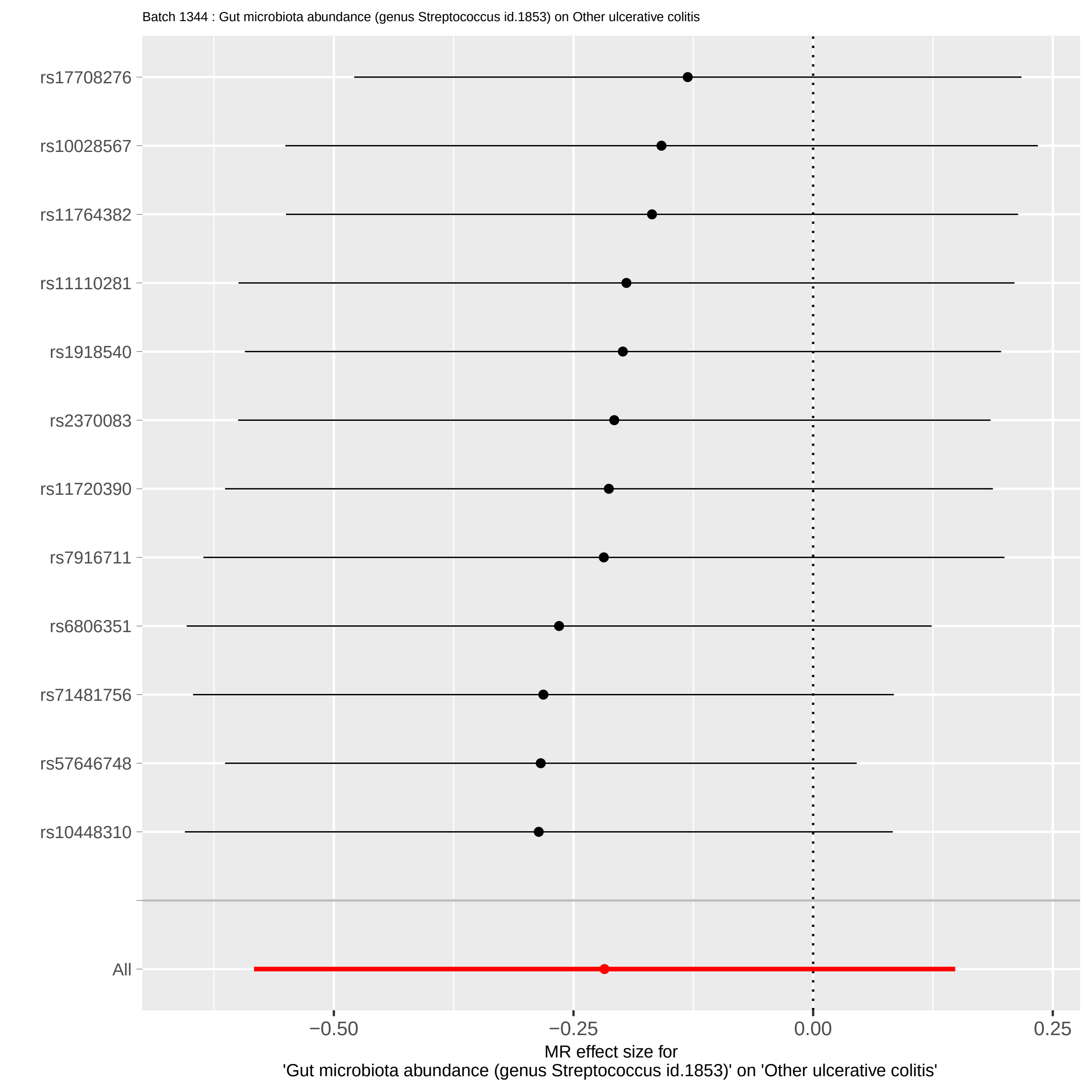

Batch 1344 : Gut microbiota abundance (genus Streptococcus id.1853) on Other ulcerative colitis
rs17708276
rs10028567
rs11764382
rs11110281
rs1918540
rs2370083
rs11720390
rs7916711
rs6806351
rs71481756
rs57646748
rs10448310
All
−0.50
−0.25
0.00
0.25
MR effect size for
'Gut microbiota abundance (genus Streptococcus id.1853)' on 'Other ulcerative colitis'

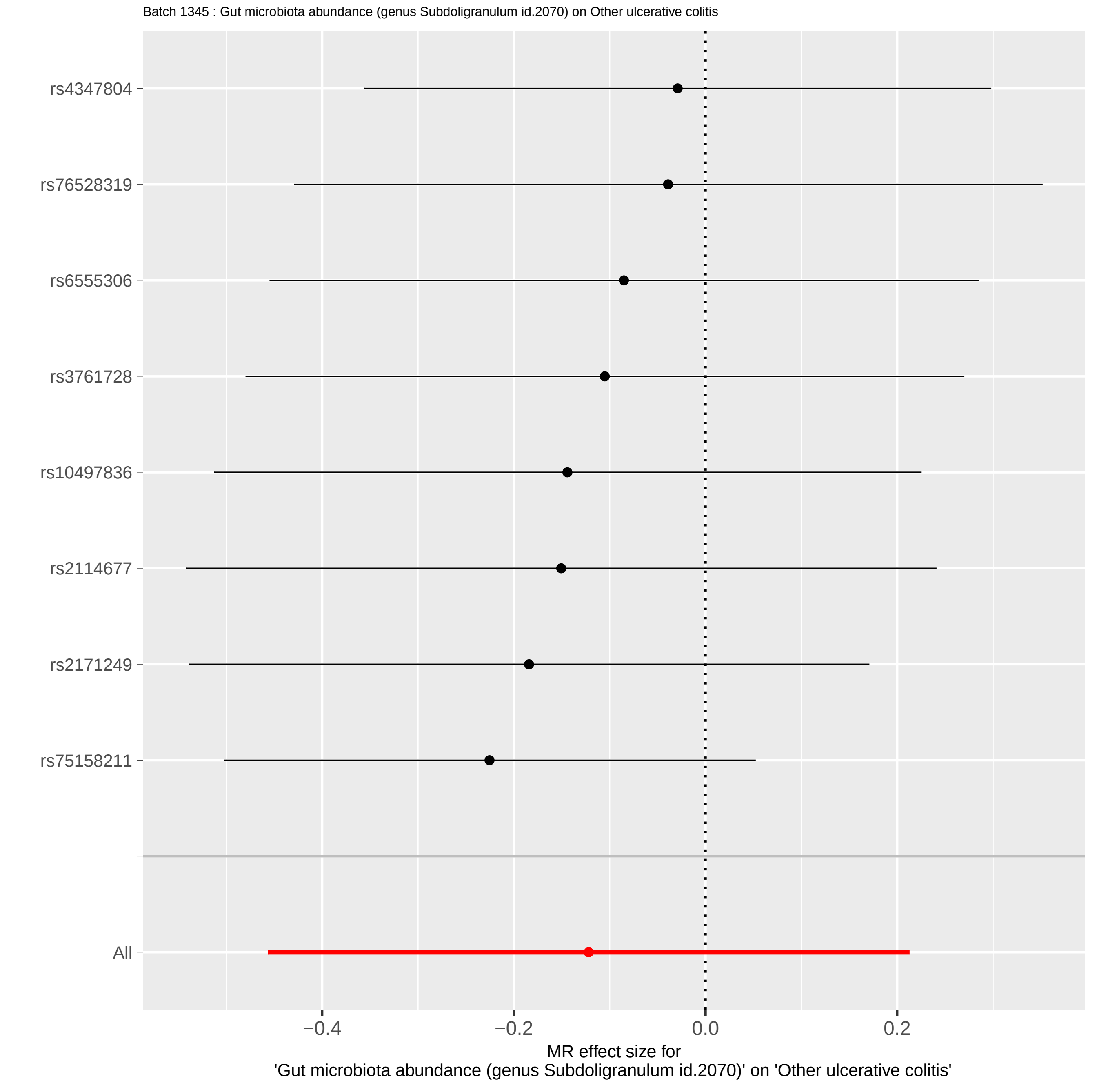
Batch 1345 : Gut microbiota abundance (genus Subdoligranulum id.2070) on Other ulcerative colitis
rs4347804
rs76528319
rs6555306
rs3761728
rs10497836
rs2114677
rs2171249
rs75158211
All
−0.4
−0.2
0.0
0.2
MR effect size for
'Gut microbiota abundance (genus Subdoligranulum id.2070)' on 'Other ulcerative colitis'

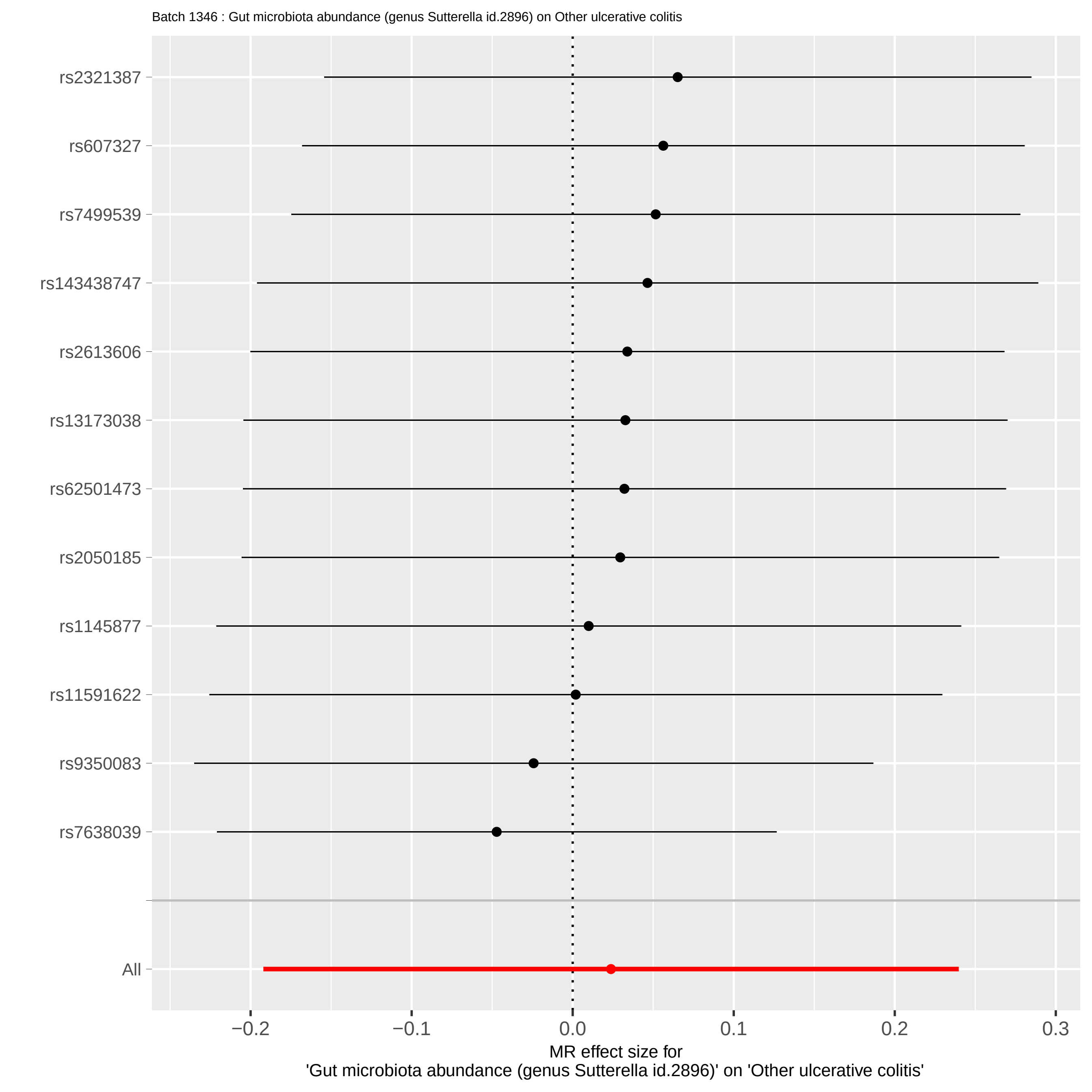

Batch 1346 : Gut microbiota abundance (genus Sutterella id.2896) on Other ulcerative colitis
rs2321387
rs607327
rs7499539
rs143438747
rs2613606
rs13173038
rs62501473
rs2050185
rs1145877
rs11591622
rs9350083
rs7638039
All
−0.2
−0.1
0.0
0.1
0.2
0.3
MR effect size for
'Gut microbiota abundance (genus Sutterella id.2896)' on 'Other ulcerative colitis'

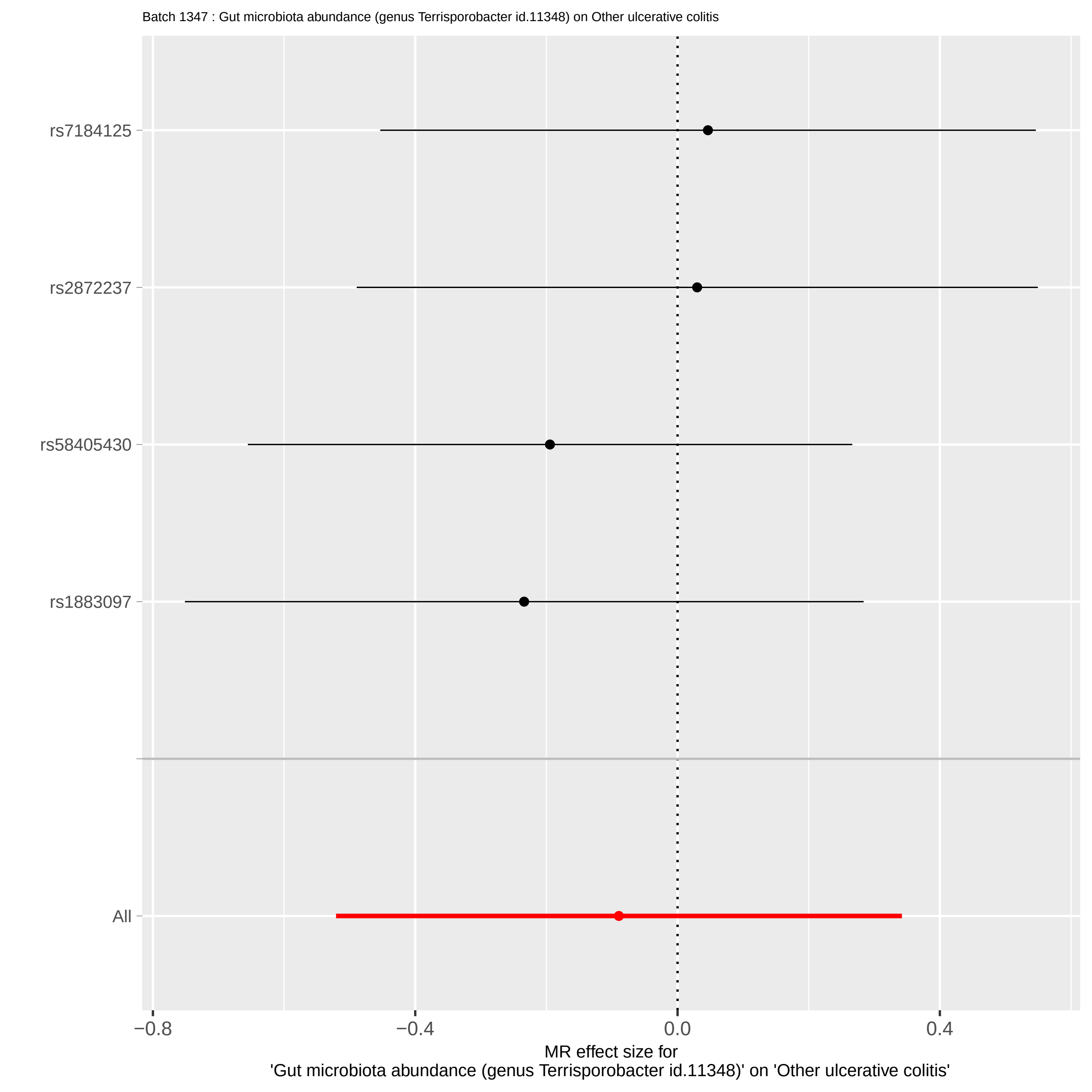
Batch 1347 : Gut microbiota abundance (genus Terrisporobacter id.11348) on Other ulcerative colitis
rs7184125
rs2872237
rs58405430
rs1883097
All
−0.8
−0.4
0.0
0.4
MR effect size for
'Gut microbiota abundance (genus Terrisporobacter id.11348)' on 'Other ulcerative colitis'

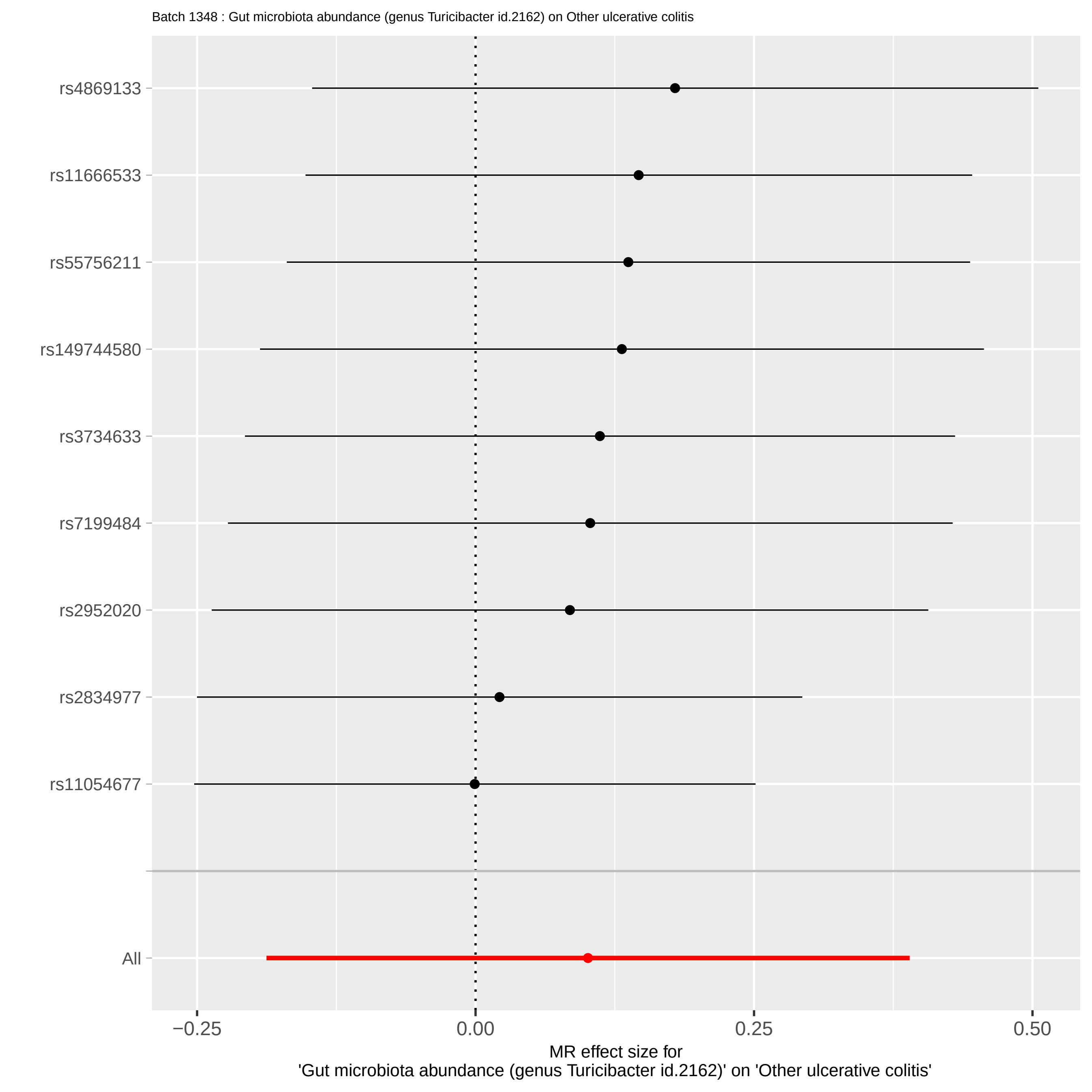

Batch 1348 : Gut microbiota abundance (genus Turicibacter id.2162) on Other ulcerative colitis
rs4869133
rs11666533
rs55756211
rs149744580
rs3734633
rs7199484
rs2952020
rs2834977
rs11054677
All
−0.25
0.00
0.25
0.50
MR effect size for
'Gut microbiota abundance (genus Turicibacter id.2162)' on 'Other ulcerative colitis'

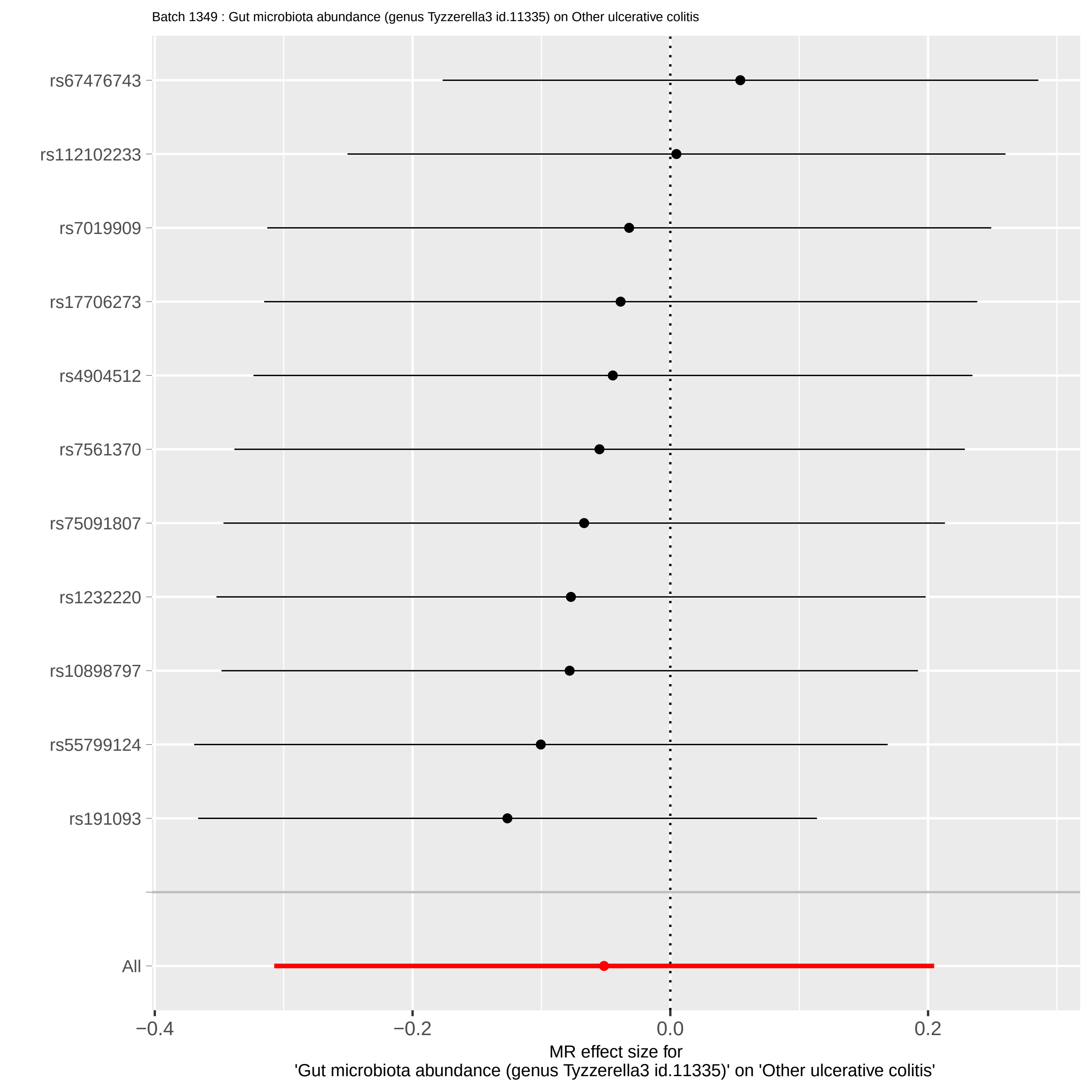

Batch 1349 : Gut microbiota abundance (genus Tyzzerella3 id.11335) on Other ulcerative colitis
rs67476743
rs112102233
rs7019909
rs17706273
rs4904512
rs7561370
rs75091807
rs1232220
rs10898797
rs55799124
rs191093
All
−0.4
−0.2
0.0
0.2
MR effect size for
'Gut microbiota abundance (genus Tyzzerella3 id.11335)' on 'Other ulcerative colitis'

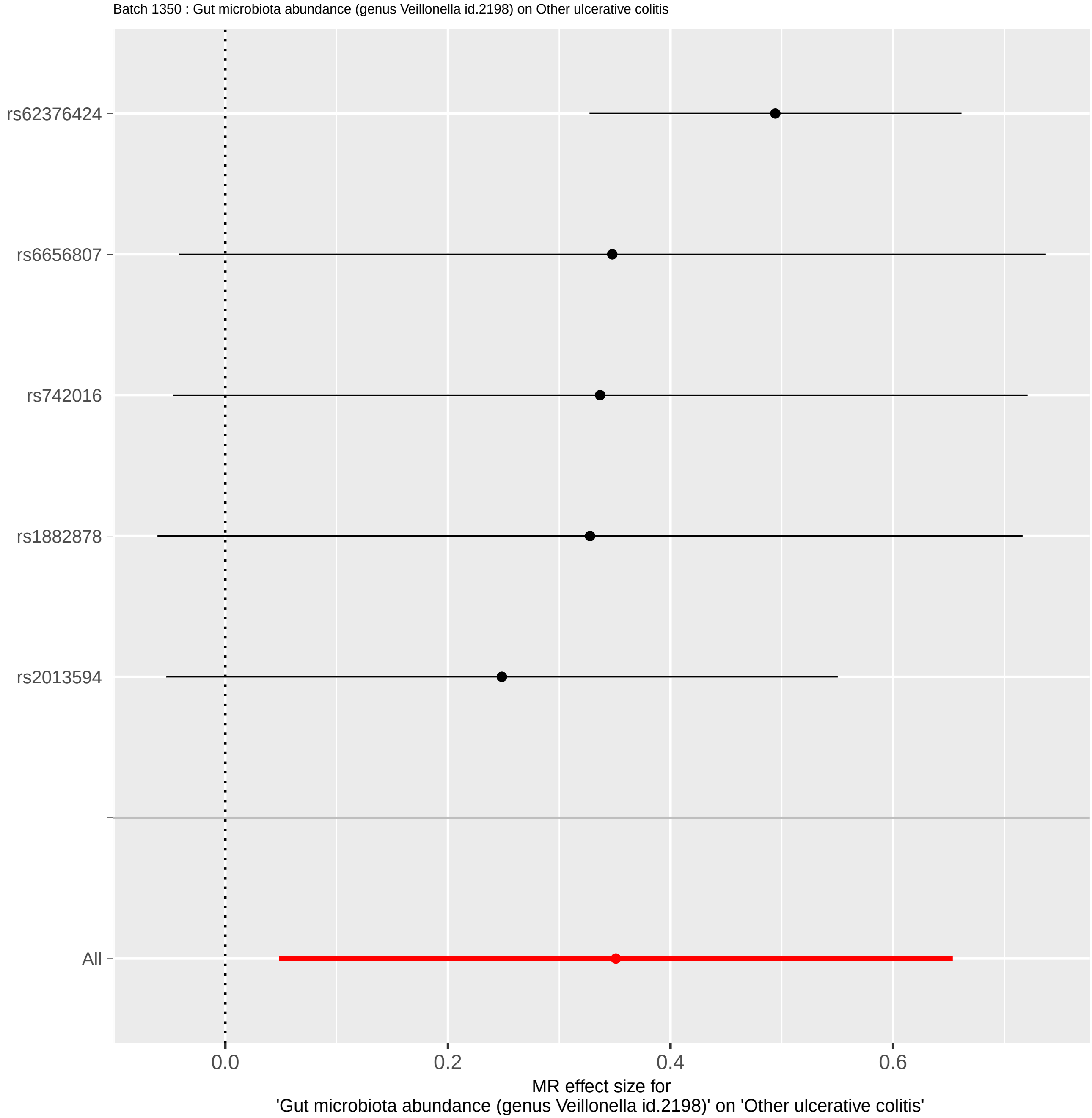

Batch 1350 : Gut microbiota abundance (genus Veillonella id.2198) on Other ulcerative colitis
rs62376424
rs6656807
rs742016
rs1882878
rs2013594
All
0.0
0.2
0.4
0.6
MR effect size for
'Gut microbiota abundance (genus Veillonella id.2198)' on 'Other ulcerative colitis'

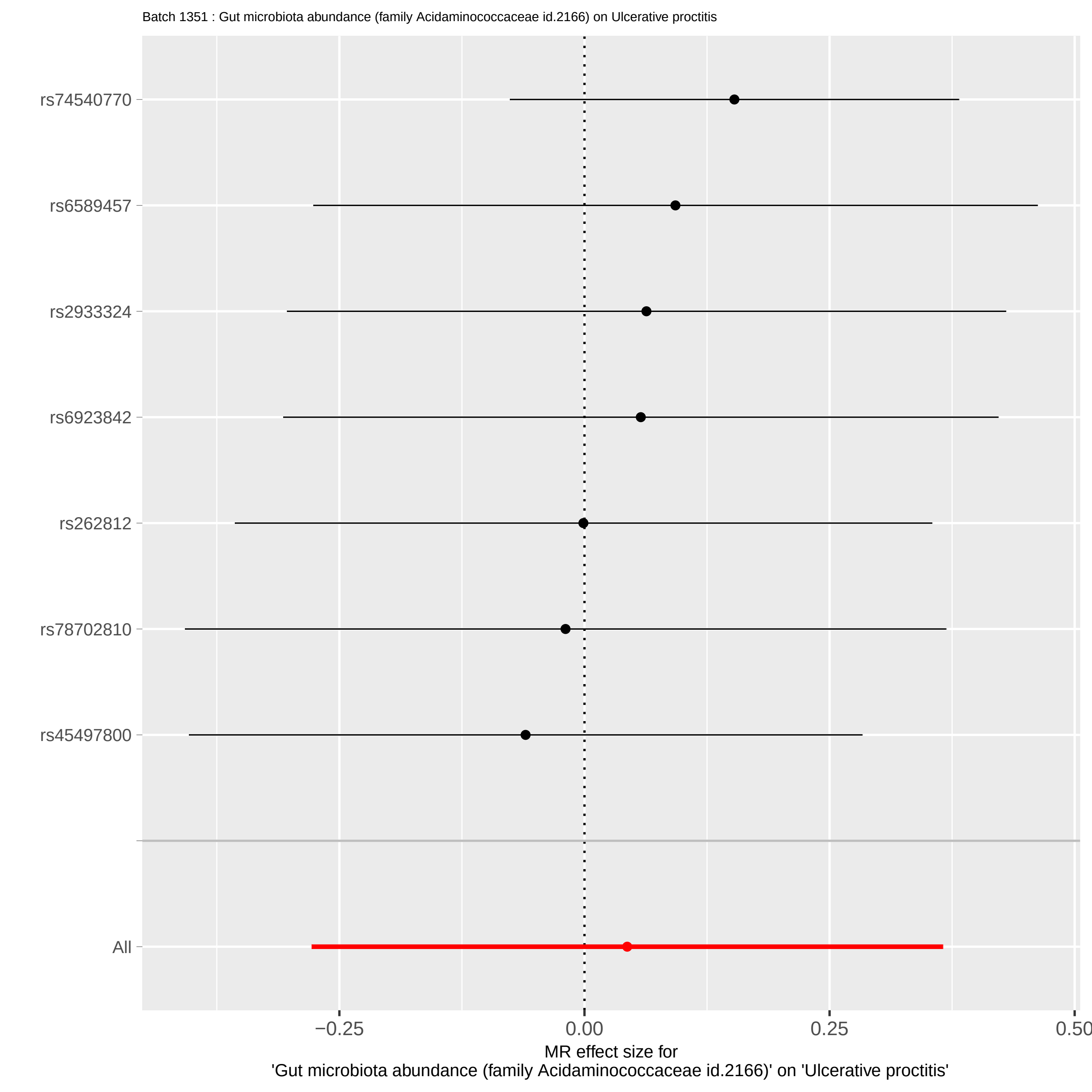

Batch 1351 : Gut microbiota abundance (family Acidaminococcaceae id.2166) on Ulcerative proctitis
rs74540770
rs6589457
rs2933324
rs6923842
rs262812
rs78702810
rs45497800
All
−0.25
0.00
0.25
0.50
MR effect size for
'Gut microbiota abundance (family Acidaminococcaceae id.2166)' on 'Ulcerative proctitis'

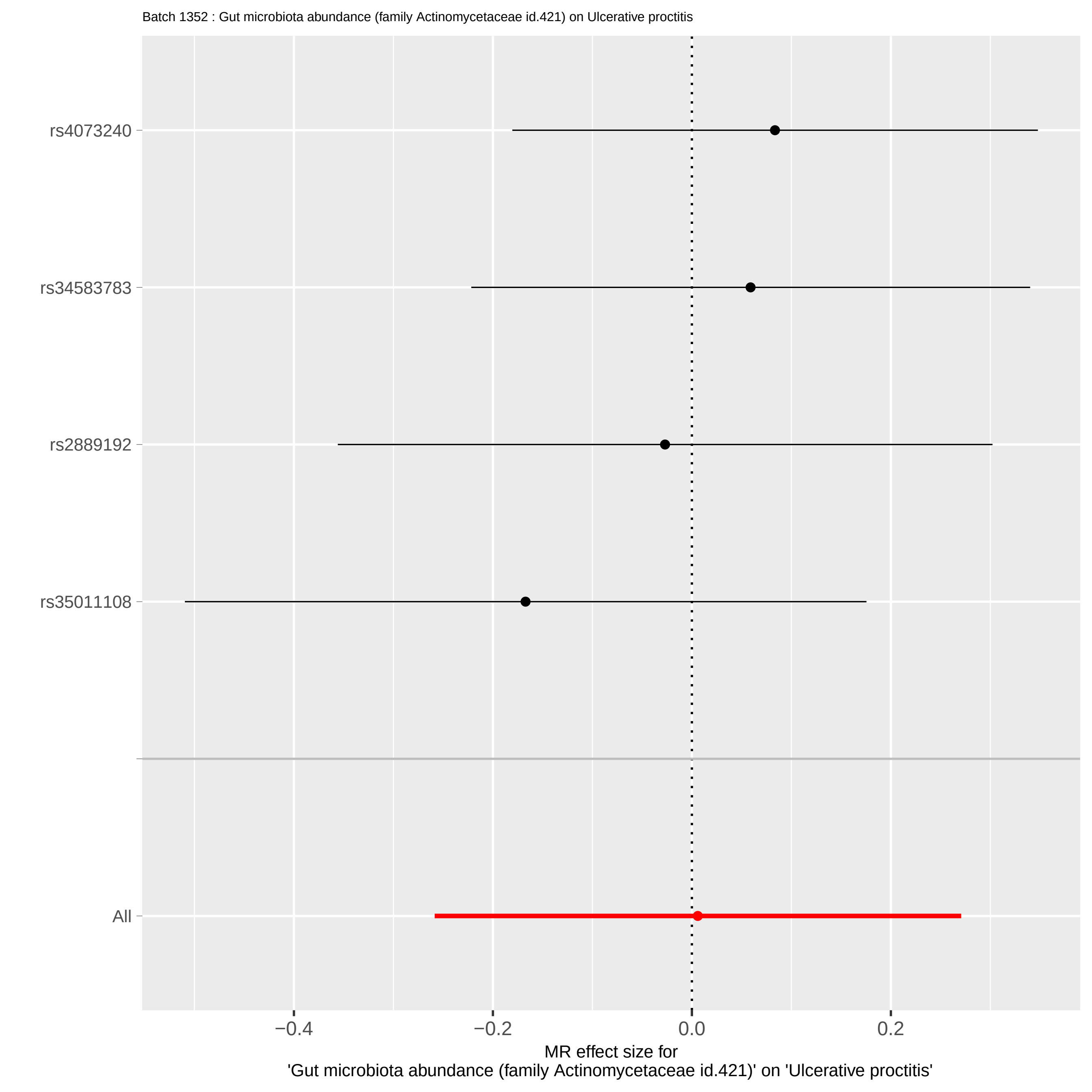

Batch 1352 : Gut microbiota abundance (family Actinomycetaceae id.421) on Ulcerative proctitis
rs4073240
rs34583783
rs2889192
rs35011108
All
−0.4
−0.2
0.0
0.2
MR effect size for
'Gut microbiota abundance (family Actinomycetaceae id.421)' on 'Ulcerative proctitis'

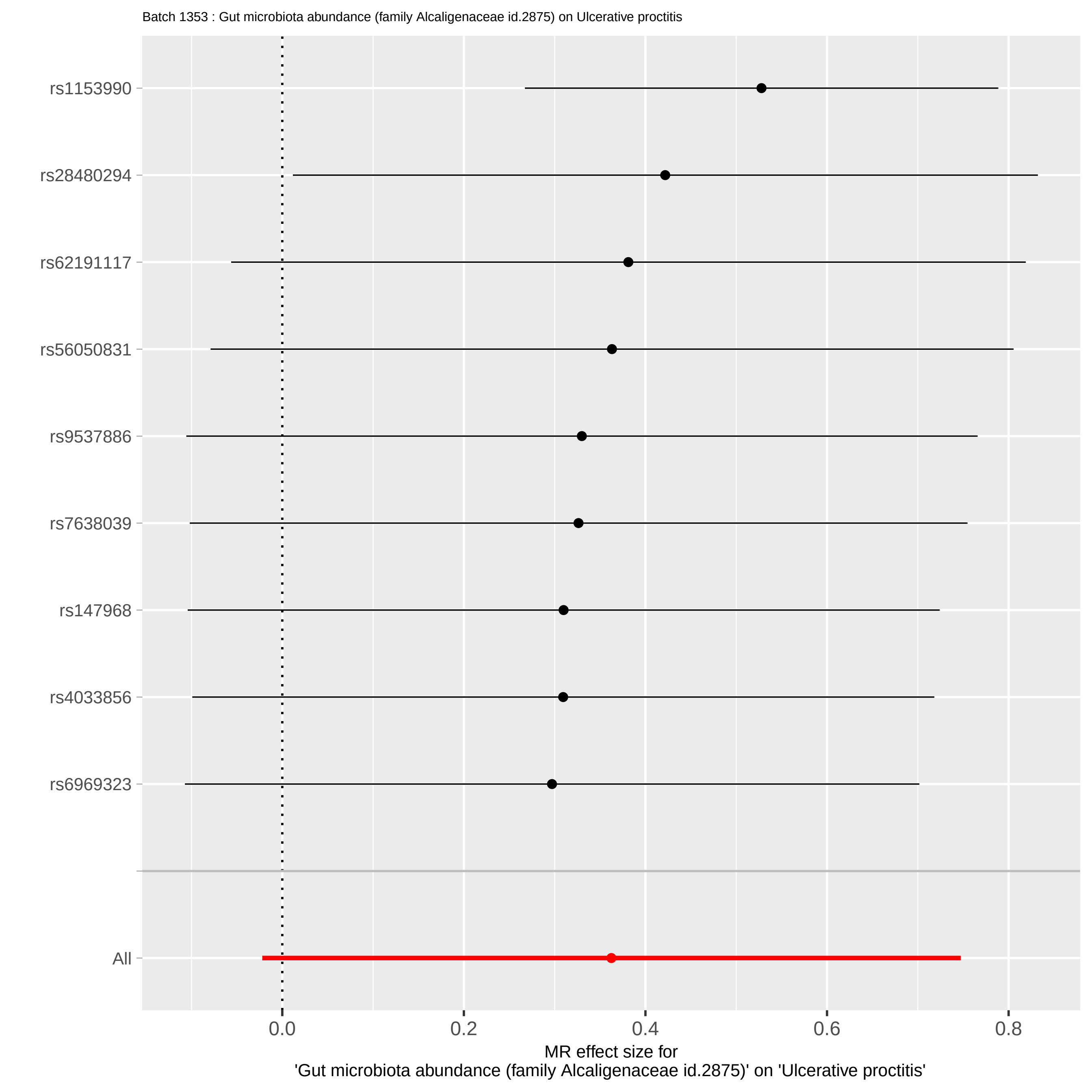

Batch 1353 : Gut microbiota abundance (family Alcaligenaceae id.2875) on Ulcerative proctitis
rs1153990
rs28480294
rs62191117
rs56050831
rs9537886
rs7638039
rs147968
rs4033856
rs6969323
All
0.0
0.2
0.4
0.6
0.8
MR effect size for
'Gut microbiota abundance (family Alcaligenaceae id.2875)' on 'Ulcerative proctitis'

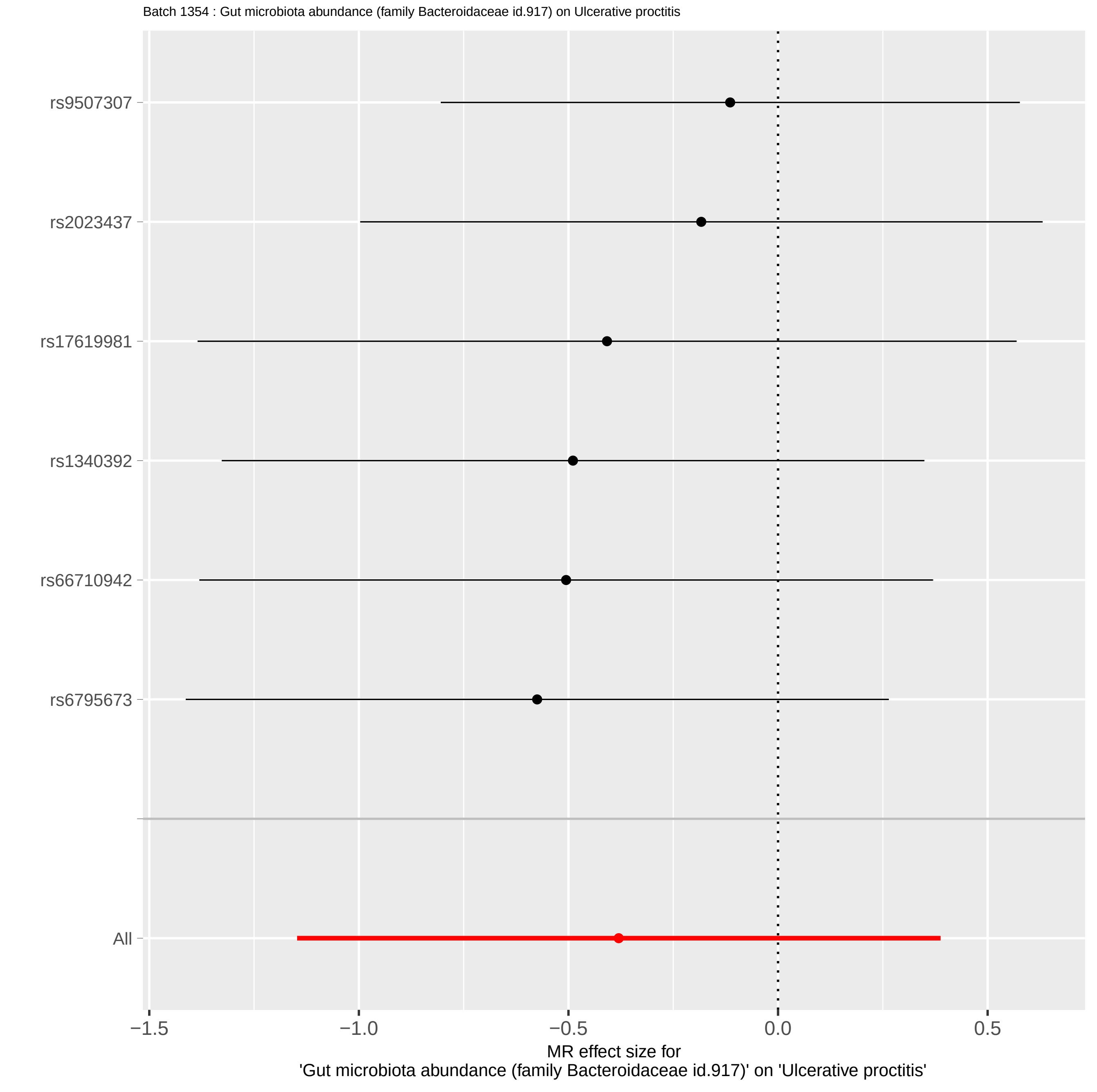

Batch 1354 : Gut microbiota abundance (family Bacteroidaceae id.917) on Ulcerative proctitis
rs9507307
rs2023437
rs17619981
rs1340392
rs66710942
rs6795673
All
−1.5
−1.0
−0.5
0.0
0.5
MR effect size for
'Gut microbiota abundance (family Bacteroidaceae id.917)' on 'Ulcerative proctitis'

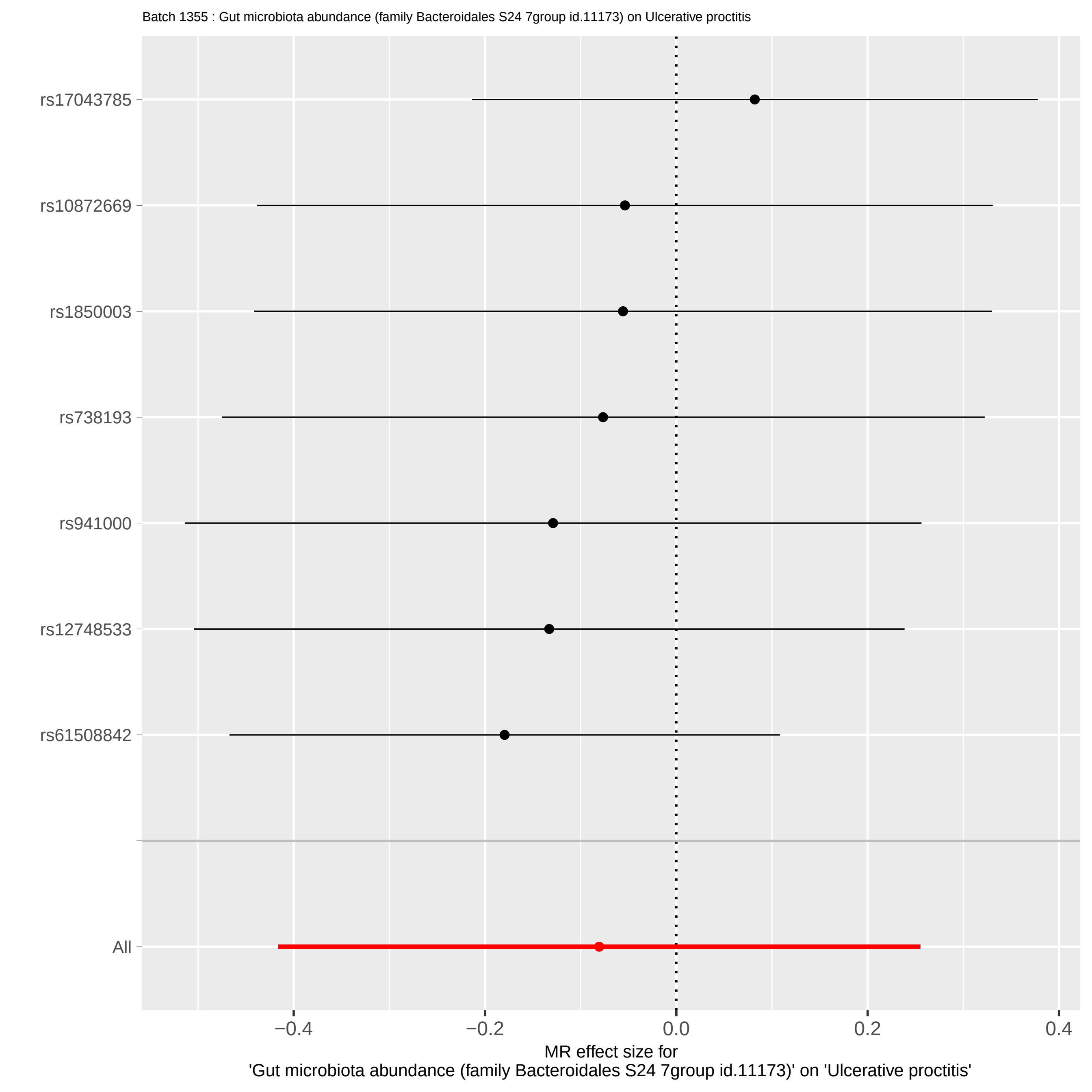

Batch 1355 : Gut microbiota abundance (family Bacteroidales S24 7group id.11173) on Ulcerative proctitis
rs17043785
rs10872669
rs1850003
rs738193
rs941000
rs12748533
rs61508842
All
−0.4
−0.2
0.0
0.2
0.4
MR effect size for
'Gut microbiota abundance (family Bacteroidales S24 7group id.11173)' on 'Ulcerative proctitis'

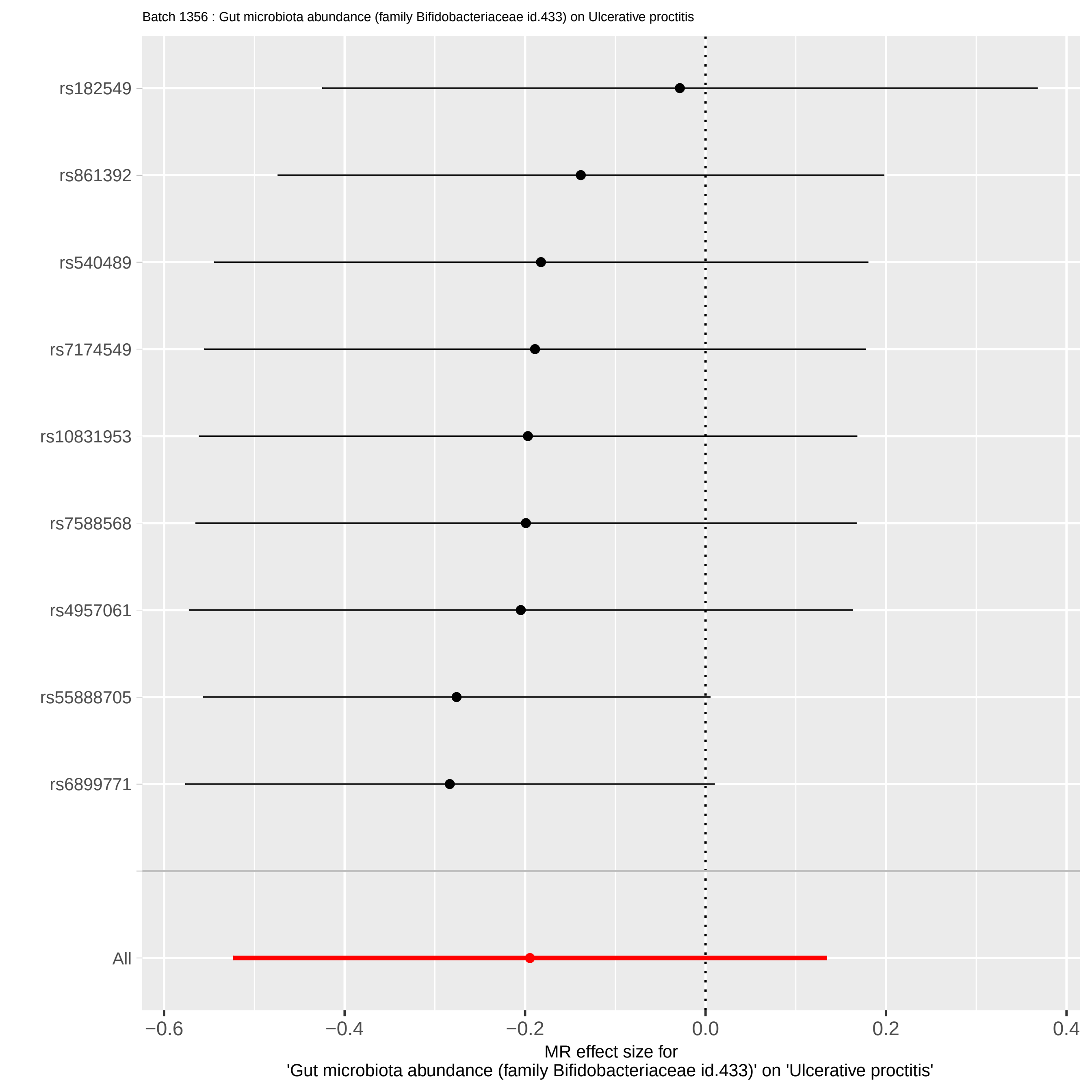
Batch 1356 : Gut microbiota abundance (family Bifidobacteriaceae id.433) on Ulcerative proctitis
rs182549
rs861392
rs540489
rs7174549
rs10831953
rs7588568
rs4957061
rs55888705
rs6899771
All
−0.6
−0.4
−0.2
0.0
0.2
0.4
MR effect size for
'Gut microbiota abundance (family Bifidobacteriaceae id.433)' on 'Ulcerative proctitis'

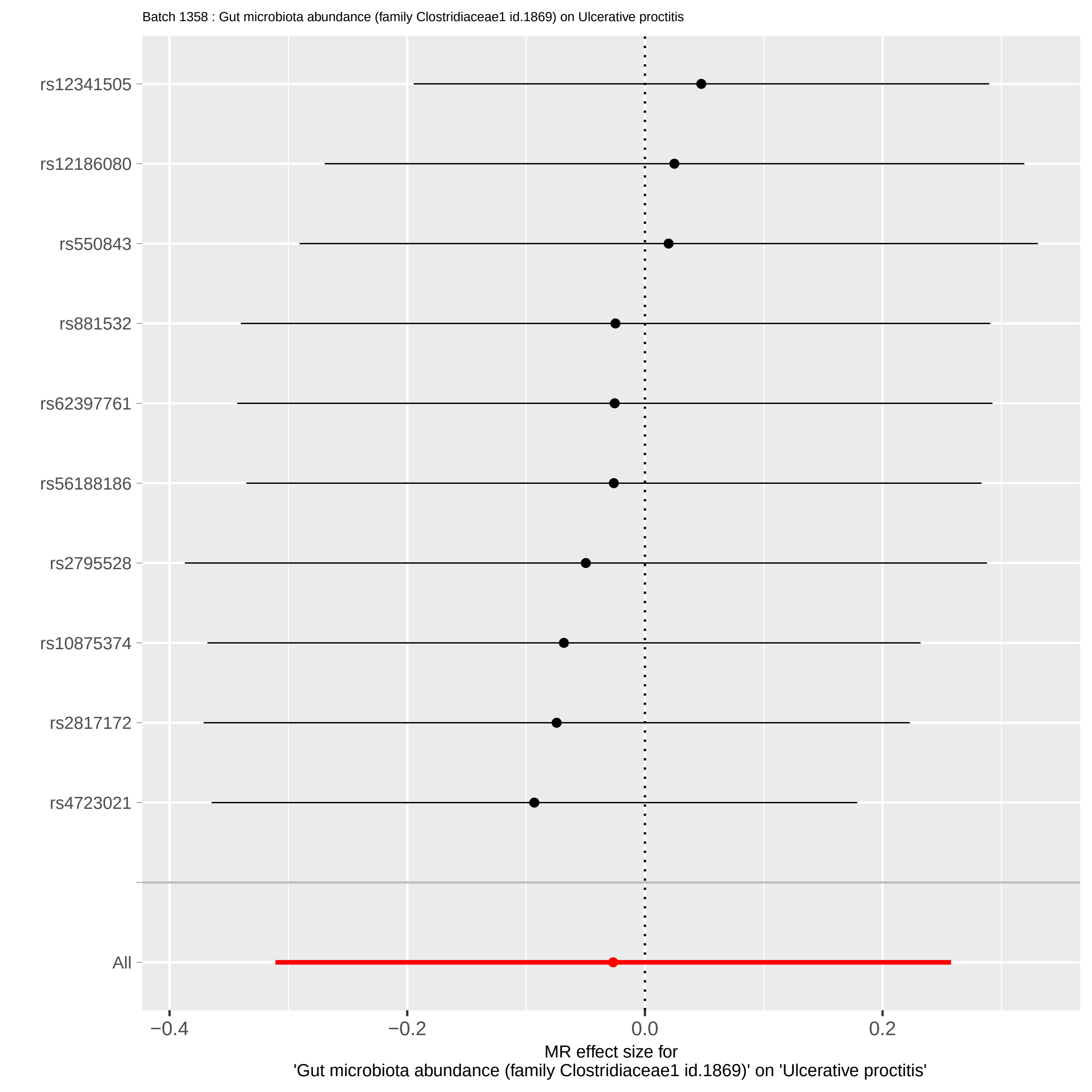

Batch 1358 : Gut microbiota abundance (family Clostridiaceae1 id.1869) on Ulcerative proctitis
rs12341505
rs12186080
rs550843
rs881532
rs62397761
rs56188186
rs2795528
rs10875374
rs2817172
rs4723021
All
−0.4
−0.2
0.0
0.2
MR effect size for
'Gut microbiota abundance (family Clostridiaceae1 id.1869)' on 'Ulcerative proctitis'

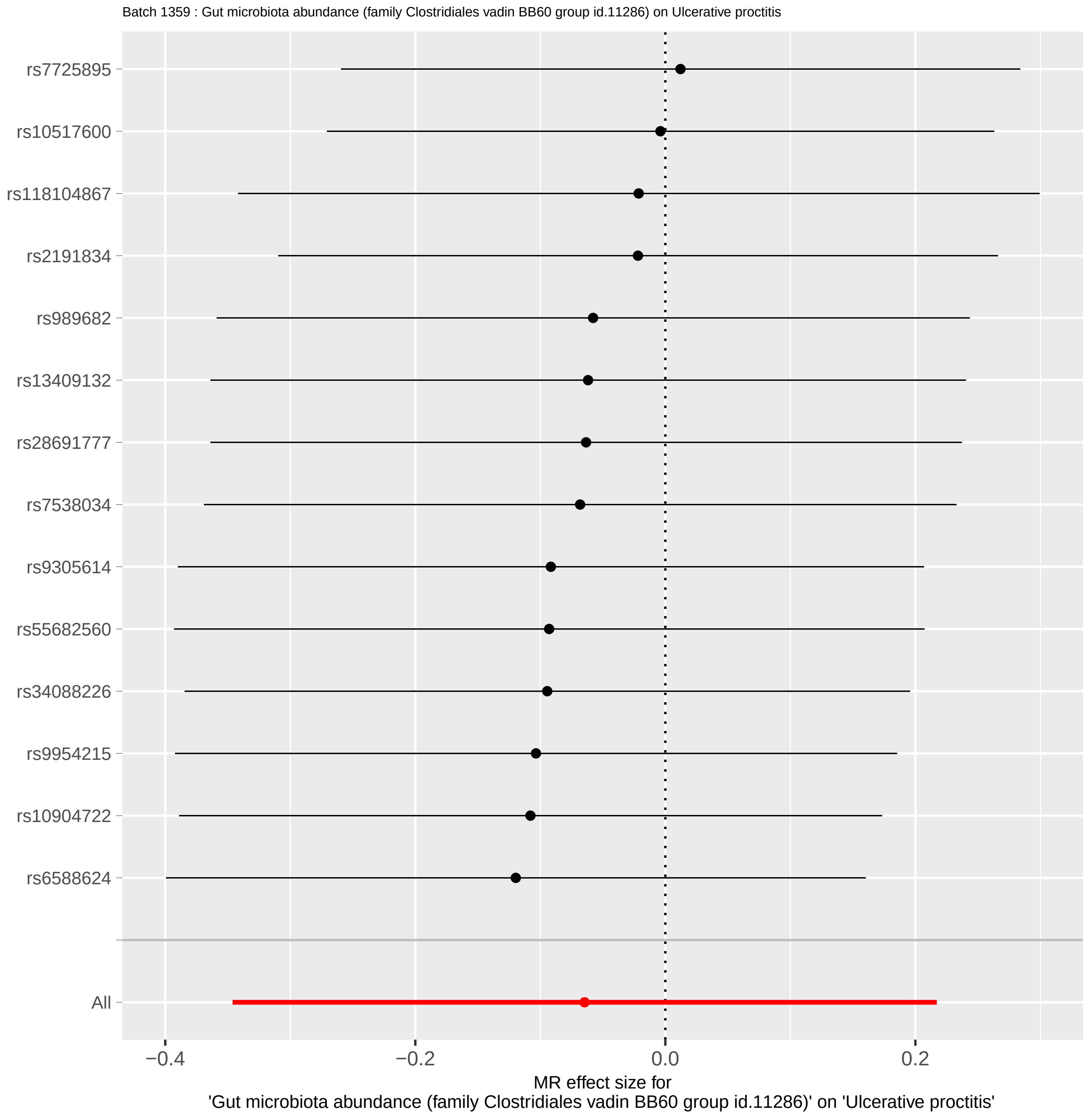

Batch 1359 : Gut microbiota abundance (family Clostridiales vadin BB60 group id.11286) on Ulcerative proctitis
rs7725895
rs10517600
rs118104867
rs2191834
rs989682
rs13409132
rs28691777
rs7538034
rs9305614
rs55682560
rs34088226
rs9954215
rs10904722
rs6588624
All
−0.4
−0.2
0.0
0.2
MR effect size for
'Gut microbiota abundance (family Clostridiales vadin BB60 group id.11286)' on 'Ulcerative proctitis'

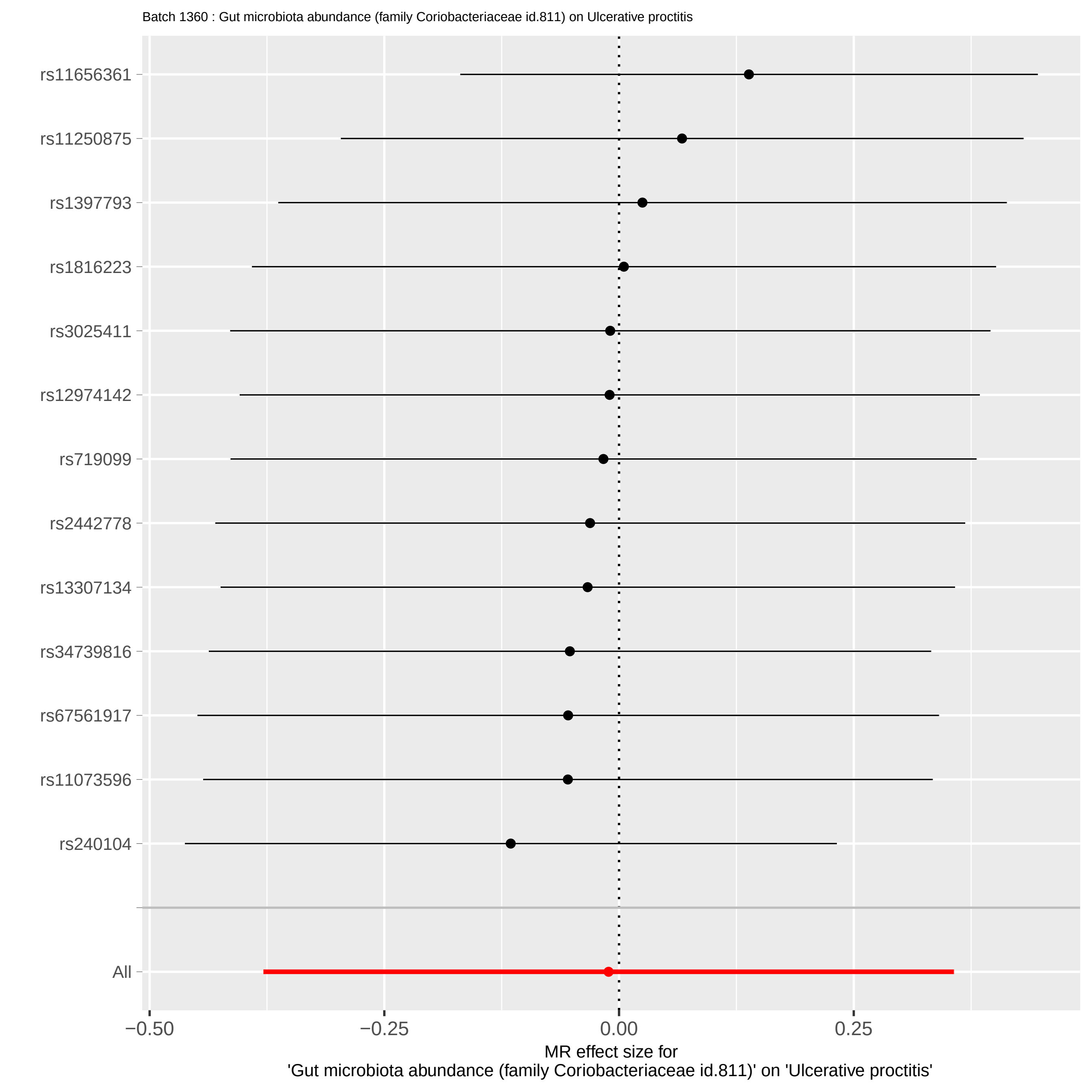

Batch 1360 : Gut microbiota abundance (family Coriobacteriaceae id.811) on Ulcerative proctitis
rs11656361
rs11250875
rs1397793
rs1816223
rs3025411
rs12974142
rs719099
rs2442778
rs13307134
rs34739816
rs67561917
rs11073596
rs240104
All
−0.50
−0.25
0.00
0.25
MR effect size for
'Gut microbiota abundance (family Coriobacteriaceae id.811)' on 'Ulcerative proctitis'

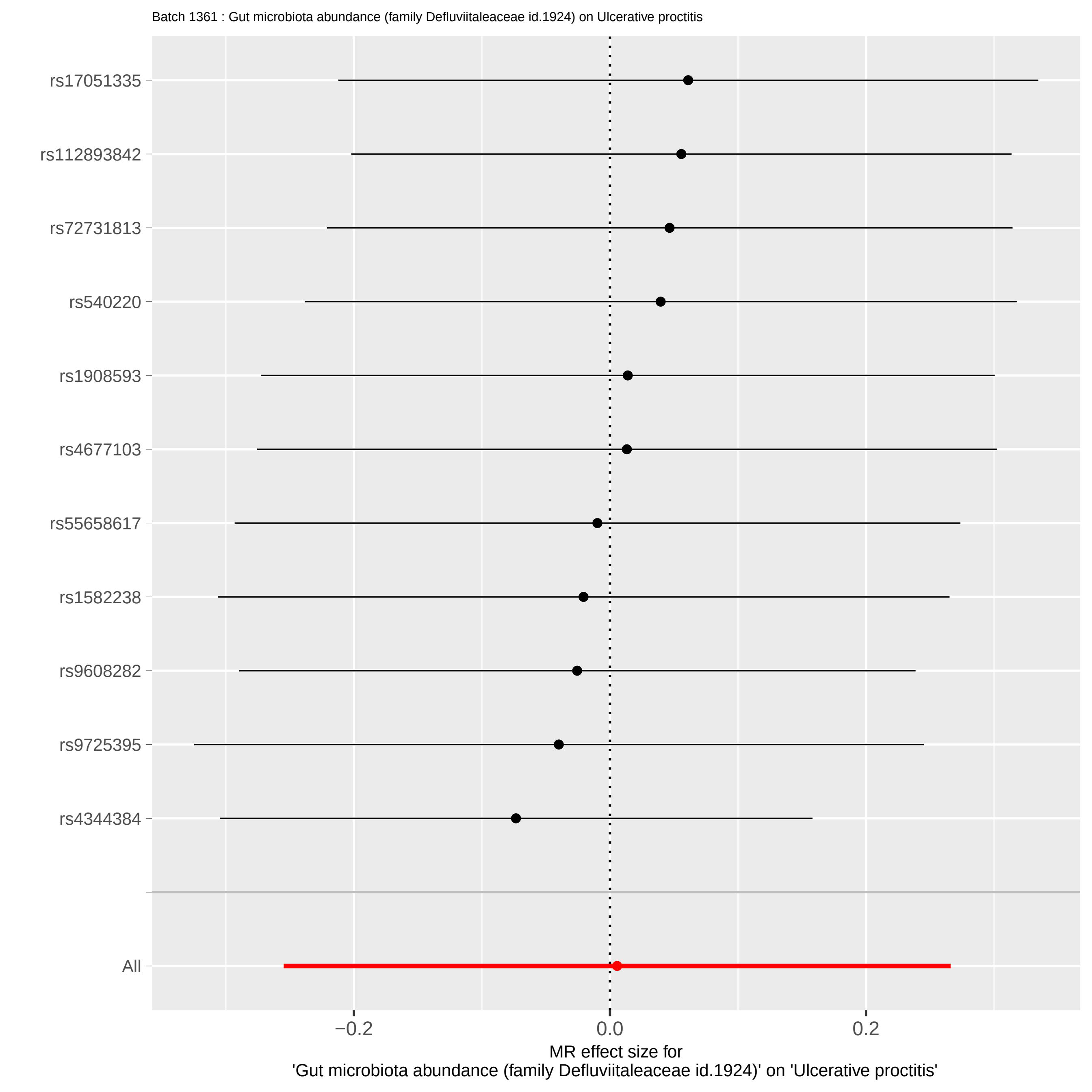
Batch 1361 : Gut microbiota abundance (family Defluviitaleaceae id.1924) on Ulcerative proctitis
rs17051335
rs112893842
rs72731813
rs540220
rs1908593
rs4677103
rs55658617
rs1582238
rs9608282
rs9725395
rs4344384
All
−0.2
0.0
0.2
MR effect size for
'Gut microbiota abundance (family Defluviitaleaceae id.1924)' on 'Ulcerative proctitis'

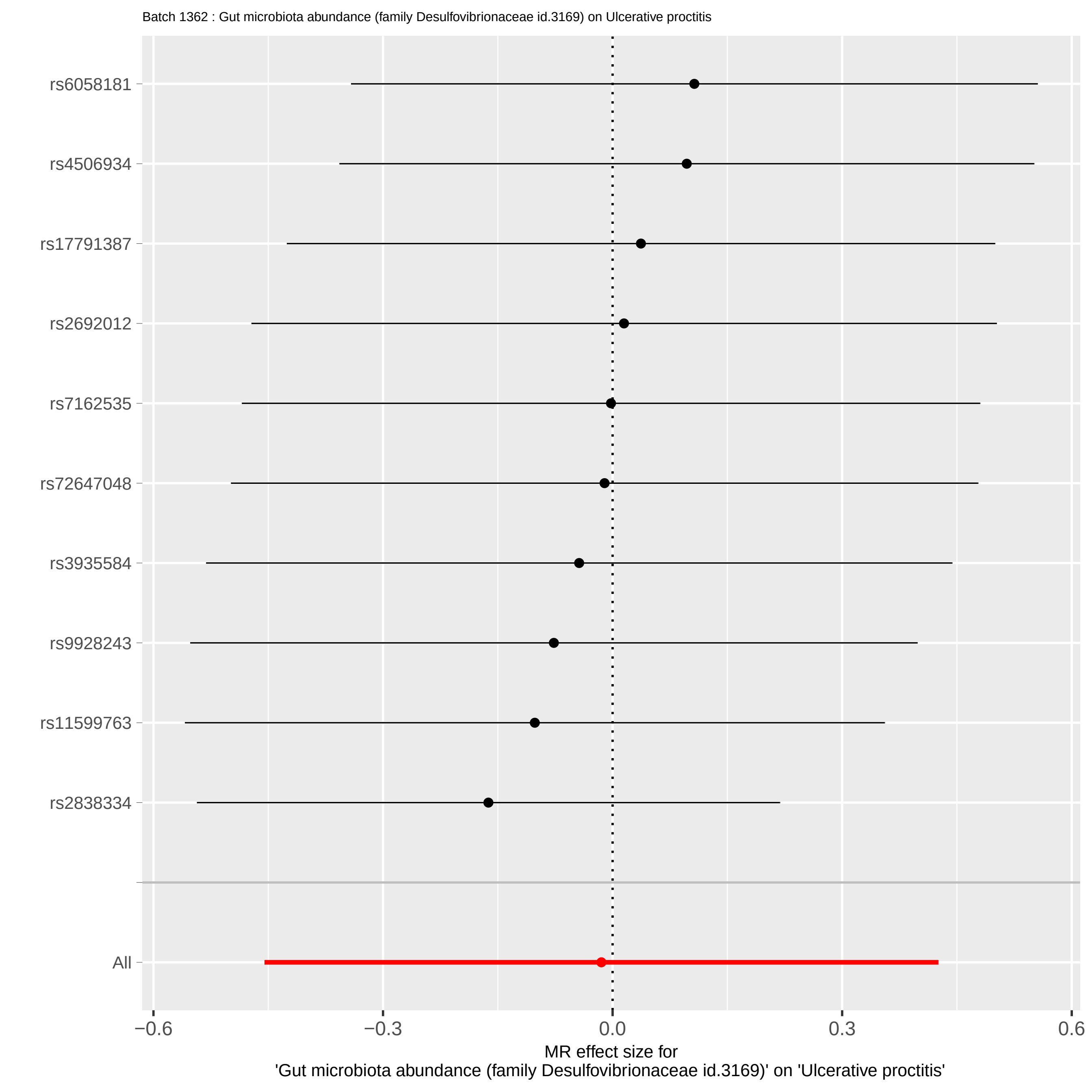
Batch 1362 : Gut microbiota abundance (family Desulfovibrionaceae id.3169) on Ulcerative proctitis
rs6058181
rs4506934
rs17791387
rs2692012
rs7162535
rs72647048
rs3935584
rs9928243
rs11599763
rs2838334
All
−0.6
−0.3
0.0
0.3
0.6
MR effect size for
'Gut microbiota abundance (family Desulfovibrionaceae id.3169)' on 'Ulcerative proctitis'

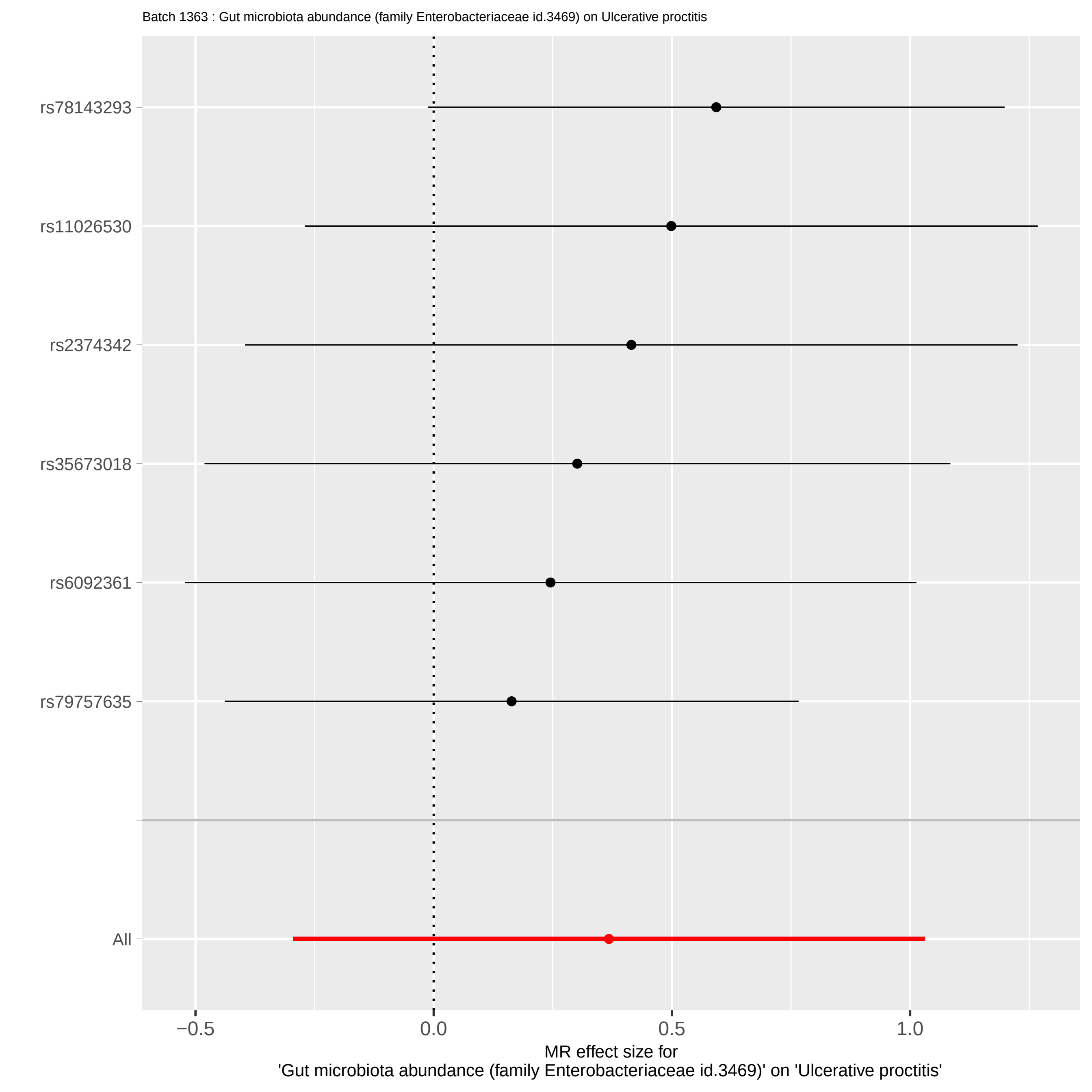
Batch 1363 : Gut microbiota abundance (family Enterobacteriaceae id.3469) on Ulcerative proctitis
rs78143293
rs11026530
rs2374342
rs35673018
rs6092361
rs79757635
All
−0.5
0.0
0.5
1.0
MR effect size for
'Gut microbiota abundance (family Enterobacteriaceae id.3469)' on 'Ulcerative proctitis'

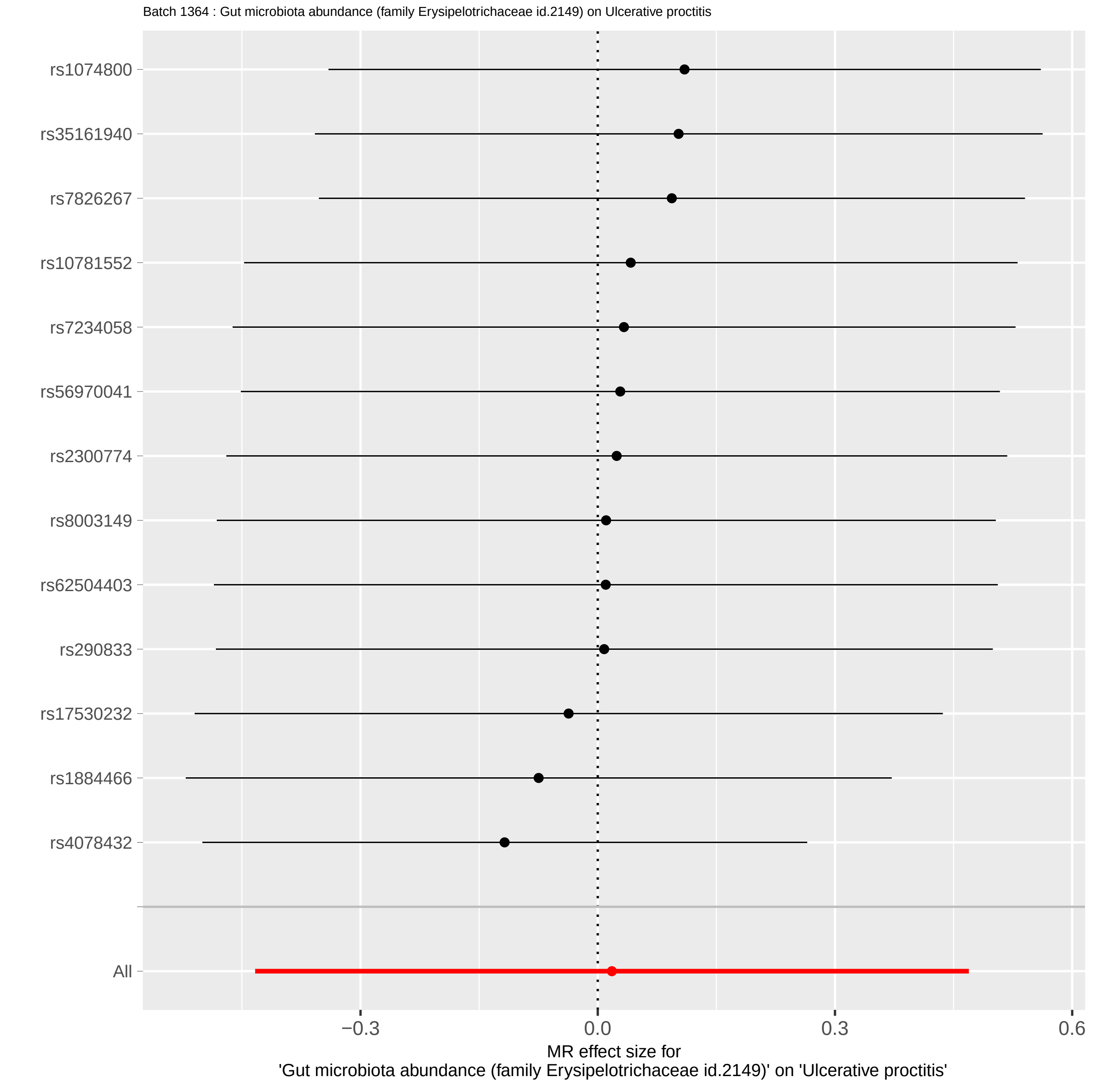
Batch 1364 : Gut microbiota abundance (family Erysipelotrichaceae id.2149) on Ulcerative proctitis
rs1074800
rs35161940
rs7826267
rs10781552
rs7234058
rs56970041
rs2300774
rs8003149
rs62504403
rs290833
rs17530232
rs1884466
rs4078432
All
−0.3
0.0
0.3
0.6
MR effect size for
'Gut microbiota abundance (family Erysipelotrichaceae id.2149)' on 'Ulcerative proctitis'

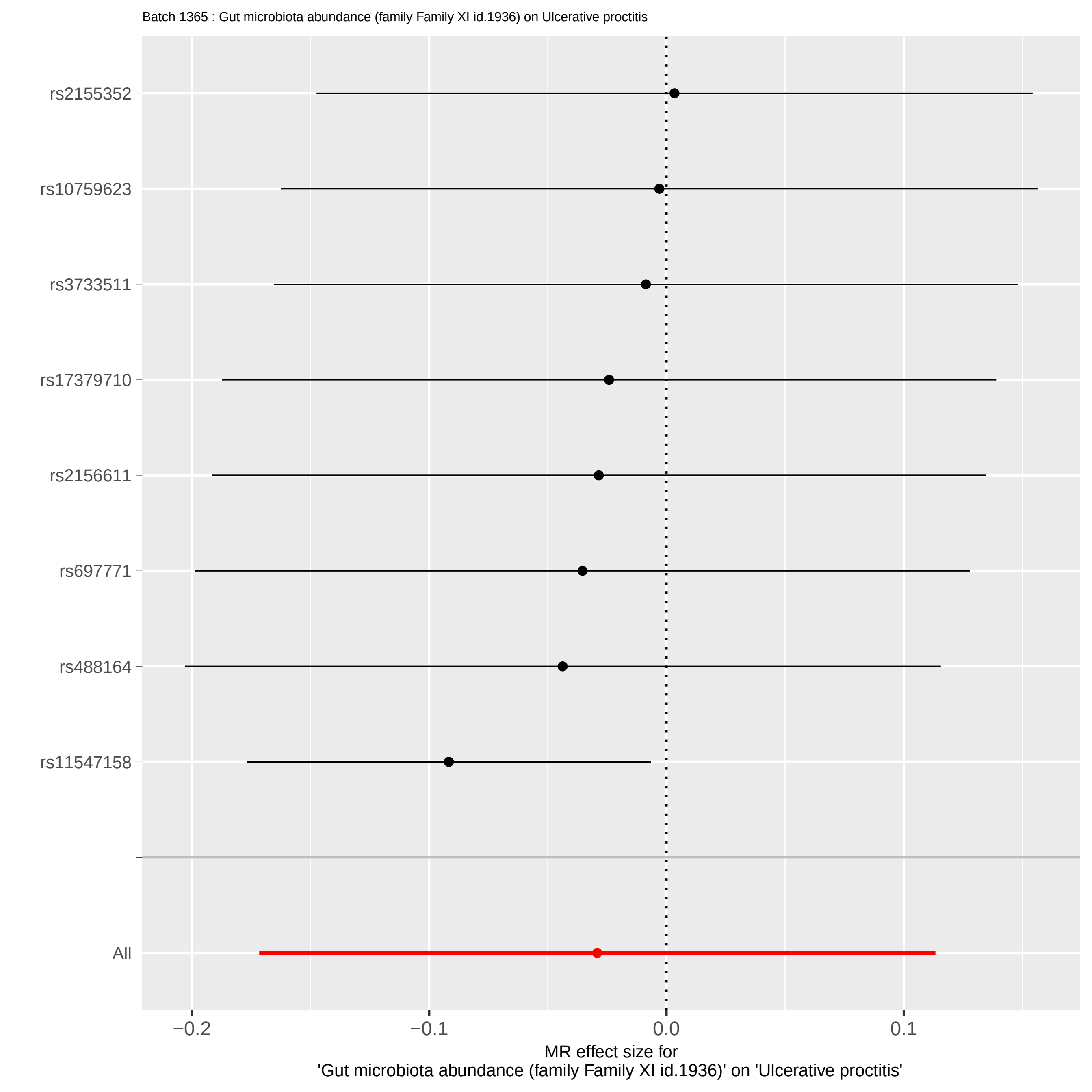

Batch 1365 : Gut microbiota abundance (family Family XI id.1936) on Ulcerative proctitis
rs2155352
rs10759623
rs3733511
rs17379710
rs2156611
rs697771
rs488164
rs11547158
All
−0.2
−0.1
0.0
0.1
MR effect size for
'Gut microbiota abundance (family Family XI id.1936)' on 'Ulcerative proctitis'

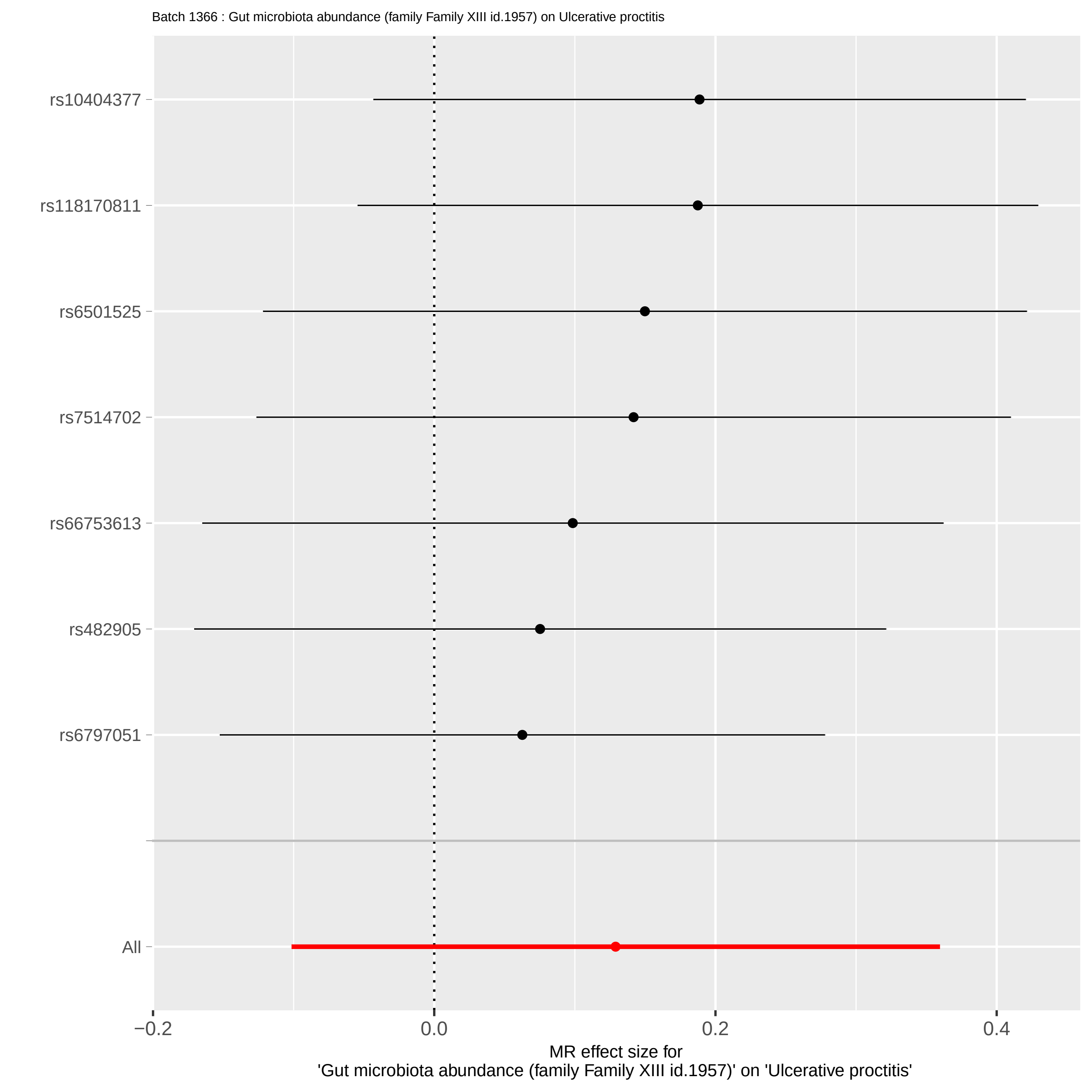

Batch 1366 : Gut microbiota abundance (family Family XIII id.1957) on Ulcerative proctitis
rs10404377
rs118170811
rs6501525
rs7514702
rs66753613
rs482905
rs6797051
All
−0.2
0.0
0.2
0.4
MR effect size for
'Gut microbiota abundance (family Family XIII id.1957)' on 'Ulcerative proctitis'

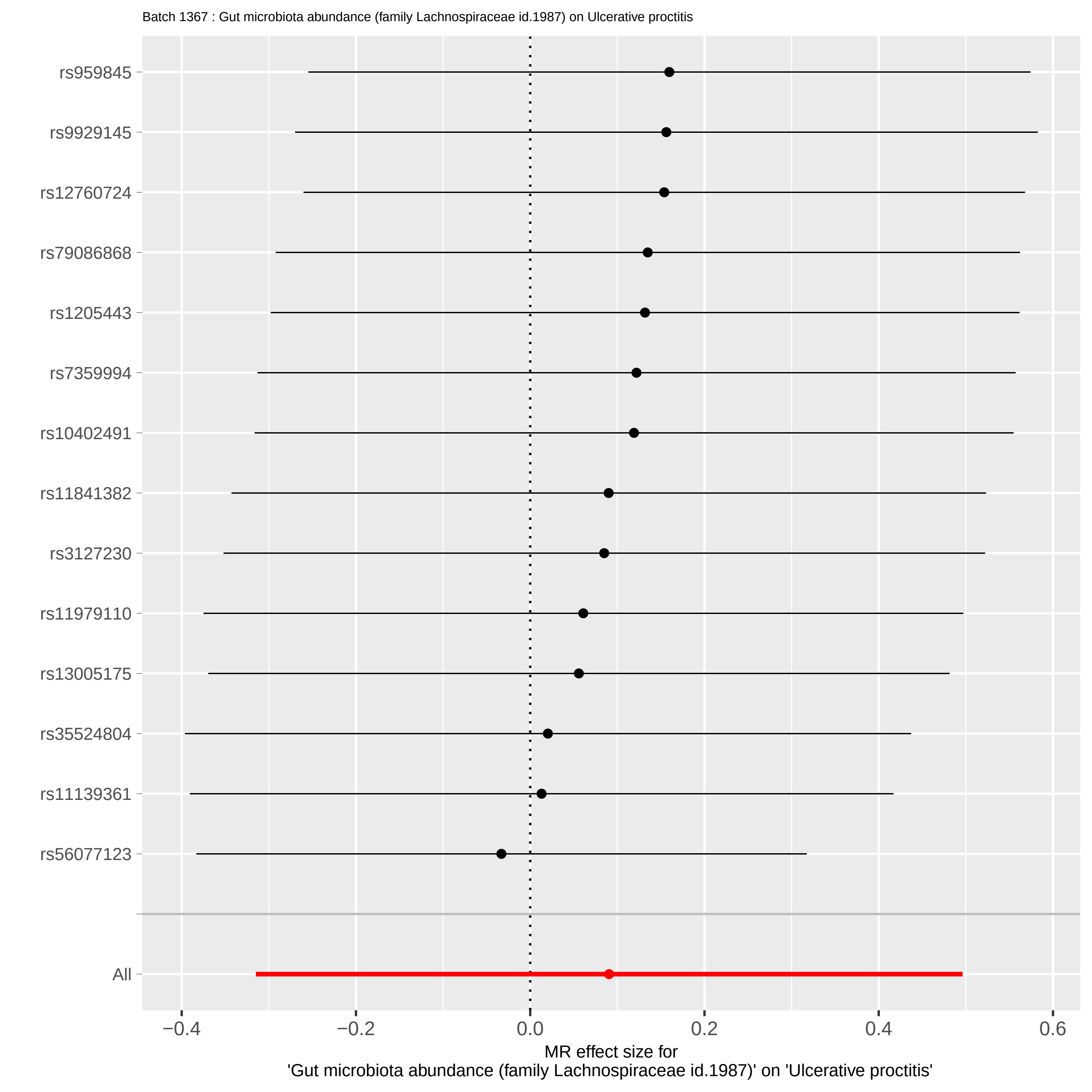
Batch 1367 : Gut microbiota abundance (family Lachnospiraceae id.1987) on Ulcerative proctitis
rs959845
rs9929145
rs12760724
rs79086868
rs1205443
rs7359994
rs10402491
rs11841382
rs3127230
rs11979110
rs13005175
rs35524804
rs11139361
rs56077123
All
−0.4
−0.2
0.0
0.2
0.4
0.6
MR effect size for
'Gut microbiota abundance (family Lachnospiraceae id.1987)' on 'Ulcerative proctitis'

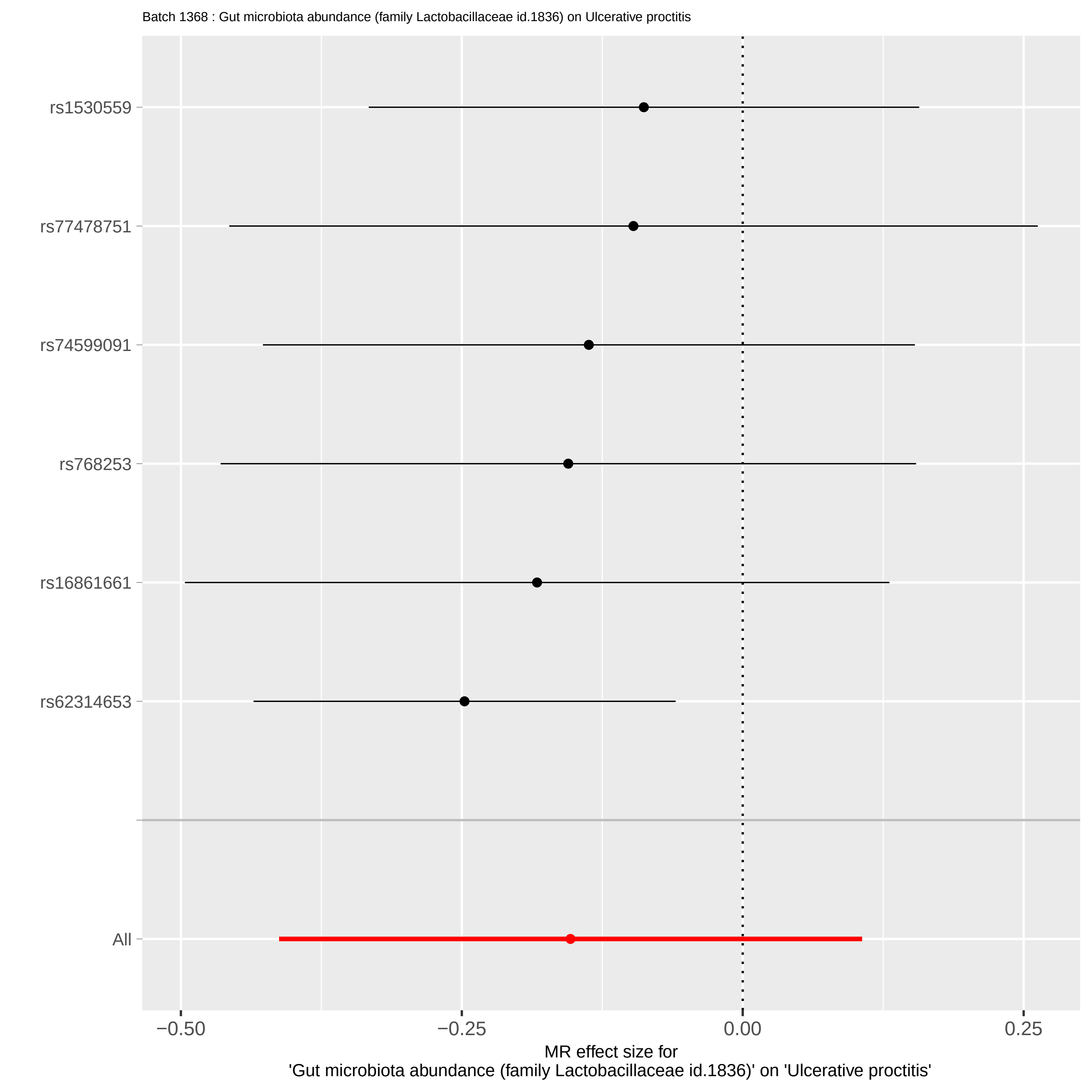

Batch 1368 : Gut microbiota abundance (family Lactobacillaceae id.1836) on Ulcerative proctitis
rs1530559
rs77478751
rs74599091
rs768253
rs16861661
rs62314653
All
−0.50
−0.25
0.00
0.25
MR effect size for
'Gut microbiota abundance (family Lactobacillaceae id.1836)' on 'Ulcerative proctitis'

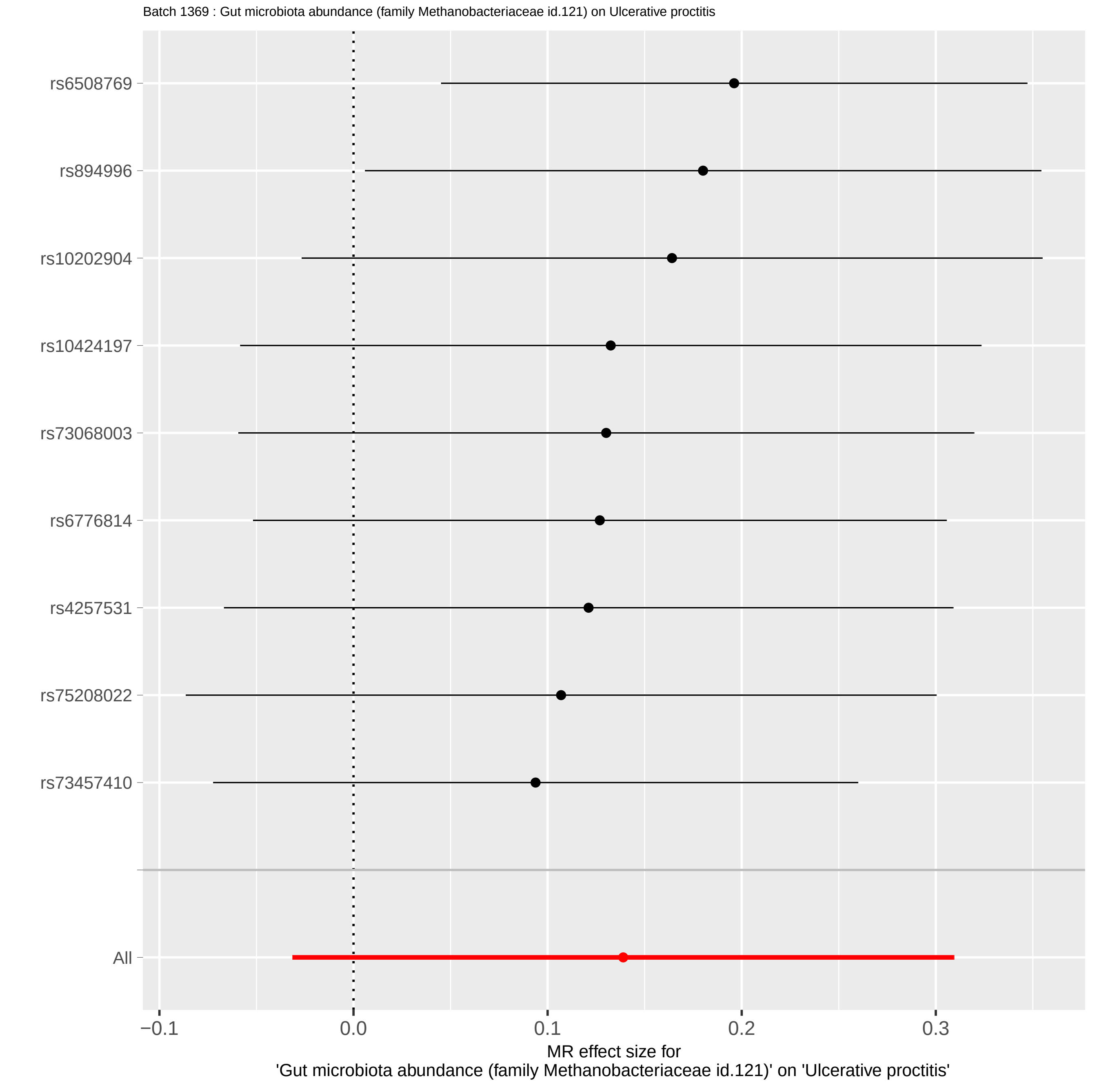
Batch 1369 : Gut microbiota abundance (family Methanobacteriaceae id.121) on Ulcerative proctitis
rs6508769
rs894996
rs10202904
rs10424197
rs73068003
rs6776814
rs4257531
rs75208022
rs73457410
All
−0.1
0.0
0.1
0.2
0.3
MR effect size for
'Gut microbiota abundance (family Methanobacteriaceae id.121)' on 'Ulcerative proctitis'

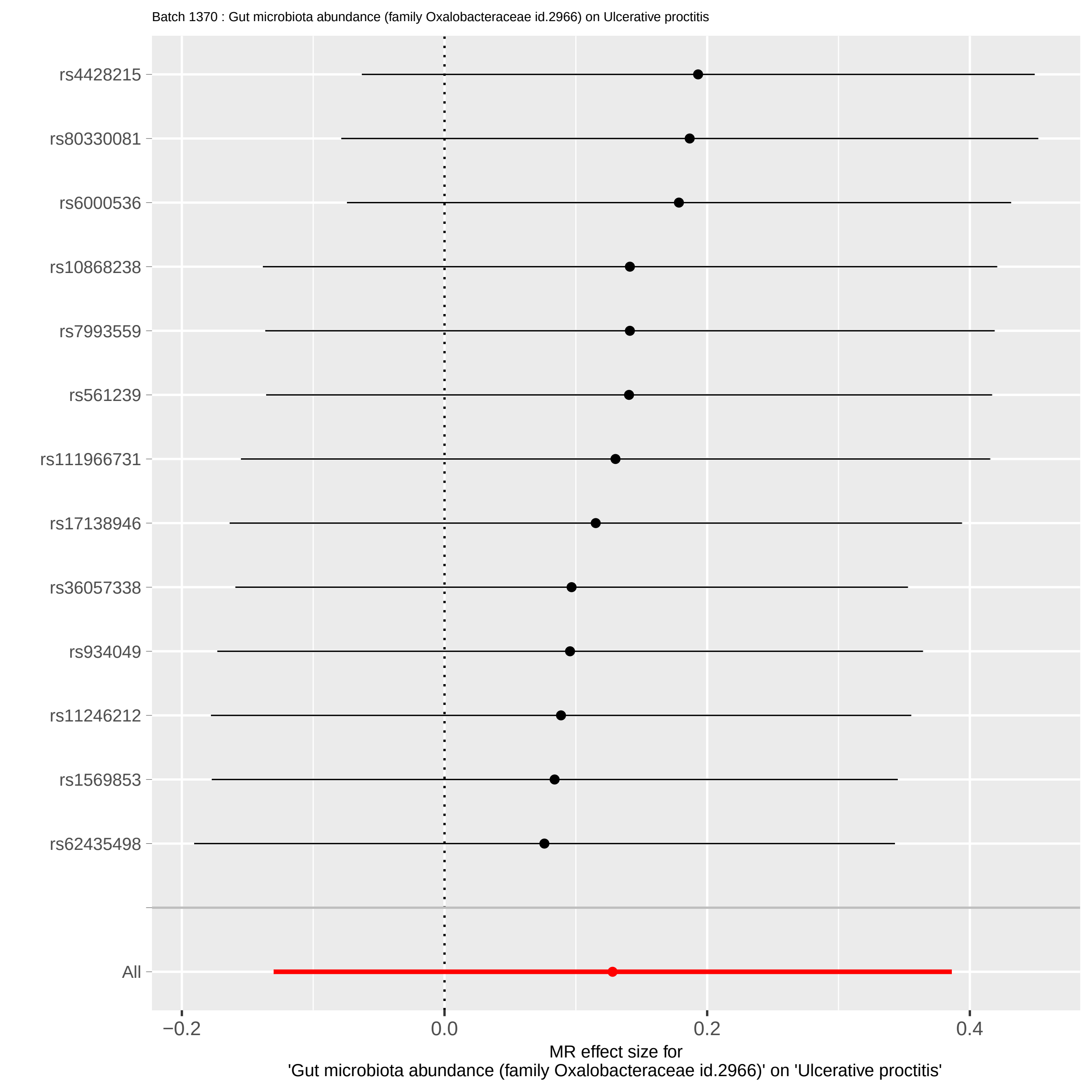

Batch 1370 : Gut microbiota abundance (family Oxalobacteraceae id.2966) on Ulcerative proctitis
rs4428215
rs80330081
rs6000536
rs10868238
rs7993559
rs561239
rs111966731
rs17138946
rs36057338
rs934049
rs11246212
rs1569853
rs62435498
All
−0.2
0.0
0.2
0.4
MR effect size for
'Gut microbiota abundance (family Oxalobacteraceae id.2966)' on 'Ulcerative proctitis'

Batch 1371 : Gut microbiota abundance (family Pasteurellaceae id.3689) on Ulcerative proctitis

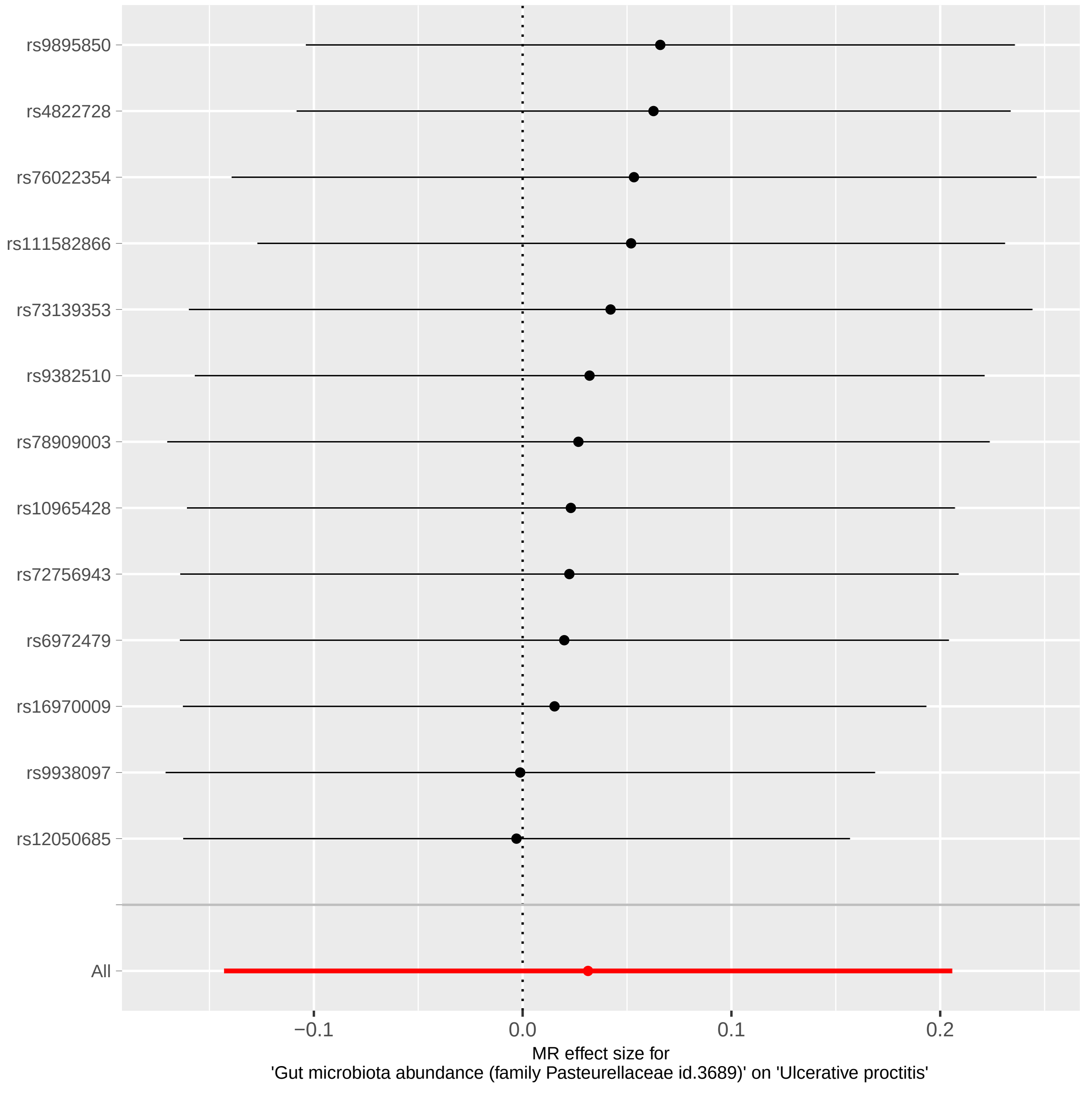

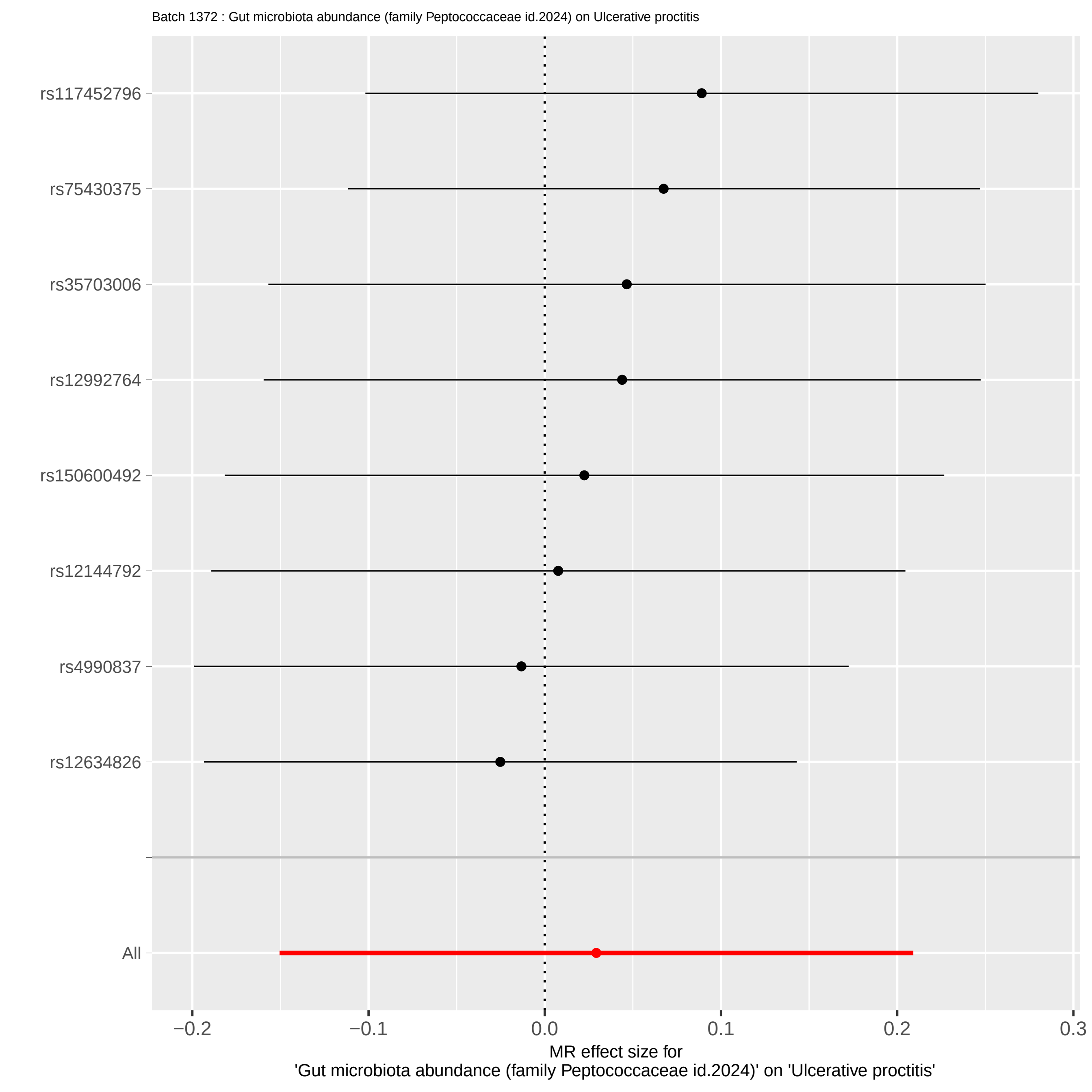
Batch 1372 : Gut microbiota abundance (family Peptococcaceae id.2024) on Ulcerative proctitis
rs117452796
rs75430375
rs35703006
rs12992764
rs150600492
rs12144792
rs4990837
rs12634826
All
−0.2
−0.1
0.0
0.1
0.2
0.3
MR effect size for
'Gut microbiota abundance (family Peptococcaceae id.2024)' on 'Ulcerative proctitis'

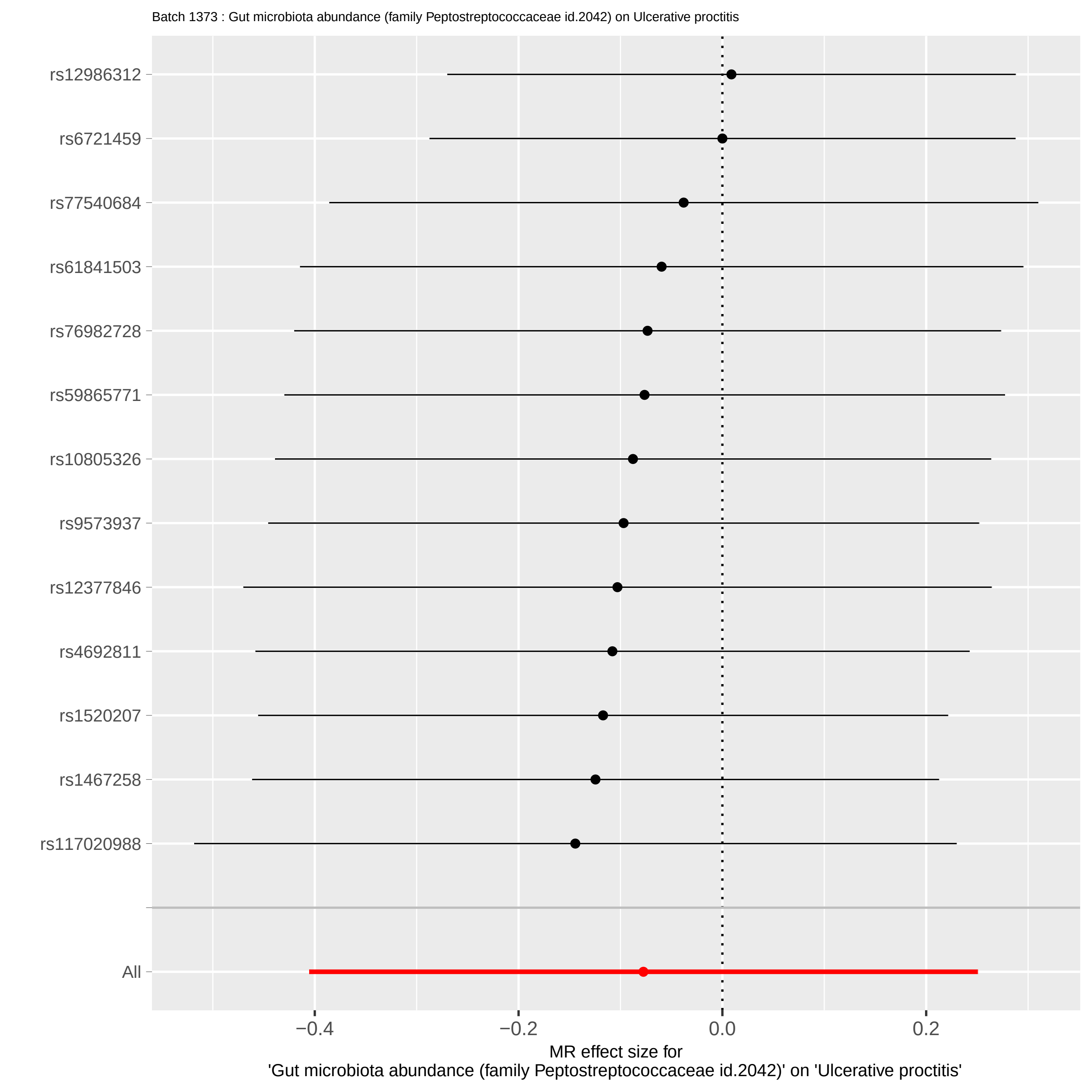

Batch 1373 : Gut microbiota abundance (family Peptostreptococcaceae id.2042) on Ulcerative proctitis
rs12986312
rs6721459
rs77540684
rs61841503
rs76982728
rs59865771
rs10805326
rs9573937
rs12377846
rs4692811
rs1520207
rs1467258
rs117020988
All
−0.4
−0.2
0.0
0.2
MR effect size for
'Gut microbiota abundance (family Peptostreptococcaceae id.2042)' on 'Ulcerative proctitis'

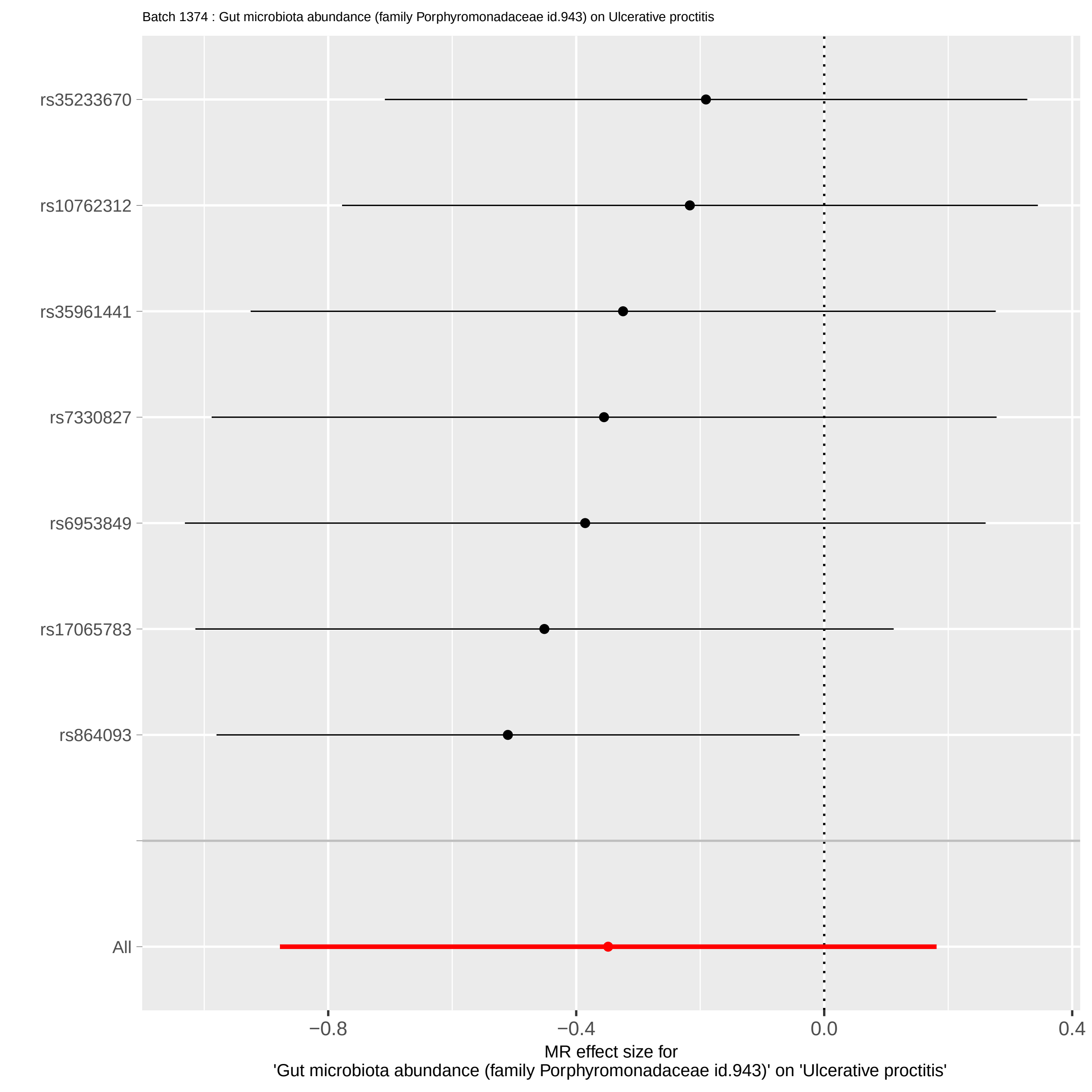

Batch 1374 : Gut microbiota abundance (family Porphyromonadaceae id.943) on Ulcerative proctitis
rs35233670
rs10762312
rs35961441
rs7330827
rs6953849
rs17065783
rs864093
All
−0.8
−0.4
0.0
0.4
MR effect size for
'Gut microbiota abundance (family Porphyromonadaceae id.943)' on 'Ulcerative proctitis'

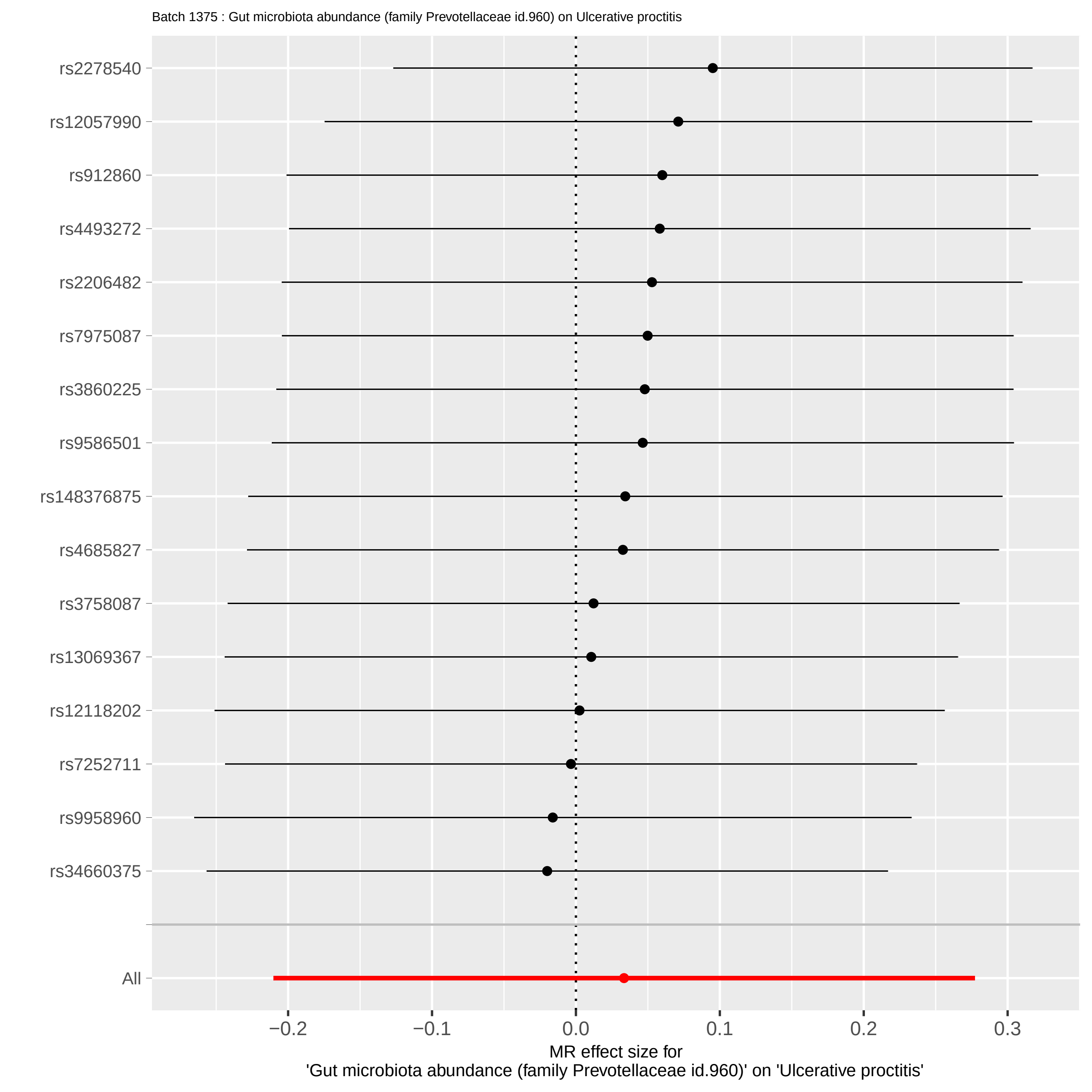
Batch 1375 : Gut microbiota abundance (family Prevotellaceae id.960) on Ulcerative proctitis
rs2278540
rs12057990
rs912860
rs4493272
rs2206482
rs7975087
rs3860225
rs9586501
rs148376875
rs4685827
rs3758087
rs13069367
rs12118202
rs7252711
rs9958960
rs34660375
All
−0.2
−0.1
0.0
0.1
0.2
0.3
MR effect size for
'Gut microbiota abundance (family Prevotellaceae id.960)' on 'Ulcerative proctitis'

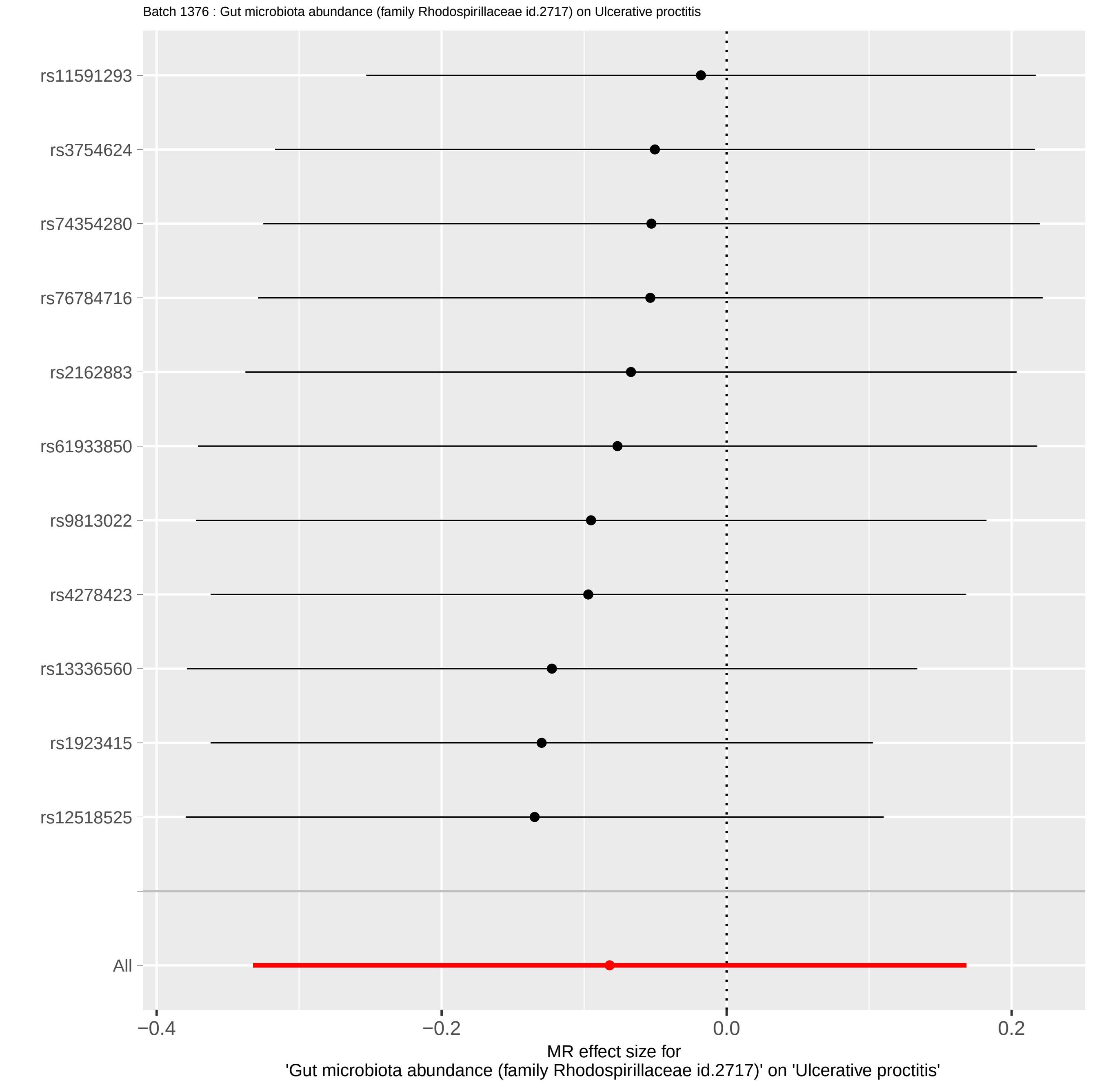

Batch 1376 : Gut microbiota abundance (family Rhodospirillaceae id.2717) on Ulcerative proctitis
rs11591293
rs3754624
rs74354280
rs76784716
rs2162883
rs61933850
rs9813022
rs4278423
rs13336560
rs1923415
rs12518525
All
−0.4
−0.2
0.0
0.2
MR effect size for
'Gut microbiota abundance (family Rhodospirillaceae id.2717)' on 'Ulcerative proctitis'

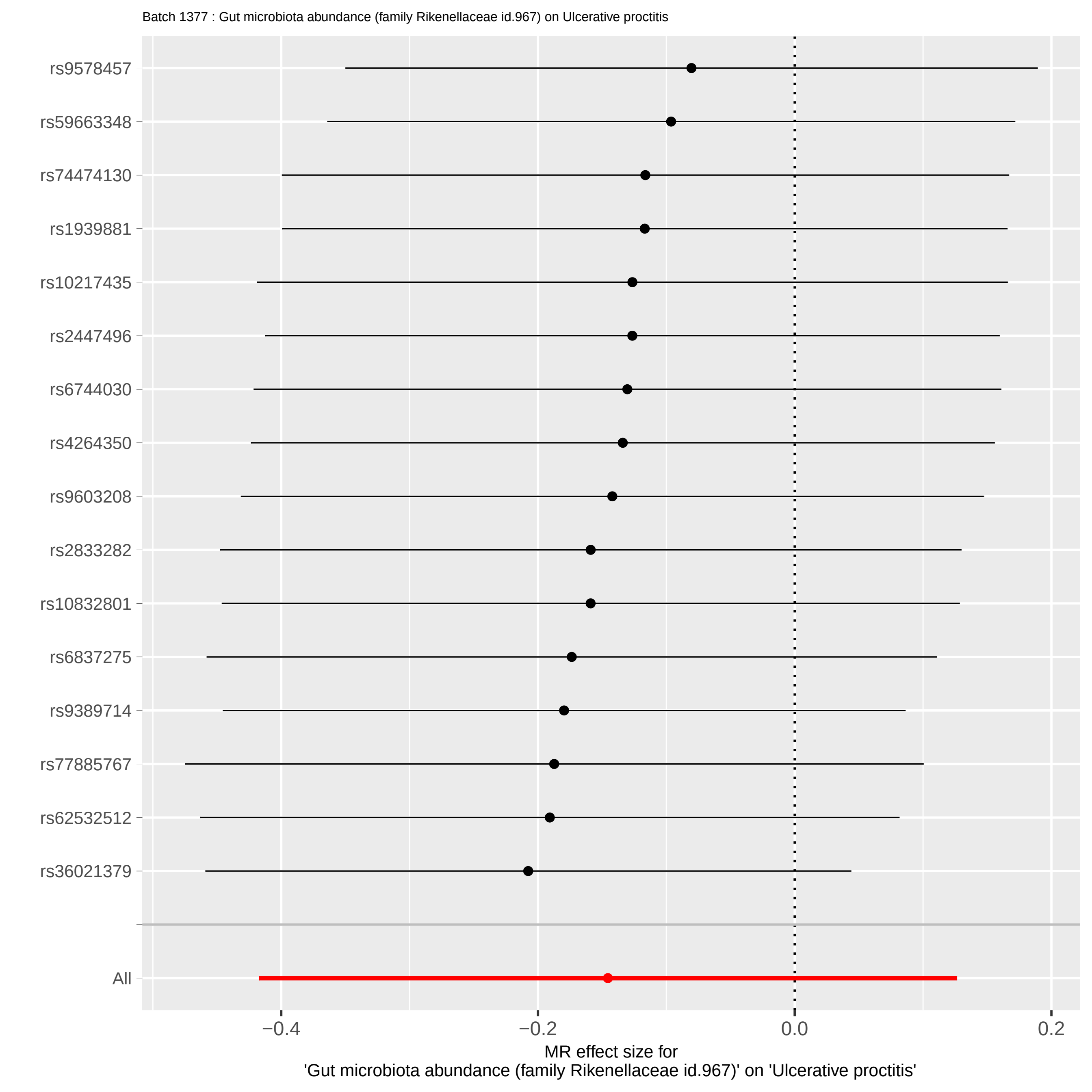

Batch 1377 : Gut microbiota abundance (family Rikenellaceae id.967) on Ulcerative proctitis
rs9578457
rs59663348
rs74474130
rs1939881
rs10217435
rs2447496
rs6744030
rs4264350
rs9603208
rs2833282
rs10832801
rs6837275
rs9389714
rs77885767
rs62532512
rs36021379
All
−0.4
−0.2
0.0
0.2
MR effect size for
'Gut microbiota abundance (family Rikenellaceae id.967)' on 'Ulcerative proctitis'

Batch 1378 : Gut microbiota abundance (family Ruminococcaceae id.2050) on Ulcerative proctitis

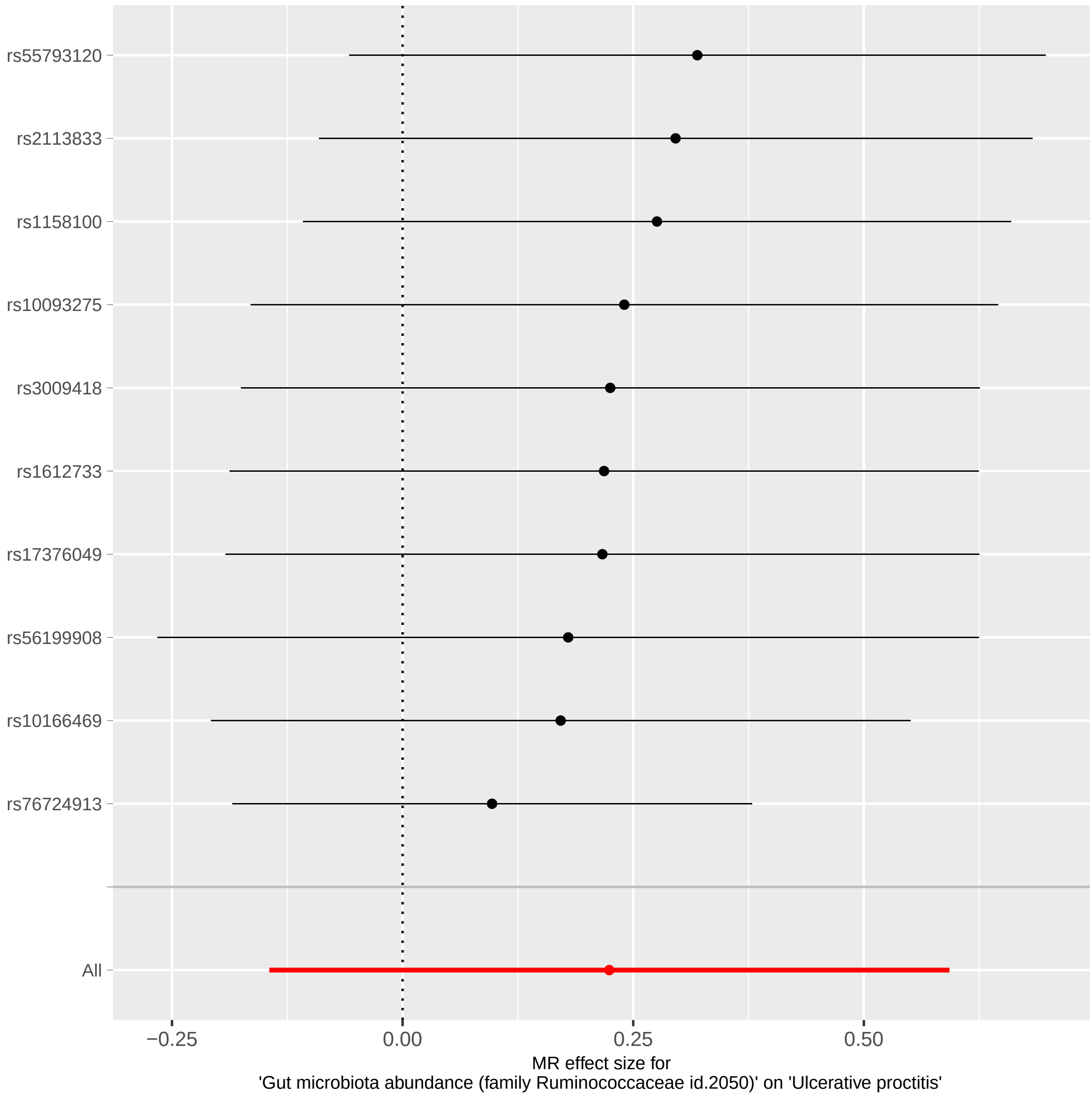

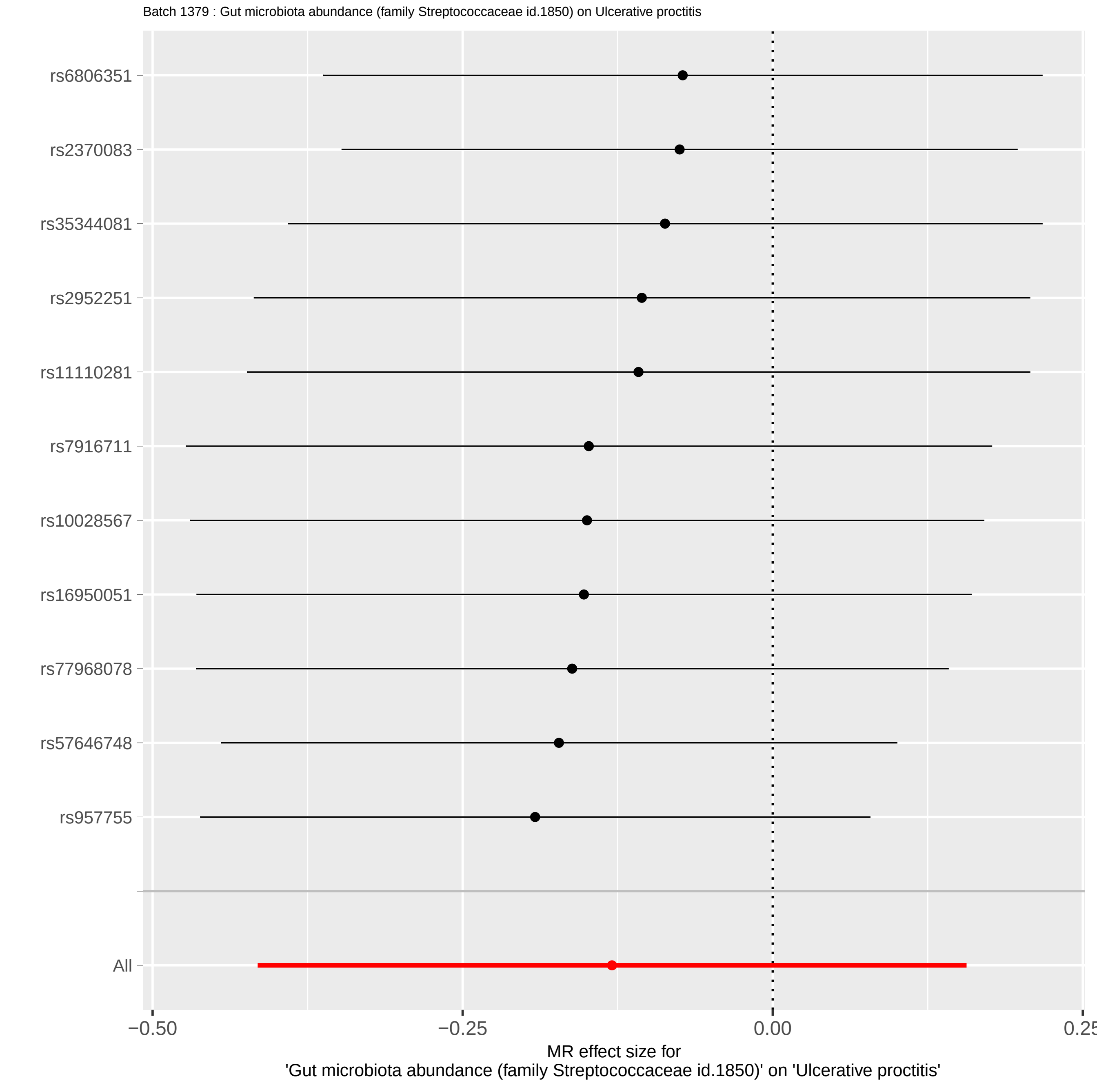
Batch 1379 : Gut microbiota abundance (family Streptococcaceae id.1850) on Ulcerative proctitis
rs6806351
rs2370083
rs35344081
rs2952251
rs11110281
rs7916711
rs10028567
rs16950051
rs77968078
rs57646748
rs957755
All
−0.50
−0.25
0.00
0.25
MR effect size for
'Gut microbiota abundance (family Streptococcaceae id.1850)' on 'Ulcerative proctitis'

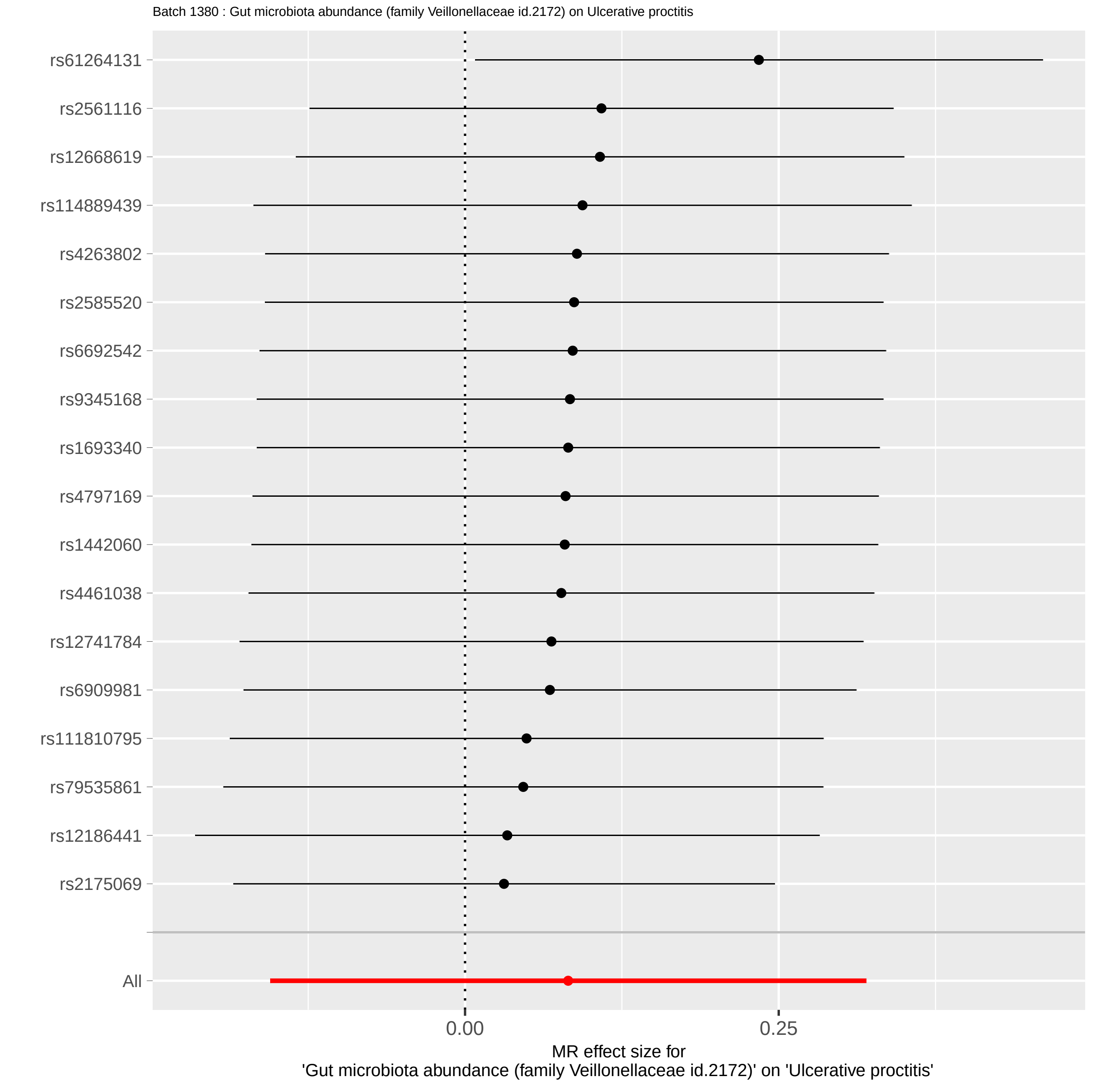
Batch 1380 : Gut microbiota abundance (family Veillonellaceae id.2172) on Ulcerative proctitis
rs61264131
rs2561116
rs12668619
rs114889439
rs4263802
rs2585520
rs6692542
rs9345168
rs1693340
rs4797169
rs1442060
rs4461038
rs12741784
rs6909981
rs111810795
rs79535861
rs12186441
rs2175069
All
0.00
0.25
MR effect size for
'Gut microbiota abundance (family Veillonellaceae id.2172)' on 'Ulcerative proctitis'

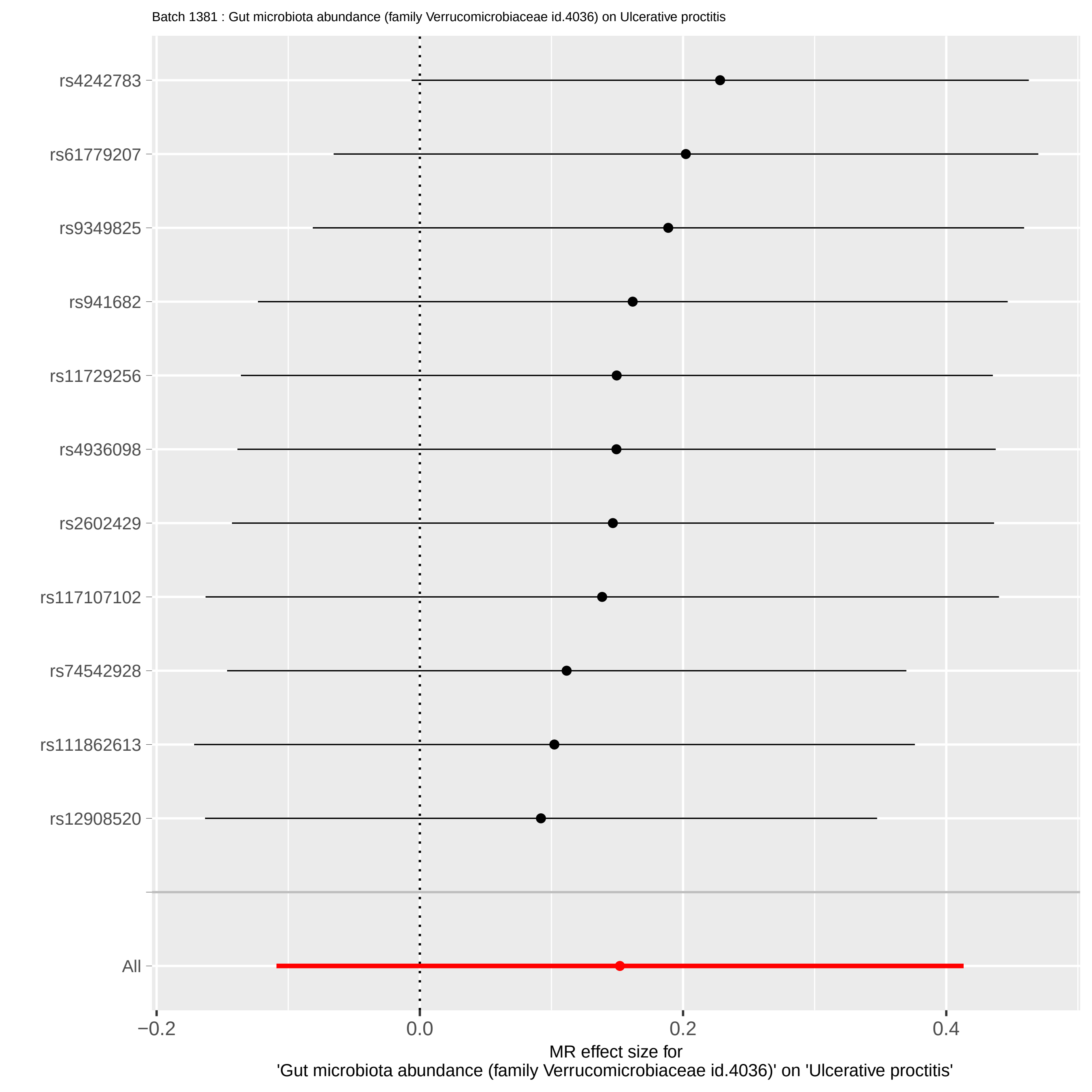

Batch 1381 : Gut microbiota abundance (family Verrucomicrobiaceae id.4036) on Ulcerative proctitis
rs4242783
rs61779207
rs9349825
rs941682
rs11729256
rs4936098
rs2602429
rs117107102
rs74542928
rs111862613
rs12908520
All
−0.2
0.0
0.2
0.4
MR effect size for
'Gut microbiota abundance (family Verrucomicrobiaceae id.4036)' on 'Ulcerative proctitis'

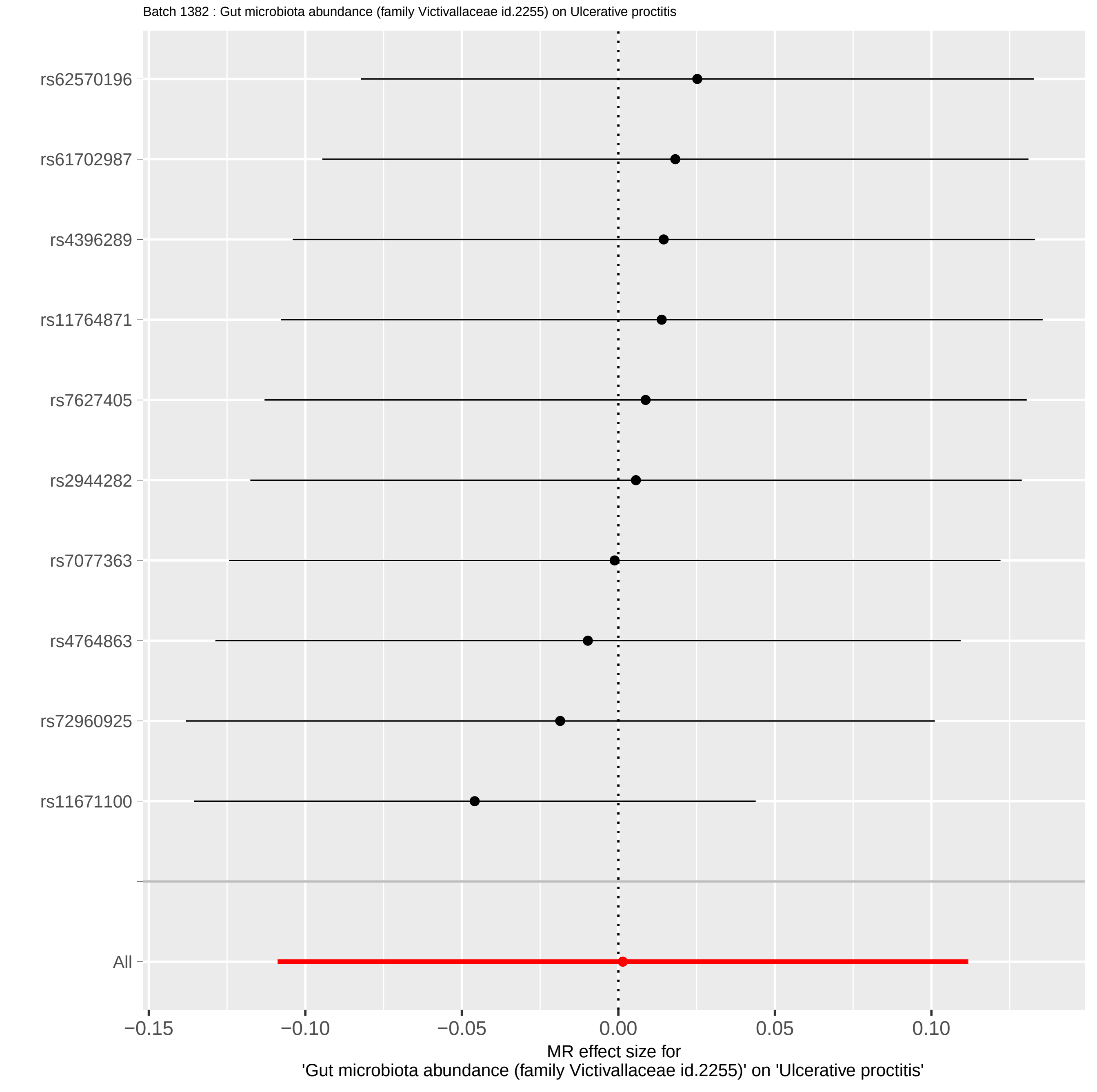

Batch 1382 : Gut microbiota abundance (family Victivallaceae id.2255) on Ulcerative proctitis
rs62570196
rs61702987
rs4396289
rs11764871
rs7627405
rs2944282
rs7077363
rs4764863
rs72960925
rs11671100
All
−0.15
−0.10
−0.05
0.00
0.05
0.10
MR effect size for
'Gut microbiota abundance (family Victivallaceae id.2255)' on 'Ulcerative proctitis'

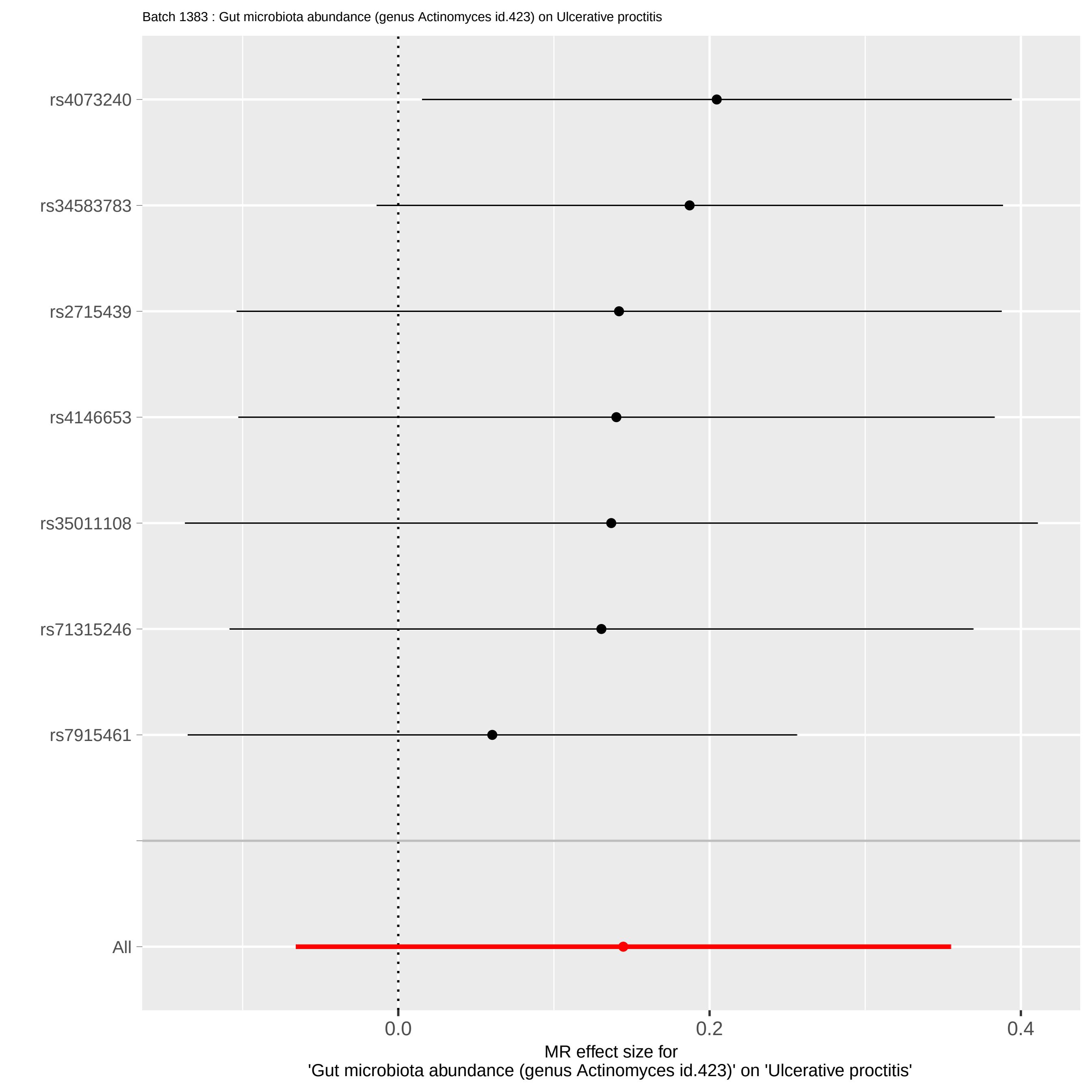

Batch 1383 : Gut microbiota abundance (genus Actinomyces id.423) on Ulcerative proctitis
rs4073240
rs34583783
rs2715439
rs4146653
rs35011108
rs71315246
rs7915461
All
0.0
0.2
0.4
MR effect size for
'Gut microbiota abundance (genus Actinomyces id.423)' on 'Ulcerative proctitis'

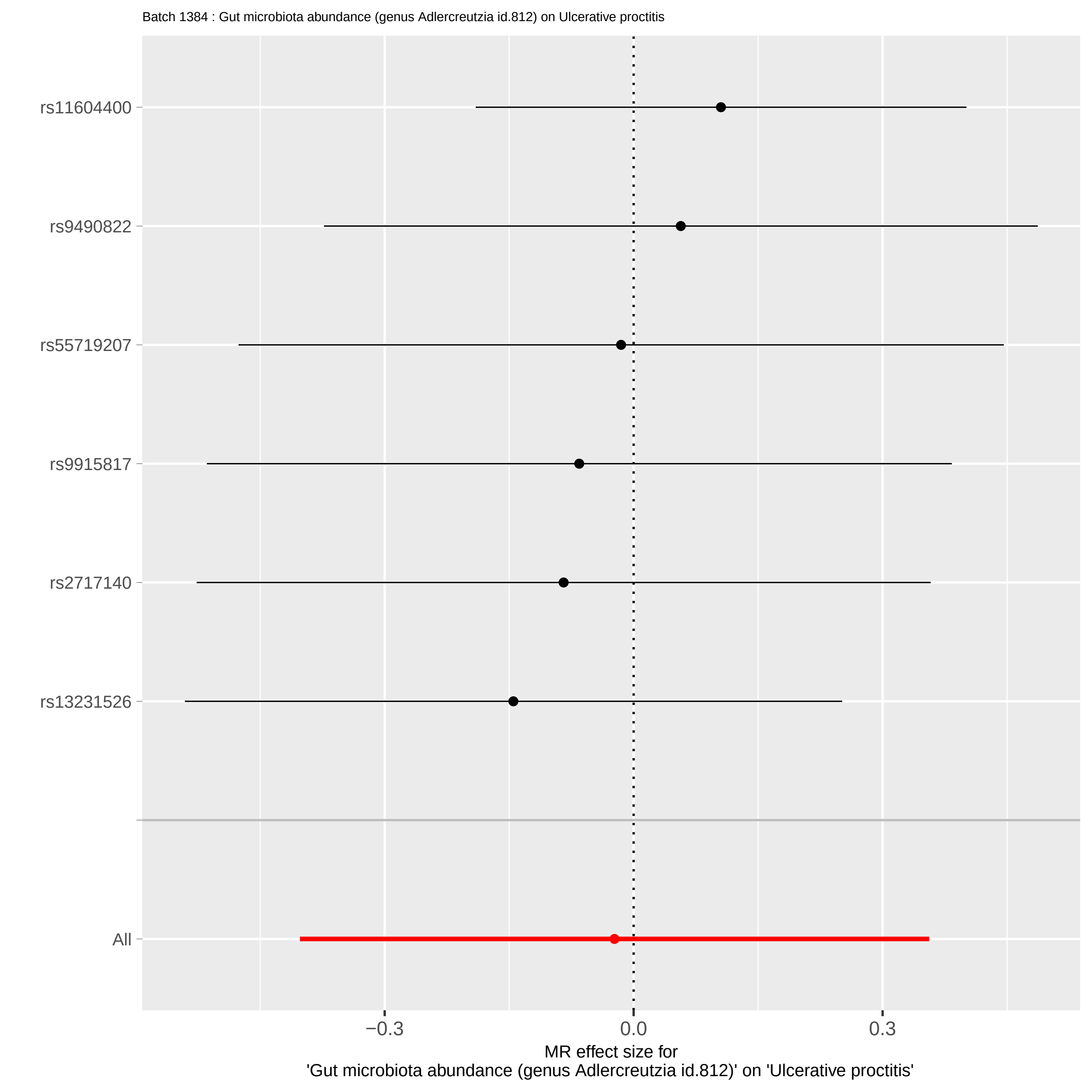
Batch 1384 : Gut microbiota abundance (genus Adlercreutzia id.812) on Ulcerative proctitis
rs11604400
rs9490822
rs55719207
rs9915817
rs2717140
rs13231526
All
−0.3
0.0
0.3
MR effect size for
'Gut microbiota abundance (genus Adlercreutzia id.812)' on 'Ulcerative proctitis'

Batch 1385 : Gut microbiota abundance (genus Akkermansia id.4037) on Ulcerative proctitis

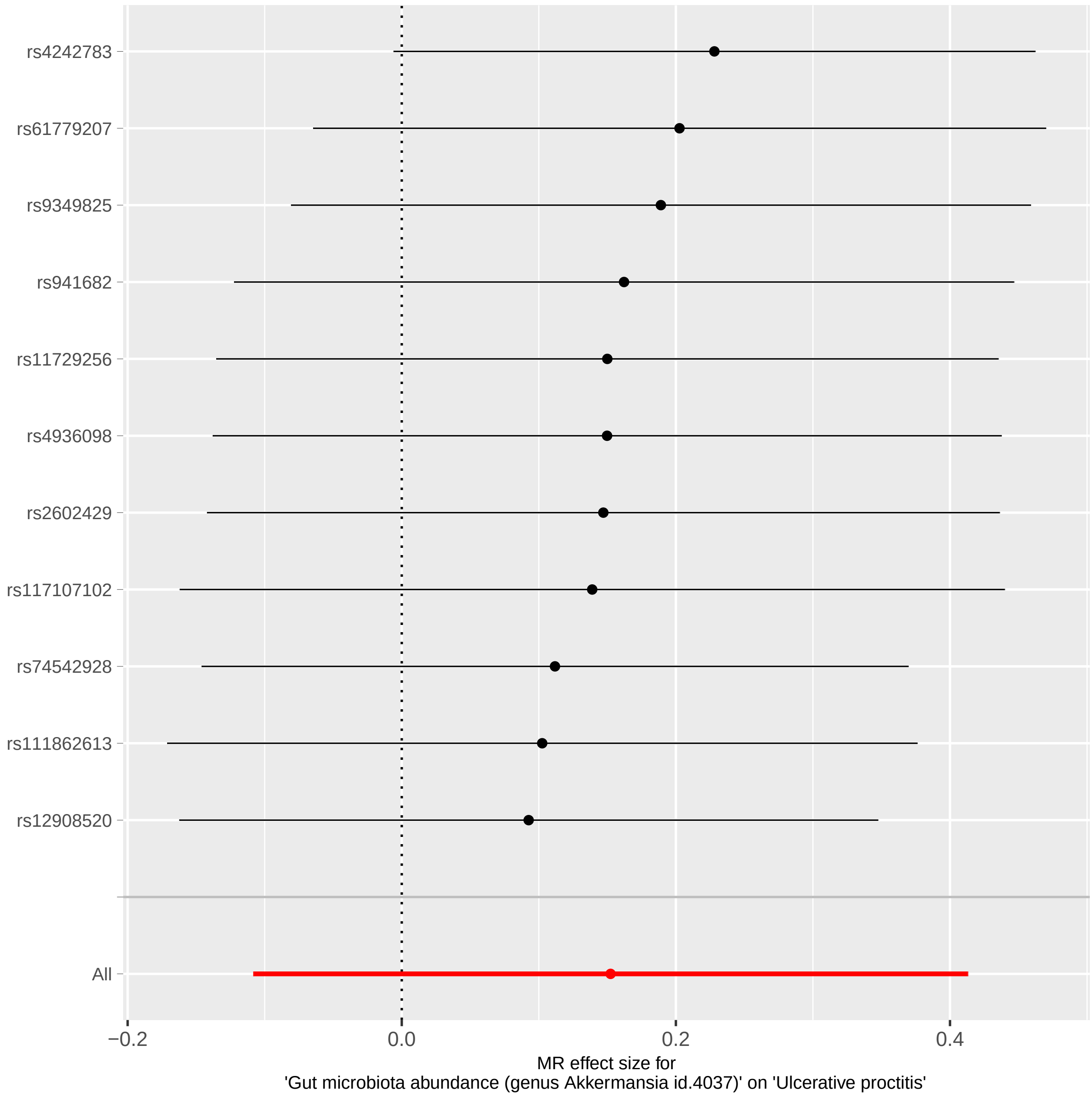

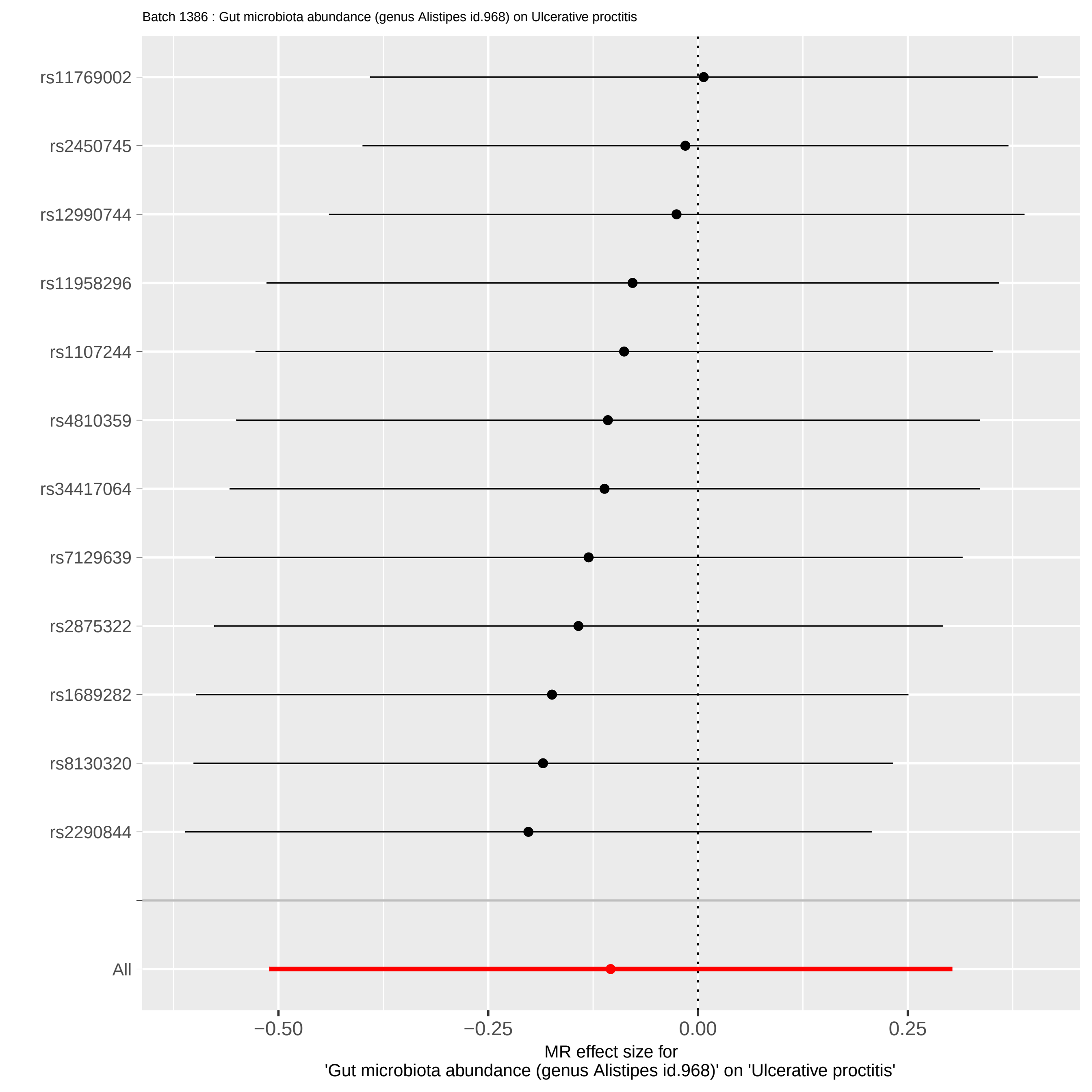

Batch 1386 : Gut microbiota abundance (genus Alistipes id.968) on Ulcerative proctitis
rs11769002
rs2450745
rs12990744
rs11958296
rs1107244
rs4810359
rs34417064
rs7129639
rs2875322
rs1689282
rs8130320
rs2290844
All
−0.50
−0.25
0.00
0.25
MR effect size for
'Gut microbiota abundance (genus Alistipes id.968)' on 'Ulcerative proctitis'

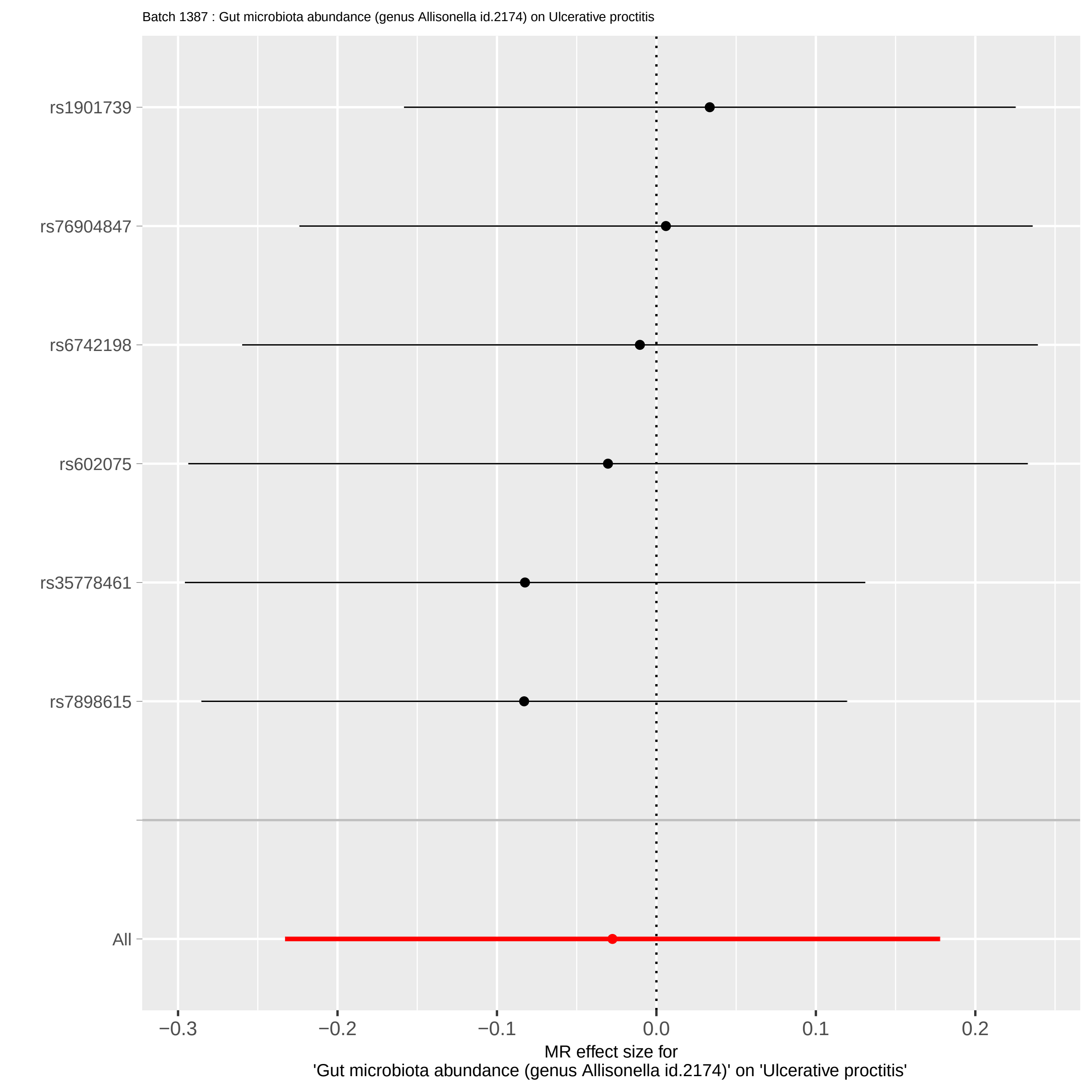
Batch 1387 : Gut microbiota abundance (genus Allisonella id.2174) on Ulcerative proctitis
rs1901739
rs76904847
rs6742198
rs602075
rs35778461
rs7898615
All
−0.3
−0.2
−0.1
0.0
0.1
0.2
MR effect size for
'Gut microbiota abundance (genus Allisonella id.2174)' on 'Ulcerative proctitis'

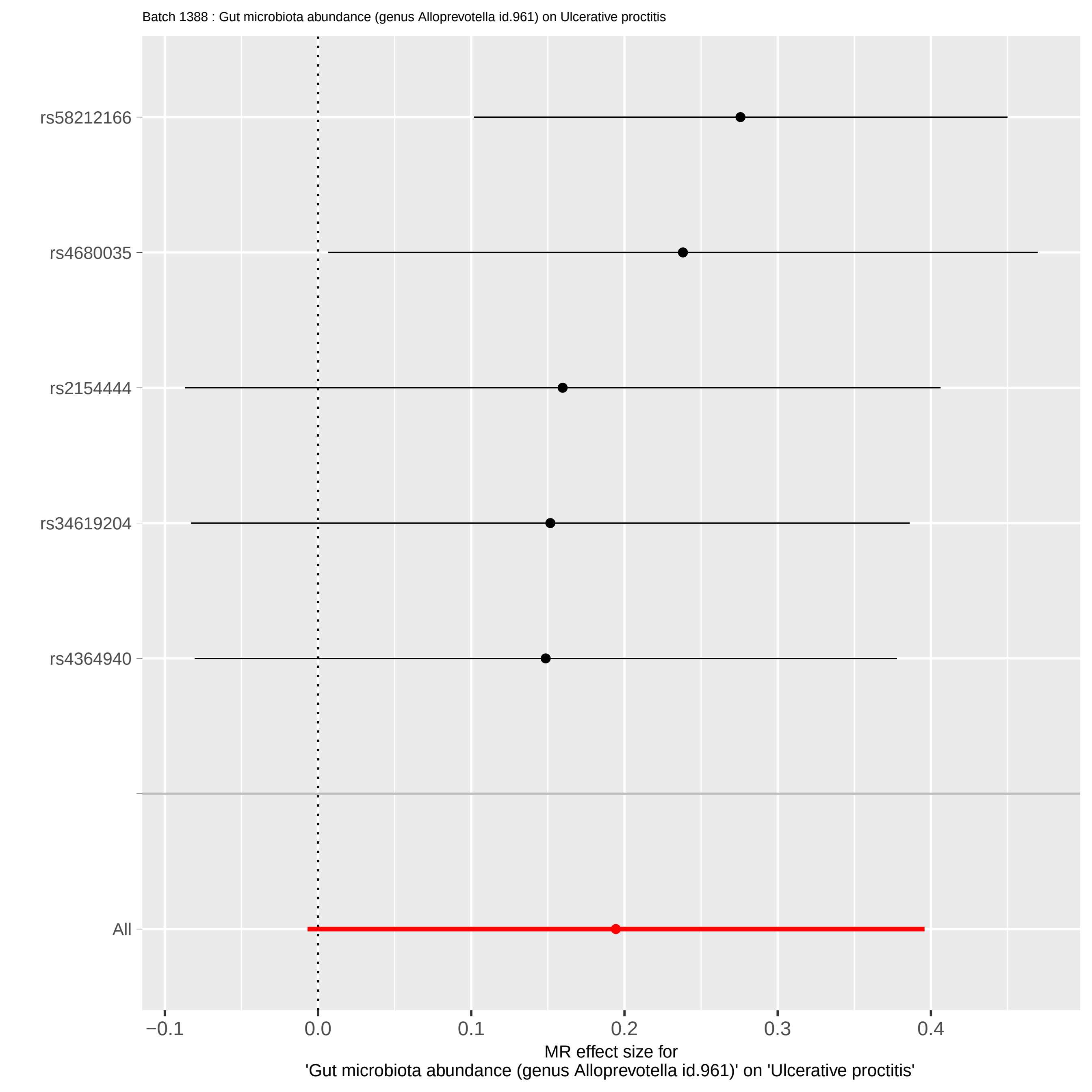

Batch 1388 : Gut microbiota abundance (genus Alloprevotella id.961) on Ulcerative proctitis
rs58212166
rs4680035
rs2154444
rs34619204
rs4364940
All
−0.1
0.0
0.1
0.2
0.3
0.4
MR effect size for
'Gut microbiota abundance (genus Alloprevotella id.961)' on 'Ulcerative proctitis'

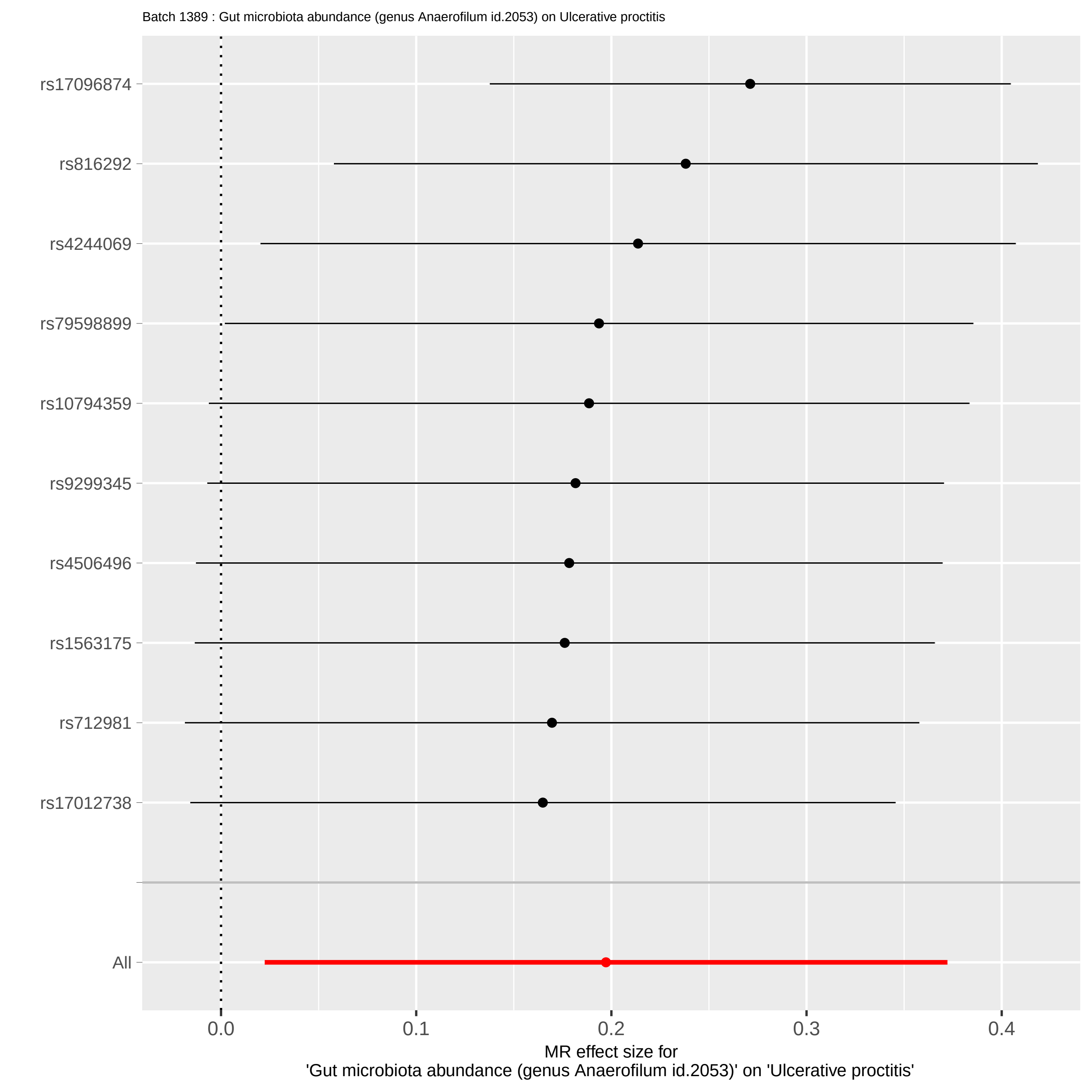

Batch 1389 : Gut microbiota abundance (genus Anaerofilum id.2053) on Ulcerative proctitis
rs17096874
rs816292
rs4244069
rs79598899
rs10794359
rs9299345
rs4506496
rs1563175
rs712981
rs17012738
All
0.0
0.1
0.2
0.3
0.4
MR effect size for
'Gut microbiota abundance (genus Anaerofilum id.2053)' on 'Ulcerative proctitis'

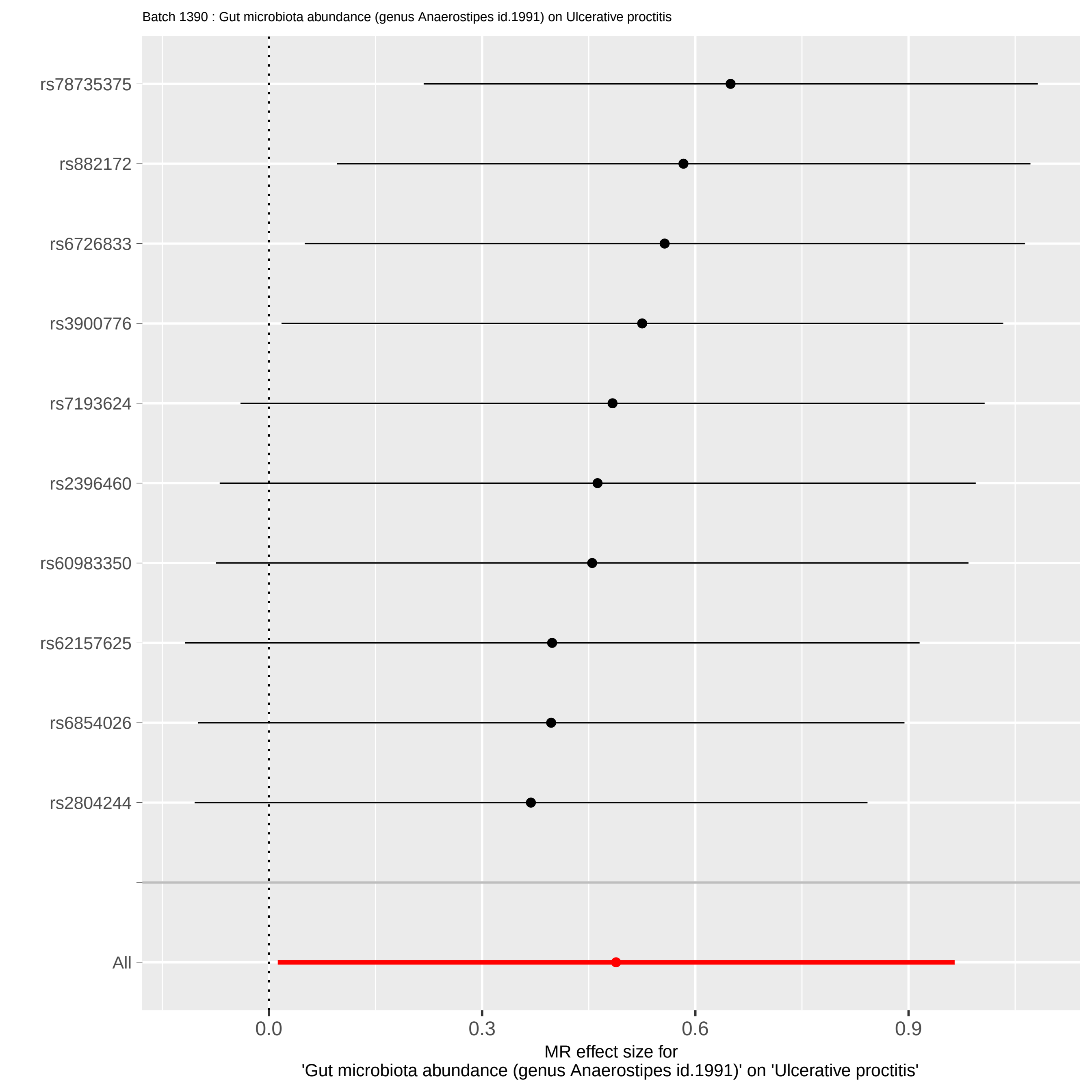

Batch 1390 : Gut microbiota abundance (genus Anaerostipes id.1991) on Ulcerative proctitis
rs78735375
rs882172
rs6726833
rs3900776
rs7193624
rs2396460
rs60983350
rs62157625
rs6854026
rs2804244
All
0.0
0.3
0.6
0.9
MR effect size for
'Gut microbiota abundance (genus Anaerostipes id.1991)' on 'Ulcerative proctitis'

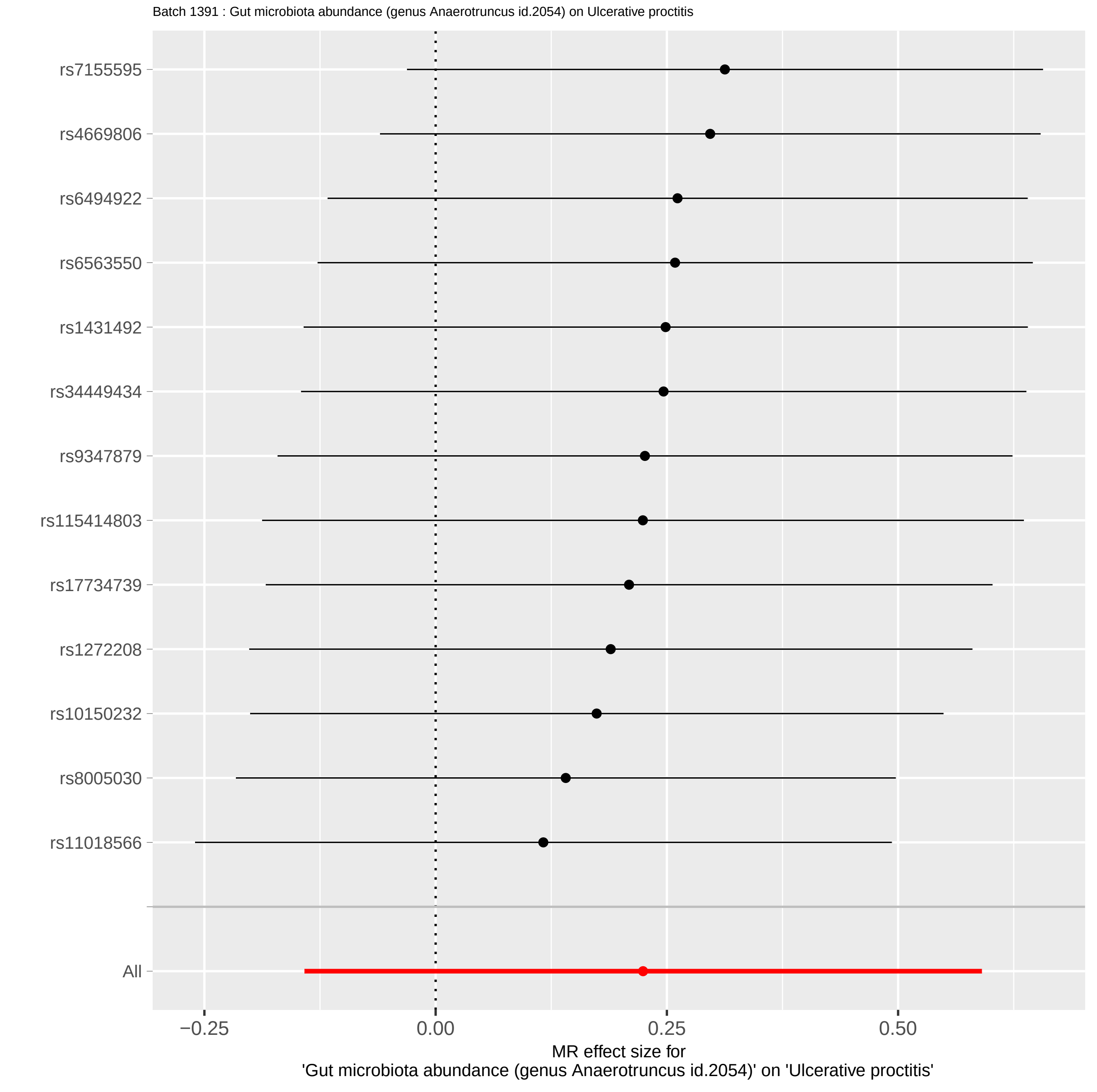

Batch 1391 : Gut microbiota abundance (genus Anaerotruncus id.2054) on Ulcerative proctitis
rs7155595
rs4669806
rs6494922
rs6563550
rs1431492
rs34449434
rs9347879
rs115414803
rs17734739
rs1272208
rs10150232
rs8005030
rs11018566
All
−0.25
0.00
0.25
0.50
MR effect size for
'Gut microbiota abundance (genus Anaerotruncus id.2054)' on 'Ulcerative proctitis'

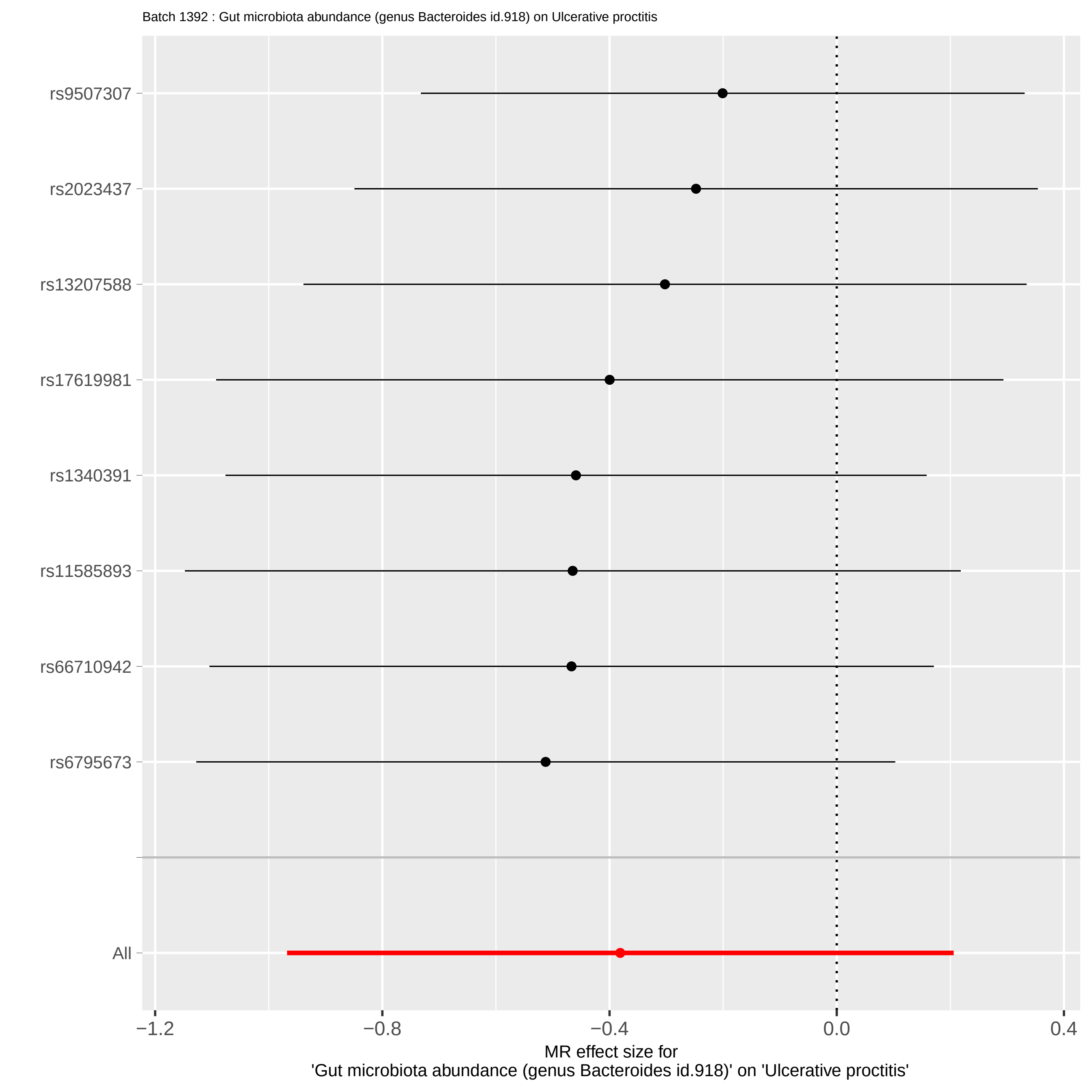
Batch 1392 : Gut microbiota abundance (genus Bacteroides id.918) on Ulcerative proctitis
rs9507307
rs2023437
rs13207588
rs17619981
rs1340391
rs11585893
rs66710942
rs6795673
All
−1.2
−0.8
−0.4
0.0
0.4
MR effect size for
'Gut microbiota abundance (genus Bacteroides id.918)' on 'Ulcerative proctitis'

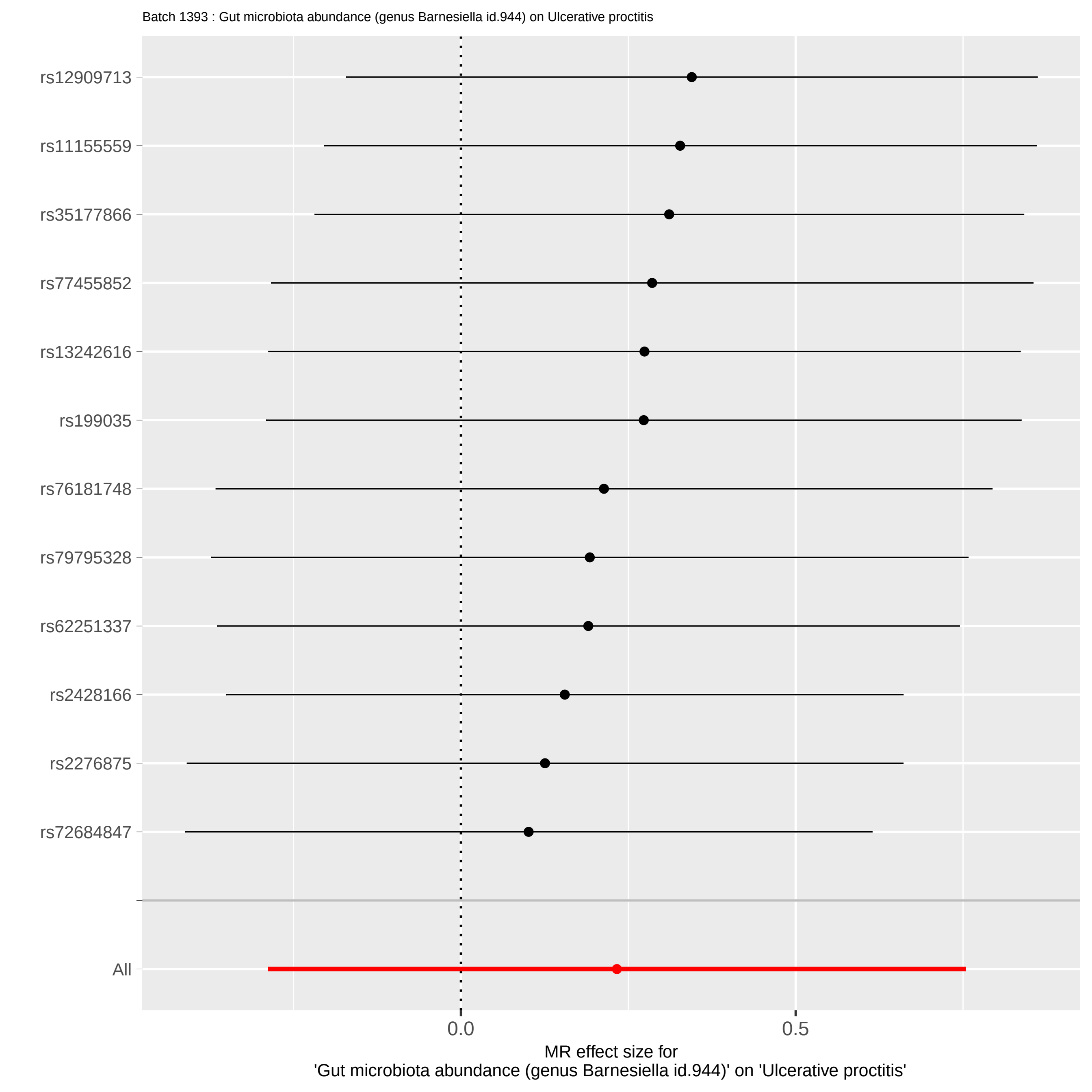

Batch 1393 : Gut microbiota abundance (genus Barnesiella id.944) on Ulcerative proctitis
rs12909713
rs11155559
rs35177866
rs77455852
rs13242616
rs199035
rs76181748
rs79795328
rs62251337
rs2428166
rs2276875
rs72684847
All
0.0
0.5
MR effect size for
'Gut microbiota abundance (genus Barnesiella id.944)' on 'Ulcerative proctitis'

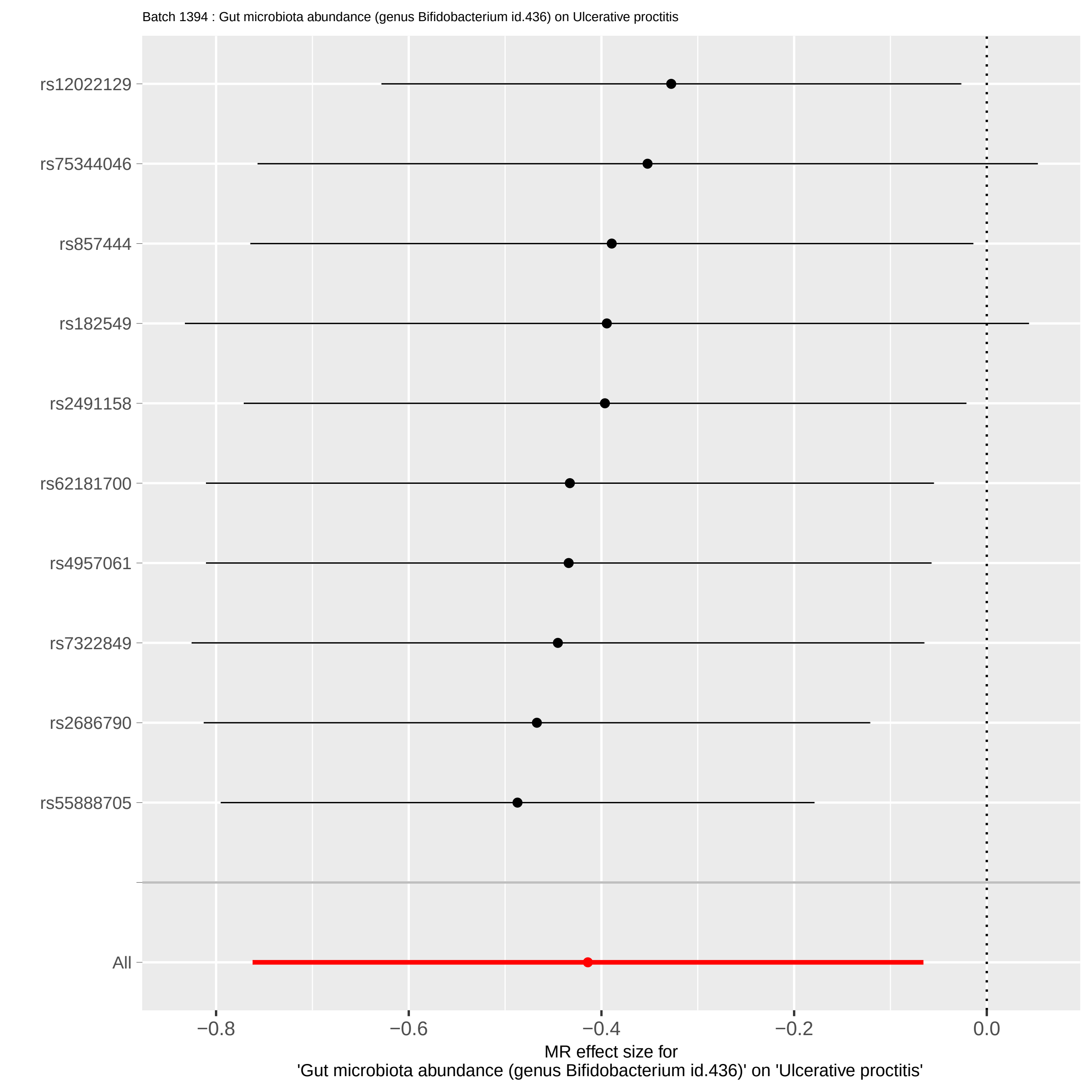
Batch 1394 : Gut microbiota abundance (genus Bifidobacterium id.436) on Ulcerative proctitis
rs12022129
rs75344046
rs857444
rs182549
rs2491158
rs62181700
rs4957061
rs7322849
rs2686790
rs55888705
All
−0.8
−0.6
−0.4
−0.2
0.0
MR effect size for
'Gut microbiota abundance (genus Bifidobacterium id.436)' on 'Ulcerative proctitis'

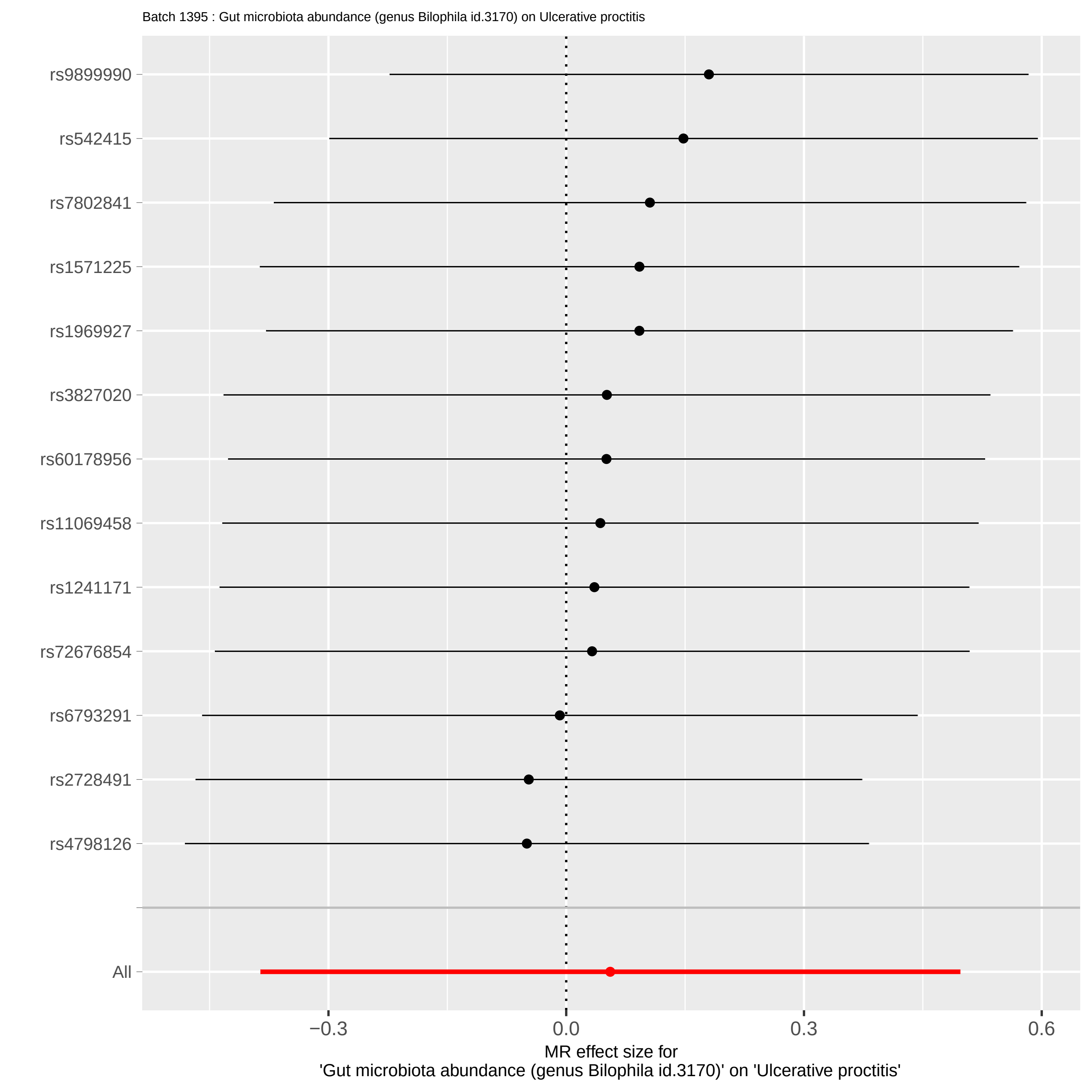
Batch 1395 : Gut microbiota abundance (genus Bilophila id.3170) on Ulcerative proctitis
rs9899990
rs542415
rs7802841
rs1571225
rs1969927
rs3827020
rs60178956
rs11069458
rs1241171
rs72676854
rs6793291
rs2728491
rs4798126
All
−0.3
0.0
0.3
0.6
MR effect size for
'Gut microbiota abundance (genus Bilophila id.3170)' on 'Ulcerative proctitis'

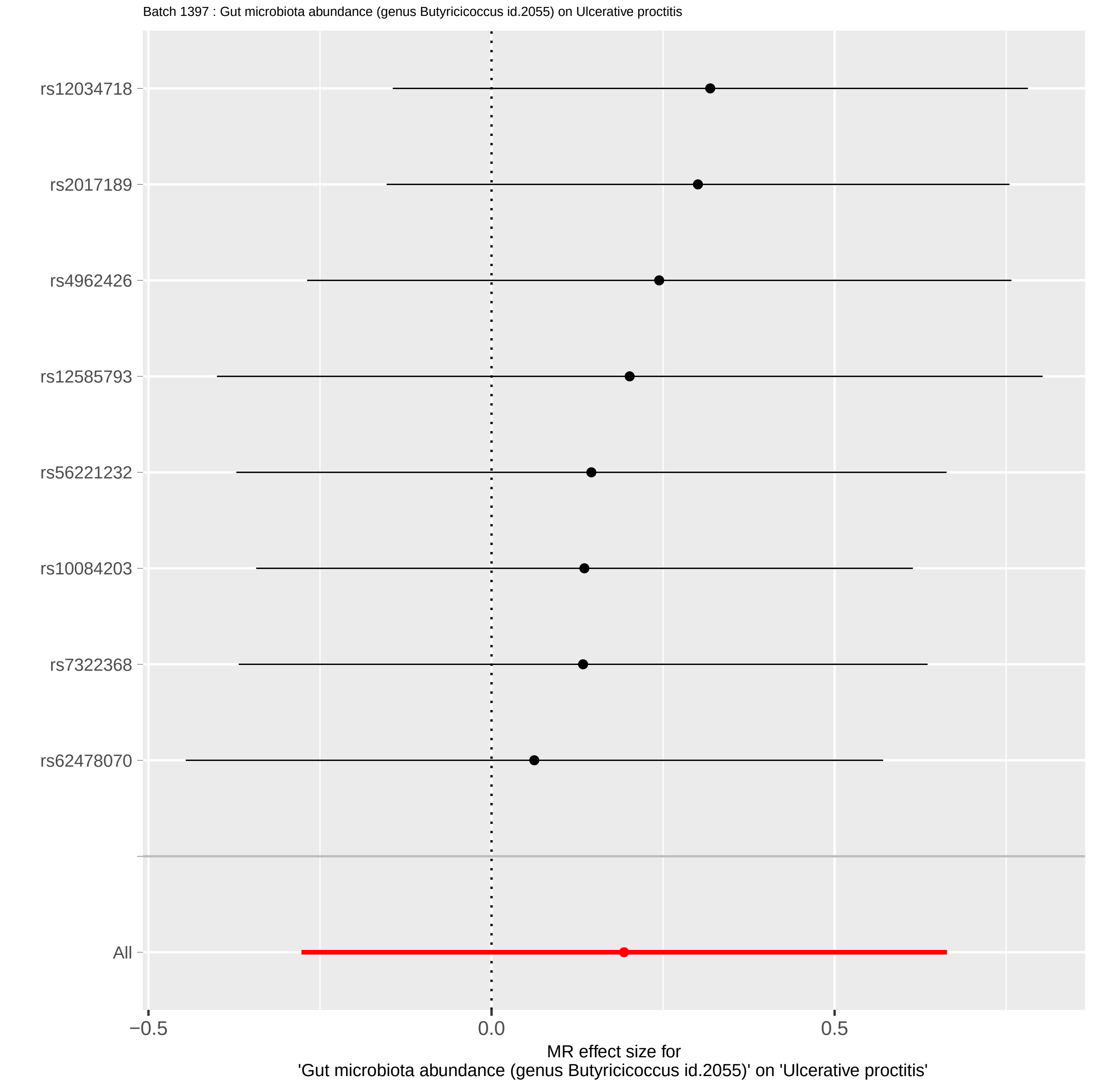

Batch 1397 : Gut microbiota abundance (genus Butyricicoccus id.2055) on Ulcerative proctitis
rs12034718
rs2017189
rs4962426
rs12585793
rs56221232
rs10084203
rs7322368
rs62478070
All
−0.5
0.0
0.5
MR effect size for
'Gut microbiota abundance (genus Butyricicoccus id.2055)' on 'Ulcerative proctitis'

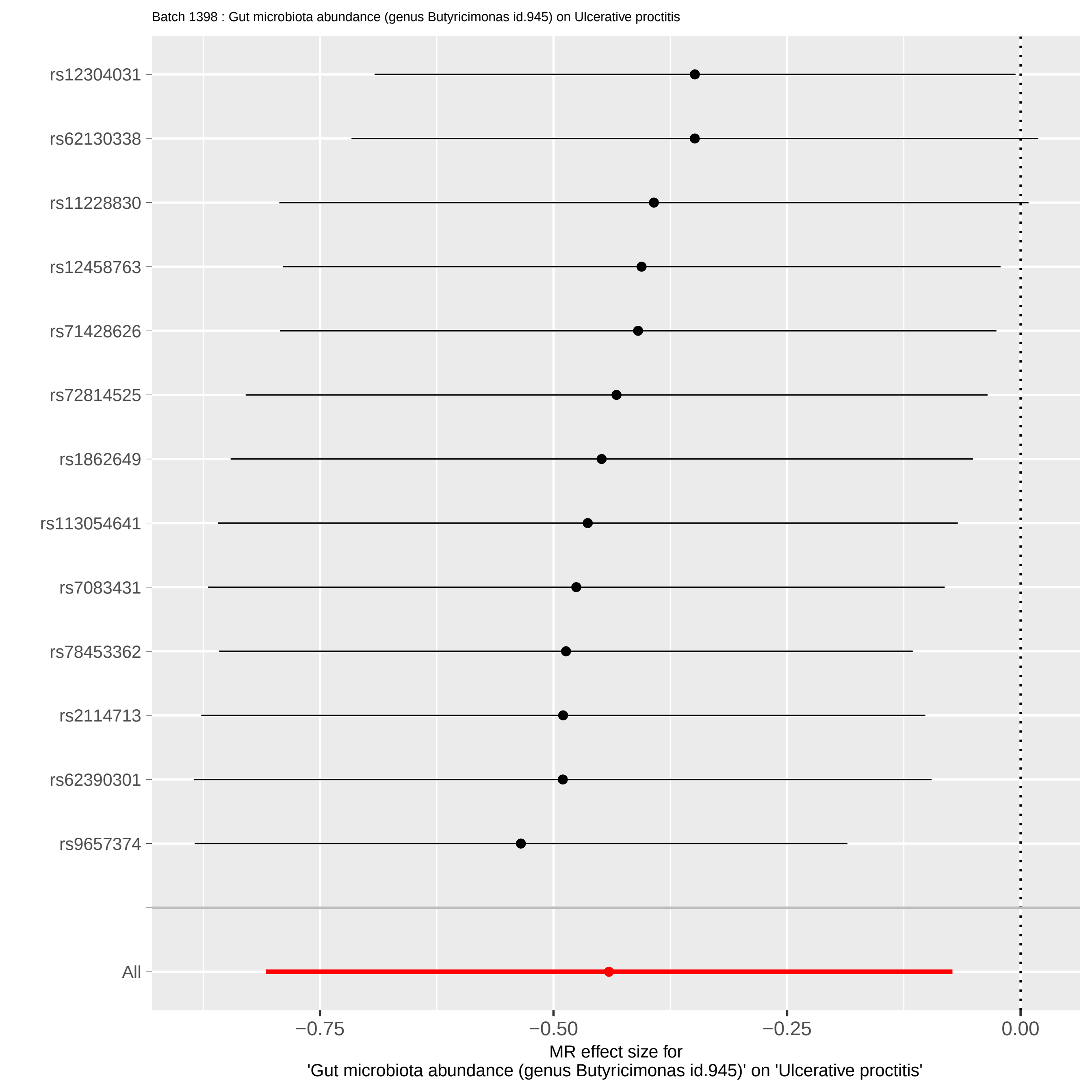

Batch 1398 : Gut microbiota abundance (genus Butyricimonas id.945) on Ulcerative proctitis
rs12304031
rs62130338
rs11228830
rs12458763
rs71428626
rs72814525
rs1862649
rs113054641
rs7083431
rs78453362
rs2114713
rs62390301
rs9657374
All
−0.75
−0.50
−0.25
0.00
MR effect size for
'Gut microbiota abundance (genus Butyricimonas id.945)' on 'Ulcerative proctitis'

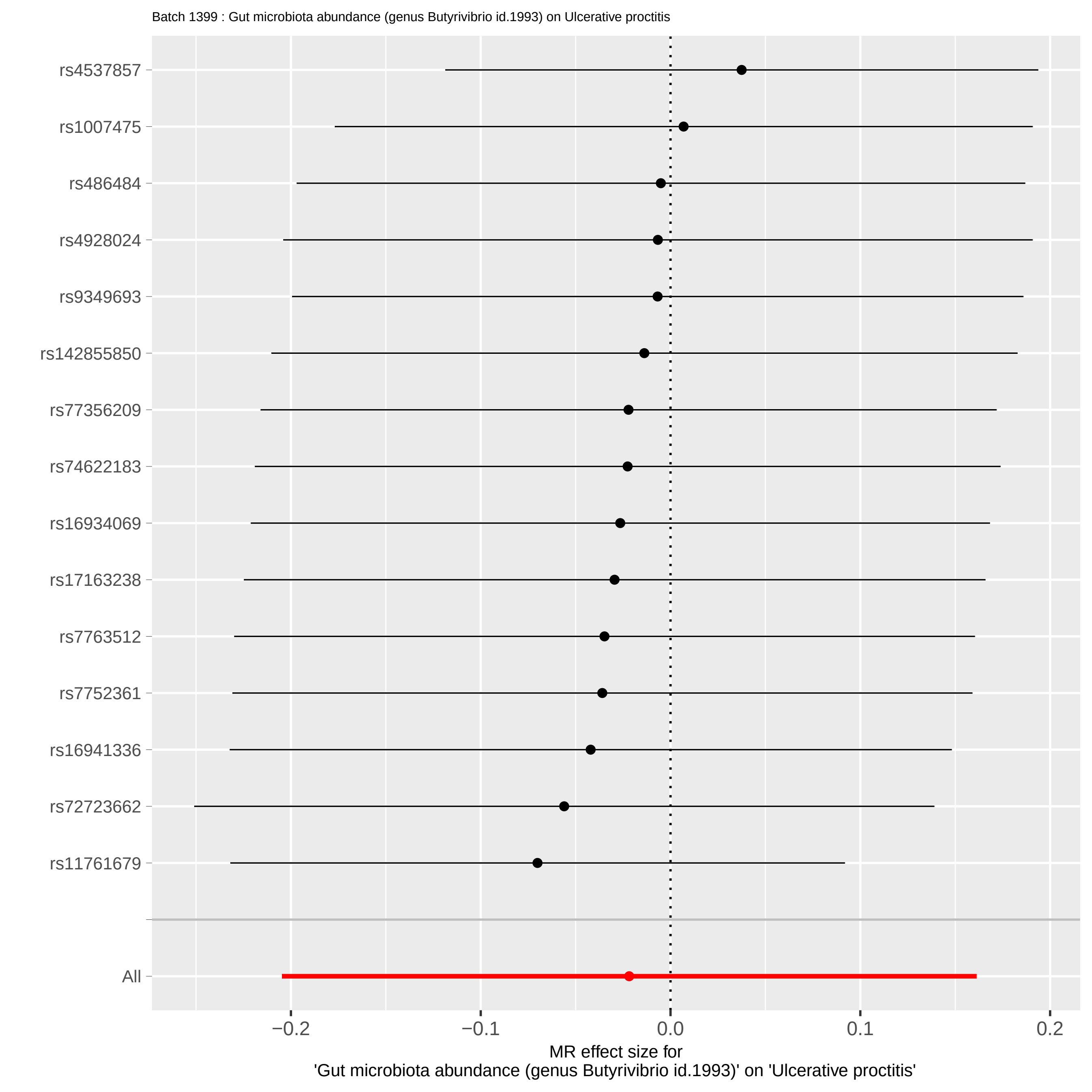

Batch 1399 : Gut microbiota abundance (genus Butyrivibrio id.1993) on Ulcerative proctitis
rs4537857
rs1007475
rs486484
rs4928024
rs9349693
rs142855850
rs77356209
rs74622183
rs16934069
rs17163238
rs7763512
rs7752361
rs16941336
rs72723662
rs11761679
All
−0.2
−0.1
0.0
0.1
0.2
MR effect size for
'Gut microbiota abundance (genus Butyrivibrio id.1993)' on 'Ulcerative proctitis'

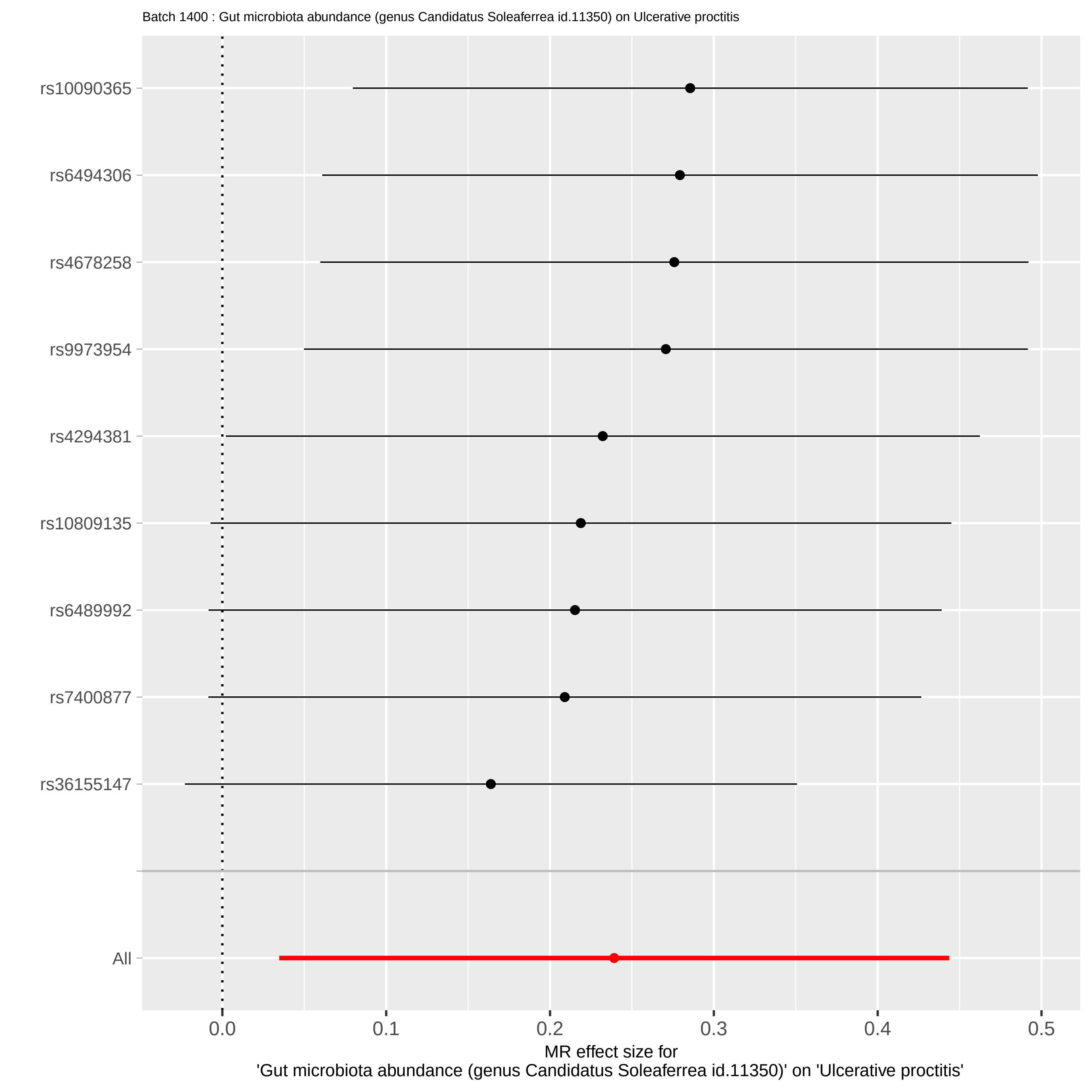
Batch 1400 : Gut microbiota abundance (genus Candidatus Soleaferrea id.11350) on Ulcerative proctitis
rs10090365
rs6494306
rs4678258
rs9973954
rs4294381
rs10809135
rs6489992
rs7400877
rs36155147
All
0.0
0.1
0.2
0.3
0.4
0.5
MR effect size for
'Gut microbiota abundance (genus Candidatus Soleaferrea id.11350)' on 'Ulcerative proctitis'

Batch 1401 : Gut microbiota abundance (genus Catenibacterium id.2153) on Ulcerative proctitis

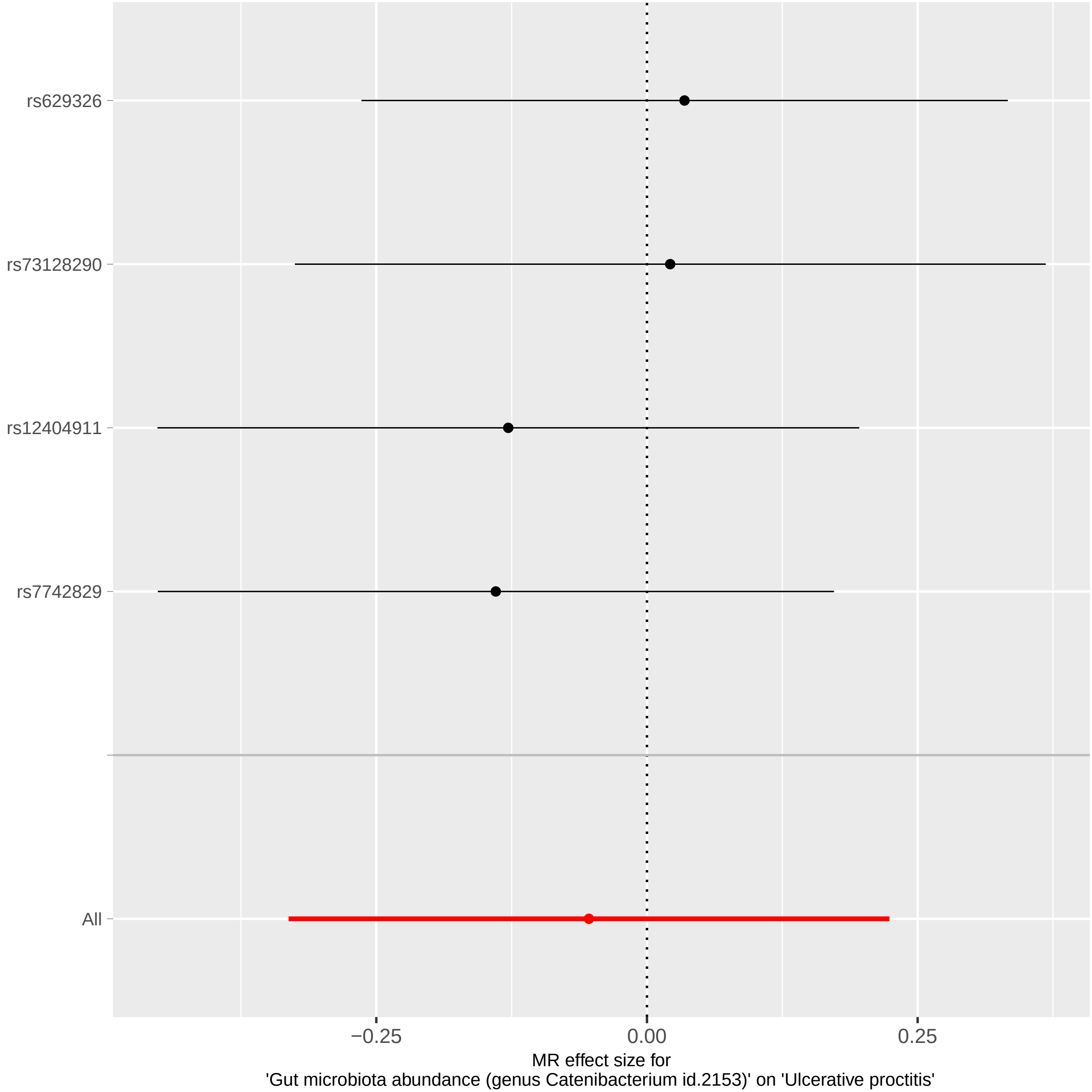

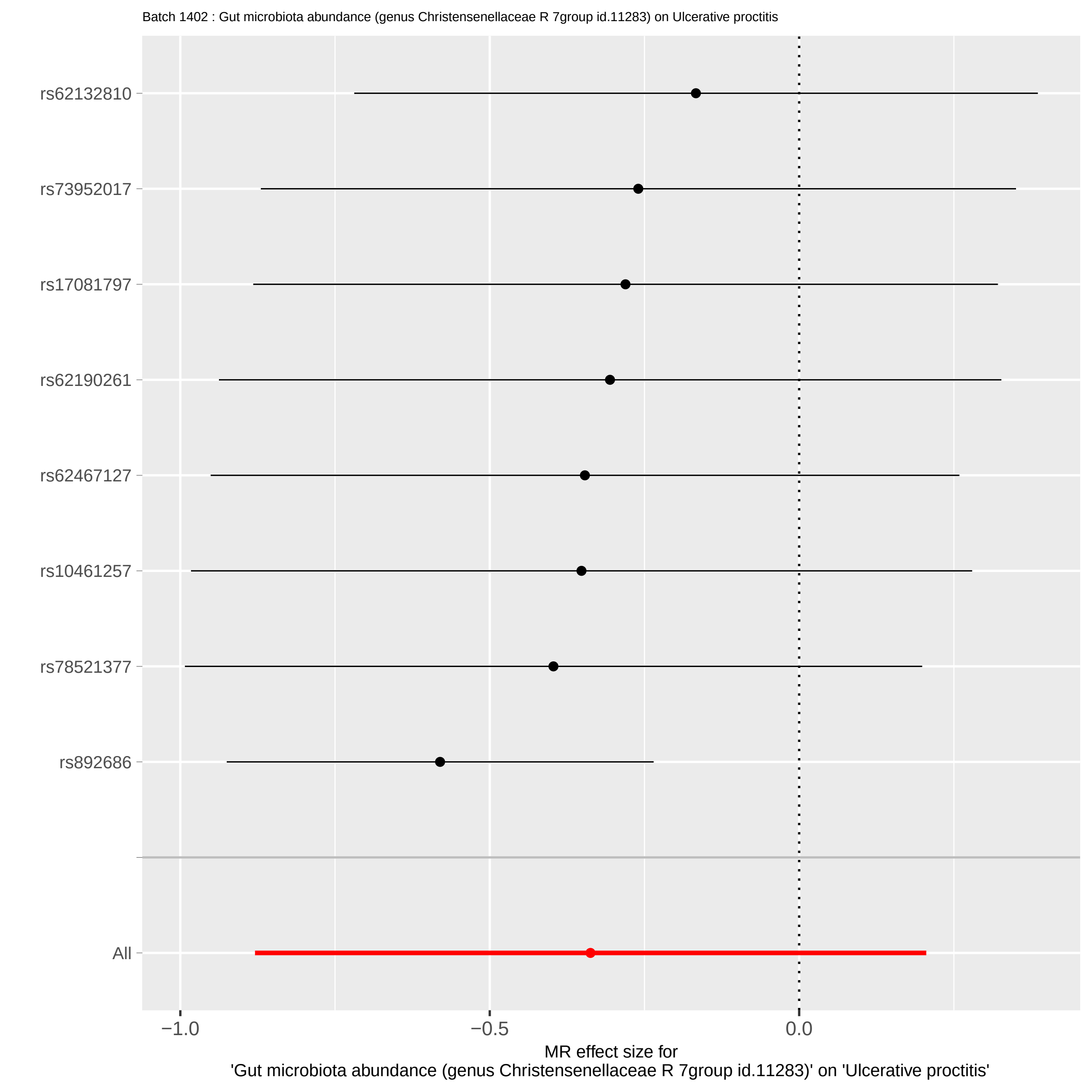

Batch 1402 : Gut microbiota abundance (genus Christensenellaceae R 7group id.11283) on Ulcerative proctitis
rs62132810
rs73952017
rs17081797
rs62190261
rs62467127
rs10461257
rs78521377
rs892686
All
−1.0
−0.5
0.0
MR effect size for
'Gut microbiota abundance (genus Christensenellaceae R 7group id.11283)' on 'Ulcerative proctitis'

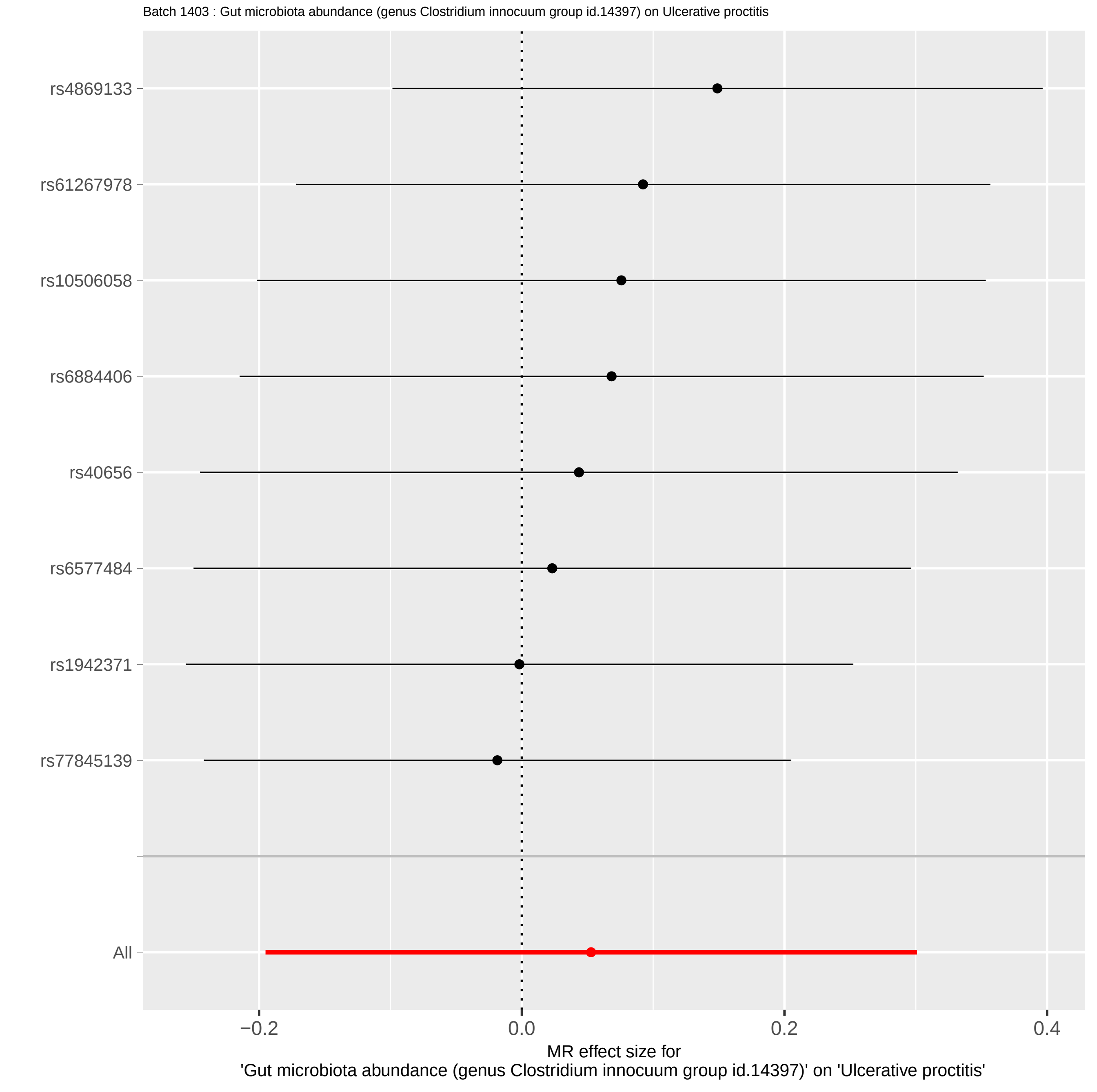
Batch 1403 : Gut microbiota abundance (genus Clostridium innocuum group id.14397) on Ulcerative proctitis
rs4869133
rs61267978
rs10506058
rs6884406
rs40656
rs6577484
rs1942371
rs77845139
All
−0.2
0.0
0.2
0.4
MR effect size for
'Gut microbiota abundance (genus Clostridium innocuum group id.14397)' on 'Ulcerative proctitis'

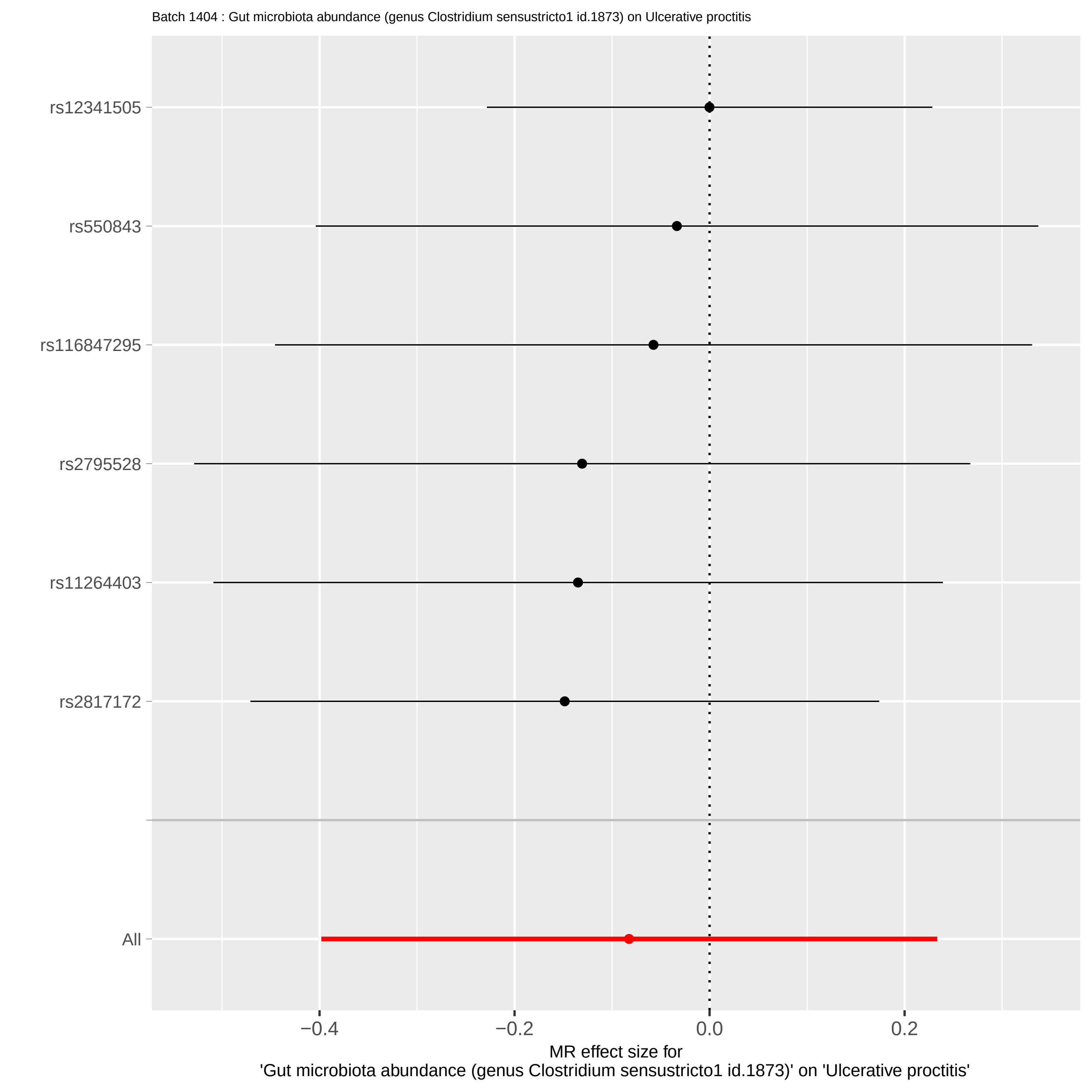

Batch 1404 : Gut microbiota abundance (genus Clostridium sensustricto1 id.1873) on Ulcerative proctitis
rs12341505
rs550843
rs116847295
rs2795528
rs11264403
rs2817172
All
−0.4
−0.2
0.0
0.2
MR effect size for
'Gut microbiota abundance (genus Clostridium sensustricto1 id.1873)' on 'Ulcerative proctitis'

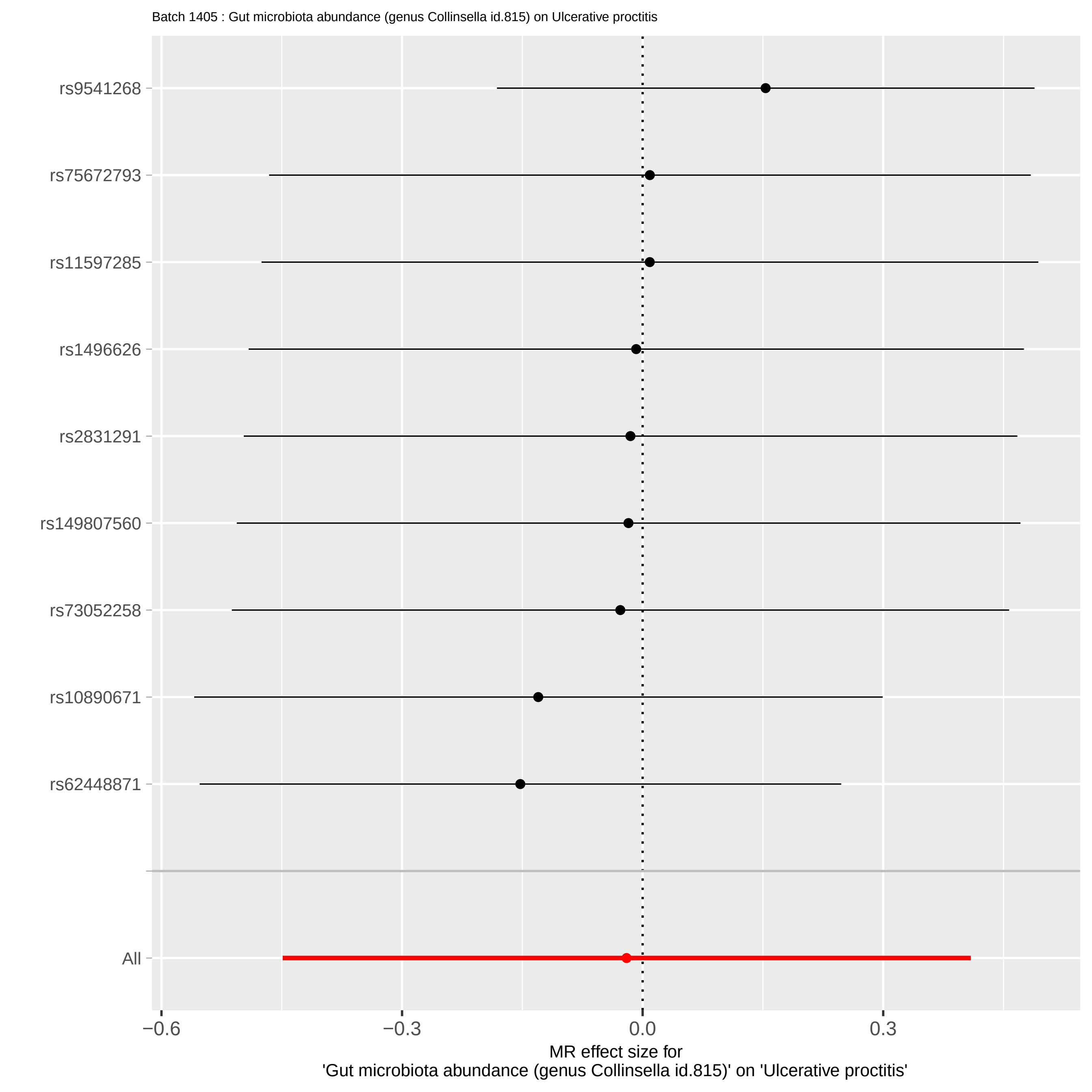

Batch 1405 : Gut microbiota abundance (genus Collinsella id.815) on Ulcerative proctitis
rs9541268
rs75672793
rs11597285
rs1496626
rs2831291
rs149807560
rs73052258
rs10890671
rs62448871
All
−0.6
−0.3
0.0
0.3
MR effect size for
'Gut microbiota abundance (genus Collinsella id.815)' on 'Ulcerative proctitis'

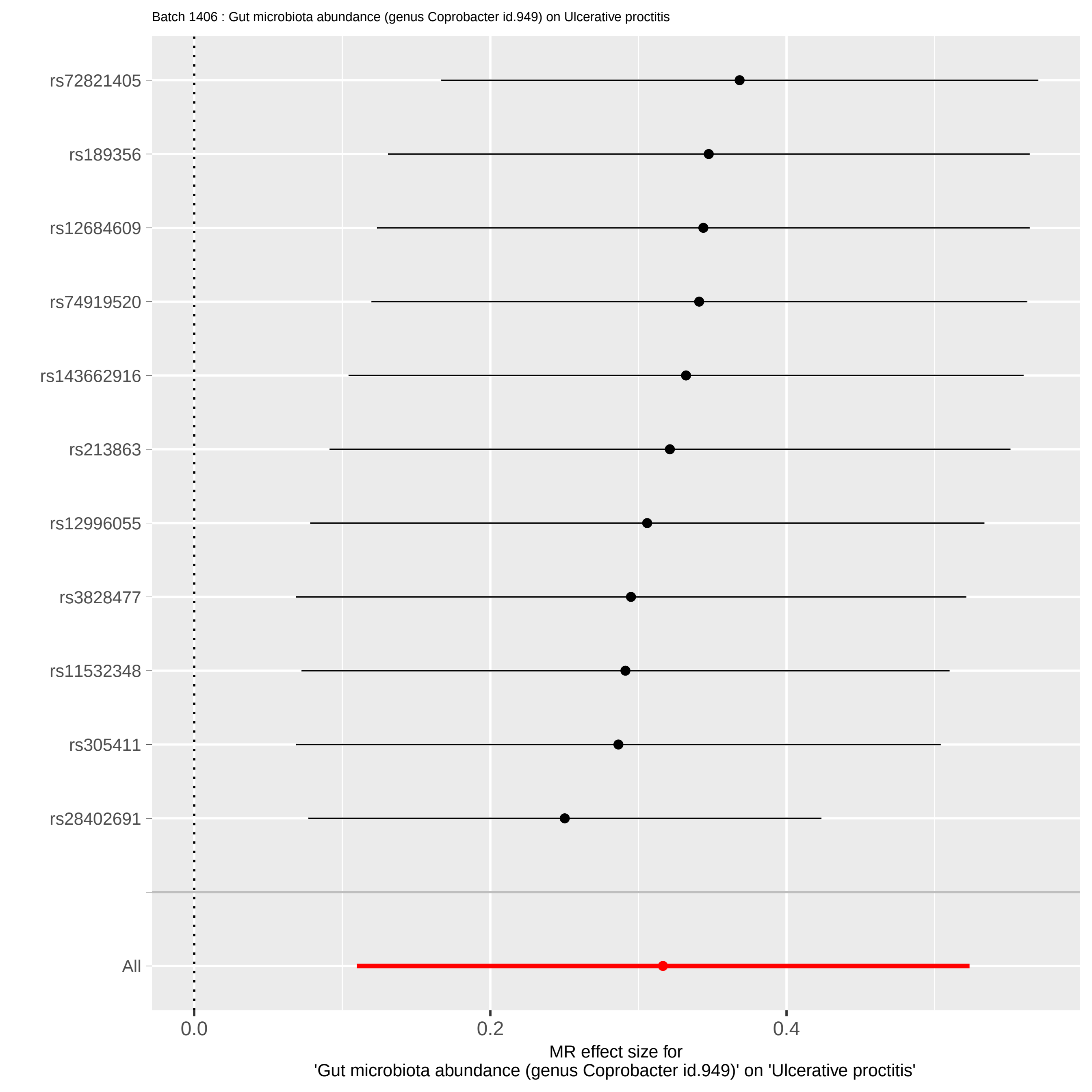
Batch 1406 : Gut microbiota abundance (genus Coprobacter id.949) on Ulcerative proctitis
rs72821405
rs189356
rs12684609
rs74919520
rs143662916
rs213863
rs12996055
rs3828477
rs11532348
rs305411
rs28402691
All
0.0
0.2
0.4
MR effect size for
'Gut microbiota abundance (genus Coprobacter id.949)' on 'Ulcerative proctitis'

Batch 1407 : Gut microbiota abundance (genus Coprococcus1 id.11301) on Ulcerative proctitis

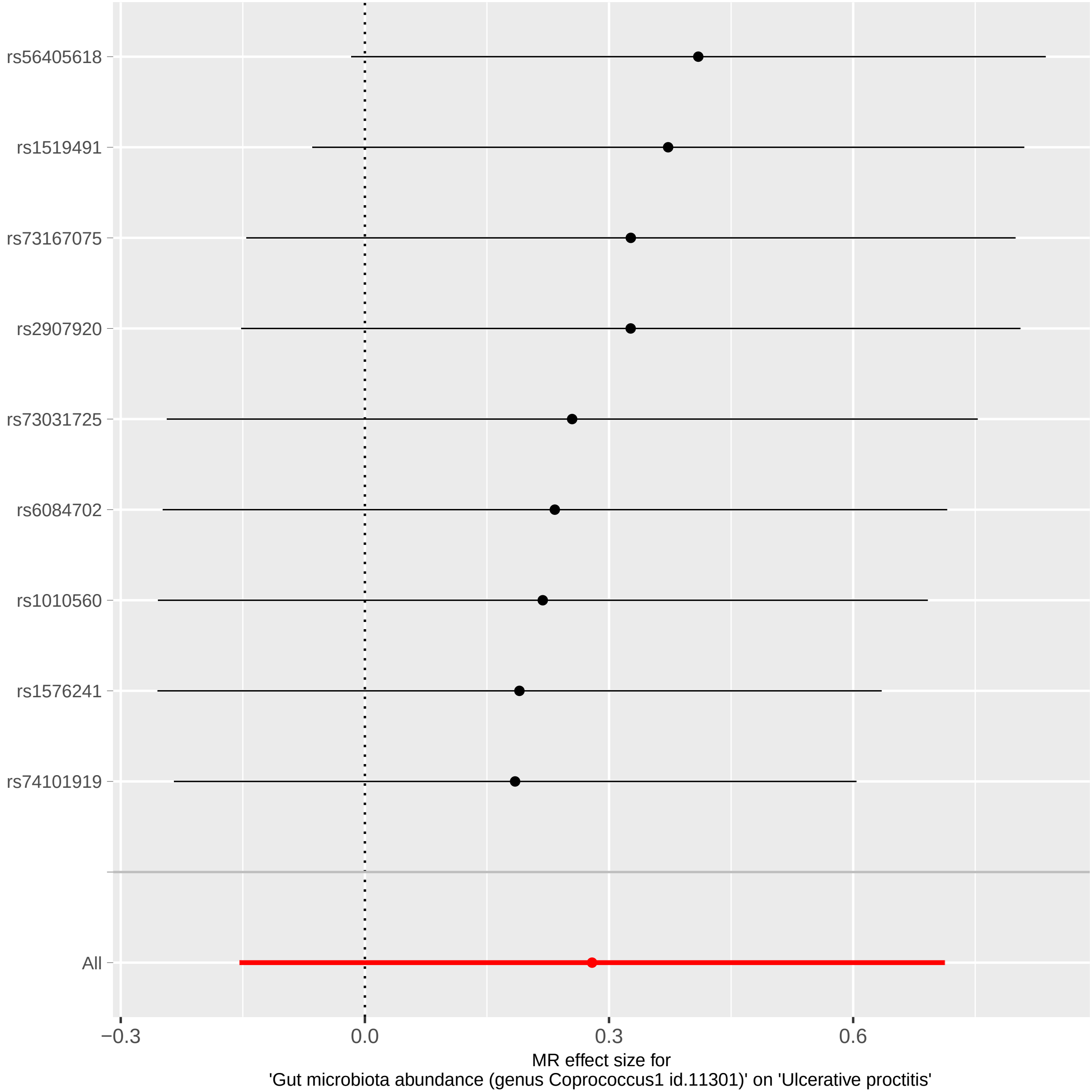

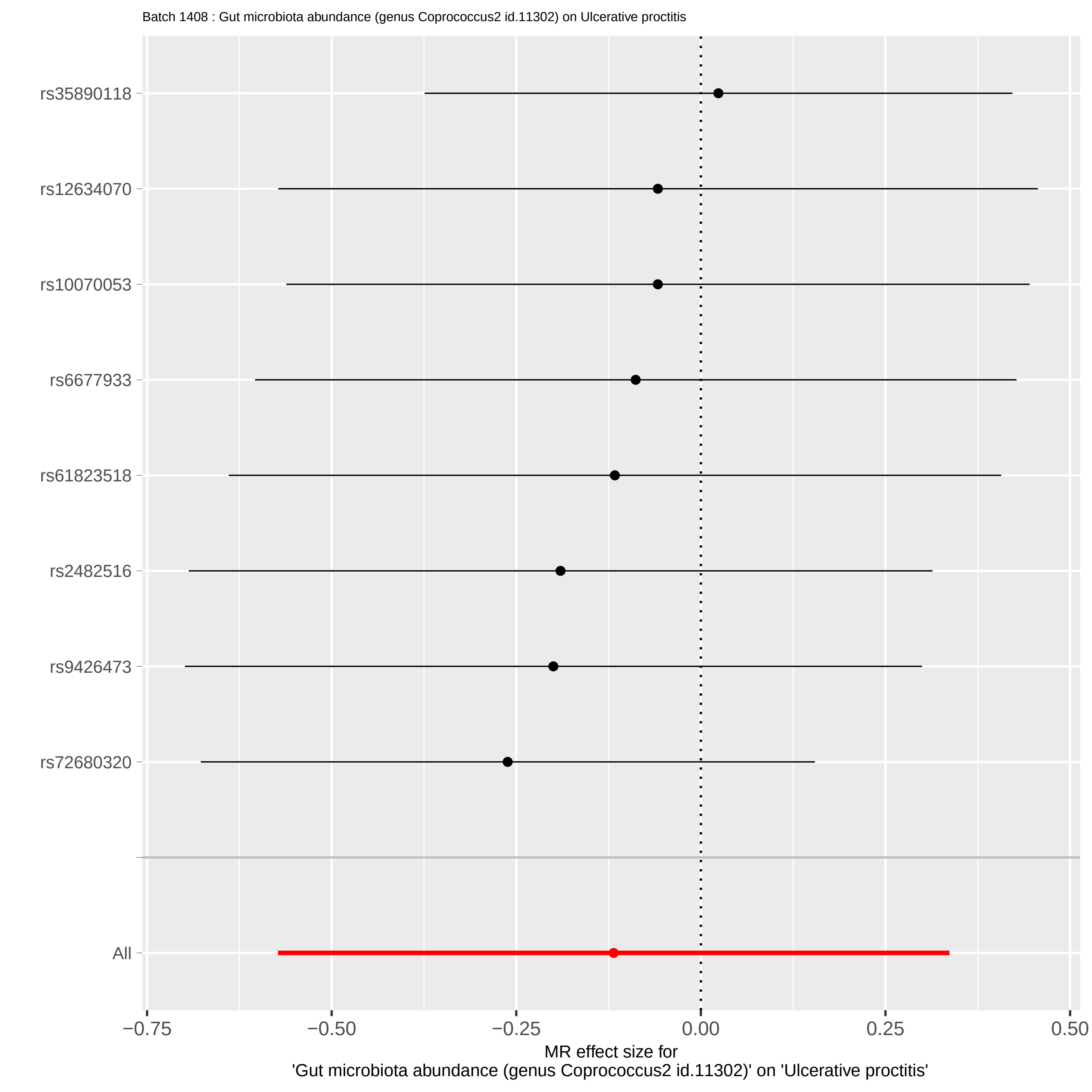

Batch 1408 : Gut microbiota abundance (genus Coprococcus2 id.11302) on Ulcerative proctitis
rs35890118
rs12634070
rs10070053
rs6677933
rs61823518
rs2482516
rs9426473
rs72680320
All
−0.75
−0.50
−0.25
0.00
0.25
0.50
MR effect size for
'Gut microbiota abundance (genus Coprococcus2 id.11302)' on 'Ulcerative proctitis'

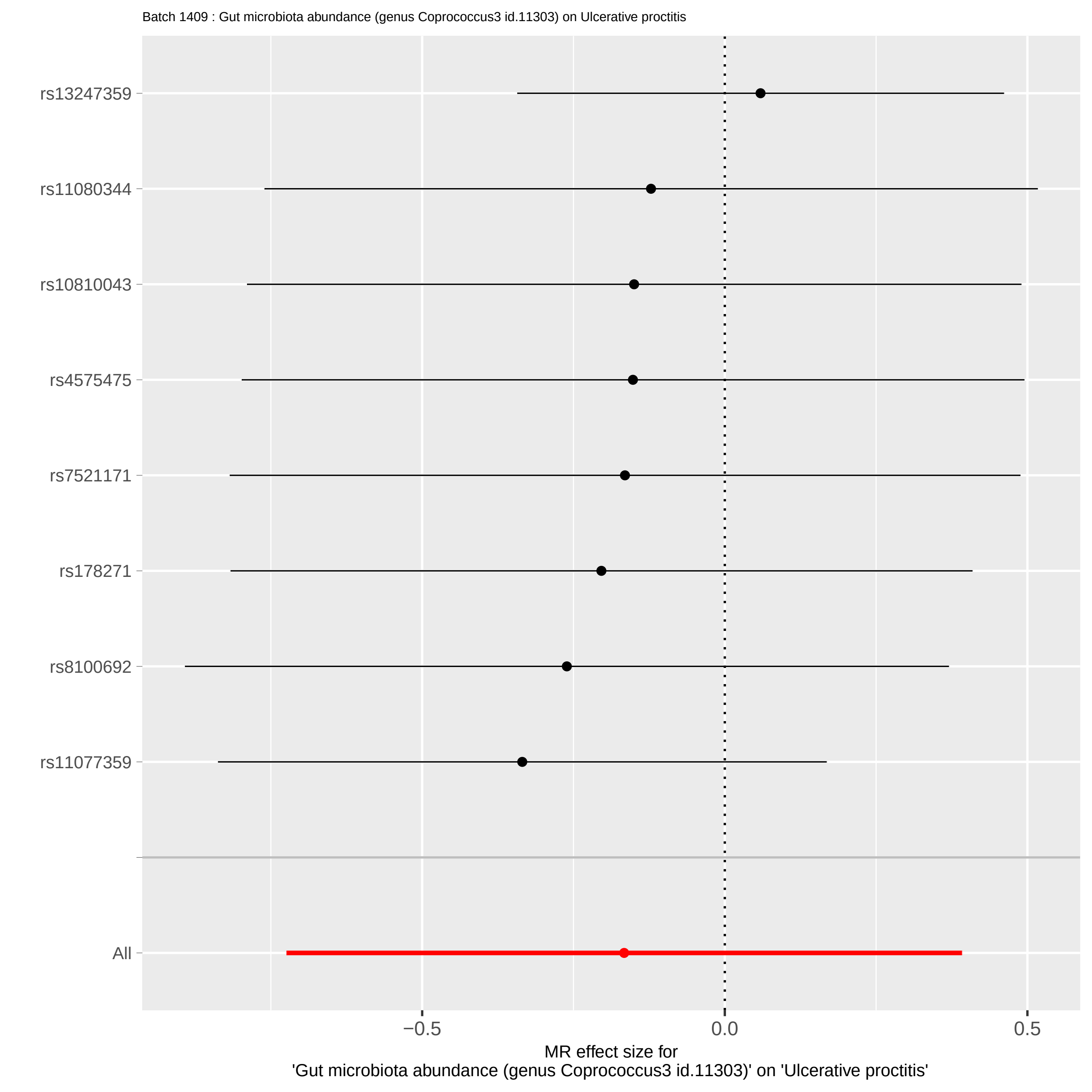

Batch 1409 : Gut microbiota abundance (genus Coprococcus3 id.11303) on Ulcerative proctitis
rs13247359
rs11080344
rs10810043
rs4575475
rs7521171
rs178271
rs8100692
rs11077359
All
−0.5
0.0
0.5
MR effect size for
'Gut microbiota abundance (genus Coprococcus3 id.11303)' on 'Ulcerative proctitis'

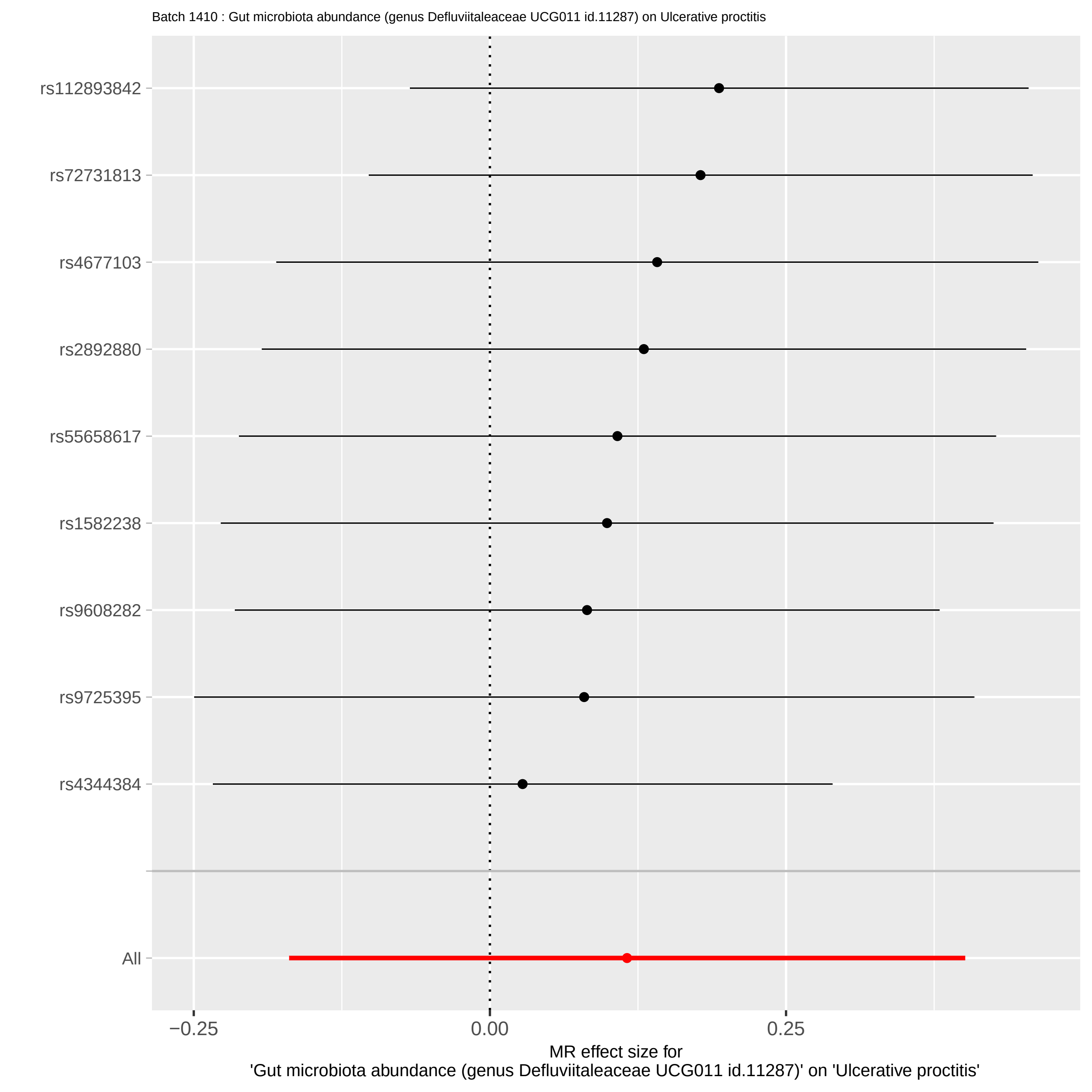

Batch 1410 : Gut microbiota abundance (genus Defluviitaleaceae UCG011 id.11287) on Ulcerative proctitis
rs112893842
rs72731813
rs4677103
rs2892880
rs55658617
rs1582238
rs9608282
rs9725395
rs4344384
All
−0.25
0.00
0.25
MR effect size for
'Gut microbiota abundance (genus Defluviitaleaceae UCG011 id.11287)' on 'Ulcerative proctitis'

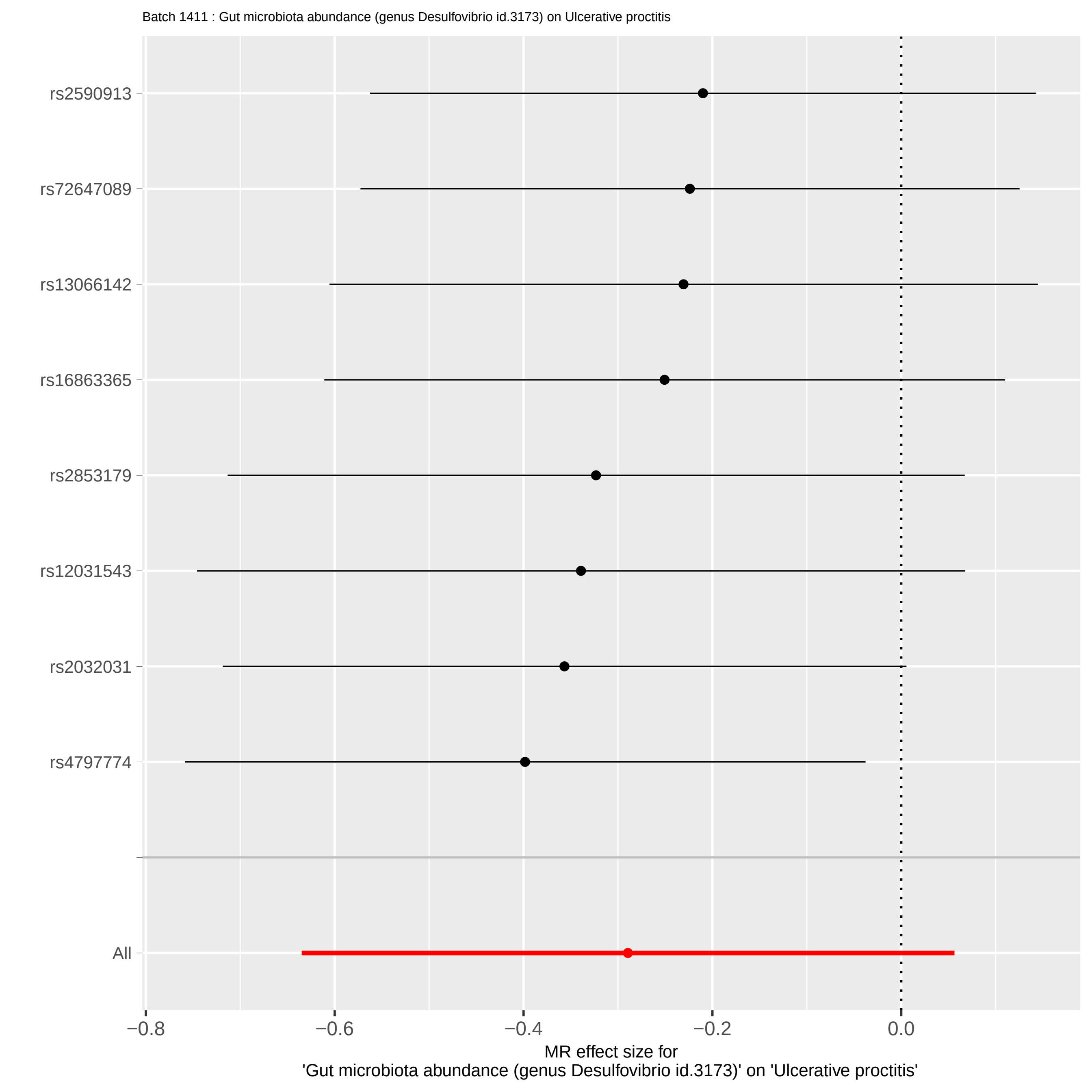
Batch 1411 : Gut microbiota abundance (genus Desulfovibrio id.3173) on Ulcerative proctitis
rs2590913
rs72647089
rs13066142
rs16863365
rs2853179
rs12031543
rs2032031
rs4797774
All
−0.8
−0.6
−0.4
−0.2
0.0
MR effect size for
'Gut microbiota abundance (genus Desulfovibrio id.3173)' on 'Ulcerative proctitis'

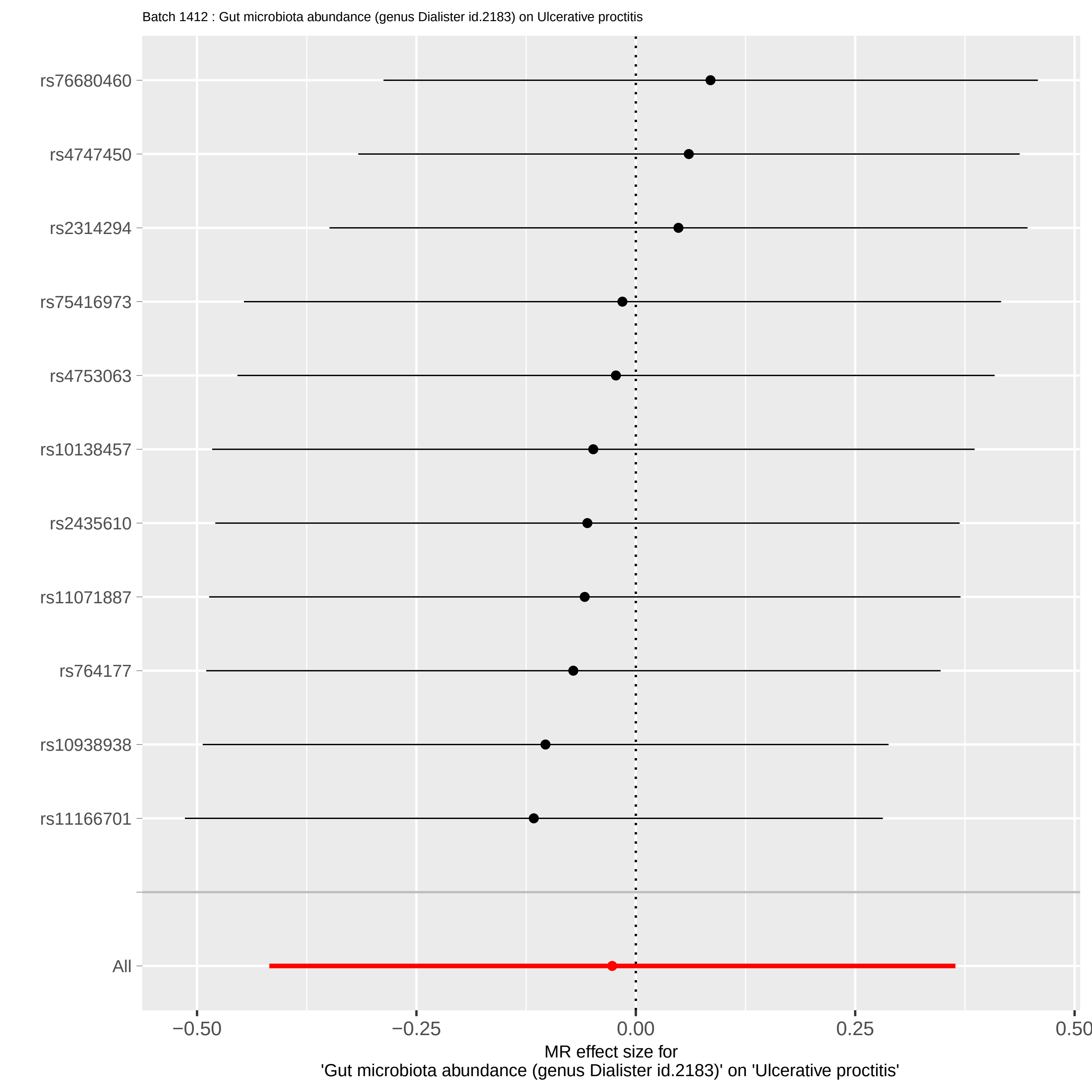
Batch 1412 : Gut microbiota abundance (genus Dialister id.2183) on Ulcerative proctitis
rs76680460
rs4747450
rs2314294
rs75416973
rs4753063
rs10138457
rs2435610
rs11071887
rs764177
rs10938938
rs11166701
All
−0.50
−0.25
0.00
0.25
0.50
MR effect size for
'Gut microbiota abundance (genus Dialister id.2183)' on 'Ulcerative proctitis'

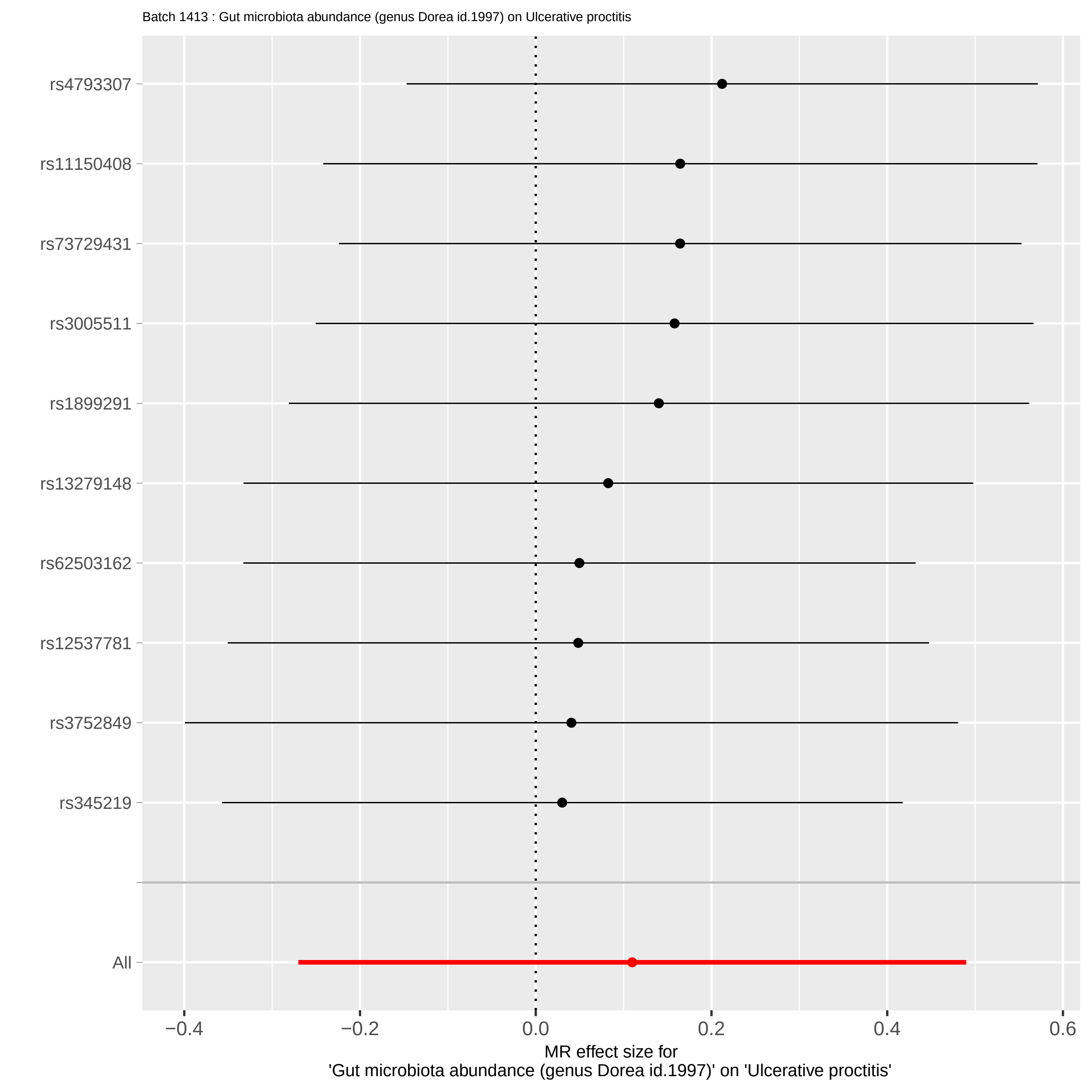

Batch 1413 : Gut microbiota abundance (genus Dorea id.1997) on Ulcerative proctitis
rs4793307
rs11150408
rs73729431
rs3005511
rs1899291
rs13279148
rs62503162
rs12537781
rs3752849
rs345219
All
−0.4
−0.2
0.0
0.2
0.4
0.6
MR effect size for
'Gut microbiota abundance (genus Dorea id.1997)' on 'Ulcerative proctitis'

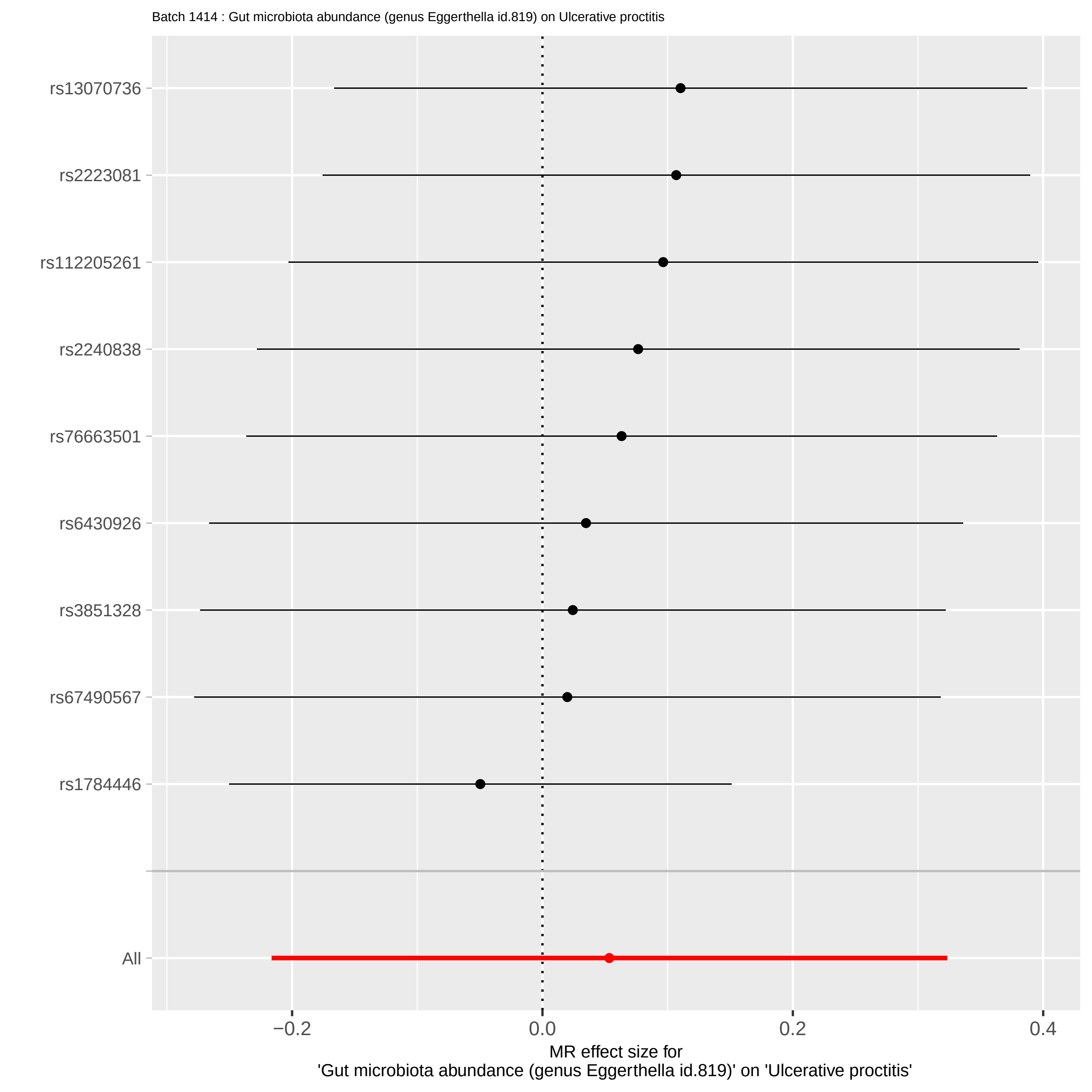

Batch 1414 : Gut microbiota abundance (genus Eggerthella id.819) on Ulcerative proctitis
rs13070736
rs2223081
rs112205261
rs2240838
rs76663501
rs6430926
rs3851328
rs67490567
rs1784446
All
−0.2
0.0
0.2
0.4
MR effect size for
'Gut microbiota abundance (genus Eggerthella id.819)' on 'Ulcerative proctitis'

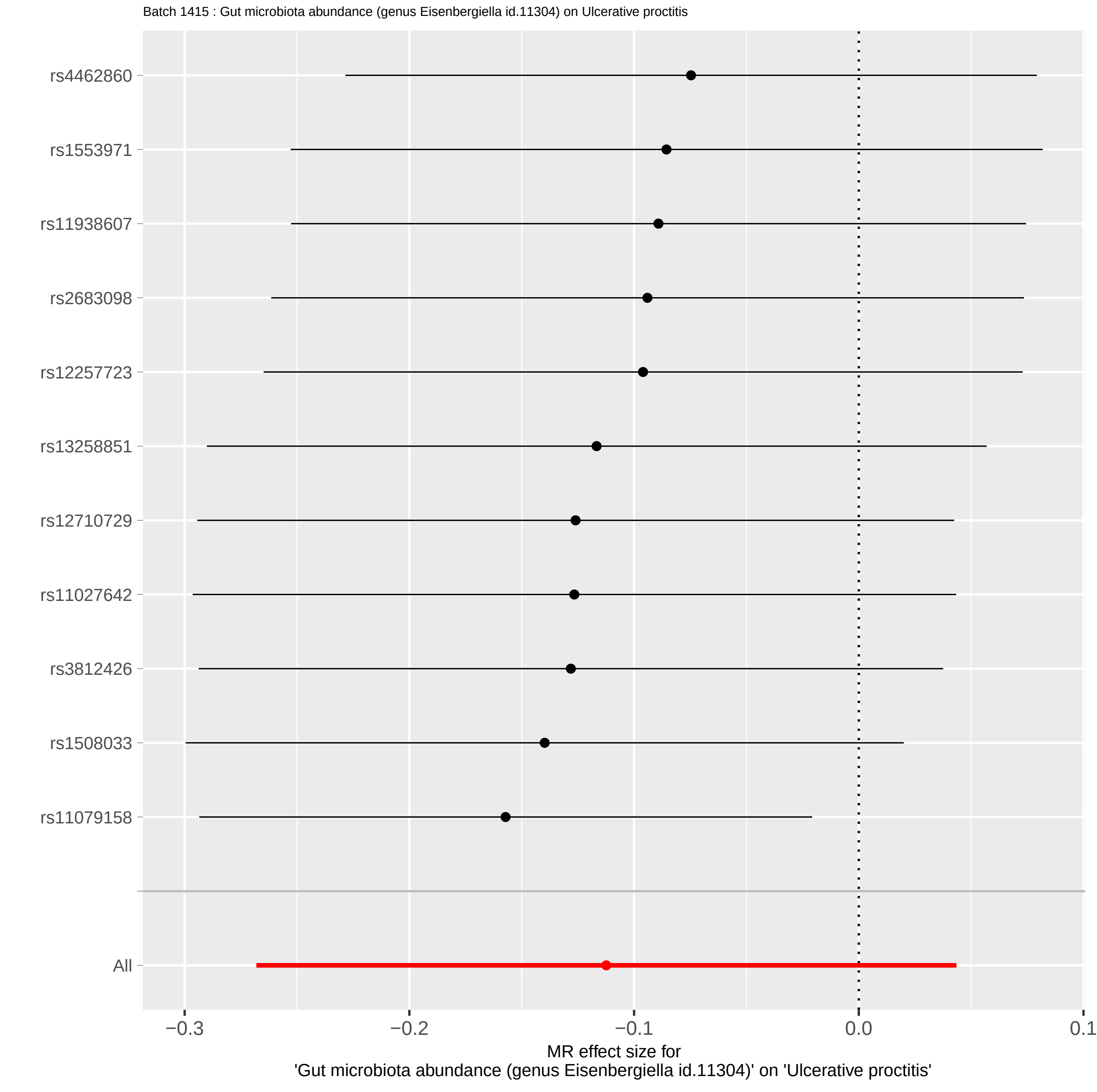

Batch 1415 : Gut microbiota abundance (genus Eisenbergiella id.11304) on Ulcerative proctitis
rs4462860
rs1553971
rs11938607
rs2683098
rs12257723
rs13258851
rs12710729
rs11027642
rs3812426
rs1508033
rs11079158
All
−0.3
−0.2
−0.1
0.0
0.1
MR effect size for
'Gut microbiota abundance (genus Eisenbergiella id.11304)' on 'Ulcerative proctitis'

Batch 1416 : Gut microbiota abundance (genus Enterorhabdus id.820) on Ulcerative proctitis

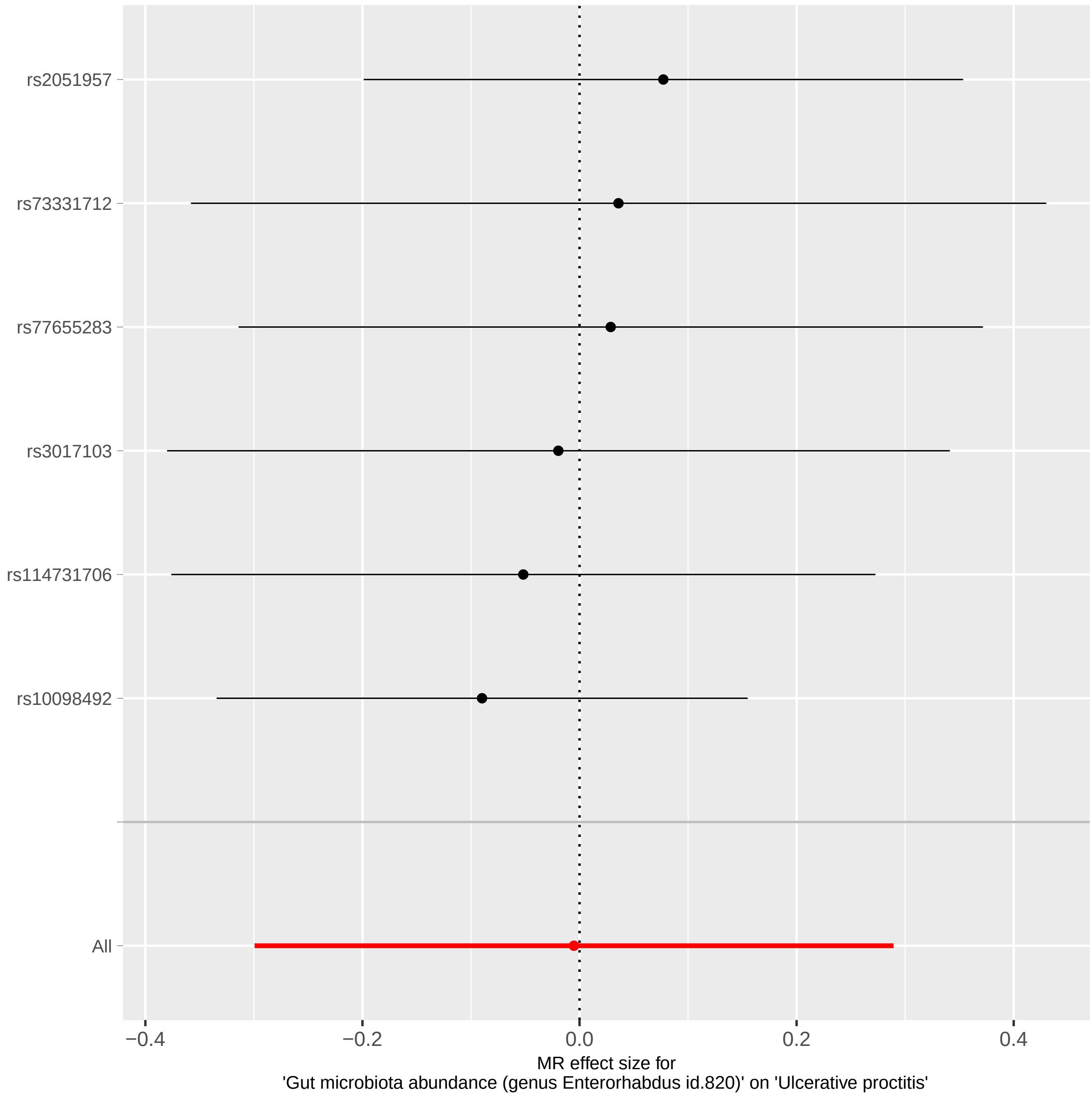

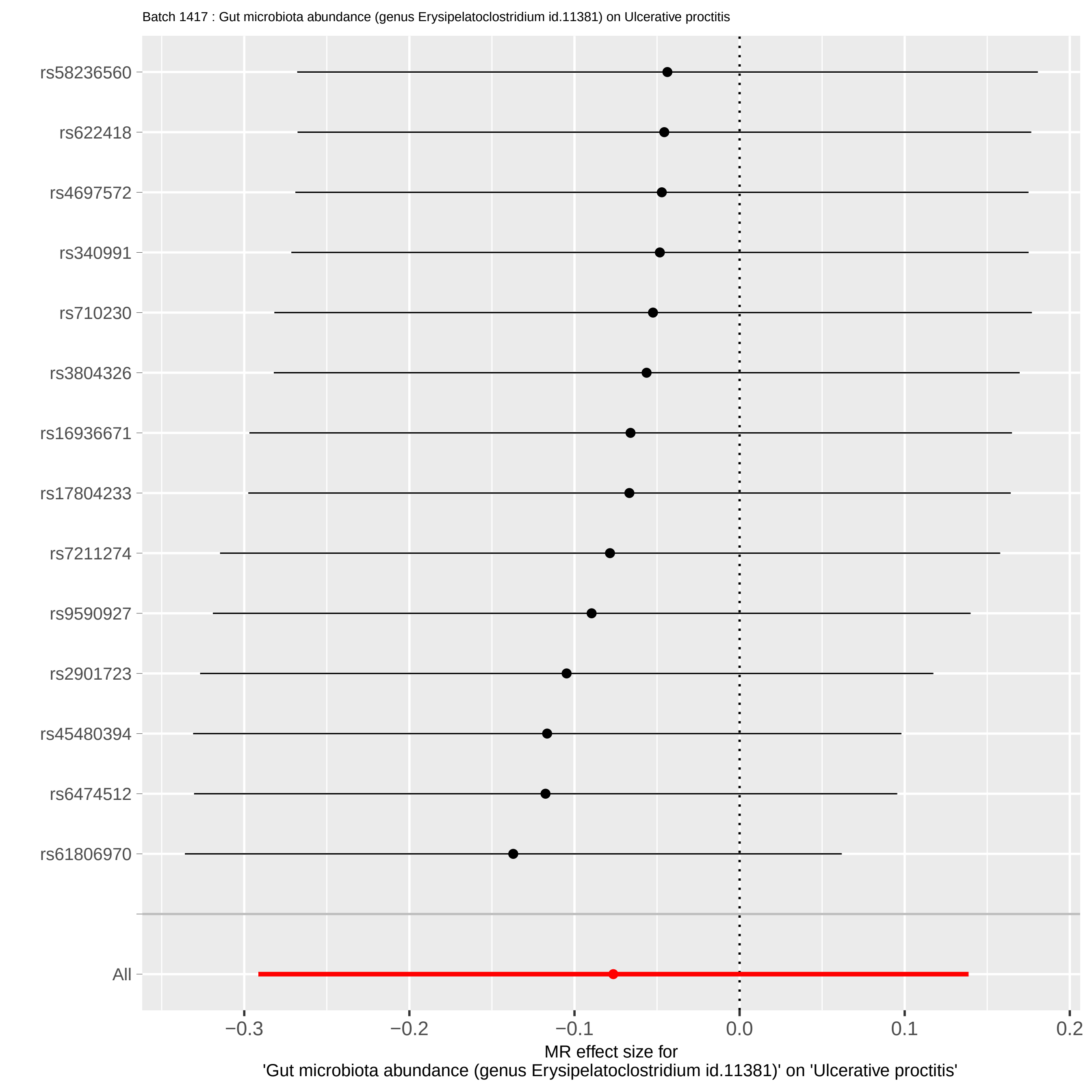
Batch 1417 : Gut microbiota abundance (genus Erysipelatoclostridium id.11381) on Ulcerative proctitis
rs58236560
rs622418
rs4697572
rs340991
rs710230
rs3804326
rs16936671
rs17804233
rs7211274
rs9590927
rs2901723
rs45480394
rs6474512
rs61806970
All
−0.3
−0.2
−0.1
0.0
0.1
0.2
MR effect size for
'Gut microbiota abundance (genus Erysipelatoclostridium id.11381)' on 'Ulcerative proctitis'

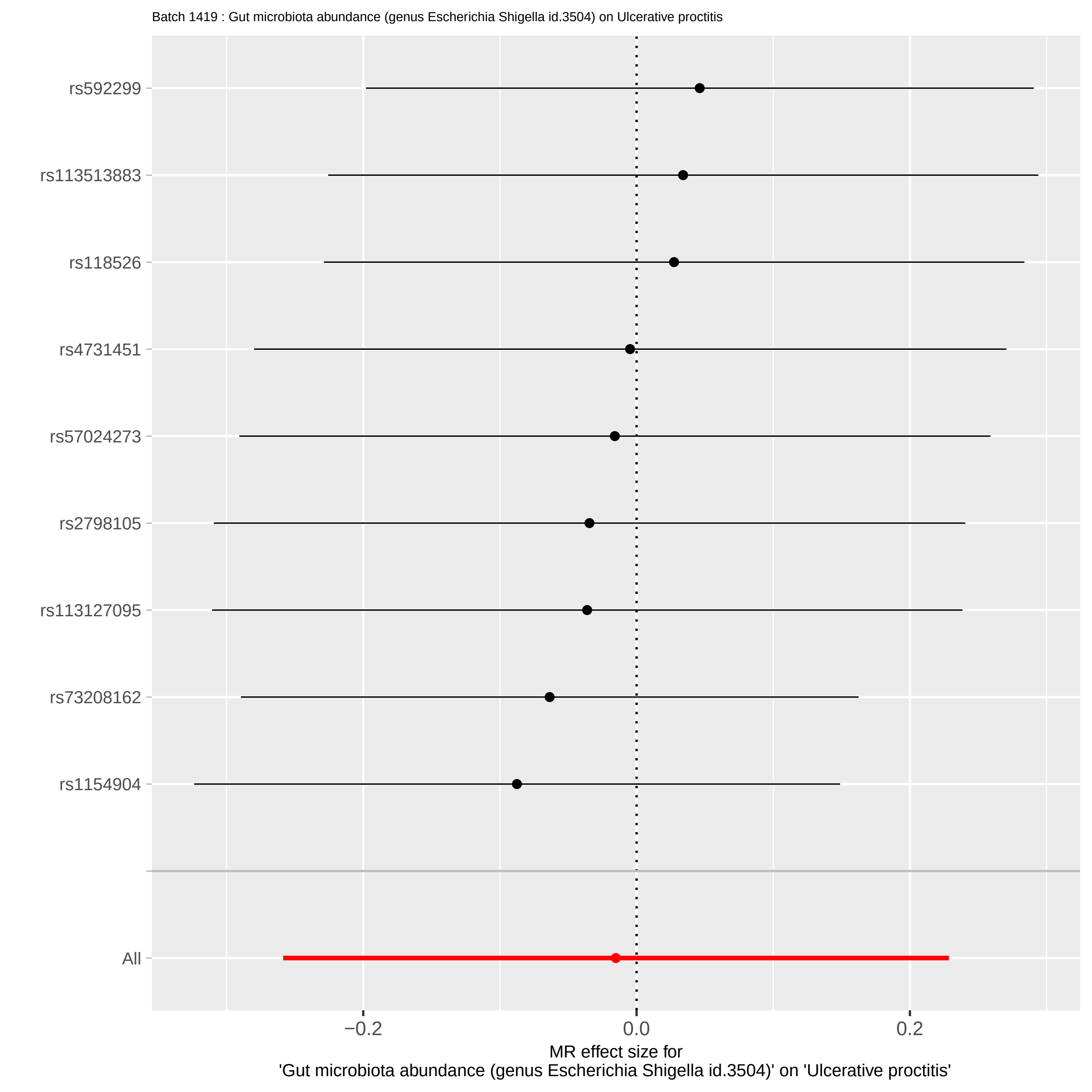
Batch 1419 : Gut microbiota abundance (genus Escherichia Shigella id.3504) on Ulcerative proctitis
rs592299
rs113513883
rs118526
rs4731451
rs57024273
rs2798105
rs113127095
rs73208162
rs1154904
All
−0.2
0.0
0.2
MR effect size for
'Gut microbiota abundance (genus Escherichia Shigella id.3504)' on 'Ulcerative proctitis'

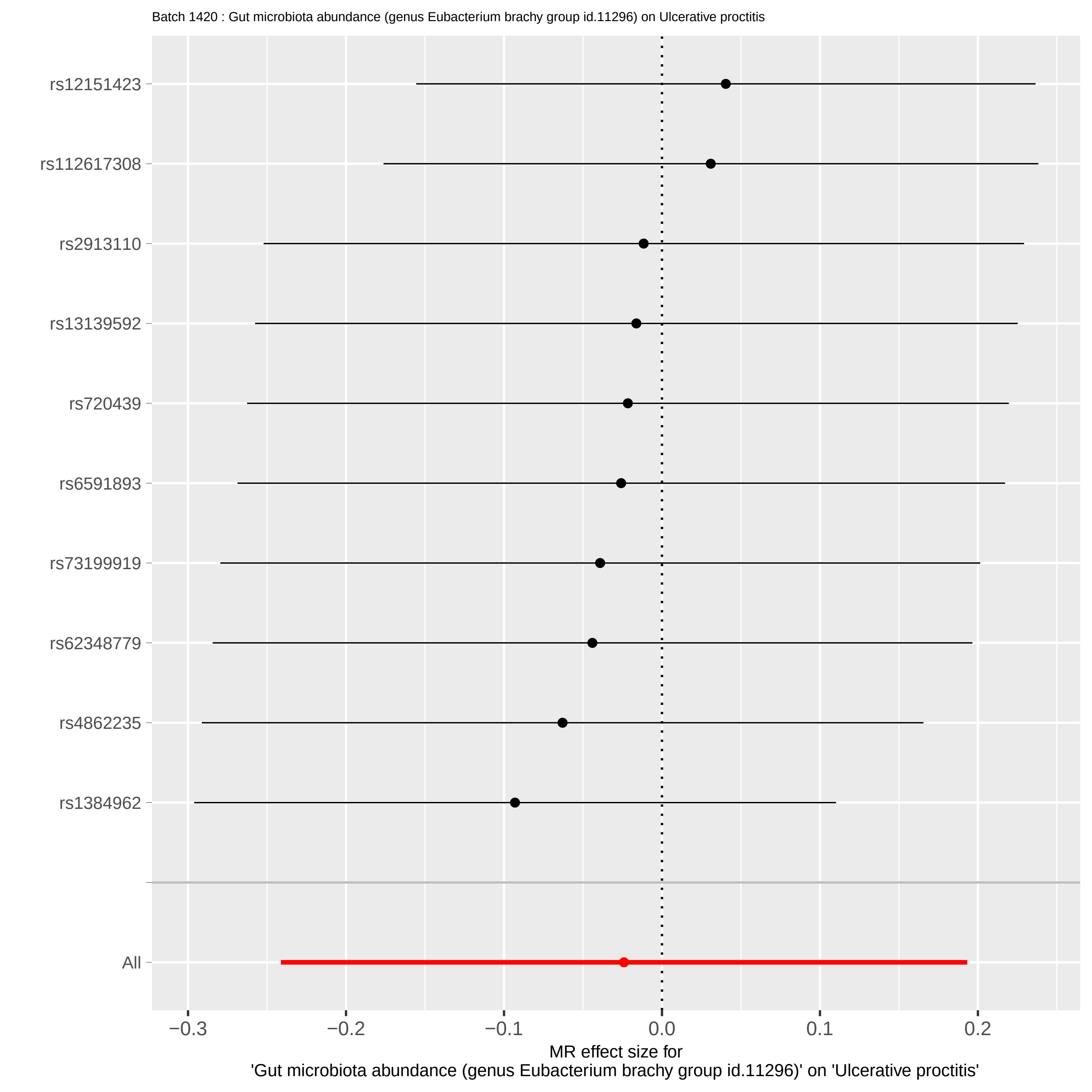
Batch 1420 : Gut microbiota abundance (genus Eubacterium brachy group id.11296) on Ulcerative proctitis
rs12151423
rs112617308
rs2913110
rs13139592
rs720439
rs6591893
rs73199919
rs62348779
rs4862235
rs1384962
All
−0.3
−0.2
−0.1
0.0
0.1
0.2
MR effect size for
'Gut microbiota abundance (genus Eubacterium brachy group id.11296)' on 'Ulcerative proctitis'

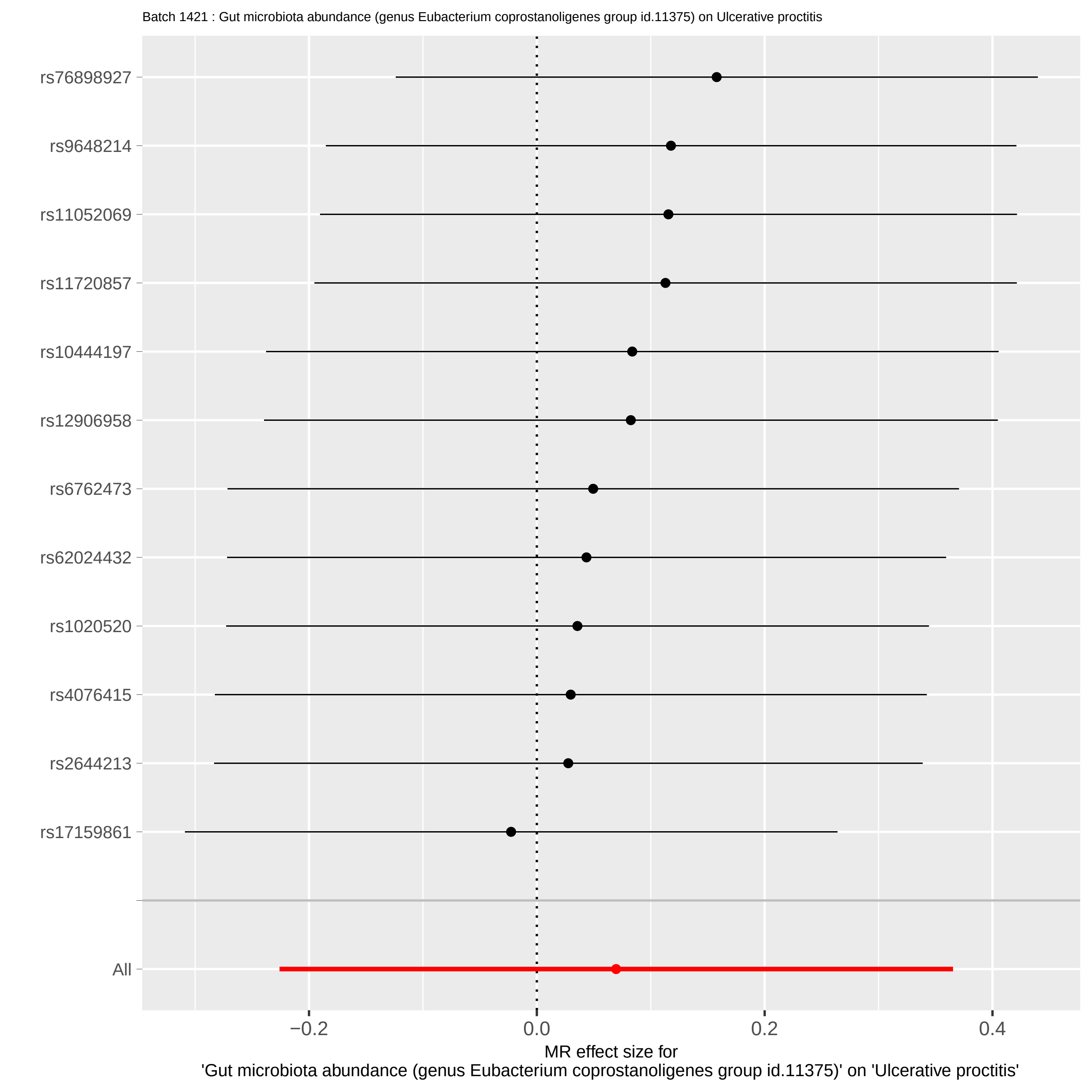

Batch 1421 : Gut microbiota abundance (genus Eubacterium coprostanoligenes group id.11375) on Ulcerative proctitis
rs76898927
rs9648214
rs11052069
rs11720857
rs10444197
rs12906958
rs6762473
rs62024432
rs1020520
rs4076415
rs2644213
rs17159861
All
−0.2
0.0
0.2
0.4
MR effect size for
'Gut microbiota abundance (genus Eubacterium coprostanoligenes group id.11375)' on 'Ulcerative proctitis'

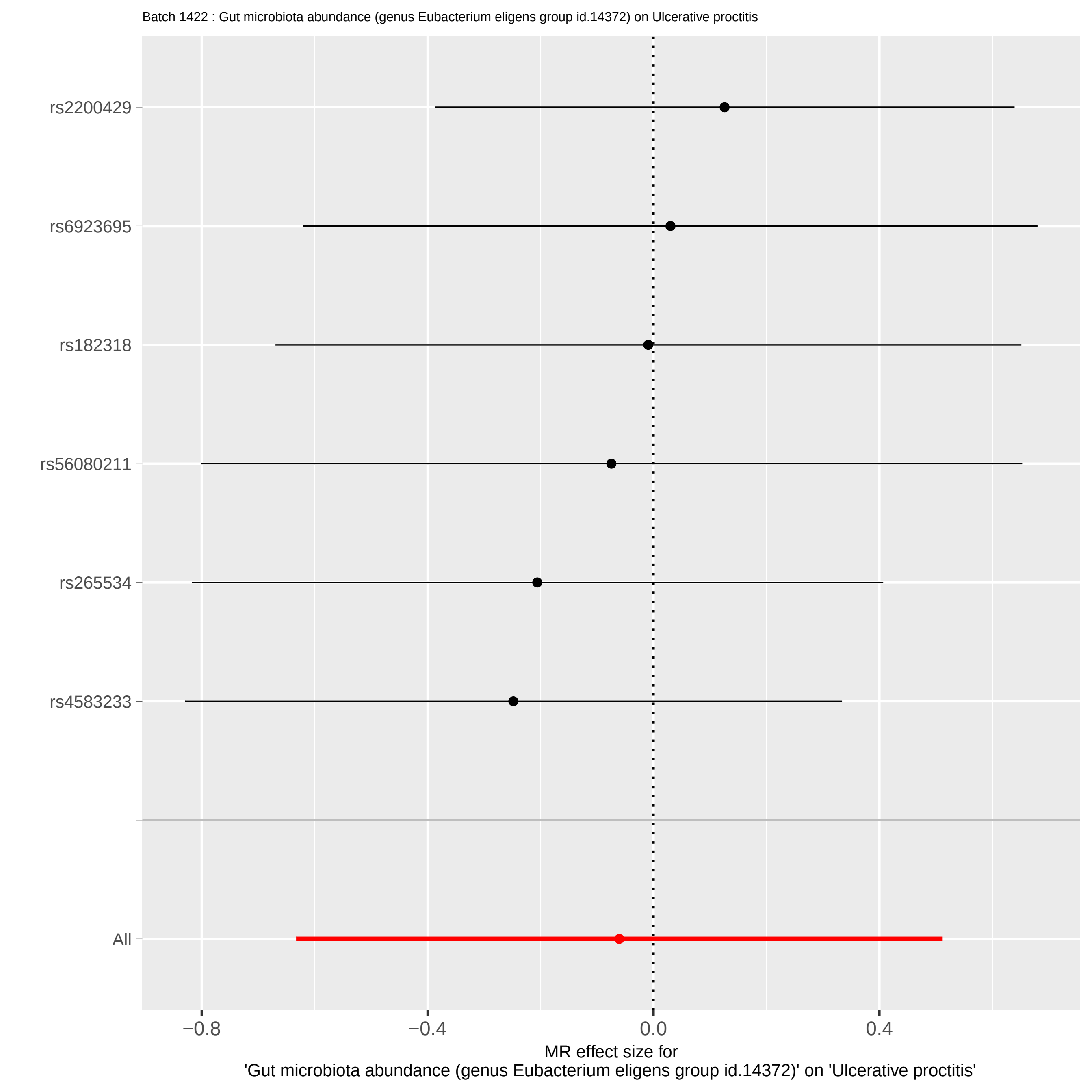

Batch 1422 : Gut microbiota abundance (genus Eubacterium eligens group id.14372) on Ulcerative proctitis
rs2200429
rs6923695
rs182318
rs56080211
rs265534
rs4583233
All
−0.8
−0.4
0.0
0.4
MR effect size for
'Gut microbiota abundance (genus Eubacterium eligens group id.14372)' on 'Ulcerative proctitis'

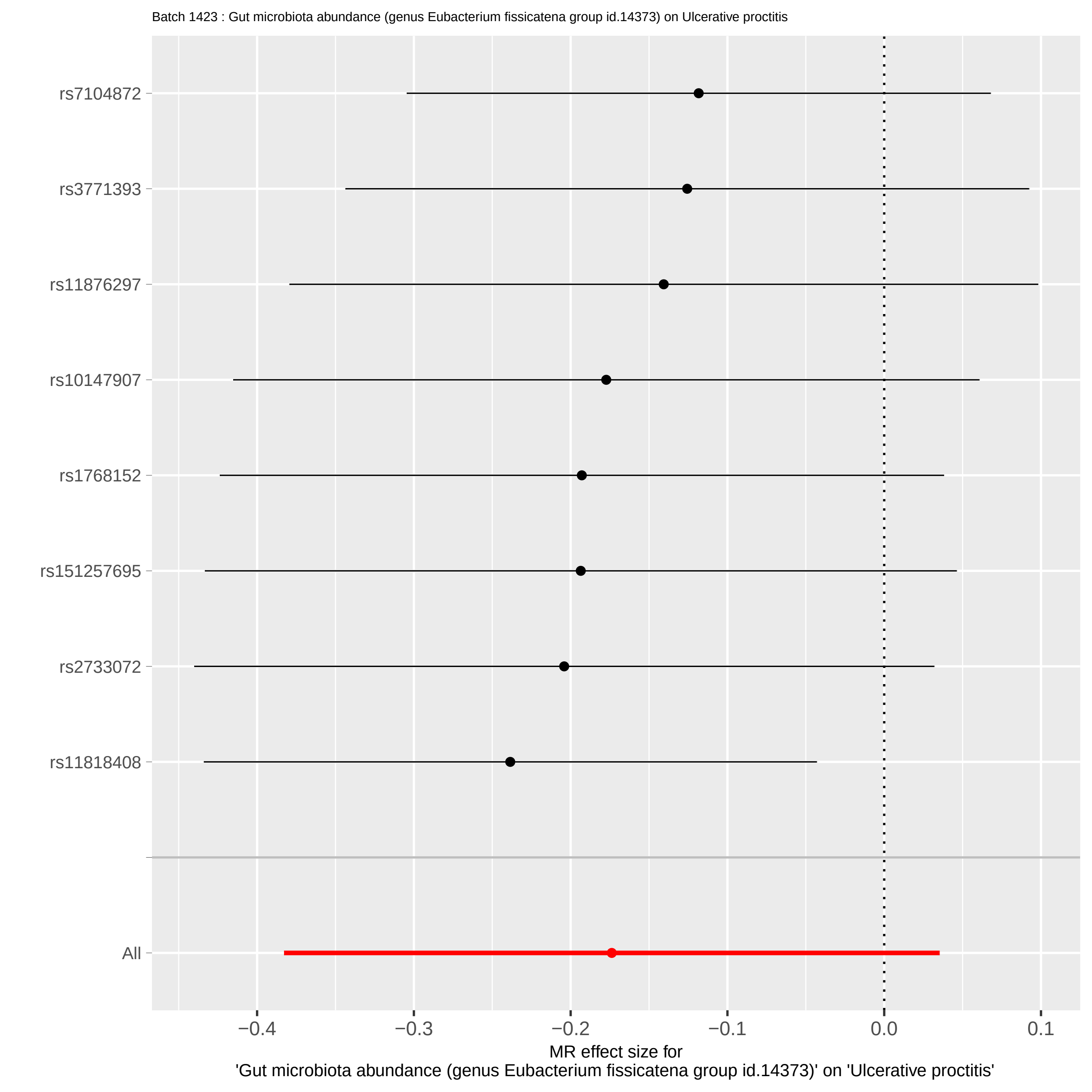

Batch 1423 : Gut microbiota abundance (genus Eubacterium fissicatena group id.14373) on Ulcerative proctitis
rs7104872
rs3771393
rs11876297
rs10147907
rs1768152
rs151257695
rs2733072
rs11818408
All
−0.4
−0.3
−0.2
−0.1
0.0
0.1
MR effect size for
'Gut microbiota abundance (genus Eubacterium fissicatena group id.14373)' on 'Ulcerative proctitis'

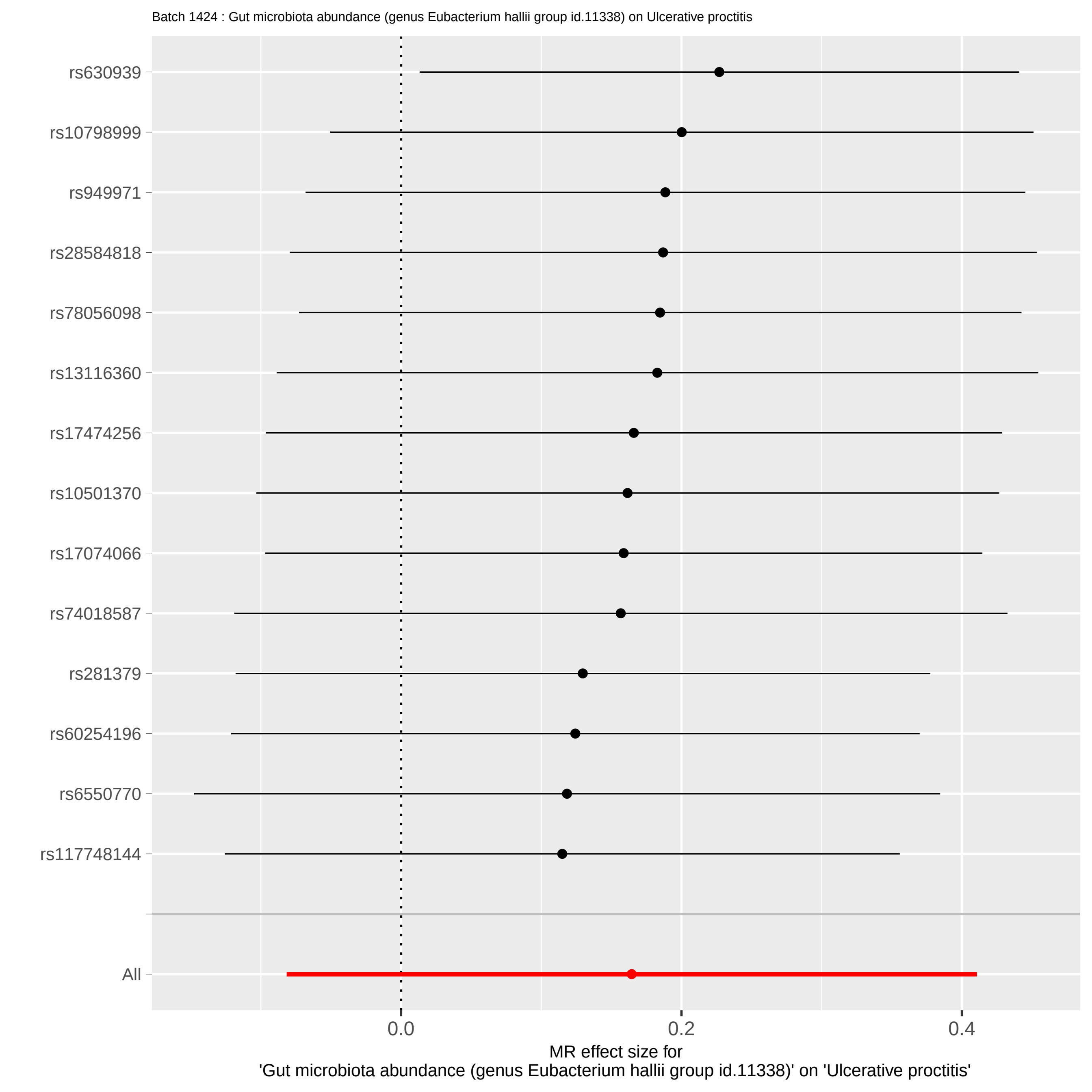
Batch 1424 : Gut microbiota abundance (genus Eubacterium hallii group id.11338) on Ulcerative proctitis
rs630939
rs10798999
rs949971
rs28584818
rs78056098
rs13116360
rs17474256
rs10501370
rs17074066
rs74018587
rs281379
rs60254196
rs6550770
rs117748144
All
0.0
0.2
0.4
MR effect size for
'Gut microbiota abundance (genus Eubacterium hallii group id.11338)' on 'Ulcerative proctitis'

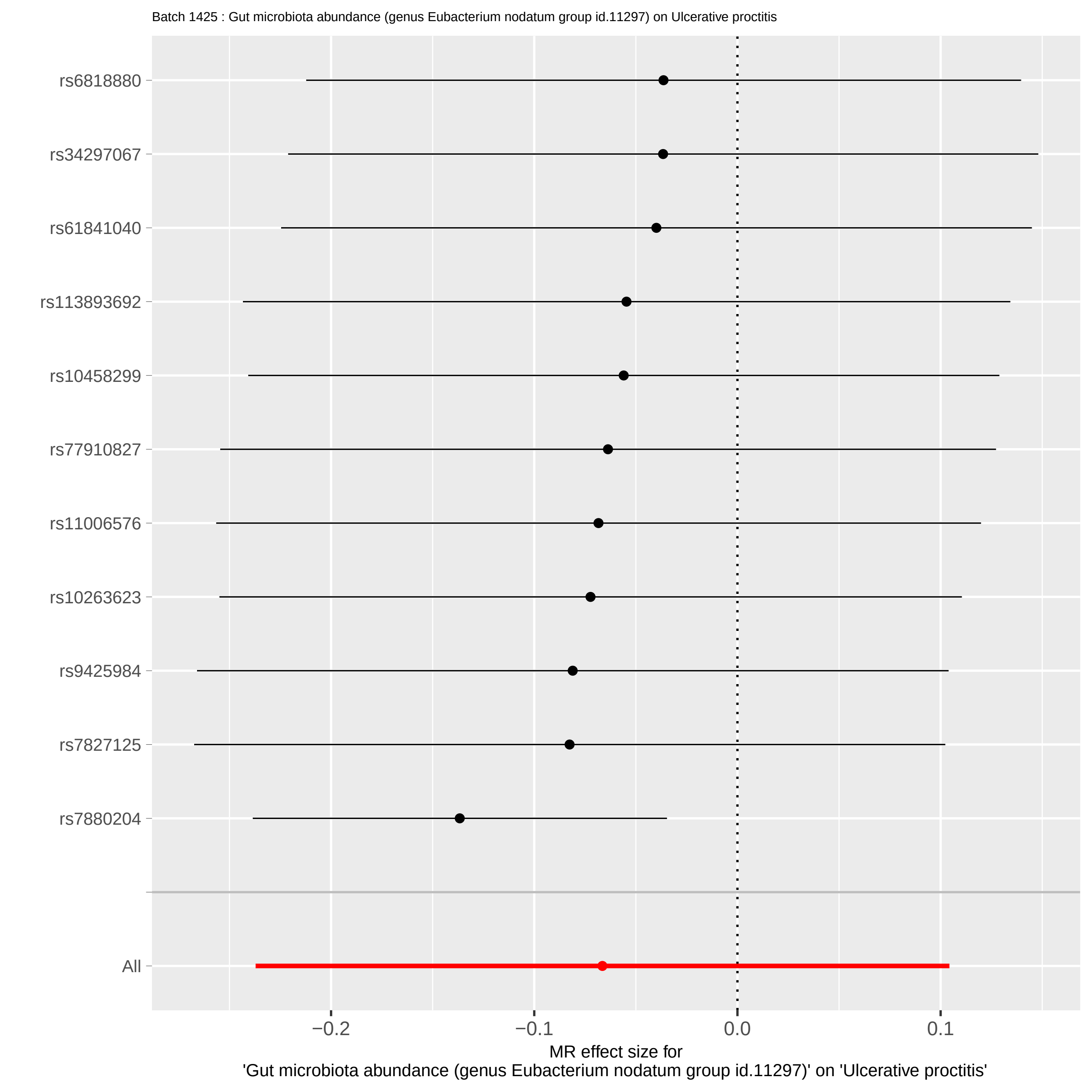

Batch 1425 : Gut microbiota abundance (genus Eubacterium nodatum group id.11297) on Ulcerative proctitis
rs6818880
rs34297067
rs61841040
rs113893692
rs10458299
rs77910827
rs11006576
rs10263623
rs9425984
rs7827125
rs7880204
All
−0.2
−0.1
0.0
0.1
MR effect size for
'Gut microbiota abundance (genus Eubacterium nodatum group id.11297)' on 'Ulcerative proctitis'

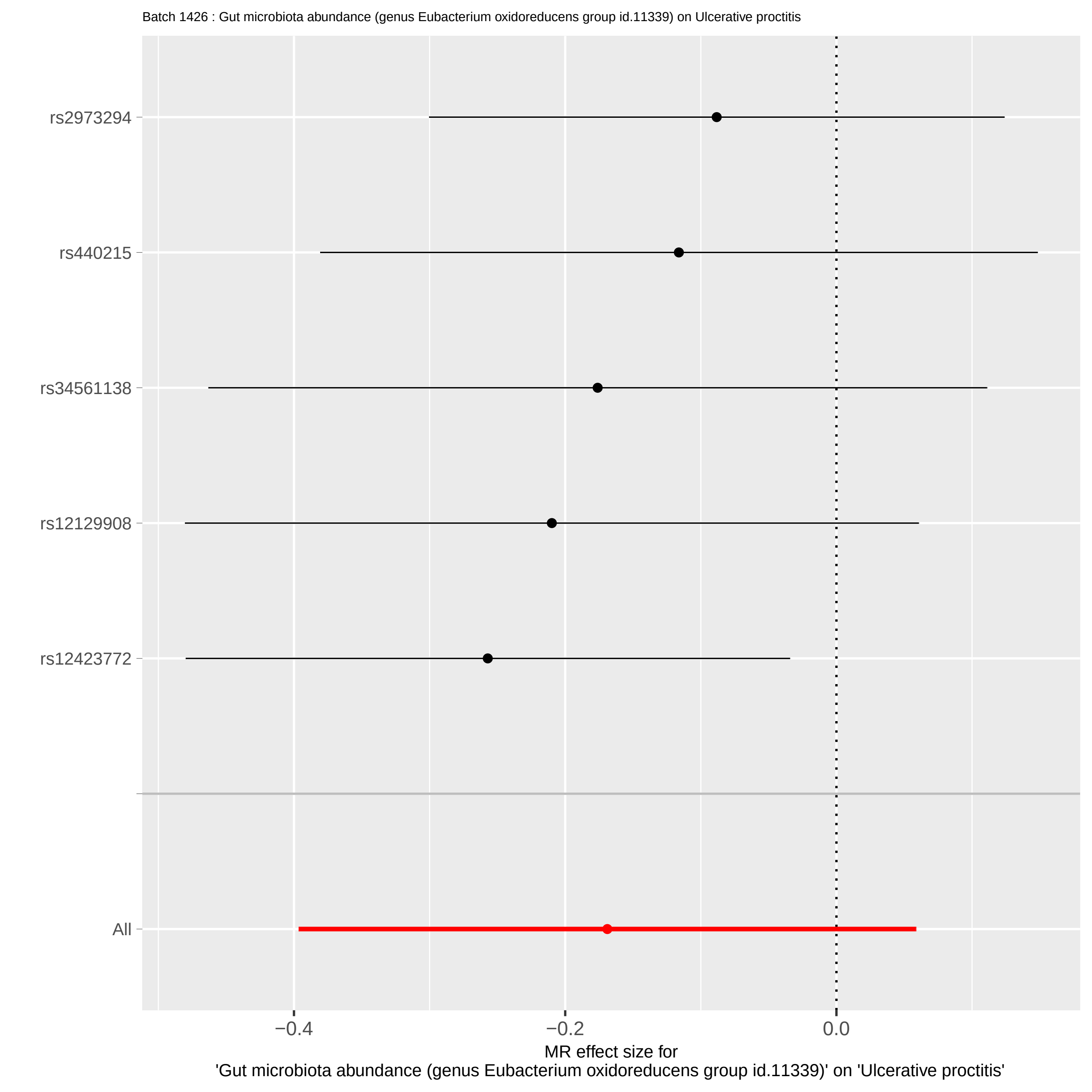

Batch 1426 : Gut microbiota abundance (genus Eubacterium oxidoreducens group id.11339) on Ulcerative proctitis
rs2973294
rs440215
rs34561138
rs12129908
rs12423772
All
−0.4
−0.2
0.0
MR effect size for
'Gut microbiota abundance (genus Eubacterium oxidoreducens group id.11339)' on 'Ulcerative proctitis'

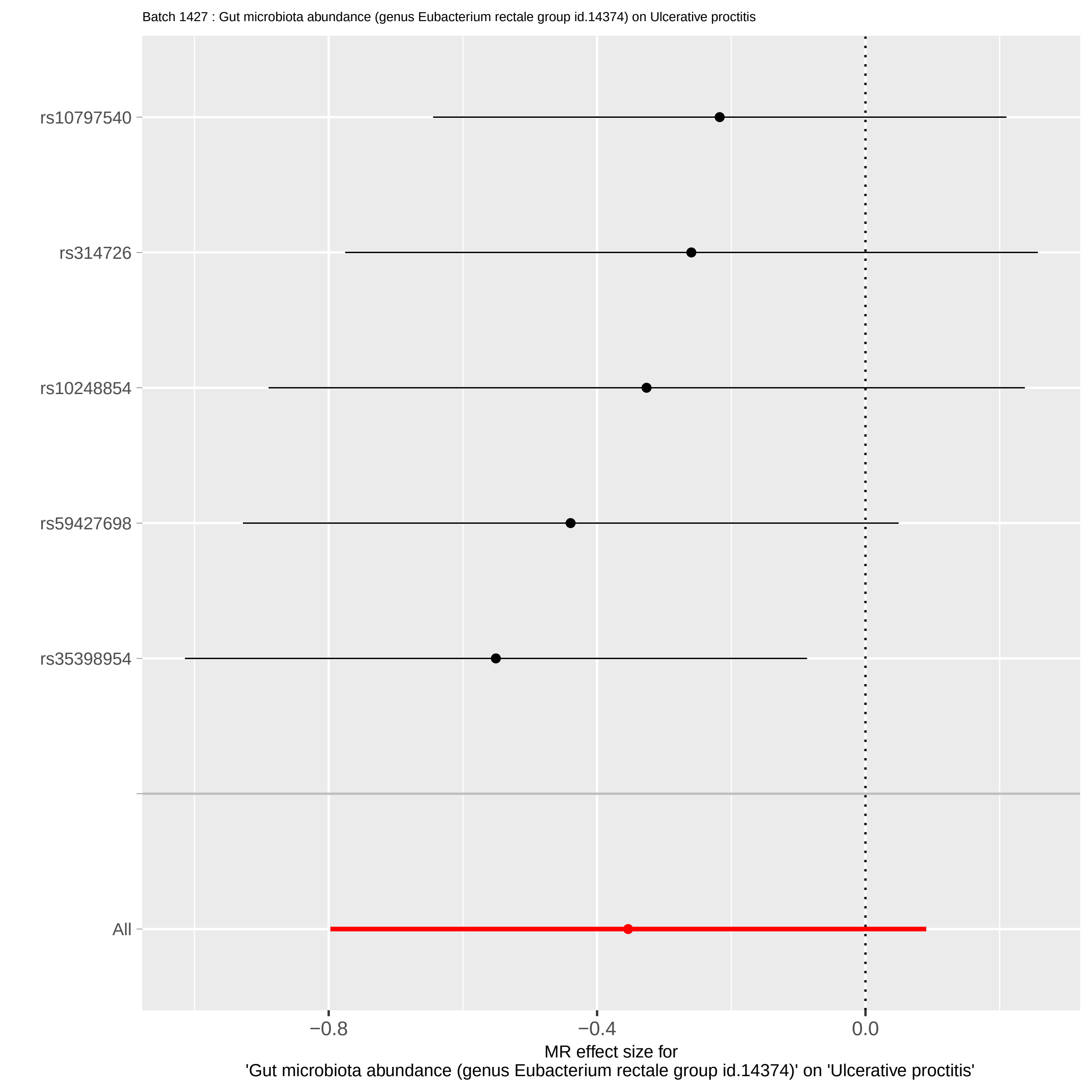

Batch 1427 : Gut microbiota abundance (genus Eubacterium rectale group id.14374) on Ulcerative proctitis
rs10797540
rs314726
rs10248854
rs59427698
rs35398954
All
−0.8
−0.4
0.0
MR effect size for
'Gut microbiota abundance (genus Eubacterium rectale group id.14374)' on 'Ulcerative proctitis'

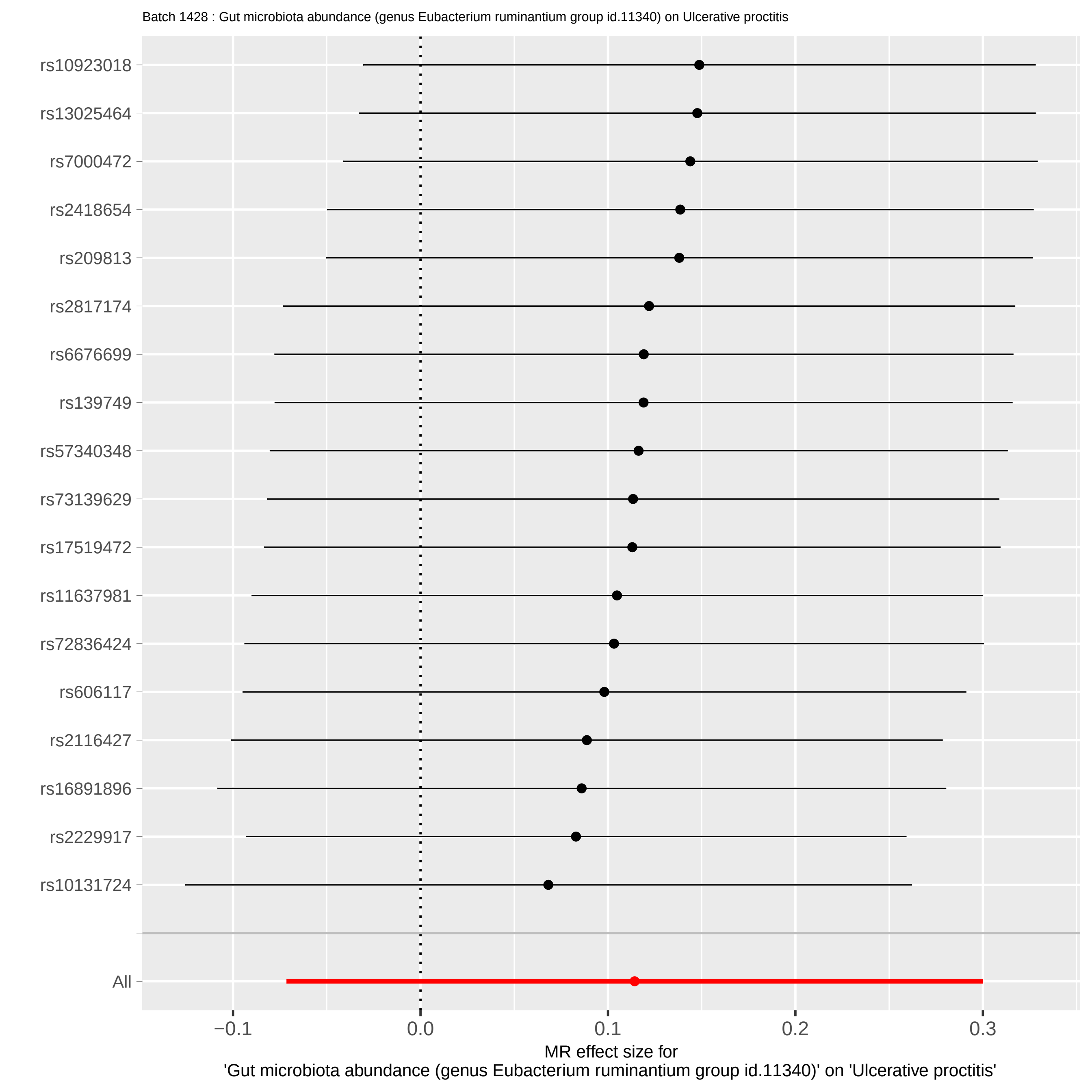

Batch 1428 : Gut microbiota abundance (genus Eubacterium ruminantium group id.11340) on Ulcerative proctitis
rs10923018
rs13025464
rs7000472
rs2418654
rs209813
rs2817174
rs6676699
rs139749
rs57340348
rs73139629
rs17519472
rs11637981
rs72836424
rs606117
rs2116427
rs16891896
rs2229917
rs10131724
All
−0.1
0.0
0.1
0.2
0.3
MR effect size for
'Gut microbiota abundance (genus Eubacterium ruminantium group id.11340)' on 'Ulcerative proctitis'

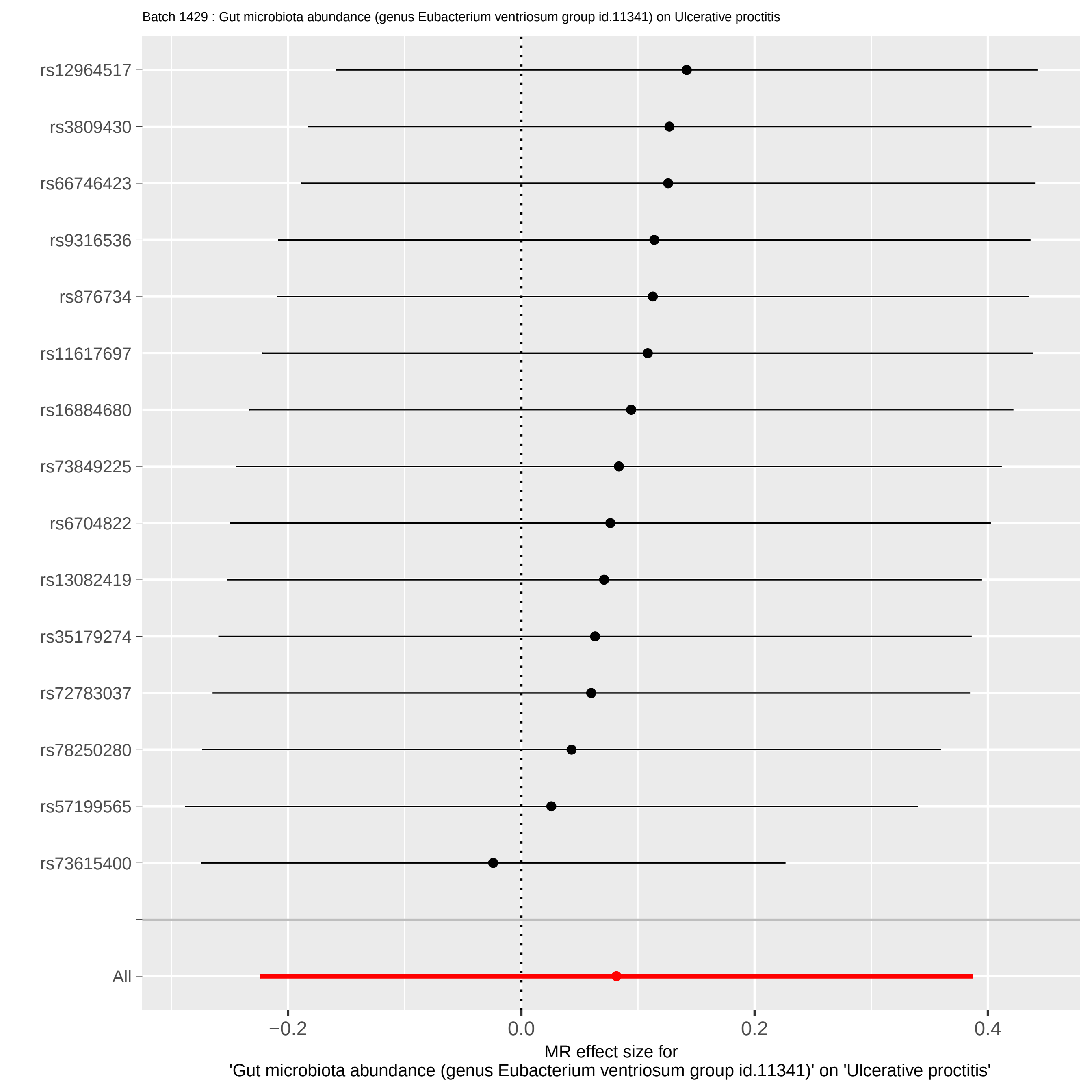

Batch 1429 : Gut microbiota abundance (genus Eubacterium ventriosum group id.11341) on Ulcerative proctitis
rs12964517
rs3809430
rs66746423
rs9316536
rs876734
rs11617697
rs16884680
rs73849225
rs6704822
rs13082419
rs35179274
rs72783037
rs78250280
rs57199565
rs73615400
All
−0.2
0.0
0.2
0.4
MR effect size for
'Gut microbiota abundance (genus Eubacterium ventriosum group id.11341)' on 'Ulcerative proctitis'

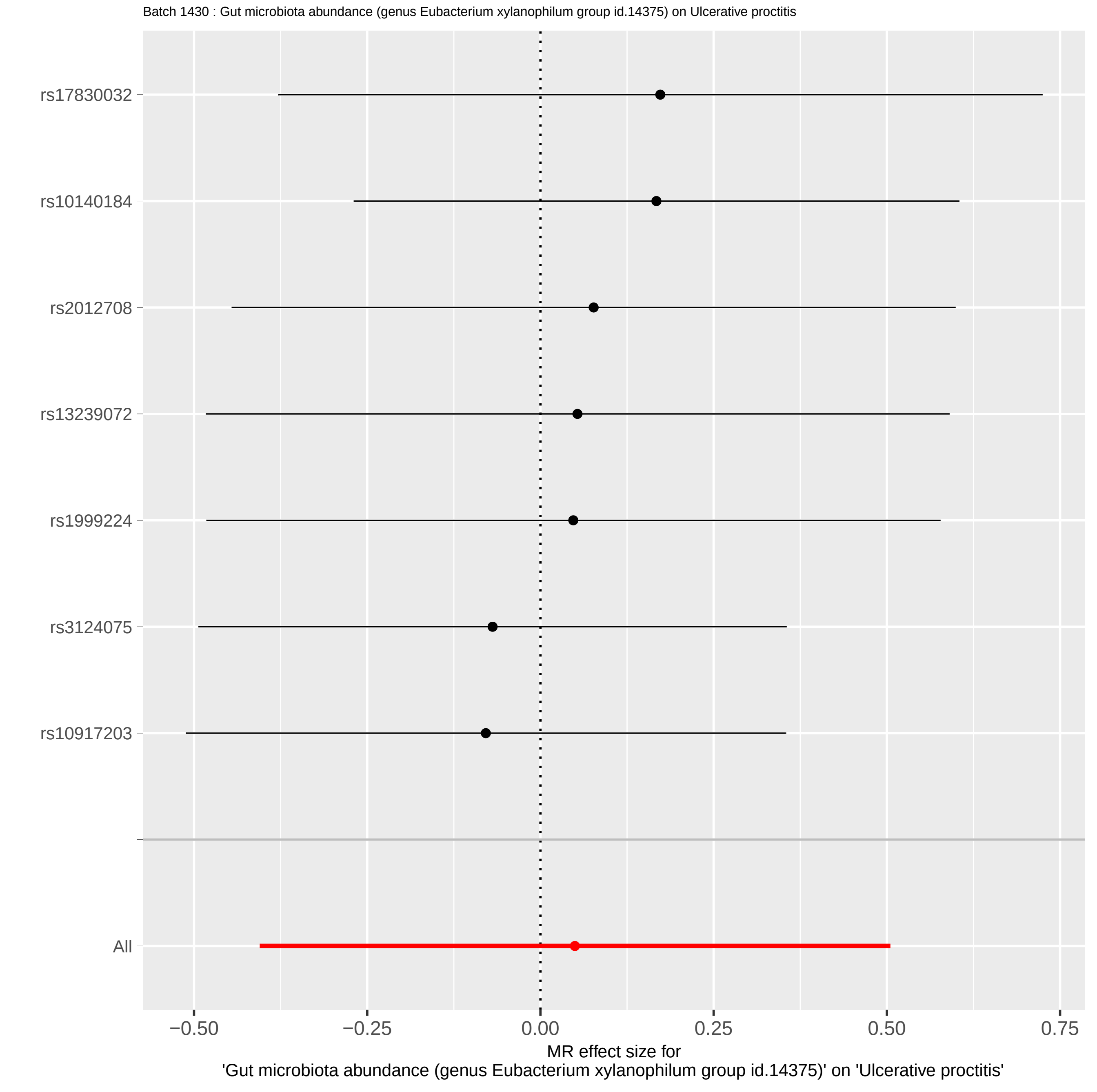

Batch 1430 : Gut microbiota abundance (genus Eubacterium xylanophilum group id.14375) on Ulcerative proctitis
rs17830032
rs10140184
rs2012708
rs13239072
rs1999224
rs3124075
rs10917203
All
−0.50
−0.25
0.00
0.25
0.50
0.75
MR effect size for
'Gut microbiota abundance (genus Eubacterium xylanophilum group id.14375)' on 'Ulcerative proctitis'

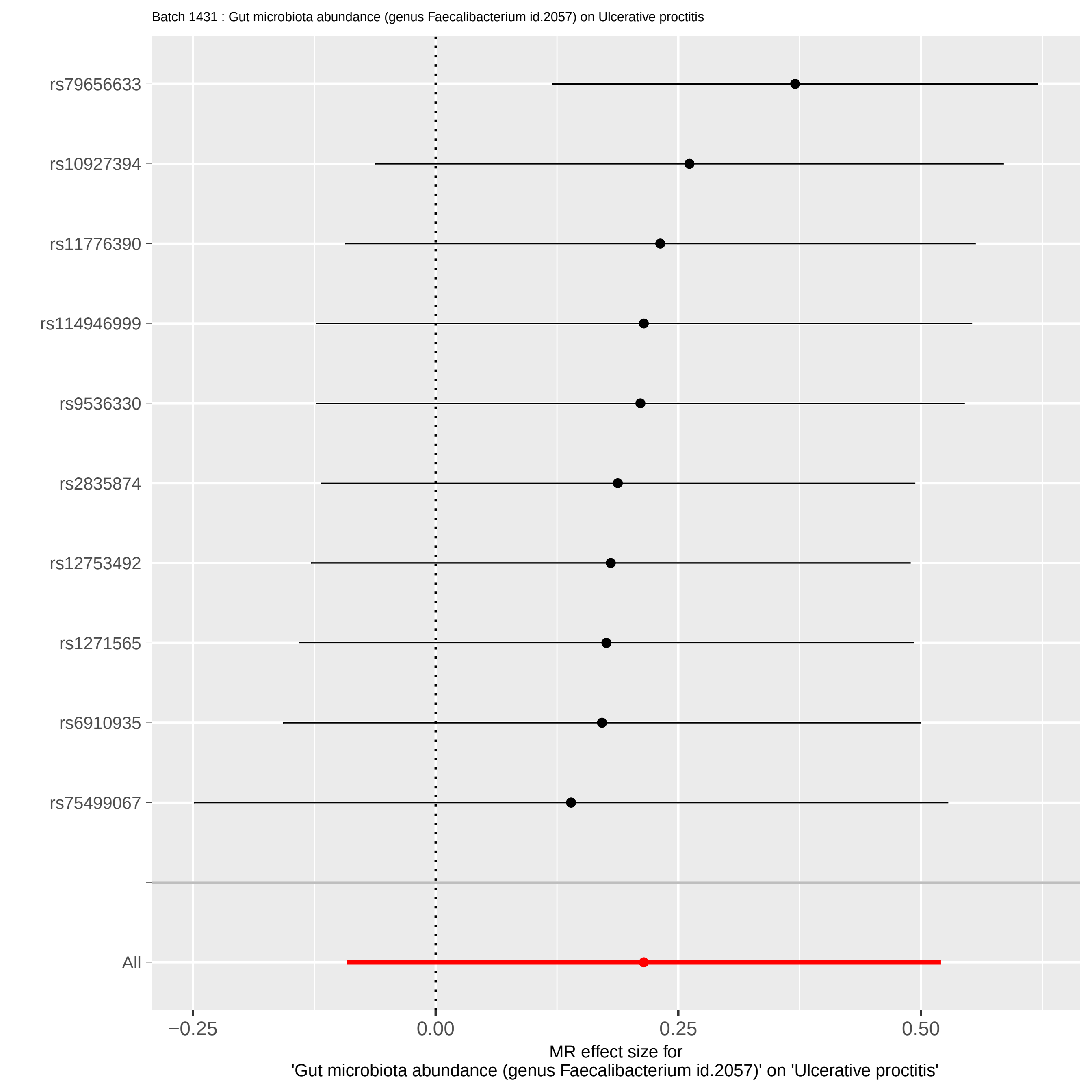

Batch 1431 : Gut microbiota abundance (genus Faecalibacterium id.2057) on Ulcerative proctitis
rs79656633
rs10927394
rs11776390
rs114946999
rs9536330
rs2835874
rs12753492
rs1271565
rs6910935
rs75499067
All
−0.25
0.00
0.25
0.50
MR effect size for
'Gut microbiota abundance (genus Faecalibacterium id.2057)' on 'Ulcerative proctitis'

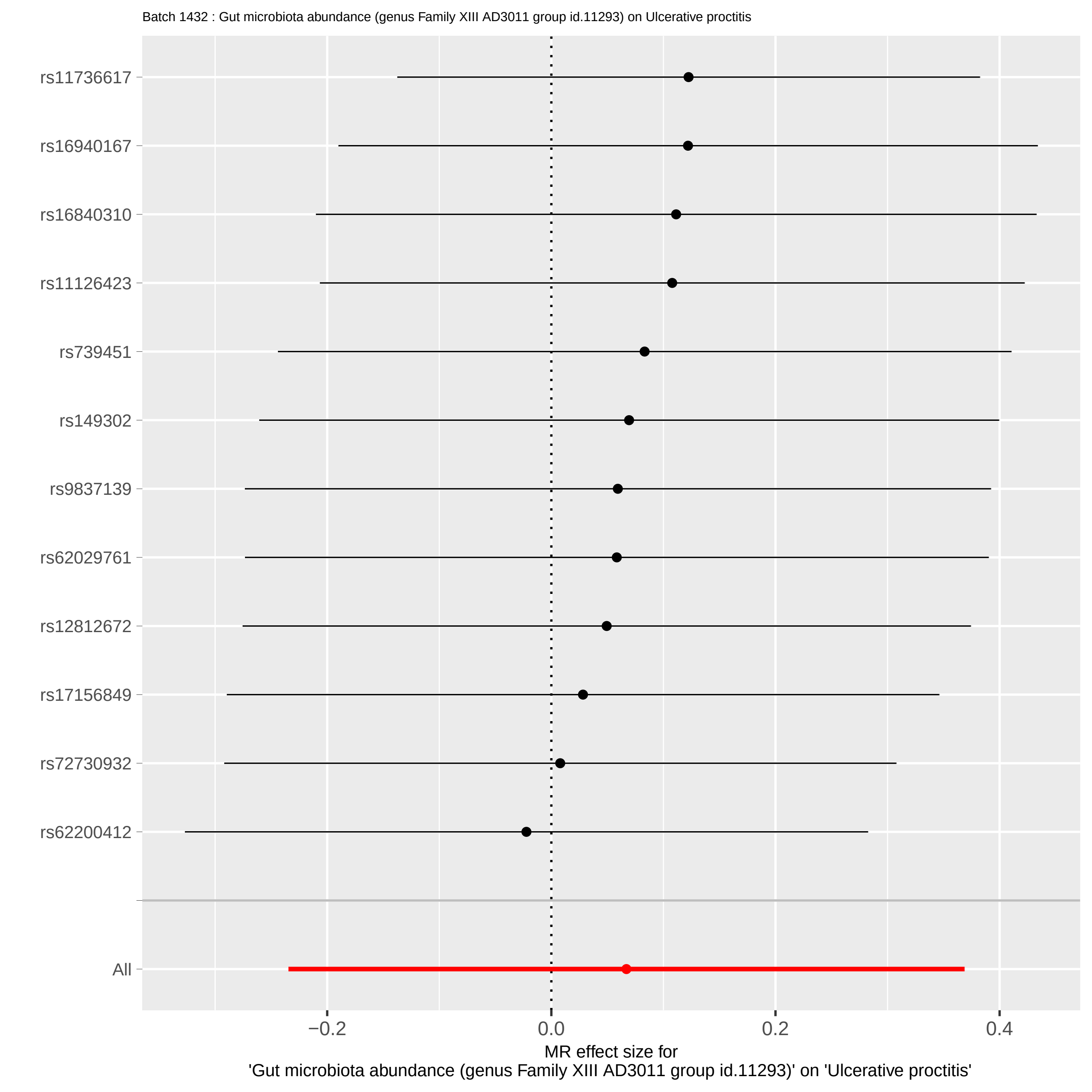

Batch 1432 : Gut microbiota abundance (genus Family XIII AD3011 group id.11293) on Ulcerative proctitis
rs11736617
rs16940167
rs16840310
rs11126423
rs739451
rs149302
rs9837139
rs62029761
rs12812672
rs17156849
rs72730932
rs62200412
All
−0.2
0.0
0.2
0.4
MR effect size for
'Gut microbiota abundance (genus Family XIII AD3011 group id.11293)' on 'Ulcerative proctitis'

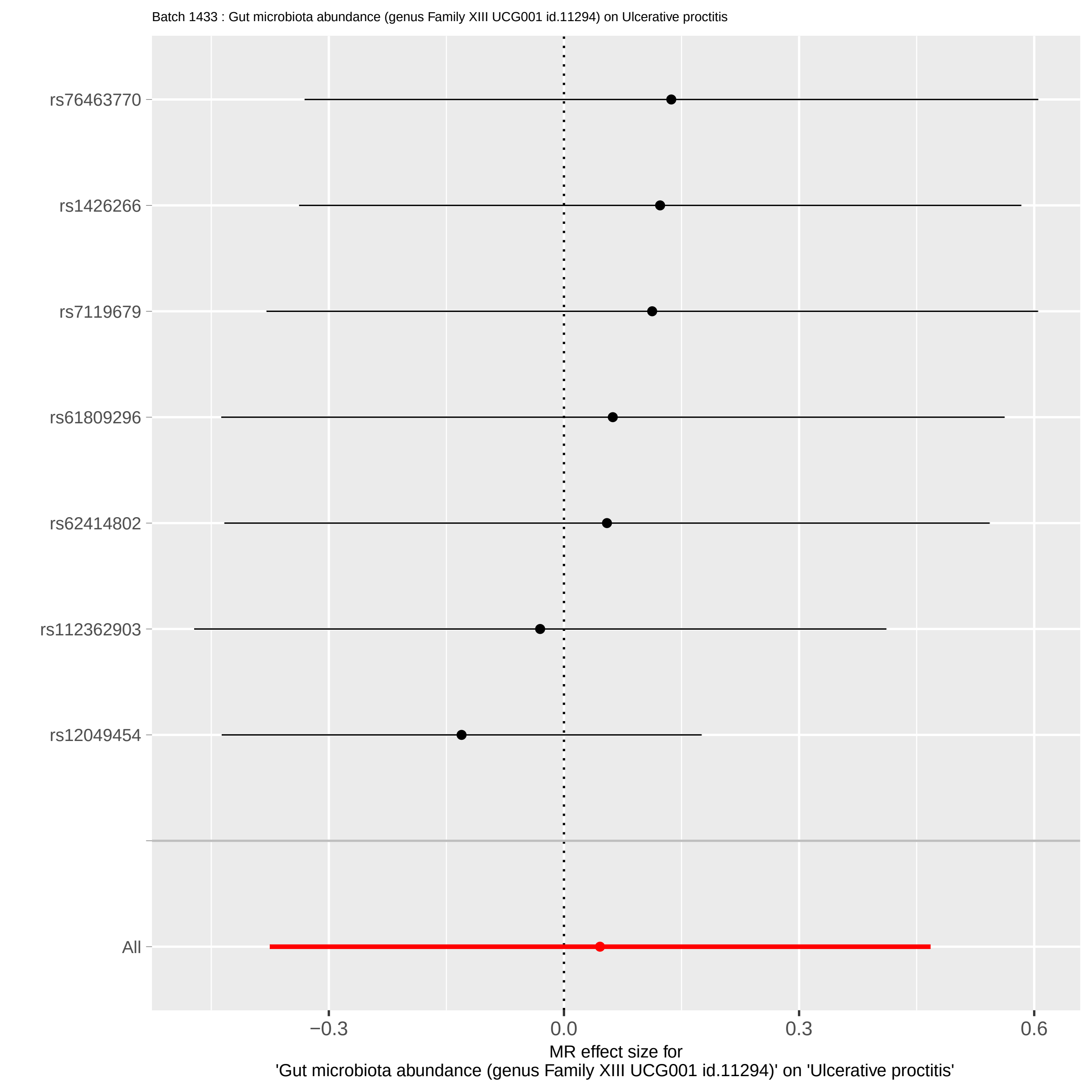
Batch 1433 : Gut microbiota abundance (genus Family XIII UCG001 id.11294) on Ulcerative proctitis
rs76463770
rs1426266
rs7119679
rs61809296
rs62414802
rs112362903
rs12049454
All
−0.3
0.0
0.3
0.6
MR effect size for
'Gut microbiota abundance (genus Family XIII UCG001 id.11294)' on 'Ulcerative proctitis'

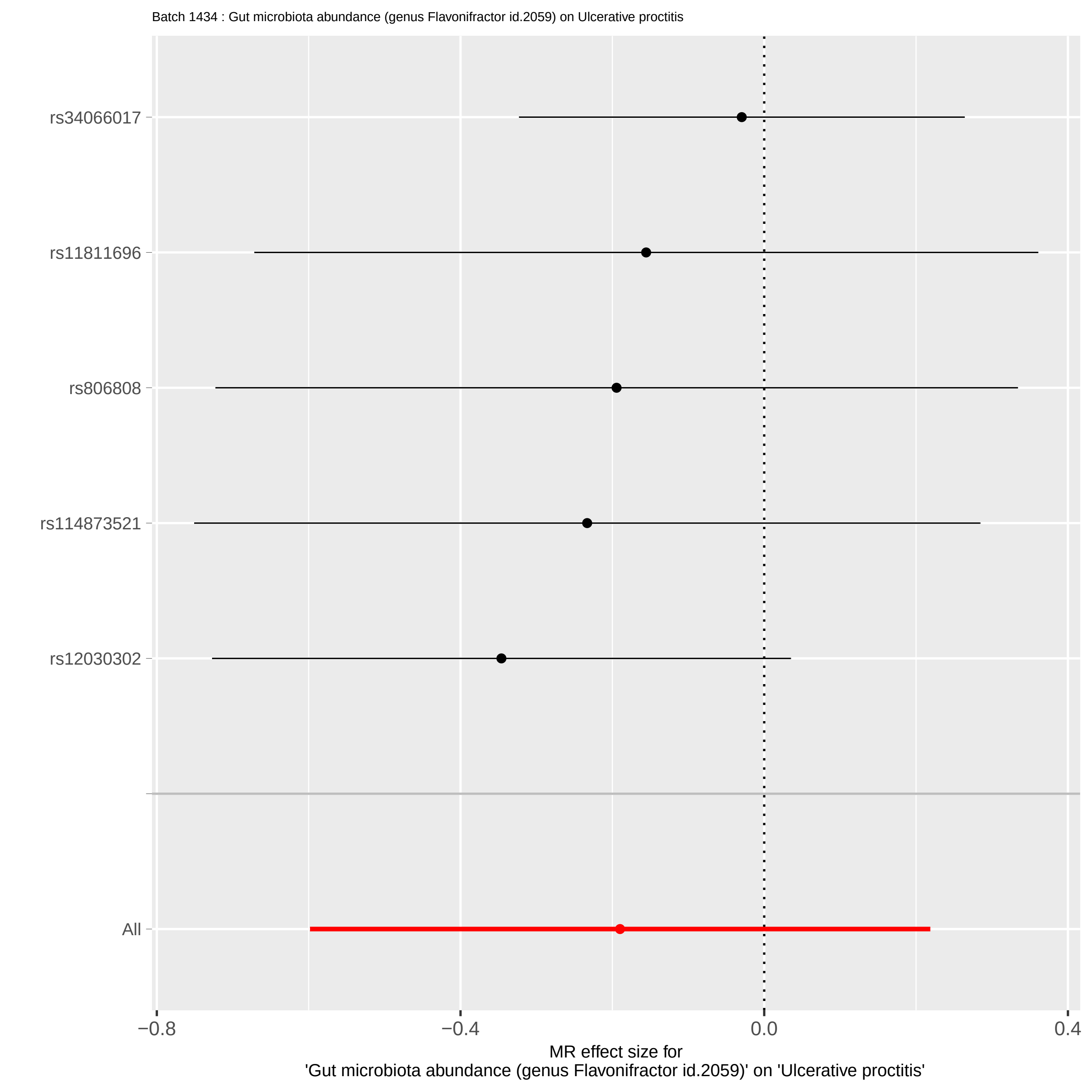

Batch 1434 : Gut microbiota abundance (genus Flavonifractor id.2059) on Ulcerative proctitis
rs34066017
rs11811696
rs806808
rs114873521
rs12030302
All
−0.8
−0.4
0.0
0.4
MR effect size for
'Gut microbiota abundance (genus Flavonifractor id.2059)' on 'Ulcerative proctitis'

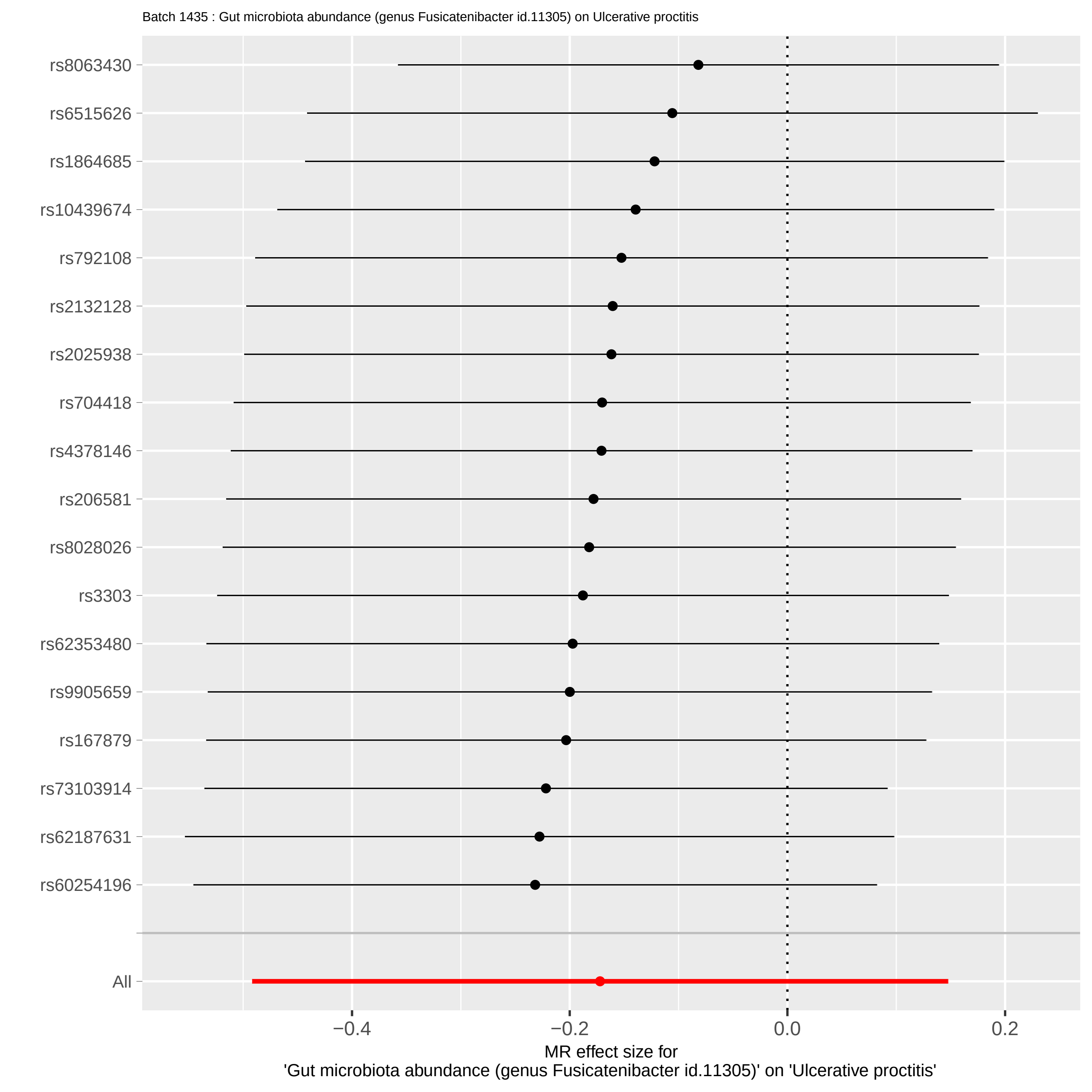
Batch 1435 : Gut microbiota abundance (genus Fusicatenibacter id.11305) on Ulcerative proctitis
rs8063430
rs6515626
rs1864685
rs10439674
rs792108
rs2132128
rs2025938
rs704418
rs4378146
rs206581
rs8028026
rs3303
rs62353480
rs9905659
rs167879
rs73103914
rs62187631
rs60254196
All
−0.4
−0.2
0.0
0.2
MR effect size for
'Gut microbiota abundance (genus Fusicatenibacter id.11305)' on 'Ulcerative proctitis'

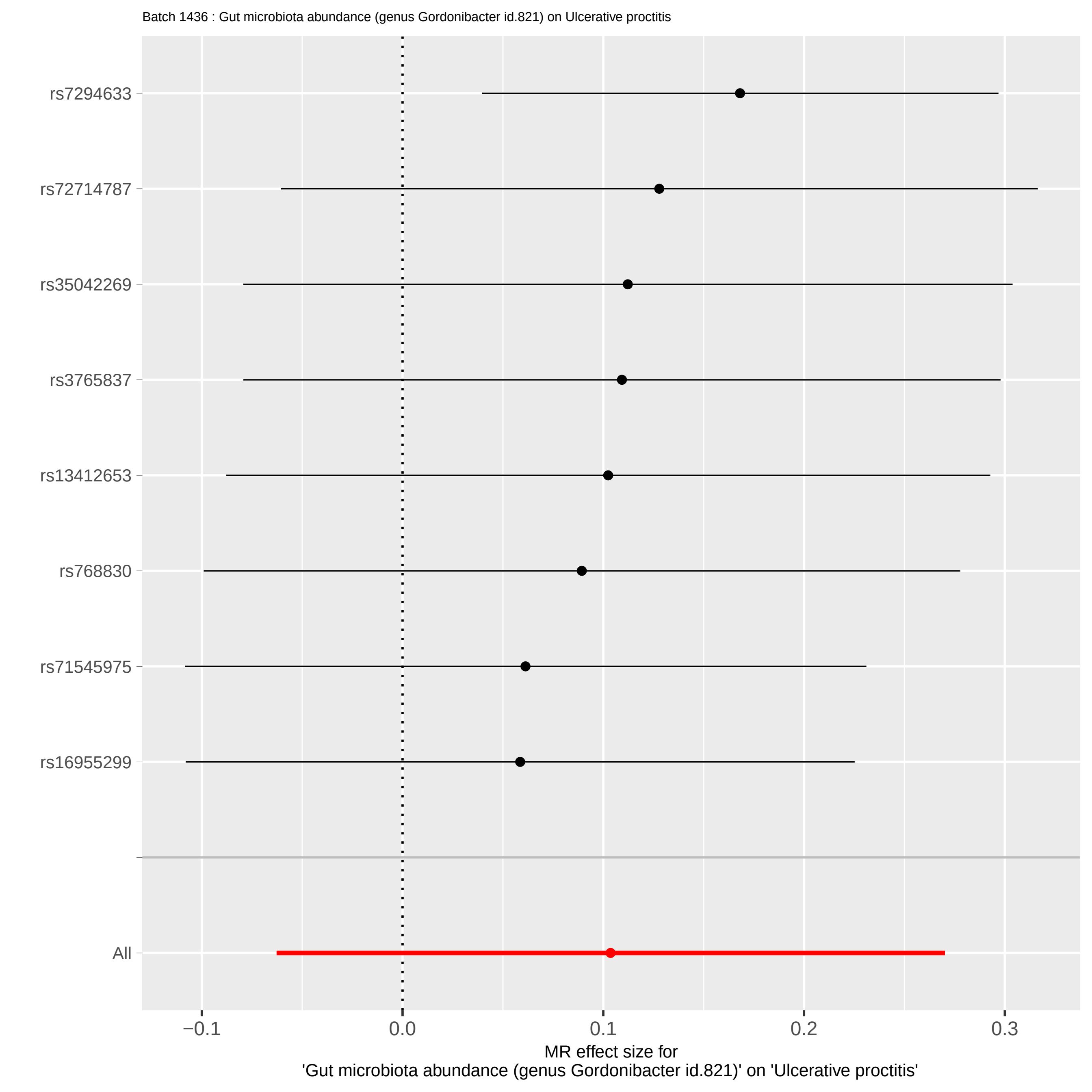

Batch 1436 : Gut microbiota abundance (genus Gordonibacter id.821) on Ulcerative proctitis
rs7294633
rs72714787
rs35042269
rs3765837
rs13412653
rs768830
rs71545975
rs16955299
All
−0.1
0.0
0.1
0.2
0.3
MR effect size for
'Gut microbiota abundance (genus Gordonibacter id.821)' on 'Ulcerative proctitis'

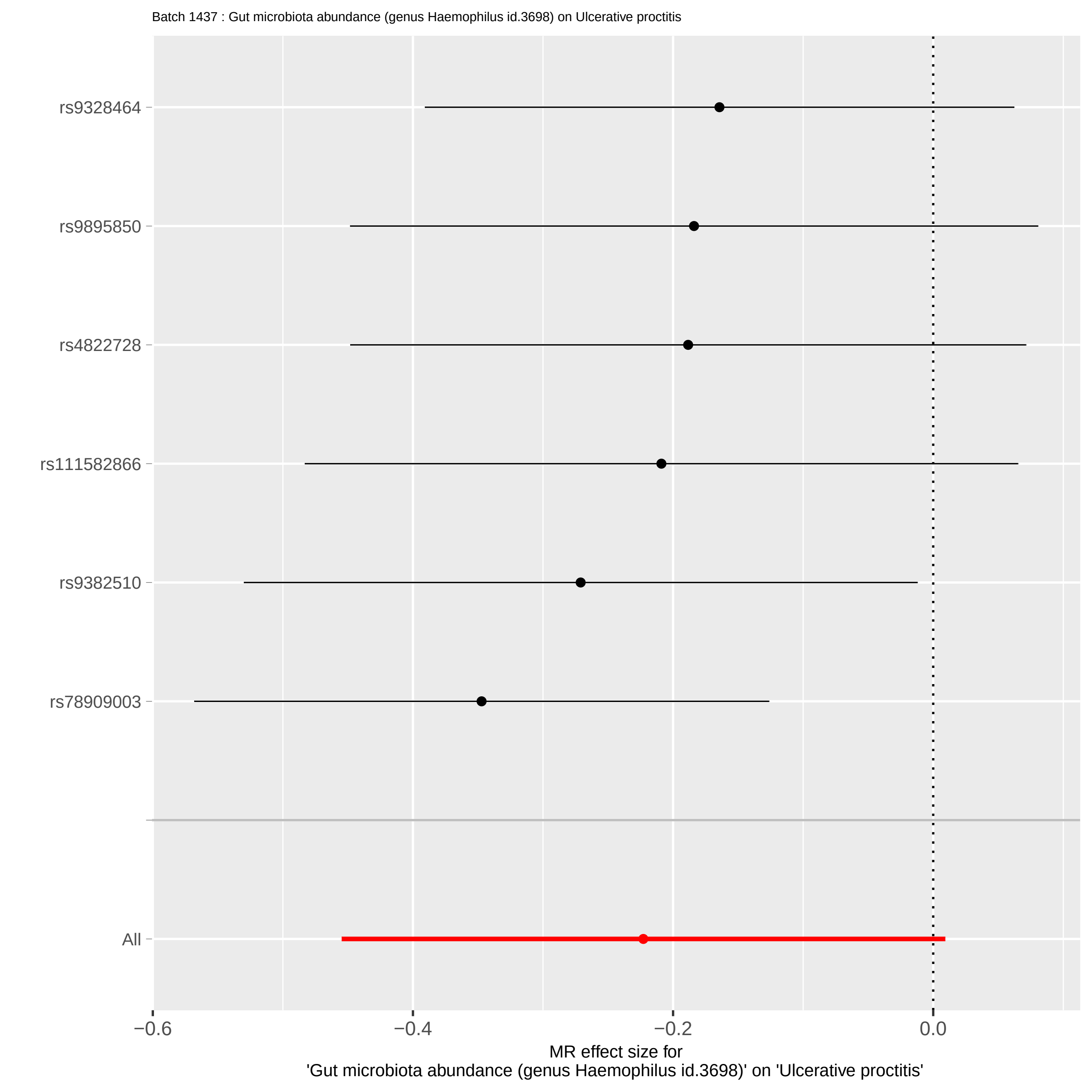
Batch 1437 : Gut microbiota abundance (genus Haemophilus id.3698) on Ulcerative proctitis
rs9328464
rs9895850
rs4822728
rs111582866
rs9382510
rs78909003
All
−0.6
−0.4
−0.2
0.0
MR effect size for
'Gut microbiota abundance (genus Haemophilus id.3698)' on 'Ulcerative proctitis'

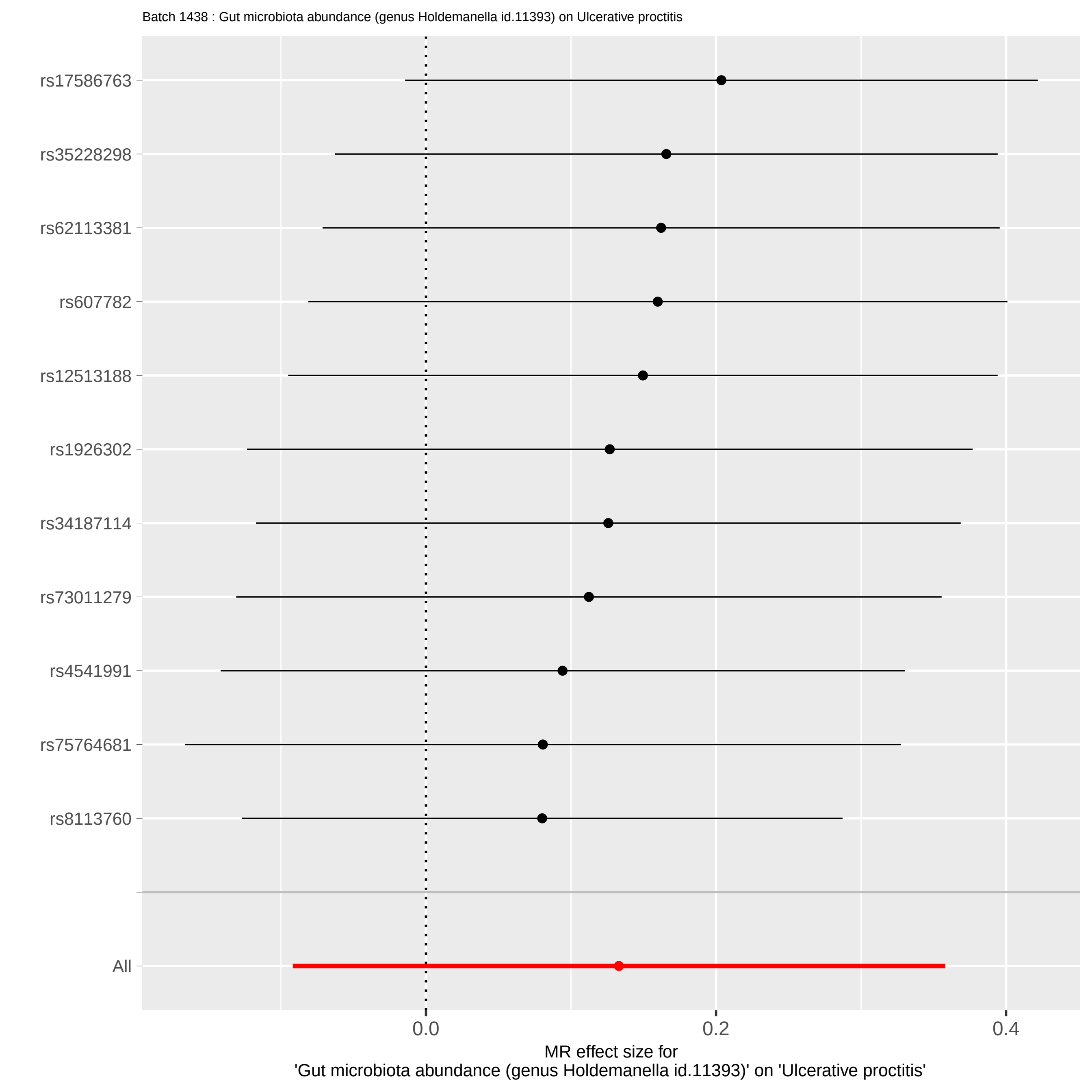
Batch 1438 : Gut microbiota abundance (genus Holdemanella id.11393) on Ulcerative proctitis
rs17586763
rs35228298
rs62113381
rs607782
rs12513188
rs1926302
rs34187114
rs73011279
rs4541991
rs75764681
rs8113760
All
0.0
0.2
0.4
MR effect size for
'Gut microbiota abundance (genus Holdemanella id.11393)' on 'Ulcerative proctitis'

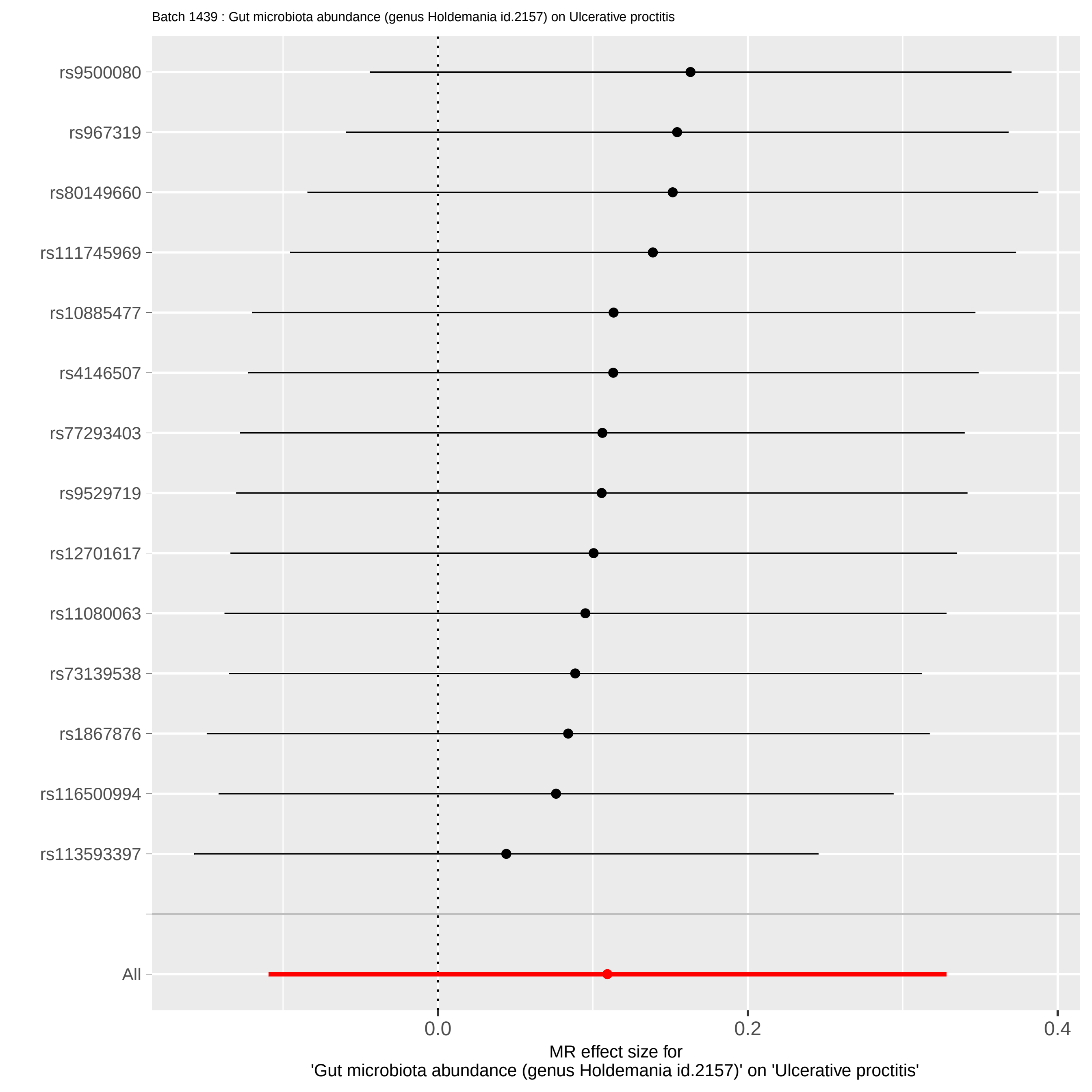
Batch 1439 : Gut microbiota abundance (genus Holdemania id.2157) on Ulcerative proctitis
rs9500080
rs967319
rs80149660
rs111745969
rs10885477
rs4146507
rs77293403
rs9529719
rs12701617
rs11080063
rs73139538
rs1867876
rs116500994
rs113593397
All
0.0
0.2
0.4
MR effect size for
'Gut microbiota abundance (genus Holdemania id.2157)' on 'Ulcerative proctitis'

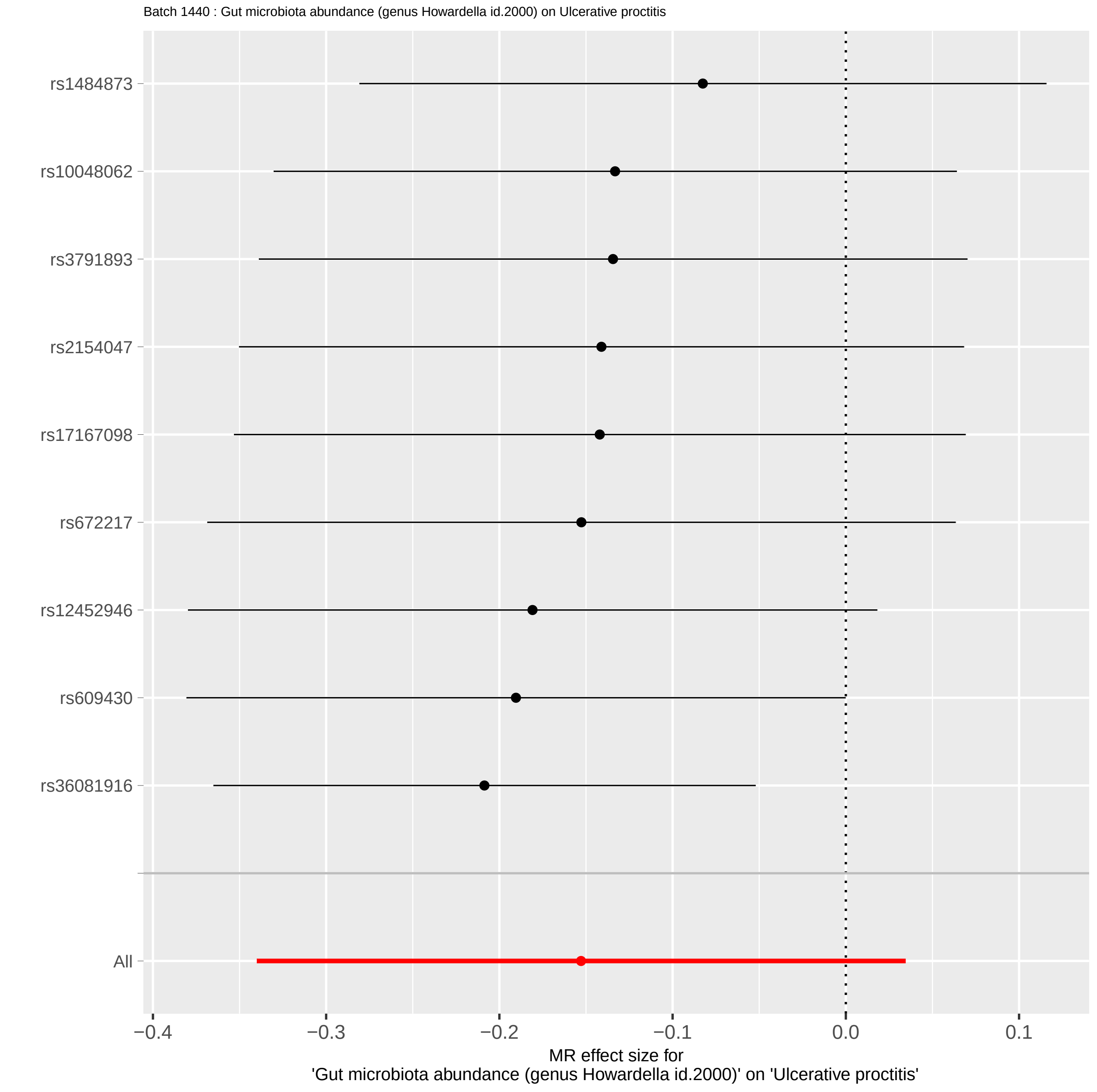
Batch 1440 : Gut microbiota abundance (genus Howardella id.2000) on Ulcerative proctitis
rs1484873
rs10048062
rs3791893
rs2154047
rs17167098
rs672217
rs12452946
rs609430
rs36081916
All
−0.4
−0.3
−0.2
−0.1
0.0
0.1
MR effect size for
'Gut microbiota abundance (genus Howardella id.2000)' on 'Ulcerative proctitis'

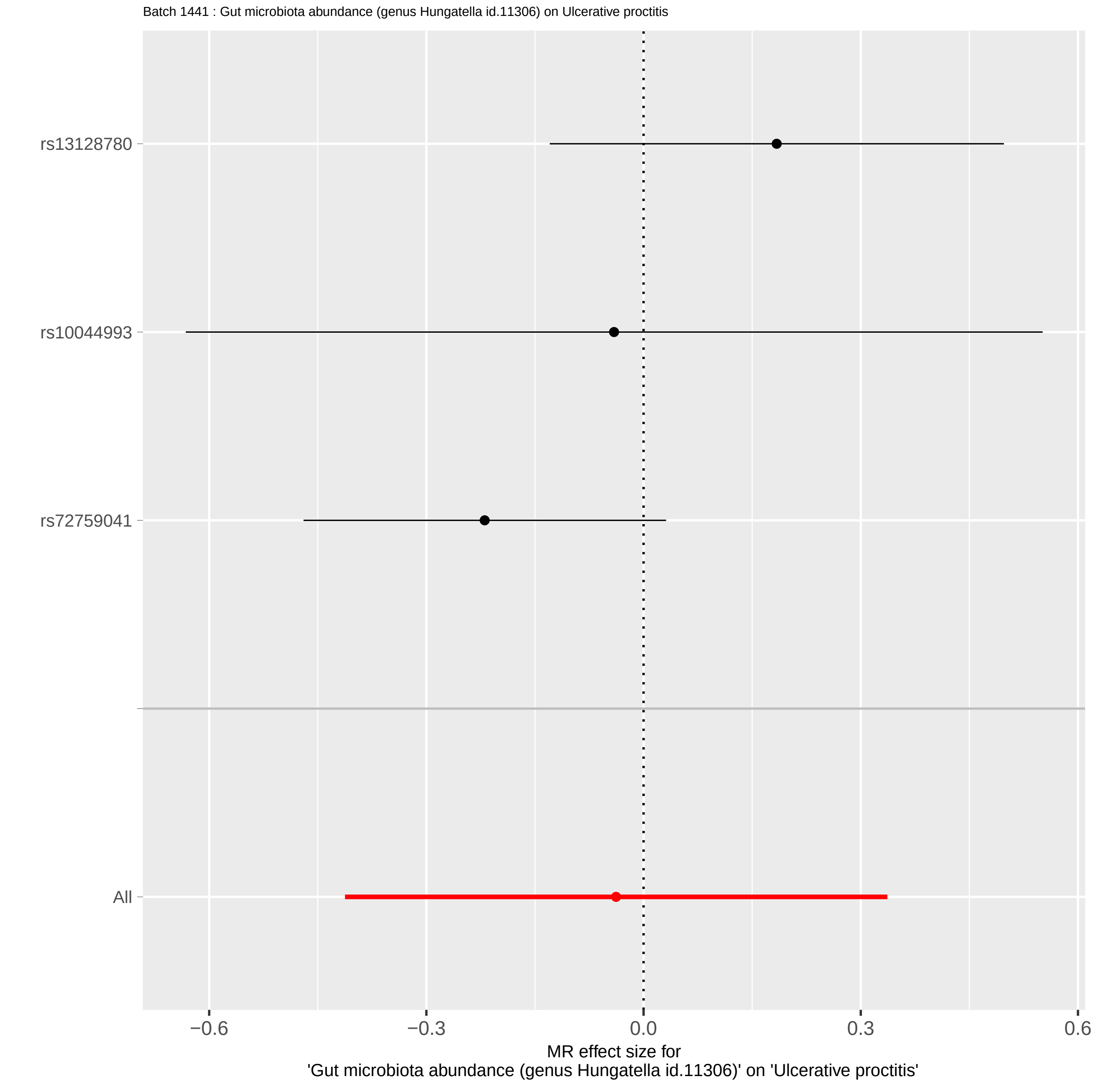

Batch 1441 : Gut microbiota abundance (genus Hungatella id.11306) on Ulcerative proctitis
rs13128780
rs10044993
rs72759041
All
−0.6
−0.3
0.0
0.3
0.6
MR effect size for
'Gut microbiota abundance (genus Hungatella id.11306)' on 'Ulcerative proctitis'

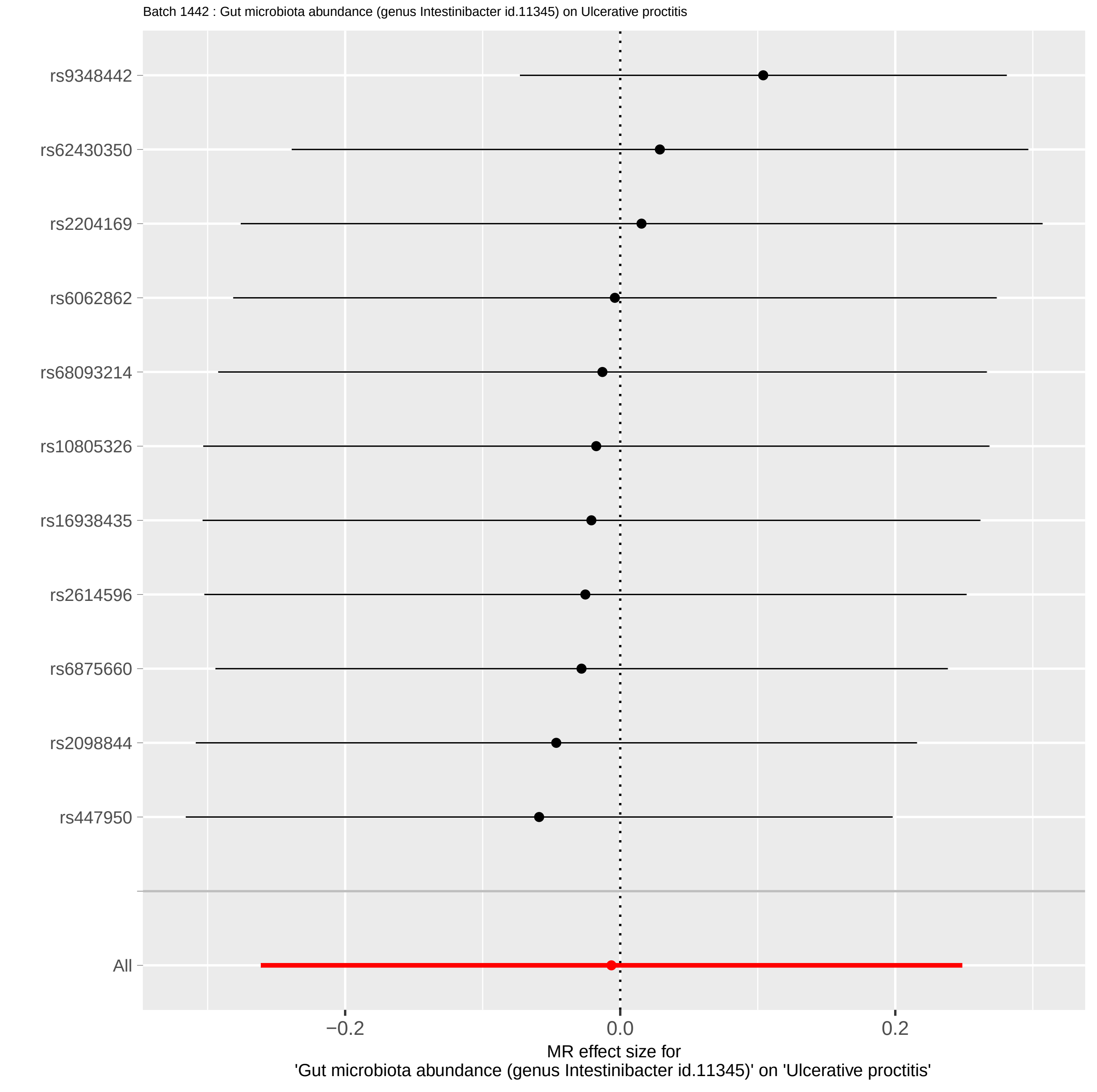

Batch 1442 : Gut microbiota abundance (genus Intestinibacter id.11345) on Ulcerative proctitis
rs9348442
rs62430350
rs2204169
rs6062862
rs68093214
rs10805326
rs16938435
rs2614596
rs6875660
rs2098844
rs447950
All
−0.2
0.0
0.2
MR effect size for
'Gut microbiota abundance (genus Intestinibacter id.11345)' on 'Ulcerative proctitis'

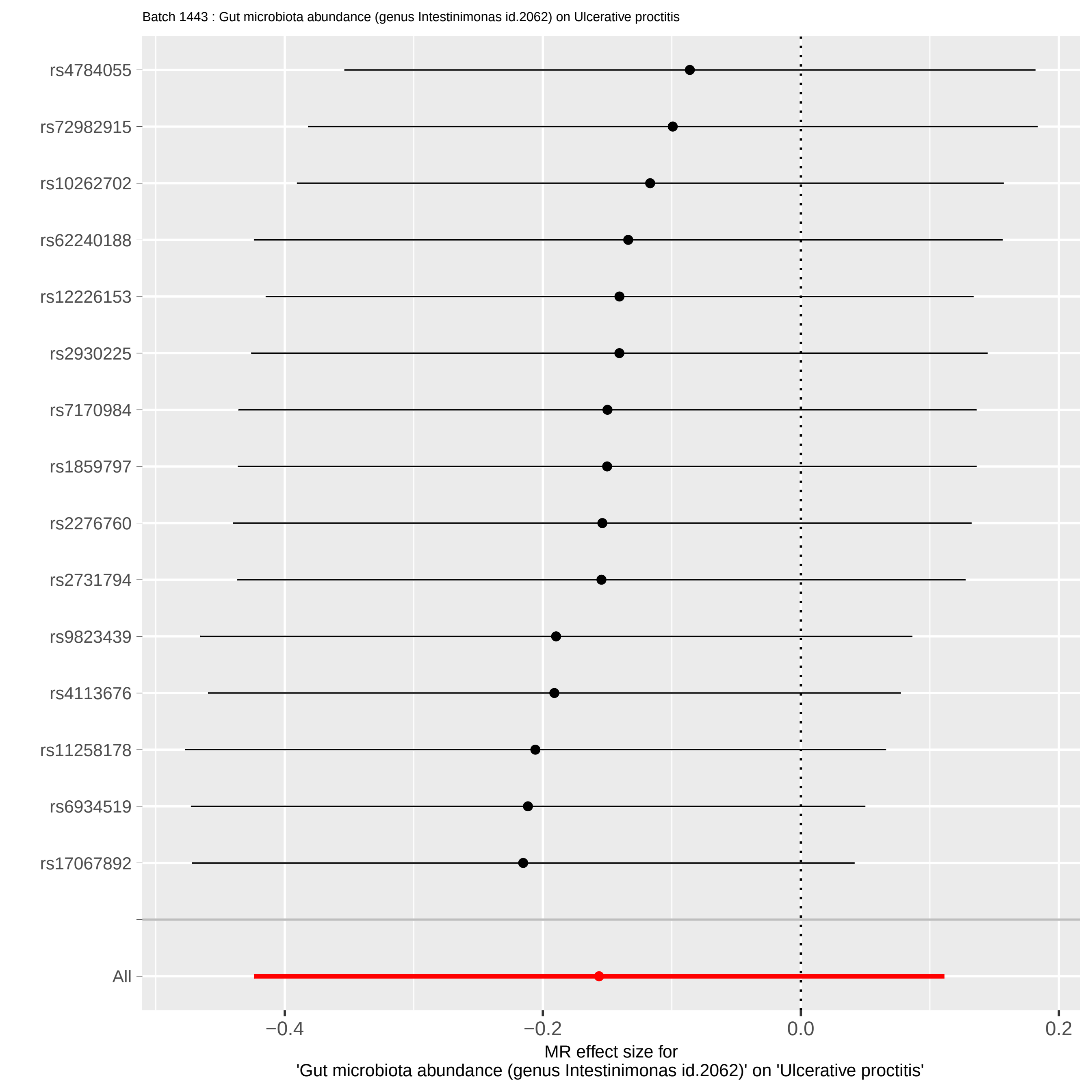

Batch 1443 : Gut microbiota abundance (genus Intestinimonas id.2062) on Ulcerative proctitis
rs4784055
rs72982915
rs10262702
rs62240188
rs12226153
rs2930225
rs7170984
rs1859797
rs2276760
rs2731794
rs9823439
rs4113676
rs11258178
rs6934519
rs17067892
All
−0.4
−0.2
0.0
0.2
MR effect size for
'Gut microbiota abundance (genus Intestinimonas id.2062)' on 'Ulcerative proctitis'

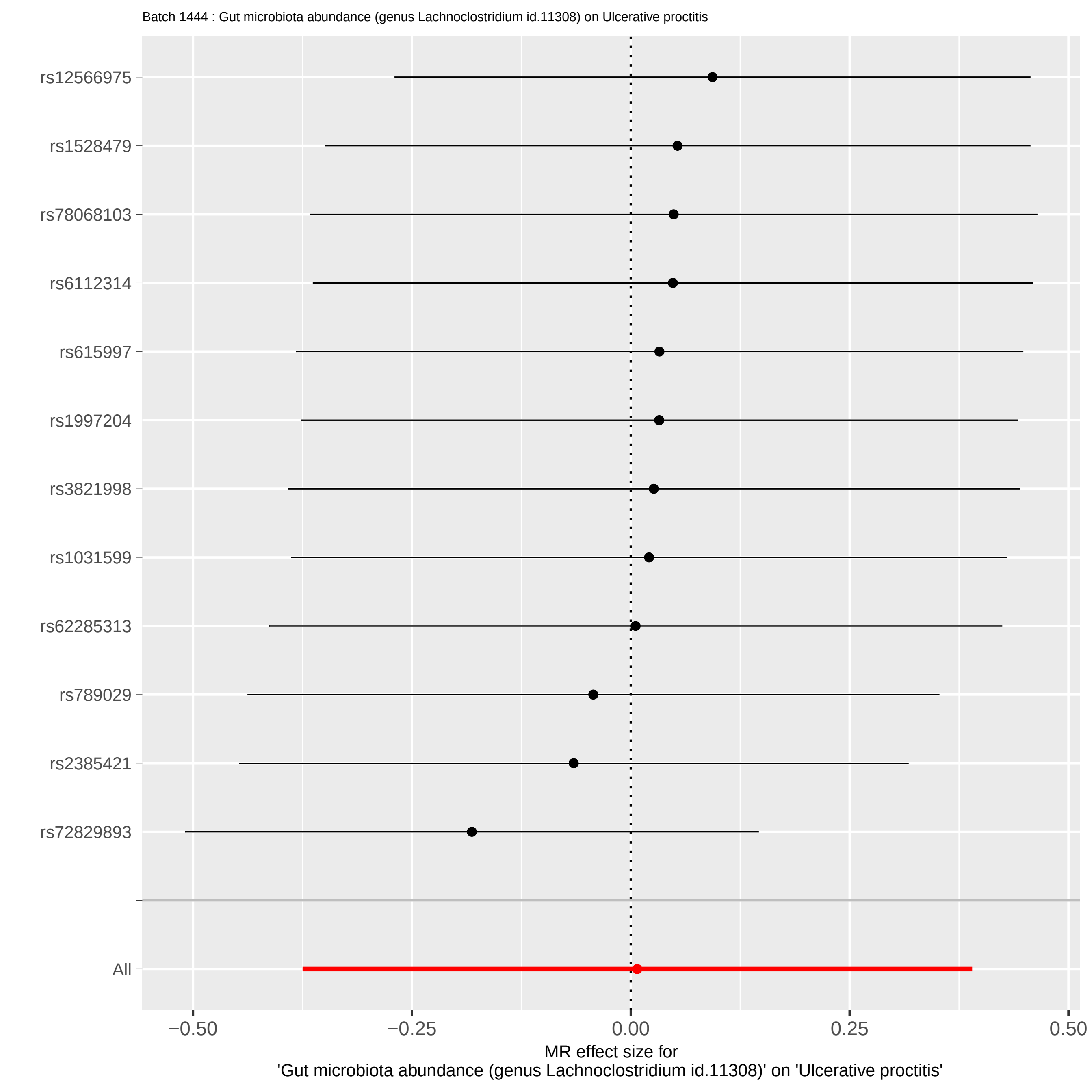

Batch 1444 : Gut microbiota abundance (genus Lachnoclostridium id.11308) on Ulcerative proctitis
rs12566975
rs1528479
rs78068103
rs6112314
rs615997
rs1997204
rs3821998
rs1031599
rs62285313
rs789029
rs2385421
rs72829893
All
−0.50
−0.25
0.00
0.25
0.50
MR effect size for
'Gut microbiota abundance (genus Lachnoclostridium id.11308)' on 'Ulcerative proctitis'

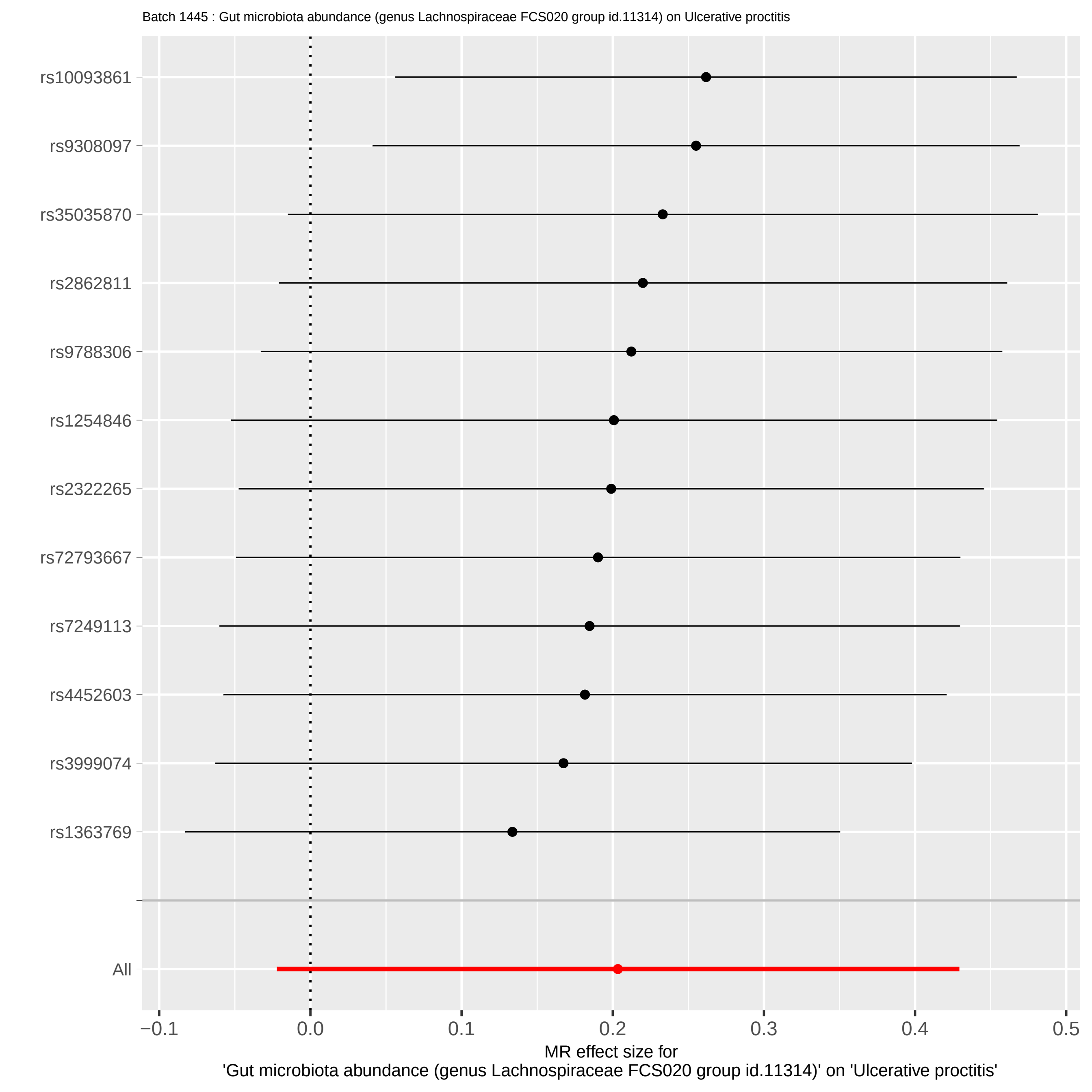

Batch 1445 : Gut microbiota abundance (genus Lachnospiraceae FCS020 group id.11314) on Ulcerative proctitis
rs10093861
rs9308097
rs35035870
rs2862811
rs9788306
rs1254846
rs2322265
rs72793667
rs7249113
rs4452603
rs3999074
rs1363769
All
−0.1
0.0
0.1
0.2
0.3
0.4
0.5
MR effect size for
'Gut microbiota abundance (genus Lachnospiraceae FCS020 group id.11314)' on 'Ulcerative proctitis'

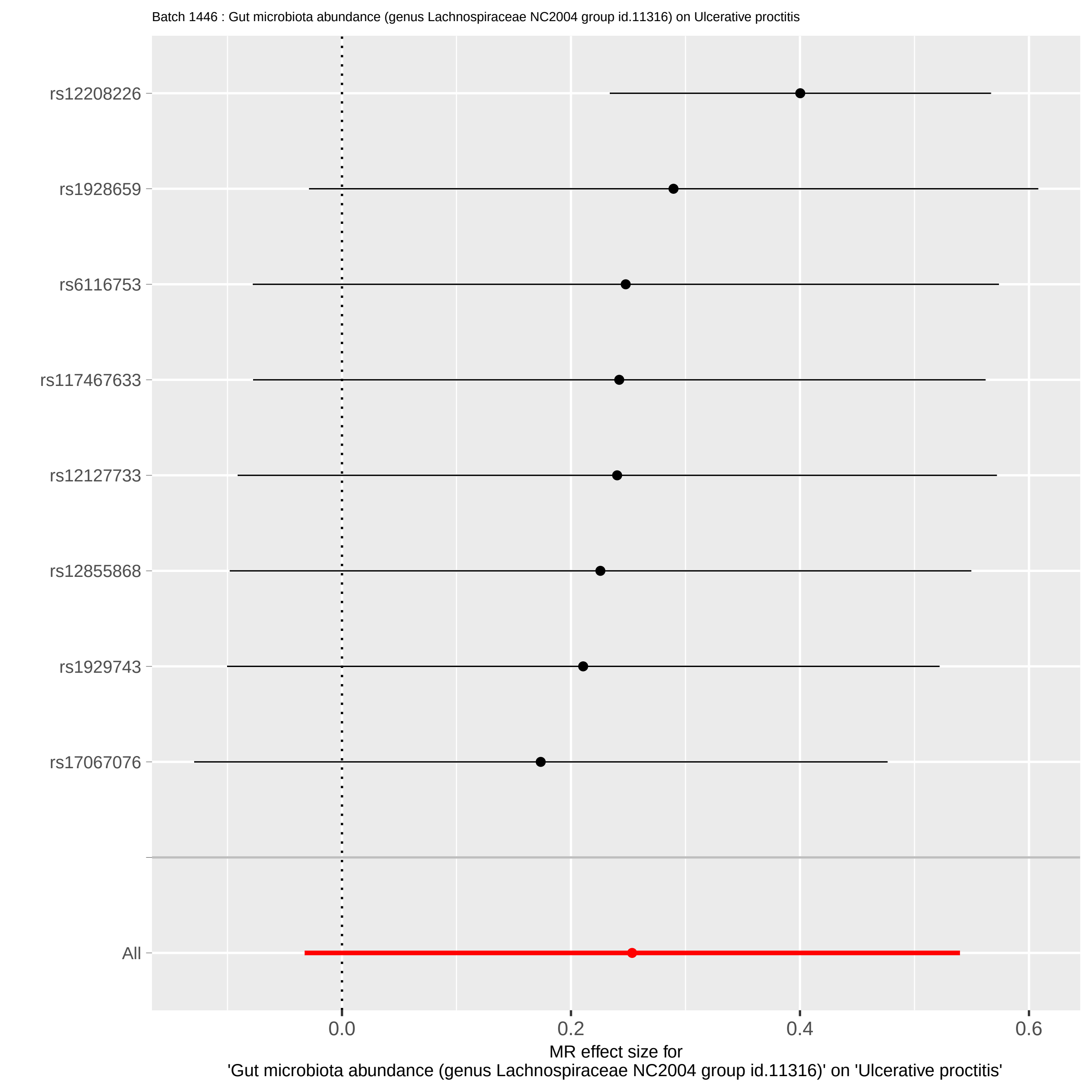

Batch 1446 : Gut microbiota abundance (genus Lachnospiraceae NC2004 group id.11316) on Ulcerative proctitis
rs12208226
rs1928659
rs6116753
rs117467633
rs12127733
rs12855868
rs1929743
rs17067076
All
0.0
0.2
0.4
0.6
MR effect size for
'Gut microbiota abundance (genus Lachnospiraceae NC2004 group id.11316)' on 'Ulcerative proctitis'

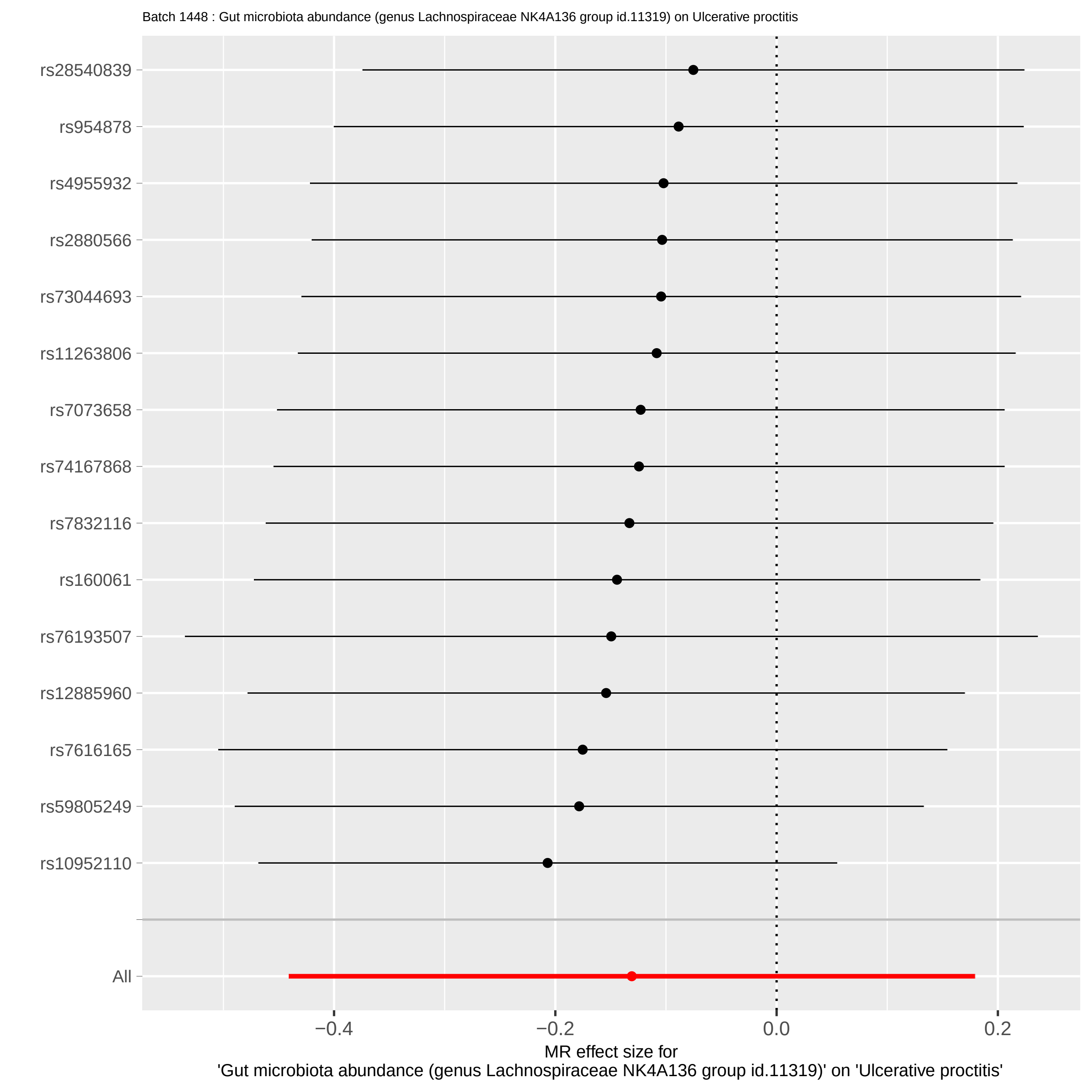

Batch 1448 : Gut microbiota abundance (genus Lachnospiraceae NK4A136 group id.11319) on Ulcerative proctitis
rs28540839
rs954878
rs4955932
rs2880566
rs73044693
rs11263806
rs7073658
rs74167868
rs7832116
rs160061
rs76193507
rs12885960
rs7616165
rs59805249
rs10952110
All
−0.4
−0.2
0.0
0.2
MR effect size for
'Gut microbiota abundance (genus Lachnospiraceae NK4A136 group id.11319)' on 'Ulcerative proctitis'

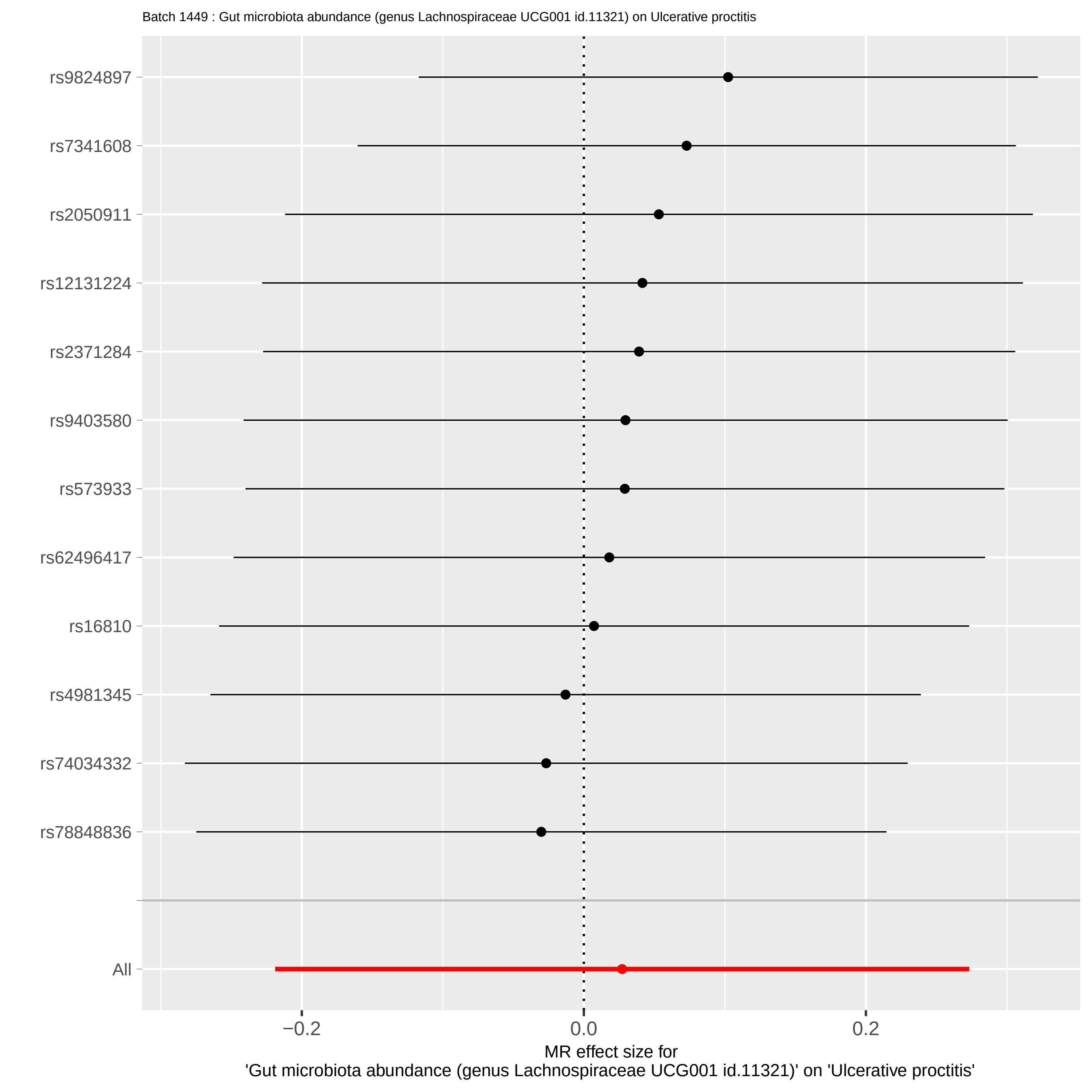
Batch 1449 : Gut microbiota abundance (genus Lachnospiraceae UCG001 id.11321) on Ulcerative proctitis
rs9824897
rs7341608
rs2050911
rs12131224
rs2371284
rs9403580
rs573933
rs62496417
rs16810
rs4981345
rs74034332
rs78848836
All
−0.2
0.0
0.2
MR effect size for
'Gut microbiota abundance (genus Lachnospiraceae UCG001 id.11321)' on 'Ulcerative proctitis'

Batch 1450 : Gut microbiota abundance (genus Lachnospiraceae UCG004 id.11324) on Ulcerative proctitis

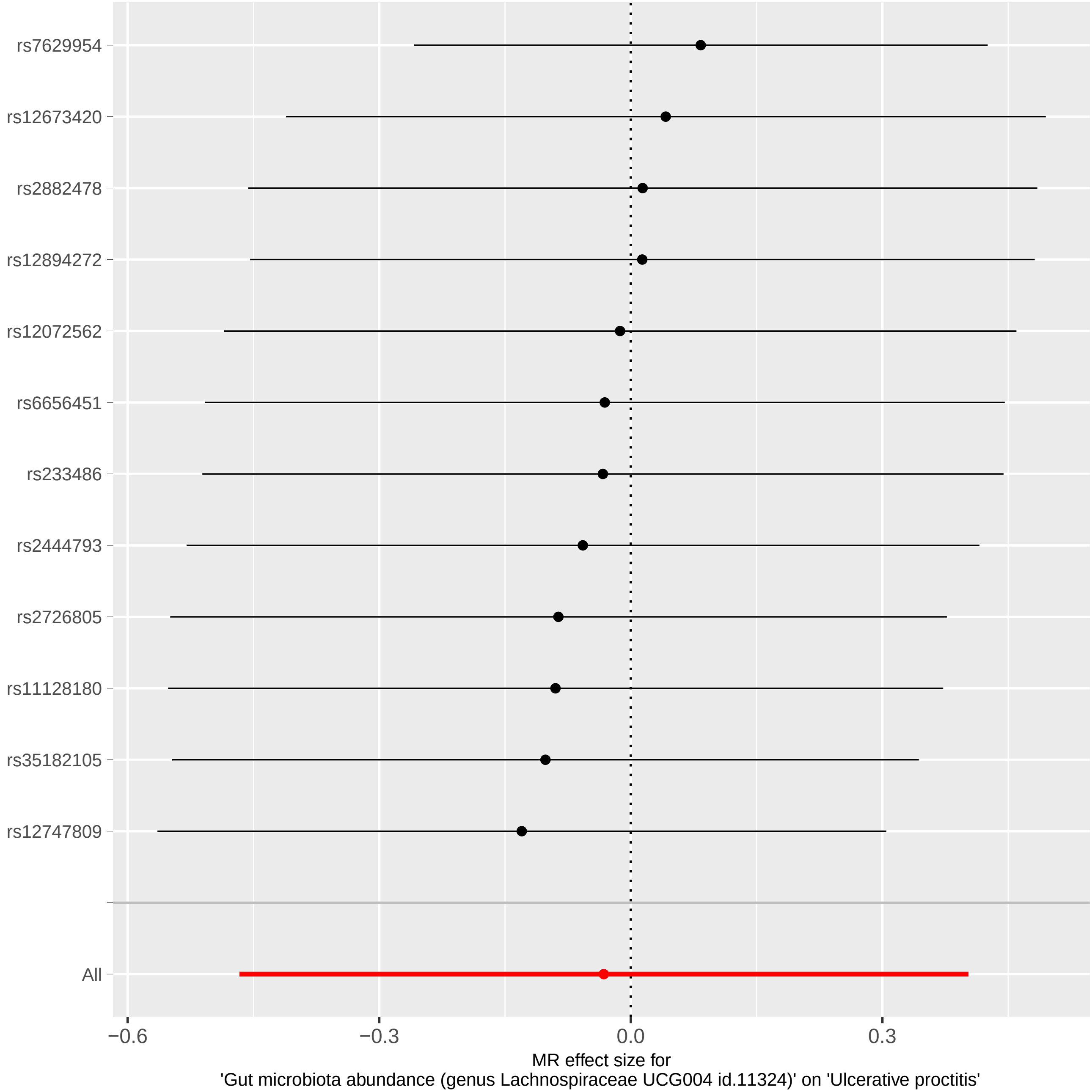

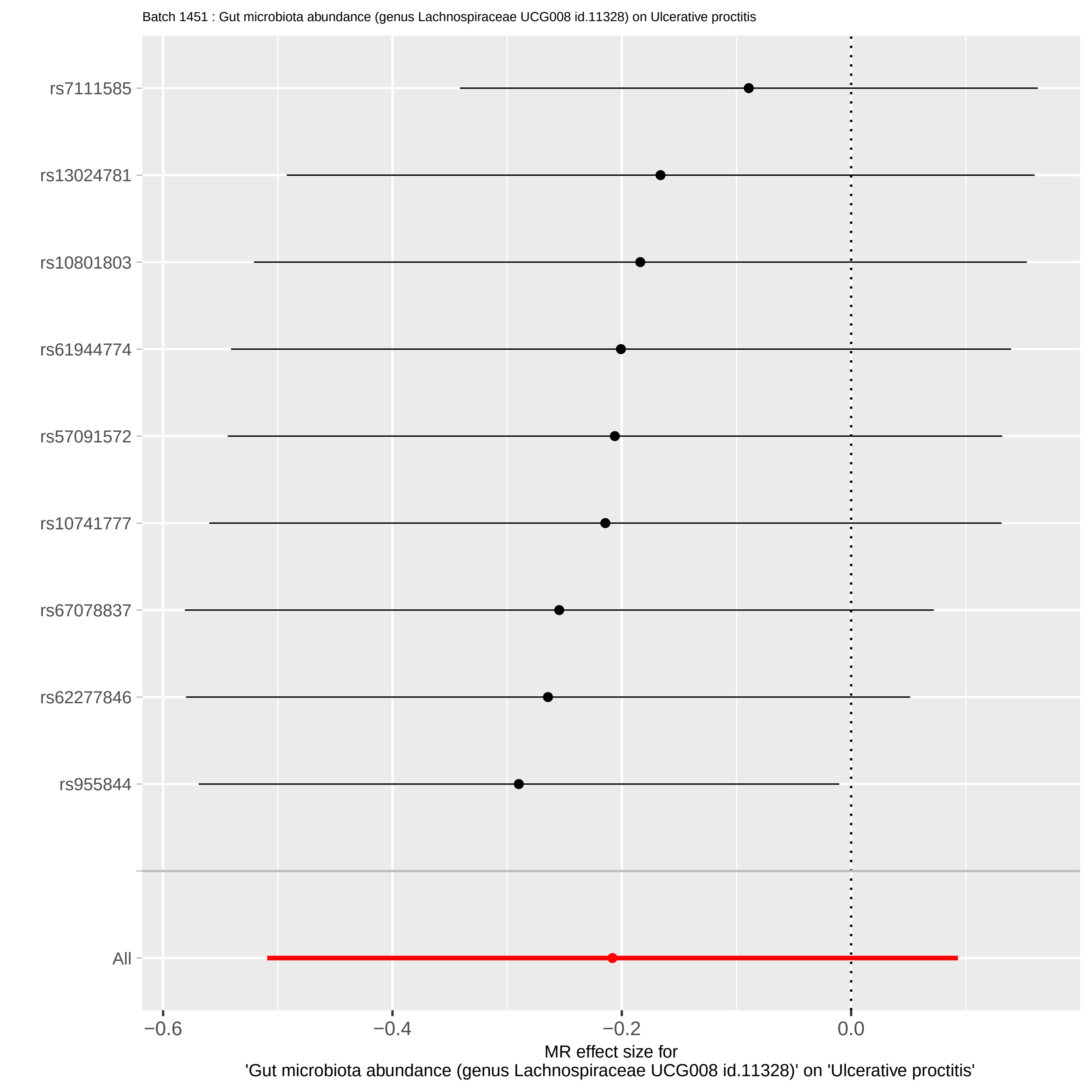
Batch 1451 : Gut microbiota abundance (genus Lachnospiraceae UCG008 id.11328) on Ulcerative proctitis
rs7111585
rs13024781
rs10801803
rs61944774
rs57091572
rs10741777
rs67078837
rs62277846
rs955844
All
−0.6
−0.4
−0.2
0.0
MR effect size for
'Gut microbiota abundance (genus Lachnospiraceae UCG008 id.11328)' on 'Ulcerative proctitis'

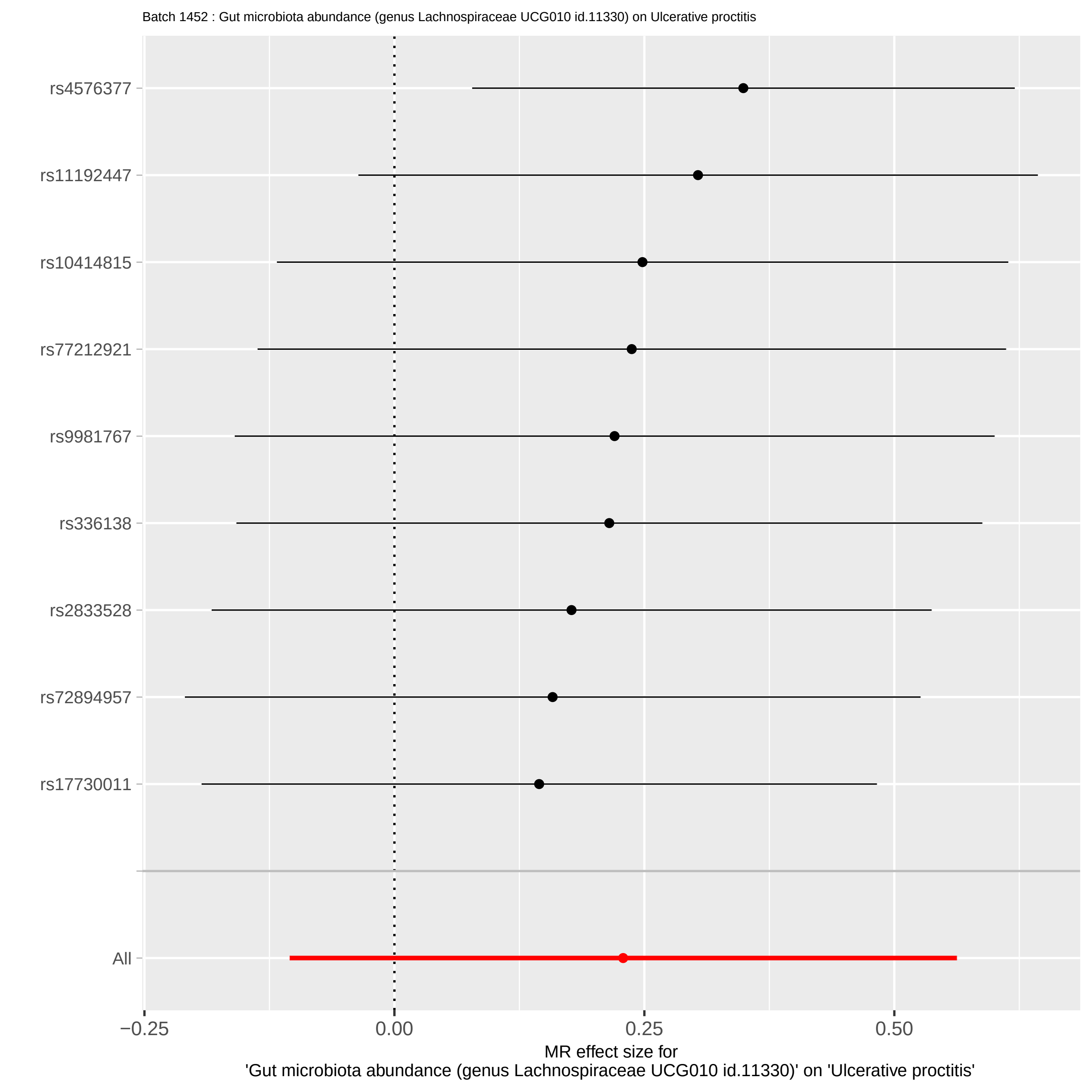

Batch 1452 : Gut microbiota abundance (genus Lachnospiraceae UCG010 id.11330) on Ulcerative proctitis
rs4576377
rs11192447
rs10414815
rs77212921
rs9981767
rs336138
rs2833528
rs72894957
rs17730011
All
−0.25
0.00
0.25
0.50
MR effect size for
'Gut microbiota abundance (genus Lachnospiraceae UCG010 id.11330)' on 'Ulcerative proctitis'

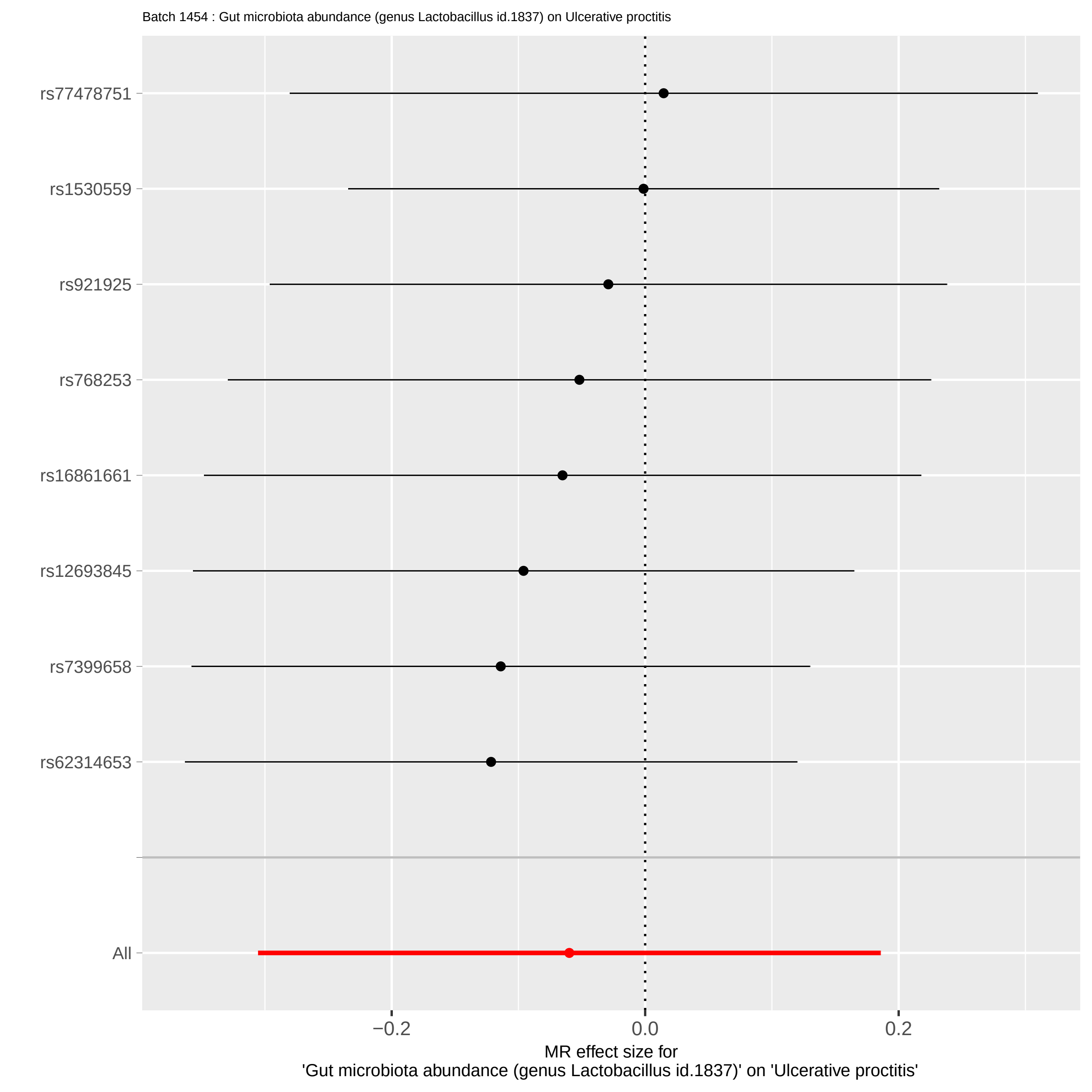
Batch 1454 : Gut microbiota abundance (genus Lactobacillus id.1837) on Ulcerative proctitis
rs77478751
rs1530559
rs921925
rs768253
rs16861661
rs12693845
rs7399658
rs62314653
All
−0.2
0.0
0.2
MR effect size for
'Gut microbiota abundance (genus Lactobacillus id.1837)' on 'Ulcerative proctitis'

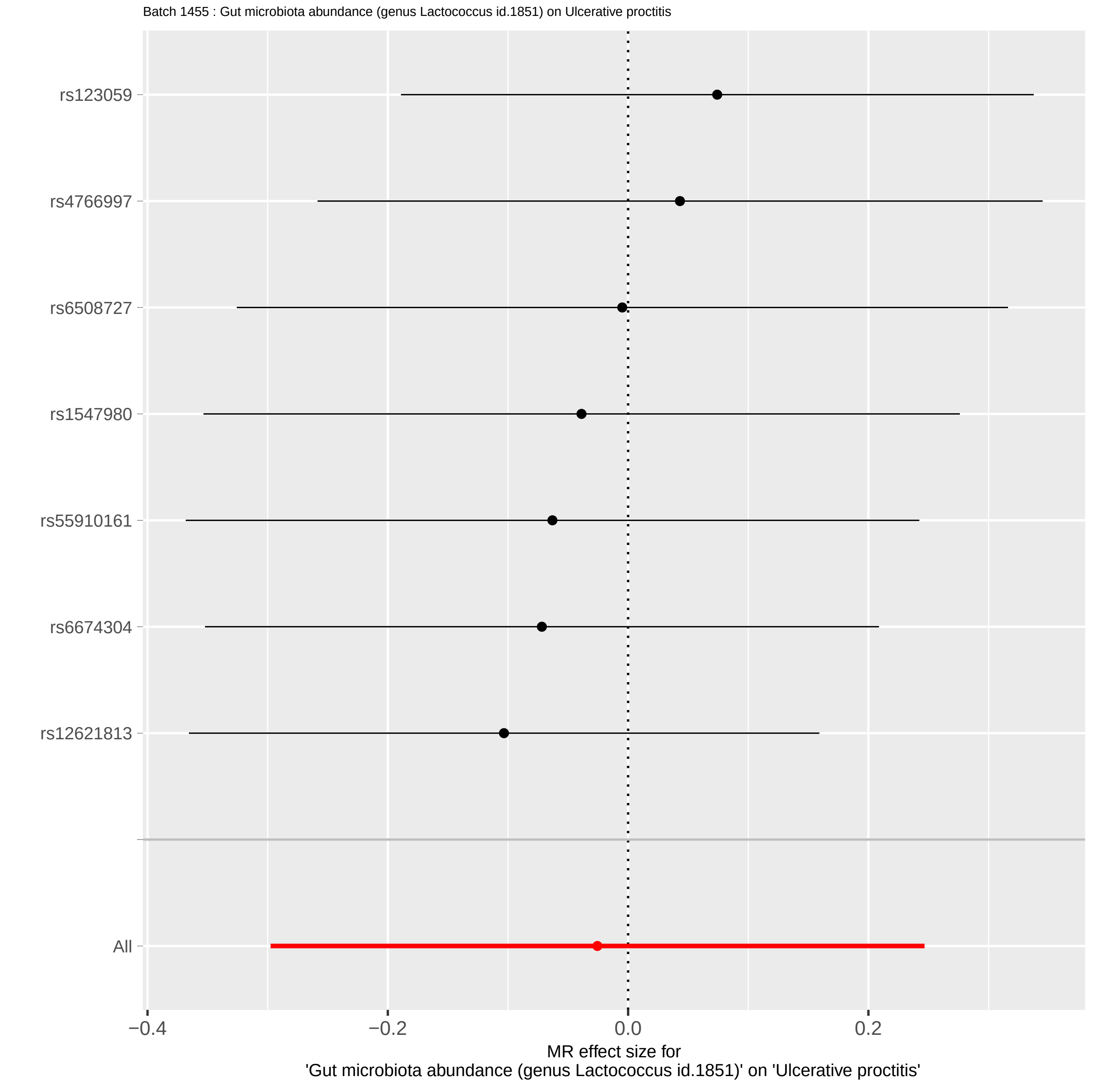
Batch 1455 : Gut microbiota abundance (genus Lactococcus id.1851) on Ulcerative proctitis
rs123059
rs4766997
rs6508727
rs1547980
rs55910161
rs6674304
rs12621813
All
−0.4
−0.2
0.0
0.2
MR effect size for
'Gut microbiota abundance (genus Lactococcus id.1851)' on 'Ulcerative proctitis'

Batch 1456 : Gut microbiota abundance (genus Marvinbryantia id.2005) on Ulcerative proctitis

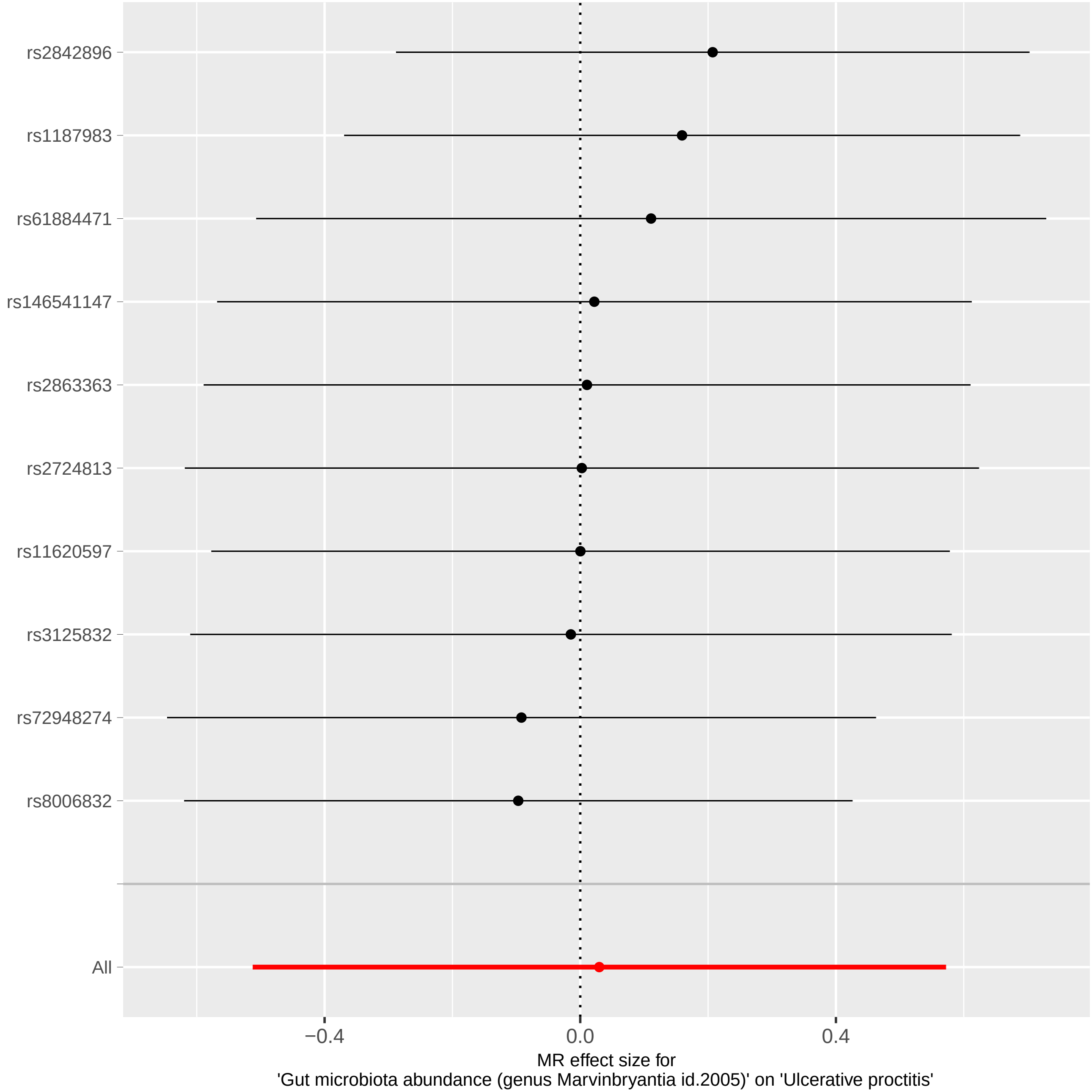

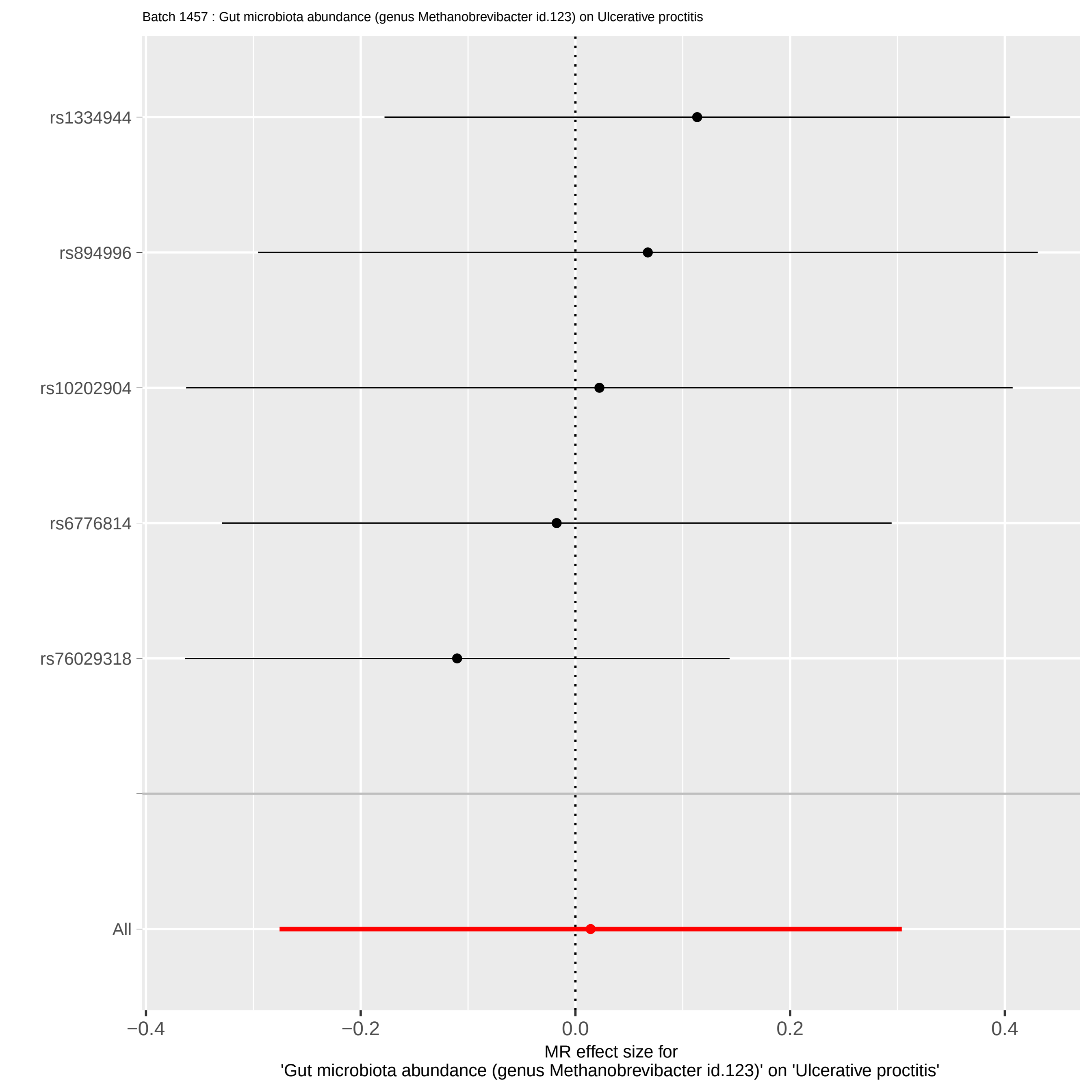
Batch 1457 : Gut microbiota abundance (genus Methanobrevibacter id.123) on Ulcerative proctitis
rs1334944
rs894996
rs10202904
rs6776814
rs76029318
All
−0.4
−0.2
0.0
0.2
0.4
MR effect size for
'Gut microbiota abundance (genus Methanobrevibacter id.123)' on 'Ulcerative proctitis'

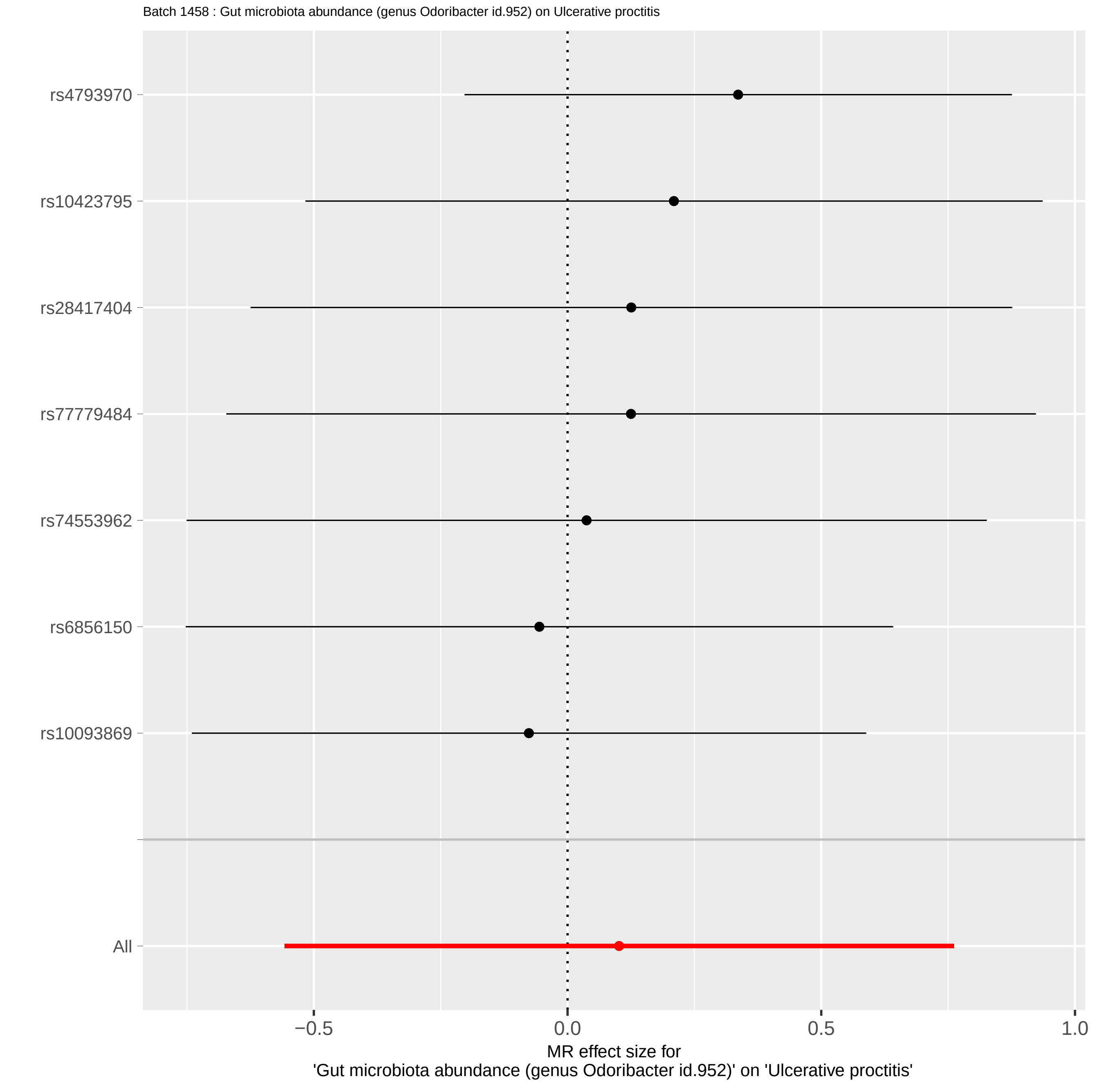
Batch 1458 : Gut microbiota abundance (genus Odoribacter id.952) on Ulcerative proctitis
rs4793970
rs10423795
rs28417404
rs77779484
rs74553962
rs6856150
rs10093869
All
−0.5
0.0
0.5
1.0
MR effect size for
'Gut microbiota abundance (genus Odoribacter id.952)' on 'Ulcerative proctitis'

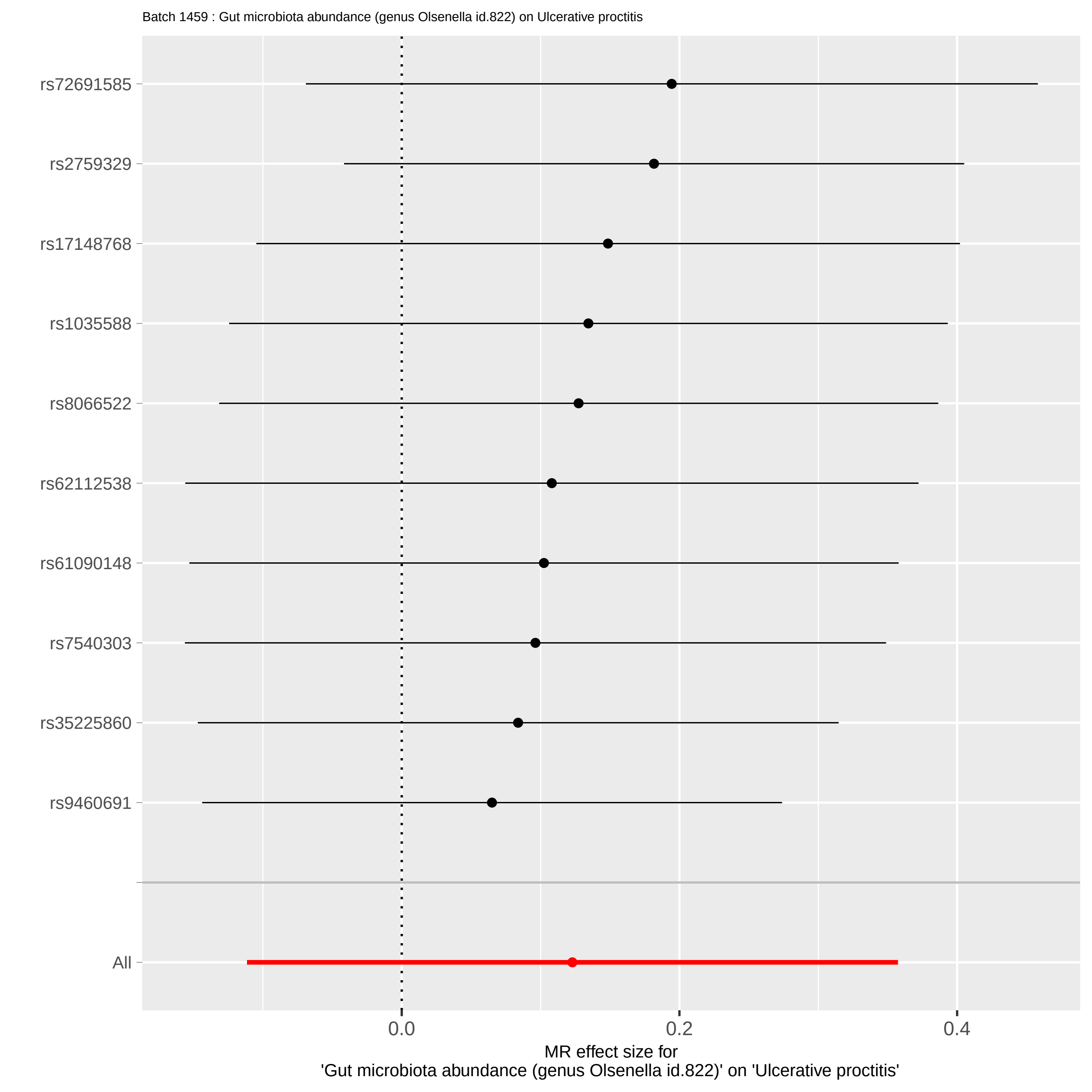
Batch 1459 : Gut microbiota abundance (genus Olsenella id.822) on Ulcerative proctitis
rs72691585
rs2759329
rs17148768
rs1035588
rs8066522
rs62112538
rs61090148
rs7540303
rs35225860
rs9460691
All
0.0
0.2
0.4
MR effect size for
'Gut microbiota abundance (genus Olsenella id.822)' on 'Ulcerative proctitis'

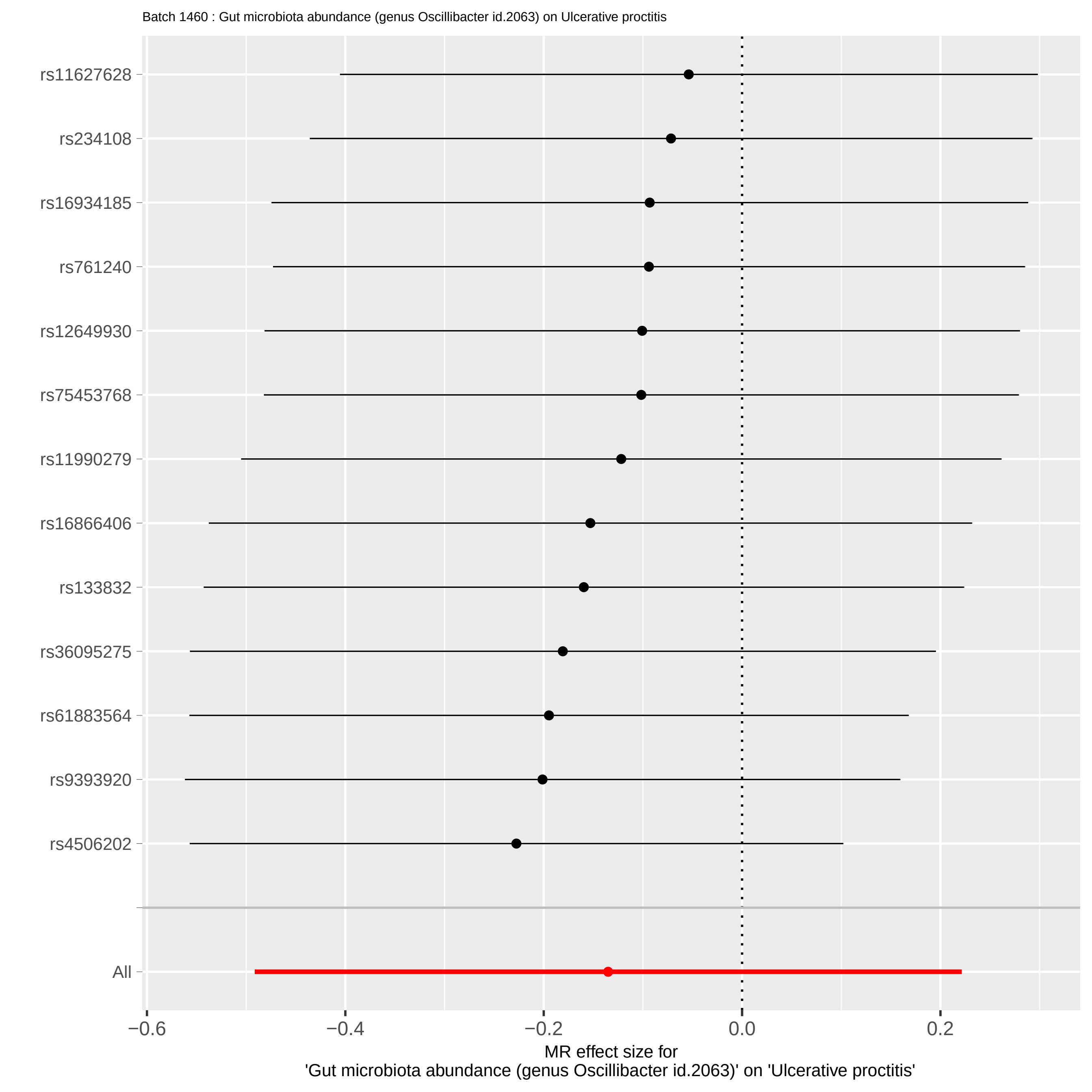

Batch 1460 : Gut microbiota abundance (genus Oscillibacter id.2063) on Ulcerative proctitis
rs11627628
rs234108
rs16934185
rs761240
rs12649930
rs75453768
rs11990279
rs16866406
rs133832
rs36095275
rs61883564
rs9393920
rs4506202
All
−0.6
−0.4
−0.2
0.0
0.2
MR effect size for
'Gut microbiota abundance (genus Oscillibacter id.2063)' on 'Ulcerative proctitis'

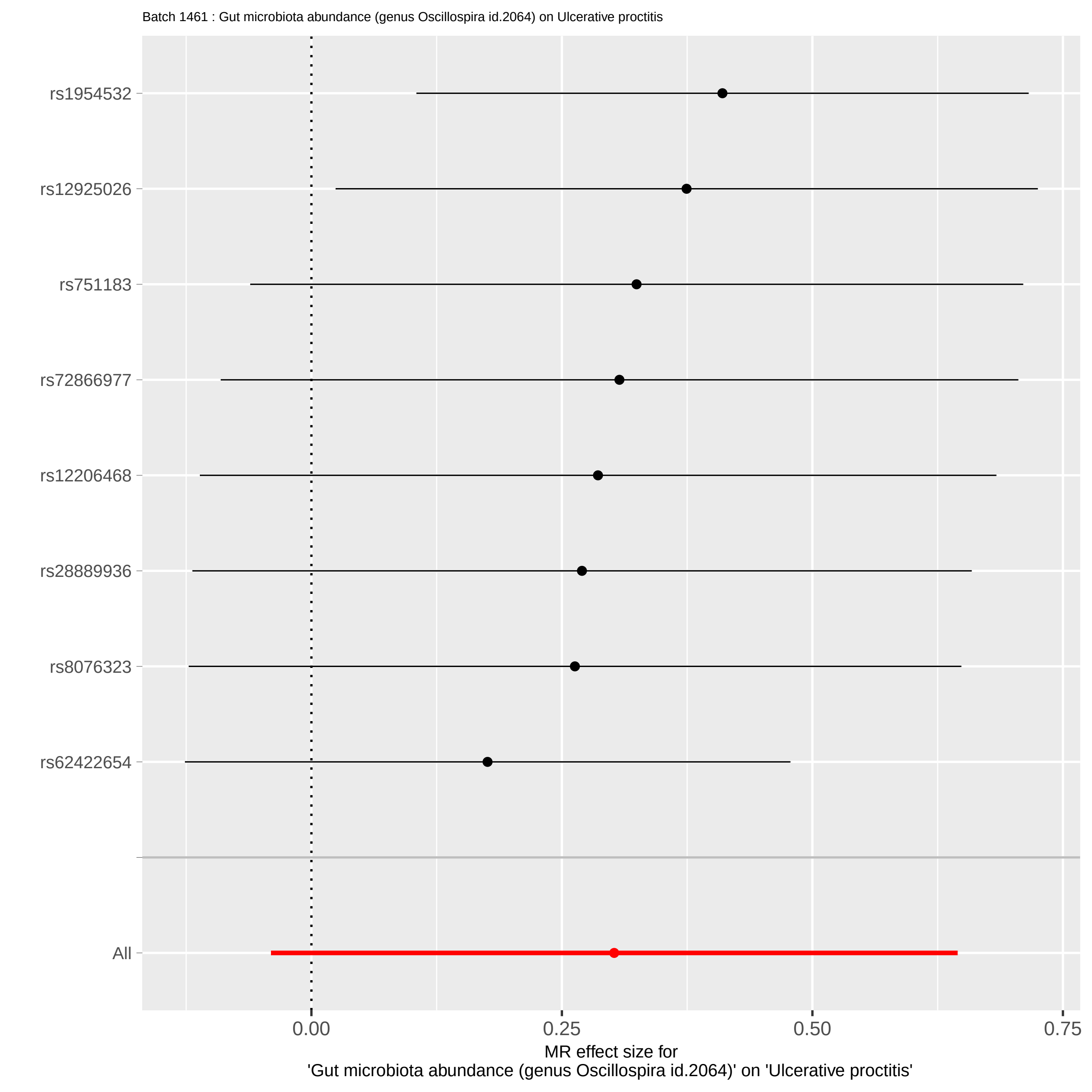
Batch 1461 : Gut microbiota abundance (genus Oscillospira id.2064) on Ulcerative proctitis
rs1954532
rs12925026
rs751183
rs72866977
rs12206468
rs28889936
rs8076323
rs62422654
All
0.00
0.25
0.50
0.75
MR effect size for
'Gut microbiota abundance (genus Oscillospira id.2064)' on 'Ulcerative proctitis'

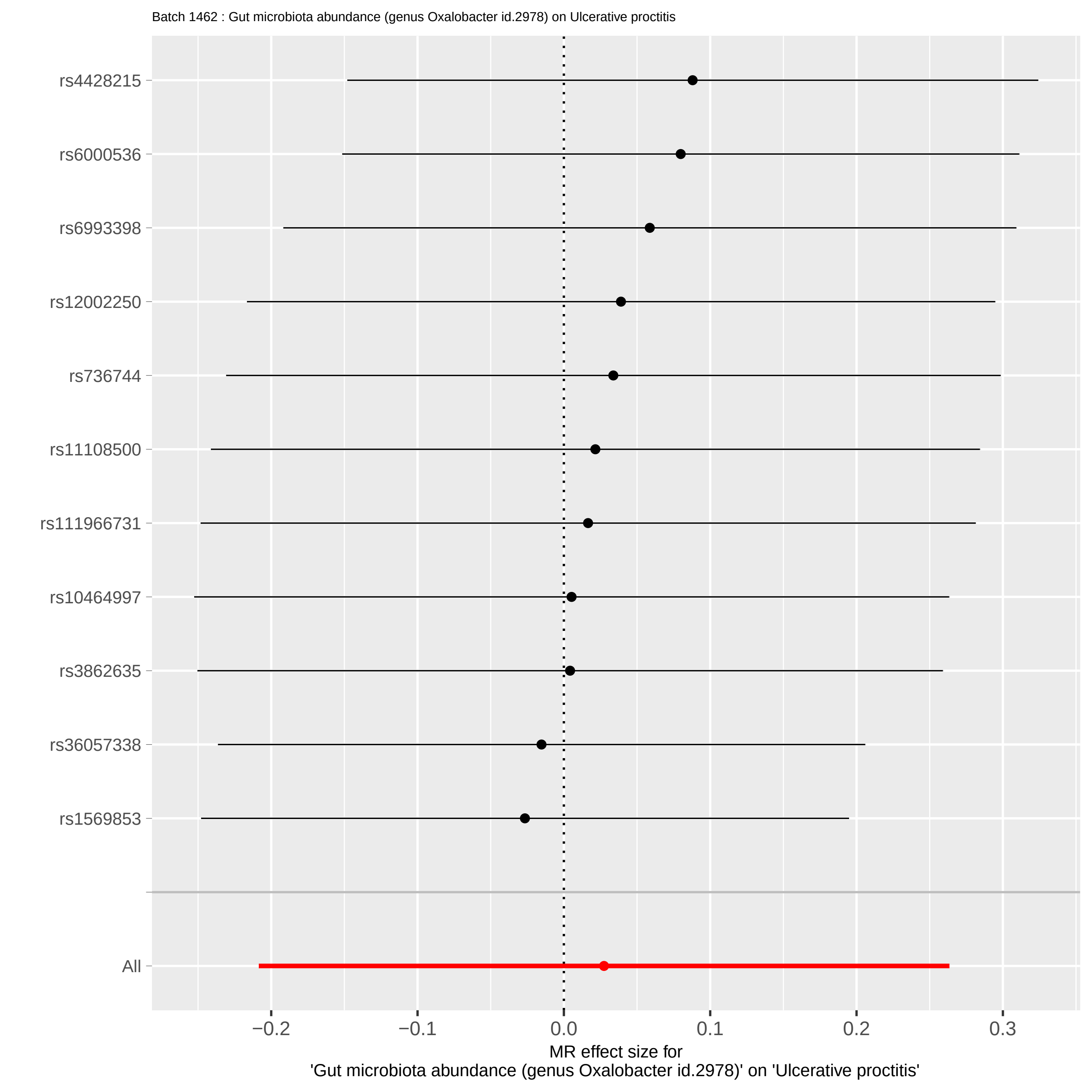
Batch 1462 : Gut microbiota abundance (genus Oxalobacter id.2978) on Ulcerative proctitis
rs4428215
rs6000536
rs6993398
rs12002250
rs736744
rs11108500
rs111966731
rs10464997
rs3862635
rs36057338
rs1569853
All
−0.2
−0.1
0.0
0.1
0.2
0.3
MR effect size for
'Gut microbiota abundance (genus Oxalobacter id.2978)' on 'Ulcerative proctitis'

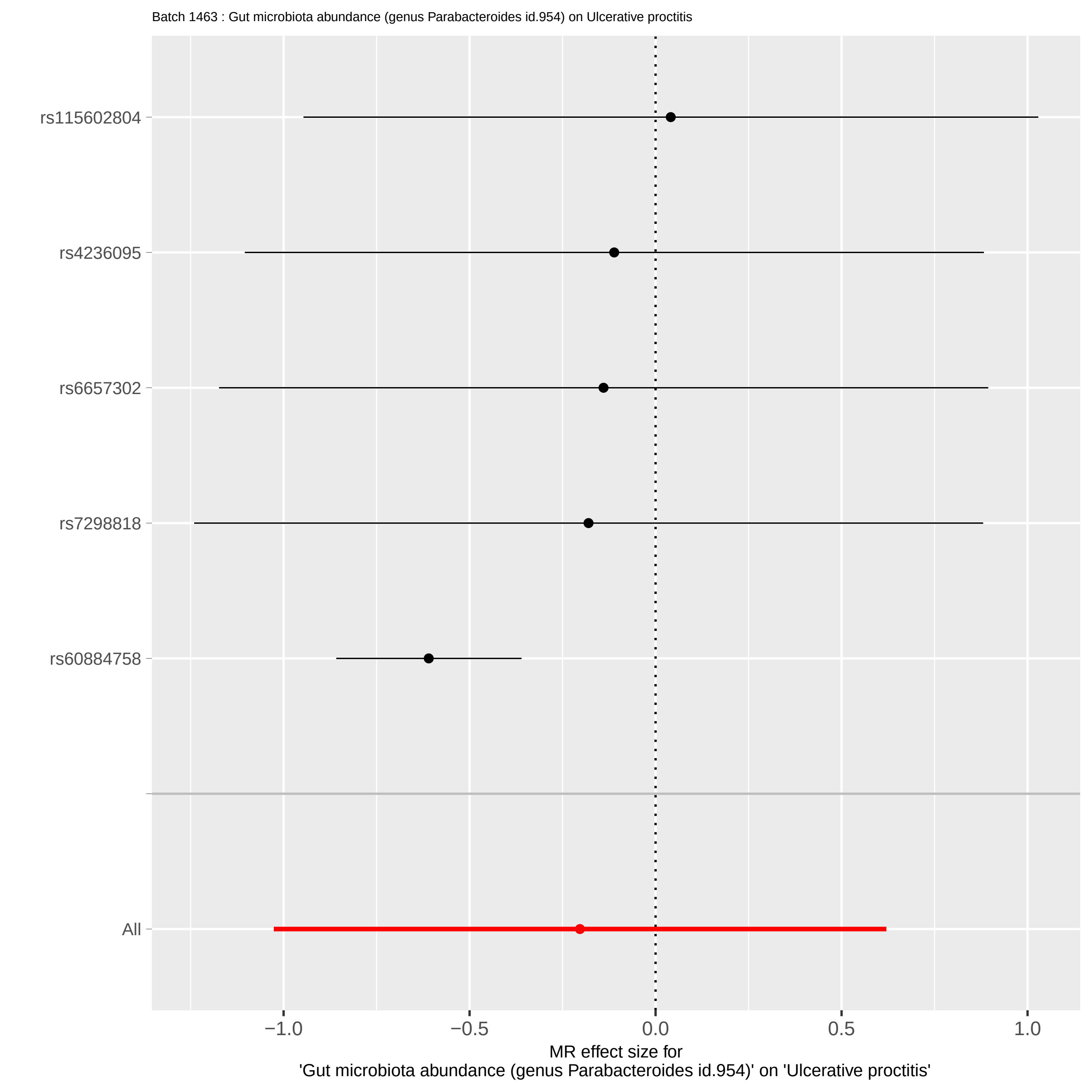
Batch 1463 : Gut microbiota abundance (genus Parabacteroides id.954) on Ulcerative proctitis
rs115602804
rs4236095
rs6657302
rs7298818
rs60884758
All
−1.0
−0.5
0.0
0.5
1.0
MR effect size for
'Gut microbiota abundance (genus Parabacteroides id.954)' on 'Ulcerative proctitis'

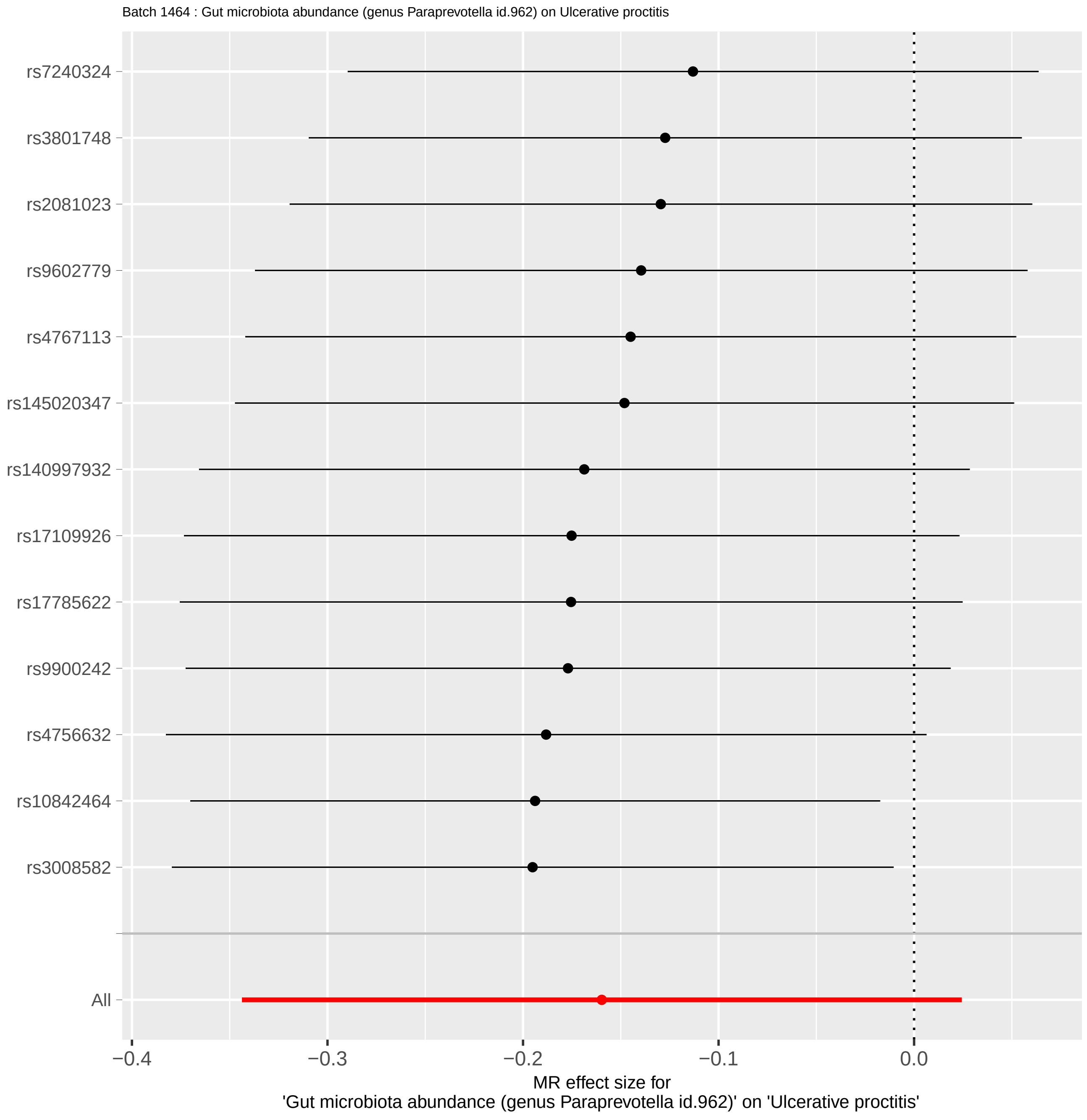
Batch 1464 : Gut microbiota abundance (genus Paraprevotella id.962) on Ulcerative proctitis
rs7240324
rs3801748
rs2081023
rs9602779
rs4767113
rs145020347
rs140997932
rs17109926
rs17785622
rs9900242
rs4756632
rs10842464
rs3008582
All
−0.4
−0.3
−0.2
−0.1
0.0
MR effect size for
'Gut microbiota abundance (genus Paraprevotella id.962)' on 'Ulcerative proctitis'

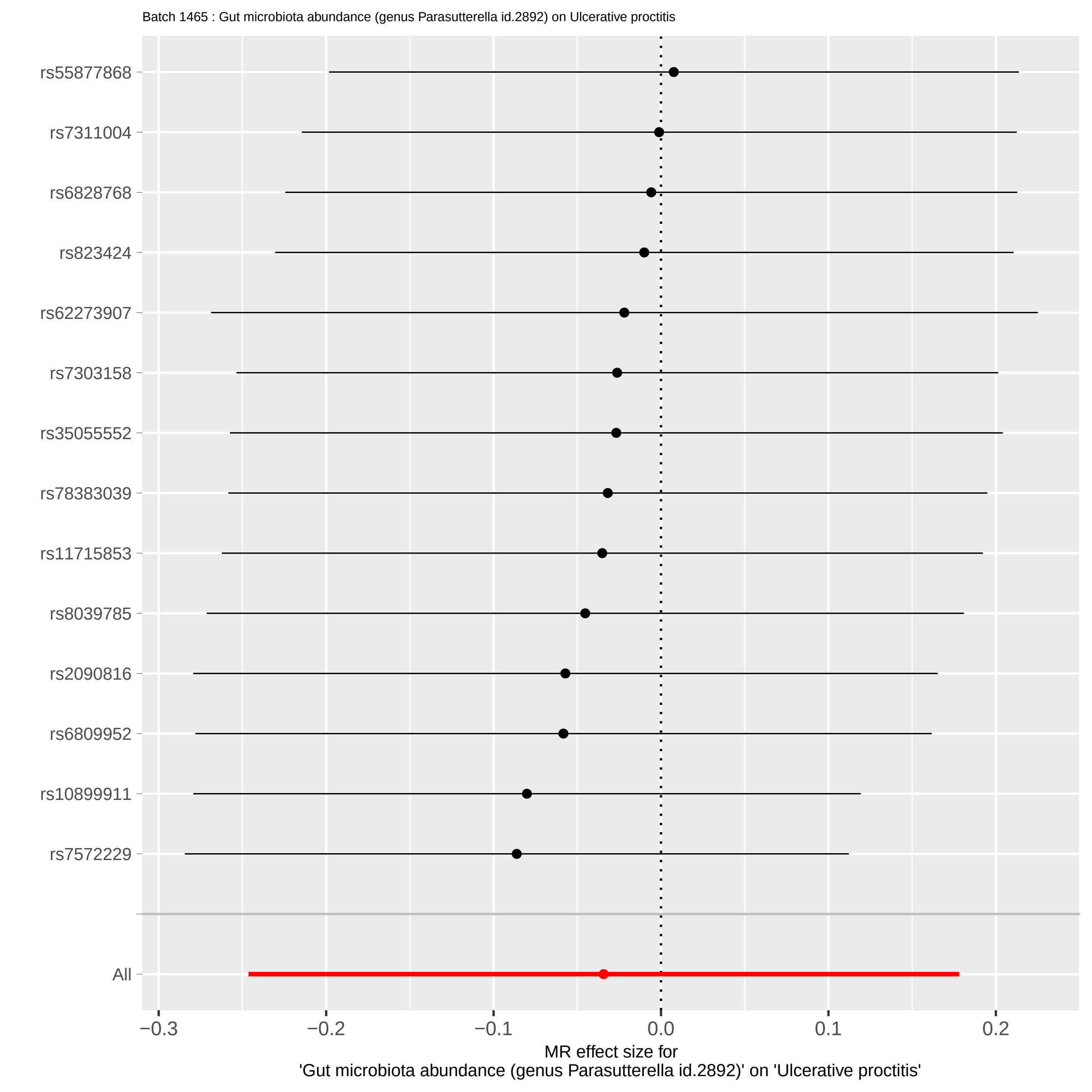

Batch 1465 : Gut microbiota abundance (genus Parasutterella id.2892) on Ulcerative proctitis
rs55877868
rs7311004
rs6828768
rs823424
rs62273907
rs7303158
rs35055552
rs78383039
rs11715853
rs8039785
rs2090816
rs6809952
rs10899911
rs7572229
All
−0.3
−0.2
−0.1
0.0
0.1
0.2
MR effect size for
'Gut microbiota abundance (genus Parasutterella id.2892)' on 'Ulcerative proctitis'

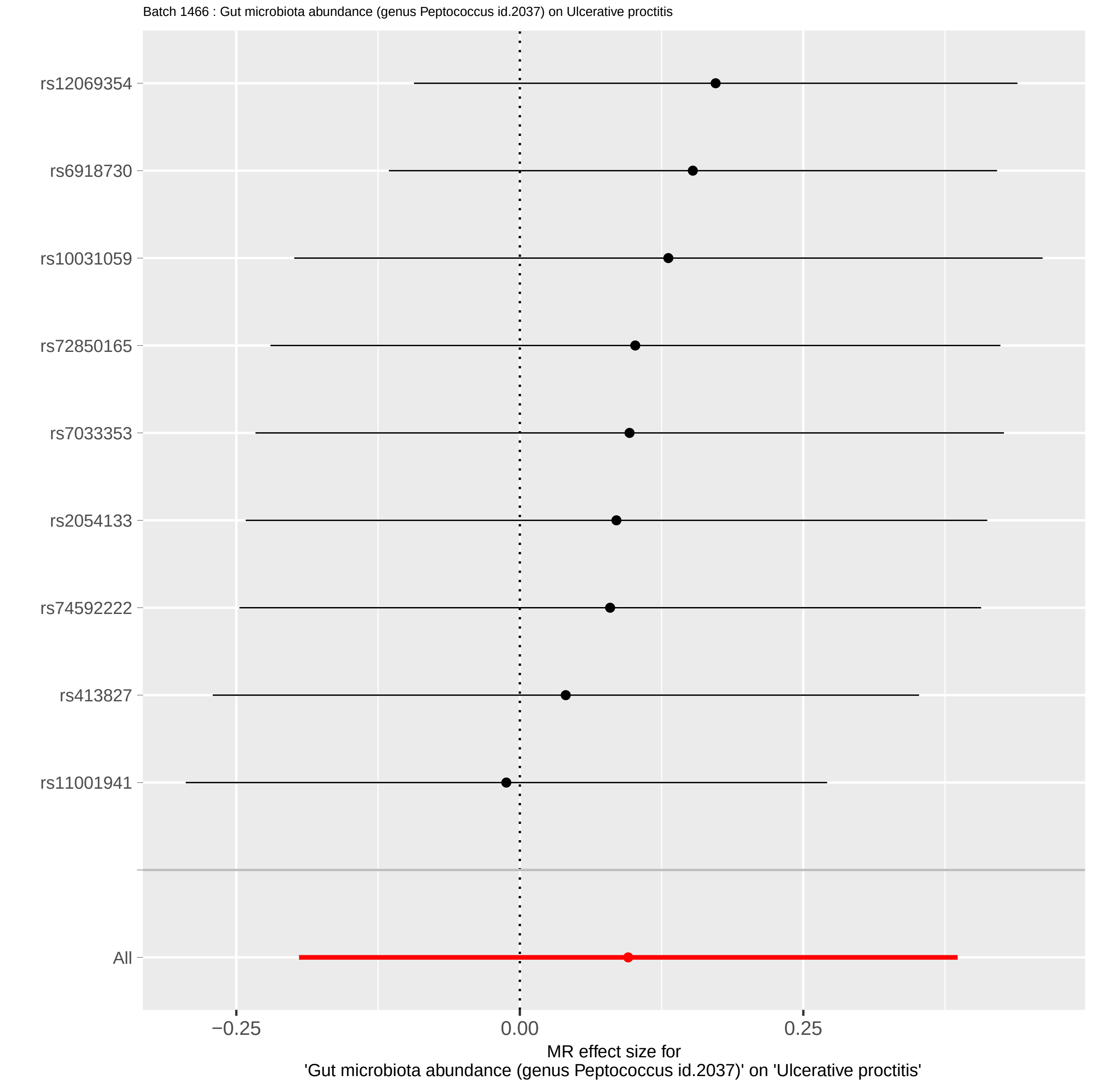
Batch 1466 : Gut microbiota abundance (genus Peptococcus id.2037) on Ulcerative proctitis
rs12069354
rs6918730
rs10031059
rs72850165
rs7033353
rs2054133
rs74592222
rs413827
rs11001941
All
−0.25
0.00
0.25
MR effect size for
'Gut microbiota abundance (genus Peptococcus id.2037)' on 'Ulcerative proctitis'

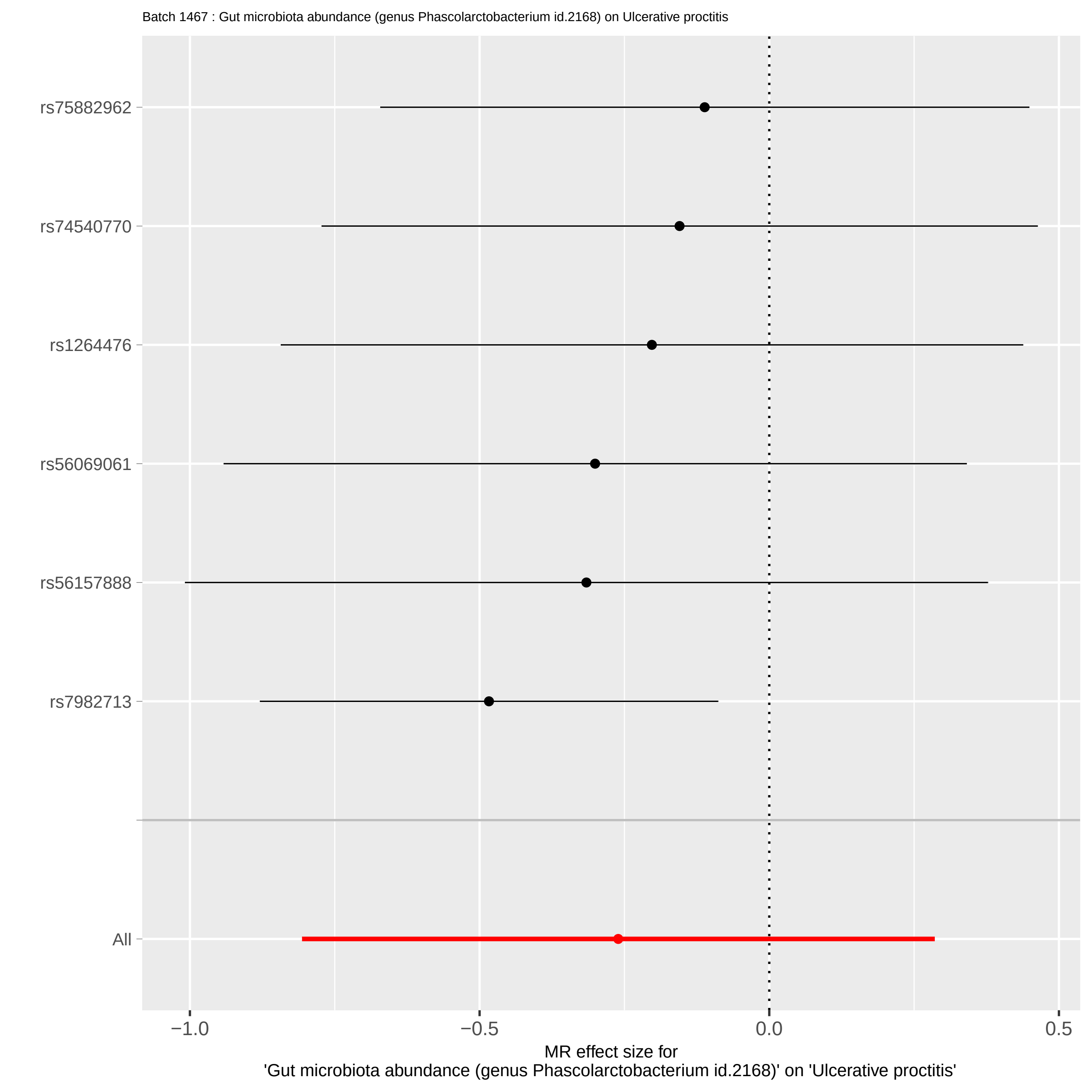

Batch 1467 : Gut microbiota abundance (genus Phascolarctobacterium id.2168) on Ulcerative proctitis
rs75882962
rs74540770
rs1264476
rs56069061
rs56157888
rs7982713
All
−1.0
−0.5
0.0
0.5
MR effect size for
'Gut microbiota abundance (genus Phascolarctobacterium id.2168)' on 'Ulcerative proctitis'

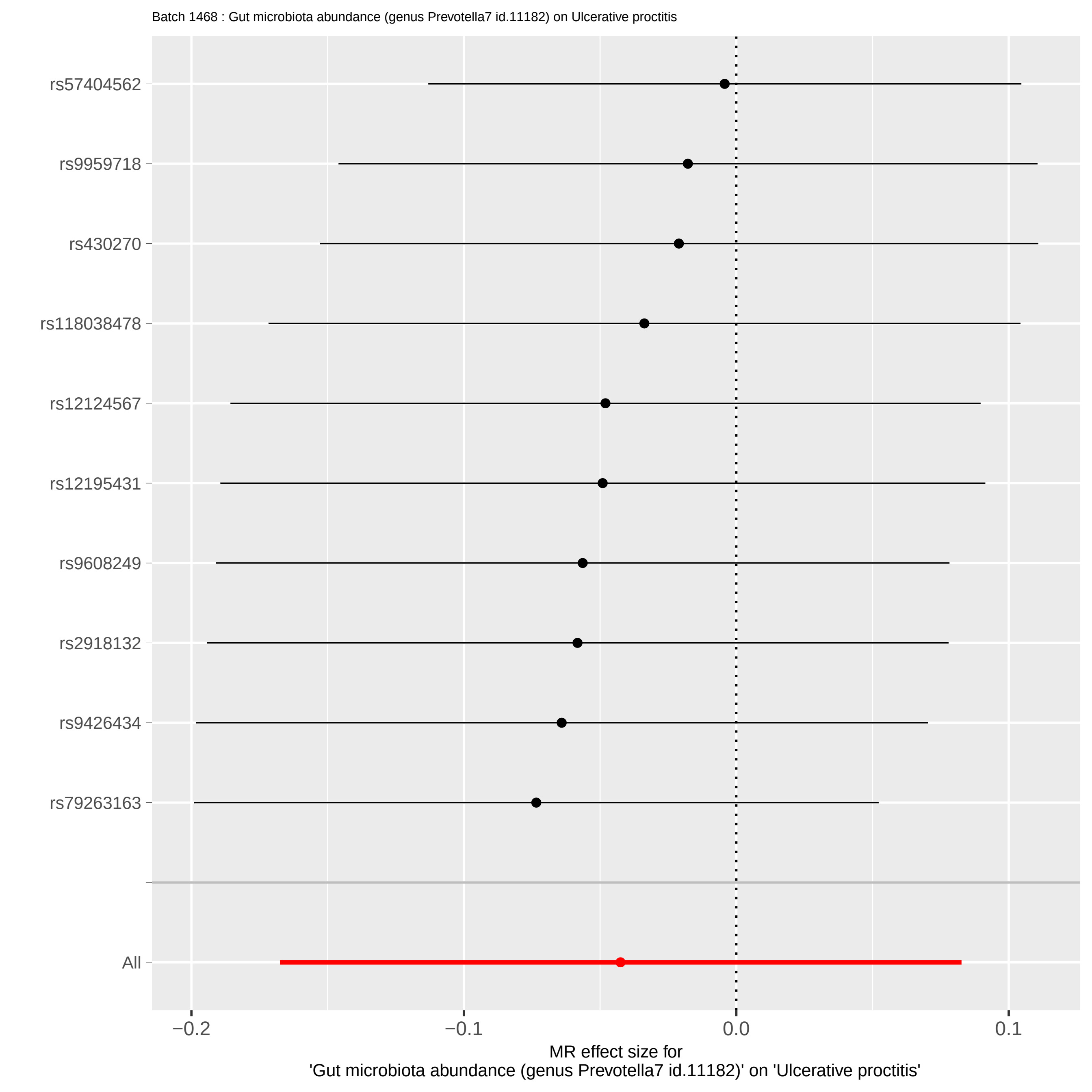

Batch 1468 : Gut microbiota abundance (genus Prevotella7 id.11182) on Ulcerative proctitis
rs57404562
rs9959718
rs430270
rs118038478
rs12124567
rs12195431
rs9608249
rs2918132
rs9426434
rs79263163
All
−0.2
−0.1
0.0
0.1
MR effect size for
'Gut microbiota abundance (genus Prevotella7 id.11182)' on 'Ulcerative proctitis'

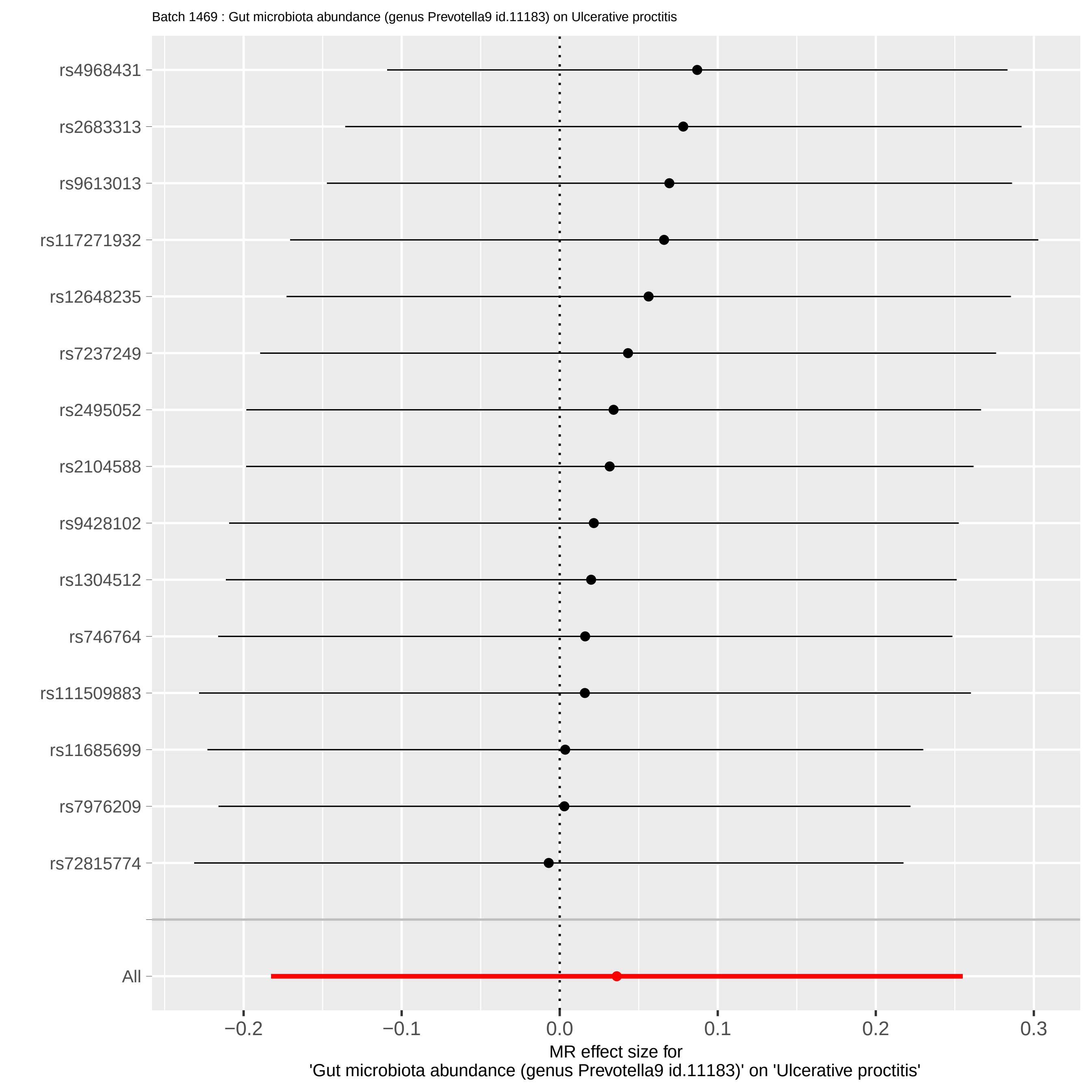

Batch 1469 : Gut microbiota abundance (genus Prevotella9 id.11183) on Ulcerative proctitis
rs4968431
rs2683313
rs9613013
rs117271932
rs12648235
rs7237249
rs2495052
rs2104588
rs9428102
rs1304512
rs746764
rs111509883
rs11685699
rs7976209
rs72815774
All
−0.2
−0.1
0.0
0.1
0.2
0.3
MR effect size for
'Gut microbiota abundance (genus Prevotella9 id.11183)' on 'Ulcerative proctitis'

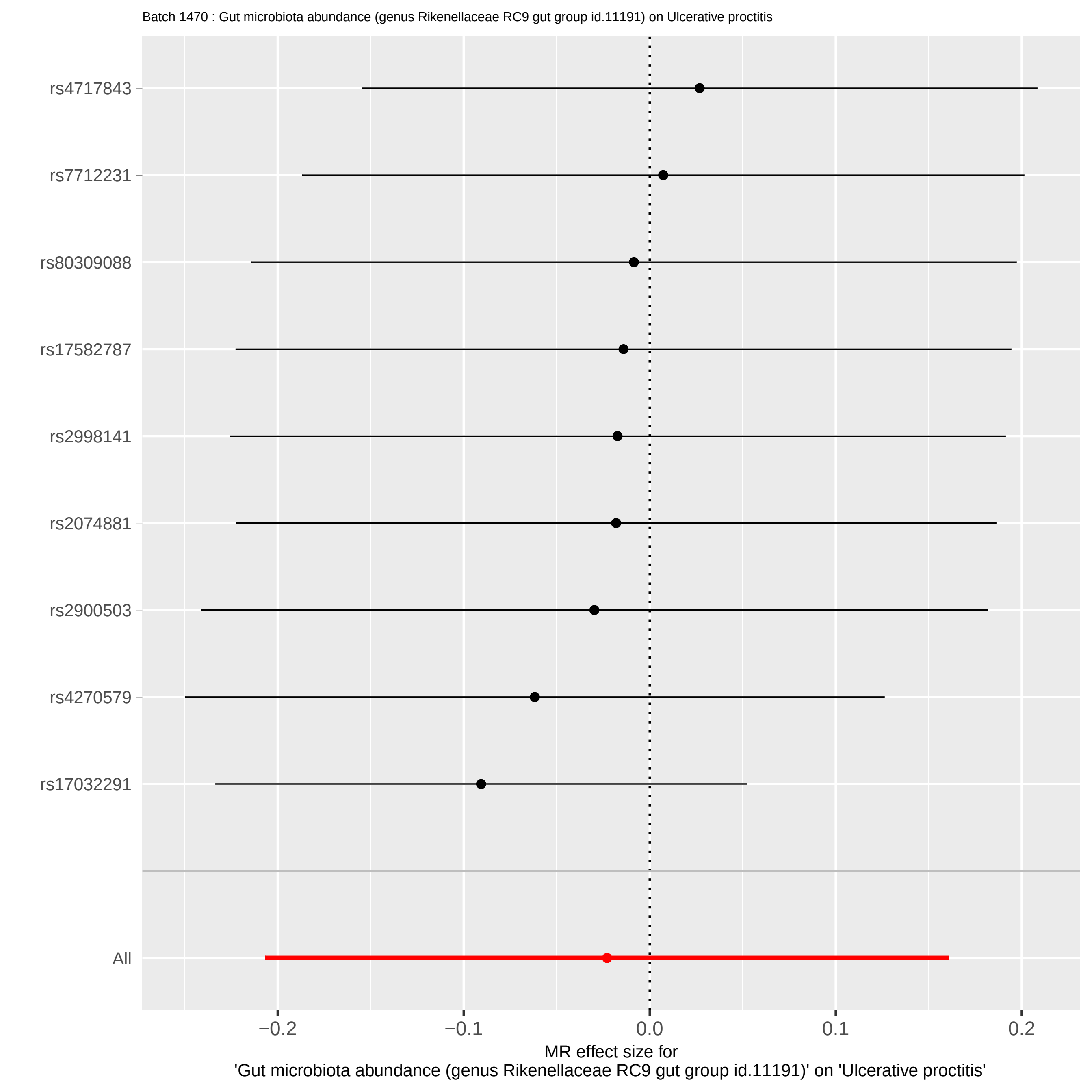
Batch 1470 : Gut microbiota abundance (genus Rikenellaceae RC9 gut group id.11191) on Ulcerative proctitis
rs4717843
rs7712231
rs80309088
rs17582787
rs2998141
rs2074881
rs2900503
rs4270579
rs17032291
All
−0.2
−0.1
0.0
0.1
0.2
MR effect size for
'Gut microbiota abundance (genus Rikenellaceae RC9 gut group id.11191)' on 'Ulcerative proctitis'

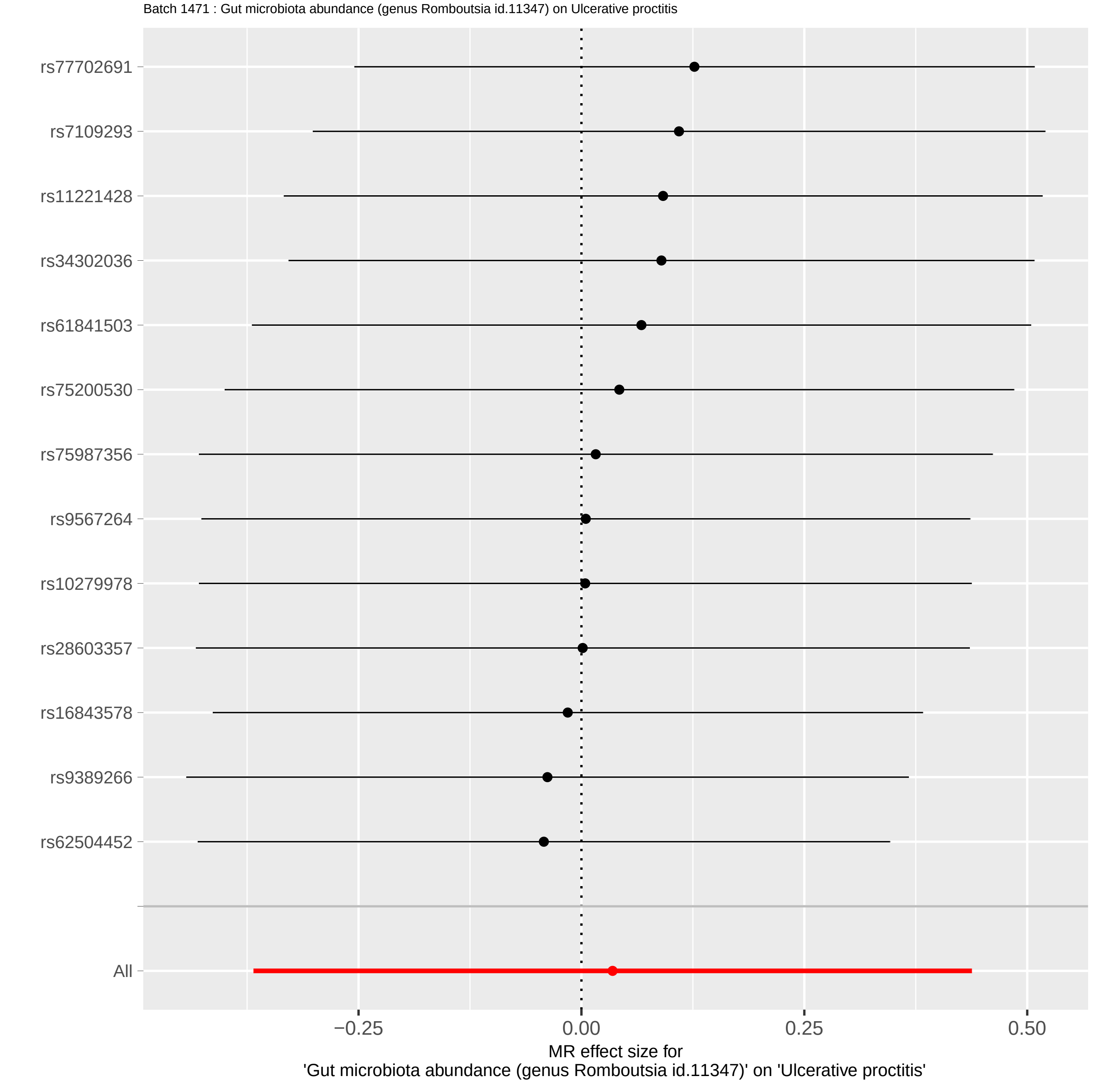

Batch 1471 : Gut microbiota abundance (genus Romboutsia id.11347) on Ulcerative proctitis
rs77702691
rs7109293
rs11221428
rs34302036
rs61841503
rs75200530
rs75987356
rs9567264
rs10279978
rs28603357
rs16843578
rs9389266
rs62504452
All
−0.25
0.00
0.25
0.50
MR effect size for
'Gut microbiota abundance (genus Romboutsia id.11347)' on 'Ulcerative proctitis'

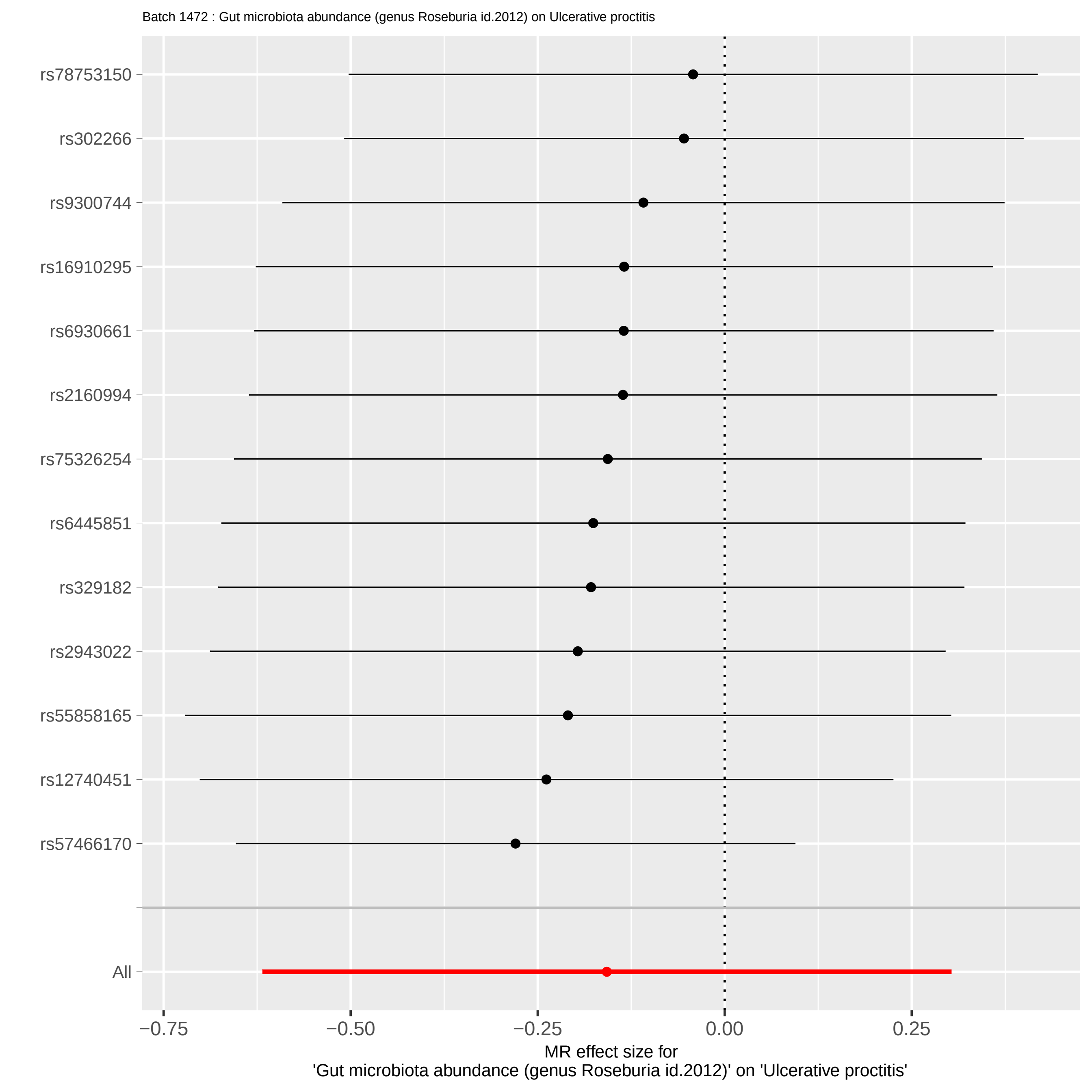

Batch 1472 : Gut microbiota abundance (genus Roseburia id.2012) on Ulcerative proctitis
rs78753150
rs302266
rs9300744
rs16910295
rs6930661
rs2160994
rs75326254
rs6445851
rs329182
rs2943022
rs55858165
rs12740451
rs57466170
All
−0.75
−0.50
−0.25
0.00
0.25
MR effect size for
'Gut microbiota abundance (genus Roseburia id.2012)' on 'Ulcerative proctitis'

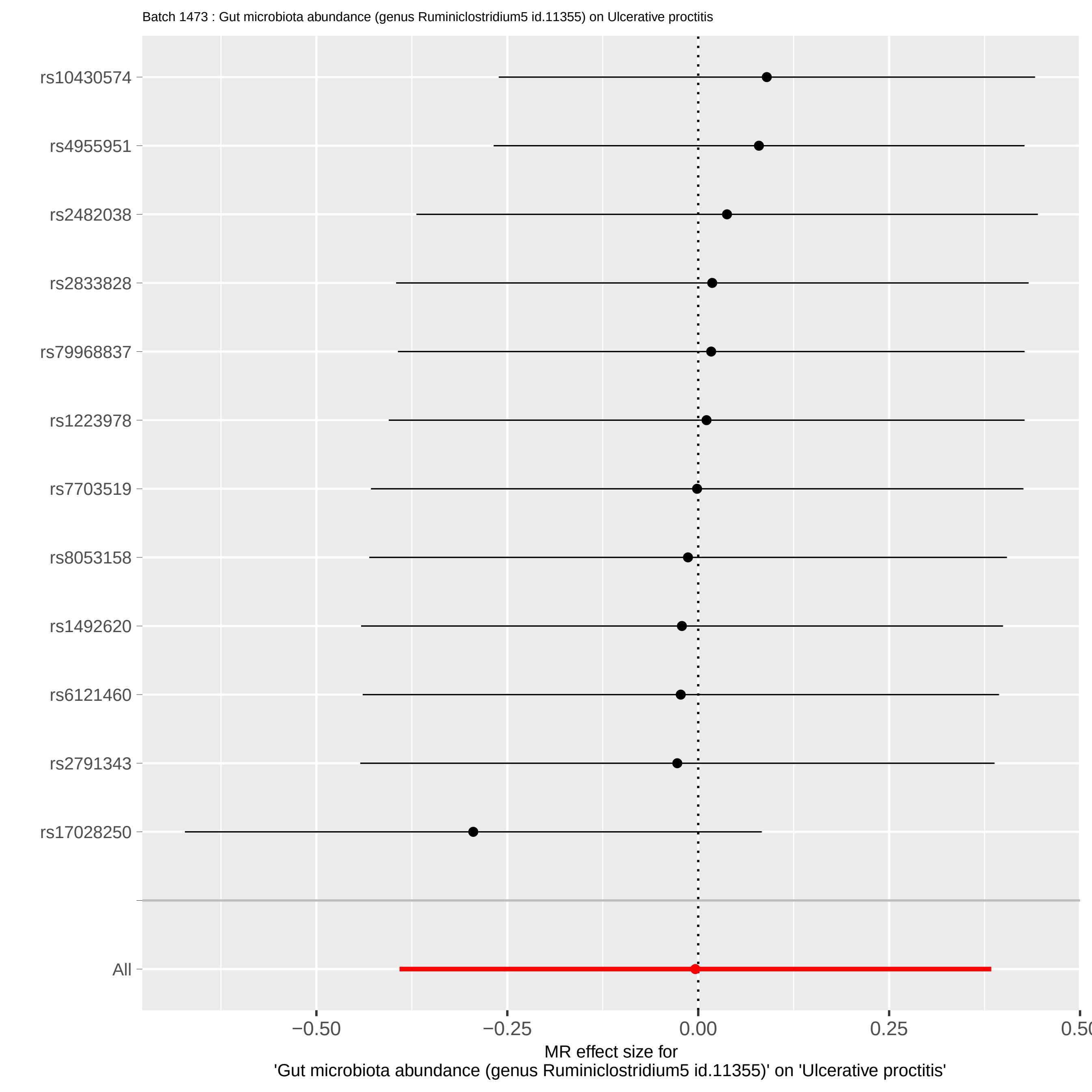
Batch 1473 : Gut microbiota abundance (genus Ruminiclostridium5 id.11355) on Ulcerative proctitis
rs10430574
rs4955951
rs2482038
rs2833828
rs79968837
rs1223978
rs7703519
rs8053158
rs1492620
rs6121460
rs2791343
rs17028250
All
−0.50
−0.25
0.00
0.25
0.50
MR effect size for
'Gut microbiota abundance (genus Ruminiclostridium5 id.11355)' on 'Ulcerative proctitis'

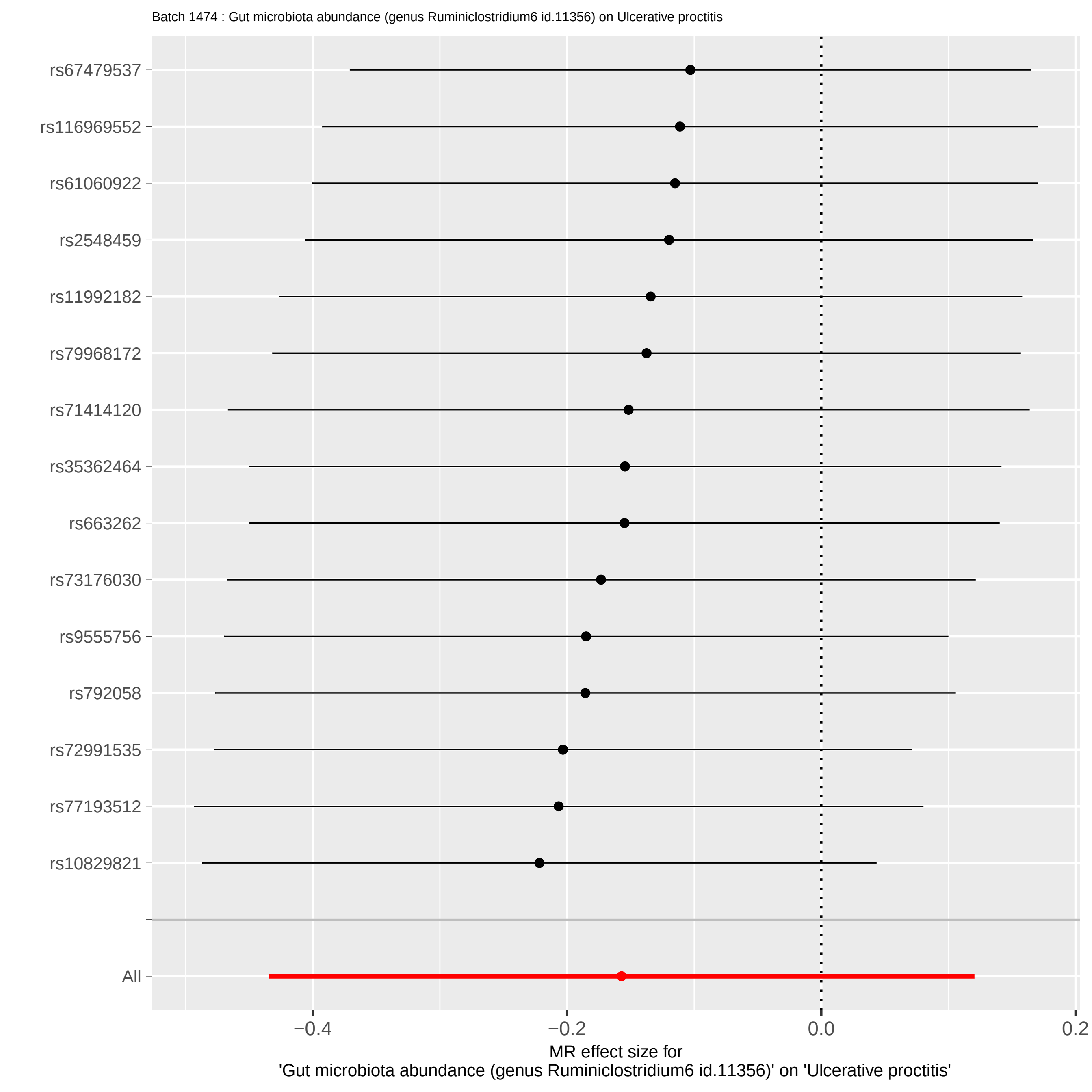
Batch 1474 : Gut microbiota abundance (genus Ruminiclostridium6 id.11356) on Ulcerative proctitis
rs67479537
rs116969552
rs61060922
rs2548459
rs11992182
rs79968172
rs71414120
rs35362464
rs663262
rs73176030
rs9555756
rs792058
rs72991535
rs77193512
rs10829821
All
−0.4
−0.2
0.0
0.2
MR effect size for
'Gut microbiota abundance (genus Ruminiclostridium6 id.11356)' on 'Ulcerative proctitis'

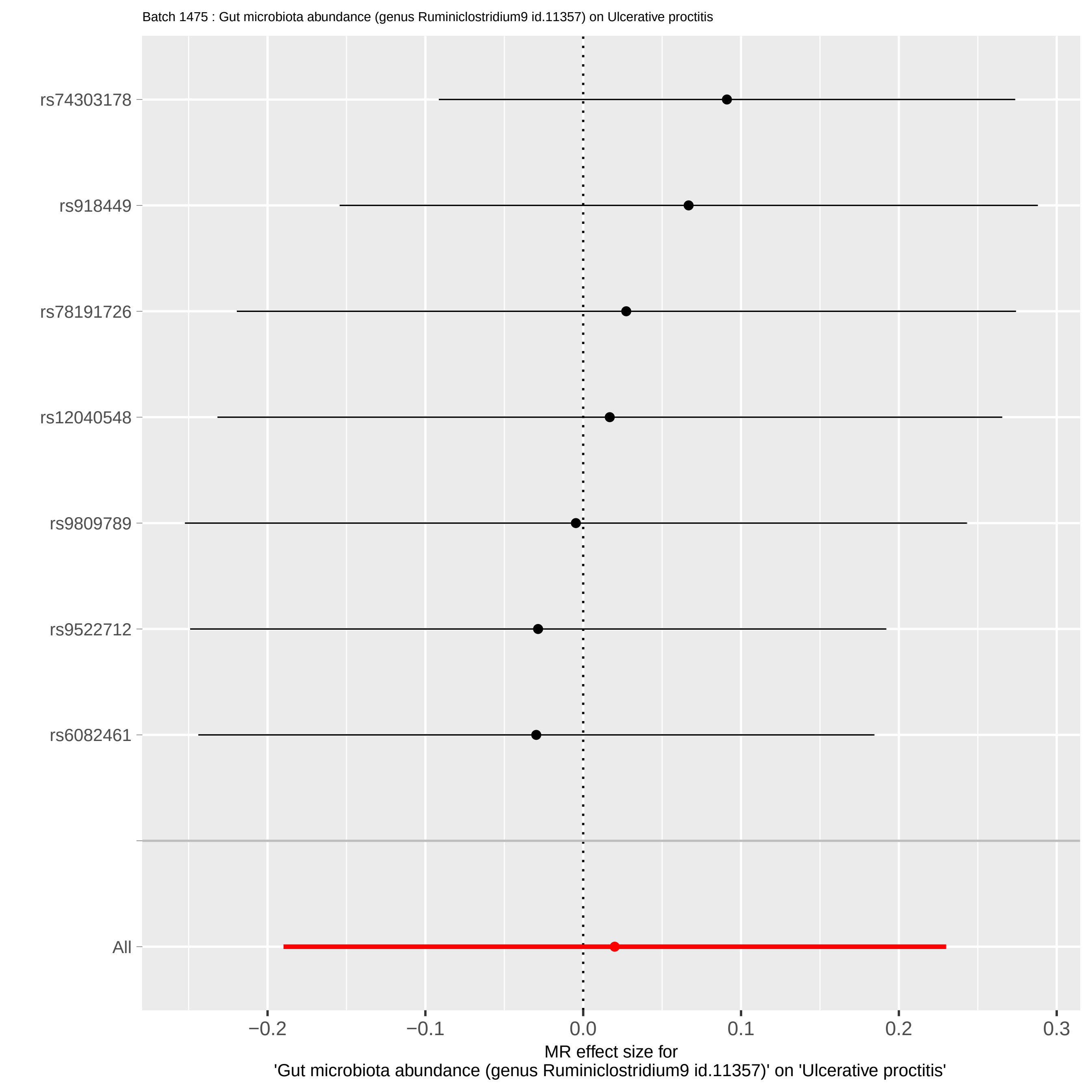

Batch 1475 : Gut microbiota abundance (genus Ruminiclostridium9 id.11357) on Ulcerative proctitis
rs74303178
rs918449
rs78191726
rs12040548
rs9809789
rs9522712
rs6082461
All
−0.2
−0.1
0.0
0.1
0.2
0.3
MR effect size for
'Gut microbiota abundance (genus Ruminiclostridium9 id.11357)' on 'Ulcerative proctitis'

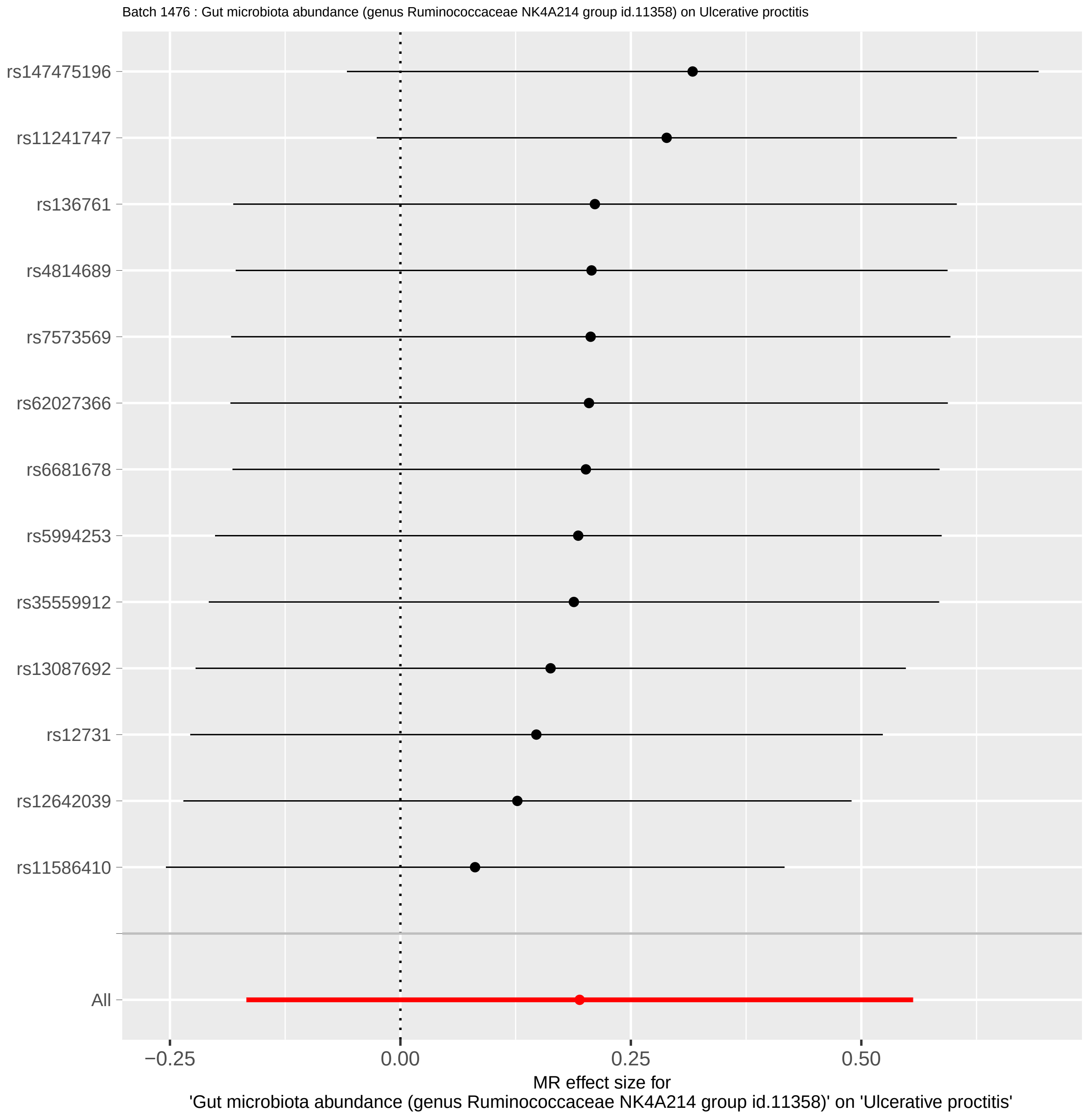

Batch 1476 : Gut microbiota abundance (genus Ruminococcaceae NK4A214 group id.11358) on Ulcerative proctitis
rs147475196
rs11241747
rs136761
rs4814689
rs7573569
rs62027366
rs6681678
rs5994253
rs35559912
rs13087692
rs12731
rs12642039
rs11586410
All
−0.25
0.00
0.25
0.50
MR effect size for
'Gut microbiota abundance (genus Ruminococcaceae NK4A214 group id.11358)' on 'Ulcerative proctitis'

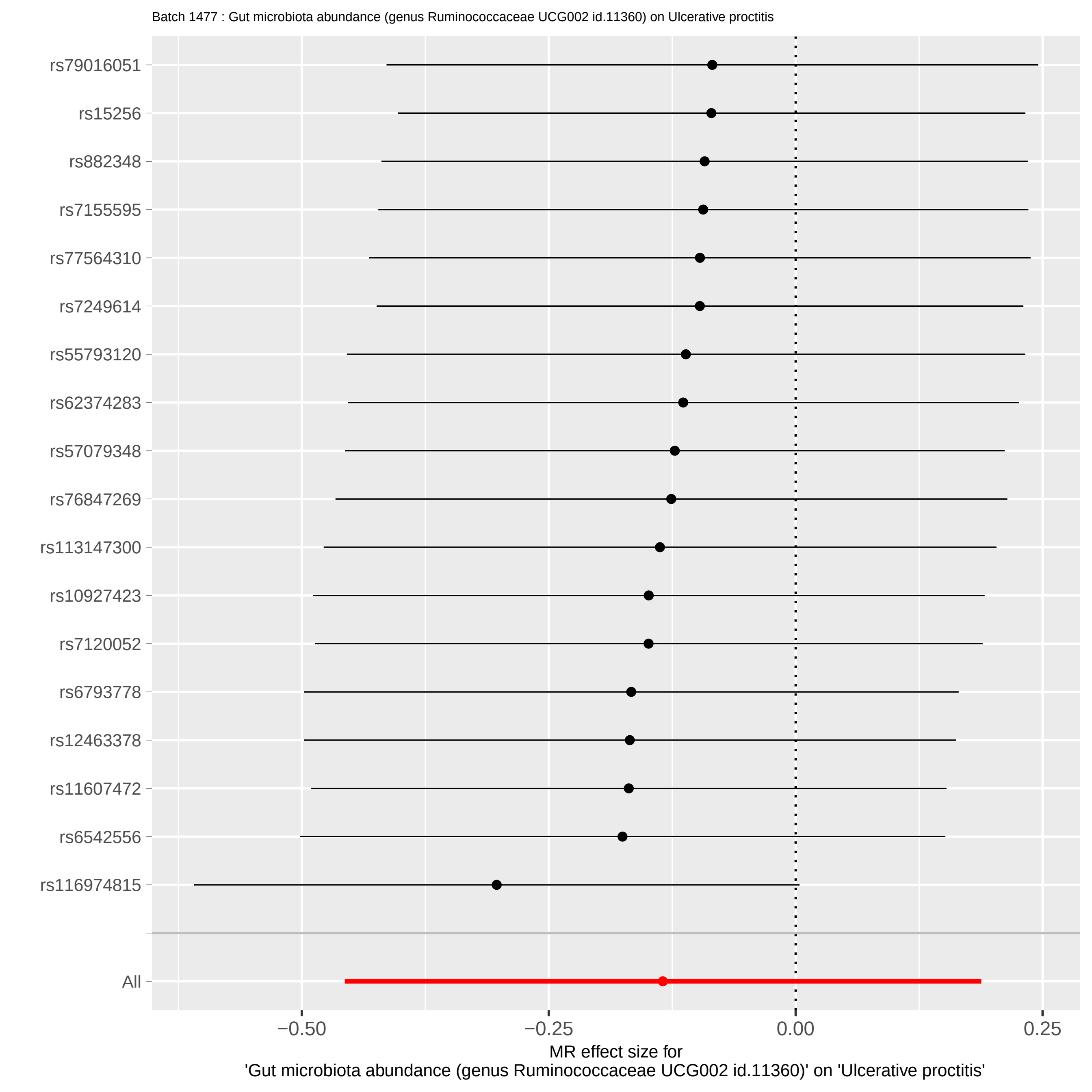

Batch 1477 : Gut microbiota abundance (genus Ruminococcaceae UCG002 id.11360) on Ulcerative proctitis
rs79016051
rs15256
rs882348
rs7155595
rs77564310
rs7249614
rs55793120
rs62374283
rs57079348
rs76847269
rs113147300
rs10927423
rs7120052
rs6793778
rs12463378
rs11607472
rs6542556
rs116974815
All
−0.50
−0.25
0.00
0.25
MR effect size for
'Gut microbiota abundance (genus Ruminococcaceae UCG002 id.11360)' on 'Ulcerative proctitis'

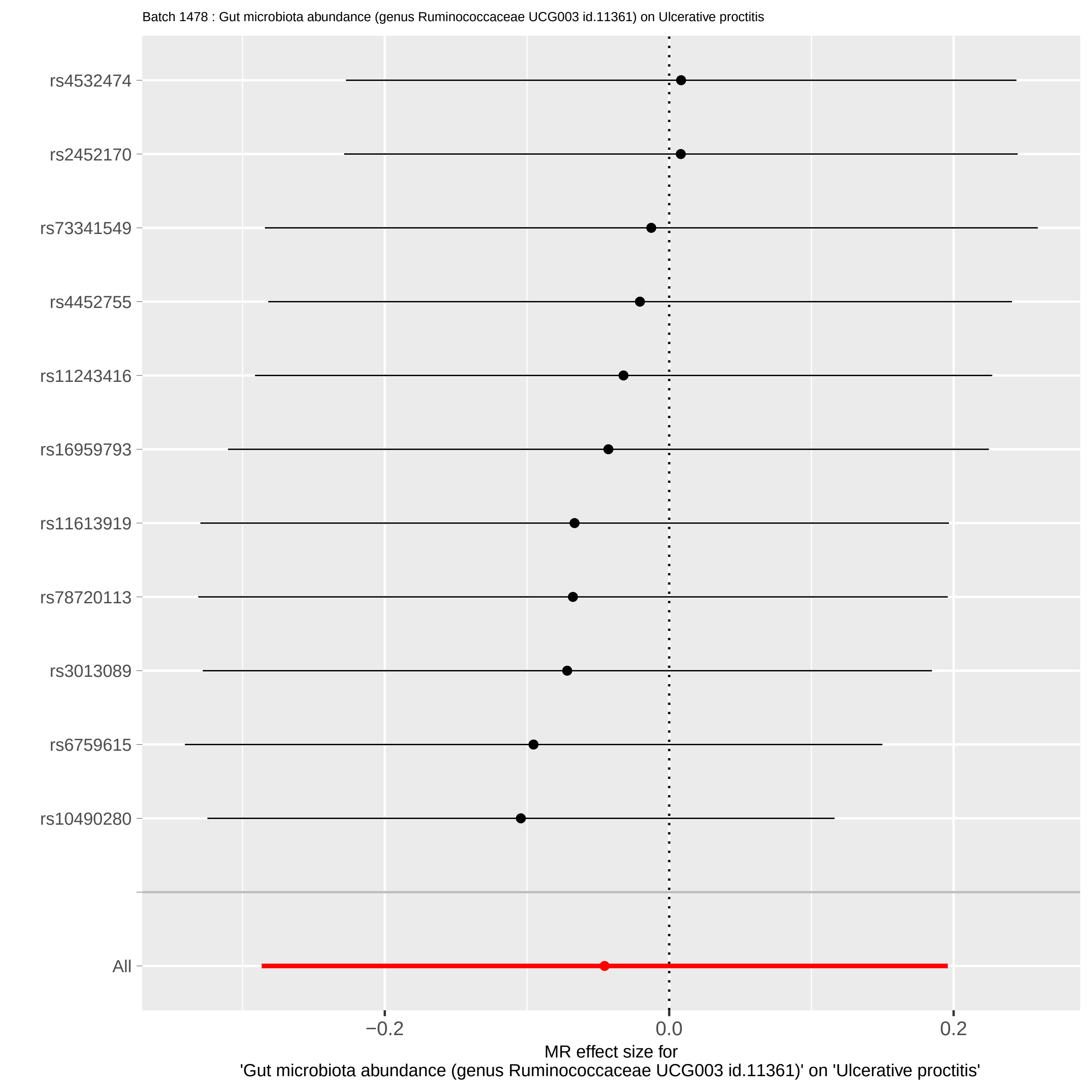

Batch 1478 : Gut microbiota abundance (genus Ruminococcaceae UCG003 id.11361) on Ulcerative proctitis
rs4532474
rs2452170
rs73341549
rs4452755
rs11243416
rs16959793
rs11613919
rs78720113
rs3013089
rs6759615
rs10490280
All
−0.2
0.0
0.2
MR effect size for
'Gut microbiota abundance (genus Ruminococcaceae UCG003 id.11361)' on 'Ulcerative proctitis'

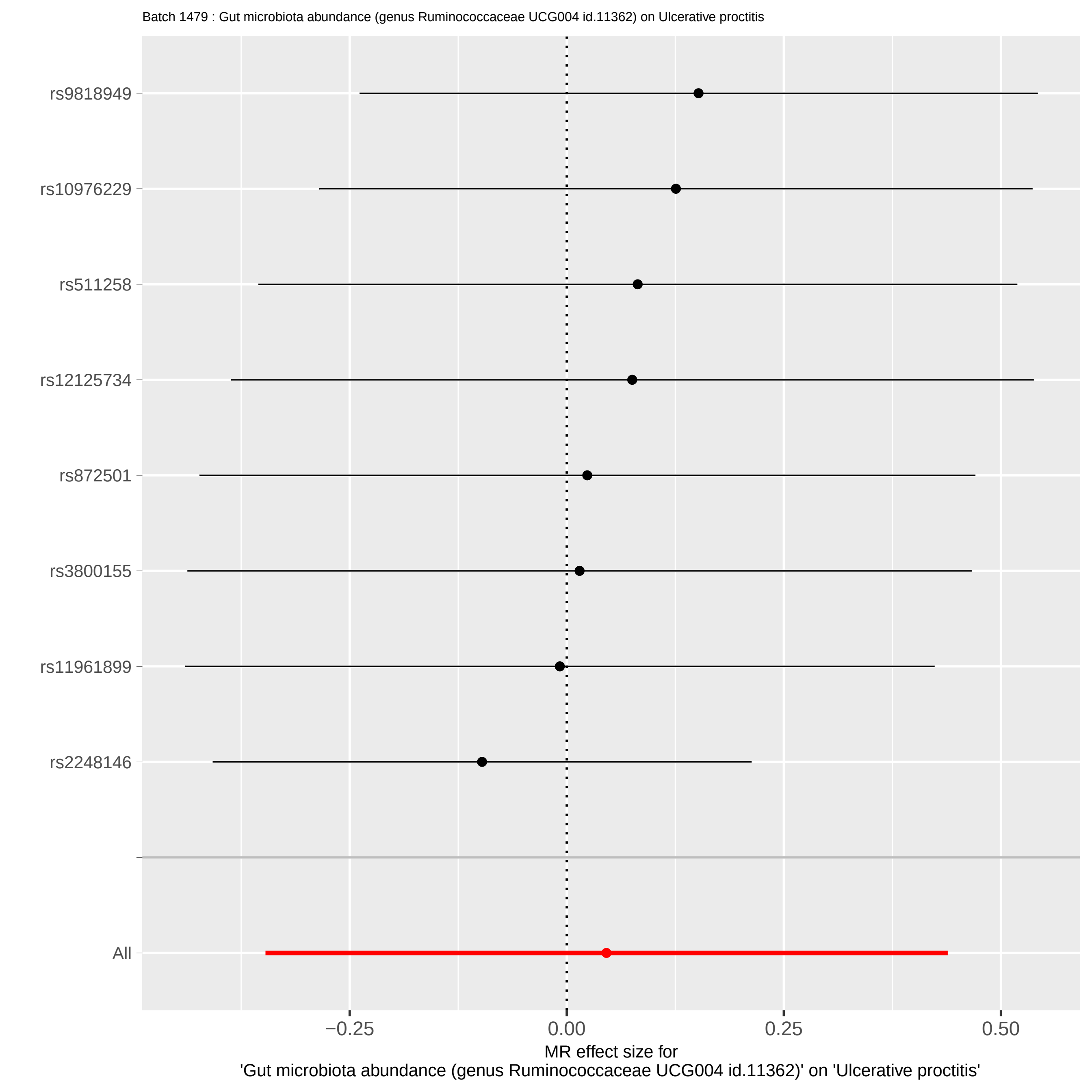

Batch 1479 : Gut microbiota abundance (genus Ruminococcaceae UCG004 id.11362) on Ulcerative proctitis
rs9818949
rs10976229
rs511258
rs12125734
rs872501
rs3800155
rs11961899
rs2248146
All
−0.25
0.00
0.25
0.50
MR effect size for
'Gut microbiota abundance (genus Ruminococcaceae UCG004 id.11362)' on 'Ulcerative proctitis'

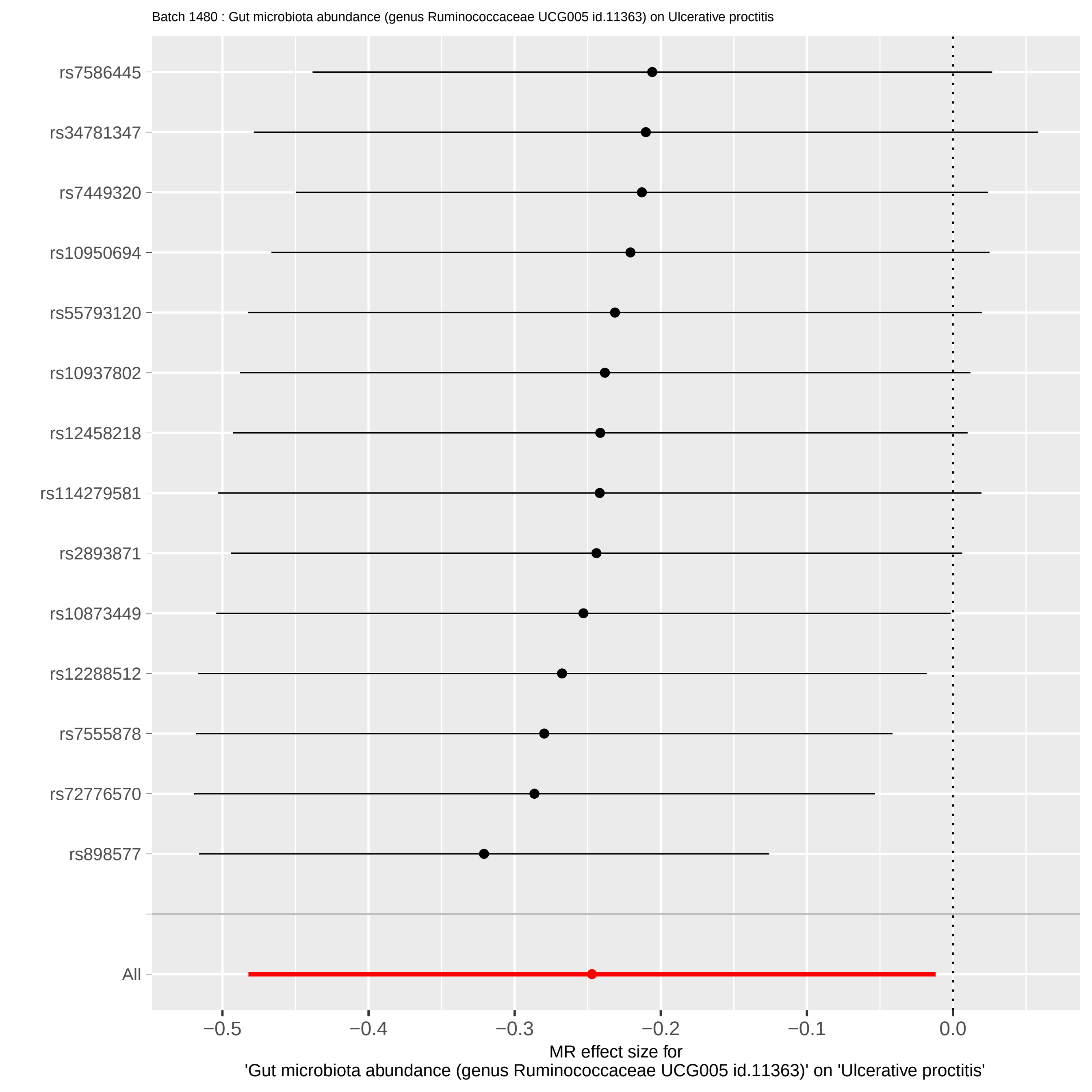
Batch 1480 : Gut microbiota abundance (genus Ruminococcaceae UCG005 id.11363) on Ulcerative proctitis
rs7586445
rs34781347
rs7449320
rs10950694
rs55793120
rs10937802
rs12458218
rs114279581
rs2893871
rs10873449
rs12288512
rs7555878
rs72776570
rs898577
All
−0.5
−0.4
−0.3
−0.2
−0.1
0.0
MR effect size for
'Gut microbiota abundance (genus Ruminococcaceae UCG005 id.11363)' on 'Ulcerative proctitis'

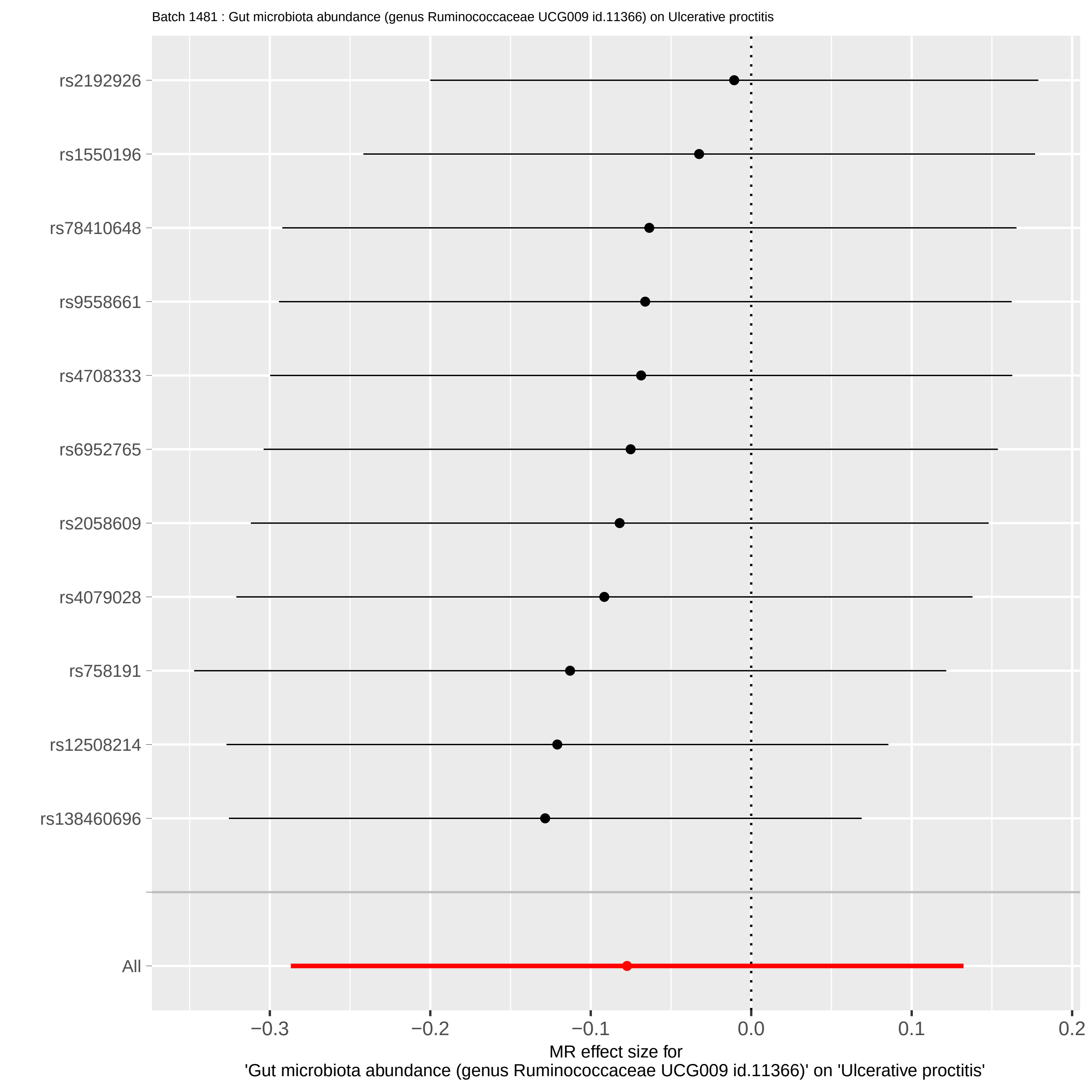

Batch 1481 : Gut microbiota abundance (genus Ruminococcaceae UCG009 id.11366) on Ulcerative proctitis
rs2192926
rs1550196
rs78410648
rs9558661
rs4708333
rs6952765
rs2058609
rs4079028
rs758191
rs12508214
rs138460696
All
−0.3
−0.2
−0.1
0.0
0.1
0.2
MR effect size for
'Gut microbiota abundance (genus Ruminococcaceae UCG009 id.11366)' on 'Ulcerative proctitis'

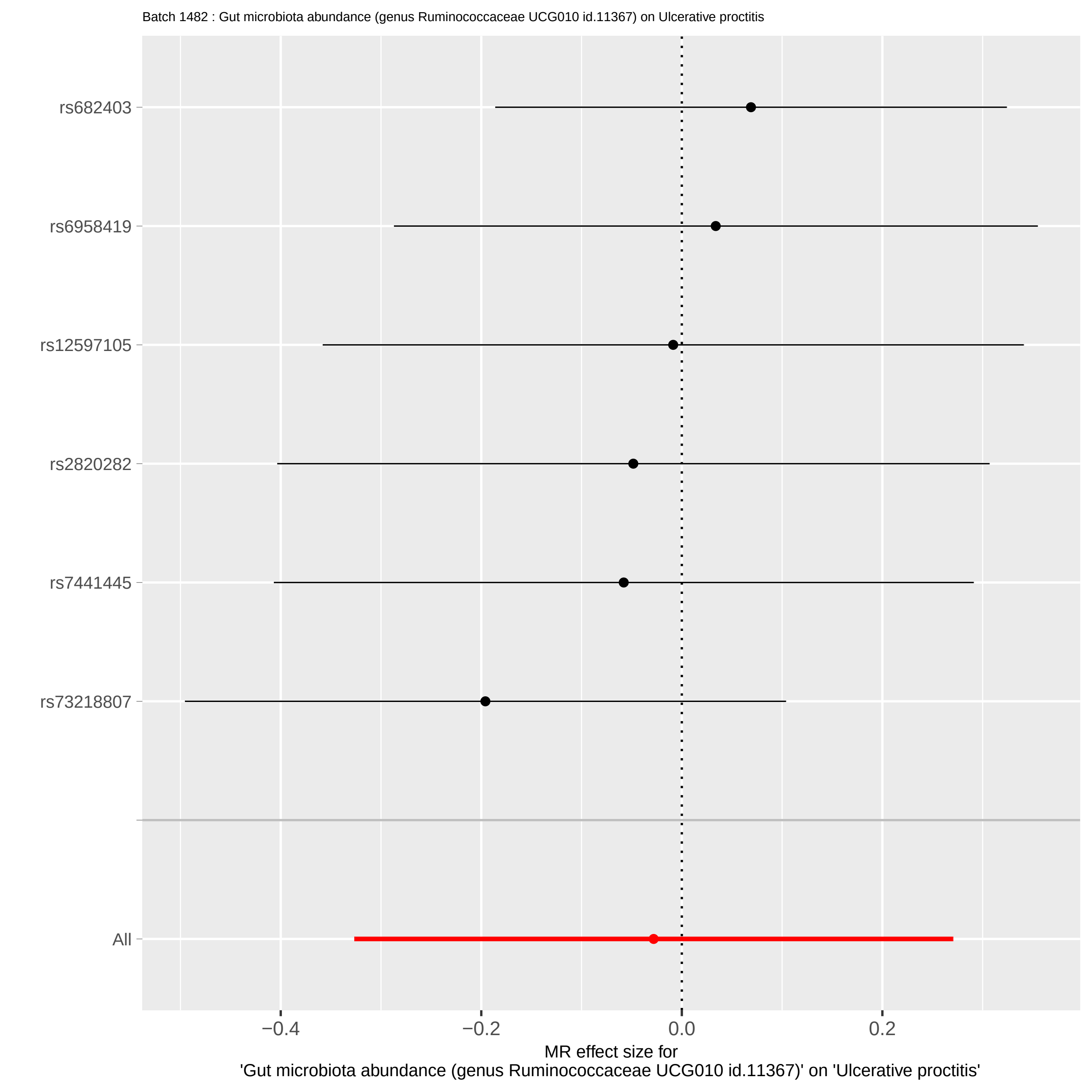

Batch 1482 : Gut microbiota abundance (genus Ruminococcaceae UCG010 id.11367) on Ulcerative proctitis
rs682403
rs6958419
rs12597105
rs2820282
rs7441445
rs73218807
All
−0.4
−0.2
0.0
0.2
MR effect size for
'Gut microbiota abundance (genus Ruminococcaceae UCG010 id.11367)' on 'Ulcerative proctitis'

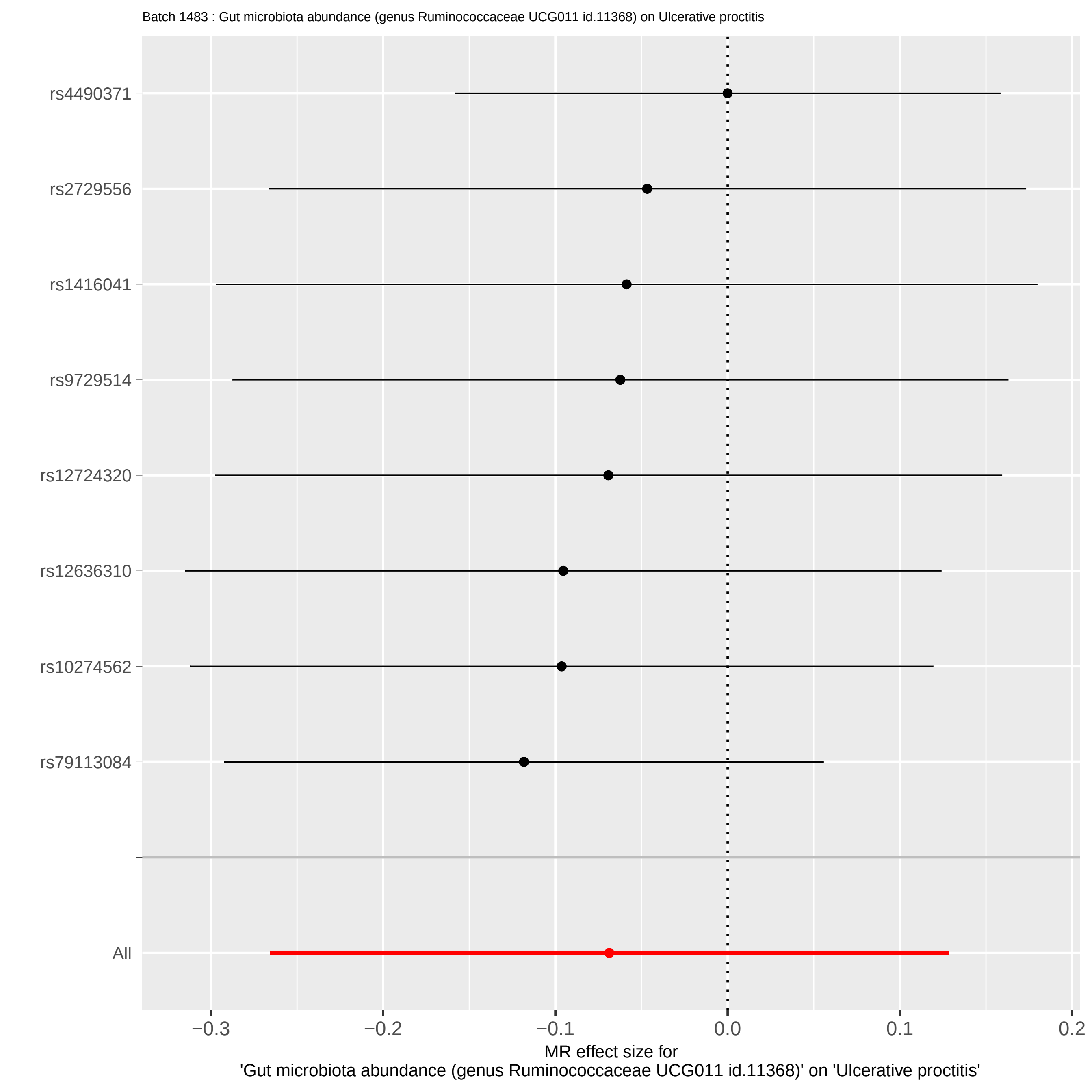

Batch 1483 : Gut microbiota abundance (genus Ruminococcaceae UCG011 id.11368) on Ulcerative proctitis
rs4490371
rs2729556
rs1416041
rs9729514
rs12724320
rs12636310
rs10274562
rs79113084
All
−0.3
−0.2
−0.1
0.0
0.1
0.2
MR effect size for
'Gut microbiota abundance (genus Ruminococcaceae UCG011 id.11368)' on 'Ulcerative proctitis'

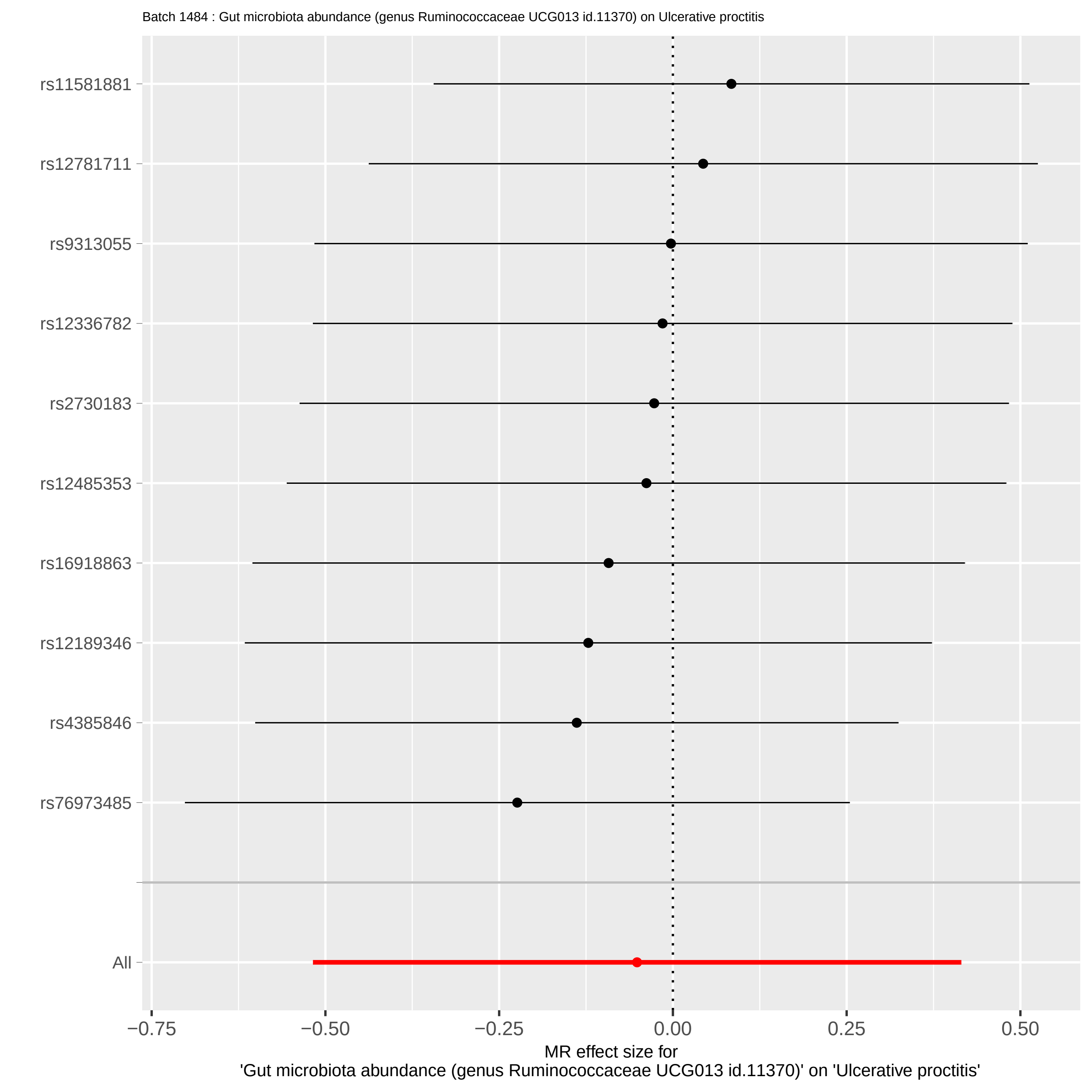

Batch 1484 : Gut microbiota abundance (genus Ruminococcaceae UCG013 id.11370) on Ulcerative proctitis
rs11581881
rs12781711
rs9313055
rs12336782
rs2730183
rs12485353
rs16918863
rs12189346
rs4385846
rs76973485
All
−0.75
−0.50
−0.25
0.00
0.25
0.50
MR effect size for
'Gut microbiota abundance (genus Ruminococcaceae UCG013 id.11370)' on 'Ulcerative proctitis'

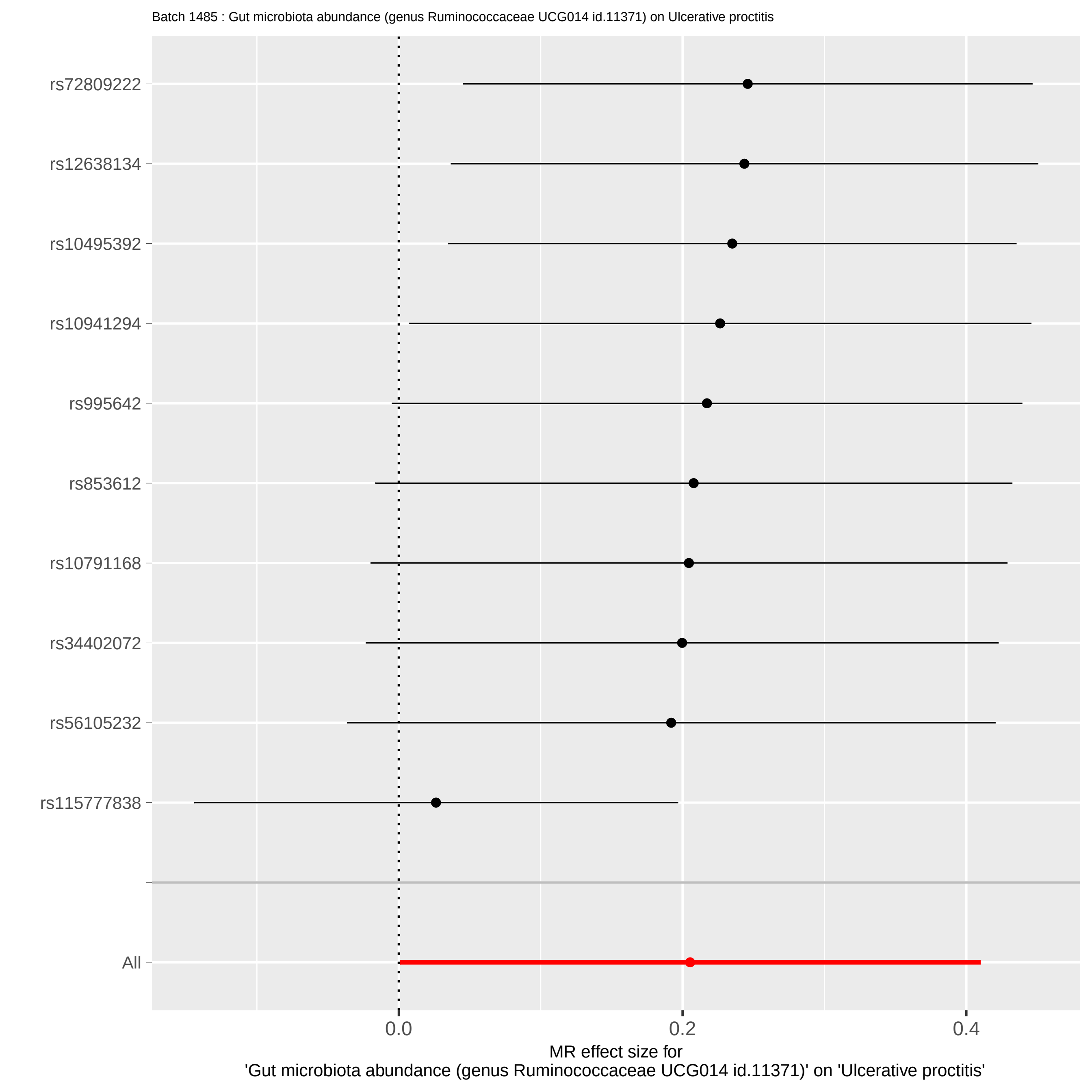

Batch 1485 : Gut microbiota abundance (genus Ruminococcaceae UCG014 id.11371) on Ulcerative proctitis
rs72809222
rs12638134
rs10495392
rs10941294
rs995642
rs853612
rs10791168
rs34402072
rs56105232
rs115777838
All
0.0
0.2
0.4
MR effect size for
'Gut microbiota abundance (genus Ruminococcaceae UCG014 id.11371)' on 'Ulcerative proctitis'

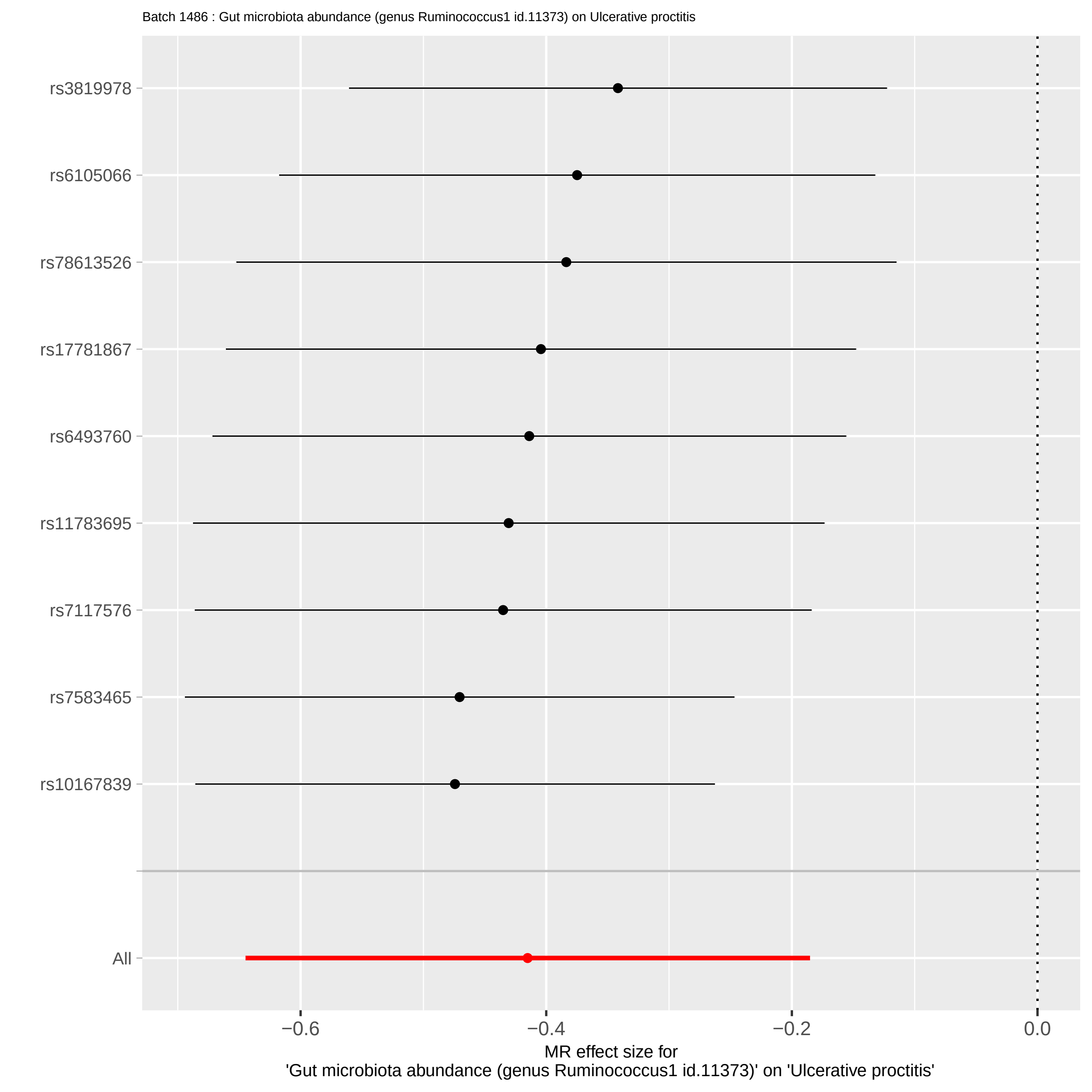

Batch 1486 : Gut microbiota abundance (genus Ruminococcus1 id.11373) on Ulcerative proctitis
rs3819978
rs6105066
rs78613526
rs17781867
rs6493760
rs11783695
rs7117576
rs7583465
rs10167839
All
−0.6
−0.4
−0.2
0.0
MR effect size for
'Gut microbiota abundance (genus Ruminococcus1 id.11373)' on 'Ulcerative proctitis'

Batch 1487 : Gut microbiota abundance (genus Ruminococcus2 id.11374) on Ulcerative proctitis

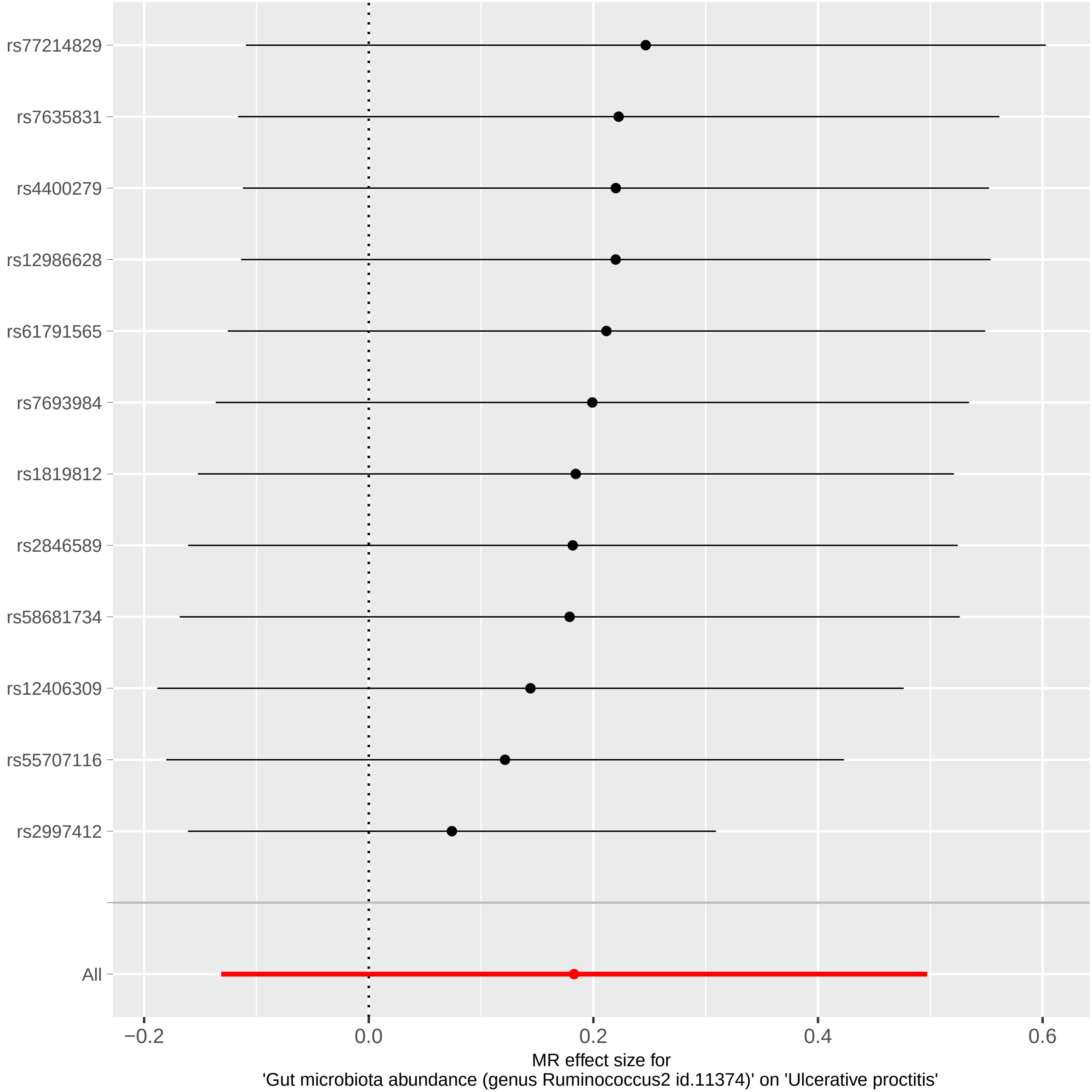

Batch 1488 : Gut microbiota abundance (genus Ruminococcus gauvreauii group id.11342) on Ulcerative proctitis

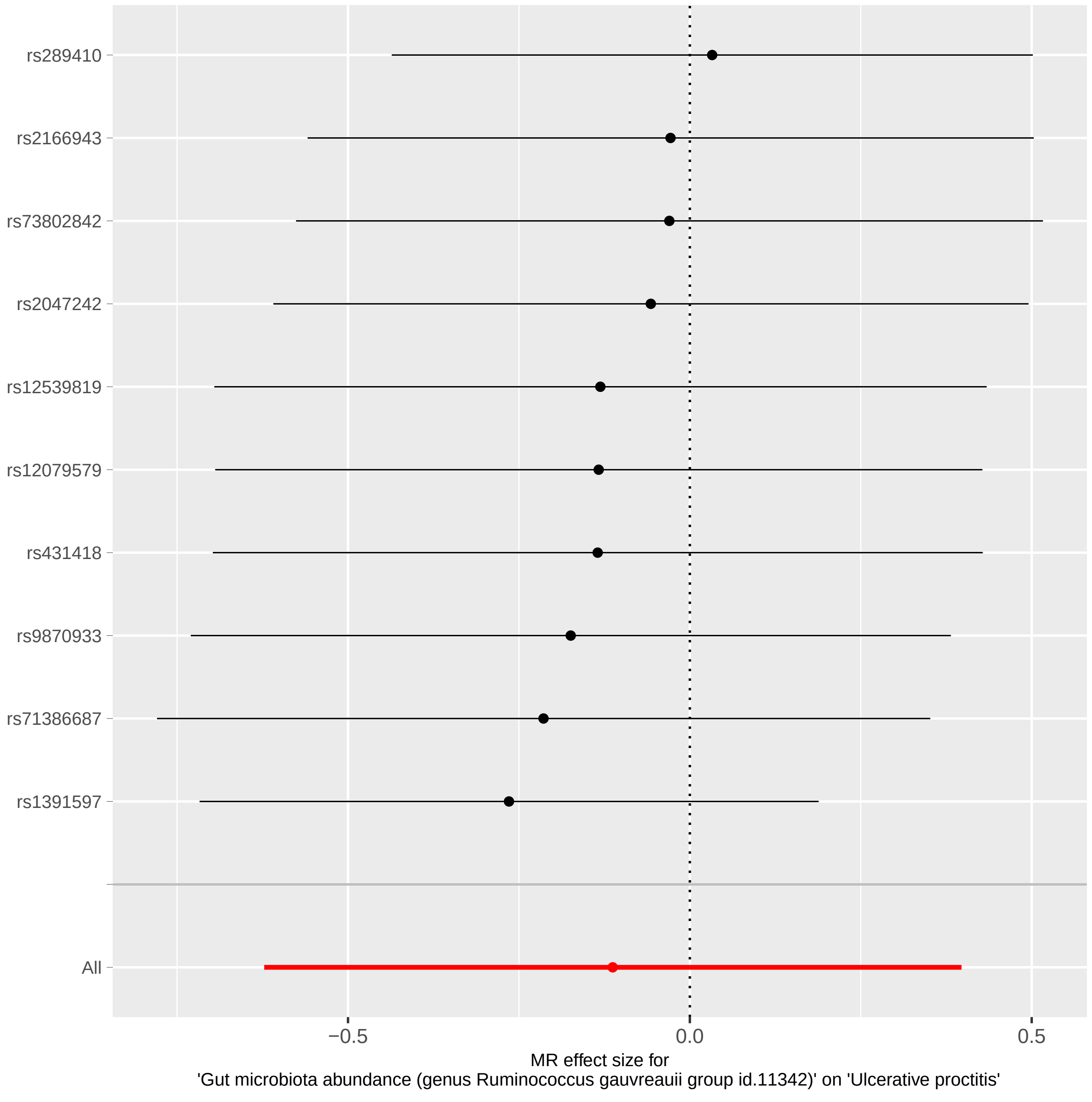

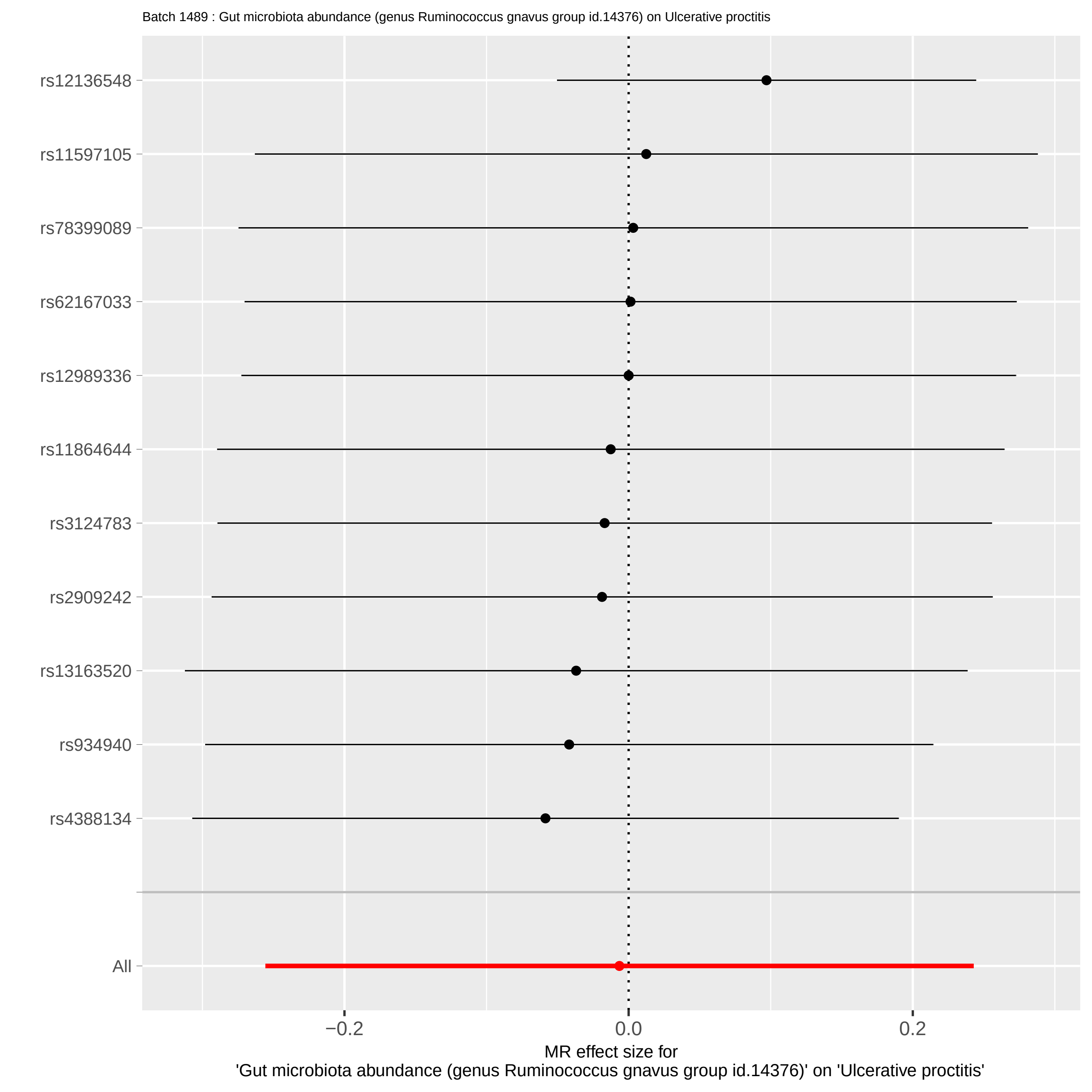
Batch 1489 : Gut microbiota abundance (genus Ruminococcus gnavus group id.14376) on Ulcerative proctitis
rs12136548
rs11597105
rs78399089
rs62167033
rs12989336
rs11864644
rs3124783
rs2909242
rs13163520
rs934940
rs4388134
All
−0.2
0.0
0.2
MR effect size for
'Gut microbiota abundance (genus Ruminococcus gnavus group id.14376)' on 'Ulcerative proctitis'

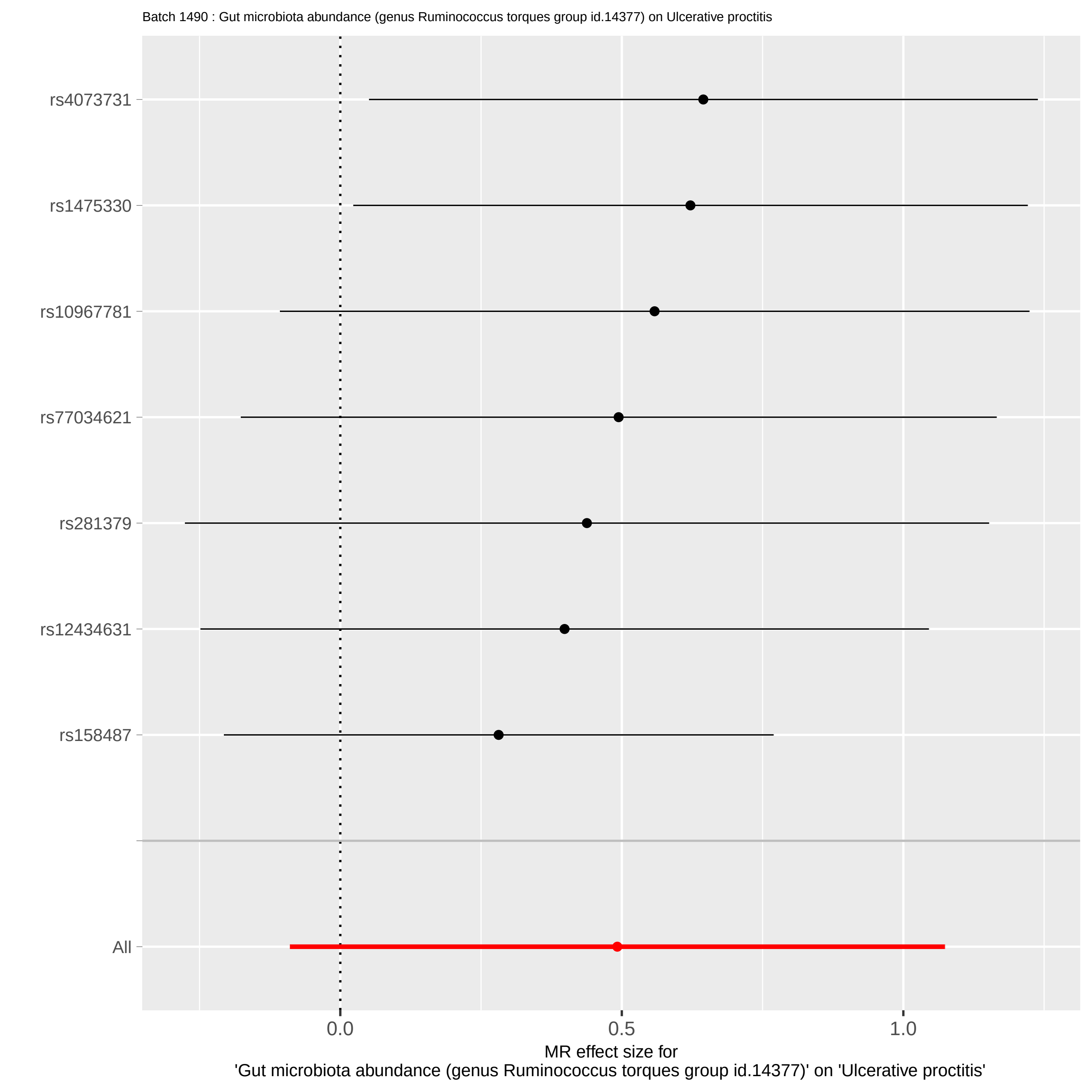

Batch 1490 : Gut microbiota abundance (genus Ruminococcus torques group id.14377) on Ulcerative proctitis
rs4073731
rs1475330
rs10967781
rs77034621
rs281379
rs12434631
rs158487
All
0.0
0.5
1.0
MR effect size for
'Gut microbiota abundance (genus Ruminococcus torques group id.14377)' on 'Ulcerative proctitis'

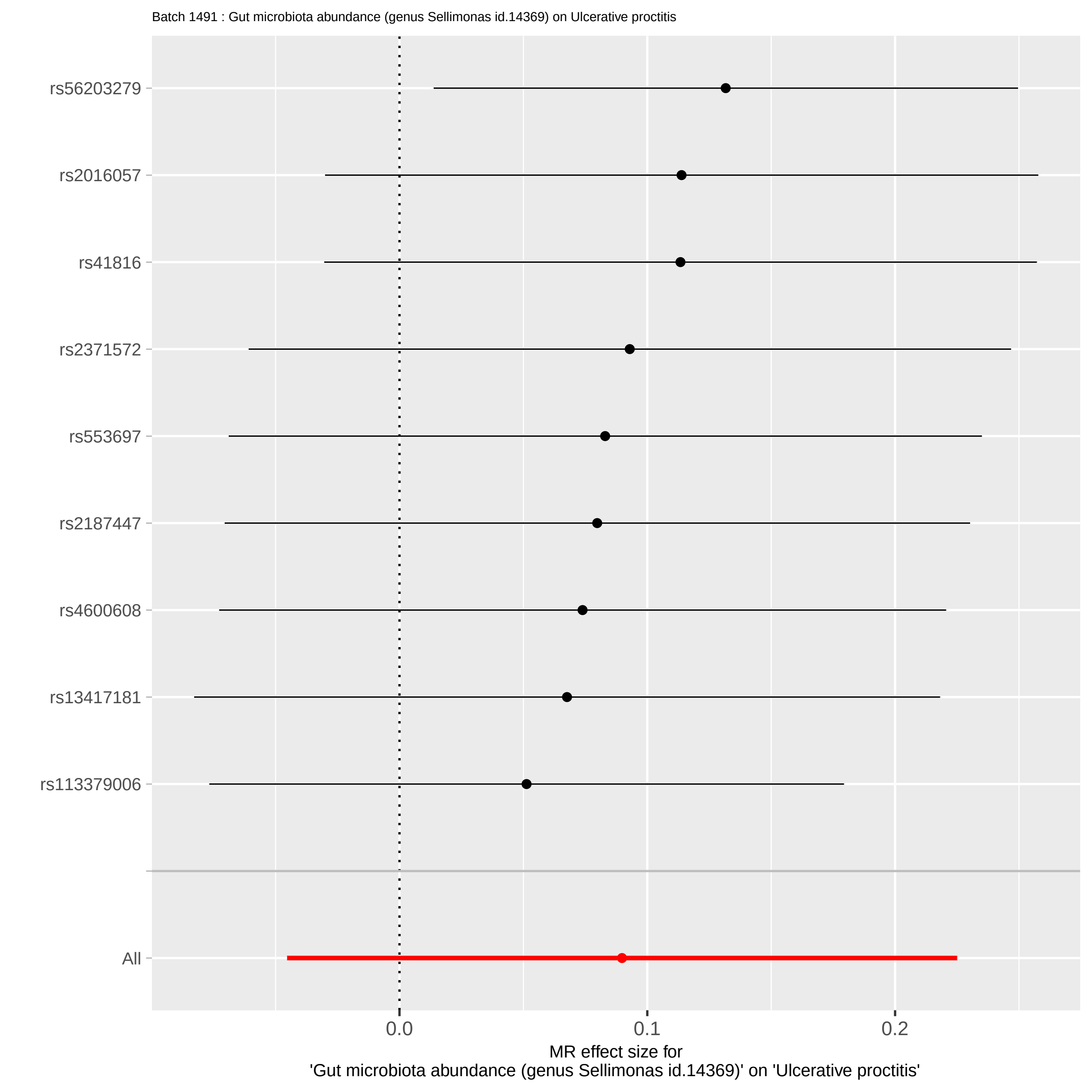

Batch 1491 : Gut microbiota abundance (genus Sellimonas id.14369) on Ulcerative proctitis
rs56203279
rs2016057
rs41816
rs2371572
rs553697
rs2187447
rs4600608
rs13417181
rs113379006
All
0.0
0.1
0.2
MR effect size for
'Gut microbiota abundance (genus Sellimonas id.14369)' on 'Ulcerative proctitis'

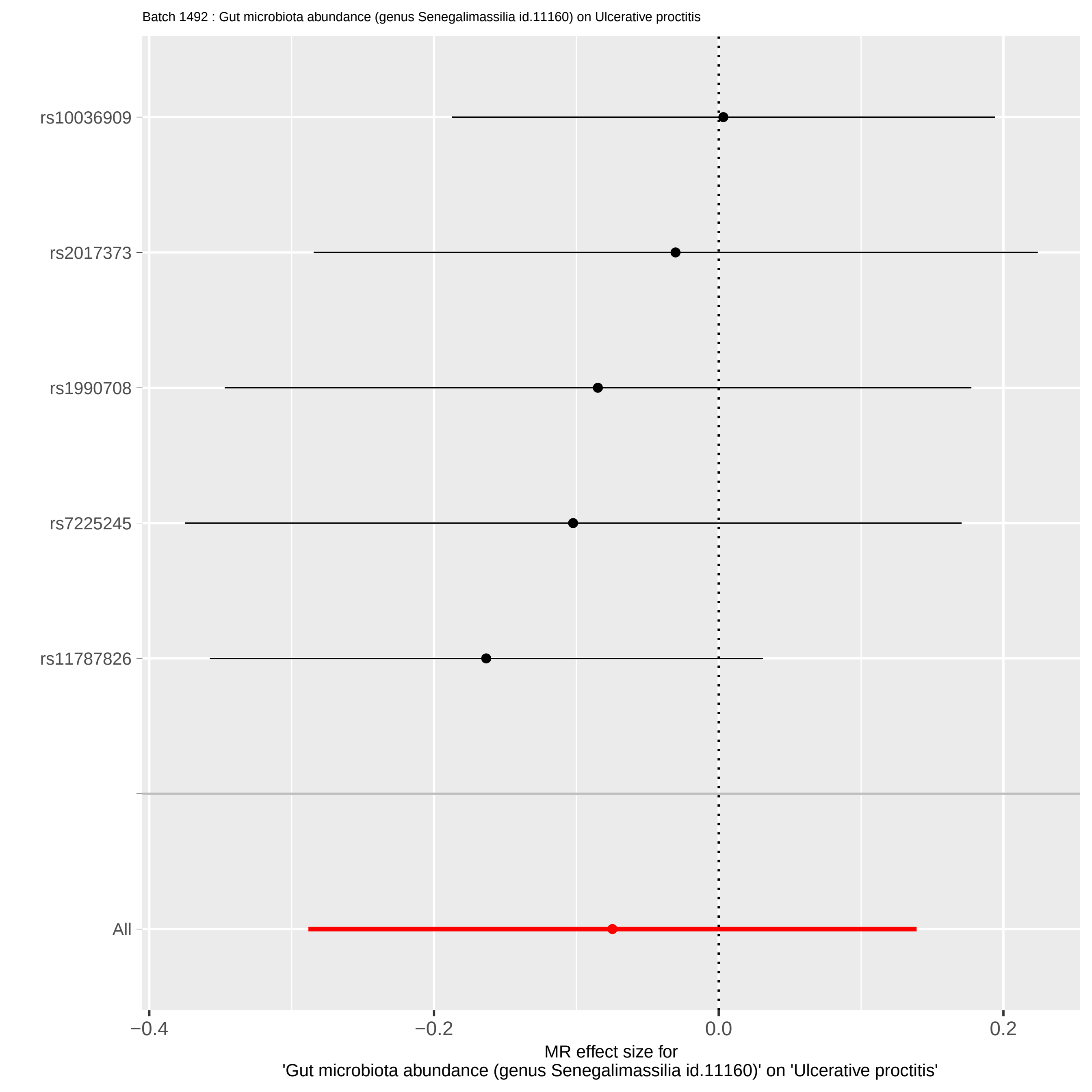

Batch 1492 : Gut microbiota abundance (genus Senegalimassilia id.11160) on Ulcerative proctitis
rs10036909
rs2017373
rs1990708
rs7225245
rs11787826
All
−0.4
−0.2
0.0
0.2
MR effect size for
'Gut microbiota abundance (genus Senegalimassilia id.11160)' on 'Ulcerative proctitis'

Batch 1493 : Gut microbiota abundance (genus Slackia id.825) on Ulcerative proctitis

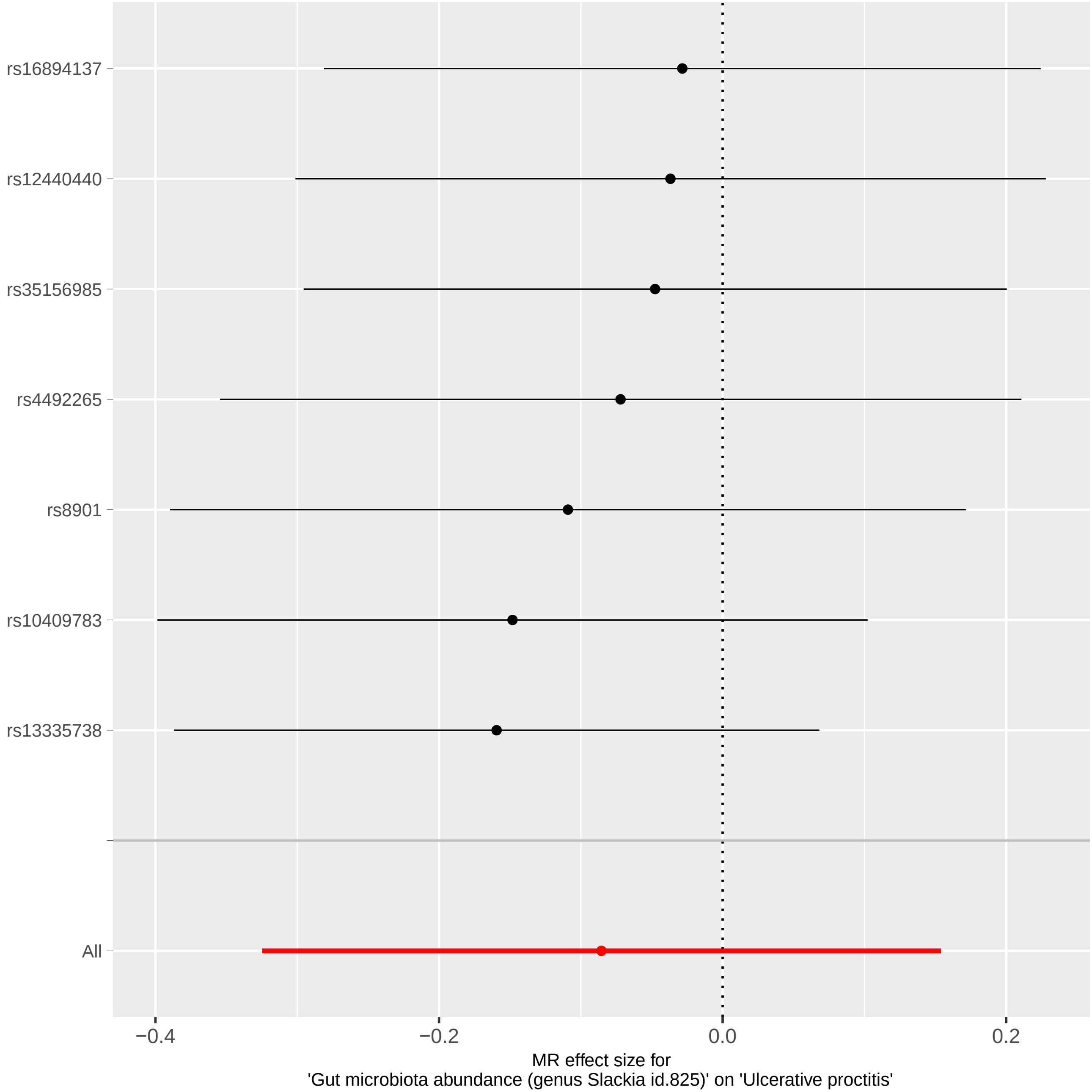

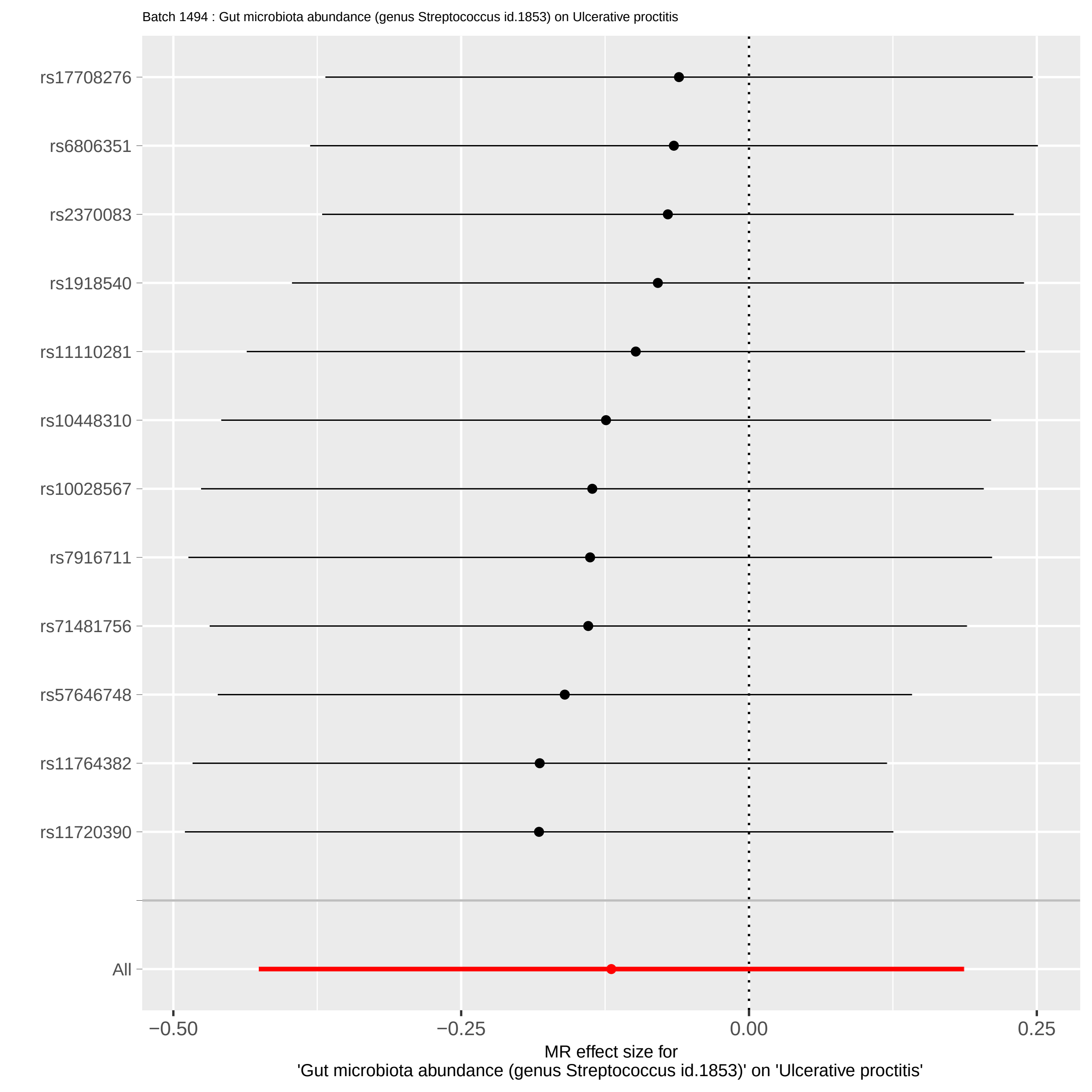

Batch 1494 : Gut microbiota abundance (genus Streptococcus id.1853) on Ulcerative proctitis
rs17708276
rs6806351
rs2370083
rs1918540
rs11110281
rs10448310
rs10028567
rs7916711
rs71481756
rs57646748
rs11764382
rs11720390
All
−0.50
−0.25
0.00
0.25
MR effect size for
'Gut microbiota abundance (genus Streptococcus id.1853)' on 'Ulcerative proctitis'

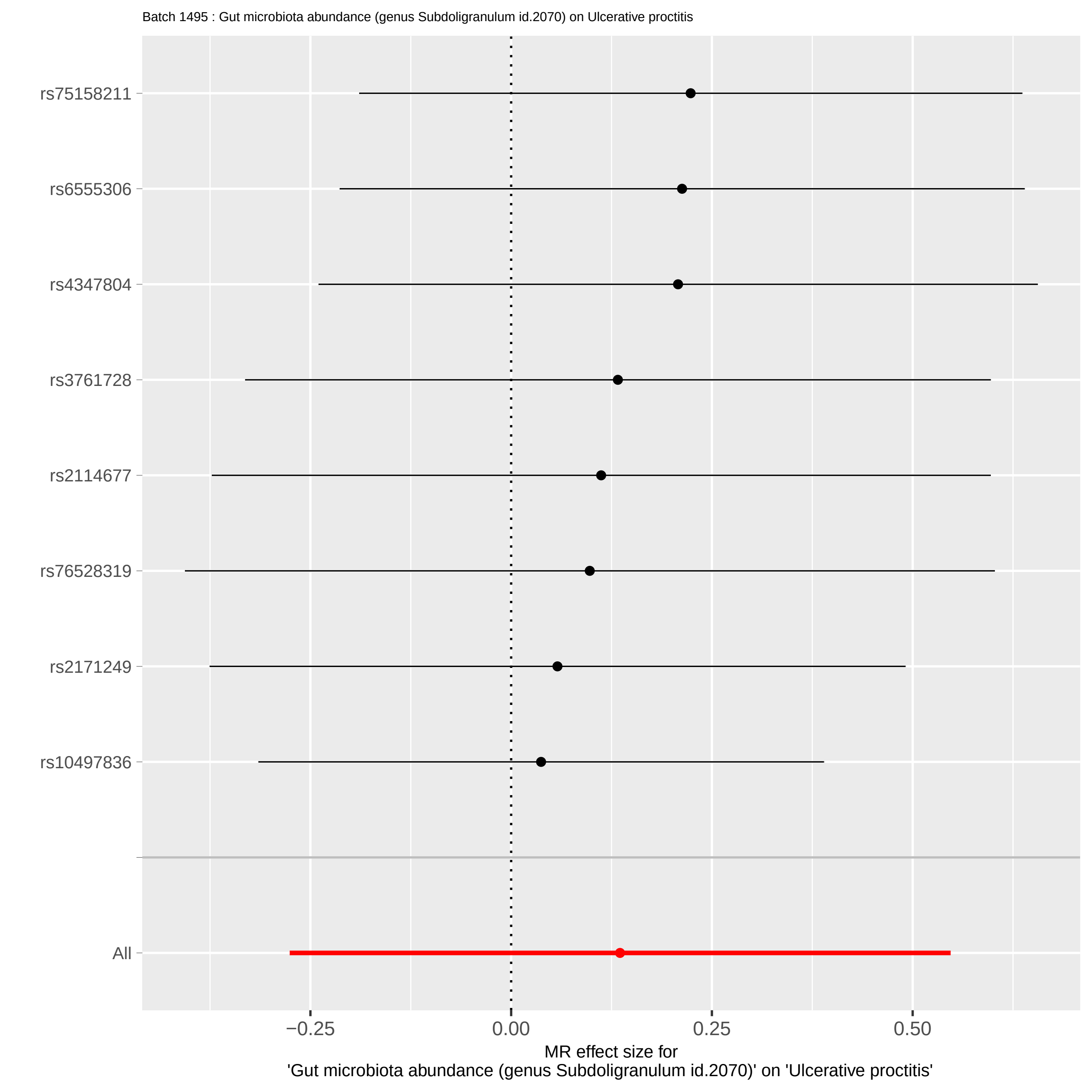
Batch 1495 : Gut microbiota abundance (genus Subdoligranulum id.2070) on Ulcerative proctitis
rs75158211
rs6555306
rs4347804
rs3761728
rs2114677
rs76528319
rs2171249
rs10497836
All
−0.25
0.00
0.25
0.50
MR effect size for
'Gut microbiota abundance (genus Subdoligranulum id.2070)' on 'Ulcerative proctitis'

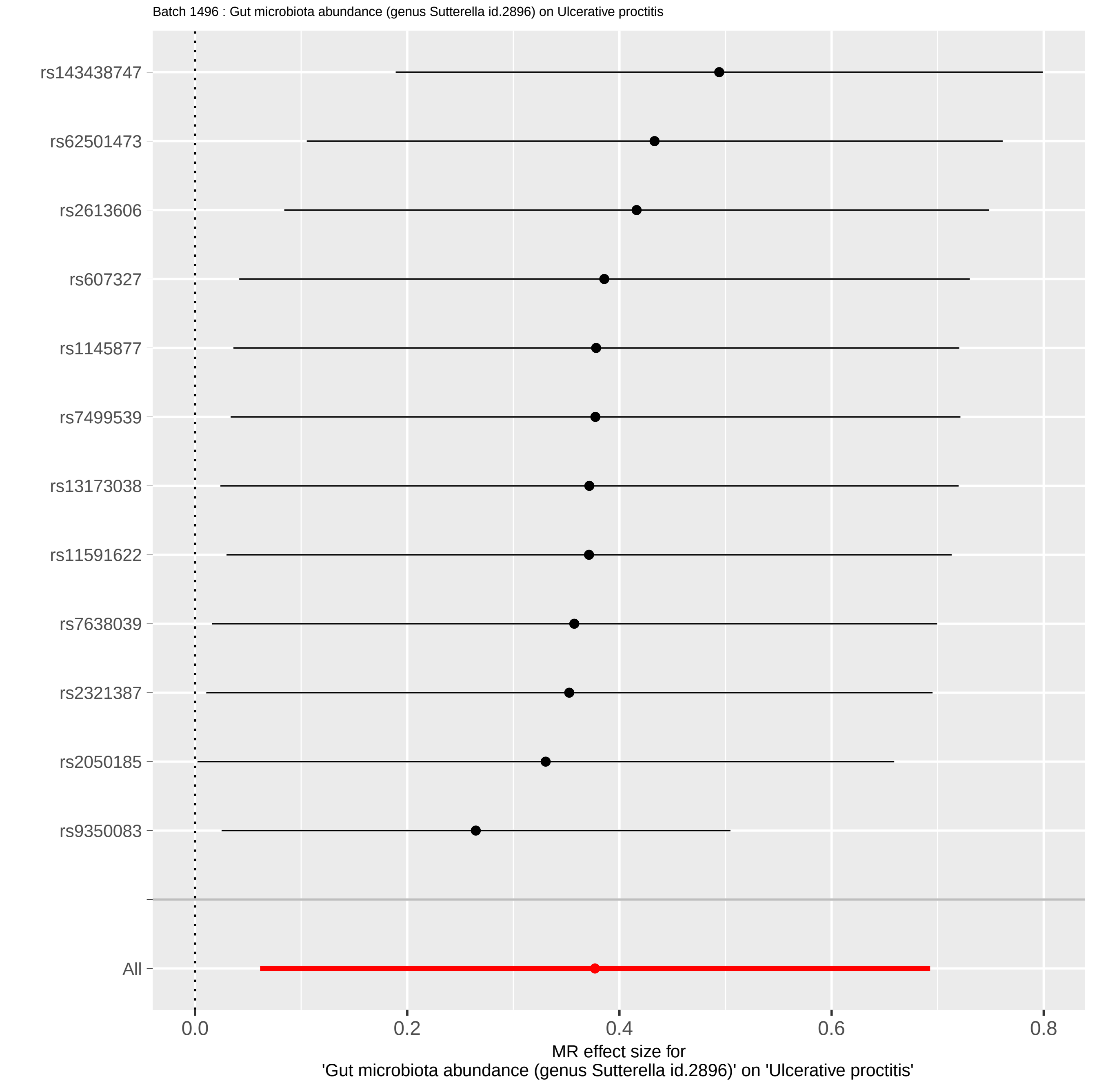

Batch 1496 : Gut microbiota abundance (genus Sutterella id.2896) on Ulcerative proctitis
rs143438747
rs62501473
rs2613606
rs607327
rs1145877
rs7499539
rs13173038
rs11591622
rs7638039
rs2321387
rs2050185
rs9350083
All
0.0
0.2
0.4
0.6
0.8
MR effect size for
'Gut microbiota abundance (genus Sutterella id.2896)' on 'Ulcerative proctitis'

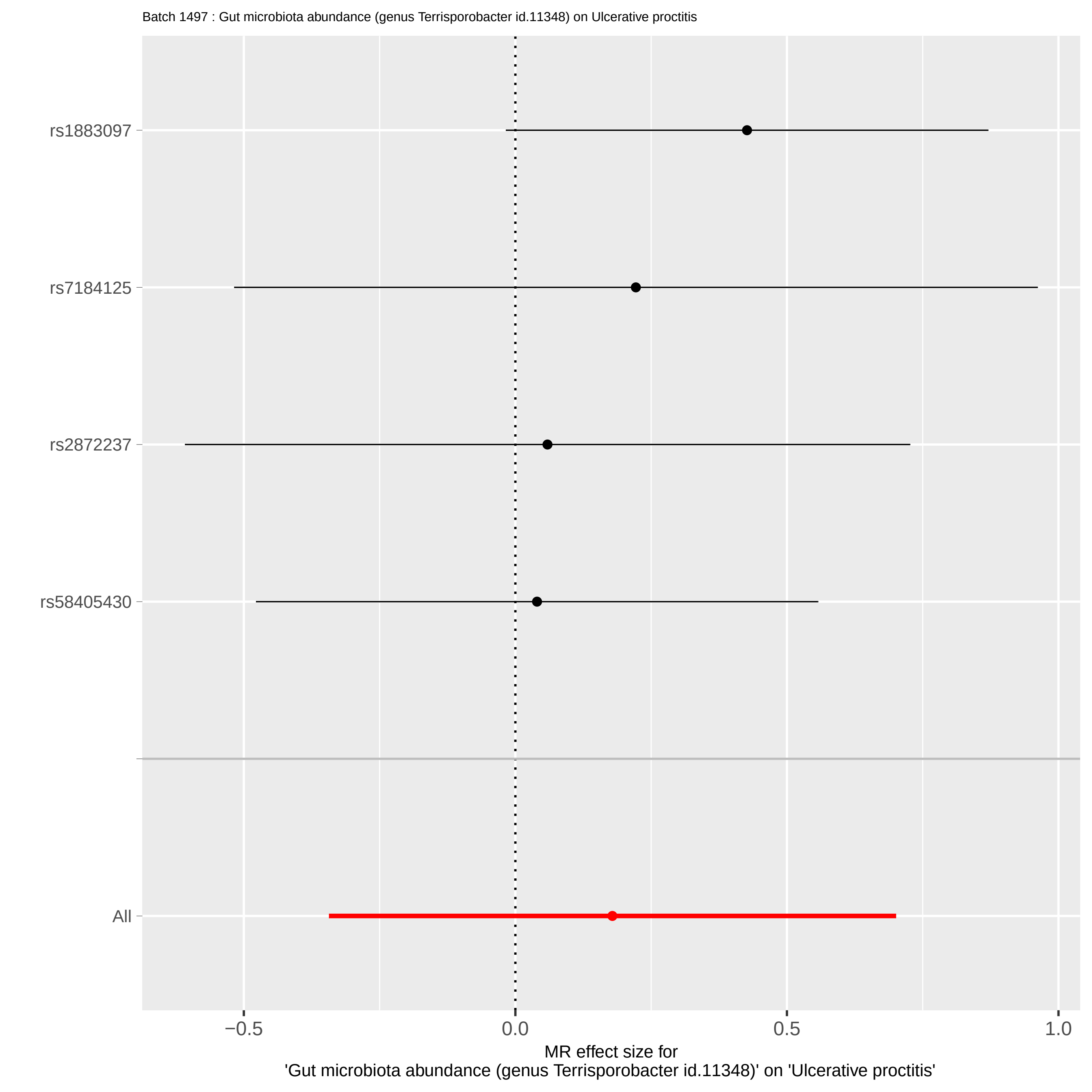

Batch 1497 : Gut microbiota abundance (genus Terrisporobacter id.11348) on Ulcerative proctitis
rs1883097
rs7184125
rs2872237
rs58405430
All
−0.5
0.0
0.5
1.0
MR effect size for
'Gut microbiota abundance (genus Terrisporobacter id.11348)' on 'Ulcerative proctitis'

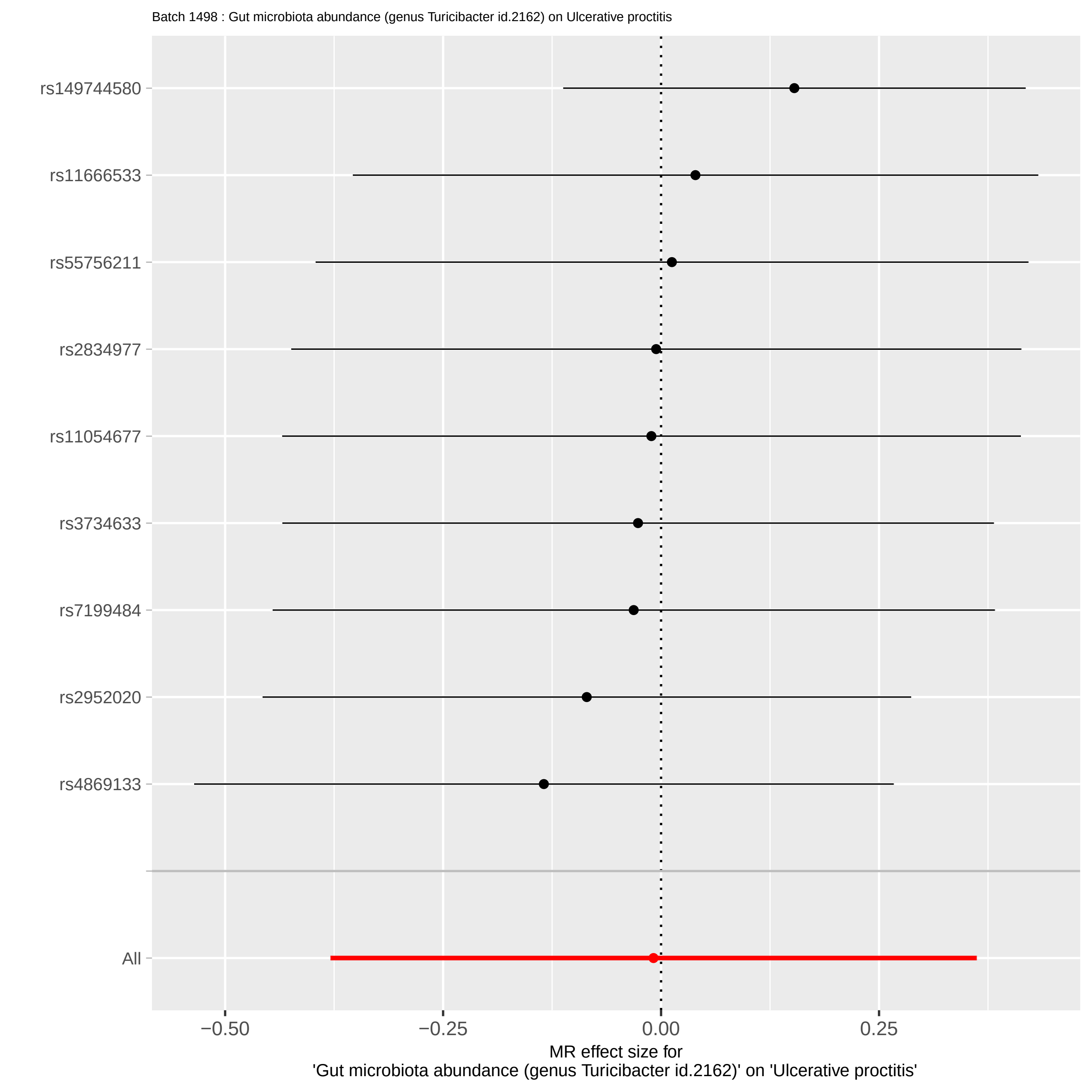

Batch 1498 : Gut microbiota abundance (genus Turicibacter id.2162) on Ulcerative proctitis
rs149744580
rs11666533
rs55756211
rs2834977
rs11054677
rs3734633
rs7199484
rs2952020
rs4869133
All
−0.50
−0.25
0.00
0.25
MR effect size for
'Gut microbiota abundance (genus Turicibacter id.2162)' on 'Ulcerative proctitis'

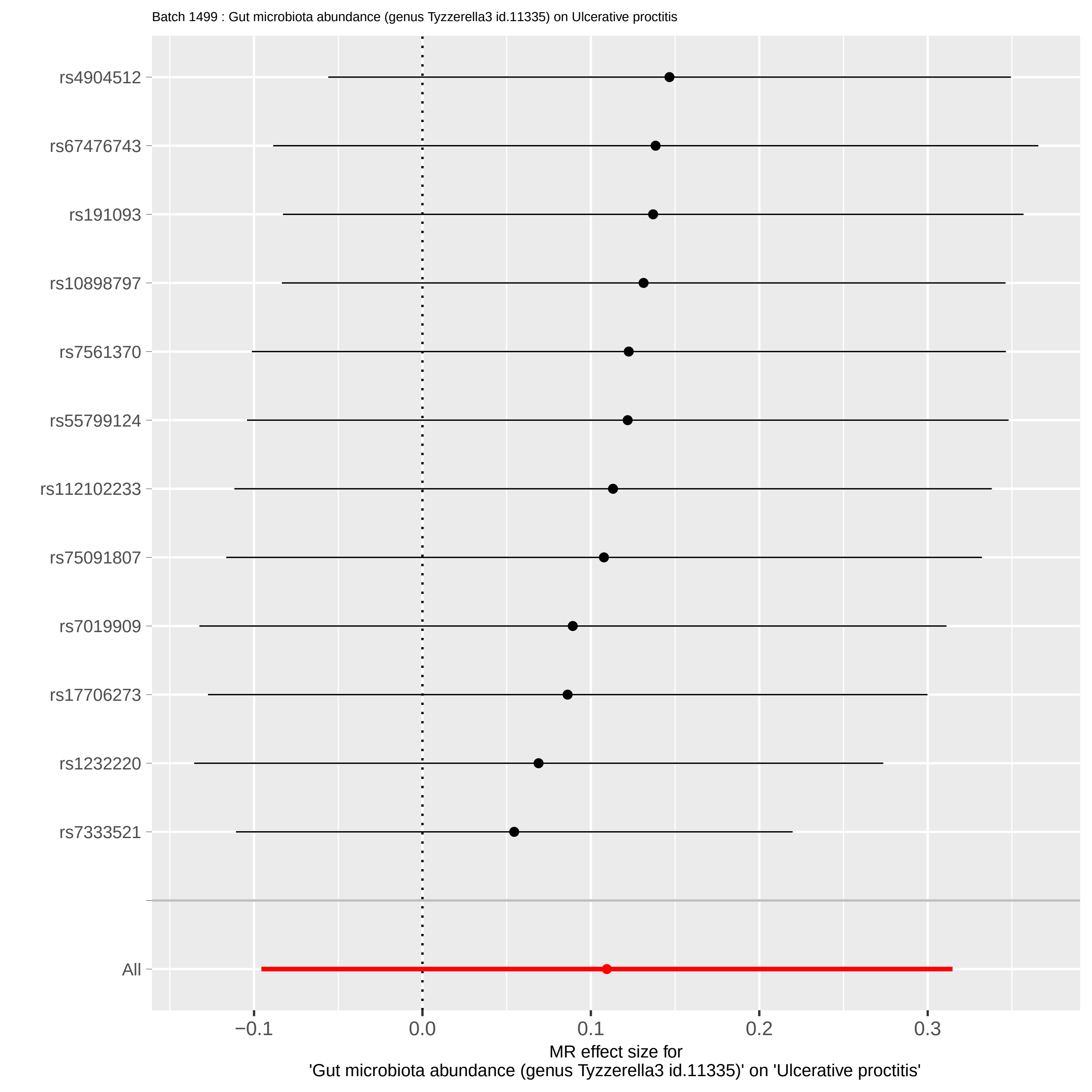
Batch 1499 : Gut microbiota abundance (genus Tyzzerella3 id.11335) on Ulcerative proctitis
rs4904512
rs67476743
rs191093
rs10898797
rs7561370
rs55799124
rs112102233
rs75091807
rs7019909
rs17706273
rs1232220
rs7333521
All
−0.1
0.0
0.1
0.2
0.3
MR effect size for
'Gut microbiota abundance (genus Tyzzerella3 id.11335)' on 'Ulcerative proctitis'

Batch 1500 : Gut microbiota abundance (genus Veillonella id.2198) on Ulcerative proctitis

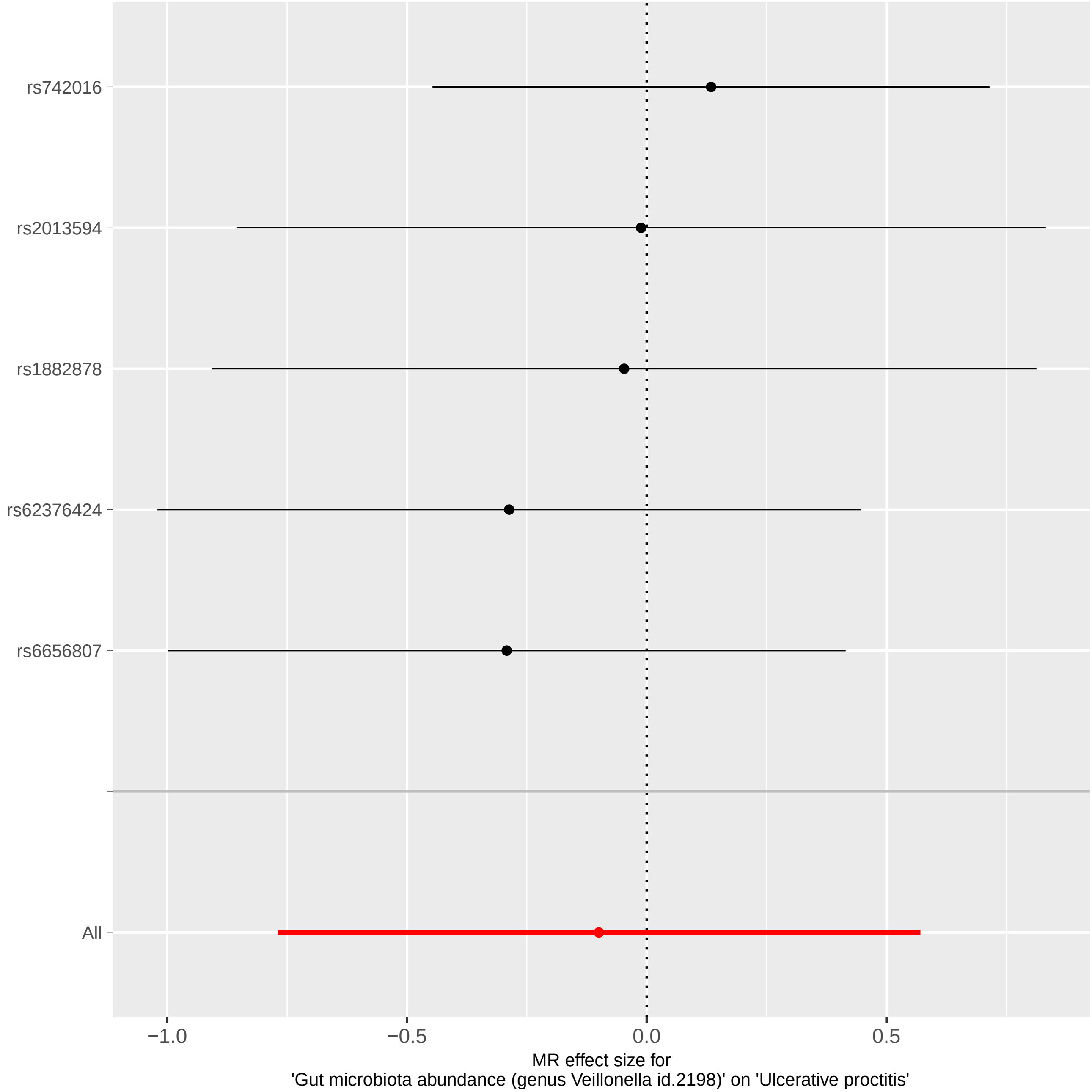

Batch 1501 : Gut microbiota abundance (family Acidaminococcaceae id.2166) on Ulcerative rectosigmoiditis

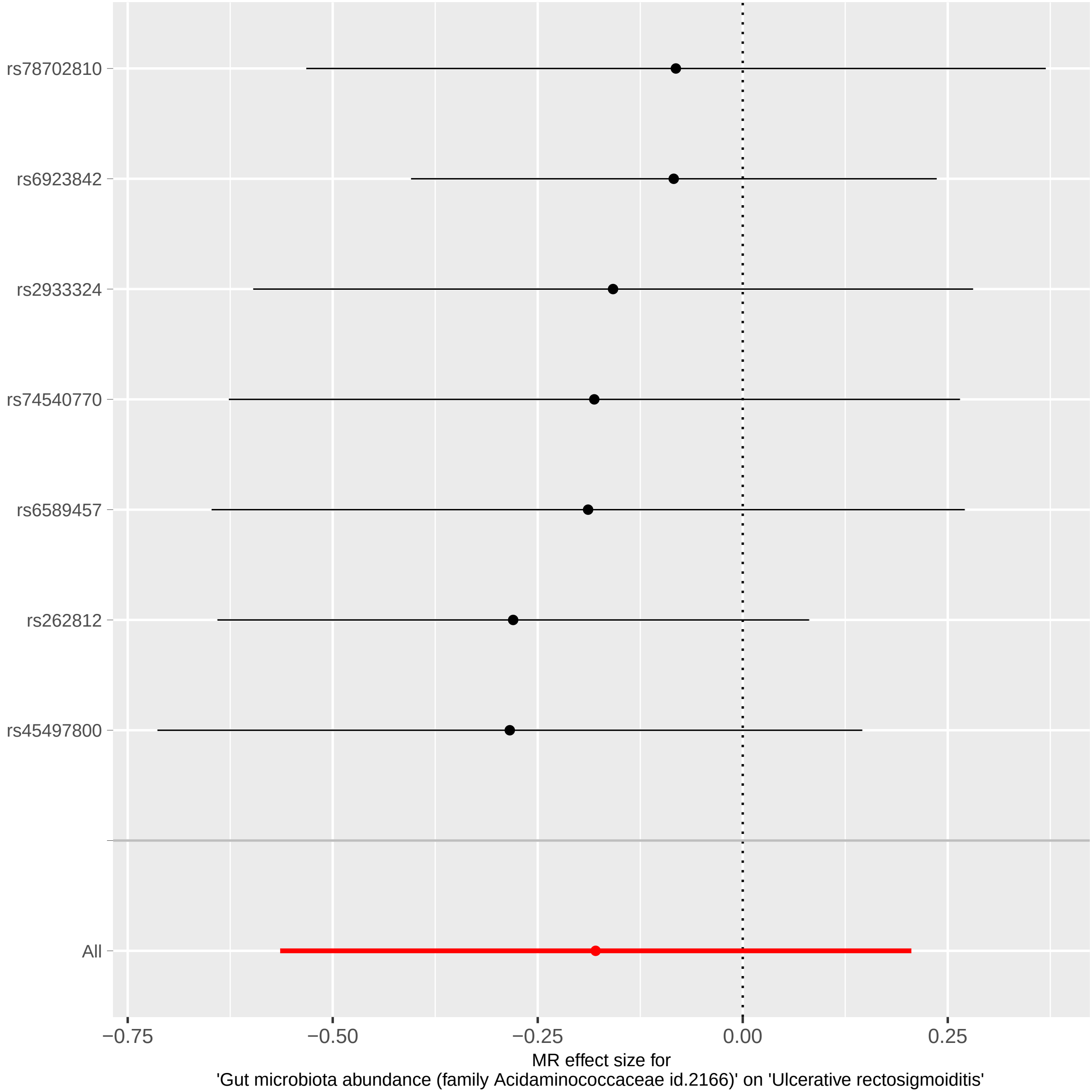

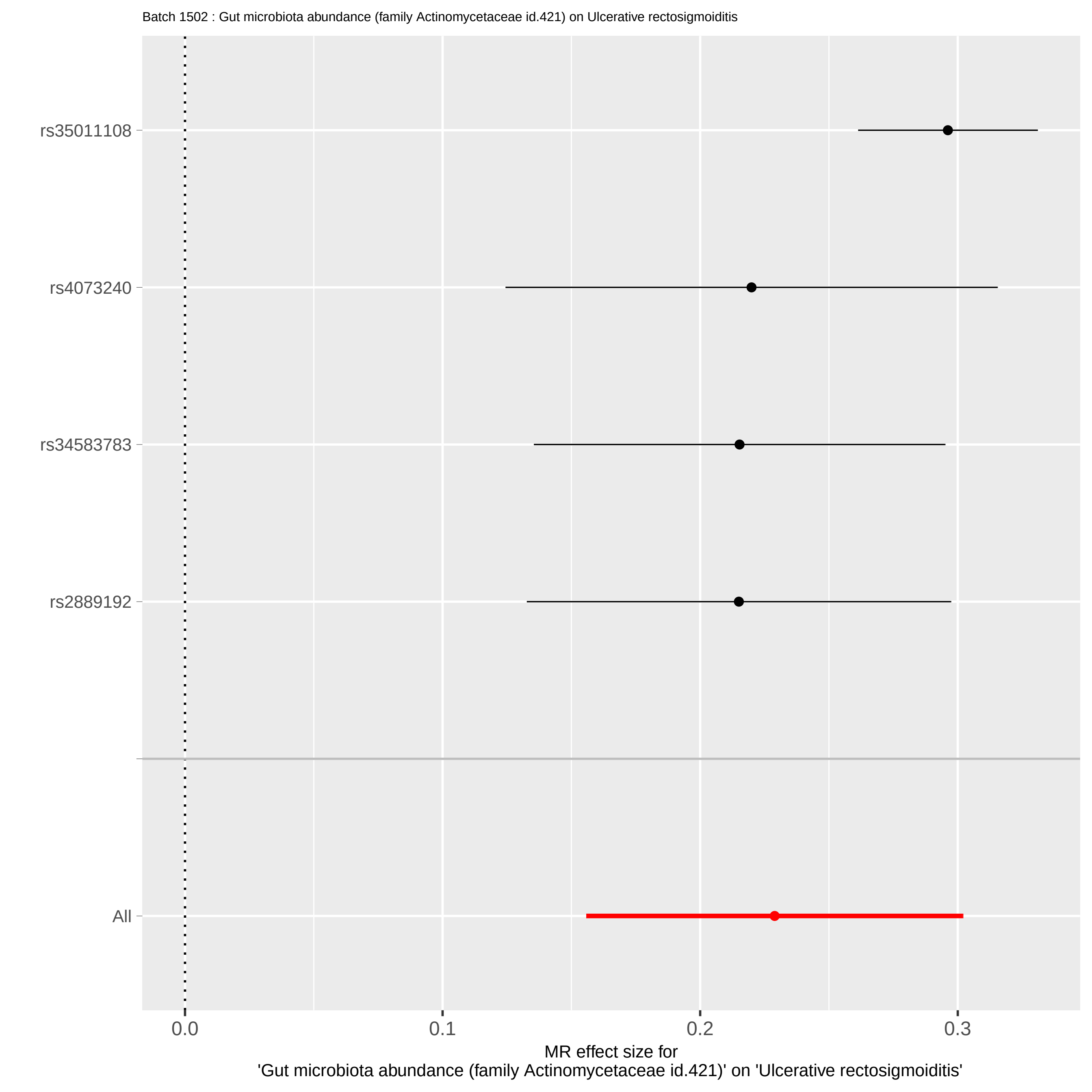

Batch 1502 : Gut microbiota abundance (family Actinomycetaceae id.421) on Ulcerative rectosigmoiditis
rs35011108
rs4073240
rs34583783
rs2889192
All
0.0
0.1
0.2
0.3
MR effect size for
'Gut microbiota abundance (family Actinomycetaceae id.421)' on 'Ulcerative rectosigmoiditis'

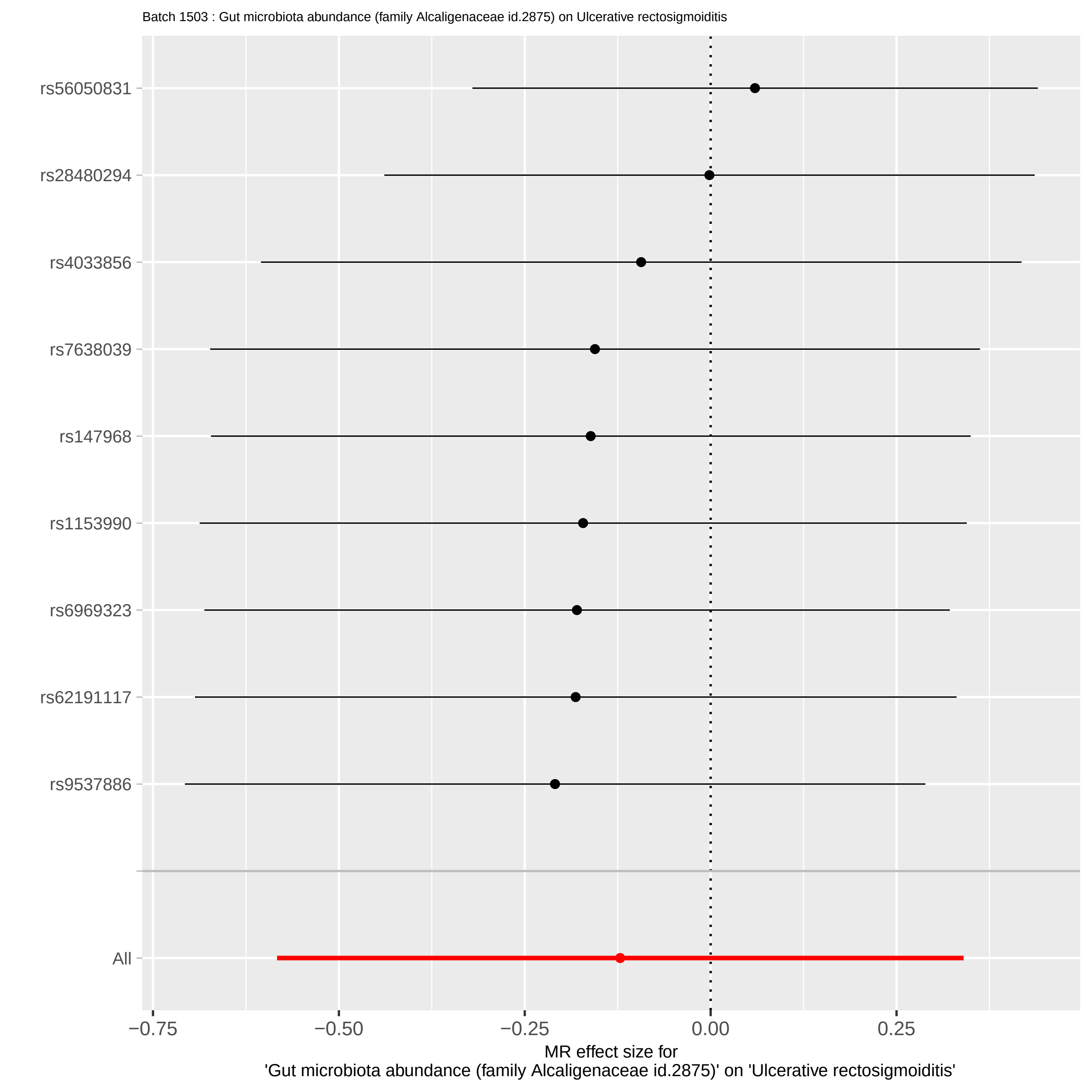

Batch 1503 : Gut microbiota abundance (family Alcaligenaceae id.2875) on Ulcerative rectosigmoiditis
rs56050831
rs28480294
rs4033856
rs7638039
rs147968
rs1153990
rs6969323
rs62191117
rs9537886
All
−0.75
−0.50
−0.25
0.00
0.25
MR effect size for
'Gut microbiota abundance (family Alcaligenaceae id.2875)' on 'Ulcerative rectosigmoiditis'

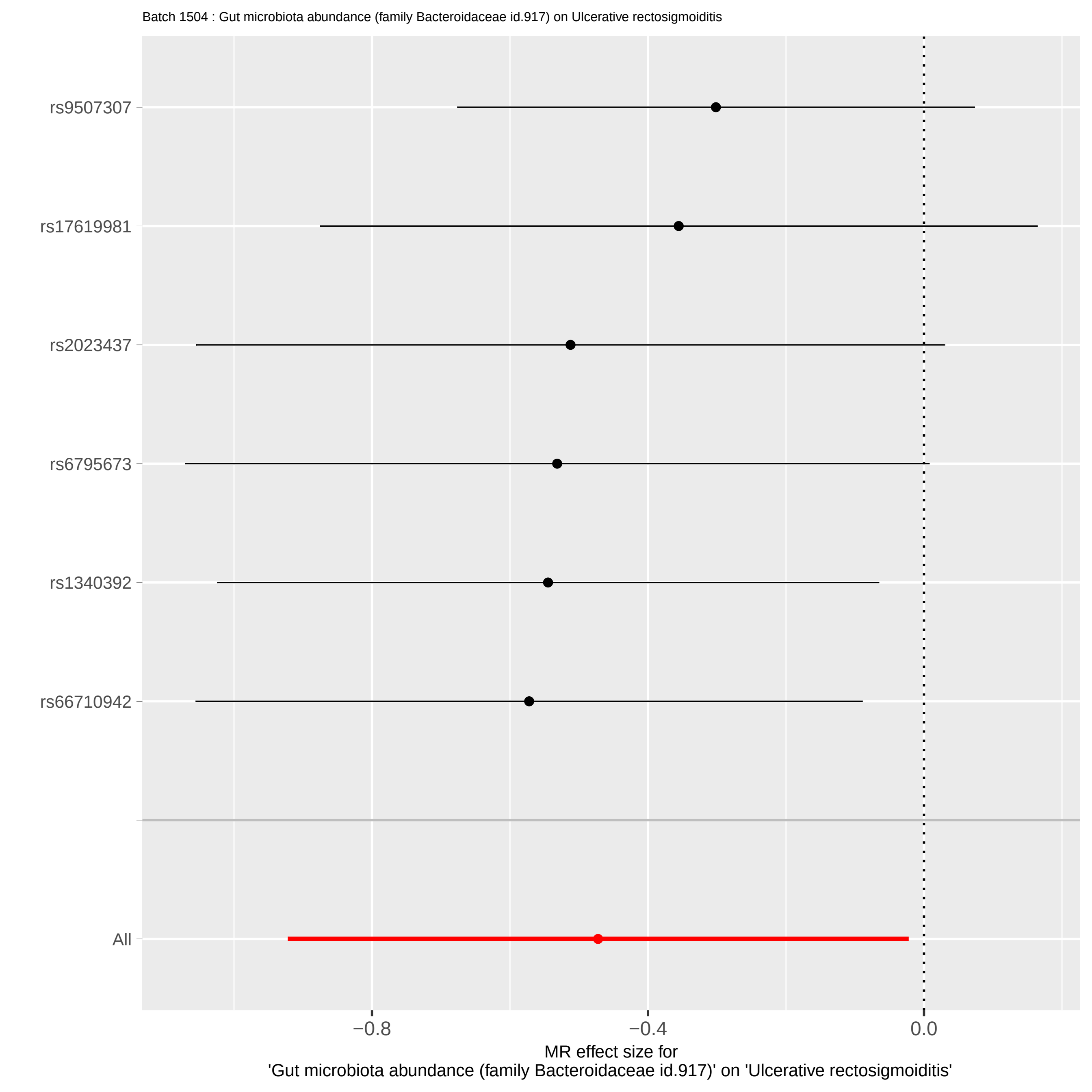

Batch 1504 : Gut microbiota abundance (family Bacteroidaceae id.917) on Ulcerative rectosigmoiditis
rs9507307
rs17619981
rs2023437
rs6795673
rs1340392
rs66710942
All
−0.8
−0.4
0.0
MR effect size for
'Gut microbiota abundance (family Bacteroidaceae id.917)' on 'Ulcerative rectosigmoiditis'

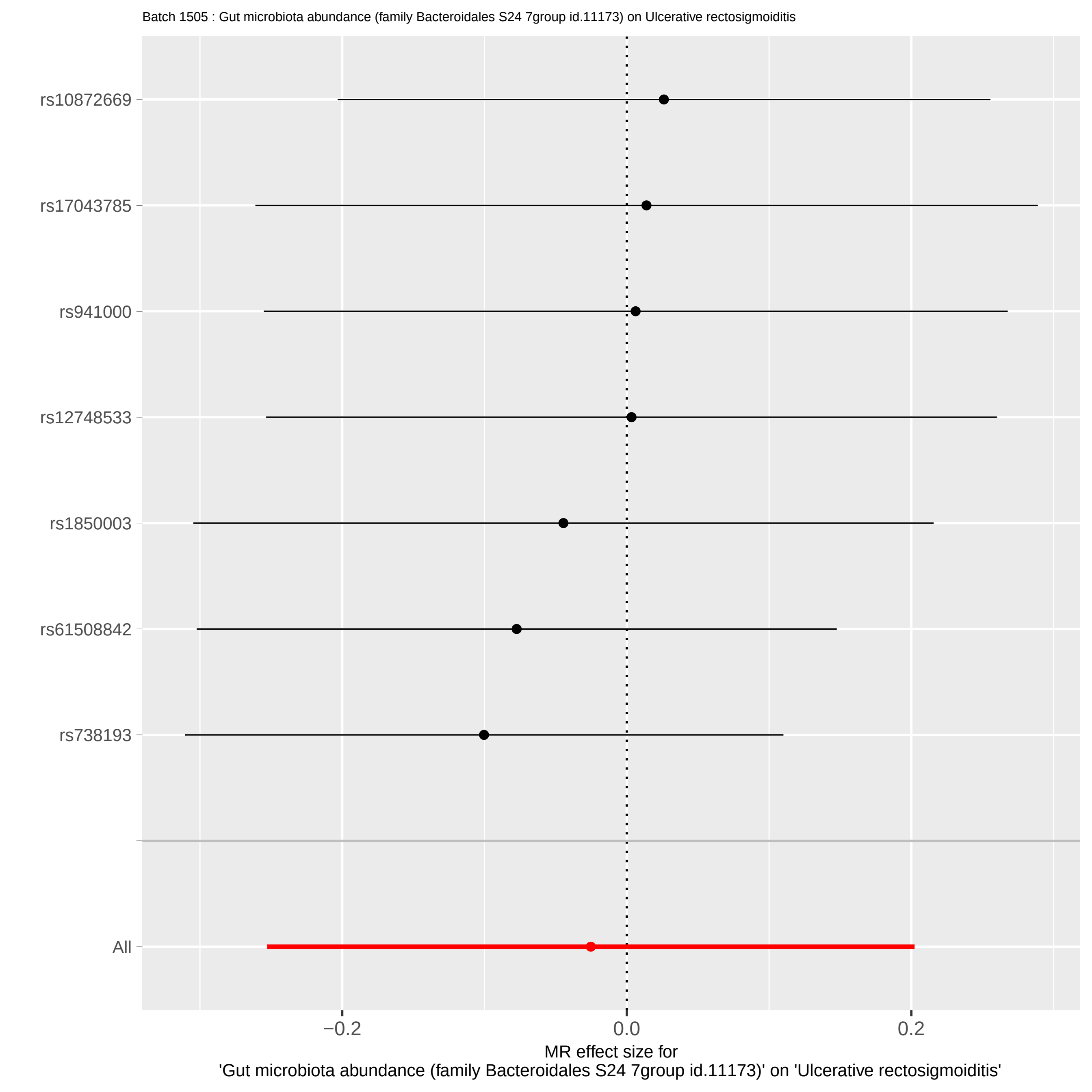

Batch 1505 : Gut microbiota abundance (family Bacteroidales S24 7group id.11173) on Ulcerative rectosigmoiditis
rs10872669
rs17043785
rs941000
rs12748533
rs1850003
rs61508842
rs738193
All
−0.2
0.0
0.2
MR effect size for
'Gut microbiota abundance (family Bacteroidales S24 7group id.11173)' on 'Ulcerative rectosigmoiditis'

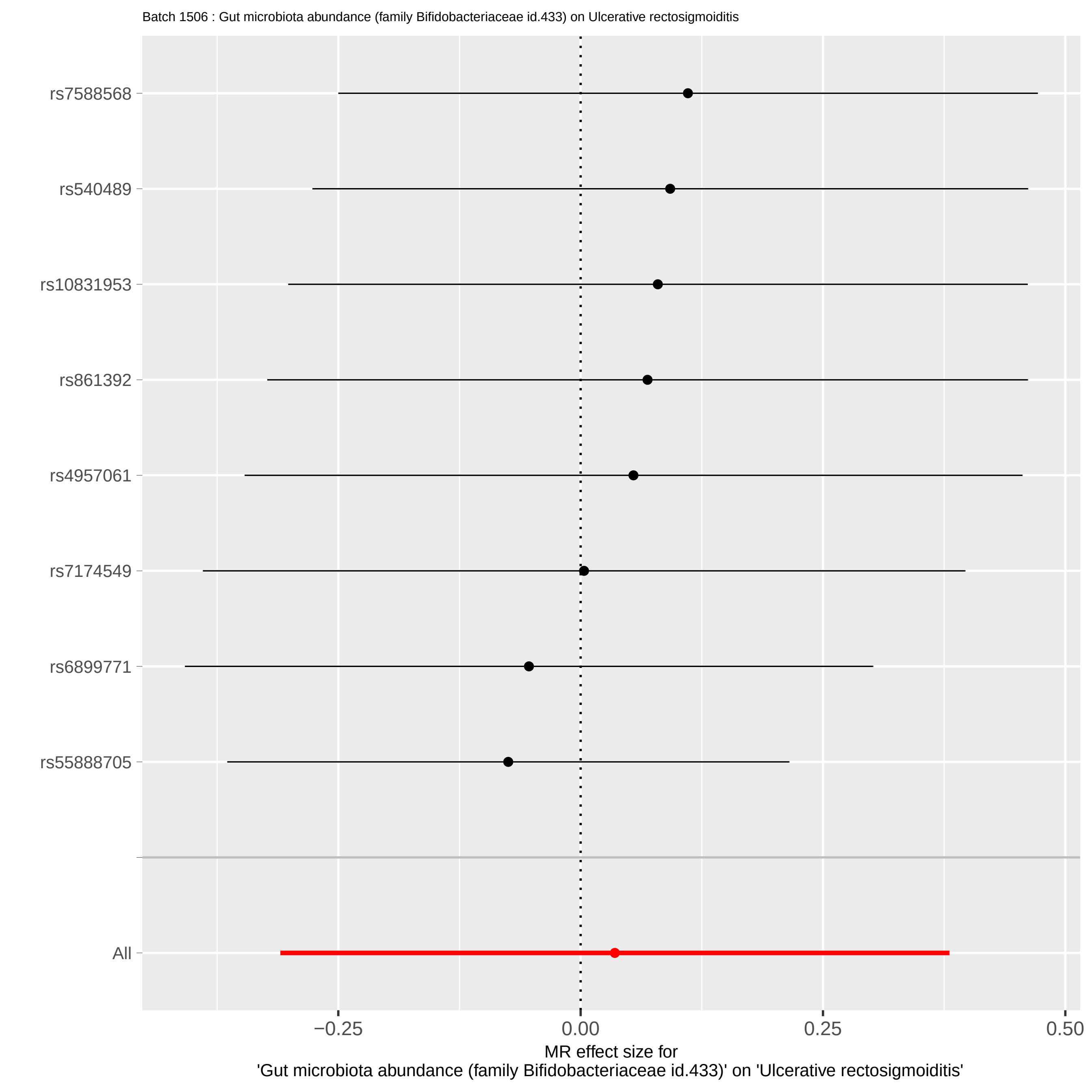

Batch 1506 : Gut microbiota abundance (family Bifidobacteriaceae id.433) on Ulcerative rectosigmoiditis
rs7588568
rs540489
rs10831953
rs861392
rs4957061
rs7174549
rs6899771
rs55888705
All
−0.25
0.00
0.25
0.50
MR effect size for
'Gut microbiota abundance (family Bifidobacteriaceae id.433)' on 'Ulcerative rectosigmoiditis'

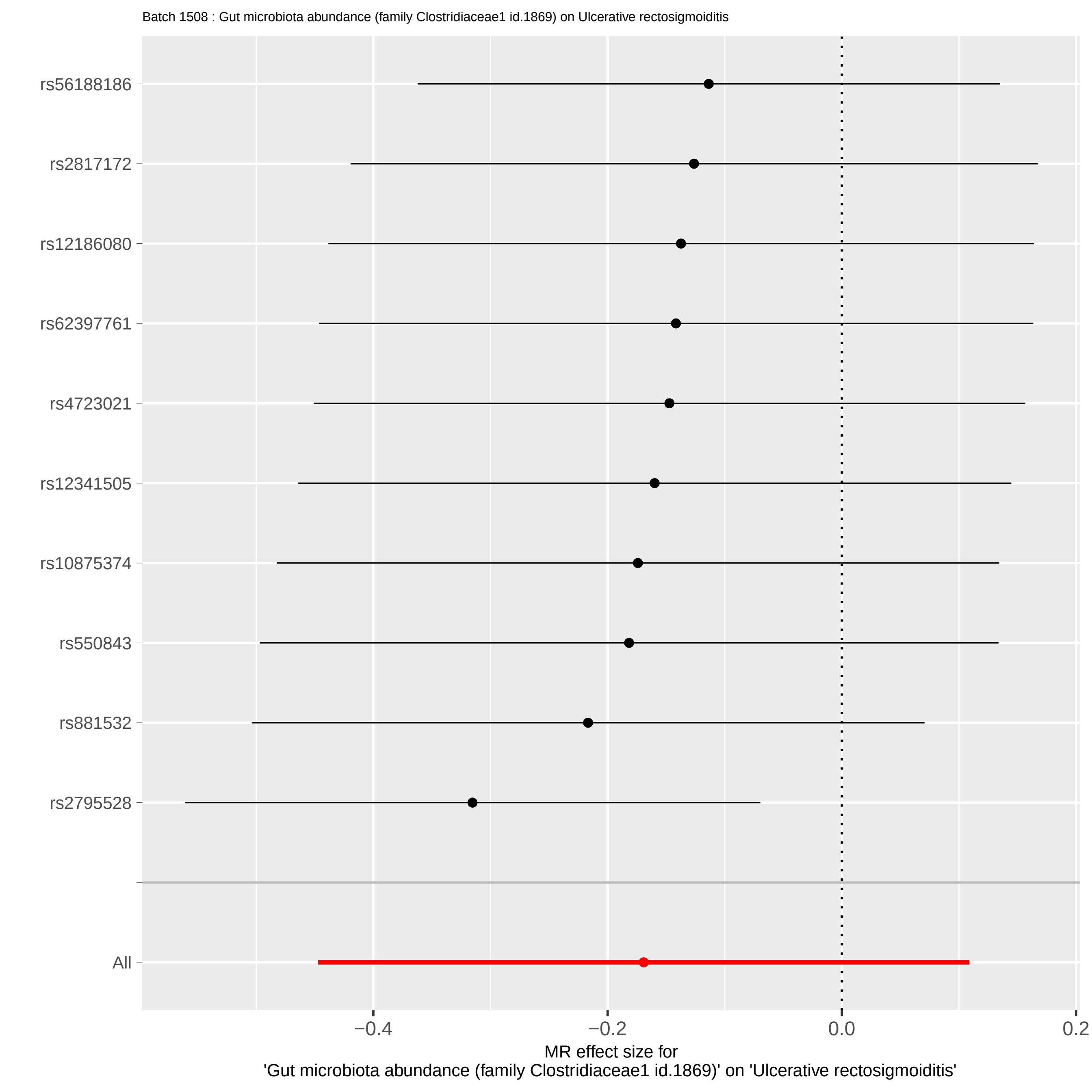

Batch 1508 : Gut microbiota abundance (family Clostridiaceae1 id.1869) on Ulcerative rectosigmoiditis
rs56188186
rs2817172
rs12186080
rs62397761
rs4723021
rs12341505
rs10875374
rs550843
rs881532
rs2795528
All
−0.4
−0.2
0.0
0.2
MR effect size for
'Gut microbiota abundance (family Clostridiaceae1 id.1869)' on 'Ulcerative rectosigmoiditis'

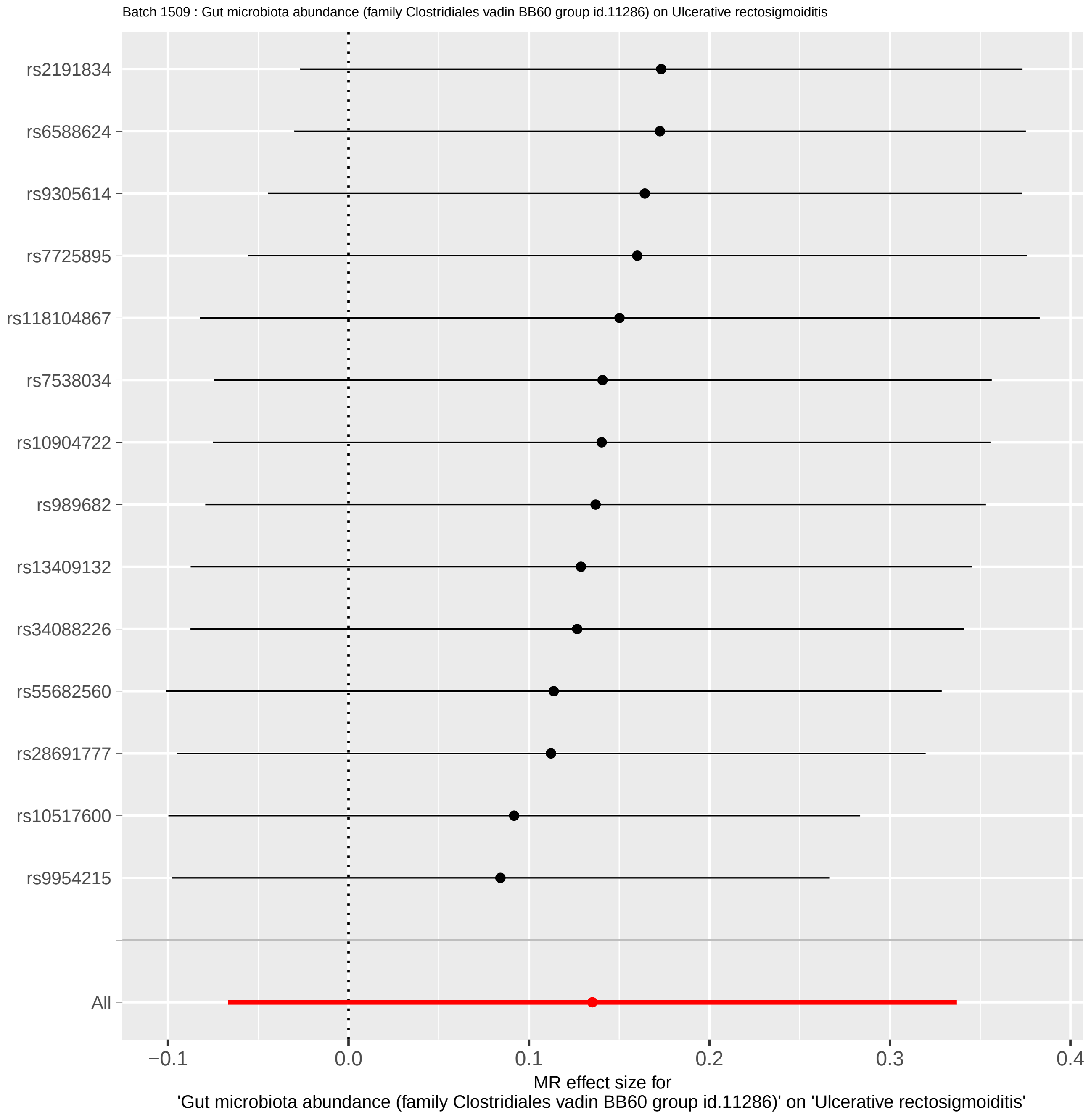
Batch 1509 : Gut microbiota abundance (family Clostridiales vadin BB60 group id.11286) on Ulcerative rectosigmoiditis
rs2191834
rs6588624
rs9305614
rs7725895
rs118104867
rs7538034
rs10904722
rs989682
rs13409132
rs34088226
rs55682560
rs28691777
rs10517600
rs9954215
All
−0.1
0.0
0.1
0.2
0.3
0.4
MR effect size for
'Gut microbiota abundance (family Clostridiales vadin BB60 group id.11286)' on 'Ulcerative rectosigmoiditis'

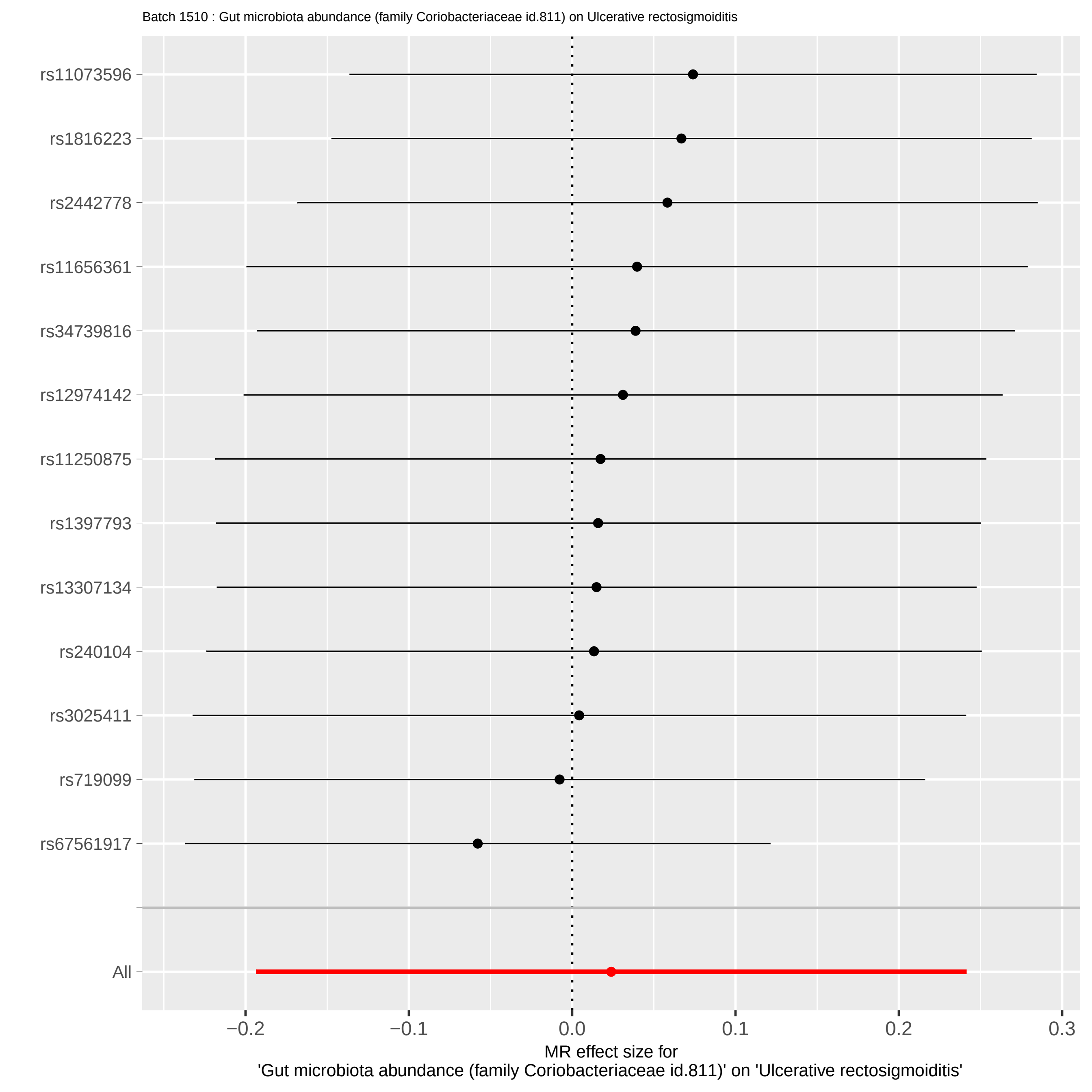

Batch 1510 : Gut microbiota abundance (family Coriobacteriaceae id.811) on Ulcerative rectosigmoiditis
rs11073596
rs1816223
rs2442778
rs11656361
rs34739816
rs12974142
rs11250875
rs1397793
rs13307134
rs240104
rs3025411
rs719099
rs67561917
All
−0.2
−0.1
0.0
0.1
0.2
0.3
MR effect size for
'Gut microbiota abundance (family Coriobacteriaceae id.811)' on 'Ulcerative rectosigmoiditis'

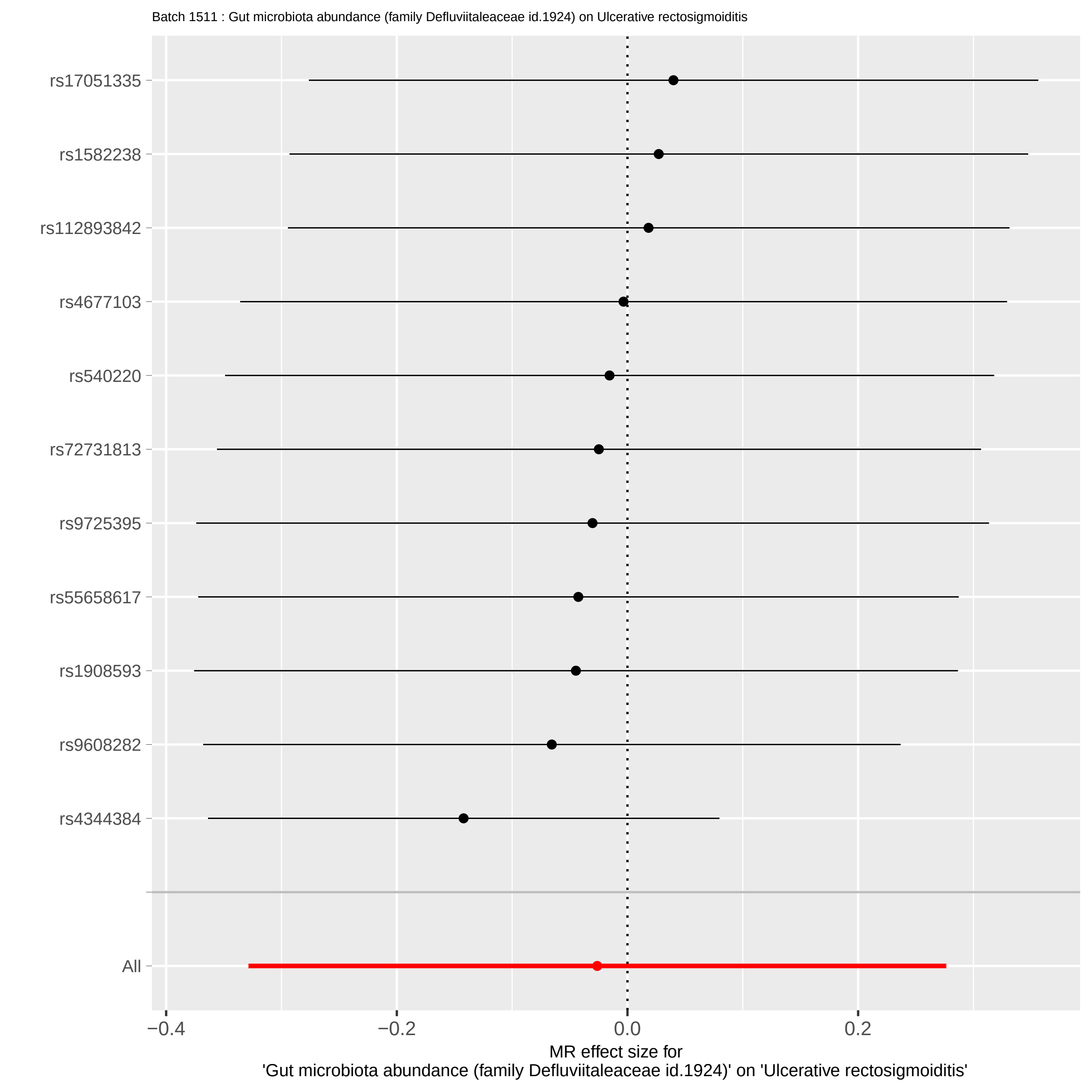

Batch 1511 : Gut microbiota abundance (family Defluviitaleaceae id.1924) on Ulcerative rectosigmoiditis
rs17051335
rs1582238
rs112893842
rs4677103
rs540220
rs72731813
rs9725395
rs55658617
rs1908593
rs9608282
rs4344384
All
−0.4
−0.2
0.0
0.2
MR effect size for
'Gut microbiota abundance (family Defluviitaleaceae id.1924)' on 'Ulcerative rectosigmoiditis'

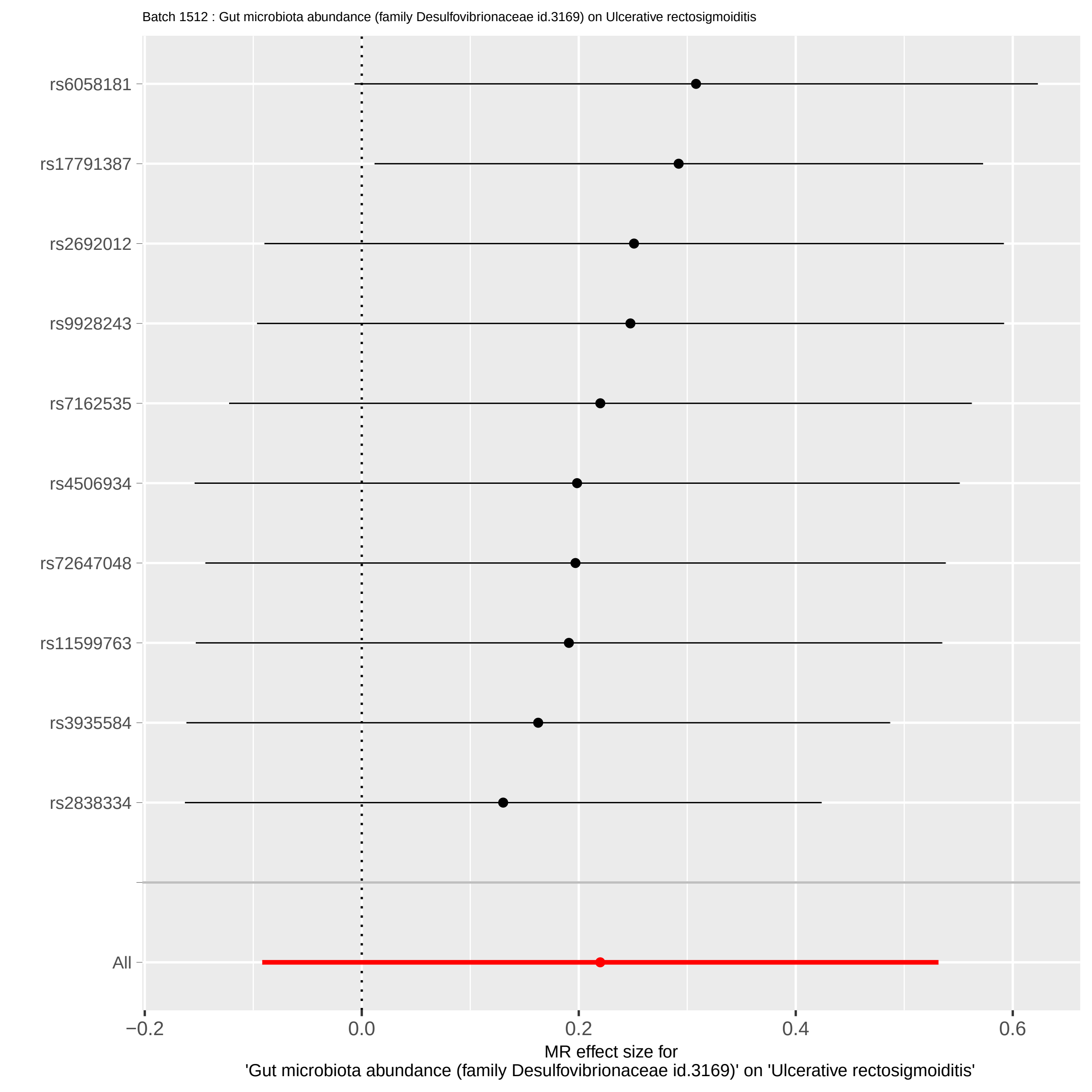
Batch 1512 : Gut microbiota abundance (family Desulfovibrionaceae id.3169) on Ulcerative rectosigmoiditis
rs6058181
rs17791387
rs2692012
rs9928243
rs7162535
rs4506934
rs72647048
rs11599763
rs3935584
rs2838334
All
−0.2
0.0
0.2
0.4
0.6
MR effect size for
'Gut microbiota abundance (family Desulfovibrionaceae id.3169)' on 'Ulcerative rectosigmoiditis'

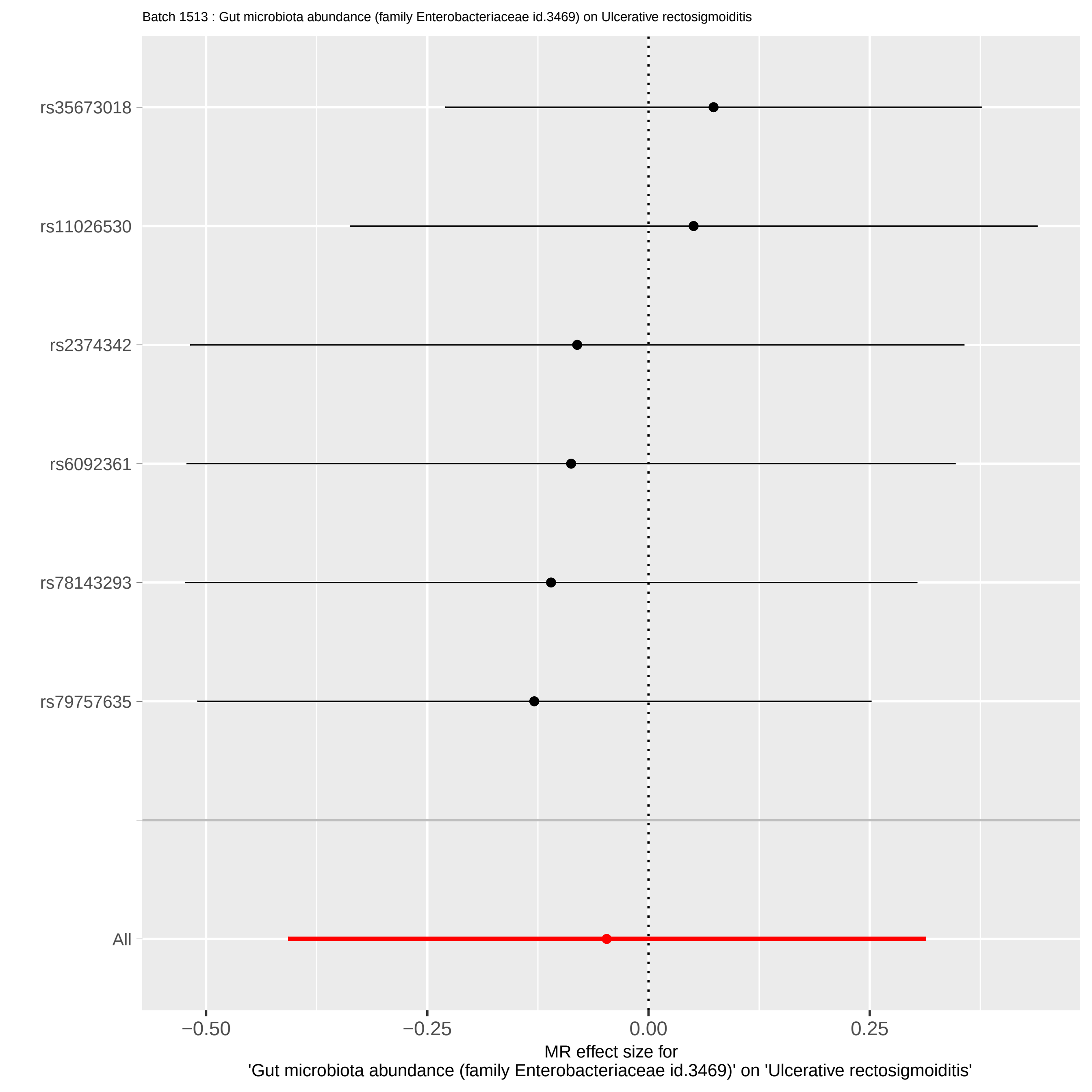

Batch 1513 : Gut microbiota abundance (family Enterobacteriaceae id.3469) on Ulcerative rectosigmoiditis
rs35673018
rs11026530
rs2374342
rs6092361
rs78143293
rs79757635
All
−0.50
−0.25
0.00
0.25
MR effect size for
'Gut microbiota abundance (family Enterobacteriaceae id.3469)' on 'Ulcerative rectosigmoiditis'

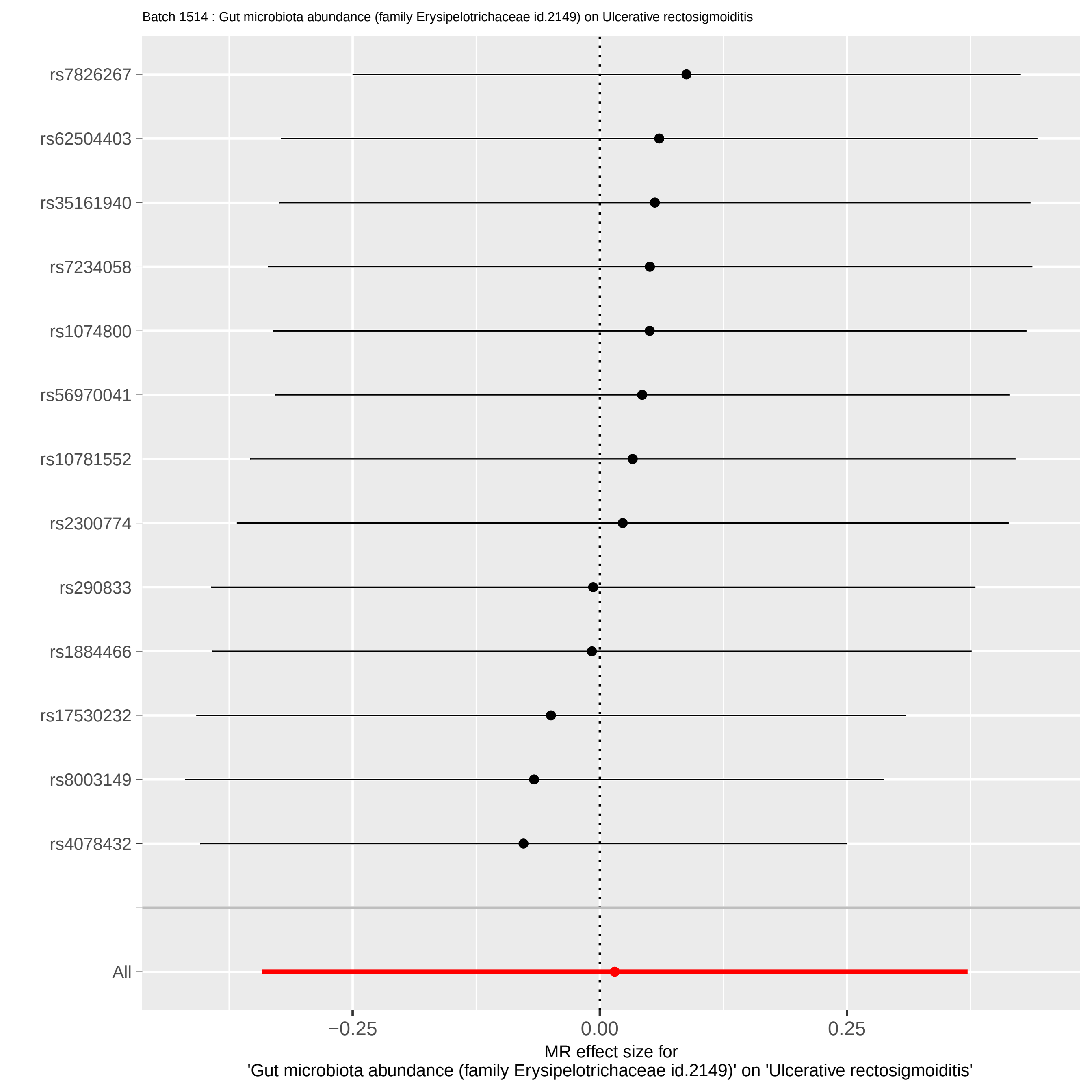

Batch 1514 : Gut microbiota abundance (family Erysipelotrichaceae id.2149) on Ulcerative rectosigmoiditis
rs7826267
rs62504403
rs35161940
rs7234058
rs1074800
rs56970041
rs10781552
rs2300774
rs290833
rs1884466
rs17530232
rs8003149
rs4078432
All
−0.25
0.00
0.25
MR effect size for
'Gut microbiota abundance (family Erysipelotrichaceae id.2149)' on 'Ulcerative rectosigmoiditis'

Batch 1515 : Gut microbiota abundance (family Family XI id.1936) on Ulcerative rectosigmoiditis

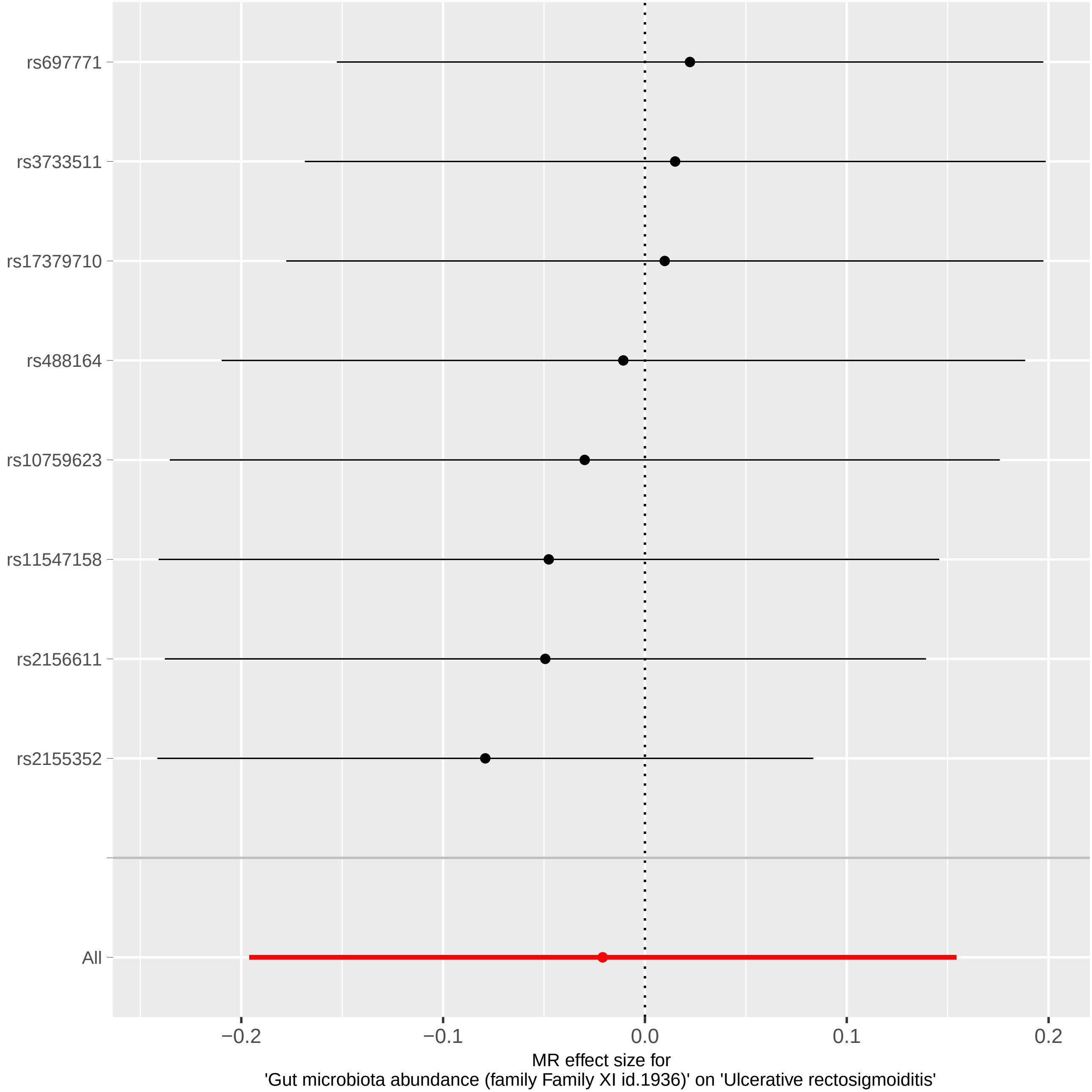

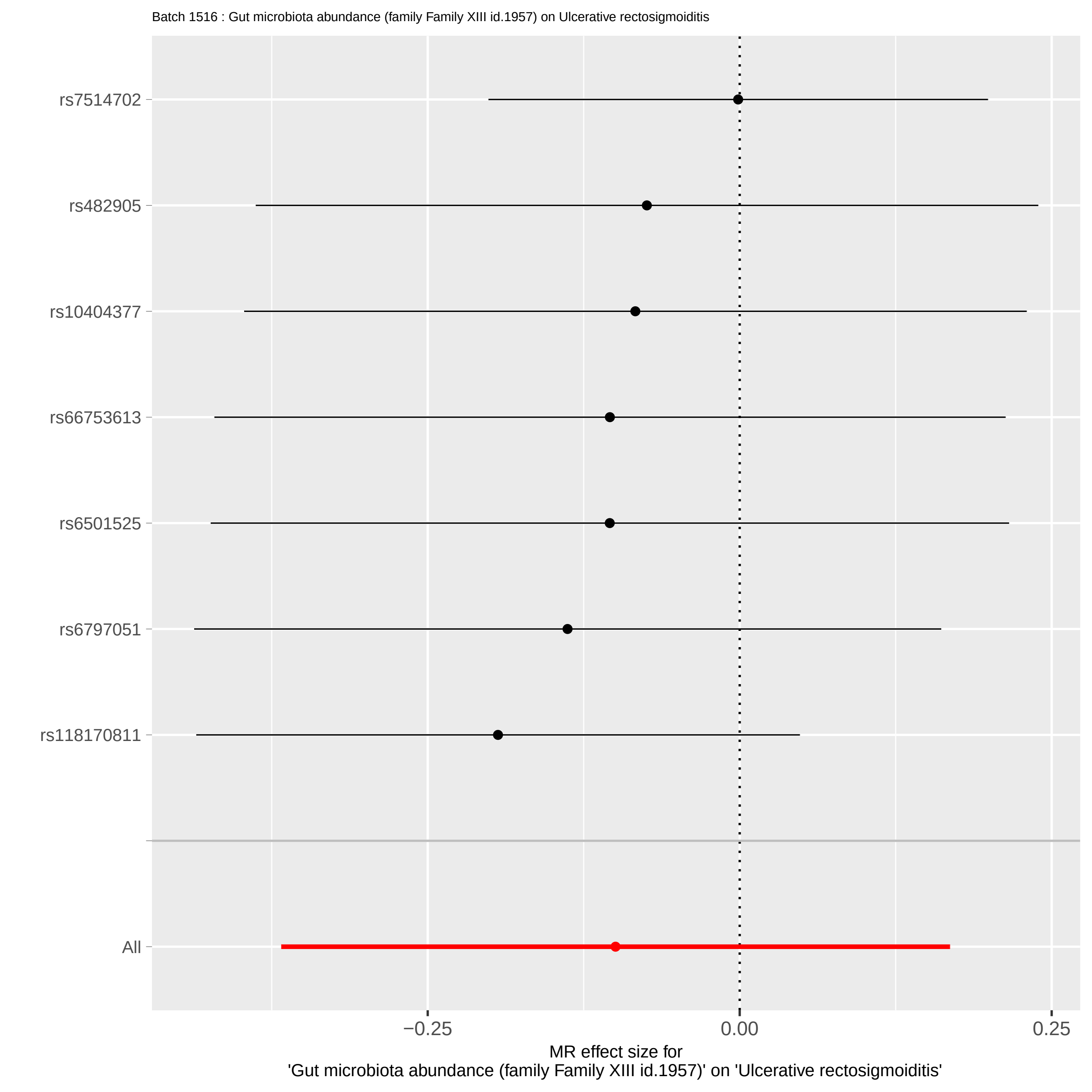

Batch 1516 : Gut microbiota abundance (family Family XIII id.1957) on Ulcerative rectosigmoiditis
rs7514702
rs482905
rs10404377
rs66753613
rs6501525
rs6797051
rs118170811
All
−0.25
0.00
0.25
MR effect size for
'Gut microbiota abundance (family Family XIII id.1957)' on 'Ulcerative rectosigmoiditis'

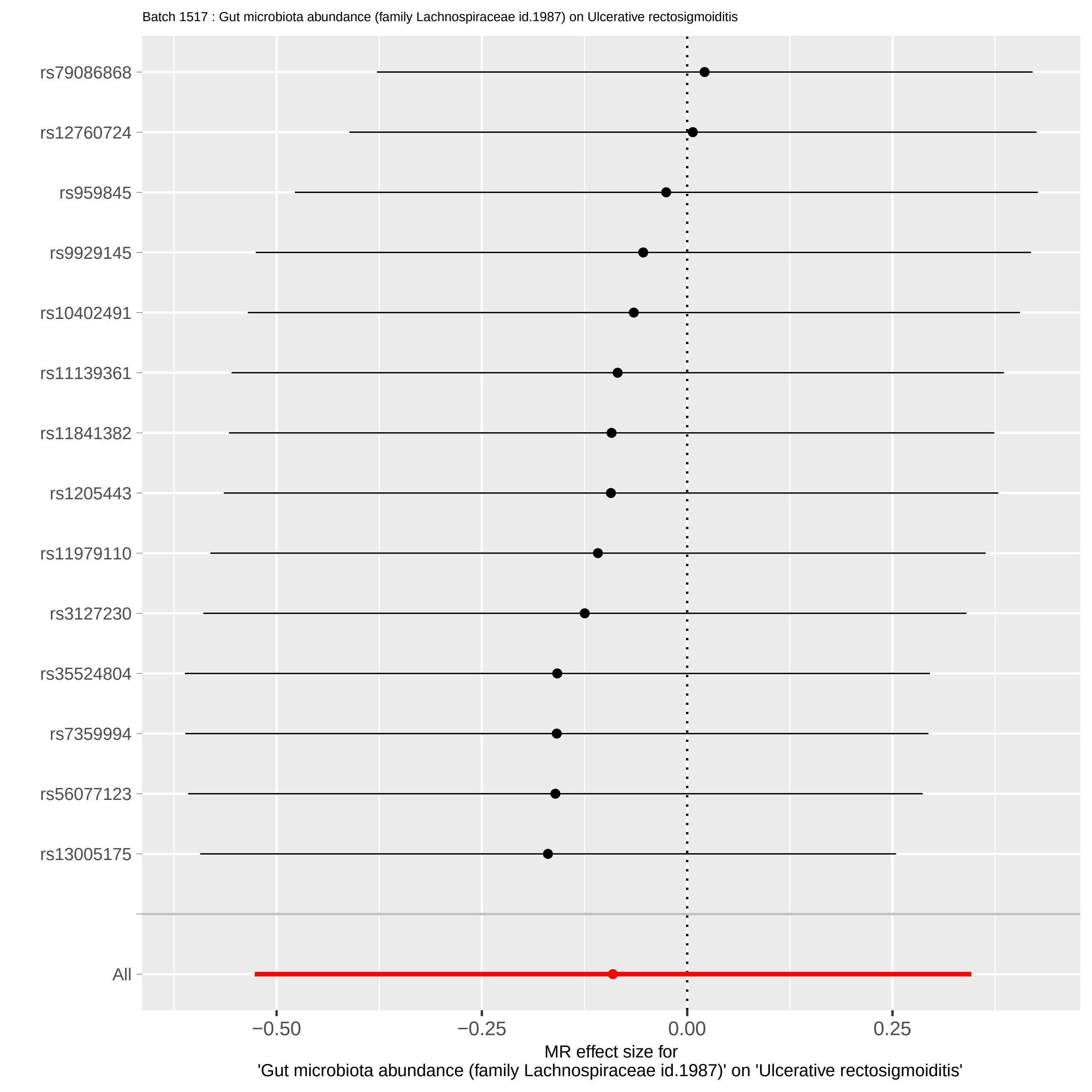

Batch 1517 : Gut microbiota abundance (family Lachnospiraceae id.1987) on Ulcerative rectosigmoiditis
rs79086868
rs12760724
rs959845
rs9929145
rs10402491
rs11139361
rs11841382
rs1205443
rs11979110
rs3127230
rs35524804
rs7359994
rs56077123
rs13005175
All
−0.50
−0.25
0.00
0.25
MR effect size for
'Gut microbiota abundance (family Lachnospiraceae id.1987)' on 'Ulcerative rectosigmoiditis'

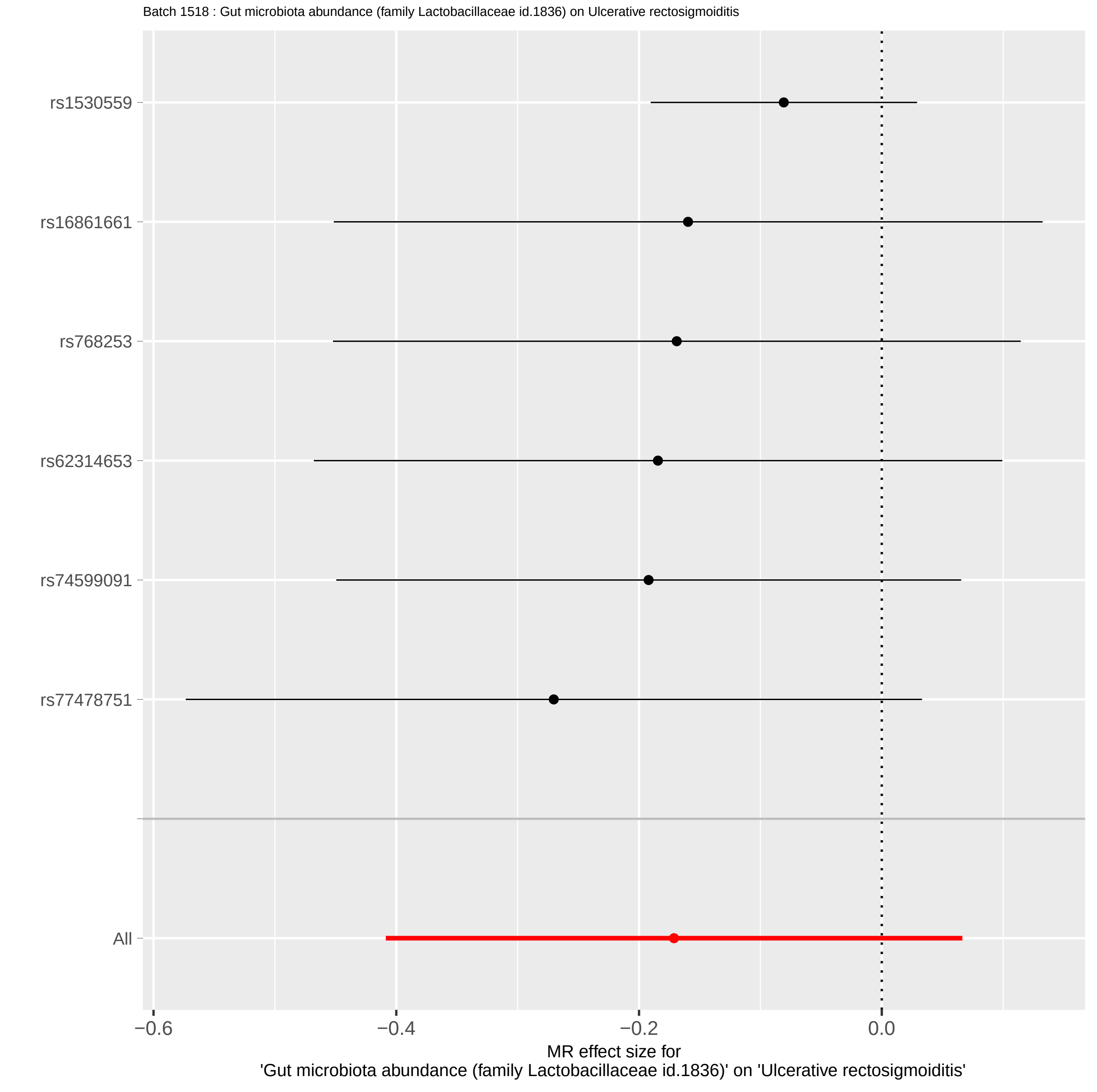

Batch 1518 : Gut microbiota abundance (family Lactobacillaceae id.1836) on Ulcerative rectosigmoiditis
rs1530559
rs16861661
rs768253
rs62314653
rs74599091
rs77478751
All
−0.6
−0.4
−0.2
0.0
MR effect size for
'Gut microbiota abundance (family Lactobacillaceae id.1836)' on 'Ulcerative rectosigmoiditis'

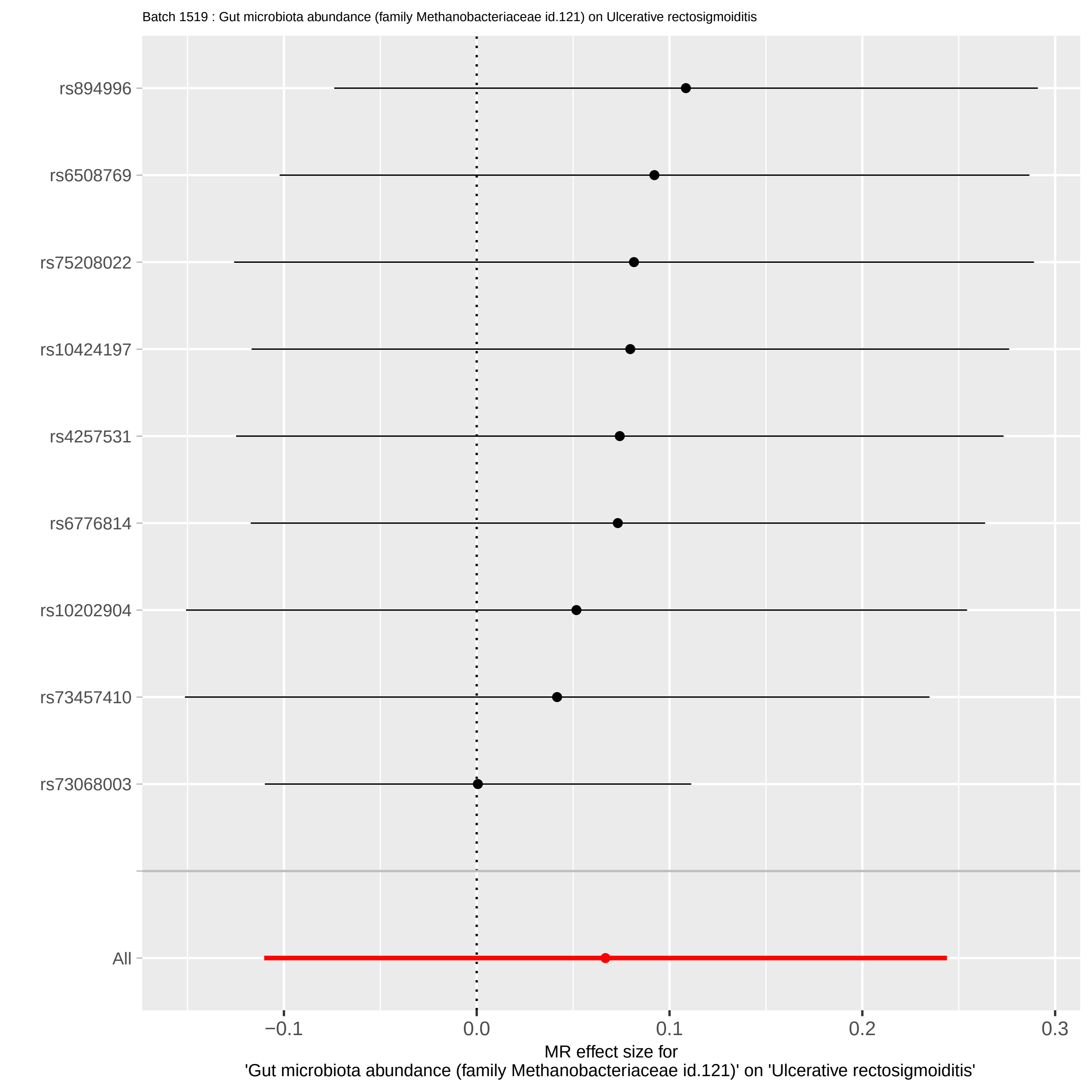

Batch 1519 : Gut microbiota abundance (family Methanobacteriaceae id.121) on Ulcerative rectosigmoiditis
rs894996
rs6508769
rs75208022
rs10424197
rs4257531
rs6776814
rs10202904
rs73457410
rs73068003
All
−0.1
0.0
0.1
0.2
0.3
MR effect size for
'Gut microbiota abundance (family Methanobacteriaceae id.121)' on 'Ulcerative rectosigmoiditis'

Batch 1520 : Gut microbiota abundance (family Oxalobacteraceae id.2966) on Ulcerative rectosigmoiditis

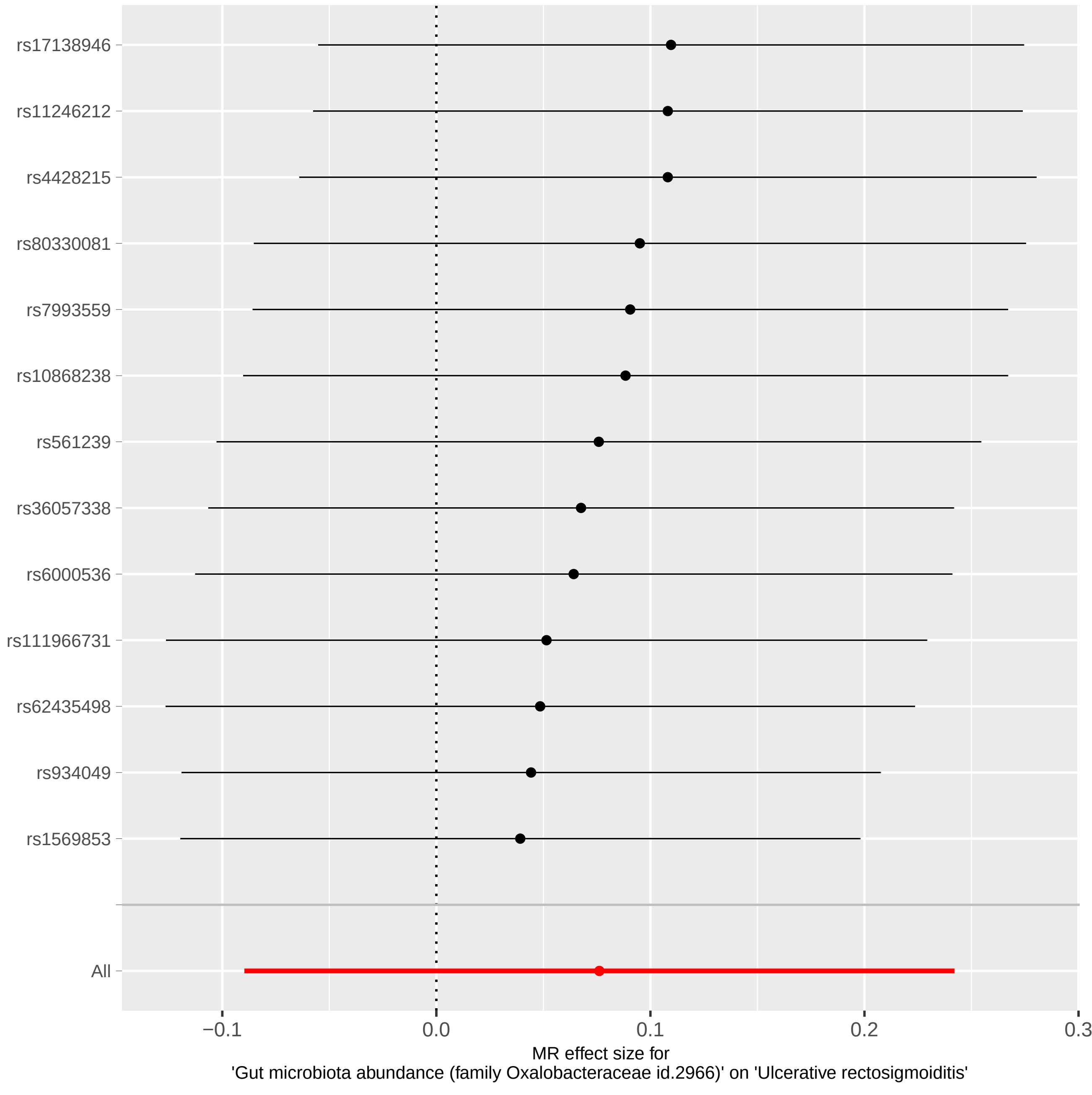

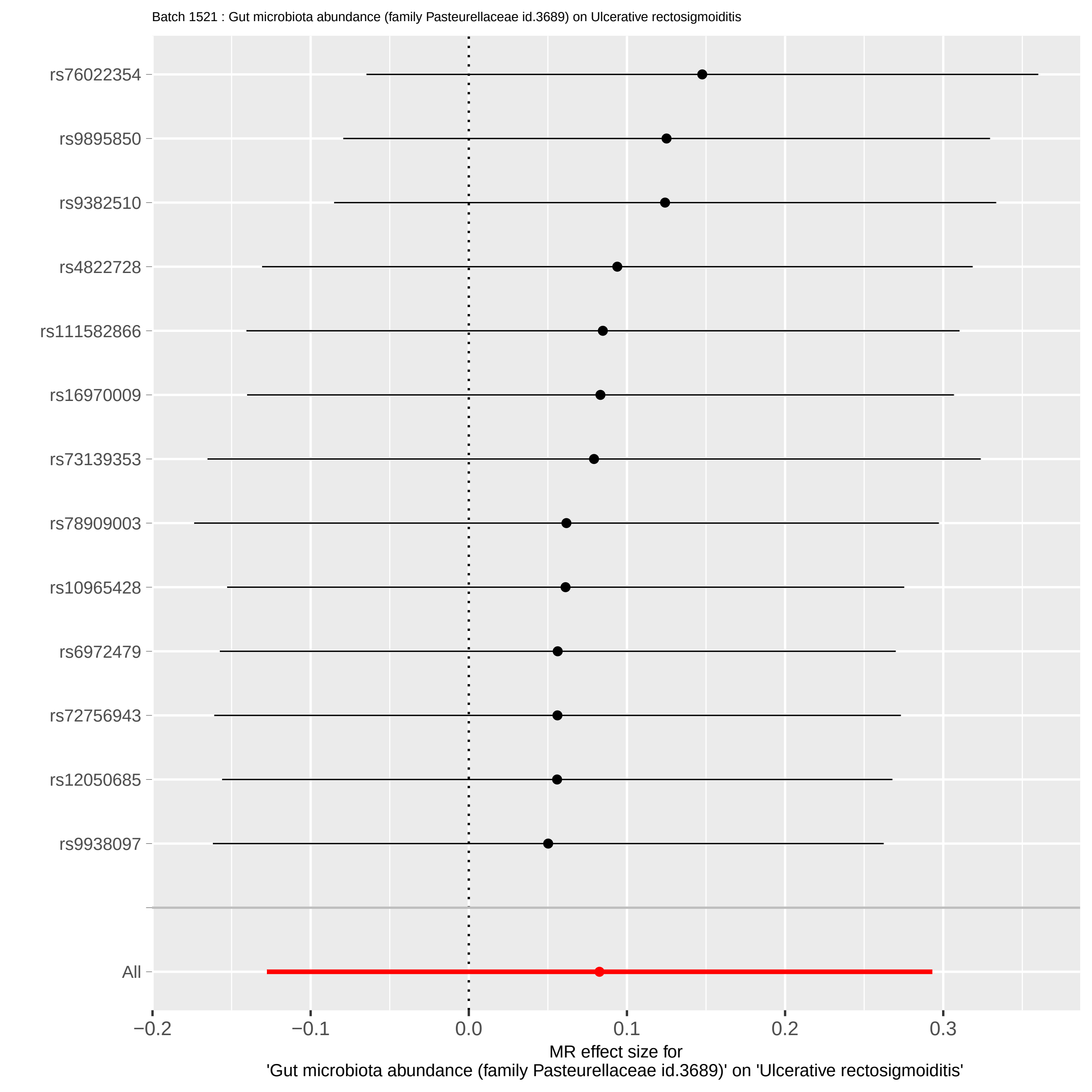

Batch 1521 : Gut microbiota abundance (family Pasteurellaceae id.3689) on Ulcerative rectosigmoiditis
rs76022354
rs9895850
rs9382510
rs4822728
rs111582866
rs16970009
rs73139353
rs78909003
rs10965428
rs6972479
rs72756943
rs12050685
rs9938097
All
−0.2
−0.1
0.0
0.1
0.2
0.3
MR effect size for
'Gut microbiota abundance (family Pasteurellaceae id.3689)' on 'Ulcerative rectosigmoiditis'

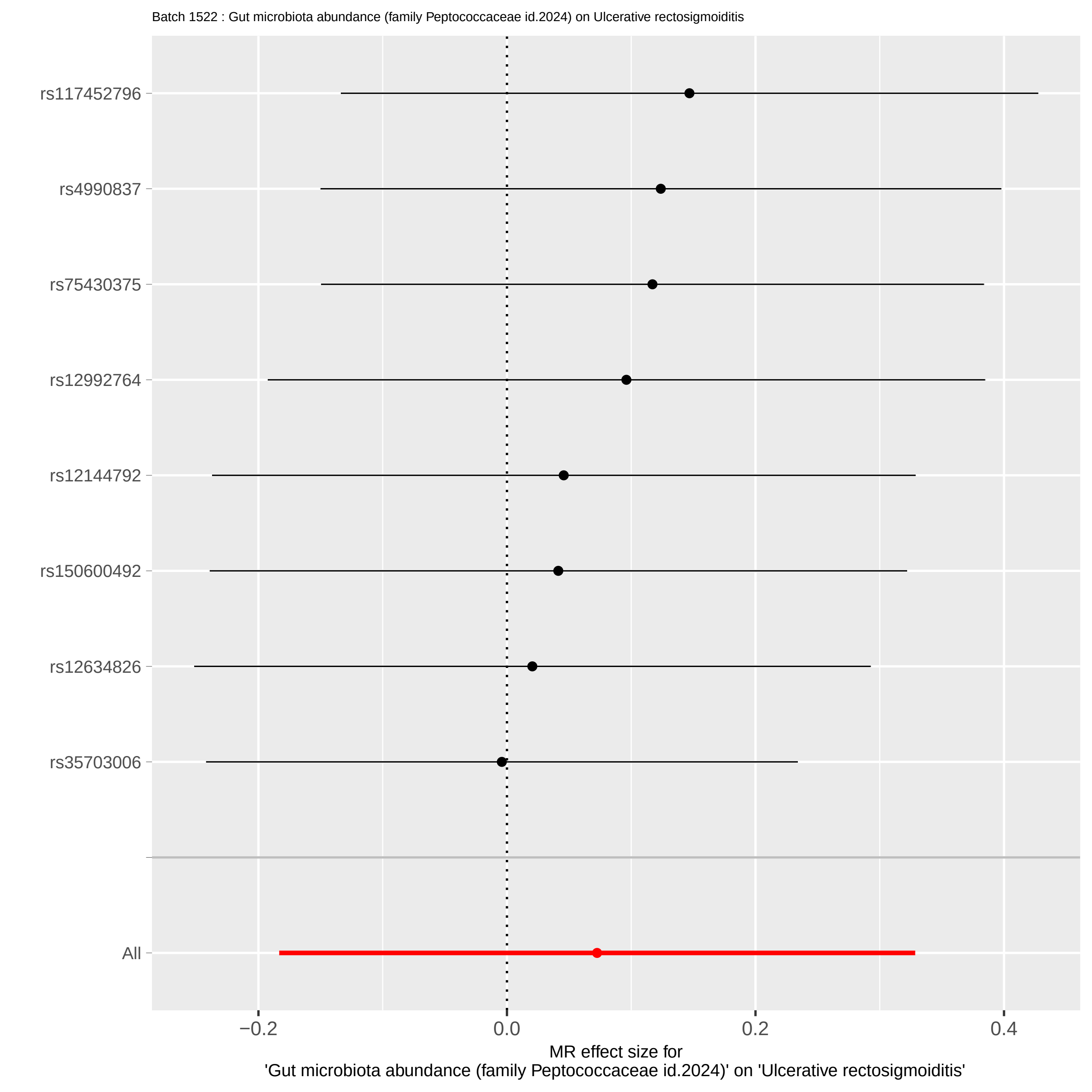

Batch 1522 : Gut microbiota abundance (family Peptococcaceae id.2024) on Ulcerative rectosigmoiditis
rs117452796
rs4990837
rs75430375
rs12992764
rs12144792
rs150600492
rs12634826
rs35703006
All
−0.2
0.0
0.2
0.4
MR effect size for
'Gut microbiota abundance (family Peptococcaceae id.2024)' on 'Ulcerative rectosigmoiditis'

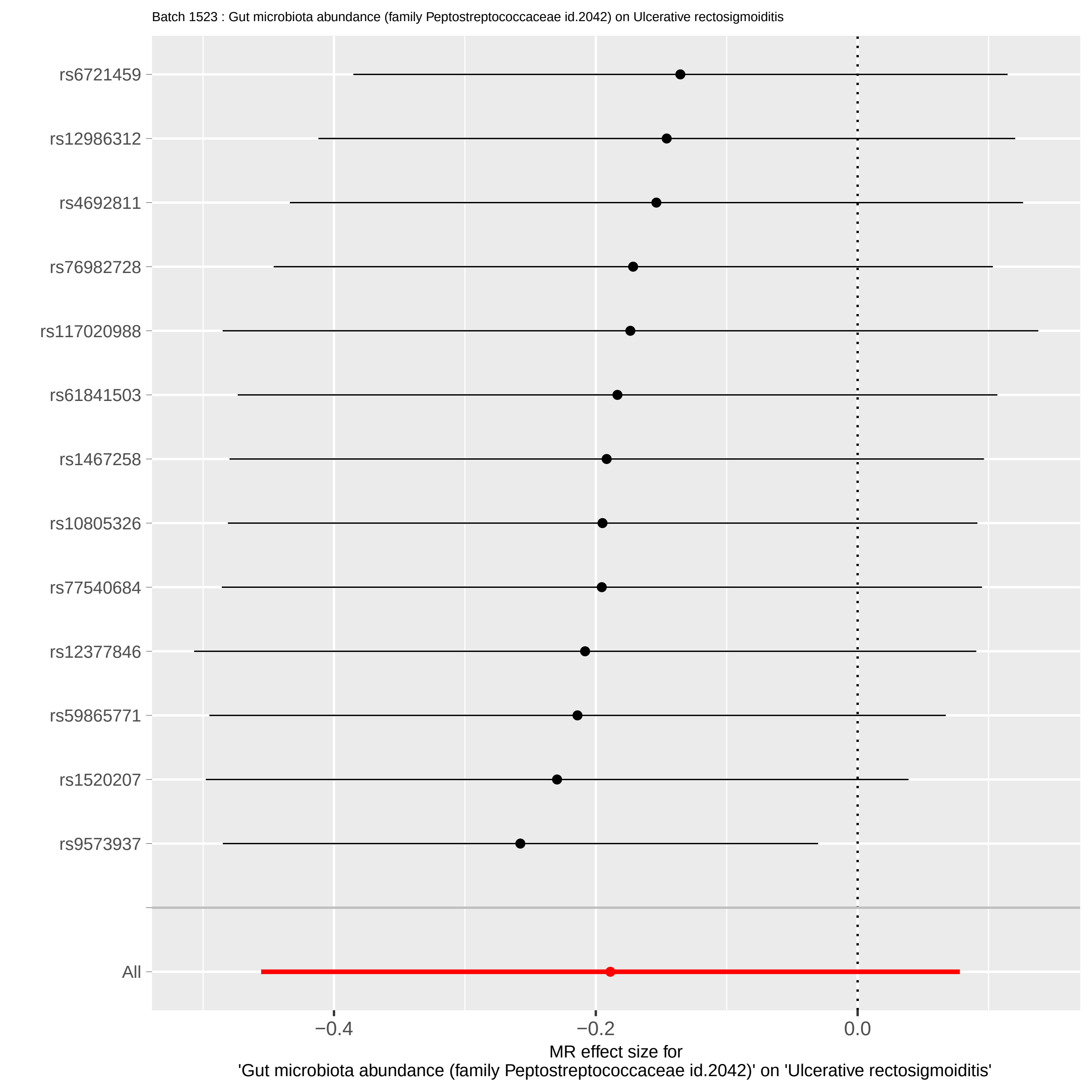

Batch 1523 : Gut microbiota abundance (family Peptostreptococcaceae id.2042) on Ulcerative rectosigmoiditis
rs6721459
rs12986312
rs4692811
rs76982728
rs117020988
rs61841503
rs1467258
rs10805326
rs77540684
rs12377846
rs59865771
rs1520207
rs9573937
All
−0.4
−0.2
0.0
MR effect size for
'Gut microbiota abundance (family Peptostreptococcaceae id.2042)' on 'Ulcerative rectosigmoiditis'

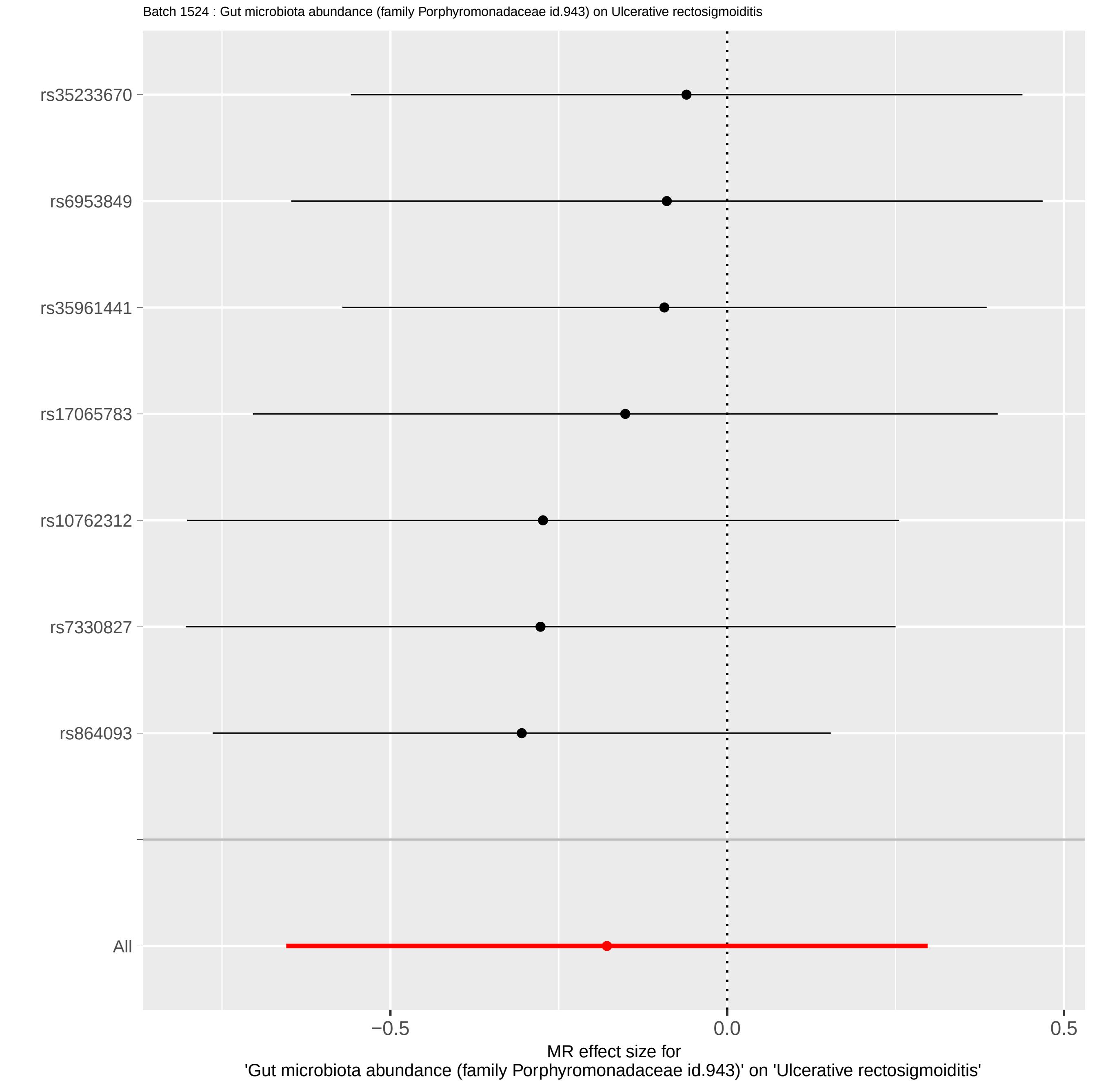

Batch 1524 : Gut microbiota abundance (family Porphyromonadaceae id.943) on Ulcerative rectosigmoiditis
rs35233670
rs6953849
rs35961441
rs17065783
rs10762312
rs7330827
rs864093
All
−0.5
0.0
0.5
MR effect size for
'Gut microbiota abundance (family Porphyromonadaceae id.943)' on 'Ulcerative rectosigmoiditis'

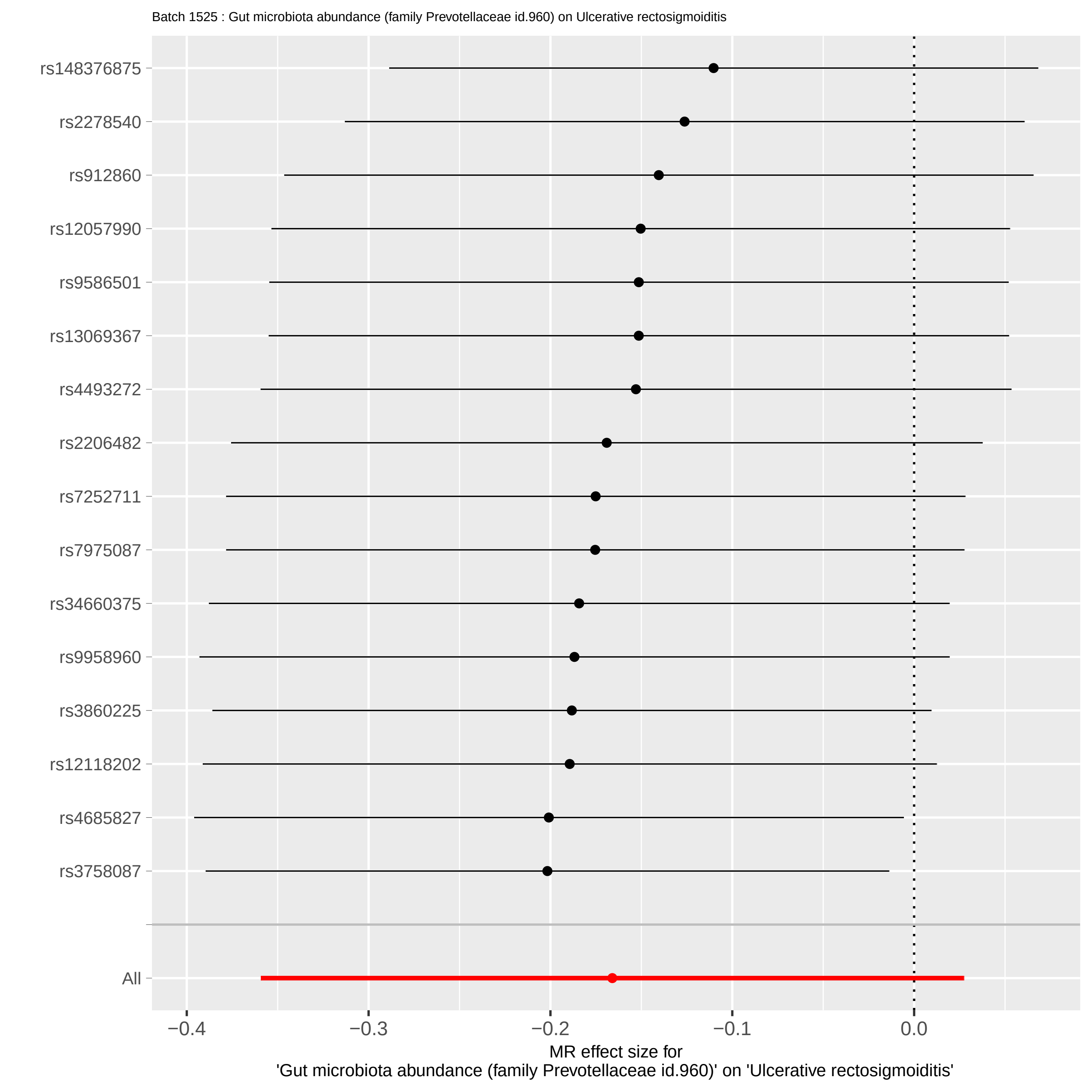

Batch 1525 : Gut microbiota abundance (family Prevotellaceae id.960) on Ulcerative rectosigmoiditis
rs148376875
rs2278540
rs912860
rs12057990
rs9586501
rs13069367
rs4493272
rs2206482
rs7252711
rs7975087
rs34660375
rs9958960
rs3860225
rs12118202
rs4685827
rs3758087
All
−0.4
−0.3
−0.2
−0.1
0.0
MR effect size for
'Gut microbiota abundance (family Prevotellaceae id.960)' on 'Ulcerative rectosigmoiditis'

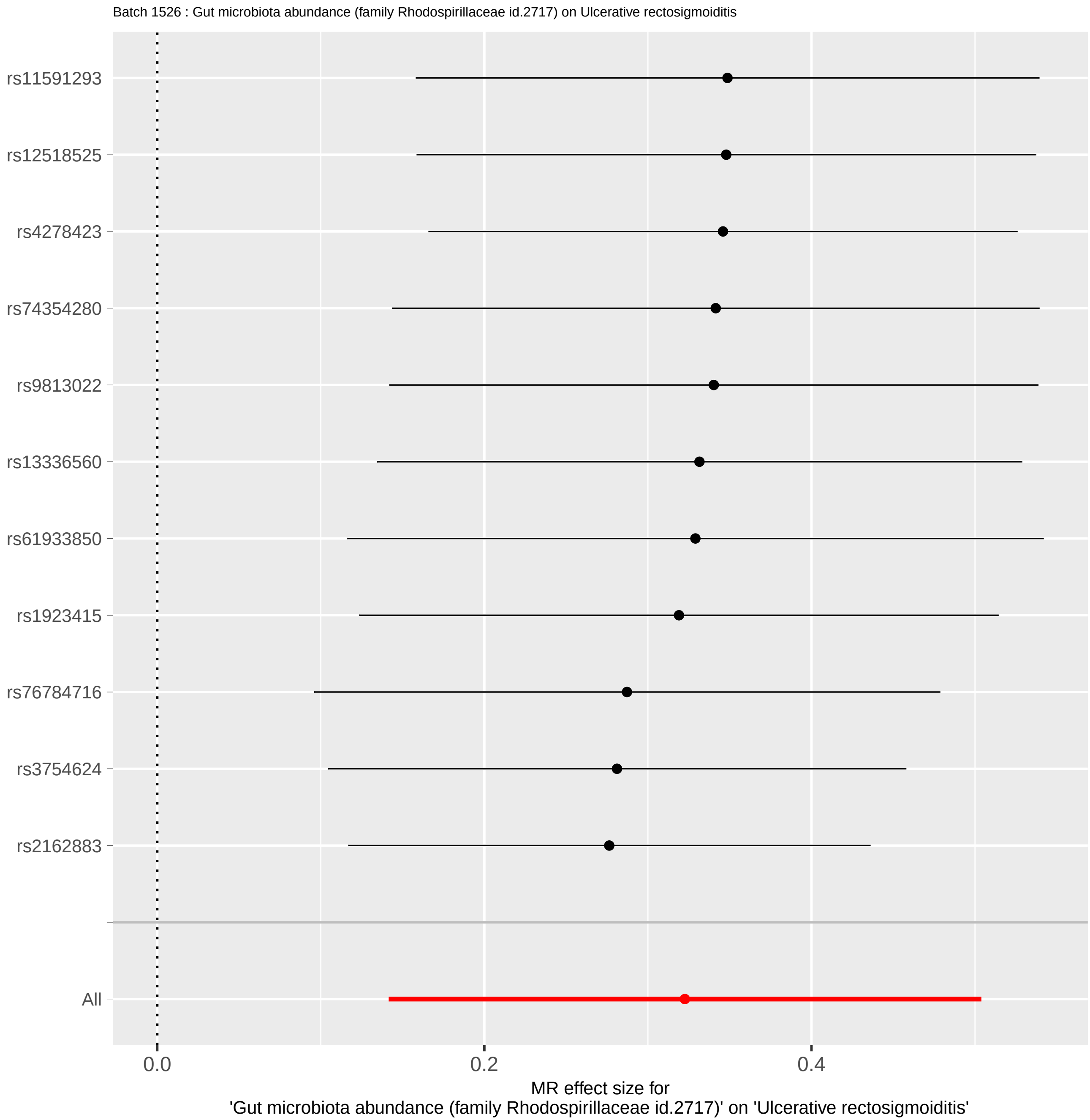
Batch 1526 : Gut microbiota abundance (family Rhodospirillaceae id.2717) on Ulcerative rectosigmoiditis
rs11591293
rs12518525
rs4278423
rs74354280
rs9813022
rs13336560
rs61933850
rs1923415
rs76784716
rs3754624
rs2162883
All
0.0
0.2
0.4
MR effect size for
'Gut microbiota abundance (family Rhodospirillaceae id.2717)' on 'Ulcerative rectosigmoiditis'

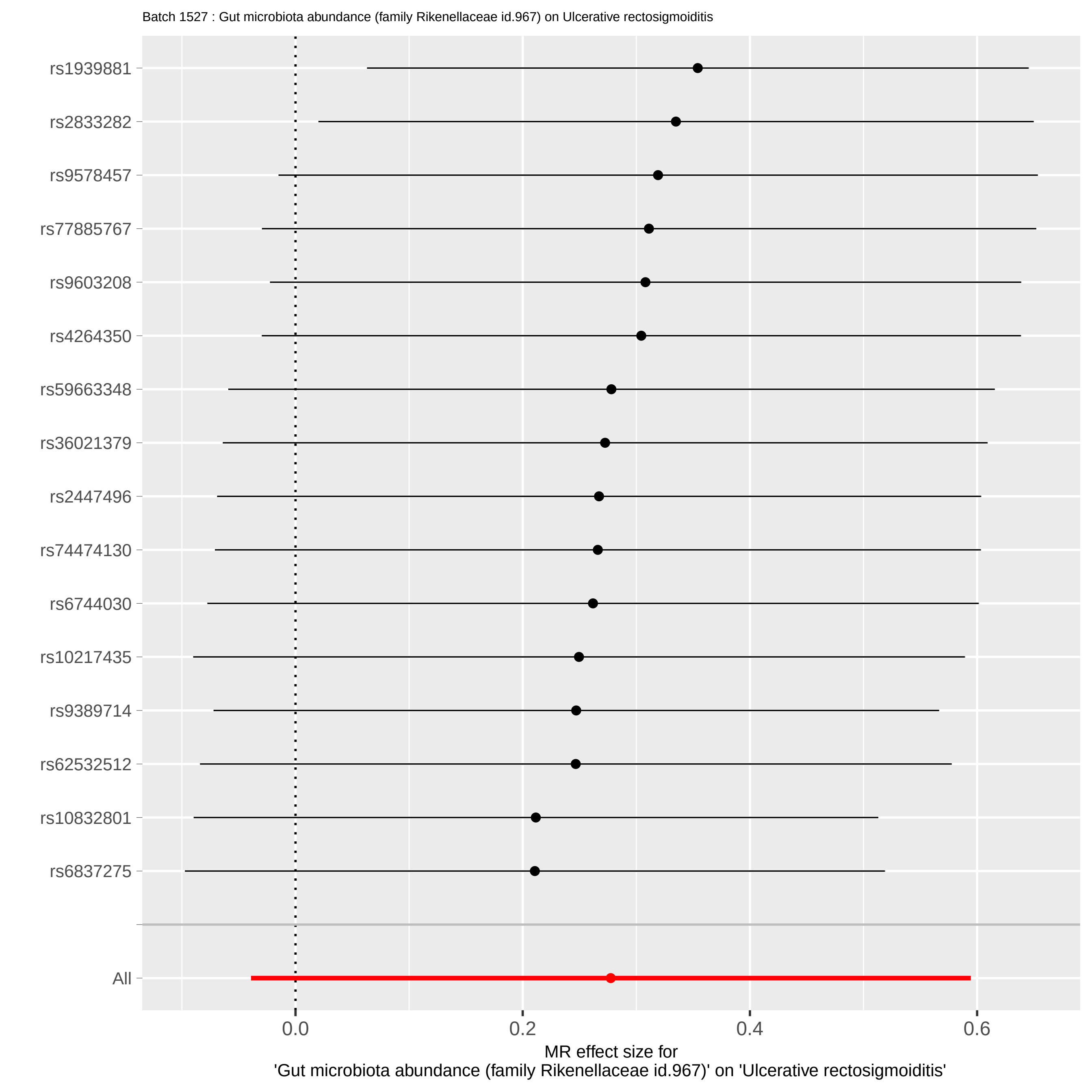
Batch 1527 : Gut microbiota abundance (family Rikenellaceae id.967) on Ulcerative rectosigmoiditis
rs1939881
rs2833282
rs9578457
rs77885767
rs9603208
rs4264350
rs59663348
rs36021379
rs2447496
rs74474130
rs6744030
rs10217435
rs9389714
rs62532512
rs10832801
rs6837275
All
0.0
0.2
0.4
0.6
MR effect size for
'Gut microbiota abundance (family Rikenellaceae id.967)' on 'Ulcerative rectosigmoiditis'

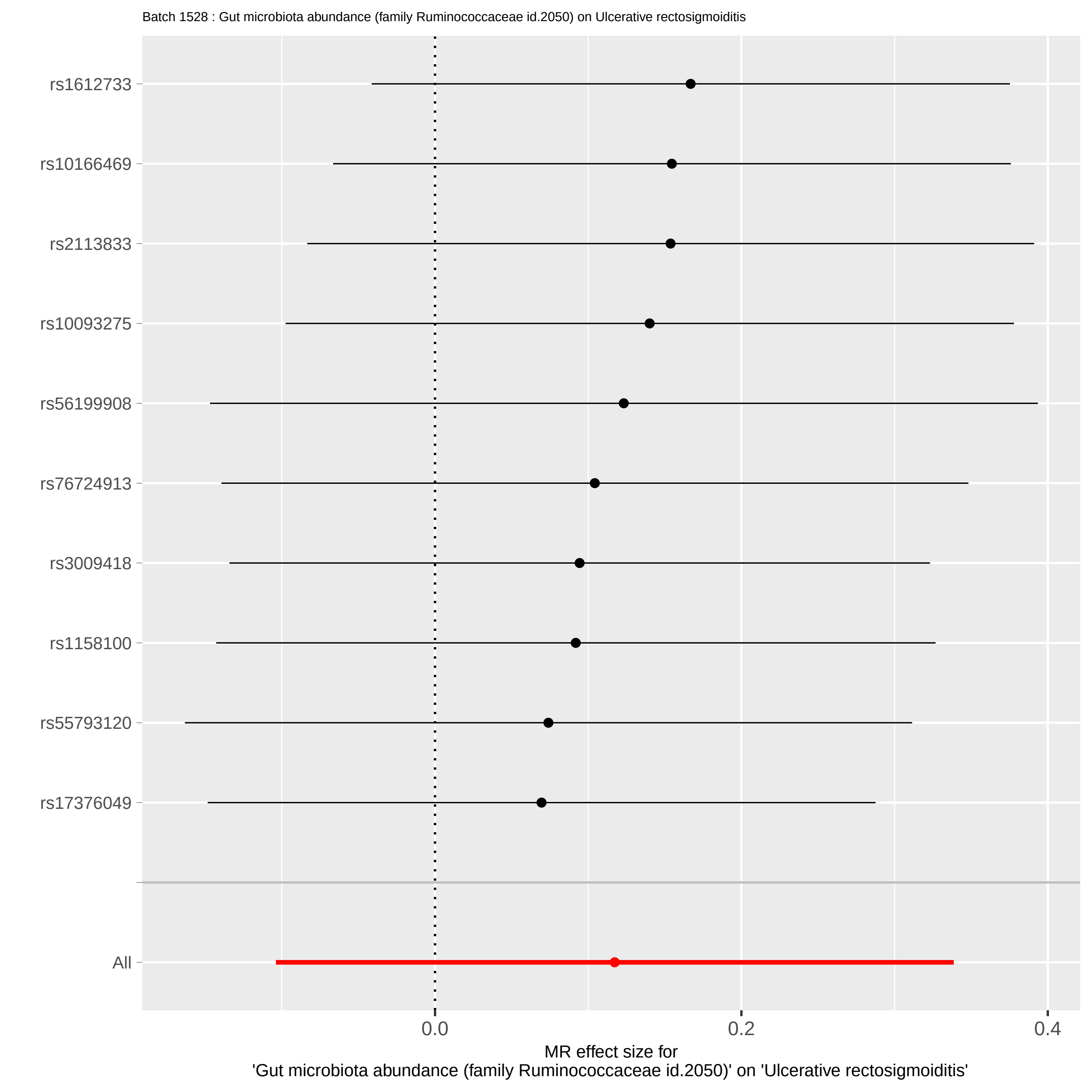

Batch 1528 : Gut microbiota abundance (family Ruminococcaceae id.2050) on Ulcerative rectosigmoiditis
rs1612733
rs10166469
rs2113833
rs10093275
rs56199908
rs76724913
rs3009418
rs1158100
rs55793120
rs17376049
All
0.0
0.2
0.4
MR effect size for
'Gut microbiota abundance (family Ruminococcaceae id.2050)' on 'Ulcerative rectosigmoiditis'

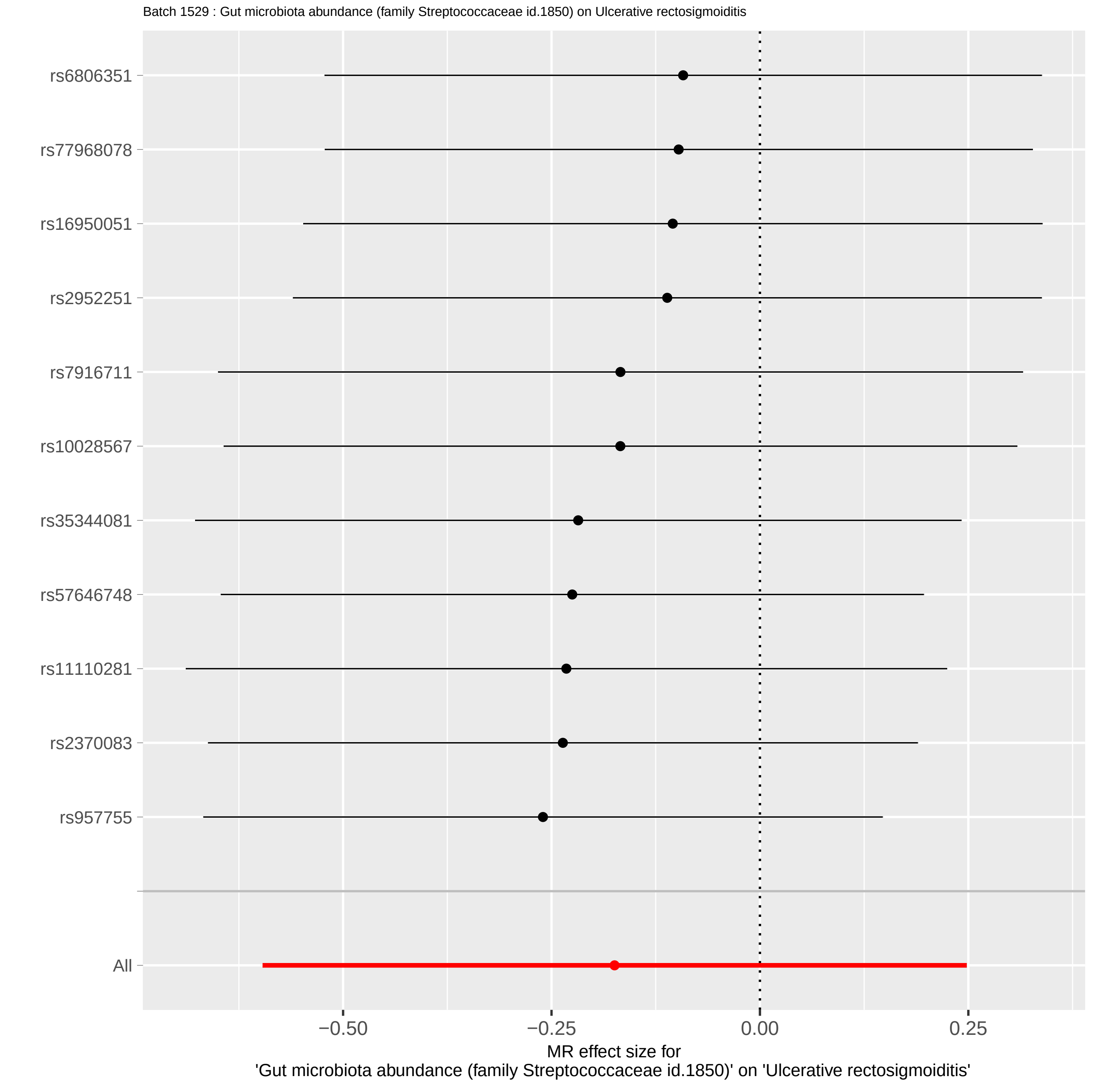
Batch 1529 : Gut microbiota abundance (family Streptococcaceae id.1850) on Ulcerative rectosigmoiditis
rs6806351
rs77968078
rs16950051
rs2952251
rs7916711
rs10028567
rs35344081
rs57646748
rs11110281
rs2370083
rs957755
All
−0.50
−0.25
0.00
0.25
MR effect size for
'Gut microbiota abundance (family Streptococcaceae id.1850)' on 'Ulcerative rectosigmoiditis'

Batch 1530 : Gut microbiota abundance (family Veillonellaceae id.2172) on Ulcerative rectosigmoiditis

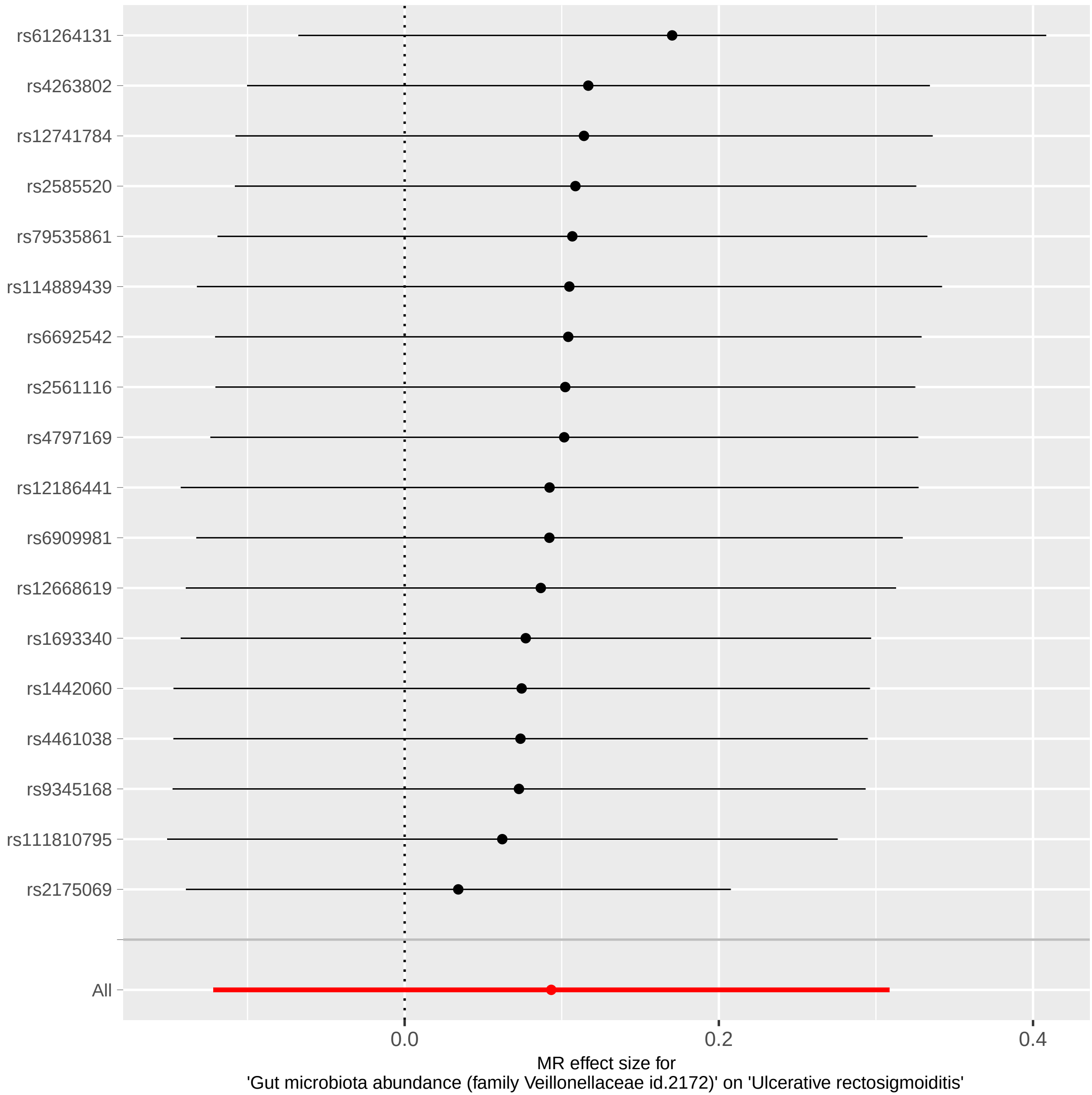

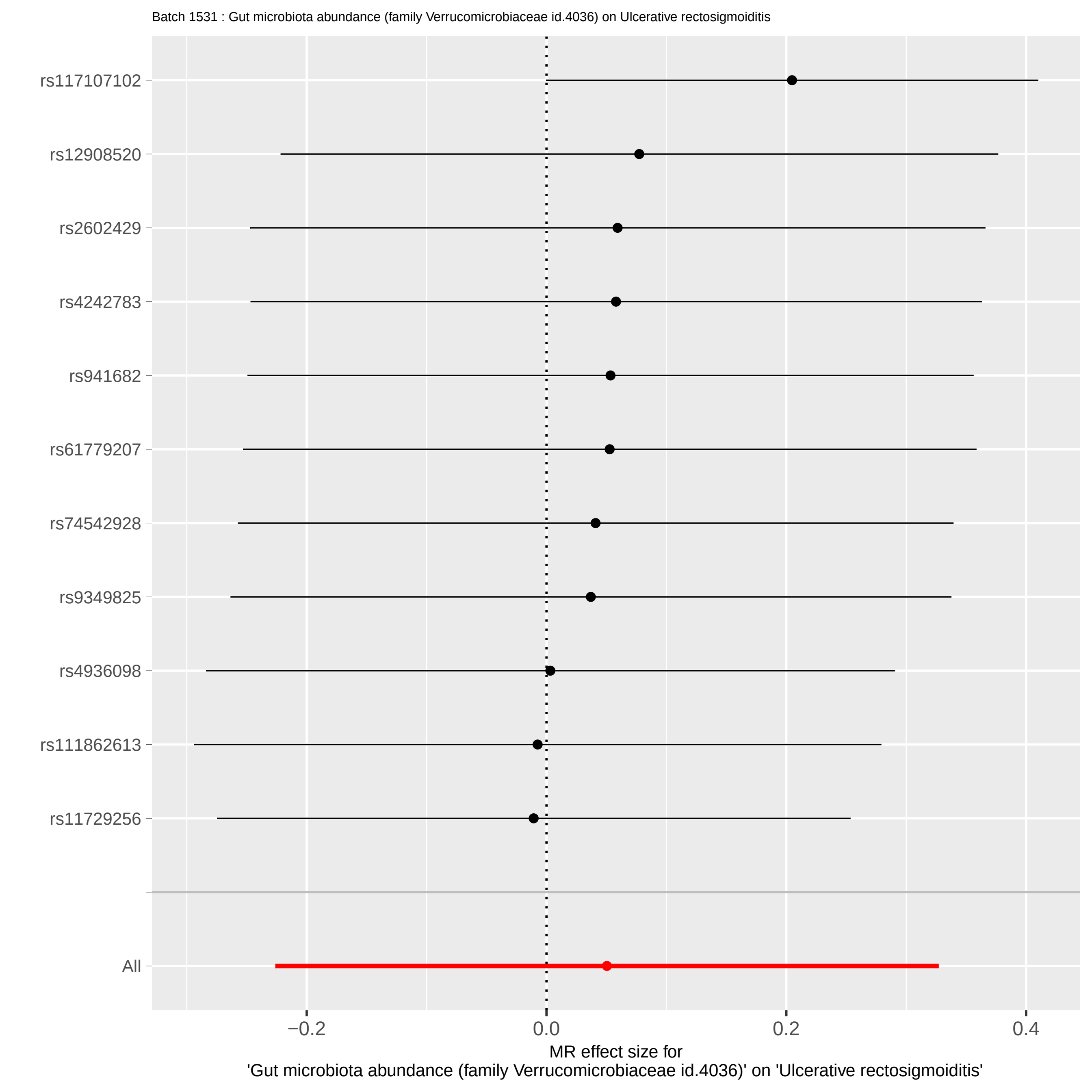

Batch 1531 : Gut microbiota abundance (family Verrucomicrobiaceae id.4036) on Ulcerative rectosigmoiditis
rs117107102
rs12908520
rs2602429
rs4242783
rs941682
rs61779207
rs74542928
rs9349825
rs4936098
rs111862613
rs11729256
All
−0.2
0.0
0.2
0.4
MR effect size for
'Gut microbiota abundance (family Verrucomicrobiaceae id.4036)' on 'Ulcerative rectosigmoiditis'

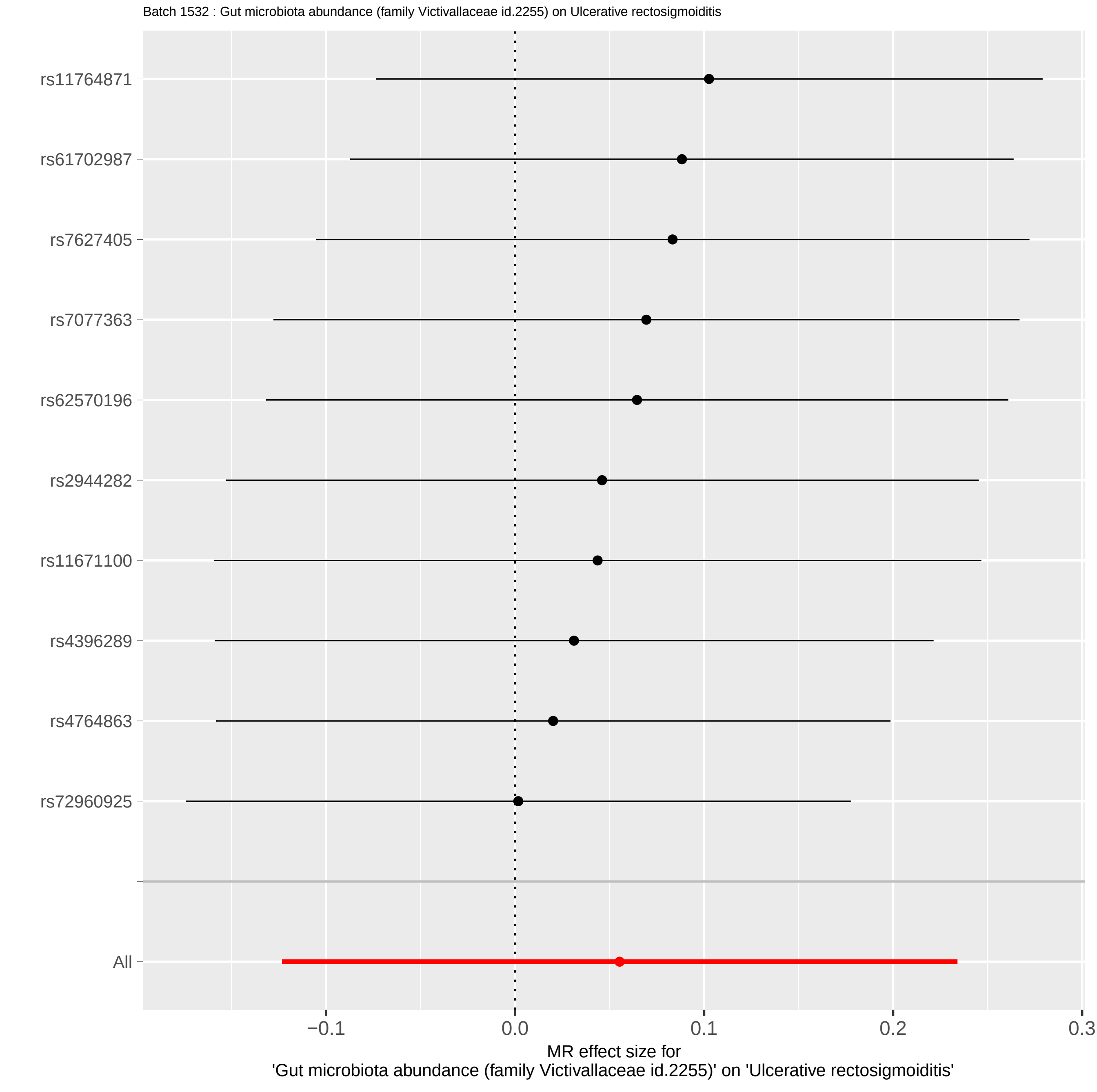

Batch 1532 : Gut microbiota abundance (family Victivallaceae id.2255) on Ulcerative rectosigmoiditis
rs11764871
rs61702987
rs7627405
rs7077363
rs62570196
rs2944282
rs11671100
rs4396289
rs4764863
rs72960925
All
−0.1
0.0
0.1
0.2
0.3
MR effect size for
'Gut microbiota abundance (family Victivallaceae id.2255)' on 'Ulcerative rectosigmoiditis'

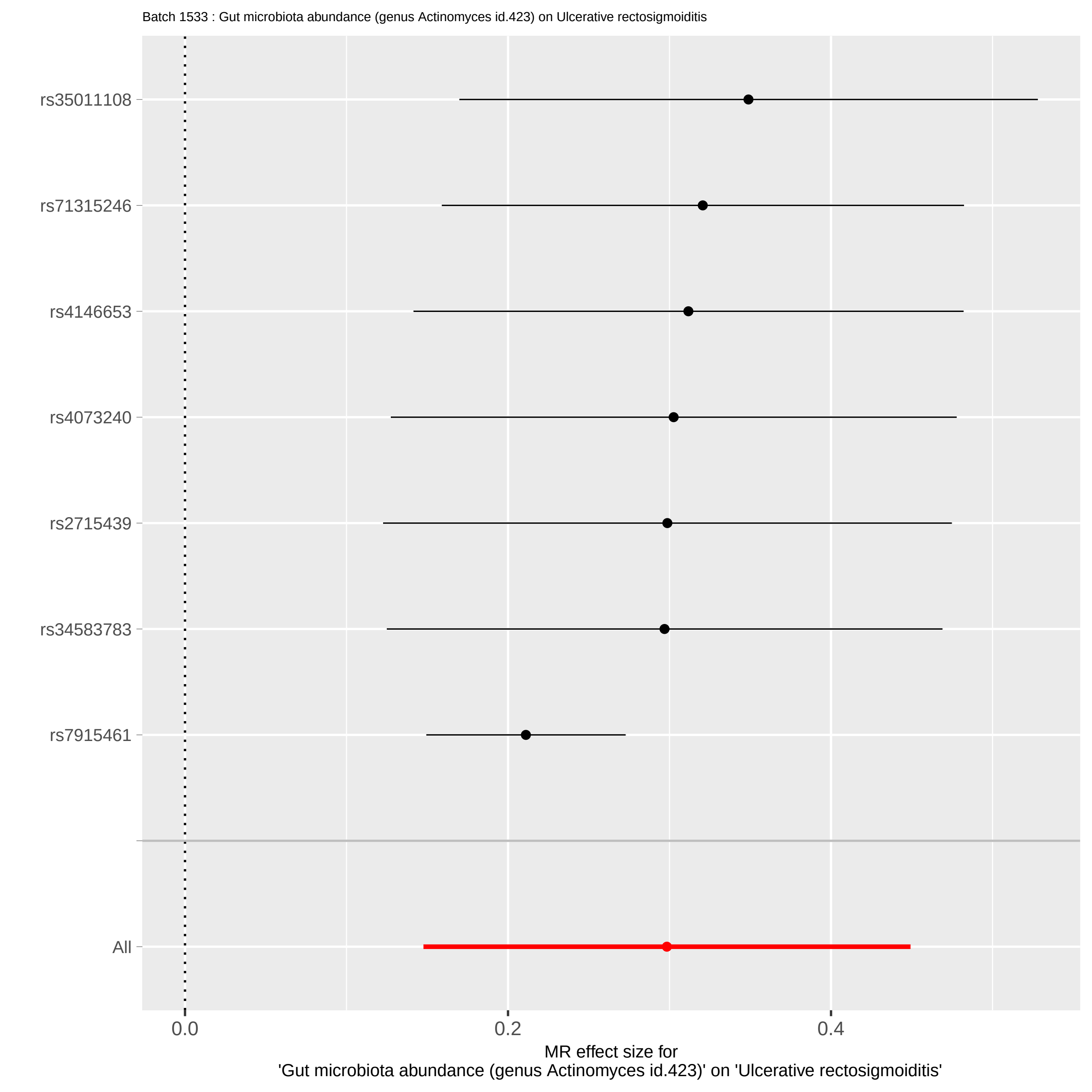

Batch 1533 : Gut microbiota abundance (genus Actinomyces id.423) on Ulcerative rectosigmoiditis
rs35011108
rs71315246
rs4146653
rs4073240
rs2715439
rs34583783
rs7915461
All
0.0
0.2
0.4
MR effect size for
'Gut microbiota abundance (genus Actinomyces id.423)' on 'Ulcerative rectosigmoiditis'

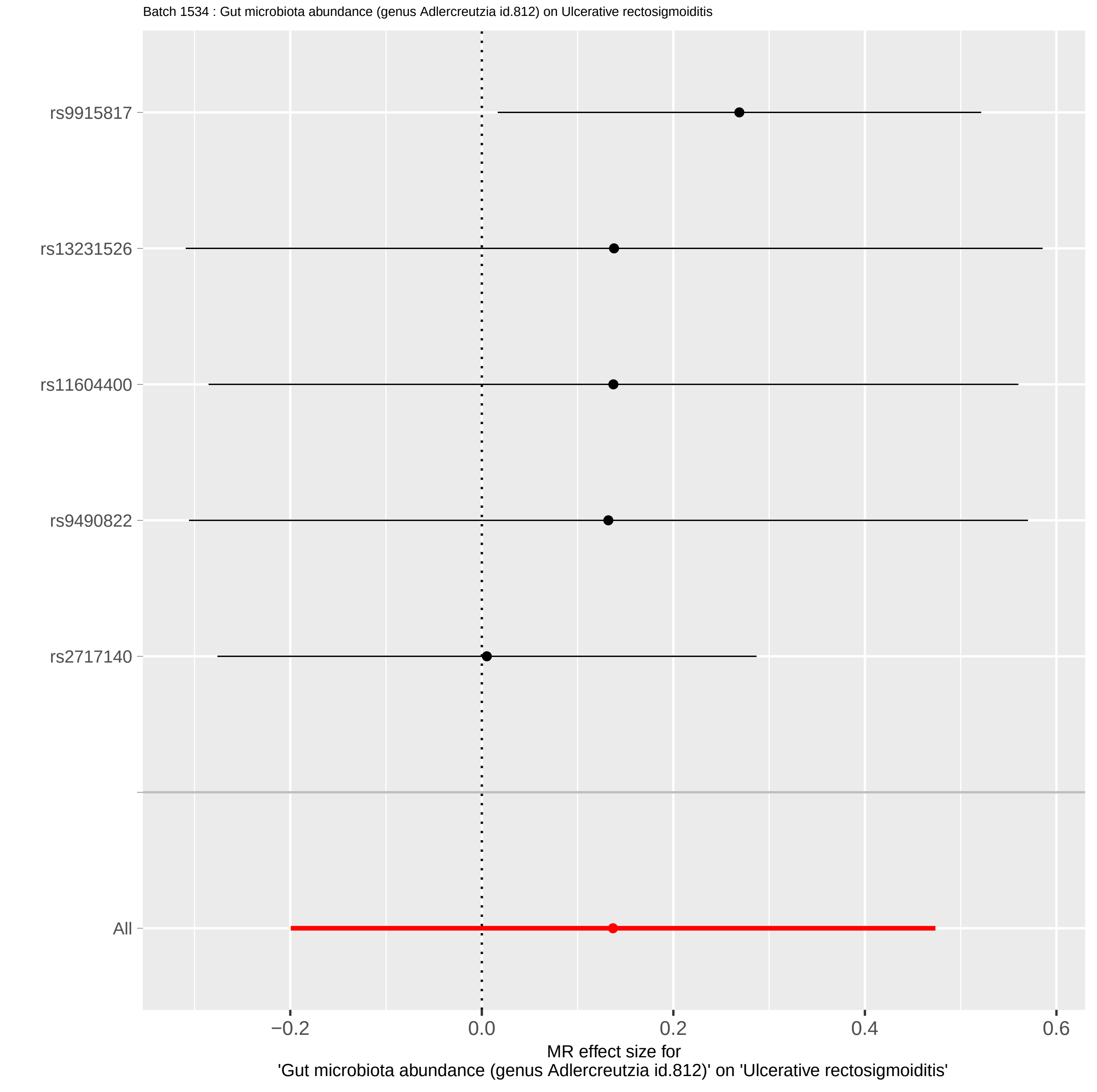

Batch 1534 : Gut microbiota abundance (genus Adlercreutzia id.812) on Ulcerative rectosigmoiditis
rs9915817
rs13231526
rs11604400
rs9490822
rs2717140
All
−0.2
0.0
0.2
0.4
0.6
MR effect size for
'Gut microbiota abundance (genus Adlercreutzia id.812)' on 'Ulcerative rectosigmoiditis'

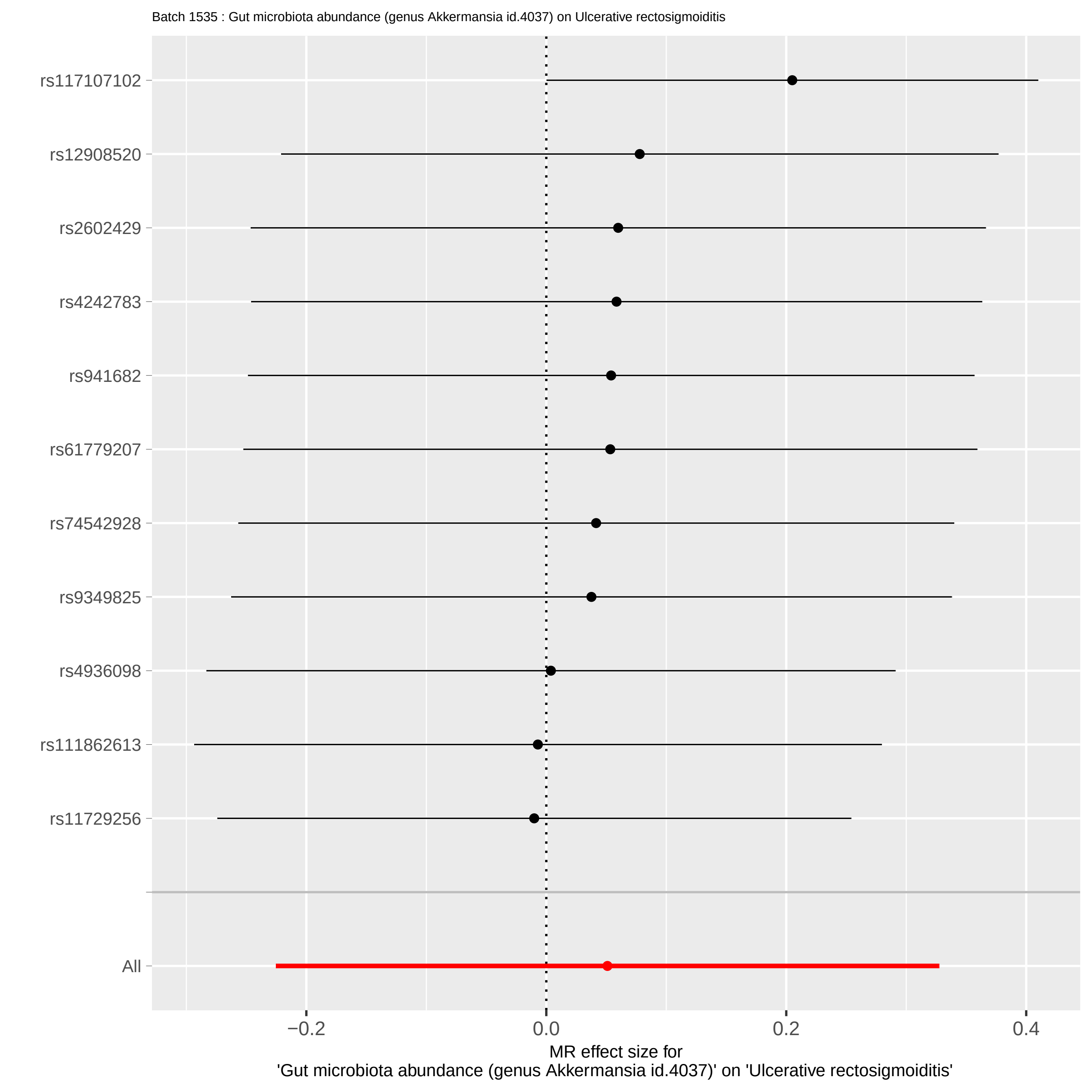

Batch 1535 : Gut microbiota abundance (genus Akkermansia id.4037) on Ulcerative rectosigmoiditis
rs117107102
rs12908520
rs2602429
rs4242783
rs941682
rs61779207
rs74542928
rs9349825
rs4936098
rs111862613
rs11729256
All
−0.2
0.0
0.2
0.4
MR effect size for
'Gut microbiota abundance (genus Akkermansia id.4037)' on 'Ulcerative rectosigmoiditis'

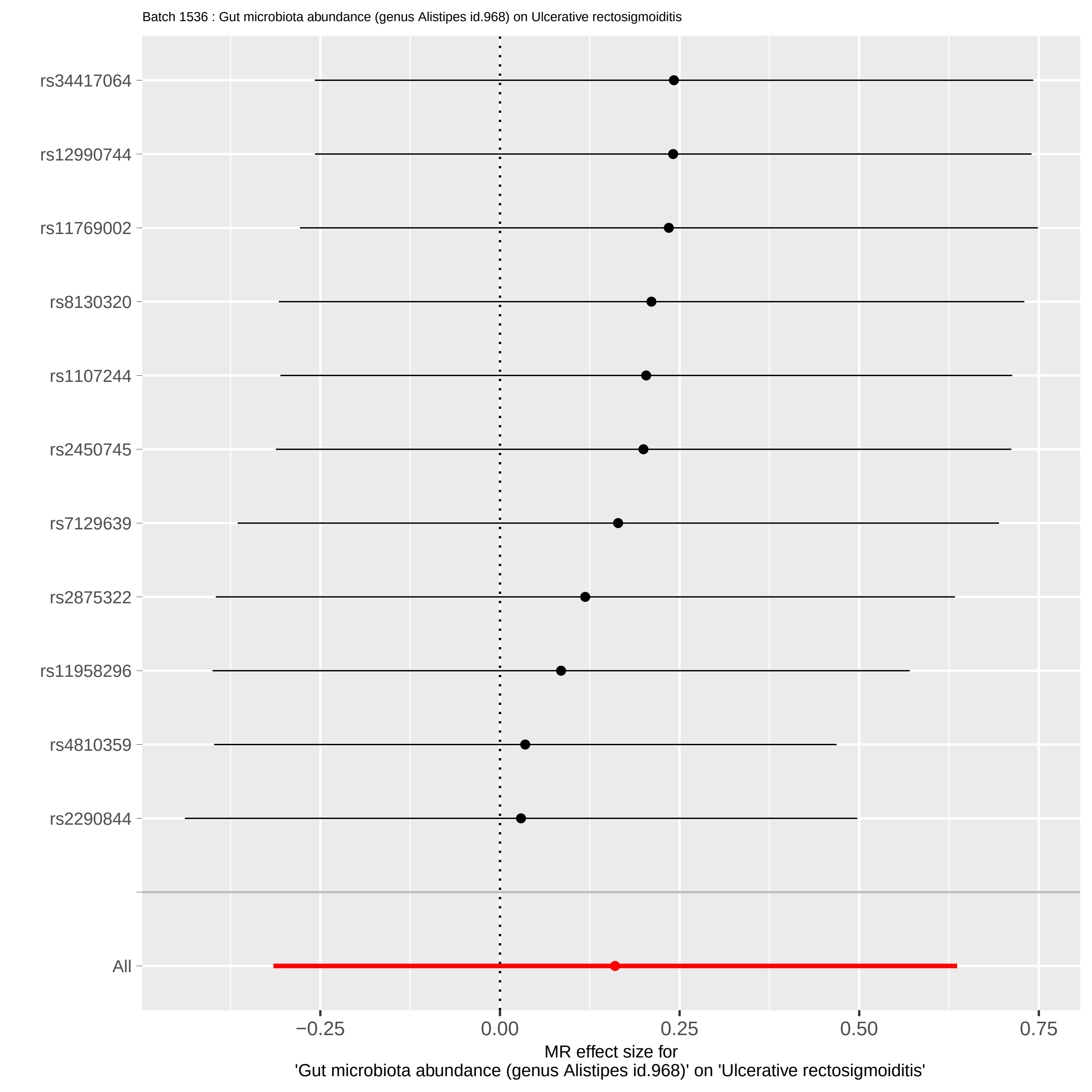

Batch 1536 : Gut microbiota abundance (genus Alistipes id.968) on Ulcerative rectosigmoiditis
rs34417064
rs12990744
rs11769002
rs8130320
rs1107244
rs2450745
rs7129639
rs2875322
rs11958296
rs4810359
rs2290844
All
−0.25
0.00
0.25
0.50
0.75
MR effect size for
'Gut microbiota abundance (genus Alistipes id.968)' on 'Ulcerative rectosigmoiditis'

Batch 1537 : Gut microbiota abundance (genus Allisonella id.2174) on Ulcerative rectosigmoiditis

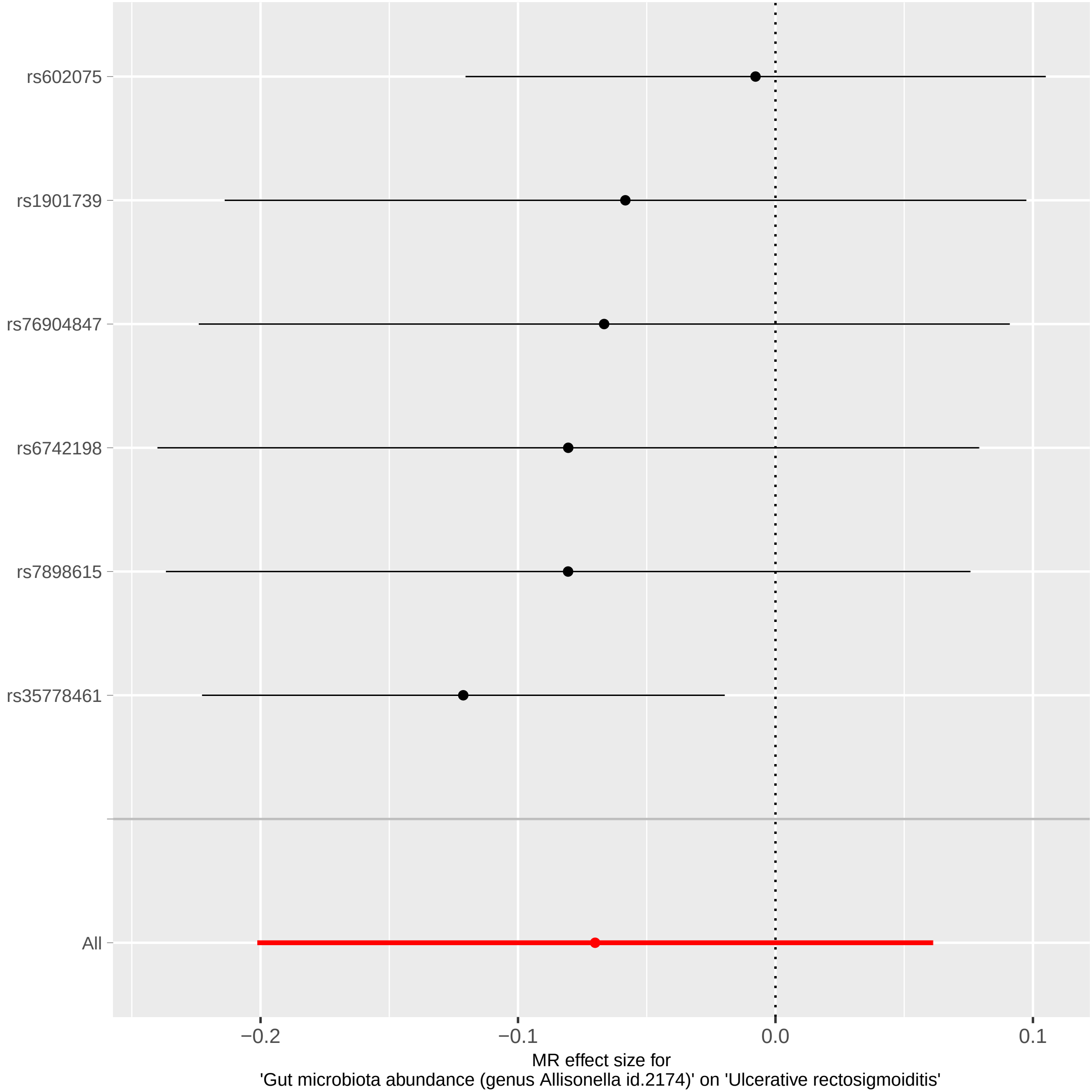

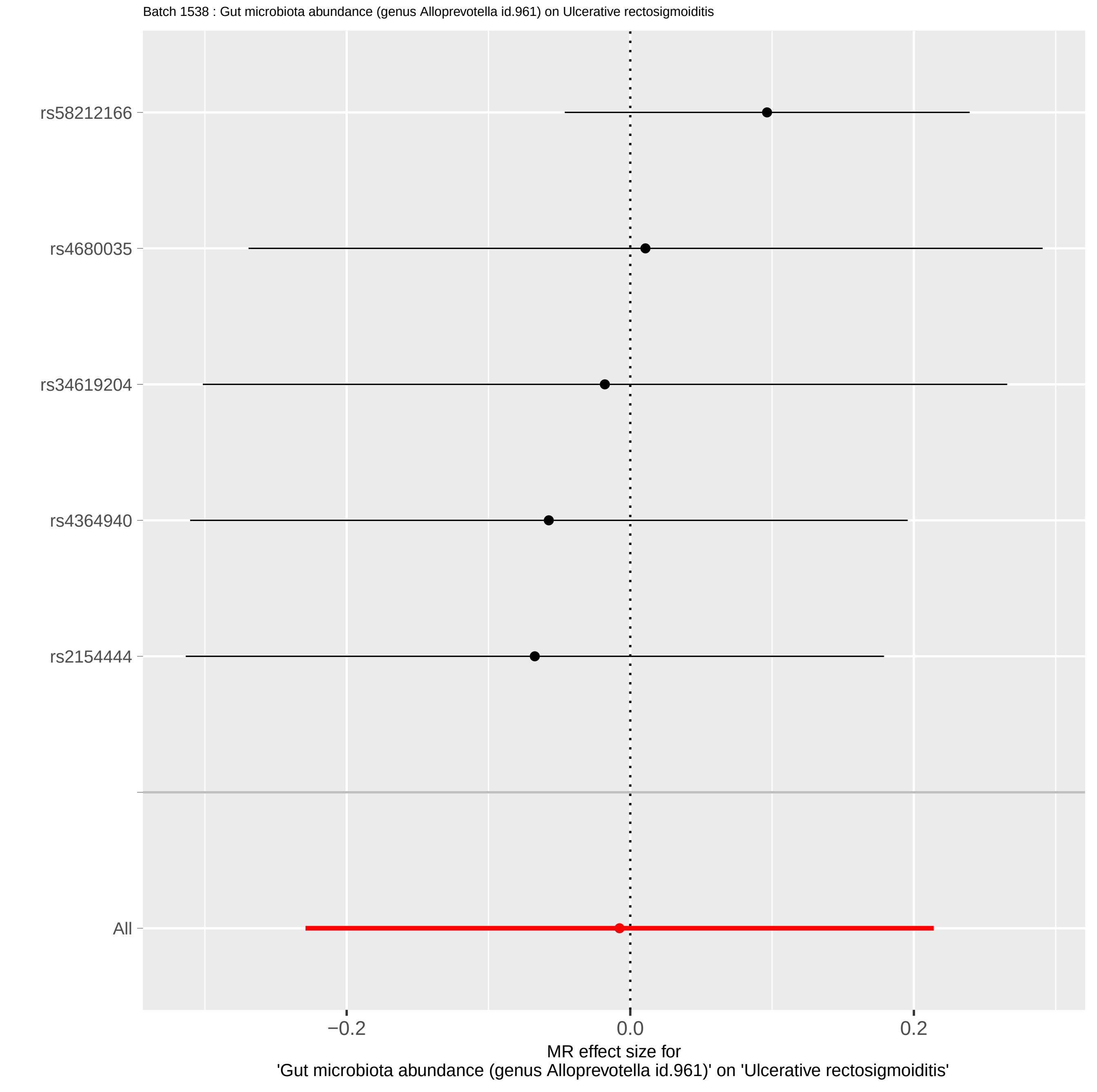
Batch 1538 : Gut microbiota abundance (genus Alloprevotella id.961) on Ulcerative rectosigmoiditis
rs58212166
rs4680035
rs34619204
rs4364940
rs2154444
All
−0.2
0.0
0.2
MR effect size for
'Gut microbiota abundance (genus Alloprevotella id.961)' on 'Ulcerative rectosigmoiditis'

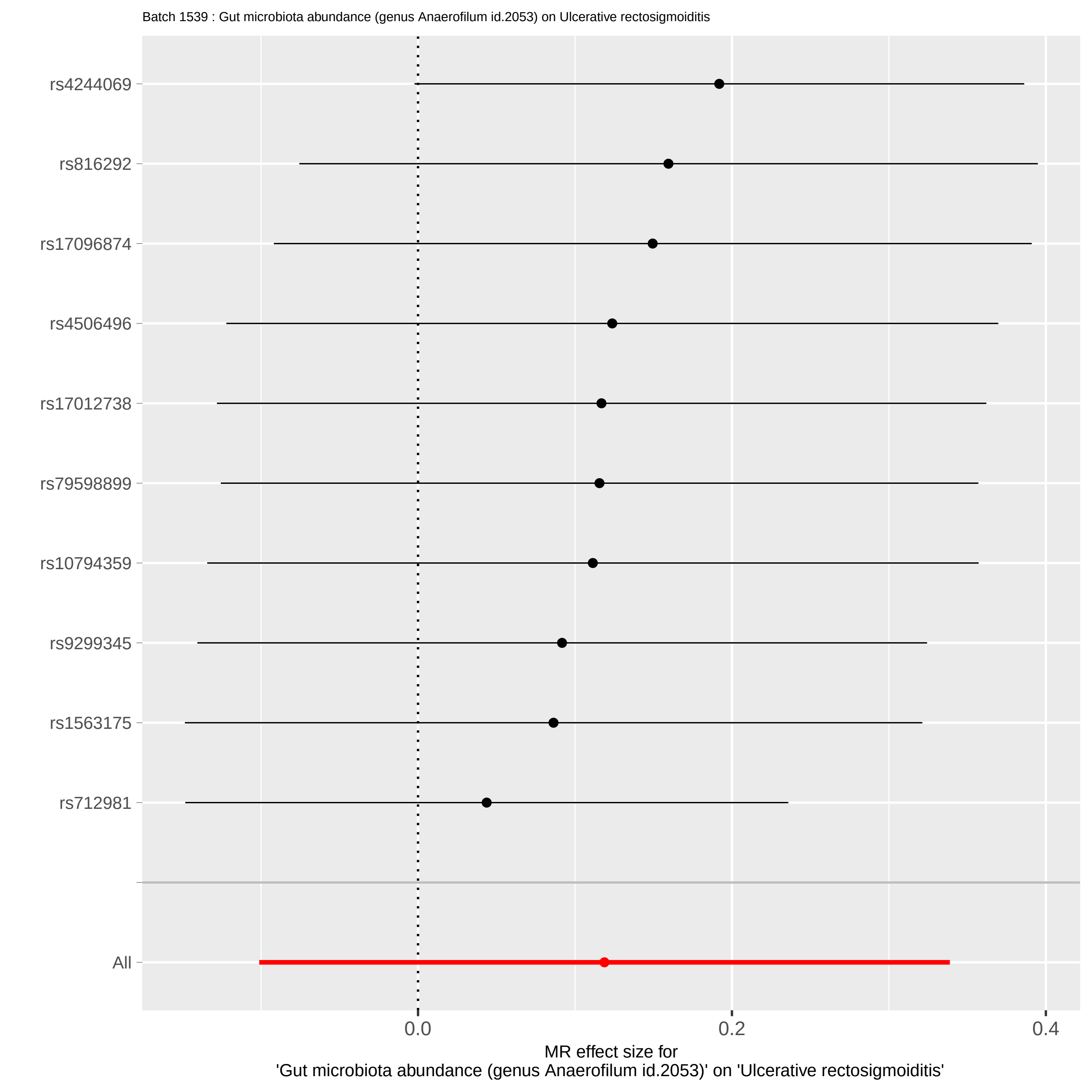
Batch 1539 : Gut microbiota abundance (genus Anaerofilum id.2053) on Ulcerative rectosigmoiditis
rs4244069
rs816292
rs17096874
rs4506496
rs17012738
rs79598899
rs10794359
rs9299345
rs1563175
rs712981
All
0.0
0.2
0.4
MR effect size for
'Gut microbiota abundance (genus Anaerofilum id.2053)' on 'Ulcerative rectosigmoiditis'

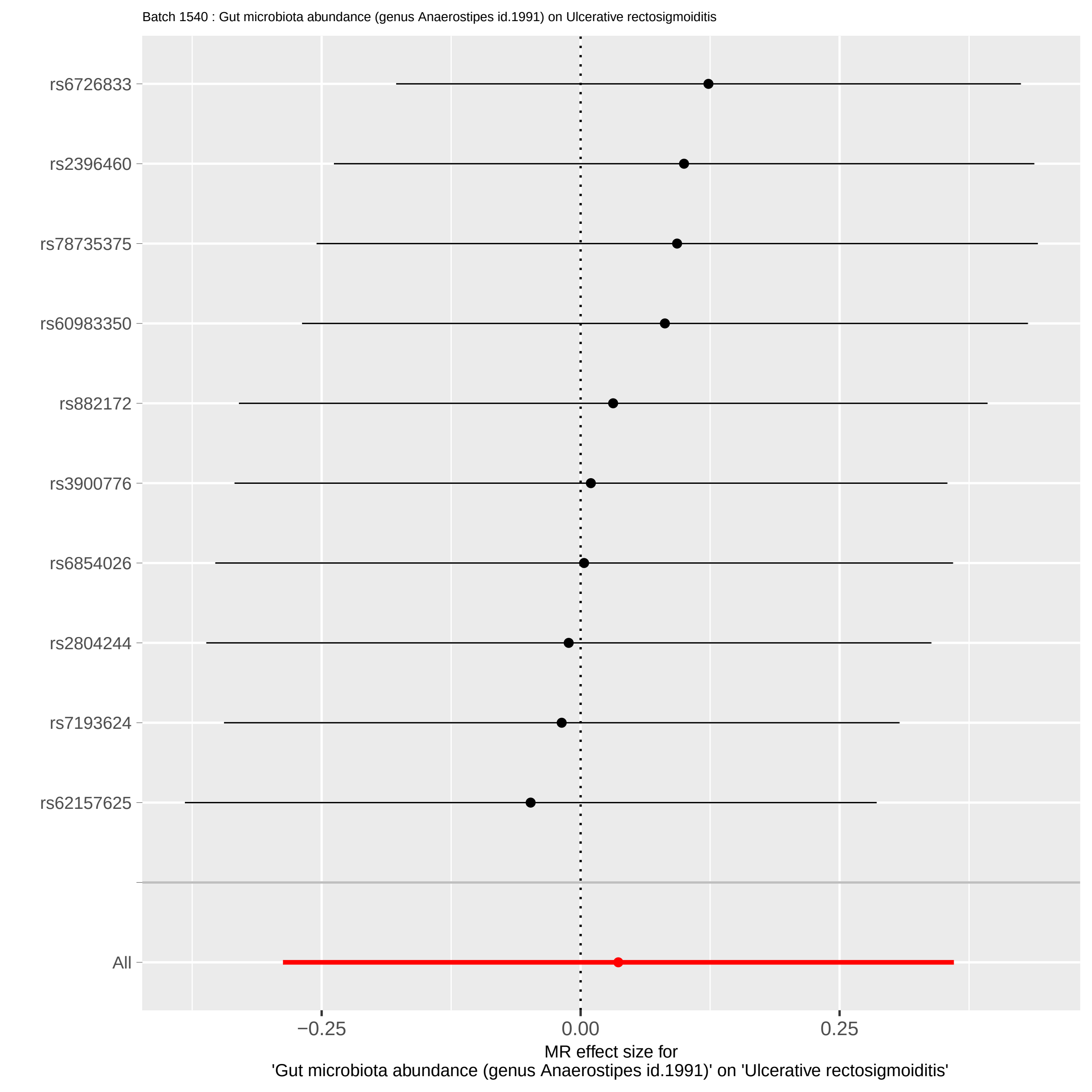

Batch 1540 : Gut microbiota abundance (genus Anaerostipes id.1991) on Ulcerative rectosigmoiditis
rs6726833
rs2396460
rs78735375
rs60983350
rs882172
rs3900776
rs6854026
rs2804244
rs7193624
rs62157625
All
−0.25
0.00
0.25
MR effect size for
'Gut microbiota abundance (genus Anaerostipes id.1991)' on 'Ulcerative rectosigmoiditis'

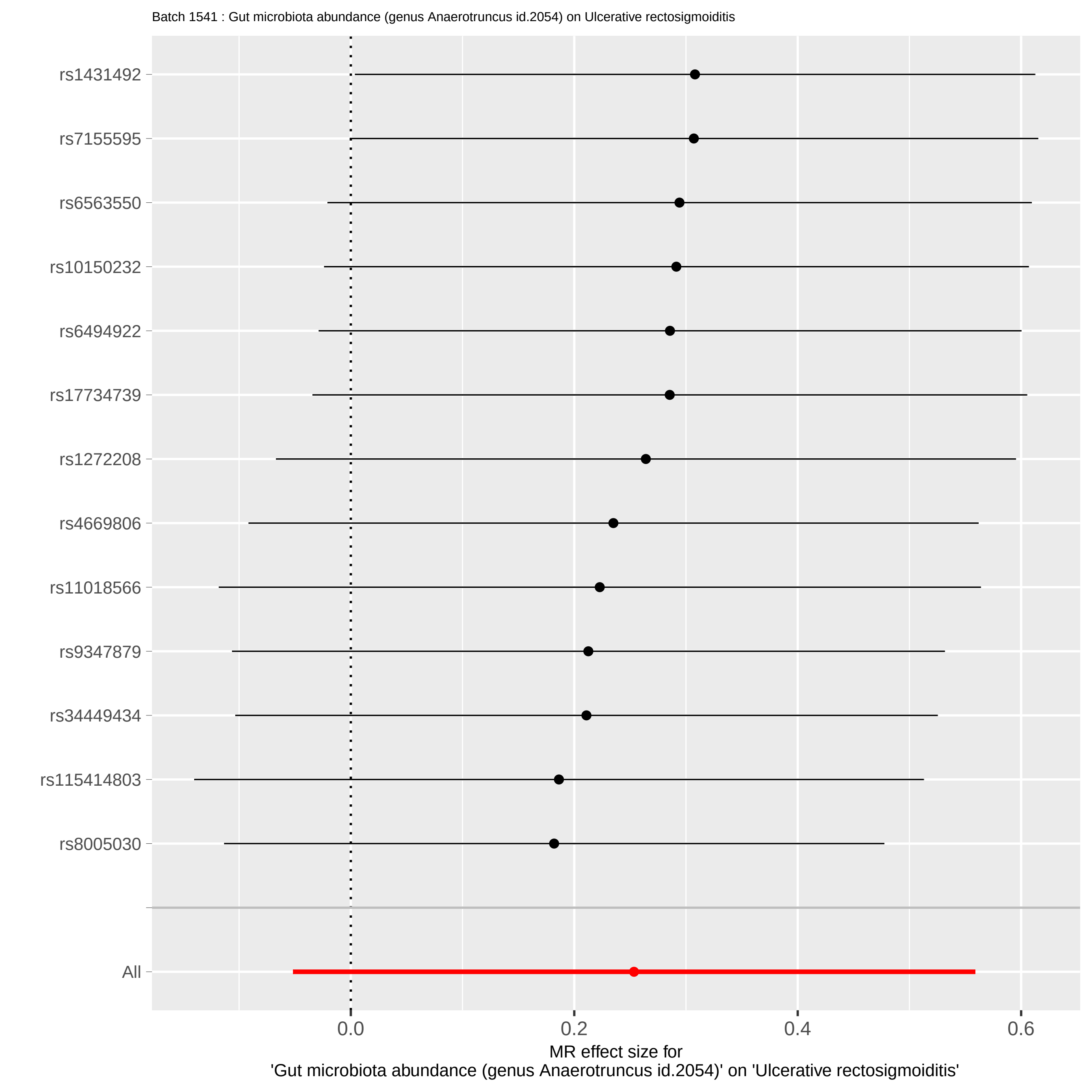

Batch 1541 : Gut microbiota abundance (genus Anaerotruncus id.2054) on Ulcerative rectosigmoiditis
rs1431492
rs7155595
rs6563550
rs10150232
rs6494922
rs17734739
rs1272208
rs4669806
rs11018566
rs9347879
rs34449434
rs115414803
rs8005030
All
0.0
0.2
0.4
0.6
MR effect size for
'Gut microbiota abundance (genus Anaerotruncus id.2054)' on 'Ulcerative rectosigmoiditis'

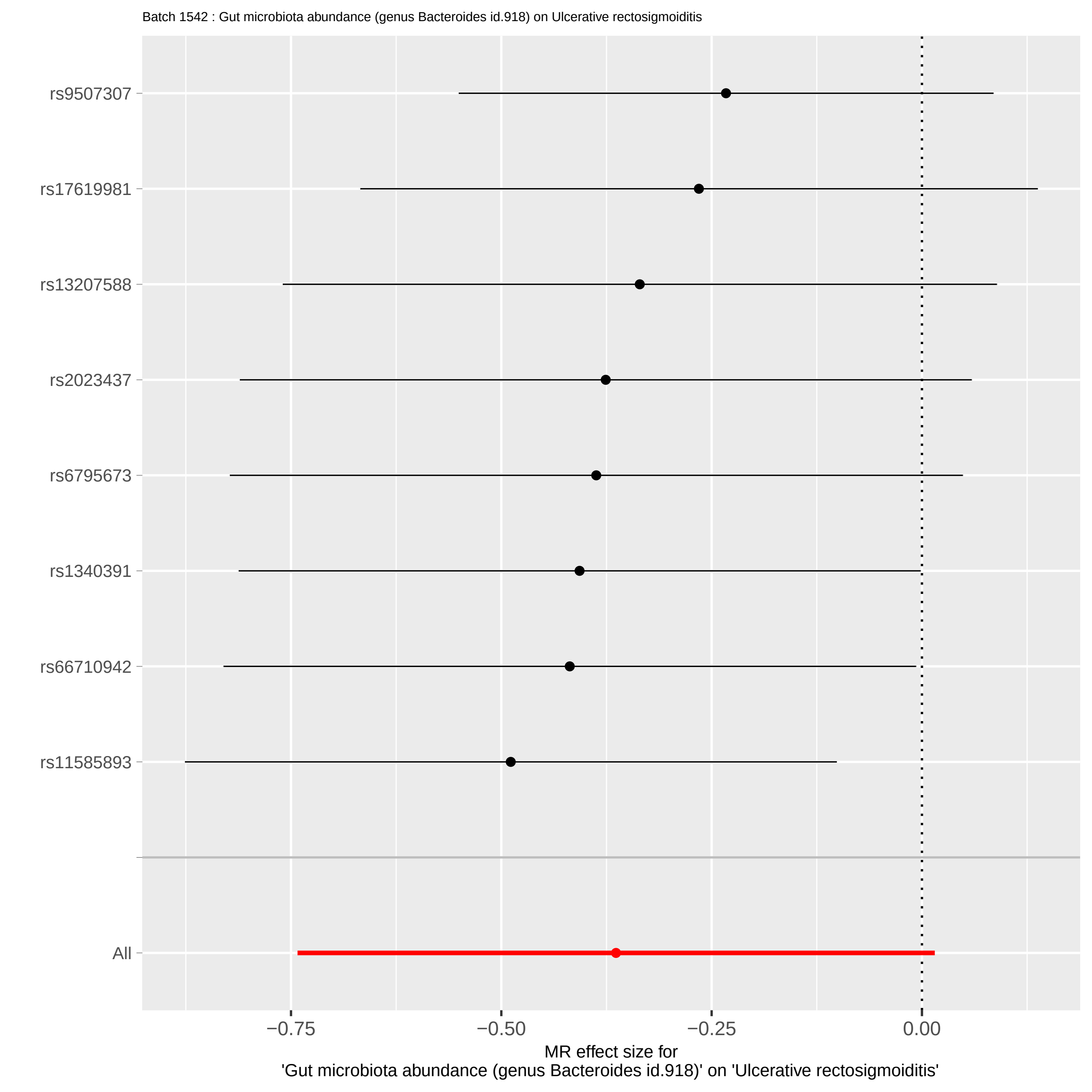

Batch 1542 : Gut microbiota abundance (genus Bacteroides id.918) on Ulcerative rectosigmoiditis
rs9507307
rs17619981
rs13207588
rs2023437
rs6795673
rs1340391
rs66710942
rs11585893
All
−0.75
−0.50
−0.25
0.00
MR effect size for
'Gut microbiota abundance (genus Bacteroides id.918)' on 'Ulcerative rectosigmoiditis'

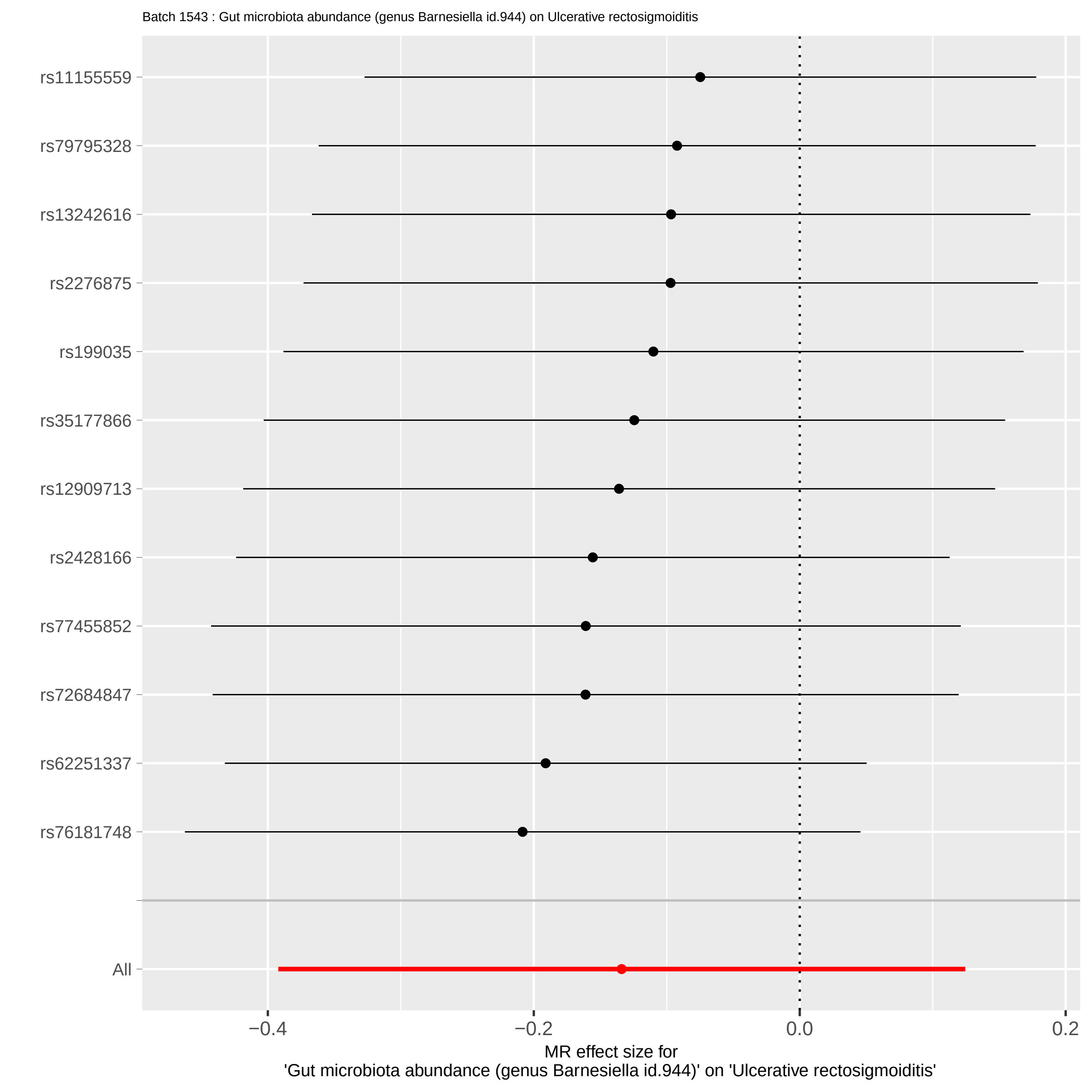

Batch 1543 : Gut microbiota abundance (genus Barnesiella id.944) on Ulcerative rectosigmoiditis
rs11155559
rs79795328
rs13242616
rs2276875
rs199035
rs35177866
rs12909713
rs2428166
rs77455852
rs72684847
rs62251337
rs76181748
All
−0.4
−0.2
0.0
0.2
MR effect size for
'Gut microbiota abundance (genus Barnesiella id.944)' on 'Ulcerative rectosigmoiditis'

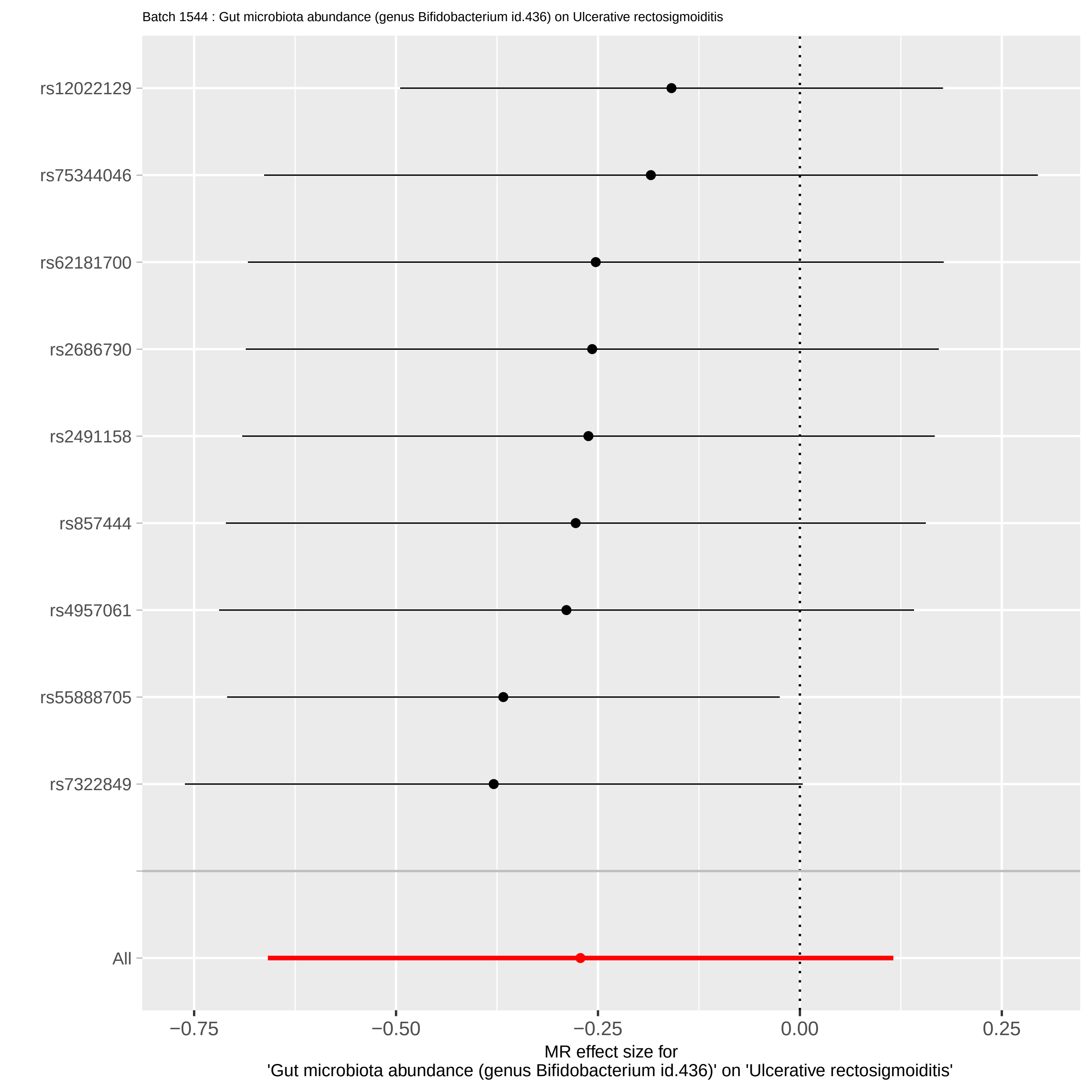
Batch 1544 : Gut microbiota abundance (genus Bifidobacterium id.436) on Ulcerative rectosigmoiditis
rs12022129
rs75344046
rs62181700
rs2686790
rs2491158
rs857444
rs4957061
rs55888705
rs7322849
All
−0.75
−0.50
−0.25
0.00
0.25
MR effect size for
'Gut microbiota abundance (genus Bifidobacterium id.436)' on 'Ulcerative rectosigmoiditis'

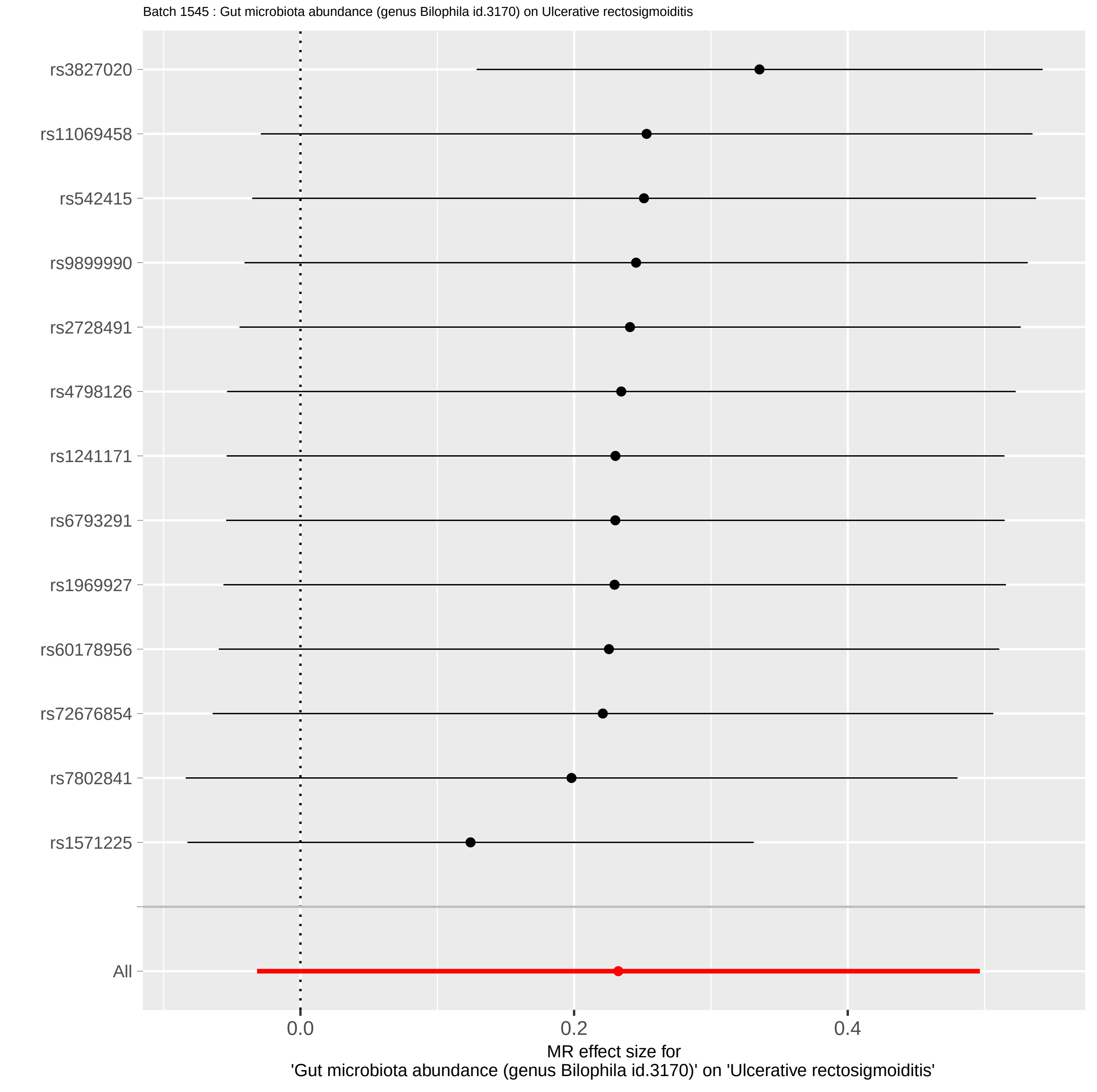
Batch 1545 : Gut microbiota abundance (genus Bilophila id.3170) on Ulcerative rectosigmoiditis
rs3827020
rs11069458
rs542415
rs9899990
rs2728491
rs4798126
rs1241171
rs6793291
rs1969927
rs60178956
rs72676854
rs7802841
rs1571225
All
0.0
0.2
0.4
MR effect size for
'Gut microbiota abundance (genus Bilophila id.3170)' on 'Ulcerative rectosigmoiditis'

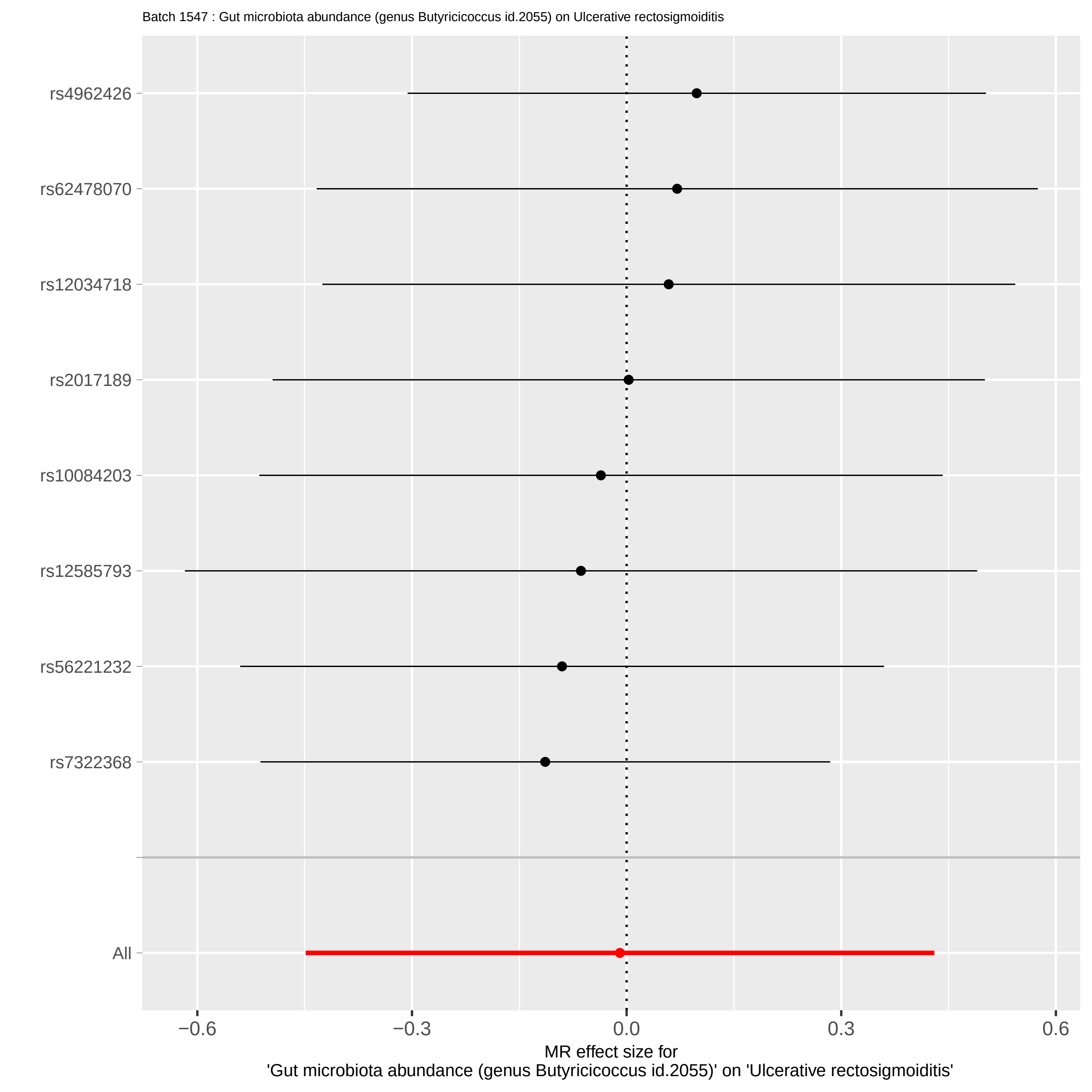

Batch 1547 : Gut microbiota abundance (genus Butyricicoccus id.2055) on Ulcerative rectosigmoiditis
rs4962426
rs62478070
rs12034718
rs2017189
rs10084203
rs12585793
rs56221232
rs7322368
All
−0.6
−0.3
0.0
0.3
0.6
MR effect size for
'Gut microbiota abundance (genus Butyricicoccus id.2055)' on 'Ulcerative rectosigmoiditis'

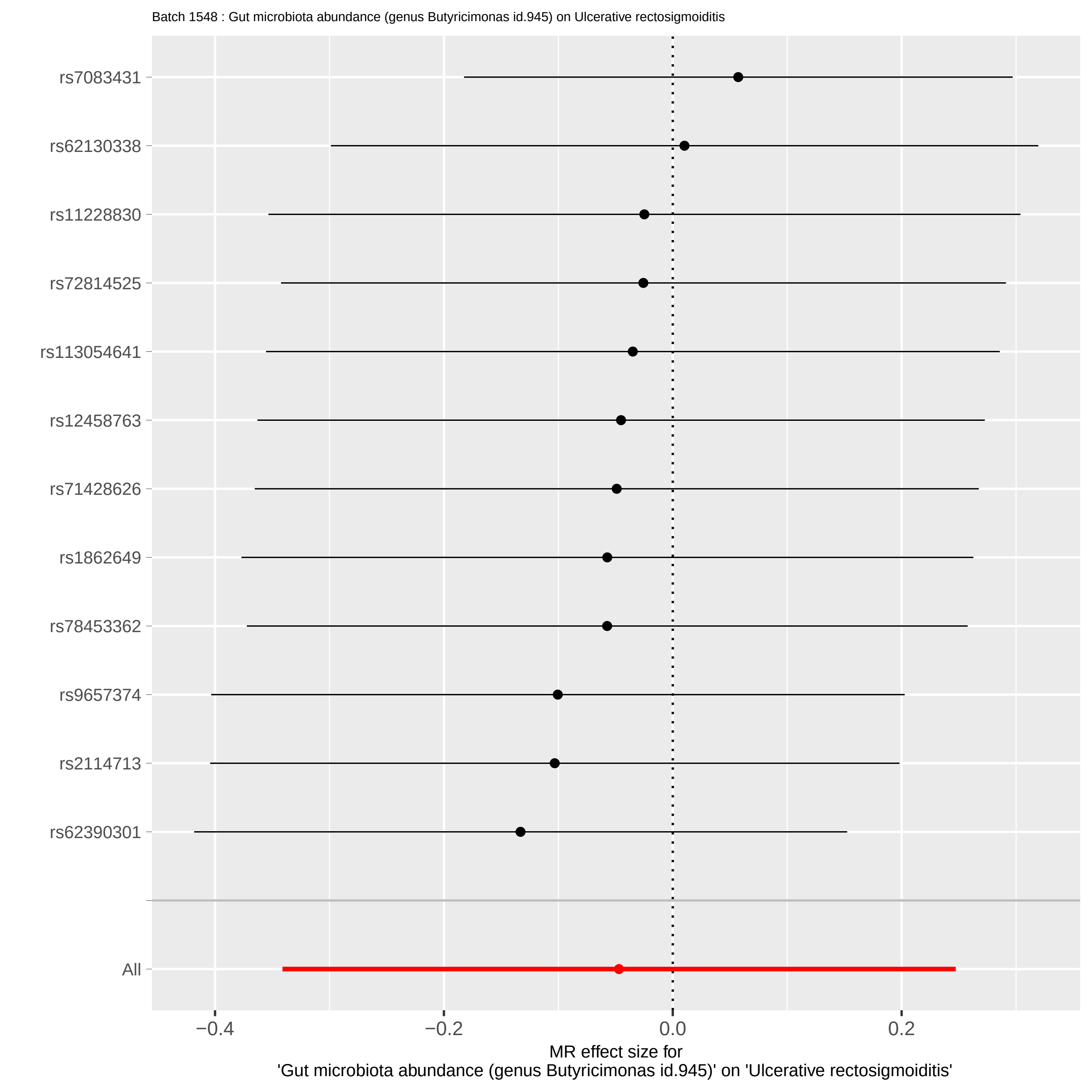

Batch 1548 : Gut microbiota abundance (genus Butyricimonas id.945) on Ulcerative rectosigmoiditis
rs7083431
rs62130338
rs11228830
rs72814525
rs113054641
rs12458763
rs71428626
rs1862649
rs78453362
rs9657374
rs2114713
rs62390301
All
−0.4
−0.2
0.0
0.2
MR effect size for
'Gut microbiota abundance (genus Butyricimonas id.945)' on 'Ulcerative rectosigmoiditis'

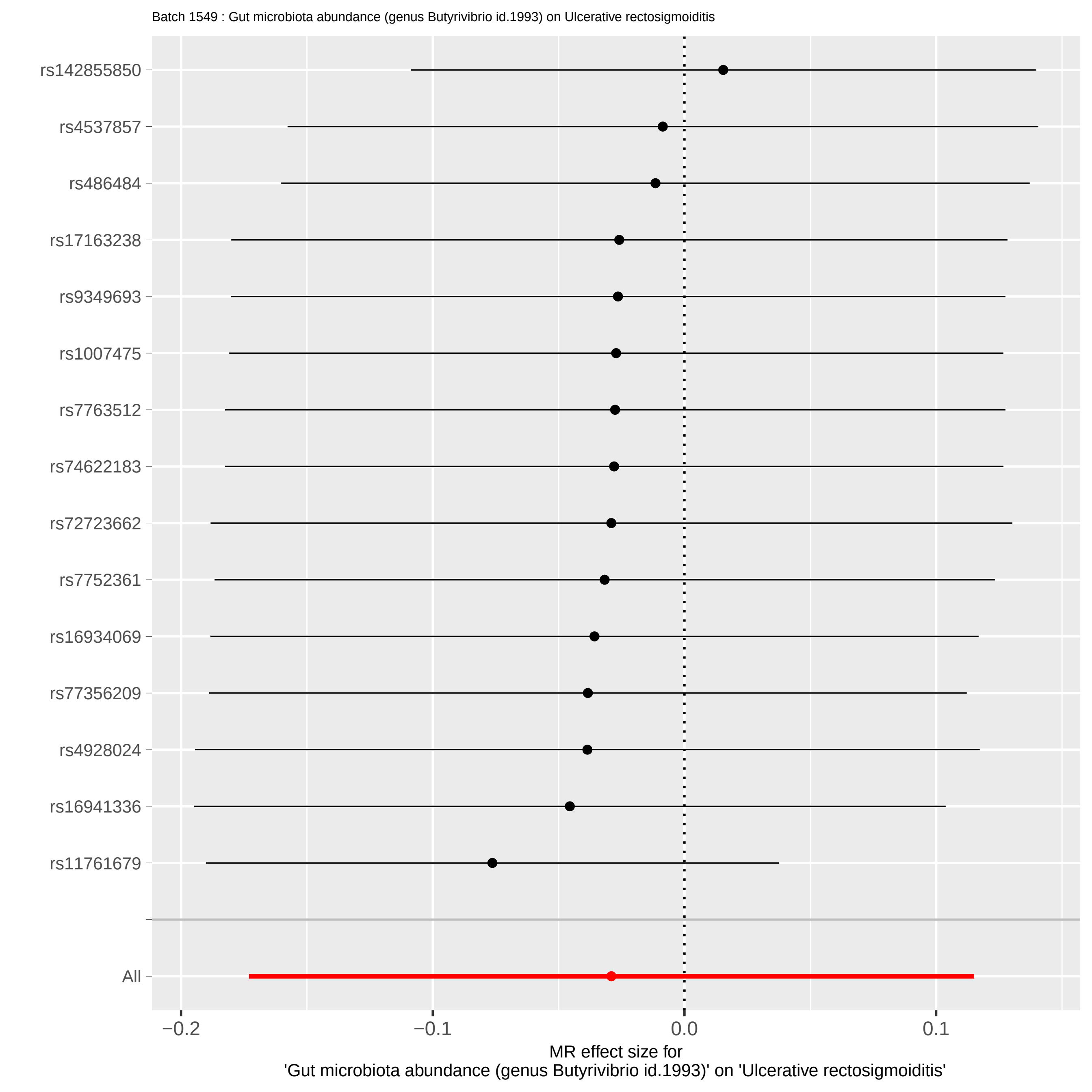

Batch 1549 : Gut microbiota abundance (genus Butyrivibrio id.1993) on Ulcerative rectosigmoiditis
rs142855850
rs4537857
rs486484
rs17163238
rs9349693
rs1007475
rs7763512
rs74622183
rs72723662
rs7752361
rs16934069
rs77356209
rs4928024
rs16941336
rs11761679
All
−0.2
−0.1
0.0
0.1
MR effect size for
'Gut microbiota abundance (genus Butyrivibrio id.1993)' on 'Ulcerative rectosigmoiditis'

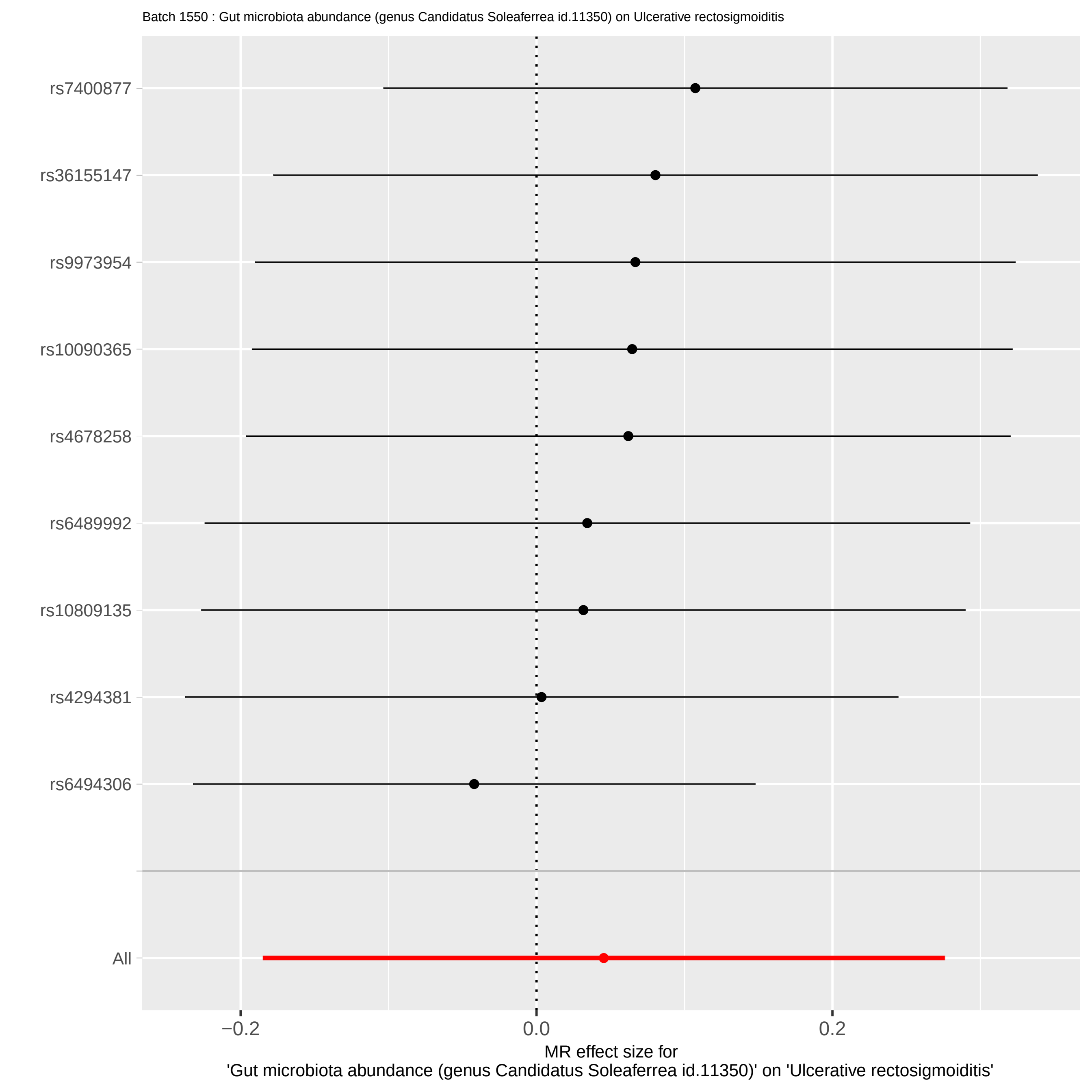

Batch 1550 : Gut microbiota abundance (genus Candidatus Soleaferrea id.11350) on Ulcerative rectosigmoiditis
rs7400877
rs36155147
rs9973954
rs10090365
rs4678258
rs6489992
rs10809135
rs4294381
rs6494306
All
−0.2
0.0
0.2
MR effect size for
'Gut microbiota abundance (genus Candidatus Soleaferrea id.11350)' on 'Ulcerative rectosigmoiditis'

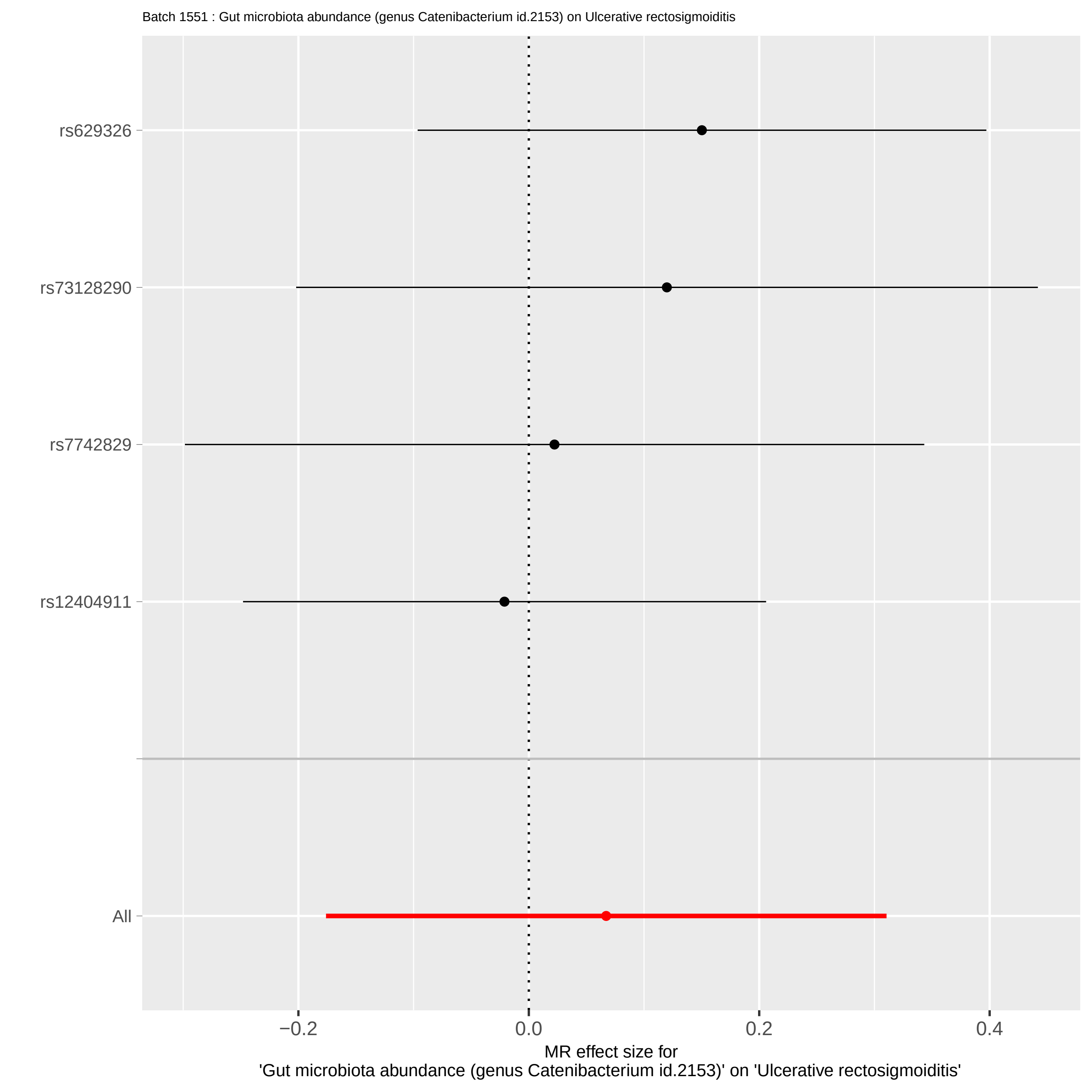

Batch 1551 : Gut microbiota abundance (genus Catenibacterium id.2153) on Ulcerative rectosigmoiditis
rs629326
rs73128290
rs7742829
rs12404911
All
−0.2
0.0
0.2
0.4
MR effect size for
'Gut microbiota abundance (genus Catenibacterium id.2153)' on 'Ulcerative rectosigmoiditis'

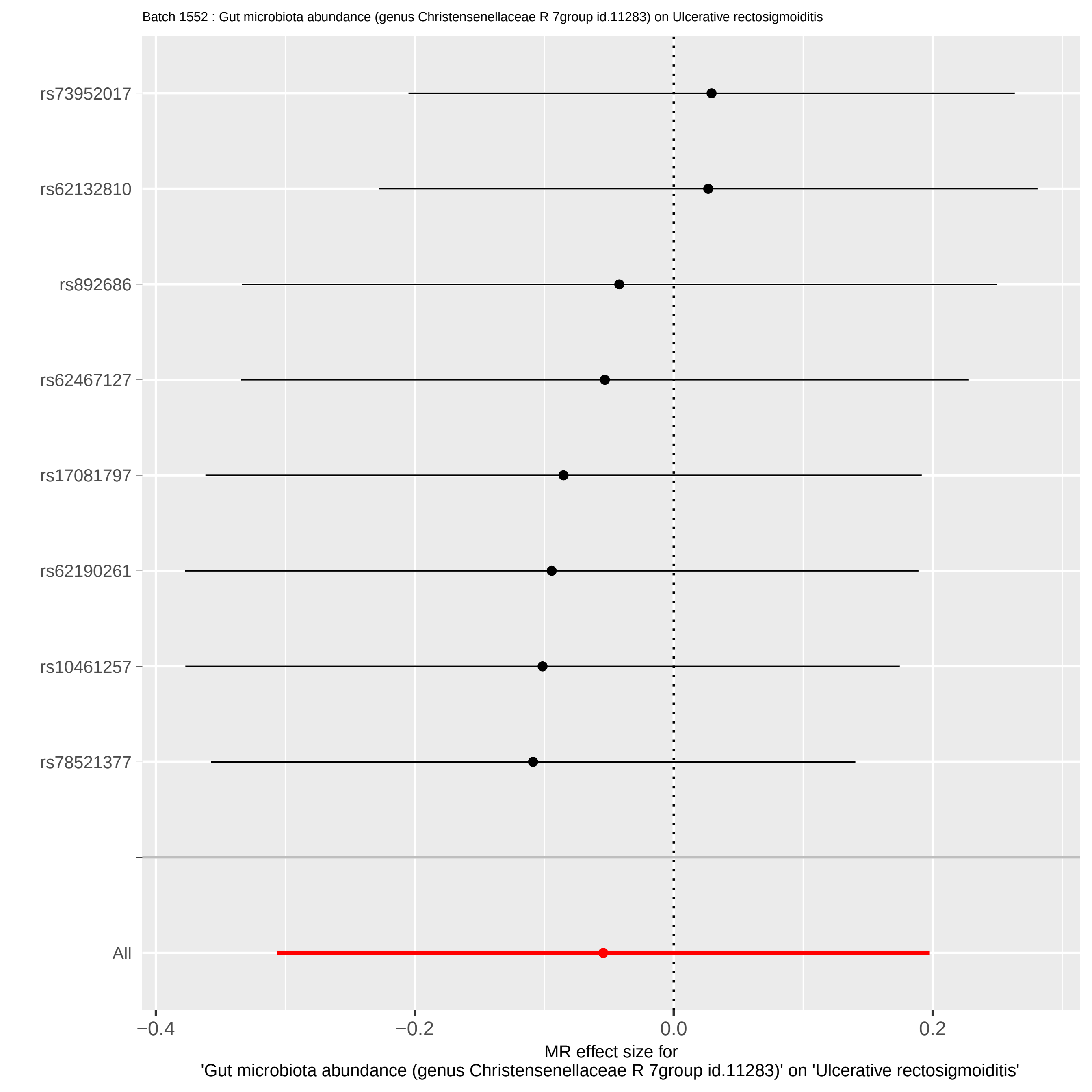
Batch 1552 : Gut microbiota abundance (genus Christensenellaceae R 7group id.11283) on Ulcerative rectosigmoiditis
rs73952017
rs62132810
rs892686
rs62467127
rs17081797
rs62190261
rs10461257
rs78521377
All
−0.4
−0.2
0.0
0.2
MR effect size for
'Gut microbiota abundance (genus Christensenellaceae R 7group id.11283)' on 'Ulcerative rectosigmoiditis'

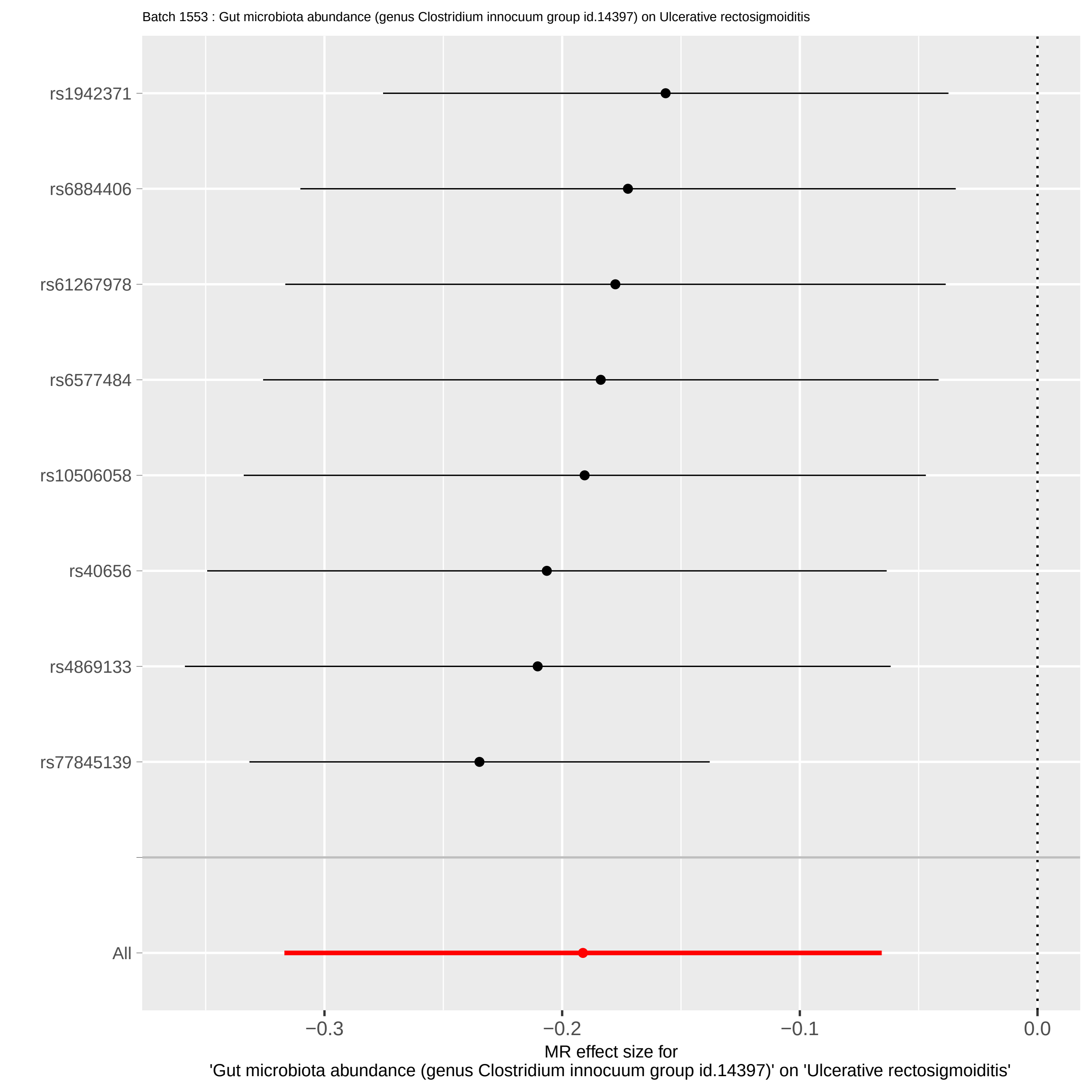

Batch 1553 : Gut microbiota abundance (genus Clostridium innocuum group id.14397) on Ulcerative rectosigmoiditis
rs1942371
rs6884406
rs61267978
rs6577484
rs10506058
rs40656
rs4869133
rs77845139
All
−0.3
−0.2
−0.1
0.0
MR effect size for
'Gut microbiota abundance (genus Clostridium innocuum group id.14397)' on 'Ulcerative rectosigmoiditis'

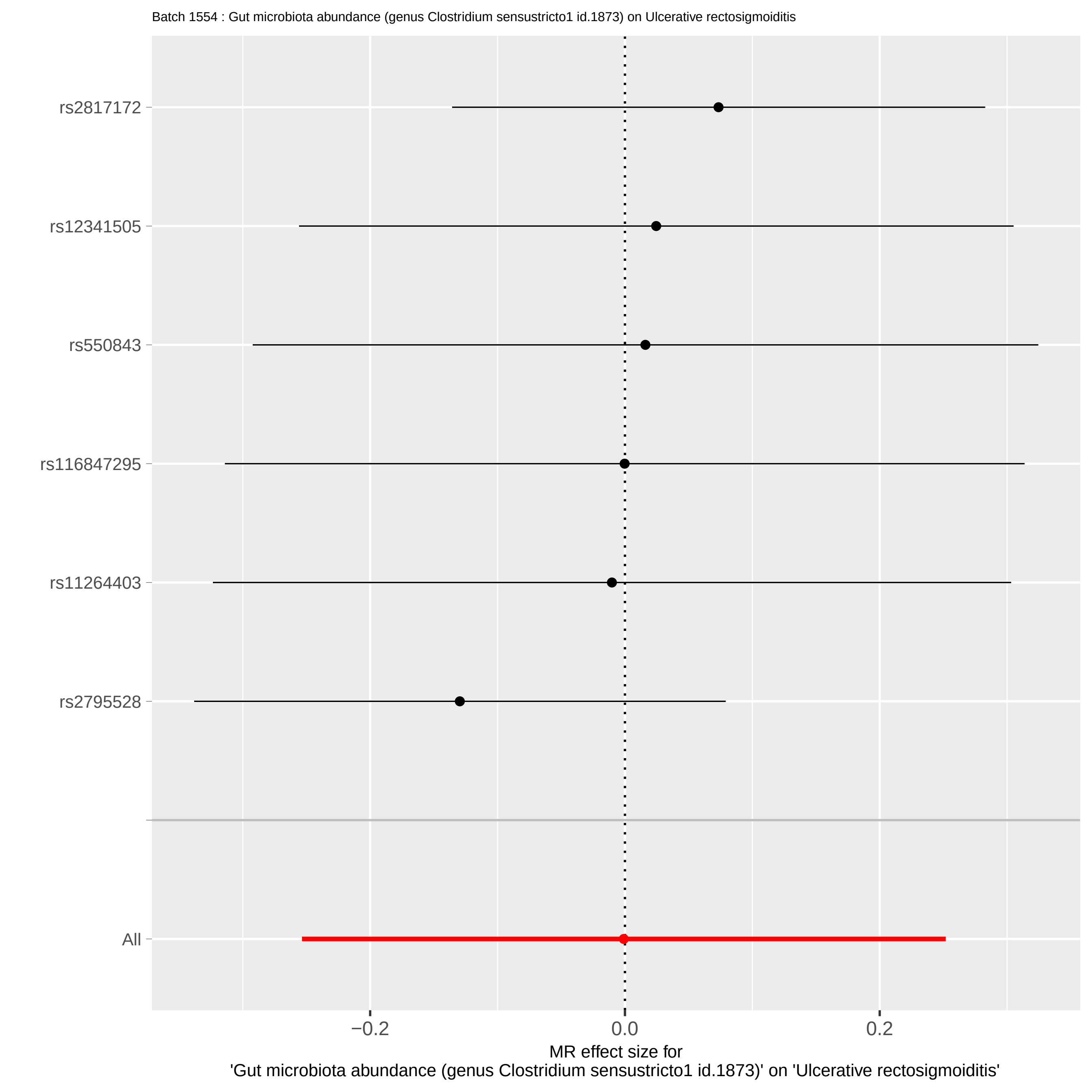

Batch 1554 : Gut microbiota abundance (genus Clostridium sensustricto1 id.1873) on Ulcerative rectosigmoiditis
rs2817172
rs12341505
rs550843
rs116847295
rs11264403
rs2795528
All
−0.2
0.0
0.2
MR effect size for
'Gut microbiota abundance (genus Clostridium sensustricto1 id.1873)' on 'Ulcerative rectosigmoiditis'

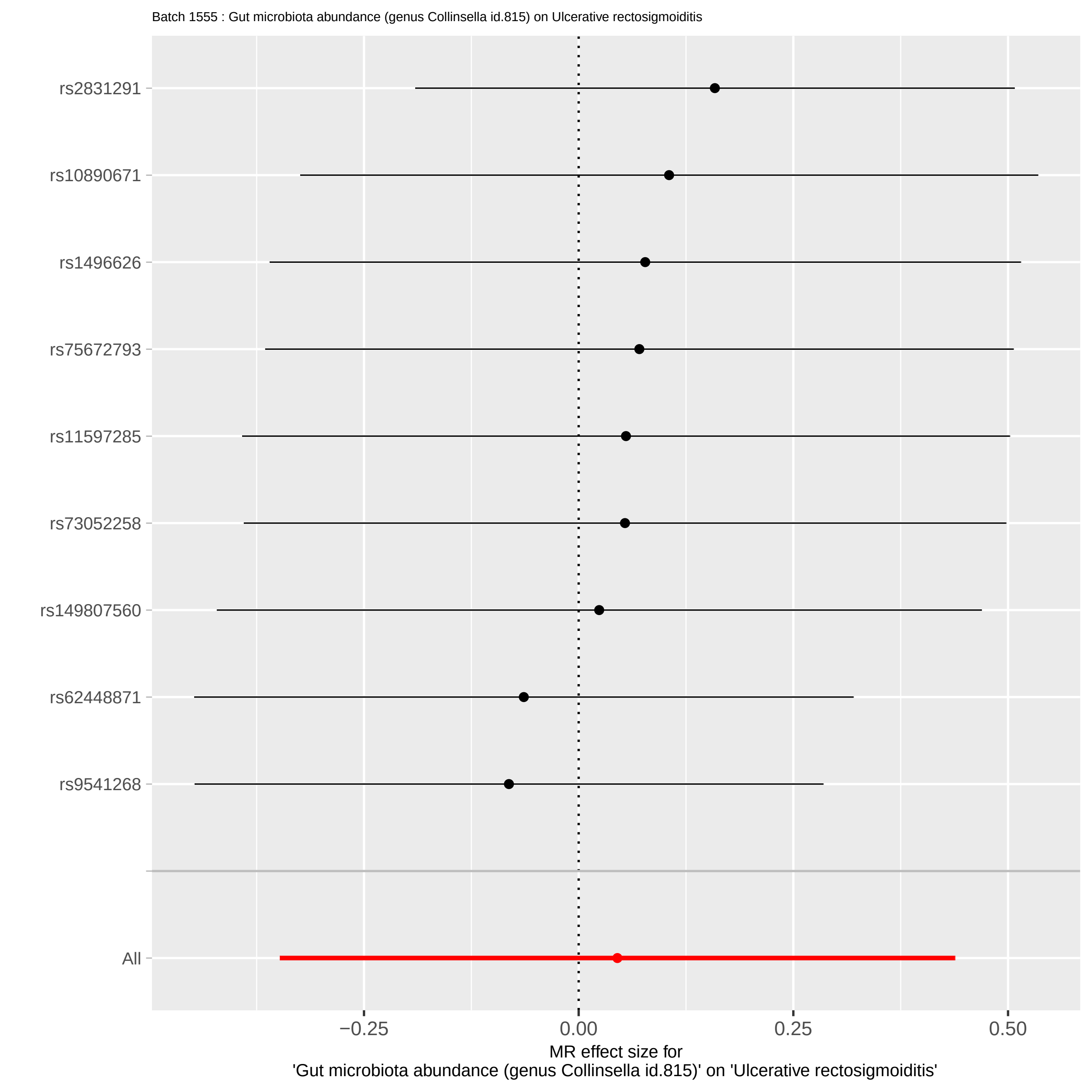
Batch 1555 : Gut microbiota abundance (genus Collinsella id.815) on Ulcerative rectosigmoiditis
rs2831291
rs10890671
rs1496626
rs75672793
rs11597285
rs73052258
rs149807560
rs62448871
rs9541268
All
−0.25
0.00
0.25
0.50
MR effect size for
'Gut microbiota abundance (genus Collinsella id.815)' on 'Ulcerative rectosigmoiditis'

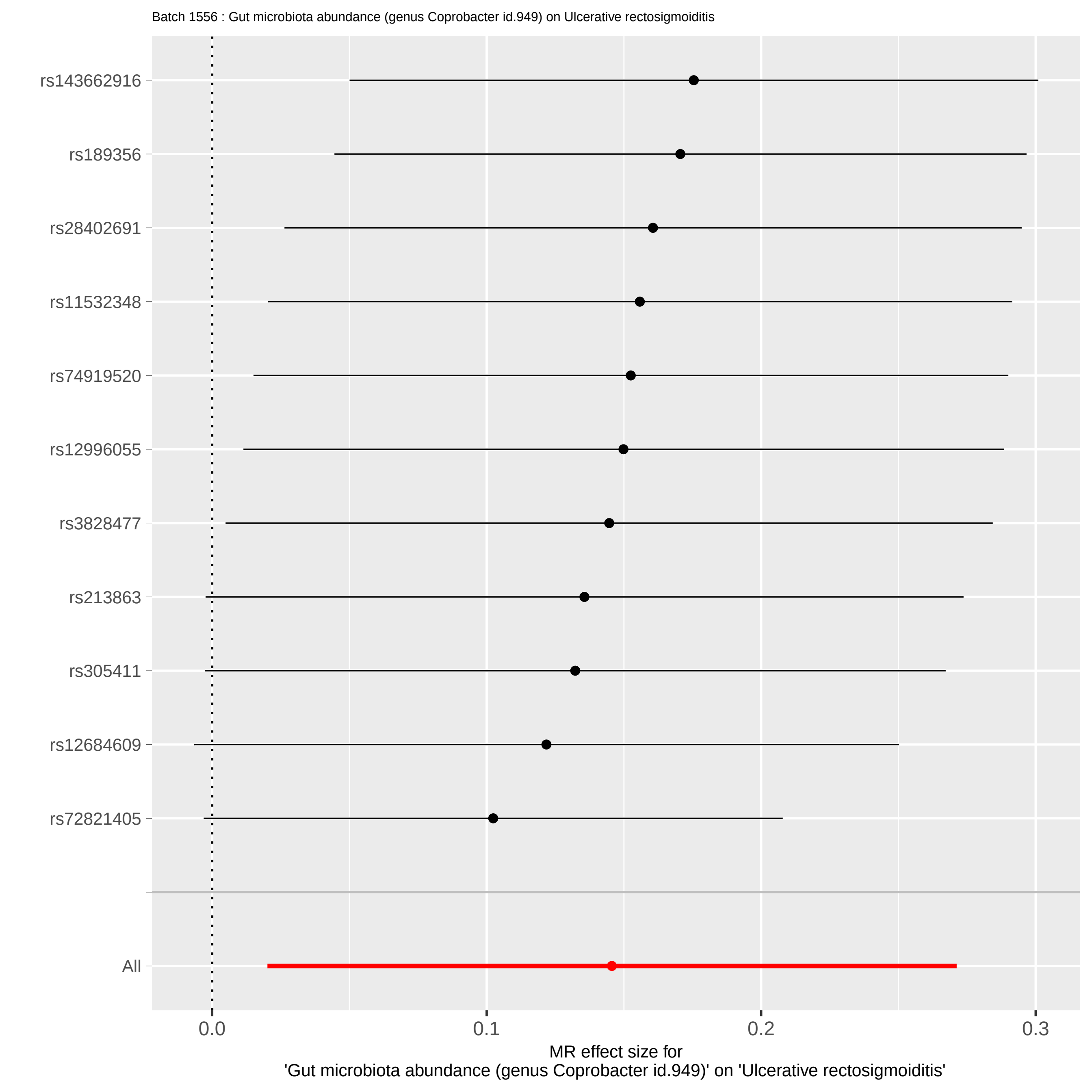

Batch 1556 : Gut microbiota abundance (genus Coprobacter id.949) on Ulcerative rectosigmoiditis
rs143662916
rs189356
rs28402691
rs11532348
rs74919520
rs12996055
rs3828477
rs213863
rs305411
rs12684609
rs72821405
All
0.0
0.1
0.2
0.3
MR effect size for
'Gut microbiota abundance (genus Coprobacter id.949)' on 'Ulcerative rectosigmoiditis'

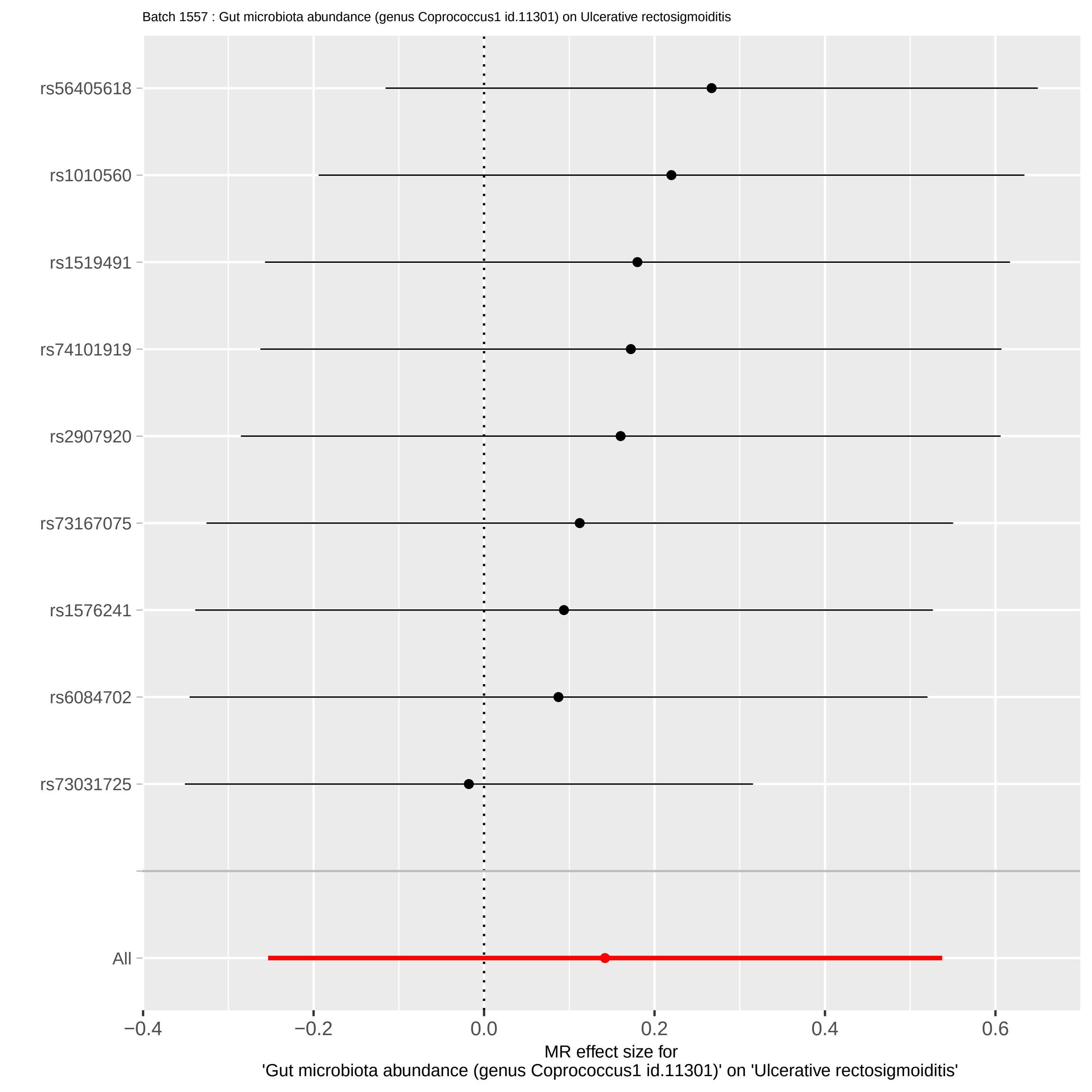

Batch 1557 : Gut microbiota abundance (genus Coprococcus1 id.11301) on Ulcerative rectosigmoiditis
rs56405618
rs1010560
rs1519491
rs74101919
rs2907920
rs73167075
rs1576241
rs6084702
rs73031725
All
−0.4
−0.2
0.0
0.2
0.4
0.6
MR effect size for
'Gut microbiota abundance (genus Coprococcus1 id.11301)' on 'Ulcerative rectosigmoiditis'

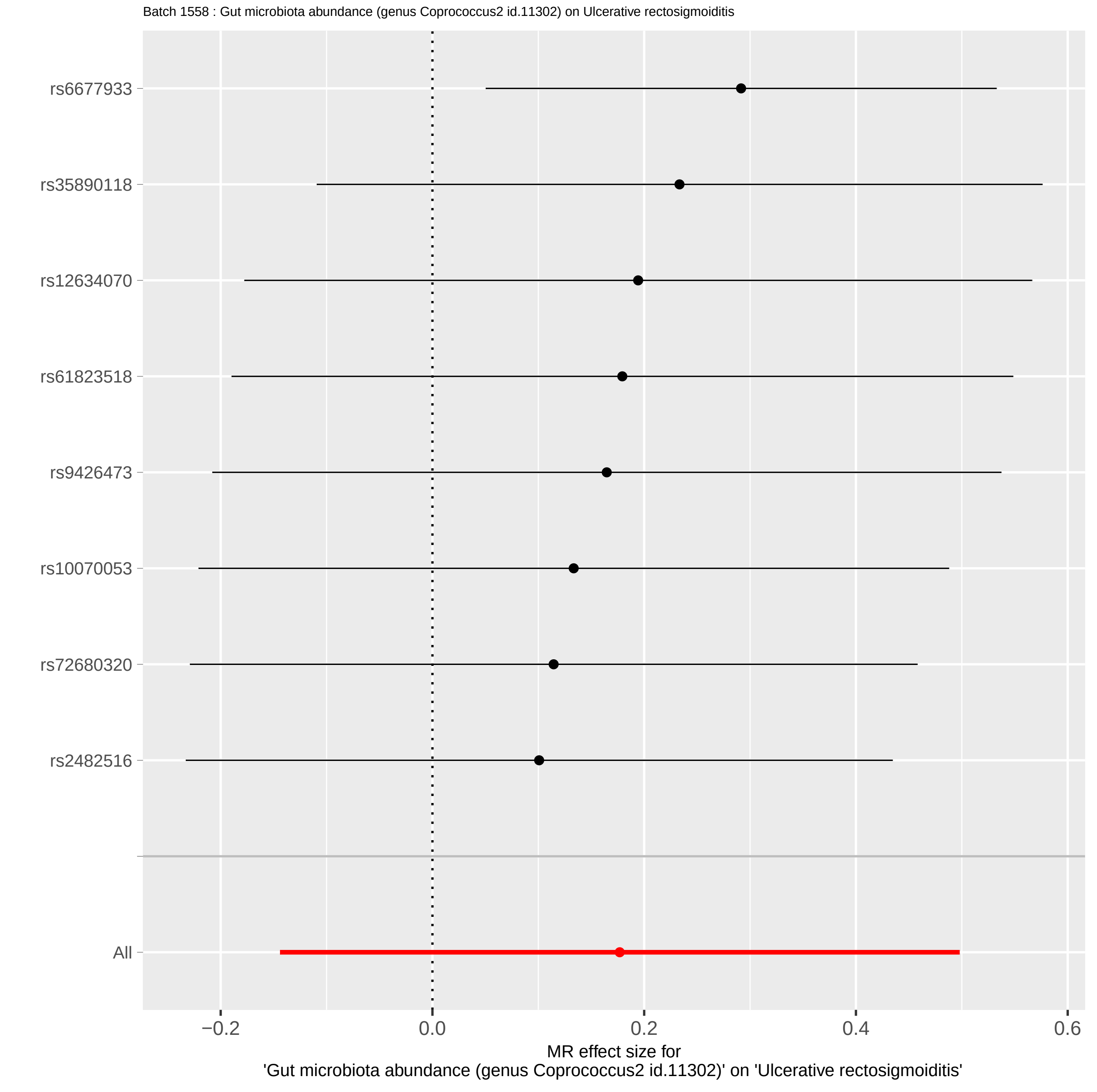

Batch 1558 : Gut microbiota abundance (genus Coprococcus2 id.11302) on Ulcerative rectosigmoiditis
rs6677933
rs35890118
rs12634070
rs61823518
rs9426473
rs10070053
rs72680320
rs2482516
All
−0.2
0.0
0.2
0.4
0.6
MR effect size for
'Gut microbiota abundance (genus Coprococcus2 id.11302)' on 'Ulcerative rectosigmoiditis'

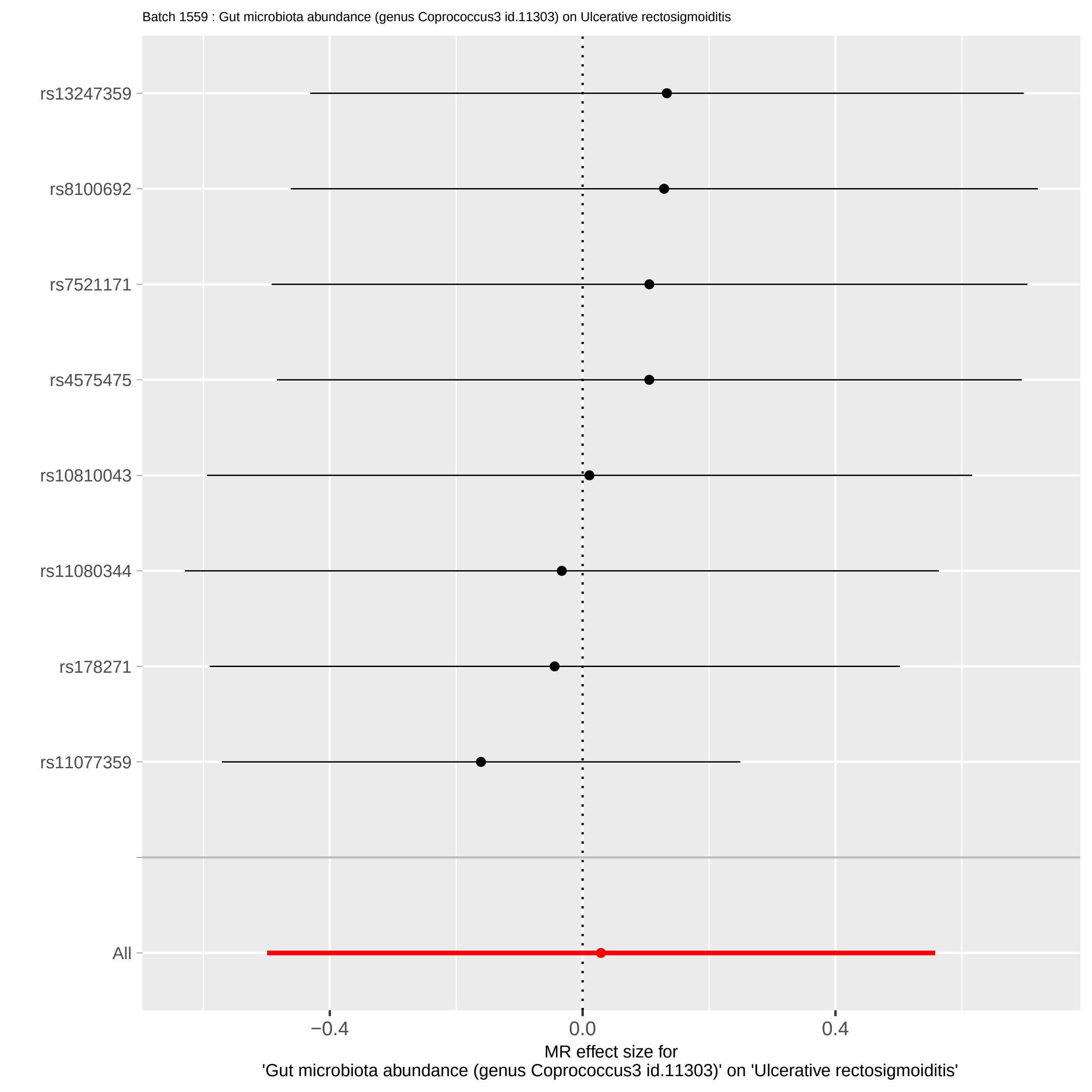

Batch 1559 : Gut microbiota abundance (genus Coprococcus3 id.11303) on Ulcerative rectosigmoiditis
rs13247359
rs8100692
rs7521171
rs4575475
rs10810043
rs11080344
rs178271
rs11077359
All
−0.4
0.0
0.4
MR effect size for
'Gut microbiota abundance (genus Coprococcus3 id.11303)' on 'Ulcerative rectosigmoiditis'

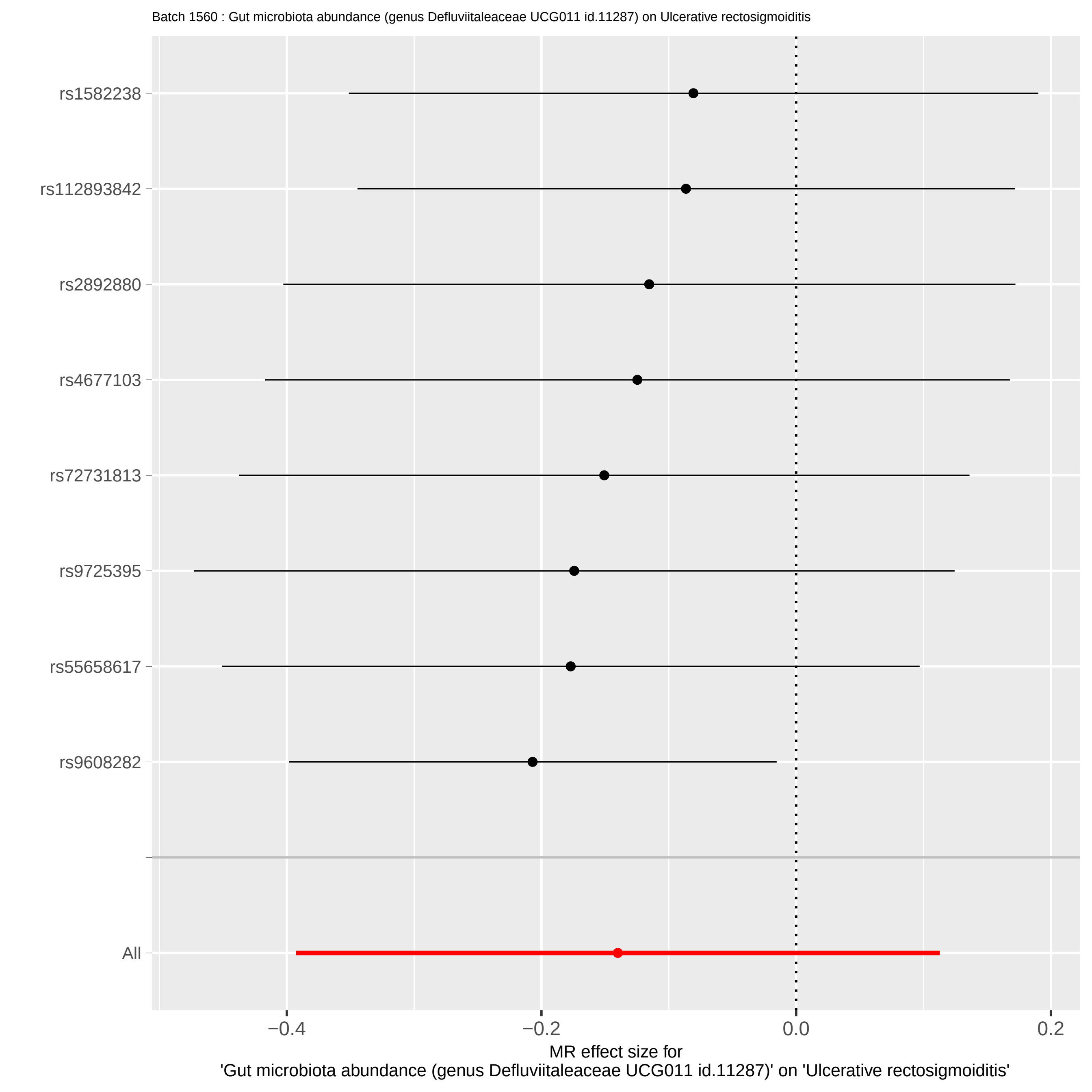

Batch 1560 : Gut microbiota abundance (genus Defluviitaleaceae UCG011 id.11287) on Ulcerative rectosigmoiditis
rs1582238
rs112893842
rs2892880
rs4677103
rs72731813
rs9725395
rs55658617
rs9608282
All
−0.4
−0.2
0.0
0.2
MR effect size for
'Gut microbiota abundance (genus Defluviitaleaceae UCG011 id.11287)' on 'Ulcerative rectosigmoiditis'

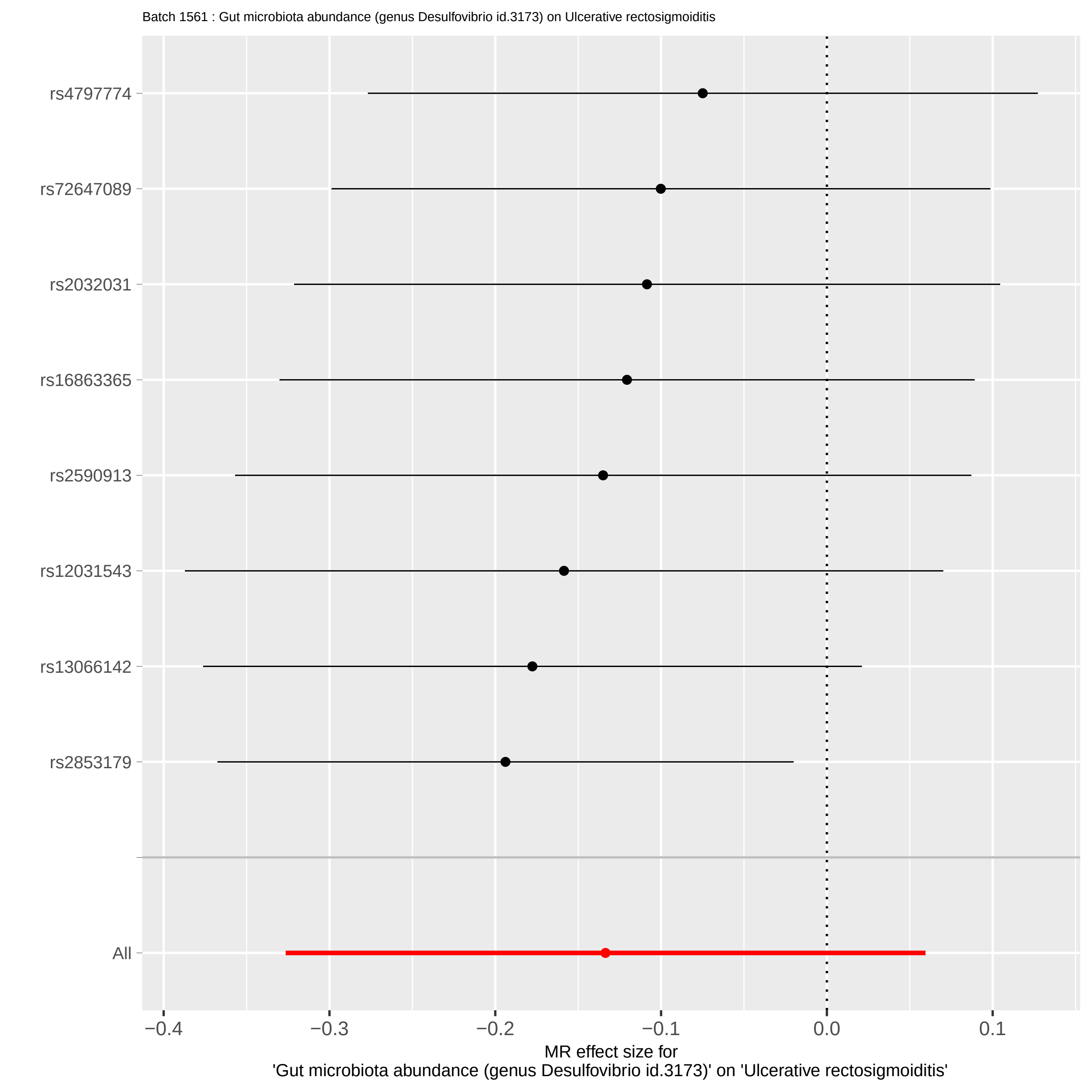

Batch 1561 : Gut microbiota abundance (genus Desulfovibrio id.3173) on Ulcerative rectosigmoiditis
rs4797774
rs72647089
rs2032031
rs16863365
rs2590913
rs12031543
rs13066142
rs2853179
All
−0.4
−0.3
−0.2
−0.1
0.0
0.1
MR effect size for
'Gut microbiota abundance (genus Desulfovibrio id.3173)' on 'Ulcerative rectosigmoiditis'

Batch 1562 : Gut microbiota abundance (genus Dialister id.2183) on Ulcerative rectosigmoiditis

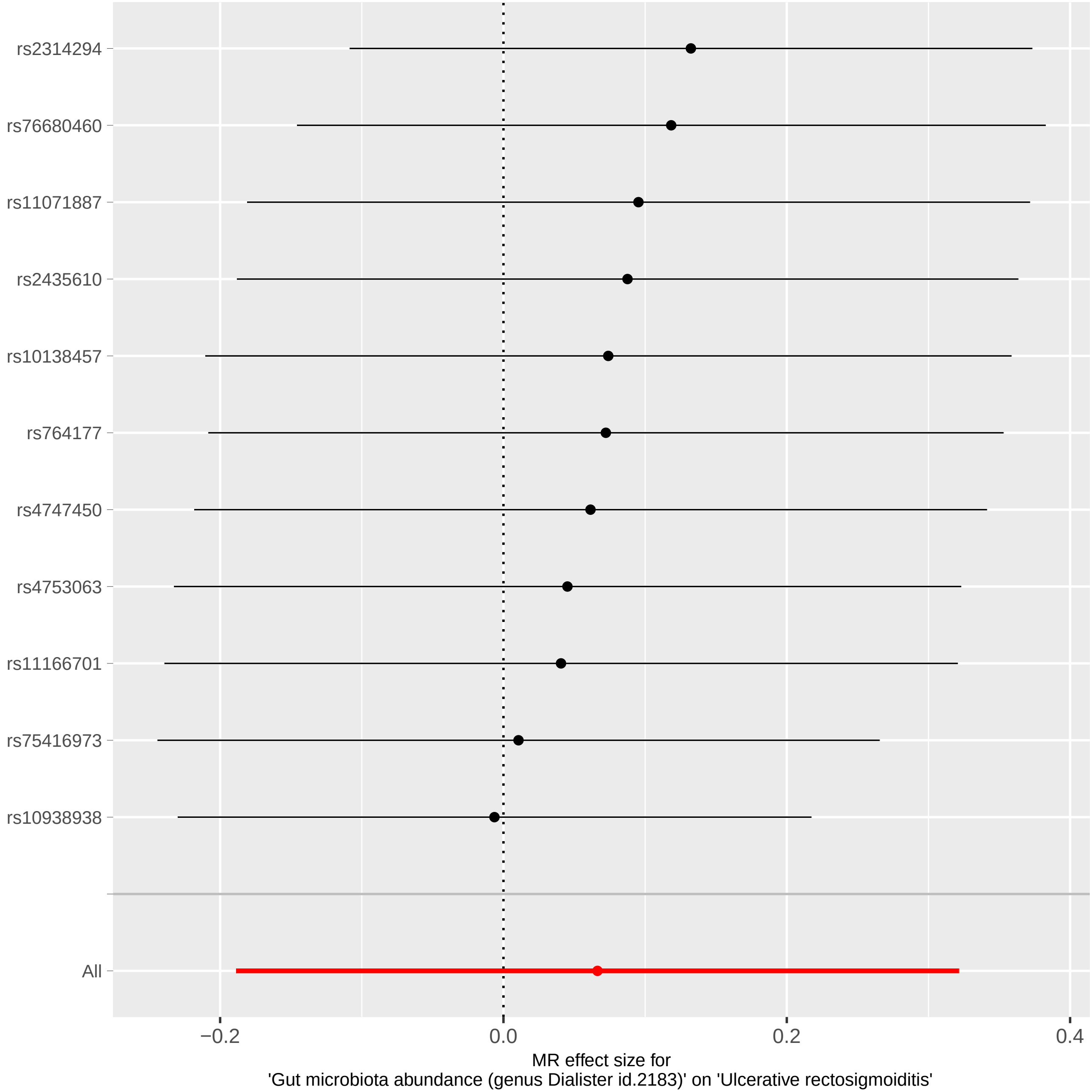

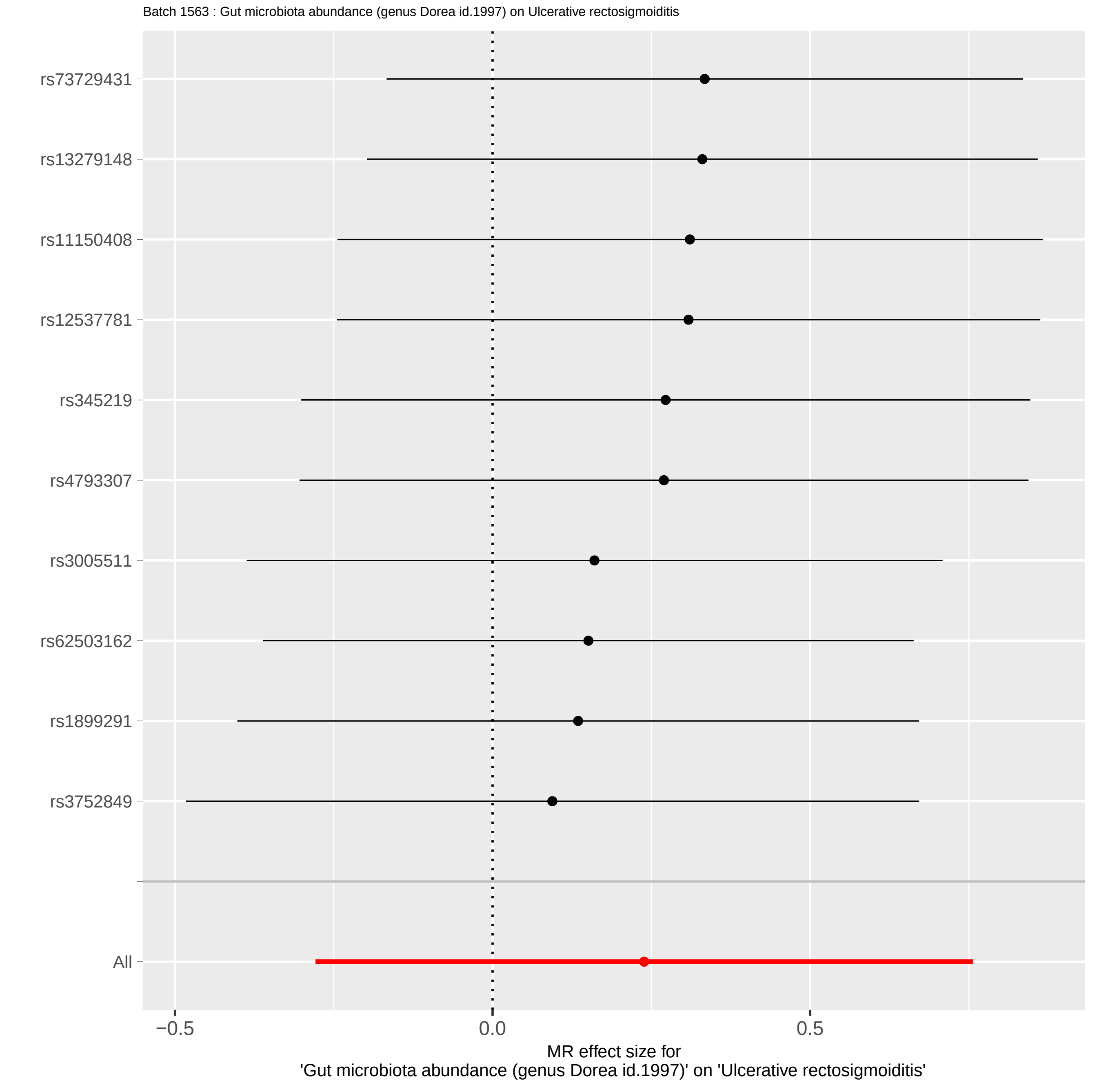

Batch 1563 : Gut microbiota abundance (genus Dorea id.1997) on Ulcerative rectosigmoiditis
rs73729431
rs13279148
rs11150408
rs12537781
rs345219
rs4793307
rs3005511
rs62503162
rs1899291
rs3752849
All
−0.5
0.0
0.5
MR effect size for
'Gut microbiota abundance (genus Dorea id.1997)' on 'Ulcerative rectosigmoiditis'

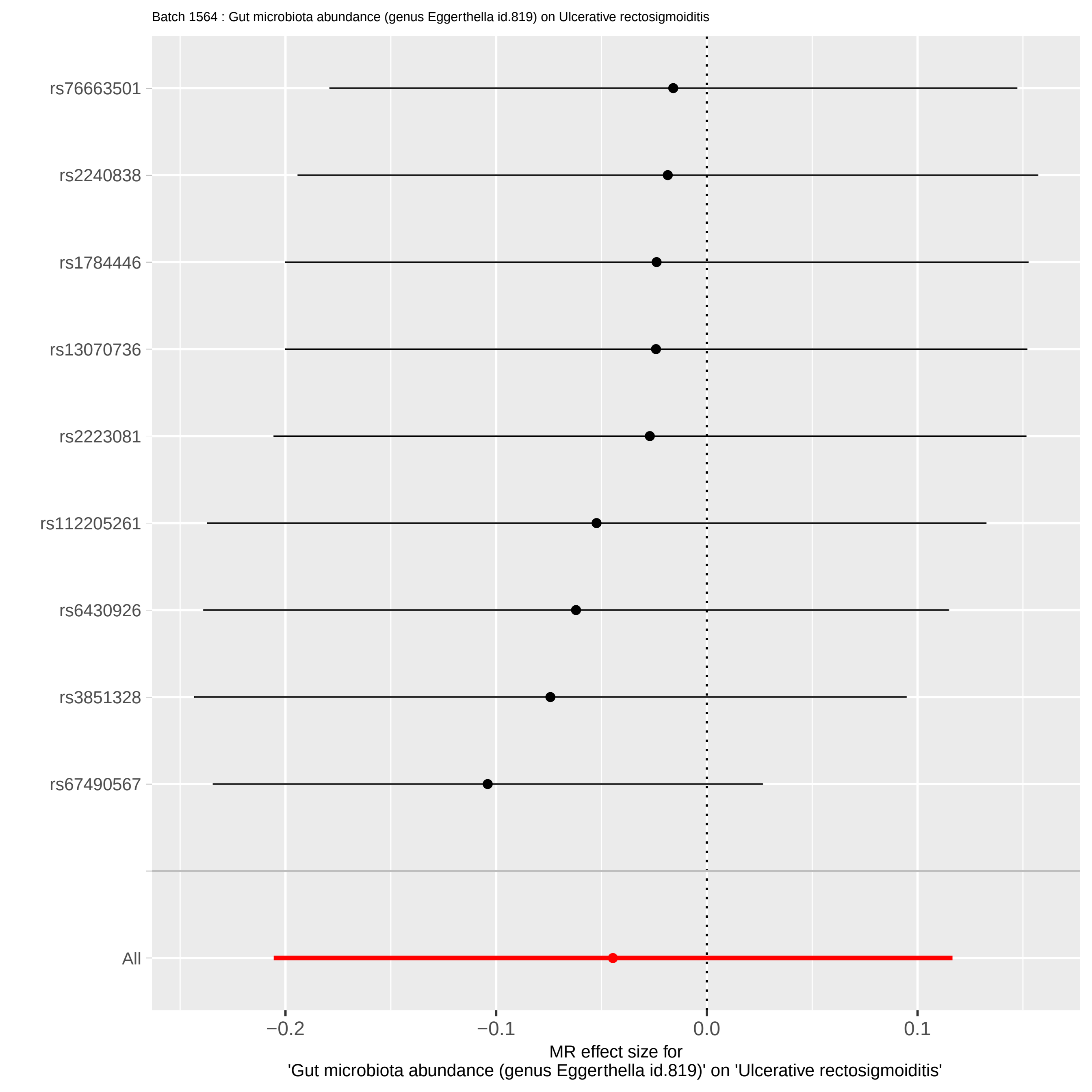
Batch 1564 : Gut microbiota abundance (genus Eggerthella id.819) on Ulcerative rectosigmoiditis
rs76663501
rs2240838
rs1784446
rs13070736
rs2223081
rs112205261
rs6430926
rs3851328
rs67490567
All
−0.2
−0.1
0.0
0.1
MR effect size for
'Gut microbiota abundance (genus Eggerthella id.819)' on 'Ulcerative rectosigmoiditis'

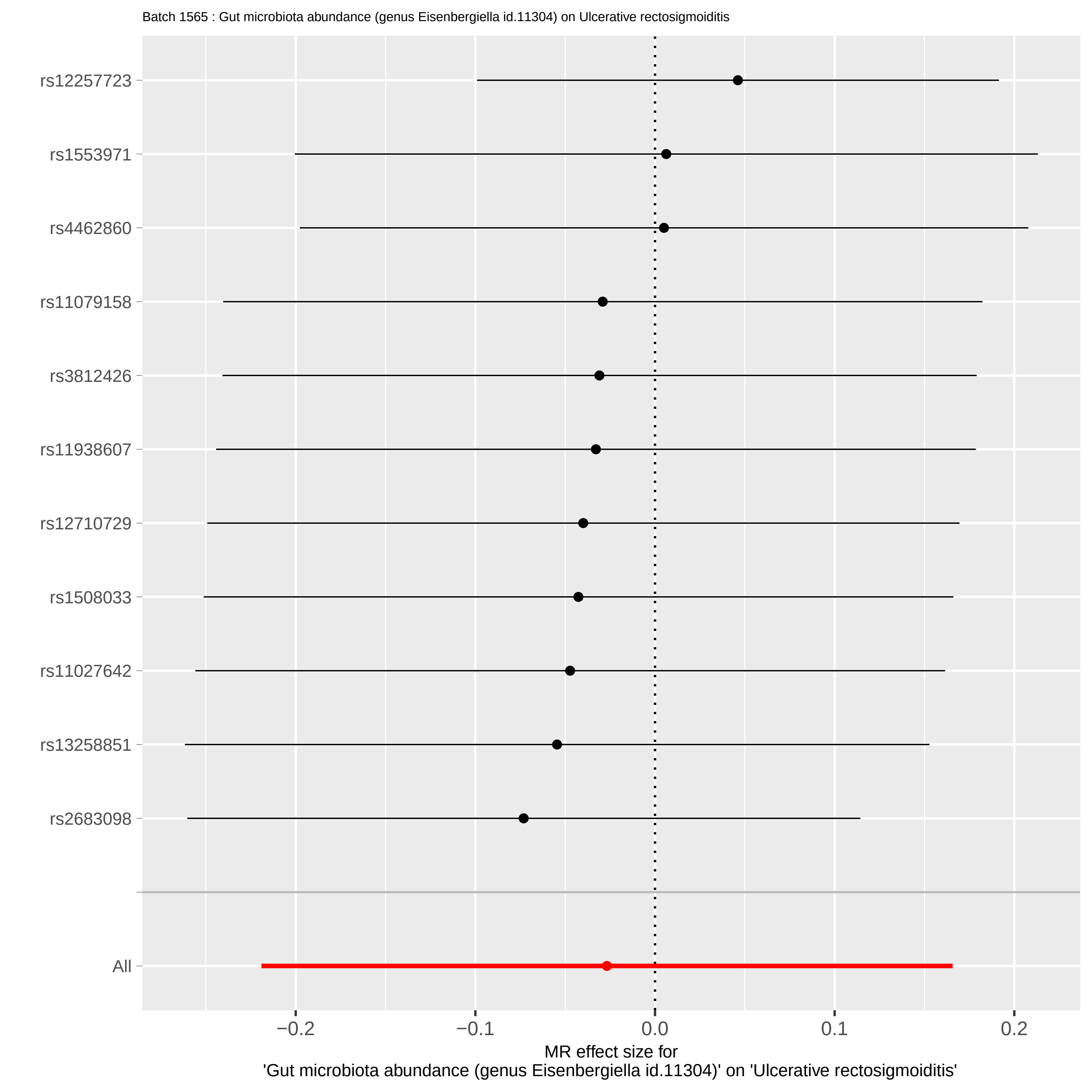

Batch 1565 : Gut microbiota abundance (genus Eisenbergiella id.11304) on Ulcerative rectosigmoiditis
rs12257723
rs1553971
rs4462860
rs11079158
rs3812426
rs11938607
rs12710729
rs1508033
rs11027642
rs13258851
rs2683098
All
−0.2
−0.1
0.0
0.1
0.2
MR effect size for
'Gut microbiota abundance (genus Eisenbergiella id.11304)' on 'Ulcerative rectosigmoiditis'

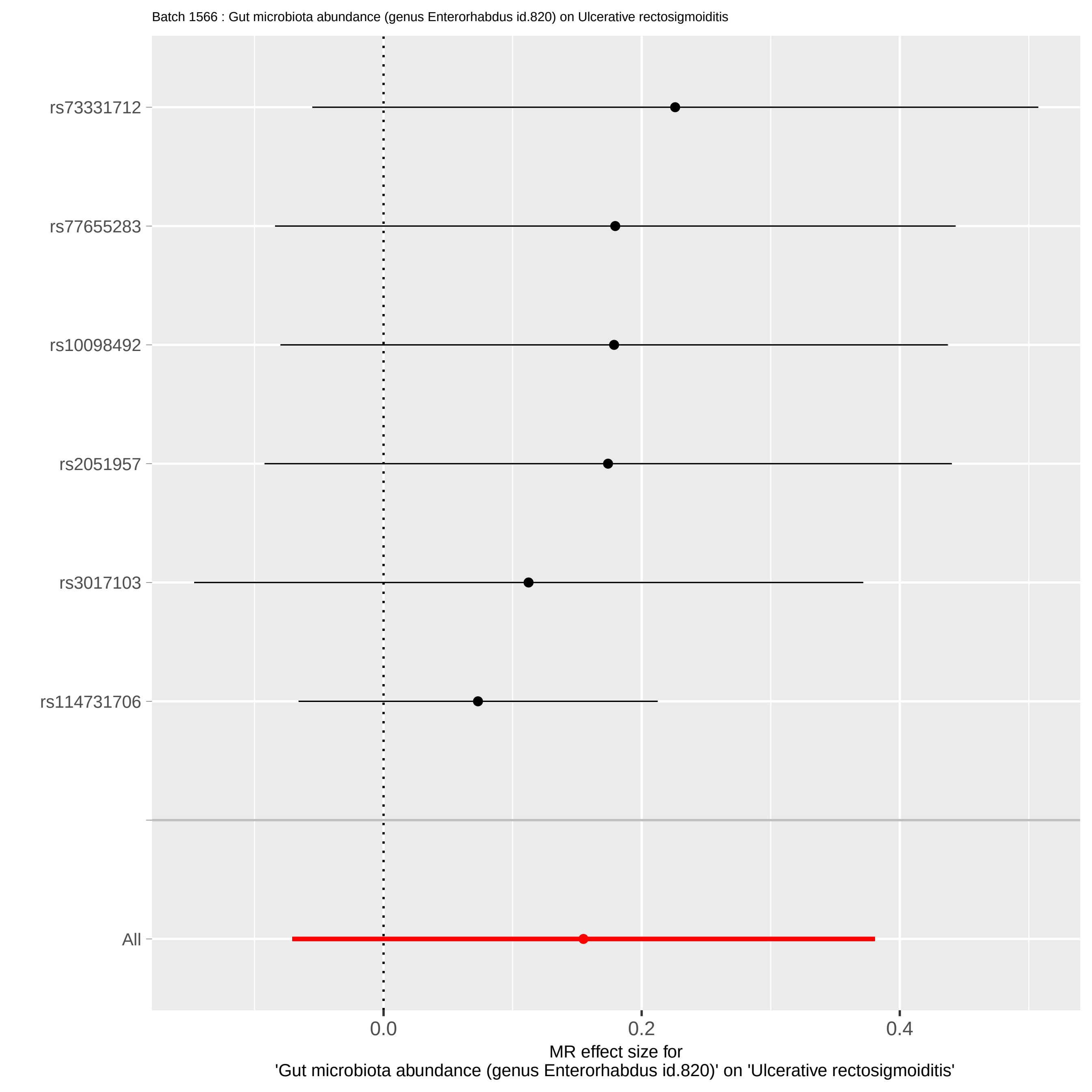

Batch 1566 : Gut microbiota abundance (genus Enterorhabdus id.820) on Ulcerative rectosigmoiditis
rs73331712
rs77655283
rs10098492
rs2051957
rs3017103
rs114731706
All
0.0
0.2
0.4
MR effect size for
'Gut microbiota abundance (genus Enterorhabdus id.820)' on 'Ulcerative rectosigmoiditis'

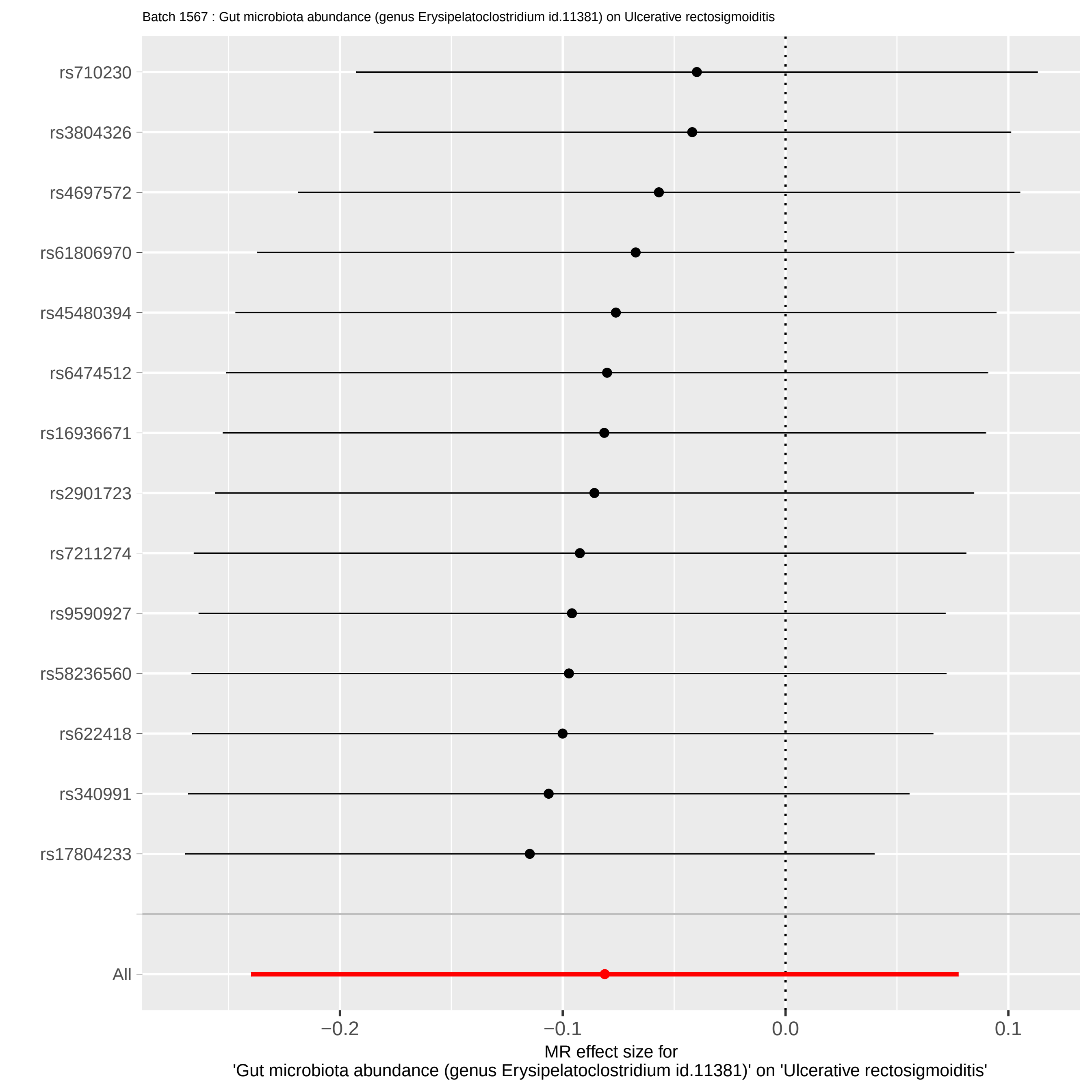

Batch 1567 : Gut microbiota abundance (genus Erysipelatoclostridium id.11381) on Ulcerative rectosigmoiditis
rs710230
rs3804326
rs4697572
rs61806970
rs45480394
rs6474512
rs16936671
rs2901723
rs7211274
rs9590927
rs58236560
rs622418
rs340991
rs17804233
All
−0.2
−0.1
0.0
0.1
MR effect size for
'Gut microbiota abundance (genus Erysipelatoclostridium id.11381)' on 'Ulcerative rectosigmoiditis'

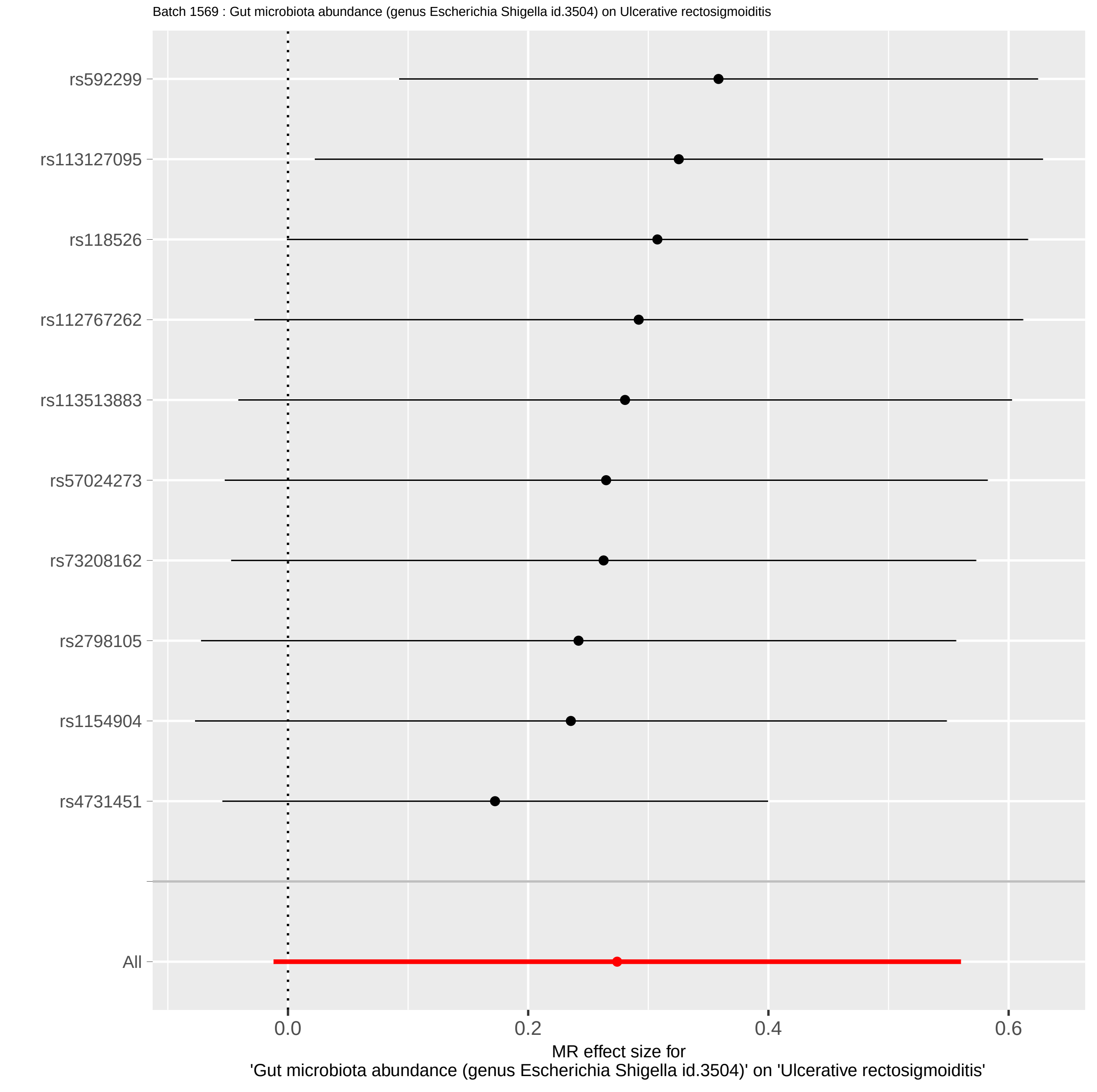

Batch 1569 : Gut microbiota abundance (genus Escherichia Shigella id.3504) on Ulcerative rectosigmoiditis
rs592299
rs113127095
rs118526
rs112767262
rs113513883
rs57024273
rs73208162
rs2798105
rs1154904
rs4731451
All
0.0
0.2
0.4
0.6
MR effect size for
'Gut microbiota abundance (genus Escherichia Shigella id.3504)' on 'Ulcerative rectosigmoiditis'

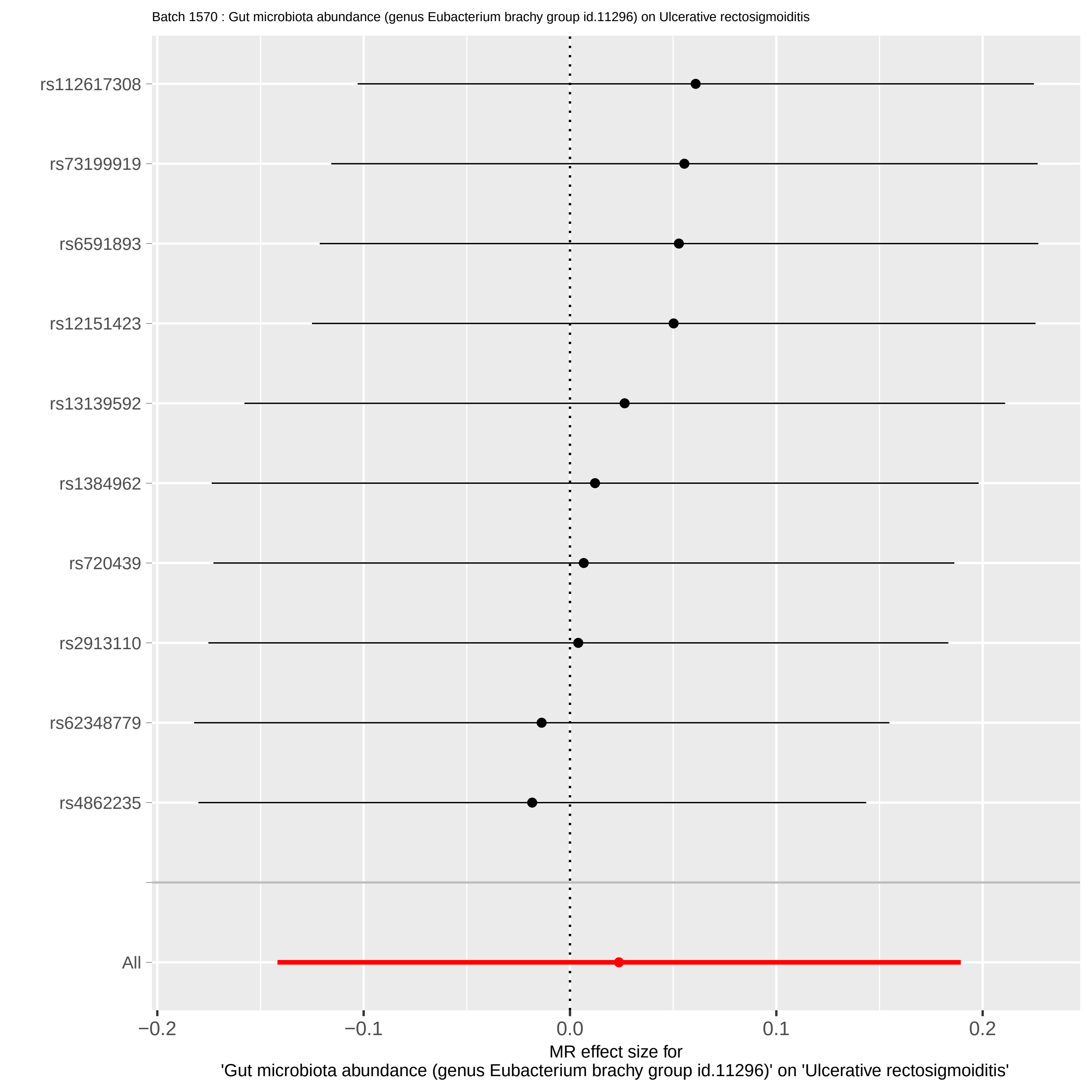

Batch 1570 : Gut microbiota abundance (genus Eubacterium brachy group id.11296) on Ulcerative rectosigmoiditis
rs112617308
rs73199919
rs6591893
rs12151423
rs13139592
rs1384962
rs720439
rs2913110
rs62348779
rs4862235
All
−0.2
−0.1
0.0
0.1
0.2
MR effect size for
'Gut microbiota abundance (genus Eubacterium brachy group id.11296)' on 'Ulcerative rectosigmoiditis'

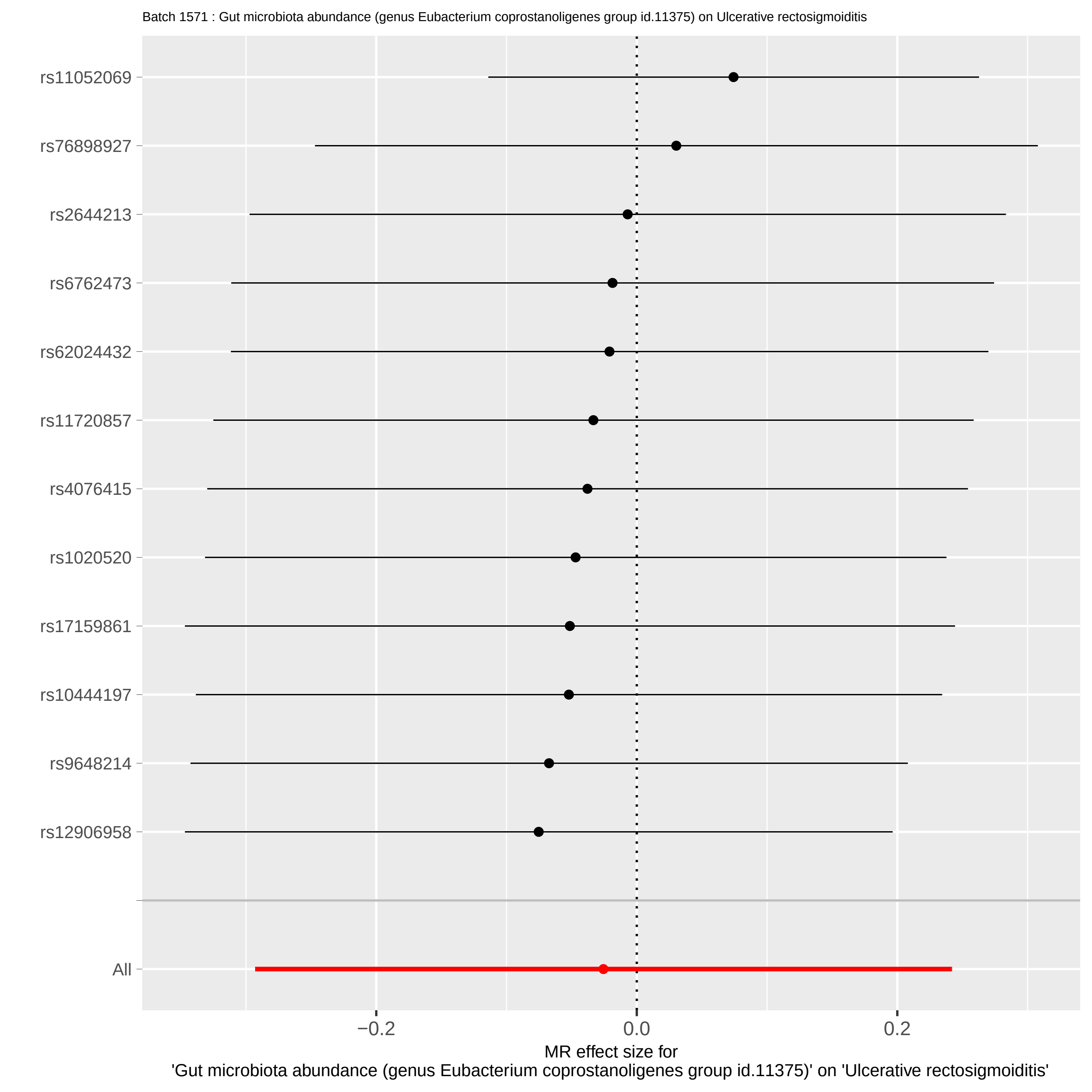

Batch 1571 : Gut microbiota abundance (genus Eubacterium coprostanoligenes group id.11375) on Ulcerative rectosigmoiditis
rs11052069
rs76898927
rs2644213
rs6762473
rs62024432
rs11720857
rs4076415
rs1020520
rs17159861
rs10444197
rs9648214
rs12906958
All
−0.2
0.0
0.2
MR effect size for
'Gut microbiota abundance (genus Eubacterium coprostanoligenes group id.11375)' on 'Ulcerative rectosigmoiditis'

Batch 1572 : Gut microbiota abundance (genus Eubacterium eligens group id.14372) on Ulcerative rectosigmoiditis

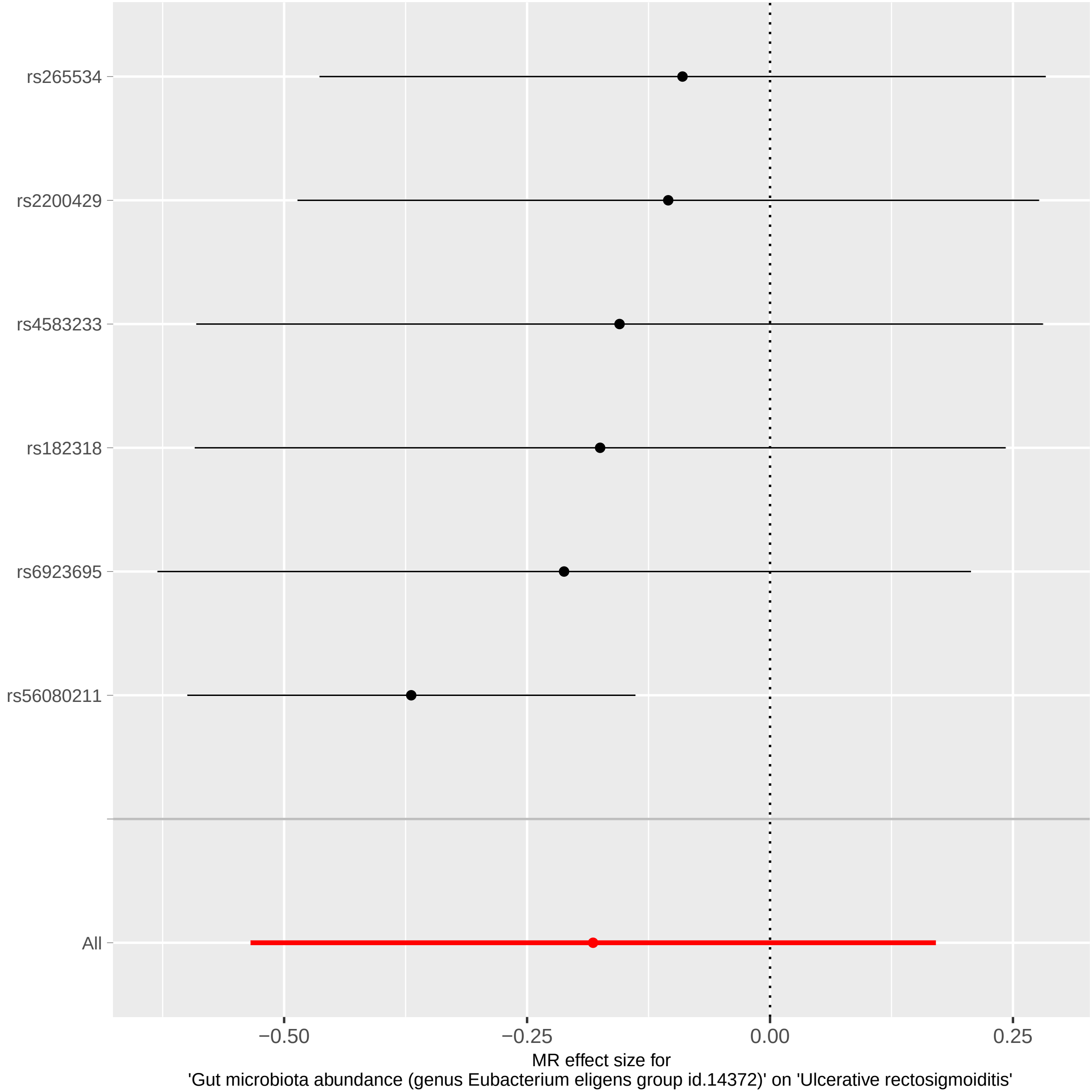

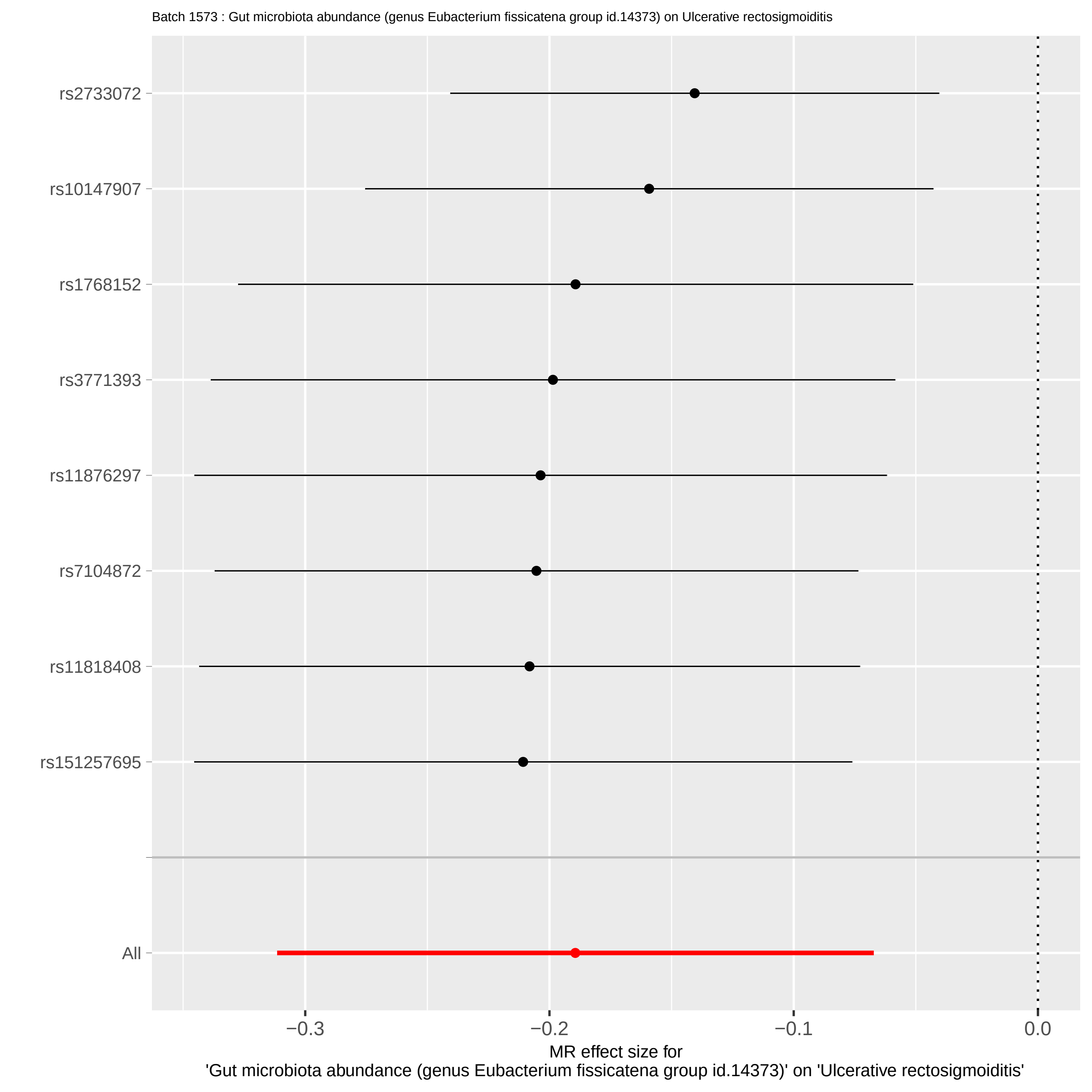
Batch 1573 : Gut microbiota abundance (genus Eubacterium fissicatena group id.14373) on Ulcerative rectosigmoiditis
rs2733072
rs10147907
rs1768152
rs3771393
rs11876297
rs7104872
rs11818408
rs151257695
All
−0.3
−0.2
−0.1
0.0
MR effect size for
'Gut microbiota abundance (genus Eubacterium fissicatena group id.14373)' on 'Ulcerative rectosigmoiditis'

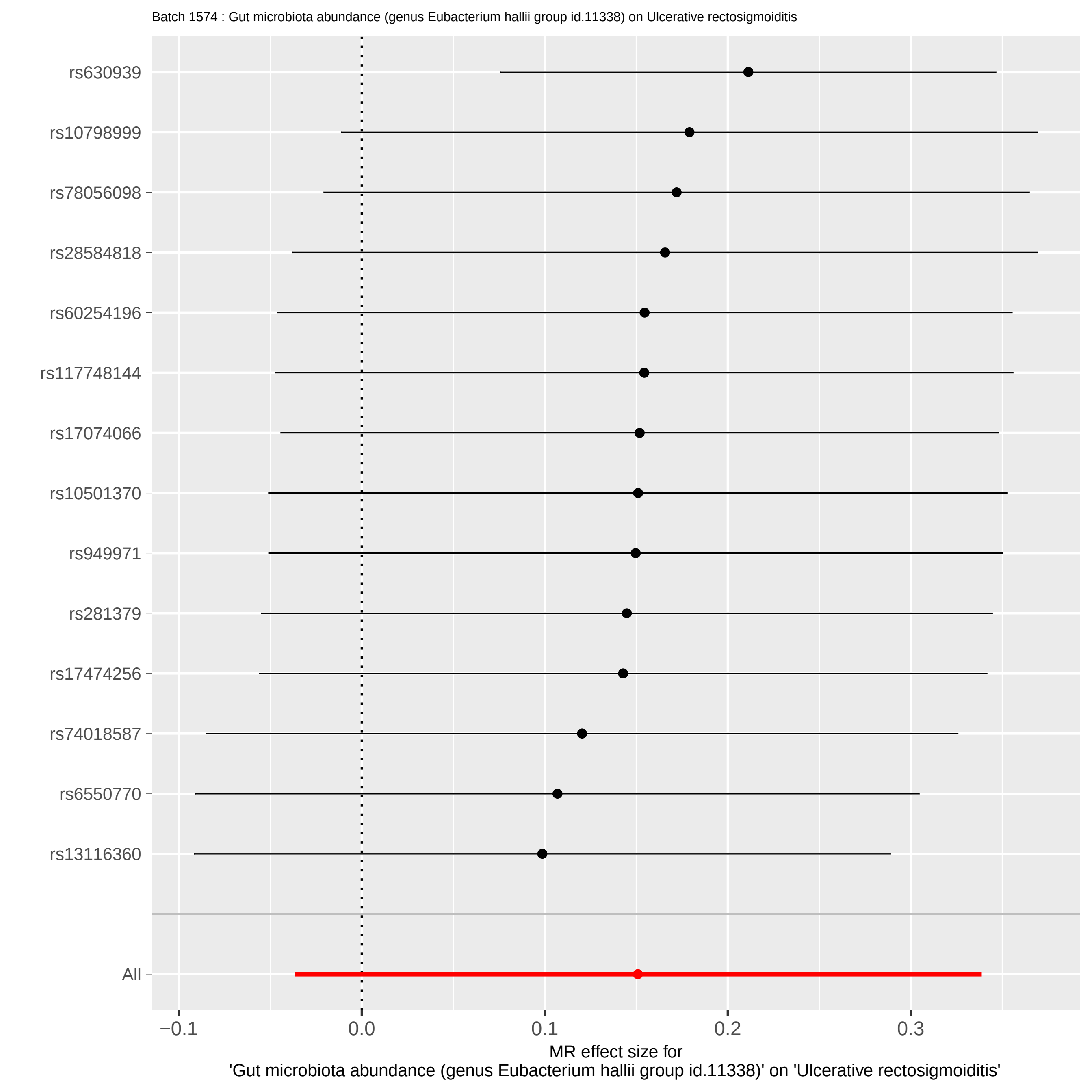

Batch 1574 : Gut microbiota abundance (genus Eubacterium hallii group id.11338) on Ulcerative rectosigmoiditis
rs630939
rs10798999
rs78056098
rs28584818
rs60254196
rs117748144
rs17074066
rs10501370
rs949971
rs281379
rs17474256
rs74018587
rs6550770
rs13116360
All
−0.1
0.0
0.1
0.2
0.3
MR effect size for
'Gut microbiota abundance (genus Eubacterium hallii group id.11338)' on 'Ulcerative rectosigmoiditis'

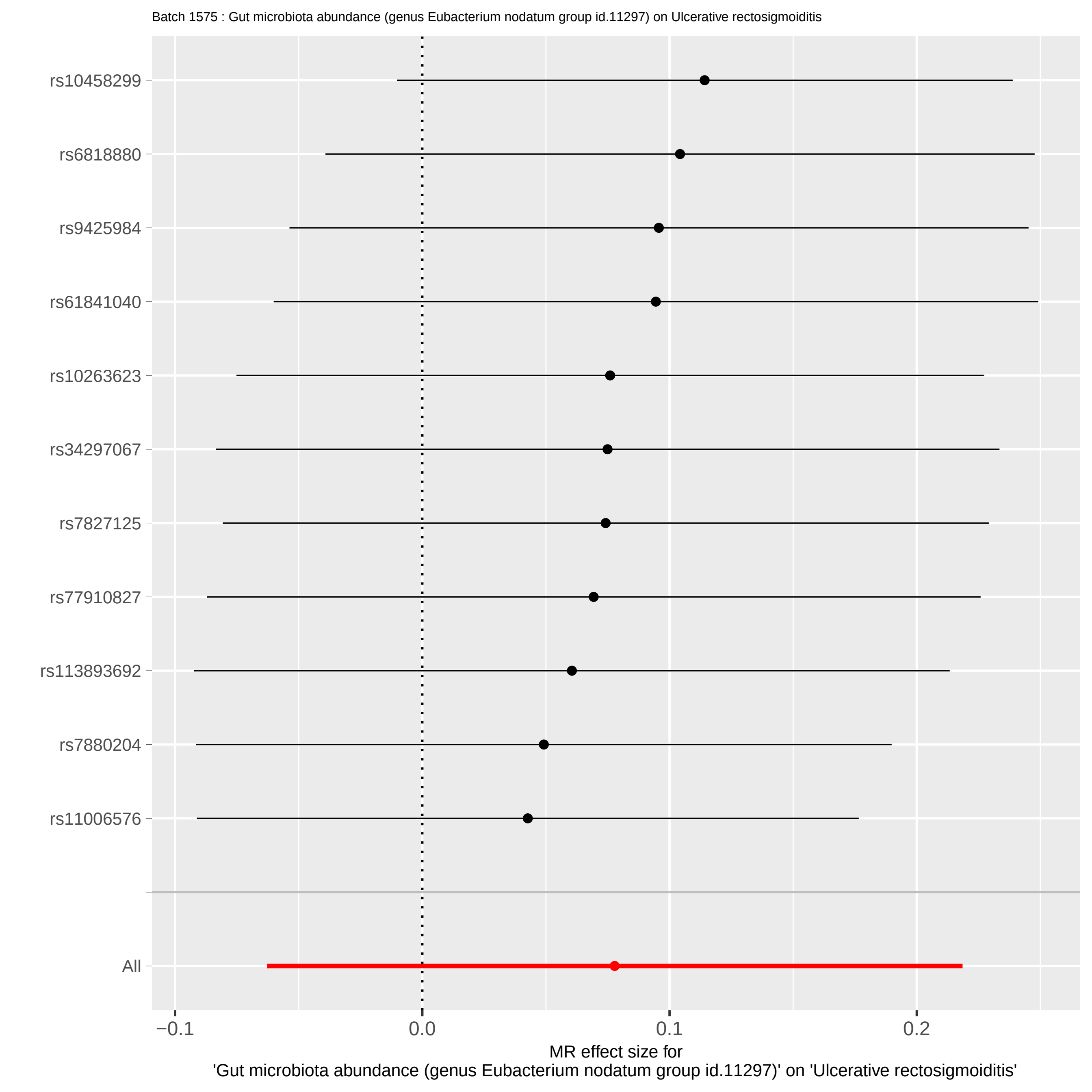
Batch 1575 : Gut microbiota abundance (genus Eubacterium nodatum group id.11297) on Ulcerative rectosigmoiditis
rs10458299
rs6818880
rs9425984
rs61841040
rs10263623
rs34297067
rs7827125
rs77910827
rs113893692
rs7880204
rs11006576
All
−0.1
0.0
0.1
0.2
MR effect size for
'Gut microbiota abundance (genus Eubacterium nodatum group id.11297)' on 'Ulcerative rectosigmoiditis'

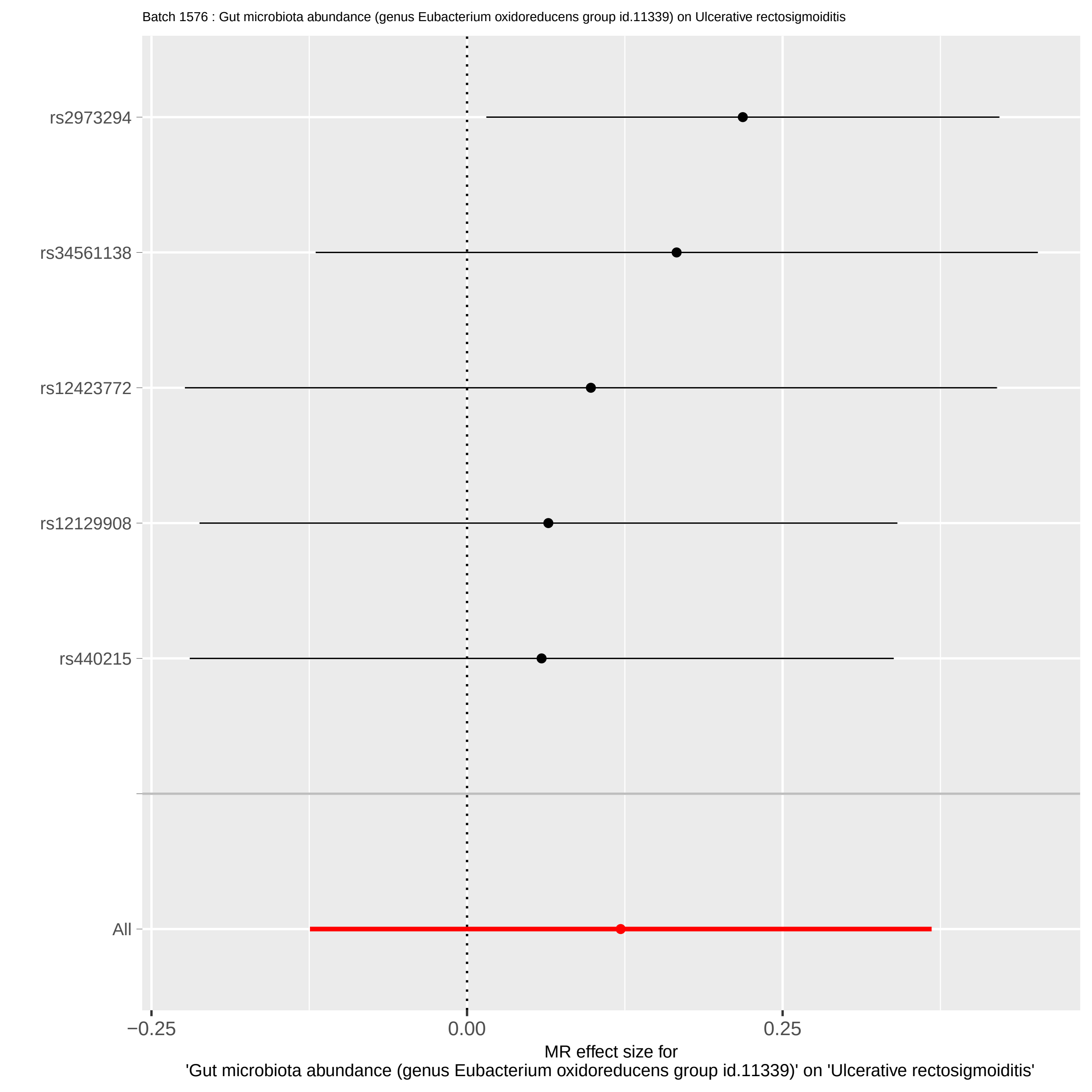

Batch 1576 : Gut microbiota abundance (genus Eubacterium oxidoreducens group id.11339) on Ulcerative rectosigmoiditis
rs2973294
rs34561138
rs12423772
rs12129908
rs440215
All
−0.25
0.00
0.25
MR effect size for
'Gut microbiota abundance (genus Eubacterium oxidoreducens group id.11339)' on 'Ulcerative rectosigmoiditis'

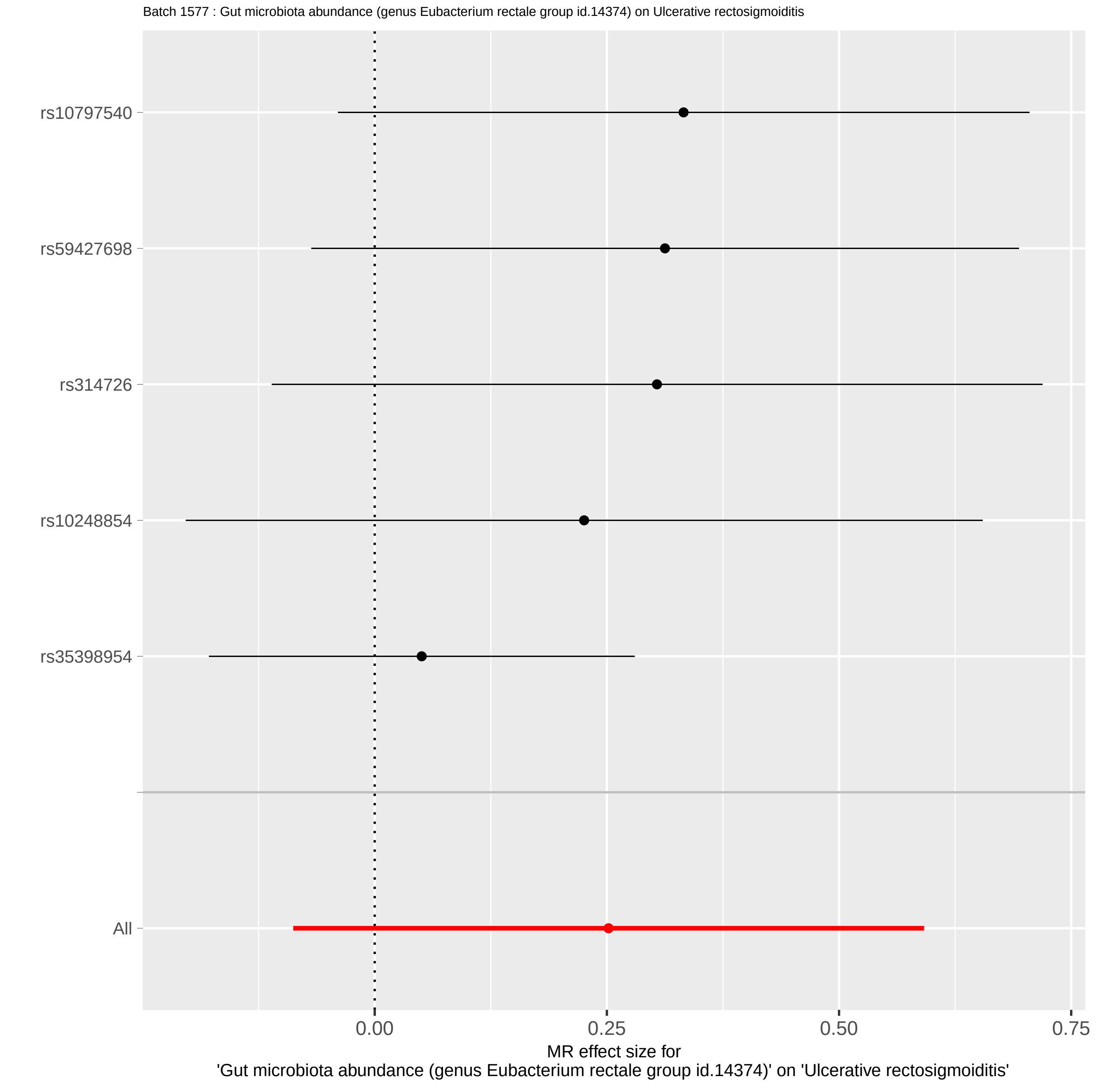

Batch 1577 : Gut microbiota abundance (genus Eubacterium rectale group id.14374) on Ulcerative rectosigmoiditis
rs10797540
rs59427698
rs314726
rs10248854
rs35398954
All
0.00
0.25
0.50
0.75
MR effect size for
'Gut microbiota abundance (genus Eubacterium rectale group id.14374)' on 'Ulcerative rectosigmoiditis'

Batch 1578 : Gut microbiota abundance (genus Eubacterium ruminantium group id.11340) on Ulcerative rectosigmoiditis

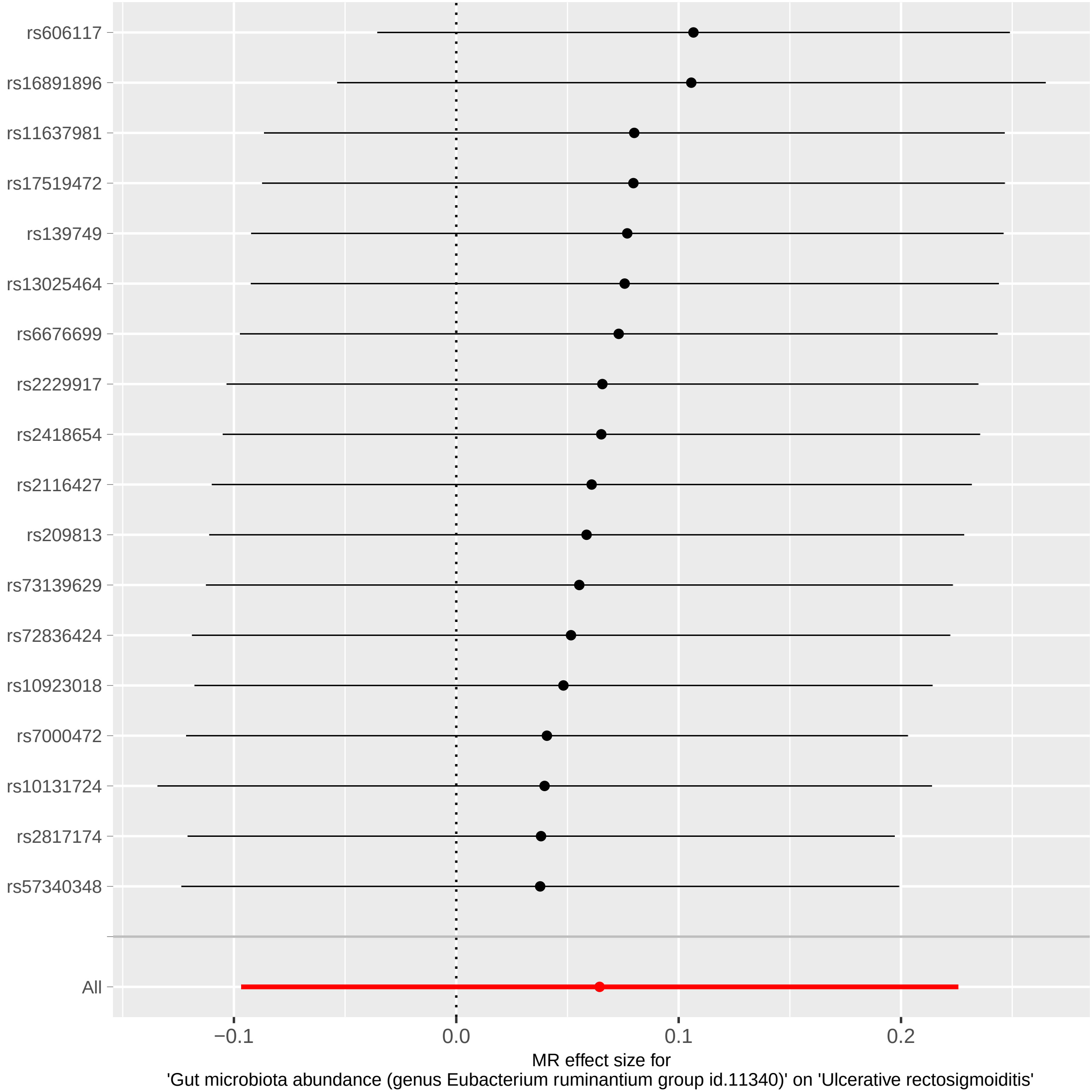

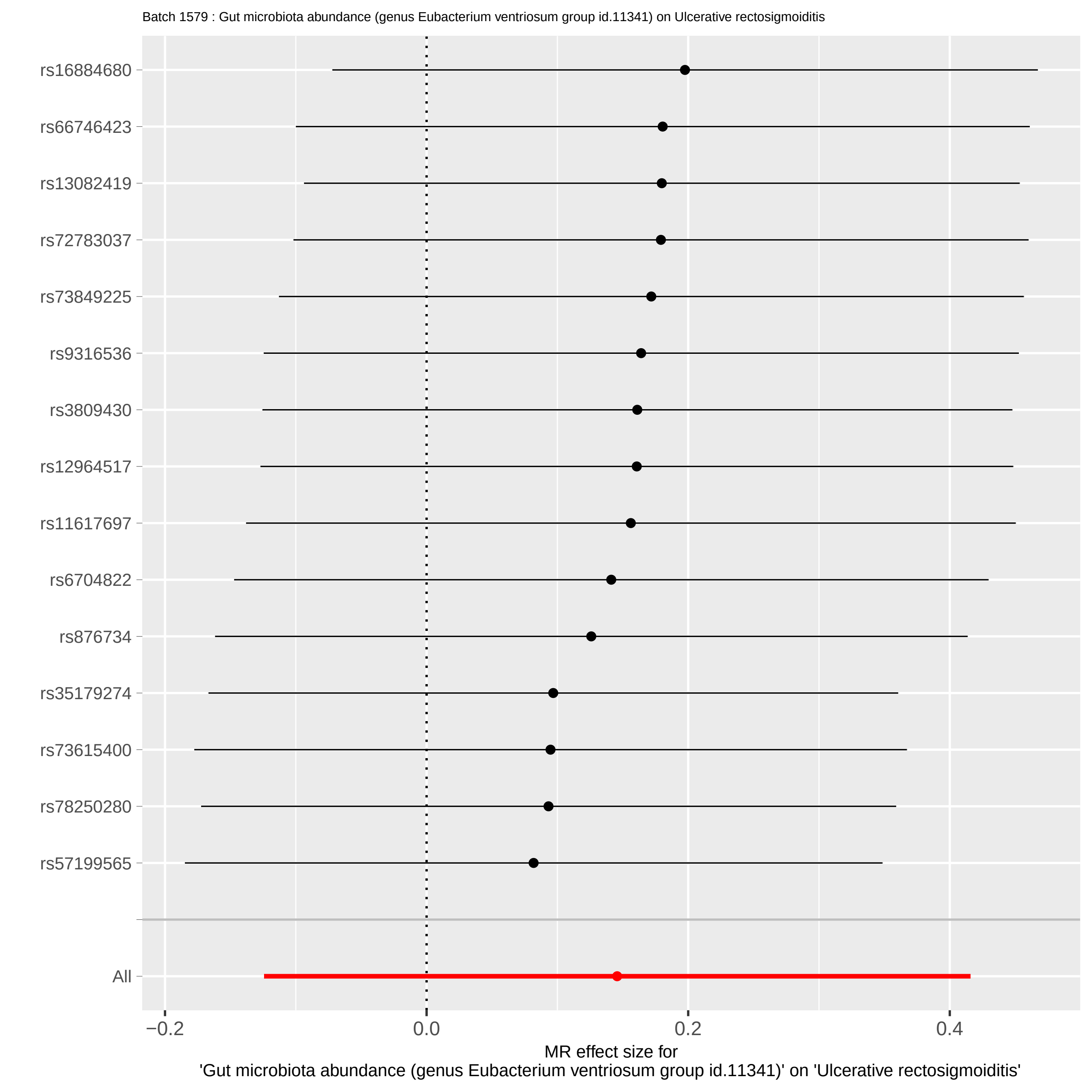

Batch 1579 : Gut microbiota abundance (genus Eubacterium ventriosum group id.11341) on Ulcerative rectosigmoiditis
rs16884680
rs66746423
rs13082419
rs72783037
rs73849225
rs9316536
rs3809430
rs12964517
rs11617697
rs6704822
rs876734
rs35179274
rs73615400
rs78250280
rs57199565
All
−0.2
0.0
0.2
0.4
MR effect size for
'Gut microbiota abundance (genus Eubacterium ventriosum group id.11341)' on 'Ulcerative rectosigmoiditis'

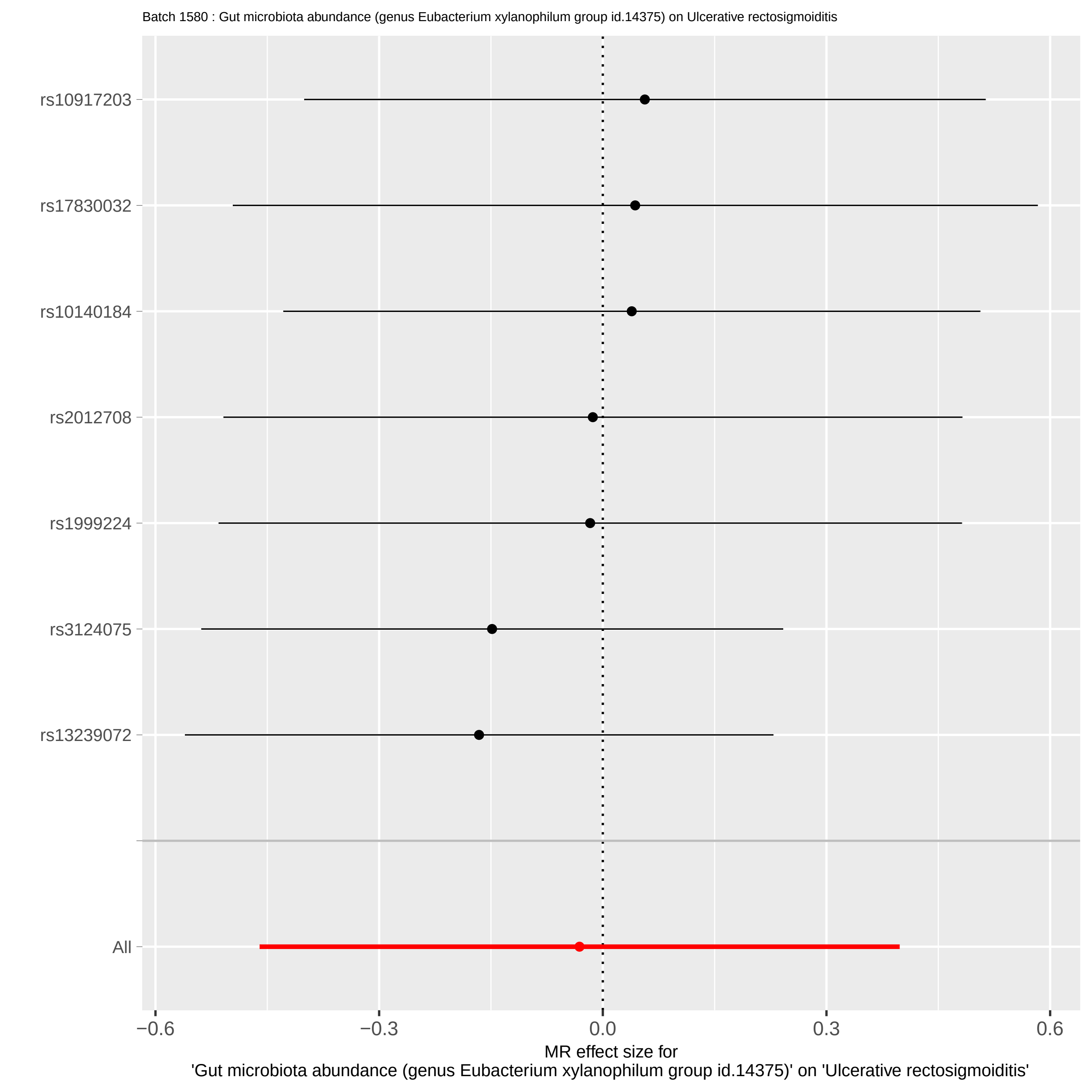

Batch 1580 : Gut microbiota abundance (genus Eubacterium xylanophilum group id.14375) on Ulcerative rectosigmoiditis
rs10917203
rs17830032
rs10140184
rs2012708
rs1999224
rs3124075
rs13239072
All
−0.6
−0.3
0.0
0.3
0.6
MR effect size for
'Gut microbiota abundance (genus Eubacterium xylanophilum group id.14375)' on 'Ulcerative rectosigmoiditis'

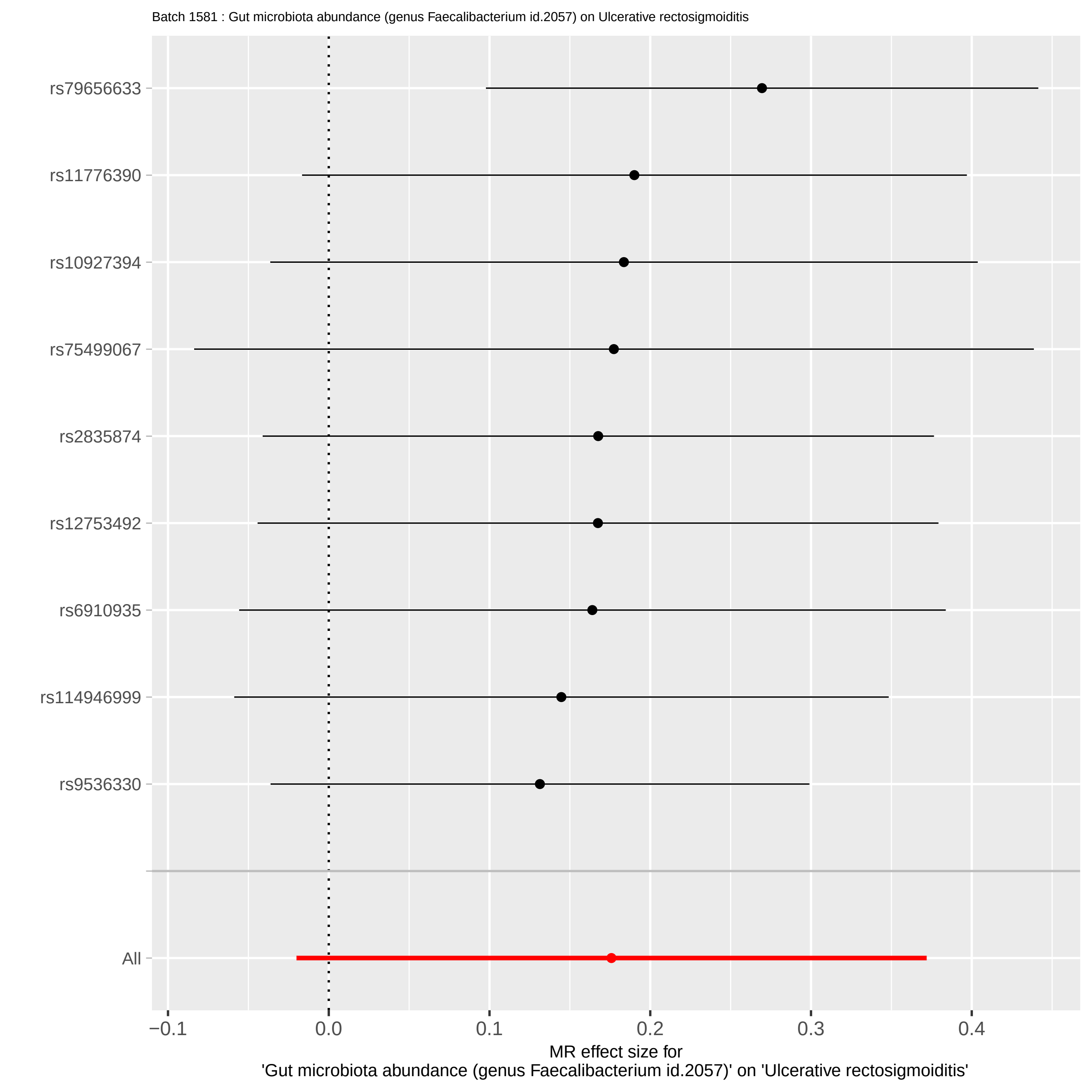

Batch 1581 : Gut microbiota abundance (genus Faecalibacterium id.2057) on Ulcerative rectosigmoiditis
rs79656633
rs11776390
rs10927394
rs75499067
rs2835874
rs12753492
rs6910935
rs114946999
rs9536330
All
−0.1
0.0
0.1
0.2
0.3
0.4
MR effect size for
'Gut microbiota abundance (genus Faecalibacterium id.2057)' on 'Ulcerative rectosigmoiditis'

Batch 1582 : Gut microbiota abundance (genus Family XIII AD3011 group id.11293) on Ulcerative rectosigmoiditis

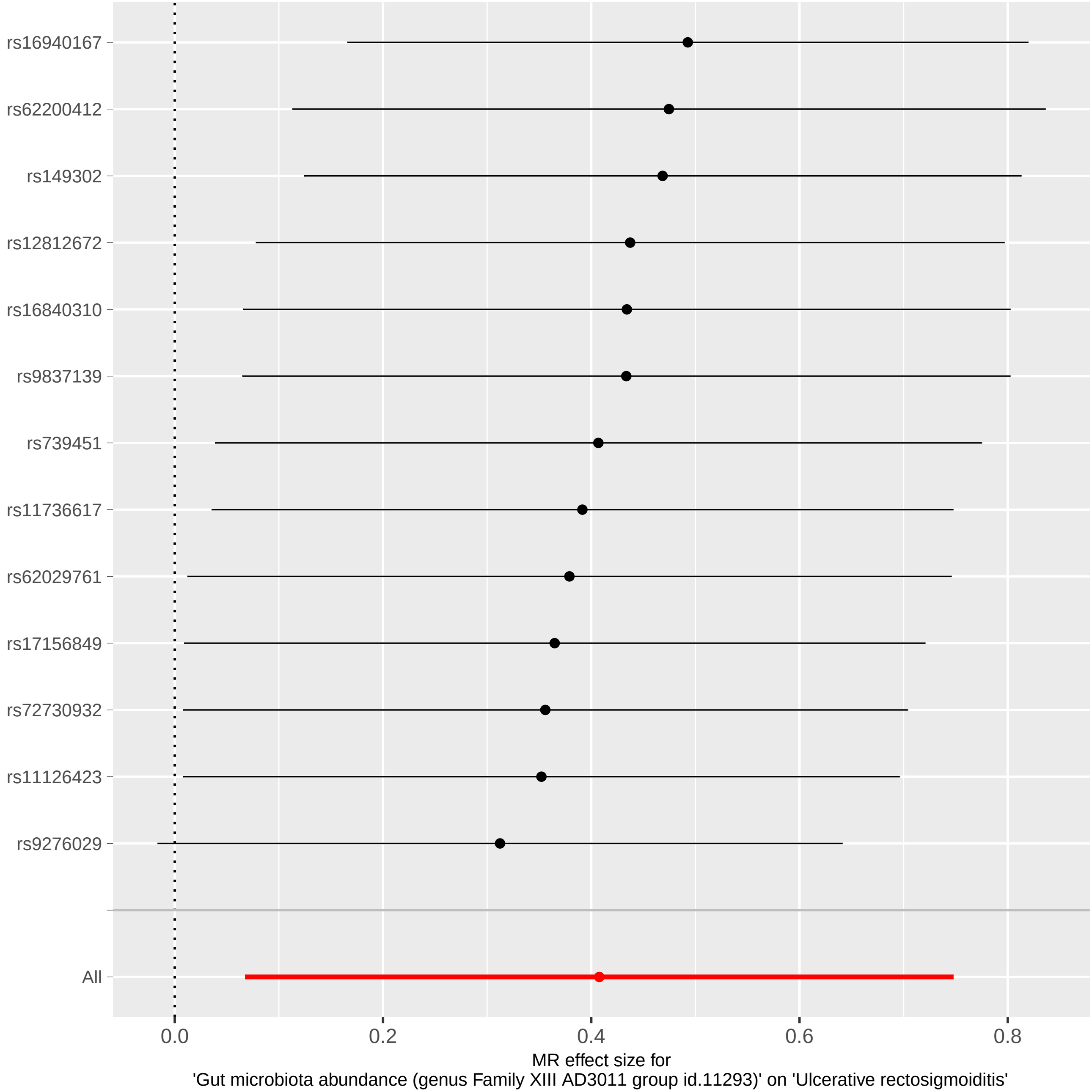

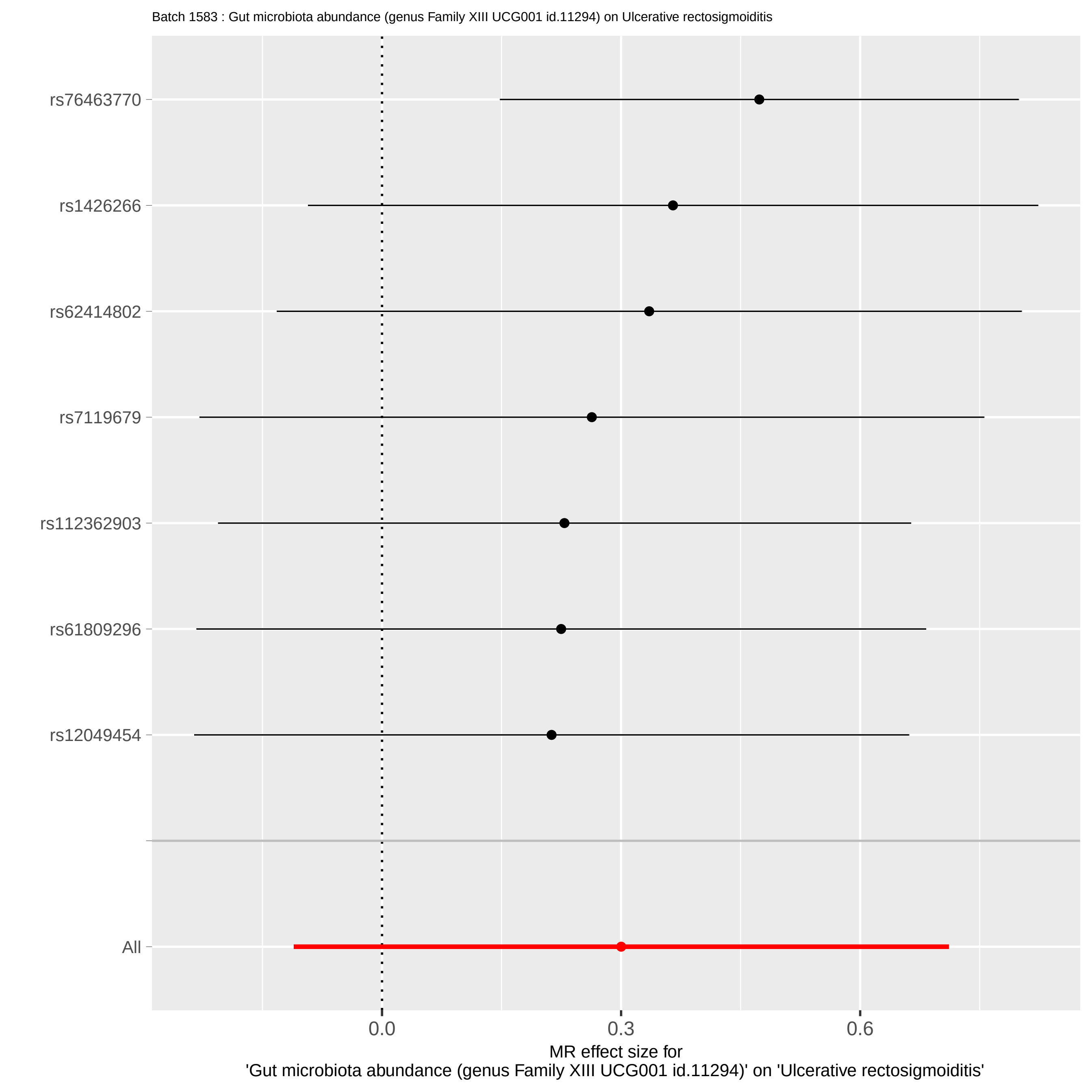

Batch 1583 : Gut microbiota abundance (genus Family XIII UCG001 id.11294) on Ulcerative rectosigmoiditis
rs76463770
rs1426266
rs62414802
rs7119679
rs112362903
rs61809296
rs12049454
All
0.0
0.3
0.6
MR effect size for
'Gut microbiota abundance (genus Family XIII UCG001 id.11294)' on 'Ulcerative rectosigmoiditis'

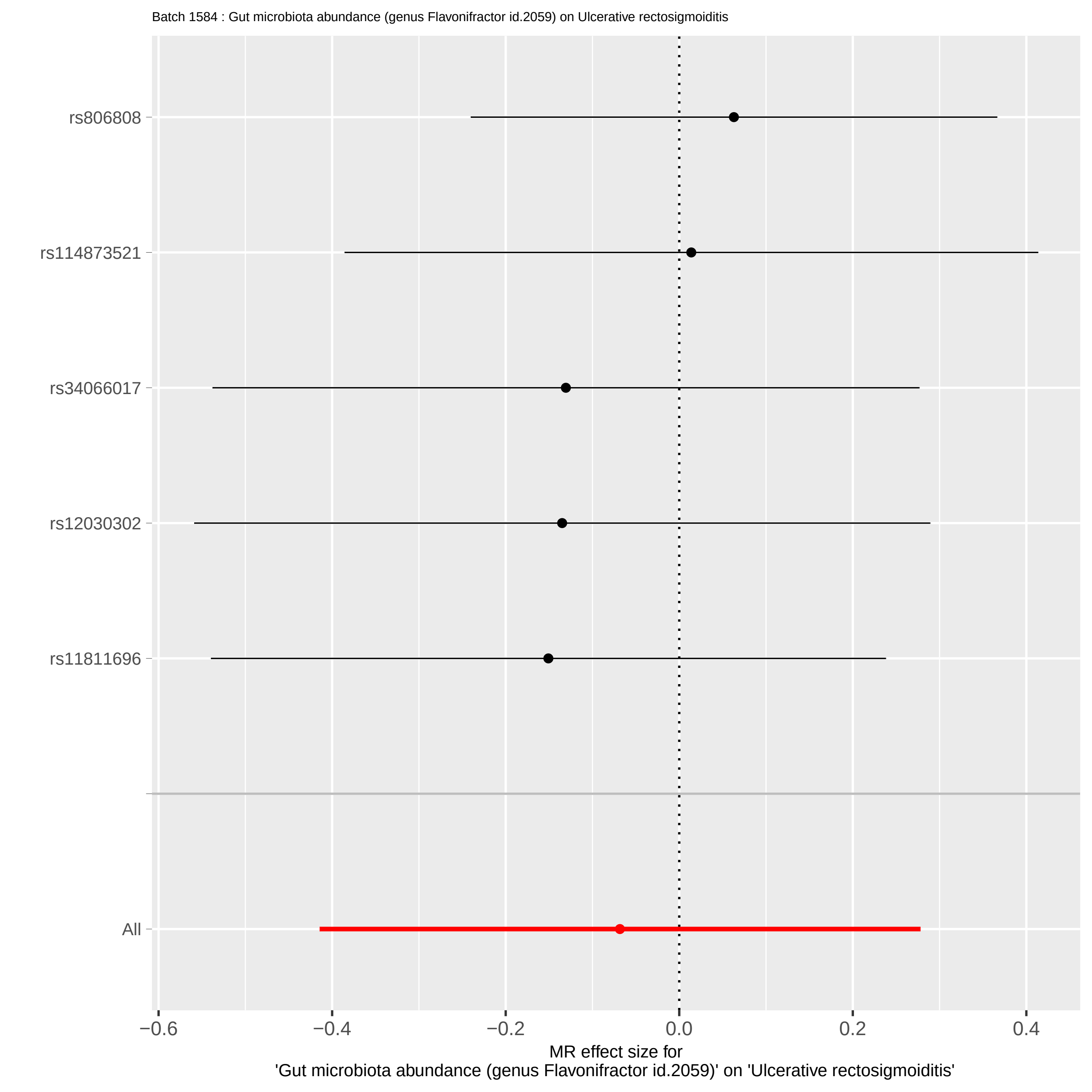
Batch 1584 : Gut microbiota abundance (genus Flavonifractor id.2059) on Ulcerative rectosigmoiditis
rs806808
rs114873521
rs34066017
rs12030302
rs11811696
All
−0.6
−0.4
−0.2
0.0
0.2
0.4
MR effect size for
'Gut microbiota abundance (genus Flavonifractor id.2059)' on 'Ulcerative rectosigmoiditis'

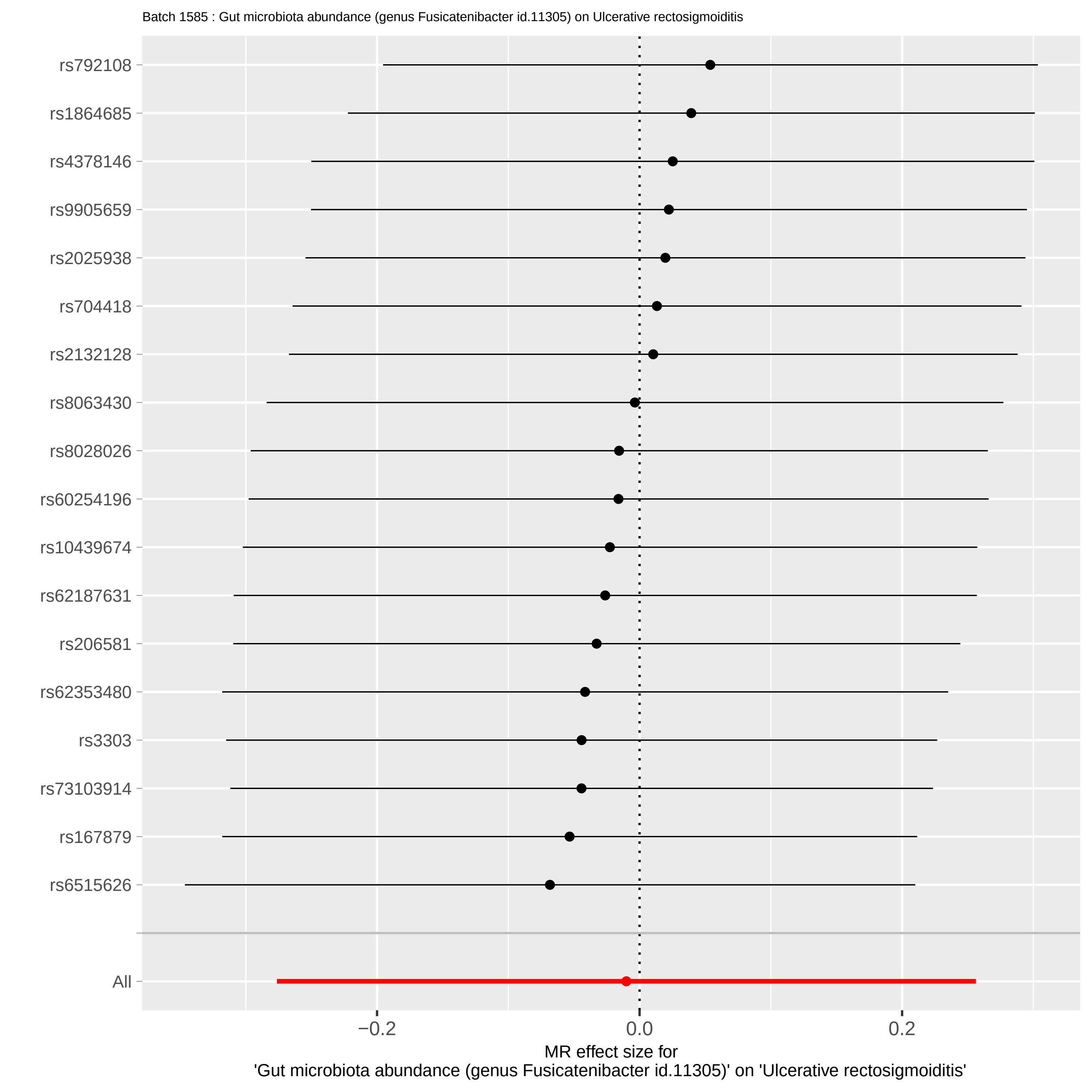

Batch 1585 : Gut microbiota abundance (genus Fusicatenibacter id.11305) on Ulcerative rectosigmoiditis
rs792108
rs1864685
rs4378146
rs9905659
rs2025938
rs704418
rs2132128
rs8063430
rs8028026
rs60254196
rs10439674
rs62187631
rs206581
rs62353480
rs3303
rs73103914
rs167879
rs6515626
All
−0.2
0.0
0.2
MR effect size for
'Gut microbiota abundance (genus Fusicatenibacter id.11305)' on 'Ulcerative rectosigmoiditis'

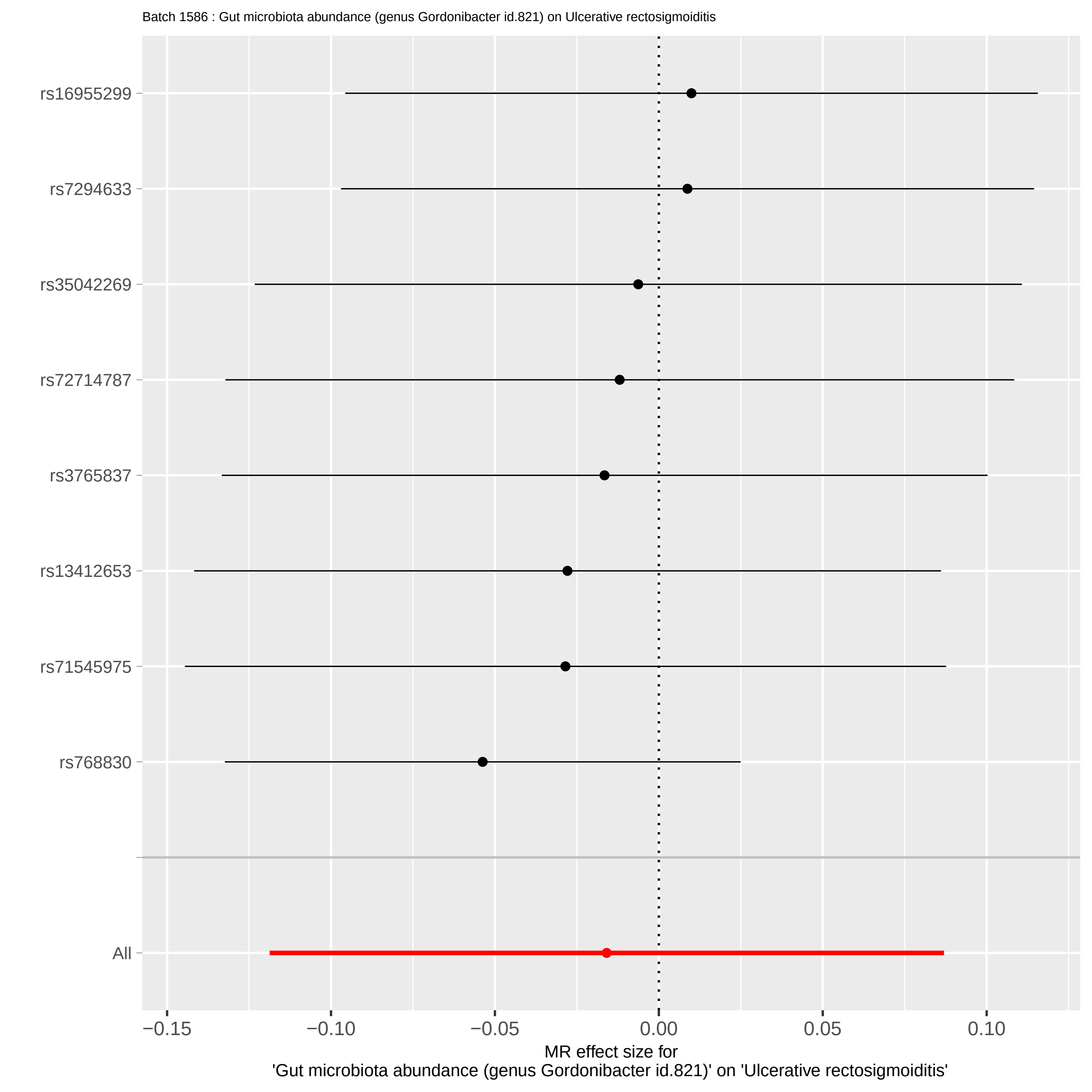

Batch 1586 : Gut microbiota abundance (genus Gordonibacter id.821) on Ulcerative rectosigmoiditis
rs16955299
rs7294633
rs35042269
rs72714787
rs3765837
rs13412653
rs71545975
rs768830
All
−0.15
−0.10
−0.05
0.00
0.05
0.10
MR effect size for
'Gut microbiota abundance (genus Gordonibacter id.821)' on 'Ulcerative rectosigmoiditis'

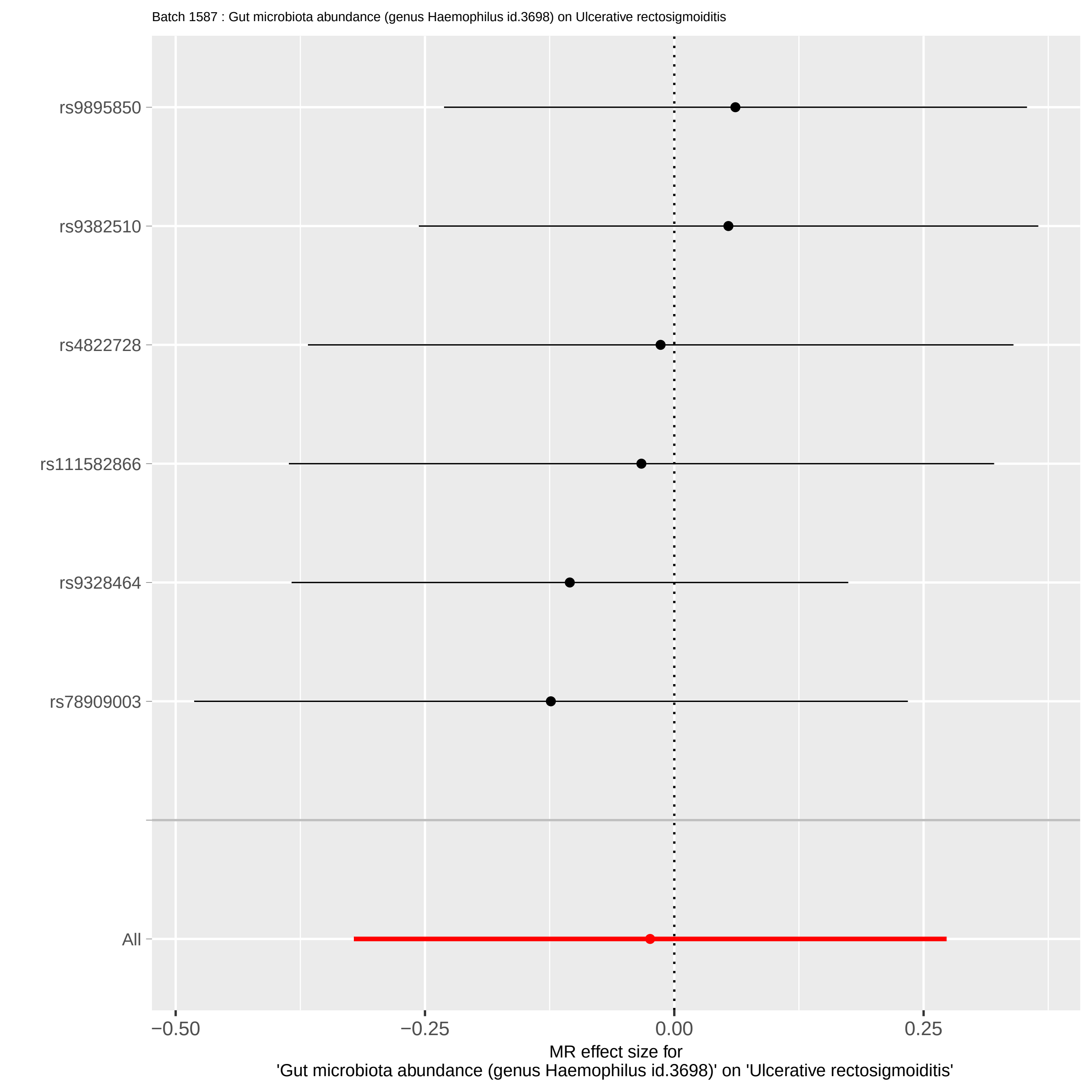

Batch 1587 : Gut microbiota abundance (genus Haemophilus id.3698) on Ulcerative rectosigmoiditis
rs9895850
rs9382510
rs4822728
rs111582866
rs9328464
rs78909003
All
−0.50
−0.25
0.00
0.25
MR effect size for
'Gut microbiota abundance (genus Haemophilus id.3698)' on 'Ulcerative rectosigmoiditis'

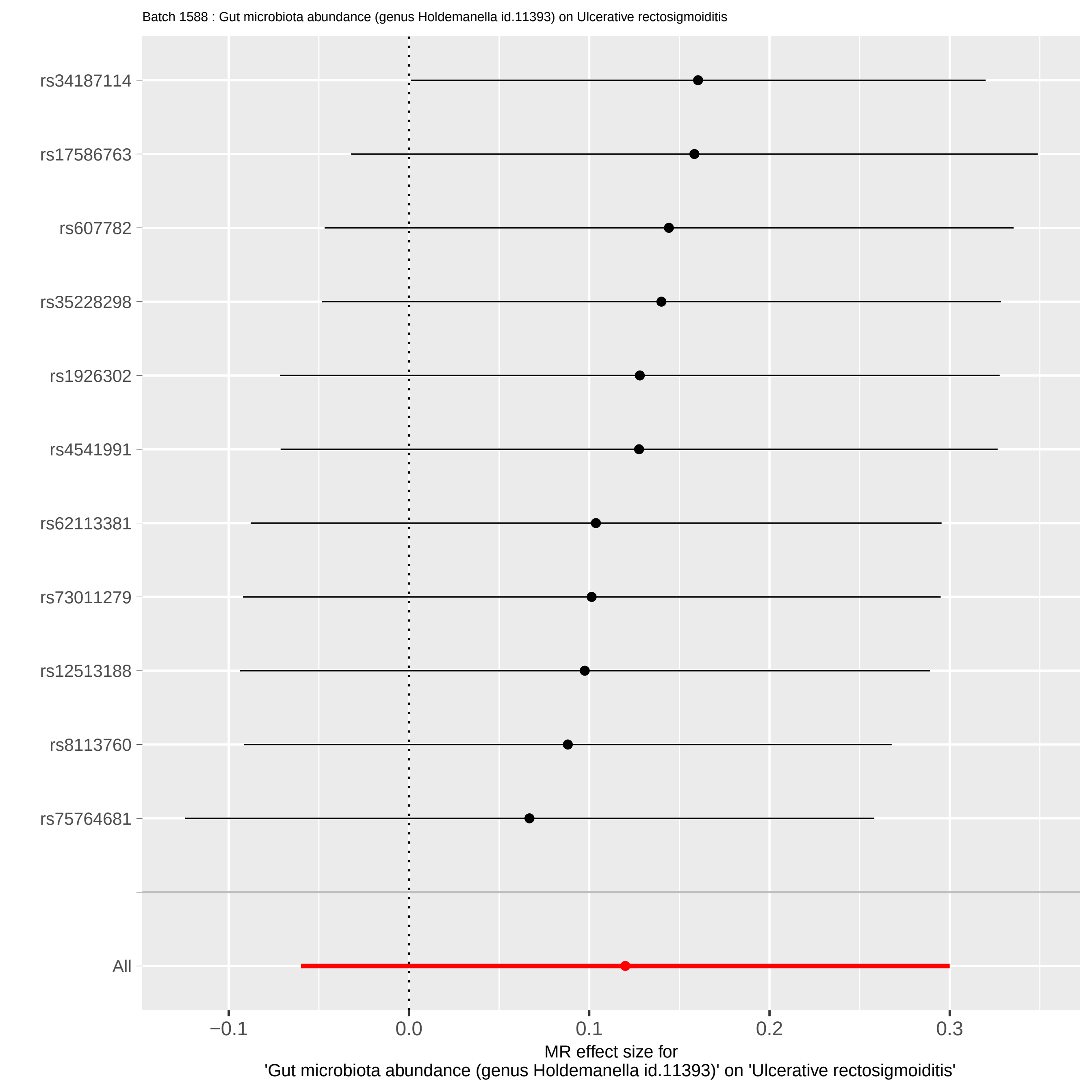

Batch 1588 : Gut microbiota abundance (genus Holdemanella id.11393) on Ulcerative rectosigmoiditis
rs34187114
rs17586763
rs607782
rs35228298
rs1926302
rs4541991
rs62113381
rs73011279
rs12513188
rs8113760
rs75764681
All
−0.1
0.0
0.1
0.2
0.3
MR effect size for
'Gut microbiota abundance (genus Holdemanella id.11393)' on 'Ulcerative rectosigmoiditis'

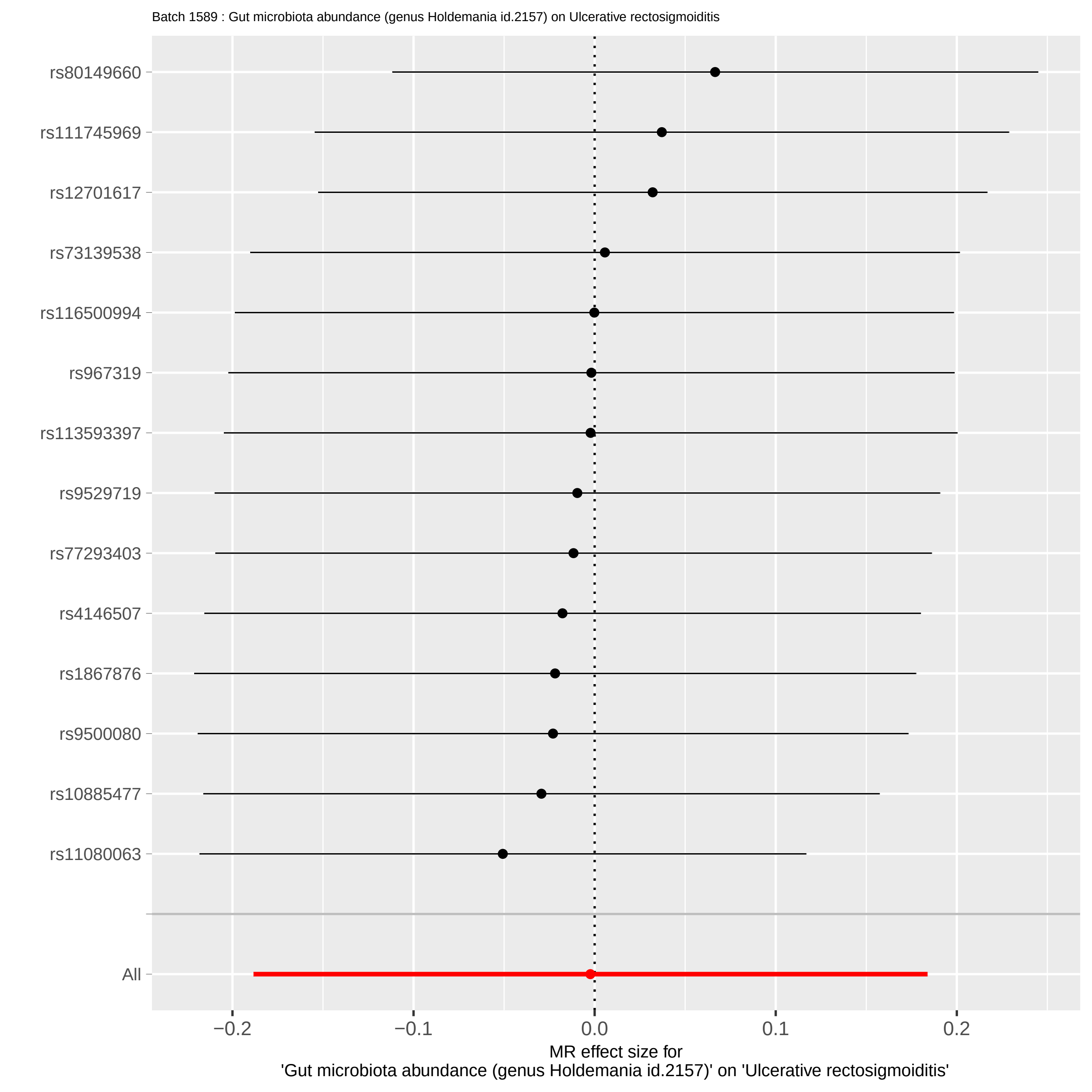

Batch 1589 : Gut microbiota abundance (genus Holdemania id.2157) on Ulcerative rectosigmoiditis
rs80149660
rs111745969
rs12701617
rs73139538
rs116500994
rs967319
rs113593397
rs9529719
rs77293403
rs4146507
rs1867876
rs9500080
rs10885477
rs11080063
All
−0.2
−0.1
0.0
0.1
0.2
MR effect size for
'Gut microbiota abundance (genus Holdemania id.2157)' on 'Ulcerative rectosigmoiditis'

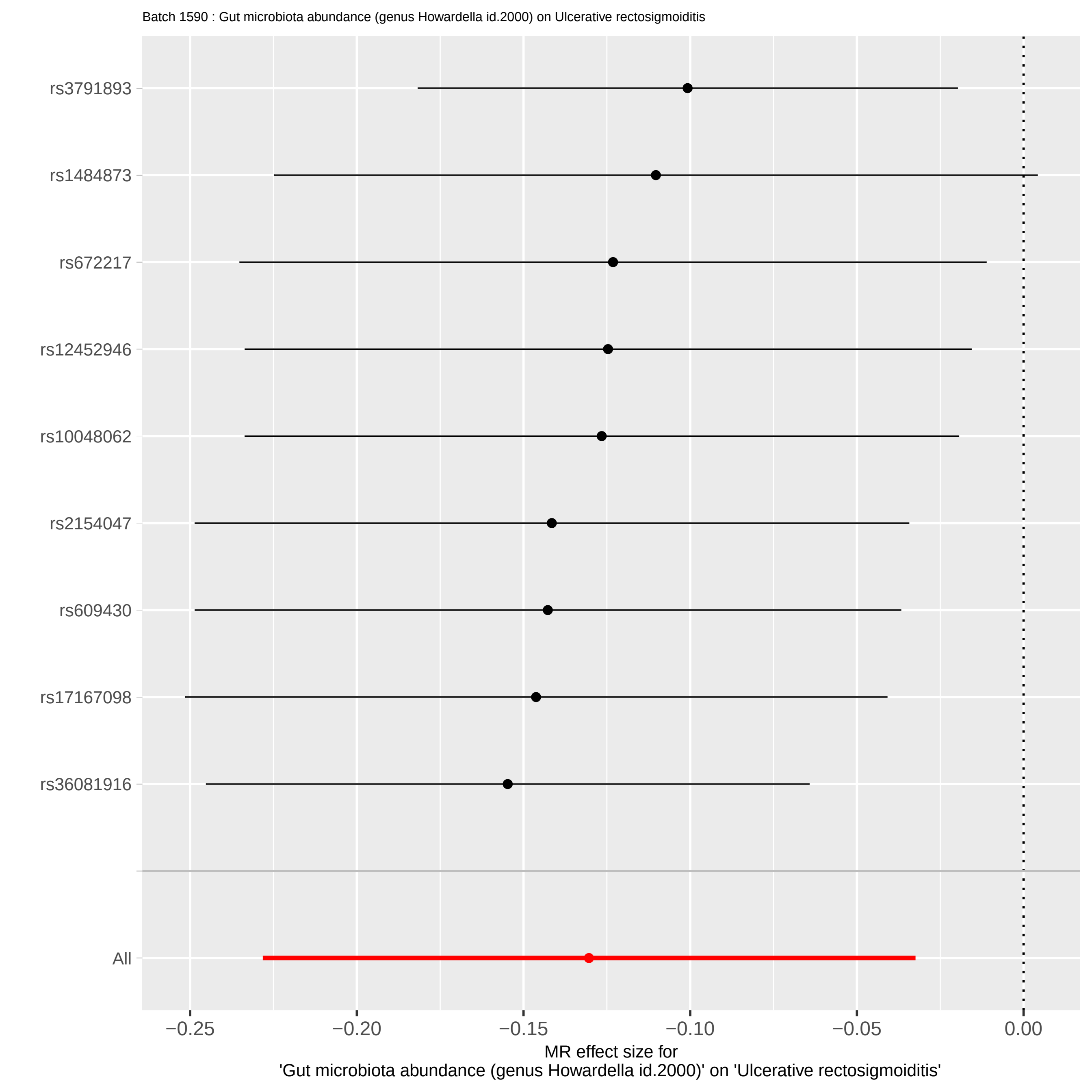

Batch 1590 : Gut microbiota abundance (genus Howardella id.2000) on Ulcerative rectosigmoiditis
rs3791893
rs1484873
rs672217
rs12452946
rs10048062
rs2154047
rs609430
rs17167098
rs36081916
All
−0.25
−0.20
−0.15
−0.10
−0.05
0.00
MR effect size for
'Gut microbiota abundance (genus Howardella id.2000)' on 'Ulcerative rectosigmoiditis'

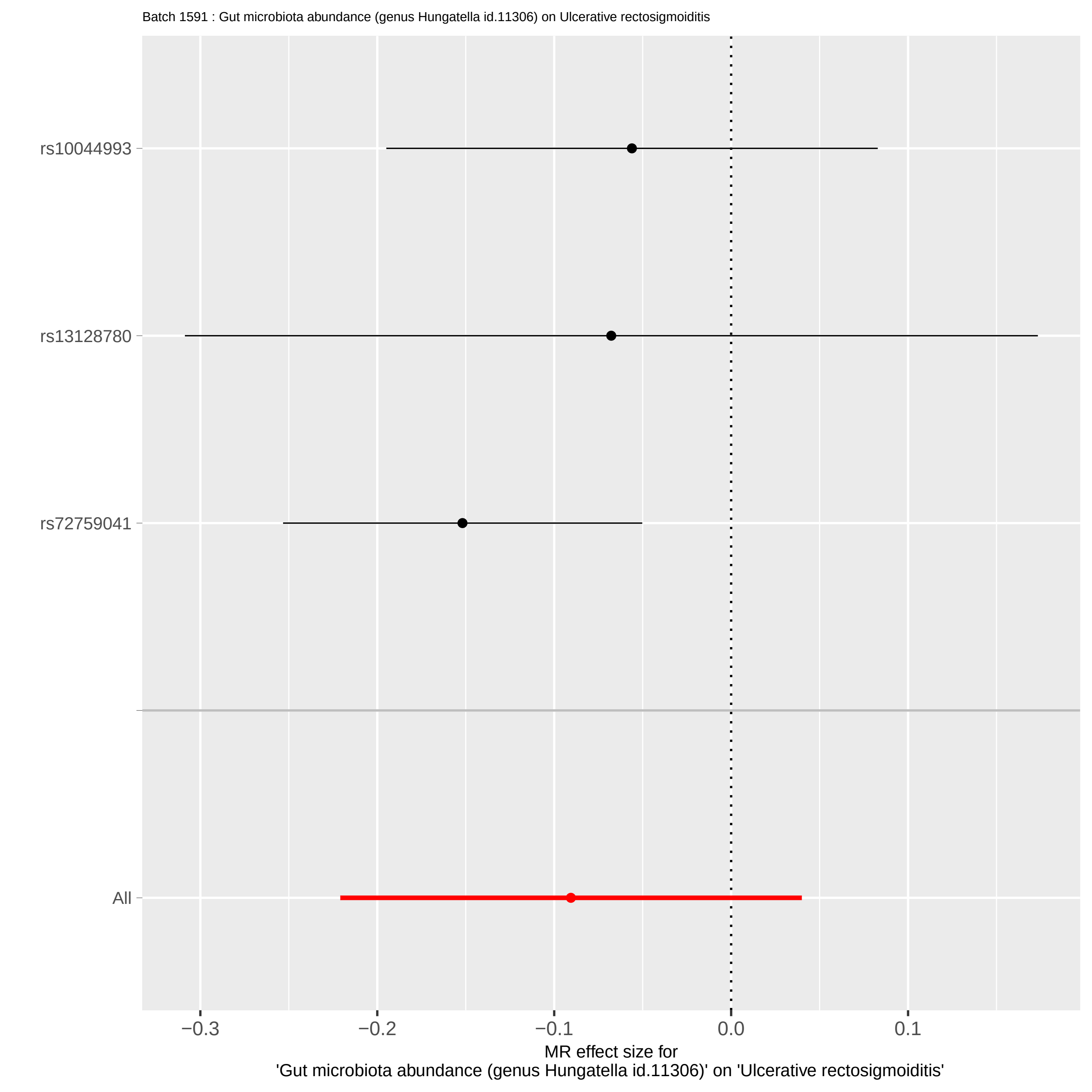

Batch 1591 : Gut microbiota abundance (genus Hungatella id.11306) on Ulcerative rectosigmoiditis
rs10044993
rs13128780
rs72759041
All
−0.3
−0.2
−0.1
0.0
0.1
MR effect size for
'Gut microbiota abundance (genus Hungatella id.11306)' on 'Ulcerative rectosigmoiditis'

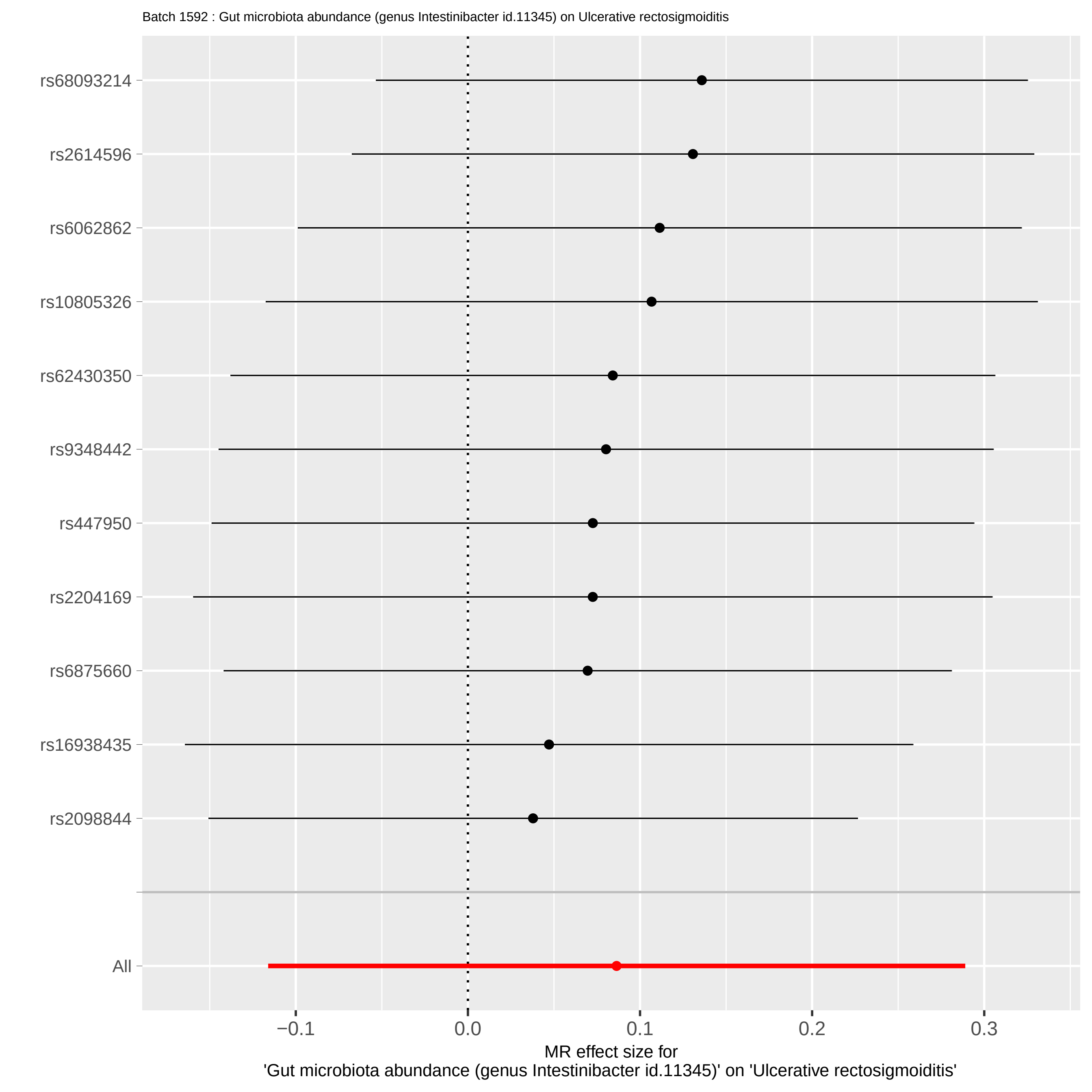

Batch 1592 : Gut microbiota abundance (genus Intestinibacter id.11345) on Ulcerative rectosigmoiditis
rs68093214
rs2614596
rs6062862
rs10805326
rs62430350
rs9348442
rs447950
rs2204169
rs6875660
rs16938435
rs2098844
All
−0.1
0.0
0.1
0.2
0.3
MR effect size for
'Gut microbiota abundance (genus Intestinibacter id.11345)' on 'Ulcerative rectosigmoiditis'

Batch 1593 : Gut microbiota abundance (genus Intestinimonas id.2062) on Ulcerative rectosigmoiditis

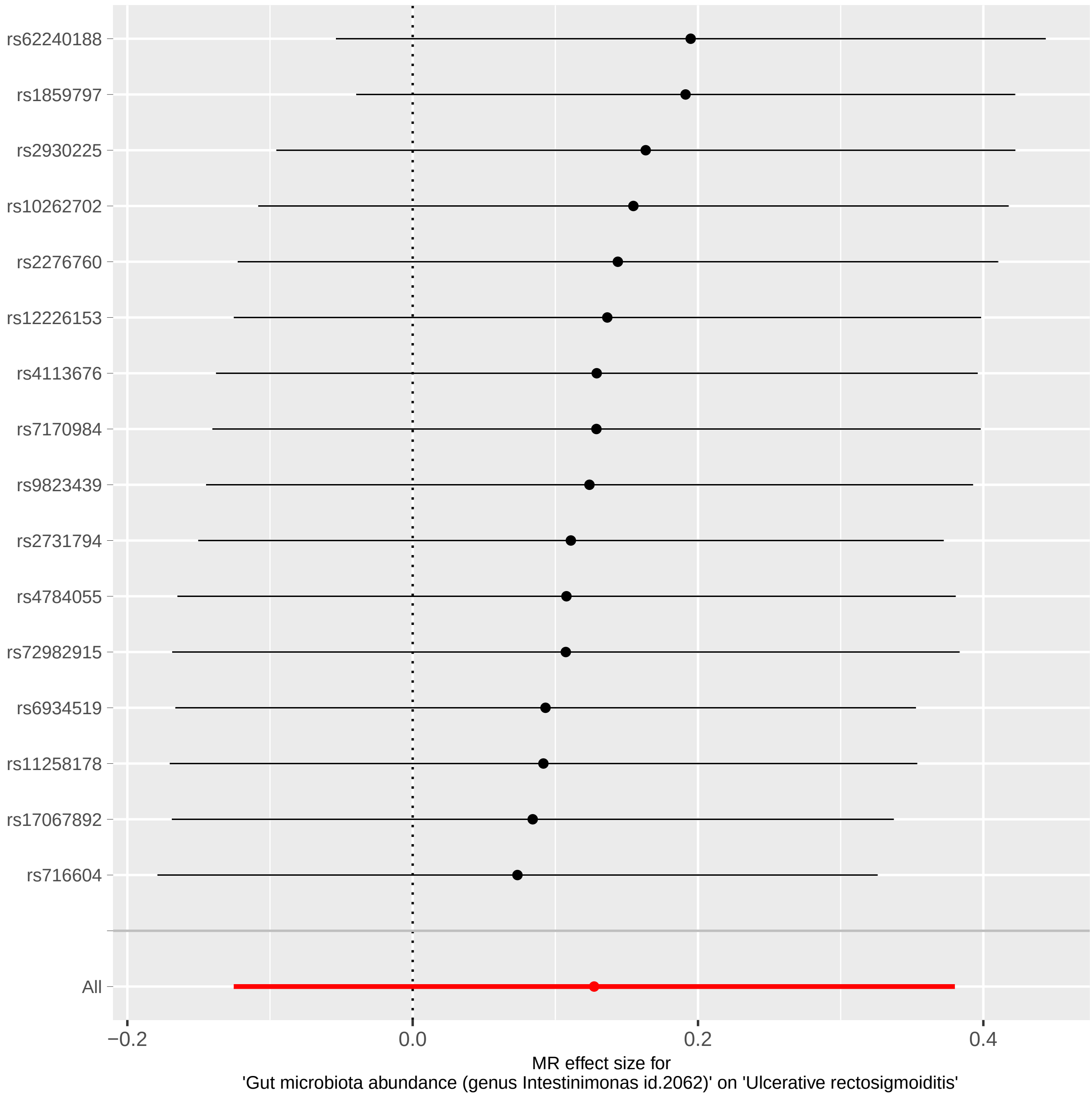

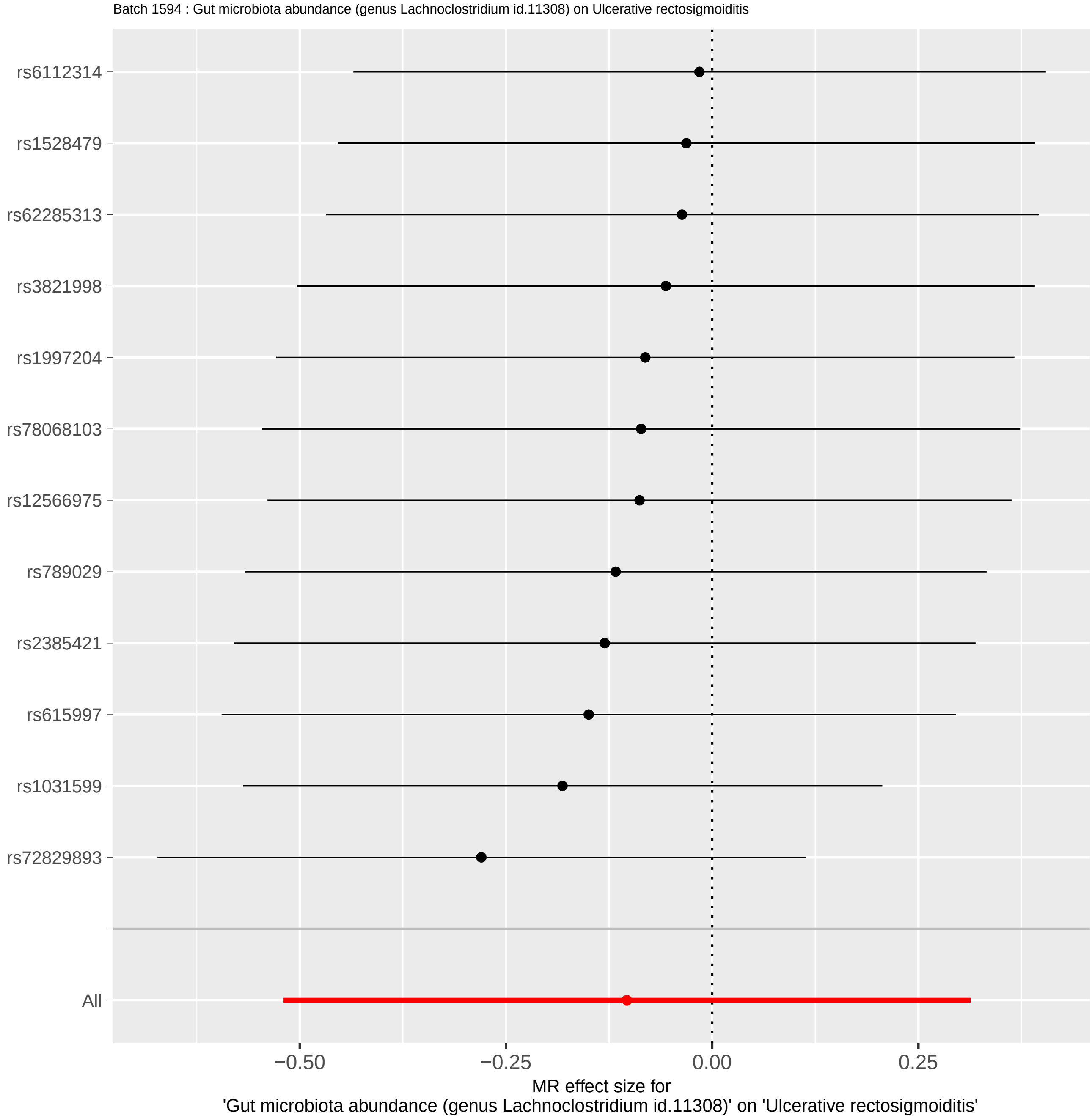

Batch 1594 : Gut microbiota abundance (genus Lachnoclostridium id.11308) on Ulcerative rectosigmoiditis
rs6112314
rs1528479
rs62285313
rs3821998
rs1997204
rs78068103
rs12566975
rs789029
rs2385421
rs615997
rs1031599
rs72829893
All
−0.50
−0.25
0.00
0.25
MR effect size for
'Gut microbiota abundance (genus Lachnoclostridium id.11308)' on 'Ulcerative rectosigmoiditis'

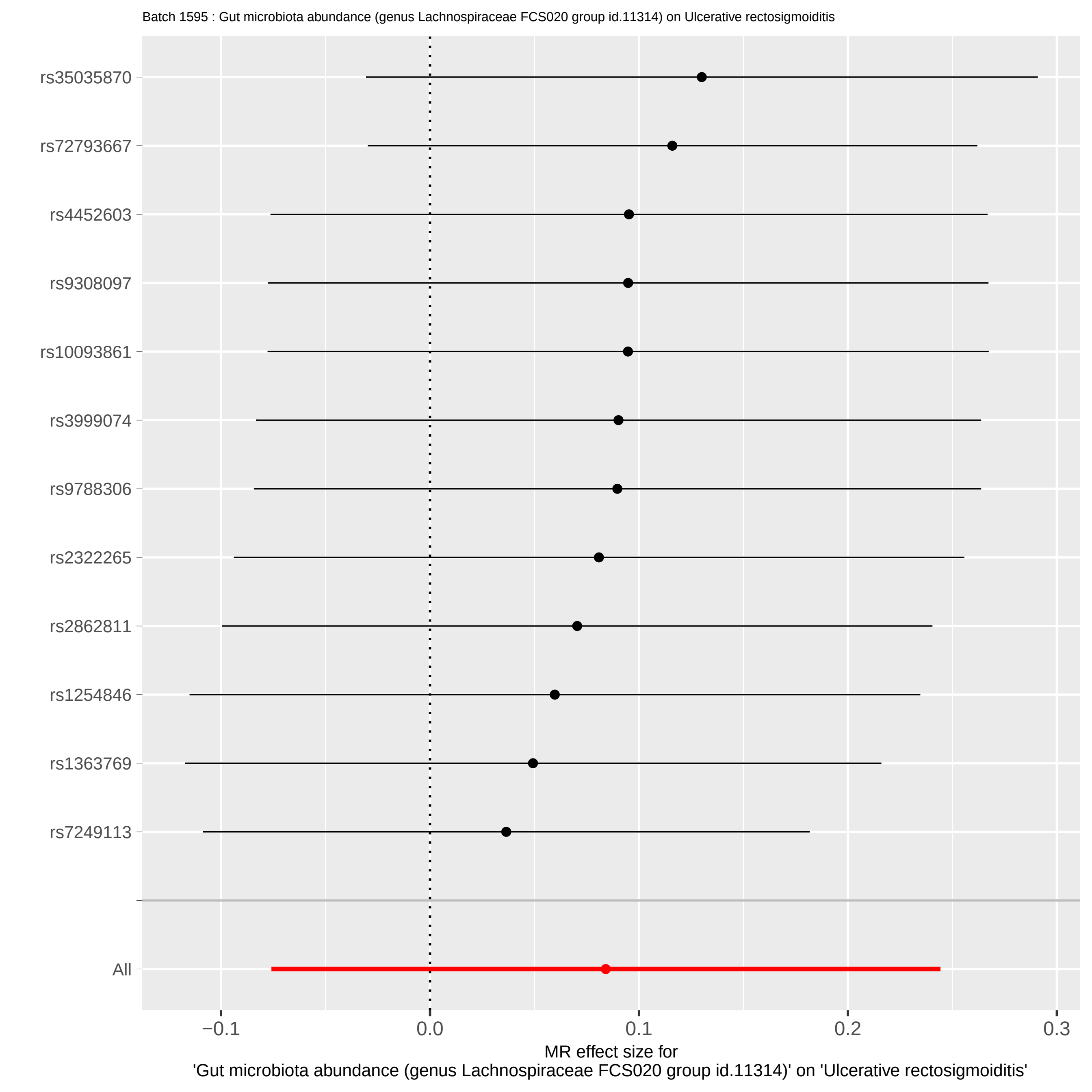
Batch 1595 : Gut microbiota abundance (genus Lachnospiraceae FCS020 group id.11314) on Ulcerative rectosigmoiditis
rs35035870
rs72793667
rs4452603
rs9308097
rs10093861
rs3999074
rs9788306
rs2322265
rs2862811
rs1254846
rs1363769
rs7249113
All
−0.1
0.0
0.1
0.2
0.3
MR effect size for
'Gut microbiota abundance (genus Lachnospiraceae FCS020 group id.11314)' on 'Ulcerative rectosigmoiditis'

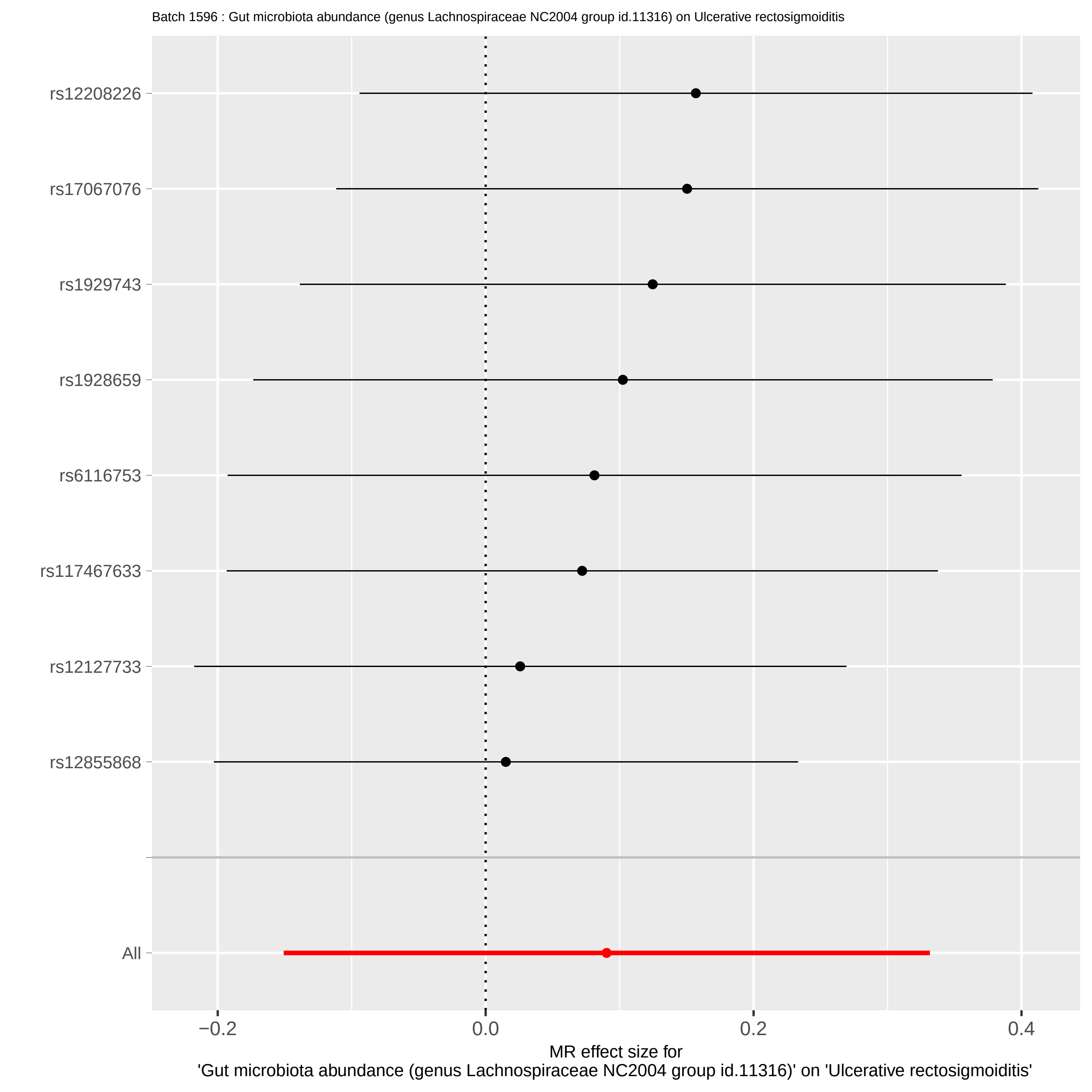

Batch 1596 : Gut microbiota abundance (genus Lachnospiraceae NC2004 group id.11316) on Ulcerative rectosigmoiditis
rs12208226
rs17067076
rs1929743
rs1928659
rs6116753
rs117467633
rs12127733
rs12855868
All
−0.2
0.0
0.2
0.4
MR effect size for
'Gut microbiota abundance (genus Lachnospiraceae NC2004 group id.11316)' on 'Ulcerative rectosigmoiditis'

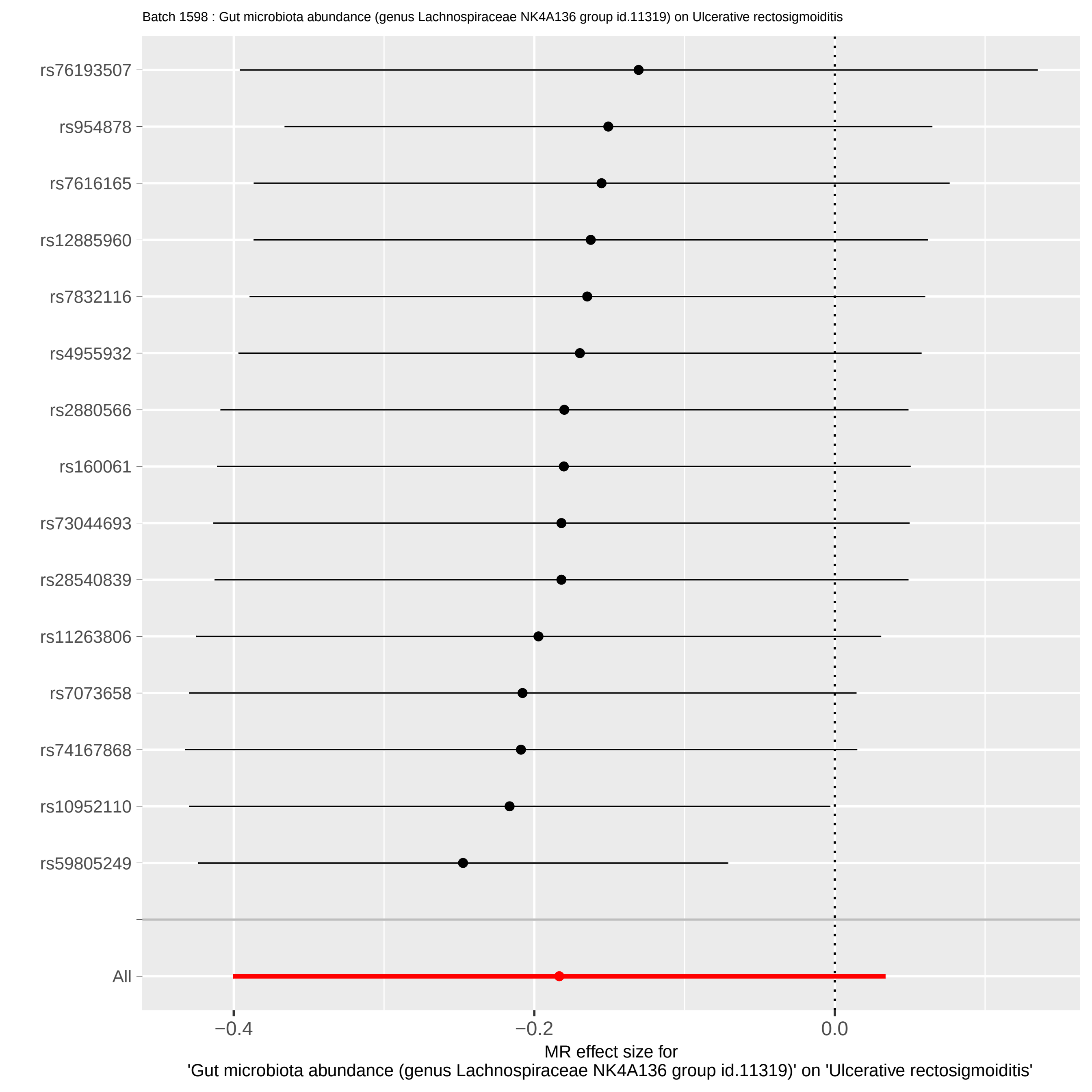

Batch 1598 : Gut microbiota abundance (genus Lachnospiraceae NK4A136 group id.11319) on Ulcerative rectosigmoiditis
rs76193507
rs954878
rs7616165
rs12885960
rs7832116
rs4955932
rs2880566
rs160061
rs73044693
rs28540839
rs11263806
rs7073658
rs74167868
rs10952110
rs59805249
All
−0.4
−0.2
0.0
MR effect size for
'Gut microbiota abundance (genus Lachnospiraceae NK4A136 group id.11319)' on 'Ulcerative rectosigmoiditis'

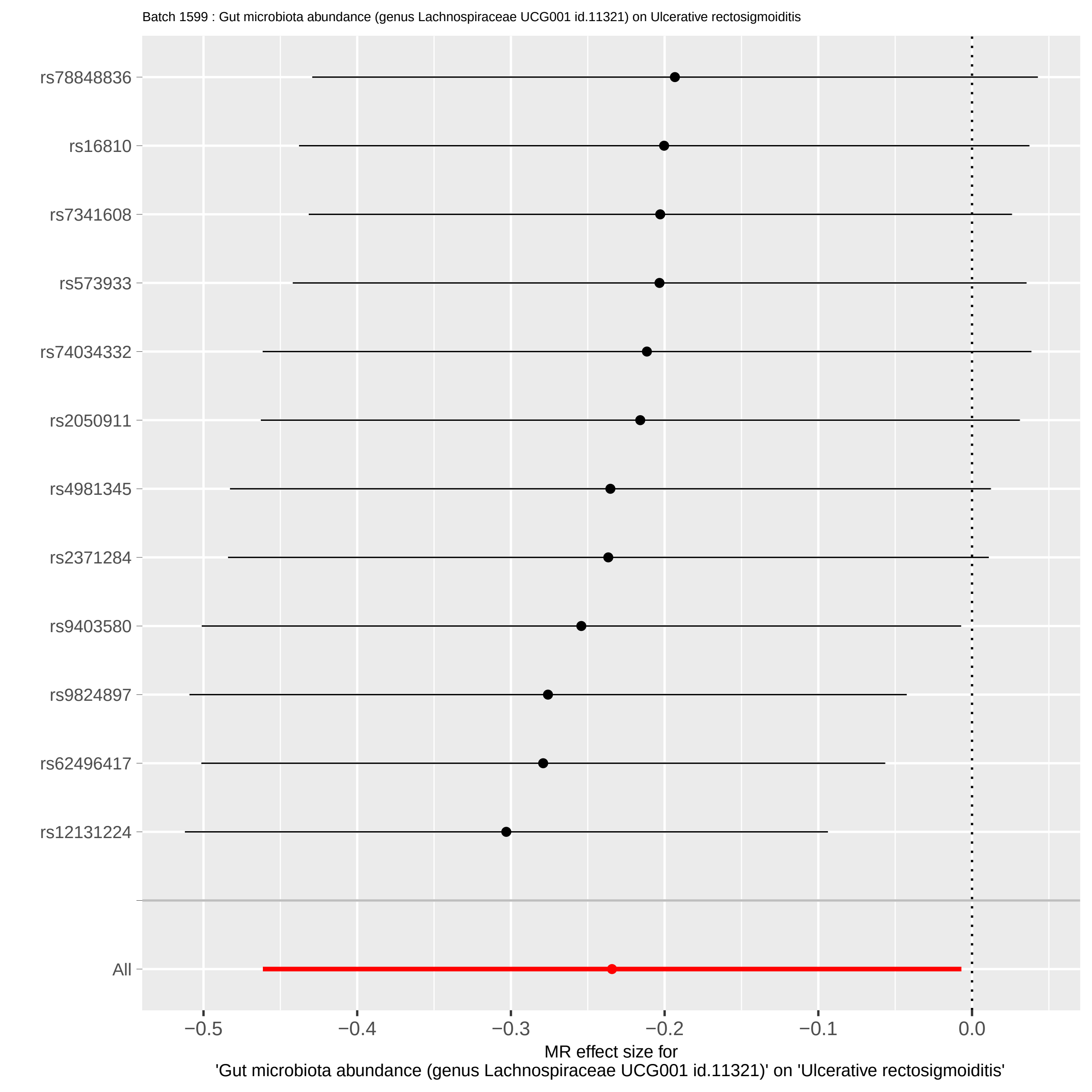

Batch 1599 : Gut microbiota abundance (genus Lachnospiraceae UCG001 id.11321) on Ulcerative rectosigmoiditis
rs78848836
rs16810
rs7341608
rs573933
rs74034332
rs2050911
rs4981345
rs2371284
rs9403580
rs9824897
rs62496417
rs12131224
All
−0.5
−0.4
−0.3
−0.2
−0.1
0.0
MR effect size for
'Gut microbiota abundance (genus Lachnospiraceae UCG001 id.11321)' on 'Ulcerative rectosigmoiditis'

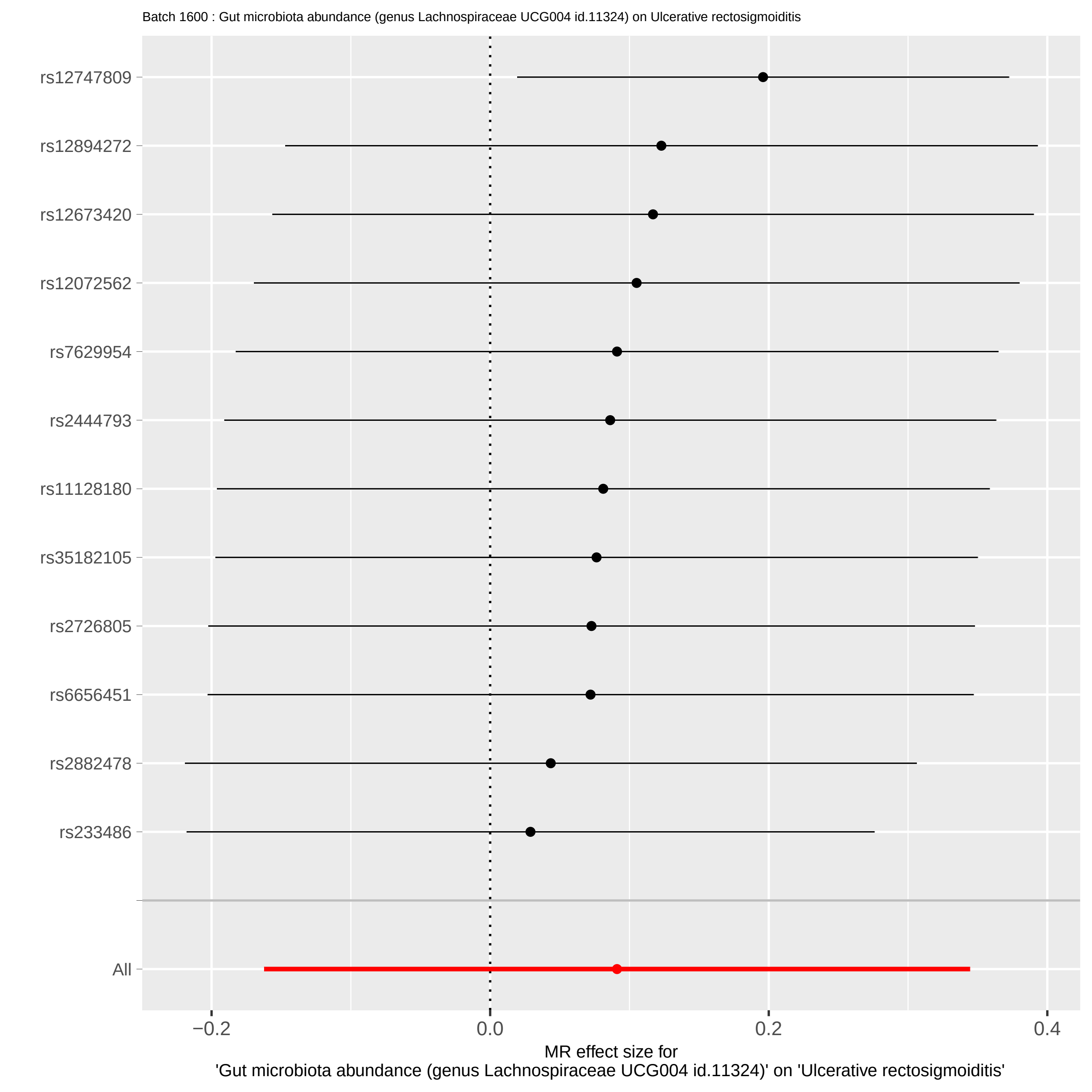

Batch 1600 : Gut microbiota abundance (genus Lachnospiraceae UCG004 id.11324) on Ulcerative rectosigmoiditis
rs12747809
rs12894272
rs12673420
rs12072562
rs7629954
rs2444793
rs11128180
rs35182105
rs2726805
rs6656451
rs2882478
rs233486
All
−0.2
0.0
0.2
0.4
MR effect size for
'Gut microbiota abundance (genus Lachnospiraceae UCG004 id.11324)' on 'Ulcerative rectosigmoiditis'

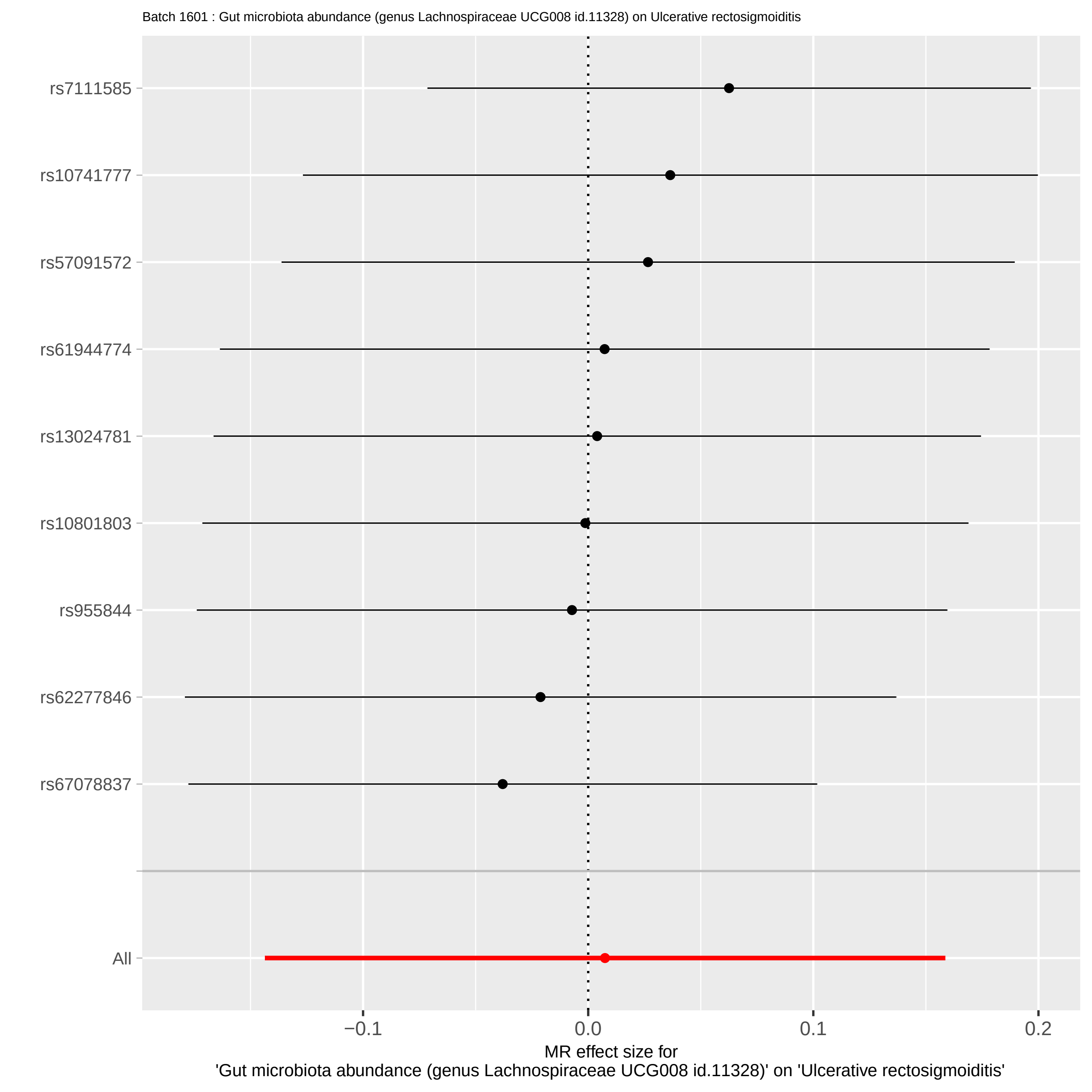

Batch 1601 : Gut microbiota abundance (genus Lachnospiraceae UCG008 id.11328) on Ulcerative rectosigmoiditis
rs7111585
rs10741777
rs57091572
rs61944774
rs13024781
rs10801803
rs955844
rs62277846
rs67078837
All
−0.1
0.0
0.1
0.2
MR effect size for
'Gut microbiota abundance (genus Lachnospiraceae UCG008 id.11328)' on 'Ulcerative rectosigmoiditis'

Batch 1602 : Gut microbiota abundance (genus Lachnospiraceae UCG010 id.11330) on Ulcerative rectosigmoiditis

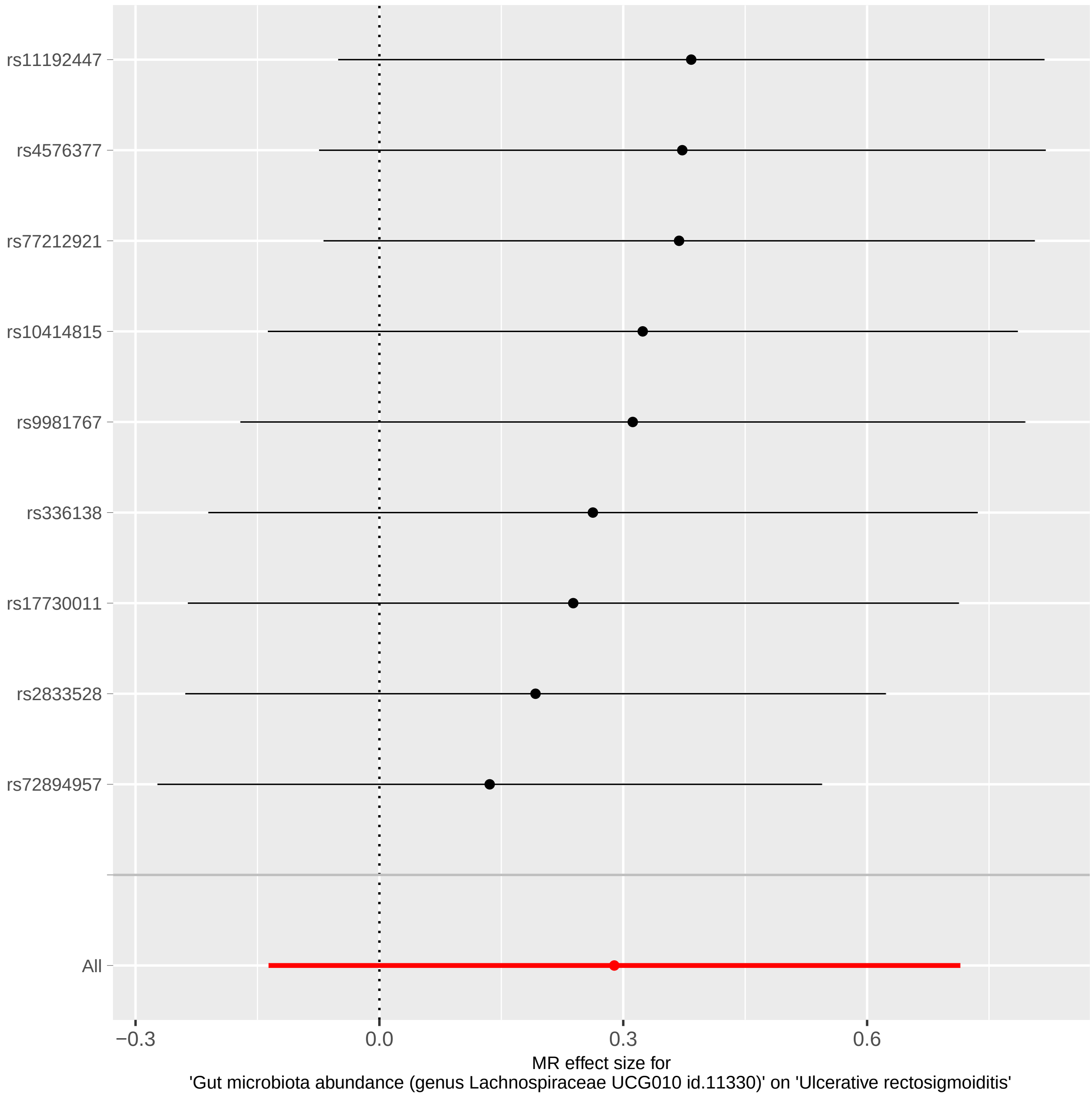

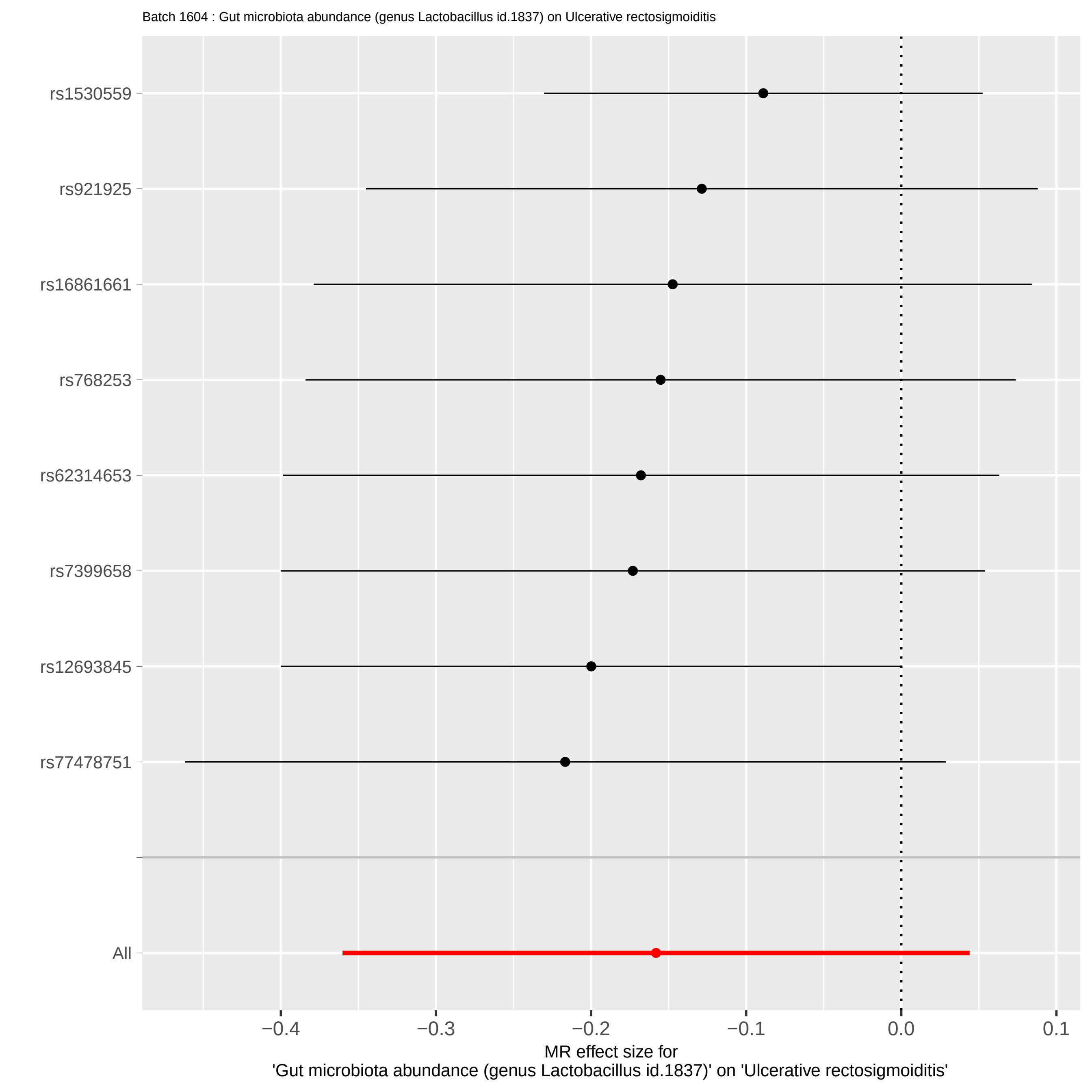

Batch 1604 : Gut microbiota abundance (genus Lactobacillus id.1837) on Ulcerative rectosigmoiditis
rs1530559
rs921925
rs16861661
rs768253
rs62314653
rs7399658
rs12693845
rs77478751
All
−0.4
−0.3
−0.2
−0.1
0.0
0.1
MR effect size for
'Gut microbiota abundance (genus Lactobacillus id.1837)' on 'Ulcerative rectosigmoiditis'

Batch 1605 : Gut microbiota abundance (genus Lactococcus id.1851) on Ulcerative rectosigmoiditis

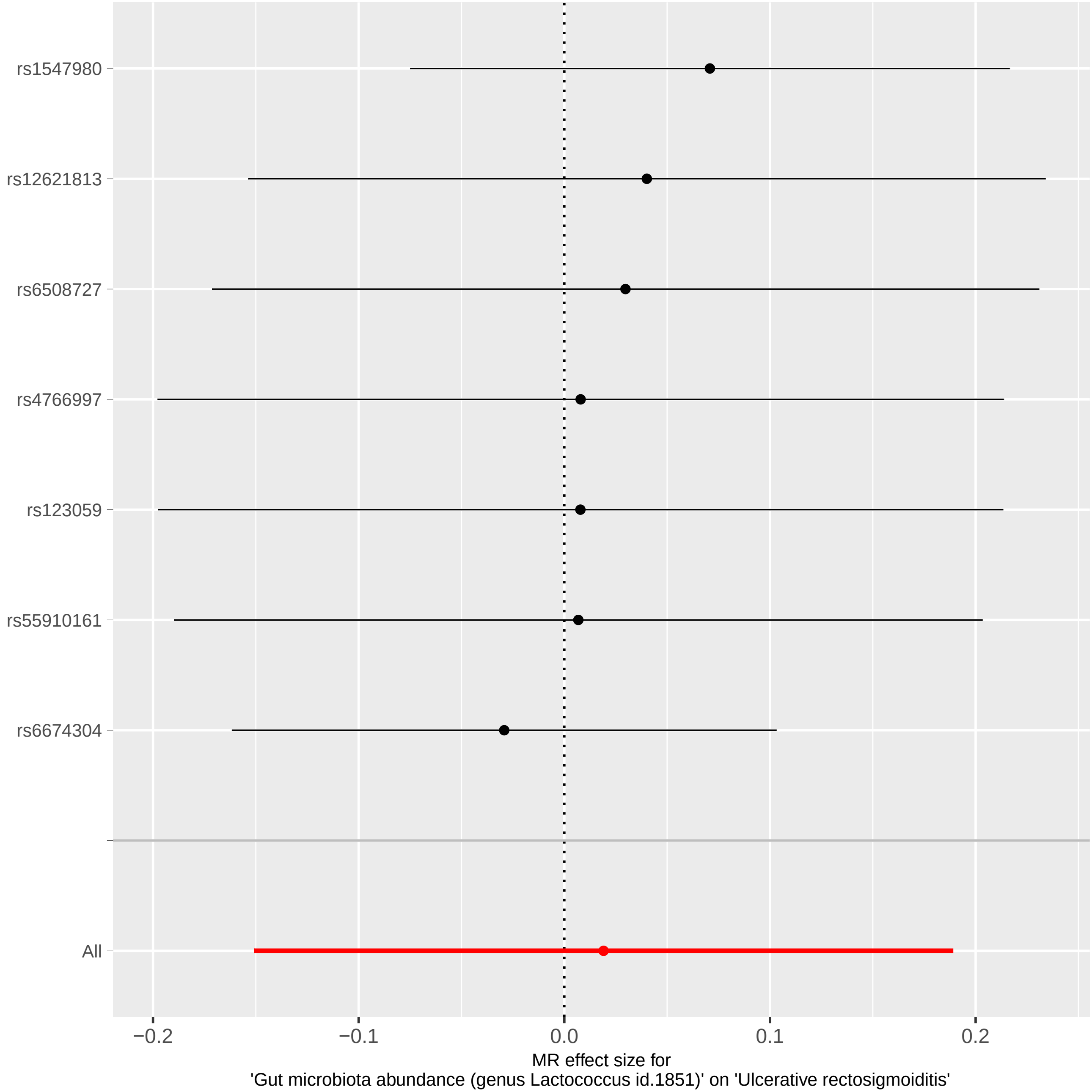

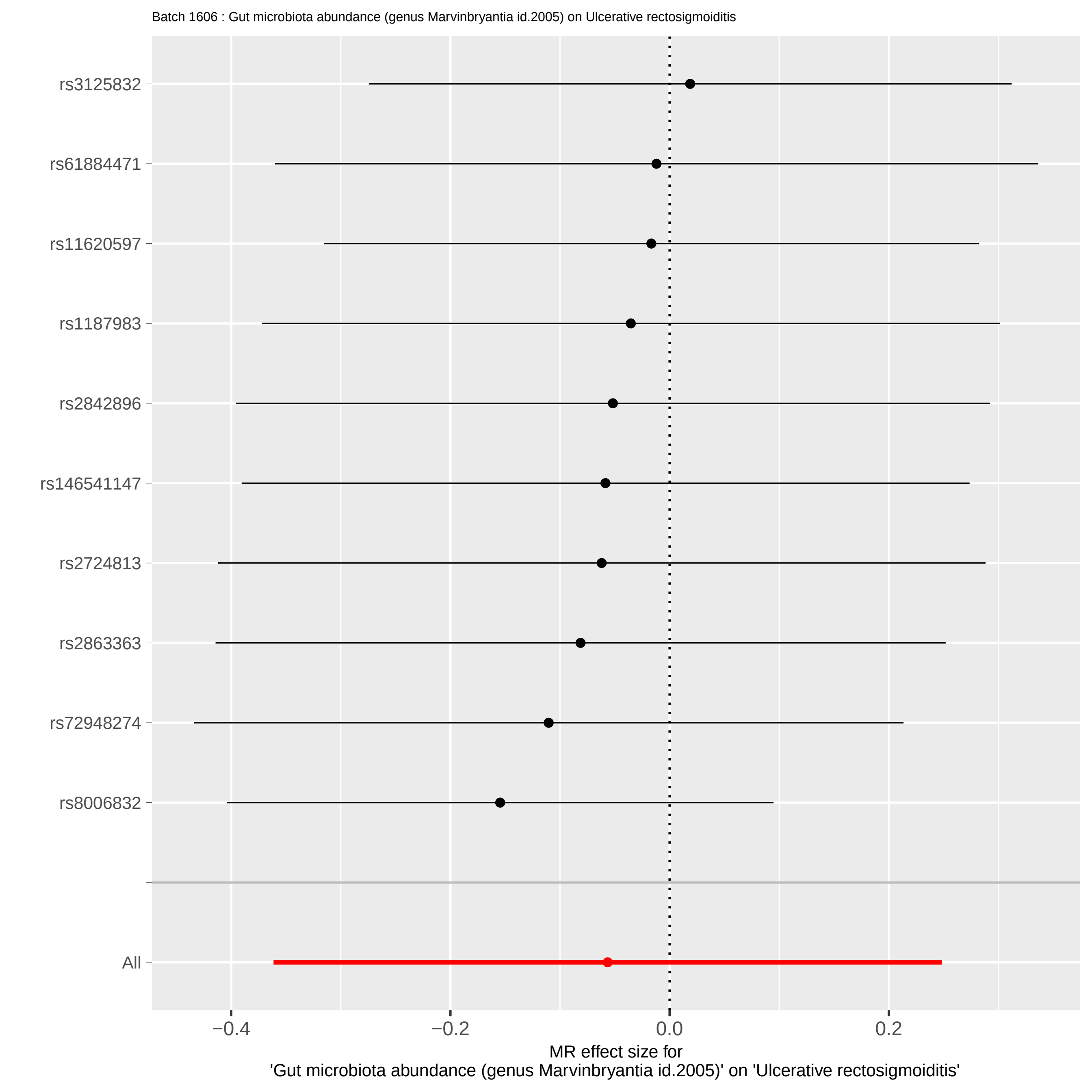

Batch 1606 : Gut microbiota abundance (genus Marvinbryantia id.2005) on Ulcerative rectosigmoiditis
rs3125832
rs61884471
rs11620597
rs1187983
rs2842896
rs146541147
rs2724813
rs2863363
rs72948274
rs8006832
All
−0.4
−0.2
0.0
0.2
MR effect size for
'Gut microbiota abundance (genus Marvinbryantia id.2005)' on 'Ulcerative rectosigmoiditis'

Batch 1607 : Gut microbiota abundance (genus Methanobrevibacter id.123) on Ulcerative rectosigmoiditis

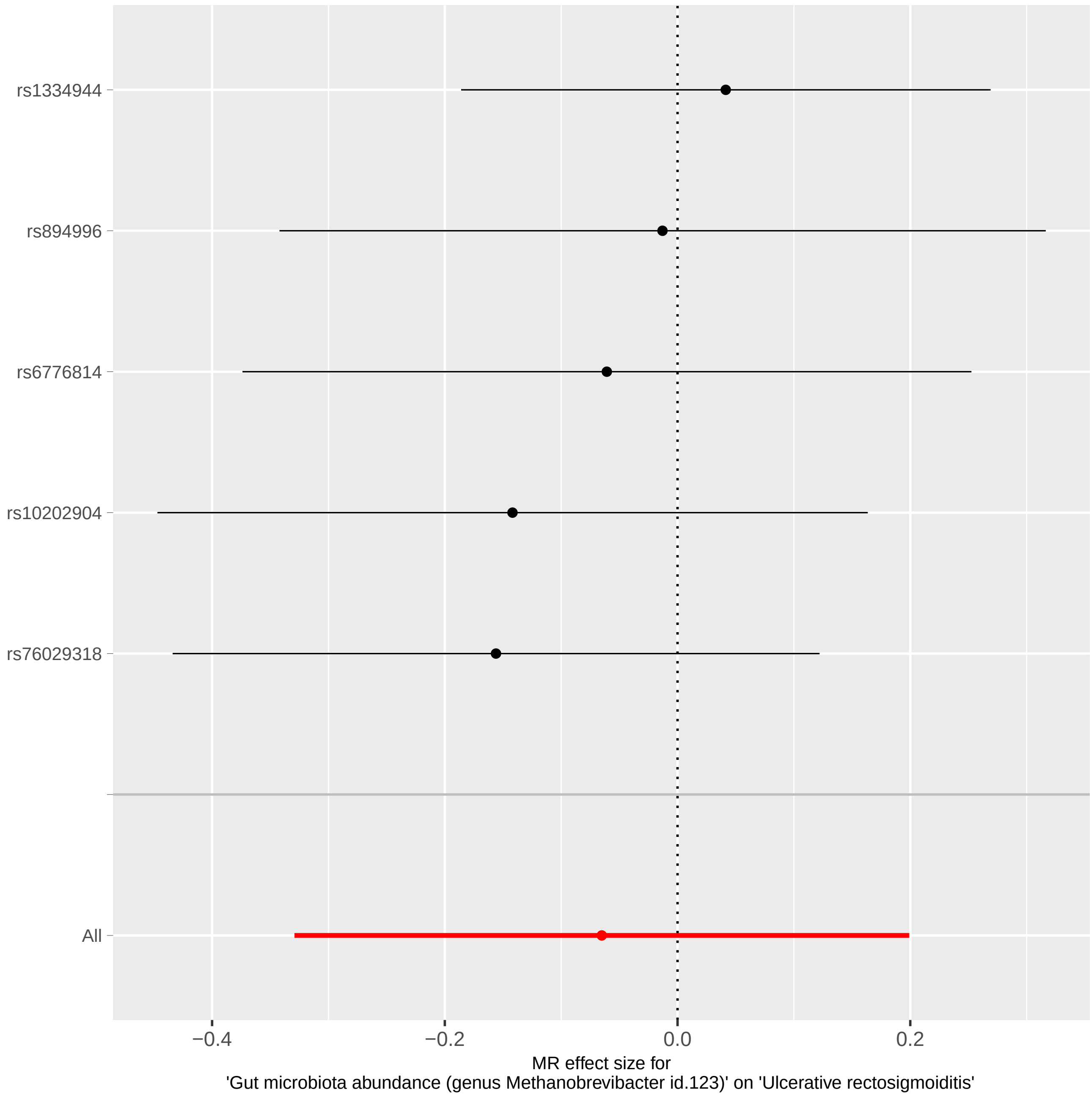

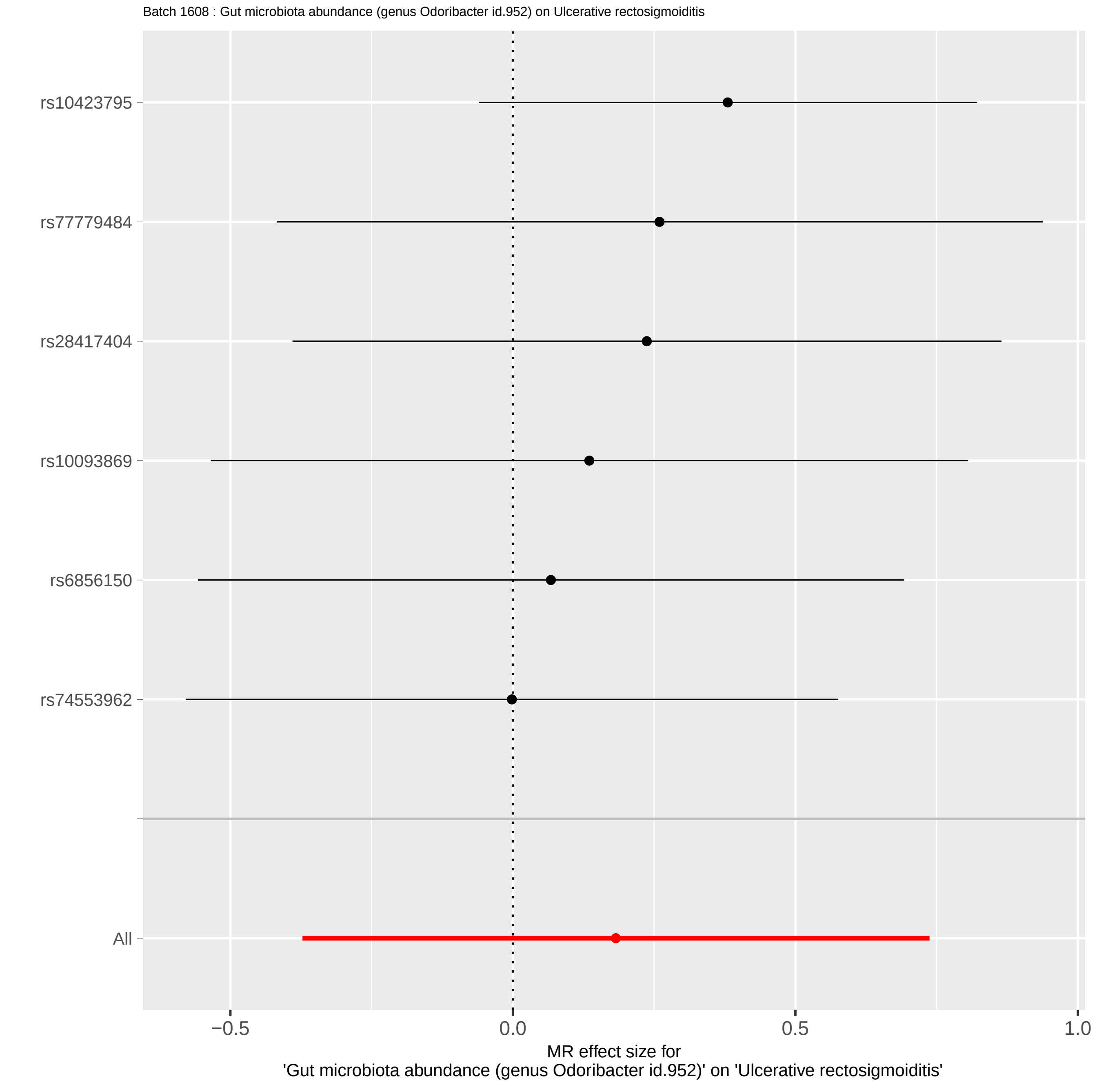
Batch 1608 : Gut microbiota abundance (genus Odoribacter id.952) on Ulcerative rectosigmoiditis
rs10423795
rs77779484
rs28417404
rs10093869
rs6856150
rs74553962
All
−0.5
0.0
0.5
1.0
MR effect size for
'Gut microbiota abundance (genus Odoribacter id.952)' on 'Ulcerative rectosigmoiditis'

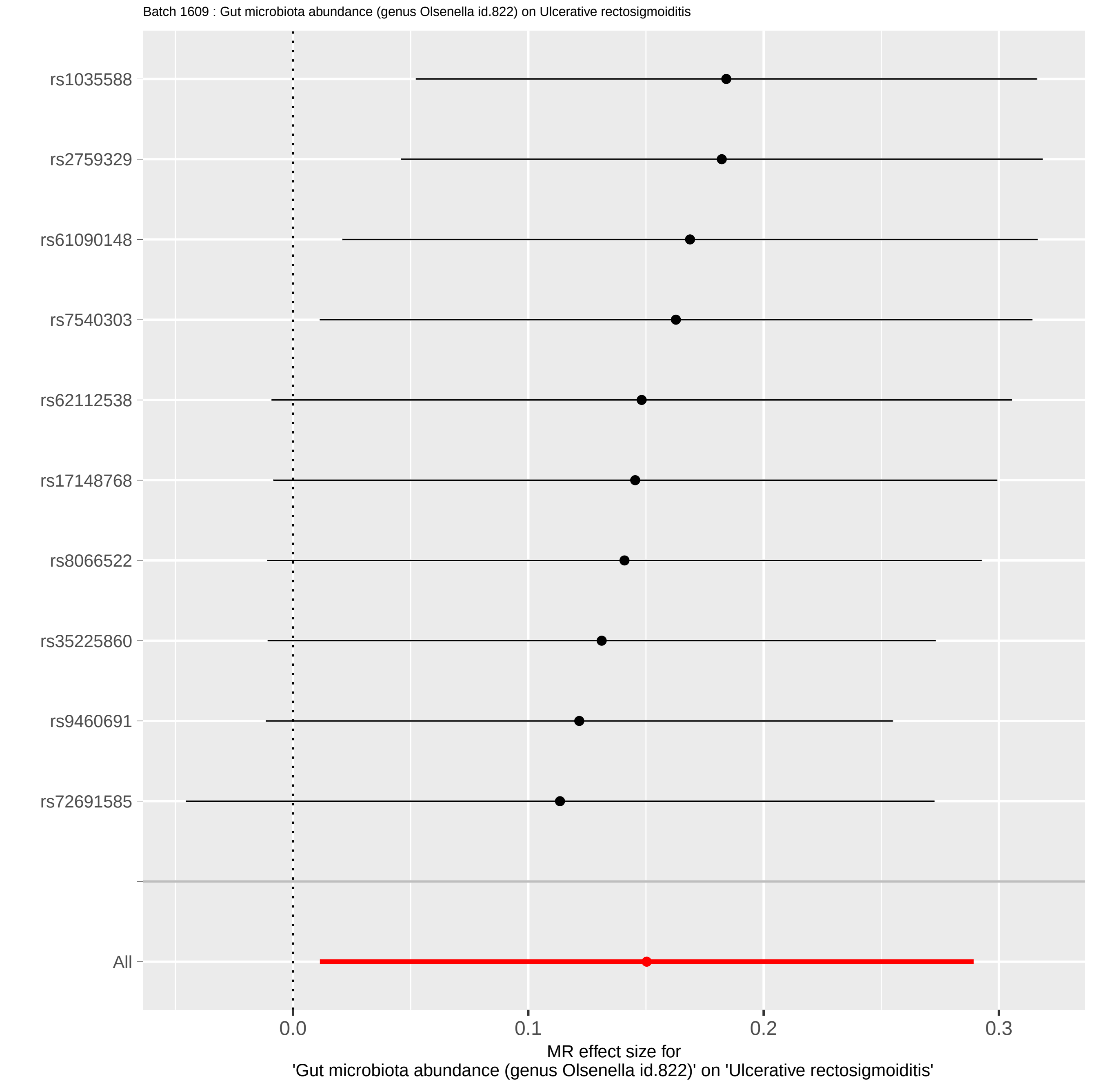
Batch 1609 : Gut microbiota abundance (genus Olsenella id.822) on Ulcerative rectosigmoiditis
rs1035588
rs2759329
rs61090148
rs7540303
rs62112538
rs17148768
rs8066522
rs35225860
rs9460691
rs72691585
All
0.0
0.1
0.2
0.3
MR effect size for
'Gut microbiota abundance (genus Olsenella id.822)' on 'Ulcerative rectosigmoiditis'

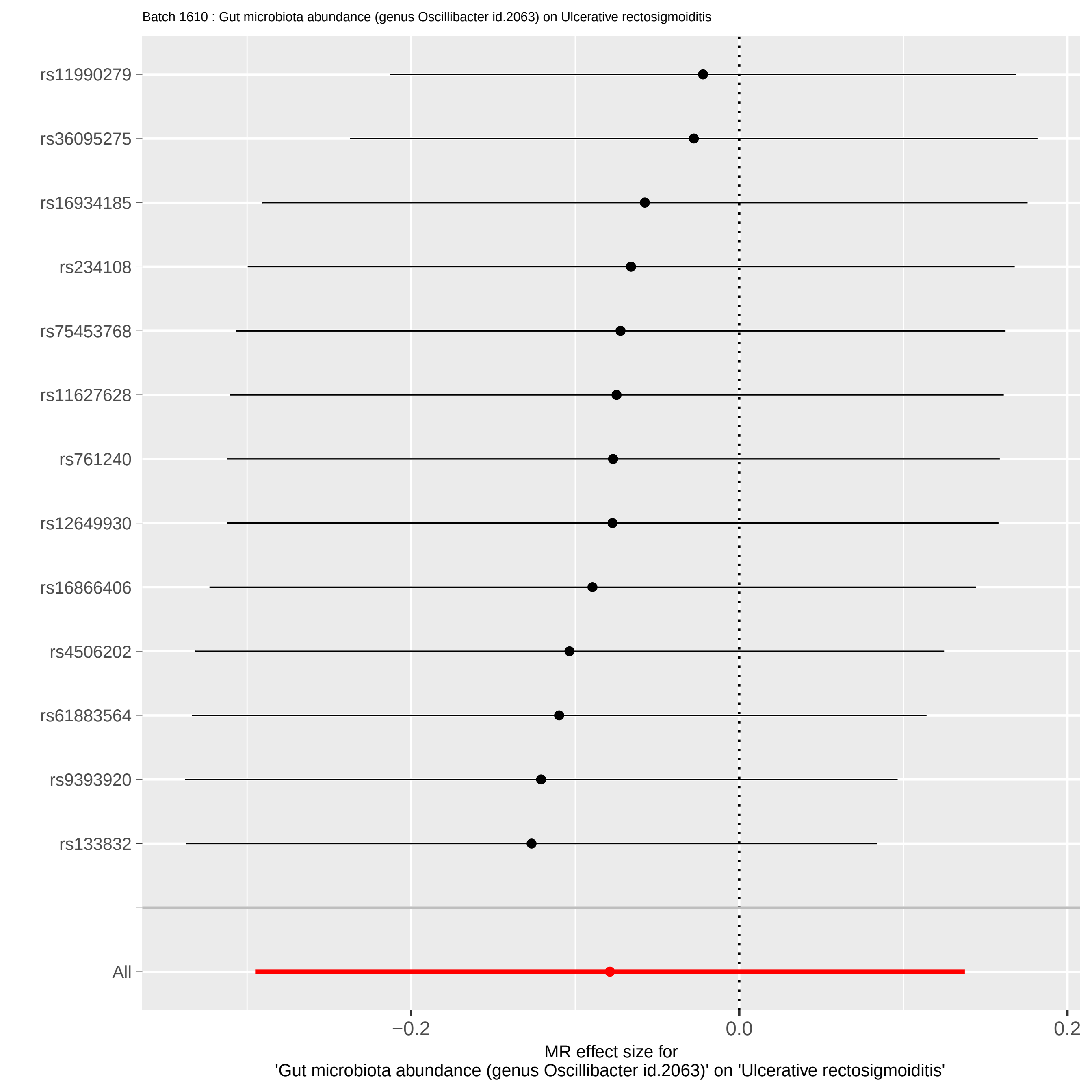
Batch 1610 : Gut microbiota abundance (genus Oscillibacter id.2063) on Ulcerative rectosigmoiditis
rs11990279
rs36095275
rs16934185
rs234108
rs75453768
rs11627628
rs761240
rs12649930
rs16866406
rs4506202
rs61883564
rs9393920
rs133832
All
−0.2
0.0
0.2
MR effect size for
'Gut microbiota abundance (genus Oscillibacter id.2063)' on 'Ulcerative rectosigmoiditis'

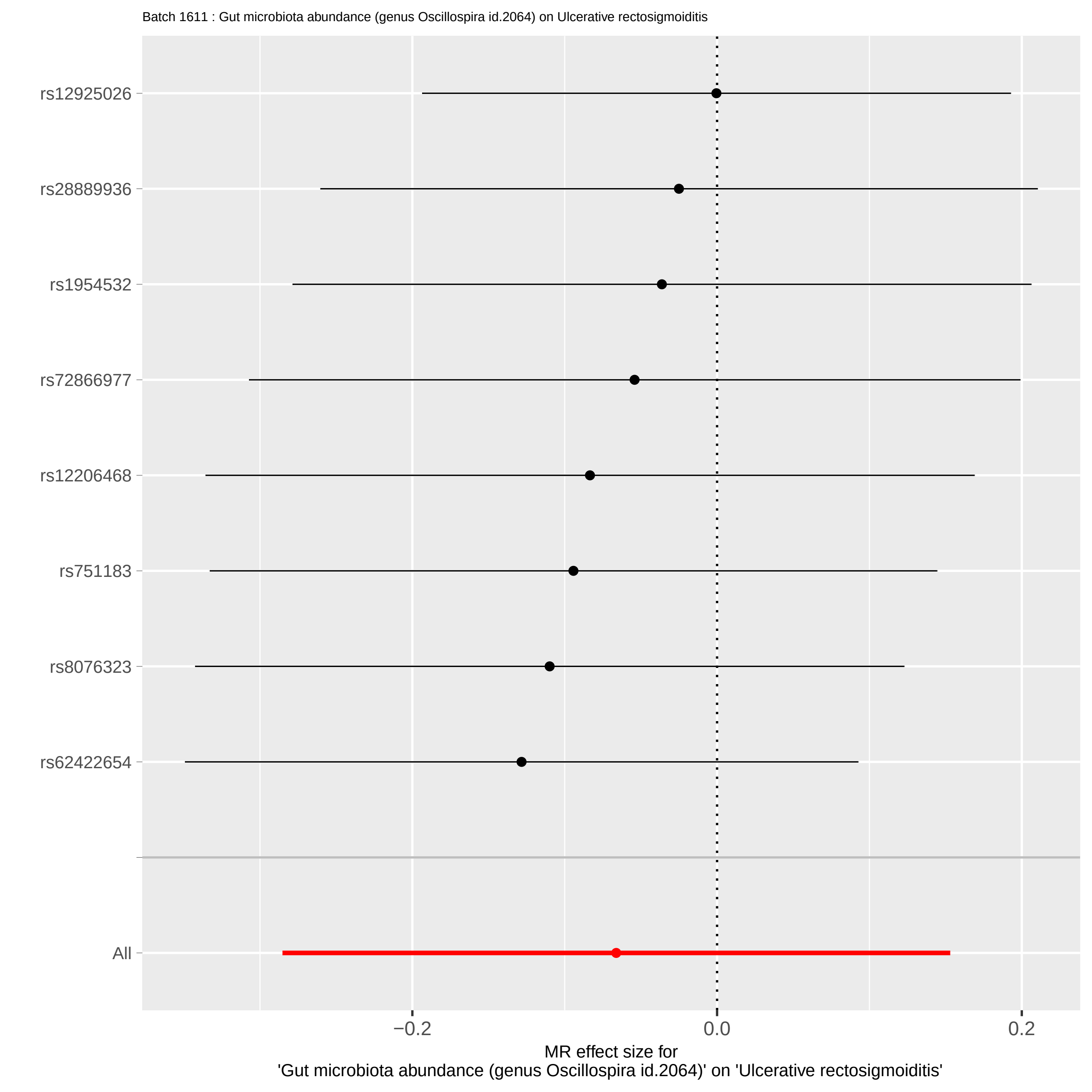

Batch 1611 : Gut microbiota abundance (genus Oscillospira id.2064) on Ulcerative rectosigmoiditis
rs12925026
rs28889936
rs1954532
rs72866977
rs12206468
rs751183
rs8076323
rs62422654
All
−0.2
0.0
0.2
MR effect size for
'Gut microbiota abundance (genus Oscillospira id.2064)' on 'Ulcerative rectosigmoiditis'

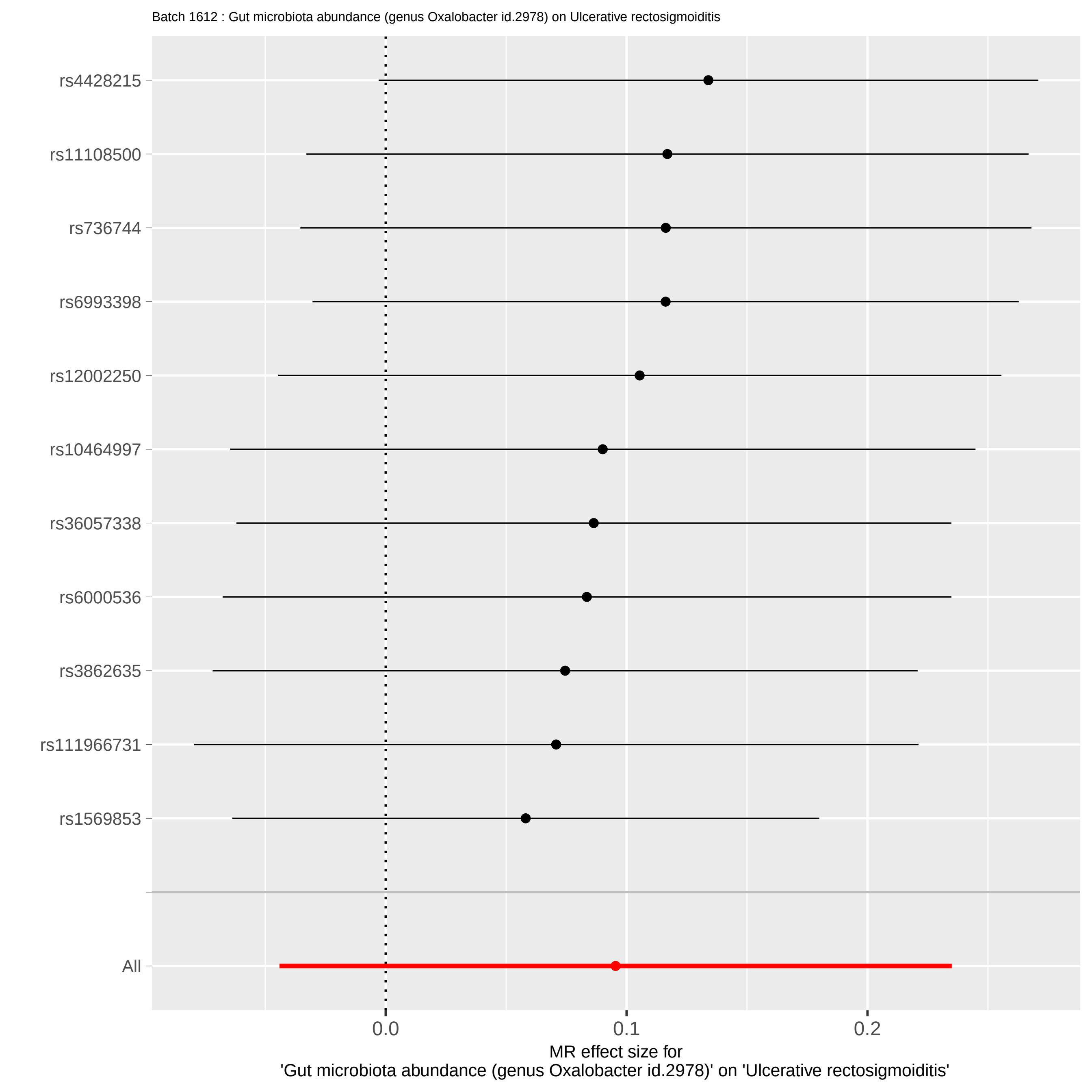

Batch 1612 : Gut microbiota abundance (genus Oxalobacter id.2978) on Ulcerative rectosigmoiditis
rs4428215
rs11108500
rs736744
rs6993398
rs12002250
rs10464997
rs36057338
rs6000536
rs3862635
rs111966731
rs1569853
All
0.0
0.1
0.2
MR effect size for
'Gut microbiota abundance (genus Oxalobacter id.2978)' on 'Ulcerative rectosigmoiditis'

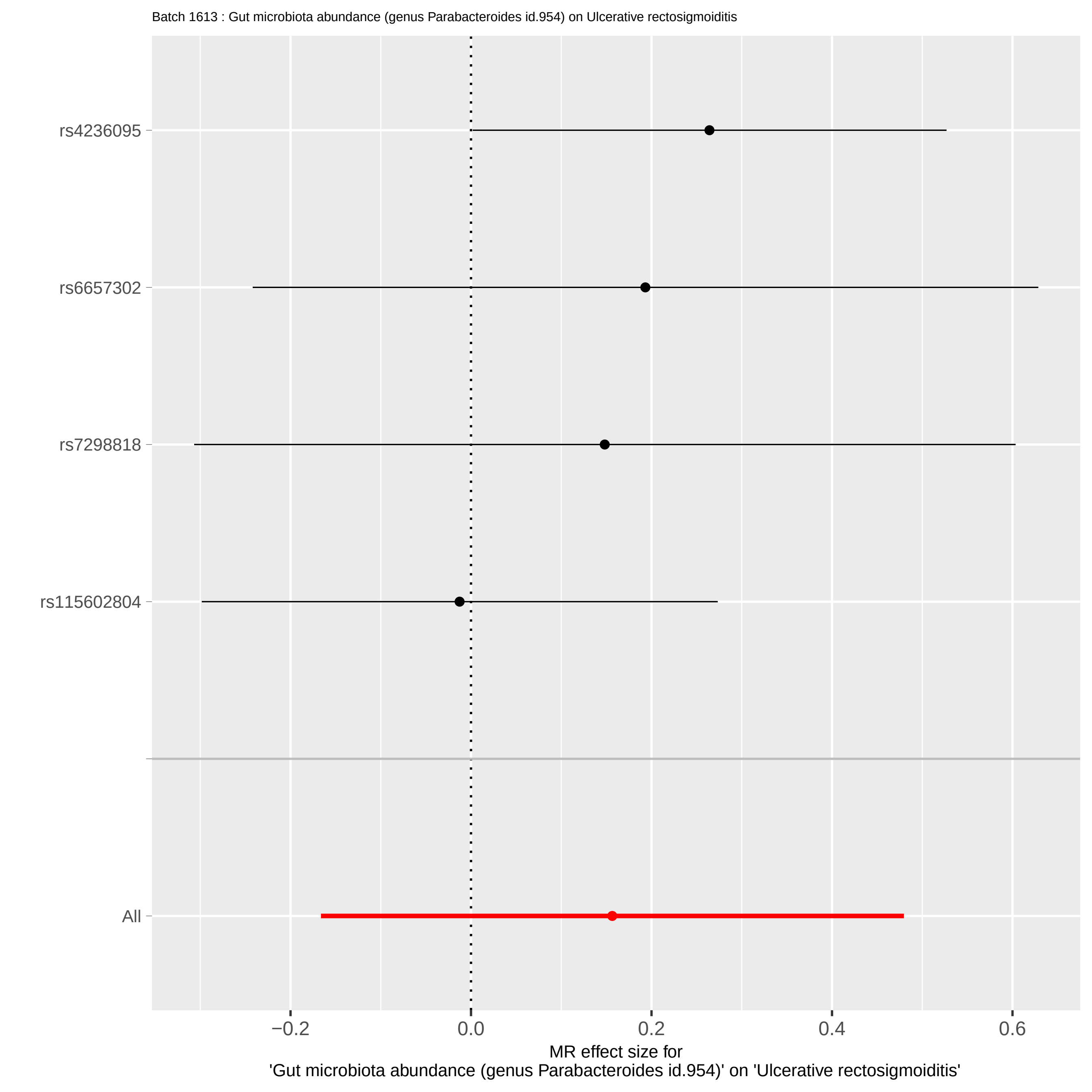

Batch 1613 : Gut microbiota abundance (genus Parabacteroides id.954) on Ulcerative rectosigmoiditis
rs4236095
rs6657302
rs7298818
rs115602804
All
−0.2
0.0
0.2
0.4
0.6
MR effect size for
'Gut microbiota abundance (genus Parabacteroides id.954)' on 'Ulcerative rectosigmoiditis'

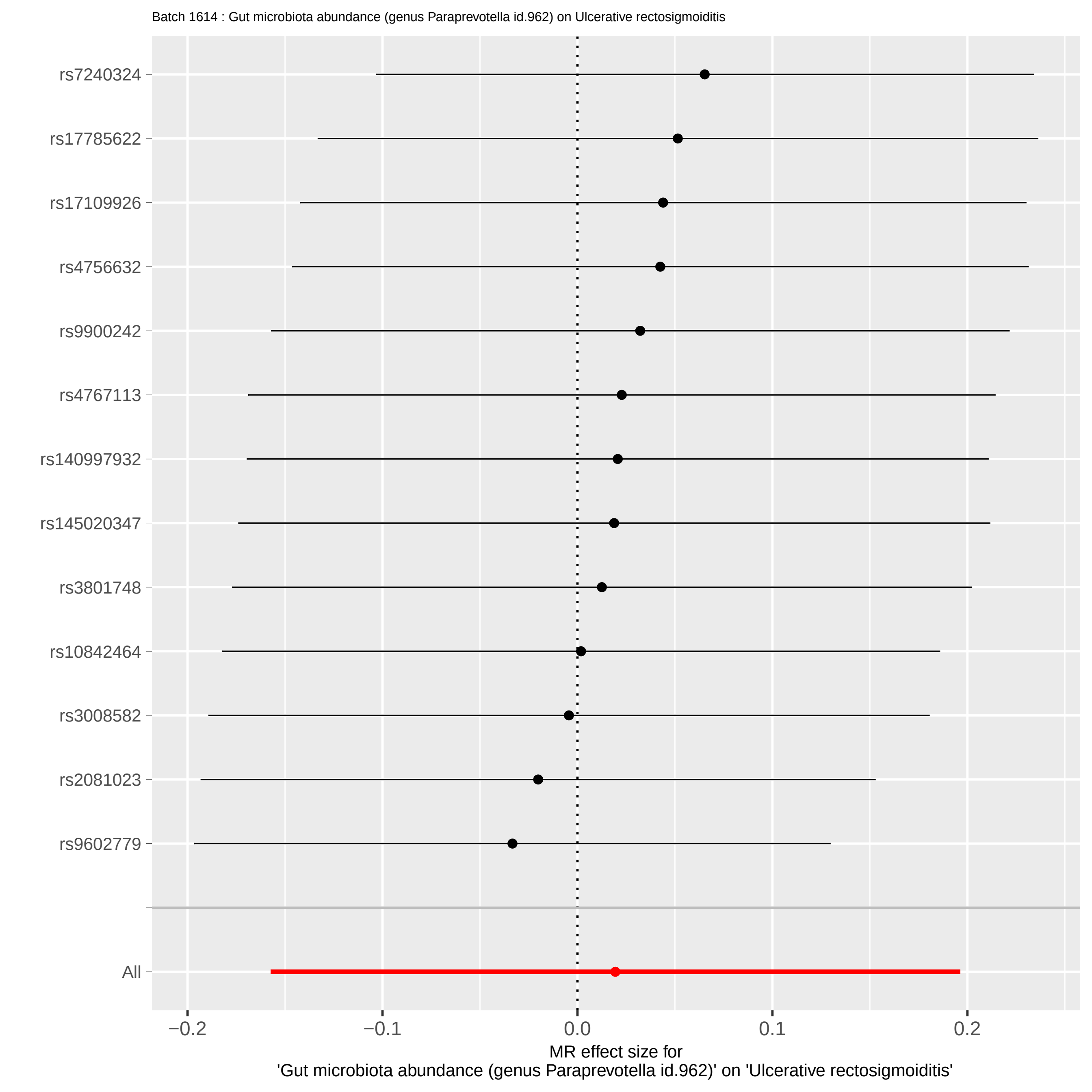
Batch 1614 : Gut microbiota abundance (genus Paraprevotella id.962) on Ulcerative rectosigmoiditis
rs7240324
rs17785622
rs17109926
rs4756632
rs9900242
rs4767113
rs140997932
rs145020347
rs3801748
rs10842464
rs3008582
rs2081023
rs9602779
All
−0.2
−0.1
0.0
0.1
0.2
MR effect size for
'Gut microbiota abundance (genus Paraprevotella id.962)' on 'Ulcerative rectosigmoiditis'

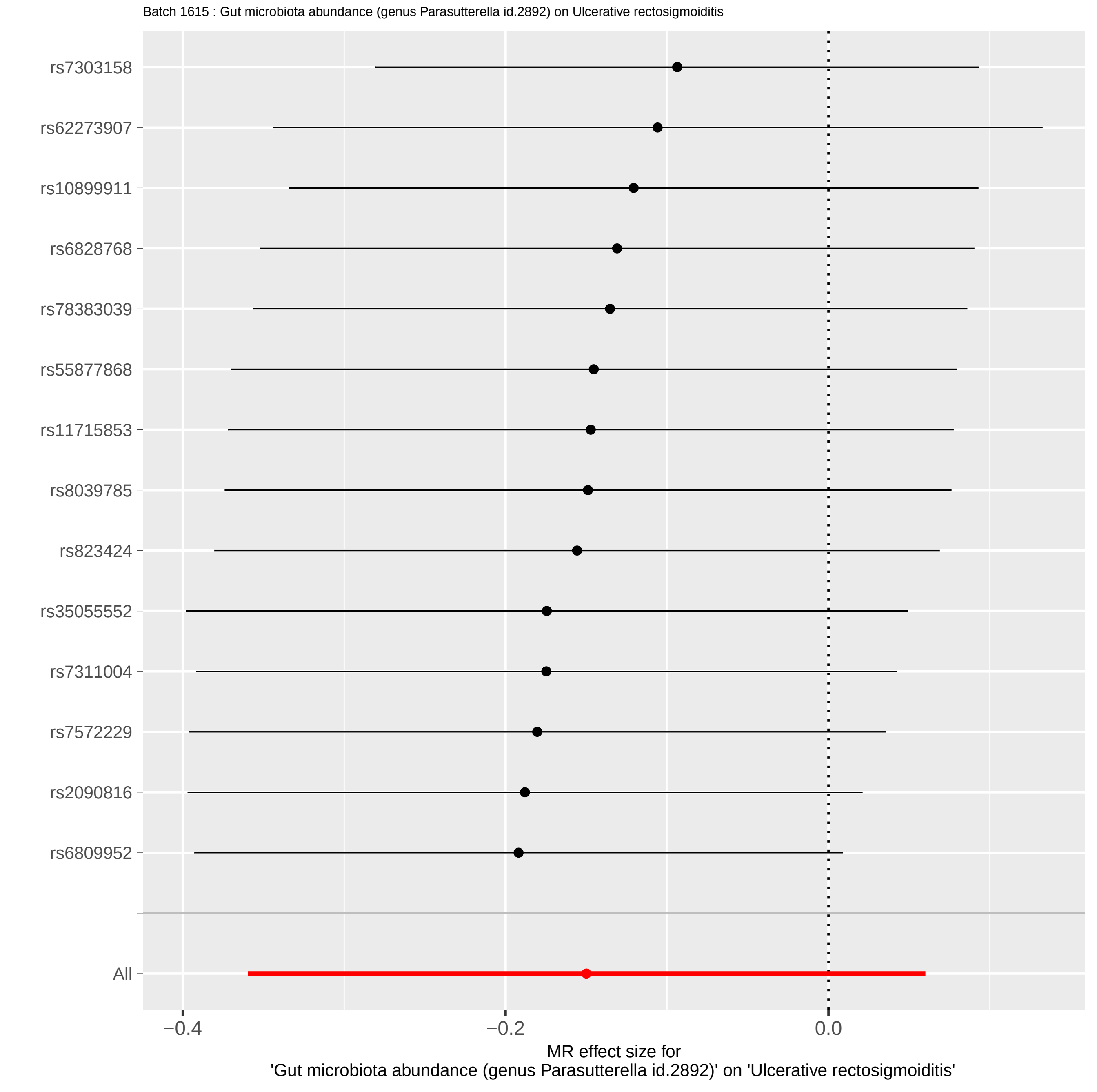

Batch 1615 : Gut microbiota abundance (genus Parasutterella id.2892) on Ulcerative rectosigmoiditis
rs7303158
rs62273907
rs10899911
rs6828768
rs78383039
rs55877868
rs11715853
rs8039785
rs823424
rs35055552
rs7311004
rs7572229
rs2090816
rs6809952
All
−0.4
−0.2
0.0
MR effect size for
'Gut microbiota abundance (genus Parasutterella id.2892)' on 'Ulcerative rectosigmoiditis'

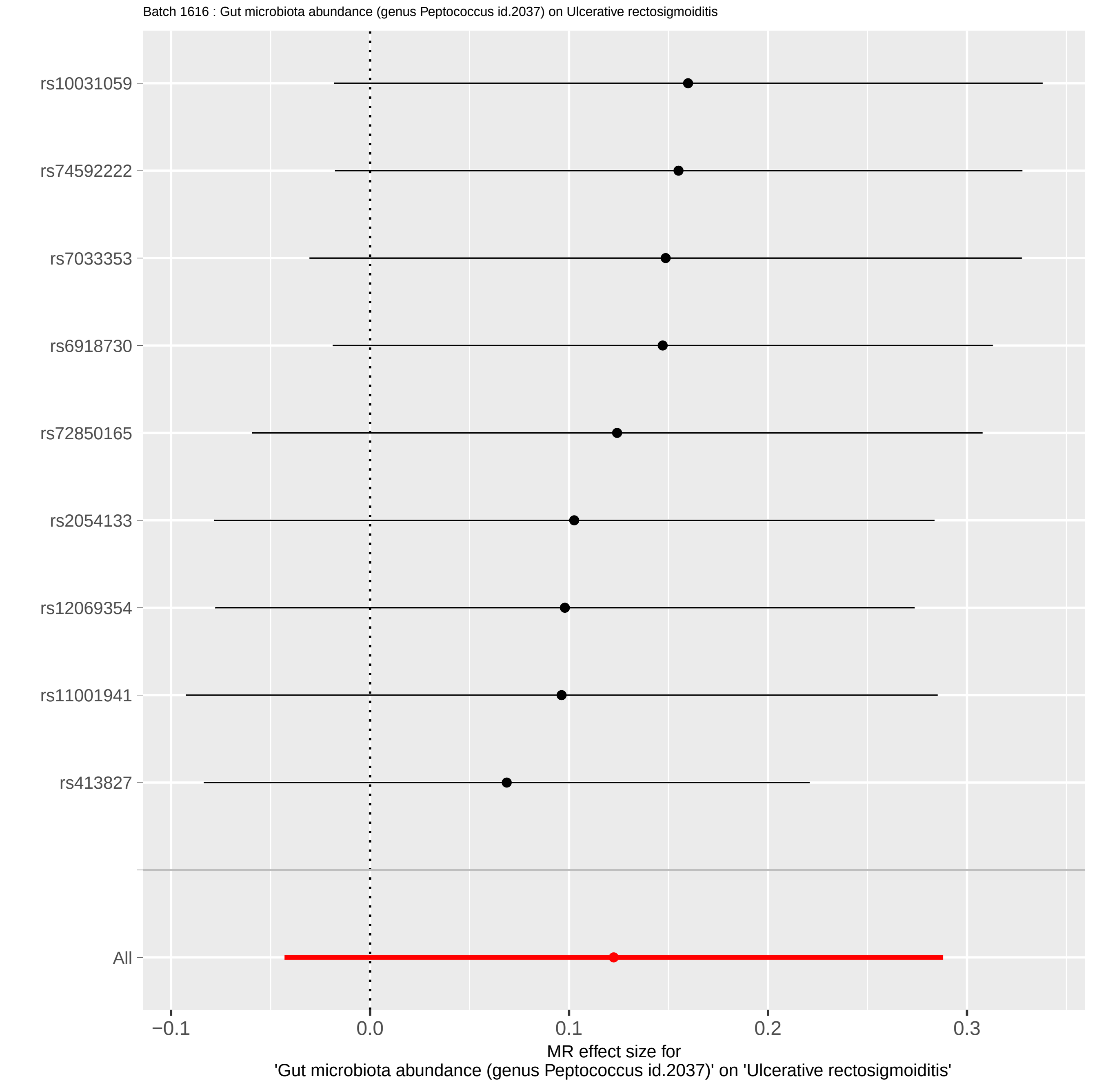

Batch 1616 : Gut microbiota abundance (genus Peptococcus id.2037) on Ulcerative rectosigmoiditis
rs10031059
rs74592222
rs7033353
rs6918730
rs72850165
rs2054133
rs12069354
rs11001941
rs413827
All
−0.1
0.0
0.1
0.2
0.3
MR effect size for
'Gut microbiota abundance (genus Peptococcus id.2037)' on 'Ulcerative rectosigmoiditis'

Batch 1617 : Gut microbiota abundance (genus Phascolarctobacterium id.2168) on Ulcerative rectosigmoiditis

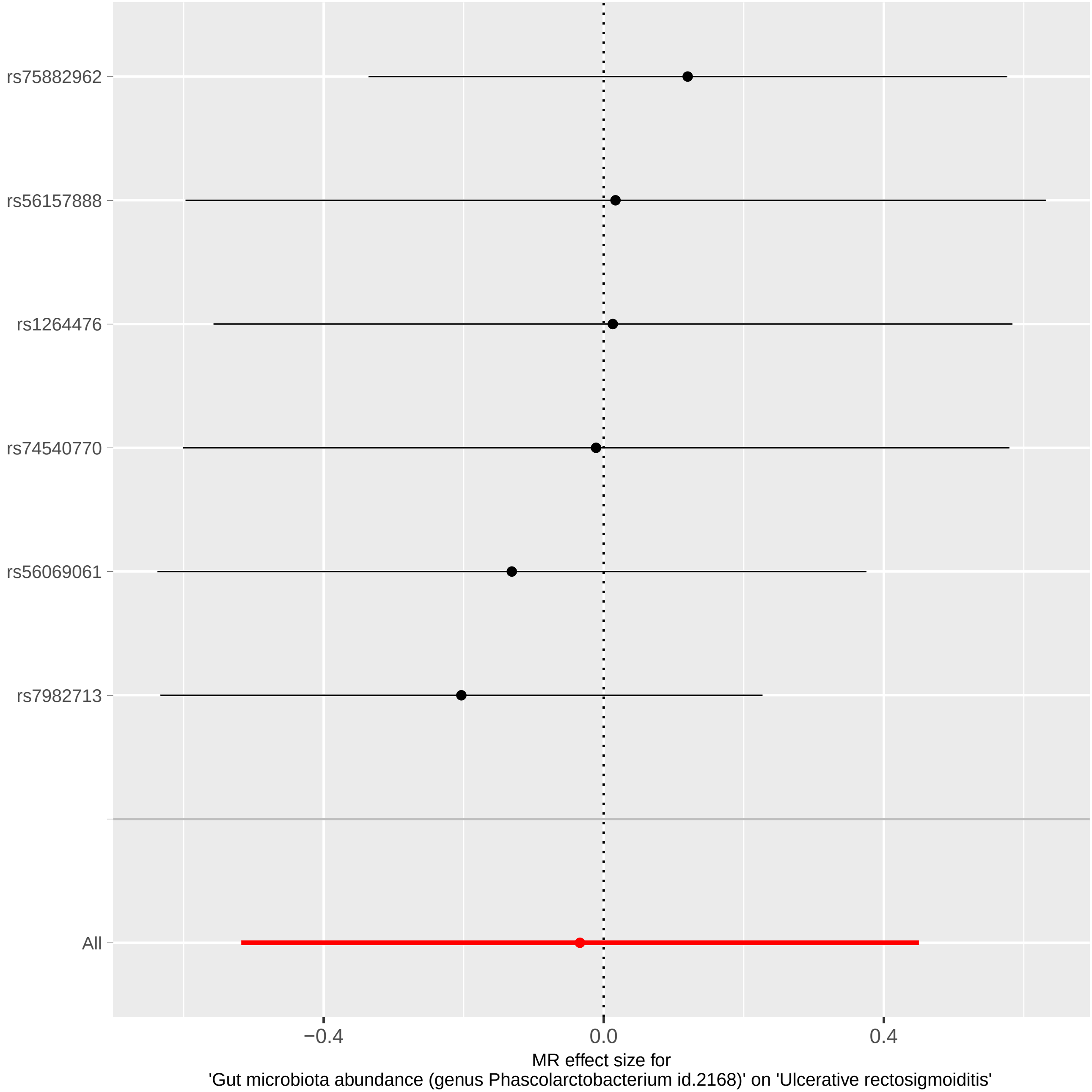

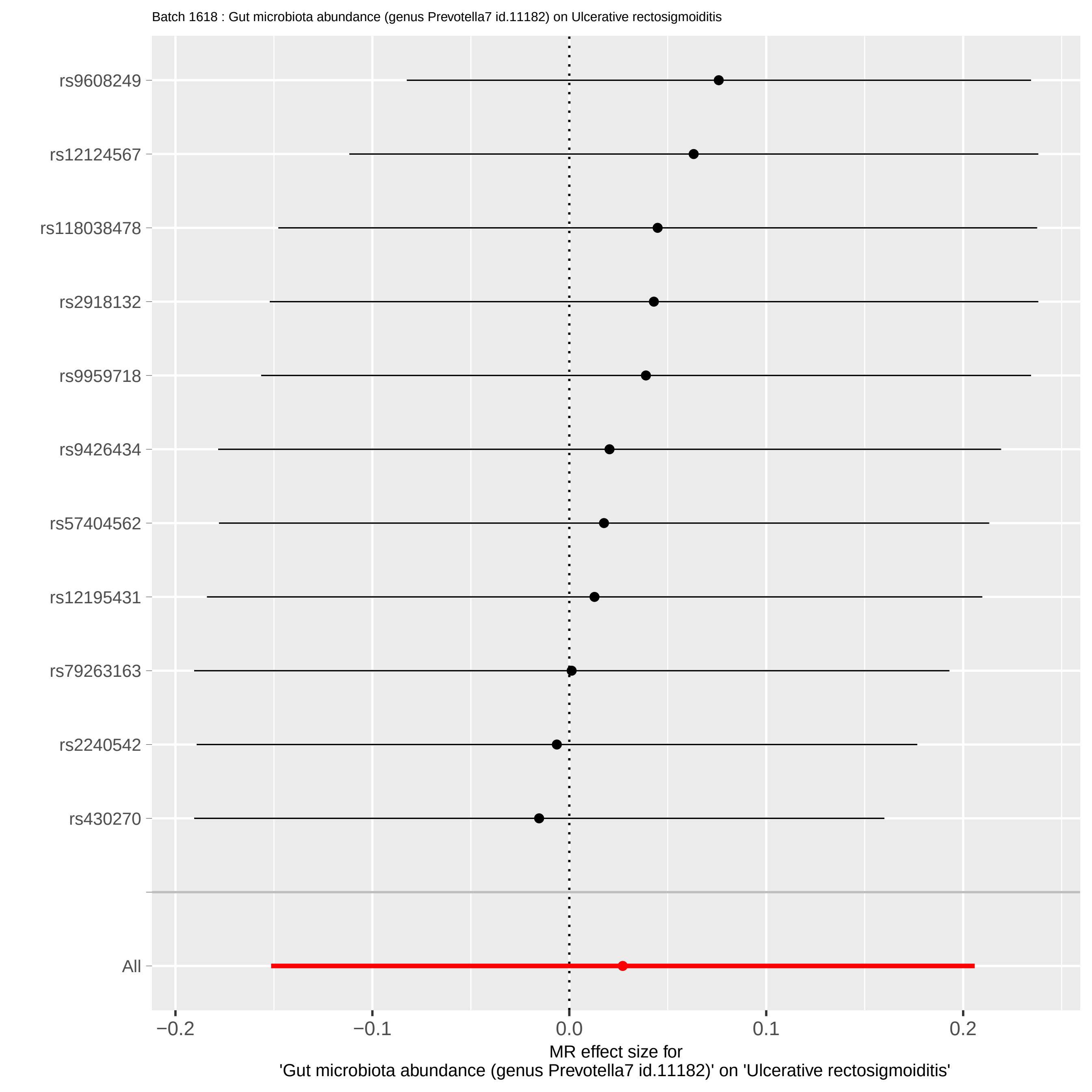
Batch 1618 : Gut microbiota abundance (genus Prevotella7 id.11182) on Ulcerative rectosigmoiditis
rs9608249
rs12124567
rs118038478
rs2918132
rs9959718
rs9426434
rs57404562
rs12195431
rs79263163
rs2240542
rs430270
All
−0.2
−0.1
0.0
0.1
0.2
MR effect size for
'Gut microbiota abundance (genus Prevotella7 id.11182)' on 'Ulcerative rectosigmoiditis'

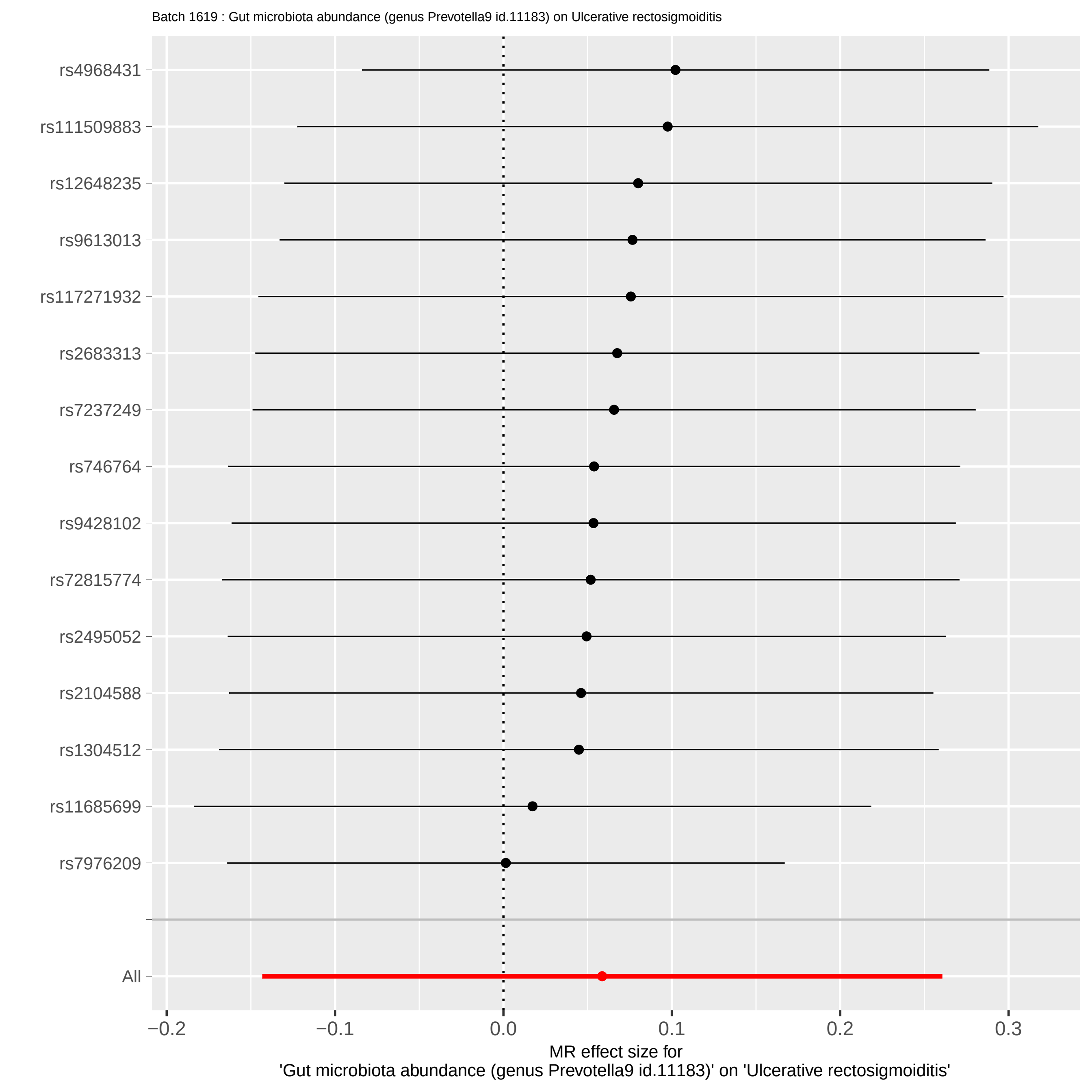
Batch 1619 : Gut microbiota abundance (genus Prevotella9 id.11183) on Ulcerative rectosigmoiditis
rs4968431
rs111509883
rs12648235
rs9613013
rs117271932
rs2683313
rs7237249
rs746764
rs9428102
rs72815774
rs2495052
rs2104588
rs1304512
rs11685699
rs7976209
All
−0.2
−0.1
0.0
0.1
0.2
0.3
MR effect size for
'Gut microbiota abundance (genus Prevotella9 id.11183)' on 'Ulcerative rectosigmoiditis'

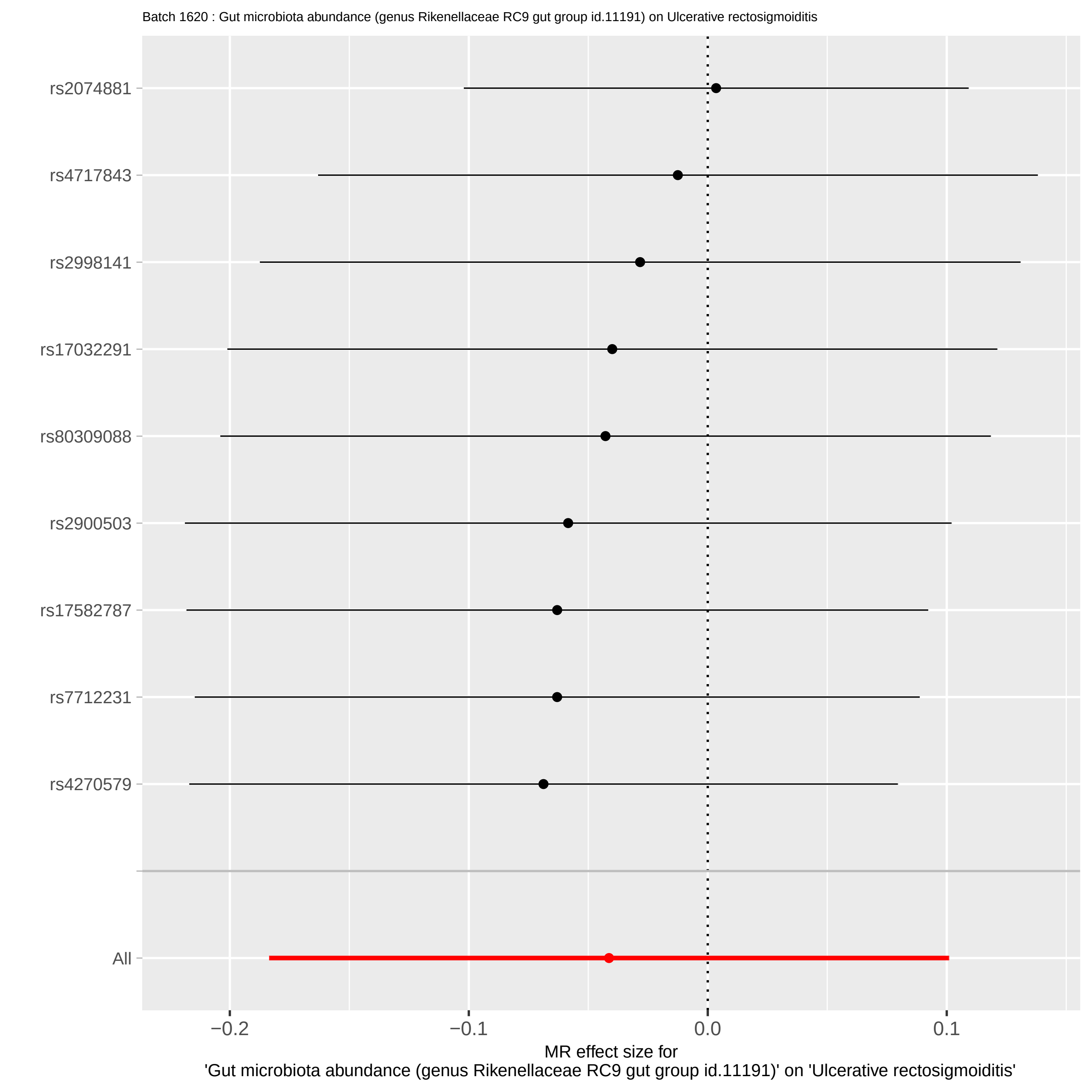

Batch 1620 : Gut microbiota abundance (genus Rikenellaceae RC9 gut group id.11191) on Ulcerative rectosigmoiditis
rs2074881
rs4717843
rs2998141
rs17032291
rs80309088
rs2900503
rs17582787
rs7712231
rs4270579
All
−0.2
−0.1
0.0
0.1
MR effect size for
'Gut microbiota abundance (genus Rikenellaceae RC9 gut group id.11191)' on 'Ulcerative rectosigmoiditis'

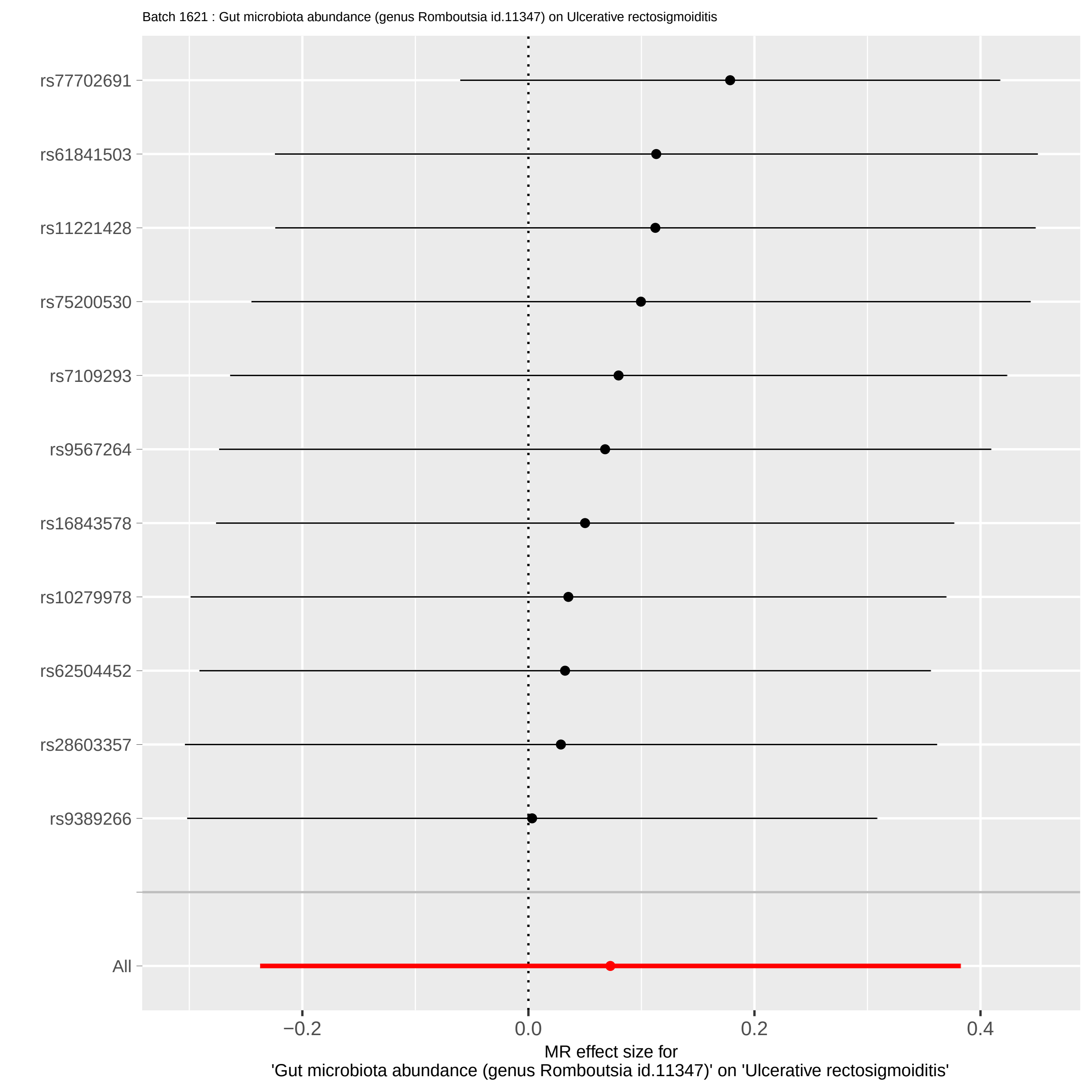
Batch 1621 : Gut microbiota abundance (genus Romboutsia id.11347) on Ulcerative rectosigmoiditis
rs77702691
rs61841503
rs11221428
rs75200530
rs7109293
rs9567264
rs16843578
rs10279978
rs62504452
rs28603357
rs9389266
All
−0.2
0.0
0.2
0.4
MR effect size for
'Gut microbiota abundance (genus Romboutsia id.11347)' on 'Ulcerative rectosigmoiditis'

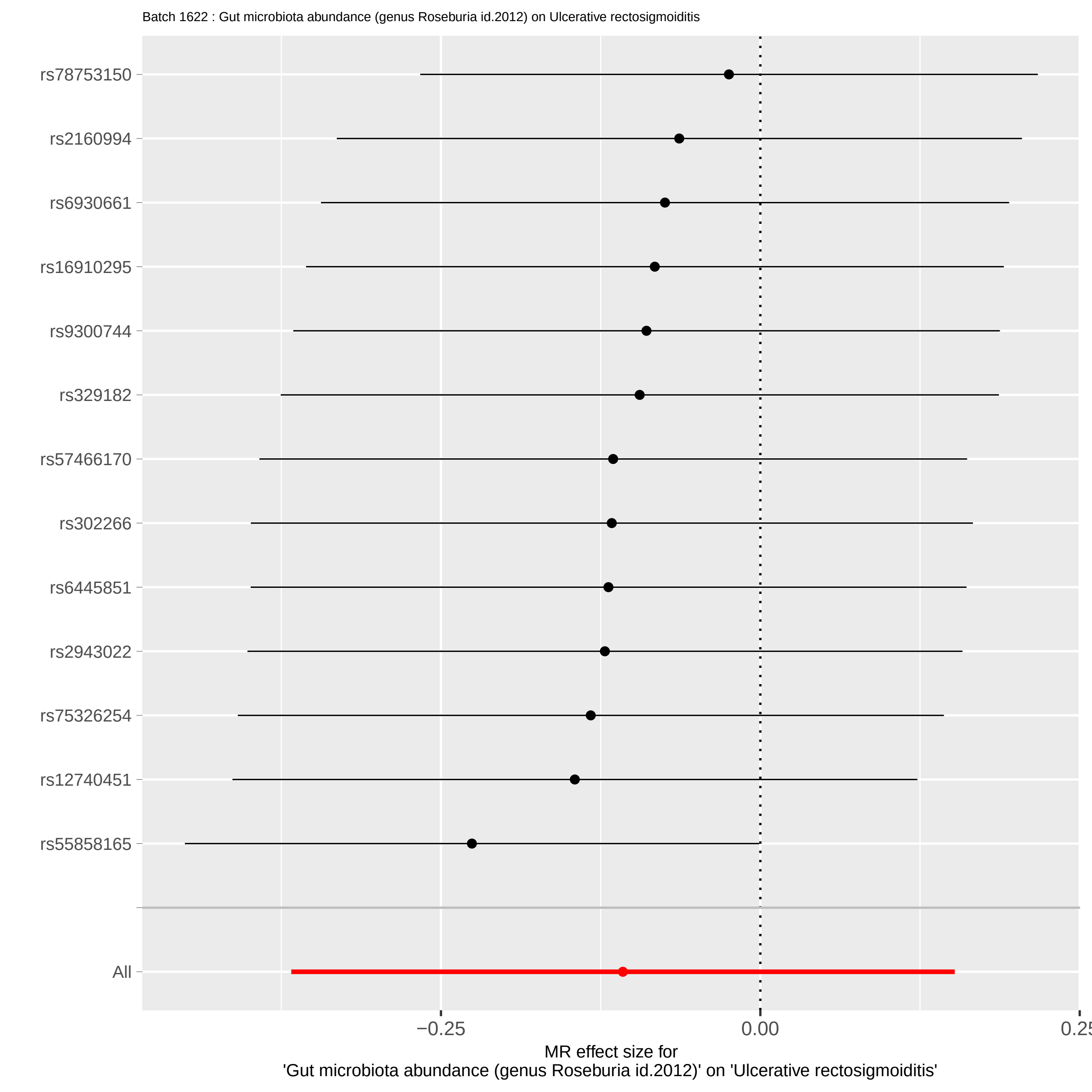

Batch 1622 : Gut microbiota abundance (genus Roseburia id.2012) on Ulcerative rectosigmoiditis
rs78753150
rs2160994
rs6930661
rs16910295
rs9300744
rs329182
rs57466170
rs302266
rs6445851
rs2943022
rs75326254
rs12740451
rs55858165
All
−0.25
0.00
0.25
MR effect size for
'Gut microbiota abundance (genus Roseburia id.2012)' on 'Ulcerative rectosigmoiditis'

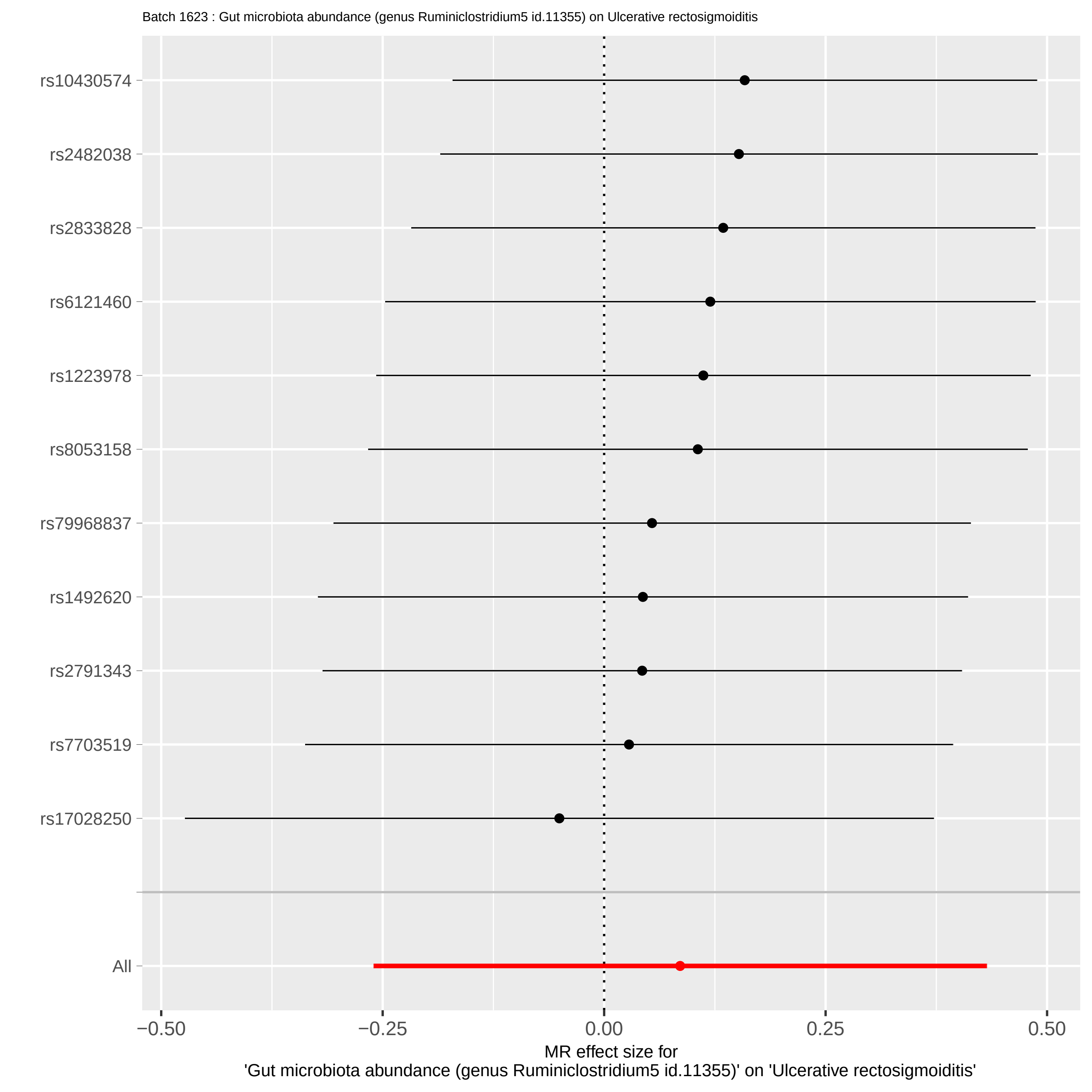
Batch 1623 : Gut microbiota abundance (genus Ruminiclostridium5 id.11355) on Ulcerative rectosigmoiditis
rs10430574
rs2482038
rs2833828
rs6121460
rs1223978
rs8053158
rs79968837
rs1492620
rs2791343
rs7703519
rs17028250
All
−0.50
−0.25
0.00
0.25
0.50
MR effect size for
'Gut microbiota abundance (genus Ruminiclostridium5 id.11355)' on 'Ulcerative rectosigmoiditis'

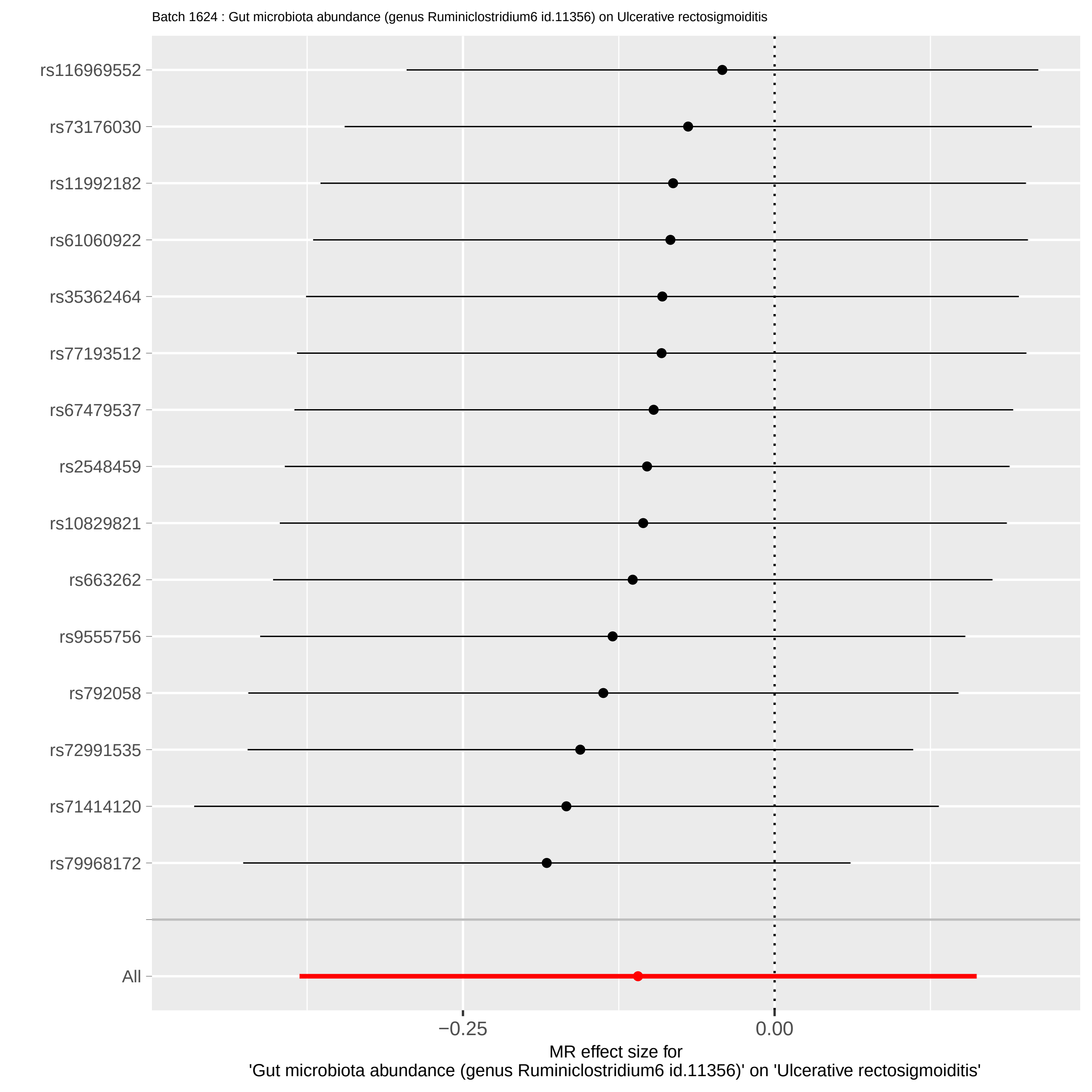

Batch 1624 : Gut microbiota abundance (genus Ruminiclostridium6 id.11356) on Ulcerative rectosigmoiditis
rs116969552
rs73176030
rs11992182
rs61060922
rs35362464
rs77193512
rs67479537
rs2548459
rs10829821
rs663262
rs9555756
rs792058
rs72991535
rs71414120
rs79968172
All
−0.25
0.00
MR effect size for
'Gut microbiota abundance (genus Ruminiclostridium6 id.11356)' on 'Ulcerative rectosigmoiditis'

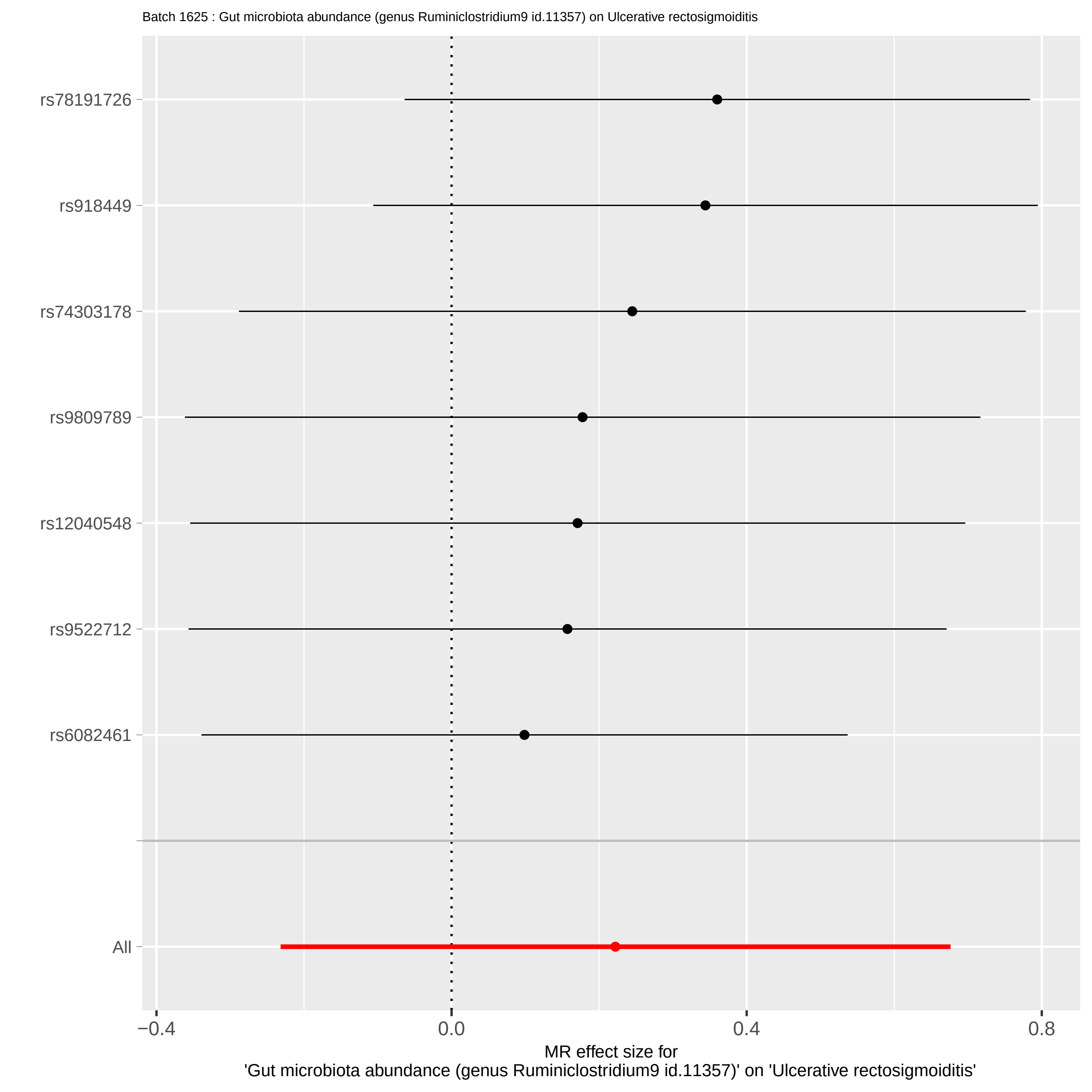
Batch 1625 : Gut microbiota abundance (genus Ruminiclostridium9 id.11357) on Ulcerative rectosigmoiditis
rs78191726
rs918449
rs74303178
rs9809789
rs12040548
rs9522712
rs6082461
All
−0.4
0.0
0.4
0.8
MR effect size for
'Gut microbiota abundance (genus Ruminiclostridium9 id.11357)' on 'Ulcerative rectosigmoiditis'

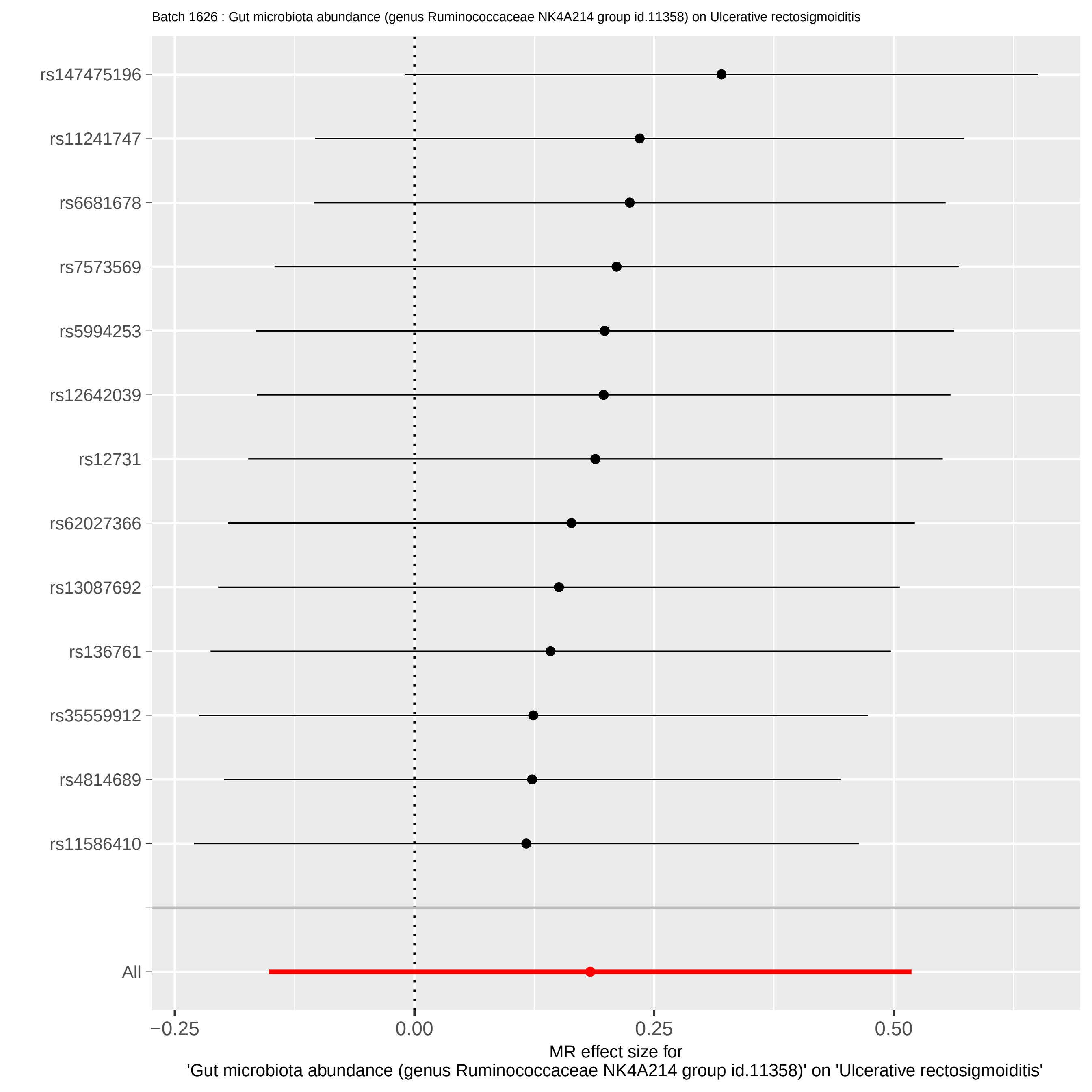

Batch 1626 : Gut microbiota abundance (genus Ruminococcaceae NK4A214 group id.11358) on Ulcerative rectosigmoiditis
rs147475196
rs11241747
rs6681678
rs7573569
rs5994253
rs12642039
rs12731
rs62027366
rs13087692
rs136761
rs35559912
rs4814689
rs11586410
All
−0.25
0.00
0.25
0.50
MR effect size for
'Gut microbiota abundance (genus Ruminococcaceae NK4A214 group id.11358)' on 'Ulcerative rectosigmoiditis'

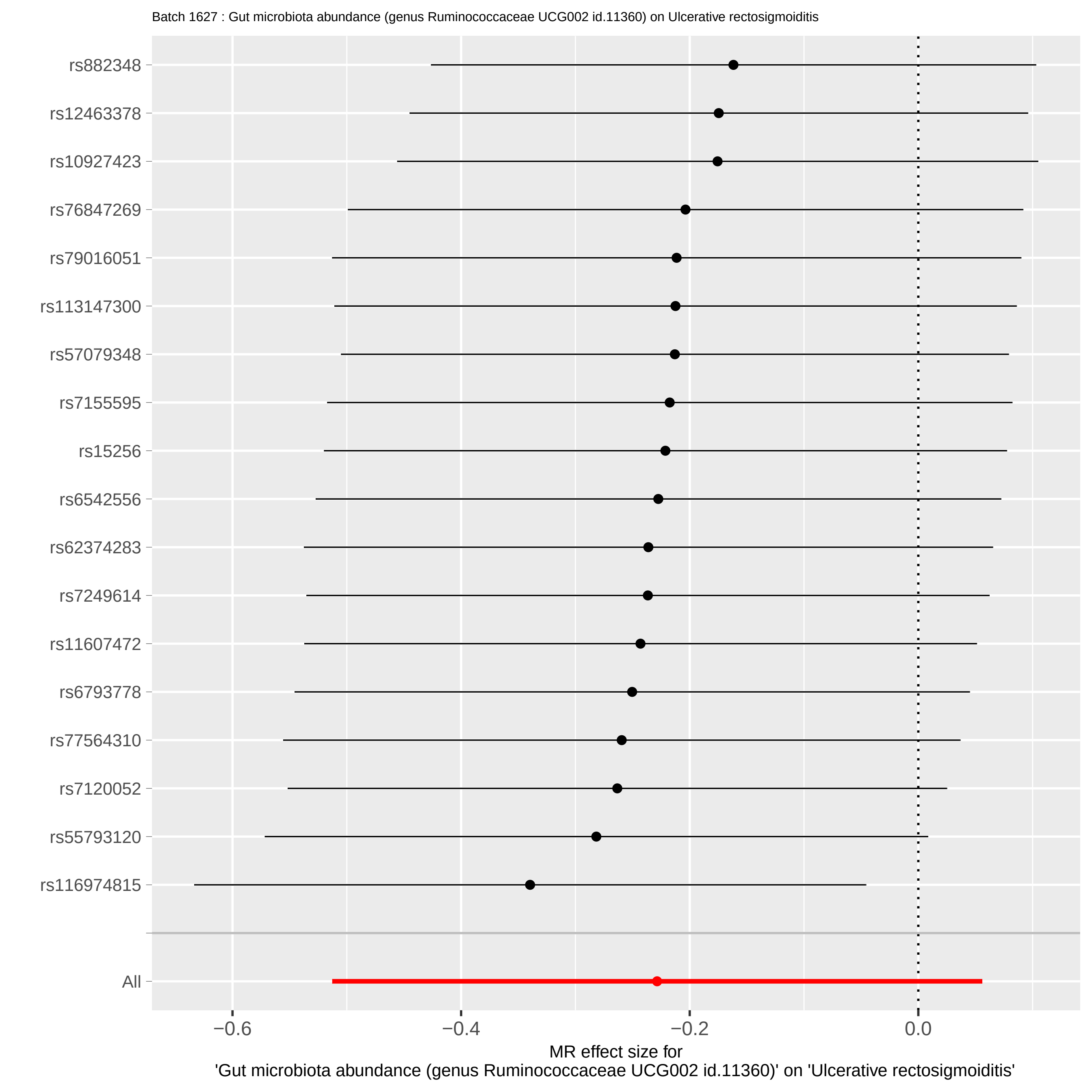
Batch 1627 : Gut microbiota abundance (genus Ruminococcaceae UCG002 id.11360) on Ulcerative rectosigmoiditis
rs882348
rs12463378
rs10927423
rs76847269
rs79016051
rs113147300
rs57079348
rs7155595
rs15256
rs6542556
rs62374283
rs7249614
rs11607472
rs6793778
rs77564310
rs7120052
rs55793120
rs116974815
All
−0.6
−0.4
−0.2
0.0
MR effect size for
'Gut microbiota abundance (genus Ruminococcaceae UCG002 id.11360)' on 'Ulcerative rectosigmoiditis'

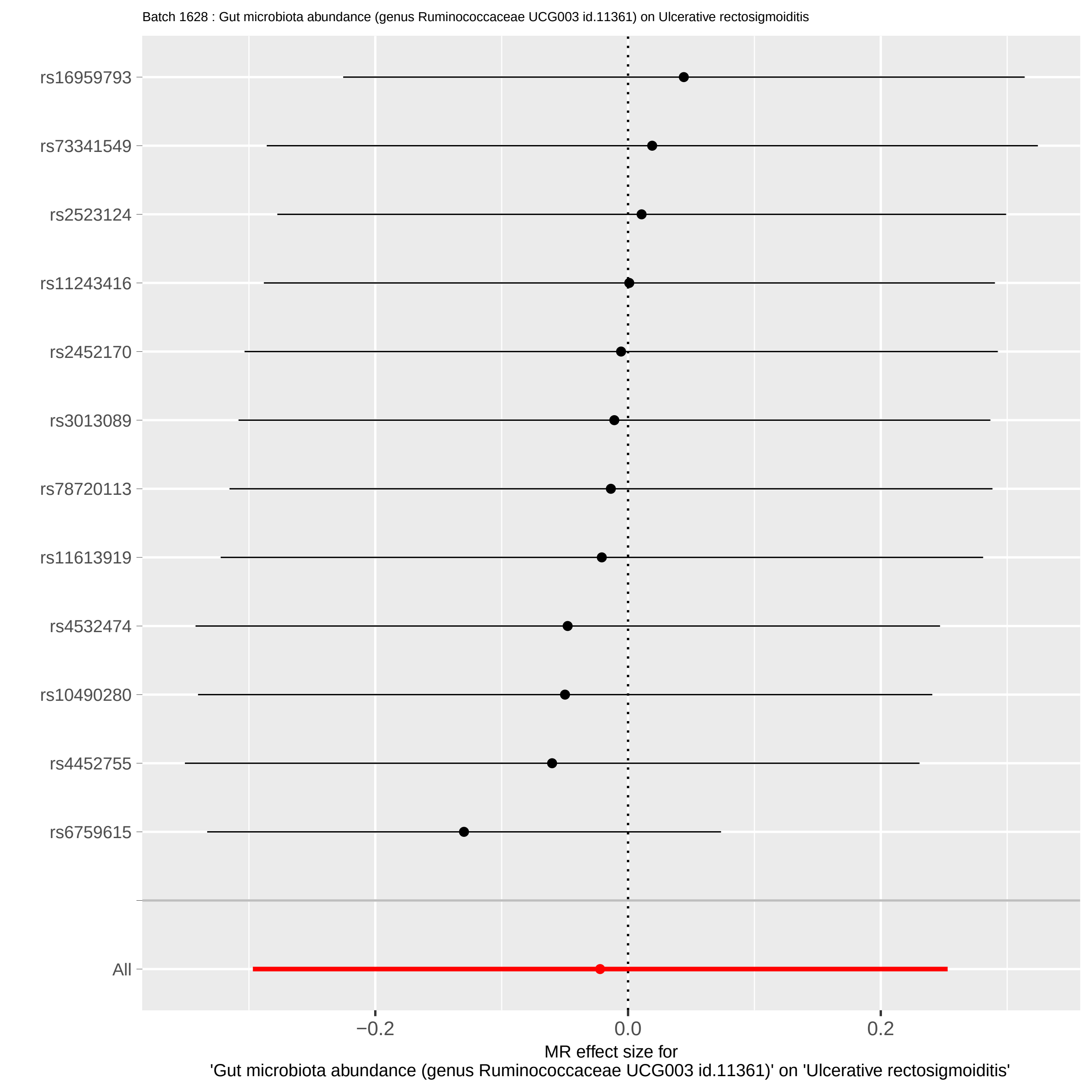

Batch 1628 : Gut microbiota abundance (genus Ruminococcaceae UCG003 id.11361) on Ulcerative rectosigmoiditis
rs16959793
rs73341549
rs2523124
rs11243416
rs2452170
rs3013089
rs78720113
rs11613919
rs4532474
rs10490280
rs4452755
rs6759615
All
−0.2
0.0
0.2
MR effect size for
'Gut microbiota abundance (genus Ruminococcaceae UCG003 id.11361)' on 'Ulcerative rectosigmoiditis'

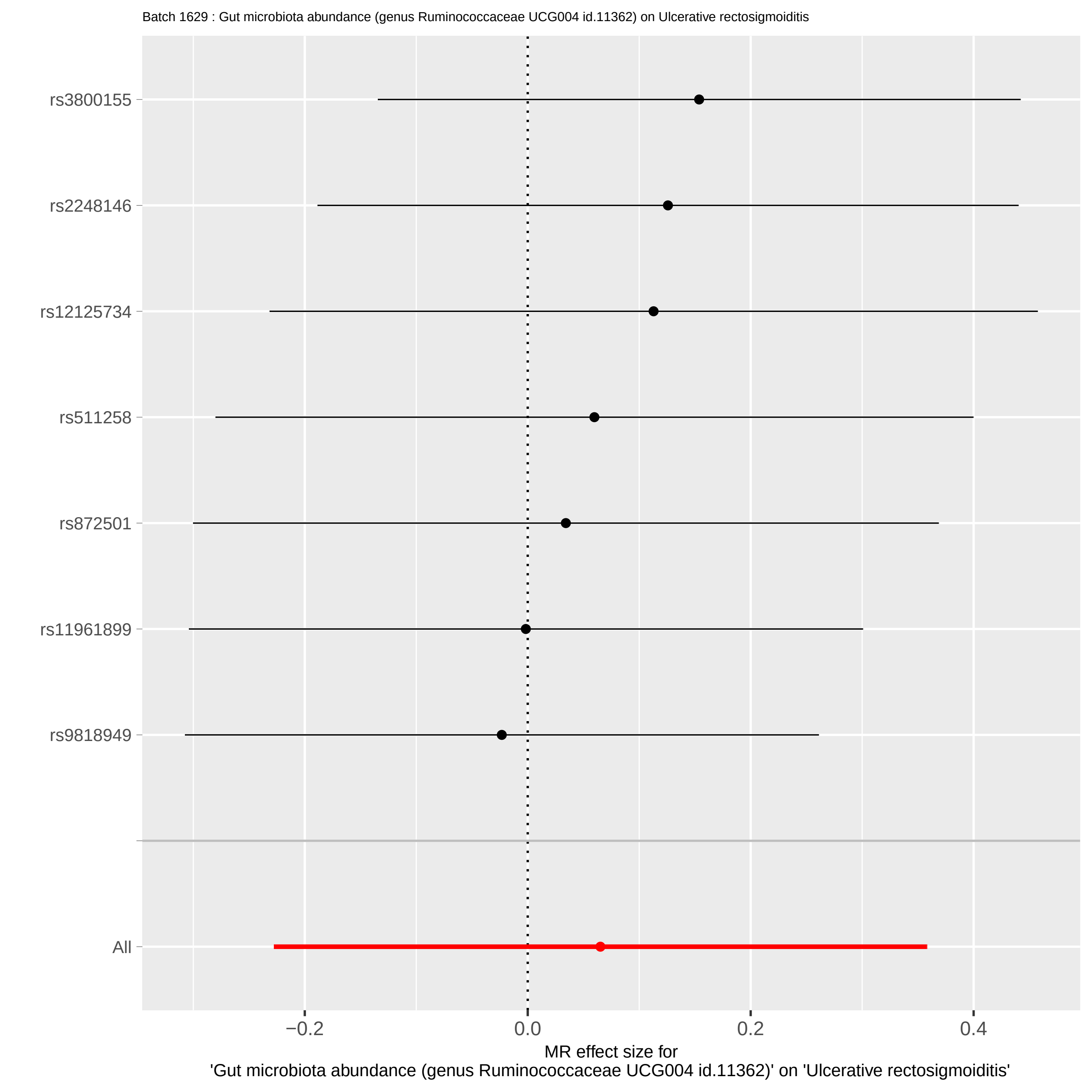

Batch 1629 : Gut microbiota abundance (genus Ruminococcaceae UCG004 id.11362) on Ulcerative rectosigmoiditis
rs3800155
rs2248146
rs12125734
rs511258
rs872501
rs11961899
rs9818949
All
−0.2
0.0
0.2
0.4
MR effect size for
'Gut microbiota abundance (genus Ruminococcaceae UCG004 id.11362)' on 'Ulcerative rectosigmoiditis'

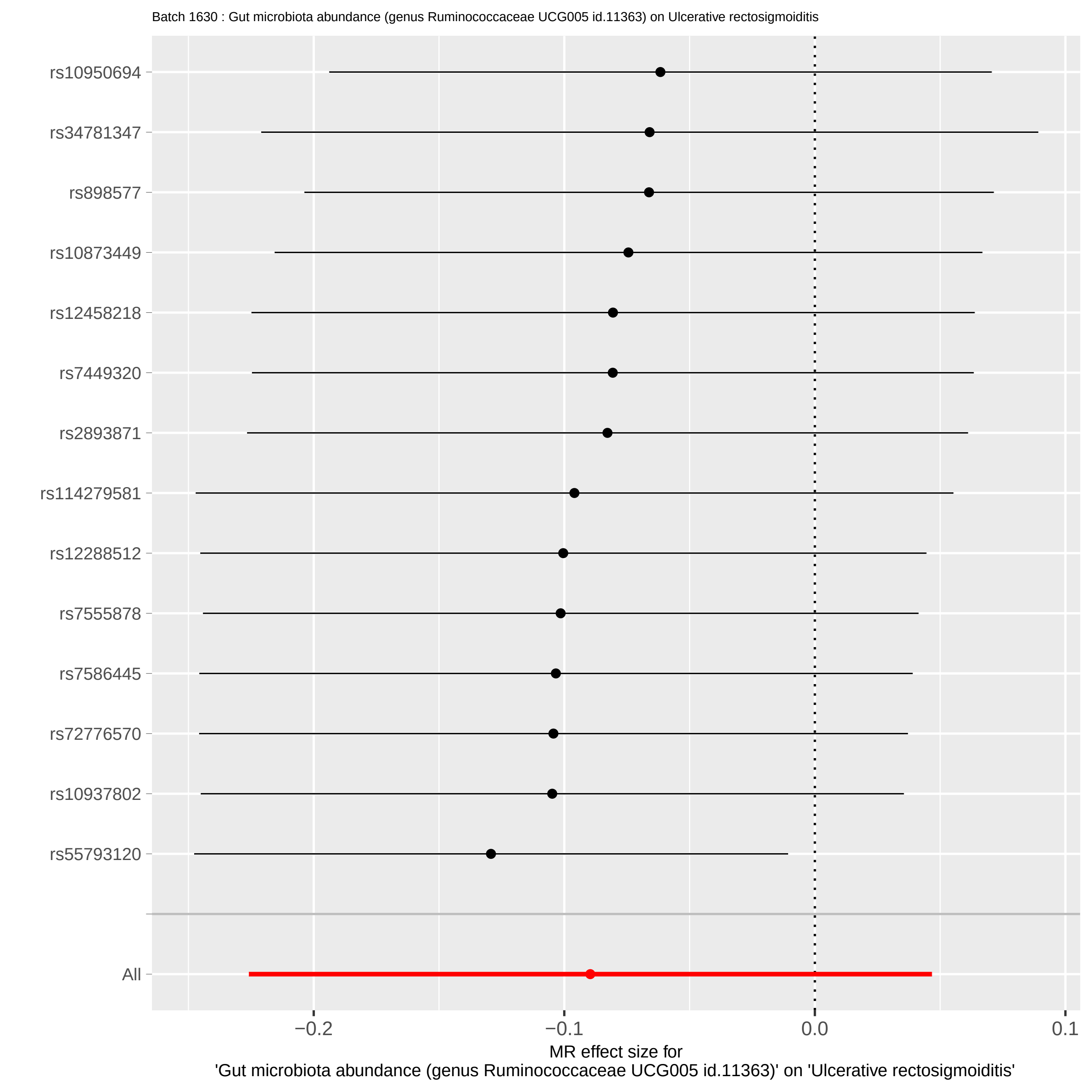

Batch 1630 : Gut microbiota abundance (genus Ruminococcaceae UCG005 id.11363) on Ulcerative rectosigmoiditis
rs10950694
rs34781347
rs898577
rs10873449
rs12458218
rs7449320
rs2893871
rs114279581
rs12288512
rs7555878
rs7586445
rs72776570
rs10937802
rs55793120
All
−0.2
−0.1
0.0
0.1
MR effect size for
'Gut microbiota abundance (genus Ruminococcaceae UCG005 id.11363)' on 'Ulcerative rectosigmoiditis'

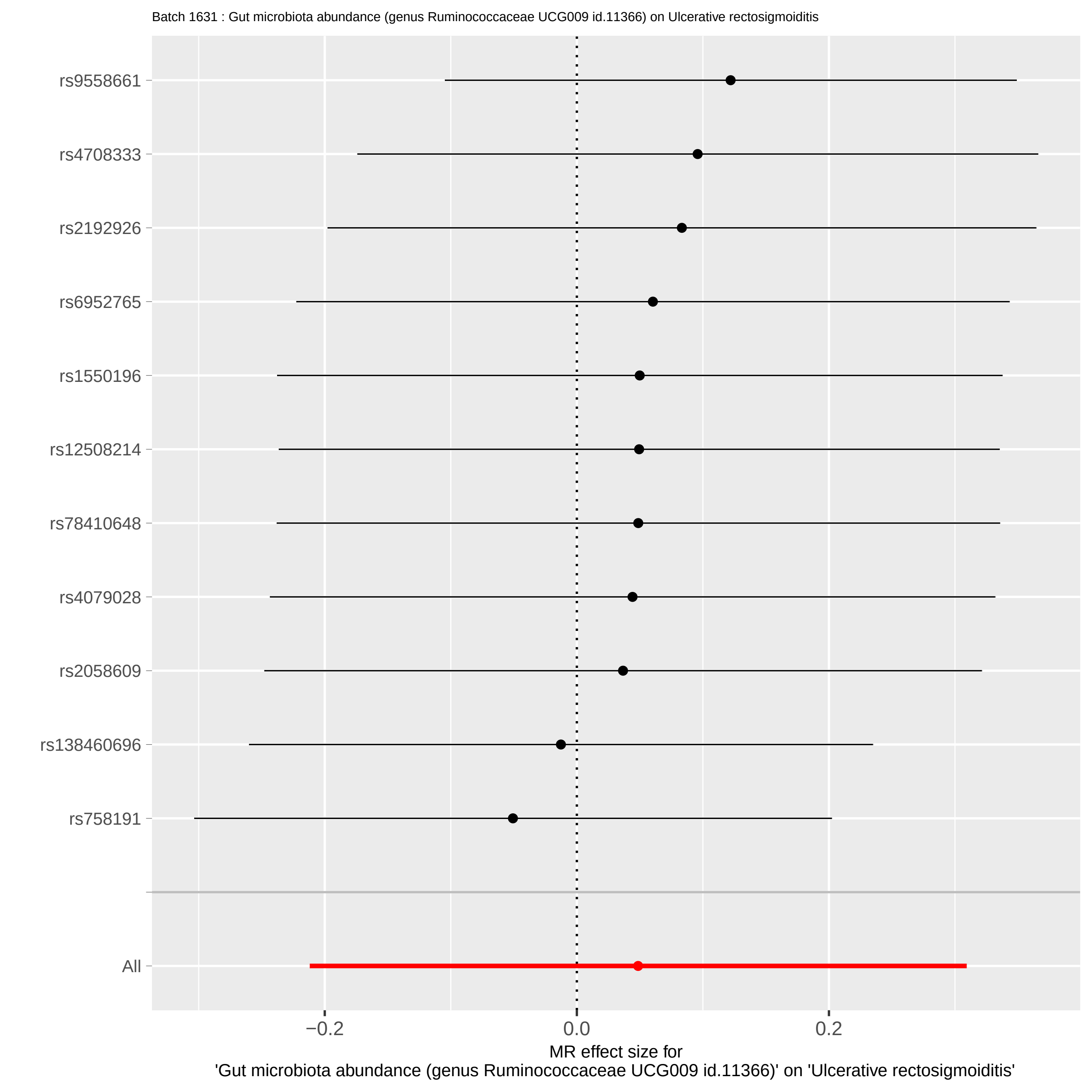

Batch 1631 : Gut microbiota abundance (genus Ruminococcaceae UCG009 id.11366) on Ulcerative rectosigmoiditis
rs9558661
rs4708333
rs2192926
rs6952765
rs1550196
rs12508214
rs78410648
rs4079028
rs2058609
rs138460696
rs758191
All
−0.2
0.0
0.2
MR effect size for
'Gut microbiota abundance (genus Ruminococcaceae UCG009 id.11366)' on 'Ulcerative rectosigmoiditis'

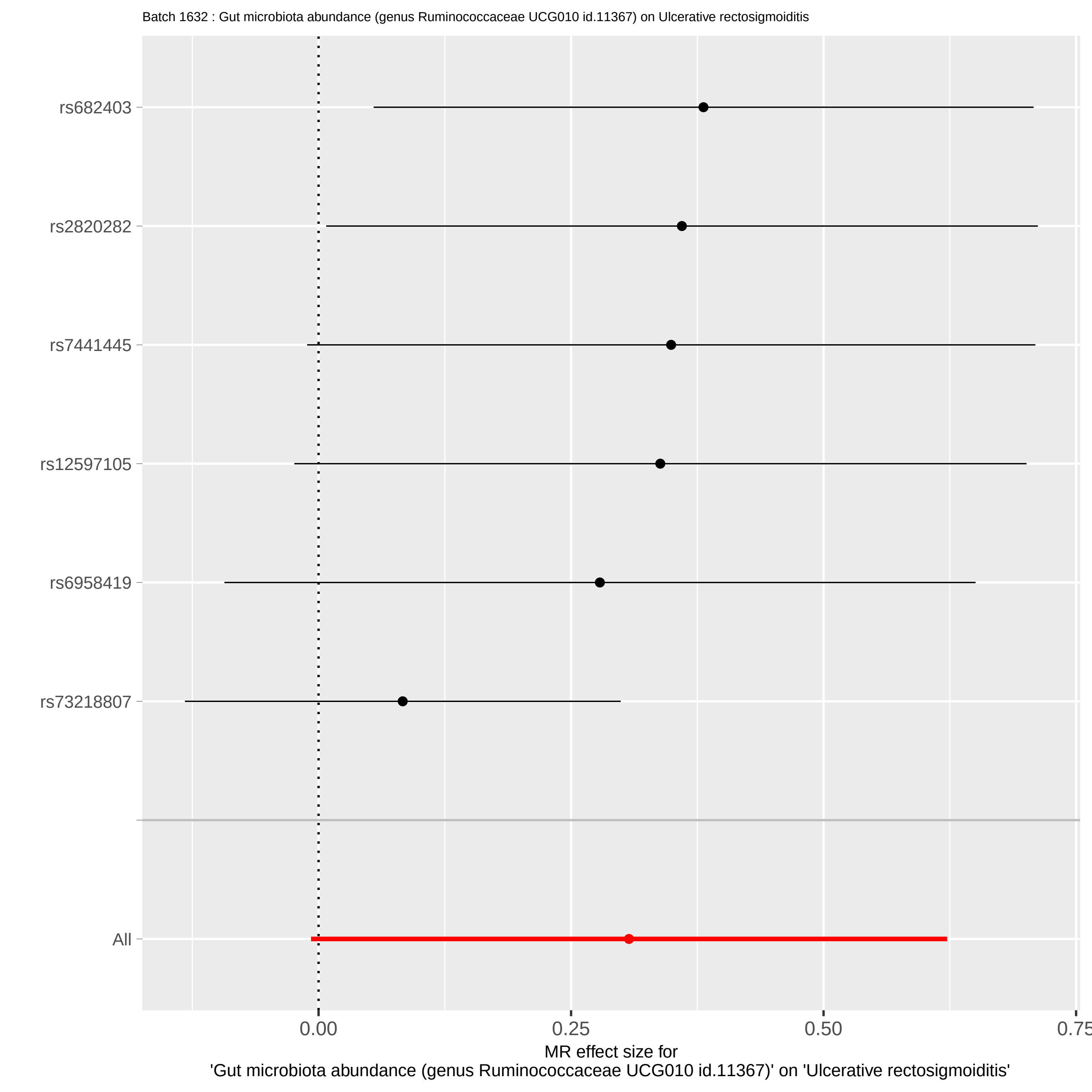

Batch 1632 : Gut microbiota abundance (genus Ruminococcaceae UCG010 id.11367) on Ulcerative rectosigmoiditis
rs682403
rs2820282
rs7441445
rs12597105
rs6958419
rs73218807
All
0.00
0.25
0.50
0.75
MR effect size for
'Gut microbiota abundance (genus Ruminococcaceae UCG010 id.11367)' on 'Ulcerative rectosigmoiditis'

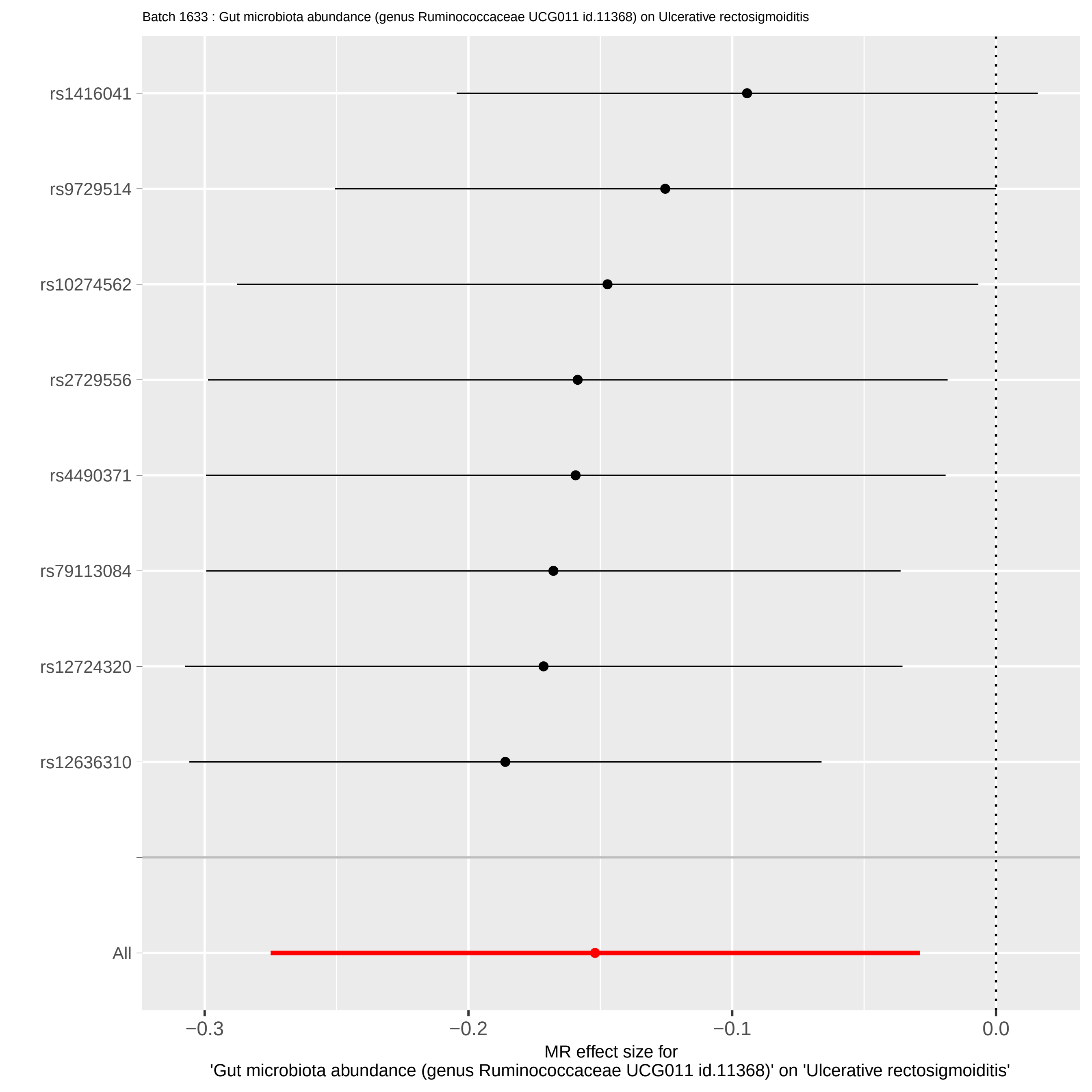

Batch 1633 : Gut microbiota abundance (genus Ruminococcaceae UCG011 id.11368) on Ulcerative rectosigmoiditis
rs1416041
rs9729514
rs10274562
rs2729556
rs4490371
rs79113084
rs12724320
rs12636310
All
−0.3
−0.2
−0.1
0.0
MR effect size for
'Gut microbiota abundance (genus Ruminococcaceae UCG011 id.11368)' on 'Ulcerative rectosigmoiditis'

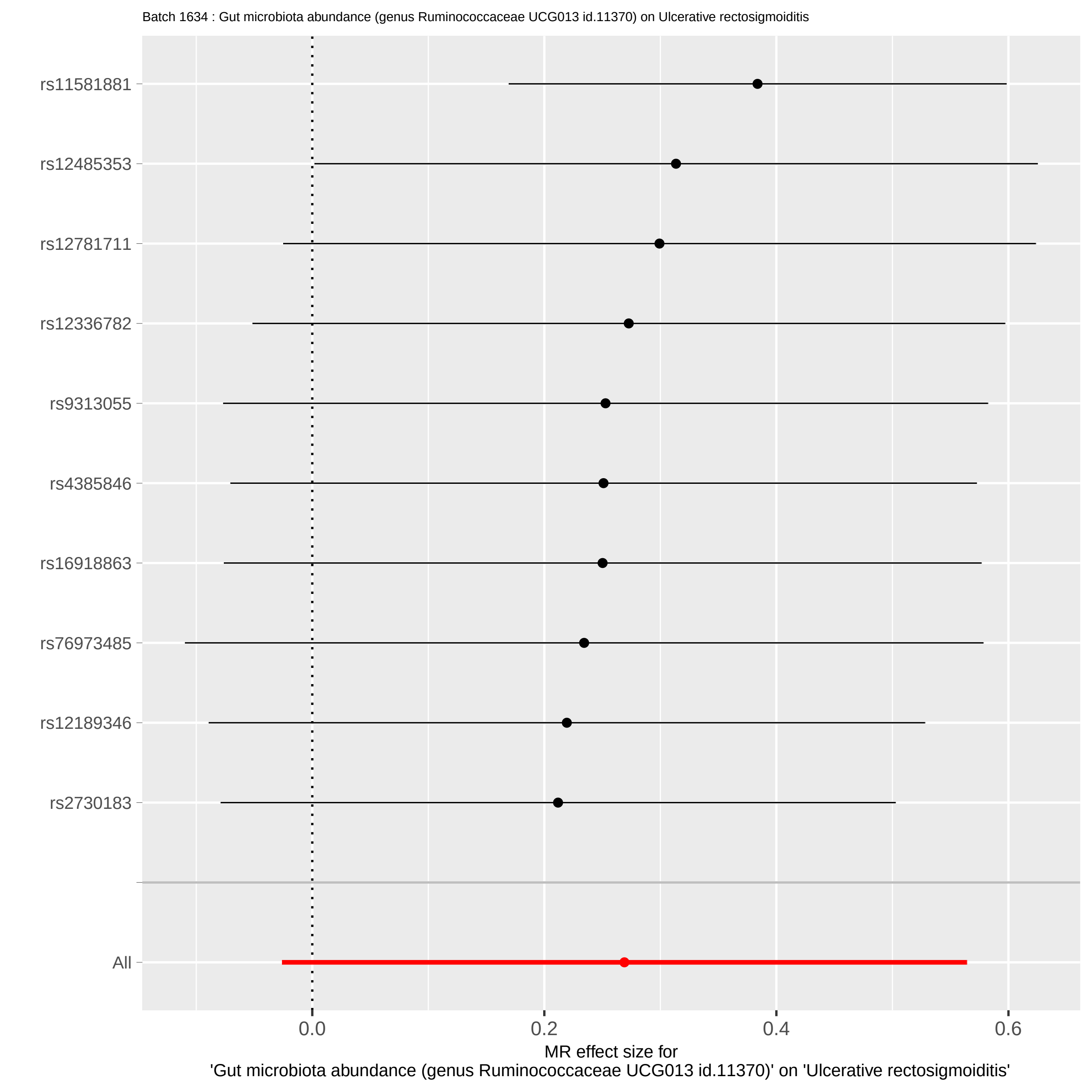
Batch 1634 : Gut microbiota abundance (genus Ruminococcaceae UCG013 id.11370) on Ulcerative rectosigmoiditis
rs11581881
rs12485353
rs12781711
rs12336782
rs9313055
rs4385846
rs16918863
rs76973485
rs12189346
rs2730183
All
0.0
0.2
0.4
0.6
MR effect size for
'Gut microbiota abundance (genus Ruminococcaceae UCG013 id.11370)' on 'Ulcerative rectosigmoiditis'

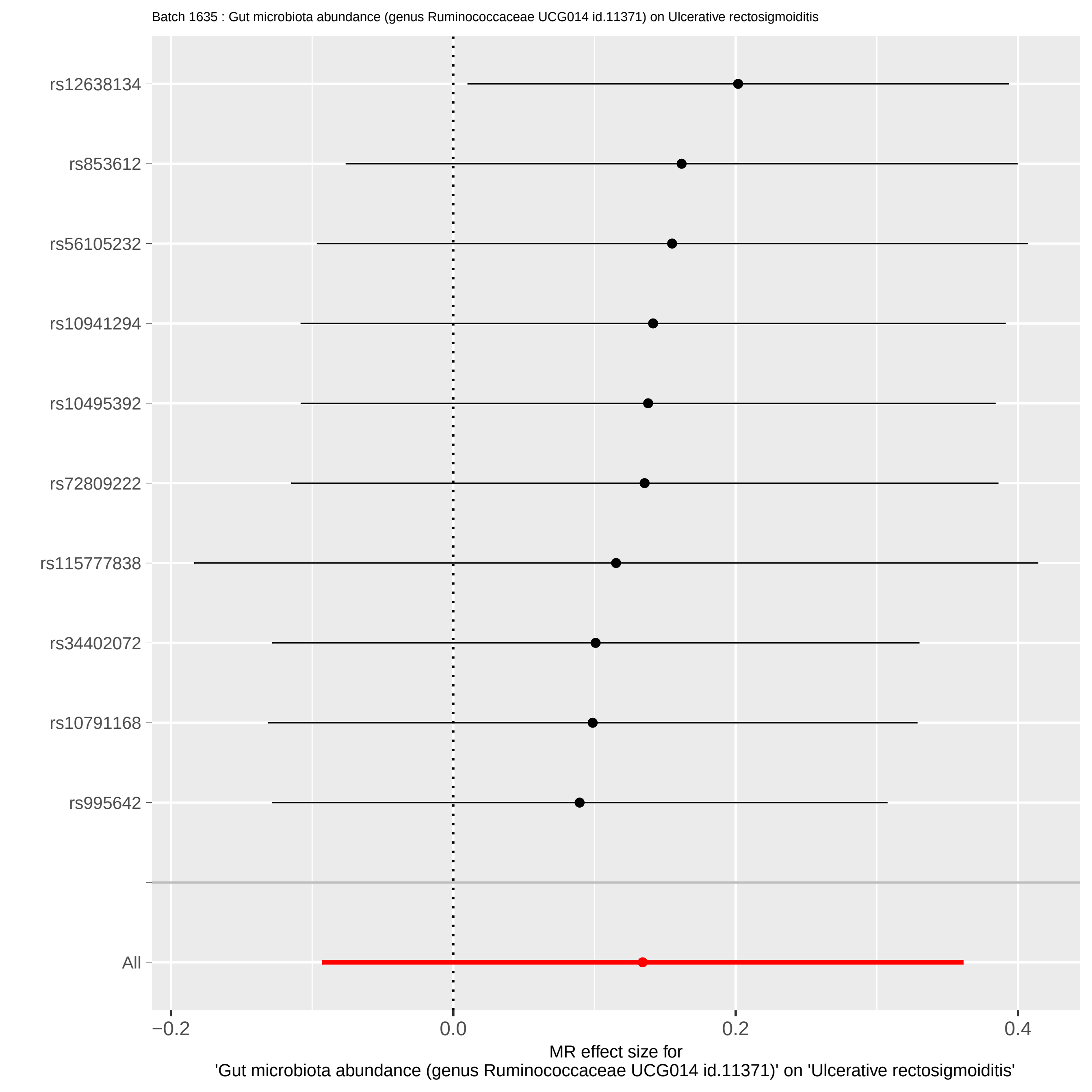
Batch 1635 : Gut microbiota abundance (genus Ruminococcaceae UCG014 id.11371) on Ulcerative rectosigmoiditis
rs12638134
rs853612
rs56105232
rs10941294
rs10495392
rs72809222
rs115777838
rs34402072
rs10791168
rs995642
All
−0.2
0.0
0.2
0.4
MR effect size for
'Gut microbiota abundance (genus Ruminococcaceae UCG014 id.11371)' on 'Ulcerative rectosigmoiditis'

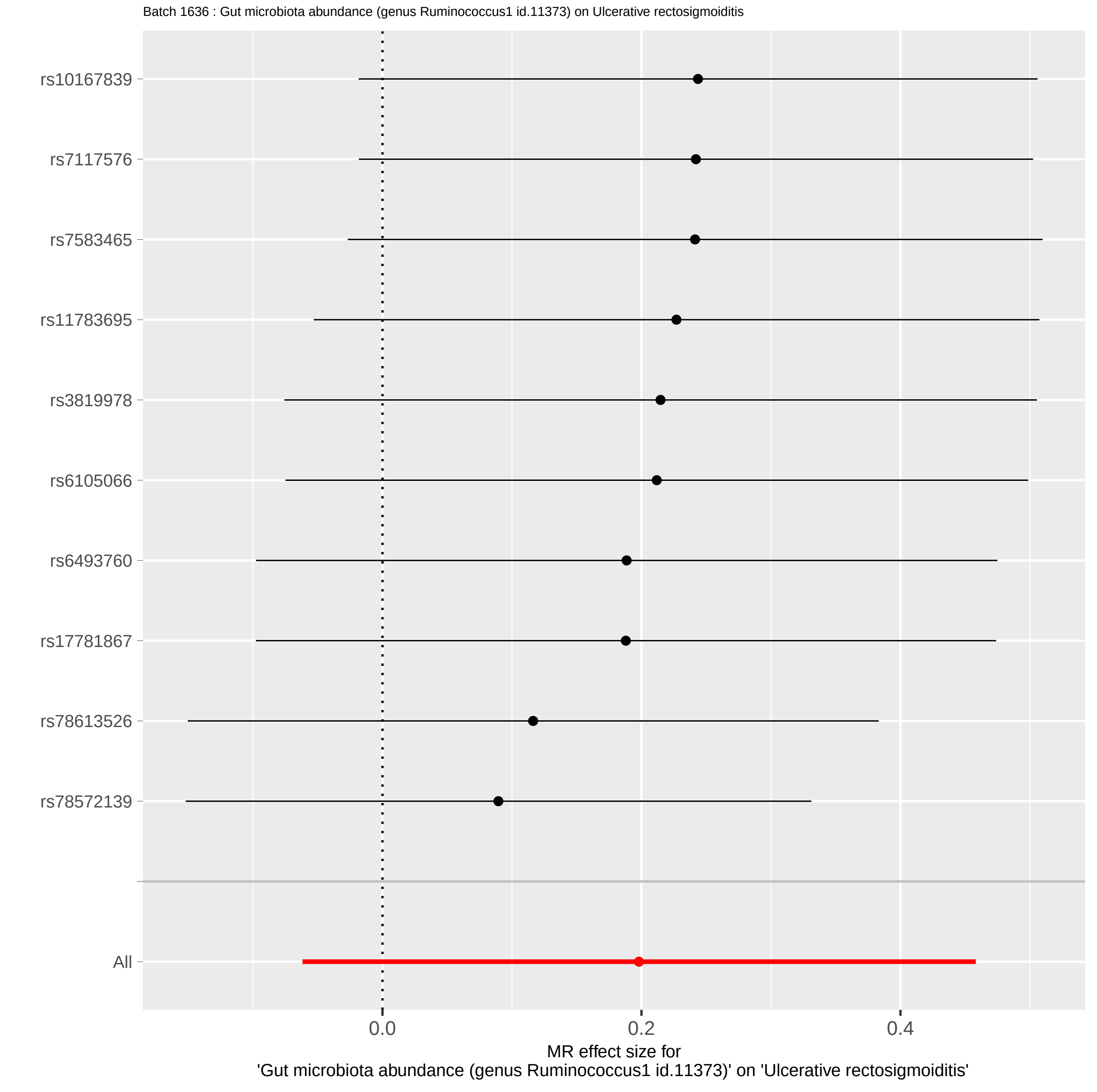

Batch 1636 : Gut microbiota abundance (genus Ruminococcus1 id.11373) on Ulcerative rectosigmoiditis
rs10167839
rs7117576
rs7583465
rs11783695
rs3819978
rs6105066
rs6493760
rs17781867
rs78613526
rs78572139
All
0.0
0.2
0.4
MR effect size for
'Gut microbiota abundance (genus Ruminococcus1 id.11373)' on 'Ulcerative rectosigmoiditis'

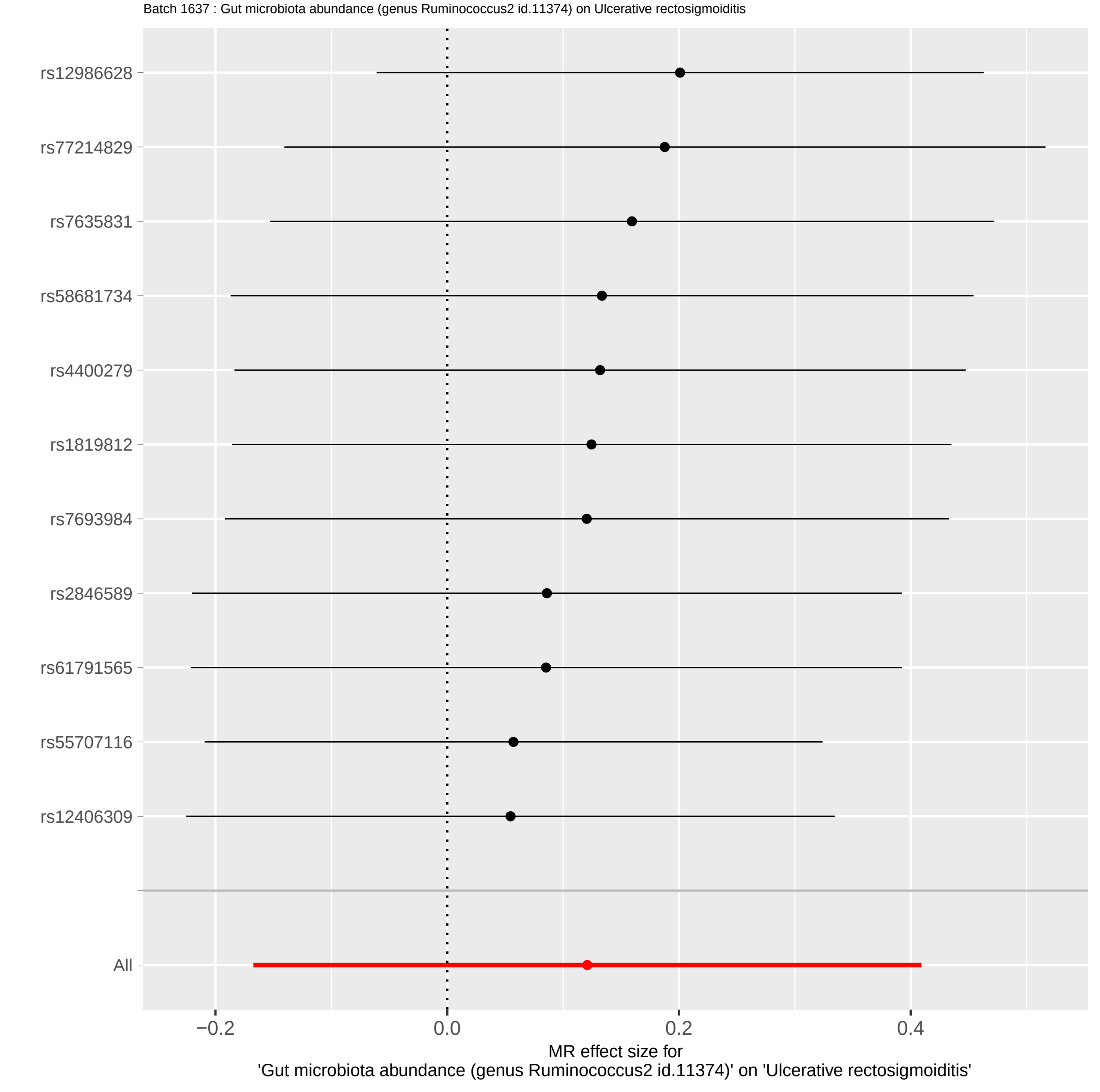
Batch 1637 : Gut microbiota abundance (genus Ruminococcus2 id.11374) on Ulcerative rectosigmoiditis
rs12986628
rs77214829
rs7635831
rs58681734
rs4400279
rs1819812
rs7693984
rs2846589
rs61791565
rs55707116
rs12406309
All
−0.2
0.0
0.2
0.4
MR effect size for
'Gut microbiota abundance (genus Ruminococcus2 id.11374)' on 'Ulcerative rectosigmoiditis'

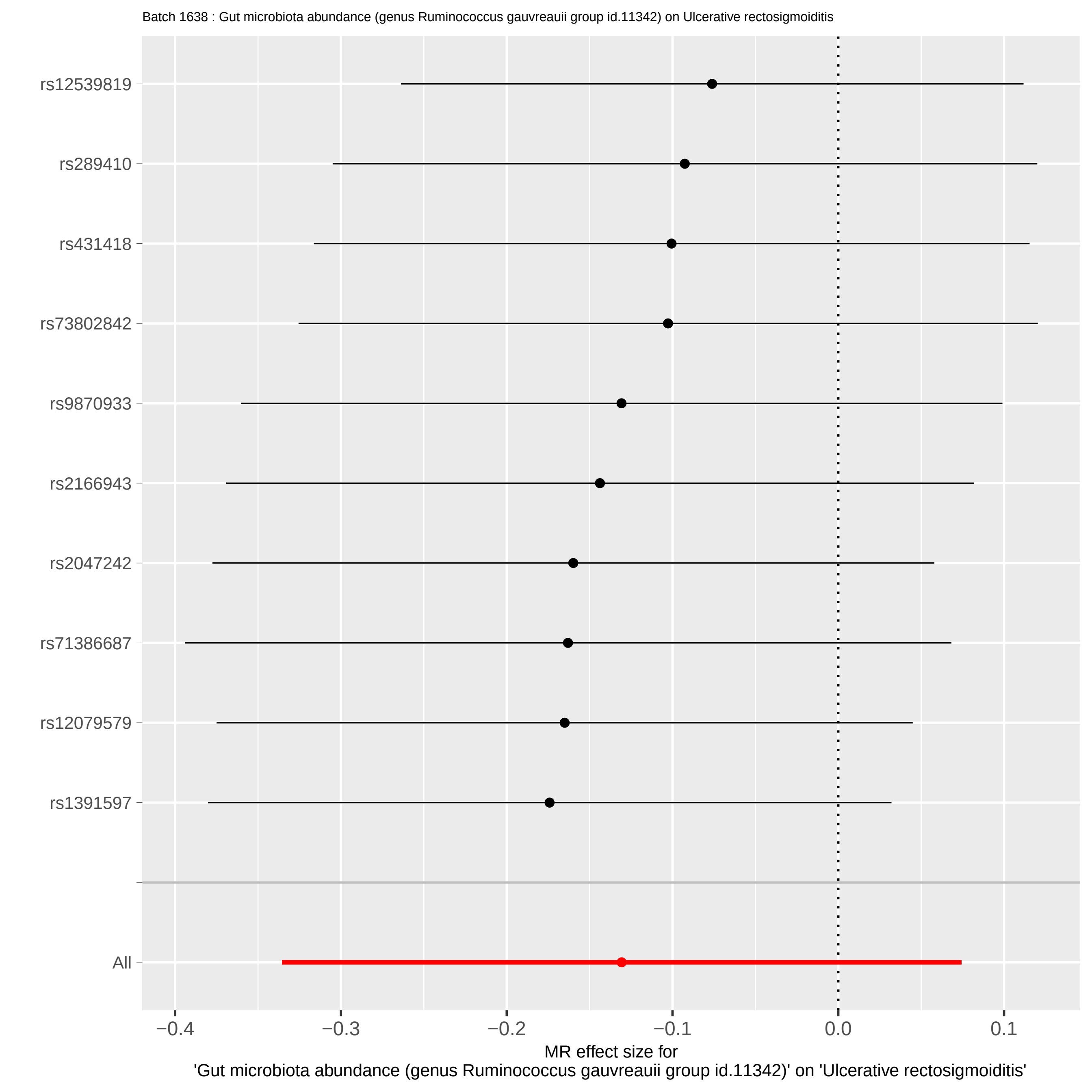

Batch 1638 : Gut microbiota abundance (genus Ruminococcus gauvreauii group id.11342) on Ulcerative rectosigmoiditis
rs12539819
rs289410
rs431418
rs73802842
rs9870933
rs2166943
rs2047242
rs71386687
rs12079579
rs1391597
All
−0.4
−0.3
−0.2
−0.1
0.0
0.1
MR effect size for
'Gut microbiota abundance (genus Ruminococcus gauvreauii group id.11342)' on 'Ulcerative rectosigmoiditis'

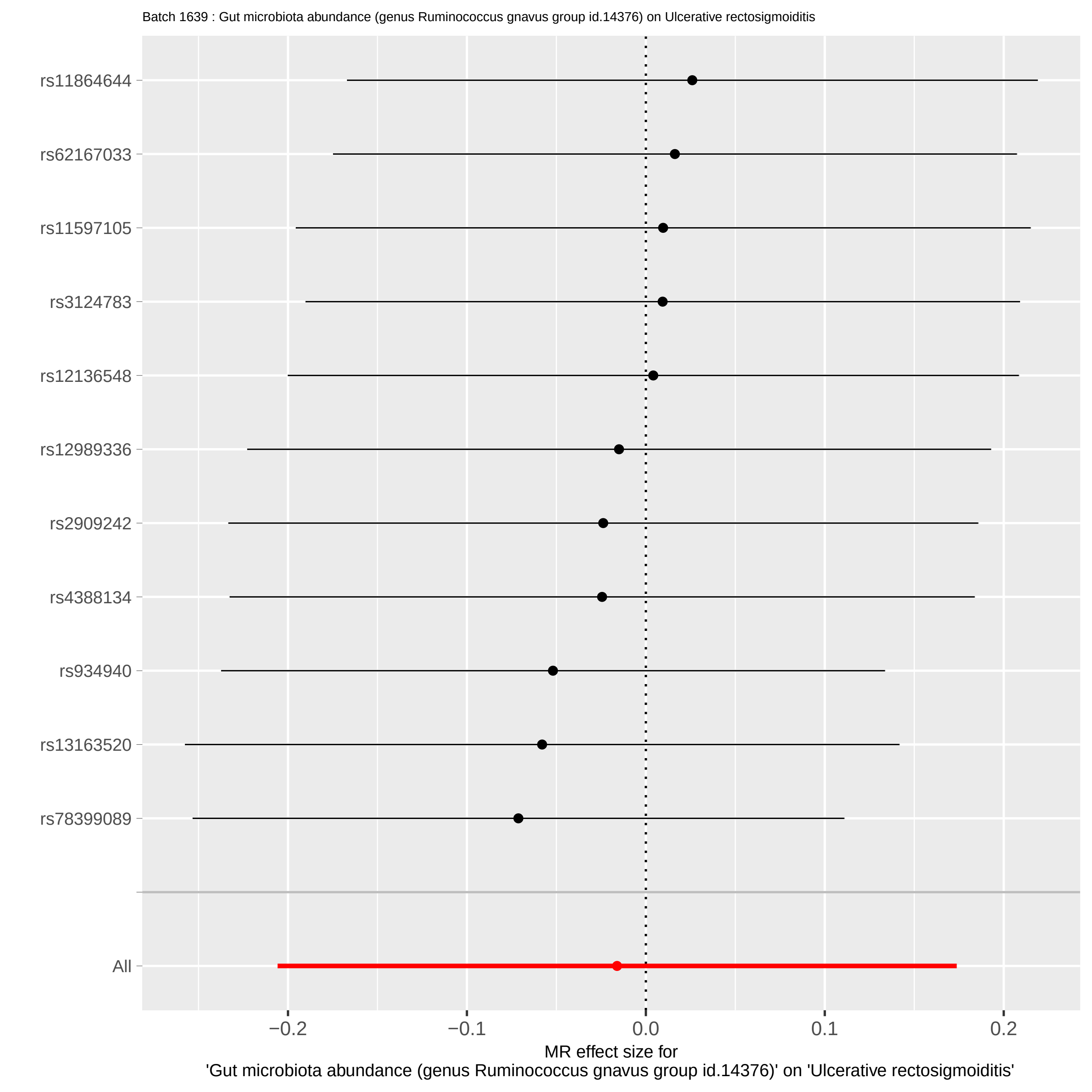

Batch 1639 : Gut microbiota abundance (genus Ruminococcus gnavus group id.14376) on Ulcerative rectosigmoiditis
rs11864644
rs62167033
rs11597105
rs3124783
rs12136548
rs12989336
rs2909242
rs4388134
rs934940
rs13163520
rs78399089
All
−0.2
−0.1
0.0
0.1
0.2
MR effect size for
'Gut microbiota abundance (genus Ruminococcus gnavus group id.14376)' on 'Ulcerative rectosigmoiditis'

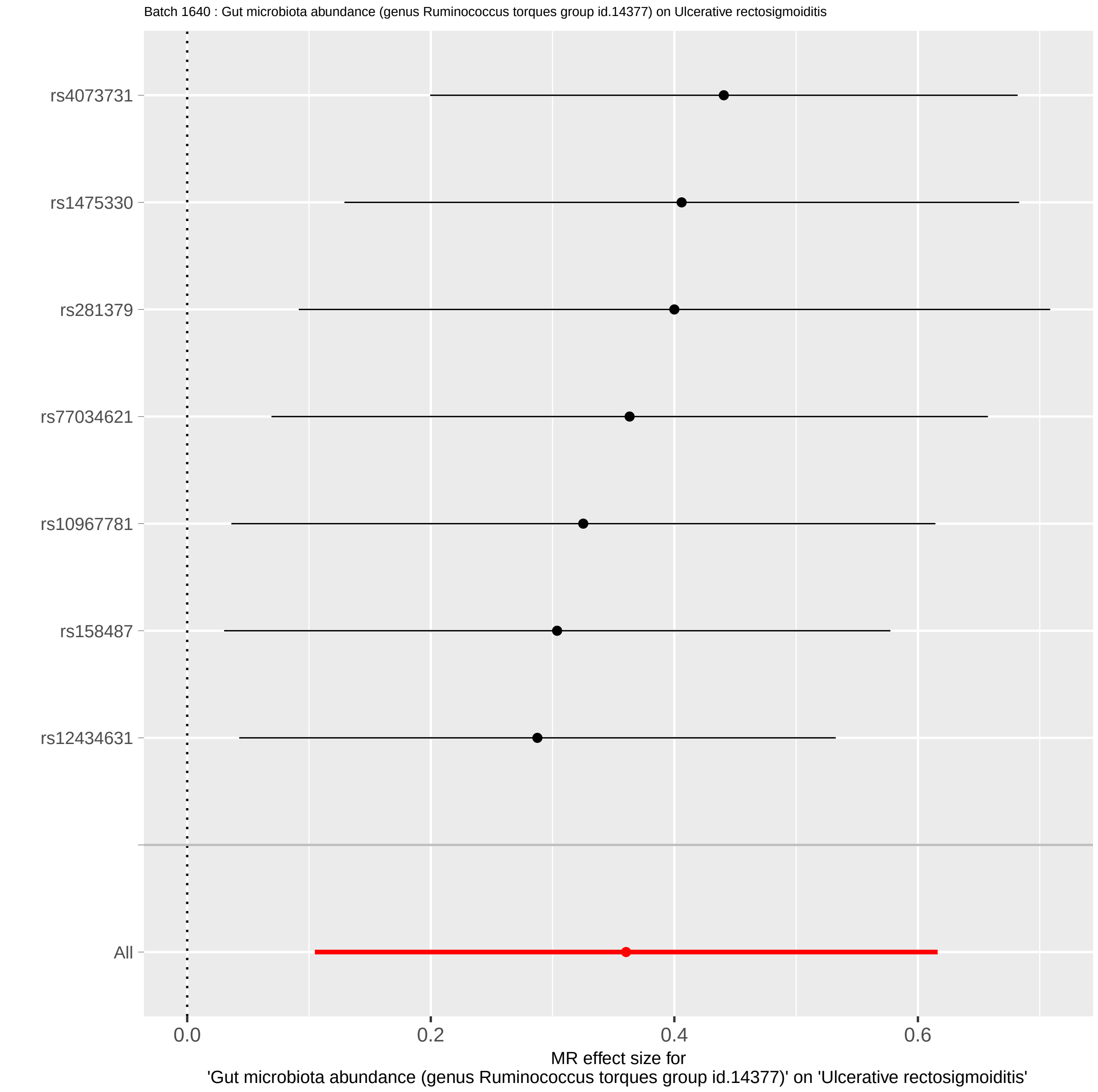

Batch 1640 : Gut microbiota abundance (genus Ruminococcus torques group id.14377) on Ulcerative rectosigmoiditis
rs4073731
rs1475330
rs281379
rs77034621
rs10967781
rs158487
rs12434631
All
0.0
0.2
0.4
0.6
MR effect size for
'Gut microbiota abundance (genus Ruminococcus torques group id.14377)' on 'Ulcerative rectosigmoiditis'

Batch 1641 : Gut microbiota abundance (genus Sellimonas id.14369) on Ulcerative rectosigmoiditis

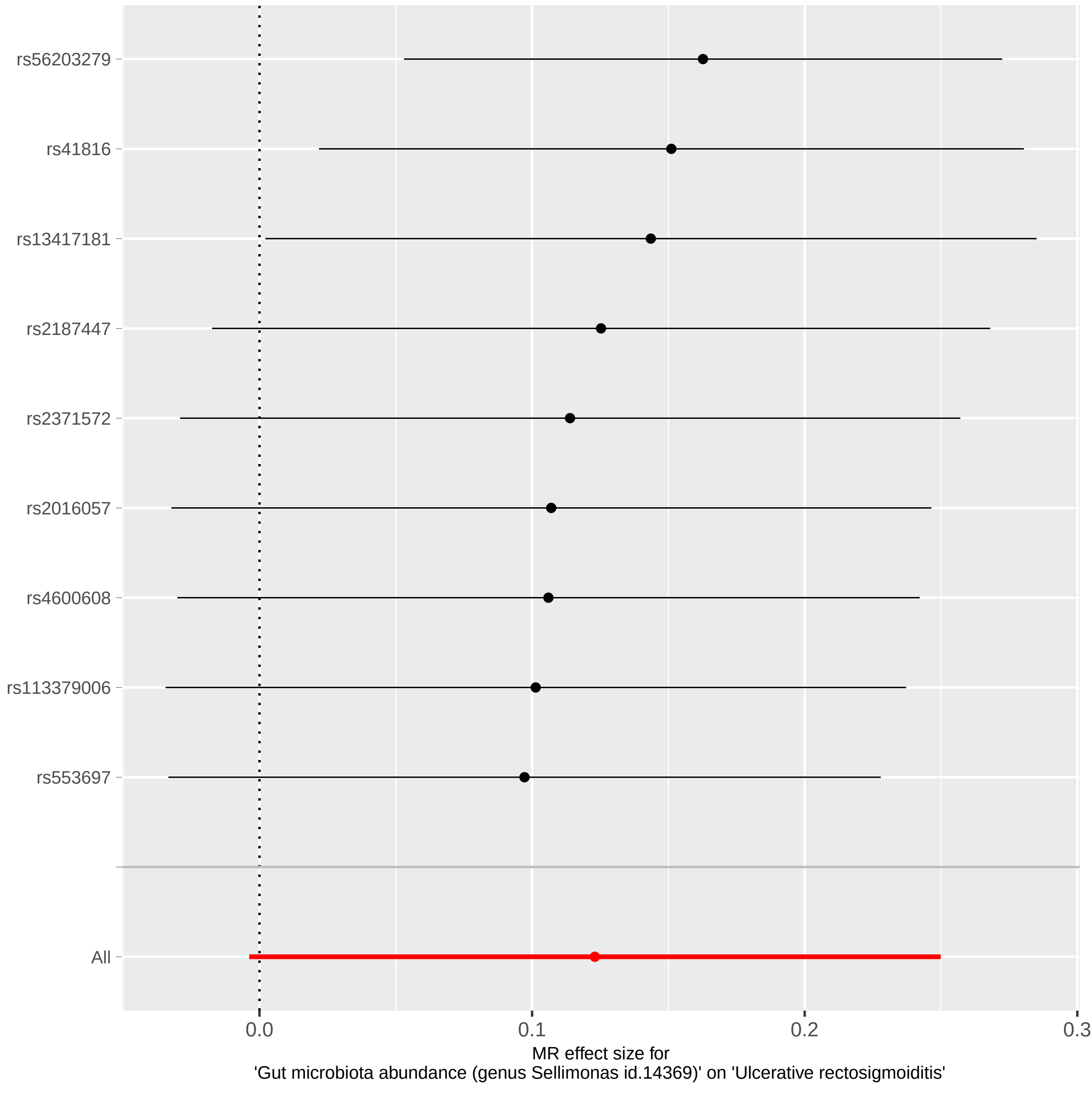

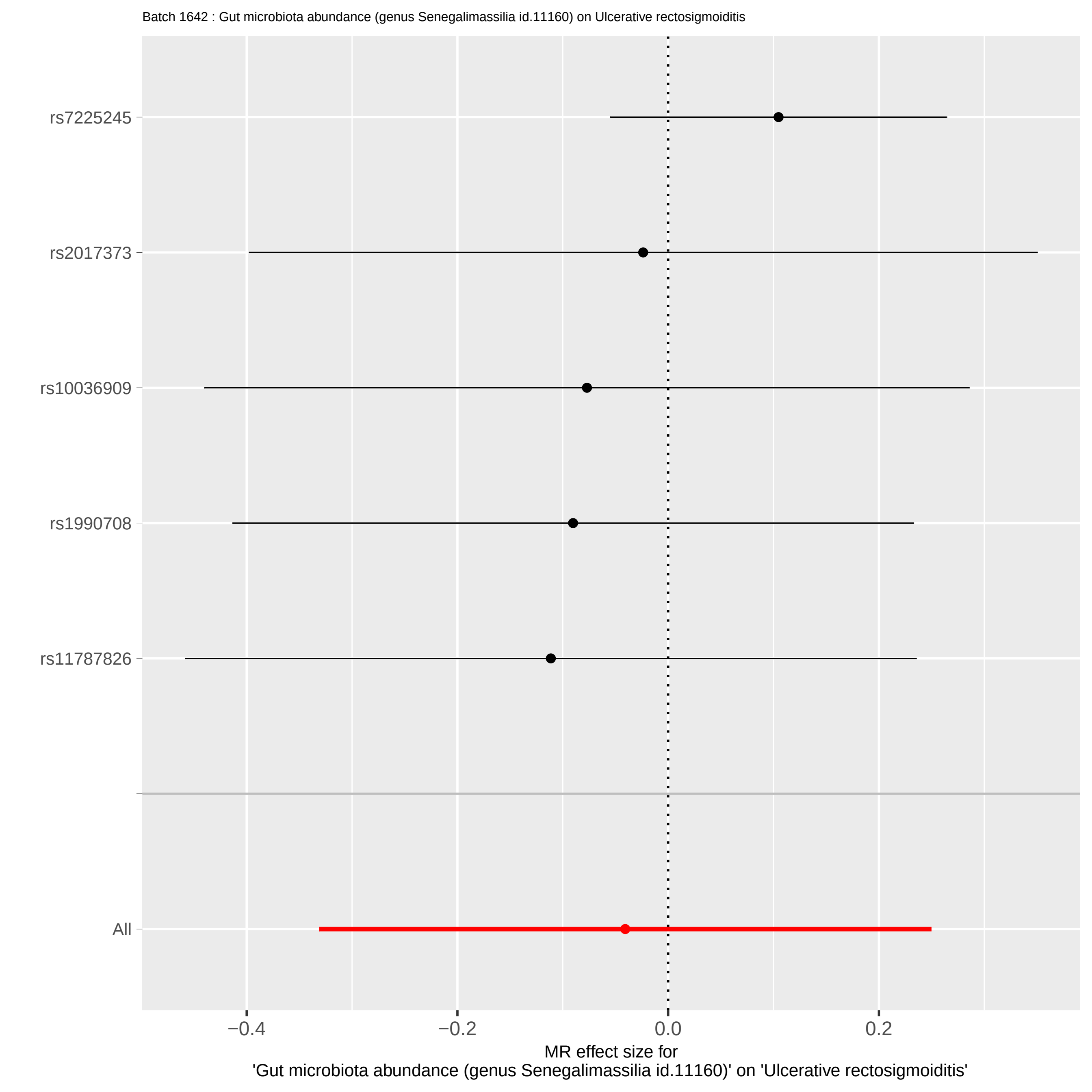

Batch 1642 : Gut microbiota abundance (genus Senegalimassilia id.11160) on Ulcerative rectosigmoiditis
rs7225245
rs2017373
rs10036909
rs1990708
rs11787826
All
−0.4
−0.2
0.0
0.2
MR effect size for
'Gut microbiota abundance (genus Senegalimassilia id.11160)' on 'Ulcerative rectosigmoiditis'

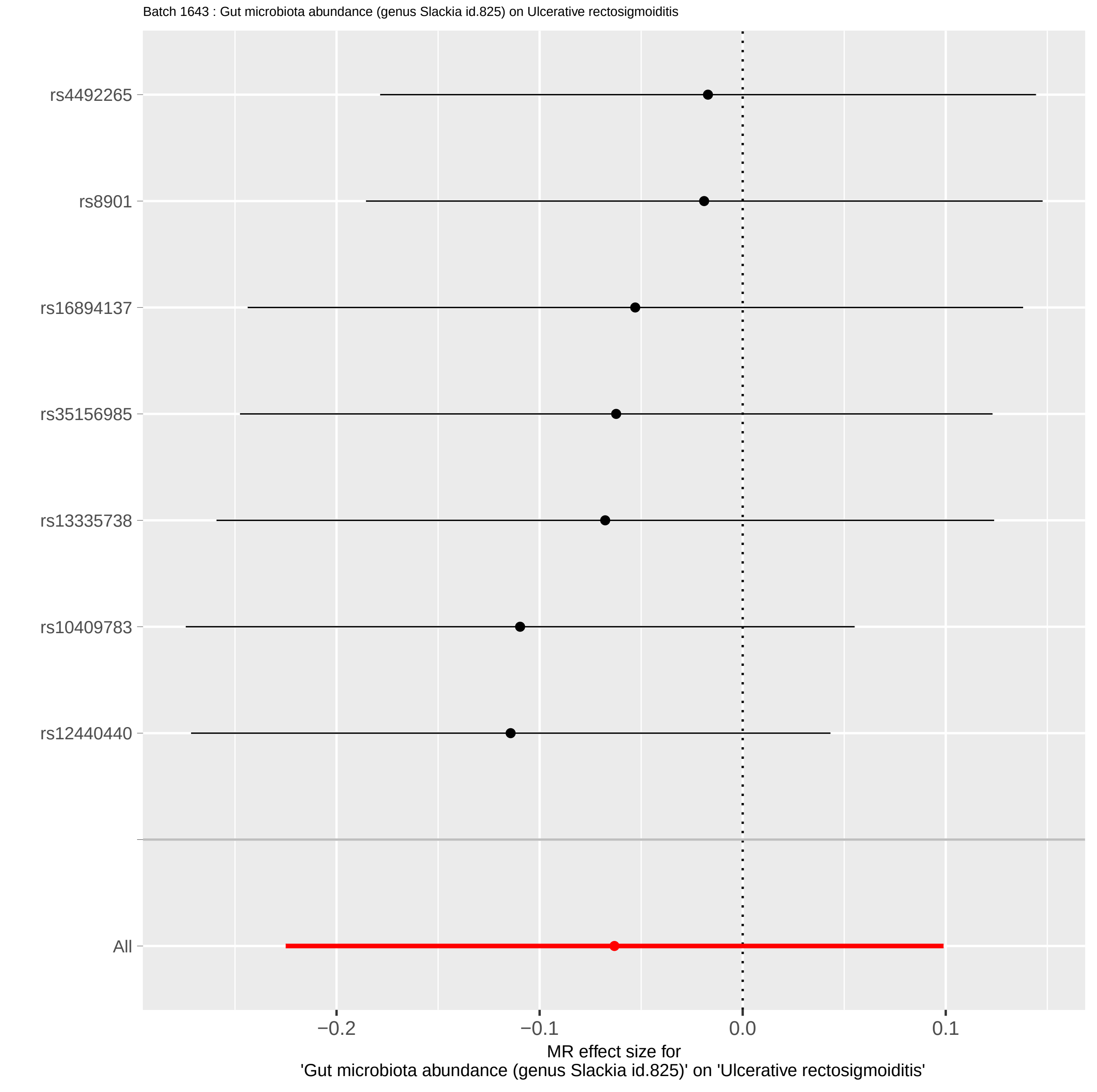

Batch 1643 : Gut microbiota abundance (genus Slackia id.825) on Ulcerative rectosigmoiditis
rs4492265
rs8901
rs16894137
rs35156985
rs13335738
rs10409783
rs12440440
All
−0.2
−0.1
0.0
0.1
MR effect size for
'Gut microbiota abundance (genus Slackia id.825)' on 'Ulcerative rectosigmoiditis'

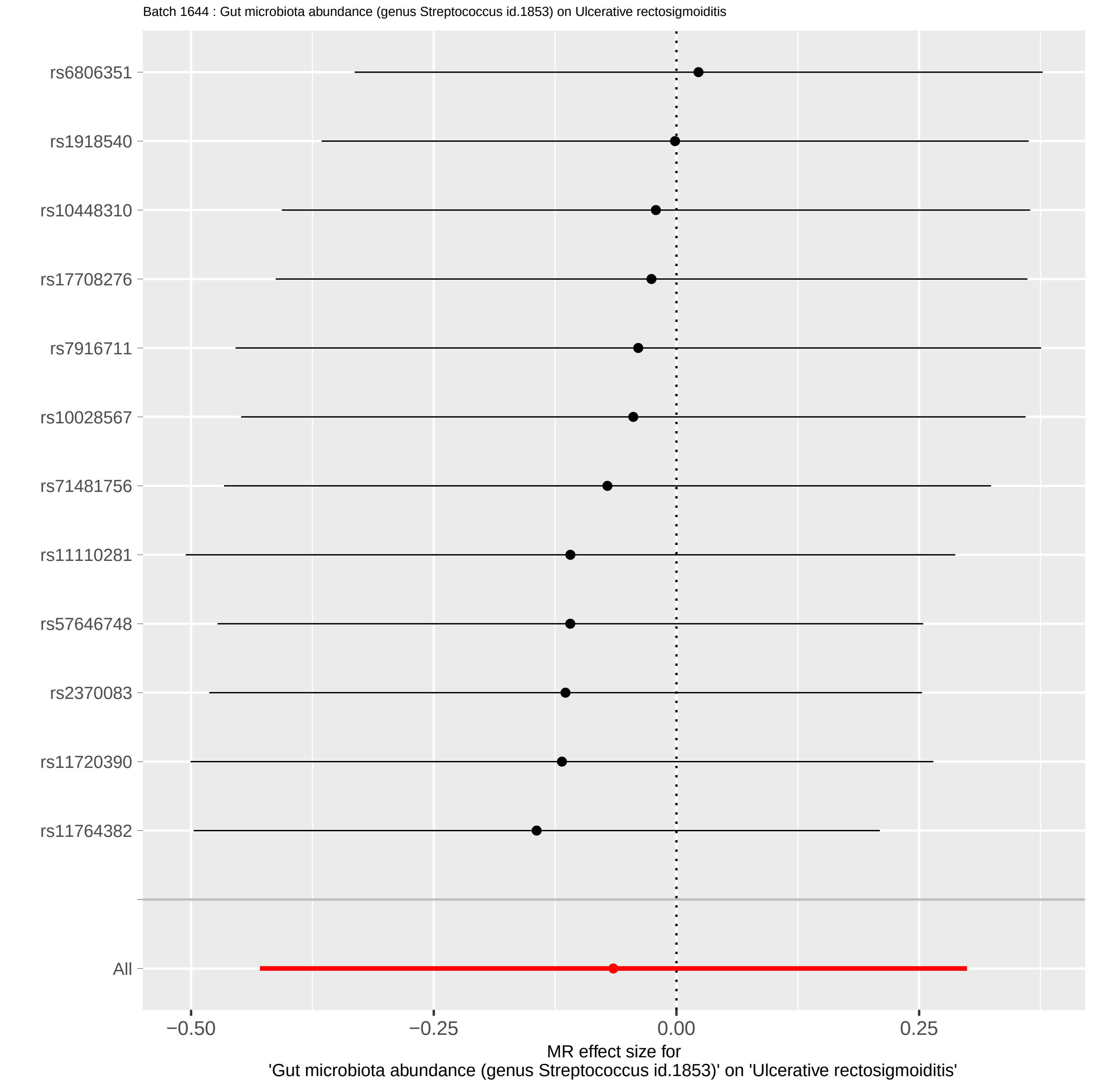

Batch 1644 : Gut microbiota abundance (genus Streptococcus id.1853) on Ulcerative rectosigmoiditis
rs6806351
rs1918540
rs10448310
rs17708276
rs7916711
rs10028567
rs71481756
rs11110281
rs57646748
rs2370083
rs11720390
rs11764382
All
−0.50
−0.25
0.00
0.25
MR effect size for
'Gut microbiota abundance (genus Streptococcus id.1853)' on 'Ulcerative rectosigmoiditis'

Batch 1645 : Gut microbiota abundance (genus Subdoligranulum id.2070) on Ulcerative rectosigmoiditis

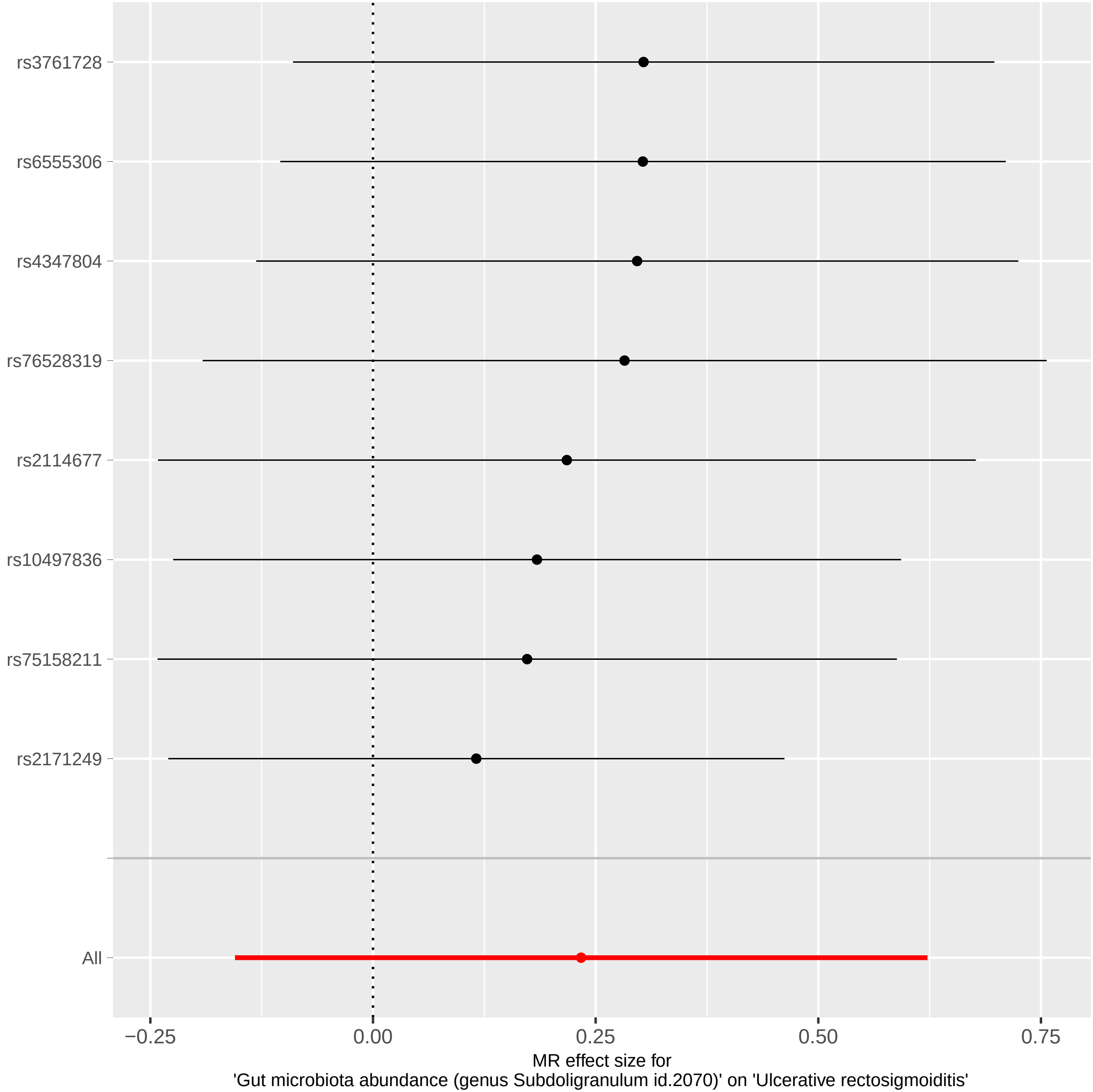

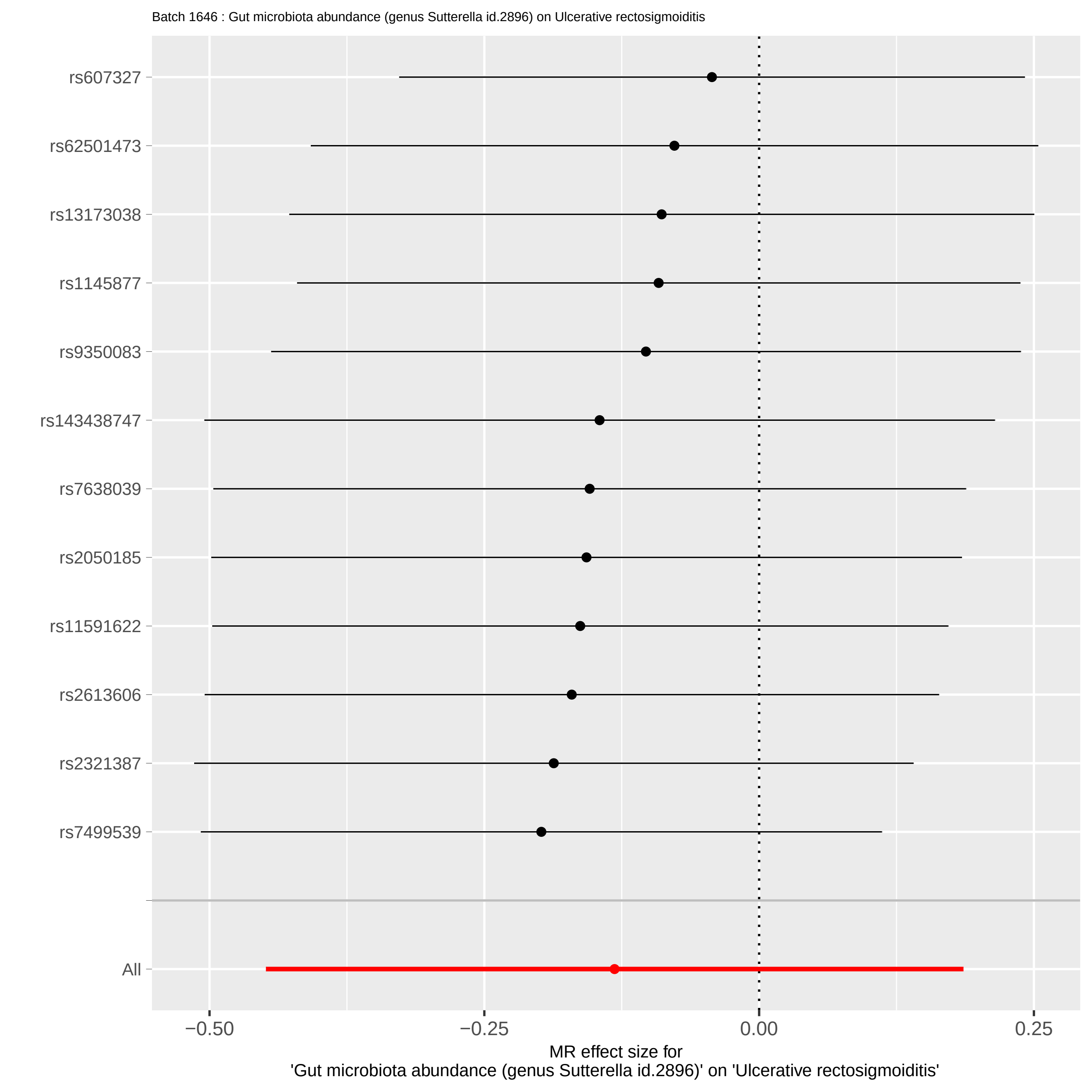
Batch 1646 : Gut microbiota abundance (genus Sutterella id.2896) on Ulcerative rectosigmoiditis
rs607327
rs62501473
rs13173038
rs1145877
rs9350083
rs143438747
rs7638039
rs2050185
rs11591622
rs2613606
rs2321387
rs7499539
All
−0.50
−0.25
0.00
0.25
MR effect size for
'Gut microbiota abundance (genus Sutterella id.2896)' on 'Ulcerative rectosigmoiditis'

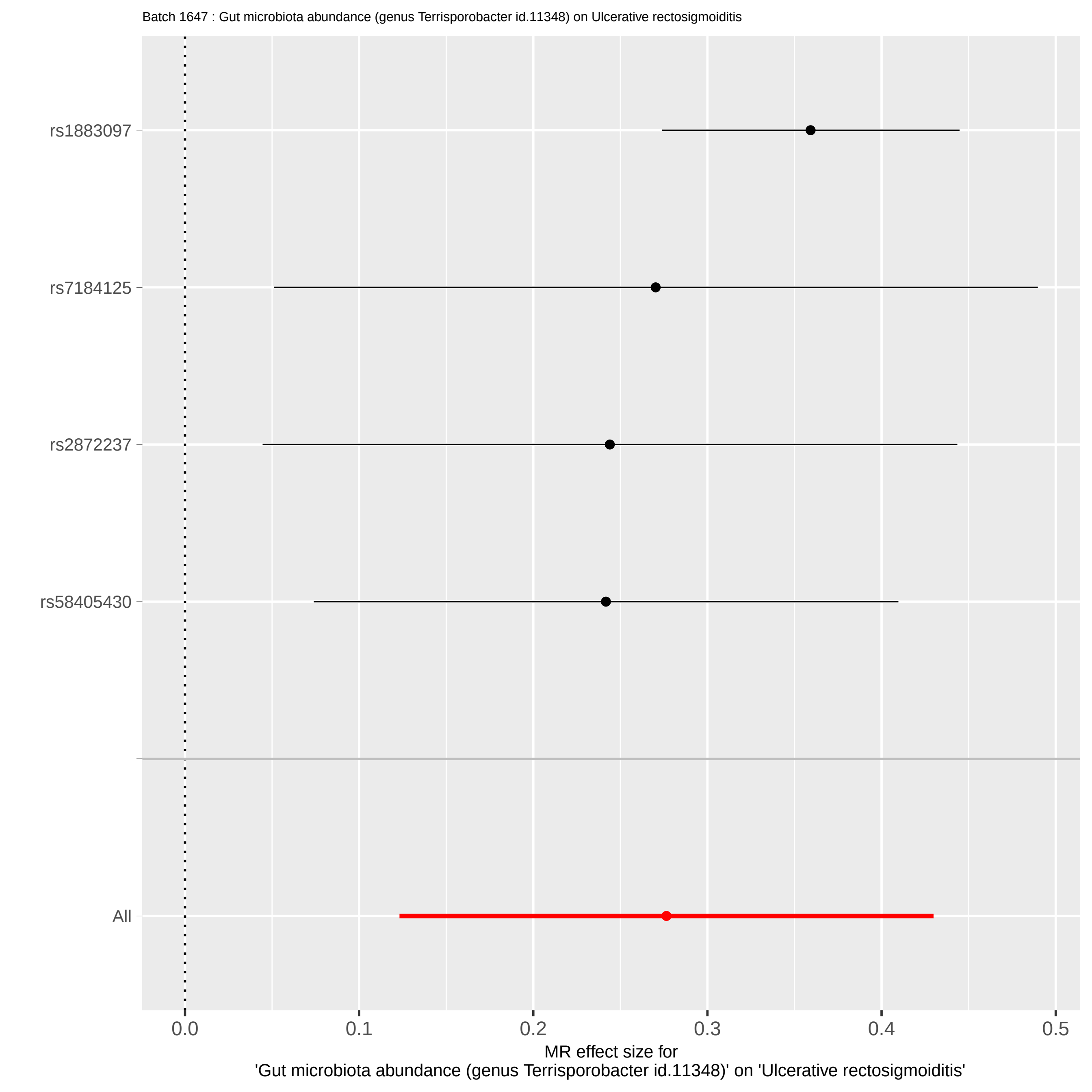
Batch 1647 : Gut microbiota abundance (genus Terrisporobacter id.11348) on Ulcerative rectosigmoiditis
rs1883097
rs7184125
rs2872237
rs58405430
All
0.0
0.1
0.2
0.3
0.4
0.5
MR effect size for
'Gut microbiota abundance (genus Terrisporobacter id.11348)' on 'Ulcerative rectosigmoiditis'

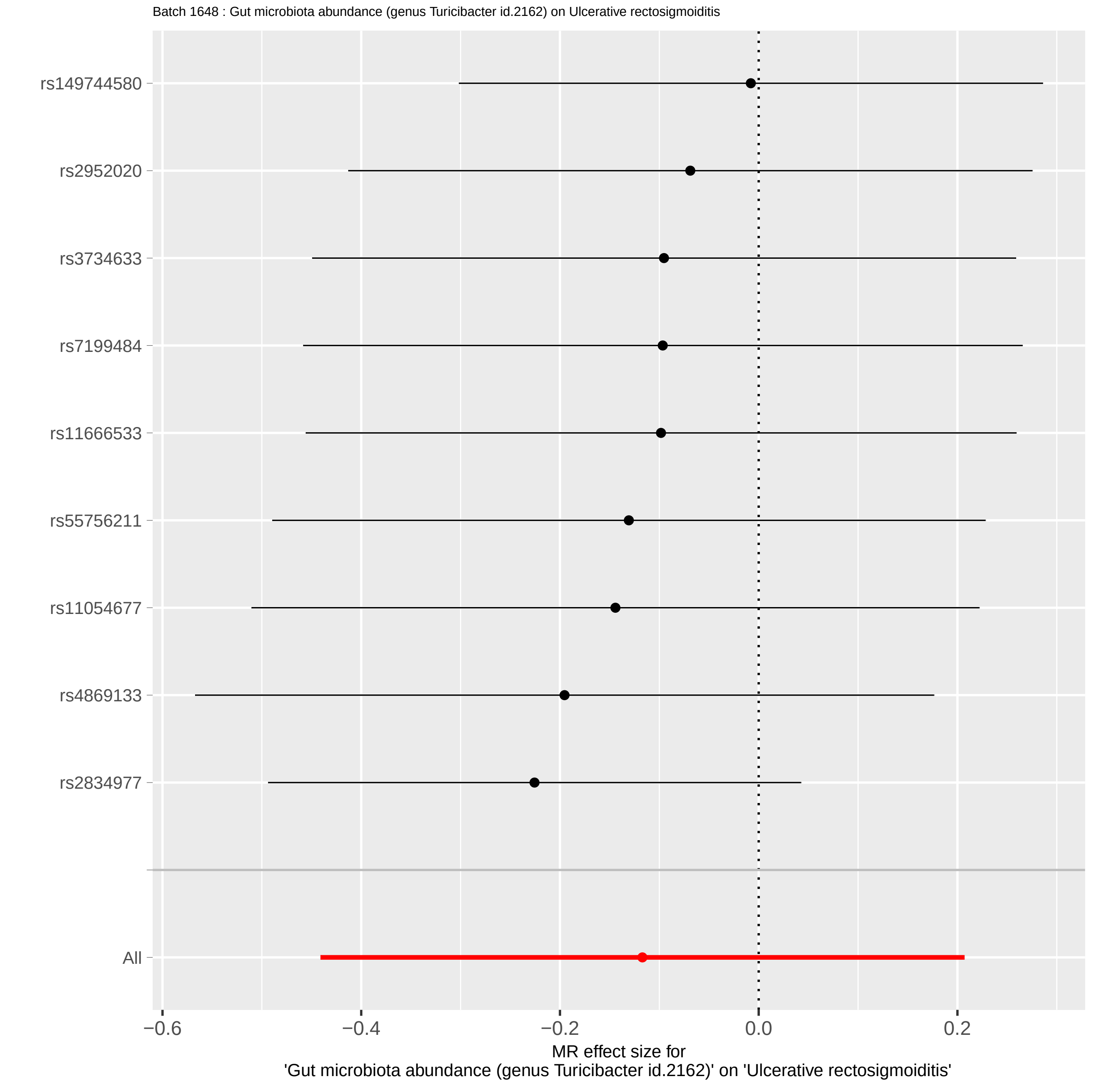

Batch 1648 : Gut microbiota abundance (genus Turicibacter id.2162) on Ulcerative rectosigmoiditis
rs149744580
rs2952020
rs3734633
rs7199484
rs11666533
rs55756211
rs11054677
rs4869133
rs2834977
All
−0.6
−0.4
−0.2
0.0
0.2
MR effect size for
'Gut microbiota abundance (genus Turicibacter id.2162)' on 'Ulcerative rectosigmoiditis'

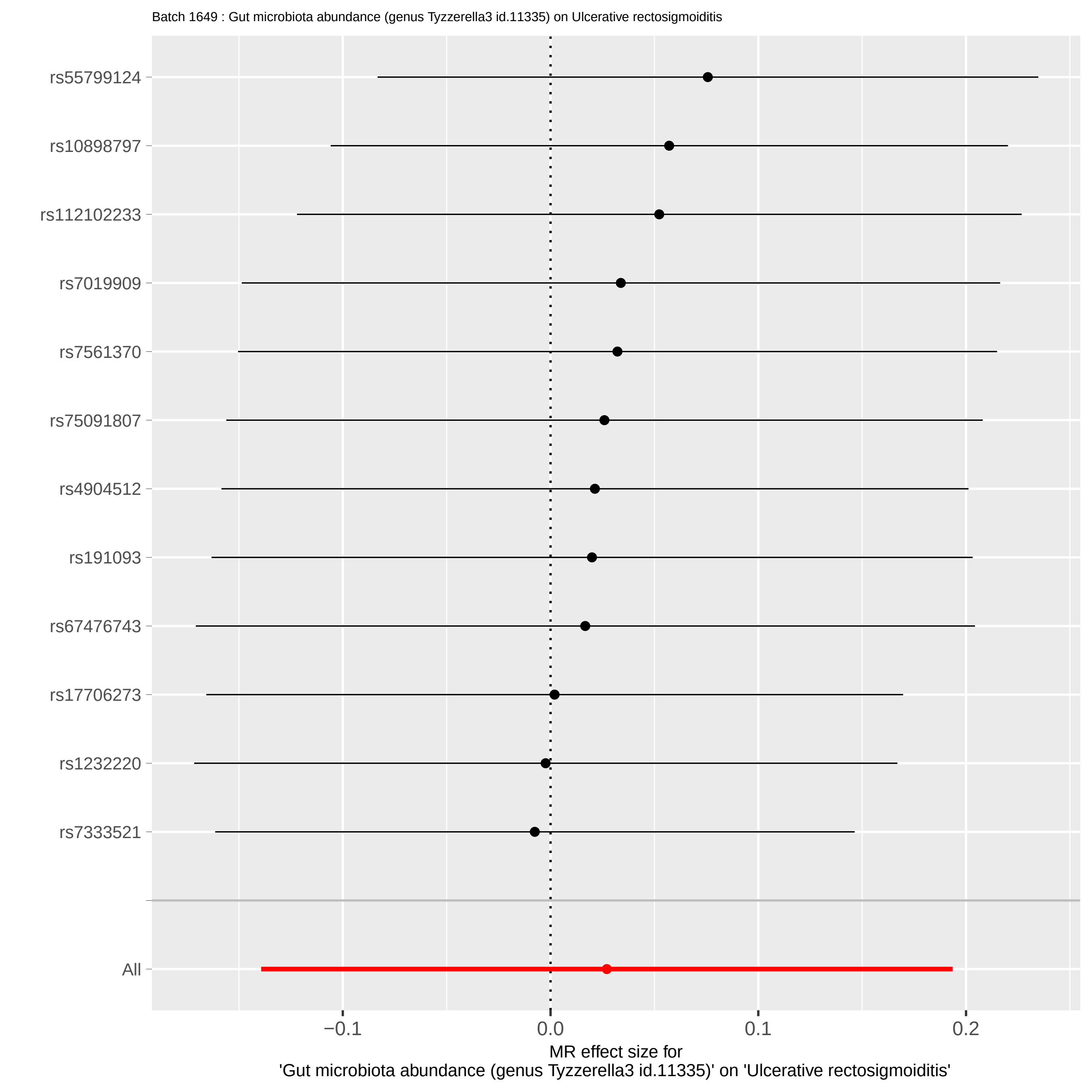

Batch 1649 : Gut microbiota abundance (genus Tyzzerella3 id.11335) on Ulcerative rectosigmoiditis
rs55799124
rs10898797
rs112102233
rs7019909
rs7561370
rs75091807
rs4904512
rs191093
rs67476743
rs17706273
rs1232220
rs7333521
All
−0.1
0.0
0.1
0.2
MR effect size for
'Gut microbiota abundance (genus Tyzzerella3 id.11335)' on 'Ulcerative rectosigmoiditis'

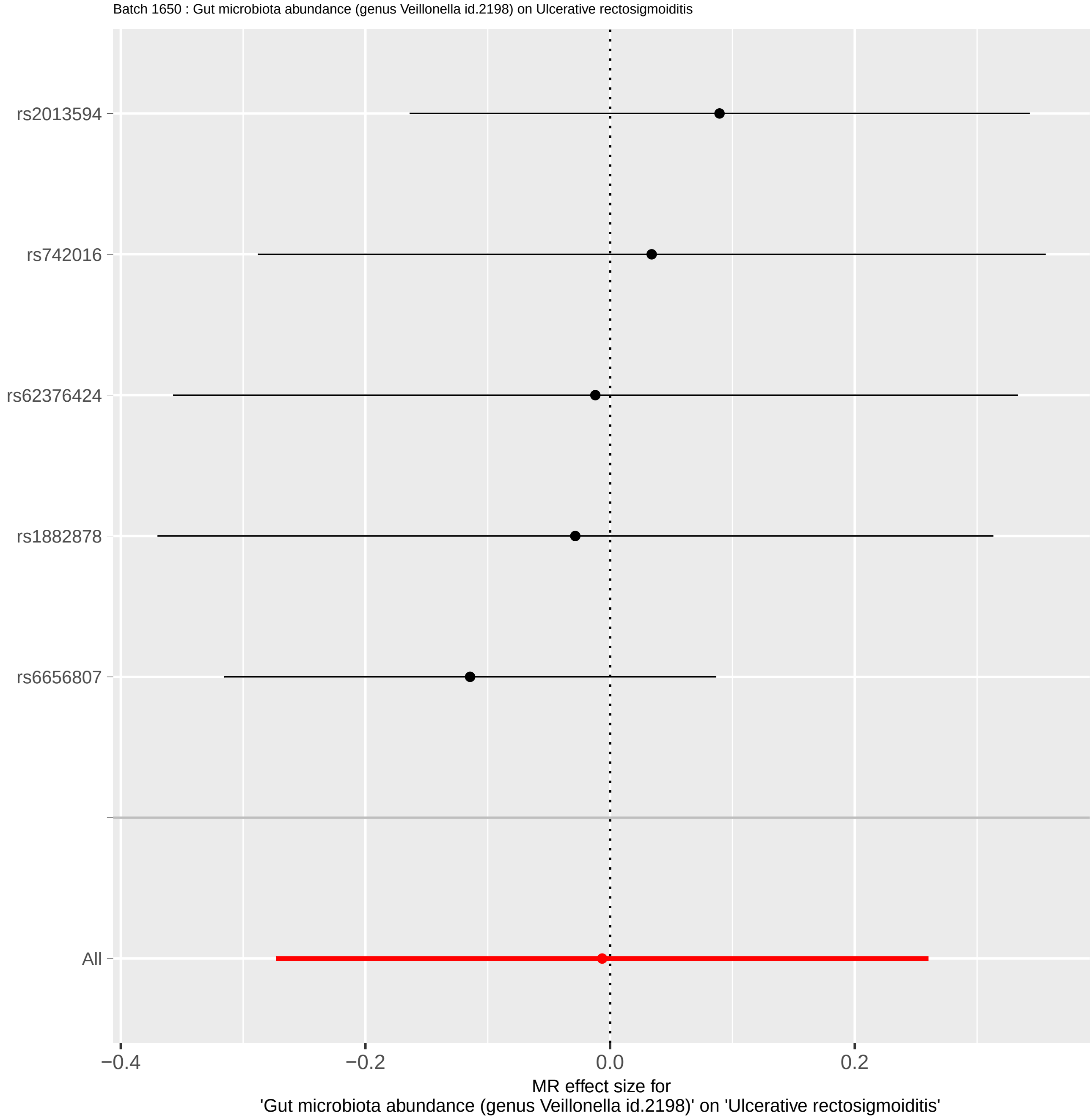

Batch 1650 : Gut microbiota abundance (genus Veillonella id.2198) on Ulcerative rectosigmoiditis
rs2013594
rs742016
rs62376424
rs1882878
rs6656807
All
−0.4
−0.2
0.0
0.2
MR effect size for
'Gut microbiota abundance (genus Veillonella id.2198)' on 'Ulcerative rectosigmoiditis'
