# Supplementary material for: Exploring the intestinal ecosystem: from gut microbiota to associations with subtypes of inflammatory bowel disease
Source: Front Cell Infect Microbiol. 2024 Jan 4;13:1304858. doi: 10.3389/fcimb.2023.1304858 (PMC10794348; doi:10.3389/fcimb.2023.1304858)

Batch 1 : Gut microbiota abundance (family Acidaminococcaceae id.2166) on Crohn's disease of large intestine

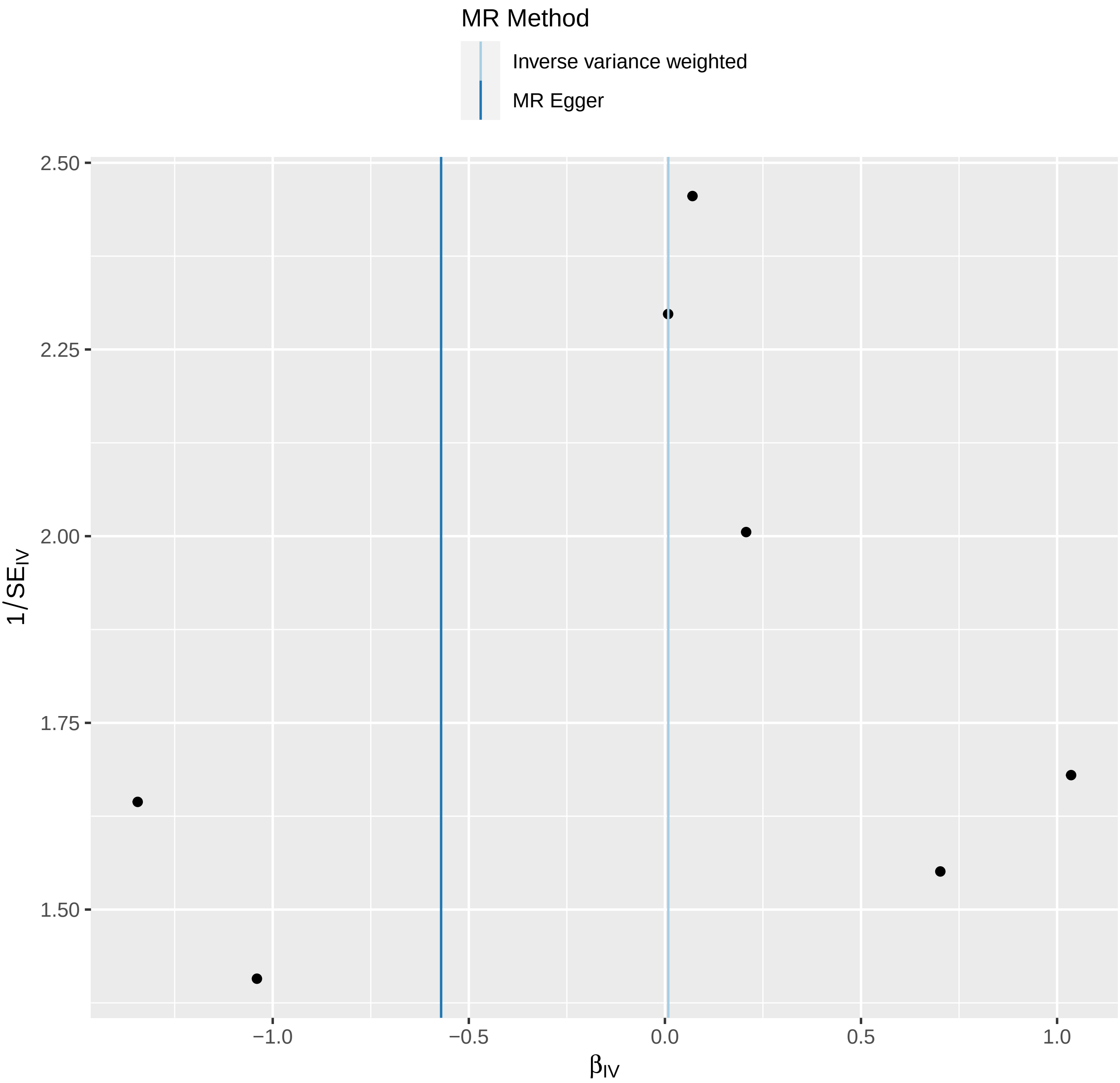

Batch 2 : Gut microbiota abundance (family Actinomycetaceae id.421) on Crohn's disease of large intestine

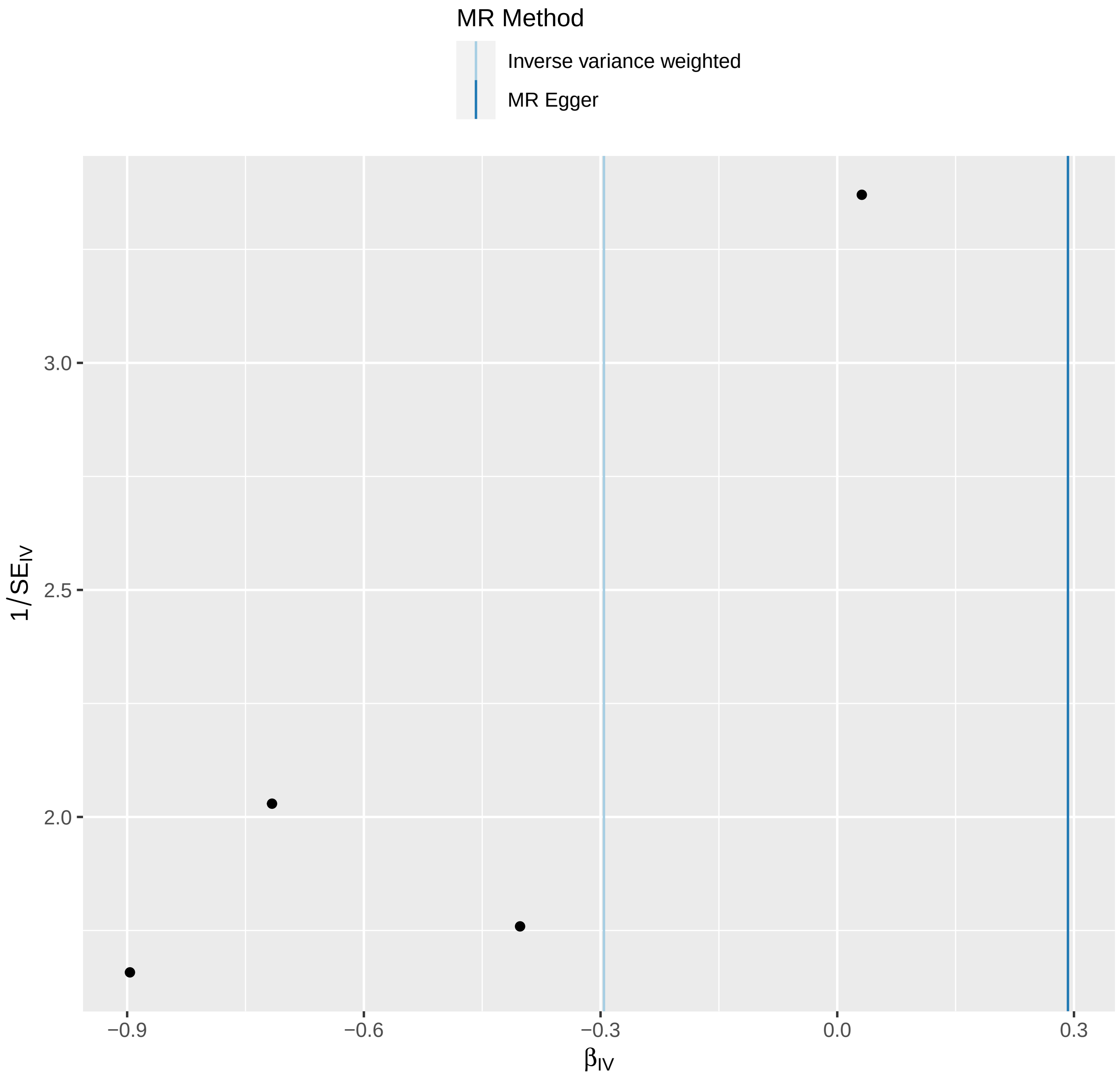

Batch 3 : Gut microbiota abundance (family Alcaligenaceae id.2875) on Crohn's disease of large intestine

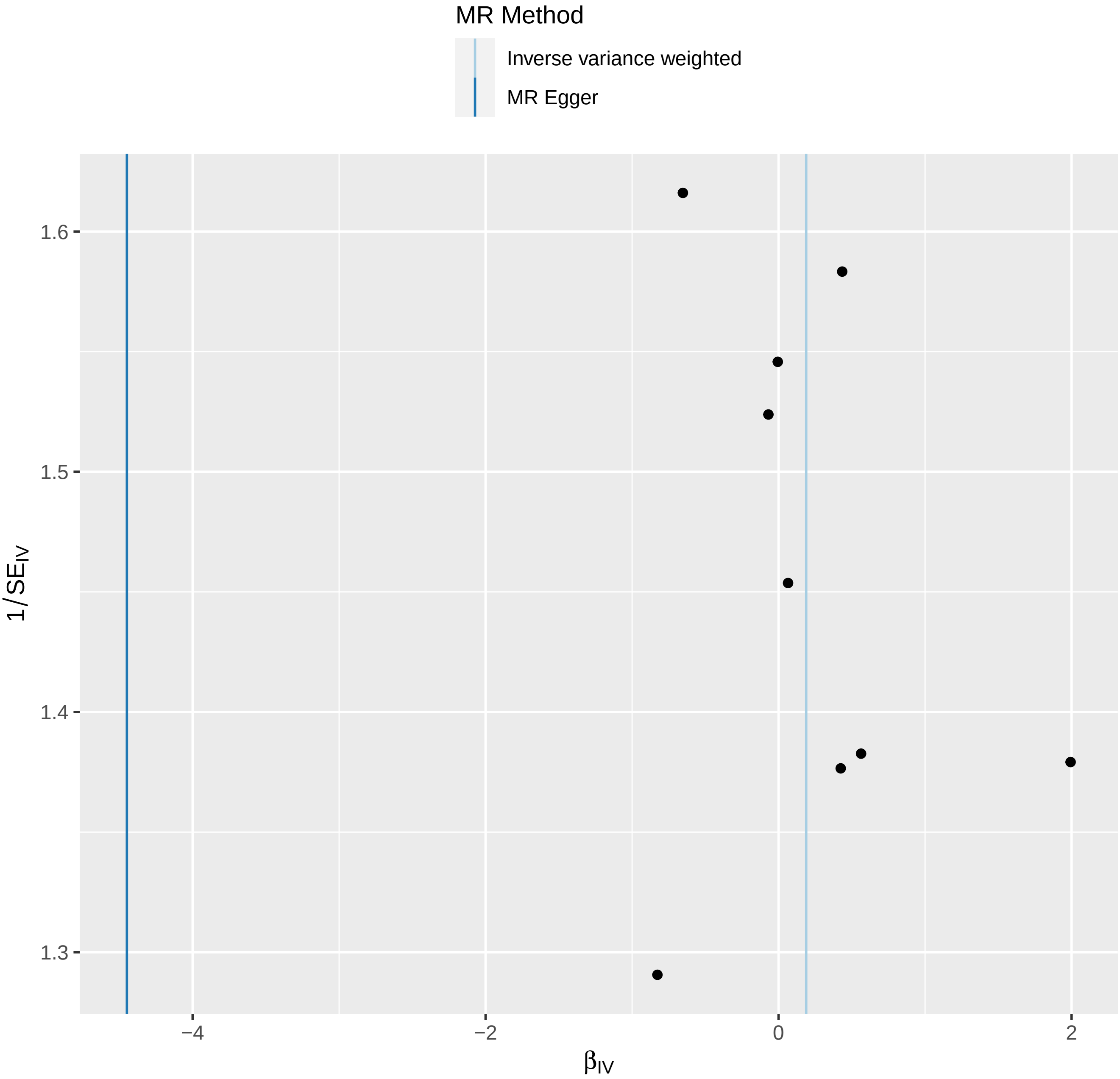

Batch 4 : Gut microbiota abundance (family Bacteroidaceae id.917) on Crohn's disease of large intestine

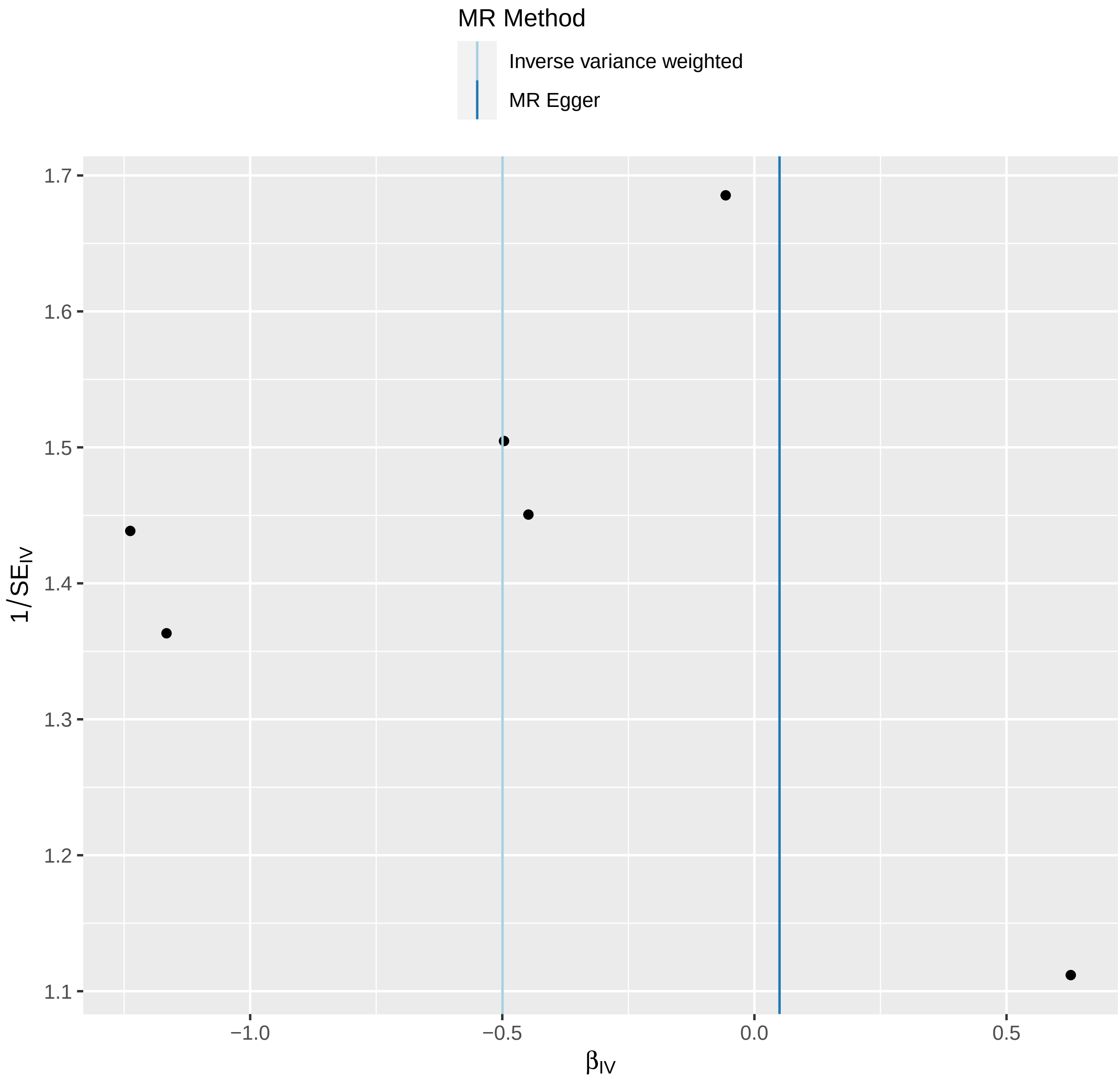

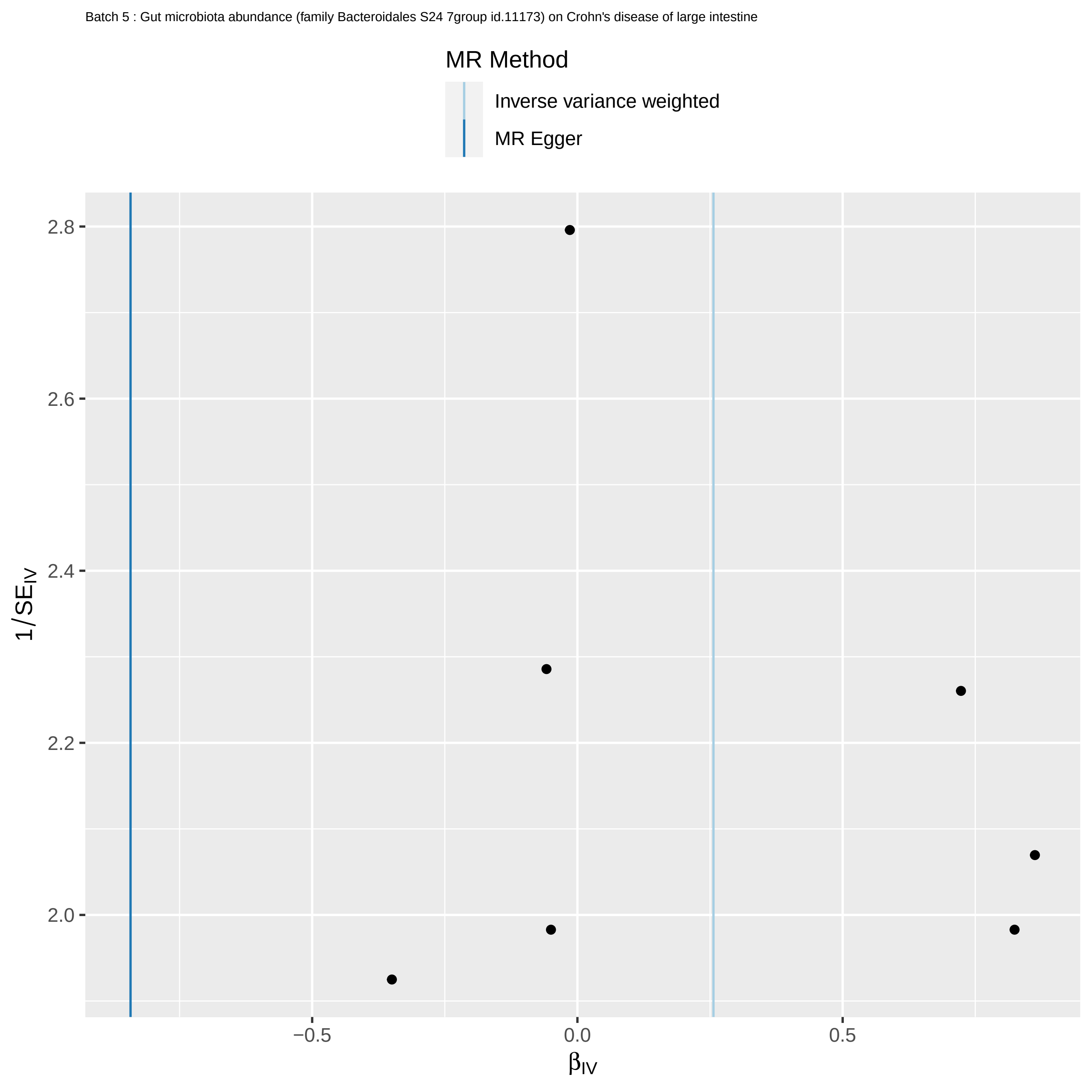

Batch 5 : Gut microbiota abundance (family Bacteroidales S24 7group id.11173) on Crohn's disease of large intestine
MR Method
Inverse variance weighted
MR Egger
2.8
2.6
2.4
2.2
2.0
1/SE_IV
−0.5
0.0
0.5
β_IV

Batch 6 : Gut microbiota abundance (family Bifidobacteriaceae id.433) on Crohn's disease of large intestine

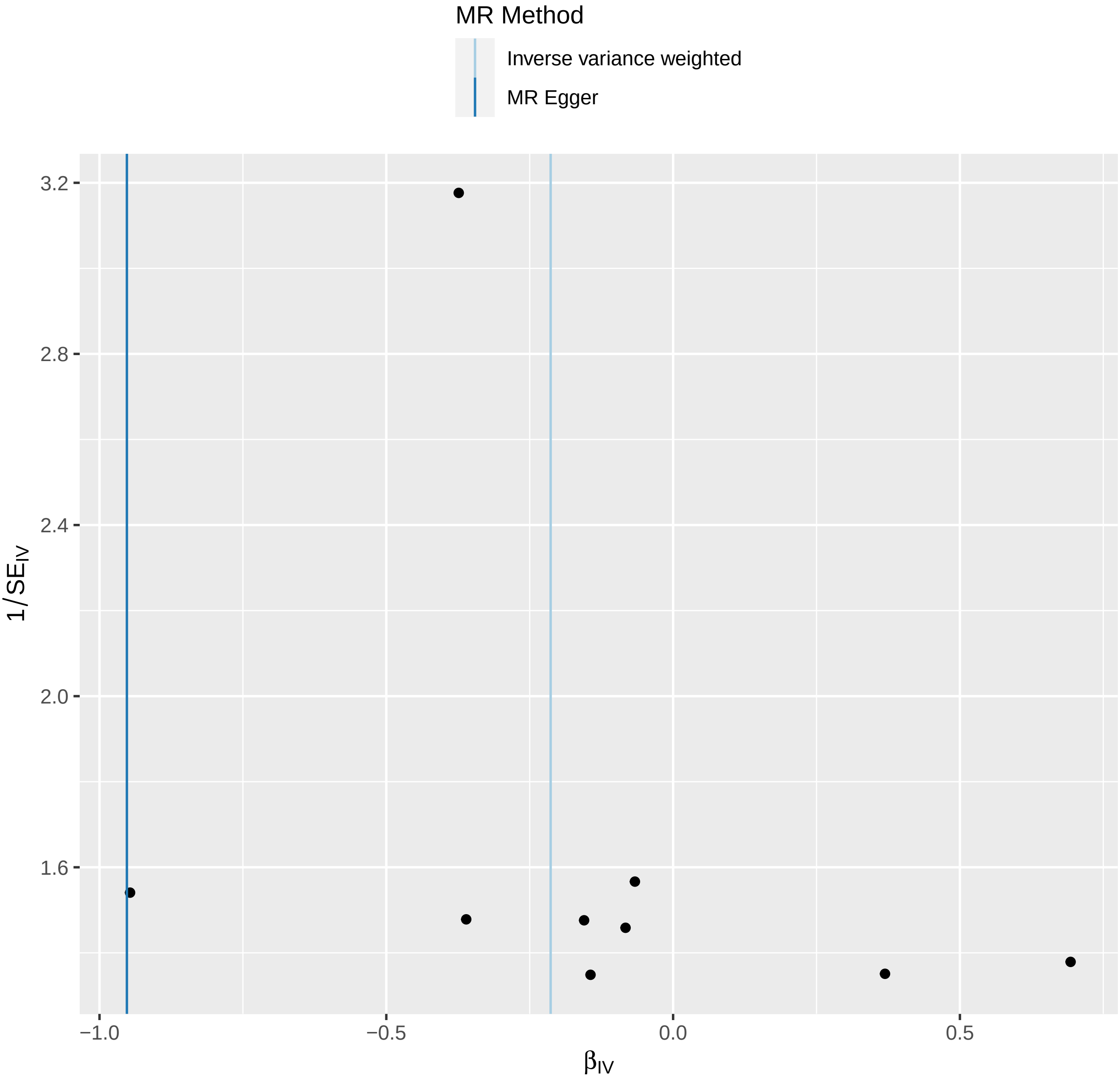

Batch 8 : Gut microbiota abundance (family Clostridiaceae1 id.1869) on Crohn's disease of large intestine

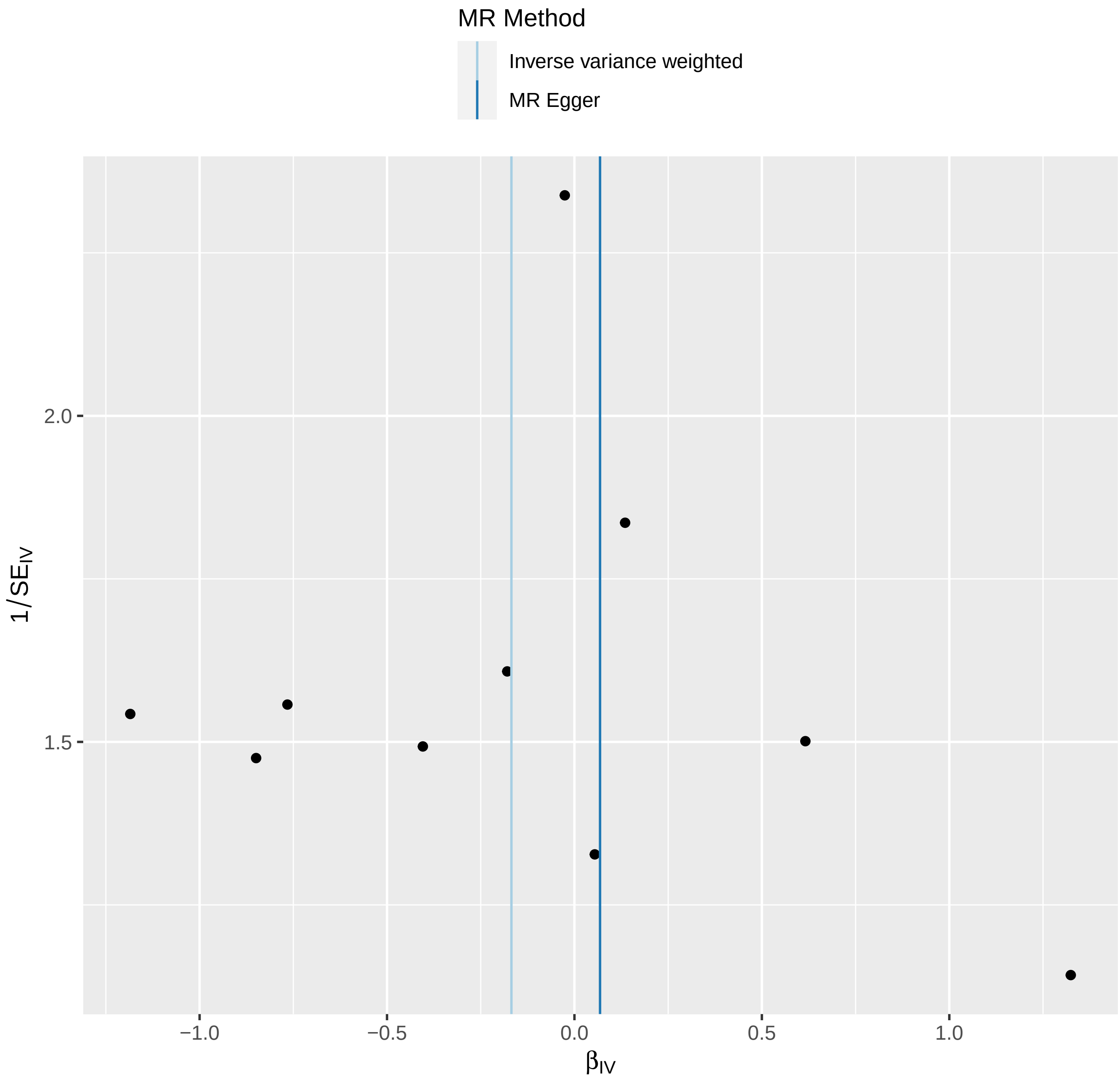

Batch 9 : Gut microbiota abundance (family Clostridiales vadin BB60 group id.11286) on Crohn's disease of large intestine

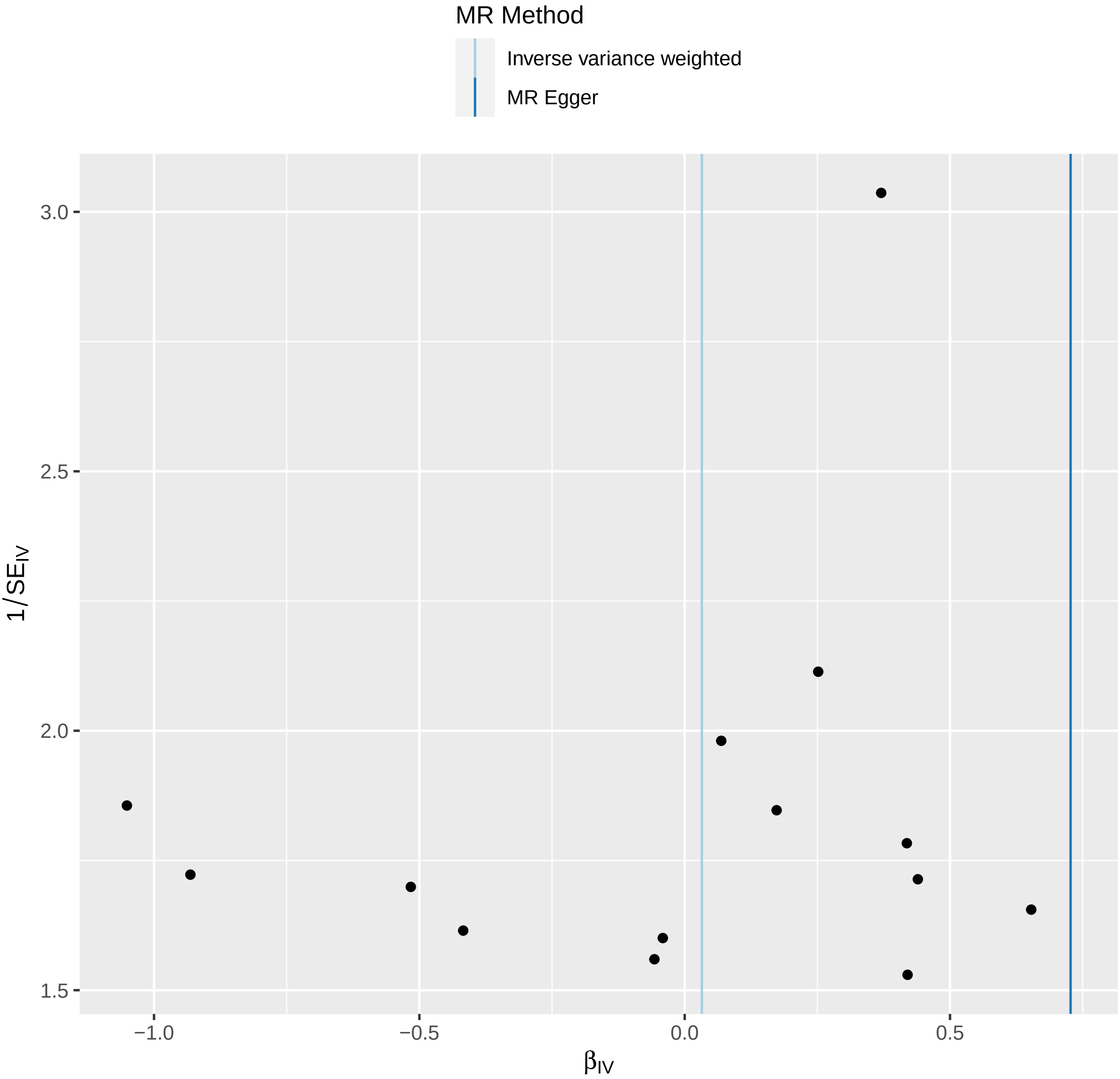

Batch 10 : Gut microbiota abundance (family Coriobacteriaceae id.811) on Crohn's disease of large intestine

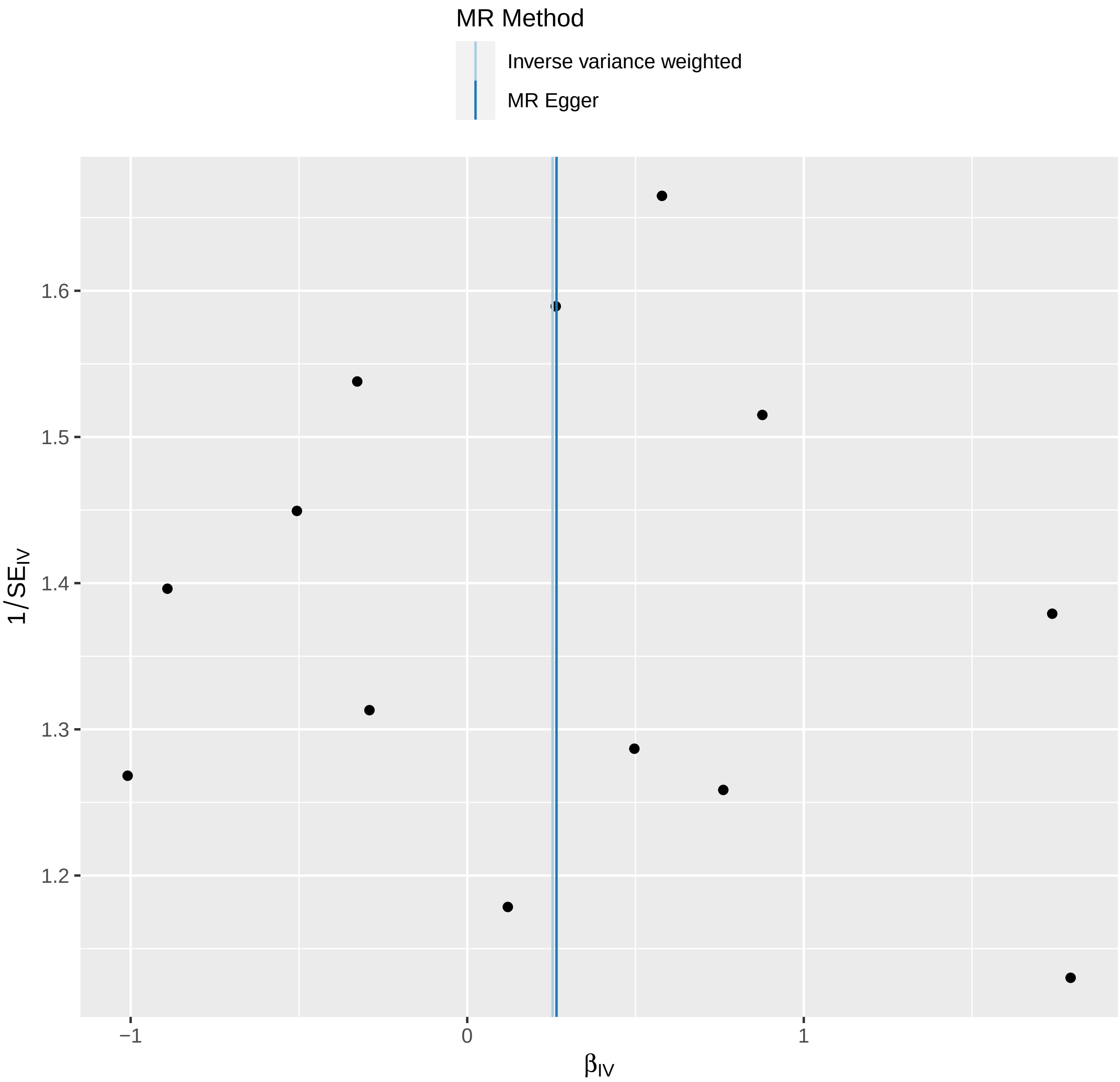

Batch 11 : Gut microbiota abundance (family Defluviitaleaceae id.1924) on Crohn's disease of large intestine

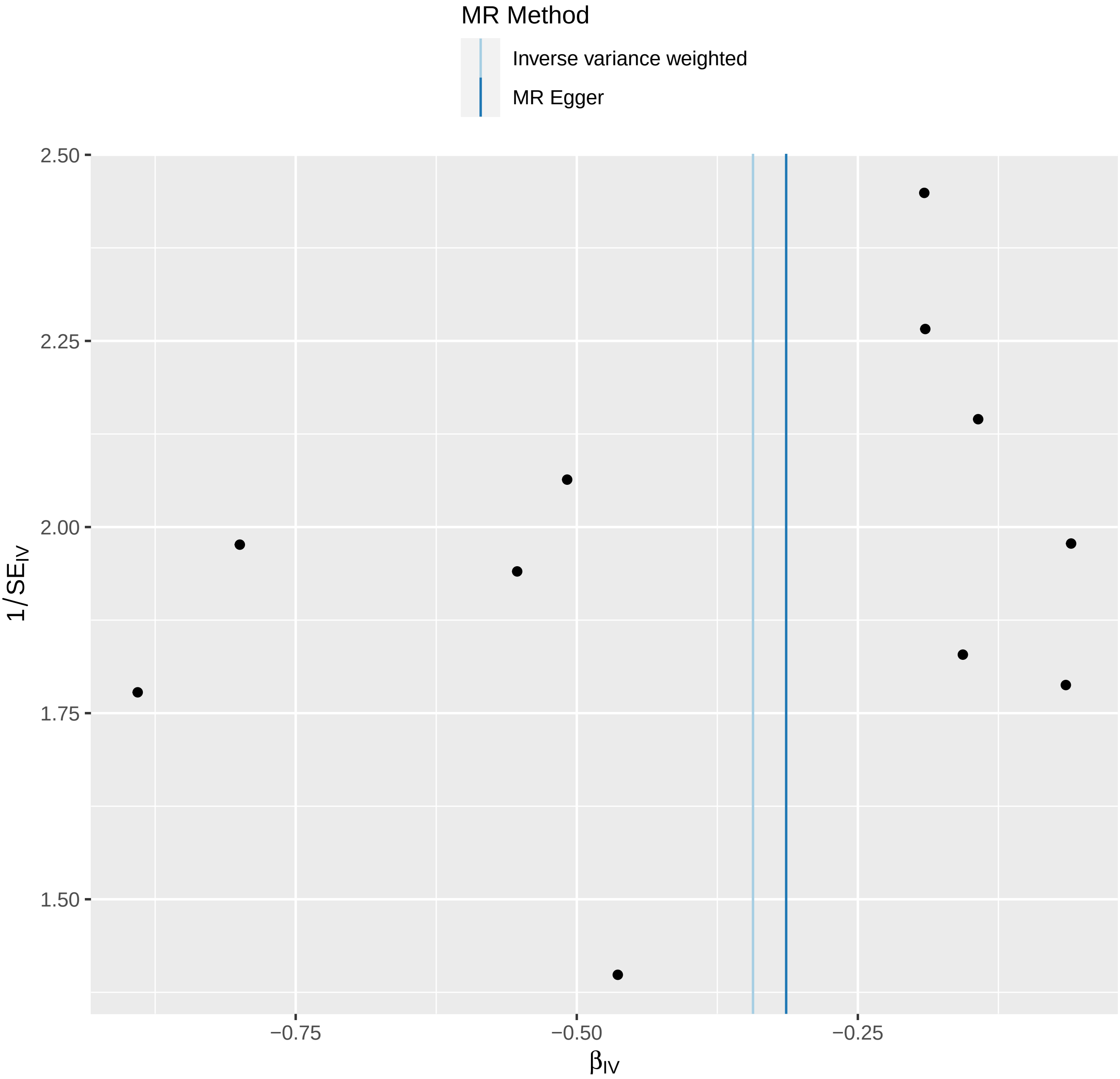

Batch 12 : Gut microbiota abundance (family Desulfovibrionaceae id.3169) on Crohn's disease of large intestine

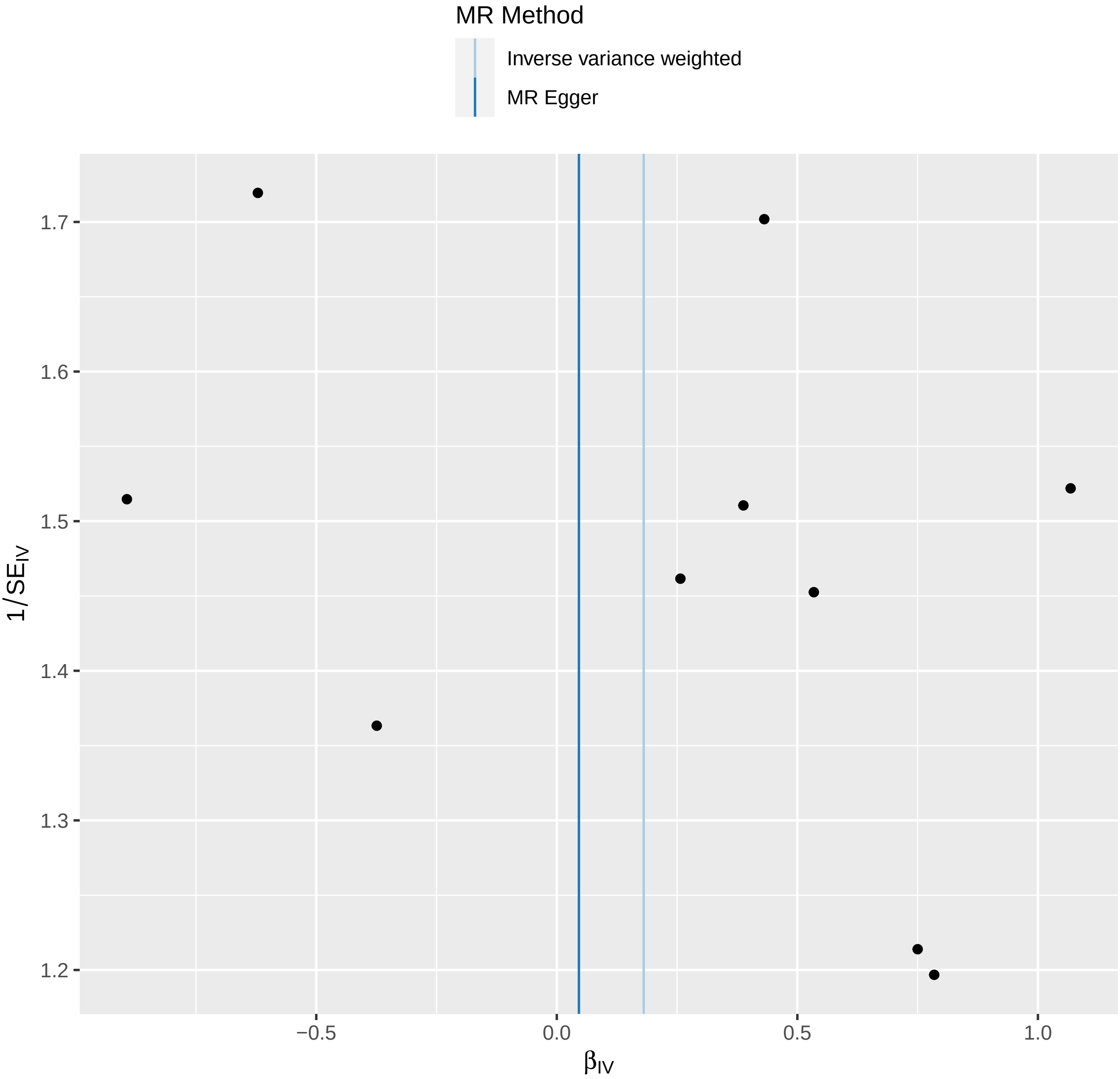

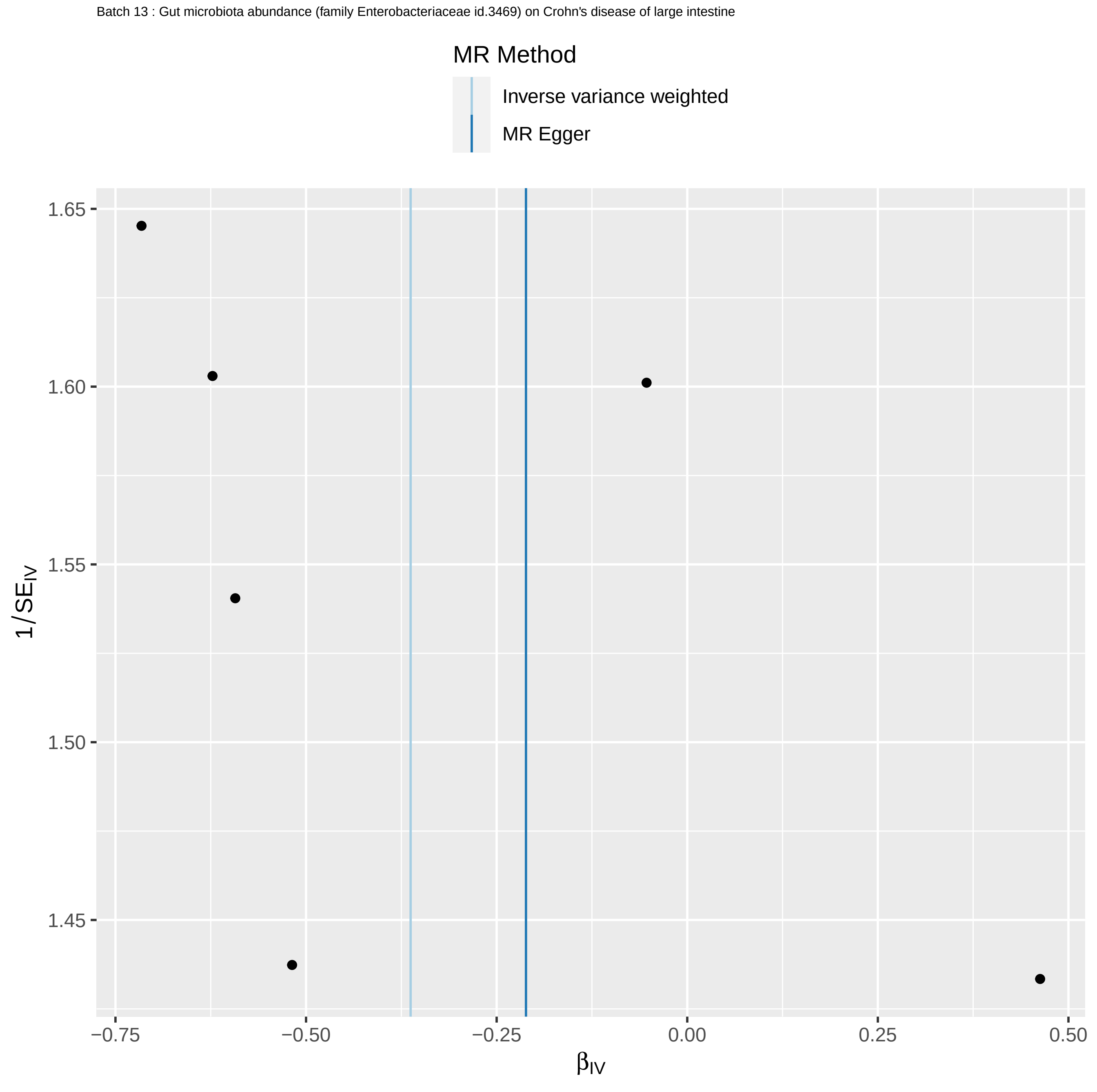
Batch 13 : Gut microbiota abundance (family Enterobacteriaceae id.3469) on Crohn's disease of large intestine
MR Method
Inverse variance weighted
MR Egger
1.65
1.60
1.55
1.50
1.45
$1/SE_{IV}$
−0.75
−0.50
−0.25
0.00
0.25
0.50
β_{IV}

Batch 14 : Gut microbiota abundance (family Erysipelotrichaceae id.2149) on Crohn's disease of large intestine

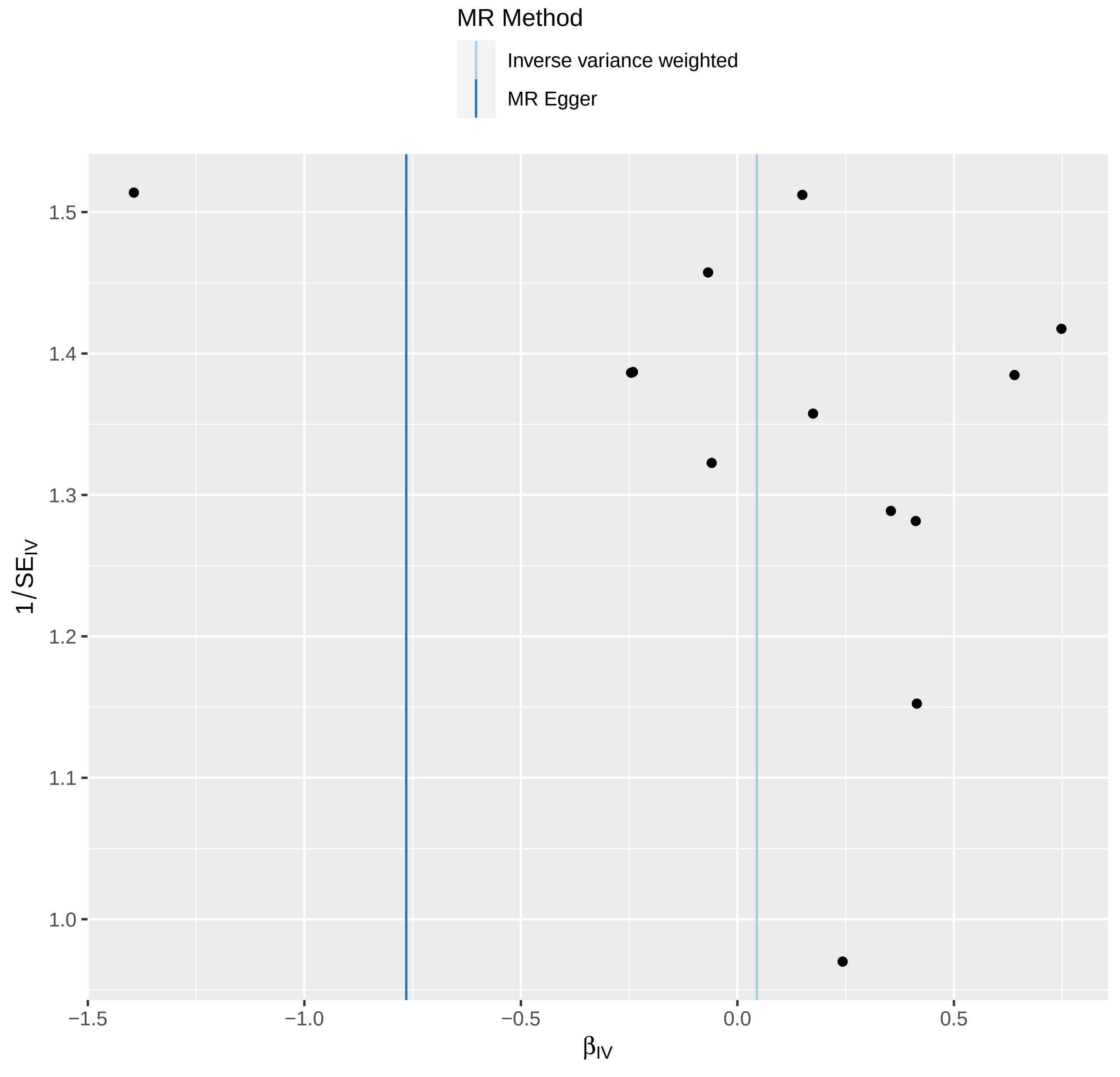

Batch 15 : Gut microbiota abundance (family Family XI id.1936) on Crohn's disease of large intestine

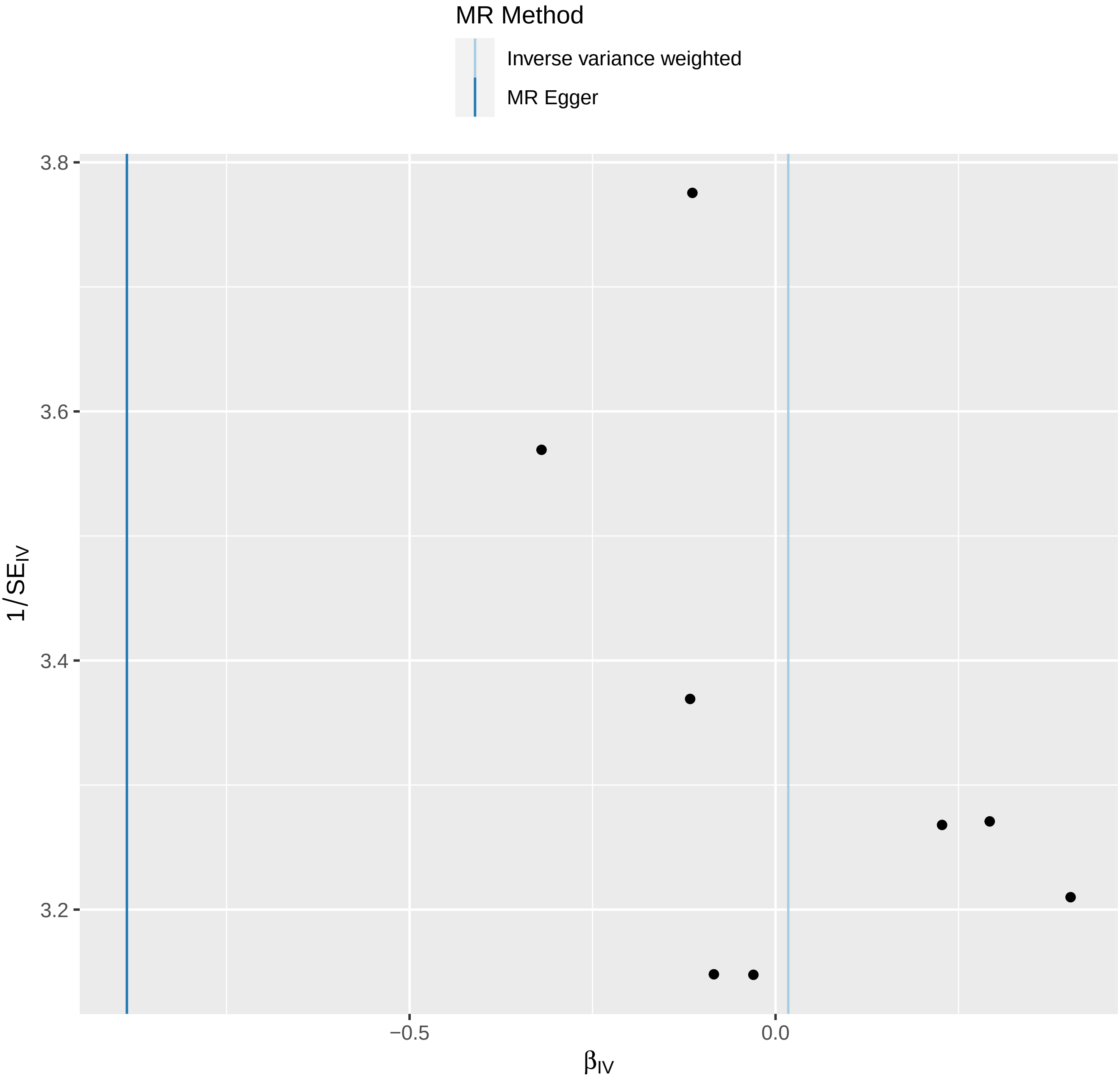

Batch 16 : Gut microbiota abundance (family Family XIII id.1957) on Crohn's disease of large intestine

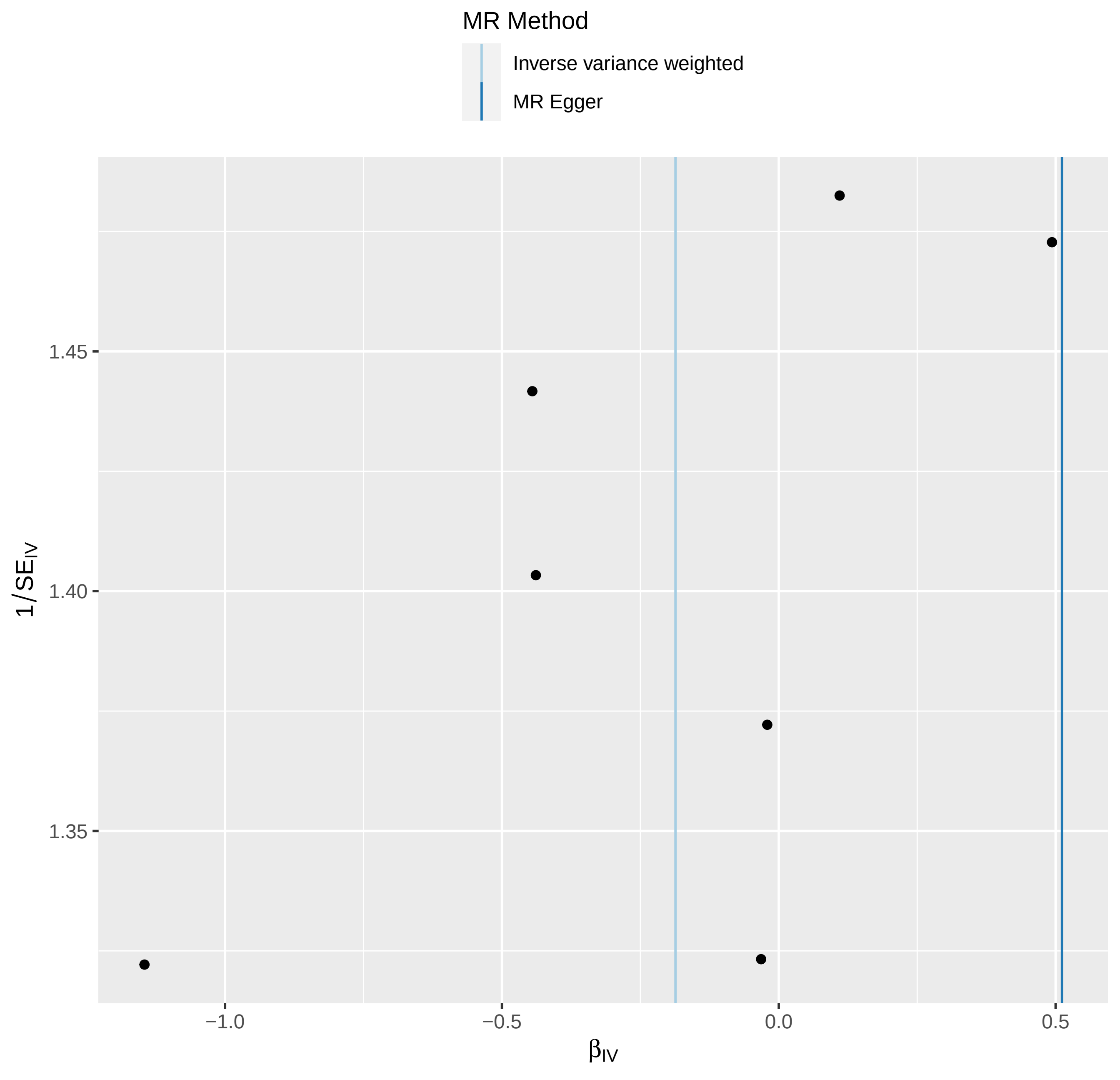

Batch 17 : Gut microbiota abundance (family Lachnospiraceae id.1987) on Crohn's disease of large intestine

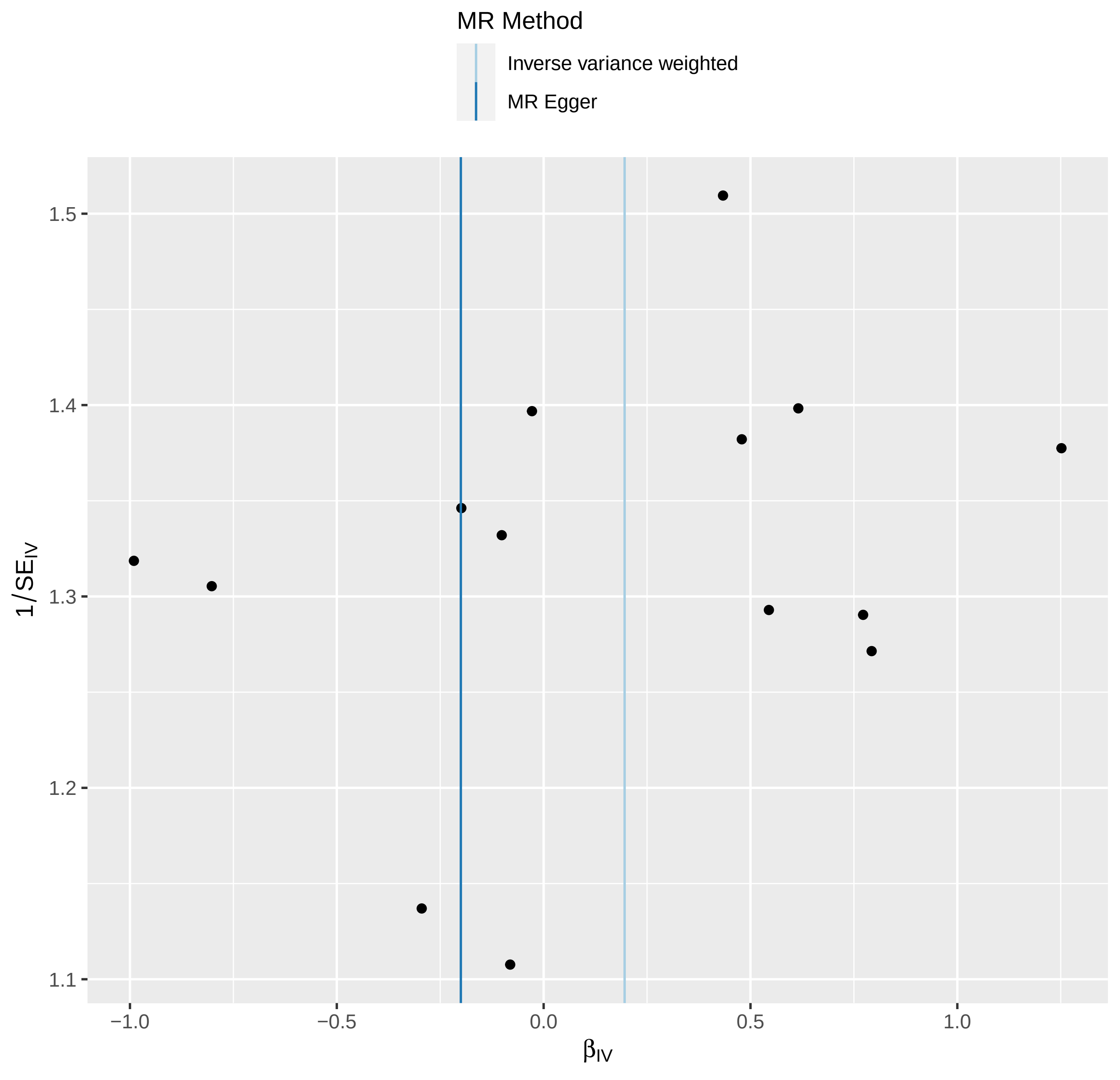

Batch 18 : Gut microbiota abundance (family Lactobacillaceae id.1836) on Crohn's disease of large intestine

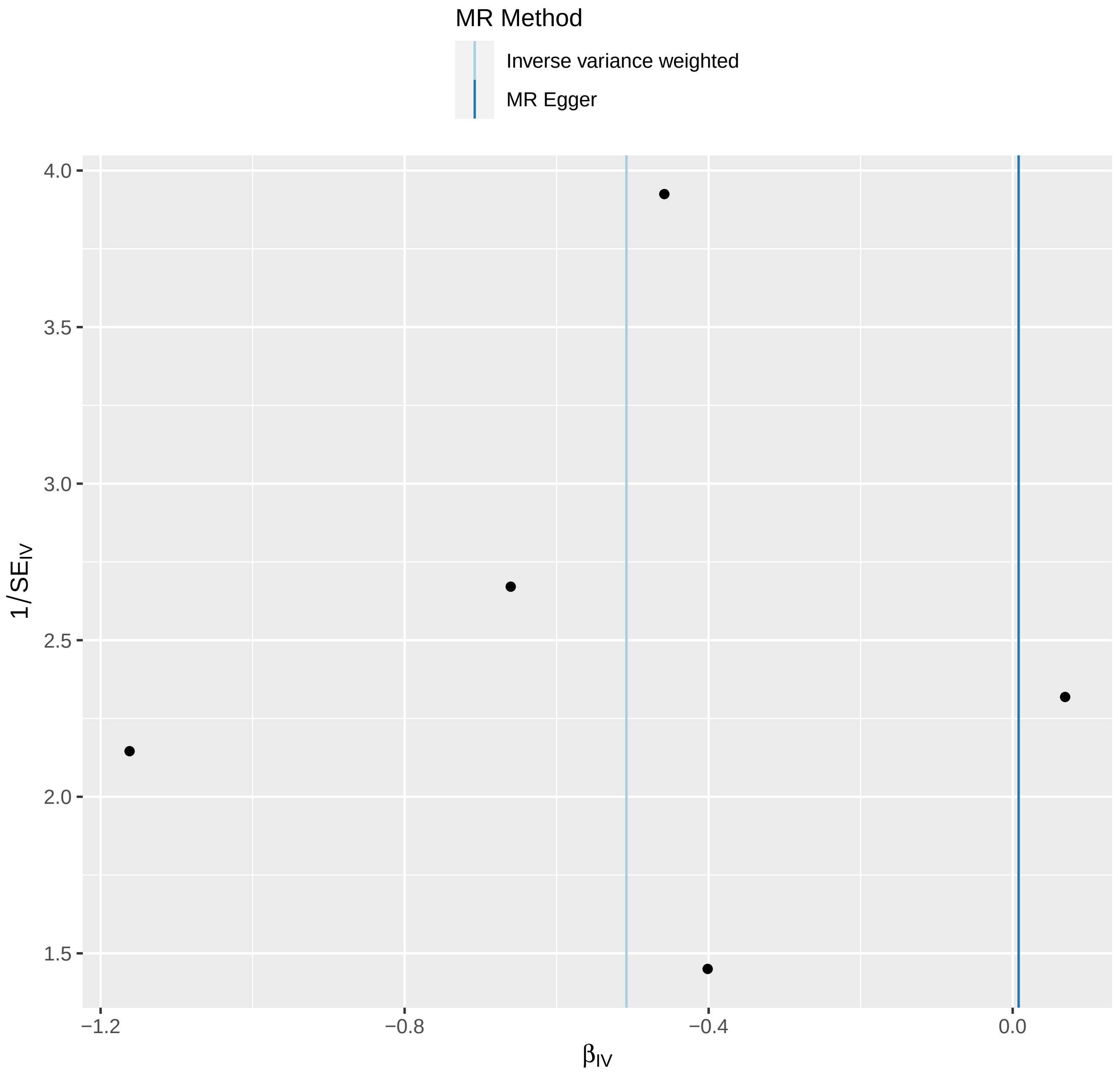

Batch 19 : Gut microbiota abundance (family Methanobacteriaceae id.121) on Crohn's disease of large intestine

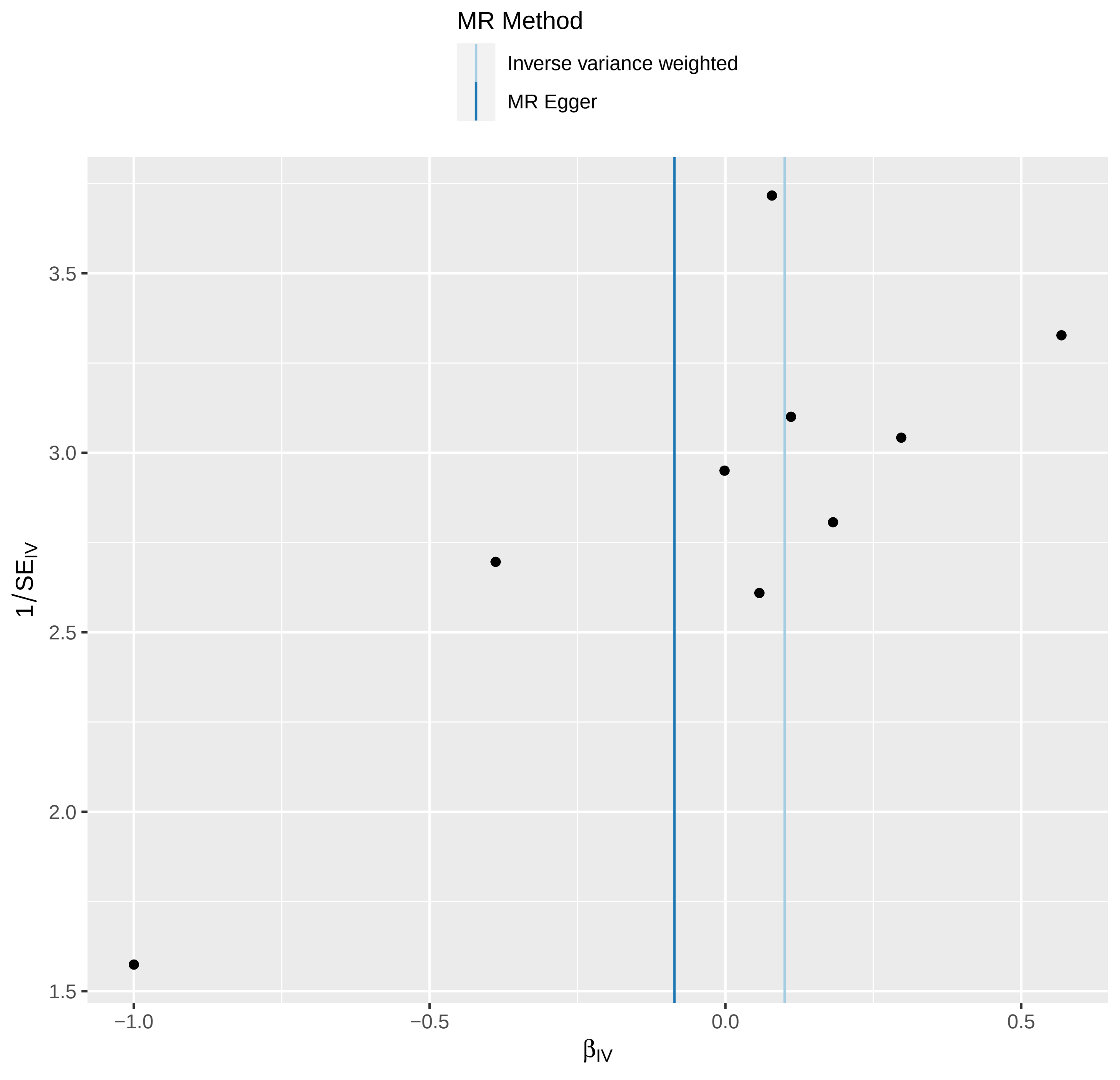

Batch 20 : Gut microbiota abundance (family Oxalobacteraceae id.2966) on Crohn's disease of large intestine

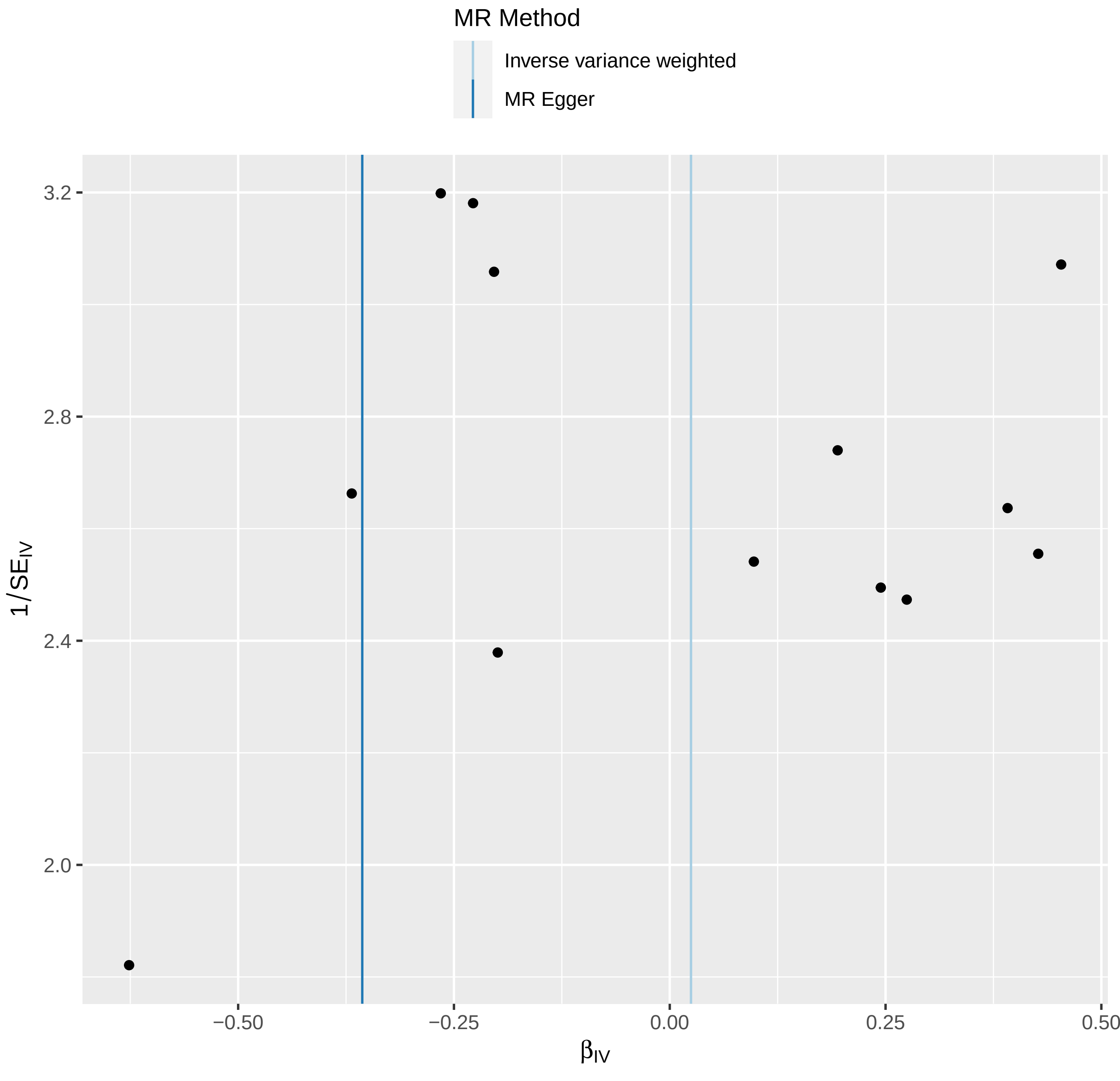

Batch 21 : Gut microbiota abundance (family Pasteurellaceae id.3689) on Crohn's disease of large intestine

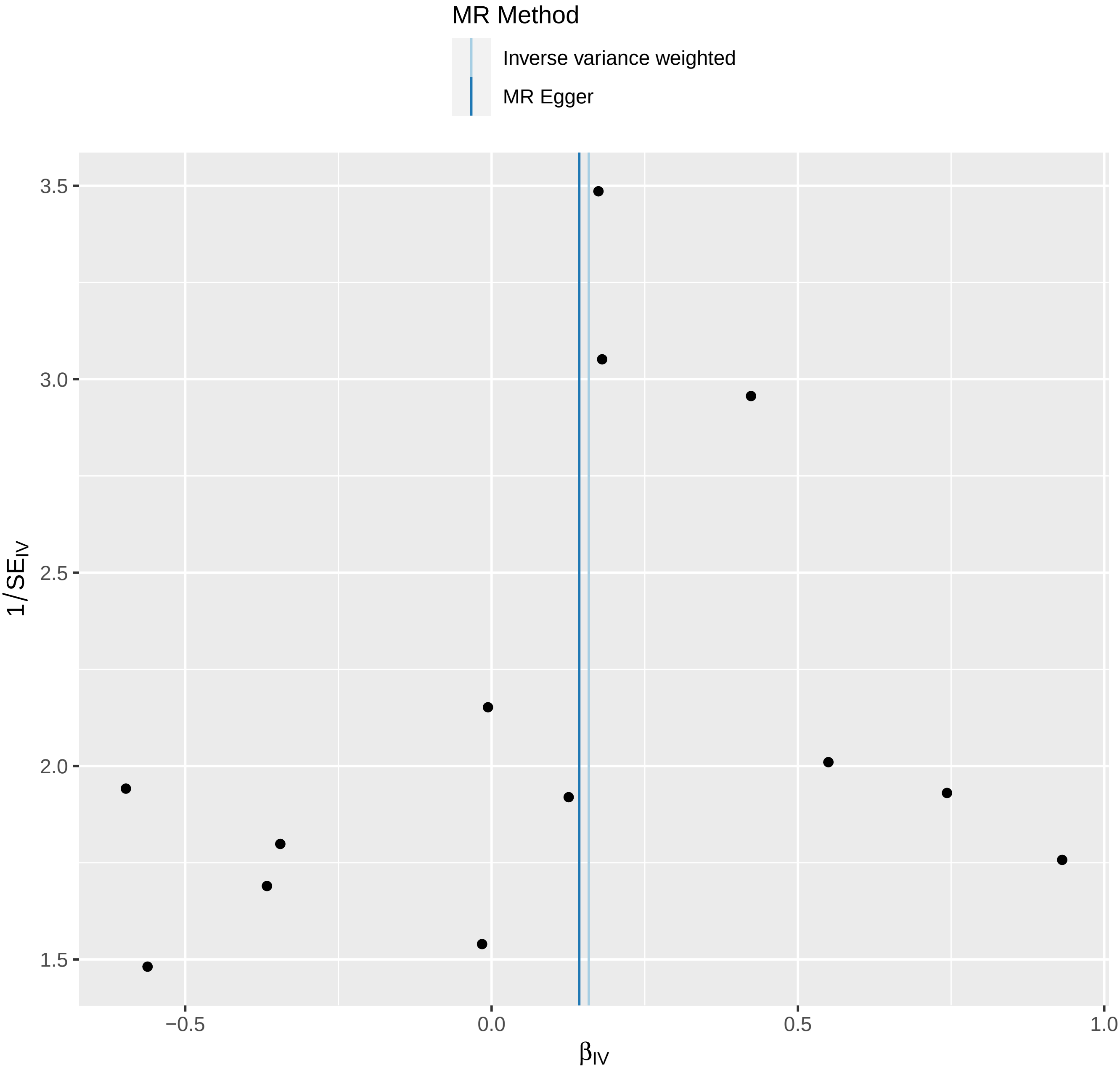

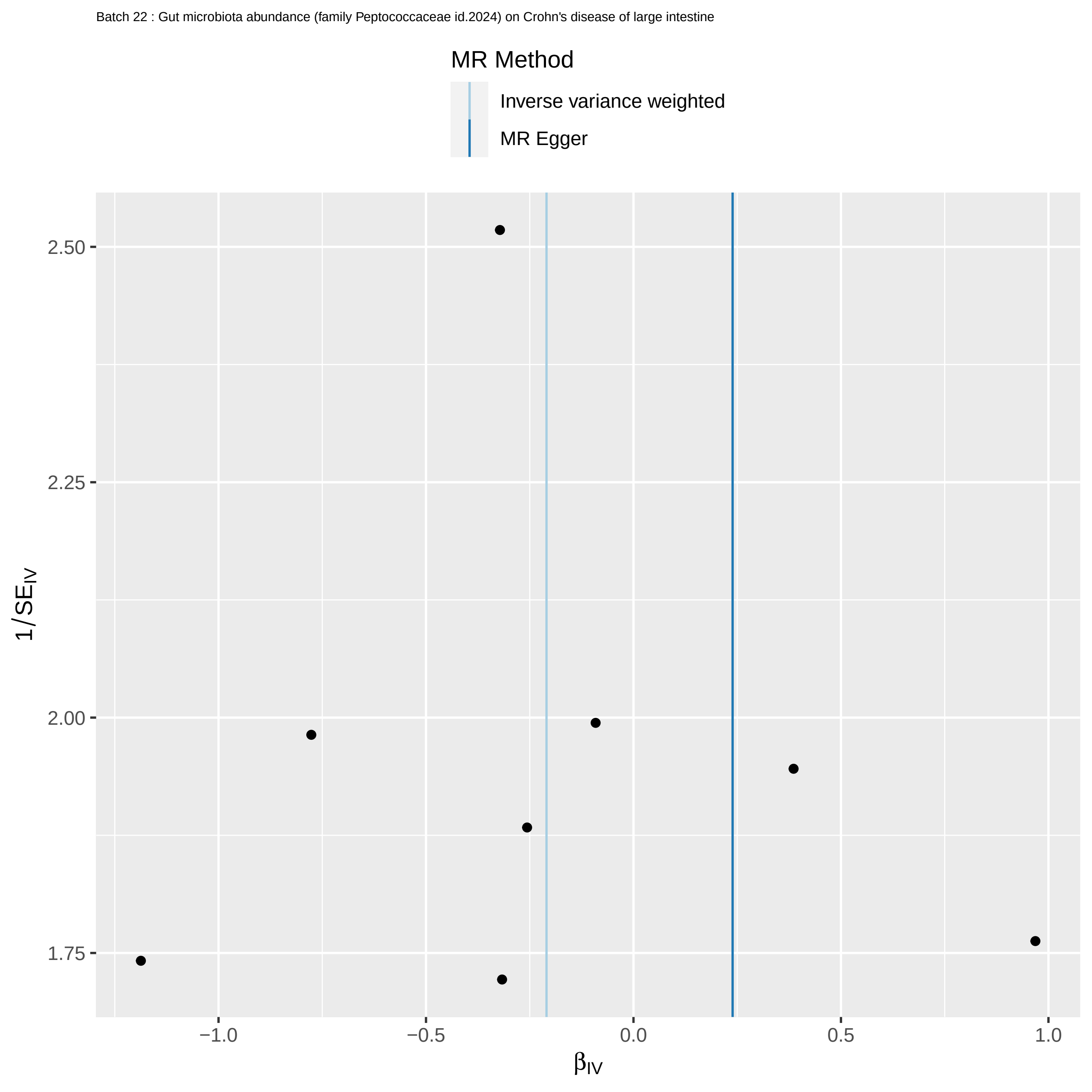

Batch 22 : Gut microbiota abundance (family Peptococcaceae id.2024) on Crohn's disease of large intestine
MR Method
Inverse variance weighted
MR Egger
2.50
2.25
2.00
1.75
$1/SE_{IV}$
−1.0
−0.5
0.0
0.5
1.0
β_{IV}

Batch 23 : Gut microbiota abundance (family Peptostreptococcaceae id.2042) on Crohn's disease of large intestine

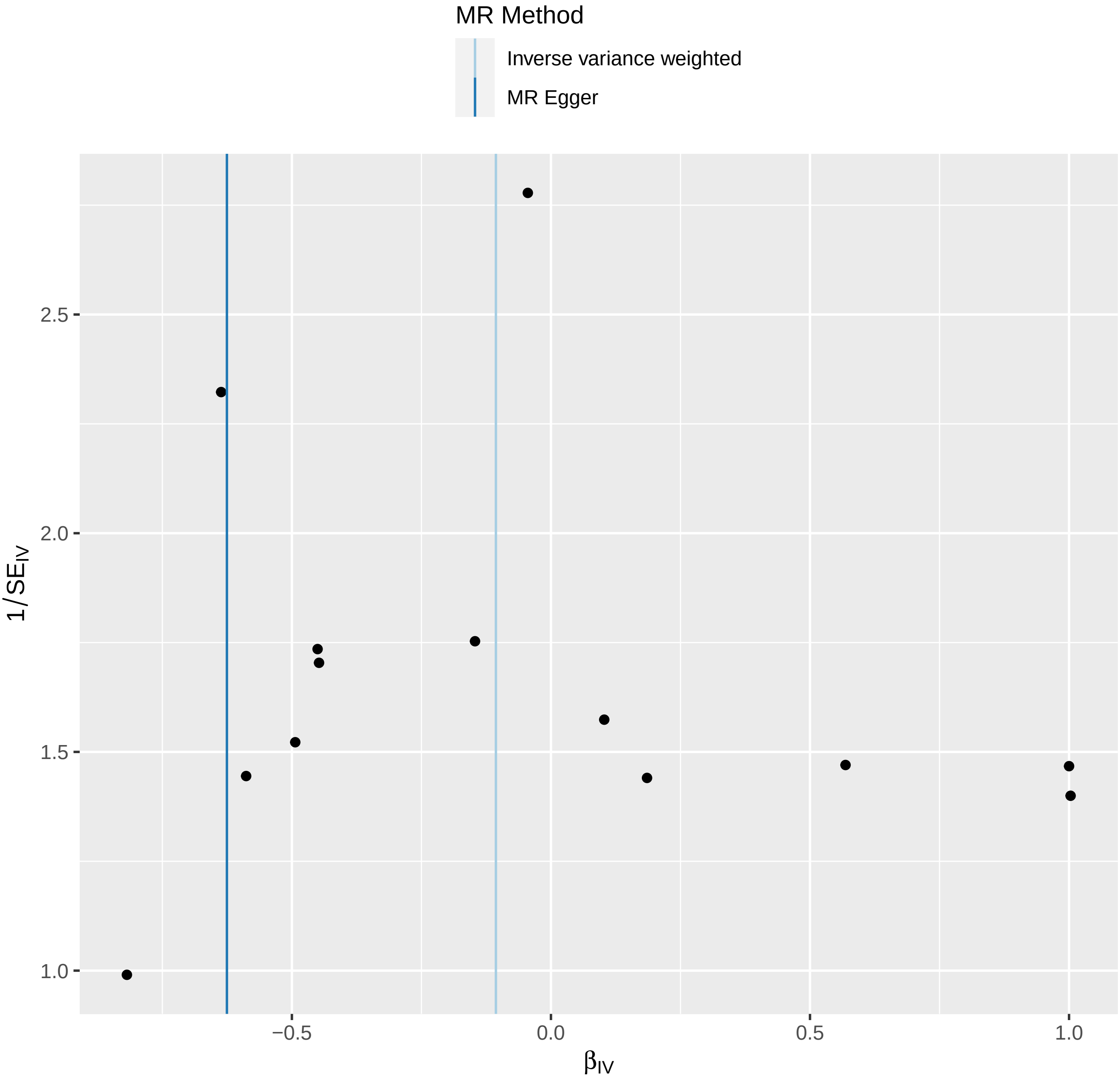

Batch 24 : Gut microbiota abundance (family Porphyromonadaceae id.943) on Crohn's disease of large intestine

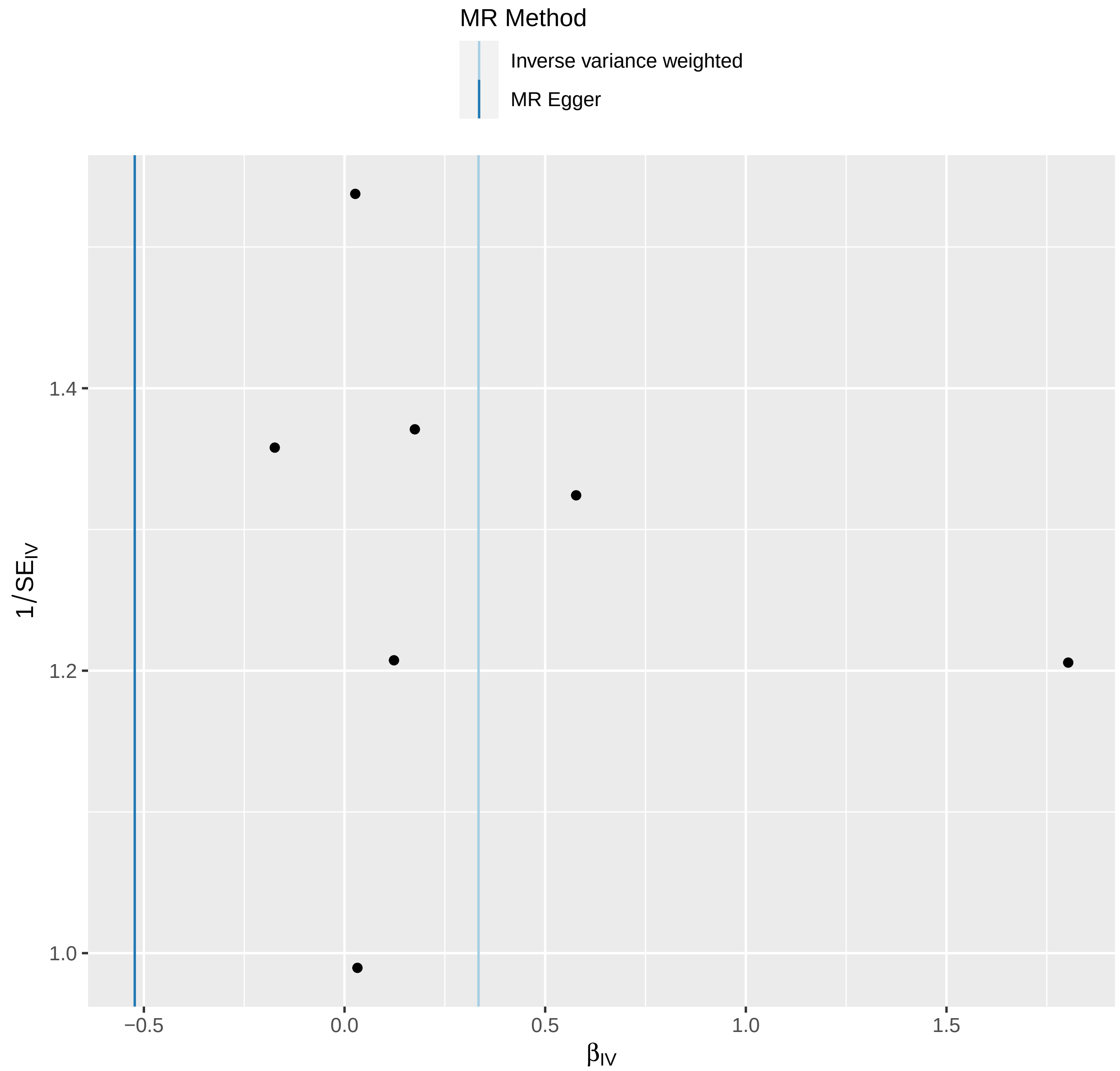

Batch 25 : Gut microbiota abundance (family Prevotellaceae id.960) on Crohn's disease of large intestine

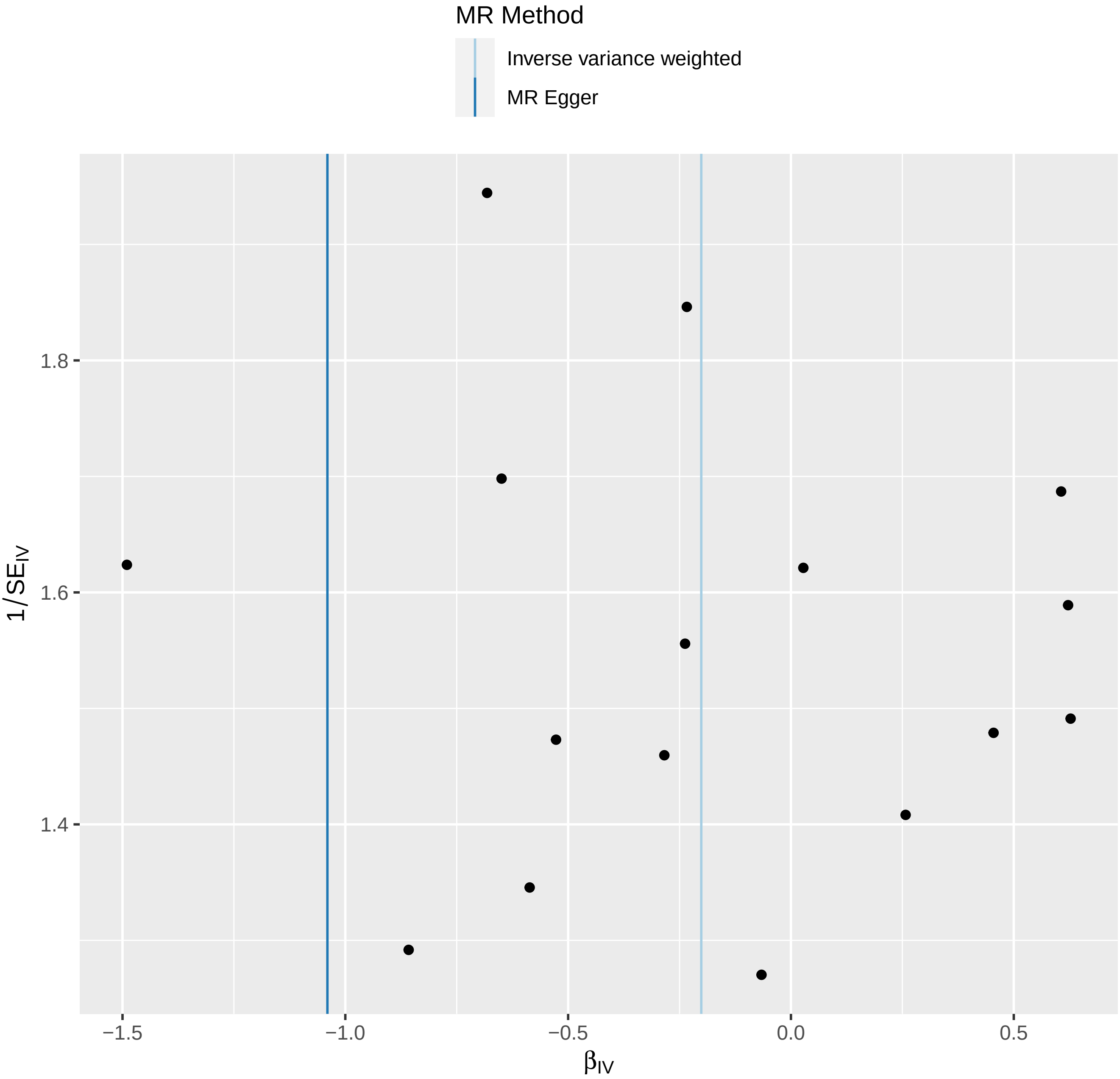

Batch 26 : Gut microbiota abundance (family Rhodospirillaceae id.2717) on Crohn's disease of large intestine

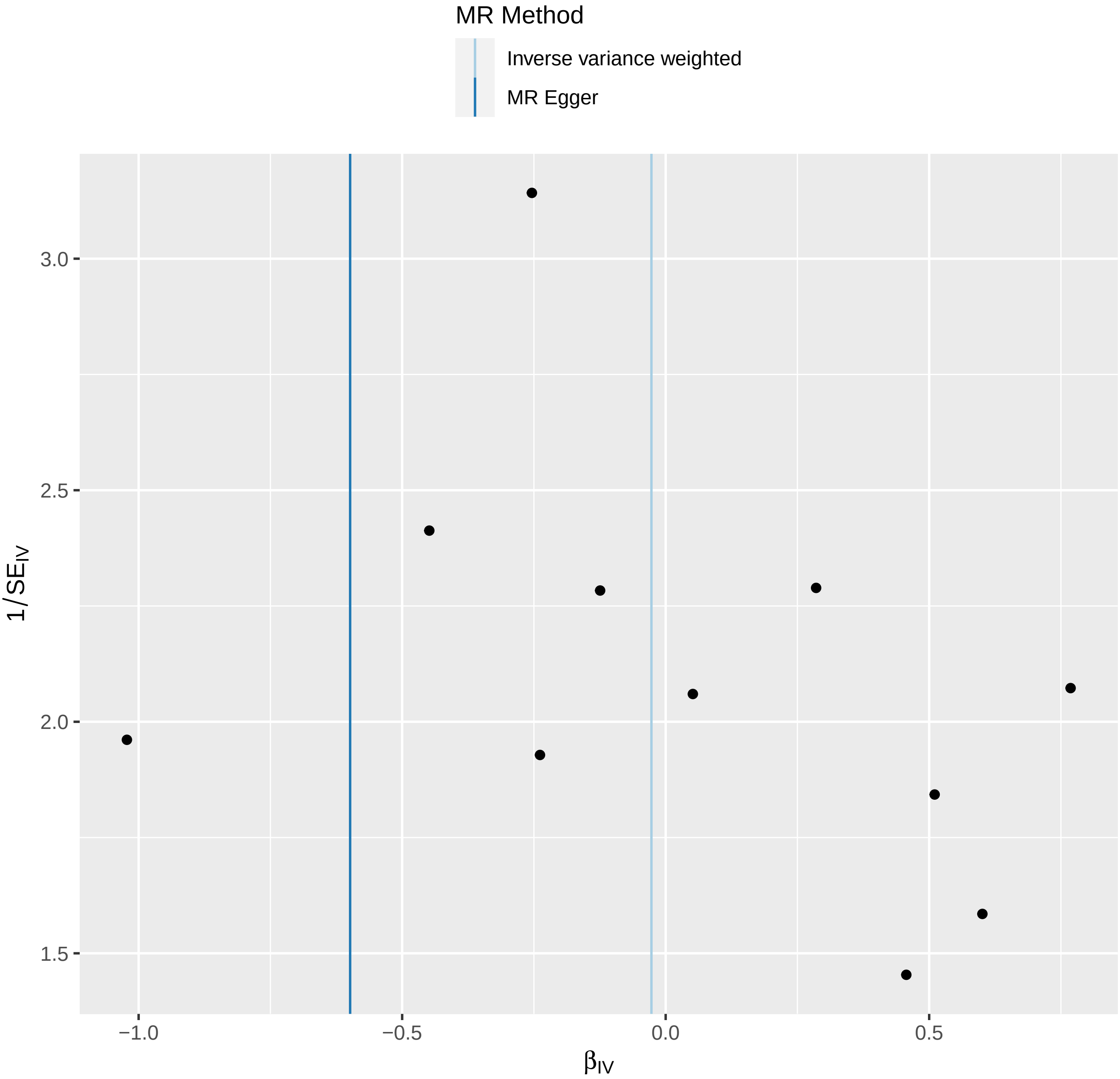

Batch 27 : Gut microbiota abundance (family Rikenellaceae id.967) on Crohn's disease of large intestine

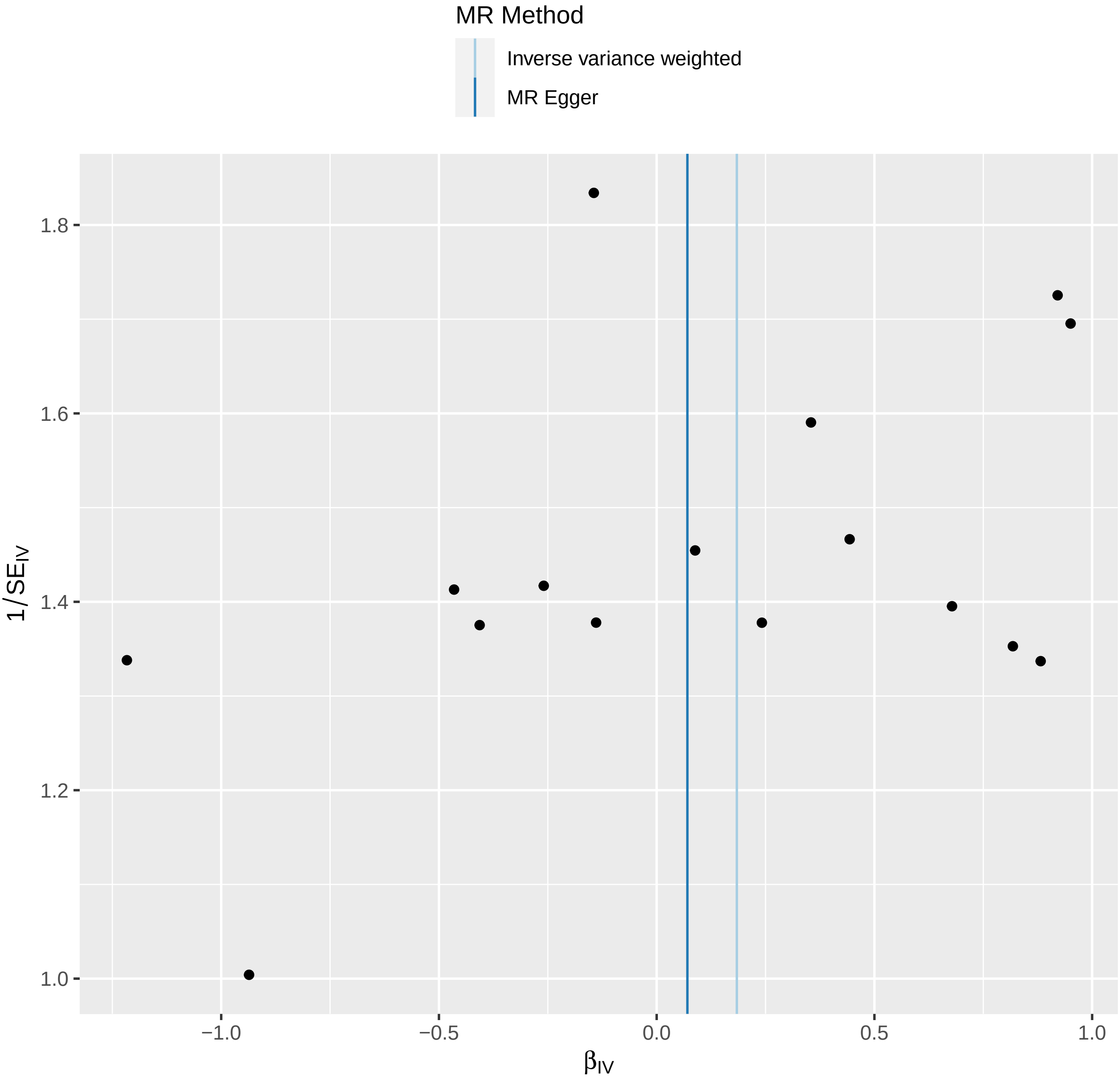

Batch 28 : Gut microbiota abundance (family Ruminococcaceae id.2050) on Crohn's disease of large intestine

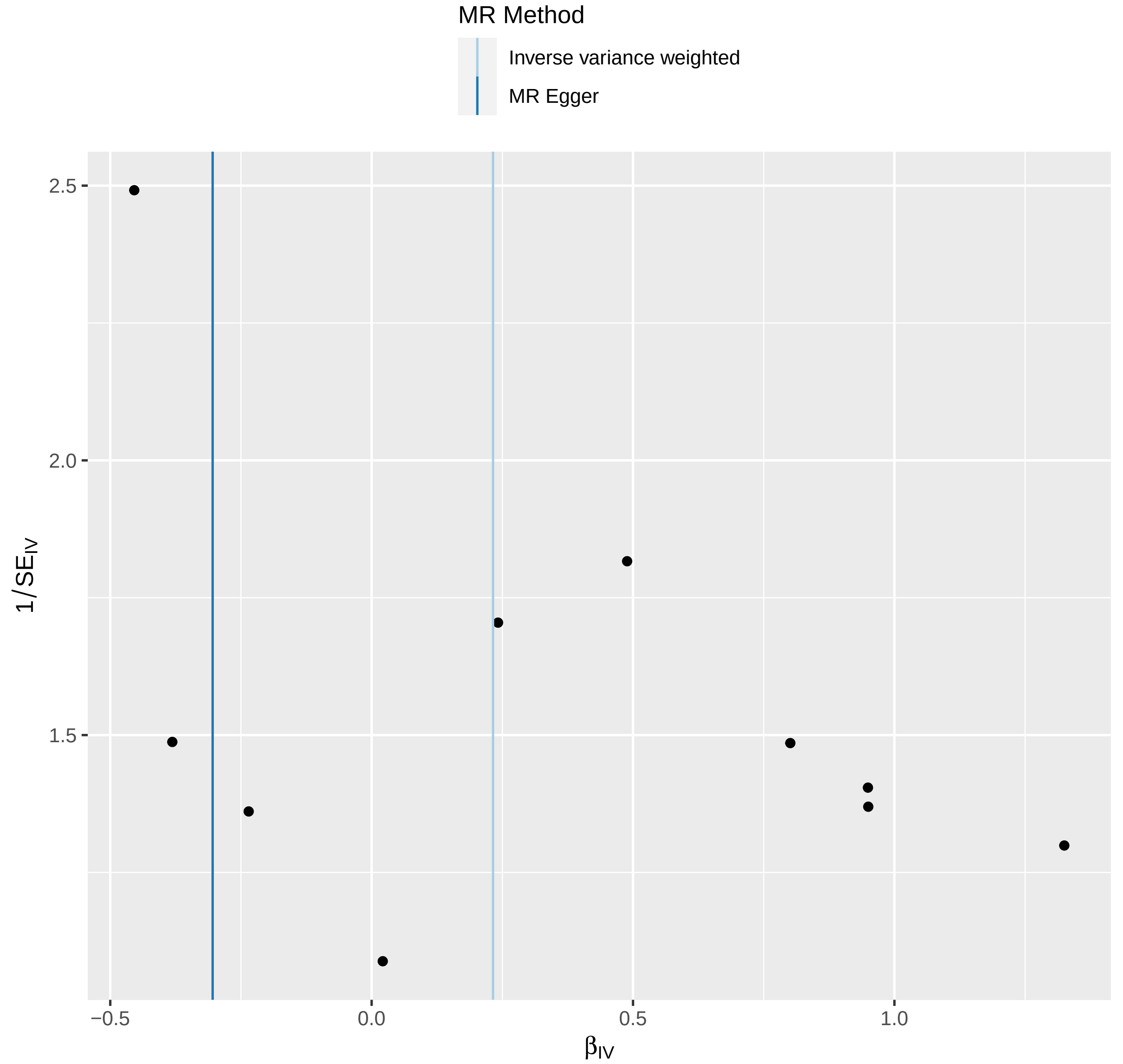

Batch 29 : Gut microbiota abundance (family Streptococcaceae id.1850) on Crohn's disease of large intestine

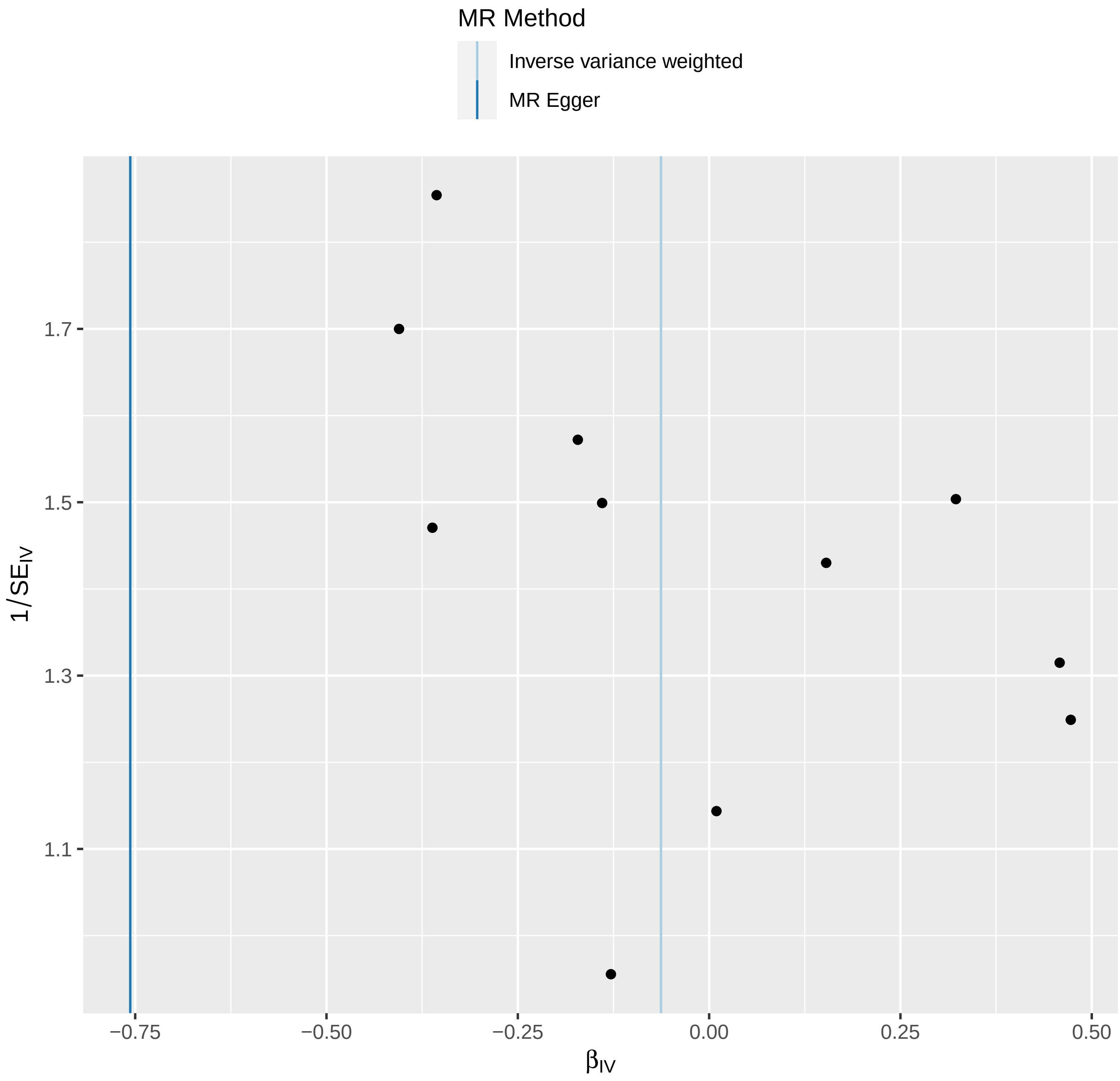

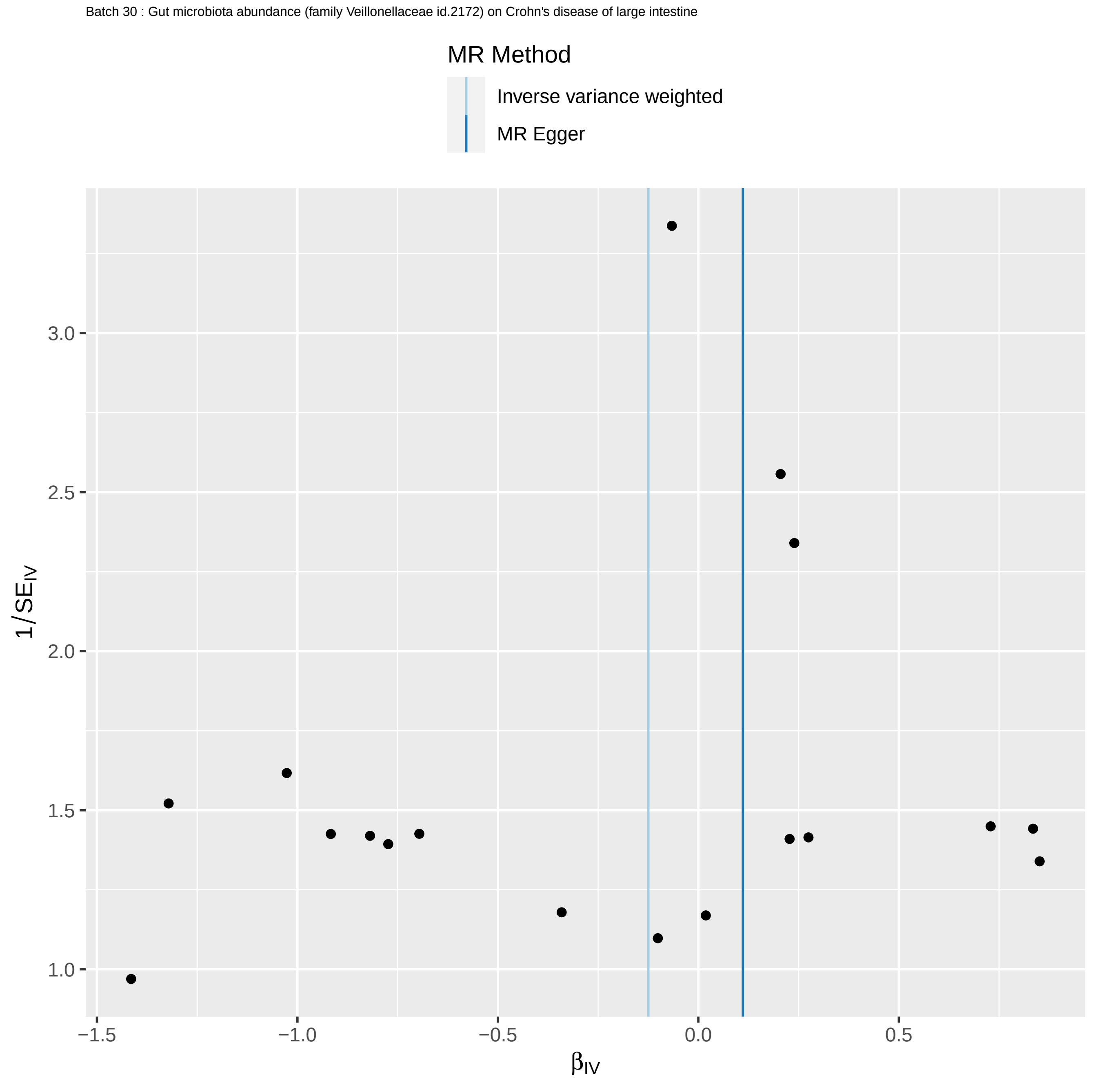
Batch 30 : Gut microbiota abundance (family Veillonellaceae id.2172) on Crohn's disease of large intestine
MR Method
Inverse variance weighted
MR Egger
1/SE_IV
β_IV
3.0
2.5
2.0
1.5
1.0
−1.5
−1.0
−0.5
0.0
0.5

Batch 31 : Gut microbiota abundance (family Verrucomicrobiaceae id.4036) on Crohn's disease of large intestine

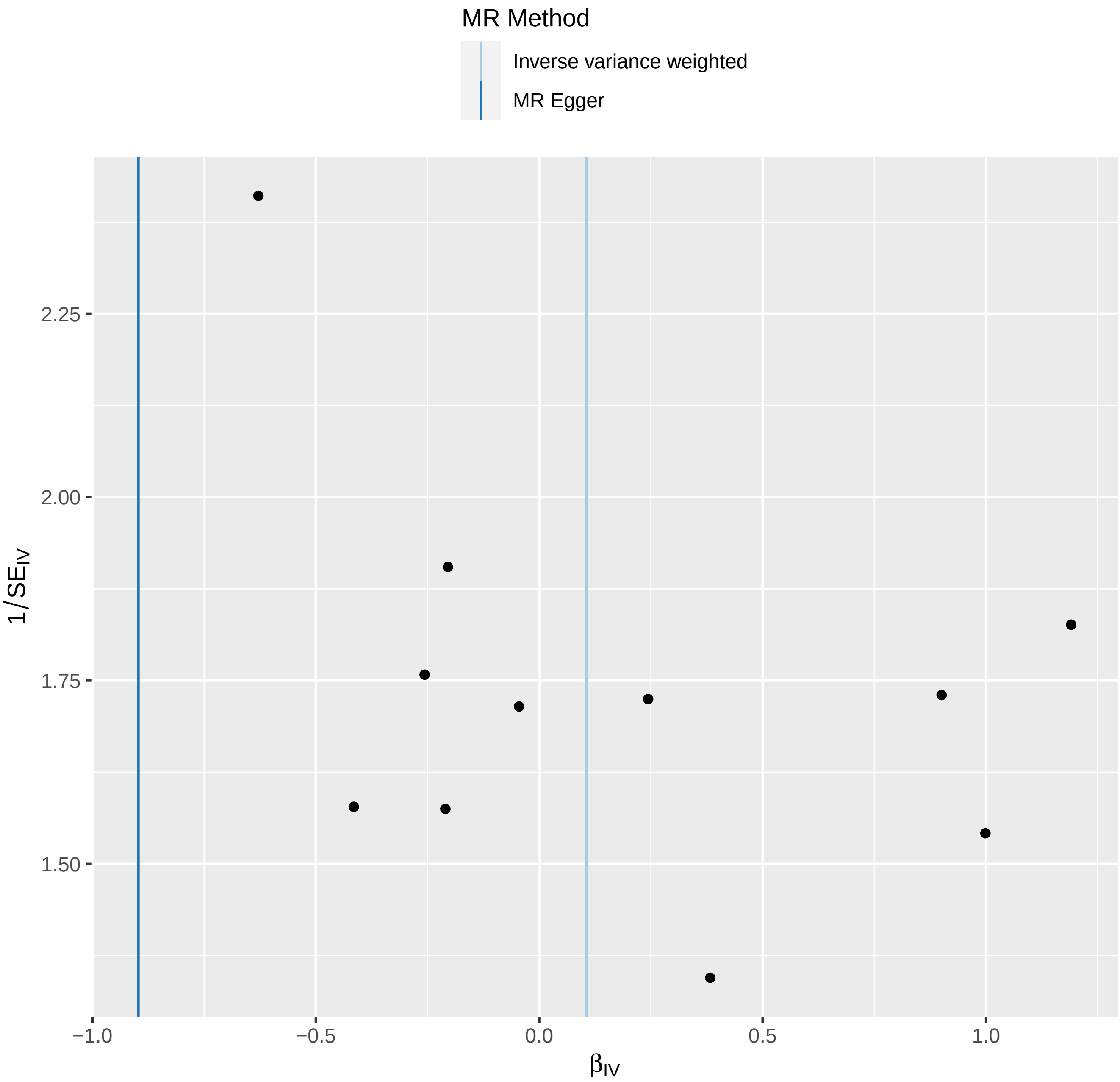

Batch 32 : Gut microbiota abundance (family Victivallaceae id.2255) on Crohn's disease of large intestine

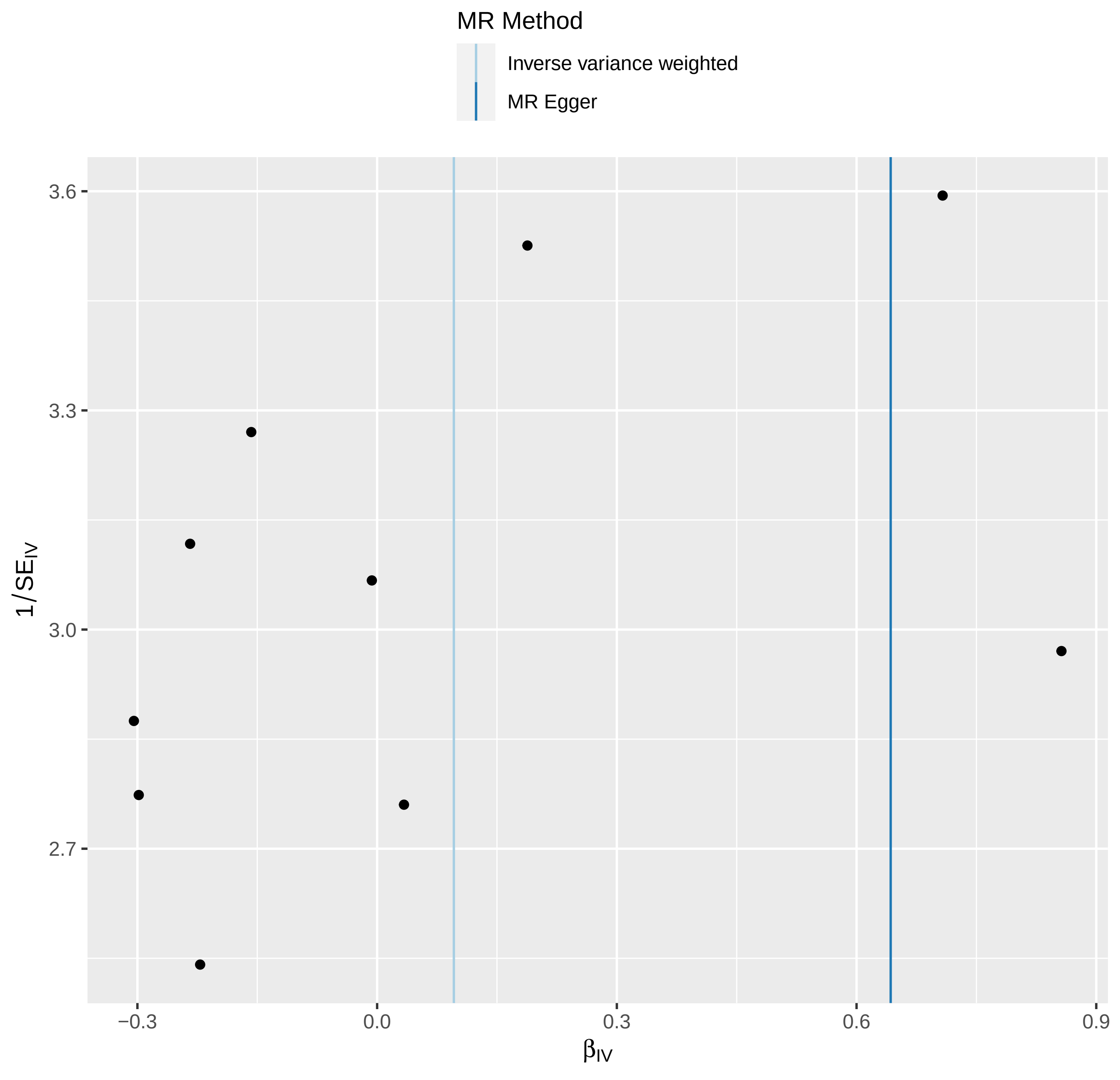

Batch 33 : Gut microbiota abundance (genus Actinomyces id.423) on Crohn's disease of large intestine

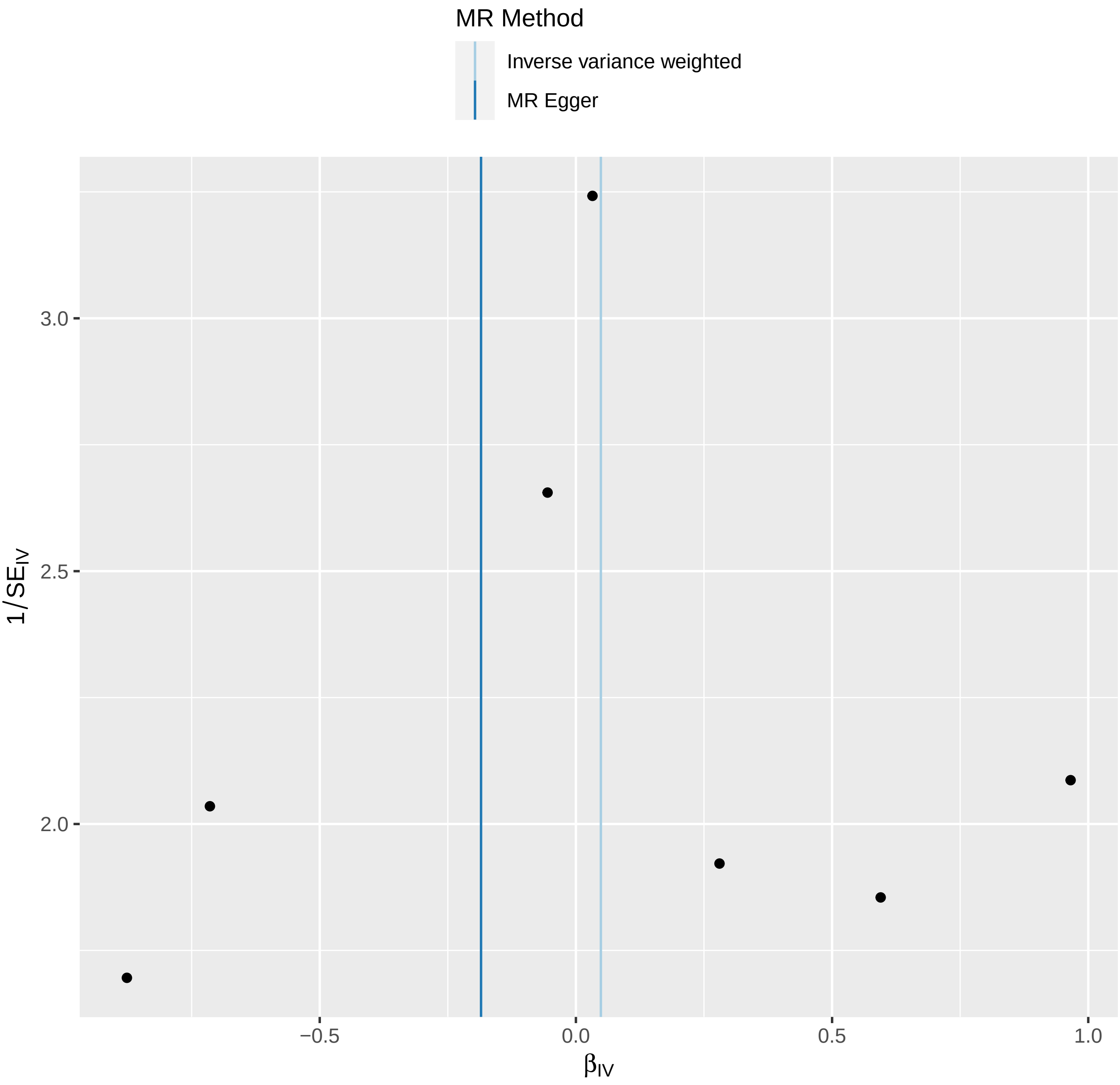

Batch 34 : Gut microbiota abundance (genus Adlercreutzia id.812) on Crohn's disease of large intestine

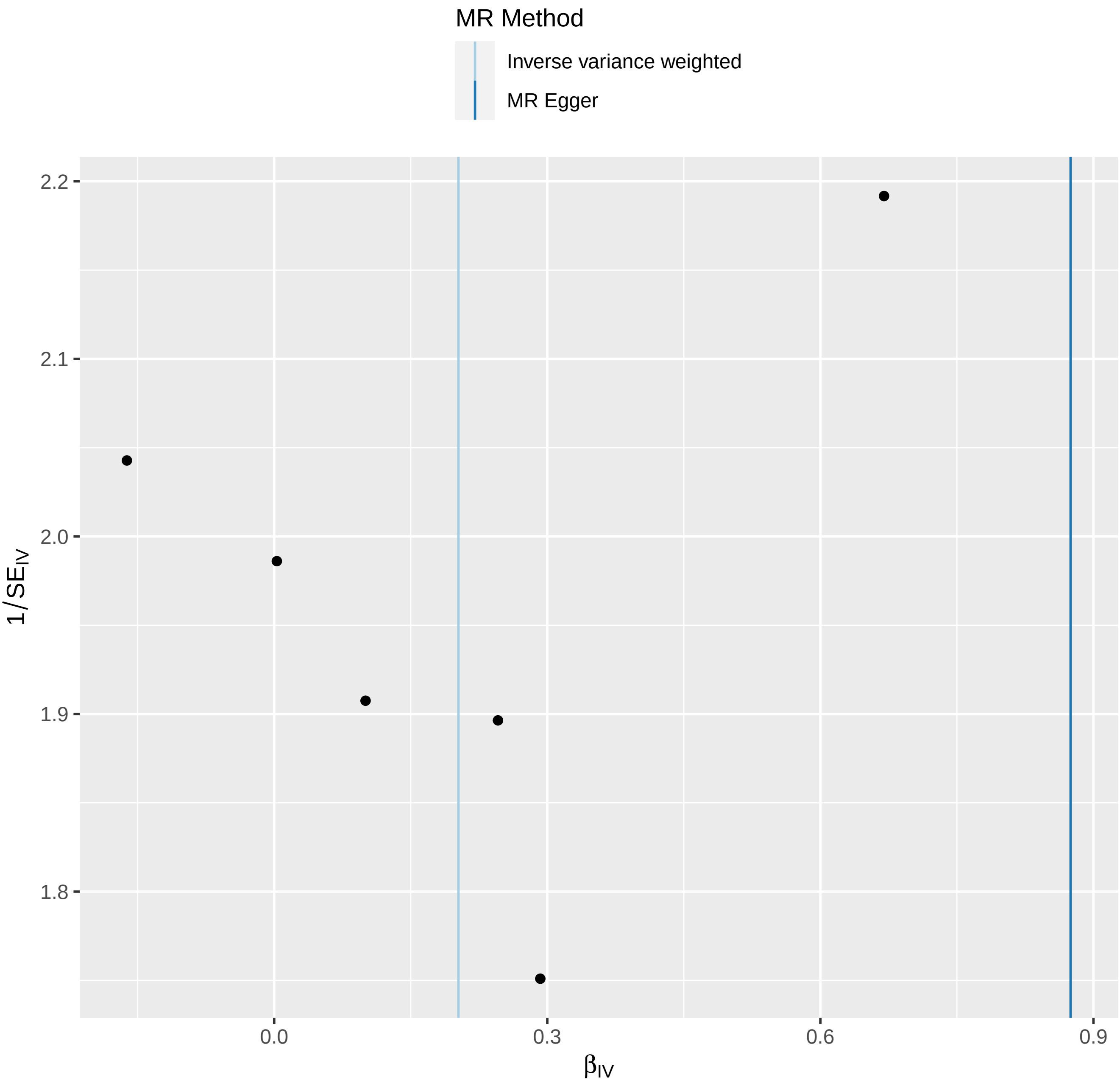

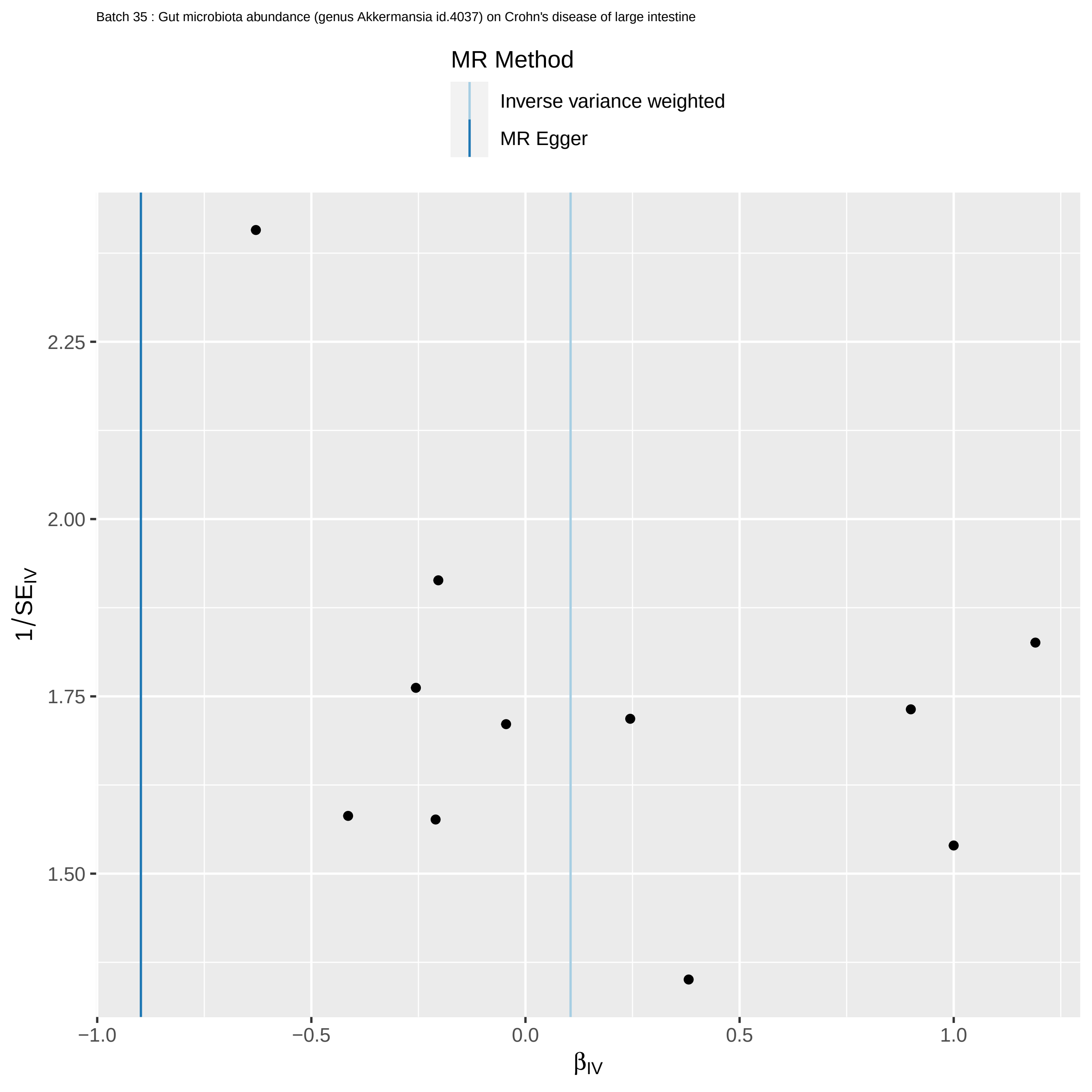

Batch 35 : Gut microbiota abundance (genus Akkermansia id.4037) on Crohn's disease of large intestine
MR Method
Inverse variance weighted
MR Egger
2.25
2.00
1.75
1.50
1/SE_IV
−1.0
−0.5
0.0
0.5
1.0
β_IV

Batch 36 : Gut microbiota abundance (genus Alistipes id.968) on Crohn's disease of large intestine

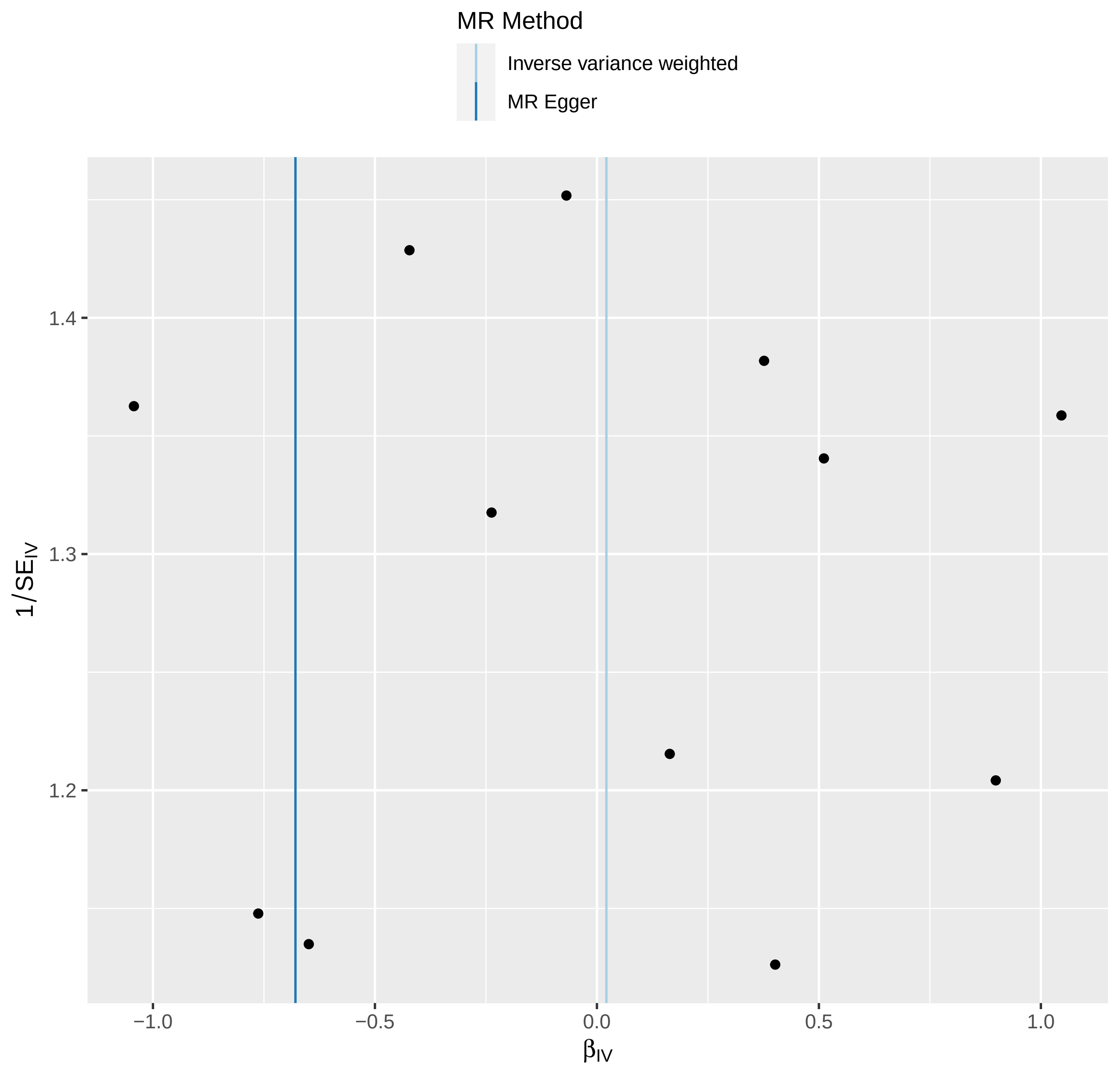

Batch 37 : Gut microbiota abundance (genus Allisonella id.2174) on Crohn's disease of large intestine

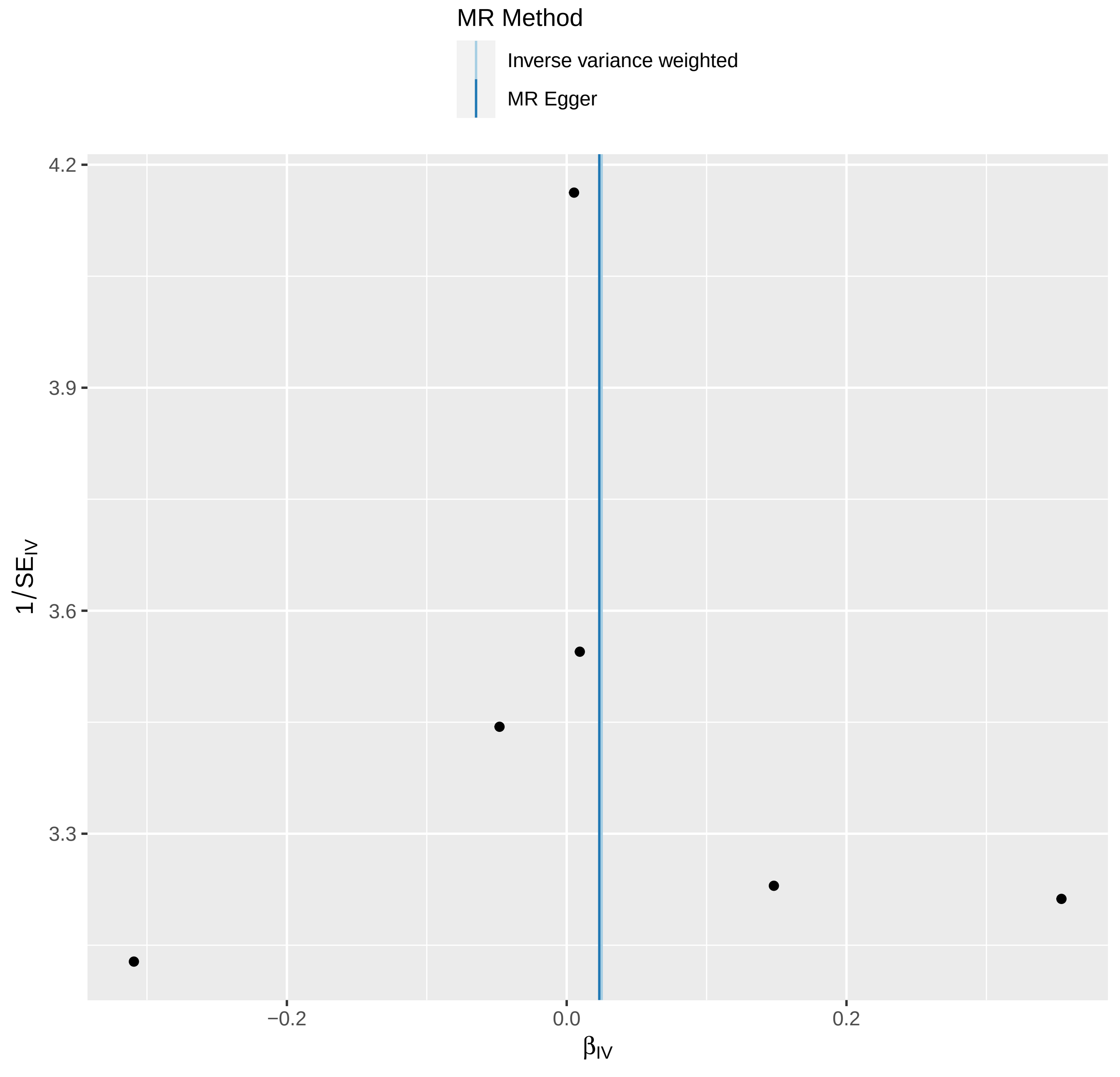

Batch 38 : Gut microbiota abundance (genus Alloprevotella id.961) on Crohn's disease of large intestine

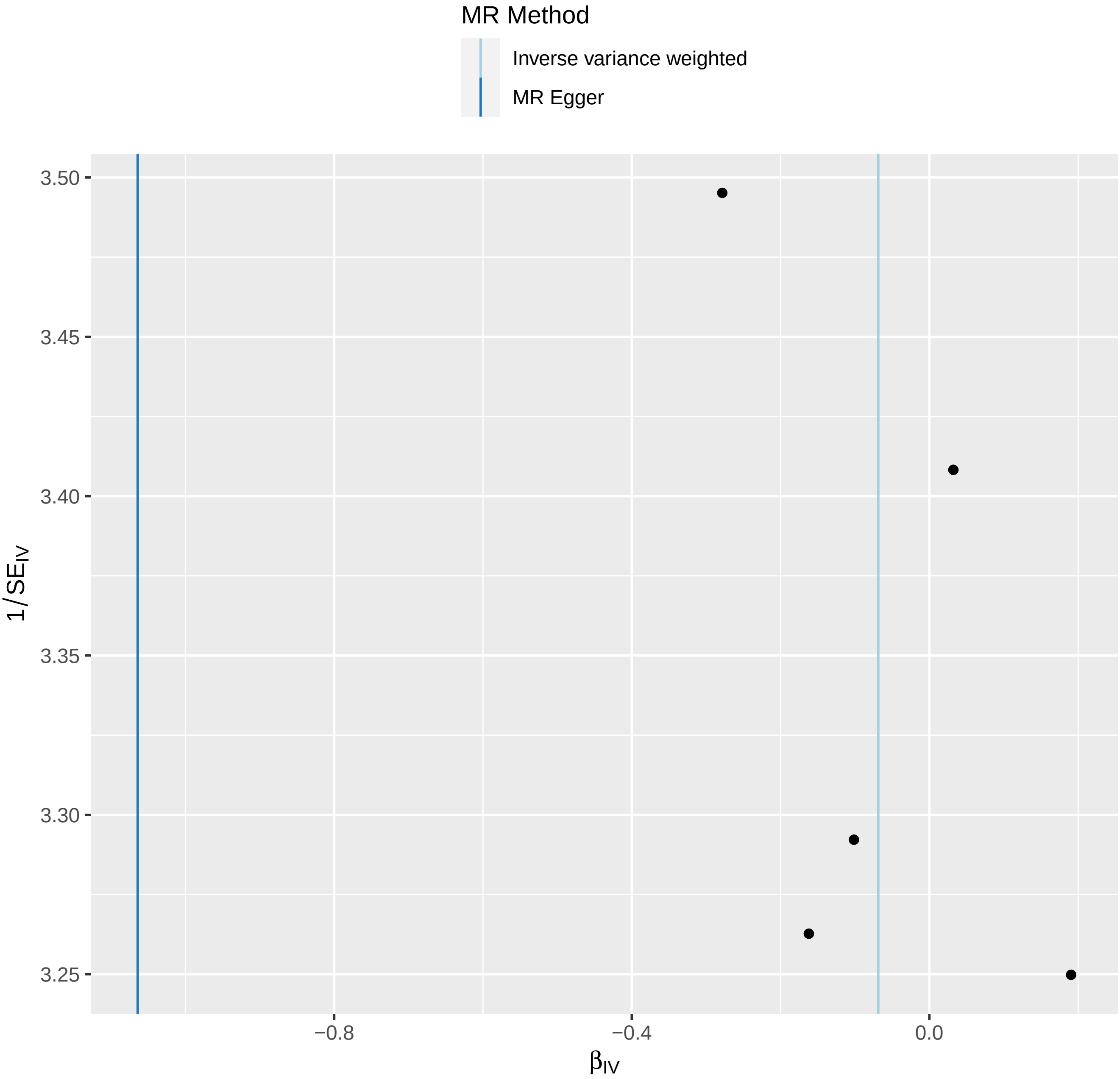

Batch 39 : Gut microbiota abundance (genus Anaerofilum id.2053) on Crohn's disease of large intestine

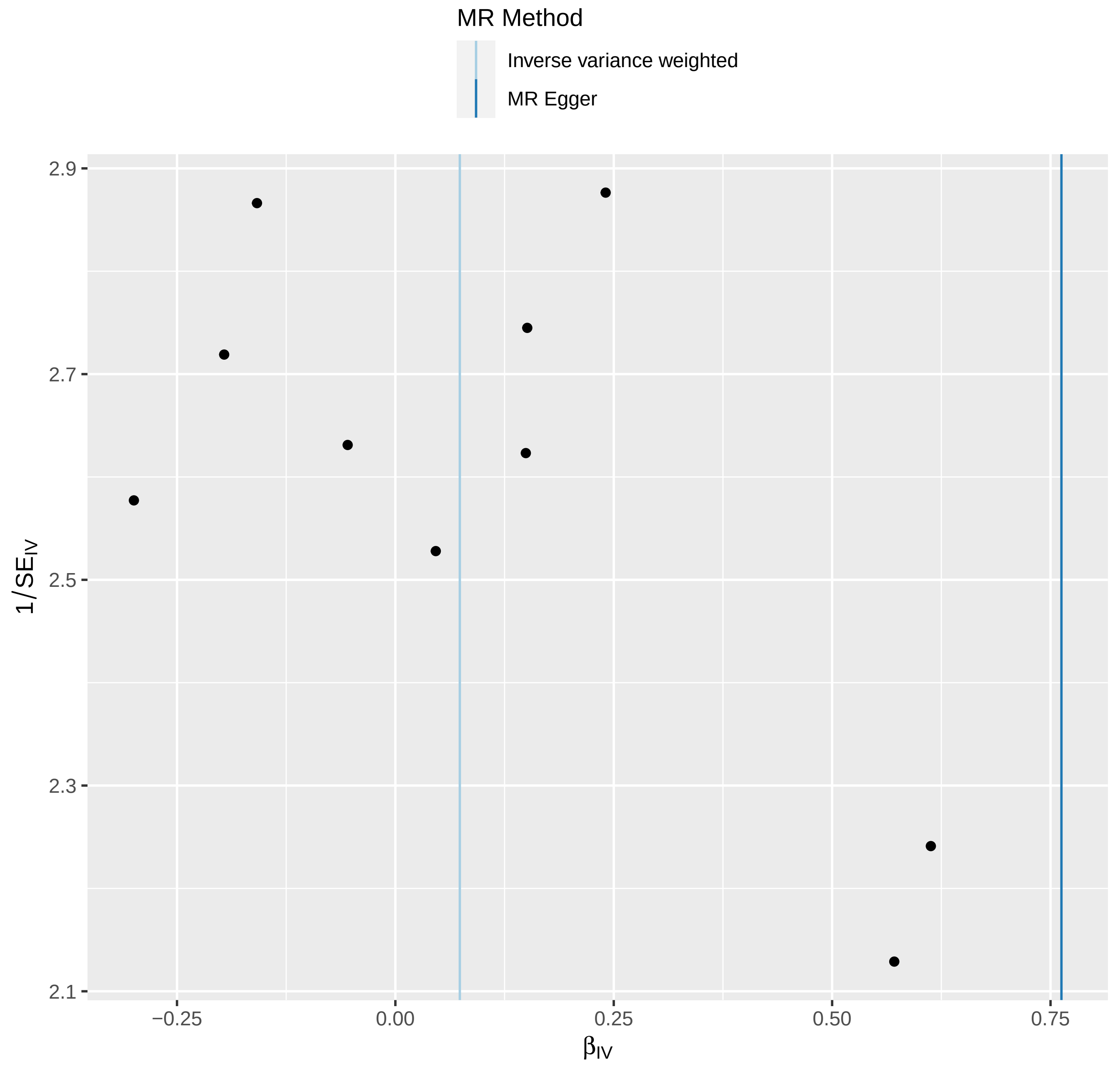

Batch 40 : Gut microbiota abundance (genus Anaerostipes id.1991) on Crohn's disease of large intestine

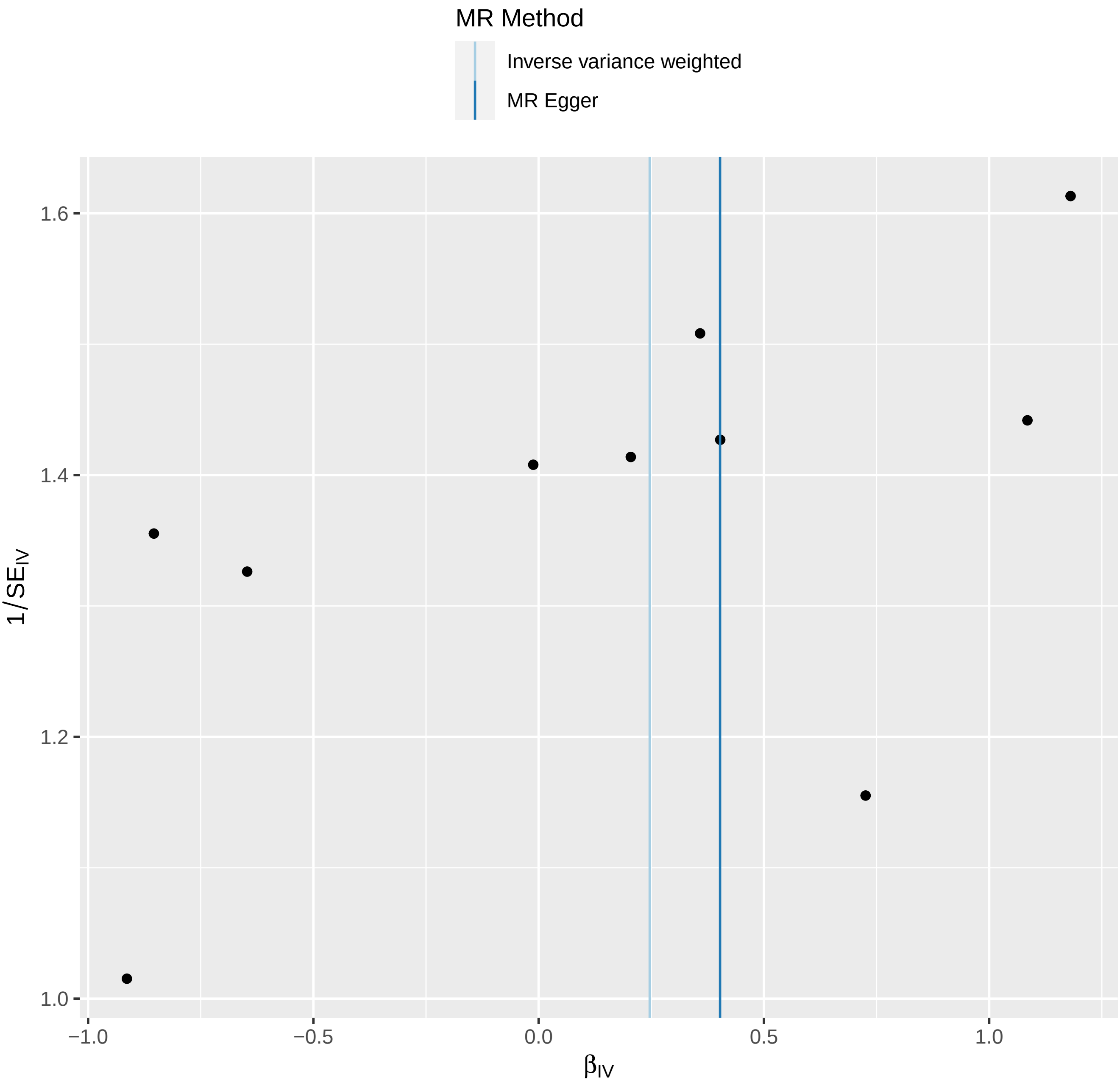

Batch 41 : Gut microbiota abundance (genus Anaerotruncus id.2054) on Crohn's disease of large intestine

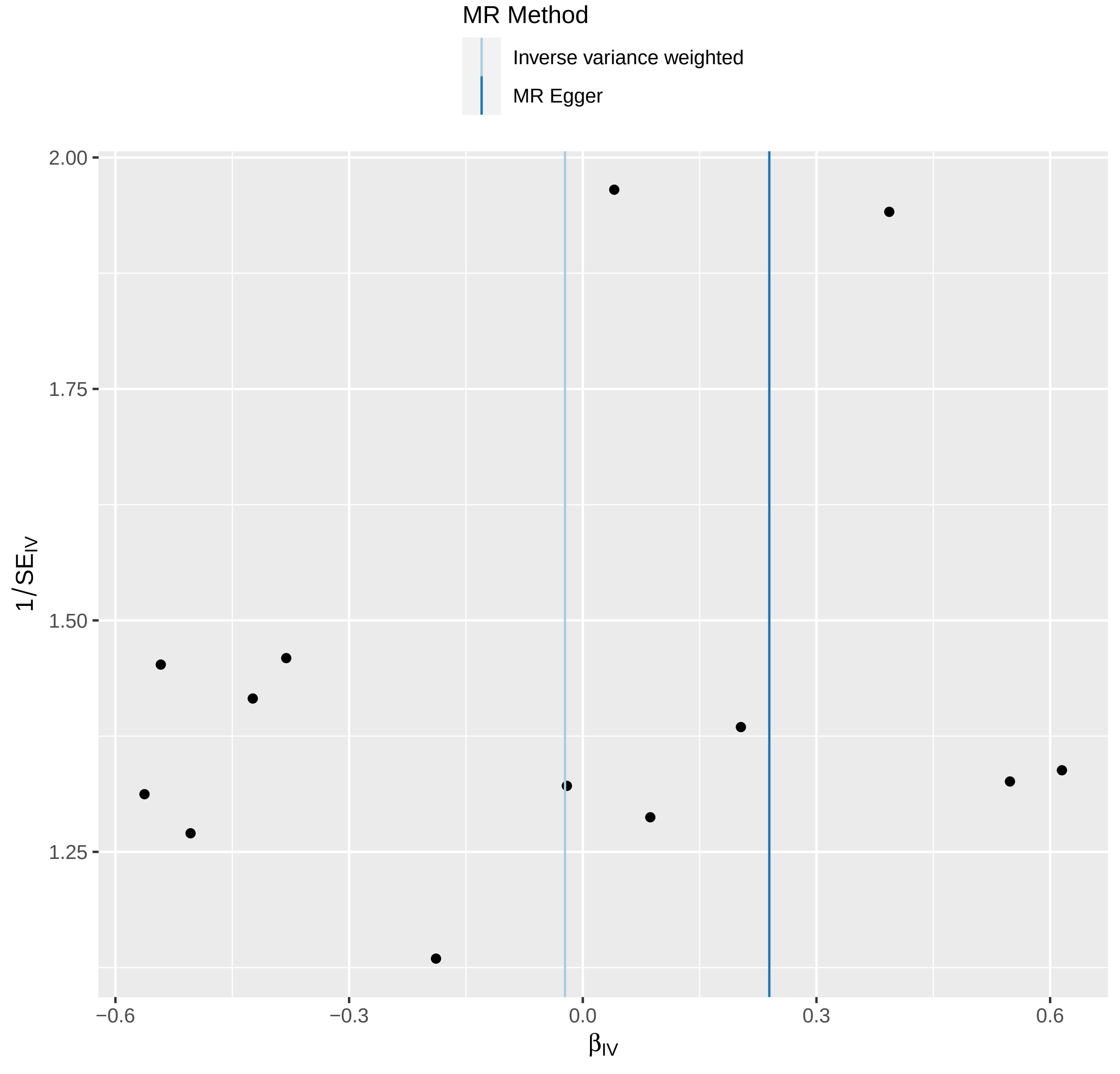

Batch 42 : Gut microbiota abundance (genus Bacteroides id.918) on Crohn's disease of large intestine

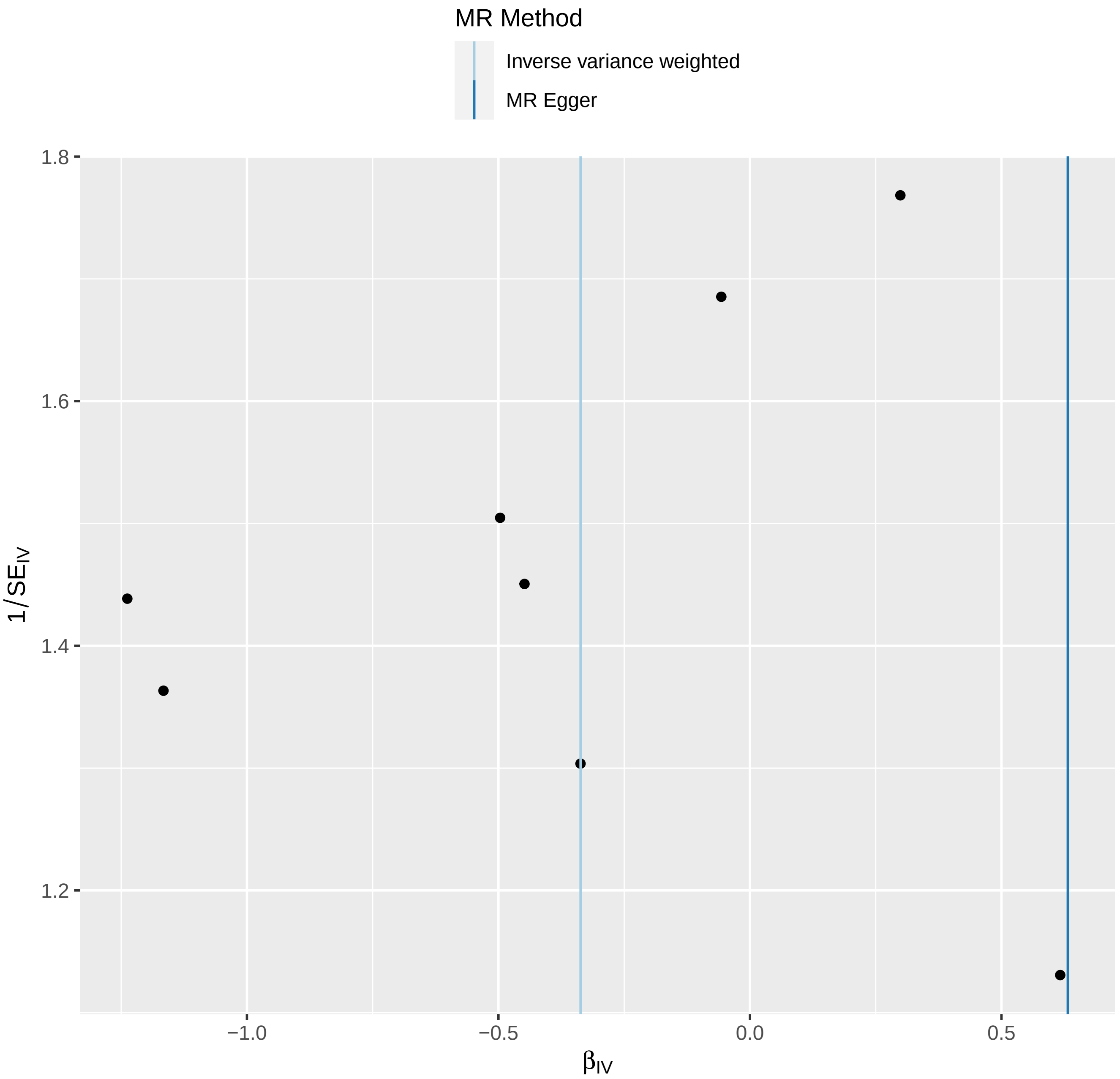

Batch 43 : Gut microbiota abundance (genus Barnesiella id.944) on Crohn's disease of large intestine

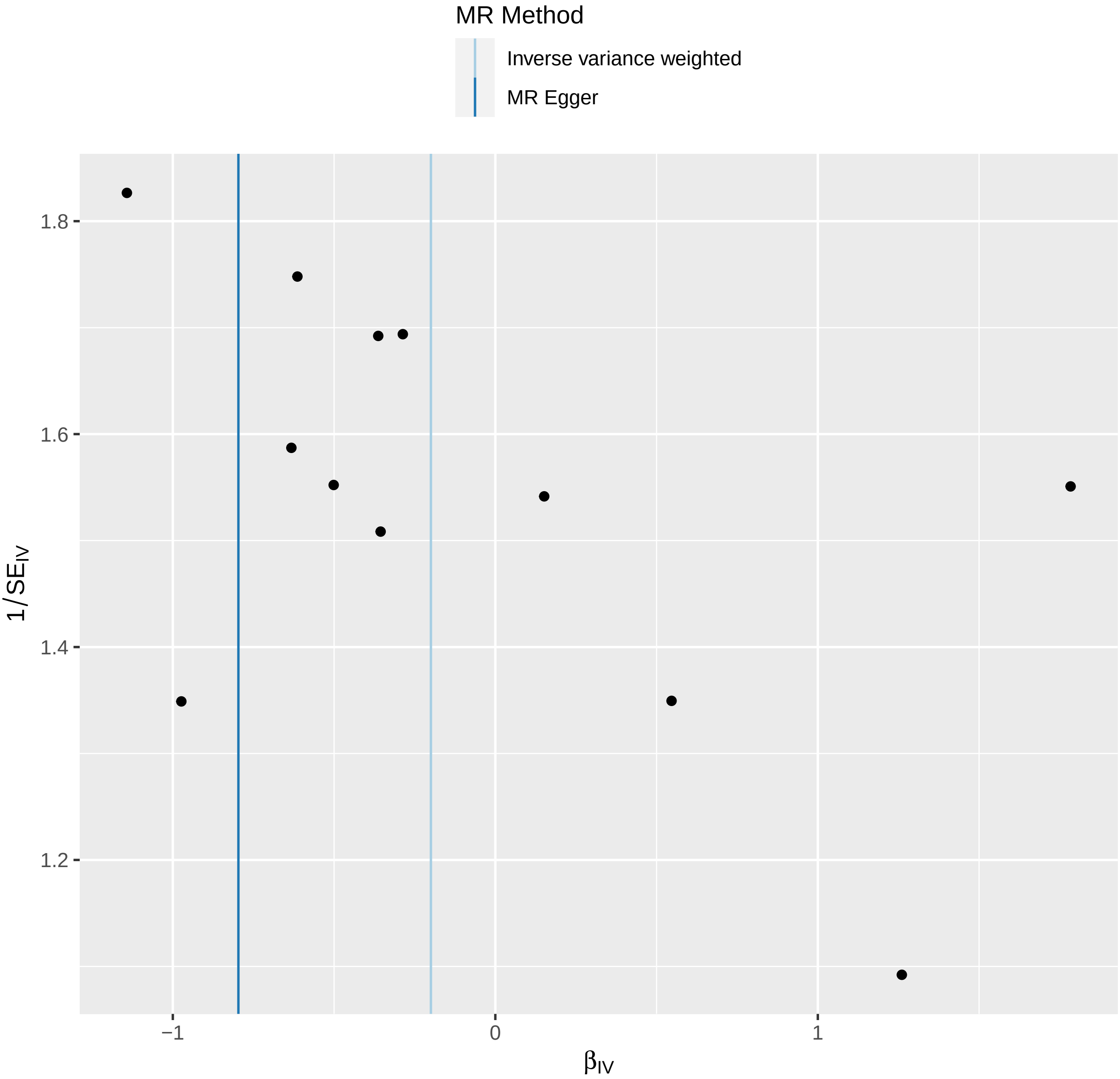

Batch 44 : Gut microbiota abundance (genus Bifidobacterium id.436) on Crohn's disease of large intestine

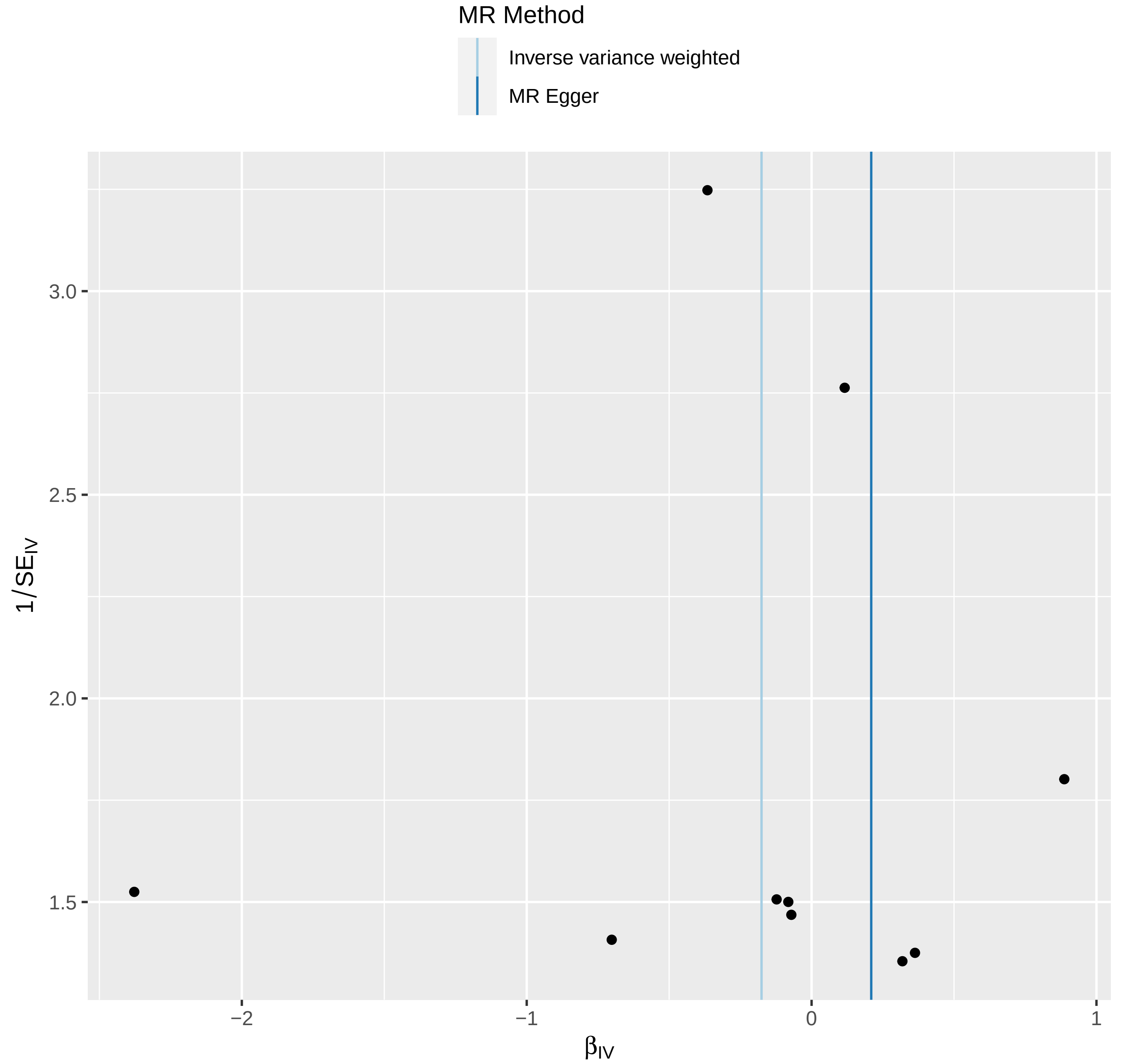

Batch 45 : Gut microbiota abundance (genus Bilophila id.3170) on Crohn's disease of large intestine

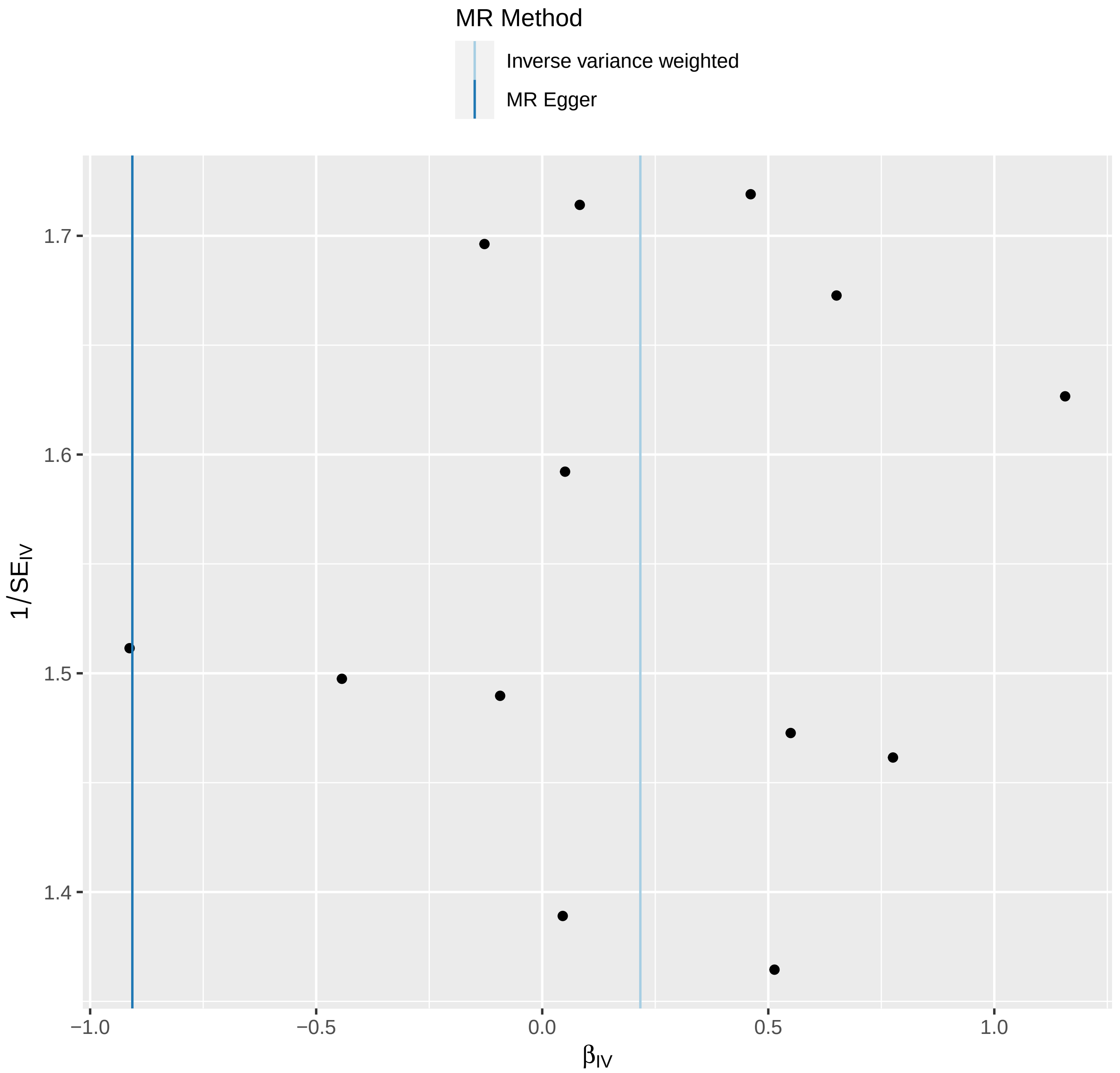

Batch 46 : Gut microbiota abundance (genus Blautia id.1992) on Crohn's disease of large intestine

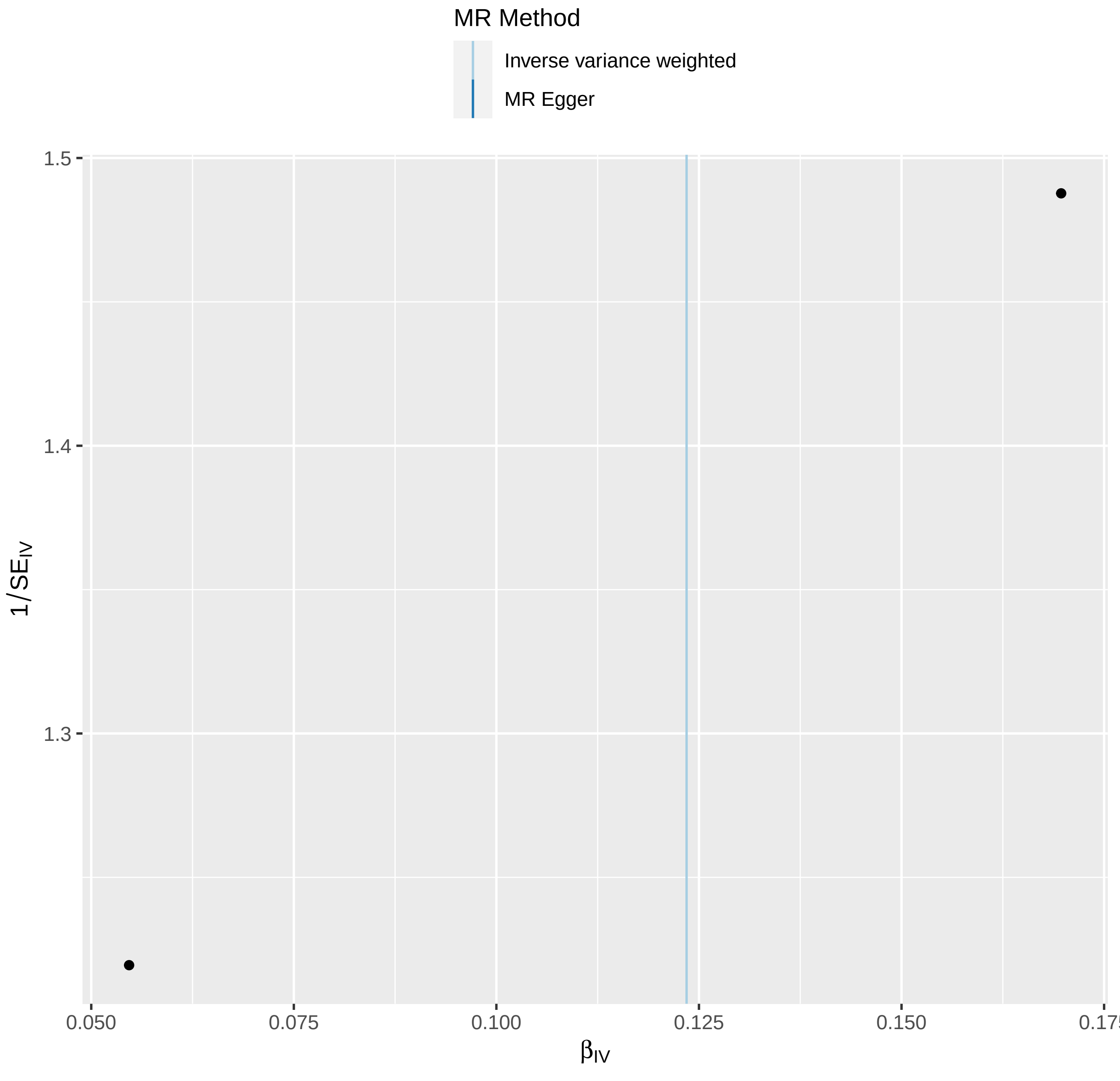

Batch 47 : Gut microbiota abundance (genus Butyricicoccus id.2055) on Crohn's disease of large intestine

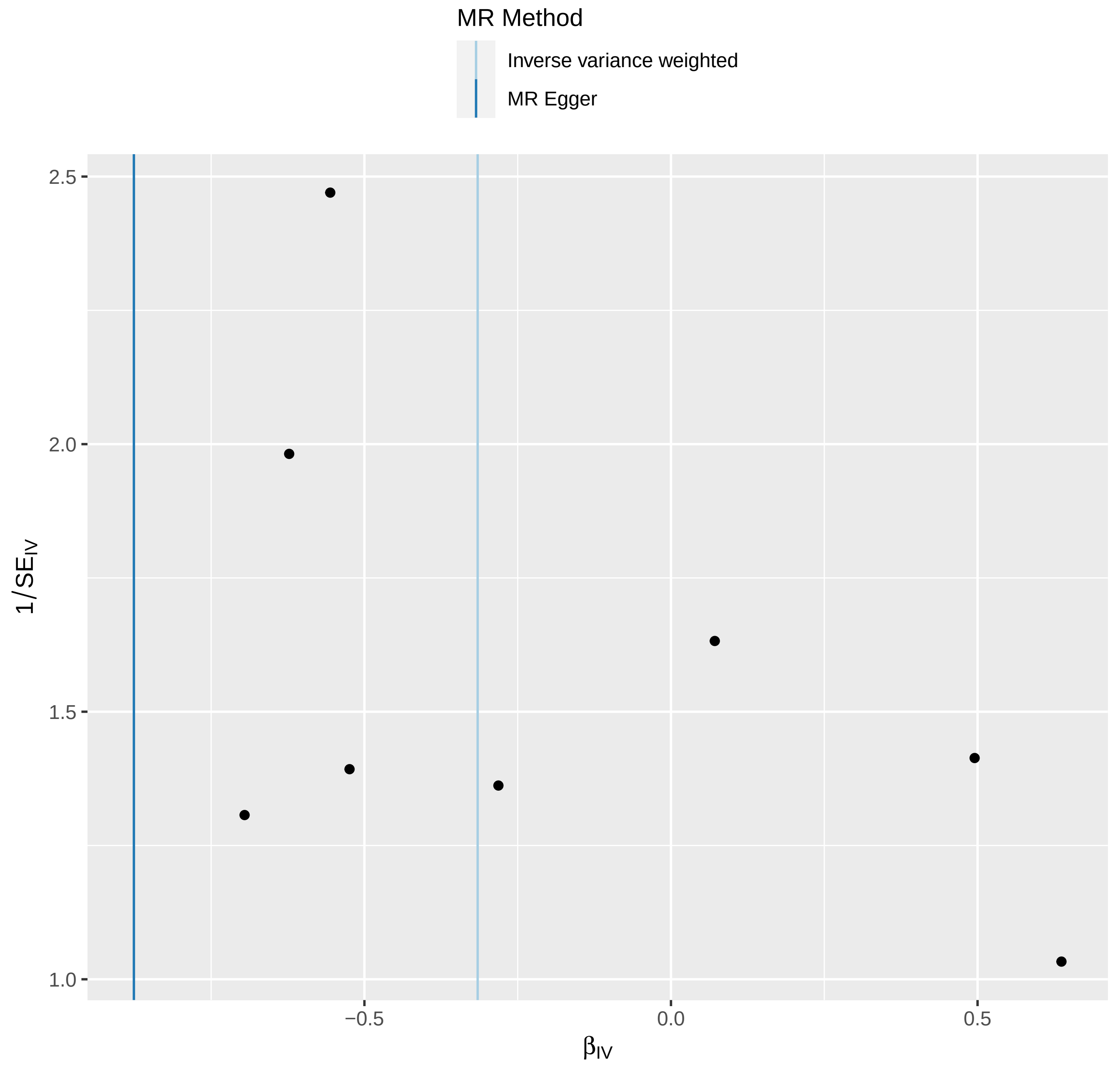

Batch 48 : Gut microbiota abundance (genus Butyricimonas id.945) on Crohn's disease of large intestine

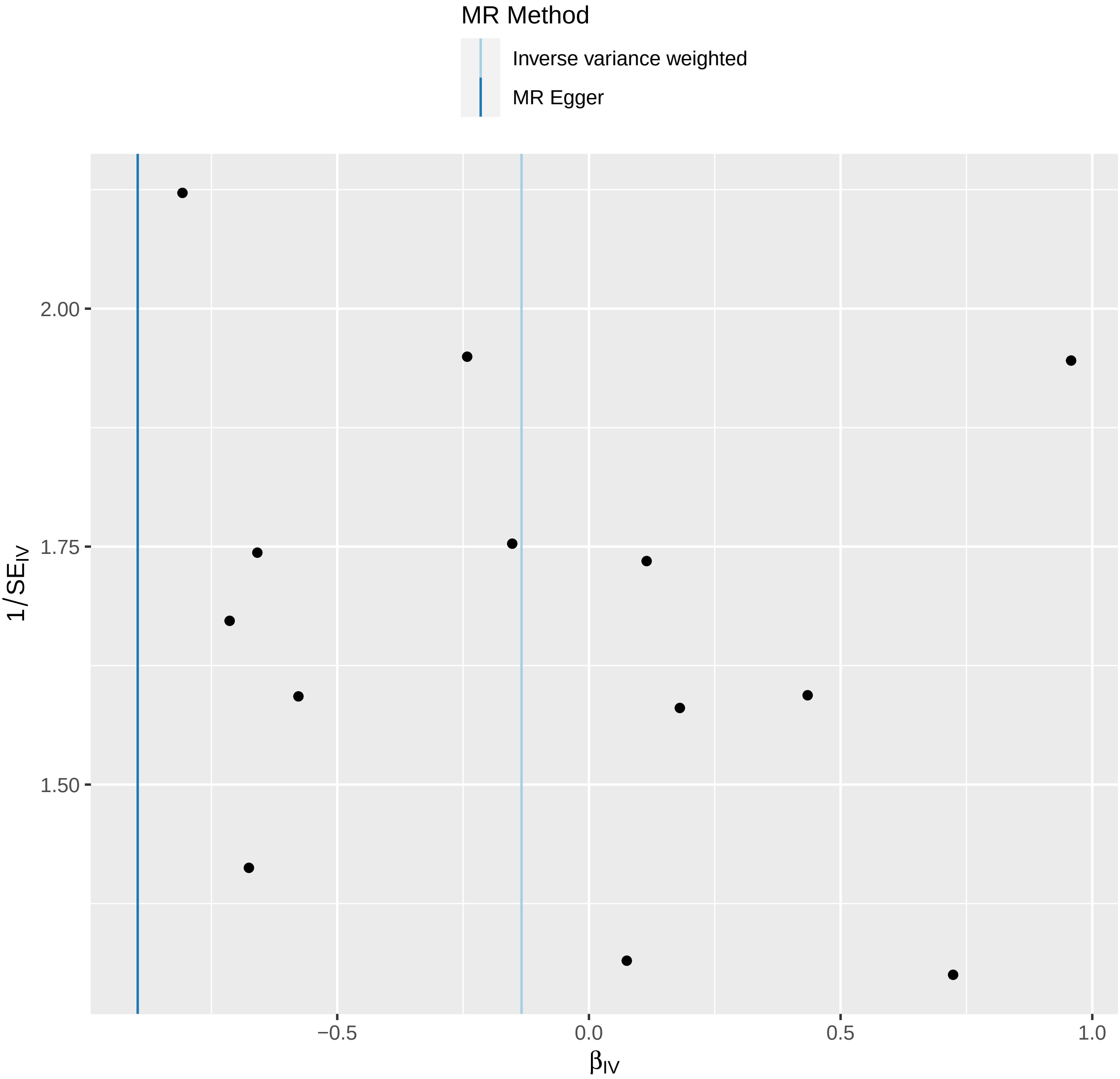

Batch 49 : Gut microbiota abundance (genus Butyrivibrio id.1993) on Crohn's disease of large intestine

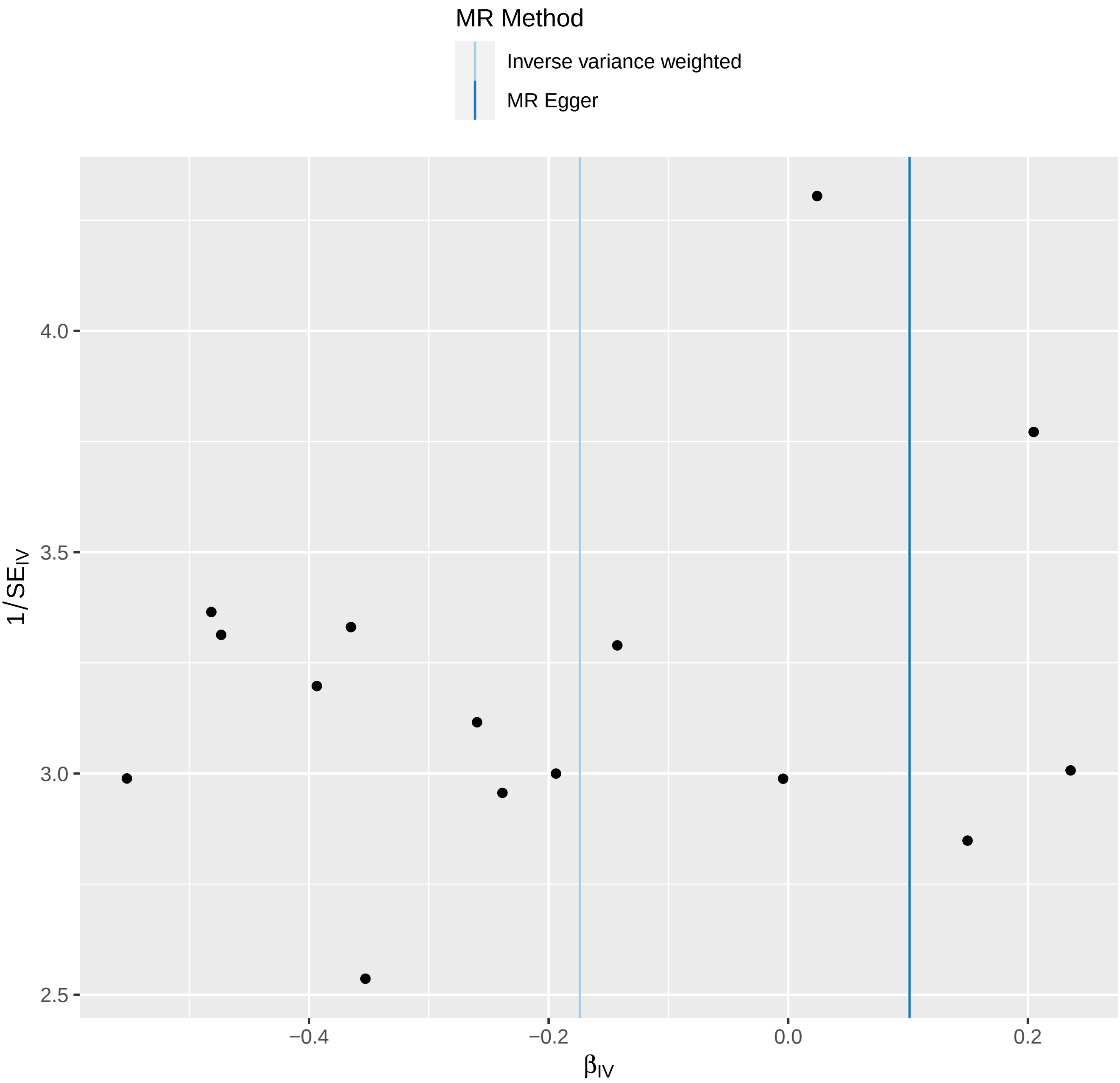

Batch 50 : Gut microbiota abundance (genus Candidatus Soleaferrea id.11350) on Crohn's disease of large intestine

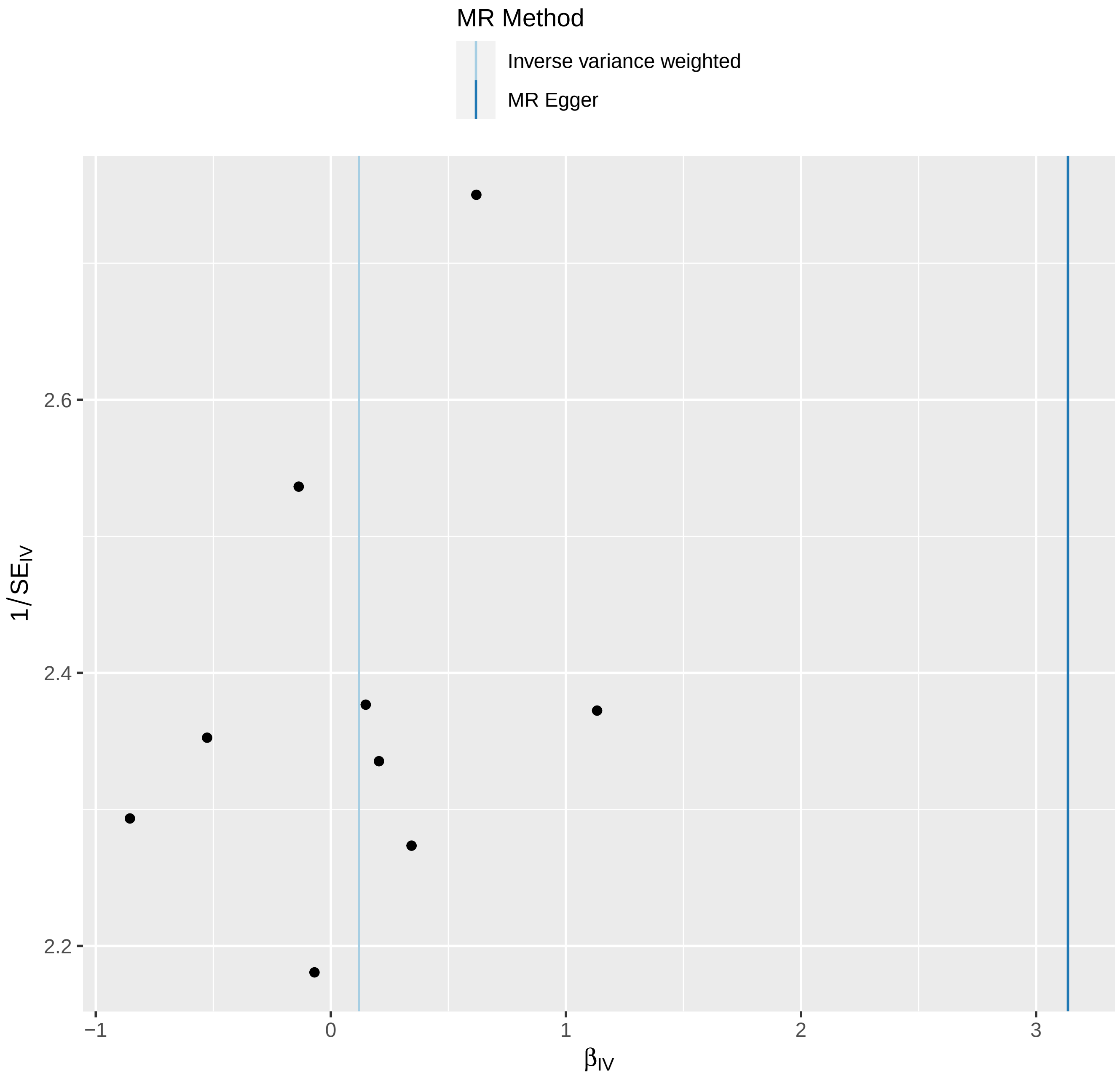

Batch 51 : Gut microbiota abundance (genus Catenibacterium id.2153) on Crohn's disease of large intestine

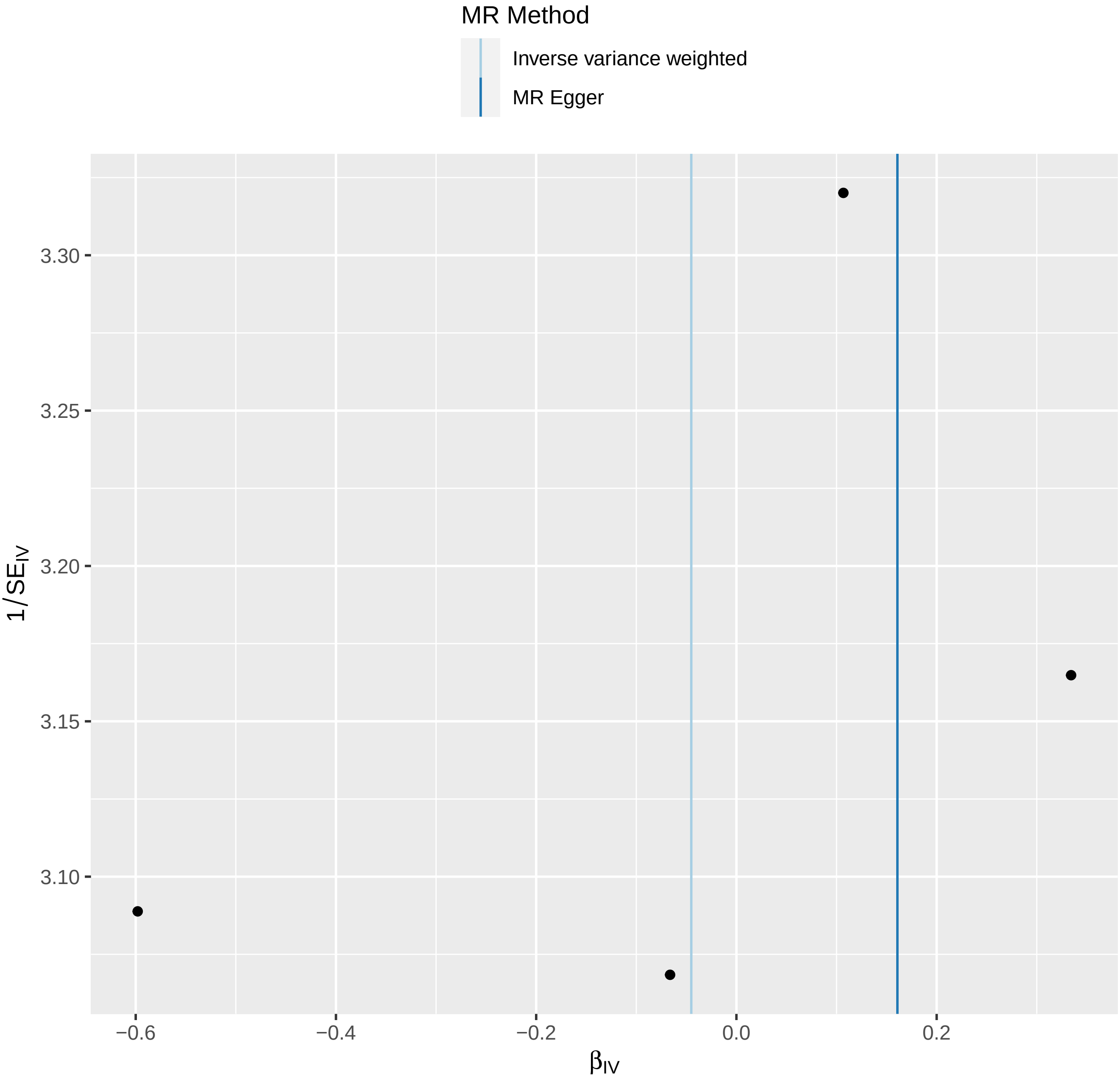

Batch 52 : Gut microbiota abundance (genus Christensenellaceae R 7group id.11283) on Crohn's disease of large intestine

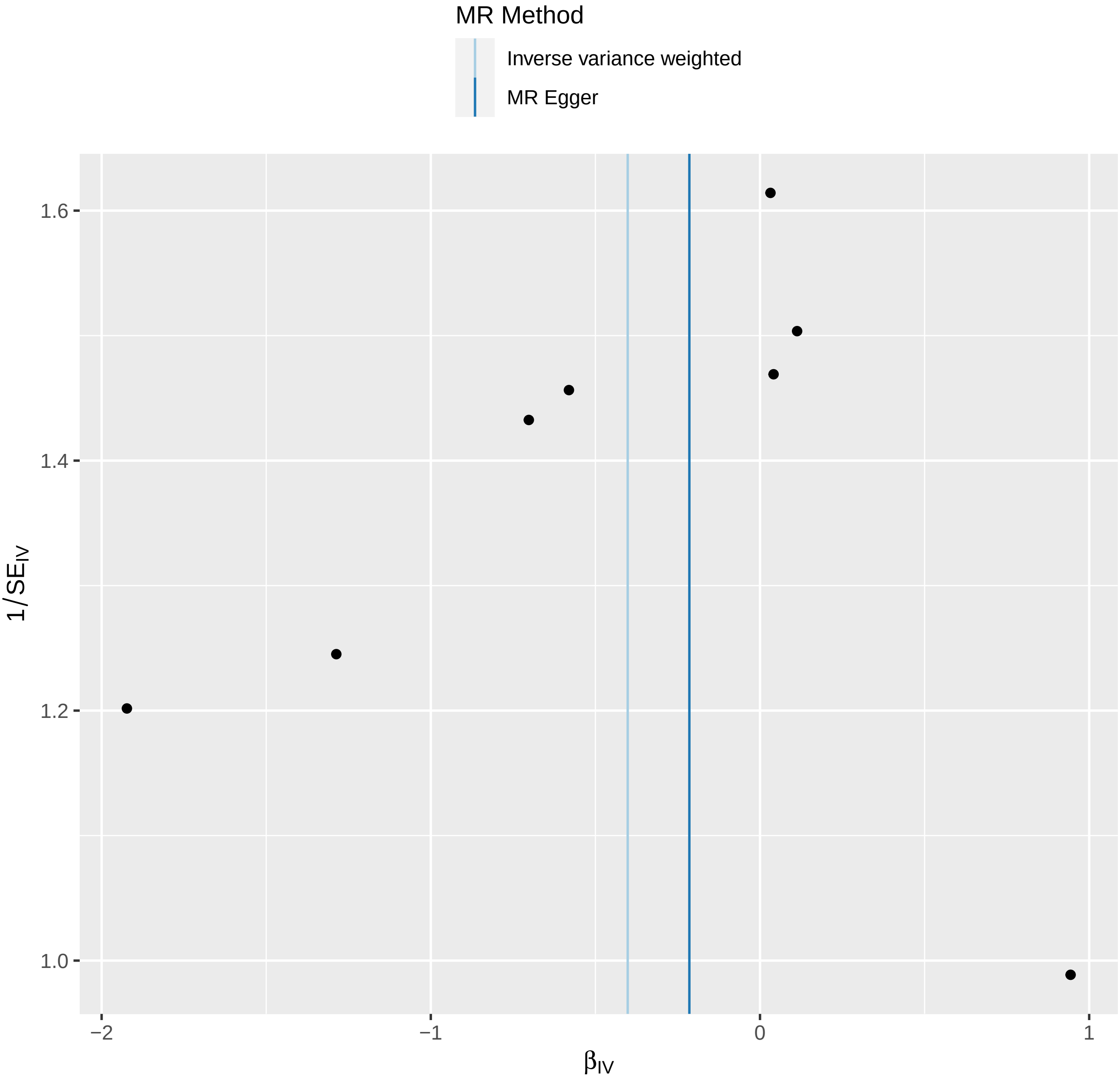

Batch 53 : Gut microbiota abundance (genus Clostridium innocuum group id.14397) on Crohn's disease of large intestine

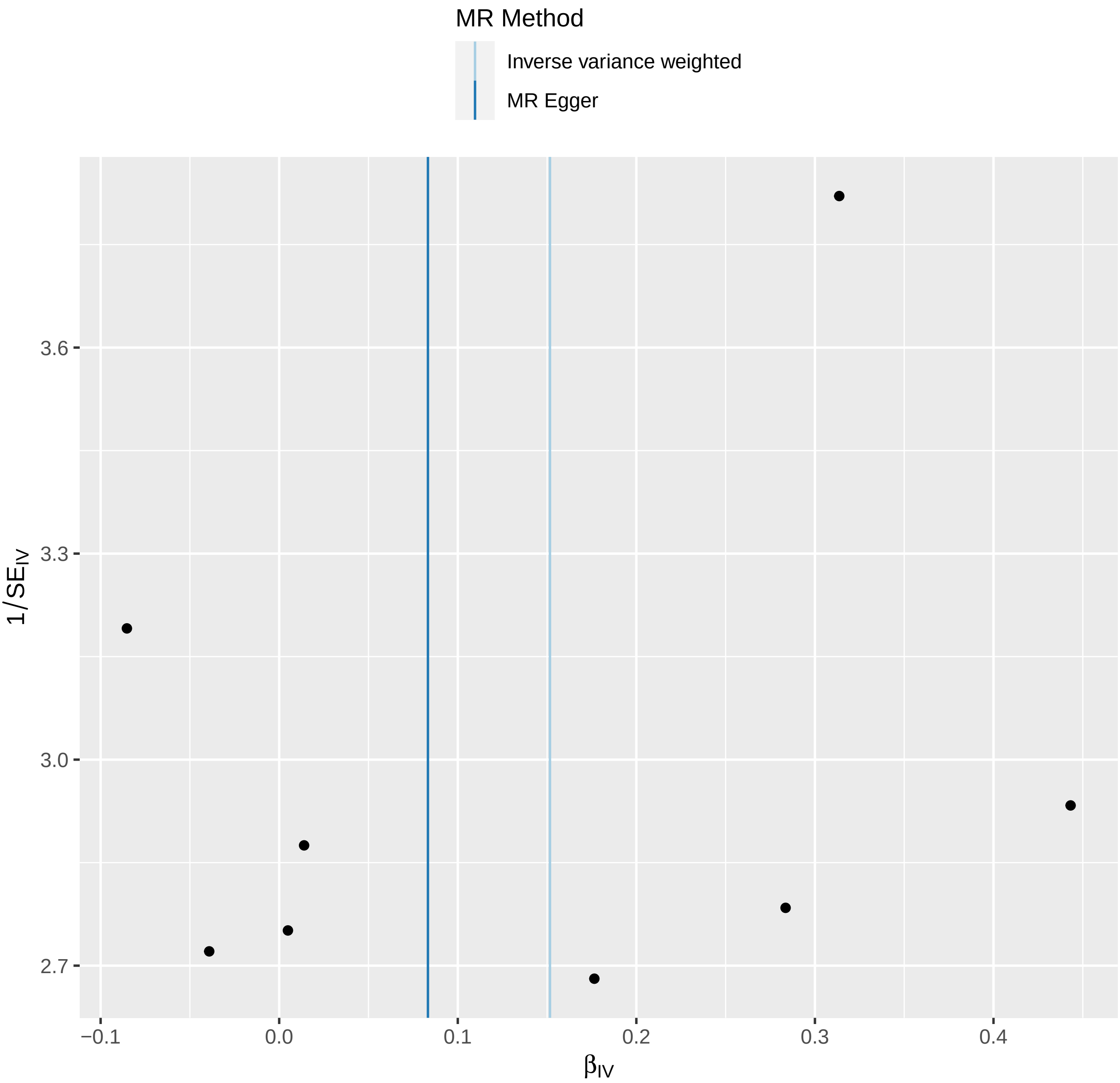

Batch 54 : Gut microbiota abundance (genus Clostridium sensustricto1 id.1873) on Crohn's disease of large intestine

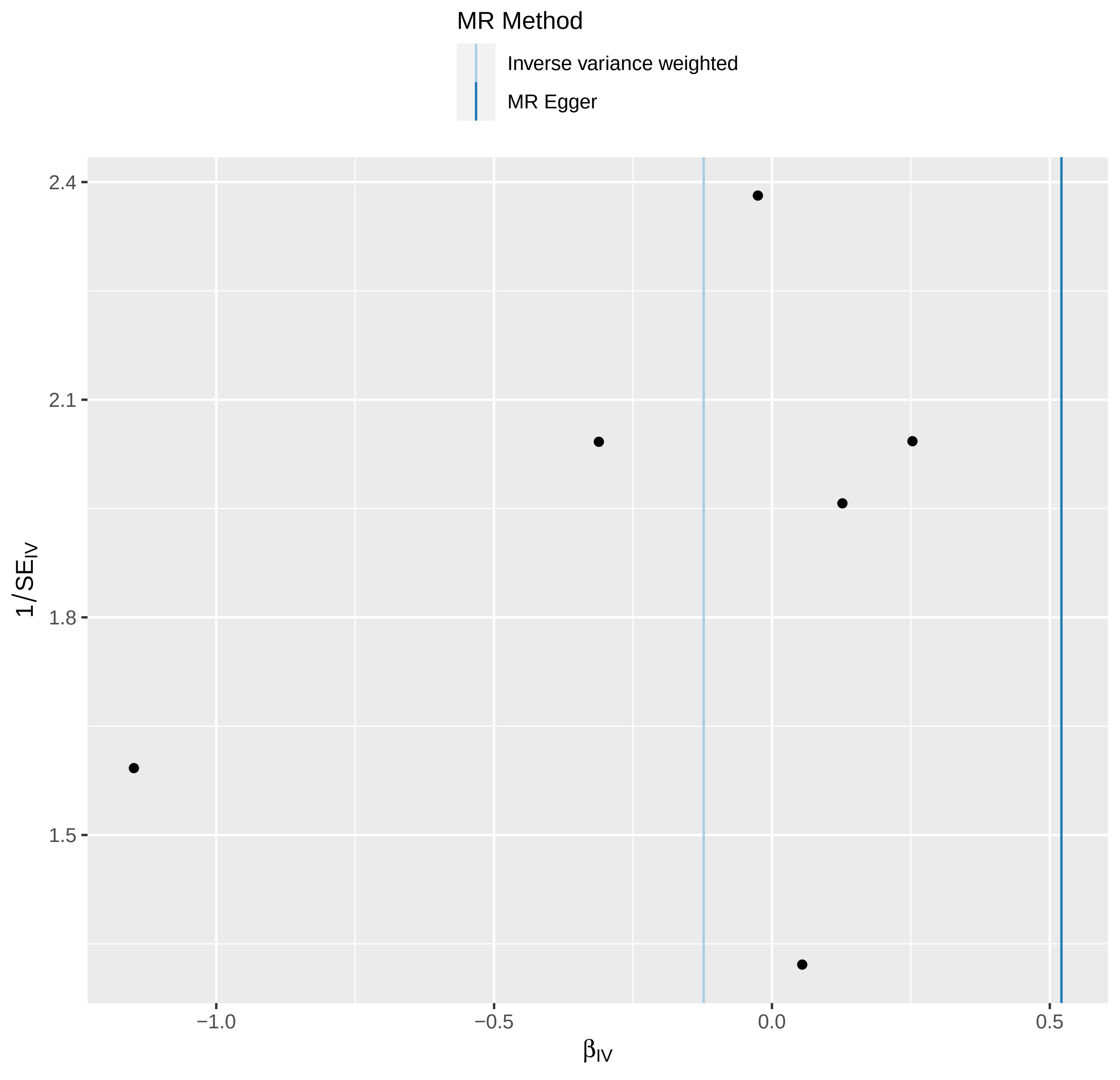

Batch 55 : Gut microbiota abundance (genus Collinsella id.815) on Crohn's disease of large intestine

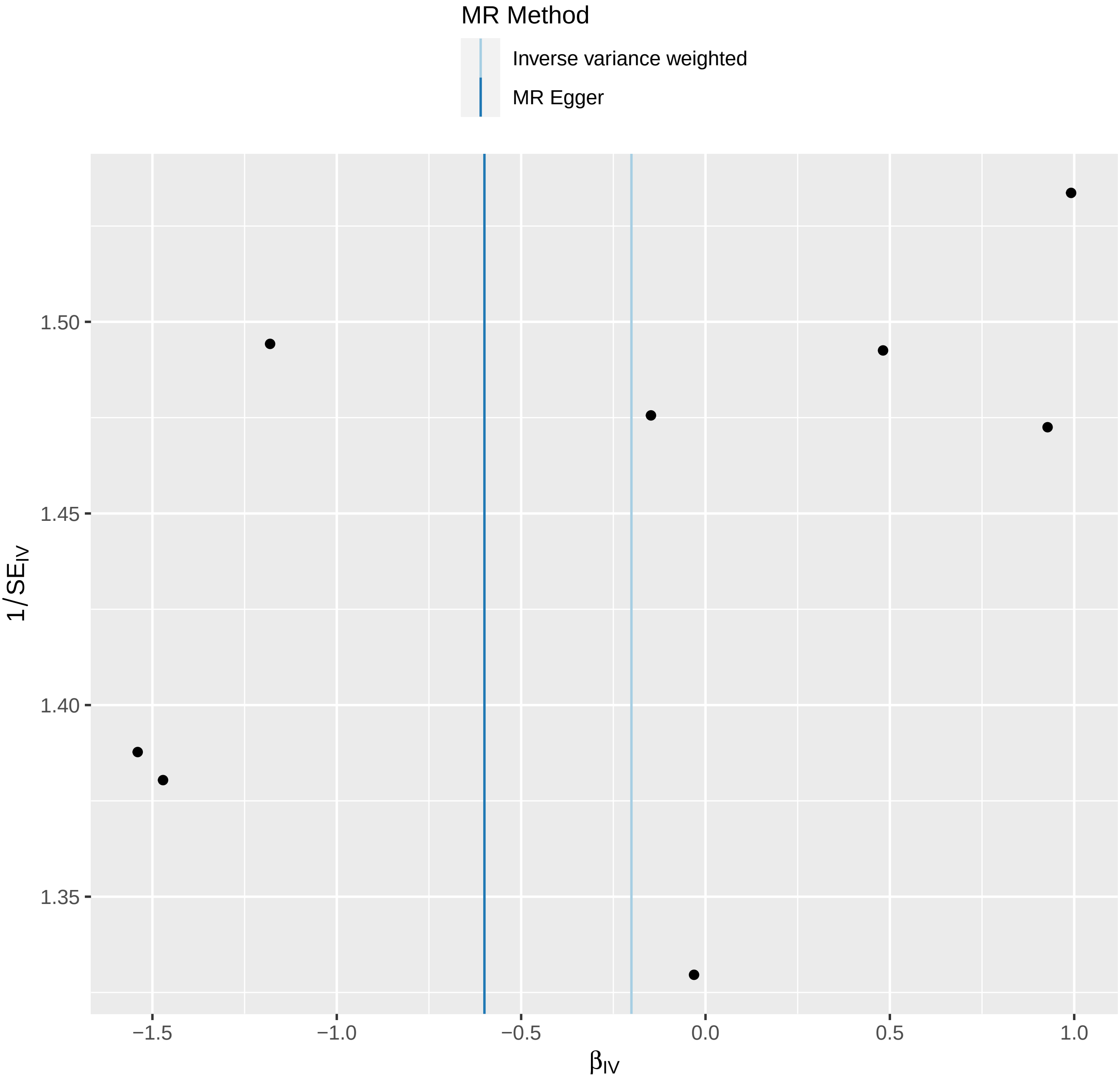

Batch 56 : Gut microbiota abundance (genus Coprobacter id.949) on Crohn's disease of large intestine

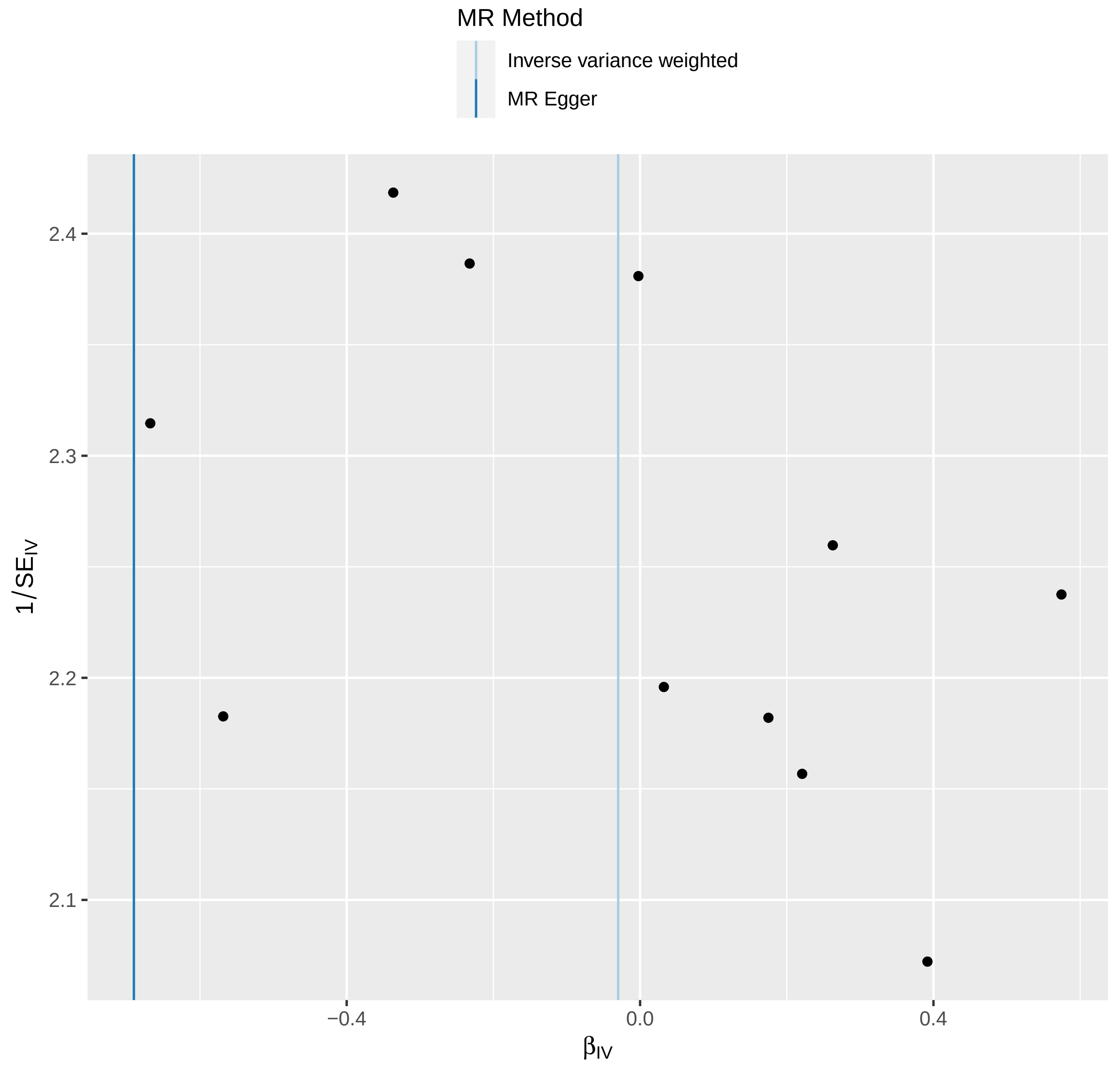

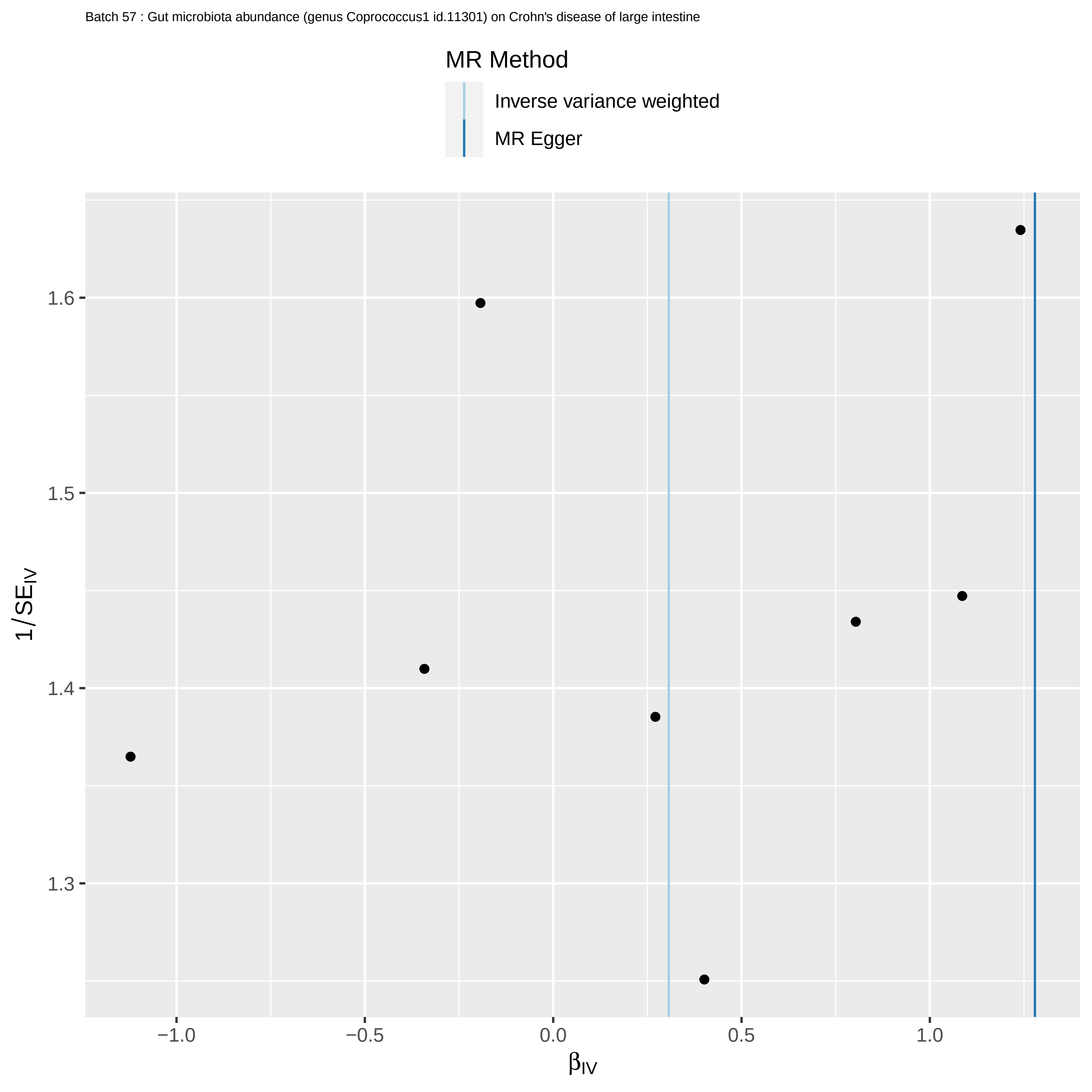
Batch 57 : Gut microbiota abundance (genus Coprococcus1 id.11301) on Crohn's disease of large intestine
MR Method
Inverse variance weighted
MR Egger
1.6
1.5
1.4
1.3
−1.0
−0.5
0.0
0.5
1.0
1/SEIV
βIV

Batch 58 : Gut microbiota abundance (genus Coprococcus2 id.11302) on Crohn's disease of large intestine

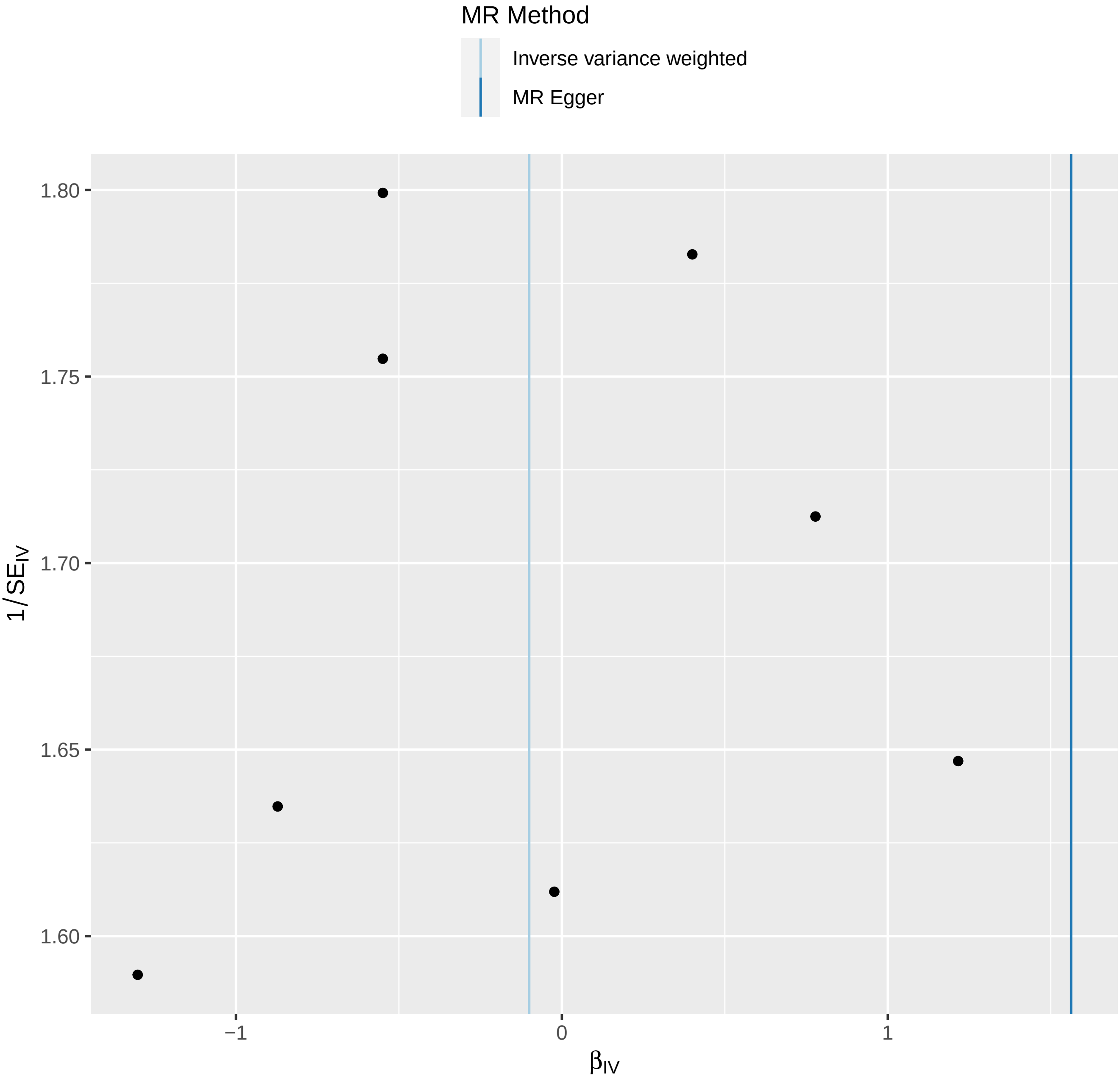

Batch 59 : Gut microbiota abundance (genus Coprococcus3 id.11303) on Crohn's disease of large intestine

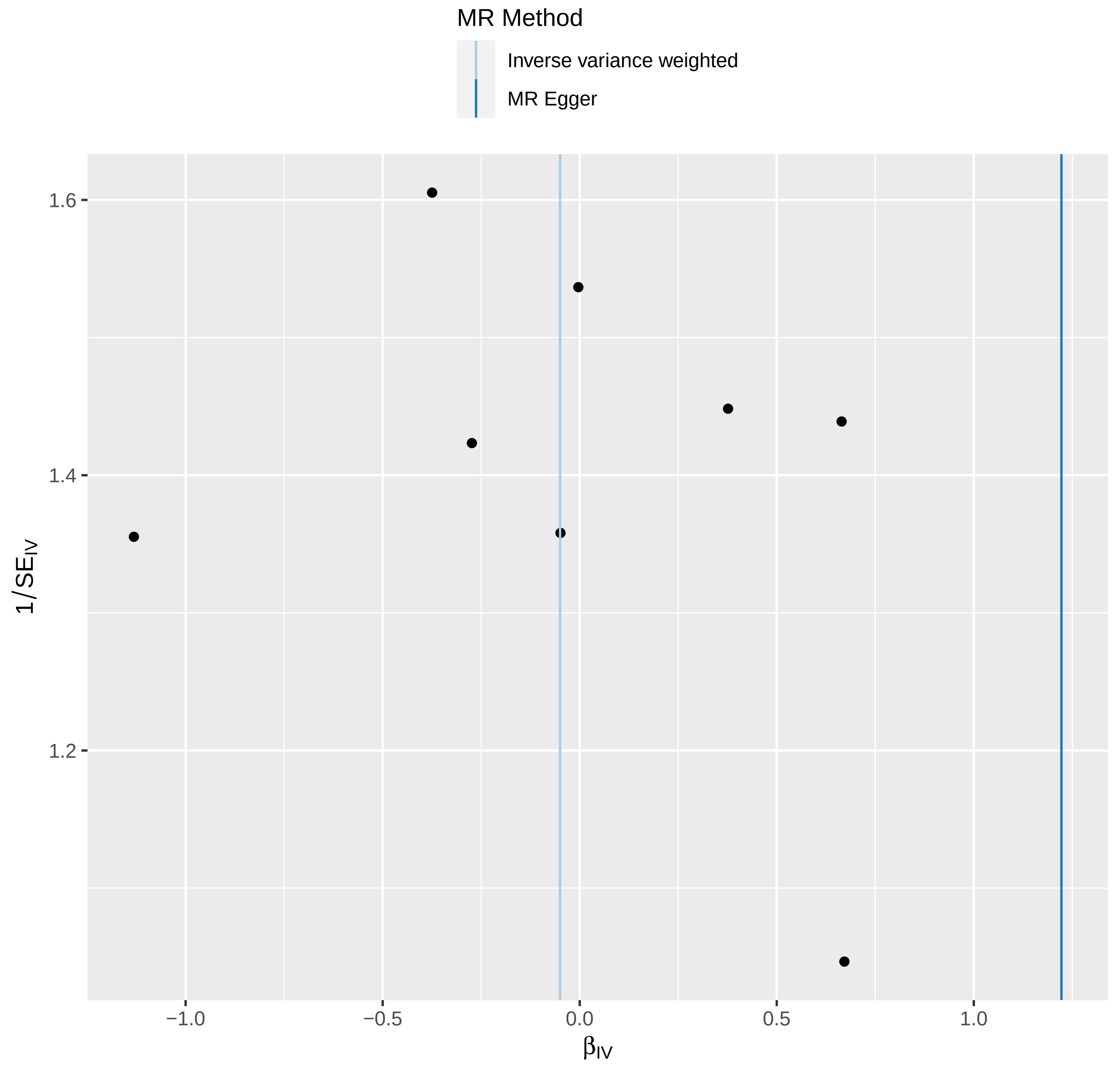

Batch 60 : Gut microbiota abundance (genus Defluviitaleaceae UCG011 id.11287) on Crohn's disease of large intestine

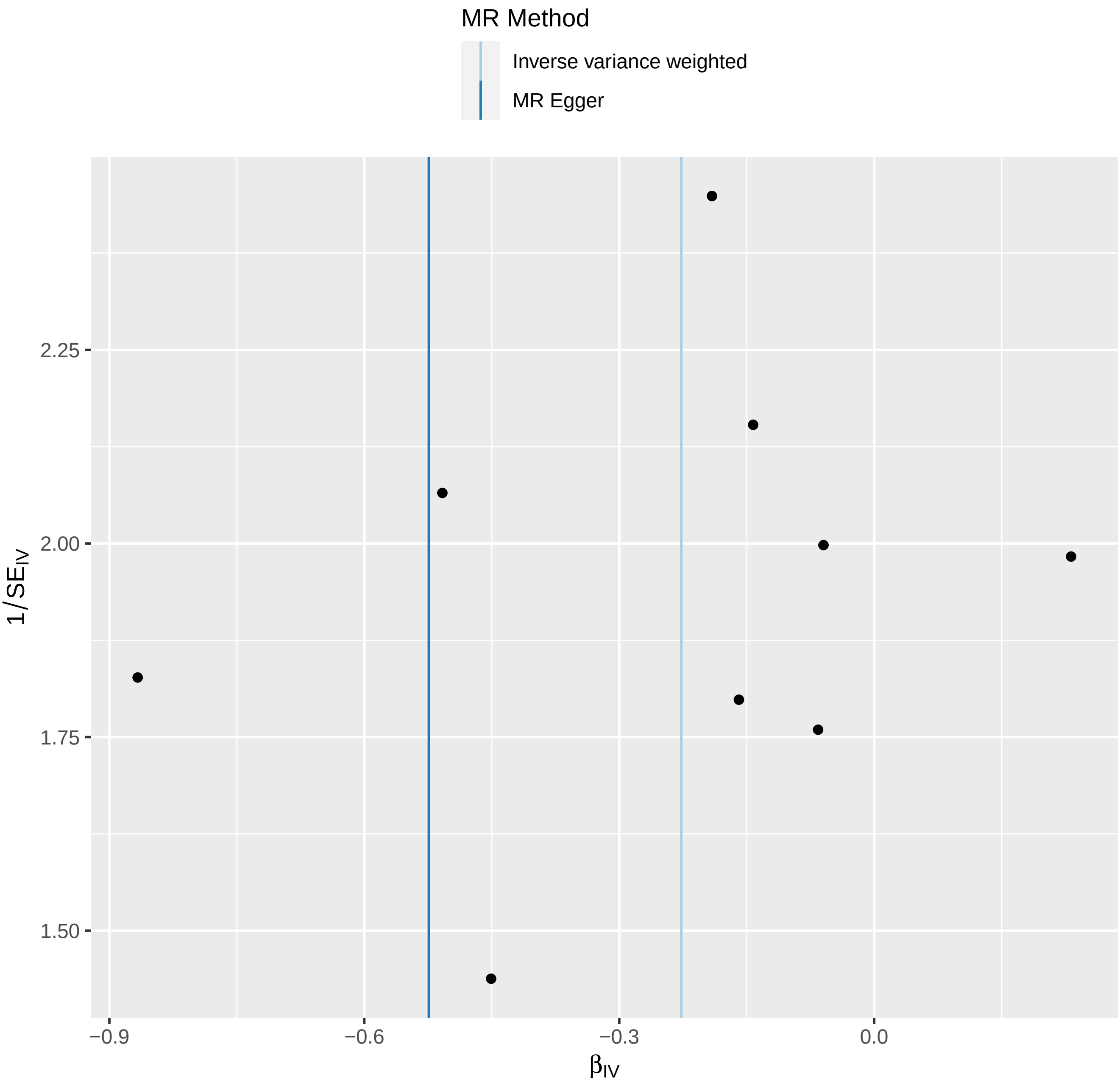

Batch 61 : Gut microbiota abundance (genus Desulfovibrio id.3173) on Crohn's disease of large intestine

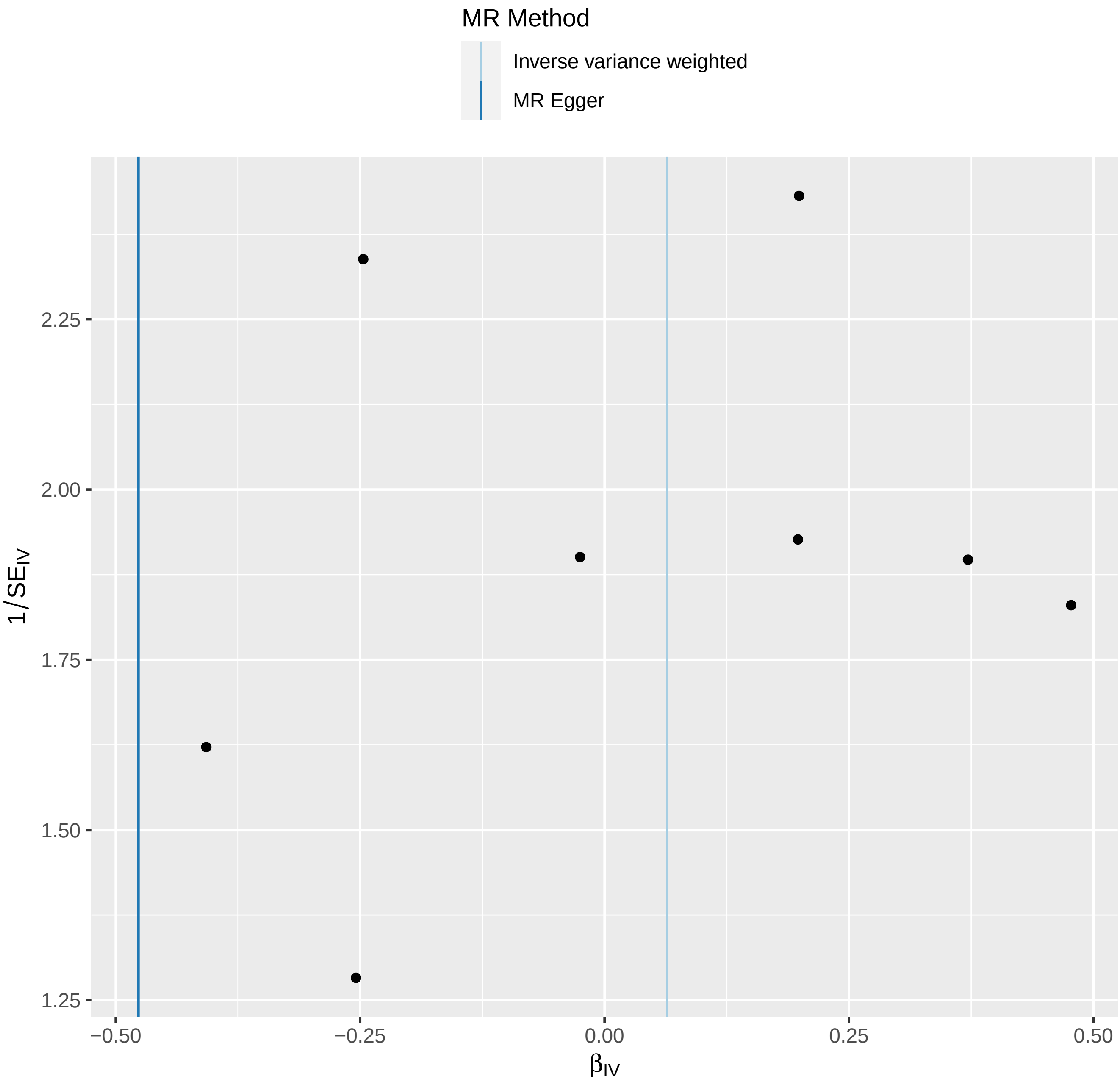

Batch 62 : Gut microbiota abundance (genus Dialister id.2183) on Crohn's disease of large intestine

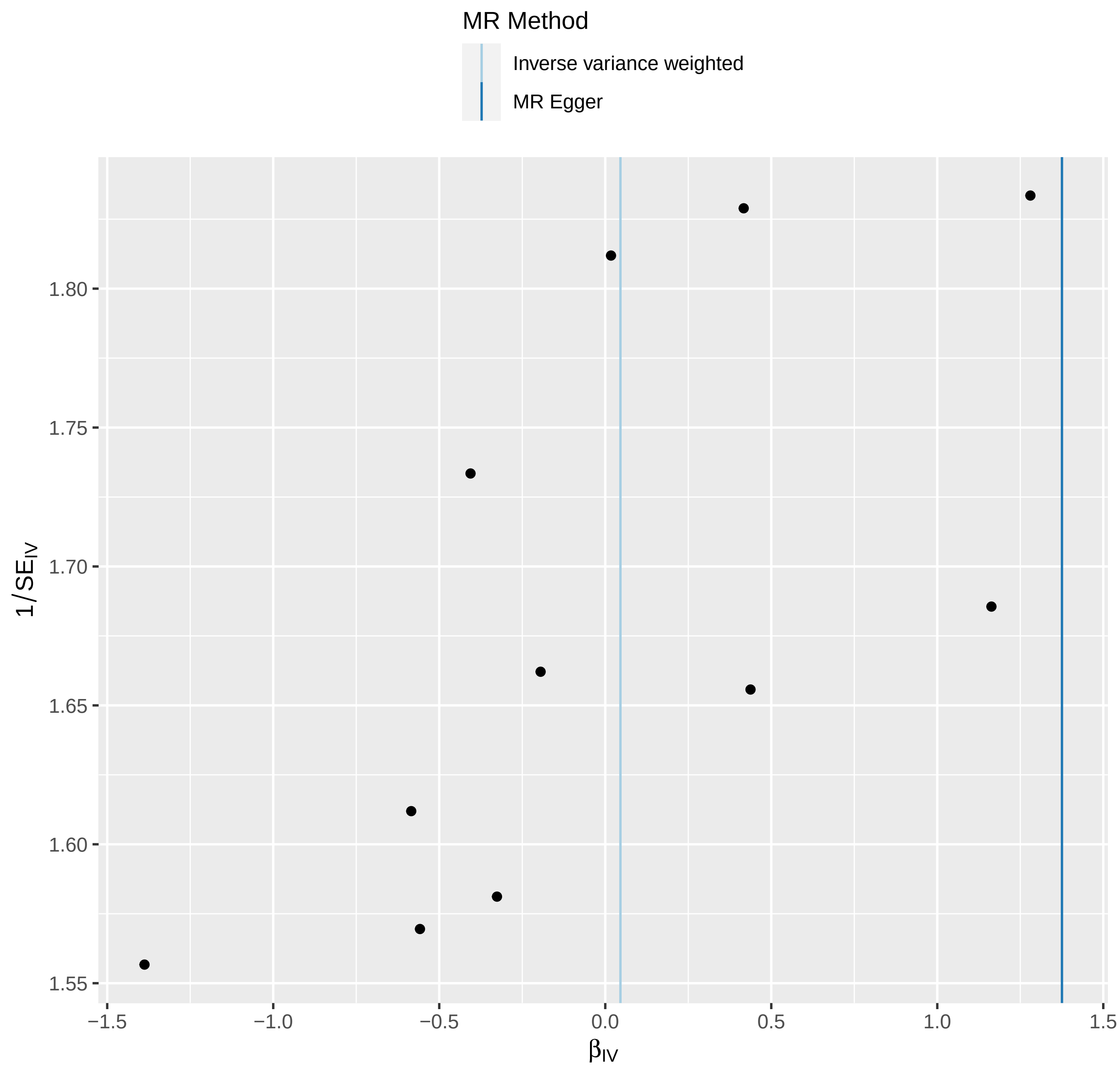

Batch 63 : Gut microbiota abundance (genus Dorea id.1997) on Crohn's disease of large intestine

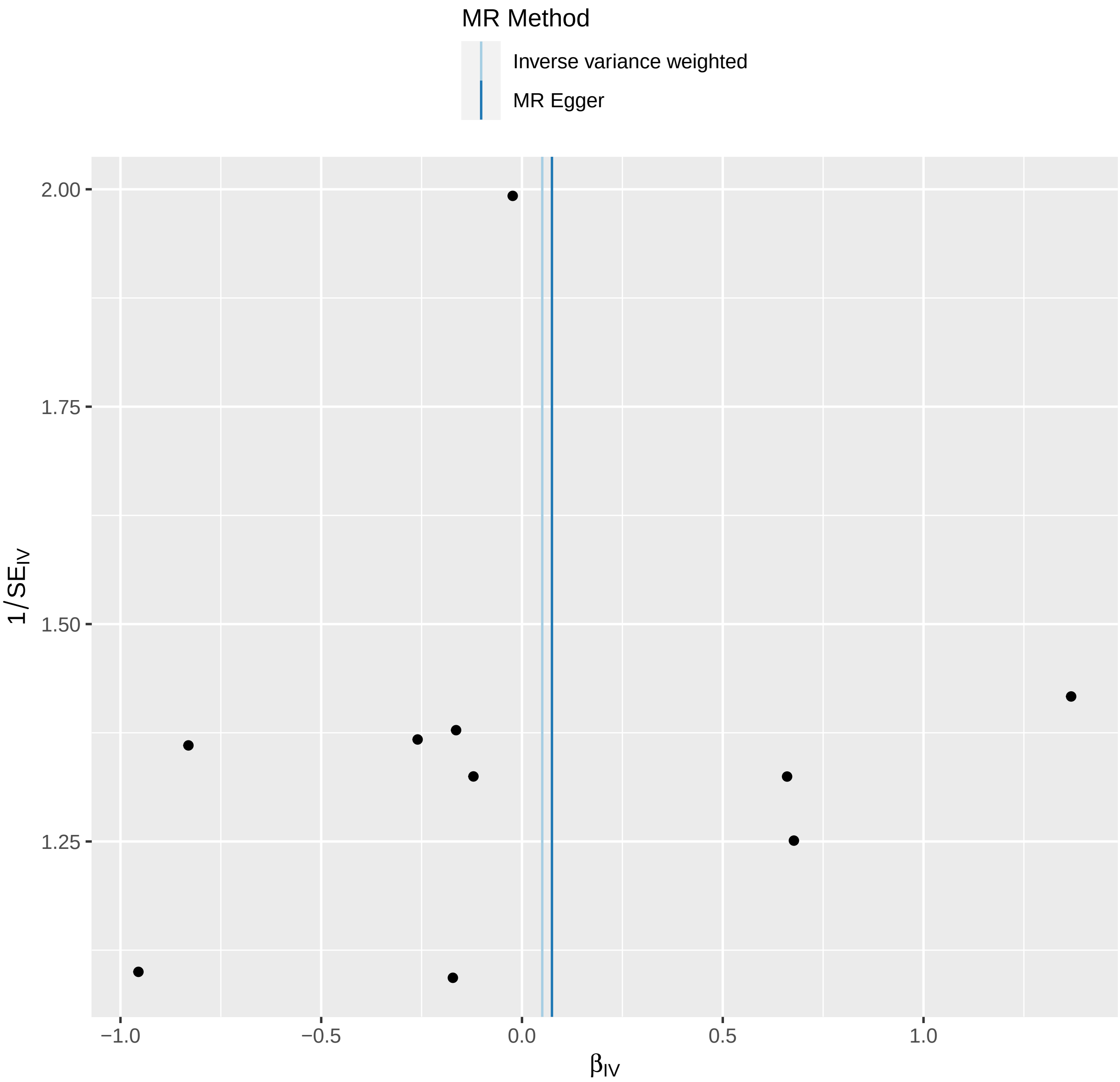

Batch 64 : Gut microbiota abundance (genus Eggerthella id.819) on Crohn's disease of large intestine

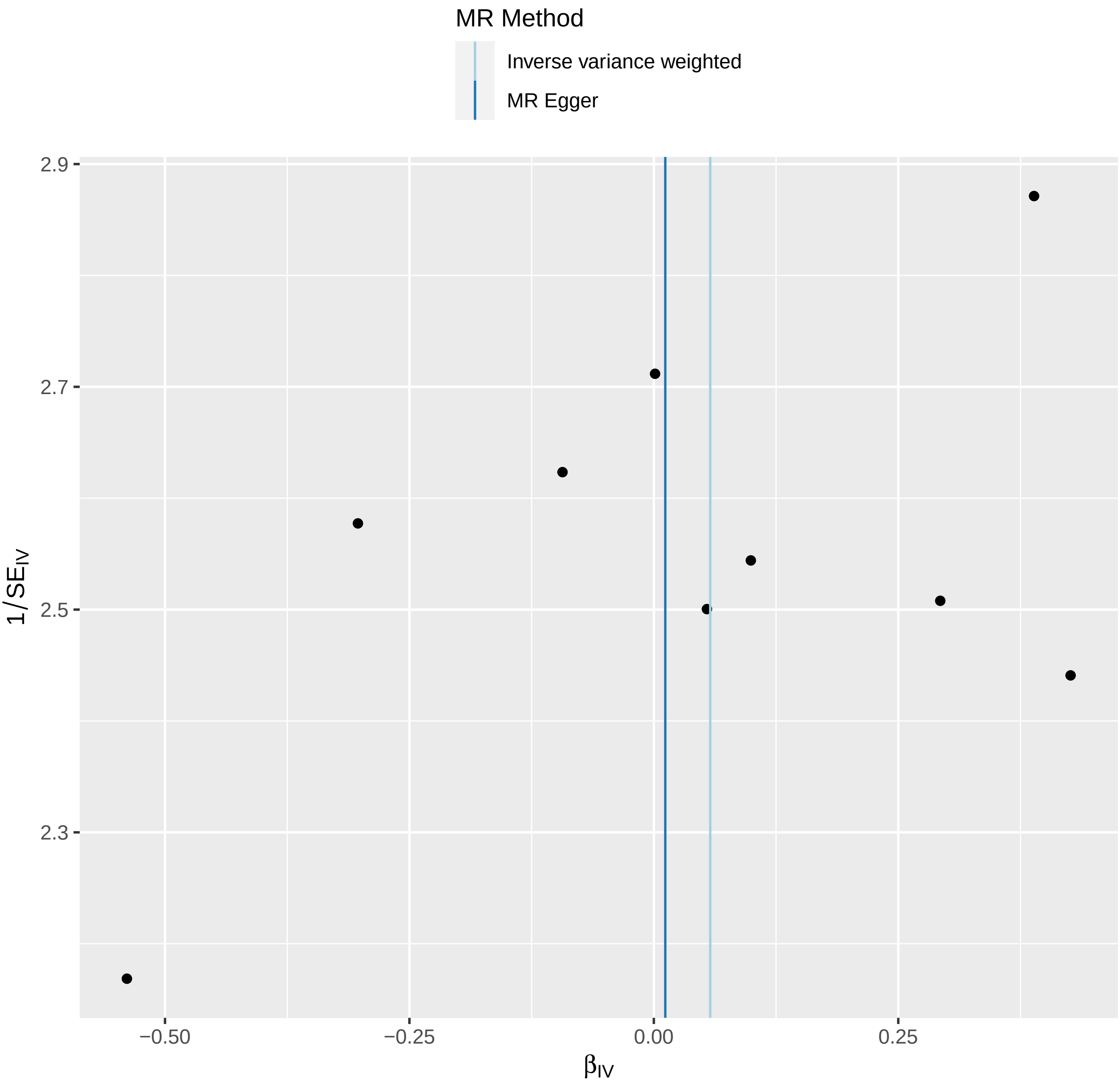

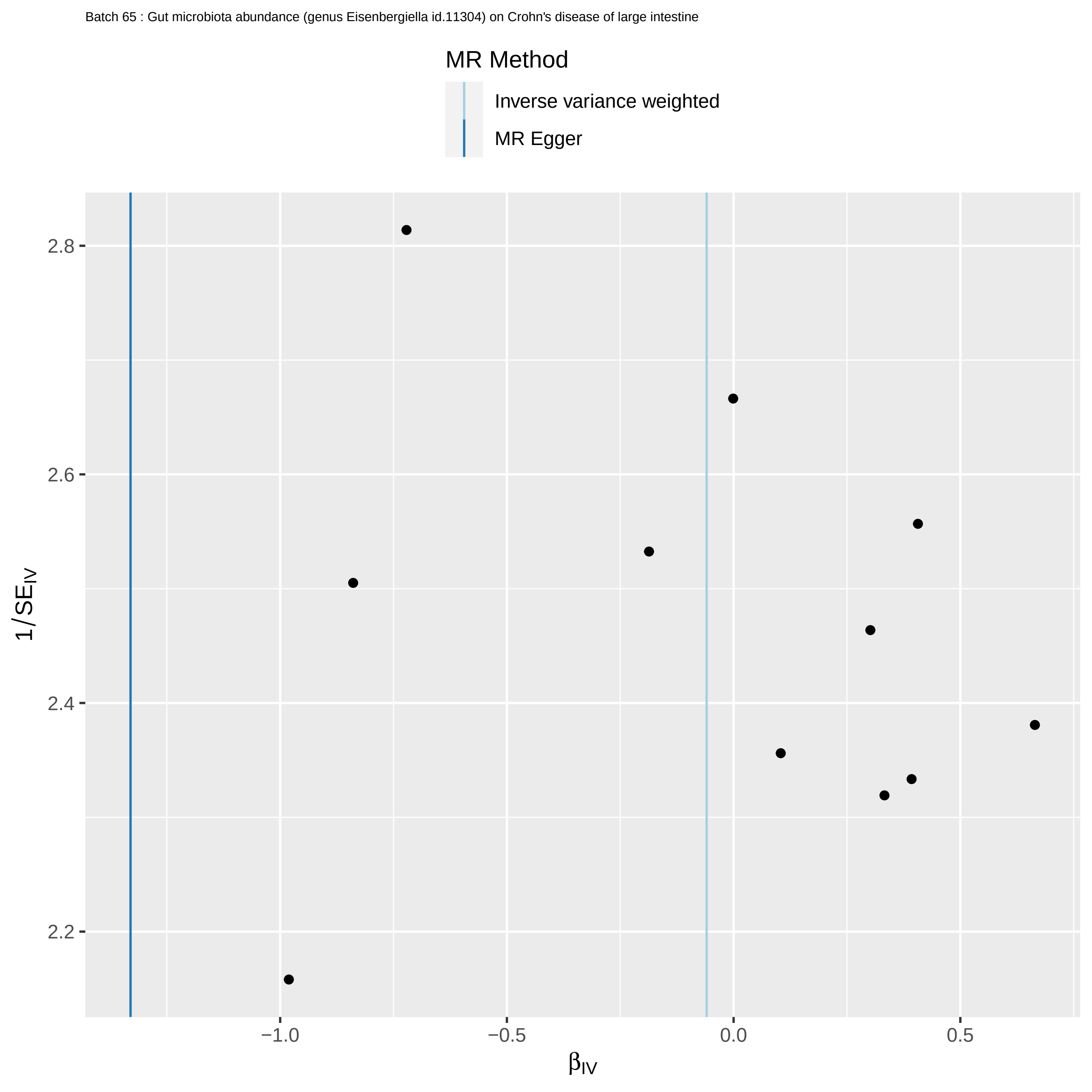
Batch 65 : Gut microbiota abundance (genus Eisenbergiella id.11304) on Crohn's disease of large intestine
MR Method
Inverse variance weighted
MR Egger
2.8
2.6
2.4
2.2
1/SE$_{IV}$
−1.0
−0.5
0.0
0.5
β$_{IV}$

Batch 66 : Gut microbiota abundance (genus Enterorhabdus id.820) on Crohn's disease of large intestine

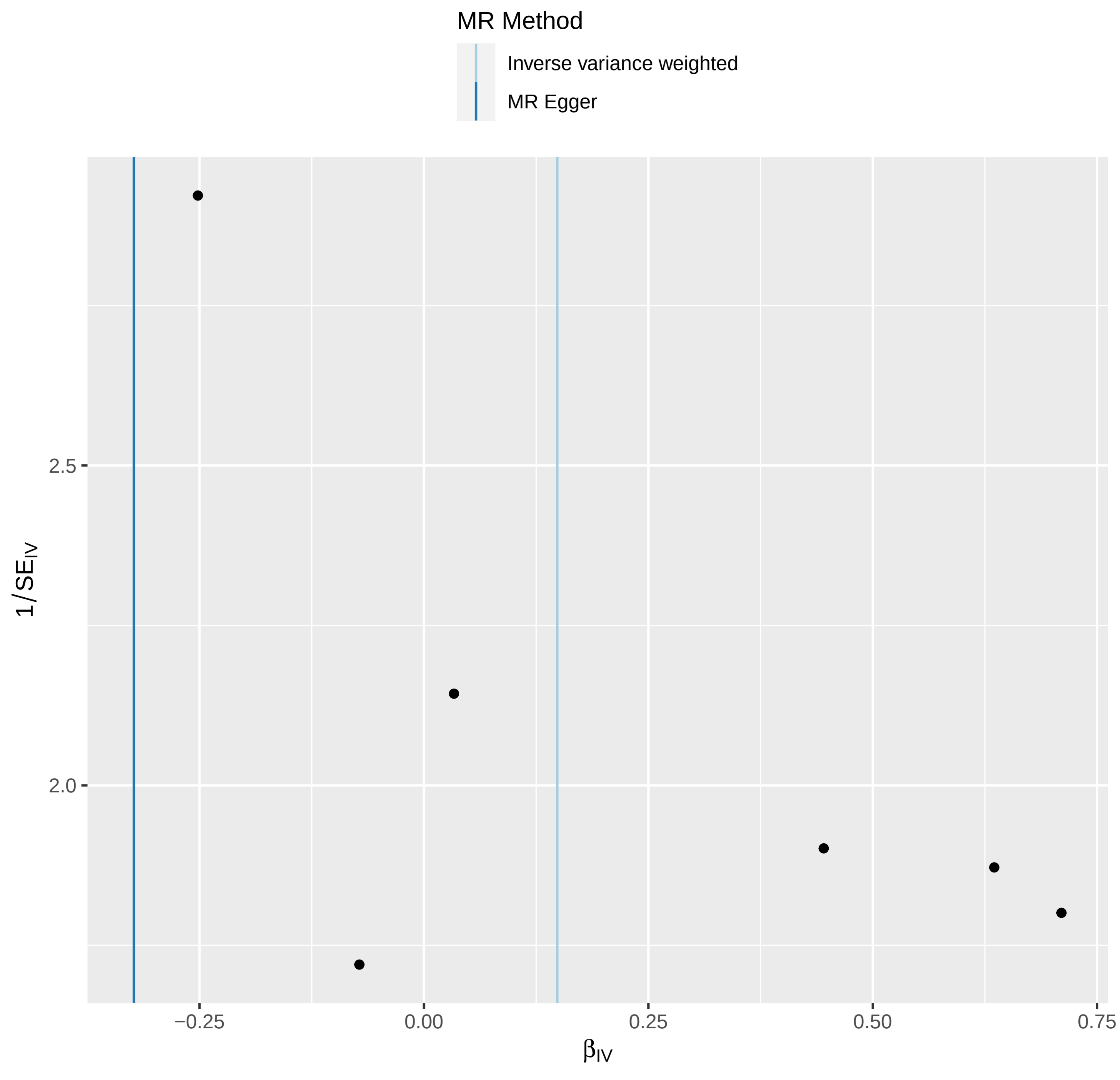

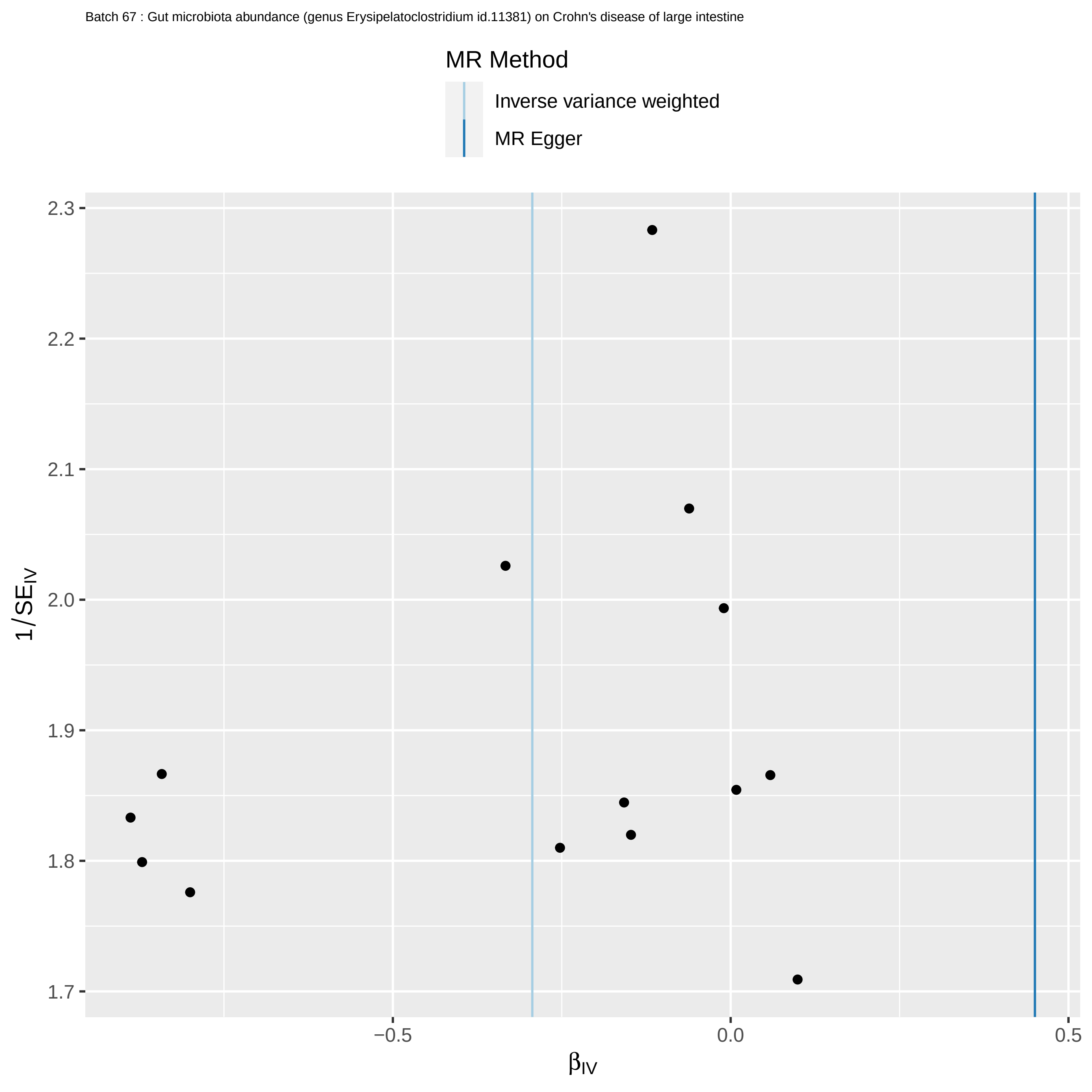

Batch 67 : Gut microbiota abundance (genus Erysipelatoclostridium id.11381) on Crohn's disease of large intestine
MR Method
Inverse variance weighted
MR Egger
2.3
2.2
2.1
2.0
1.9
1.8
1.7
1/SE_IV
−0.5
0.0
0.5
β_IV

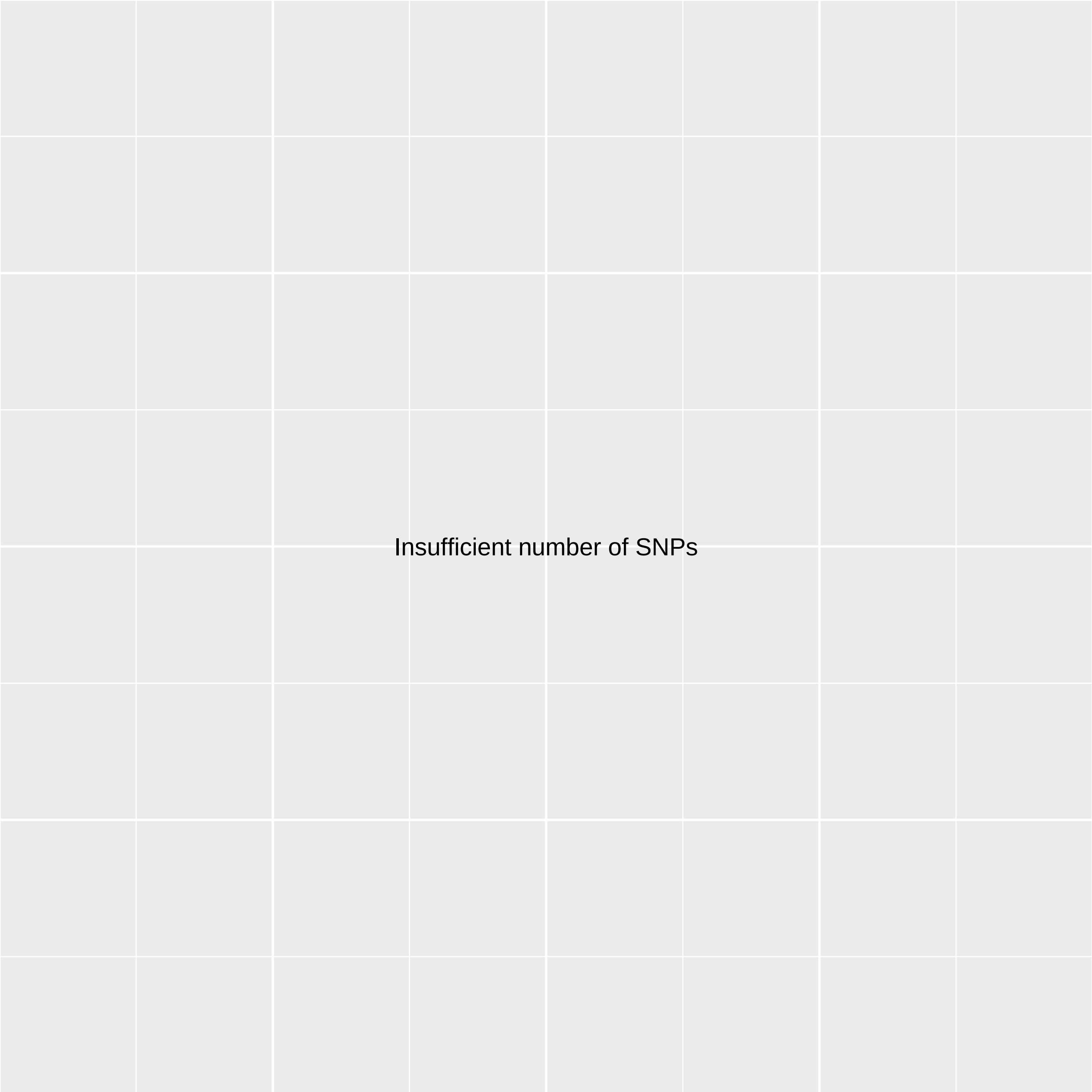
Insufficient number of SNPs

Batch 69 : Gut microbiota abundance (genus Escherichia Shigella id.3504) on Crohn's disease of large intestine

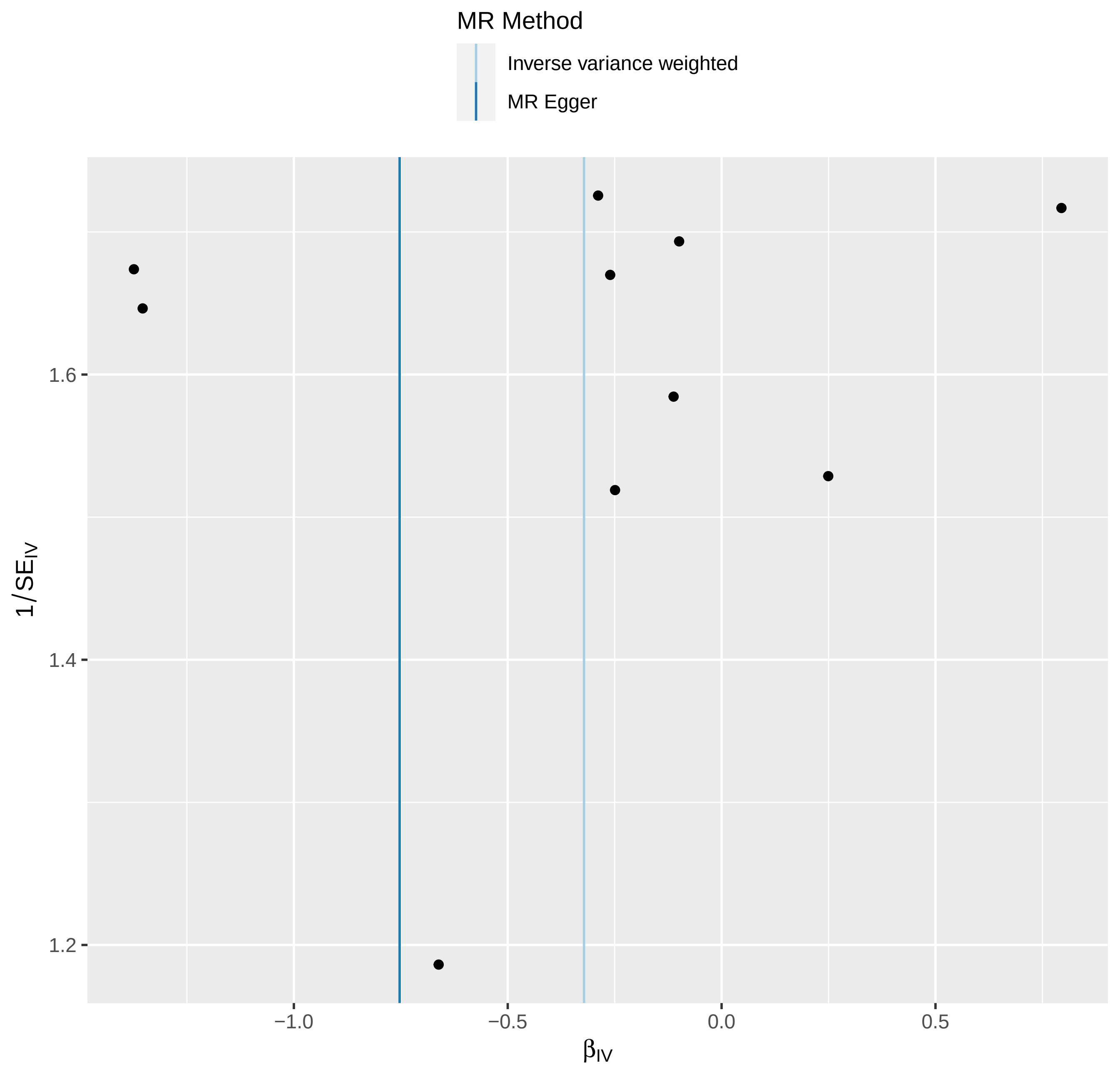

Batch 70 : Gut microbiota abundance (genus Eubacterium brachy group id.11296) on Crohn's disease of large intestine

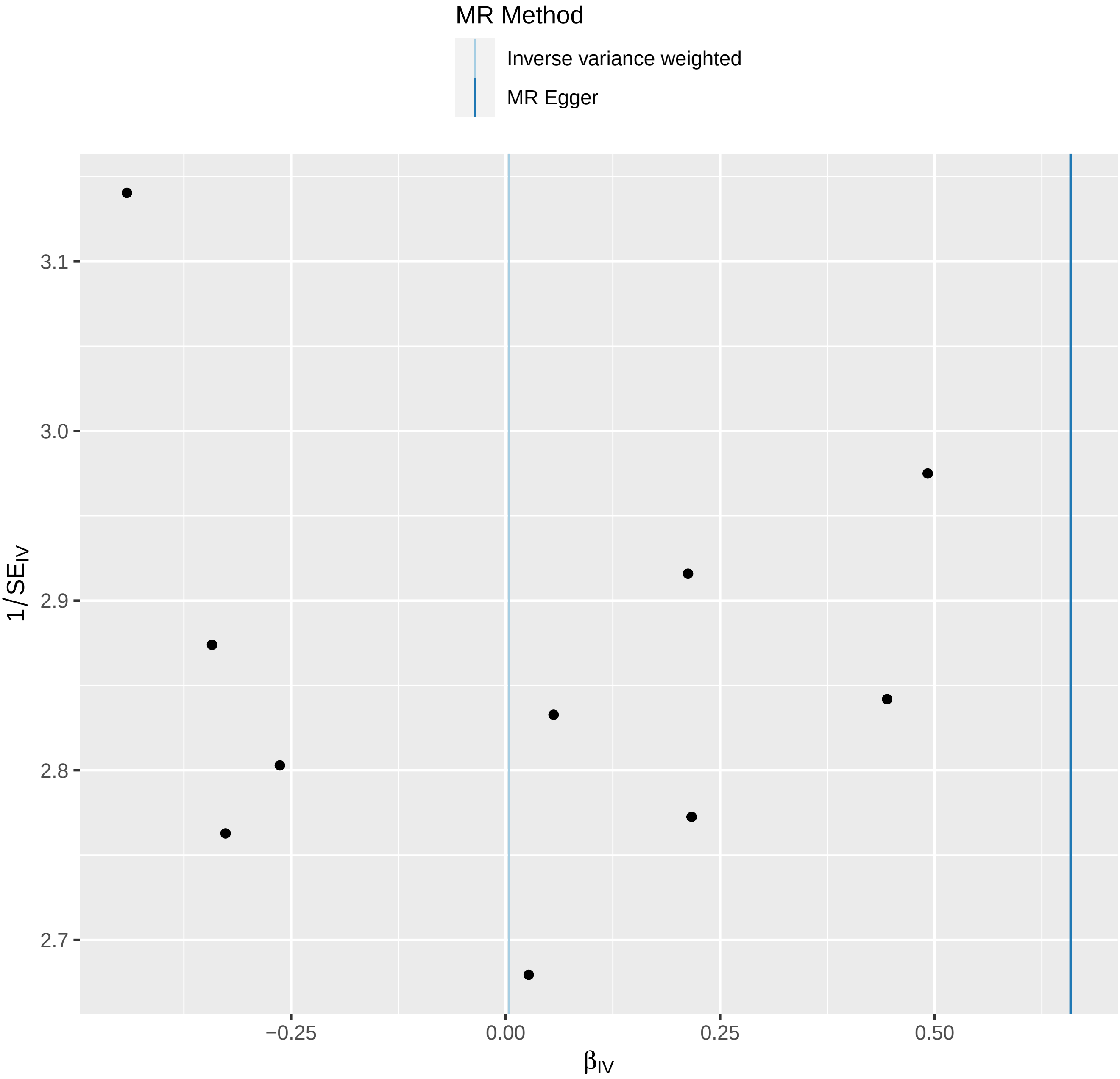

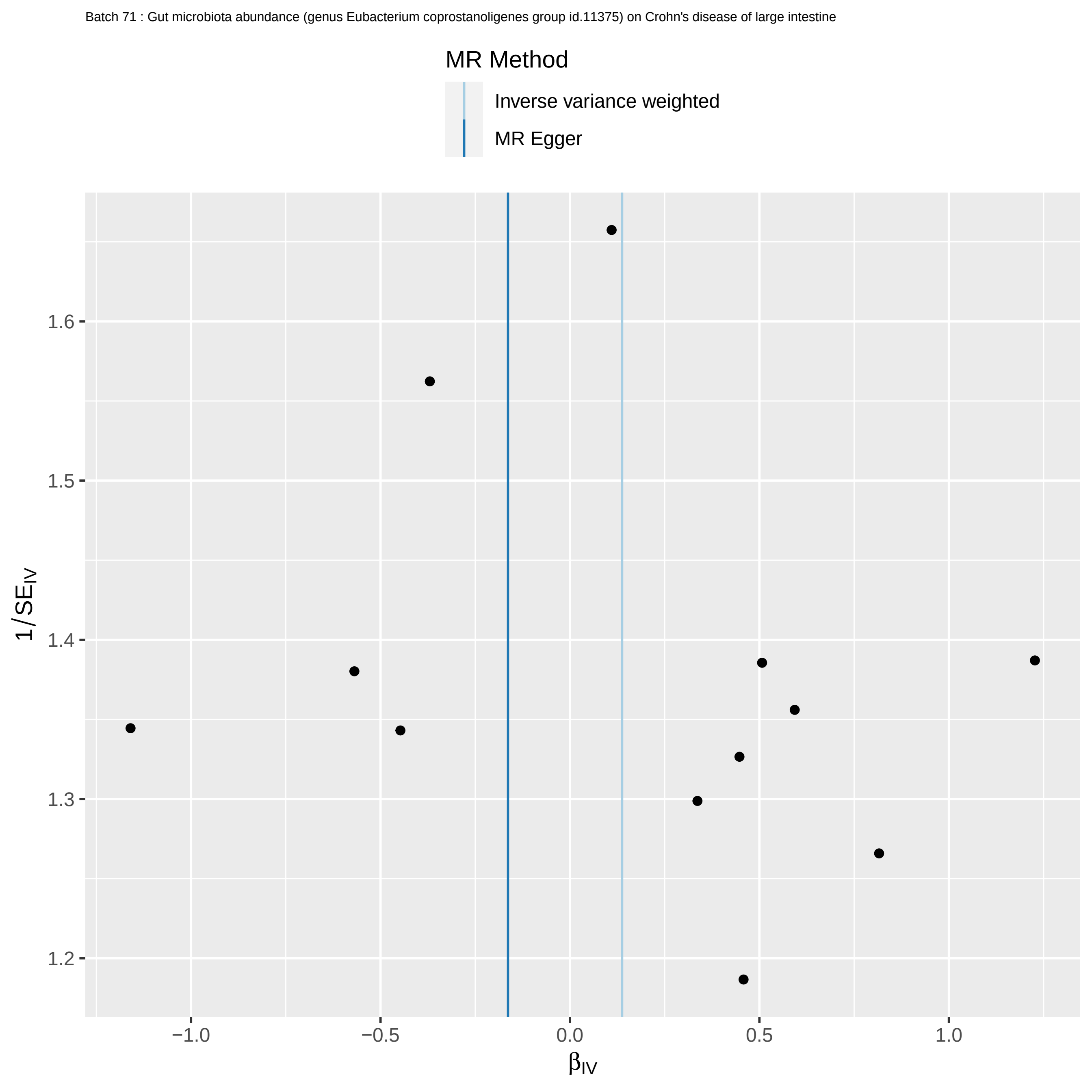

Batch 71 : Gut microbiota abundance (genus Eubacterium coprostanoligenes group id.11375) on Crohn's disease of large intestine
MR Method
Inverse variance weighted
MR Egger
1.6
1.5
1.4
1.3
1.2
1/SE_IV
−1.0
−0.5
0.0
0.5
1.0
β_IV

Batch 72 : Gut microbiota abundance (genus Eubacterium eligens group id.14372) on Crohn's disease of large intestine

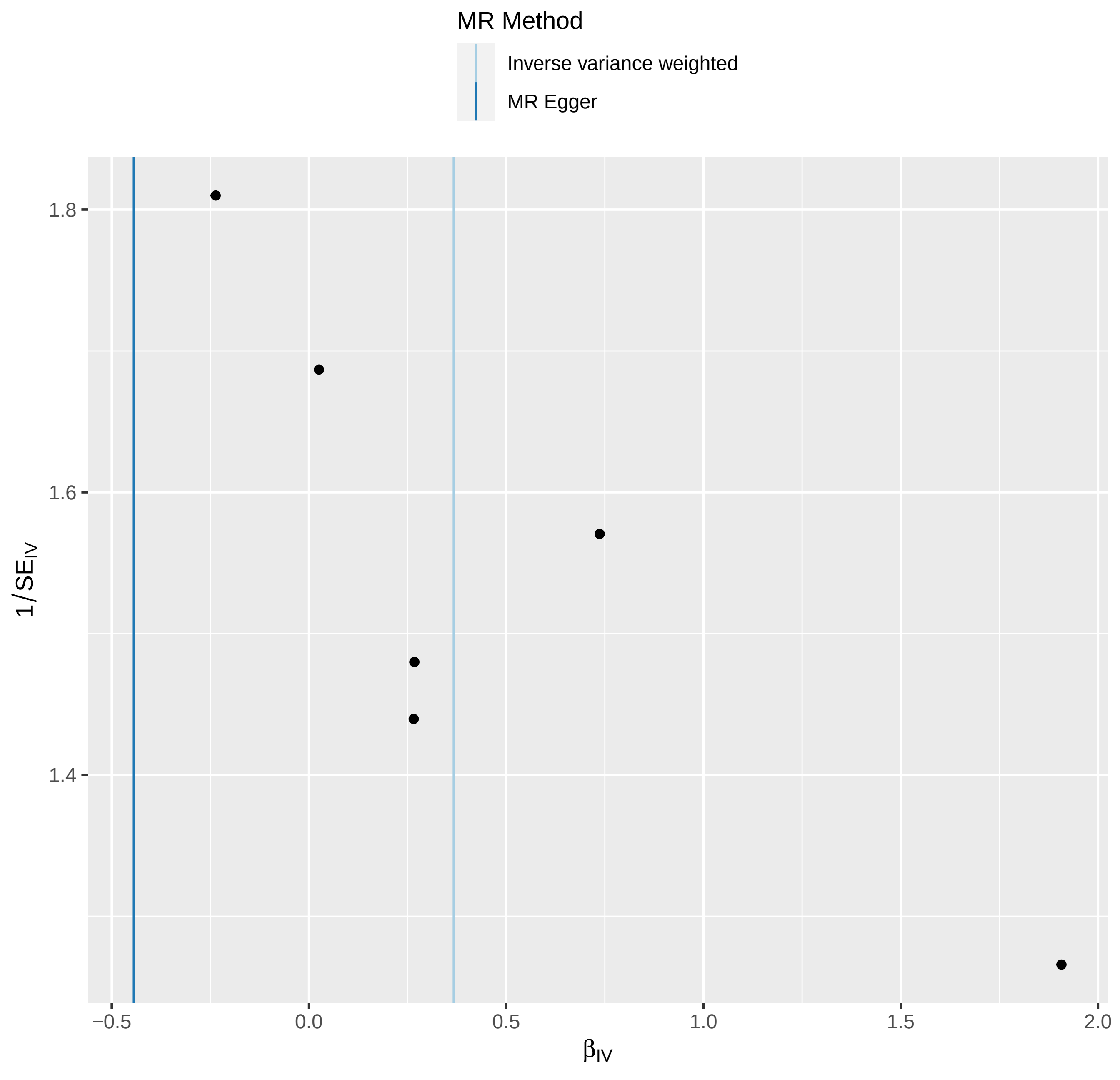

Batch 73 : Gut microbiota abundance (genus Eubacterium fissicatena group id.14373) on Crohn's disease of large intestine

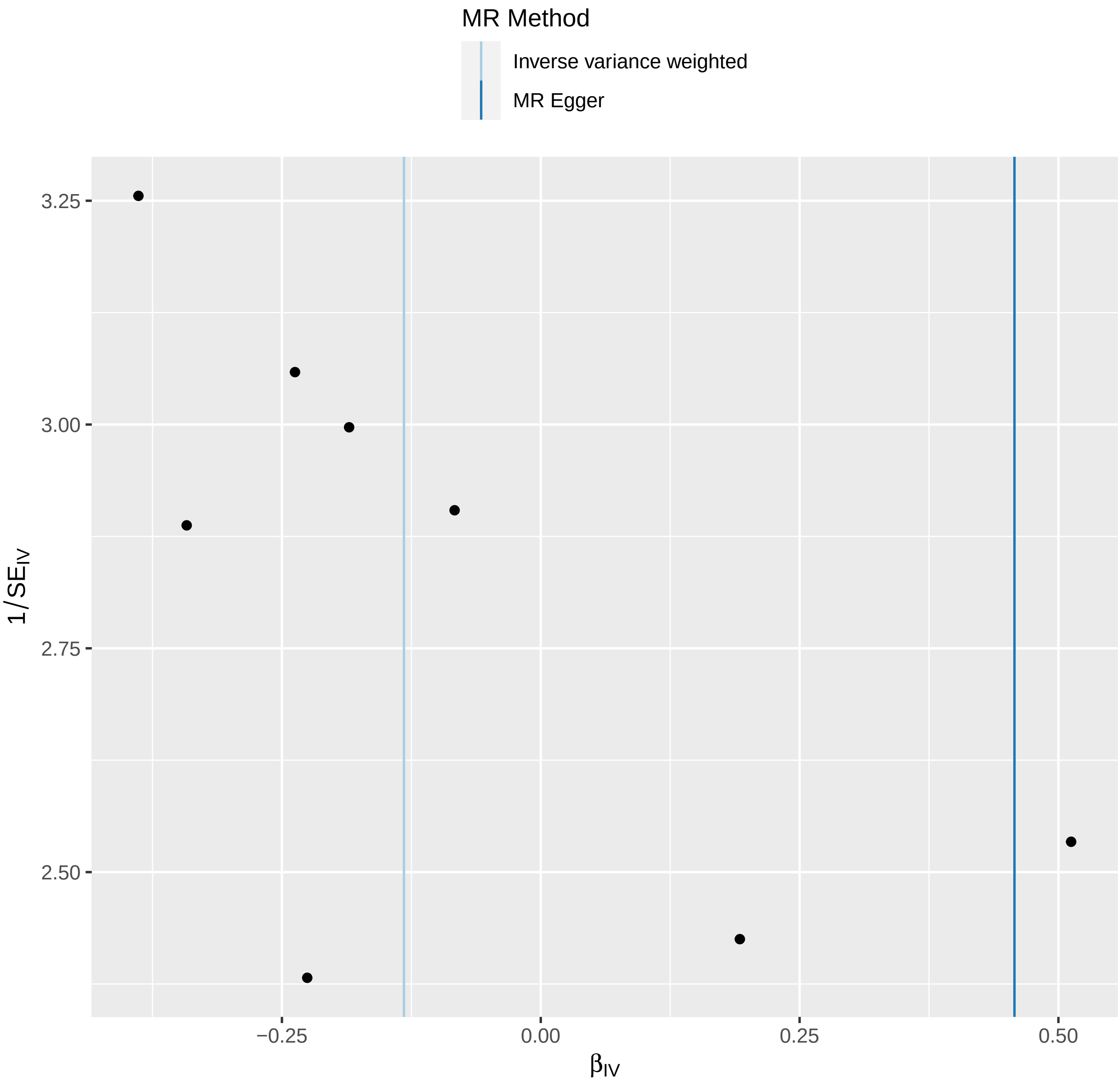

Batch 74 : Gut microbiota abundance (genus Eubacterium hallii group id.11338) on Crohn's disease of large intestine

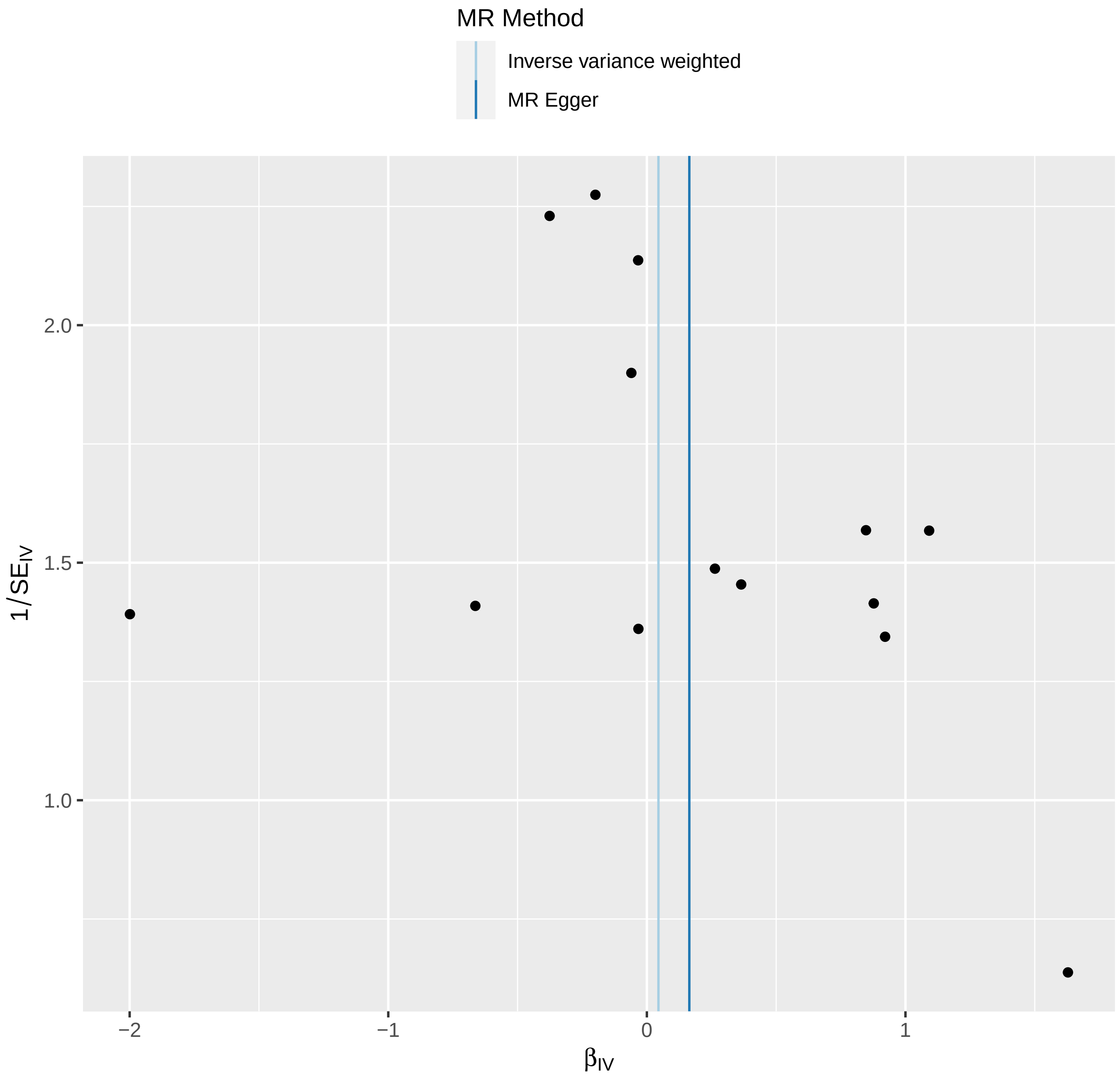

Batch 75 : Gut microbiota abundance (genus Eubacterium nodatum group id.11297) on Crohn's disease of large intestine

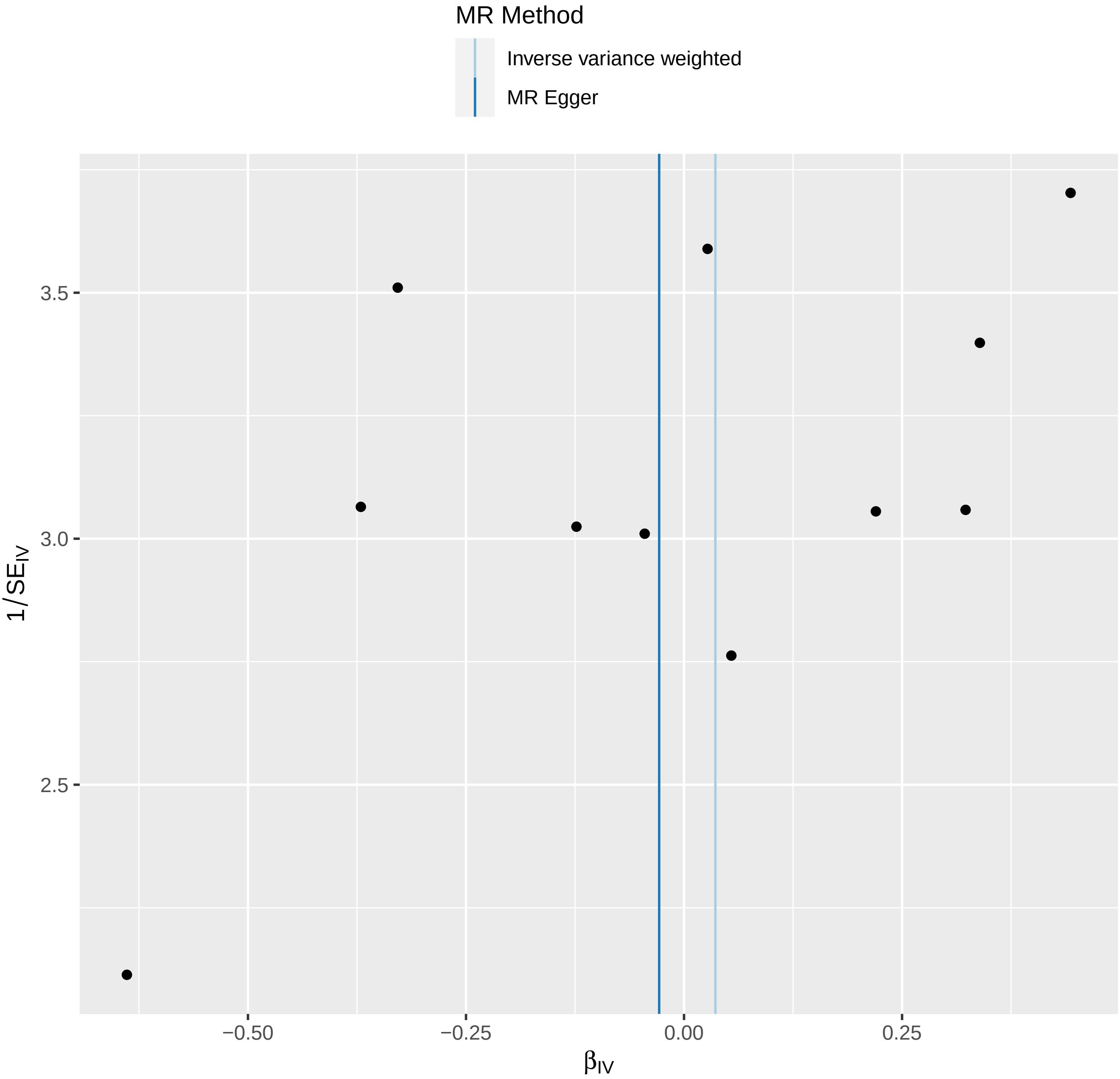

Batch 76 : Gut microbiota abundance (genus Eubacterium oxidoreducens group id.11339) on Crohn's disease of large intestine

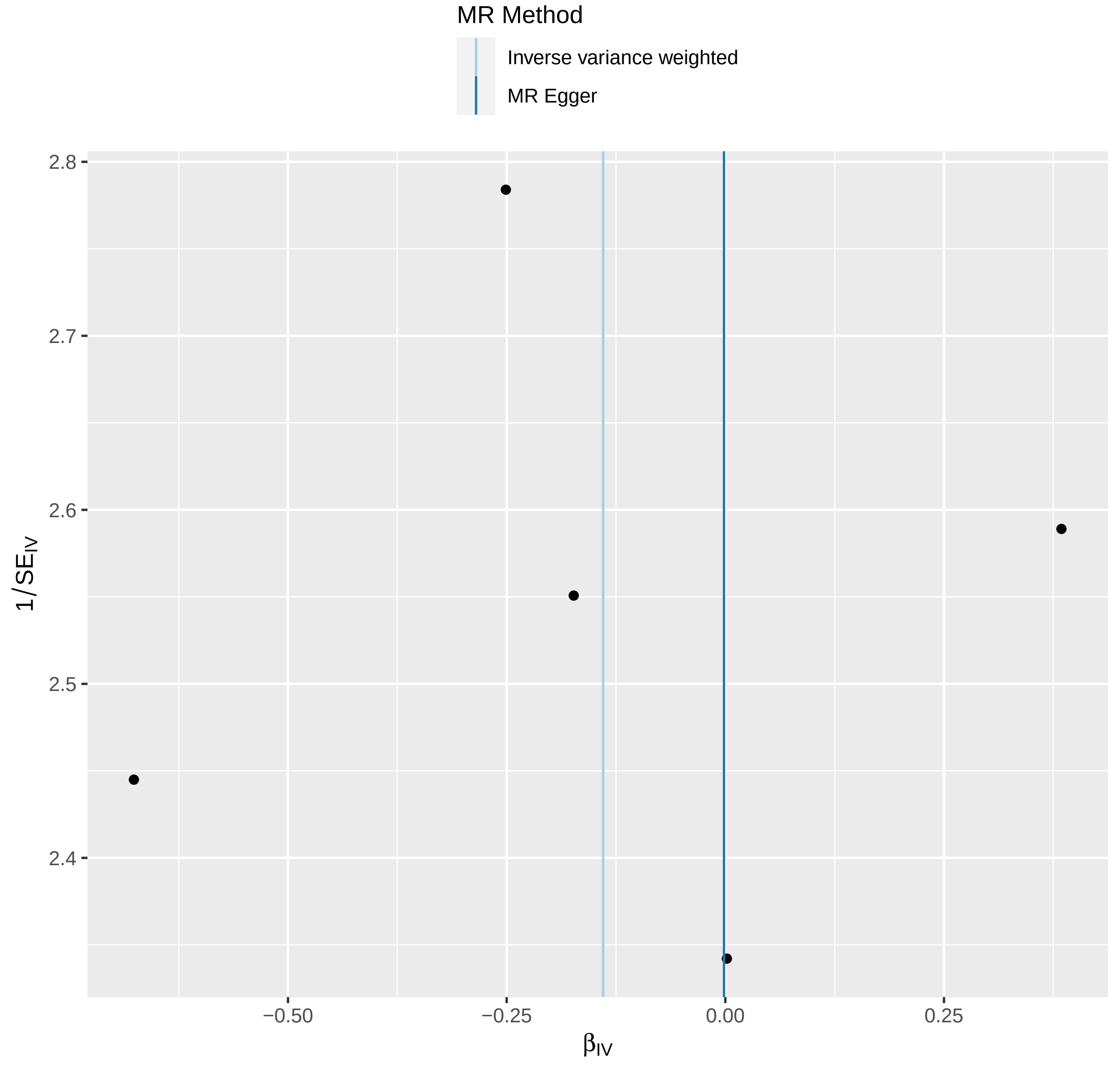

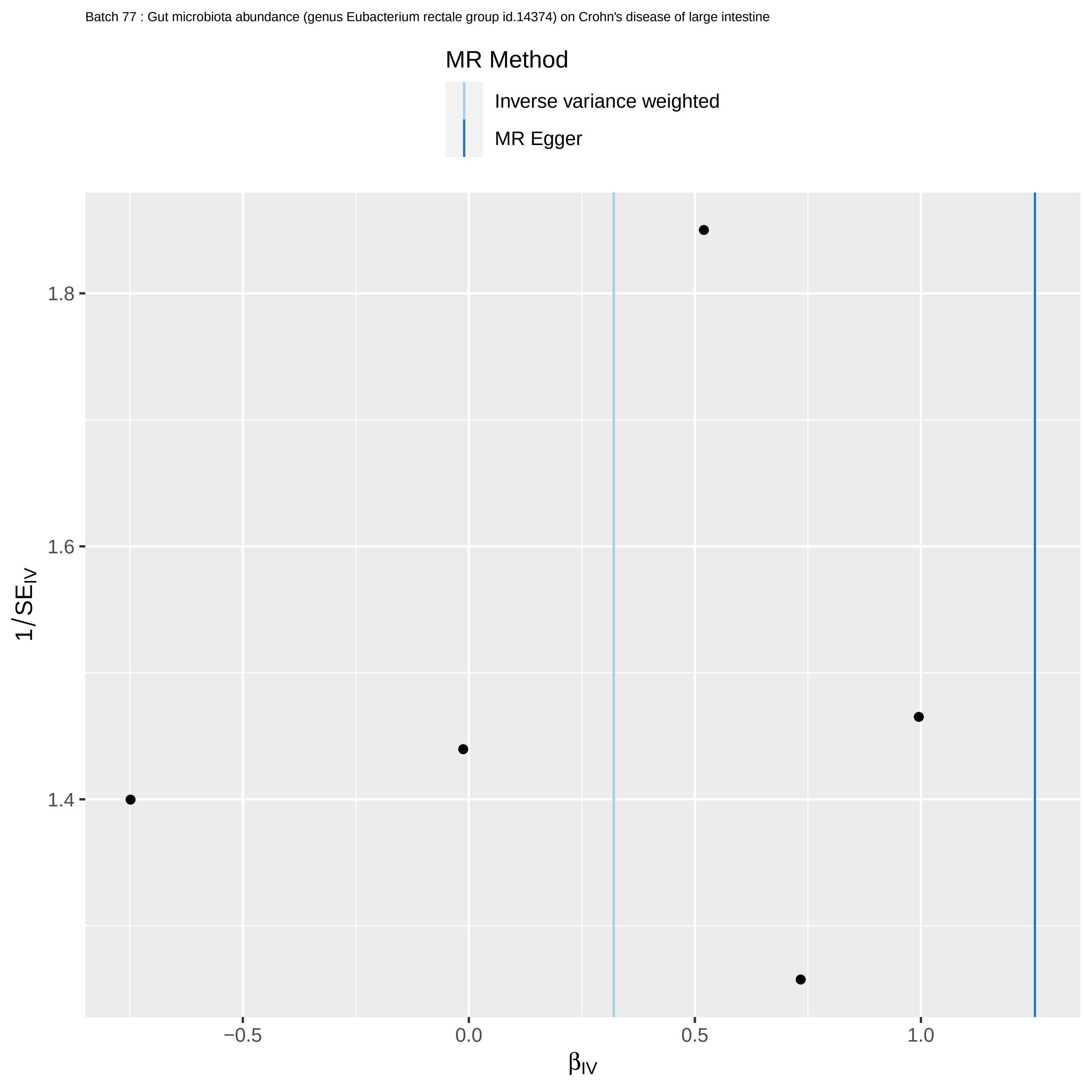

Batch 77 : Gut microbiota abundance (genus Eubacterium rectale group id.14374) on Crohn's disease of large intestine
MR Method
Inverse variance weighted
MR Egger
1.8
1.6
1.4
1/SE_IV
−0.5
0.0
0.5
1.0
β_IV

Batch 78 : Gut microbiota abundance (genus Eubacterium ruminantium group id.11340) on Crohn's disease of large intestine

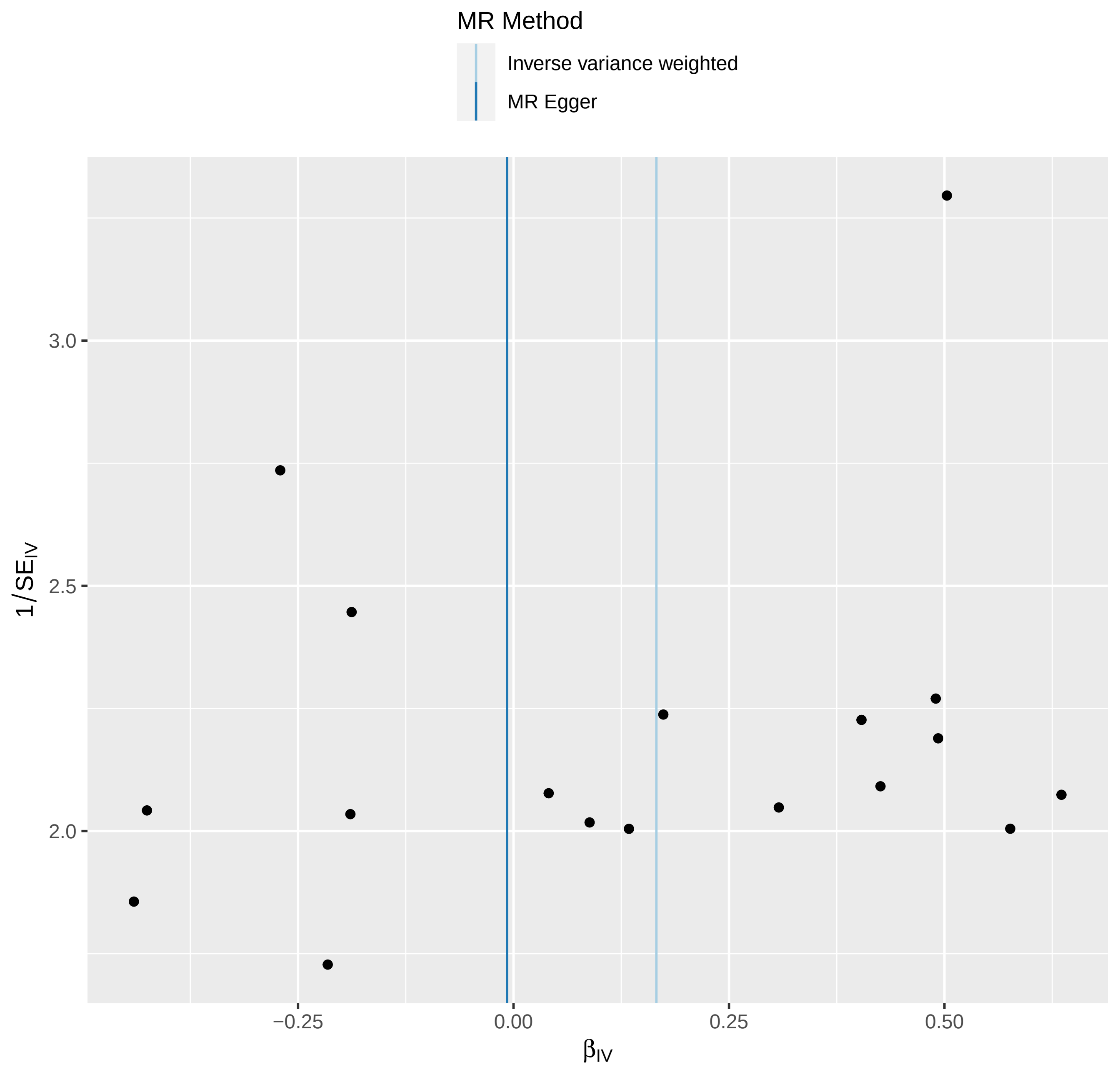

Batch 79 : Gut microbiota abundance (genus Eubacterium ventriosum group id.11341) on Crohn's disease of large intestine

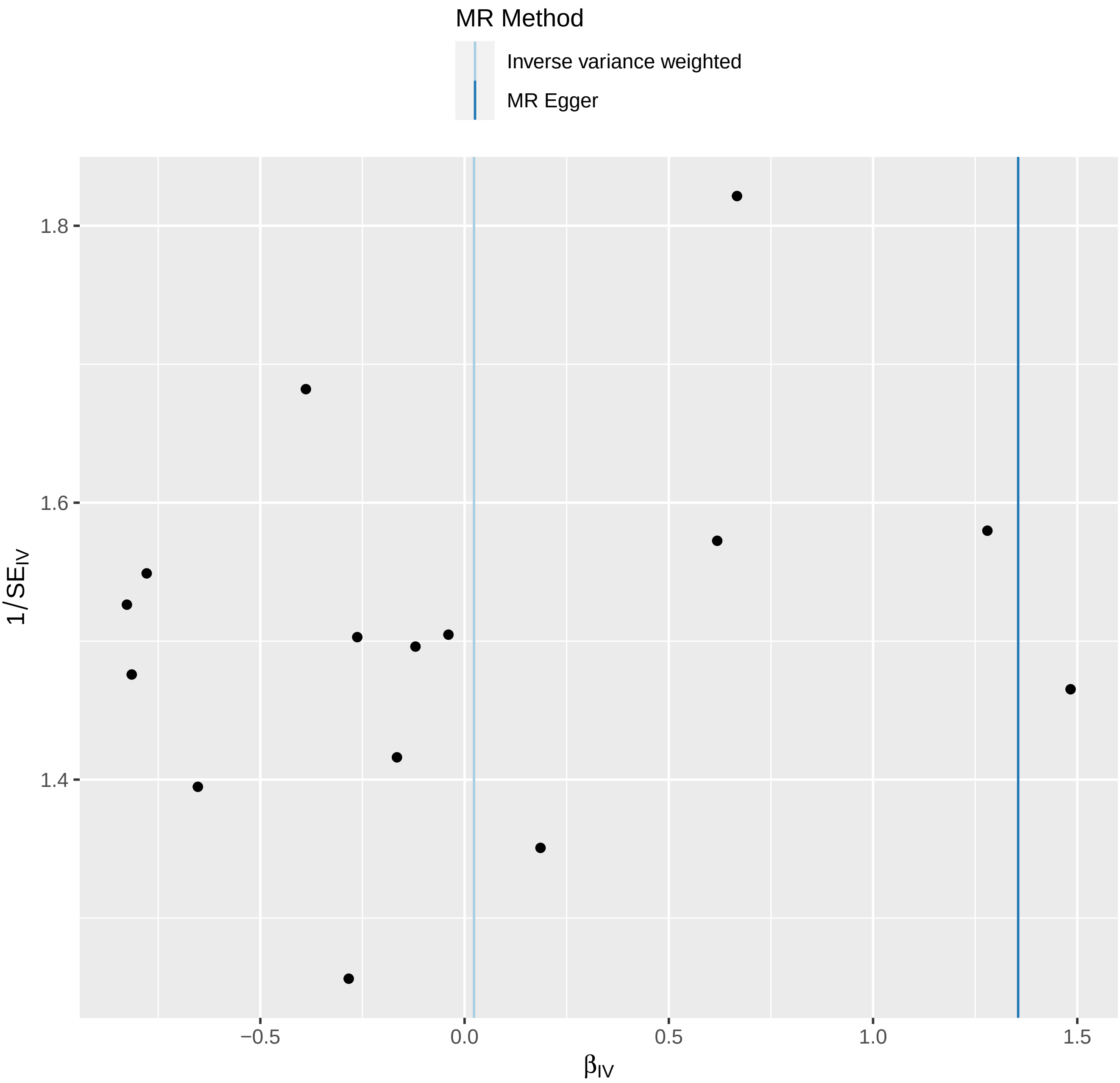

Batch 80 : Gut microbiota abundance (genus Eubacterium xylanophilum group id.14375) on Crohn's disease of large intestine

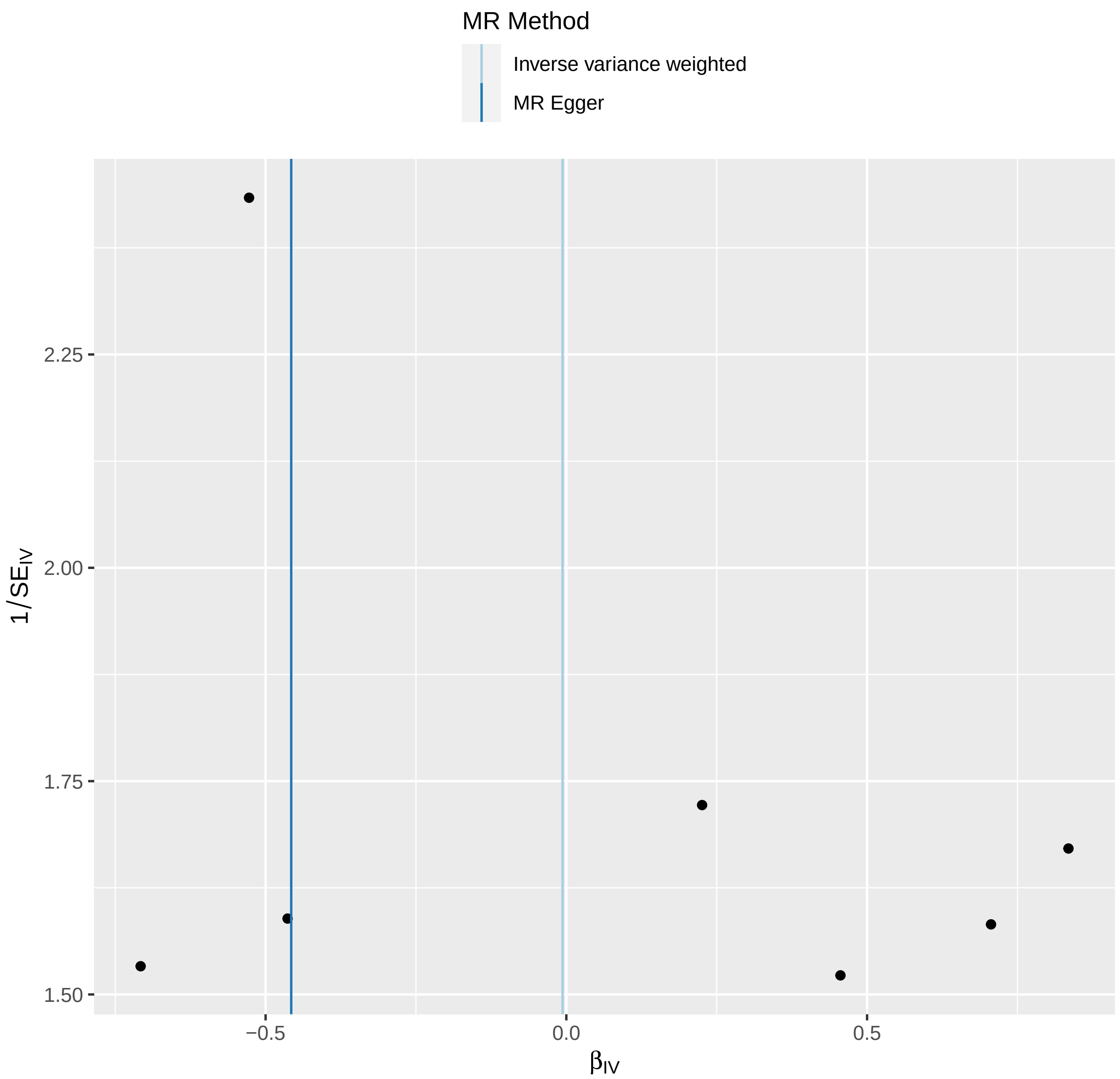

Batch 81 : Gut microbiota abundance (genus Faecalibacterium id.2057) on Crohn's disease of large intestine

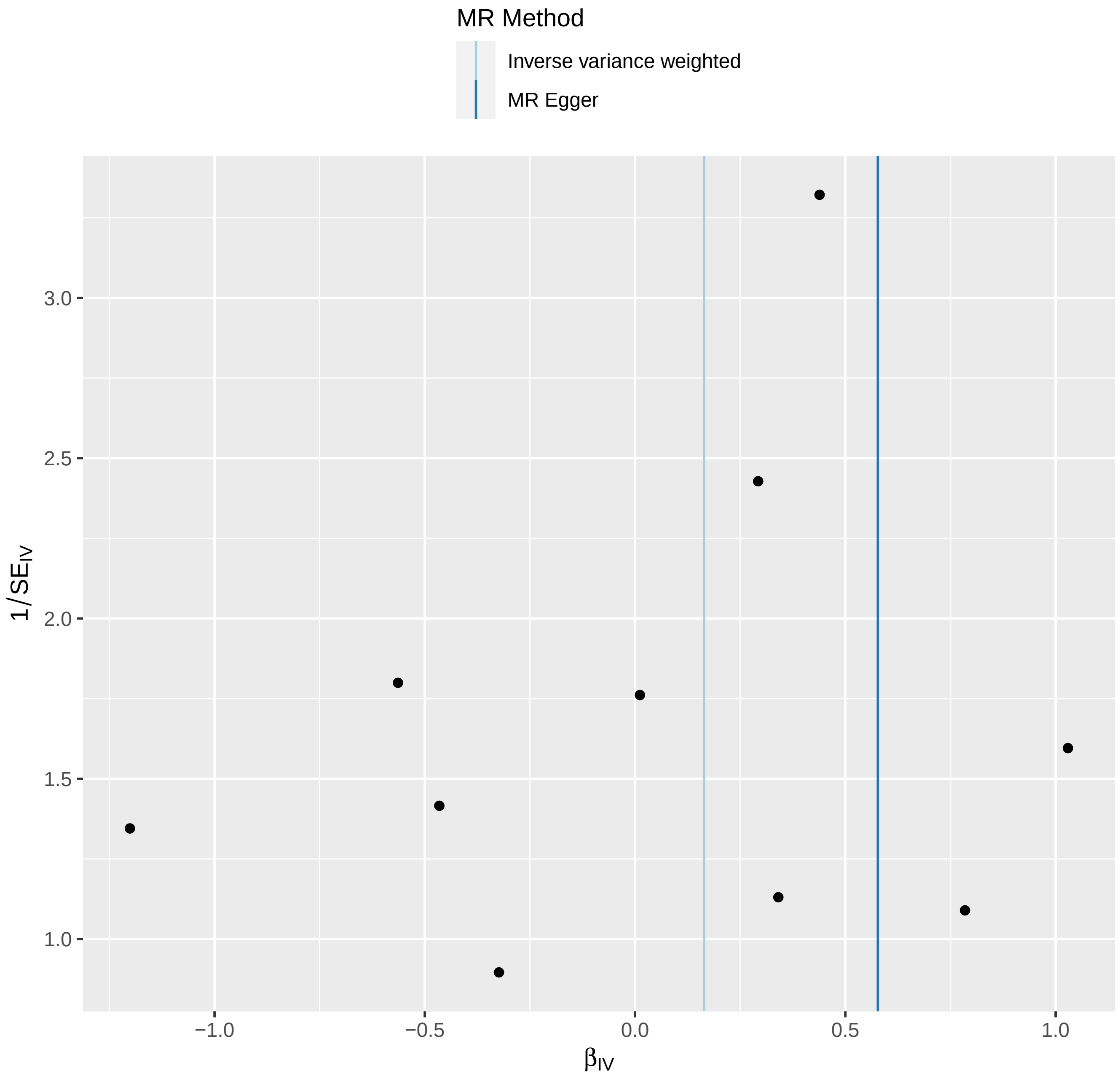

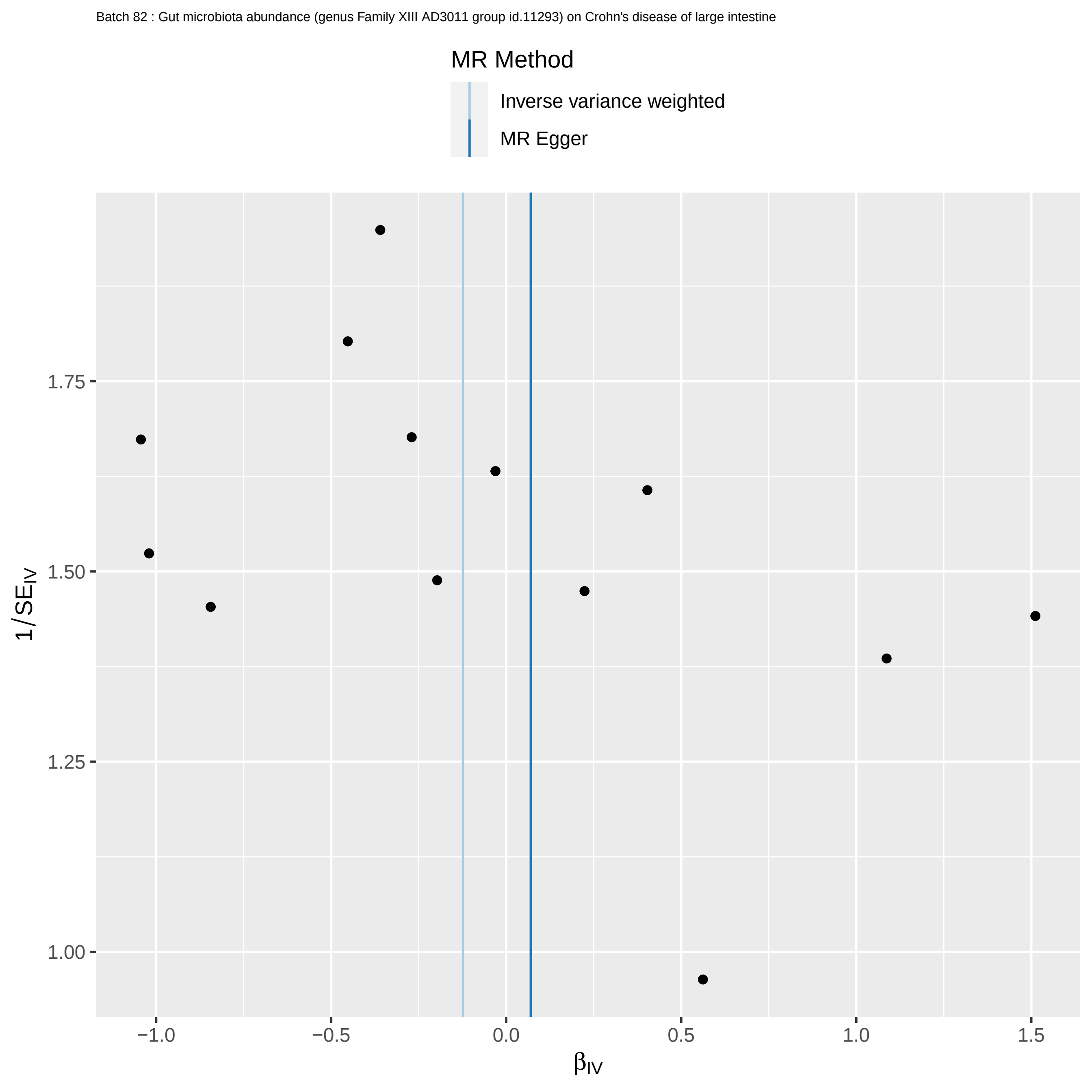

Batch 82 : Gut microbiota abundance (genus Family XIII AD3011 group id.11293) on Crohn's disease of large intestine
MR Method
Inverse variance weighted
MR Egger
1.75
1.50
1.25
1.00
1/SE_IV
−1.0
−0.5
0.0
0.5
1.0
1.5
β_IV

Batch 83 : Gut microbiota abundance (genus Family XIII UCG001 id.11294) on Crohn's disease of large intestine

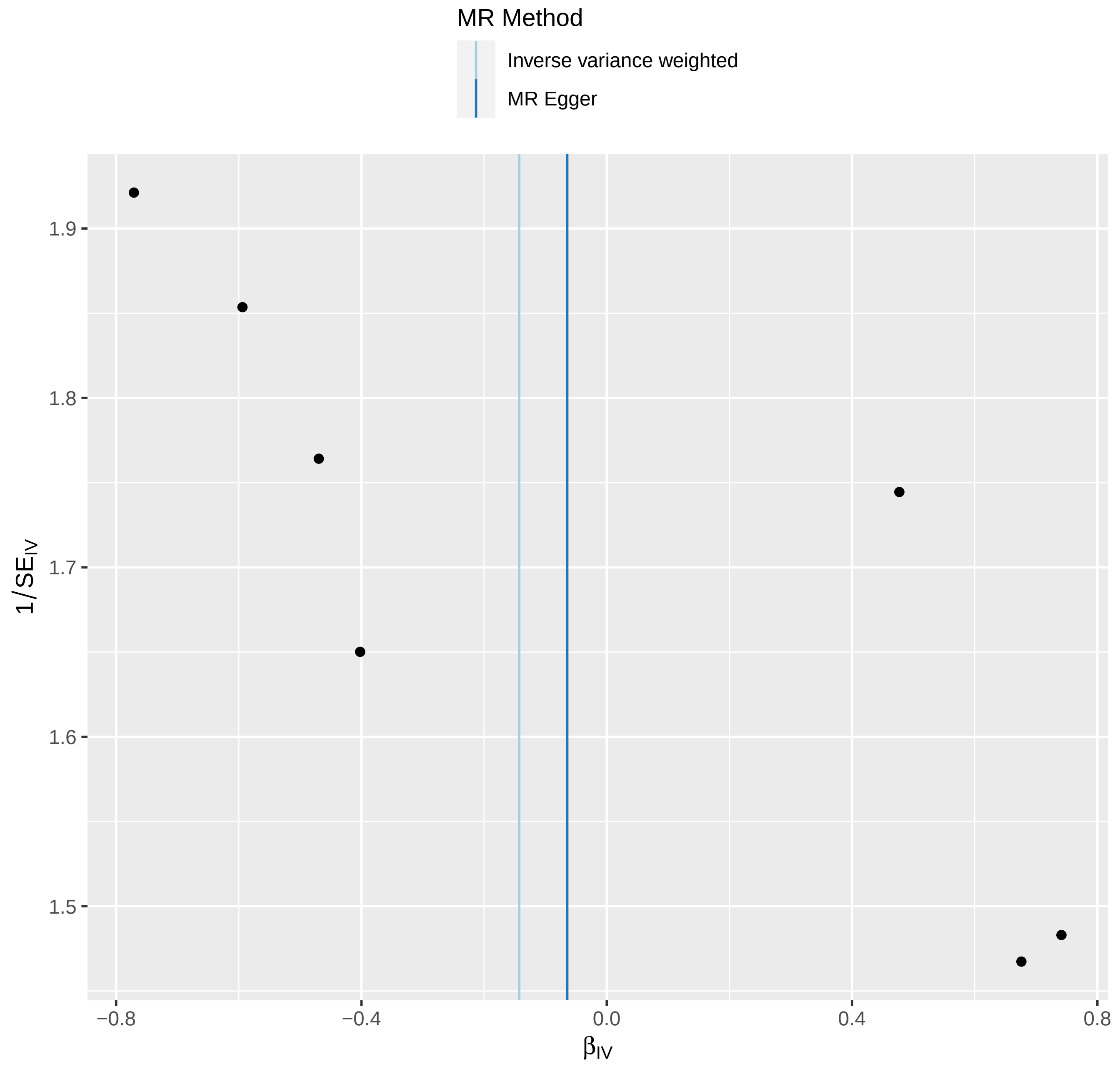

Batch 84 : Gut microbiota abundance (genus Flavonifractor id.2059) on Crohn's disease of large intestine

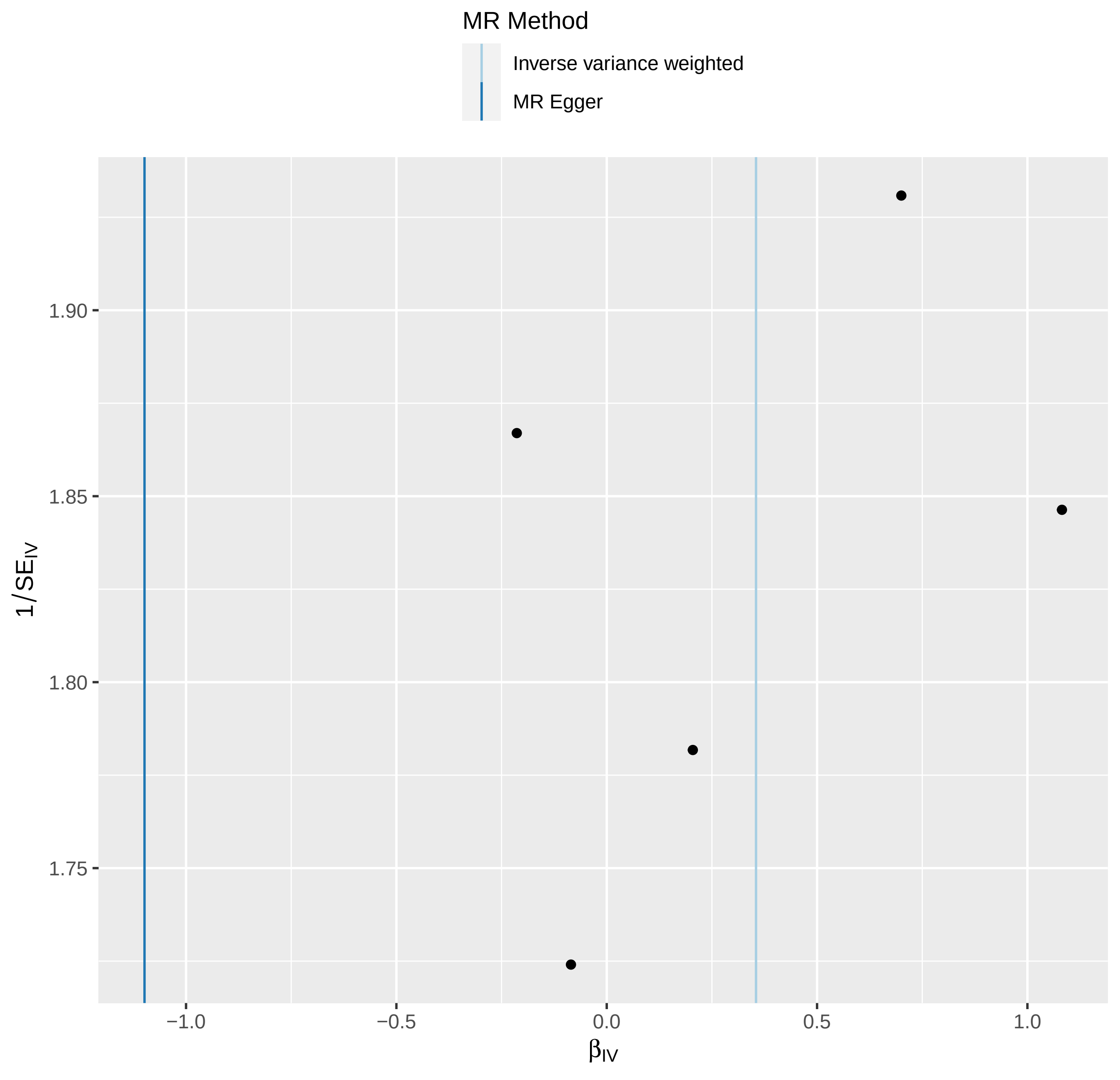

Batch 85 : Gut microbiota abundance (genus Fusicatenibacter id.11305) on Crohn's disease of large intestine

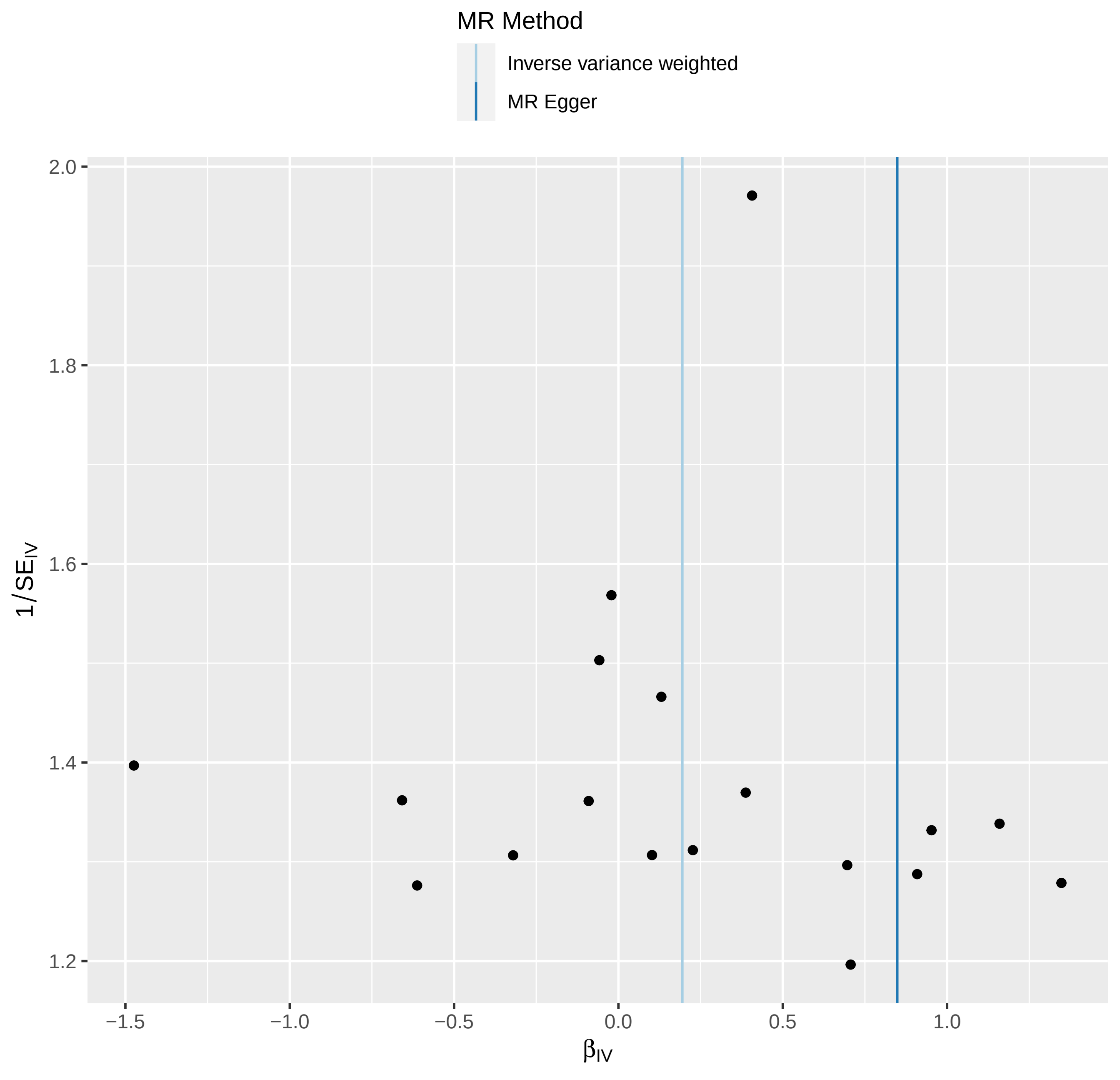

Batch 86 : Gut microbiota abundance (genus Gordonibacter id.821) on Crohn's disease of large intestine

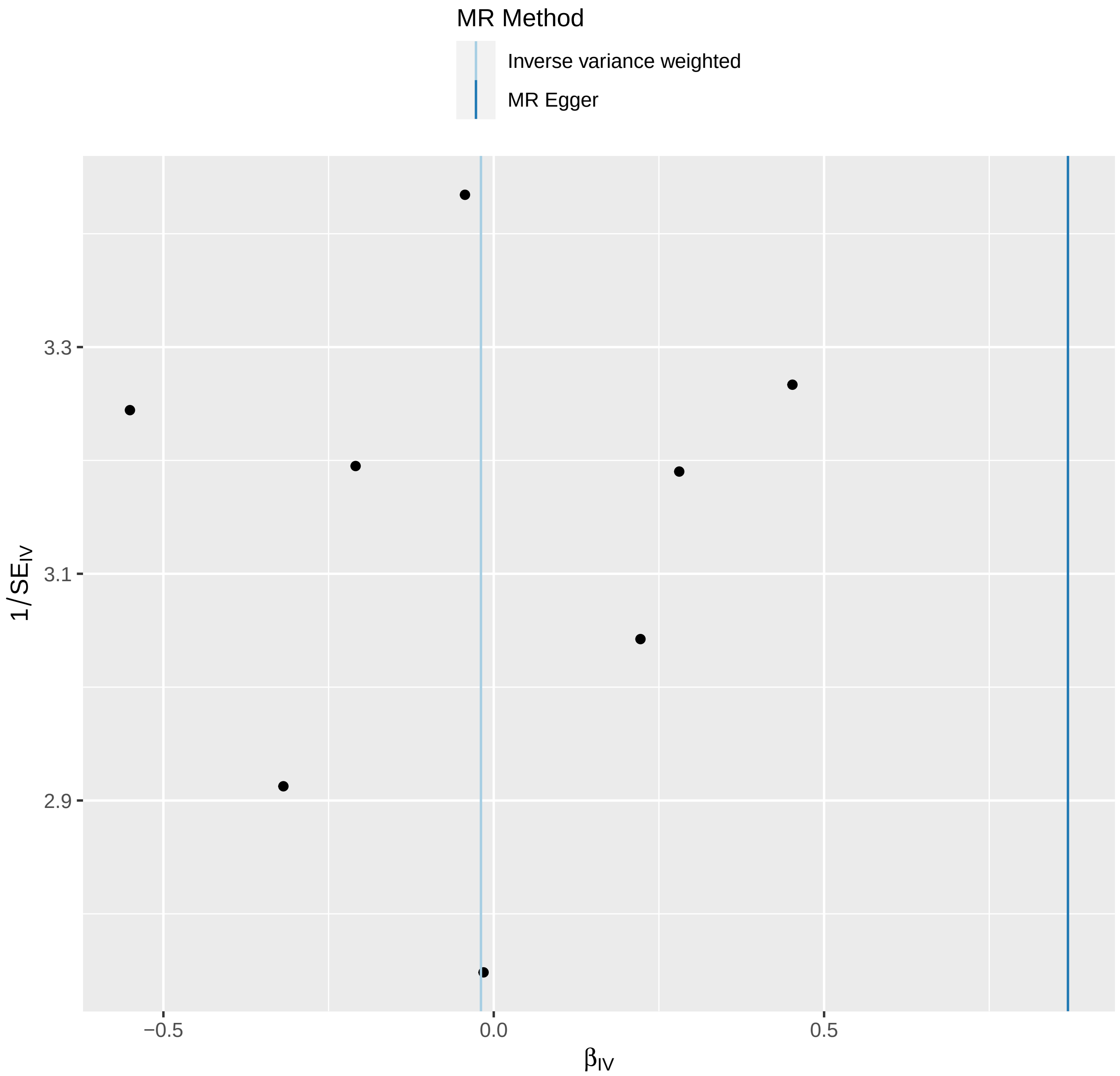

Batch 87 : Gut microbiota abundance (genus Haemophilus id.3698) on Crohn's disease of large intestine

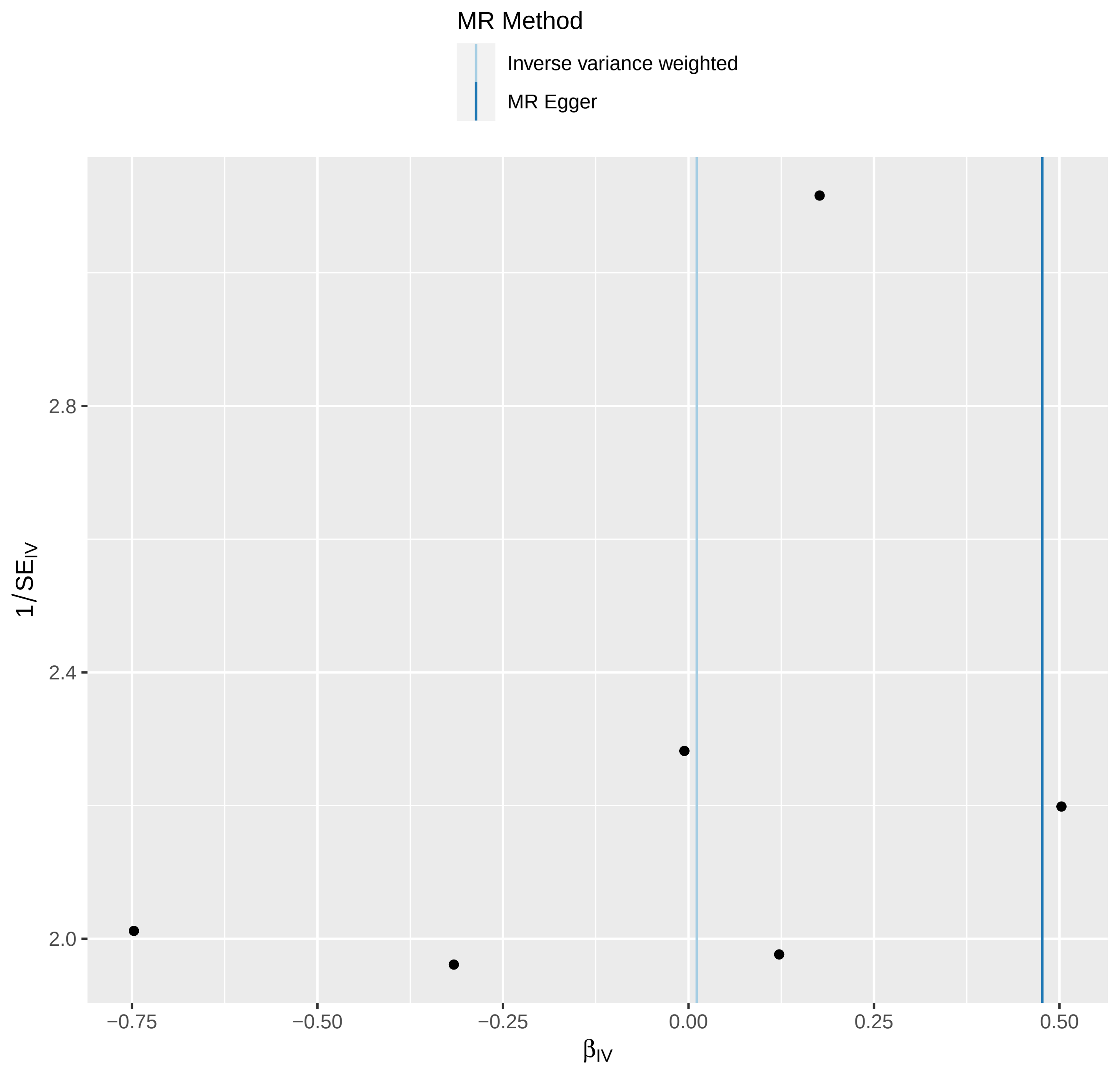

Batch 88 : Gut microbiota abundance (genus Holdemanella id.11393) on Crohn's disease of large intestine

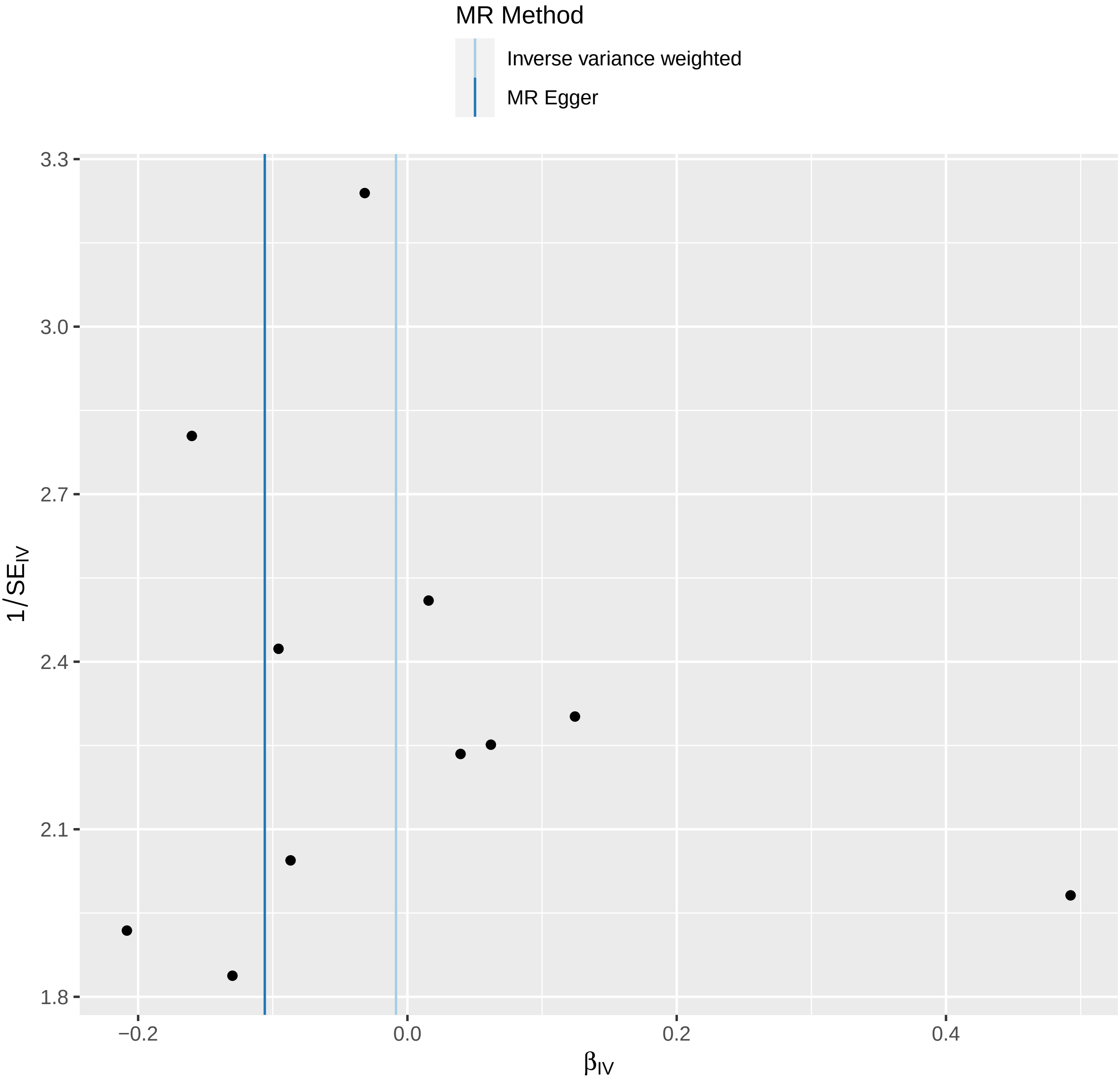

Batch 89 : Gut microbiota abundance (genus Holdemania id.2157) on Crohn's disease of large intestine

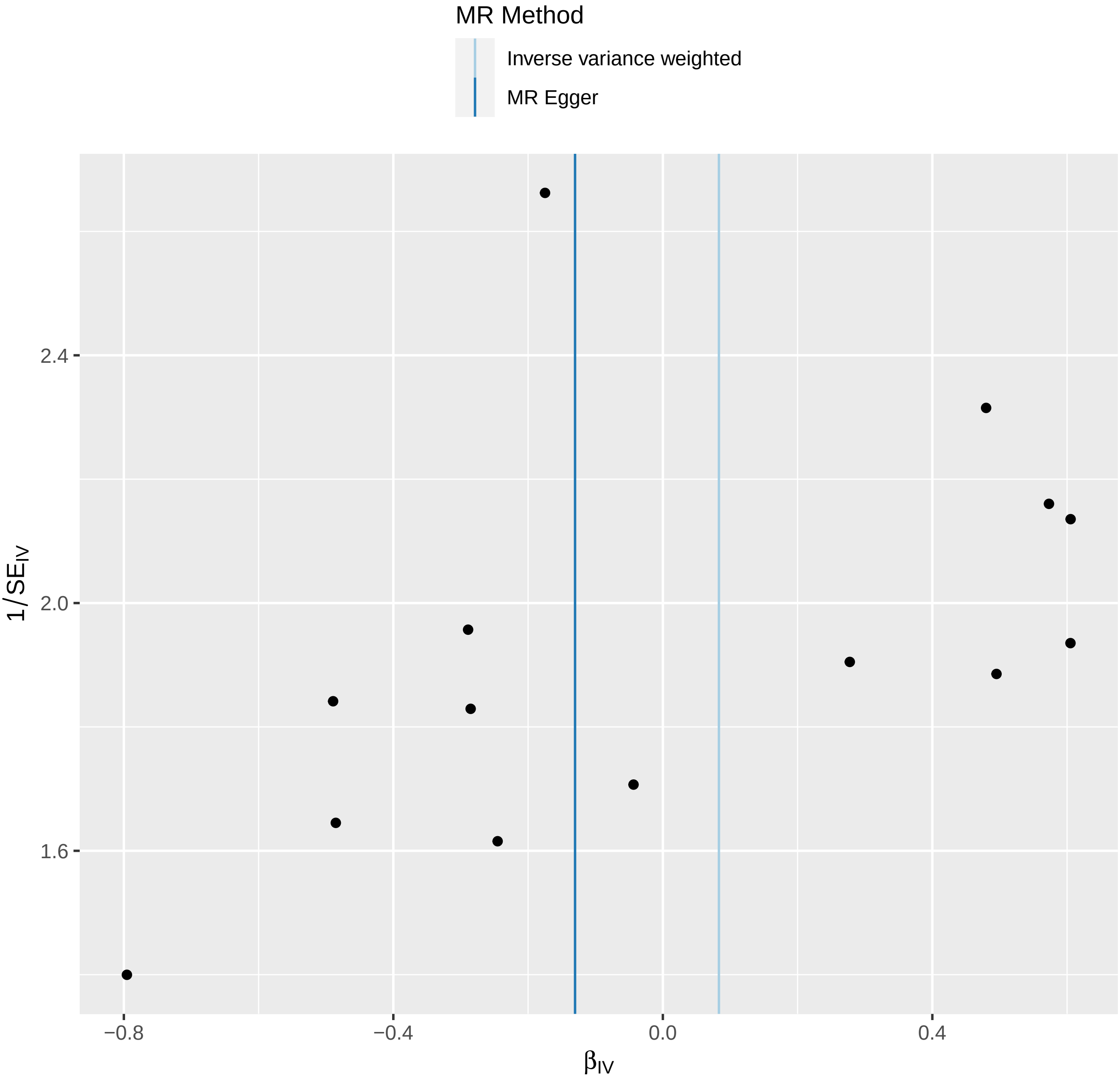

Batch 90 : Gut microbiota abundance (genus Howardella id.2000) on Crohn's disease of large intestine

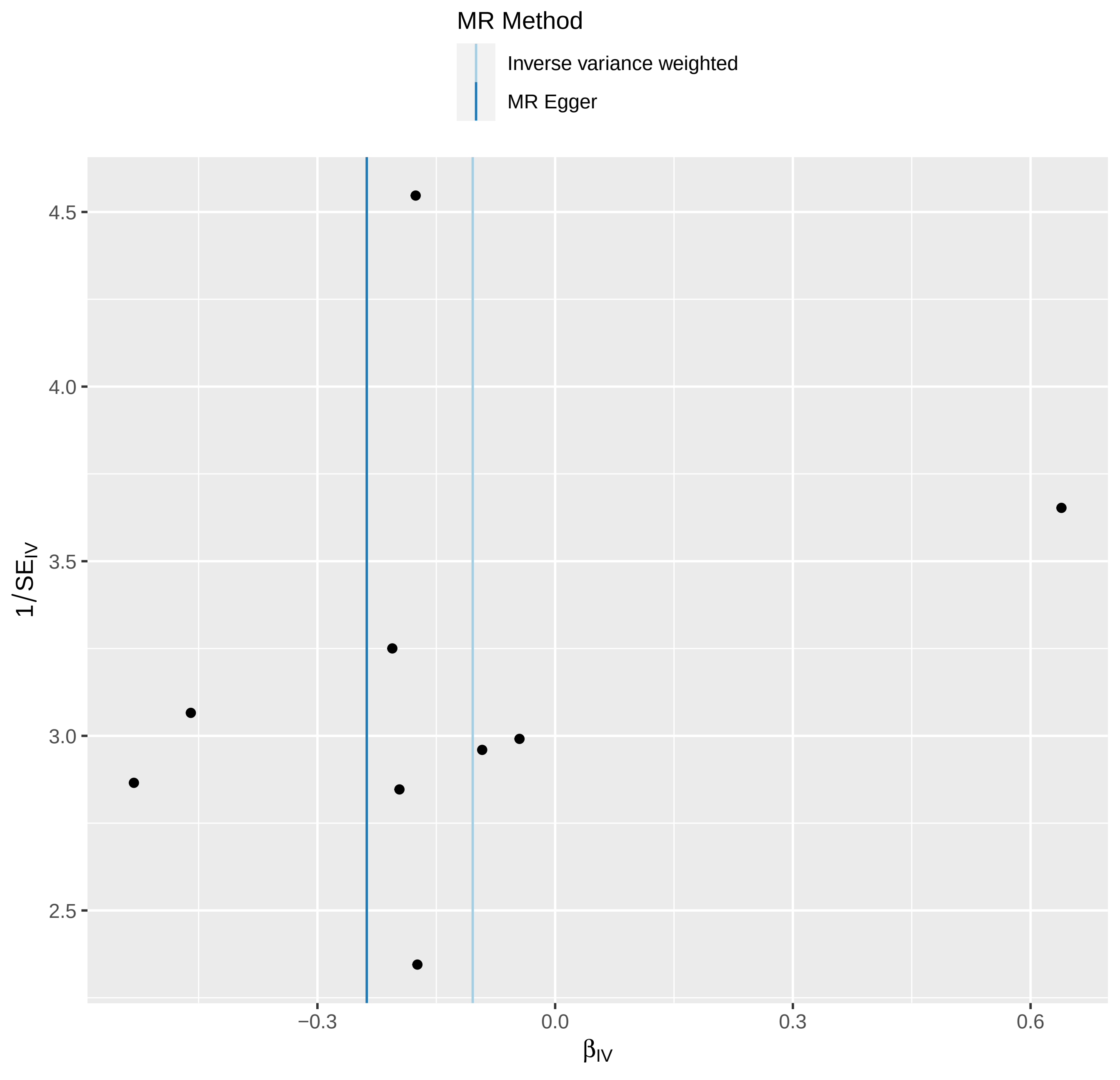

Batch 91 : Gut microbiota abundance (genus Hungatella id.11306) on Crohn's disease of large intestine

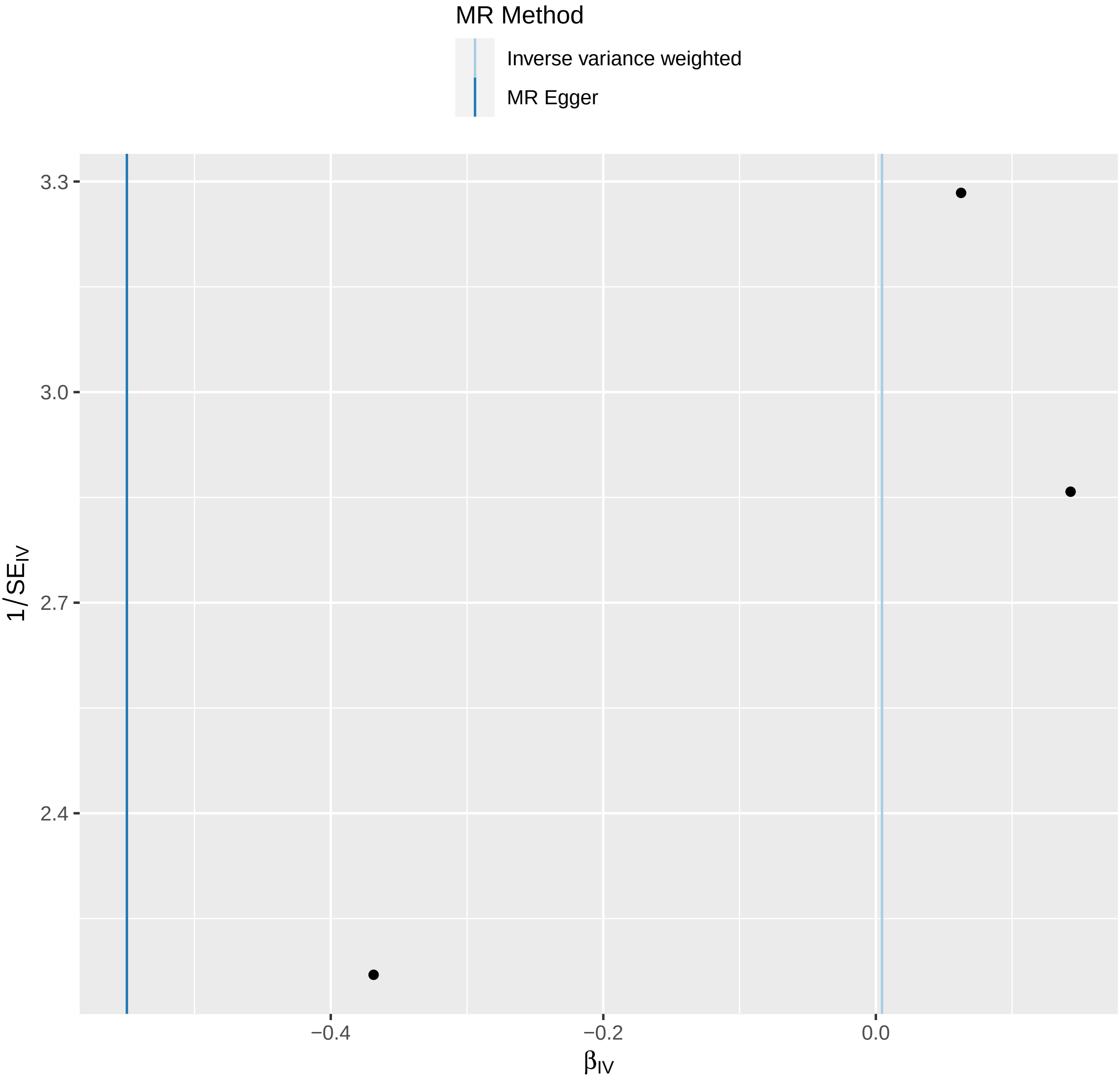

Batch 92 : Gut microbiota abundance (genus Intestinibacter id.11345) on Crohn's disease of large intestine

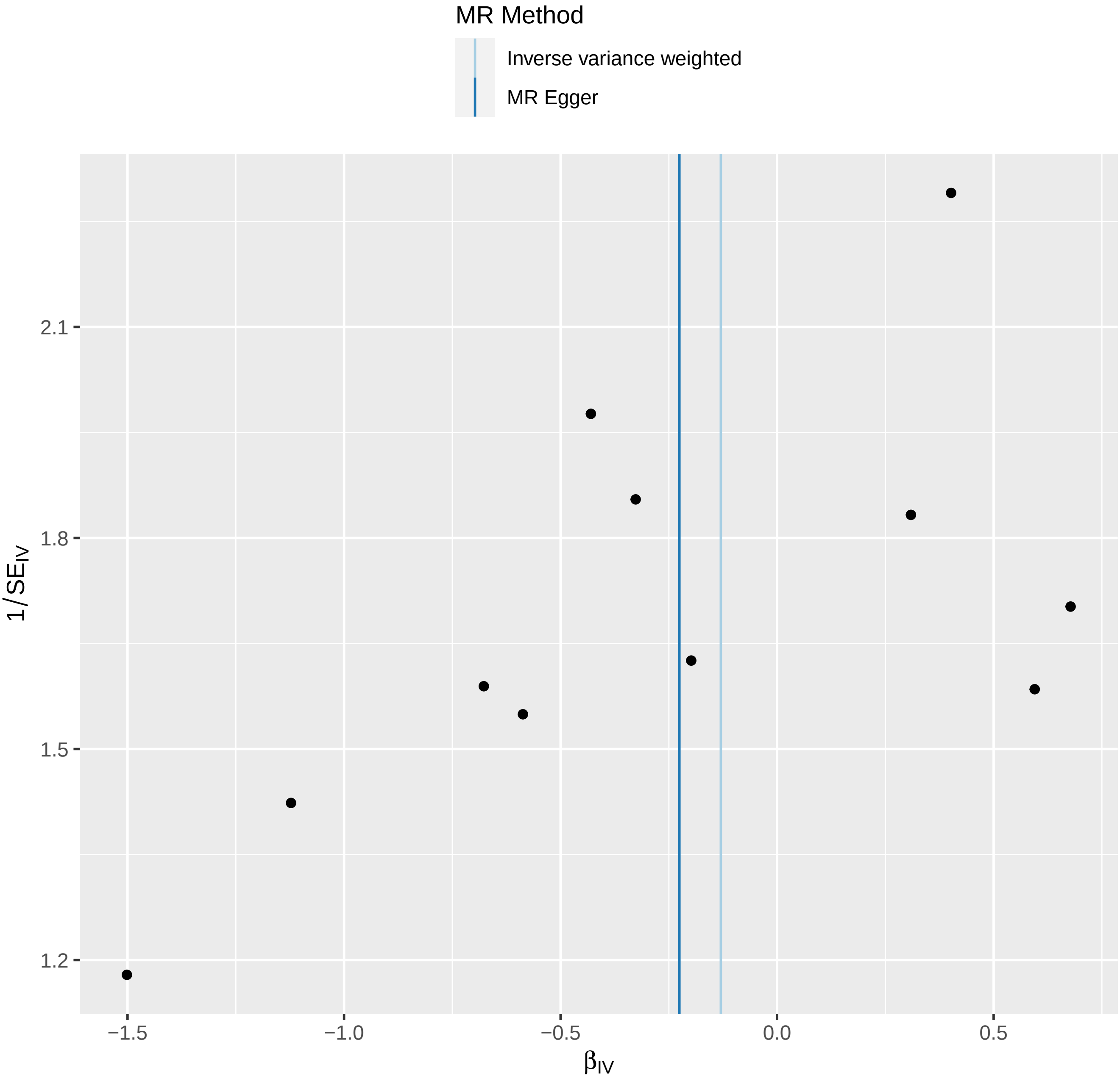

Batch 93 : Gut microbiota abundance (genus Intestinimonas id.2062) on Crohn's disease of large intestine

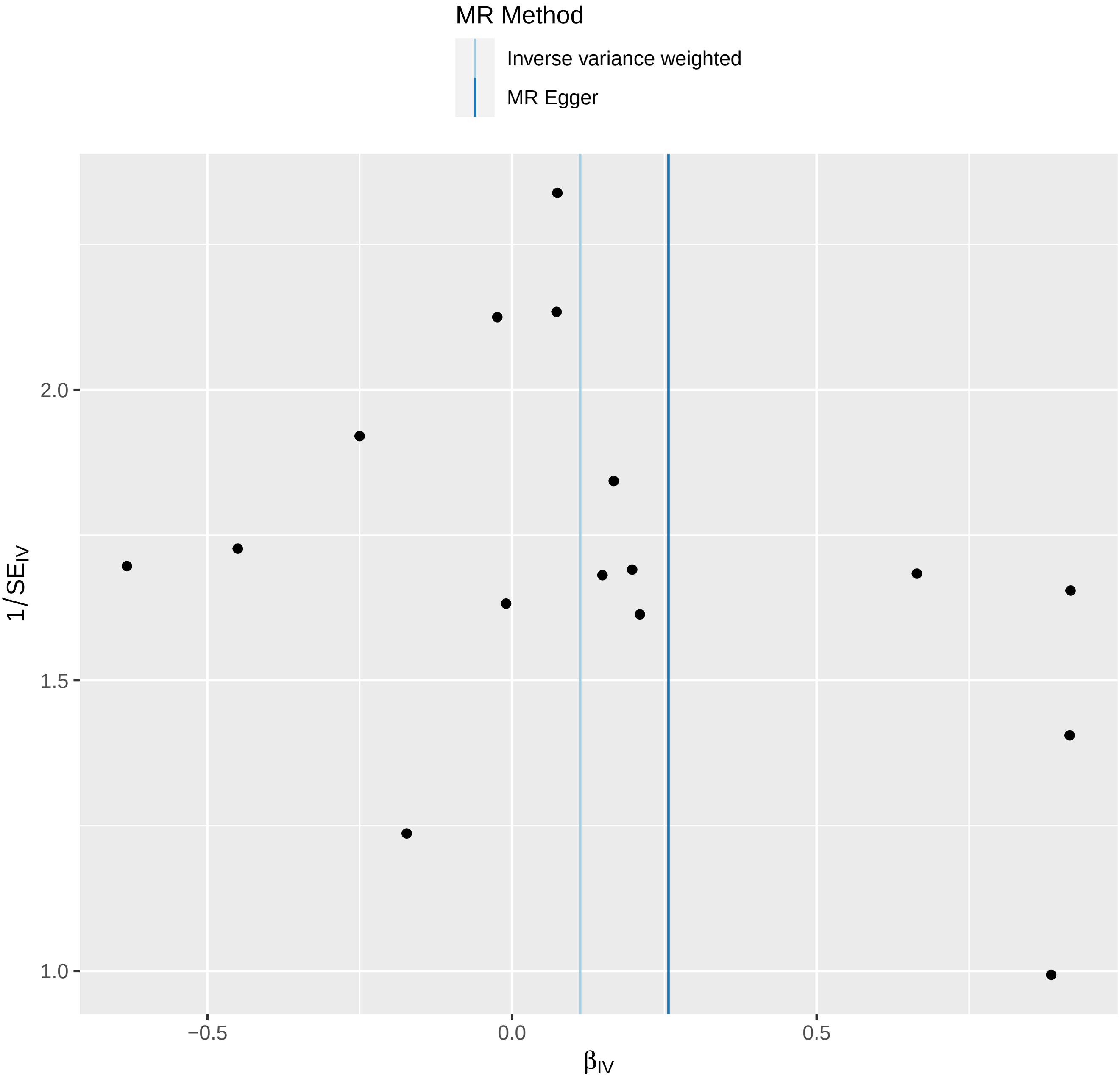

Batch 94 : Gut microbiota abundance (genus Lachnoclostridium id.11308) on Crohn's disease of large intestine

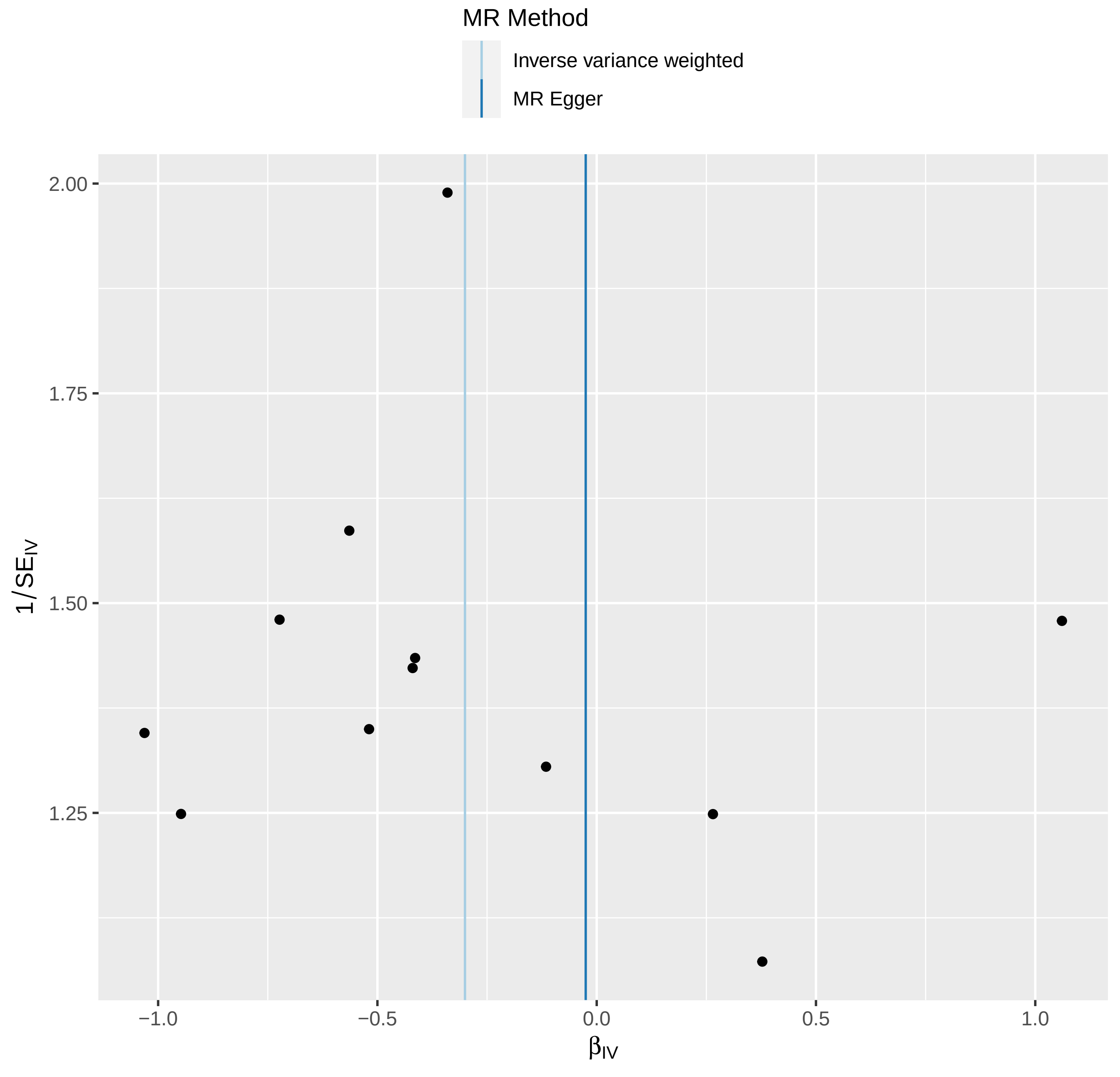

Batch 95 : Gut microbiota abundance (genus Lachnospiraceae FCS020 group id.11314) on Crohn's disease of large intestine

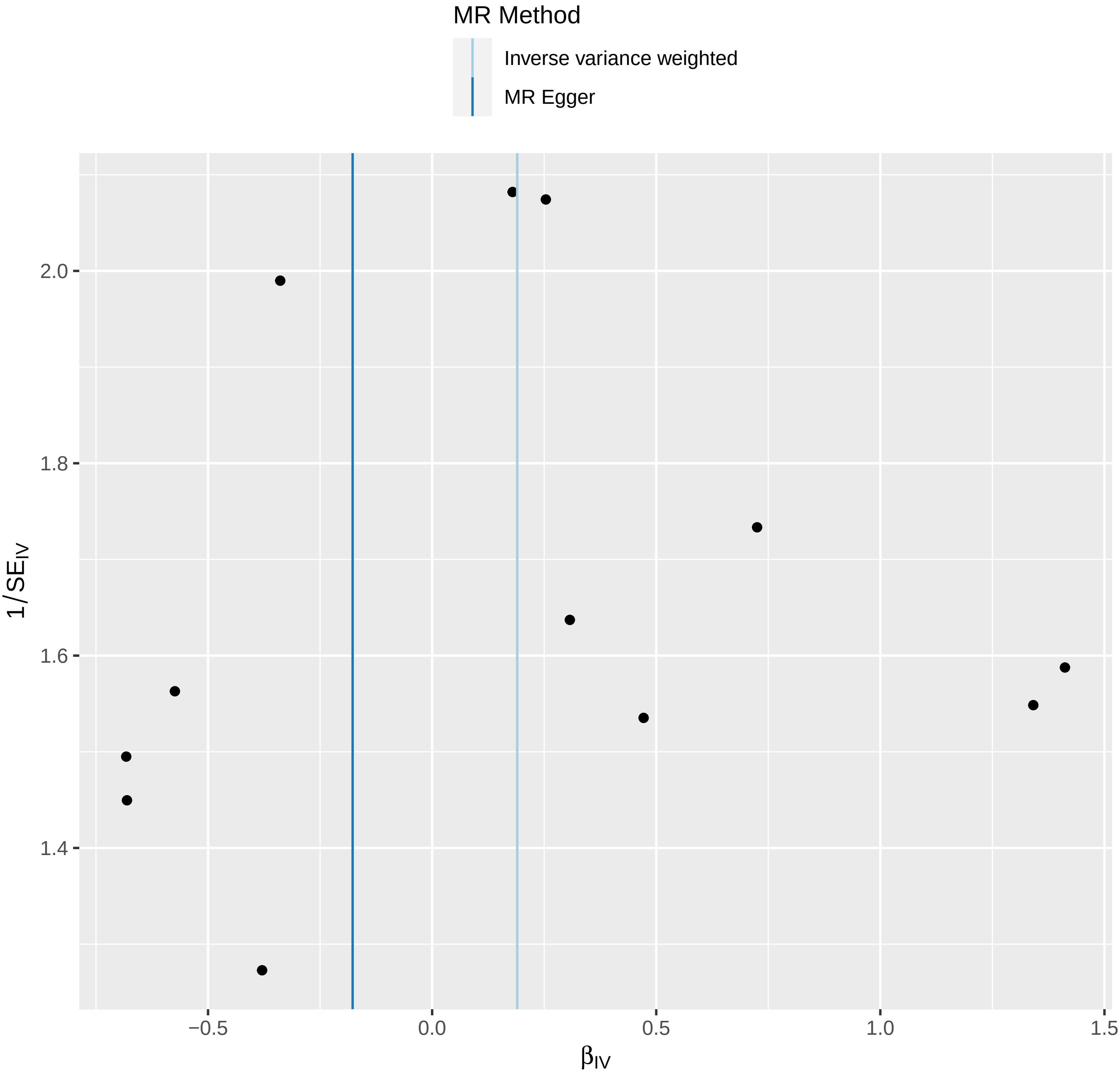

Batch 96 : Gut microbiota abundance (genus Lachnospiraceae NC2004 group id.11316) on Crohn's disease of large intestine

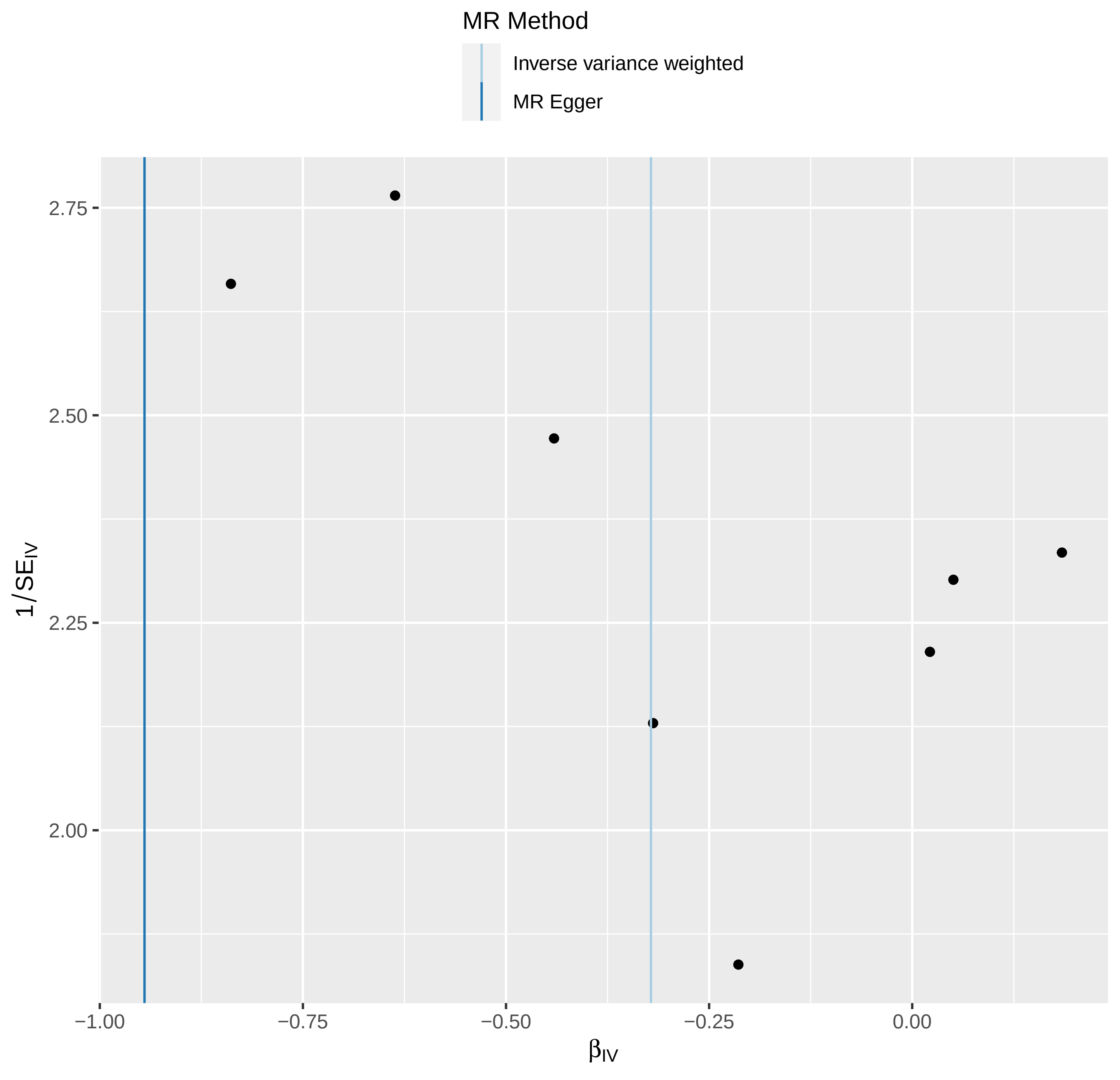

Batch 97 : Gut microbiota abundance (genus Lachnospiraceae ND3007 group id.11317) on Crohn's disease of large intestine

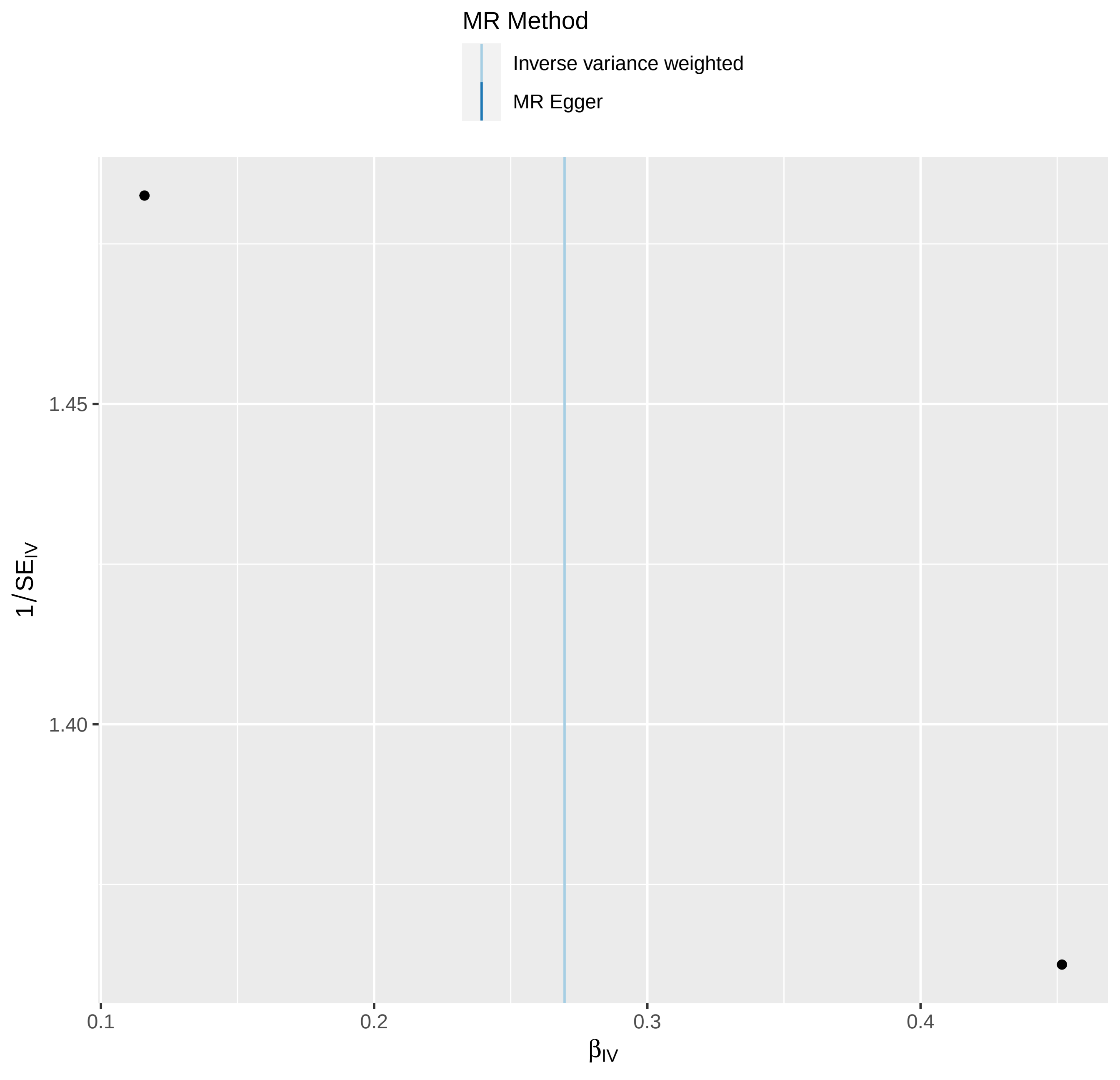

Batch 98 : Gut microbiota abundance (genus Lachnospiraceae NK4A136 group id.11319) on Crohn's disease of large intestine

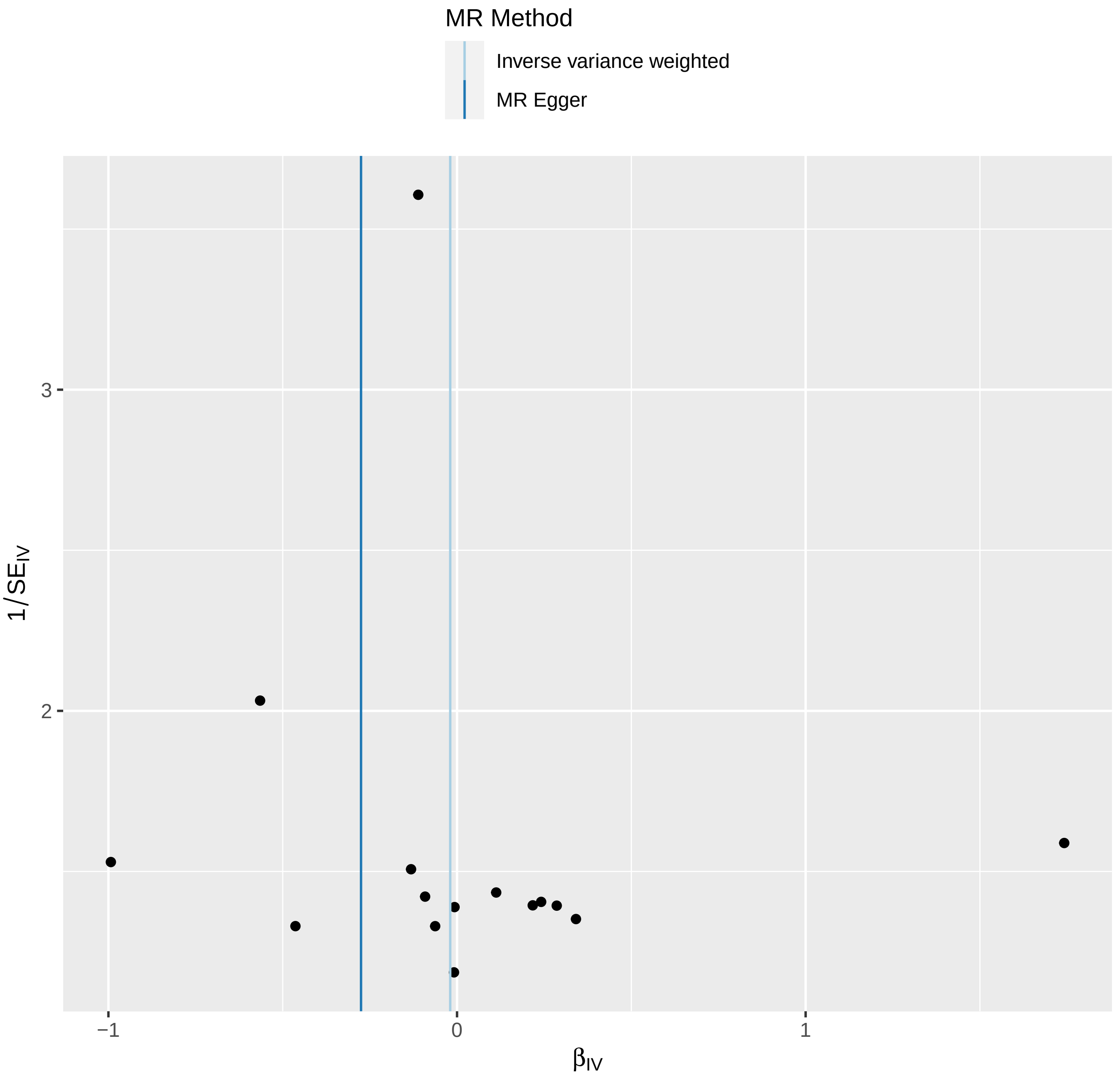

Batch 99 : Gut microbiota abundance (genus Lachnospiraceae UCG001 id.11321) on Crohn's disease of large intestine

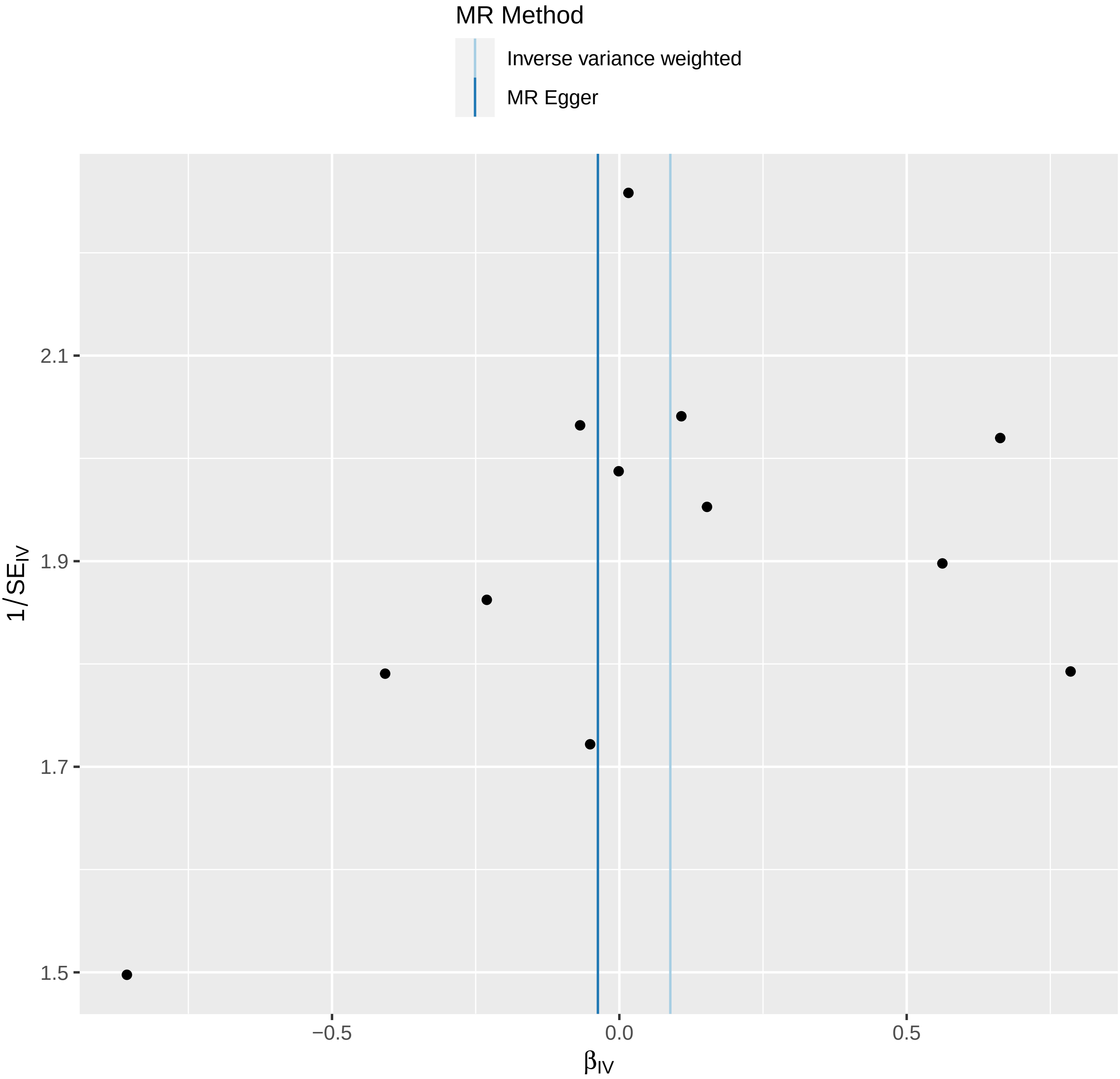

Batch 100 : Gut microbiota abundance (genus Lachnospiraceae UCG004 id.11324) on Crohn's disease of large intestine

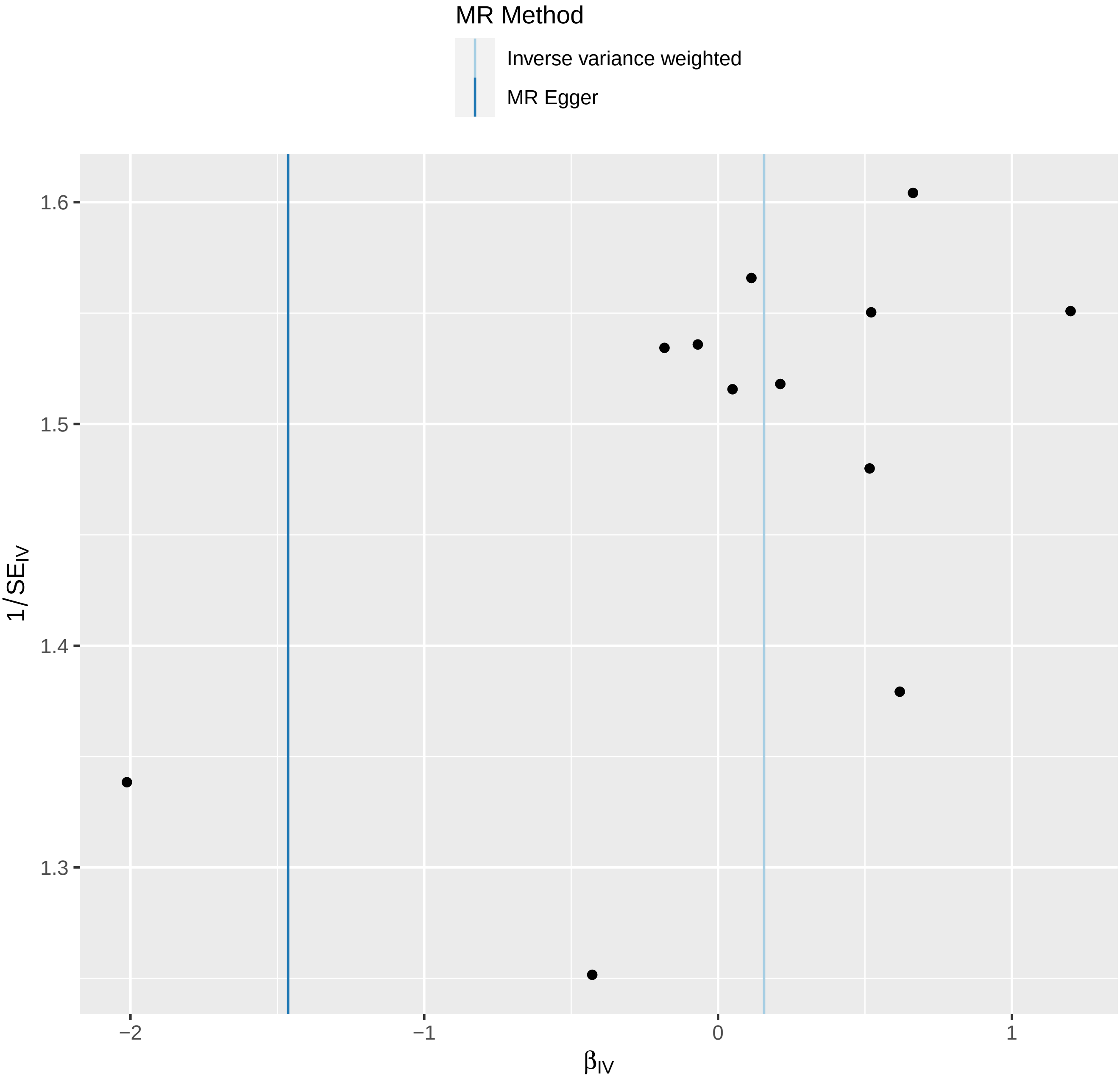

Batch 101 : Gut microbiota abundance (genus Lachnospiraceae UCG008 id.11328) on Crohn's disease of large intestine

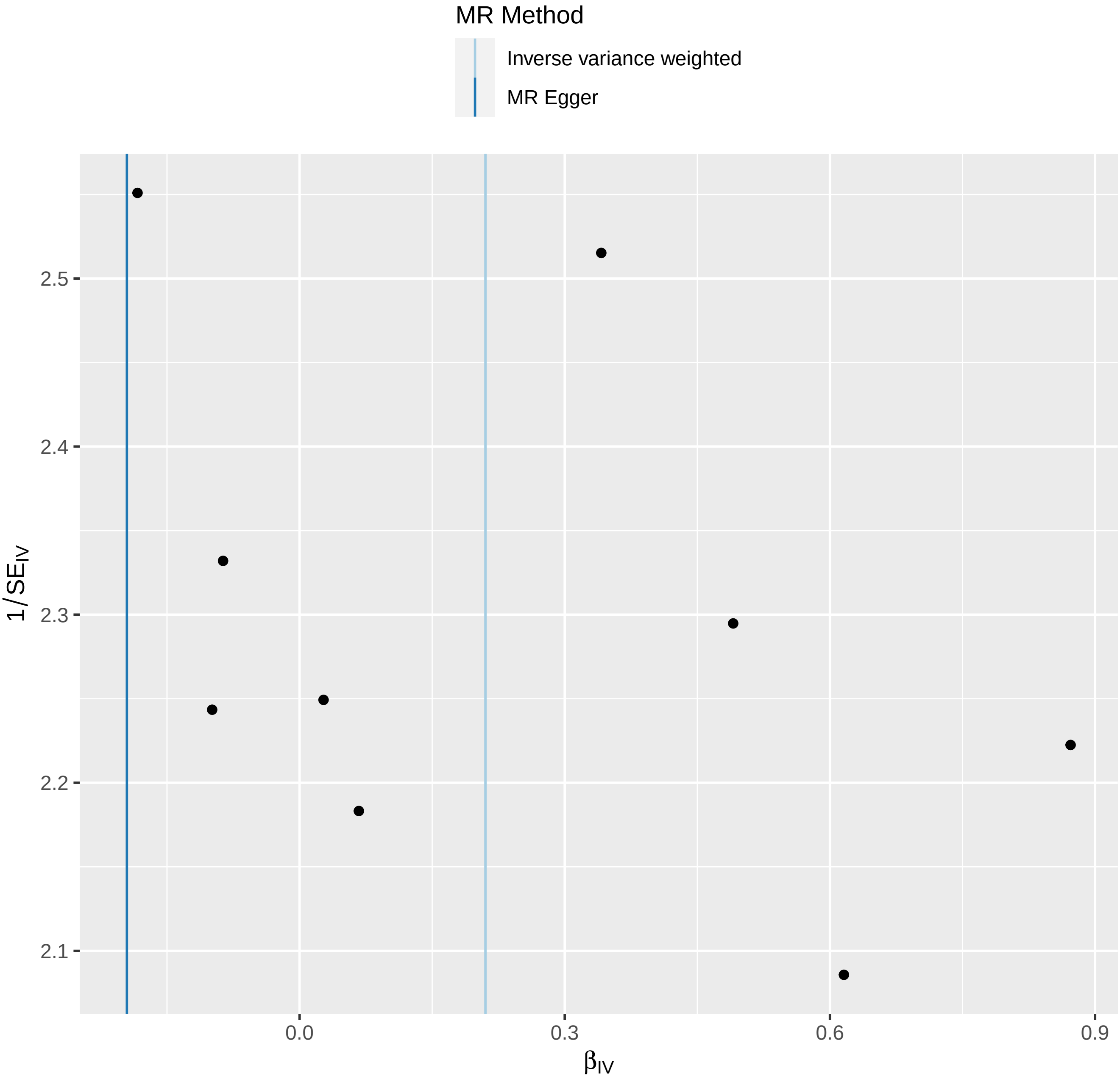

Batch 102 : Gut microbiota abundance (genus Lachnospiraceae UCG010 id.11330) on Crohn's disease of large intestine

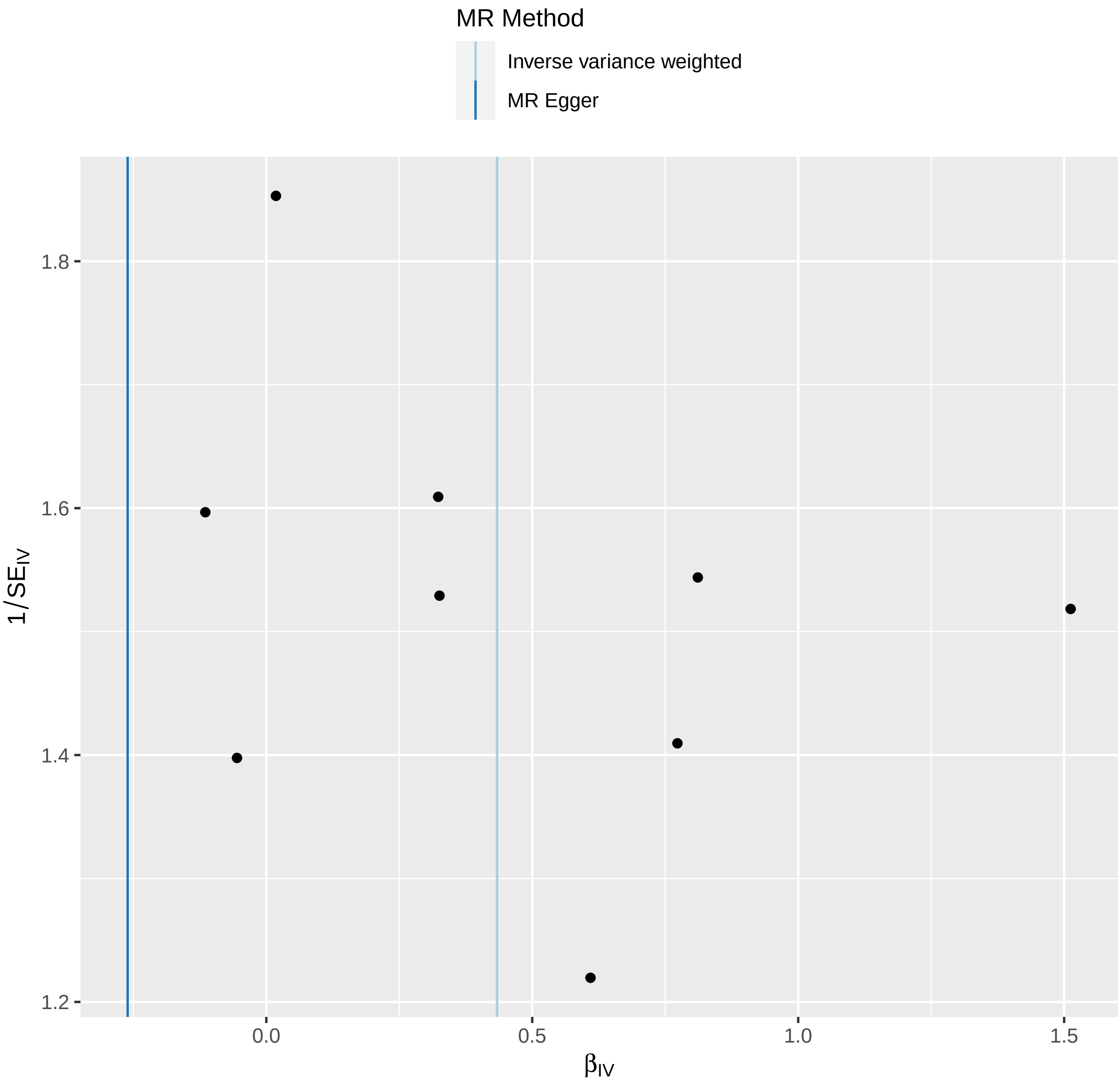

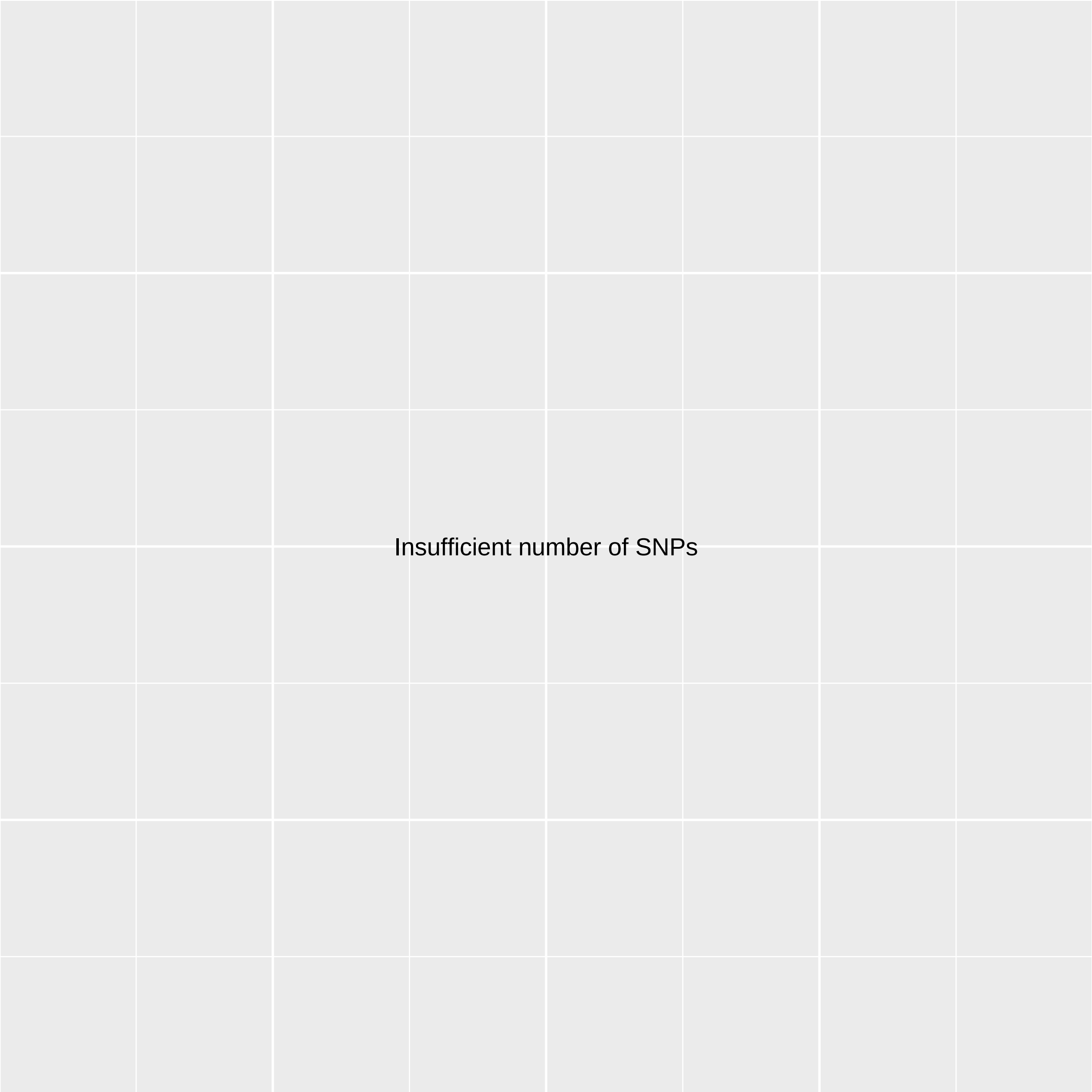
Insufficient number of SNPs

Batch 104 : Gut microbiota abundance (genus Lactobacillus id.1837) on Crohn's disease of large intestine

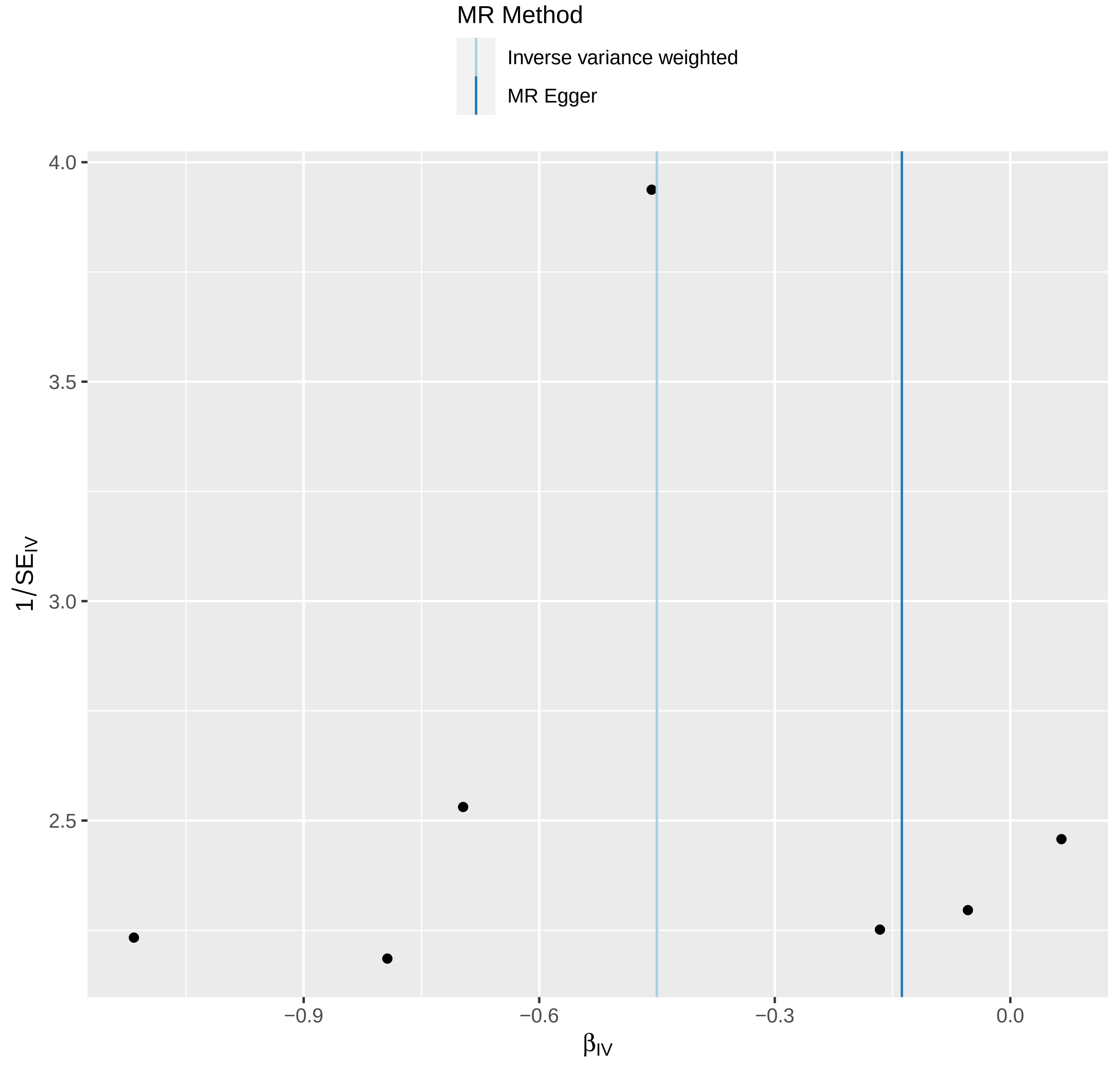

Batch 105 : Gut microbiota abundance (genus Lactococcus id.1851) on Crohn's disease of large intestine

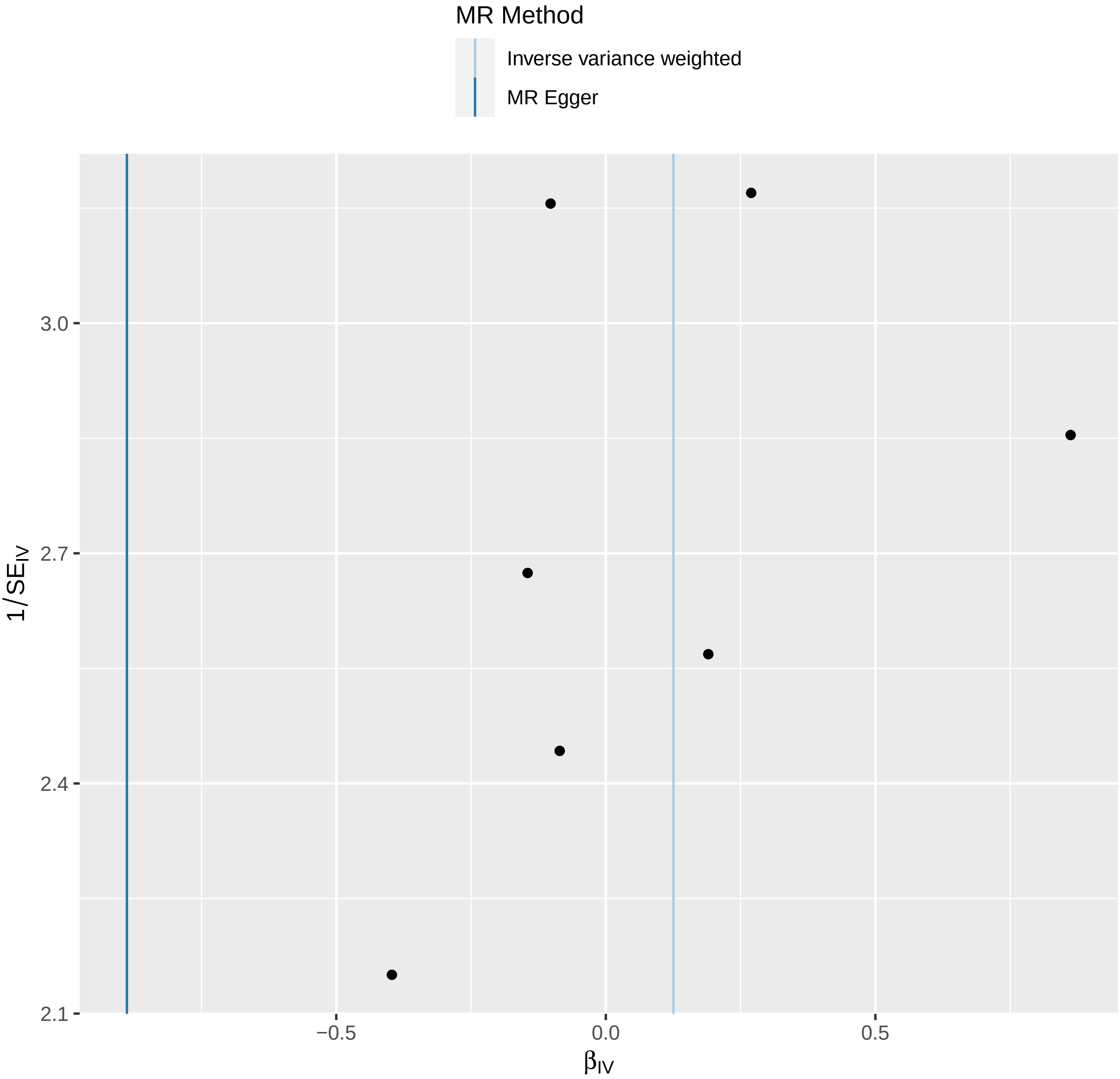

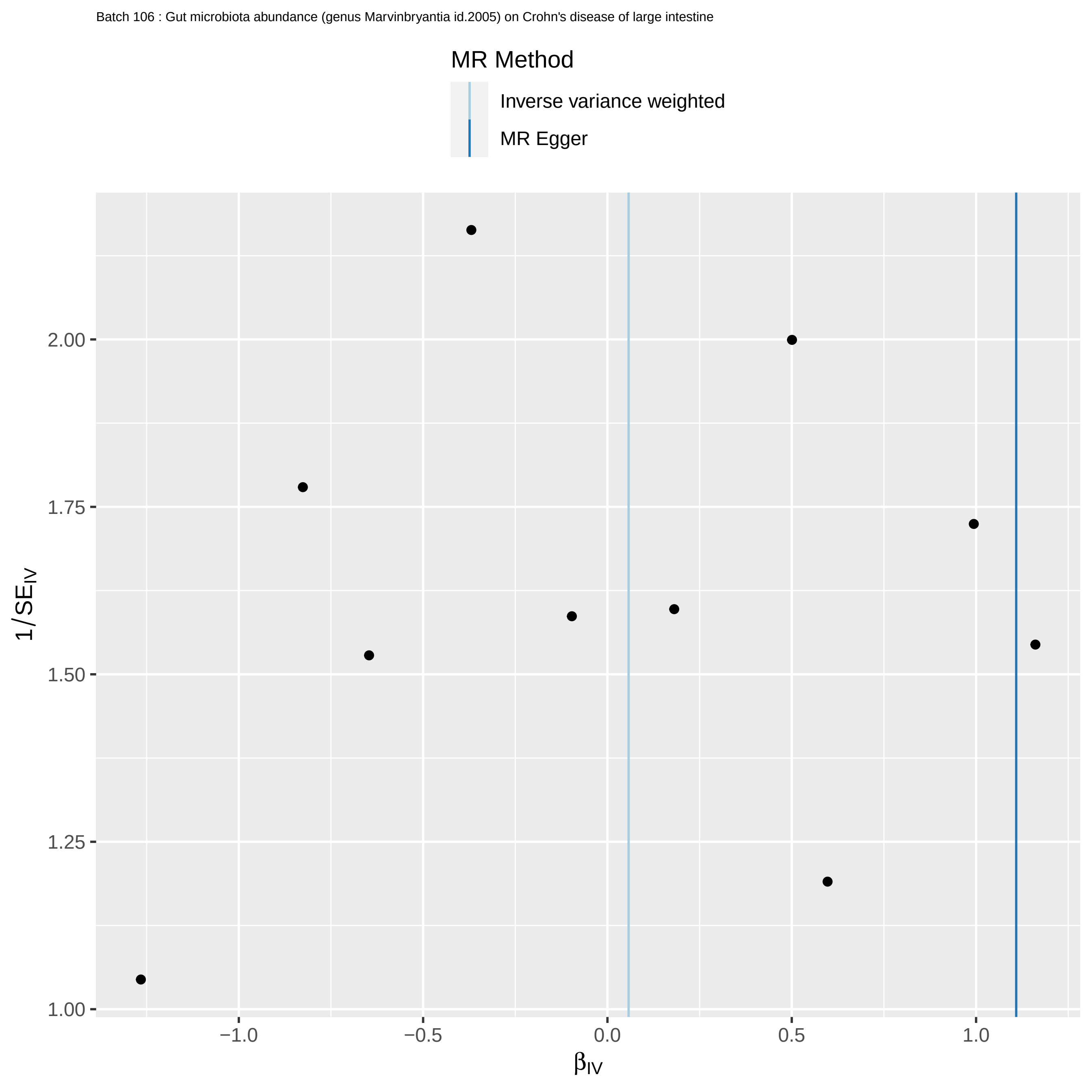
Batch 106 : Gut microbiota abundance (genus Marvinbryantia id.2005) on Crohn's disease of large intestine
MR Method
Inverse variance weighted
MR Egger
2.00
1.75
1.50
1.25
1.00
1/SE_IV
−1.0
−0.5
0.0
0.5
1.0
β_IV

Batch 107 : Gut microbiota abundance (genus Methanobrevibacter id.123) on Crohn's disease of large intestine

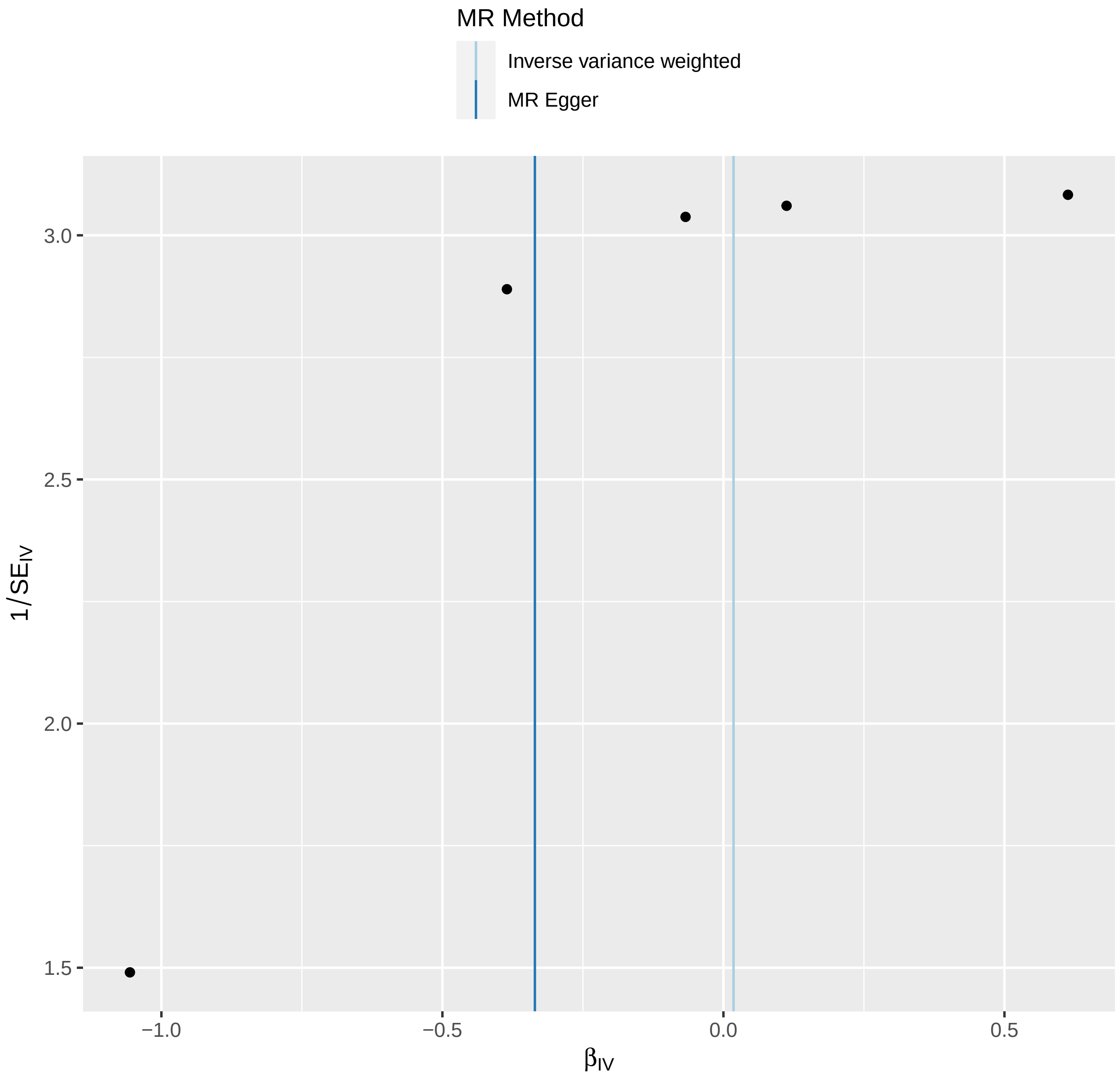

Batch 108 : Gut microbiota abundance (genus Odoribacter id.952) on Crohn's disease of large intestine

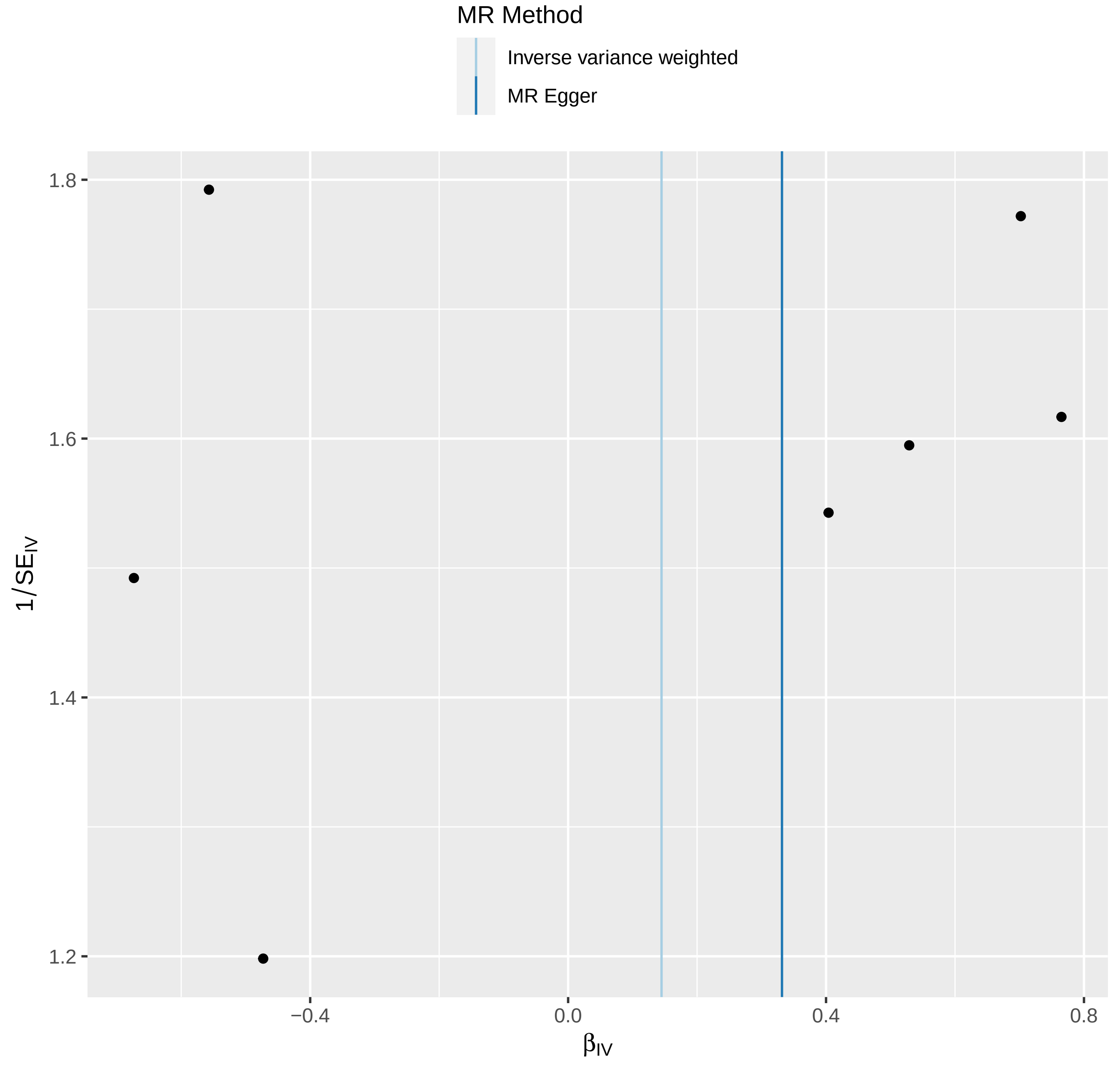

Batch 109 : Gut microbiota abundance (genus Olsenella id.822) on Crohn's disease of large intestine

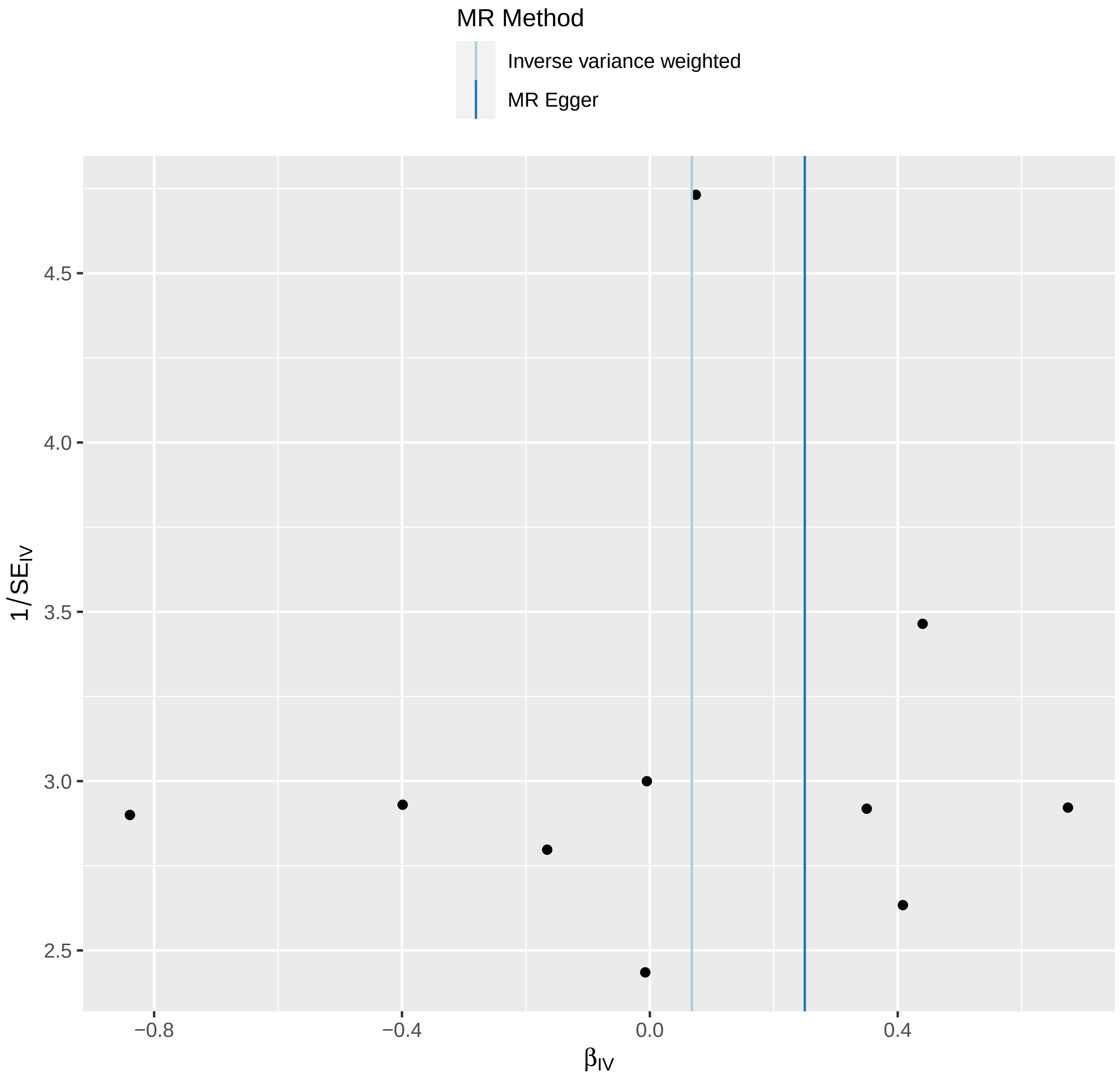

Batch 110 : Gut microbiota abundance (genus Oscillibacter id.2063) on Crohn's disease of large intestine

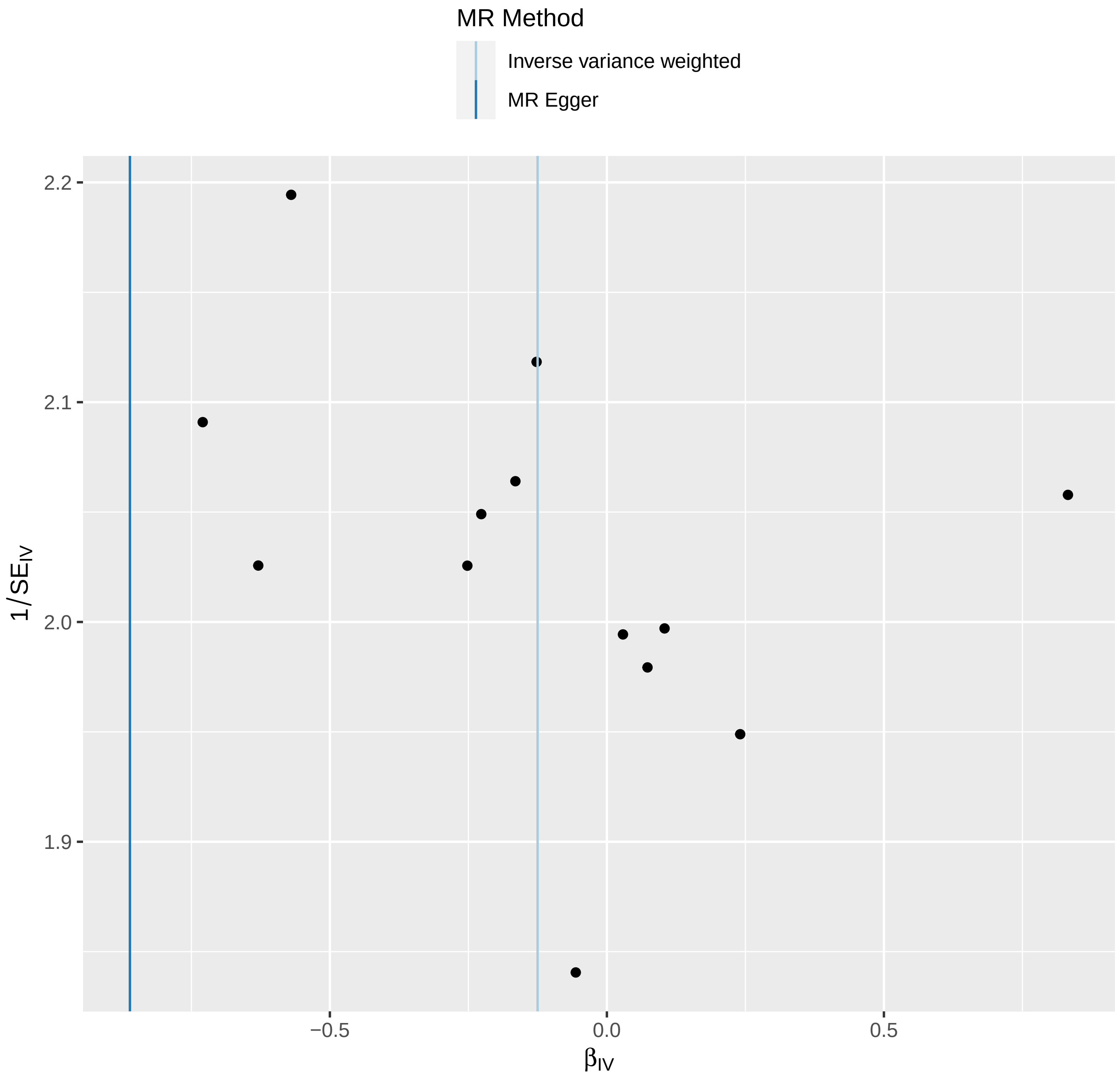

Batch 111 : Gut microbiota abundance (genus Oscillospira id.2064) on Crohn's disease of large intestine

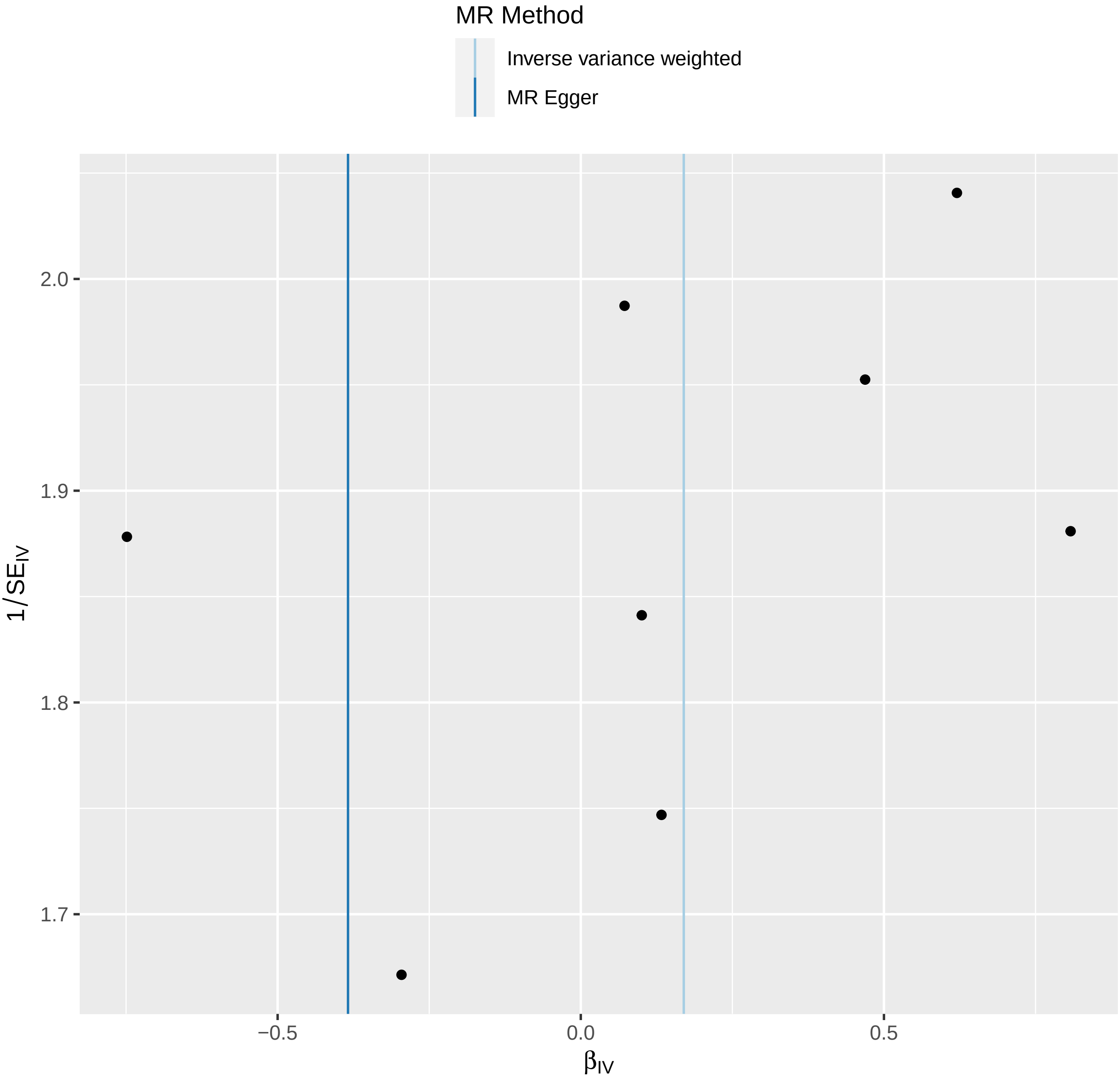

Batch 112 : Gut microbiota abundance (genus Oxalobacter id.2978) on Crohn's disease of large intestine

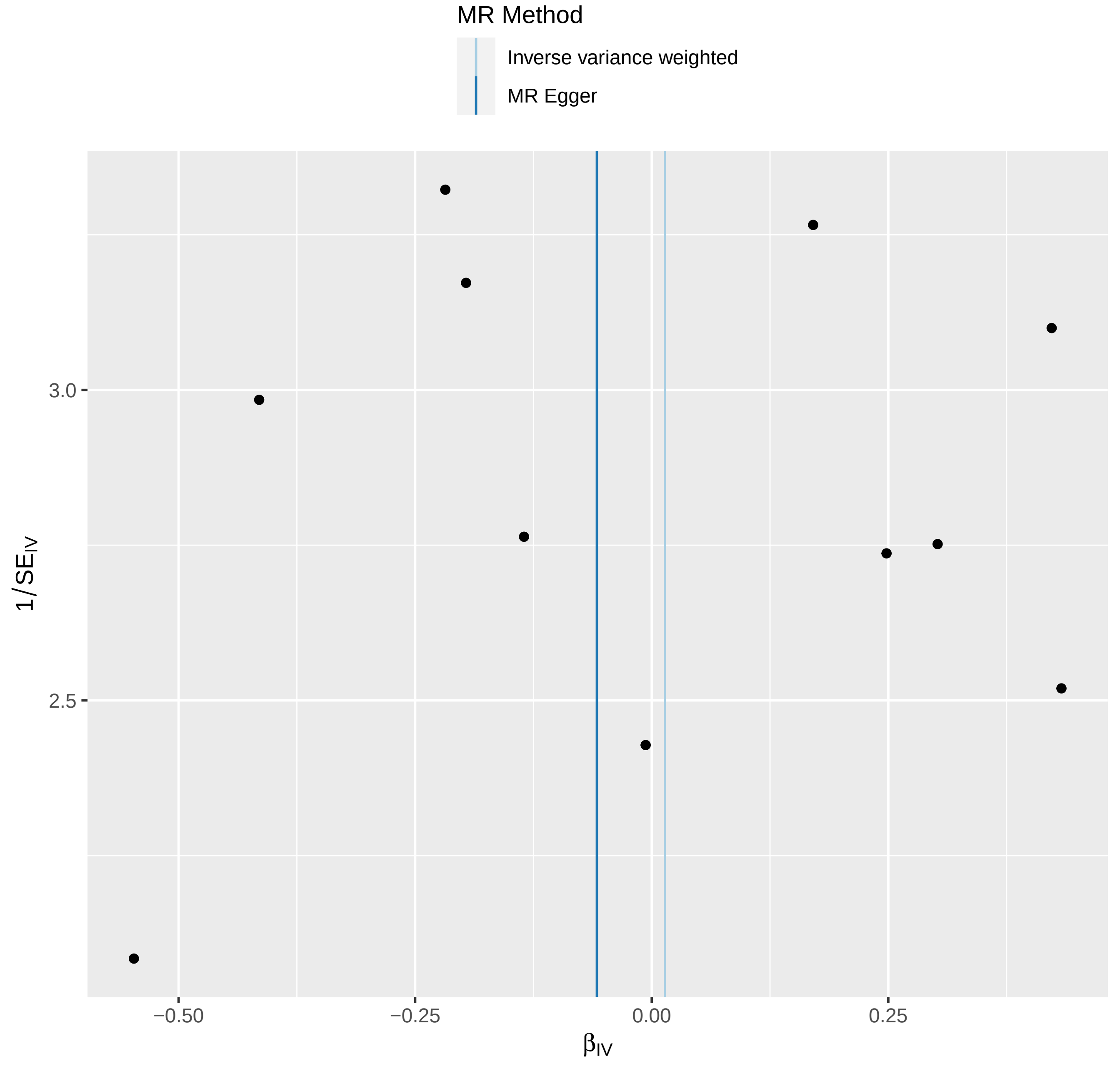

Batch 113 : Gut microbiota abundance (genus Parabacteroides id.954) on Crohn's disease of large intestine

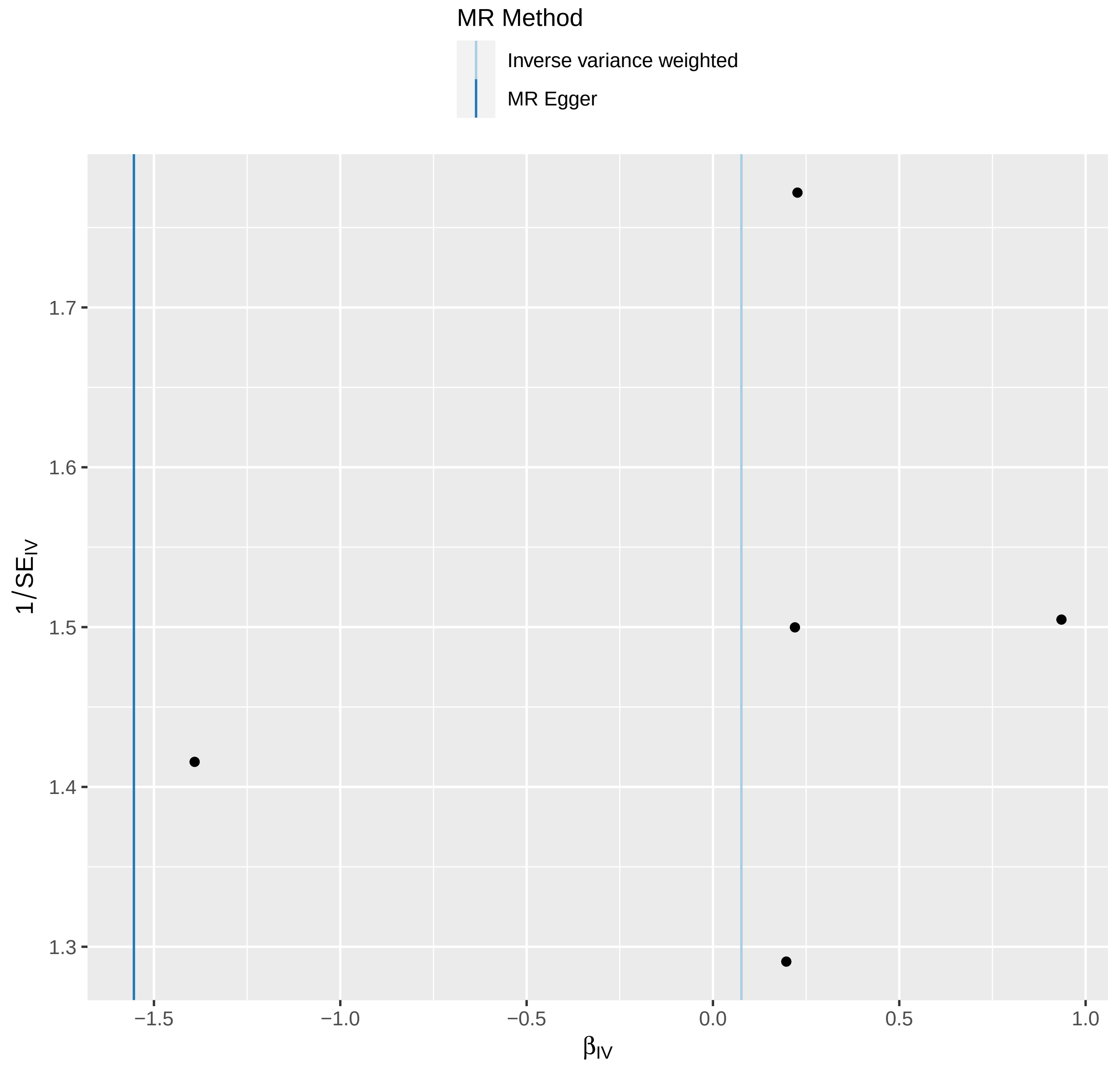

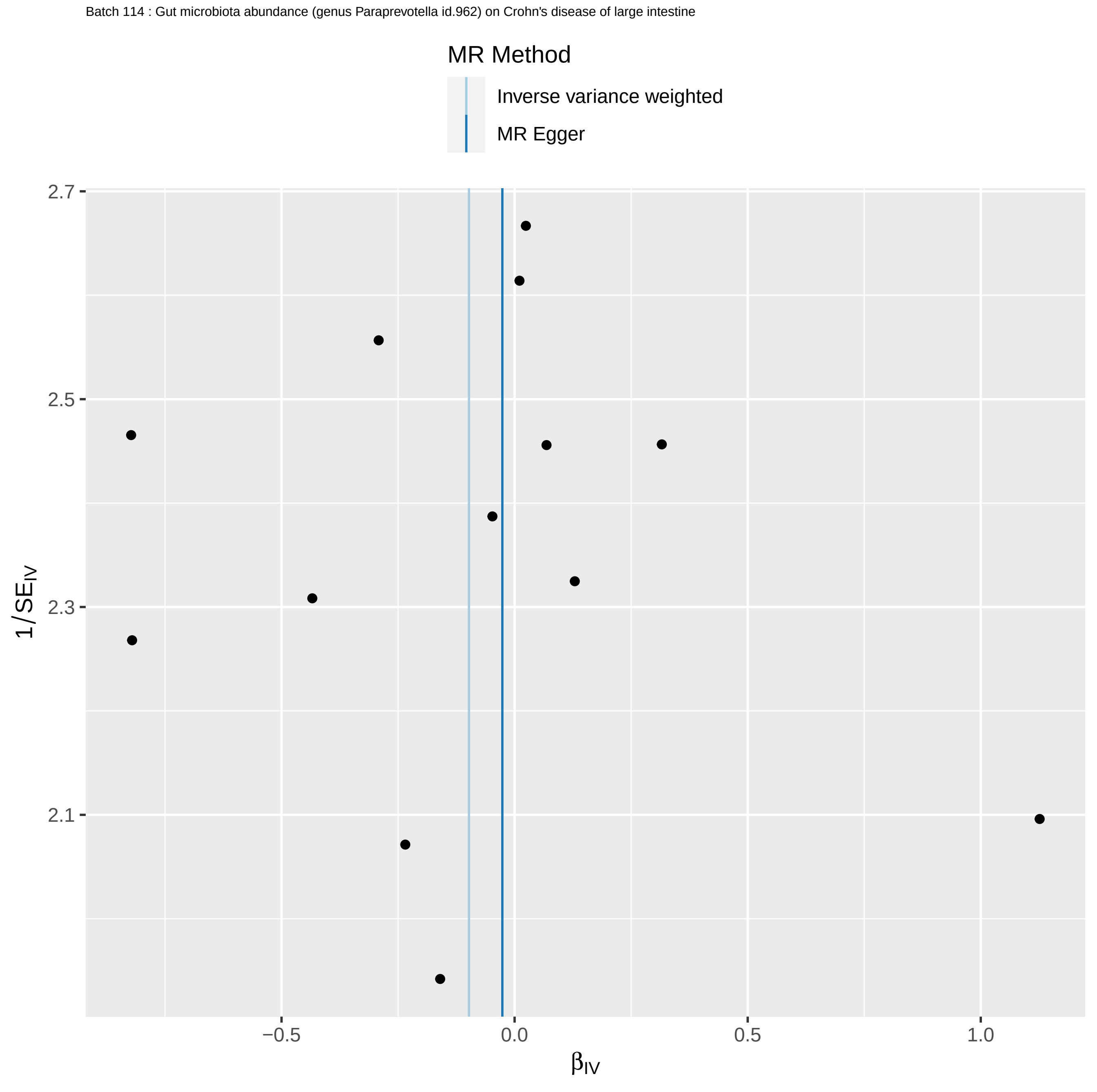

Batch 114 : Gut microbiota abundance (genus Paraprevotella id.962) on Crohn's disease of large intestine
MR Method
Inverse variance weighted
MR Egger
2.7
2.5
2.3
2.1
1/SE_IV
−0.5
0.0
0.5
1.0
β_IV

Batch 115 : Gut microbiota abundance (genus Parasutterella id.2892) on Crohn's disease of large intestine

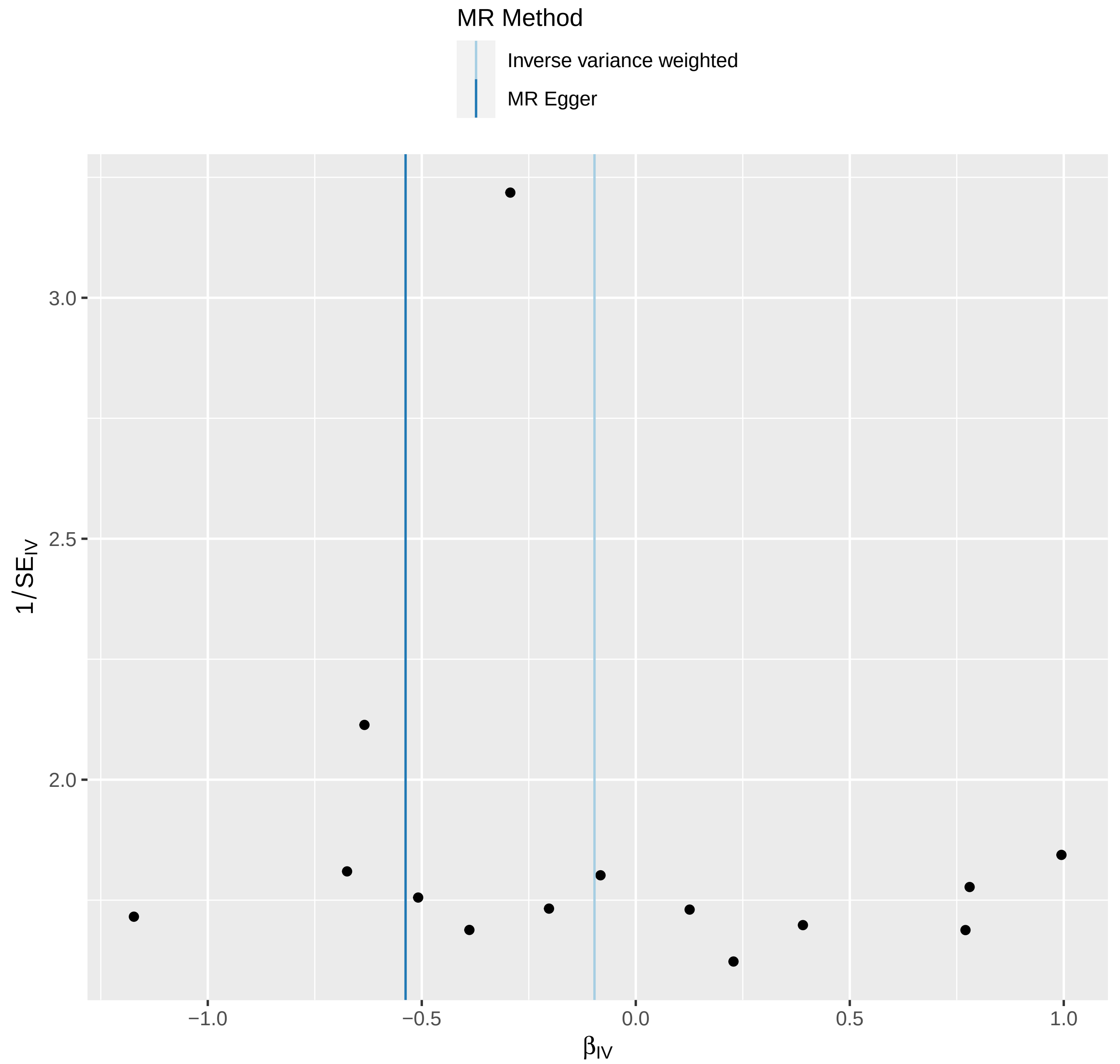

Batch 116 : Gut microbiota abundance (genus Peptococcus id.2037) on Crohn's disease of large intestine

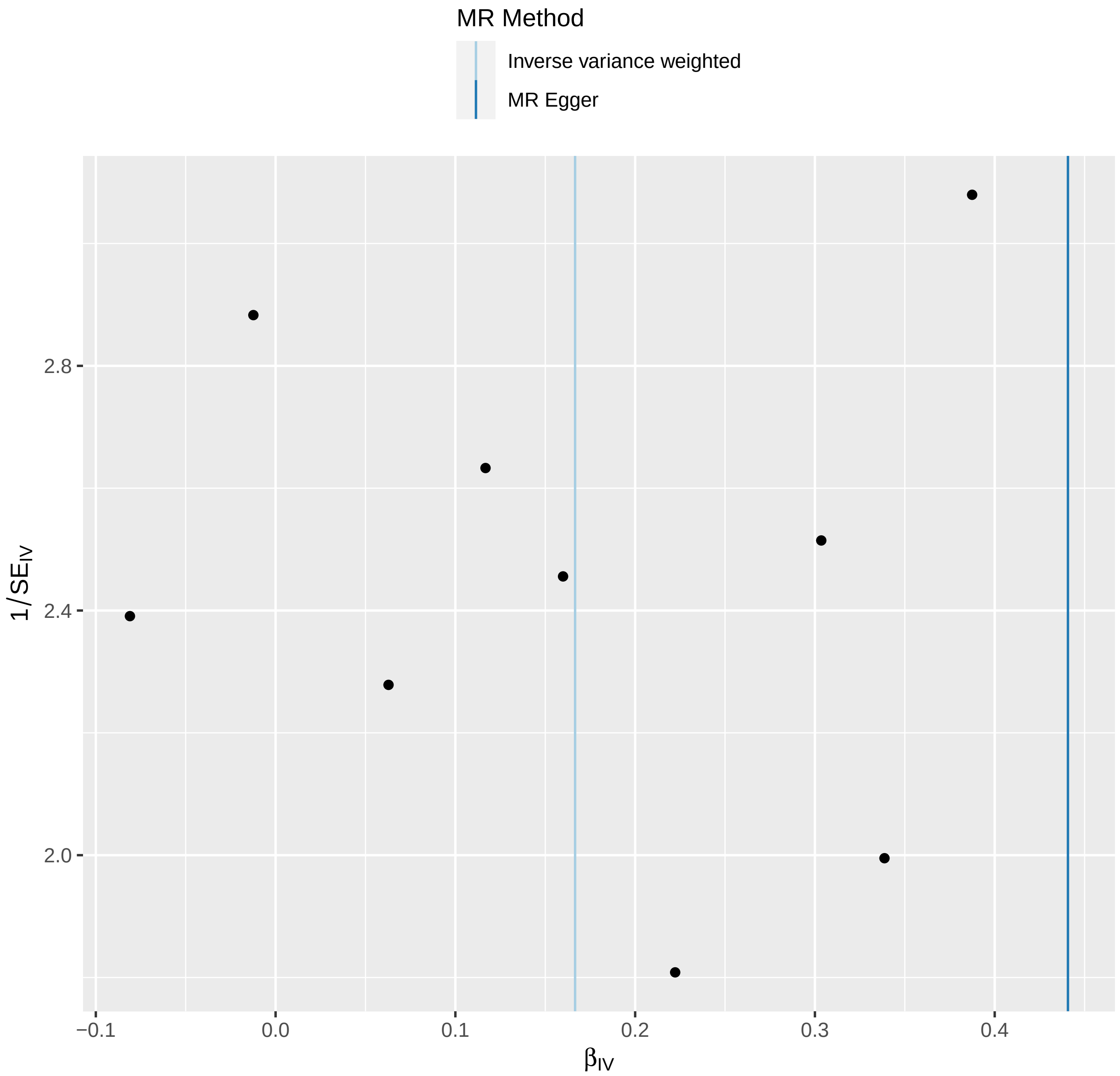

Batch 117 : Gut microbiota abundance (genus Phascolarctobacterium id.2168) on Crohn's disease of large intestine

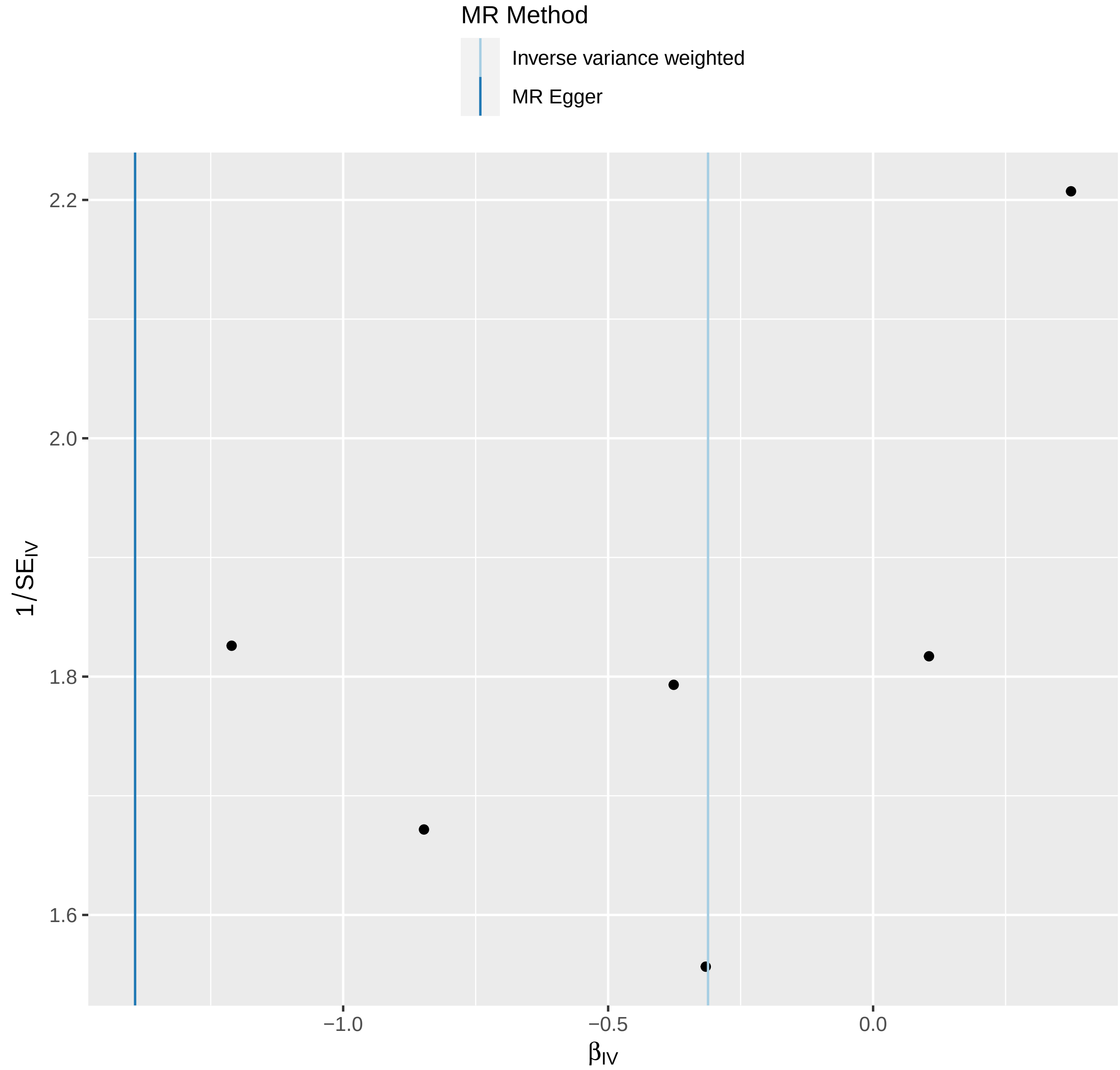

Batch 118 : Gut microbiota abundance (genus Prevotella7 id.11182) on Crohn's disease of large intestine

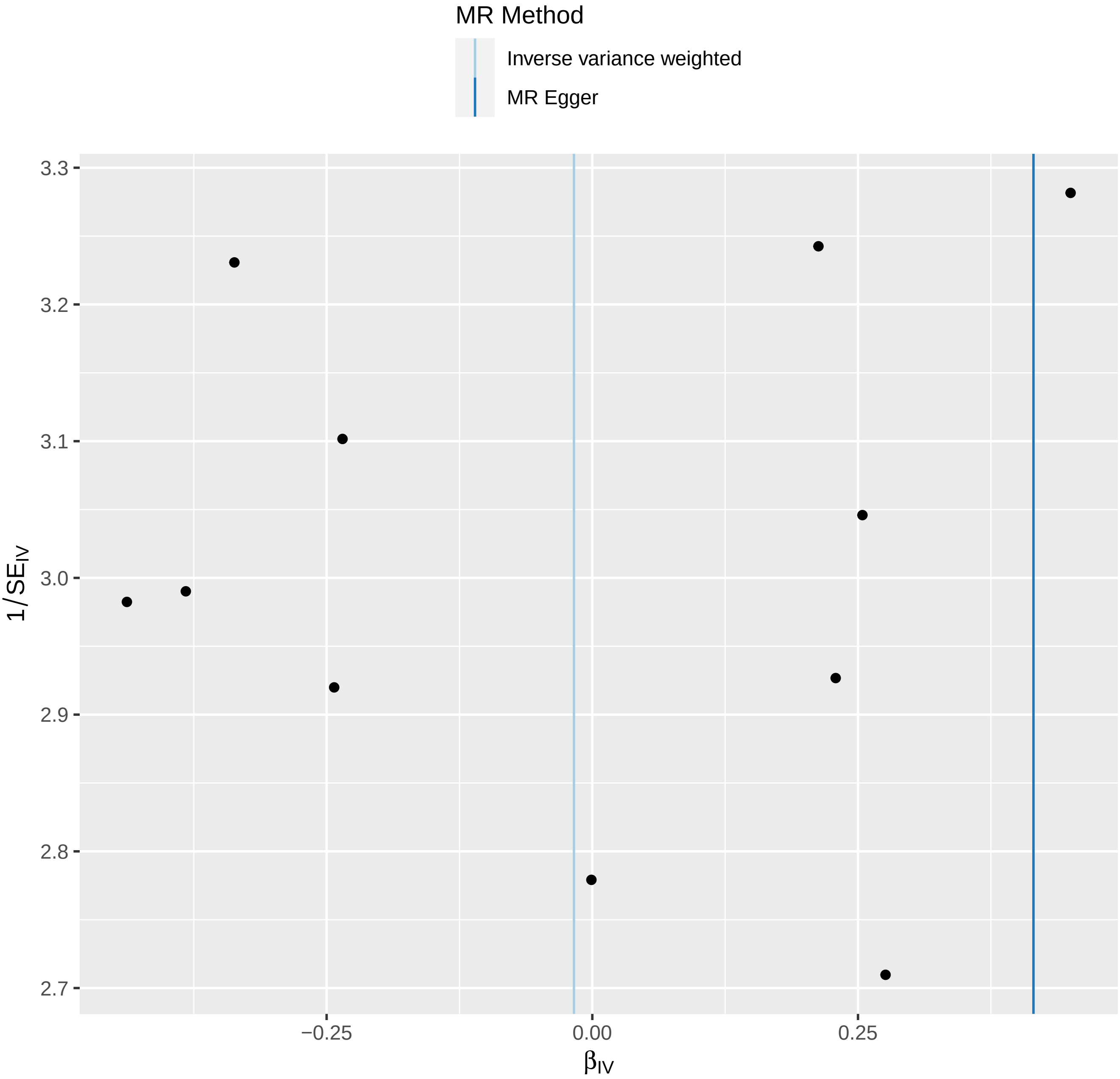

Batch 119 : Gut microbiota abundance (genus Prevotella9 id.11183) on Crohn's disease of large intestine

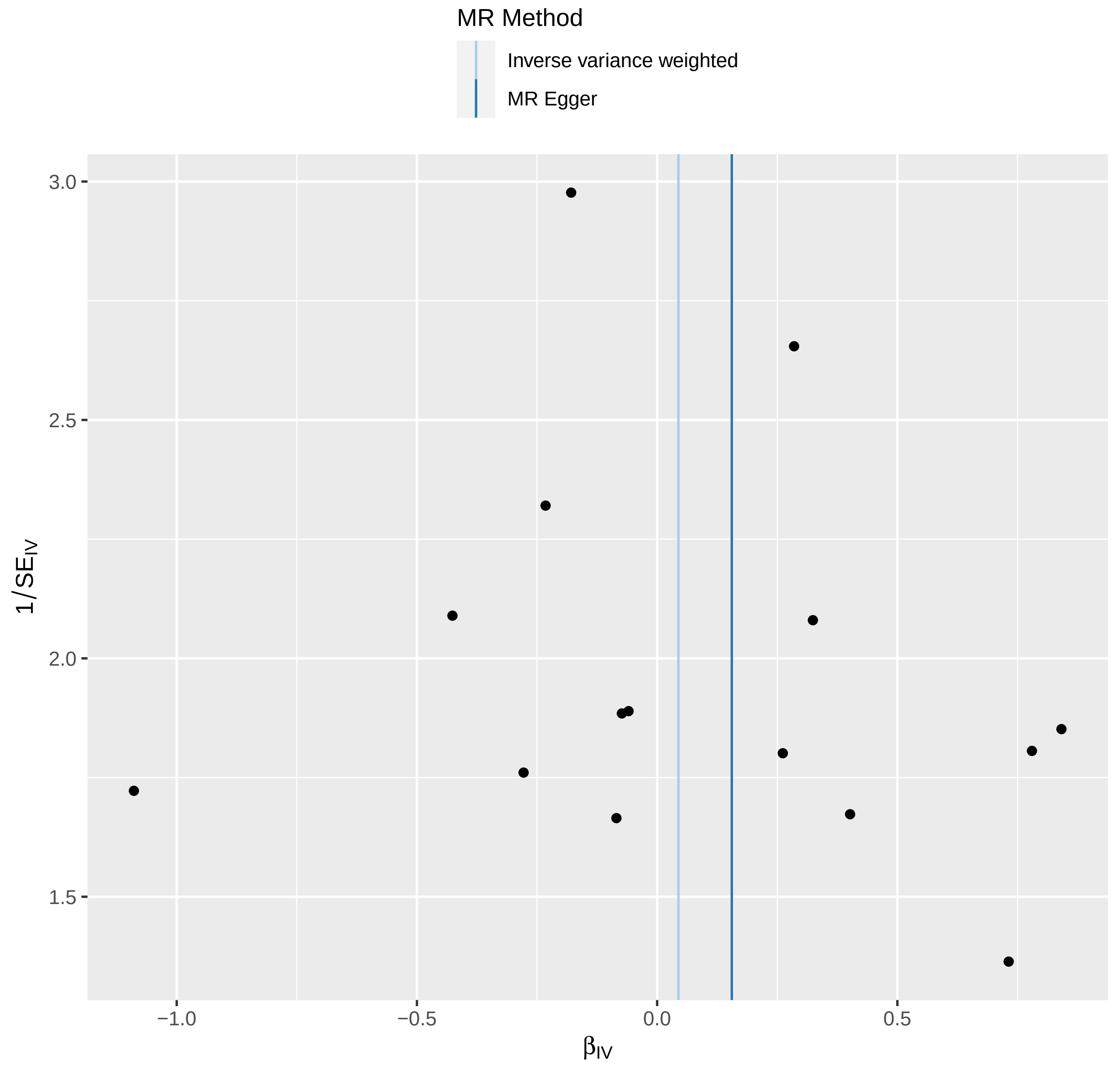

Batch 120 : Gut microbiota abundance (genus Rikenellaceae RC9 gut group id.11191) on Crohn's disease of large intestine

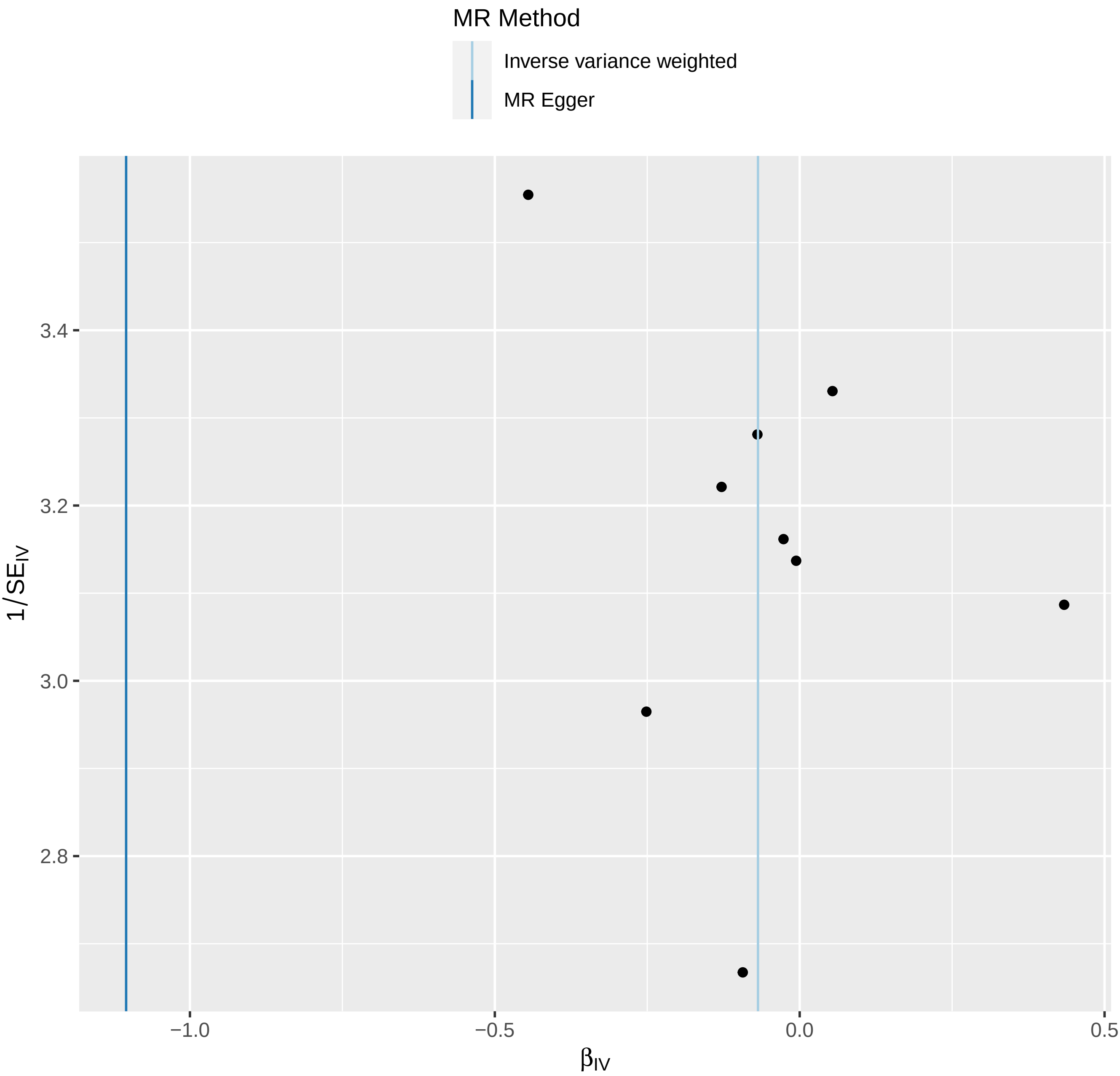

Batch 121 : Gut microbiota abundance (genus Romboutsia id.11347) on Crohn's disease of large intestine

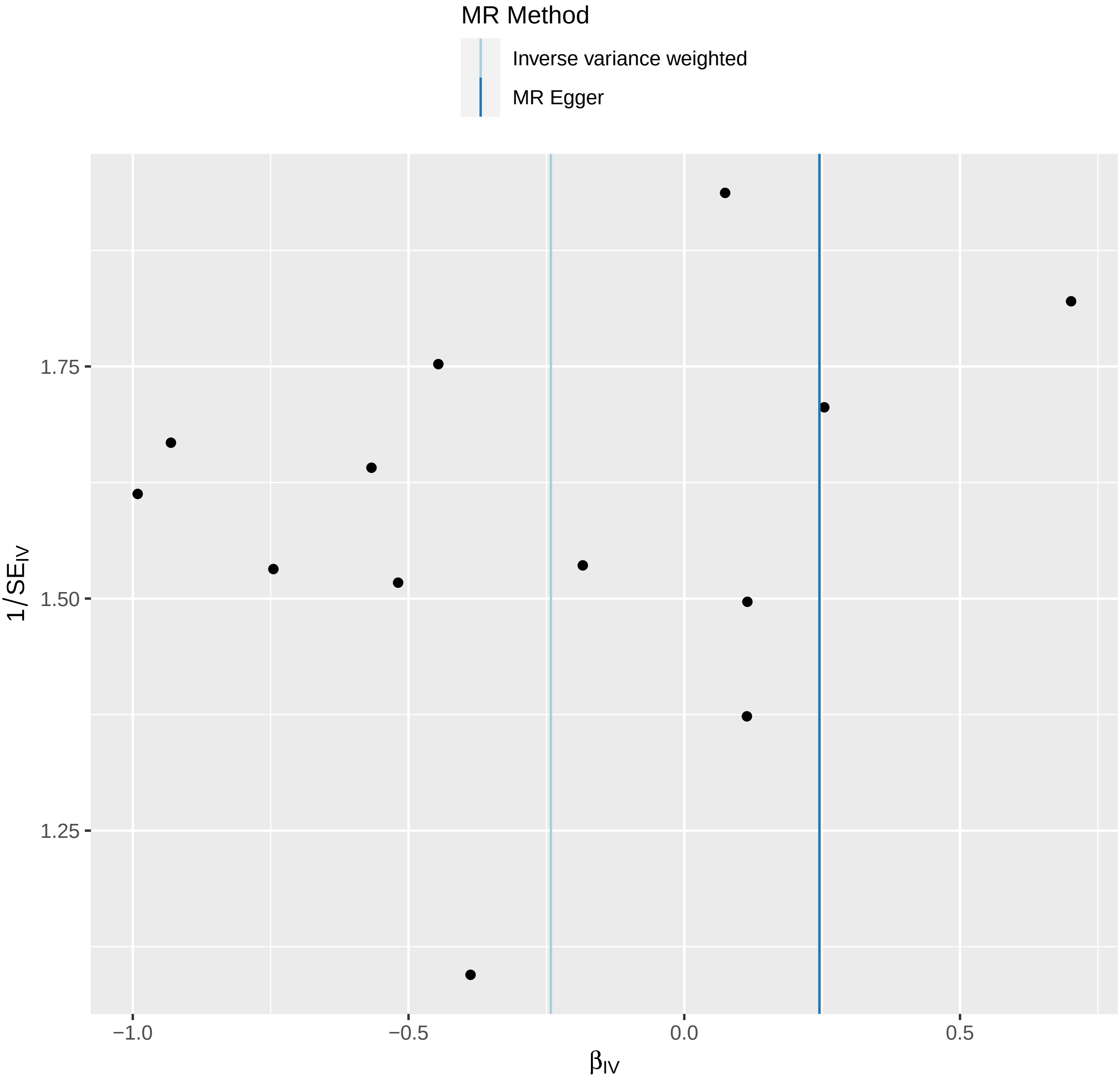

Batch 122 : Gut microbiota abundance (genus Roseburia id.2012) on Crohn's disease of large intestine

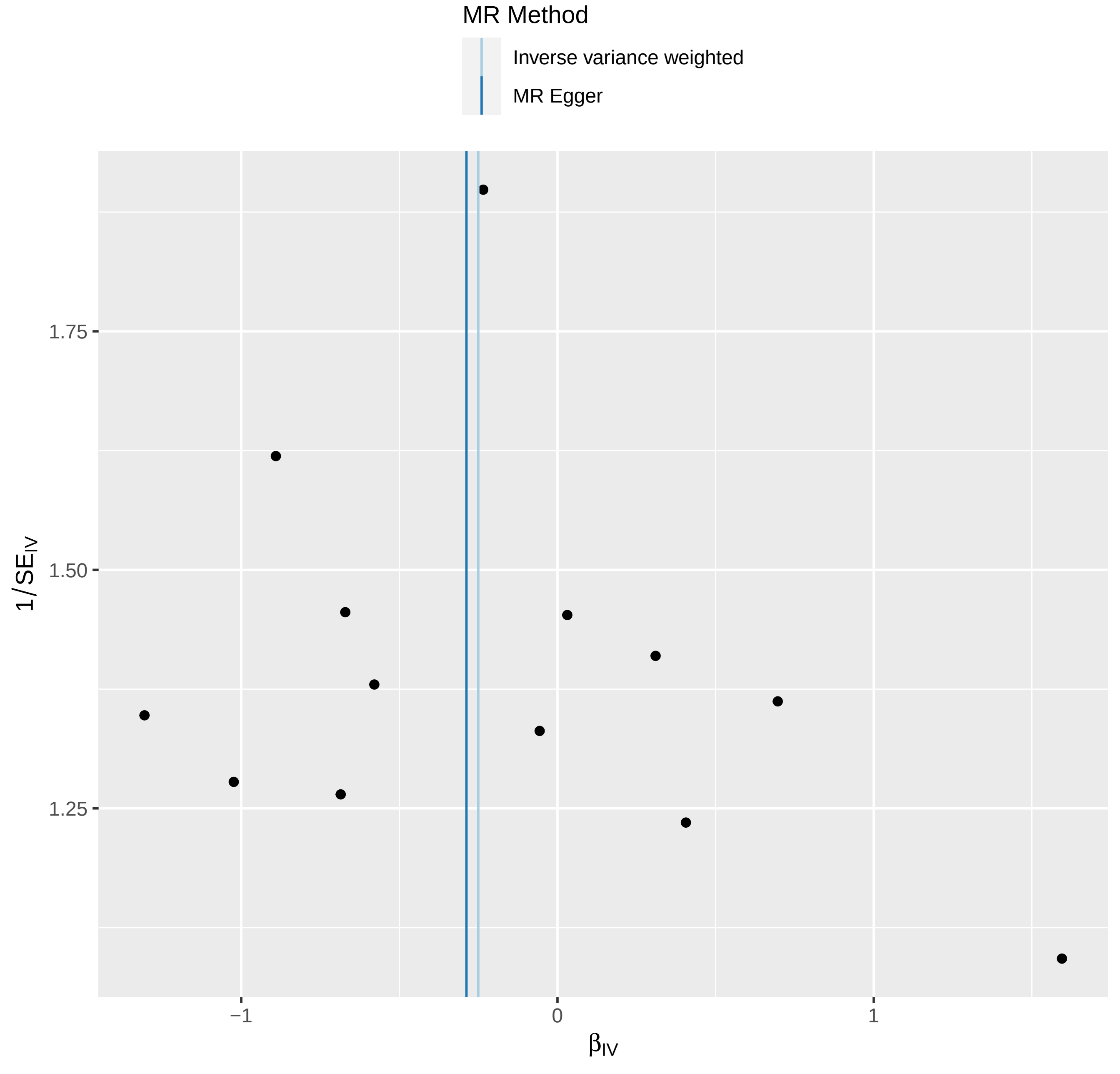

Batch 123 : Gut microbiota abundance (genus Ruminiclostridium5 id.11355) on Crohn's disease of large intestine

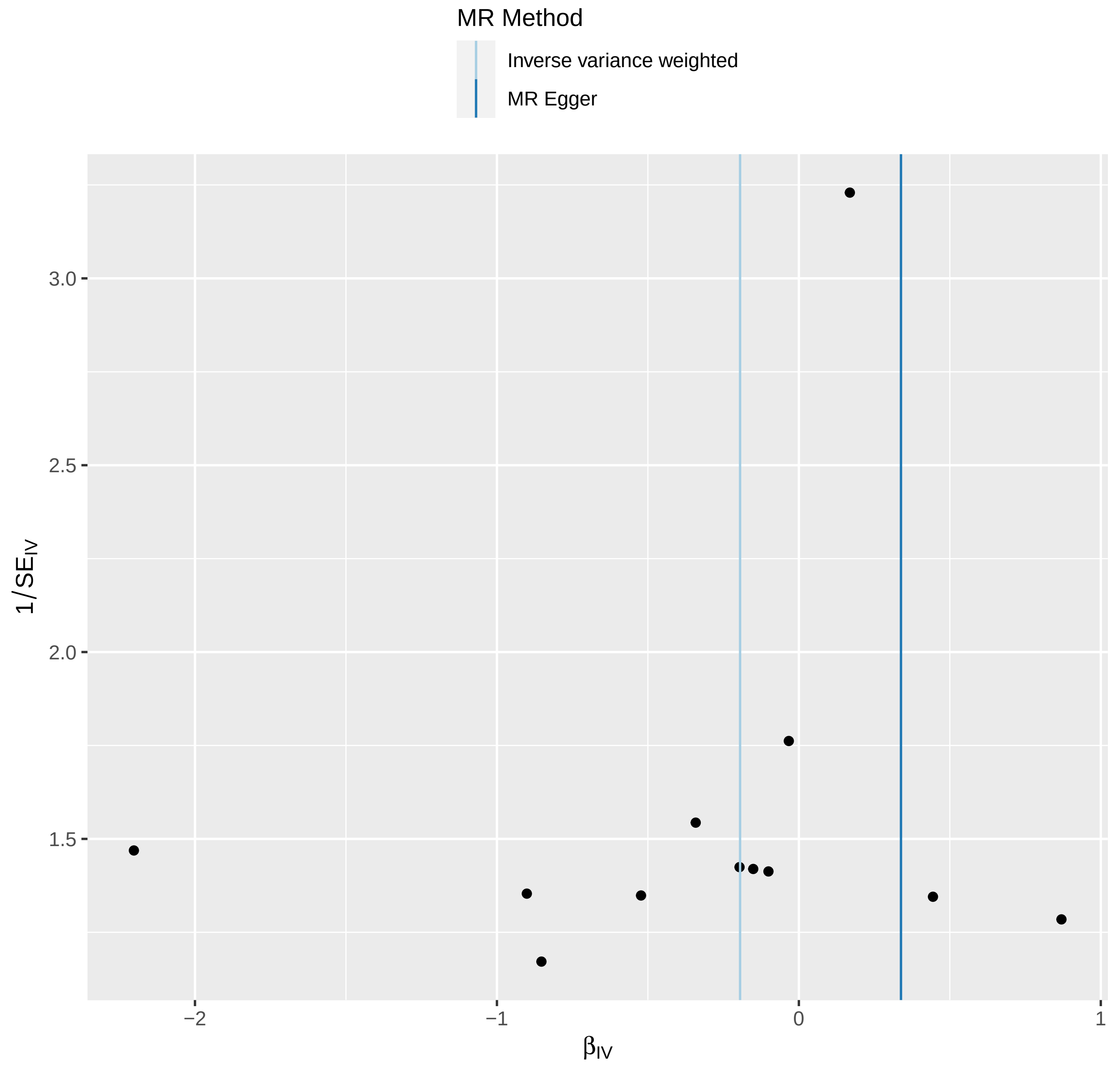

Batch 124 : Gut microbiota abundance (genus Ruminiclostridium6 id.11356) on Crohn's disease of large intestine

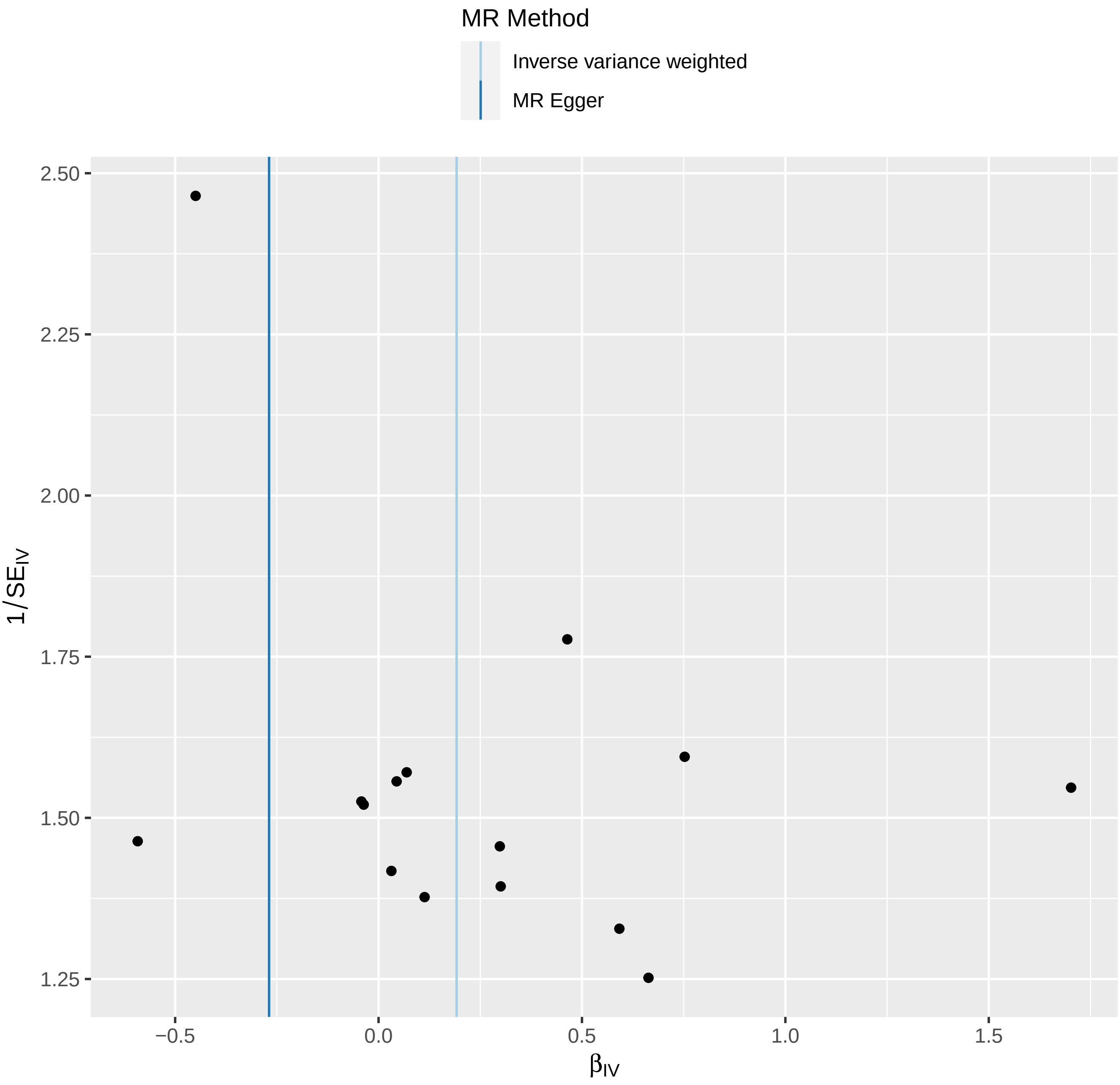

Batch 125 : Gut microbiota abundance (genus Ruminiclostridium9 id.11357) on Crohn's disease of large intestine

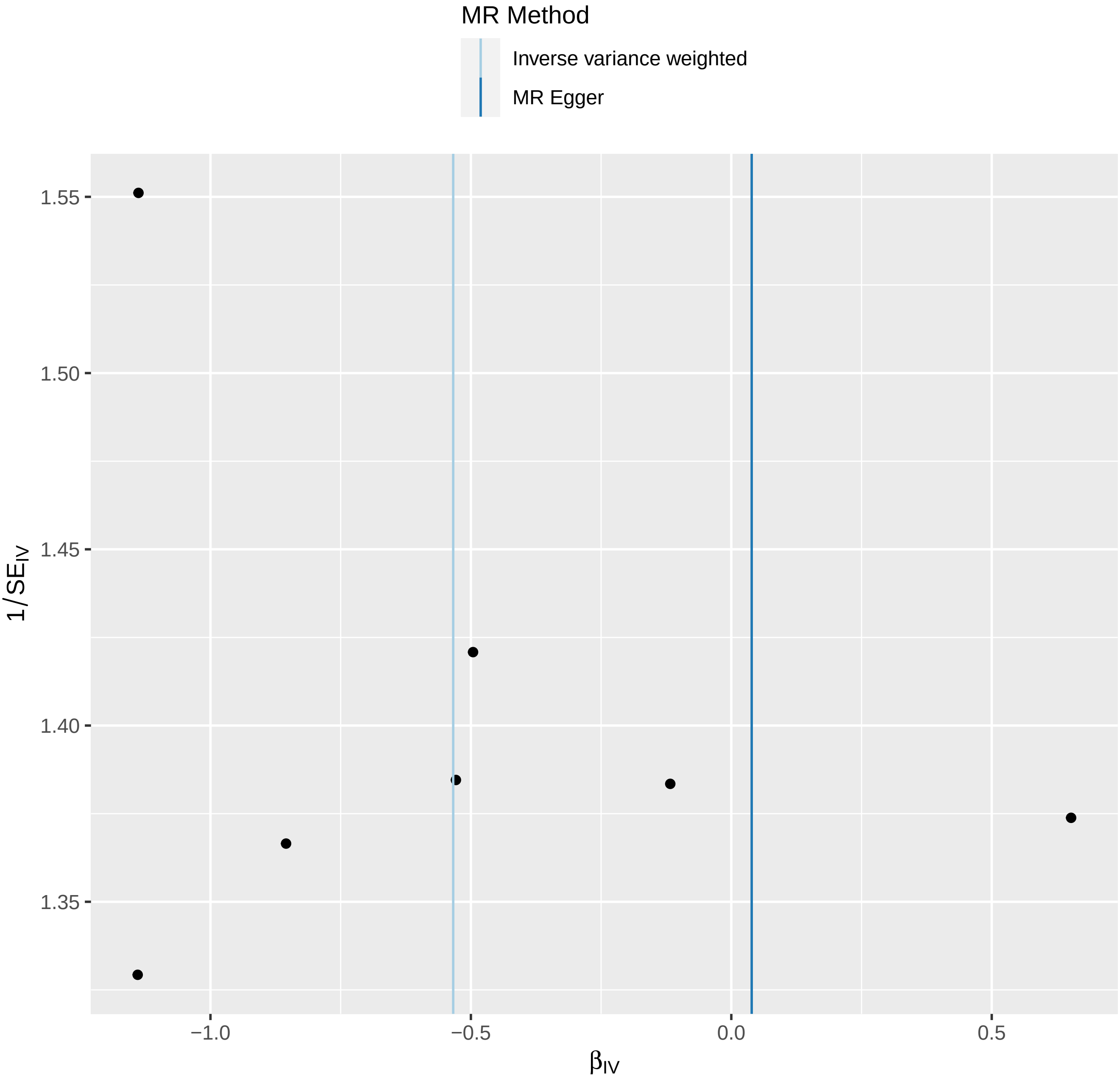

Batch 126 : Gut microbiota abundance (genus Ruminococcaceae NK4A214 group id.11358) on Crohn's disease of large intestine

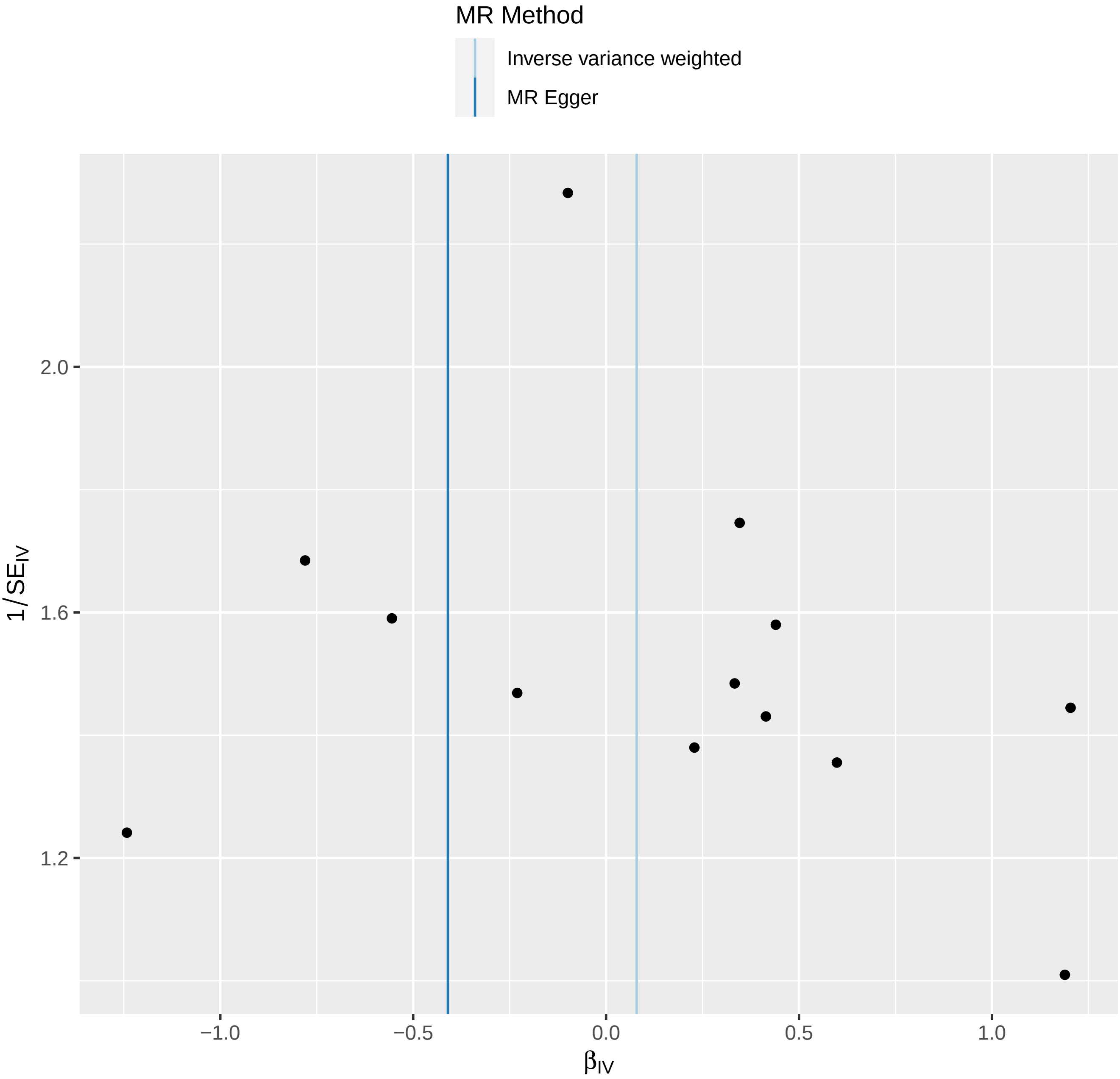

Batch 127 : Gut microbiota abundance (genus Ruminococcaceae UCG002 id.11360) on Crohn's disease of large intestine

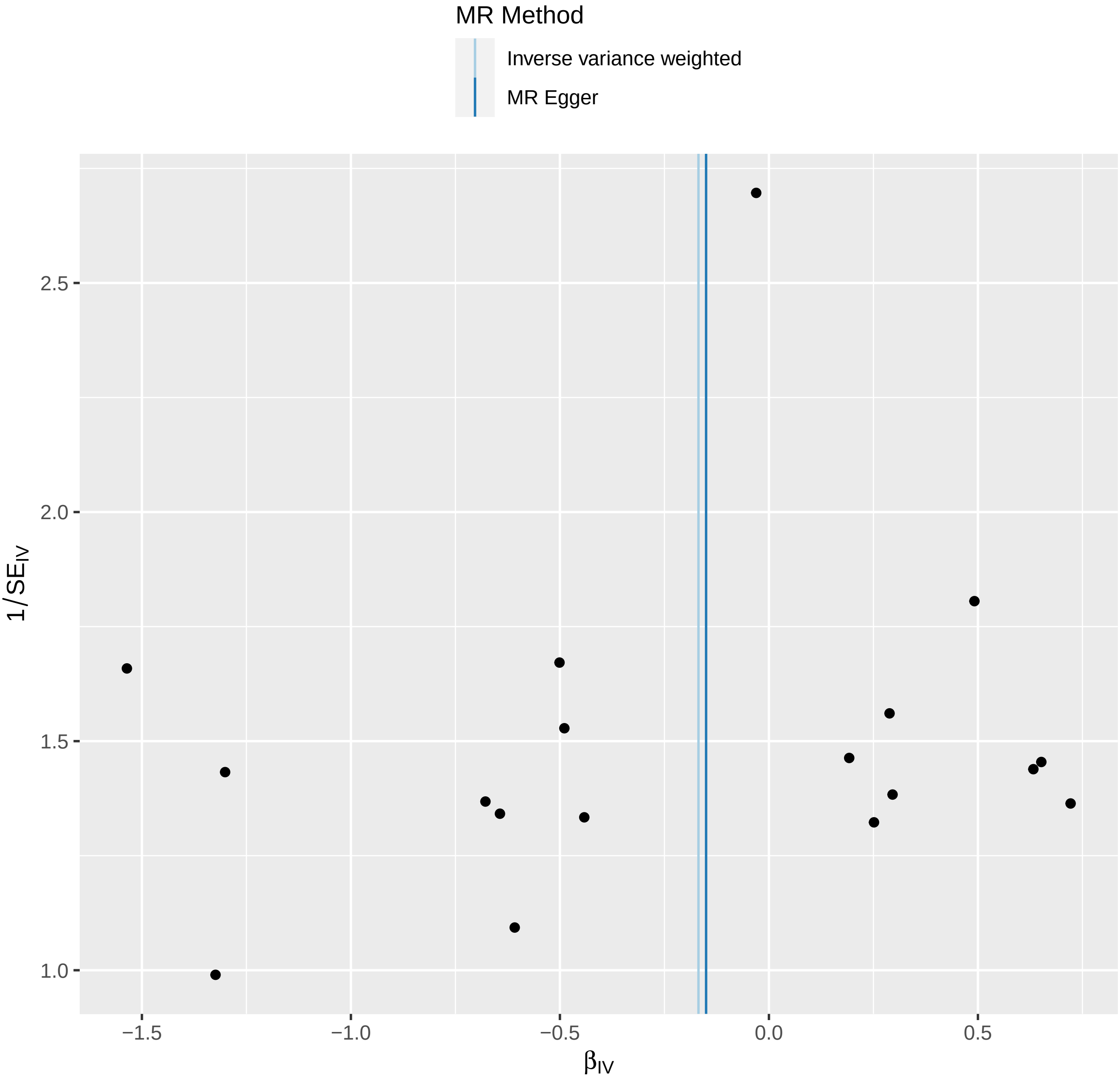

Batch 128 : Gut microbiota abundance (genus Ruminococcaceae UCG003 id.11361) on Crohn's disease of large intestine

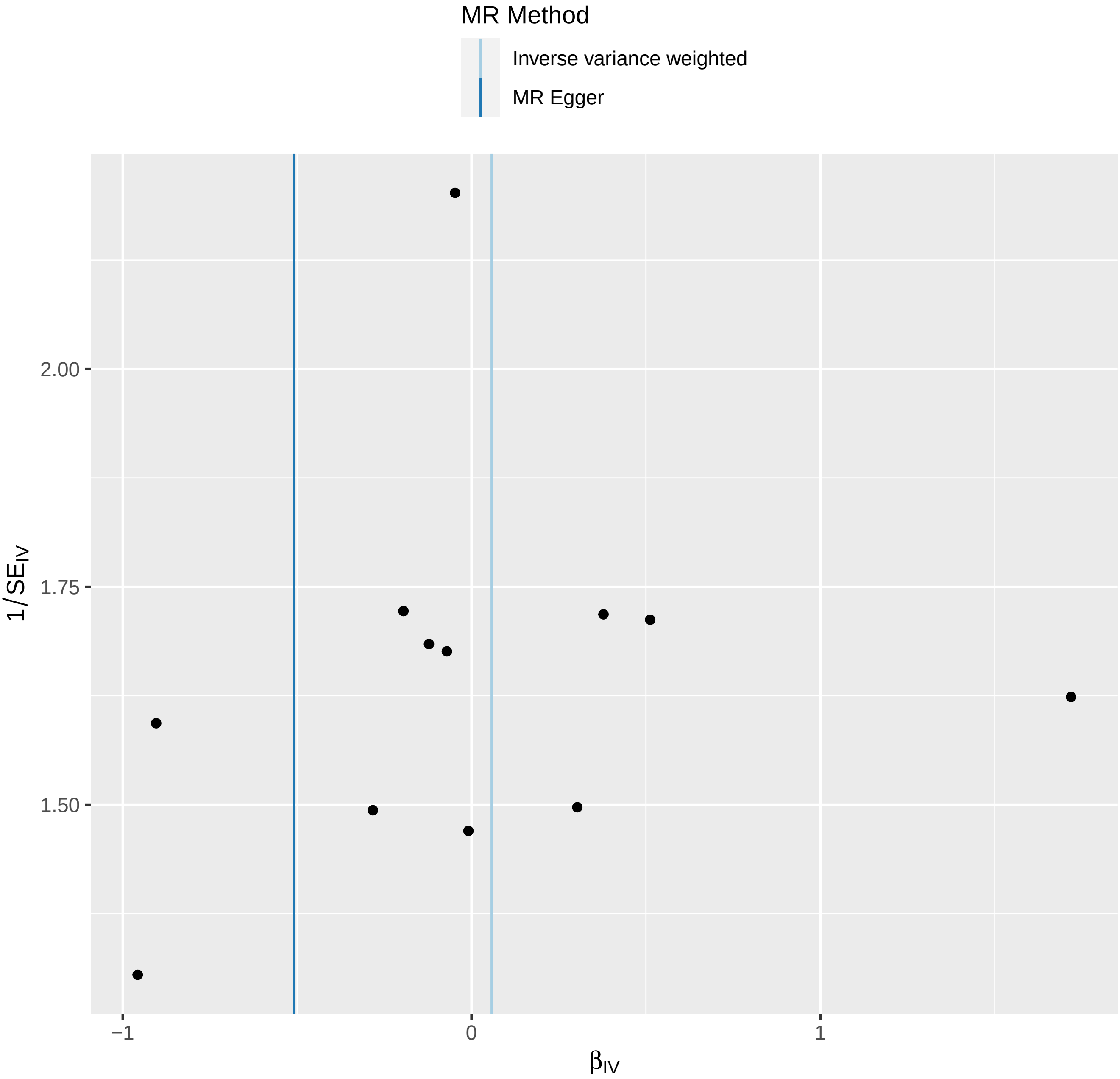

Batch 129 : Gut microbiota abundance (genus Ruminococcaceae UCG004 id.11362) on Crohn's disease of large intestine

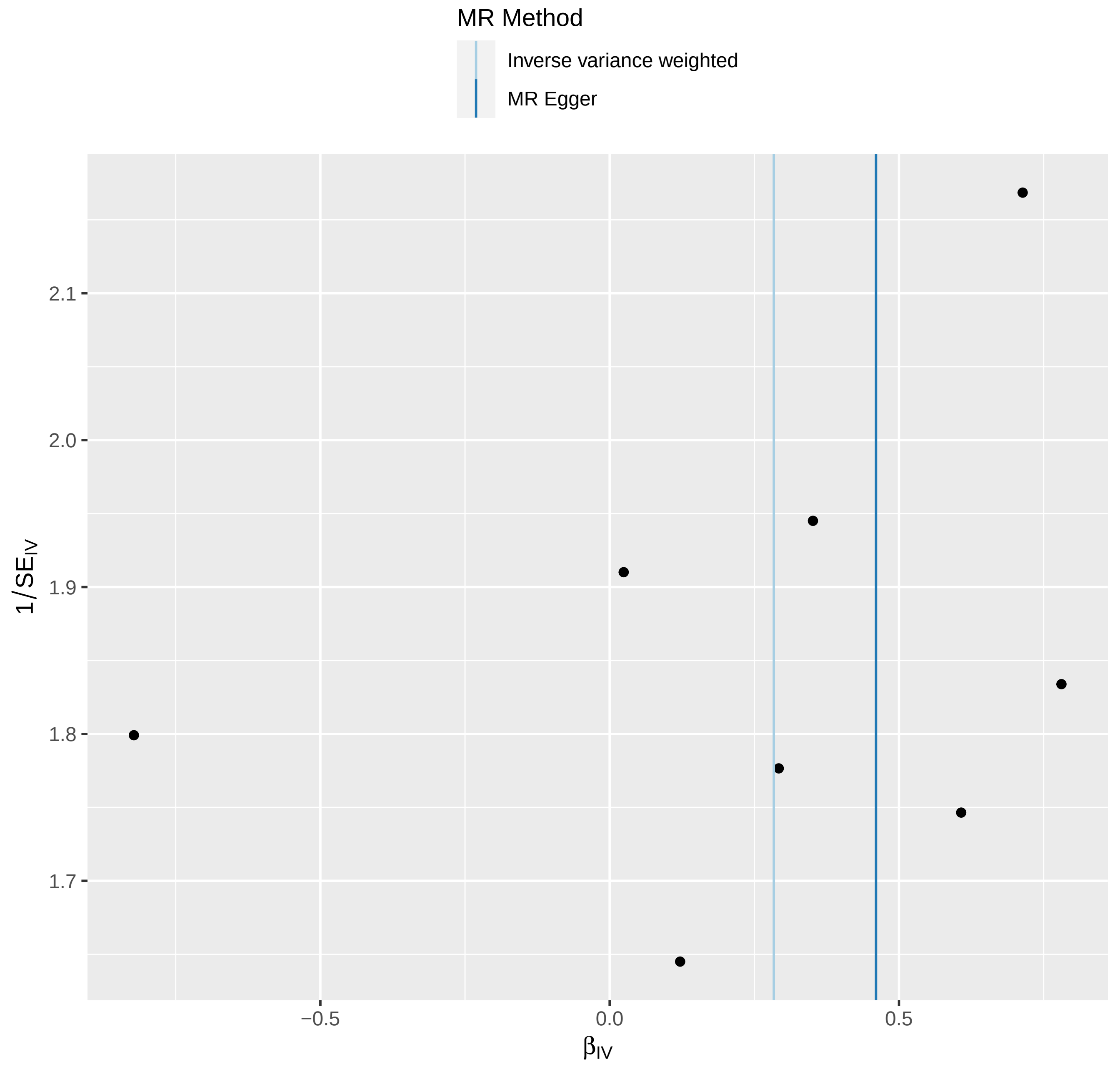

Batch 130 : Gut microbiota abundance (genus Ruminococcaceae UCG005 id.11363) on Crohn's disease of large intestine

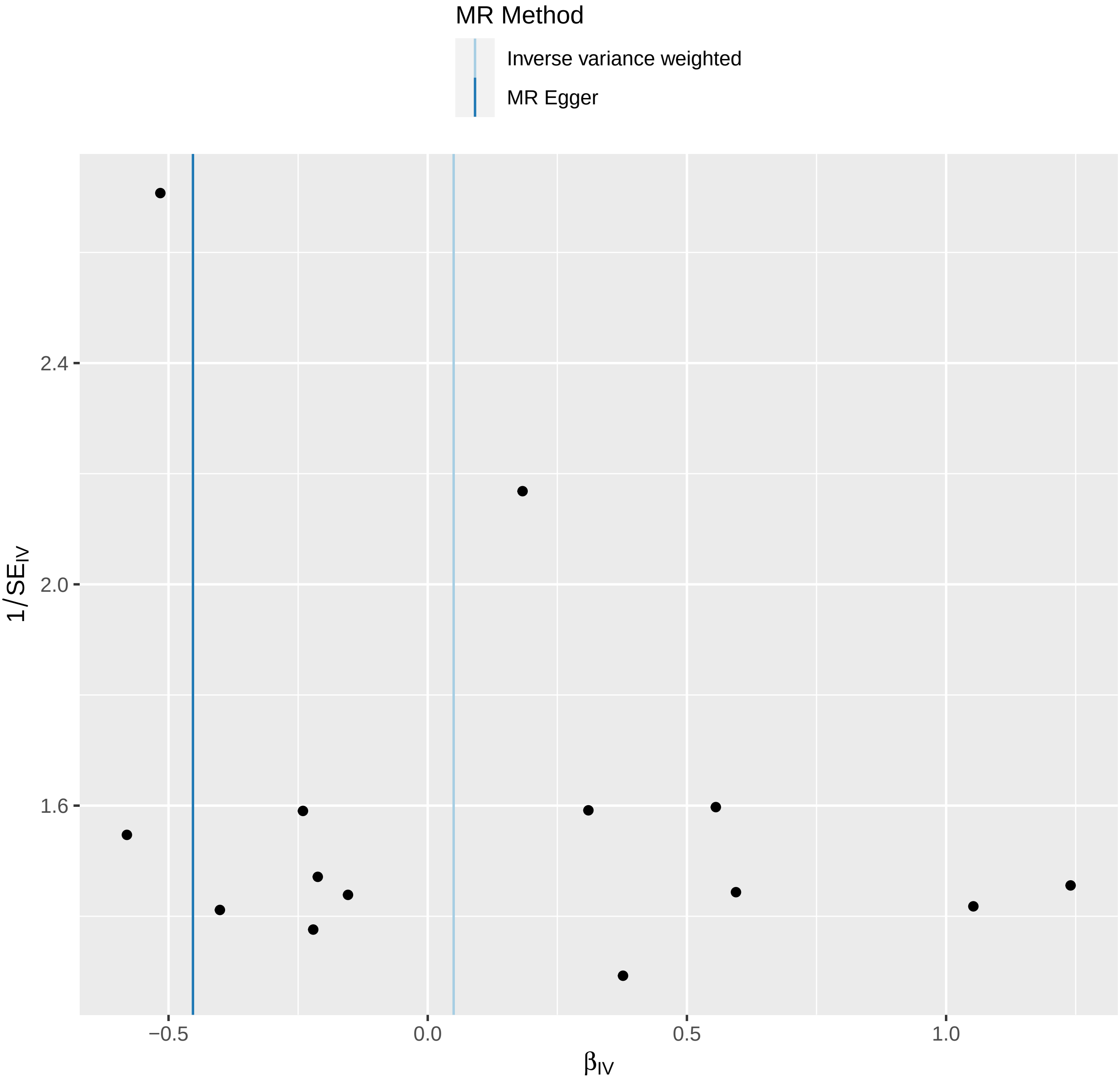

Batch 131 : Gut microbiota abundance (genus Ruminococcaceae UCG009 id.11366) on Crohn's disease of large intestine

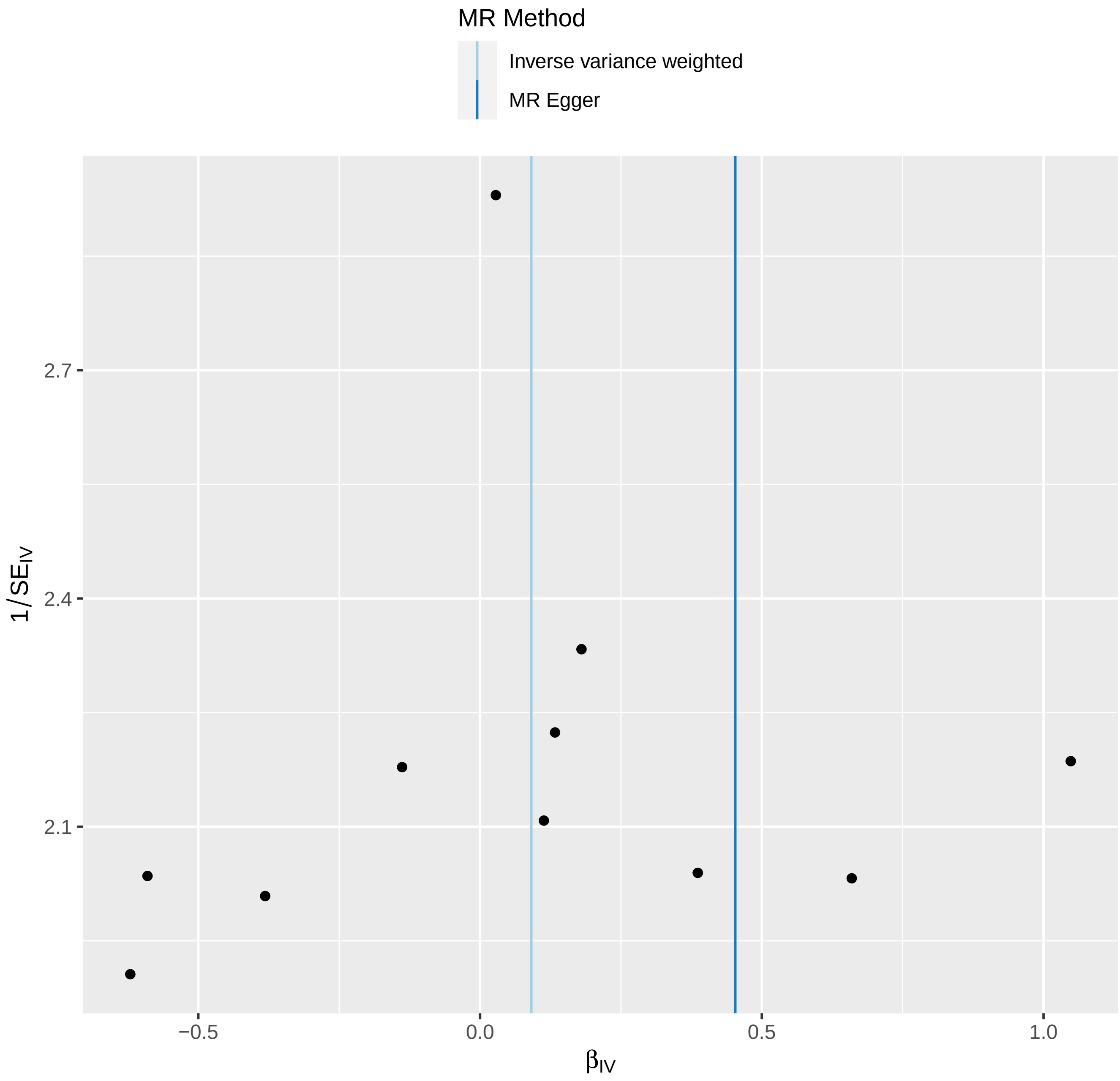

Batch 132 : Gut microbiota abundance (genus Ruminococcaceae UCG010 id.11367) on Crohn's disease of large intestine

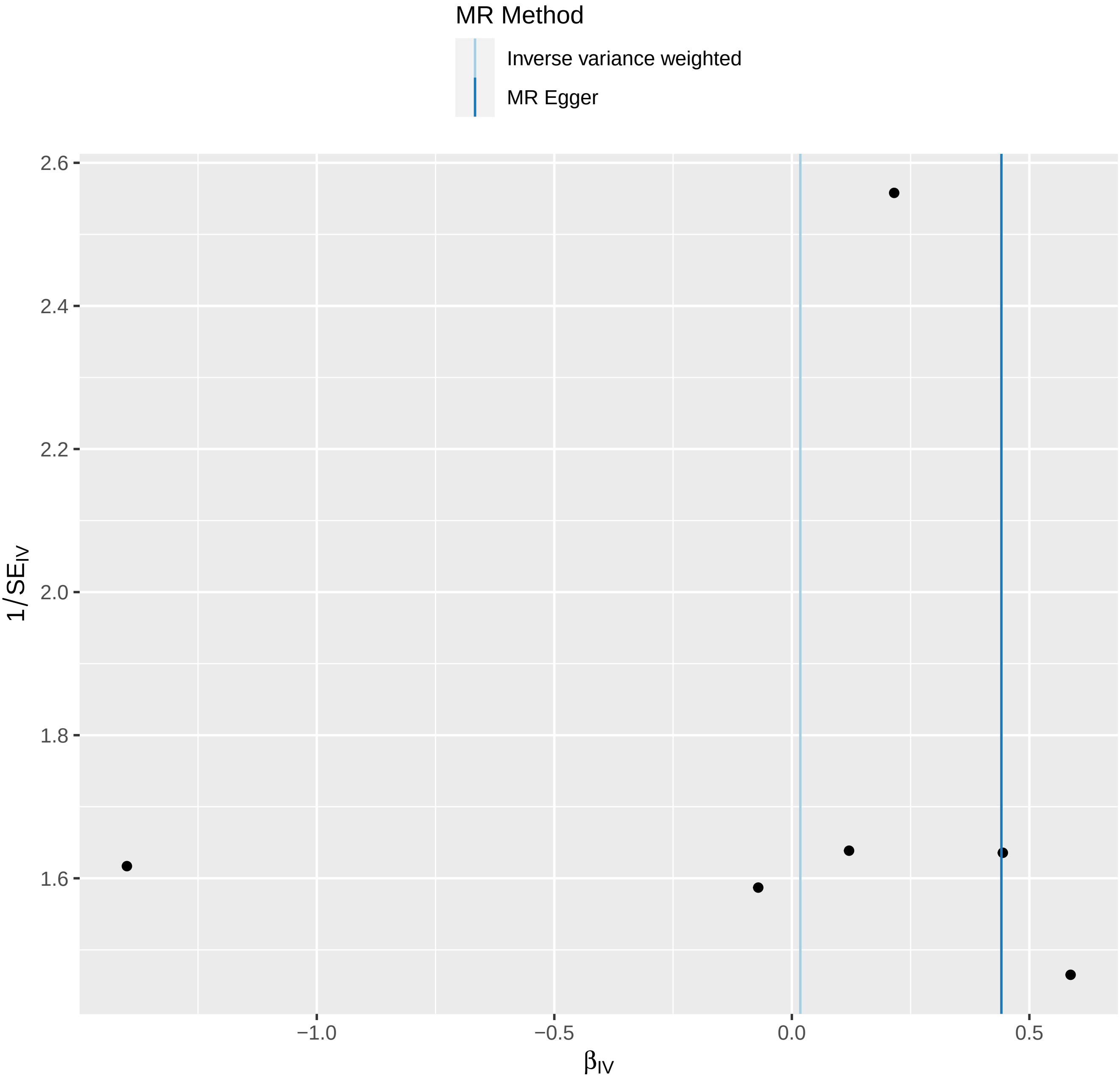

Batch 133 : Gut microbiota abundance (genus Ruminococcaceae UCG011 id.11368) on Crohn's disease of large intestine

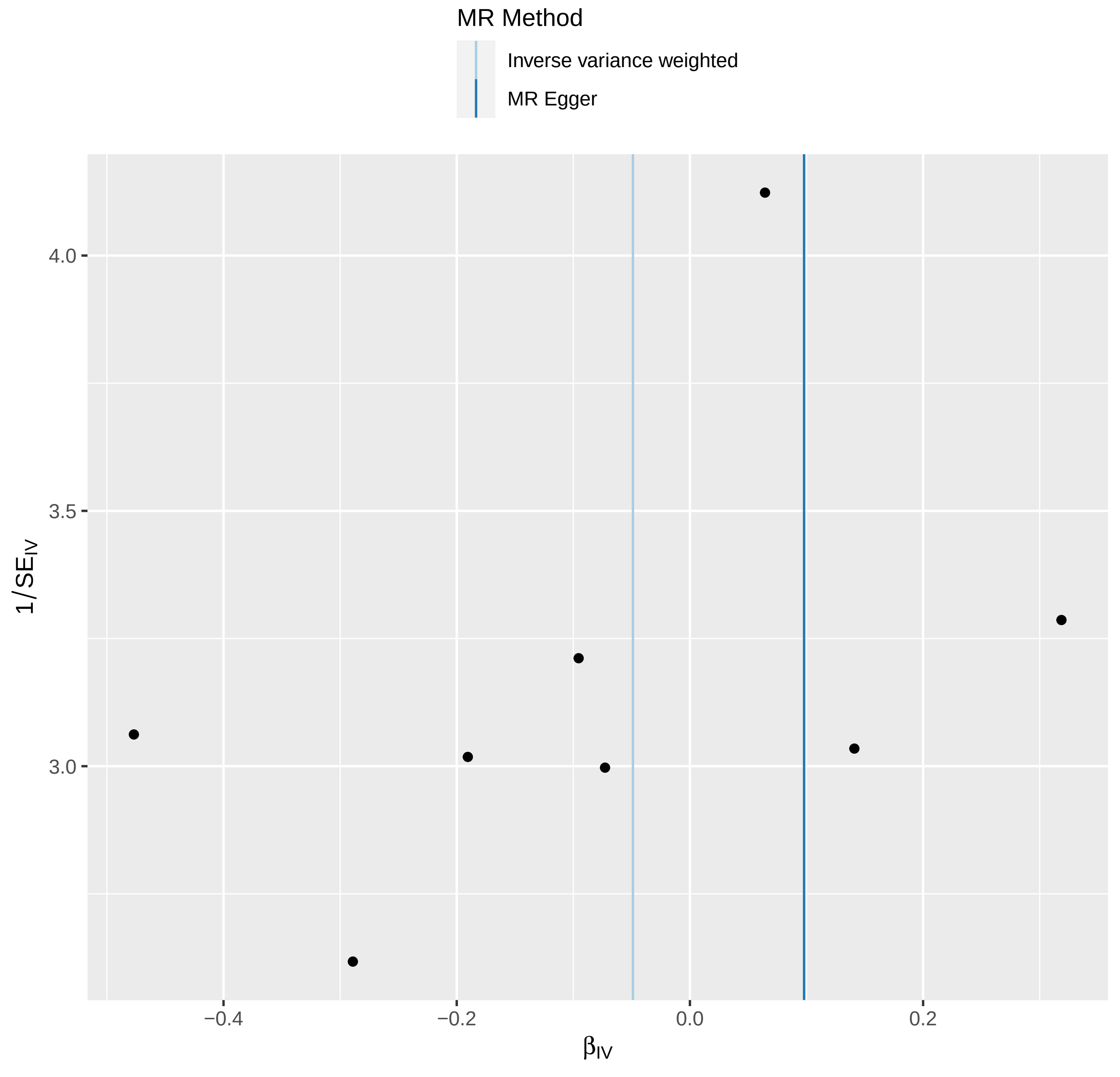

Batch 134 : Gut microbiota abundance (genus Ruminococcaceae UCG013 id.11370) on Crohn's disease of large intestine

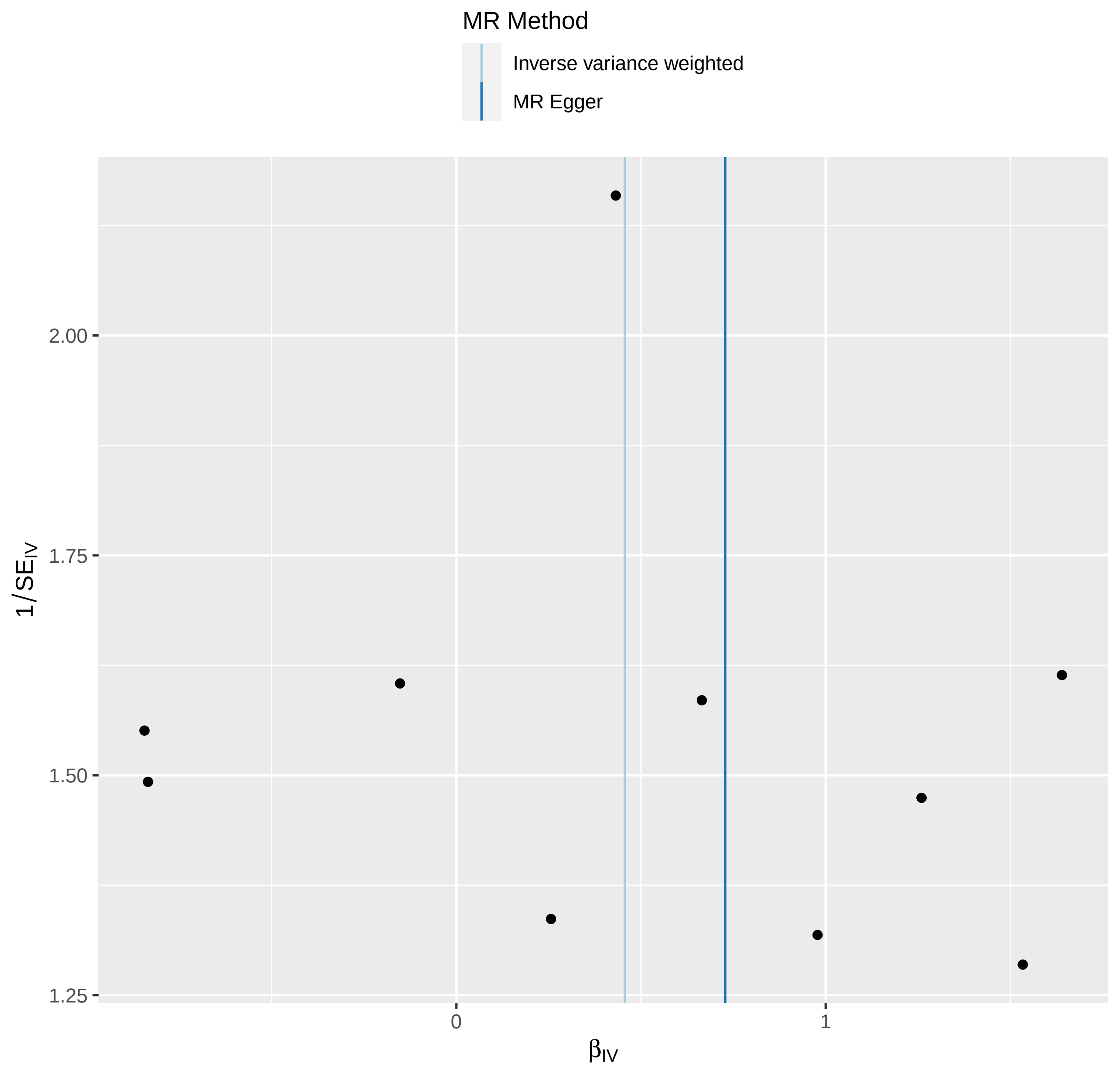

Batch 135 : Gut microbiota abundance (genus Ruminococcaceae UCG014 id.11371) on Crohn's disease of large intestine

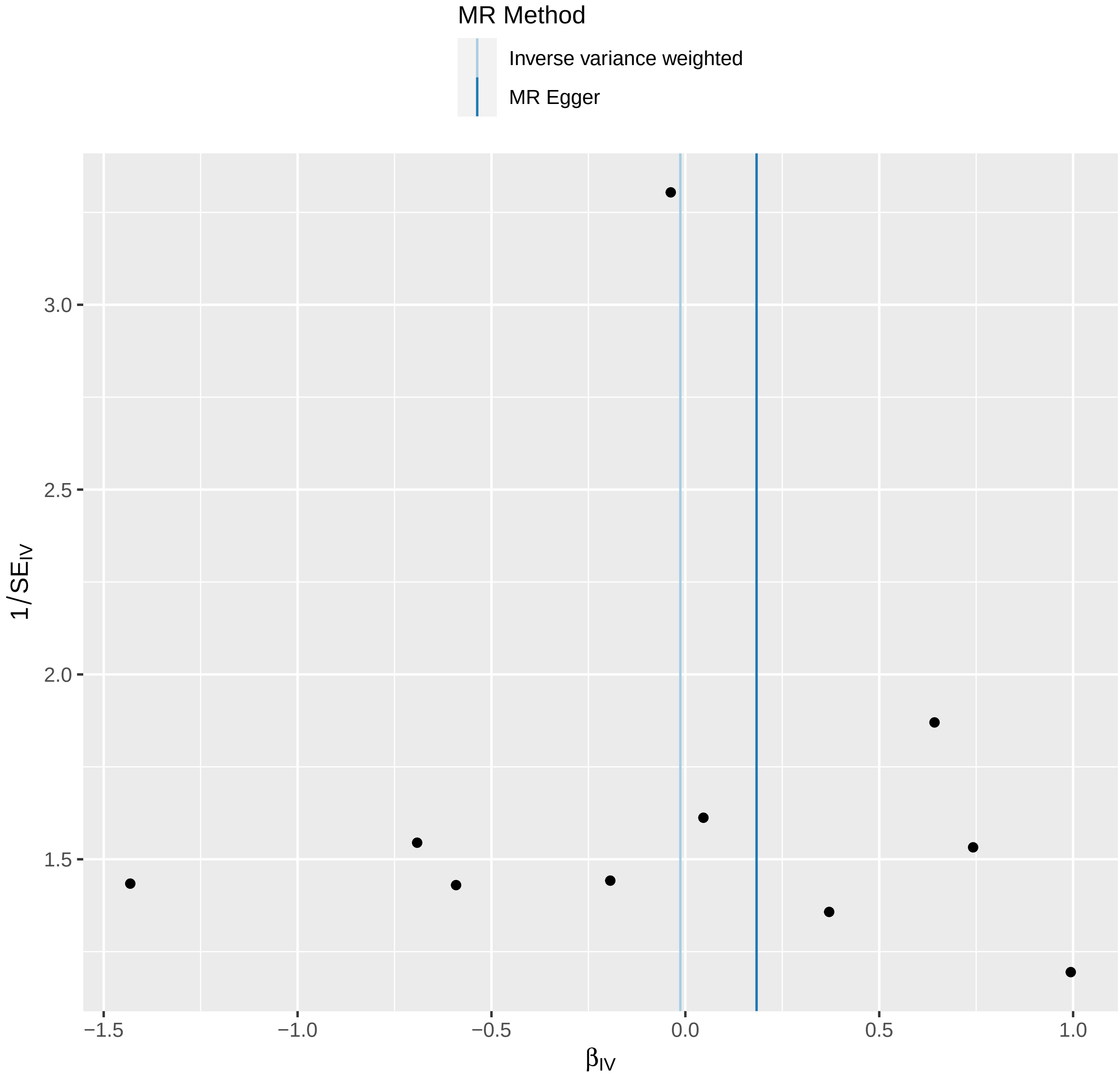

Batch 136 : Gut microbiota abundance (genus Ruminococcus1 id.11373) on Crohn's disease of large intestine

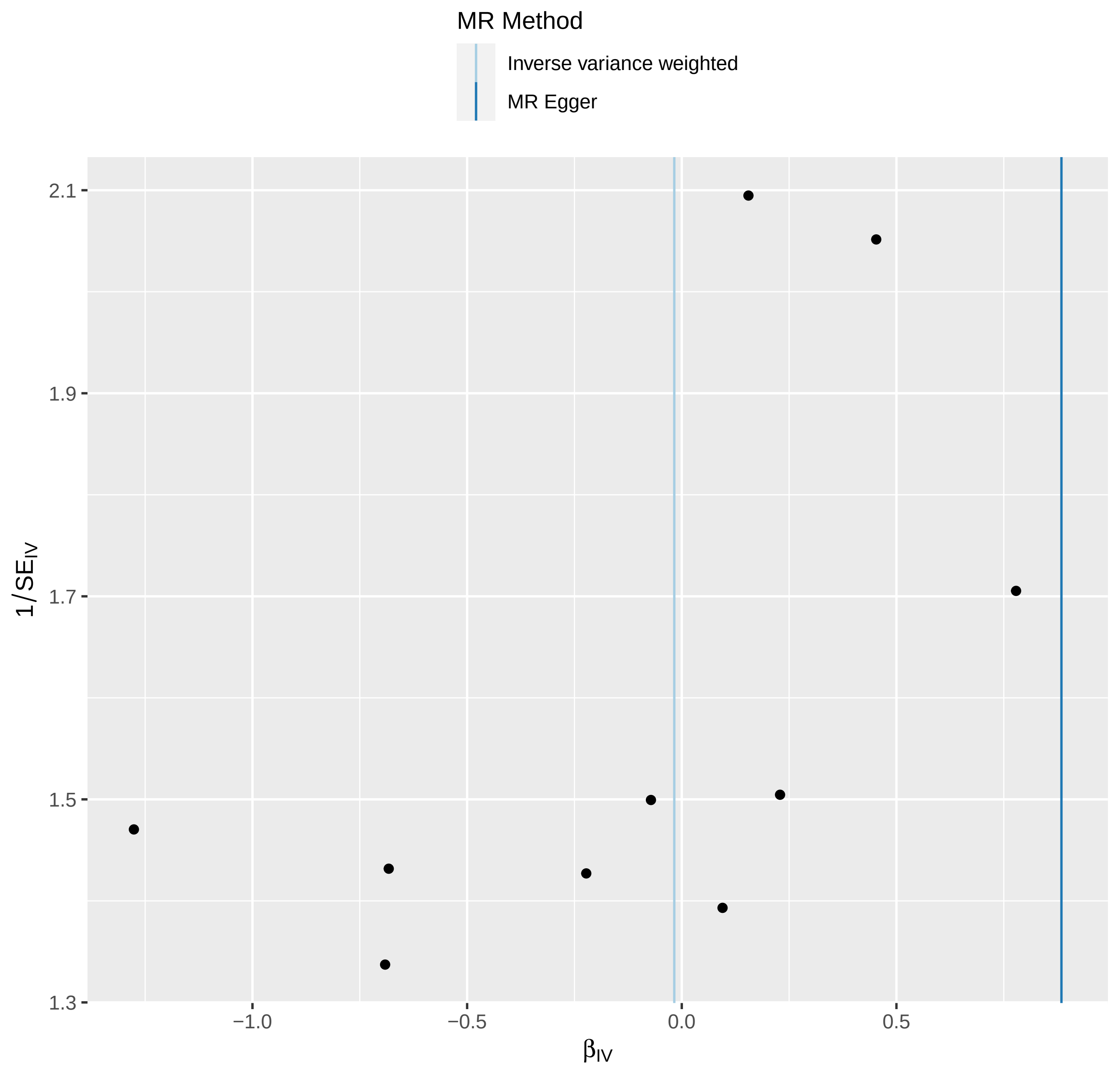

Batch 137 : Gut microbiota abundance (genus Ruminococcus2 id.11374) on Crohn's disease of large intestine

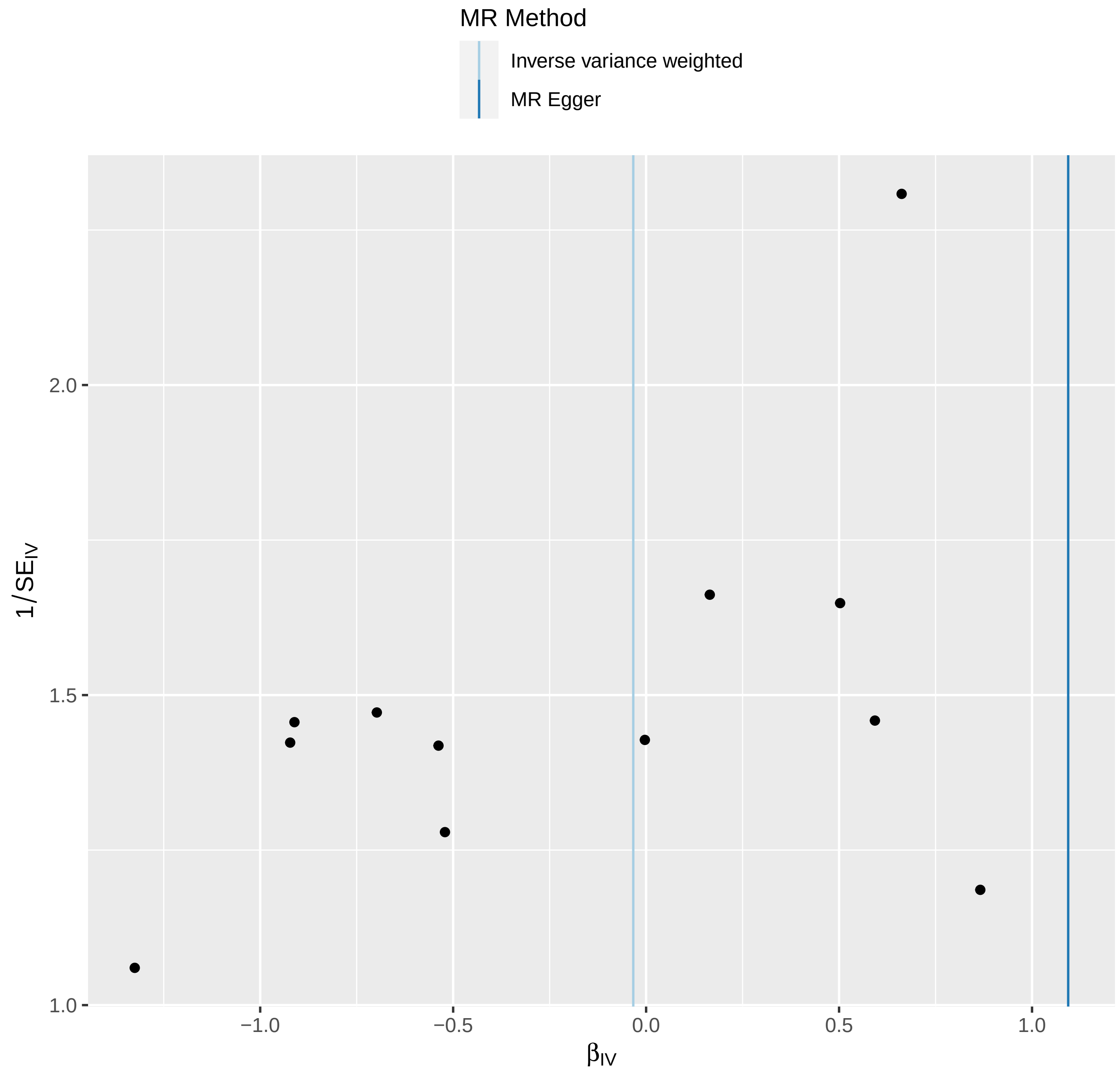

Batch 138 : Gut microbiota abundance (genus Ruminococcus gauvreauii group id.11342) on Crohn's disease of large intestine

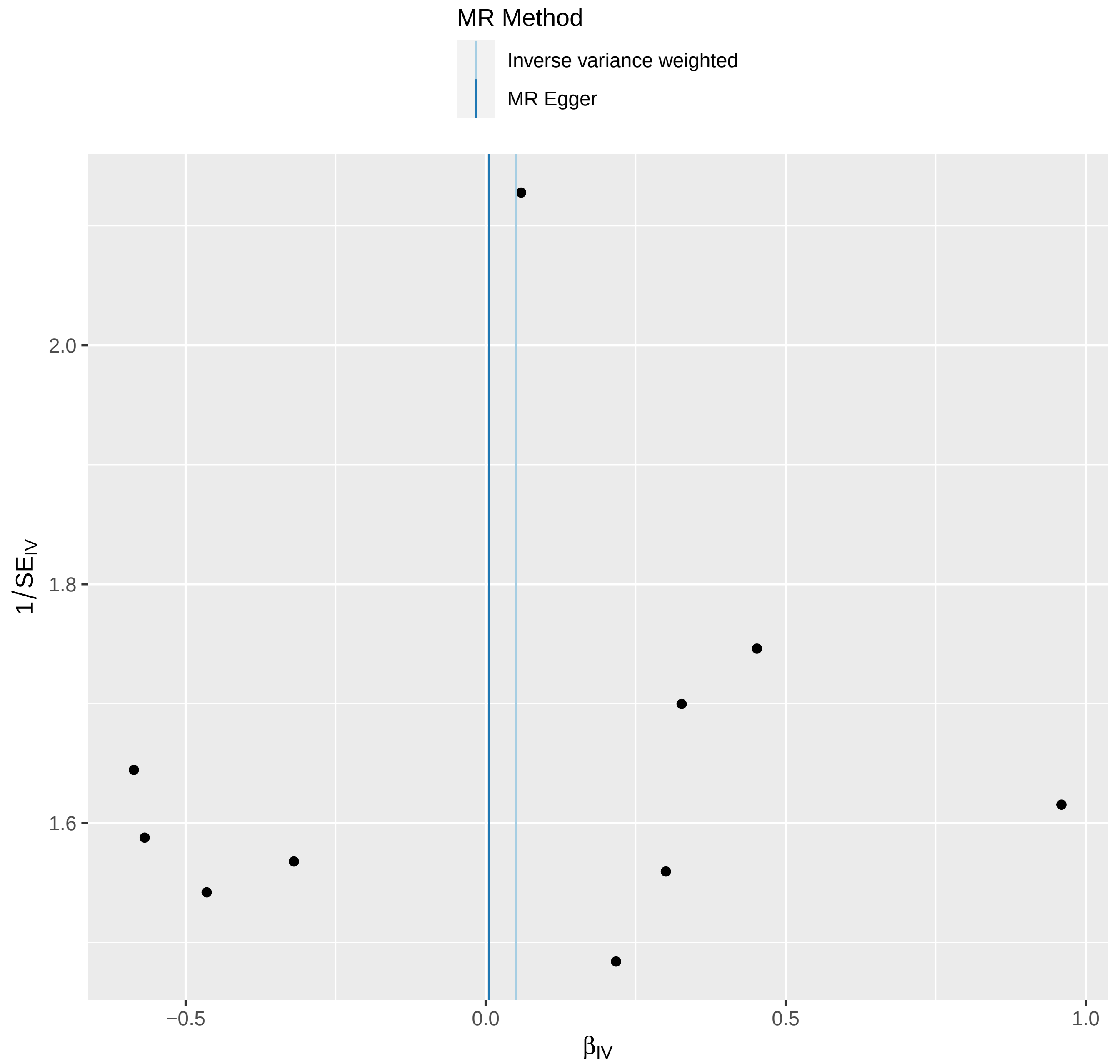

Batch 139 : Gut microbiota abundance (genus Ruminococcus gnavus group id.14376) on Crohn's disease of large intestine

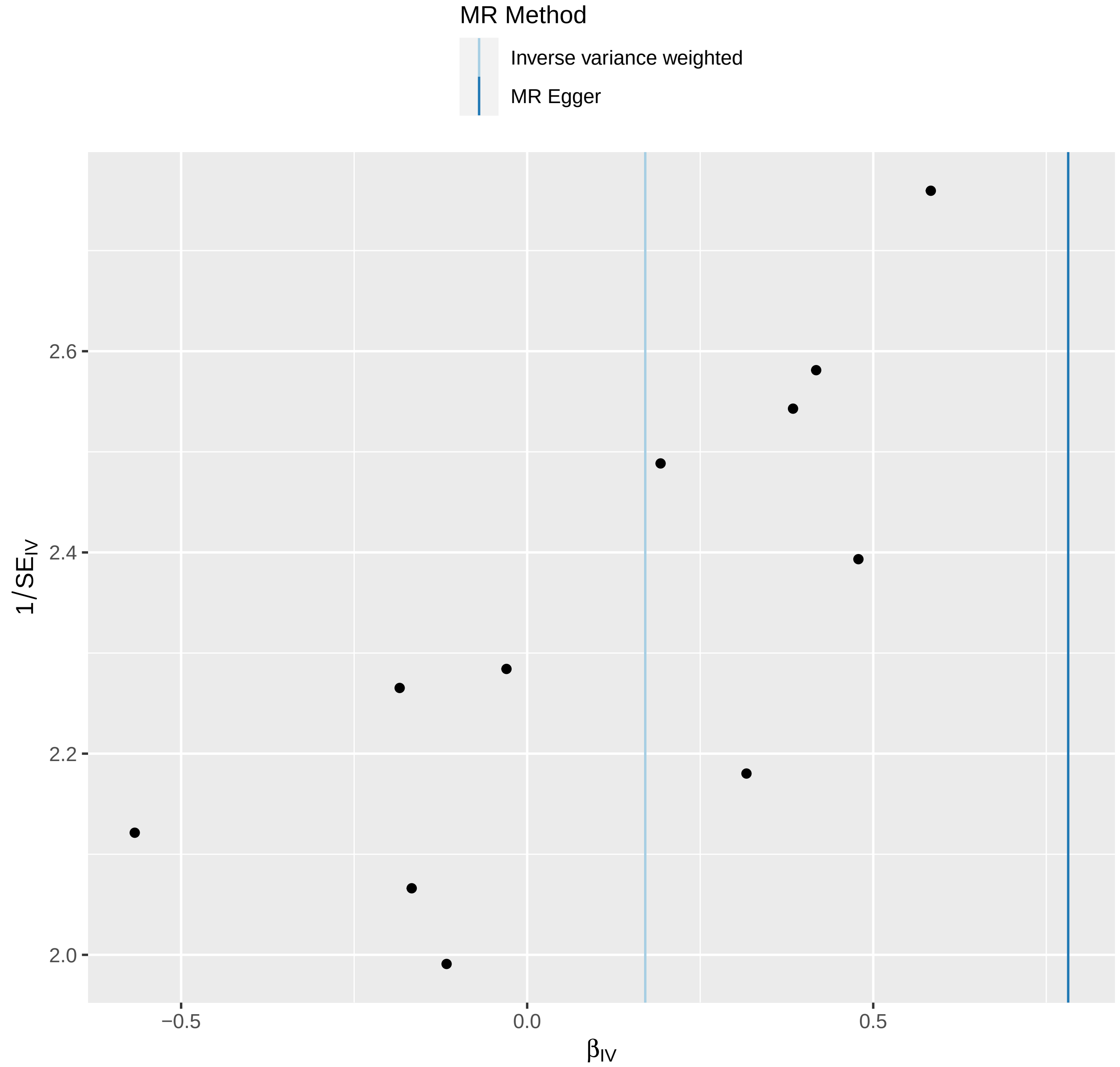

Batch 140 : Gut microbiota abundance (genus Ruminococcus torques group id.14377) on Crohn's disease of large intestine

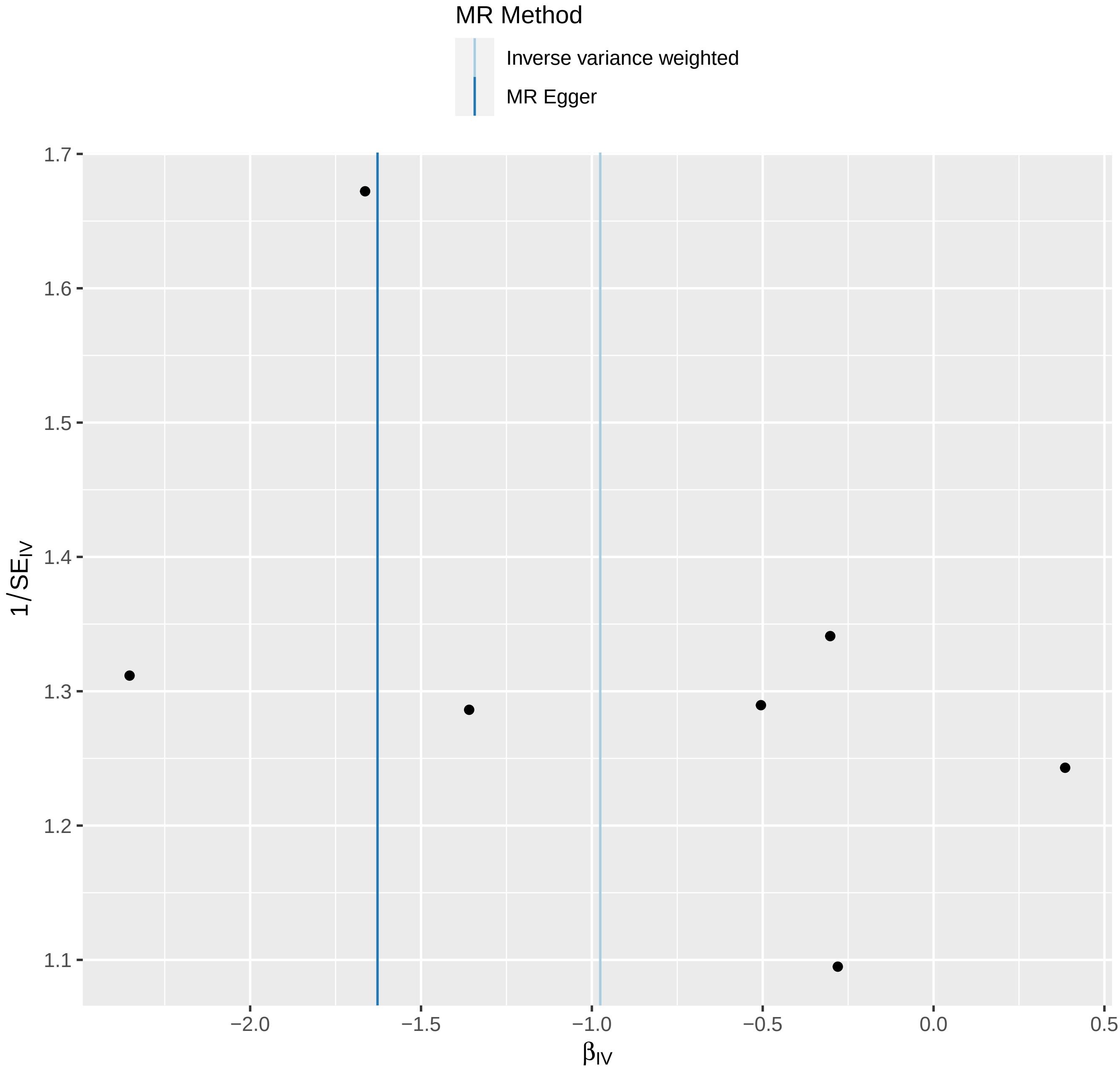

Batch 141 : Gut microbiota abundance (genus Sellimonas id.14369) on Crohn's disease of large intestine

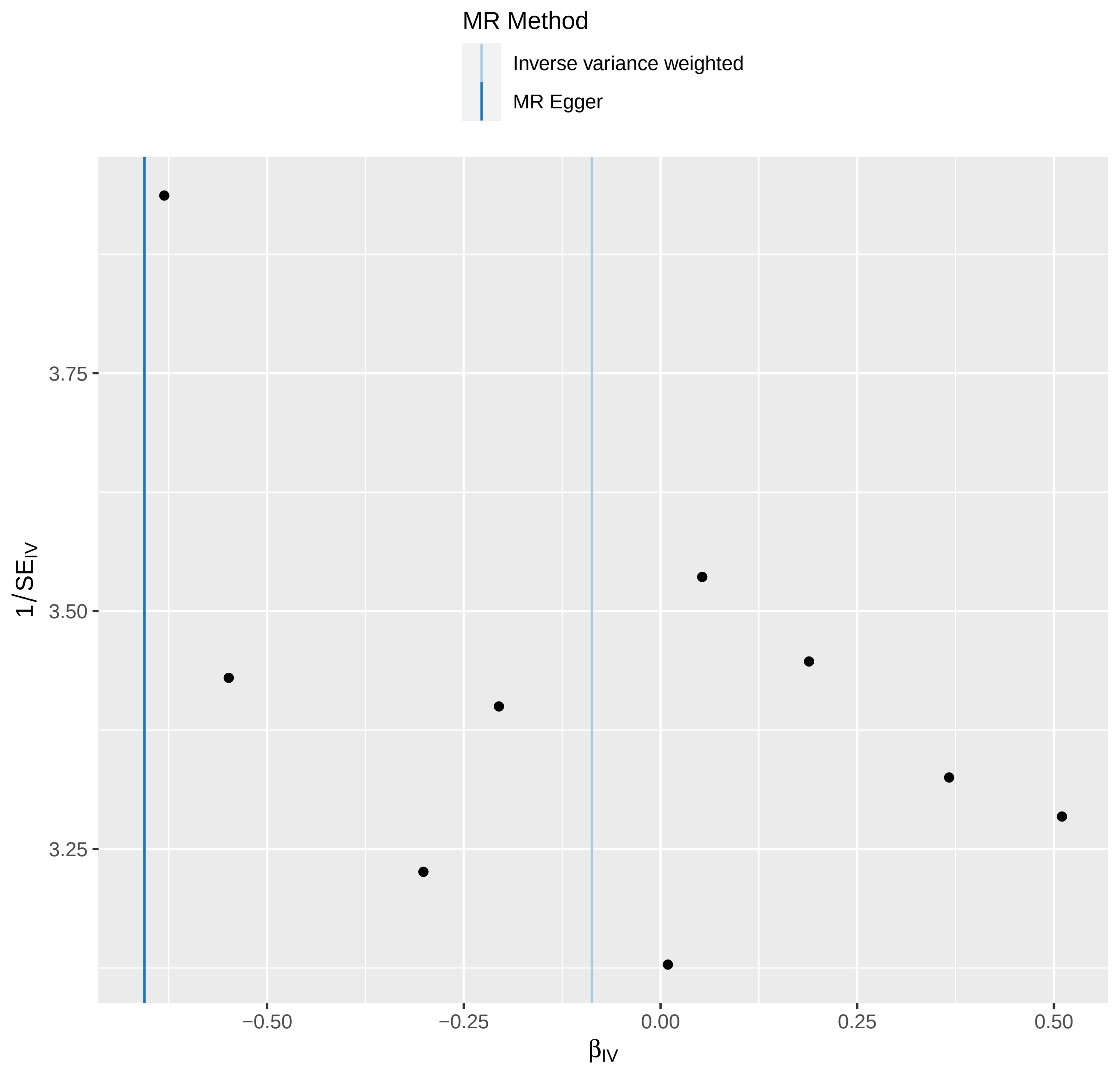

Batch 142 : Gut microbiota abundance (genus Senegalimassilia id.11160) on Crohn's disease of large intestine

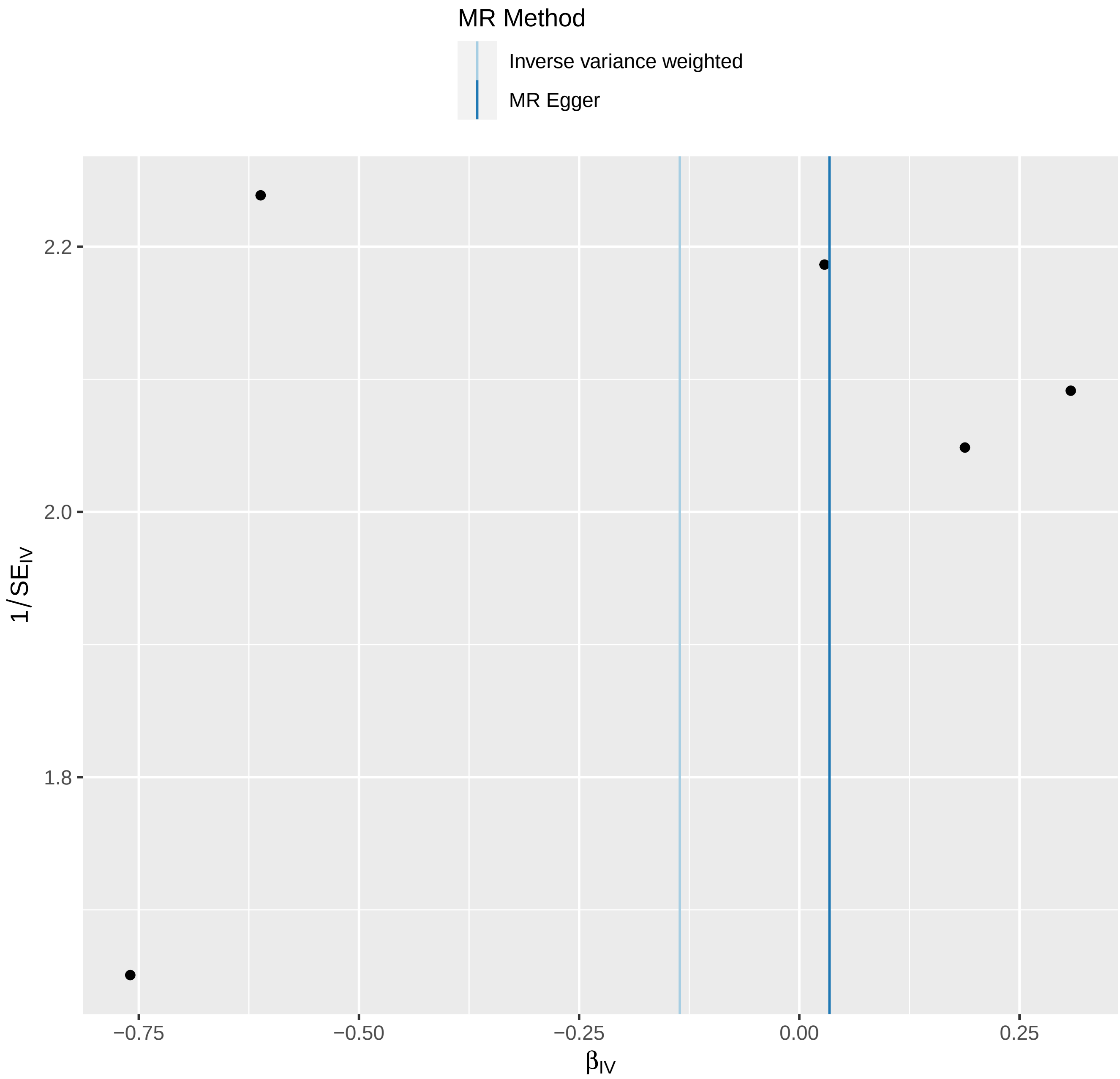

Batch 143 : Gut microbiota abundance (genus Slackia id.825) on Crohn's disease of large intestine

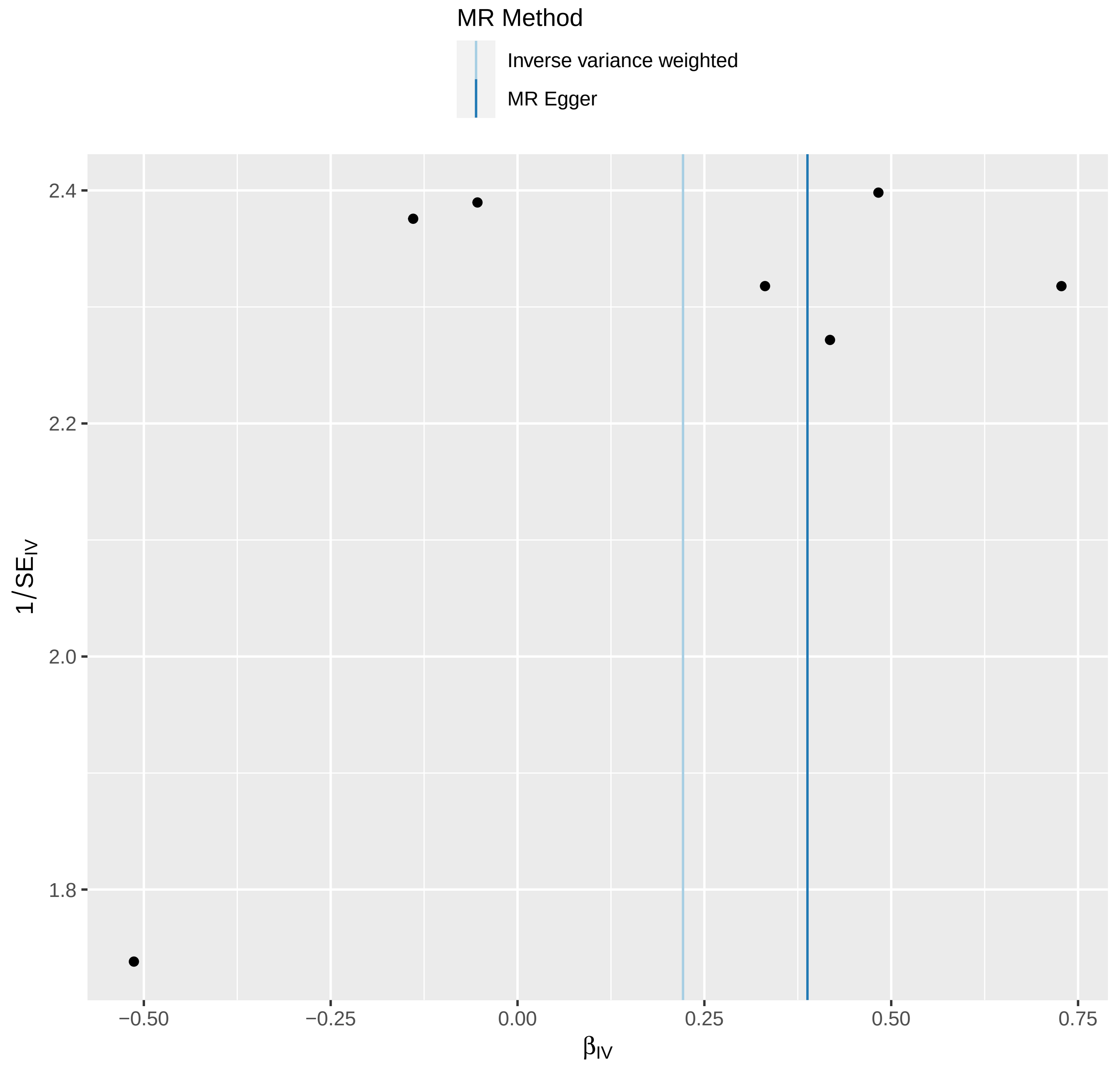

Batch 144 : Gut microbiota abundance (genus Streptococcus id.1853) on Crohn's disease of large intestine

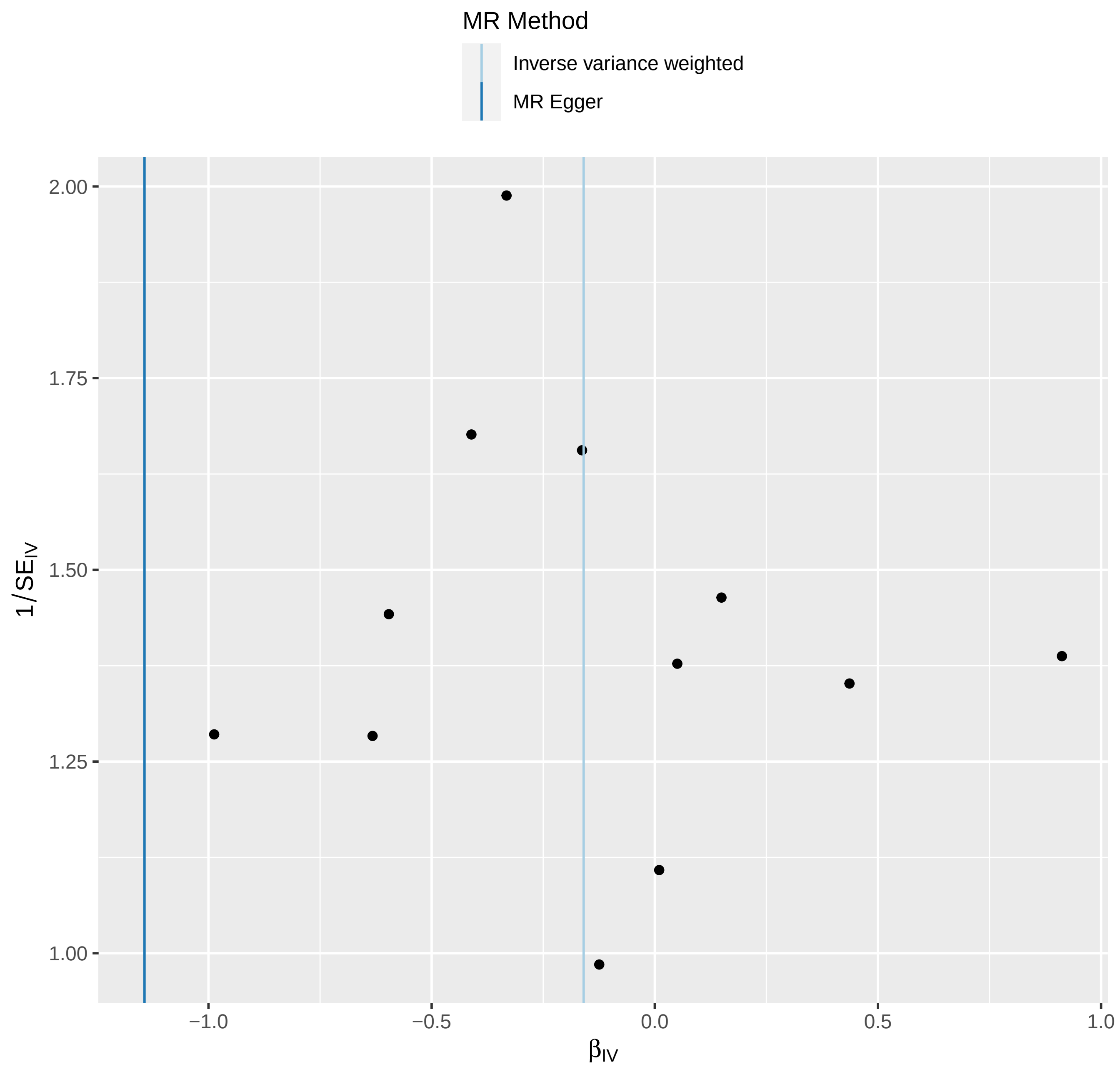

Batch 145 : Gut microbiota abundance (genus Subdoligranulum id.2070) on Crohn's disease of large intestine

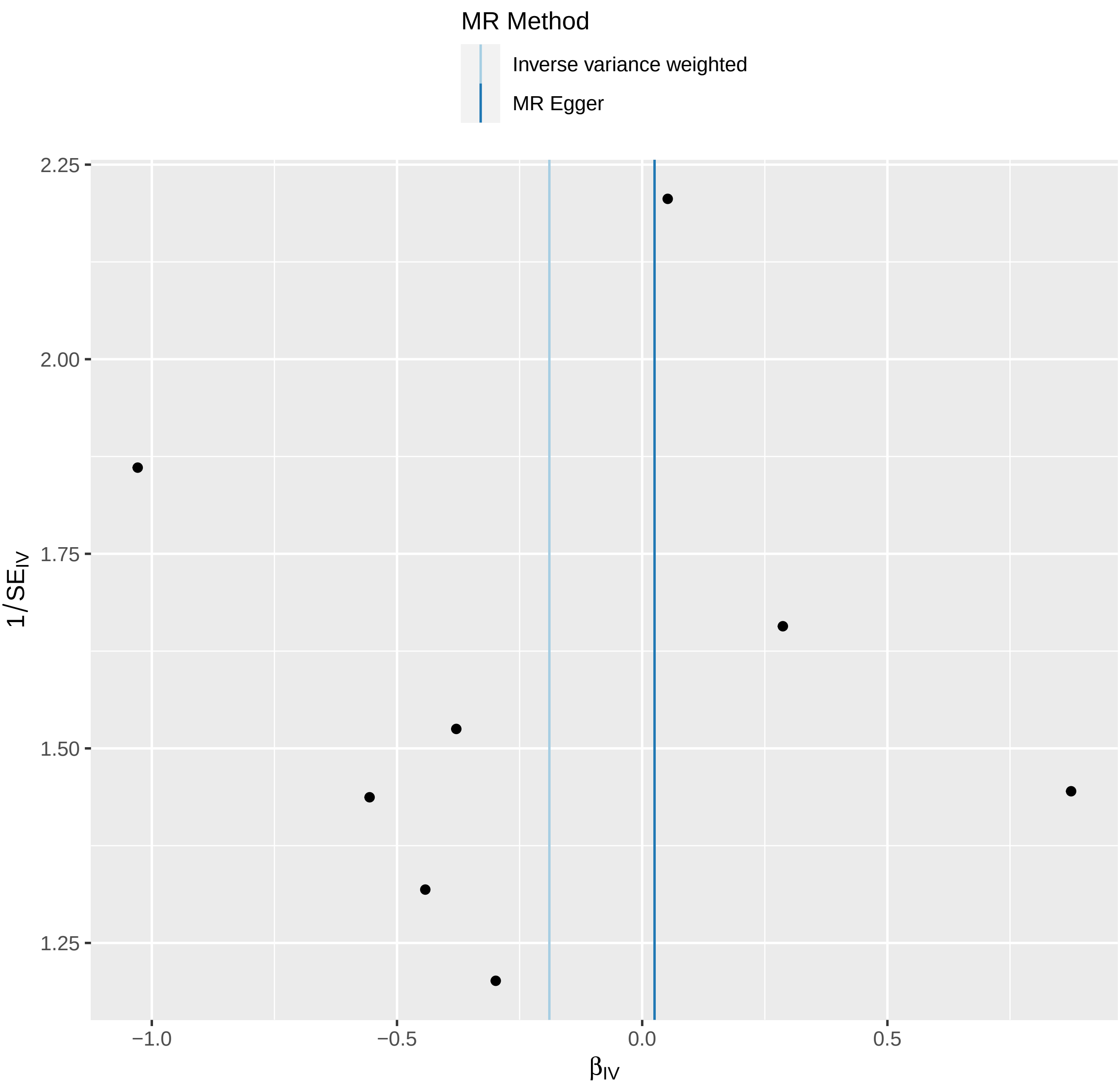

Batch 146 : Gut microbiota abundance (genus Sutterella id.2896) on Crohn's disease of large intestine

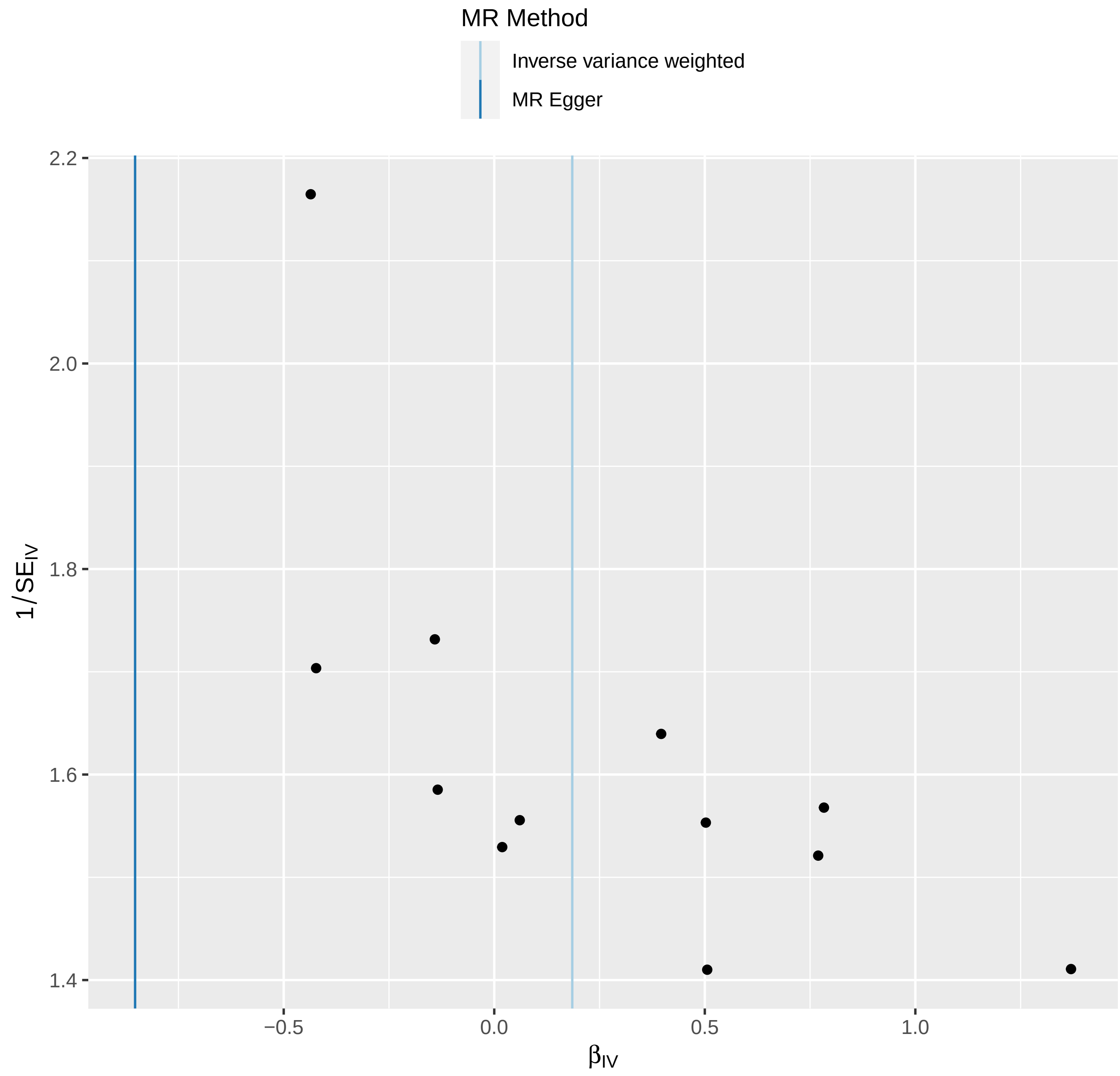

Batch 147 : Gut microbiota abundance (genus Terrisporobacter id.11348) on Crohn's disease of large intestine

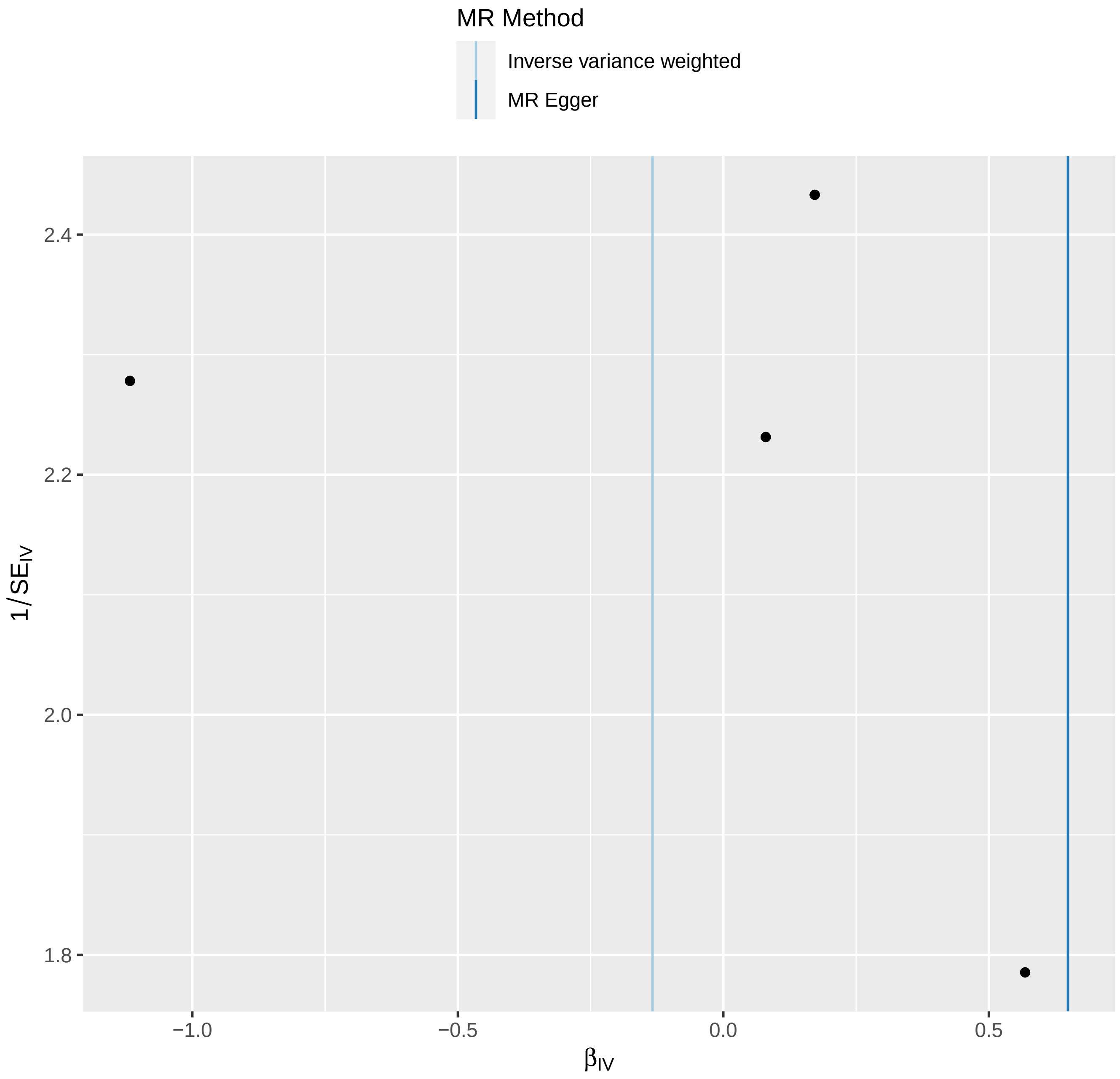

Batch 148 : Gut microbiota abundance (genus Turicibacter id.2162) on Crohn's disease of large intestine

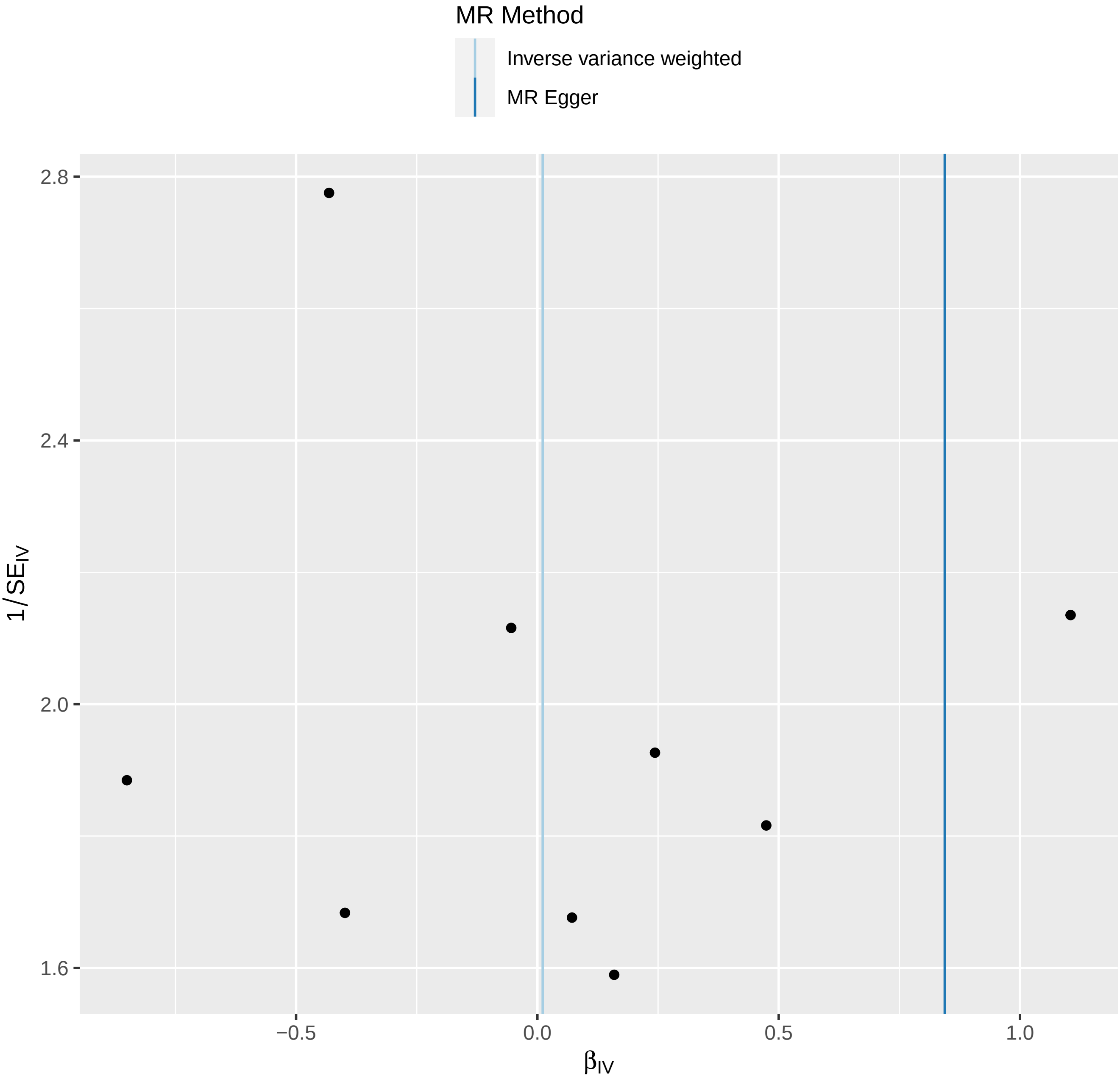

Batch 149 : Gut microbiota abundance (genus Tyzzerella3 id.11335) on Crohn's disease of large intestine

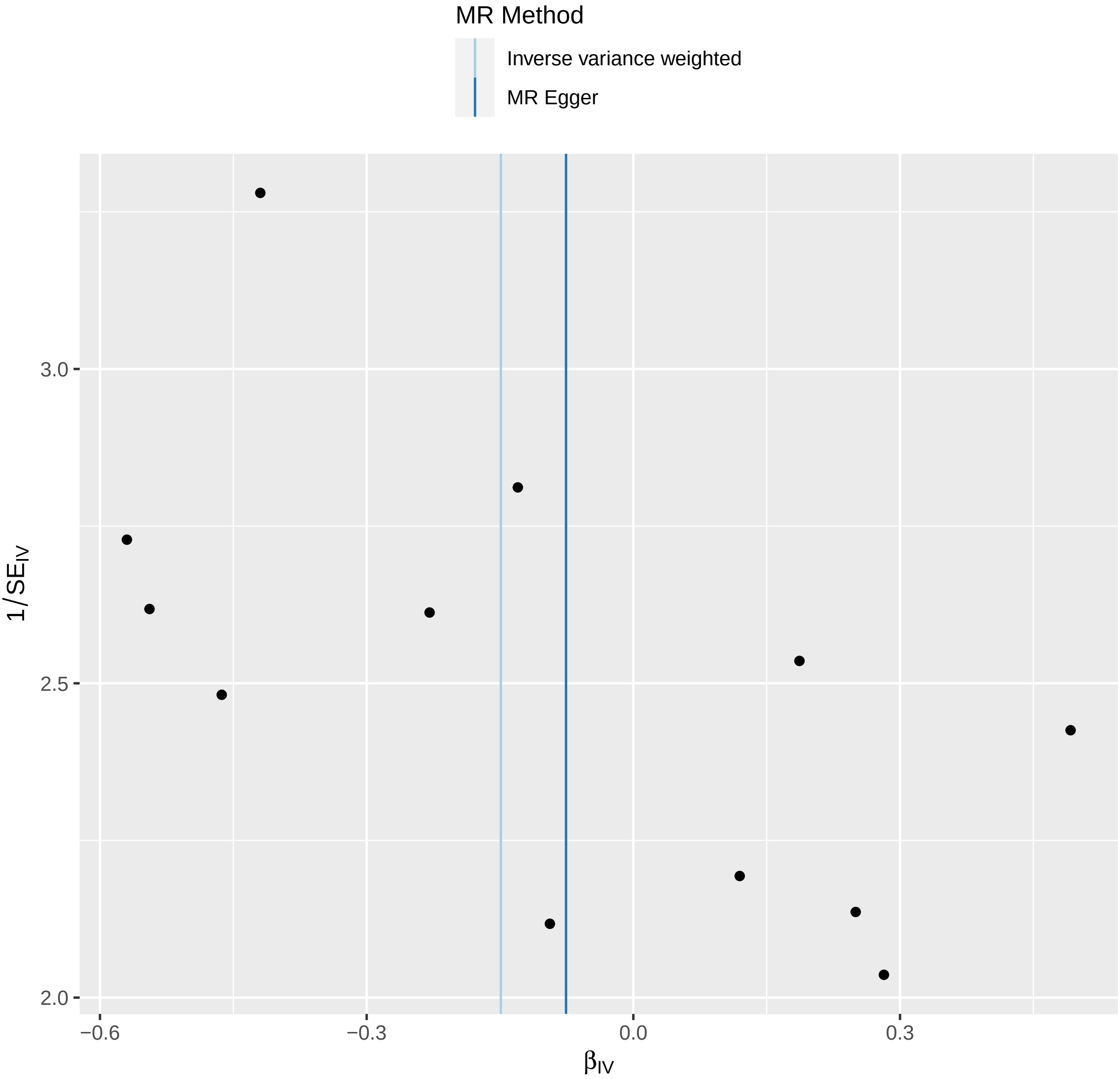

Batch 150 : Gut microbiota abundance (genus Veillonella id.2198) on Crohn's disease of large intestine

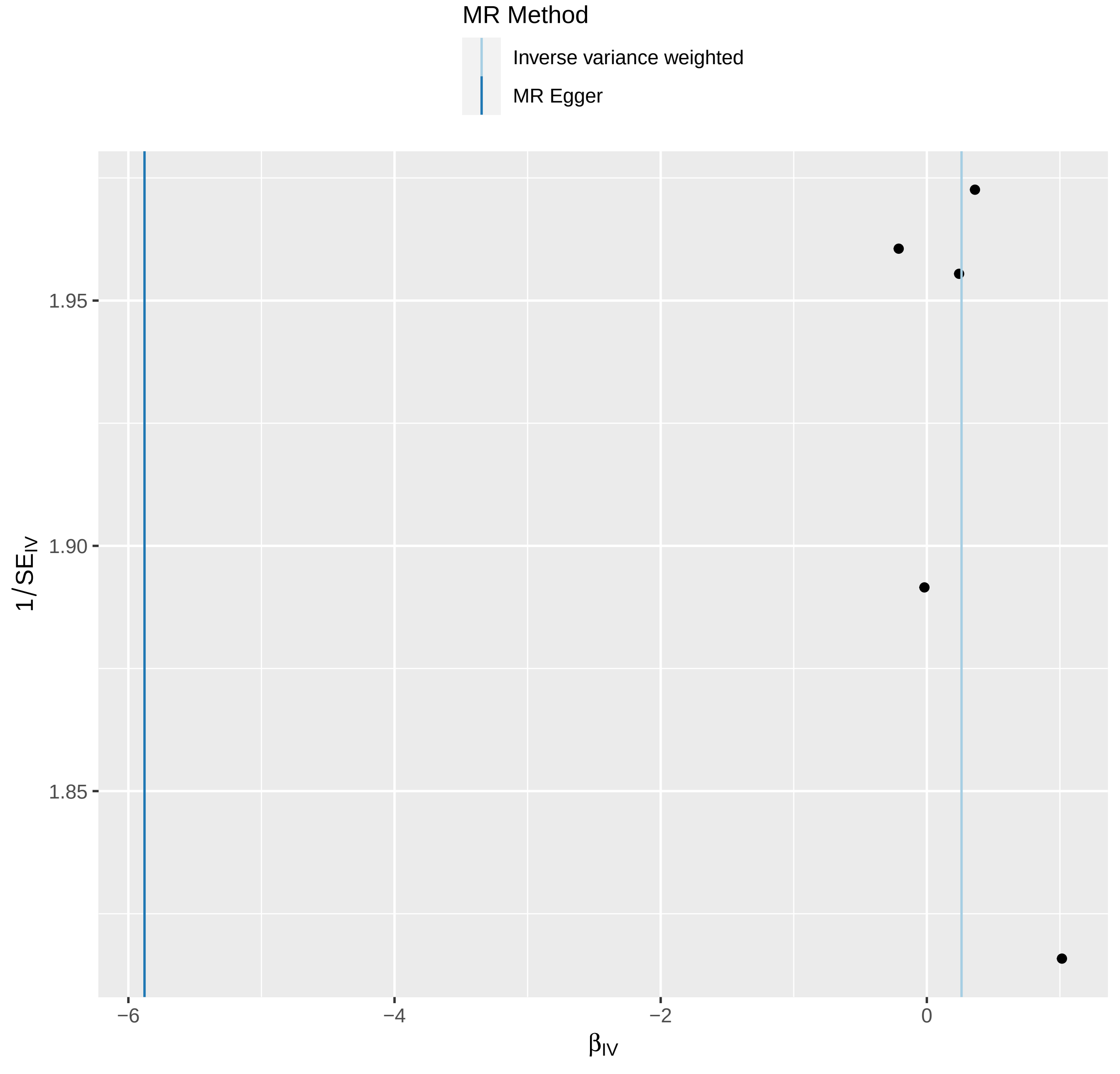

Batch 151 : Gut microbiota abundance (family Acidaminococcaceae id.2166) on Other Crohn's disease

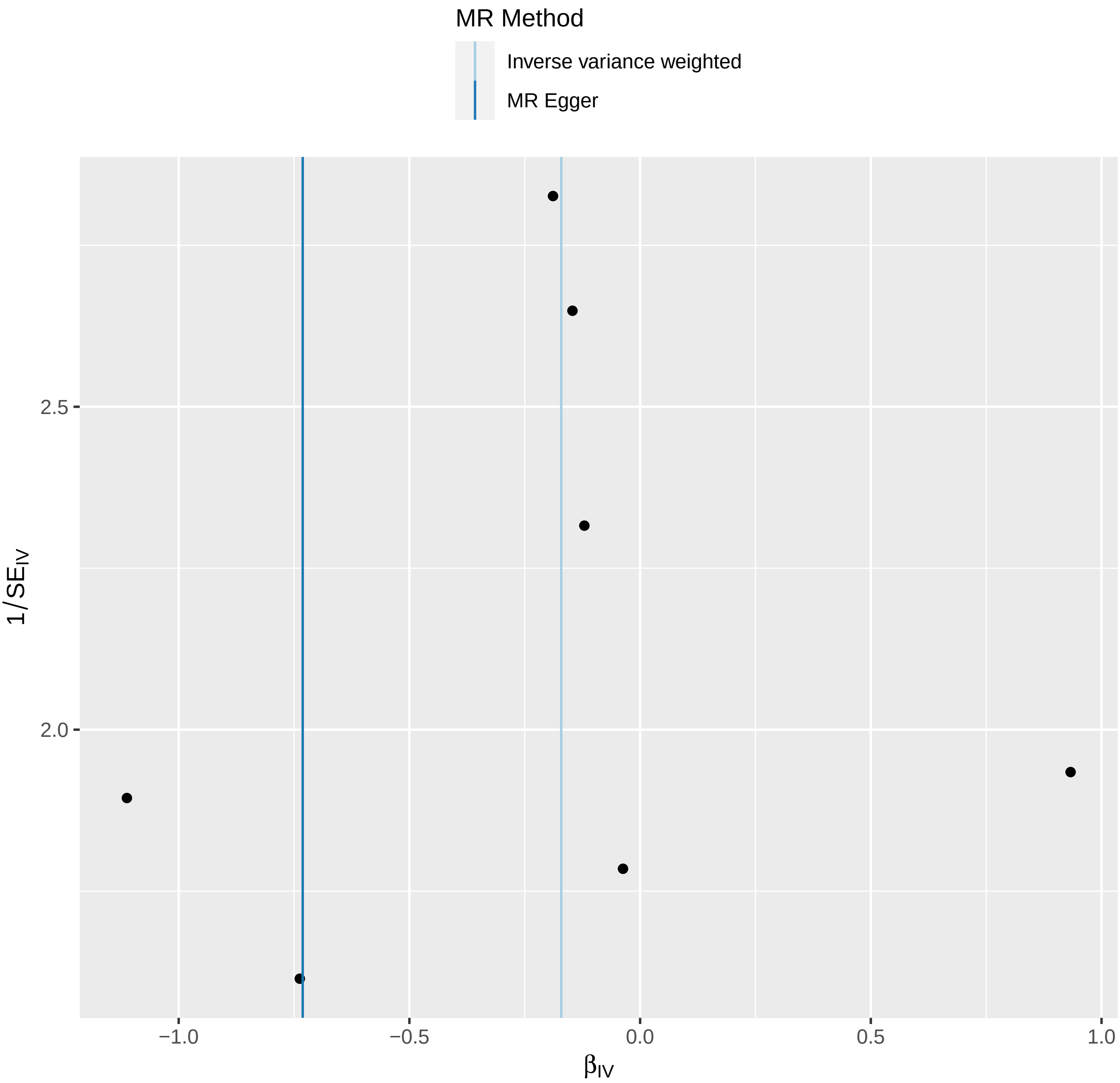

Batch 152 : Gut microbiota abundance (family Actinomycetaceae id.421) on Other Crohn's disease

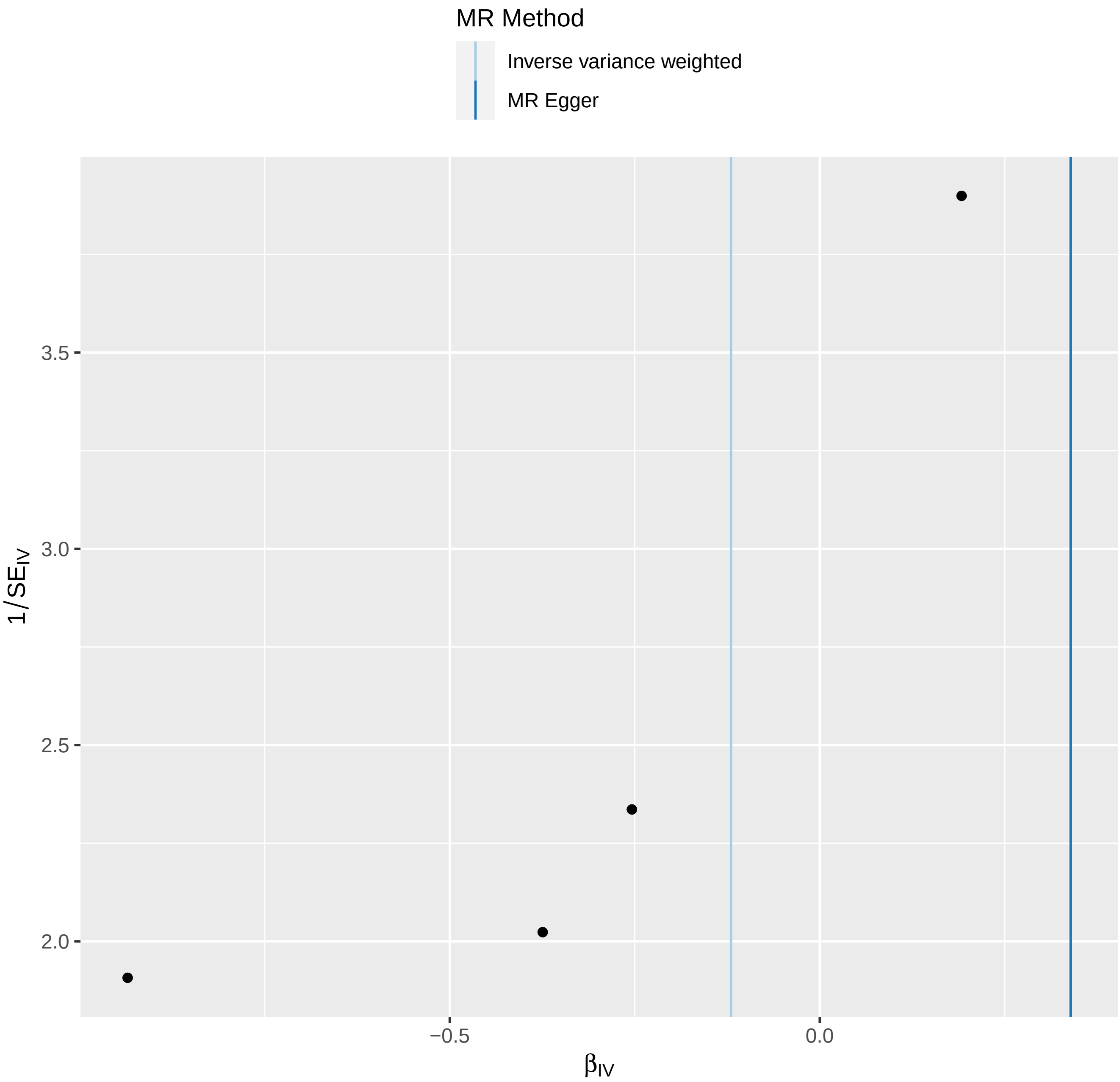

Batch 153 : Gut microbiota abundance (family Alcaligenaceae id.2875) on Other Crohn's disease

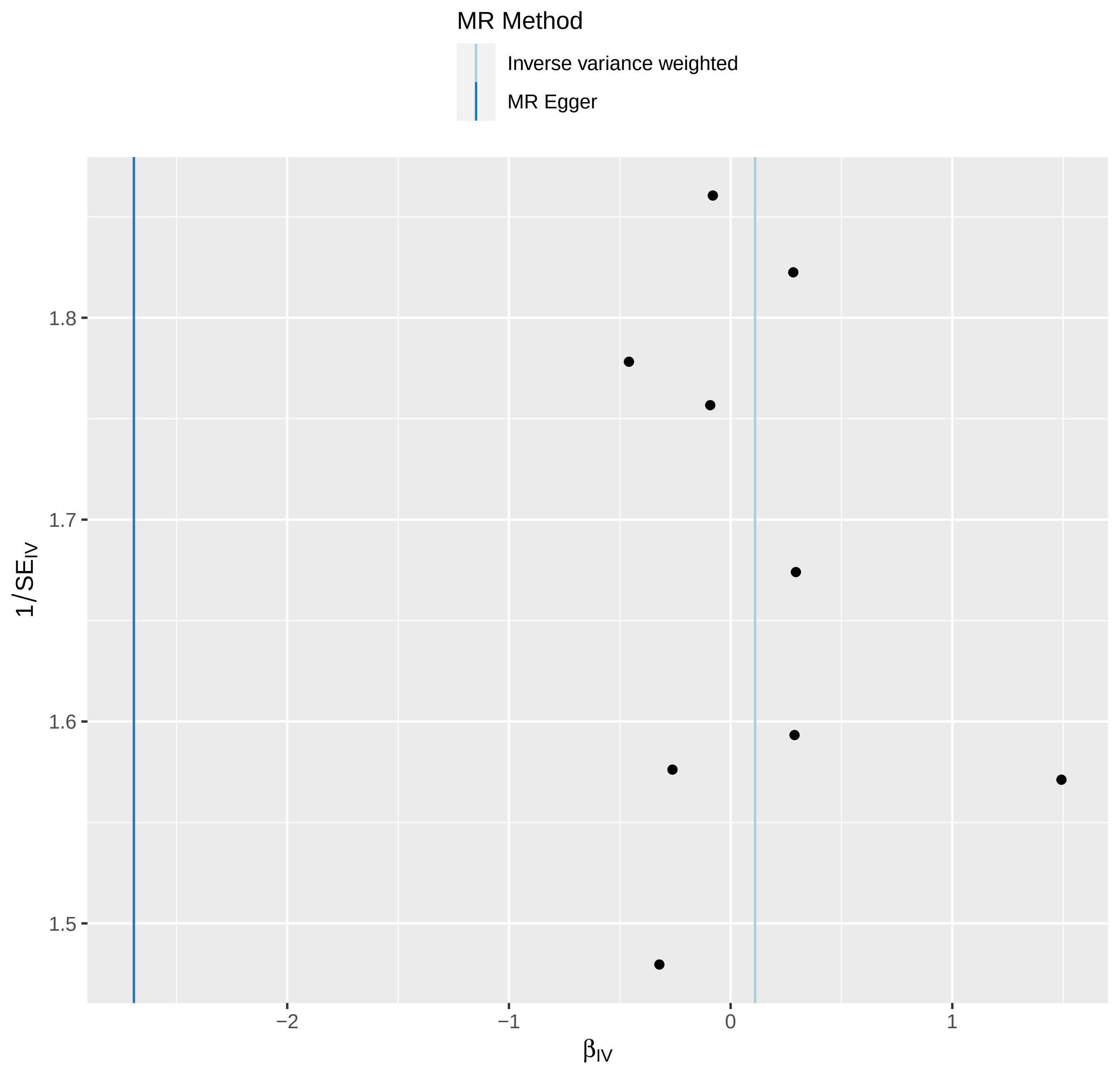

Batch 154 : Gut microbiota abundance (family Bacteroidaceae id.917) on Other Crohn's disease

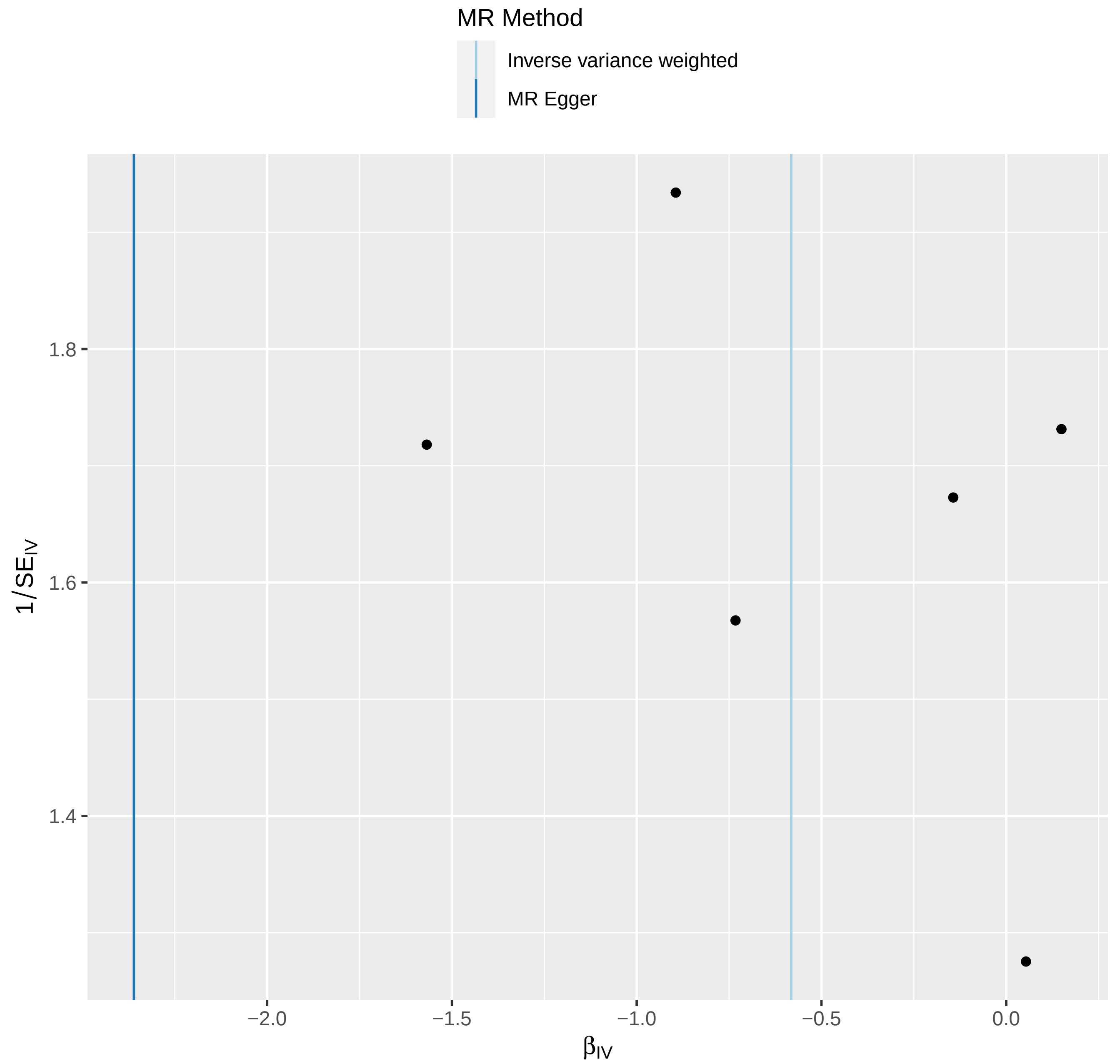

Batch 155 : Gut microbiota abundance (family Bacteroidales S24 7group id.11173) on Other Crohn's disease

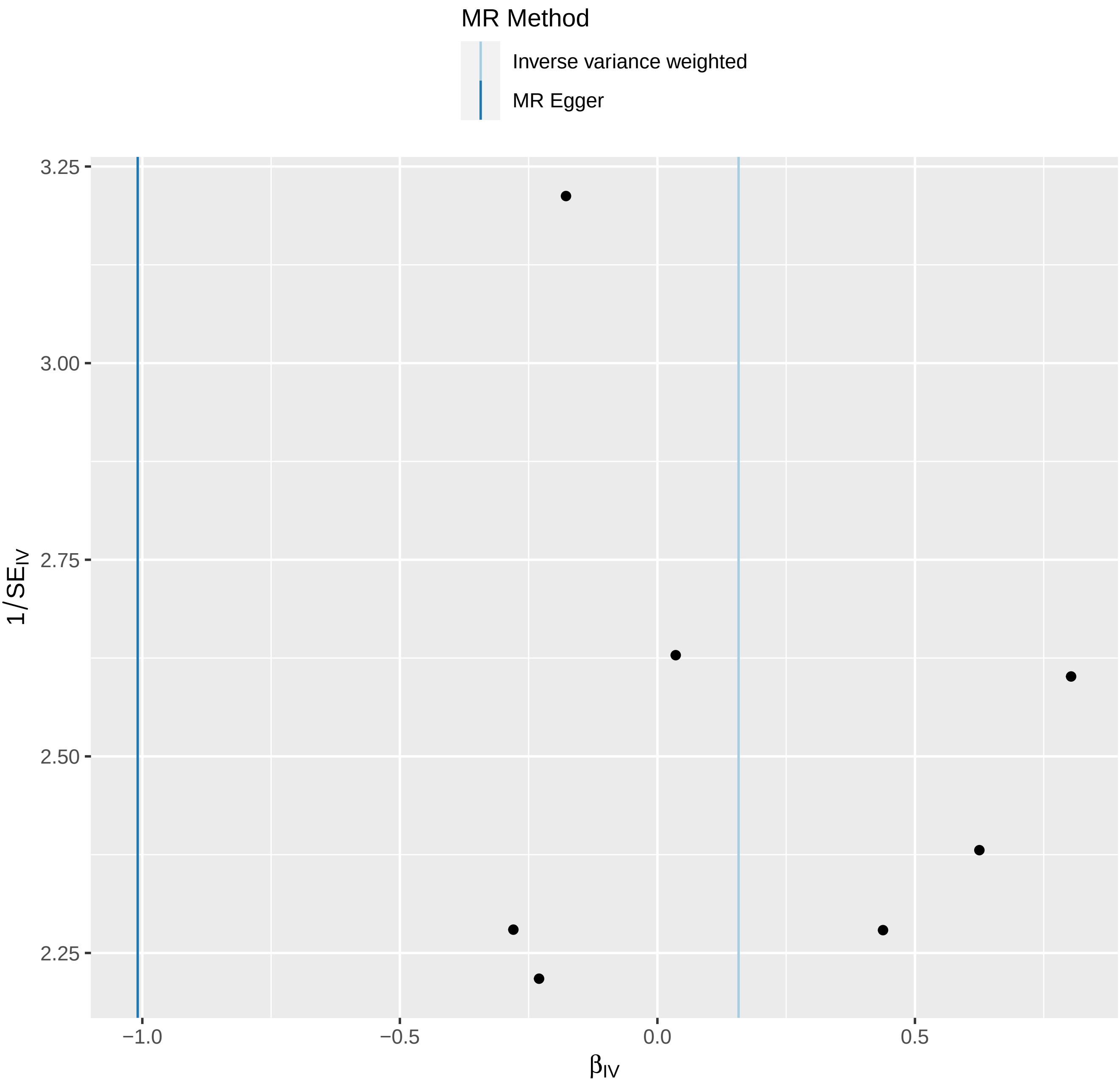

Batch 156 : Gut microbiota abundance (family Bifidobacteriaceae id.433) on Other Crohn's disease

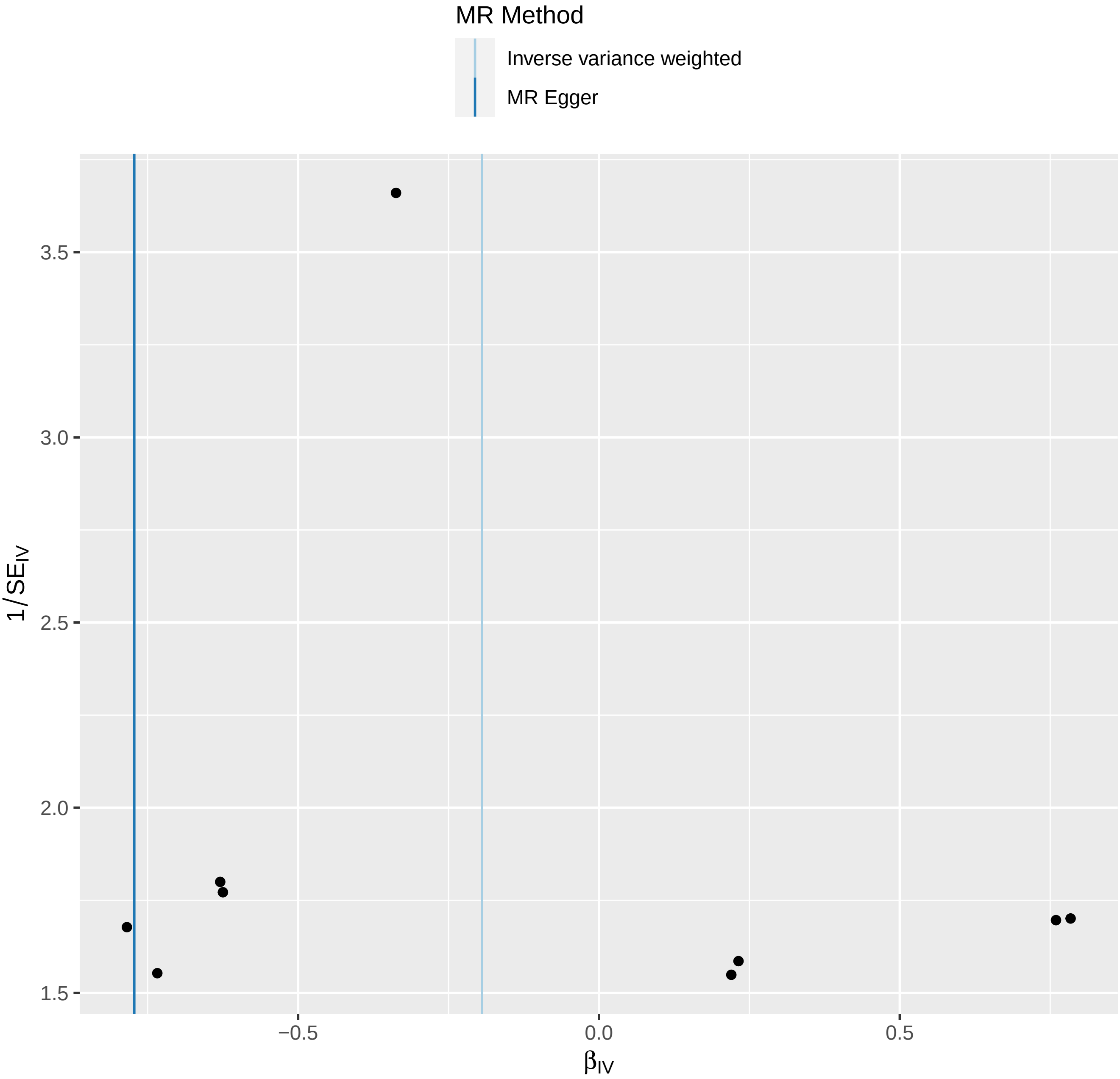

Batch 158 : Gut microbiota abundance (family Clostridiaceae1 id.1869) on Other Crohn's disease

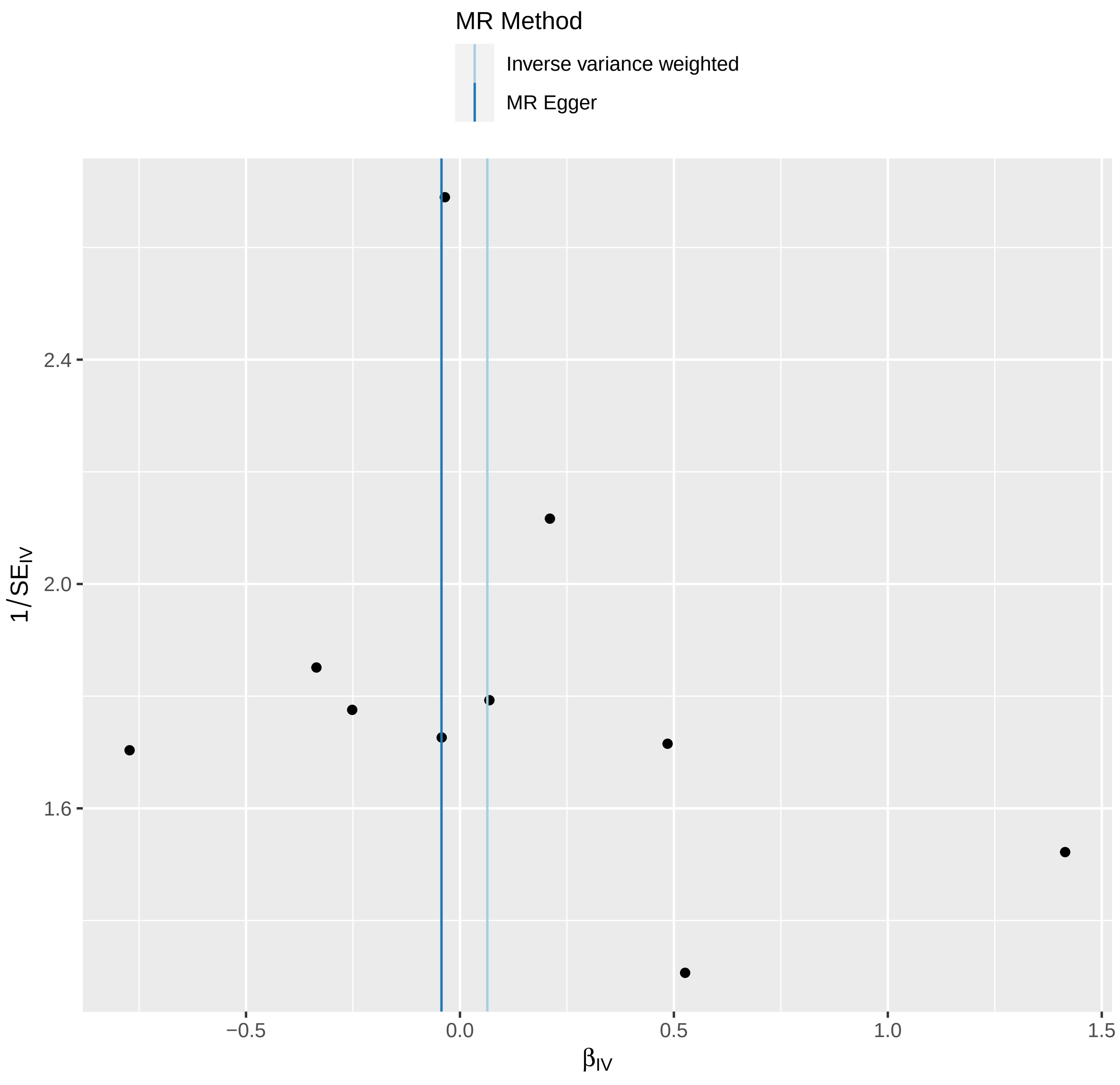

Batch 159 : Gut microbiota abundance (family Clostridiales vadin BB60 group id.11286) on Other Crohn's disease

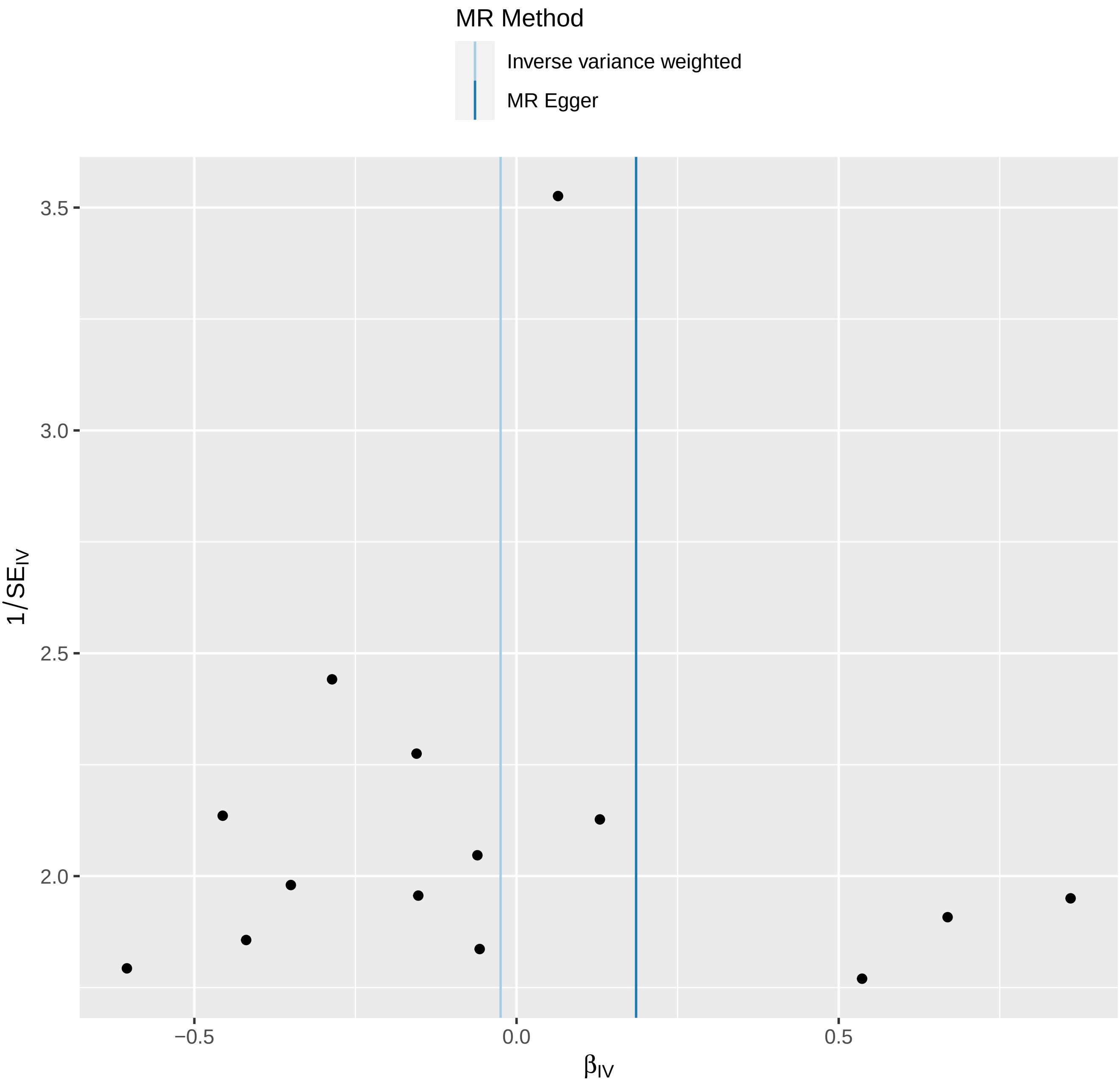

Batch 160 : Gut microbiota abundance (family Coriobacteriaceae id.811) on Other Crohn's disease

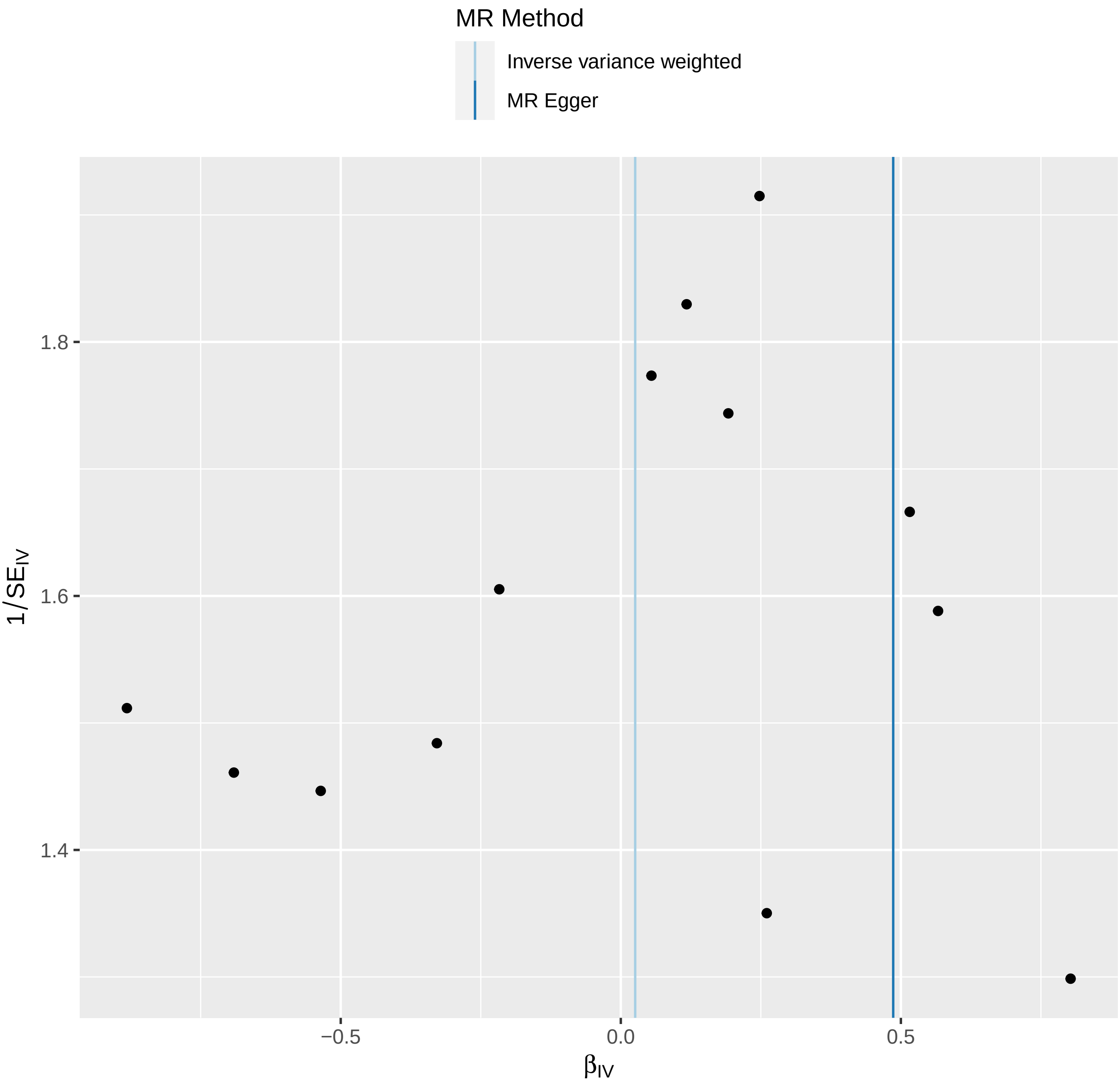

Batch 161 : Gut microbiota abundance (family Defluviitaleaceae id.1924) on Other Crohn's disease

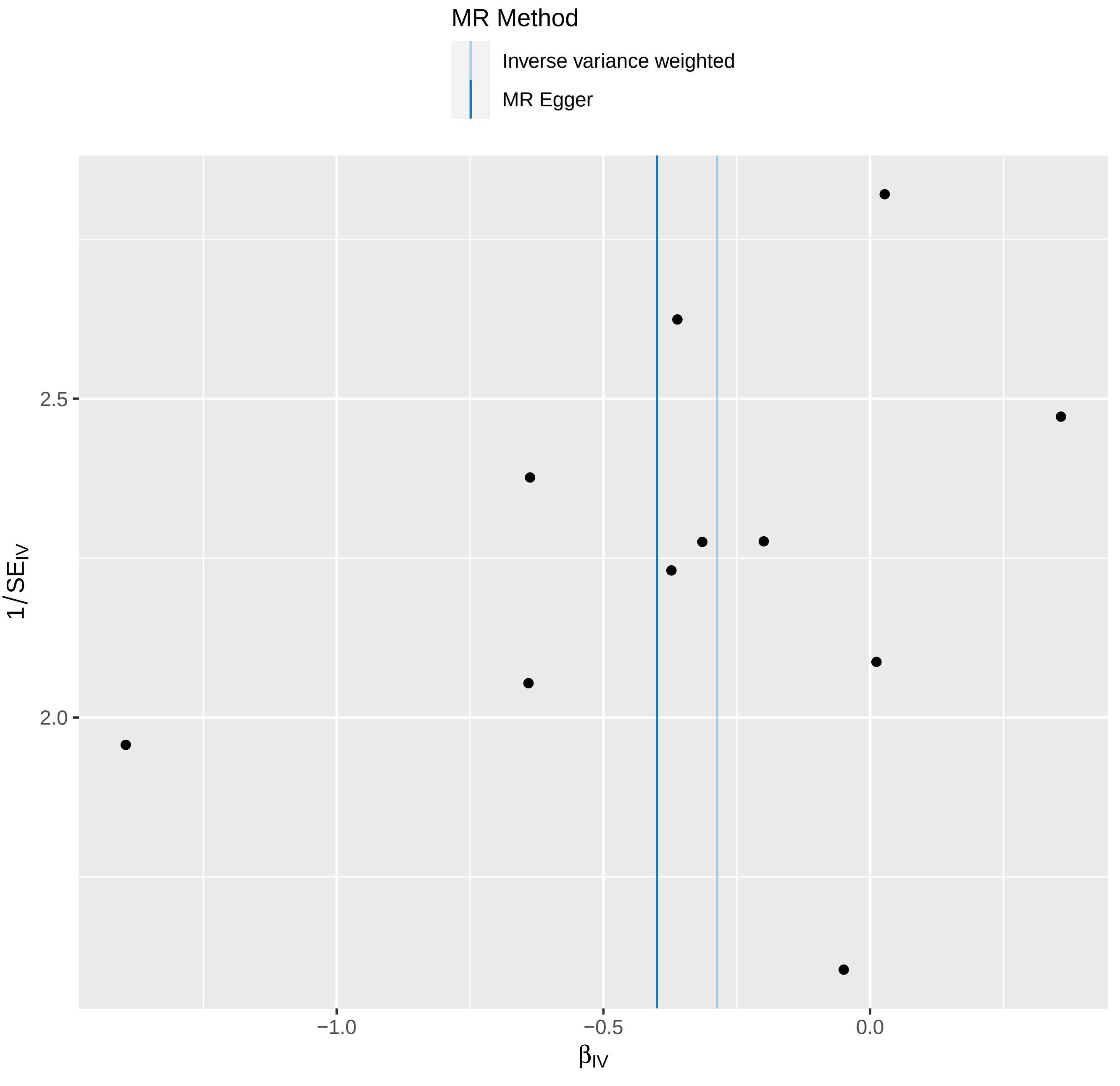

Batch 162 : Gut microbiota abundance (family Desulfovibrionaceae id.3169) on Other Crohn's disease

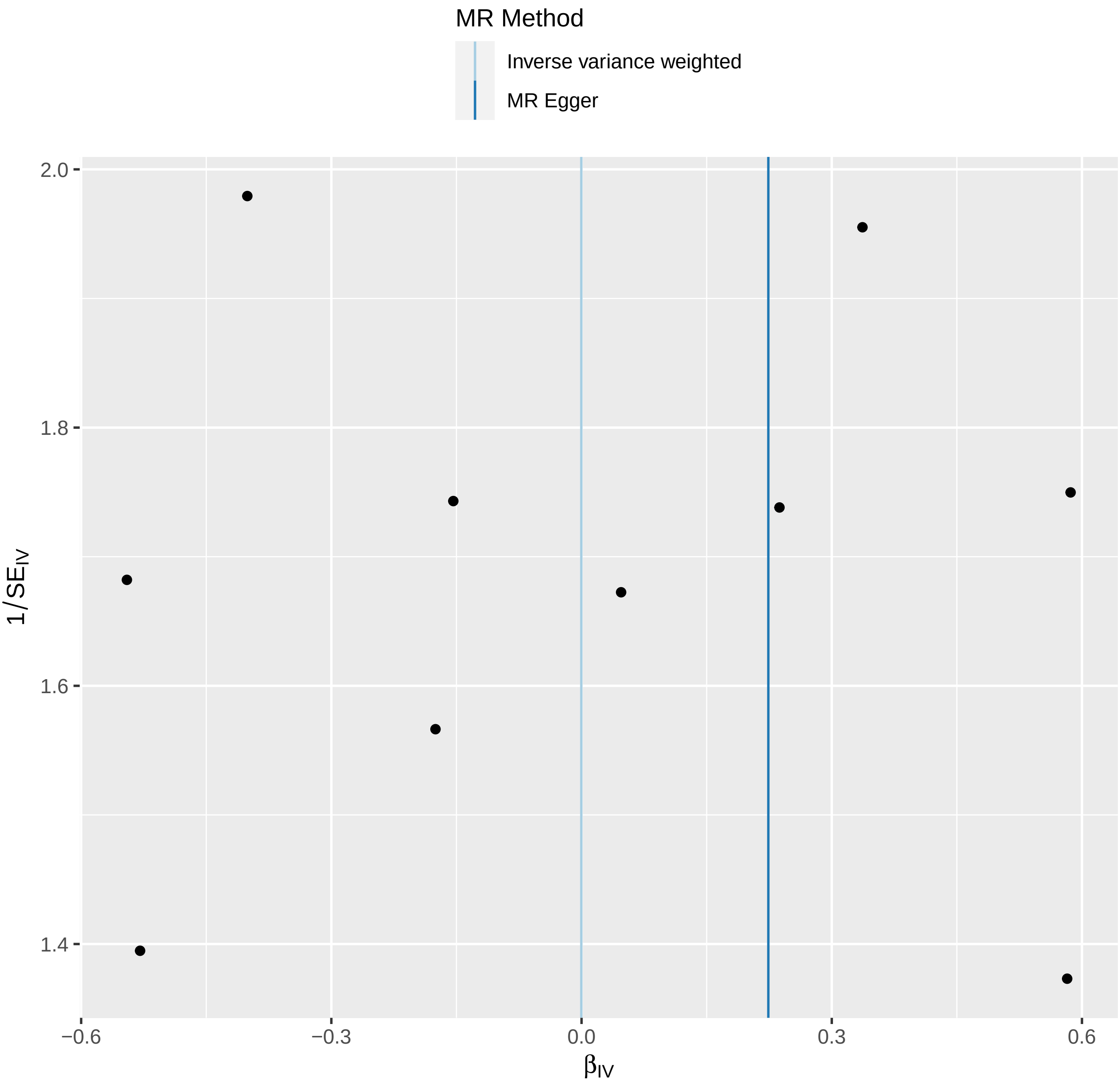

Batch 163 : Gut microbiota abundance (family Enterobacteriaceae id.3469) on Other Crohn's disease

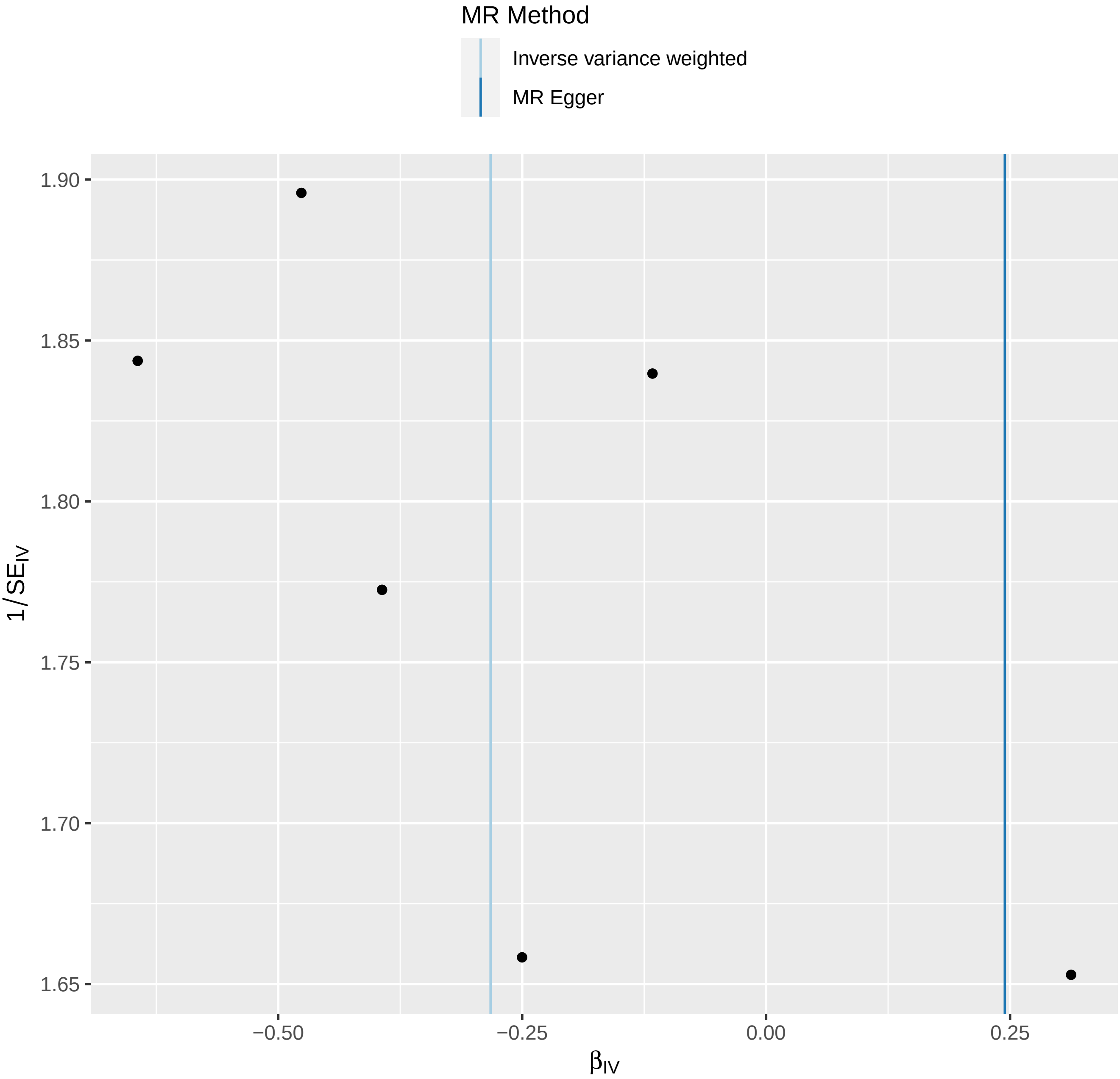

Batch 164 : Gut microbiota abundance (family Erysipelotrichaceae id.2149) on Other Crohn's disease

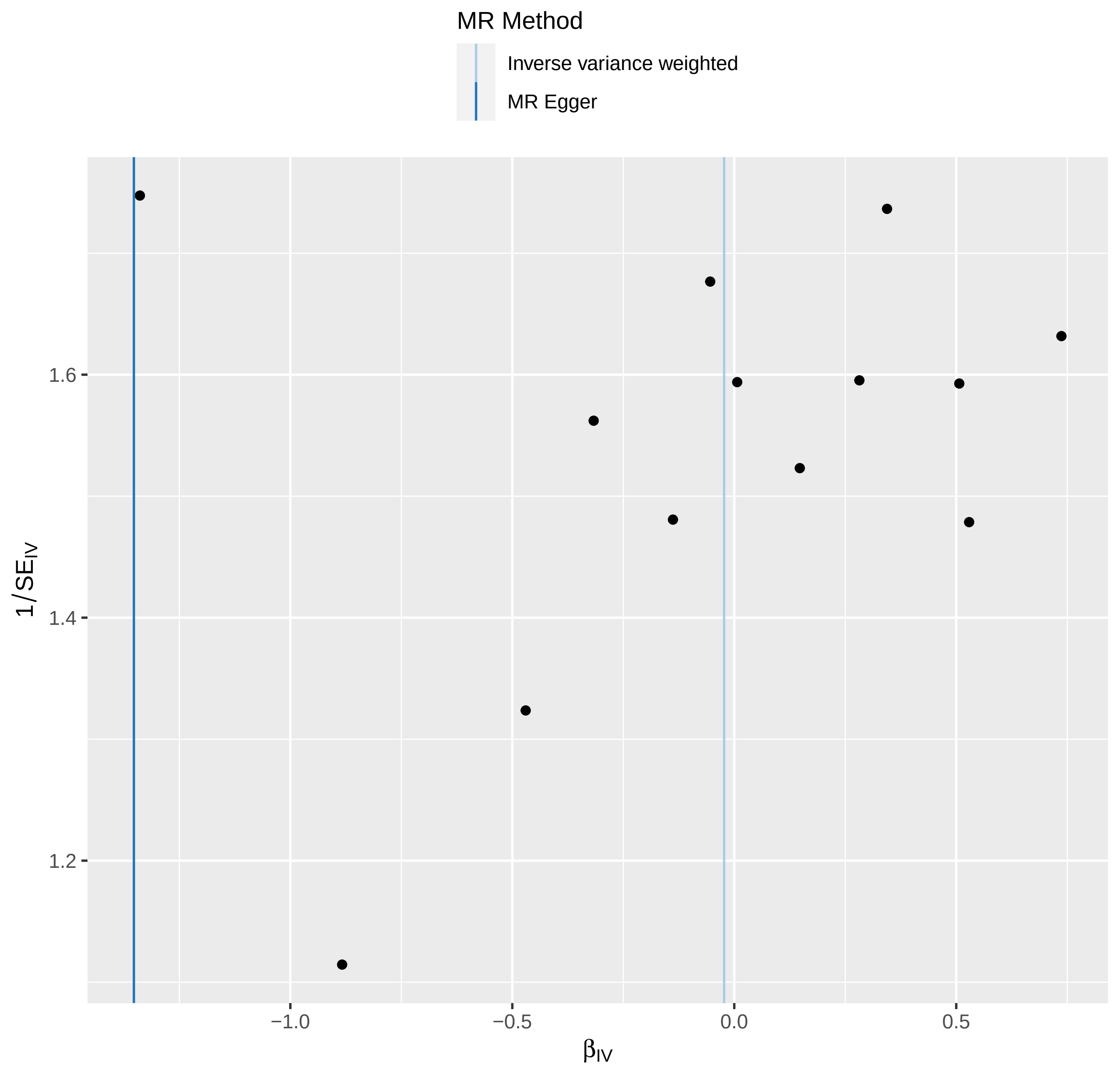

Batch 165 : Gut microbiota abundance (family Family XI id.1936) on Other Crohn's disease

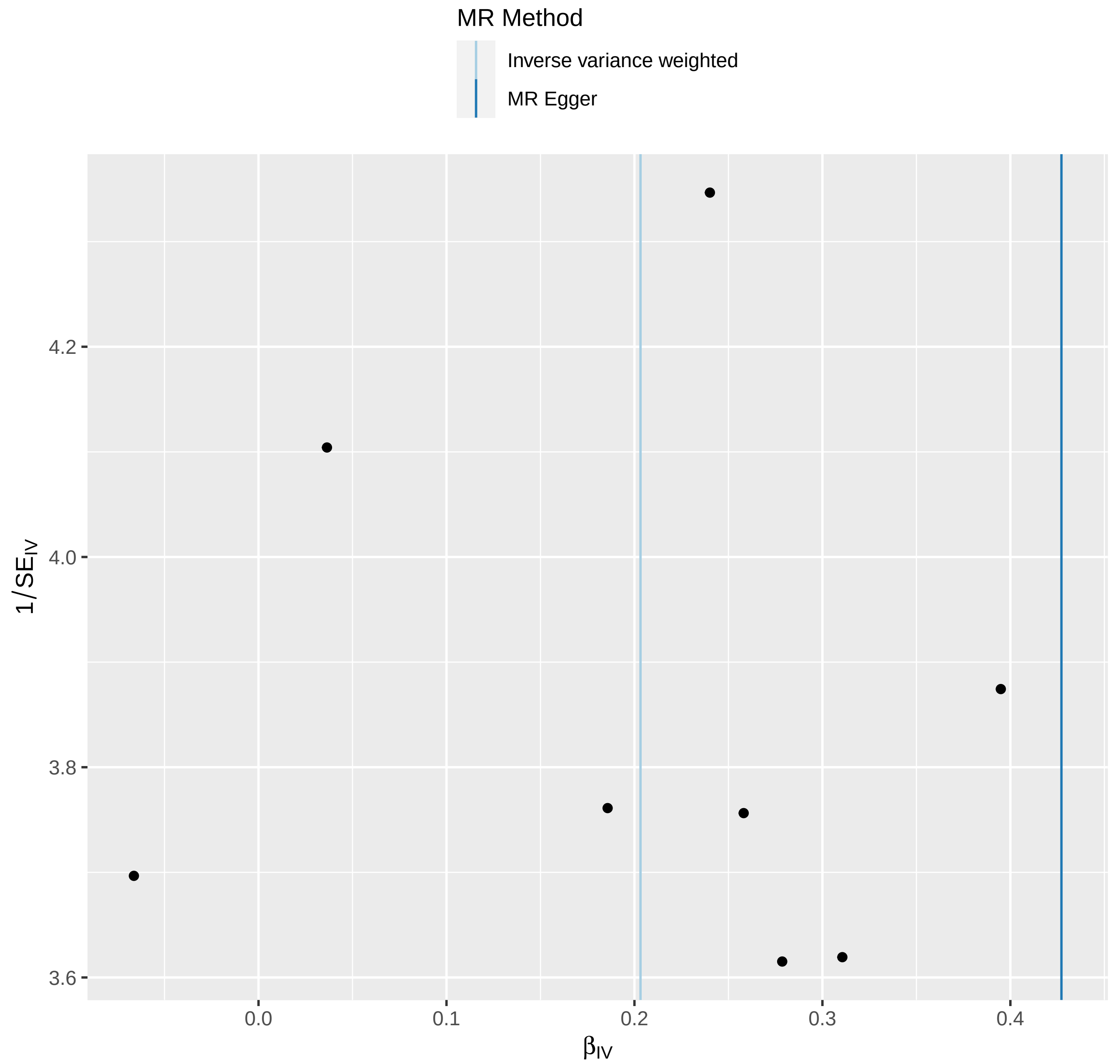

Batch 166 : Gut microbiota abundance (family Family XIII id.1957) on Other Crohn's disease

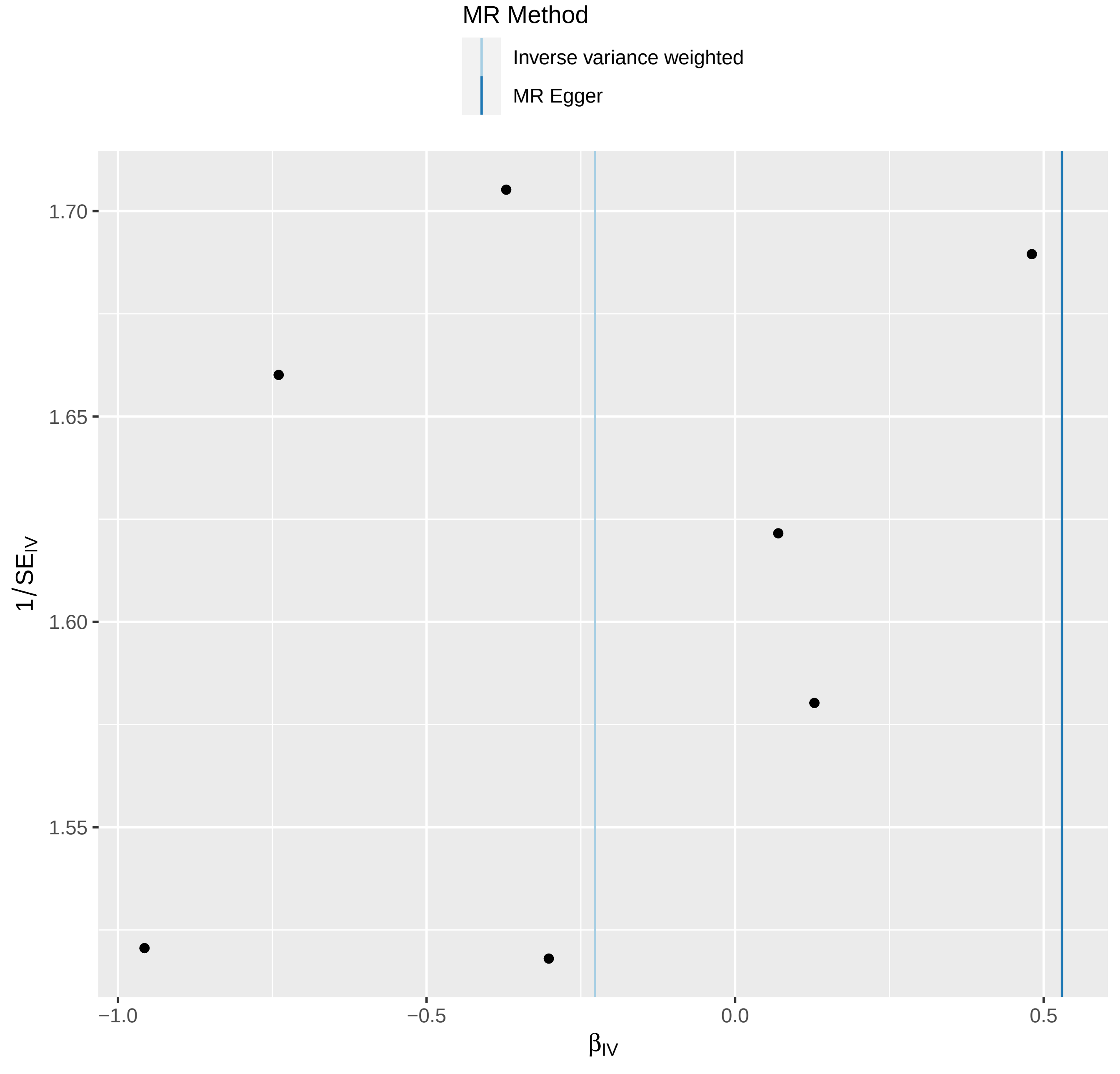

Batch 167 : Gut microbiota abundance (family Lachnospiraceae id.1987) on Other Crohn's disease

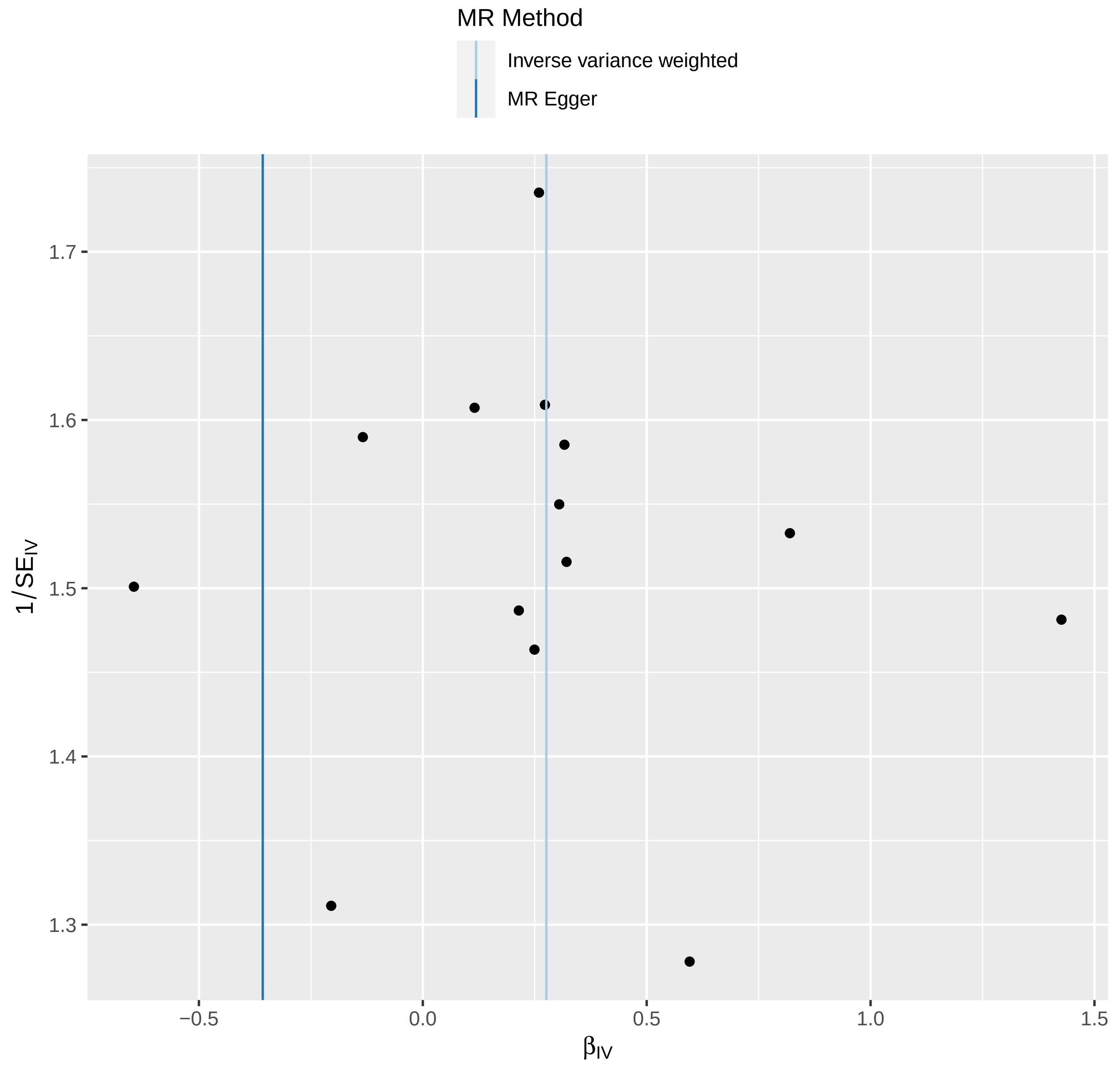

Batch 168 : Gut microbiota abundance (family Lactobacillaceae id.1836) on Other Crohn's disease

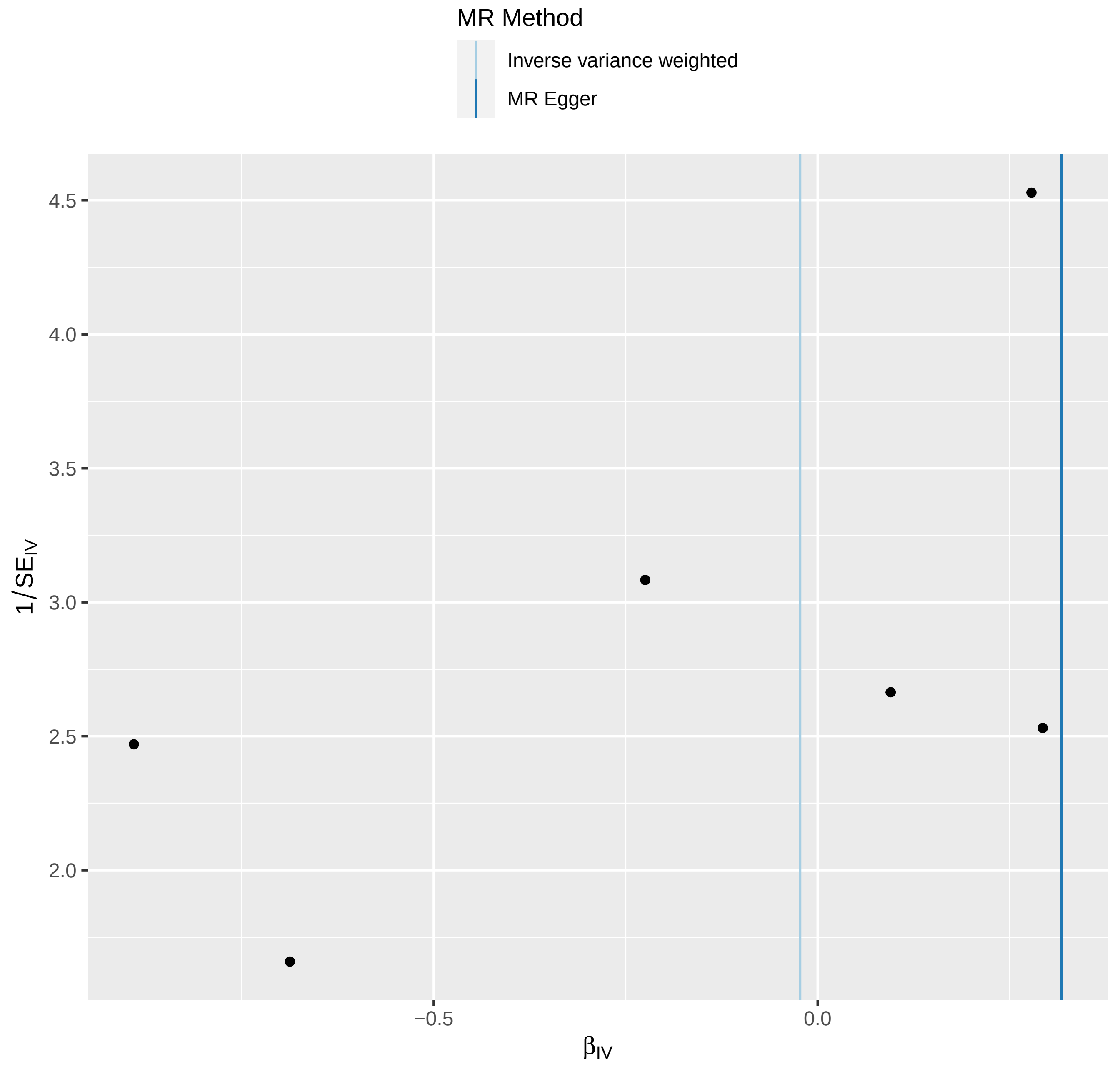

Batch 169 : Gut microbiota abundance (family Methanobacteriaceae id.121) on Other Crohn's disease

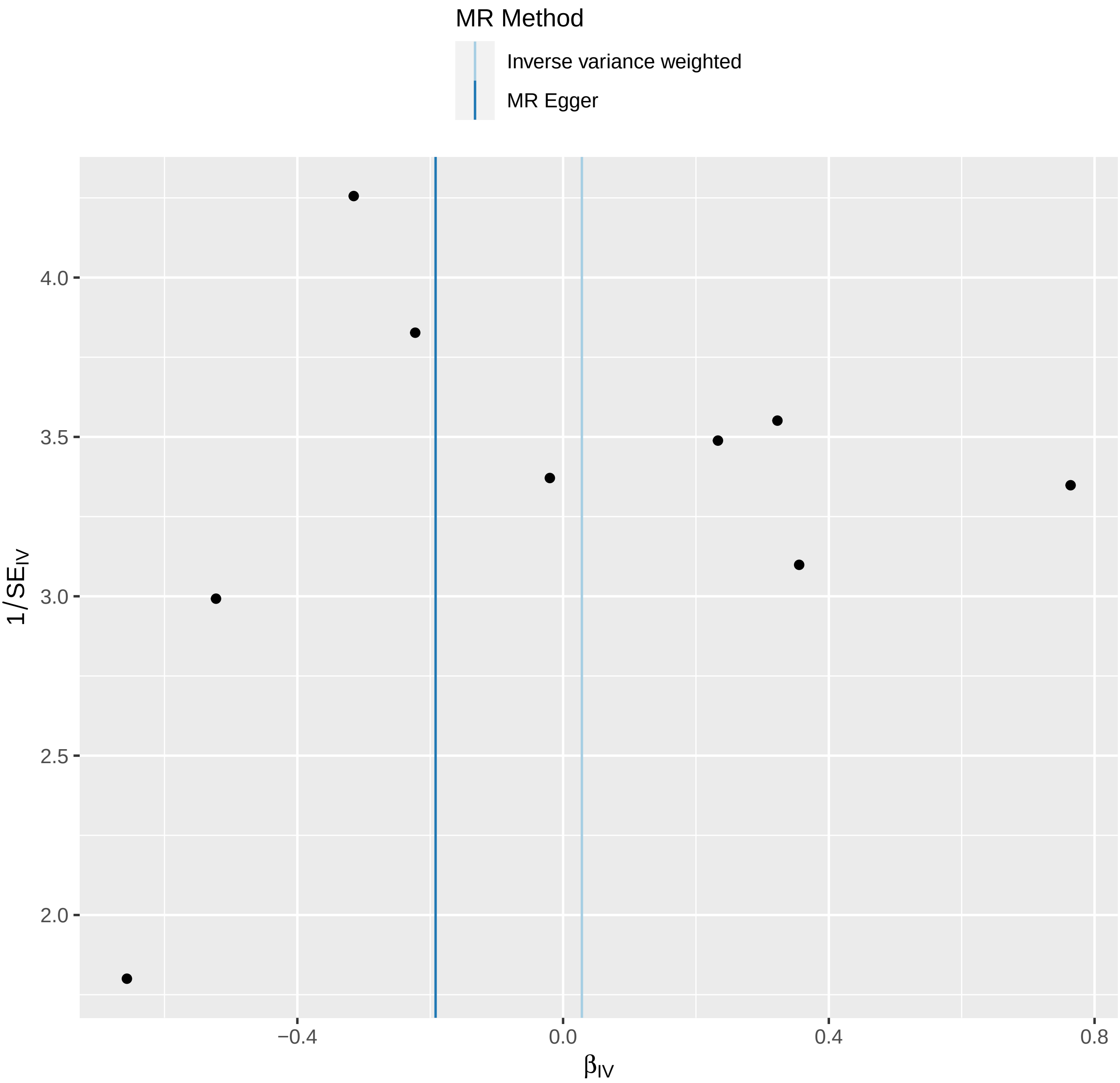

Batch 170 : Gut microbiota abundance (family Oxalobacteraceae id.2966) on Other Crohn's disease

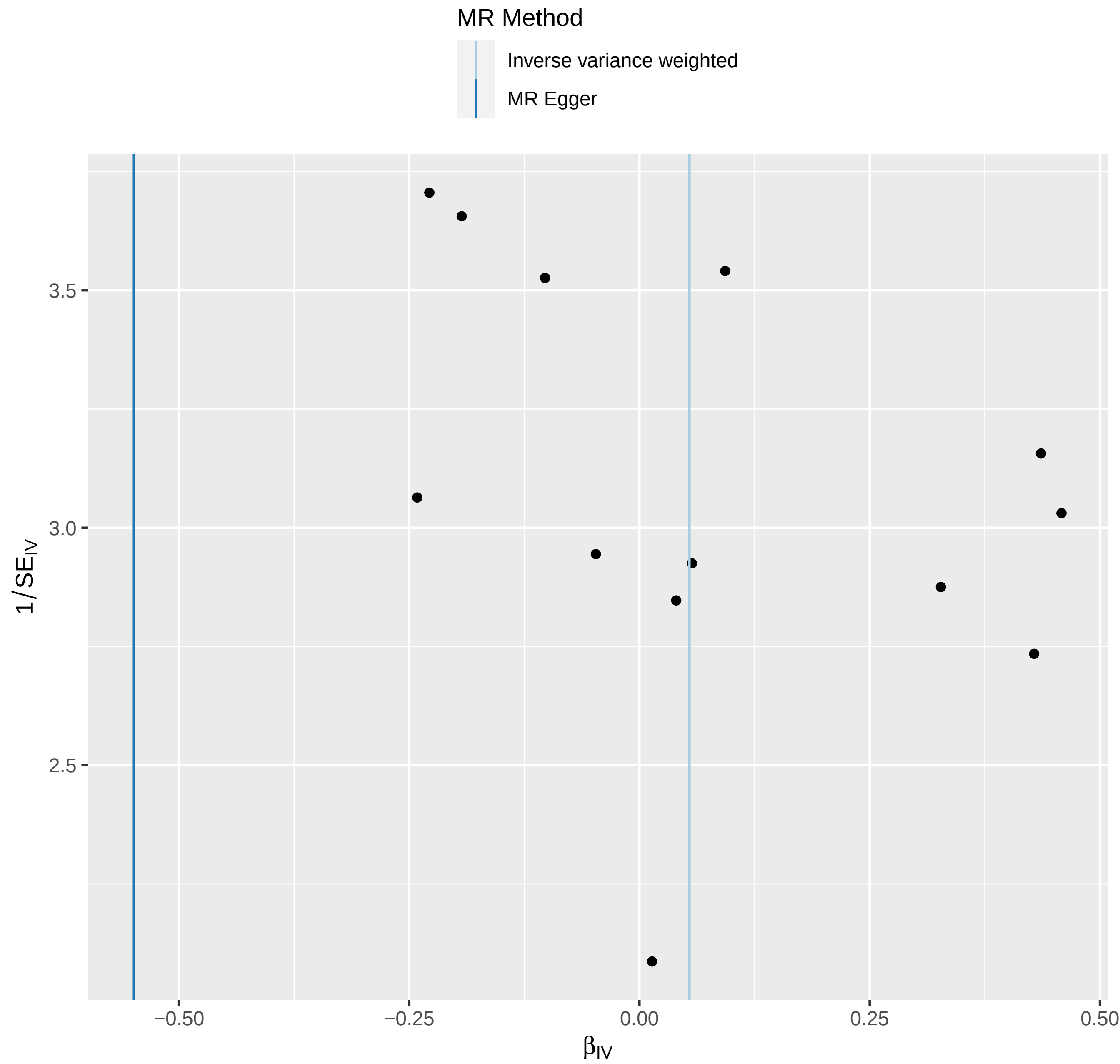

Batch 171 : Gut microbiota abundance (family Pasteurellaceae id.3689) on Other Crohn's disease

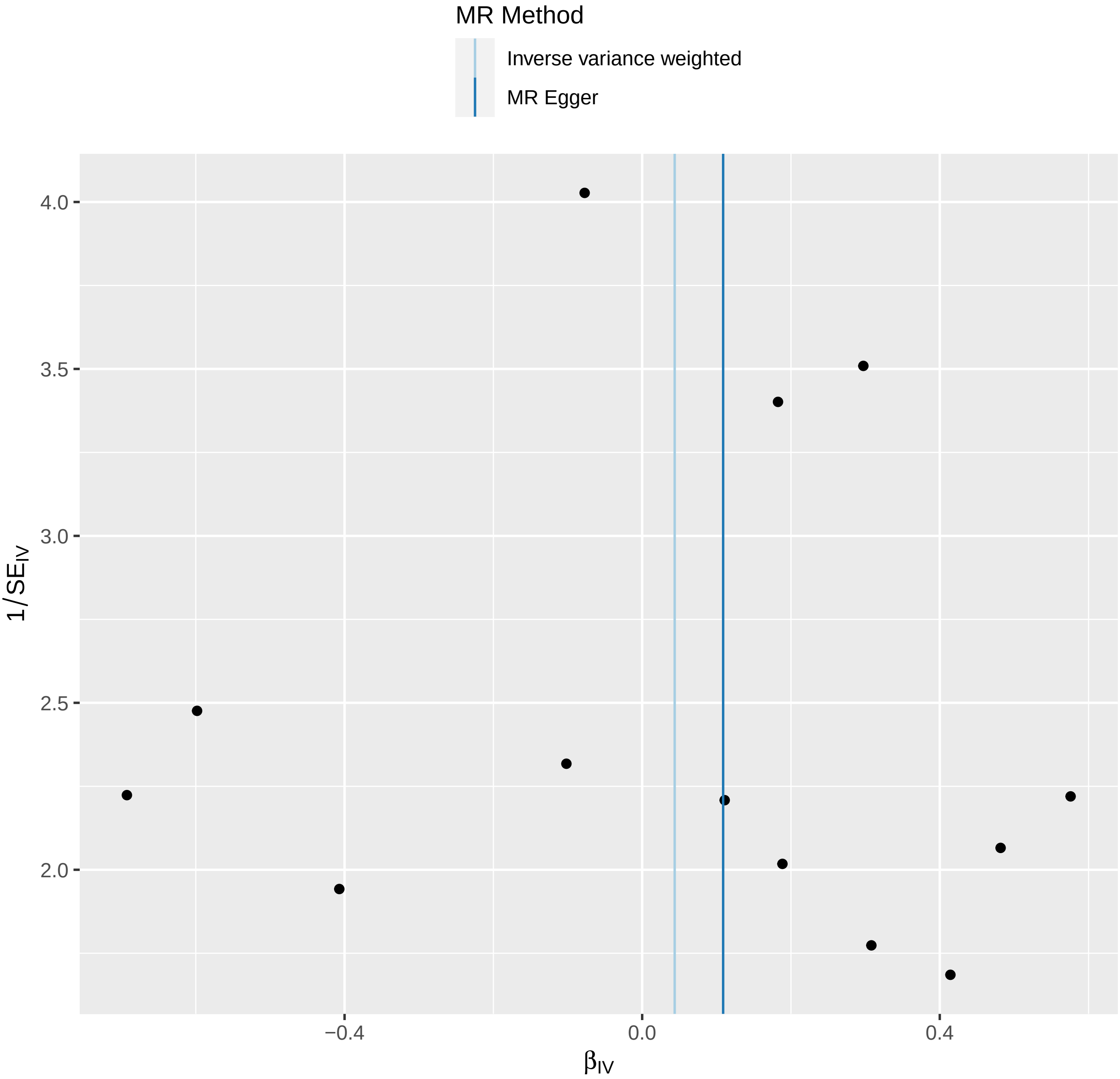

Batch 172 : Gut microbiota abundance (family Peptococcaceae id.2024) on Other Crohn's disease

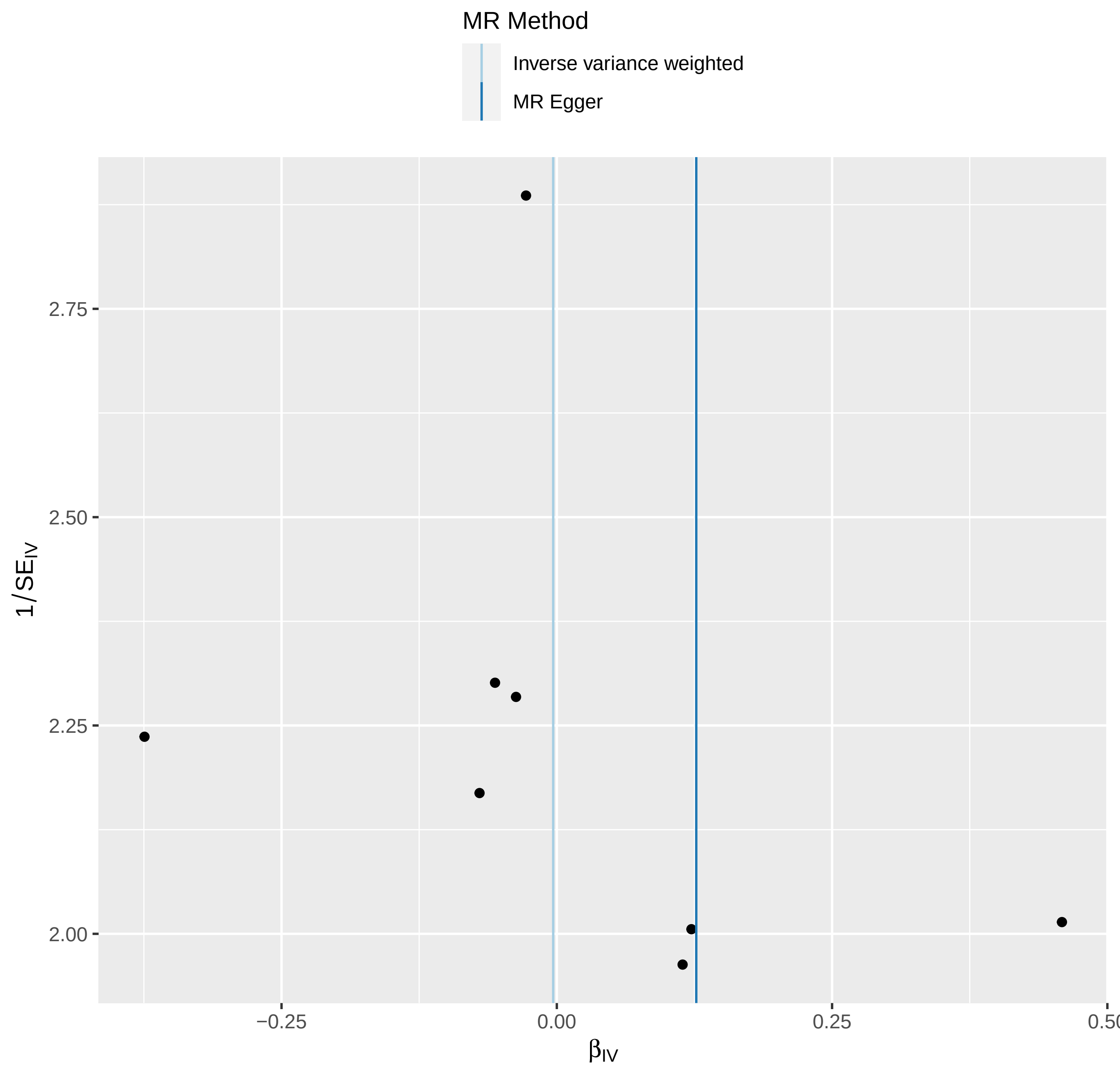

Batch 173 : Gut microbiota abundance (family Peptostreptococcaceae id.2042) on Other Crohn's disease

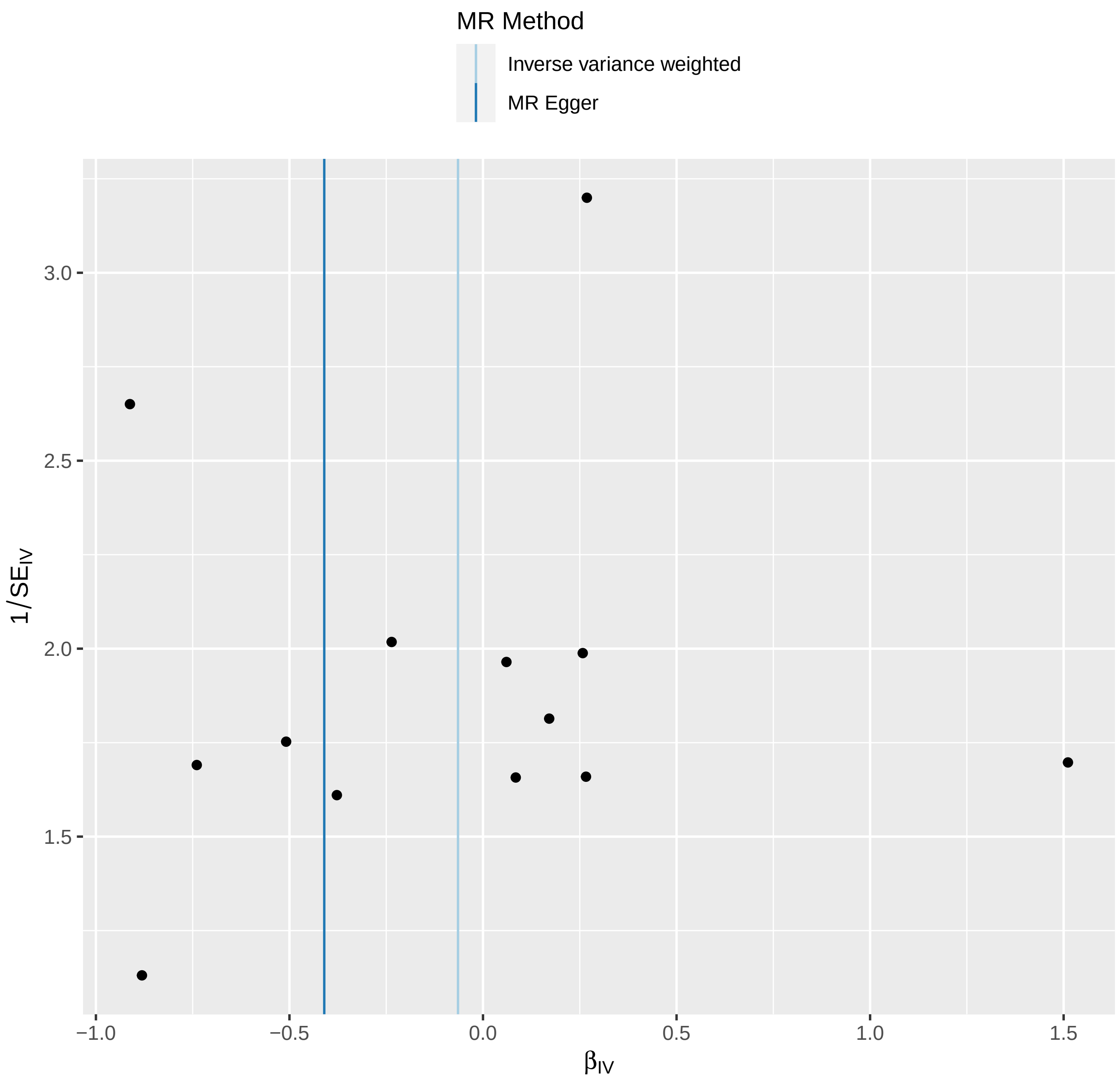

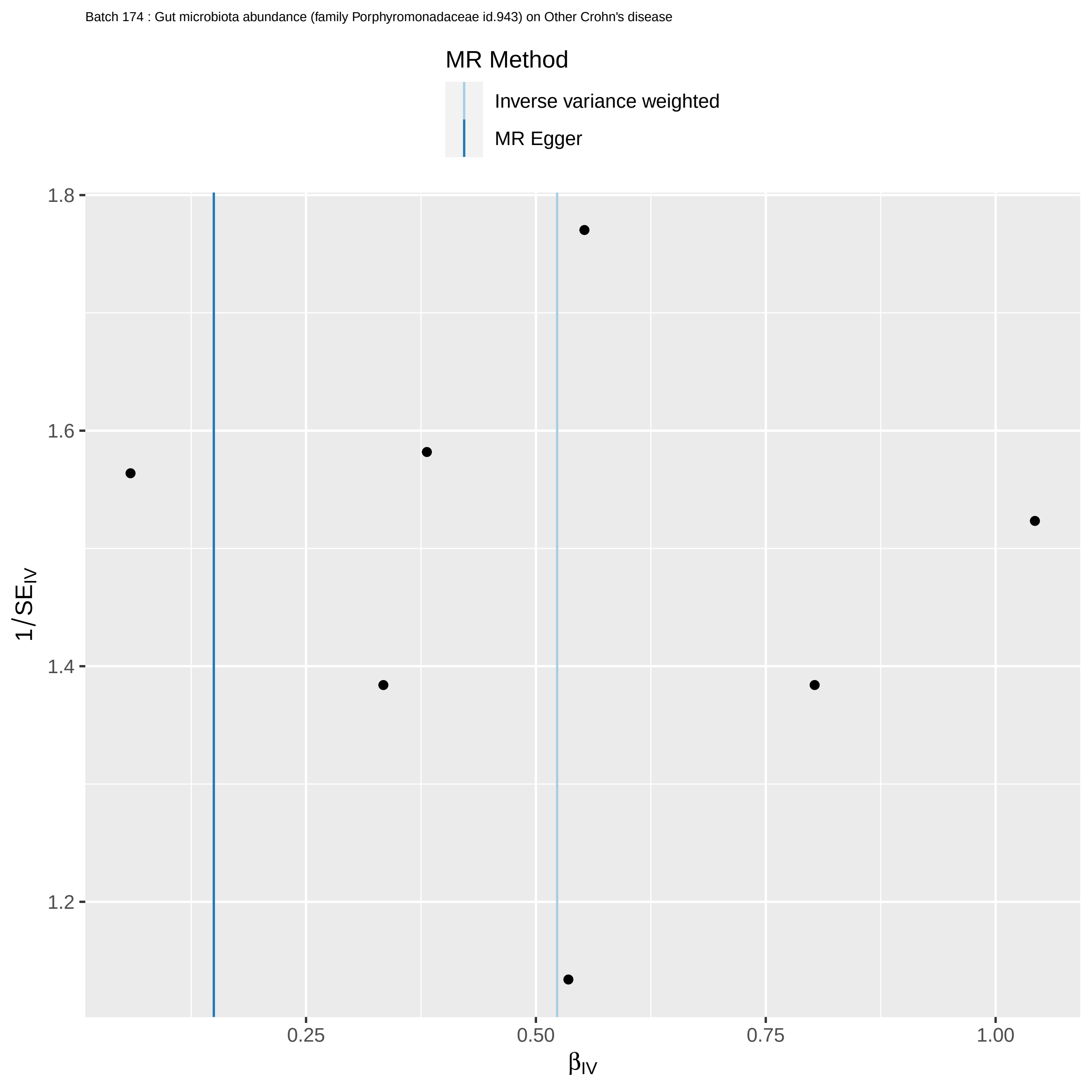

Batch 174 : Gut microbiota abundance (family Porphyromonadaceae id.943) on Other Crohn's disease
MR Method
Inverse variance weighted
MR Egger
1.8
1.6
1.4
1.2
$1/SE_{IV}$
0.25
0.50
0.75
1.00
β_{IV}

Batch 175 : Gut microbiota abundance (family Prevotellaceae id.960) on Other Crohn's disease

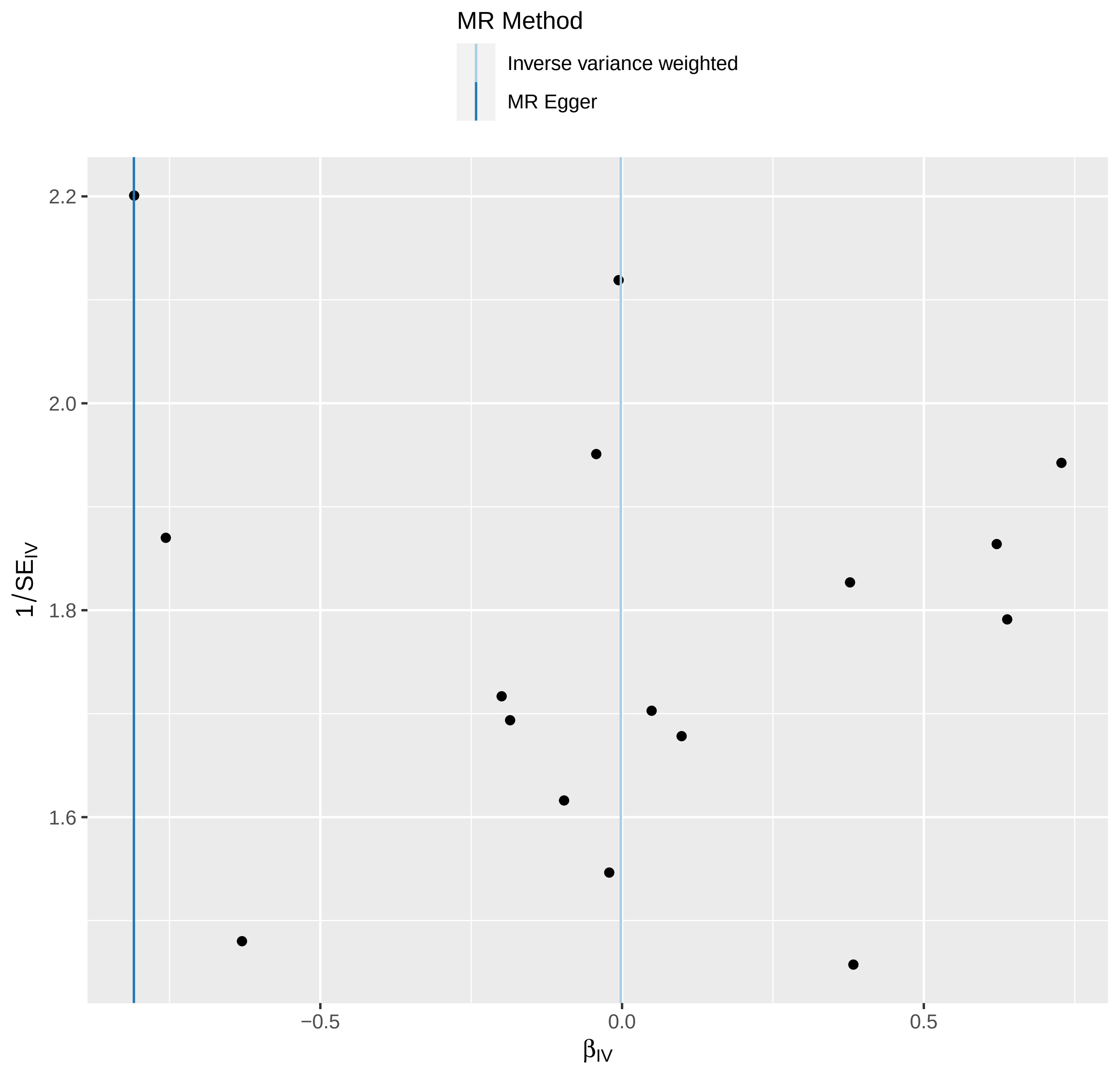

Batch 176 : Gut microbiota abundance (family Rhodospirillaceae id.2717) on Other Crohn's disease

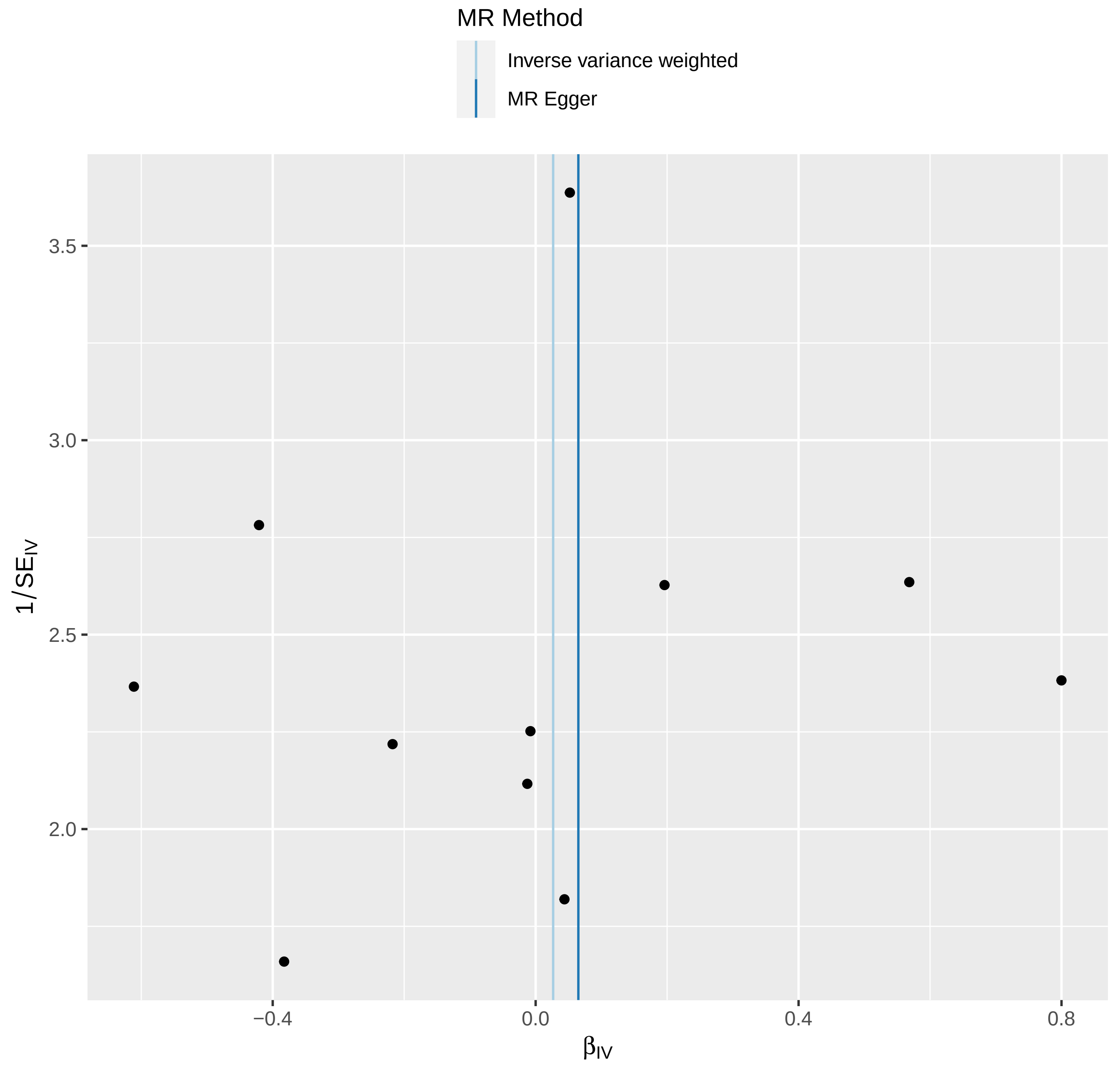

Batch 177 : Gut microbiota abundance (family Rikenellaceae id.967) on Other Crohn's disease

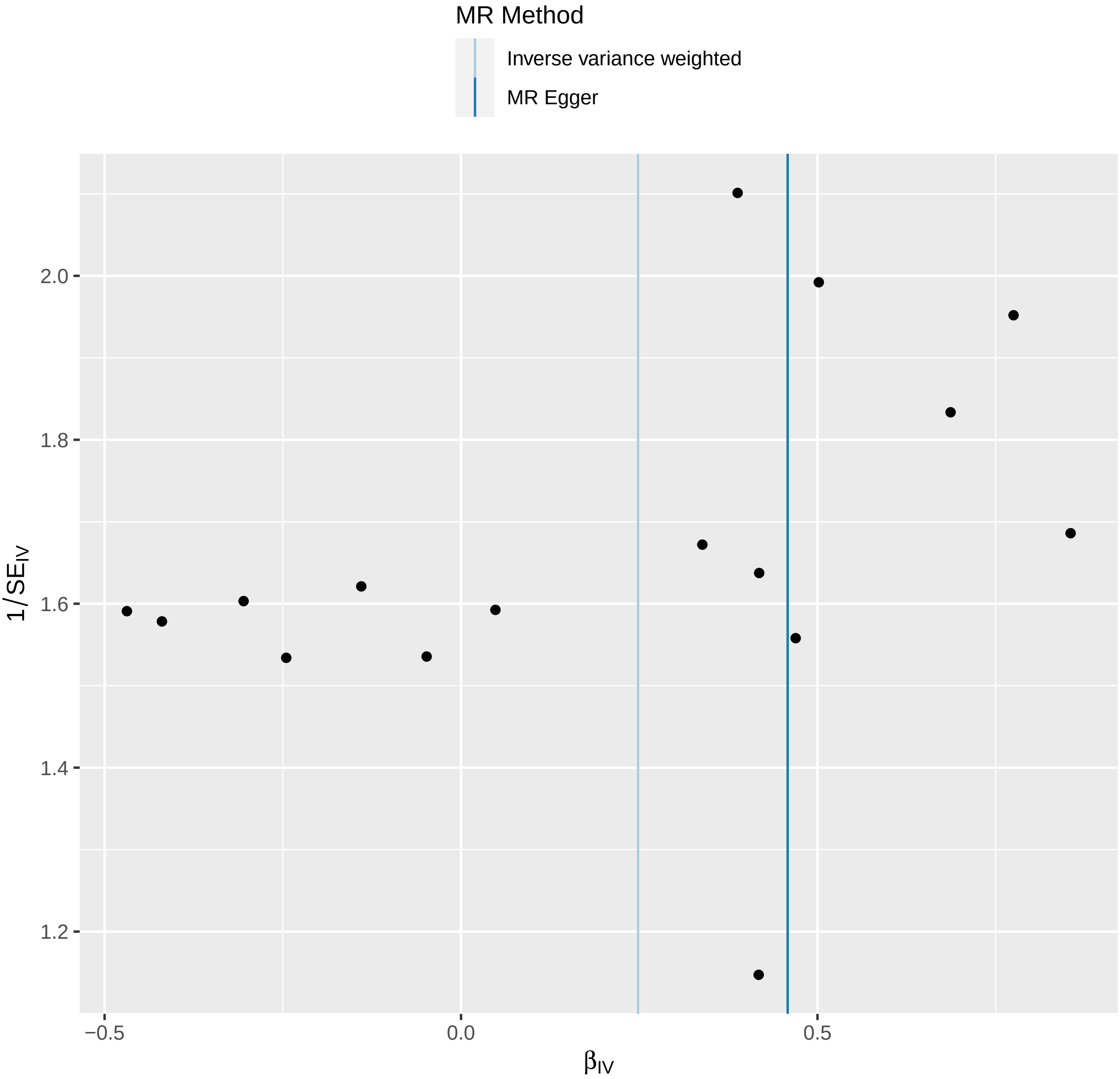

Batch 178 : Gut microbiota abundance (family Ruminococcaceae id.2050) on Other Crohn's disease

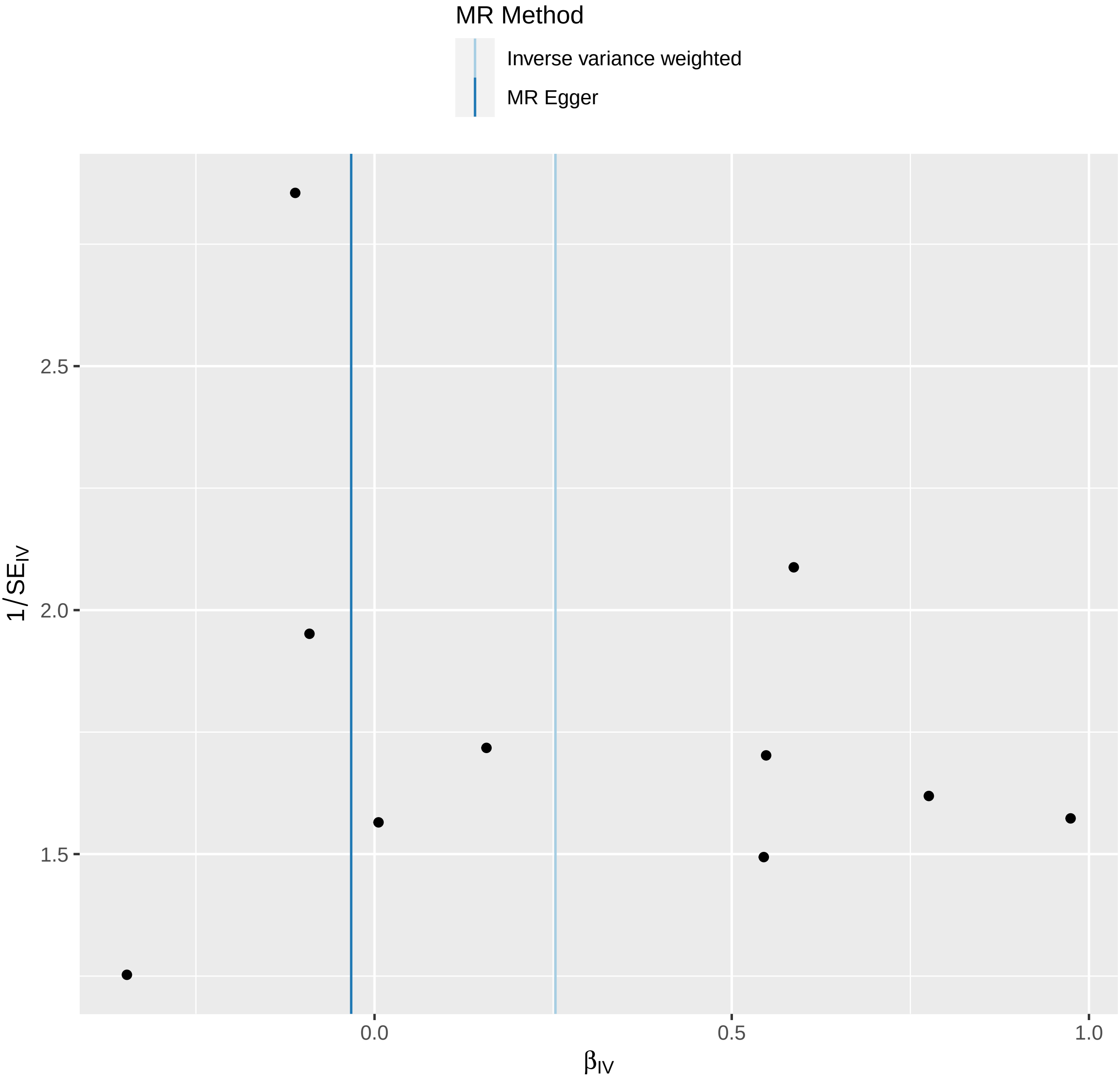

Batch 179 : Gut microbiota abundance (family Streptococcaceae id.1850) on Other Crohn's disease

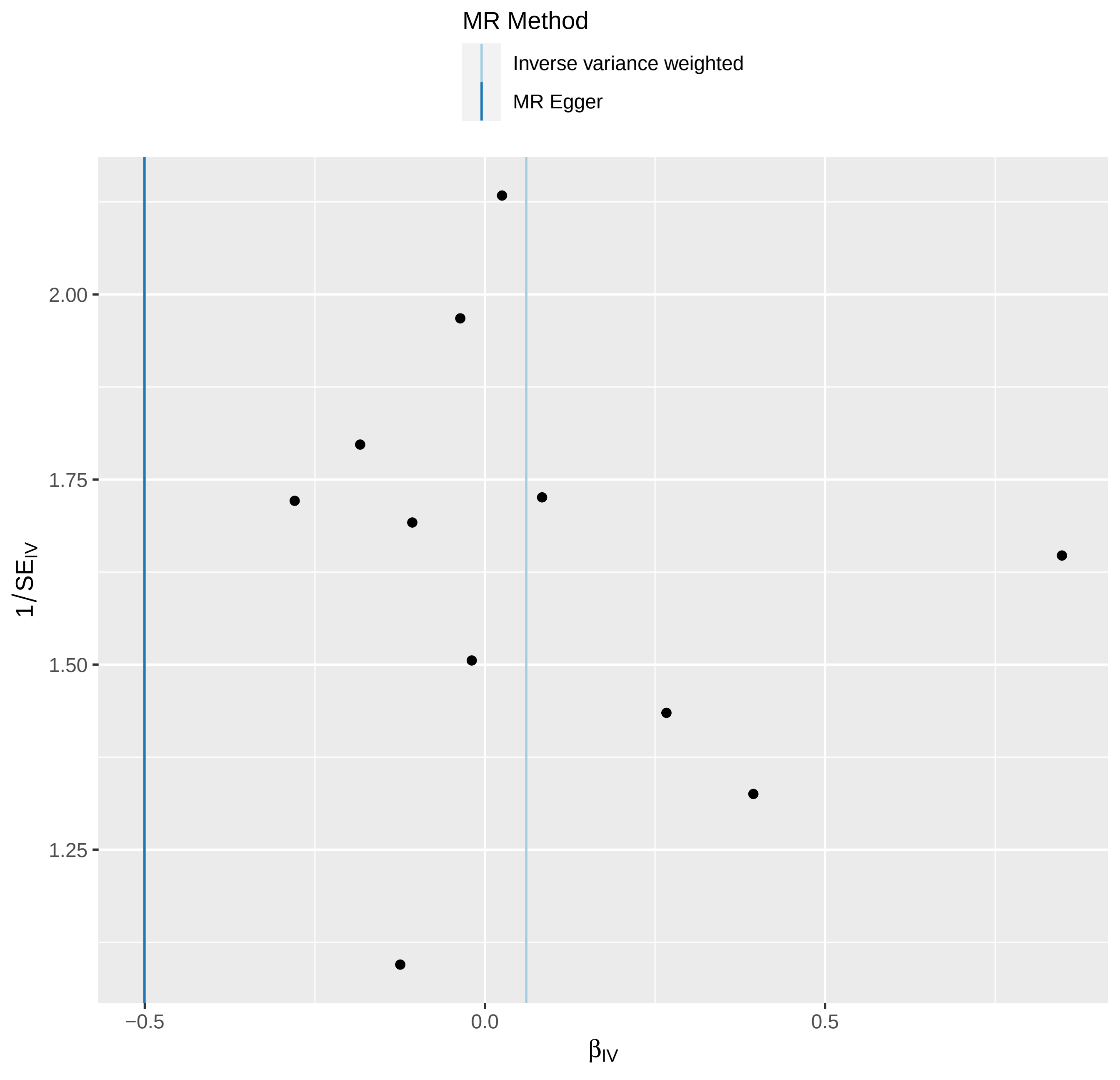

Batch 180 : Gut microbiota abundance (family Veillonellaceae id.2172) on Other Crohn's disease

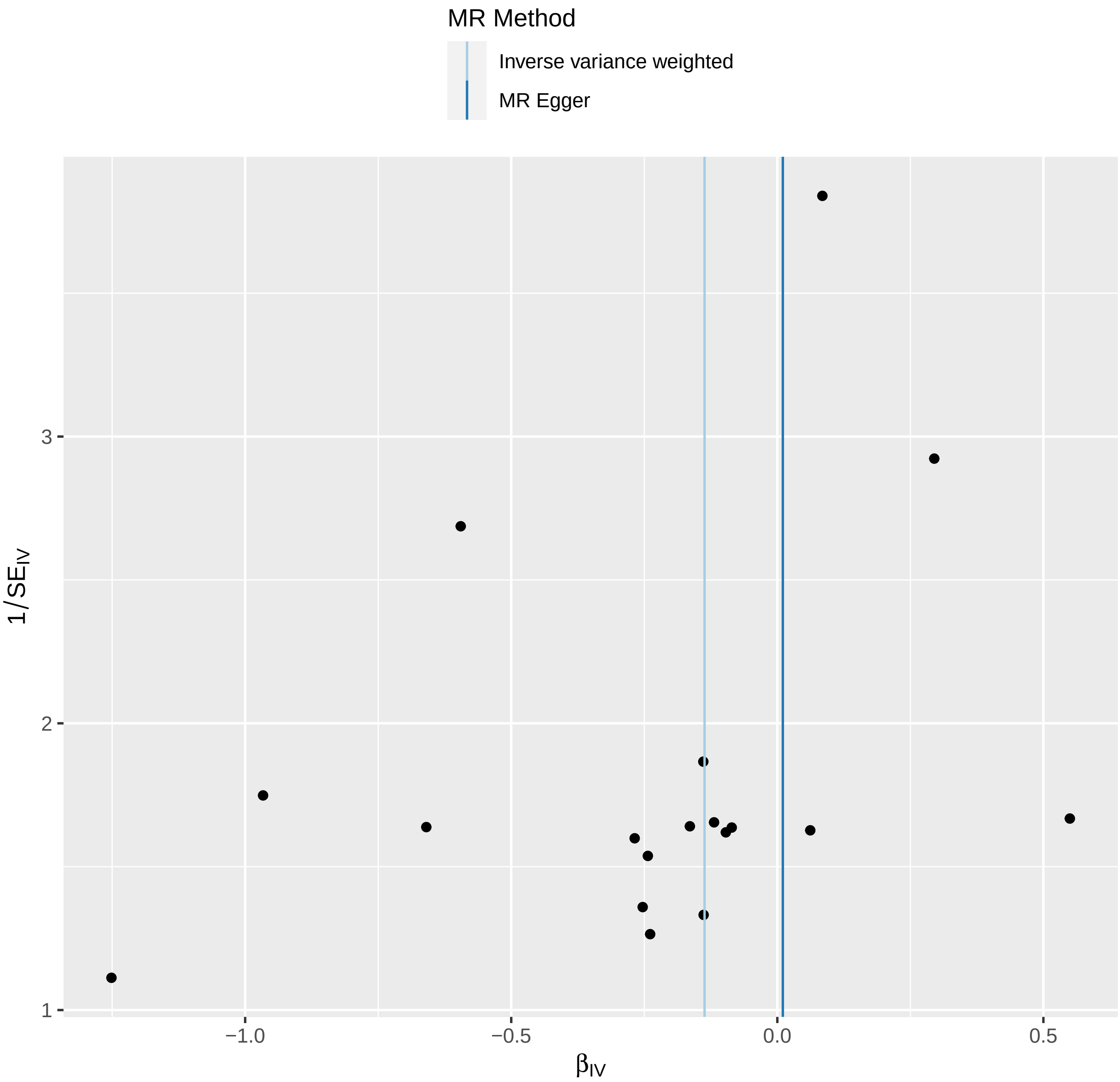

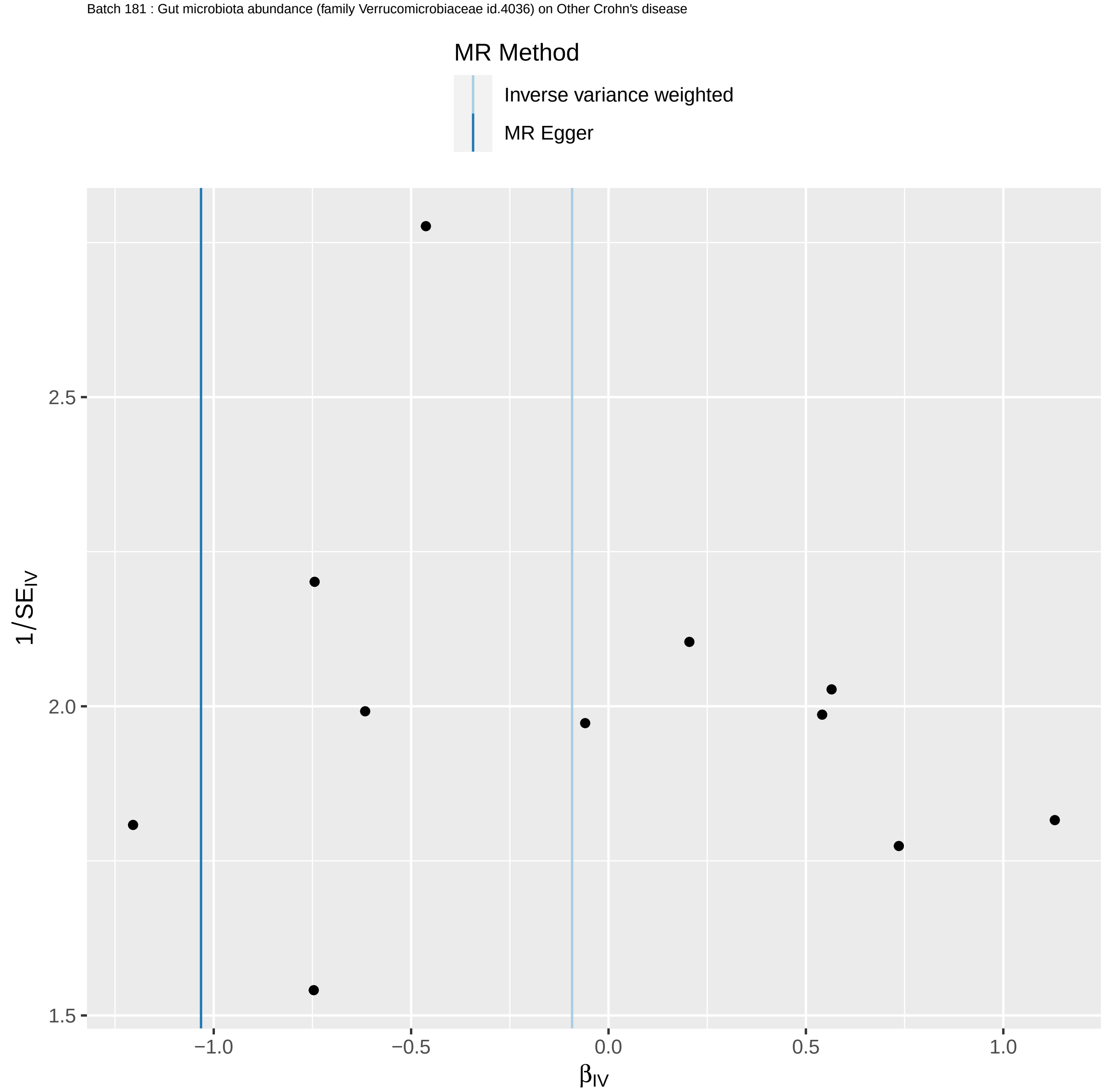
Batch 181 : Gut microbiota abundance (family Verrucomicrobiaceae id.4036) on Other Crohn's disease
MR Method
Inverse variance weighted
MR Egger
2.5
2.0
1.5
$1/SE_{IV}$
−1.0
−0.5
0.0
0.5
1.0
β_{IV}

Batch 182 : Gut microbiota abundance (family Victivallaceae id.2255) on Other Crohn's disease

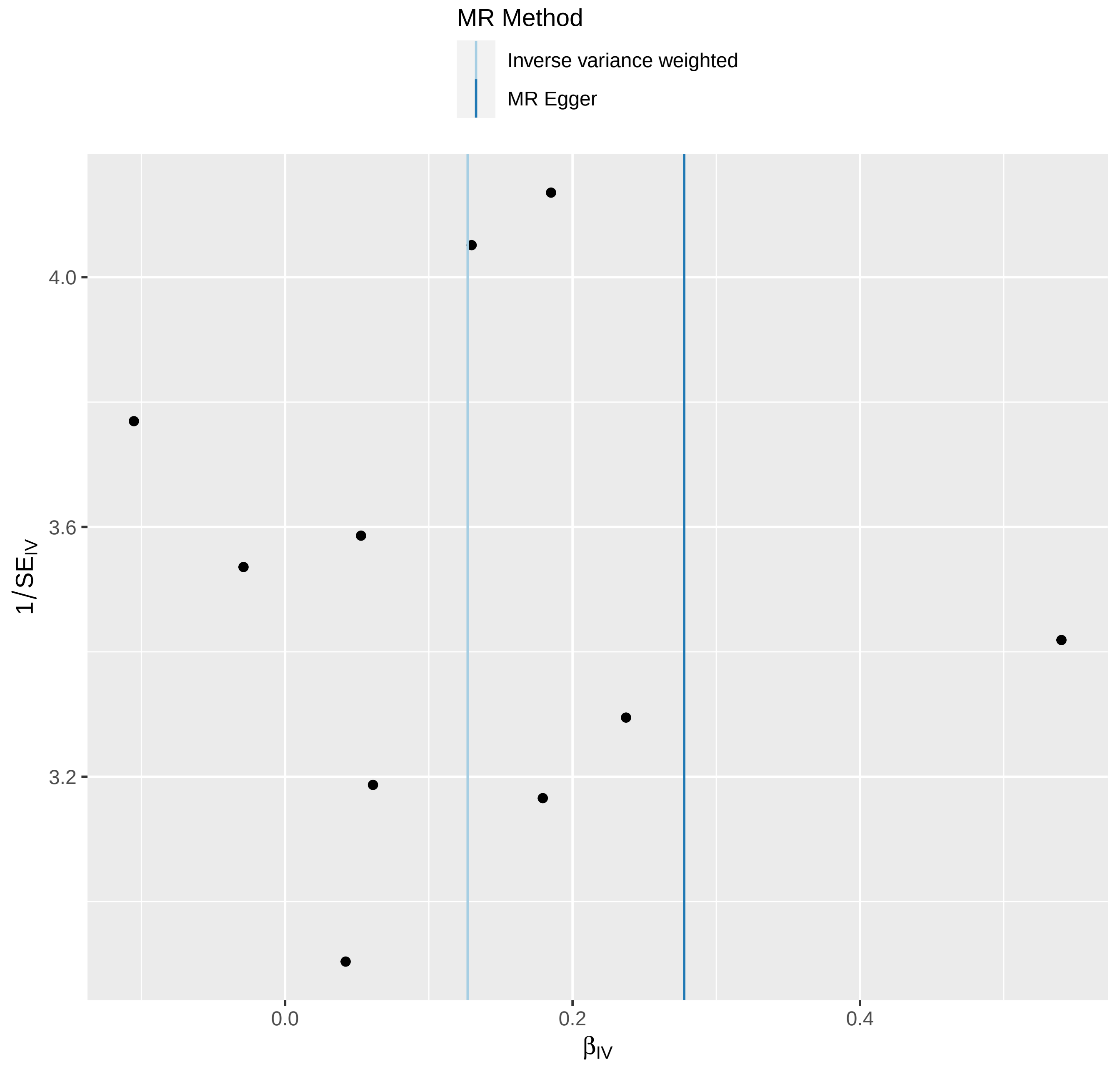

Batch 183 : Gut microbiota abundance (genus Actinomyces id.423) on Other Crohn's disease

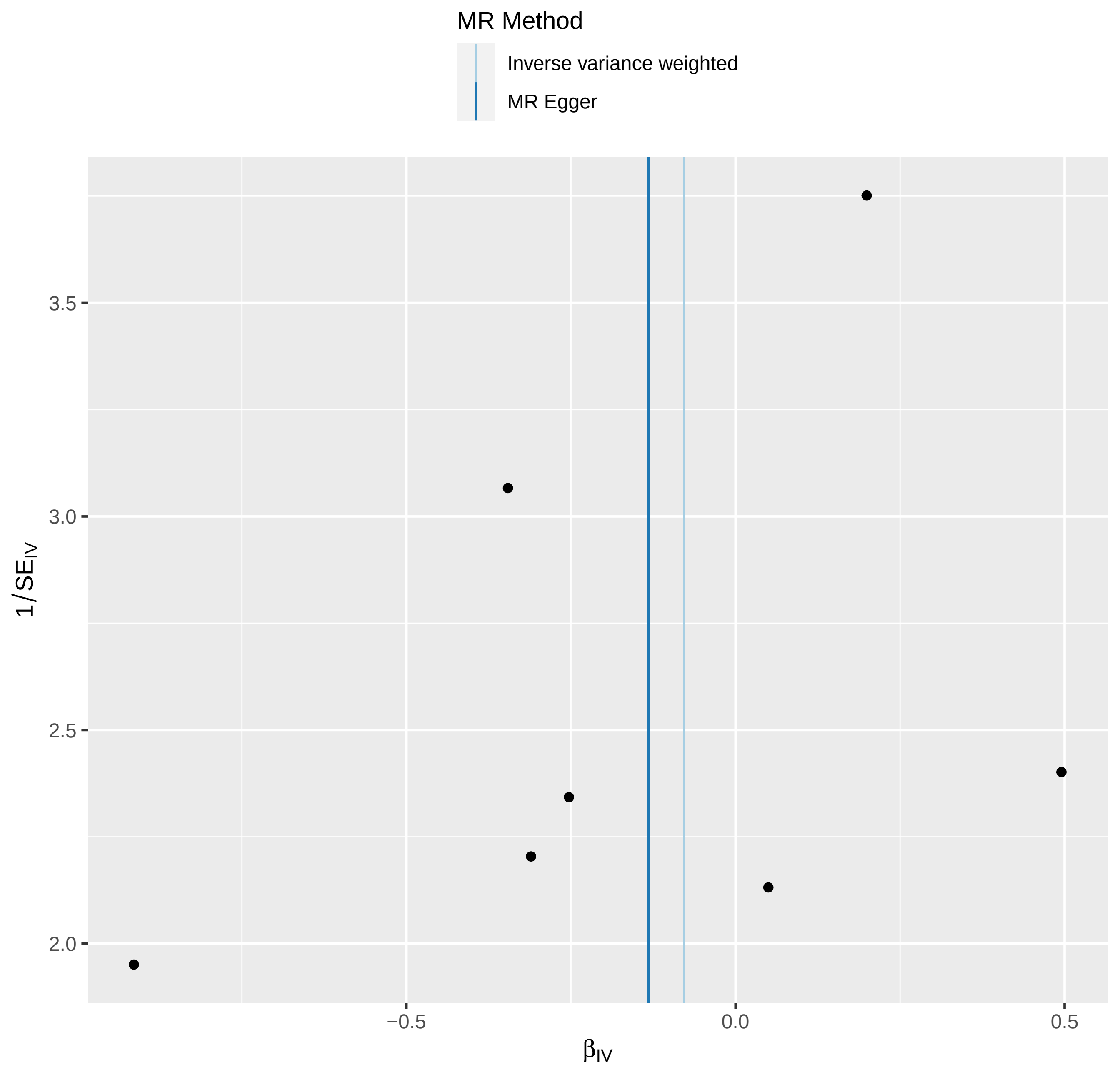

Batch 184 : Gut microbiota abundance (genus Adlercreutzia id.812) on Other Crohn's disease

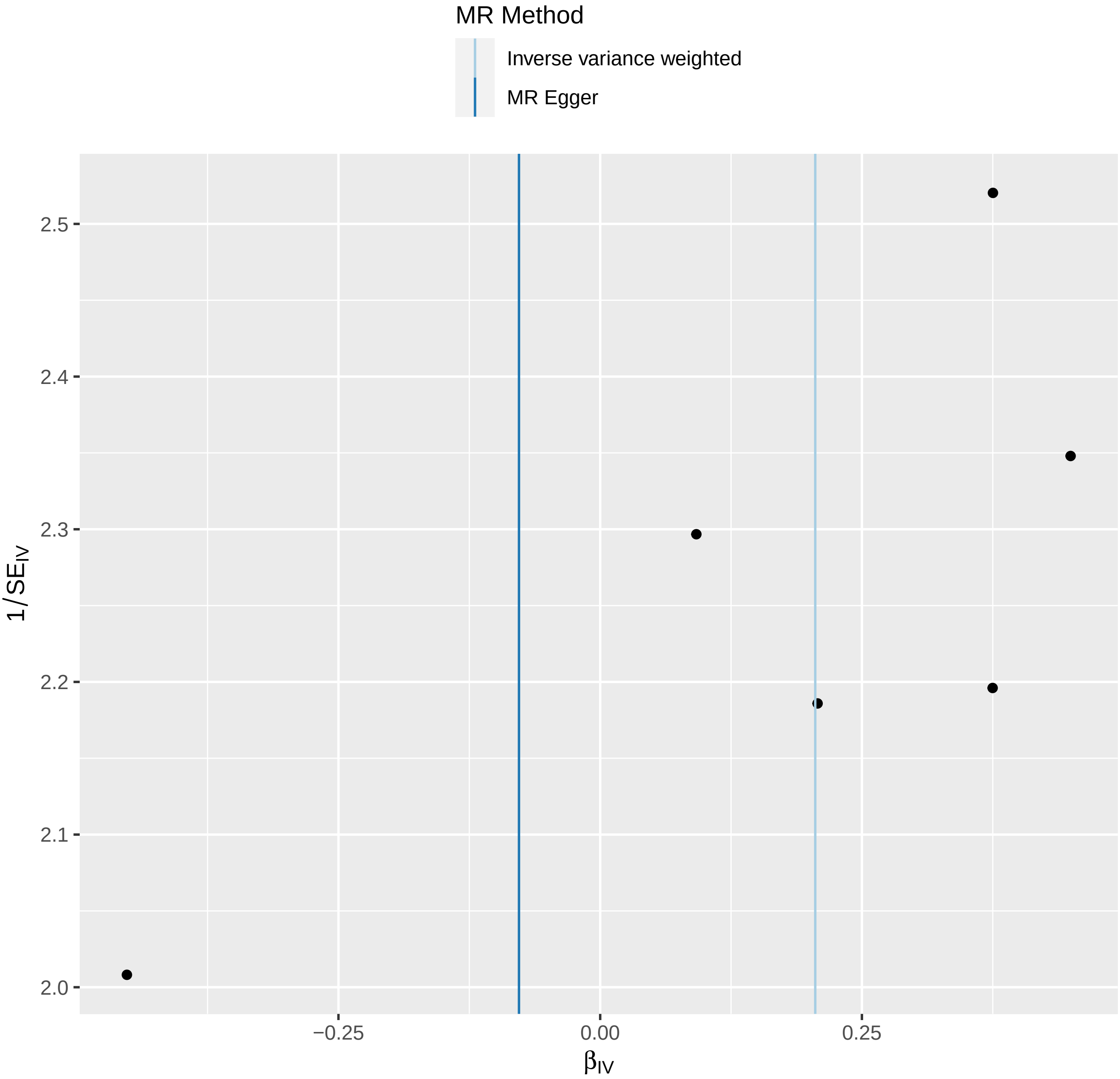

Batch 185 : Gut microbiota abundance (genus Akkermansia id.4037) on Other Crohn's disease

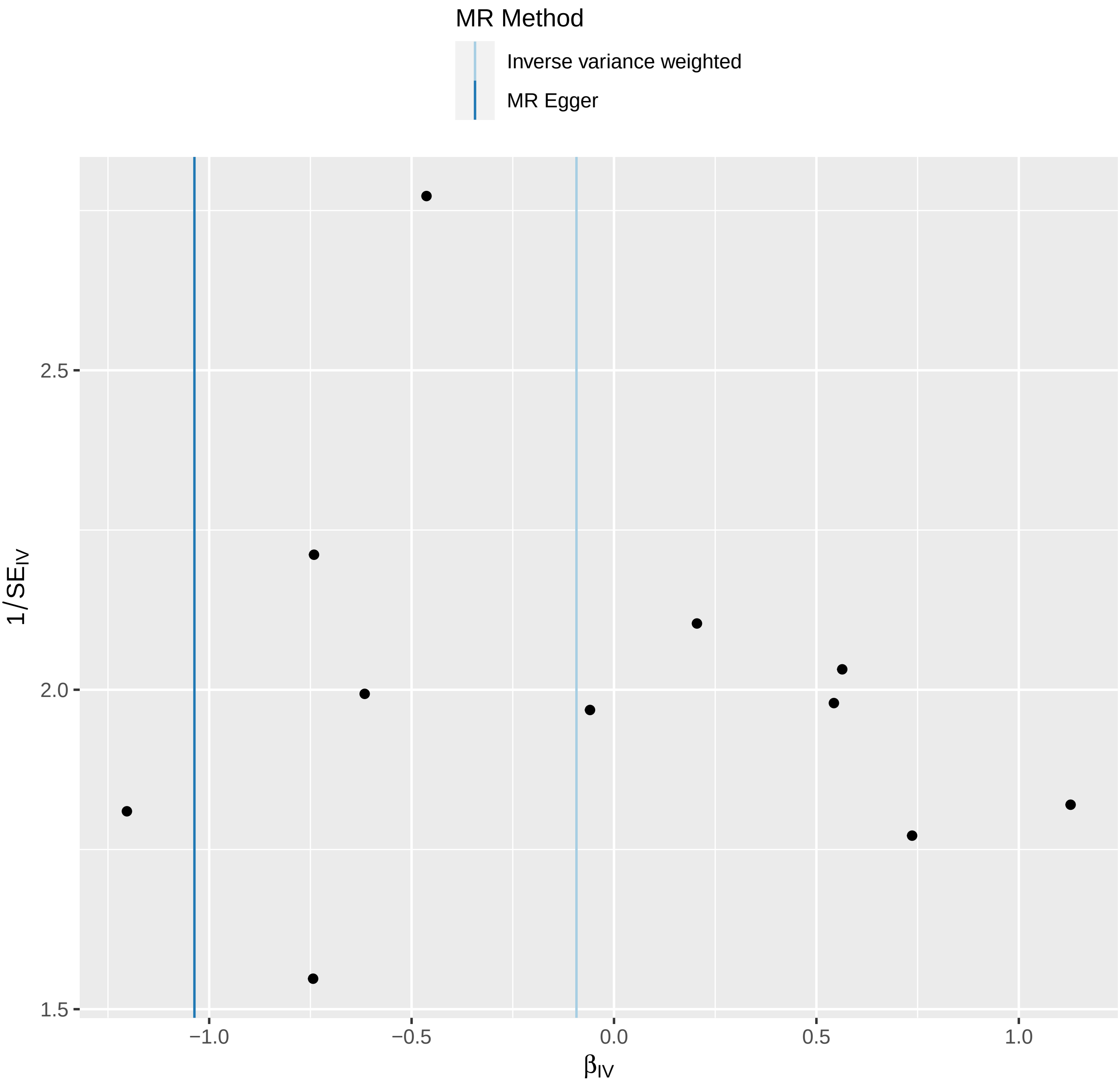

Batch 186 : Gut microbiota abundance (genus Alistipes id.968) on Other Crohn's disease

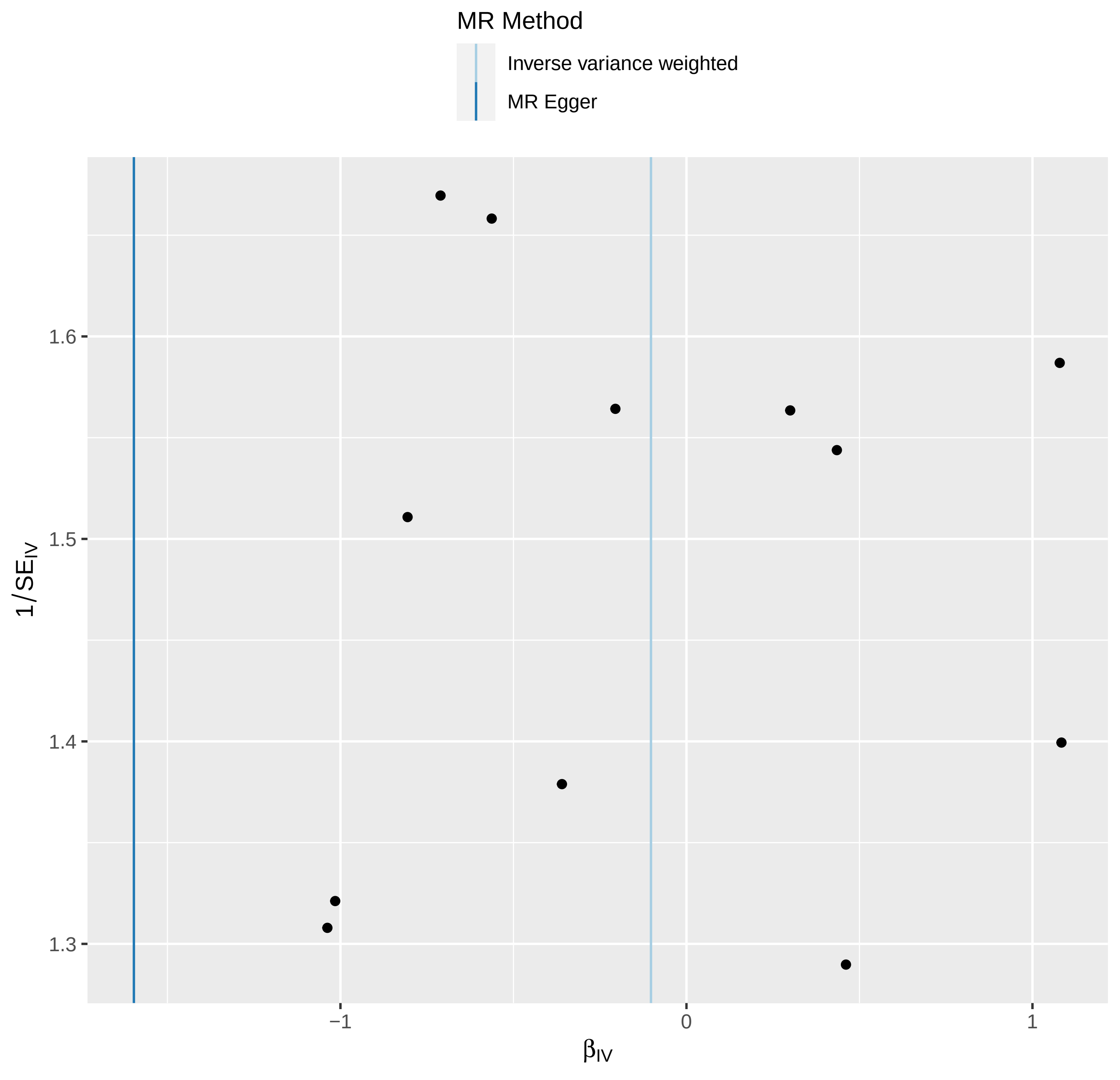

Batch 187 : Gut microbiota abundance (genus Allisonella id.2174) on Other Crohn's disease

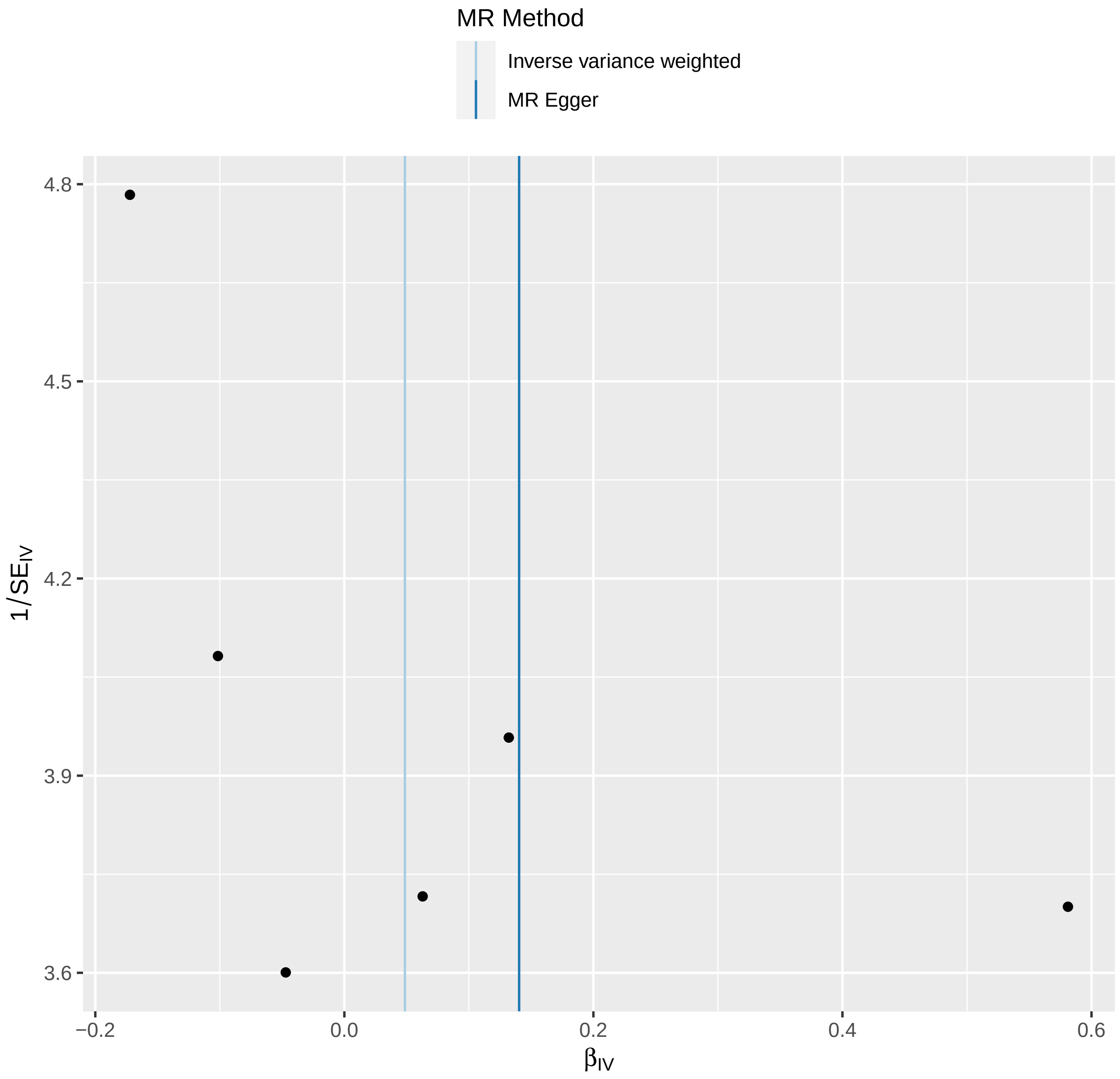

Batch 188 : Gut microbiota abundance (genus Alloprevotella id.961) on Other Crohn's disease

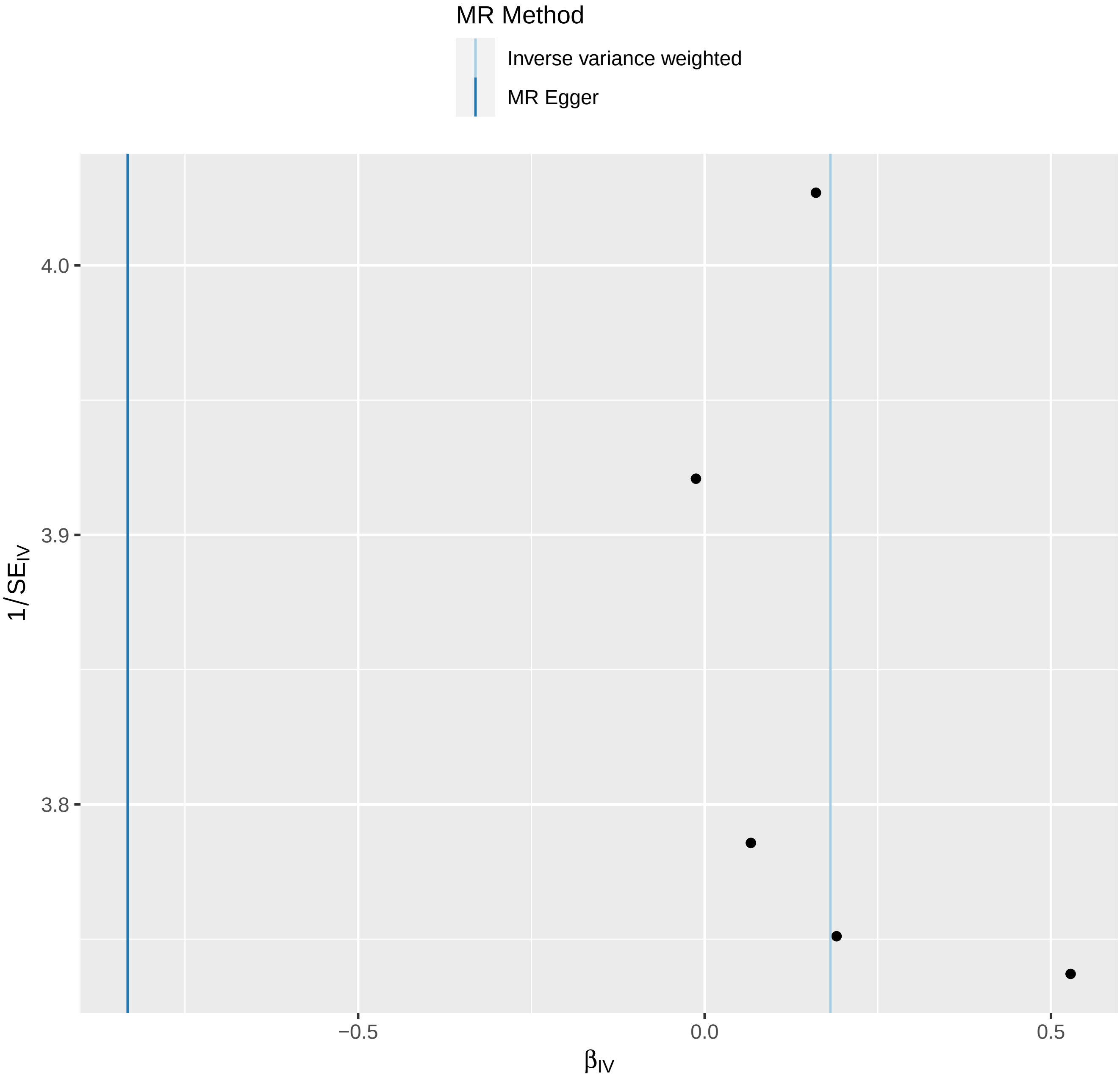

Batch 189 : Gut microbiota abundance (genus Anaerofilum id.2053) on Other Crohn's disease

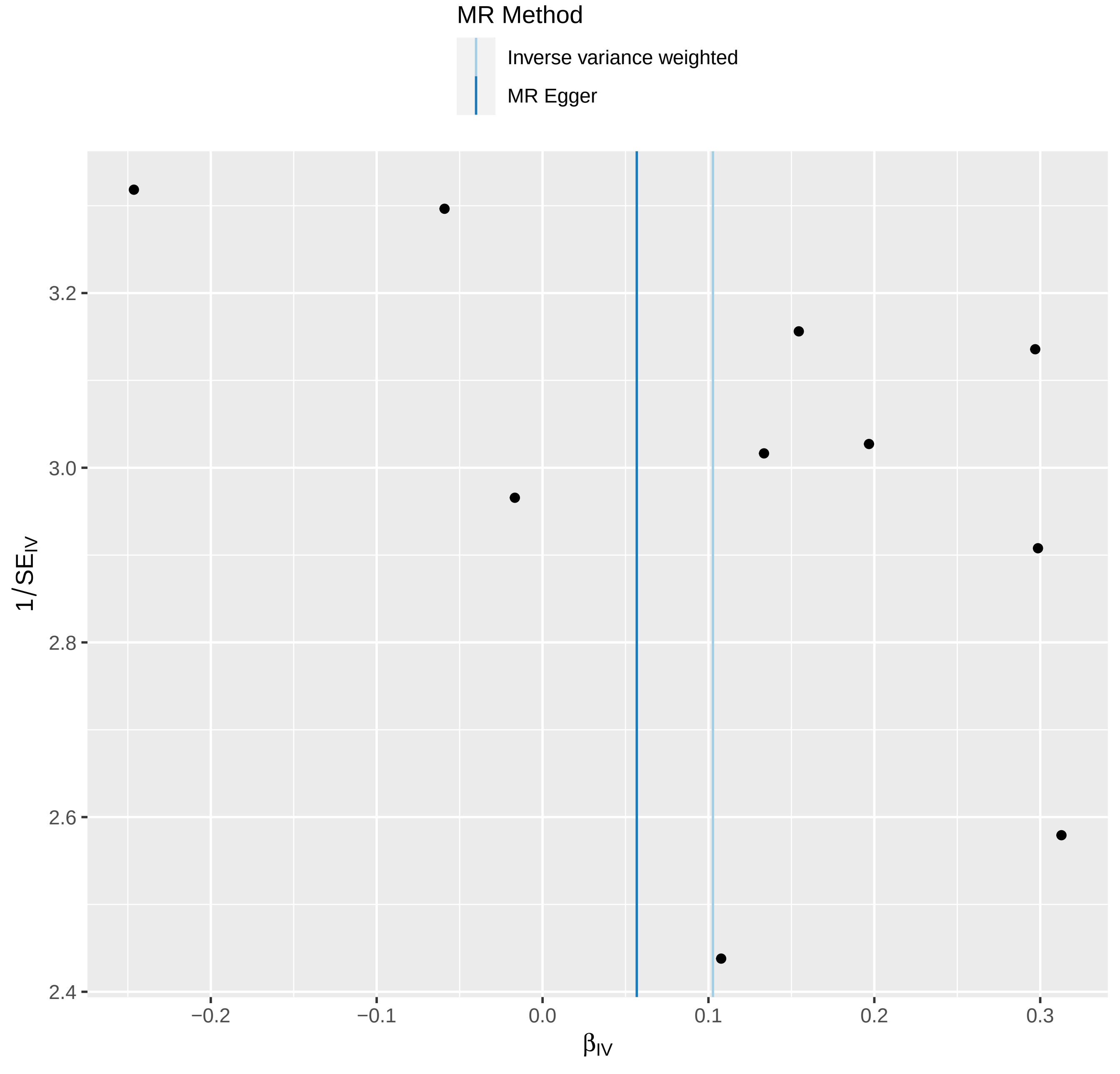

Batch 190 : Gut microbiota abundance (genus Anaerostipes id.1991) on Other Crohn's disease

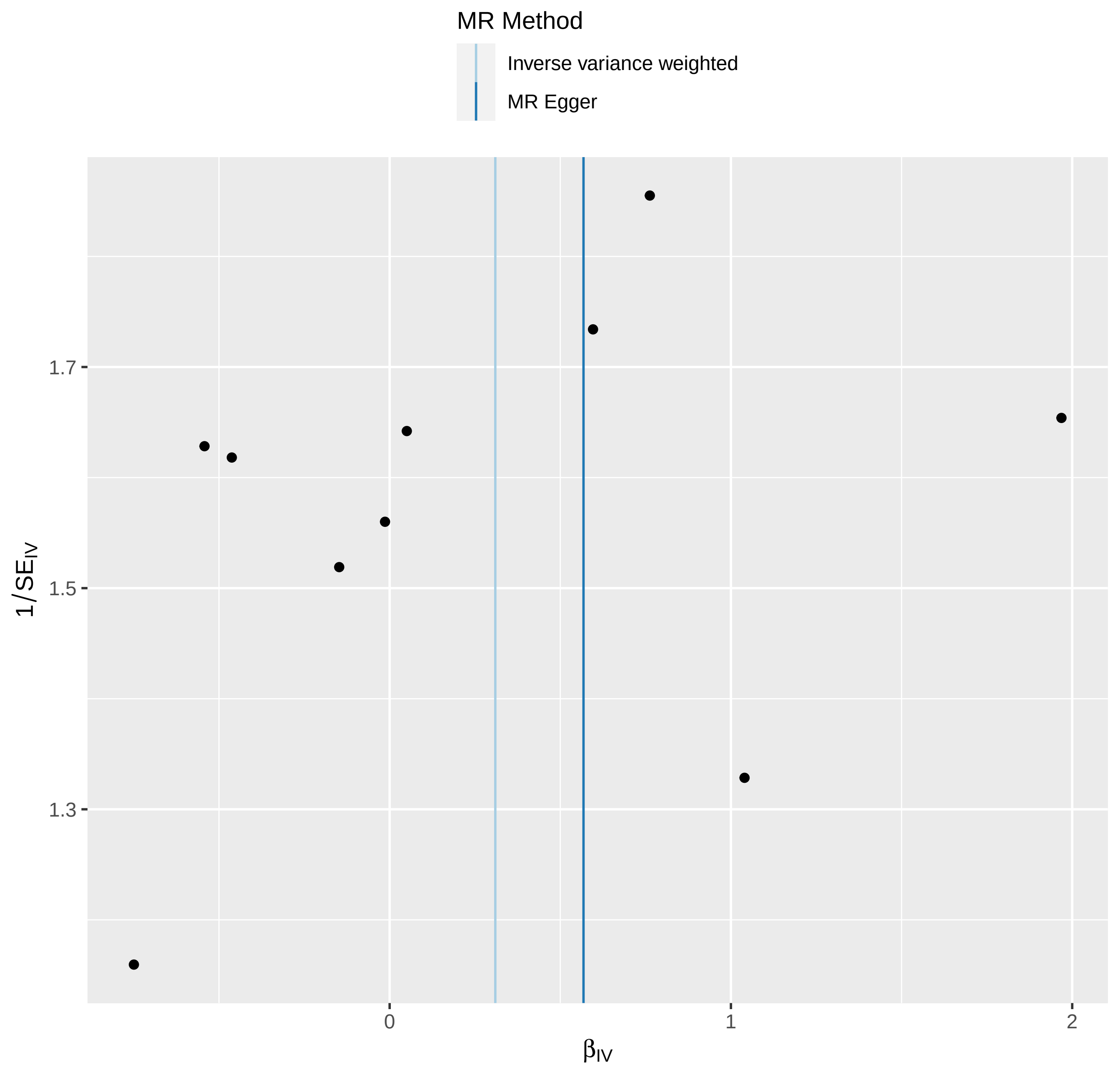

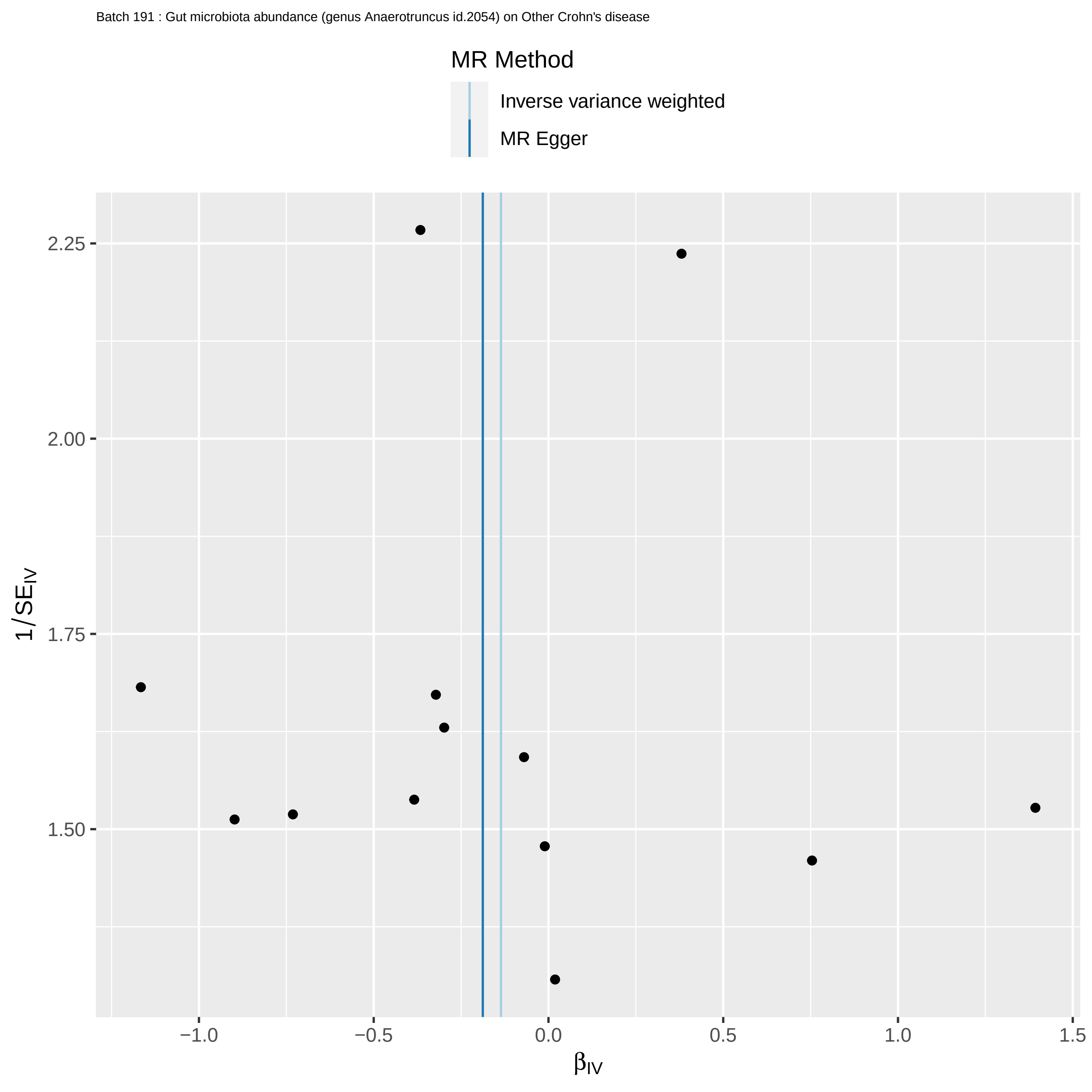
Batch 191 : Gut microbiota abundance (genus Anaerotruncus id.2054) on Other Crohn's disease
MR Method
Inverse variance weighted
MR Egger
2.25
2.00
1.75
1.50
1/SE_IV
−1.0
−0.5
0.0
0.5
1.0
1.5
β_IV

Batch 192 : Gut microbiota abundance (genus Bacteroides id.918) on Other Crohn's disease

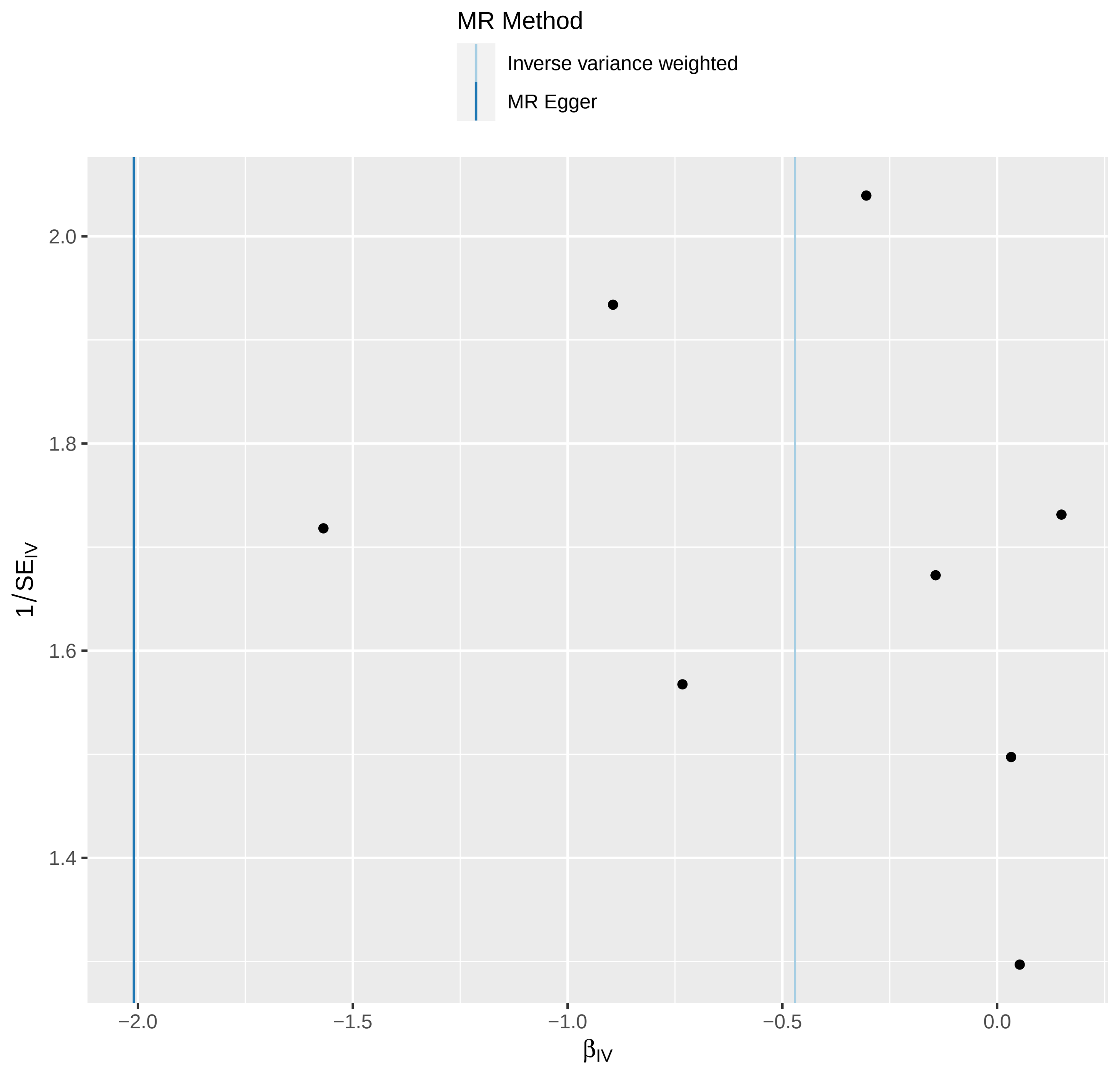

Batch 193 : Gut microbiota abundance (genus Barnesiella id.944) on Other Crohn's disease

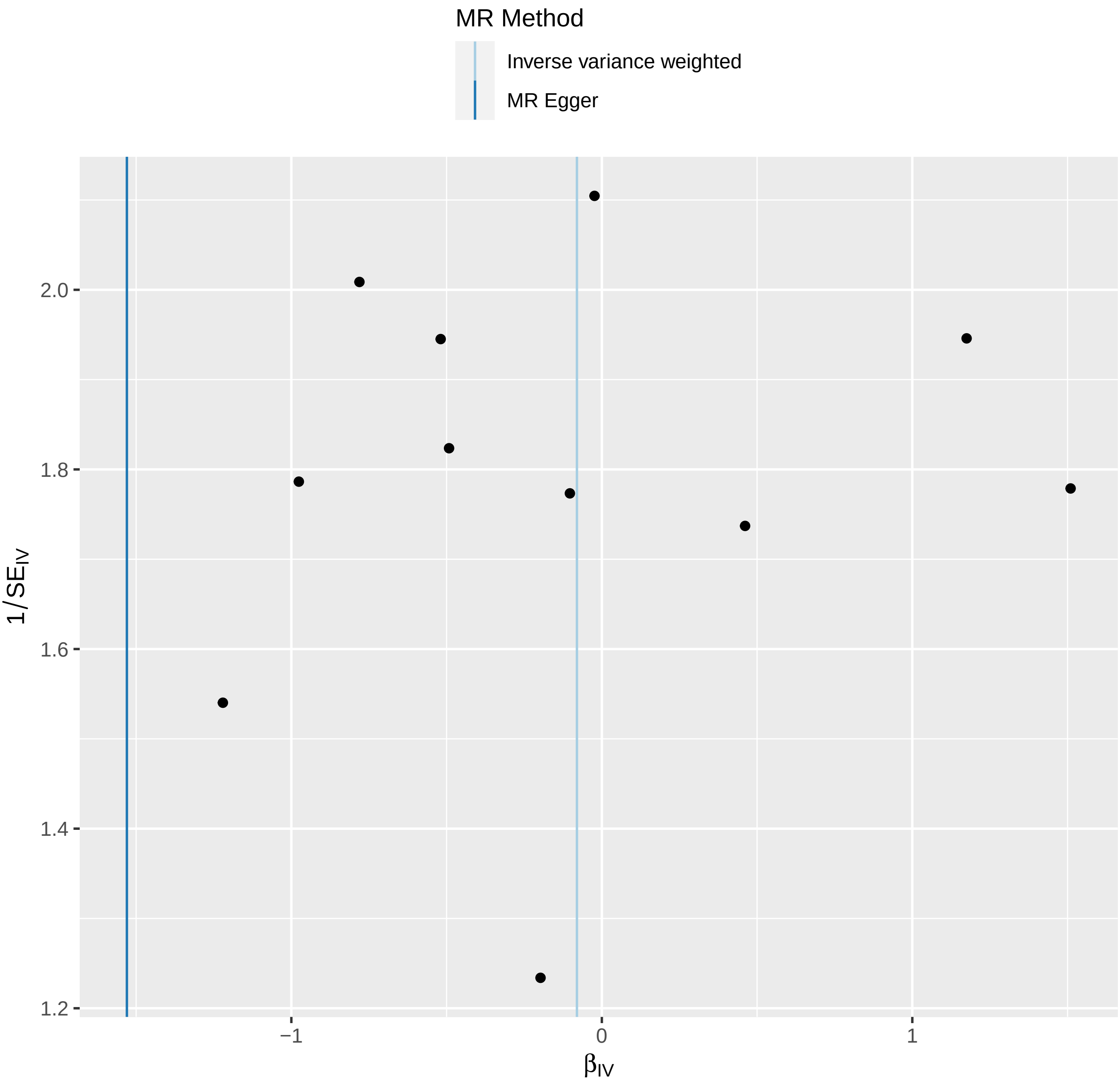

Batch 194 : Gut microbiota abundance (genus Bifidobacterium id.436) on Other Crohn's disease

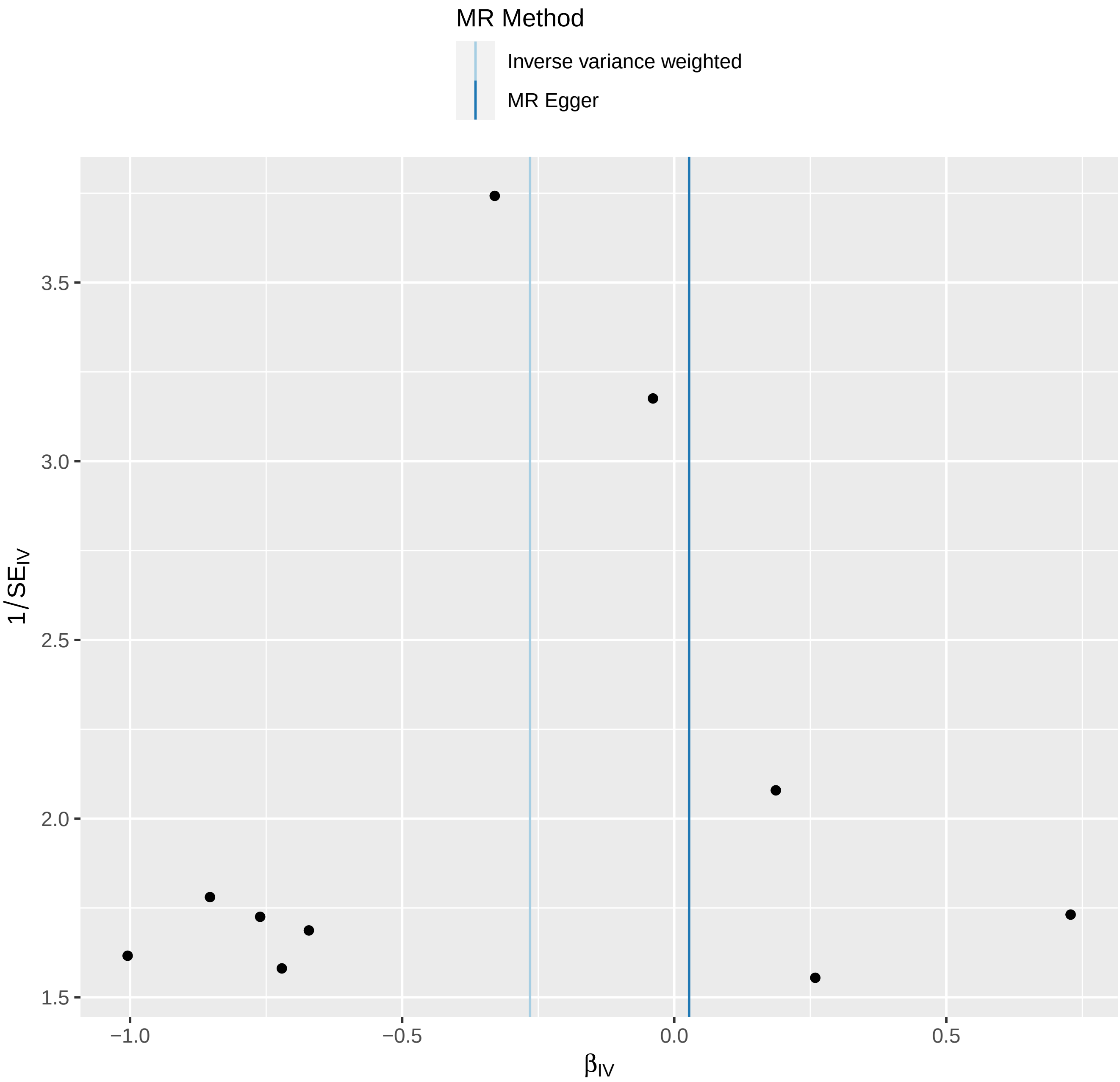

Batch 195 : Gut microbiota abundance (genus Bilophila id.3170) on Other Crohn's disease

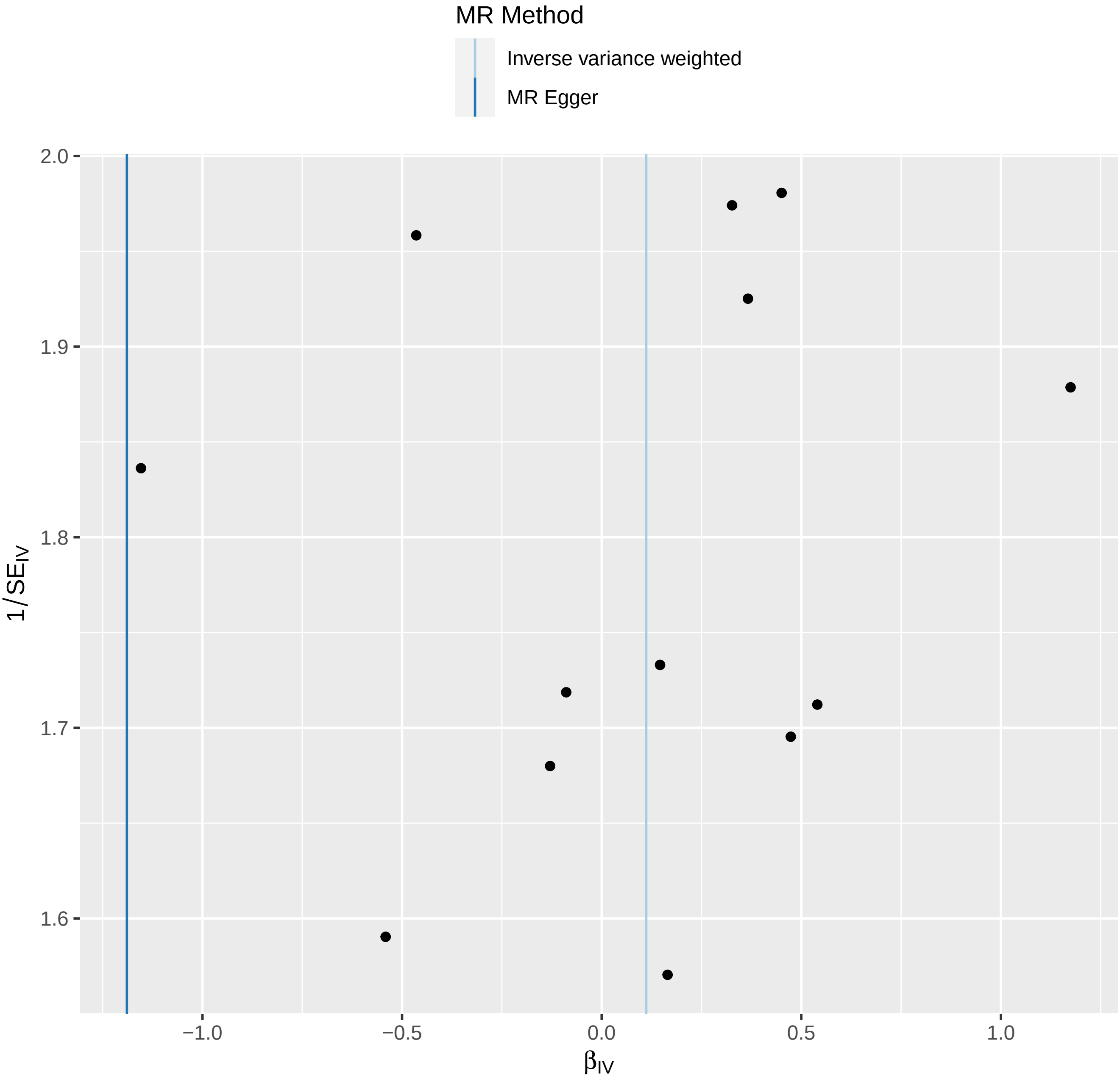

Batch 196 : Gut microbiota abundance (genus Blautia id.1992) on Other Crohn's disease

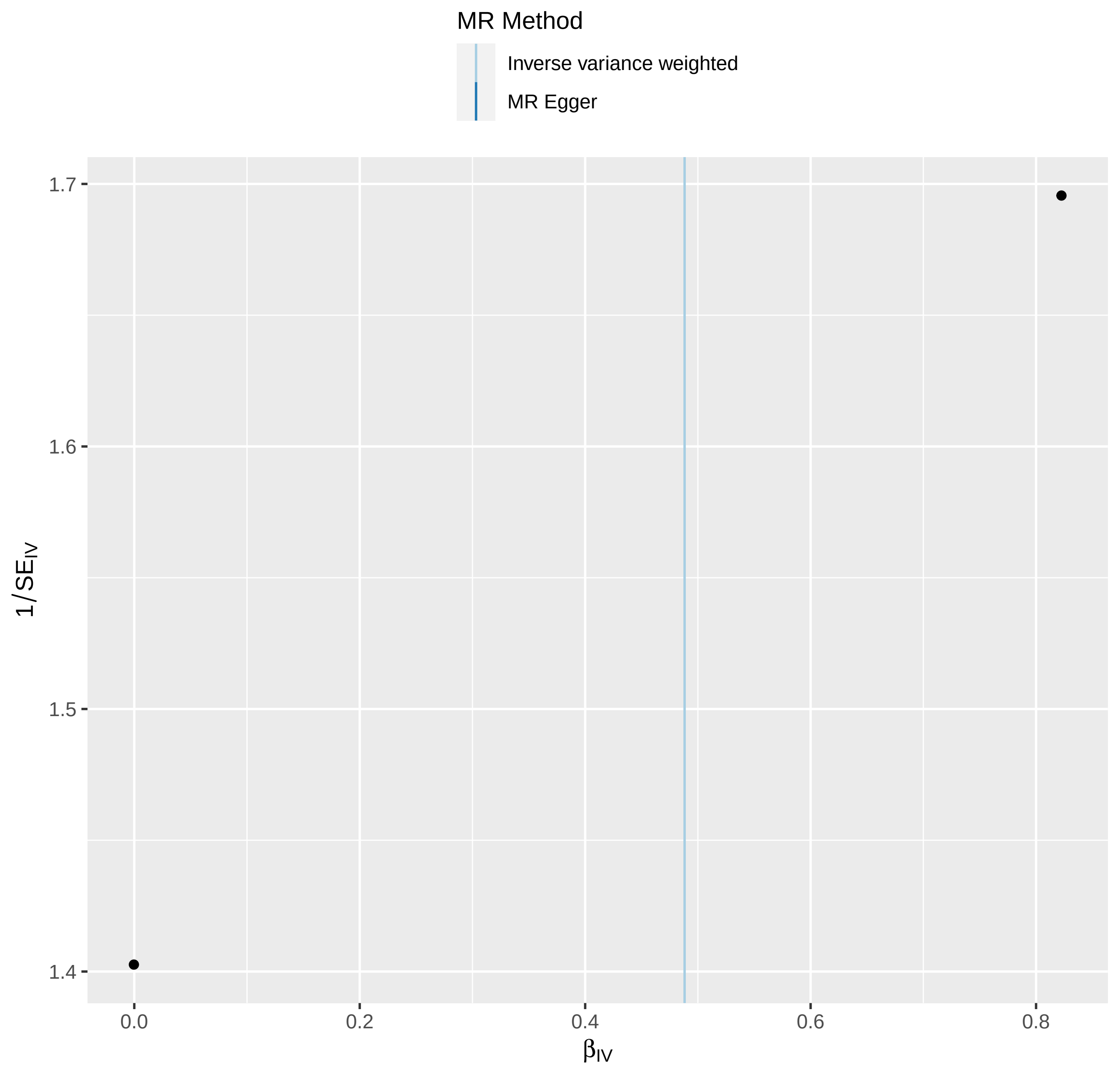

Batch 197 : Gut microbiota abundance (genus Butyricicoccus id.2055) on Other Crohn's disease

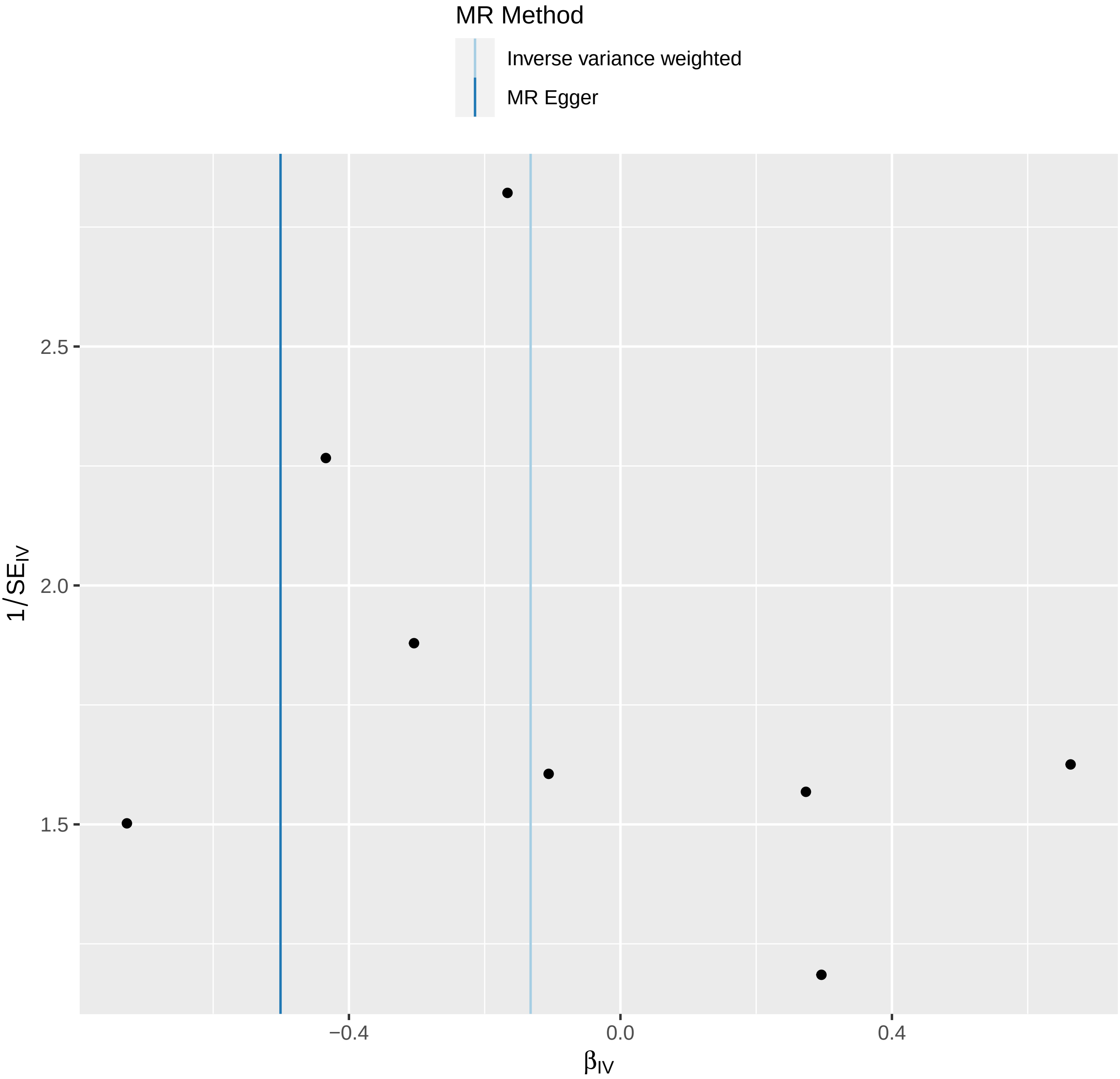

Batch 198 : Gut microbiota abundance (genus Butyricimonas id.945) on Other Crohn's disease

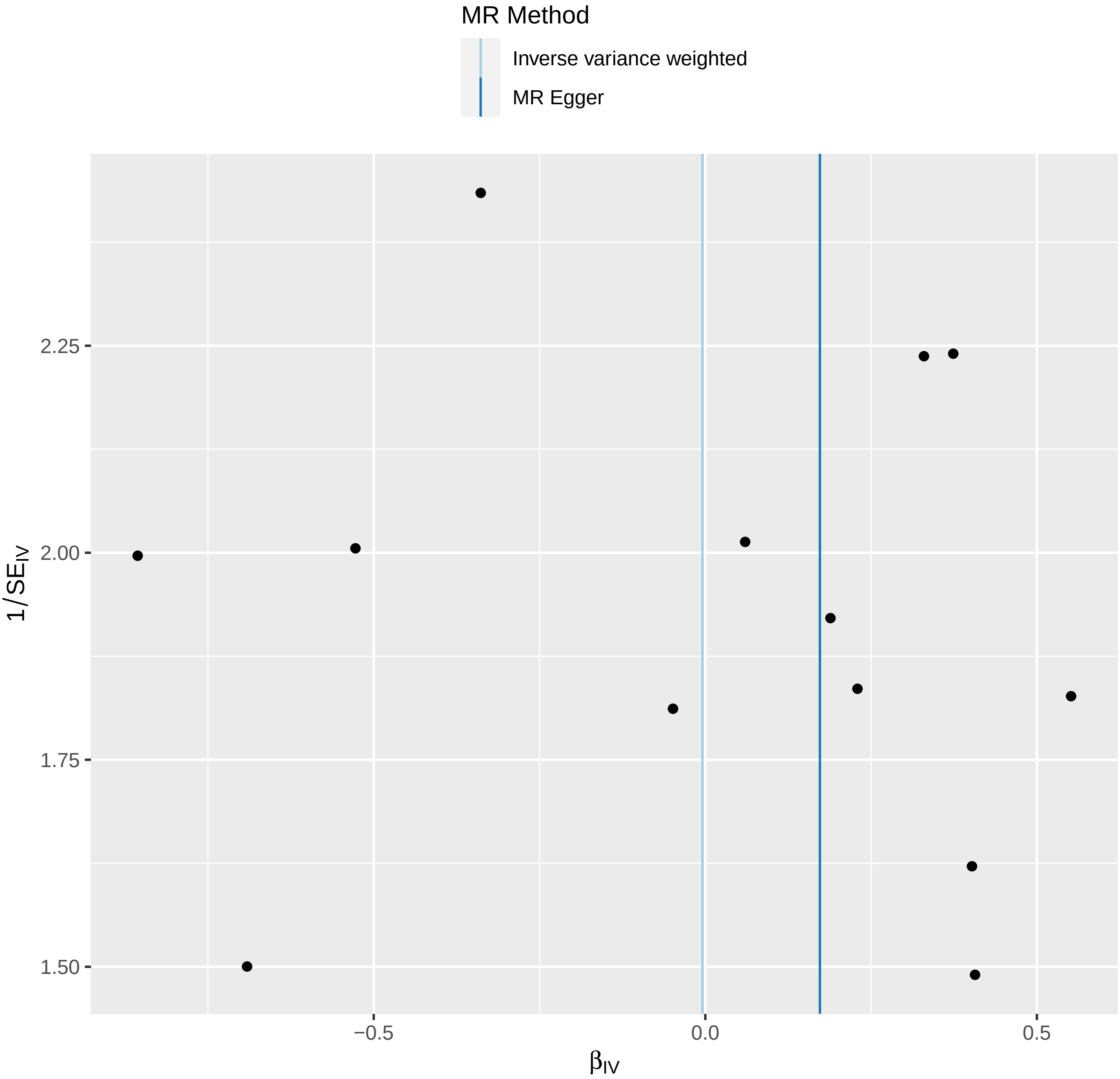

Batch 199 : Gut microbiota abundance (genus Butyrivibrio id.1993) on Other Crohn's disease

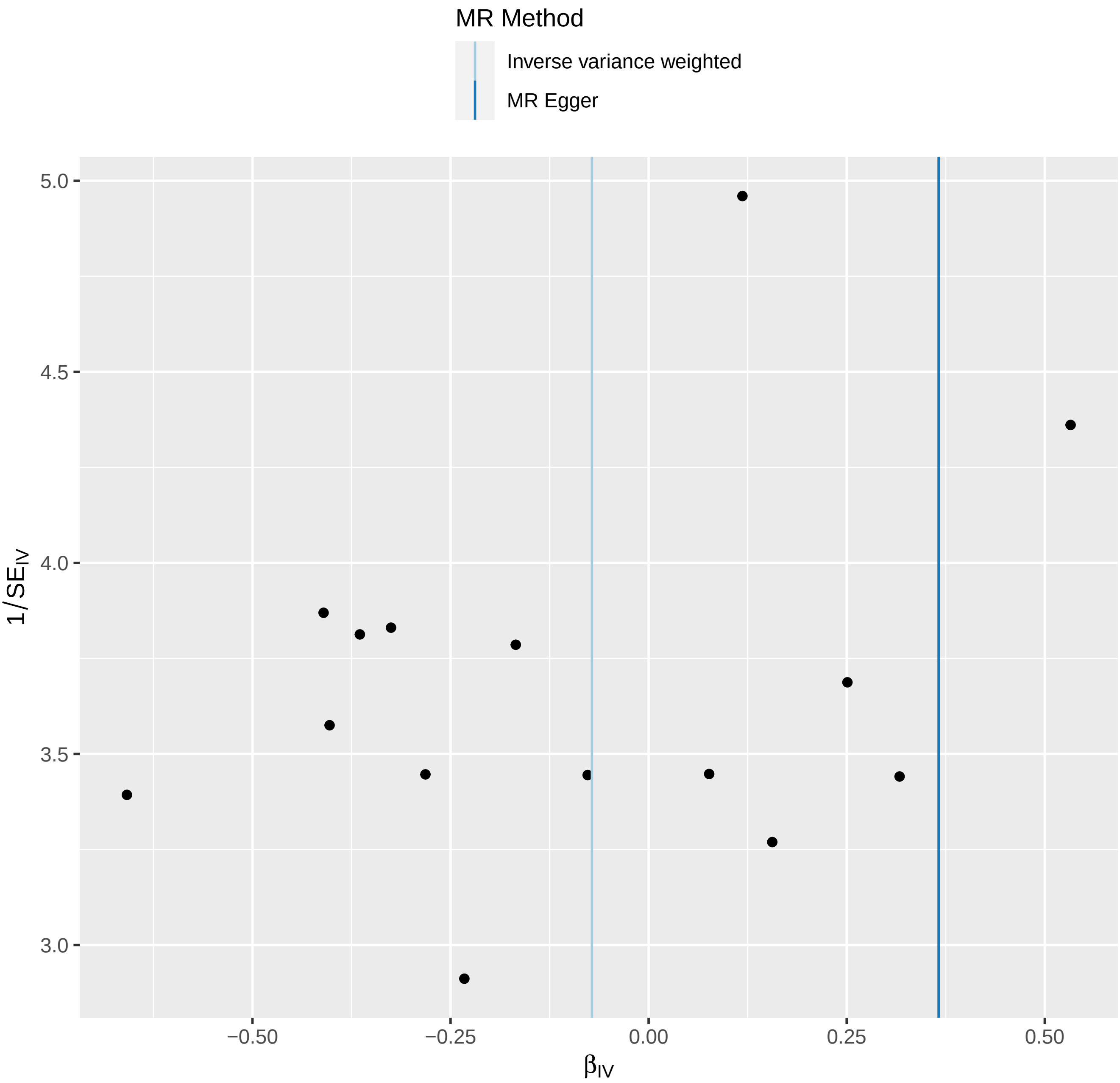

Batch 200 : Gut microbiota abundance (genus Candidatus Soleaferrea id.11350) on Other Crohn's disease

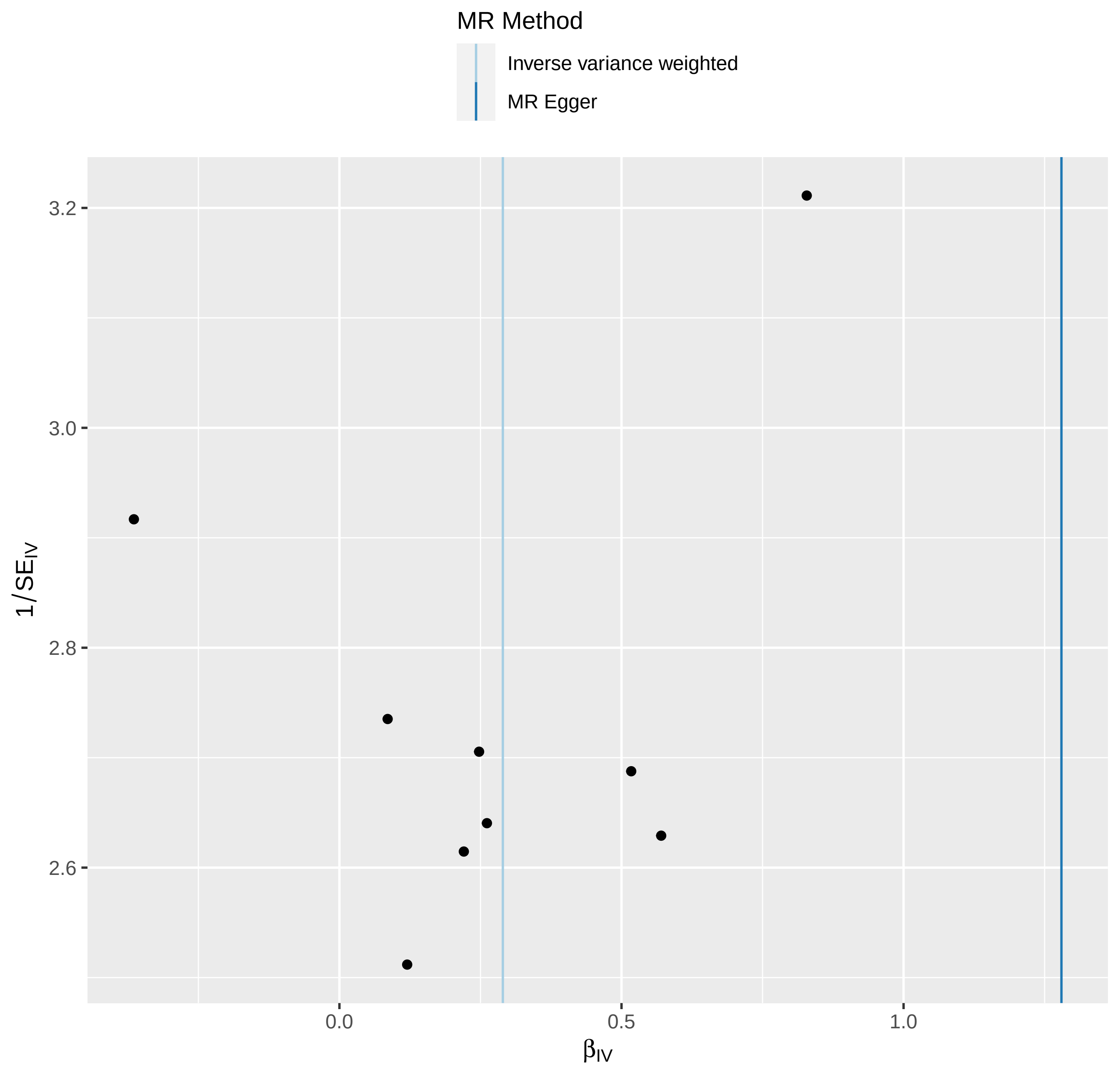

Batch 201 : Gut microbiota abundance (genus Catenibacterium id.2153) on Other Crohn's disease

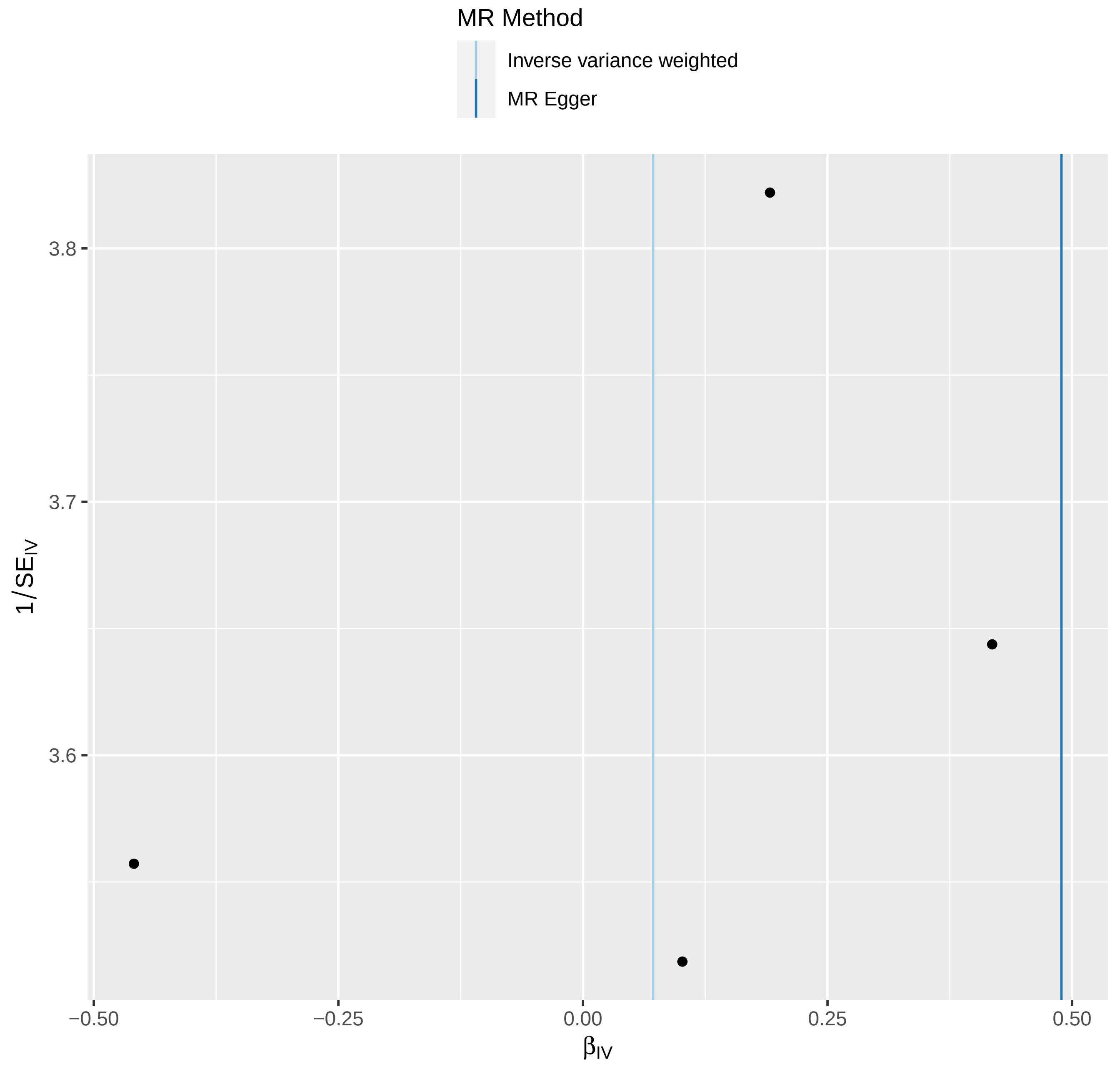

Batch 202 : Gut microbiota abundance (genus Christensenellaceae R 7group id.11283) on Other Crohn's disease

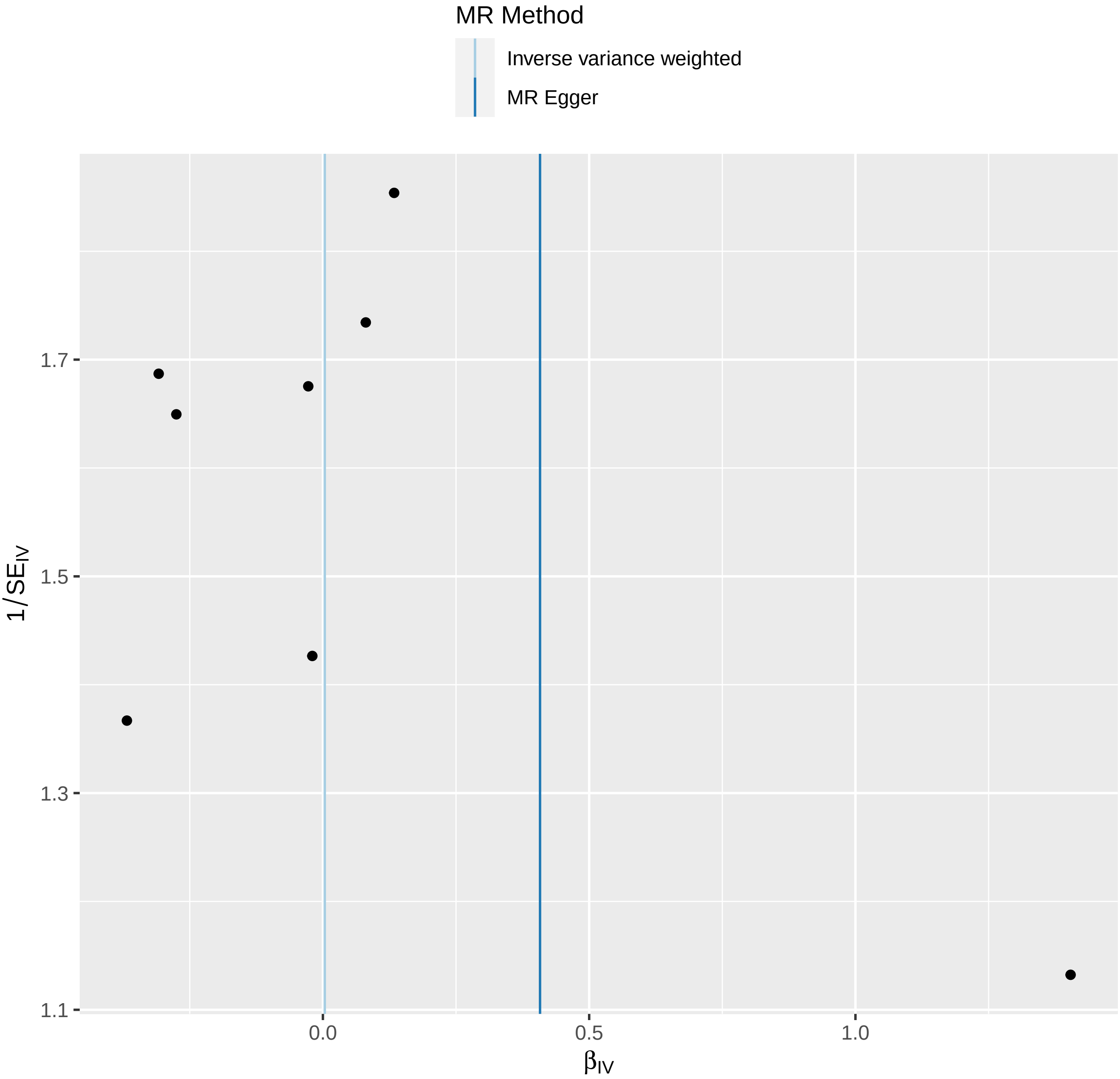

Batch 203 : Gut microbiota abundance (genus Clostridium innocuum group id.14397) on Other Crohn's disease

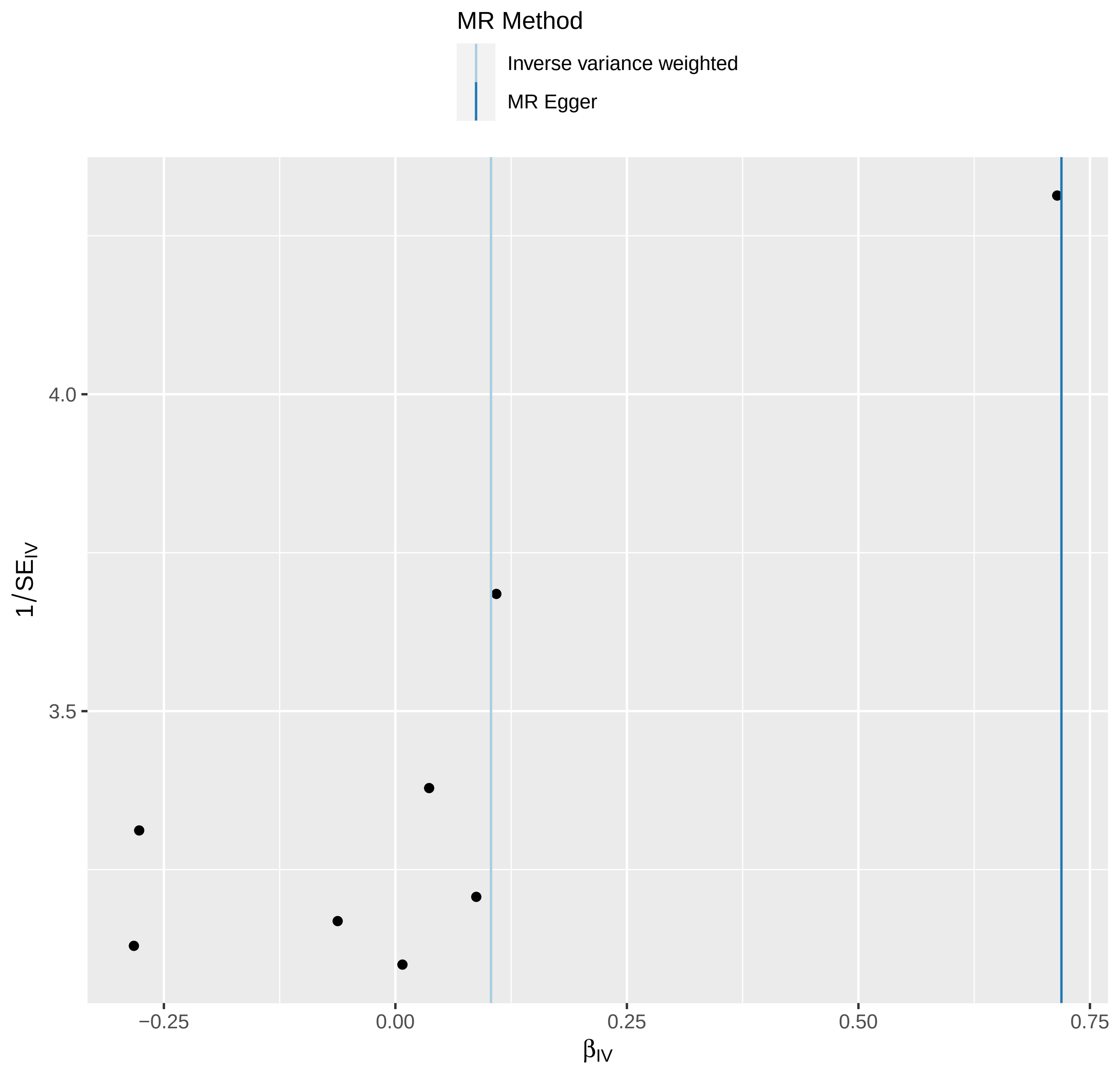

Batch 204 : Gut microbiota abundance (genus Clostridium sensustricto1 id.1873) on Other Crohn's disease

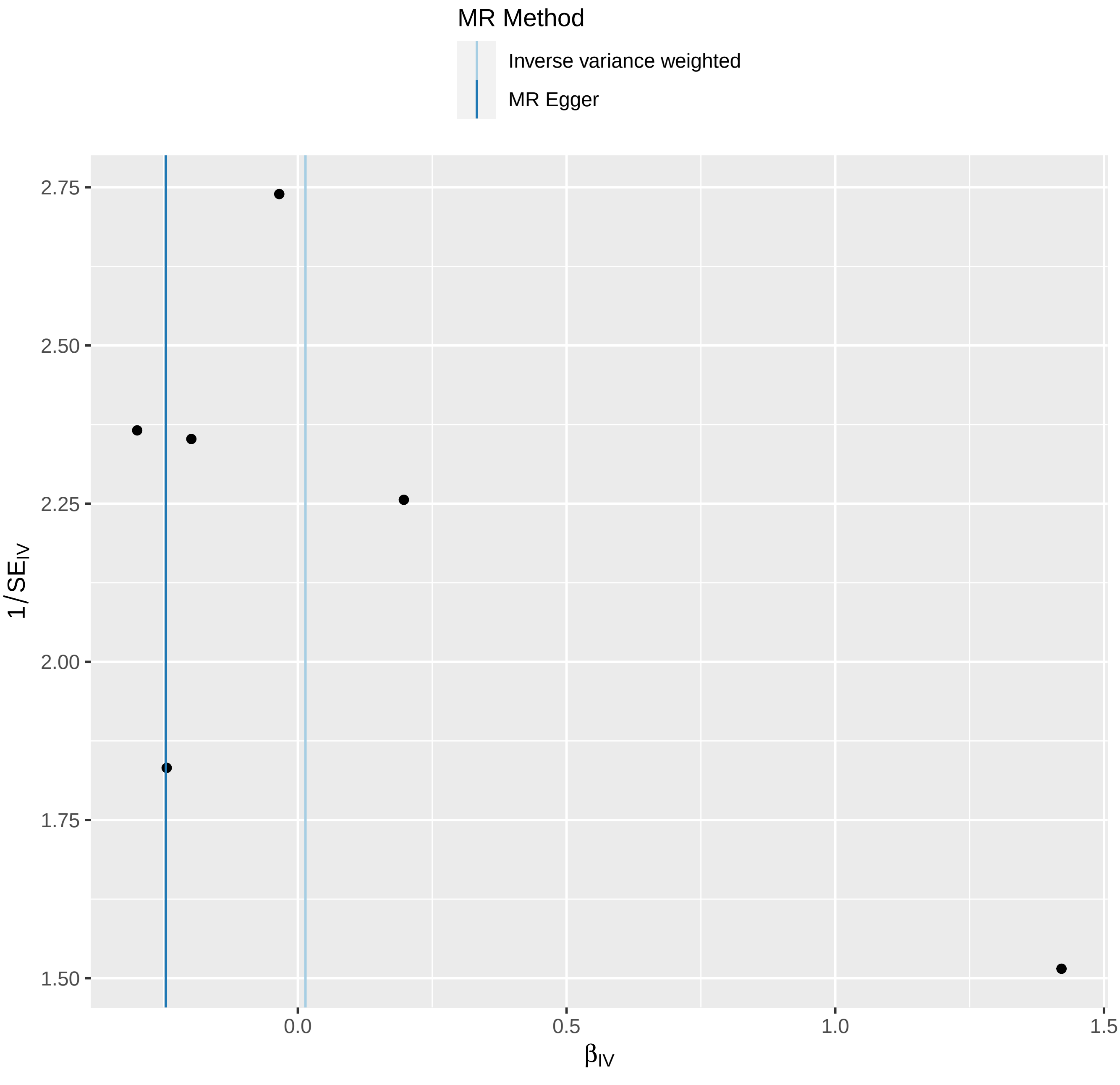

Batch 205 : Gut microbiota abundance (genus Collinsella id.815) on Other Crohn's disease

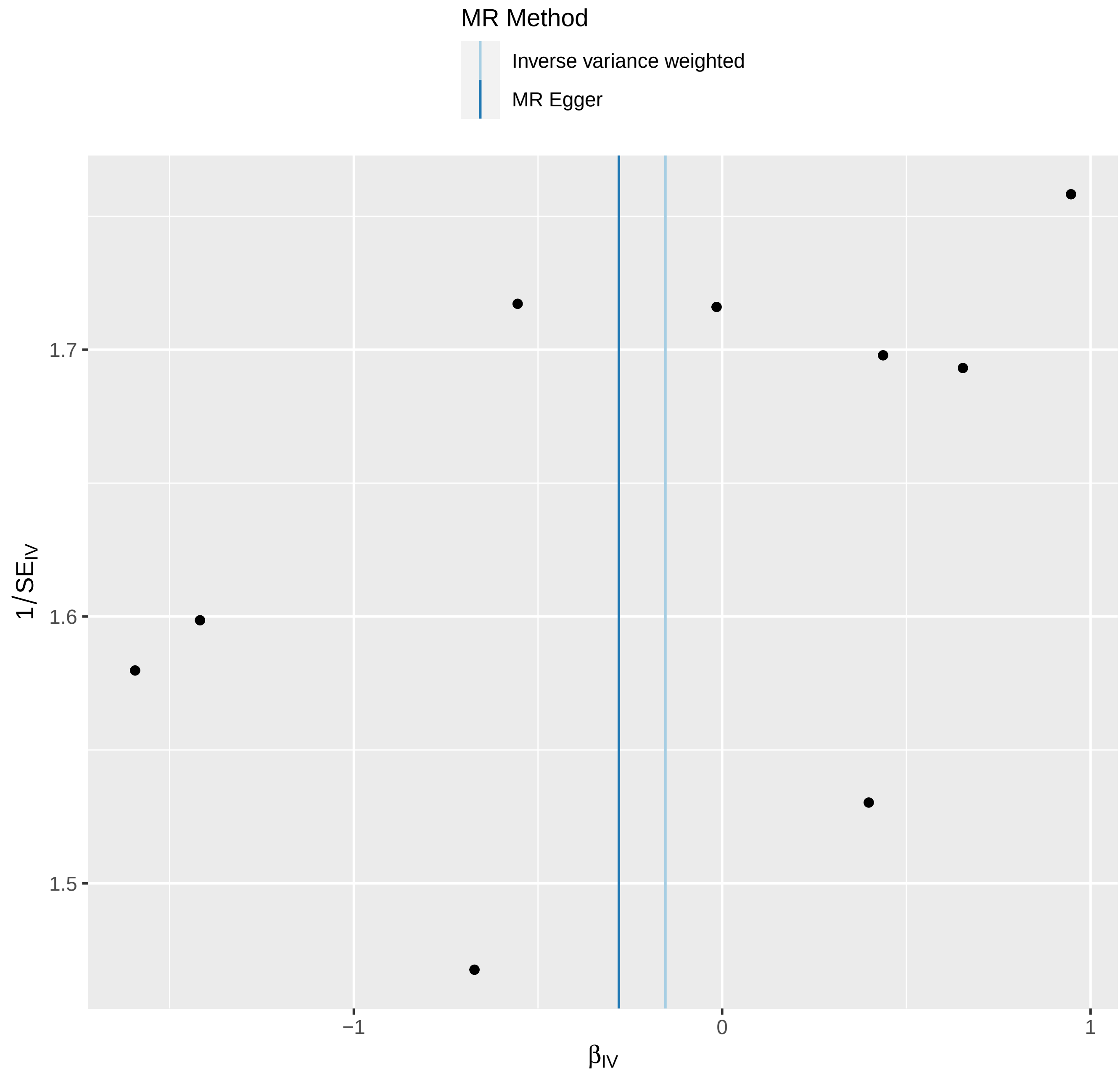

Batch 206 : Gut microbiota abundance (genus Coprobacter id.949) on Other Crohn's disease

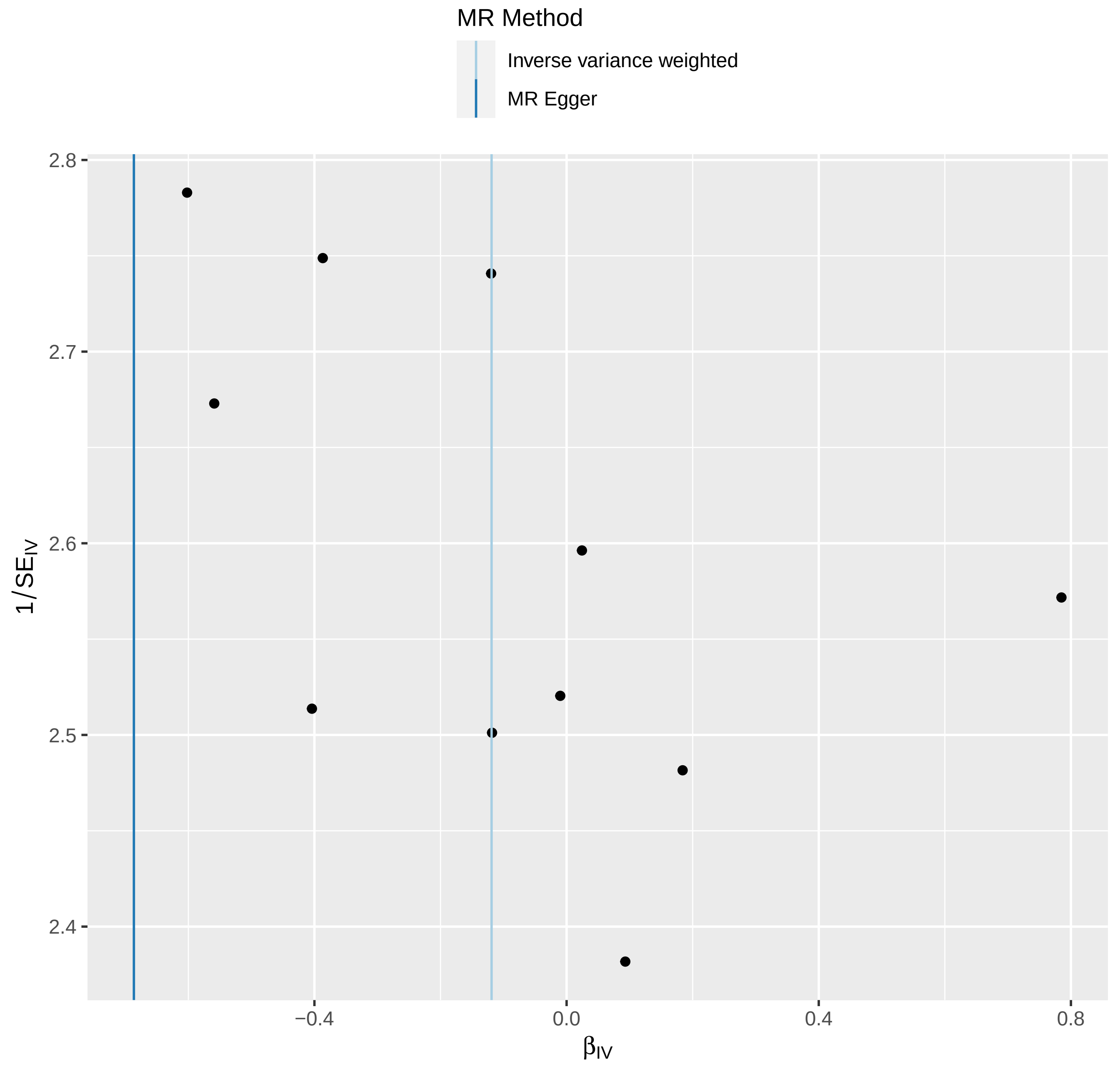

Batch 207 : Gut microbiota abundance (genus Coprococcus1 id.11301) on Other Crohn's disease

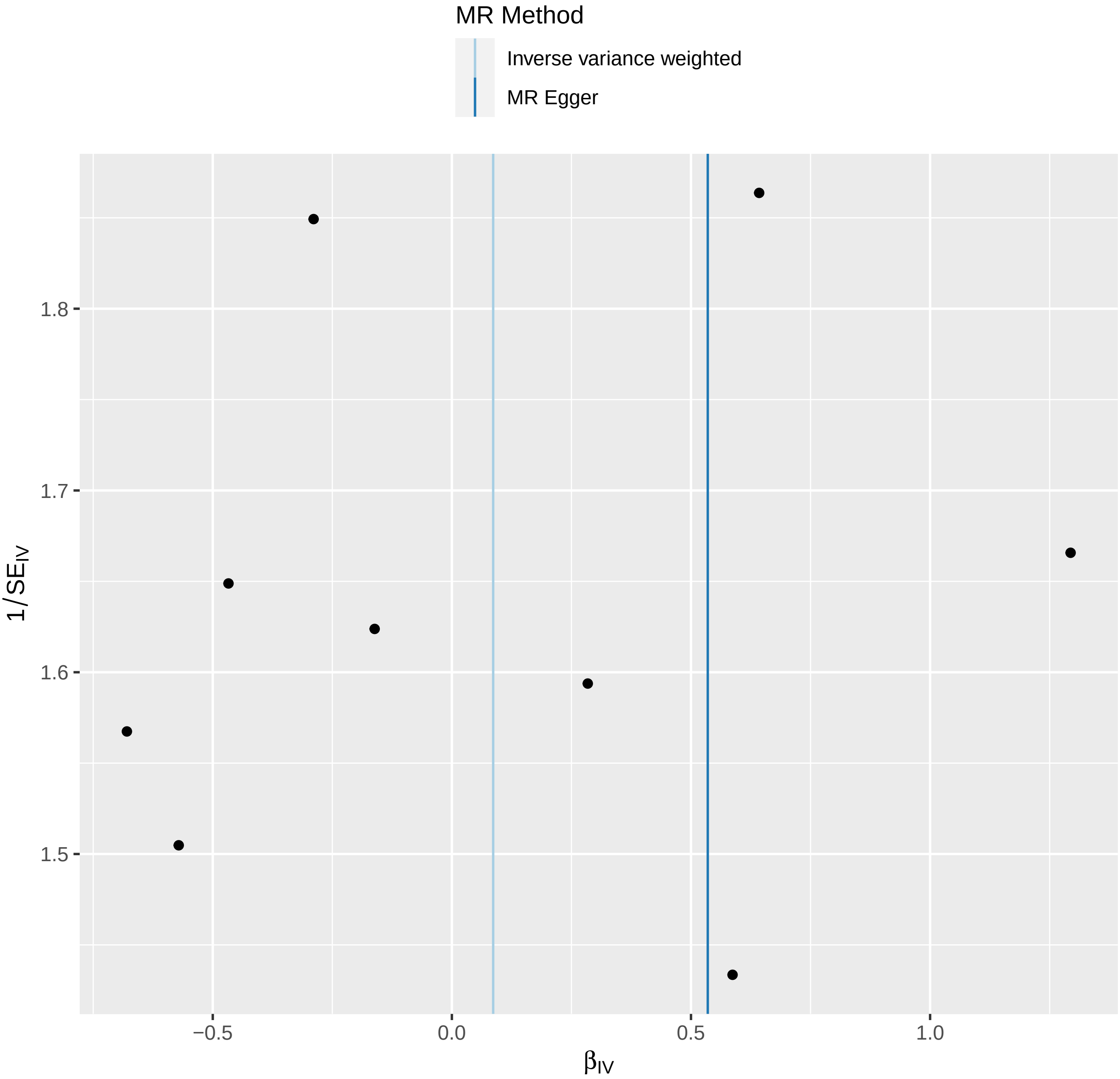

Batch 208 : Gut microbiota abundance (genus Coprococcus2 id.11302) on Other Crohn's disease

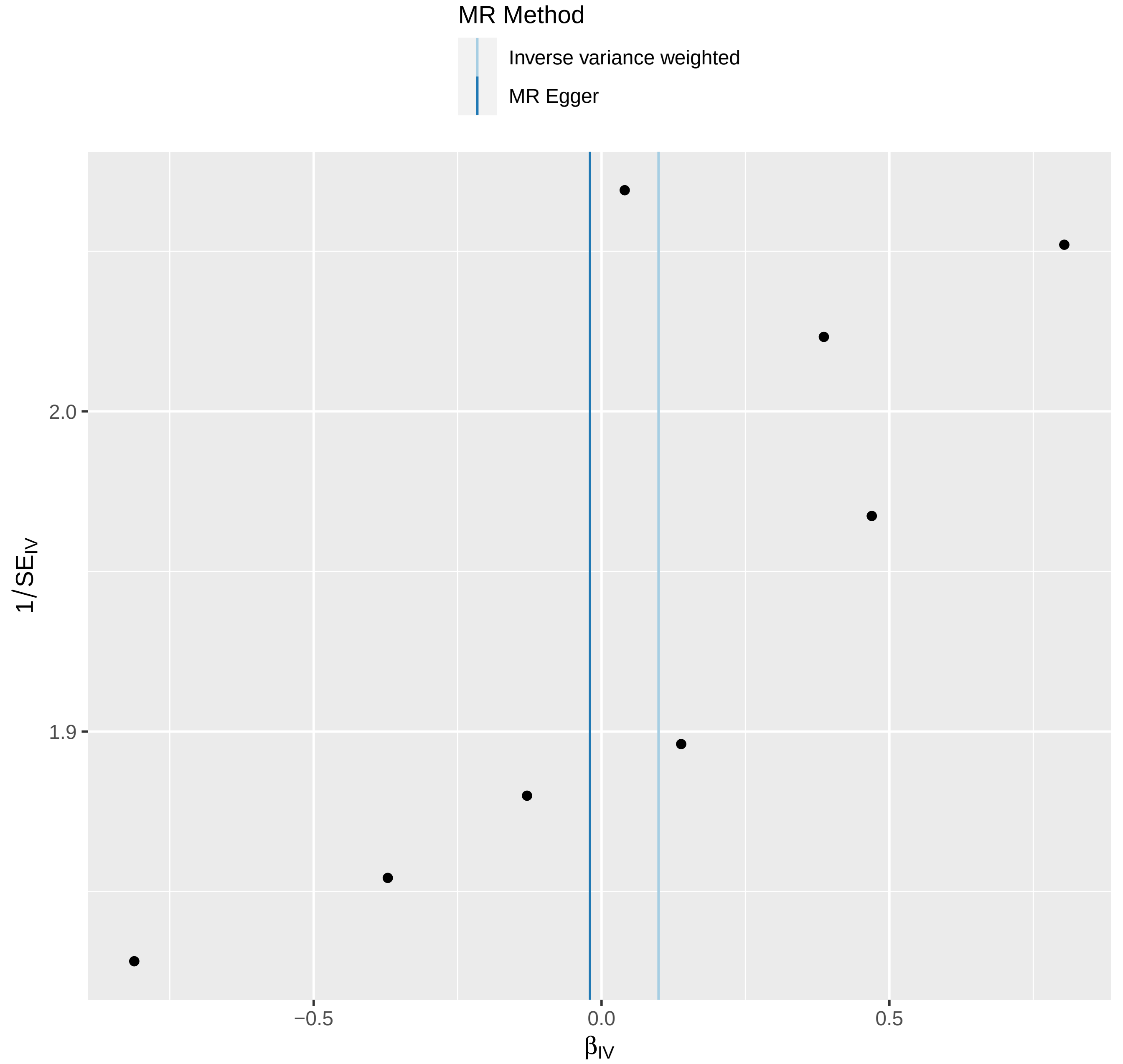

Batch 209 : Gut microbiota abundance (genus Coprococcus3 id.11303) on Other Crohn's disease

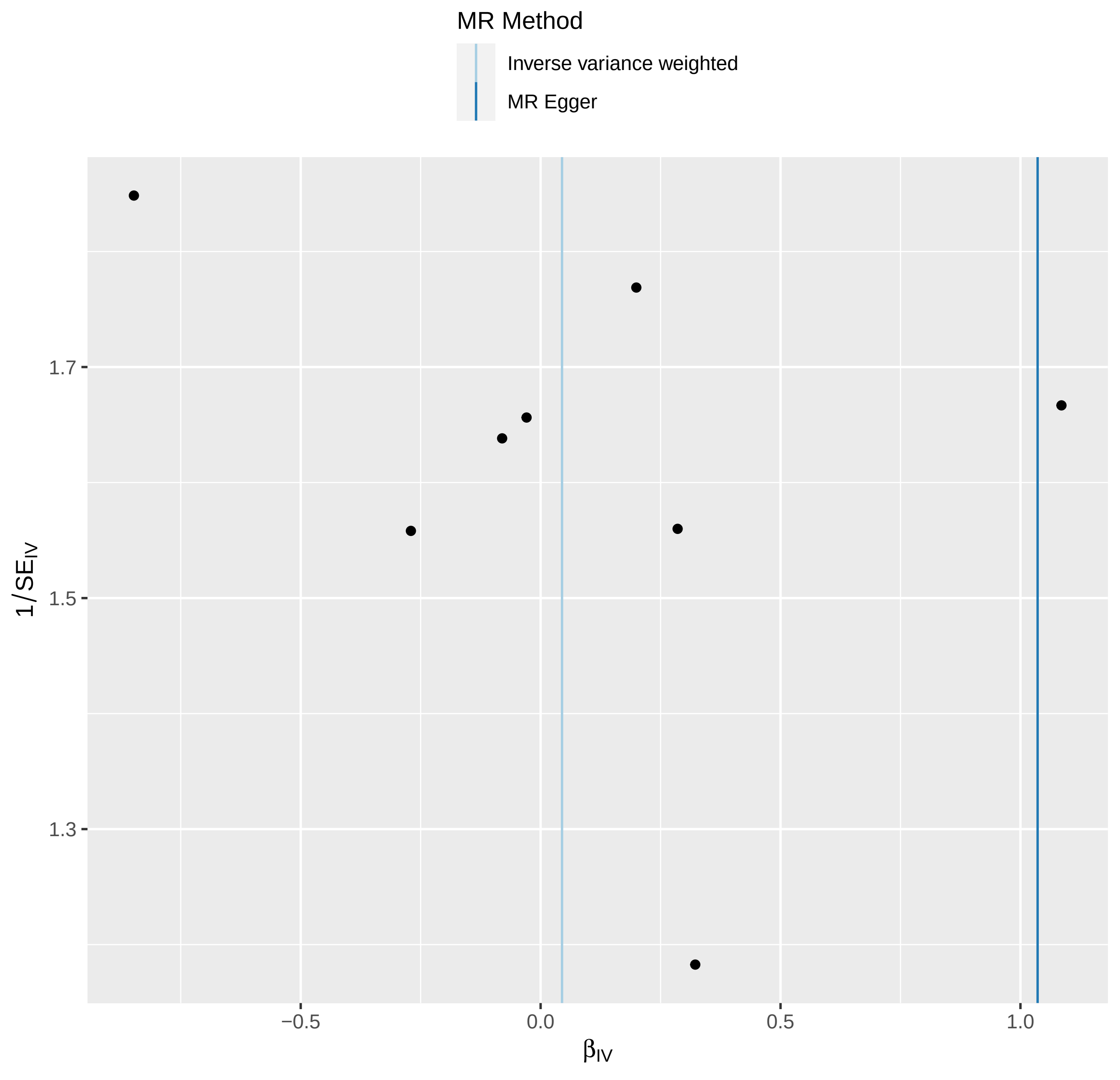

Batch 210 : Gut microbiota abundance (genus Defluviitaleaceae UCG011 id.11287) on Other Crohn's disease

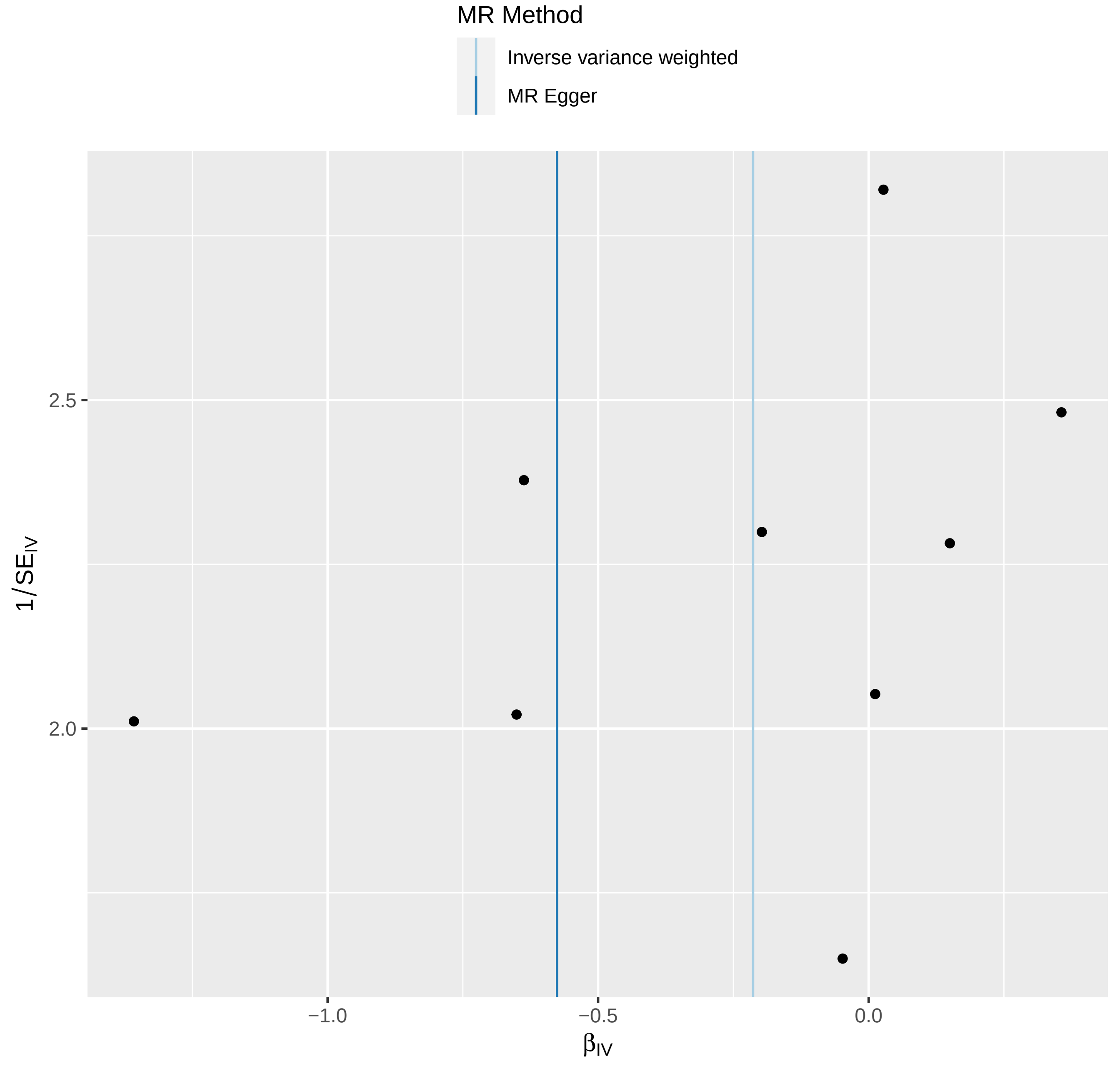

Batch 211 : Gut microbiota abundance (genus Desulfovibrio id.3173) on Other Crohn's disease

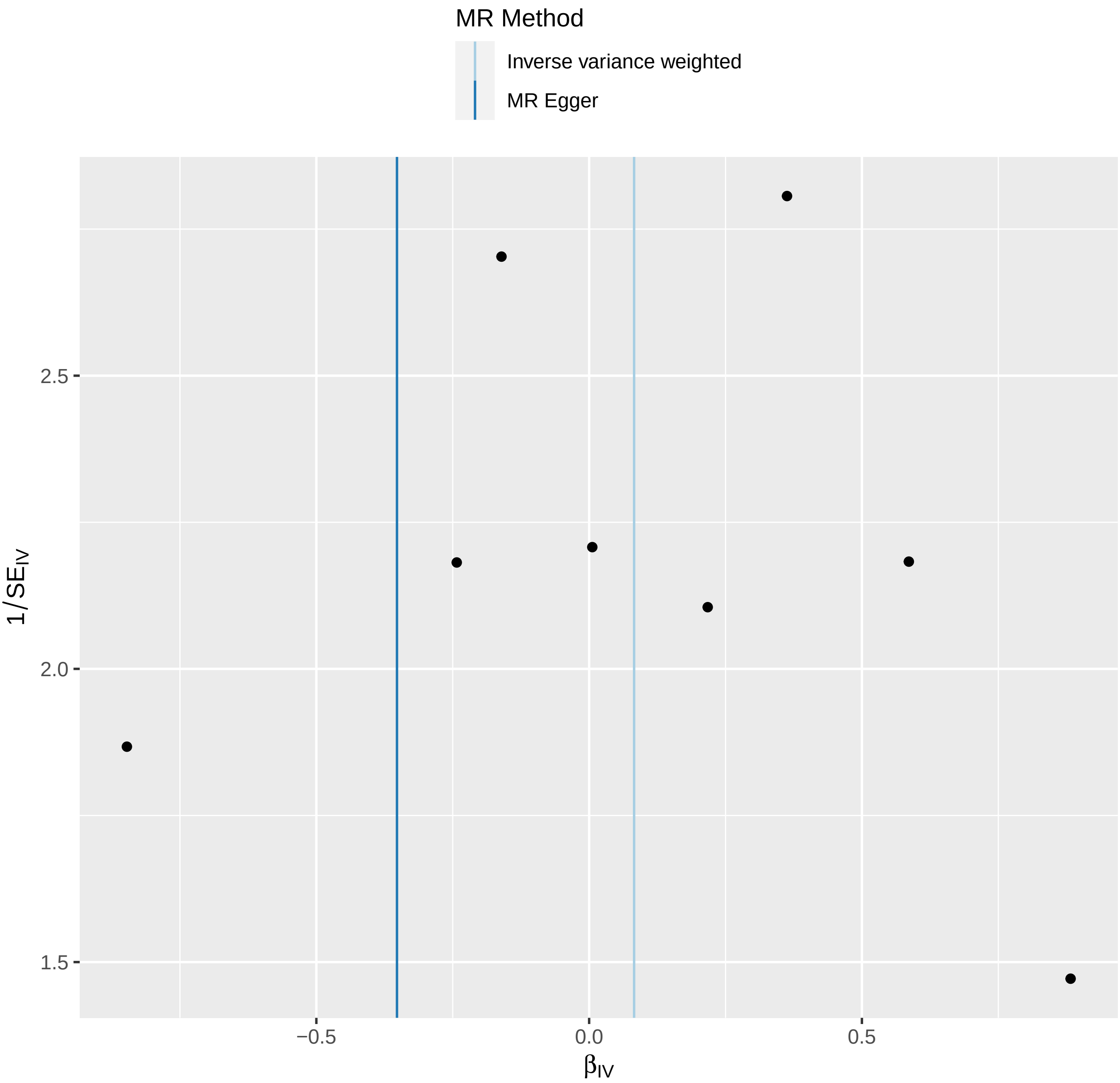

Batch 212 : Gut microbiota abundance (genus Dialister id.2183) on Other Crohn's disease

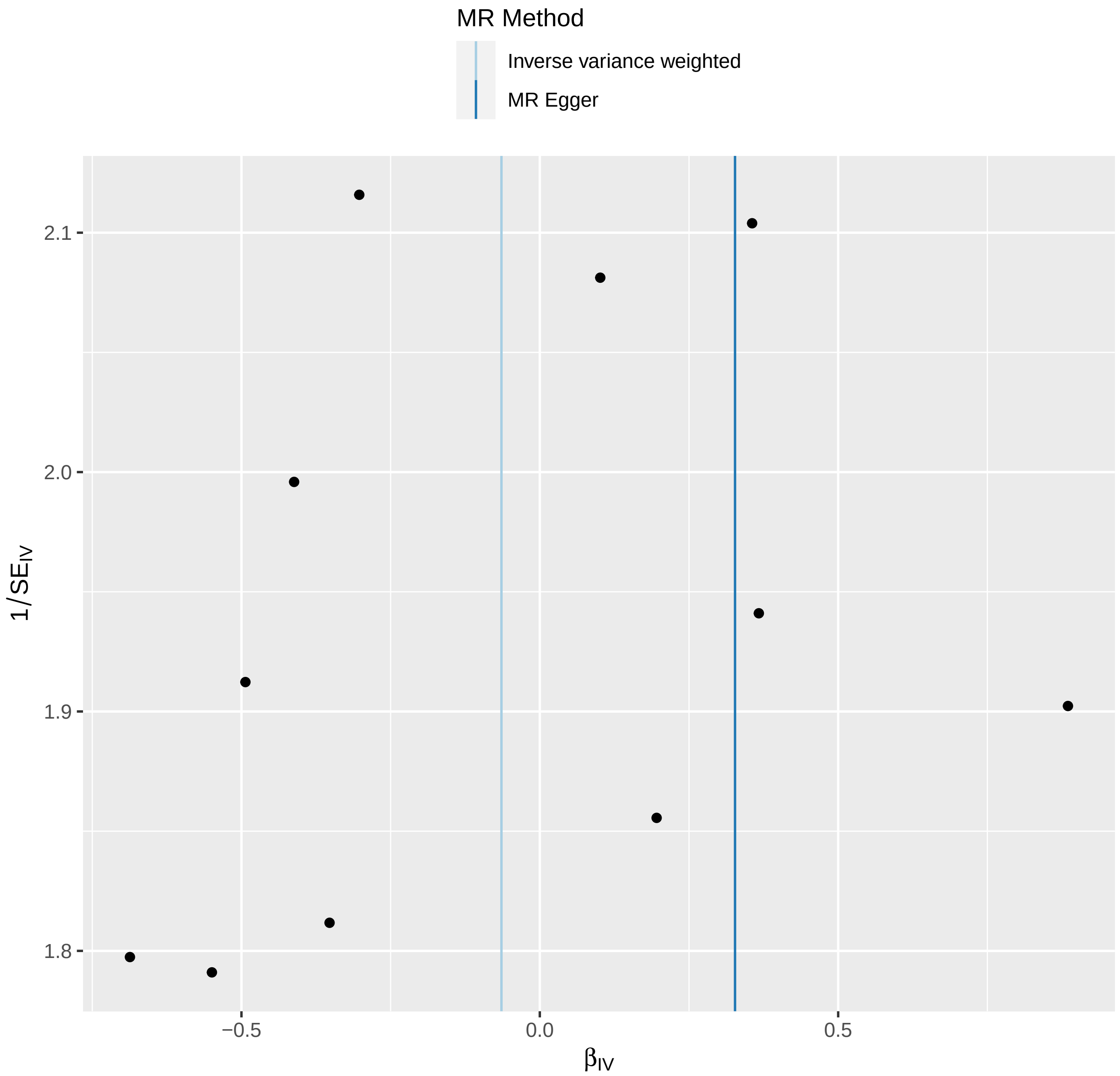

Batch 213 : Gut microbiota abundance (genus Dorea id.1997) on Other Crohn's disease

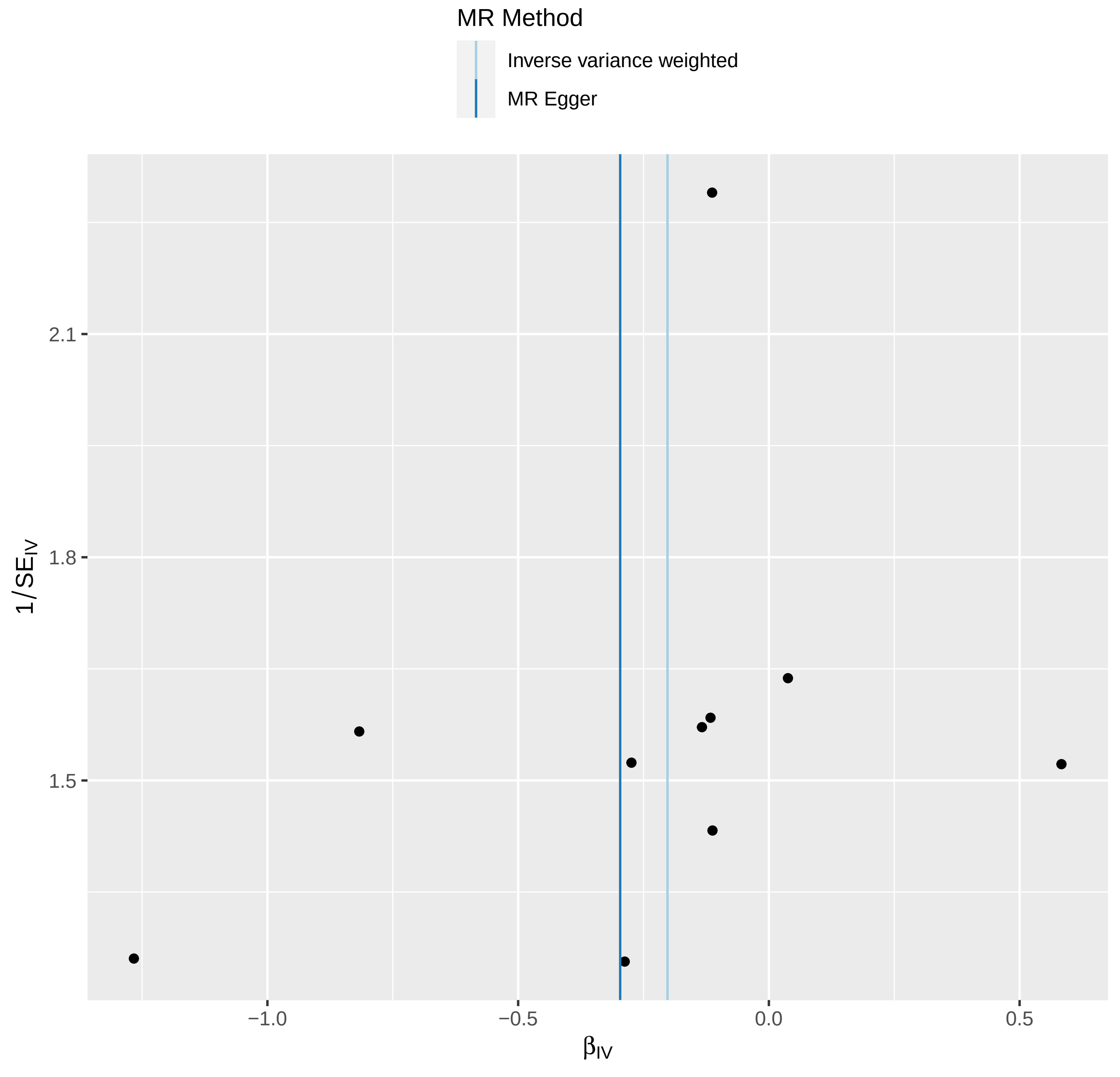

Batch 214 : Gut microbiota abundance (genus Eggerthella id.819) on Other Crohn's disease

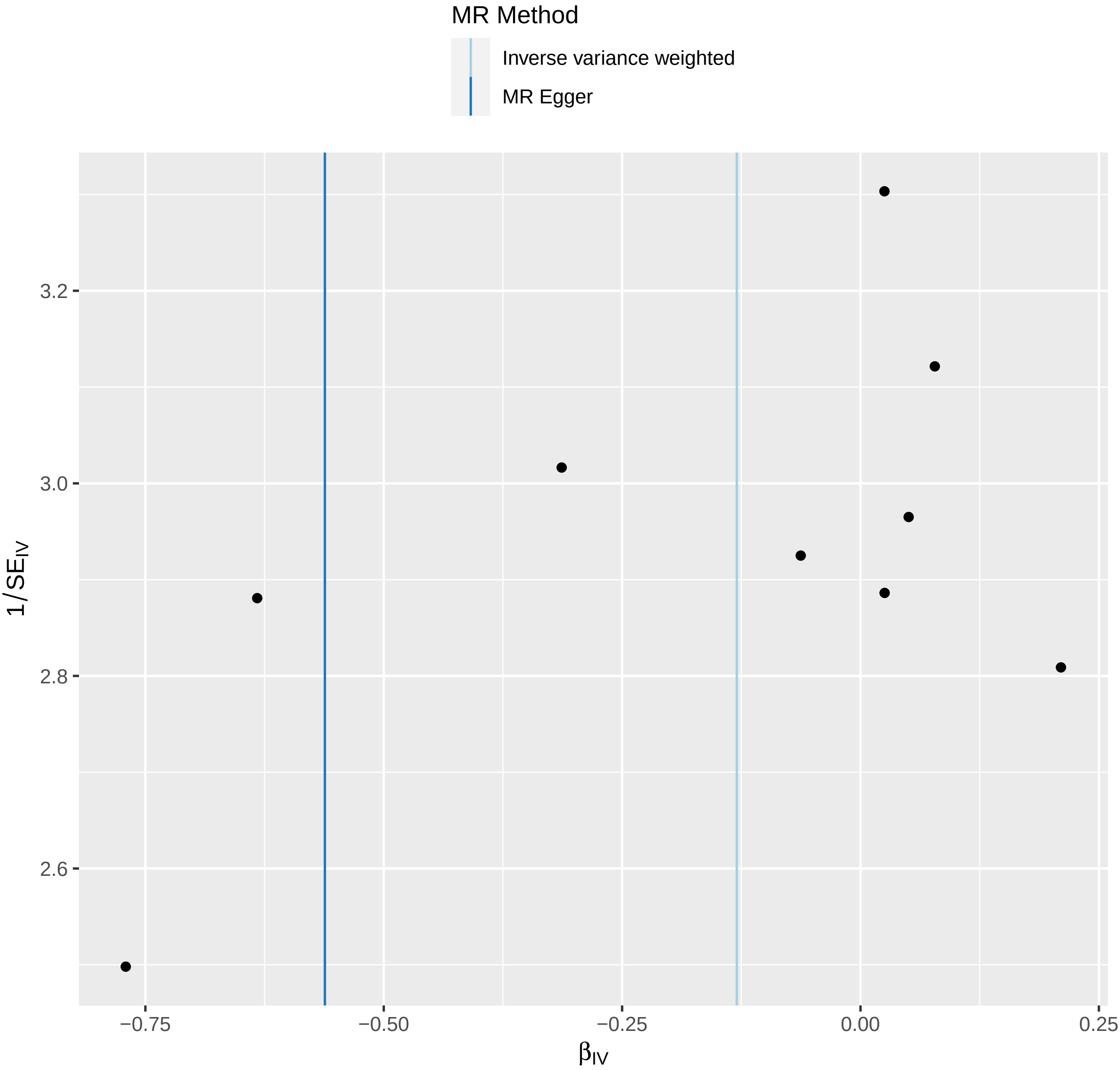

Batch 215 : Gut microbiota abundance (genus Eisenbergiella id.11304) on Other Crohn's disease

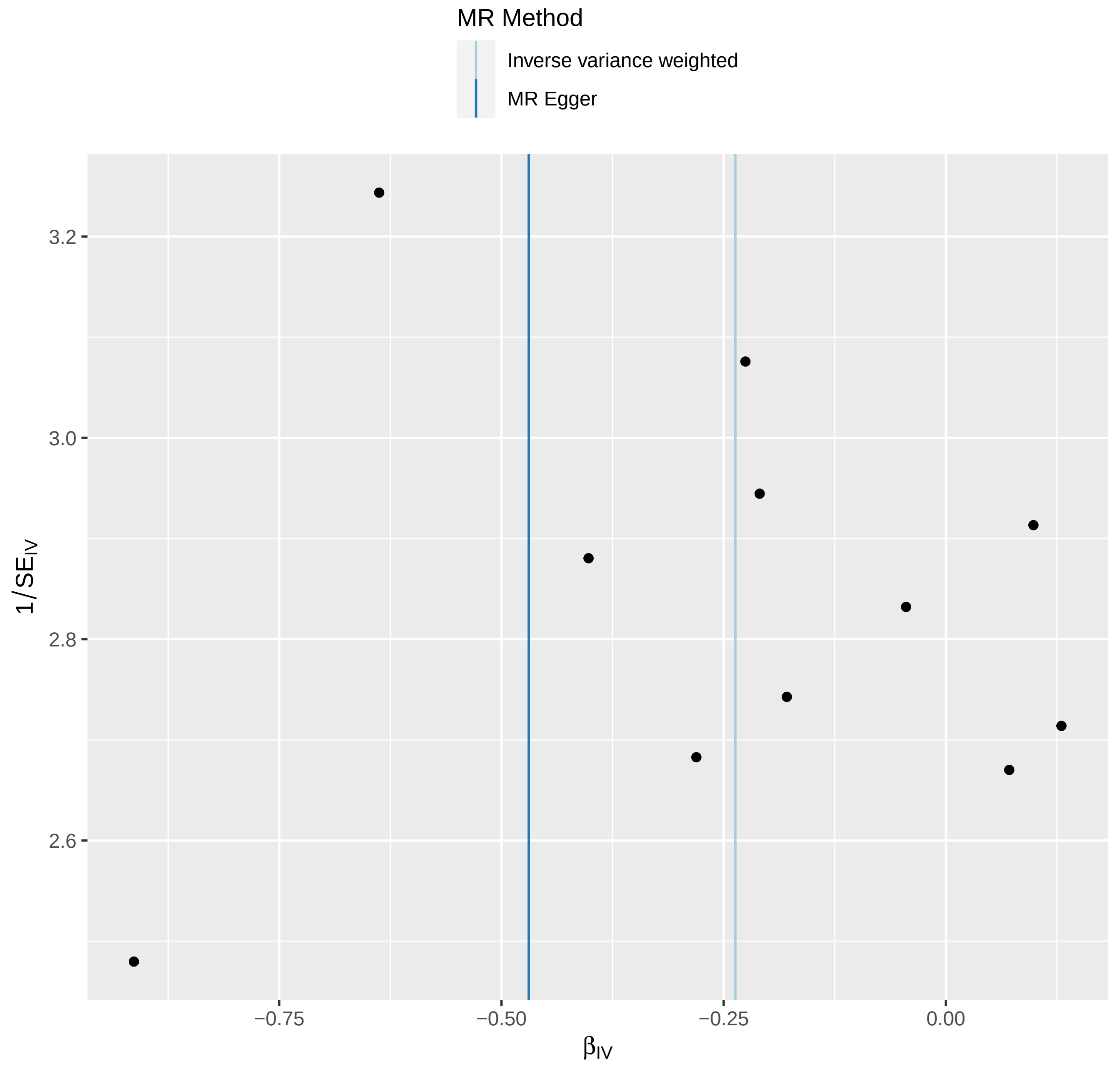

Batch 216 : Gut microbiota abundance (genus Enterorhabdus id.820) on Other Crohn's disease

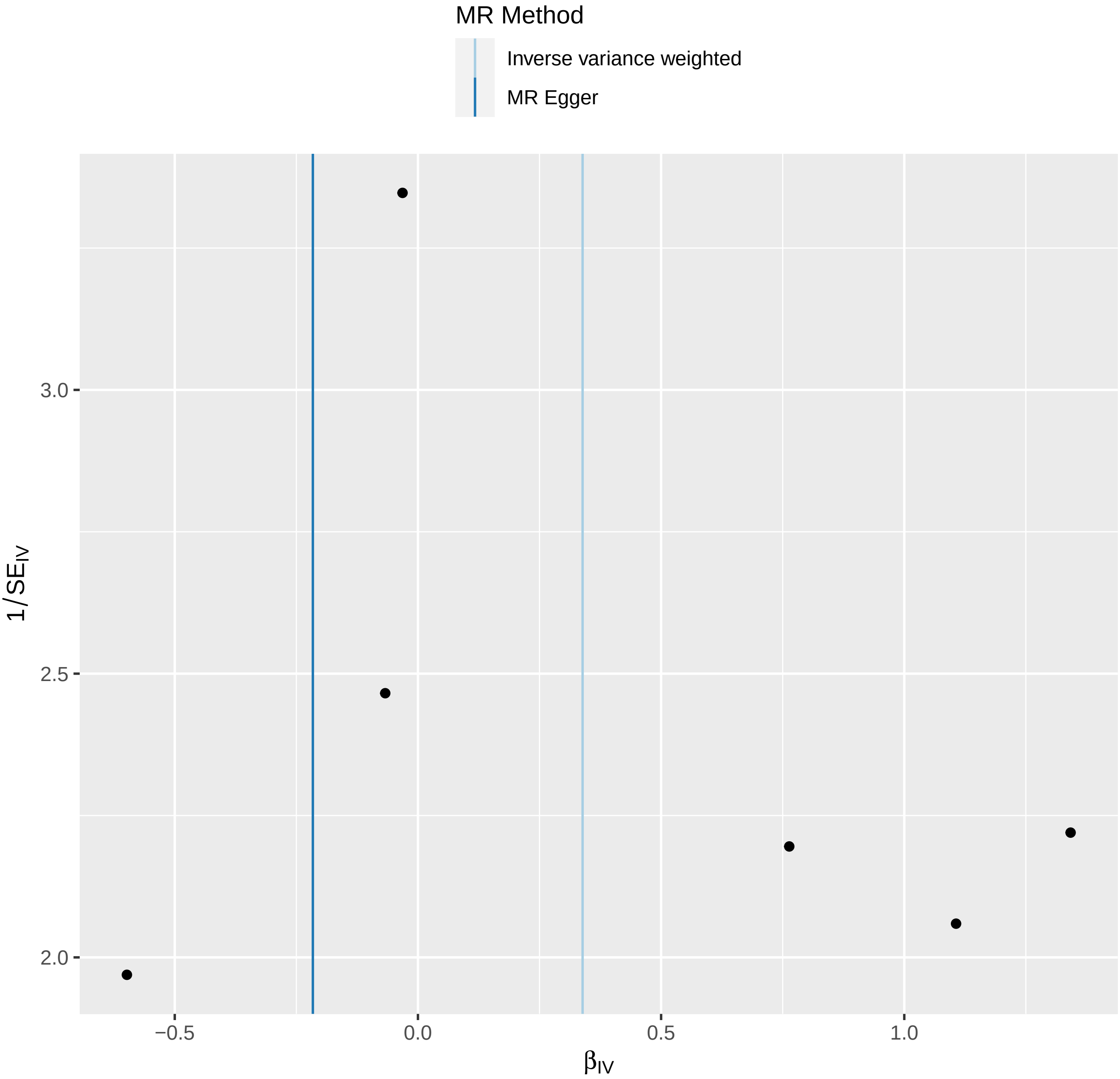

Batch 217 : Gut microbiota abundance (genus Erysipelatoclostridium id.11381) on Other Crohn's disease

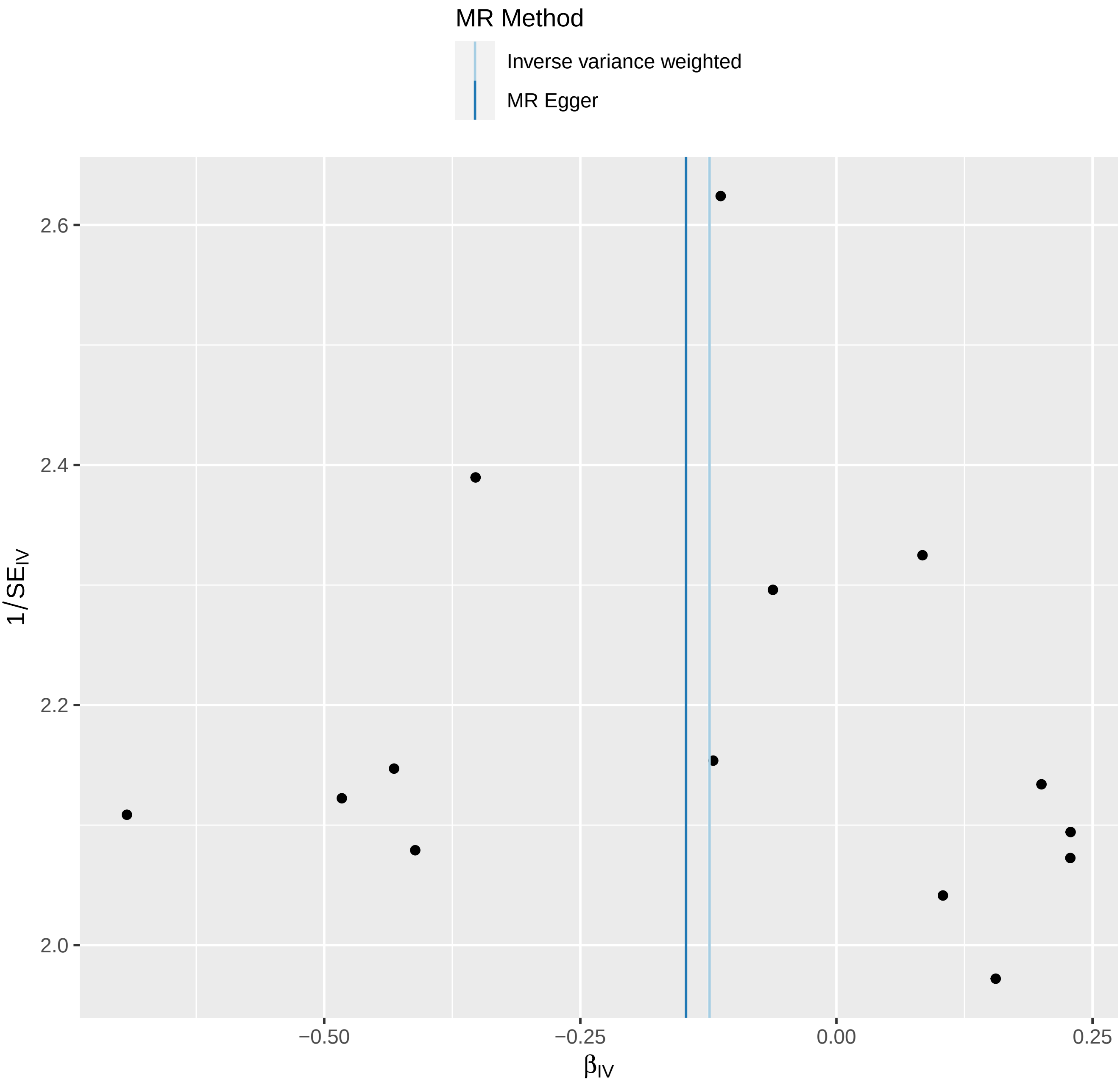

Insufficient number of SNPs

Batch 219 : Gut microbiota abundance (genus Escherichia Shigella id.3504) on Other Crohn's disease

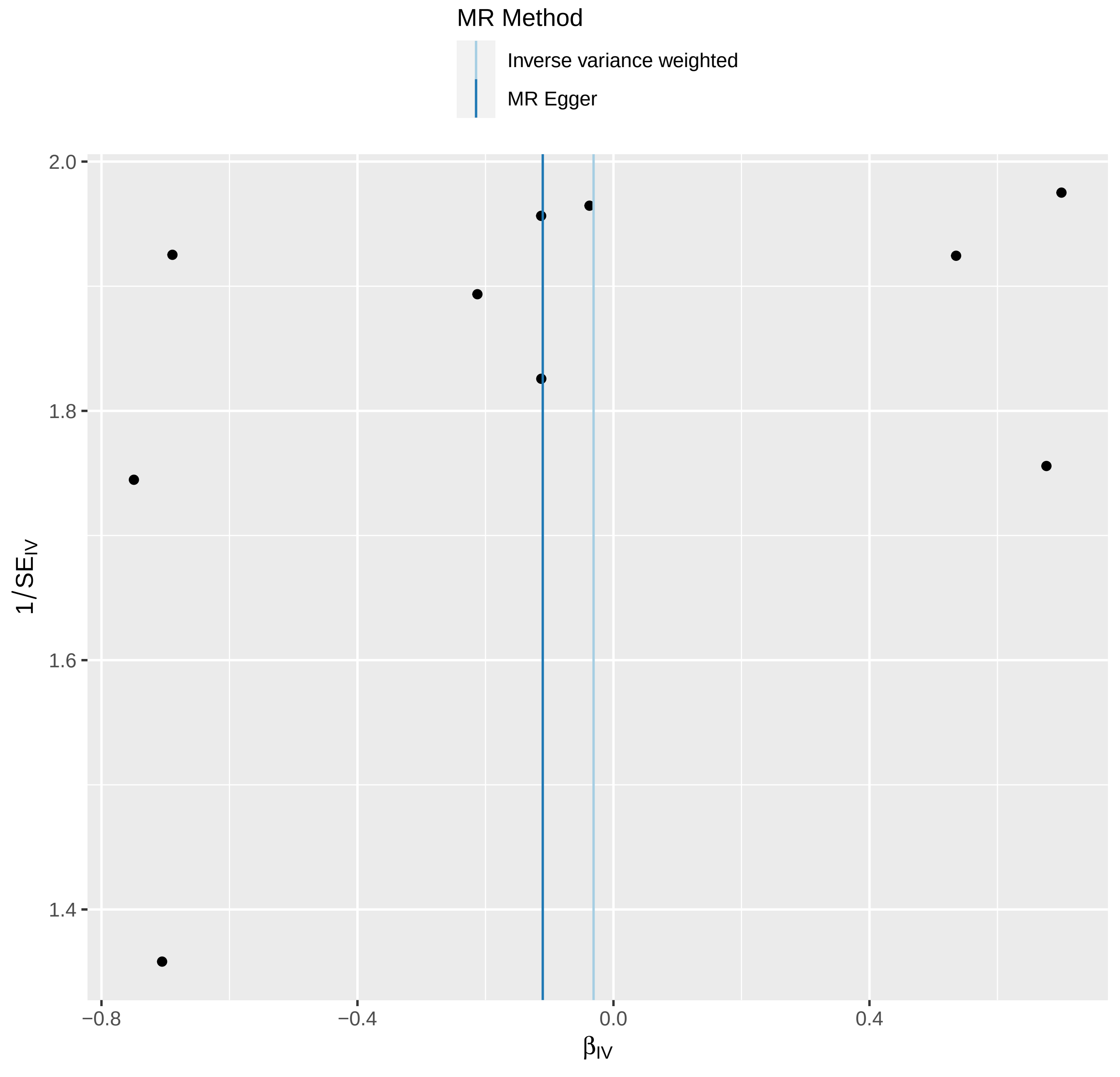

Batch 220 : Gut microbiota abundance (genus Eubacterium brachy group id.11296) on Other Crohn's disease

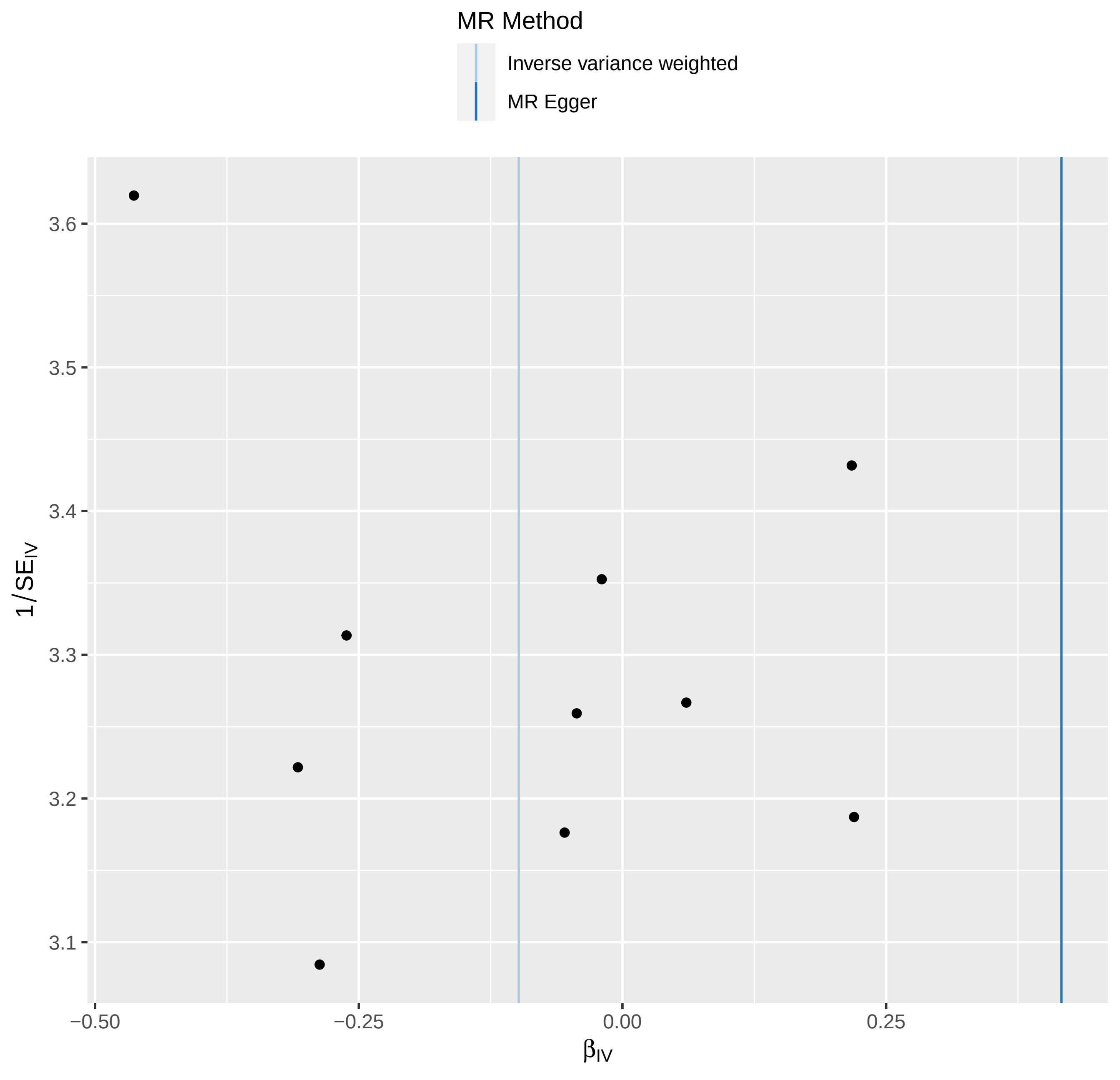

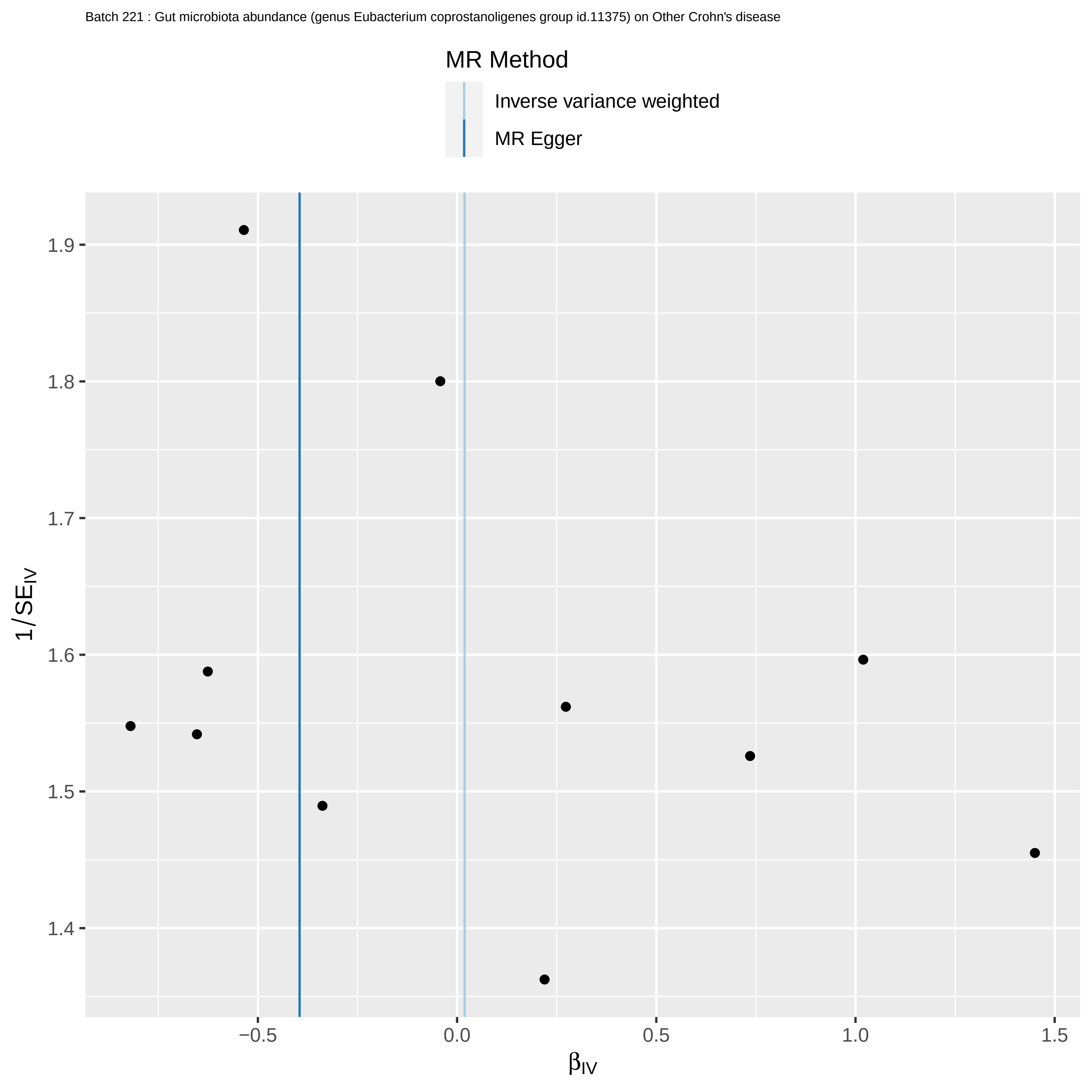

Batch 221 : Gut microbiota abundance (genus Eubacterium coprostanoligenes group id.11375) on Other Crohn's disease
MR Method
Inverse variance weighted
MR Egger
1.9
1.8
1.7
1.6
1.5
1.4
1/SE_IV
−0.5
0.0
0.5
1.0
1.5
β_IV

Batch 222 : Gut microbiota abundance (genus Eubacterium eligens group id.14372) on Other Crohn's disease

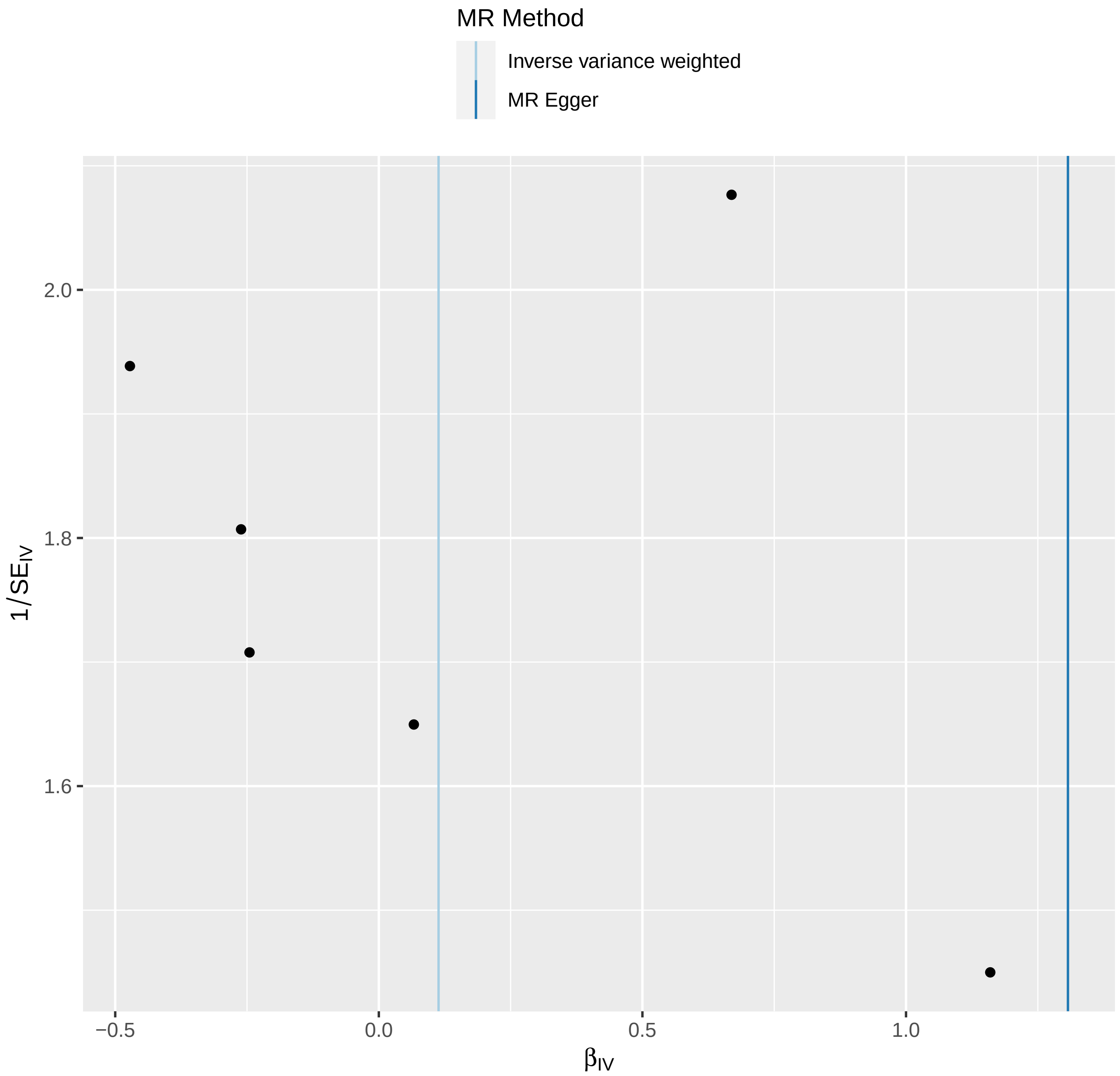

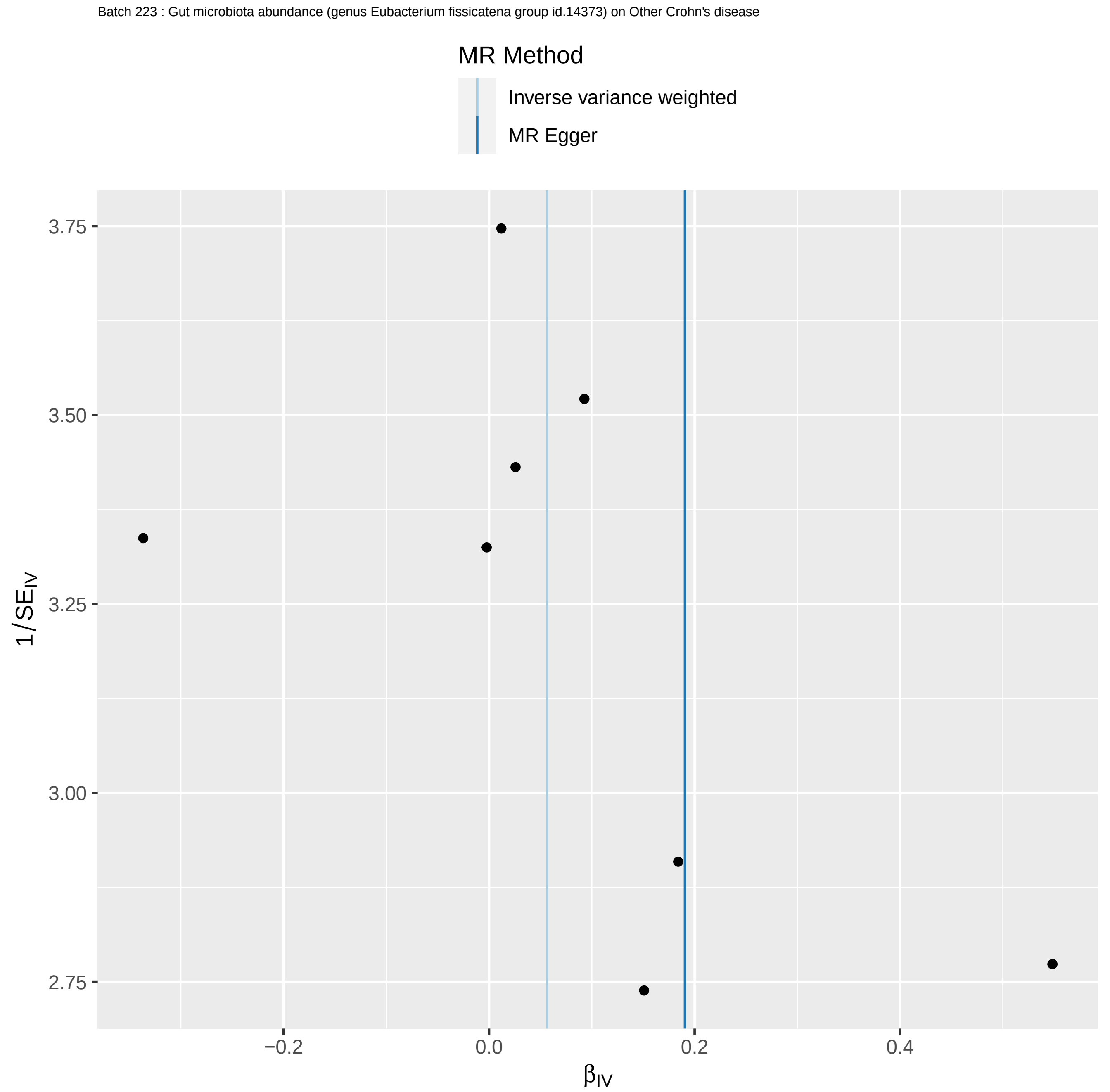

Batch 223 : Gut microbiota abundance (genus Eubacterium fissicatena group id.14373) on Other Crohn's disease
MR Method
Inverse variance weighted
MR Egger
3.75
3.50
3.25
3.00
2.75
1/SE_IV
−0.2
0.0
0.2
0.4
β_IV

Batch 224 : Gut microbiota abundance (genus Eubacterium hallii group id.11338) on Other Crohn's disease

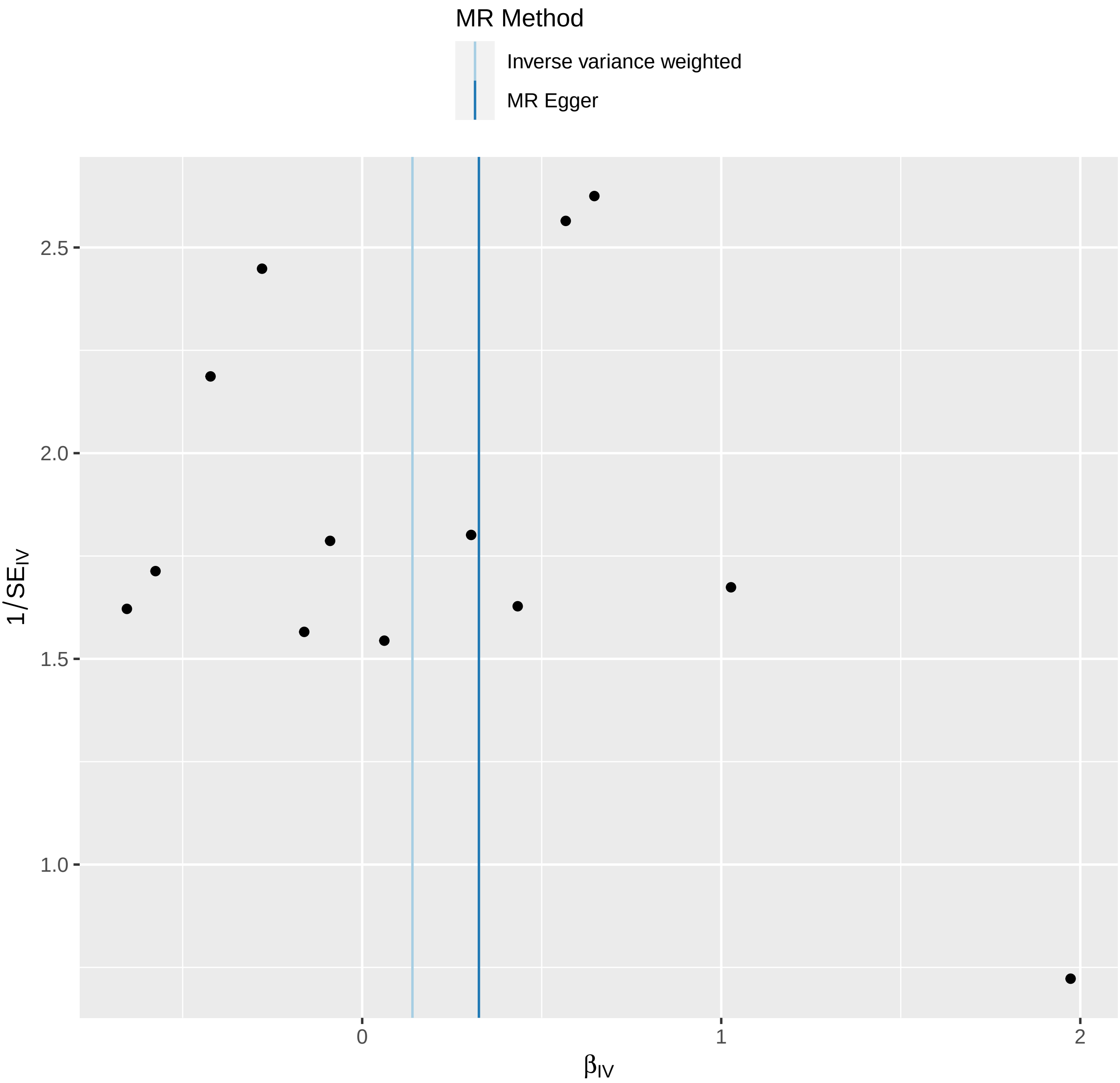

Batch 225 : Gut microbiota abundance (genus Eubacterium nodatum group id.11297) on Other Crohn's disease

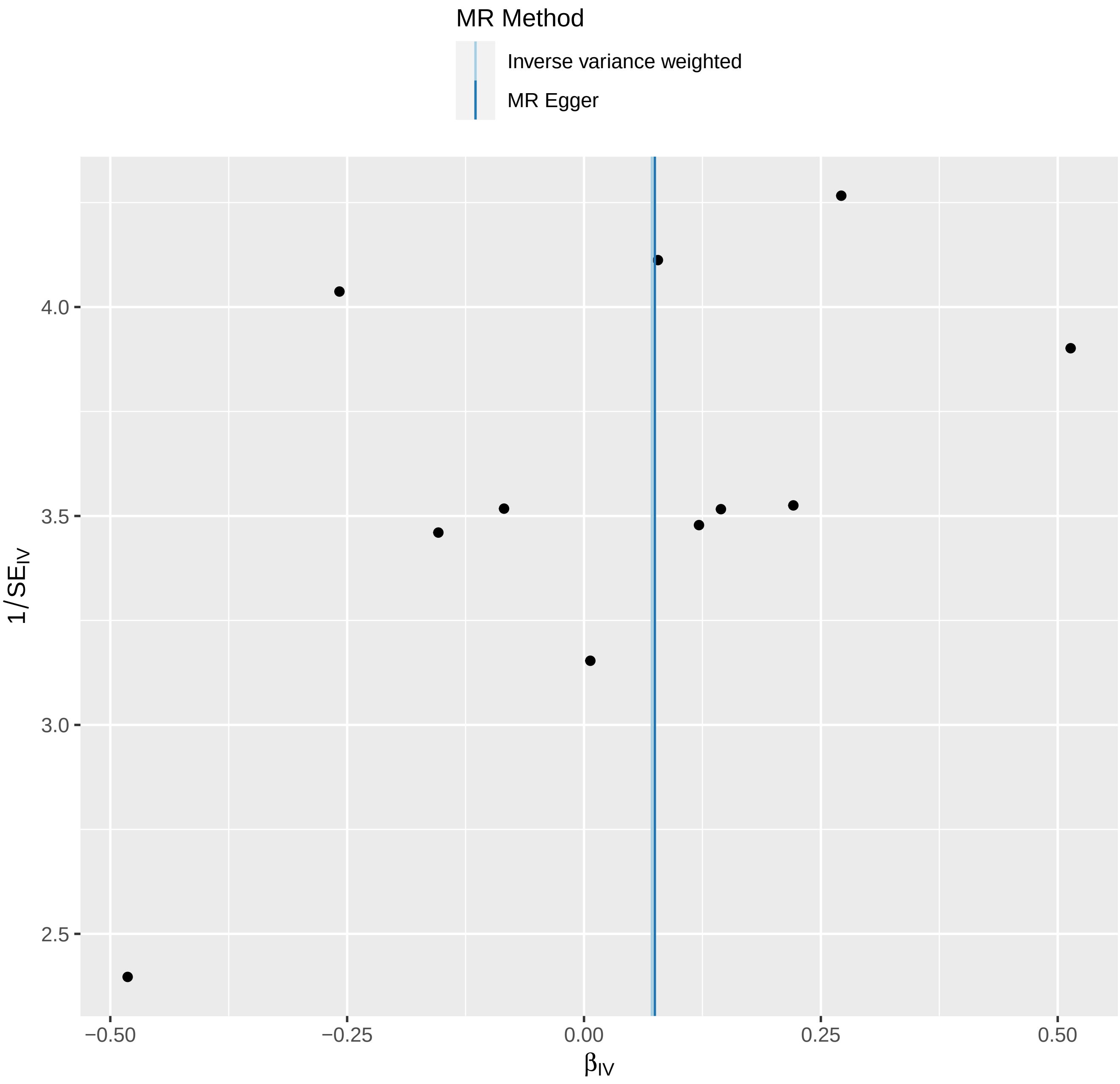

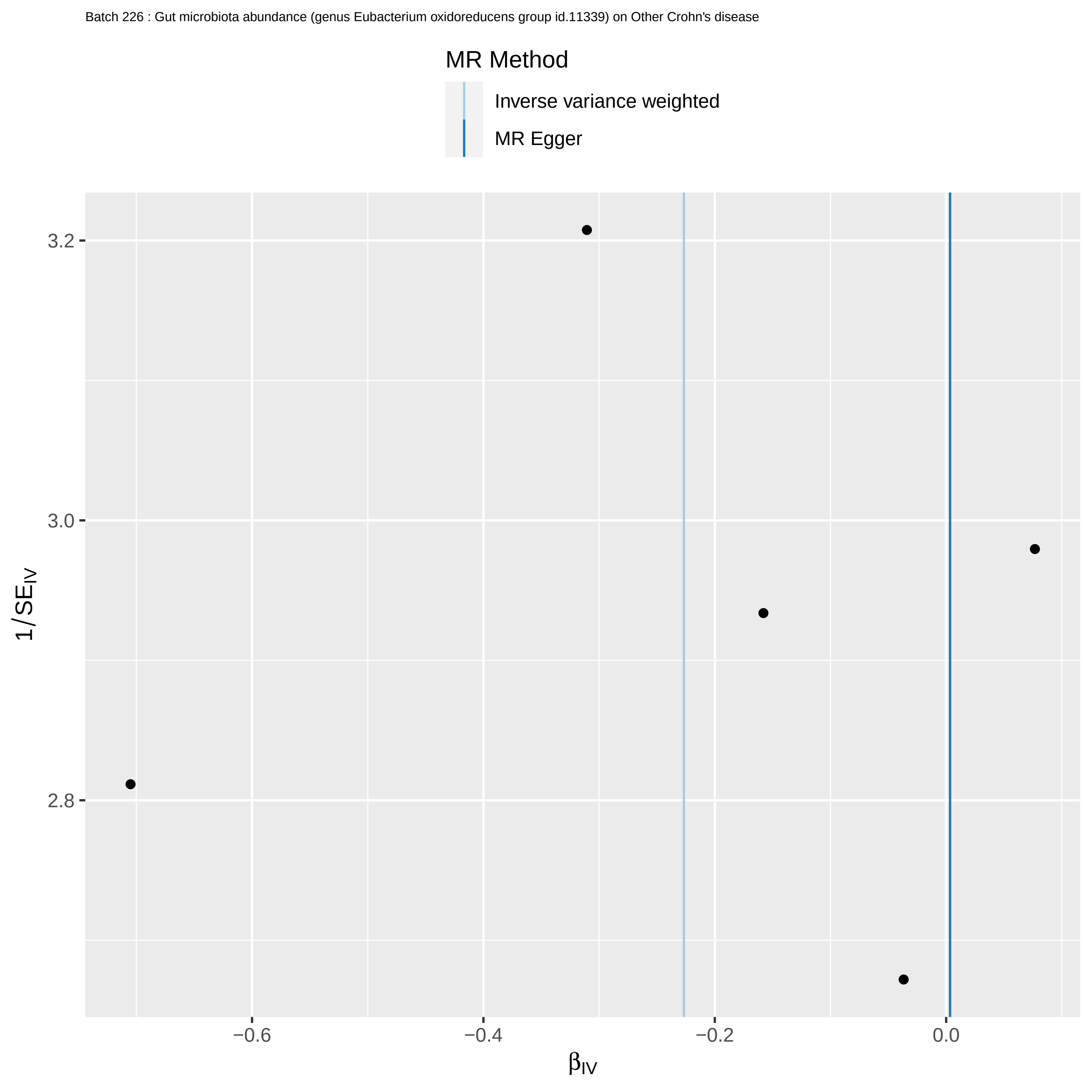

Batch 226 : Gut microbiota abundance (genus Eubacterium oxidoreducens group id.11339) on Other Crohn's disease
MR Method
Inverse variance weighted
MR Egger
3.2
3.0
2.8
1/SE_IV
−0.6
−0.4
−0.2
0.0
β_IV

Batch 227 : Gut microbiota abundance (genus Eubacterium rectale group id.14374) on Other Crohn's disease

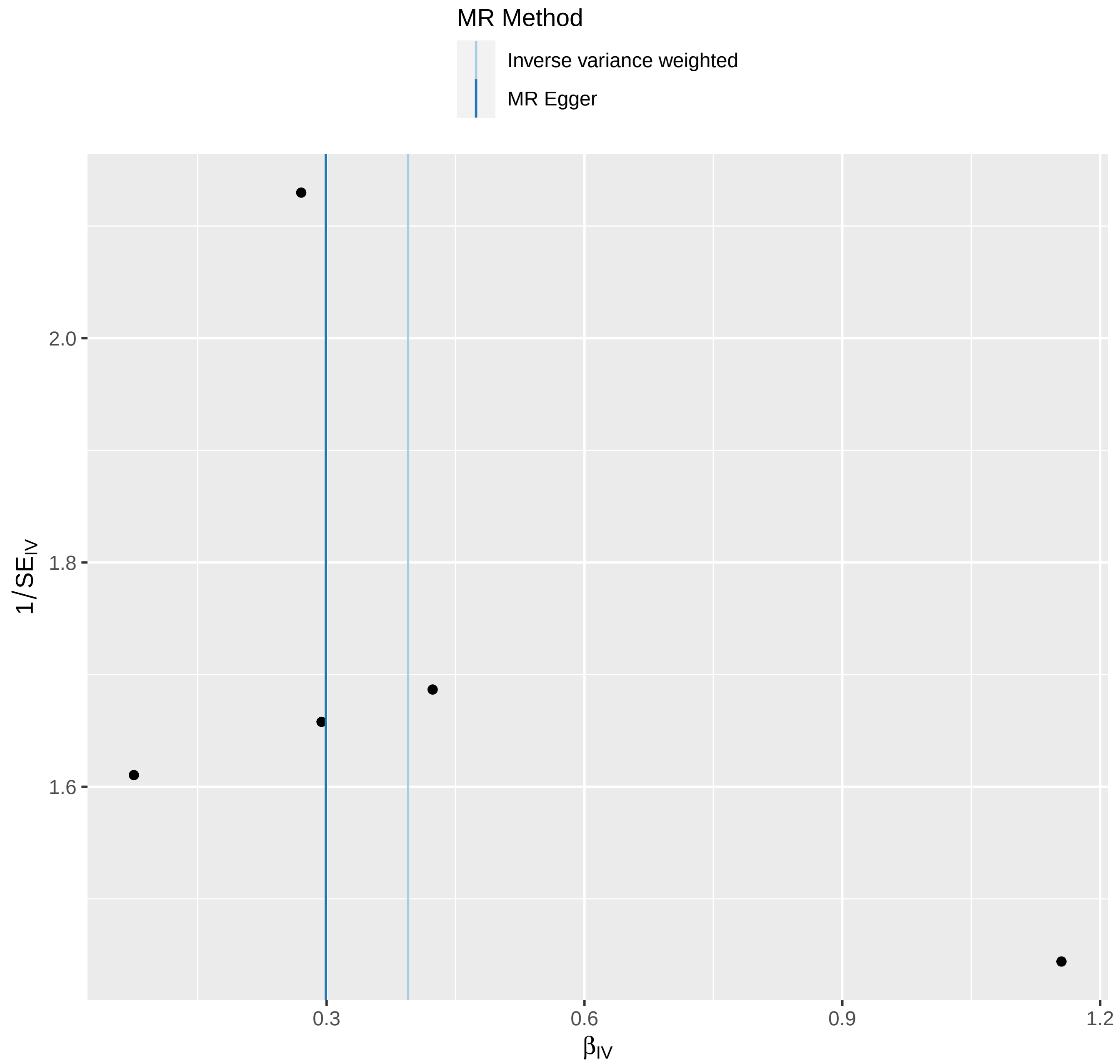

Batch 228 : Gut microbiota abundance (genus Eubacterium ruminantium group id.11340) on Other Crohn's disease

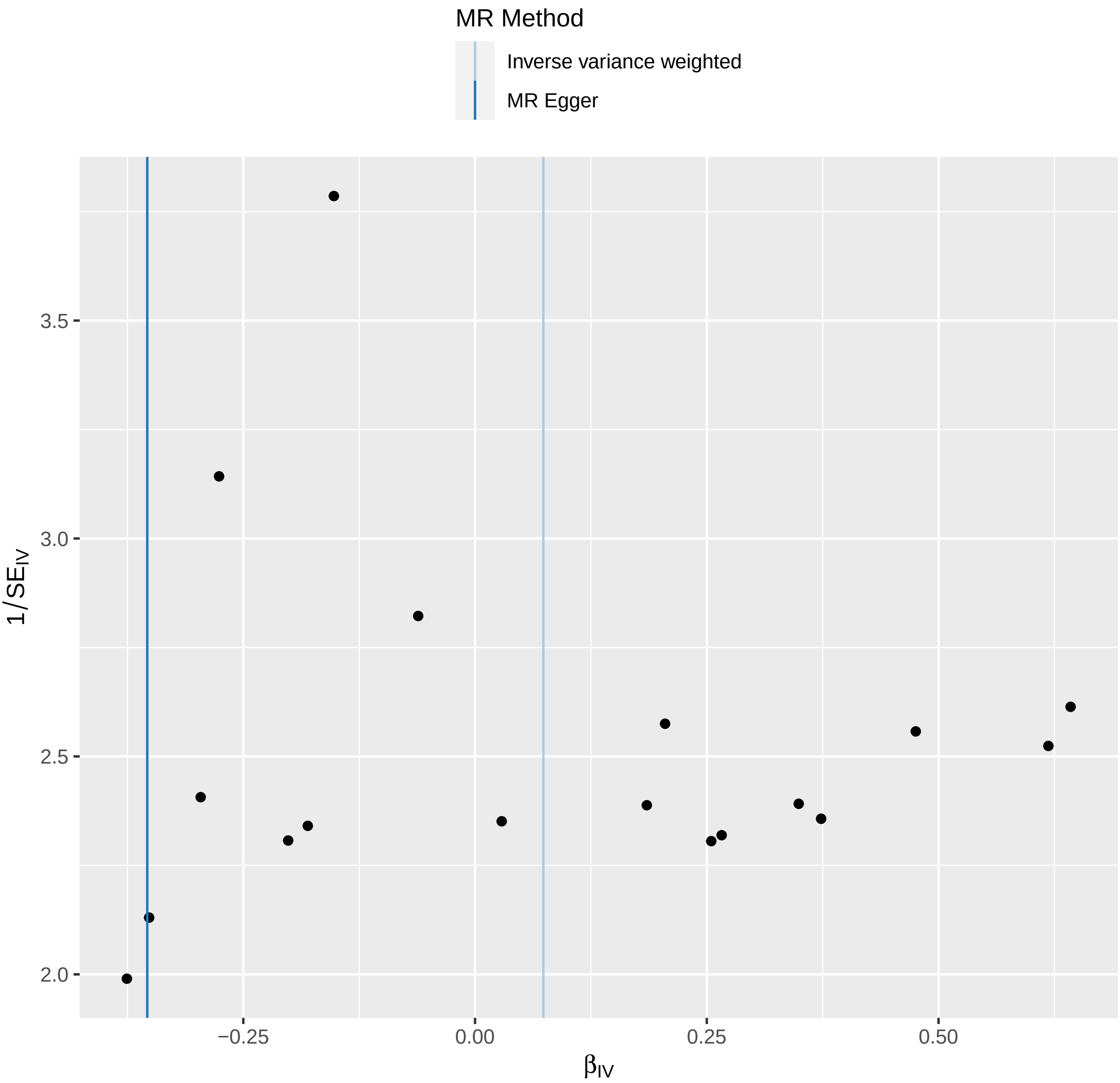

Batch 229 : Gut microbiota abundance (genus Eubacterium ventriosum group id.11341) on Other Crohn's disease

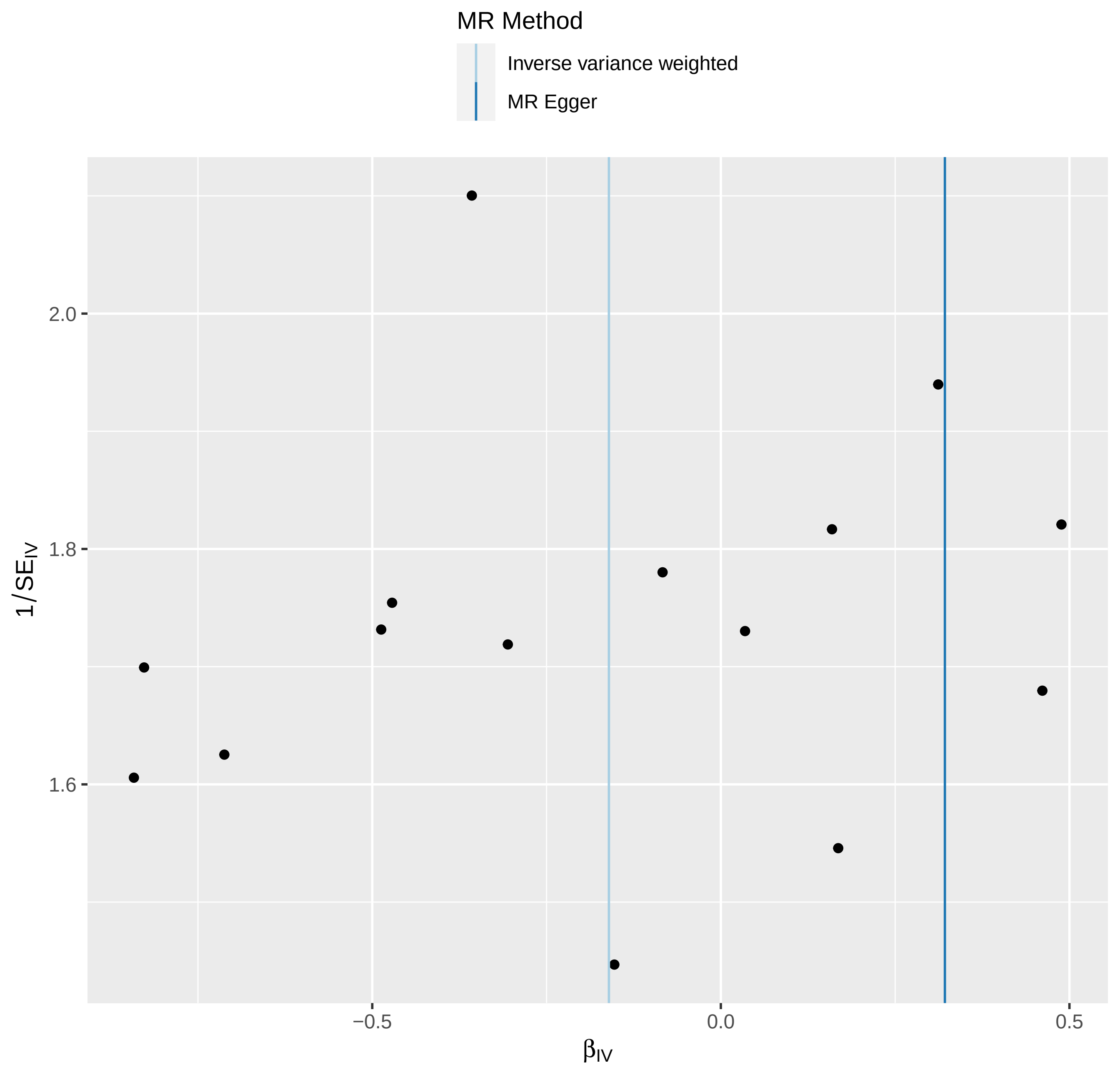

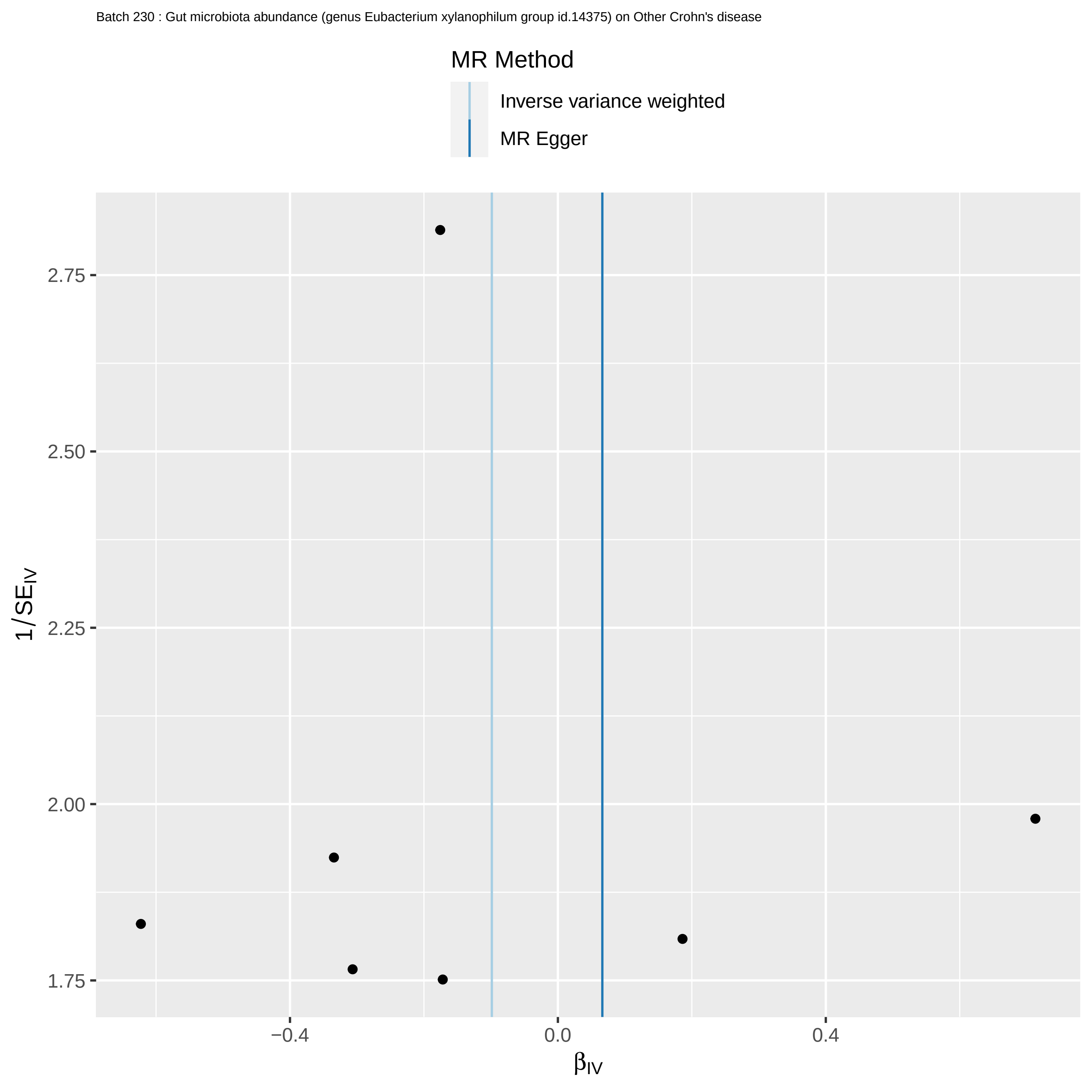
Batch 230 : Gut microbiota abundance (genus Eubacterium xylanophilum group id.14375) on Other Crohn's disease
MR Method
Inverse variance weighted
MR Egger
2.75
2.50
2.25
2.00
1.75
1/SE_IV
−0.4
0.0
0.4
β_IV

Batch 231 : Gut microbiota abundance (genus Faecalibacterium id.2057) on Other Crohn's disease

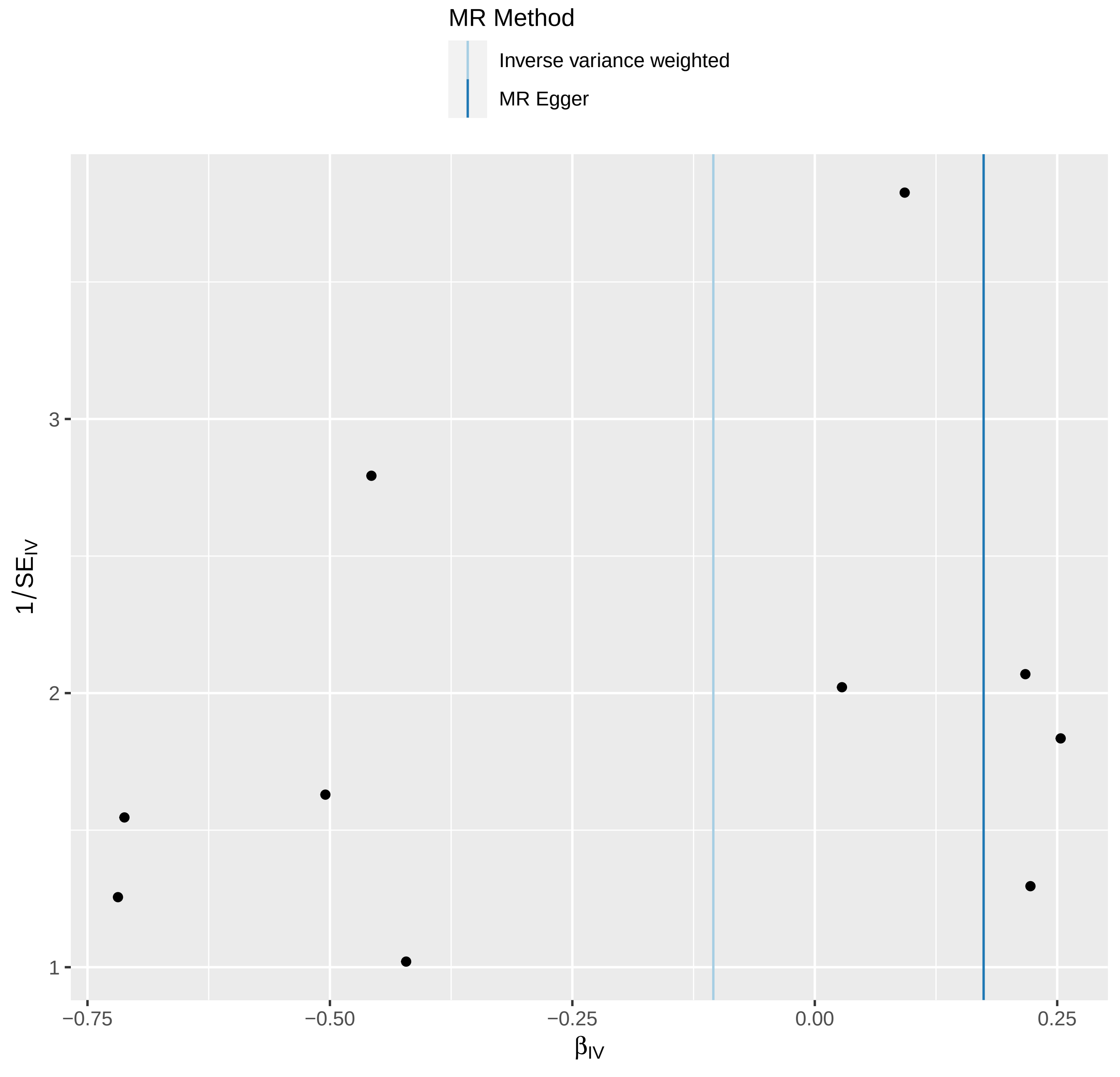

Batch 232 : Gut microbiota abundance (genus Family XIII AD3011 group id.11293) on Other Crohn's disease

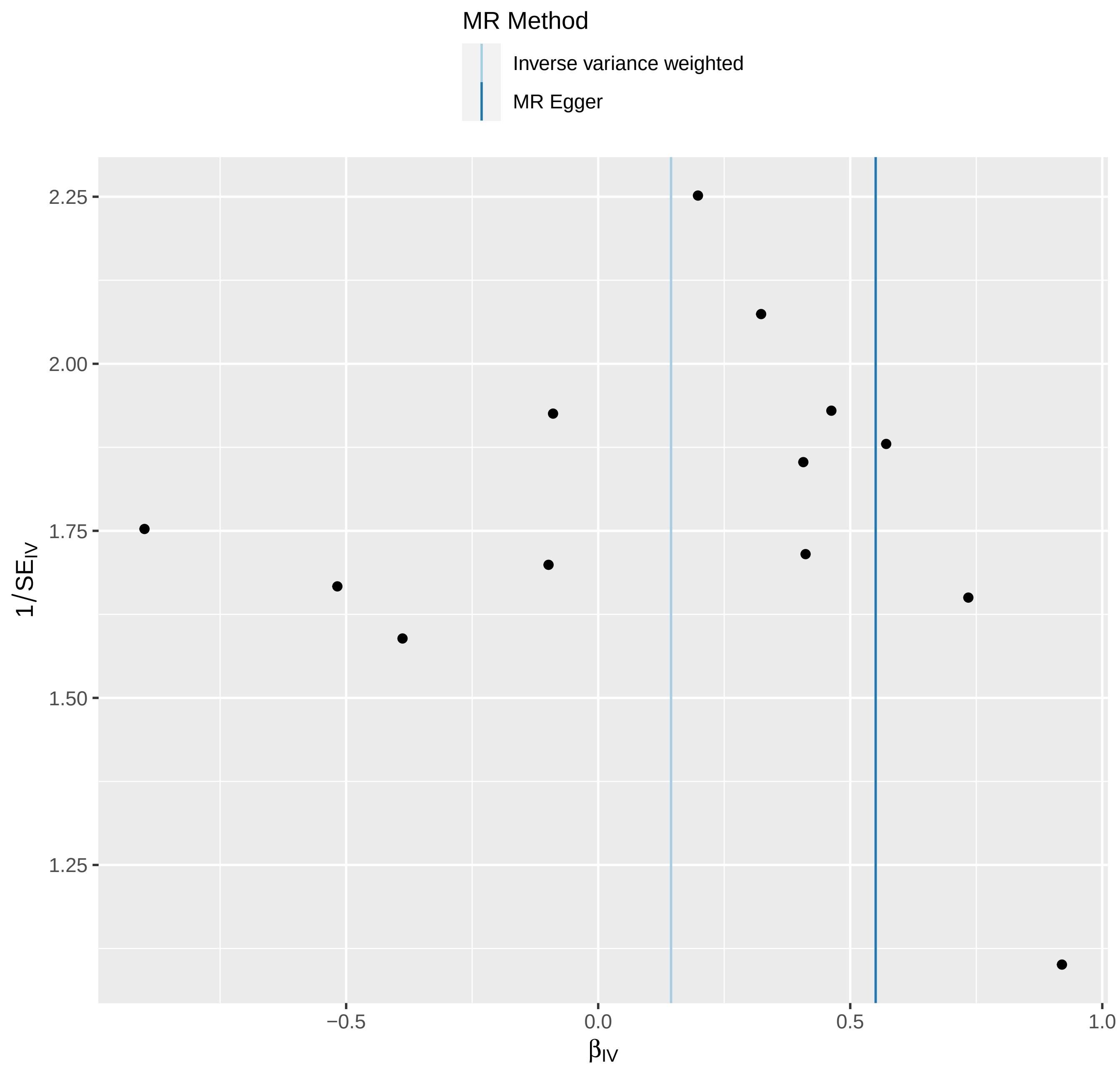

Batch 233 : Gut microbiota abundance (genus Family XIII UCG001 id.11294) on Other Crohn's disease

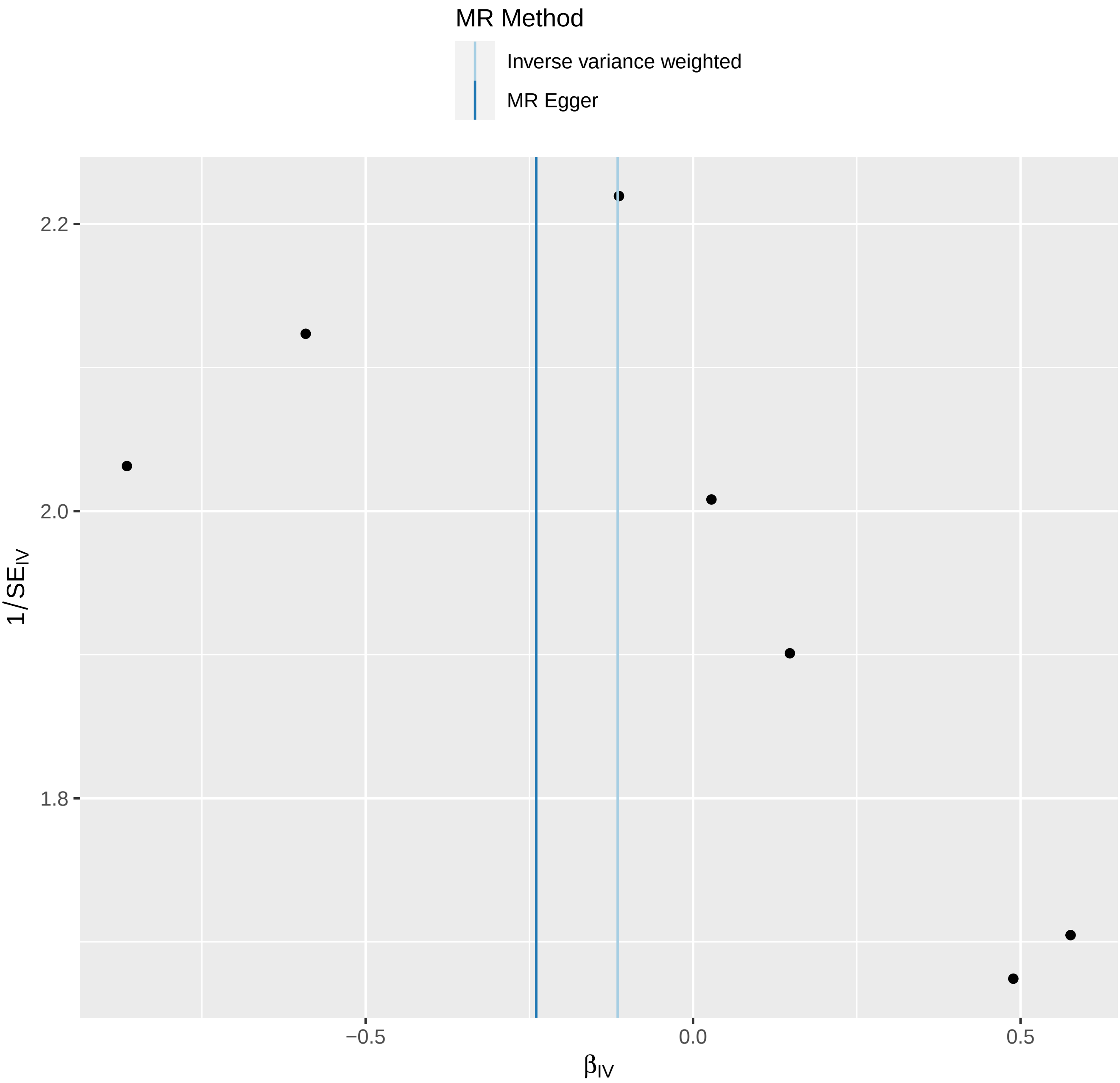

Batch 234 : Gut microbiota abundance (genus Flavonifractor id.2059) on Other Crohn's disease

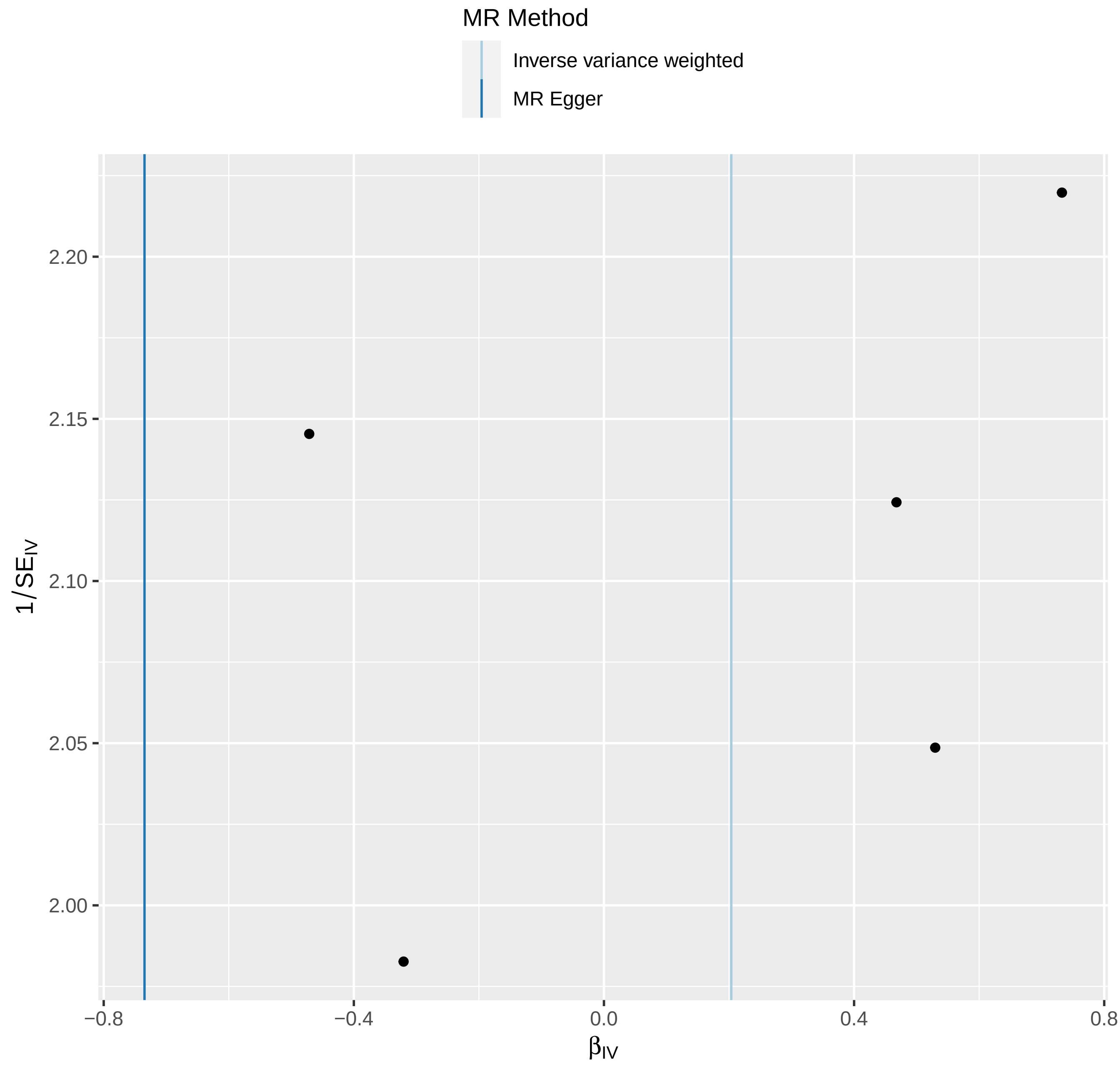

Batch 235 : Gut microbiota abundance (genus Fusicatenibacter id.11305) on Other Crohn's disease

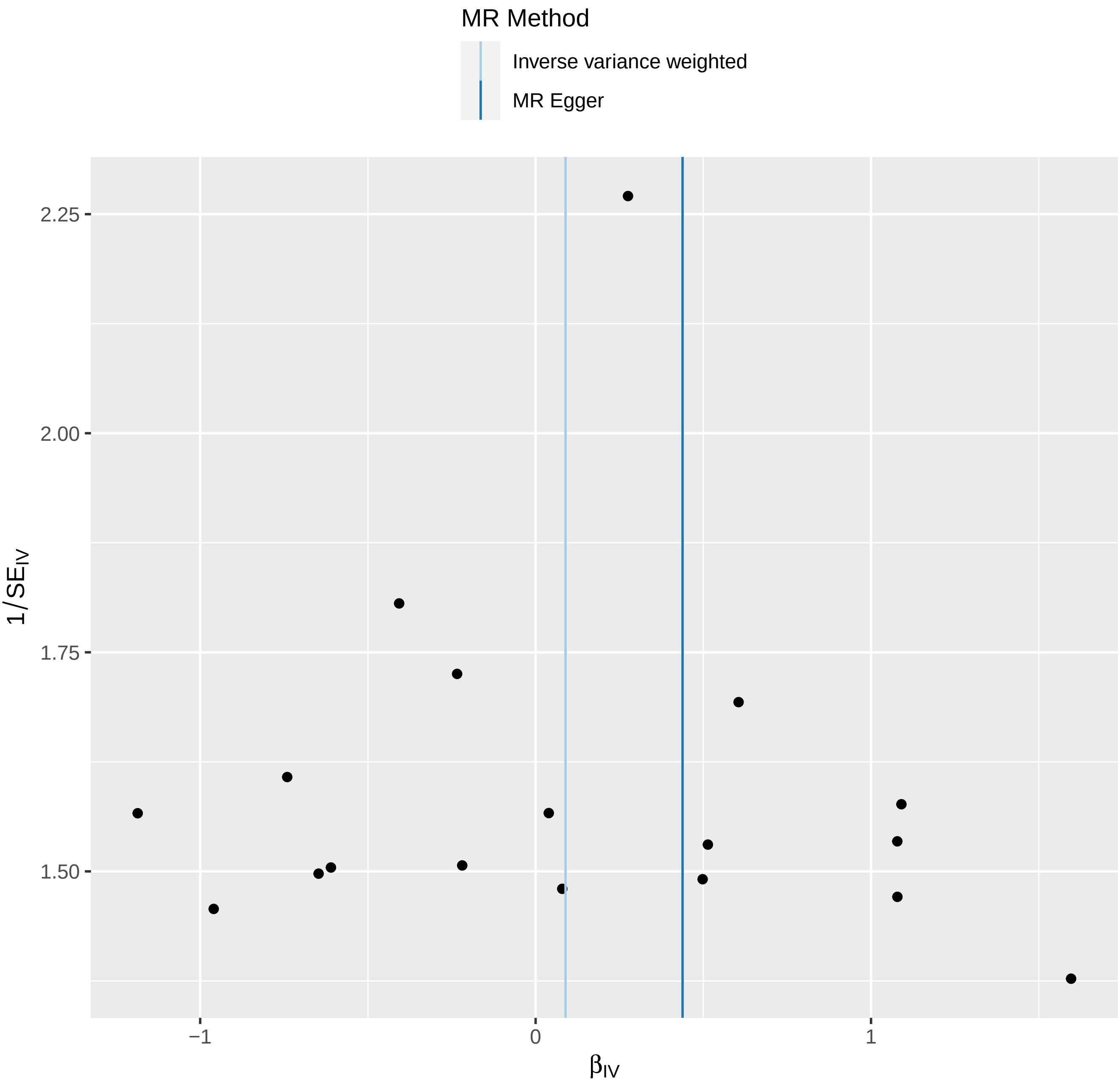

Batch 236 : Gut microbiota abundance (genus Gordonibacter id.821) on Other Crohn's disease

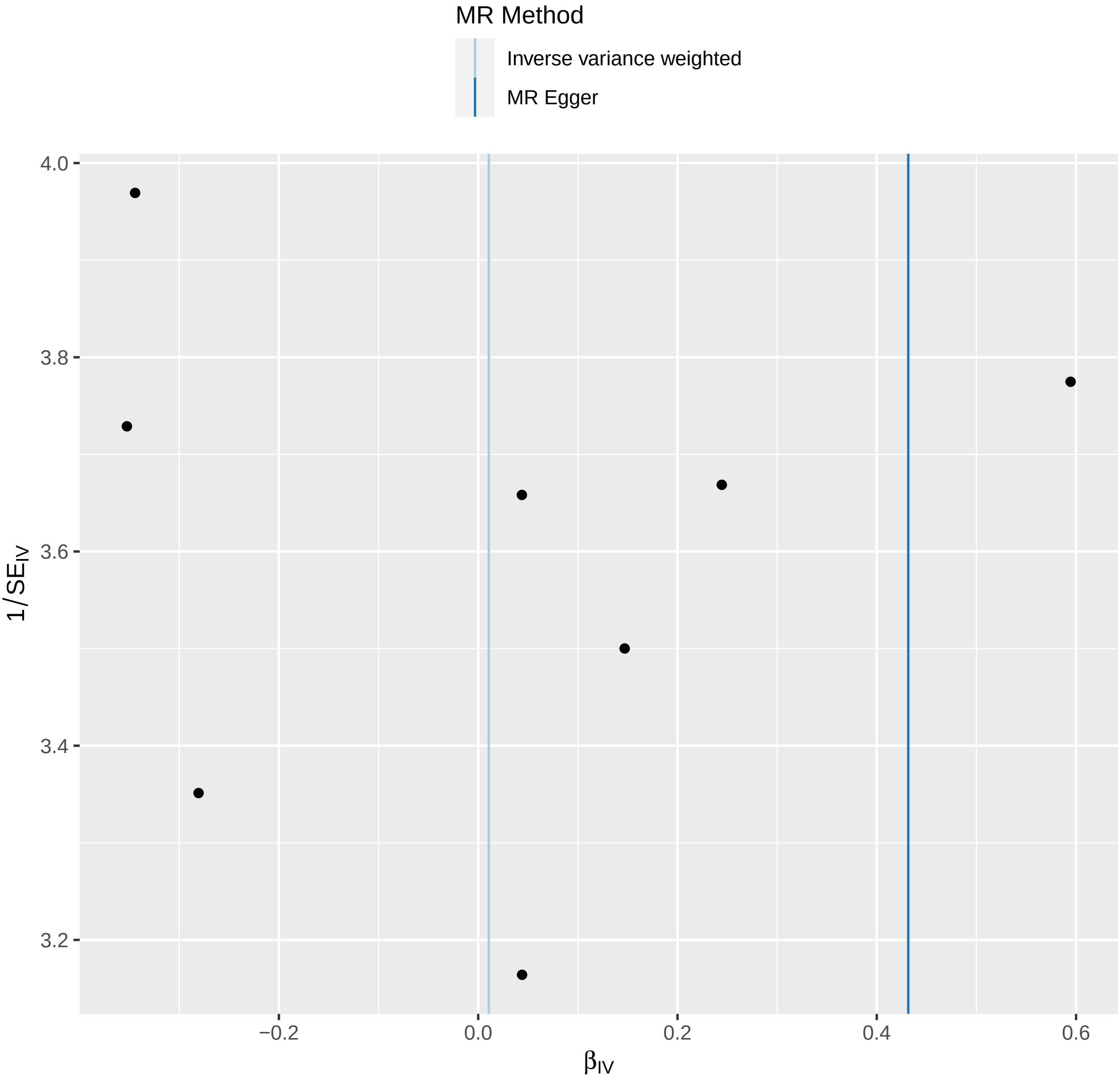

Batch 237 : Gut microbiota abundance (genus Haemophilus id.3698) on Other Crohn's disease

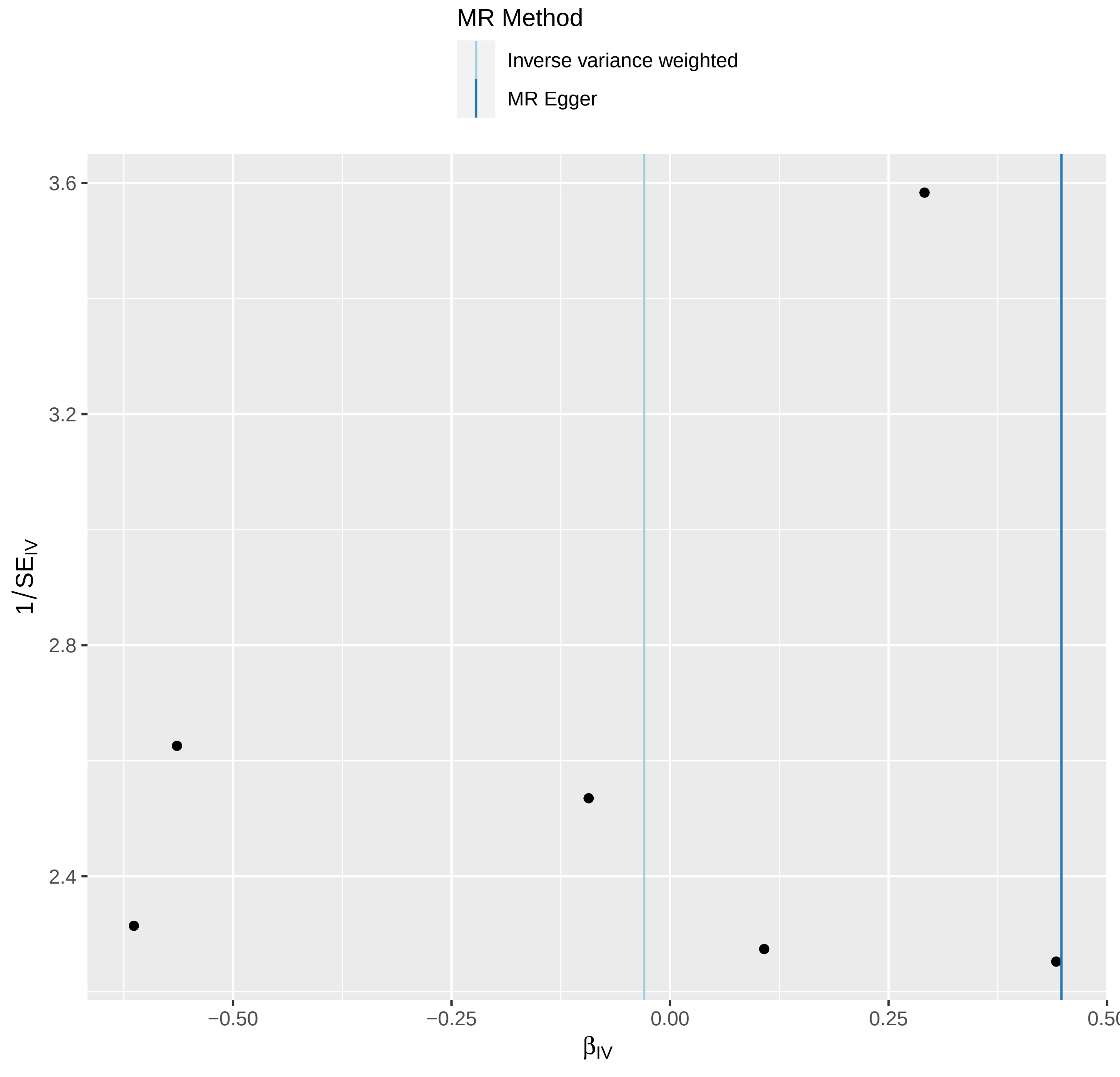

Batch 238 : Gut microbiota abundance (genus Holdemanella id.11393) on Other Crohn's disease

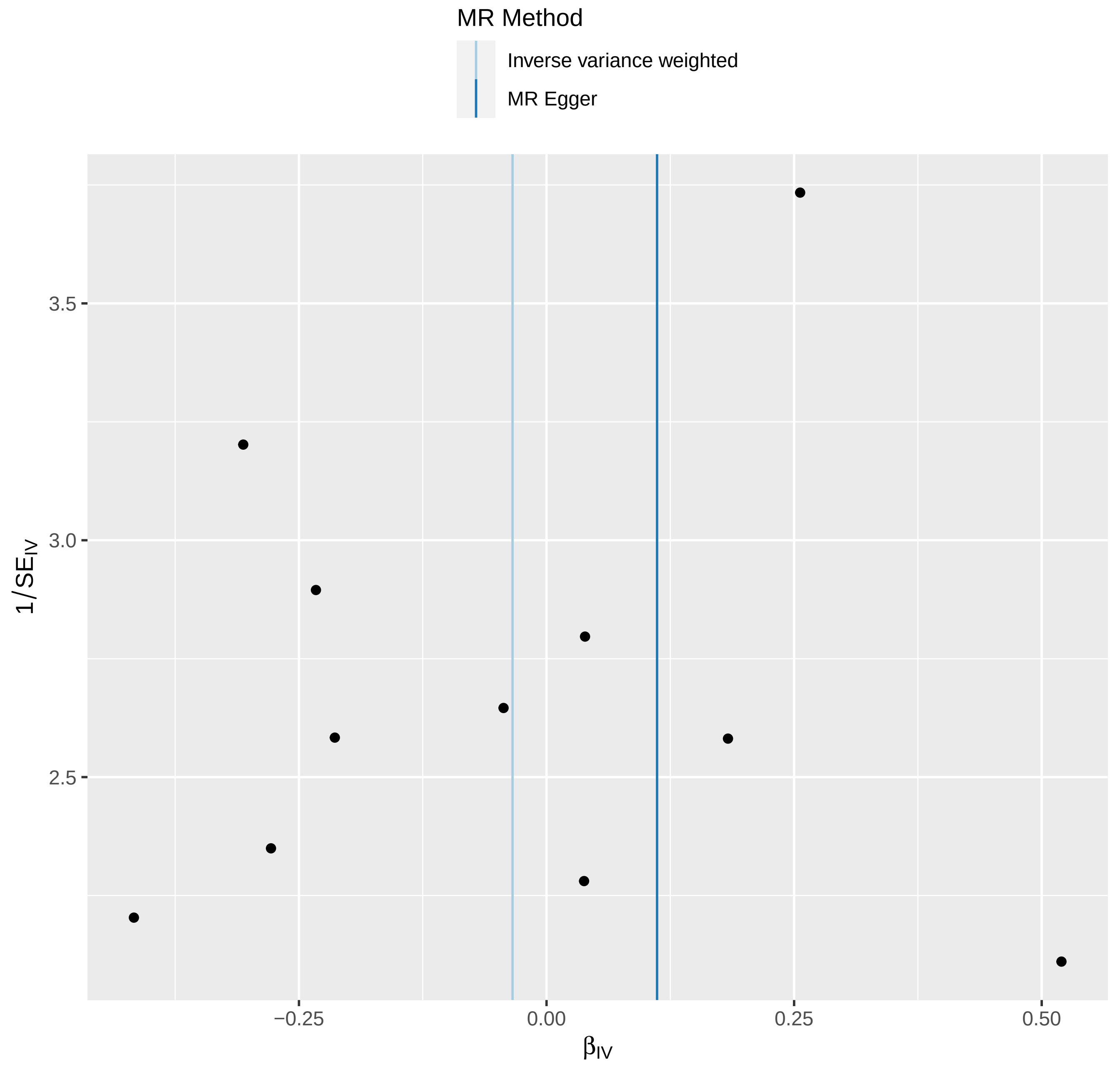

Batch 239 : Gut microbiota abundance (genus Holdemania id.2157) on Other Crohn's disease

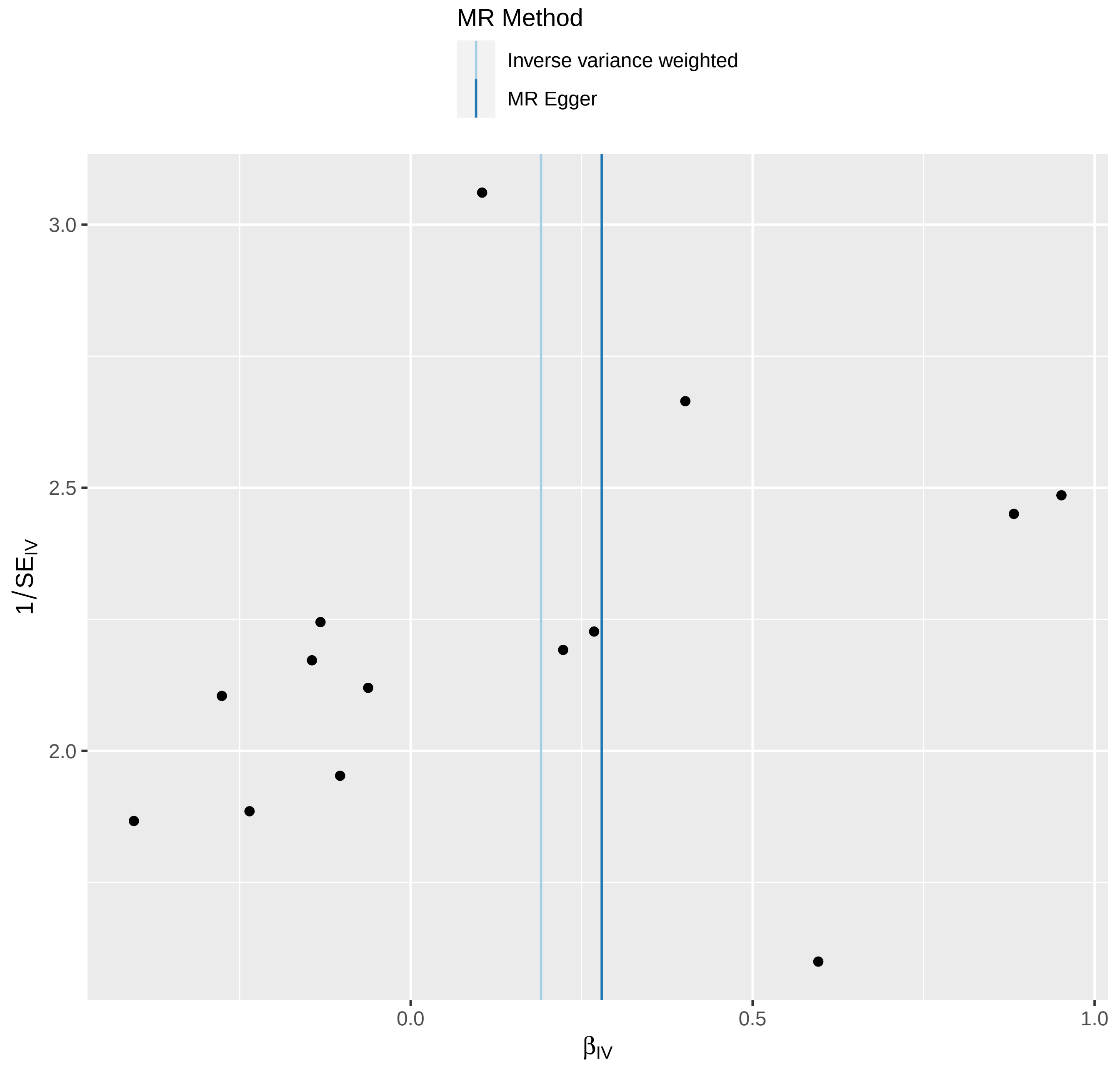

Batch 240 : Gut microbiota abundance (genus Howardella id.2000) on Other Crohn's disease

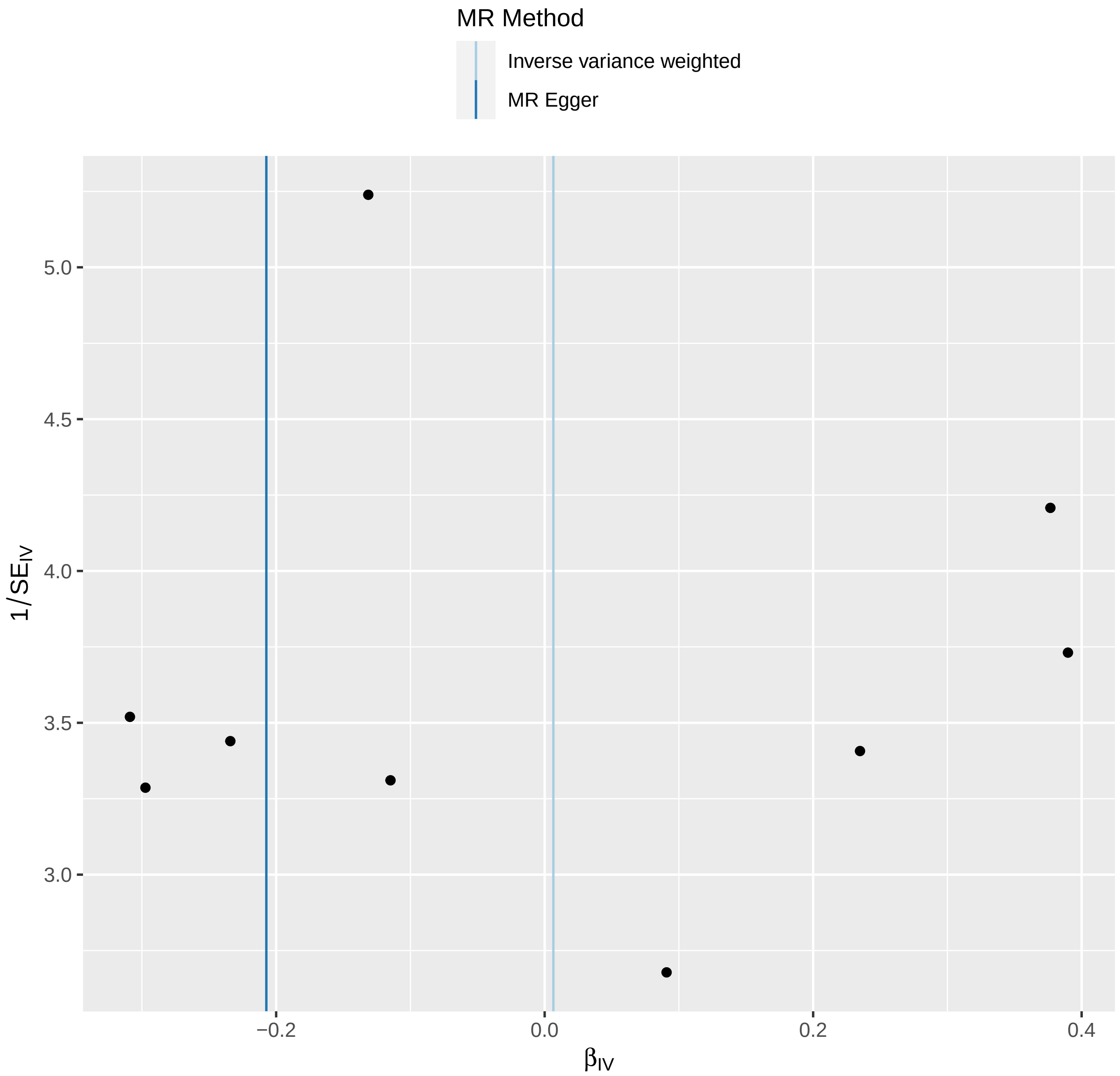

Batch 241 : Gut microbiota abundance (genus Hungatella id.11306) on Other Crohn's disease

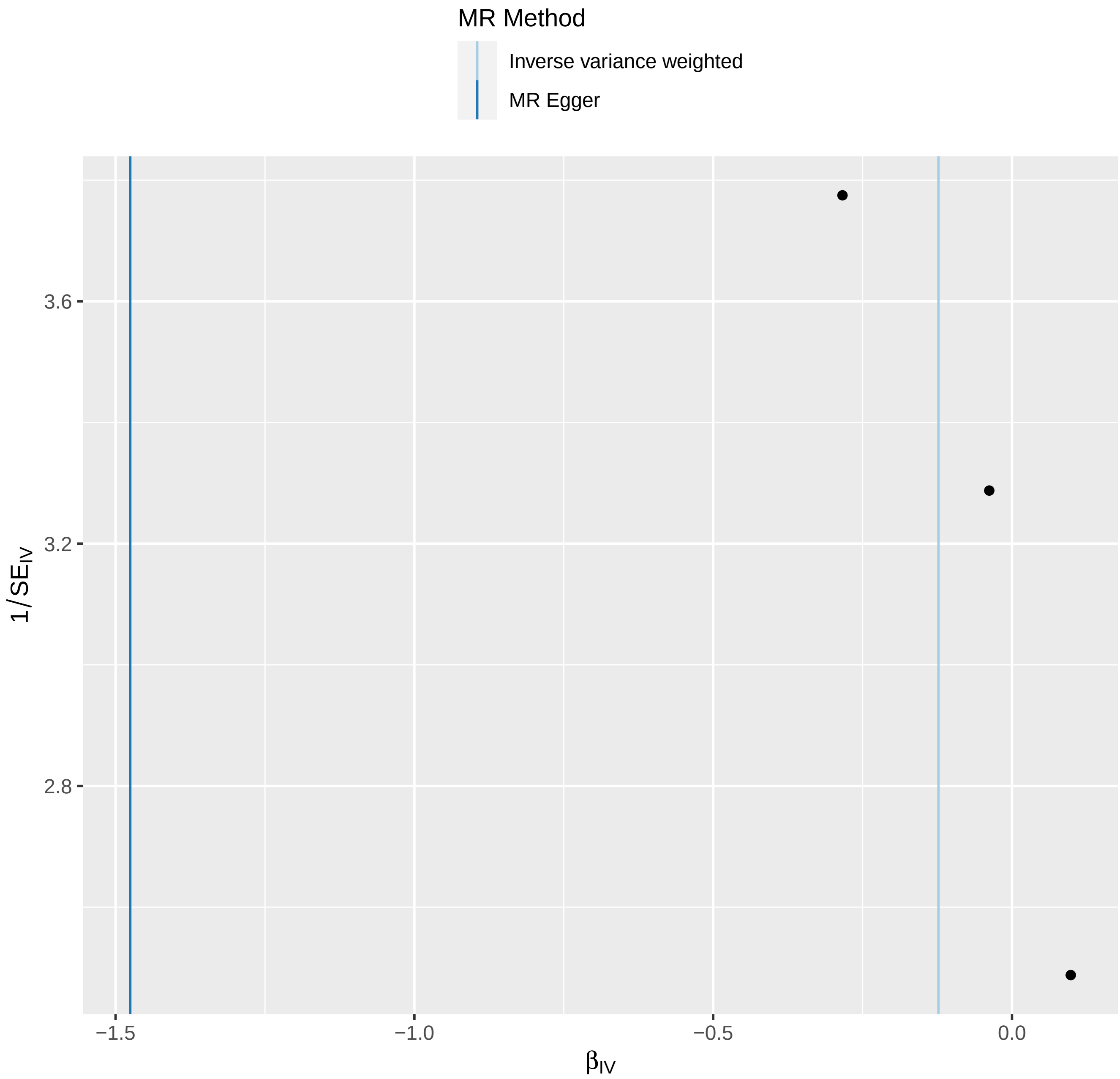

Batch 242 : Gut microbiota abundance (genus Intestinibacter id.11345) on Other Crohn's disease

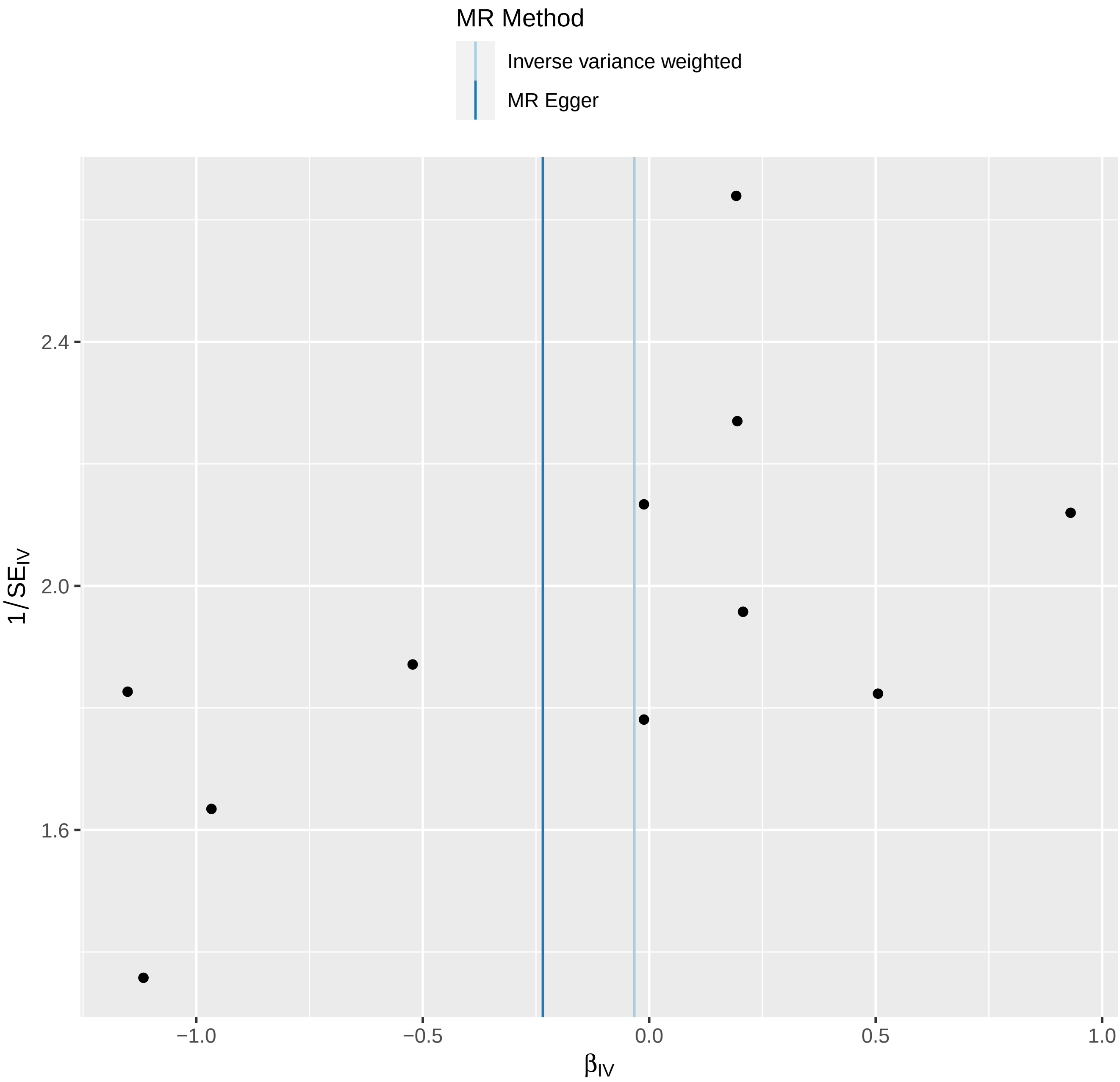

Batch 243 : Gut microbiota abundance (genus Intestinimonas id.2062) on Other Crohn's disease

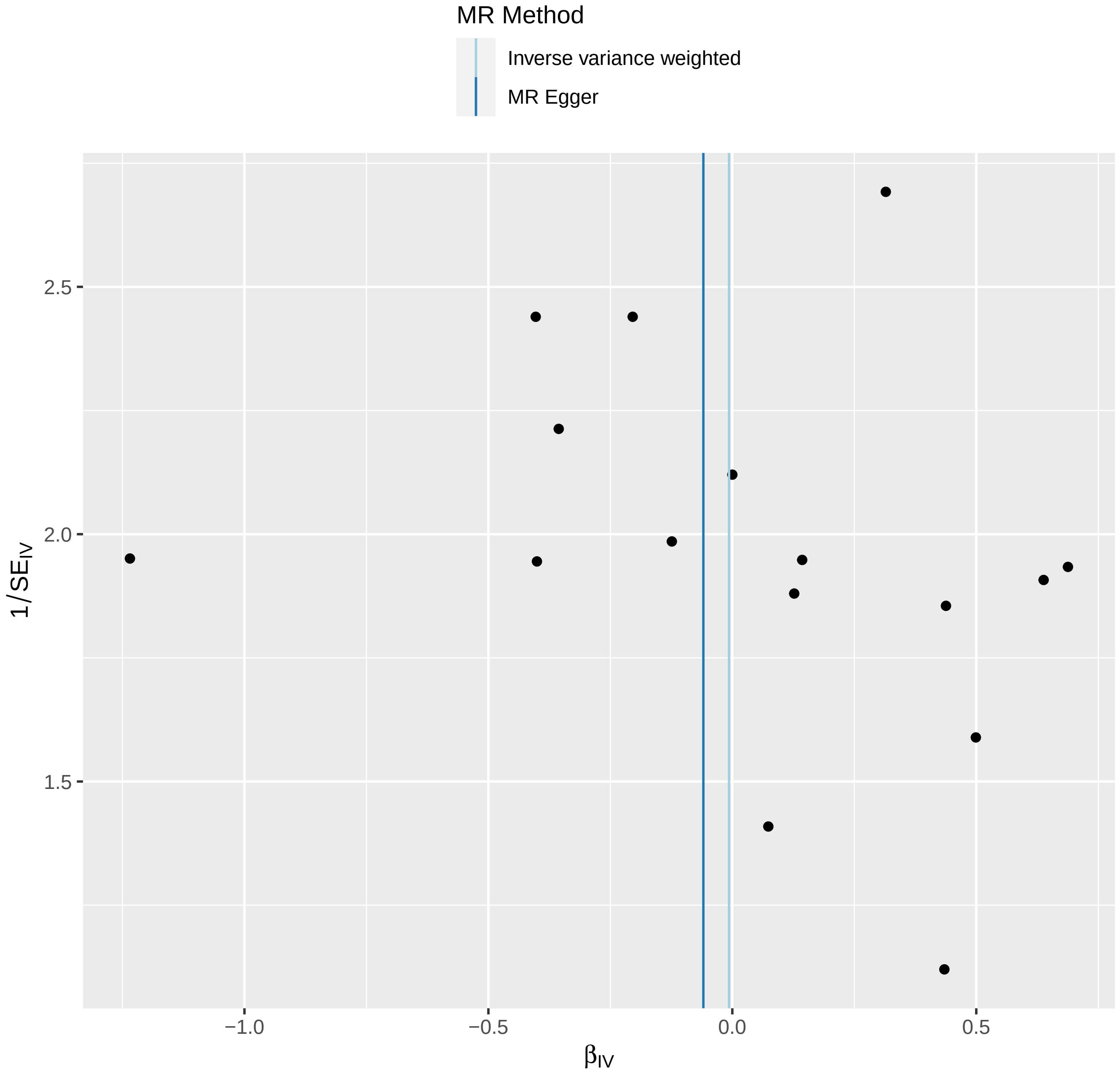

Batch 244 : Gut microbiota abundance (genus Lachnoclostridium id.11308) on Other Crohn's disease

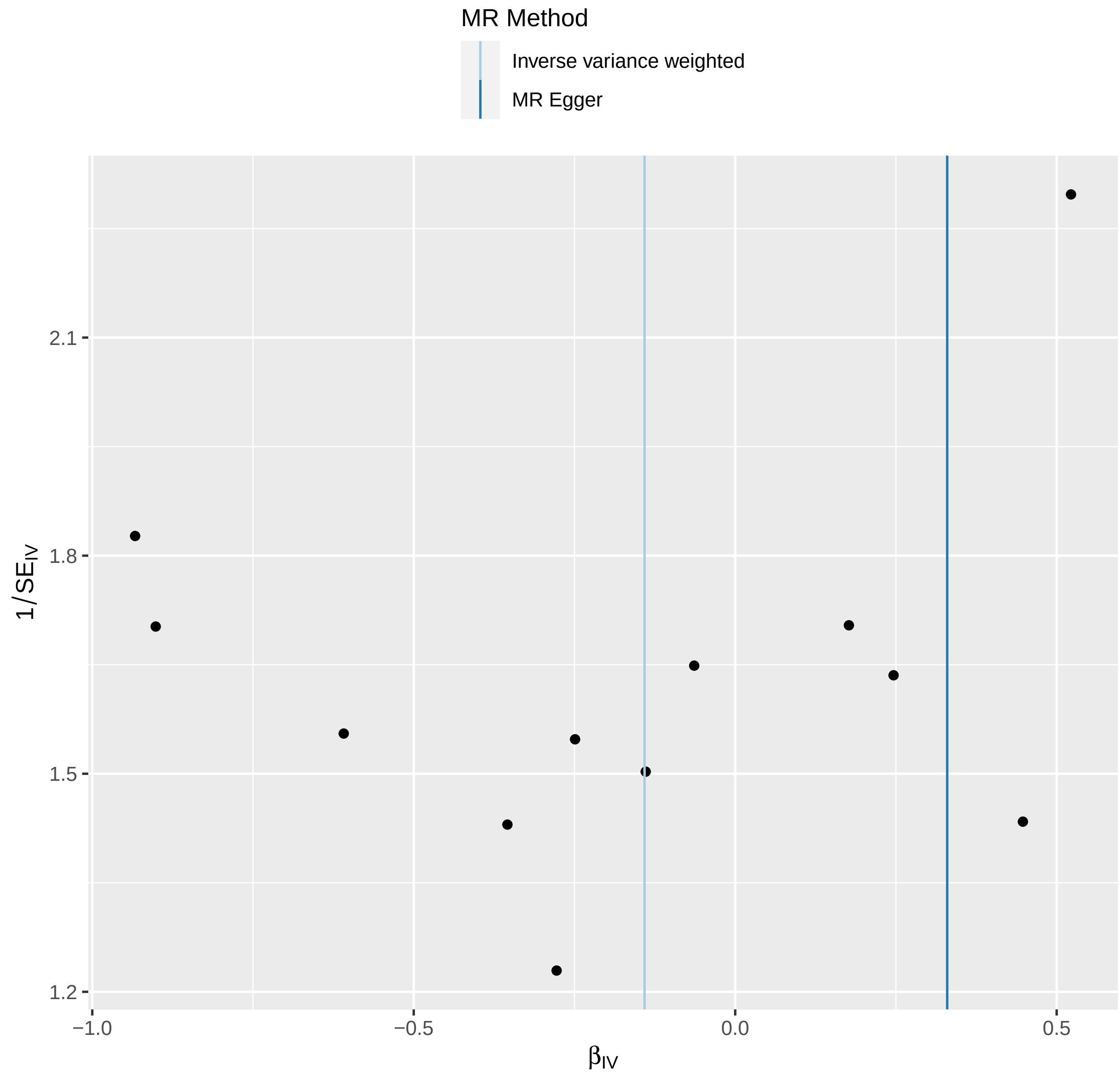

Batch 245 : Gut microbiota abundance (genus Lachnospiraceae FCS020 group id.11314) on Other Crohn's disease

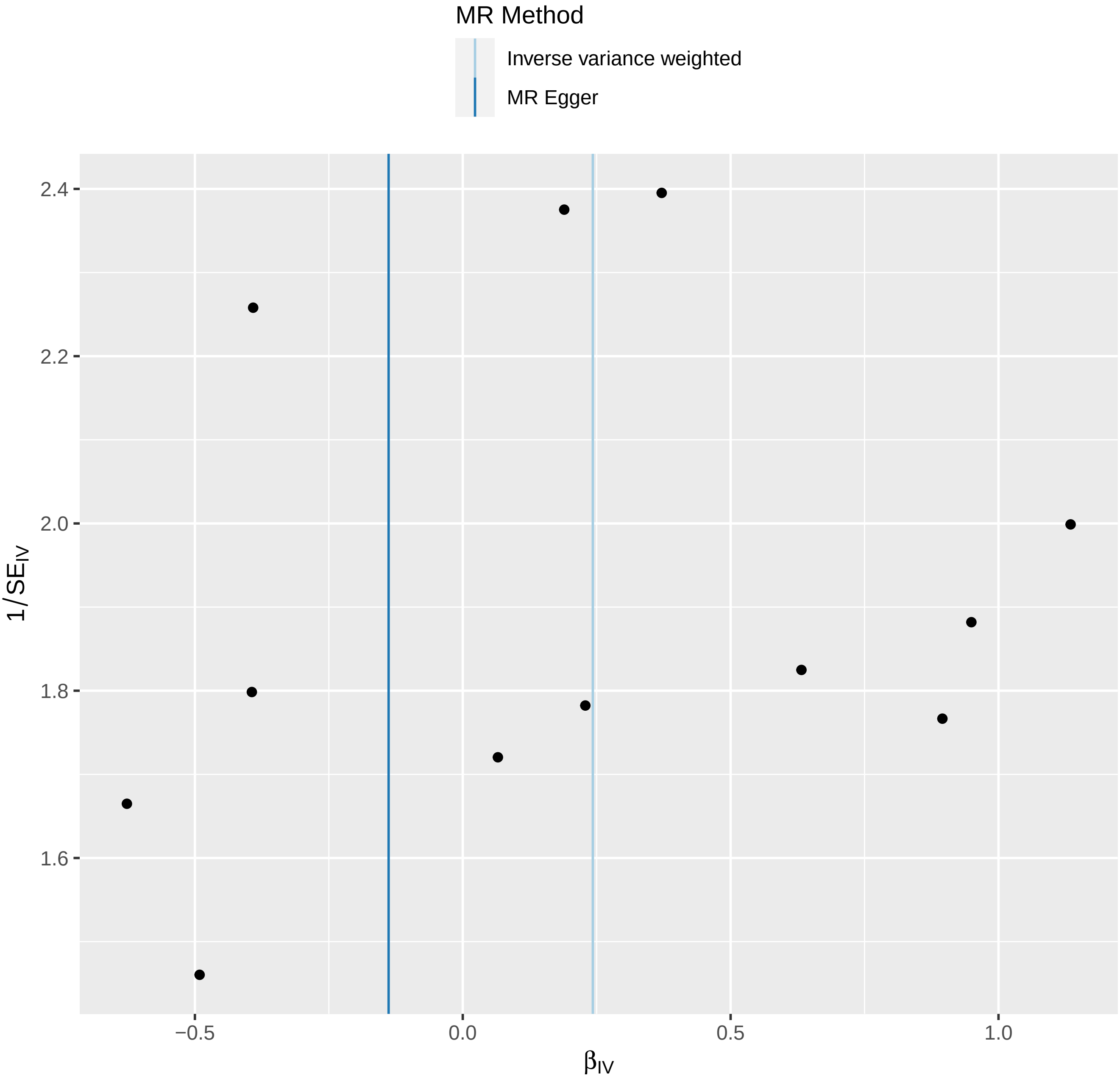

Batch 246 : Gut microbiota abundance (genus Lachnospiraceae NC2004 group id.11316) on Other Crohn's disease

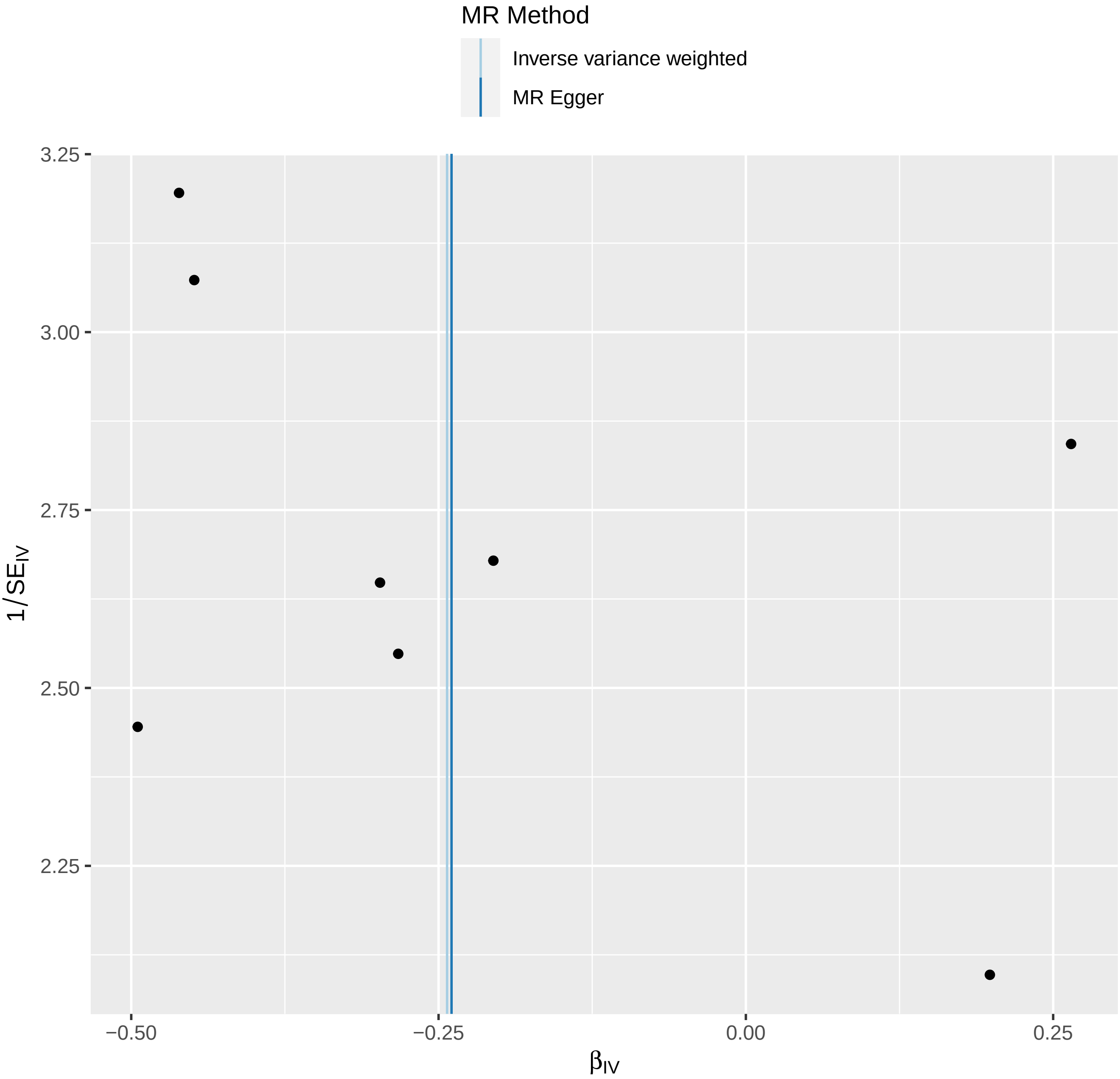

Batch 247 : Gut microbiota abundance (genus Lachnospiraceae ND3007 group id.11317) on Other Crohn's disease

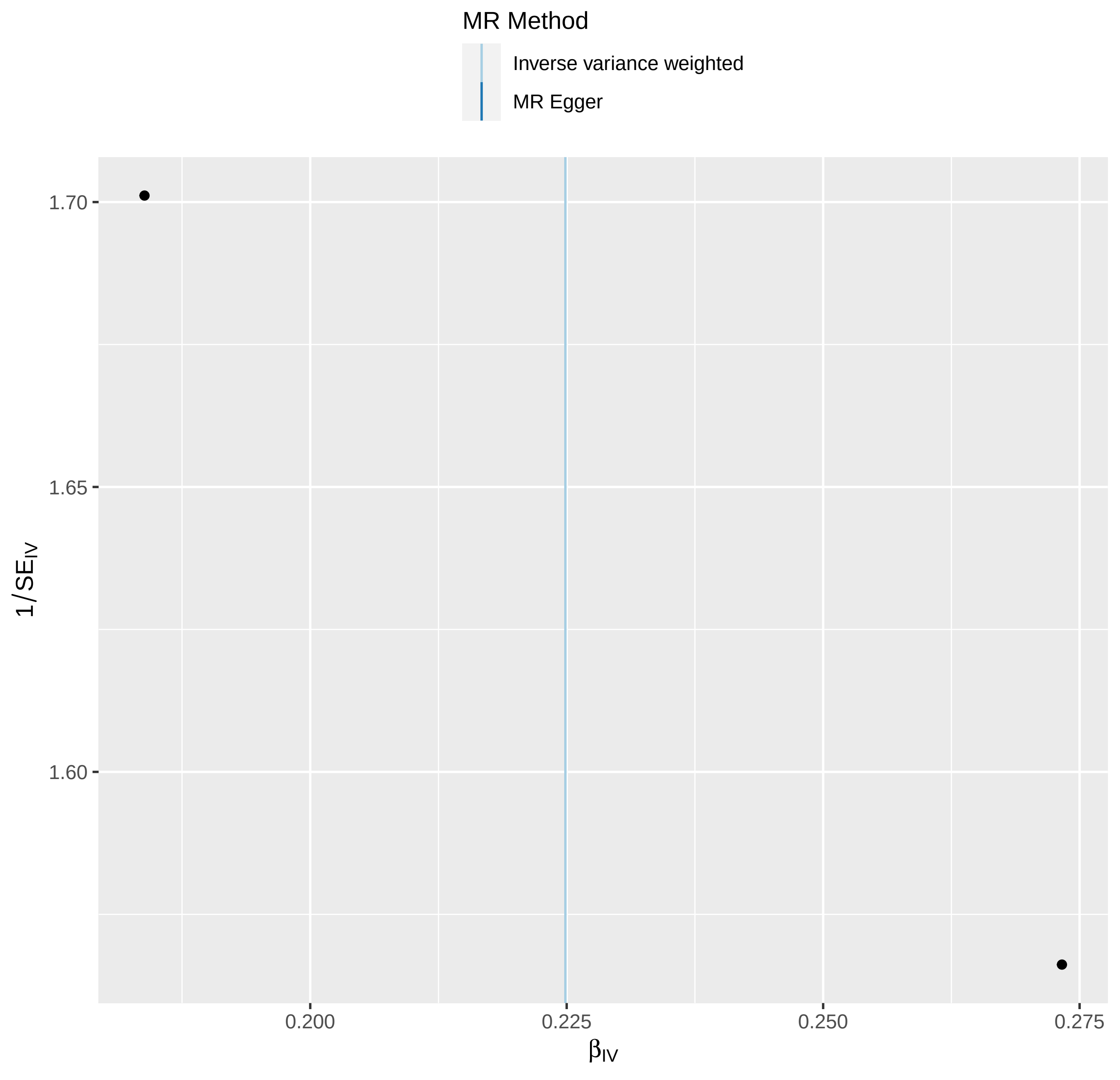

Batch 248 : Gut microbiota abundance (genus Lachnospiraceae NK4A136 group id.11319) on Other Crohn's disease

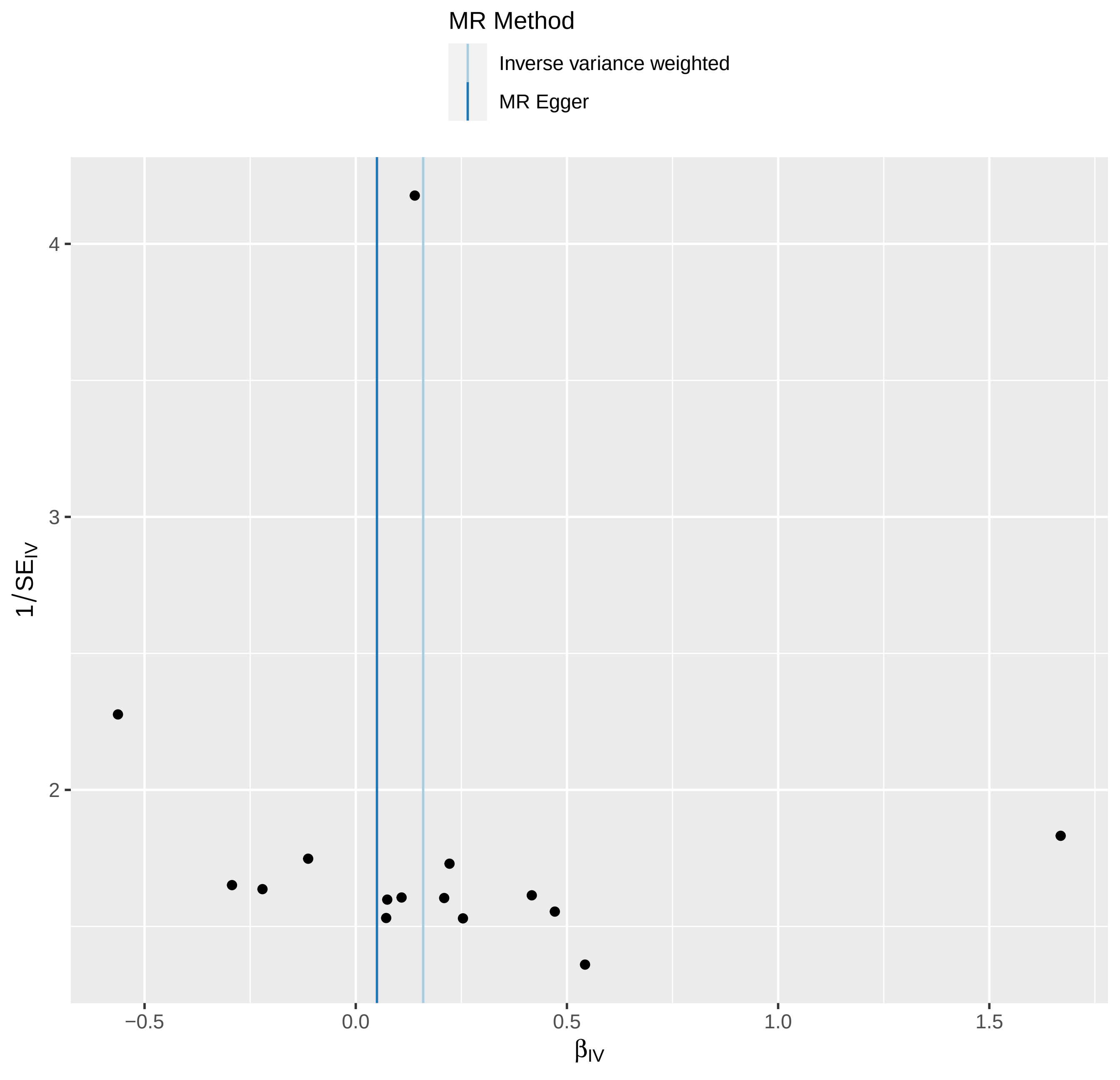

Batch 249 : Gut microbiota abundance (genus Lachnospiraceae UCG001 id.11321) on Other Crohn's disease

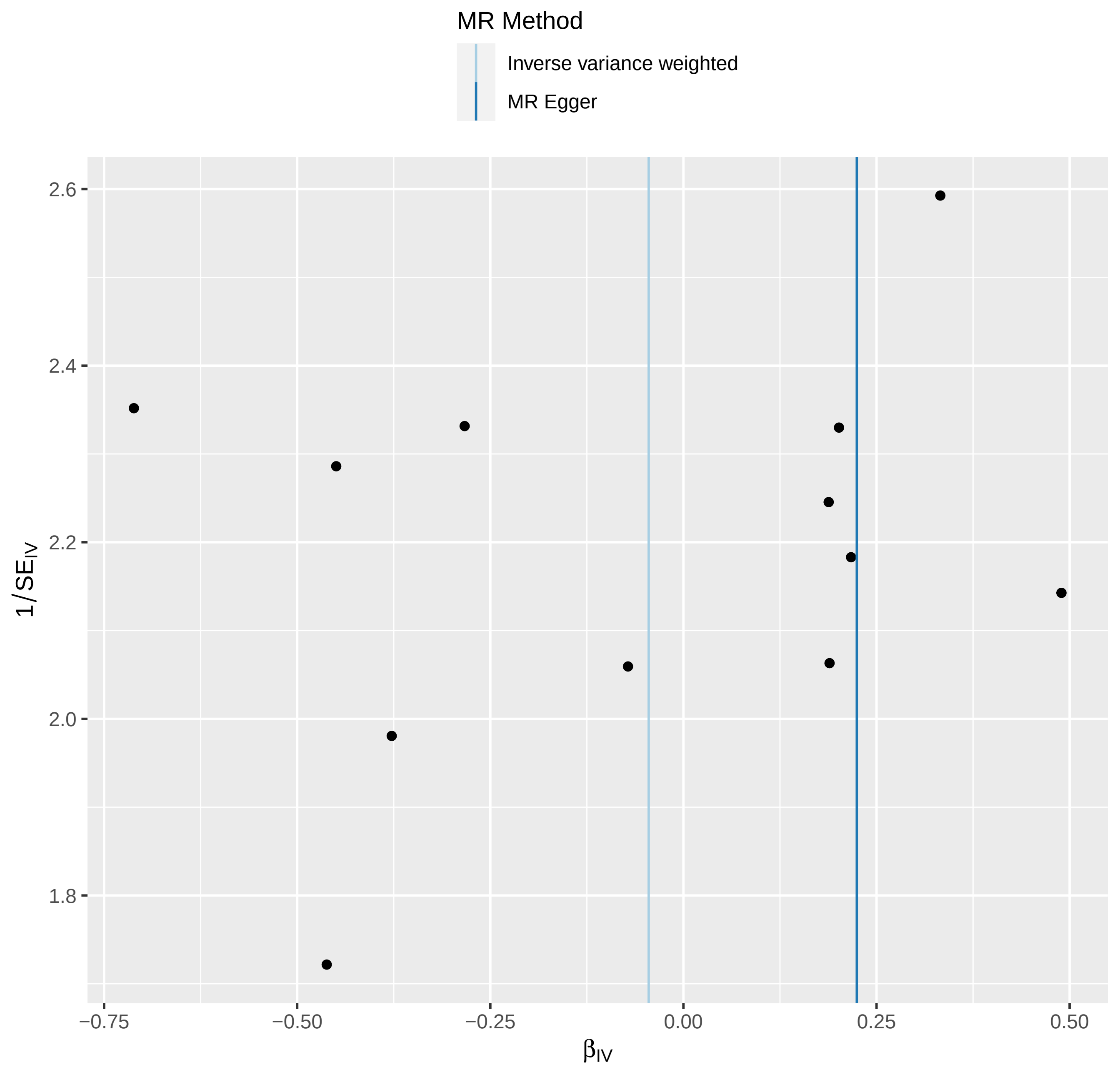

Batch 250 : Gut microbiota abundance (genus Lachnospiraceae UCG004 id.11324) on Other Crohn's disease

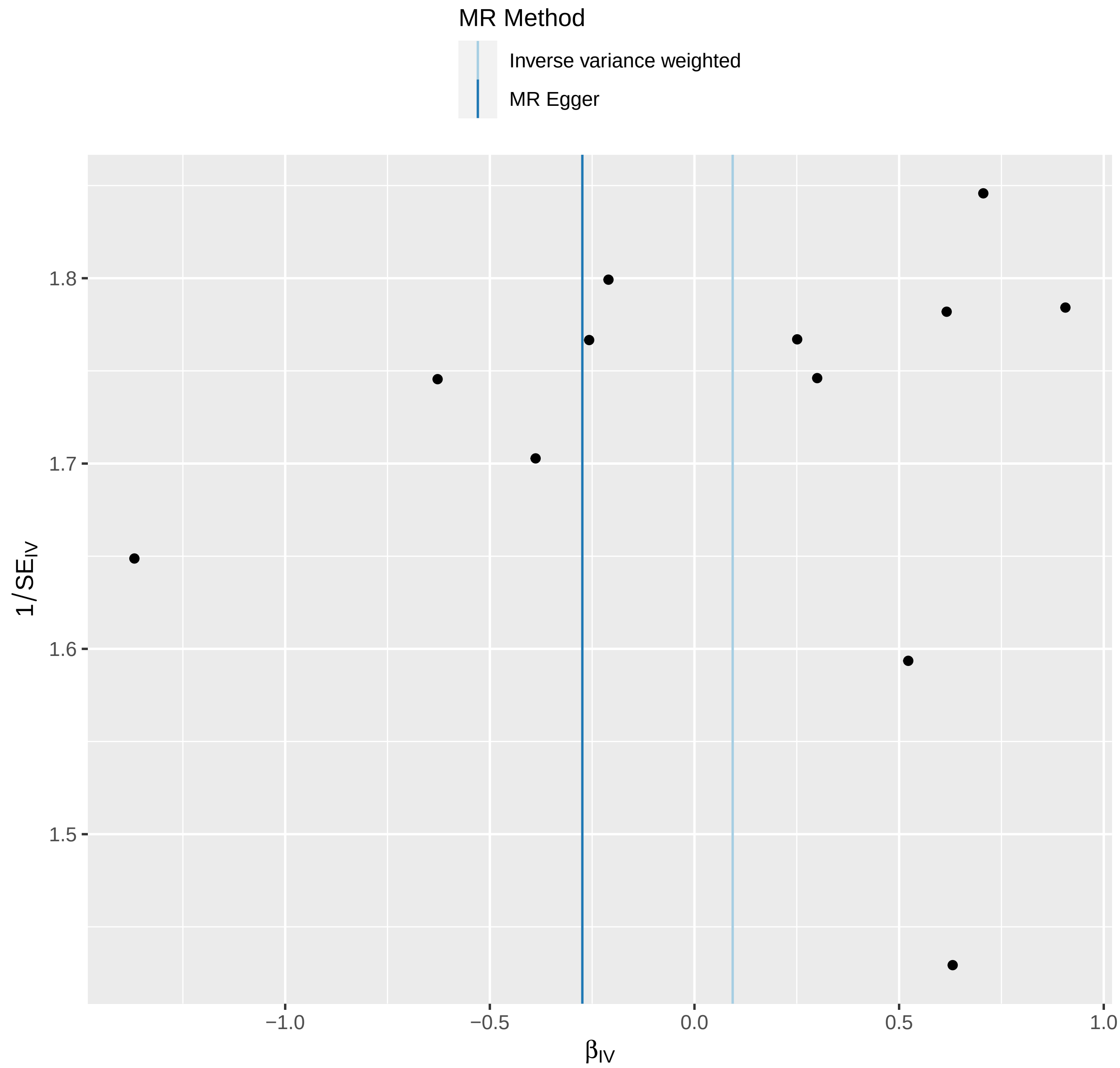

Batch 251 : Gut microbiota abundance (genus Lachnospiraceae UCG008 id.11328) on Other Crohn's disease

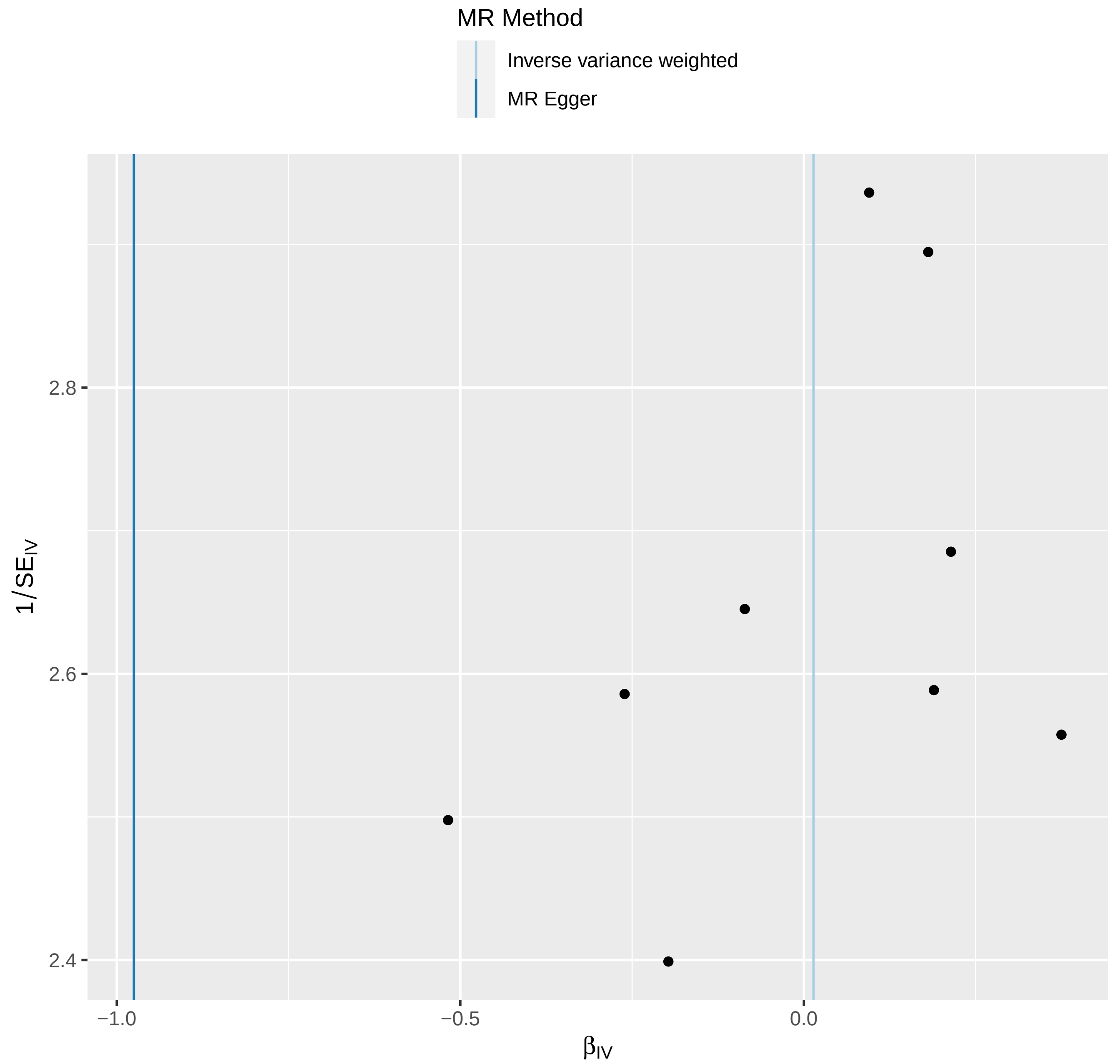

Batch 252 : Gut microbiota abundance (genus Lachnospiraceae UCG010 id.11330) on Other Crohn's disease

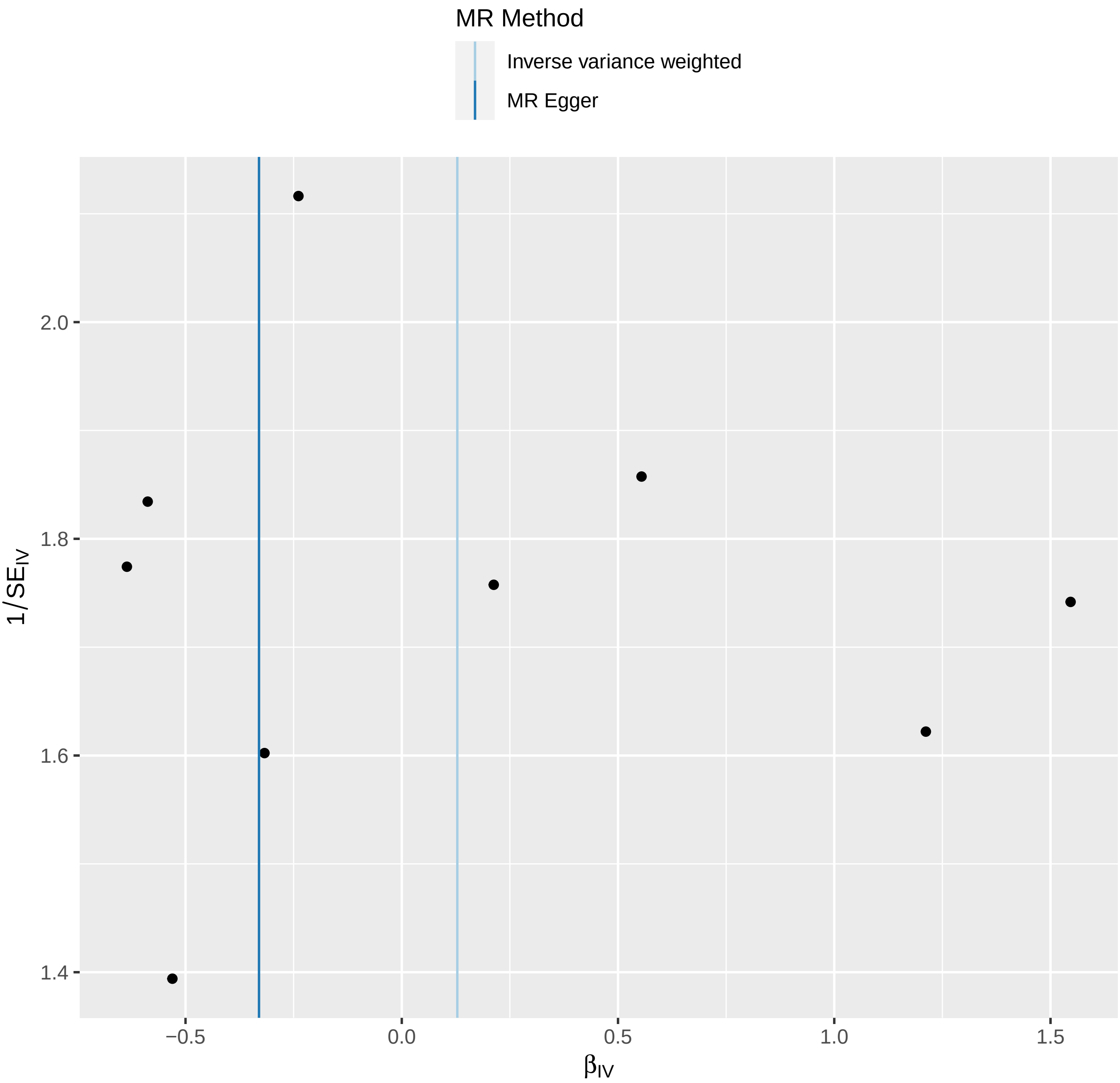

Insufficient number of SNPs

Batch 254 : Gut microbiota abundance (genus Lactobacillus id.1837) on Other Crohn's disease

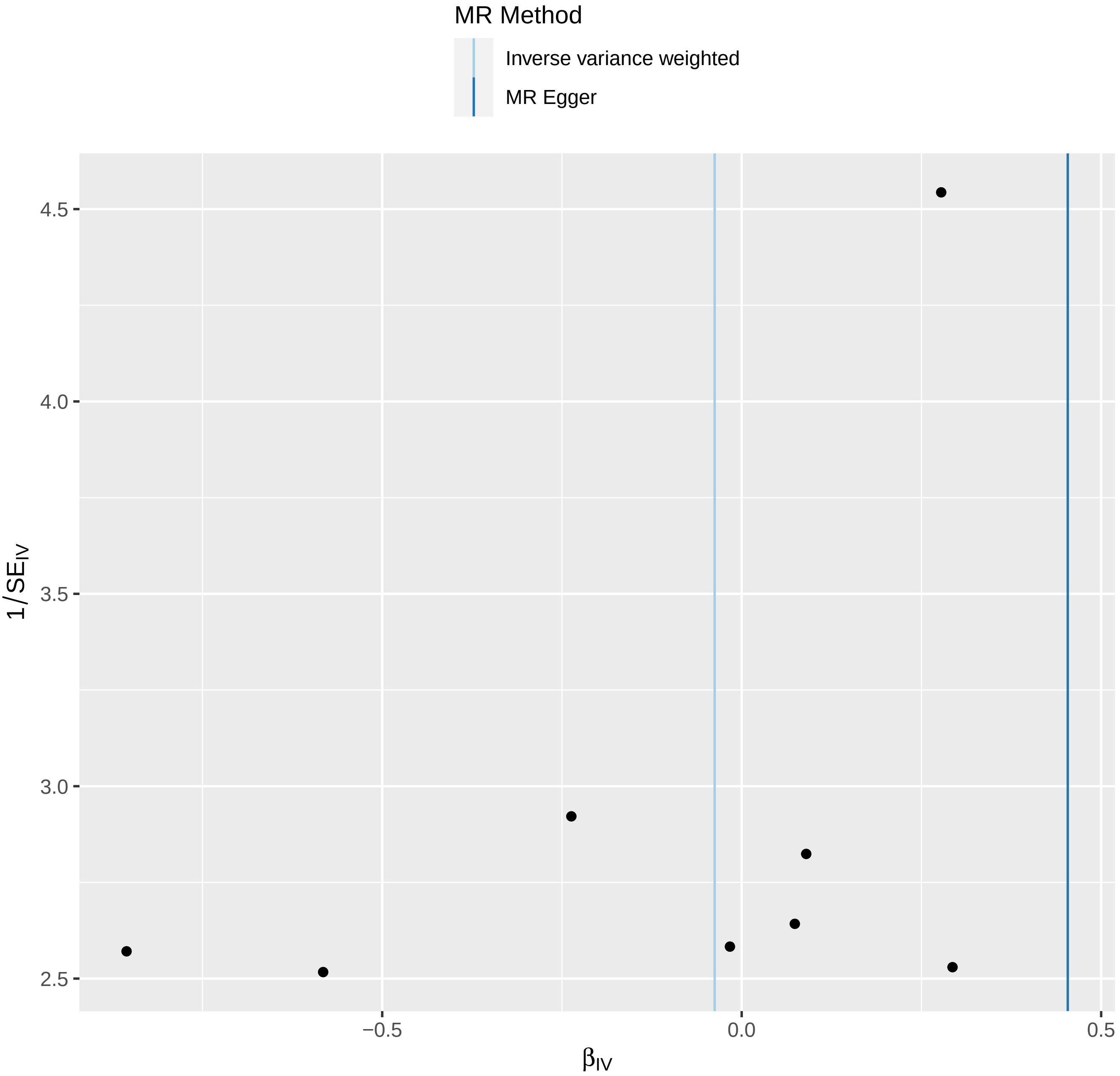

Batch 255 : Gut microbiota abundance (genus Lactococcus id.1851) on Other Crohn's disease

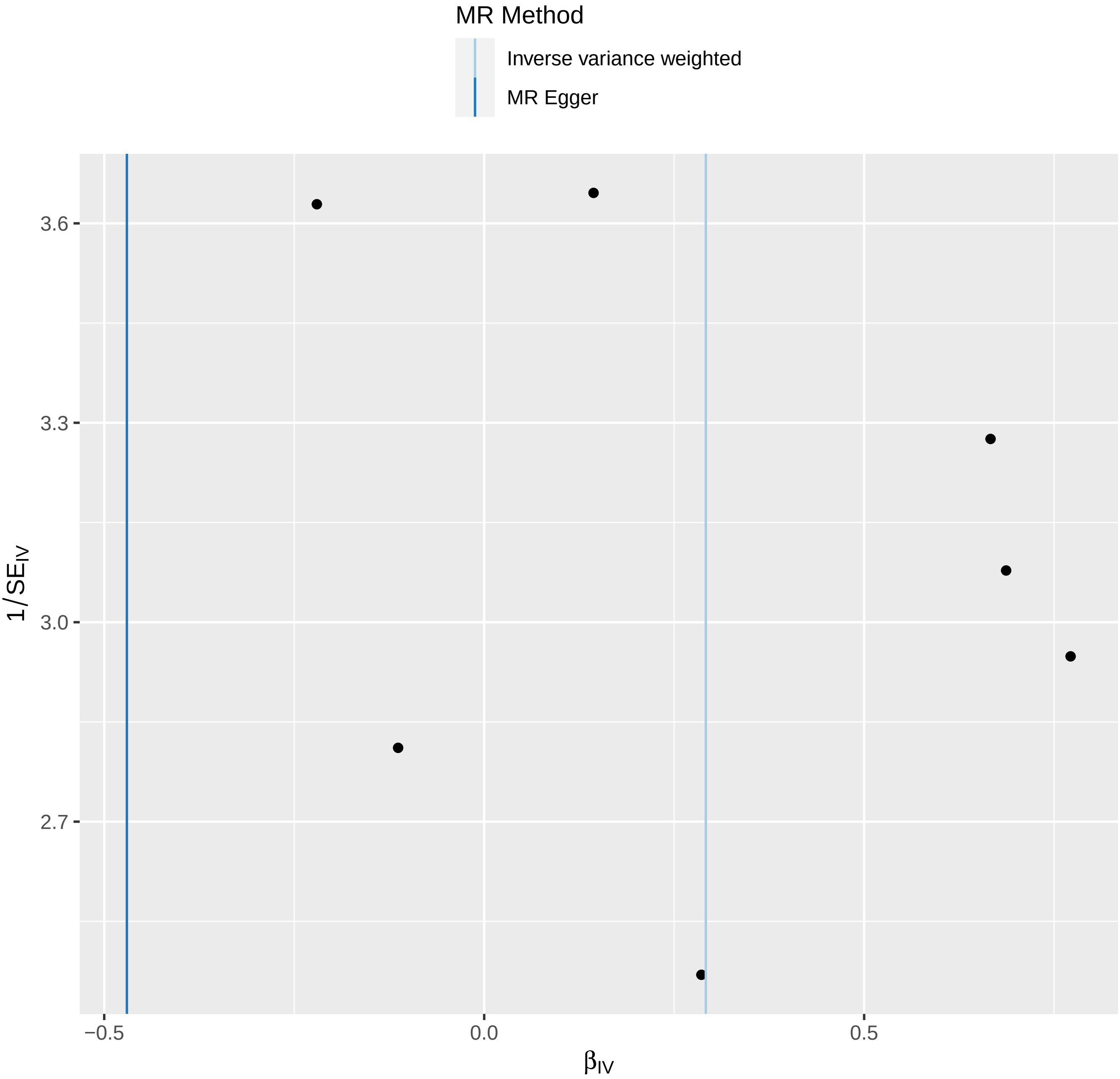

Batch 256 : Gut microbiota abundance (genus Marvinbryantia id.2005) on Other Crohn's disease

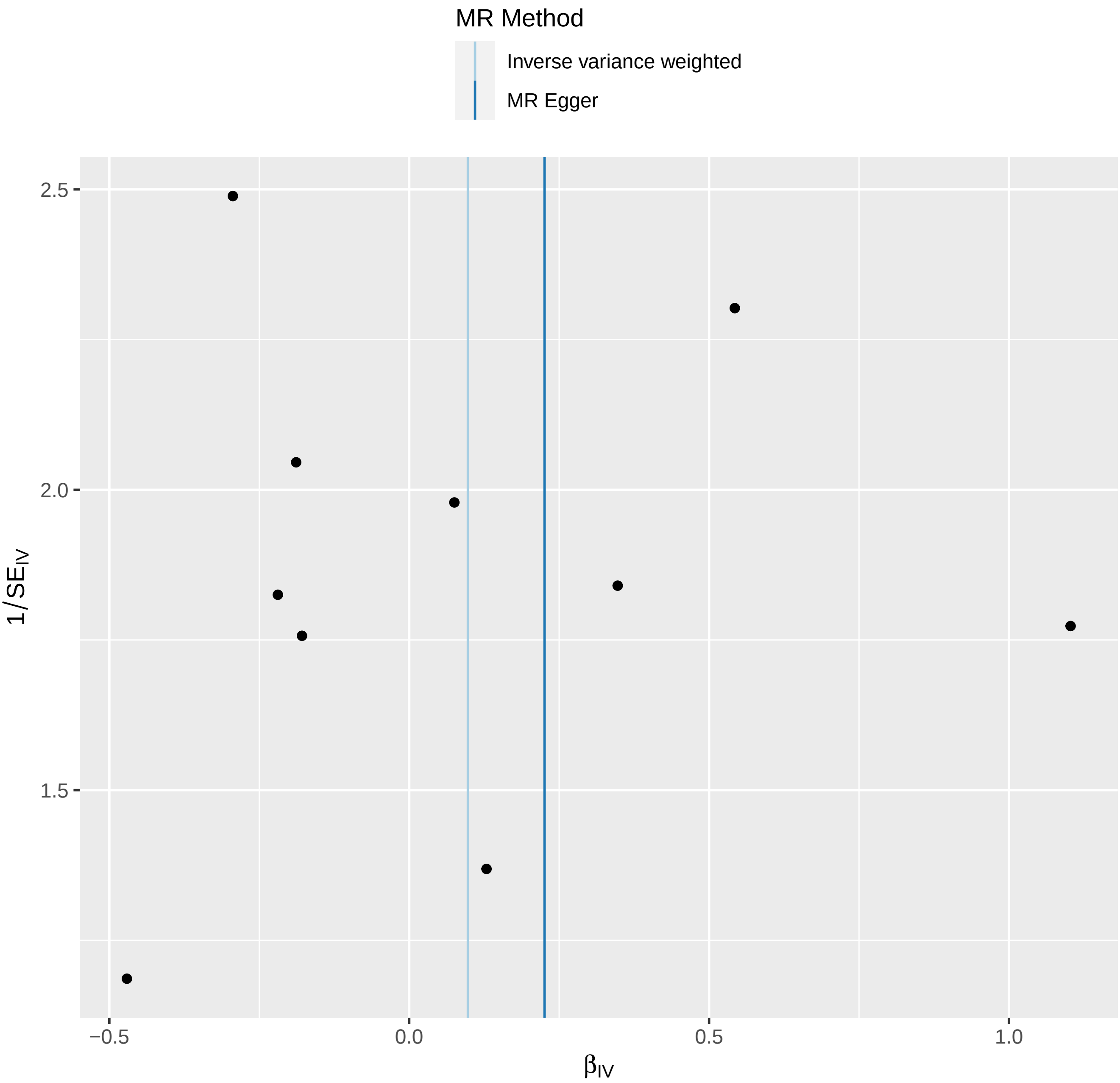

Batch 257 : Gut microbiota abundance (genus Methanobrevibacter id.123) on Other Crohn's disease

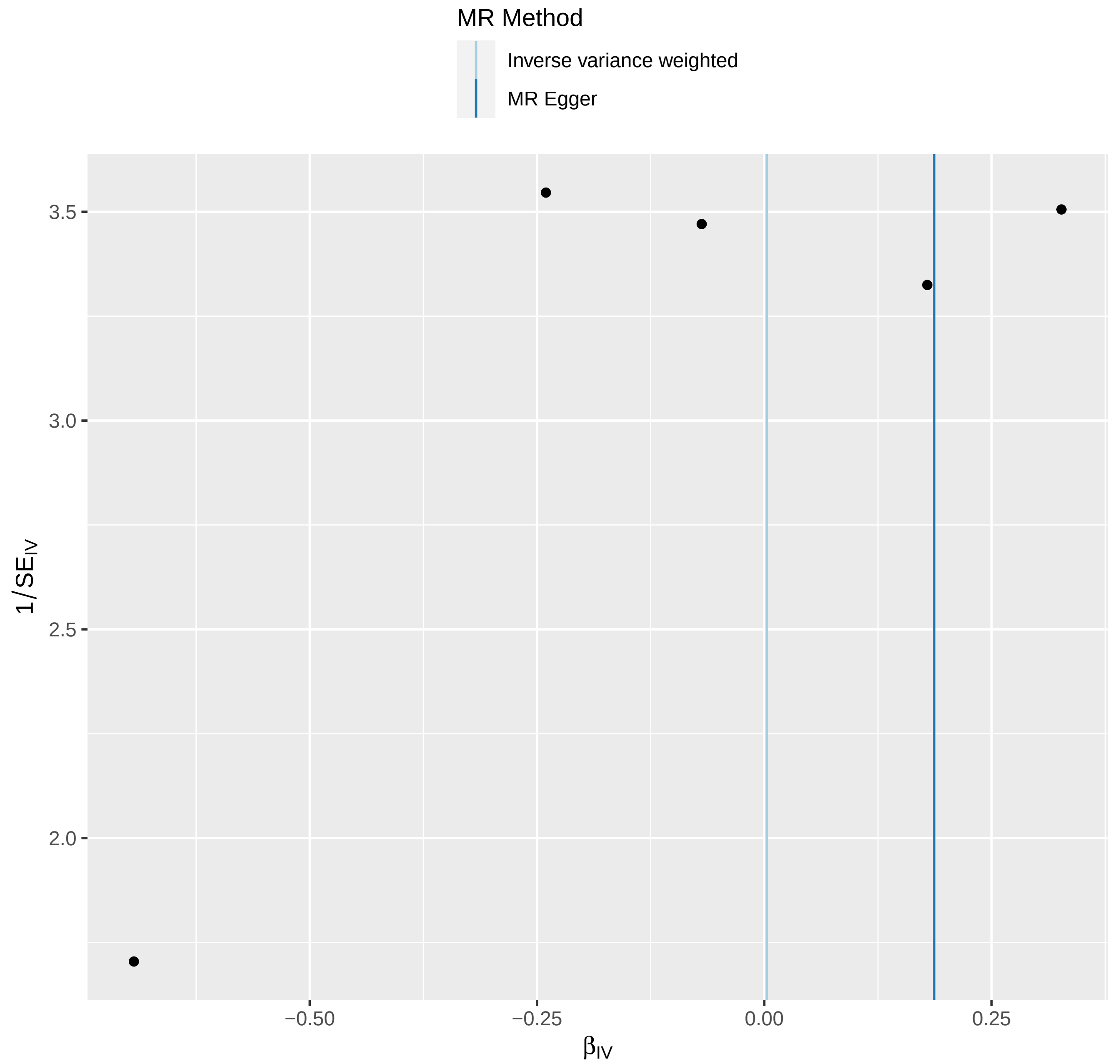

Batch 258 : Gut microbiota abundance (genus Odoribacter id.952) on Other Crohn's disease

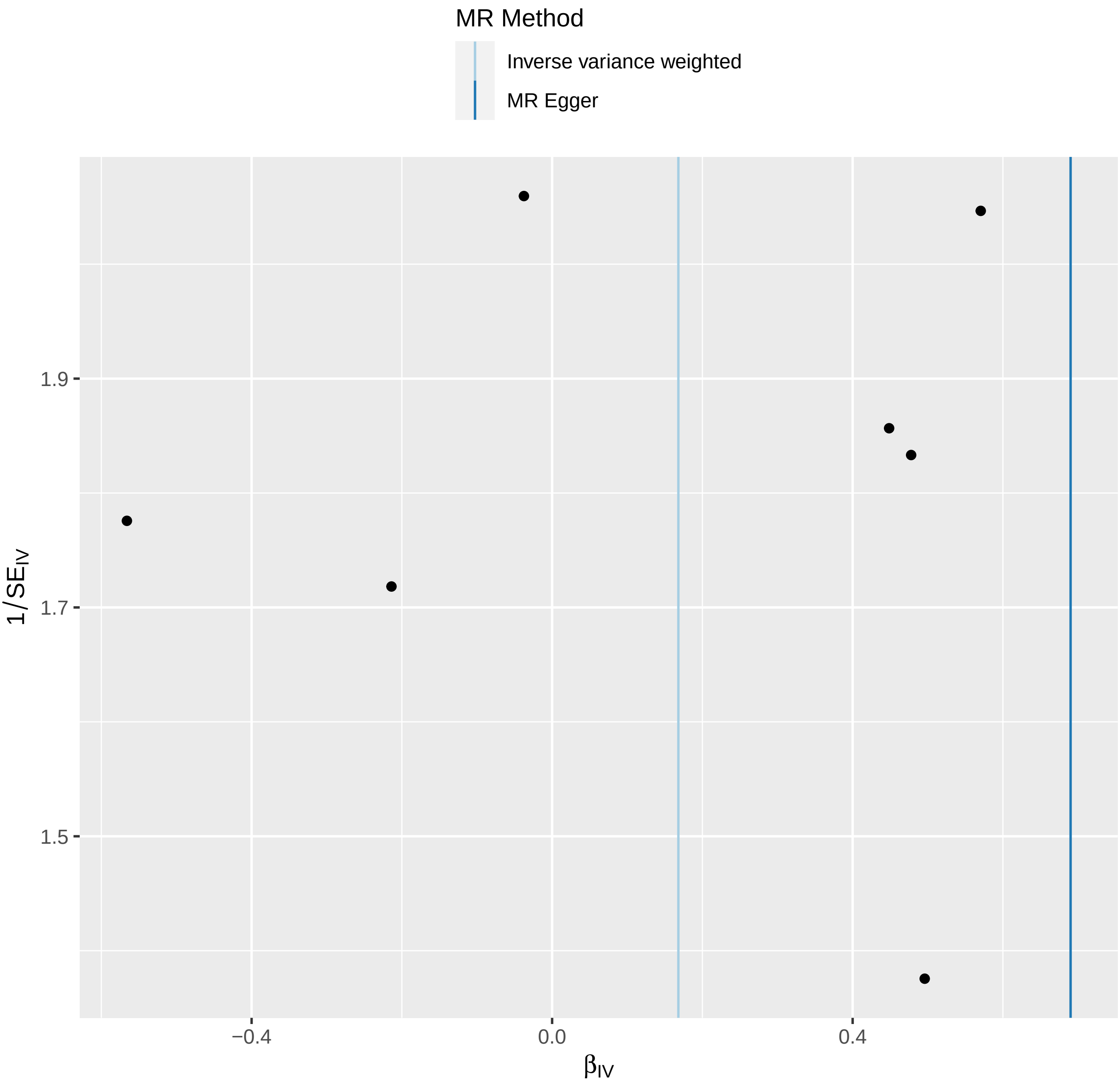

Batch 259 : Gut microbiota abundance (genus Olsenella id.822) on Other Crohn's disease

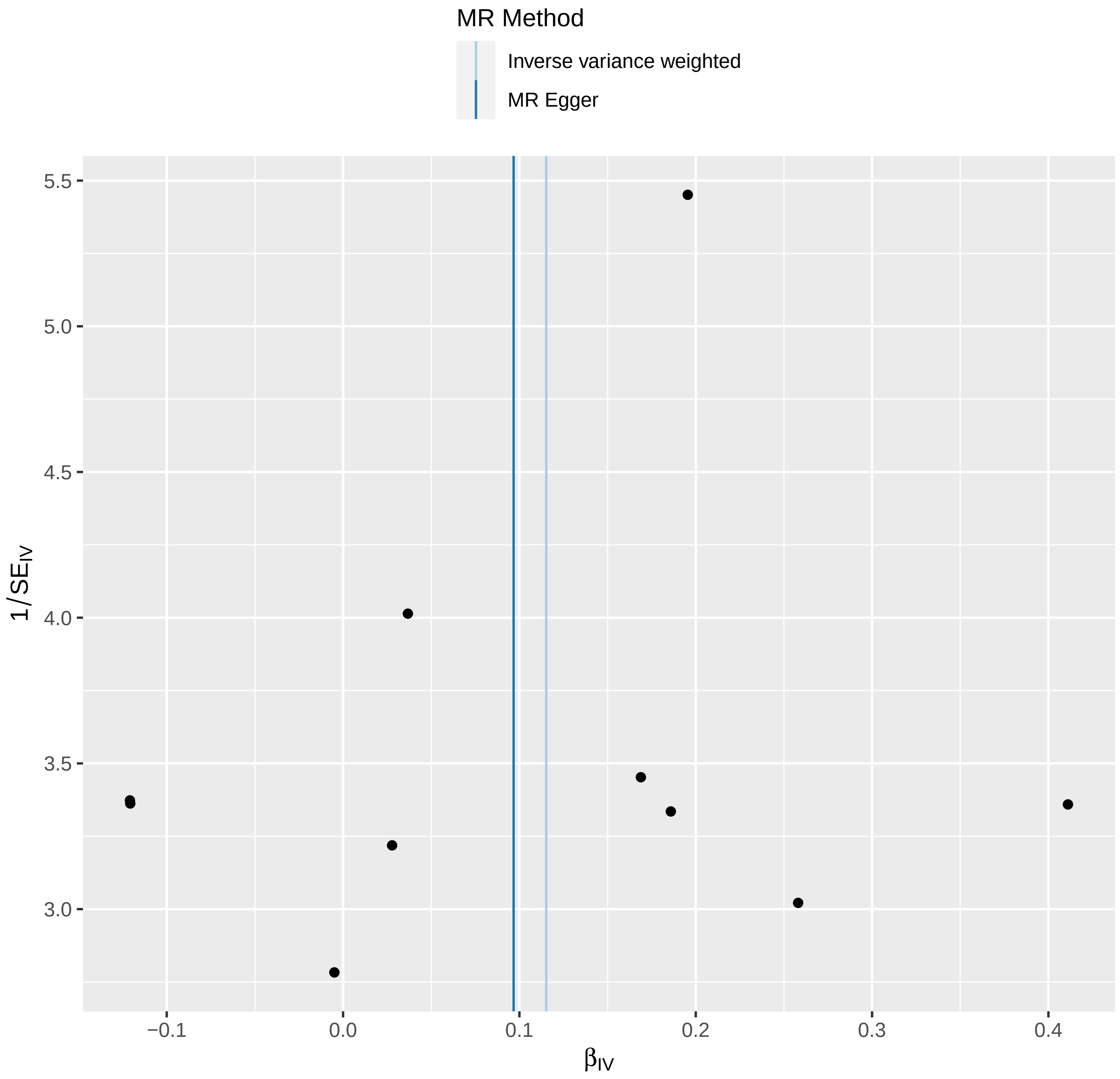

Batch 260 : Gut microbiota abundance (genus Oscillibacter id.2063) on Other Crohn's disease

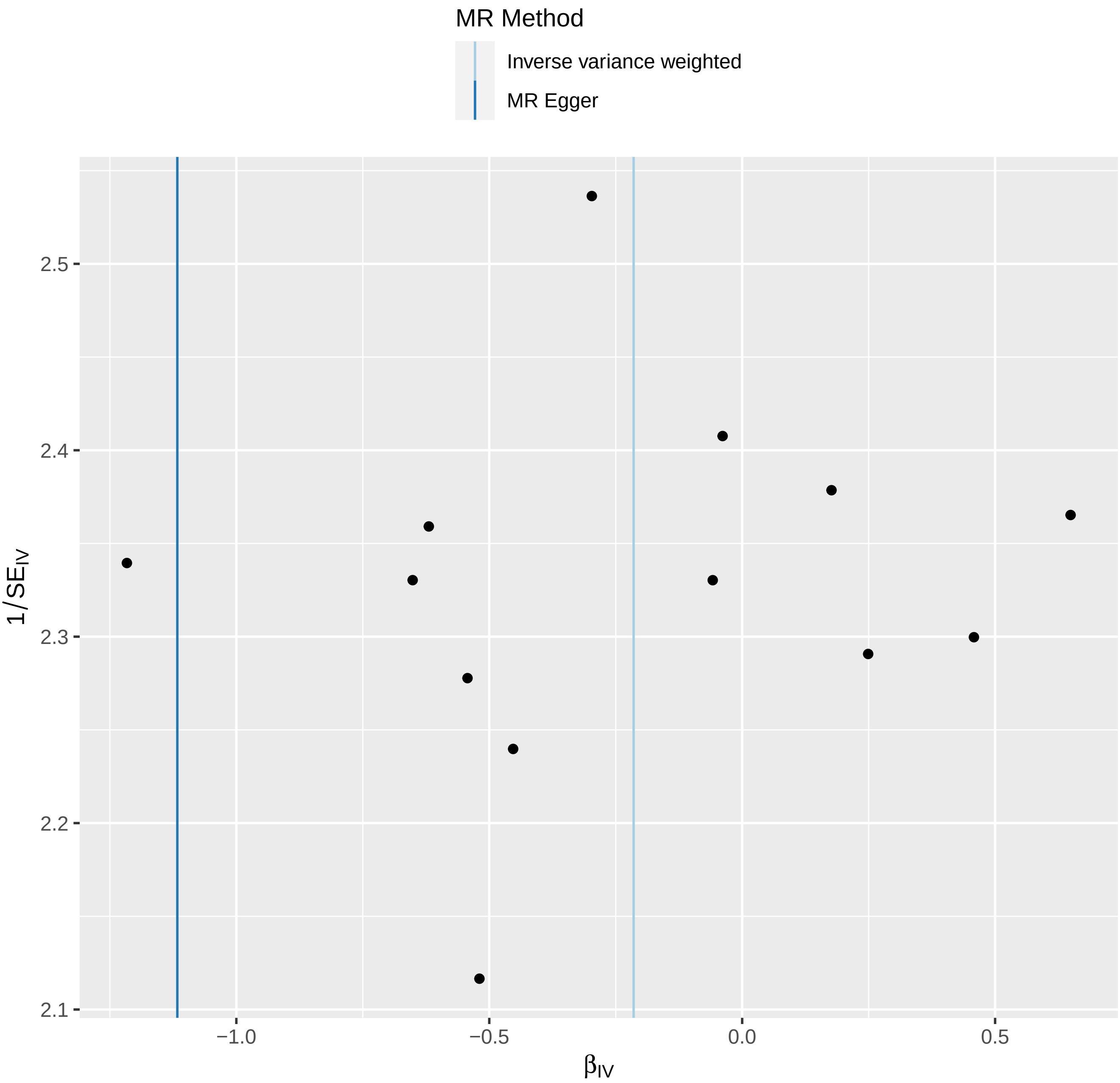

Batch 261 : Gut microbiota abundance (genus Oscillospira id.2064) on Other Crohn's disease

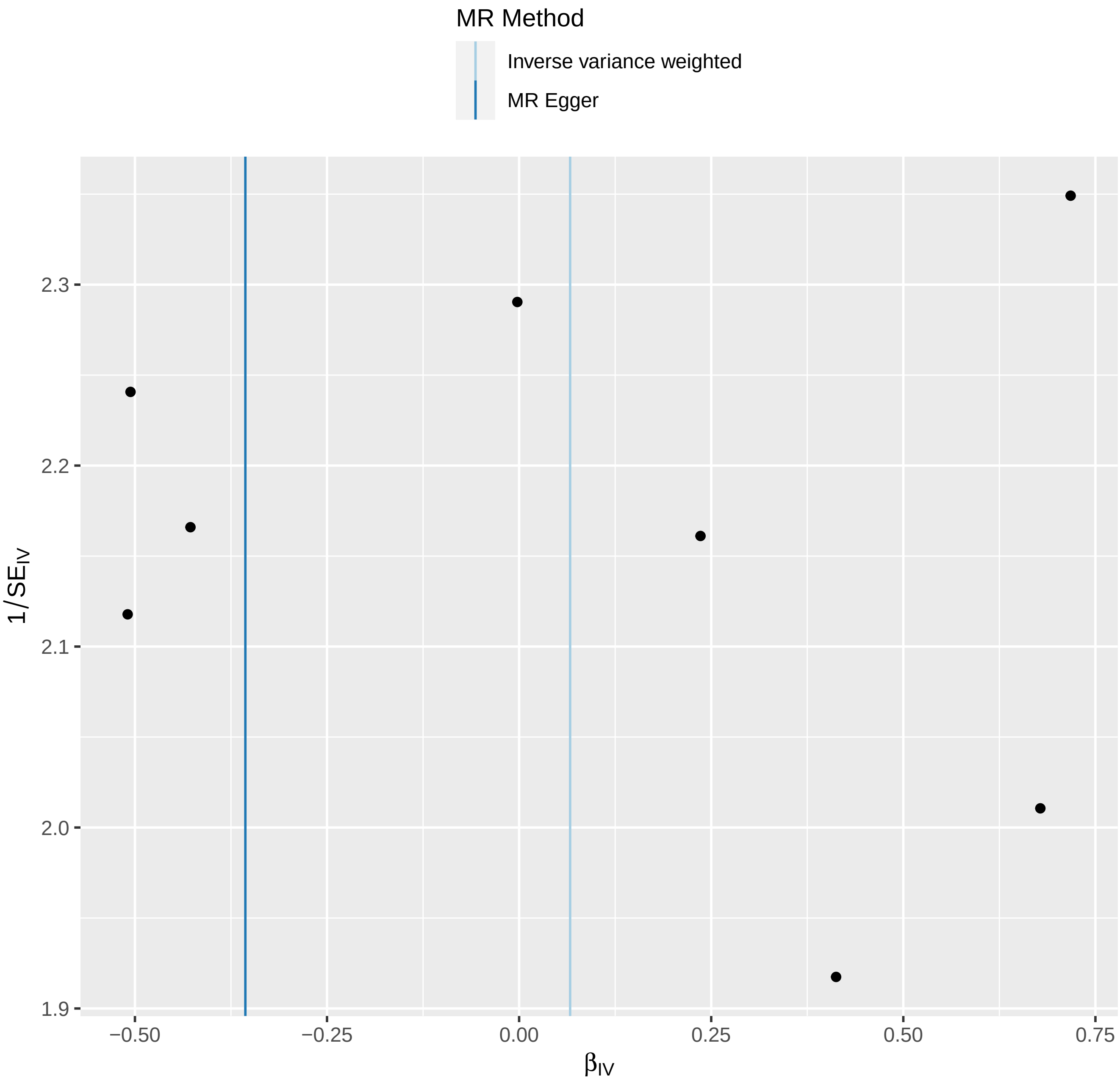

Batch 262 : Gut microbiota abundance (genus Oxalobacter id.2978) on Other Crohn's disease

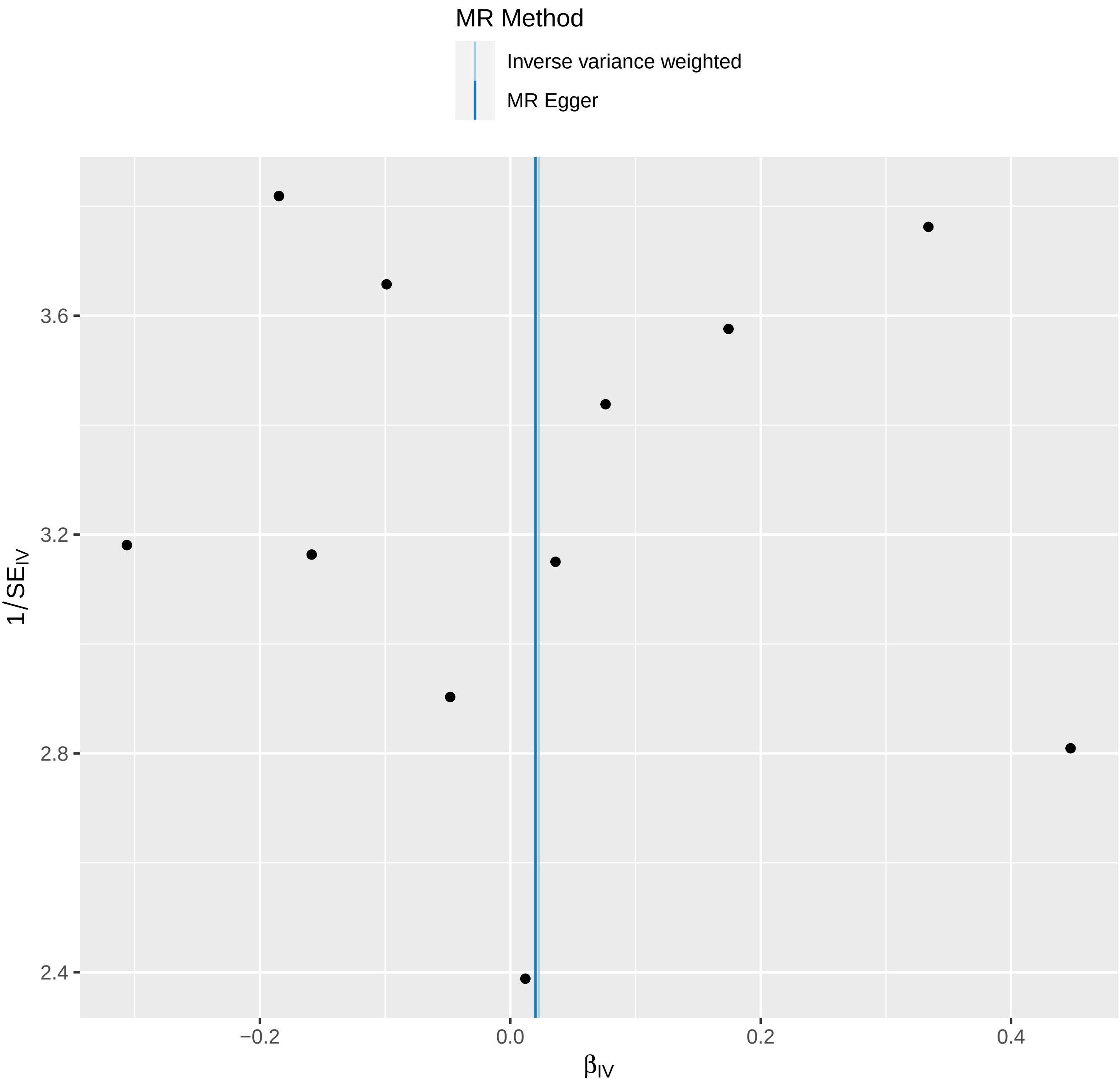

Batch 263 : Gut microbiota abundance (genus Parabacteroides id.954) on Other Crohn's disease

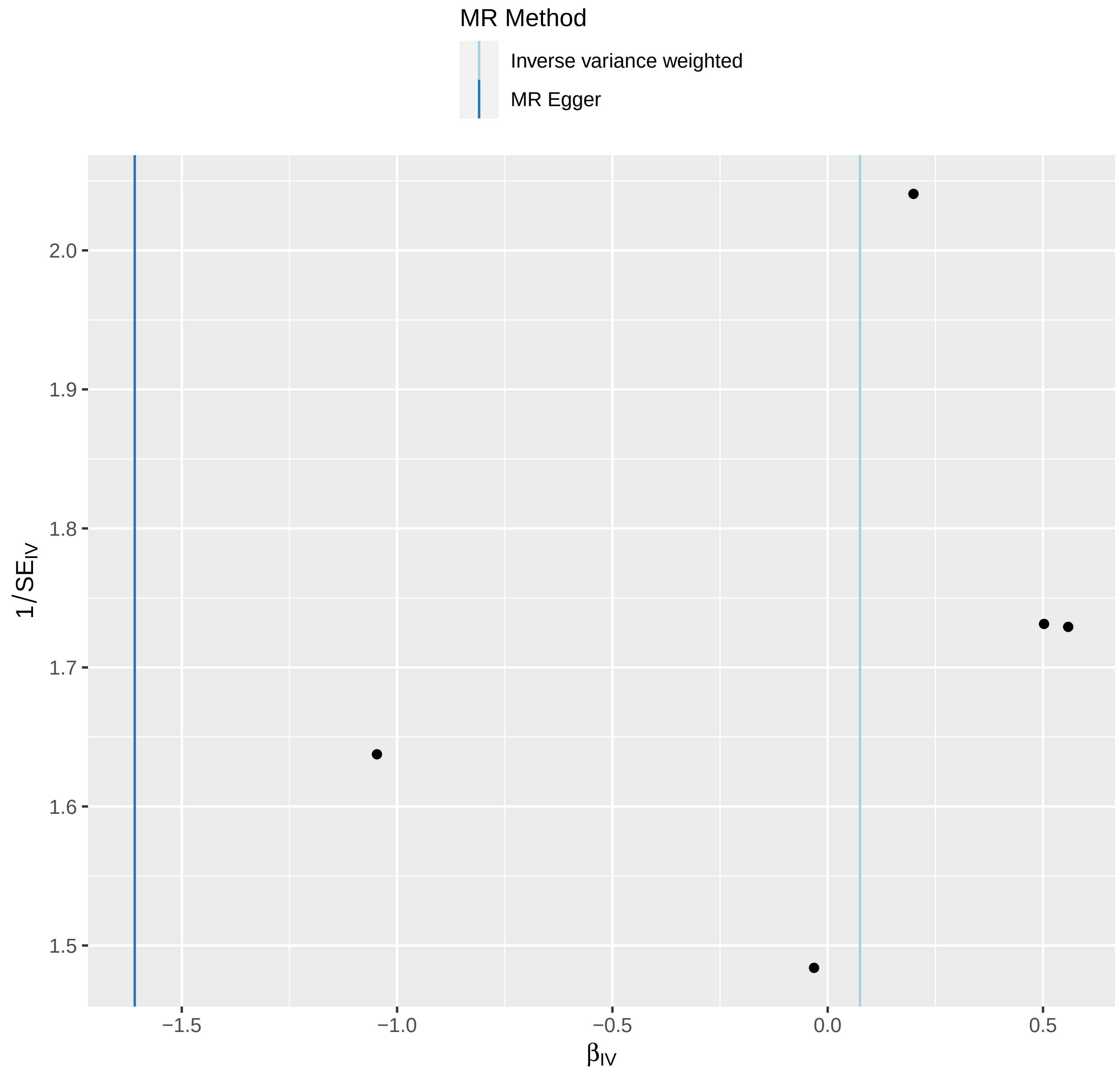

Batch 264 : Gut microbiota abundance (genus Paraprevotella id.962) on Other Crohn's disease

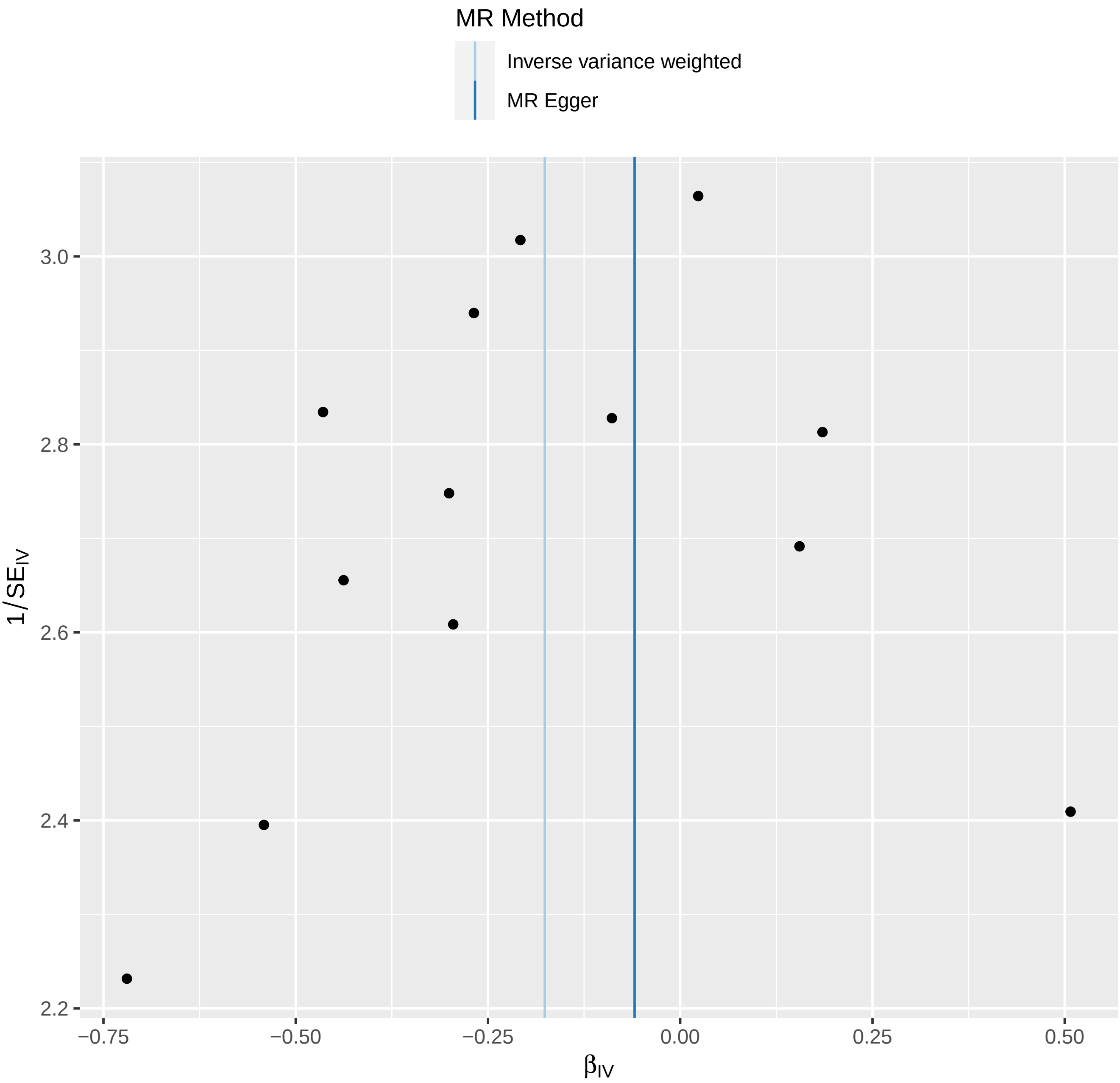

Batch 265 : Gut microbiota abundance (genus Parasutterella id.2892) on Other Crohn's disease

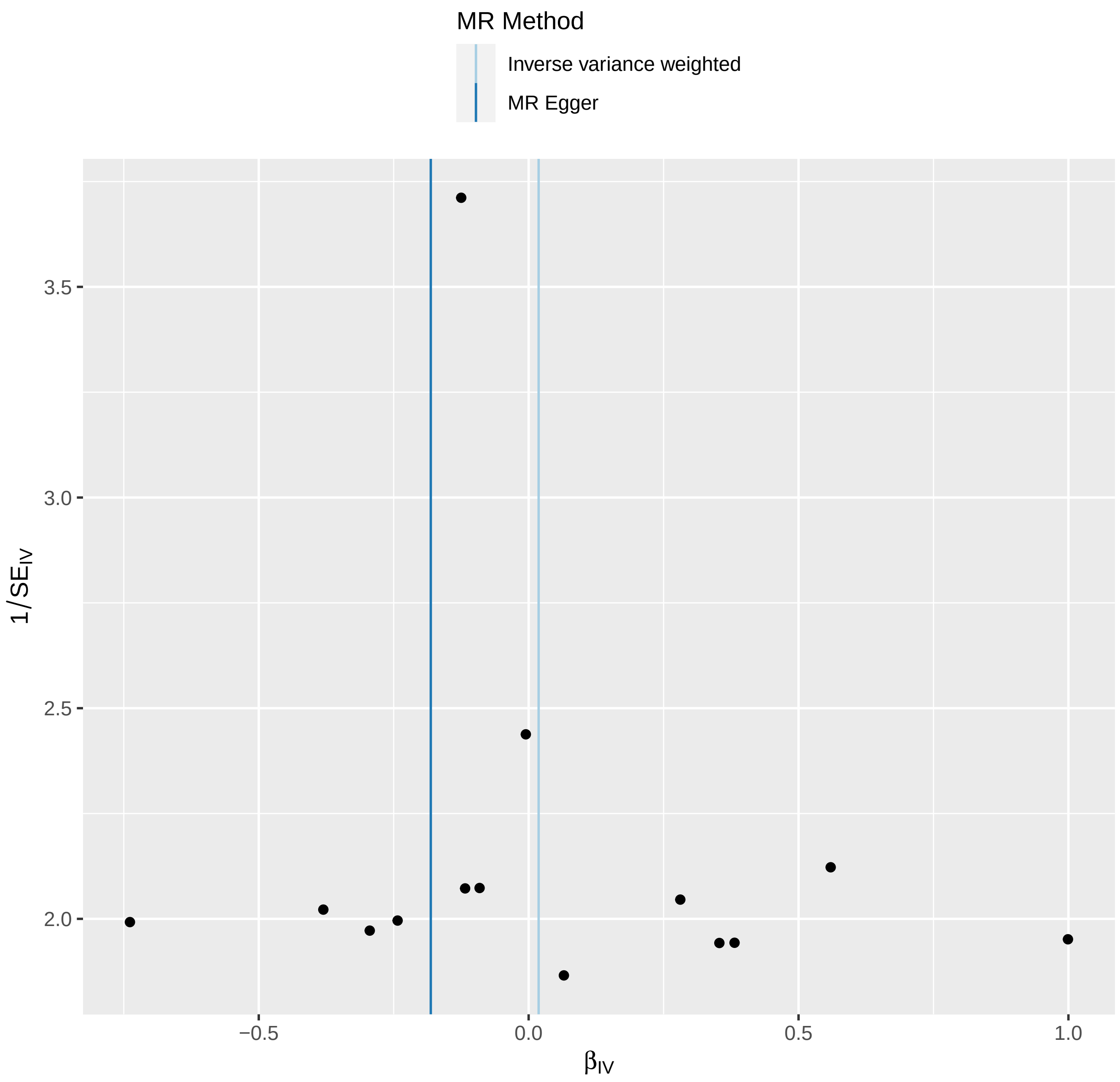

Batch 266 : Gut microbiota abundance (genus Peptococcus id.2037) on Other Crohn's disease

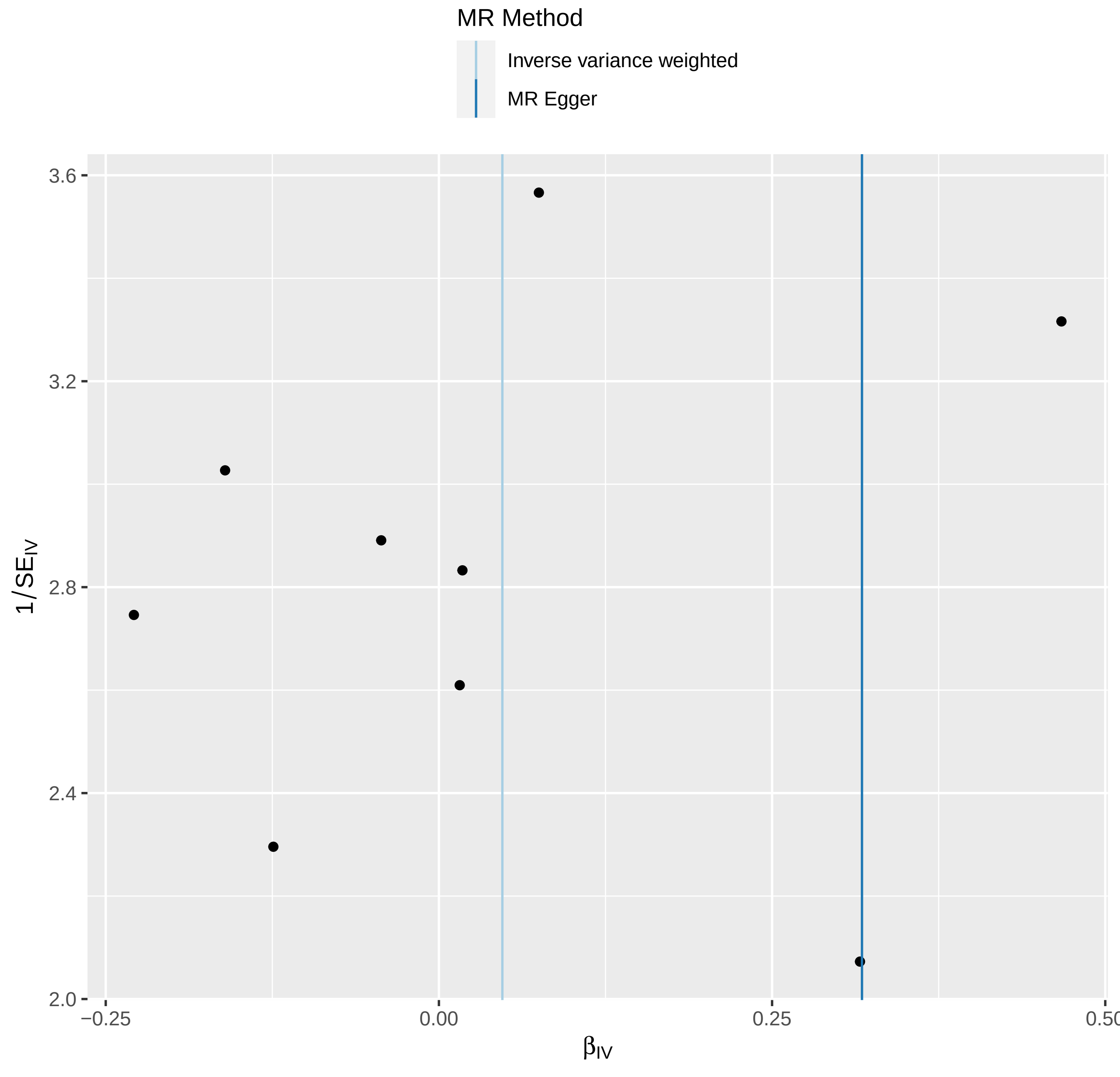

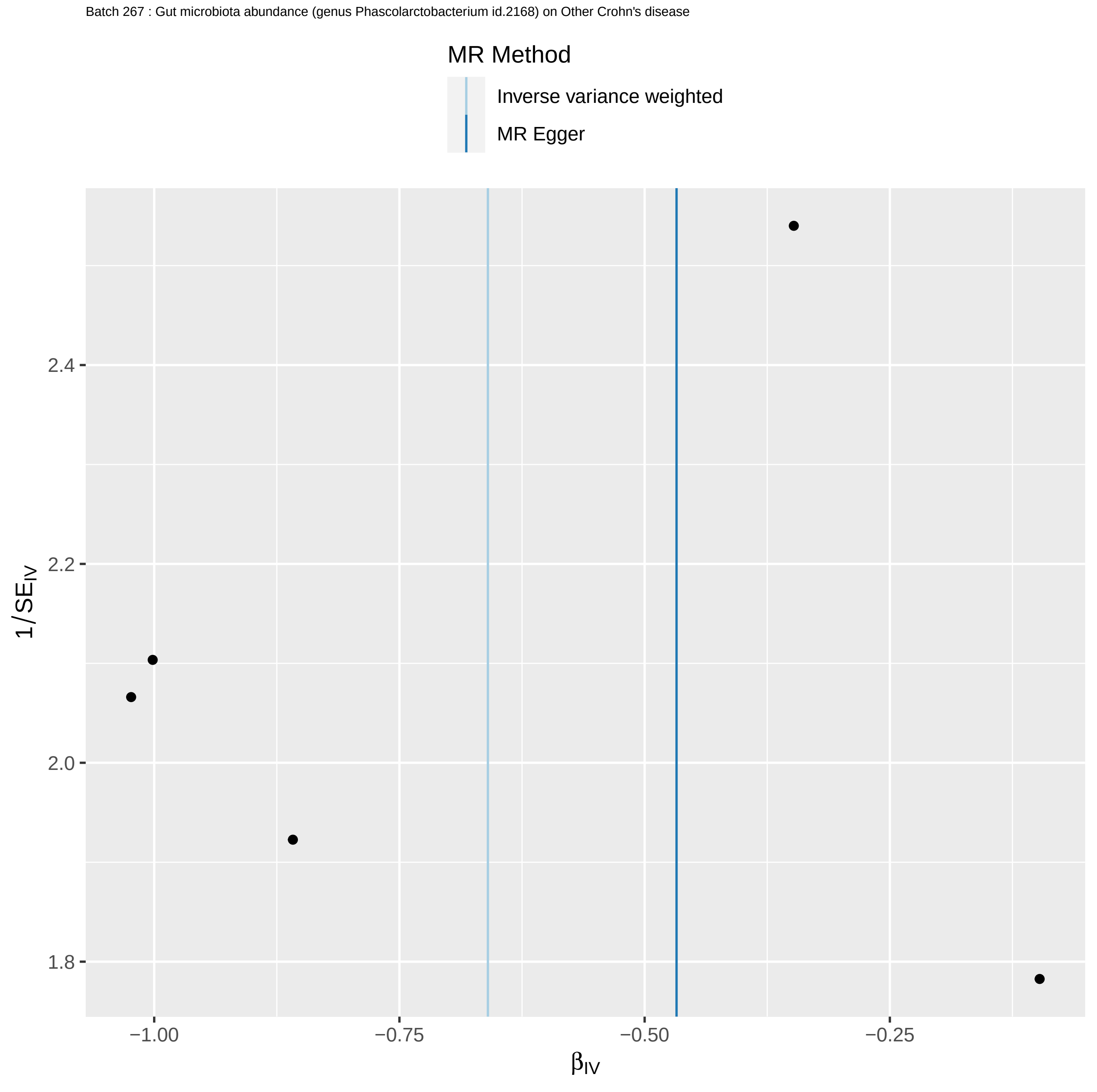
Batch 267 : Gut microbiota abundance (genus Phascolarctobacterium id.2168) on Other Crohn's disease
MR Method
Inverse variance weighted
MR Egger
2.4
2.2
2.0
1.8
1/SE_IV
−1.00
−0.75
−0.50
−0.25
β_IV

Batch 268 : Gut microbiota abundance (genus Prevotella7 id.11182) on Other Crohn's disease

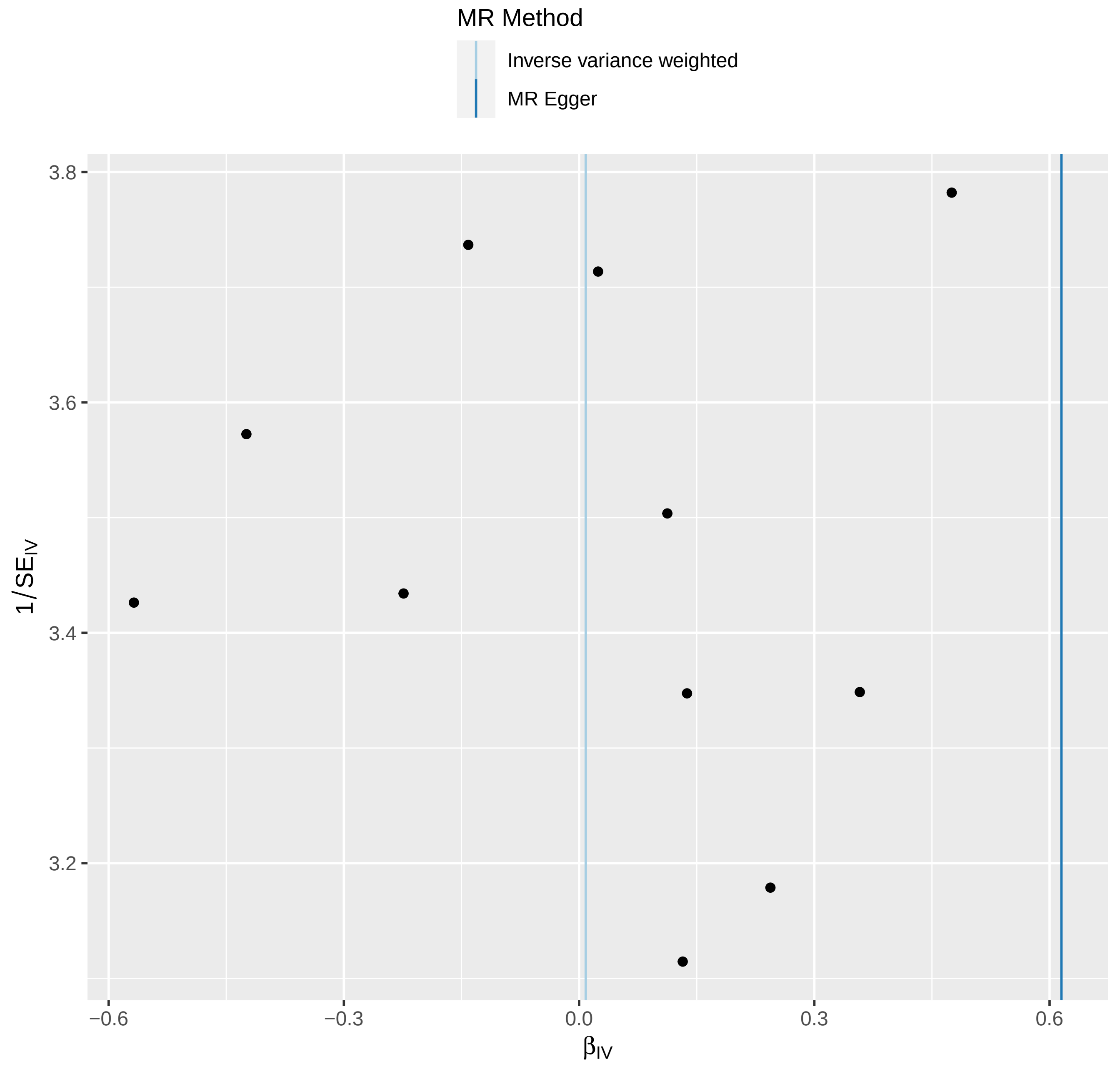

Batch 269 : Gut microbiota abundance (genus Prevotella9 id.11183) on Other Crohn's disease

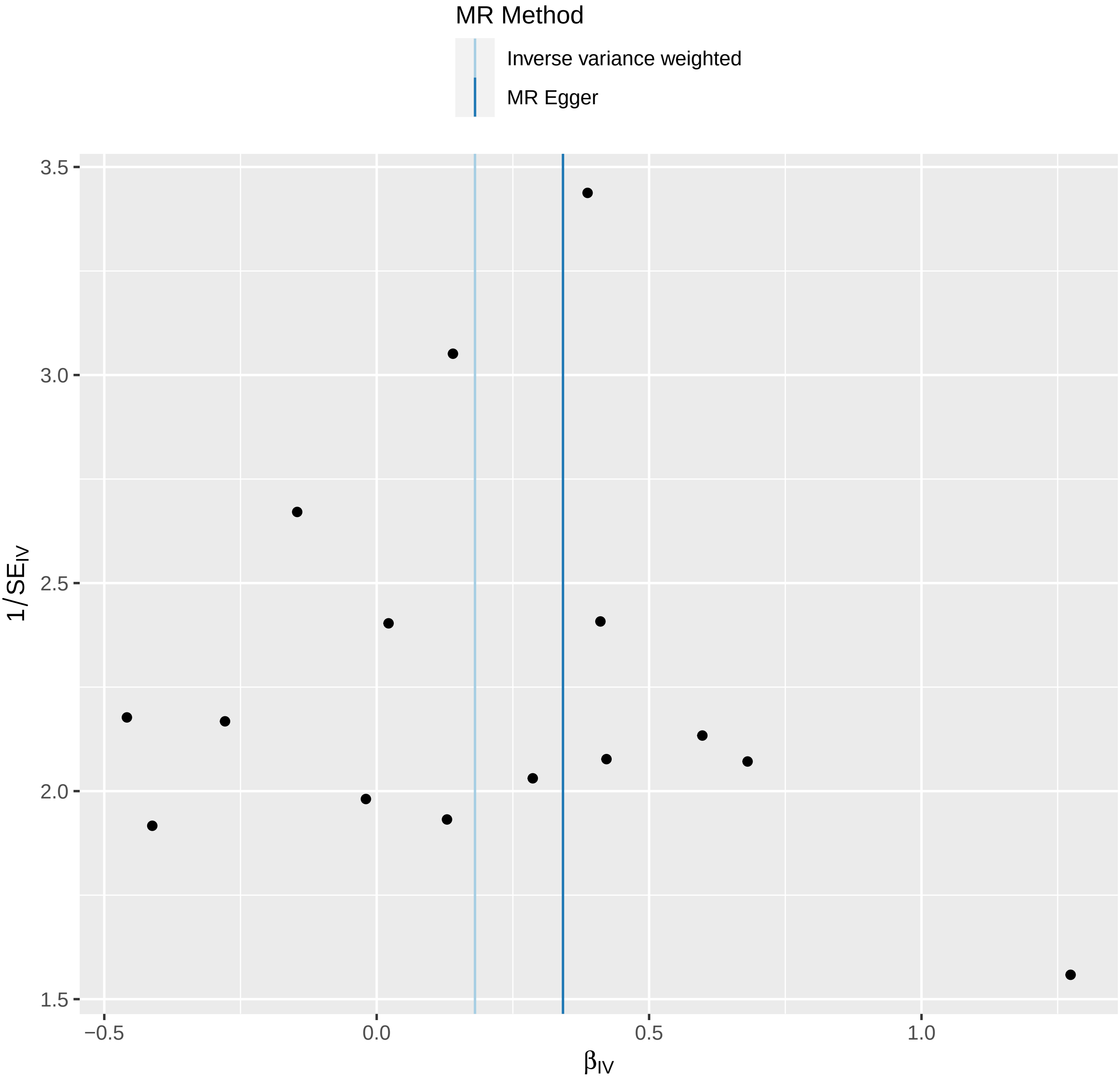

Batch 270 : Gut microbiota abundance (genus Rikenellaceae RC9 gut group id.11191) on Other Crohn's disease

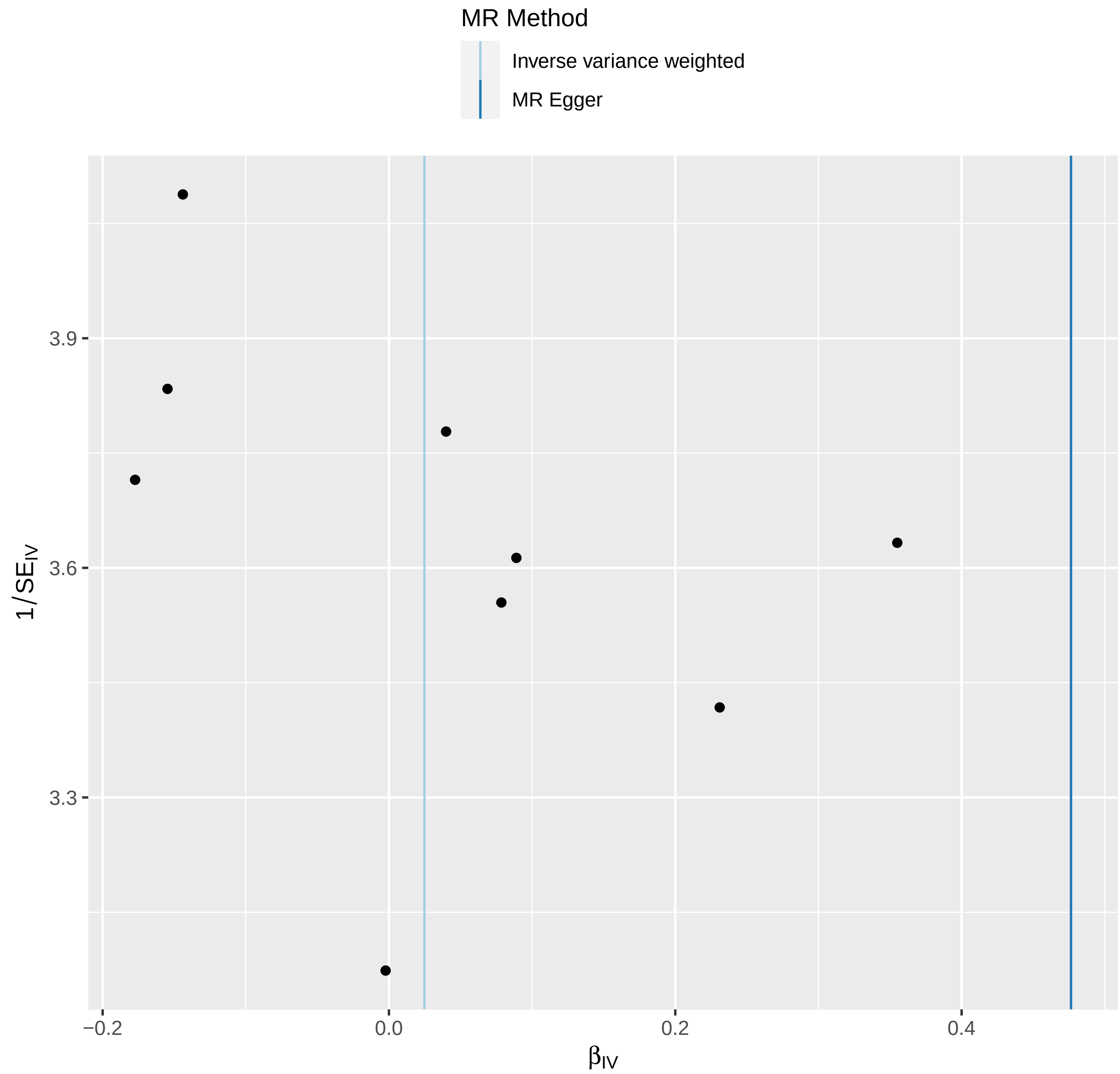

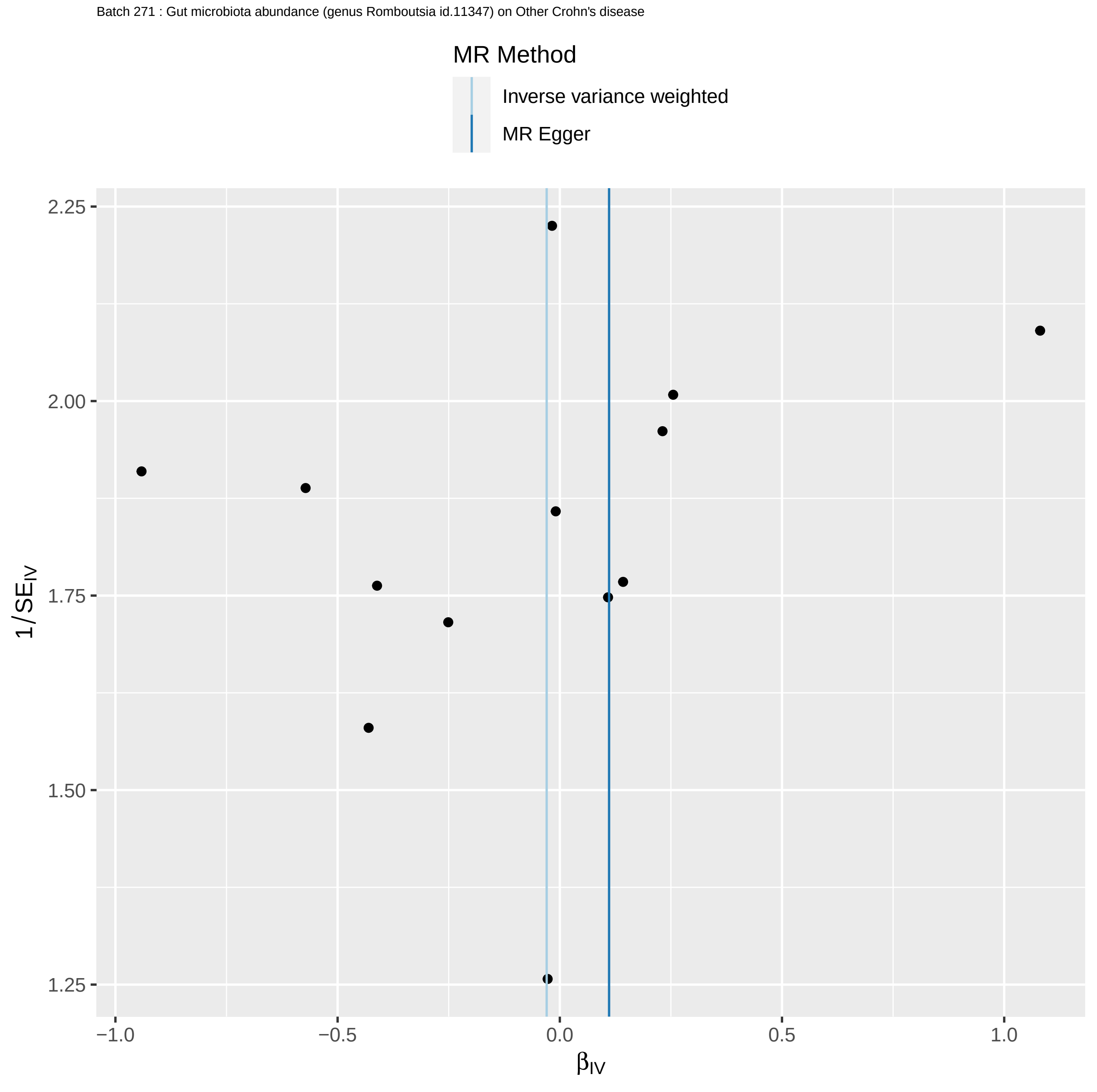

Batch 271 : Gut microbiota abundance (genus Romboutsia id.11347) on Other Crohn's disease
MR Method
Inverse variance weighted
MR Egger
2.25
2.00
1.75
1.50
1.25
1/SE_IV
−1.0
−0.5
0.0
0.5
1.0
β_IV

Batch 272 : Gut microbiota abundance (genus Roseburia id.2012) on Other Crohn's disease

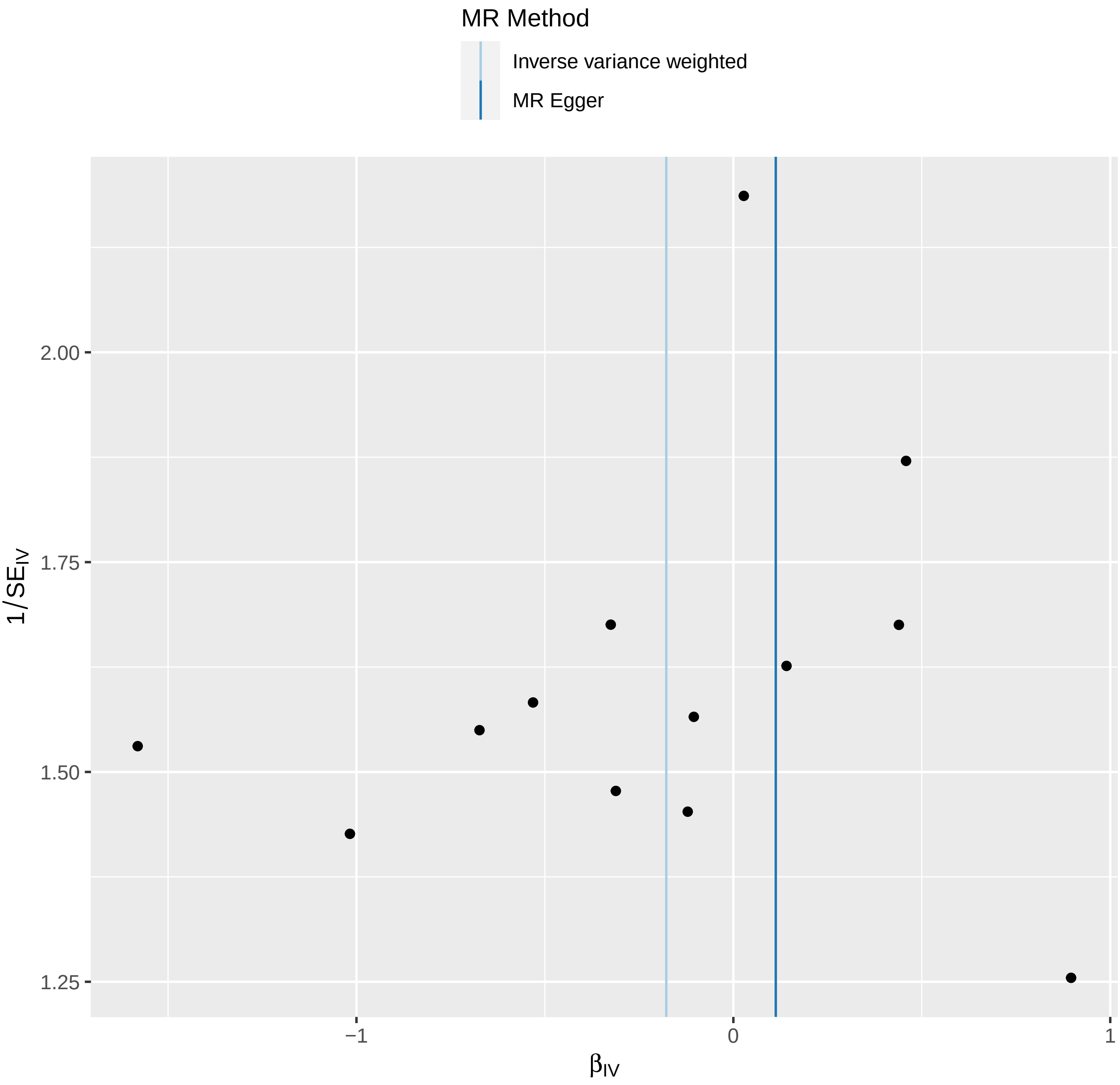

Batch 273 : Gut microbiota abundance (genus Ruminiclostridium5 id.11355) on Other Crohn's disease

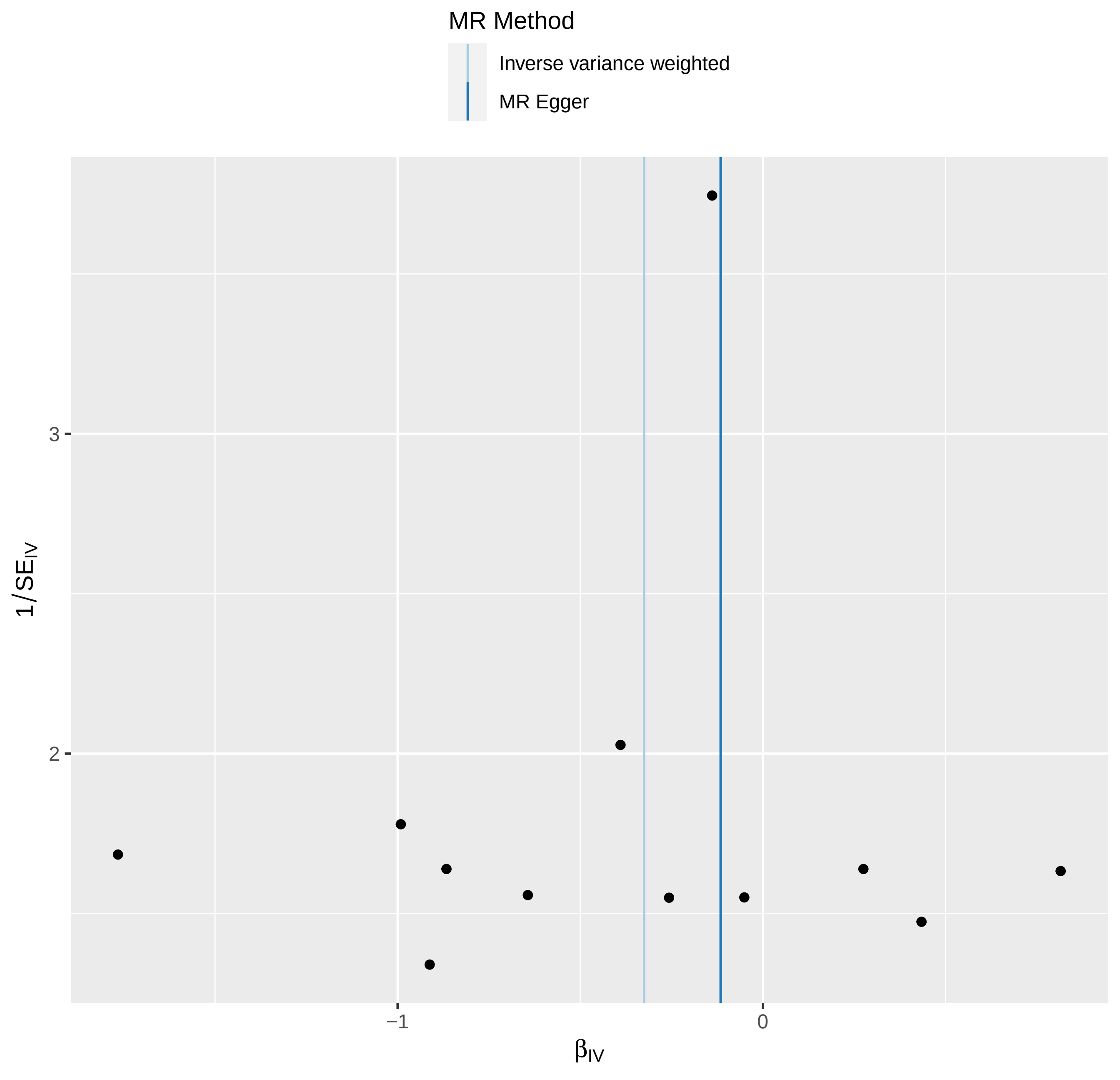

Batch 274 : Gut microbiota abundance (genus Ruminiclostridium6 id.11356) on Other Crohn's disease

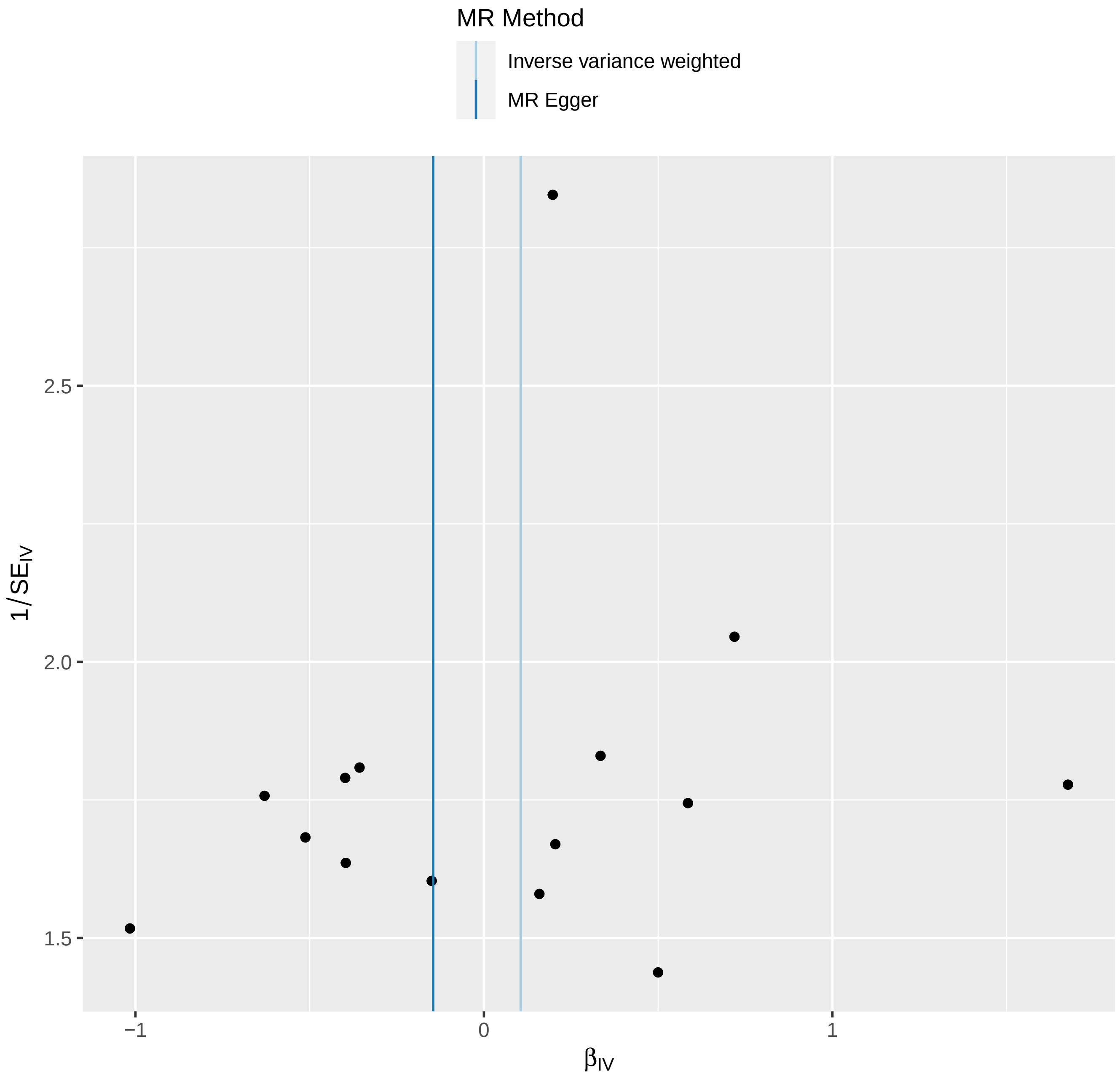

Batch 275 : Gut microbiota abundance (genus Ruminiclostridium9 id.11357) on Other Crohn's disease

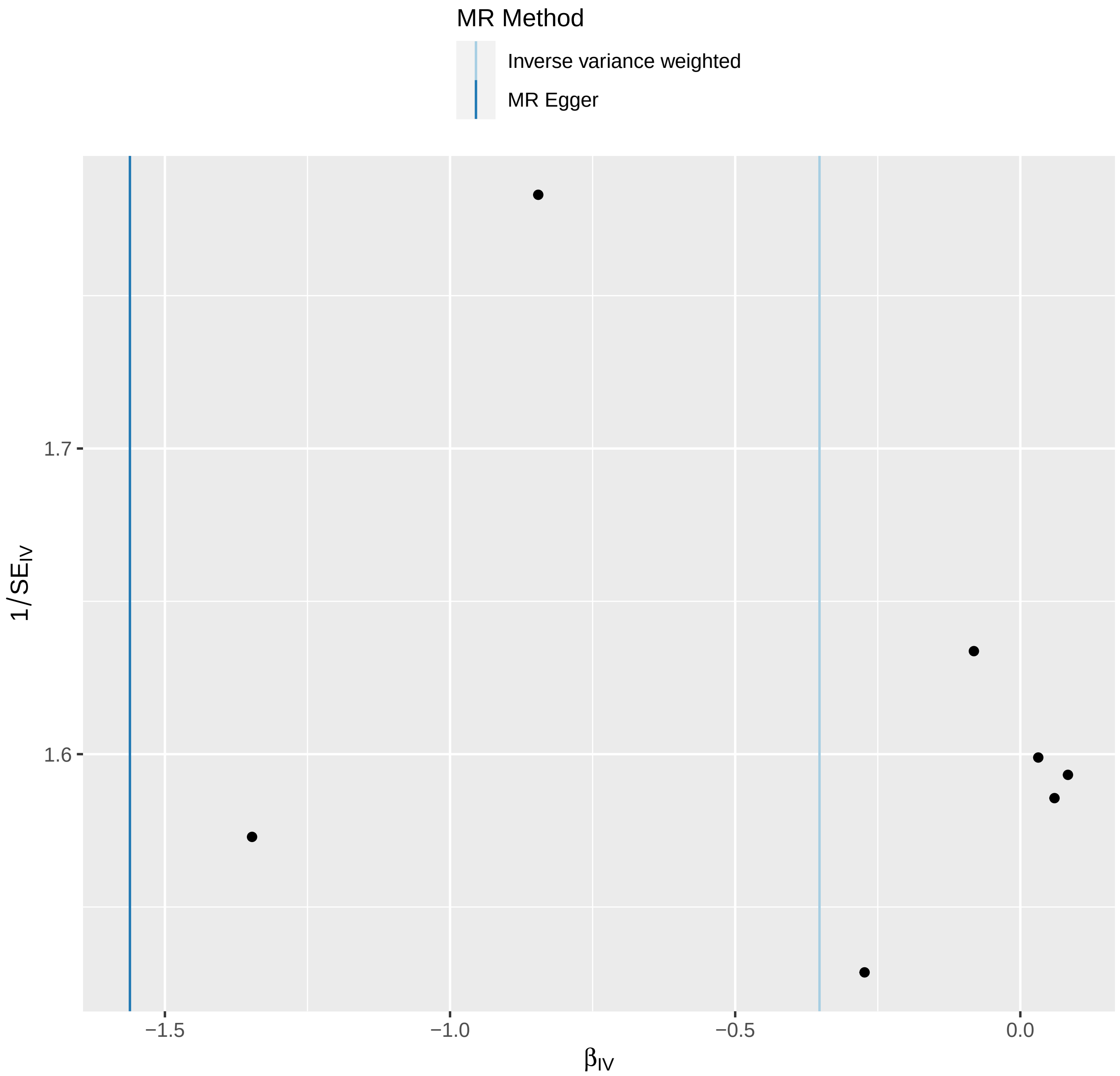

Batch 276 : Gut microbiota abundance (genus Ruminococcaceae NK4A214 group id.11358) on Other Crohn's disease

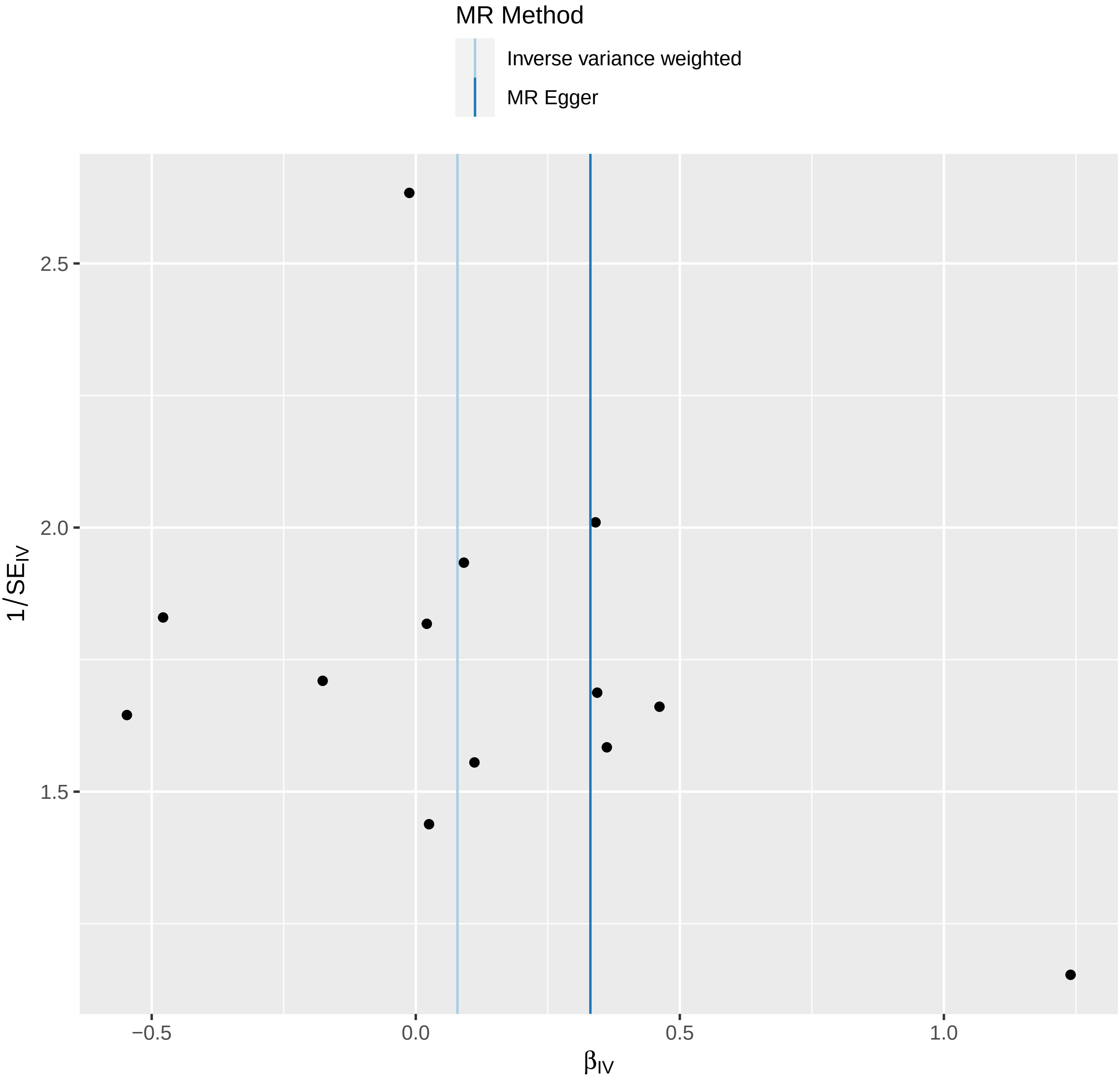

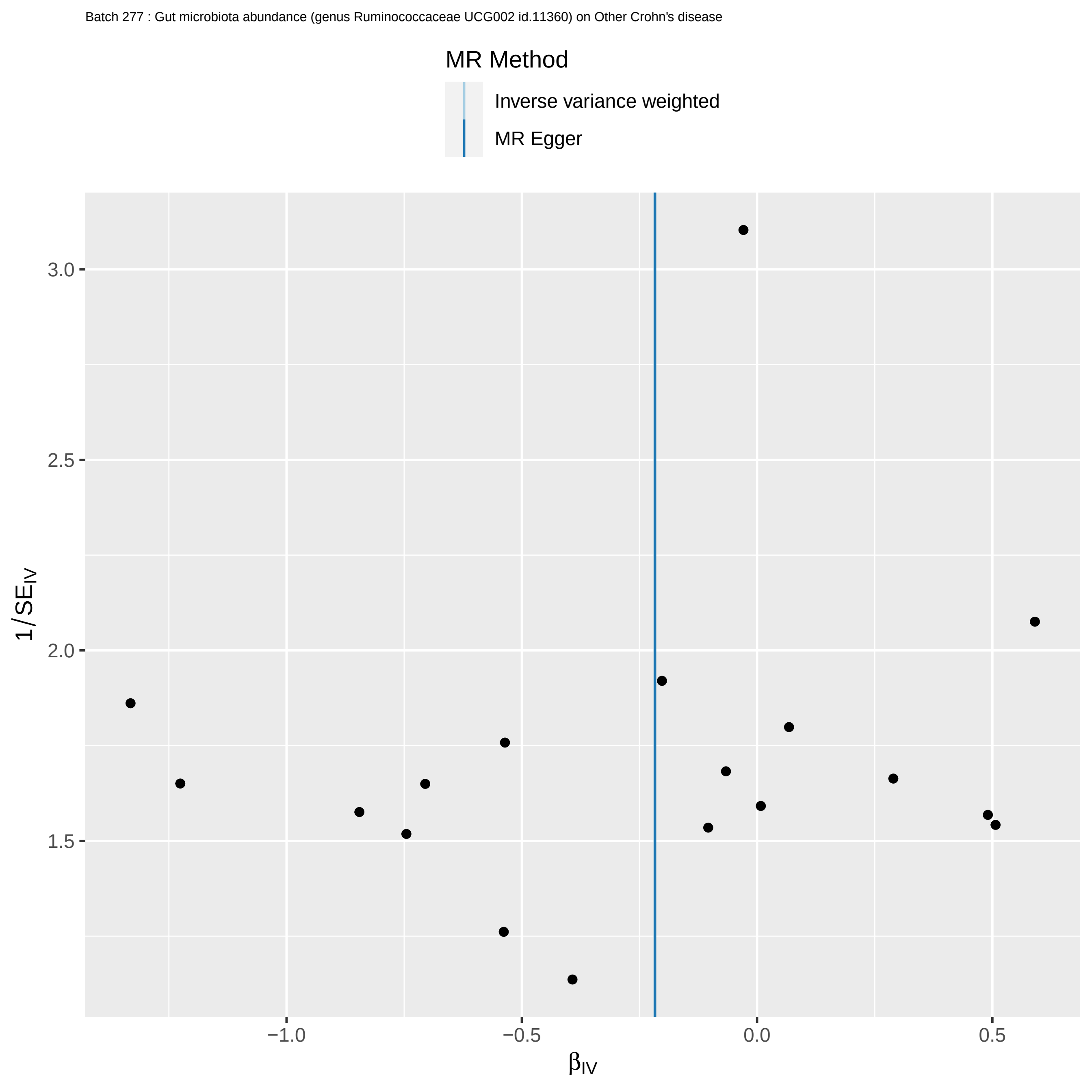
Batch 277 : Gut microbiota abundance (genus Ruminococcaceae UCG002 id.11360) on Other Crohn's disease
MR Method
Inverse variance weighted
MR Egger
3.0
2.5
2.0
1.5
1/SE_IV
−1.0
−0.5
0.0
0.5
β_IV

Batch 278 : Gut microbiota abundance (genus Ruminococcaceae UCG003 id.11361) on Other Crohn's disease

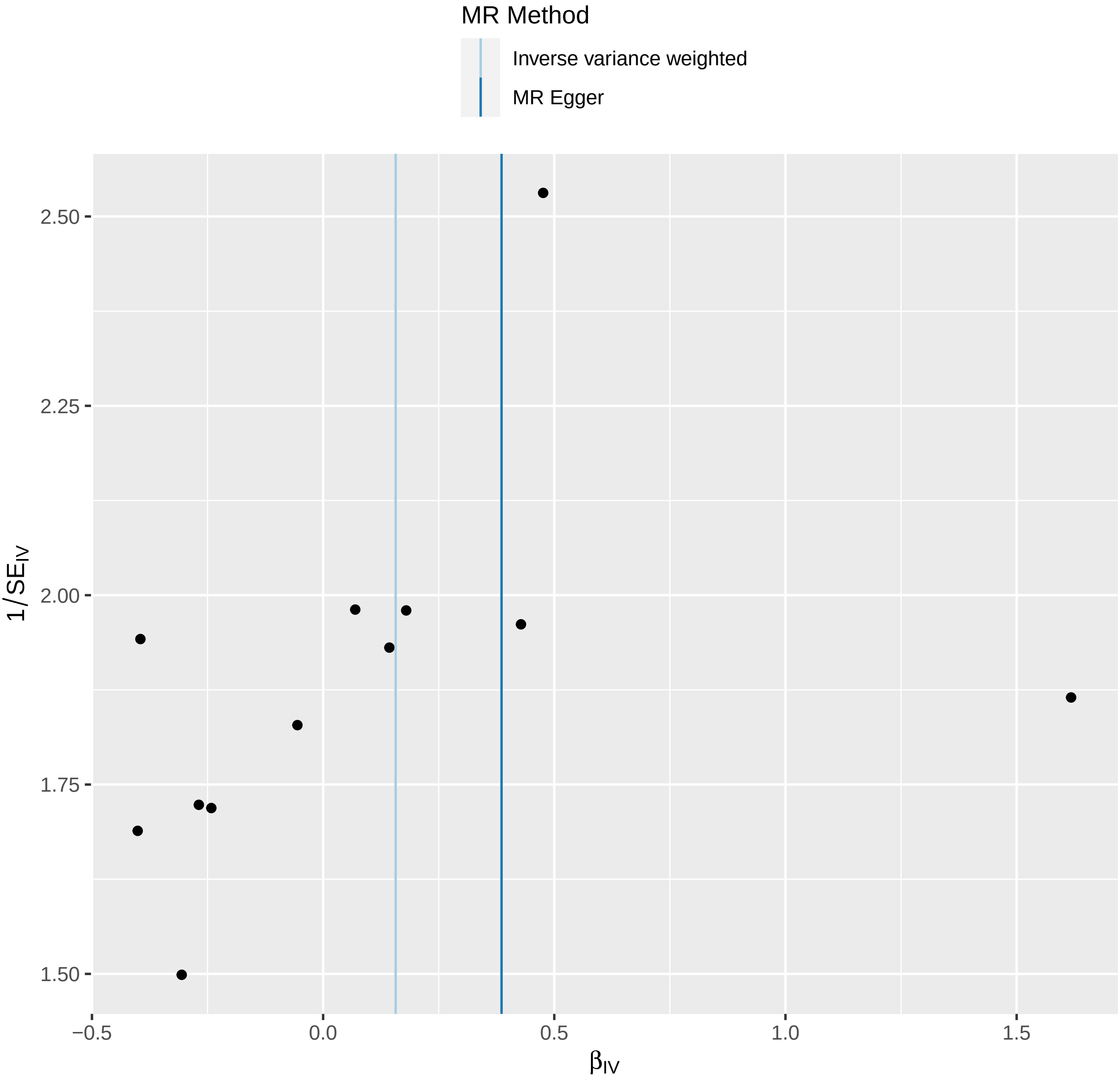

Batch 279 : Gut microbiota abundance (genus Ruminococcaceae UCG004 id.11362) on Other Crohn's disease

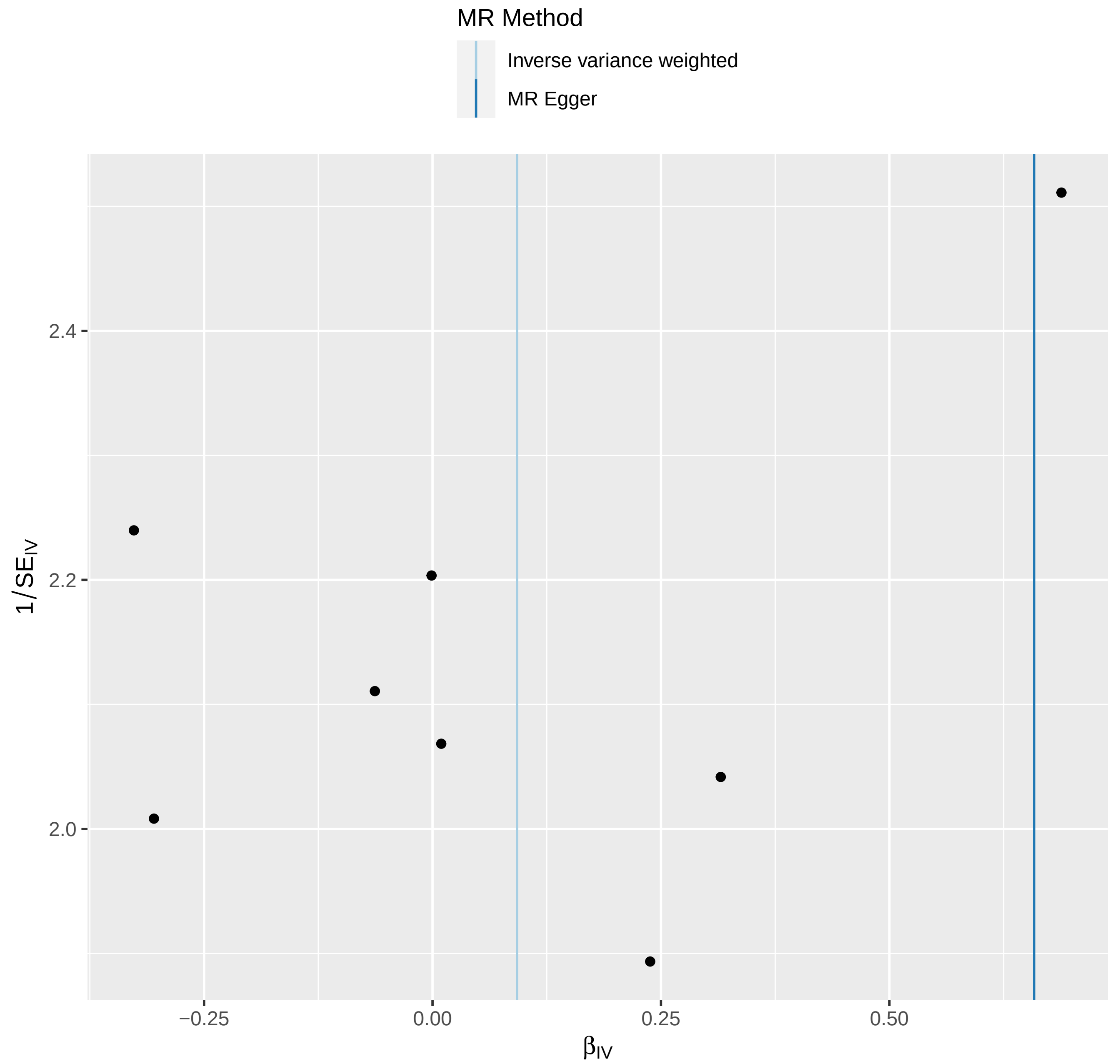

Batch 280 : Gut microbiota abundance (genus Ruminococcaceae UCG005 id.11363) on Other Crohn's disease

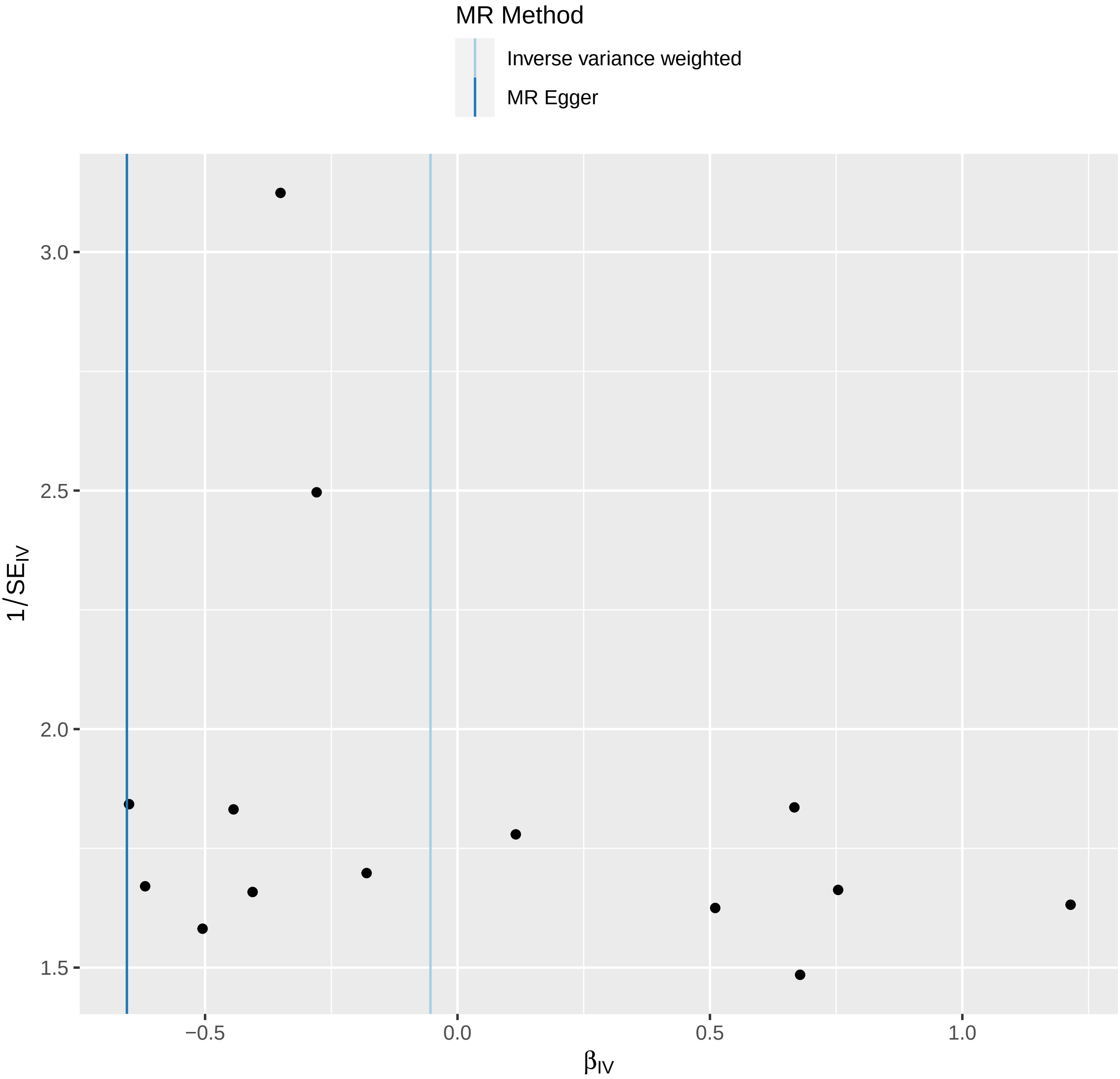

Batch 281 : Gut microbiota abundance (genus Ruminococcaceae UCG009 id.11366) on Other Crohn's disease

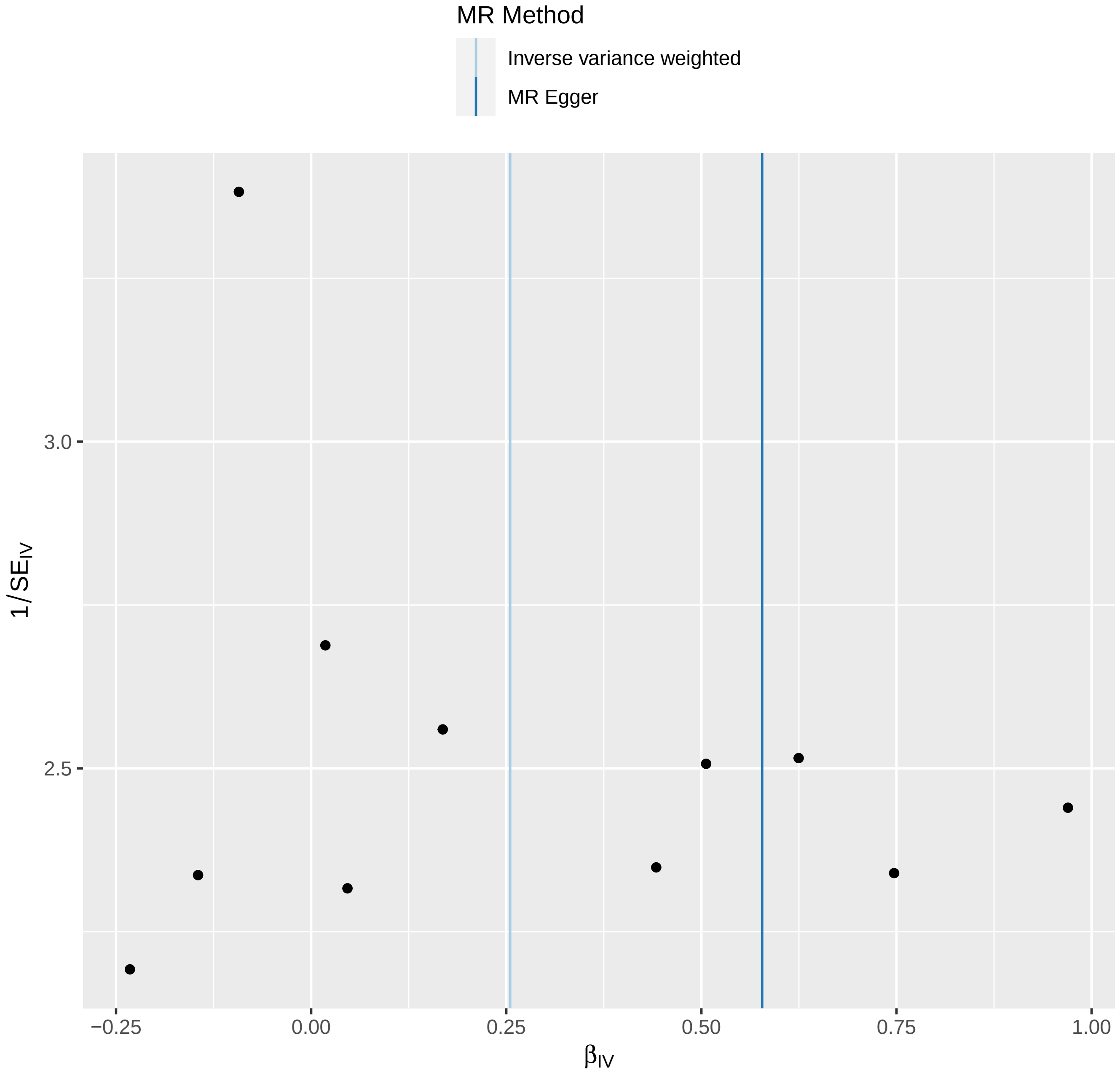

Batch 282 : Gut microbiota abundance (genus Ruminococcaceae UCG010 id.11367) on Other Crohn's disease

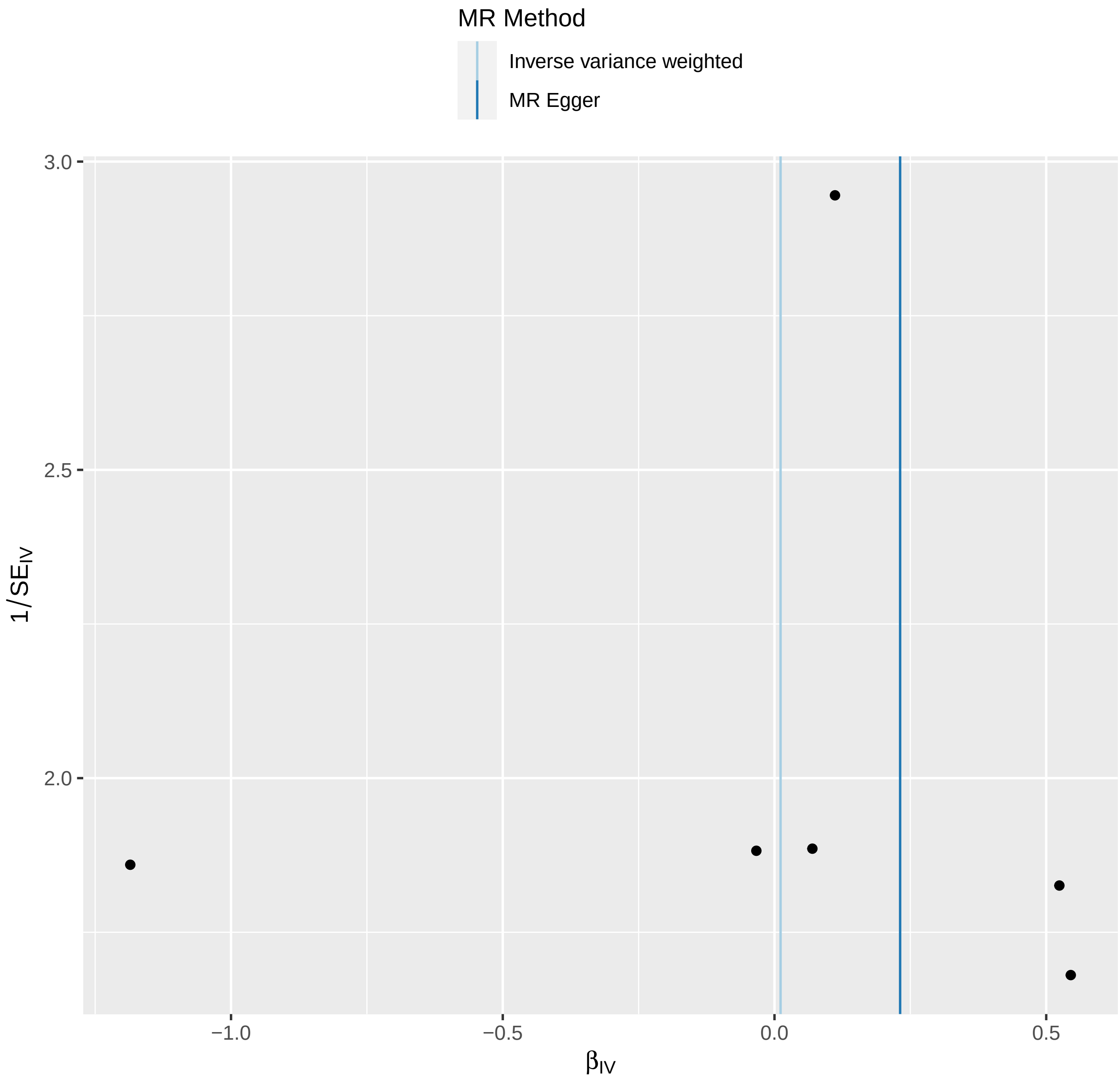

Batch 283 : Gut microbiota abundance (genus Ruminococcaceae UCG011 id.11368) on Other Crohn's disease

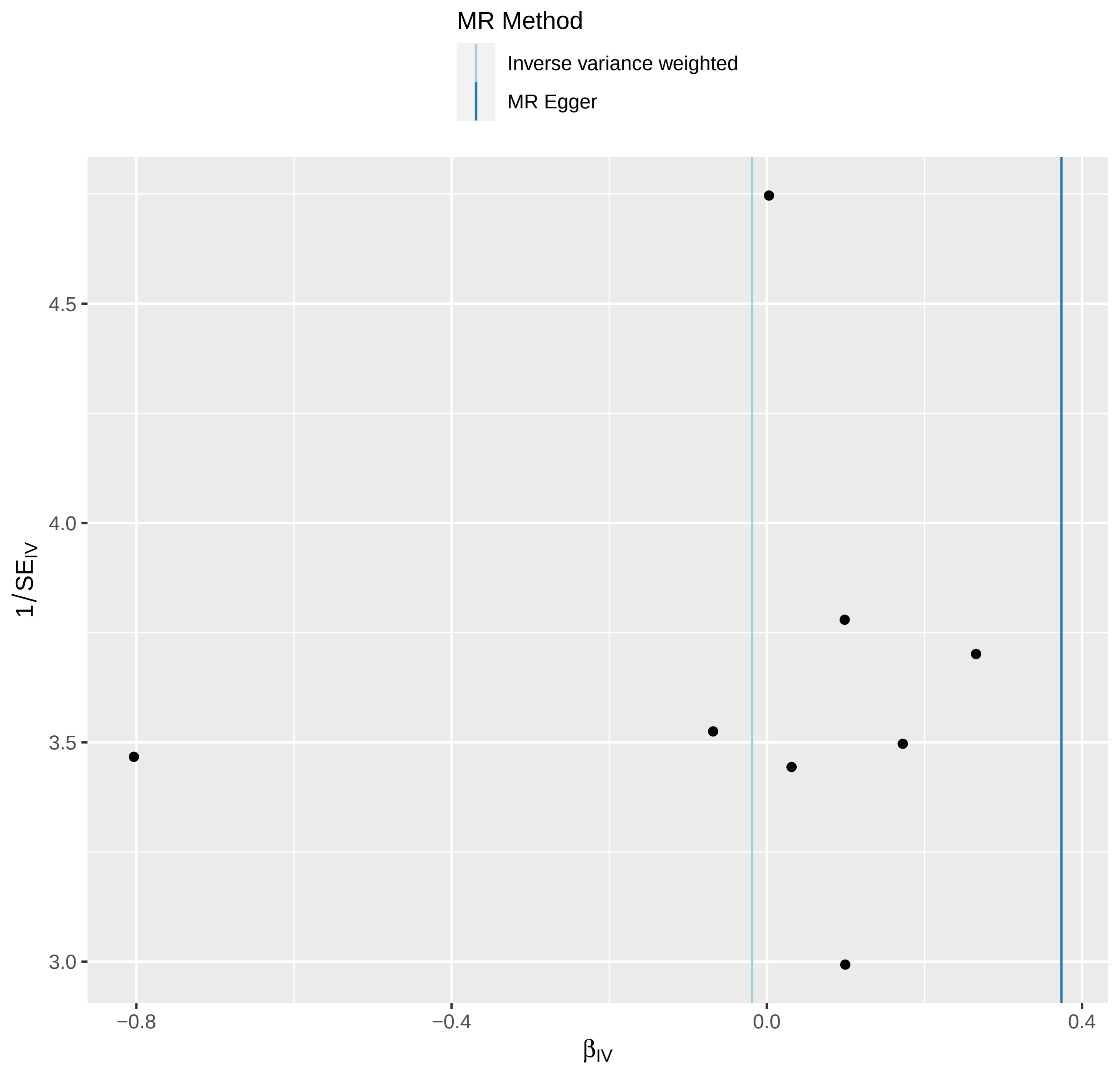

Batch 284 : Gut microbiota abundance (genus Ruminococcaceae UCG013 id.11370) on Other Crohn's disease

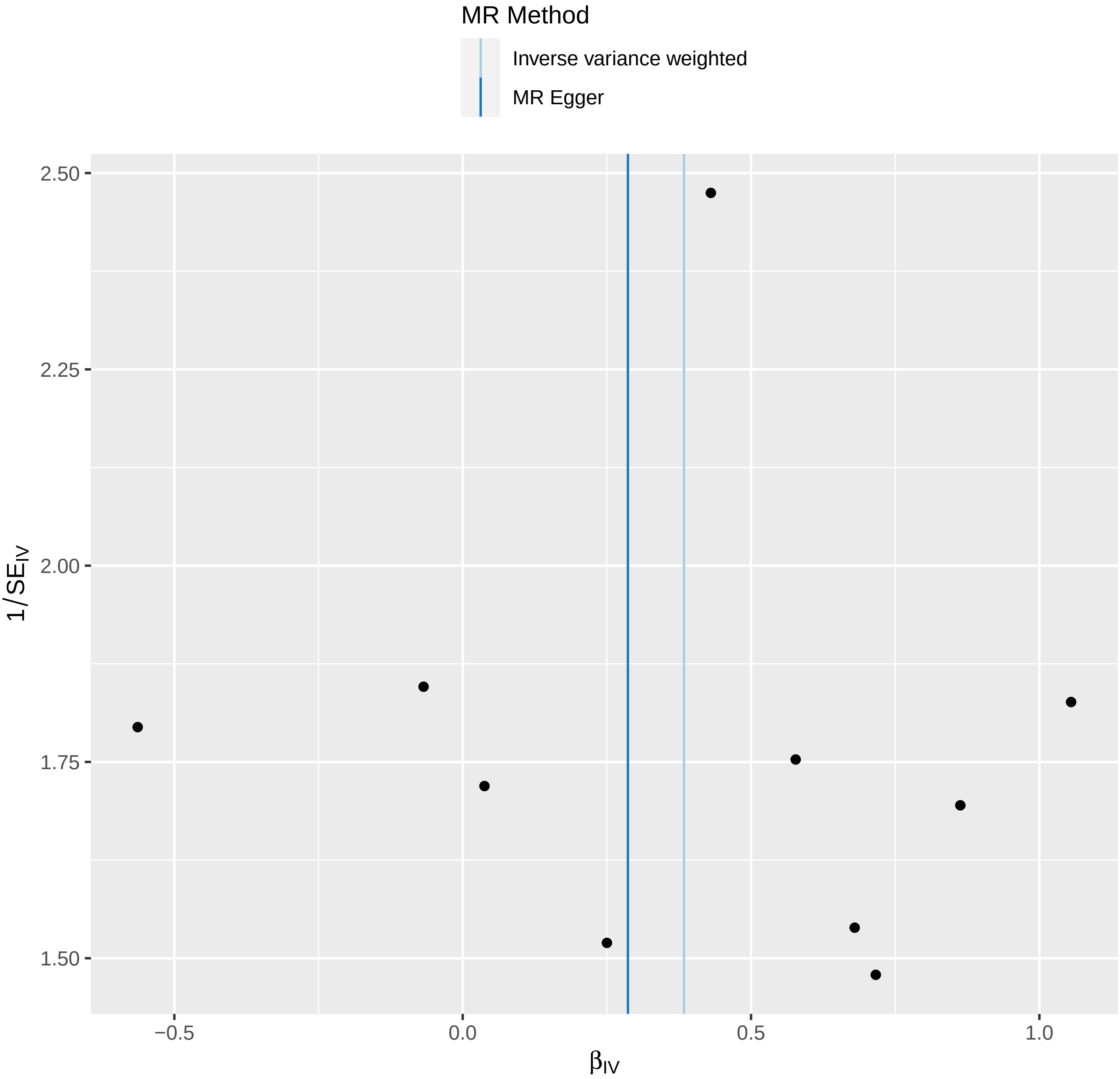

Batch 285 : Gut microbiota abundance (genus Ruminococcaceae UCG014 id.11371) on Other Crohn's disease

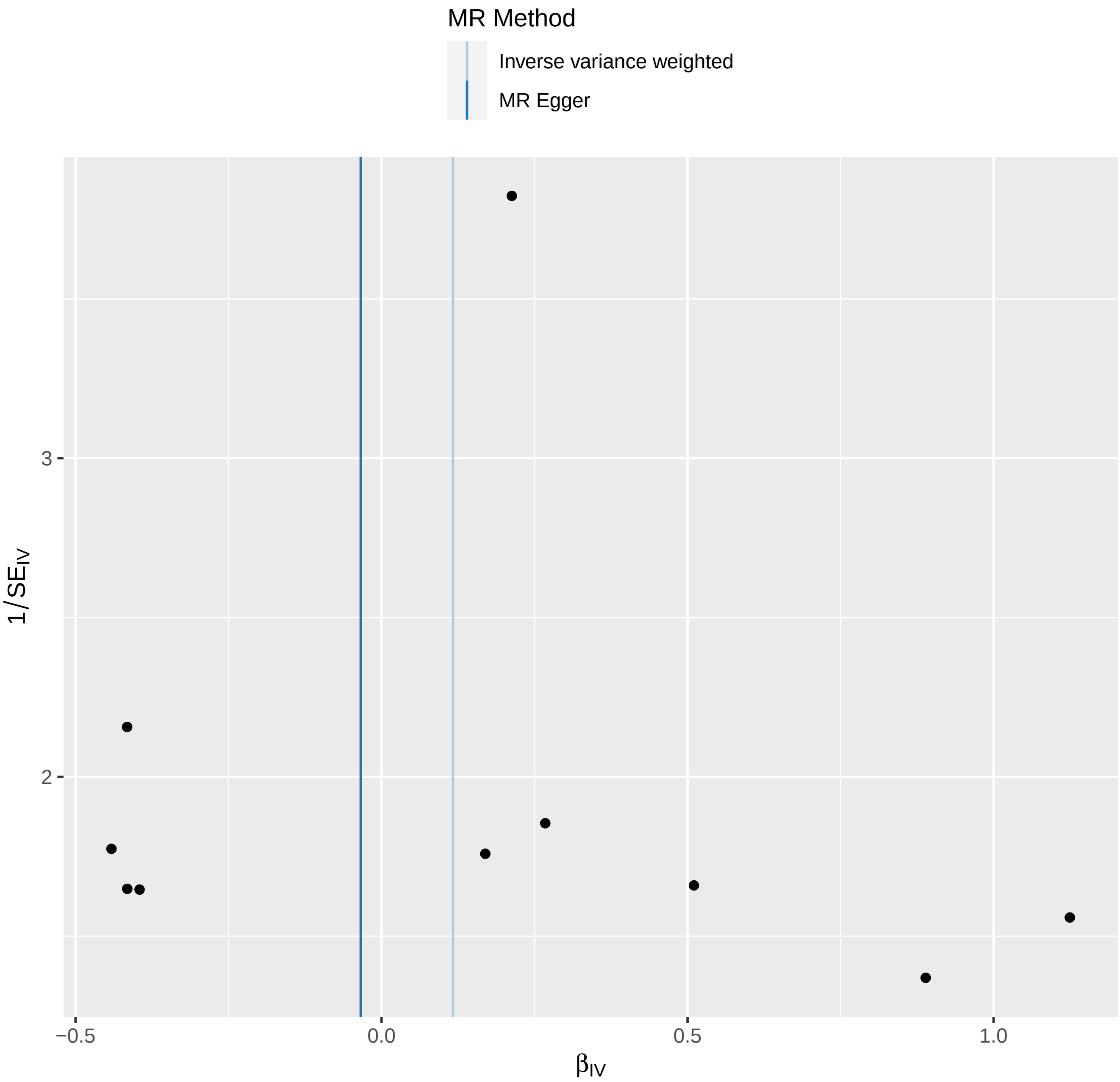

Batch 286 : Gut microbiota abundance (genus Ruminococcus1 id.11373) on Other Crohn's disease

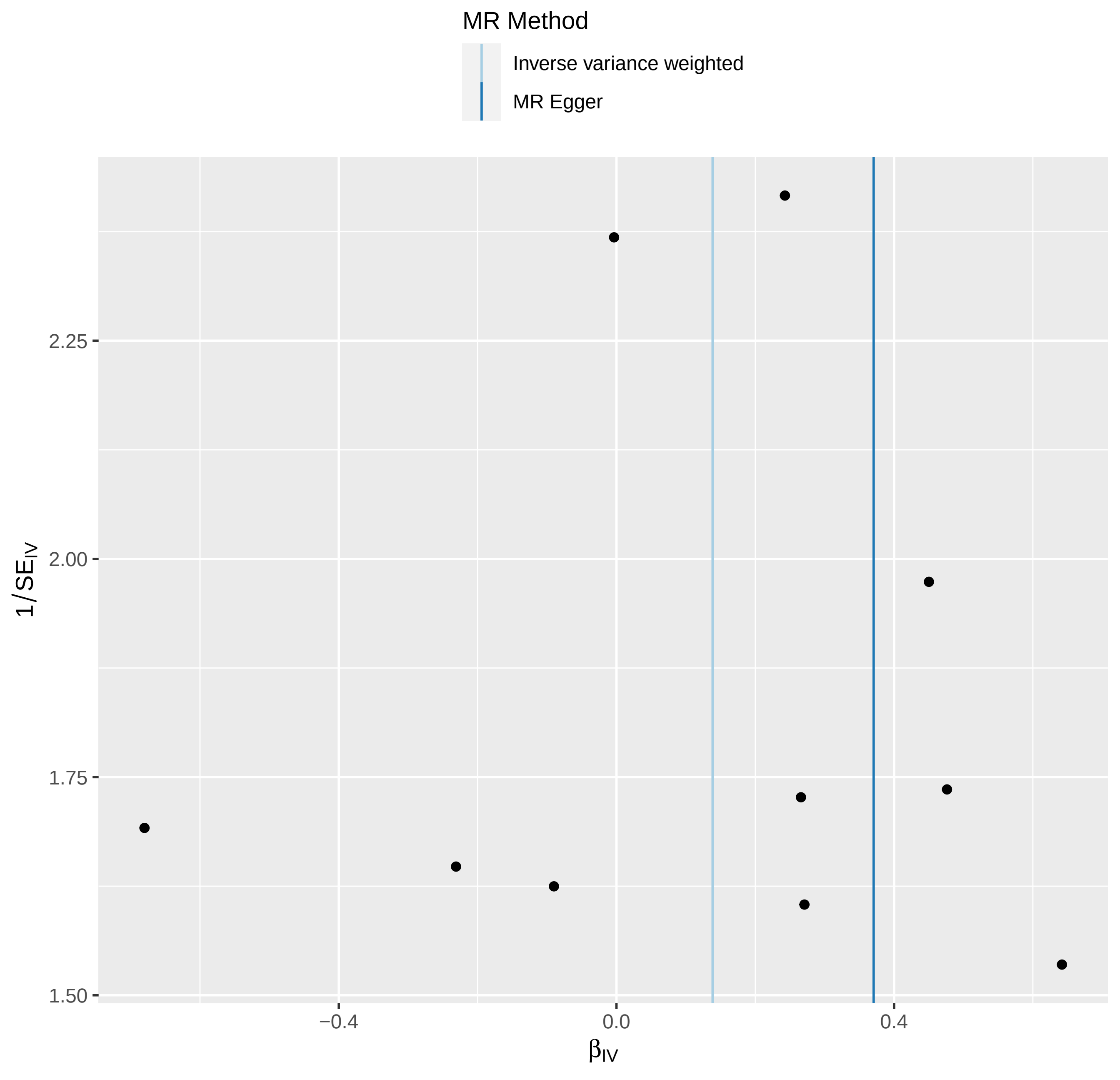

Batch 287 : Gut microbiota abundance (genus Ruminococcus2 id.11374) on Other Crohn's disease

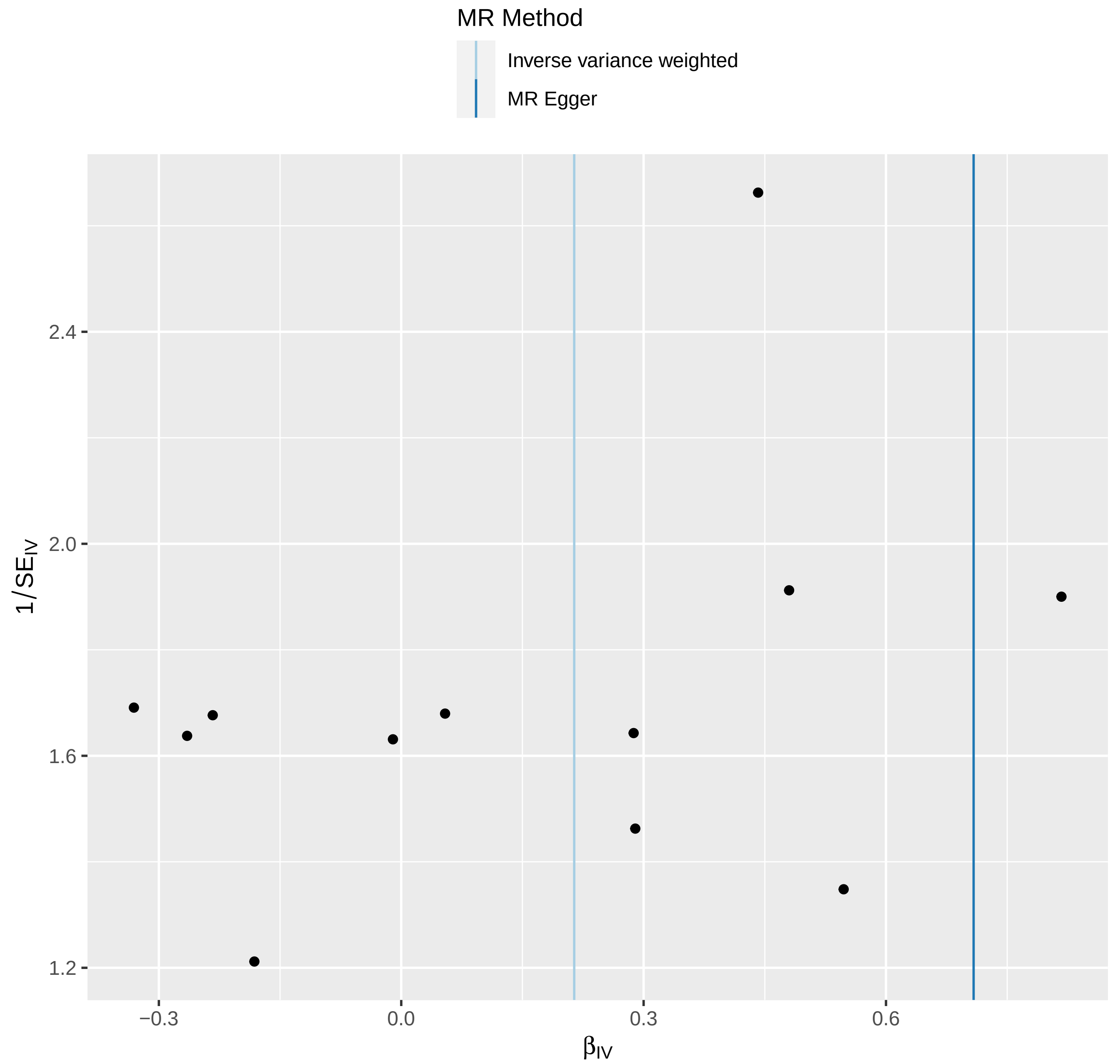

Batch 288 : Gut microbiota abundance (genus Ruminococcus gauvreauii group id.11342) on Other Crohn's disease

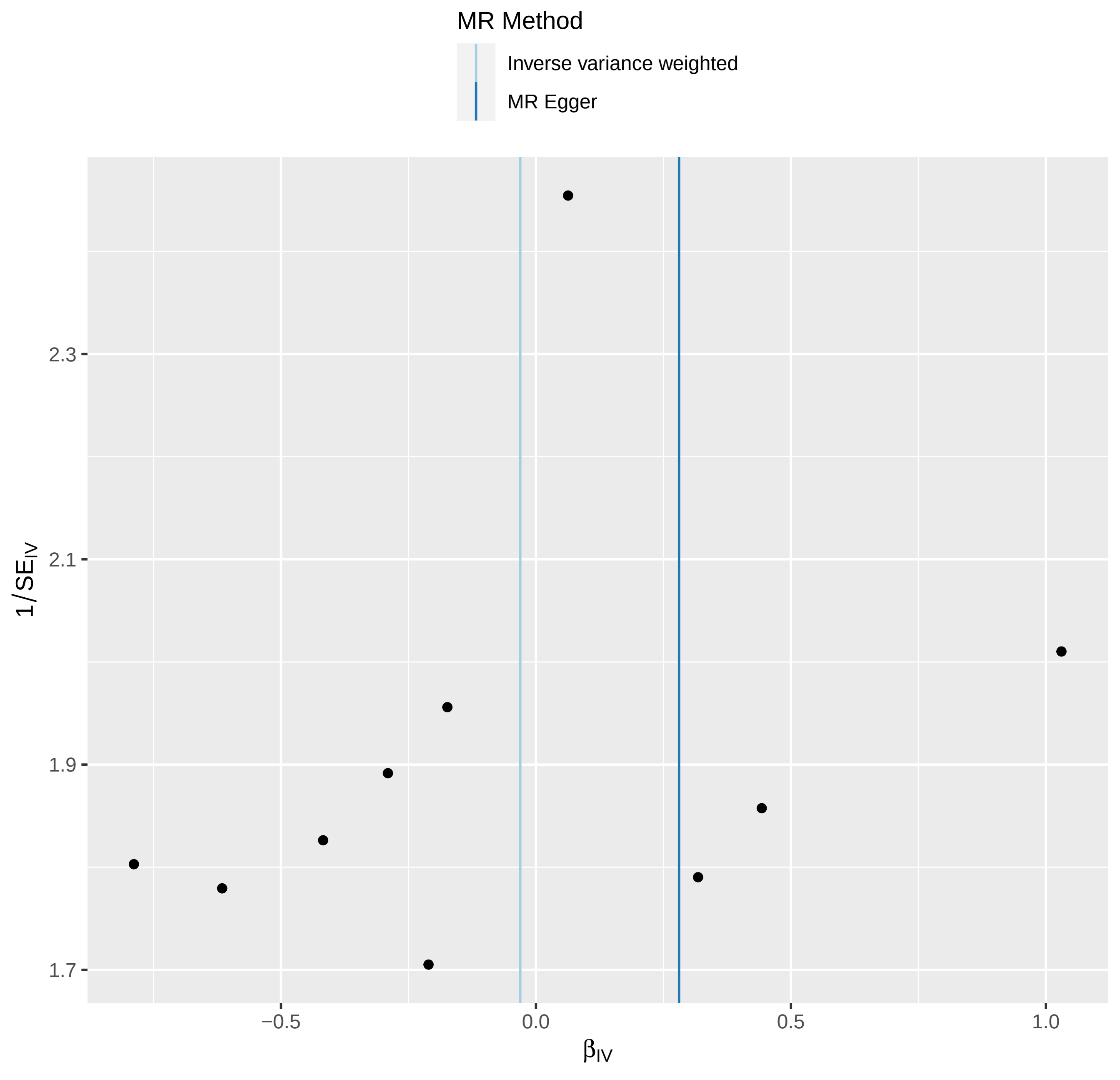

Batch 289 : Gut microbiota abundance (genus Ruminococcus gnavus group id.14376) on Other Crohn's disease

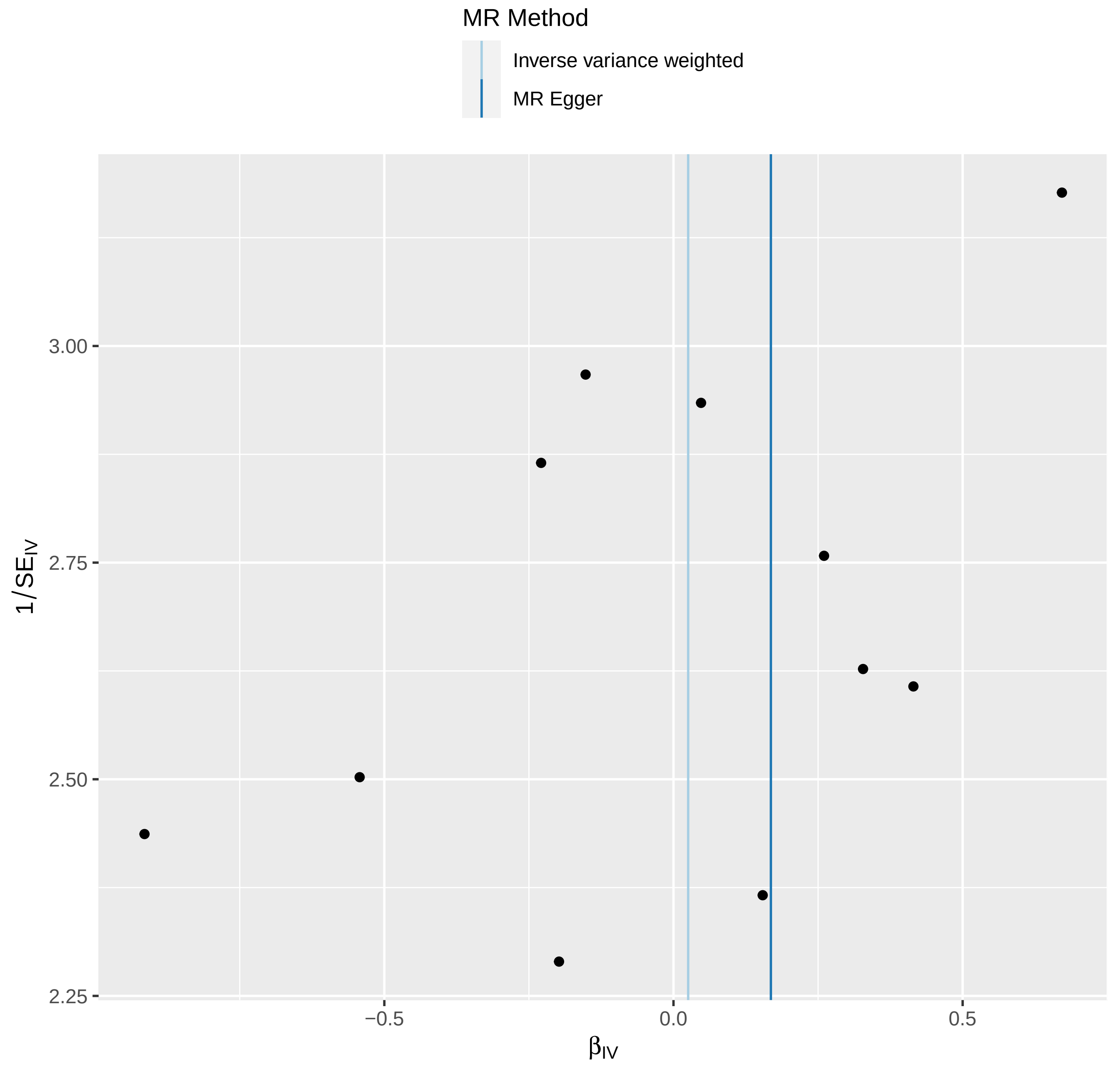

Batch 290 : Gut microbiota abundance (genus Ruminococcus torques group id.14377) on Other Crohn's disease

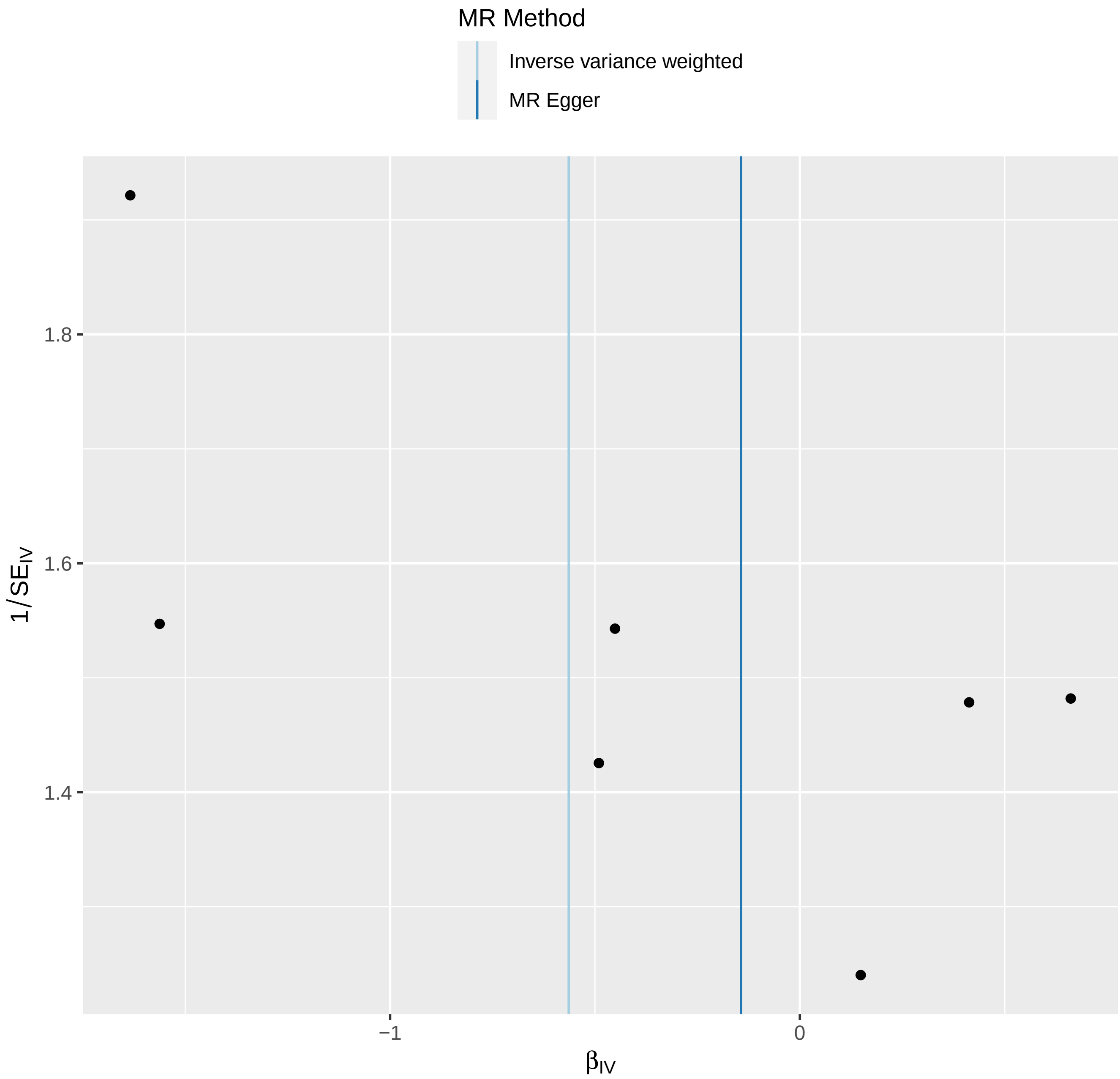

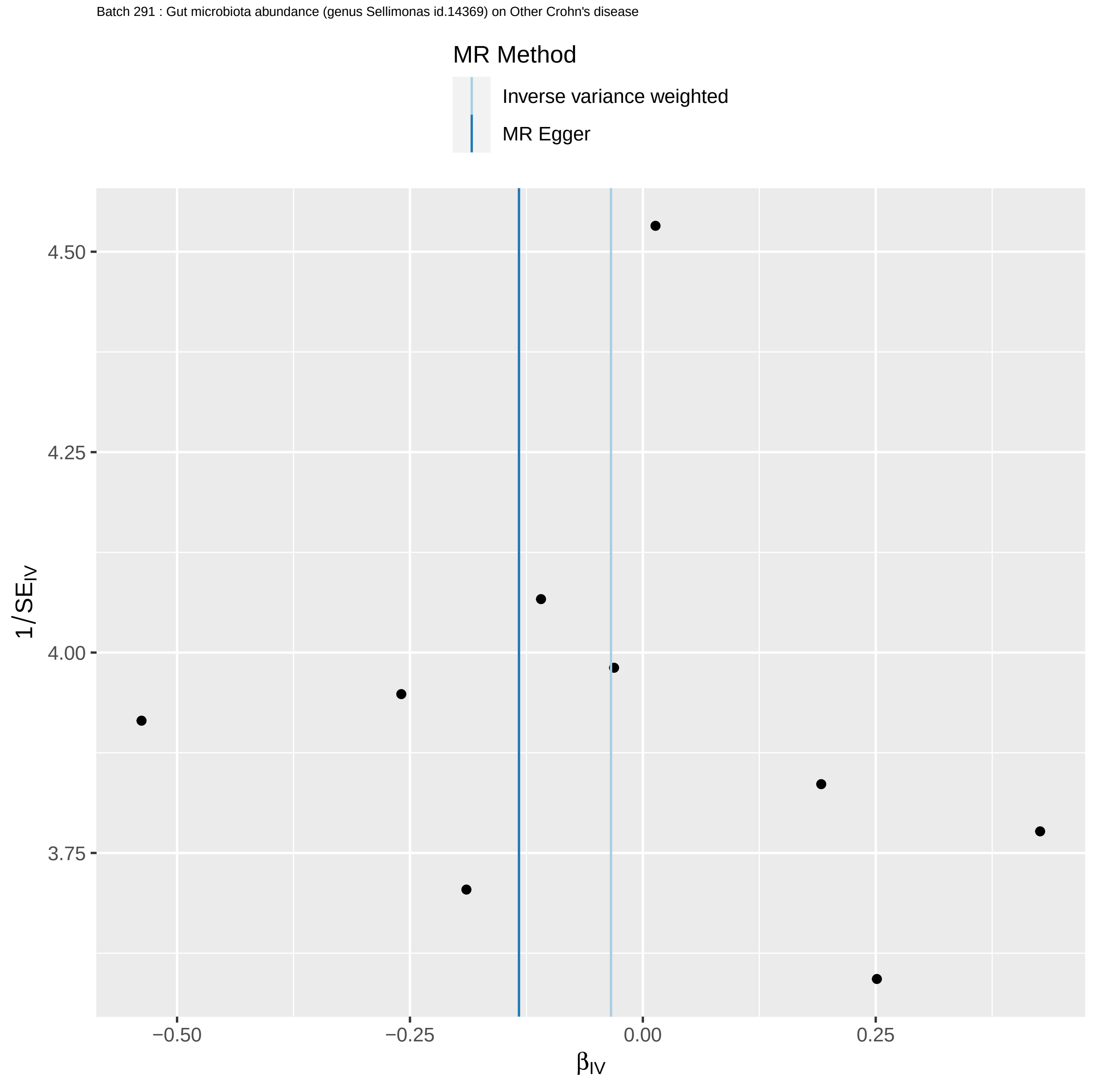
Batch 291 : Gut microbiota abundance (genus Sellimonas id.14369) on Other Crohn's disease
MR Method
Inverse variance weighted
MR Egger
4.50
4.25
4.00
3.75
1/SE_IV
−0.50
−0.25
0.00
0.25
β_IV

Batch 292 : Gut microbiota abundance (genus Senegalimassilia id.11160) on Other Crohn's disease

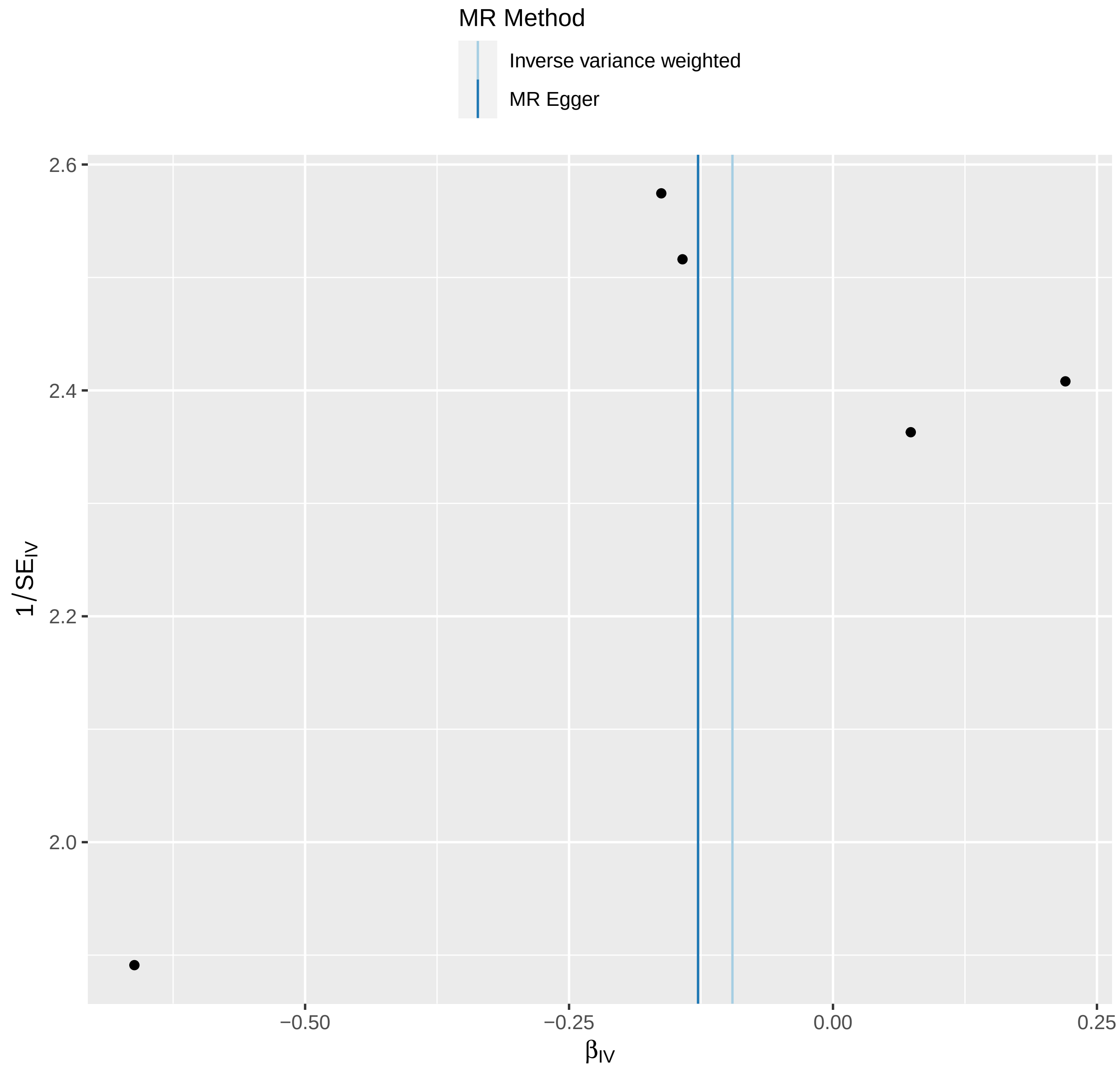

Batch 293 : Gut microbiota abundance (genus Slackia id.825) on Other Crohn's disease

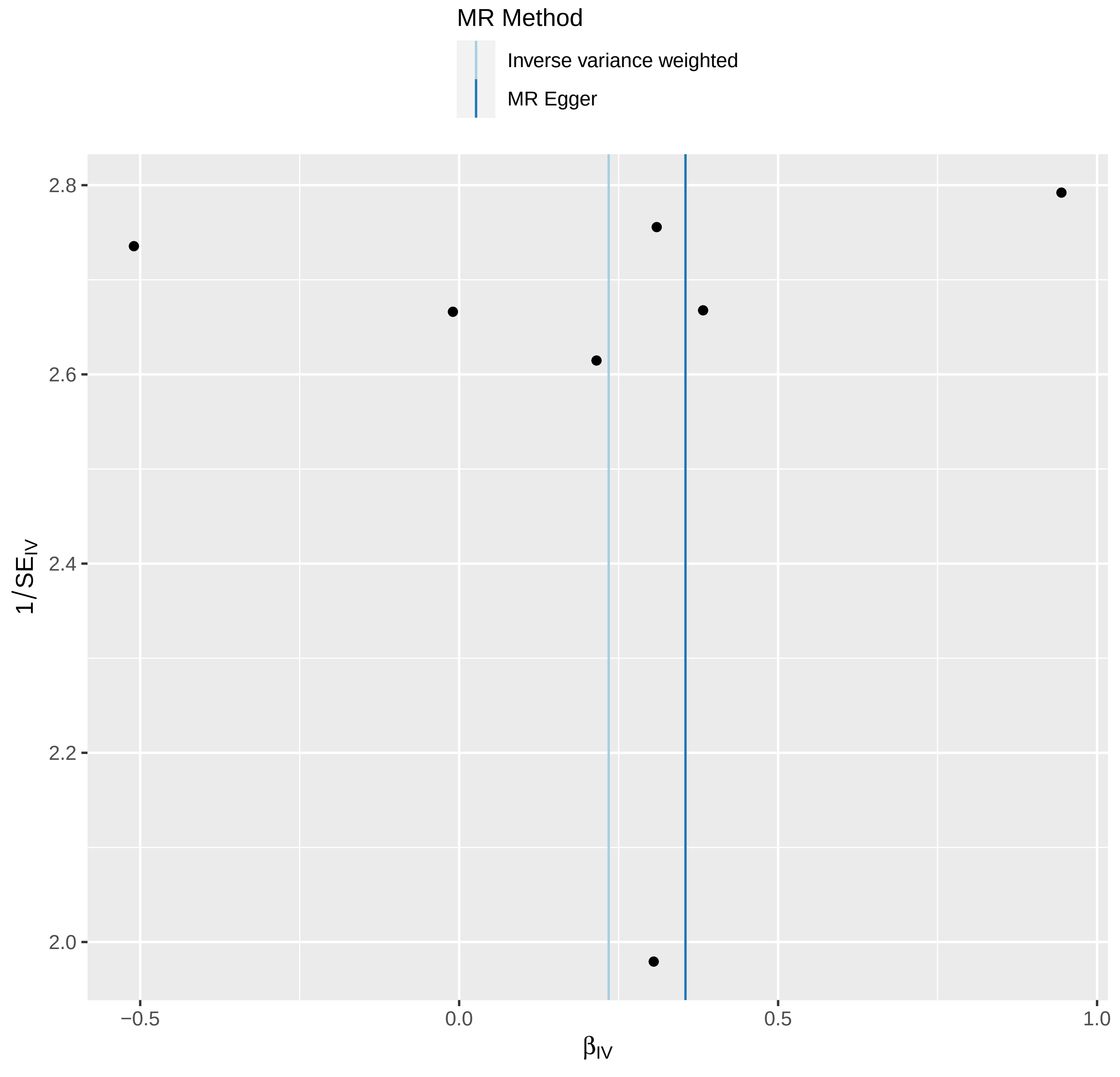

Batch 294 : Gut microbiota abundance (genus Streptococcus id.1853) on Other Crohn's disease

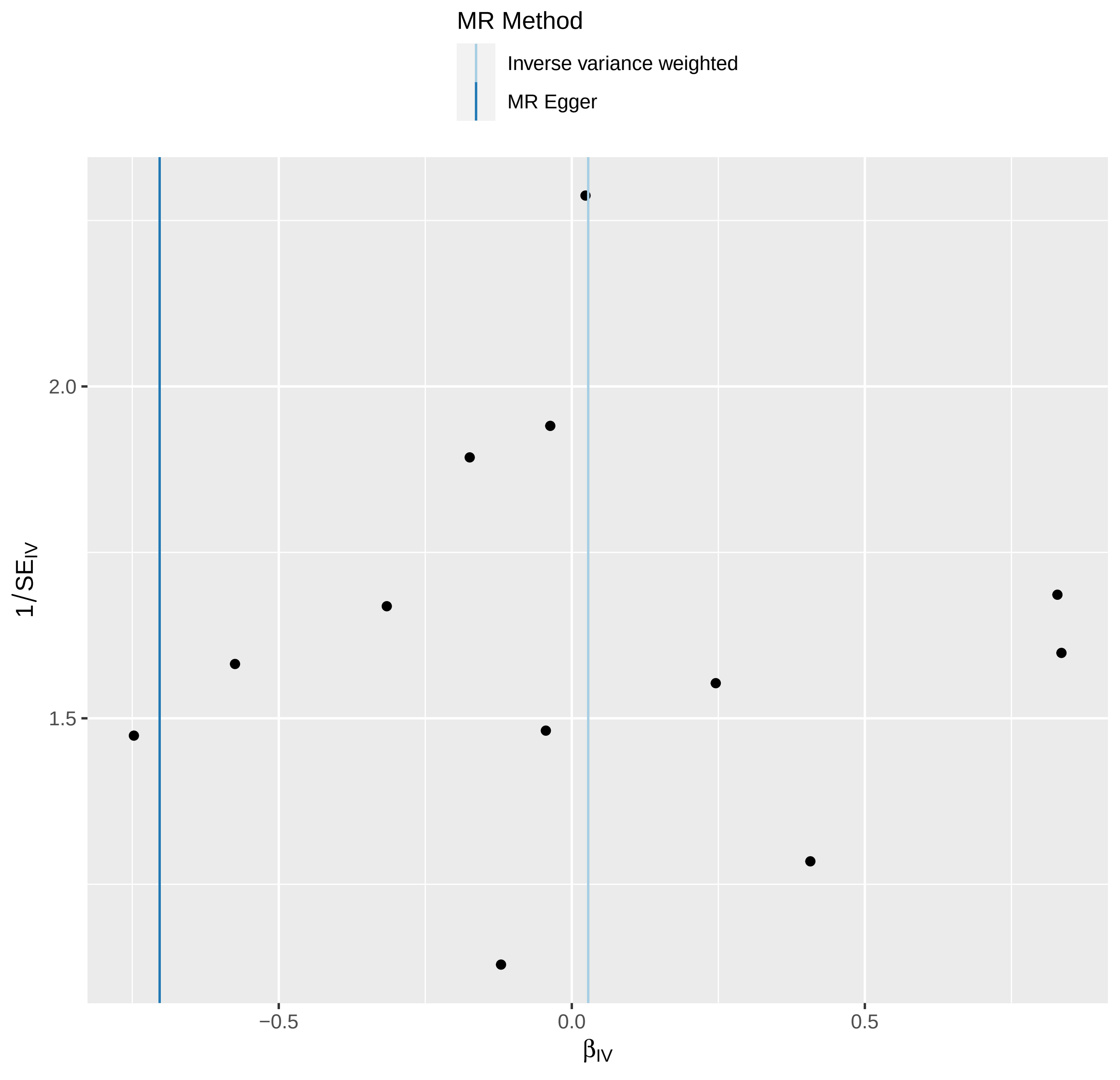

Batch 295 : Gut microbiota abundance (genus Subdoligranulum id.2070) on Other Crohn's disease

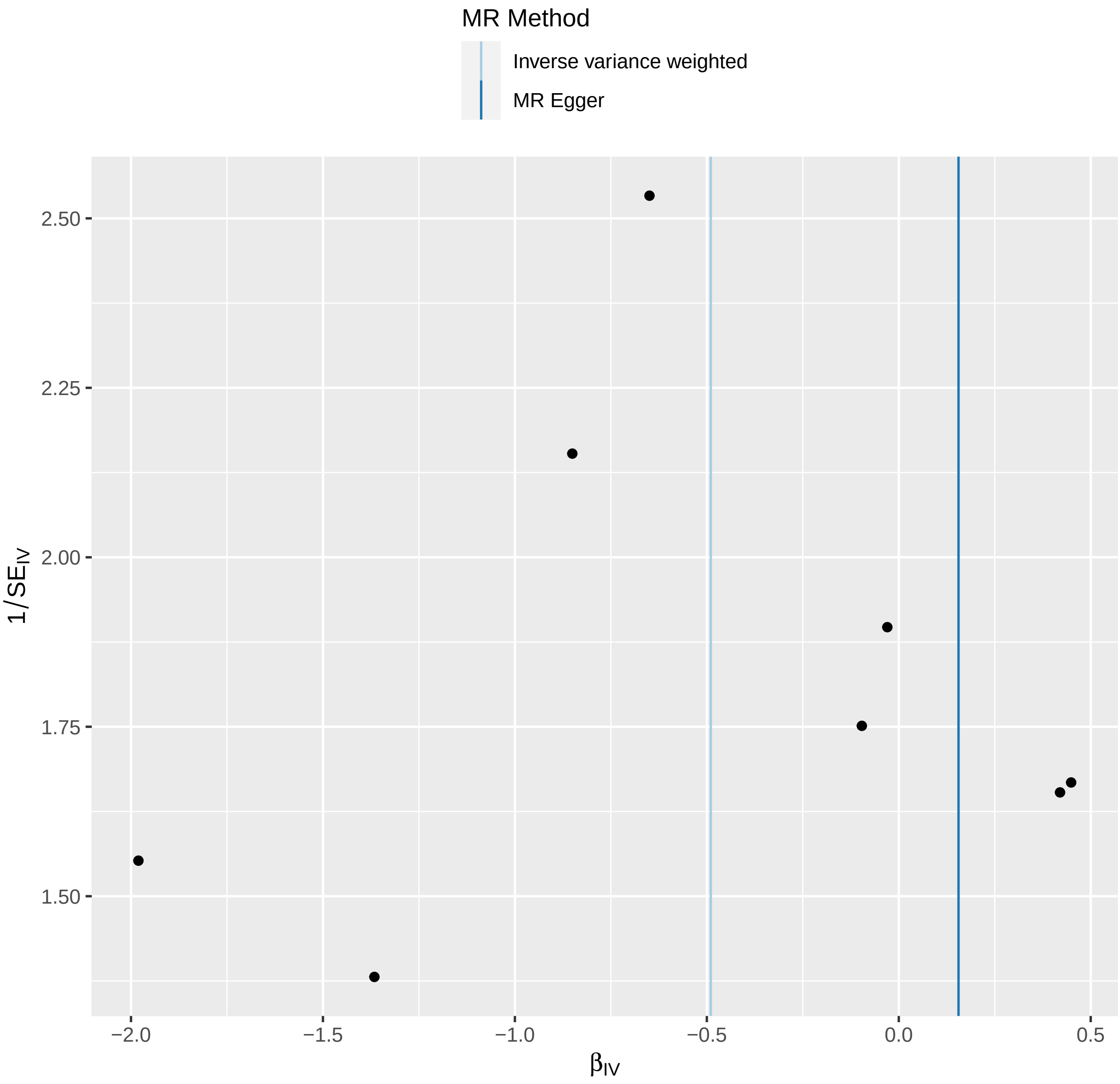

Batch 296 : Gut microbiota abundance (genus Sutterella id.2896) on Other Crohn's disease

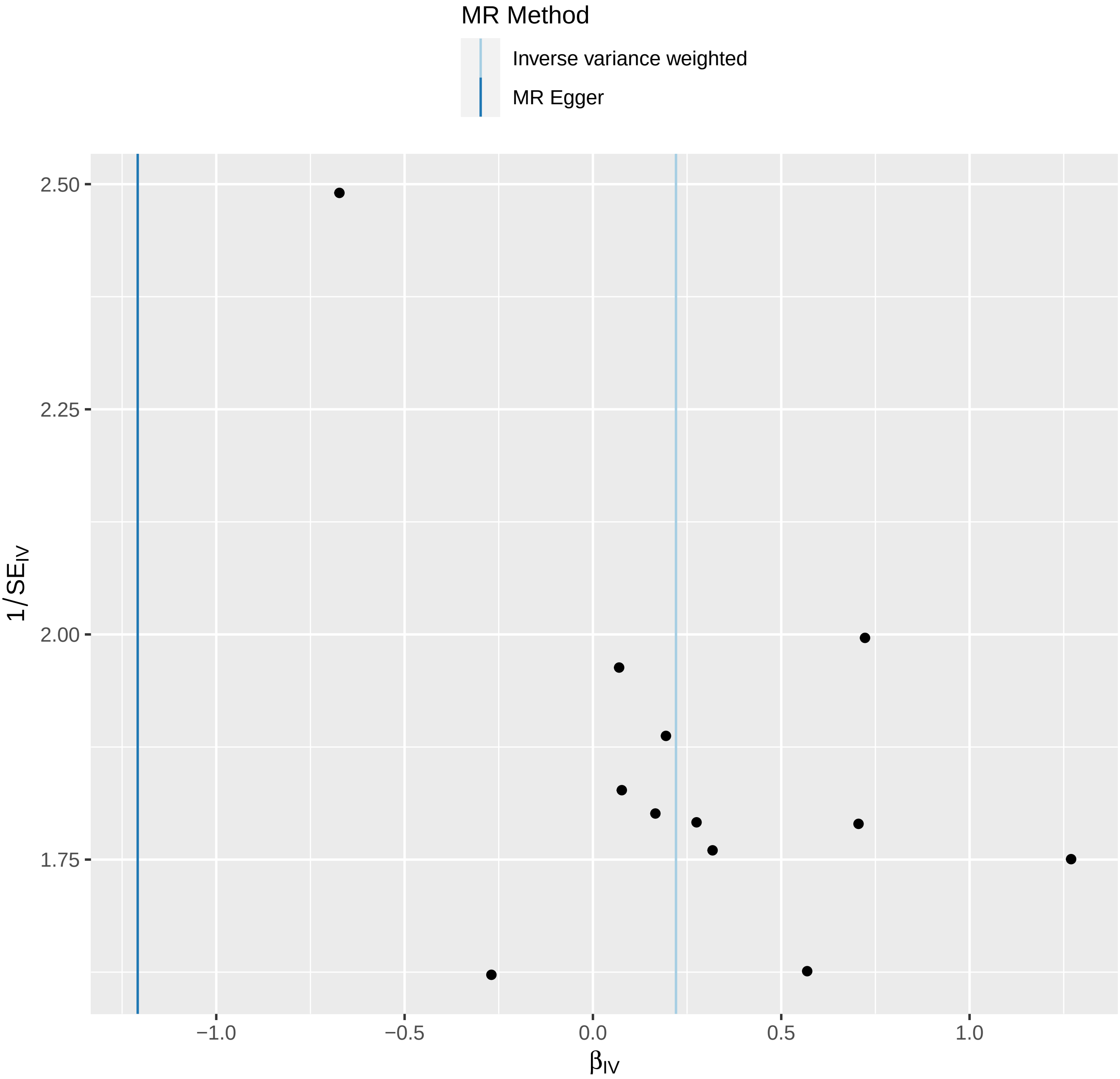

Batch 297 : Gut microbiota abundance (genus Terrisporobacter id.11348) on Other Crohn's disease

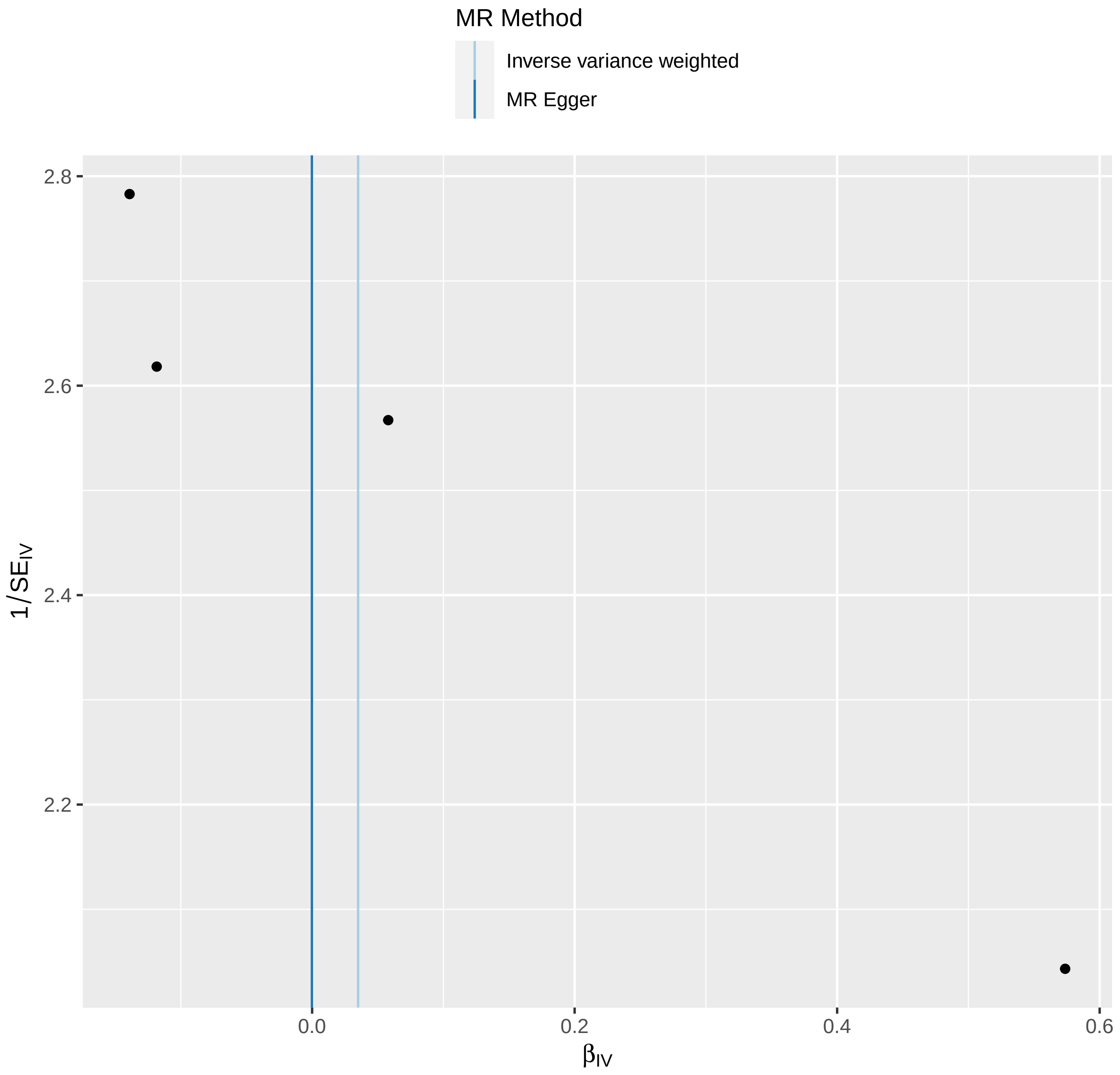

Batch 298 : Gut microbiota abundance (genus Turicibacter id.2162) on Other Crohn's disease

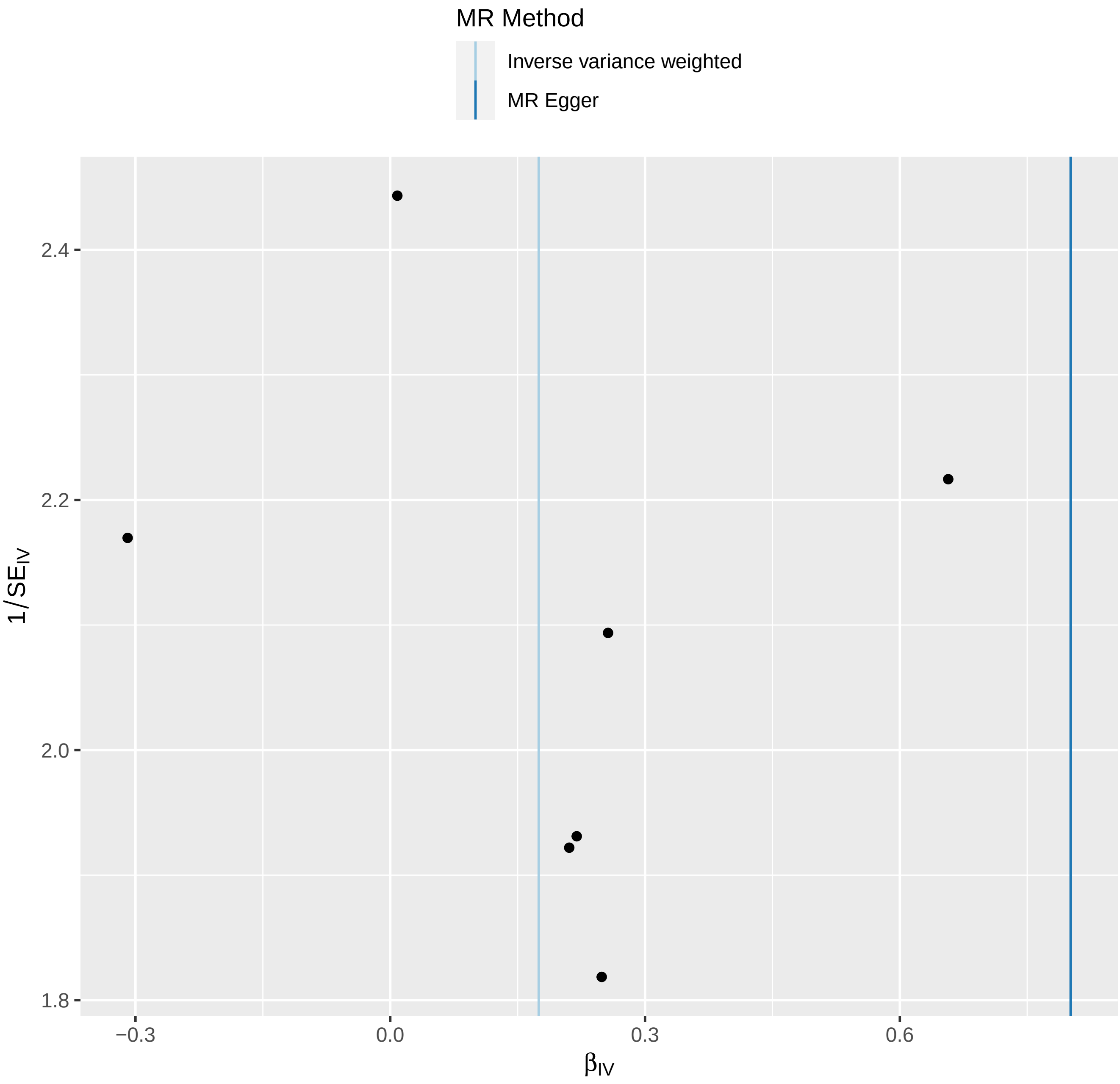

Batch 299 : Gut microbiota abundance (genus Tyzzerella3 id.11335) on Other Crohn's disease

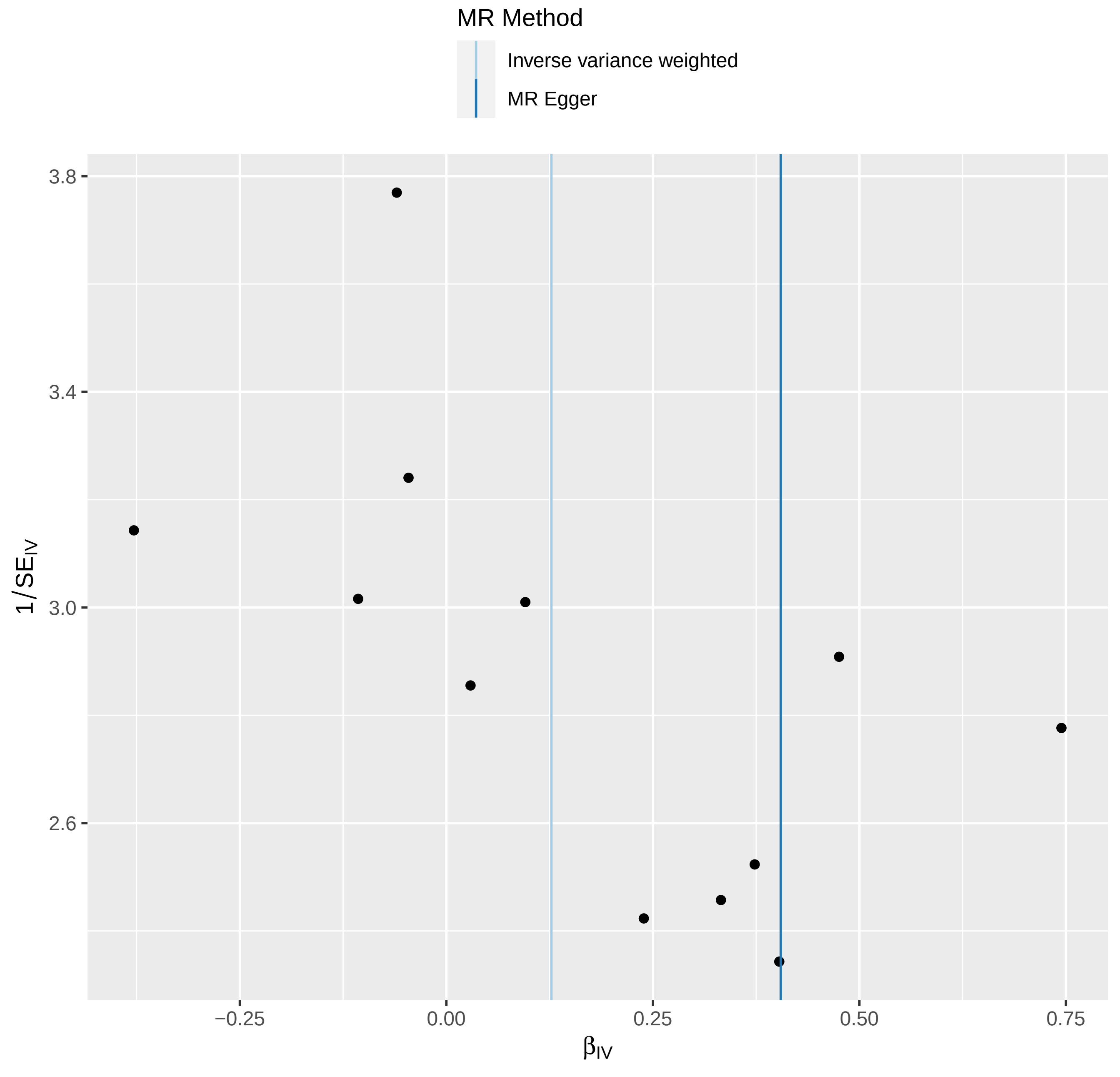

Batch 300 : Gut microbiota abundance (genus Veillonella id.2198) on Other Crohn's disease

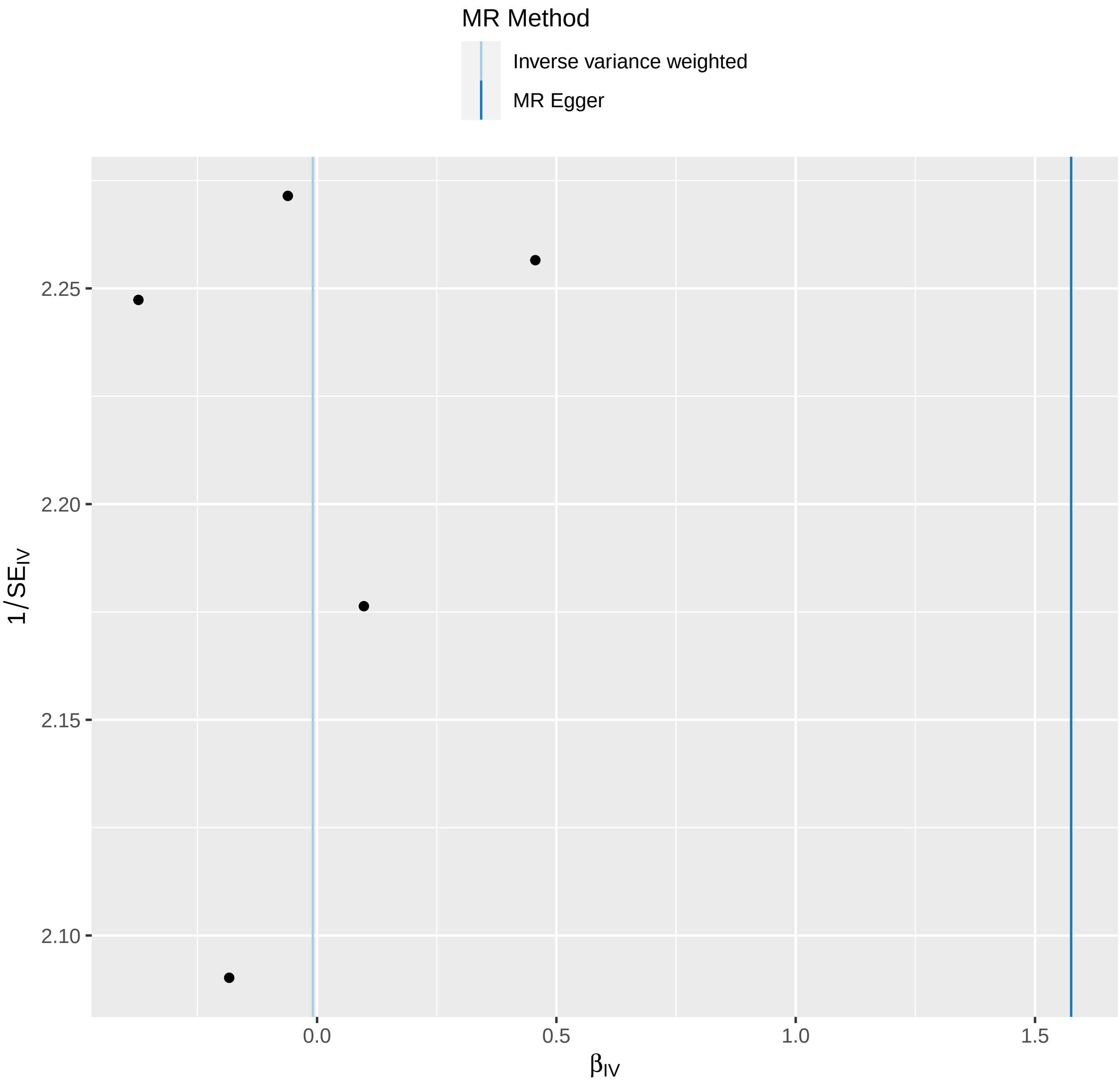

Batch 301 : Gut microbiota abundance (family Acidaminococcaceae id.2166) on Crohn's disease of small intestine

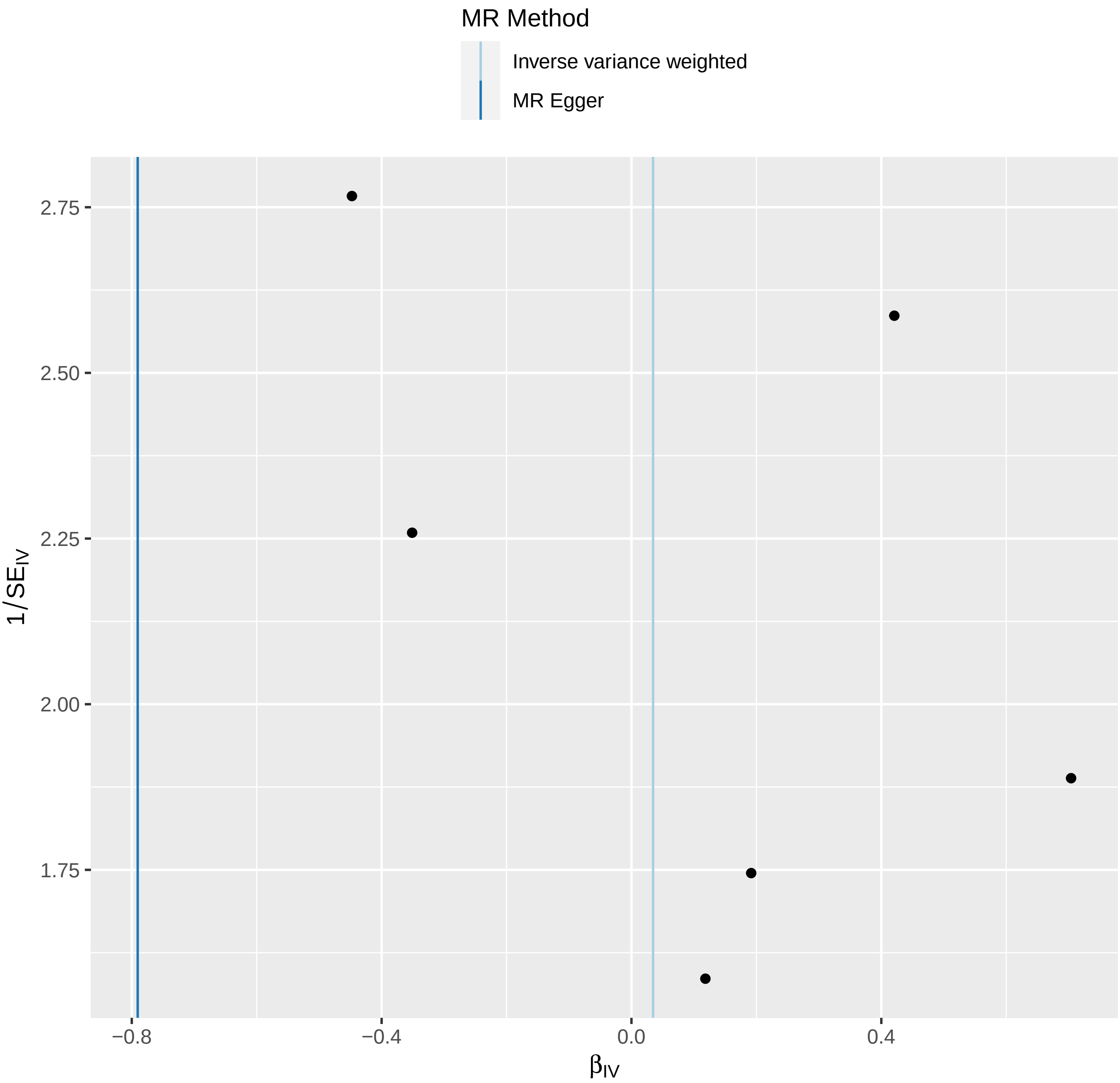

Batch 302 : Gut microbiota abundance (family Actinomycetaceae id.421) on Crohn's disease of small intestine

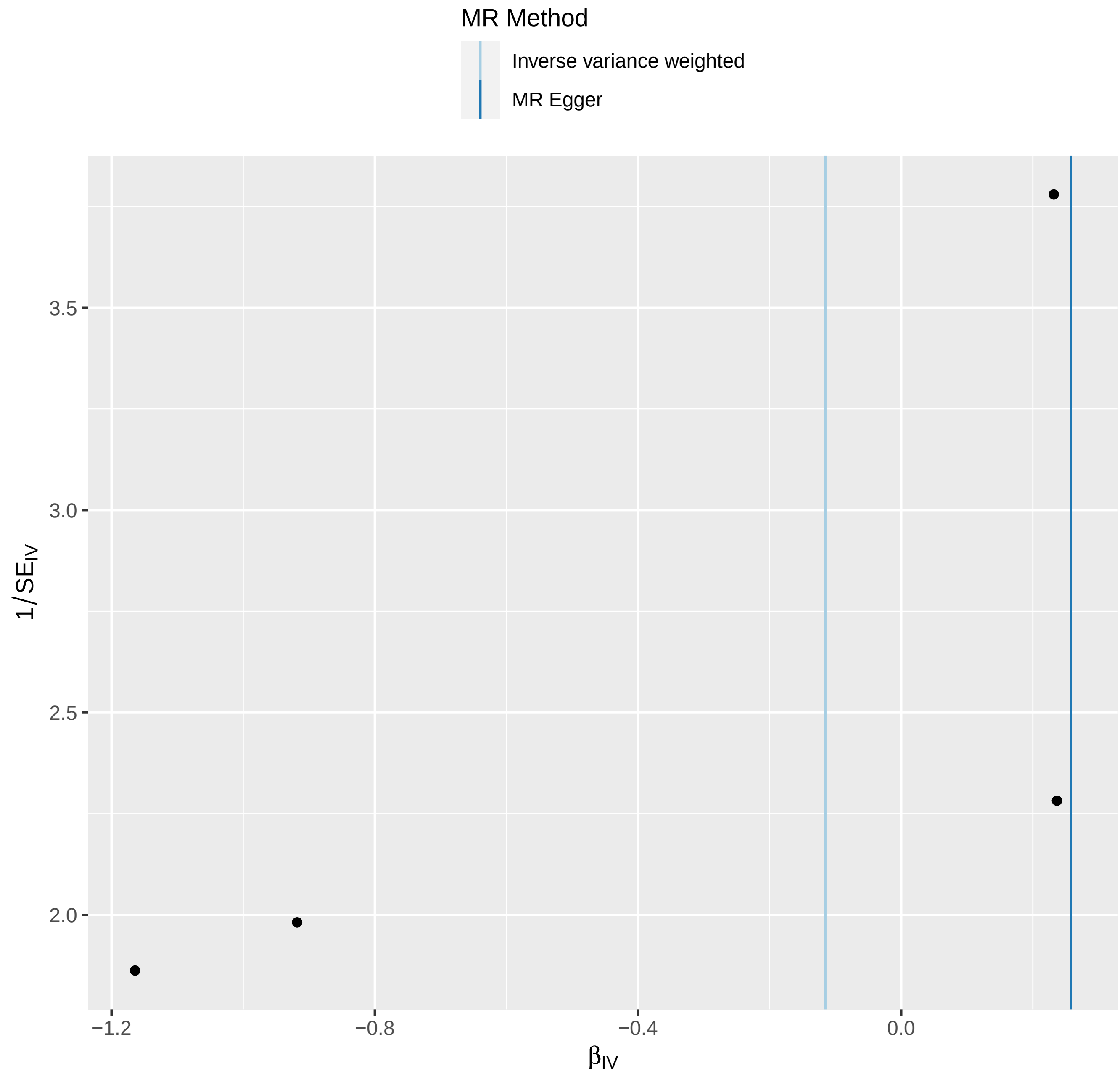

Batch 303 : Gut microbiota abundance (family Alcaligenaceae id.2875) on Crohn's disease of small intestine

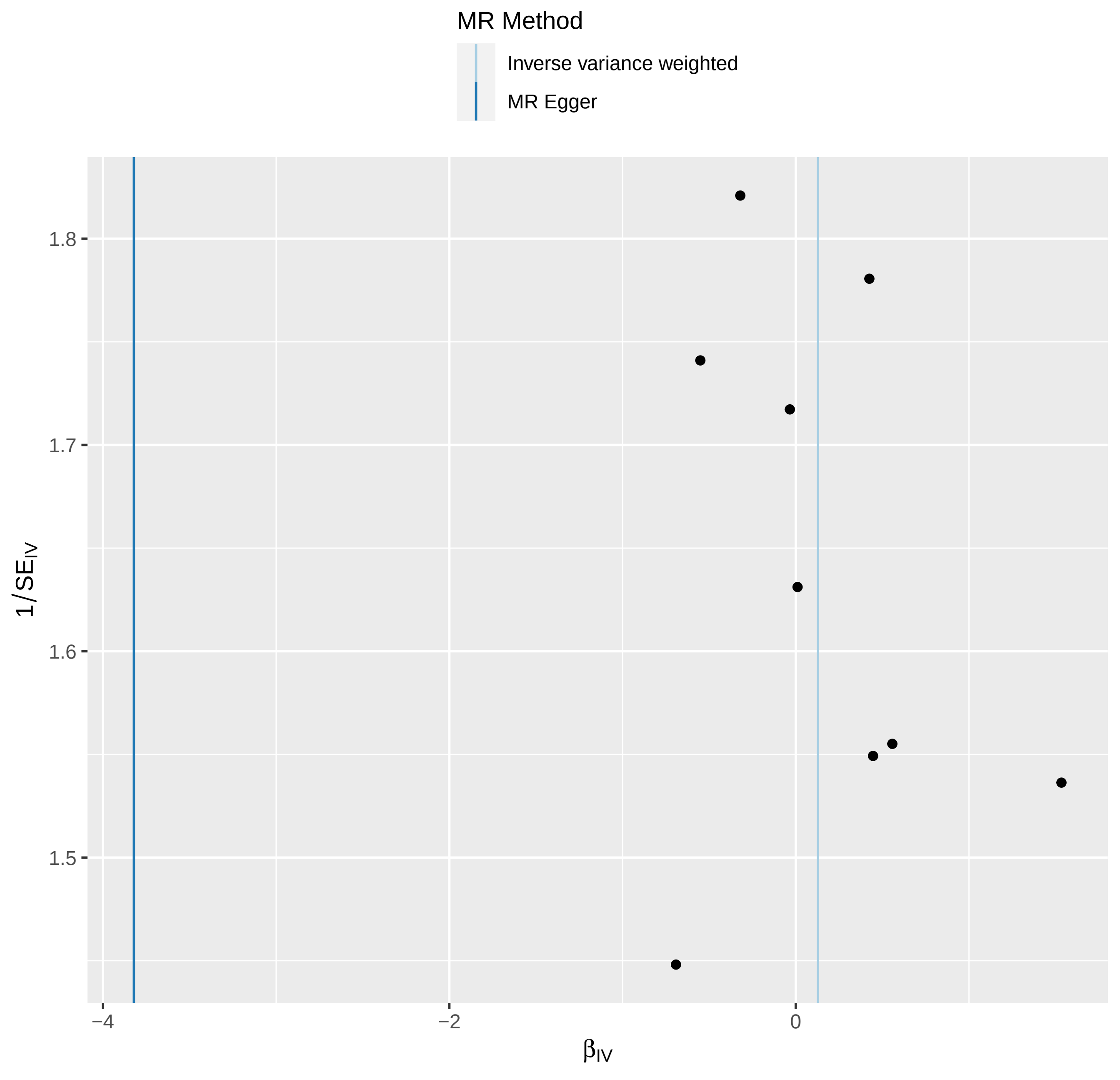

Batch 304 : Gut microbiota abundance (family Bacteroidaceae id.917) on Crohn's disease of small intestine

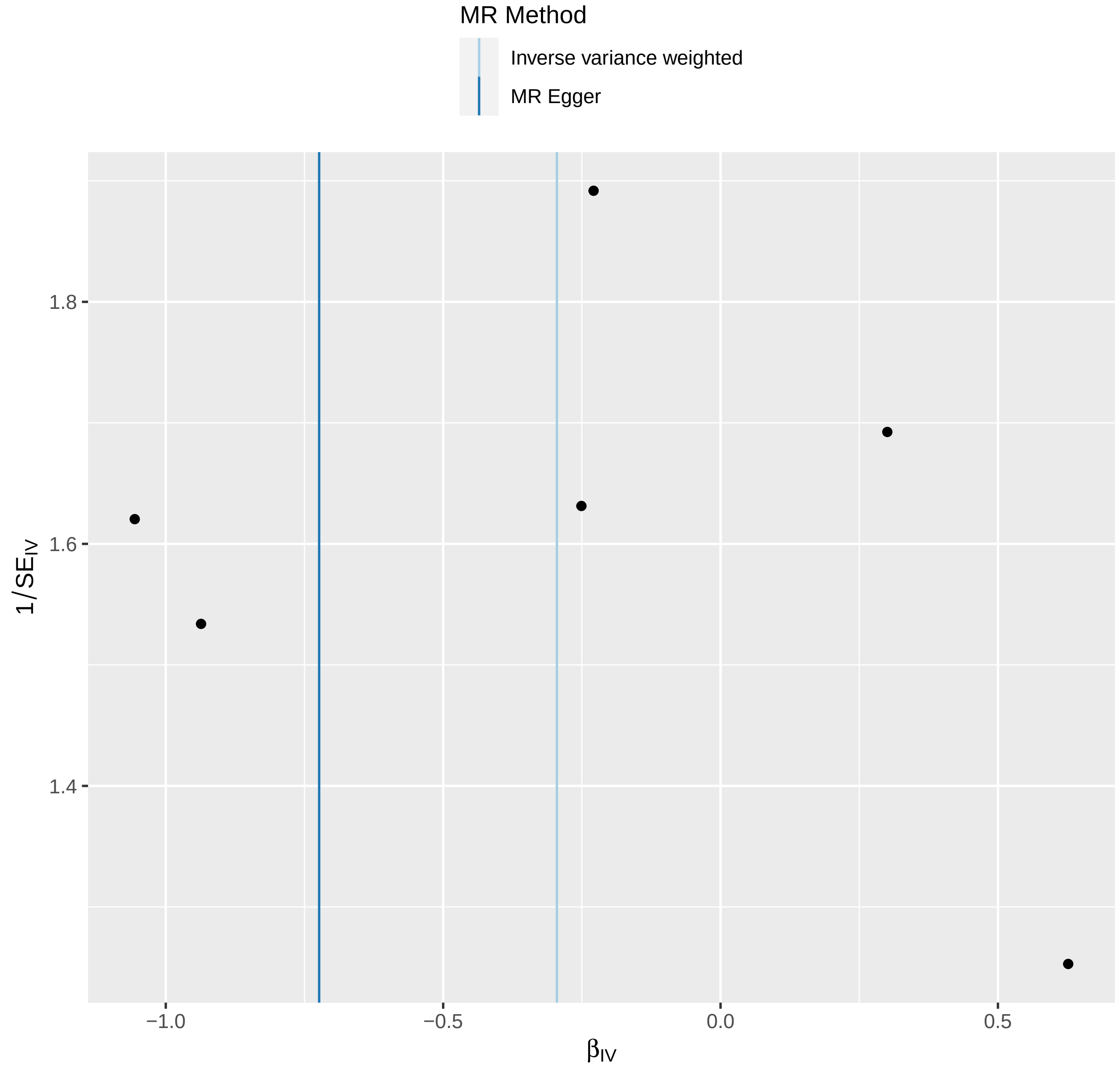

Batch 305 : Gut microbiota abundance (family Bacteroidales S24 7group id.11173) on Crohn's disease of small intestine

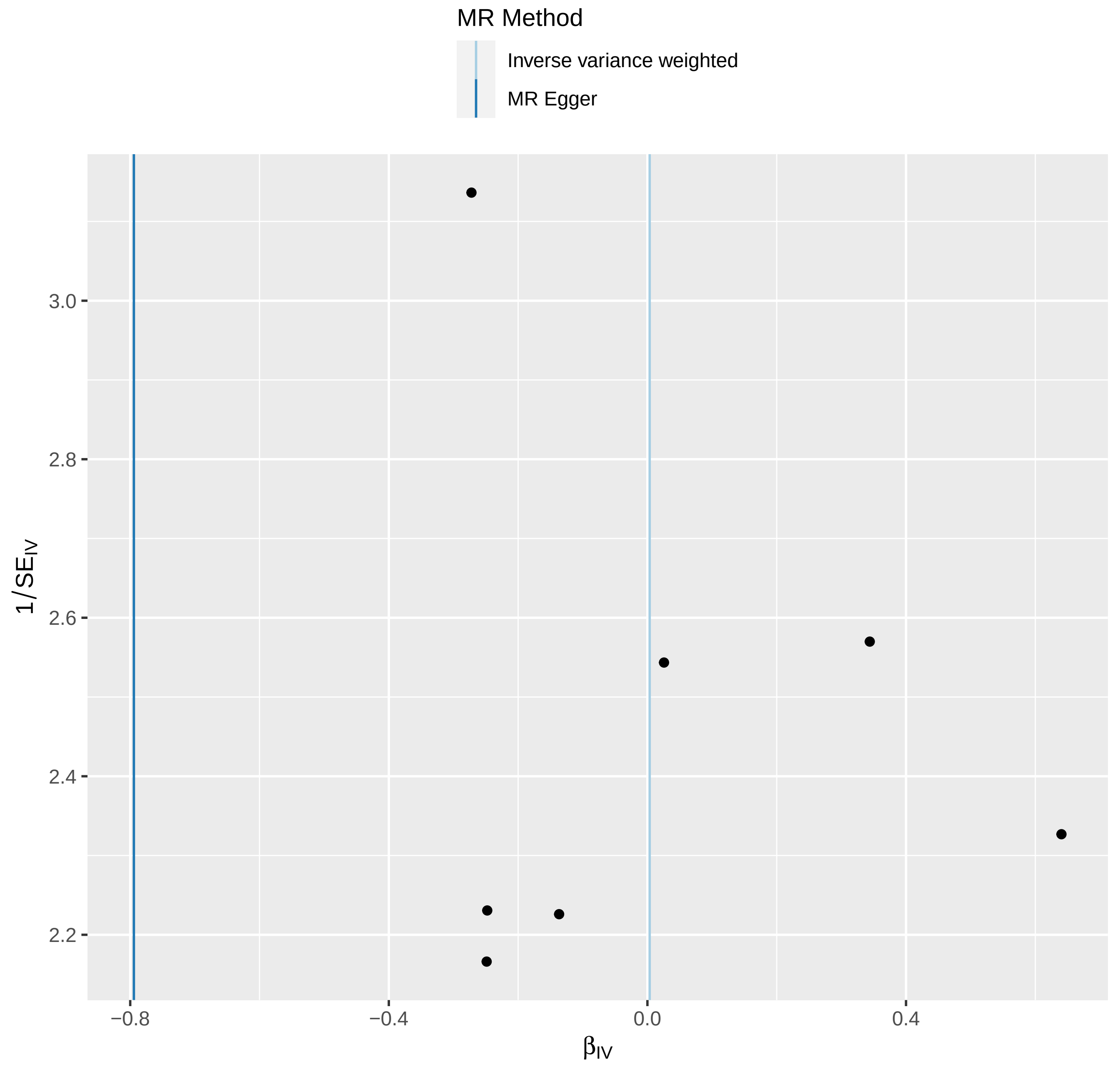

Batch 306 : Gut microbiota abundance (family Bifidobacteriaceae id.433) on Crohn's disease of small intestine

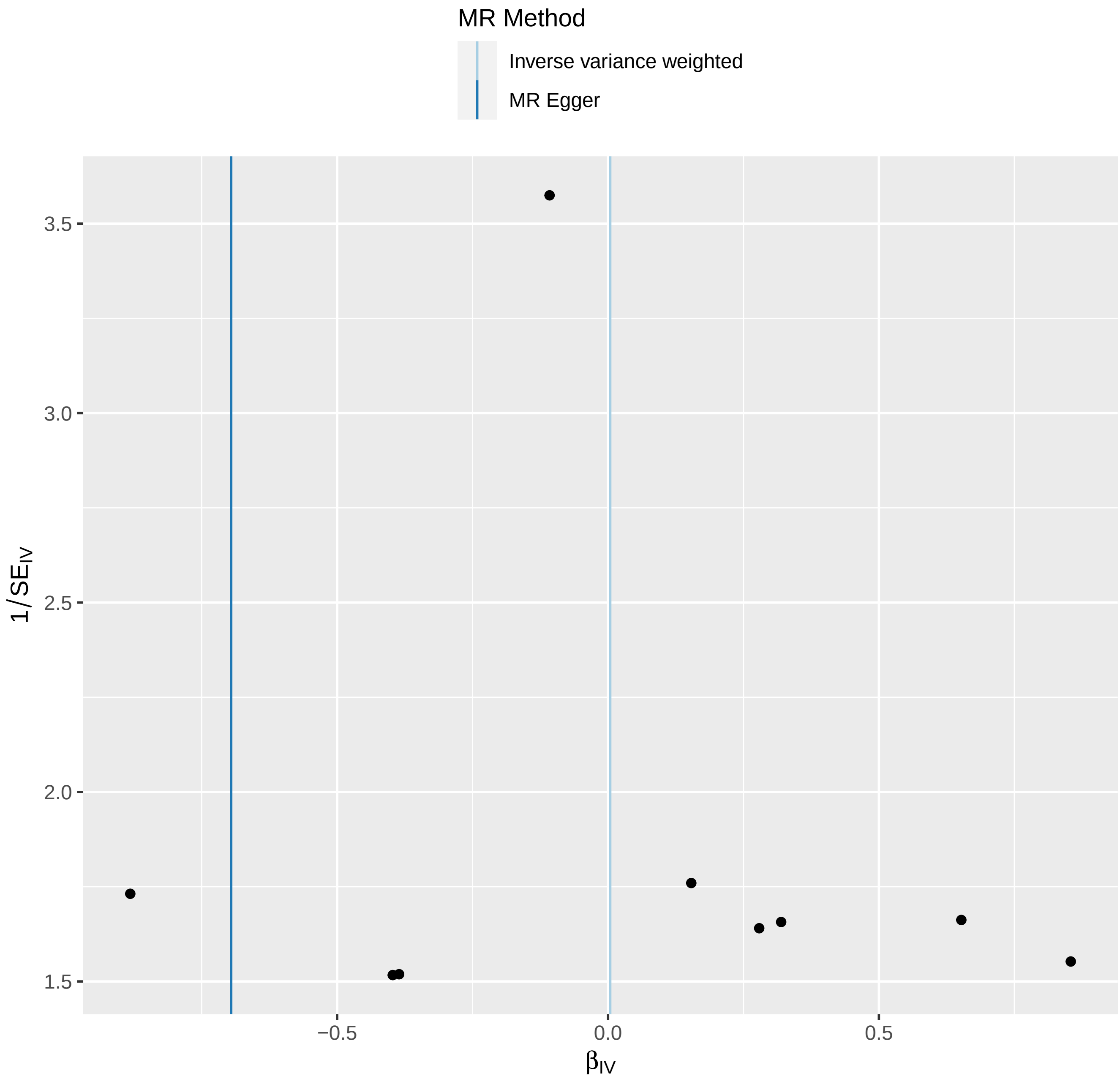

Batch 308 : Gut microbiota abundance (family Clostridiaceae1 id.1869) on Crohn's disease of small intestine

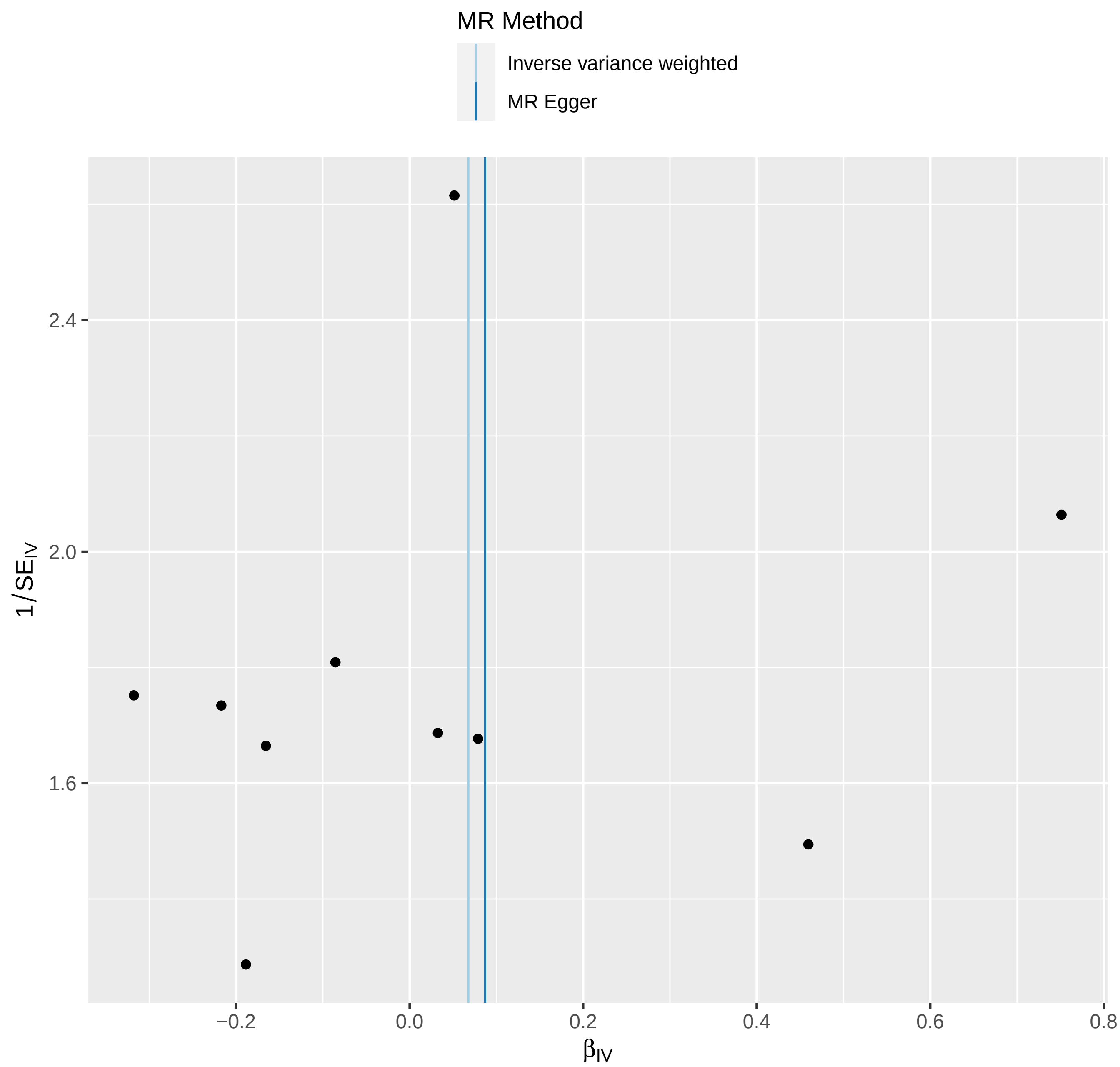

Batch 309 : Gut microbiota abundance (family Clostridiales vadin BB60 group id.11286) on Crohn's disease of small intestine

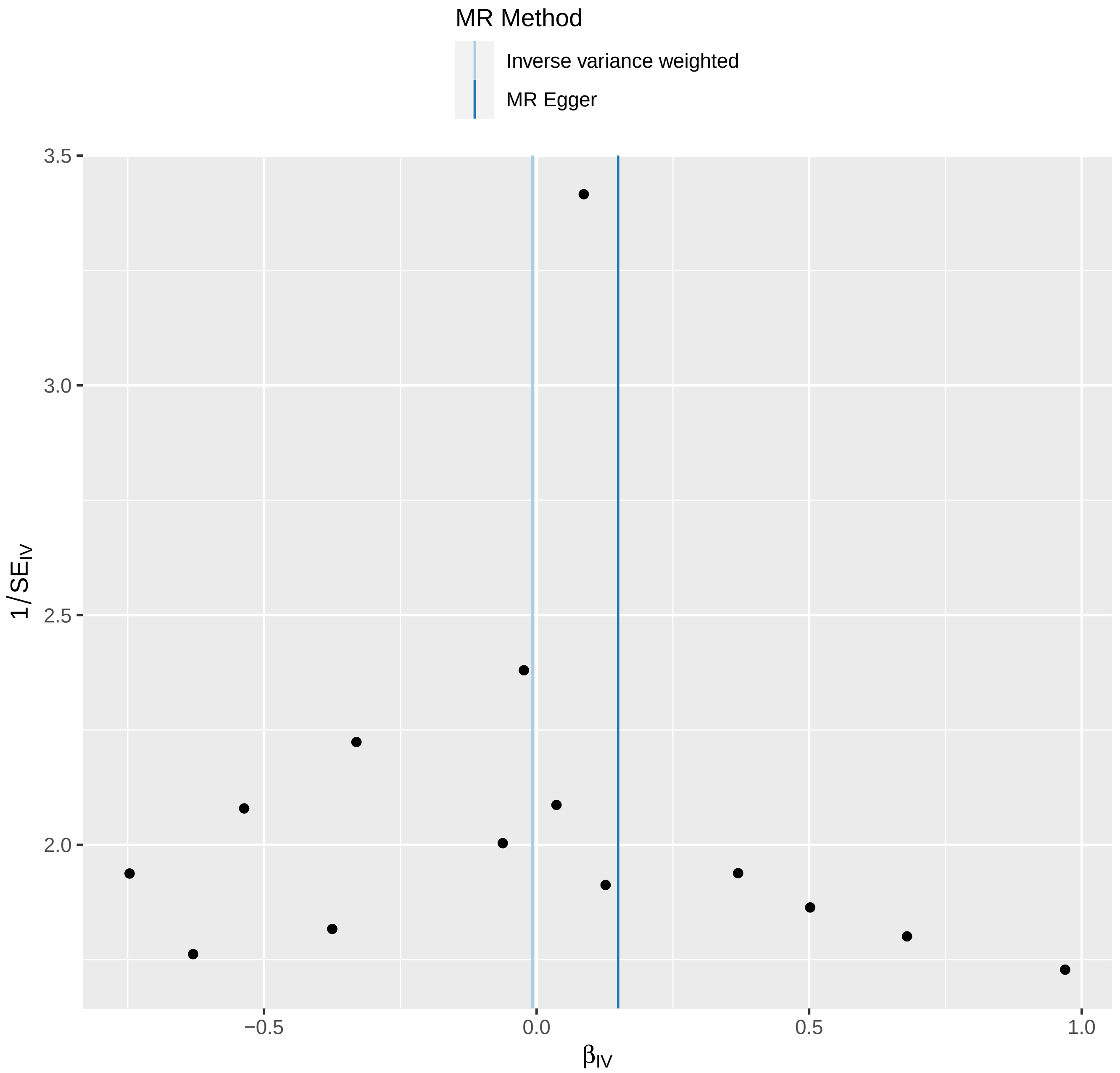

Batch 310 : Gut microbiota abundance (family Coriobacteriaceae id.811) on Crohn's disease of small intestine

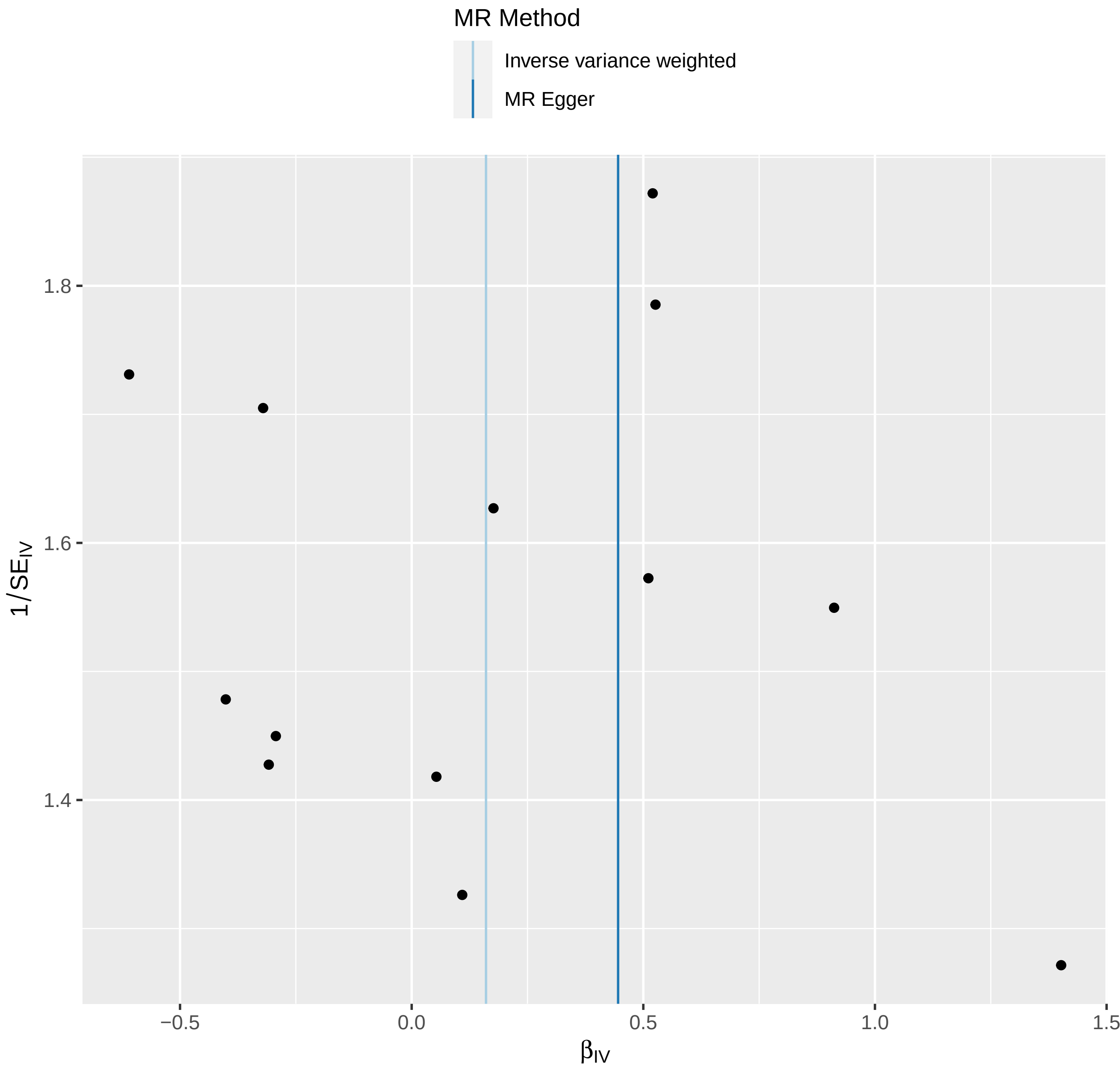

Batch 311 : Gut microbiota abundance (family Defluviitaleaceae id.1924) on Crohn's disease of small intestine

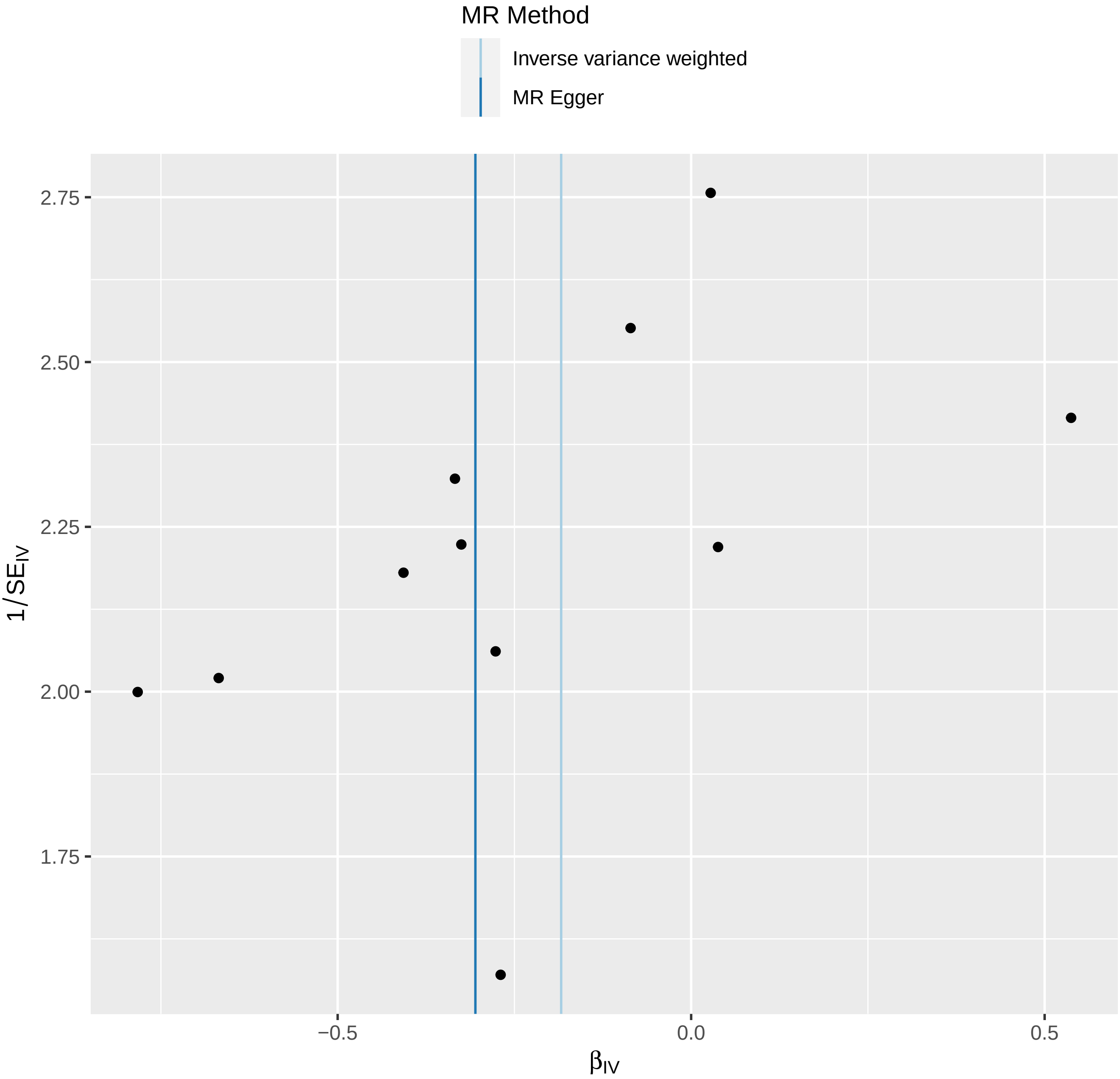

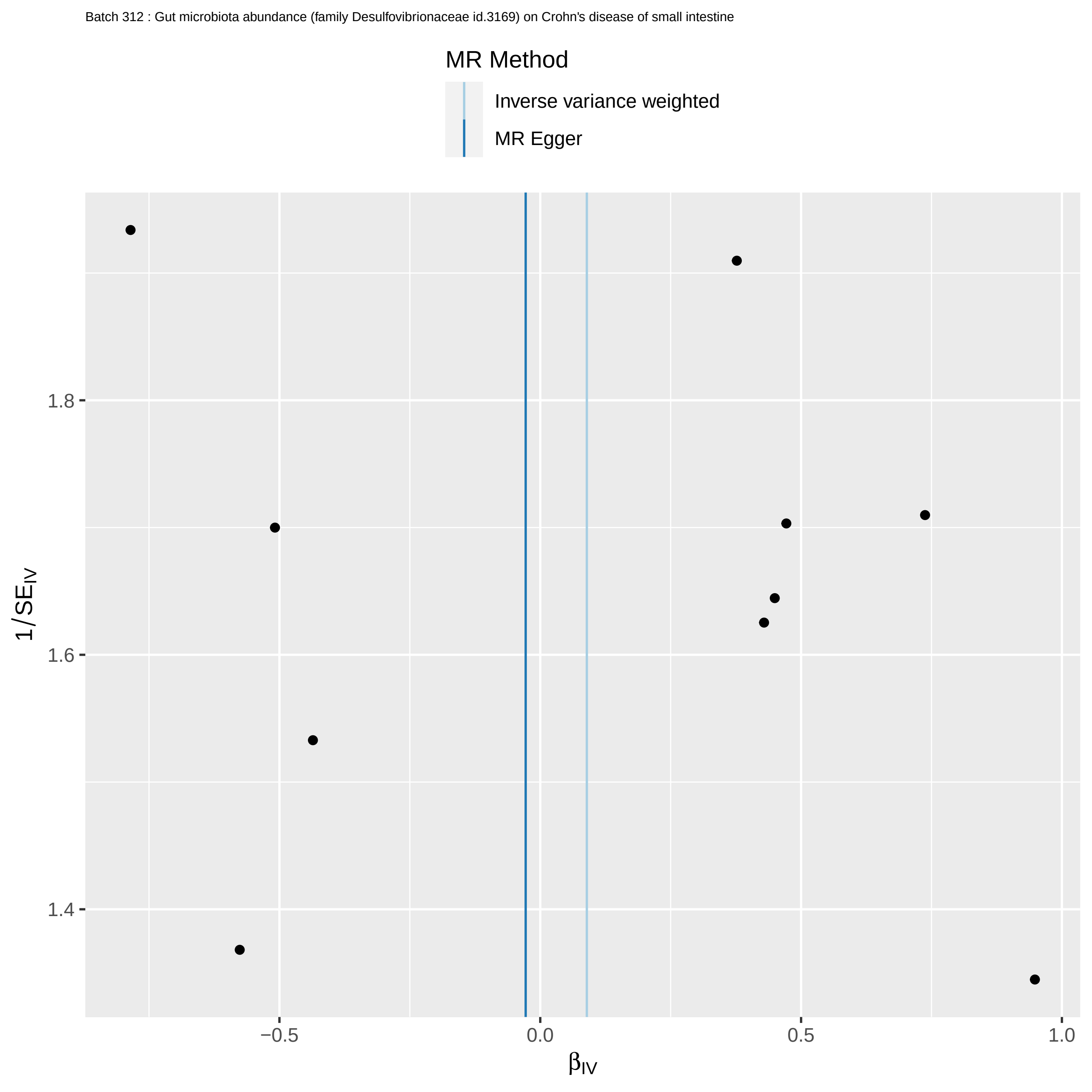
Batch 312 : Gut microbiota abundance (family Desulfovibrionaceae id.3169) on Crohn's disease of small intestine
MR Method
Inverse variance weighted
MR Egger
1.8
1.6
1.4
$1/SE_{IV}$
−0.5
0.0
0.5
1.0
β_{IV}

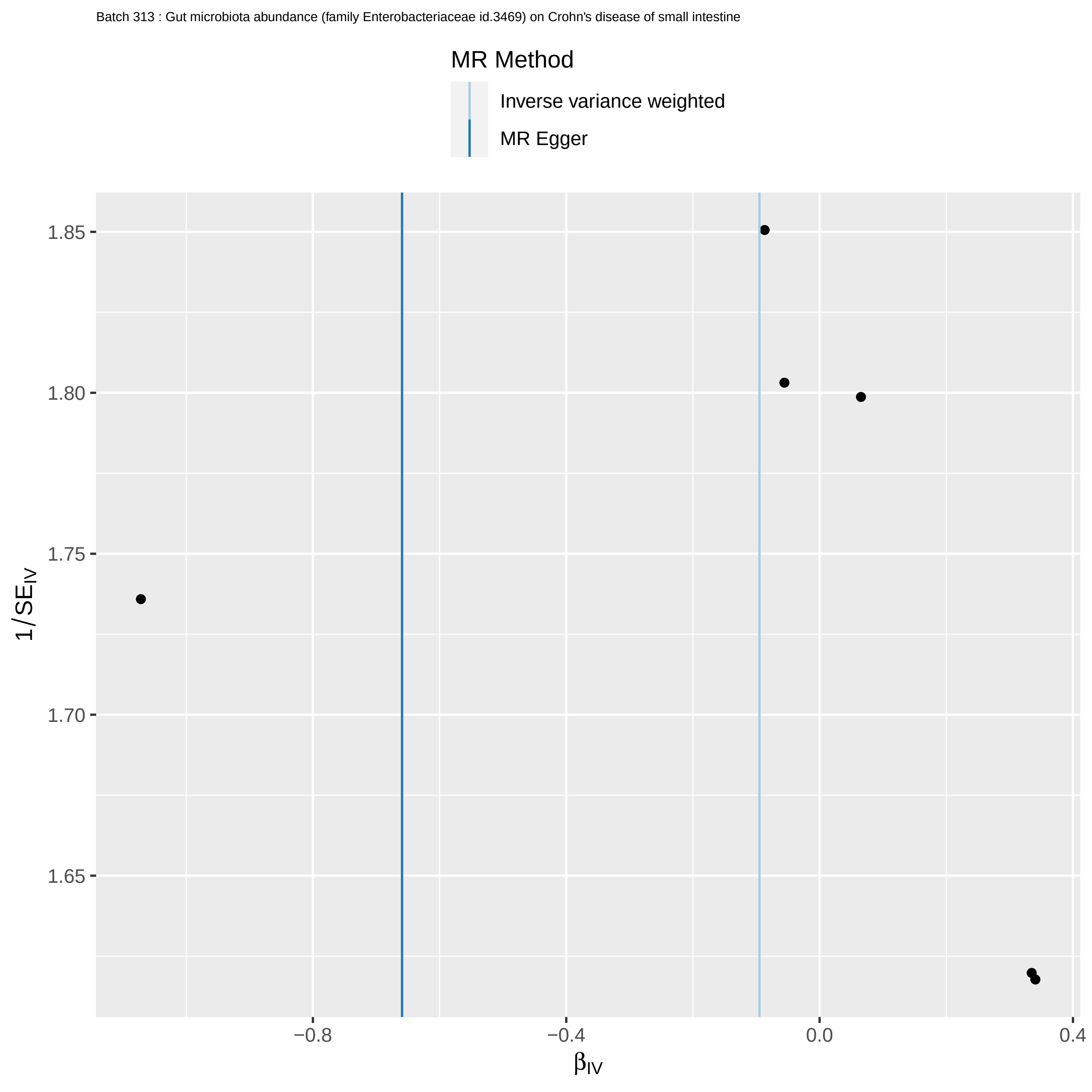

Batch 313 : Gut microbiota abundance (family Enterobacteriaceae id.3469) on Crohn's disease of small intestine
MR Method
Inverse variance weighted
MR Egger
1.85
1.80
1.75
1.70
1.65
1/SE_IV
−0.8
−0.4
0.0
0.4
β_IV

Batch 314 : Gut microbiota abundance (family Erysipelotrichaceae id.2149) on Crohn's disease of small intestine

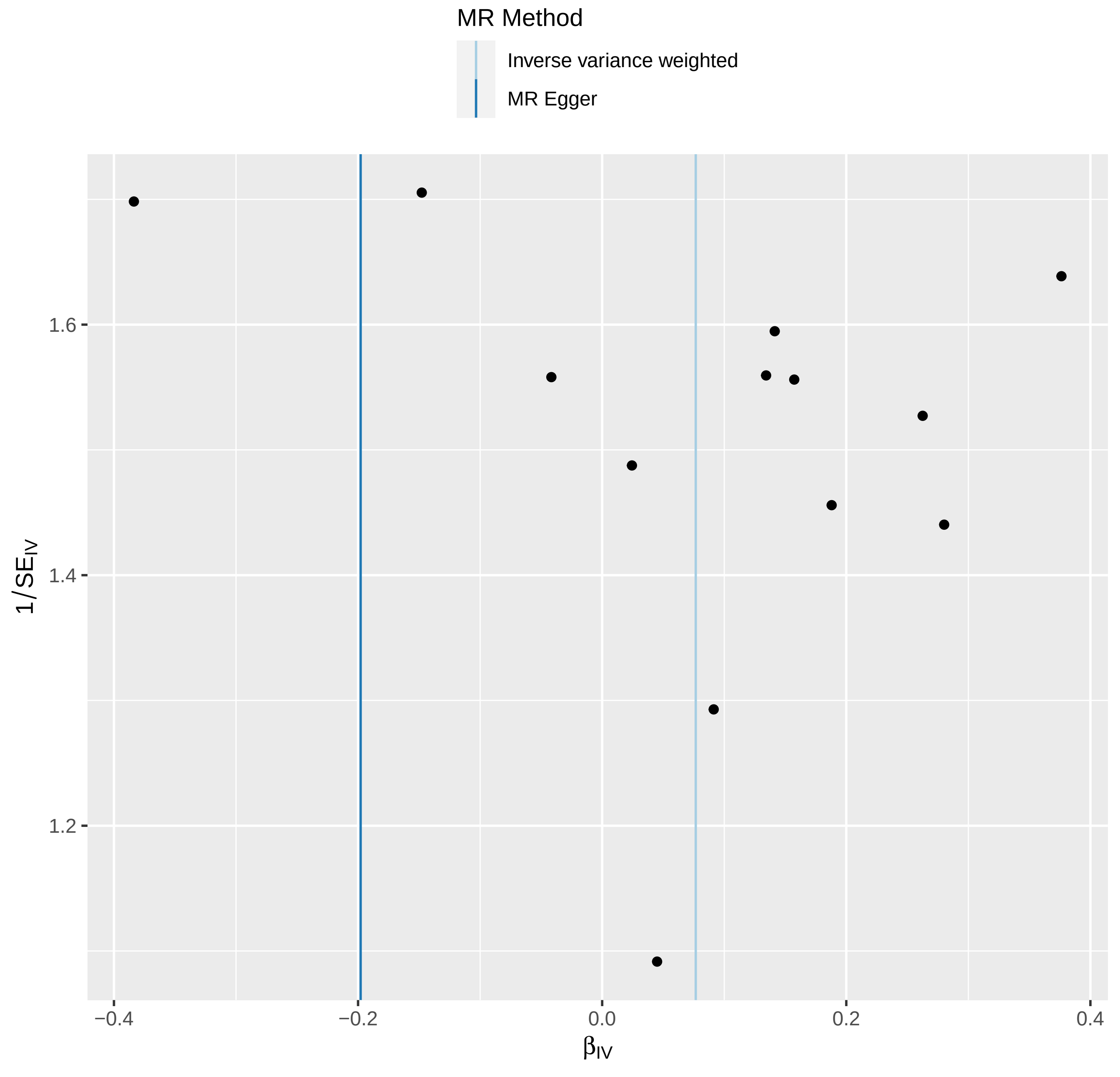

Batch 315 : Gut microbiota abundance (family Family XI id.1936) on Crohn's disease of small intestine

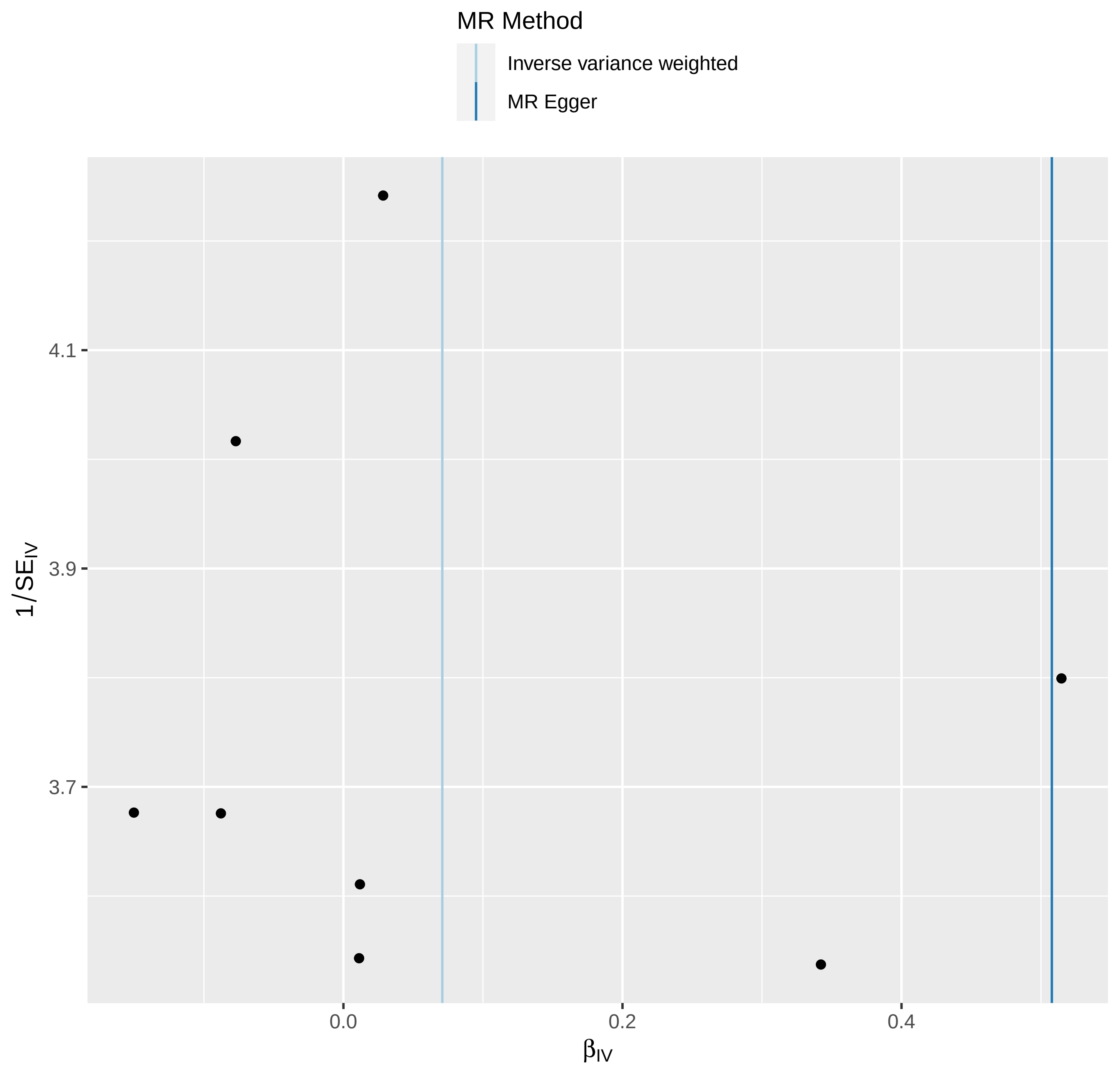

Batch 316 : Gut microbiota abundance (family Family XIII id.1957) on Crohn's disease of small intestine

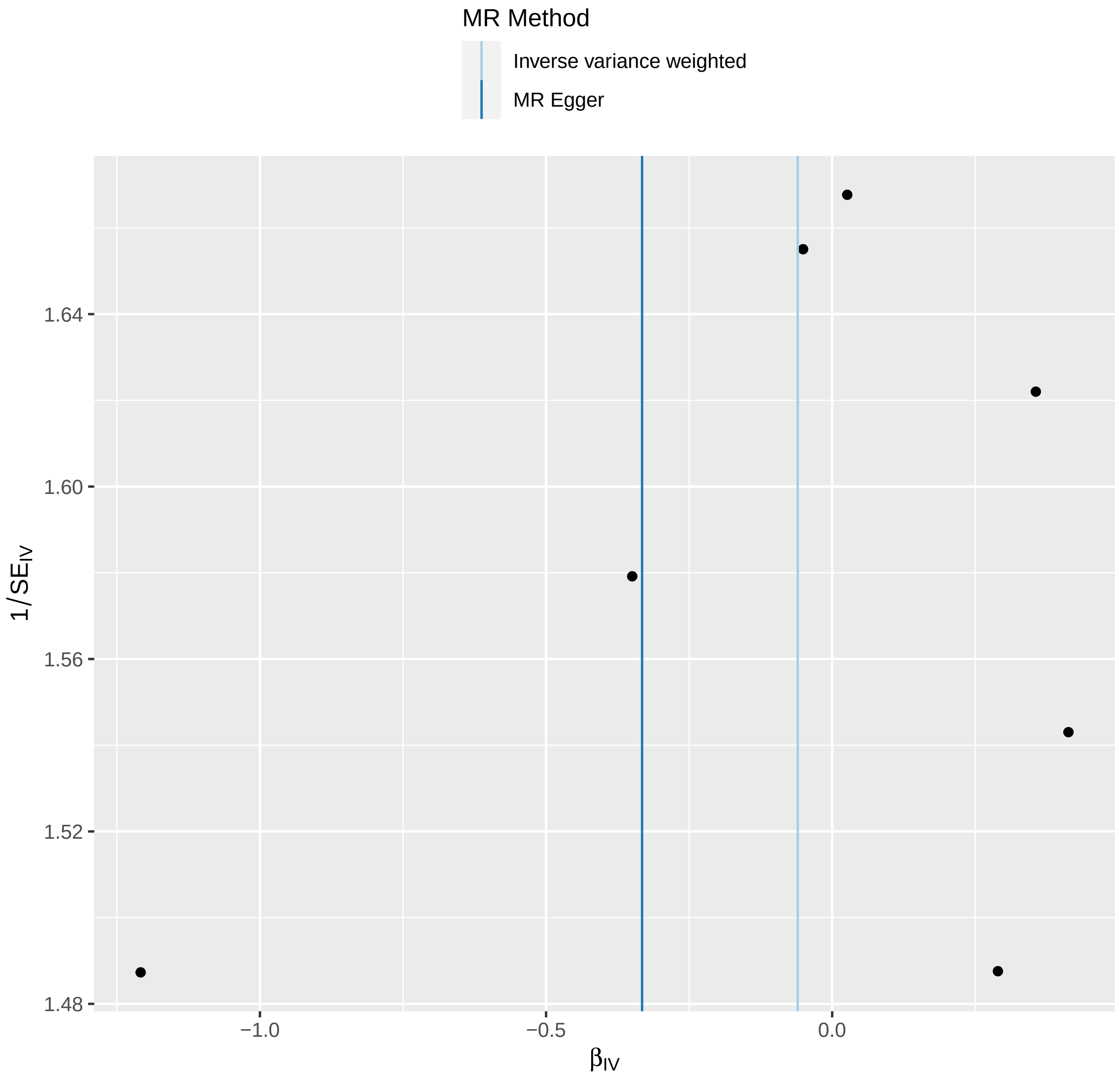

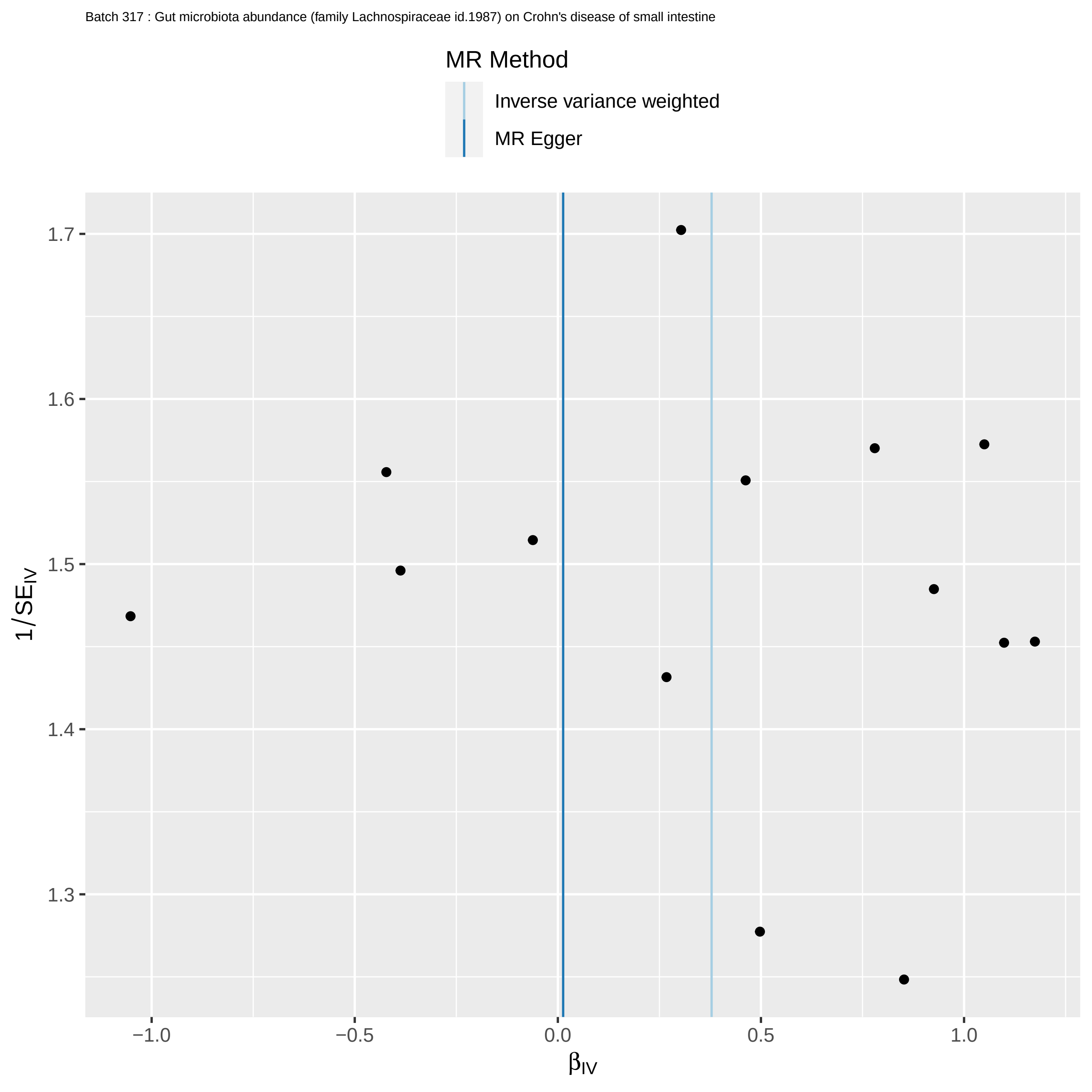
Batch 317 : Gut microbiota abundance (family Lachnospiraceae id.1987) on Crohn's disease of small intestine
MR Method
Inverse variance weighted
MR Egger
1.7
1.6
1.5
1.4
1.3
1/SE_IV
−1.0
−0.5
0.0
0.5
1.0
β_IV

Batch 318 : Gut microbiota abundance (family Lactobacillaceae id.1836) on Crohn's disease of small intestine

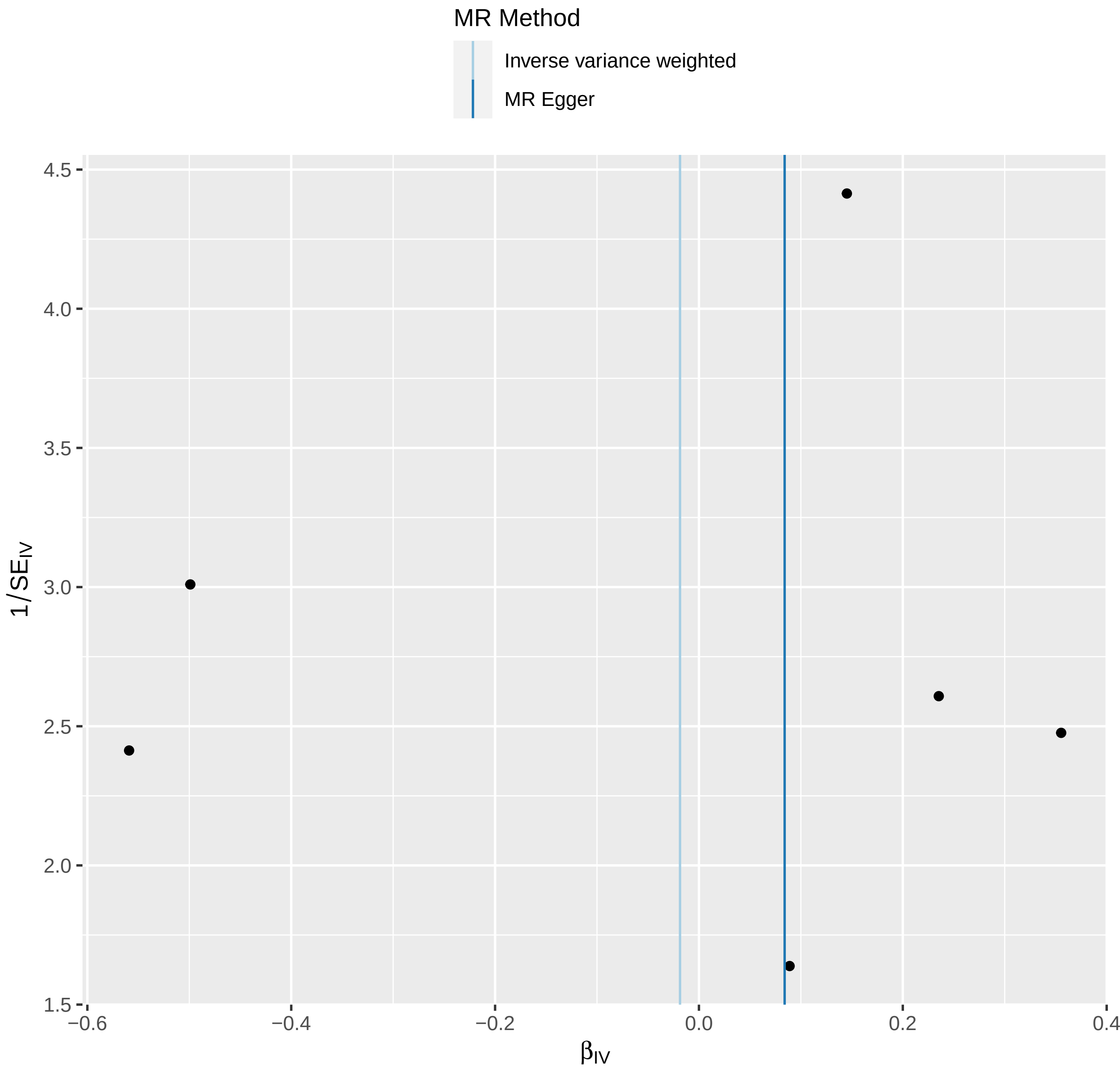

Batch 319 : Gut microbiota abundance (family Methanobacteriaceae id.121) on Crohn's disease of small intestine

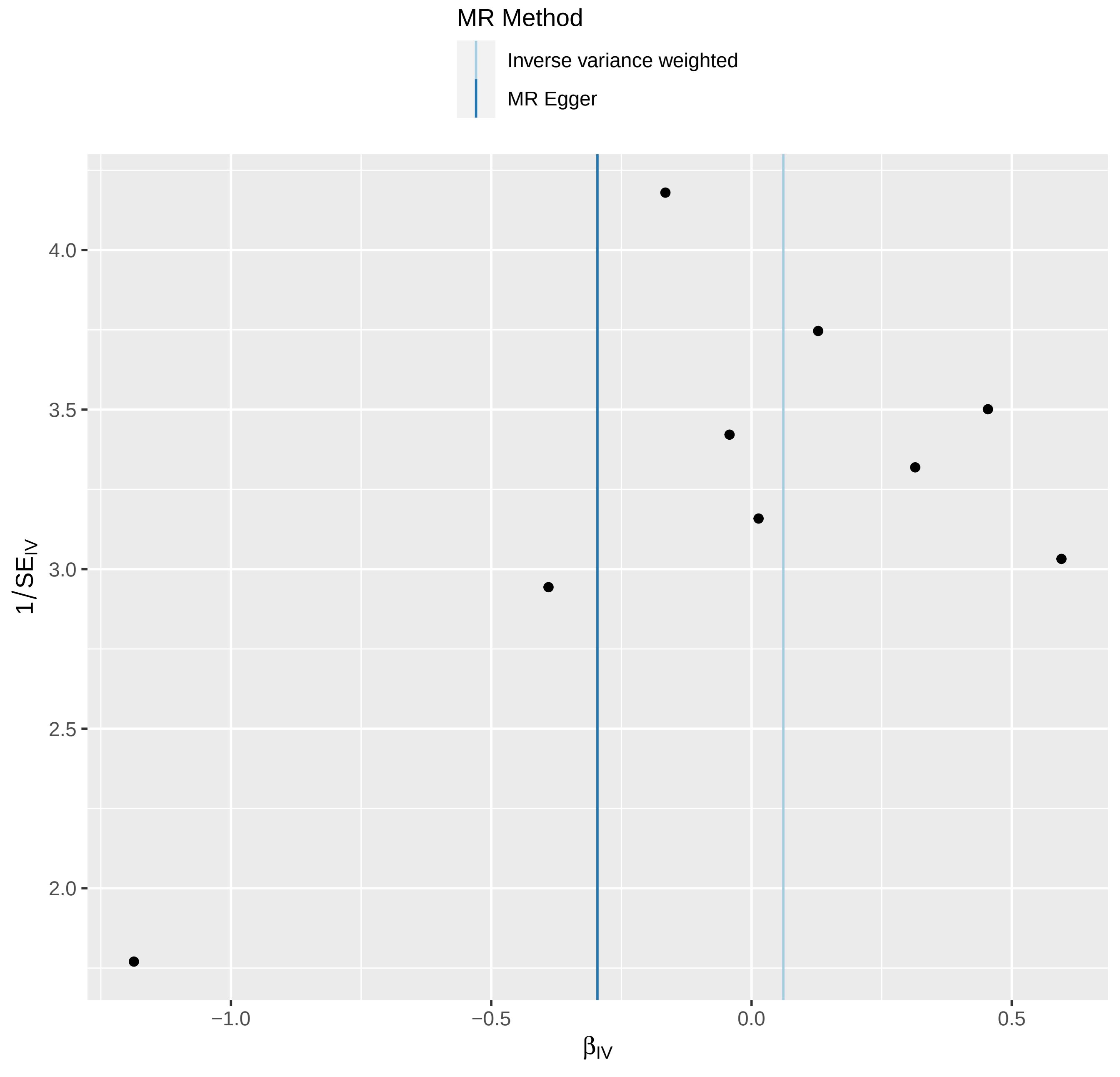

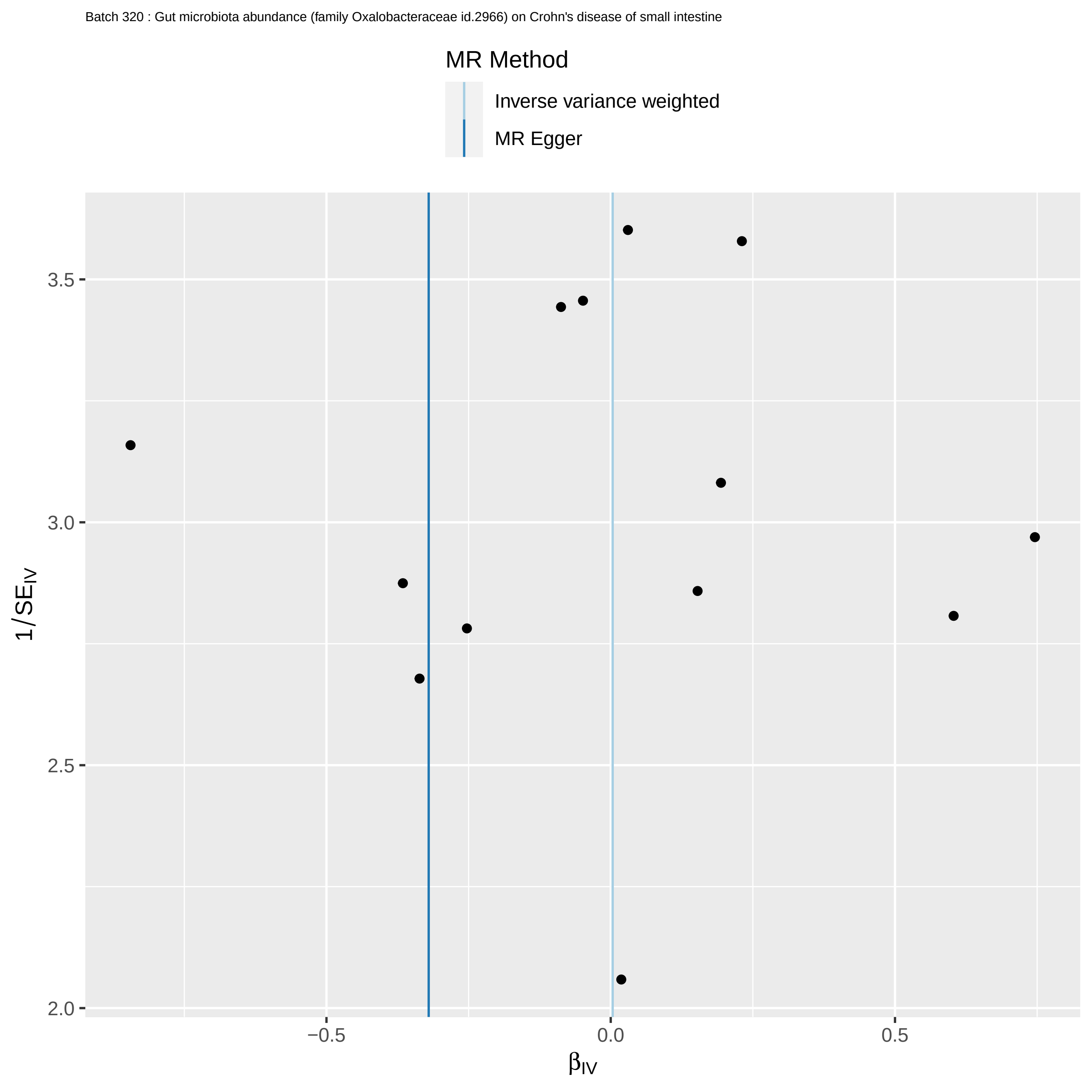
Batch 320 : Gut microbiota abundance (family Oxalobacteraceae id.2966) on Crohn's disease of small intestine
MR Method
Inverse variance weighted
MR Egger
3.5
3.0
2.5
2.0
−0.5
0.0
0.5
1/SE_IV
β_IV

Batch 321 : Gut microbiota abundance (family Pasteurellaceae id.3689) on Crohn's disease of small intestine

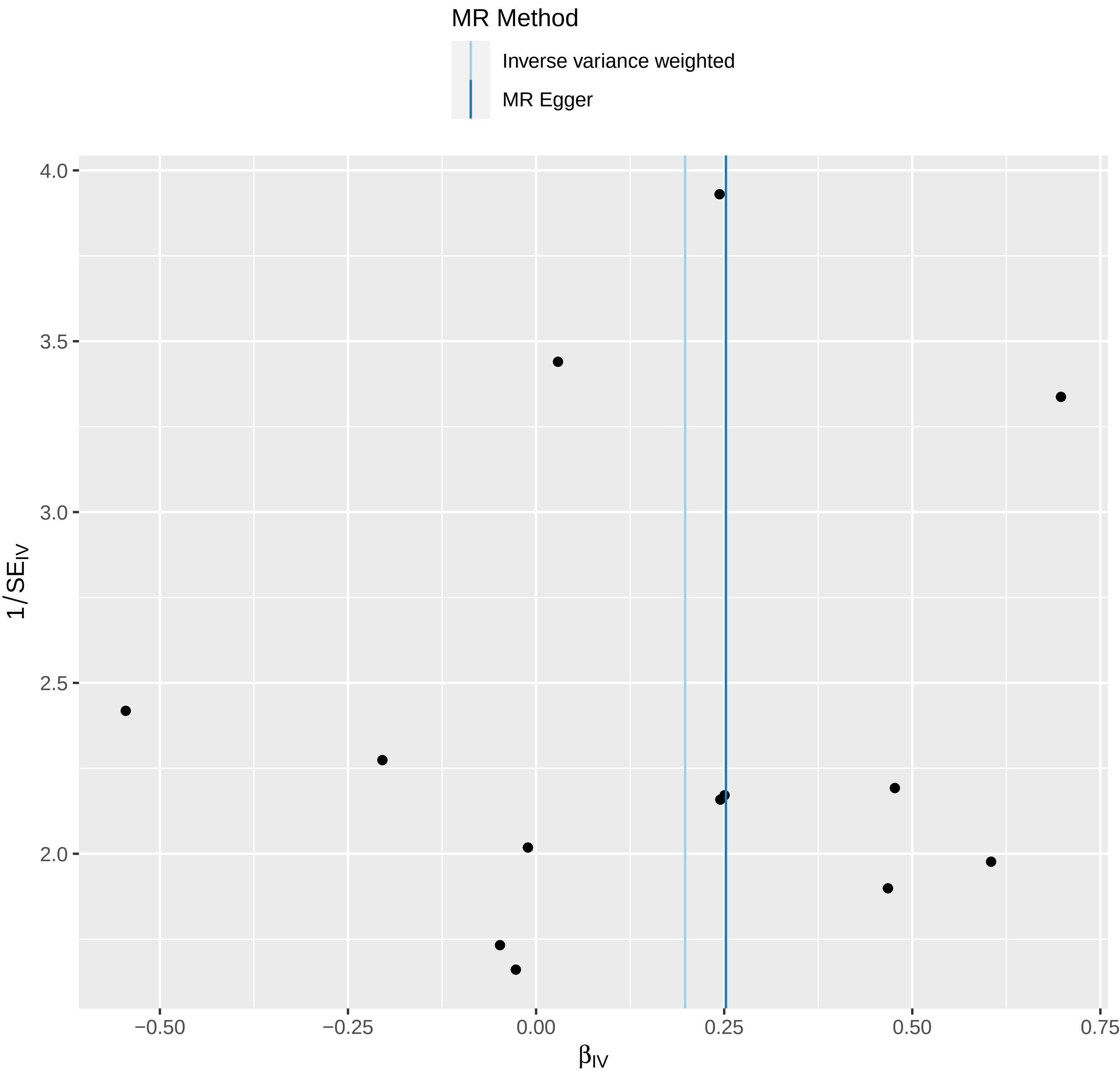

Batch 322 : Gut microbiota abundance (family Peptococcaceae id.2024) on Crohn's disease of small intestine

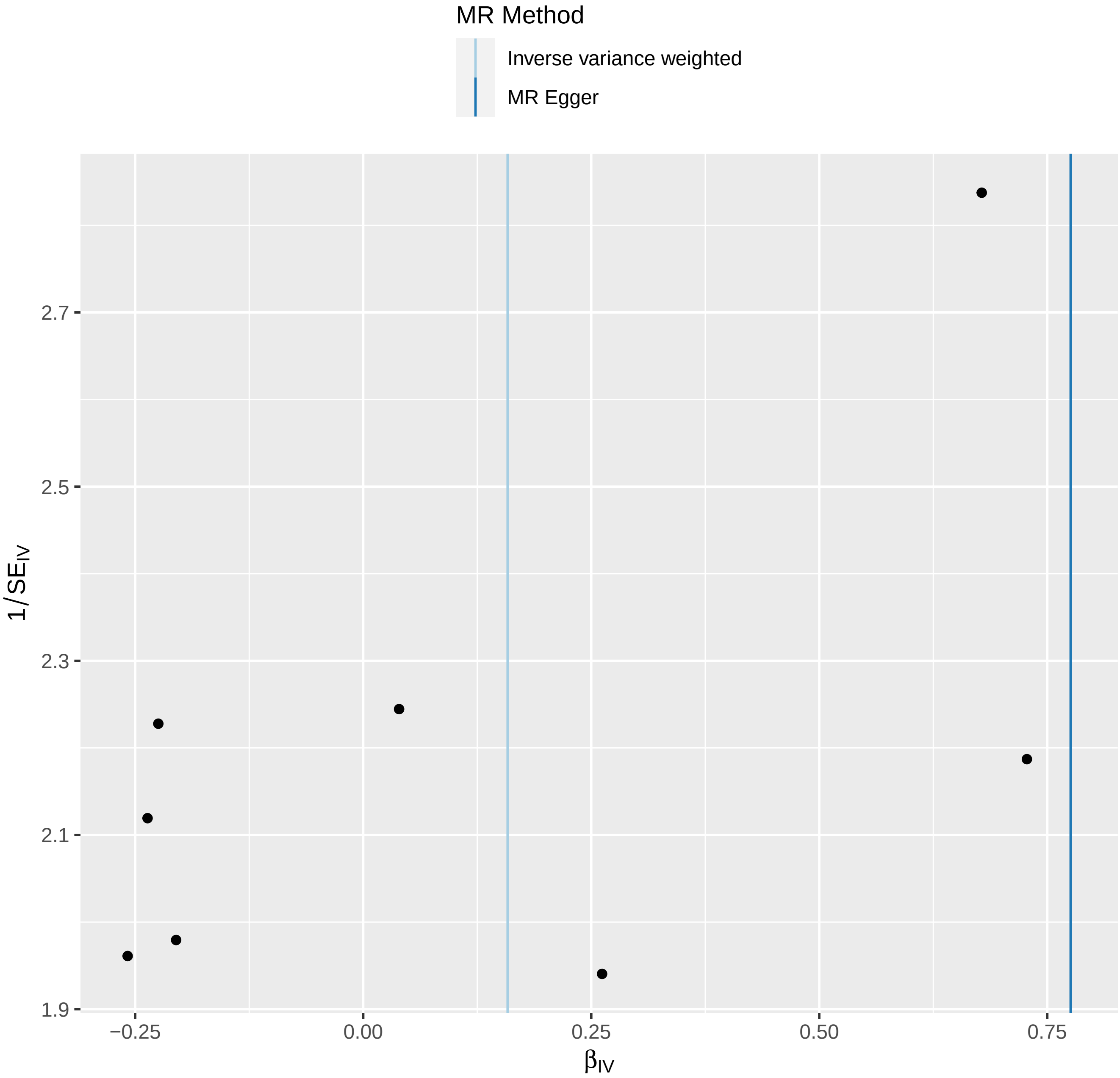

Batch 323 : Gut microbiota abundance (family Peptostreptococcaceae id.2042) on Crohn's disease of small intestine

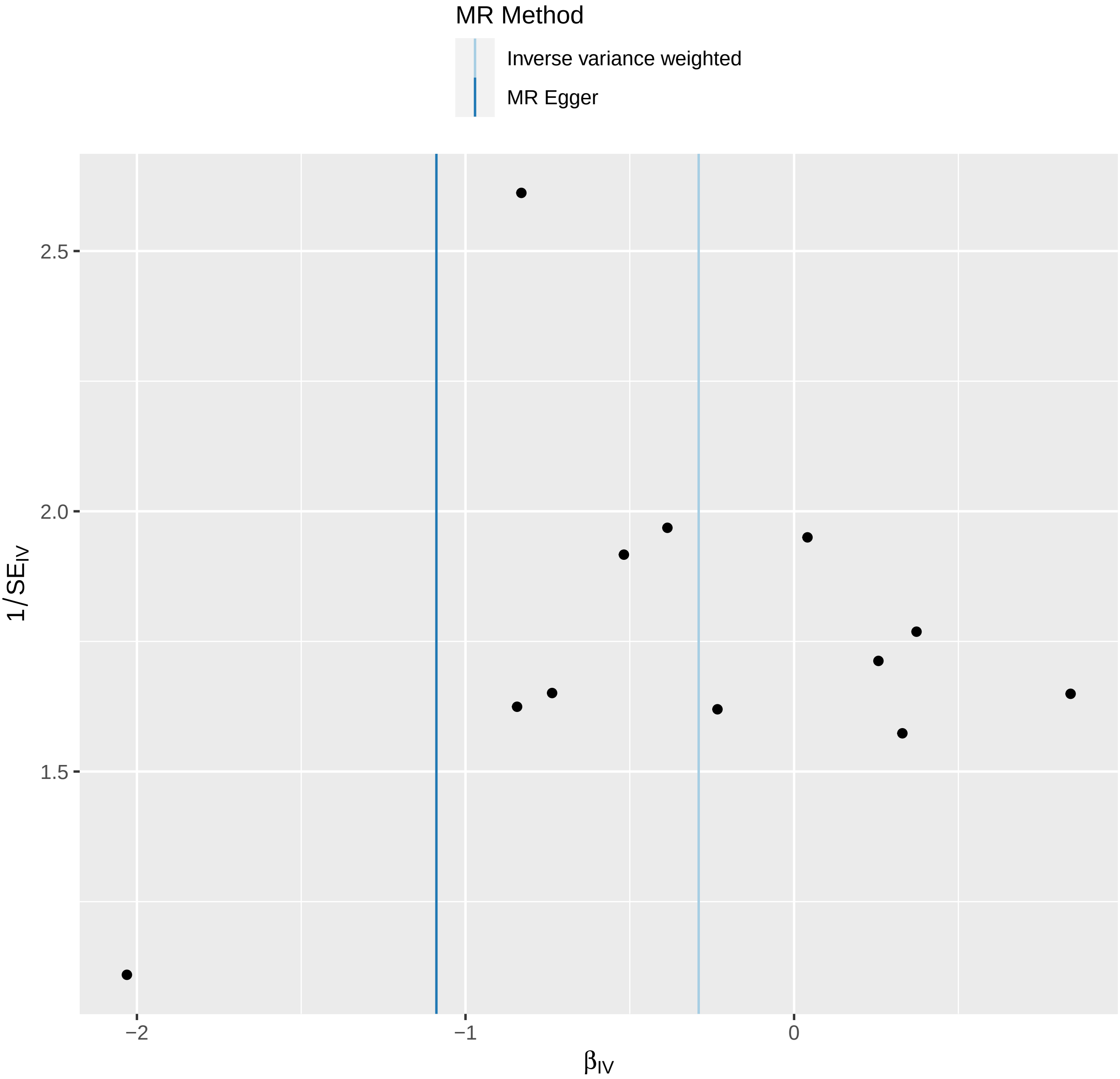

Batch 324 : Gut microbiota abundance (family Porphyromonadaceae id.943) on Crohn's disease of small intestine

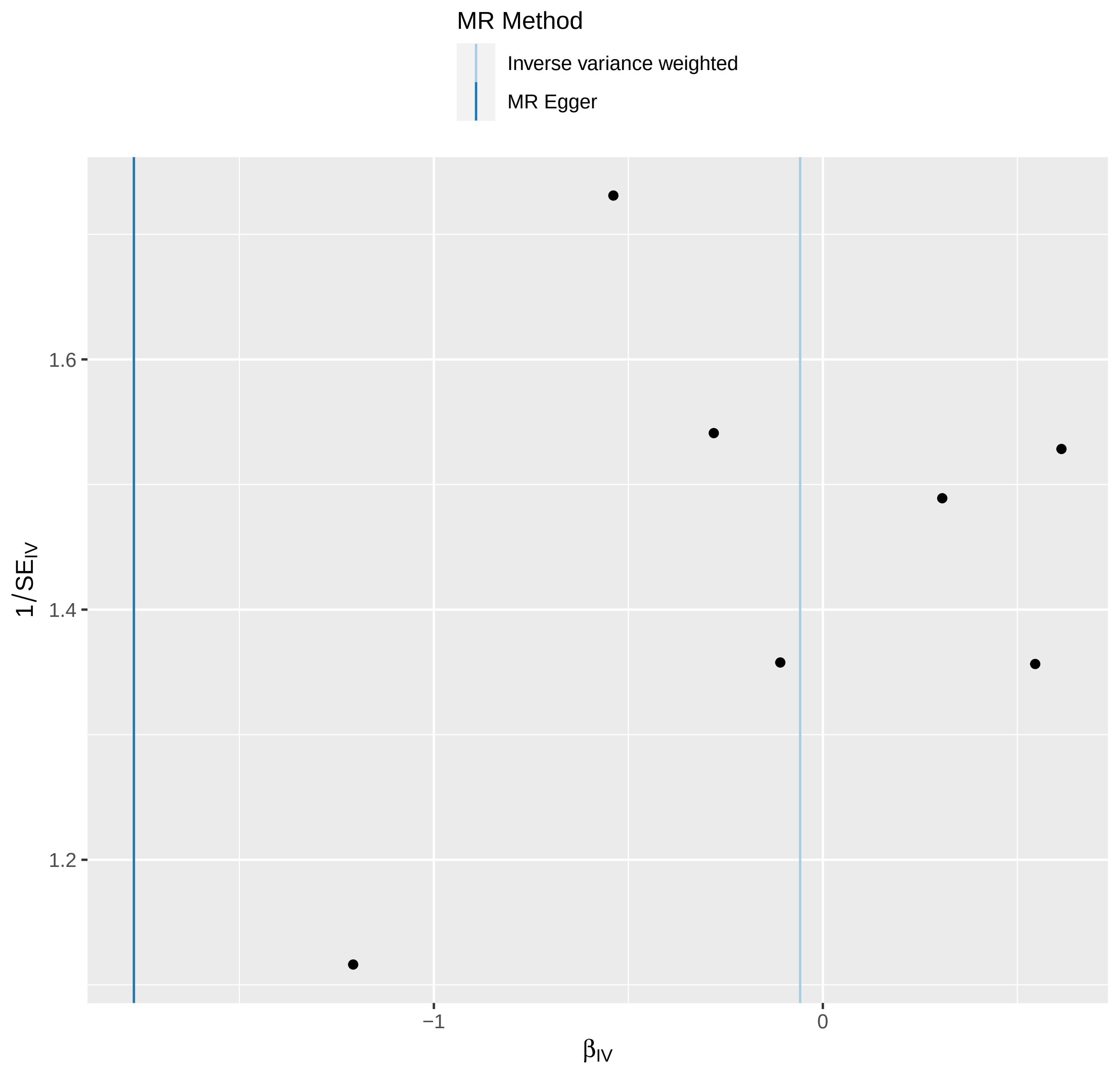

Batch 325 : Gut microbiota abundance (family Prevotellaceae id.960) on Crohn's disease of small intestine

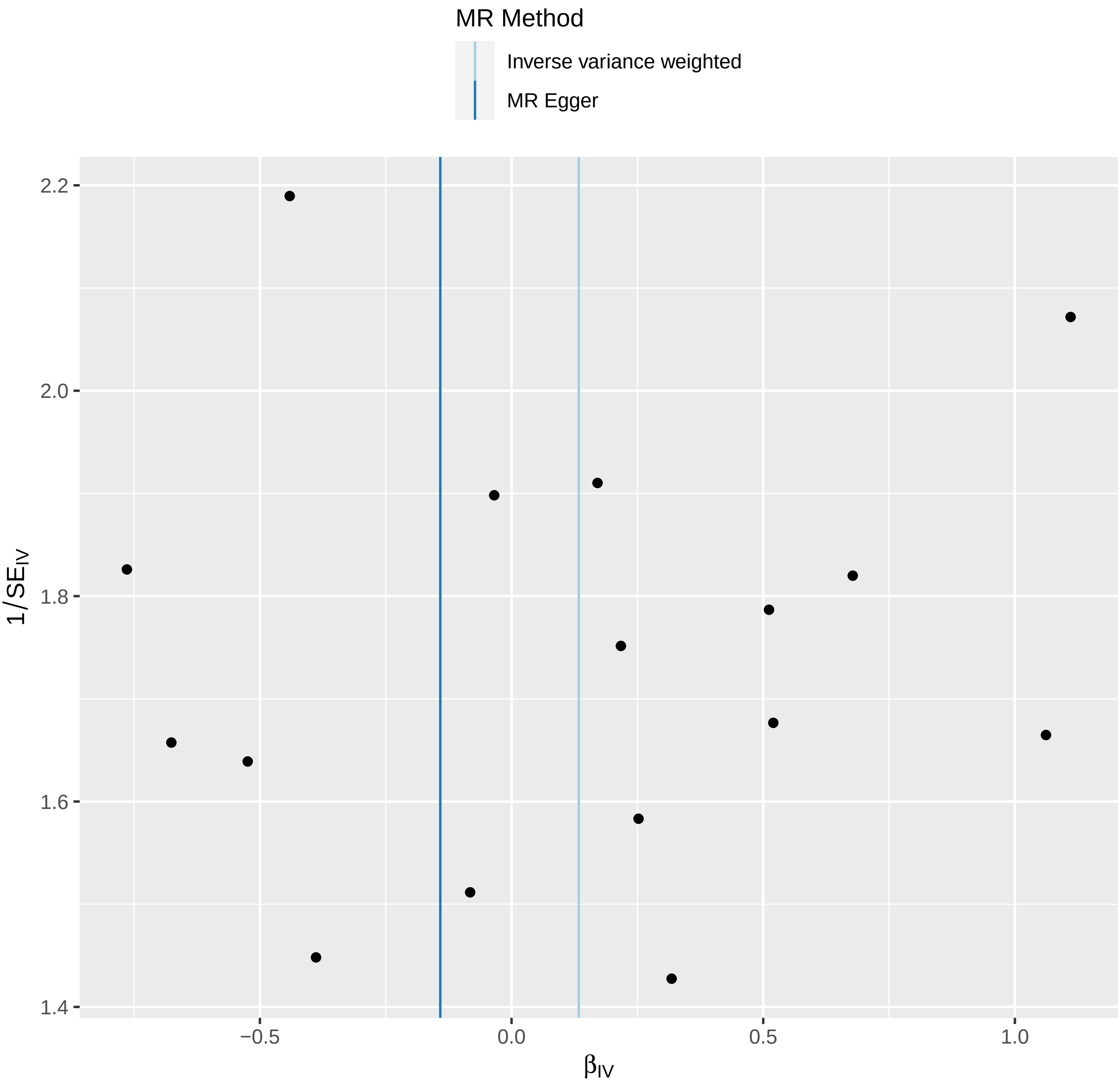

Batch 326 : Gut microbiota abundance (family Rhodospirillaceae id.2717) on Crohn's disease of small intestine

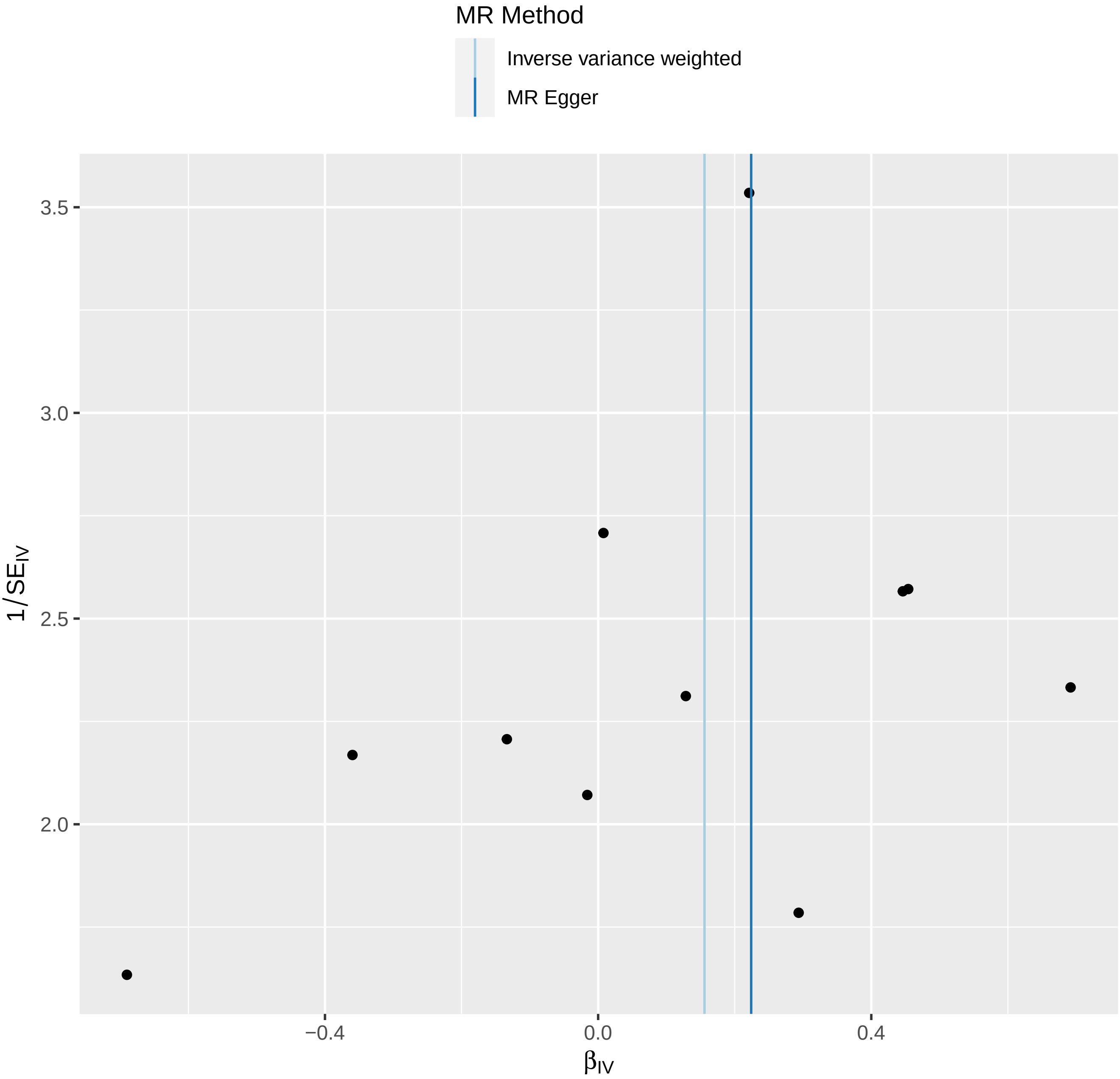

Batch 327 : Gut microbiota abundance (family Rikenellaceae id.967) on Crohn's disease of small intestine

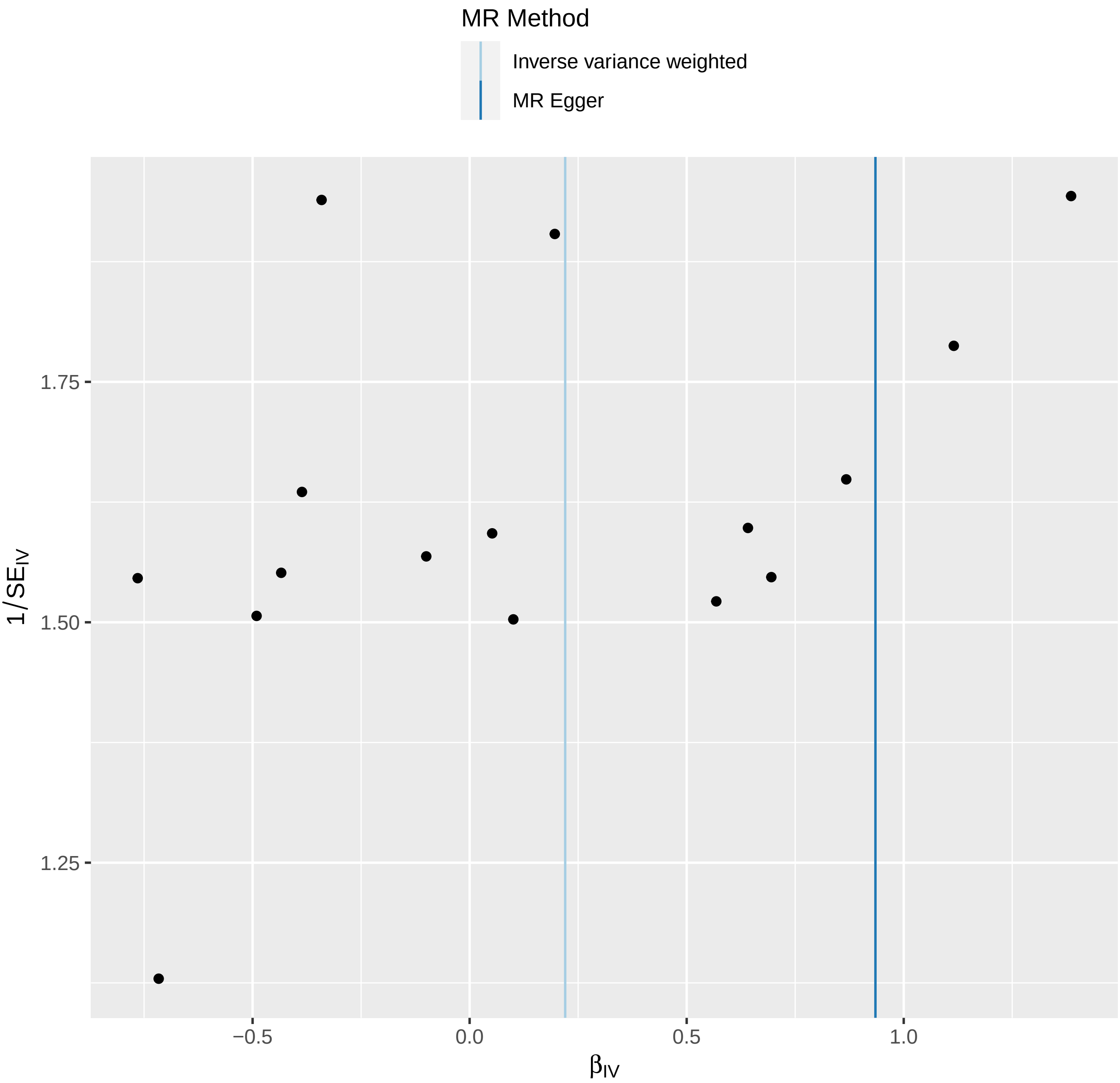

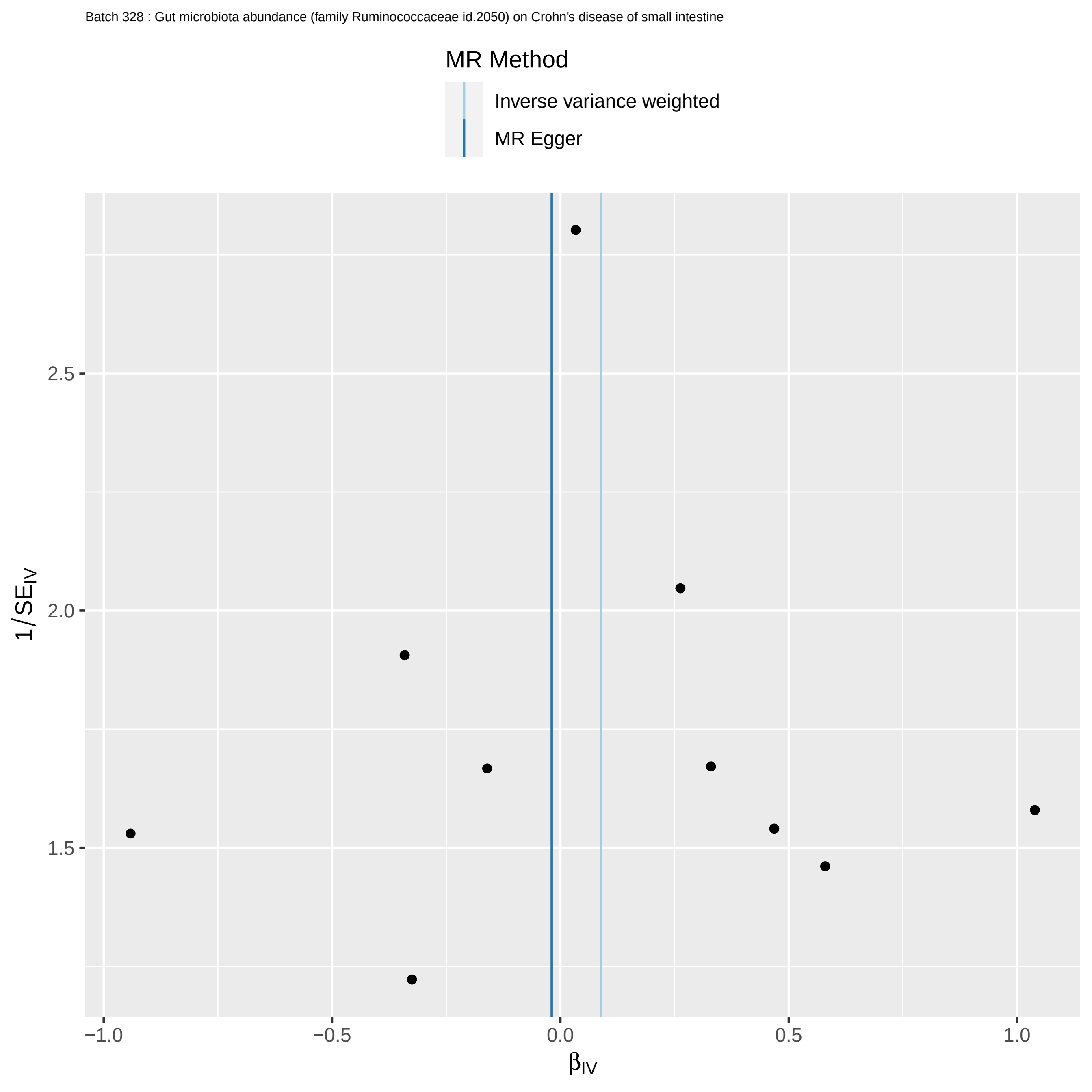
Batch 328 : Gut microbiota abundance (family Ruminococcaceae id.2050) on Crohn's disease of small intestine
MR Method
Inverse variance weighted
MR Egger
2.5
2.0
1.5
1/SE_IV
−1.0
−0.5
0.0
0.5
1.0
β_IV

Batch 329 : Gut microbiota abundance (family Streptococcaceae id.1850) on Crohn's disease of small intestine

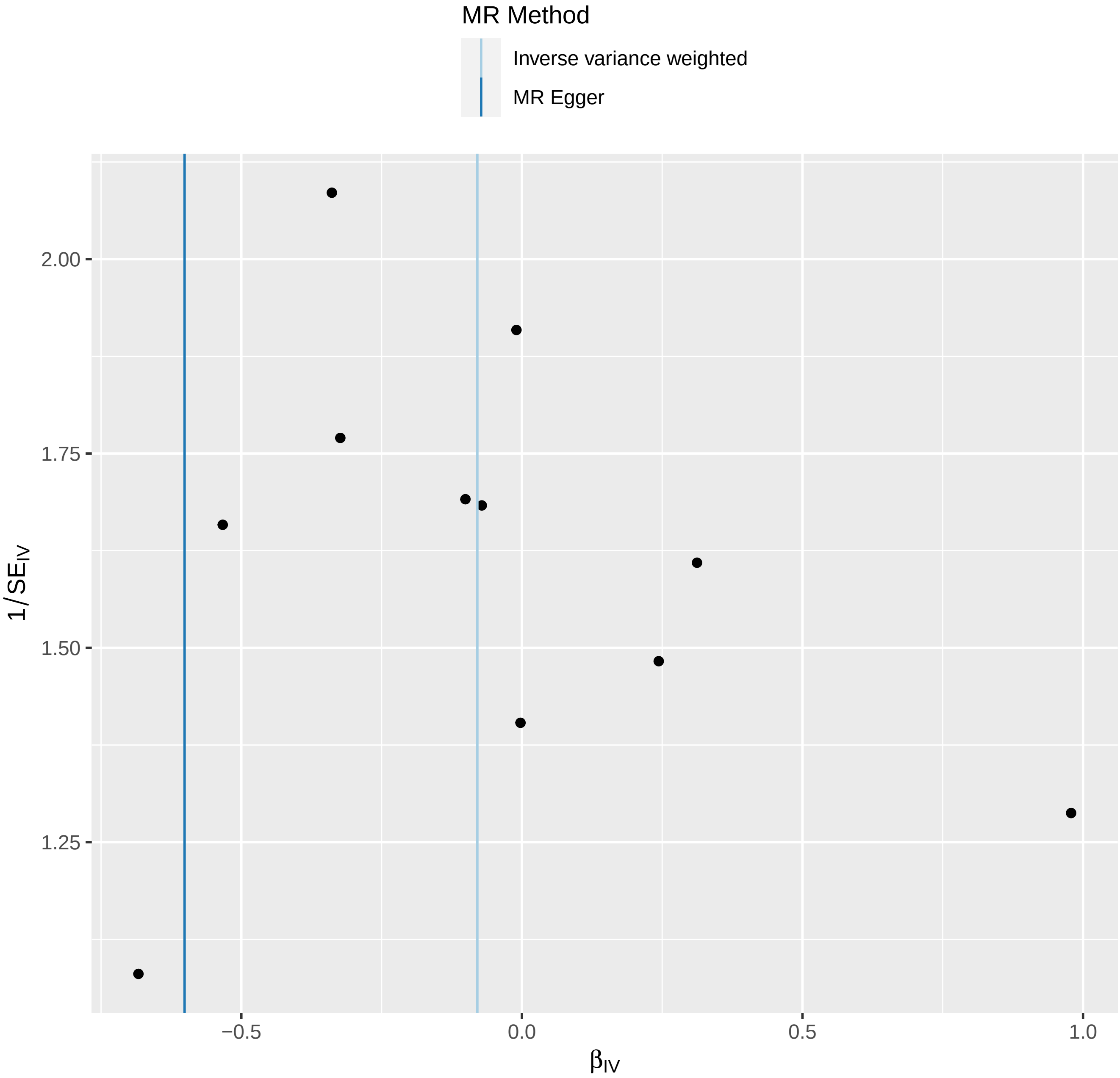

Batch 330 : Gut microbiota abundance (family Veillonellaceae id.2172) on Crohn's disease of small intestine

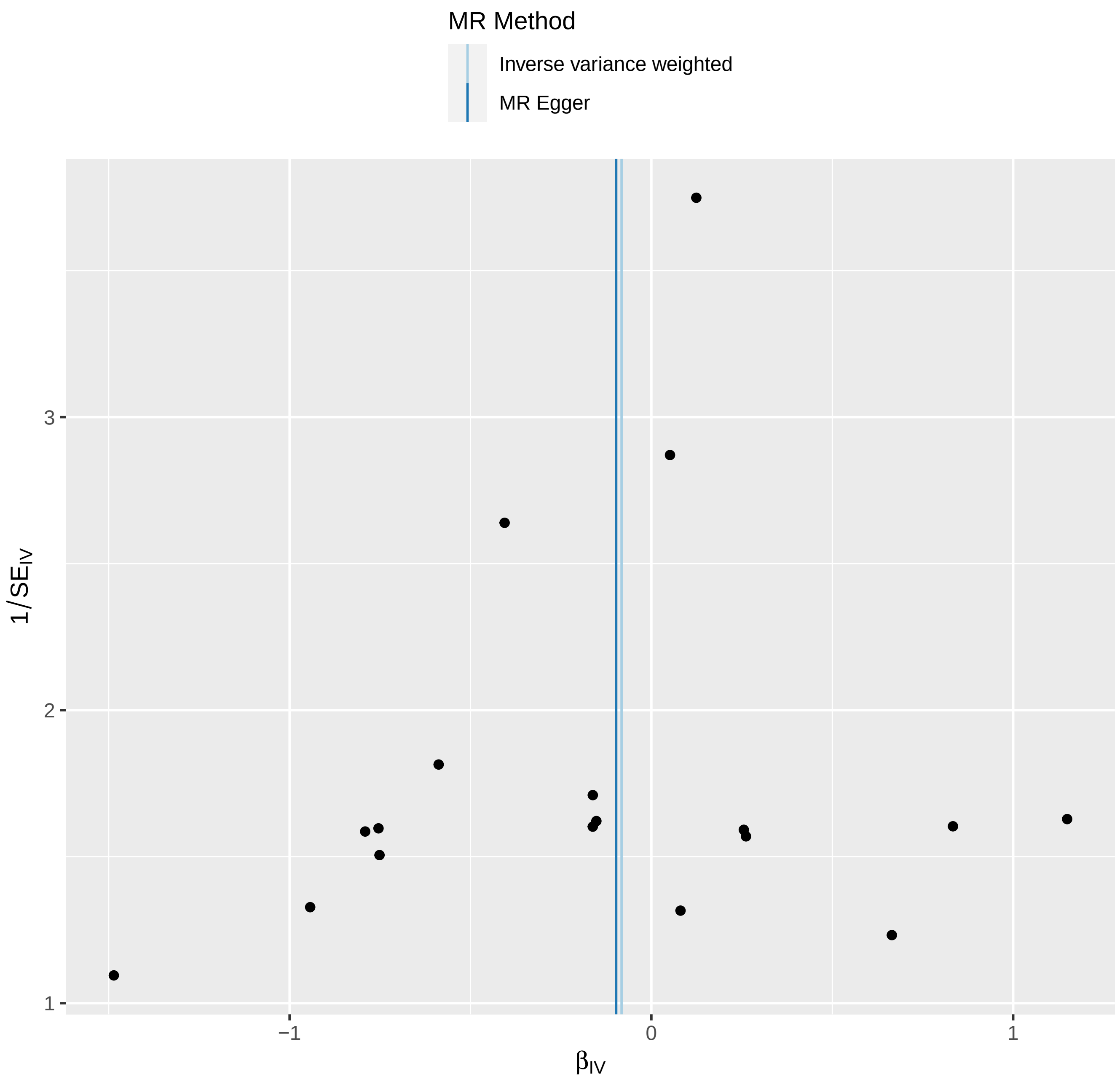

Batch 331 : Gut microbiota abundance (family Verrucomicrobiaceae id.4036) on Crohn's disease of small intestine

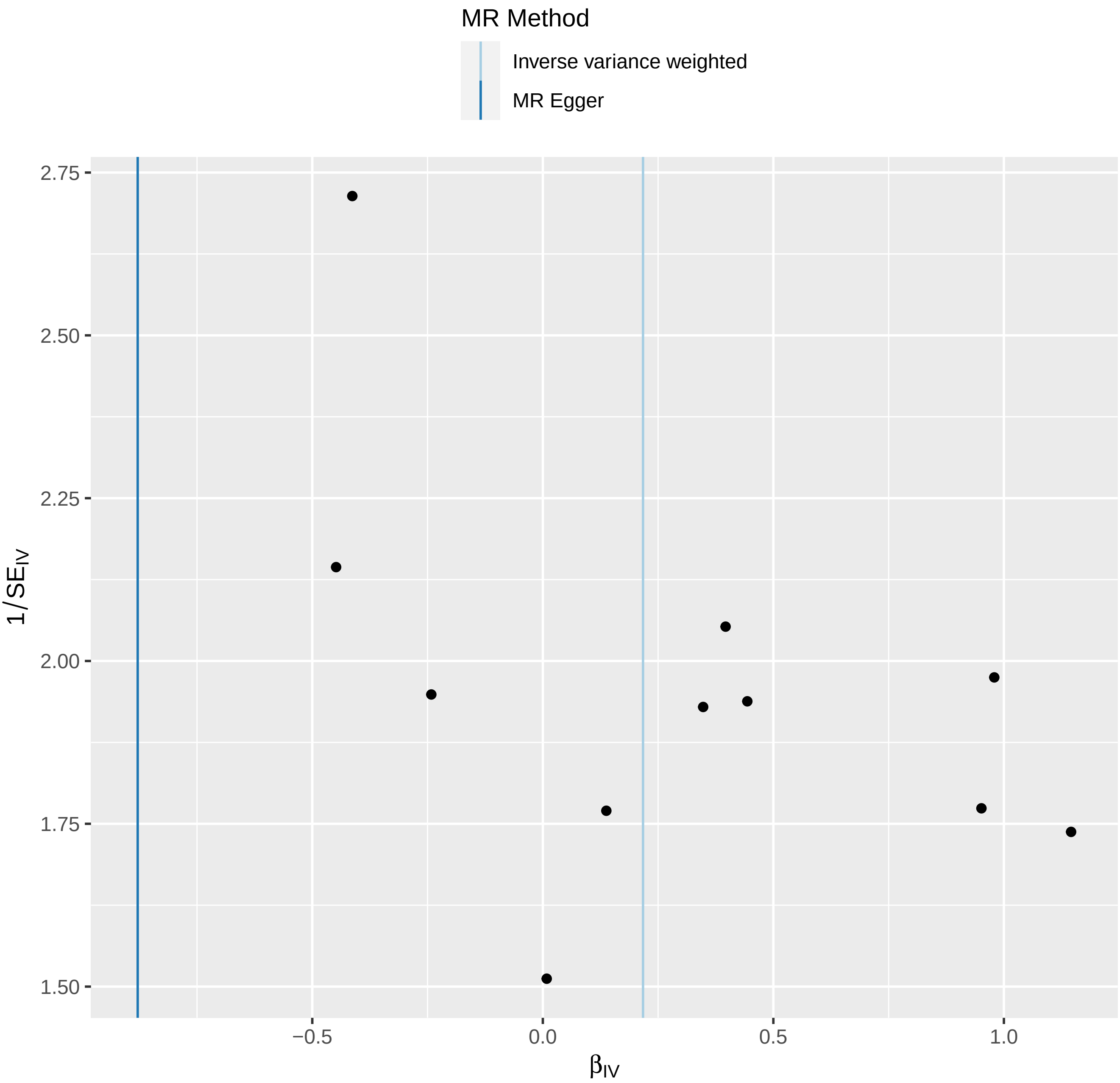

Batch 332 : Gut microbiota abundance (family Victivallaceae id.2255) on Crohn's disease of small intestine

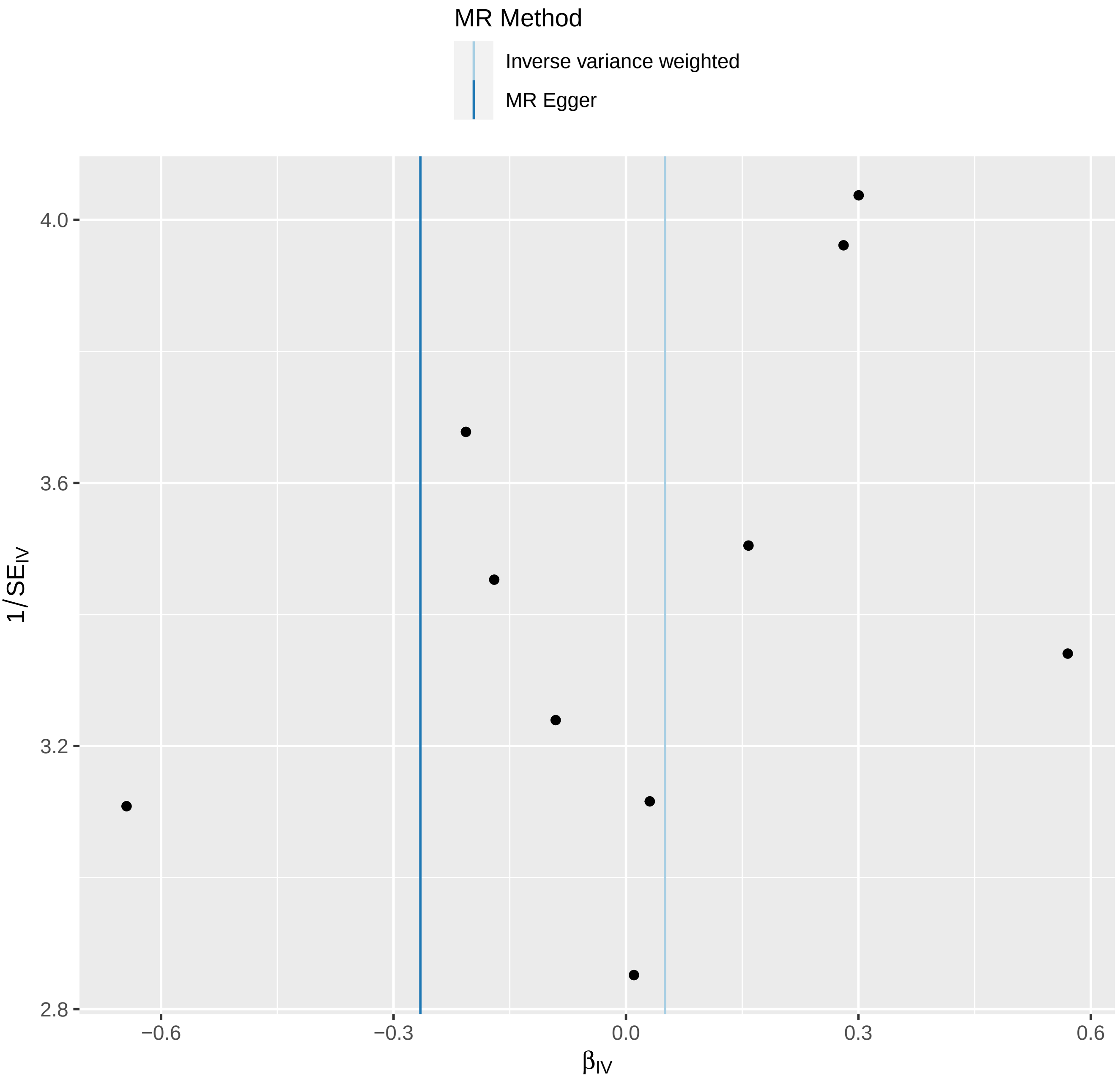

Batch 333 : Gut microbiota abundance (genus Actinomyces id.423) on Crohn's disease of small intestine

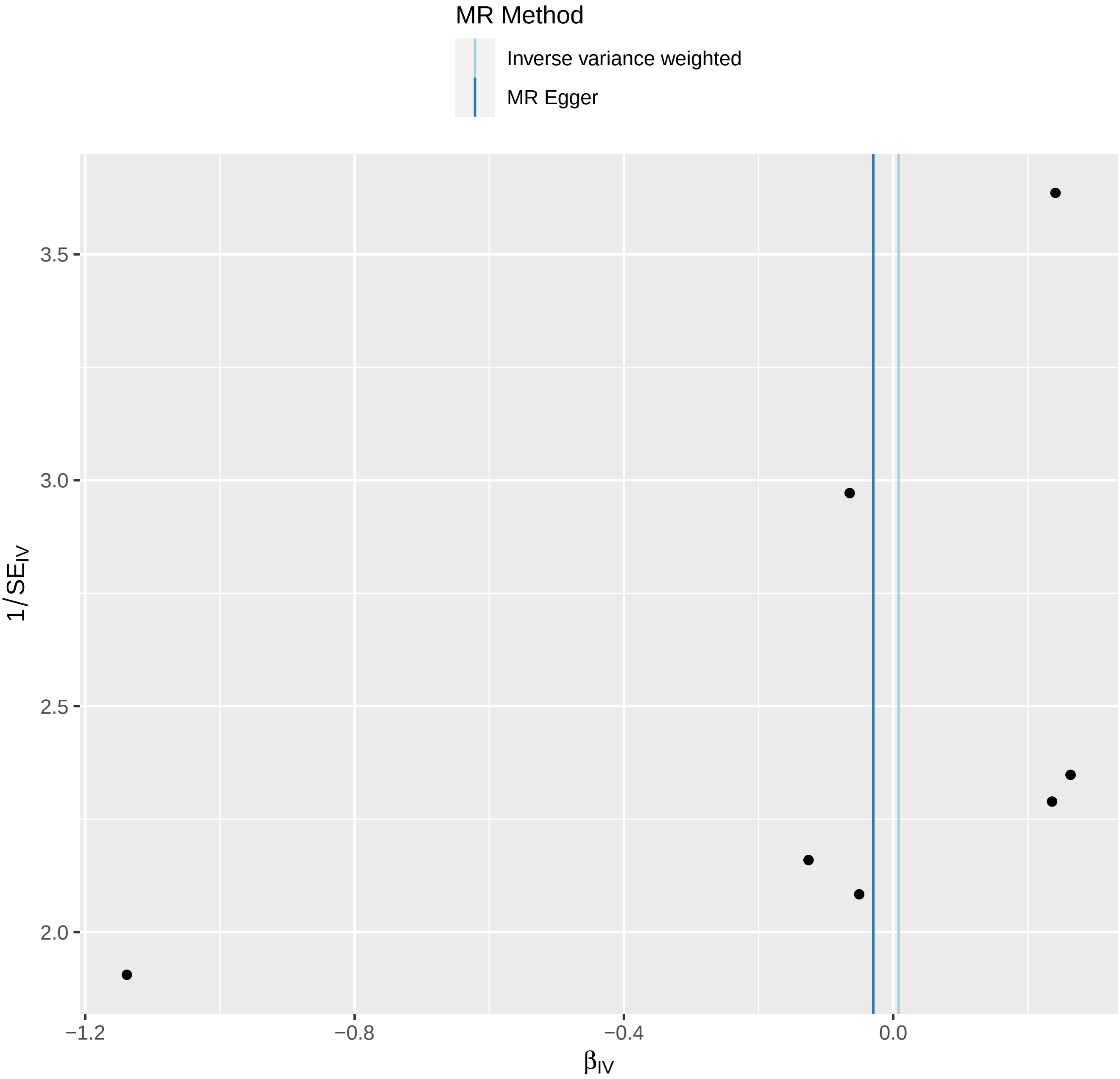

Batch 334 : Gut microbiota abundance (genus Adlercreutzia id.812) on Crohn's disease of small intestine

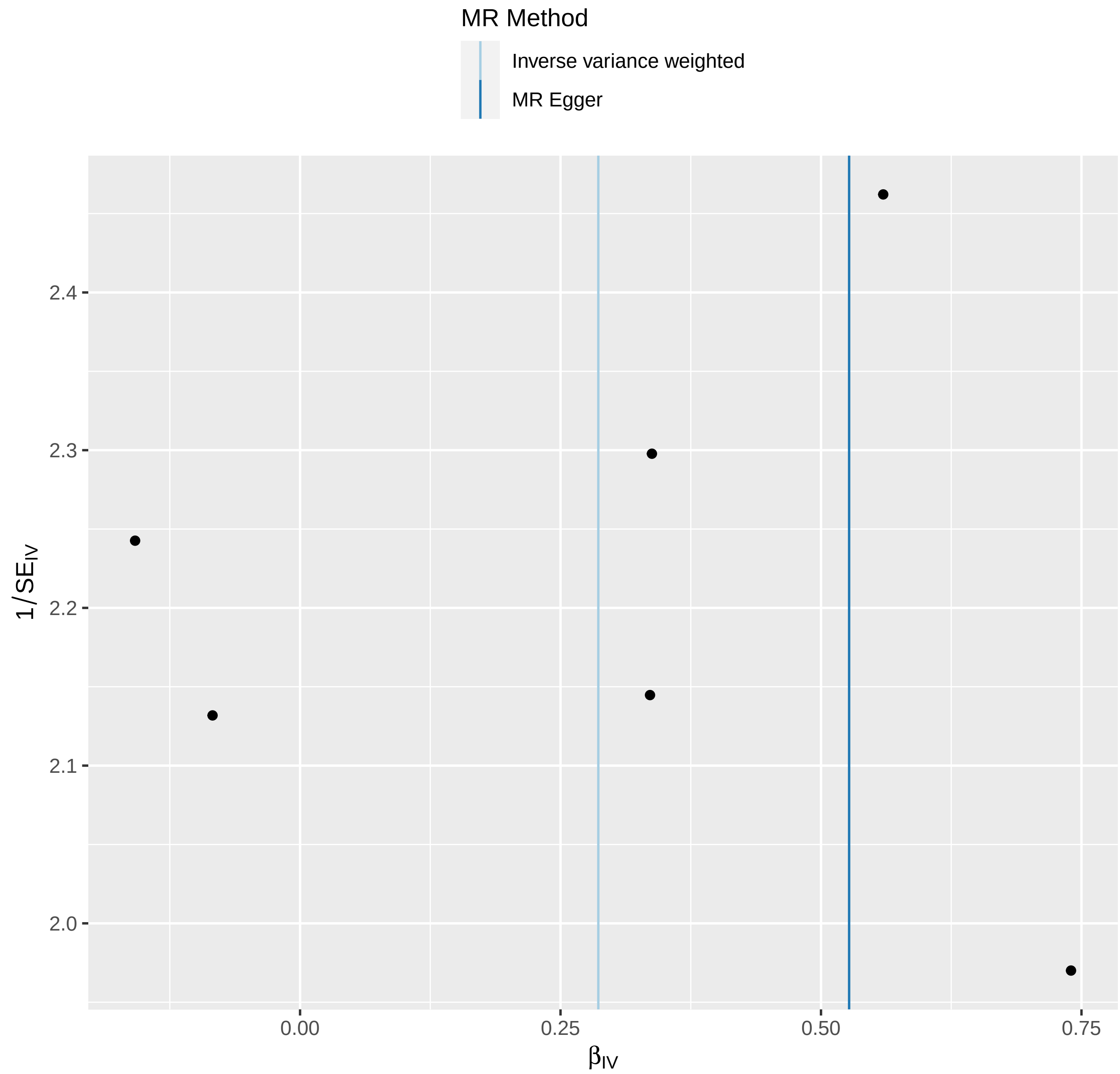

Batch 335 : Gut microbiota abundance (genus Akkermansia id.4037) on Crohn's disease of small intestine

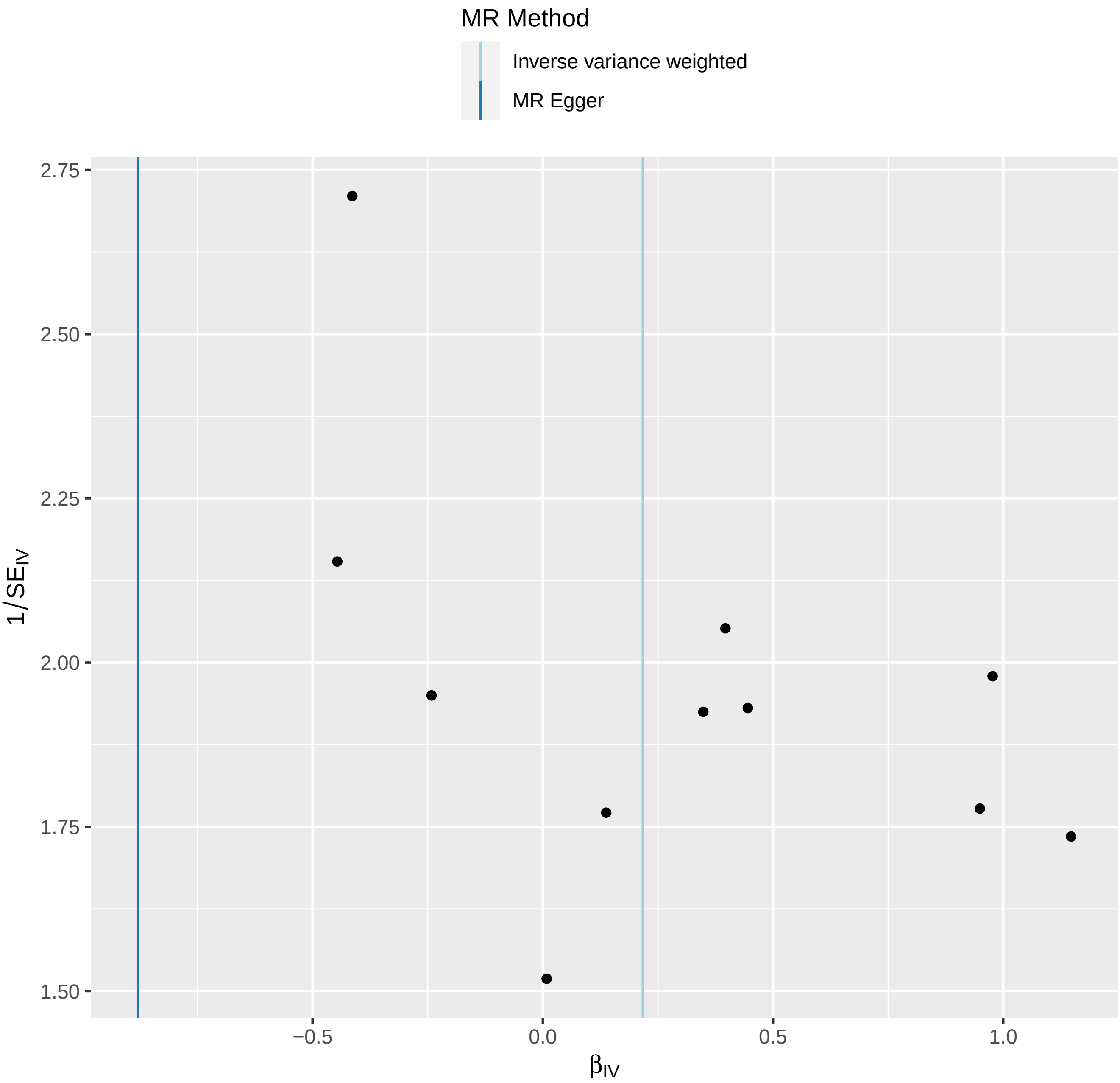

Batch 336 : Gut microbiota abundance (genus Alistipes id.968) on Crohn's disease of small intestine

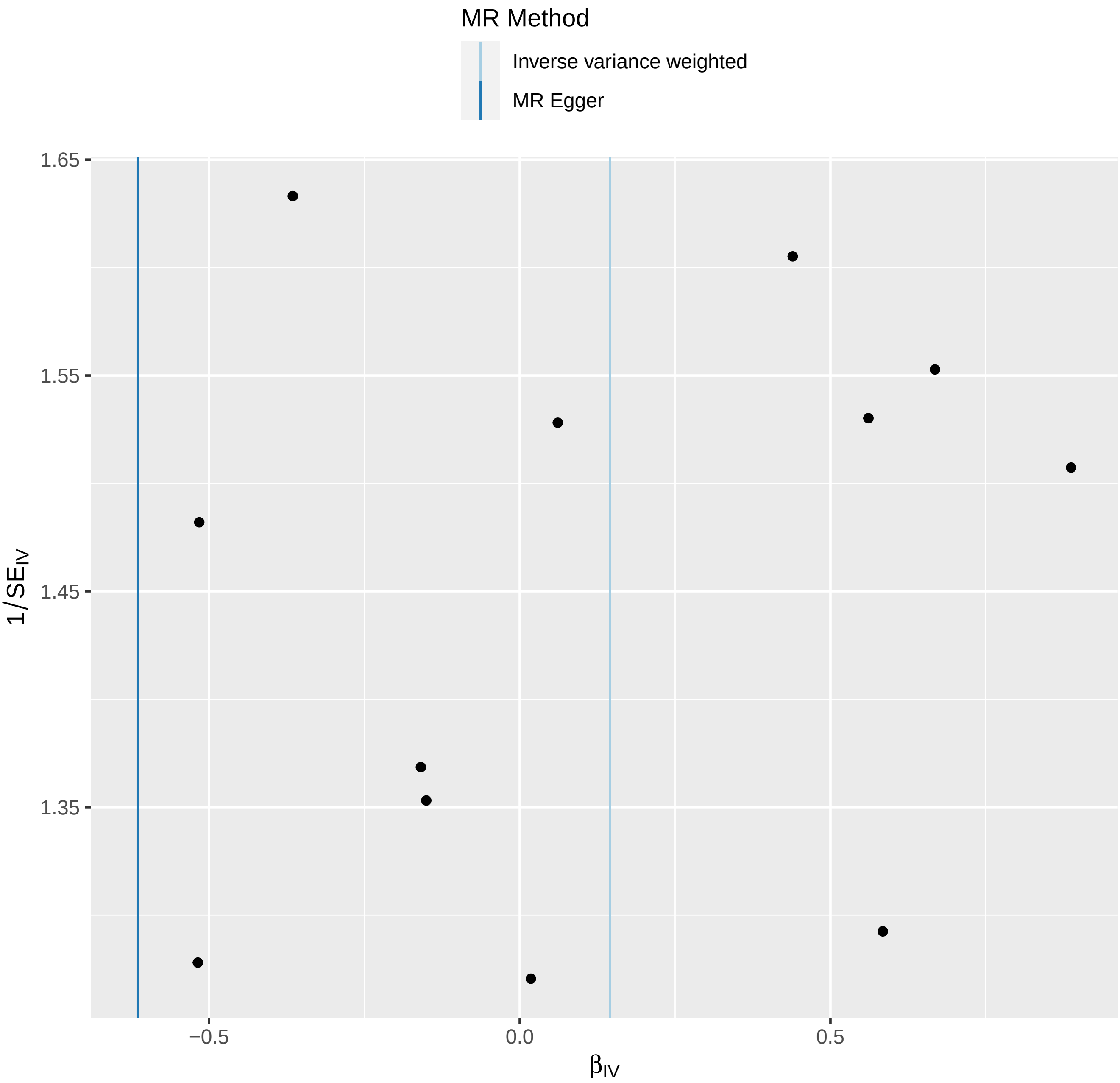

Batch 337 : Gut microbiota abundance (genus Allisonella id.2174) on Crohn's disease of small intestine

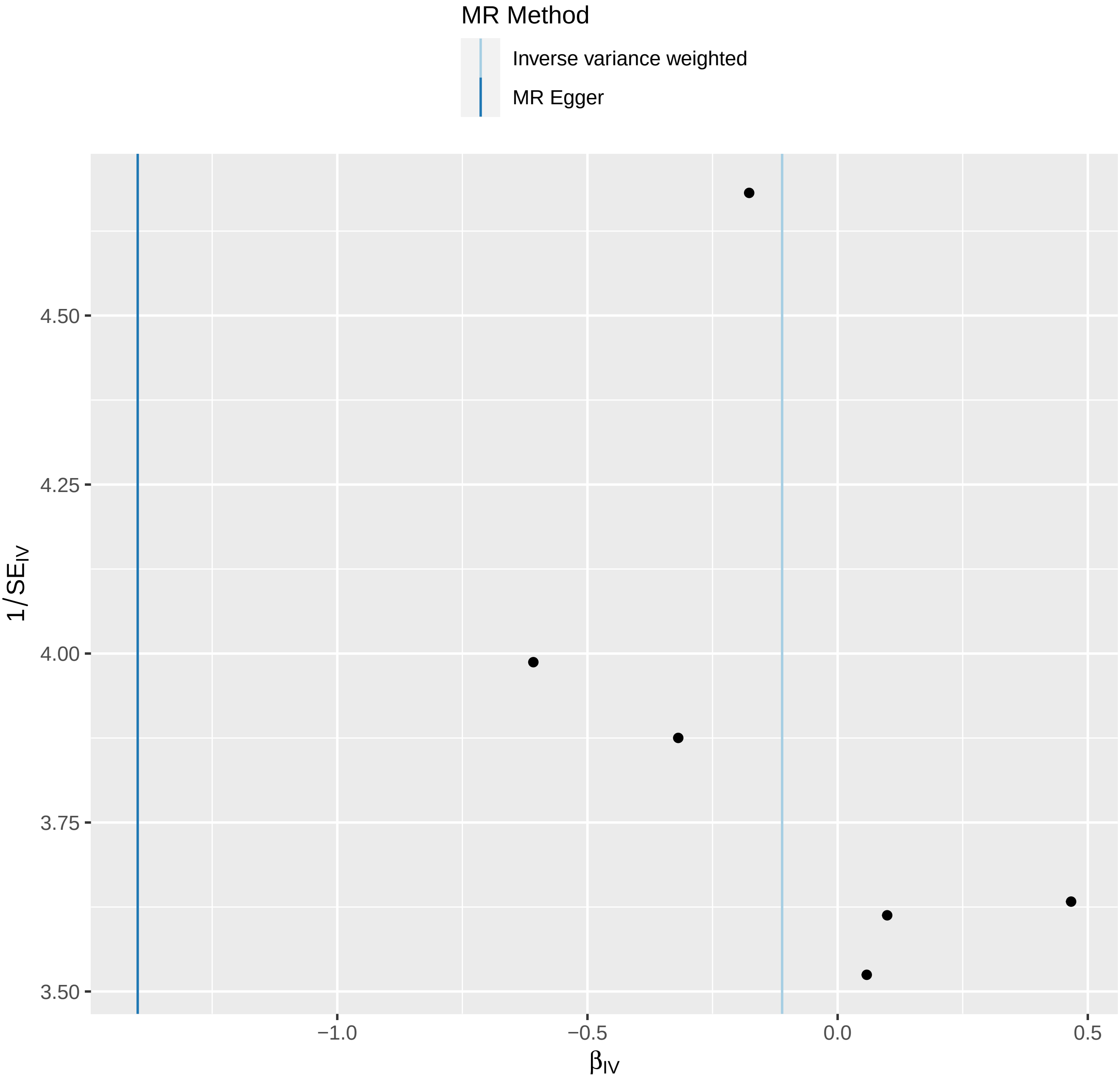

Batch 338 : Gut microbiota abundance (genus Alloprevotella id.961) on Crohn's disease of small intestine

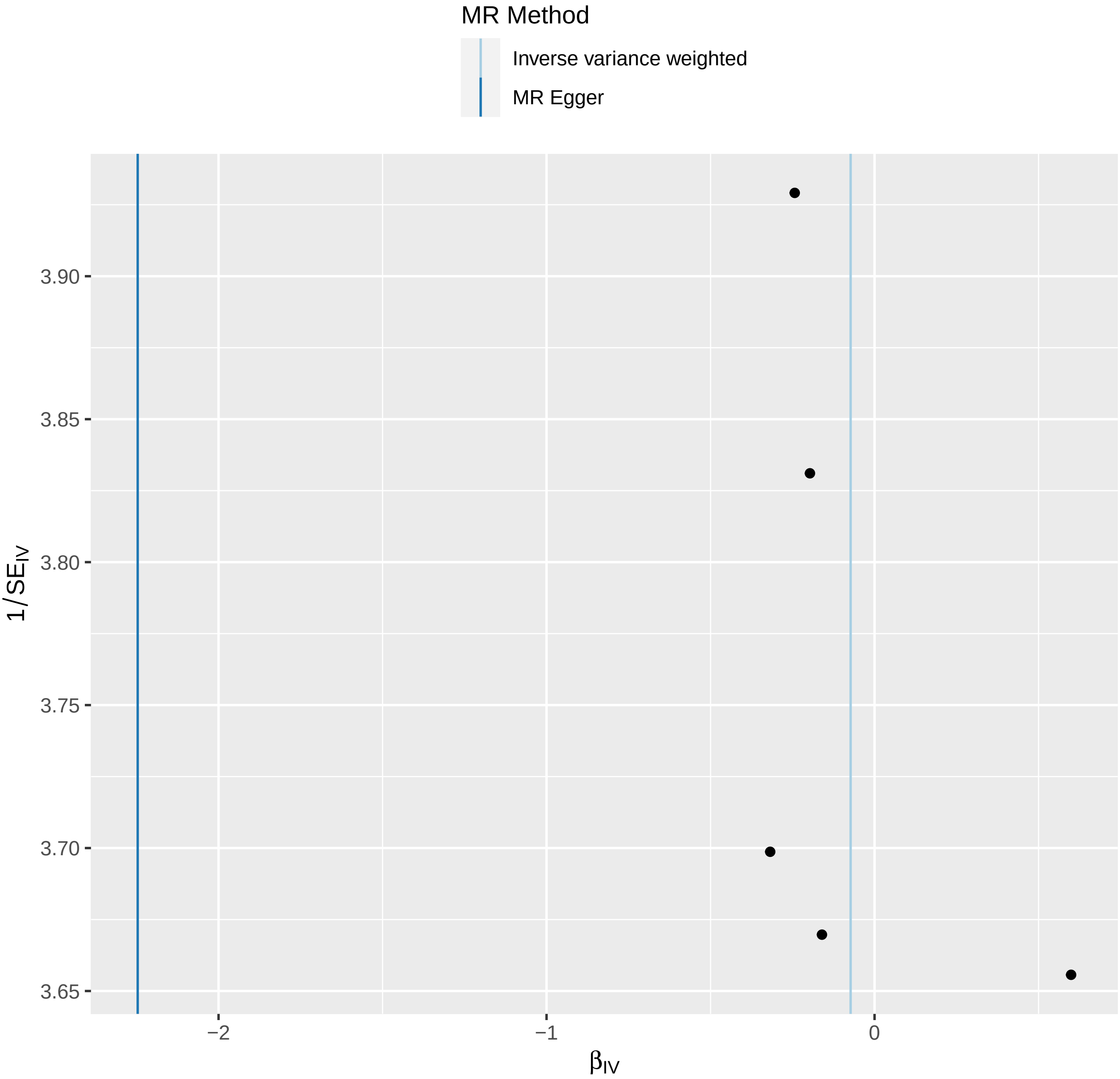

Batch 339 : Gut microbiota abundance (genus Anaerofilum id.2053) on Crohn's disease of small intestine

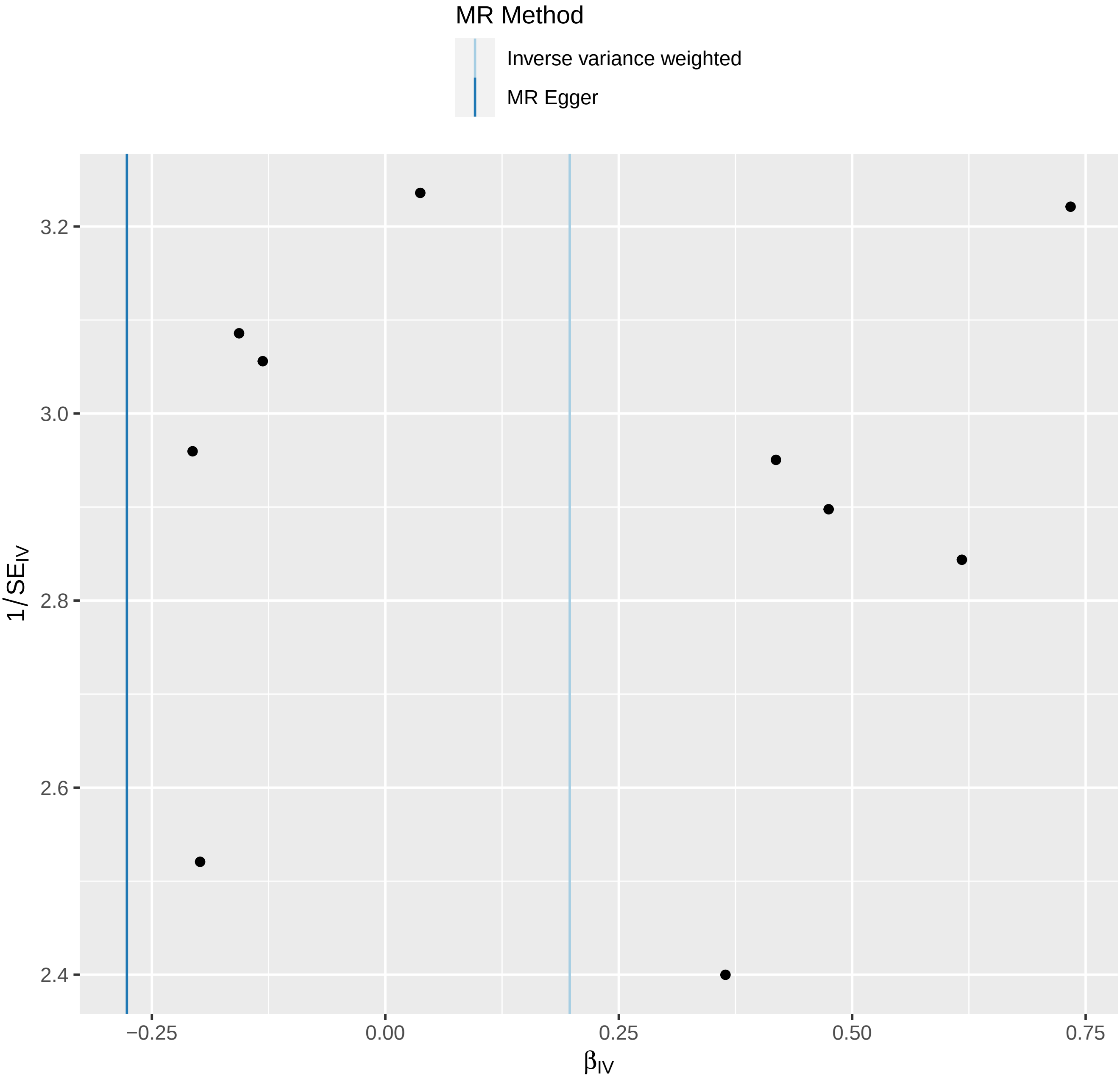

Batch 340 : Gut microbiota abundance (genus Anaerostipes id.1991) on Crohn's disease of small intestine

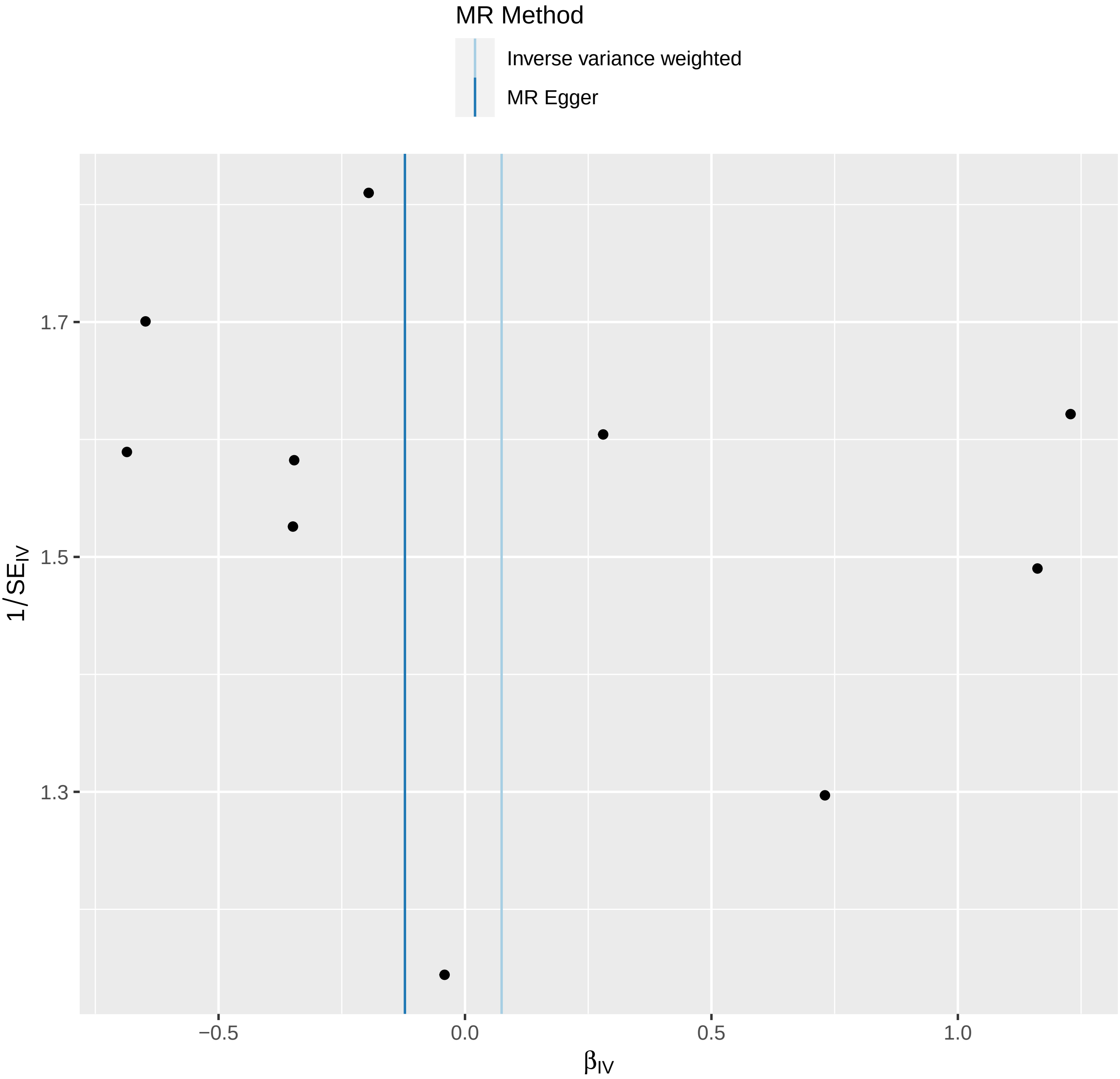

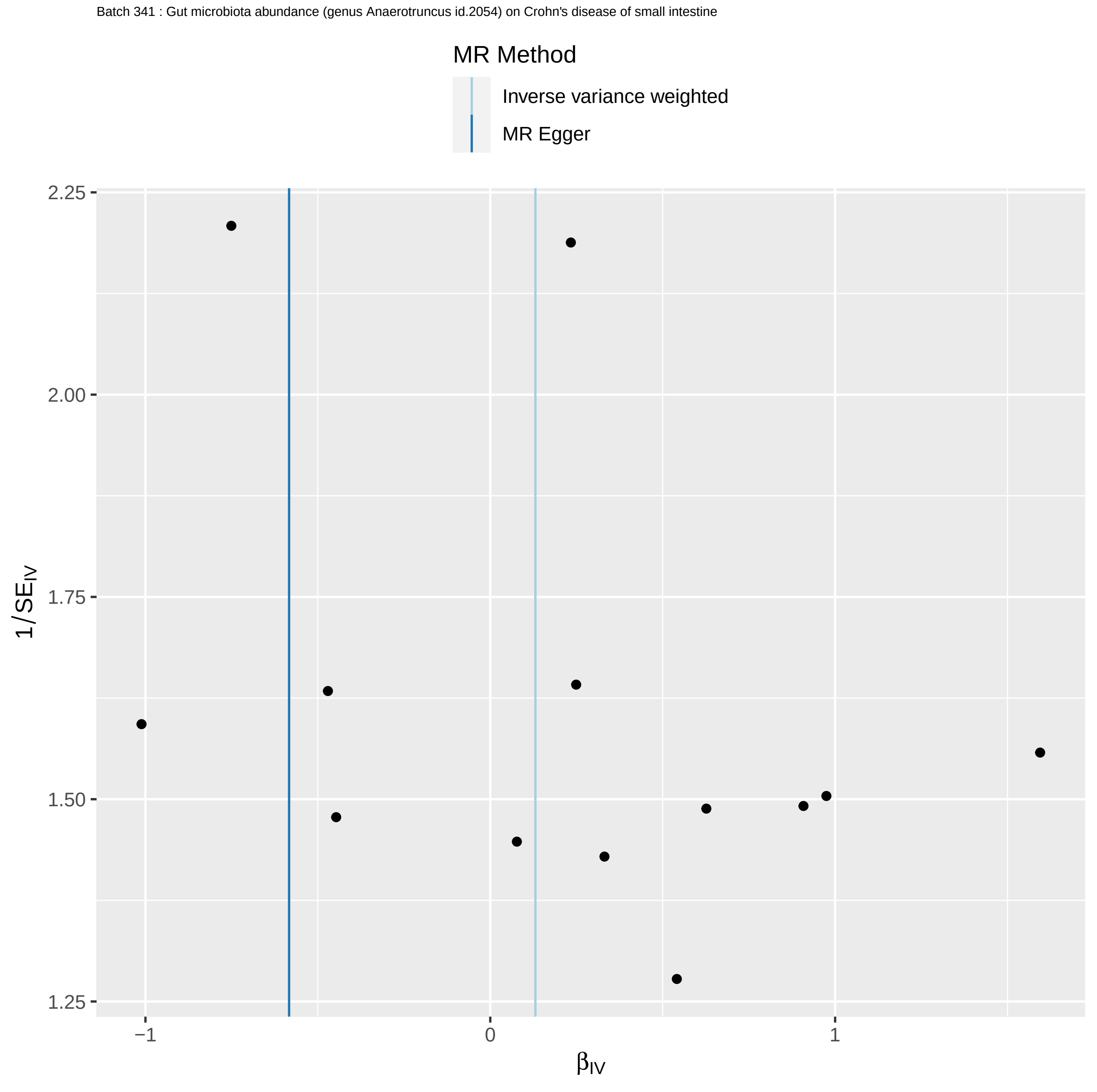

Batch 341 : Gut microbiota abundance (genus Anaerotruncus id.2054) on Crohn's disease of small intestine
MR Method
Inverse variance weighted
MR Egger
2.25
2.00
1.75
1.50
1.25
1/SE_IV
−1
0
1
β_IV

Batch 342 : Gut microbiota abundance (genus Bacteroides id.918) on Crohn's disease of small intestine

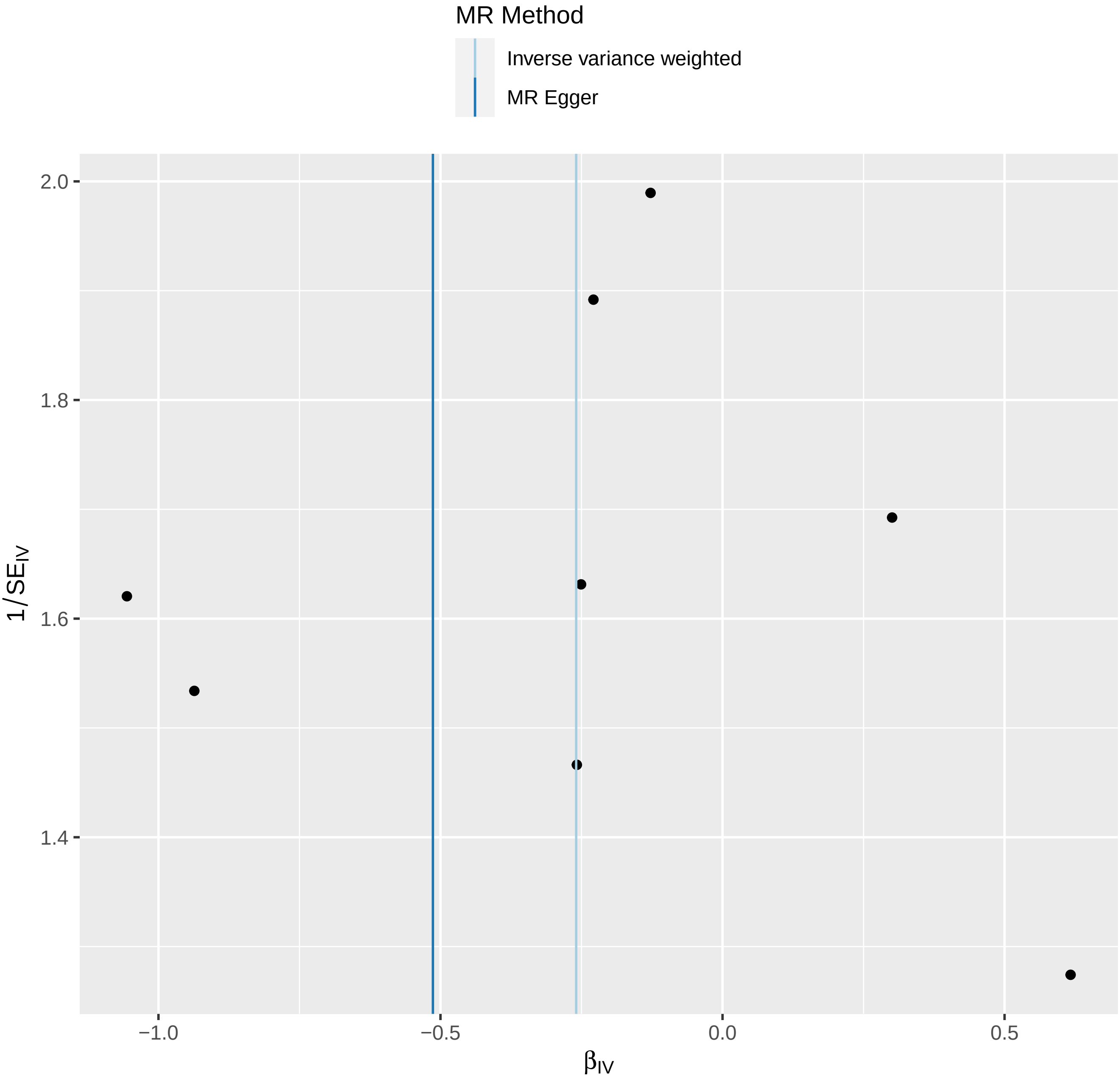

Batch 343 : Gut microbiota abundance (genus Barnesiella id.944) on Crohn's disease of small intestine

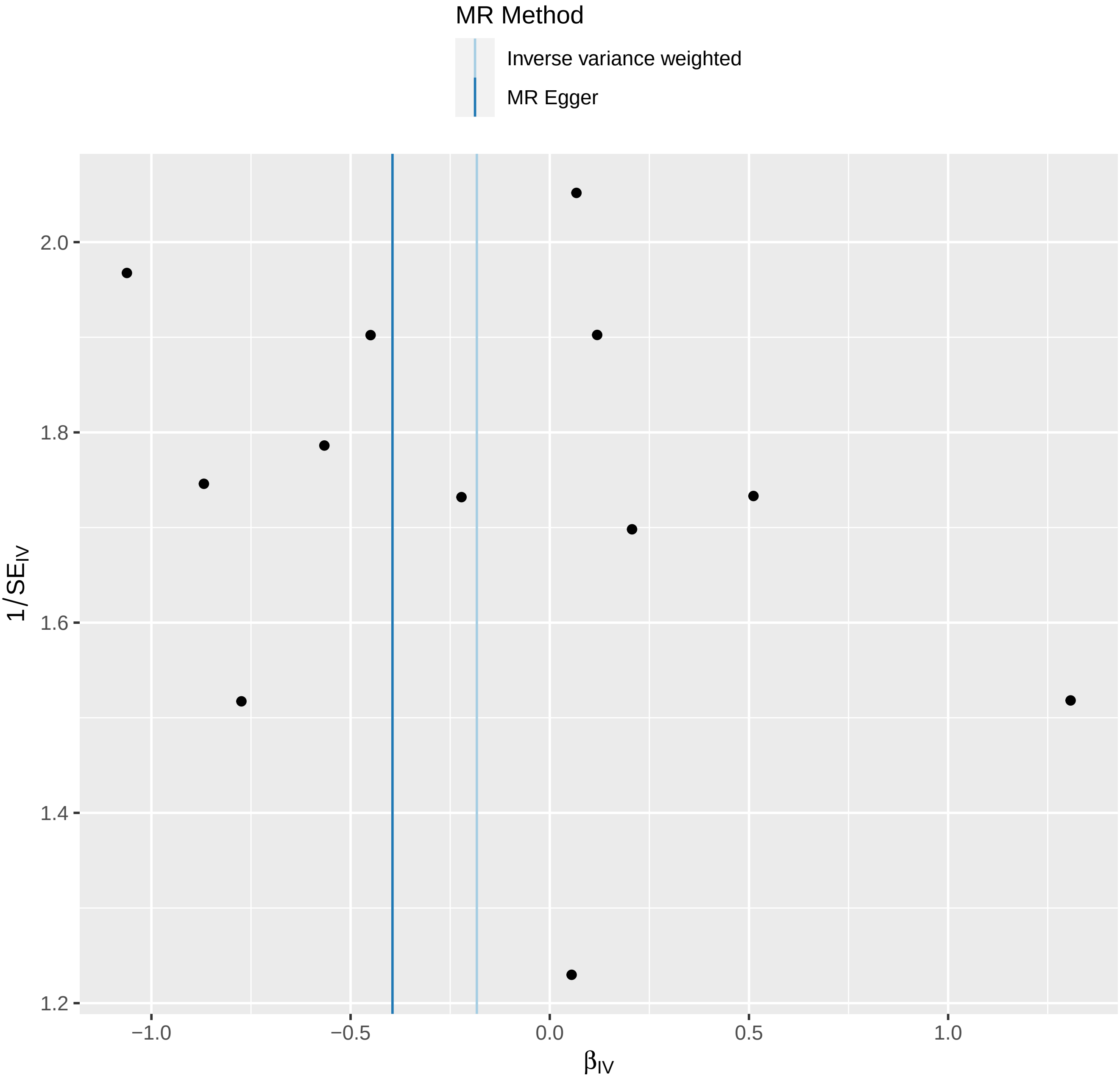

Batch 344 : Gut microbiota abundance (genus Bifidobacterium id.436) on Crohn's disease of small intestine

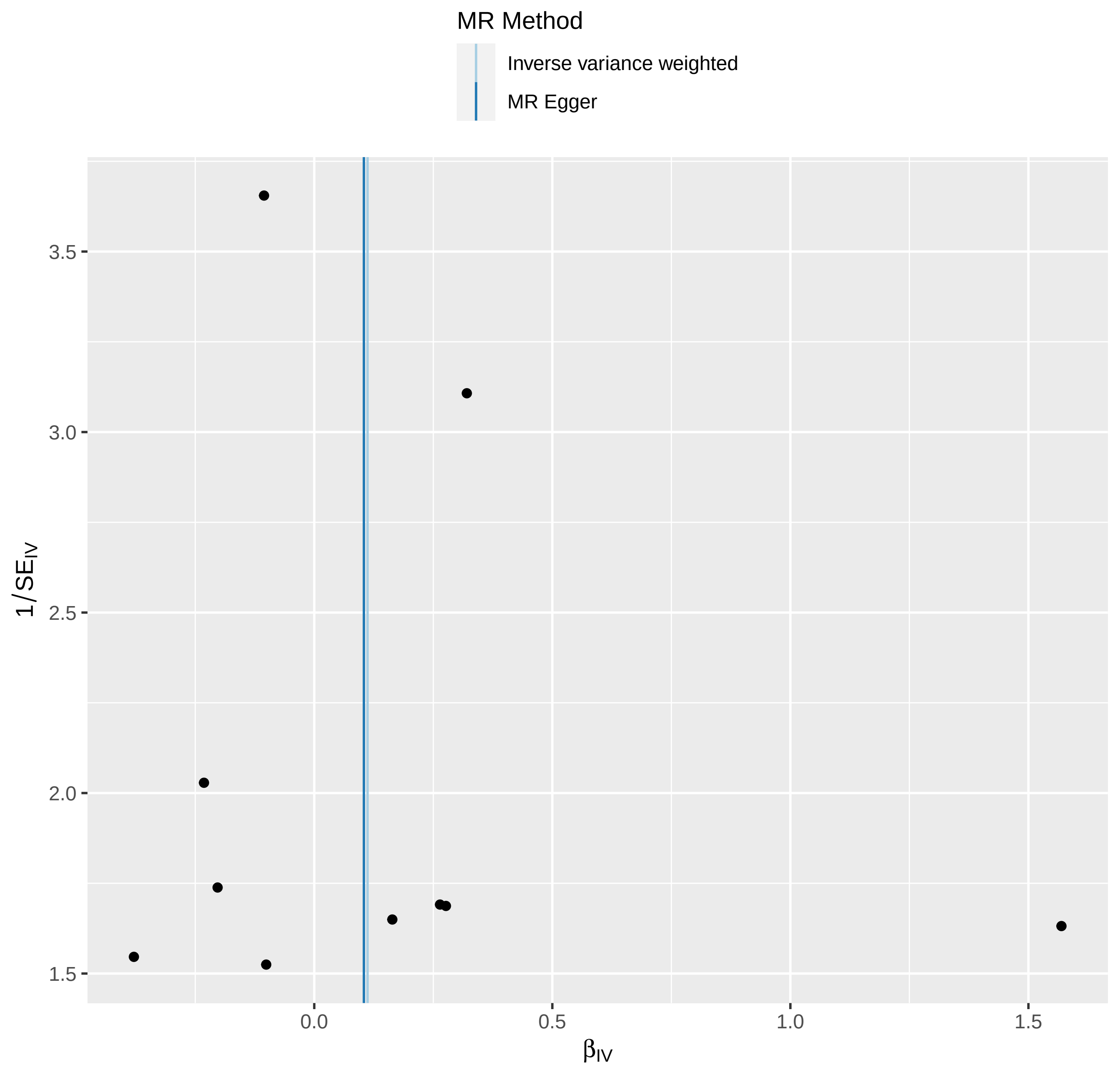

Batch 345 : Gut microbiota abundance (genus Bilophila id.3170) on Crohn's disease of small intestine

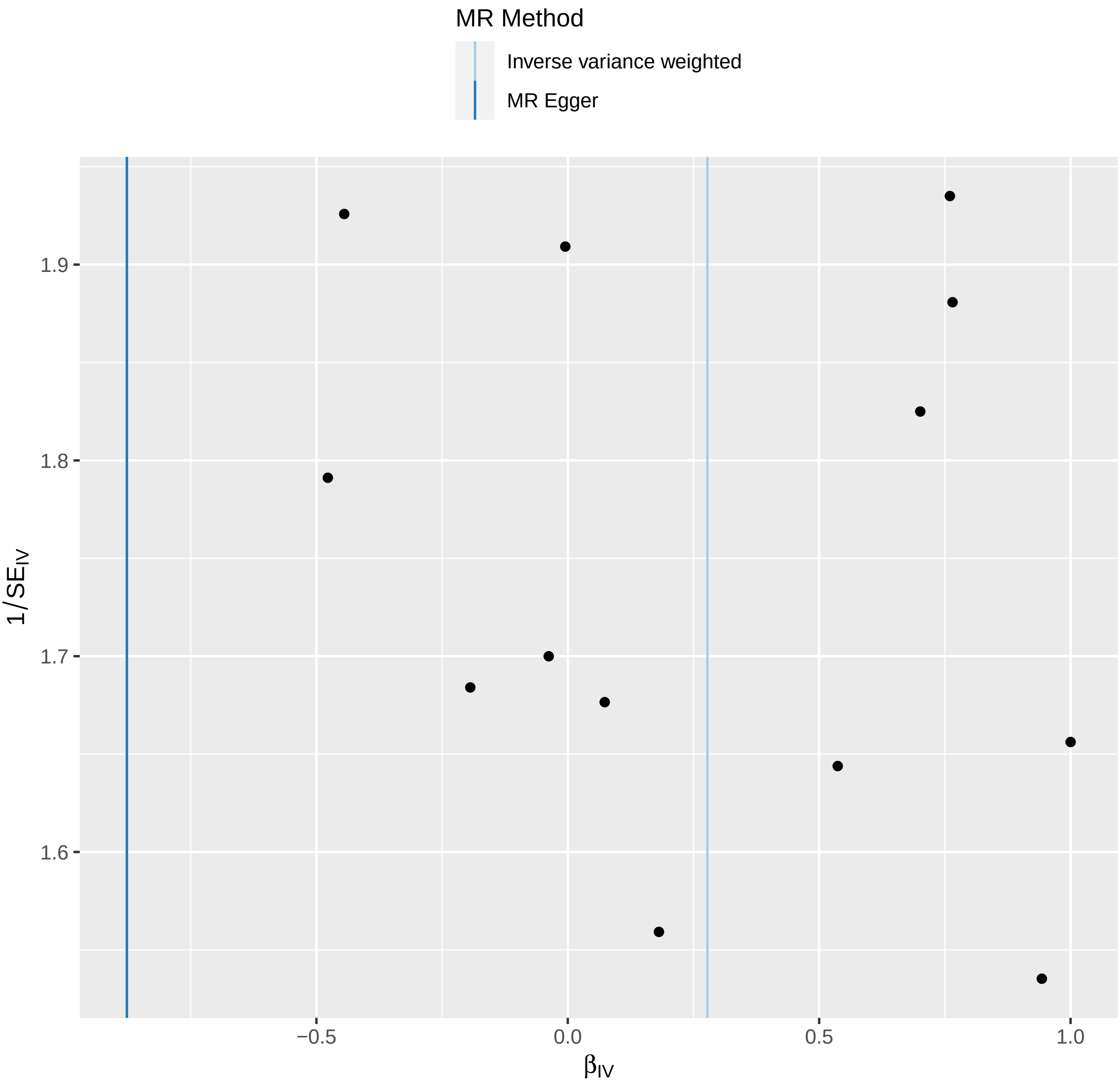

Batch 346 : Gut microbiota abundance (genus Blautia id.1992) on Crohn's disease of small intestine

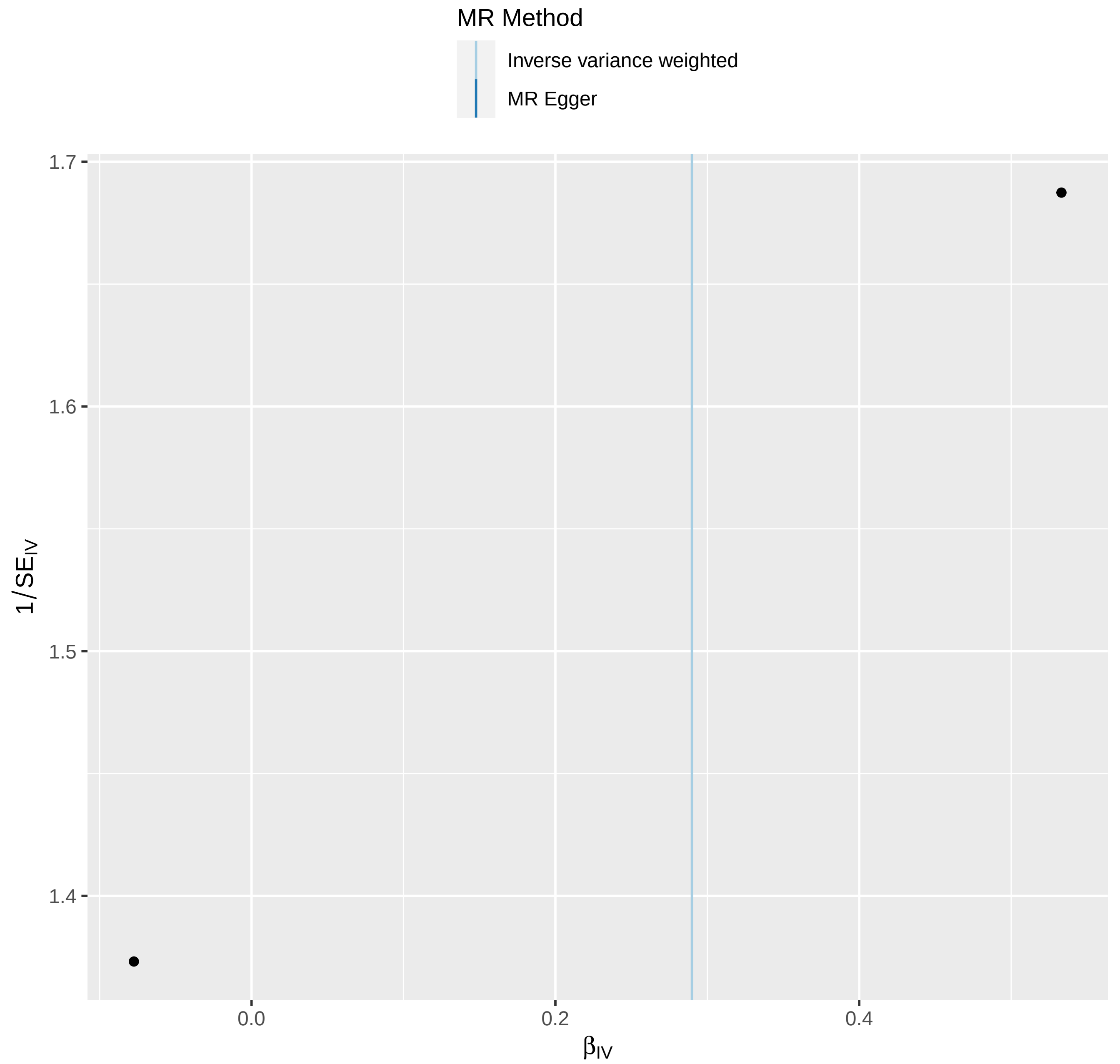

Batch 347 : Gut microbiota abundance (genus Butyricicoccus id.2055) on Crohn's disease of small intestine

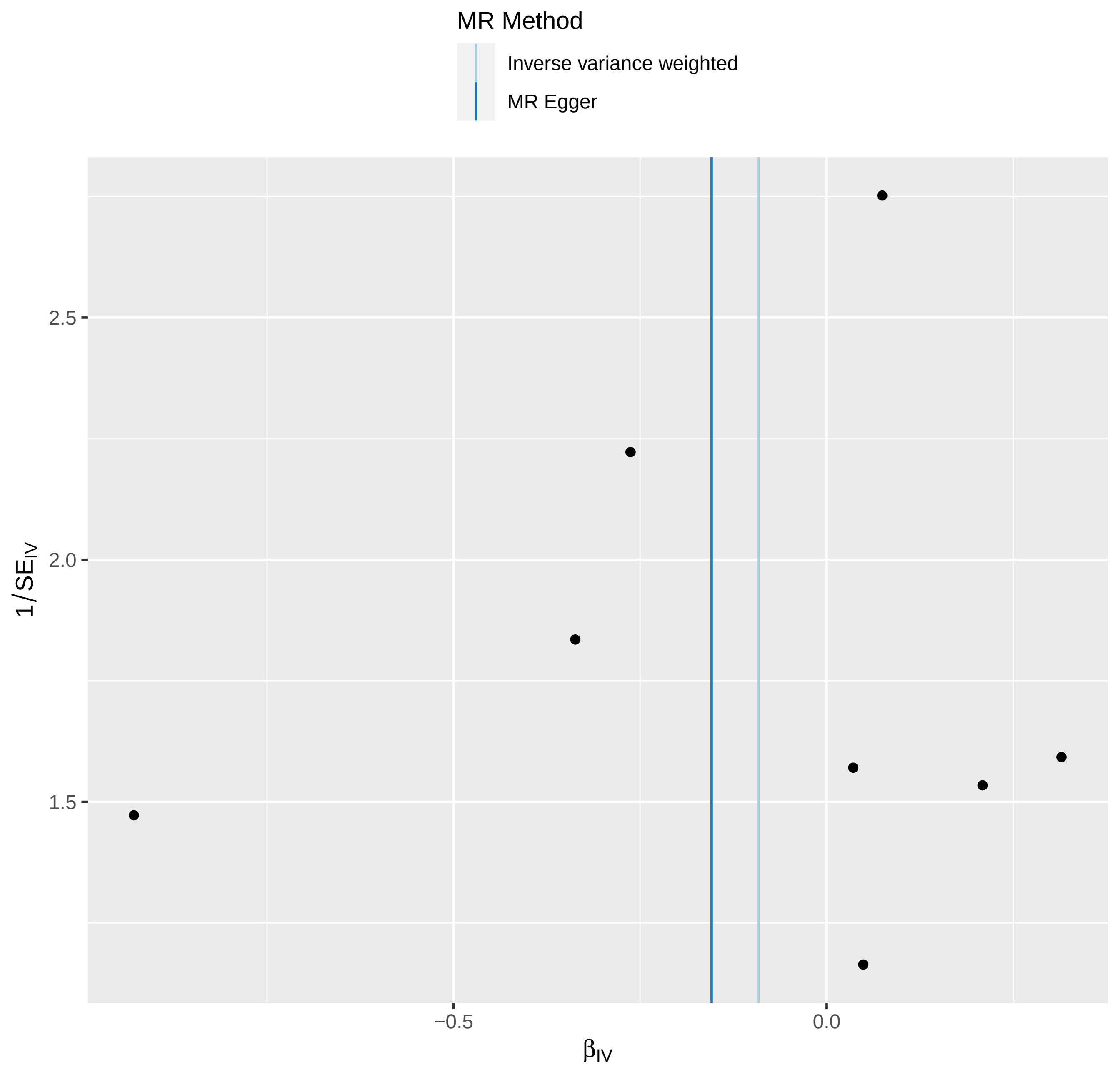

Batch 348 : Gut microbiota abundance (genus Butyricimonas id.945) on Crohn's disease of small intestine

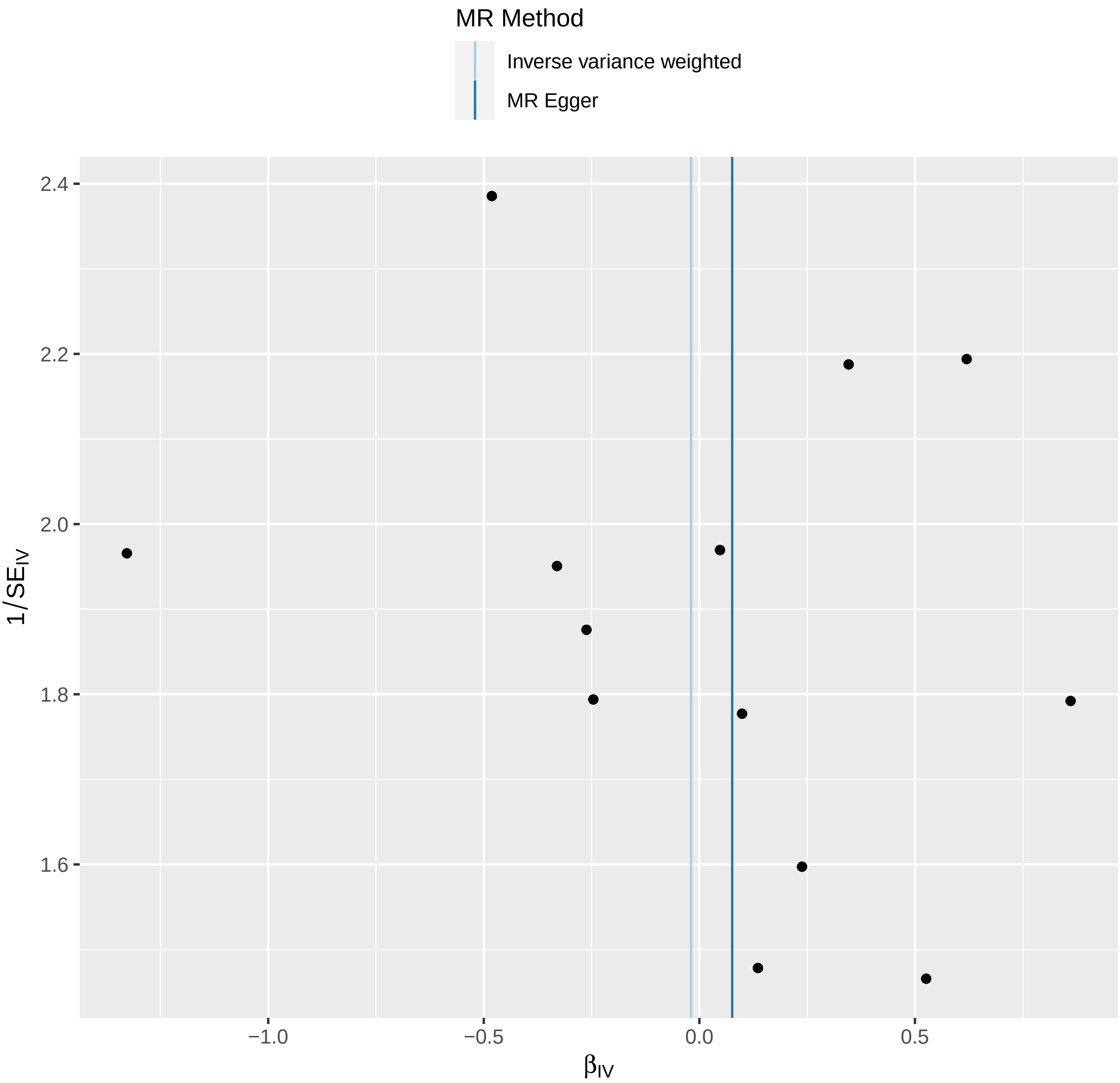

Batch 349 : Gut microbiota abundance (genus Butyrivibrio id.1993) on Crohn's disease of small intestine

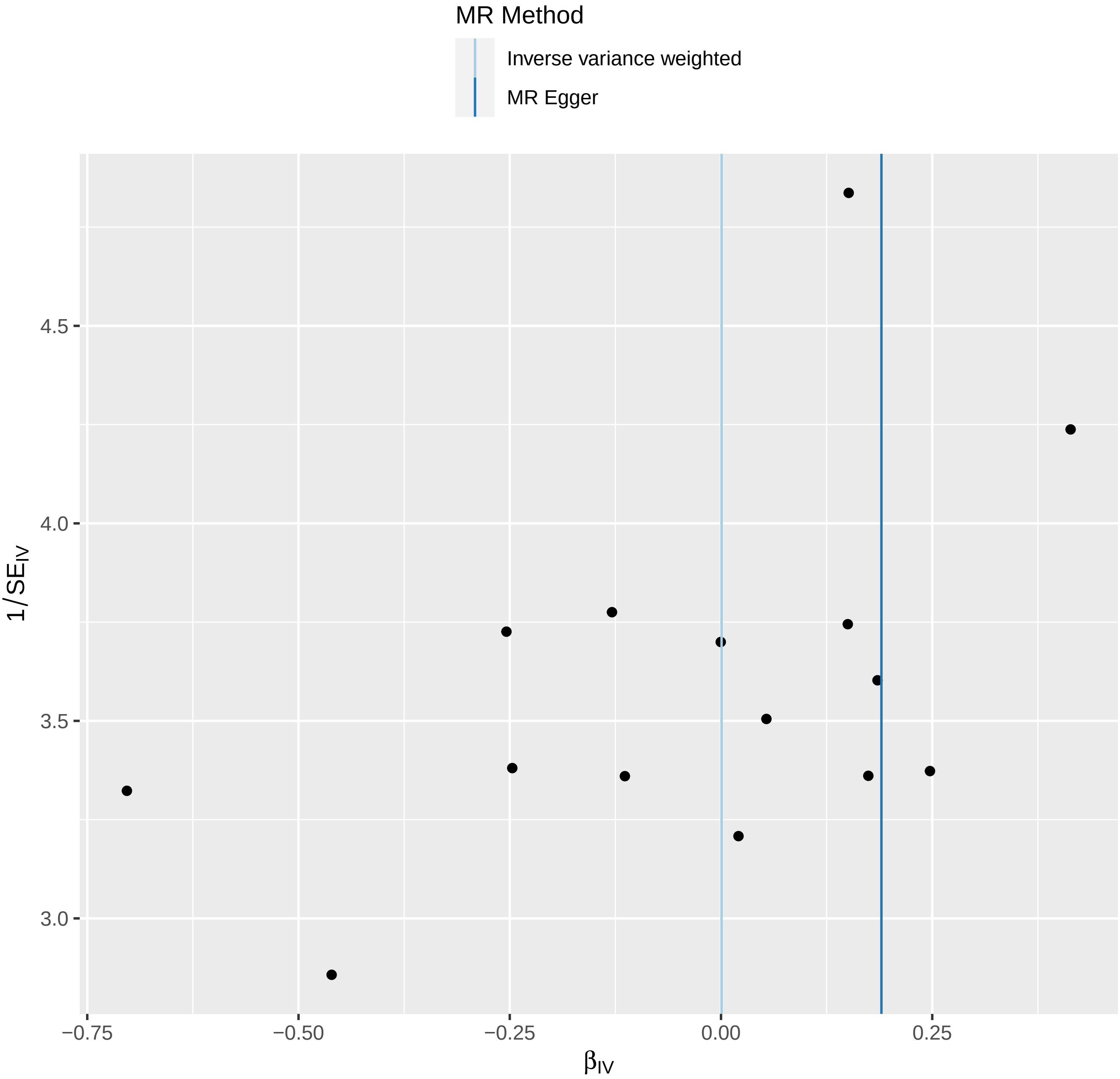

Batch 350 : Gut microbiota abundance (genus Candidatus Soleaferrea id.11350) on Crohn's disease of small intestine

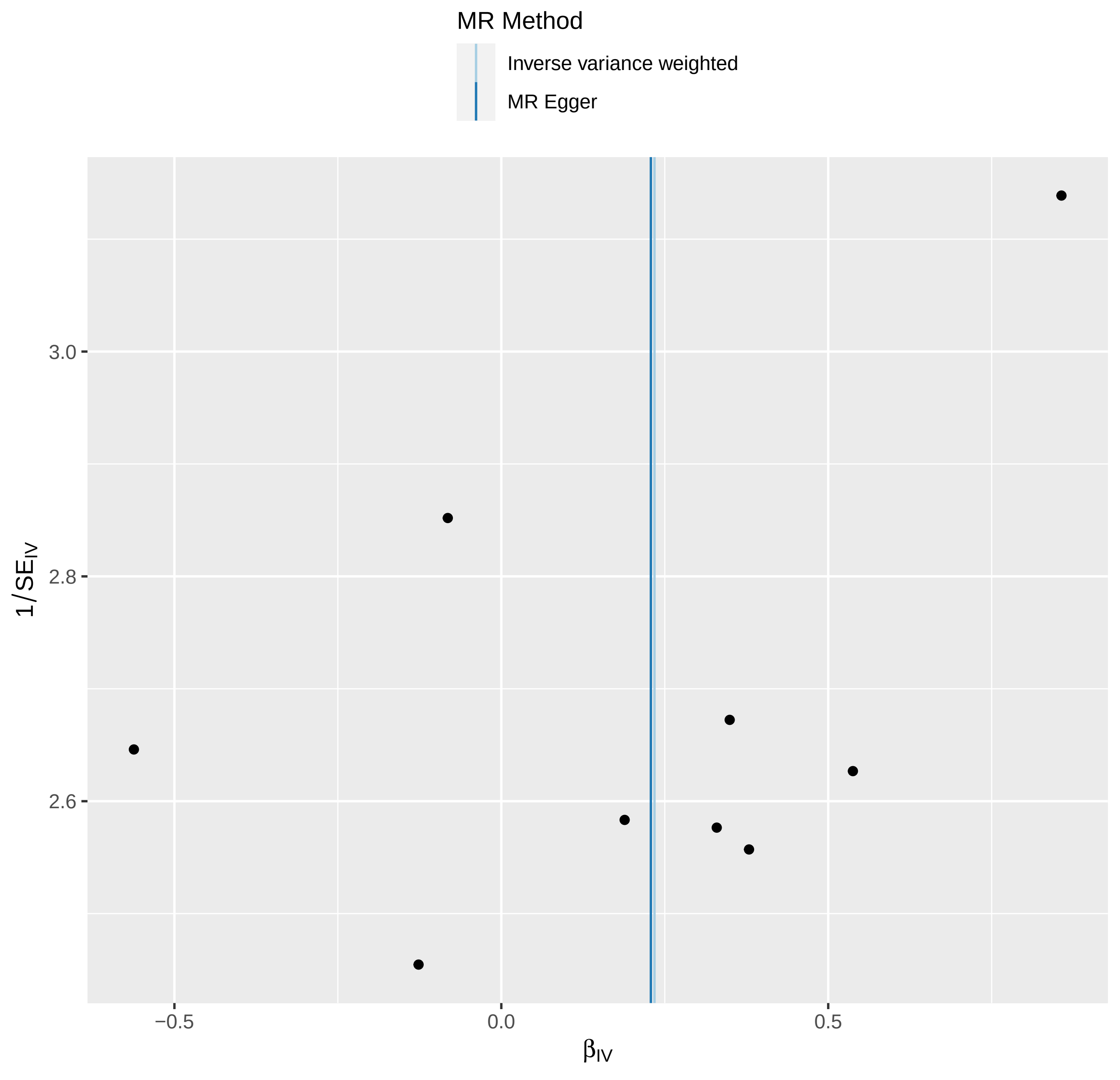

Batch 351 : Gut microbiota abundance (genus Catenibacterium id.2153) on Crohn's disease of small intestine

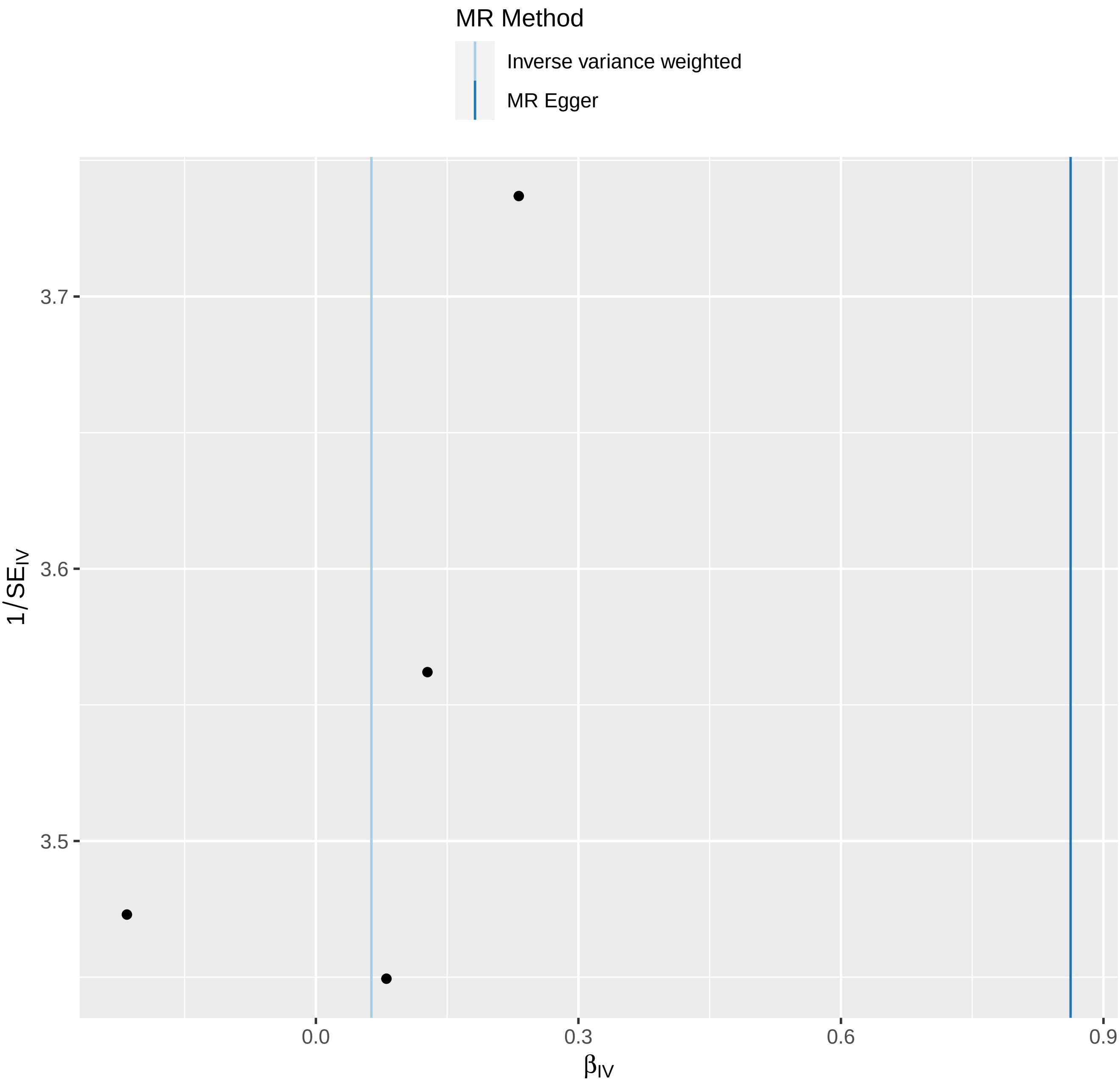

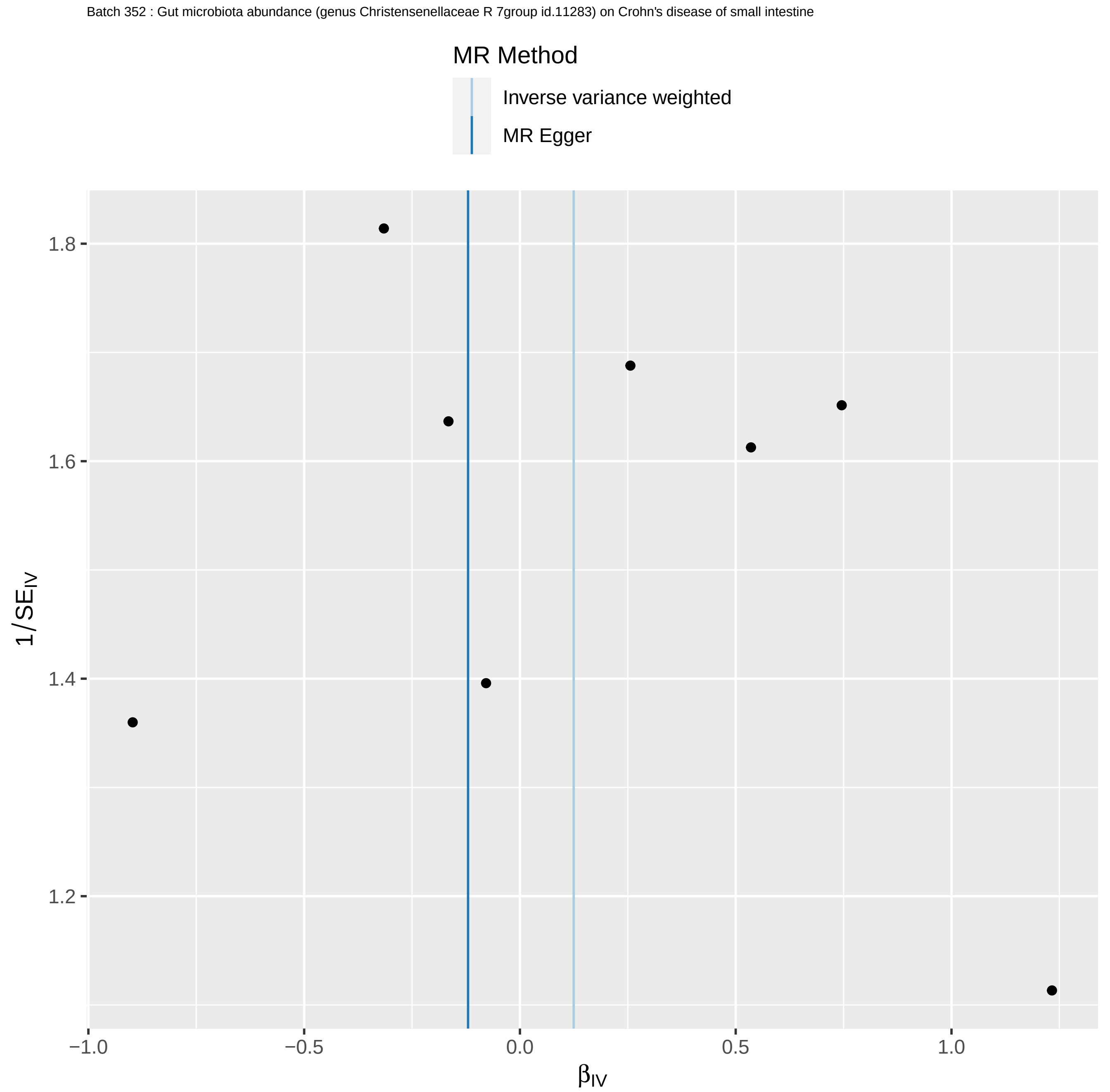
Batch 352 : Gut microbiota abundance (genus Christensenellaceae R 7group id.11283) on Crohn's disease of small intestine
MR Method
Inverse variance weighted
MR Egger
1.8
1.6
1.4
1.2
1/SE_IV
−1.0
−0.5
0.0
0.5
1.0
β_IV

Batch 353 : Gut microbiota abundance (genus Clostridium innocuum group id.14397) on Crohn's disease of small intestine

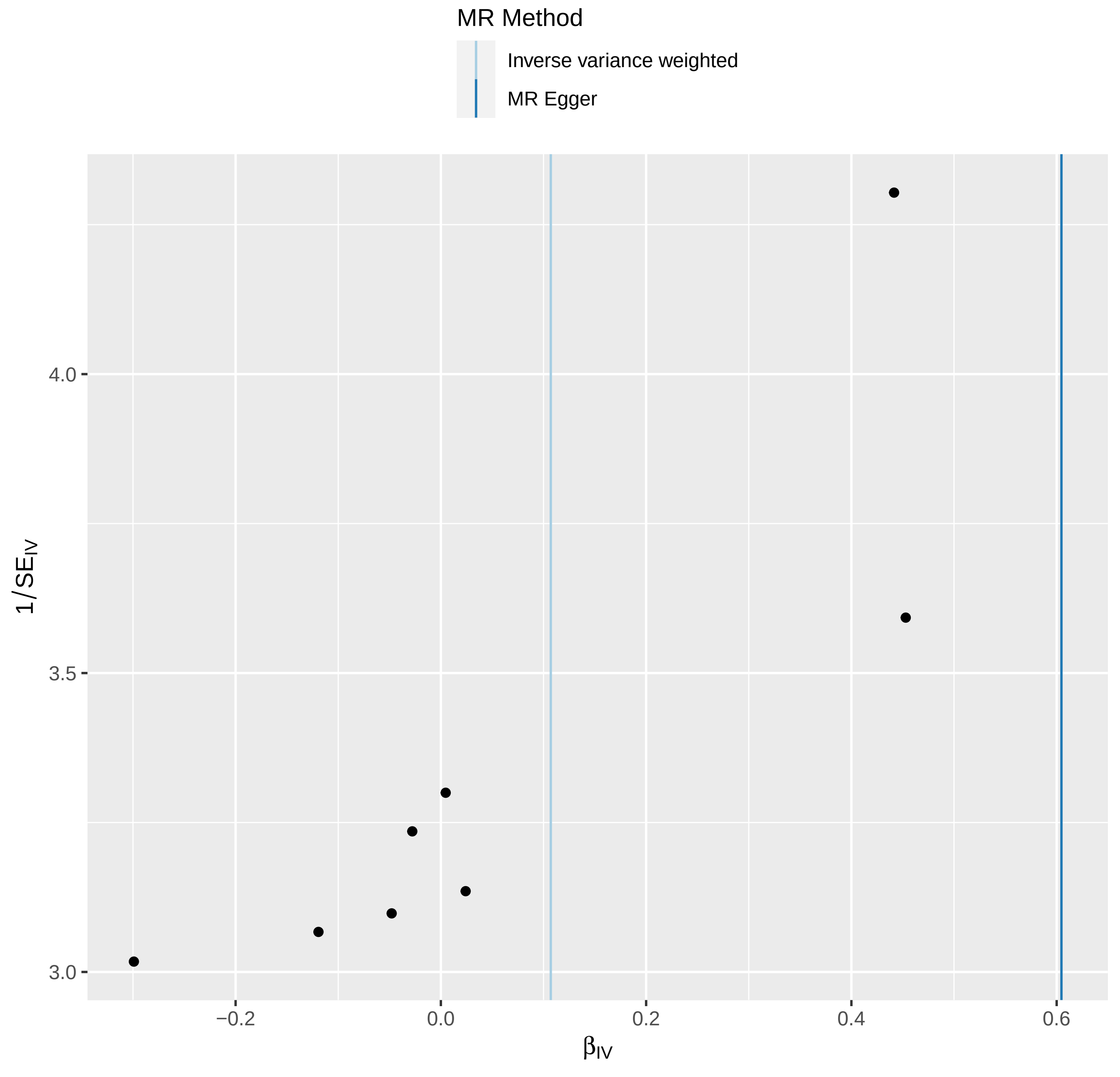

Batch 354 : Gut microbiota abundance (genus Clostridium sensustricto1 id.1873) on Crohn's disease of small intestine

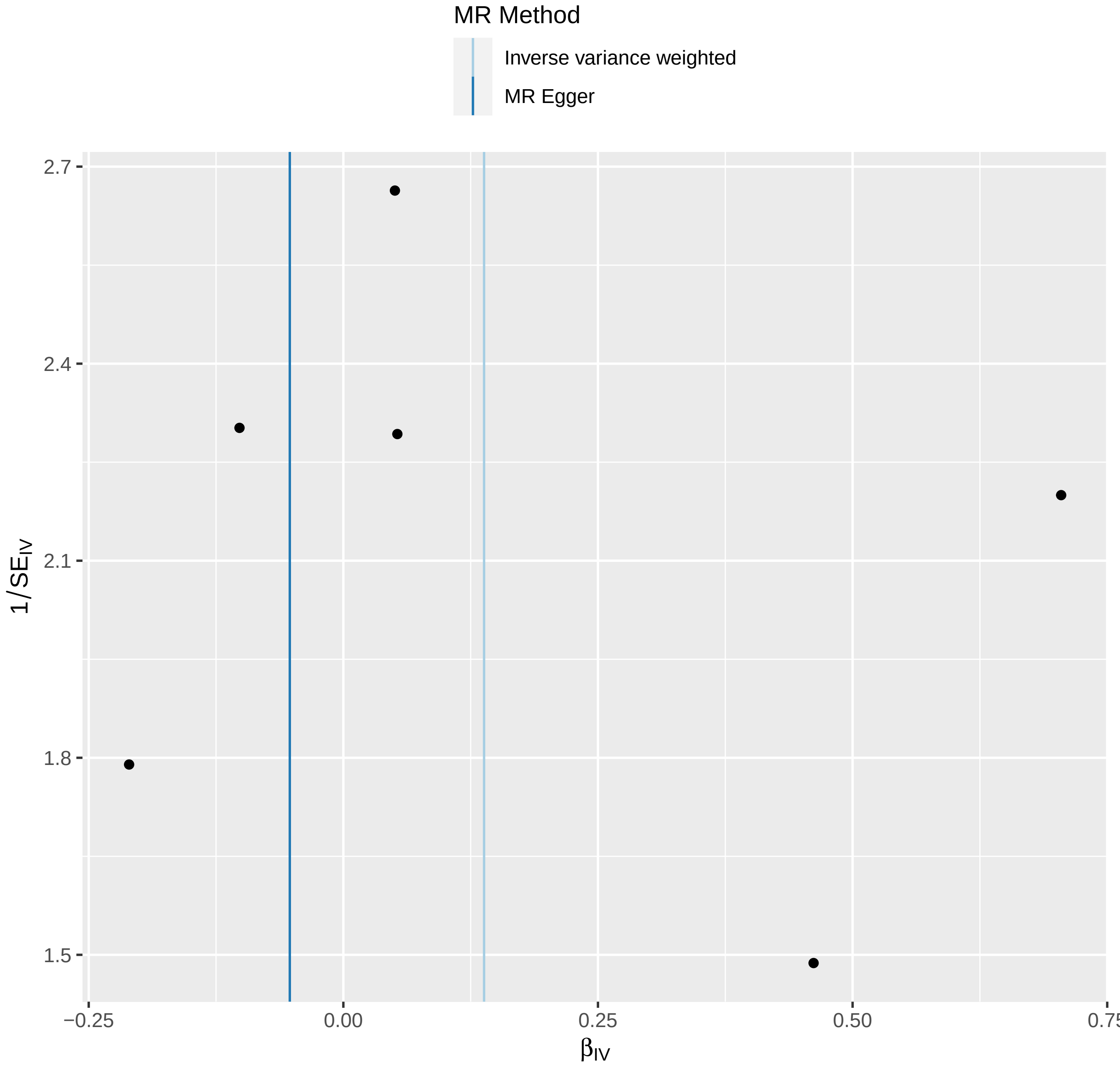

Batch 355 : Gut microbiota abundance (genus Collinsella id.815) on Crohn's disease of small intestine

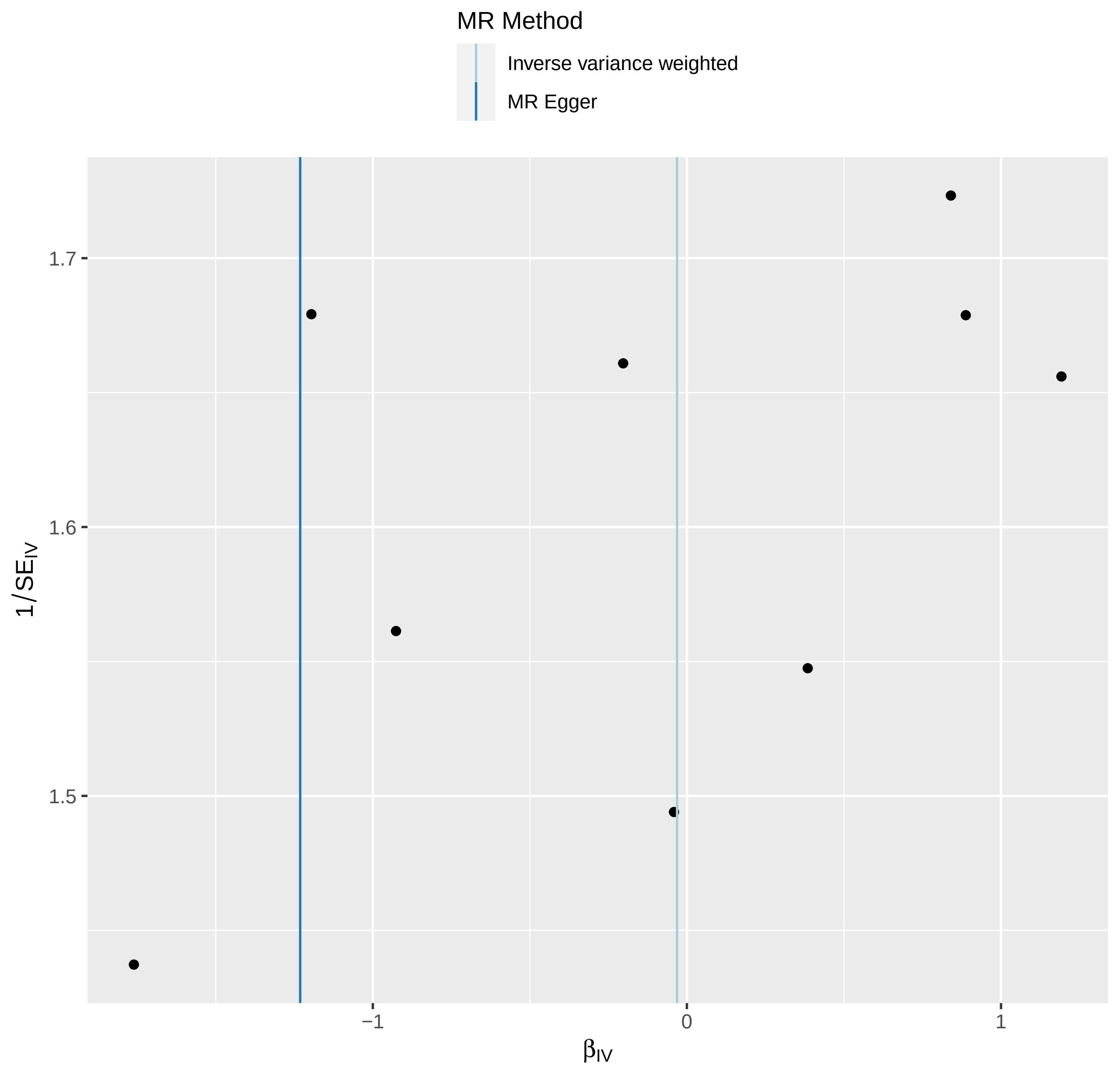

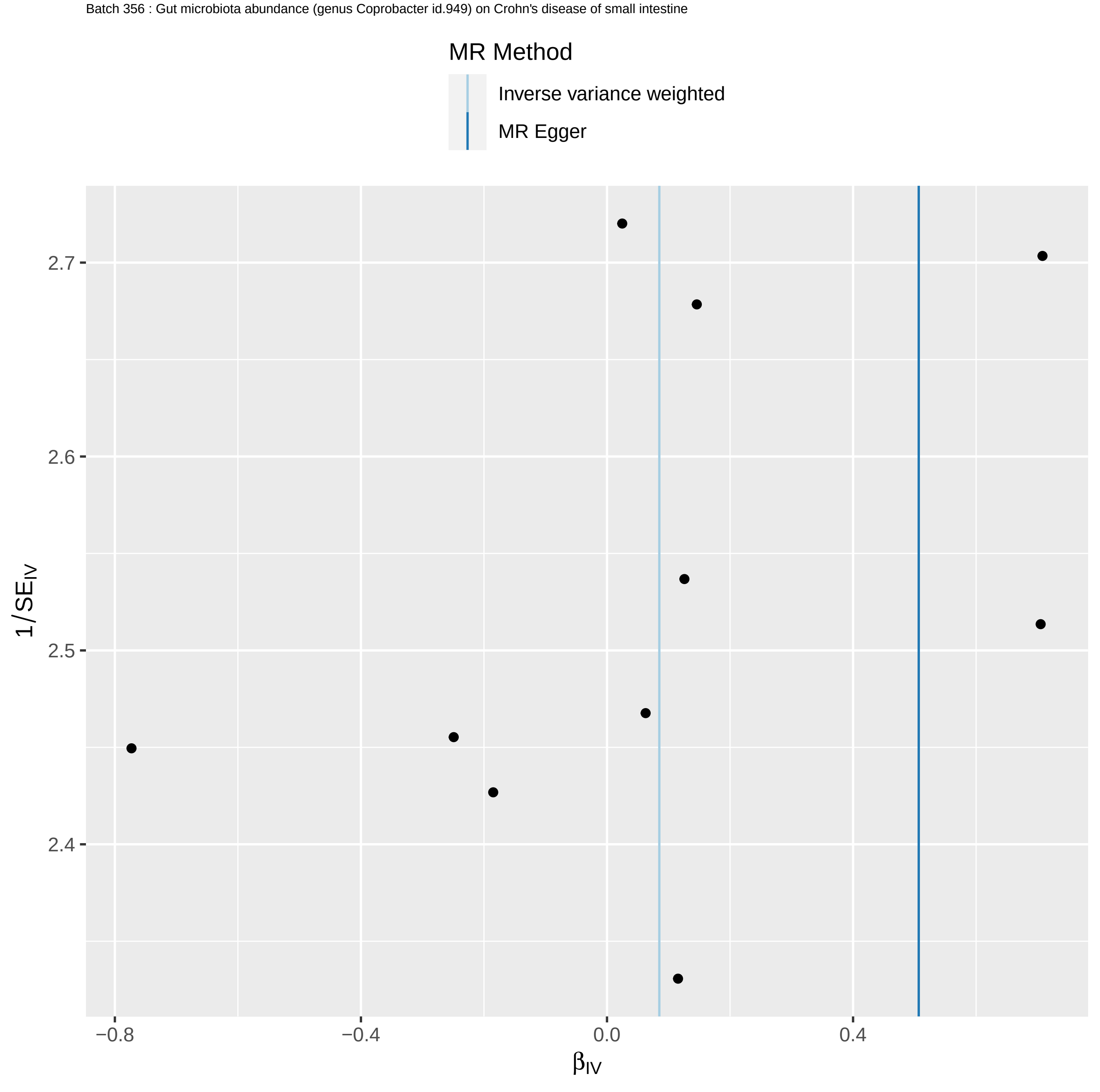
Batch 356 : Gut microbiota abundance (genus Coprobacter id.949) on Crohn's disease of small intestine
MR Method
Inverse variance weighted
MR Egger
2.7
2.6
2.5
2.4
1/SE_IV
−0.8
−0.4
0.0
0.4
β_IV

Batch 357 : Gut microbiota abundance (genus Coprococcus1 id.11301) on Crohn's disease of small intestine

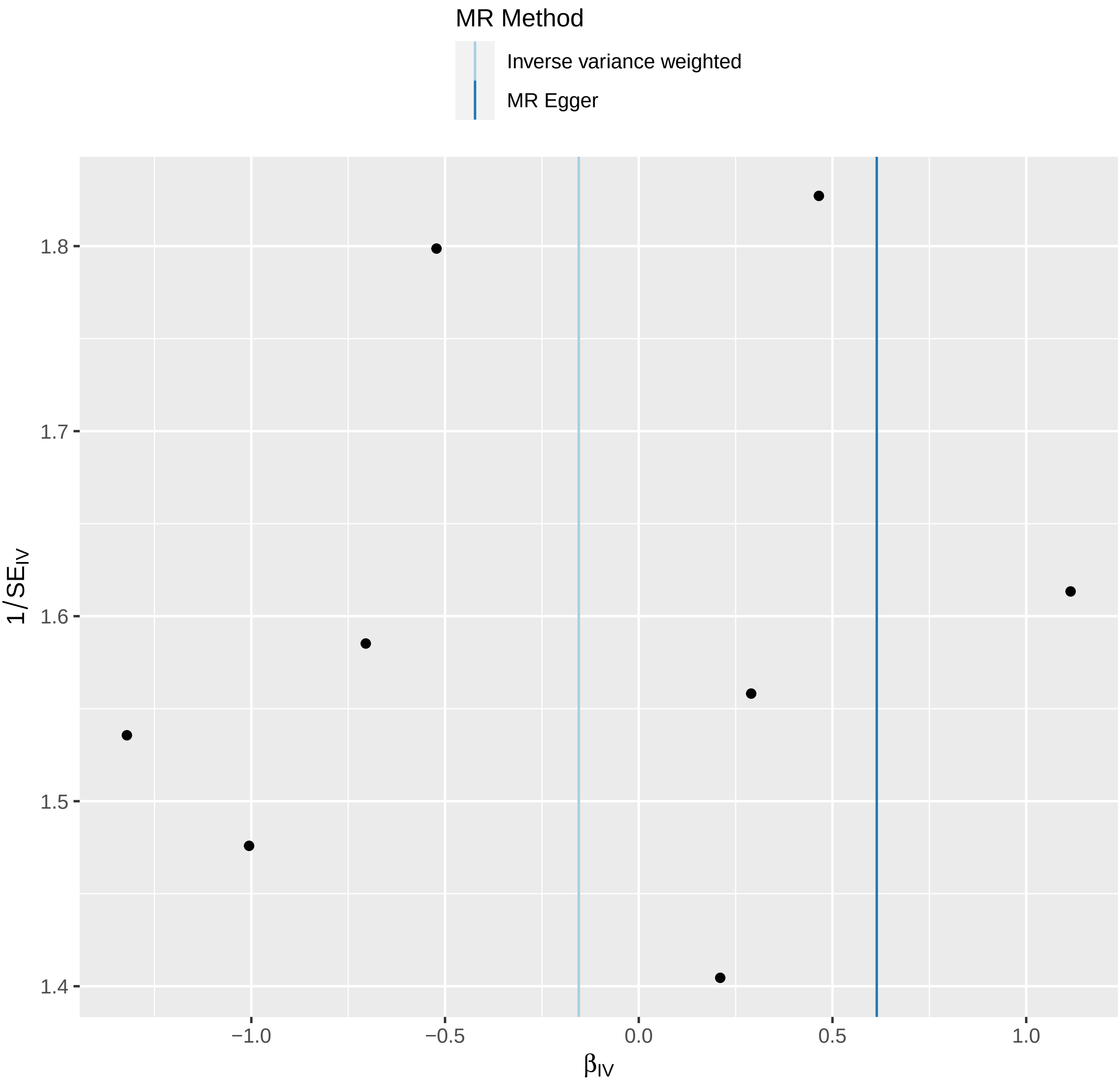

Batch 358 : Gut microbiota abundance (genus Coprococcus2 id.11302) on Crohn's disease of small intestine

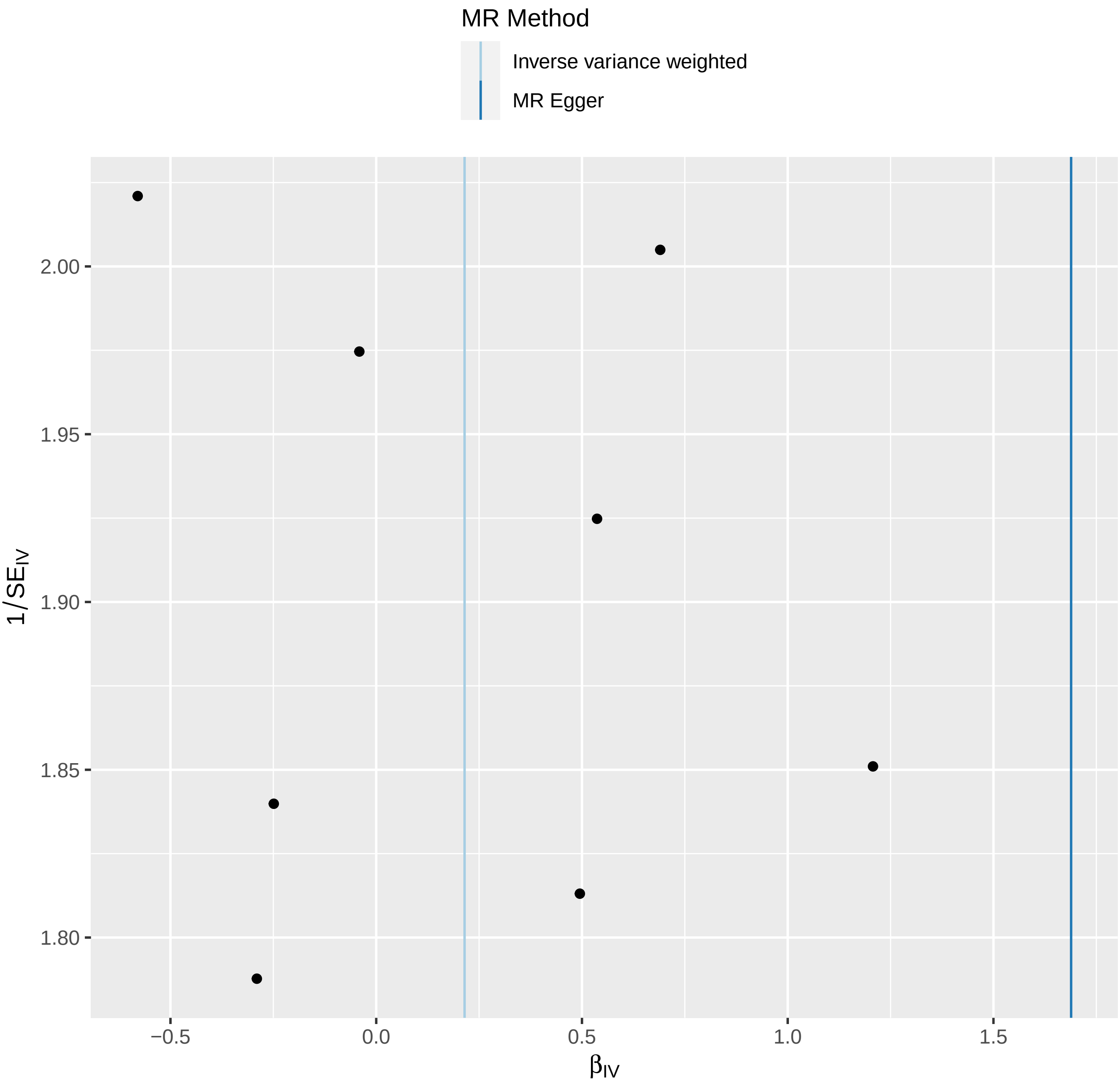

Batch 359 : Gut microbiota abundance (genus Coprococcus3 id.11303) on Crohn's disease of small intestine

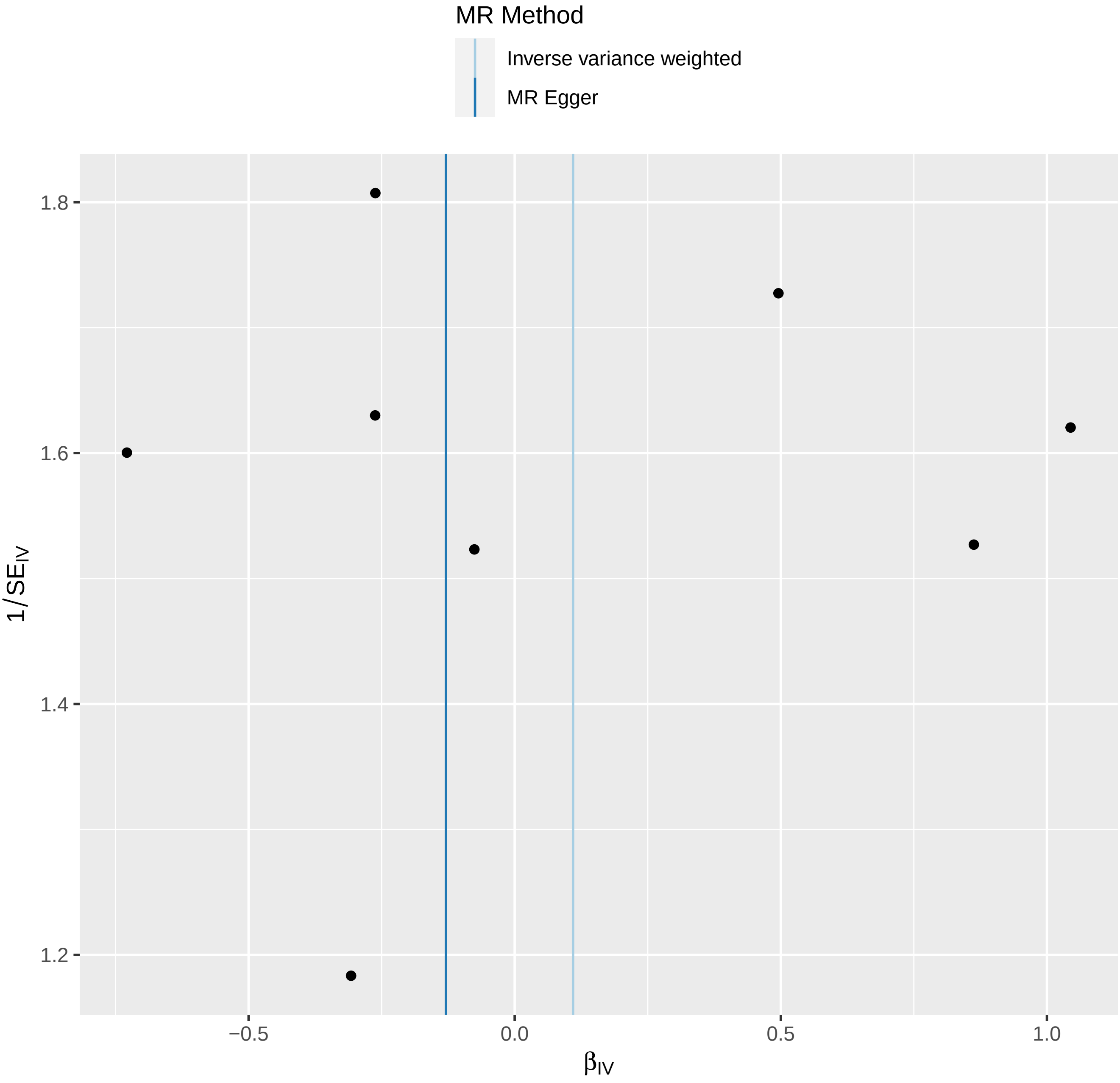

Batch 360 : Gut microbiota abundance (genus Defluviitaleaceae UCG011 id.11287) on Crohn's disease of small intestine

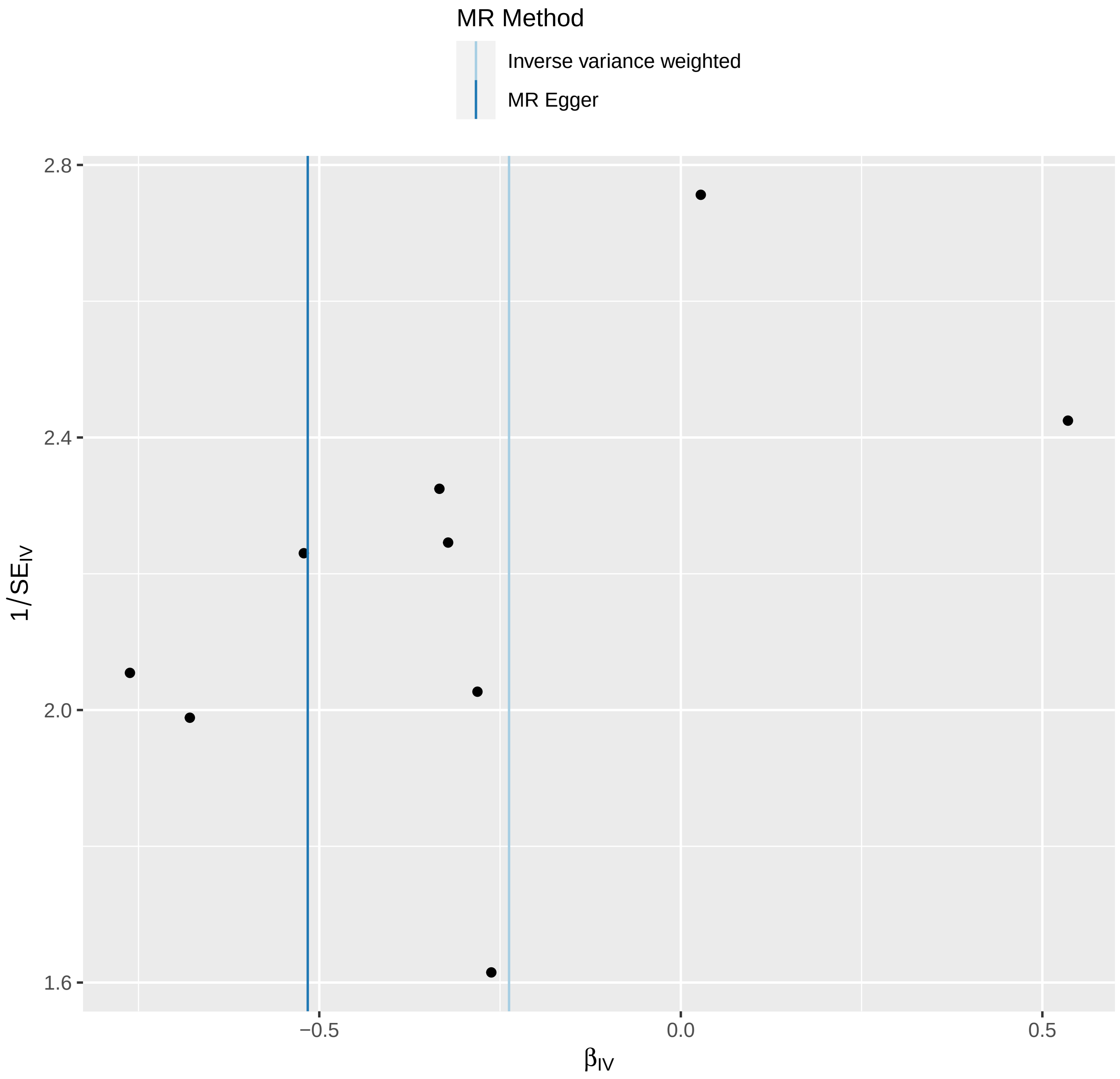

Batch 361 : Gut microbiota abundance (genus Desulfovibrio id.3173) on Crohn's disease of small intestine

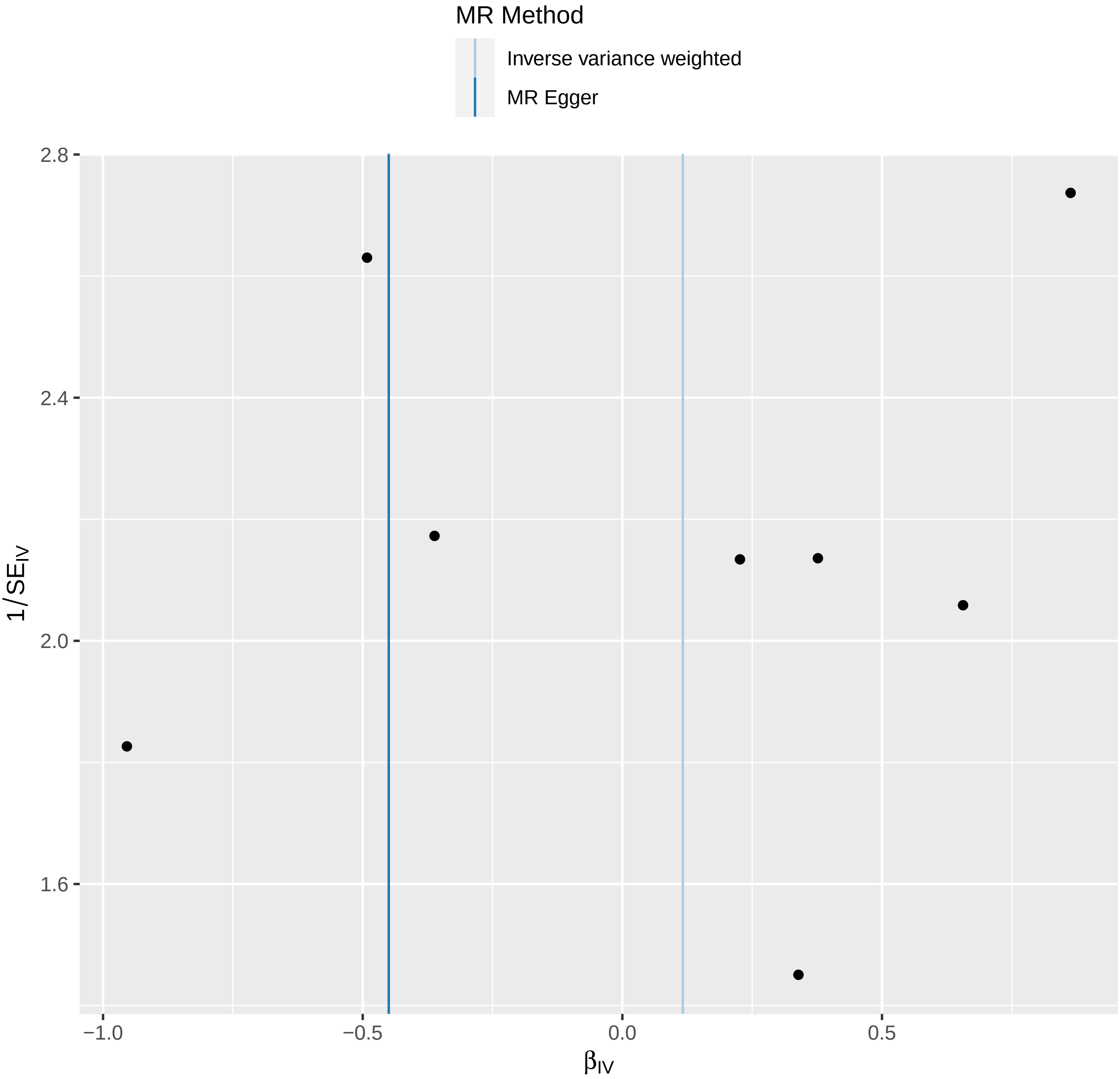

Batch 362 : Gut microbiota abundance (genus Dialister id.2183) on Crohn's disease of small intestine

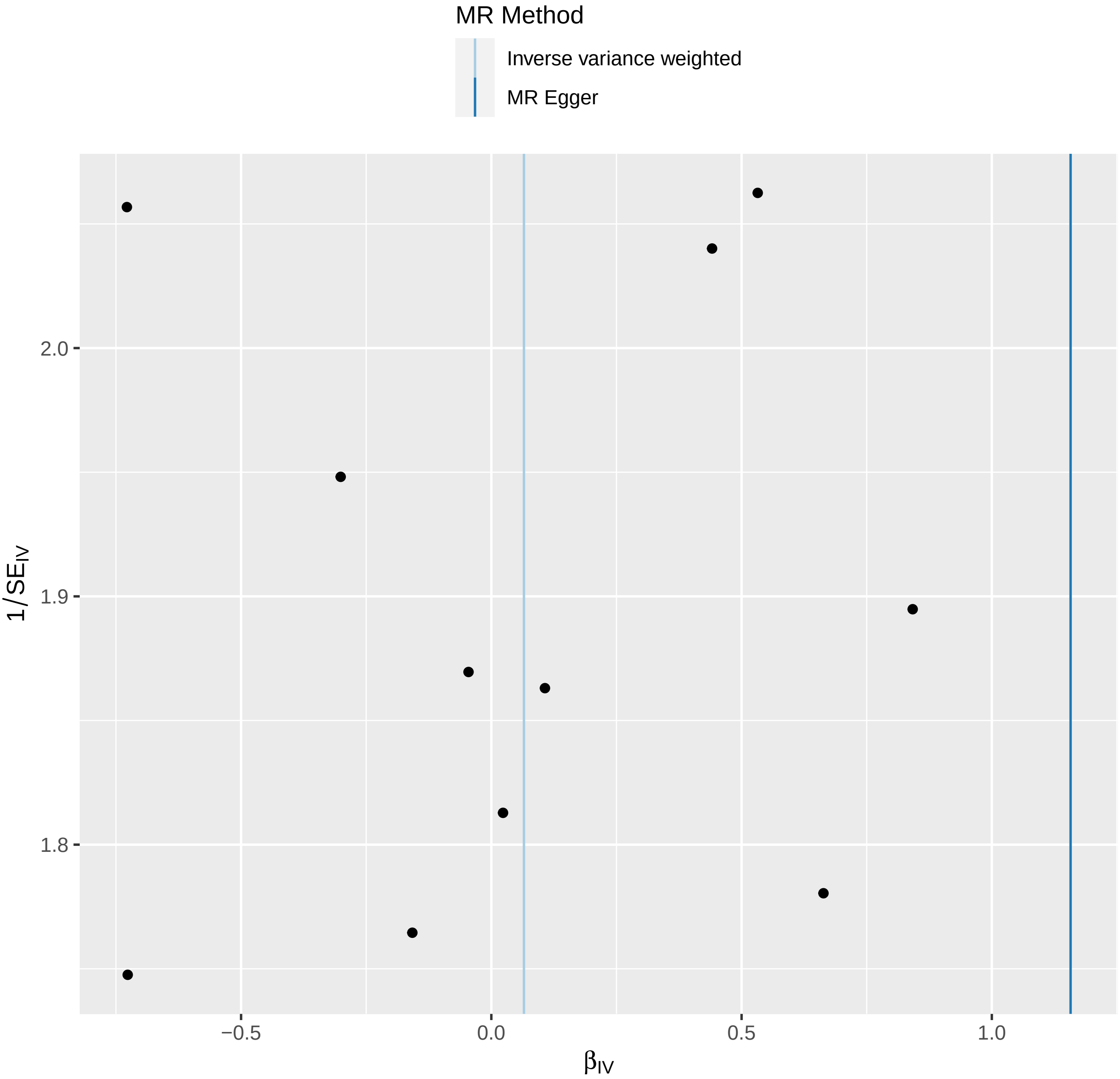

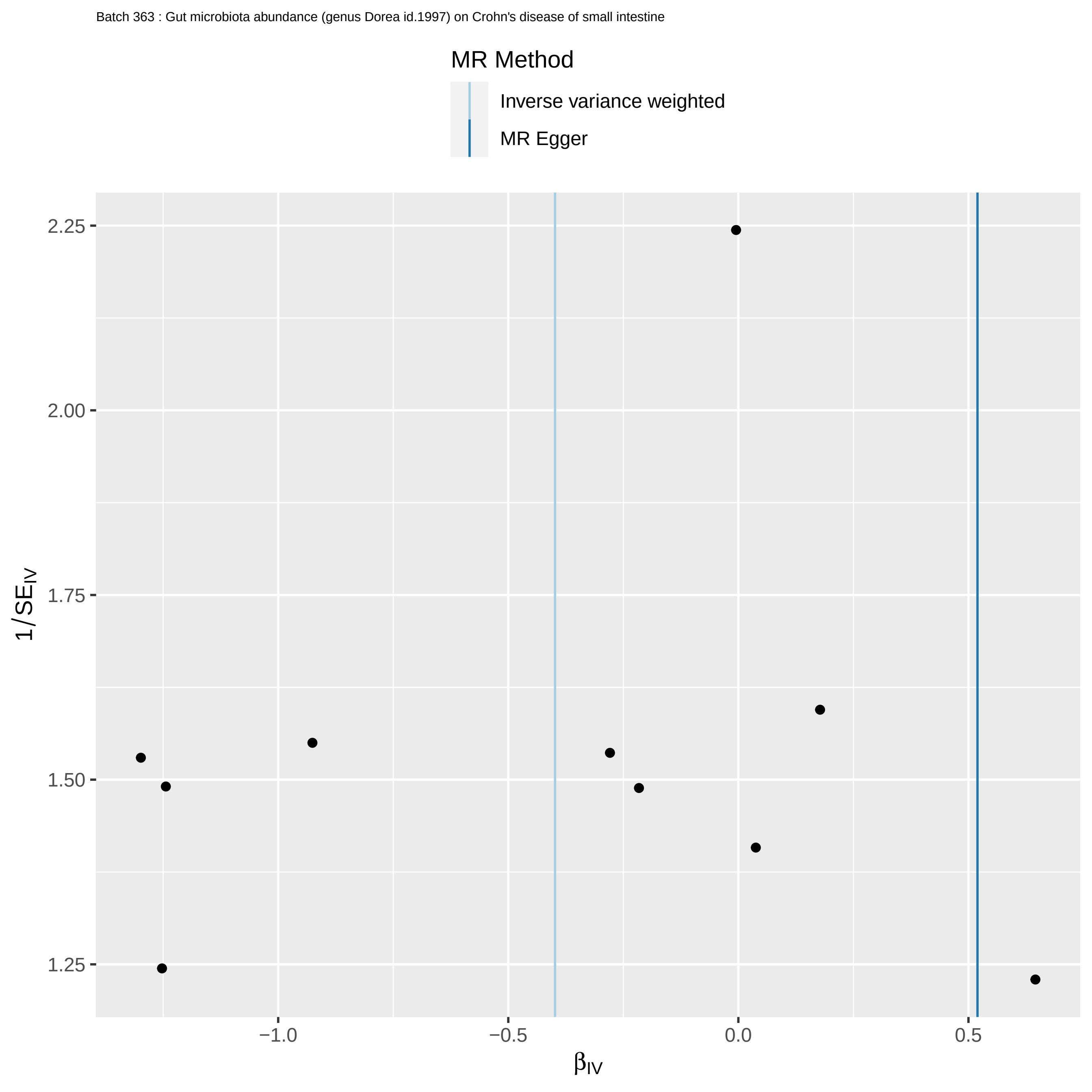

Batch 363 : Gut microbiota abundance (genus Dorea id.1997) on Crohn's disease of small intestine
MR Method
Inverse variance weighted
MR Egger
2.25
2.00
1.75
1.50
1.25
1/SE$_{IV}$
−1.0
−0.5
0.0
0.5
β$_{IV}$

Batch 364 : Gut microbiota abundance (genus Eggerthella id.819) on Crohn's disease of small intestine

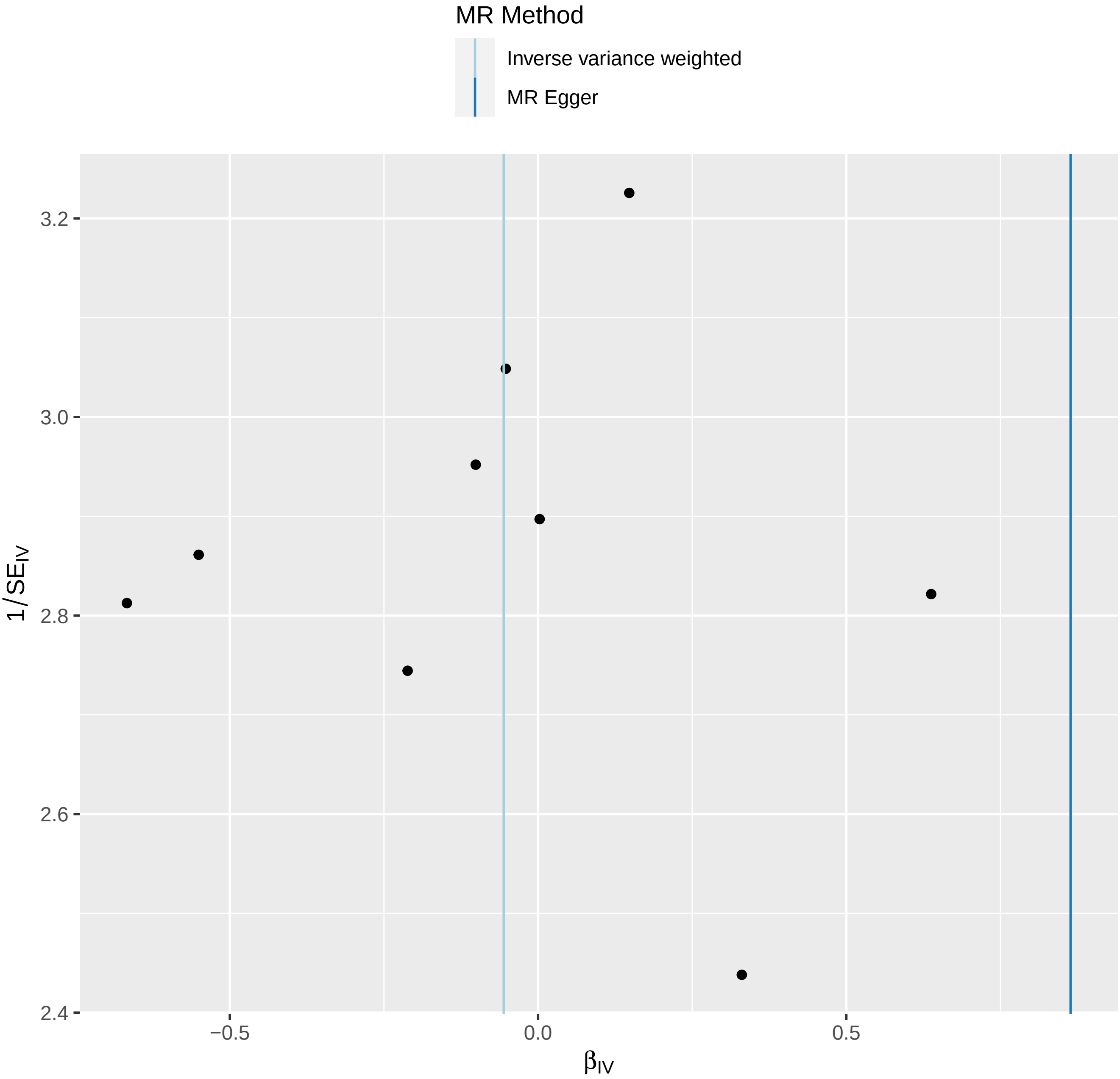

Batch 365 : Gut microbiota abundance (genus Eisenbergiella id.11304) on Crohn's disease of small intestine

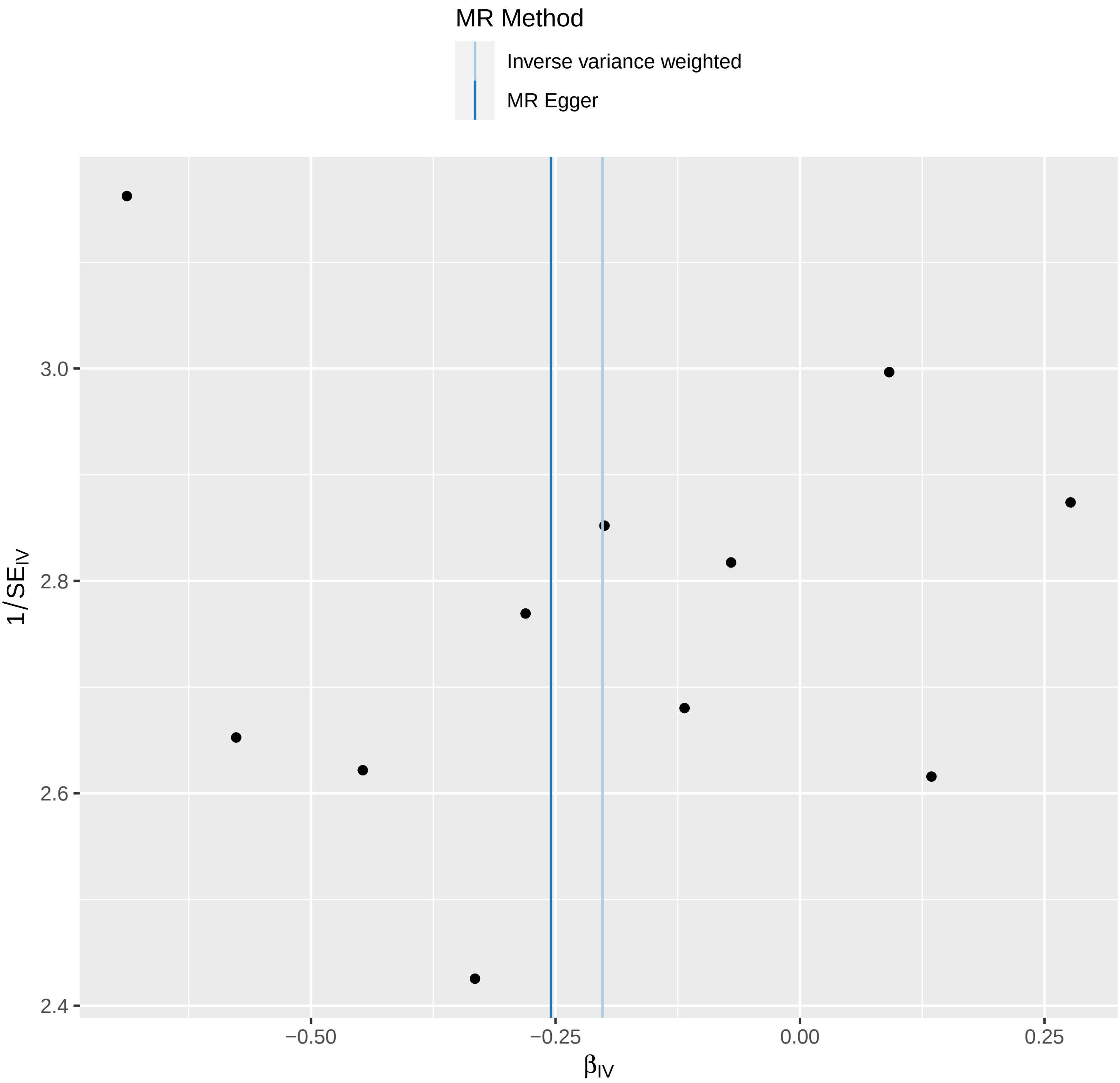

Batch 366 : Gut microbiota abundance (genus Enterorhabdus id.820) on Crohn's disease of small intestine

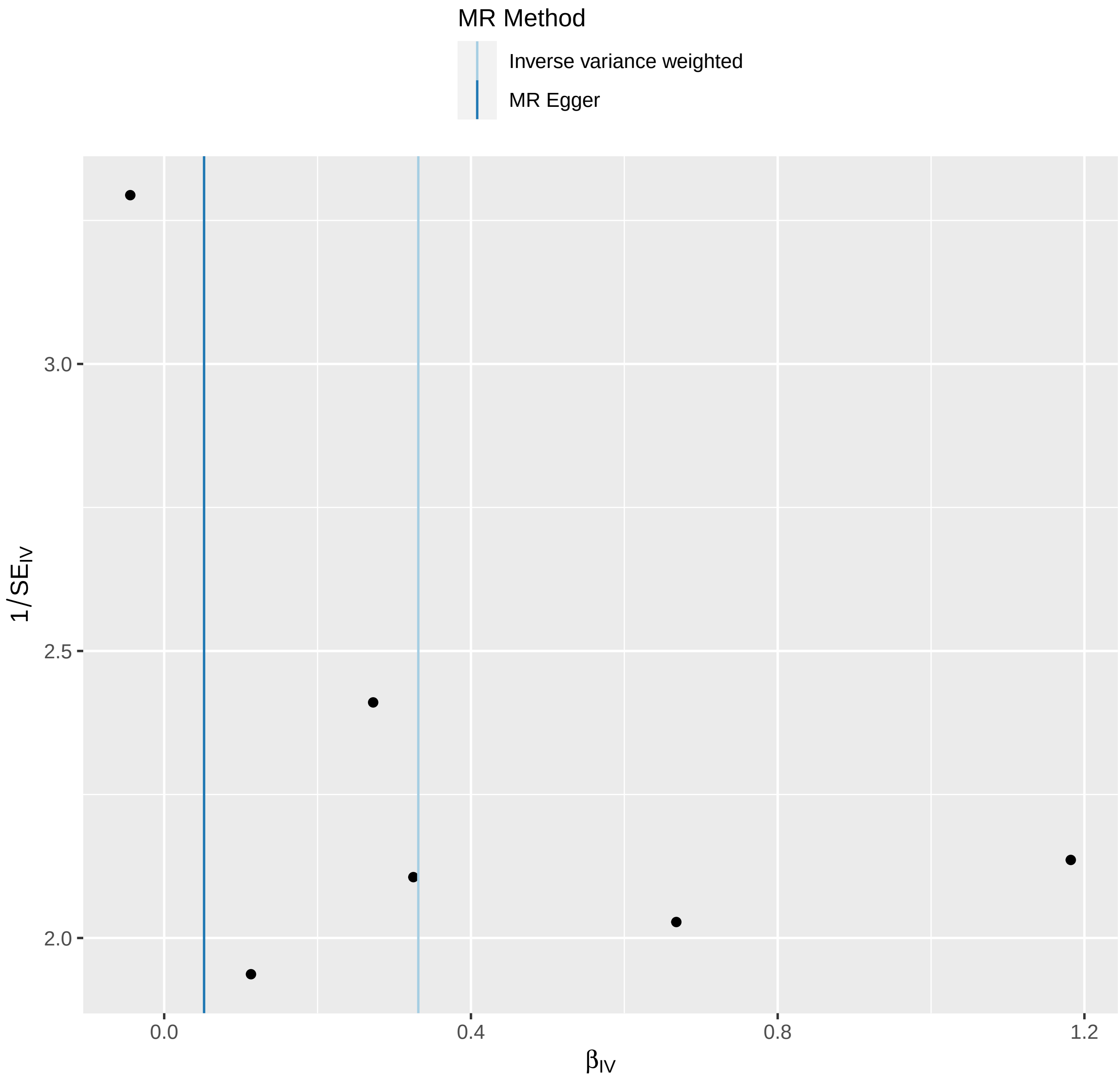

Batch 367 : Gut microbiota abundance (genus Erysipelatoclostridium id.11381) on Crohn's disease of small intestine

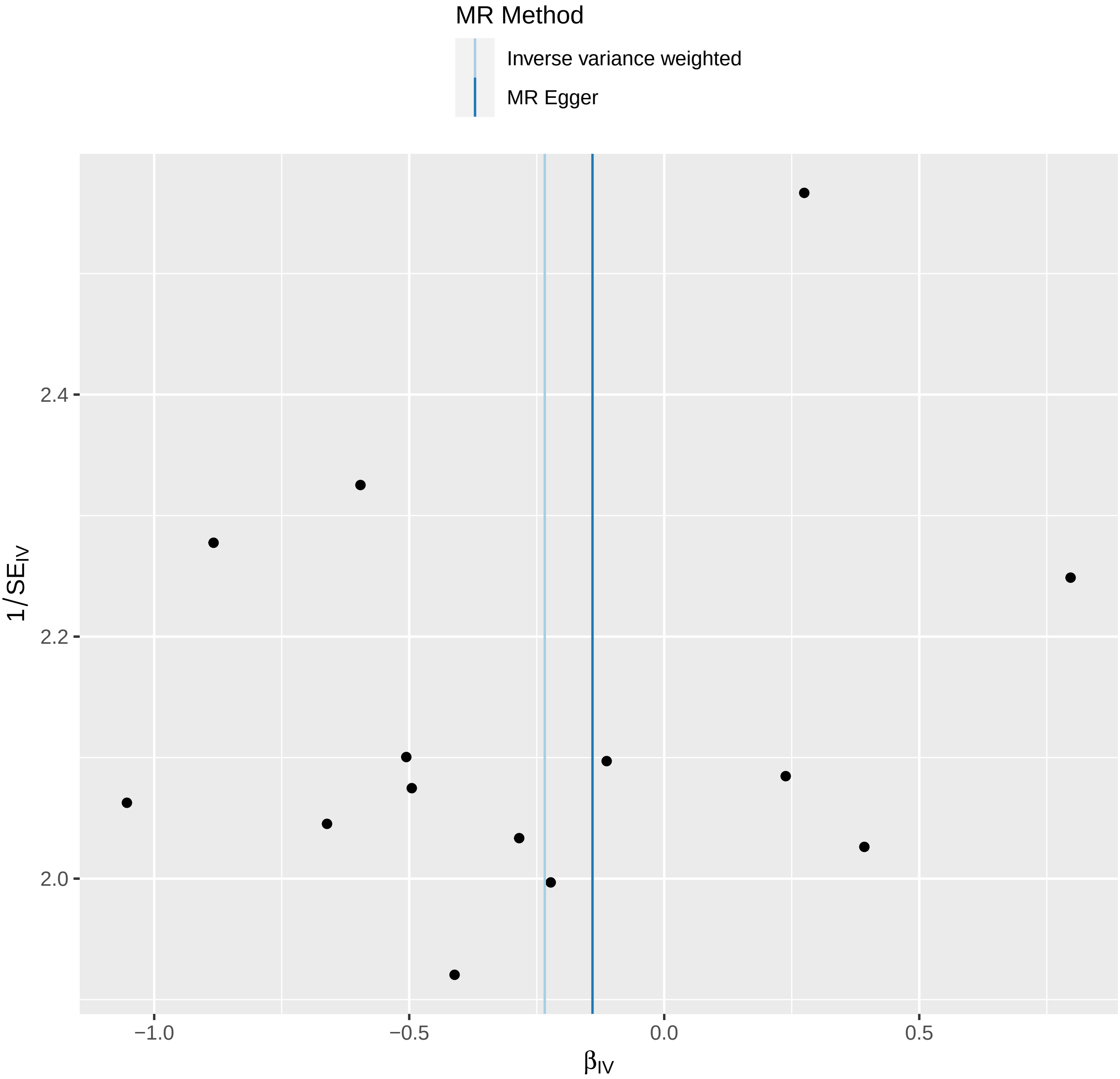

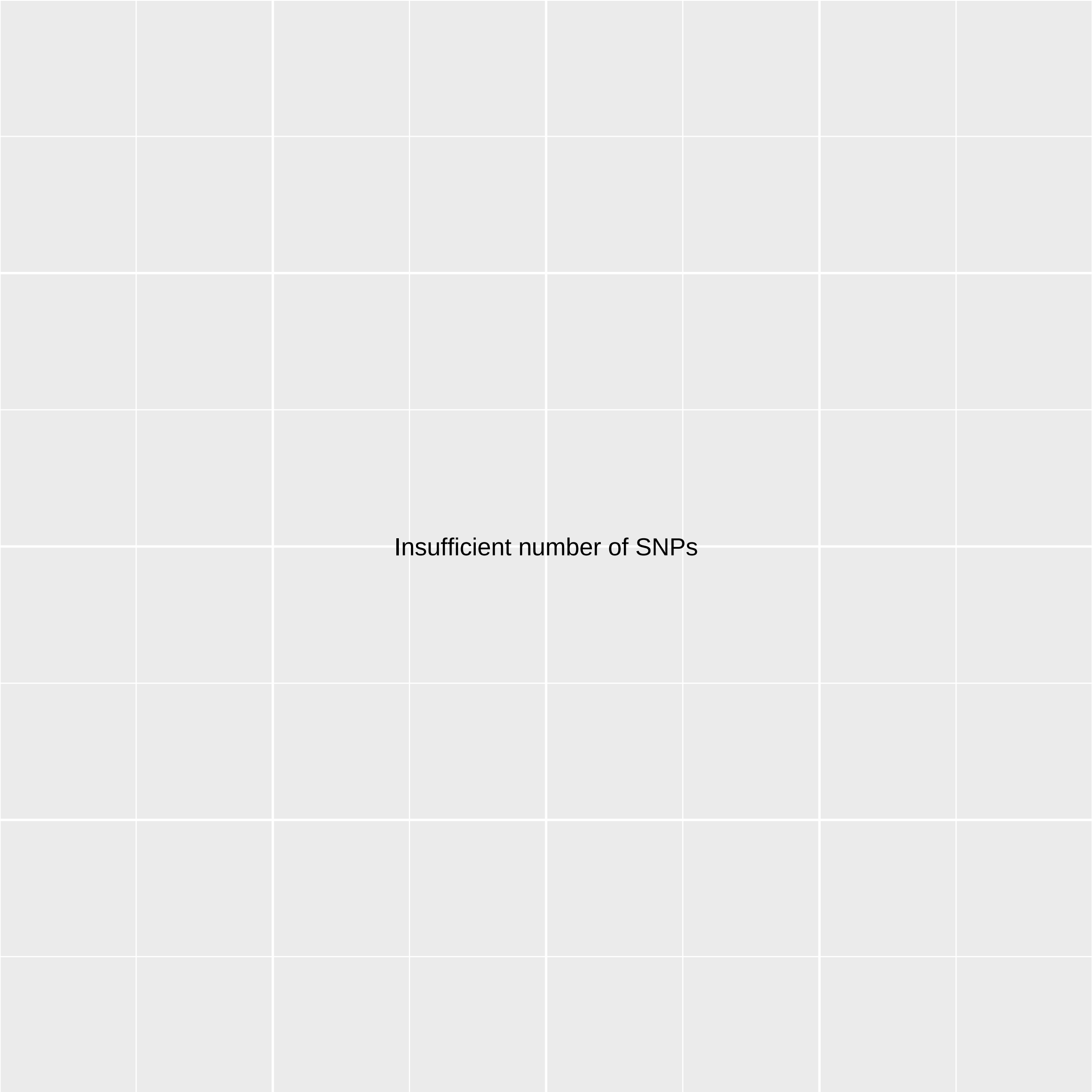
Insufficient number of SNPs

Batch 369 : Gut microbiota abundance (genus Escherichia Shigella id.3504) on Crohn's disease of small intestine

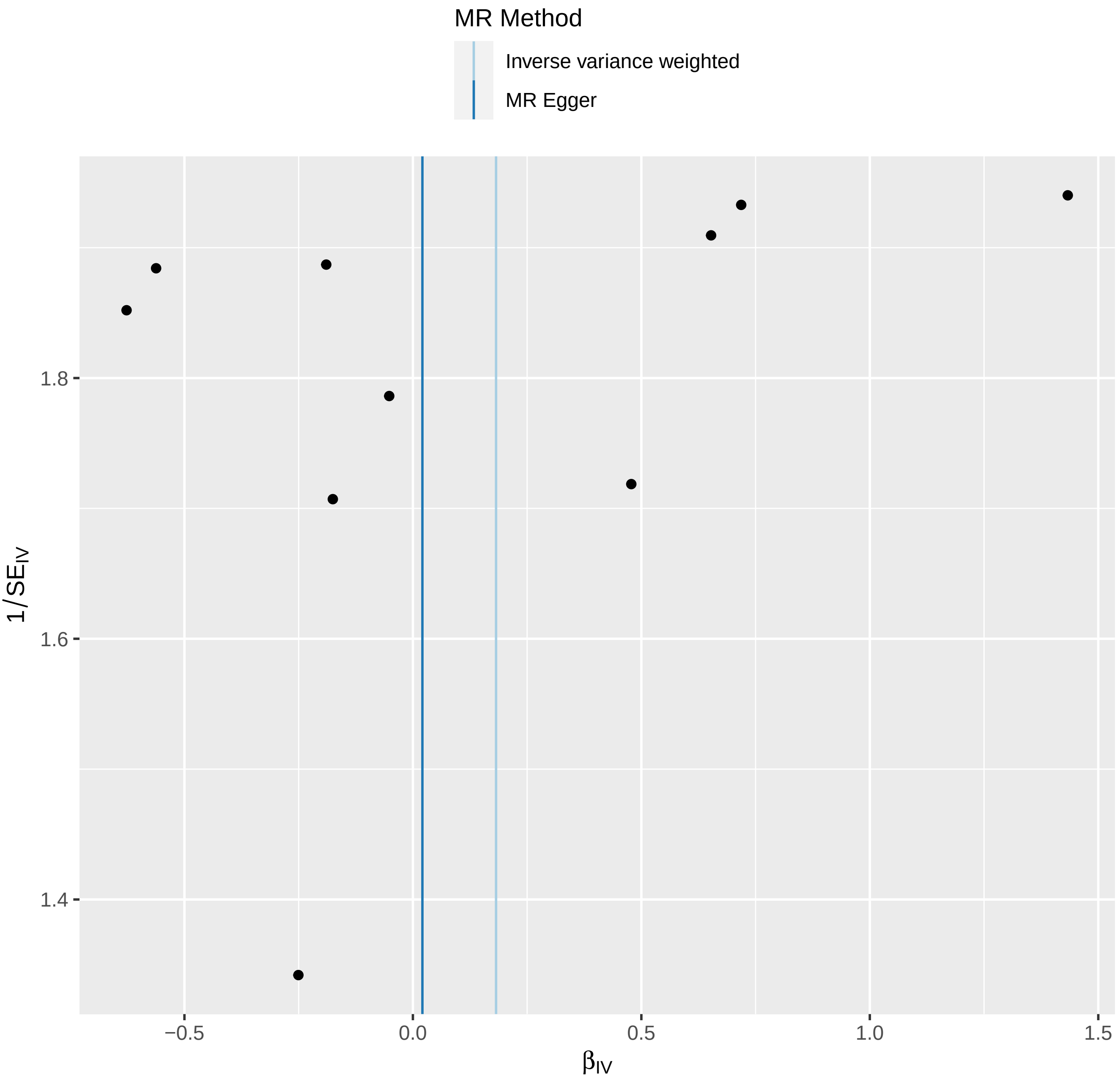

Batch 370 : Gut microbiota abundance (genus Eubacterium brachy group id.11296) on Crohn's disease of small intestine

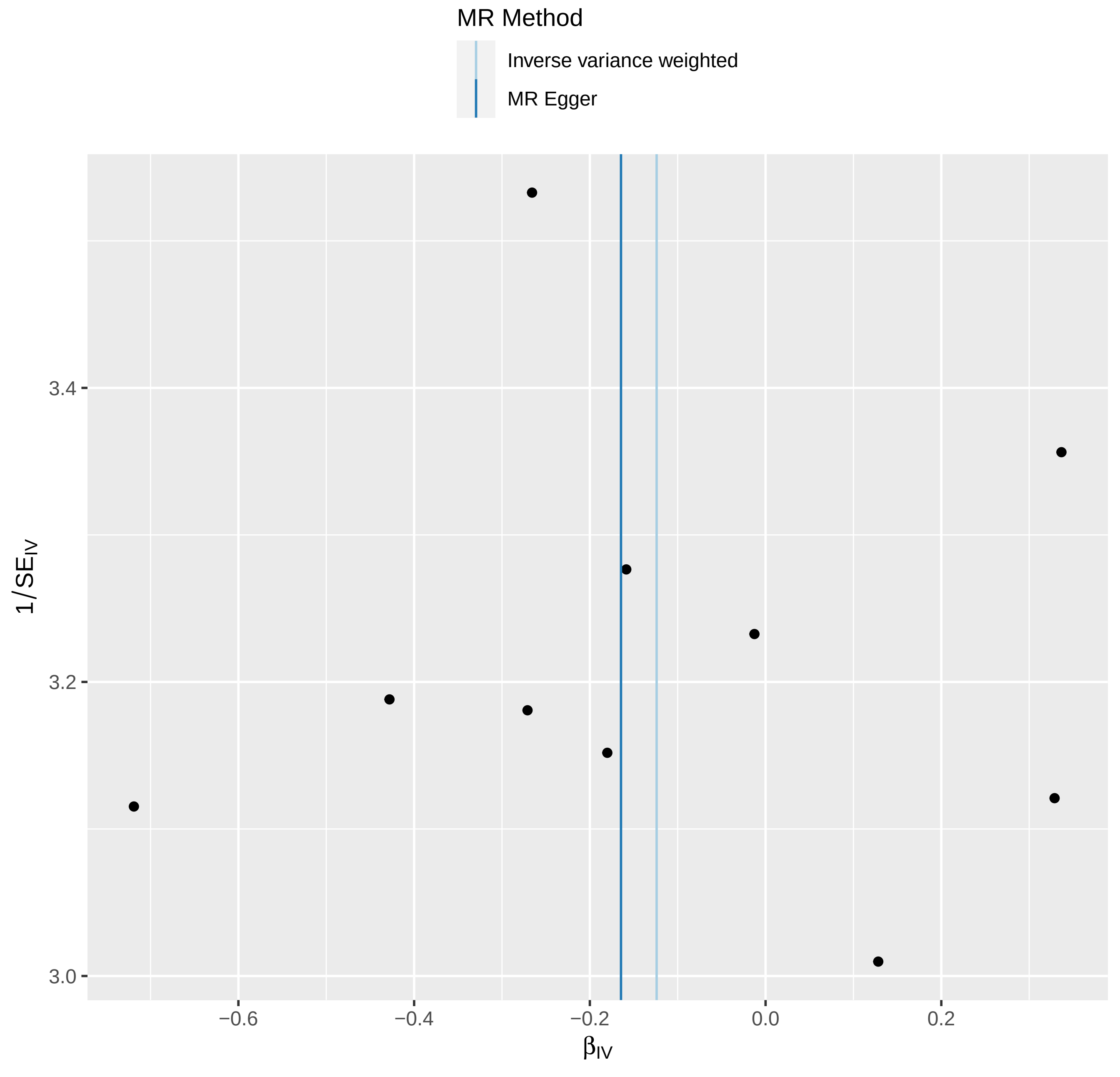

Batch 371 : Gut microbiota abundance (genus Eubacterium coprostanoligenes group id.11375) on Crohn's disease of small intestine

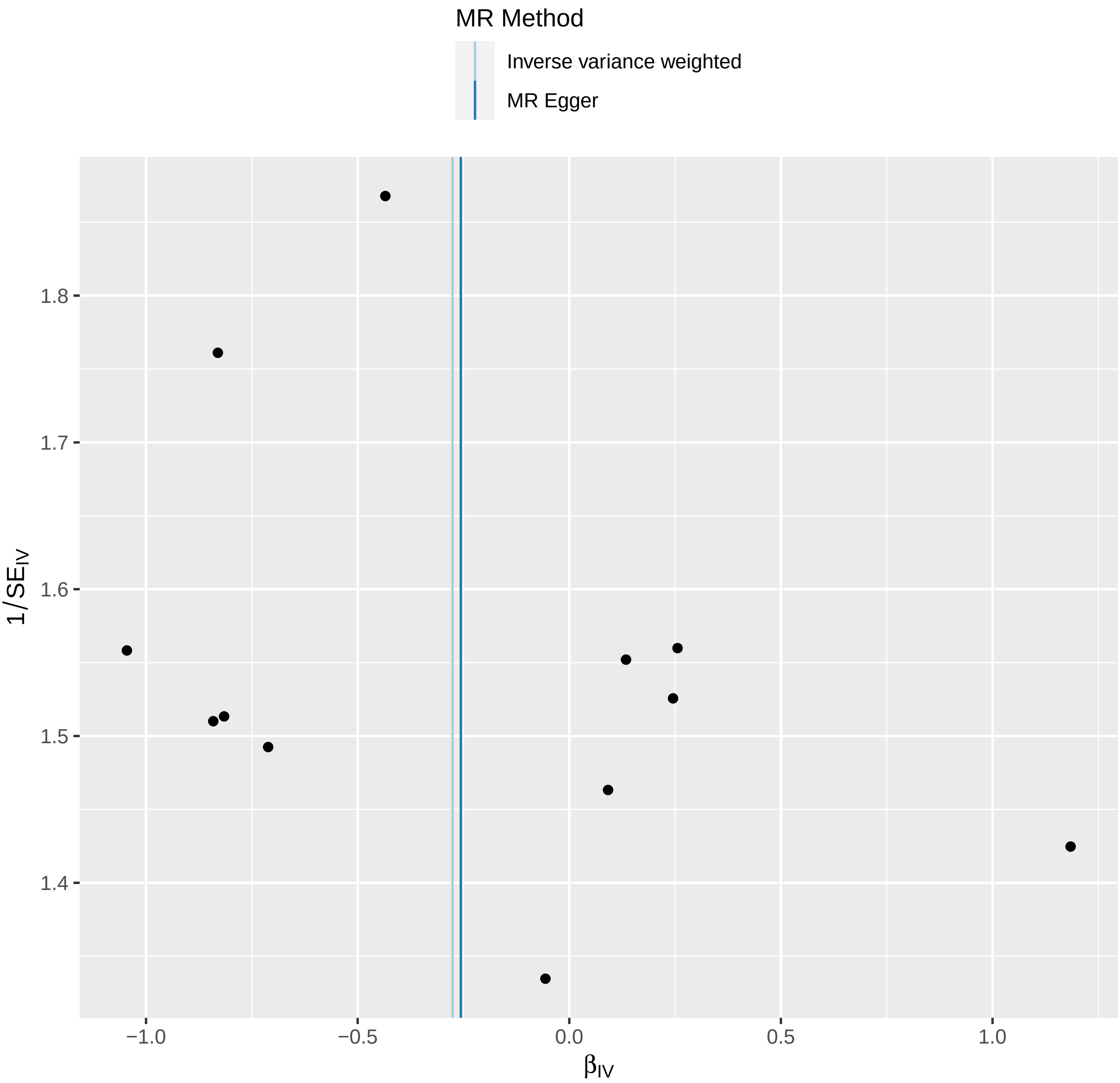

Batch 372 : Gut microbiota abundance (genus Eubacterium eligens group id.14372) on Crohn's disease of small intestine

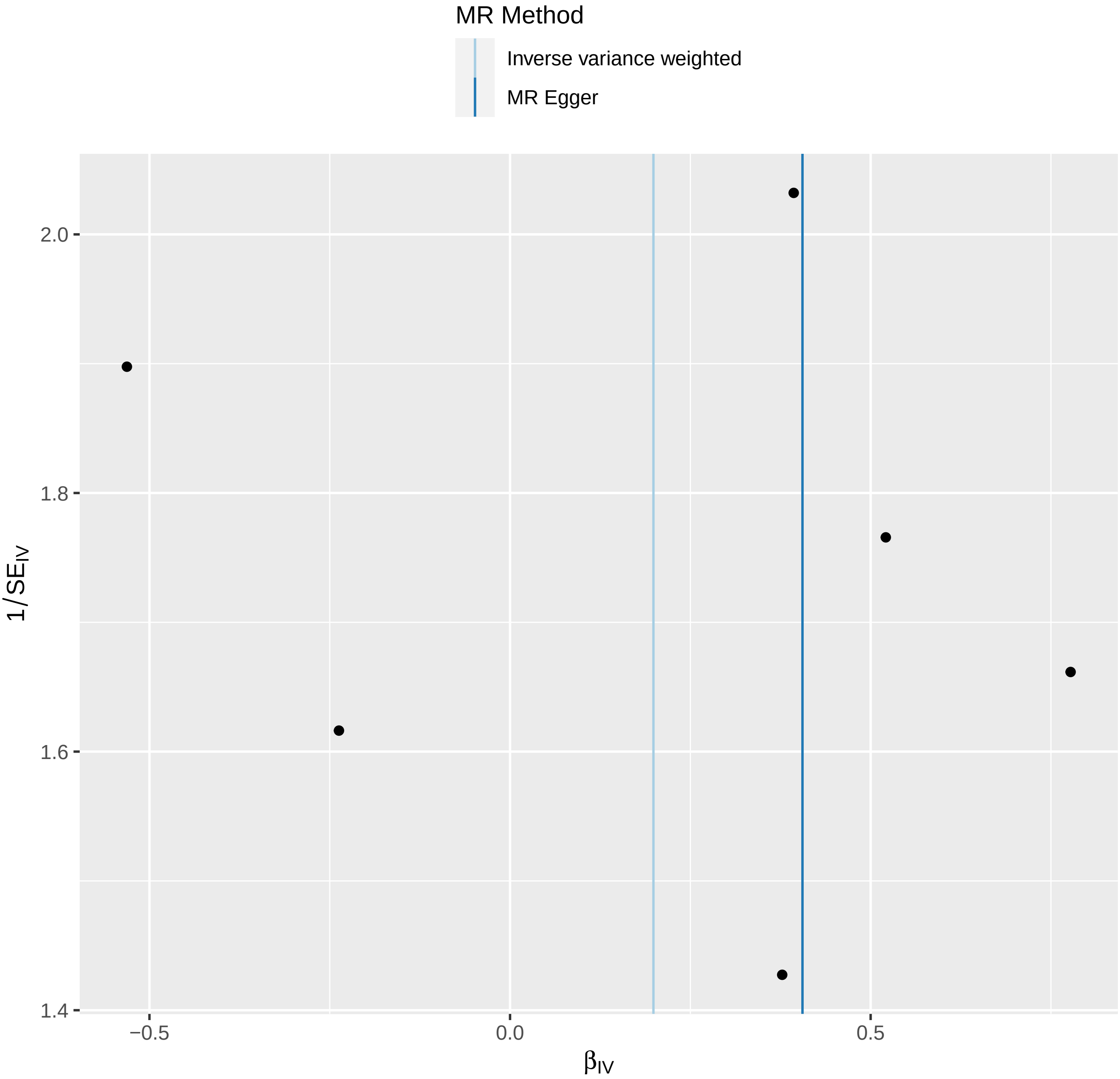

Batch 373 : Gut microbiota abundance (genus Eubacterium fissicatena group id.14373) on Crohn's disease of small intestine

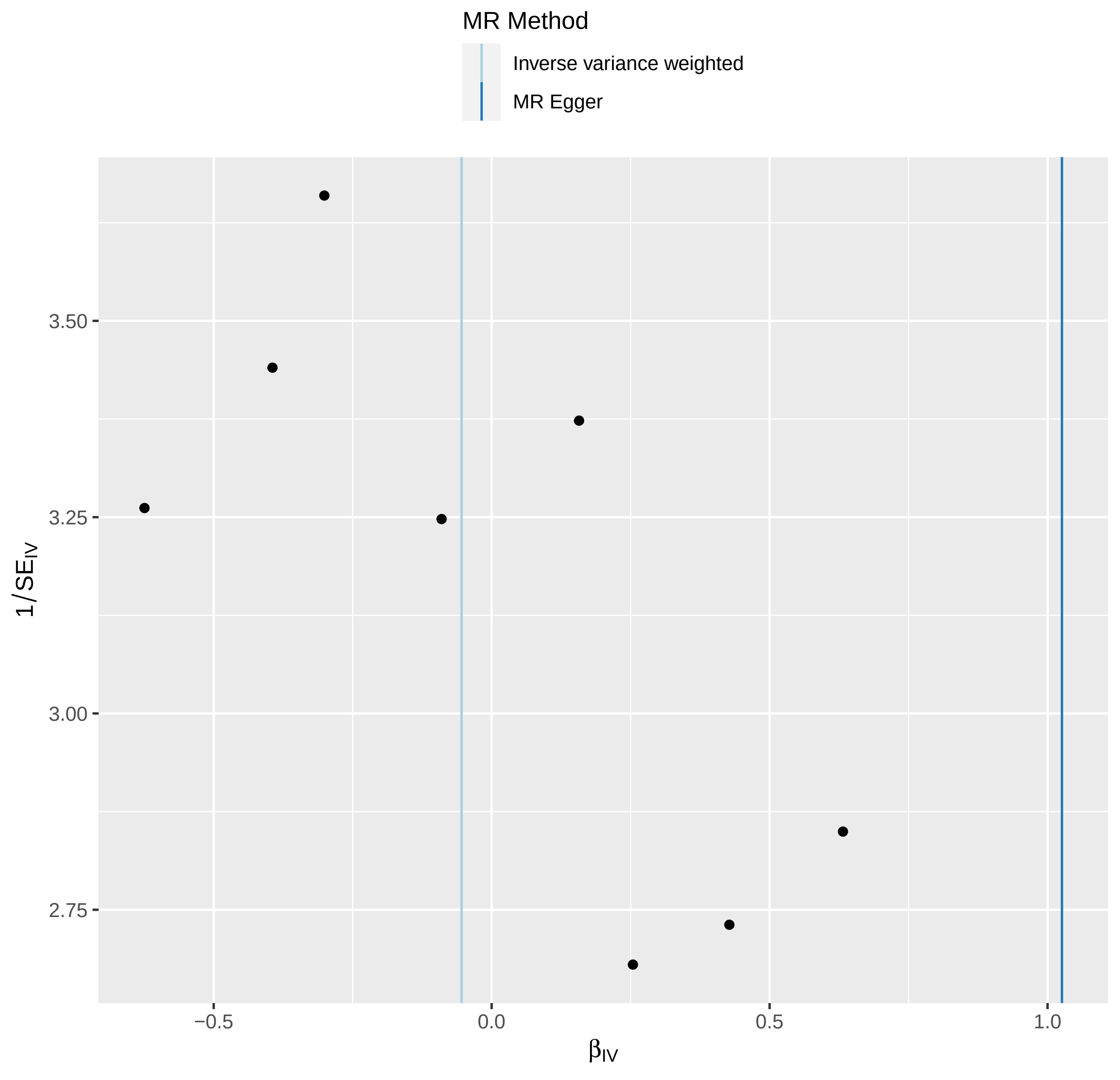

Batch 374 : Gut microbiota abundance (genus Eubacterium hallii group id.11338) on Crohn's disease of small intestine

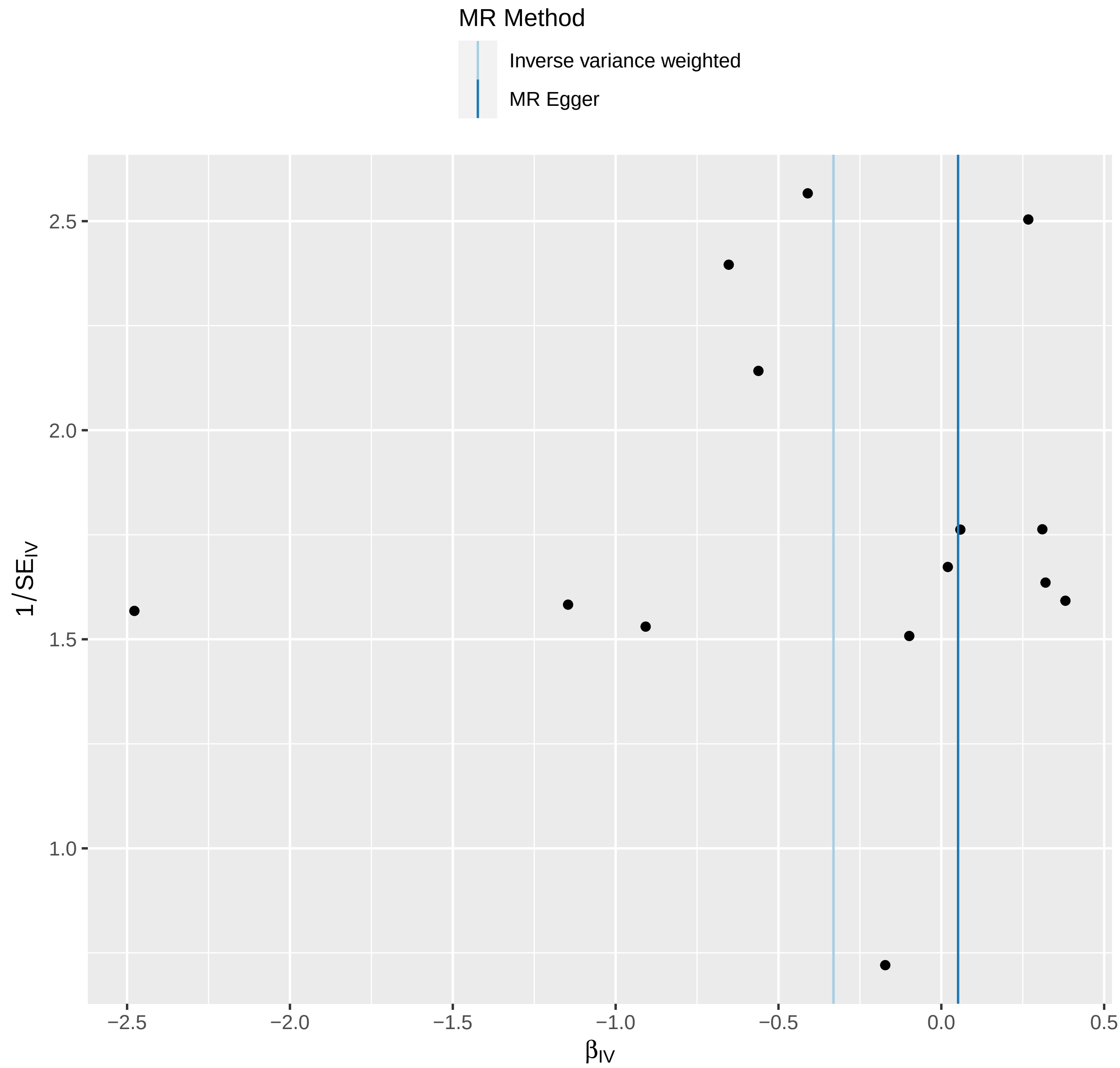

Batch 375 : Gut microbiota abundance (genus Eubacterium nodatum group id.11297) on Crohn's disease of small intestine

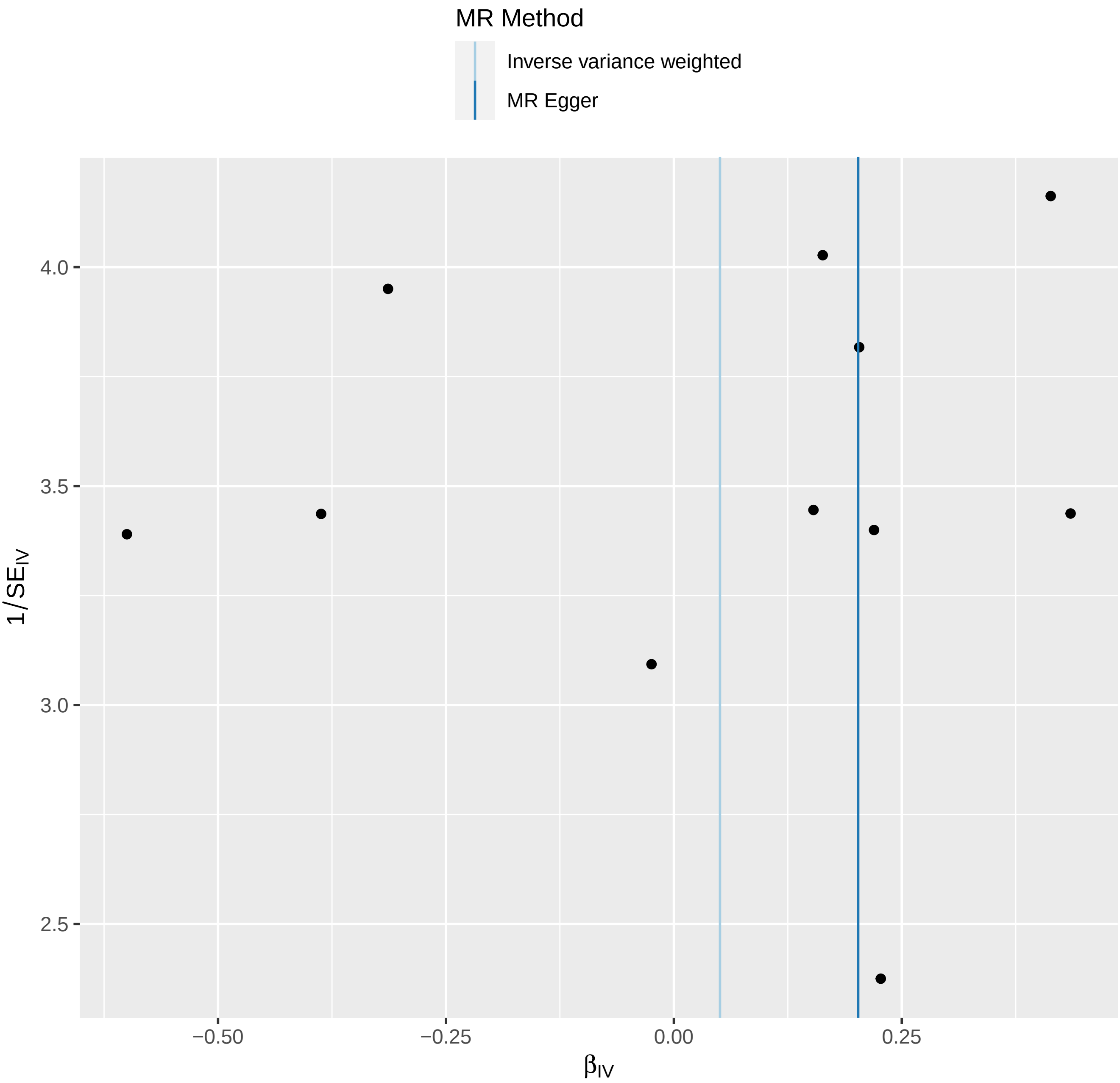

Batch 376 : Gut microbiota abundance (genus Eubacterium oxidoreducens group id.11339) on Crohn's disease of small intestine

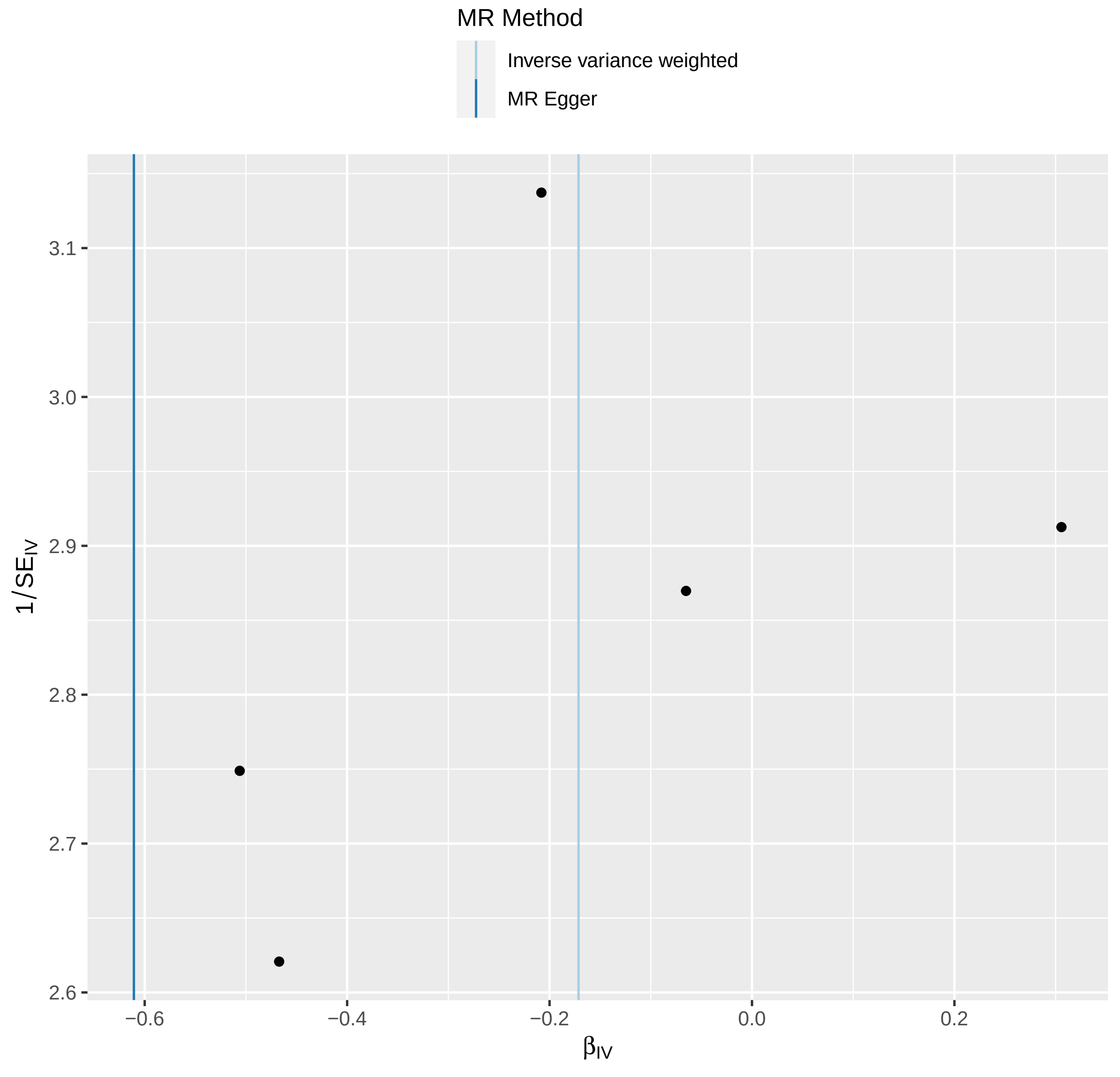

Batch 377 : Gut microbiota abundance (genus Eubacterium rectale group id.14374) on Crohn's disease of small intestine

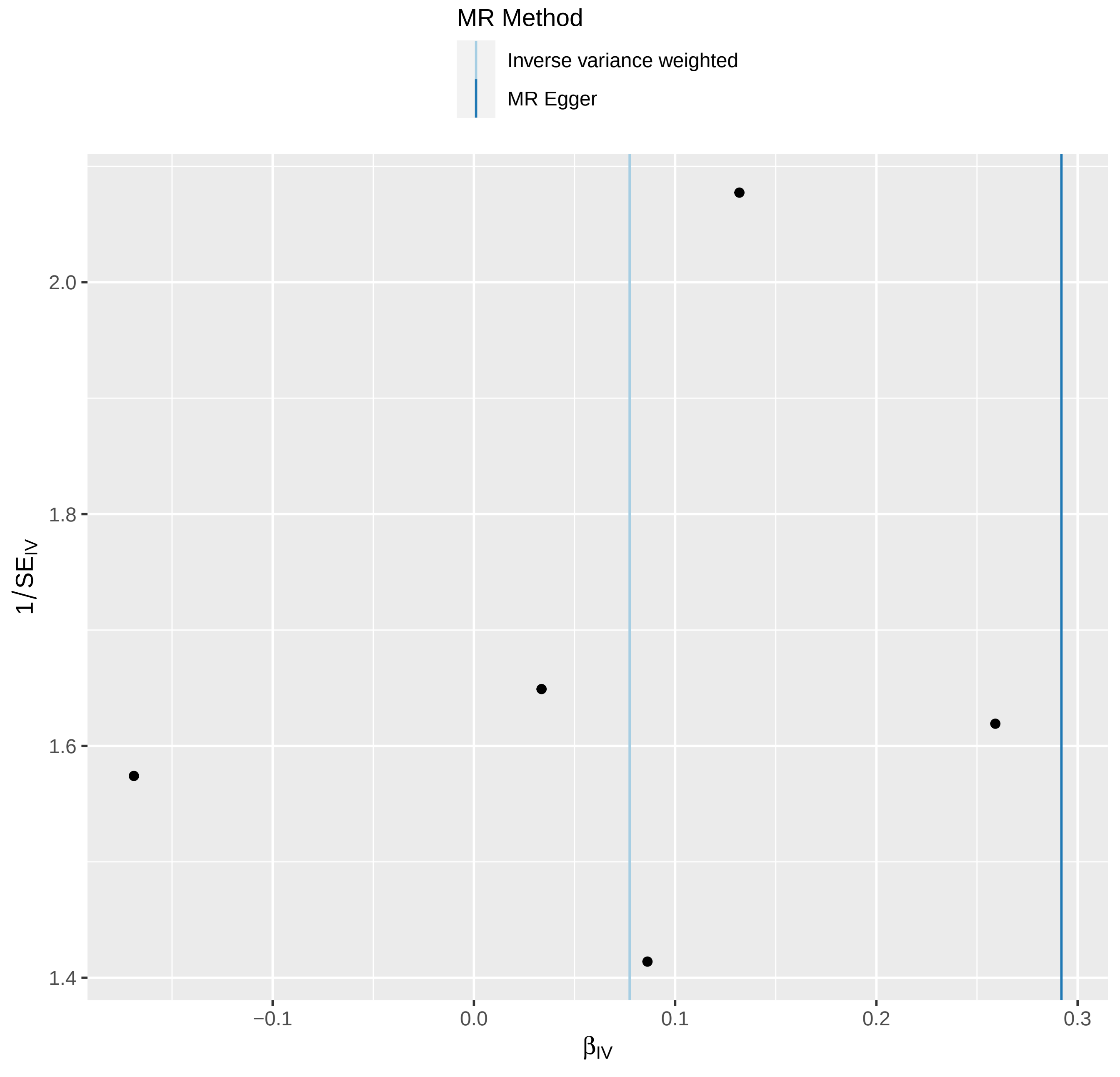

Batch 378 : Gut microbiota abundance (genus Eubacterium ruminantium group id.11340) on Crohn's disease of small intestine

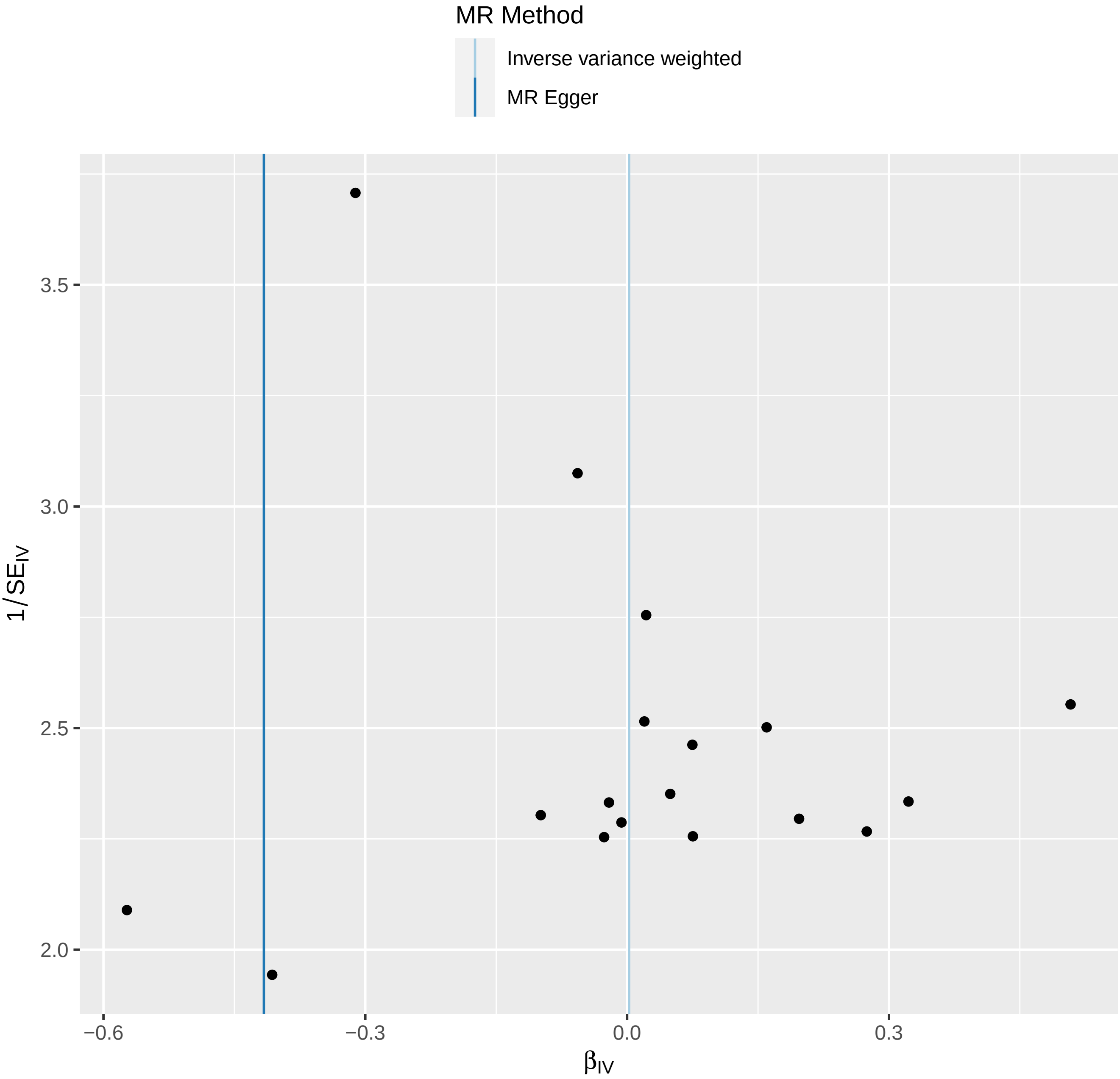

Batch 379 : Gut microbiota abundance (genus Eubacterium ventriosum group id.11341) on Crohn's disease of small intestine

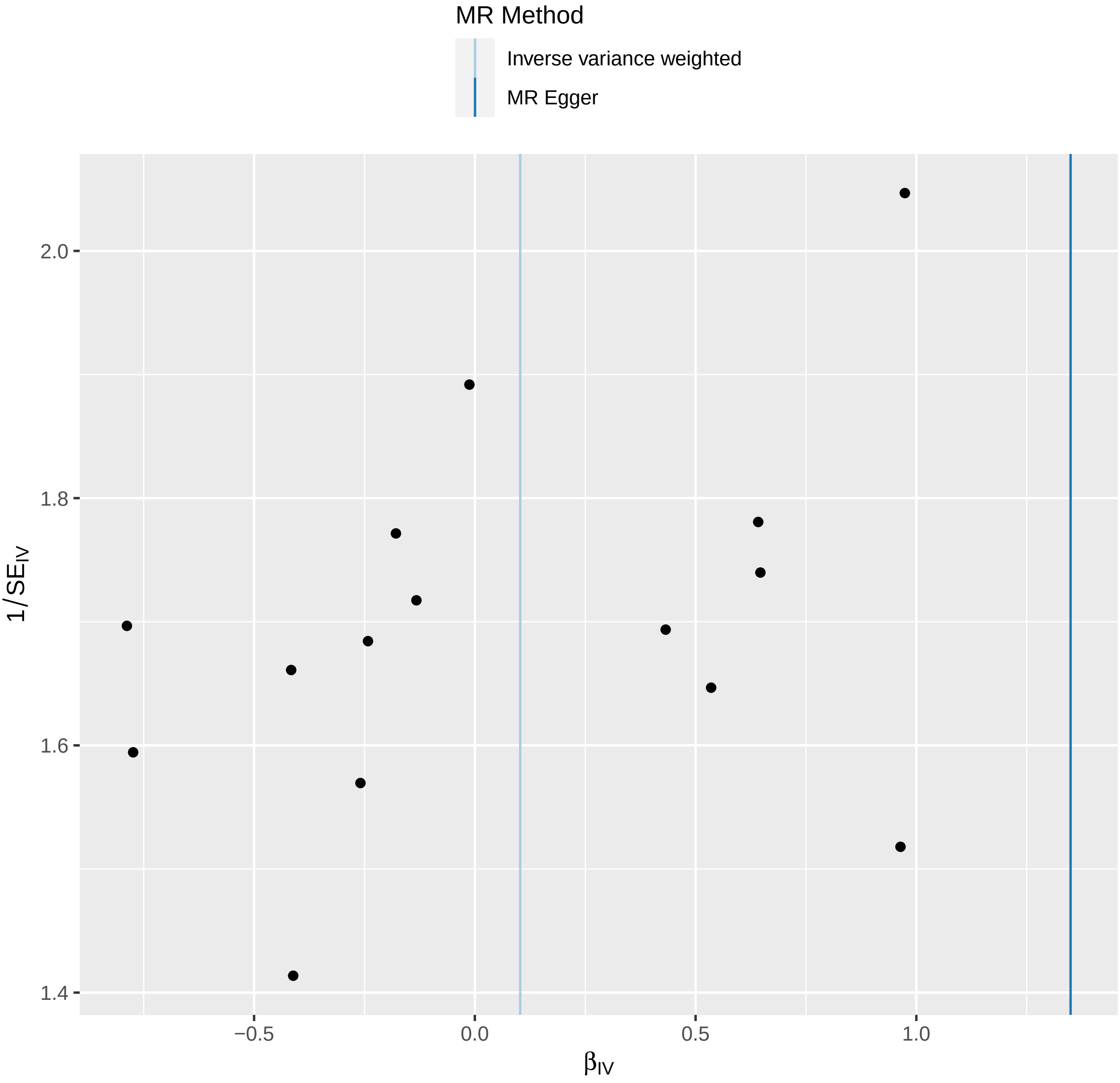

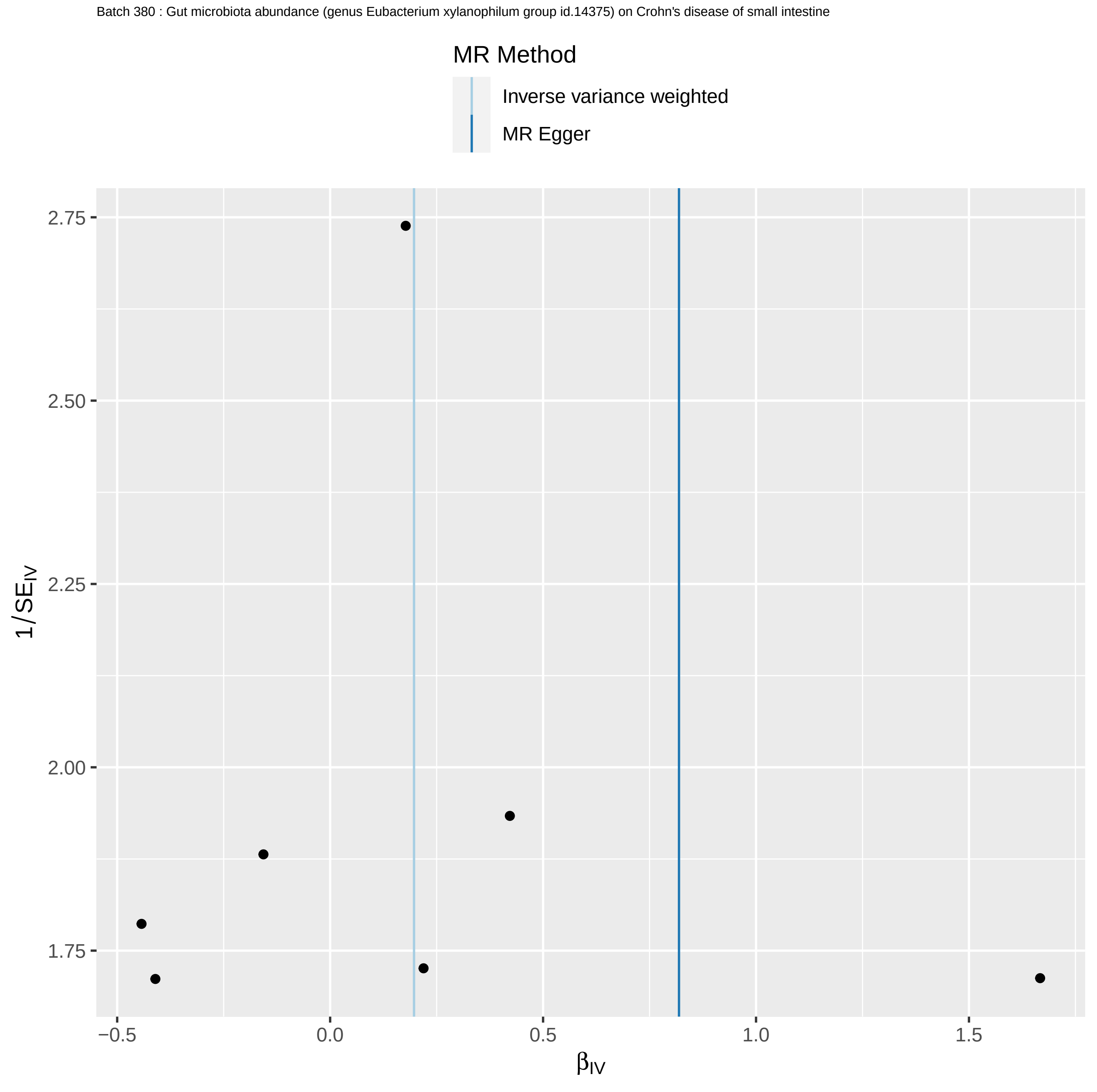

Batch 380 : Gut microbiota abundance (genus Eubacterium xylanophilum group id.14375) on Crohn's disease of small intestine
MR Method
Inverse variance weighted
MR Egger
2.75
2.50
2.25
2.00
1.75
1/SE_IV
−0.5
0.0
0.5
1.0
1.5
β_IV

Batch 381 : Gut microbiota abundance (genus Faecalibacterium id.2057) on Crohn's disease of small intestine

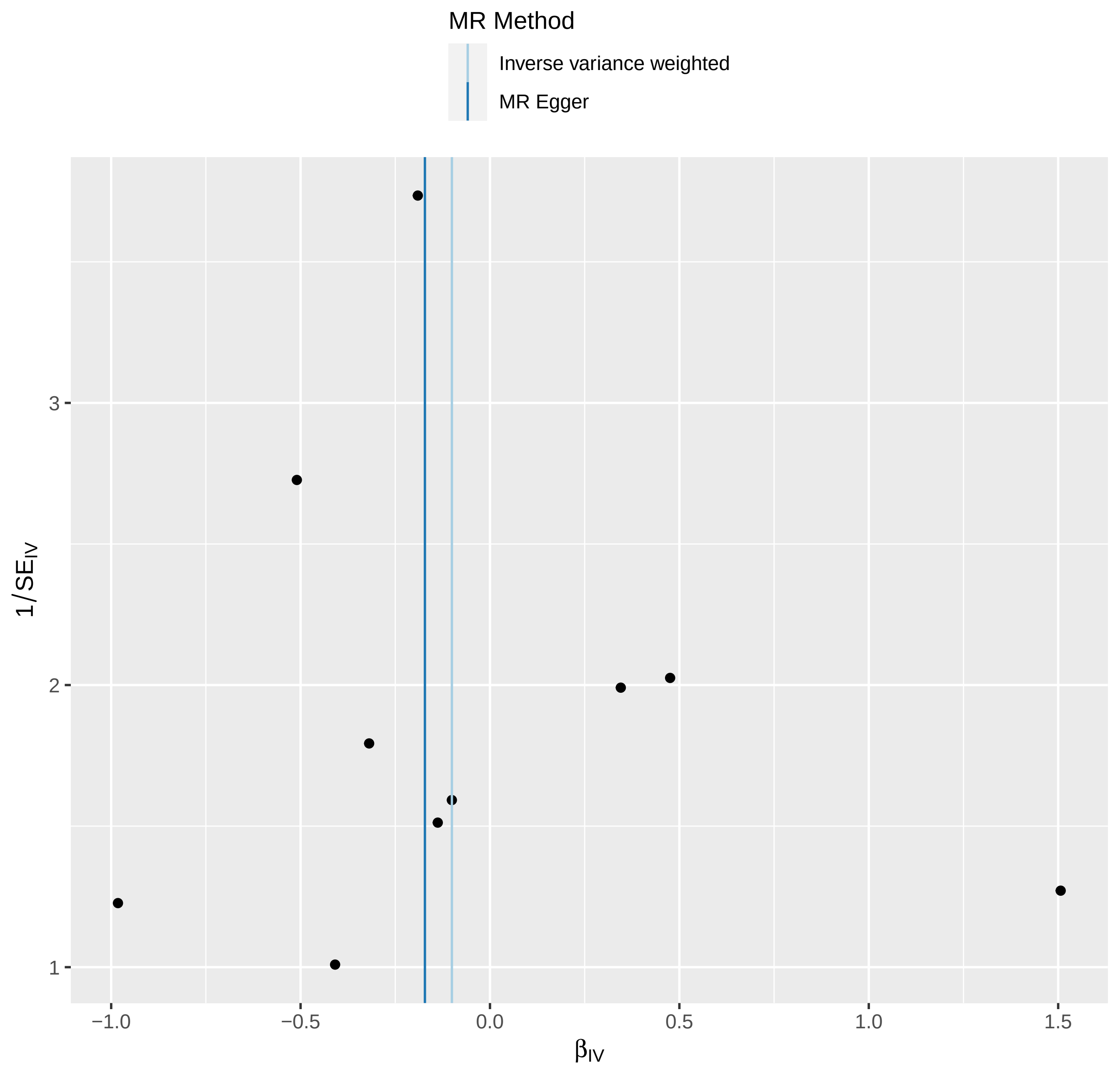

Batch 382 : Gut microbiota abundance (genus Family XIII AD3011 group id.11293) on Crohn's disease of small intestine

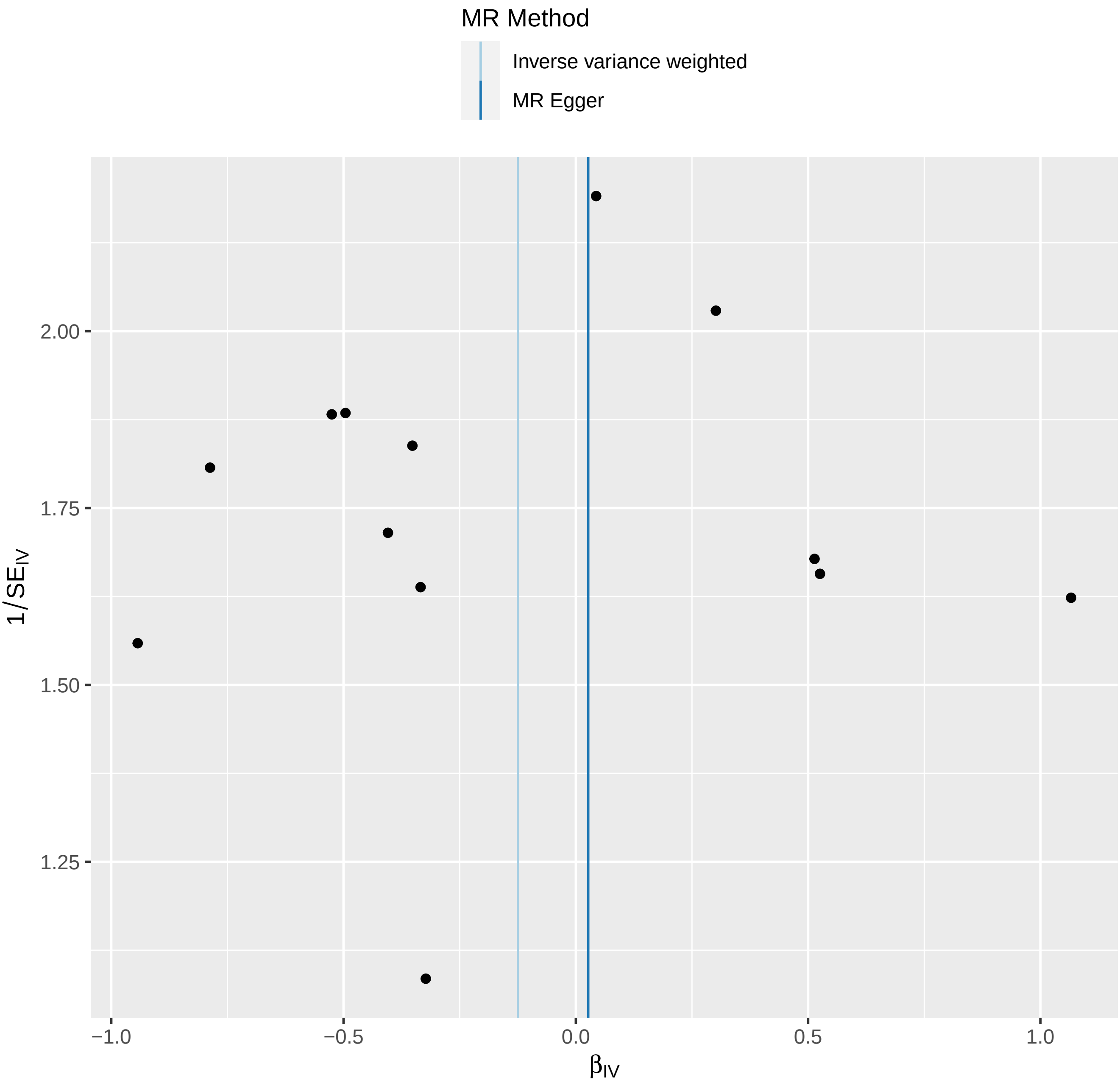

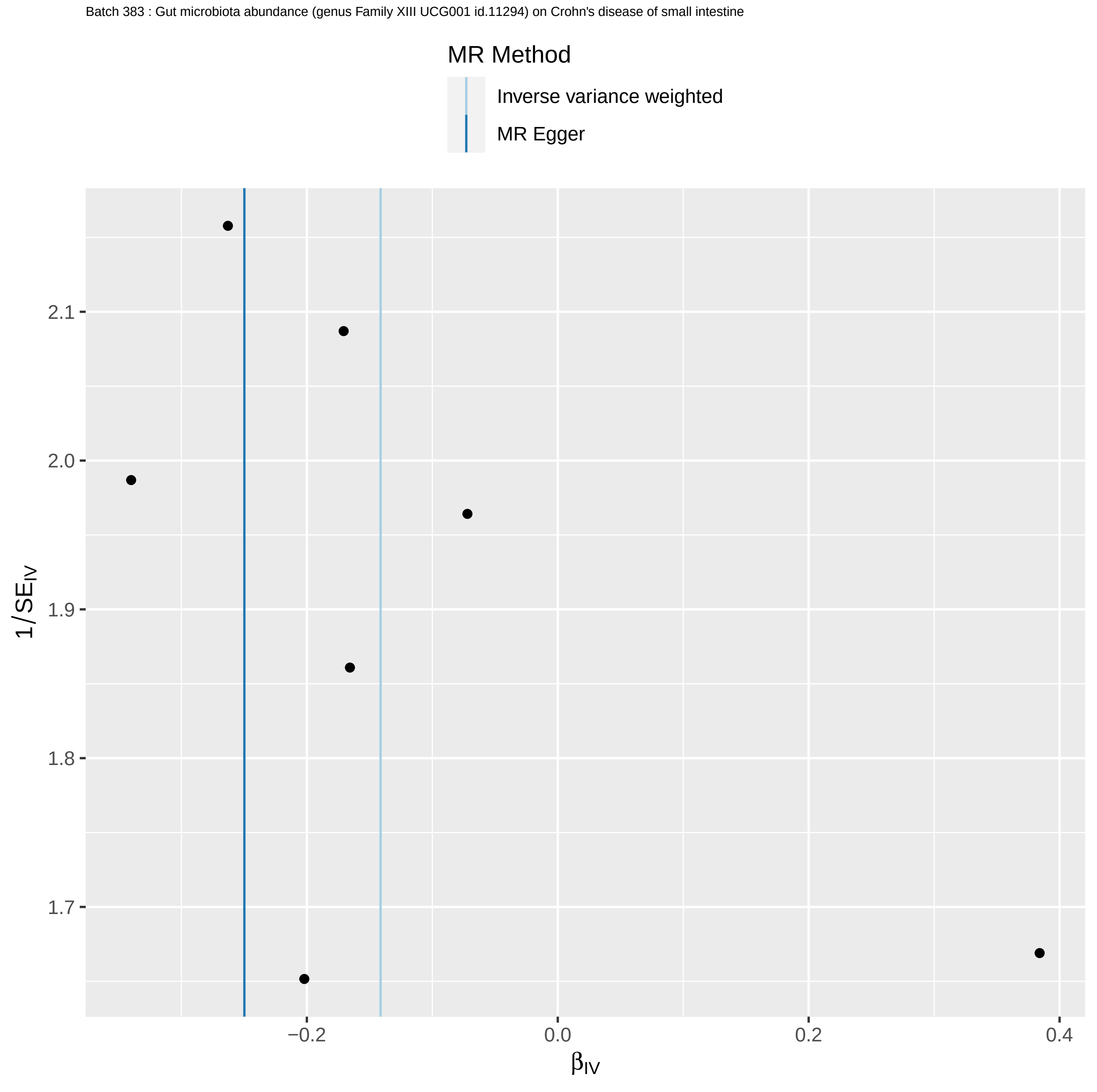

Batch 383 : Gut microbiota abundance (genus Family XIII UCG001 id.11294) on Crohn's disease of small intestine
MR Method
Inverse variance weighted
MR Egger
1/SE$_{IV}$
2.1
2.0
1.9
1.8
1.7
−0.2
0.0
0.2
0.4
β_{IV}

Batch 384 : Gut microbiota abundance (genus Flavonifractor id.2059) on Crohn's disease of small intestine

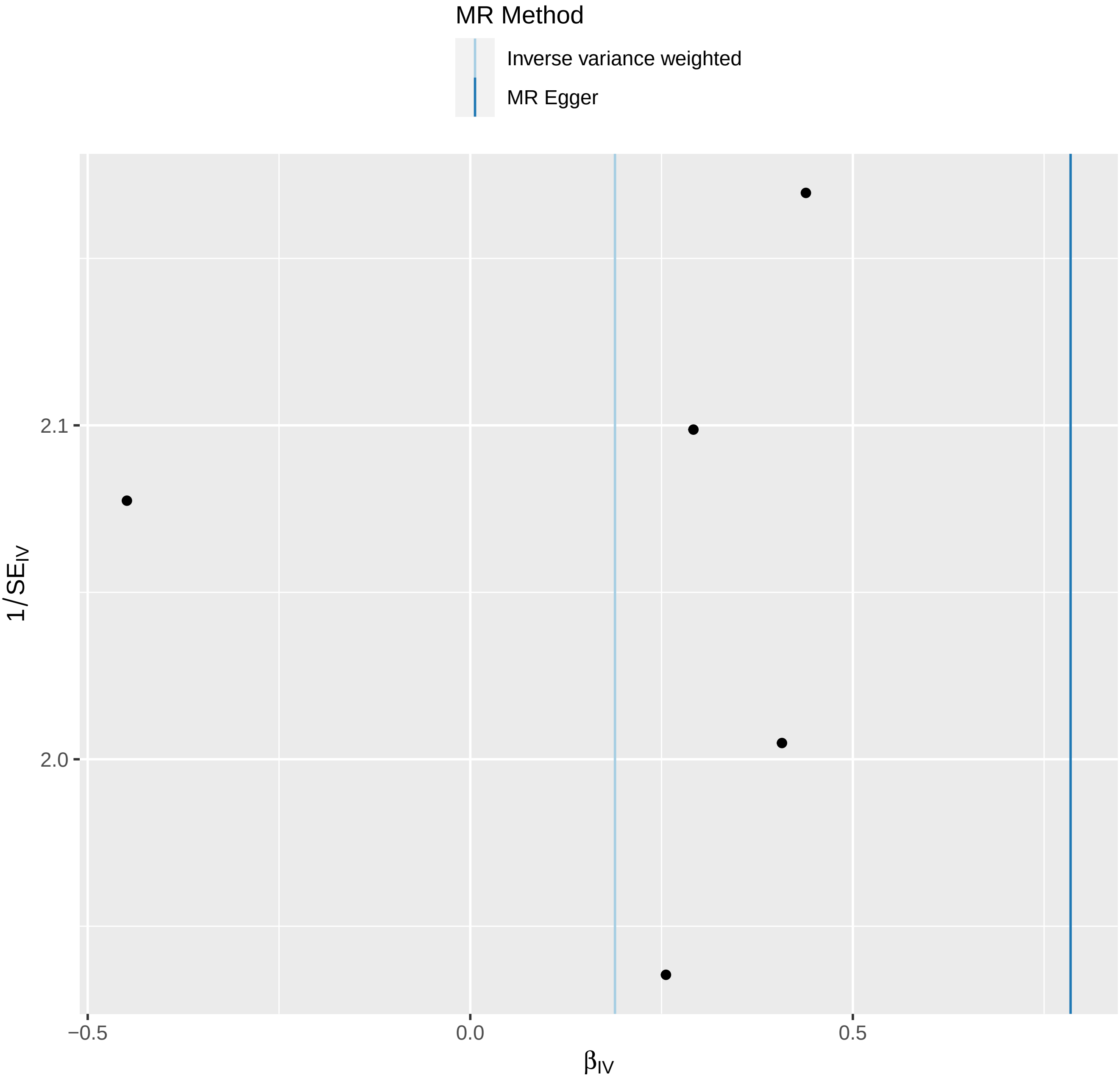

Batch 385 : Gut microbiota abundance (genus Fusicatenibacter id.11305) on Crohn's disease of small intestine

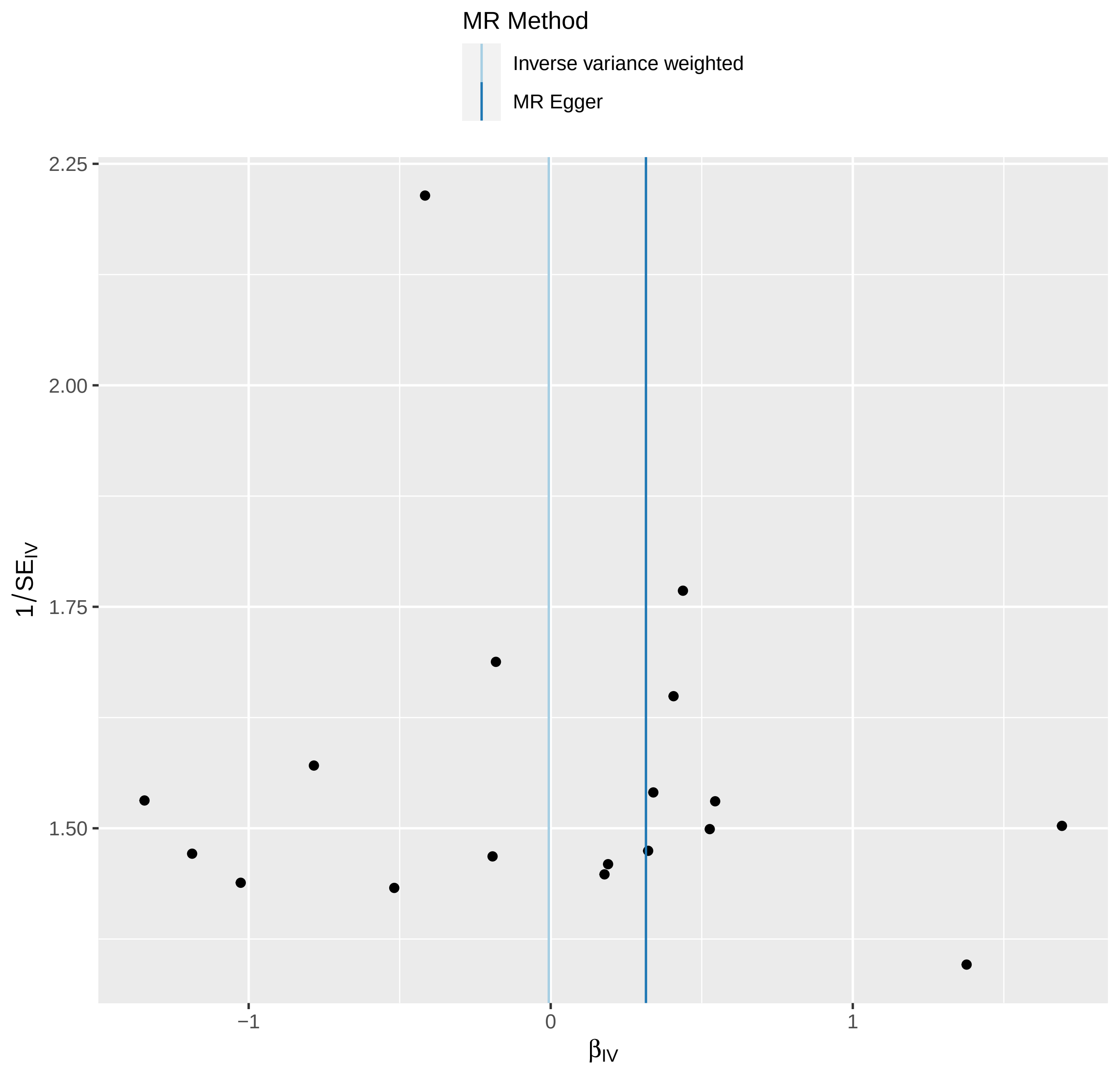

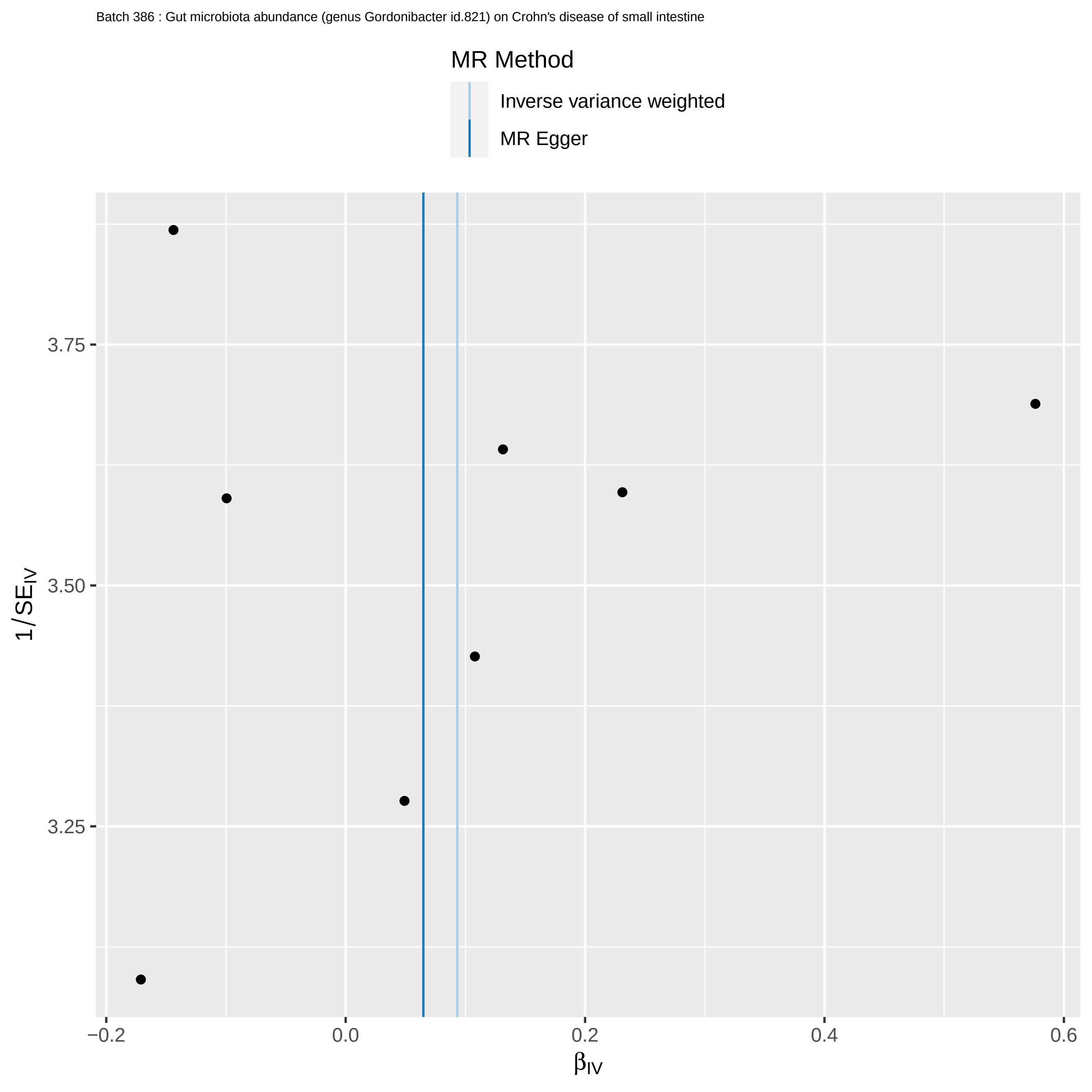
Batch 386 : Gut microbiota abundance (genus Gordonibacter id.821) on Crohn's disease of small intestine
MR Method
Inverse variance weighted
MR Egger
3.75
3.50
3.25
1/SE_IV
−0.2
0.0
0.2
0.4
0.6
β_IV

Batch 387 : Gut microbiota abundance (genus Haemophilus id.3698) on Crohn's disease of small intestine

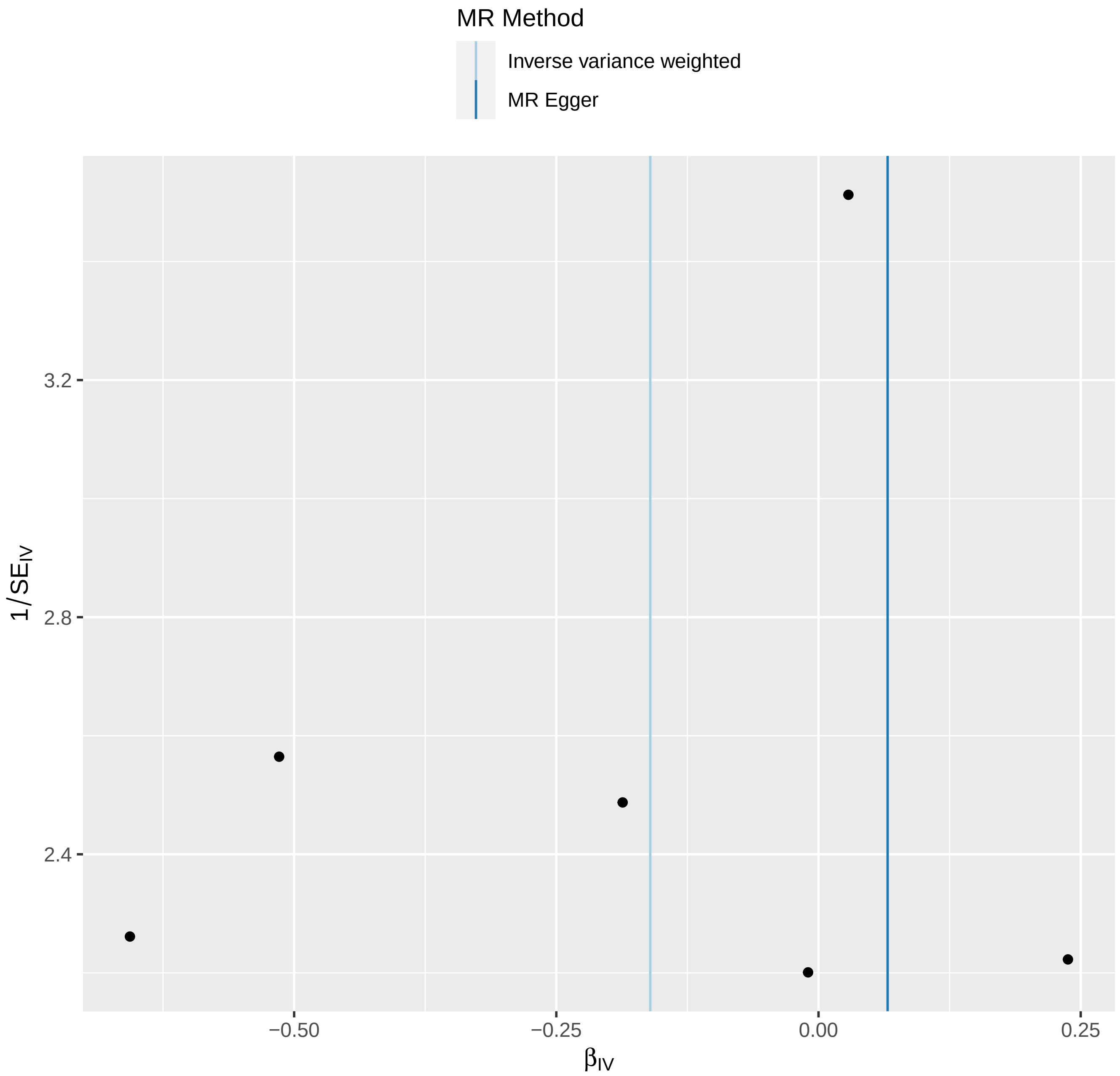

Batch 388 : Gut microbiota abundance (genus Holdemanella id.11393) on Crohn's disease of small intestine

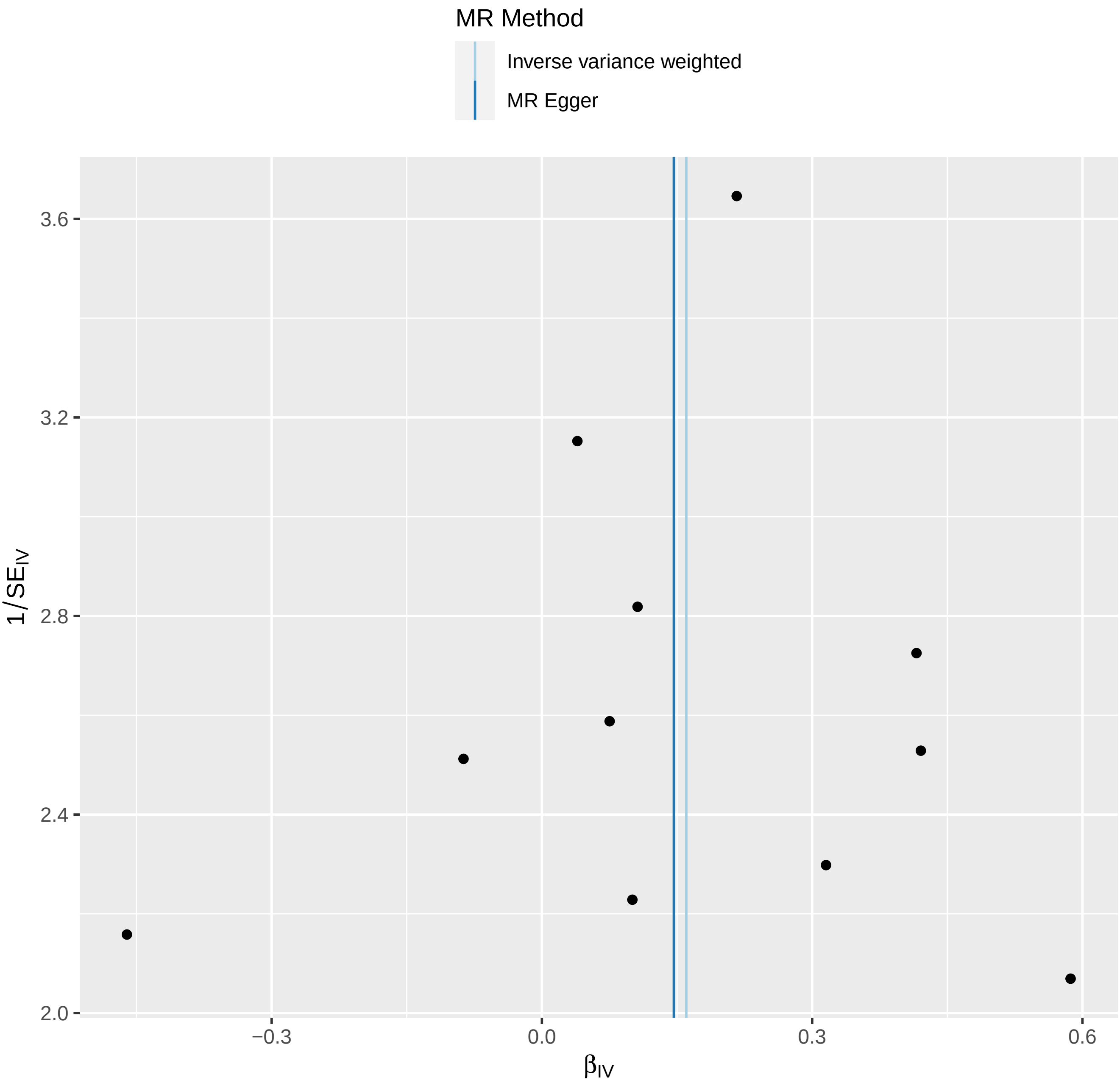

Batch 389 : Gut microbiota abundance (genus Holdemania id.2157) on Crohn's disease of small intestine

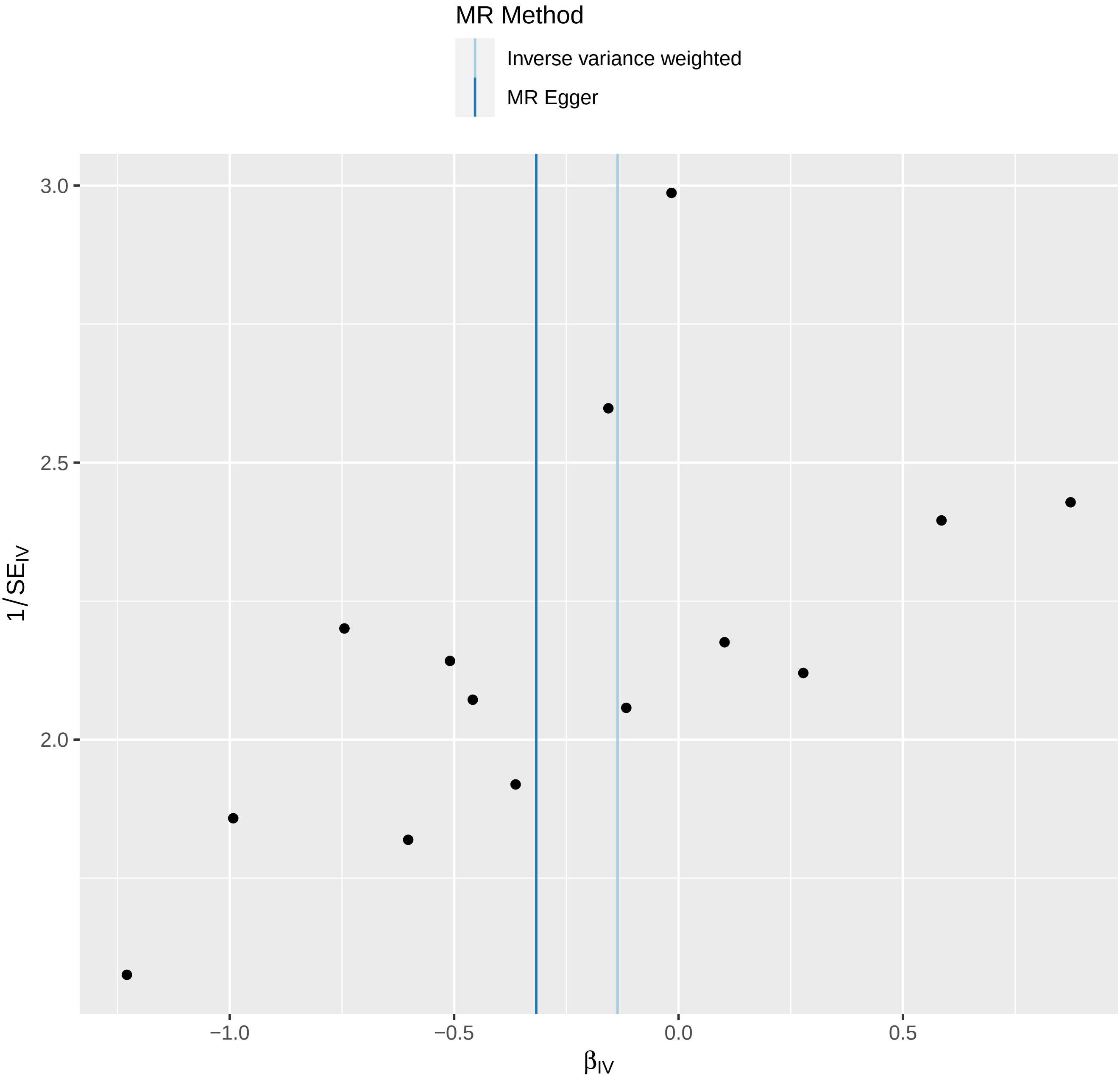

Batch 390 : Gut microbiota abundance (genus Howardella id.2000) on Crohn's disease of small intestine

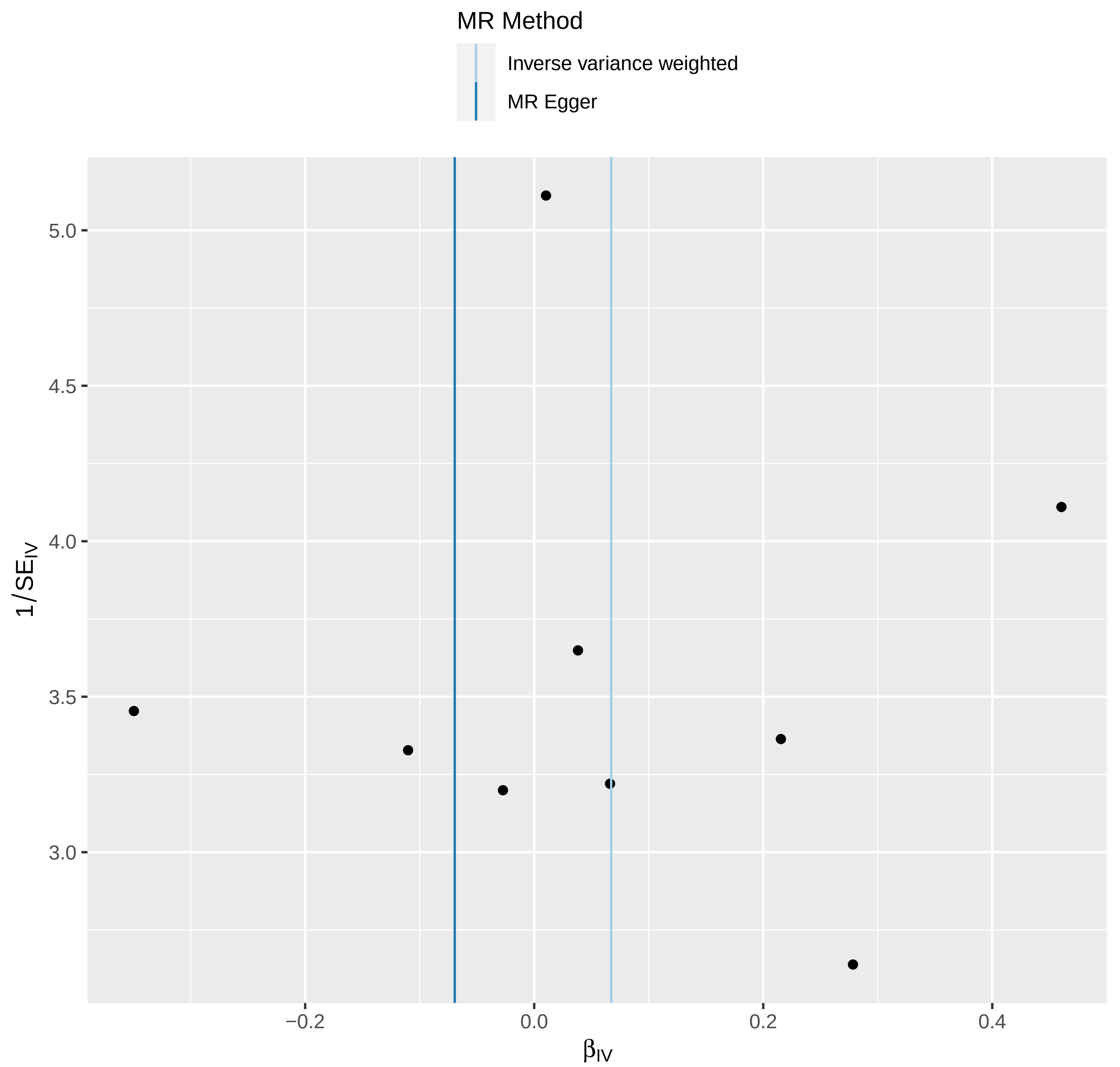

Batch 391 : Gut microbiota abundance (genus Hungatella id.11306) on Crohn's disease of small intestine

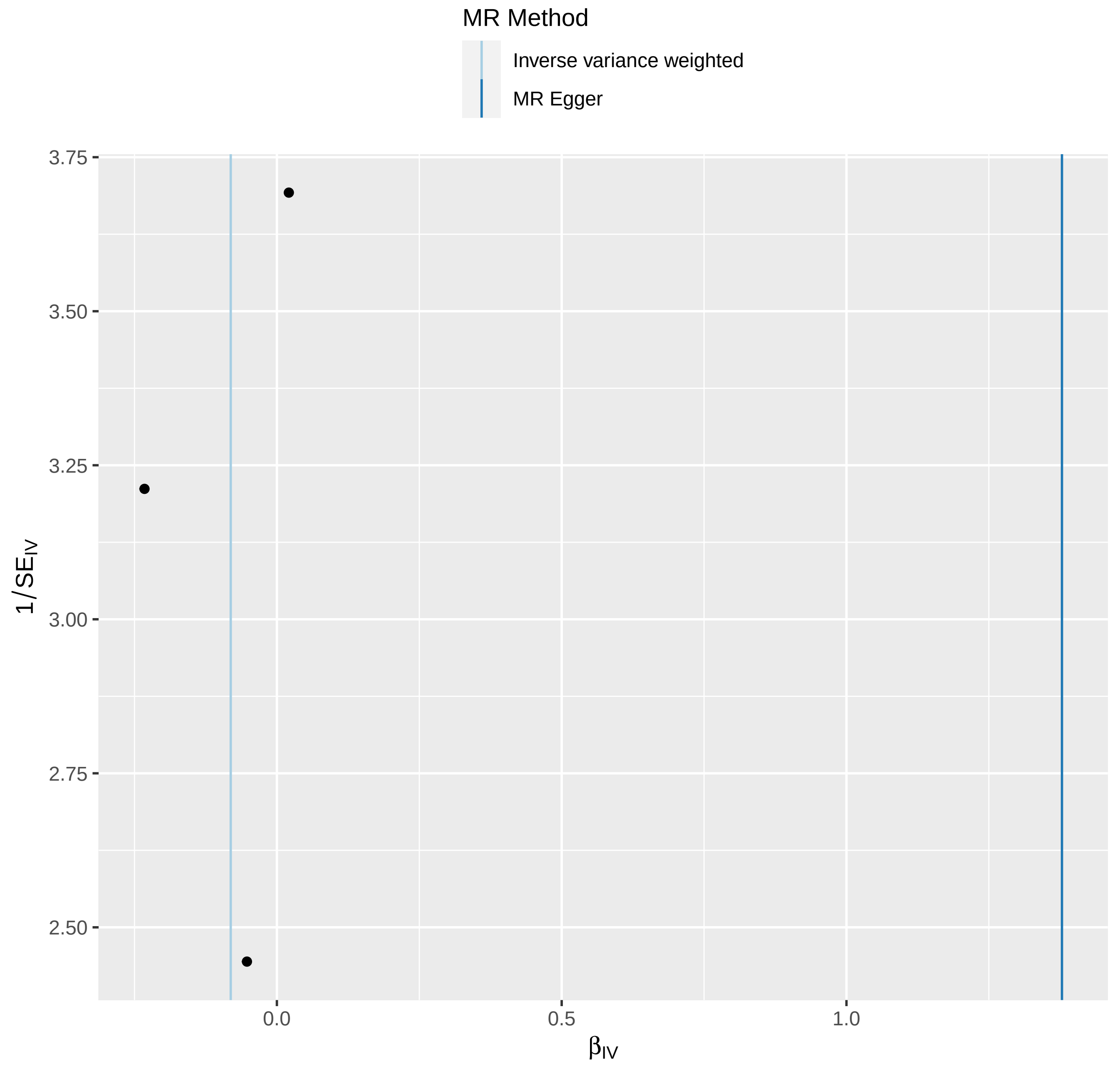

Batch 392 : Gut microbiota abundance (genus Intestinibacter id.11345) on Crohn's disease of small intestine

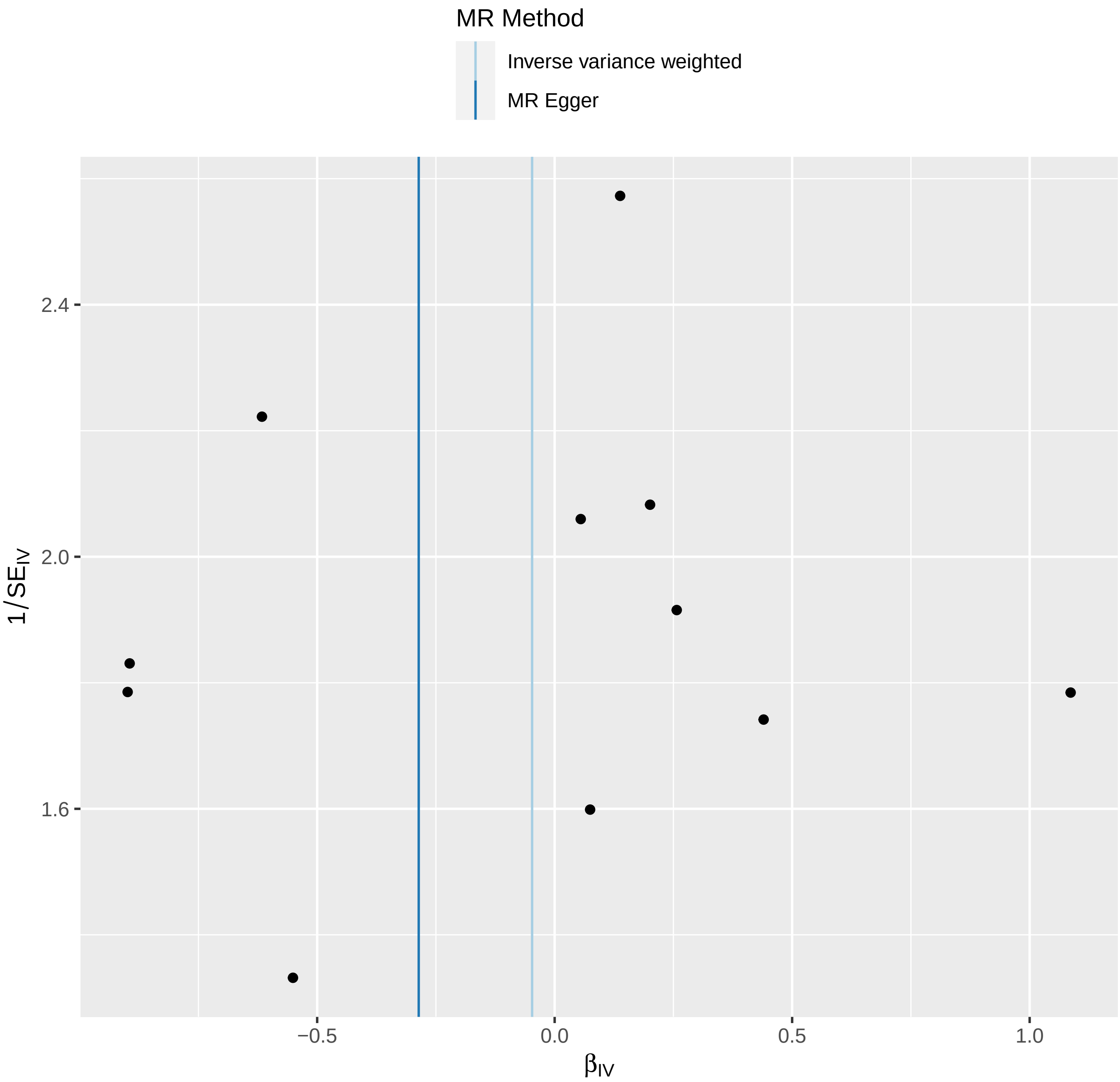

Batch 393 : Gut microbiota abundance (genus Intestinimonas id.2062) on Crohn's disease of small intestine

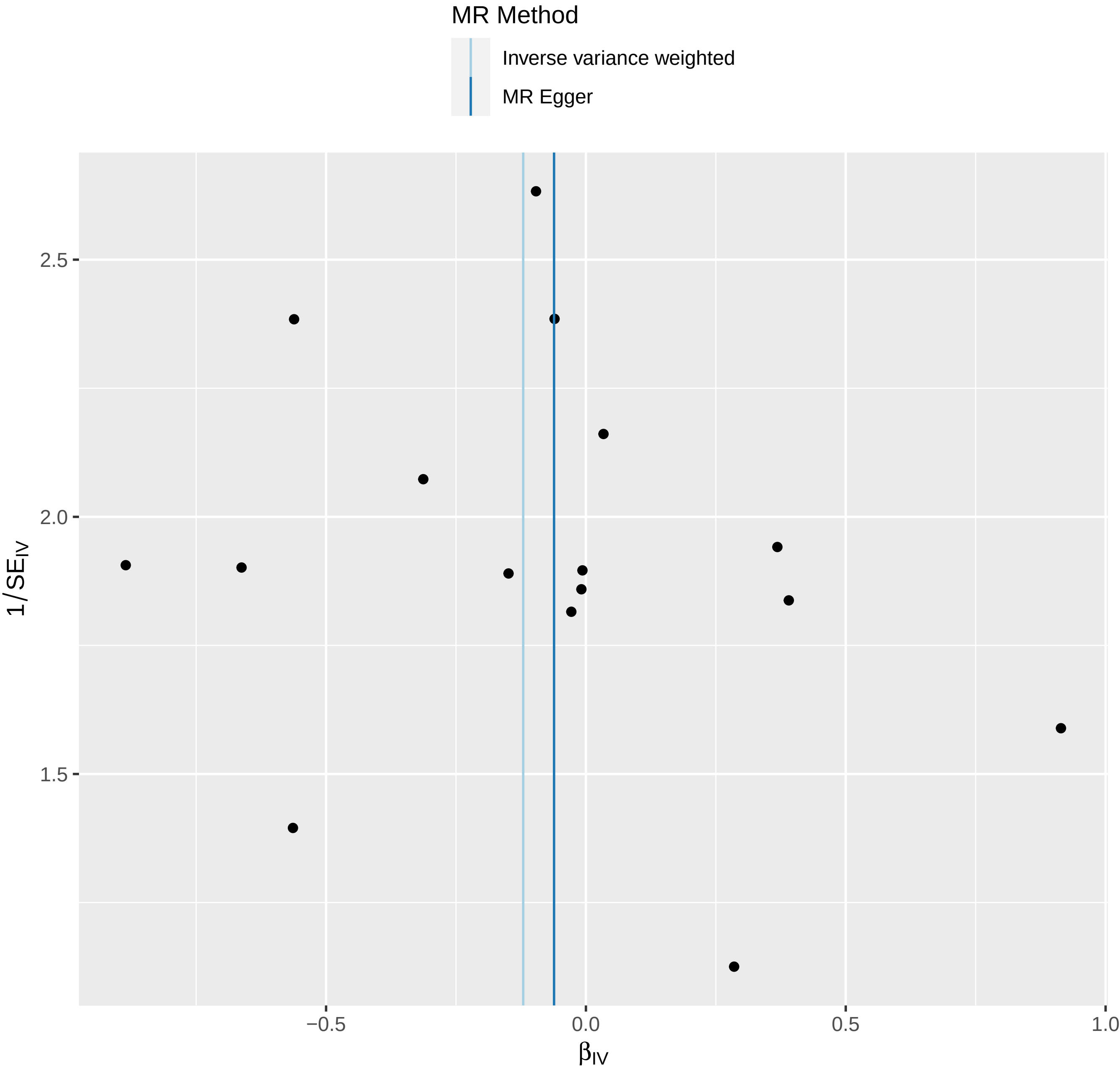

Batch 394 : Gut microbiota abundance (genus Lachnoclostridium id.11308) on Crohn's disease of small intestine

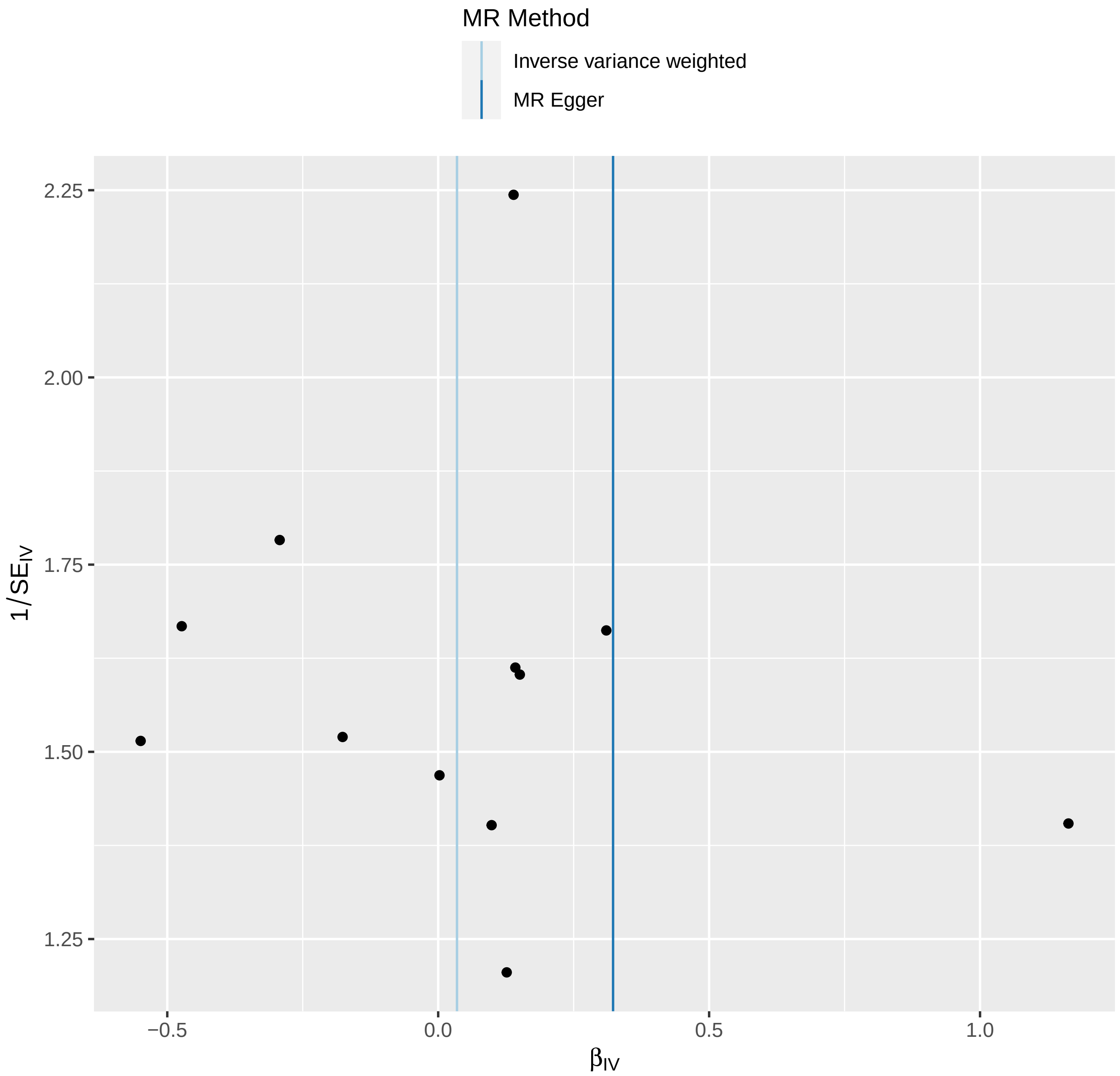

Batch 395 : Gut microbiota abundance (genus Lachnospiraceae FCS020 group id.11314) on Crohn's disease of small intestine

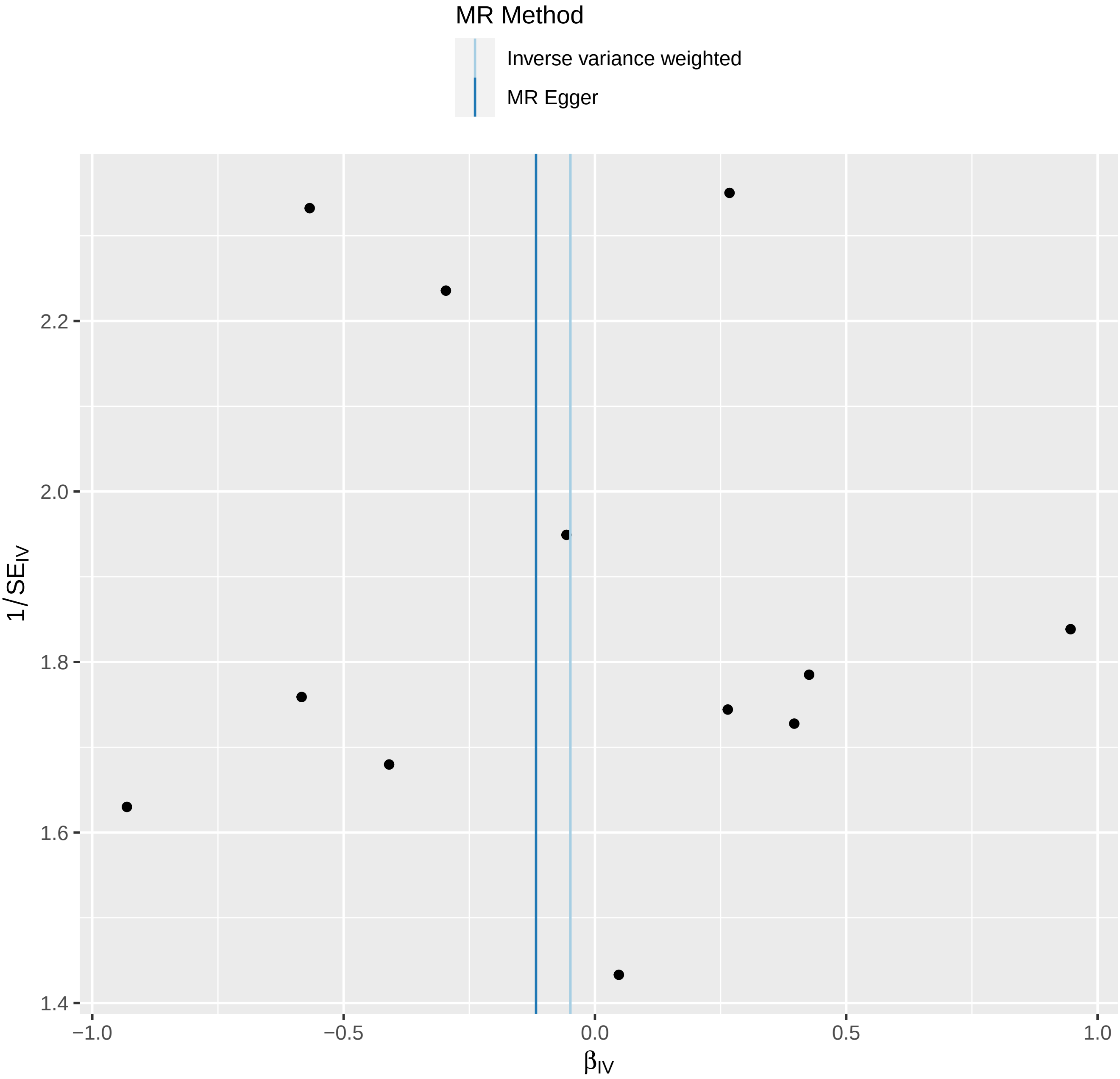

Batch 396 : Gut microbiota abundance (genus Lachnospiraceae NC2004 group id.11316) on Crohn's disease of small intestine

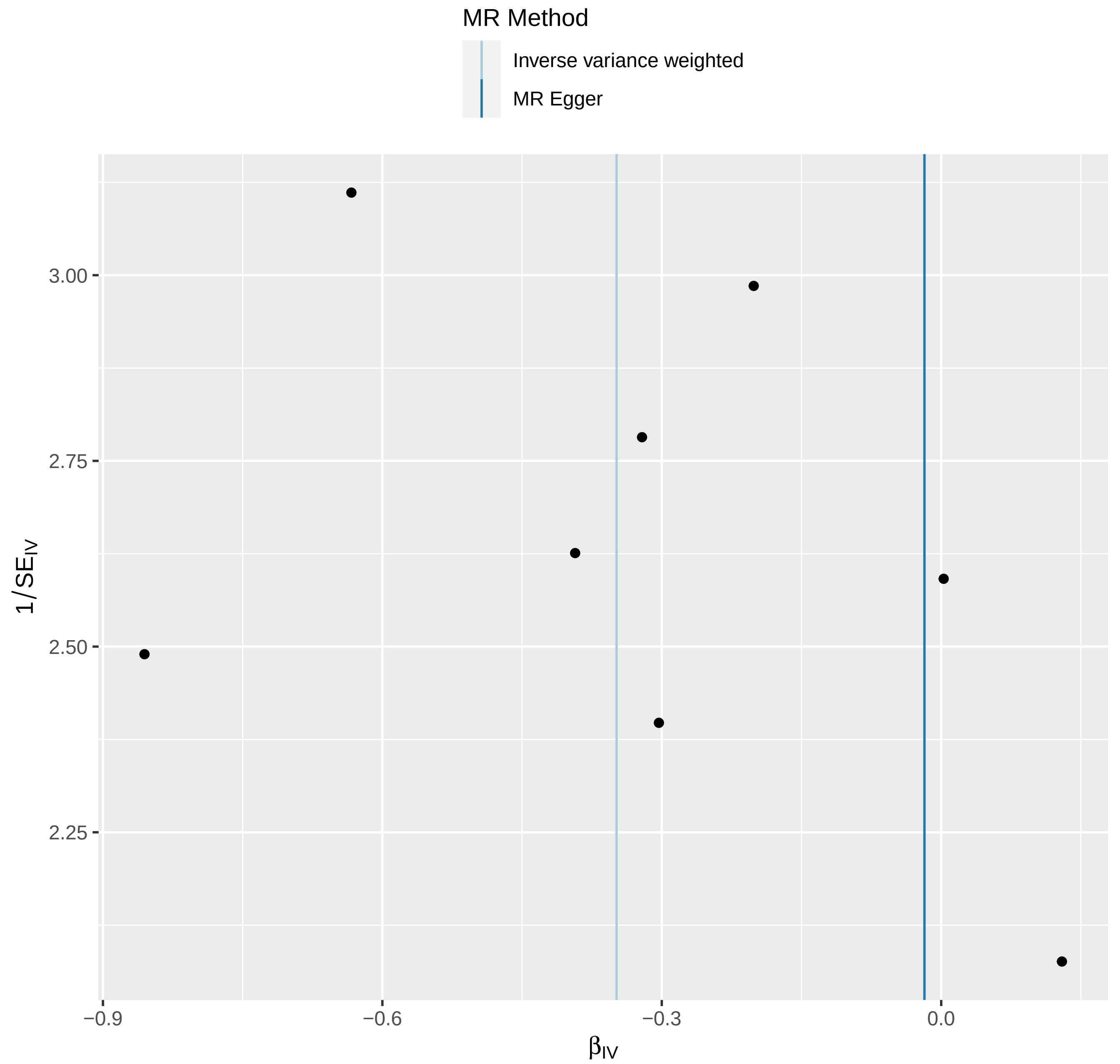

Batch 397 : Gut microbiota abundance (genus Lachnospiraceae ND3007 group id.11317) on Crohn's disease of small intestine

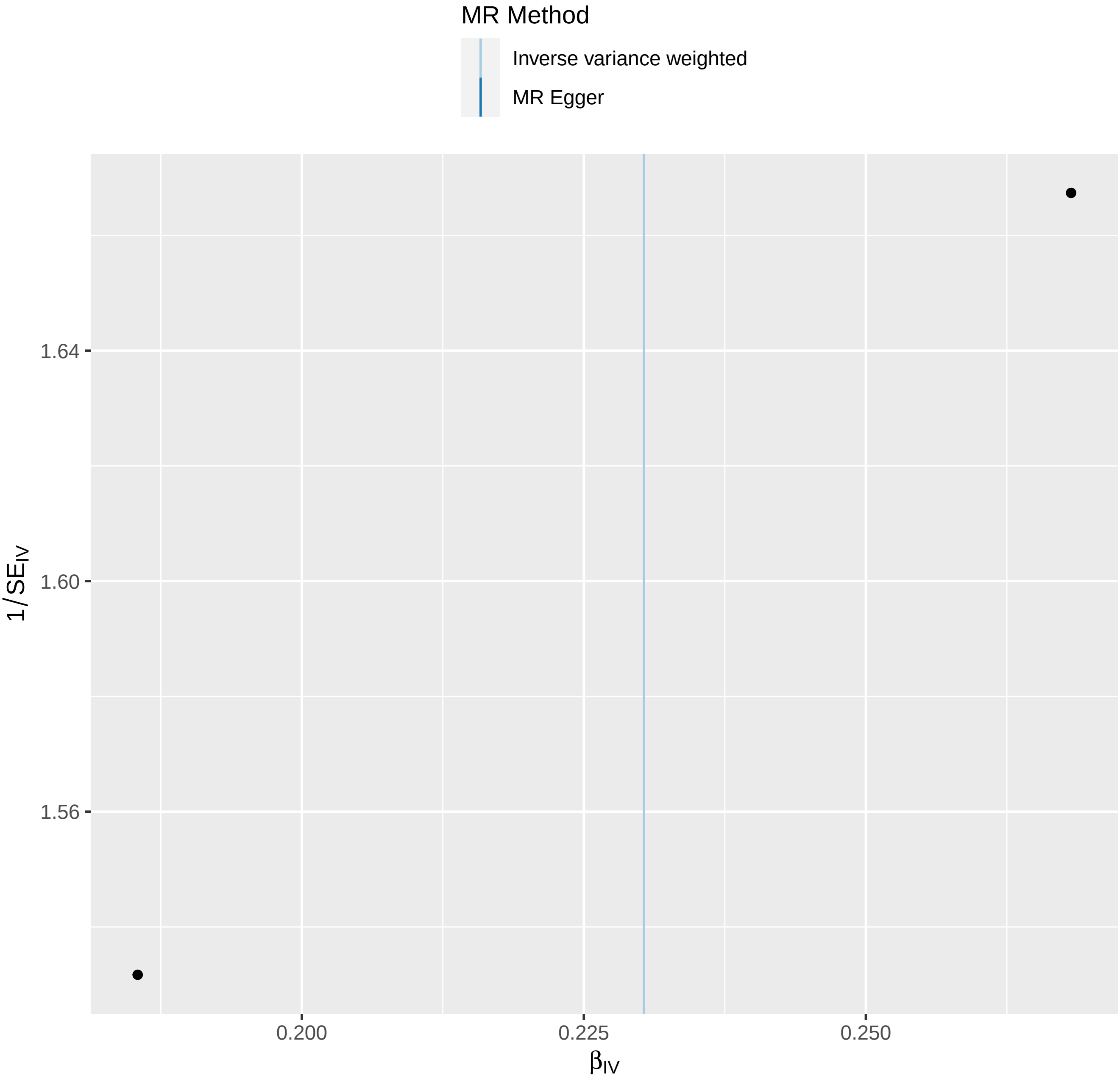

Batch 398 : Gut microbiota abundance (genus Lachnospiraceae NK4A136 group id.11319) on Crohn's disease of small intestine

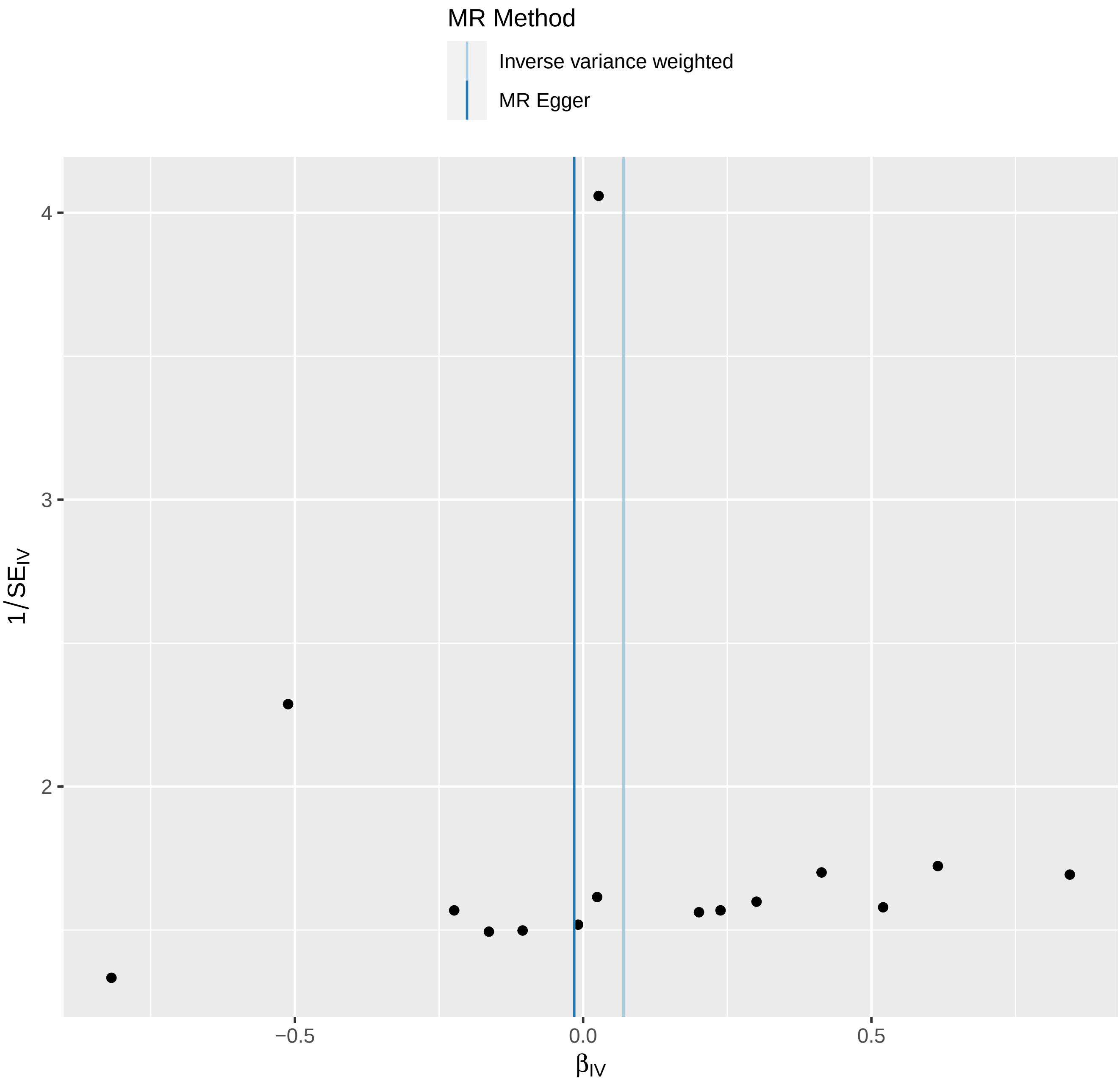

Batch 399 : Gut microbiota abundance (genus Lachnospiraceae UCG001 id.11321) on Crohn's disease of small intestine

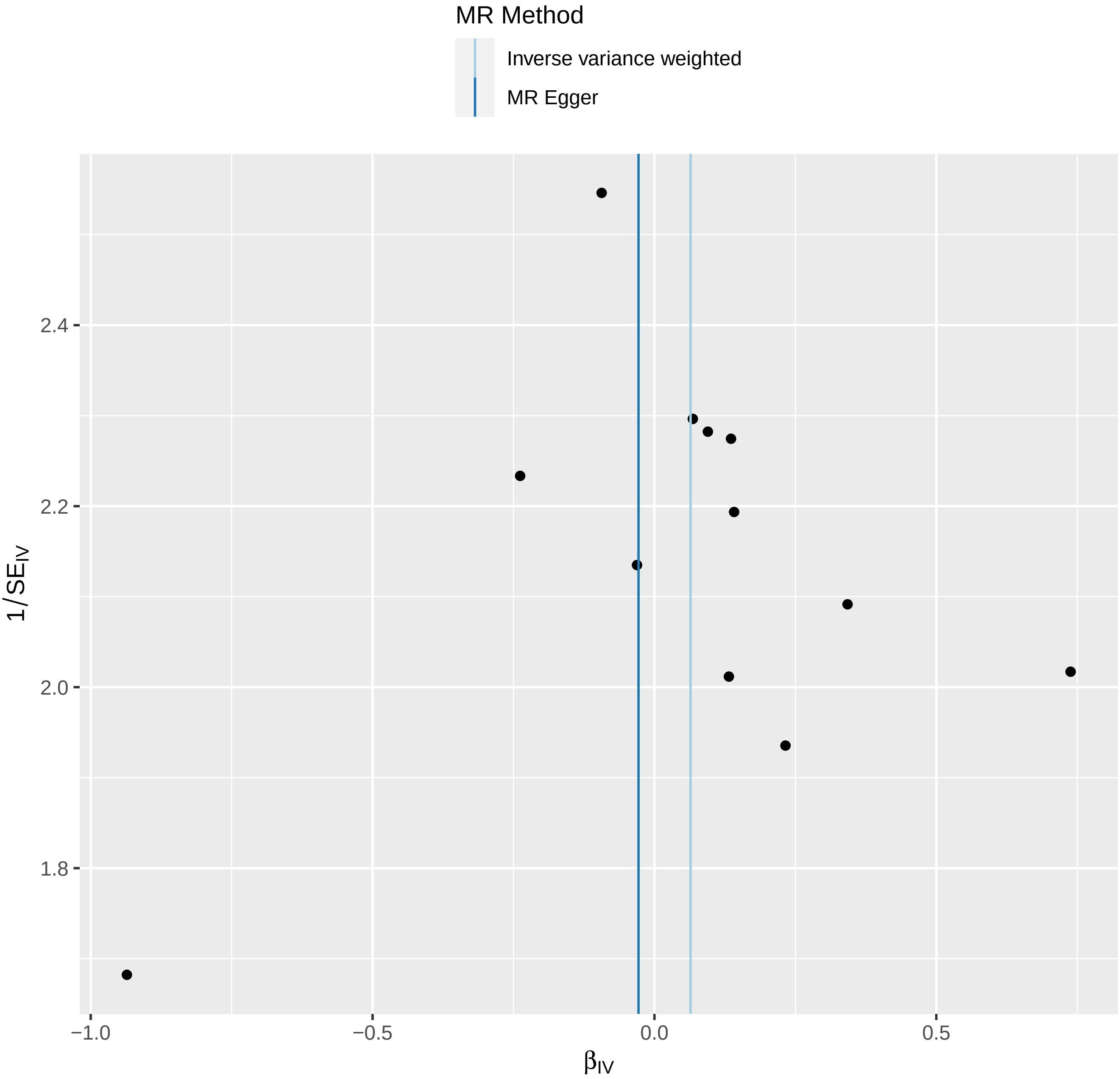

Batch 400 : Gut microbiota abundance (genus Lachnospiraceae UCG004 id.11324) on Crohn's disease of small intestine

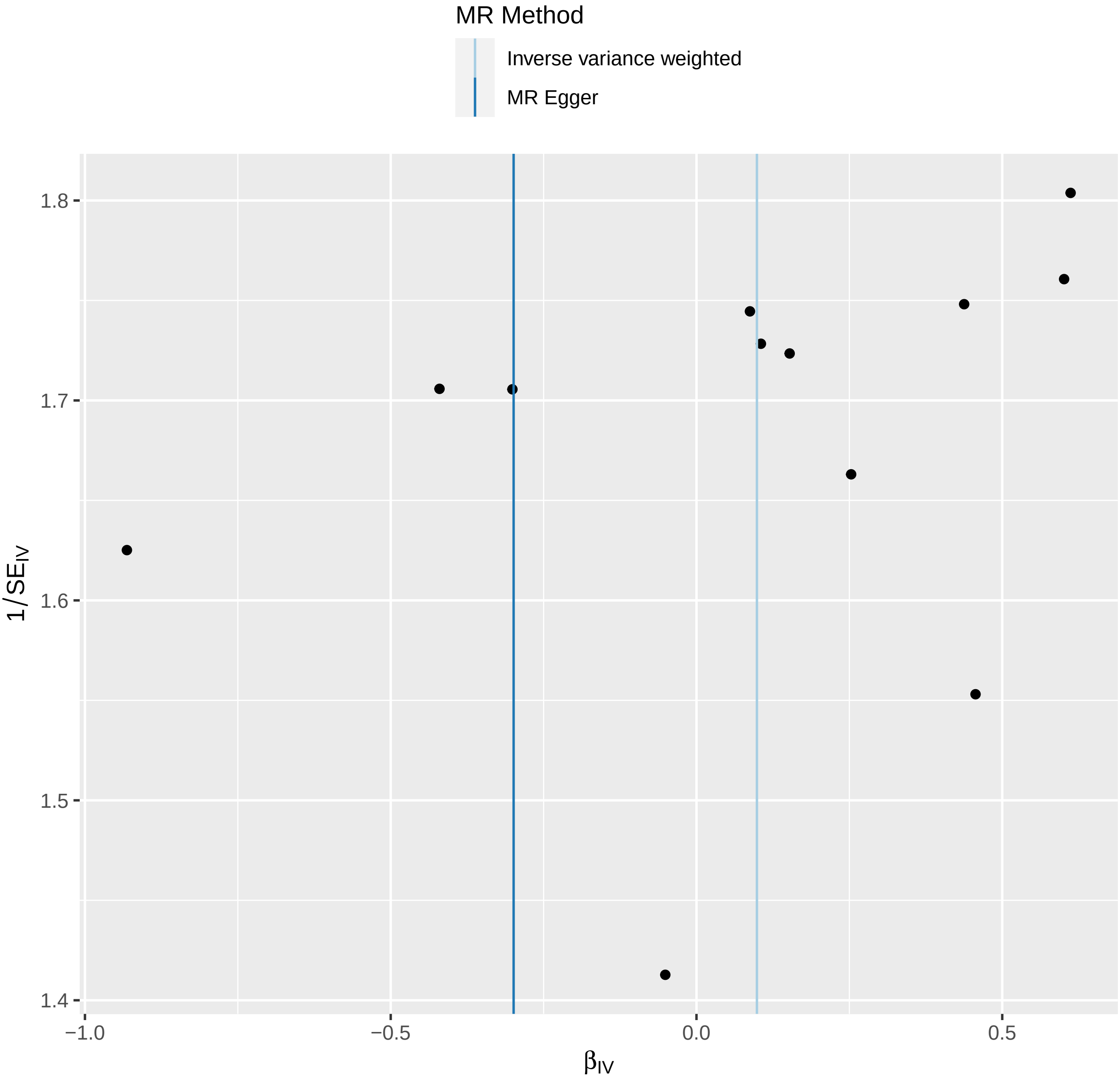

Batch 401 : Gut microbiota abundance (genus Lachnospiraceae UCG008 id.11328) on Crohn's disease of small intestine

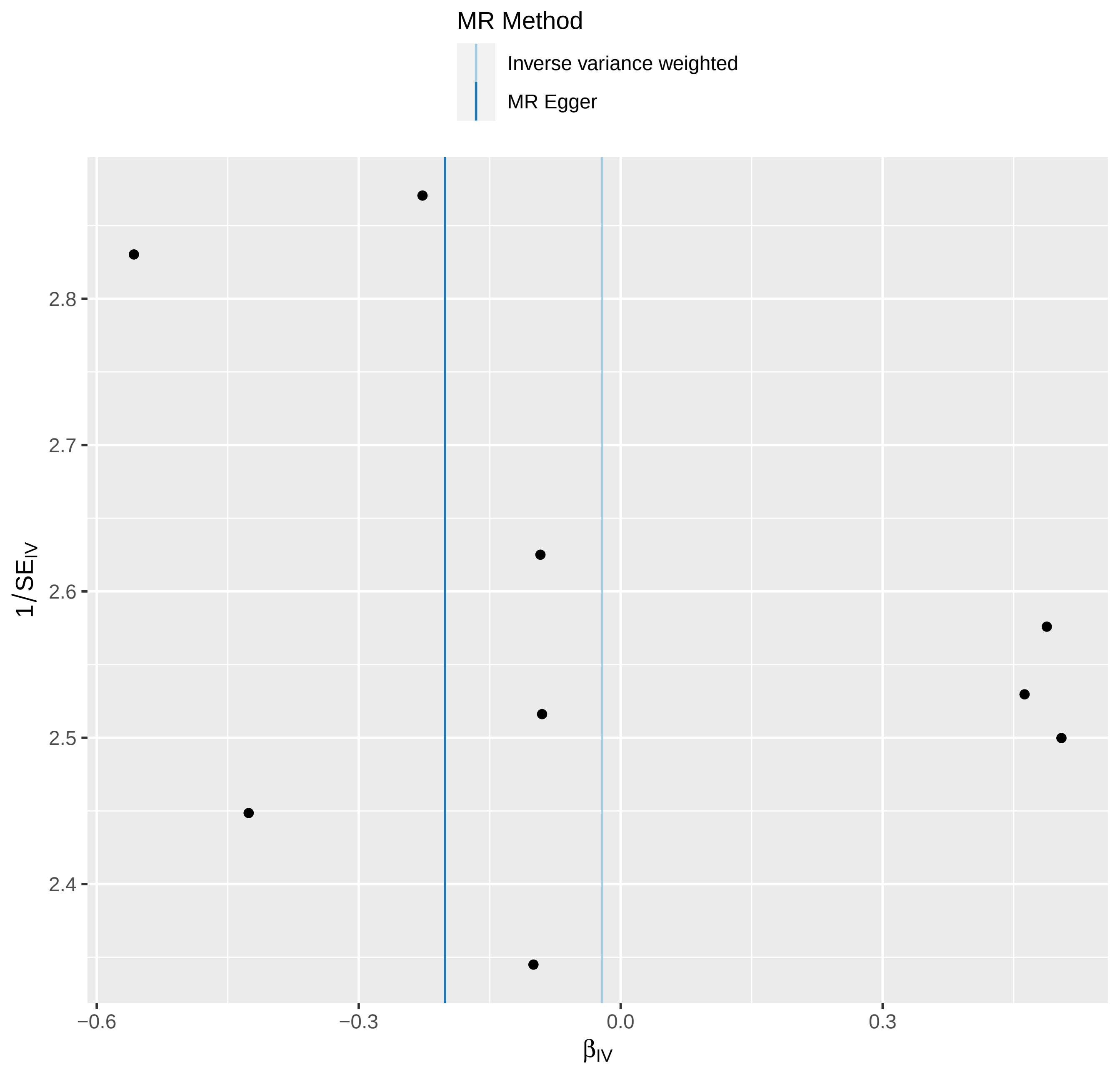

Batch 402 : Gut microbiota abundance (genus Lachnospiraceae UCG010 id.11330) on Crohn's disease of small intestine

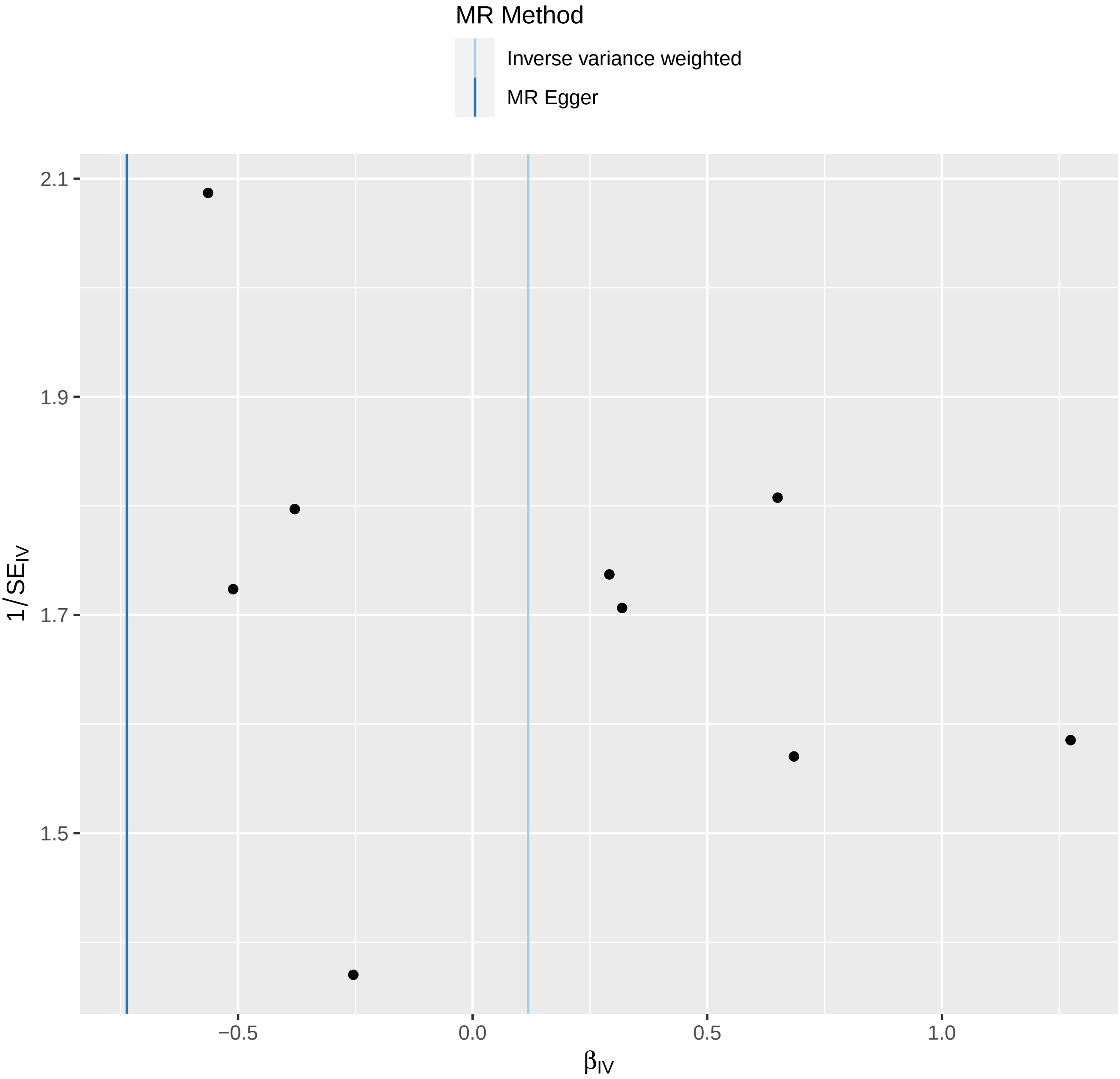

Insufficient number of SNPs

Batch 404 : Gut microbiota abundance (genus Lactobacillus id.1837) on Crohn's disease of small intestine

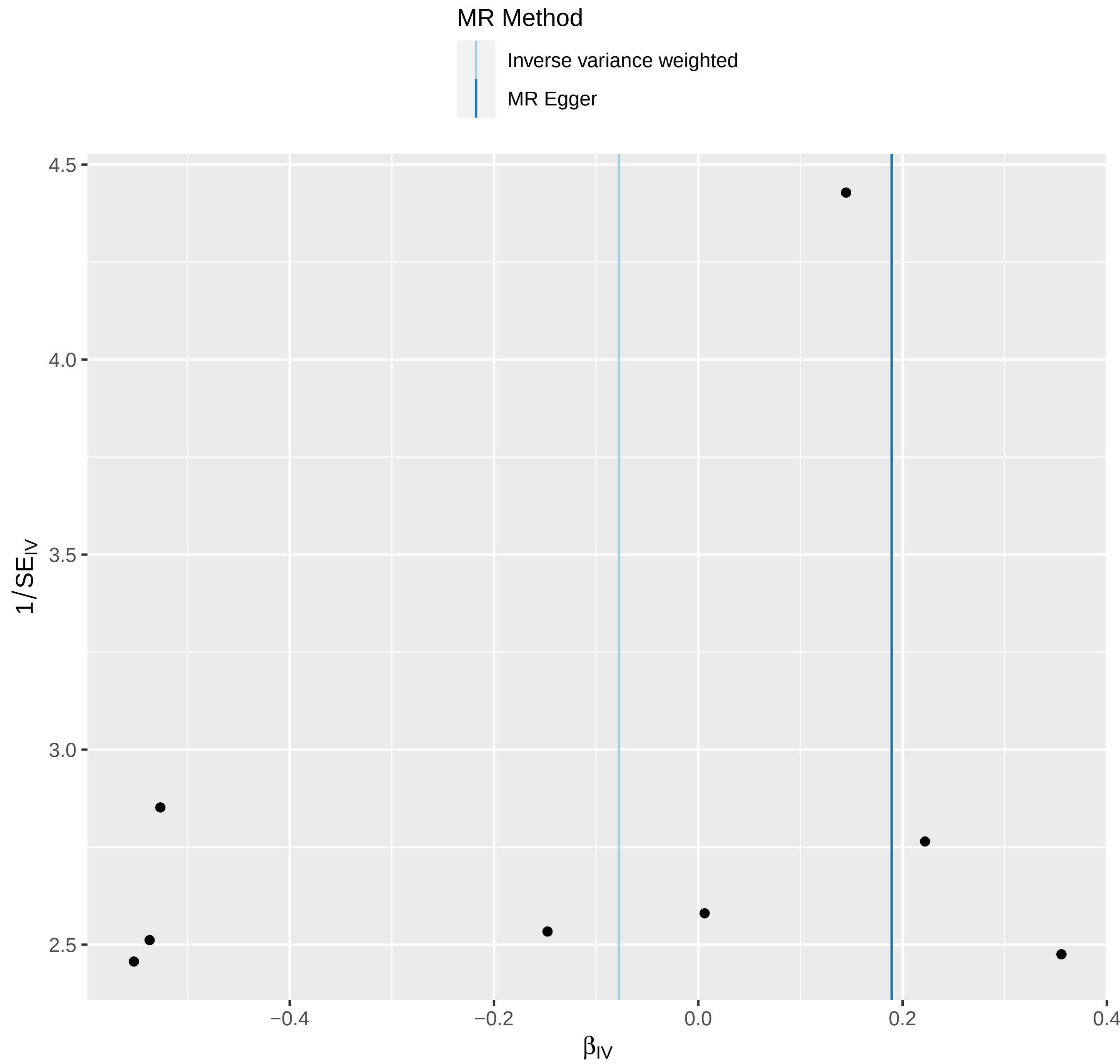

Batch 405 : Gut microbiota abundance (genus Lactococcus id.1851) on Crohn's disease of small intestine

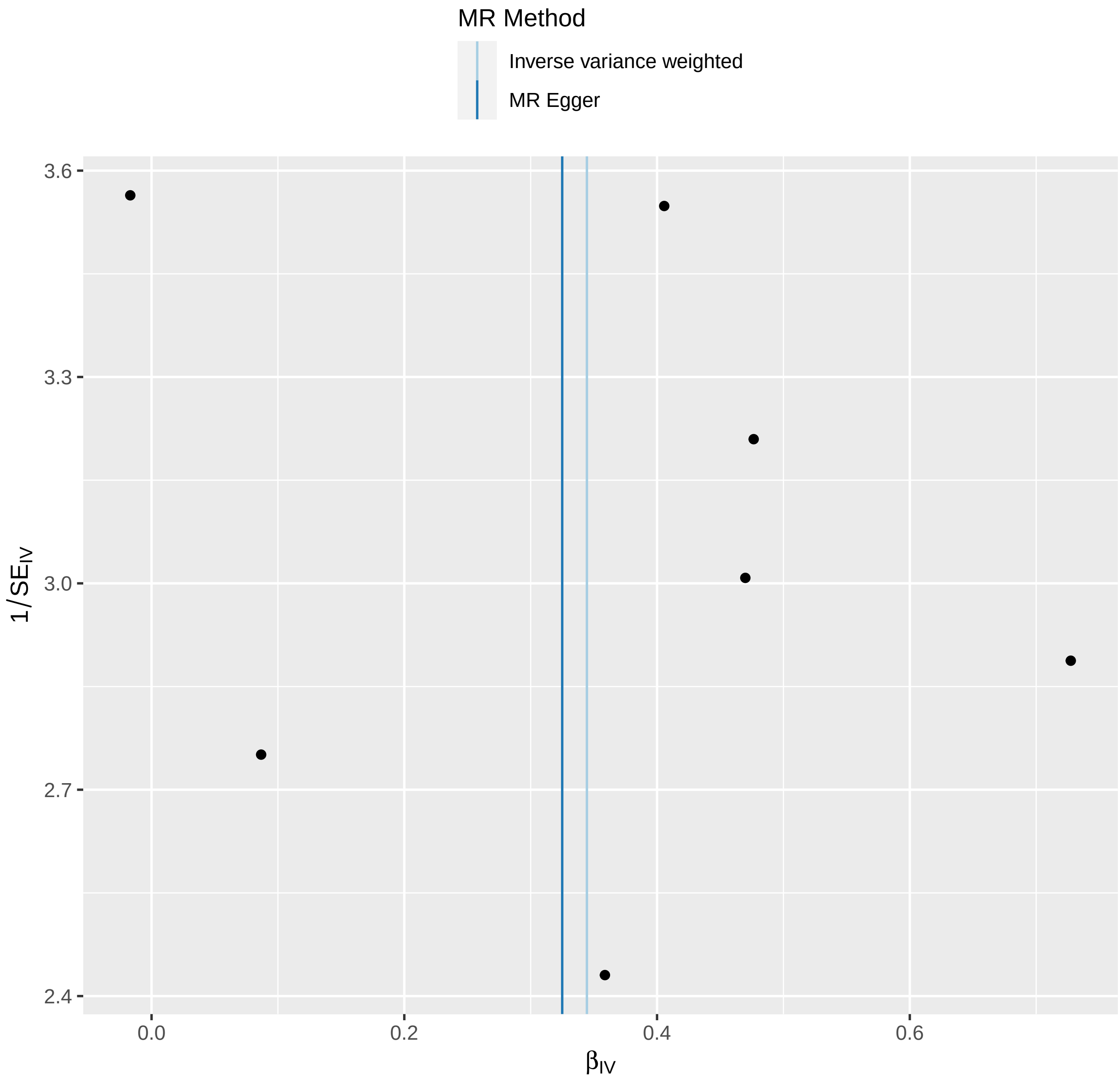

Batch 406 : Gut microbiota abundance (genus Marvinbryantia id.2005) on Crohn's disease of small intestine

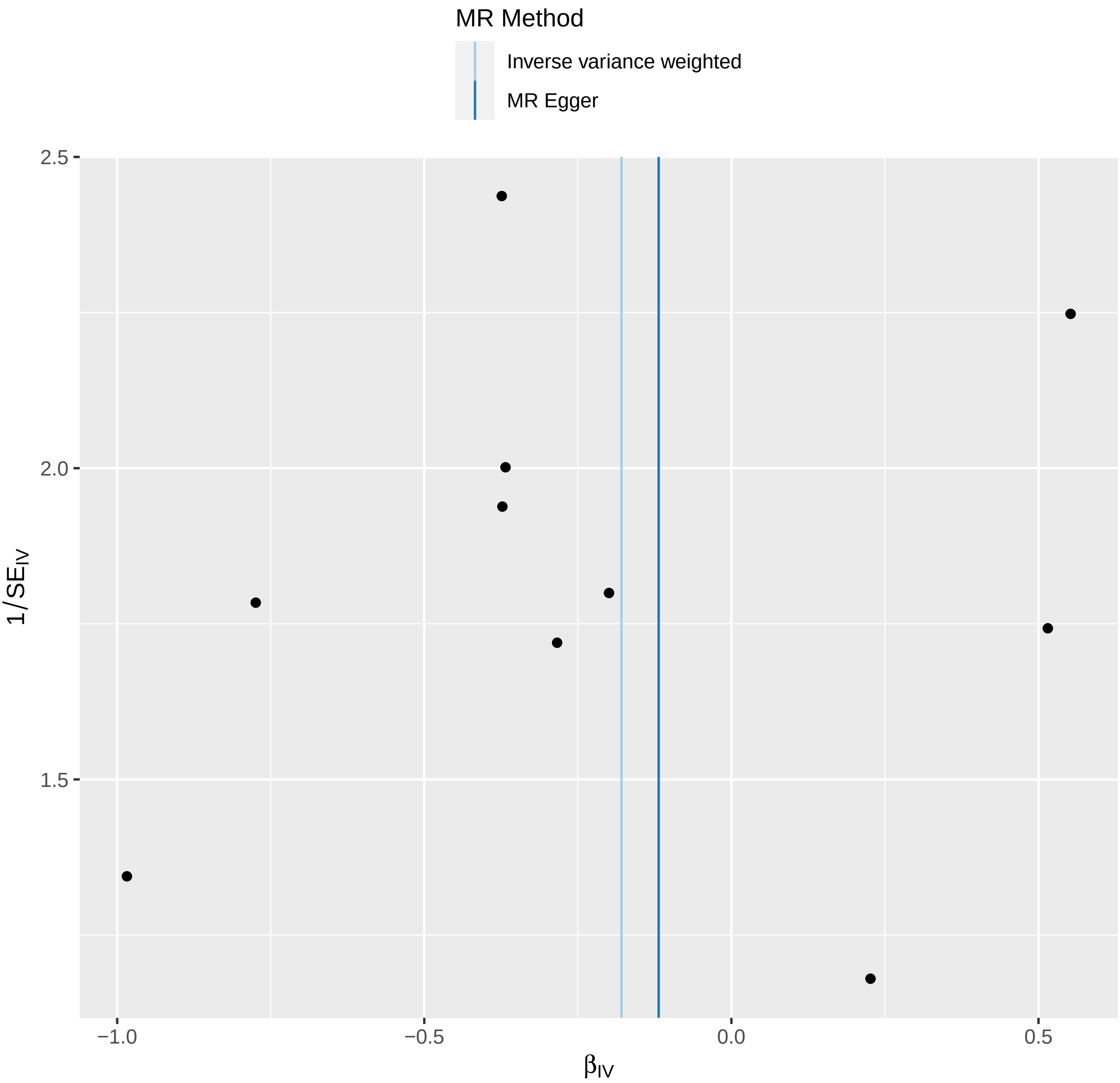

Batch 407 : Gut microbiota abundance (genus Methanobrevibacter id.123) on Crohn's disease of small intestine

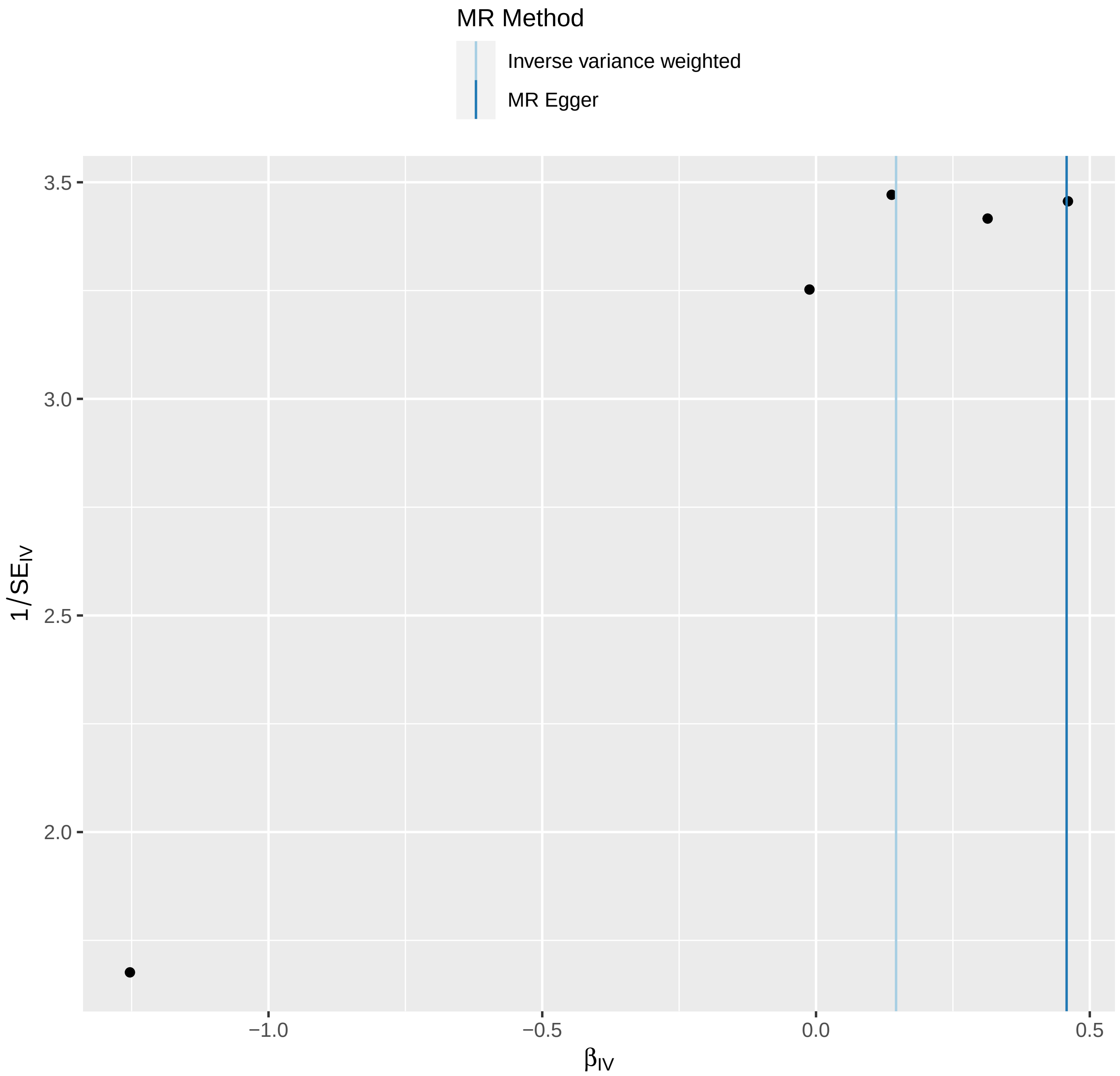

Batch 408 : Gut microbiota abundance (genus Odoribacter id.952) on Crohn's disease of small intestine

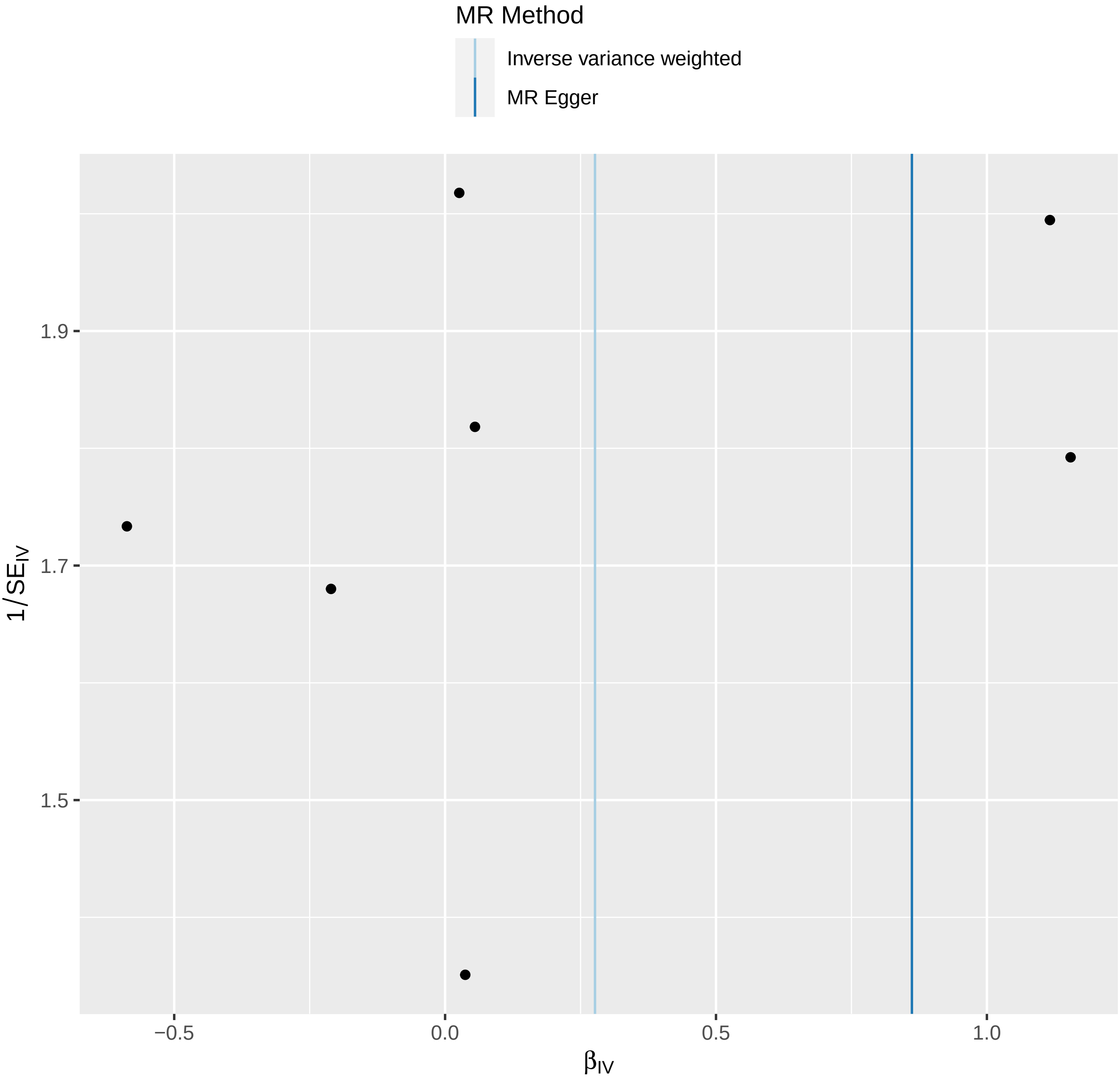

Batch 409 : Gut microbiota abundance (genus Olsenella id.822) on Crohn's disease of small intestine

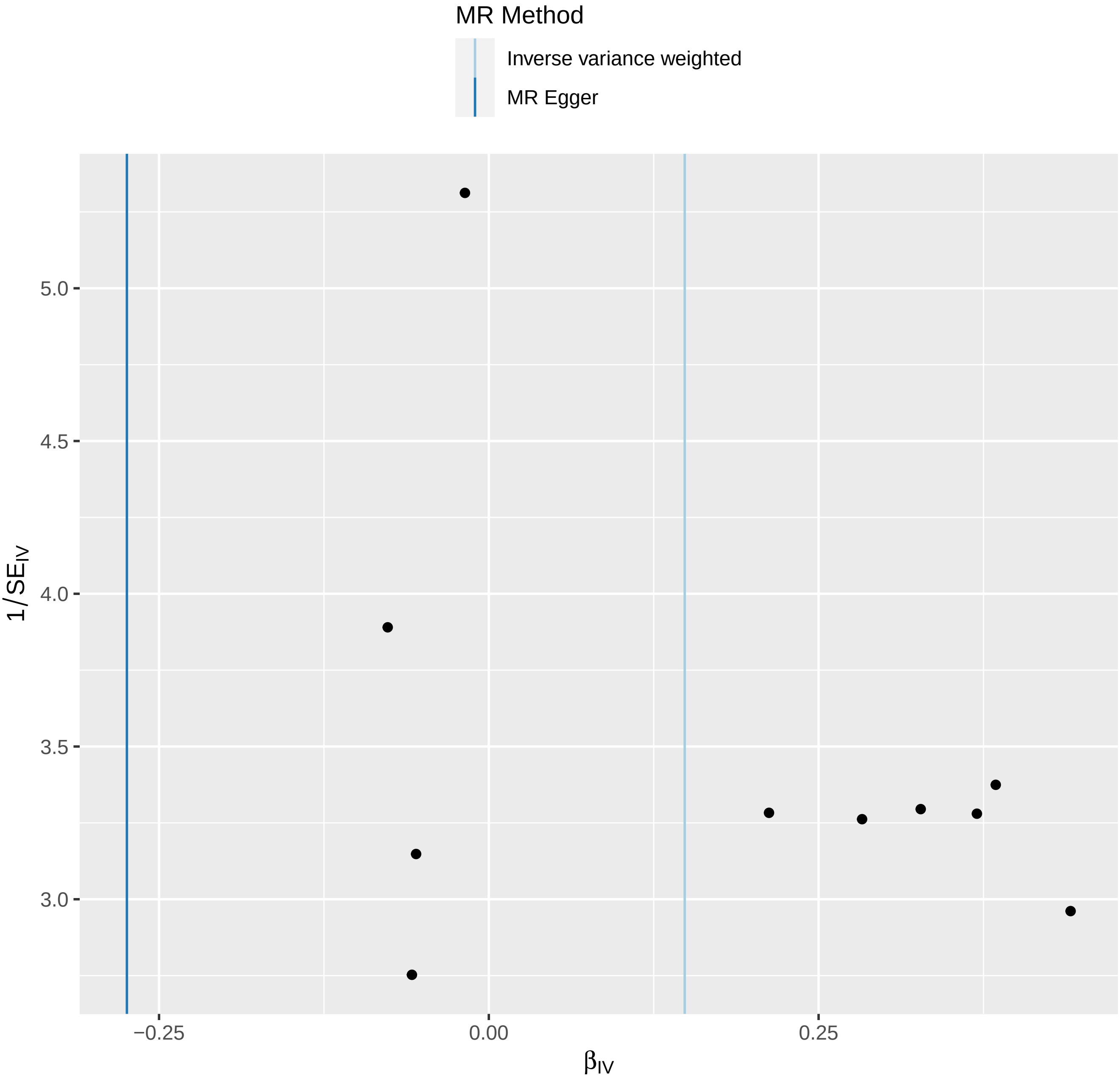

Batch 410 : Gut microbiota abundance (genus Oscillibacter id.2063) on Crohn's disease of small intestine

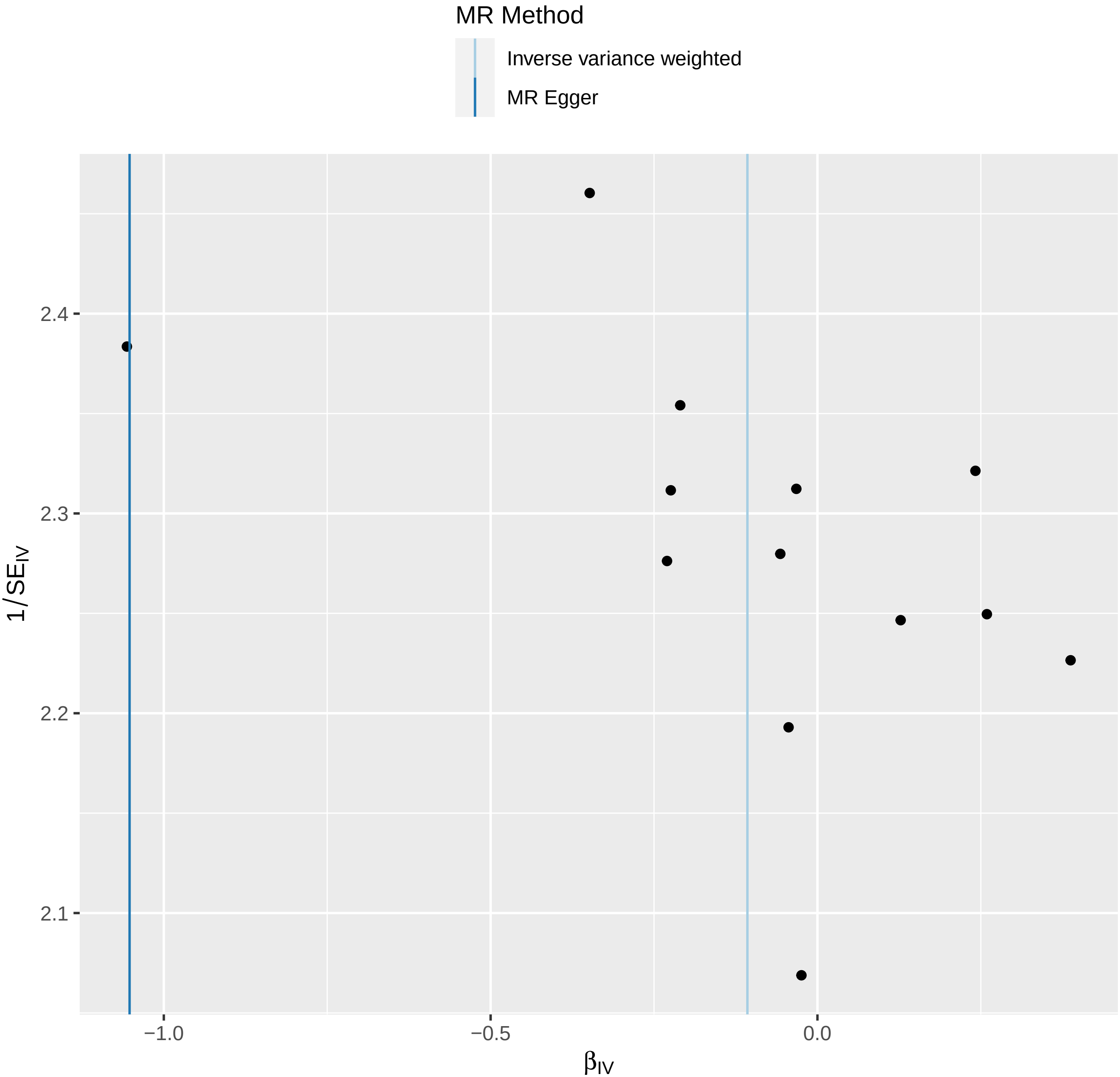

Batch 411 : Gut microbiota abundance (genus Oscillospira id.2064) on Crohn's disease of small intestine

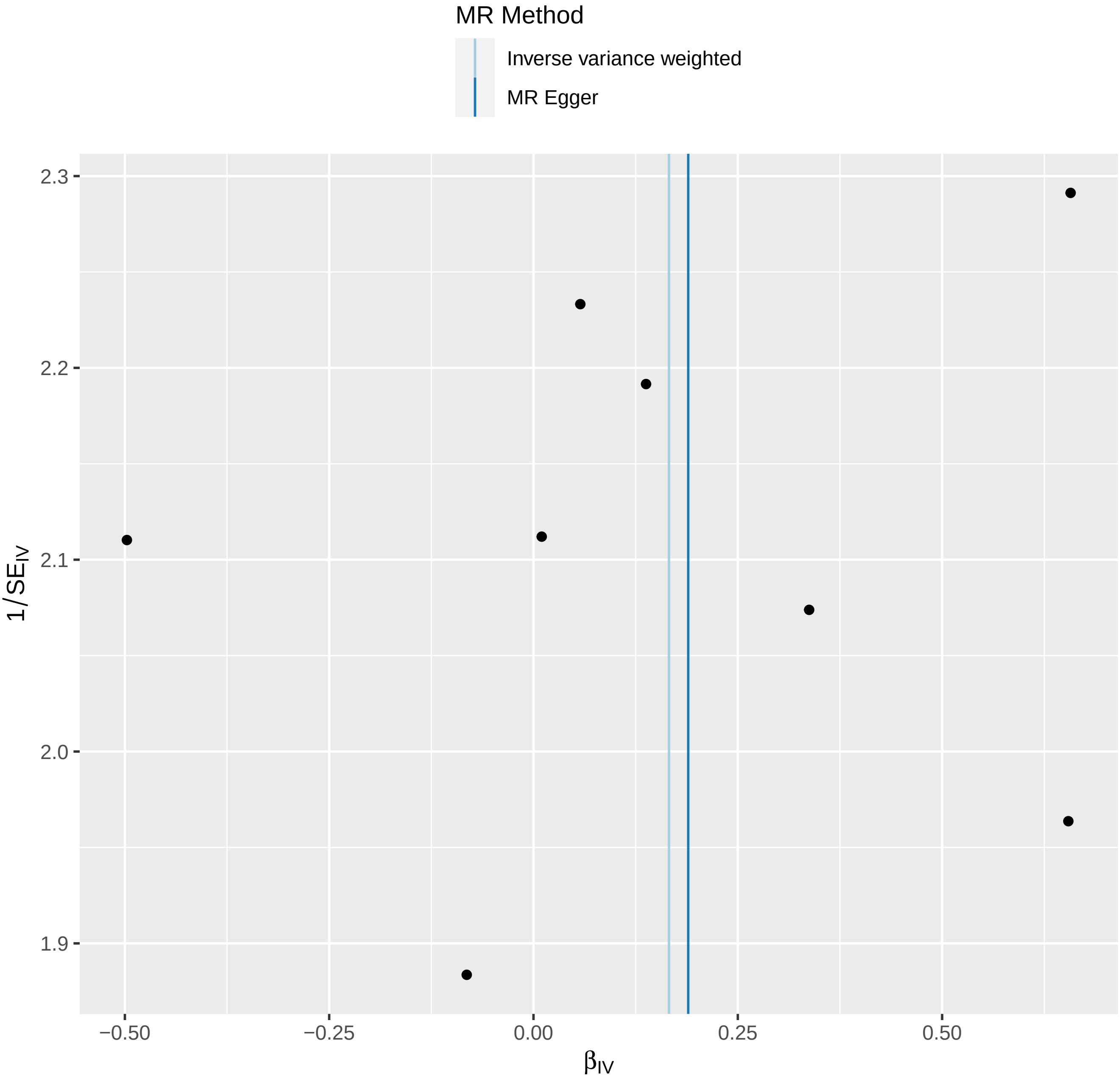

Batch 412 : Gut microbiota abundance (genus Oxalobacter id.2978) on Crohn's disease of small intestine

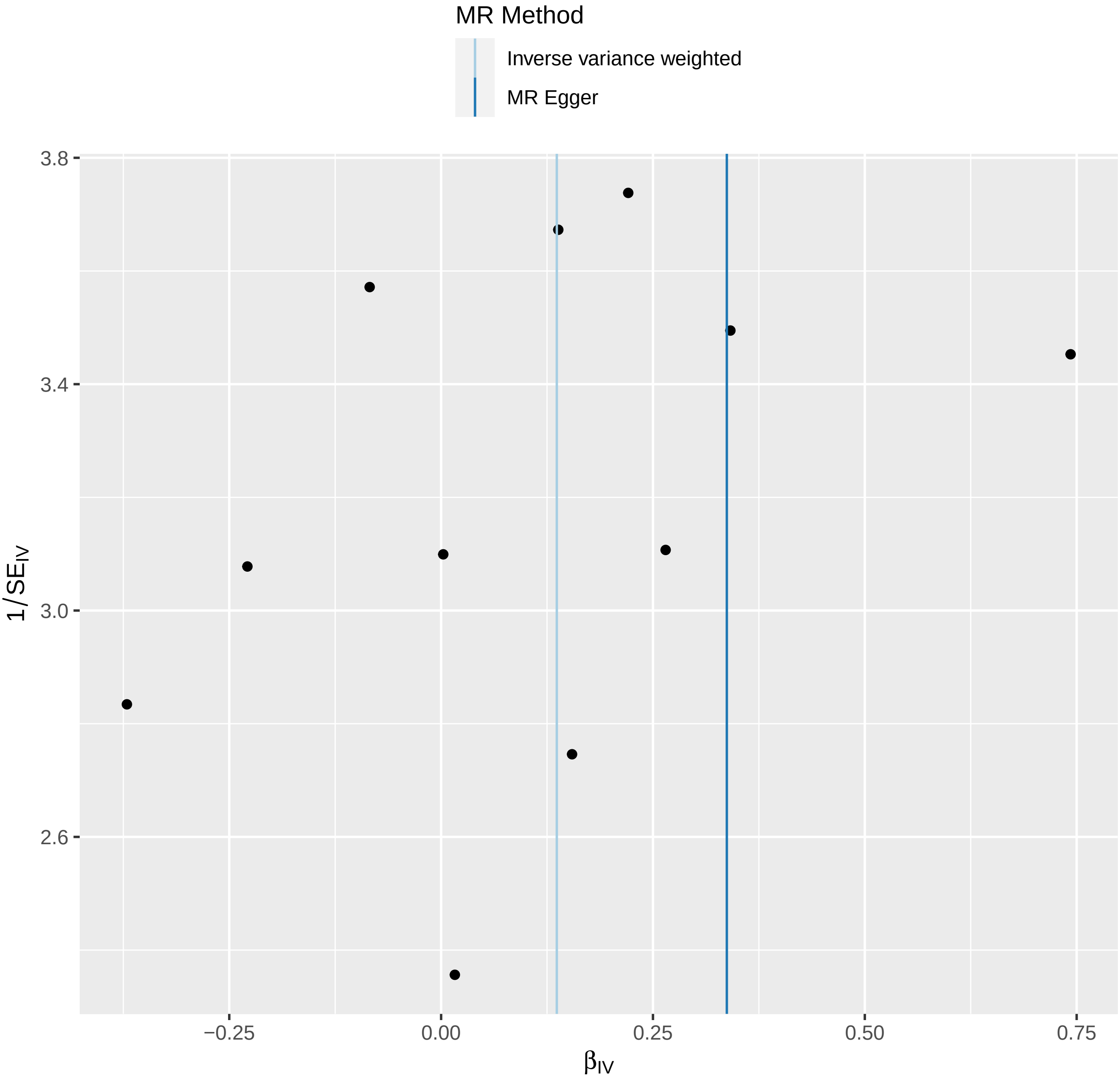

Batch 413 : Gut microbiota abundance (genus Parabacteroides id.954) on Crohn's disease of small intestine

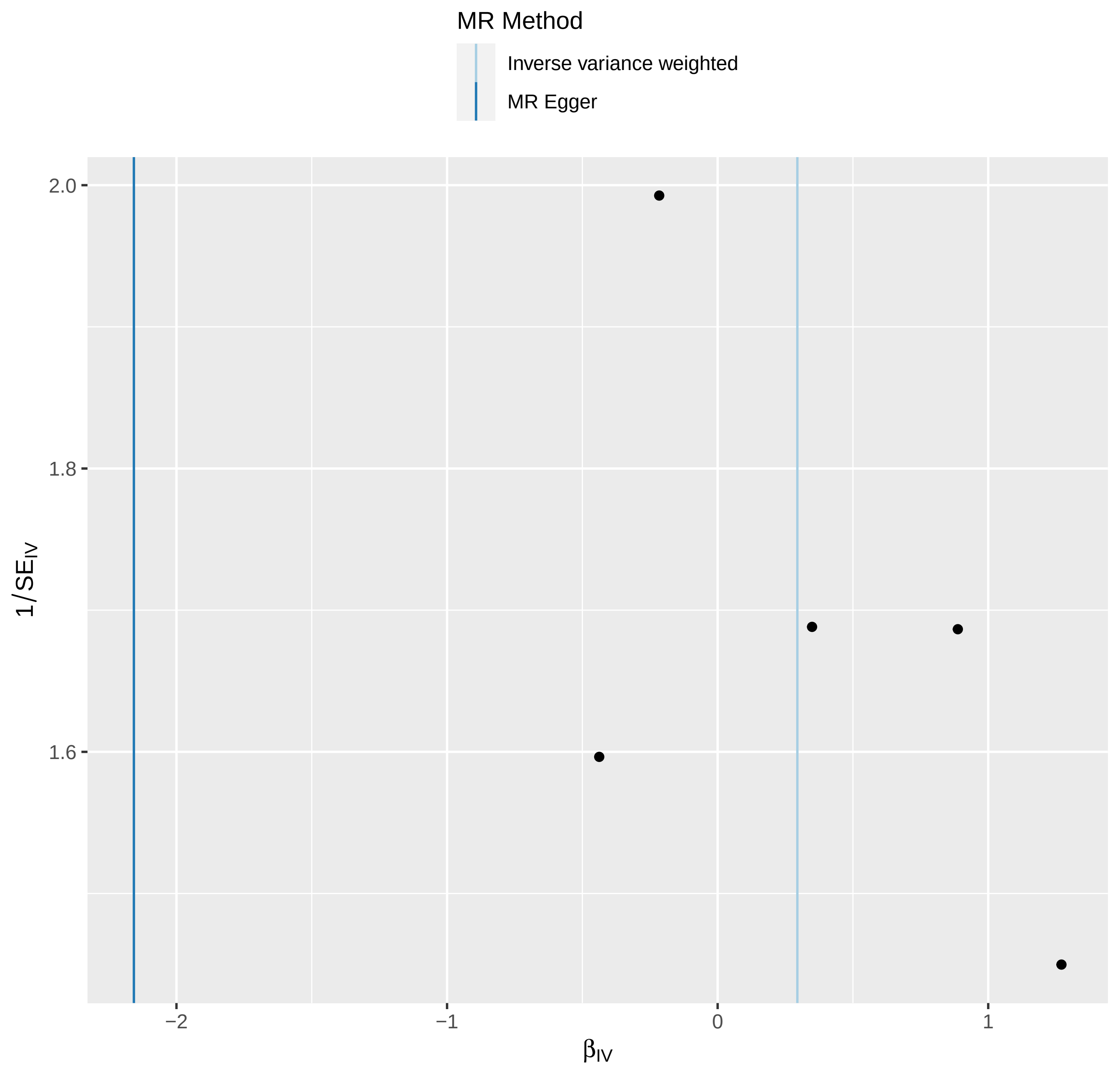

Batch 414 : Gut microbiota abundance (genus Paraprevotella id.962) on Crohn's disease of small intestine

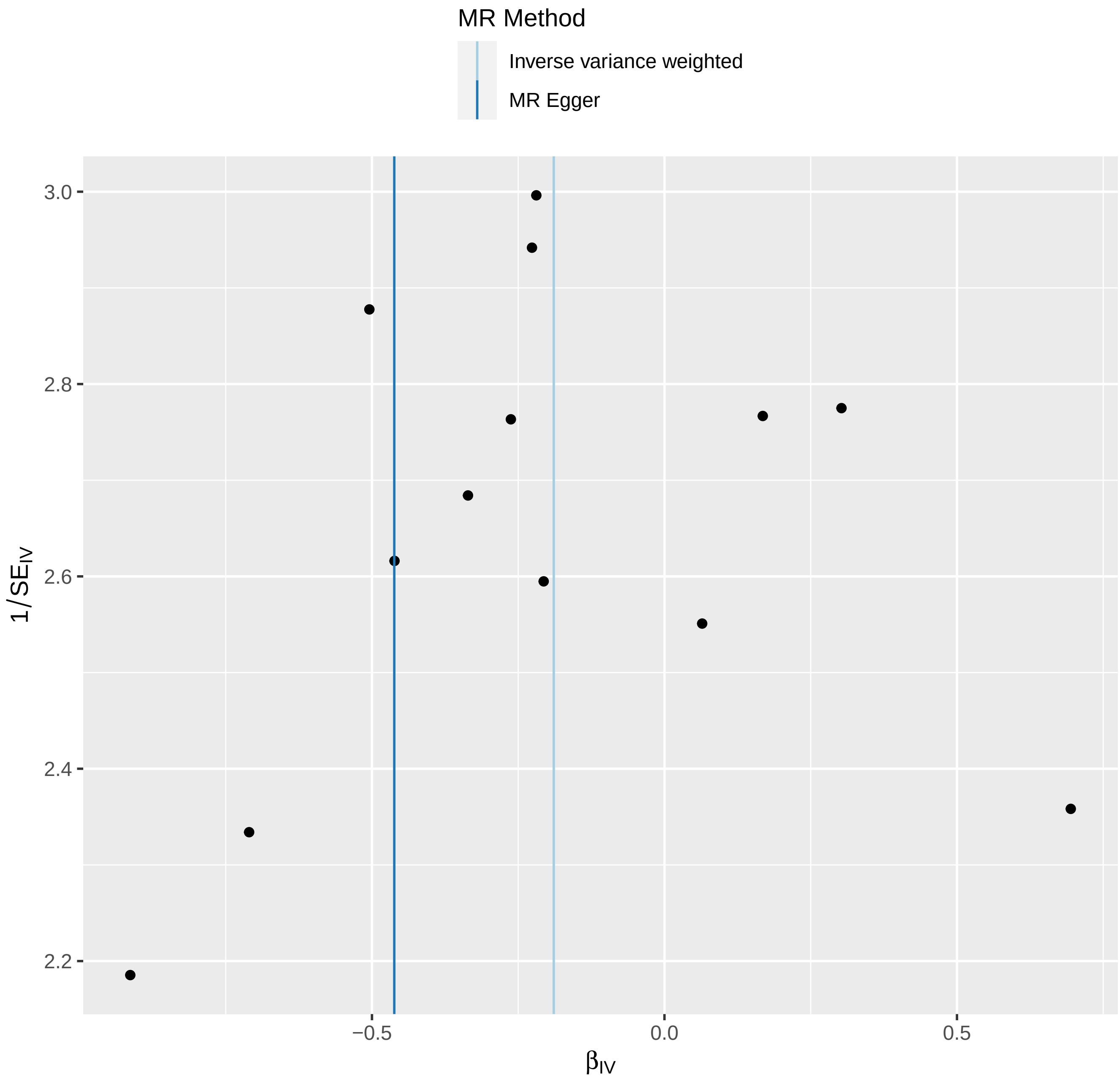

Batch 415 : Gut microbiota abundance (genus Parasutterella id.2892) on Crohn's disease of small intestine

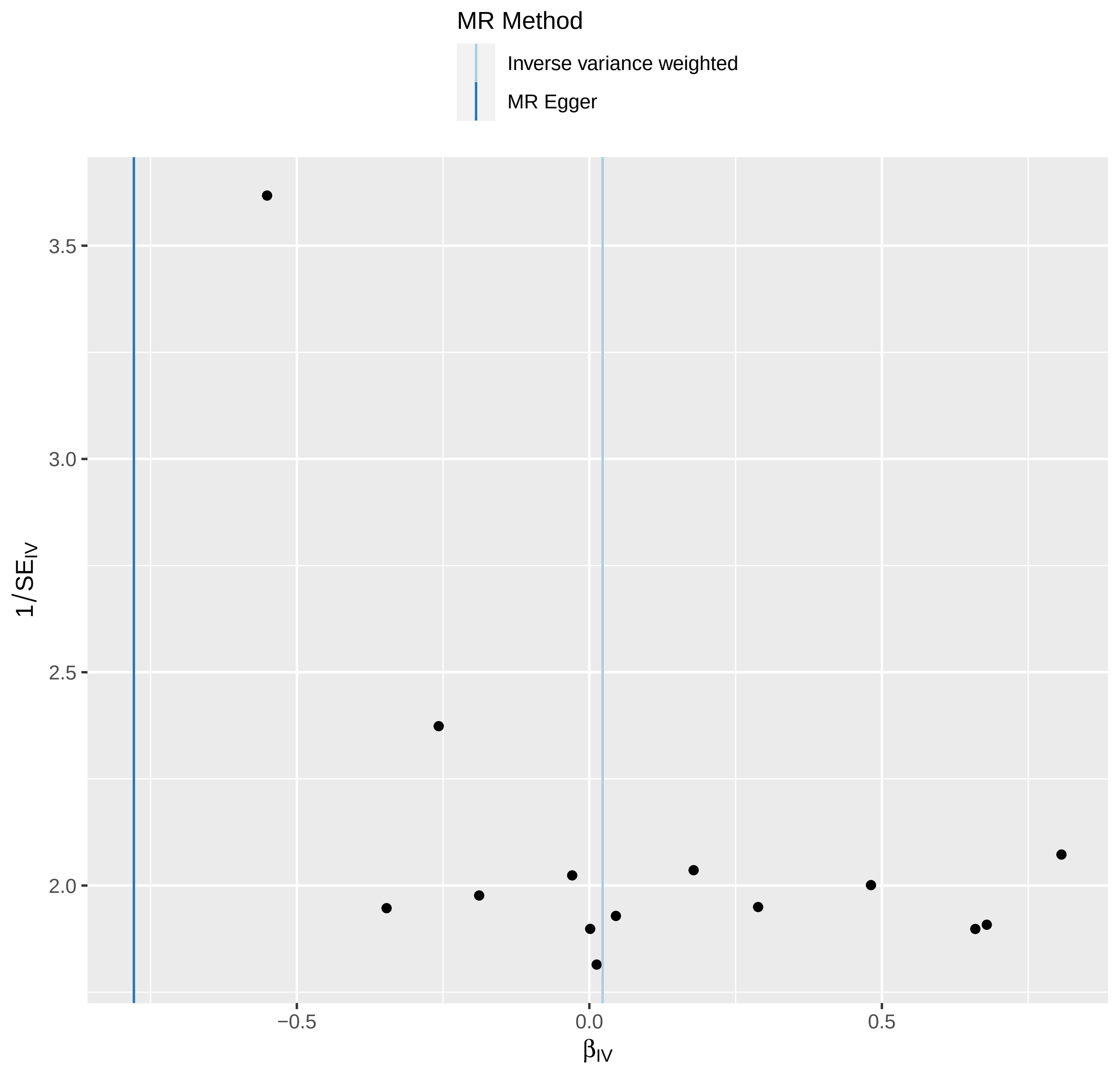

Batch 416 : Gut microbiota abundance (genus Peptococcus id.2037) on Crohn's disease of small intestine

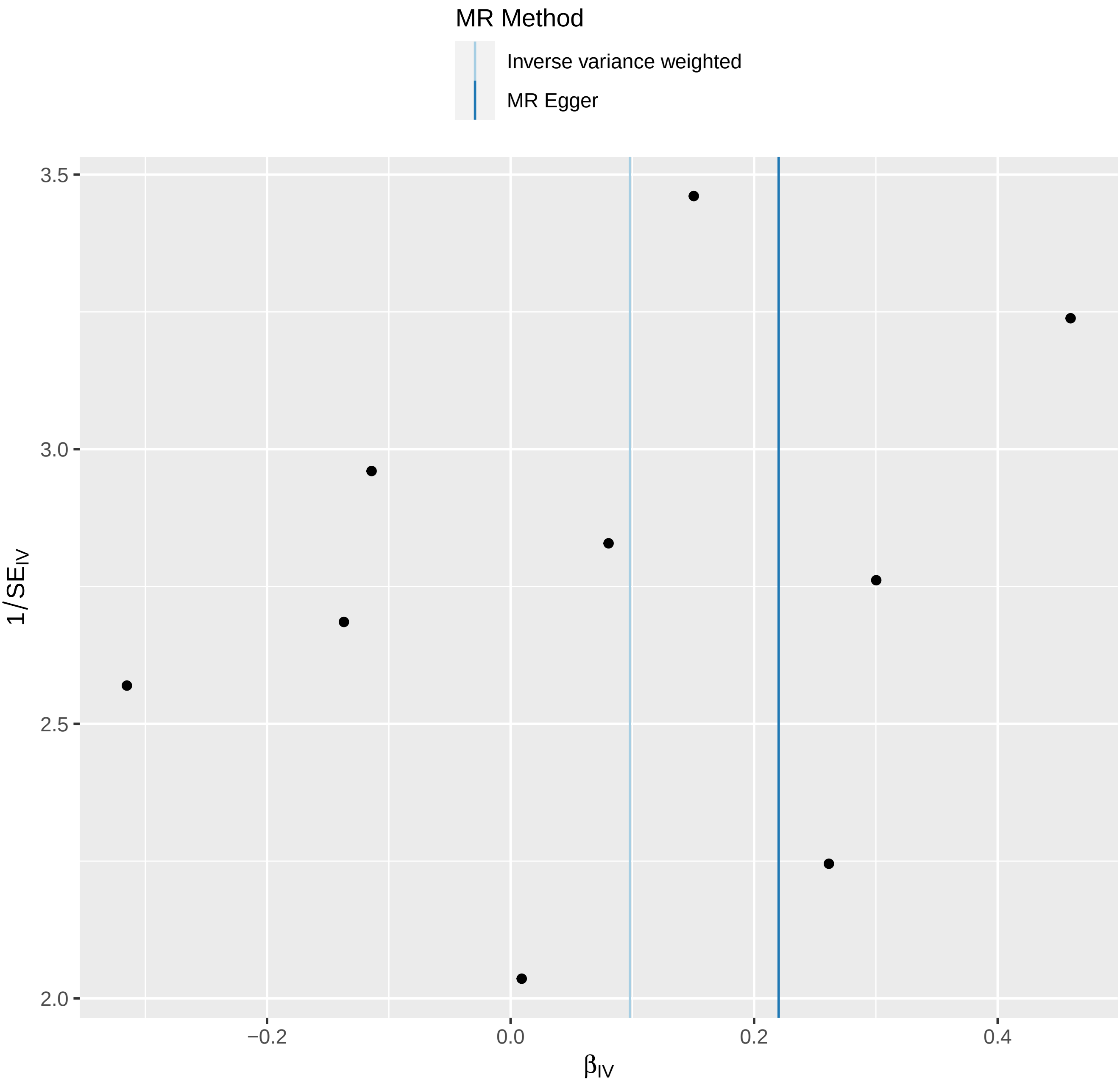

Batch 417 : Gut microbiota abundance (genus Phascolarctobacterium id.2168) on Crohn's disease of small intestine

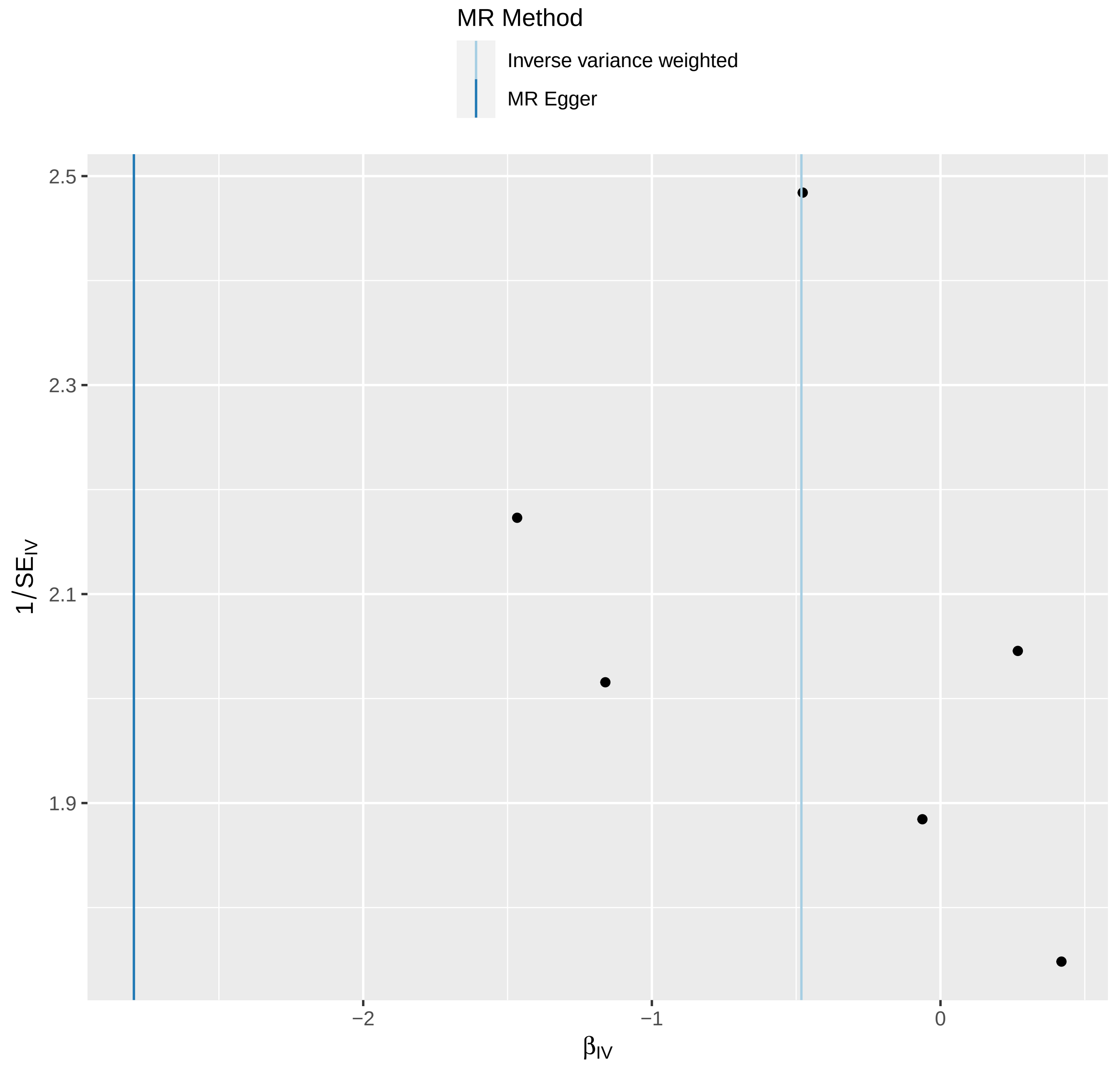

Batch 418 : Gut microbiota abundance (genus Prevotella7 id.11182) on Crohn's disease of small intestine

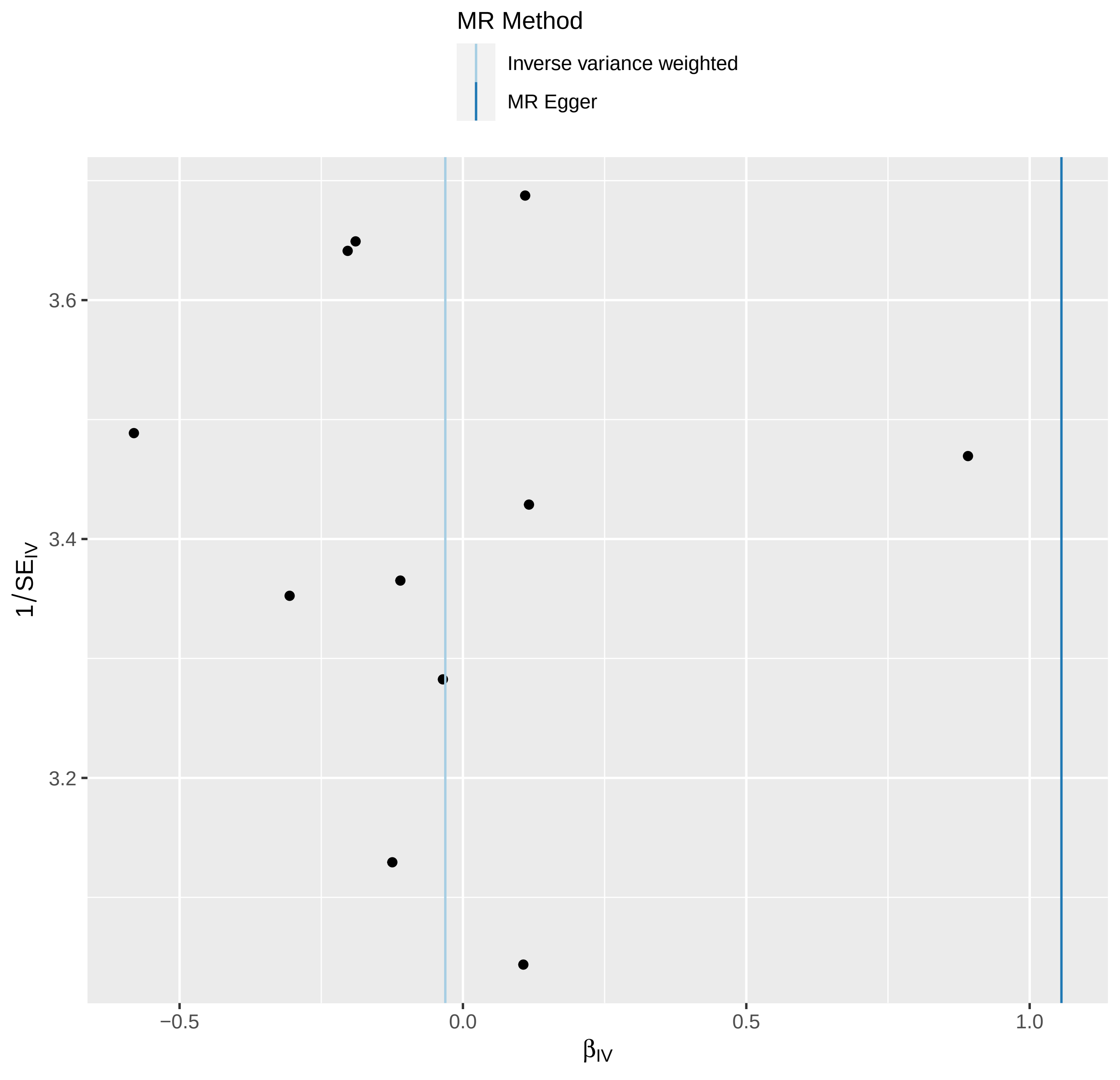

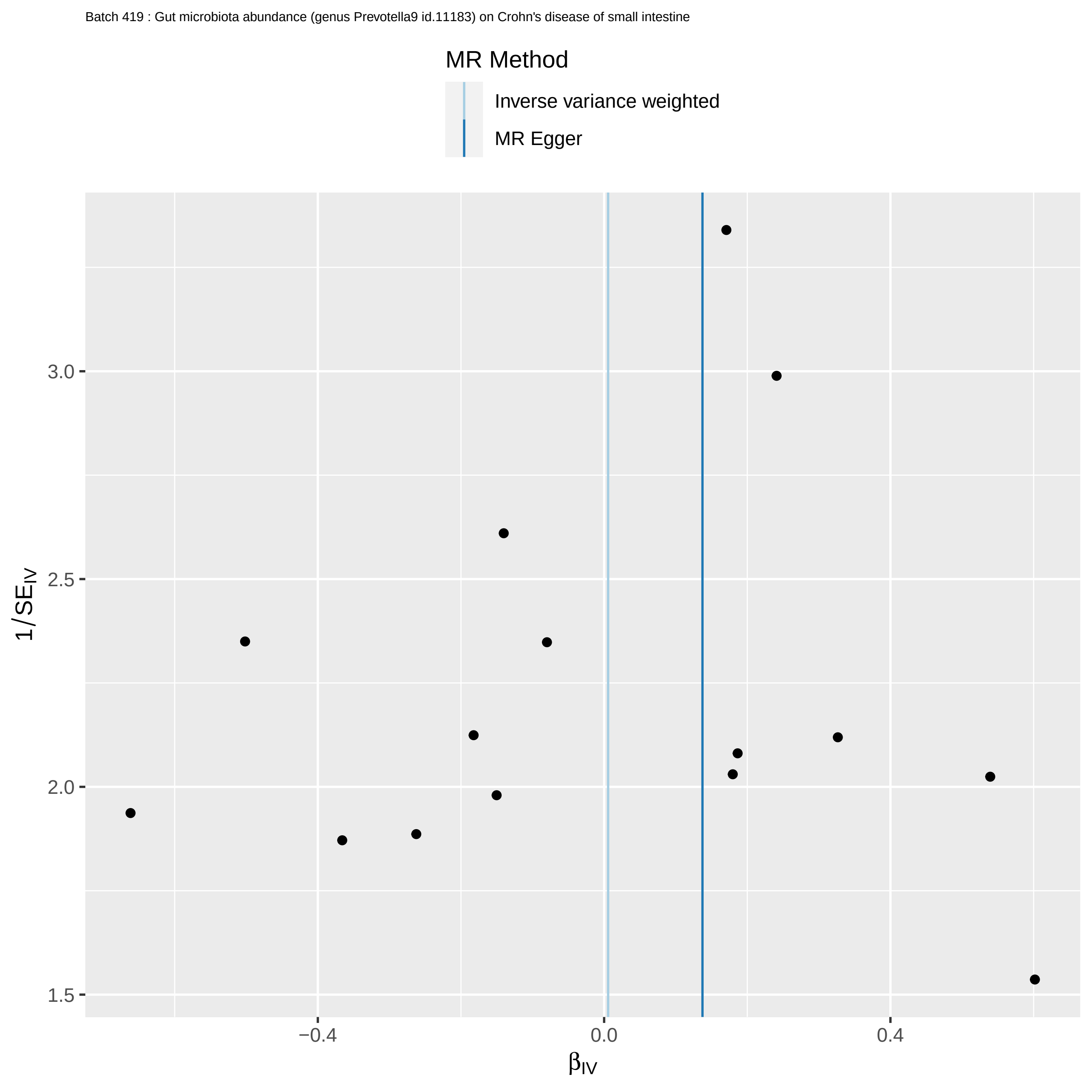

Batch 419 : Gut microbiota abundance (genus Prevotella9 id.11183) on Crohn's disease of small intestine
MR Method
Inverse variance weighted
MR Egger
3.0
2.5
2.0
1.5
1/SE$_{IV}$
−0.4
0.0
0.4
β$_{IV}$

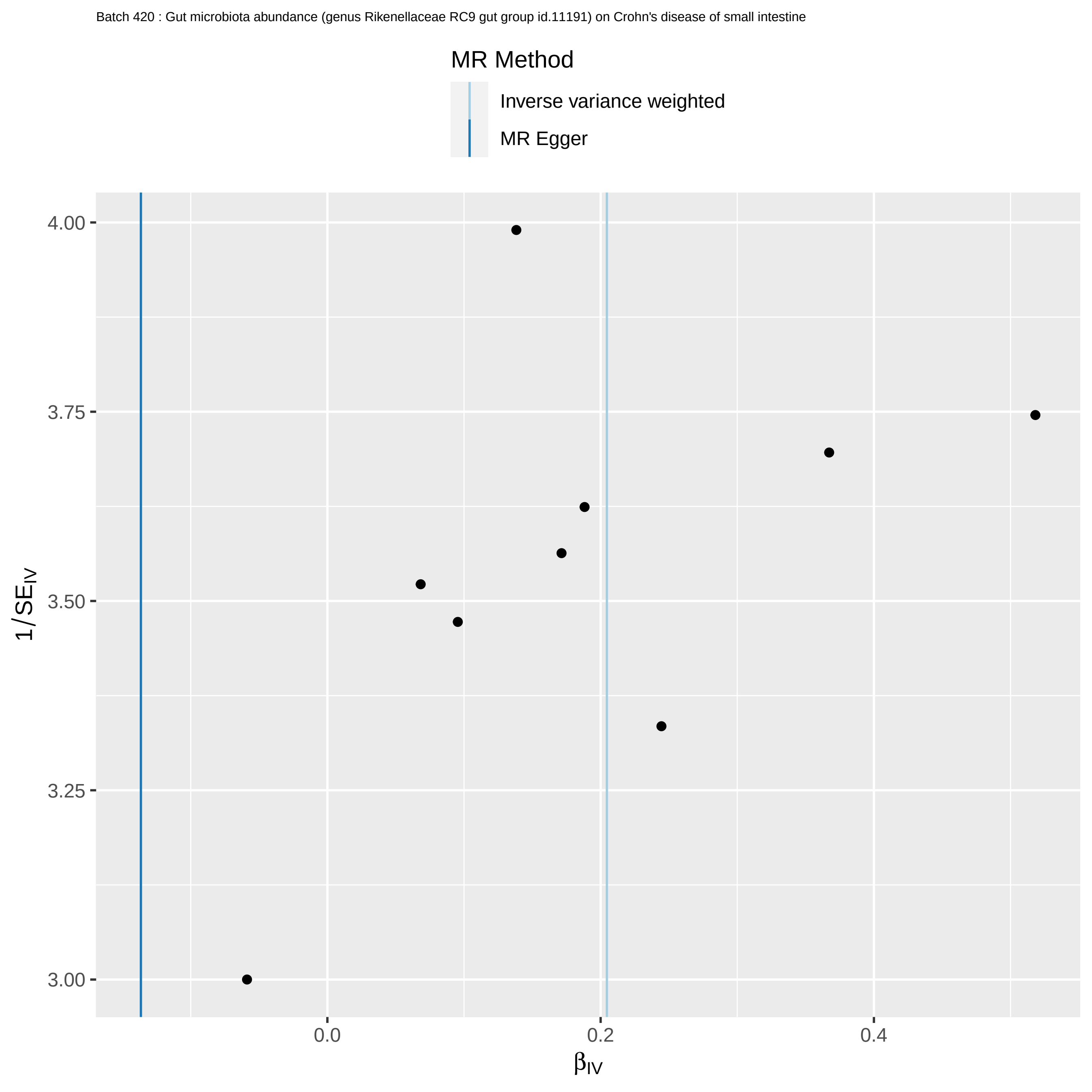
Batch 420 : Gut microbiota abundance (genus Rikenellaceae RC9 gut group id.11191) on Crohn's disease of small intestine
MR Method
Inverse variance weighted
MR Egger
4.00
3.75
3.50
3.25
3.00
1/SE$_{IV}$
0.0
0.2
0.4
β_{IV}

Batch 421 : Gut microbiota abundance (genus Romboutsia id.11347) on Crohn's disease of small intestine

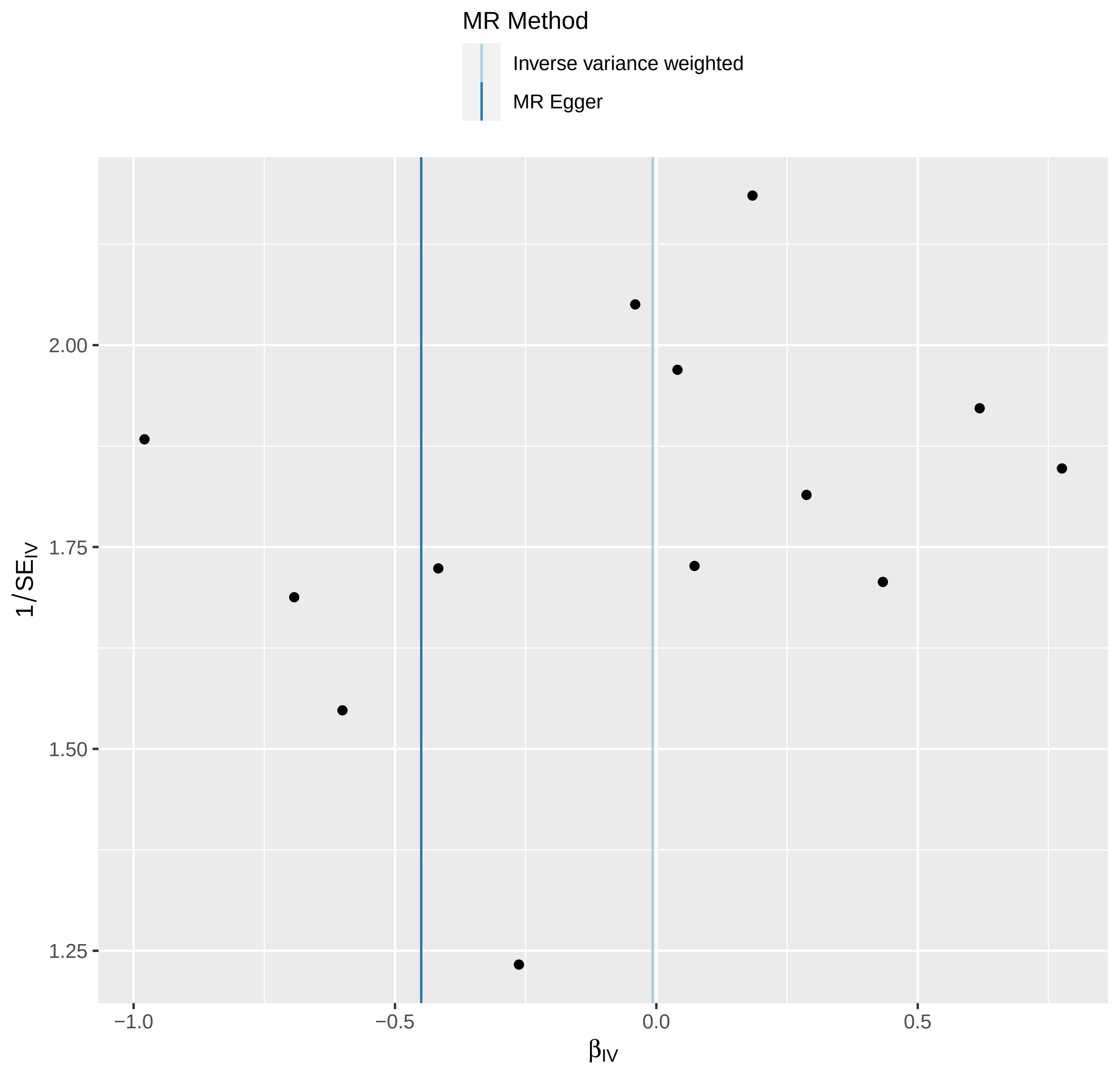

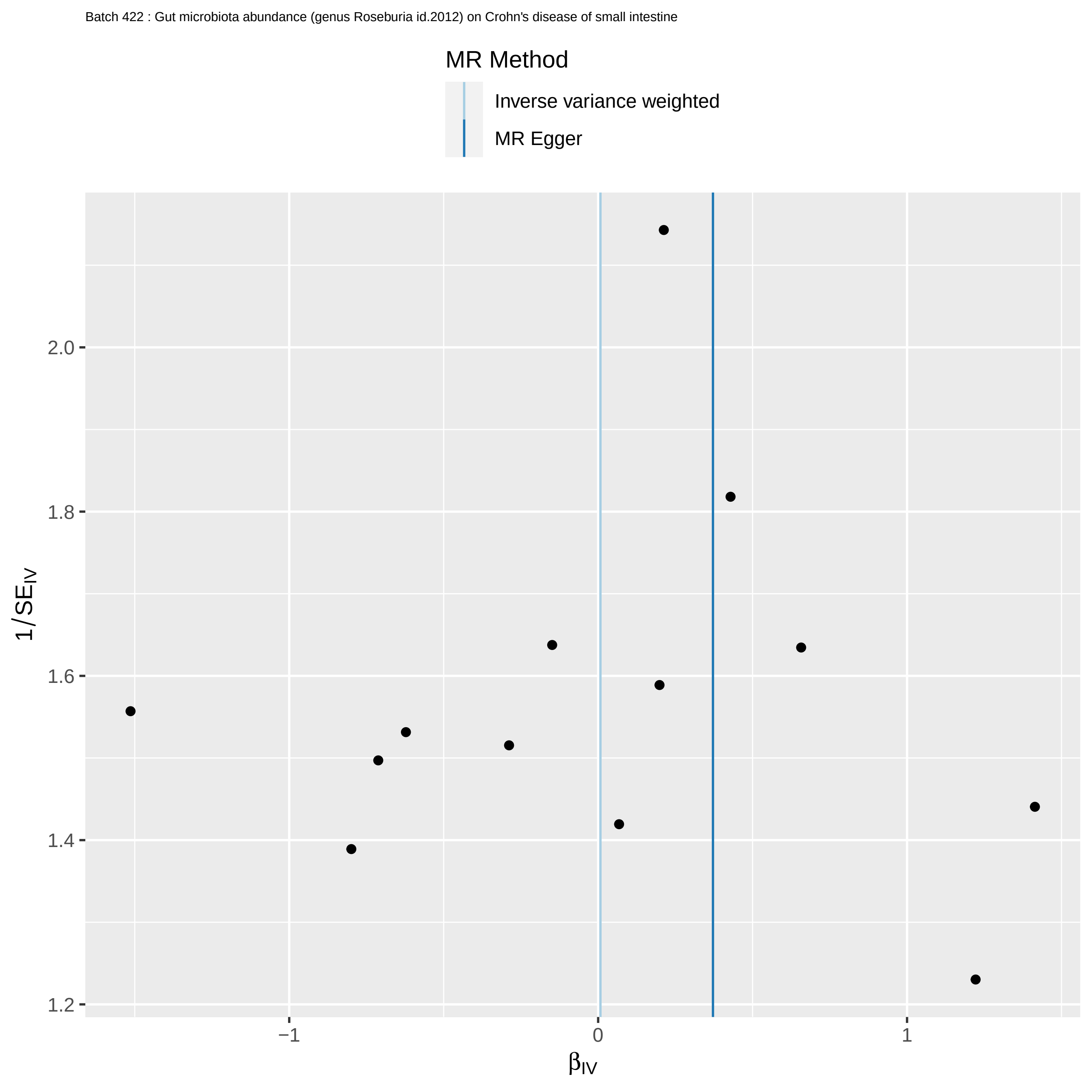

Batch 422 : Gut microbiota abundance (genus Roseburia id.2012) on Crohn's disease of small intestine
MR Method
Inverse variance weighted
MR Egger
2.0
1.8
1.6
1.4
1.2
1/SE_IV
−1
0
1
β_IV

Batch 423 : Gut microbiota abundance (genus Ruminiclostridium5 id.11355) on Crohn's disease of small intestine

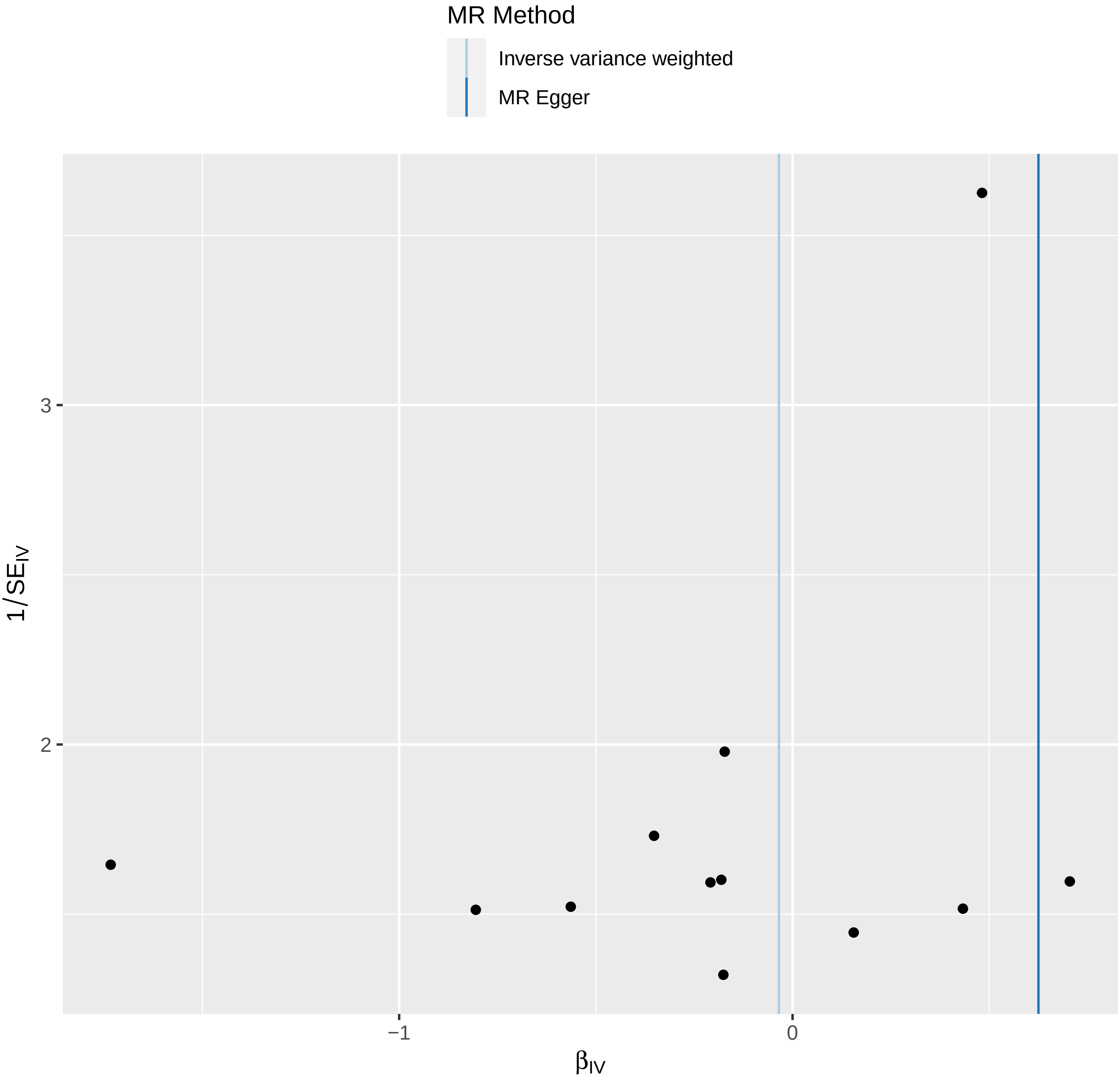

Batch 424 : Gut microbiota abundance (genus Ruminiclostridium6 id.11356) on Crohn's disease of small intestine

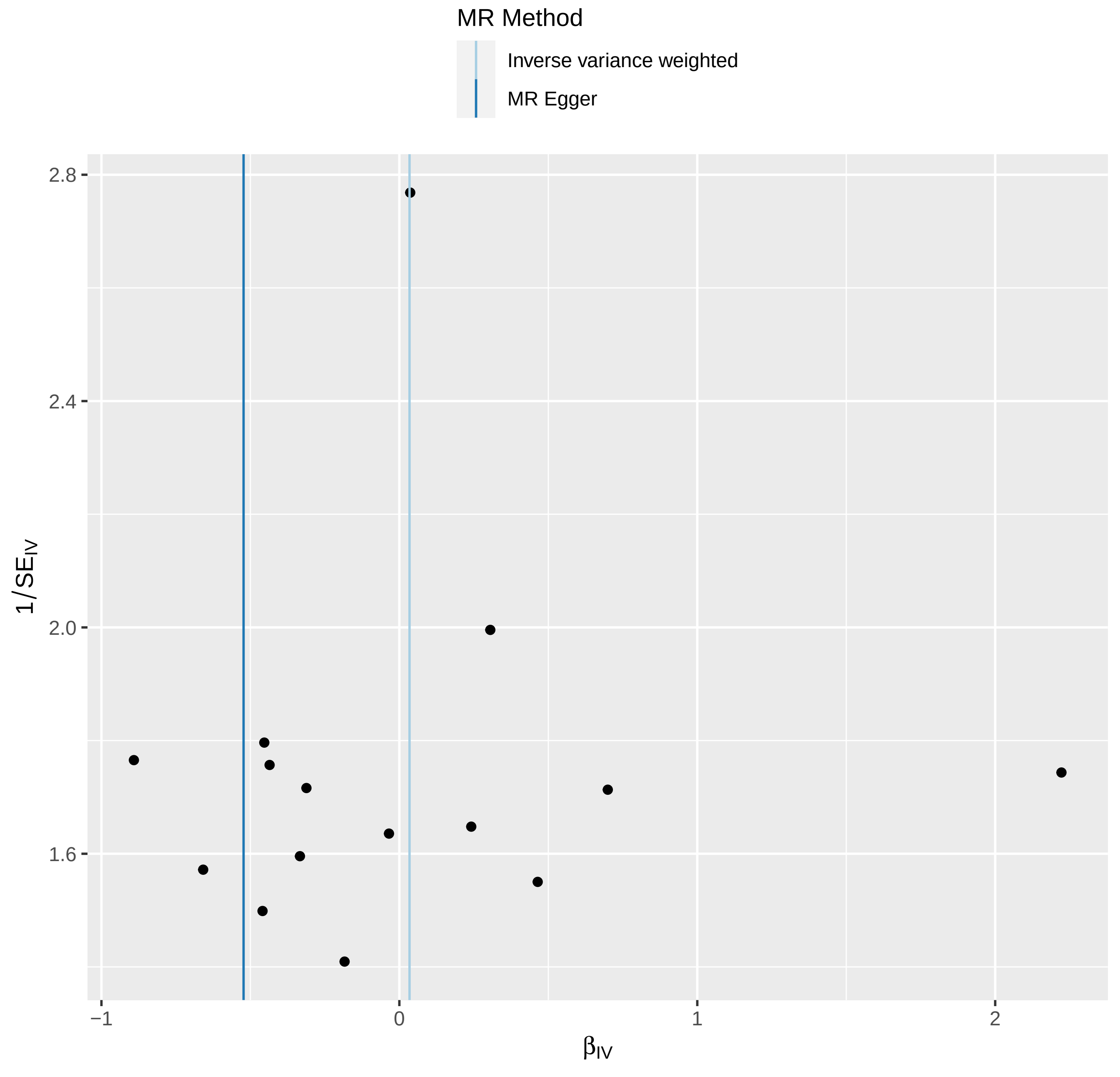

Batch 425 : Gut microbiota abundance (genus Ruminiclostridium9 id.11357) on Crohn's disease of small intestine

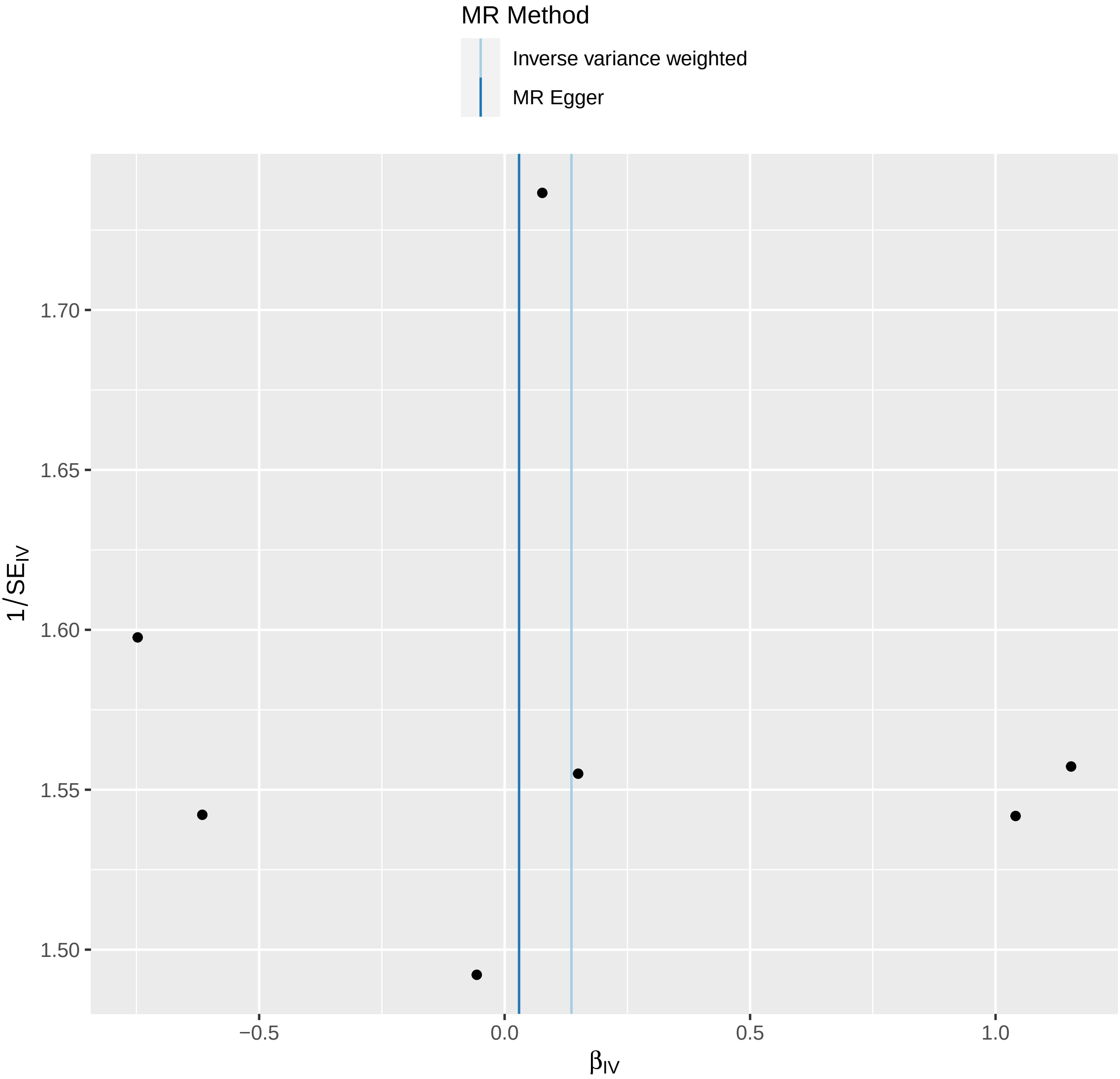

Batch 426 : Gut microbiota abundance (genus Ruminococcaceae NK4A214 group id.11358) on Crohn's disease of small intestine

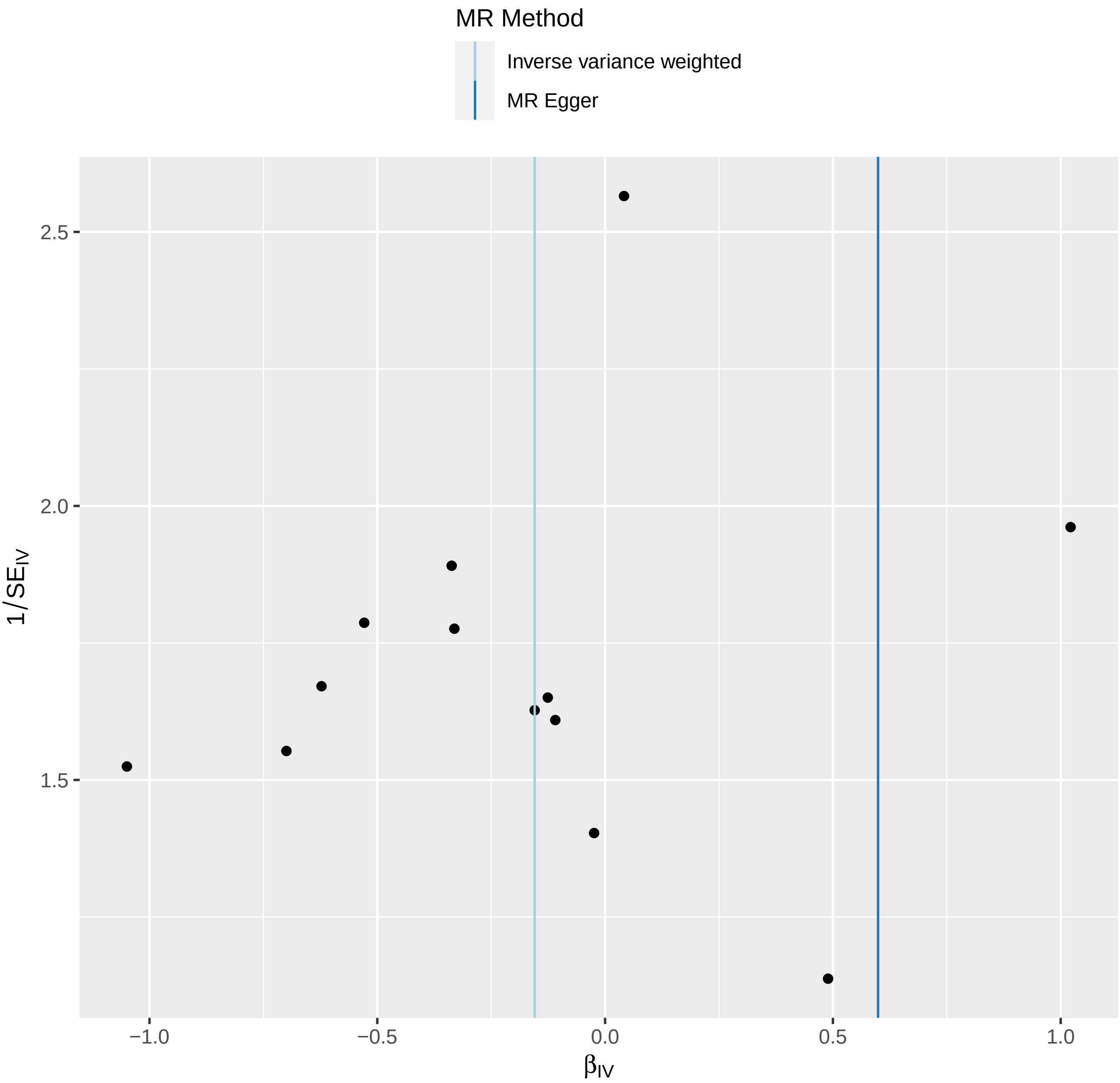

Batch 427 : Gut microbiota abundance (genus Ruminococcaceae UCG002 id.11360) on Crohn's disease of small intestine

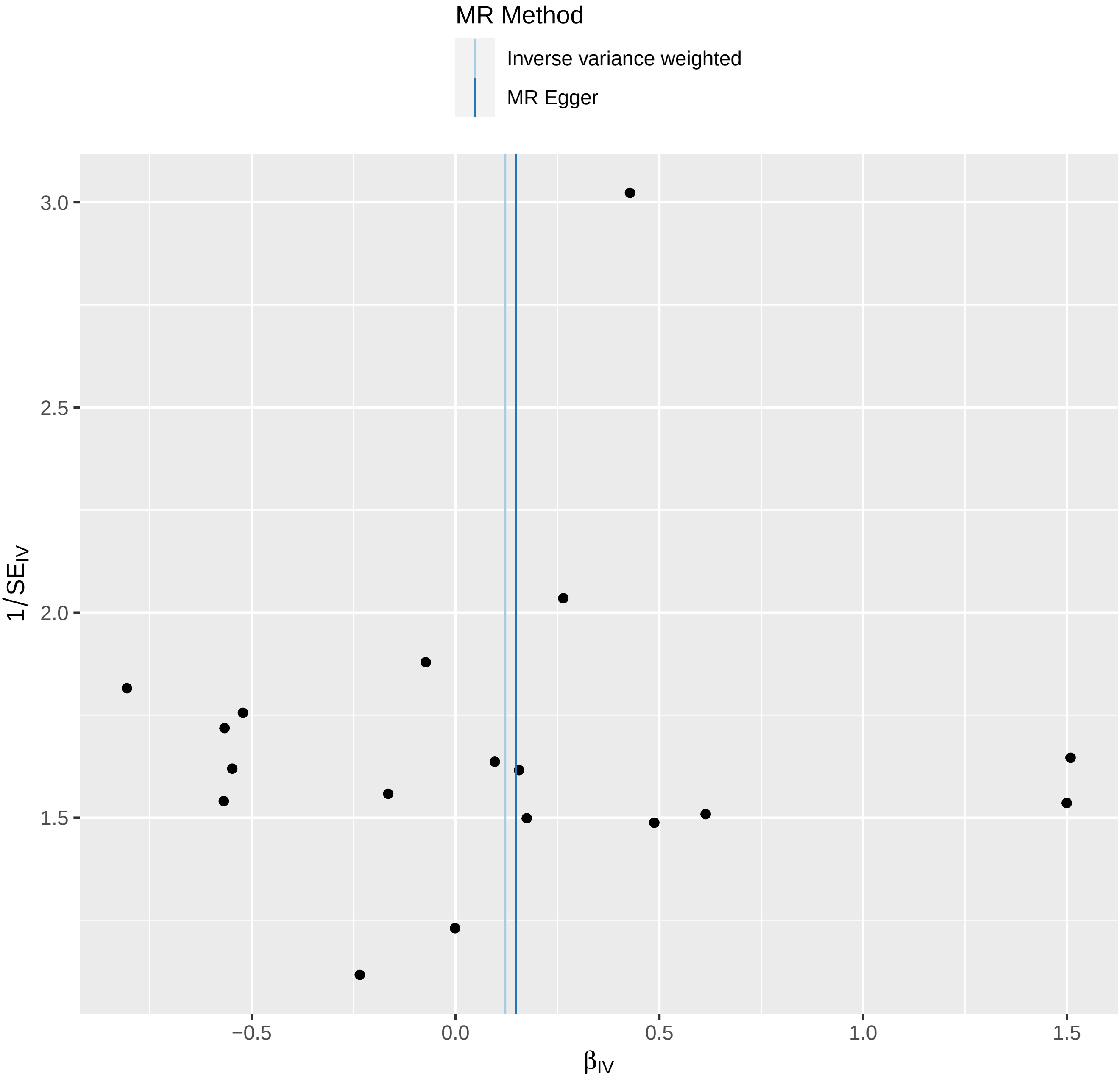

Batch 428 : Gut microbiota abundance (genus Ruminococcaceae UCG003 id.11361) on Crohn's disease of small intestine

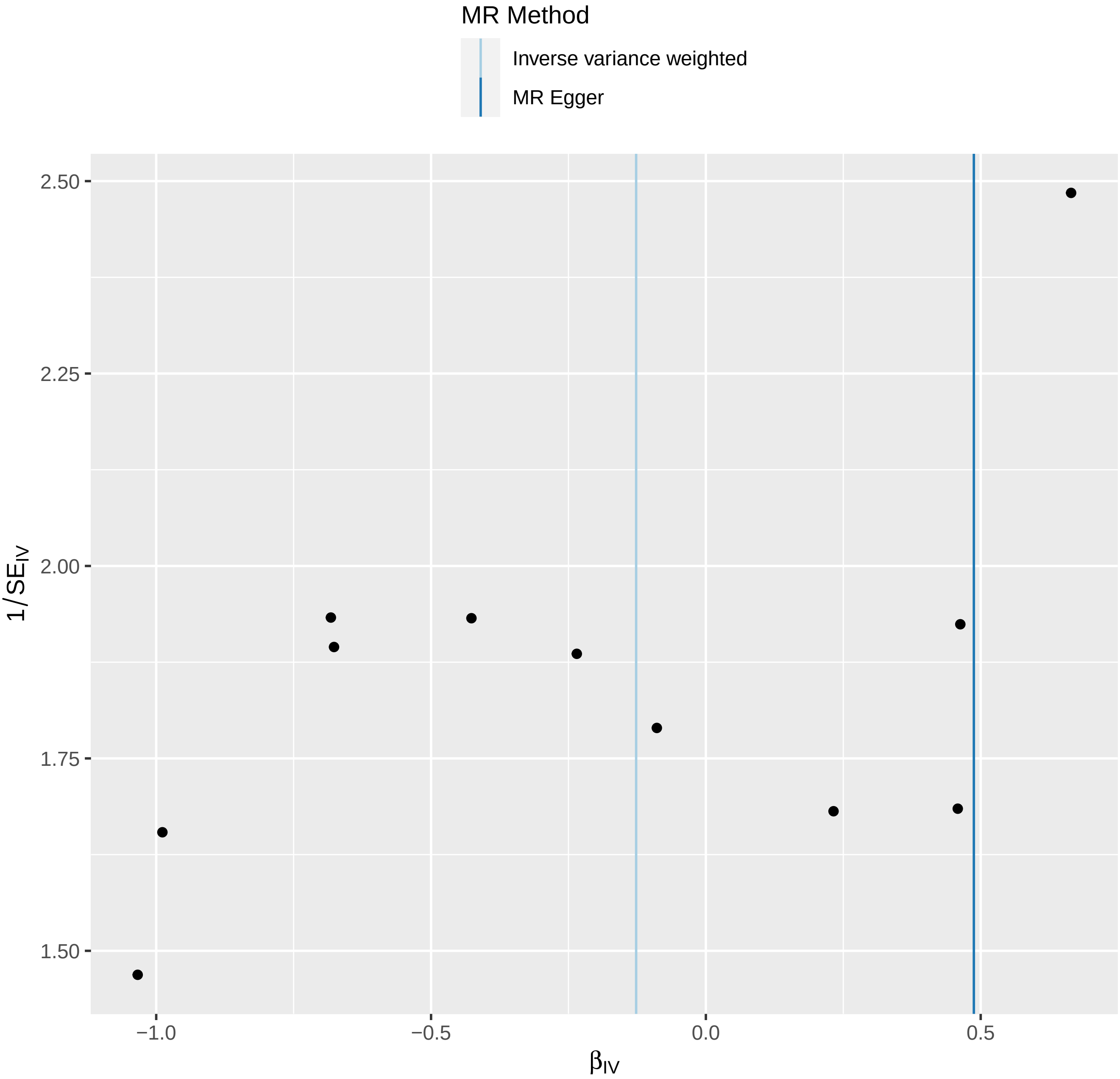

Batch 429 : Gut microbiota abundance (genus Ruminococcaceae UCG004 id.11362) on Crohn's disease of small intestine

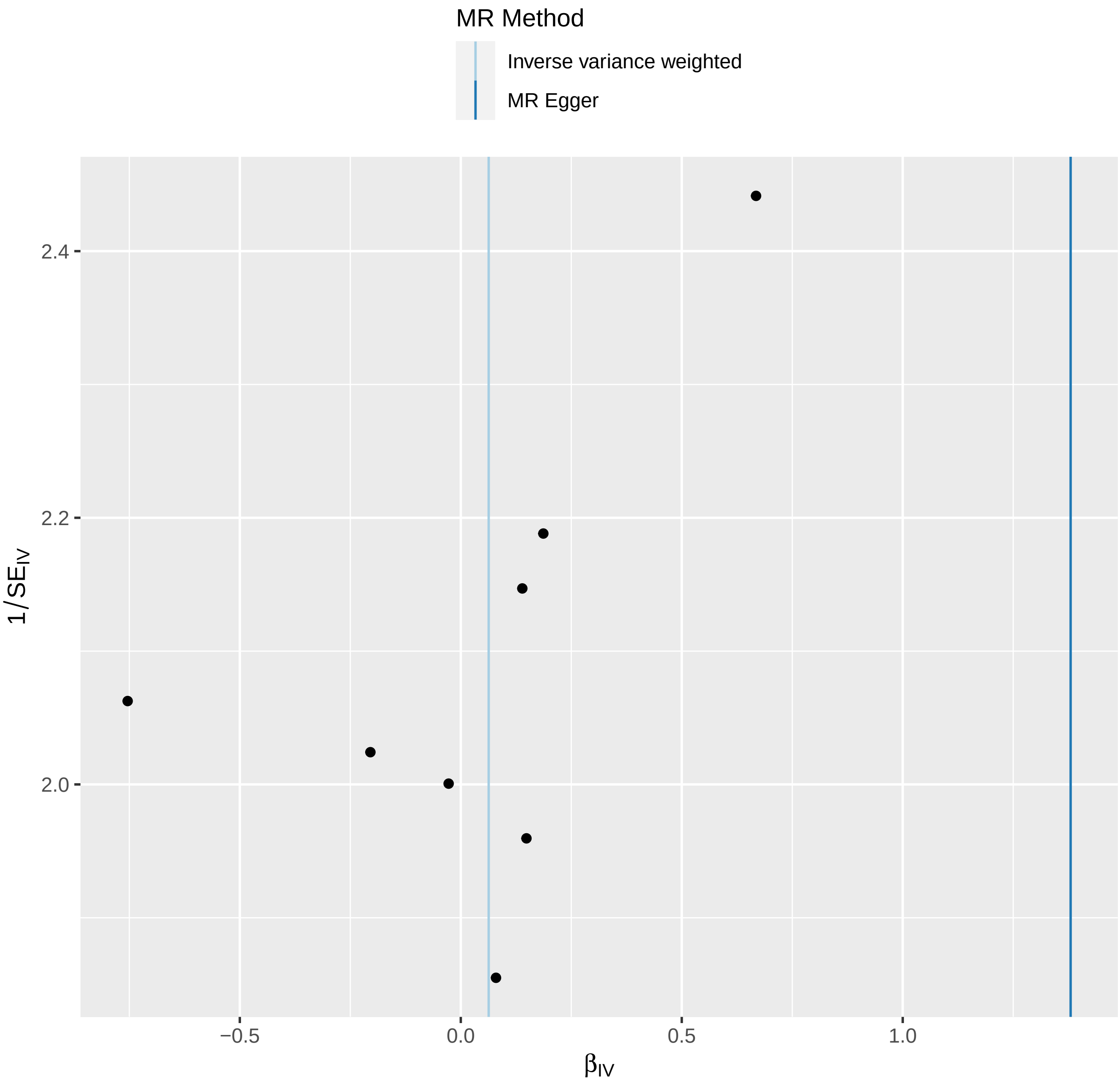

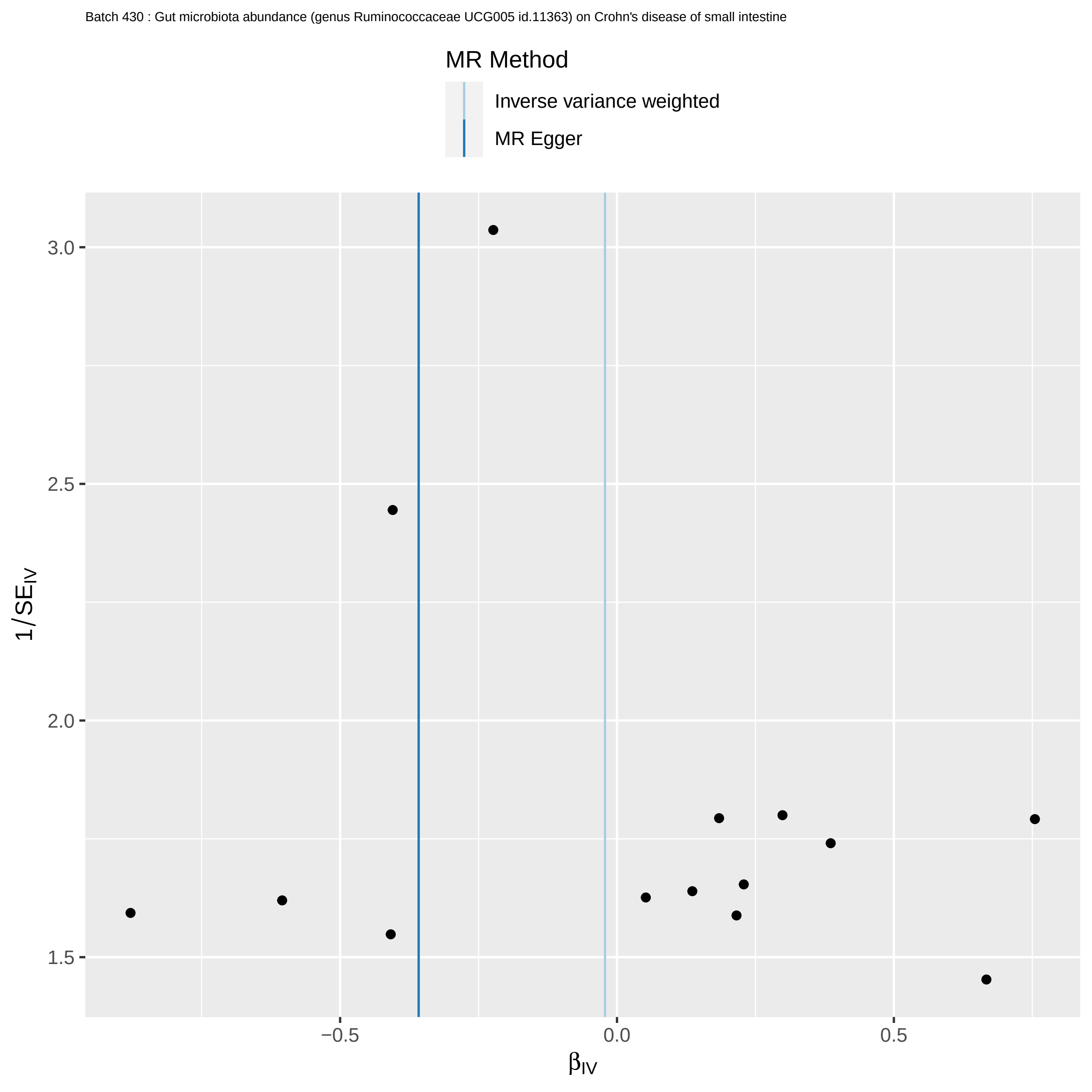
Batch 430 : Gut microbiota abundance (genus Ruminococcaceae UCG005 id.11363) on Crohn's disease of small intestine
MR Method
Inverse variance weighted
MR Egger
3.0
2.5
2.0
1.5
1/SE_IV
−0.5
0.0
0.5
β_IV

Batch 431 : Gut microbiota abundance (genus Ruminococcaceae UCG009 id.11366) on Crohn's disease of small intestine

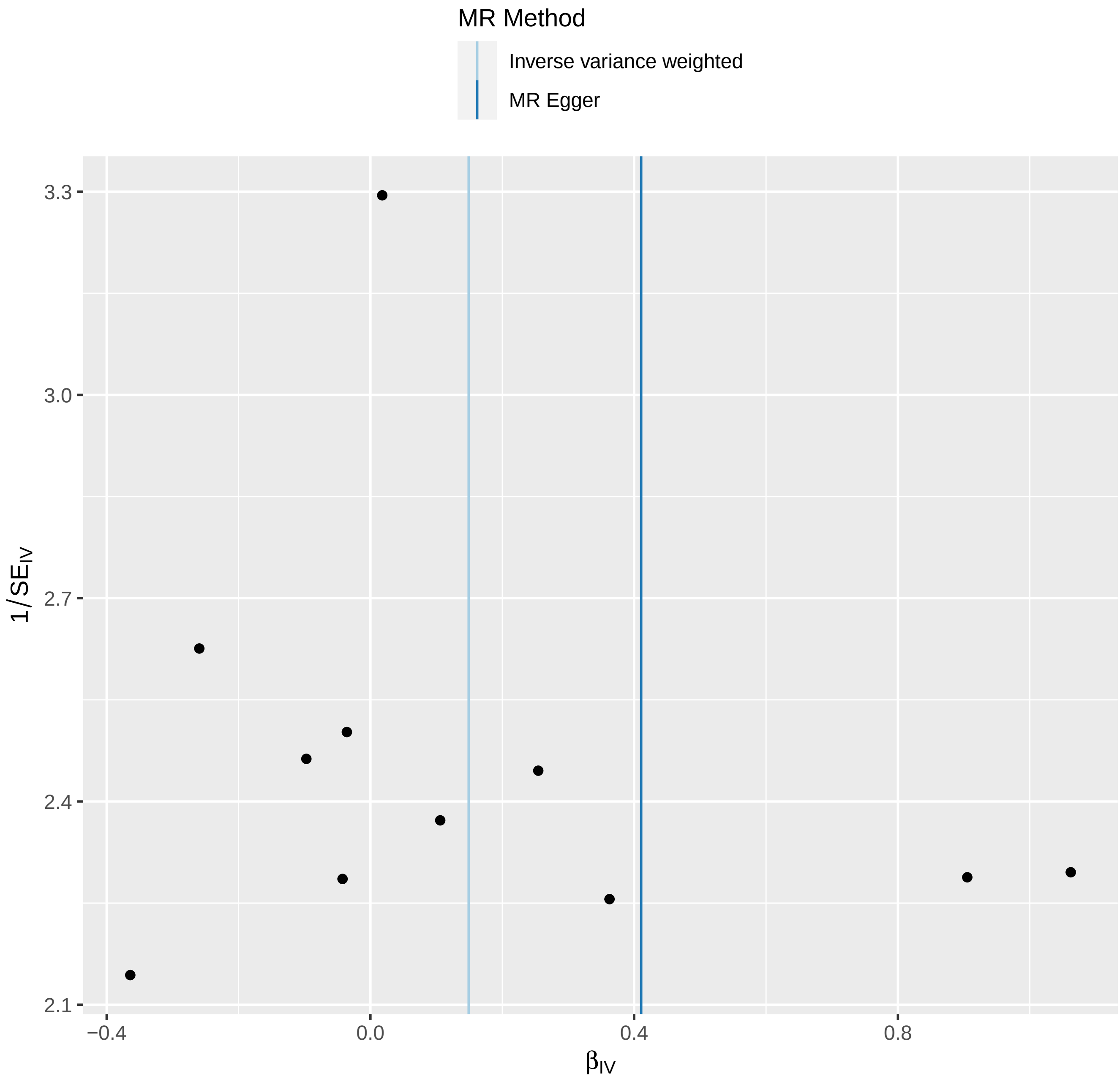

Batch 432 : Gut microbiota abundance (genus Ruminococcaceae UCG010 id.11367) on Crohn's disease of small intestine

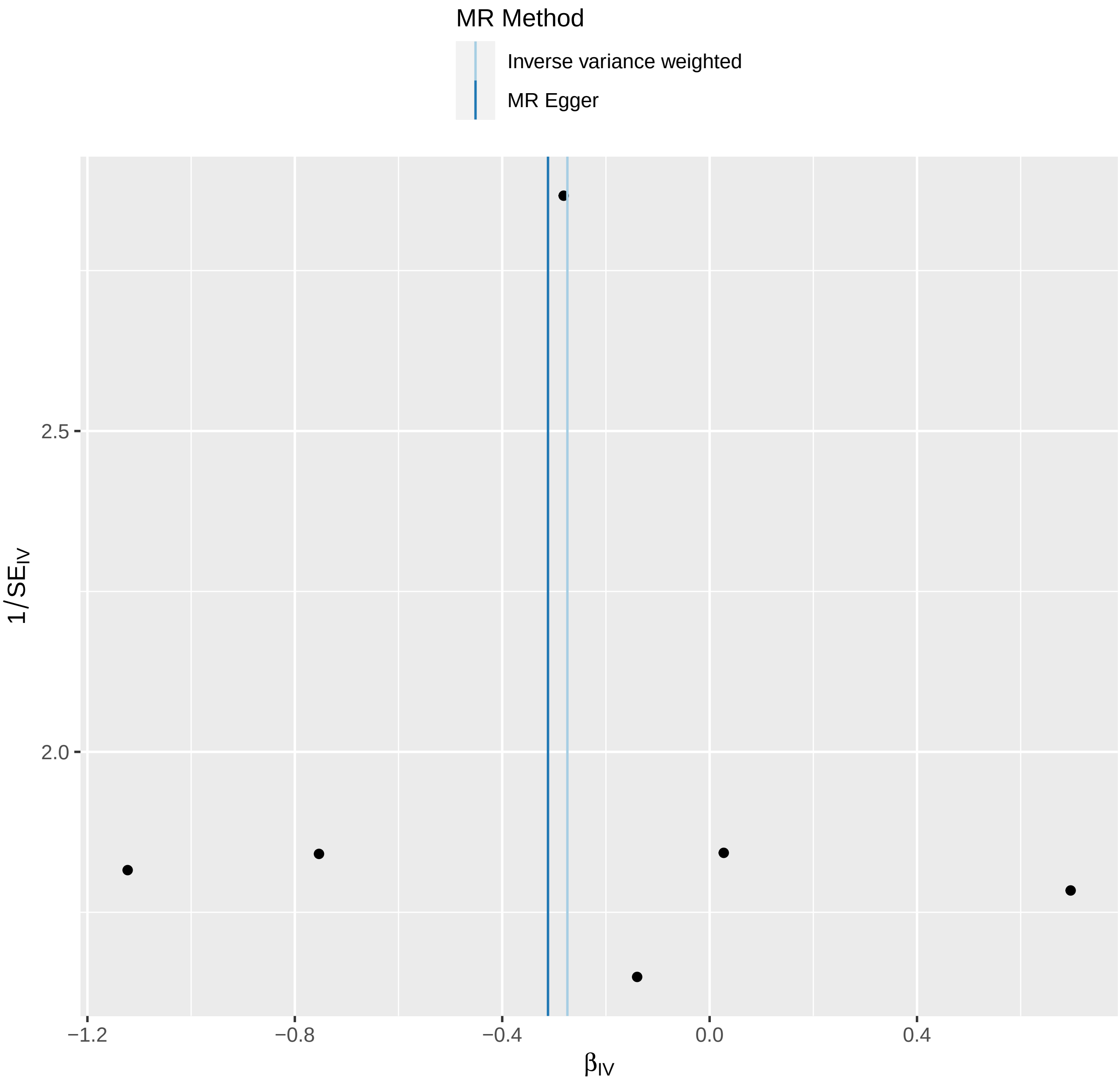

Batch 433 : Gut microbiota abundance (genus Ruminococcaceae UCG011 id.11368) on Crohn's disease of small intestine

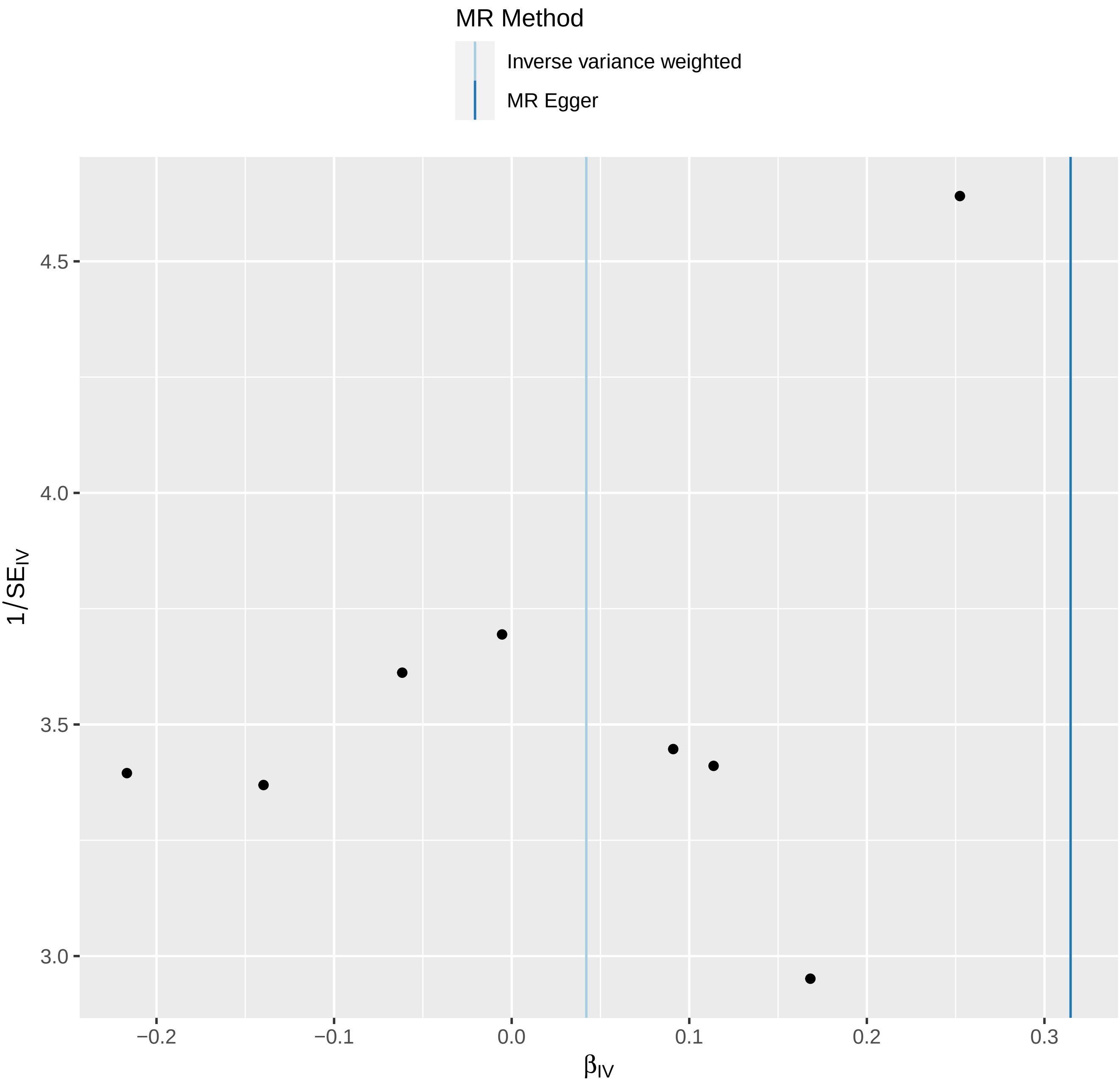

Batch 434 : Gut microbiota abundance (genus Ruminococcaceae UCG013 id.11370) on Crohn's disease of small intestine

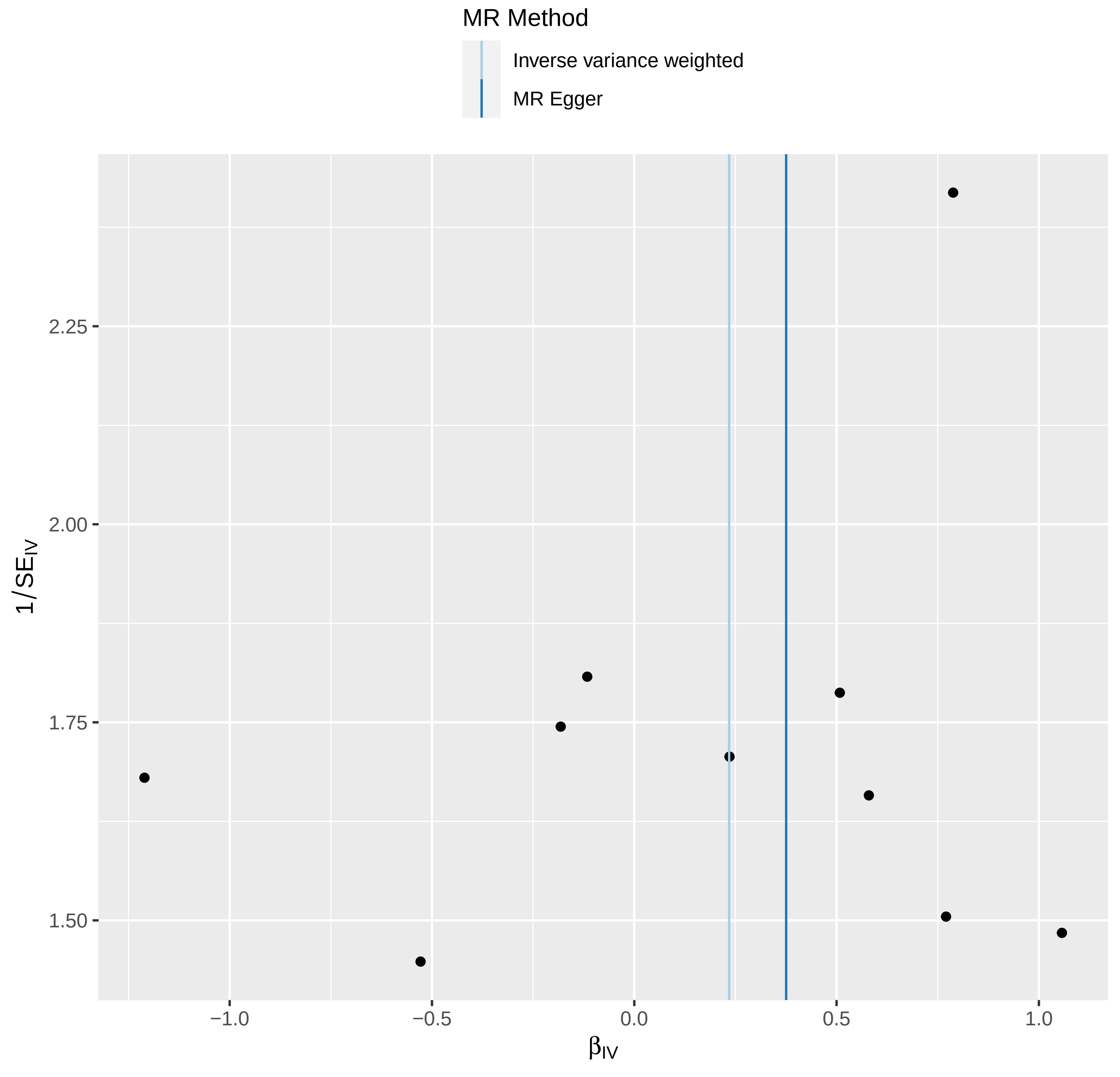

Batch 435 : Gut microbiota abundance (genus Ruminococcaceae UCG014 id.11371) on Crohn's disease of small intestine

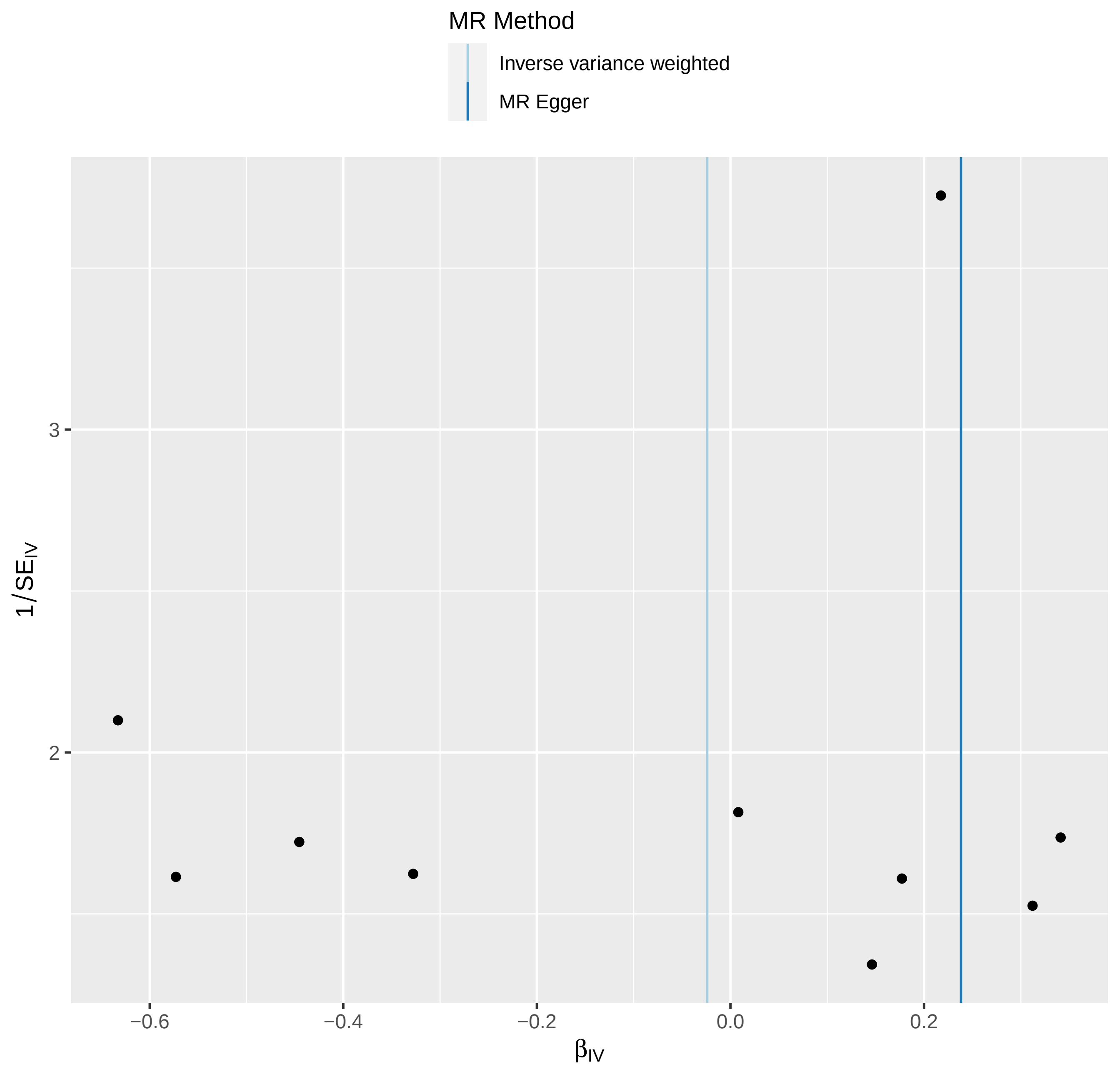

Batch 436 : Gut microbiota abundance (genus Ruminococcus1 id.11373) on Crohn's disease of small intestine

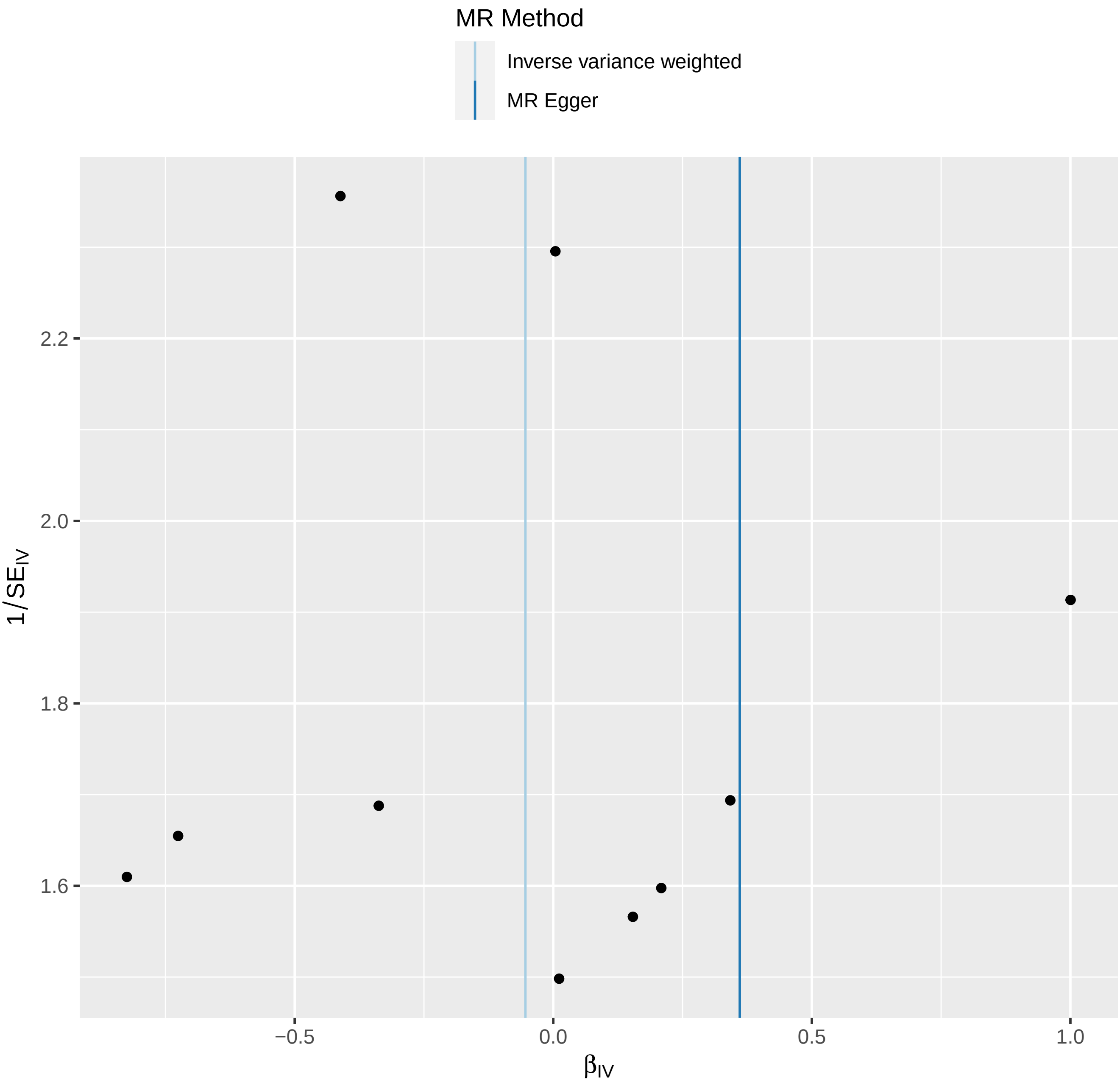

Batch 437 : Gut microbiota abundance (genus Ruminococcus2 id.11374) on Crohn's disease of small intestine

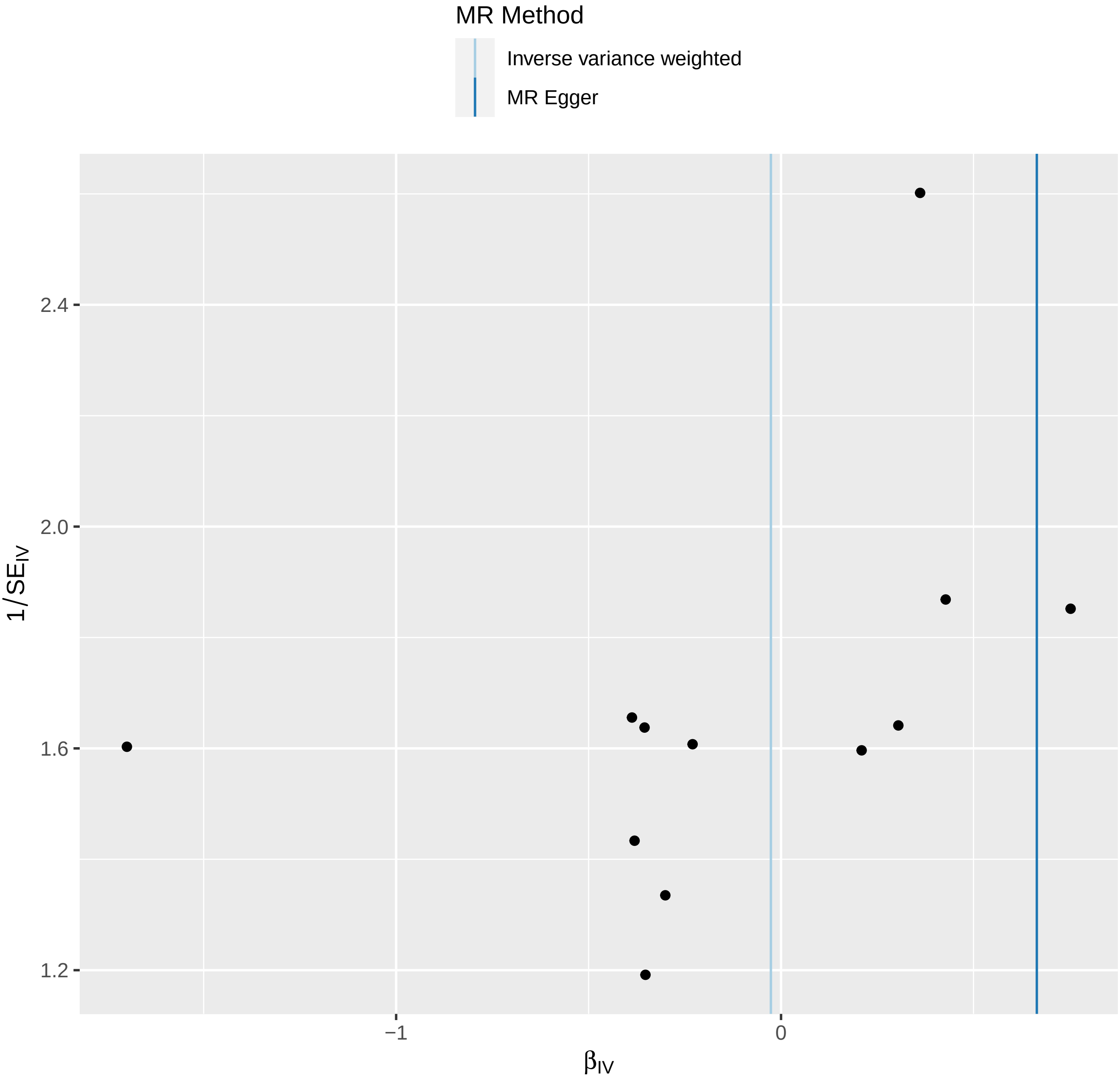

Batch 438 : Gut microbiota abundance (genus Ruminococcus gauvreauii group id.11342) on Crohn's disease of small intestine

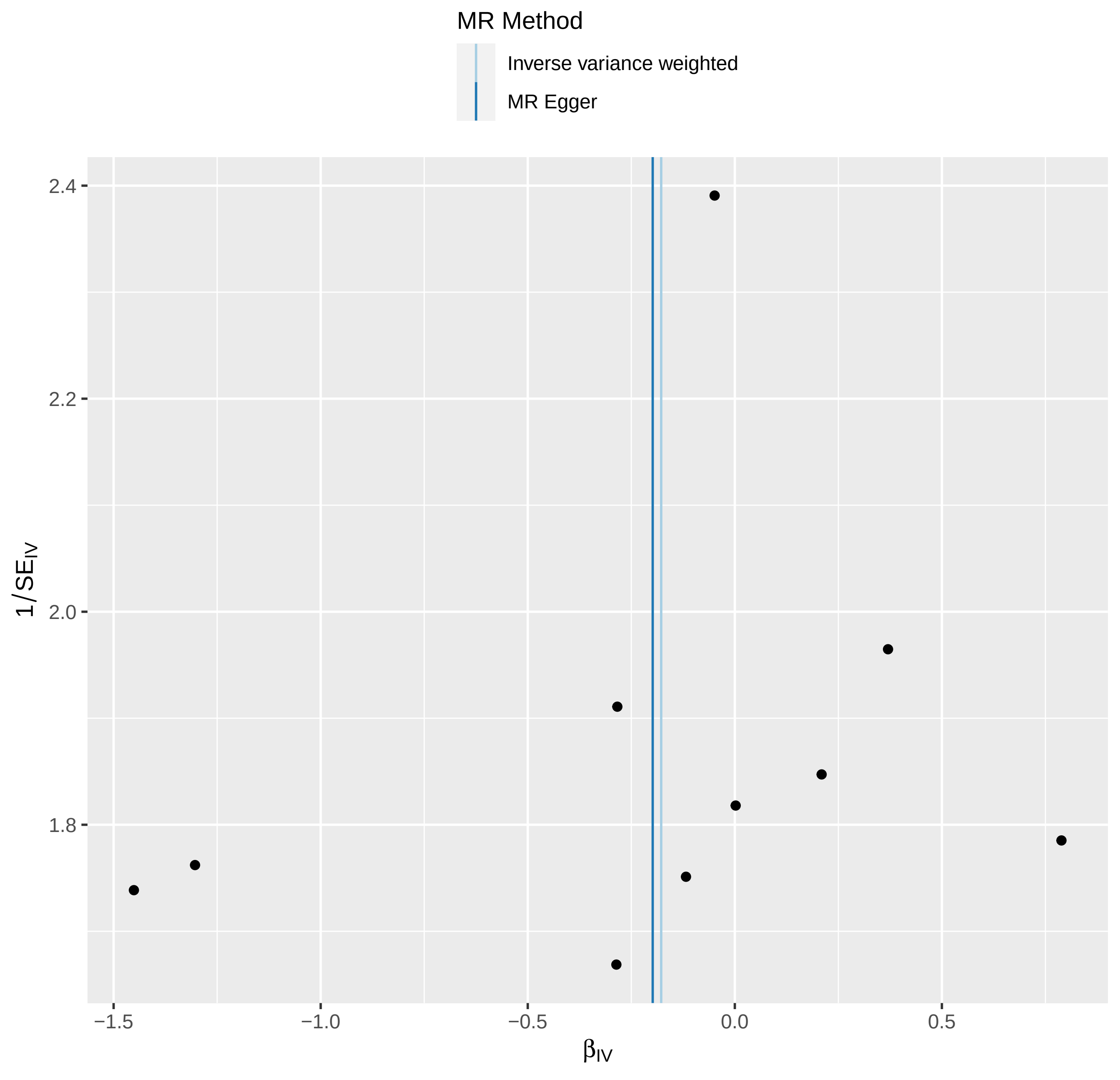

Batch 439 : Gut microbiota abundance (genus Ruminococcus gnavus group id.14376) on Crohn's disease of small intestine

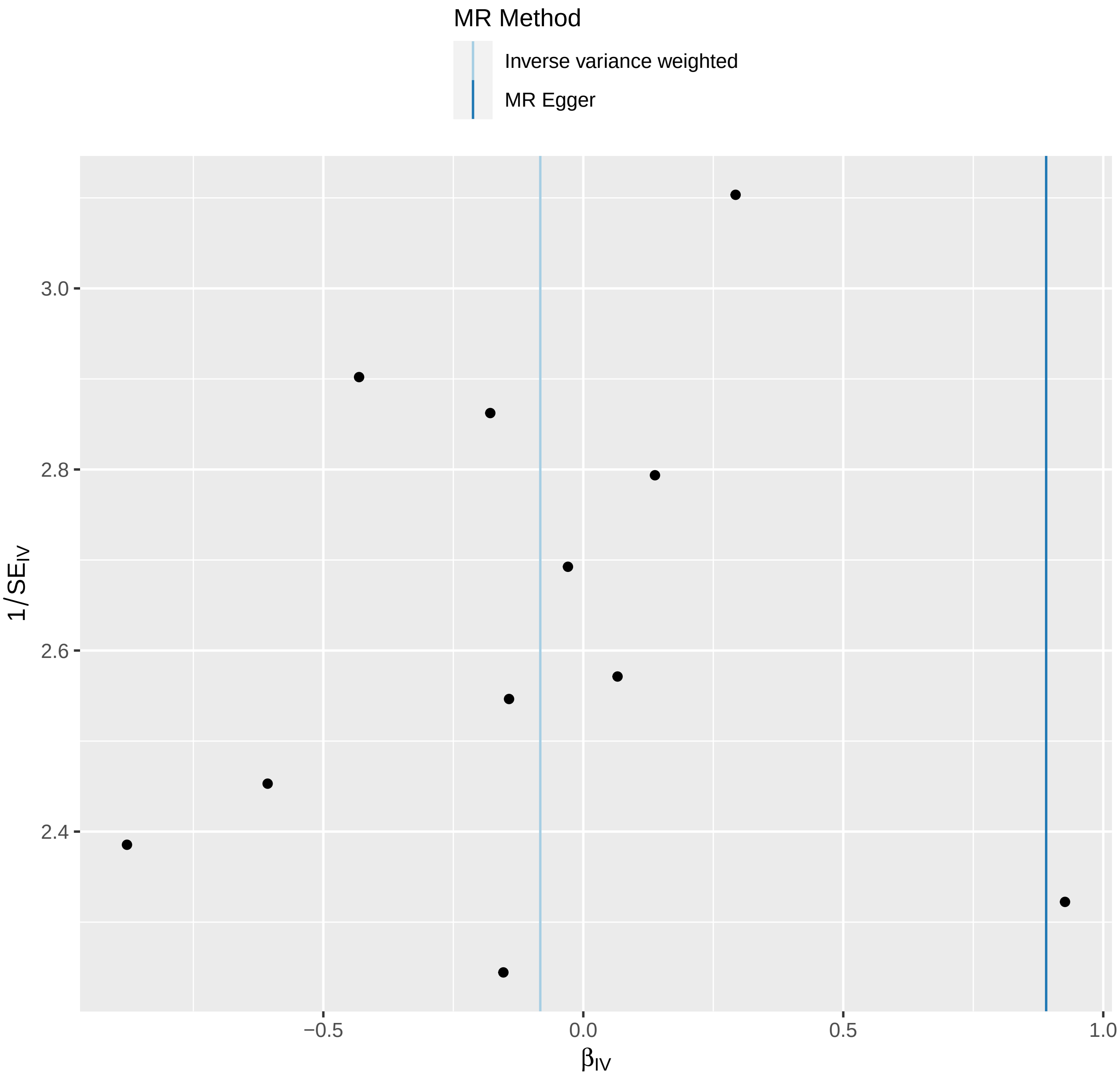

Batch 440 : Gut microbiota abundance (genus Ruminococcus torques group id.14377) on Crohn's disease of small intestine

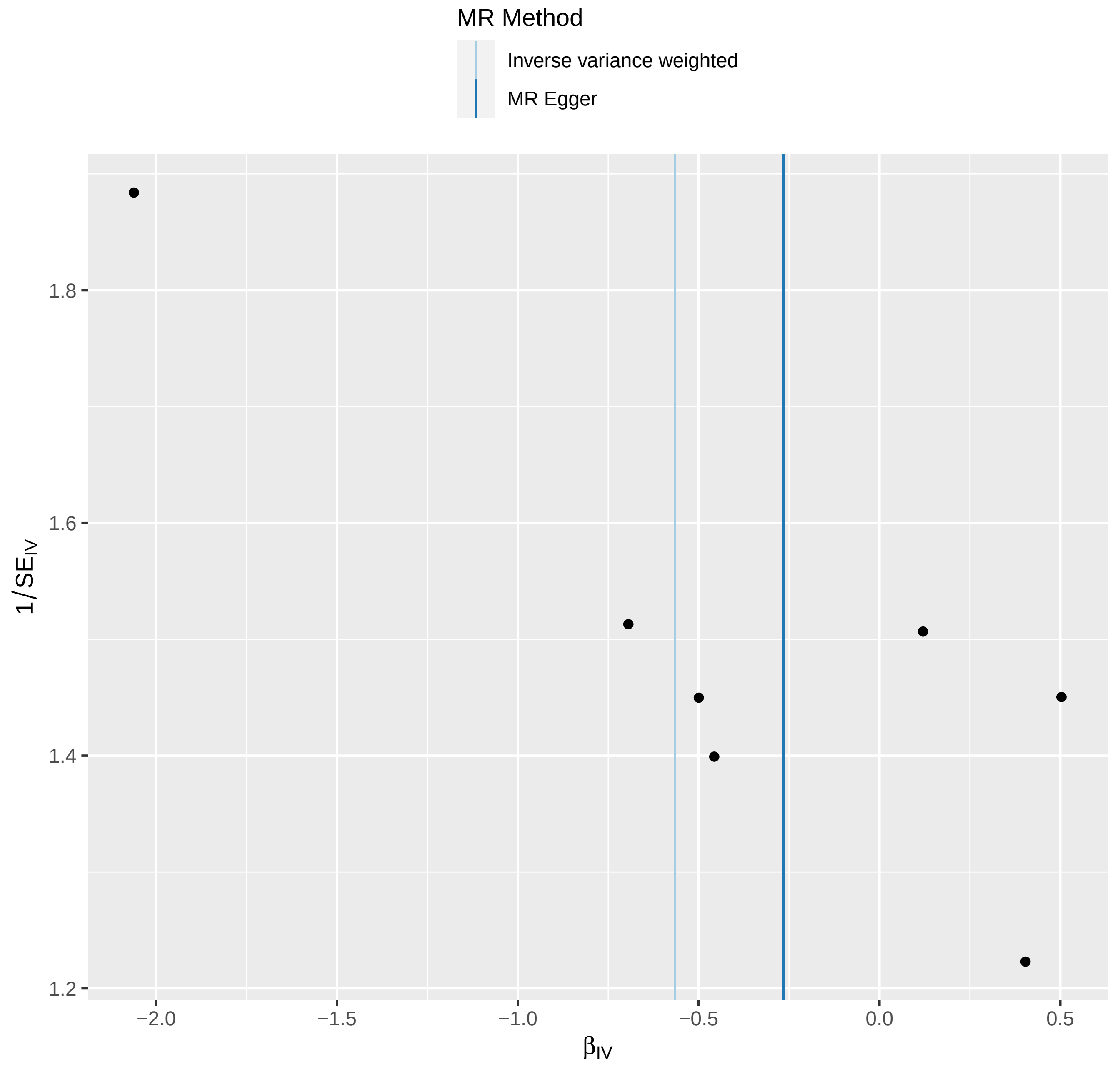

Batch 441 : Gut microbiota abundance (genus Sellimonas id.14369) on Crohn's disease of small intestine

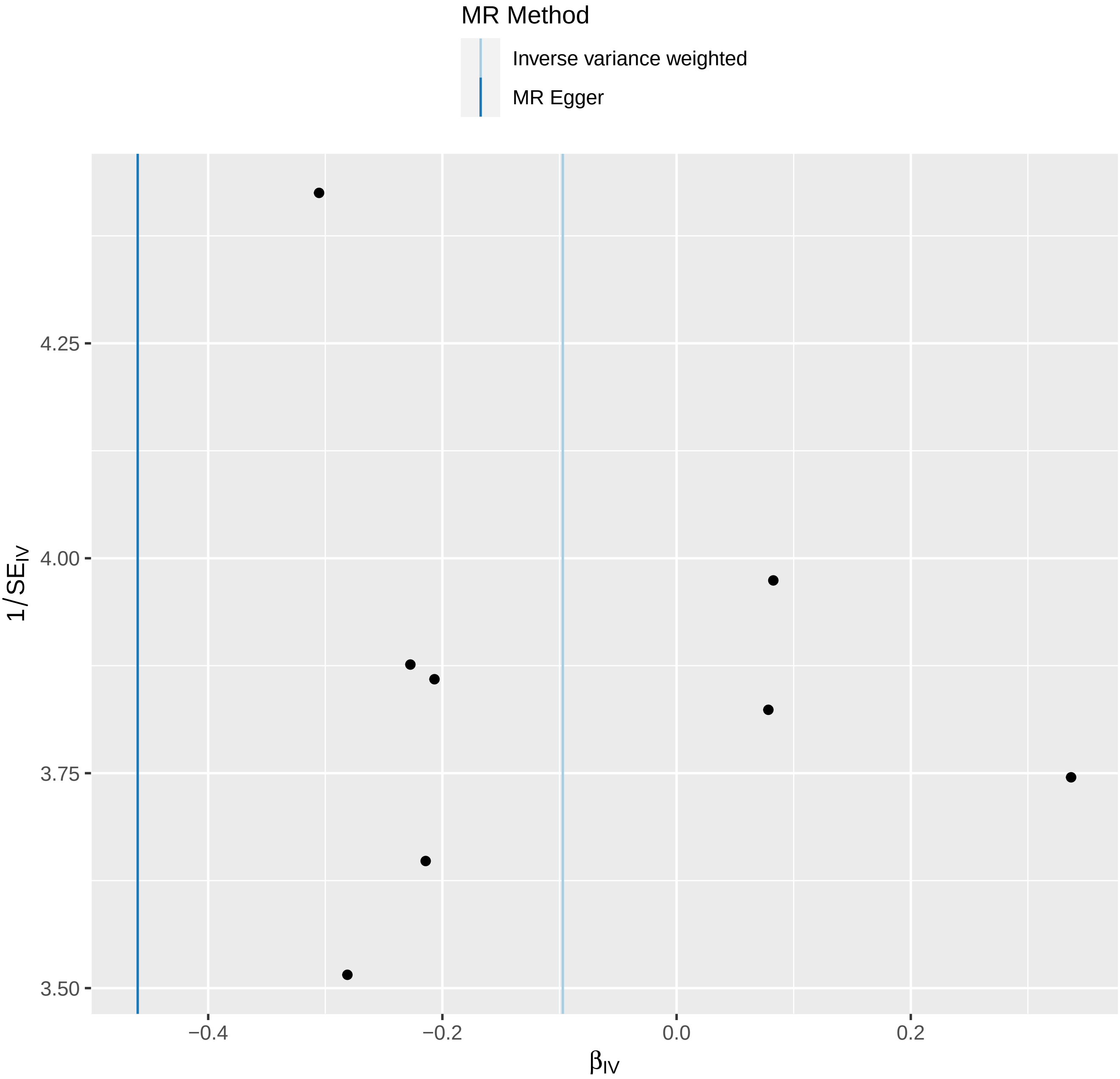

Batch 442 : Gut microbiota abundance (genus Senegalimassilia id.11160) on Crohn's disease of small intestine

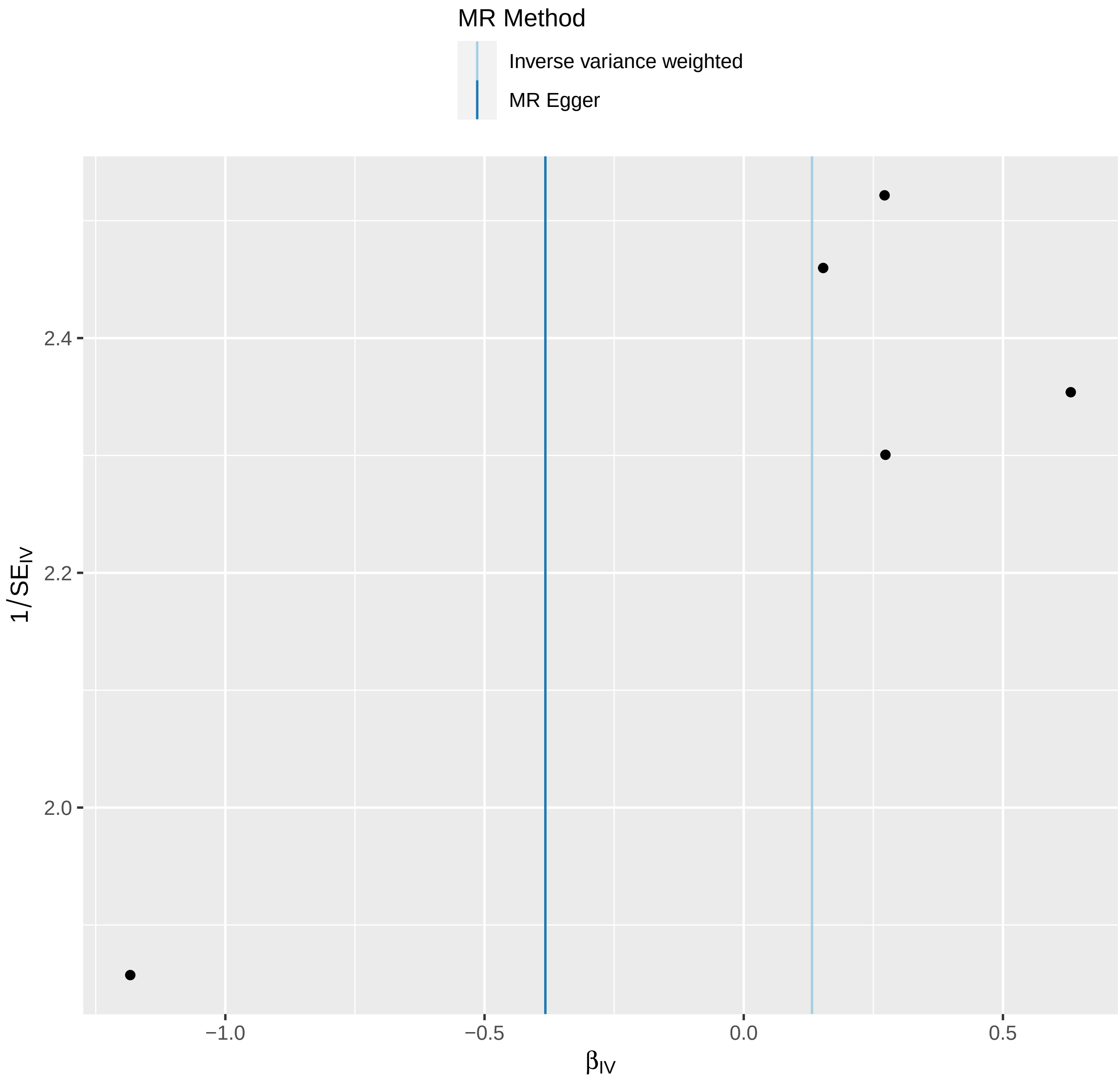

Batch 443 : Gut microbiota abundance (genus Slackia id.825) on Crohn's disease of small intestine

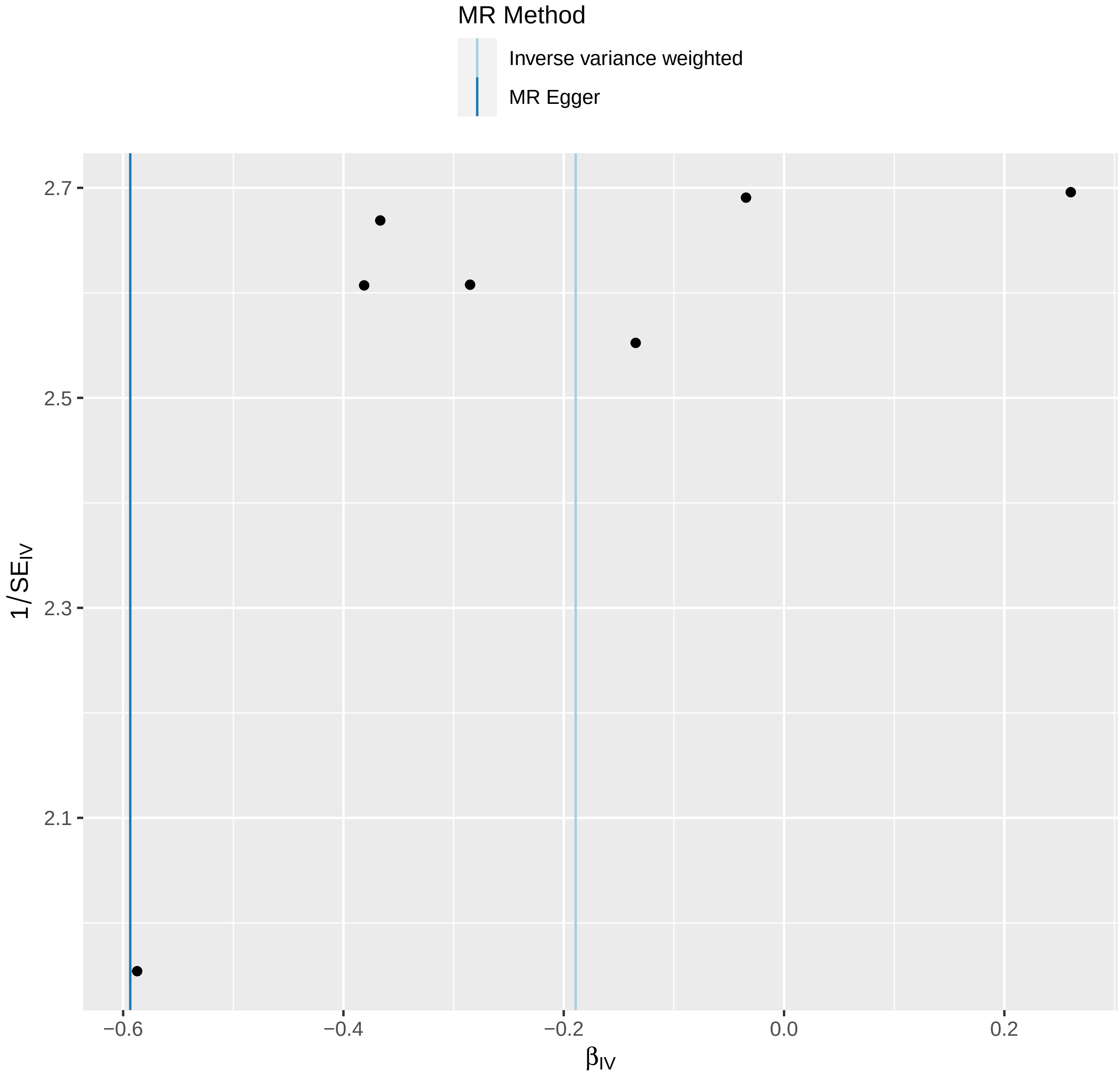

Batch 444 : Gut microbiota abundance (genus Streptococcus id.1853) on Crohn's disease of small intestine

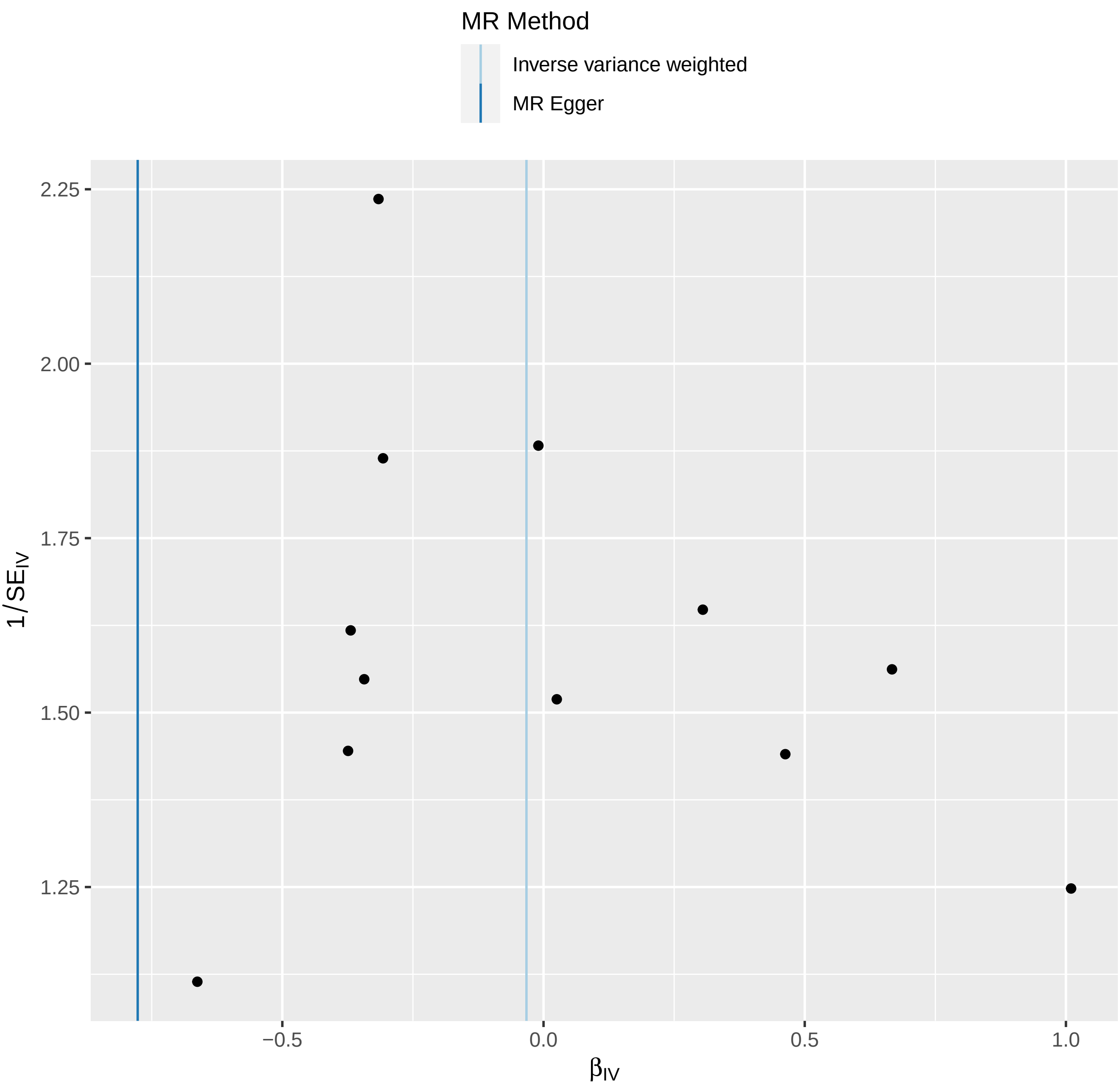

Batch 445 : Gut microbiota abundance (genus Subdoligranulum id.2070) on Crohn's disease of small intestine

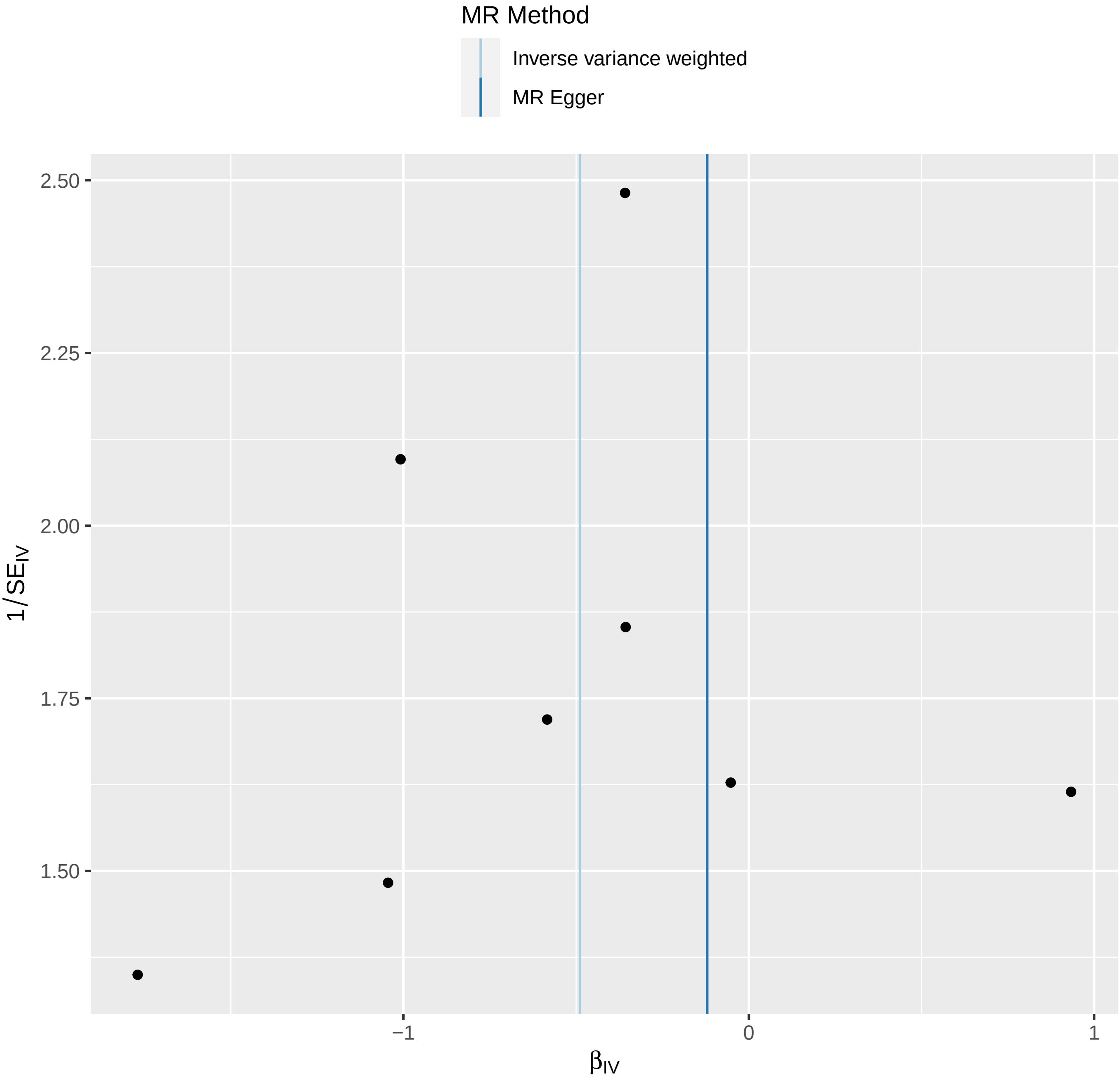

Batch 446 : Gut microbiota abundance (genus Sutterella id.2896) on Crohn's disease of small intestine

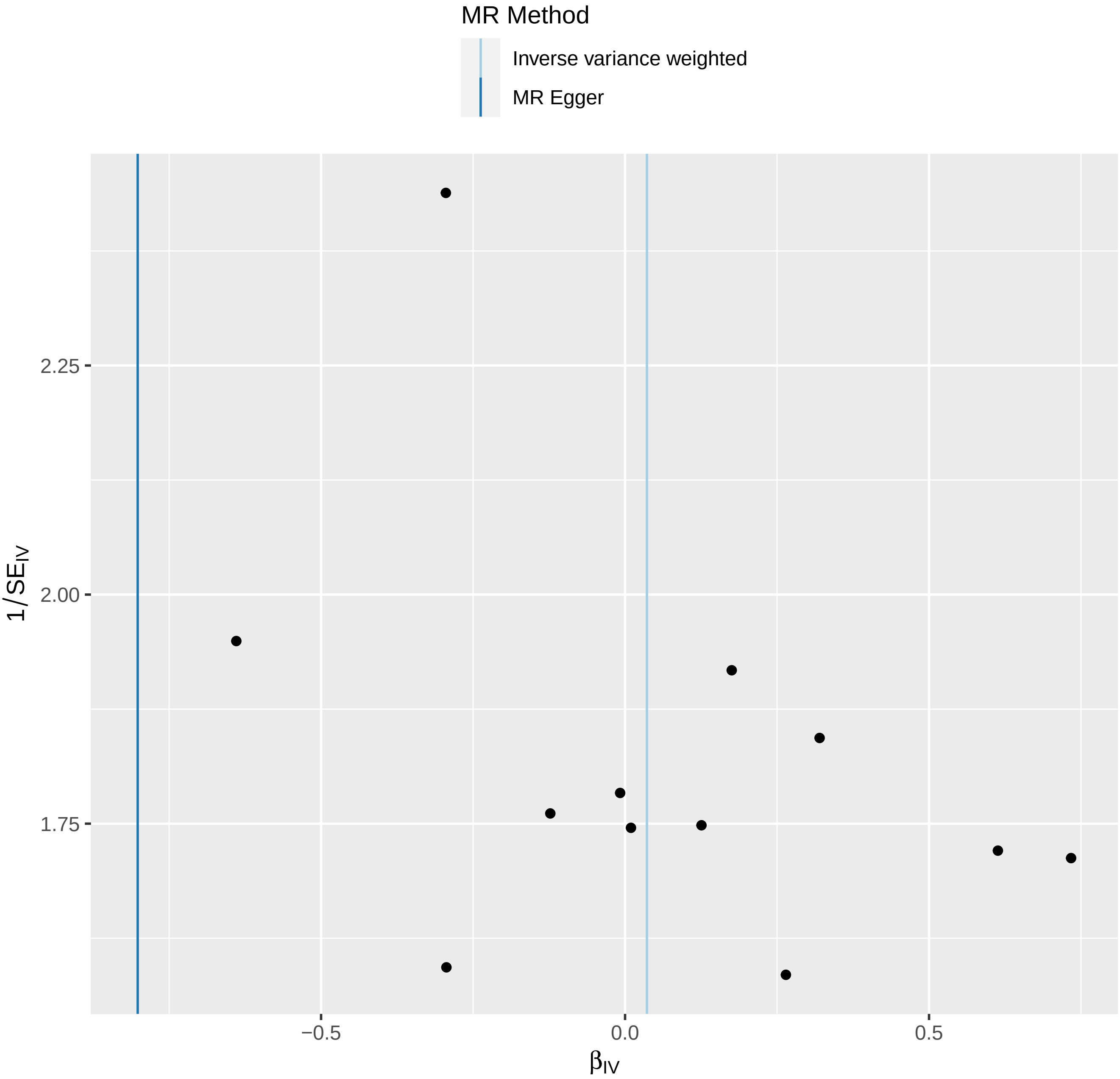

Batch 447 : Gut microbiota abundance (genus Terrisporobacter id.11348) on Crohn's disease of small intestine

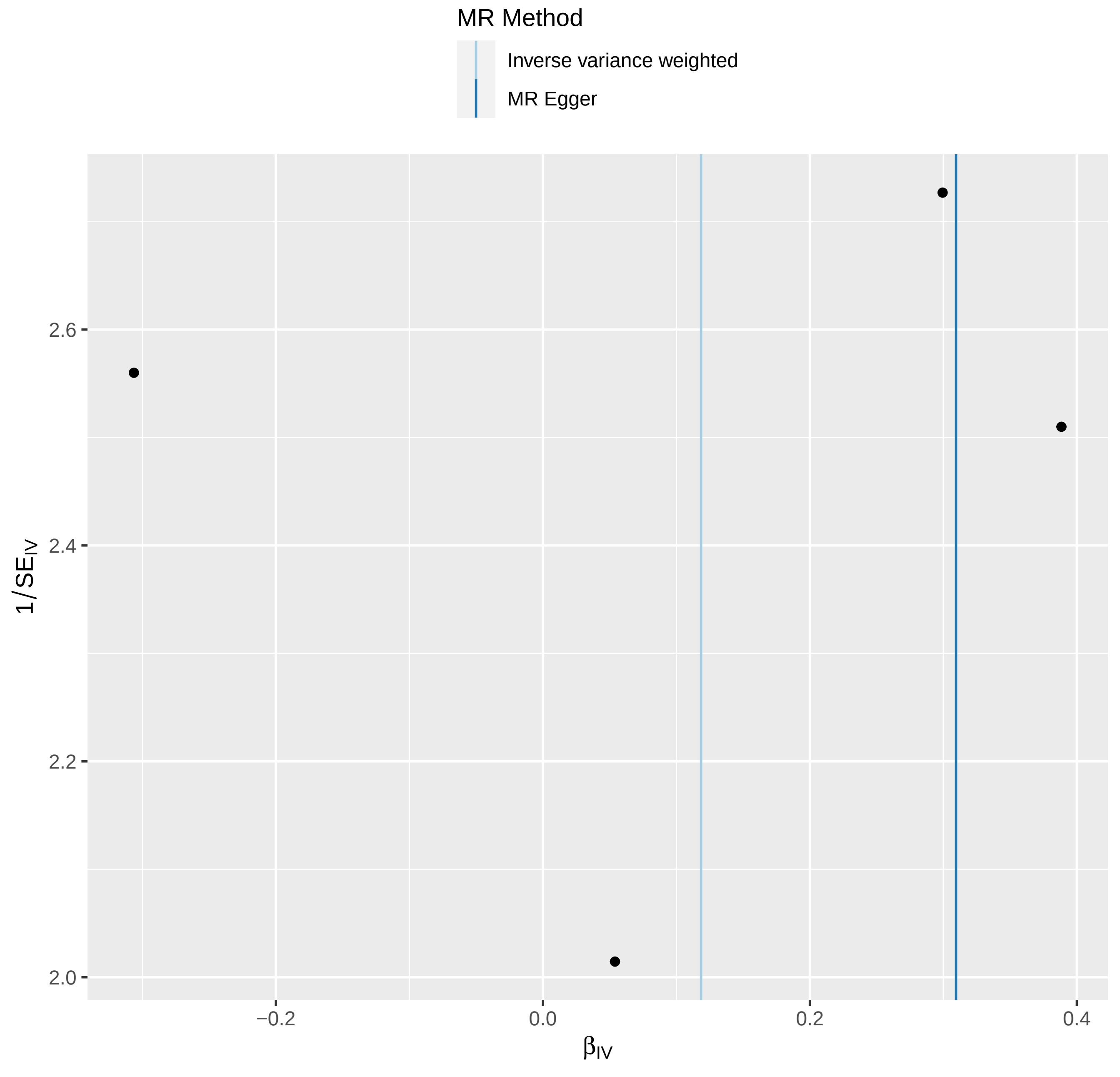

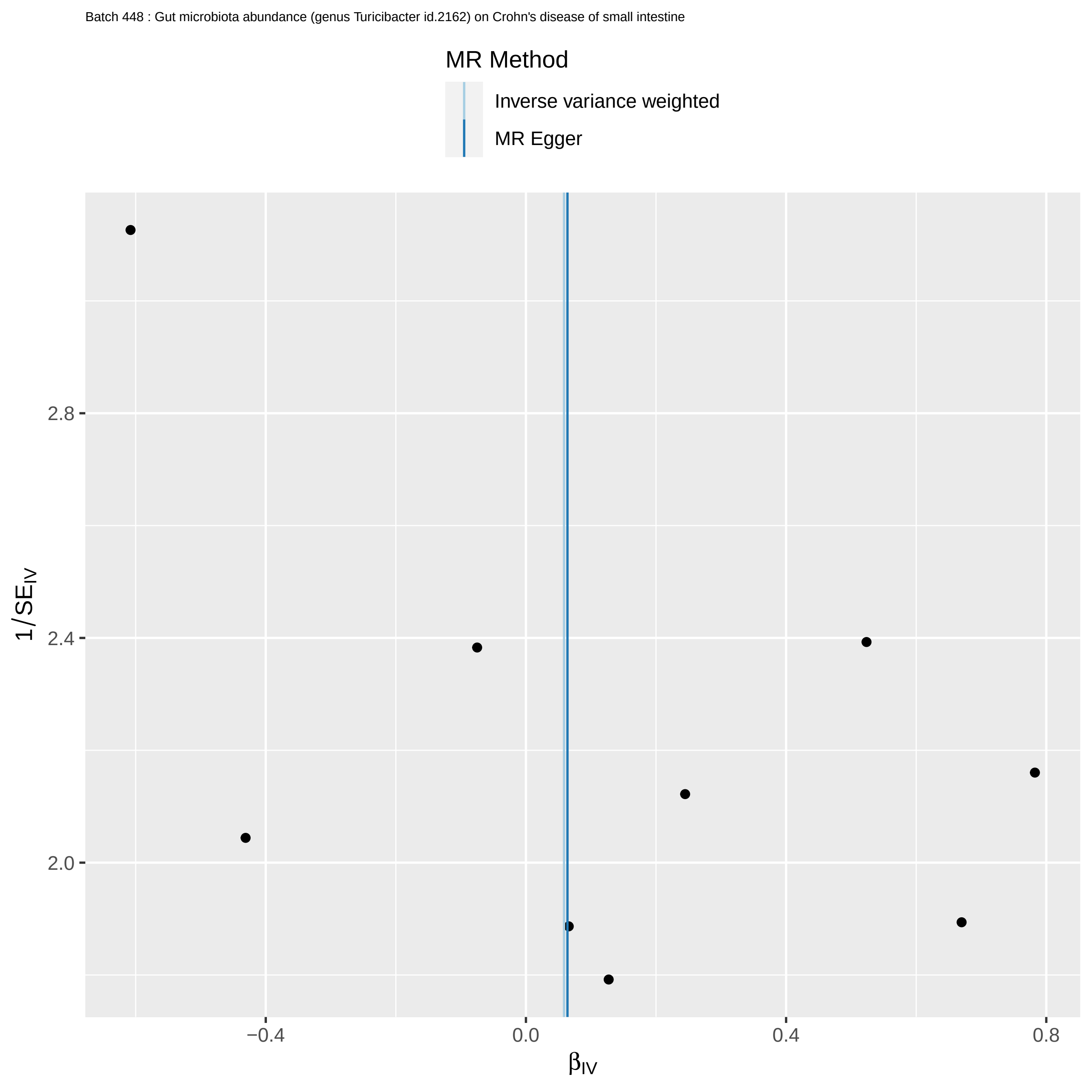

Batch 448 : Gut microbiota abundance (genus Turicibacter id.2162) on Crohn's disease of small intestine
MR Method
Inverse variance weighted
MR Egger
2.8
2.4
2.0
1/SE_IV
−0.4
0.0
0.4
0.8
β_IV

Batch 449 : Gut microbiota abundance (genus Tyzzerella3 id.11335) on Crohn's disease of small intestine

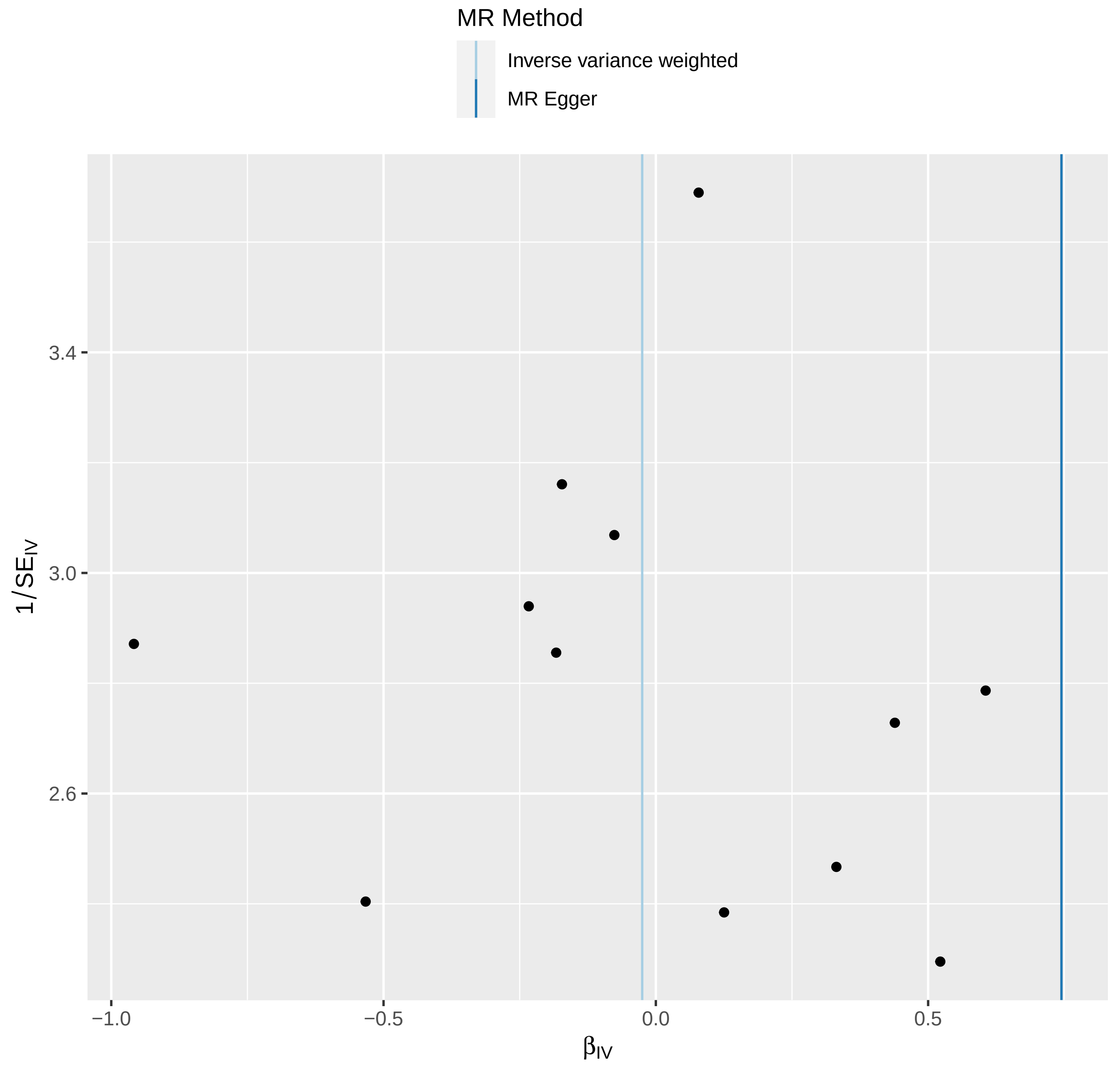

Batch 450 : Gut microbiota abundance (genus Veillonella id.2198) on Crohn's disease of small intestine

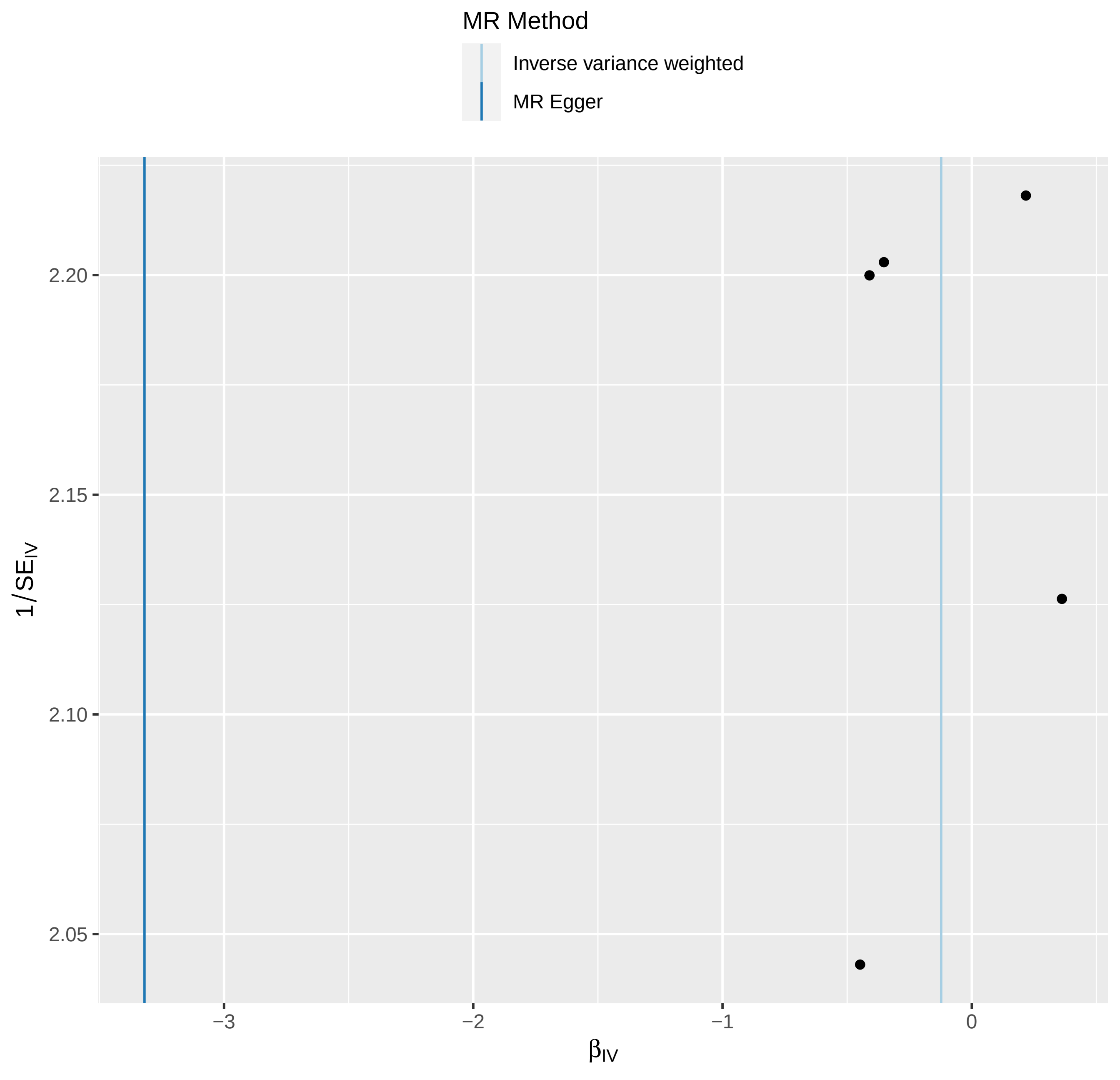

Batch 451 : Gut microbiota abundance (family Acidaminococcaceae id.2166) on Arthropathy in Crohn disease

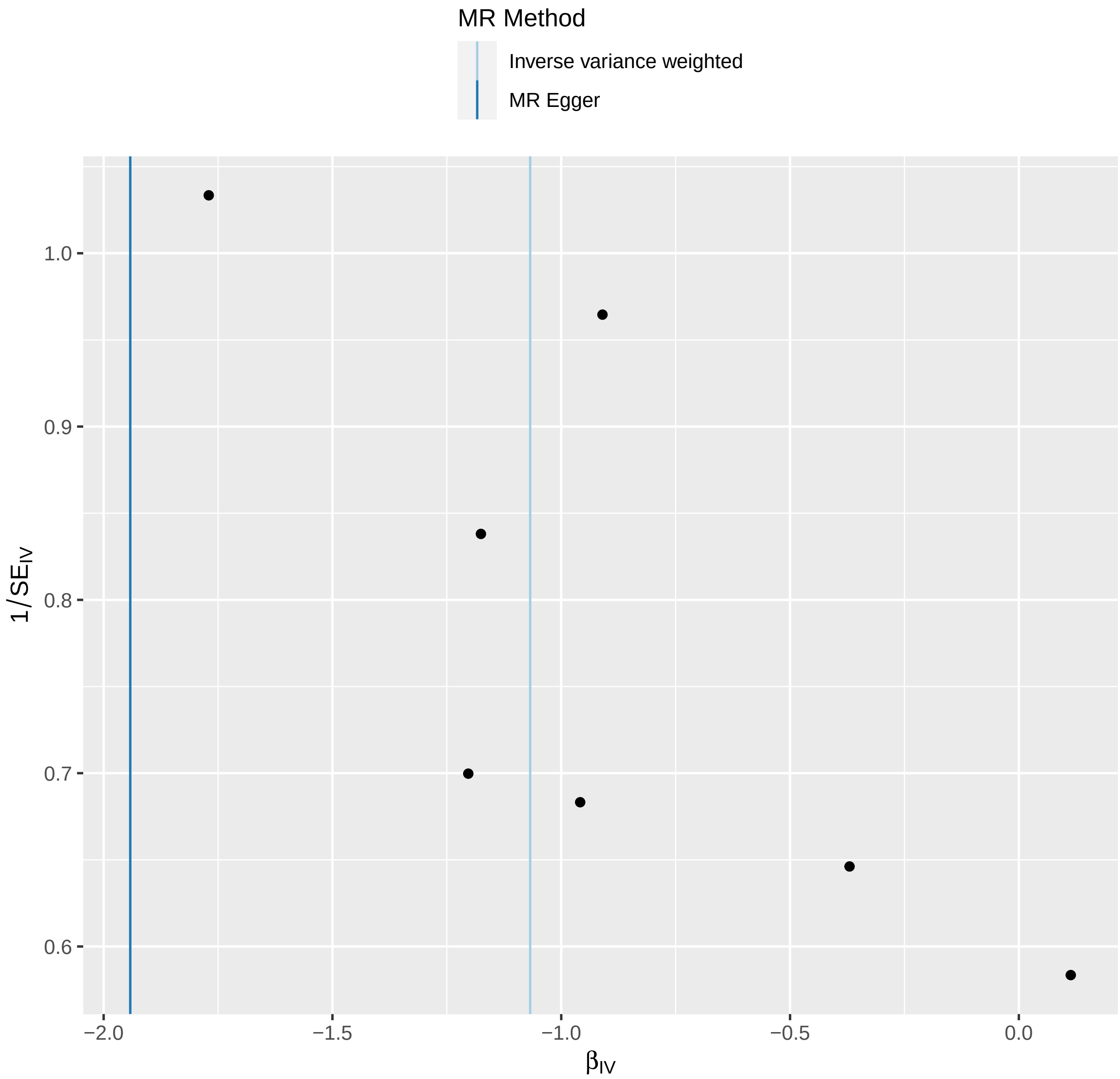

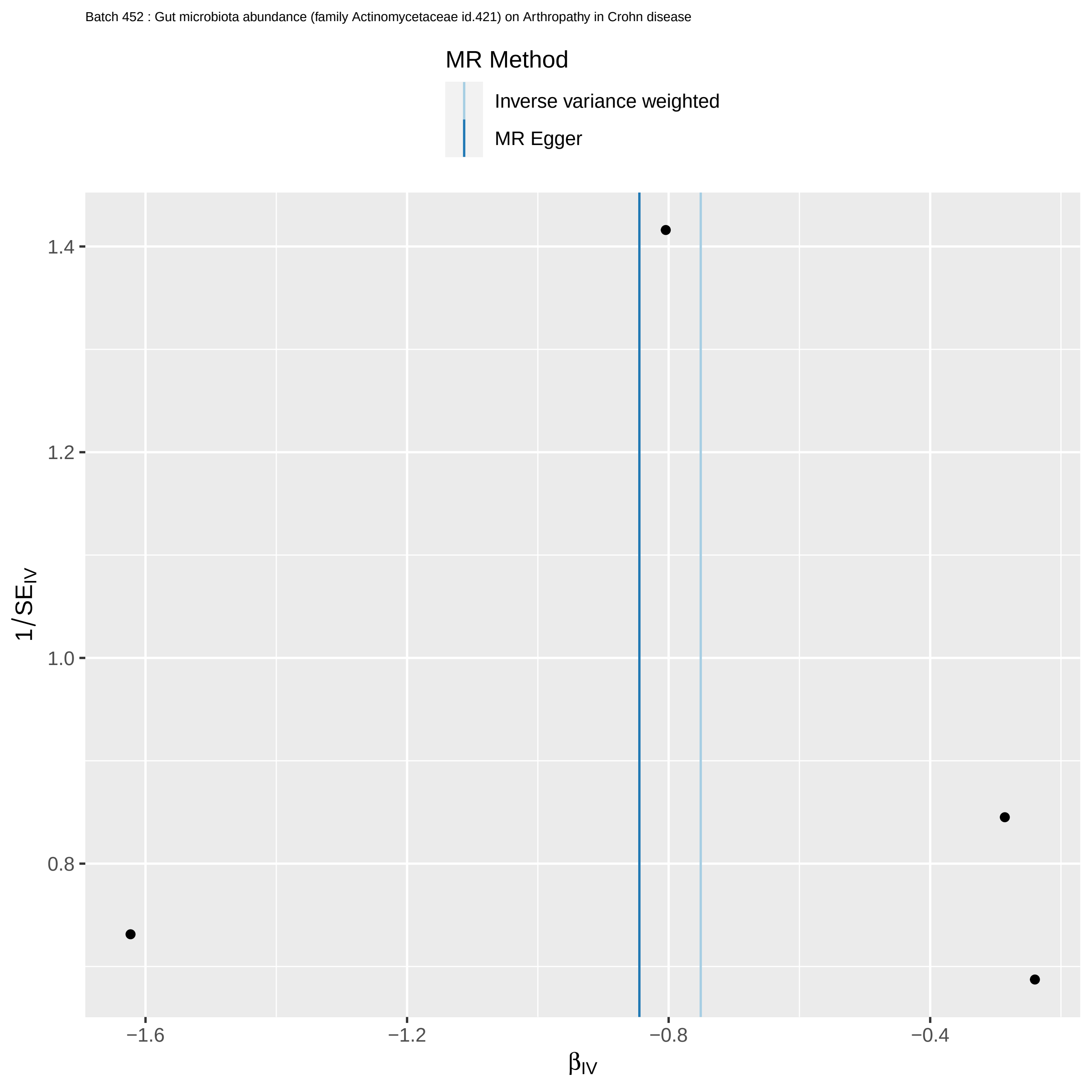

Batch 452 : Gut microbiota abundance (family Actinomycetaceae id.421) on Arthropathy in Crohn disease
MR Method
Inverse variance weighted
MR Egger
1.4
1.2
1.0
0.8
$1/SE_{IV}$
−1.6
−1.2
−0.8
−0.4
β_{IV}

Batch 453 : Gut microbiota abundance (family Alcaligenaceae id.2875) on Arthropathy in Crohn disease

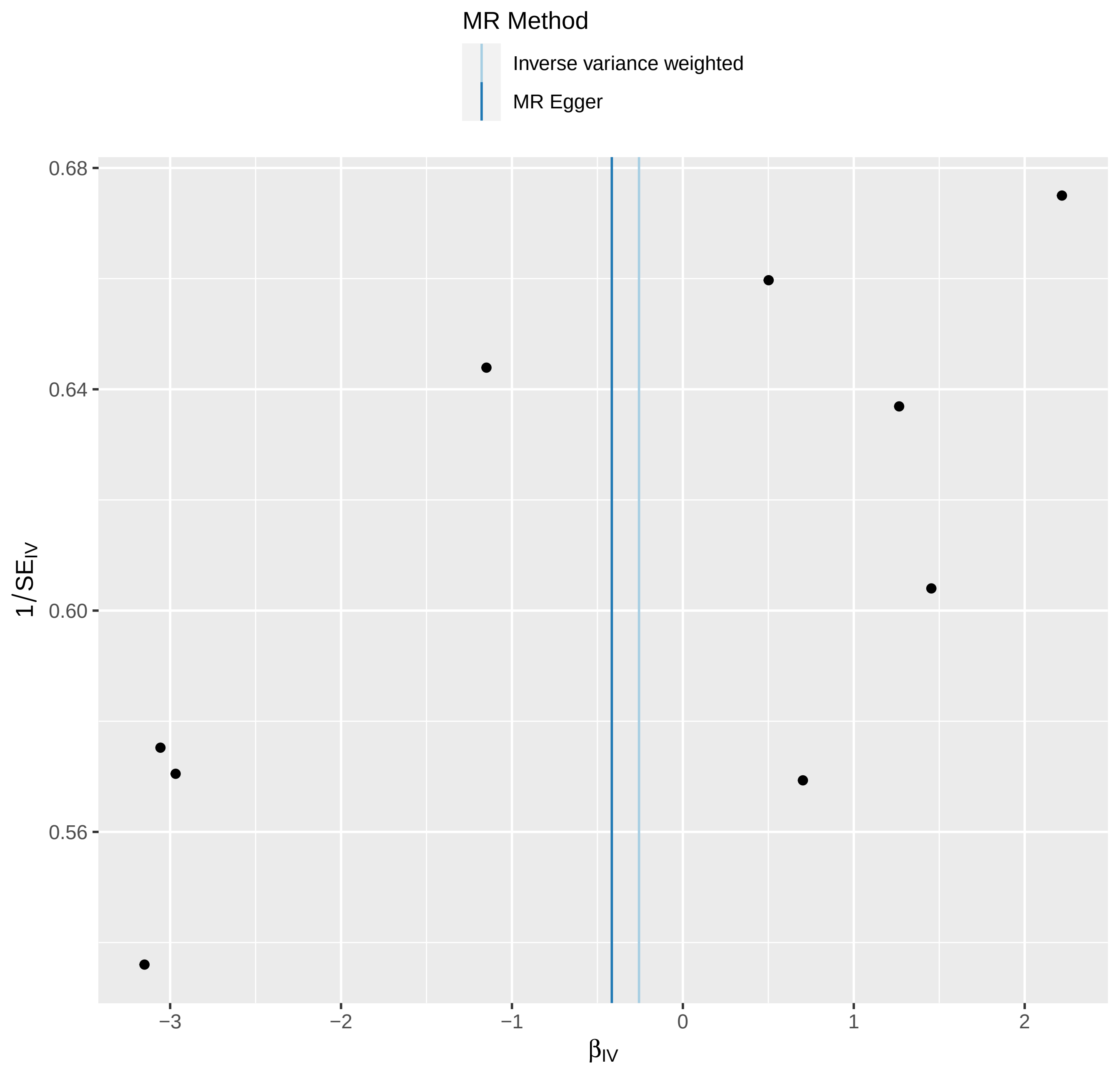

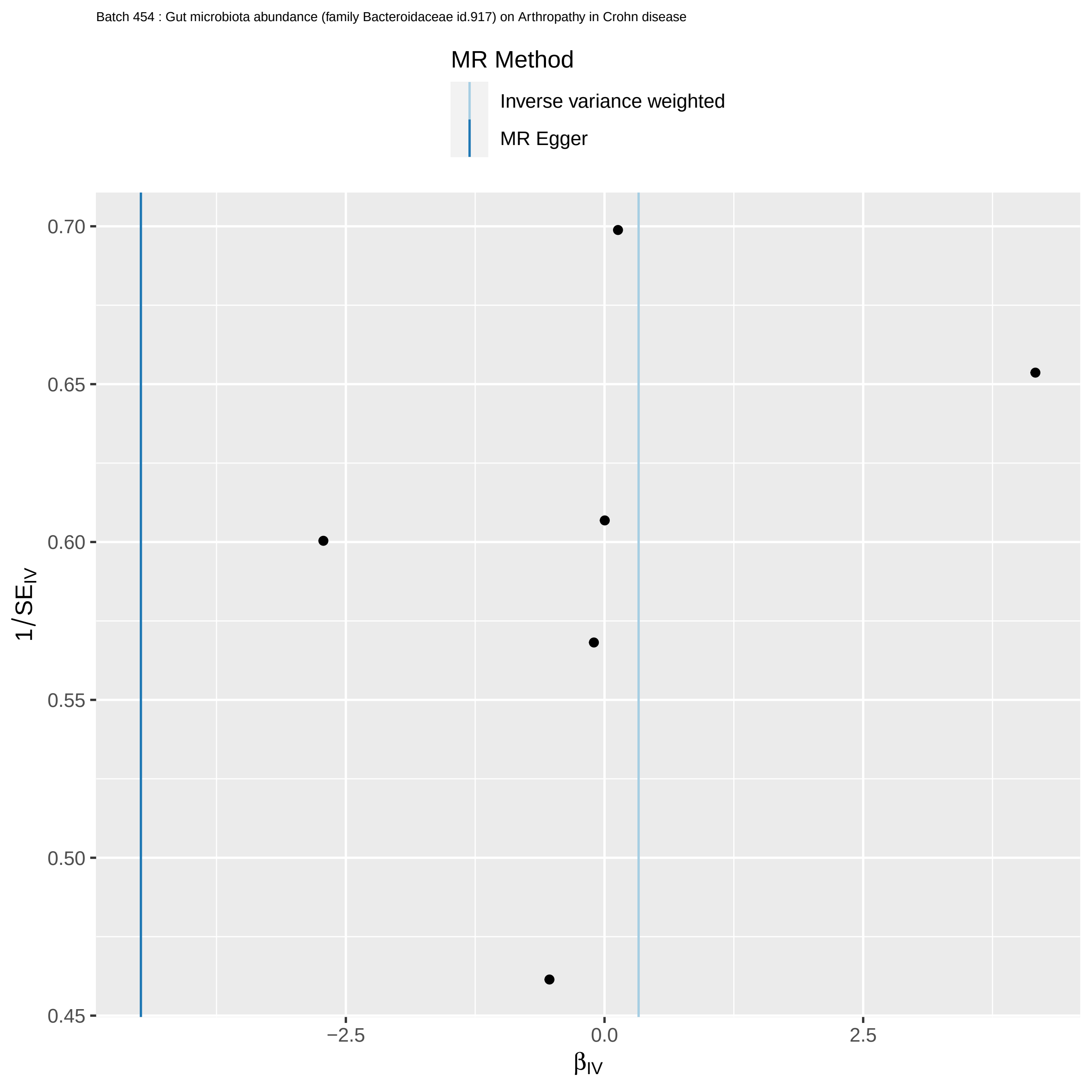

Batch 454 : Gut microbiota abundance (family Bacteroidaceae id.917) on Arthropathy in Crohn disease
MR Method
Inverse variance weighted
MR Egger
0.70
0.65
0.60
0.55
0.50
0.45
1/SE_IV
−2.5
0.0
2.5
β_IV

Batch 455 : Gut microbiota abundance (family Bacteroidales S24 7group id.11173) on Arthropathy in Crohn disease

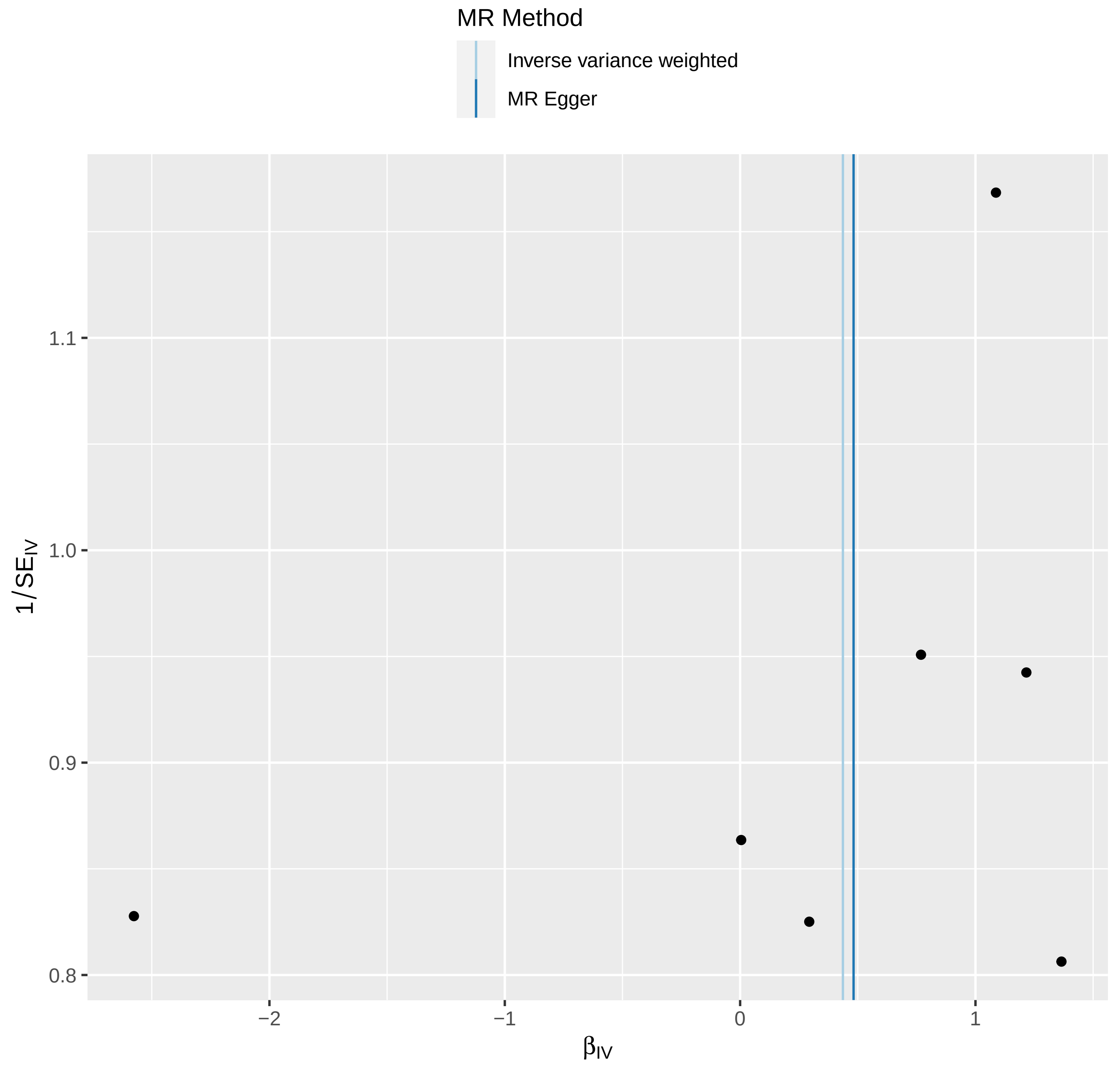

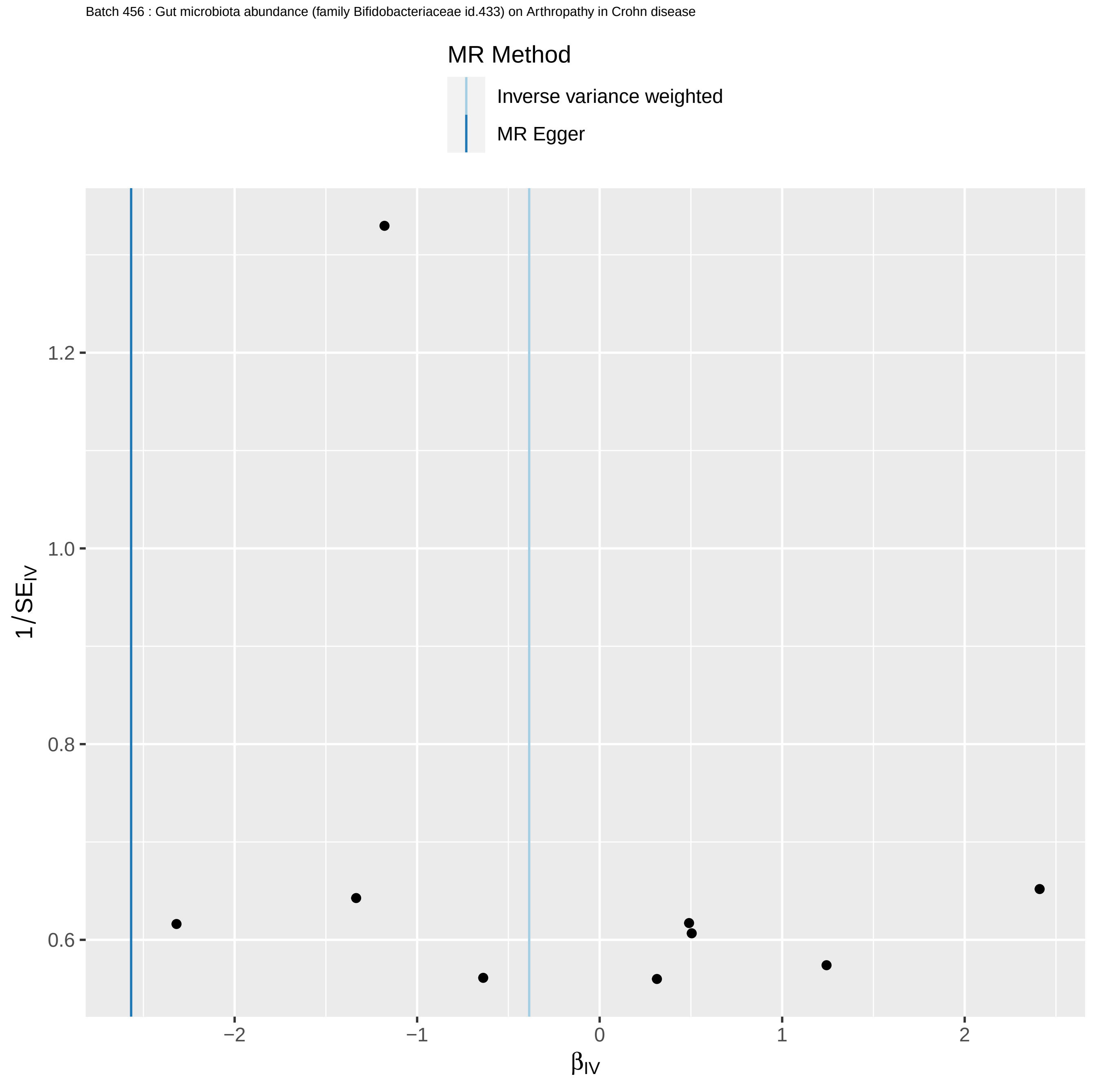

Batch 456 : Gut microbiota abundance (family Bifidobacteriaceae id.433) on Arthropathy in Crohn disease
MR Method
Inverse variance weighted
MR Egger
1.2
1.0
0.8
0.6
1/SE_IV
−2
−1
0
1
2
β_IV

Batch 458 : Gut microbiota abundance (family Clostridiaceae1 id.1869) on Arthropathy in Crohn disease

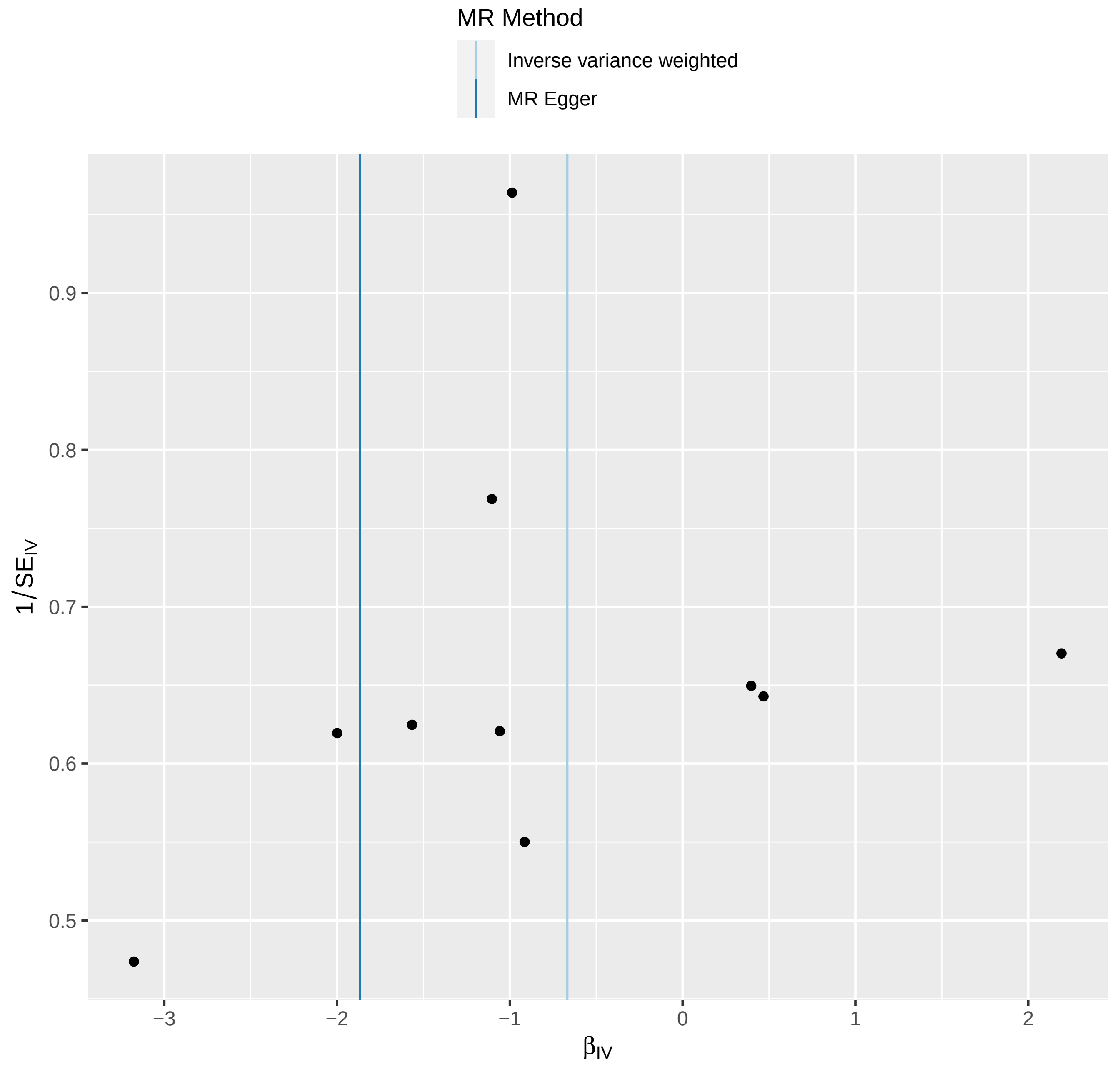

Batch 459 : Gut microbiota abundance (family Clostridiales vadin BB60 group id.11286) on Arthropathy in Crohn disease

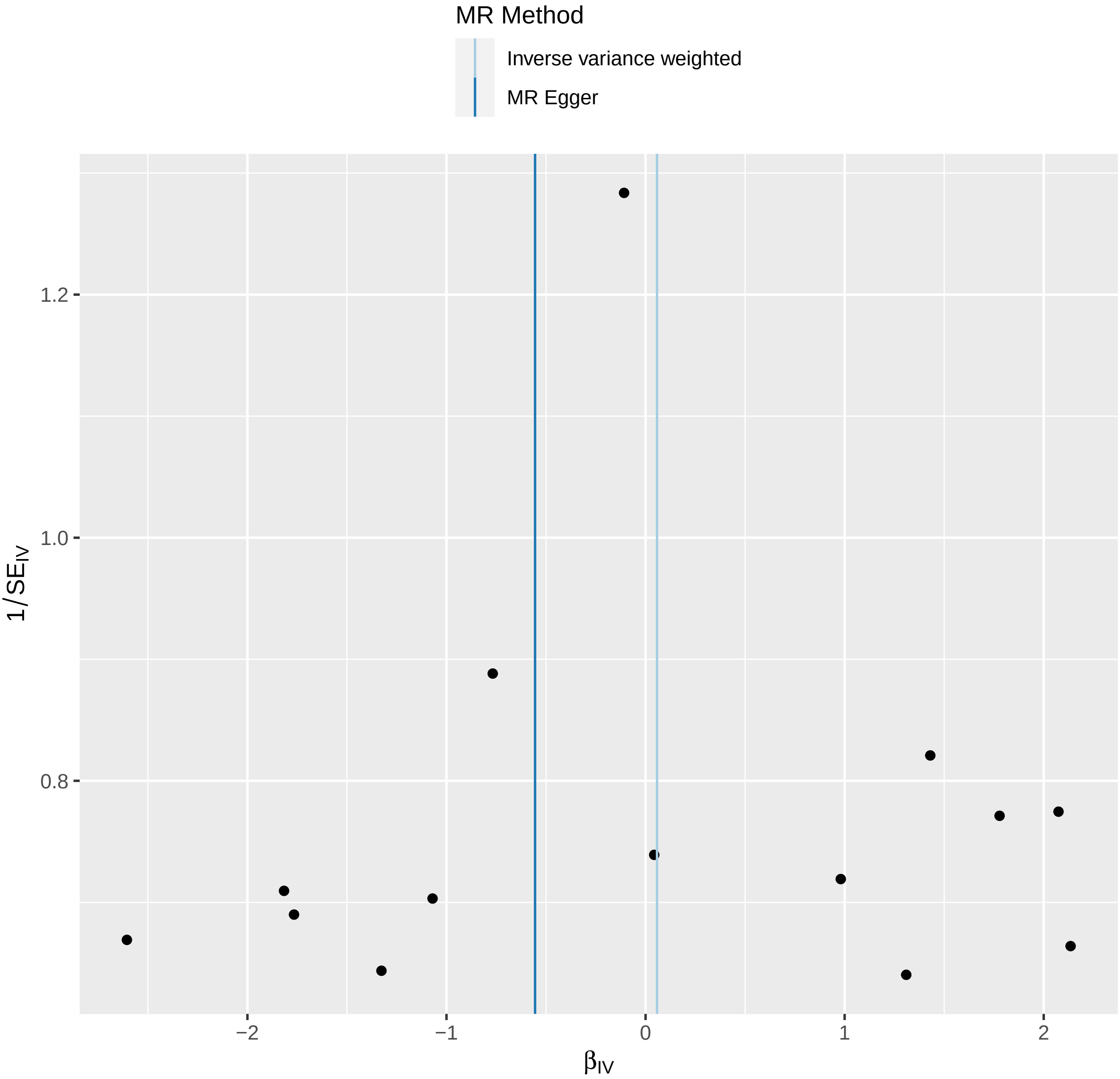

Batch 460 : Gut microbiota abundance (family Coriobacteriaceae id.811) on Arthropathy in Crohn disease

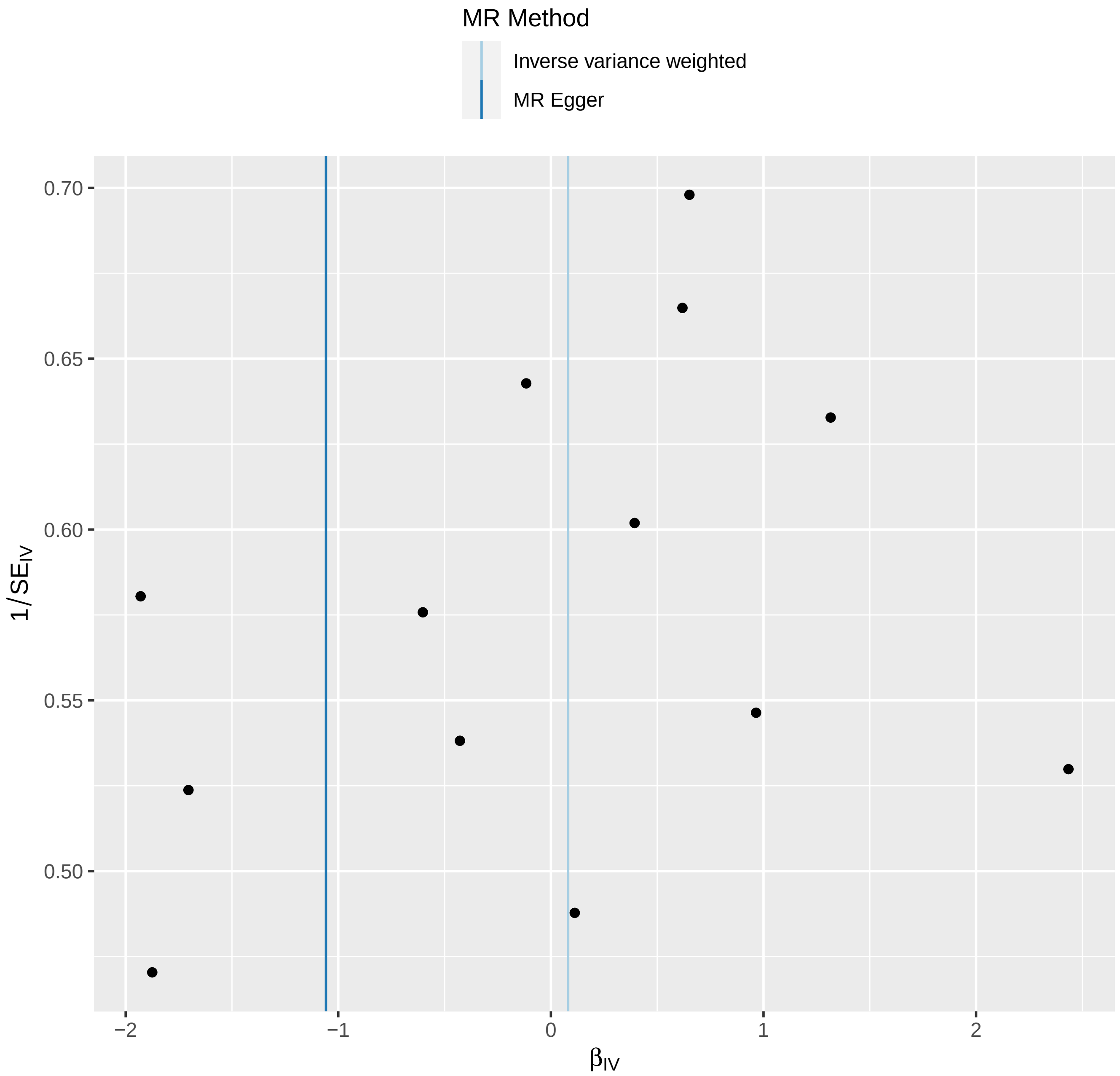

Batch 461 : Gut microbiota abundance (family Defluviitaleaceae id.1924) on Arthropathy in Crohn disease

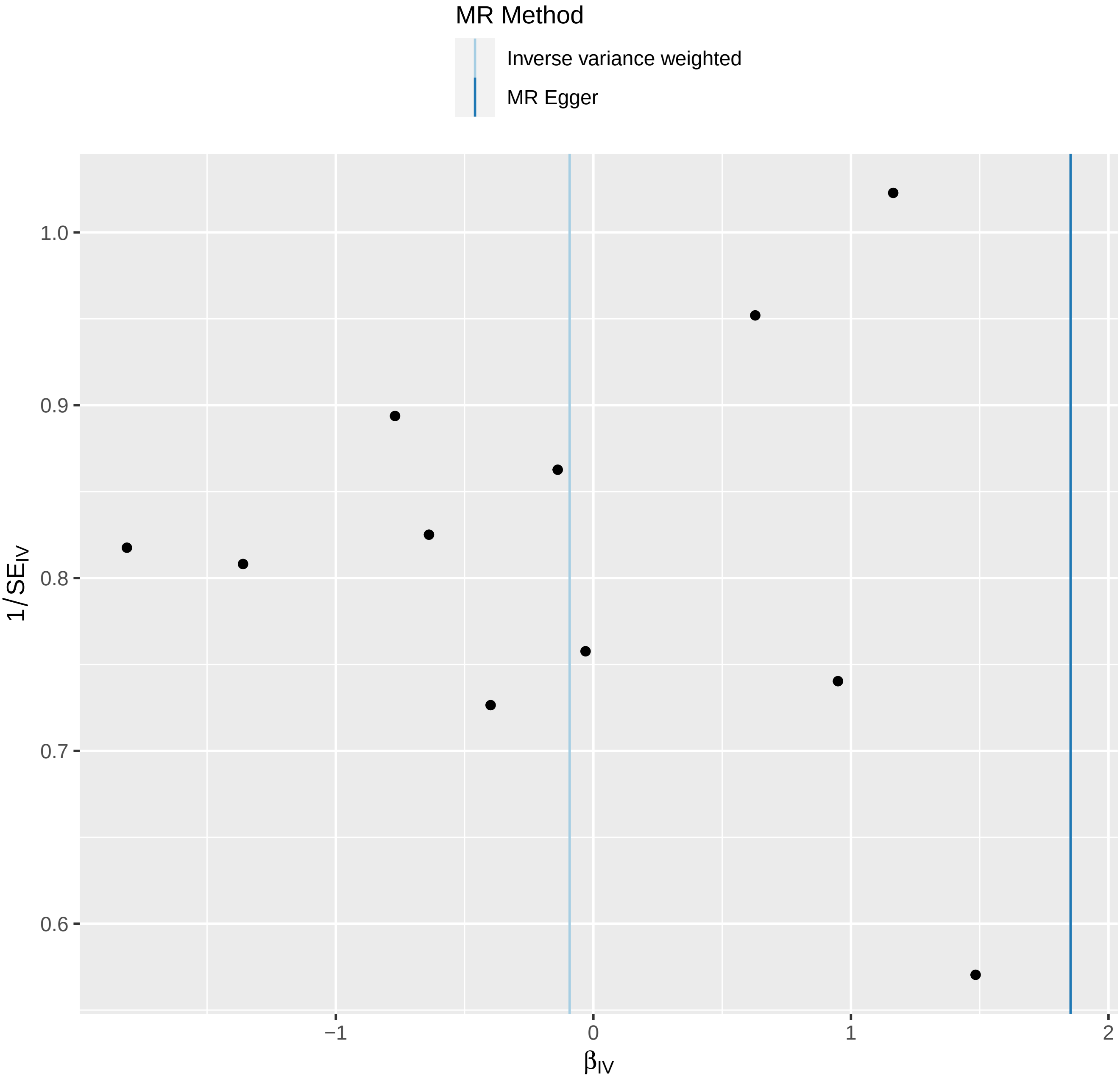

Batch 462 : Gut microbiota abundance (family Desulfovibrionaceae id.3169) on Arthropathy in Crohn disease

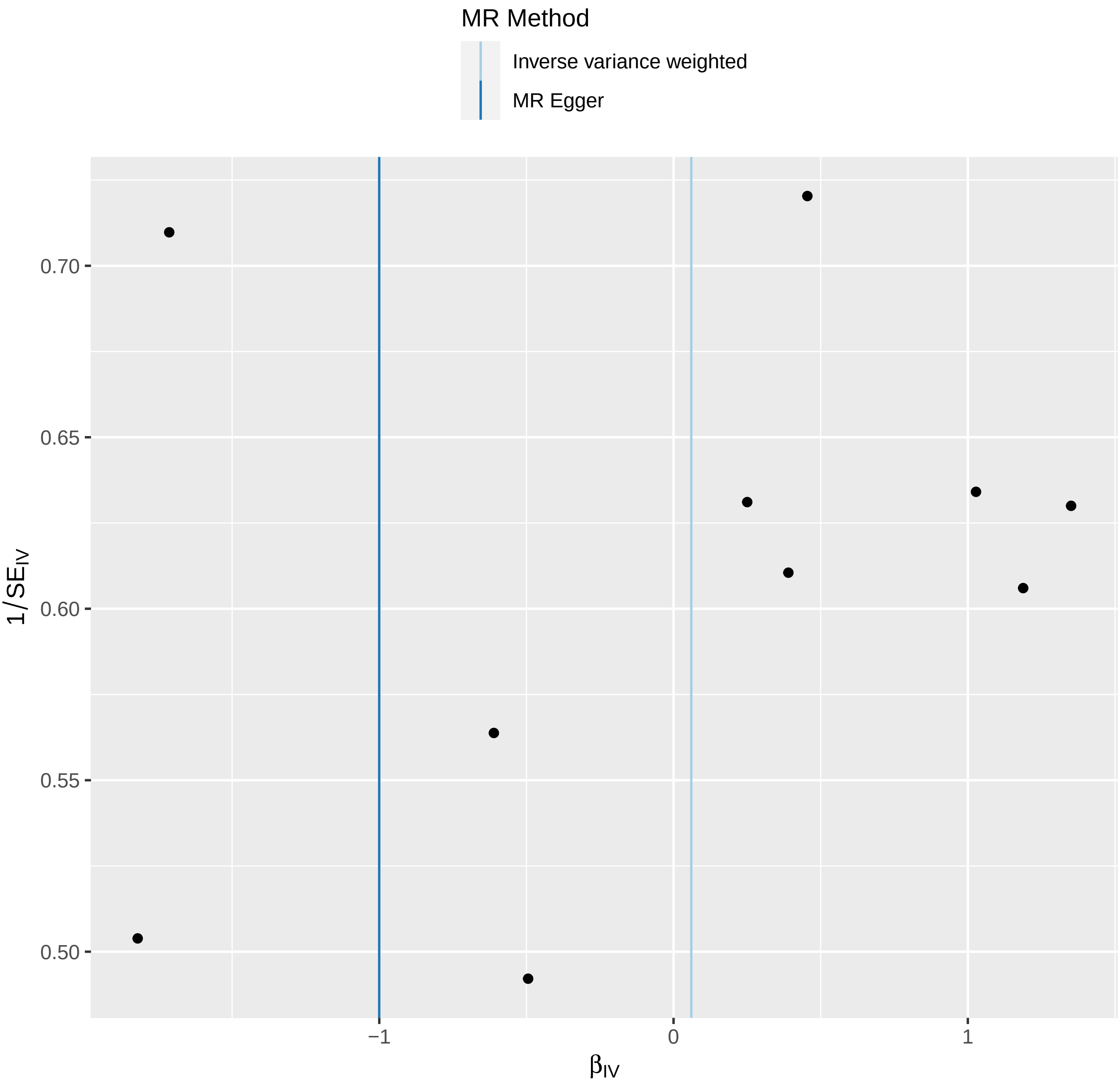

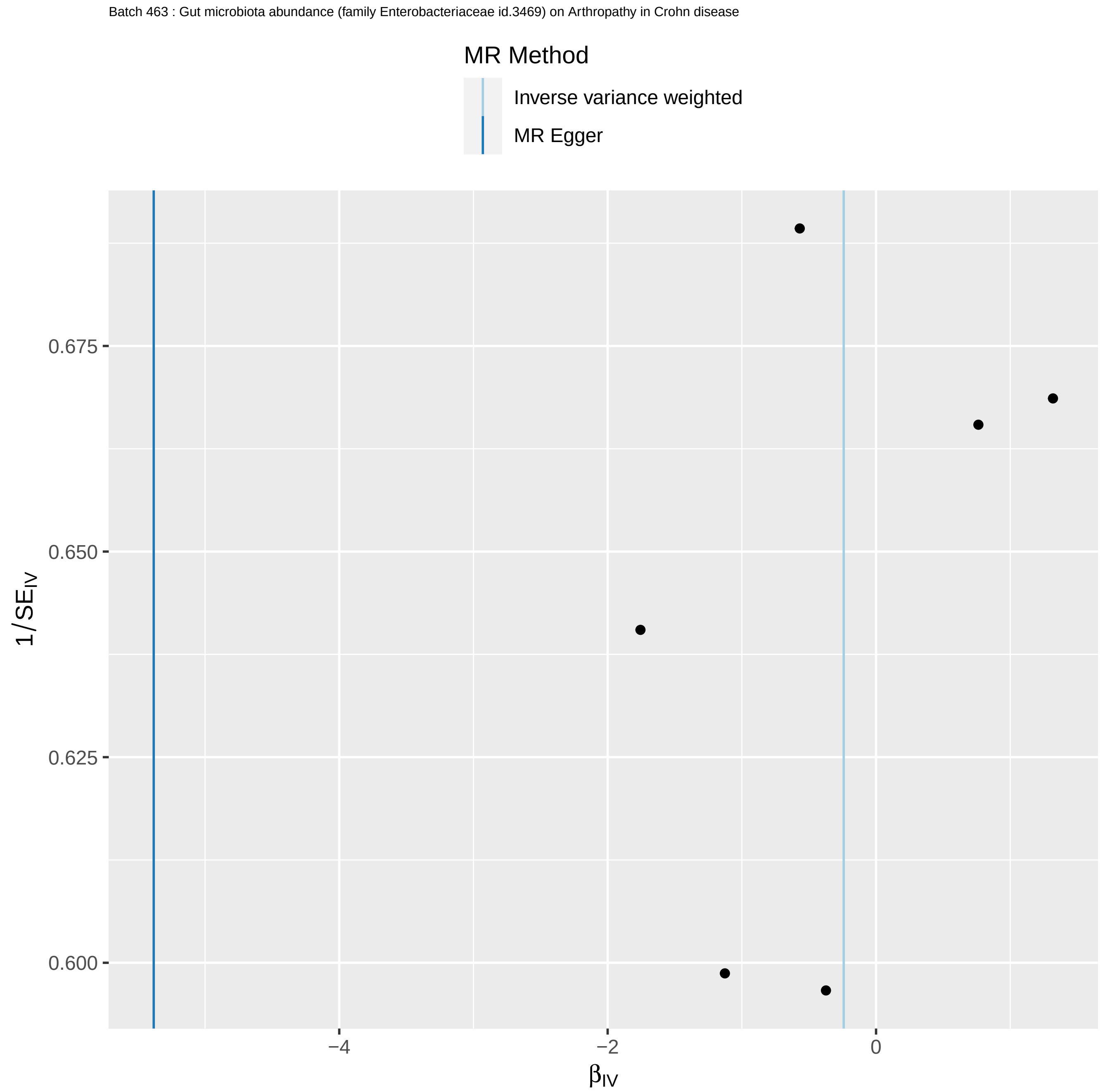
Batch 463 : Gut microbiota abundance (family Enterobacteriaceae id.3469) on Arthropathy in Crohn disease
MR Method
Inverse variance weighted
MR Egger
0.675
0.650
0.625
0.600
1/SE_IV
−4
−2
0
β_IV

Batch 464 : Gut microbiota abundance (family Erysipelotrichaceae id.2149) on Arthropathy in Crohn disease

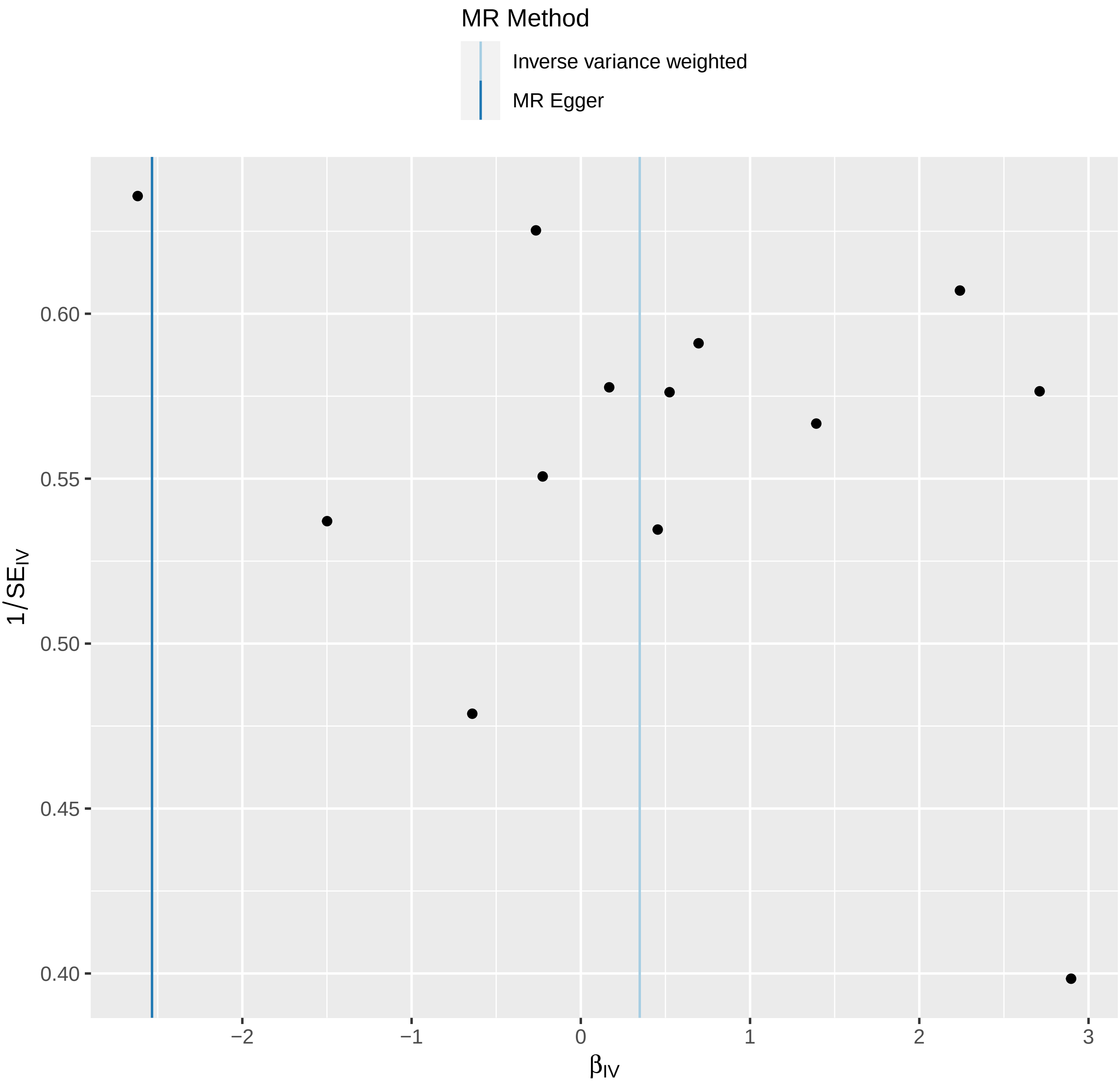

Batch 465 : Gut microbiota abundance (family Family XI id.1936) on Arthropathy in Crohn disease

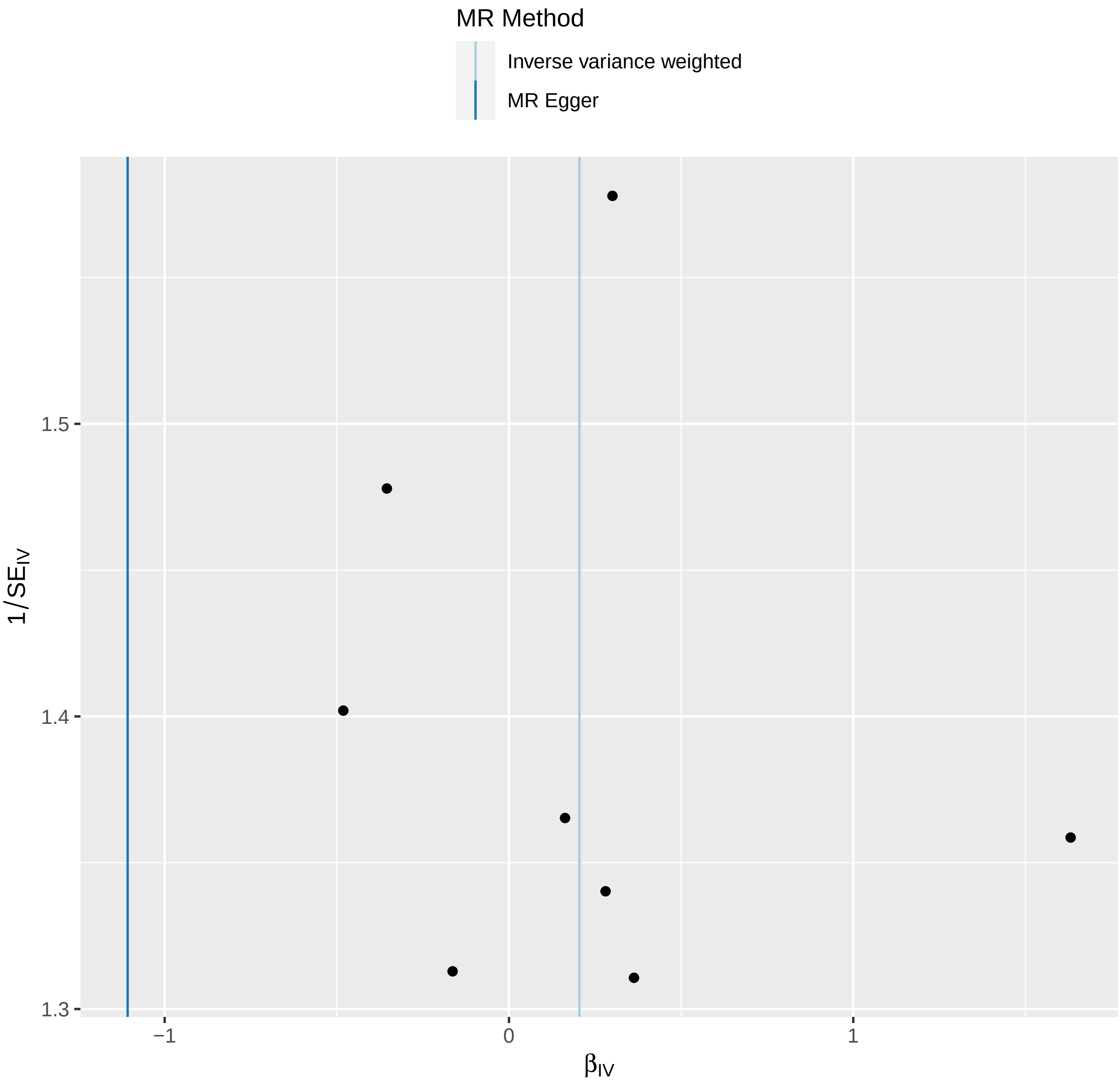

Batch 466 : Gut microbiota abundance (family Family XIII id.1957) on Arthropathy in Crohn disease

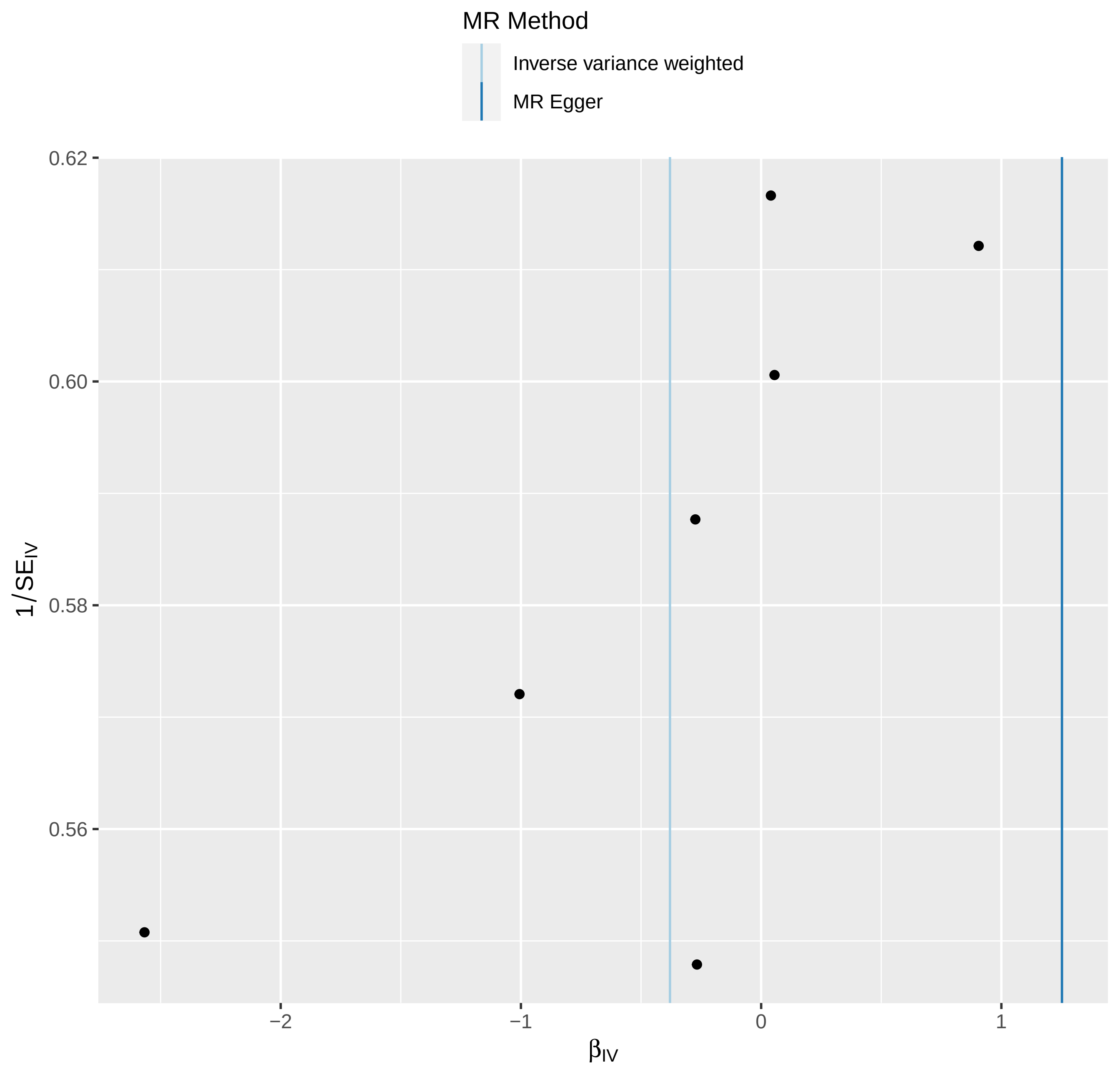

Batch 467 : Gut microbiota abundance (family Lachnospiraceae id.1987) on Arthropathy in Crohn disease

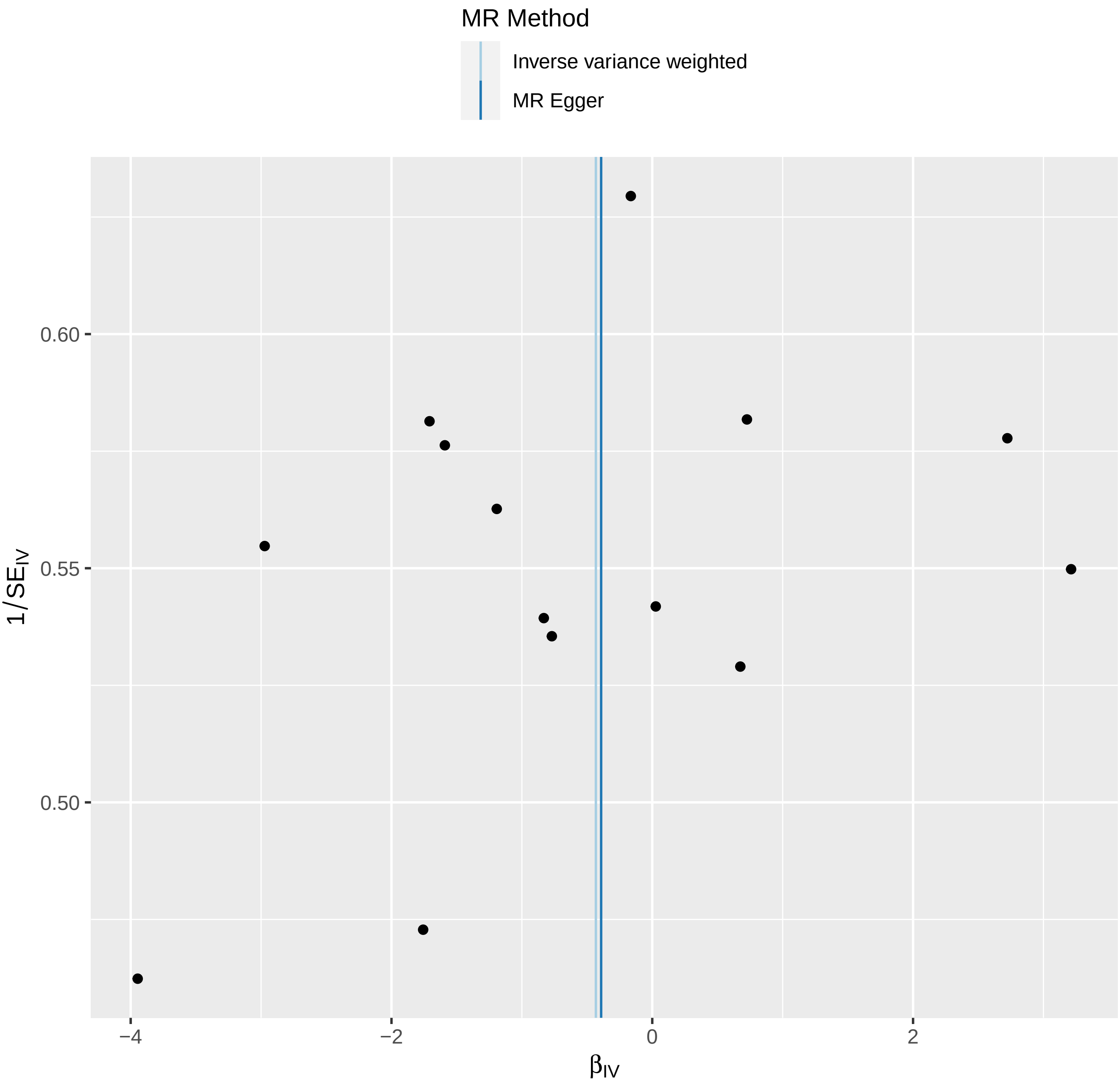

Batch 468 : Gut microbiota abundance (family Lactobacillaceae id.1836) on Arthropathy in Crohn disease

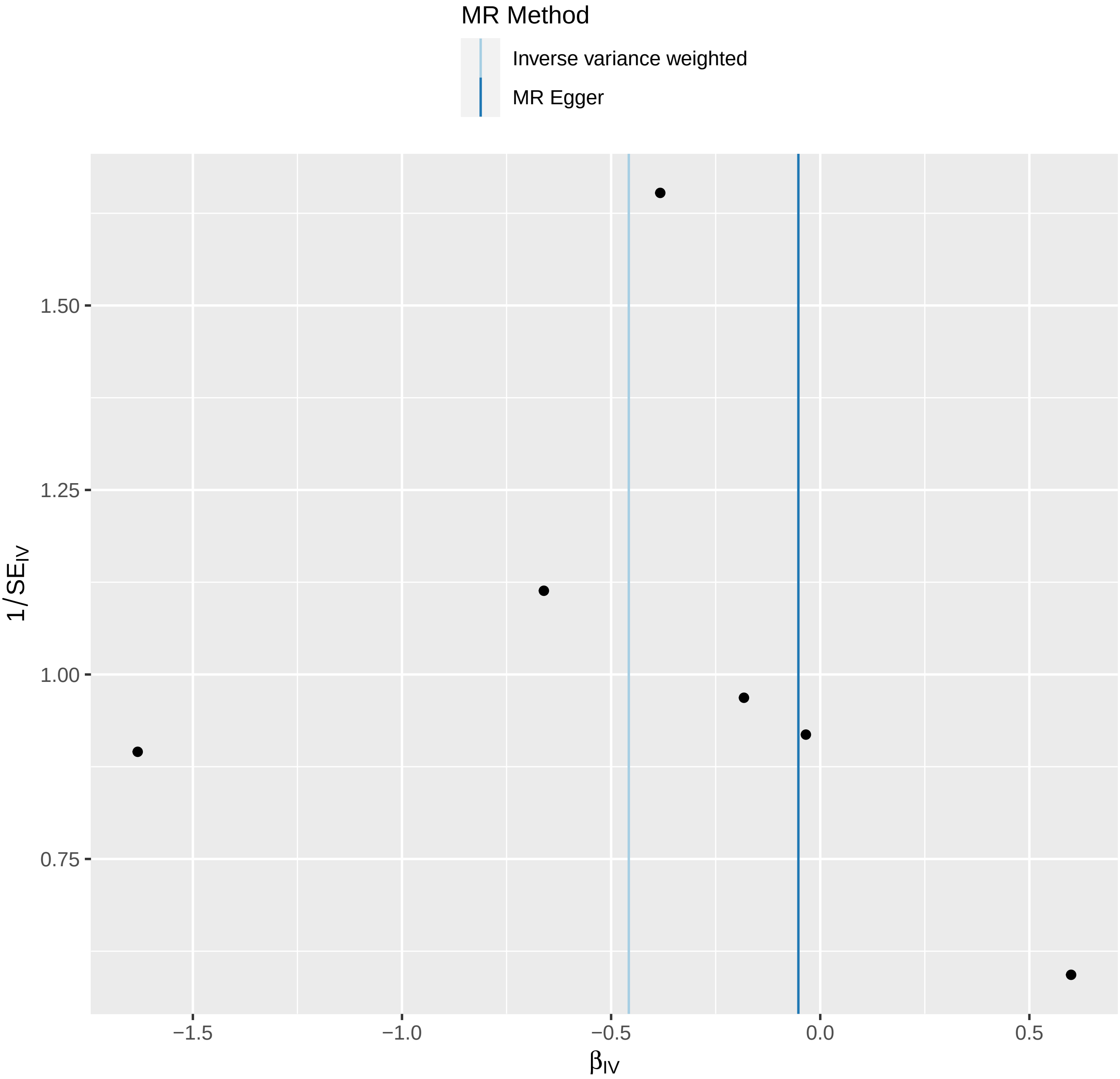

Batch 469 : Gut microbiota abundance (family Methanobacteriaceae id.121) on Arthropathy in Crohn disease

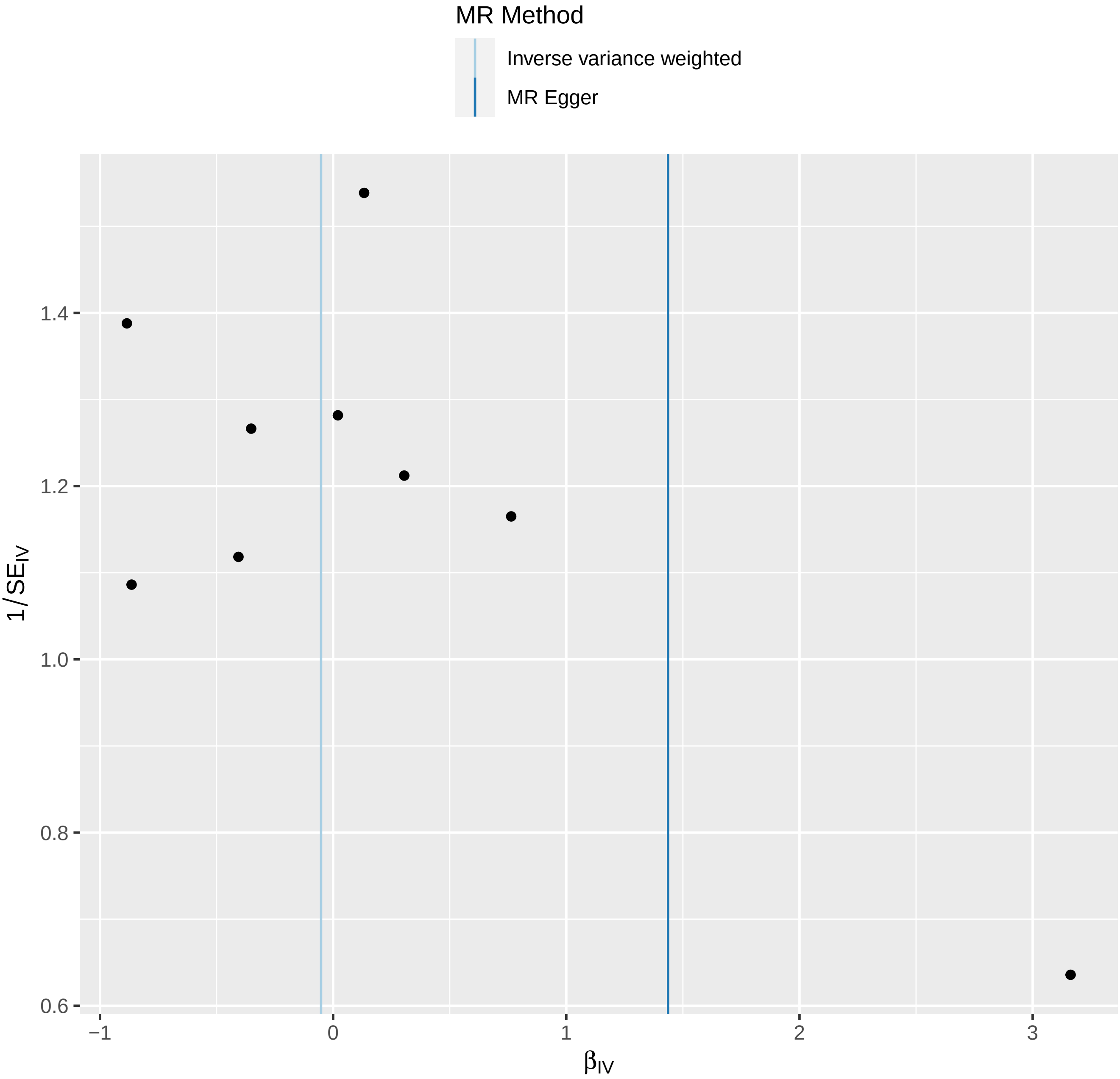

Batch 470 : Gut microbiota abundance (family Oxalobacteraceae id.2966) on Arthropathy in Crohn disease

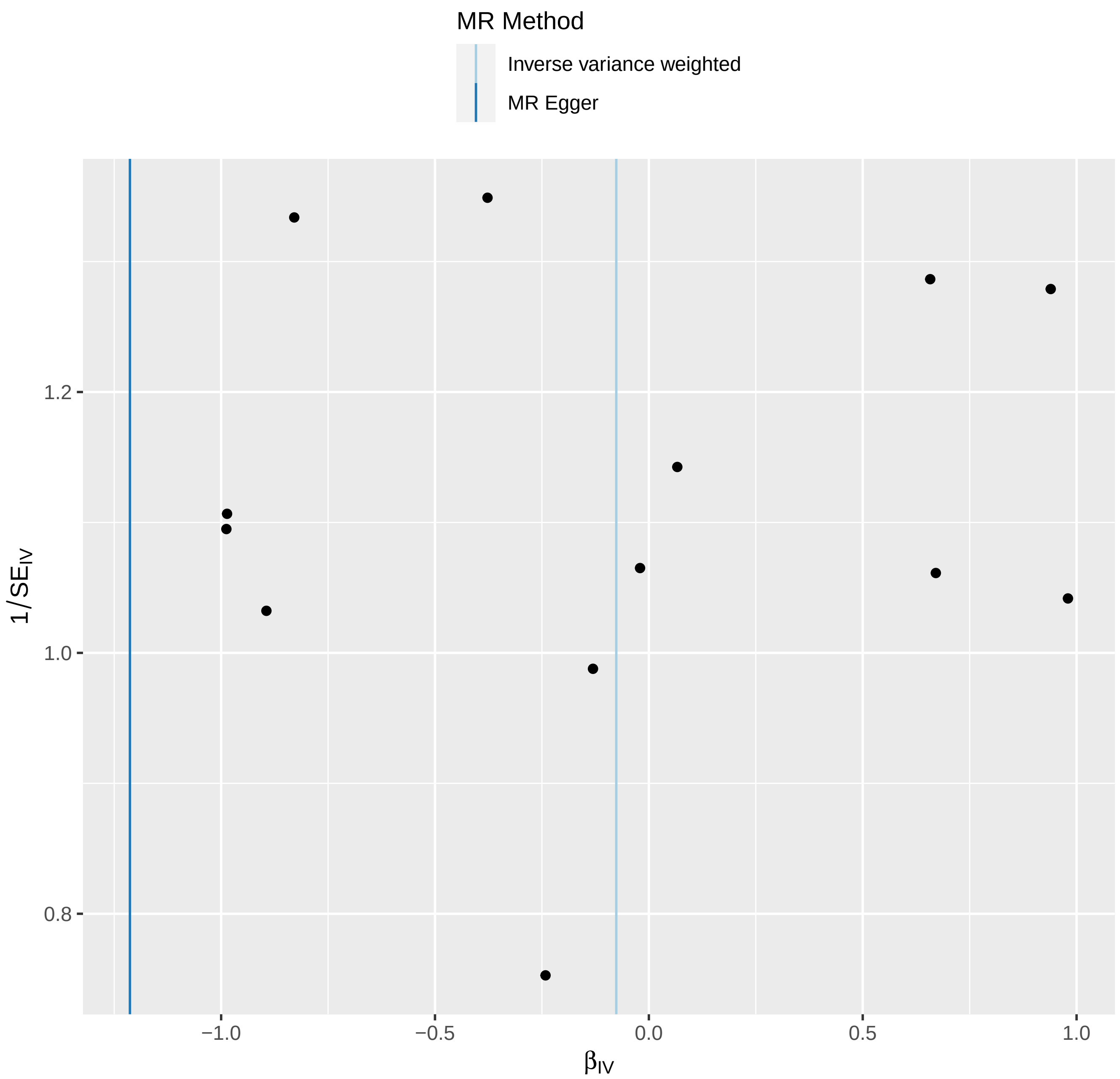

Batch 471 : Gut microbiota abundance (family Pasteurellaceae id.3689) on Arthropathy in Crohn disease

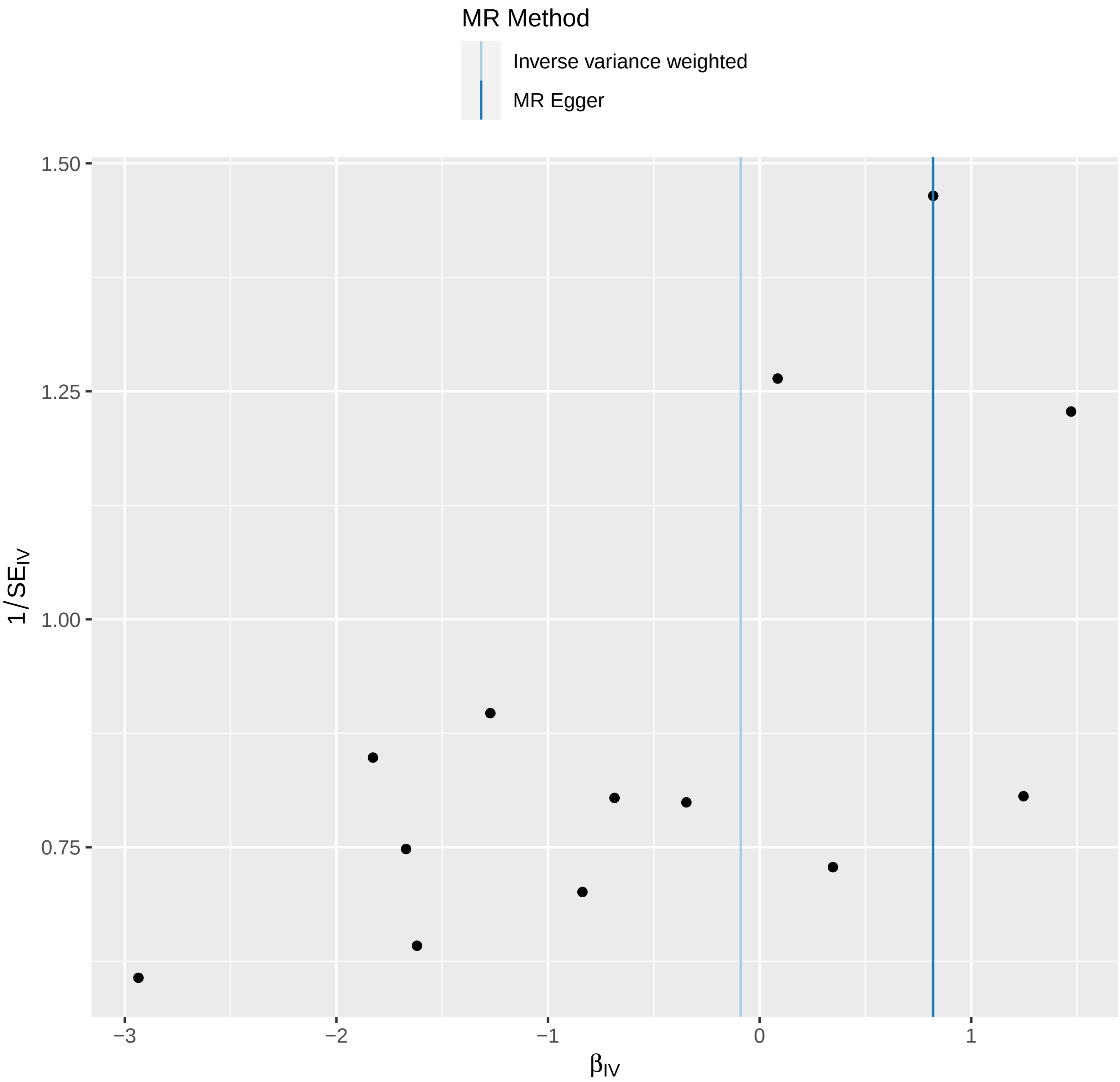

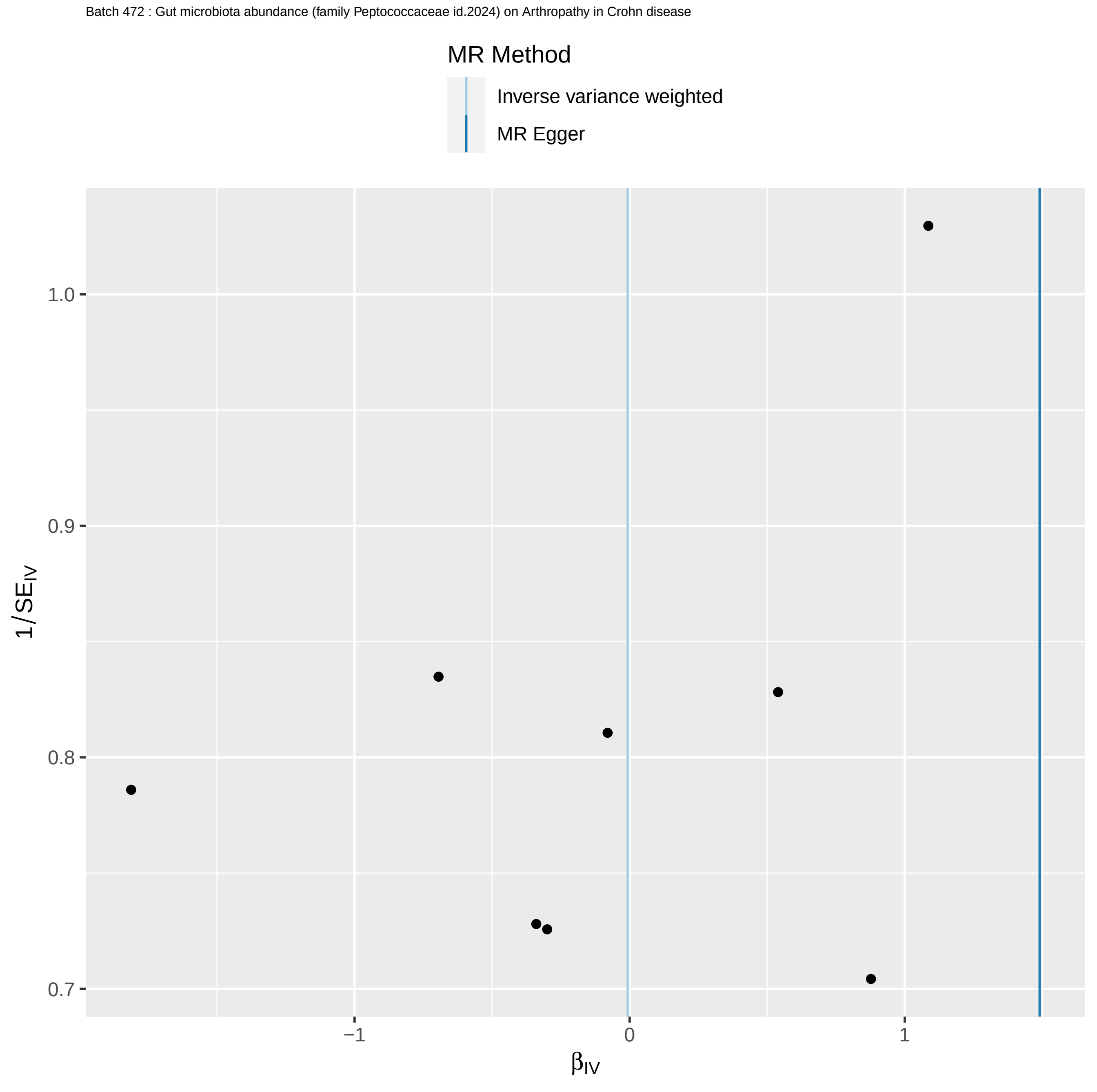
Batch 472 : Gut microbiota abundance (family Peptococcaceae id.2024) on Arthropathy in Crohn disease
MR Method
Inverse variance weighted
MR Egger
1.0
0.9
0.8
0.7
1/SE_IV
−1
0
1
β_IV

Batch 473 : Gut microbiota abundance (family Peptostreptococcaceae id.2042) on Arthropathy in Crohn disease

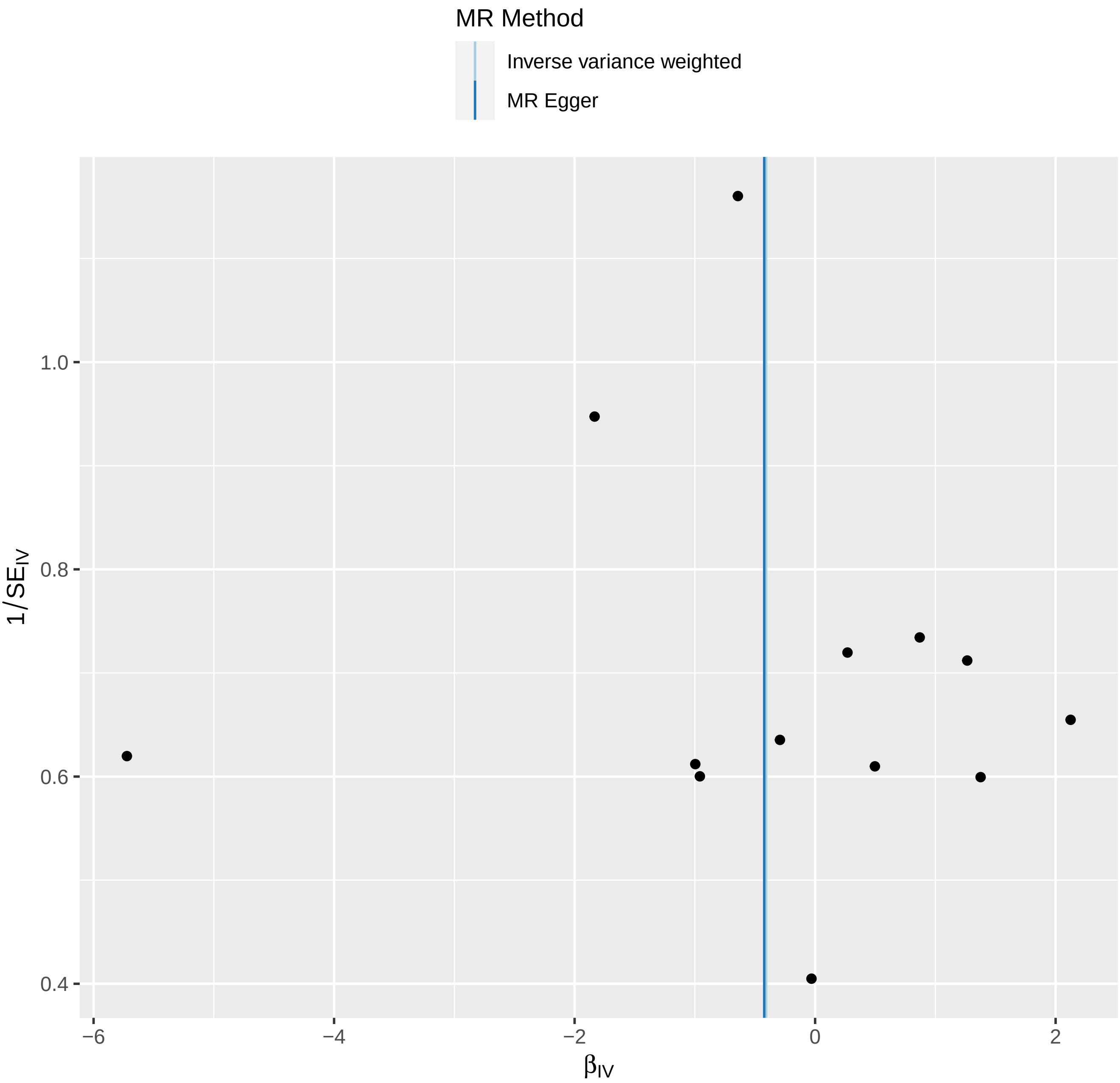

Batch 474 : Gut microbiota abundance (family Porphyromonadaceae id.943) on Arthropathy in Crohn disease

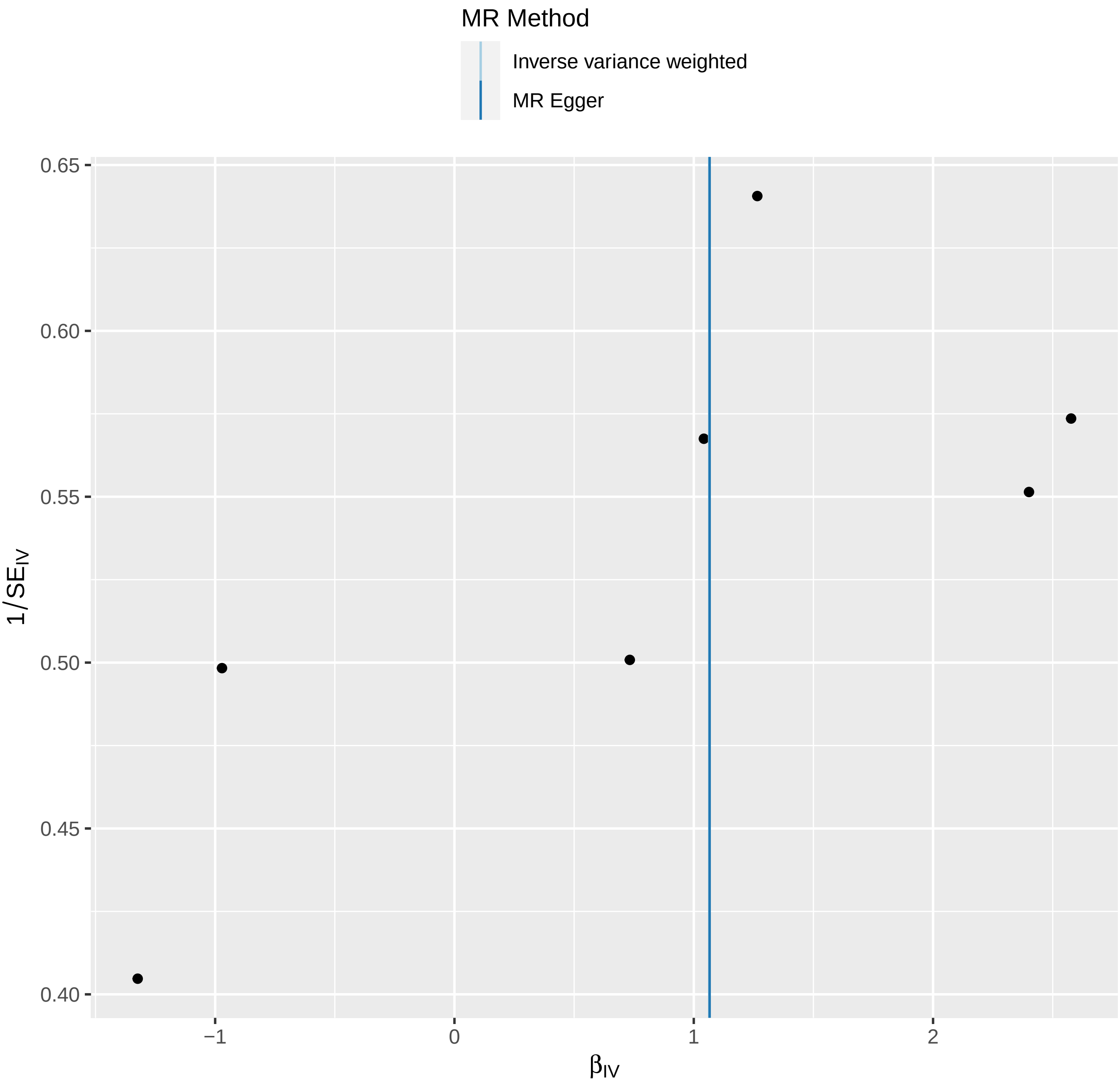

Batch 475 : Gut microbiota abundance (family Prevotellaceae id.960) on Arthropathy in Crohn disease

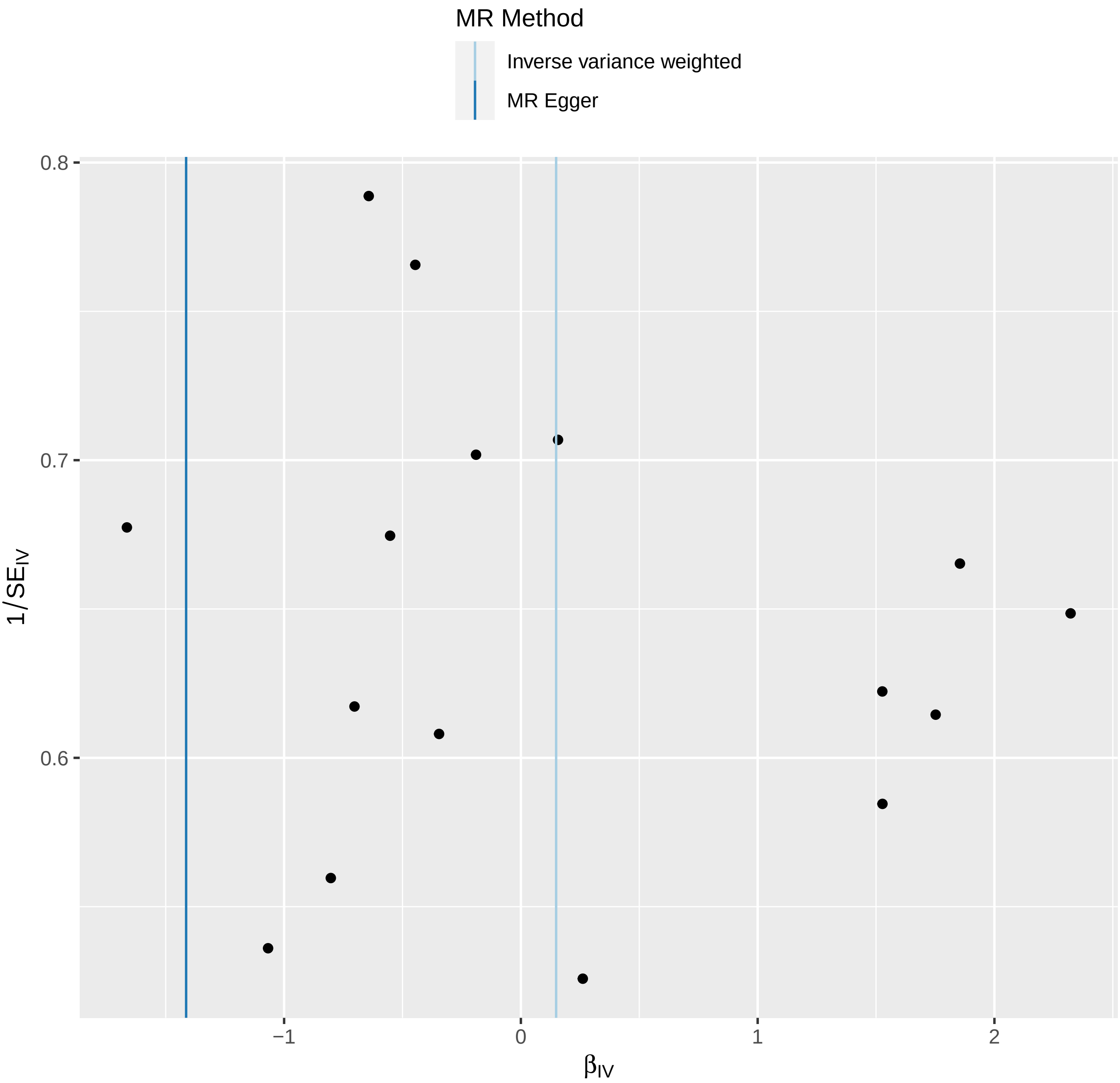

Batch 476 : Gut microbiota abundance (family Rhodospirillaceae id.2717) on Arthropathy in Crohn disease

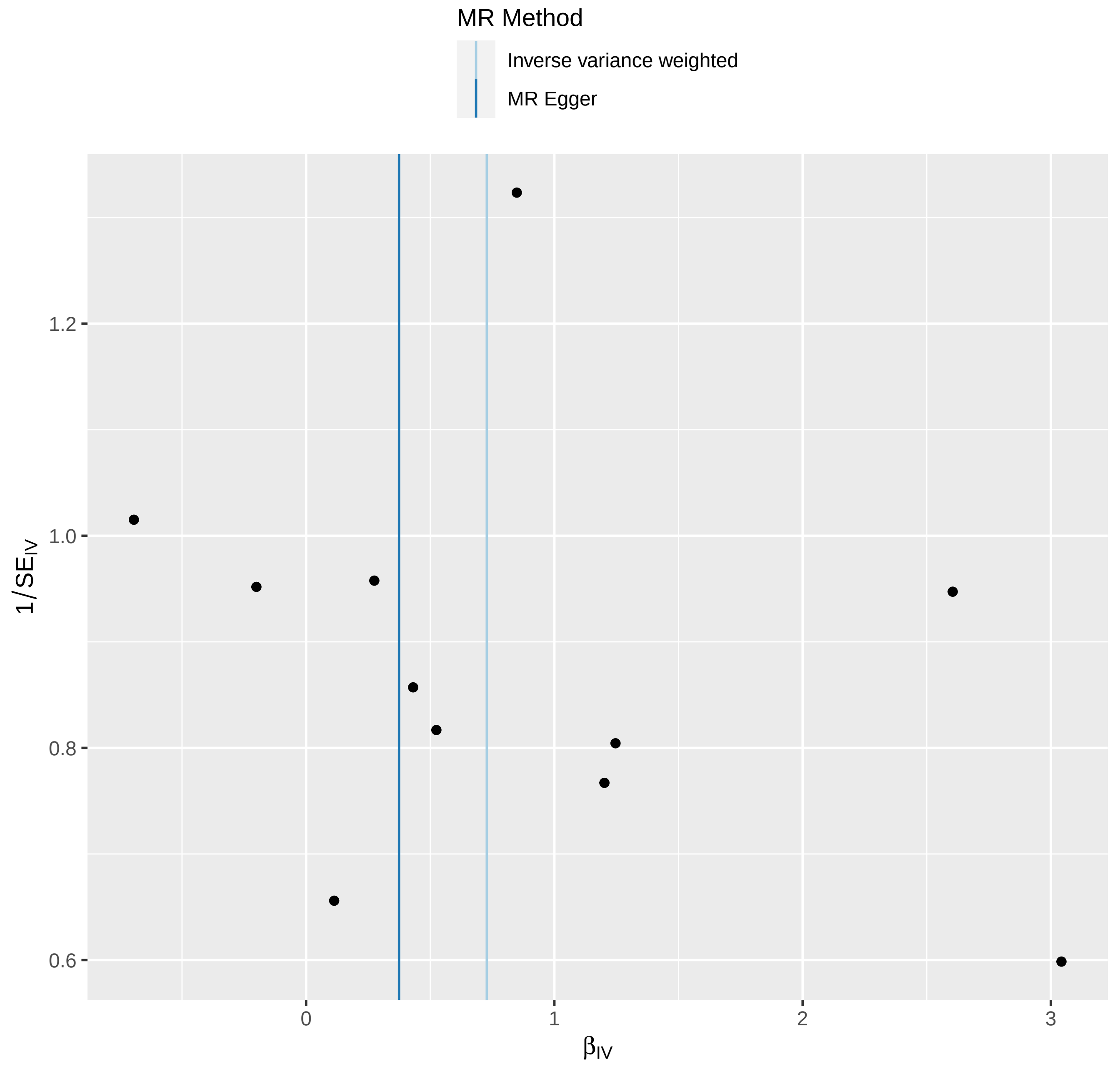

Batch 477 : Gut microbiota abundance (family Rikenellaceae id.967) on Arthropathy in Crohn disease

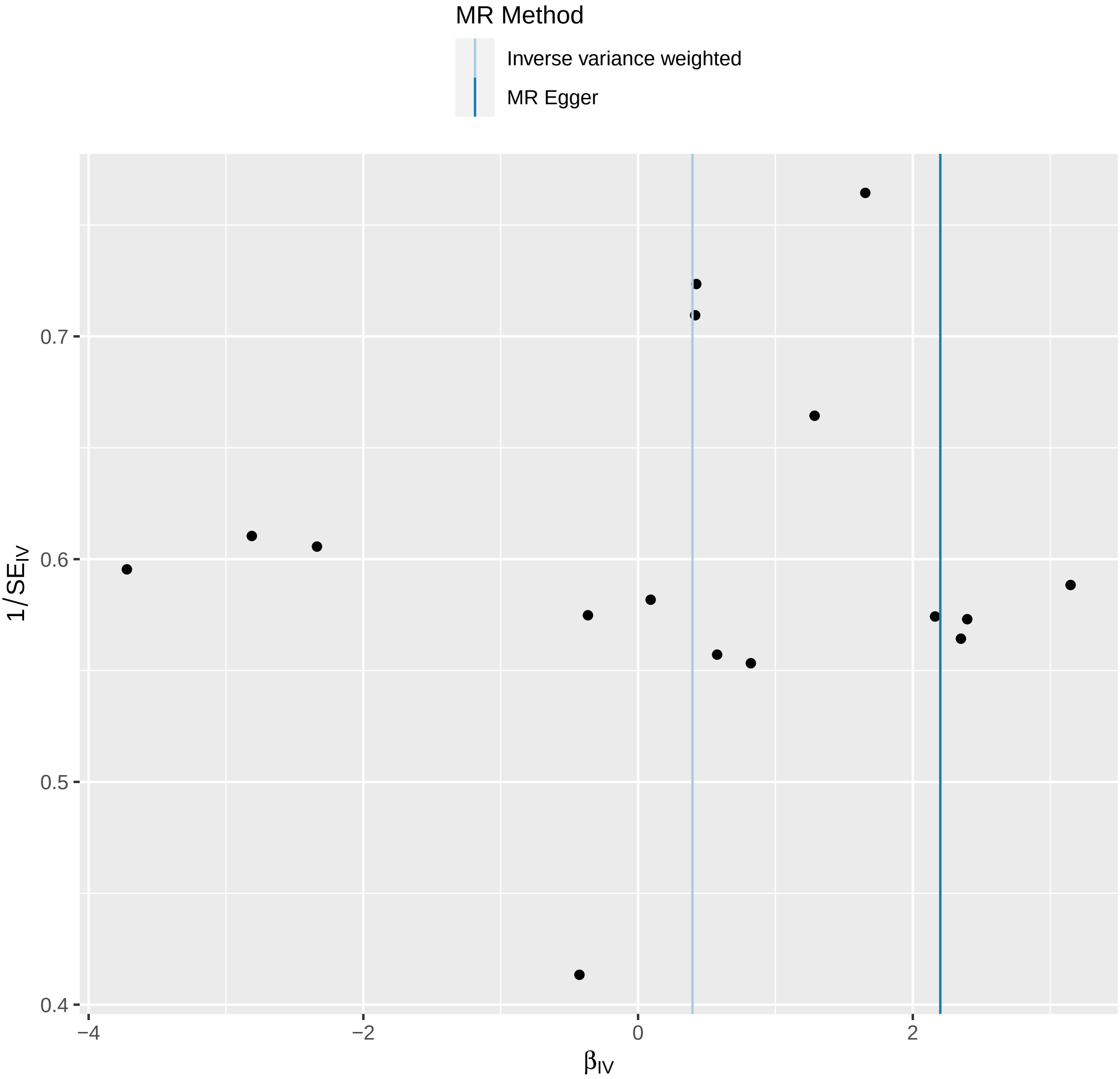

Batch 478 : Gut microbiota abundance (family Ruminococcaceae id.2050) on Arthropathy in Crohn disease

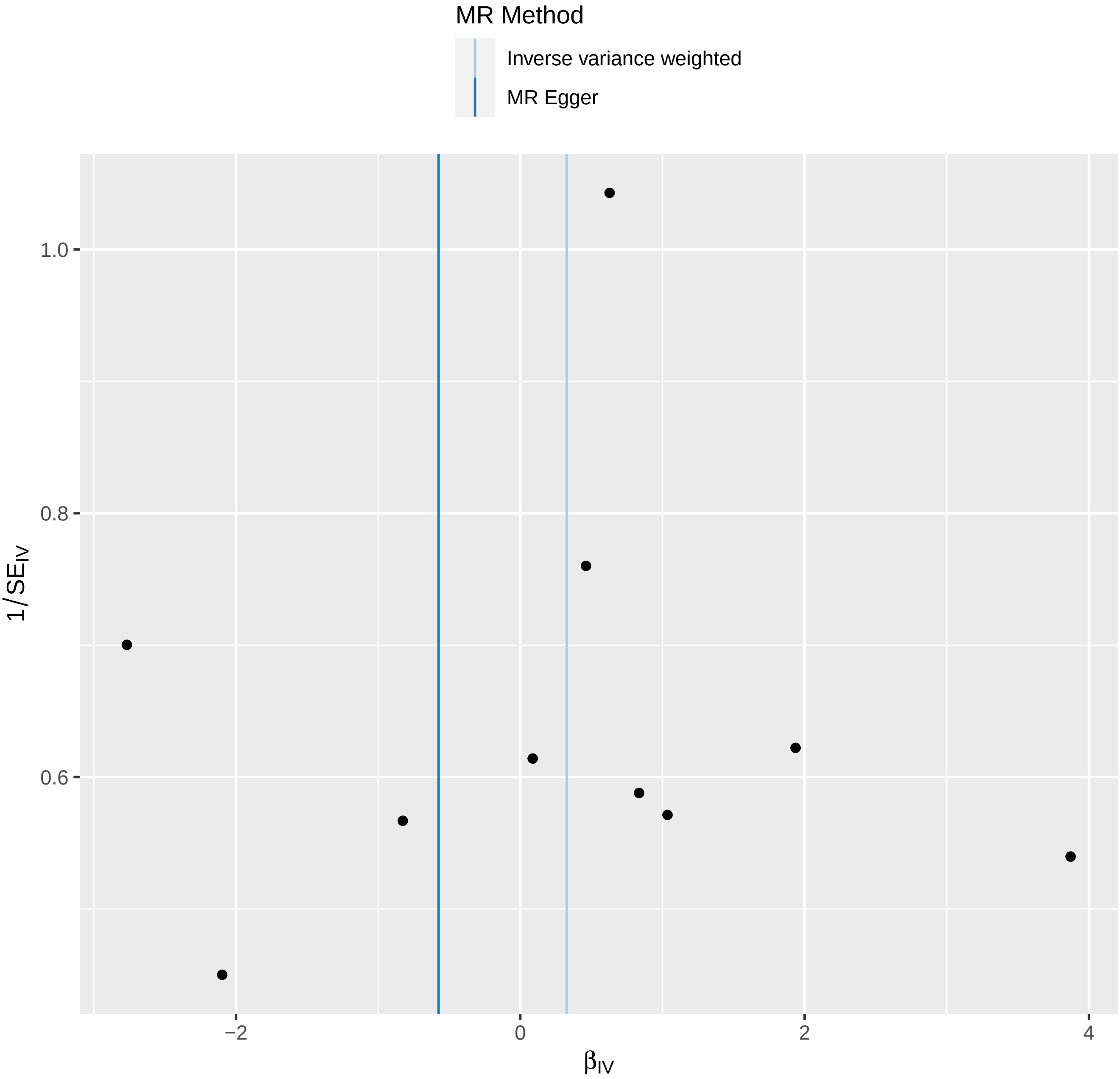

Batch 479 : Gut microbiota abundance (family Streptococcaceae id.1850) on Arthropathy in Crohn disease

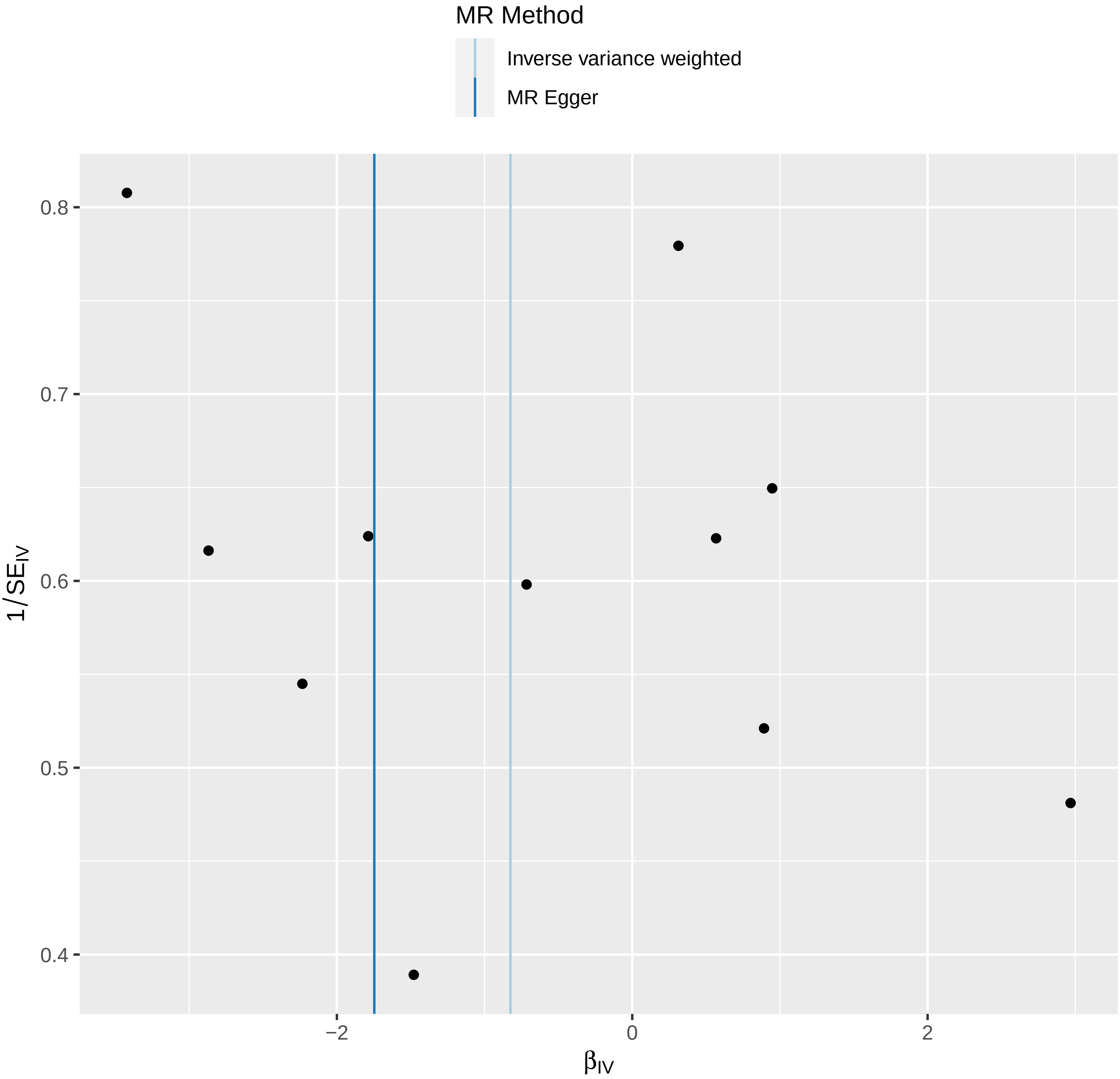

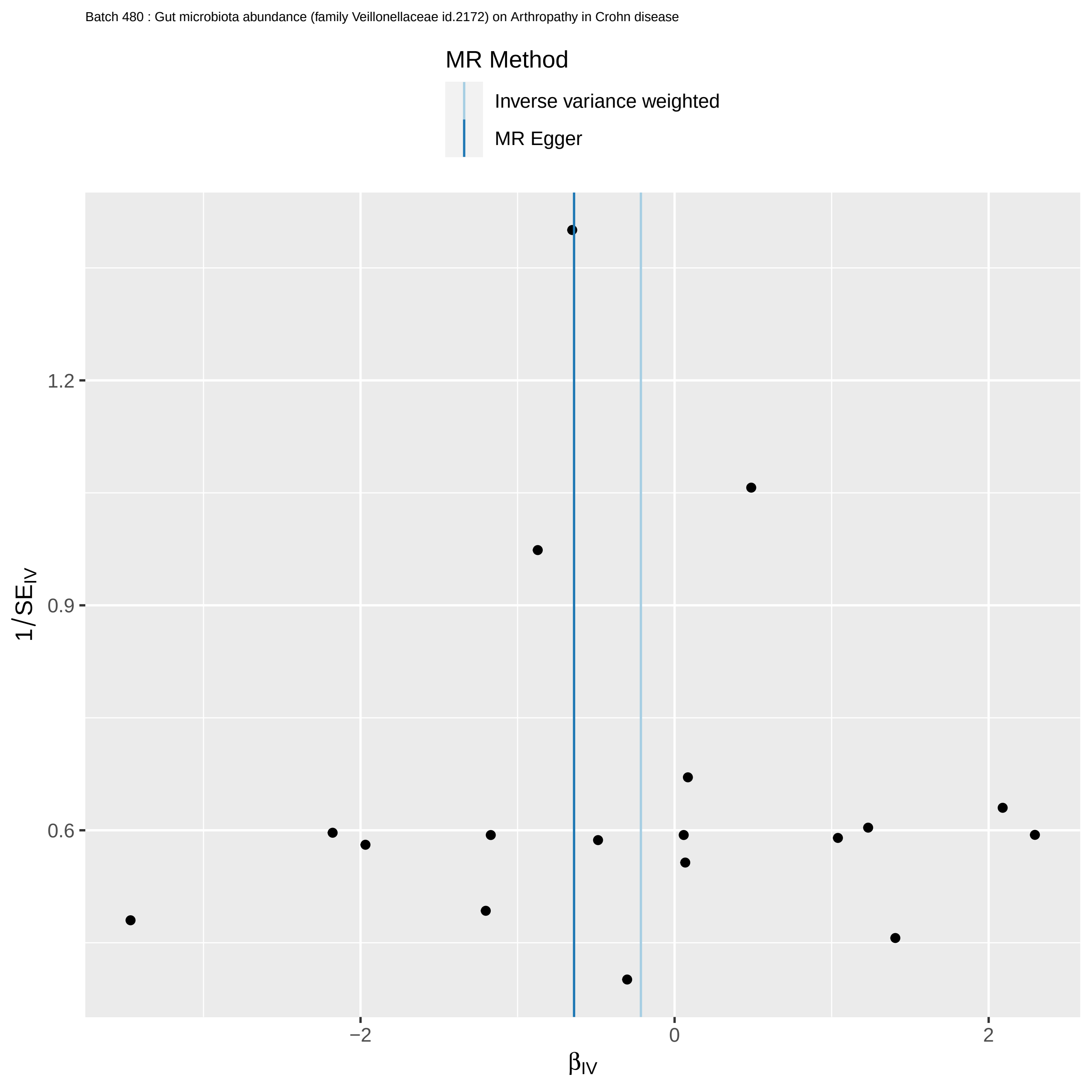

Batch 480 : Gut microbiota abundance (family Veillonellaceae id.2172) on Arthropathy in Crohn disease
MR Method
Inverse variance weighted
MR Egger
1.2
0.9
0.6
1/SE_IV
−2
0
2
β_IV

Batch 481 : Gut microbiota abundance (family Verrucomicrobiaceae id.4036) on Arthropathy in Crohn disease

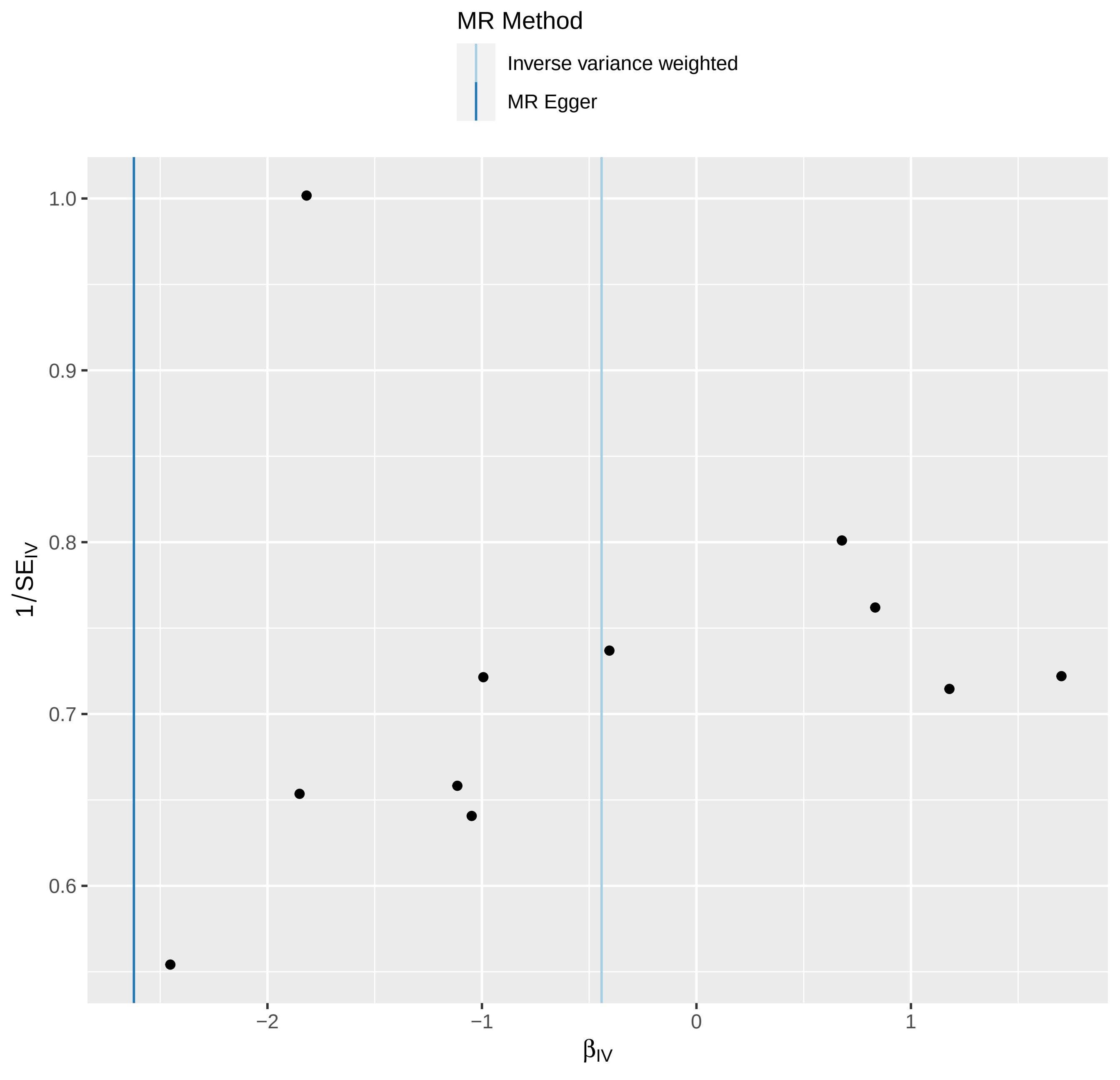

Batch 482 : Gut microbiota abundance (family Victivallaceae id.2255) on Arthropathy in Crohn disease

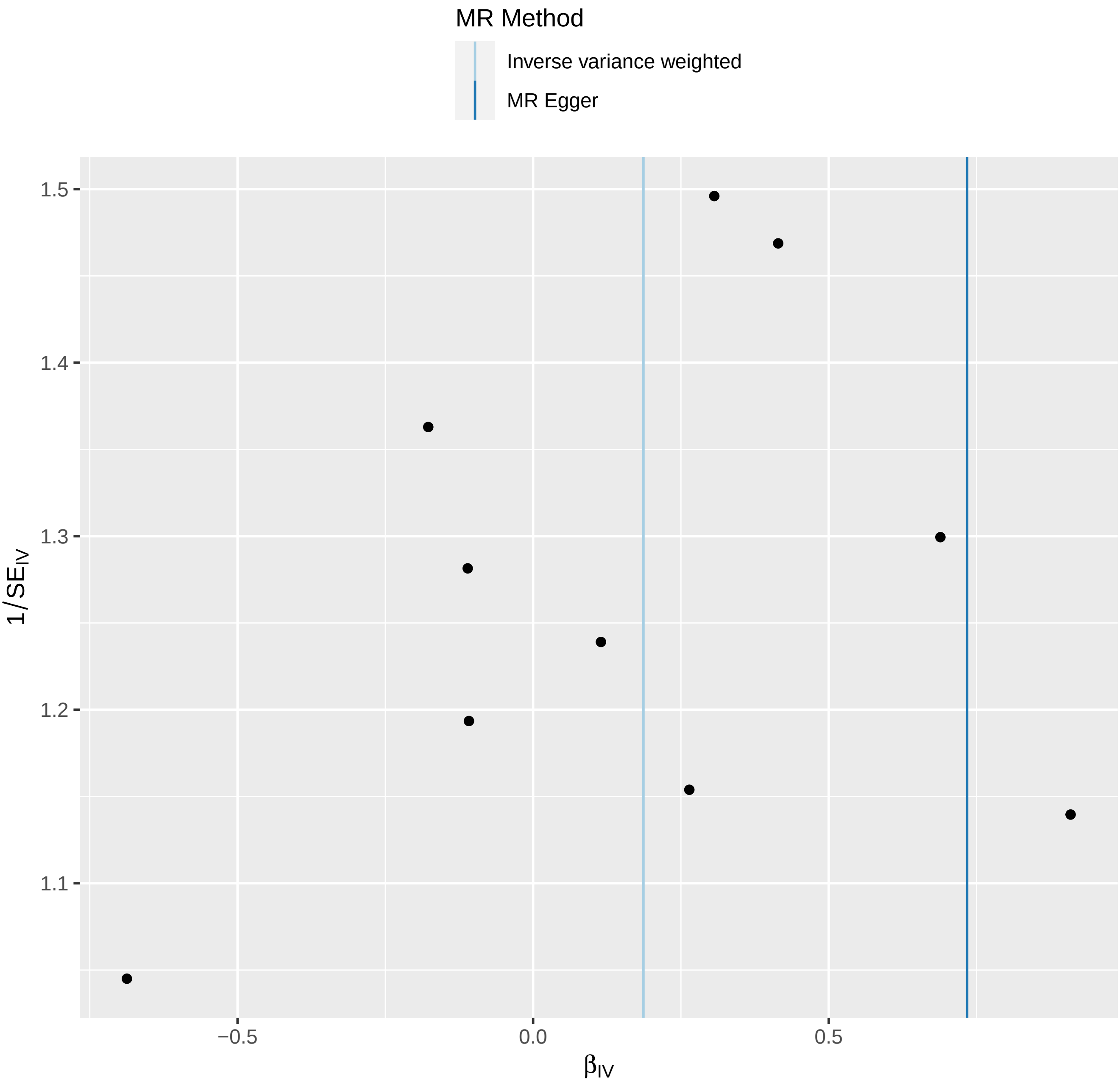

Batch 483 : Gut microbiota abundance (genus Actinomyces id.423) on Arthropathy in Crohn disease

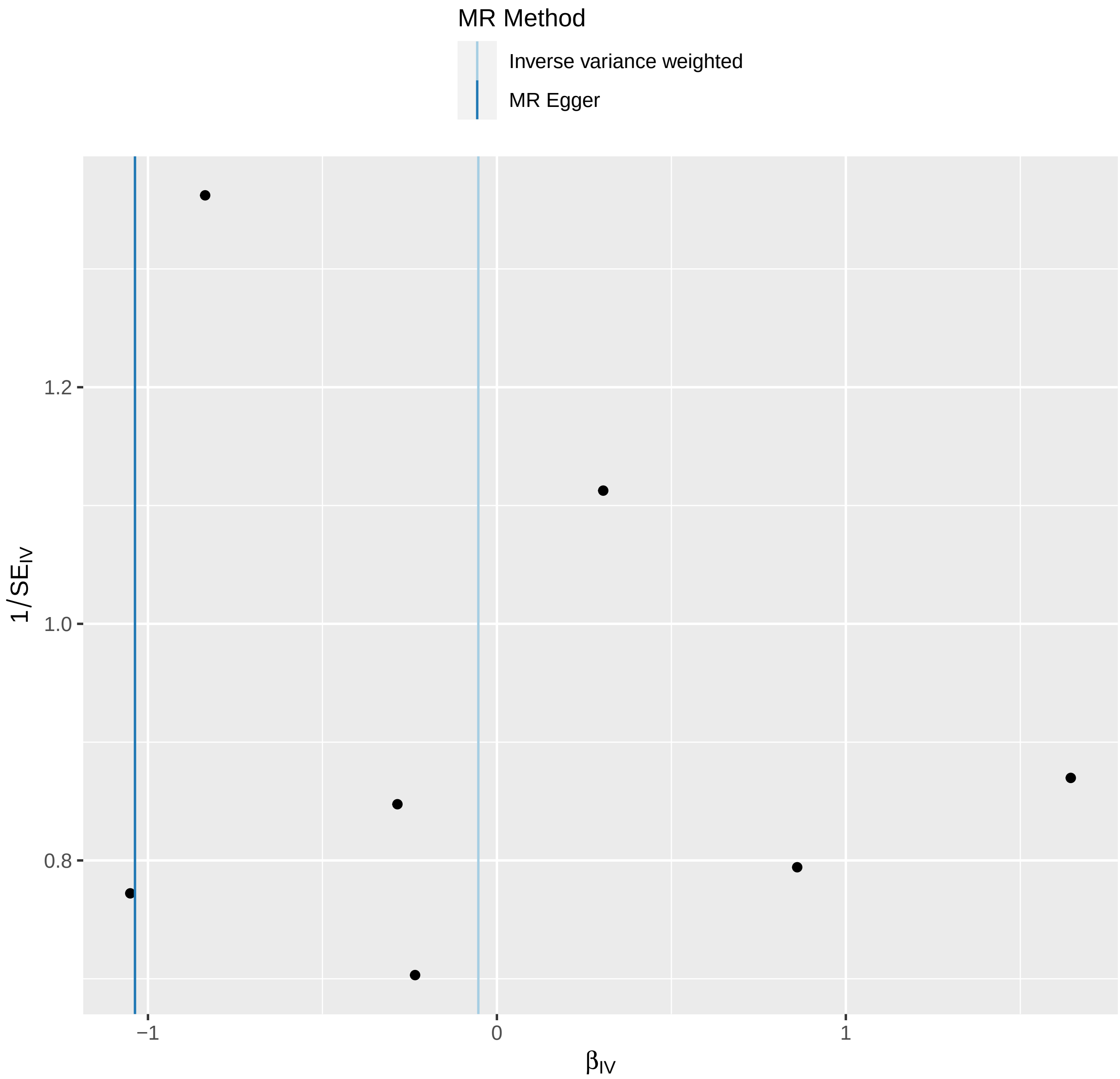

Batch 484 : Gut microbiota abundance (genus Adlercreutzia id.812) on Arthropathy in Crohn disease

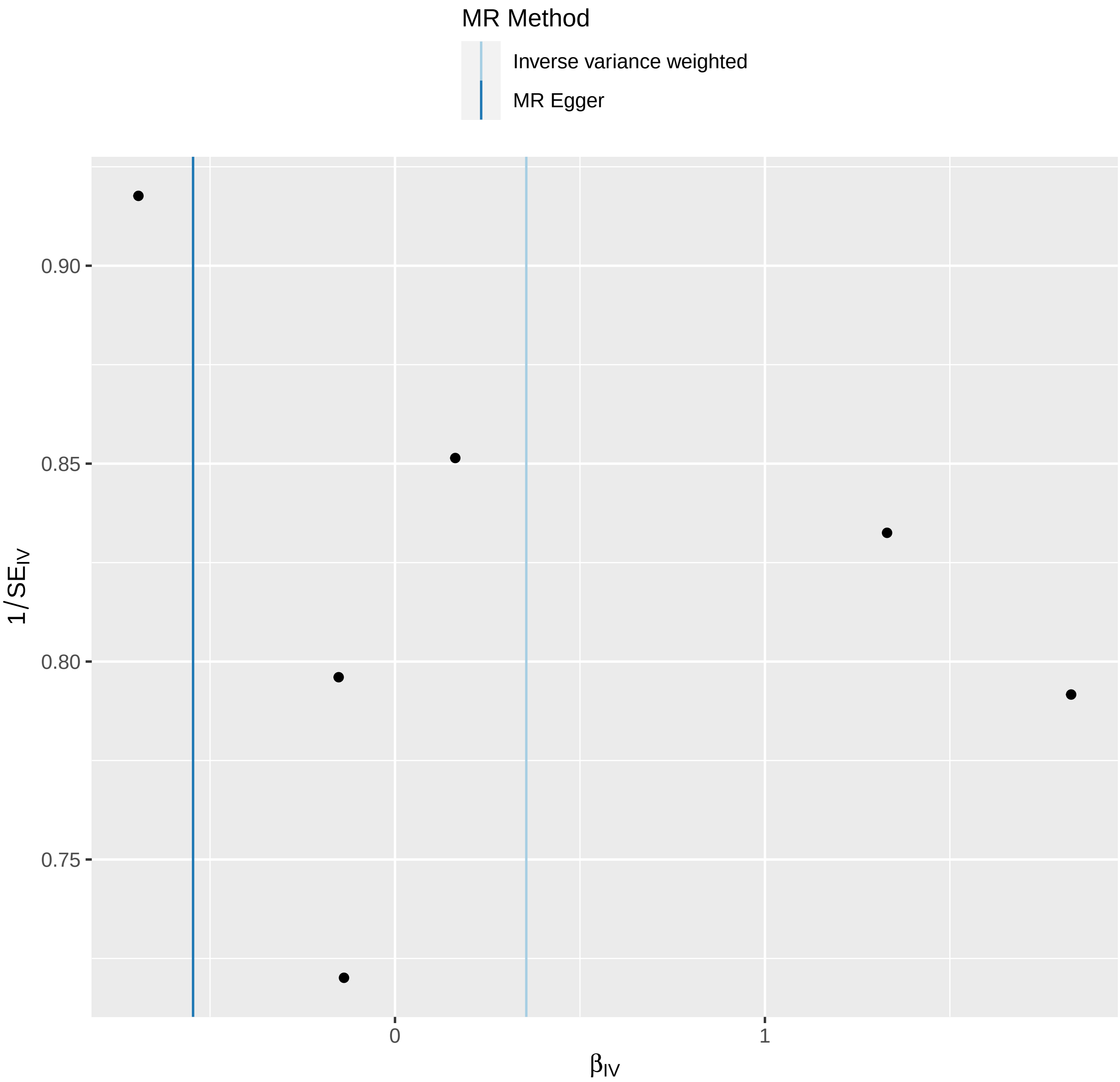

Batch 485 : Gut microbiota abundance (genus Akkermansia id.4037) on Arthropathy in Crohn disease

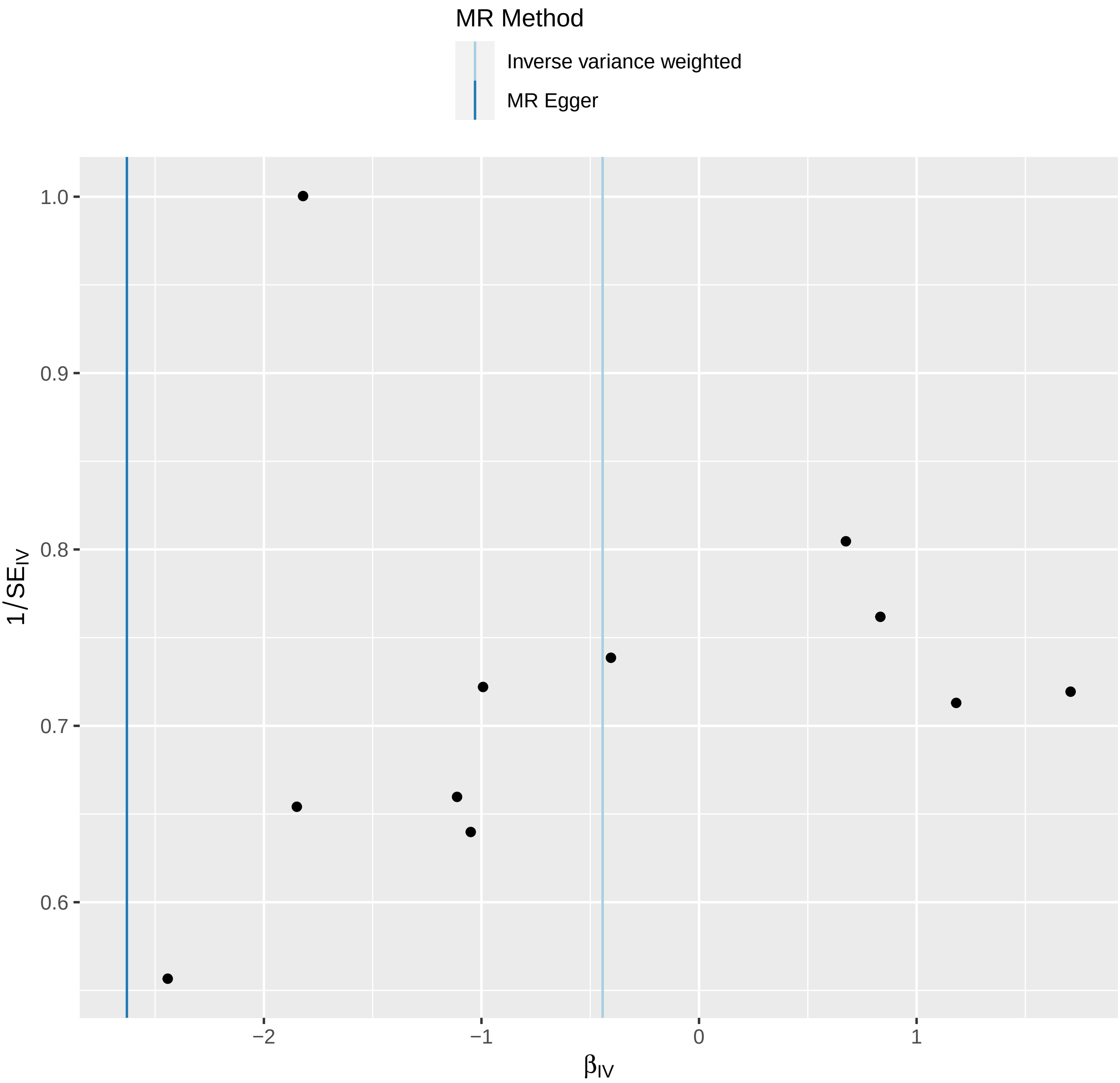

Batch 486 : Gut microbiota abundance (genus Alistipes id.968) on Arthropathy in Crohn disease

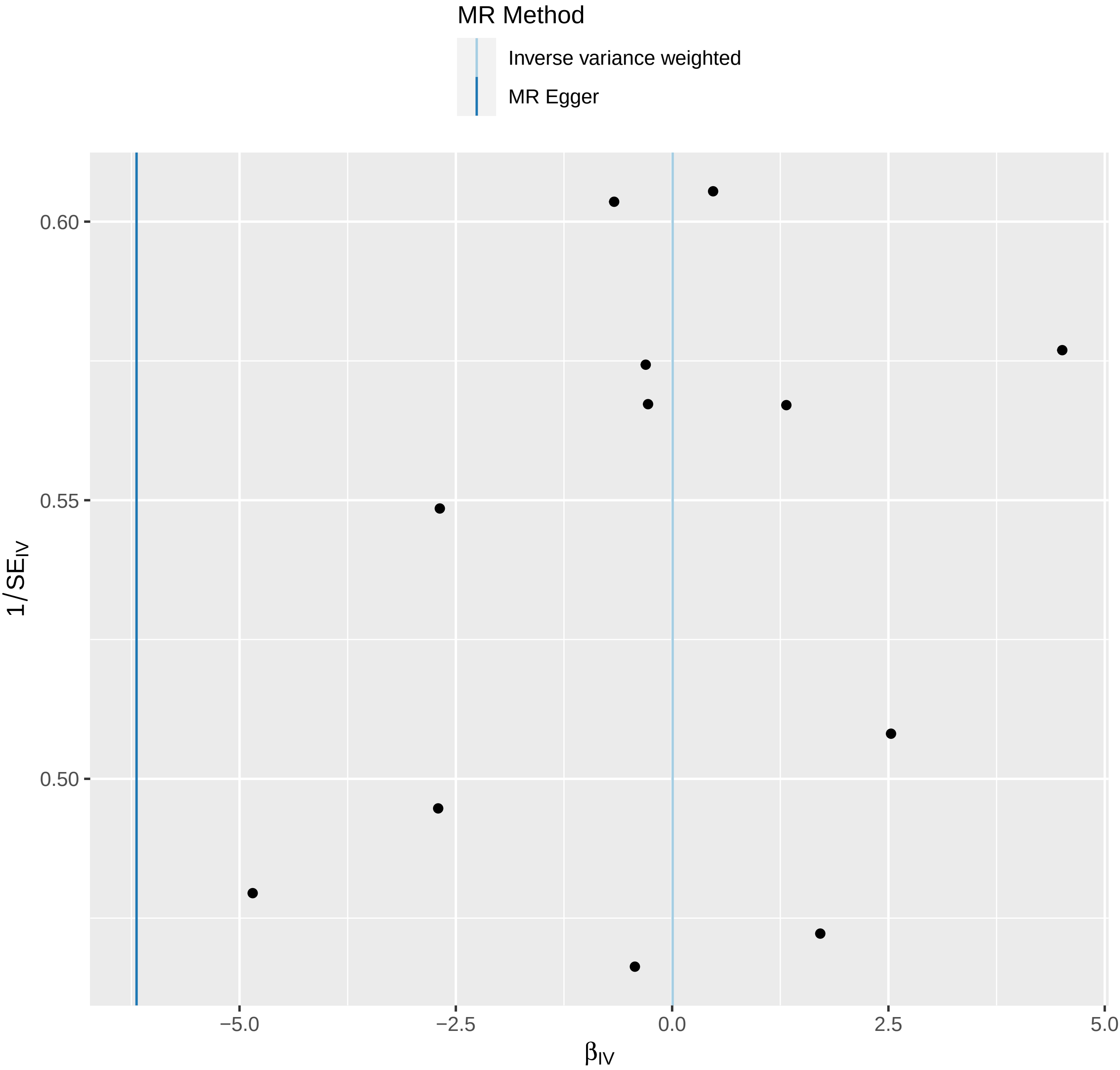

Batch 487 : Gut microbiota abundance (genus Allisonella id.2174) on Arthropathy in Crohn disease

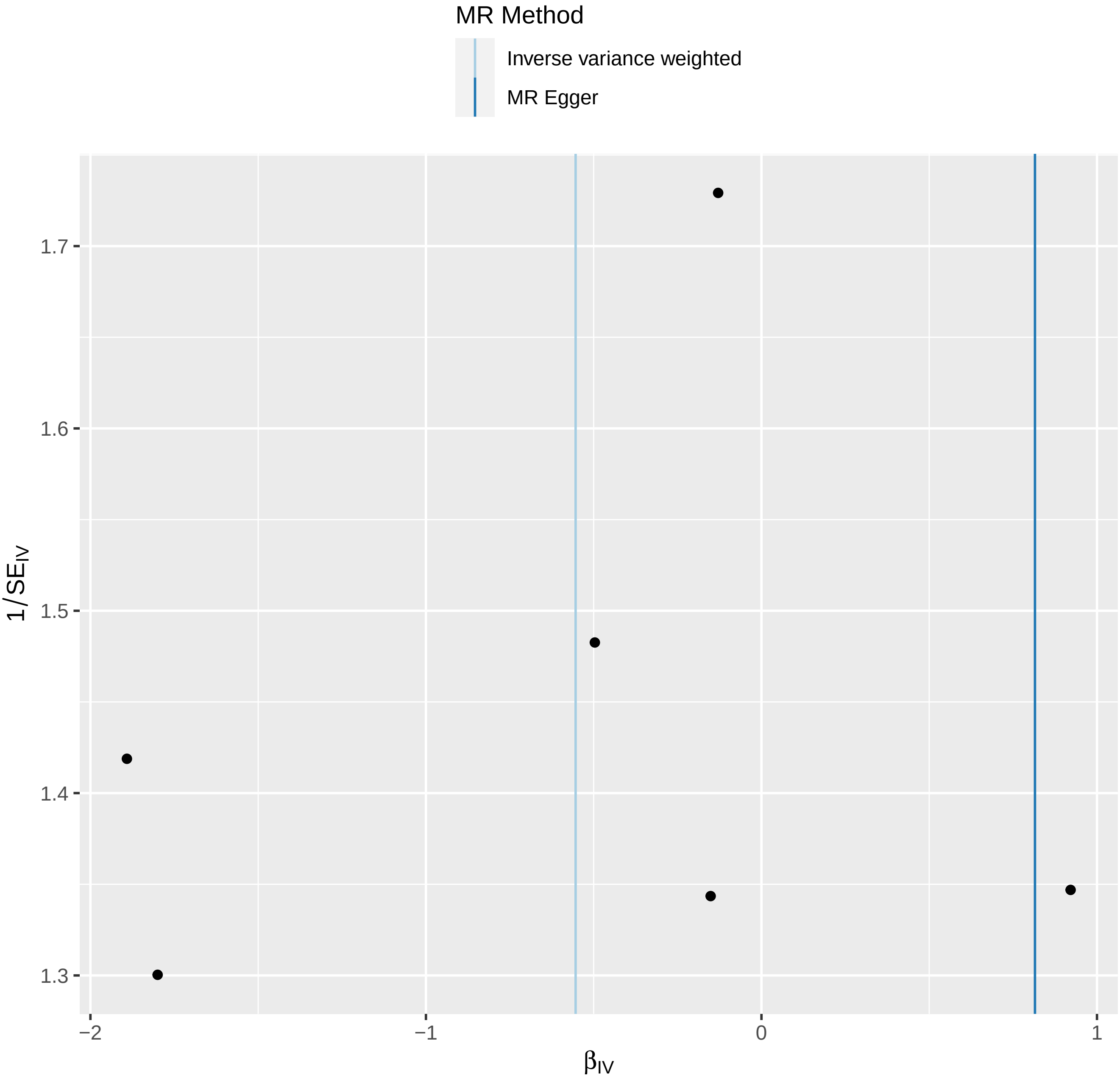

Batch 488 : Gut microbiota abundance (genus Alloprevotella id.961) on Arthropathy in Crohn disease

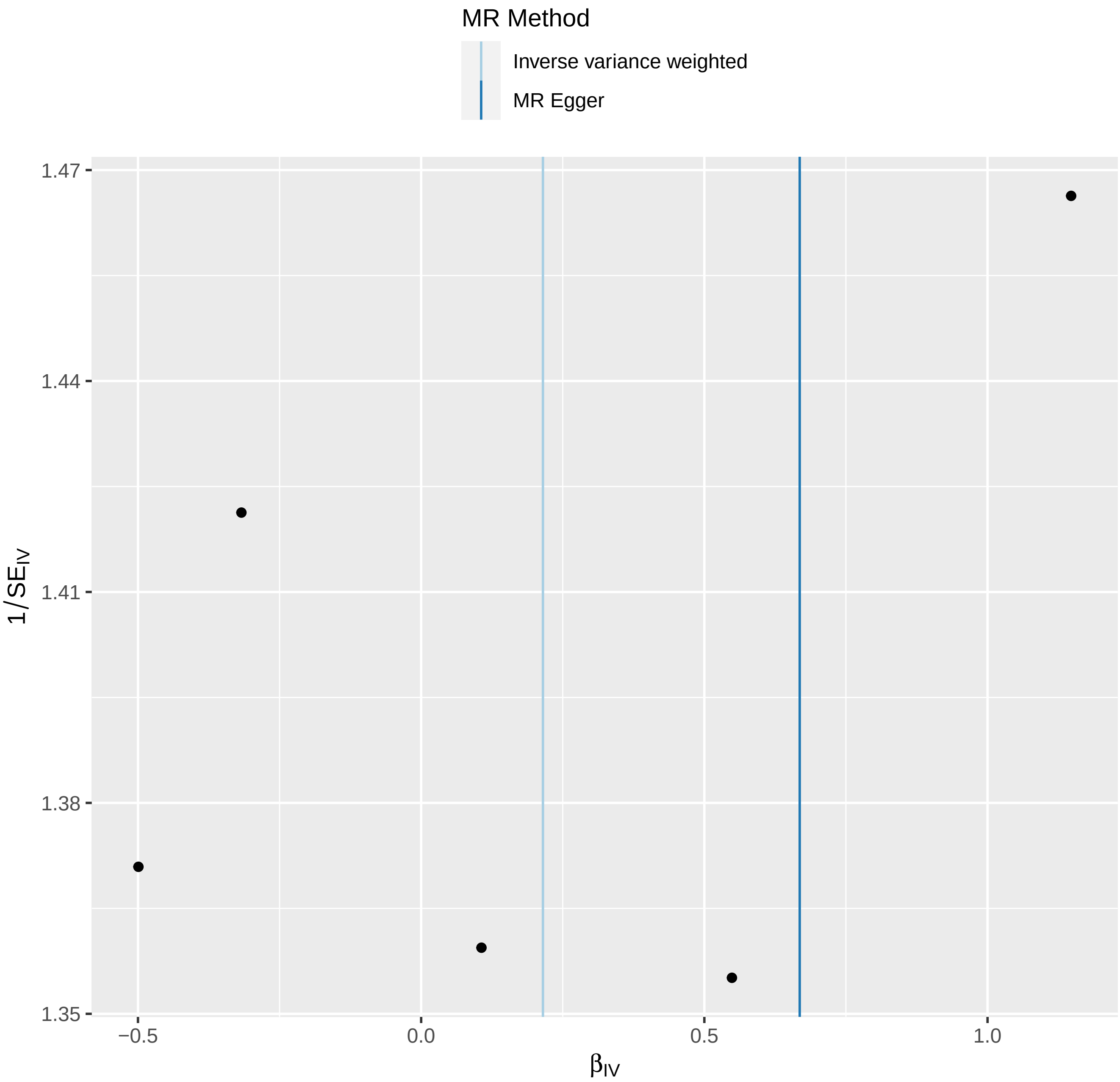

Batch 489 : Gut microbiota abundance (genus Anaerofilum id.2053) on Arthropathy in Crohn disease

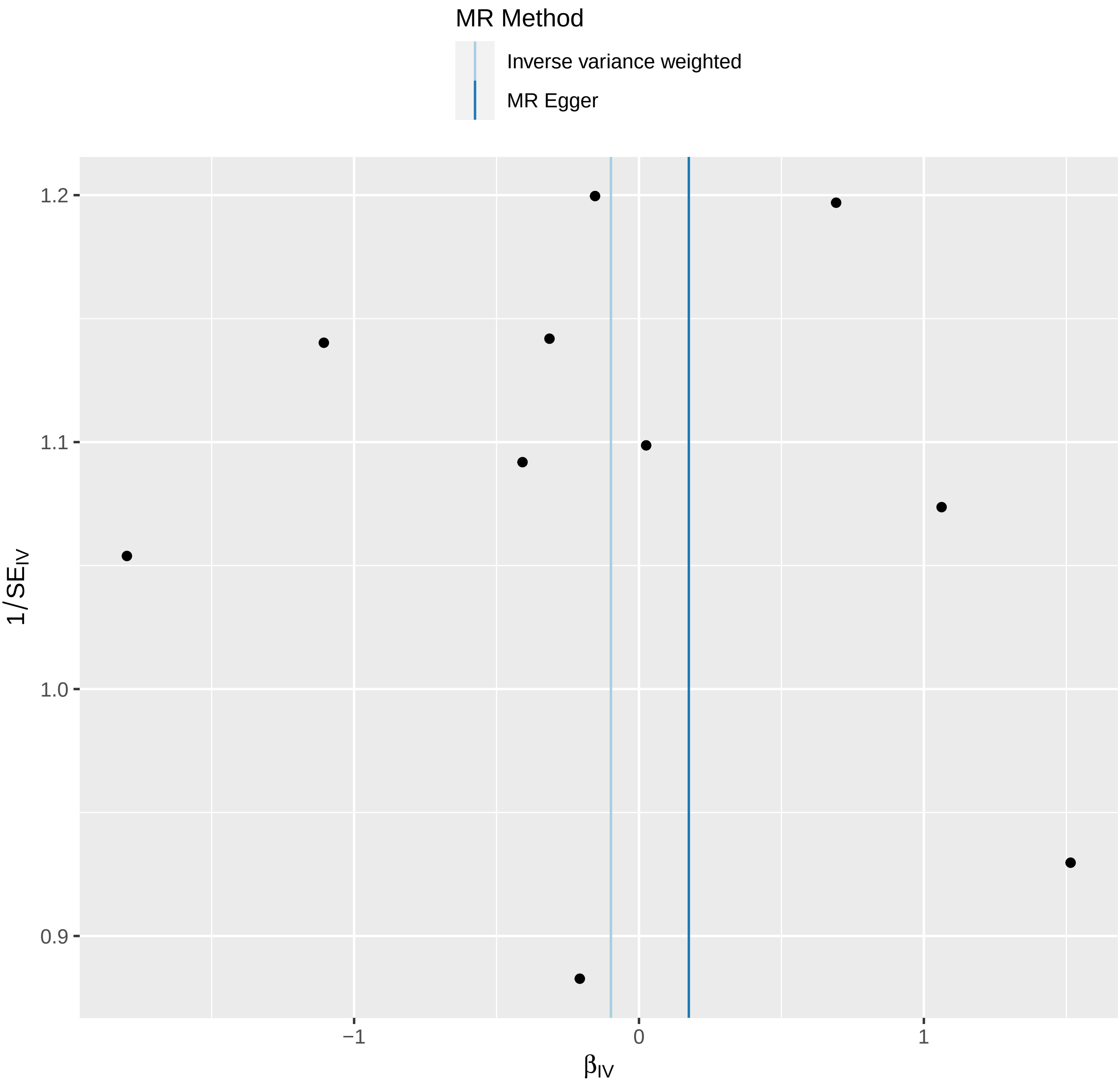

Batch 490 : Gut microbiota abundance (genus Anaerostipes id.1991) on Arthropathy in Crohn disease

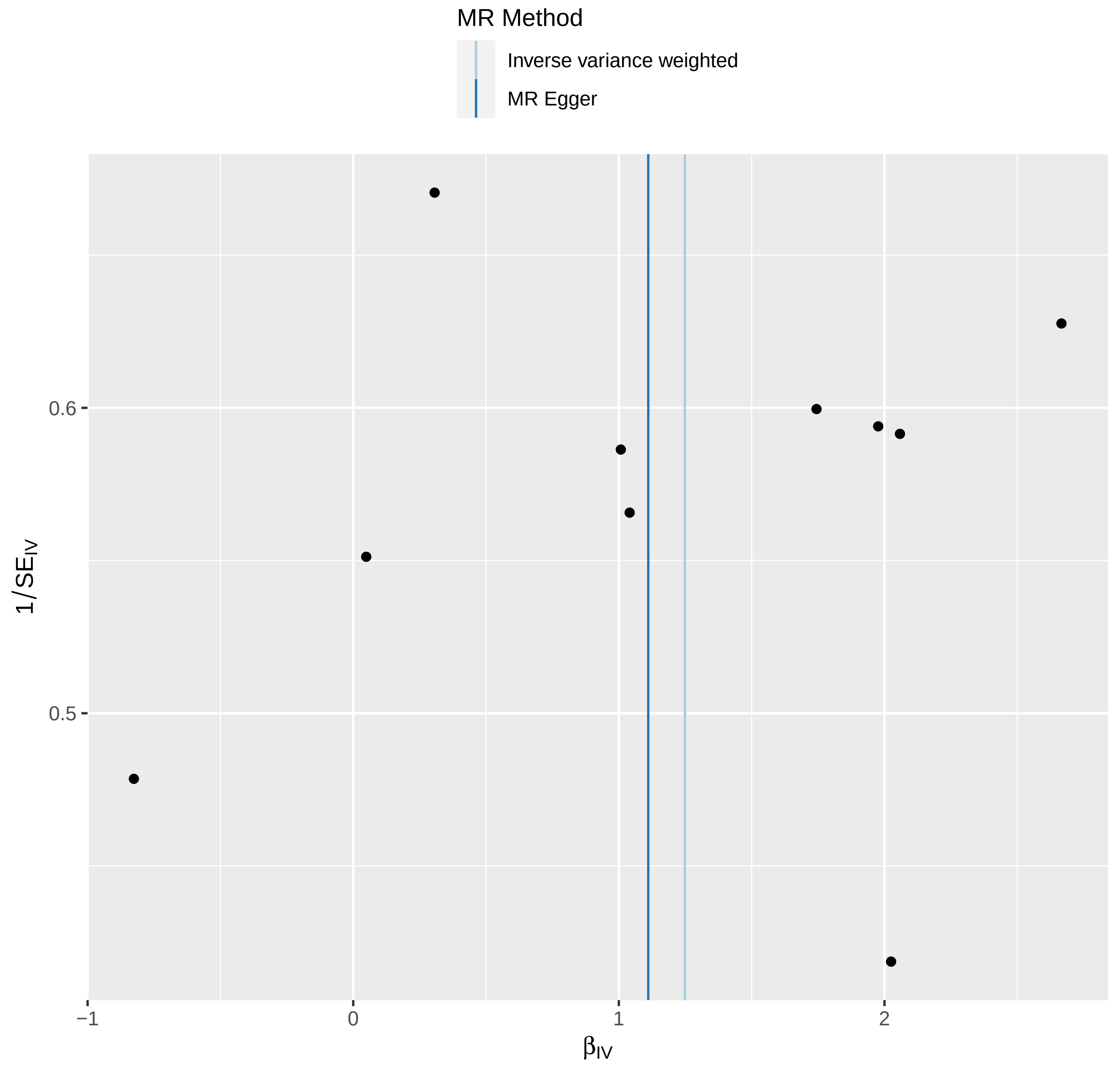

Batch 491 : Gut microbiota abundance (genus Anaerotruncus id.2054) on Arthropathy in Crohn disease

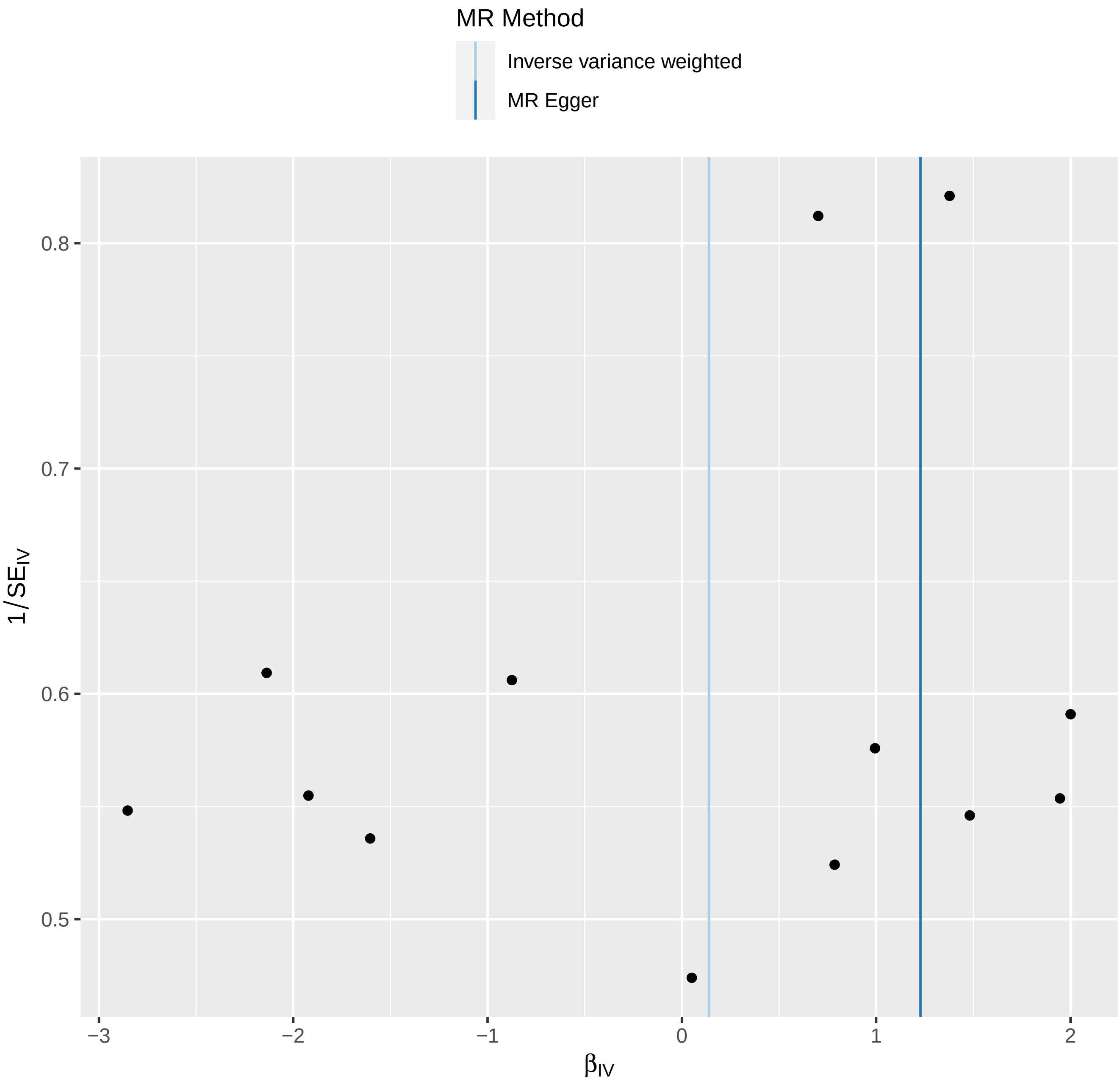

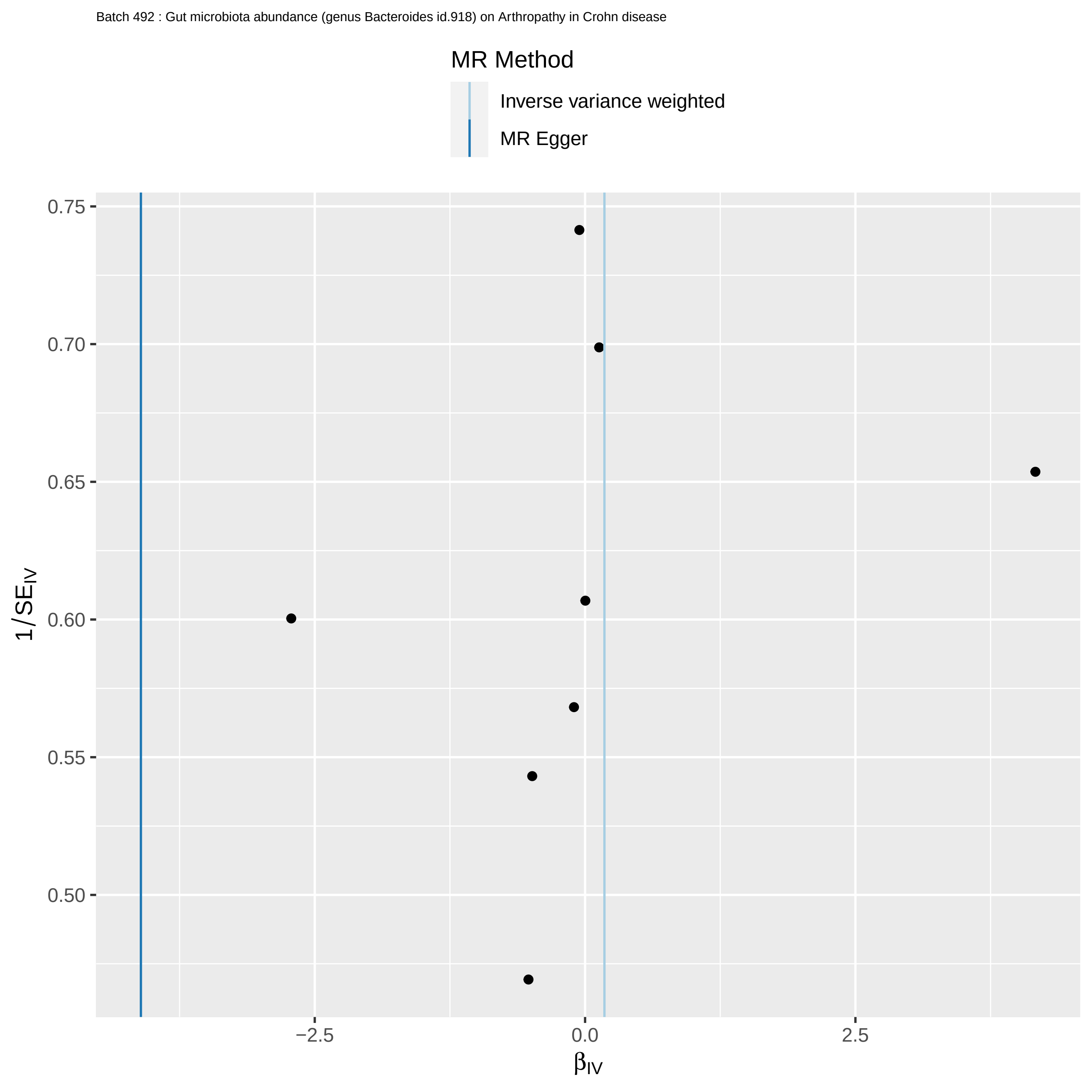
Batch 492 : Gut microbiota abundance (genus Bacteroides id.918) on Arthropathy in Crohn disease
MR Method
Inverse variance weighted
MR Egger
0.75
0.70
0.65
0.60
0.55
0.50
1/SE_IV
−2.5
0.0
2.5
β_IV

Batch 493 : Gut microbiota abundance (genus Barnesiella id.944) on Arthropathy in Crohn disease

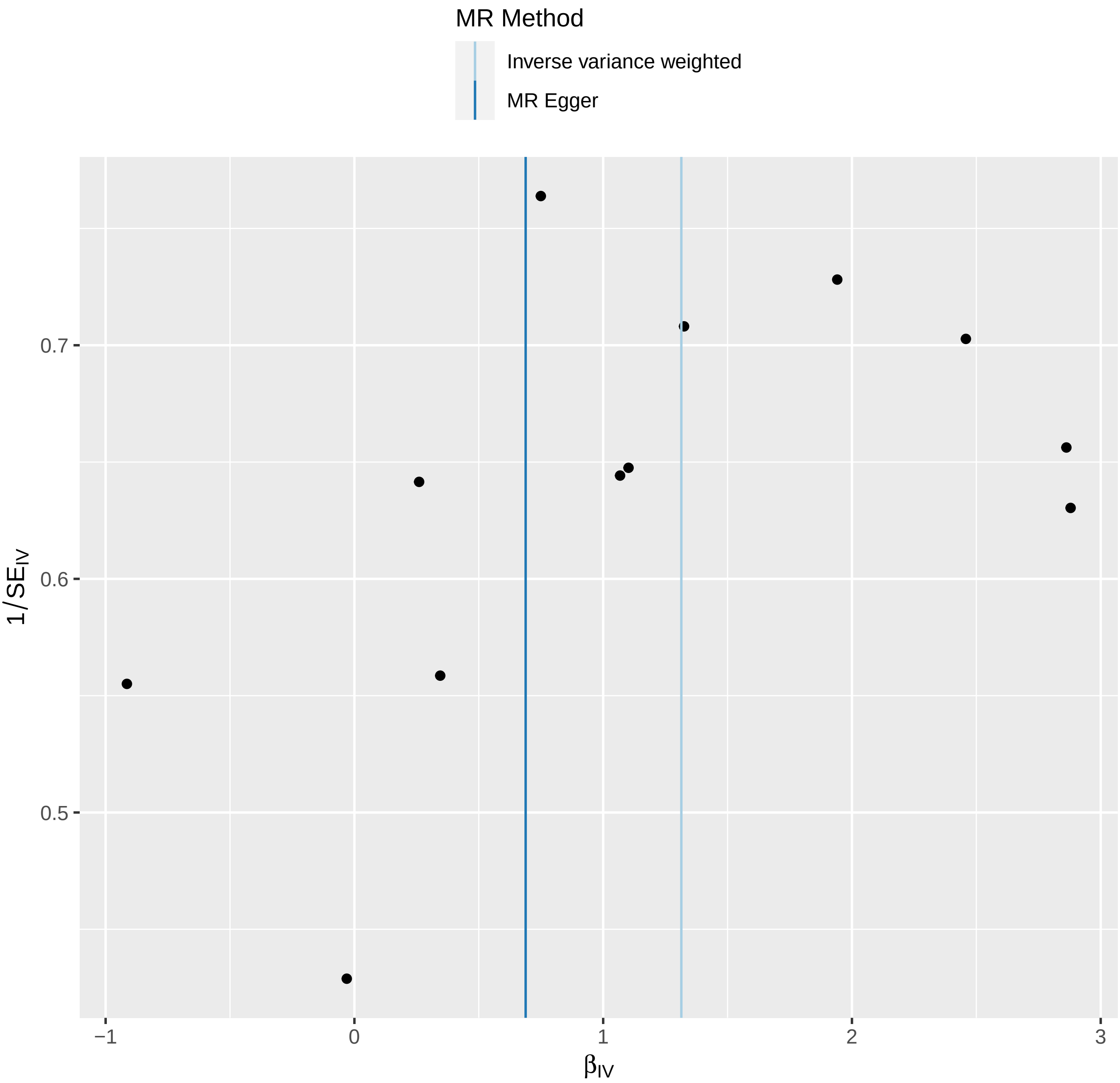

Batch 494 : Gut microbiota abundance (genus Bifidobacterium id.436) on Arthropathy in Crohn disease

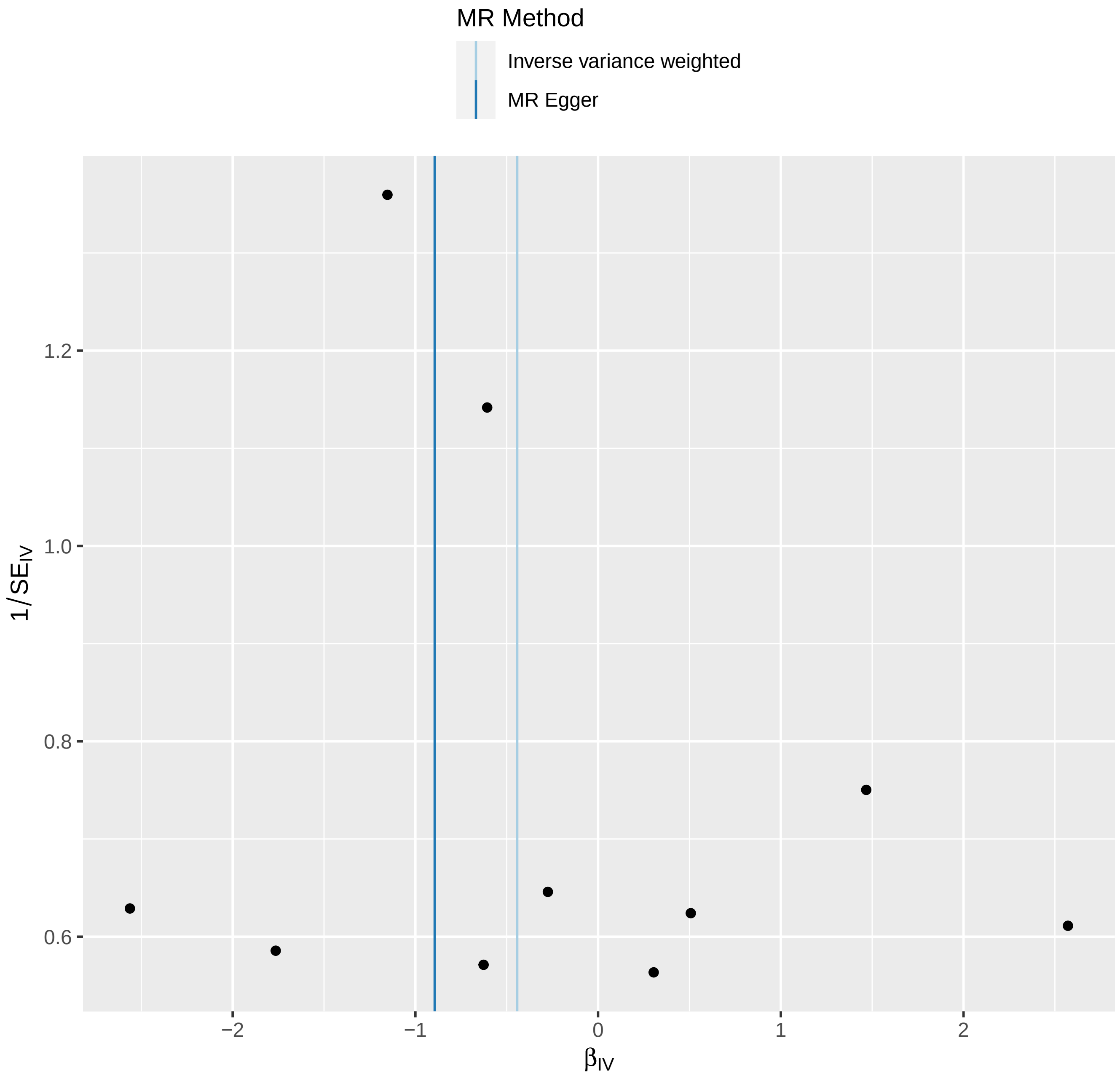

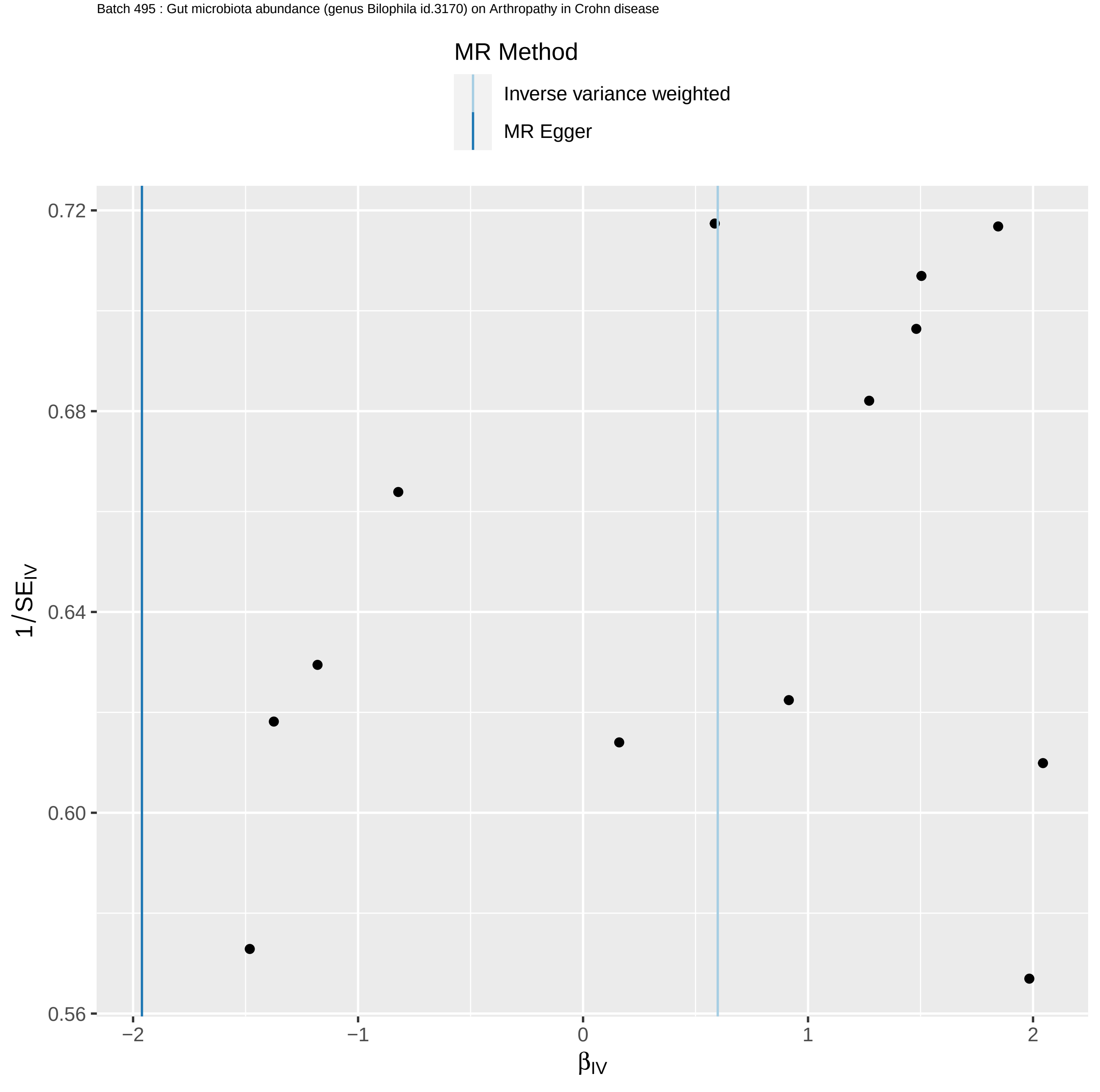

Batch 495 : Gut microbiota abundance (genus Bilophila id.3170) on Arthropathy in Crohn disease
MR Method
Inverse variance weighted
MR Egger
0.72
0.68
0.64
0.60
0.56
$1/SE_{IV}$
−2
−1
0
1
2
β_{IV}

Batch 496 : Gut microbiota abundance (genus Blautia id.1992) on Arthropathy in Crohn disease

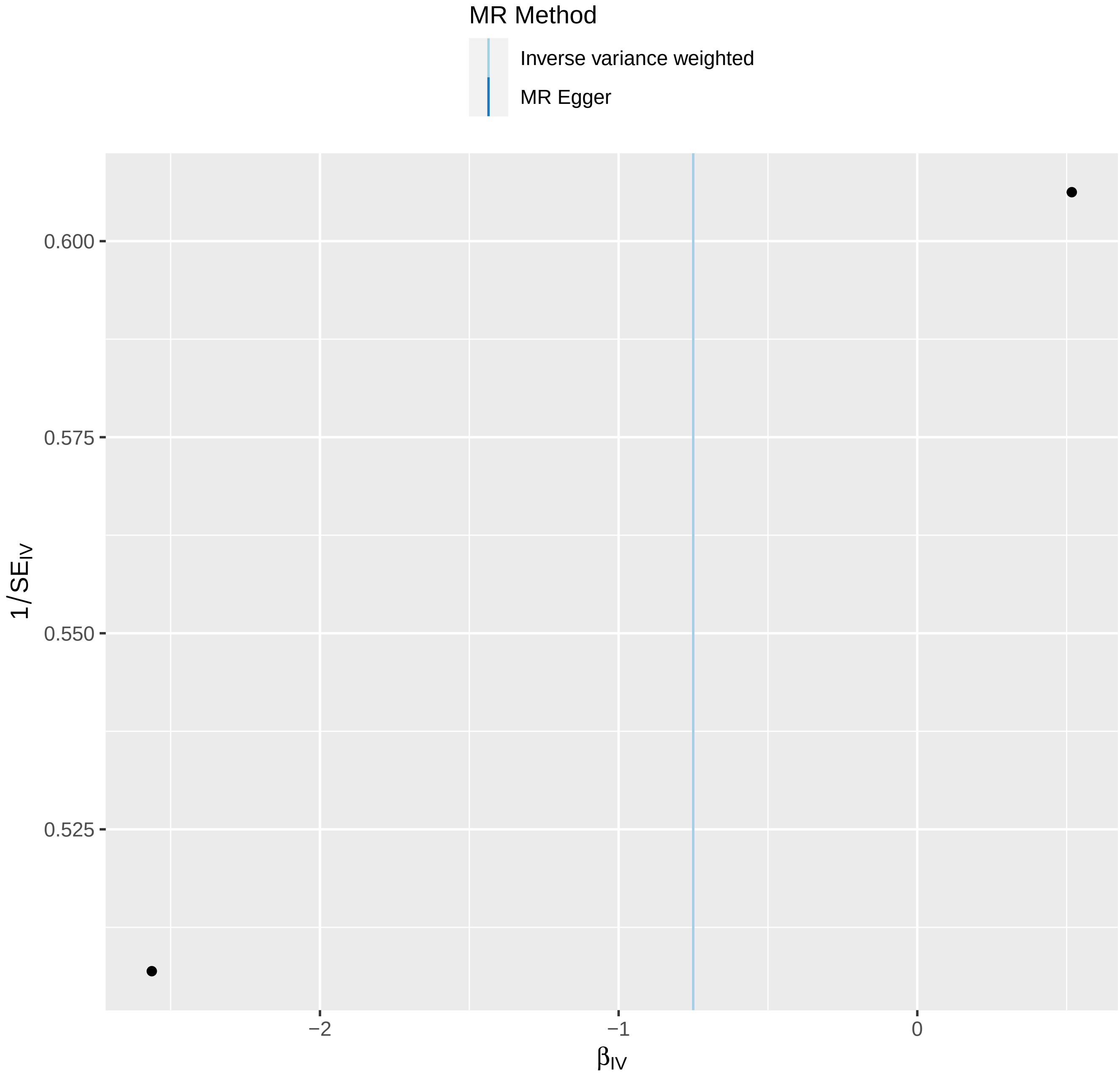

Batch 497 : Gut microbiota abundance (genus Butyricicoccus id.2055) on Arthropathy in Crohn disease

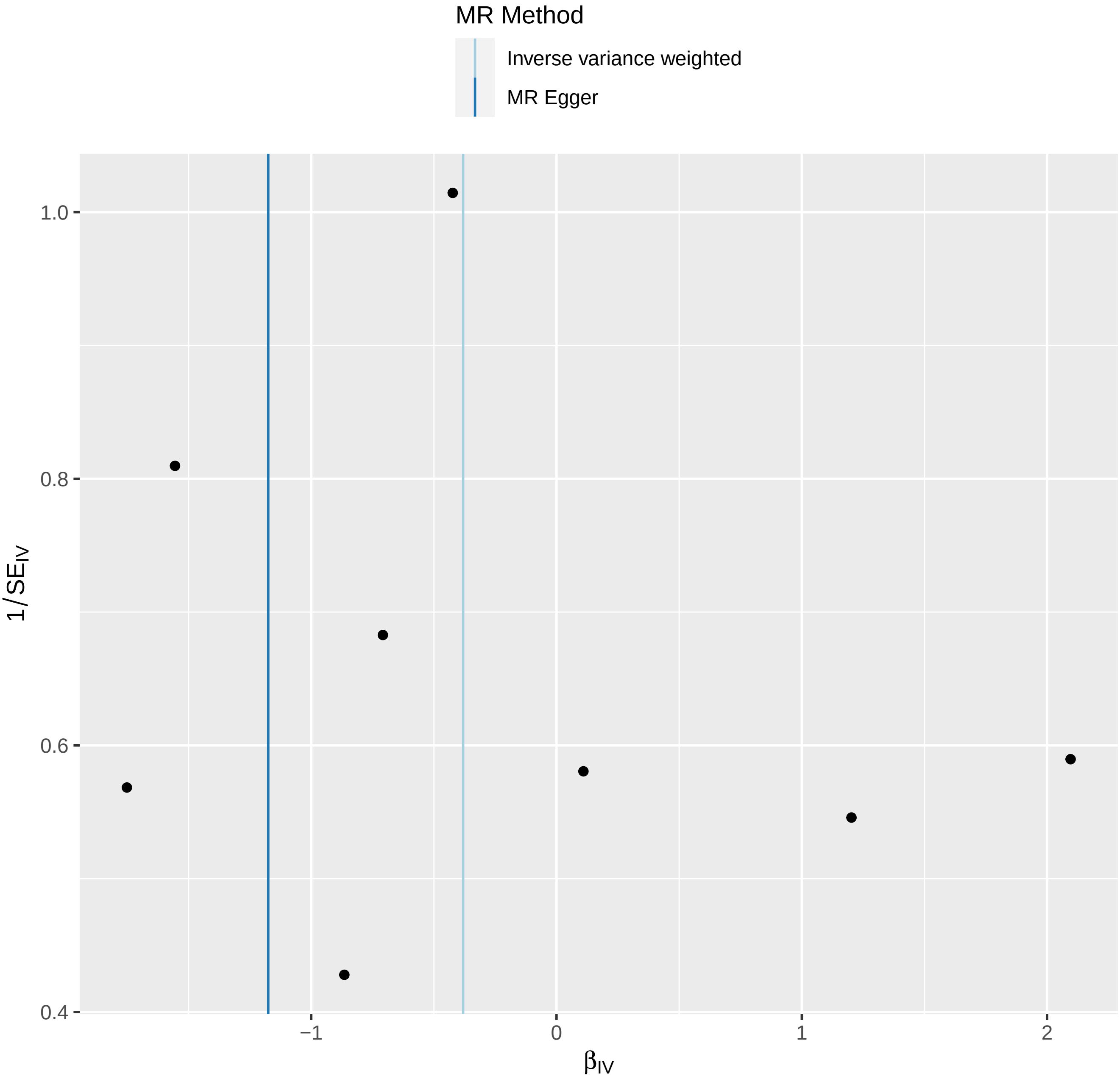

Batch 498 : Gut microbiota abundance (genus Butyricimonas id.945) on Arthropathy in Crohn disease

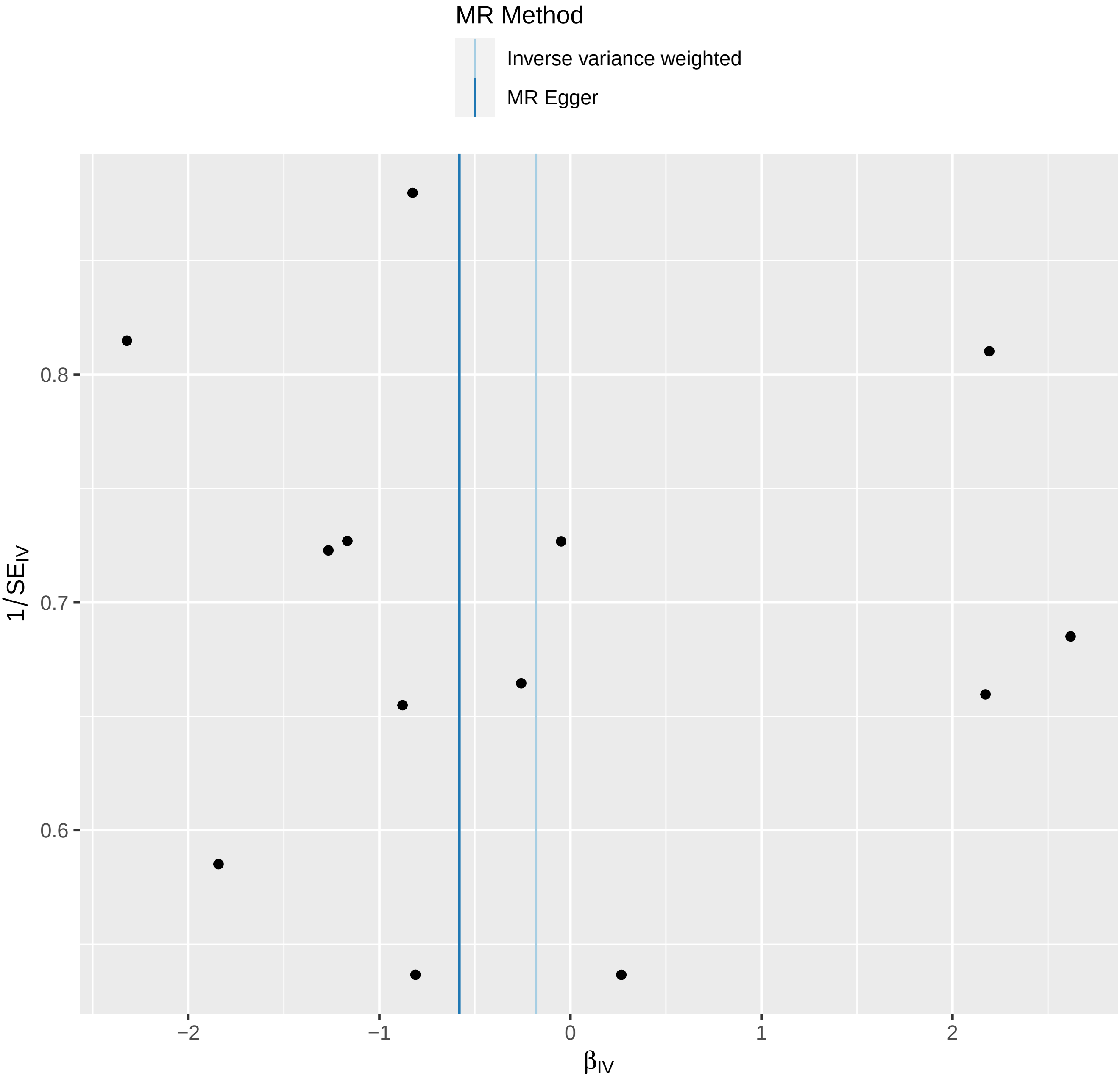

Batch 499 : Gut microbiota abundance (genus Butyrivibrio id.1993) on Arthropathy in Crohn disease

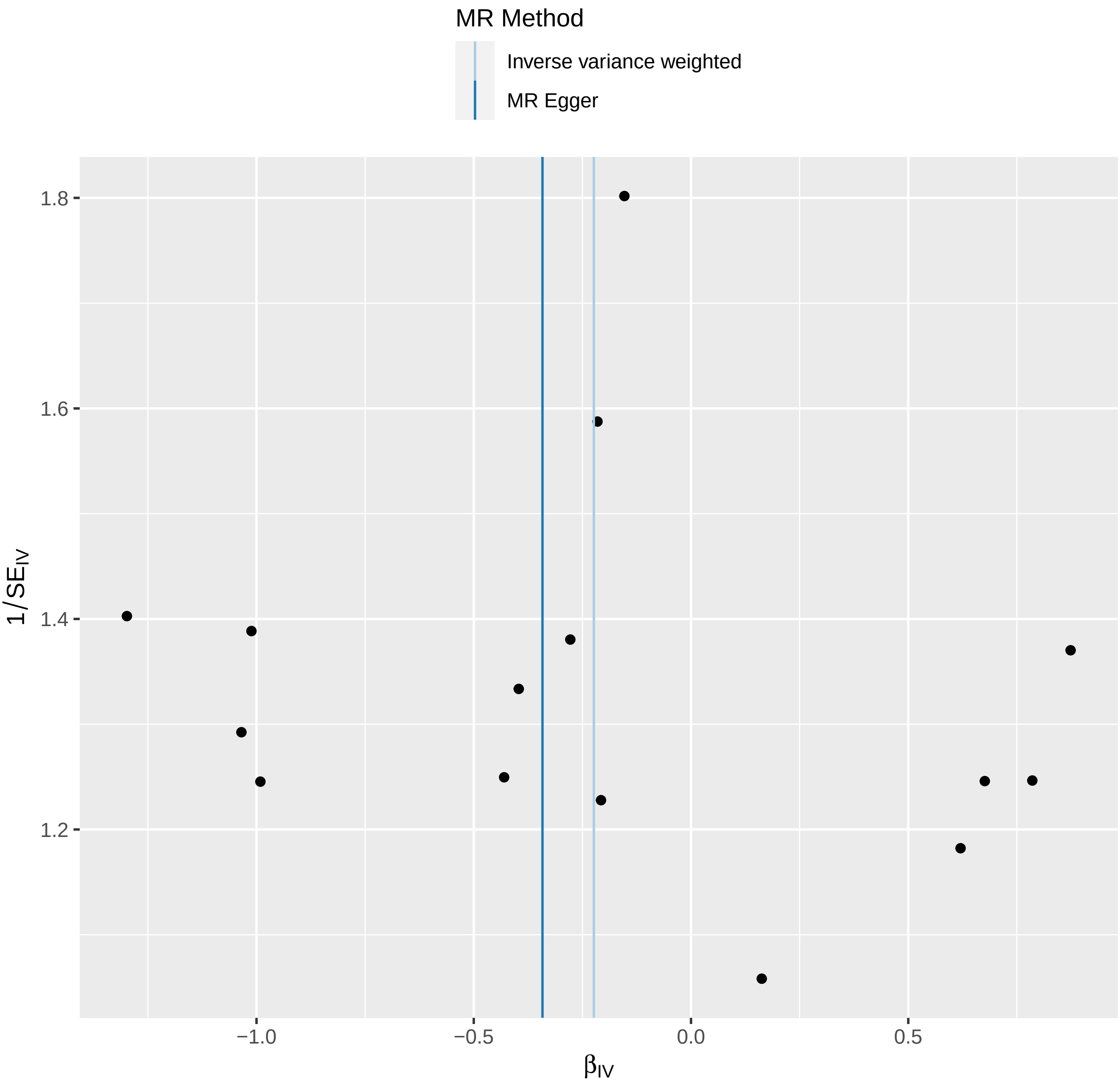

Batch 500 : Gut microbiota abundance (genus Candidatus Soleaferrea id.11350) on Arthropathy in Crohn disease

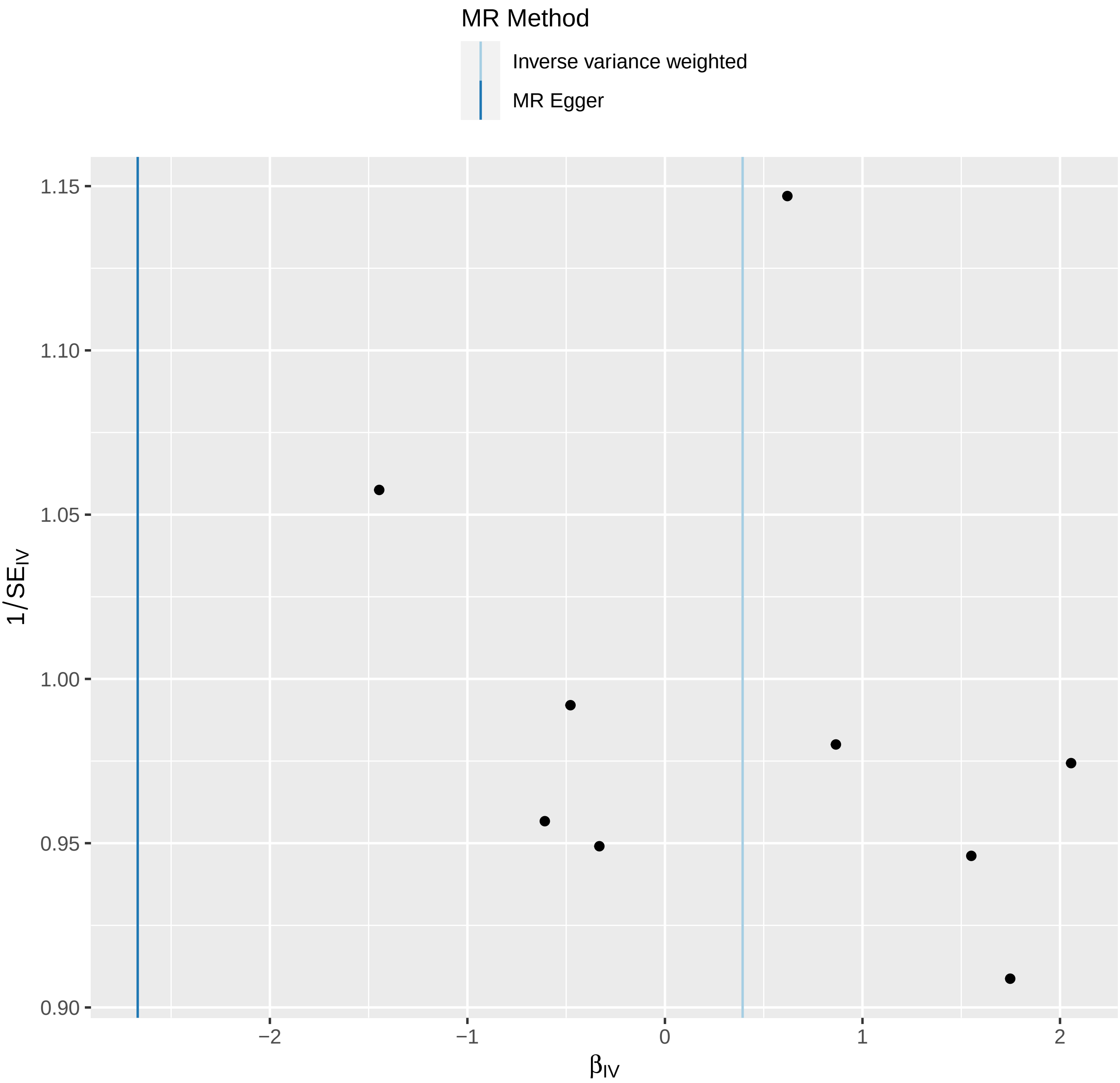

Batch 501 : Gut microbiota abundance (genus Catenibacterium id.2153) on Arthropathy in Crohn disease

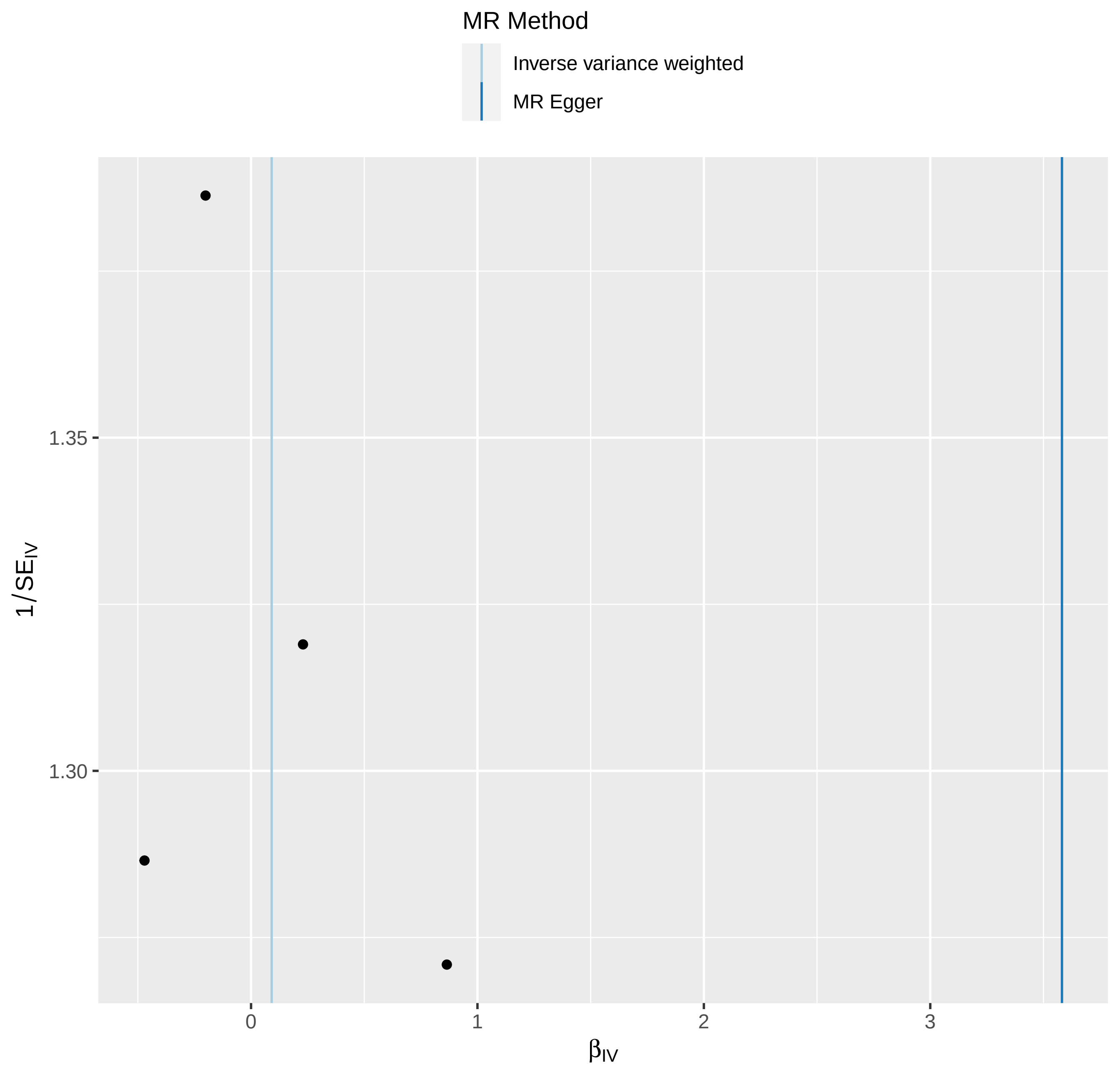

Batch 502 : Gut microbiota abundance (genus Christensenellaceae R 7group id.11283) on Arthropathy in Crohn disease

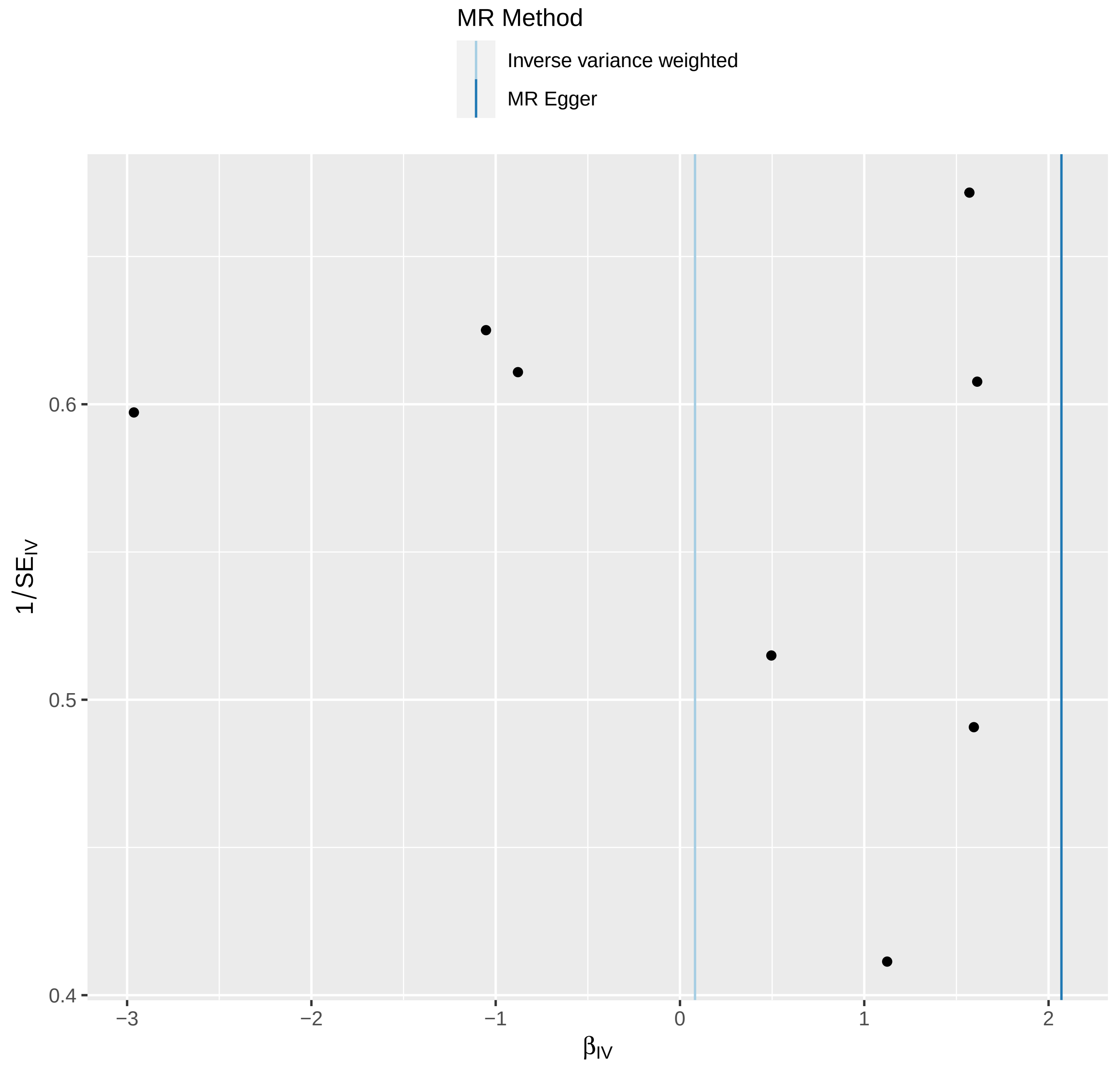

Batch 503 : Gut microbiota abundance (genus Clostridium innocuum group id.14397) on Arthropathy in Crohn disease

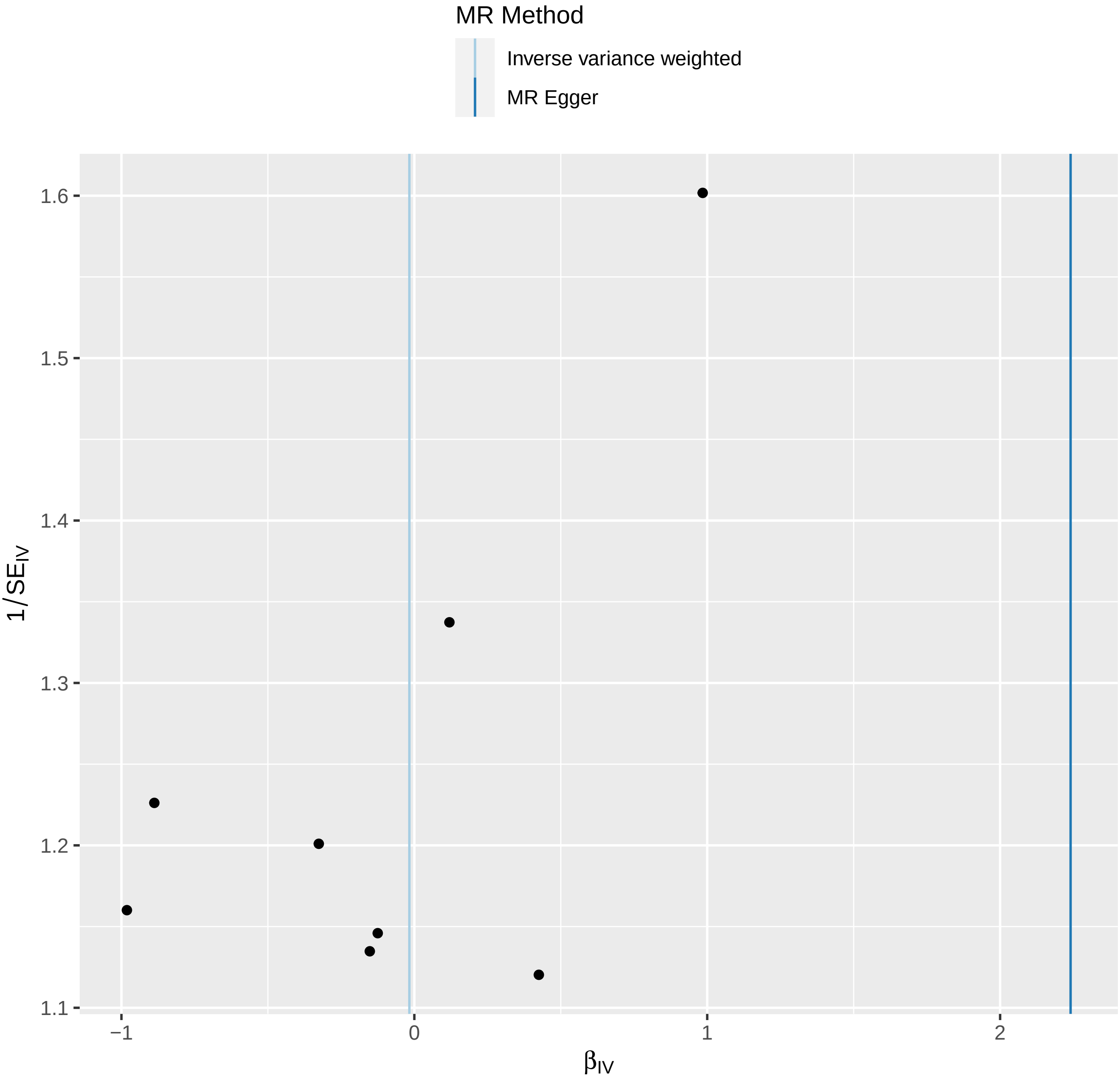

Batch 504 : Gut microbiota abundance (genus Clostridium sensustricto1 id.1873) on Arthropathy in Crohn disease

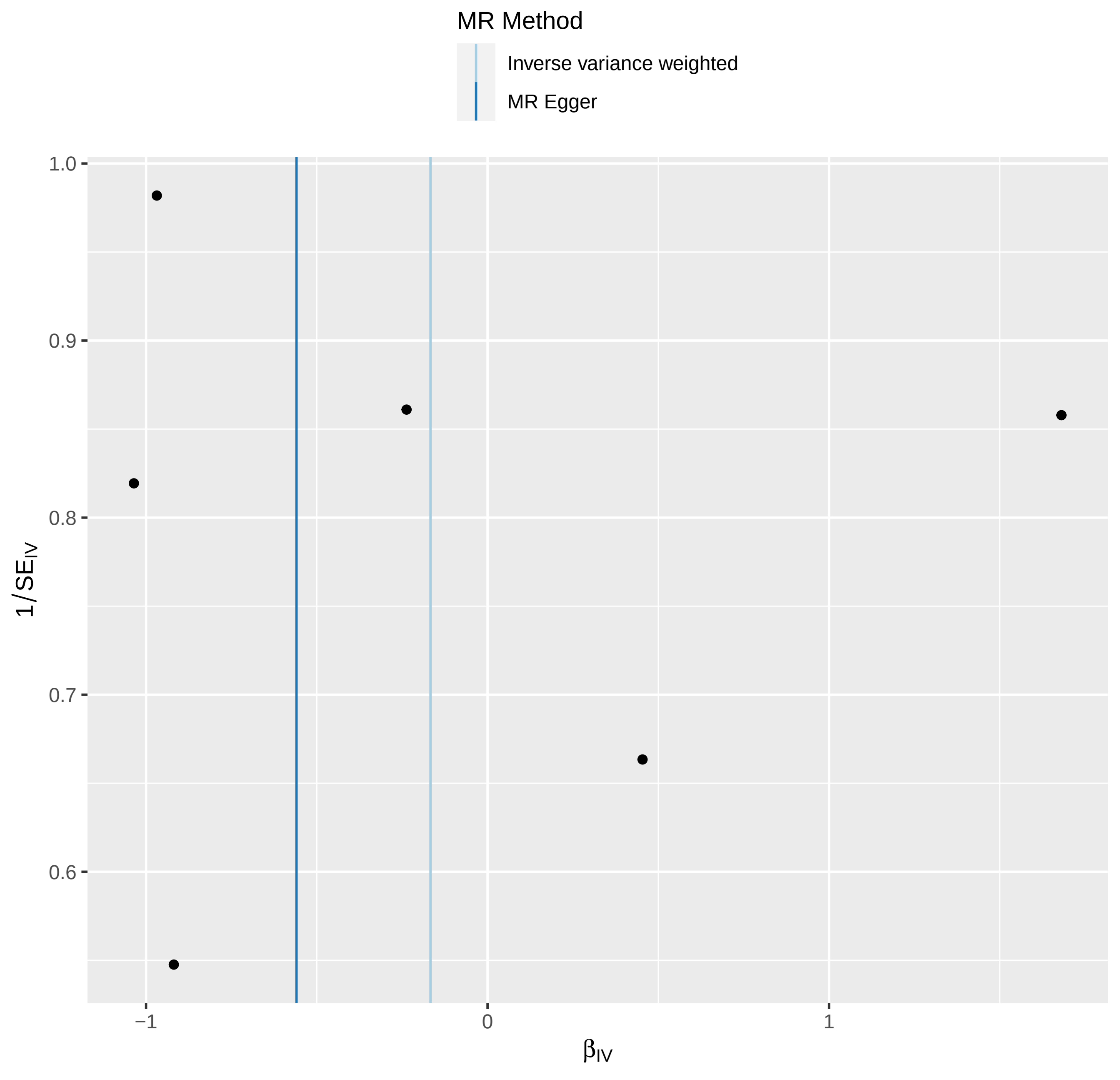

Batch 505 : Gut microbiota abundance (genus Collinsella id.815) on Arthropathy in Crohn disease

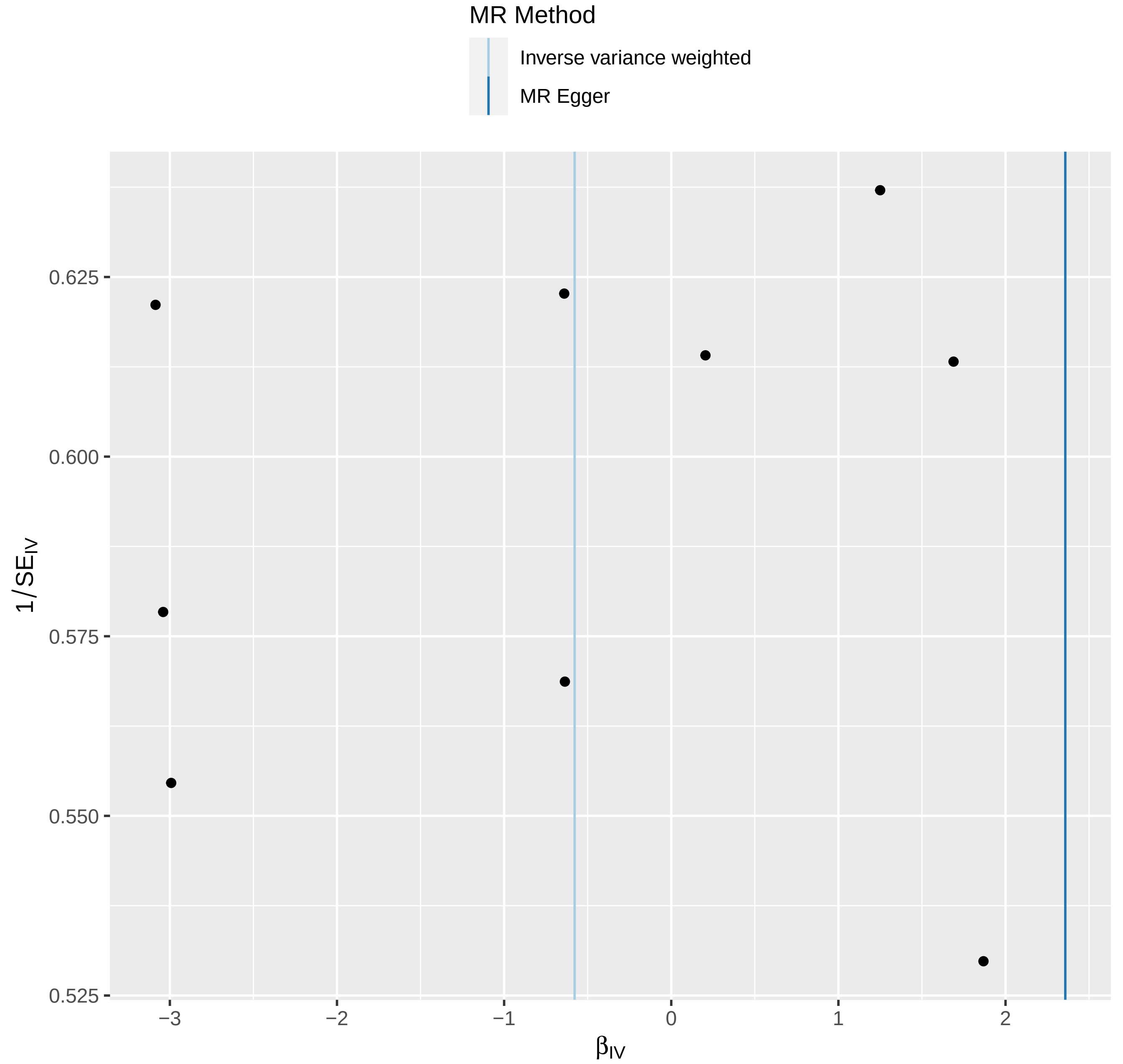

Batch 506 : Gut microbiota abundance (genus Coprobacter id.949) on Arthropathy in Crohn disease

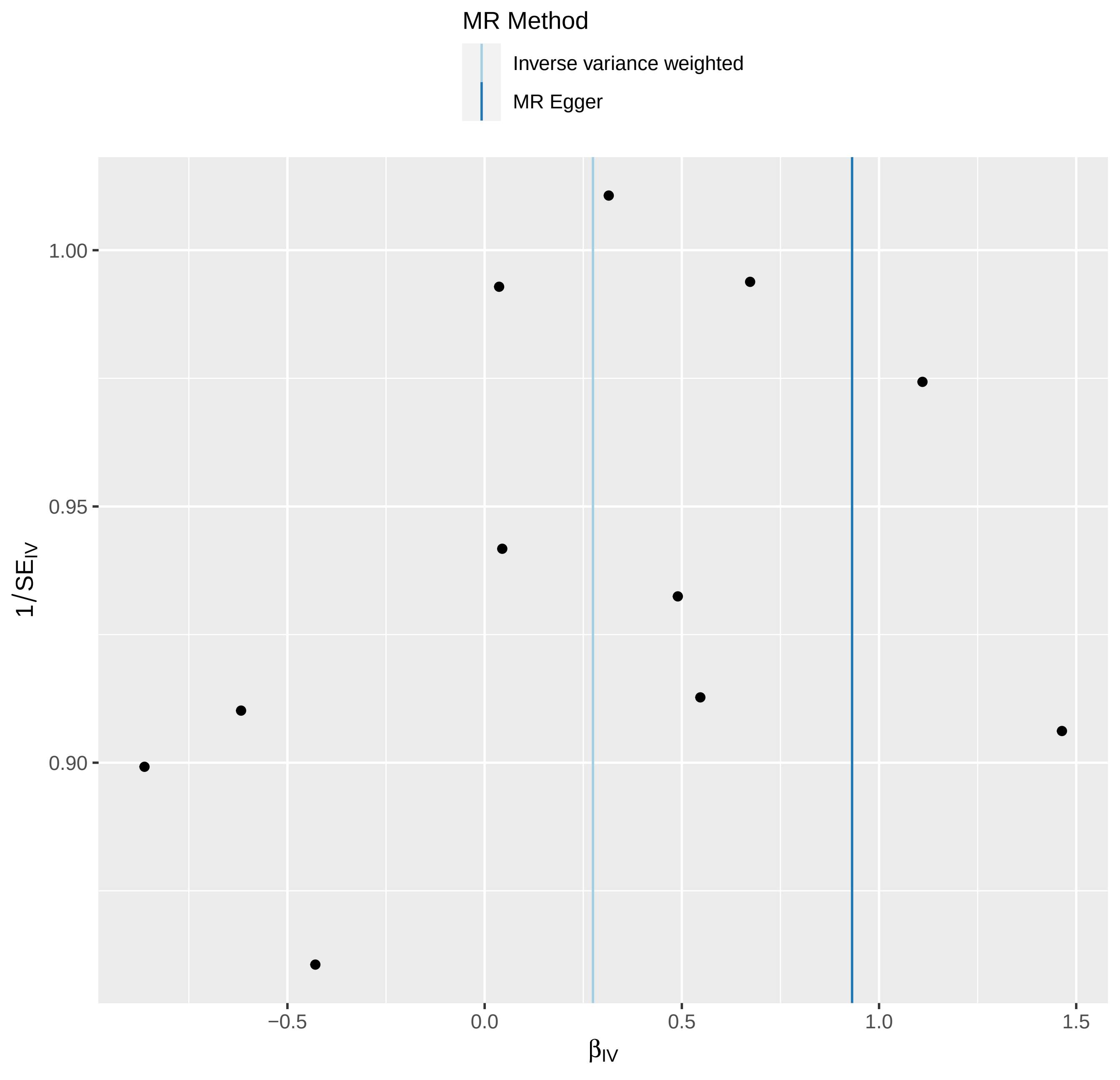

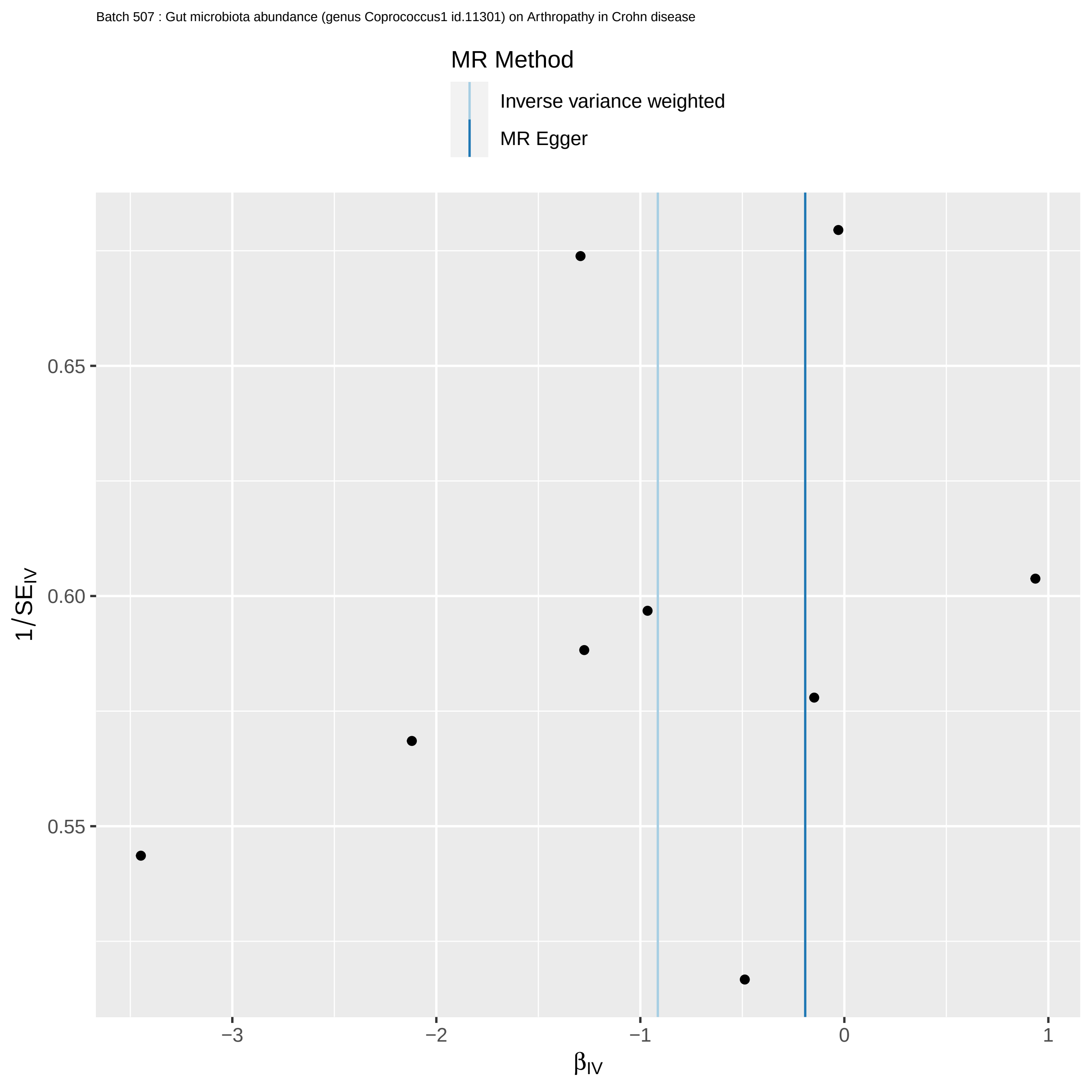
Batch 507 : Gut microbiota abundance (genus Coprococcus1 id.11301) on Arthropathy in Crohn disease
MR Method
Inverse variance weighted
MR Egger
0.65
0.60
0.55
1/SE_IV
−3
−2
−1
0
1
β_IV

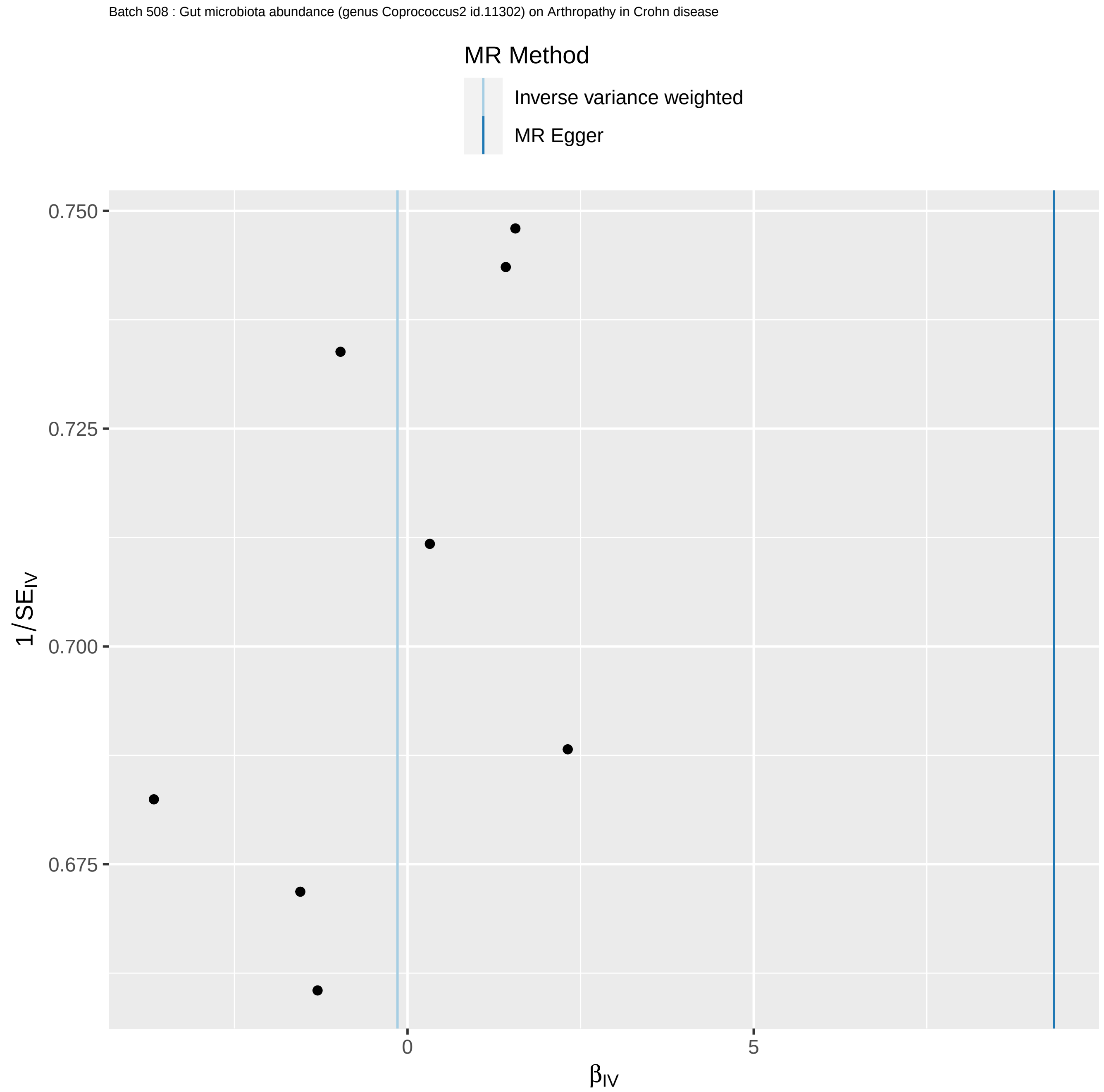

Batch 508 : Gut microbiota abundance (genus Coprococcus2 id.11302) on Arthropathy in Crohn disease
MR Method
Inverse variance weighted
MR Egger
0.750
0.725
0.700
0.675
1/SE_IV
0
5
β_IV

Batch 509 : Gut microbiota abundance (genus Coprococcus3 id.11303) on Arthropathy in Crohn disease

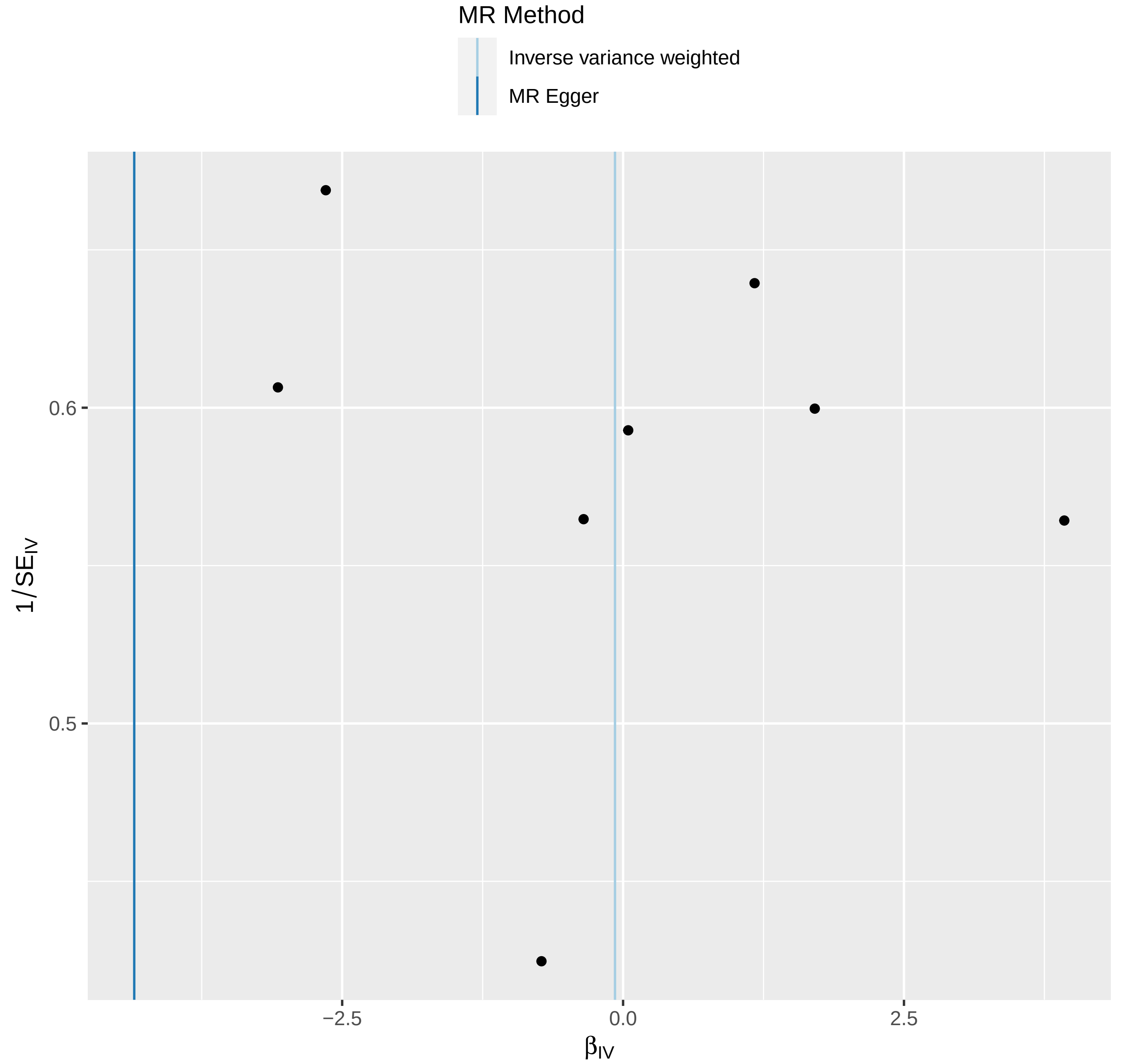

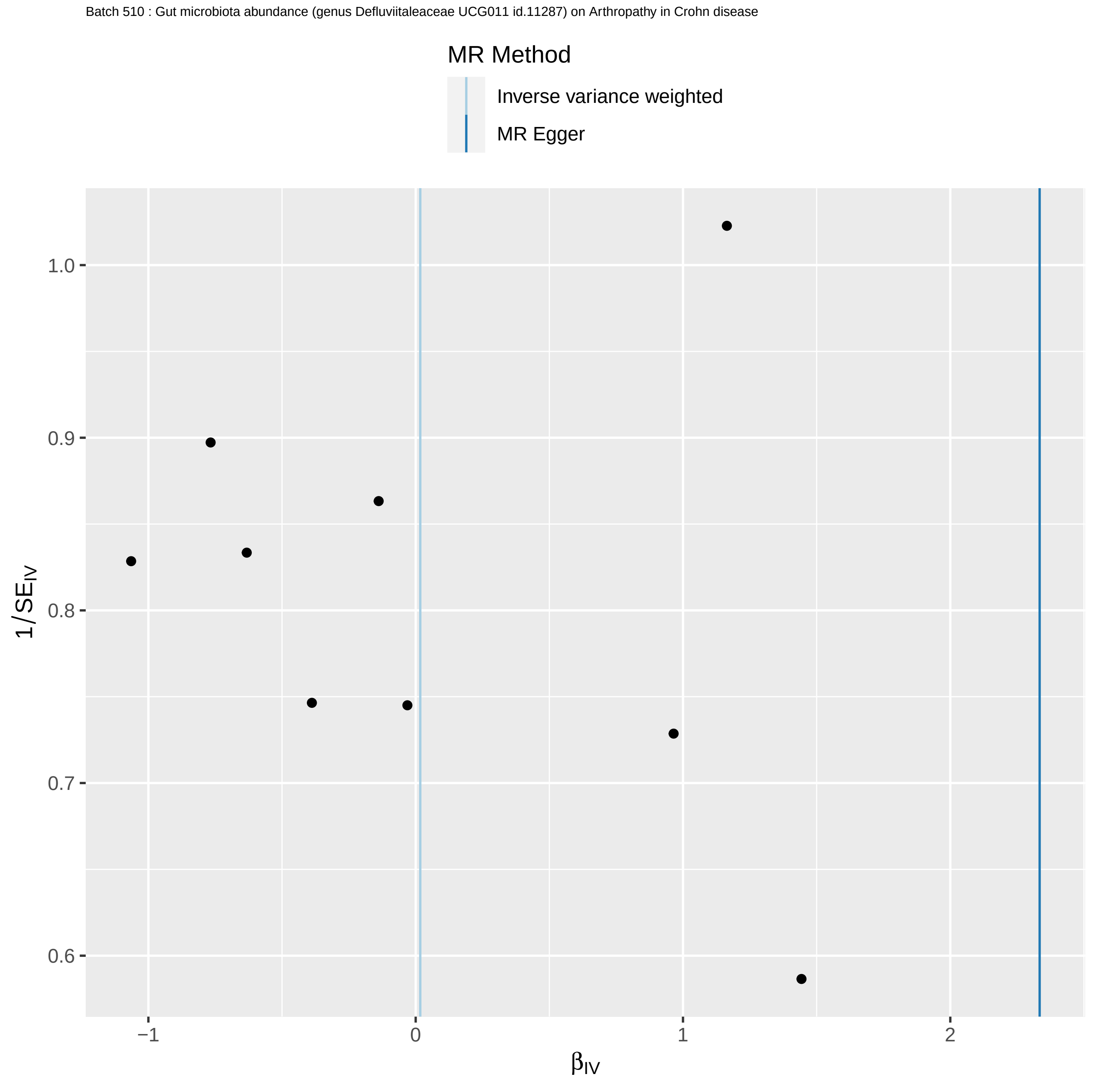

Batch 510 : Gut microbiota abundance (genus Defluviitaleaceae UCG011 id.11287) on Arthropathy in Crohn disease
MR Method
Inverse variance weighted
MR Egger
1.0
0.9
0.8
0.7
0.6
$1/SE_{IV}$
−1
0
1
2
β_{IV}

Batch 511 : Gut microbiota abundance (genus Desulfovibrio id.3173) on Arthropathy in Crohn disease

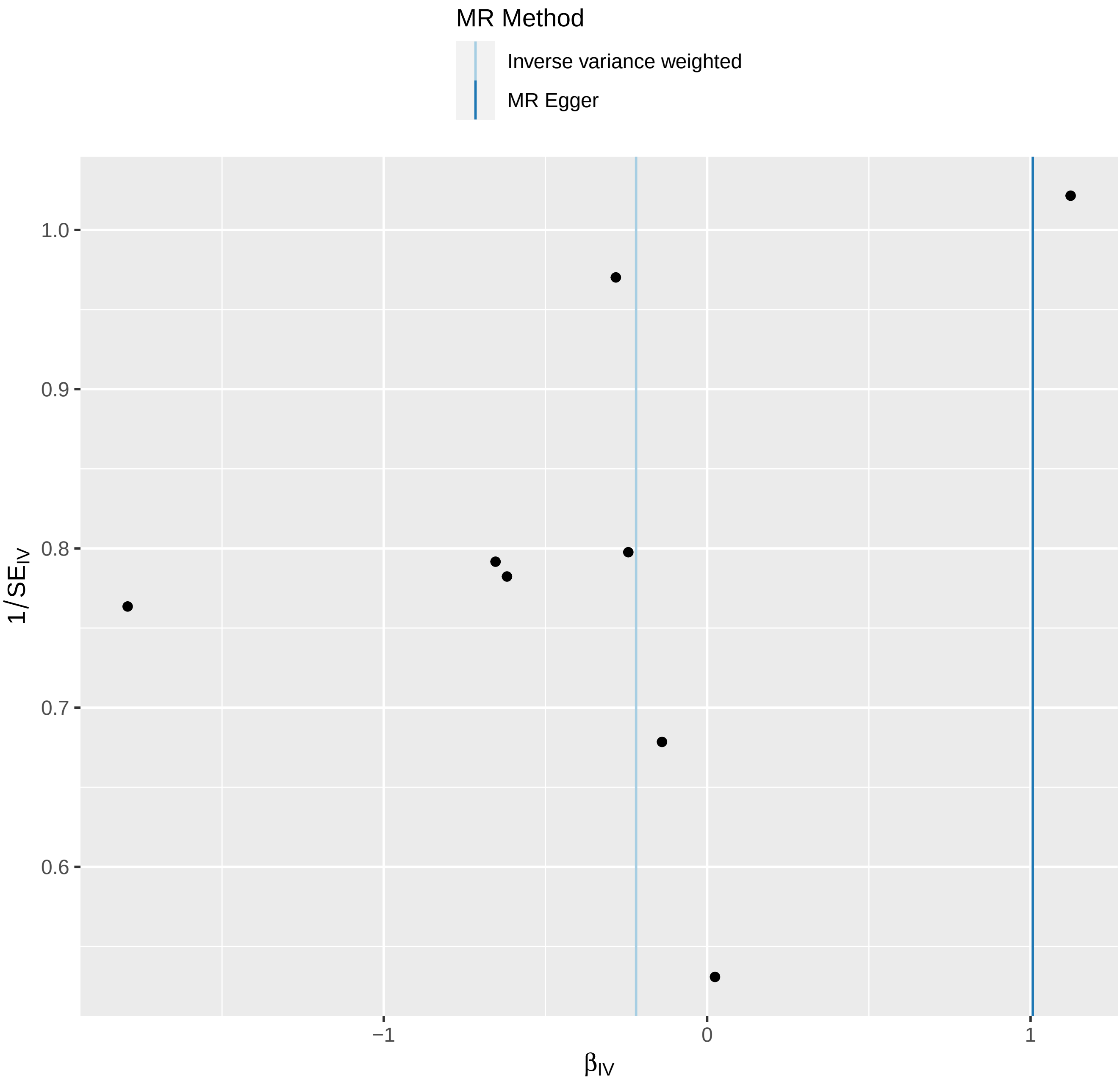

Batch 512 : Gut microbiota abundance (genus Dialister id.2183) on Arthropathy in Crohn disease

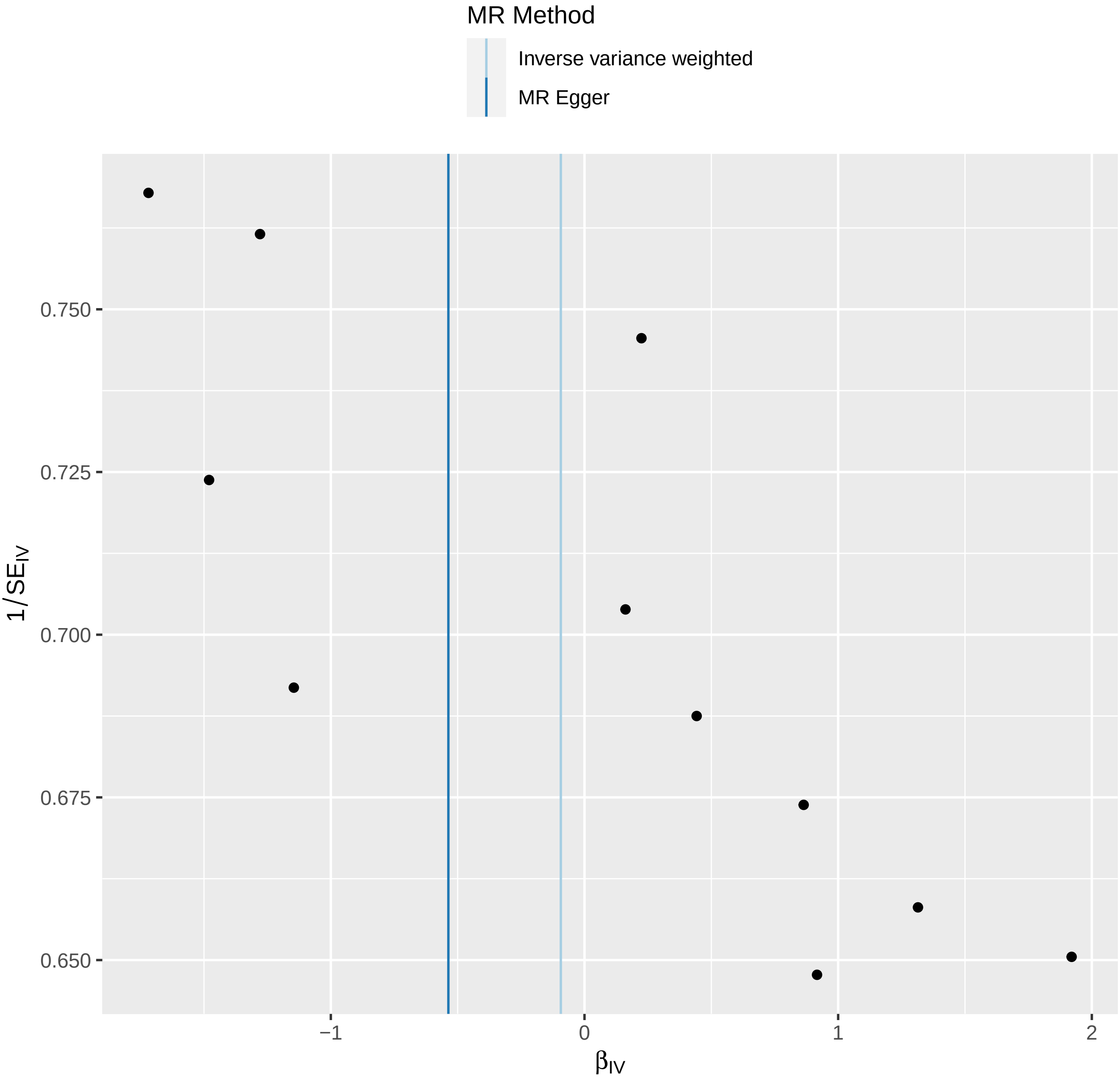

Batch 513 : Gut microbiota abundance (genus Dorea id.1997) on Arthropathy in Crohn disease

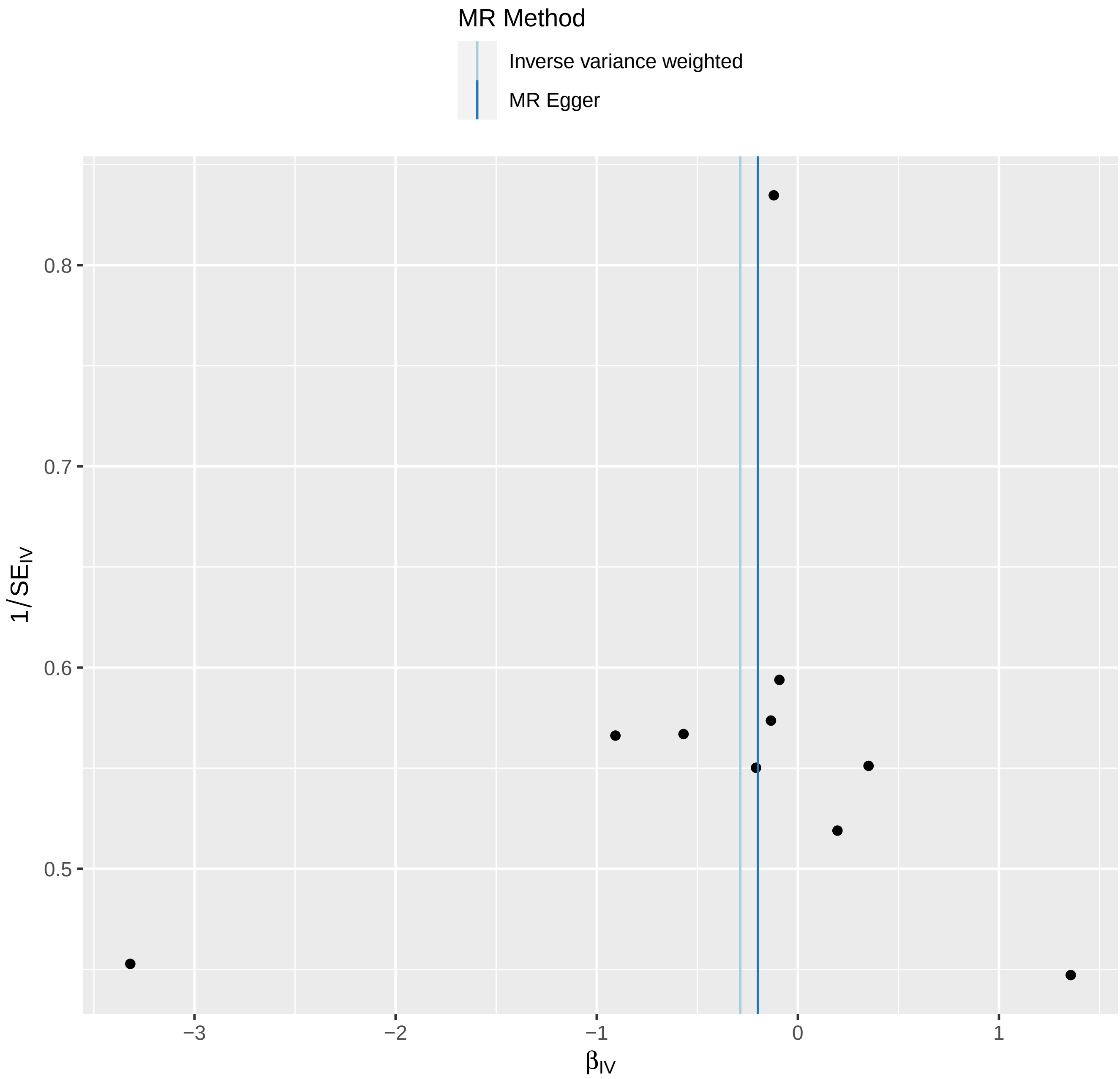

Batch 514 : Gut microbiota abundance (genus Eggerthella id.819) on Arthropathy in Crohn disease

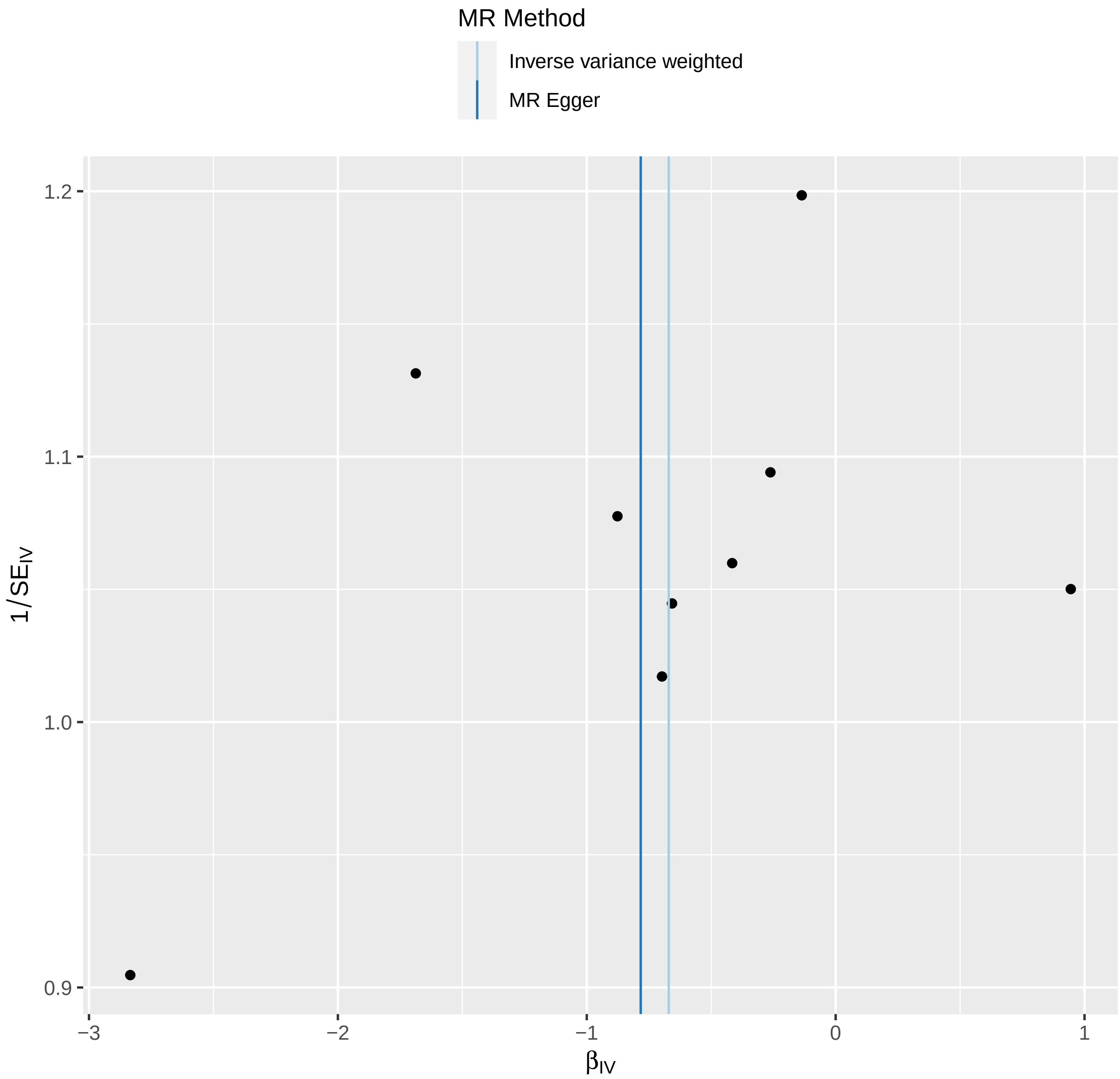

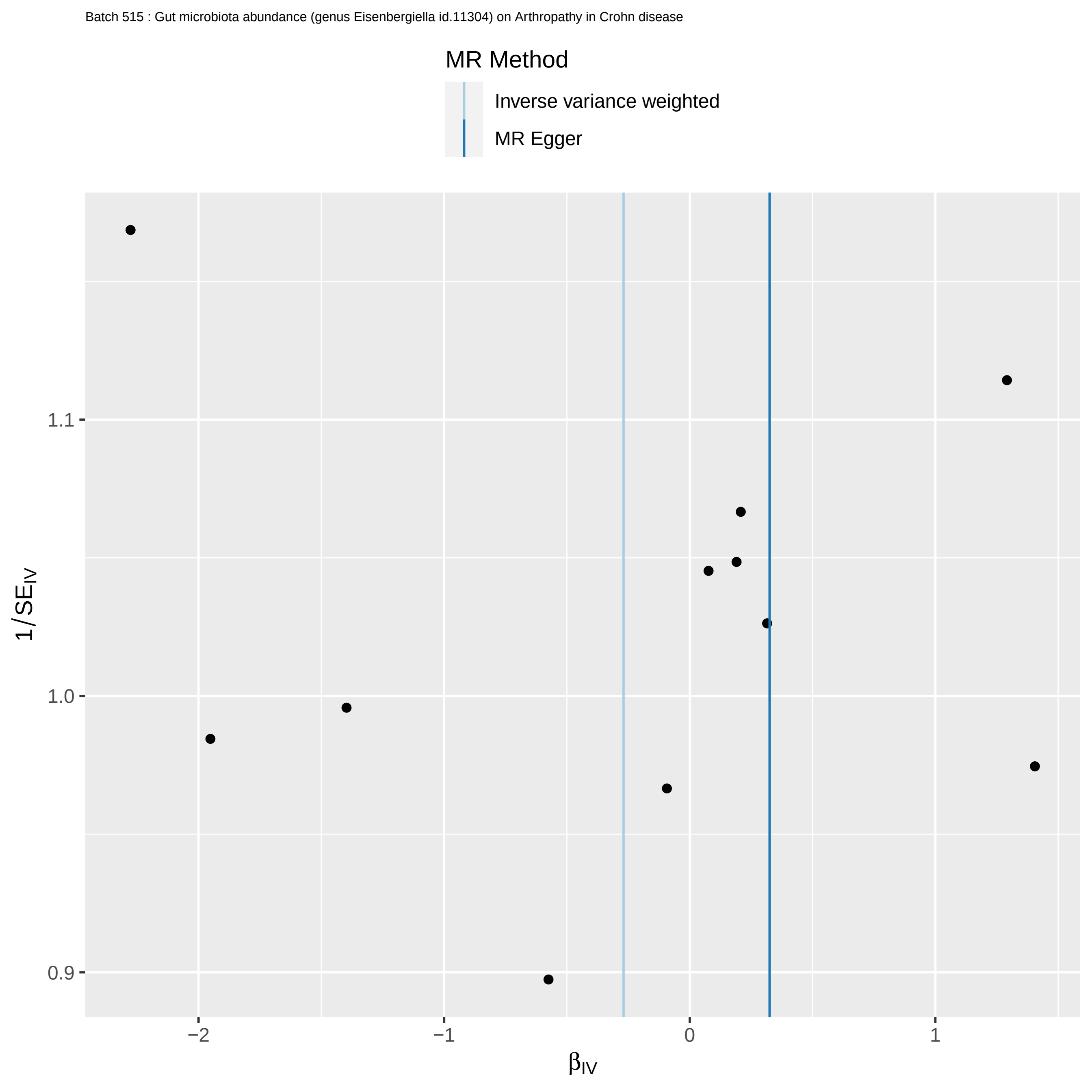

Batch 515 : Gut microbiota abundance (genus Eisenbergiella id.11304) on Arthropathy in Crohn disease
MR Method
Inverse variance weighted
MR Egger
1.1
1.0
0.9
1/SE$_{IV}$
−2
−1
0
1
β_{IV}

Batch 516 : Gut microbiota abundance (genus Enterorhabdus id.820) on Arthropathy in Crohn disease

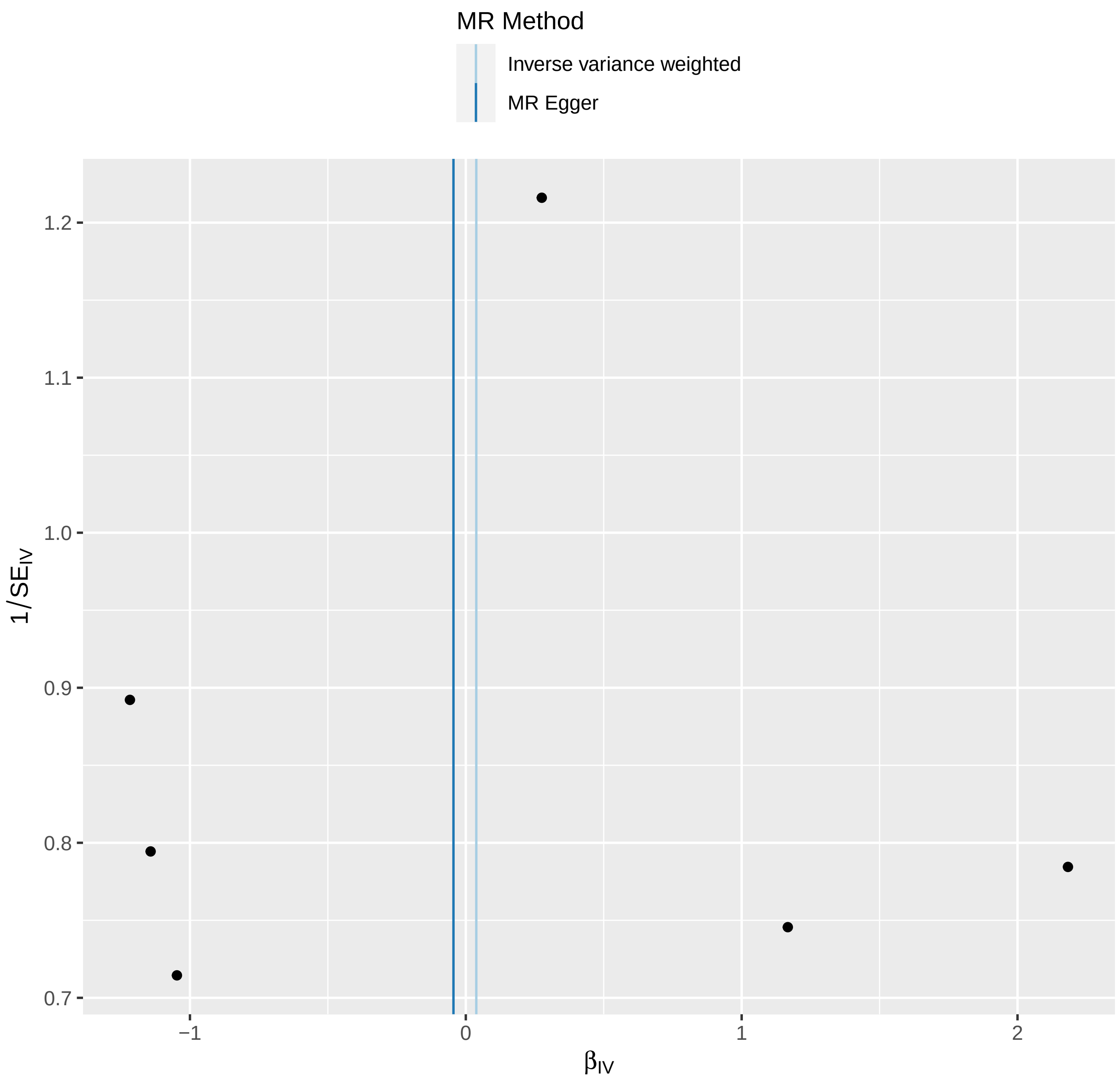

Batch 517 : Gut microbiota abundance (genus Erysipelatoclostridium id.11381) on Arthropathy in Crohn disease

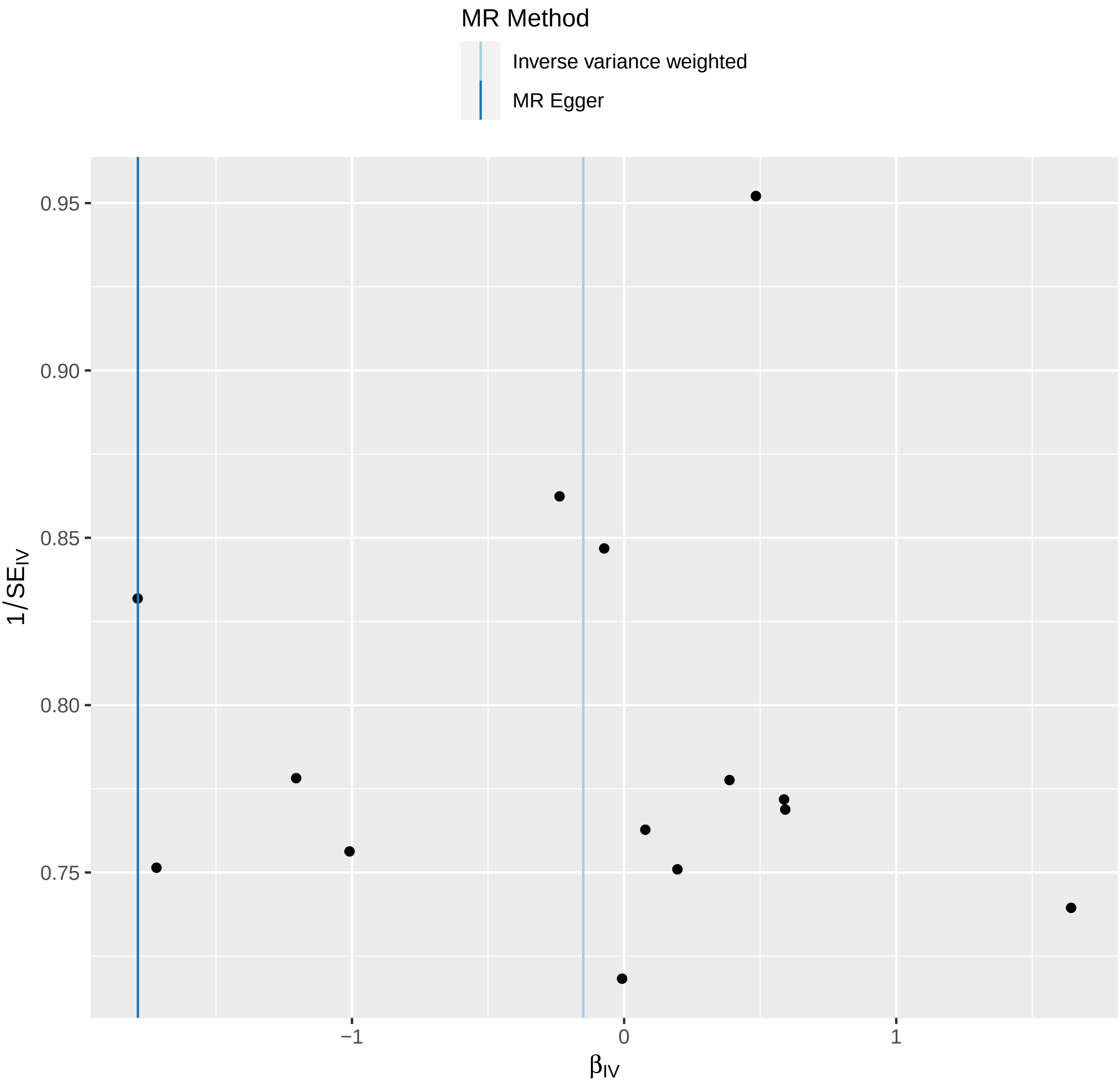

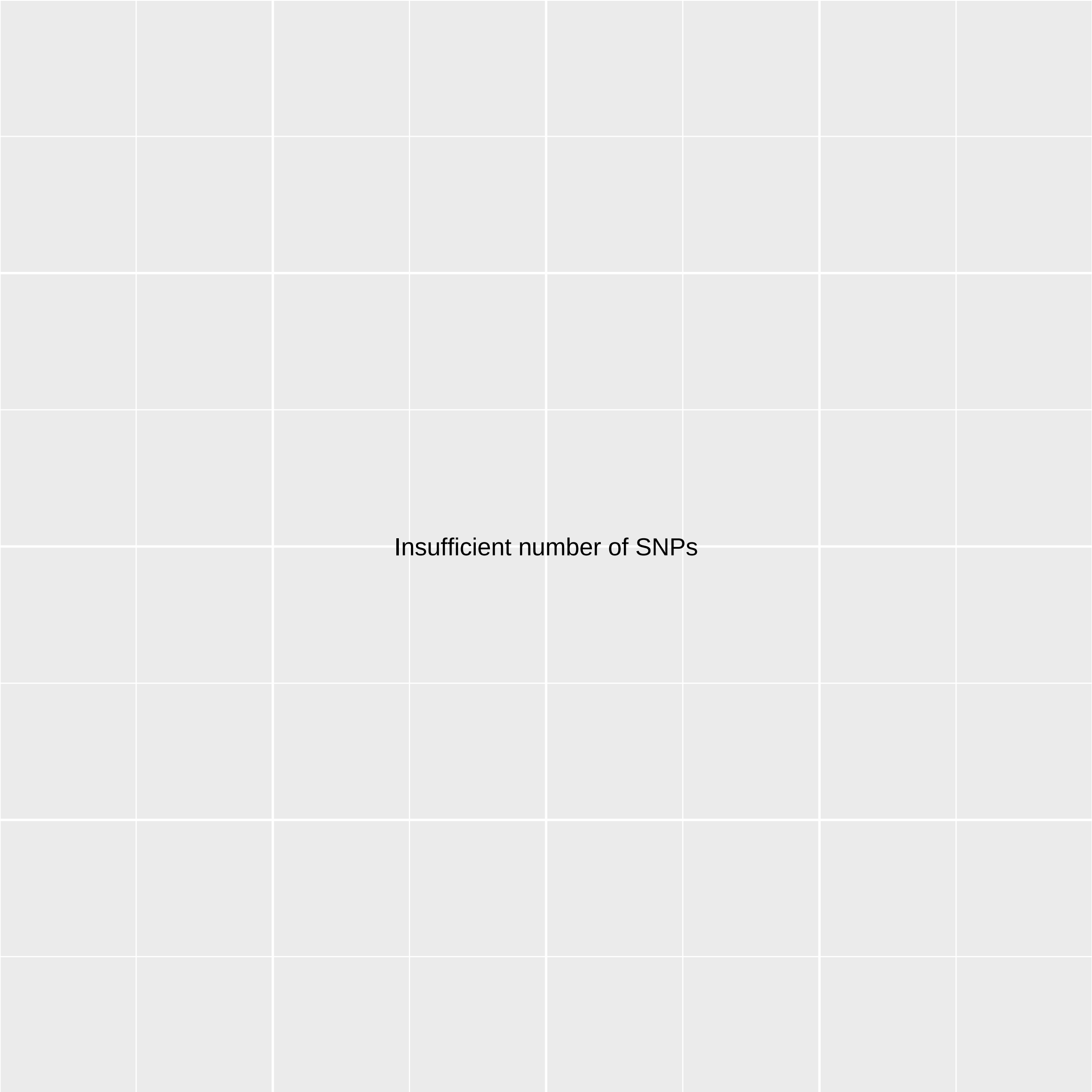
Insufficient number of SNPs

Batch 519 : Gut microbiota abundance (genus Escherichia Shigella id.3504) on Arthropathy in Crohn disease

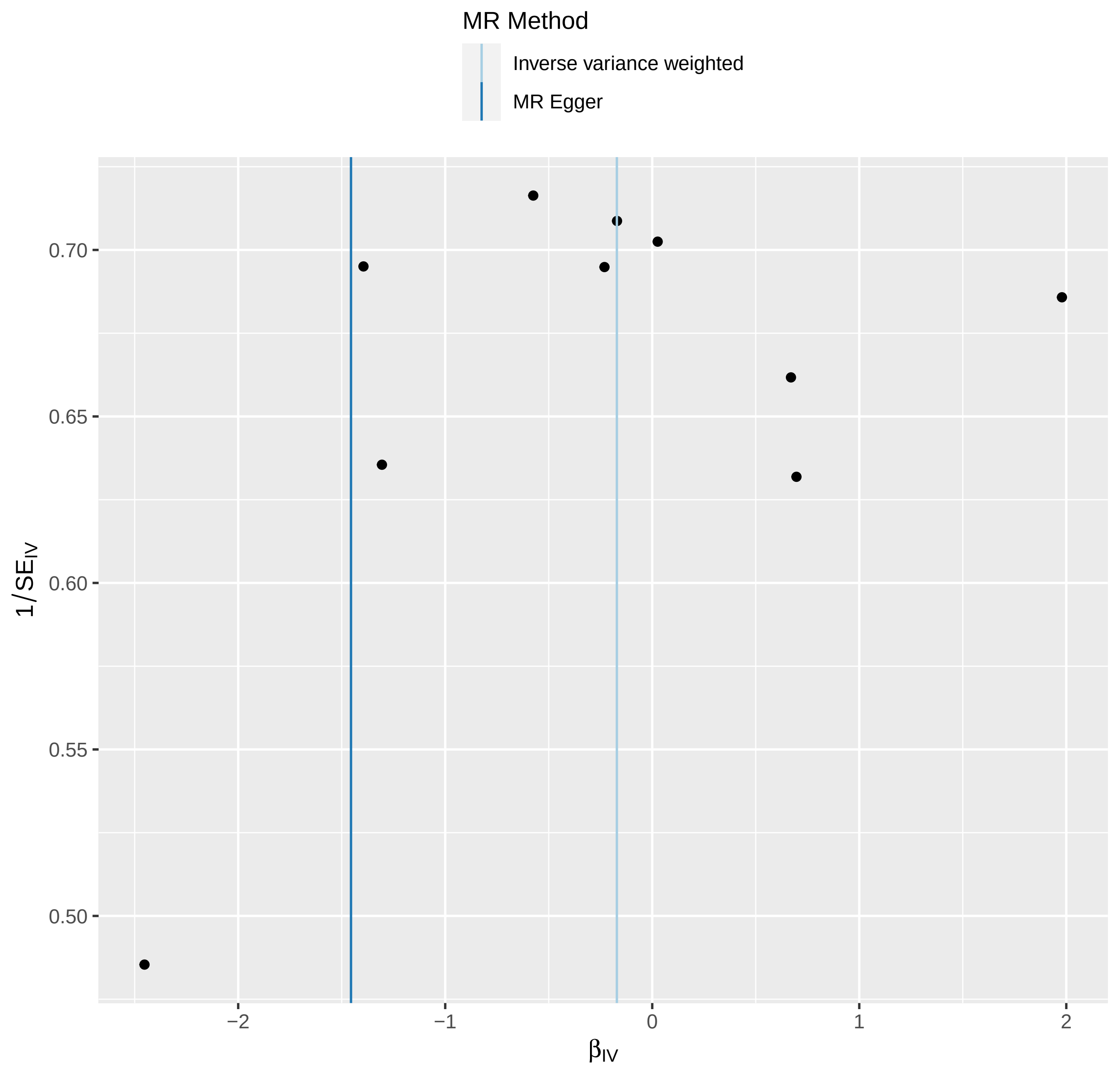

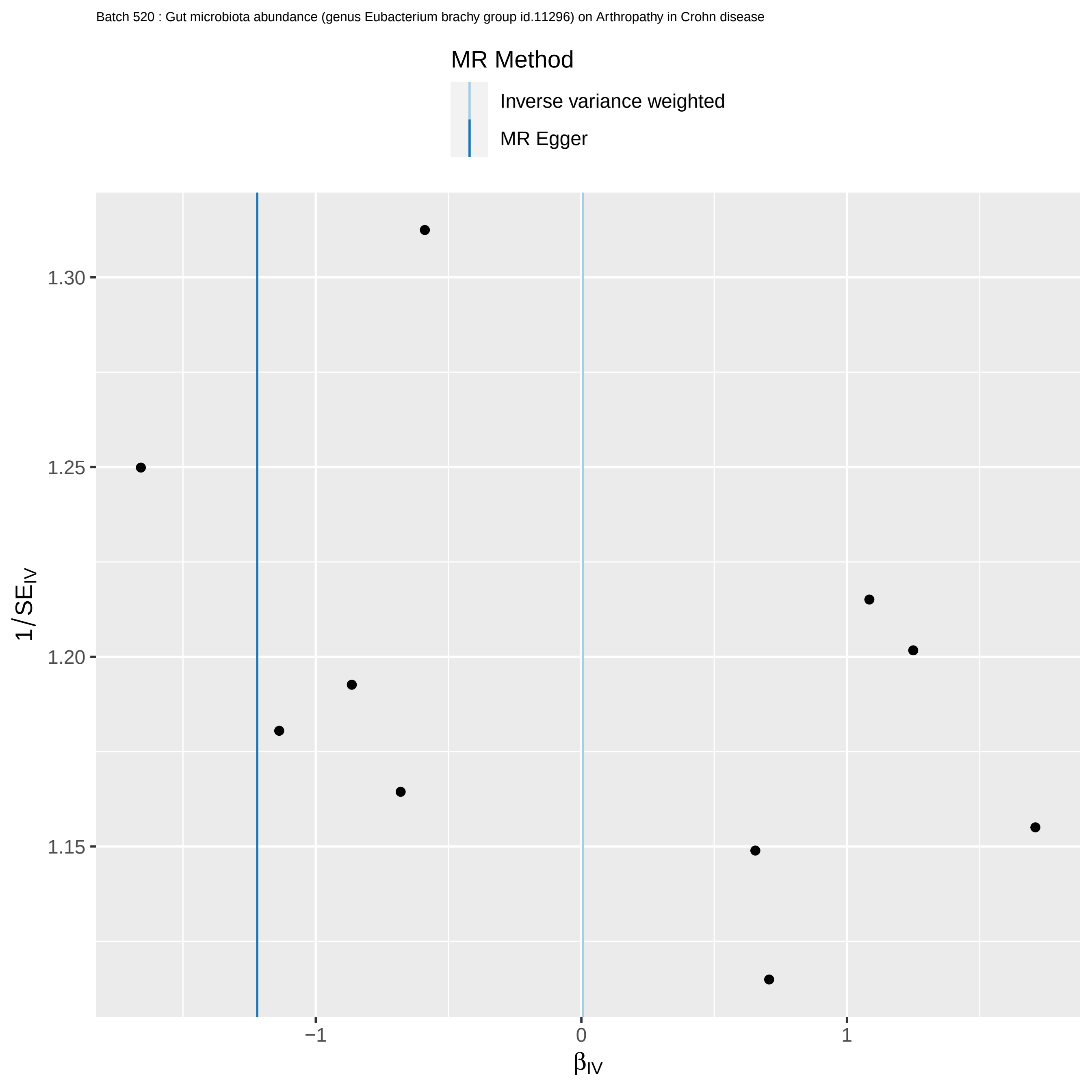

Batch 520 : Gut microbiota abundance (genus Eubacterium brachy group id.11296) on Arthropathy in Crohn disease
MR Method
Inverse variance weighted
MR Egger
1.30
1.25
1.20
1.15
1/SE_IV
−1
0
1
β_IV

Batch 521 : Gut microbiota abundance (genus Eubacterium coprostanoligenes group id.11375) on Arthropathy in Crohn disease

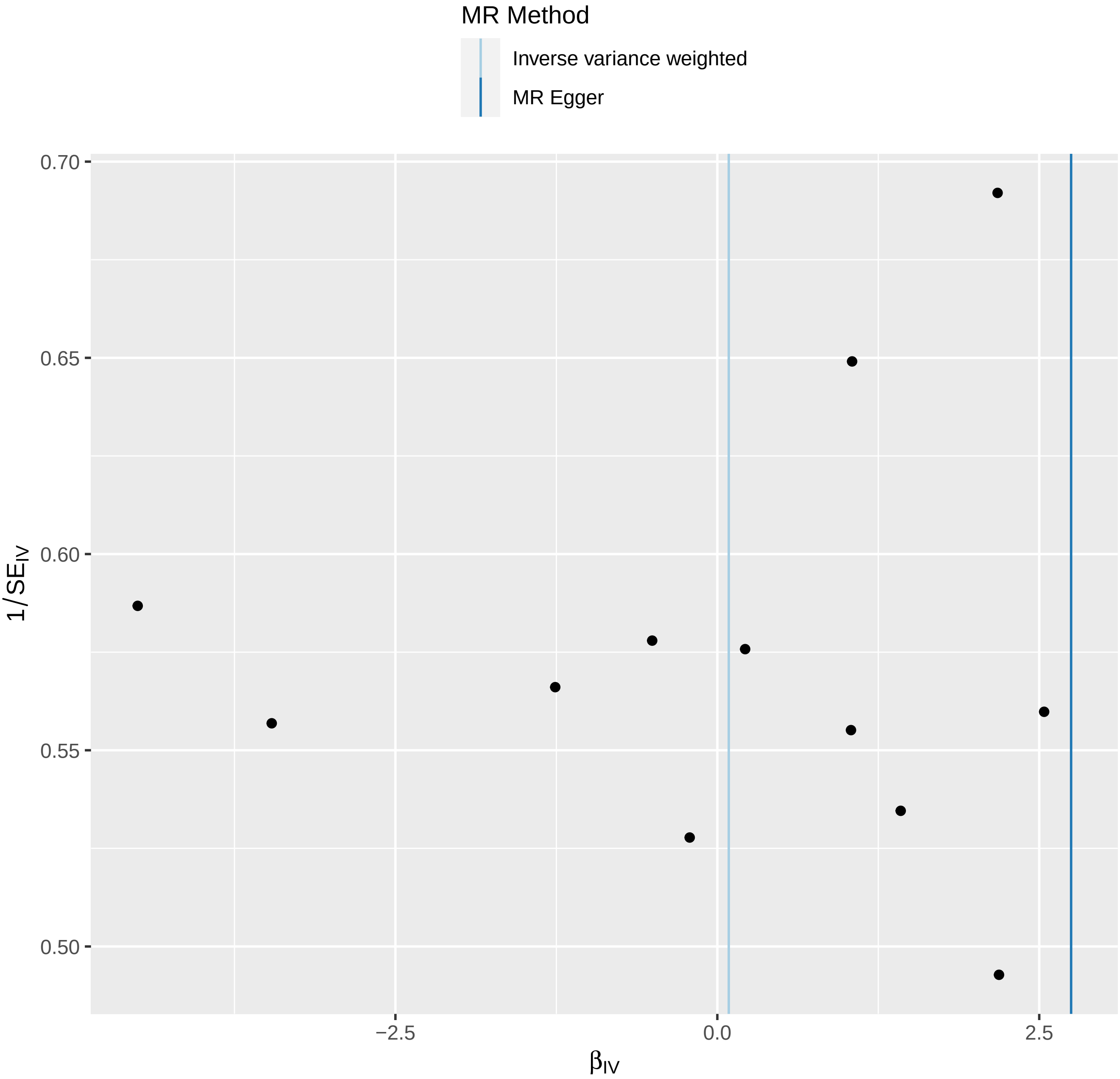

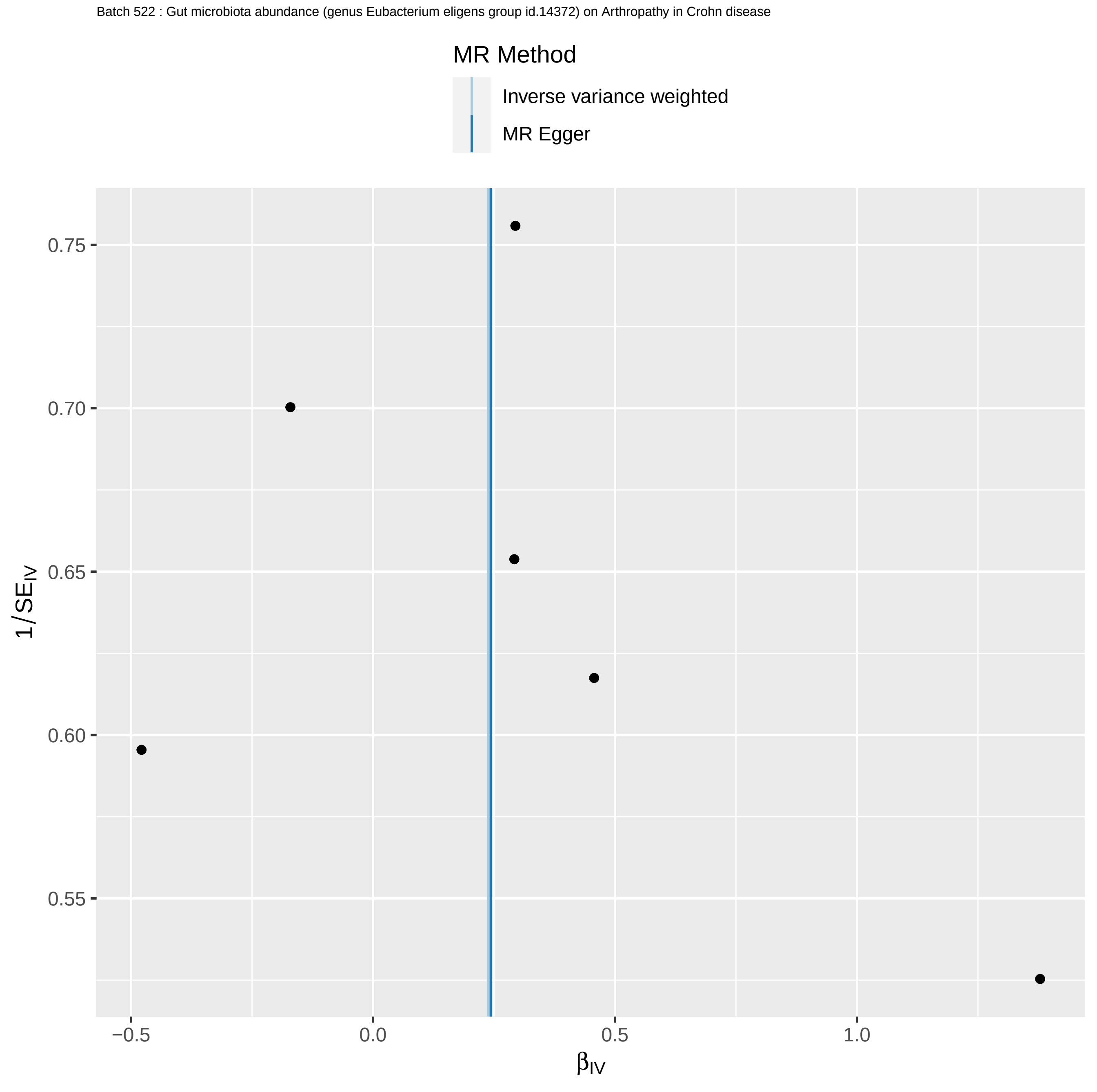

Batch 522 : Gut microbiota abundance (genus Eubacterium eligens group id.14372) on Arthropathy in Crohn disease
MR Method
Inverse variance weighted
MR Egger
1/SE_IV
0.75
0.70
0.65
0.60
0.55
β_IV
−0.5
0.0
0.5
1.0

Batch 523 : Gut microbiota abundance (genus Eubacterium fissicatena group id.14373) on Arthropathy in Crohn disease

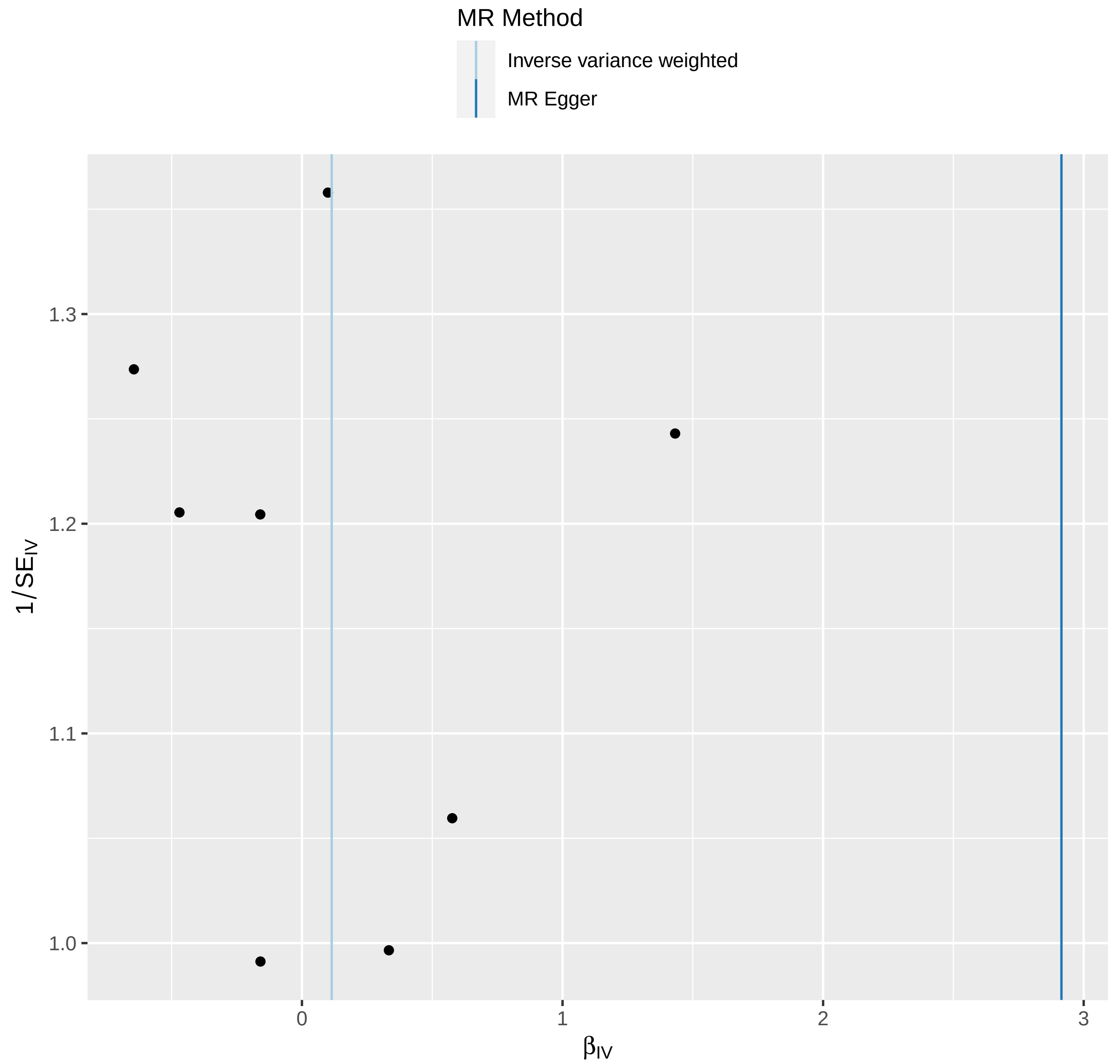

Batch 524 : Gut microbiota abundance (genus Eubacterium hallii group id.11338) on Arthropathy in Crohn disease

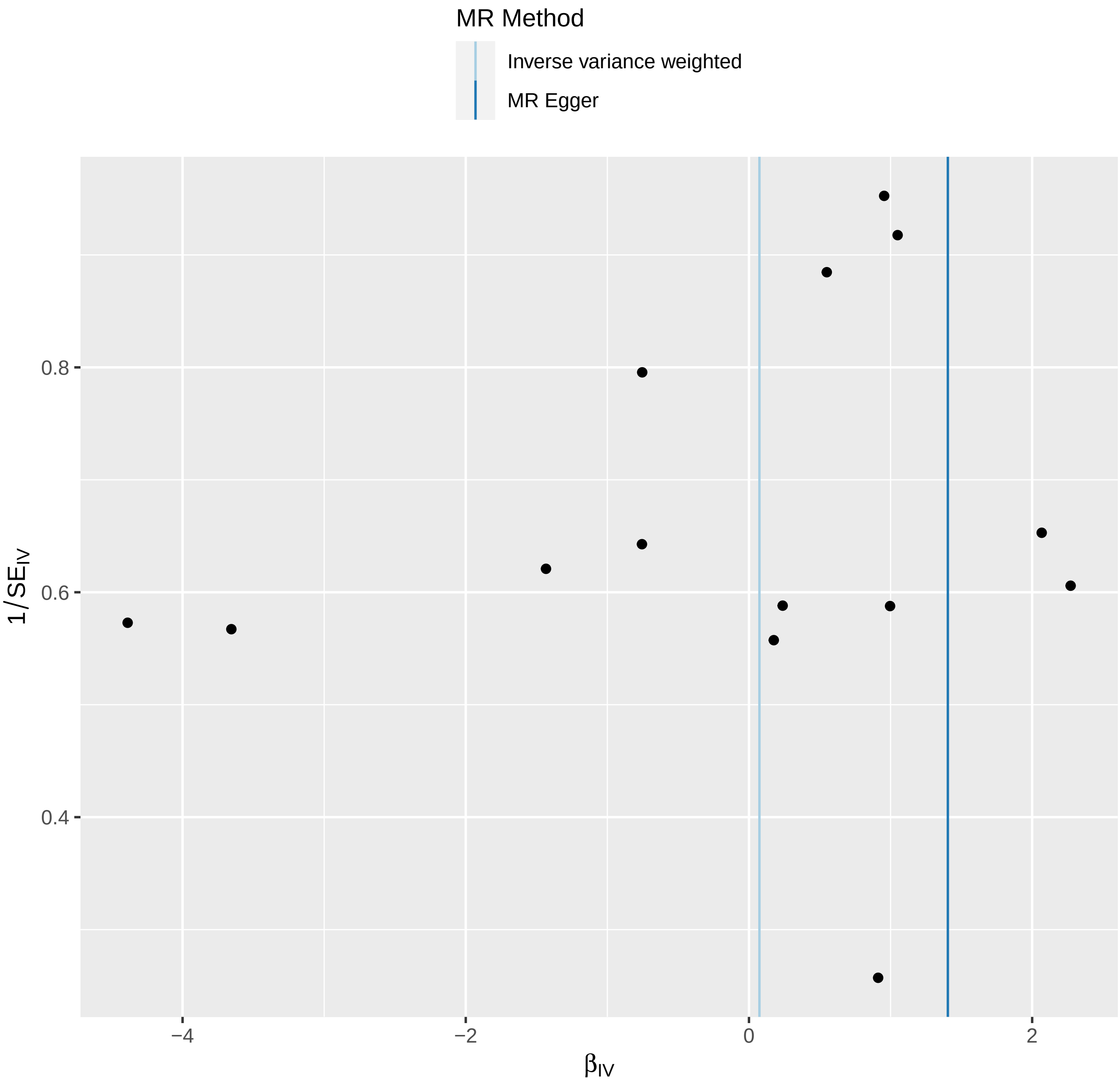

Batch 525 : Gut microbiota abundance (genus Eubacterium nodatum group id.11297) on Arthropathy in Crohn disease

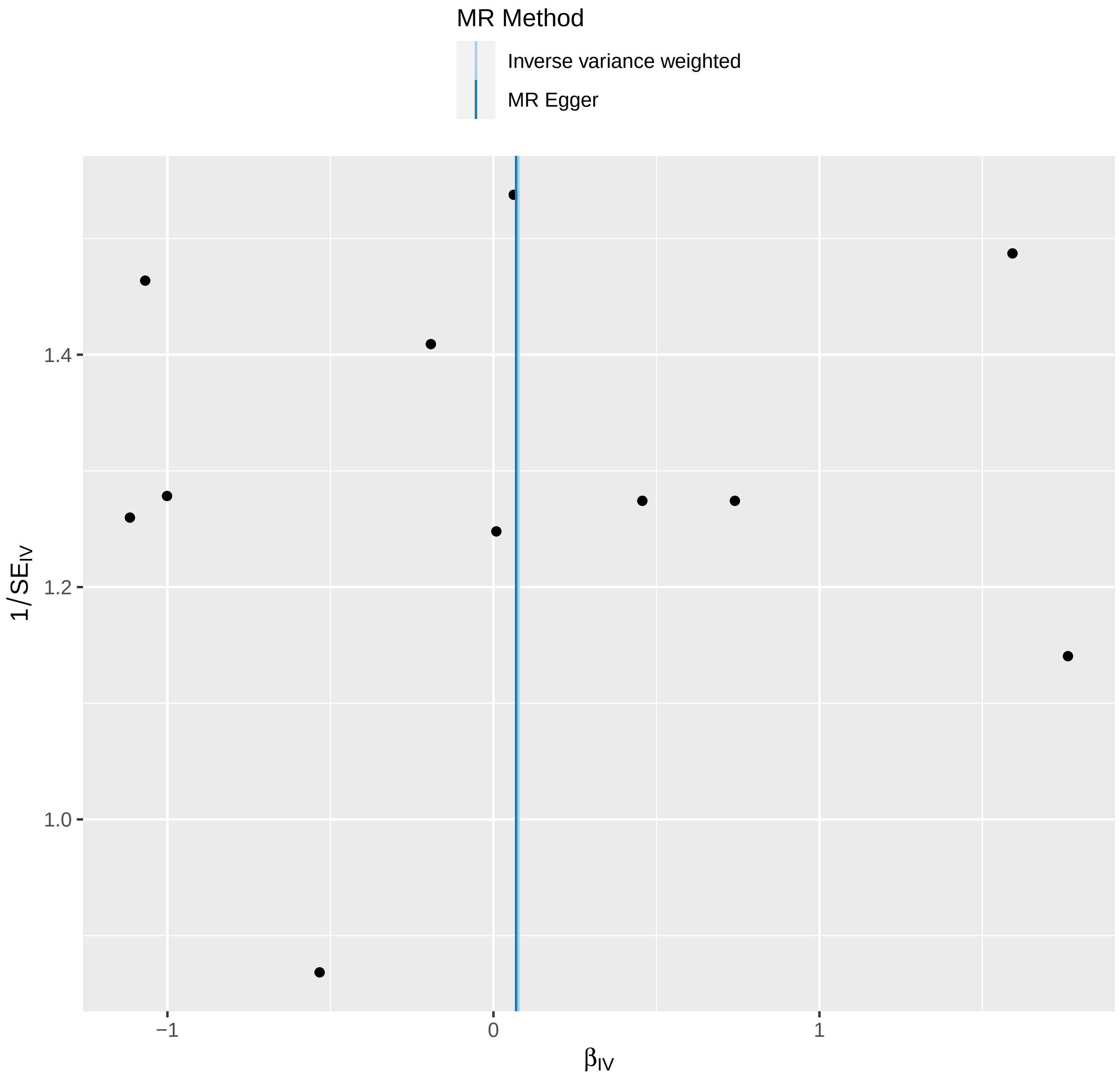

Batch 526 : Gut microbiota abundance (genus Eubacterium oxidoreducens group id.11339) on Arthropathy in Crohn disease

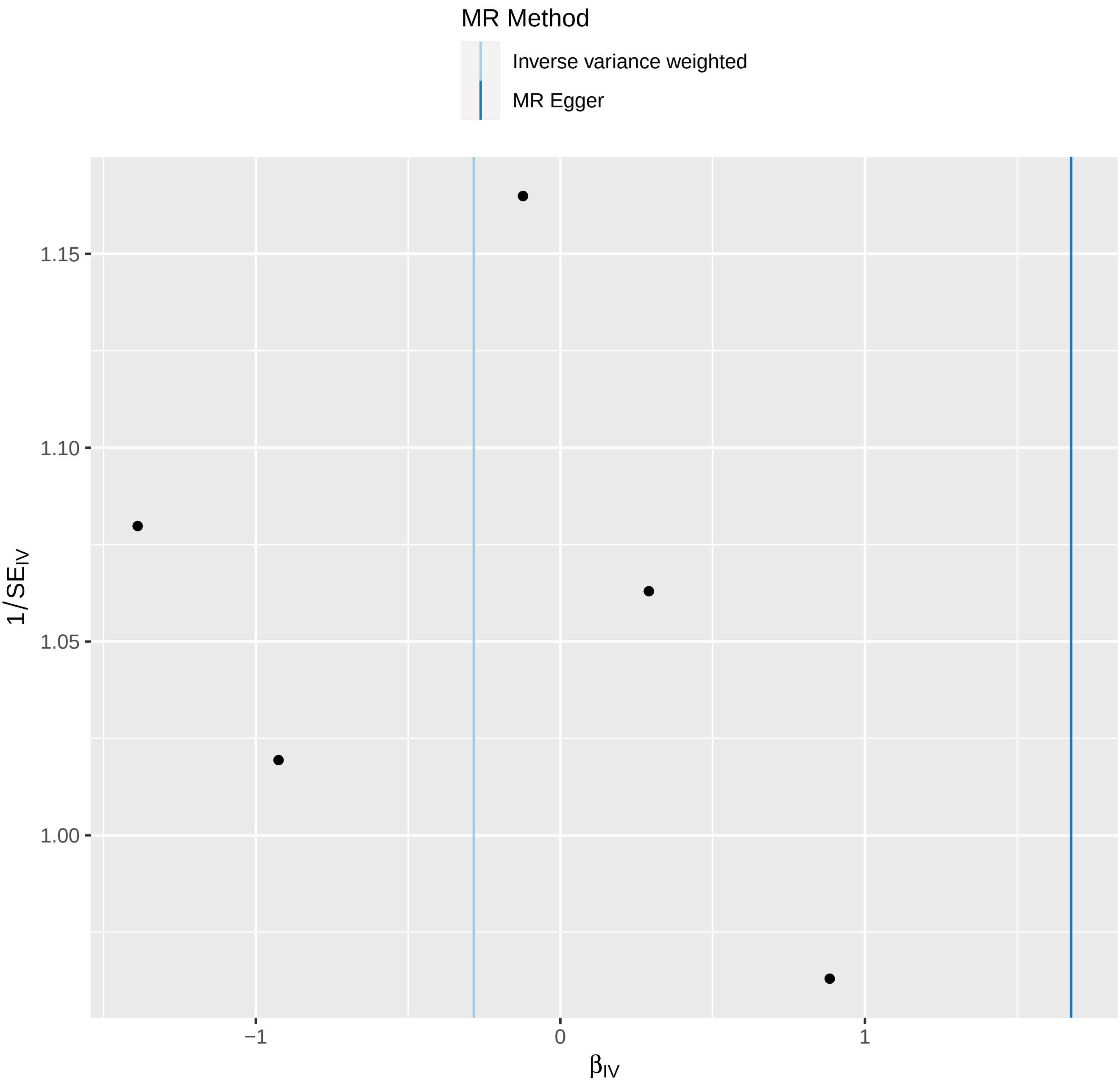

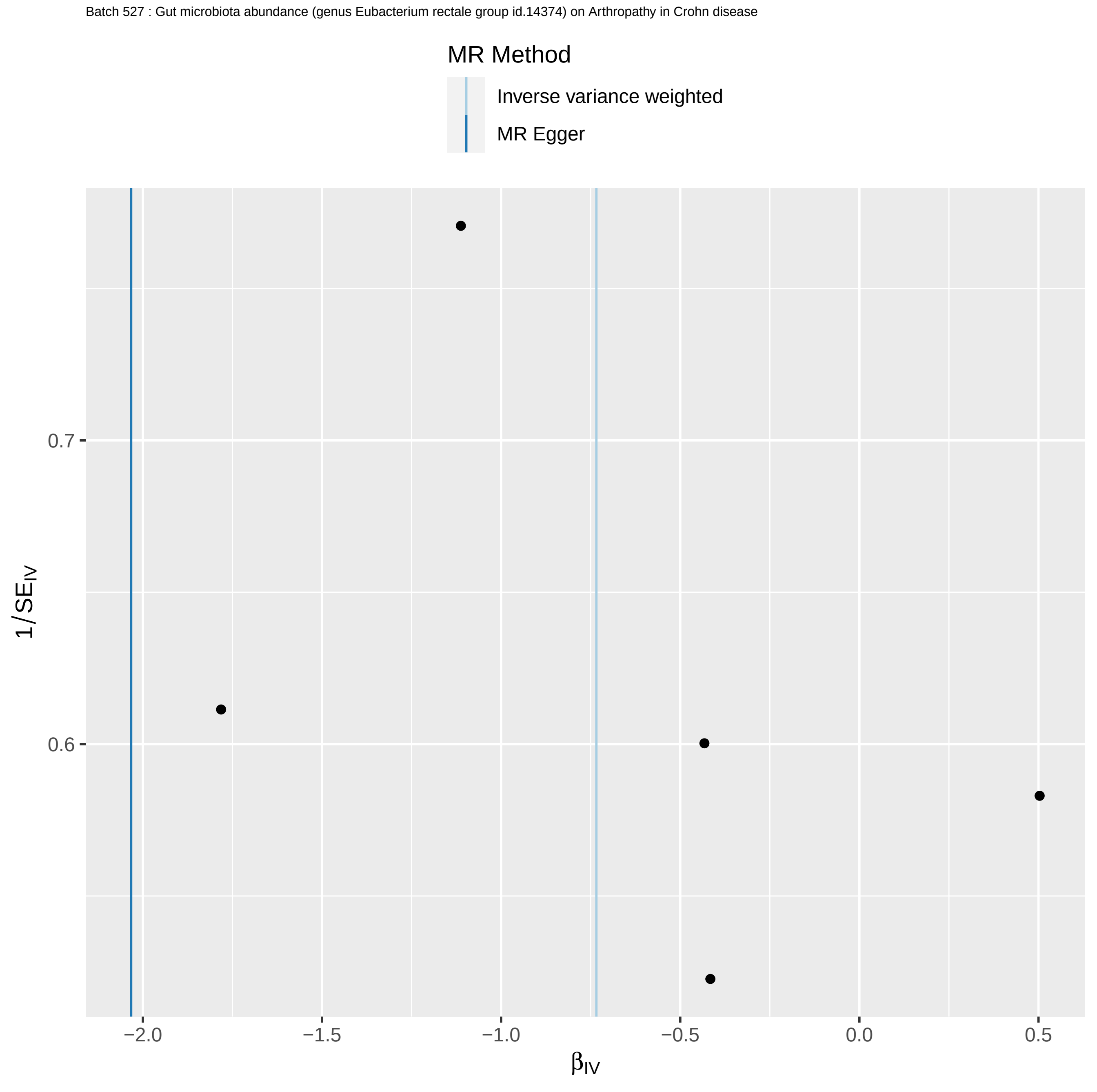

Batch 527 : Gut microbiota abundance (genus Eubacterium rectale group id.14374) on Arthropathy in Crohn disease
MR Method
Inverse variance weighted
MR Egger
0.7
0.6
1/SE_IV
−2.0
−1.5
−1.0
−0.5
0.0
0.5
β_IV

Batch 528 : Gut microbiota abundance (genus Eubacterium ruminantium group id.11340) on Arthropathy in Crohn disease

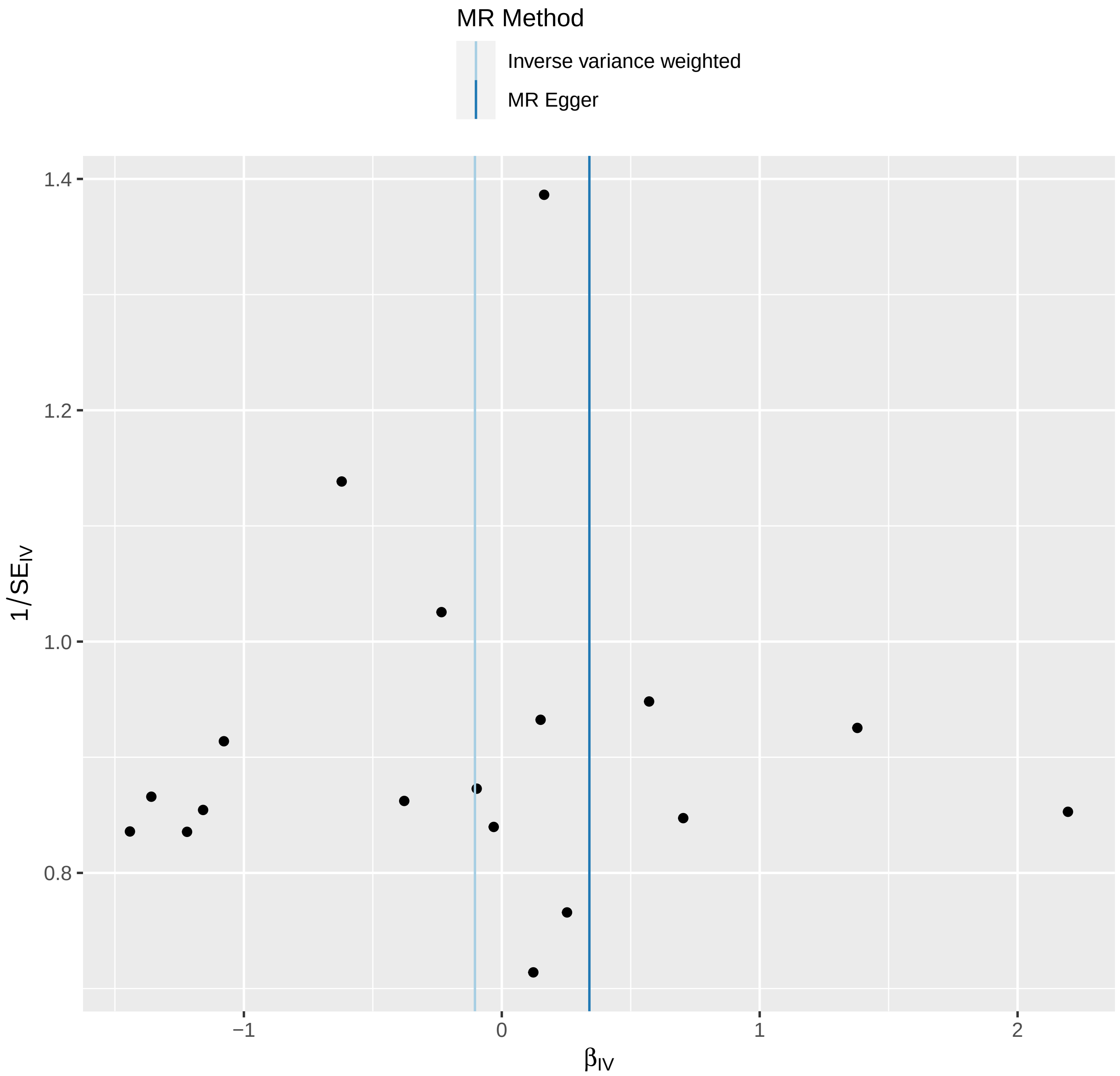

Batch 529 : Gut microbiota abundance (genus Eubacterium ventriosum group id.11341) on Arthropathy in Crohn disease

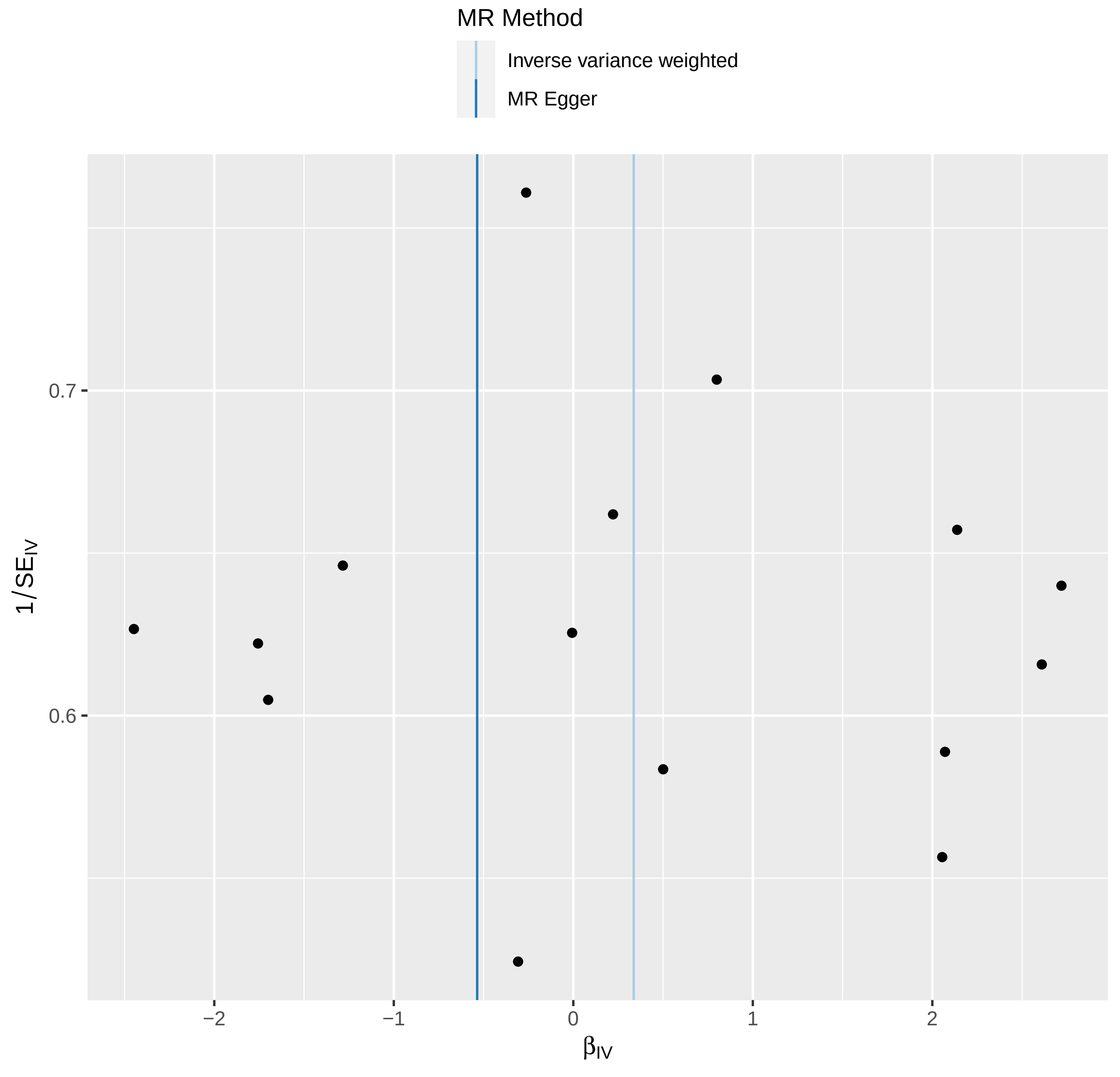

Batch 530 : Gut microbiota abundance (genus Eubacterium xylanophilum group id.14375) on Arthropathy in Crohn disease

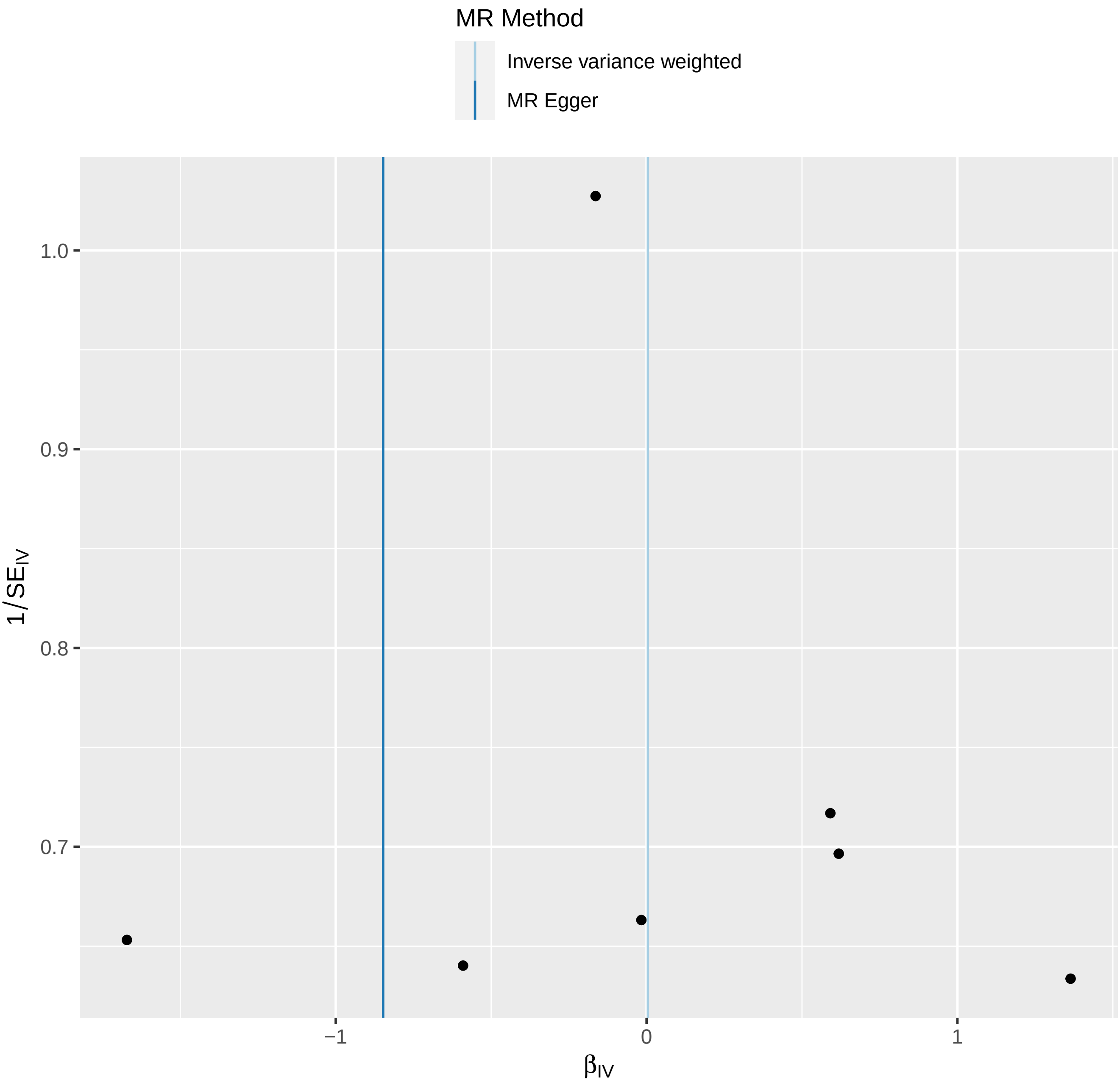

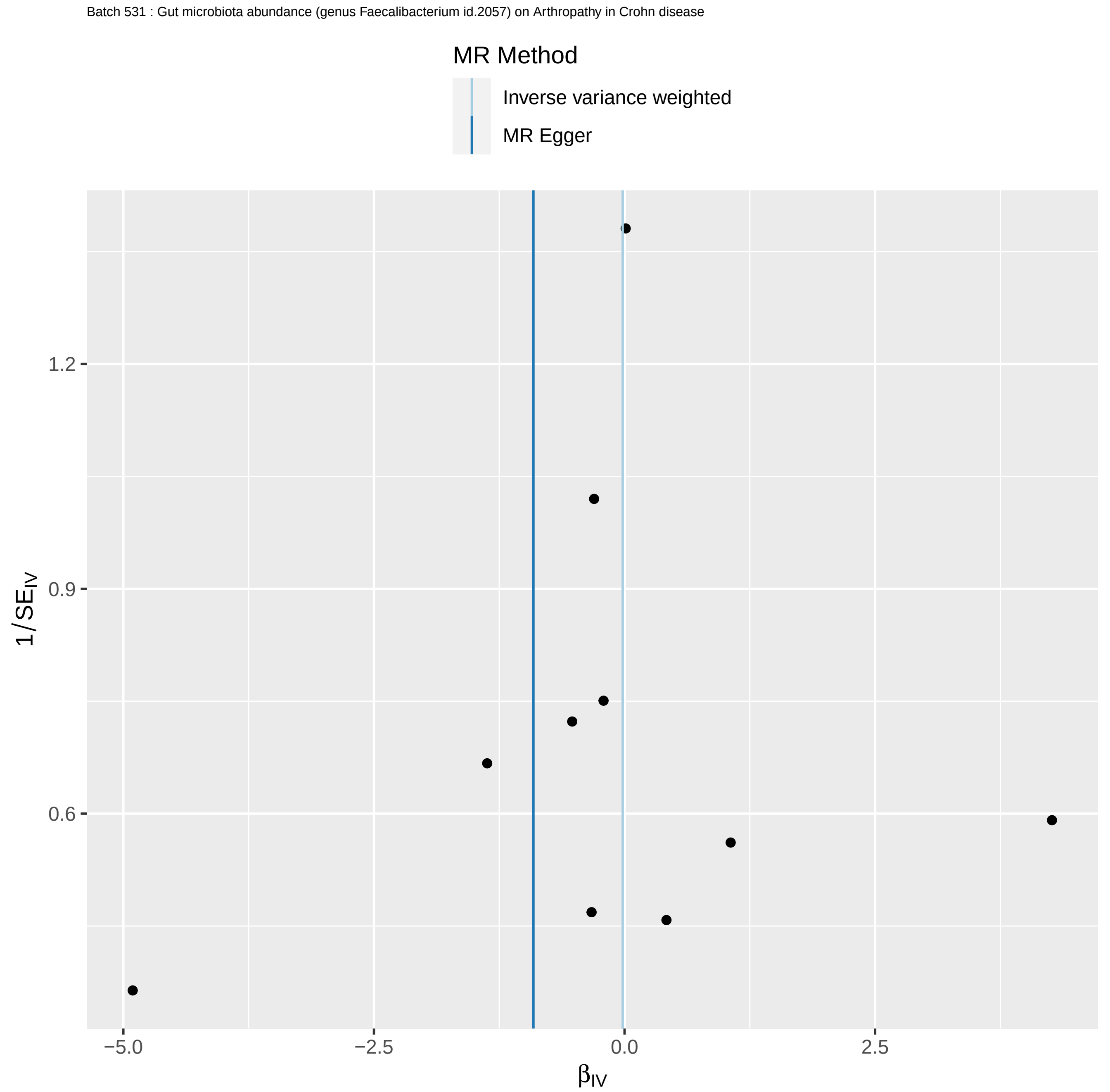
Batch 531 : Gut microbiota abundance (genus Faecalibacterium id.2057) on Arthropathy in Crohn disease
MR Method
Inverse variance weighted
MR Egger
1.2
0.9
0.6
$1/SE_{IV}$
−5.0
−2.5
0.0
2.5
β_{IV}

Batch 532 : Gut microbiota abundance (genus Family XIII AD3011 group id.11293) on Arthropathy in Crohn disease

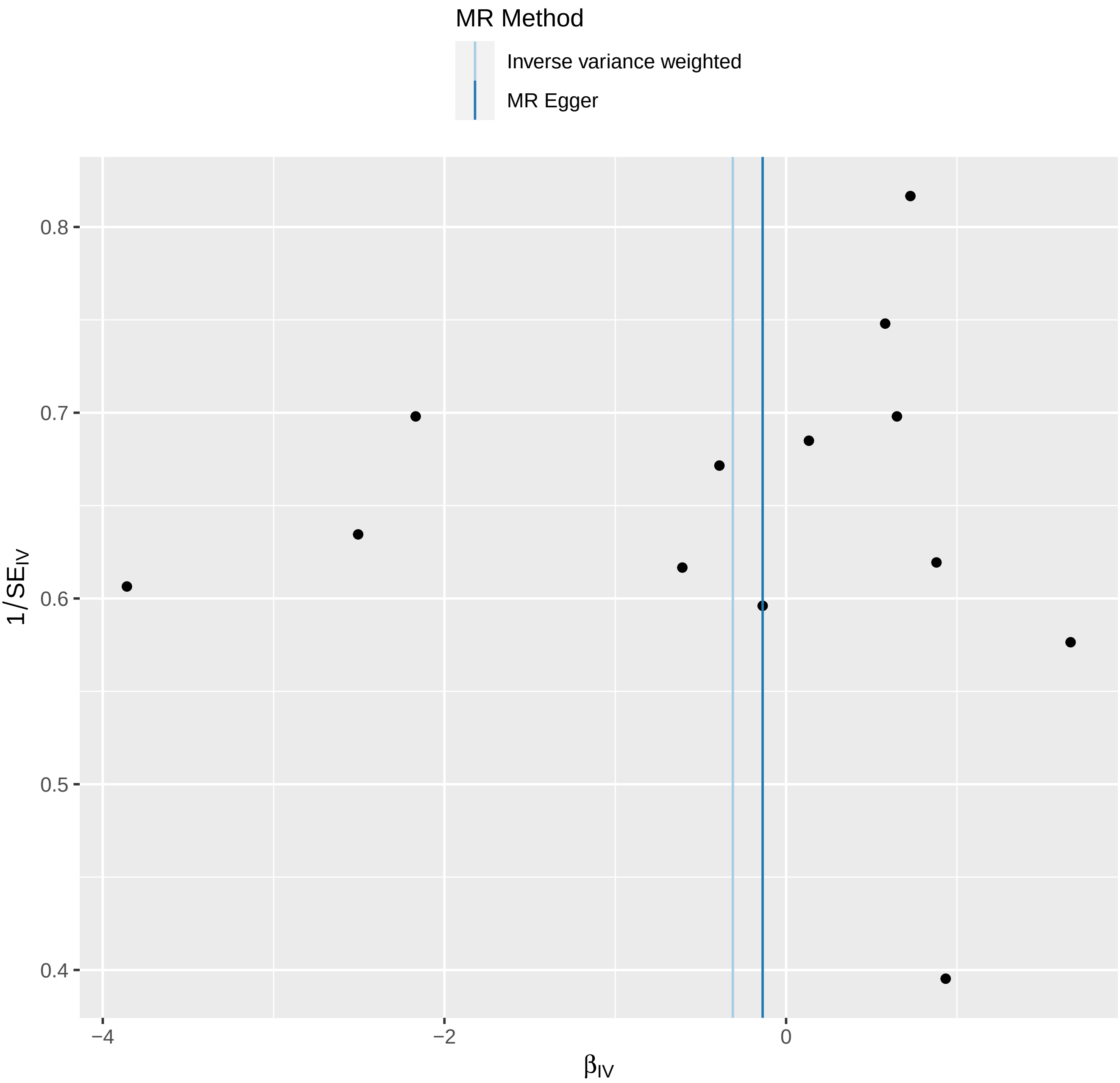

Batch 533 : Gut microbiota abundance (genus Family XIII UCG001 id.11294) on Arthropathy in Crohn disease

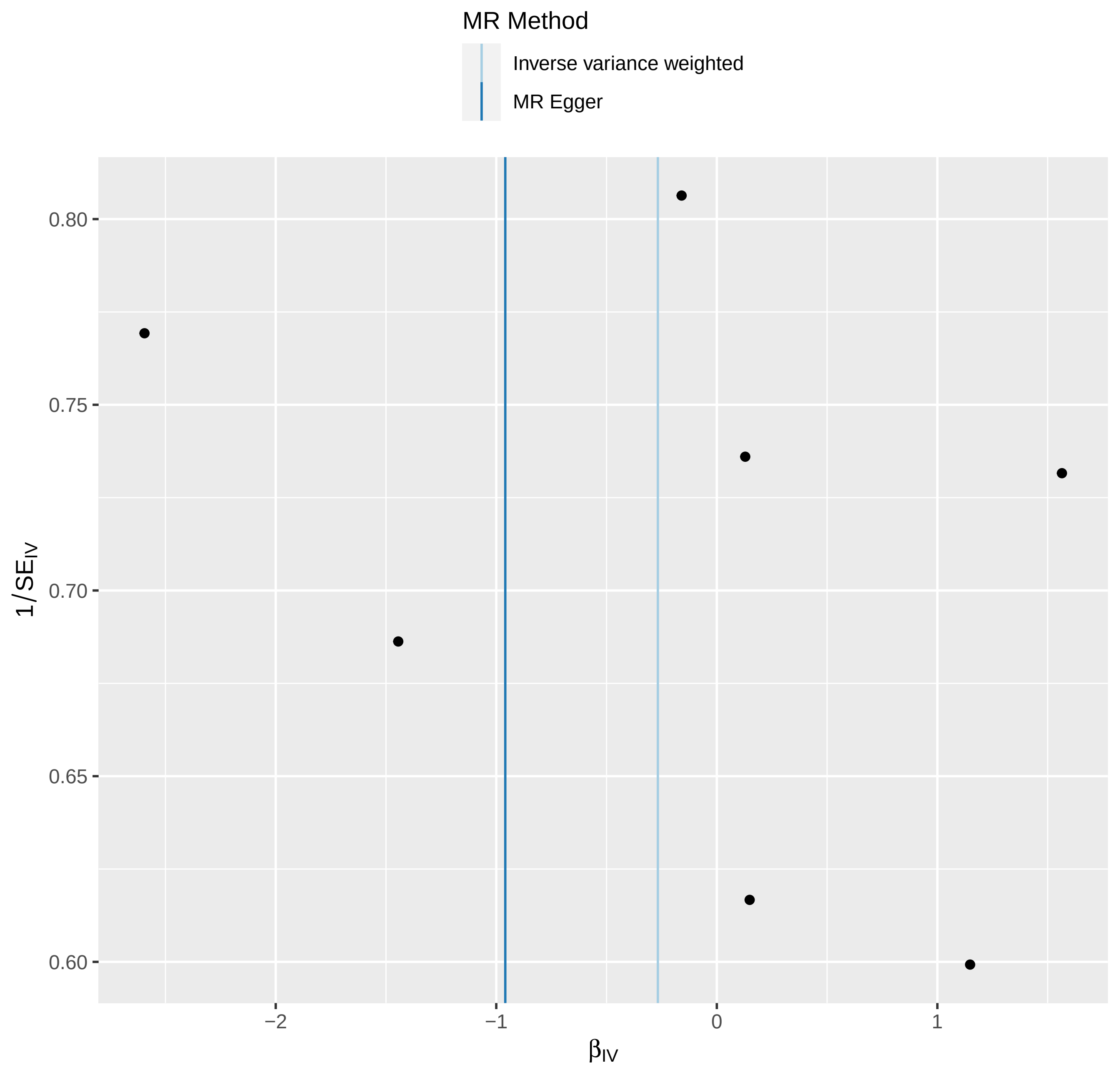

Batch 534 : Gut microbiota abundance (genus Flavonifractor id.2059) on Arthropathy in Crohn disease

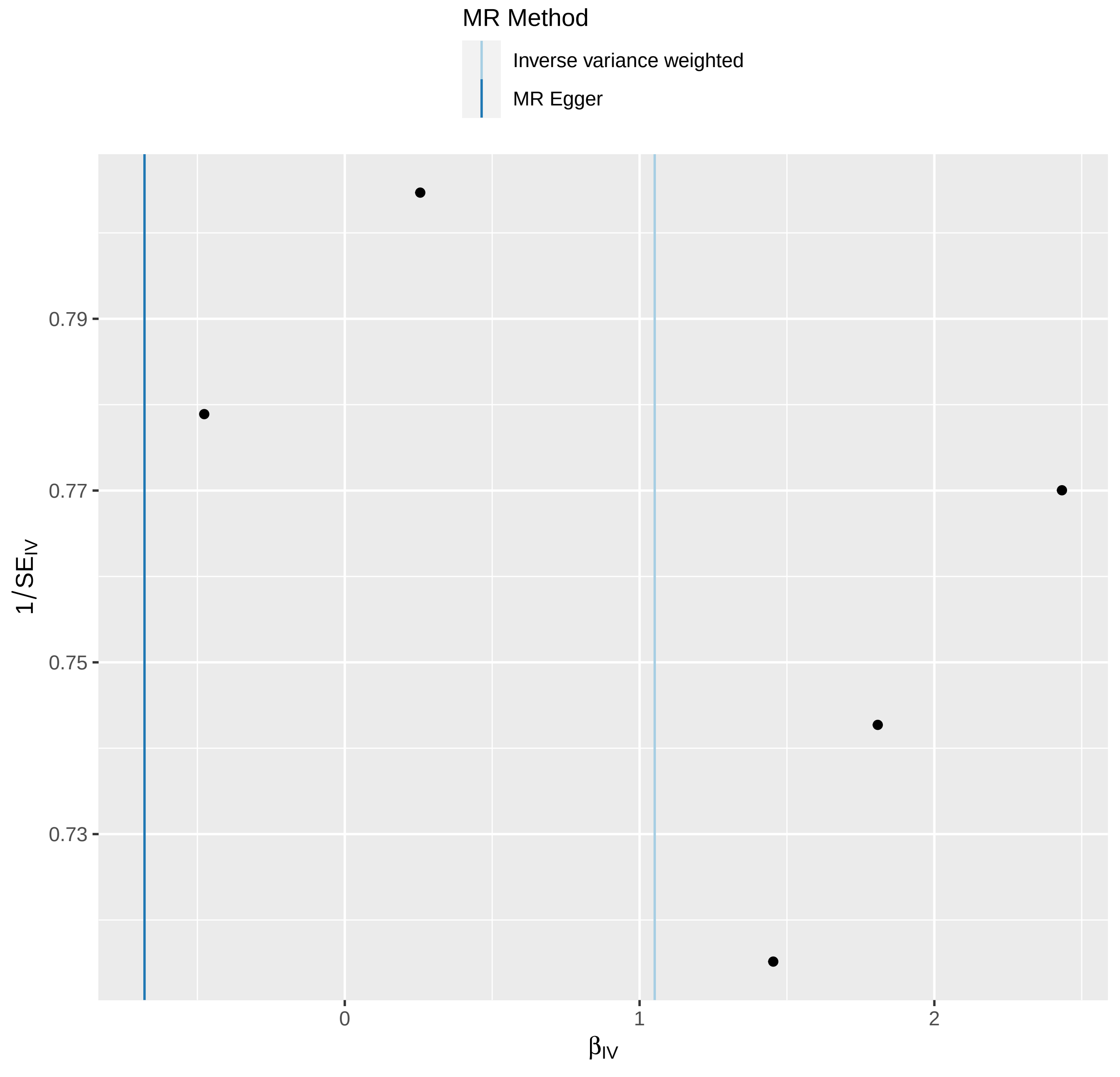

Batch 535 : Gut microbiota abundance (genus Fusicatenibacter id.11305) on Arthropathy in Crohn disease

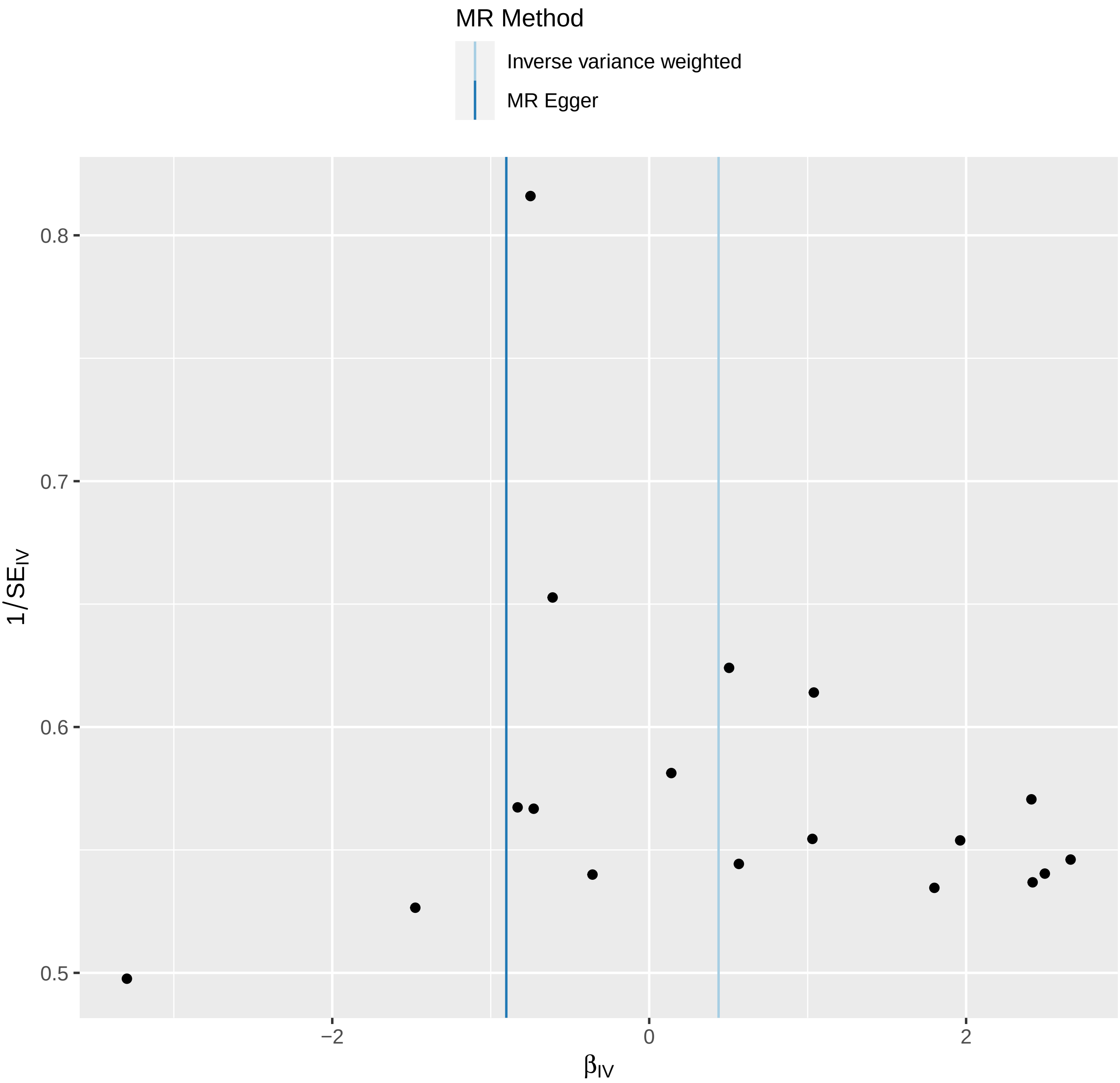

Batch 536 : Gut microbiota abundance (genus Gordonibacter id.821) on Arthropathy in Crohn disease

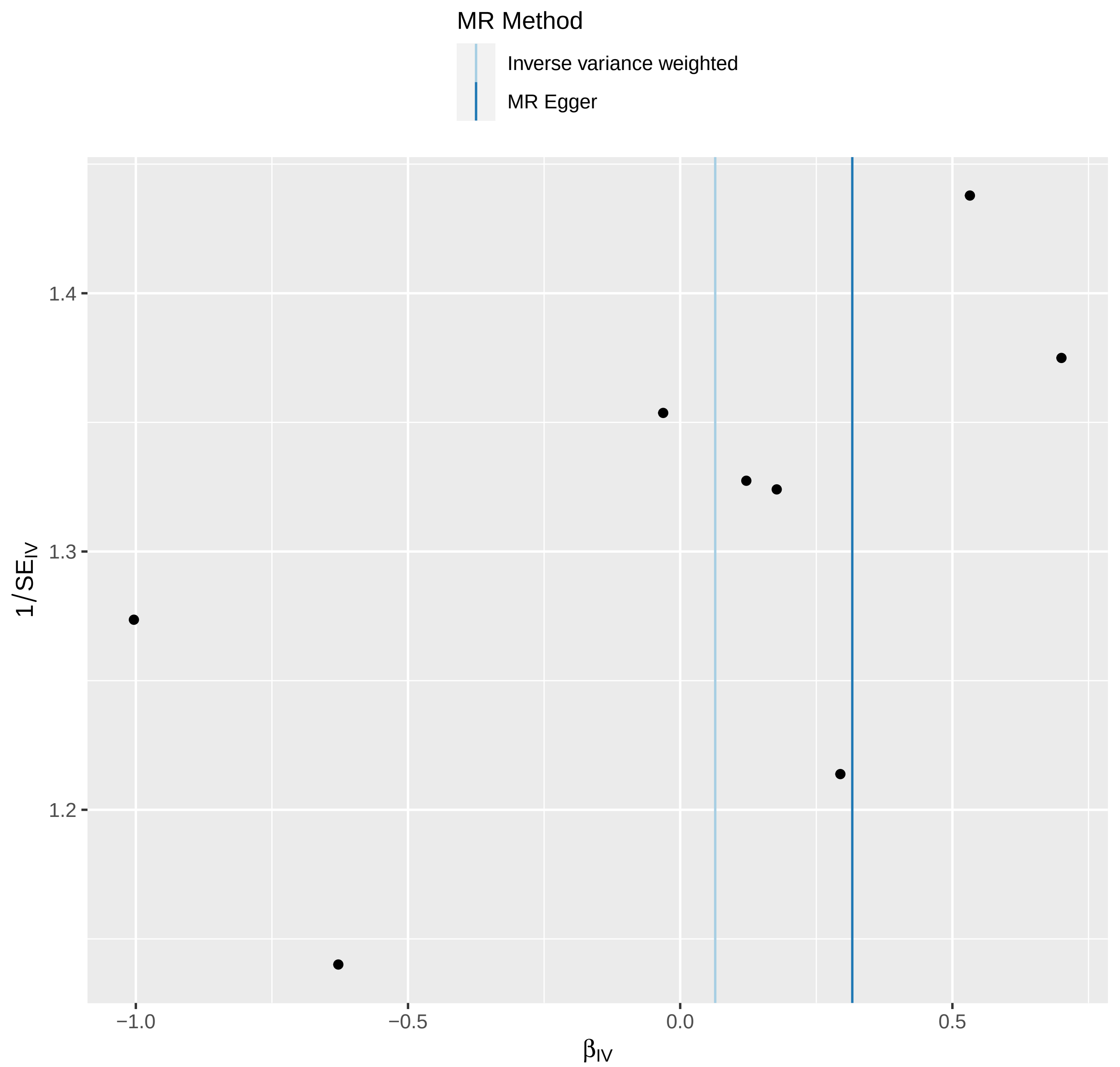

Batch 537 : Gut microbiota abundance (genus Haemophilus id.3698) on Arthropathy in Crohn disease

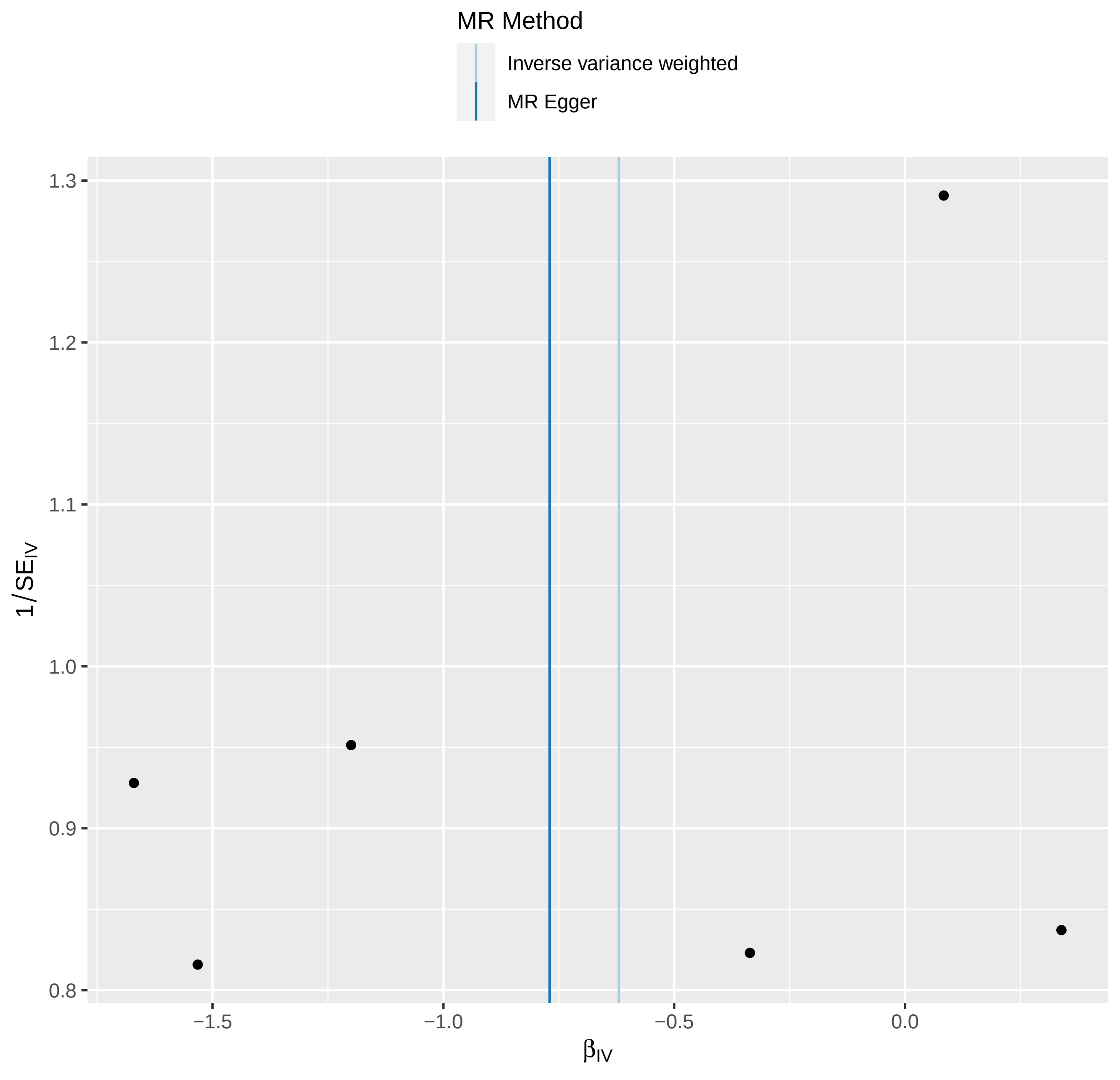

Batch 538 : Gut microbiota abundance (genus Holdemanella id.11393) on Arthropathy in Crohn disease

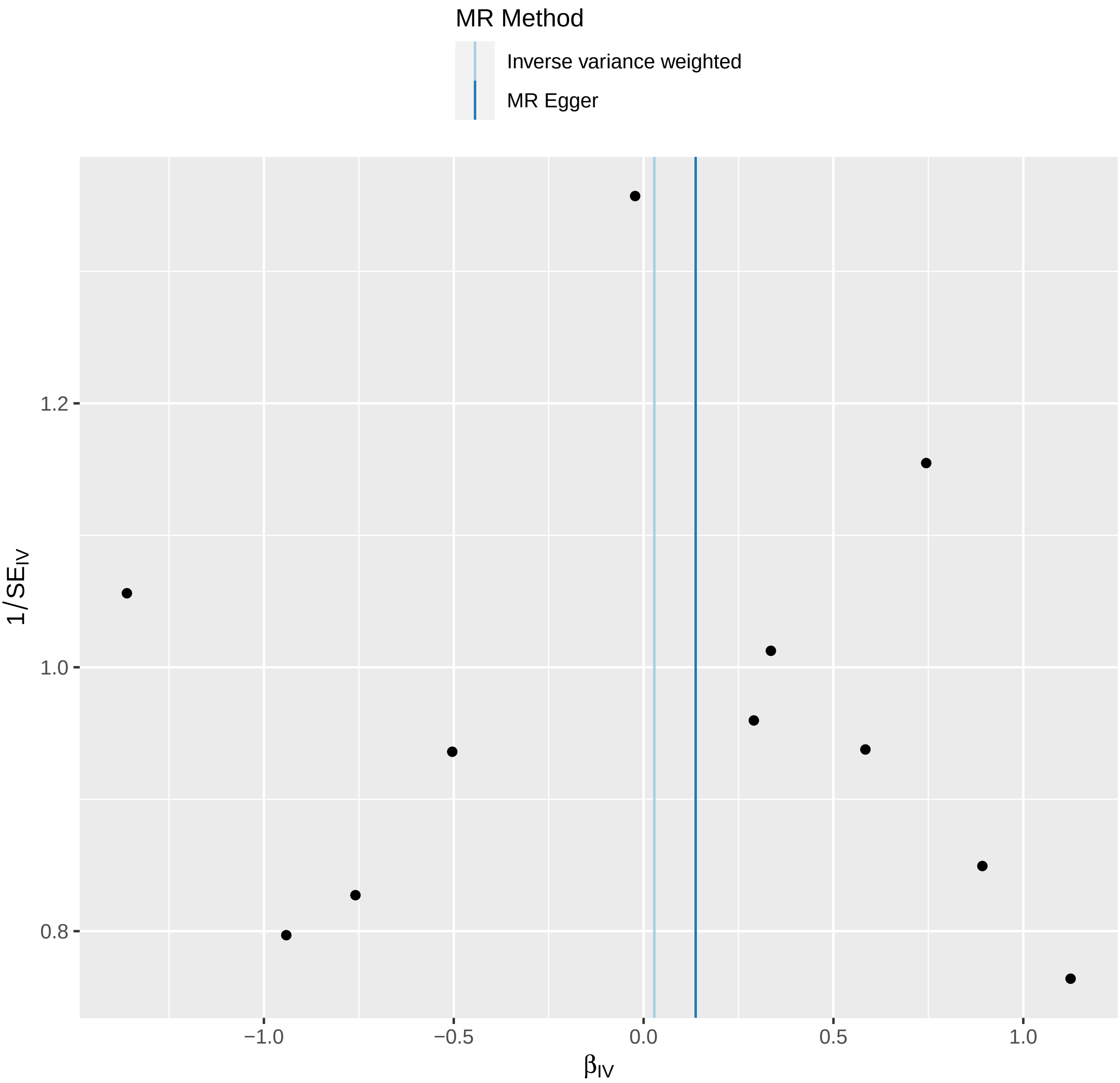

Batch 539 : Gut microbiota abundance (genus Holdemania id.2157) on Arthropathy in Crohn disease

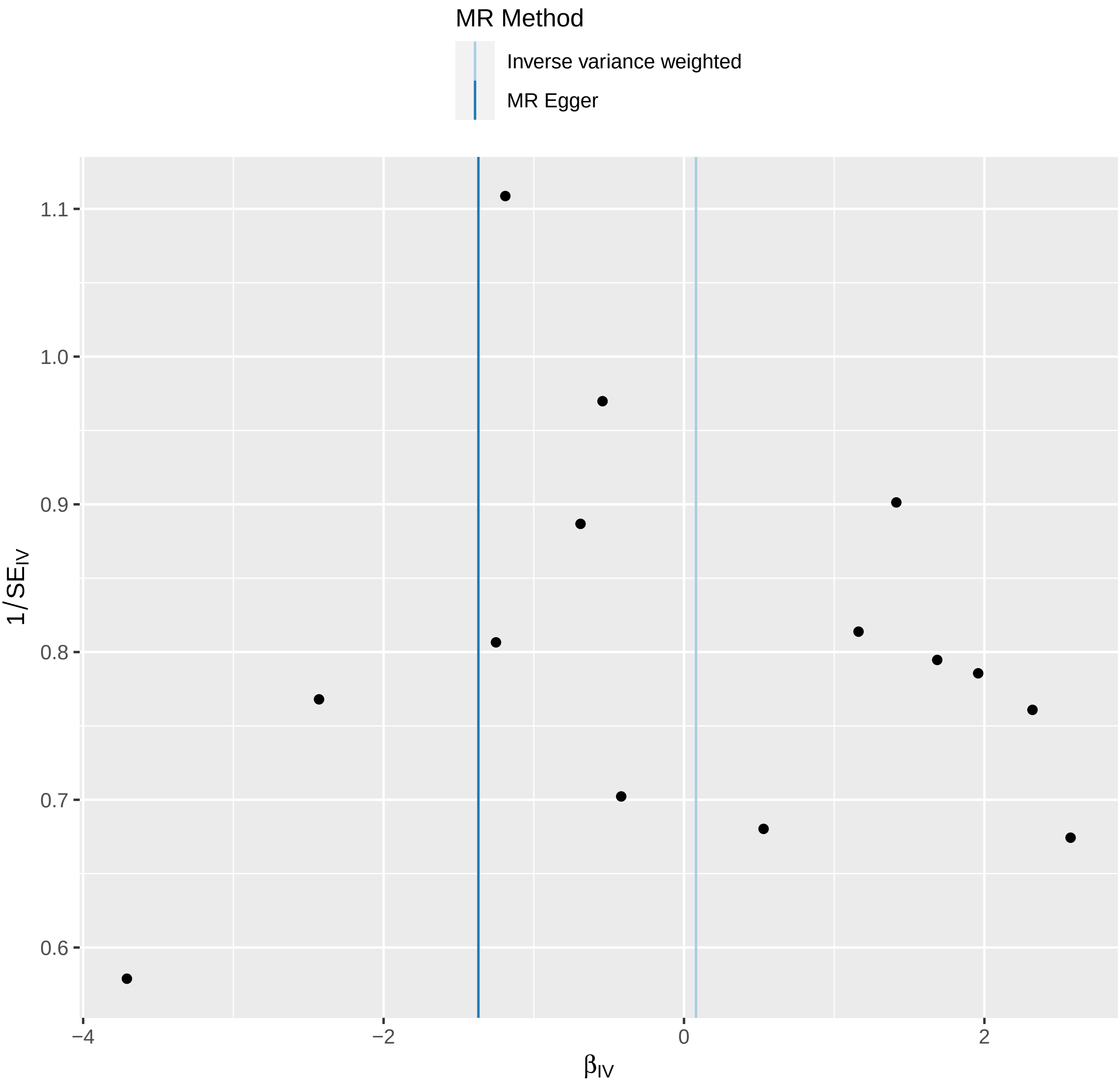

Batch 540 : Gut microbiota abundance (genus Howardella id.2000) on Arthropathy in Crohn disease

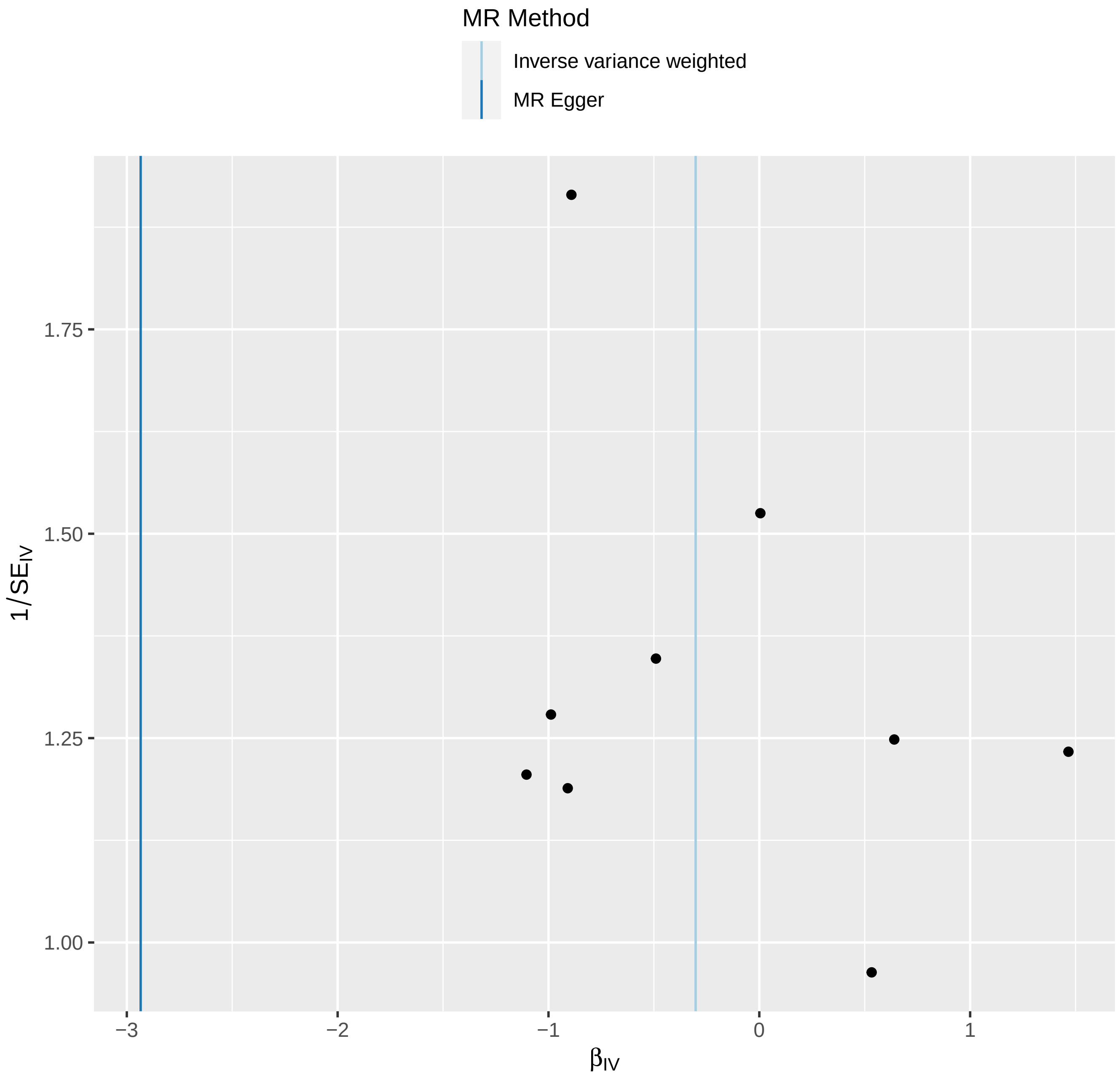

Batch 541 : Gut microbiota abundance (genus Hungatella id.11306) on Arthropathy in Crohn disease

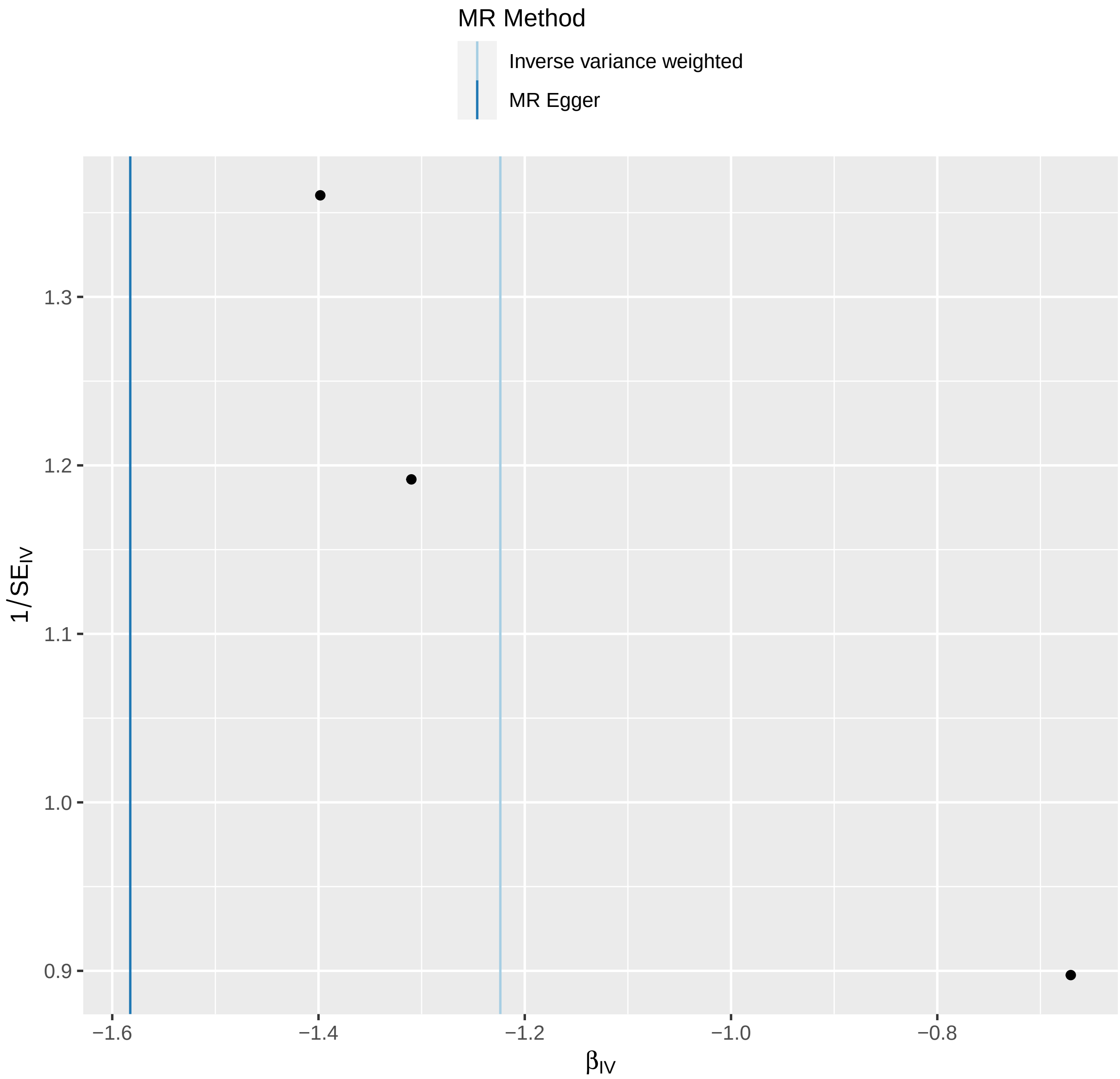

Batch 542 : Gut microbiota abundance (genus Intestinibacter id.11345) on Arthropathy in Crohn disease

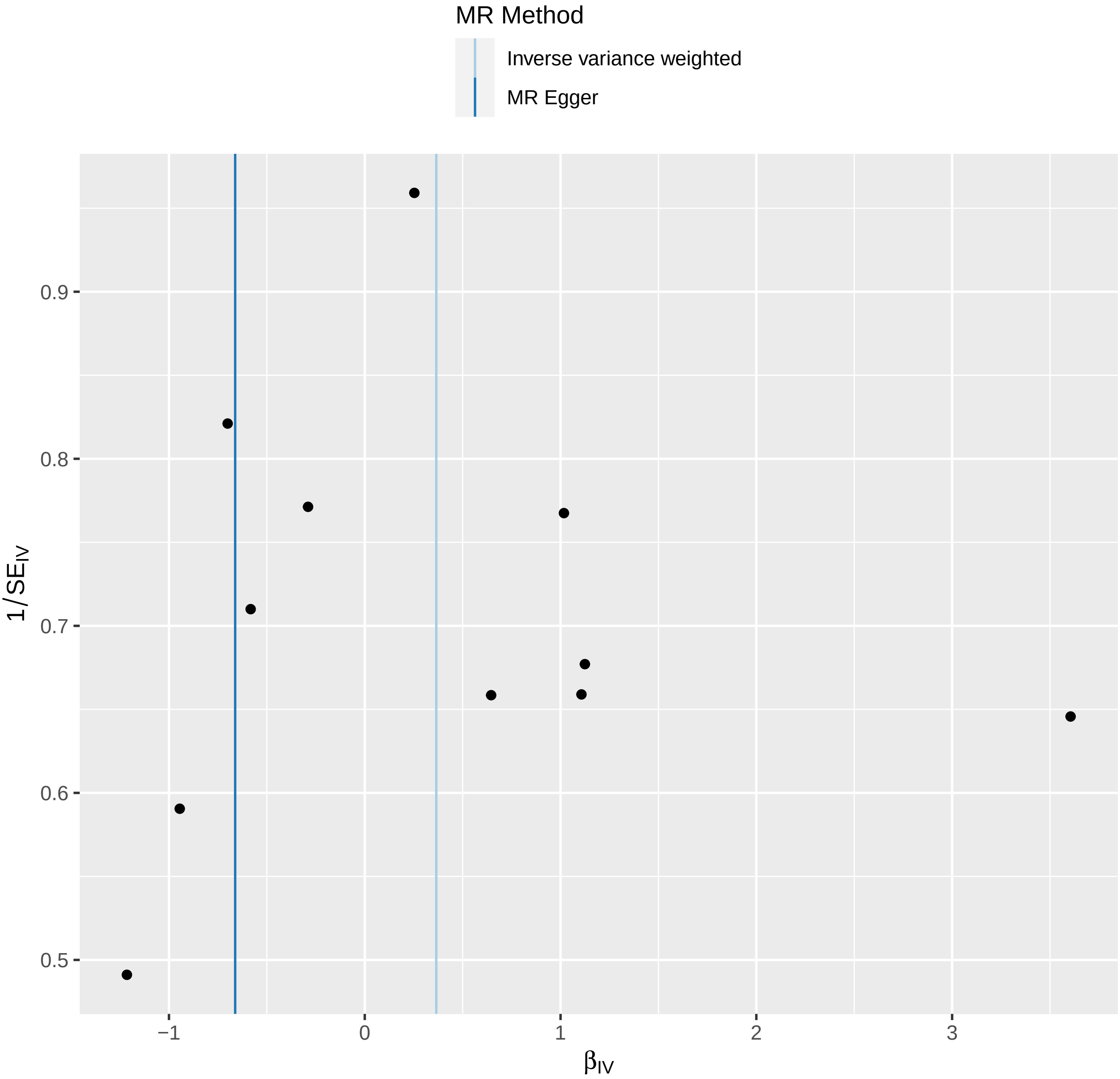

Batch 543 : Gut microbiota abundance (genus Intestinimonas id.2062) on Arthropathy in Crohn disease

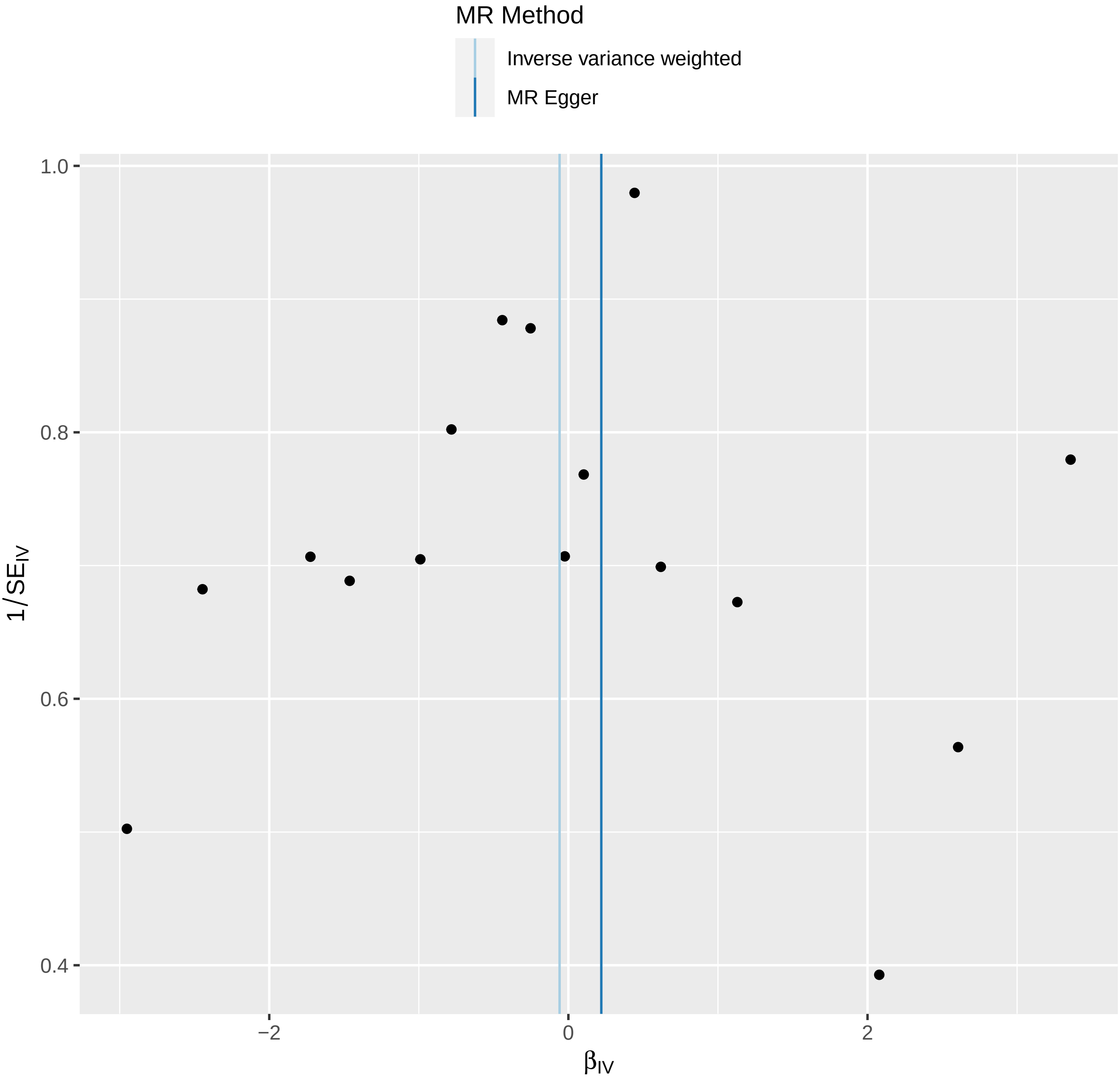

Batch 544 : Gut microbiota abundance (genus Lachnoclostridium id.11308) on Arthropathy in Crohn disease

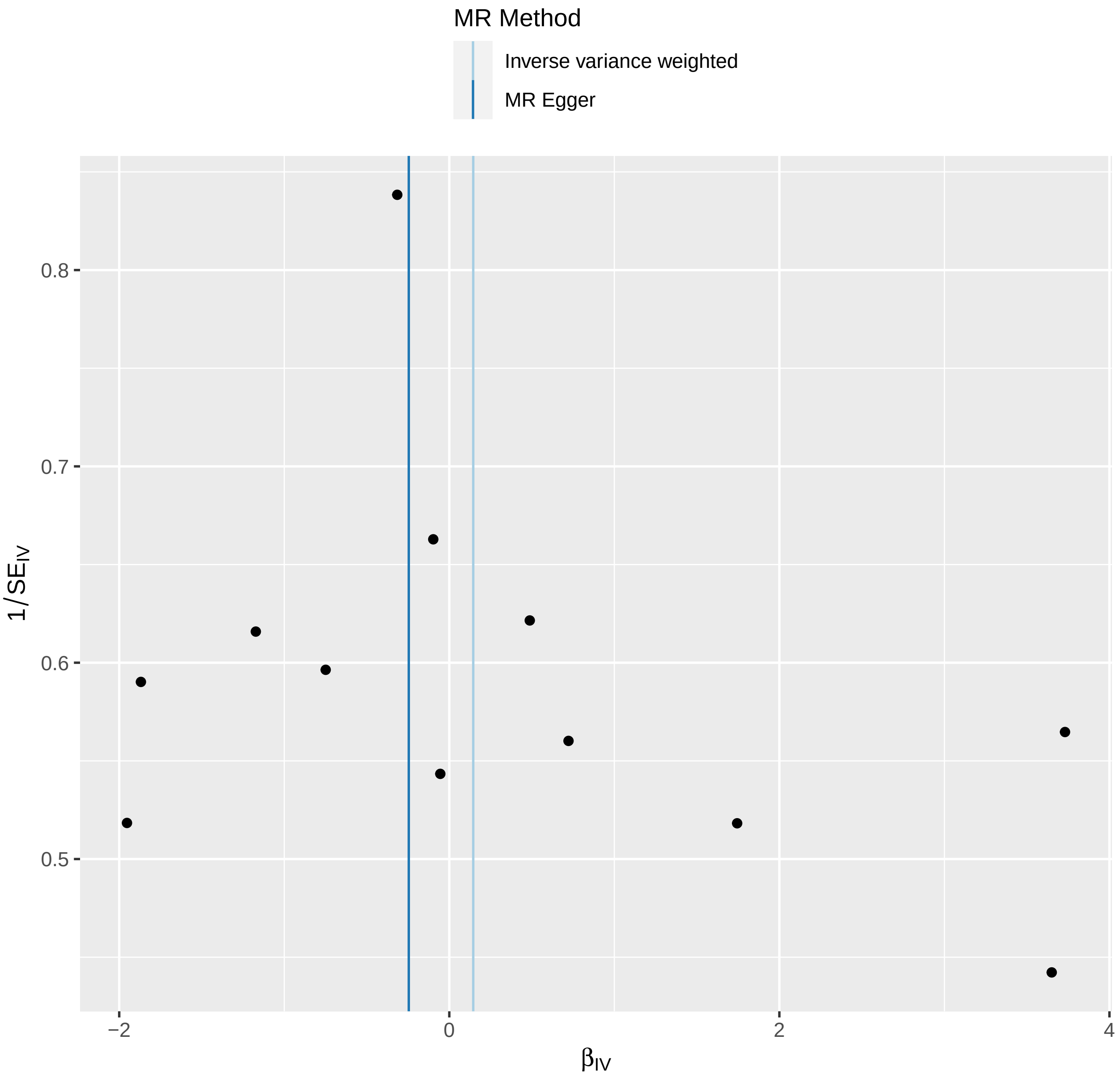

Batch 545 : Gut microbiota abundance (genus Lachnospiraceae FCS020 group id.11314) on Arthropathy in Crohn disease

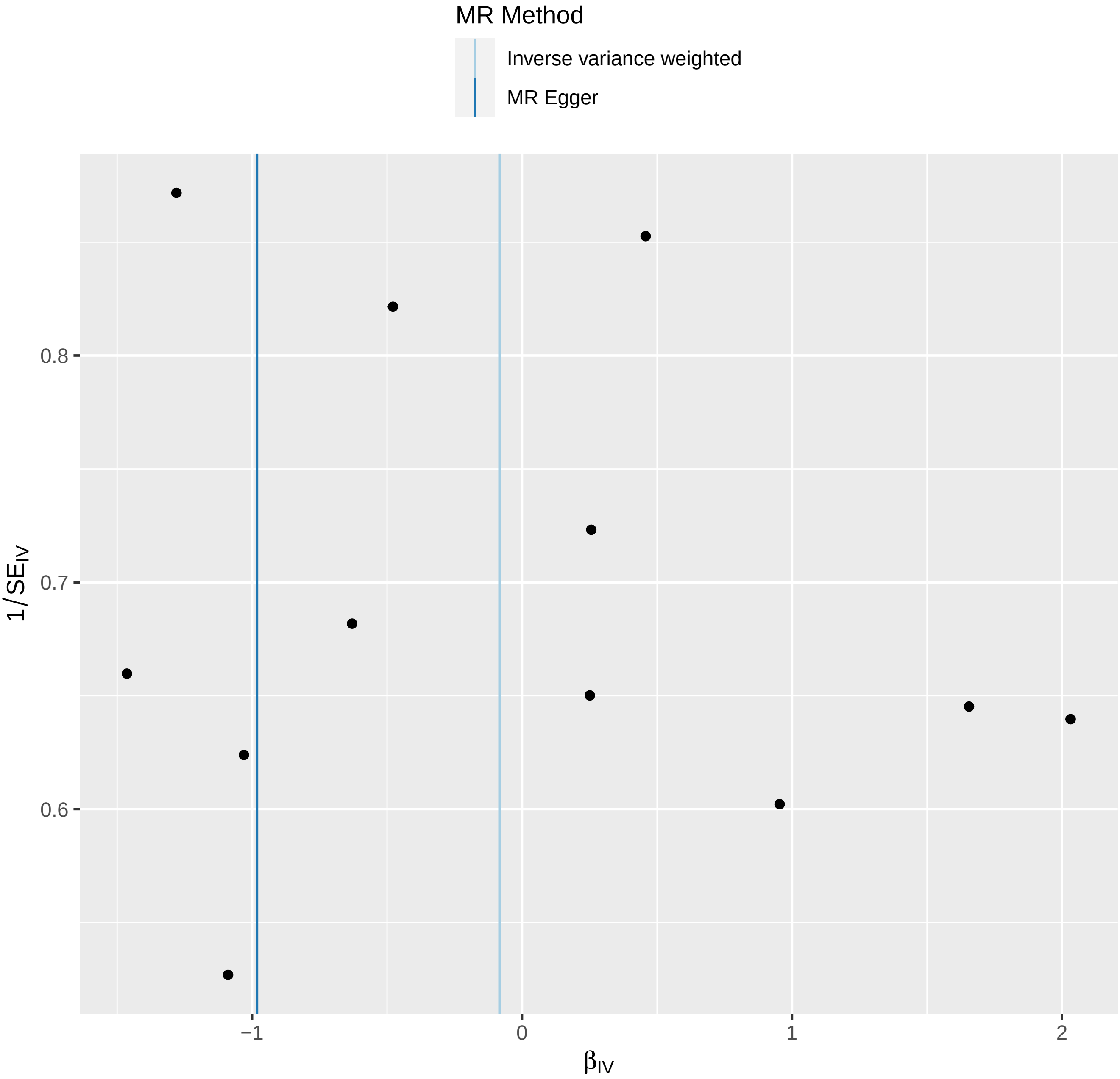

Batch 546 : Gut microbiota abundance (genus Lachnospiraceae NC2004 group id.11316) on Arthropathy in Crohn disease

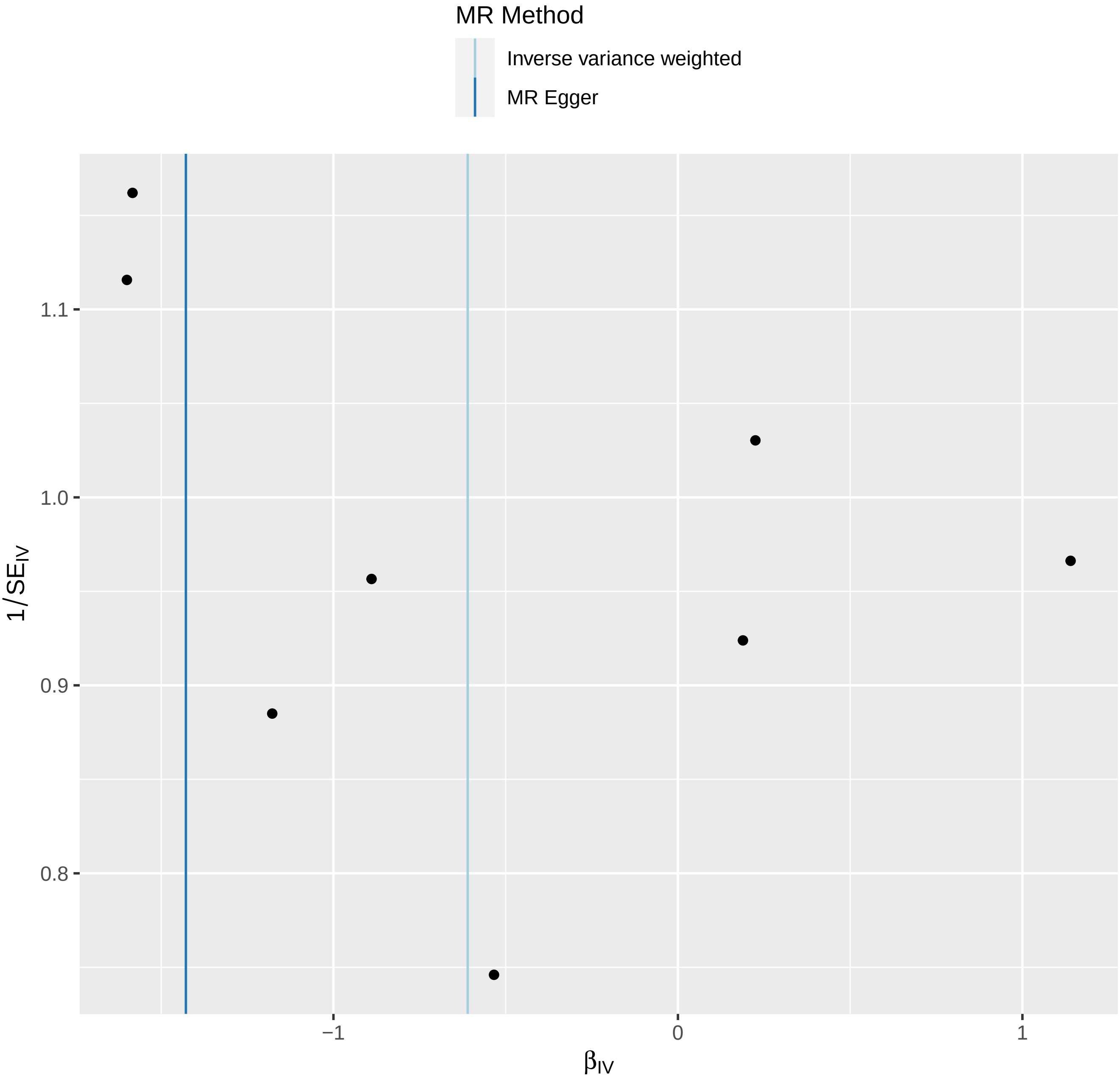

Batch 547 : Gut microbiota abundance (genus Lachnospiraceae ND3007 group id.11317) on Arthropathy in Crohn disease

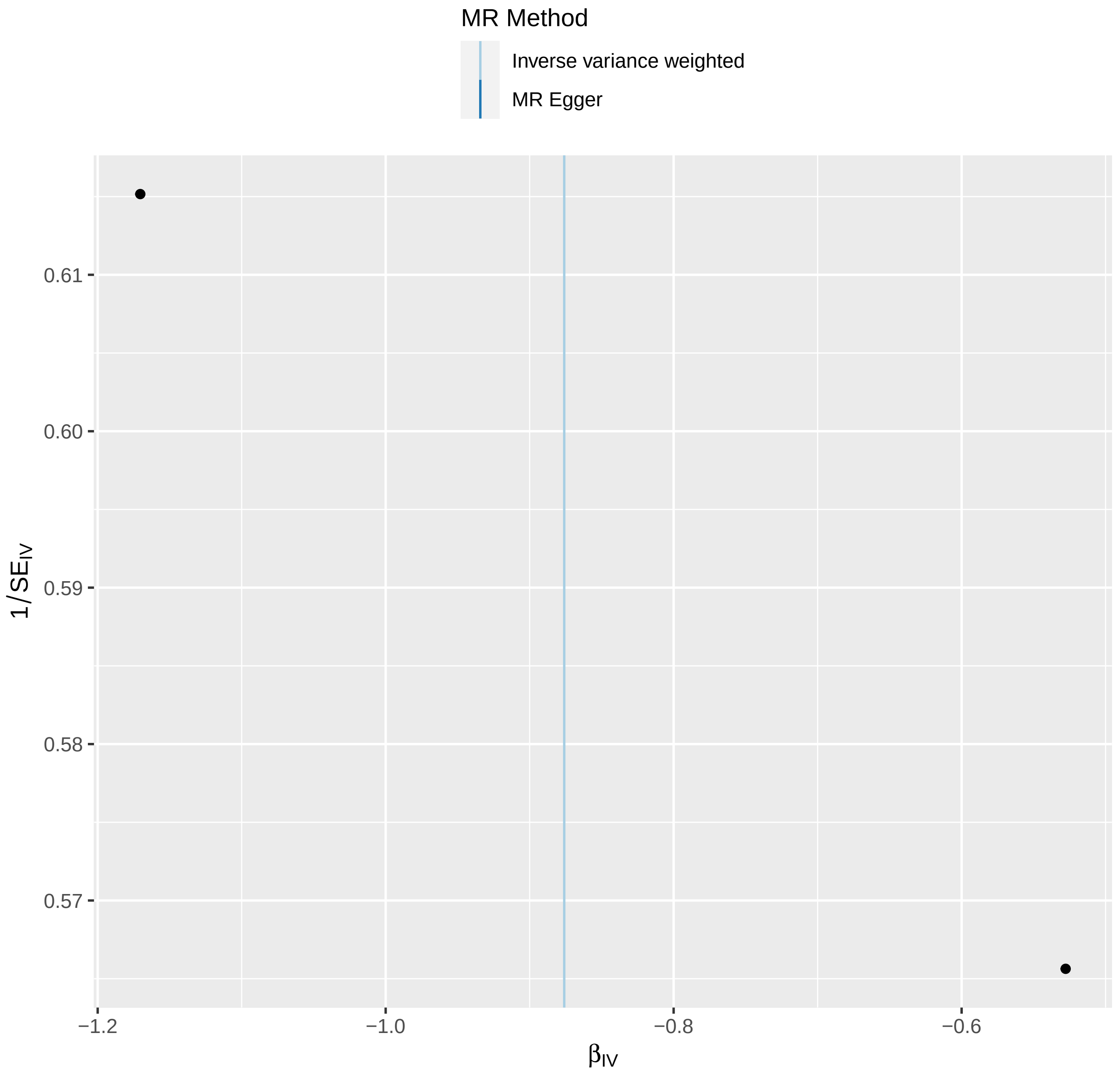

Batch 548 : Gut microbiota abundance (genus Lachnospiraceae NK4A136 group id.11319) on Arthropathy in Crohn disease

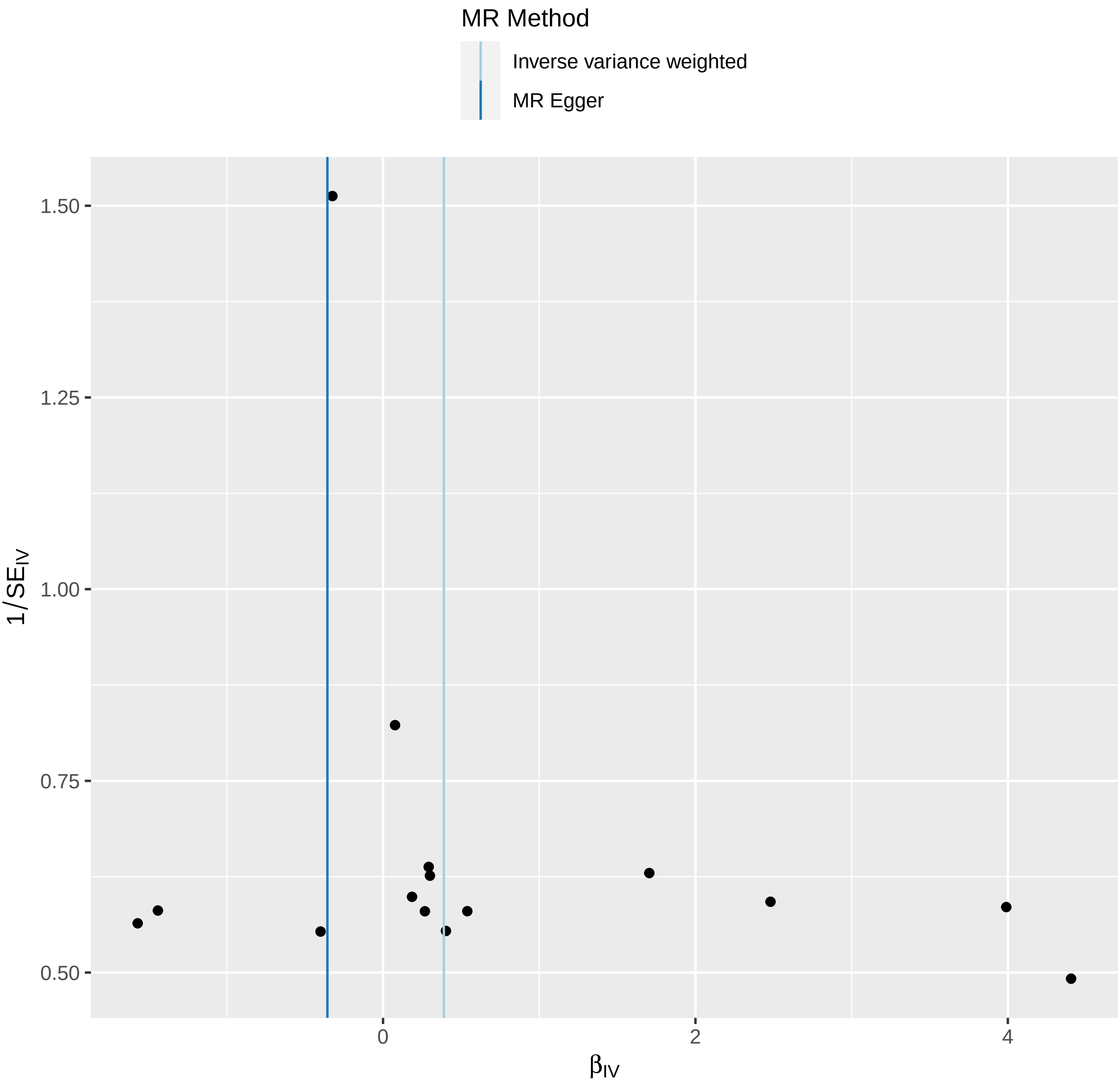

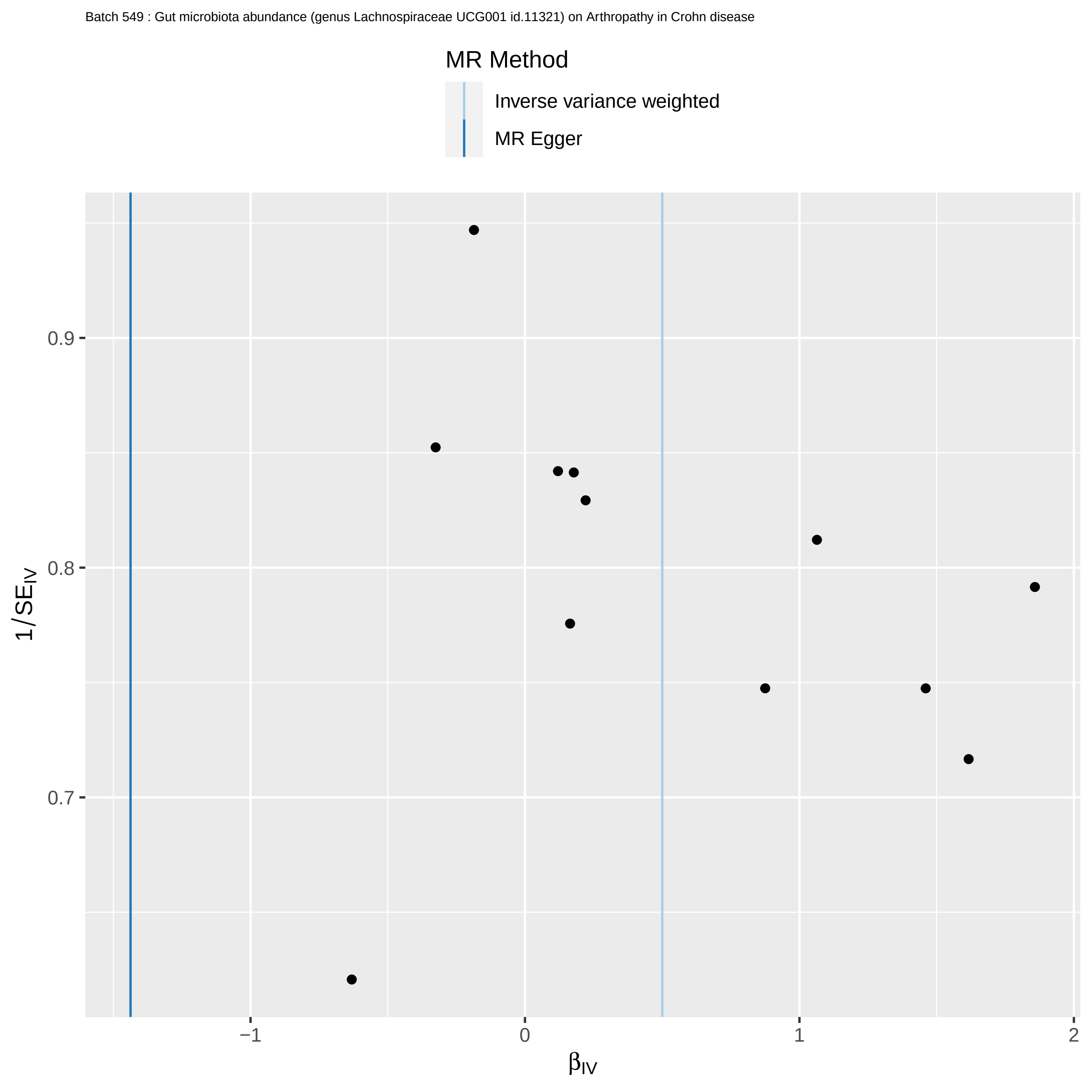

Batch 549 : Gut microbiota abundance (genus Lachnospiraceae UCG001 id.11321) on Arthropathy in Crohn disease
MR Method
Inverse variance weighted
MR Egger
0.9
0.8
0.7
1/SE_IV
−1
0
1
2
β_IV

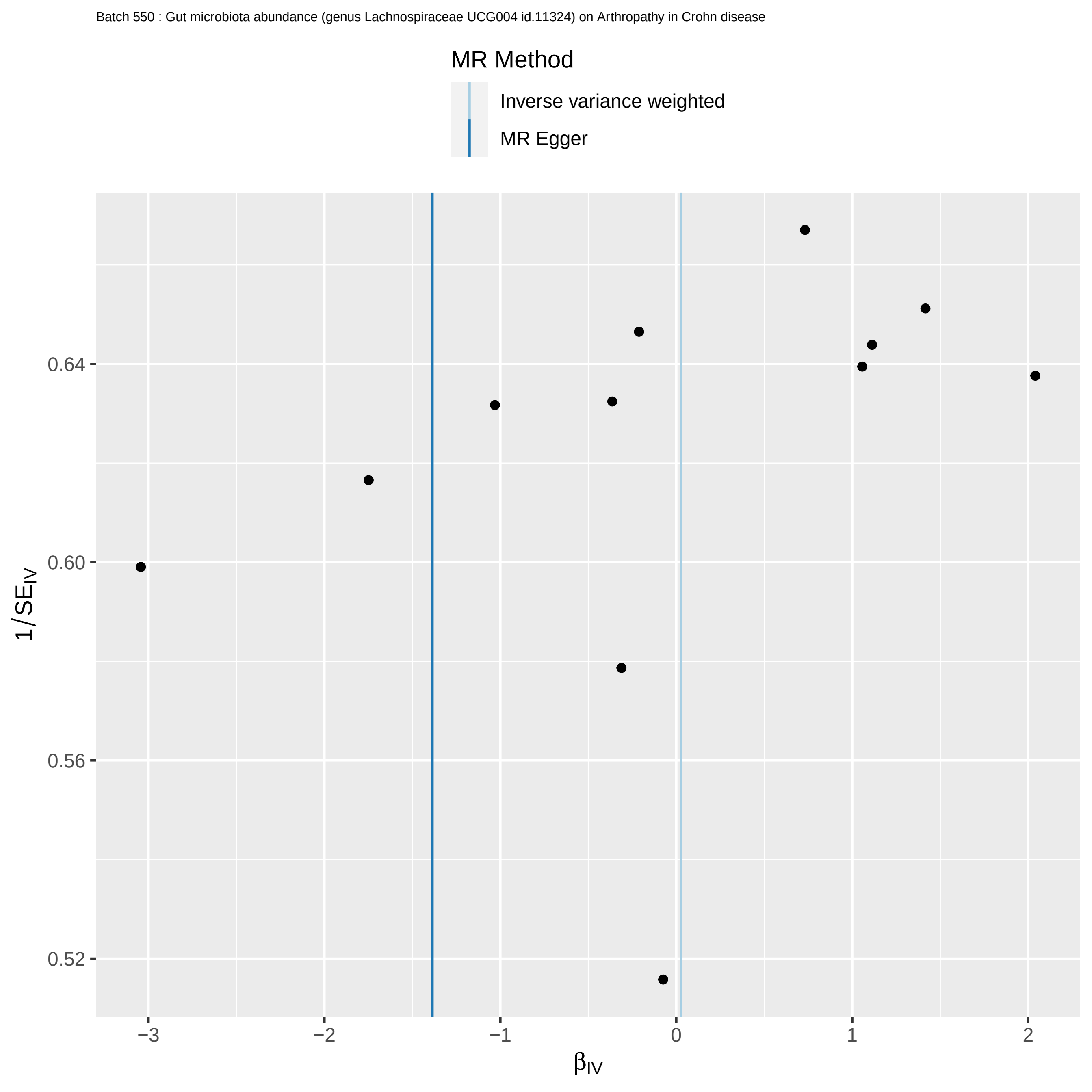
Batch 550 : Gut microbiota abundance (genus Lachnospiraceae UCG004 id.11324) on Arthropathy in Crohn disease
MR Method
Inverse variance weighted
MR Egger
0.64
0.60
0.56
0.52
1/SE_IV
−3
−2
−1
0
1
2
β_IV

Batch 551 : Gut microbiota abundance (genus Lachnospiraceae UCG008 id.11328) on Arthropathy in Crohn disease

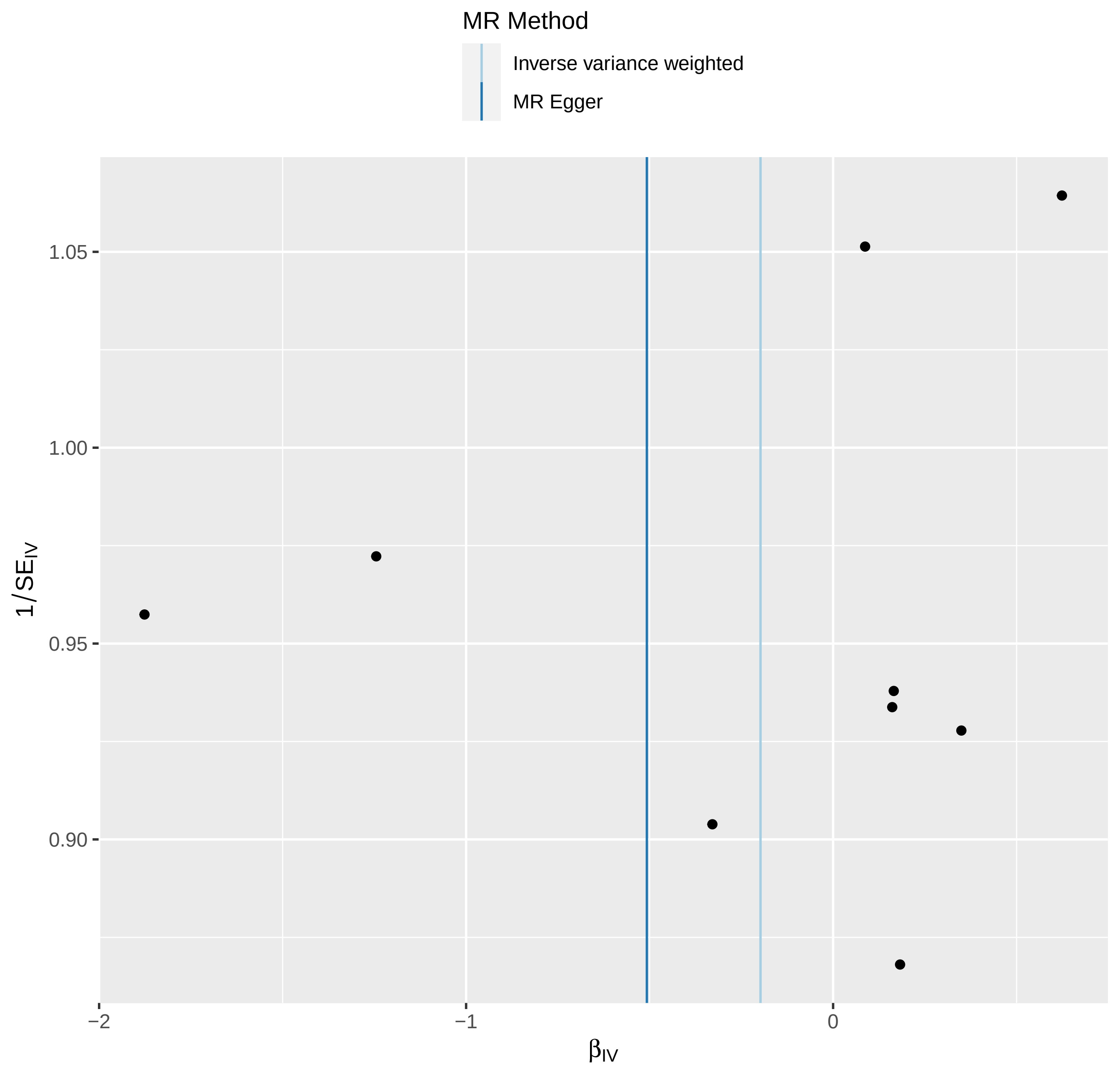

Batch 552 : Gut microbiota abundance (genus Lachnospiraceae UCG010 id.11330) on Arthropathy in Crohn disease

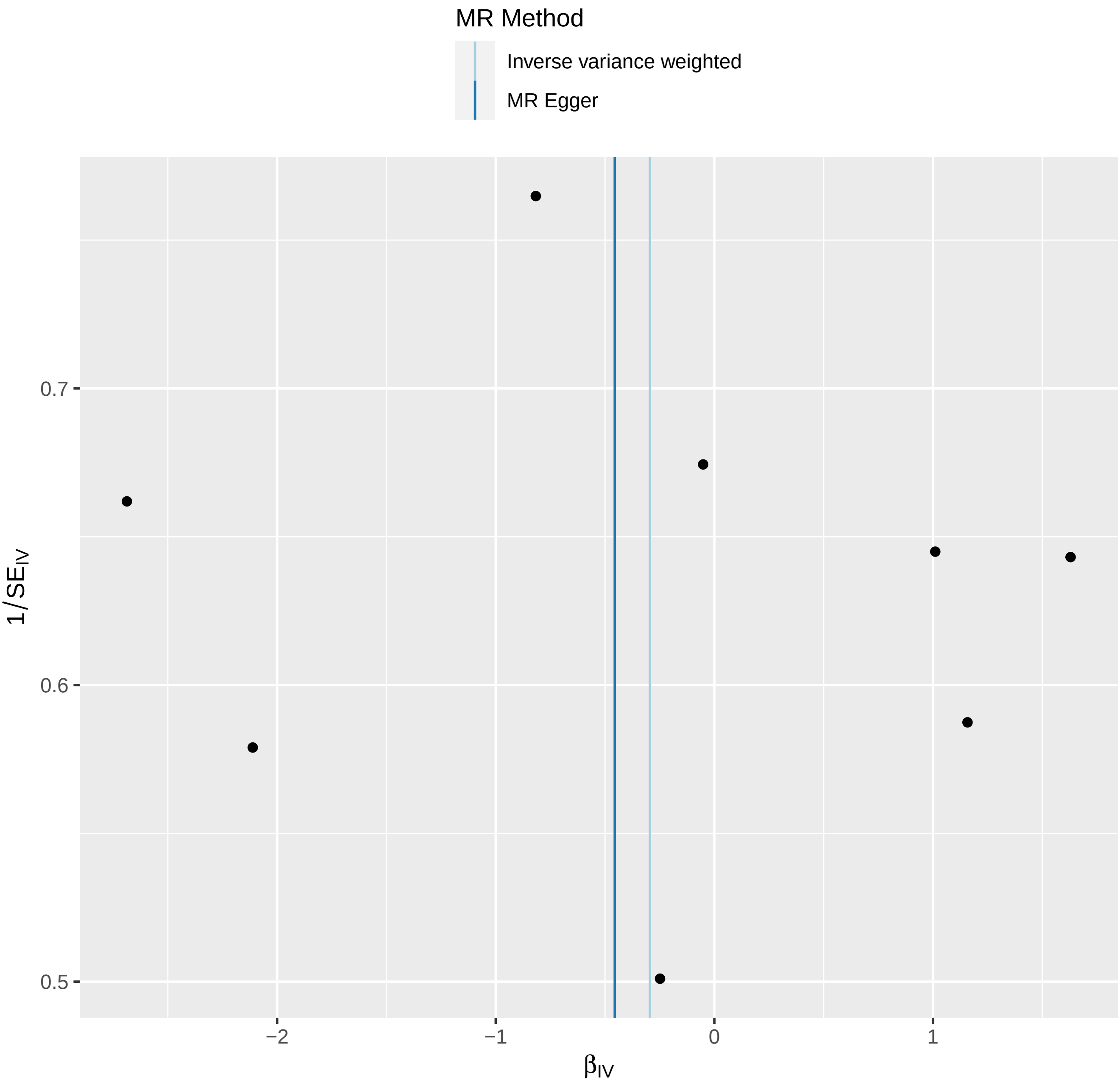

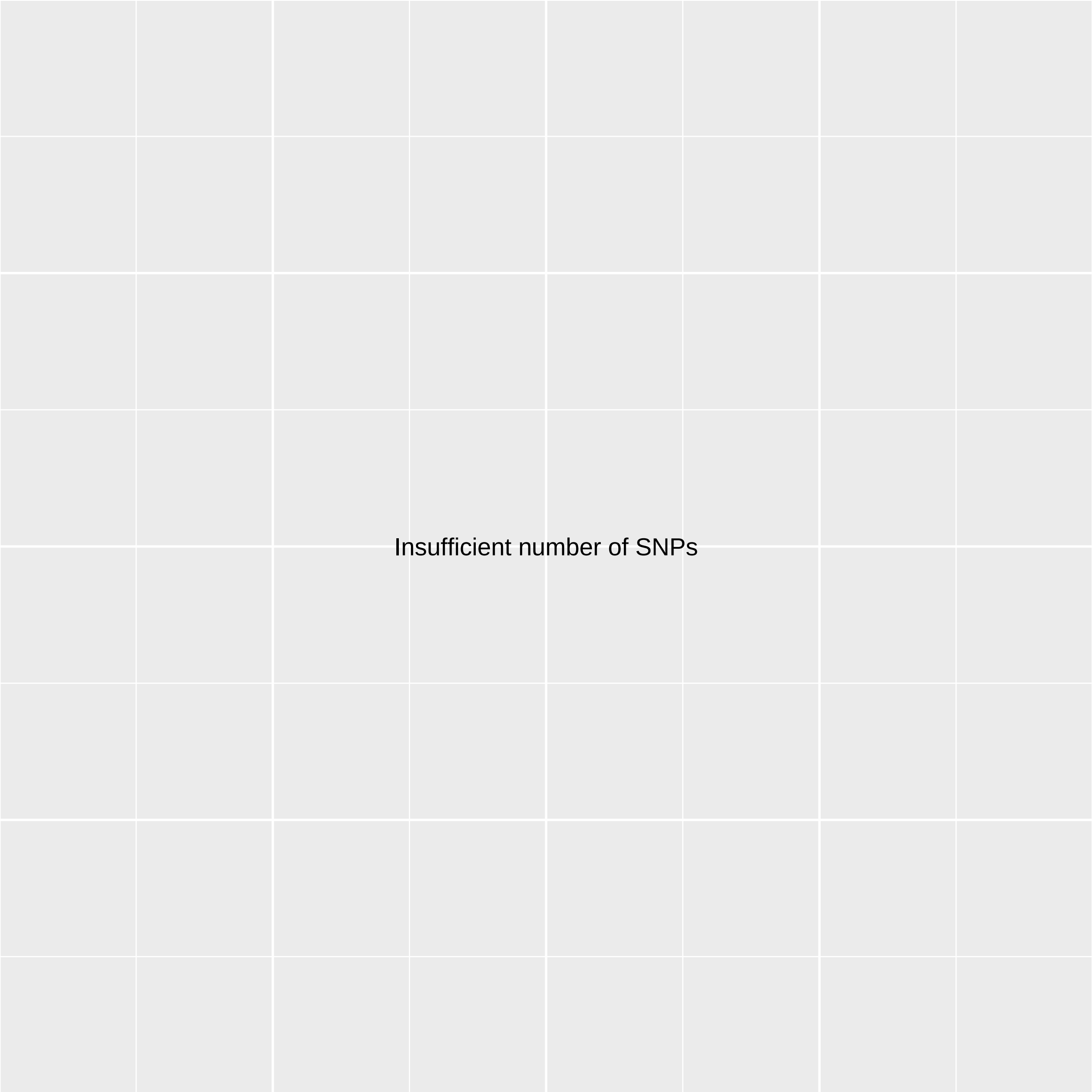
Insufficient number of SNPs

Batch 554 : Gut microbiota abundance (genus Lactobacillus id.1837) on Arthropathy in Crohn disease

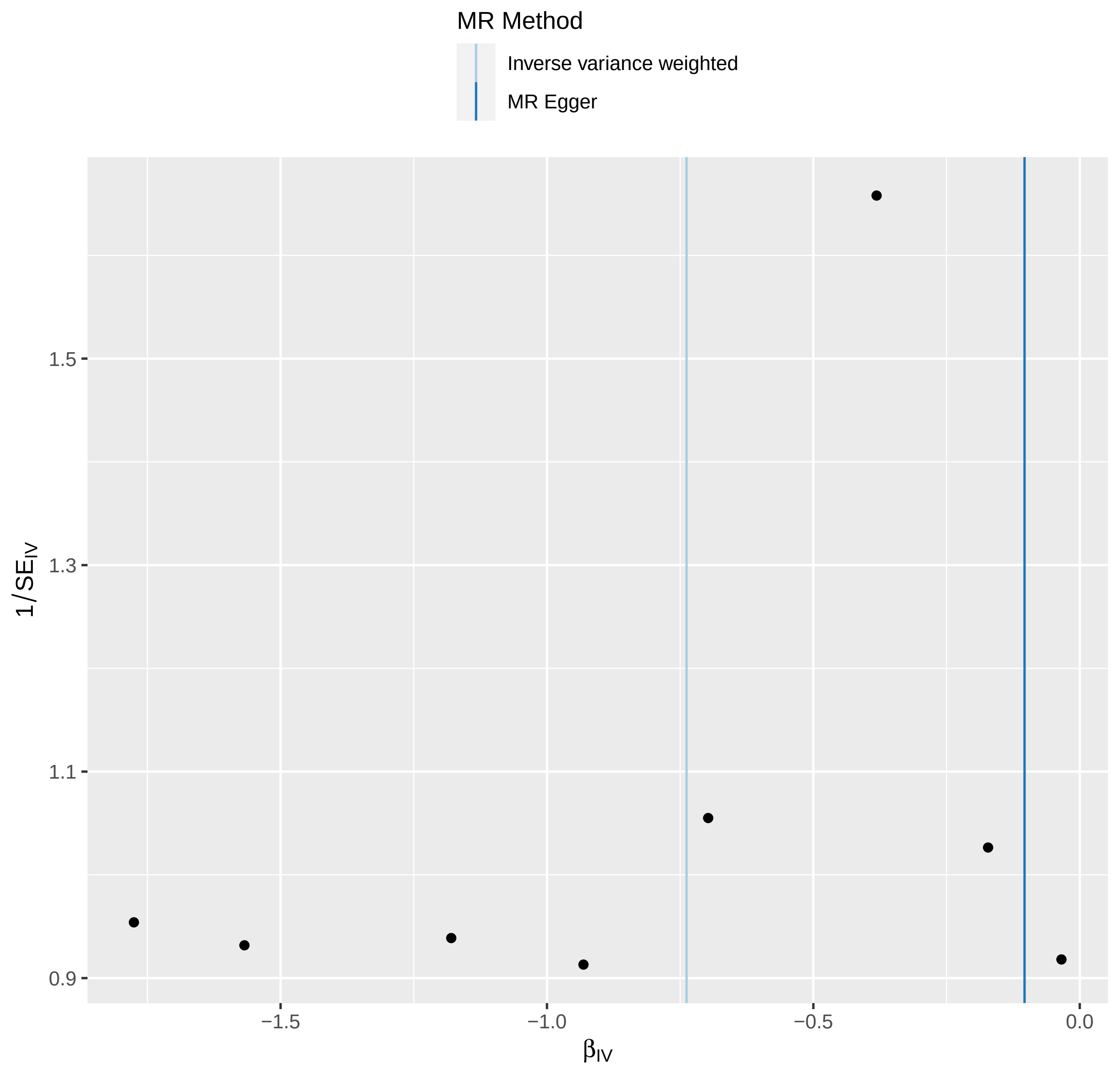

Batch 555 : Gut microbiota abundance (genus Lactococcus id.1851) on Arthropathy in Crohn disease

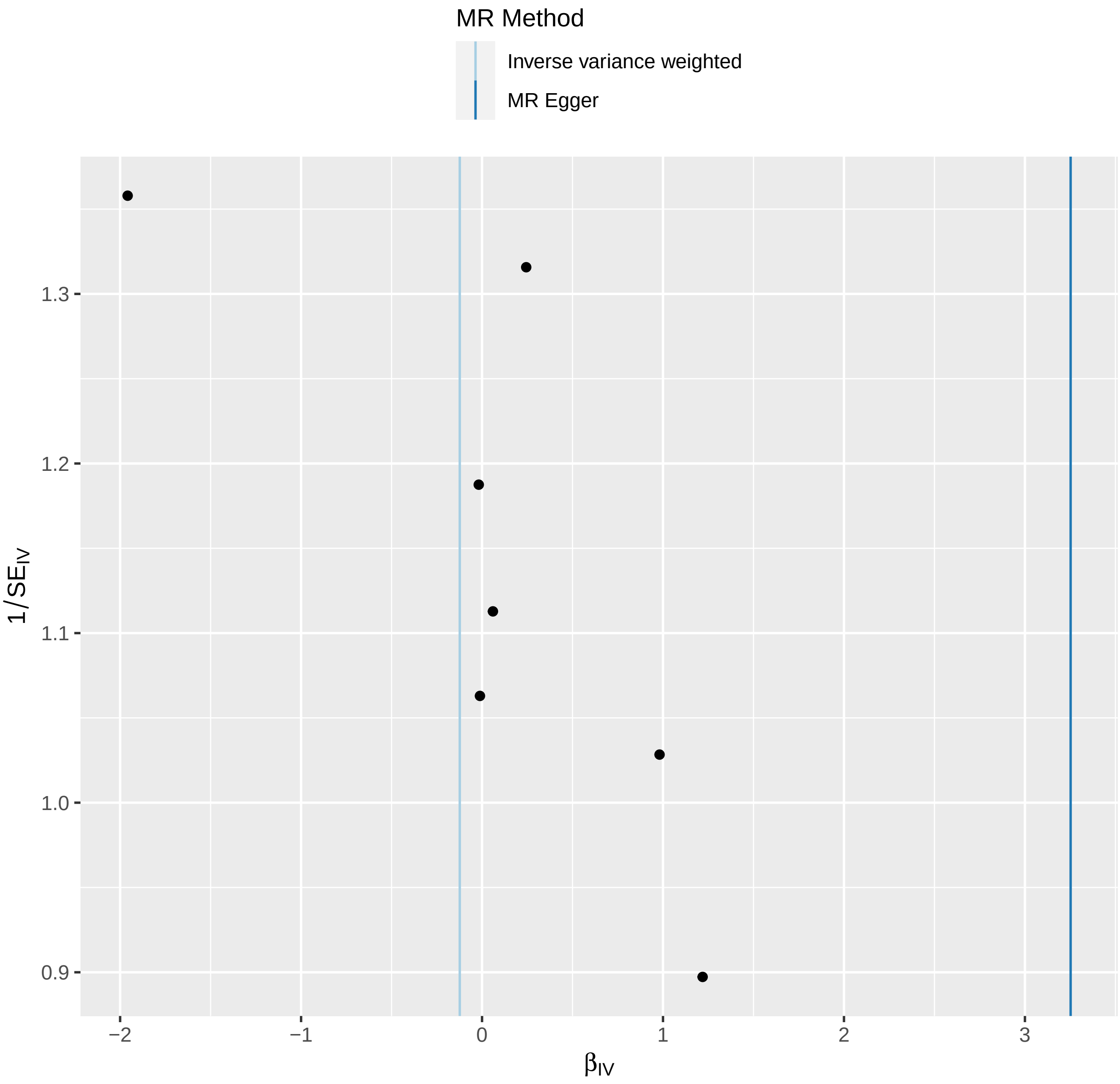

Batch 556 : Gut microbiota abundance (genus Marvinbryantia id.2005) on Arthropathy in Crohn disease

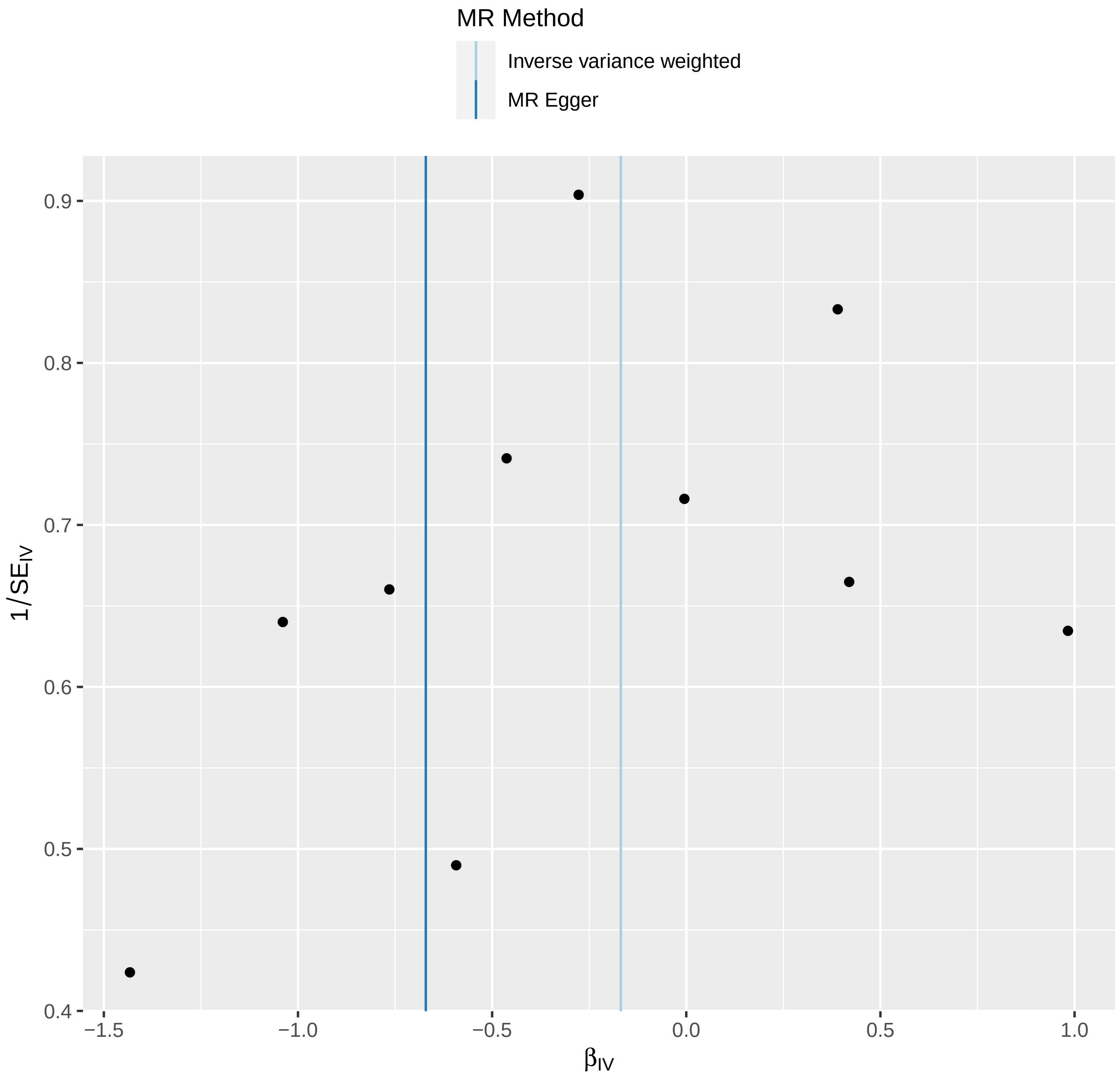

Batch 557 : Gut microbiota abundance (genus Methanobrevibacter id.123) on Arthropathy in Crohn disease

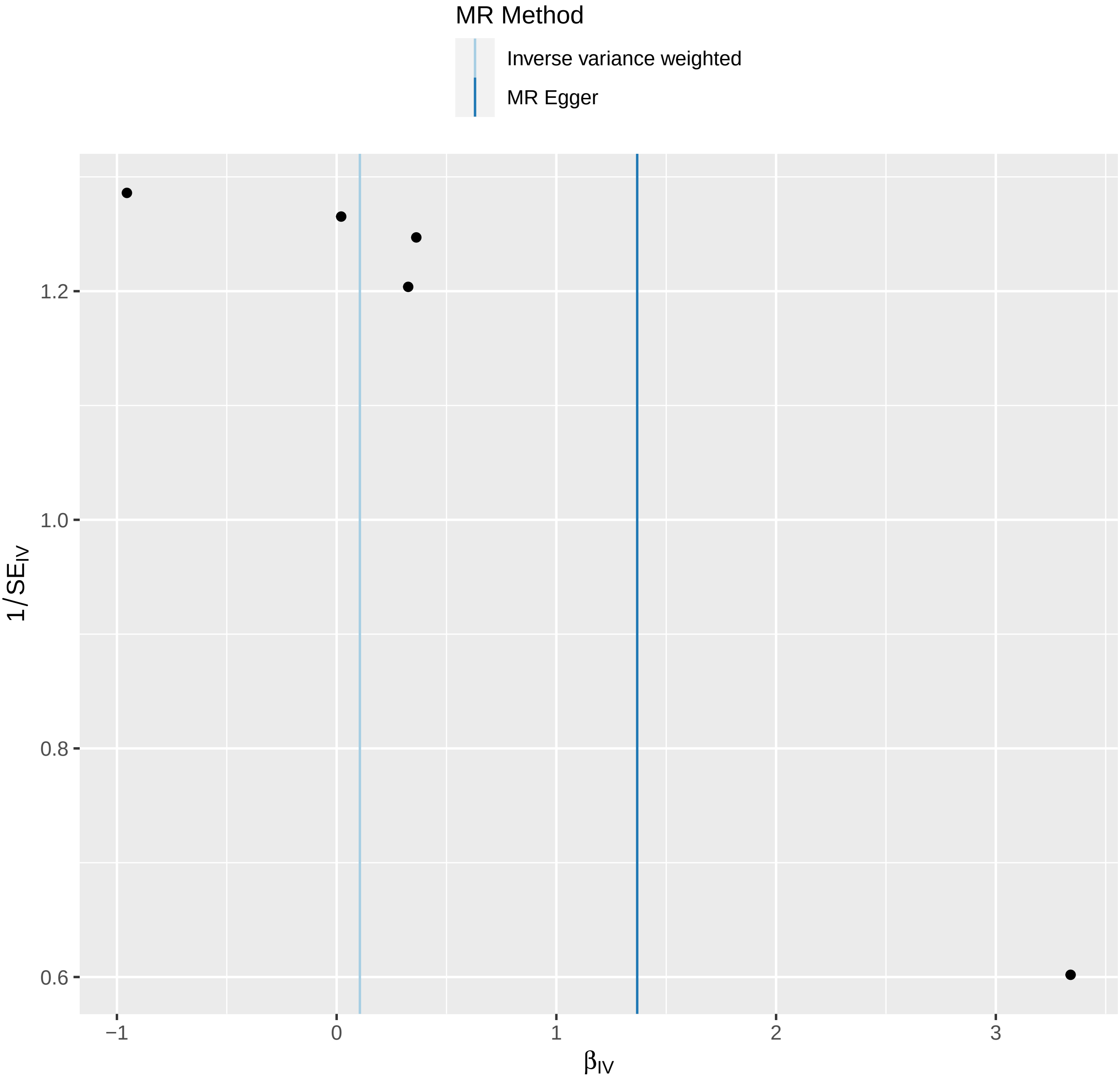

Batch 558 : Gut microbiota abundance (genus Odoribacter id.952) on Arthropathy in Crohn disease

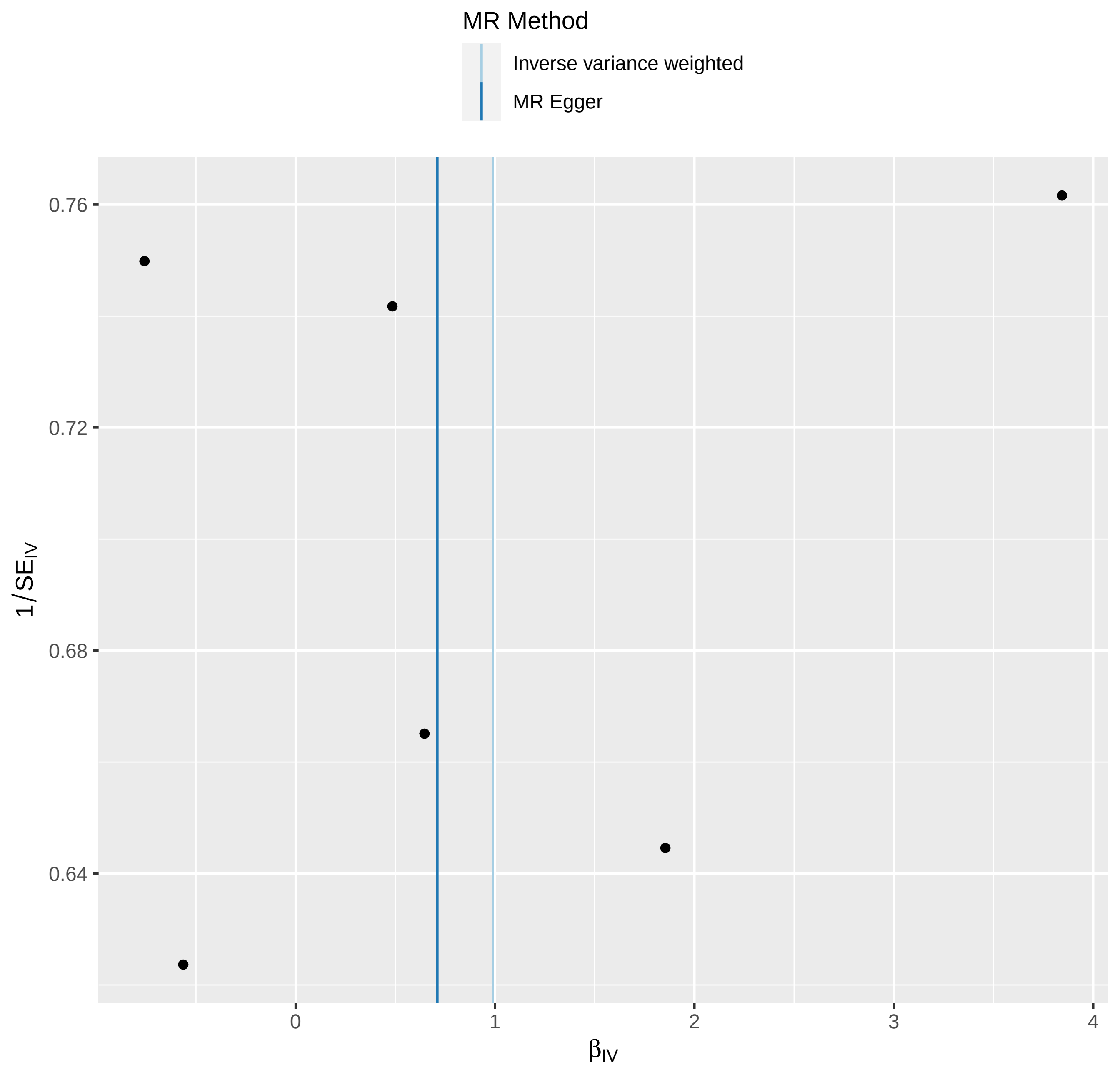

Batch 559 : Gut microbiota abundance (genus Olsenella id.822) on Arthropathy in Crohn disease

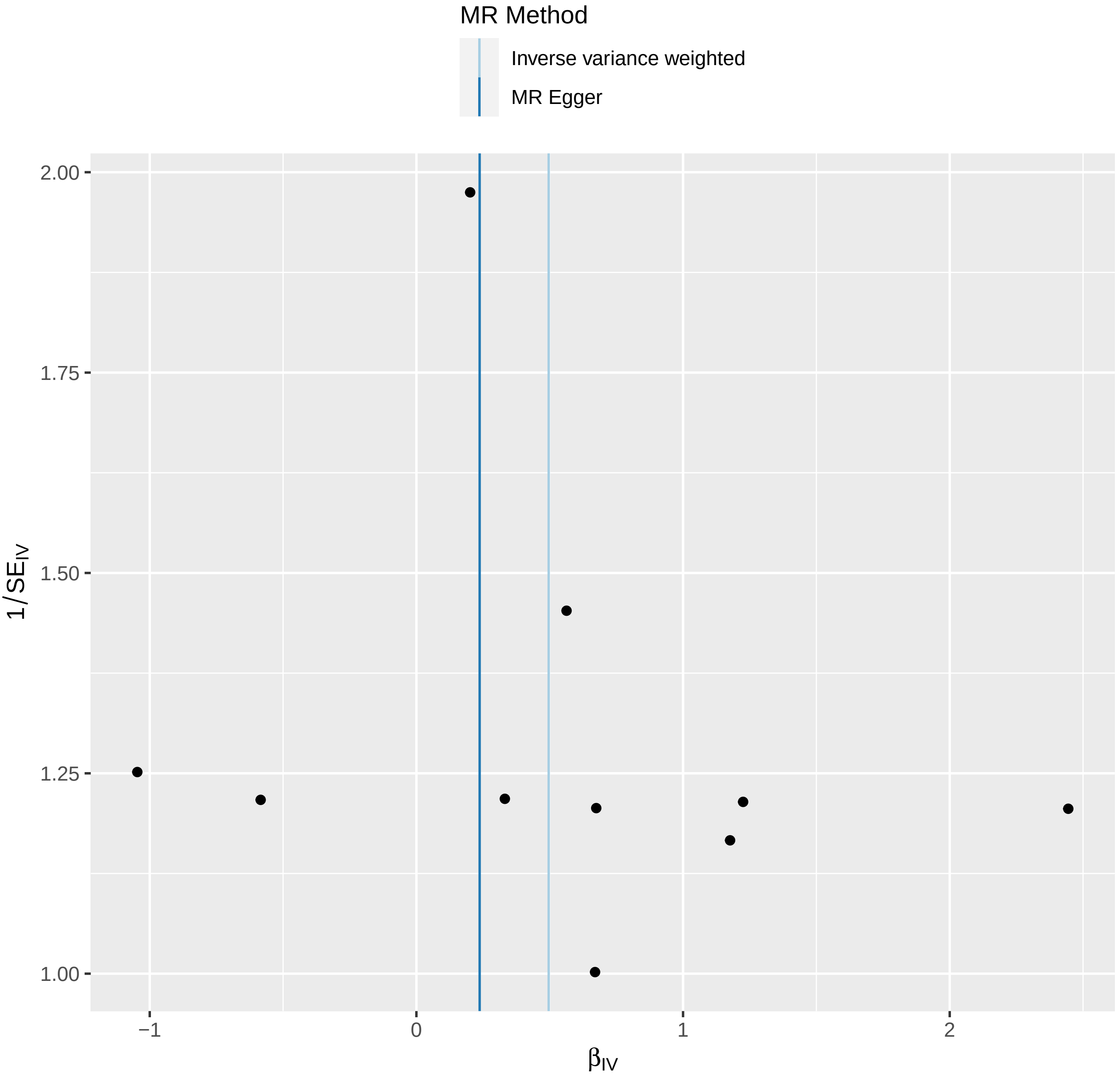

Batch 560 : Gut microbiota abundance (genus Oscillibacter id.2063) on Arthropathy in Crohn disease

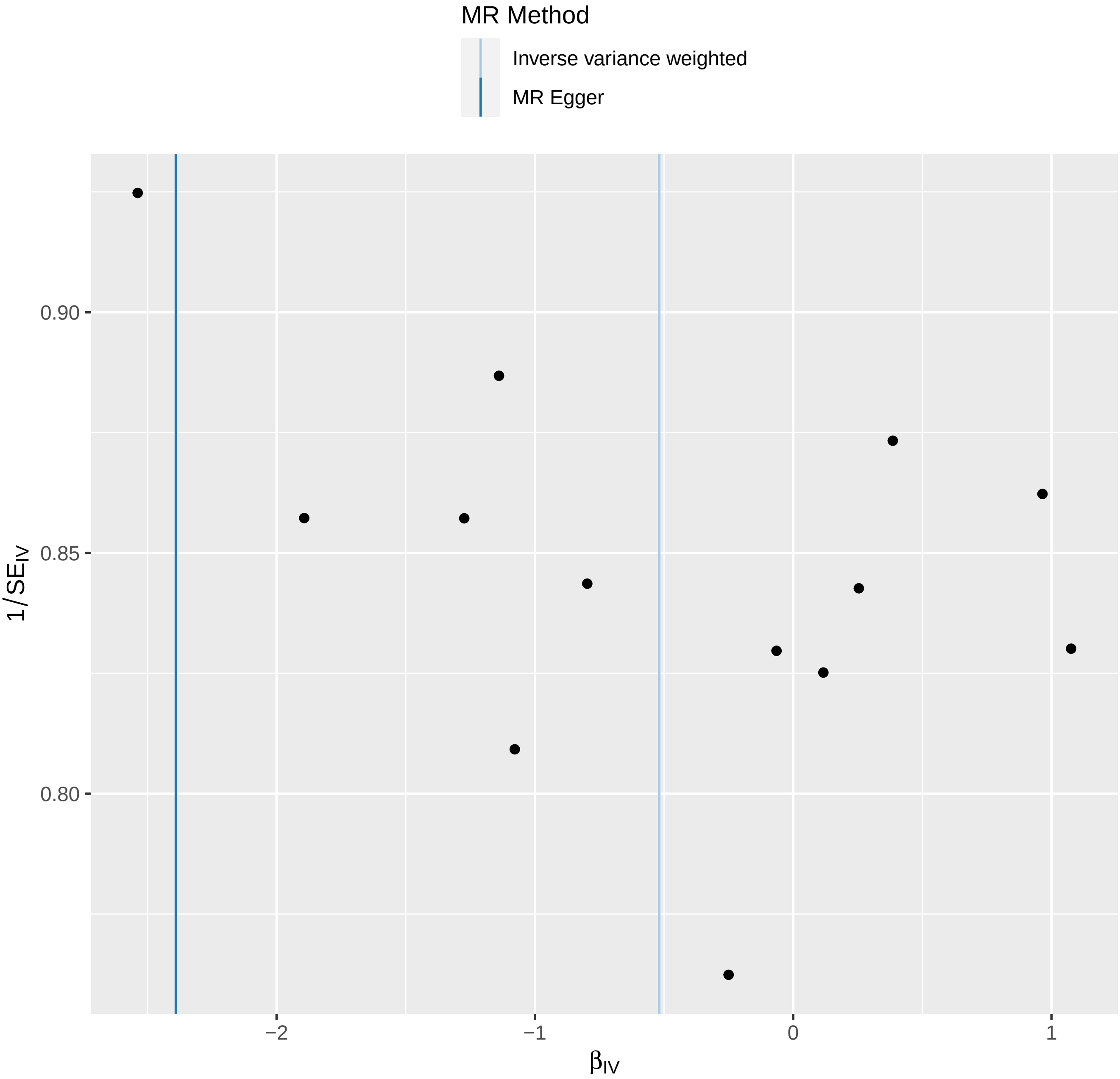

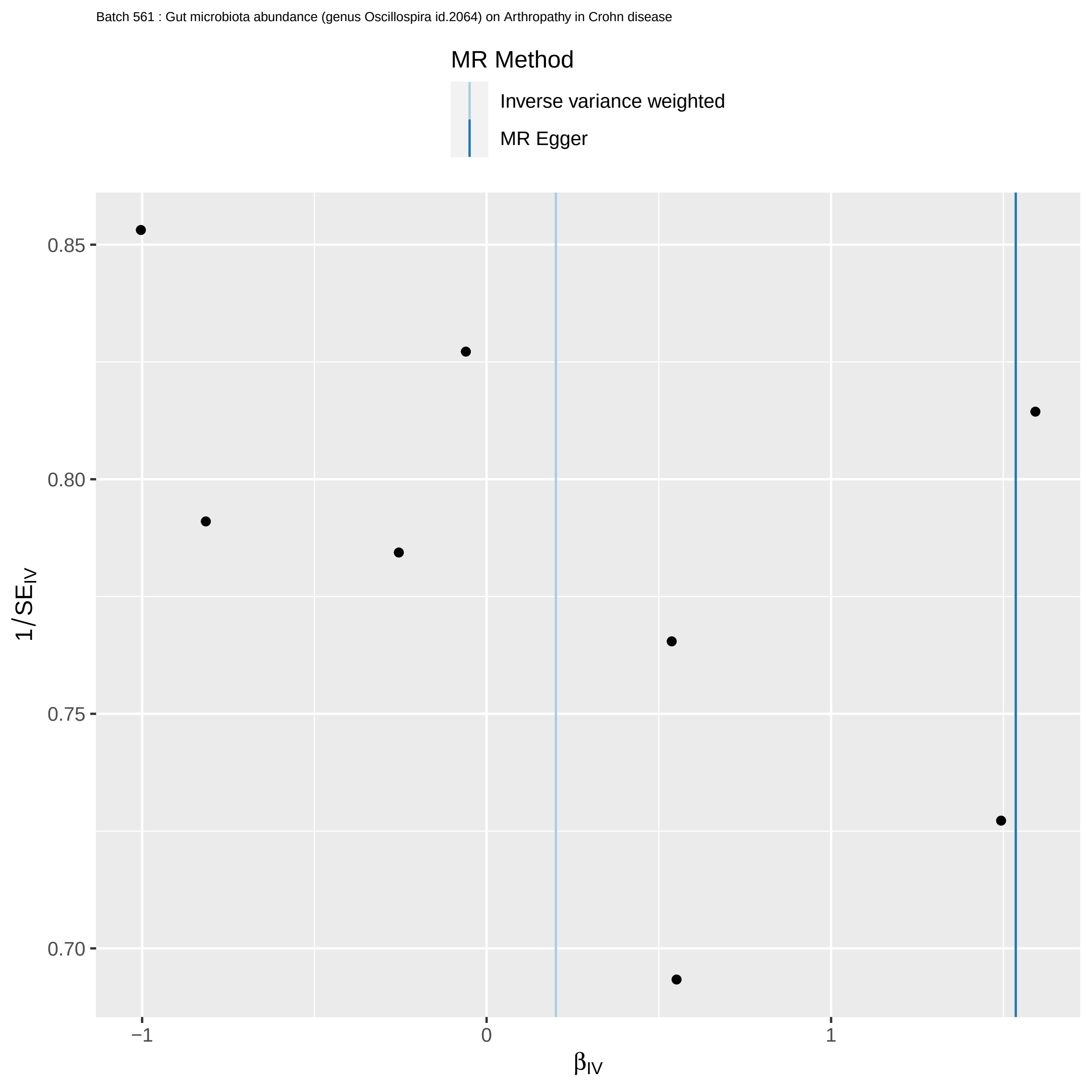
Batch 561 : Gut microbiota abundance (genus Oscillospira id.2064) on Arthropathy in Crohn disease
MR Method
Inverse variance weighted
MR Egger
0.85
0.80
0.75
0.70
1/SE_IV
−1
0
1
β_IV

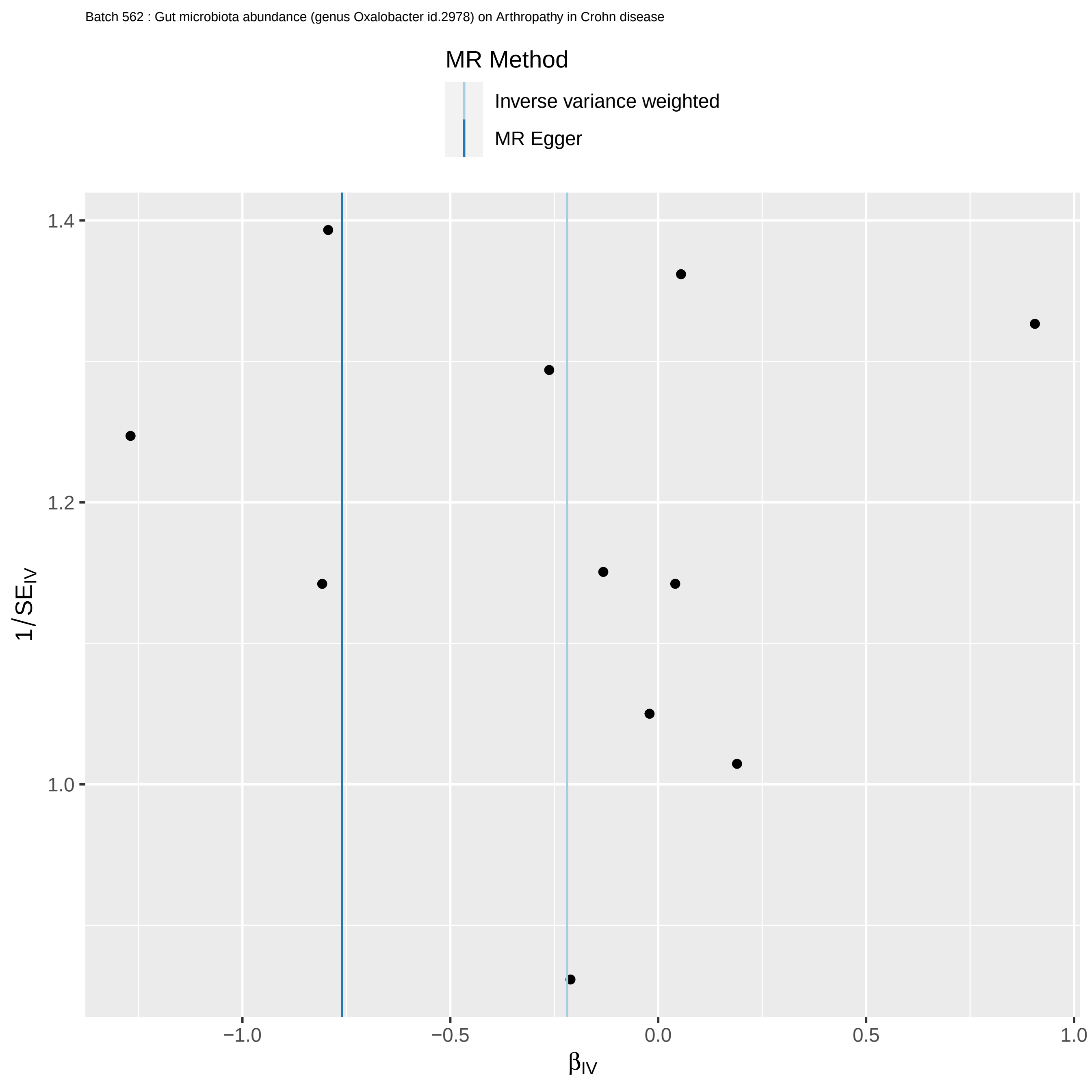
Batch 562 : Gut microbiota abundance (genus Oxalobacter id.2978) on Arthropathy in Crohn disease
MR Method
Inverse variance weighted
MR Egger
1.4
1.2
1.0
1/SE_IV
−1.0
−0.5
0.0
0.5
1.0
β_IV

Batch 563 : Gut microbiota abundance (genus Parabacteroides id.954) on Arthropathy in Crohn disease

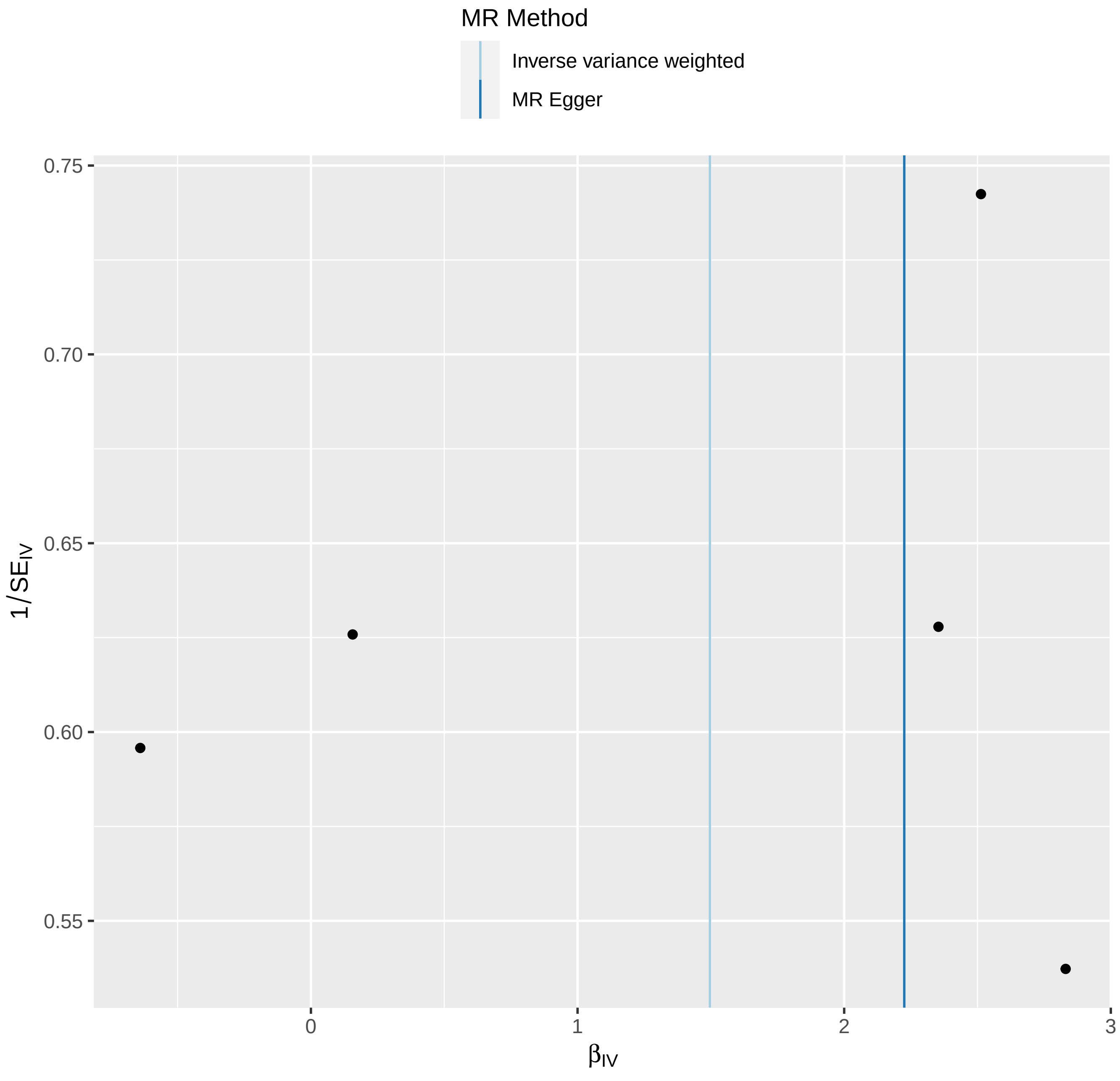

Batch 564 : Gut microbiota abundance (genus Paraprevotella id.962) on Arthropathy in Crohn disease

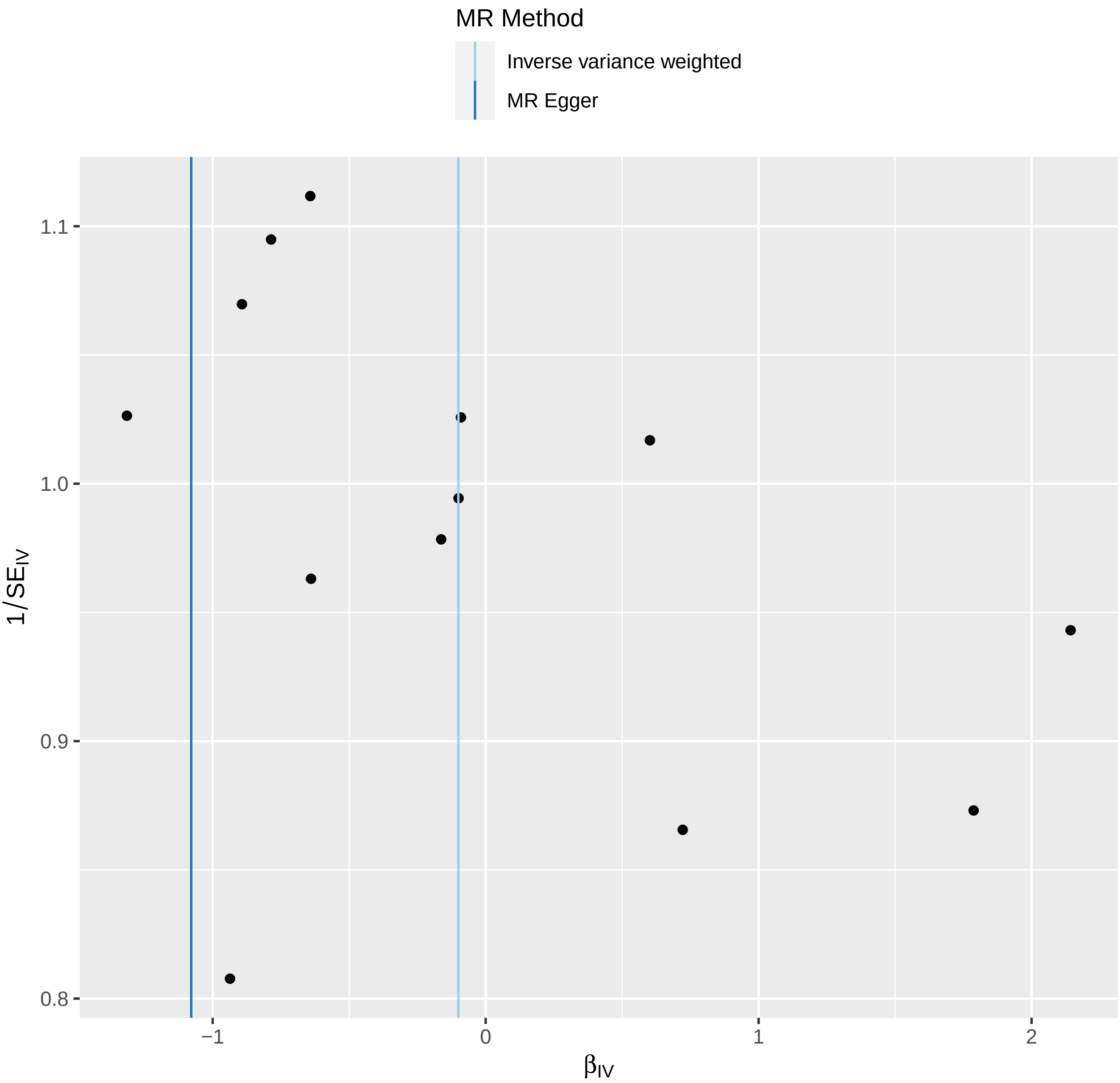

Batch 565 : Gut microbiota abundance (genus Parasutterella id.2892) on Arthropathy in Crohn disease

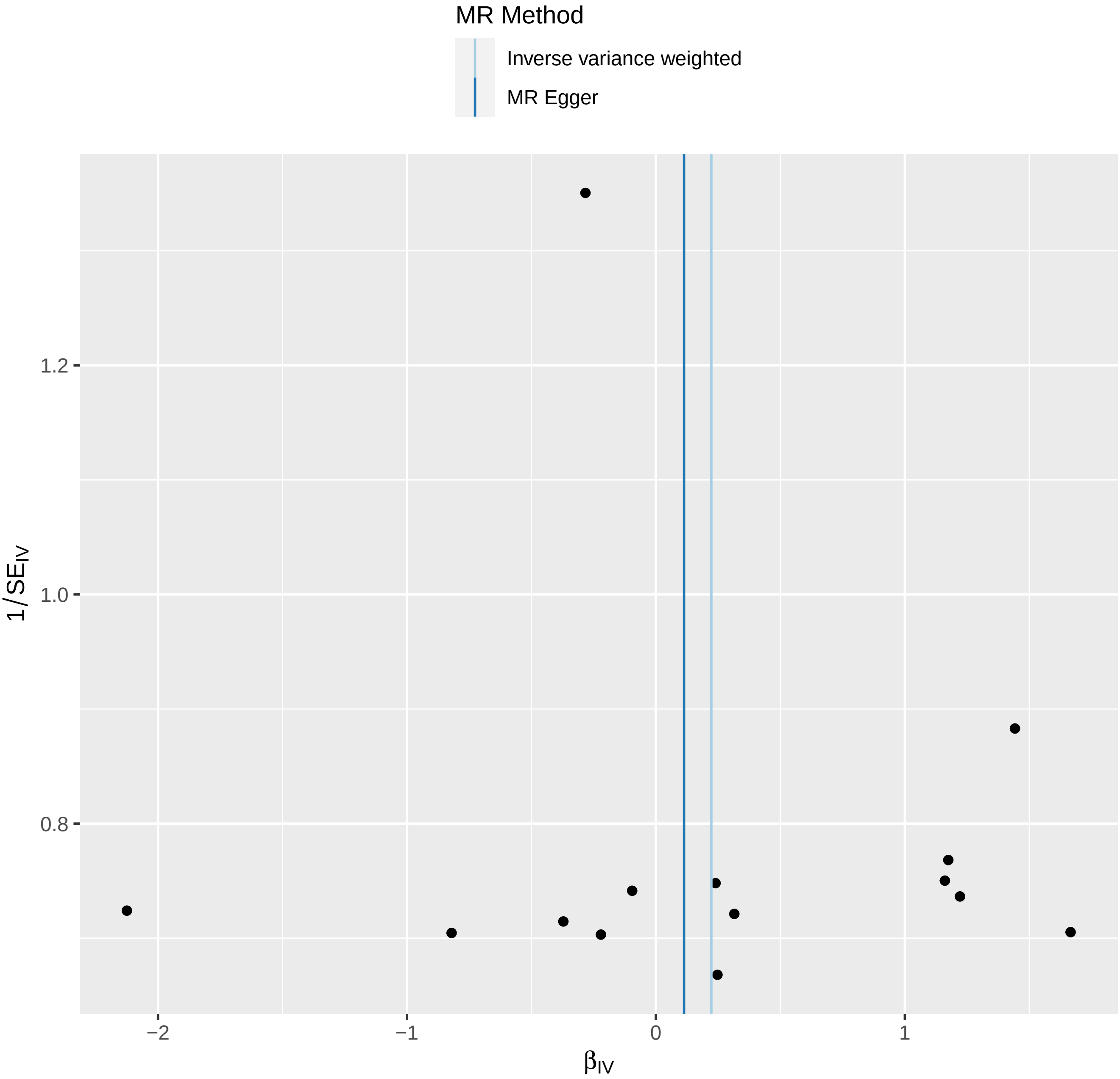

Batch 566 : Gut microbiota abundance (genus Peptococcus id.2037) on Arthropathy in Crohn disease

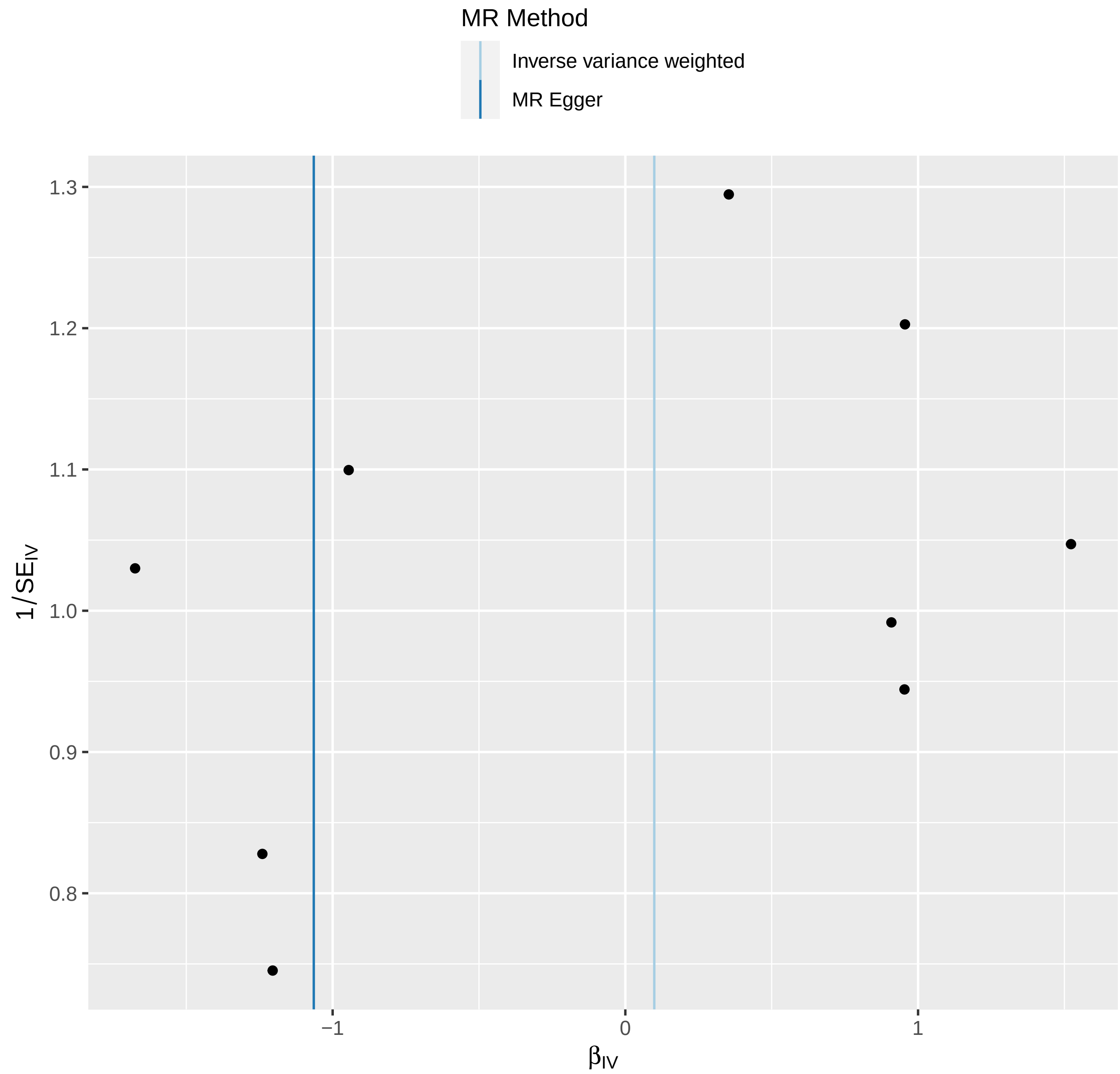

Batch 567 : Gut microbiota abundance (genus Phascolarctobacterium id.2168) on Arthropathy in Crohn disease

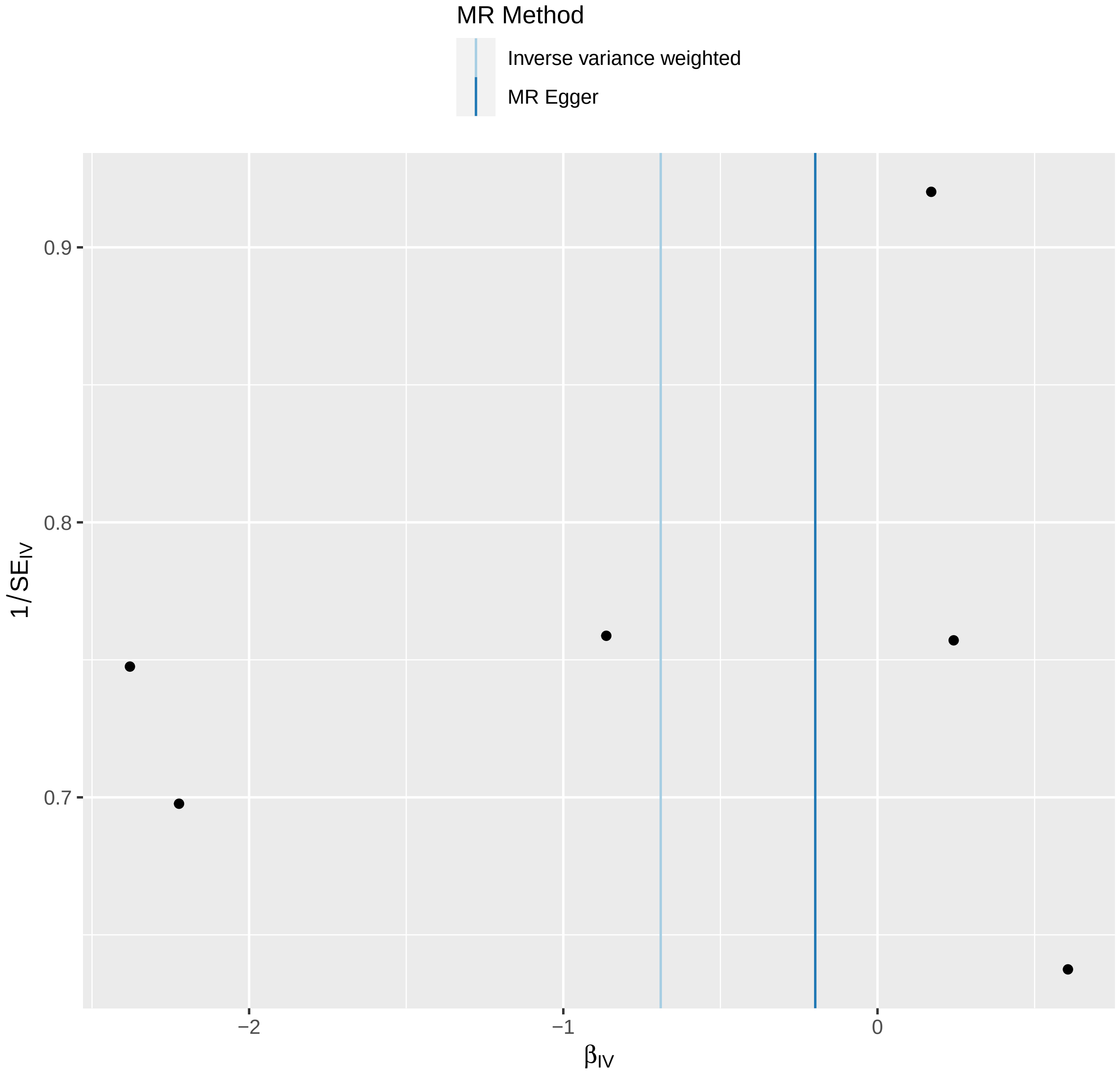

Batch 568 : Gut microbiota abundance (genus Prevotella7 id.11182) on Arthropathy in Crohn disease

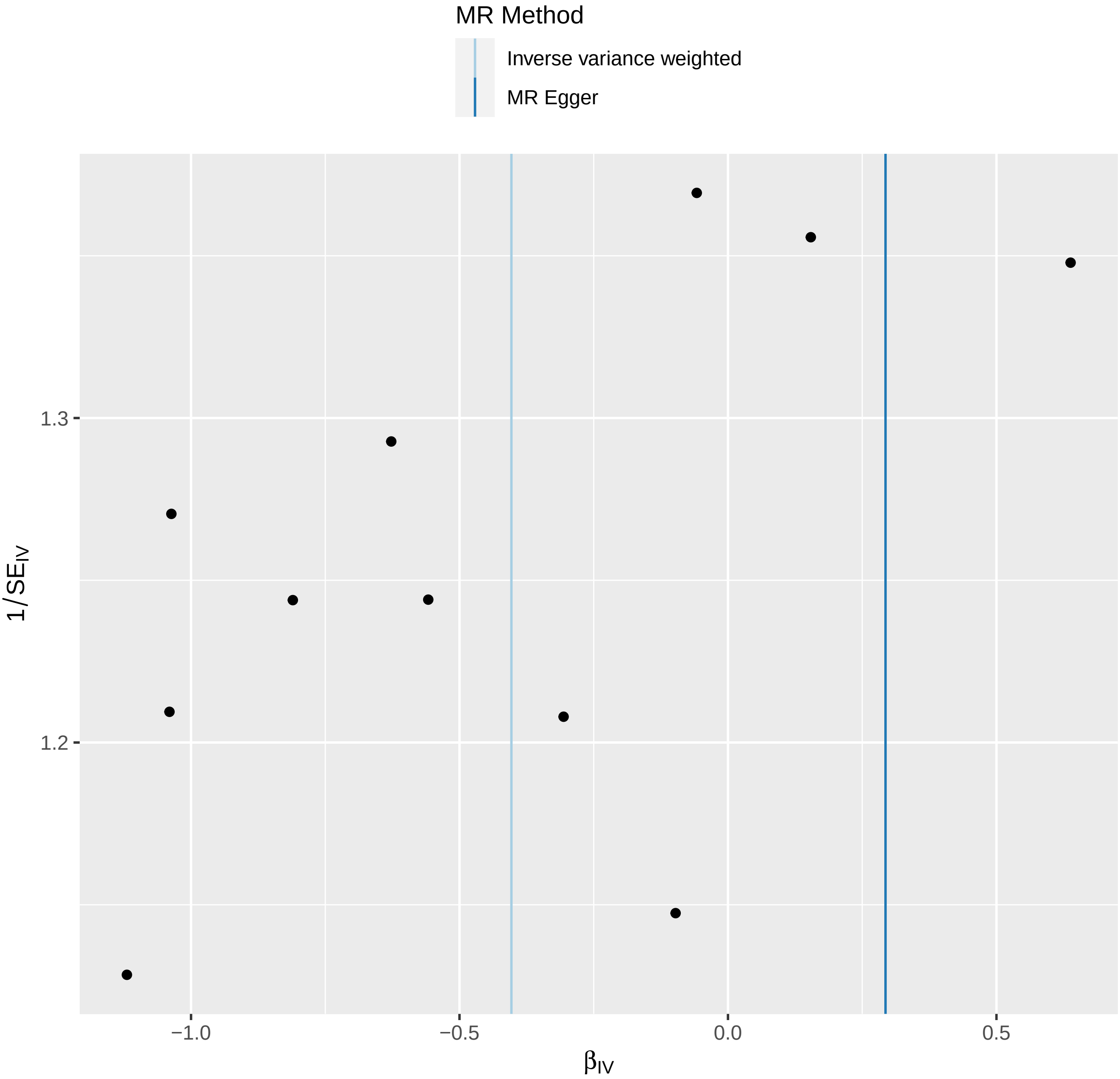

Batch 569 : Gut microbiota abundance (genus Prevotella9 id.11183) on Arthropathy in Crohn disease

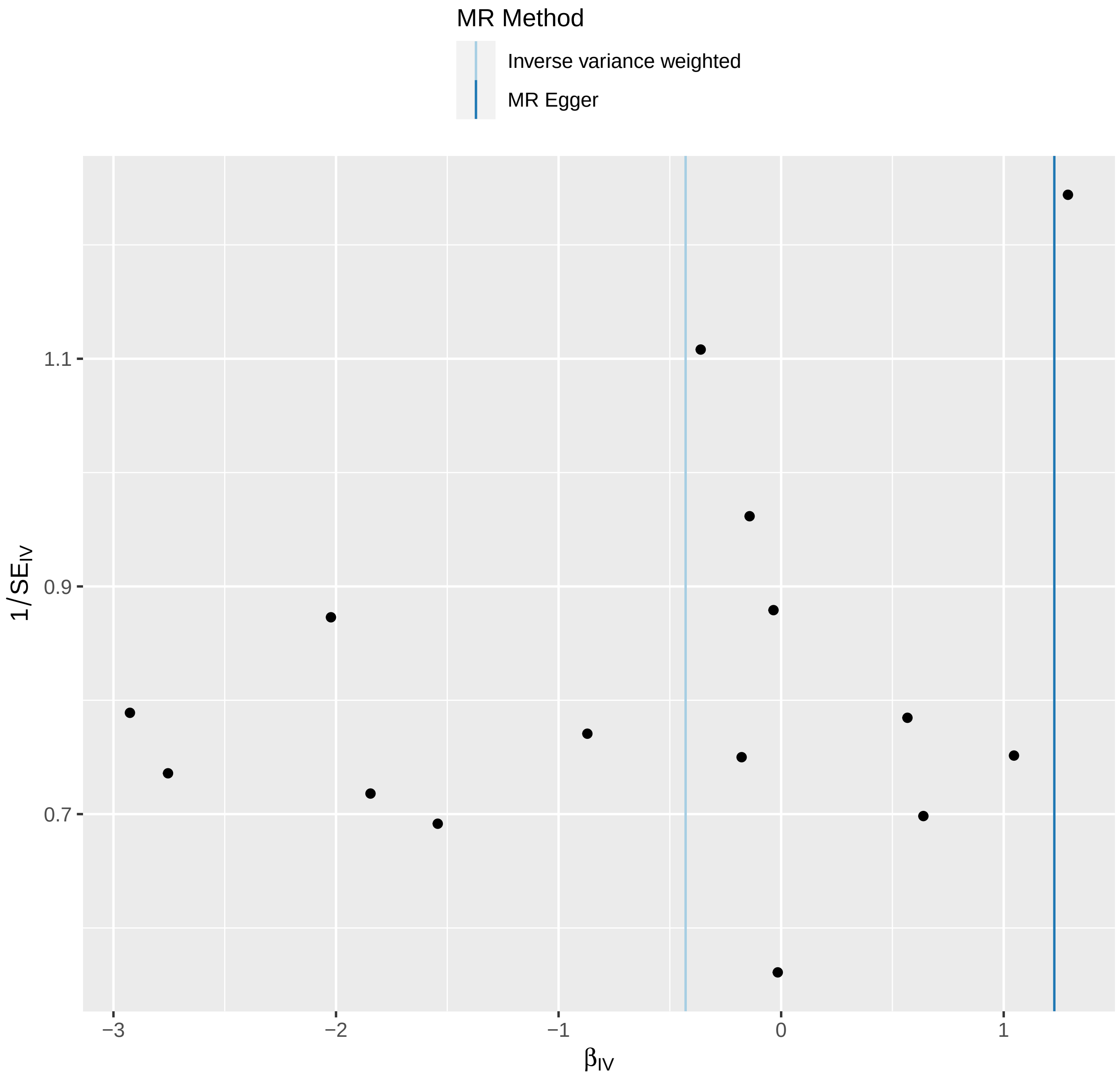

Batch 570 : Gut microbiota abundance (genus Rikenellaceae RC9 gut group id.11191) on Arthropathy in Crohn disease

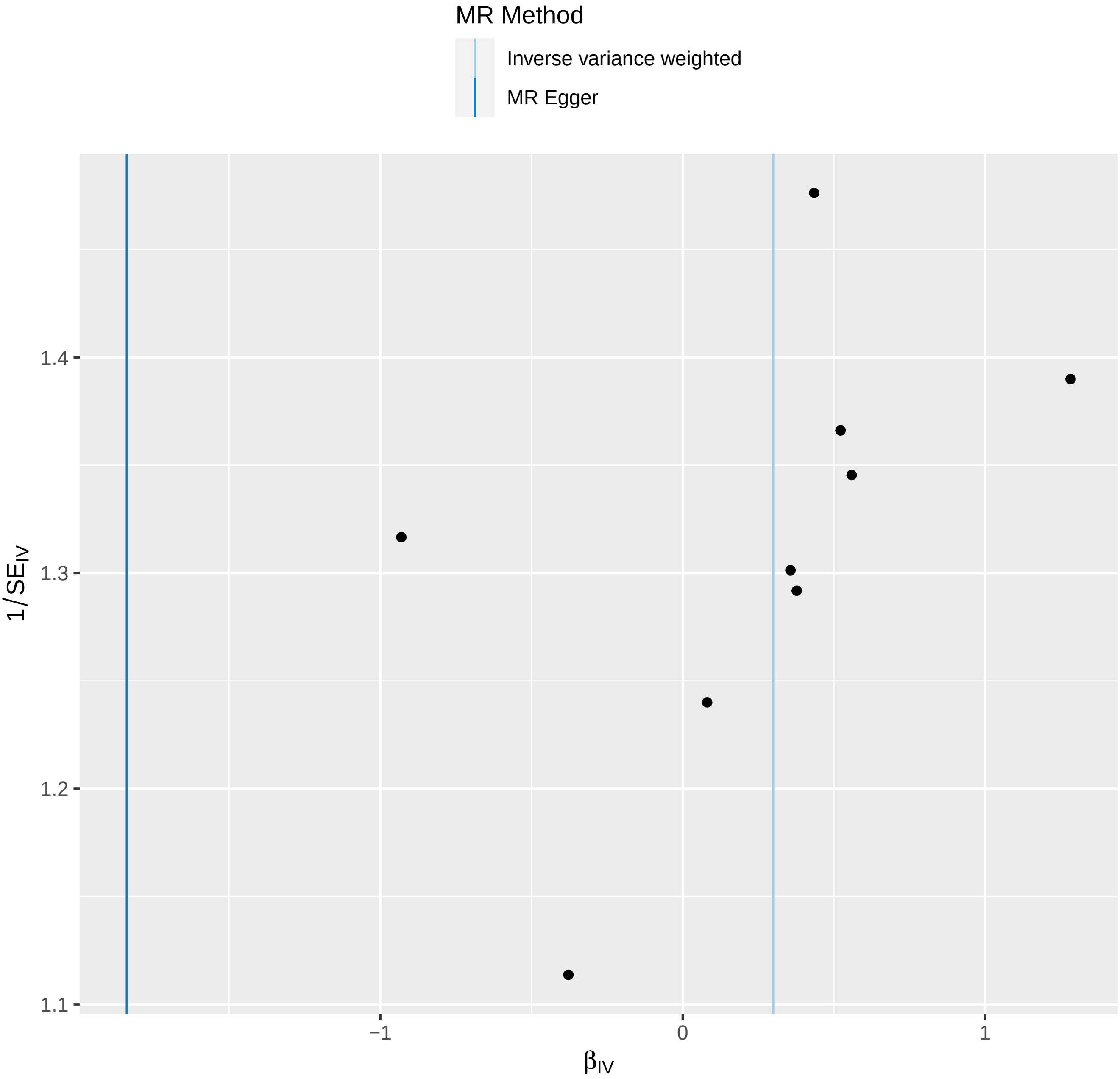

Batch 571 : Gut microbiota abundance (genus Romboutsia id.11347) on Arthropathy in Crohn disease

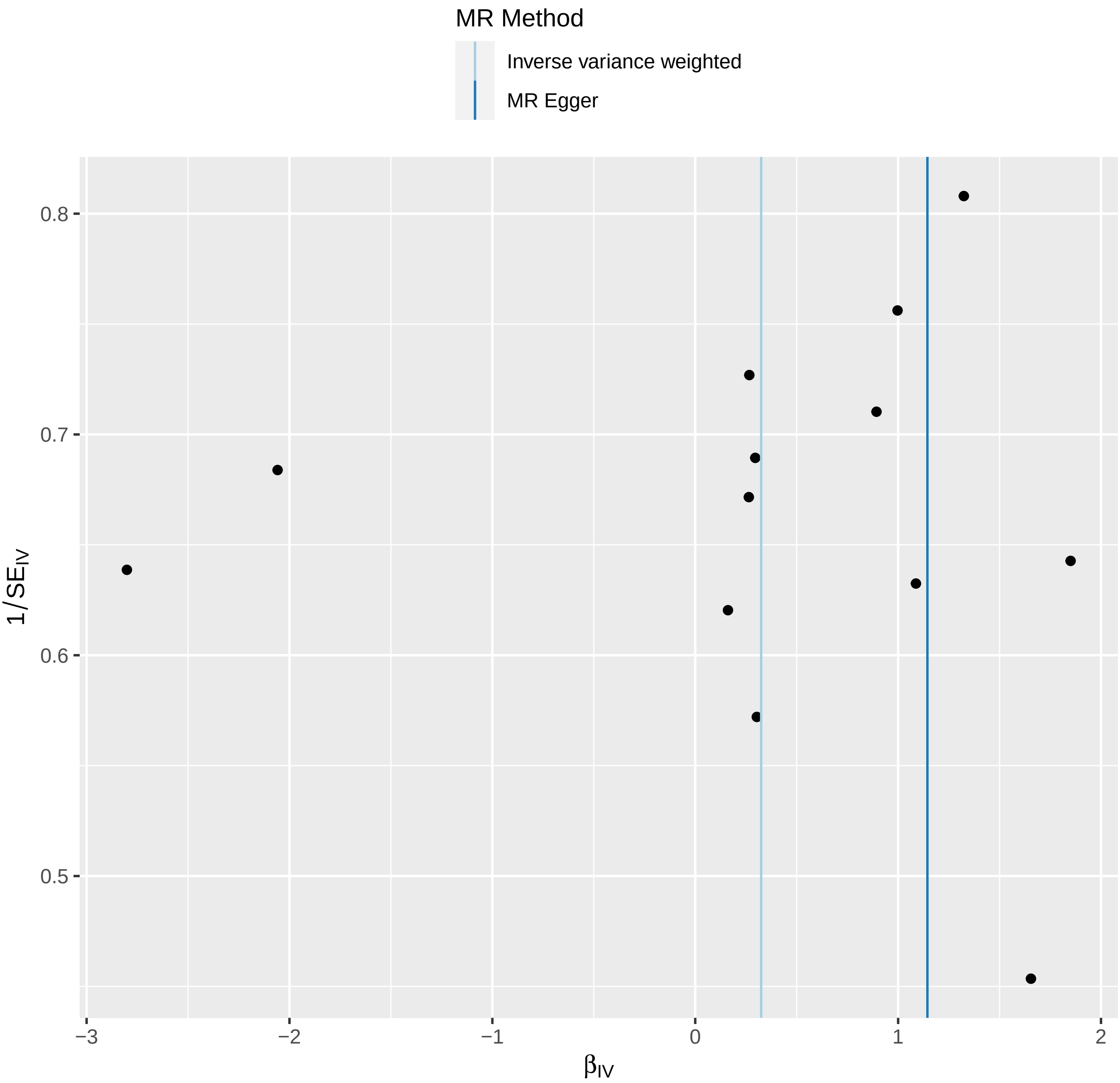

Batch 572 : Gut microbiota abundance (genus Roseburia id.2012) on Arthropathy in Crohn disease

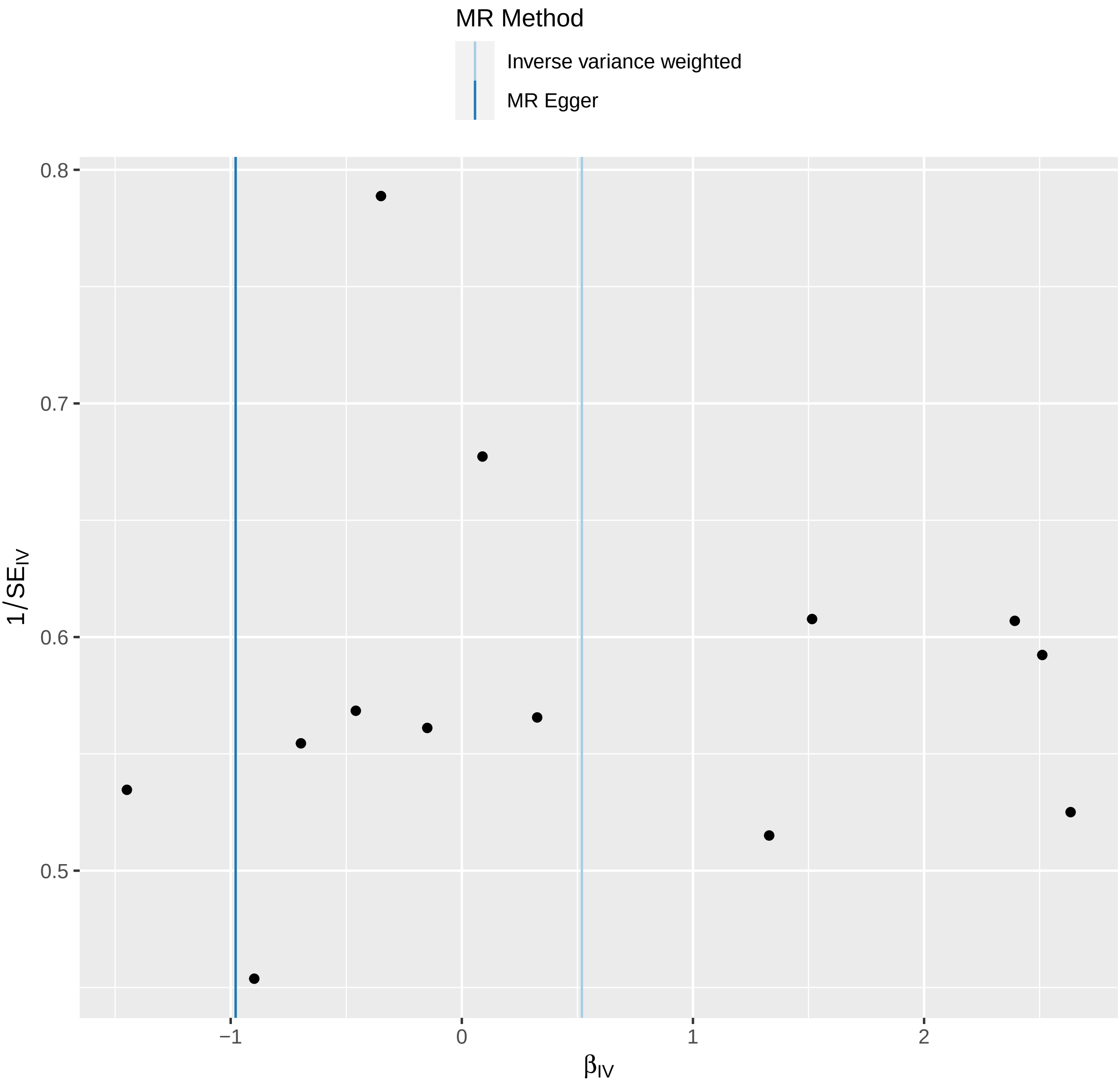

Batch 573 : Gut microbiota abundance (genus Ruminiclostridium5 id.11355) on Arthropathy in Crohn disease

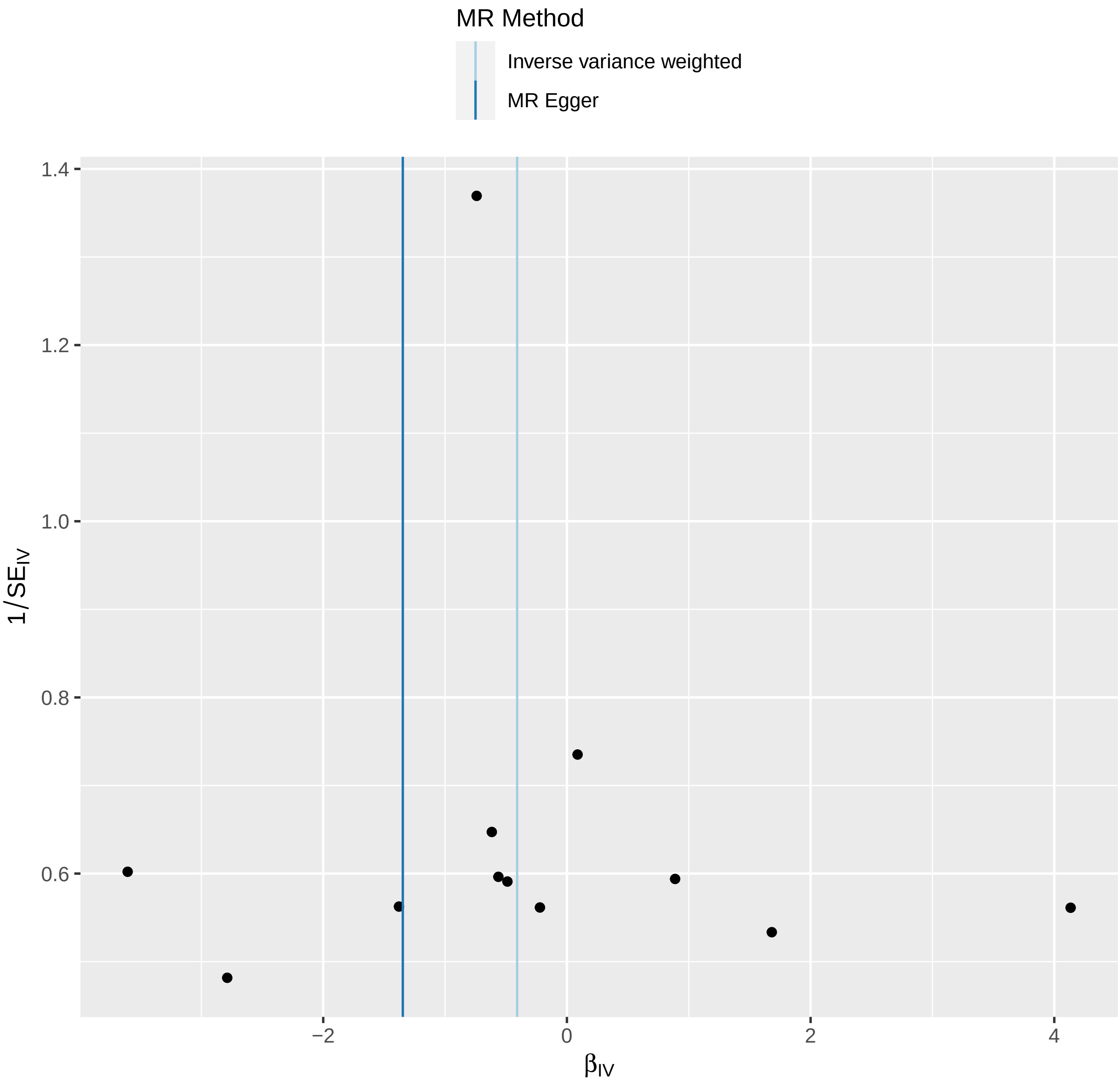

Batch 574 : Gut microbiota abundance (genus Ruminiclostridium6 id.11356) on Arthropathy in Crohn disease

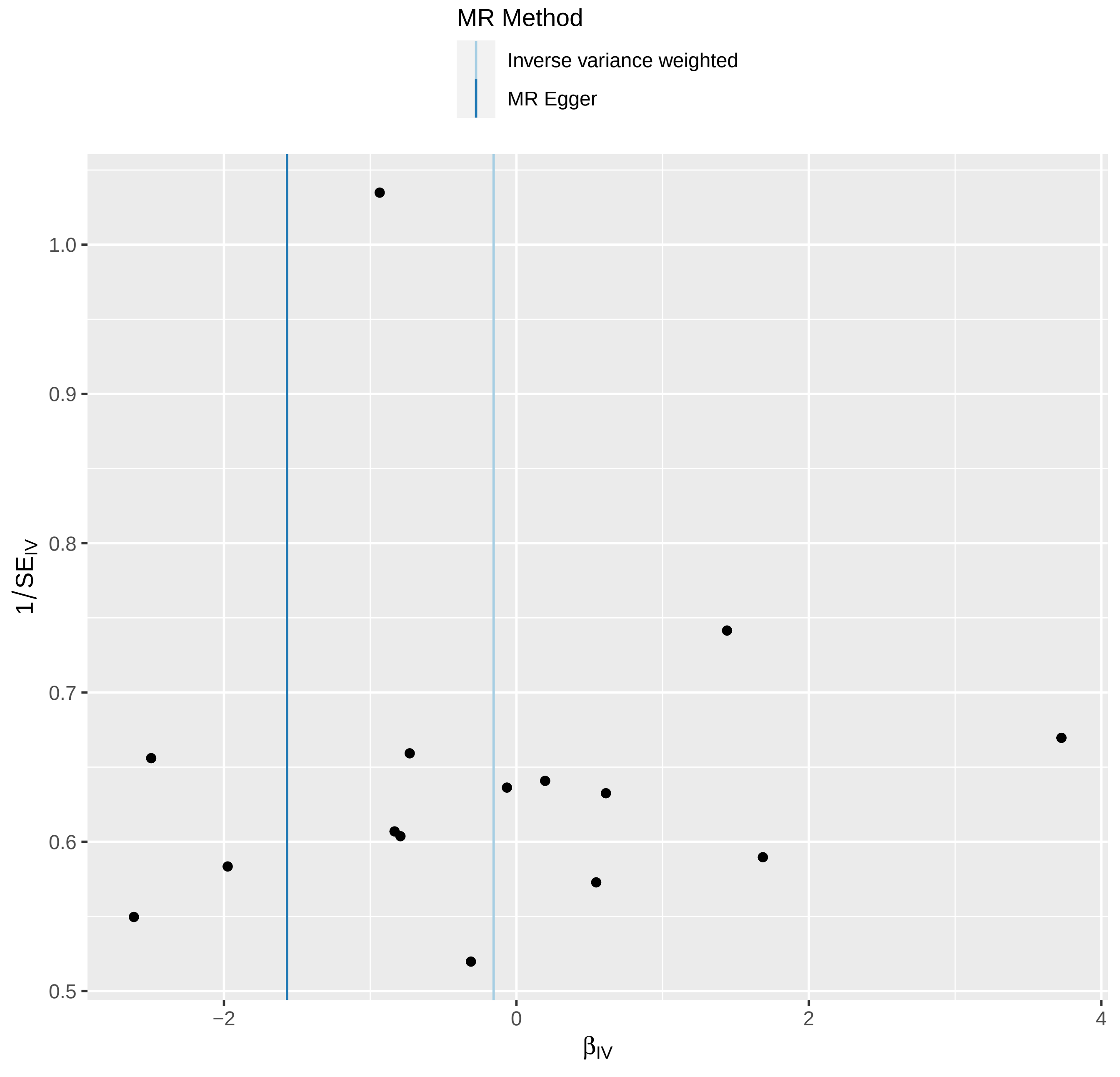

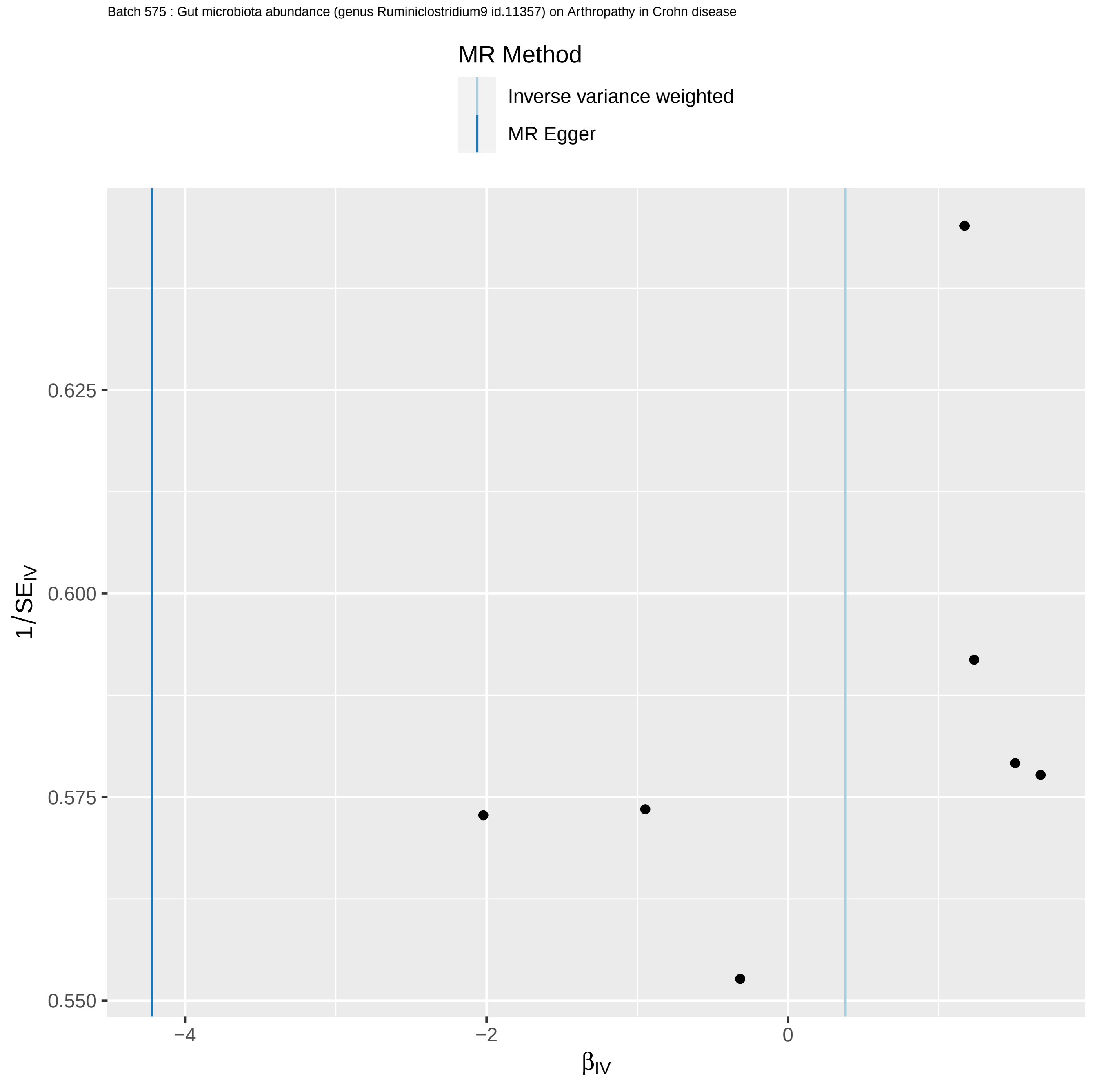

Batch 575 : Gut microbiota abundance (genus Ruminiclostridium9 id.11357) on Arthropathy in Crohn disease
MR Method
Inverse variance weighted
MR Egger
0.625
0.600
0.575
0.550
1/SE_IV
−4
−2
0
β_IV

Batch 576 : Gut microbiota abundance (genus Ruminococcaceae NK4A214 group id.11358) on Arthropathy in Crohn disease

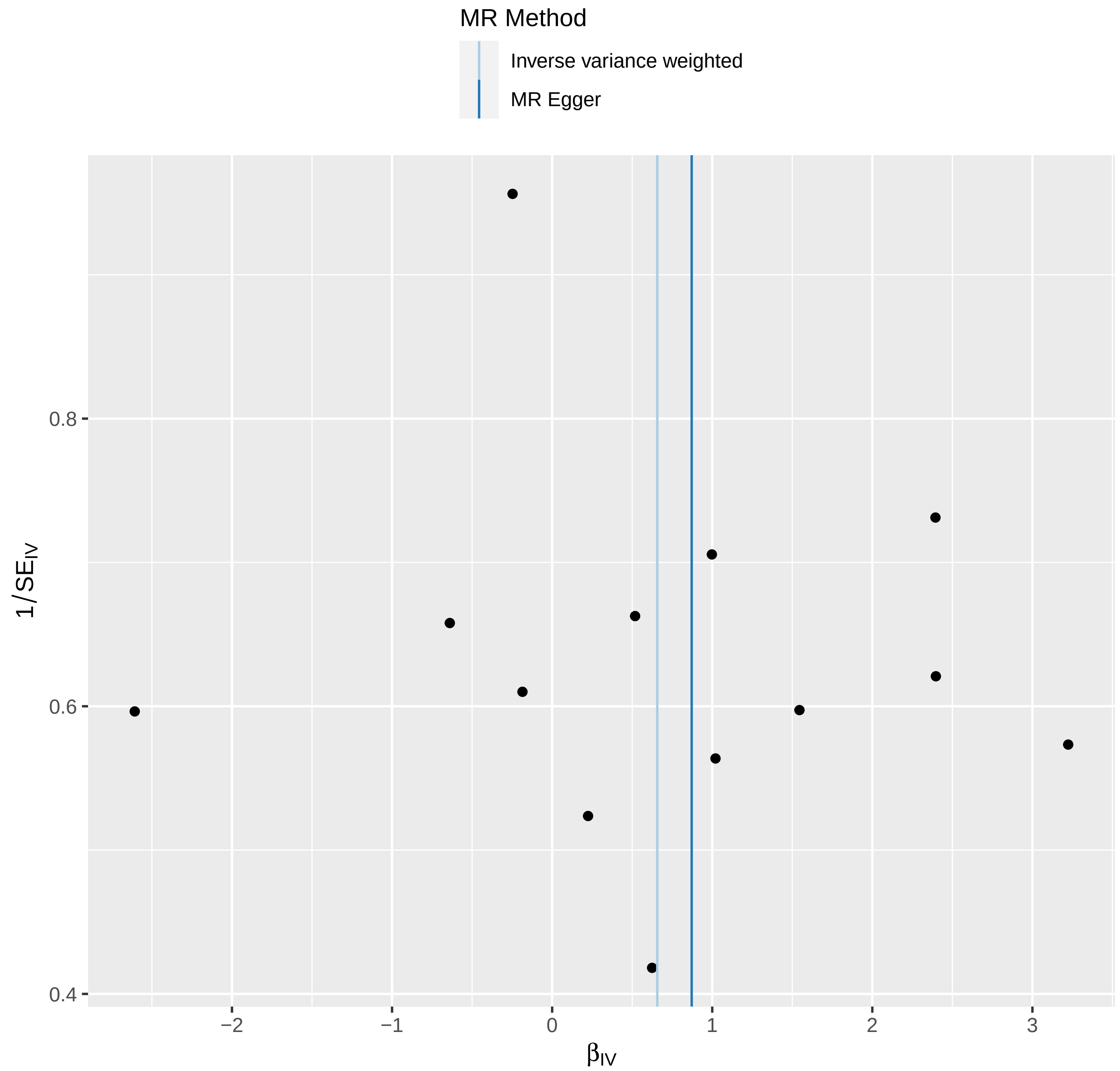

Batch 577 : Gut microbiota abundance (genus Ruminococcaceae UCG002 id.11360) on Arthropathy in Crohn disease

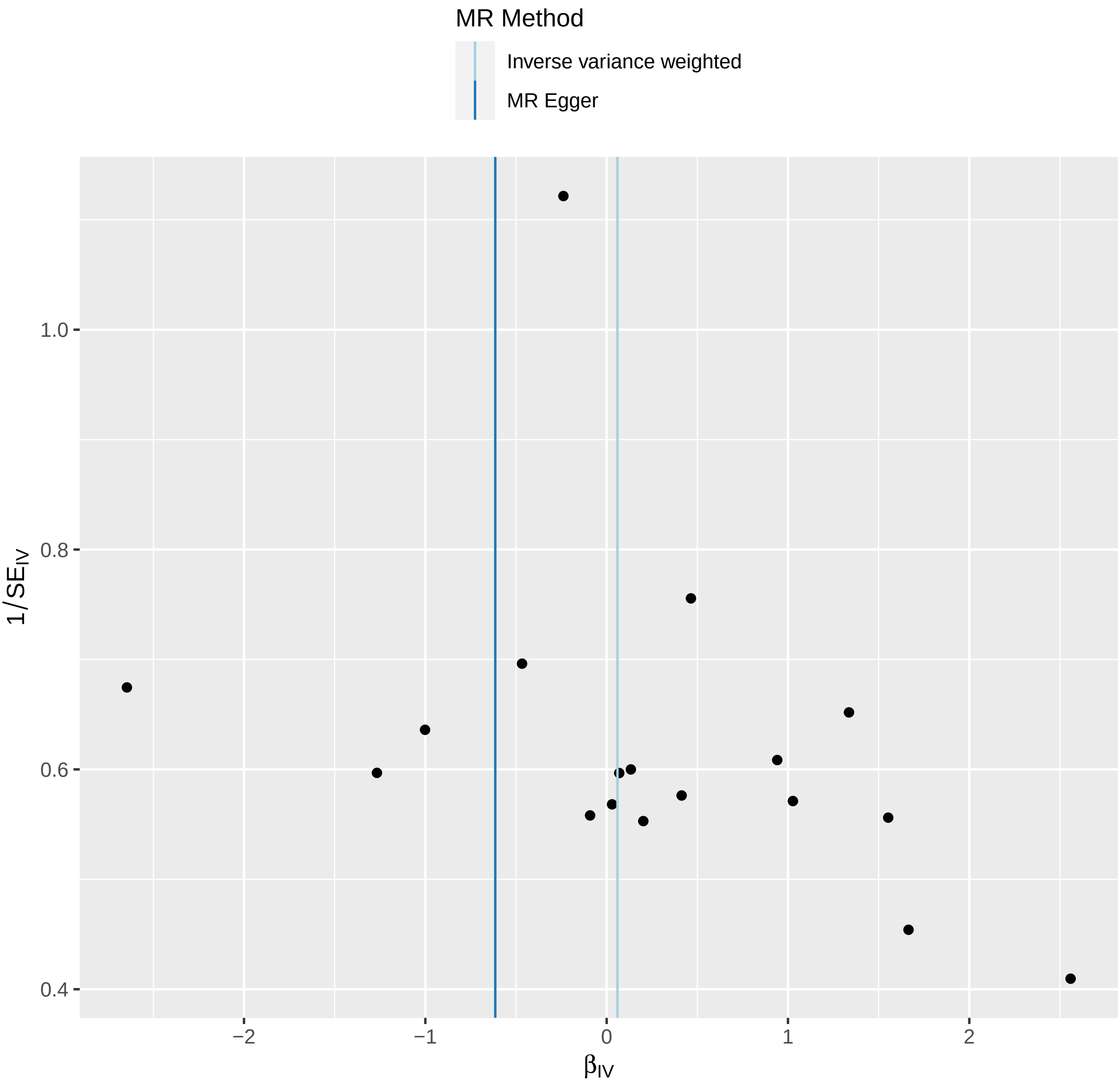

Batch 578 : Gut microbiota abundance (genus Ruminococcaceae UCG003 id.11361) on Arthropathy in Crohn disease

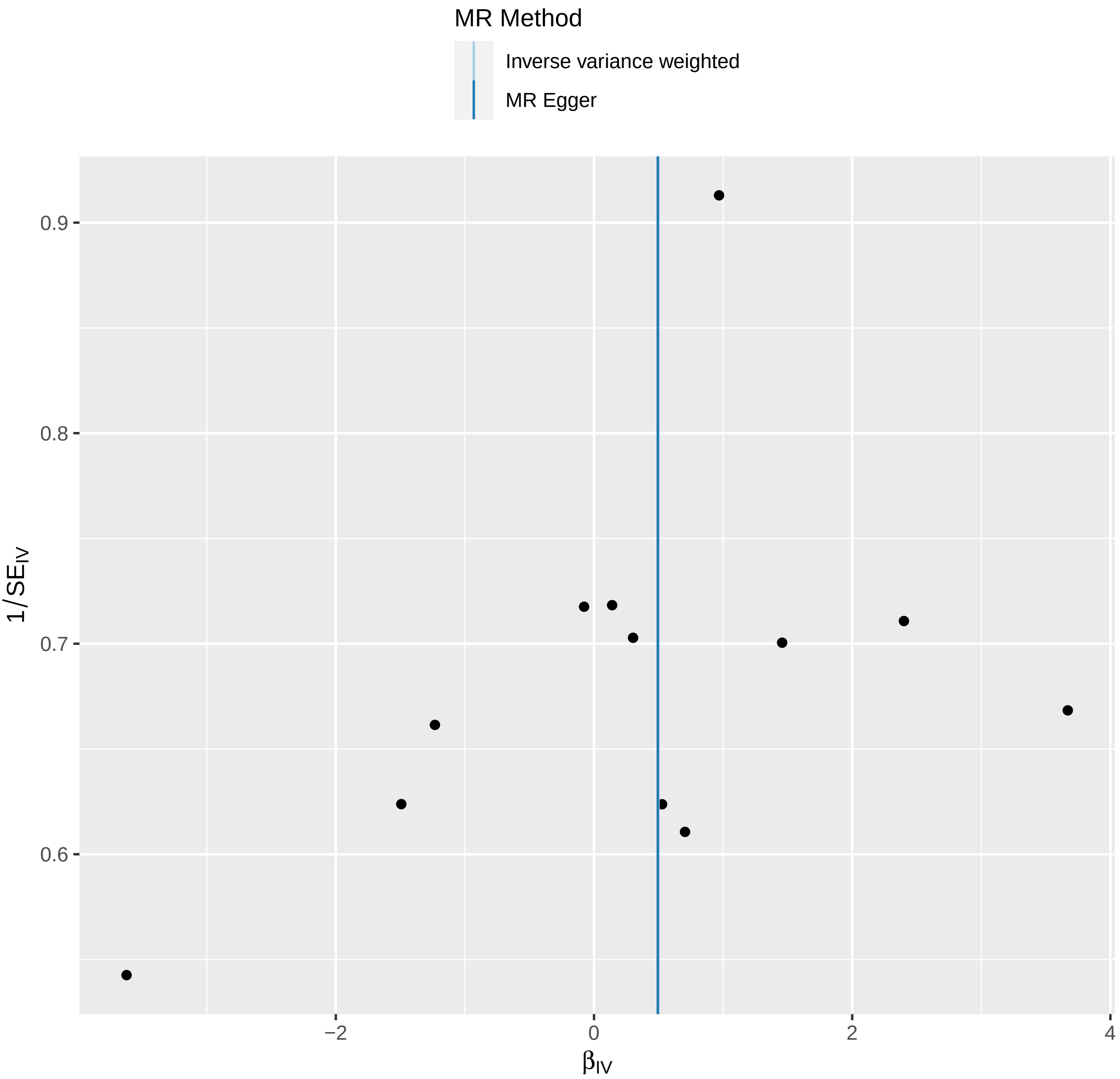

Batch 579 : Gut microbiota abundance (genus Ruminococcaceae UCG004 id.11362) on Arthropathy in Crohn disease

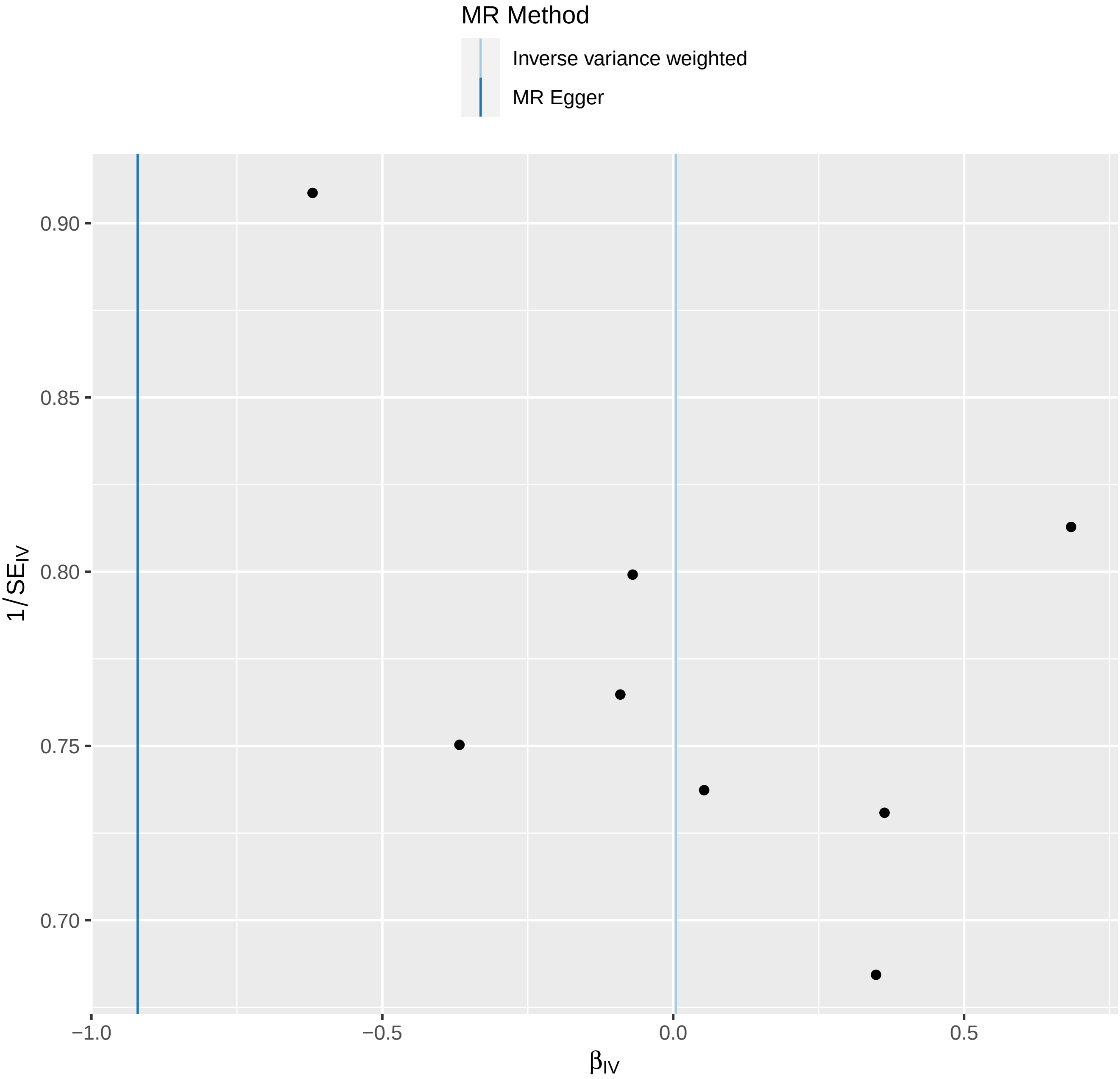

Batch 580 : Gut microbiota abundance (genus Ruminococcaceae UCG005 id.11363) on Arthropathy in Crohn disease

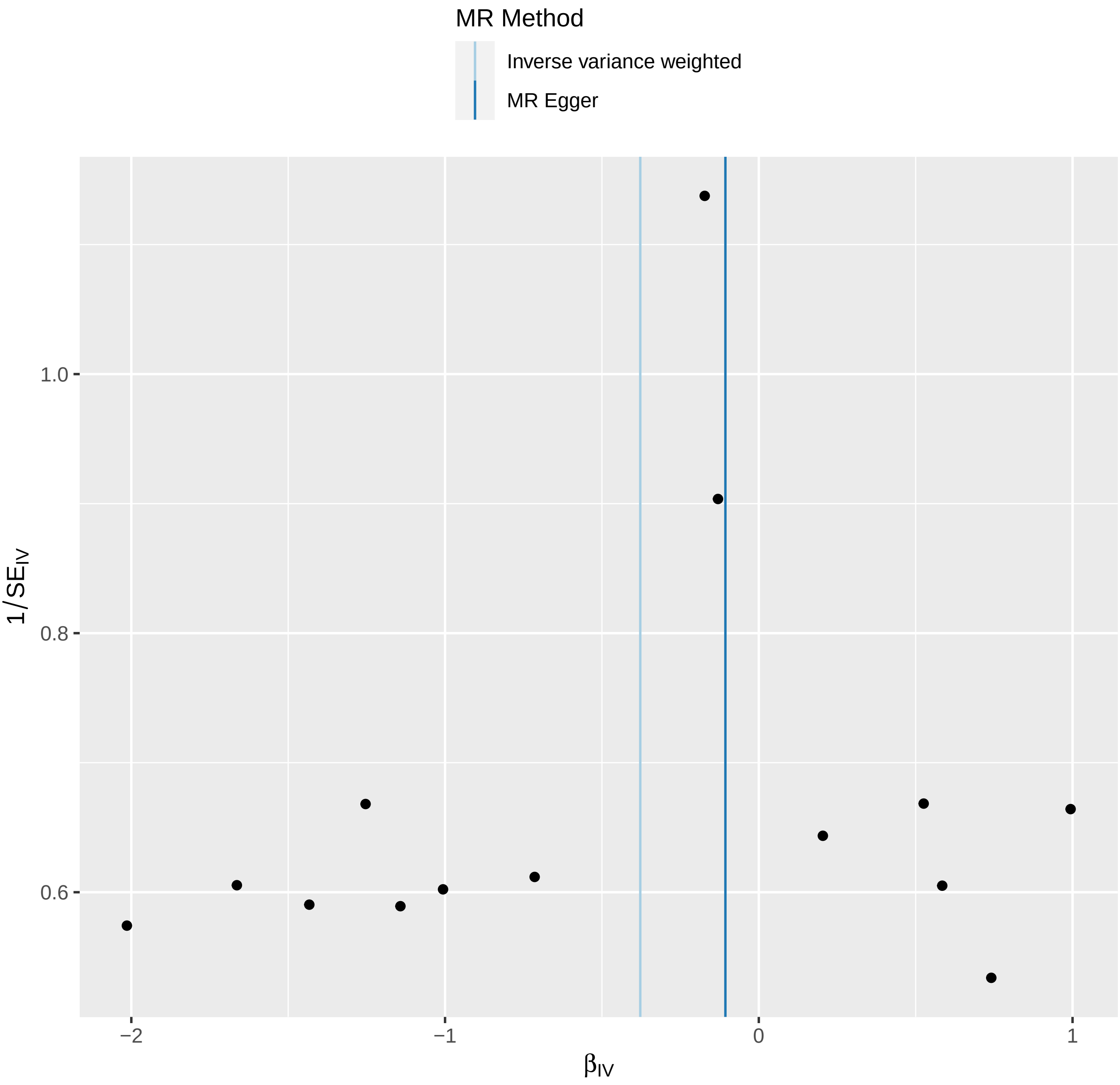

Batch 581 : Gut microbiota abundance (genus Ruminococcaceae UCG009 id.11366) on Arthropathy in Crohn disease

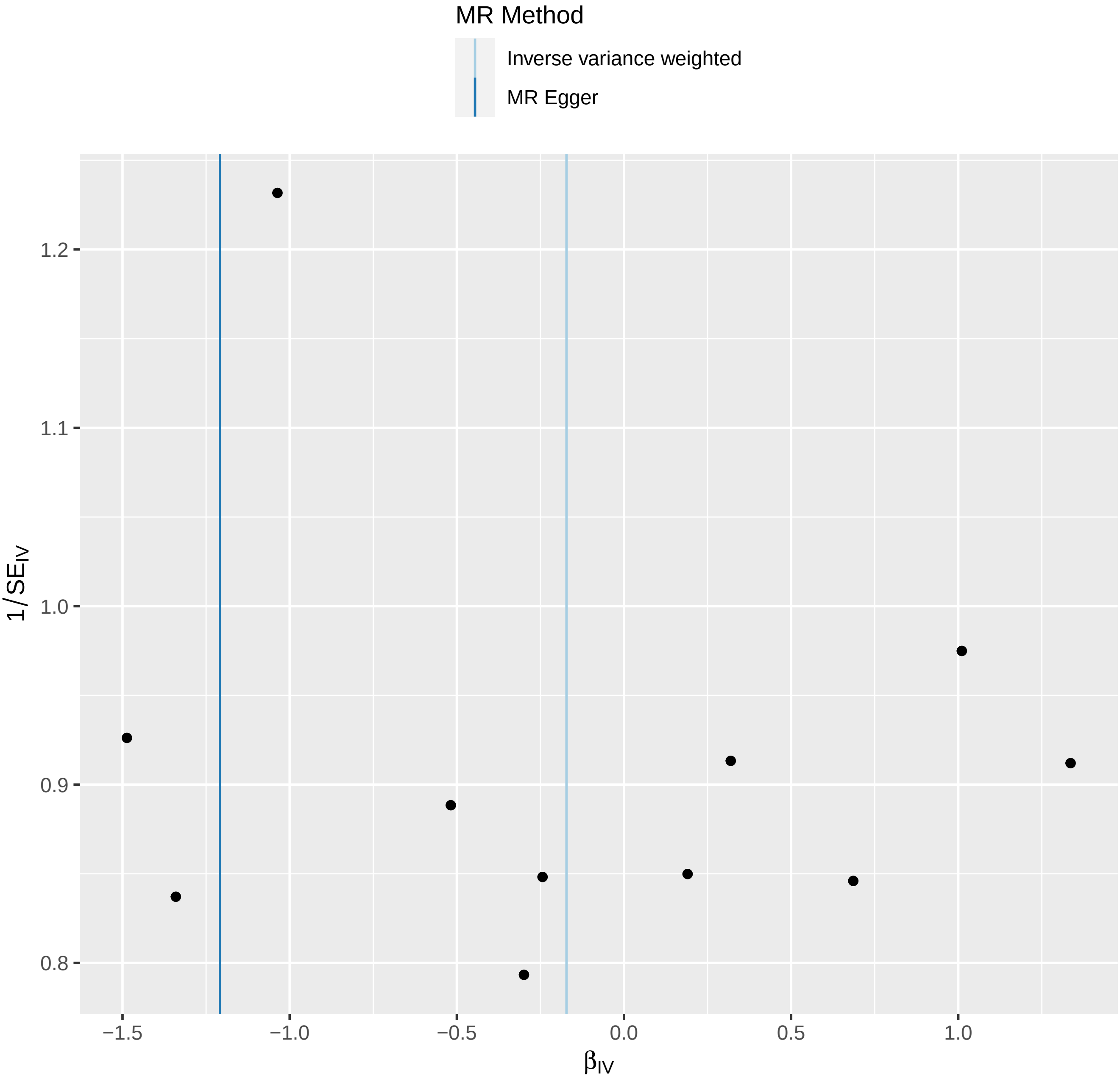

Batch 582 : Gut microbiota abundance (genus Ruminococcaceae UCG010 id.11367) on Arthropathy in Crohn disease

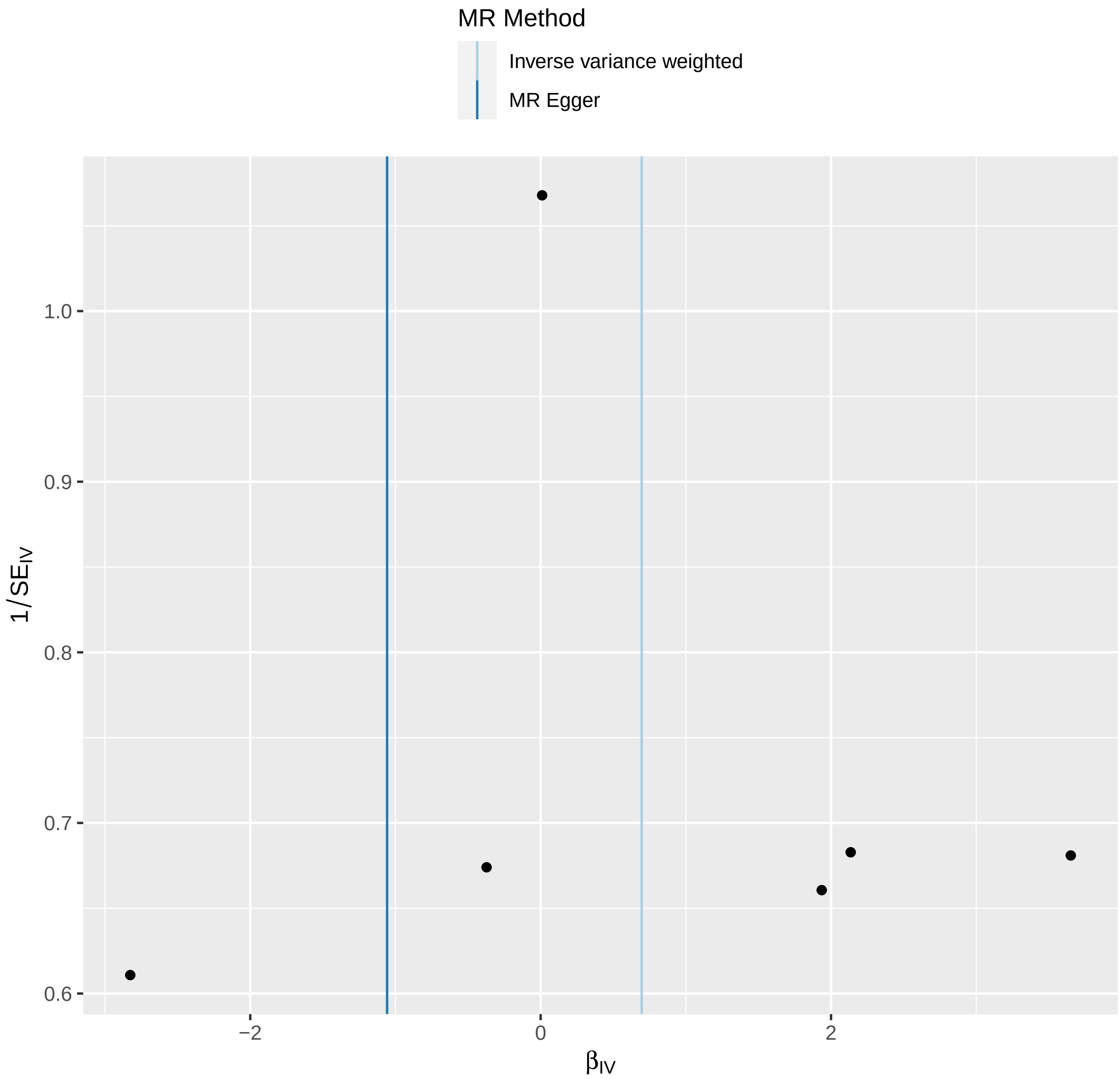

Batch 583 : Gut microbiota abundance (genus Ruminococcaceae UCG011 id.11368) on Arthropathy in Crohn disease

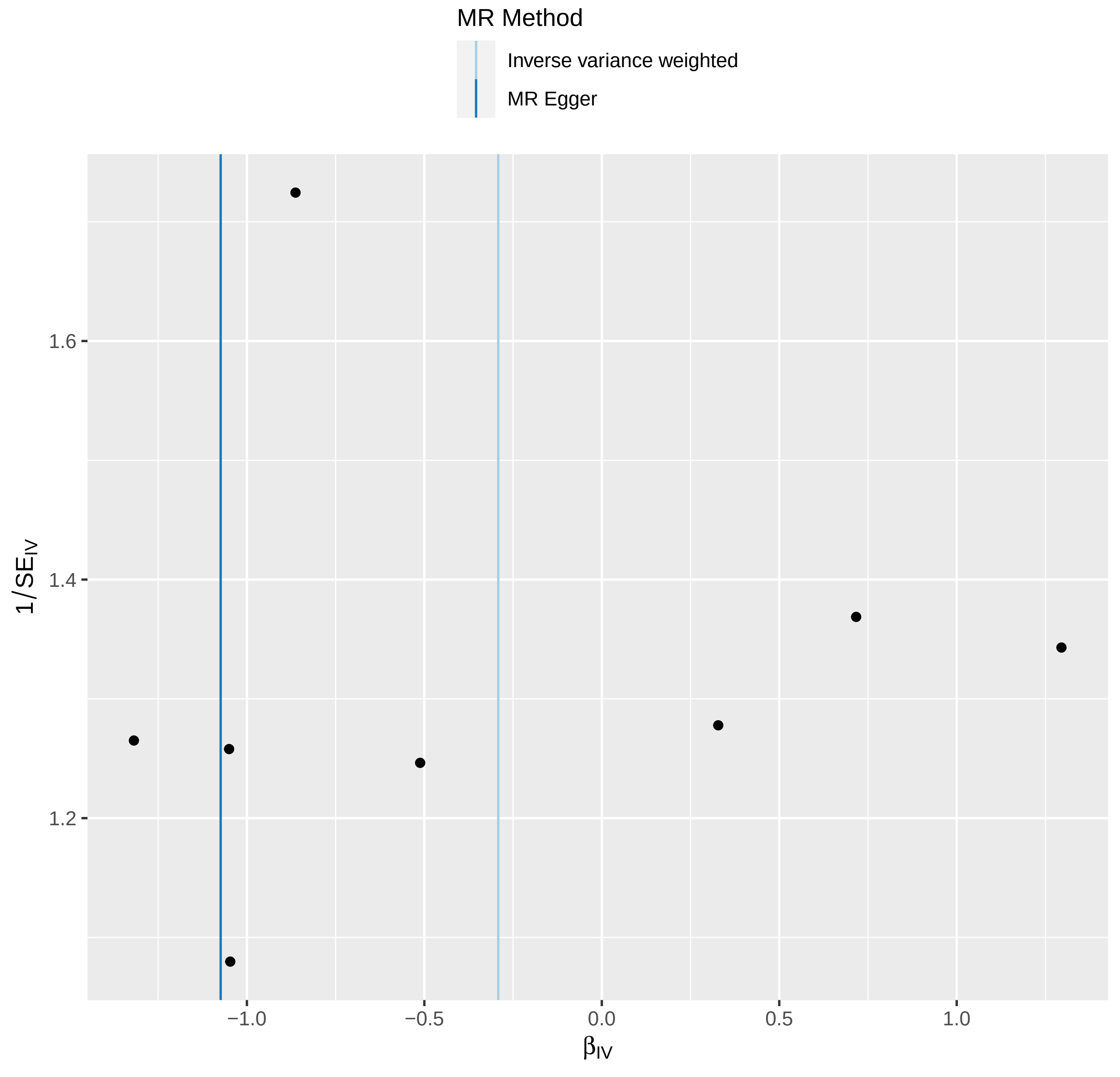

Batch 584 : Gut microbiota abundance (genus Ruminococcaceae UCG013 id.11370) on Arthropathy in Crohn disease

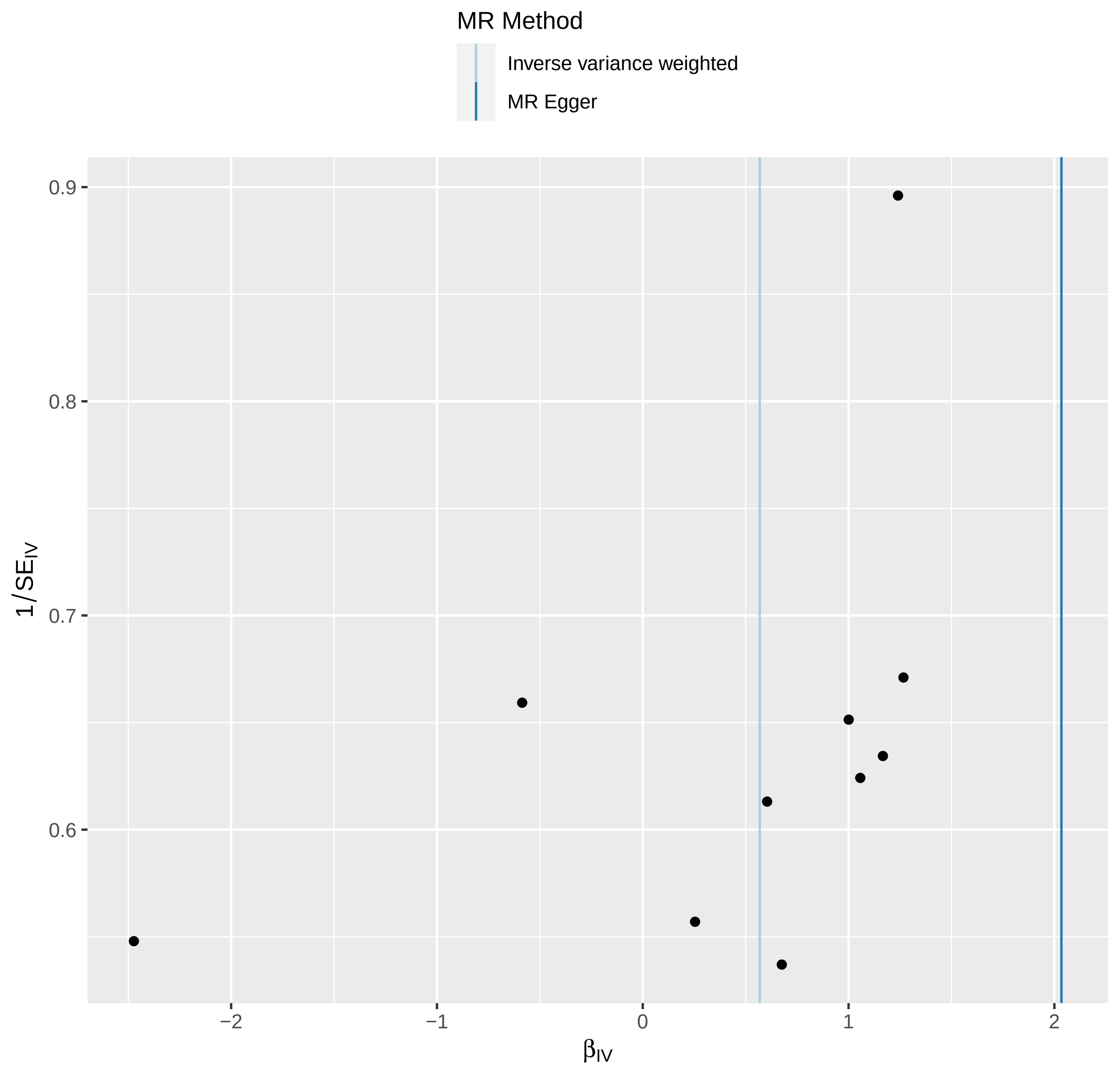

Batch 585 : Gut microbiota abundance (genus Ruminococcaceae UCG014 id.11371) on Arthropathy in Crohn disease

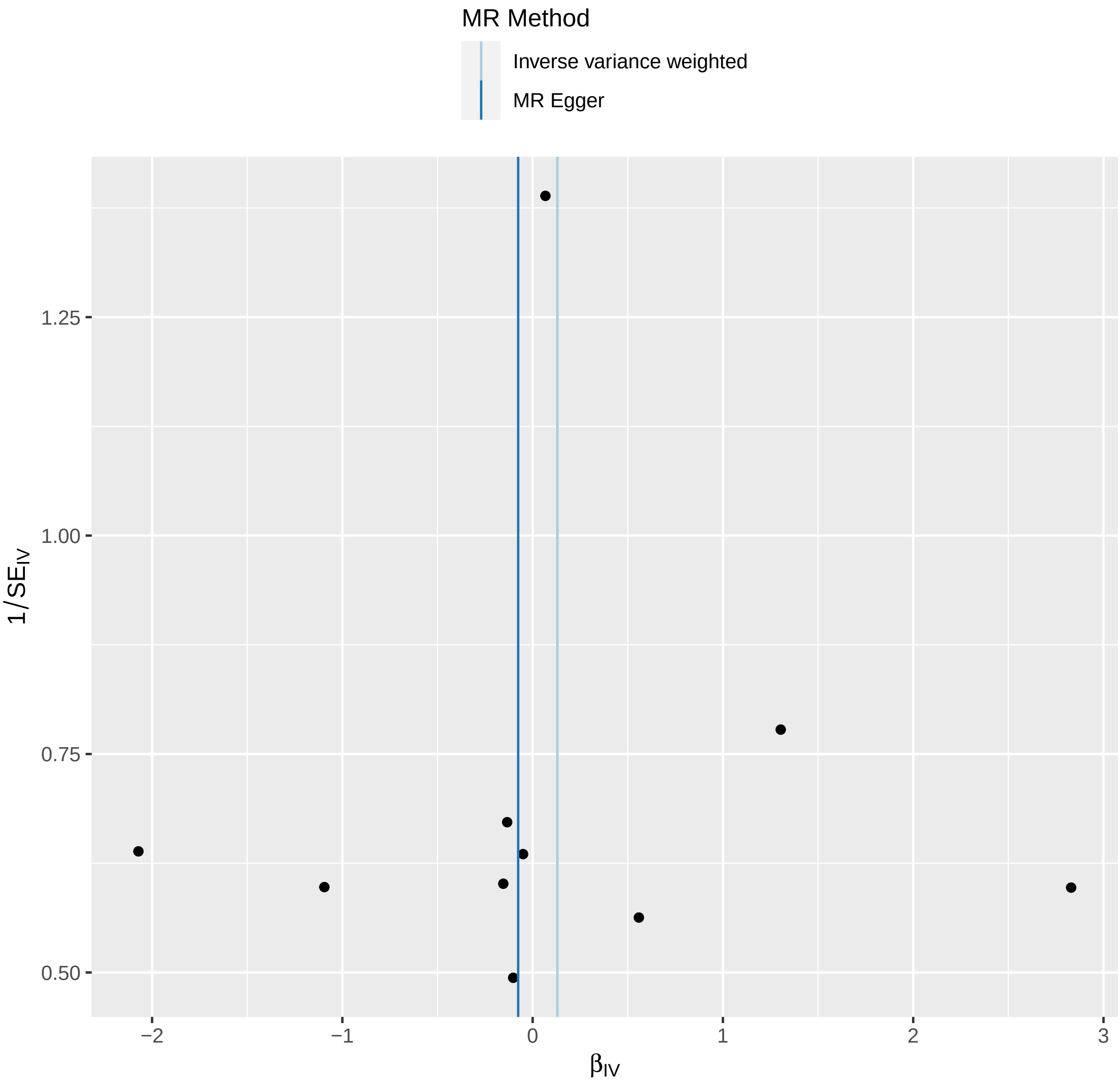

Batch 586 : Gut microbiota abundance (genus Ruminococcus1 id.11373) on Arthropathy in Crohn disease

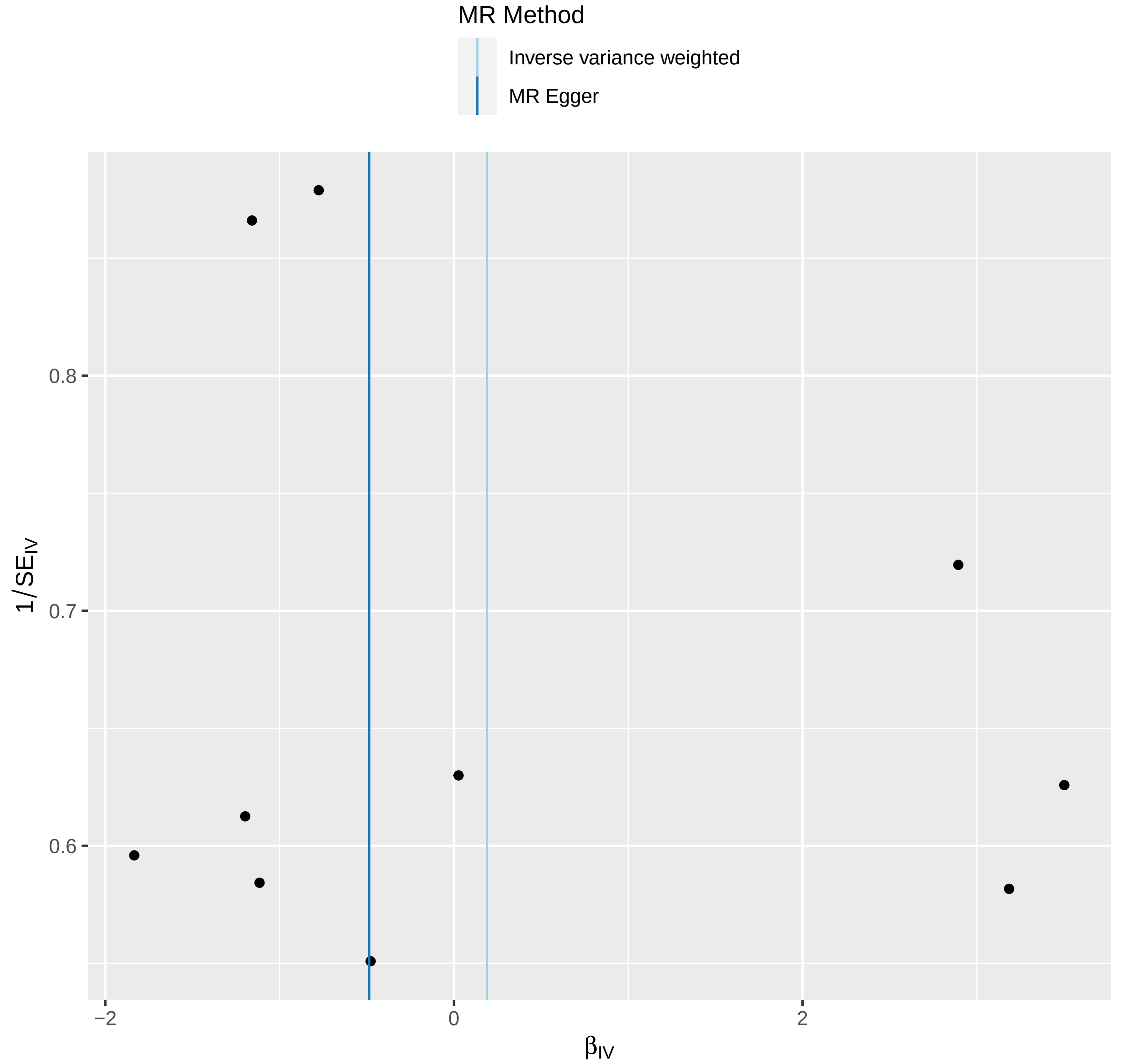

Batch 587 : Gut microbiota abundance (genus Ruminococcus2 id.11374) on Arthropathy in Crohn disease

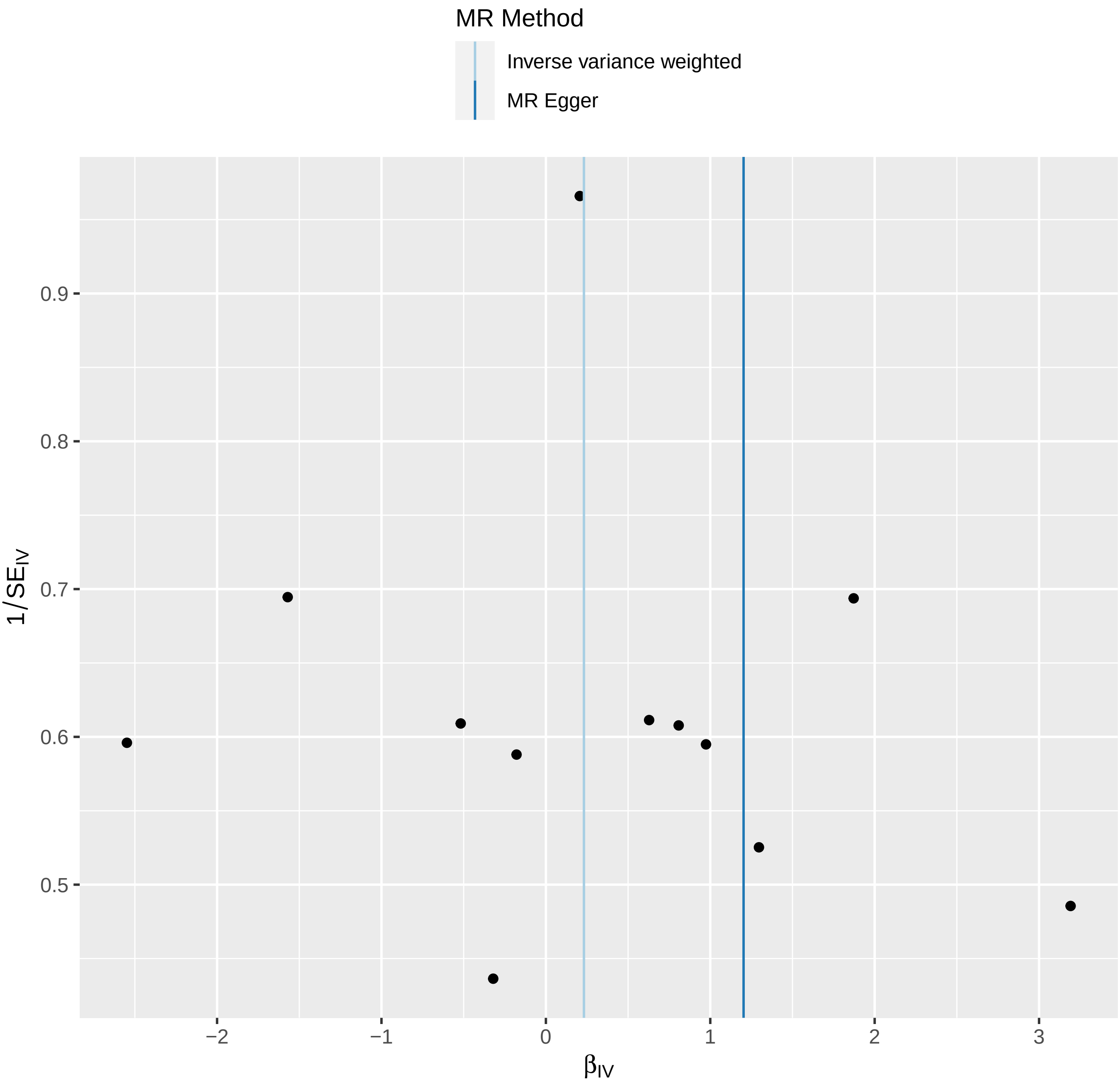

Batch 588 : Gut microbiota abundance (genus Ruminococcus gauvreauii group id.11342) on Arthropathy in Crohn disease

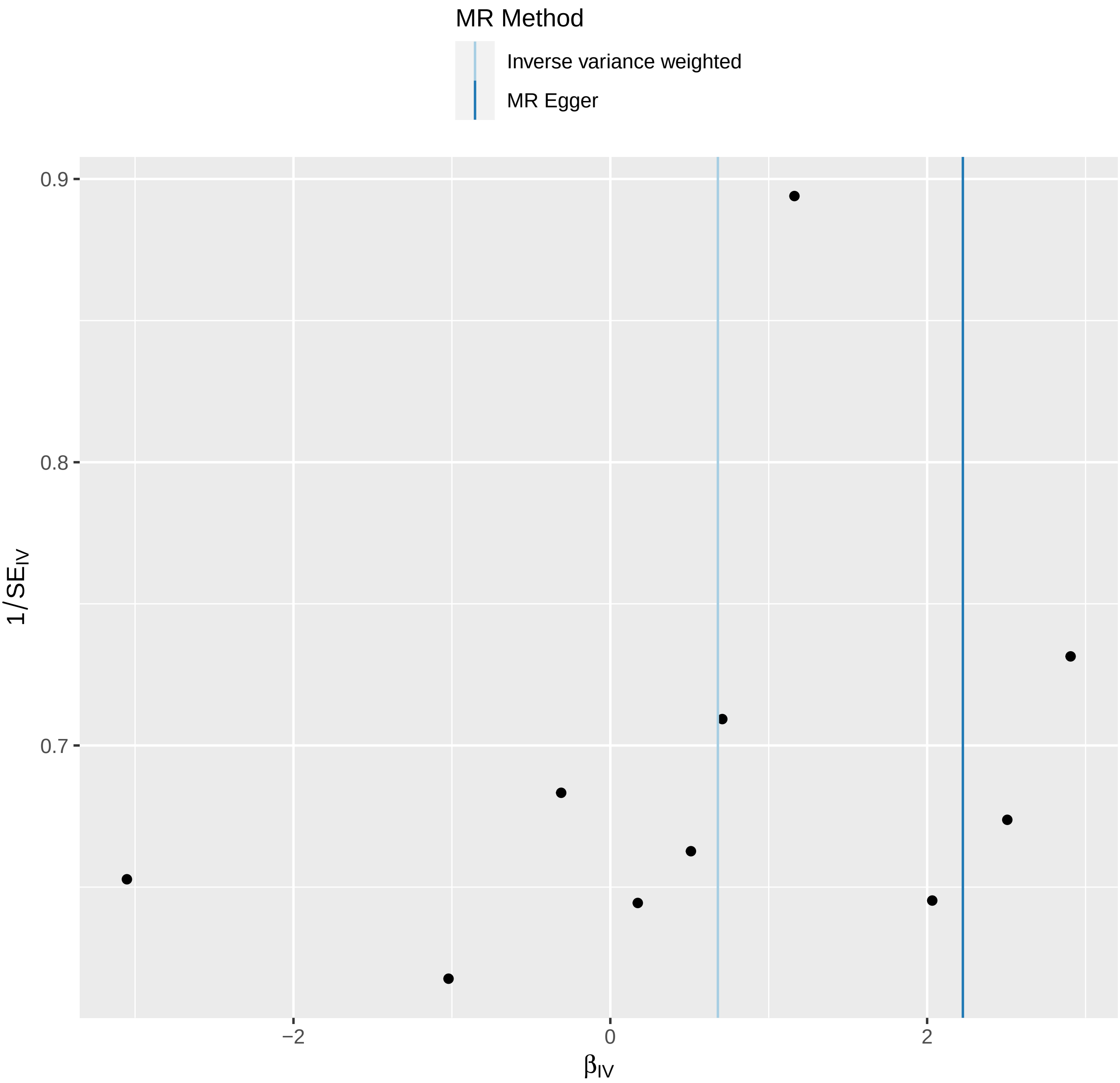

Batch 589 : Gut microbiota abundance (genus Ruminococcus gnavus group id.14376) on Arthropathy in Crohn disease

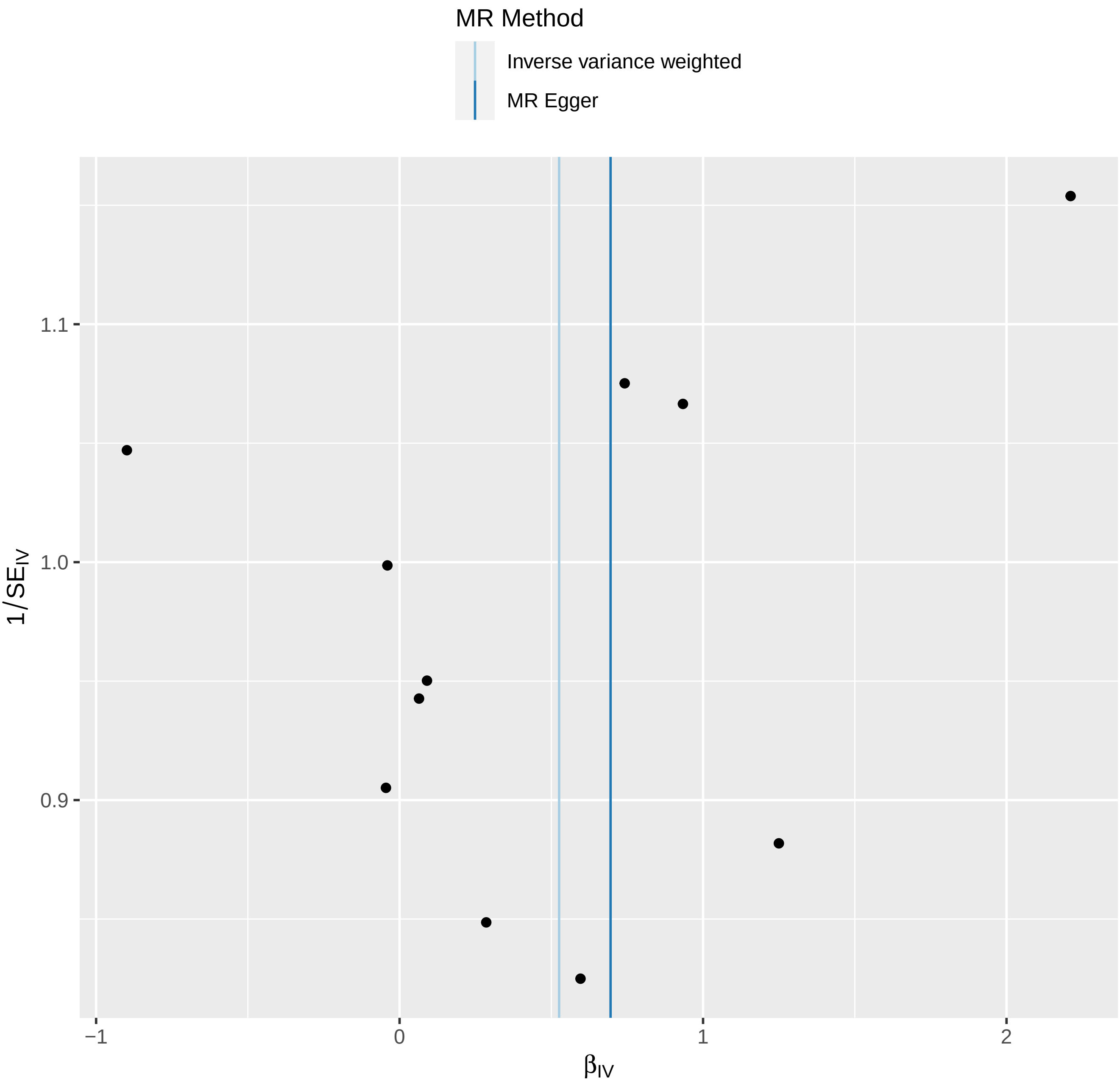

Batch 590 : Gut microbiota abundance (genus Ruminococcus torques group id.14377) on Arthropathy in Crohn disease

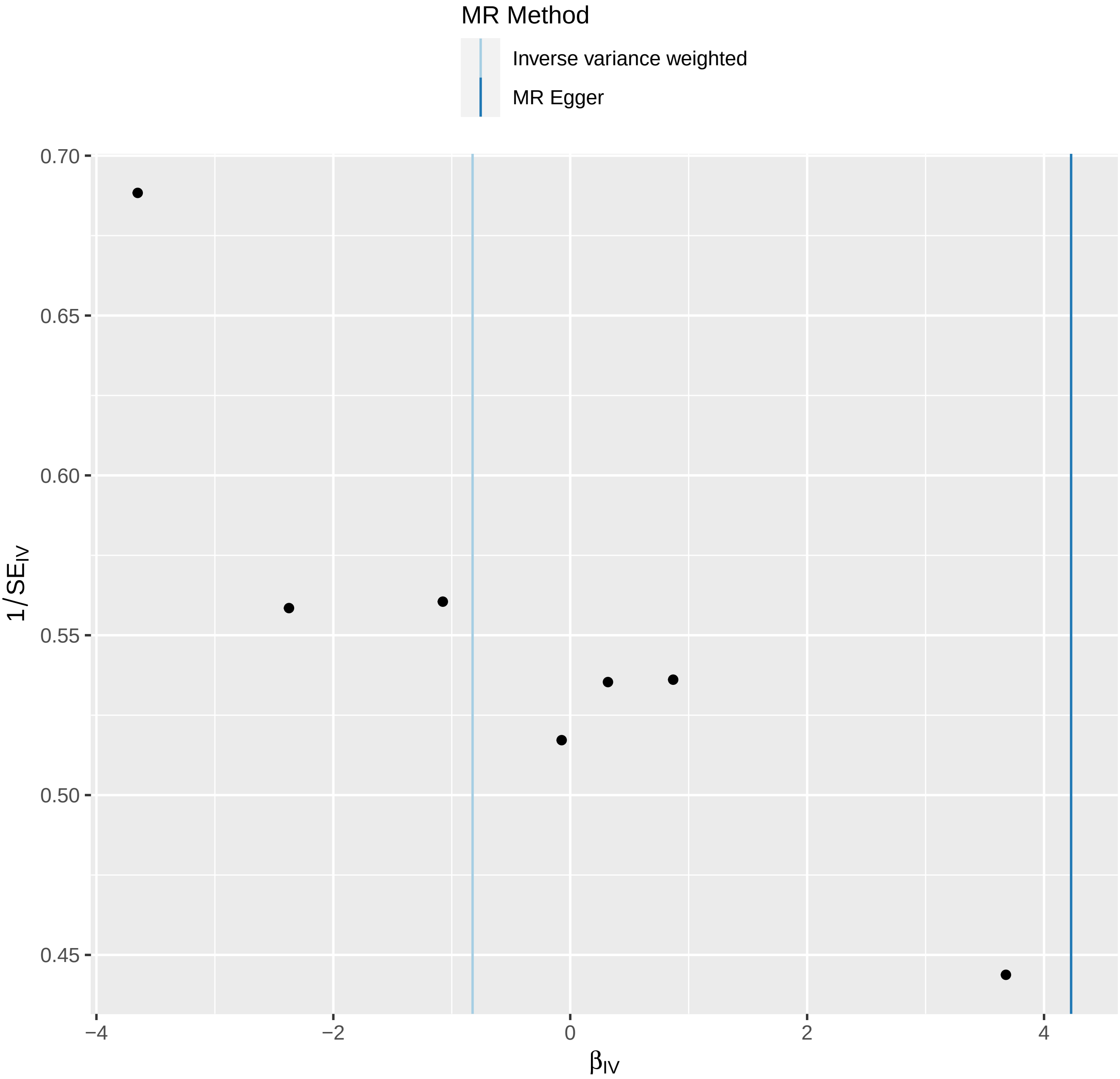

Batch 591 : Gut microbiota abundance (genus Sellimonas id.14369) on Arthropathy in Crohn disease

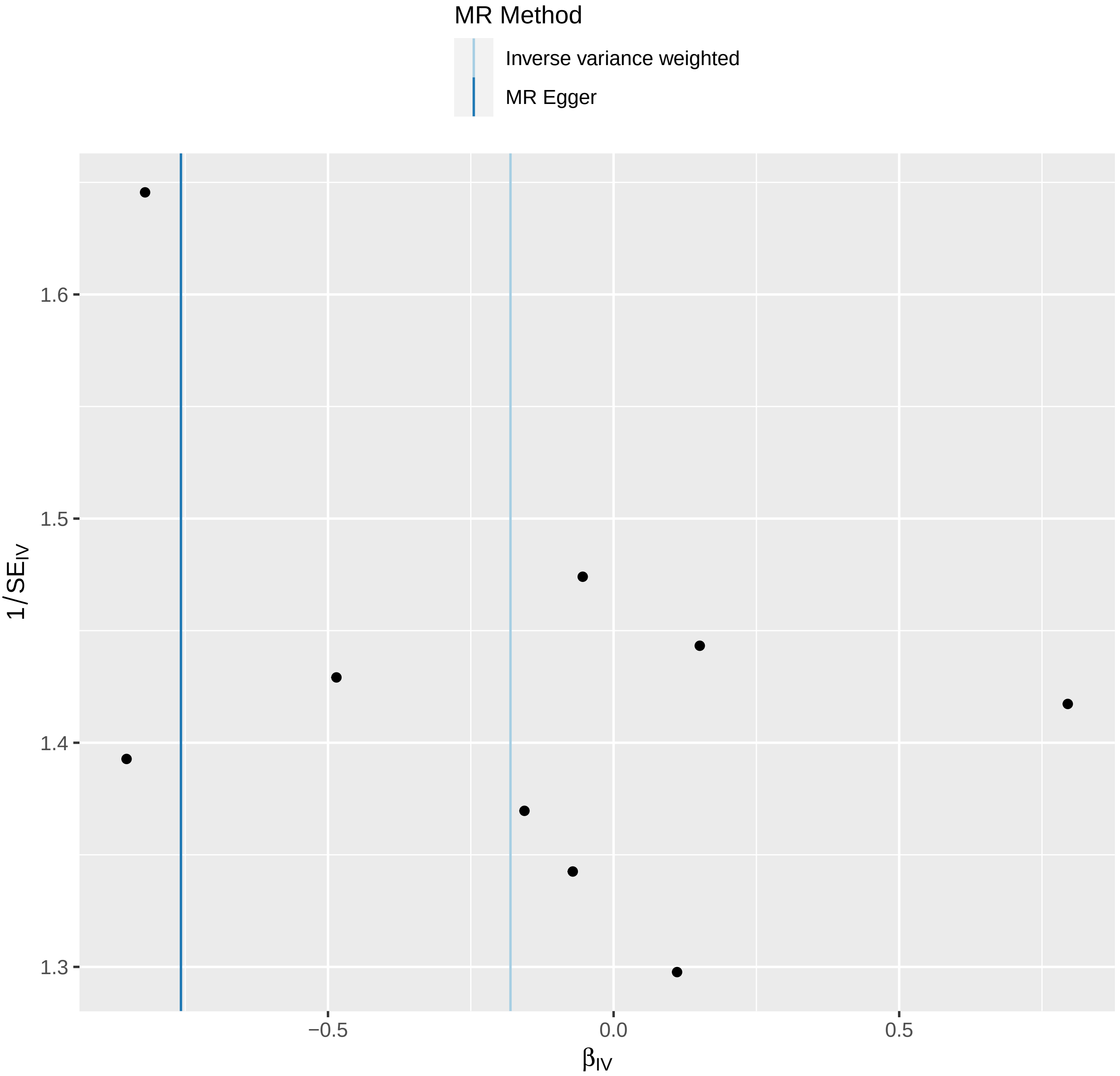

Batch 592 : Gut microbiota abundance (genus Senegalimassilia id.11160) on Arthropathy in Crohn disease

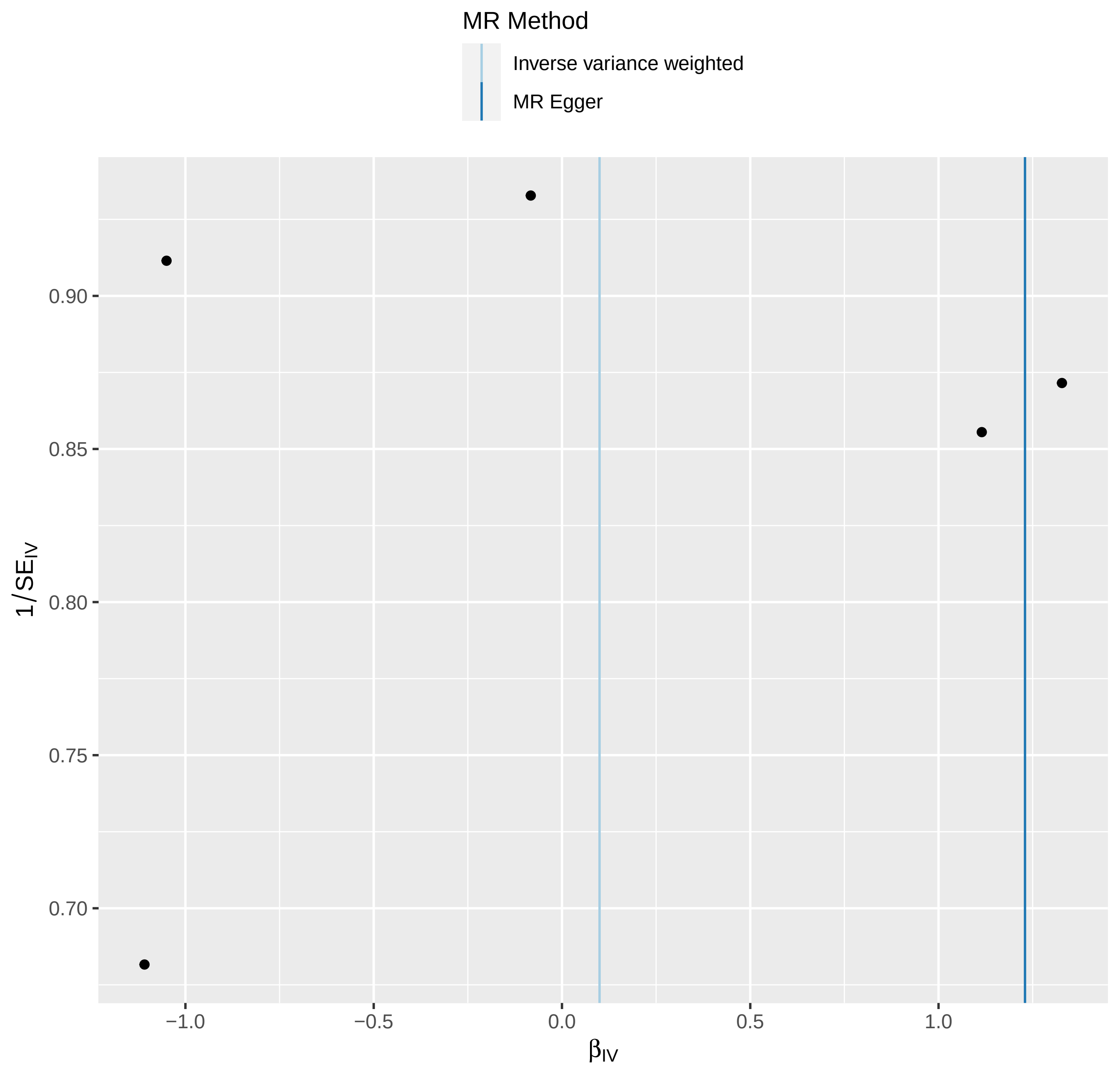

Batch 593 : Gut microbiota abundance (genus Slackia id.825) on Arthropathy in Crohn disease

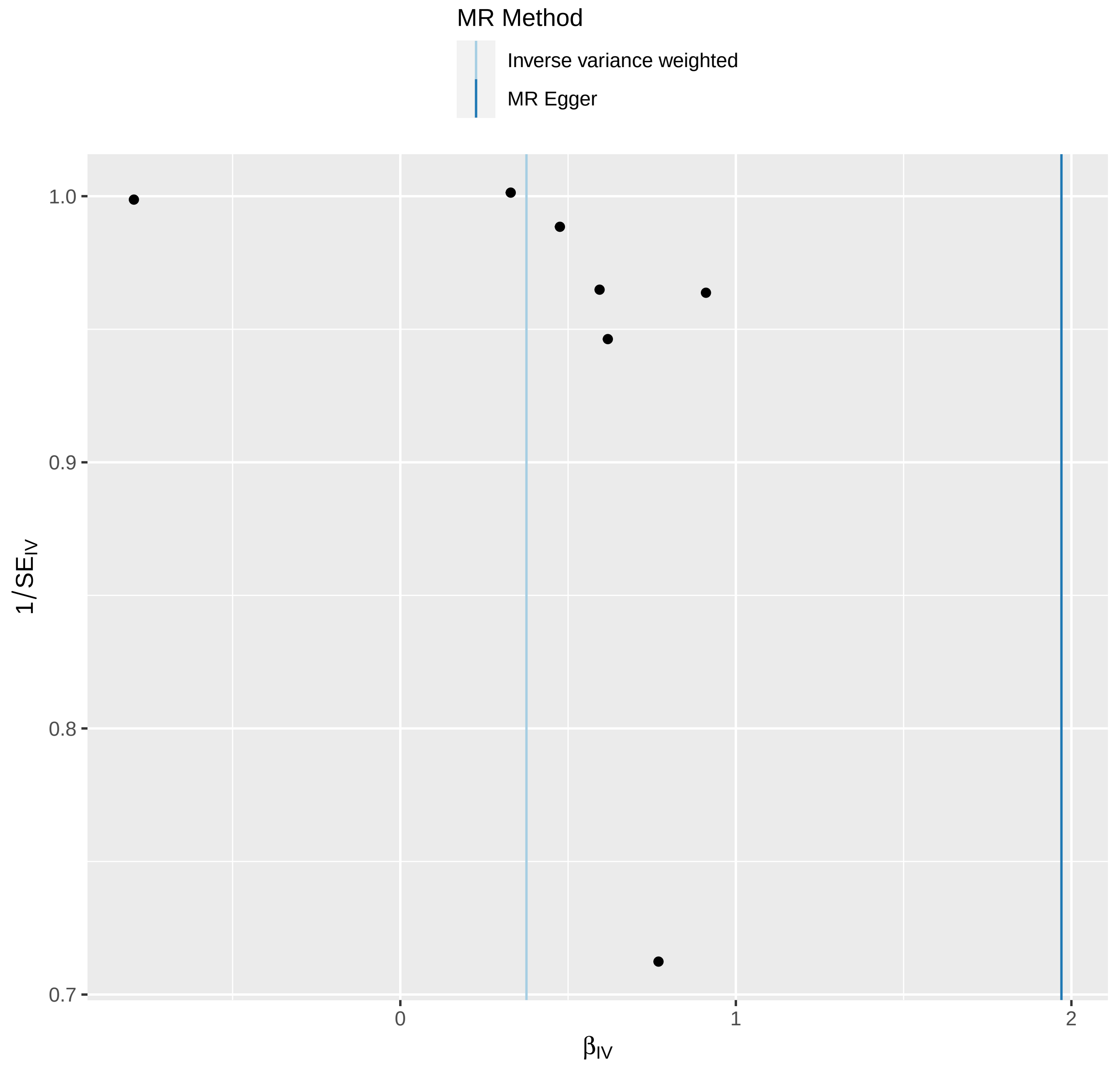

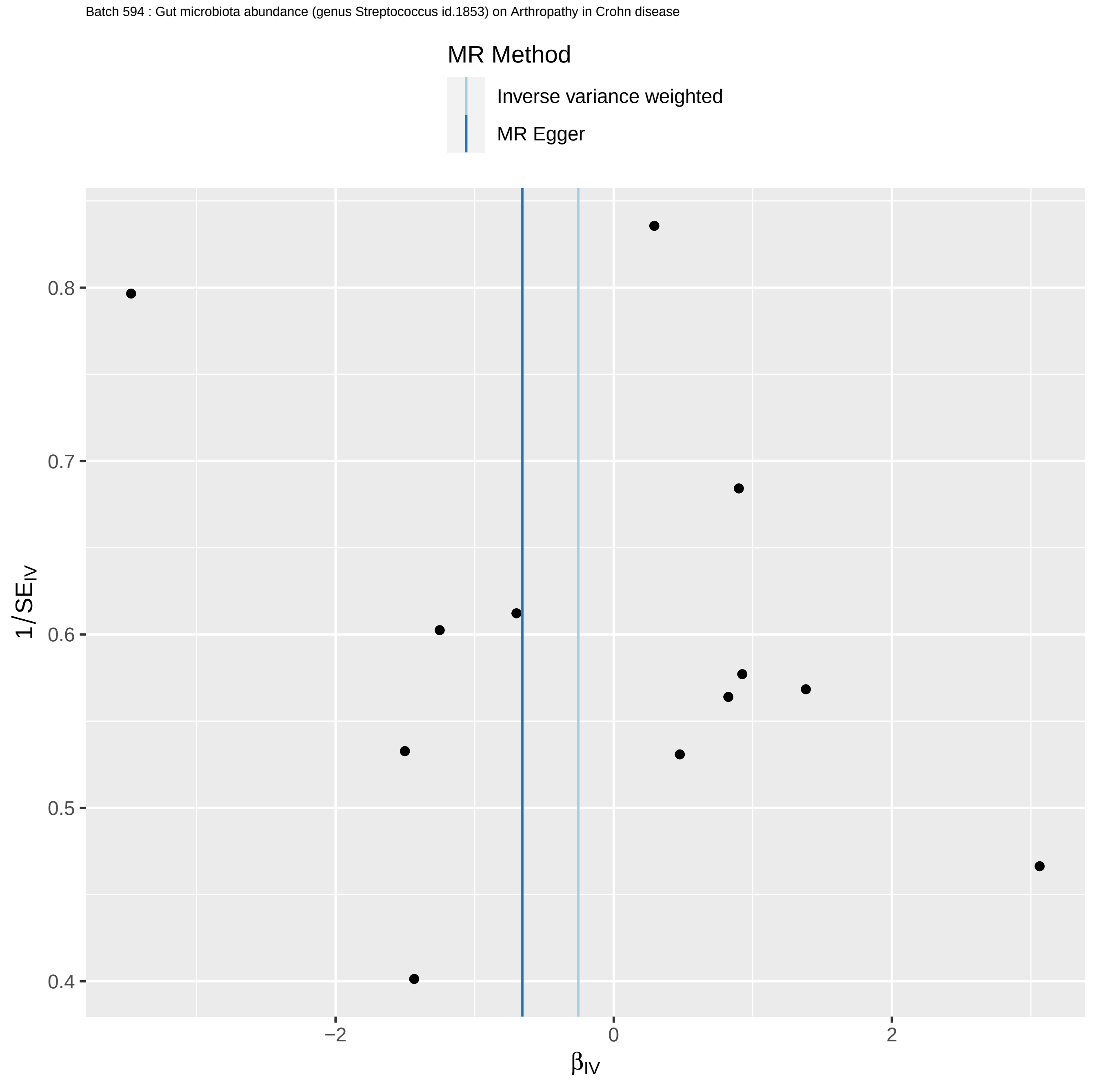
Batch 594 : Gut microbiota abundance (genus Streptococcus id.1853) on Arthropathy in Crohn disease
MR Method
Inverse variance weighted
MR Egger
0.8
0.7
0.6
0.5
0.4
−2
0
2
$1/SE_{IV}$
β_{IV}

Batch 595 : Gut microbiota abundance (genus Subdoligranulum id.2070) on Arthropathy in Crohn disease

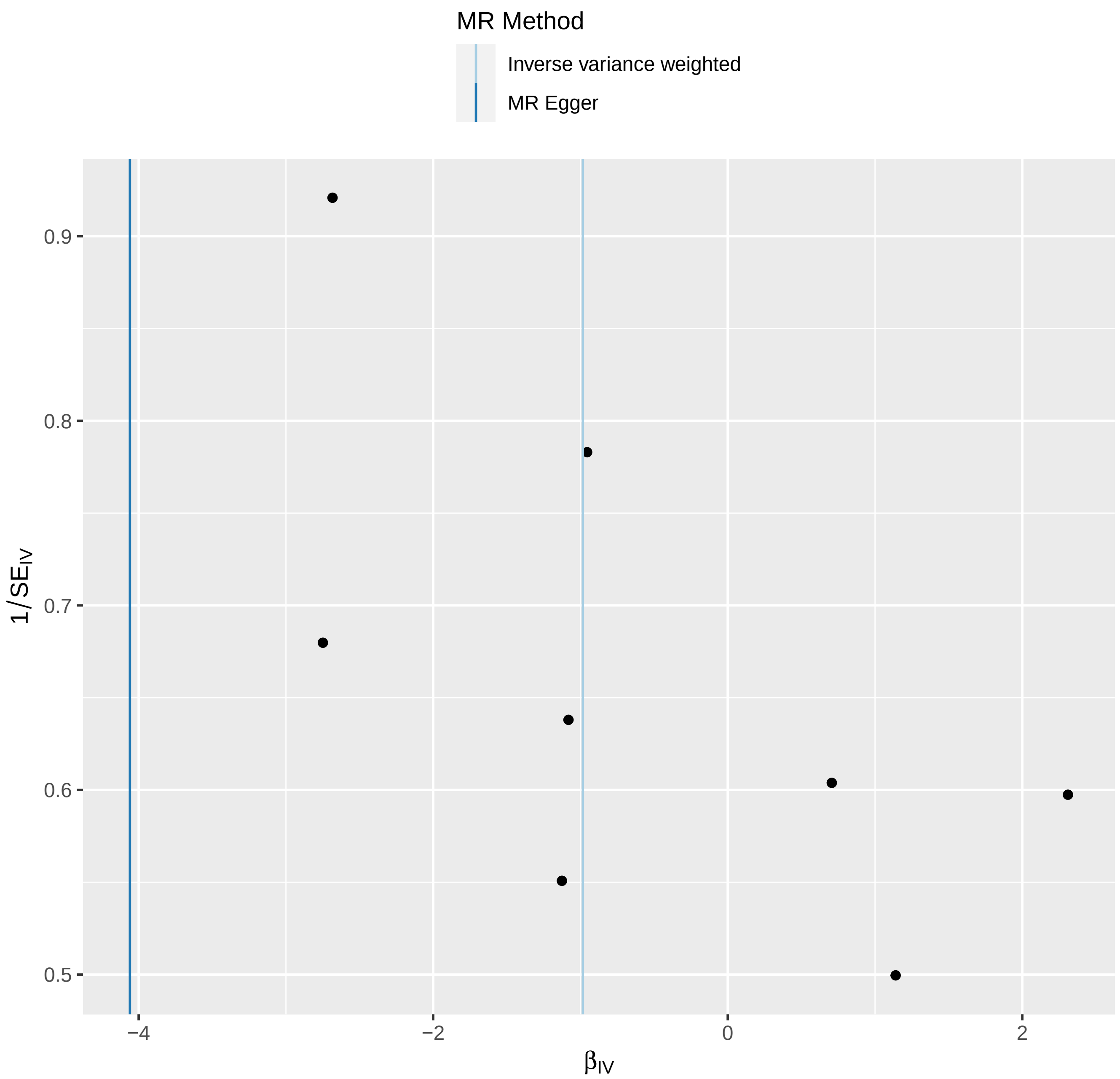

Batch 596 : Gut microbiota abundance (genus Sutterella id.2896) on Arthropathy in Crohn disease

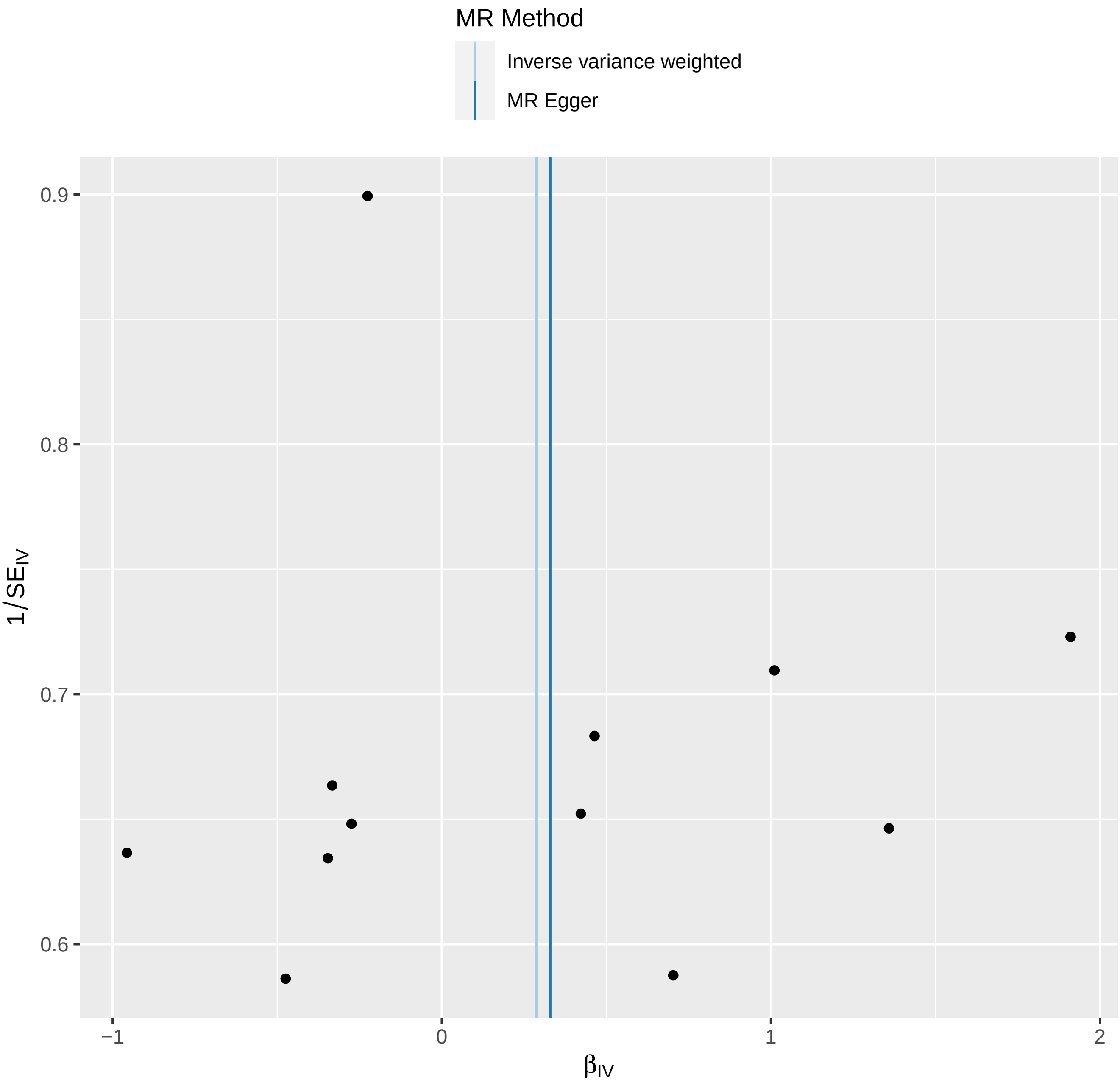

Batch 597 : Gut microbiota abundance (genus Terrisporobacter id.11348) on Arthropathy in Crohn disease

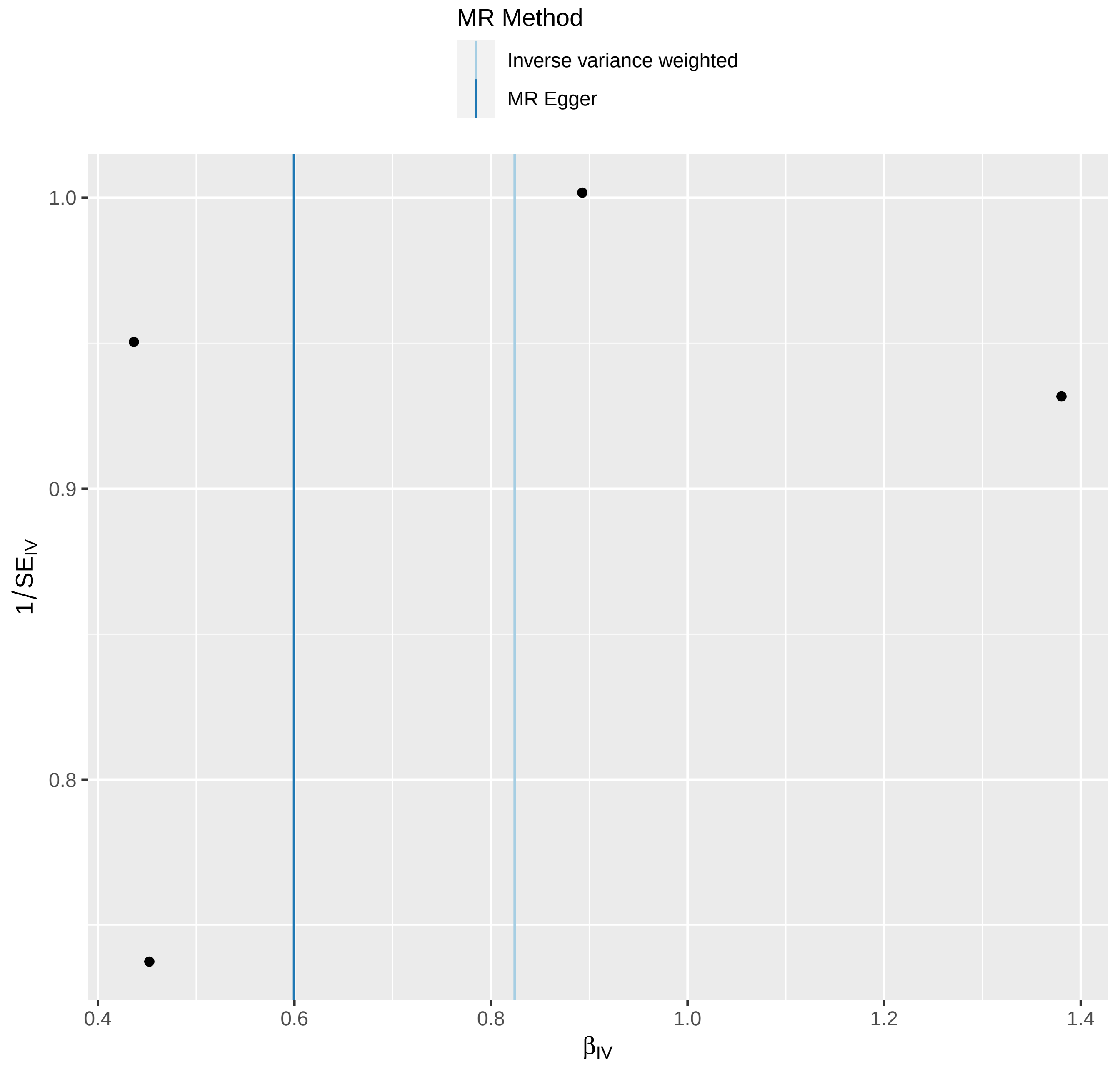

Batch 598 : Gut microbiota abundance (genus Turicibacter id.2162) on Arthropathy in Crohn disease

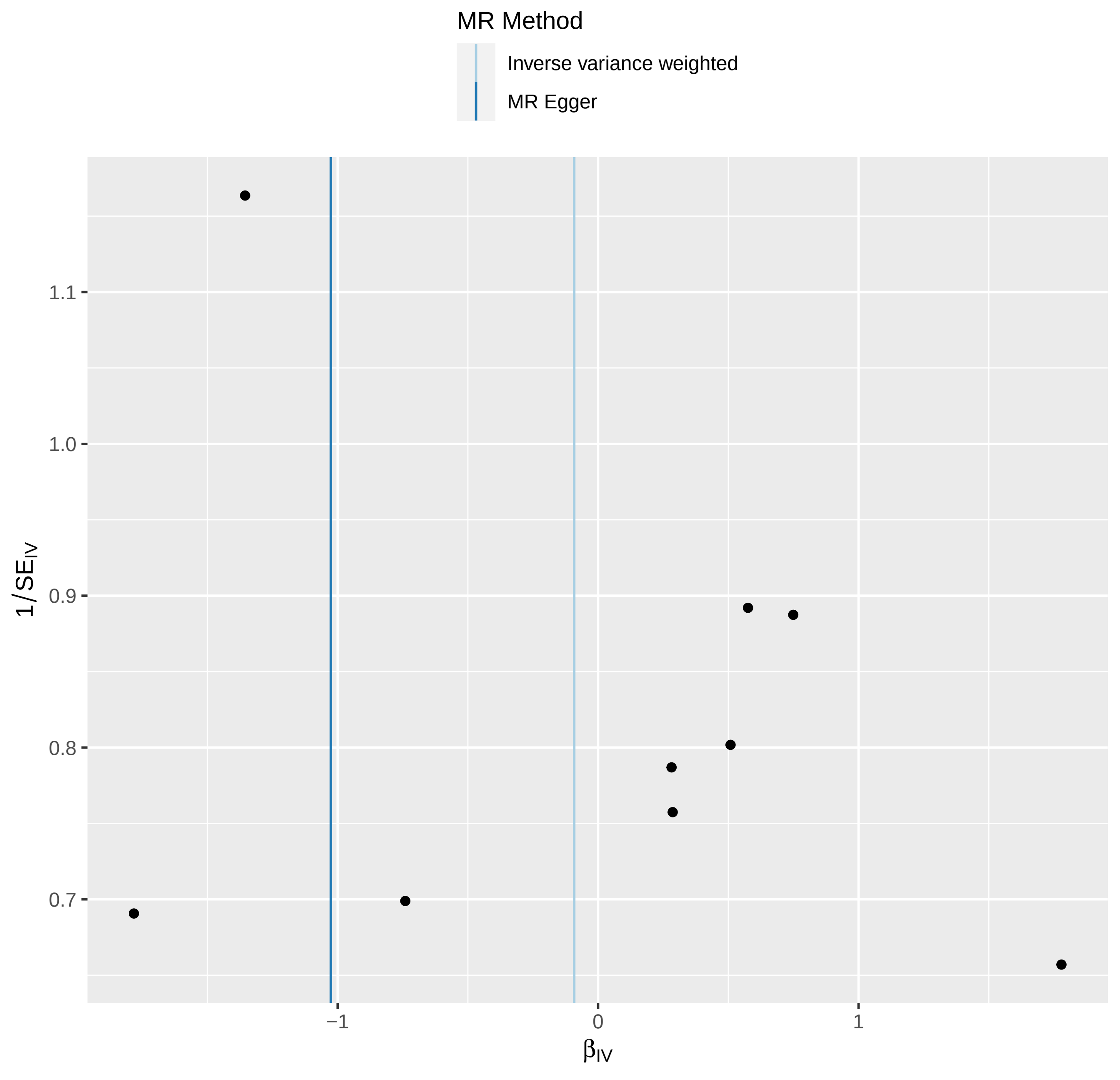

Batch 599 : Gut microbiota abundance (genus Tyzzerella3 id.11335) on Arthropathy in Crohn disease

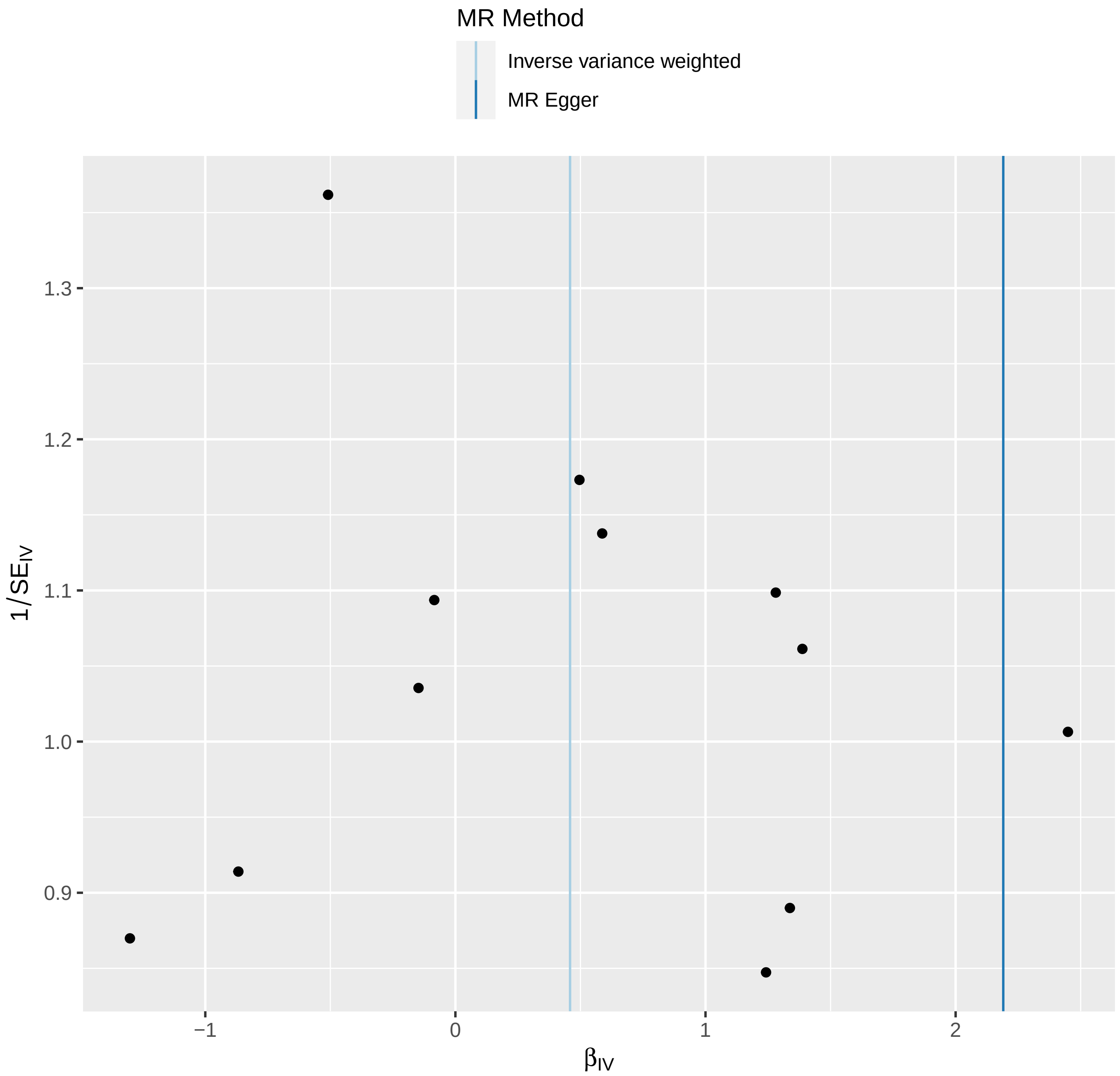

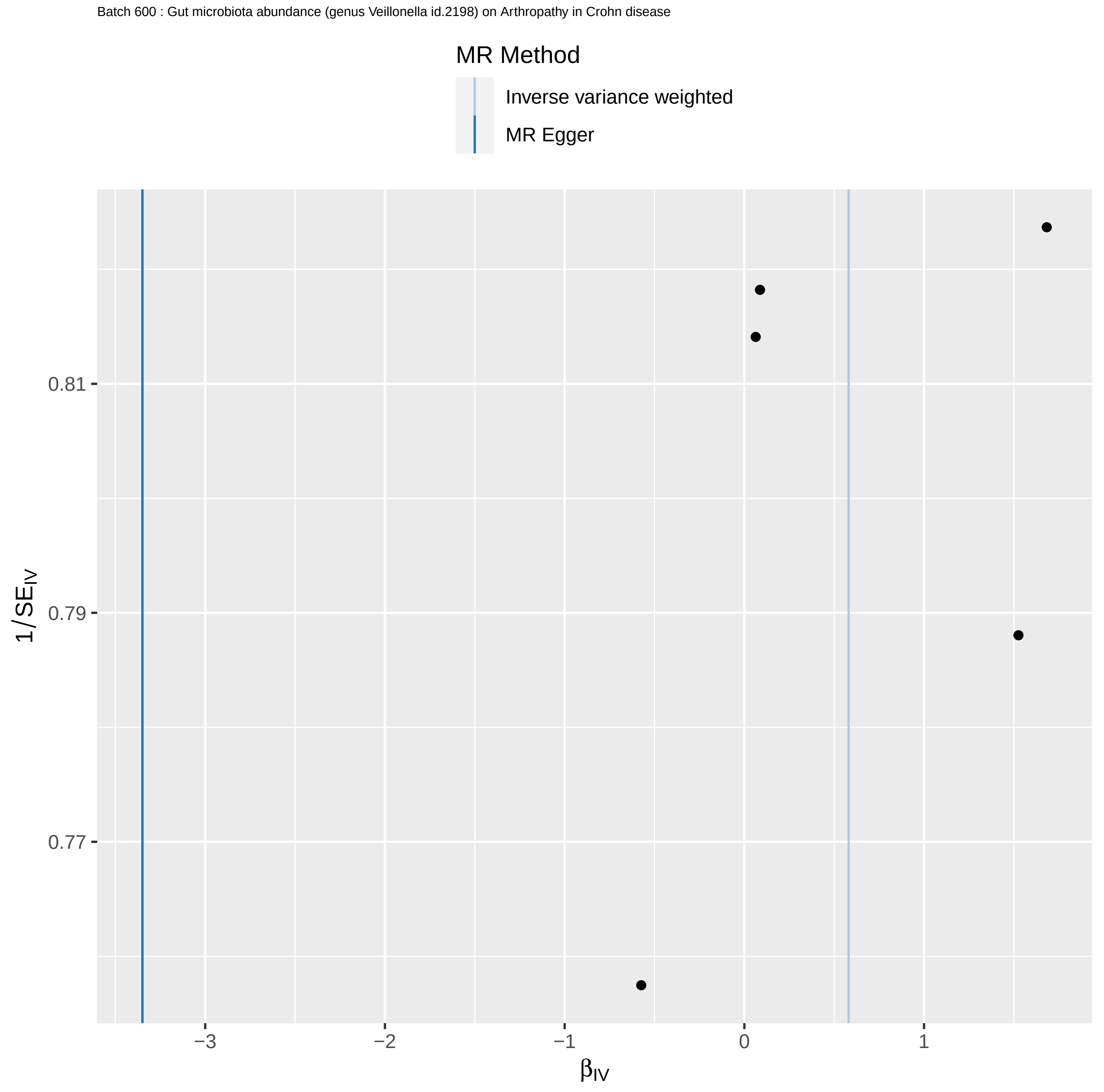
Batch 600 : Gut microbiota abundance (genus Veillonella id.2198) on Arthropathy in Crohn disease
MR Method
Inverse variance weighted
MR Egger
0.81
0.79
0.77
1/SE_IV
−3
−2
−1
0
1
β_IV

Batch 601 : Gut microbiota abundance (family Acidaminococcaceae id.2166) on Ulcerative colitis (strict) with PSC

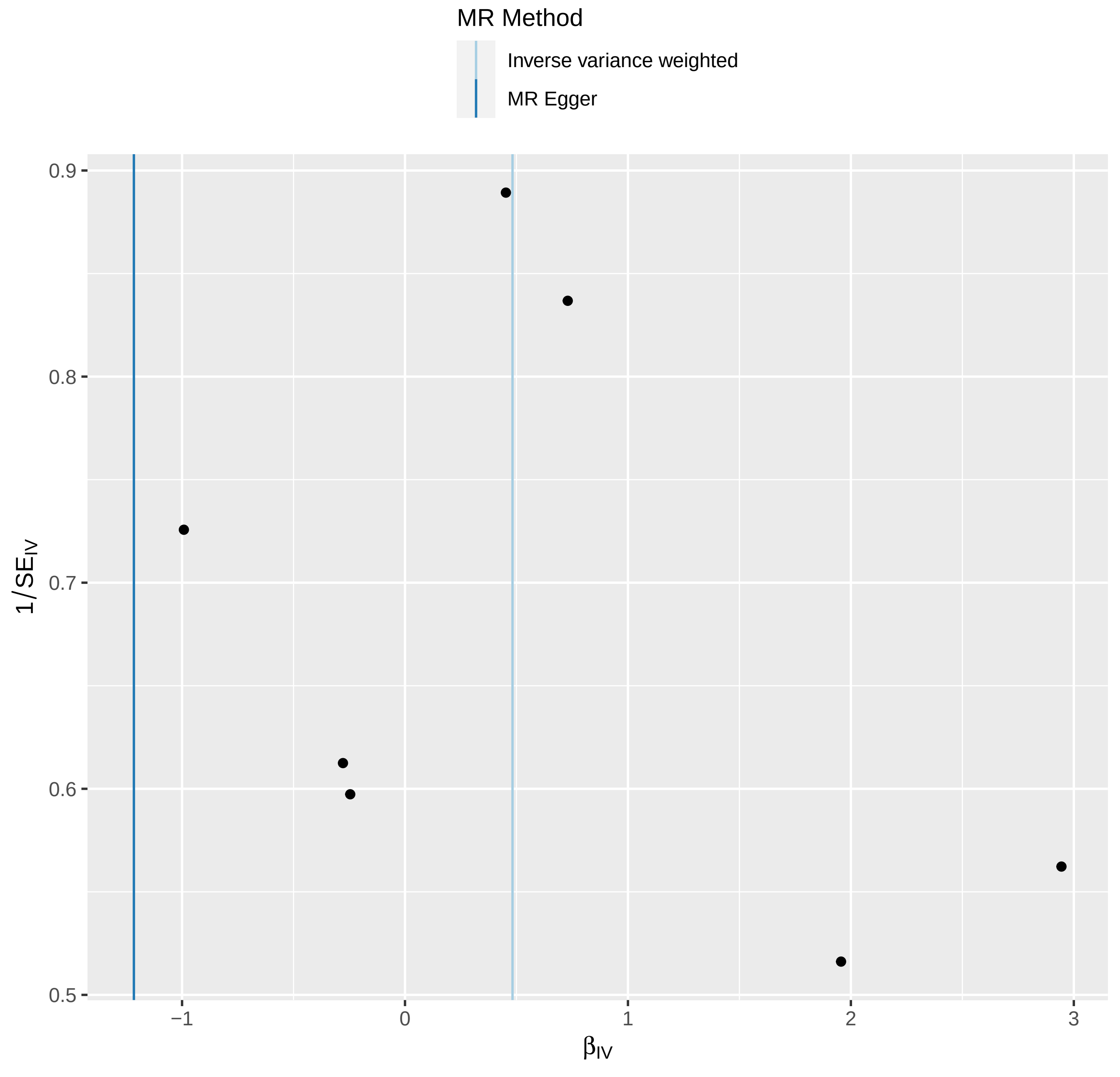

Batch 602 : Gut microbiota abundance (family Actinomycetaceae id.421) on Ulcerative colitis (strict) with PSC

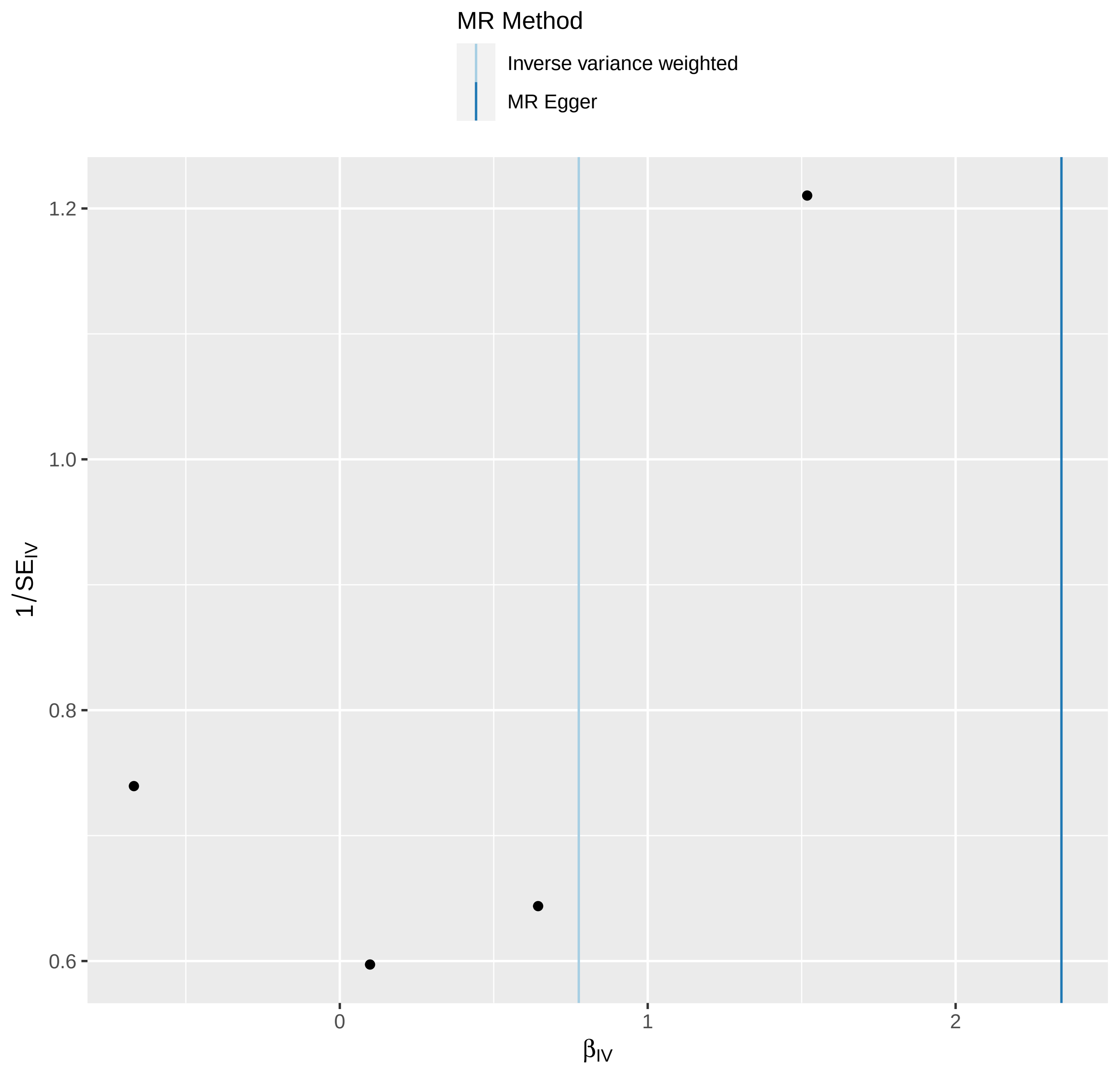

Batch 603 : Gut microbiota abundance (family Alcaligenaceae id.2875) on Ulcerative colitis (strict) with PSC

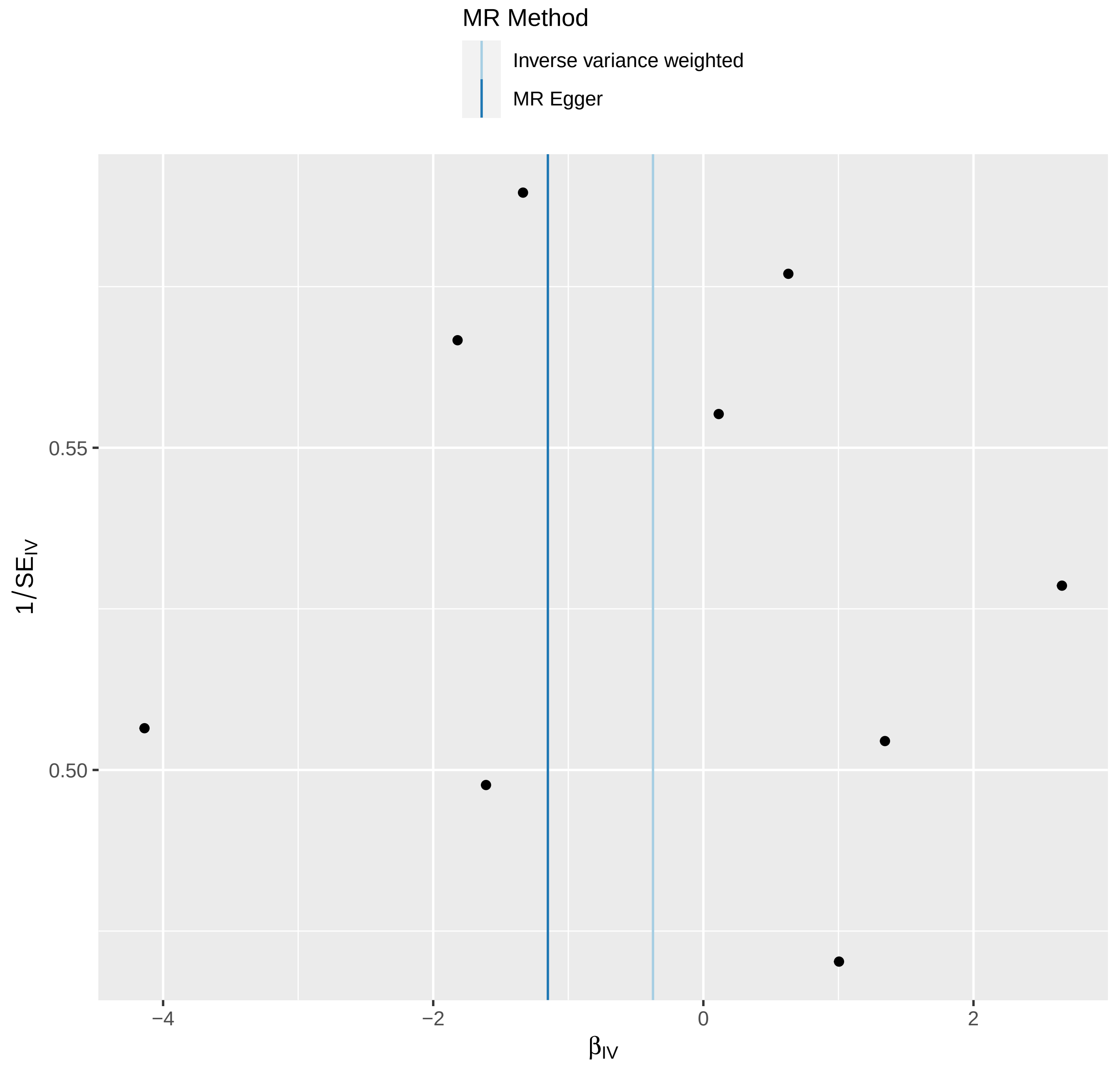

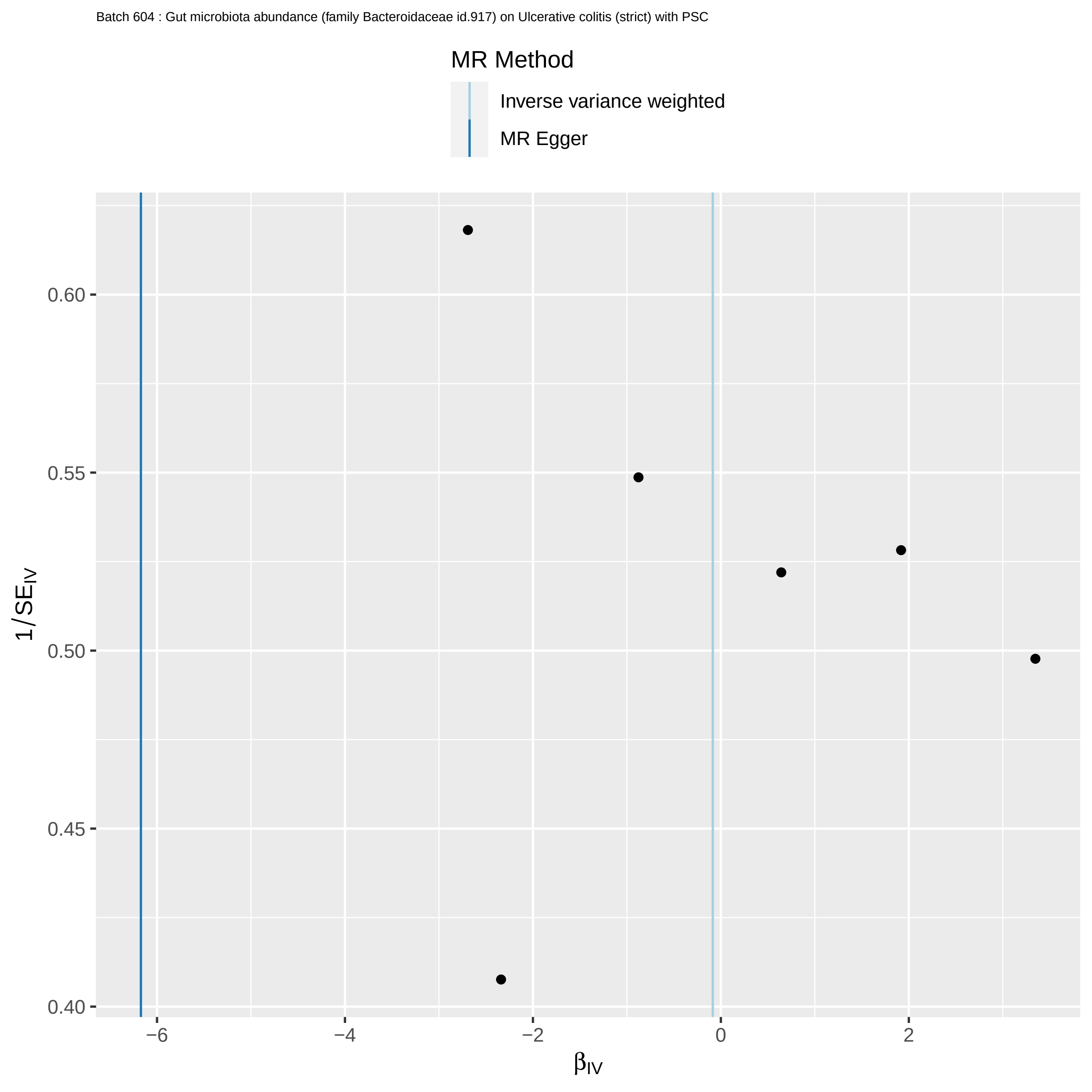
Batch 604 : Gut microbiota abundance (family Bacteroidaceae id.917) on Ulcerative colitis (strict) with PSC
MR Method
Inverse variance weighted
MR Egger
0.60
0.55
0.50
0.45
0.40
1/SE_IV
−6
−4
−2
0
2
β_IV

Batch 605 : Gut microbiota abundance (family Bacteroidales S24 7group id.11173) on Ulcerative colitis (strict) with PSC

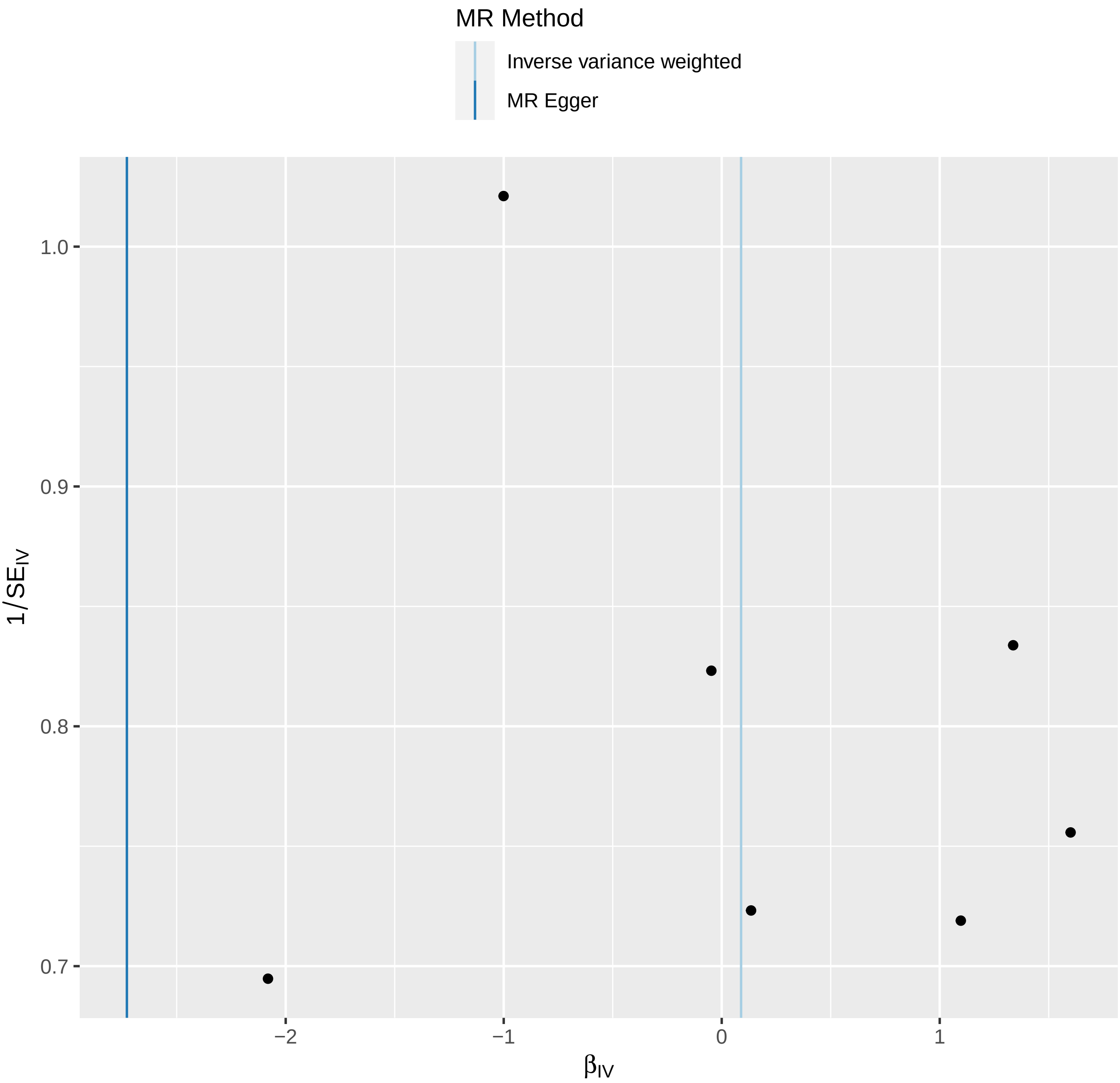

Batch 606 : Gut microbiota abundance (family Bifidobacteriaceae id.433) on Ulcerative colitis (strict) with PSC

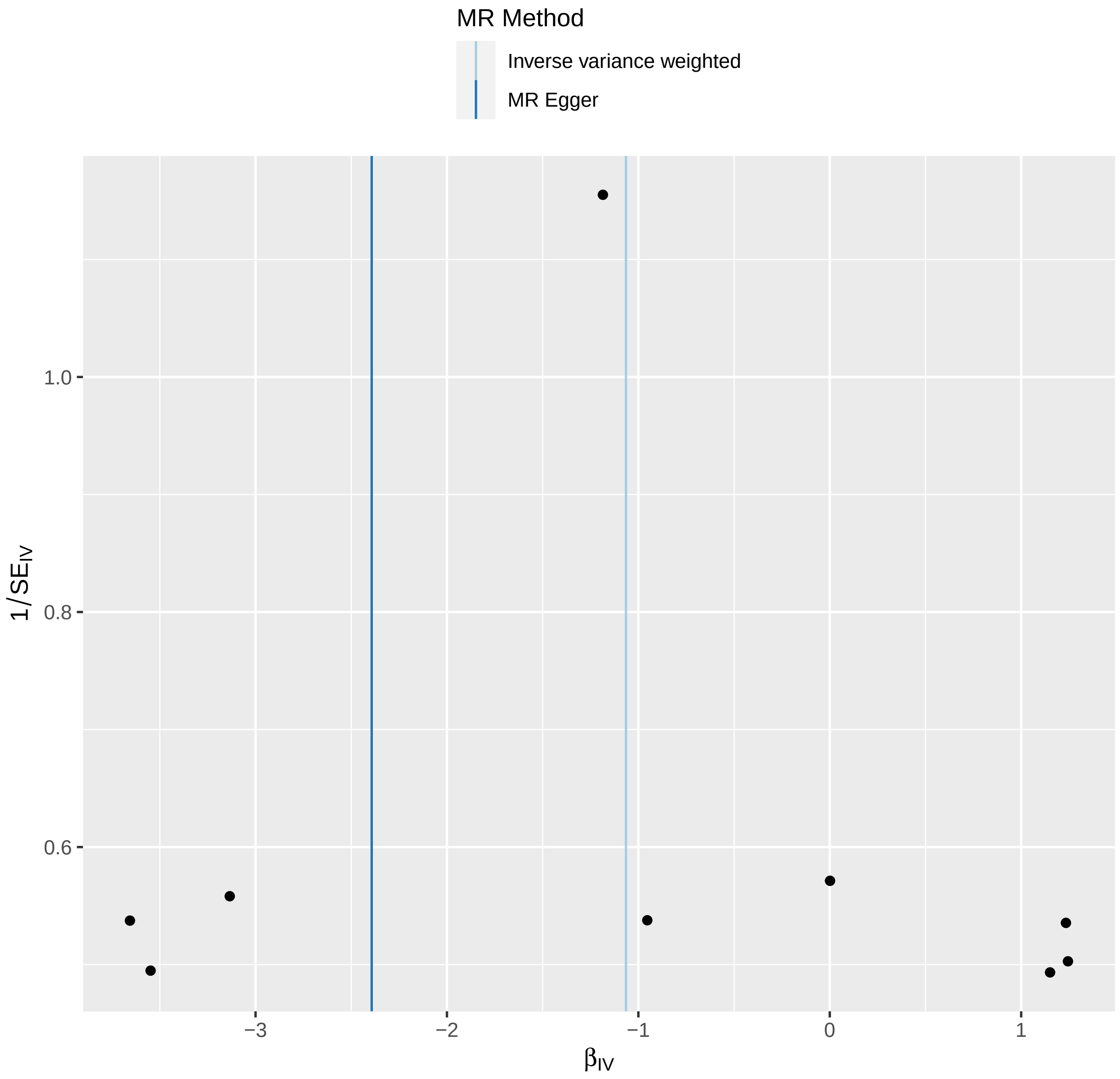

Batch 608 : Gut microbiota abundance (family Clostridiaceae1 id.1869) on Ulcerative colitis (strict) with PSC

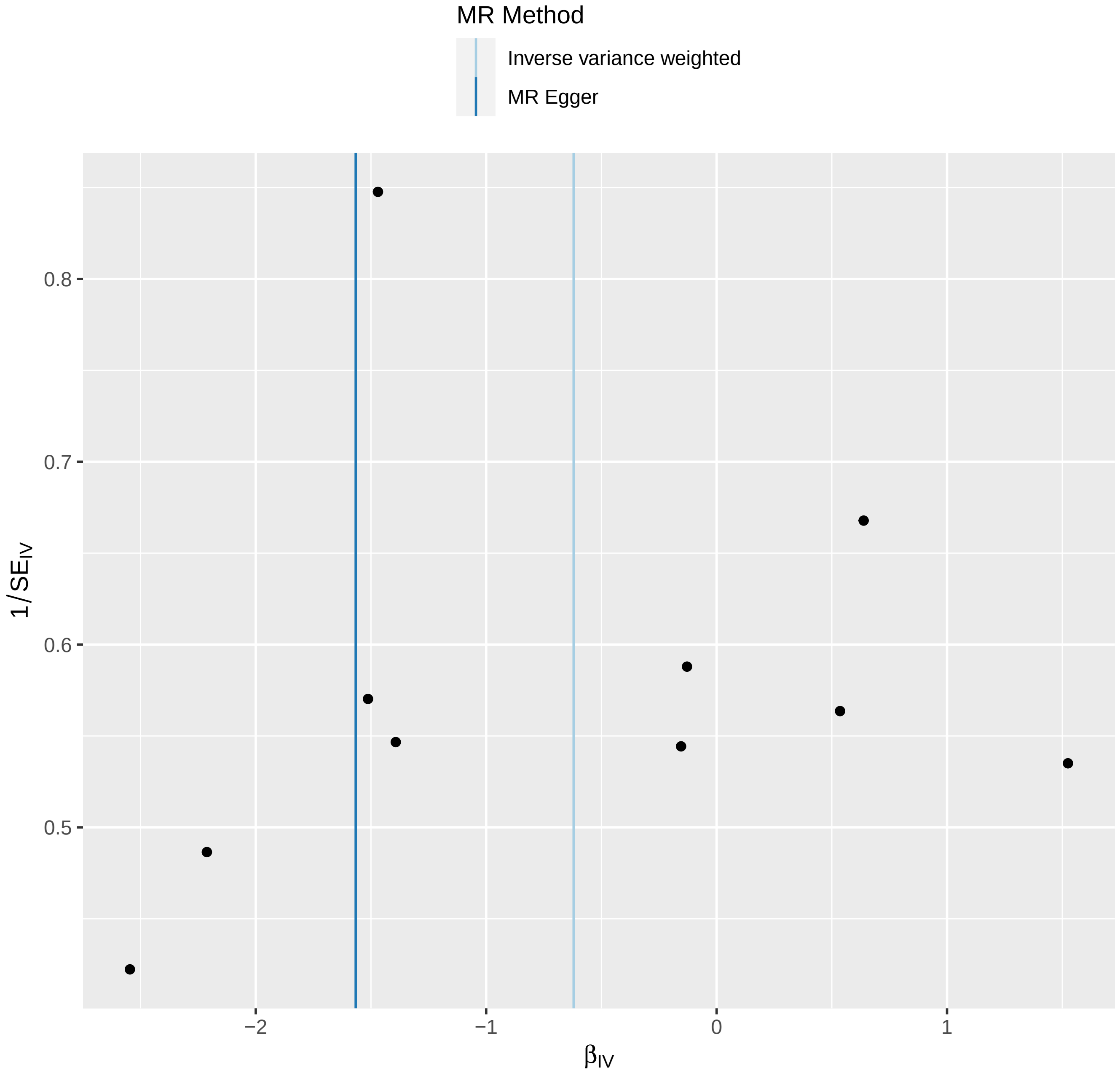

Batch 609 : Gut microbiota abundance (family Clostridiales vadin BB60 group id.11286) on Ulcerative colitis (strict) with PSC

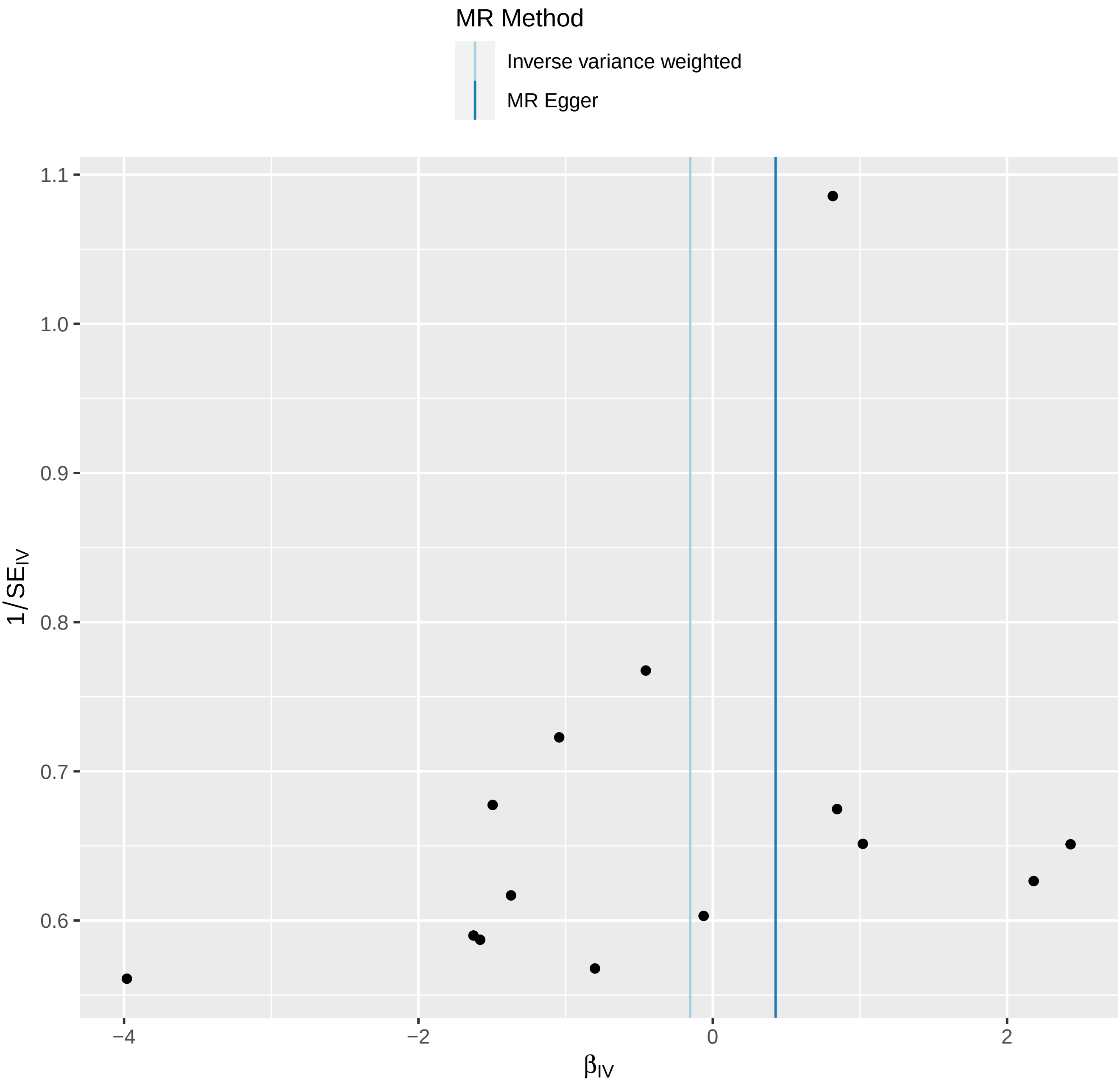

Batch 610 : Gut microbiota abundance (family Coriobacteriaceae id.811) on Ulcerative colitis (strict) with PSC

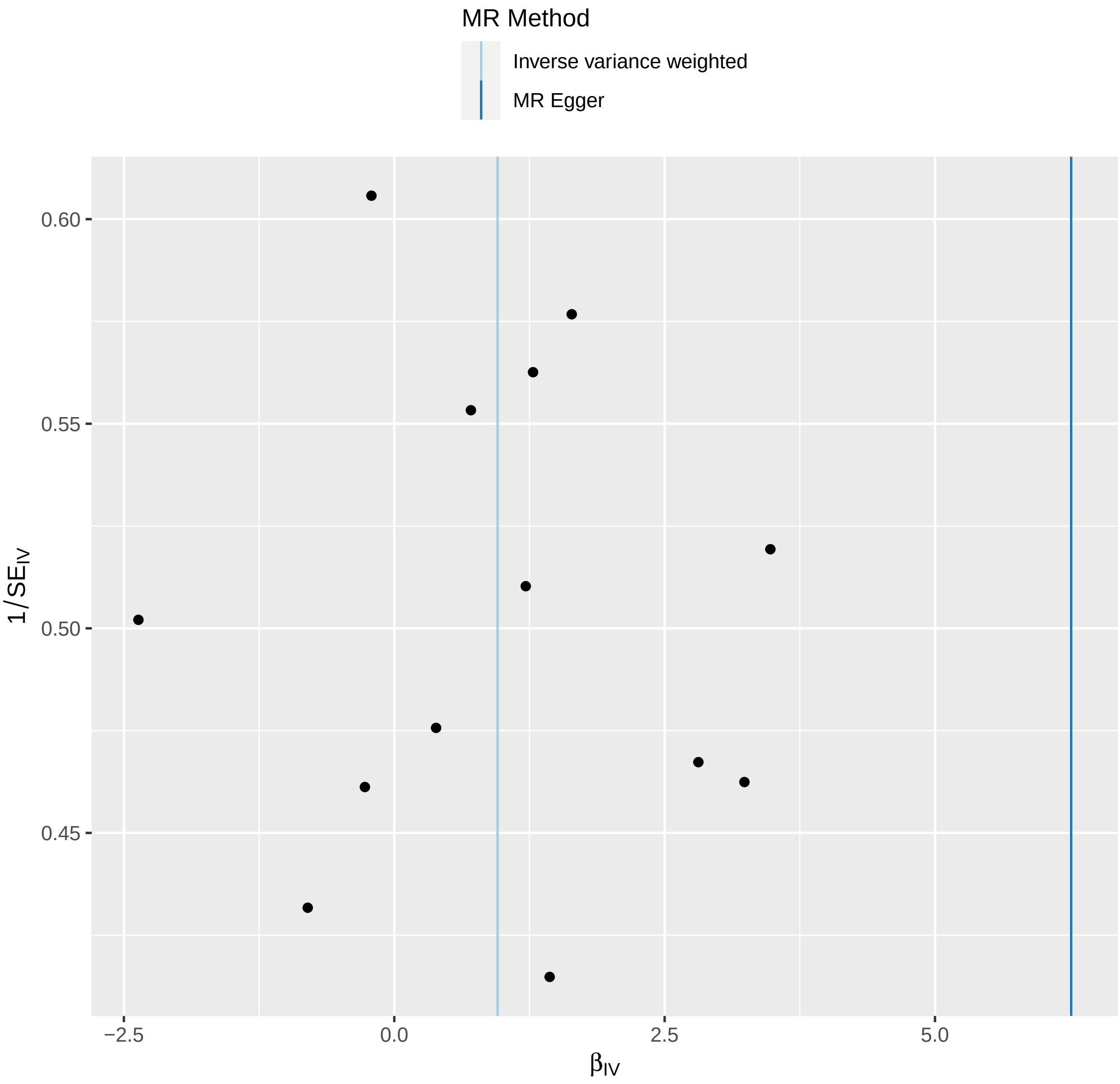

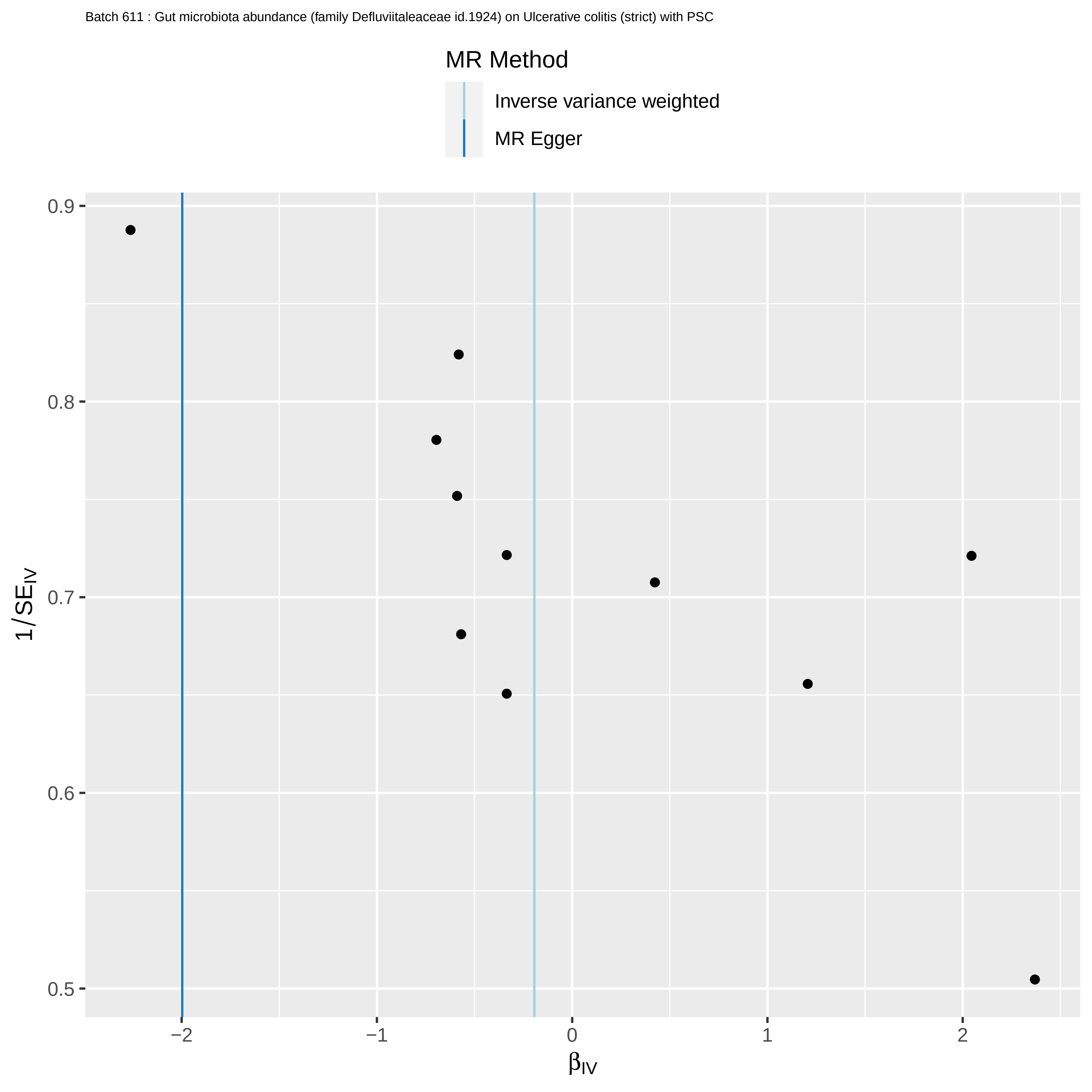

Batch 611 : Gut microbiota abundance (family Defluviitaleaceae id.1924) on Ulcerative colitis (strict) with PSC
MR Method
Inverse variance weighted
MR Egger
0.9
0.8
0.7
0.6
0.5
1/SE_IV
−2
−1
0
1
2
β_IV

Batch 612 : Gut microbiota abundance (family Desulfovibrionaceae id.3169) on Ulcerative colitis (strict) with PSC

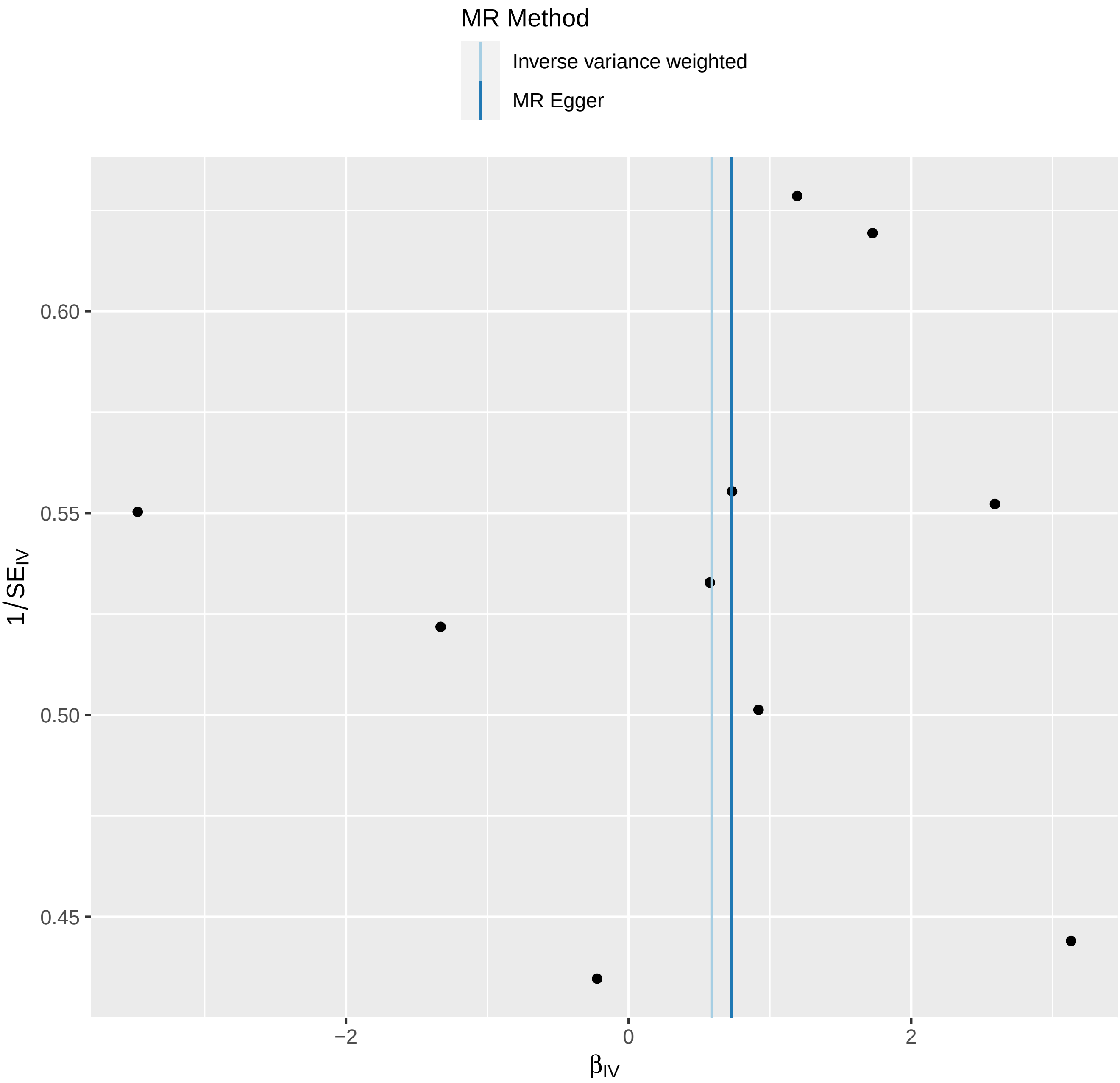

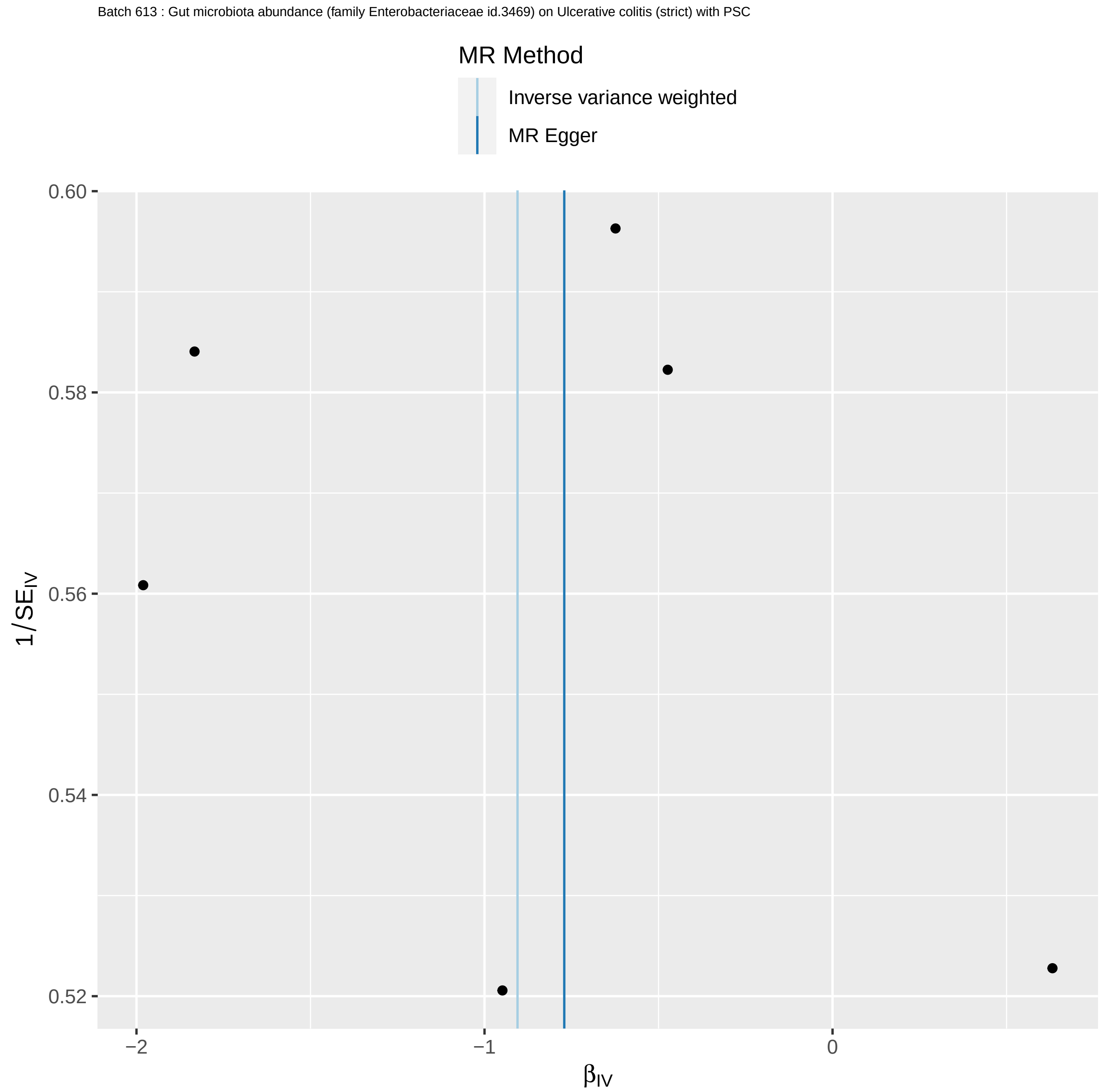

Batch 613 : Gut microbiota abundance (family Enterobacteriaceae id.3469) on Ulcerative colitis (strict) with PSC
MR Method
Inverse variance weighted
MR Egger
0.60
0.58
0.56
0.54
0.52
1/SE_IV
−2
−1
0
β_IV

Batch 614 : Gut microbiota abundance (family Erysipelotrichaceae id.2149) on Ulcerative colitis (strict) with PSC

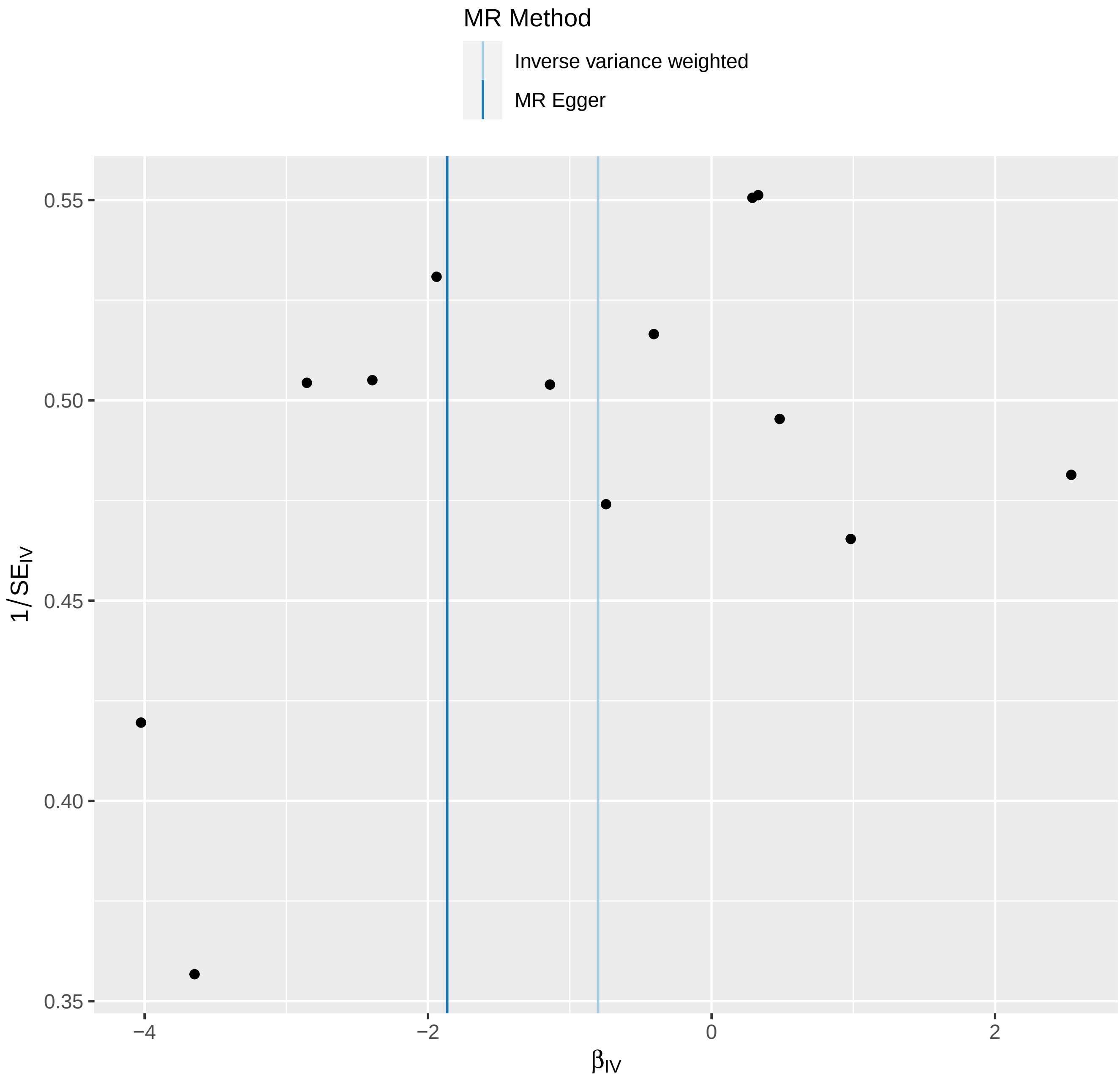

Batch 615 : Gut microbiota abundance (family Family XI id.1936) on Ulcerative colitis (strict) with PSC

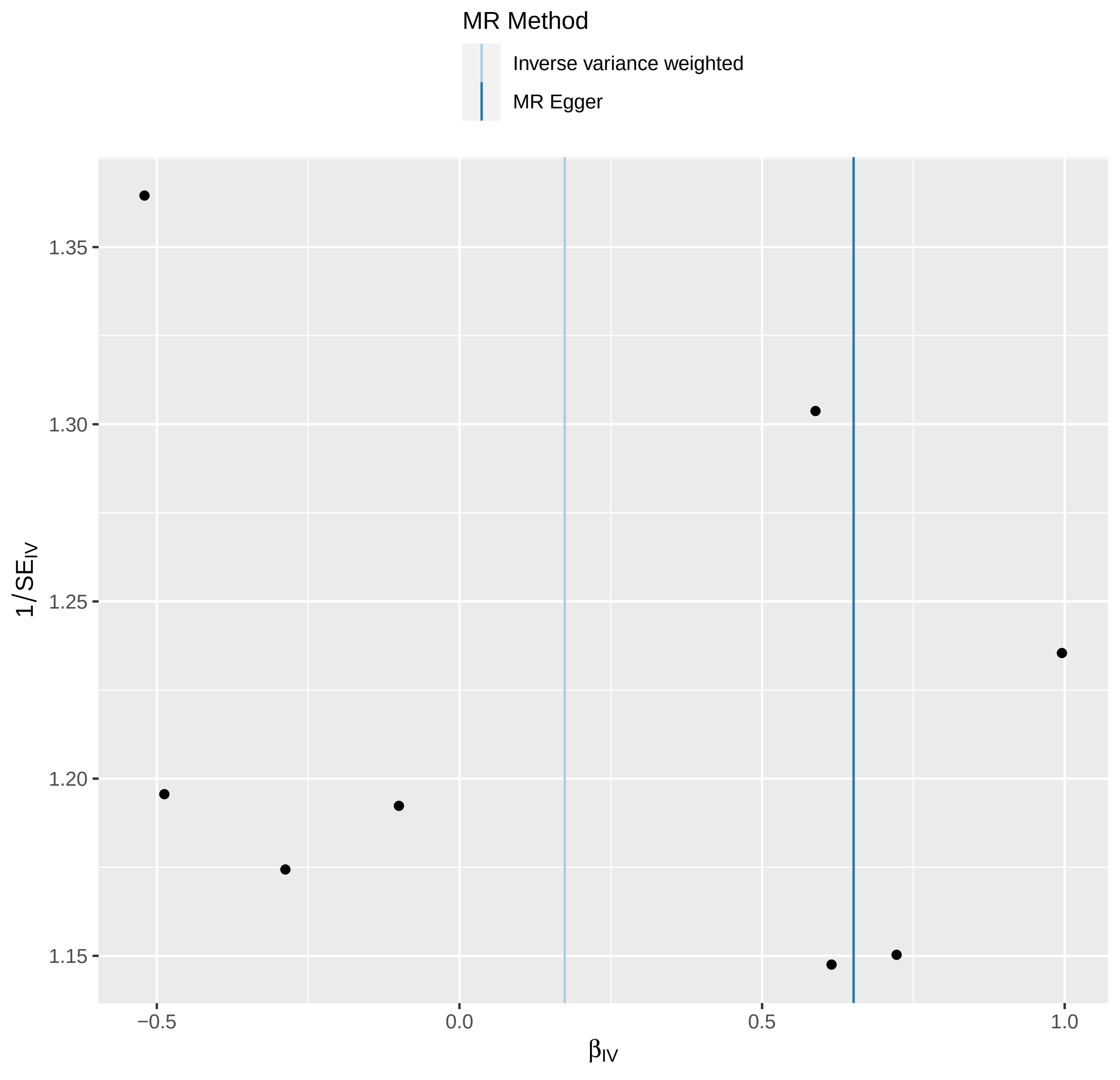

Batch 616 : Gut microbiota abundance (family Family XIII id.1957) on Ulcerative colitis (strict) with PSC

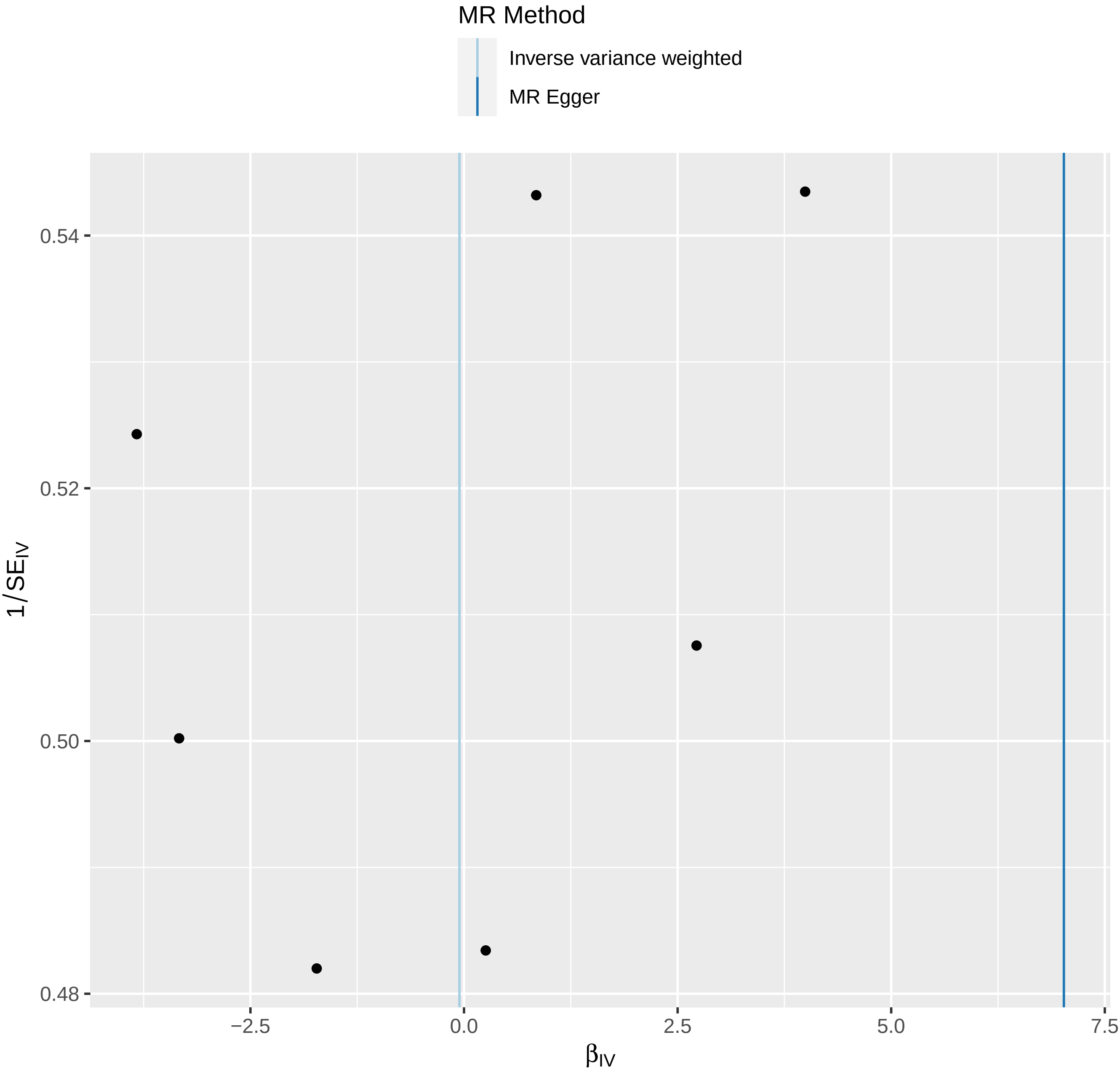

Batch 617 : Gut microbiota abundance (family Lachnospiraceae id.1987) on Ulcerative colitis (strict) with PSC

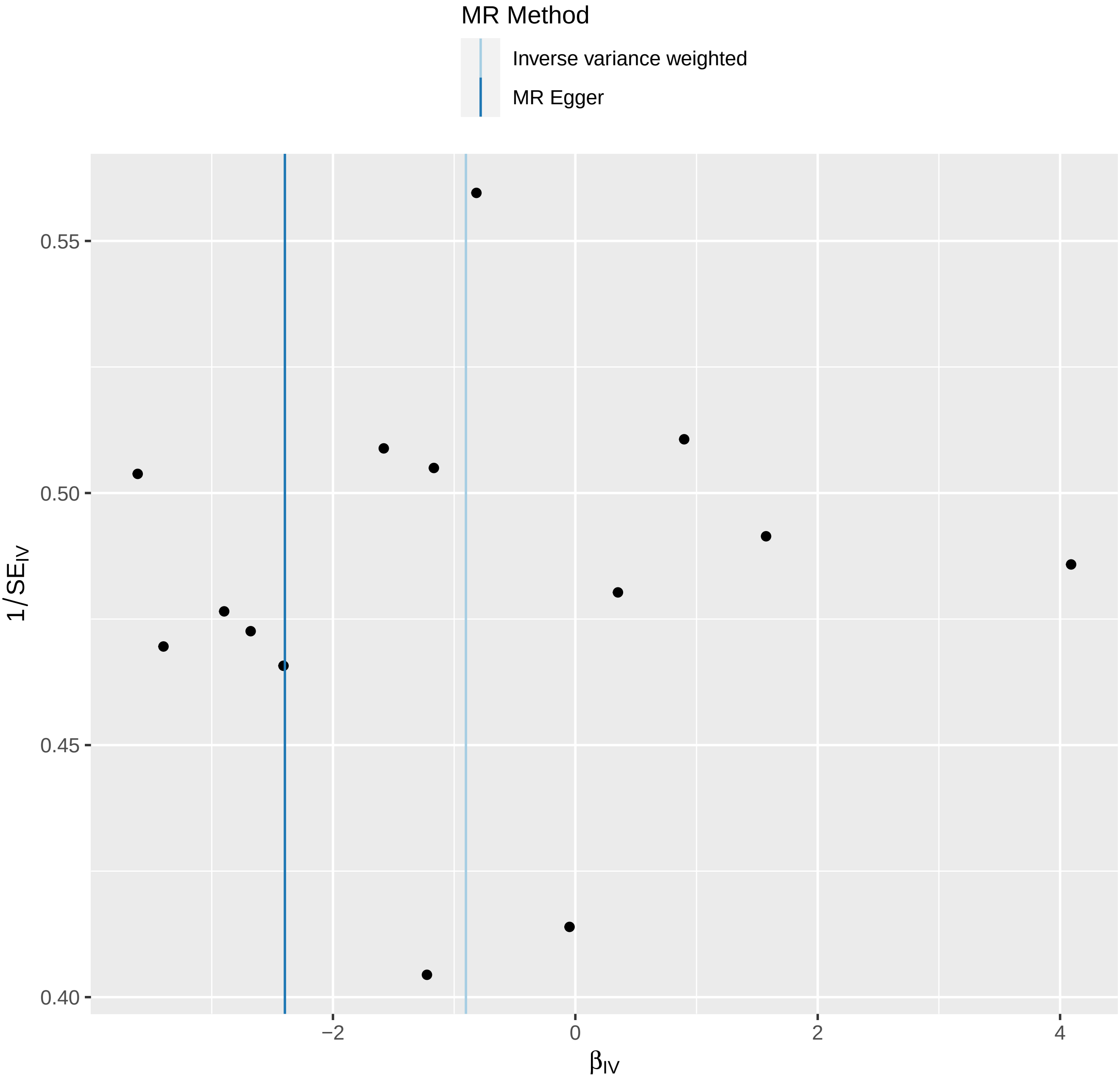

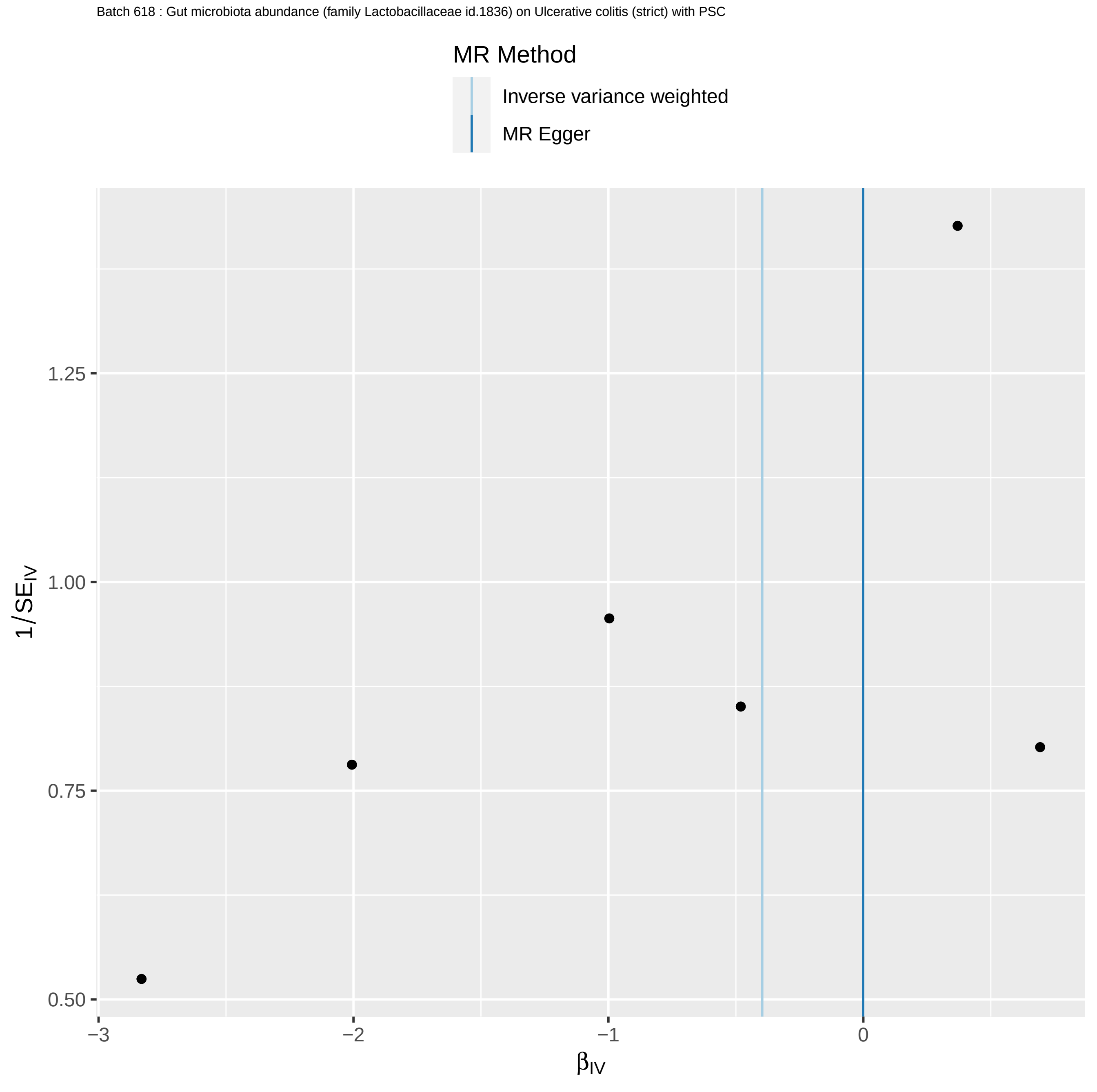
Batch 618 : Gut microbiota abundance (family Lactobacillaceae id.1836) on Ulcerative colitis (strict) with PSC
MR Method
Inverse variance weighted
MR Egger
1.25
1.00
0.75
0.50
$1/SE_{IV}$
−3
−2
−1
0
β_{IV}

Batch 619 : Gut microbiota abundance (family Methanobacteriaceae id.121) on Ulcerative colitis (strict) with PSC

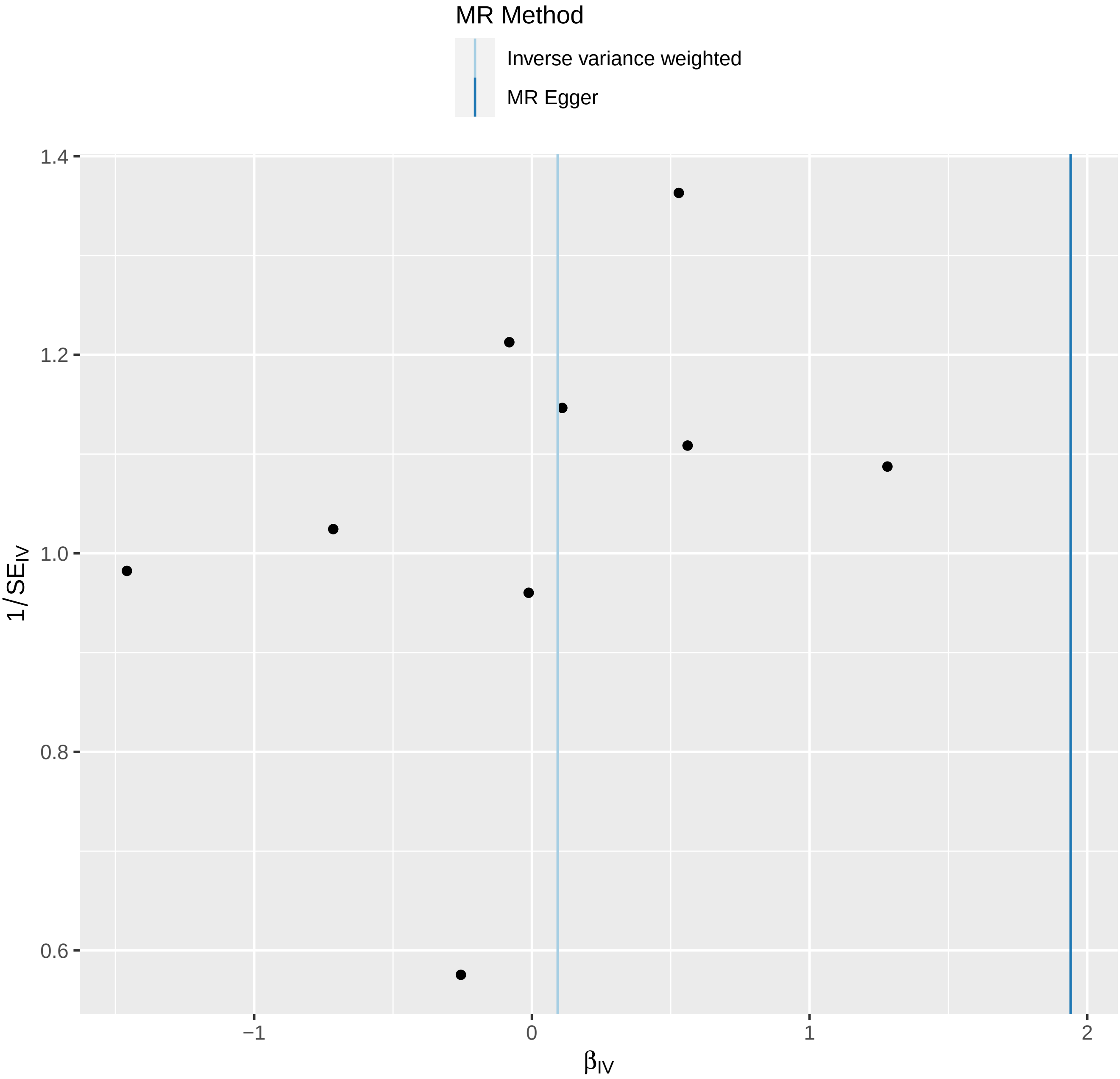

Batch 620 : Gut microbiota abundance (family Oxalobacteraceae id.2966) on Ulcerative colitis (strict) with PSC

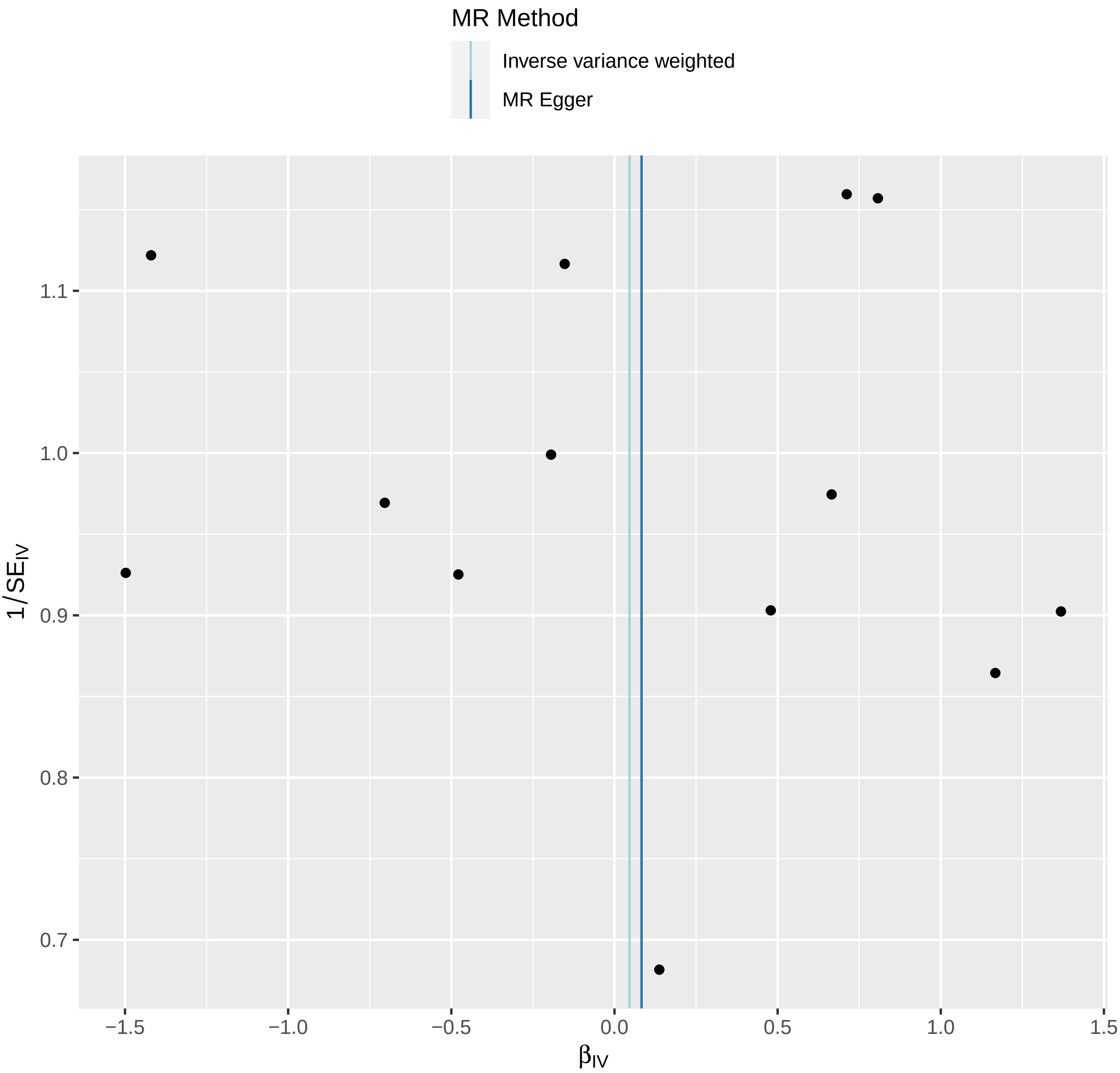

Batch 621 : Gut microbiota abundance (family Pasteurellaceae id.3689) on Ulcerative colitis (strict) with PSC

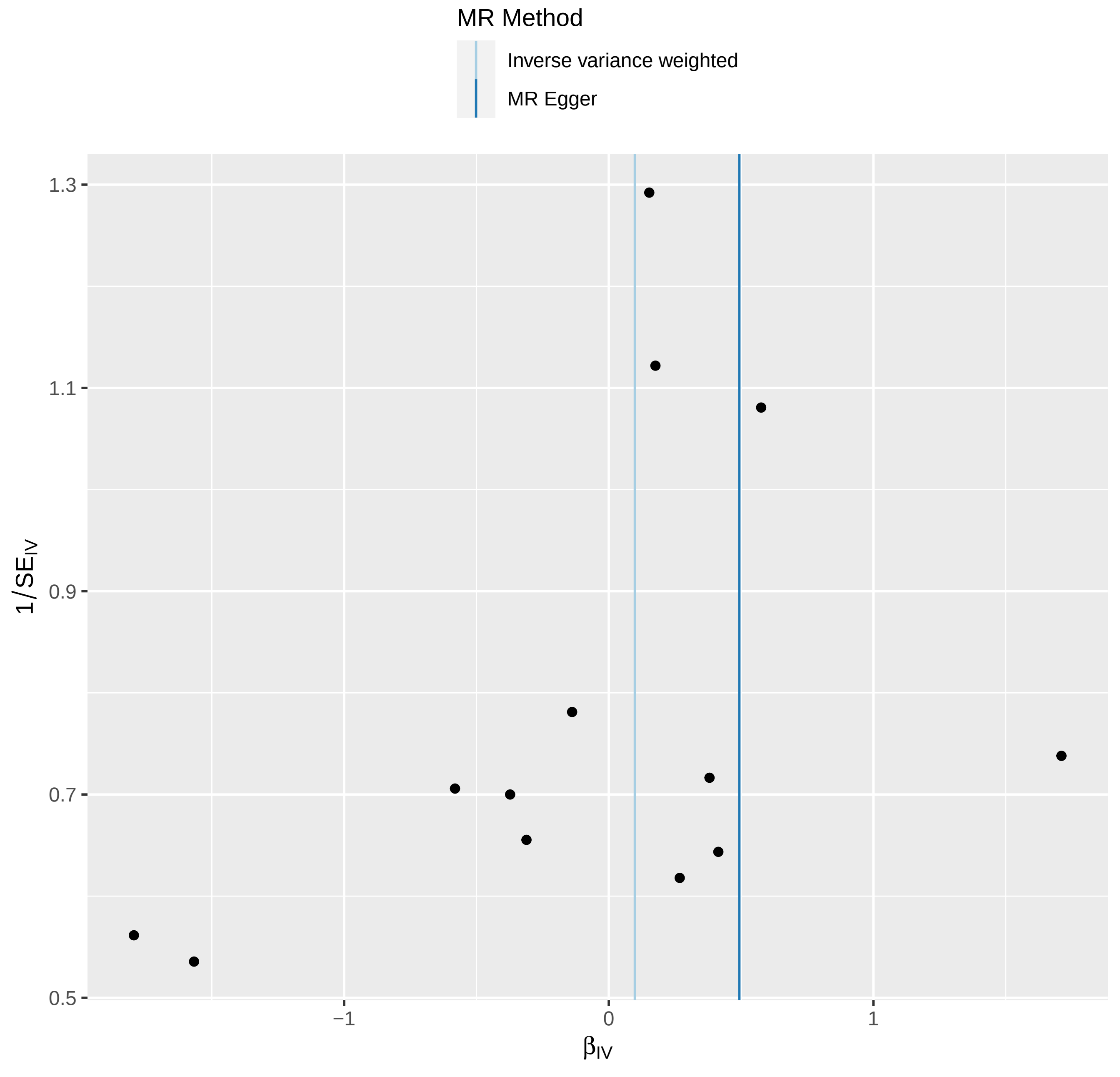

Batch 622 : Gut microbiota abundance (family Peptococcaceae id.2024) on Ulcerative colitis (strict) with PSC

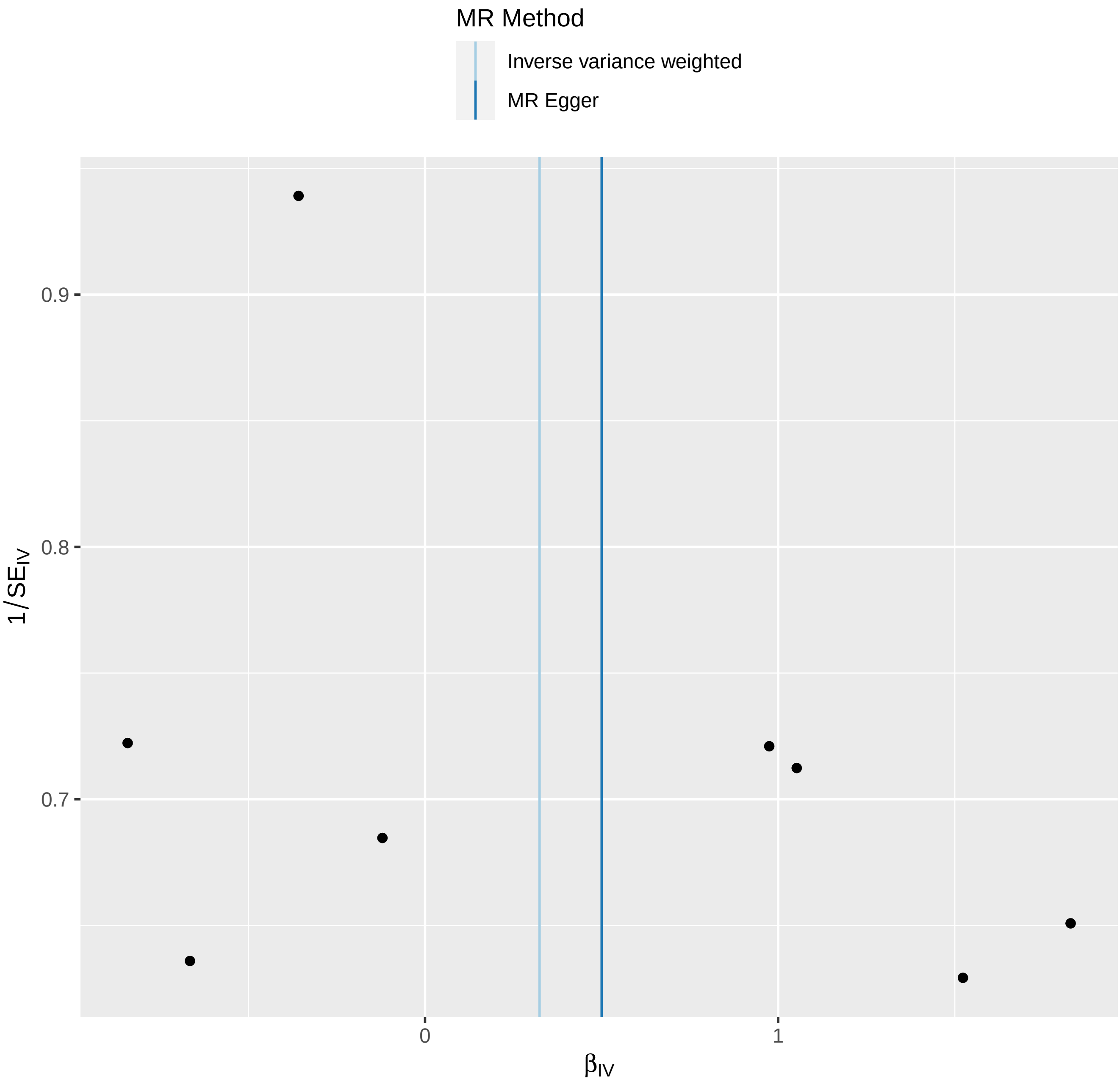

Batch 623 : Gut microbiota abundance (family Peptostreptococcaceae id.2042) on Ulcerative colitis (strict) with PSC

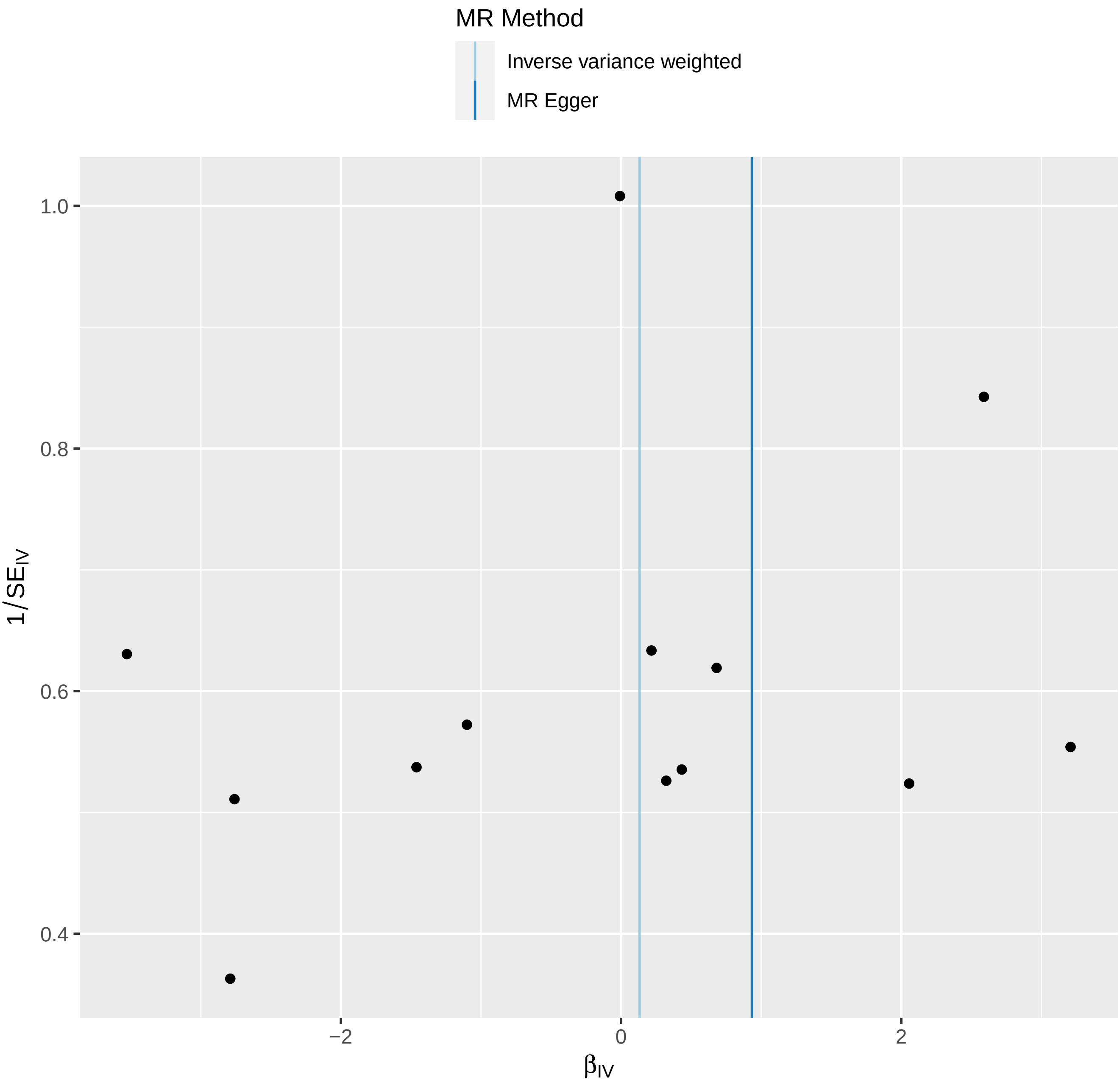

Batch 624 : Gut microbiota abundance (family Porphyromonadaceae id.943) on Ulcerative colitis (strict) with PSC

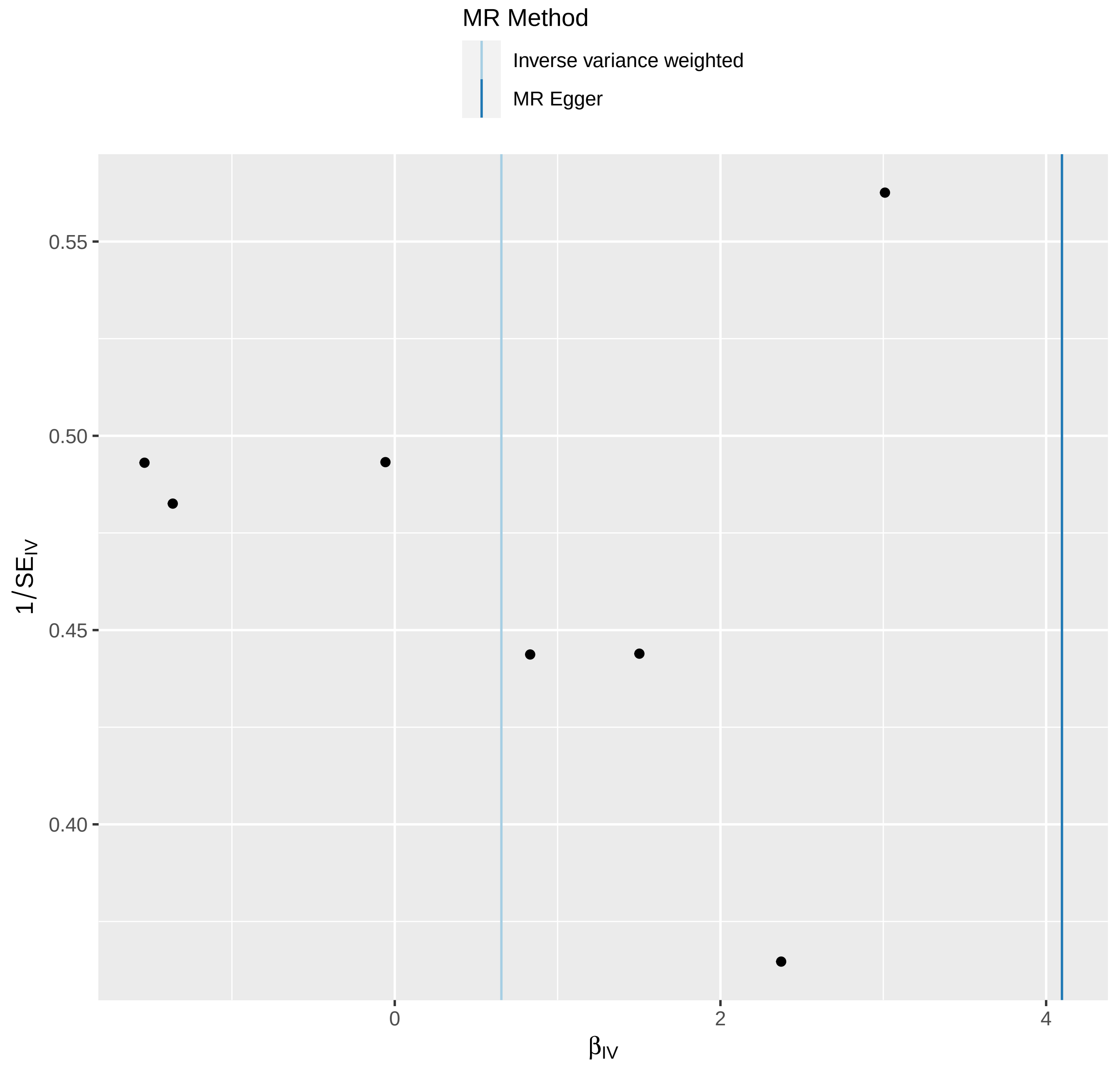

Batch 625 : Gut microbiota abundance (family Prevotellaceae id.960) on Ulcerative colitis (strict) with PSC

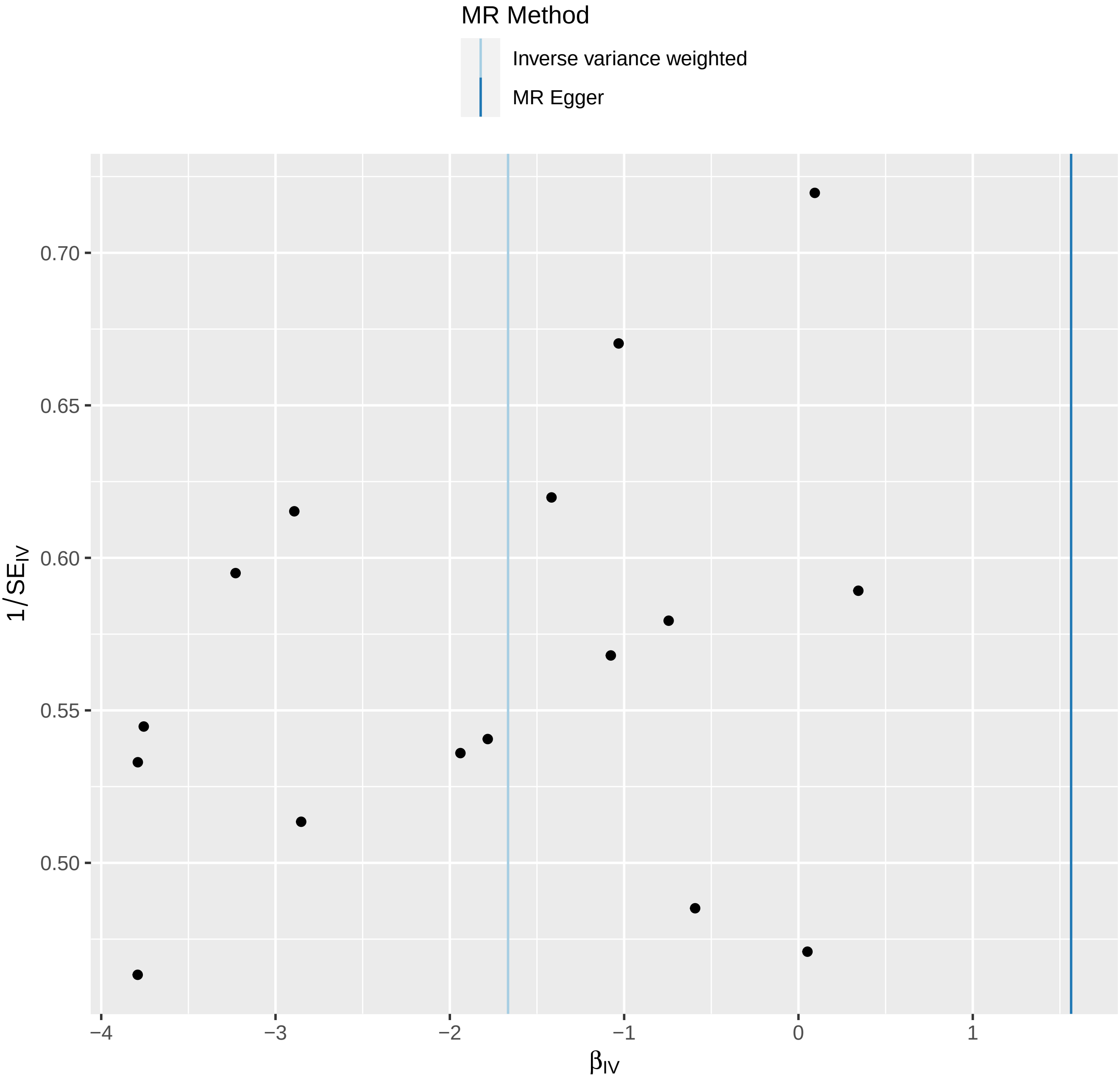

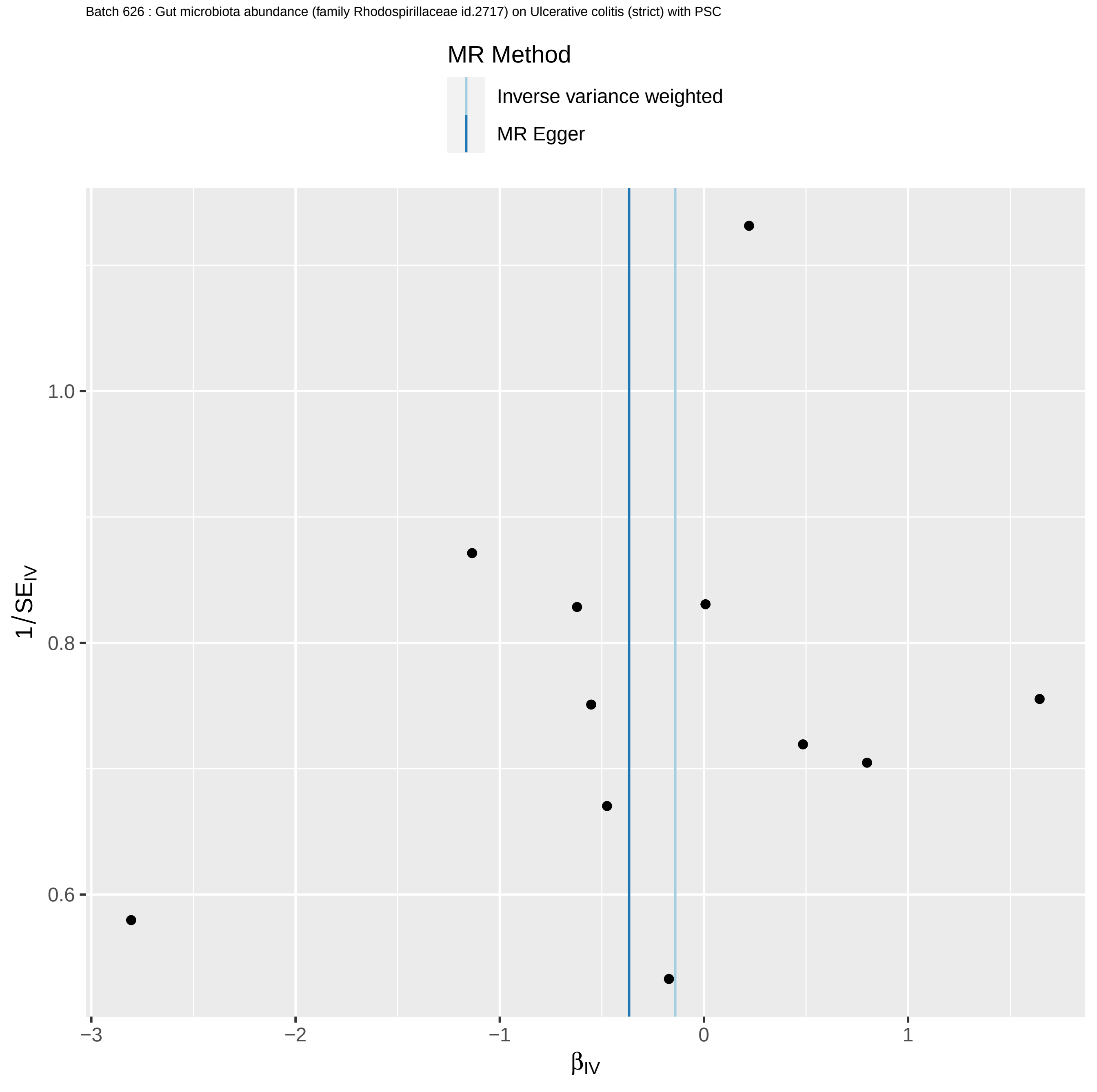

Batch 626 : Gut microbiota abundance (family Rhodospirillaceae id.2717) on Ulcerative colitis (strict) with PSC
MR Method
Inverse variance weighted
MR Egger
1.0
0.8
0.6
1/SE_IV
−3
−2
−1
0
1
β_IV

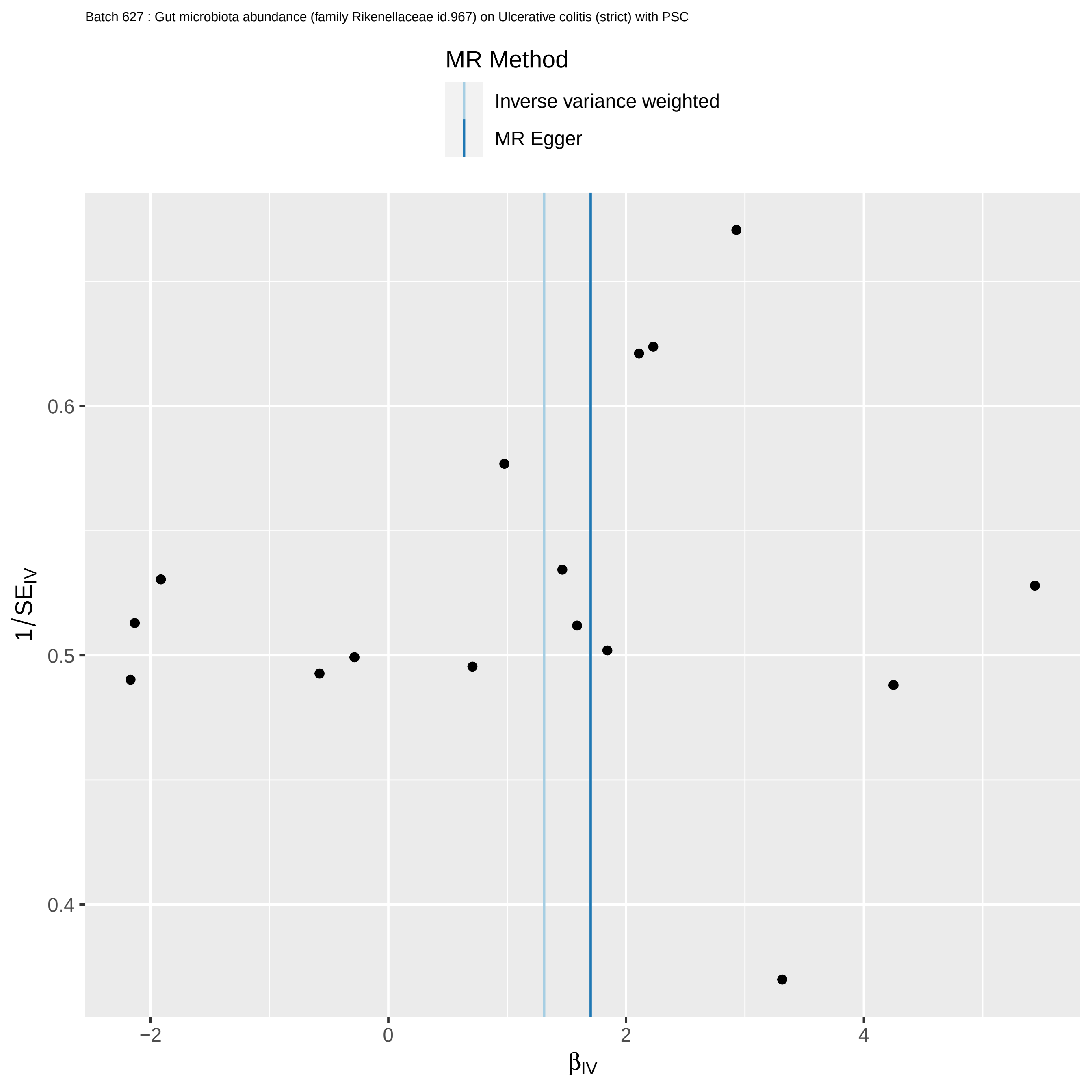

Batch 627 : Gut microbiota abundance (family Rikenellaceae id.967) on Ulcerative colitis (strict) with PSC
MR Method
Inverse variance weighted
MR Egger
0.6
0.5
0.4
−2
0
2
4
1/SE_IV
β_IV

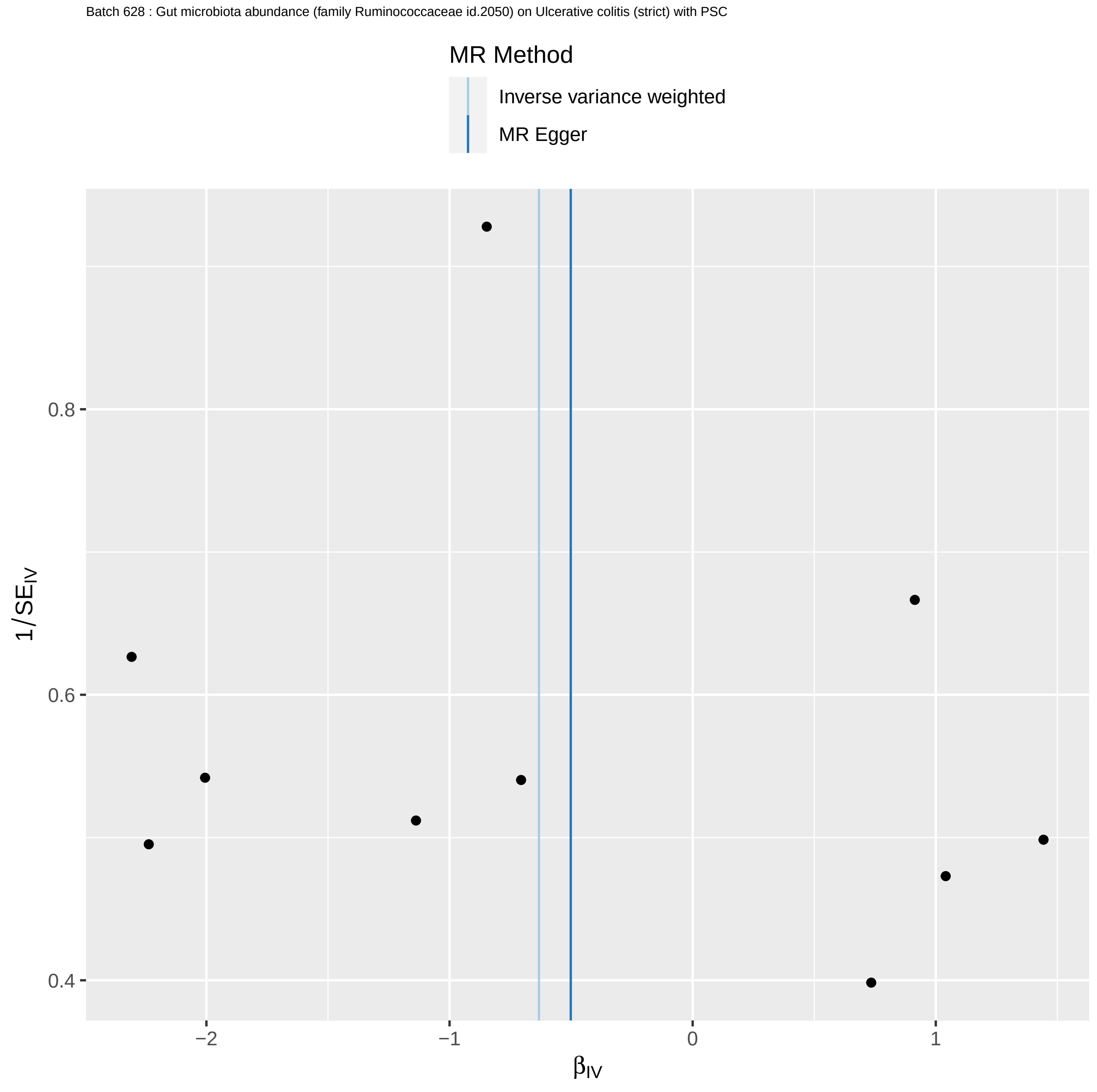
Batch 628 : Gut microbiota abundance (family Ruminococcaceae id.2050) on Ulcerative colitis (strict) with PSC
MR Method
Inverse variance weighted
MR Egger
0.8
0.6
0.4
$1/SE_{IV}$
−2
−1
0
1
β_{IV}

Batch 629 : Gut microbiota abundance (family Streptococcaceae id.1850) on Ulcerative colitis (strict) with PSC

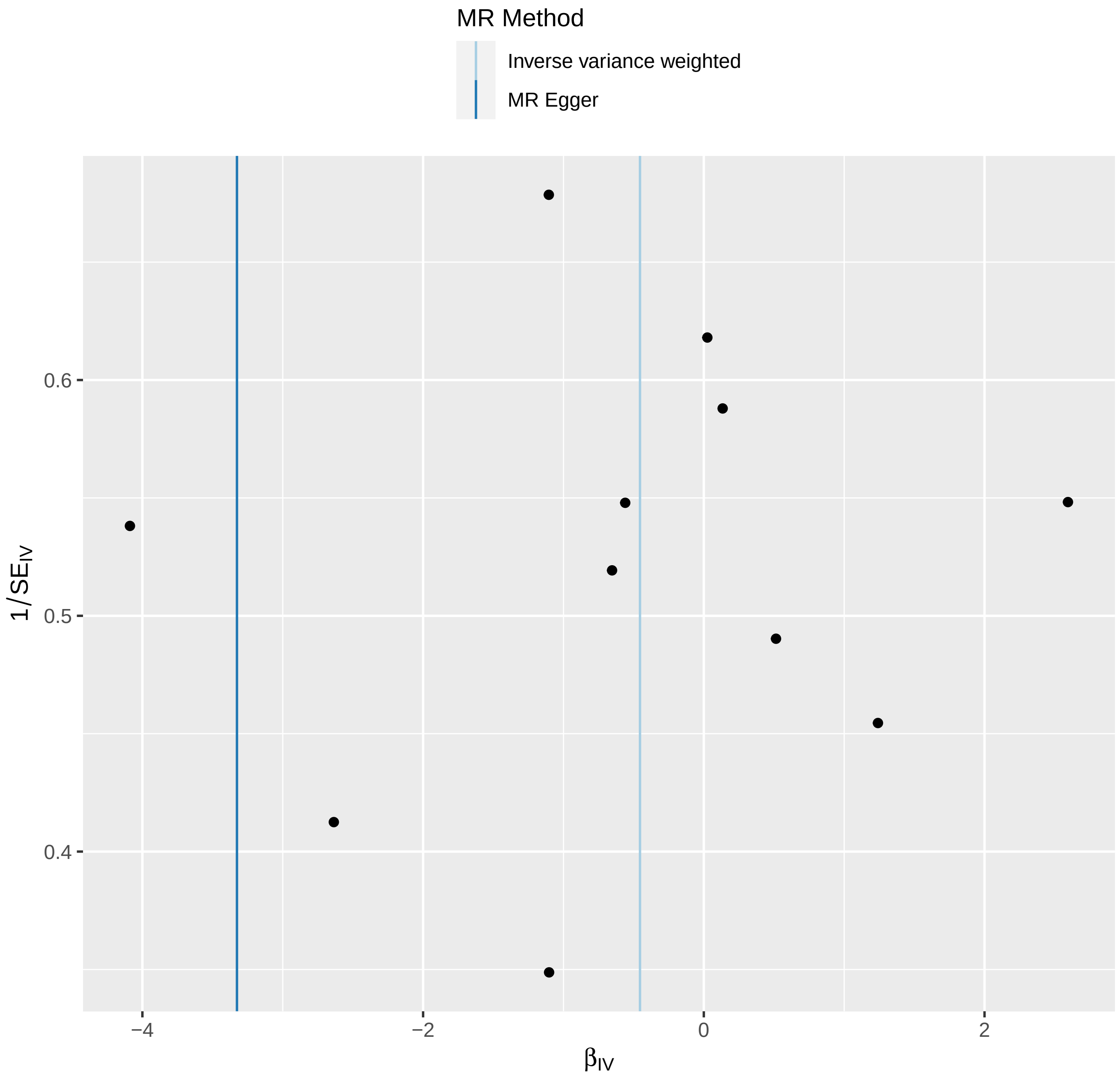

Batch 630 : Gut microbiota abundance (family Veillonellaceae id.2172) on Ulcerative colitis (strict) with PSC

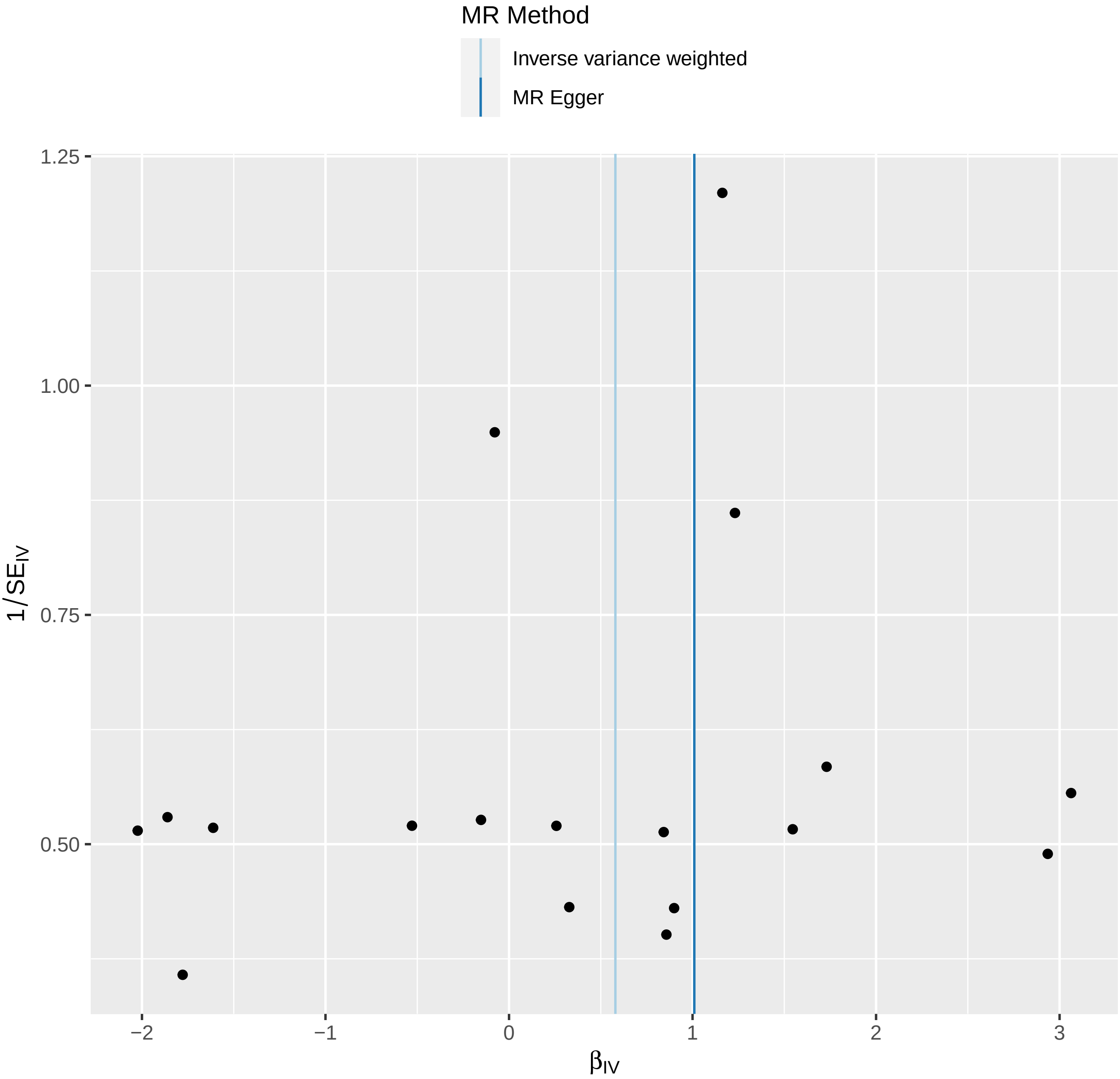

Batch 631 : Gut microbiota abundance (family Verrucomicrobiaceae id.4036) on Ulcerative colitis (strict) with PSC

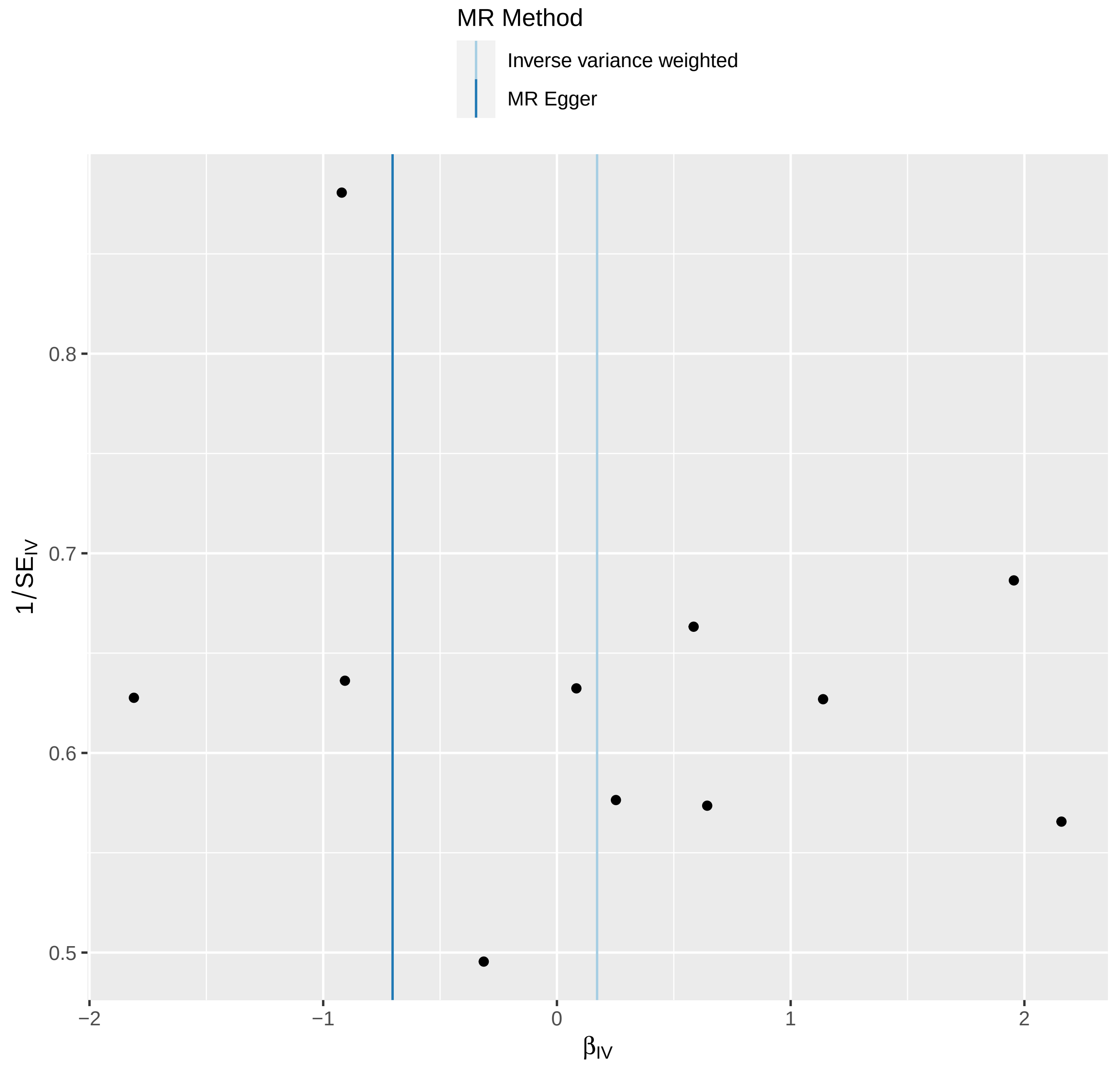

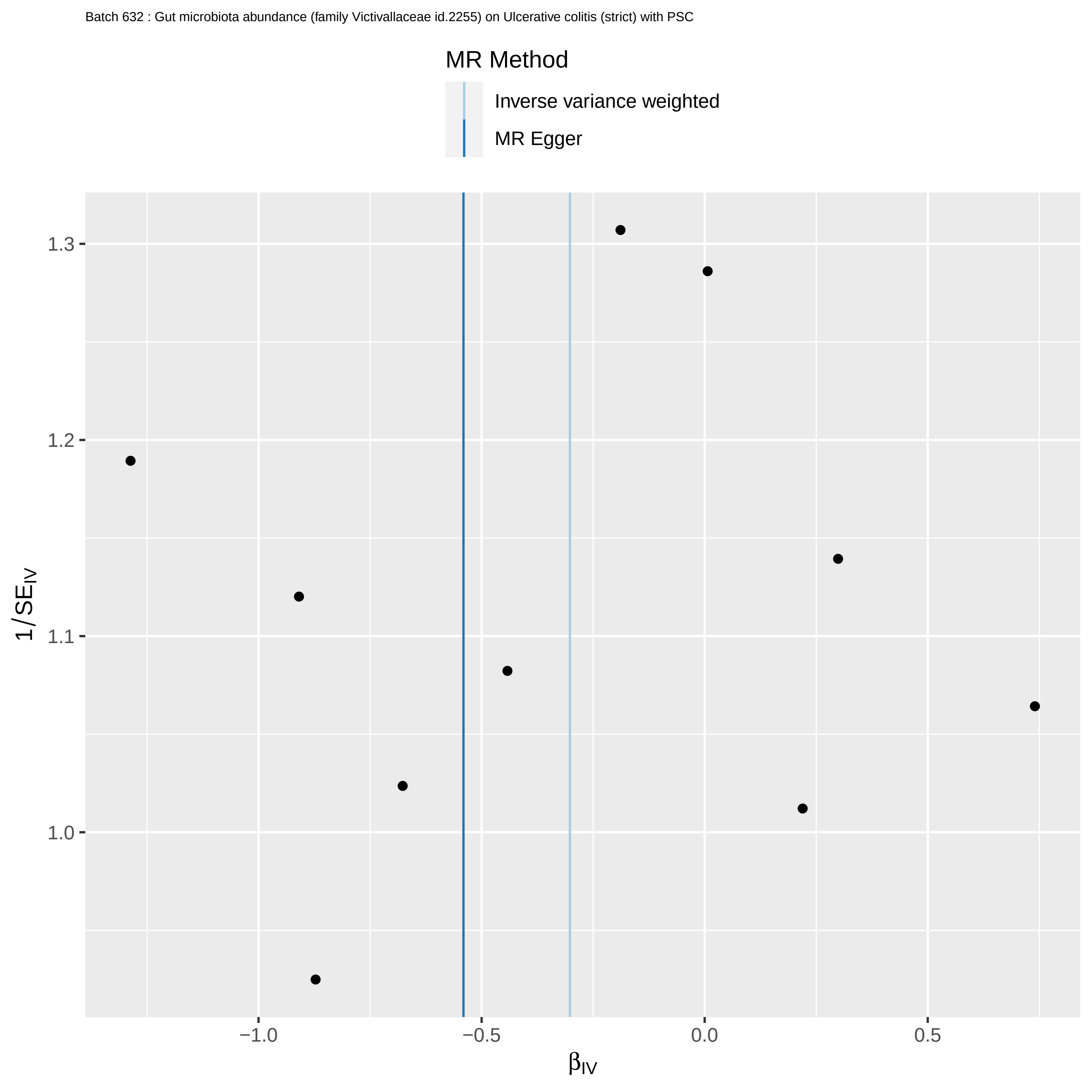
Batch 632 : Gut microbiota abundance (family Victivallaceae id.2255) on Ulcerative colitis (strict) with PSC
MR Method
Inverse variance weighted
MR Egger
1.3
1.2
1.1
1.0
1/SE_IV
−1.0
−0.5
0.0
0.5
β_IV

Batch 633 : Gut microbiota abundance (genus Actinomyces id.423) on Ulcerative colitis (strict) with PSC

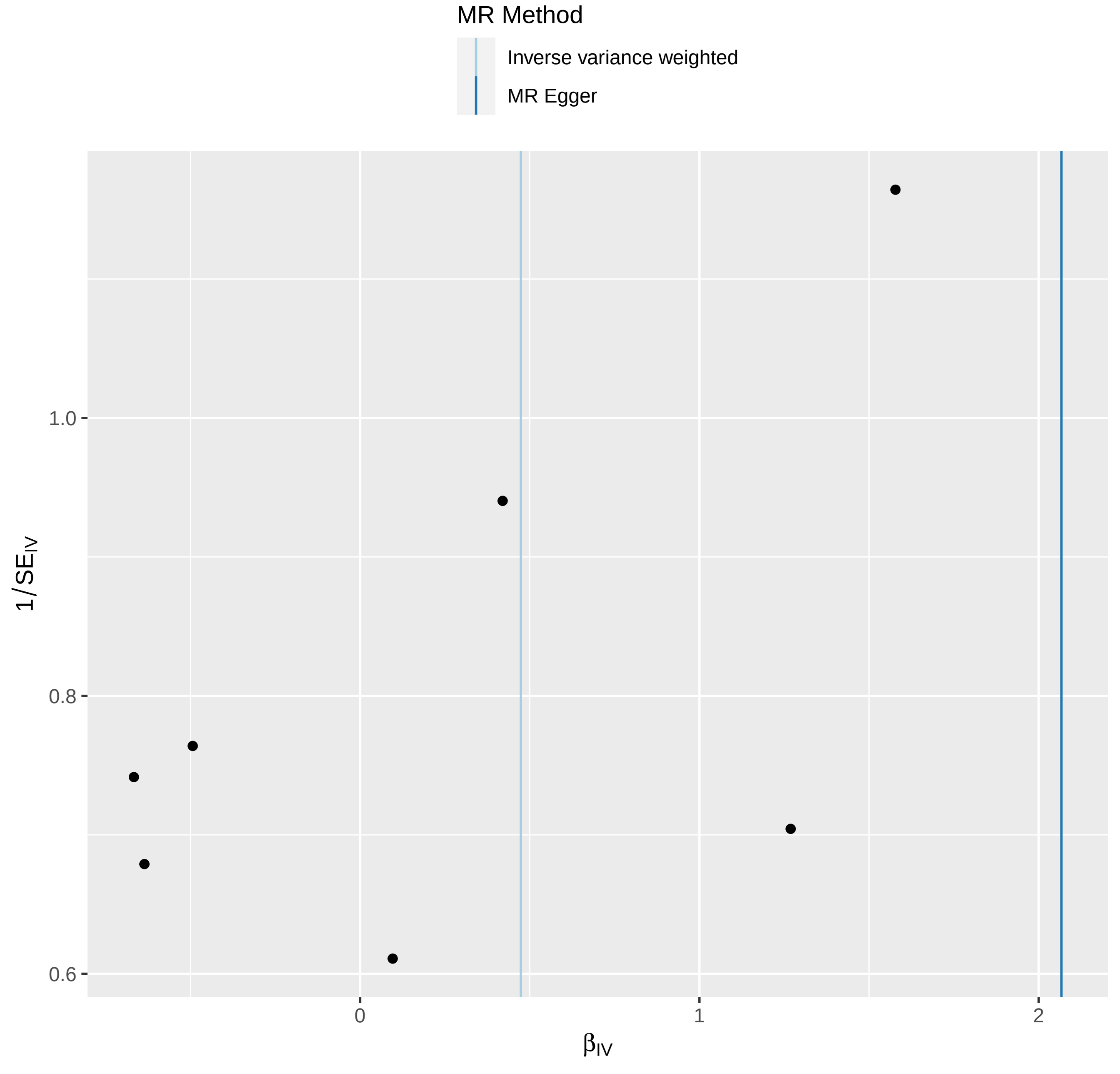

Batch 634 : Gut microbiota abundance (genus Adlercreutzia id.812) on Ulcerative colitis (strict) with PSC

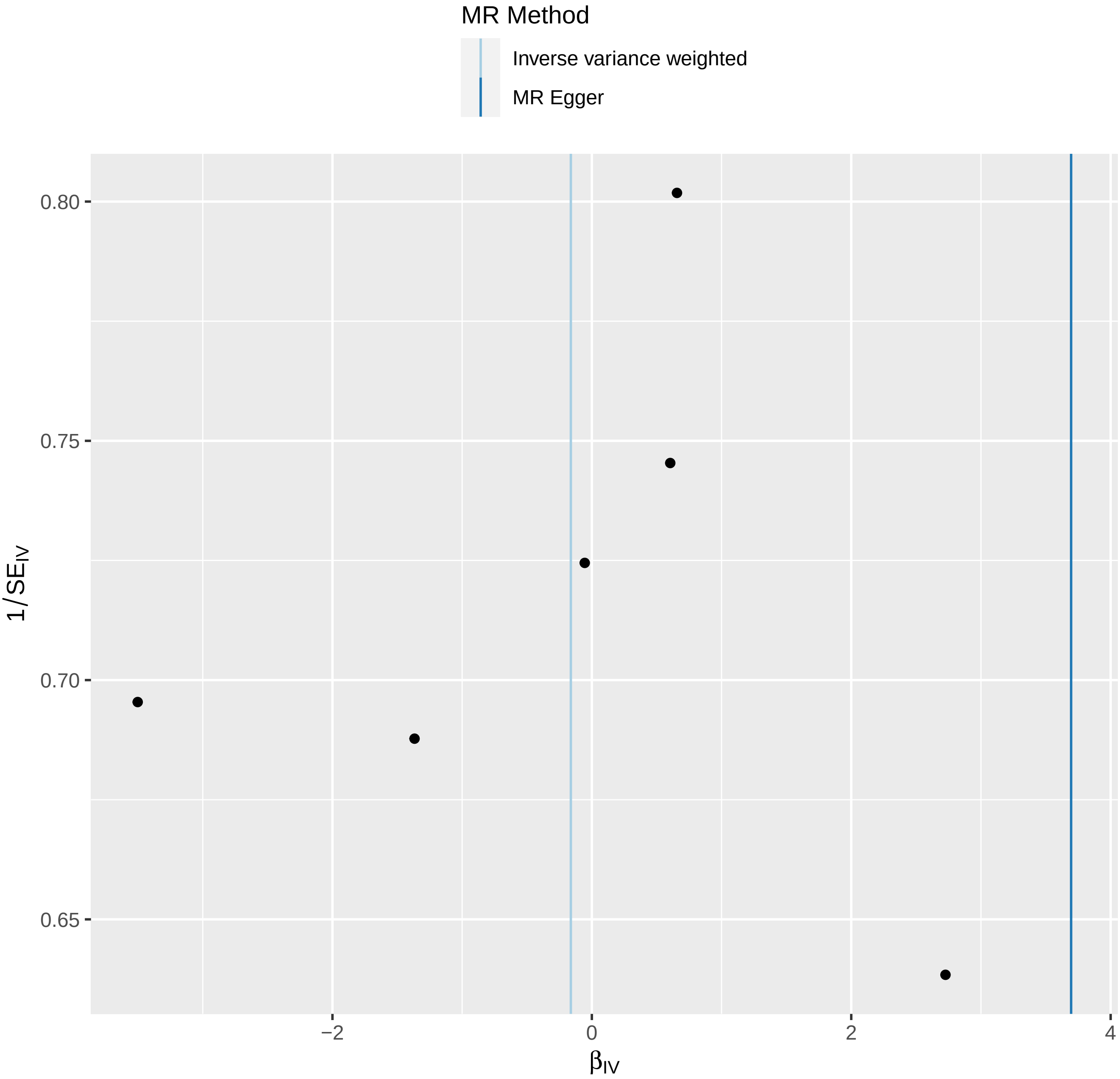

Batch 635 : Gut microbiota abundance (genus Akkermansia id.4037) on Ulcerative colitis (strict) with PSC

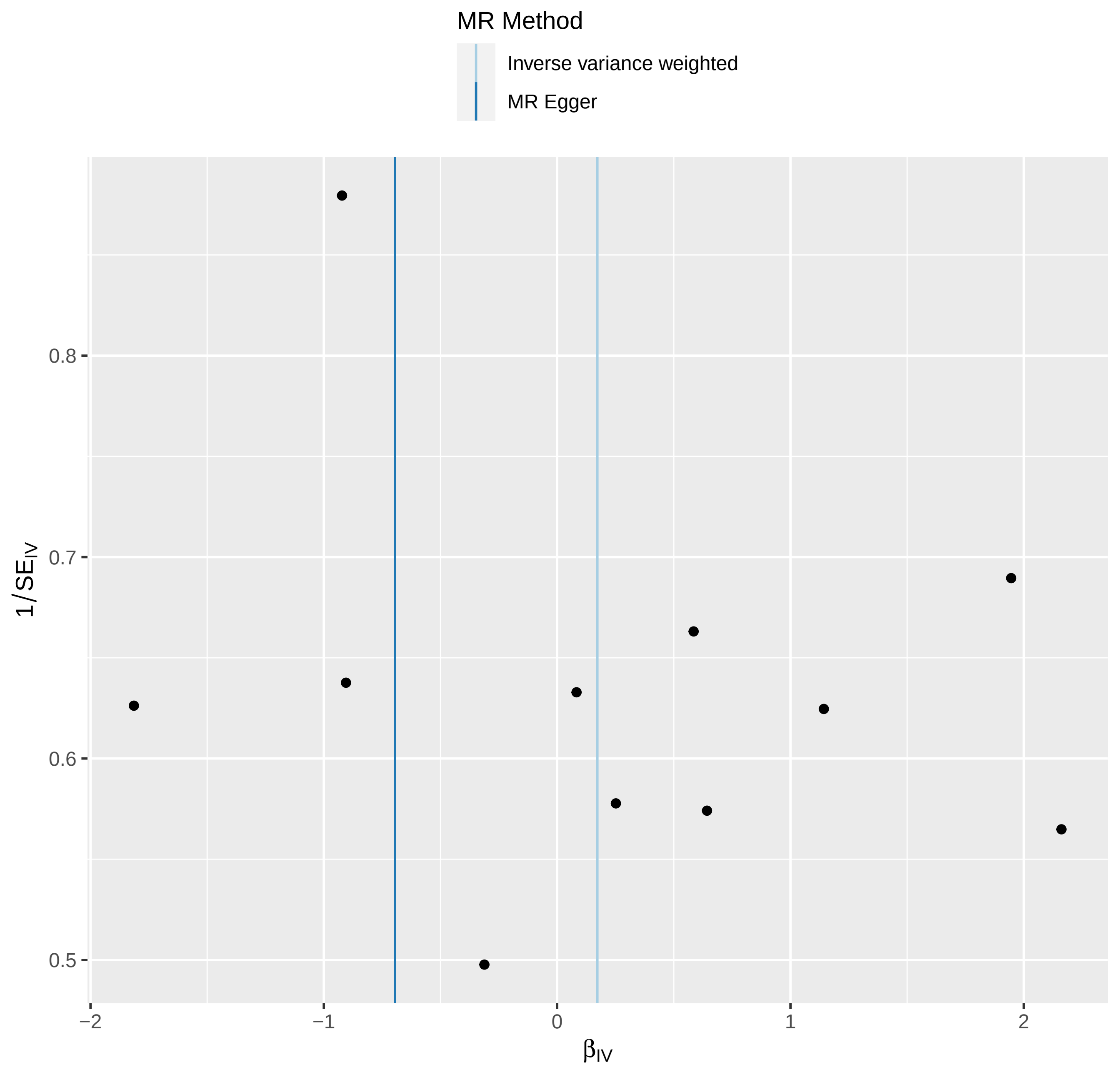

Batch 636 : Gut microbiota abundance (genus Alistipes id.968) on Ulcerative colitis (strict) with PSC

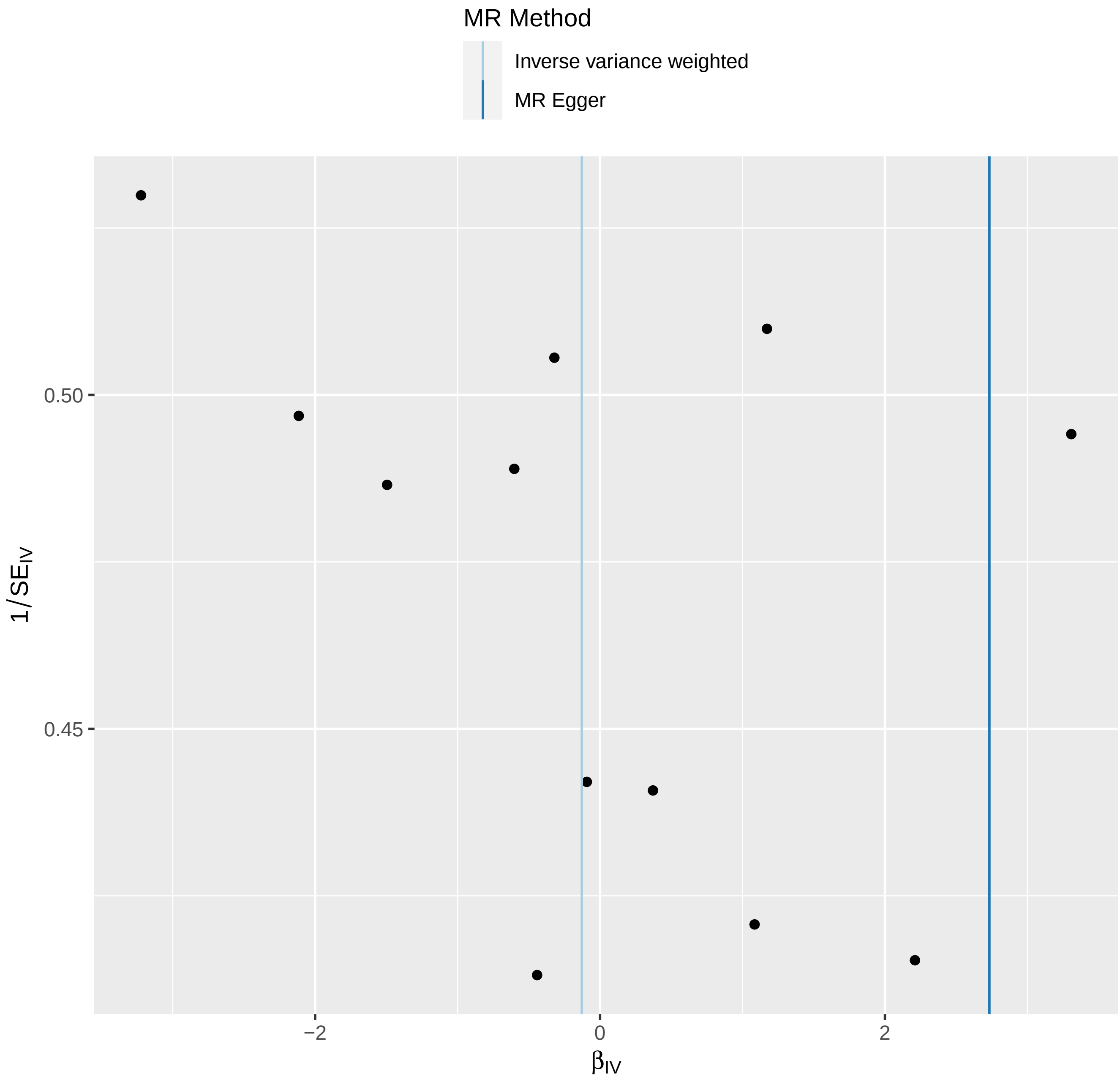

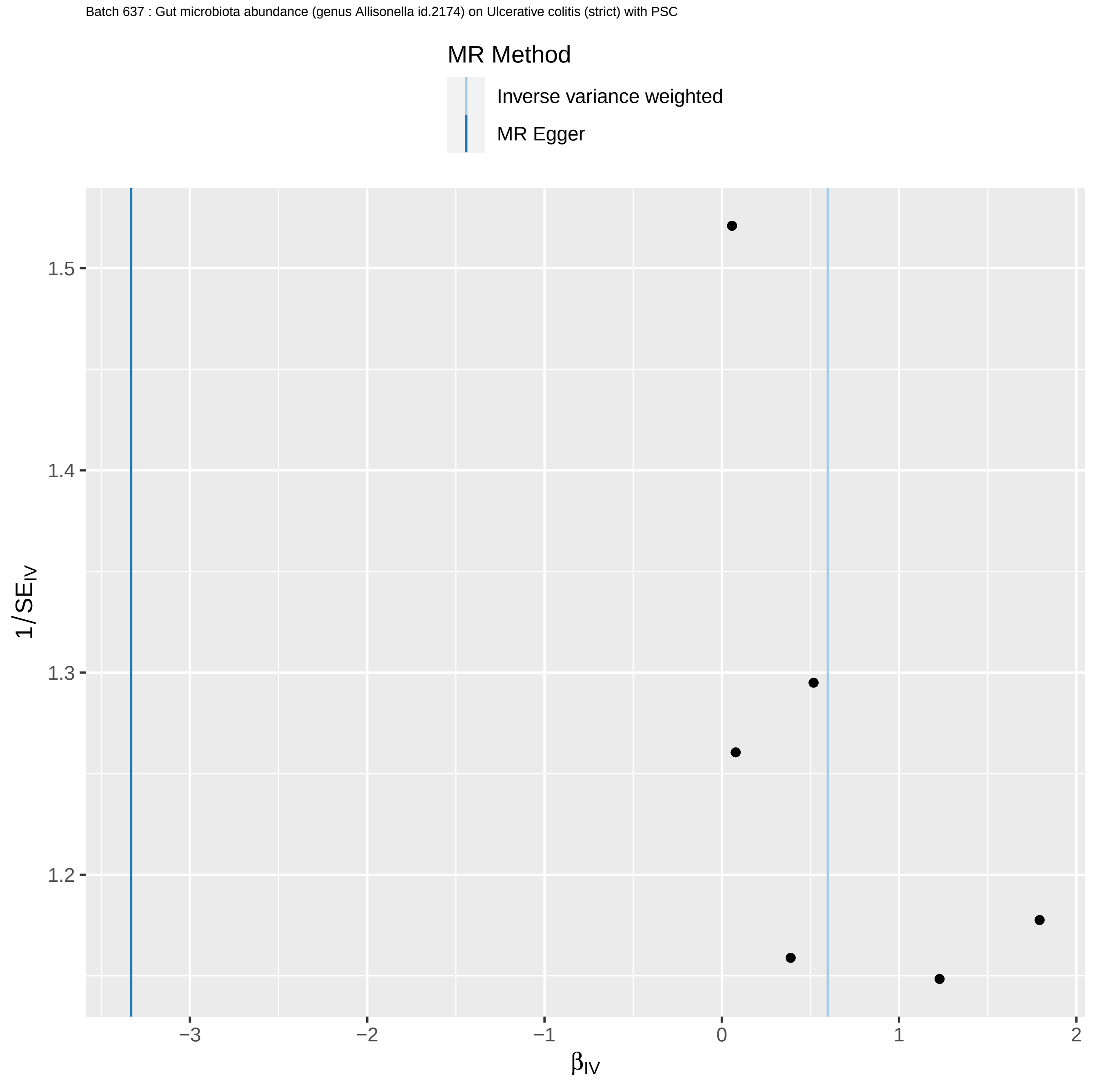
Batch 637 : Gut microbiota abundance (genus Allisonella id.2174) on Ulcerative colitis (strict) with PSC
MR Method
Inverse variance weighted
MR Egger
1.5
1.4
1.3
1.2
1/SE_IV
−3
−2
−1
0
1
2
β_IV

Batch 638 : Gut microbiota abundance (genus Alloprevotella id.961) on Ulcerative colitis (strict) with PSC

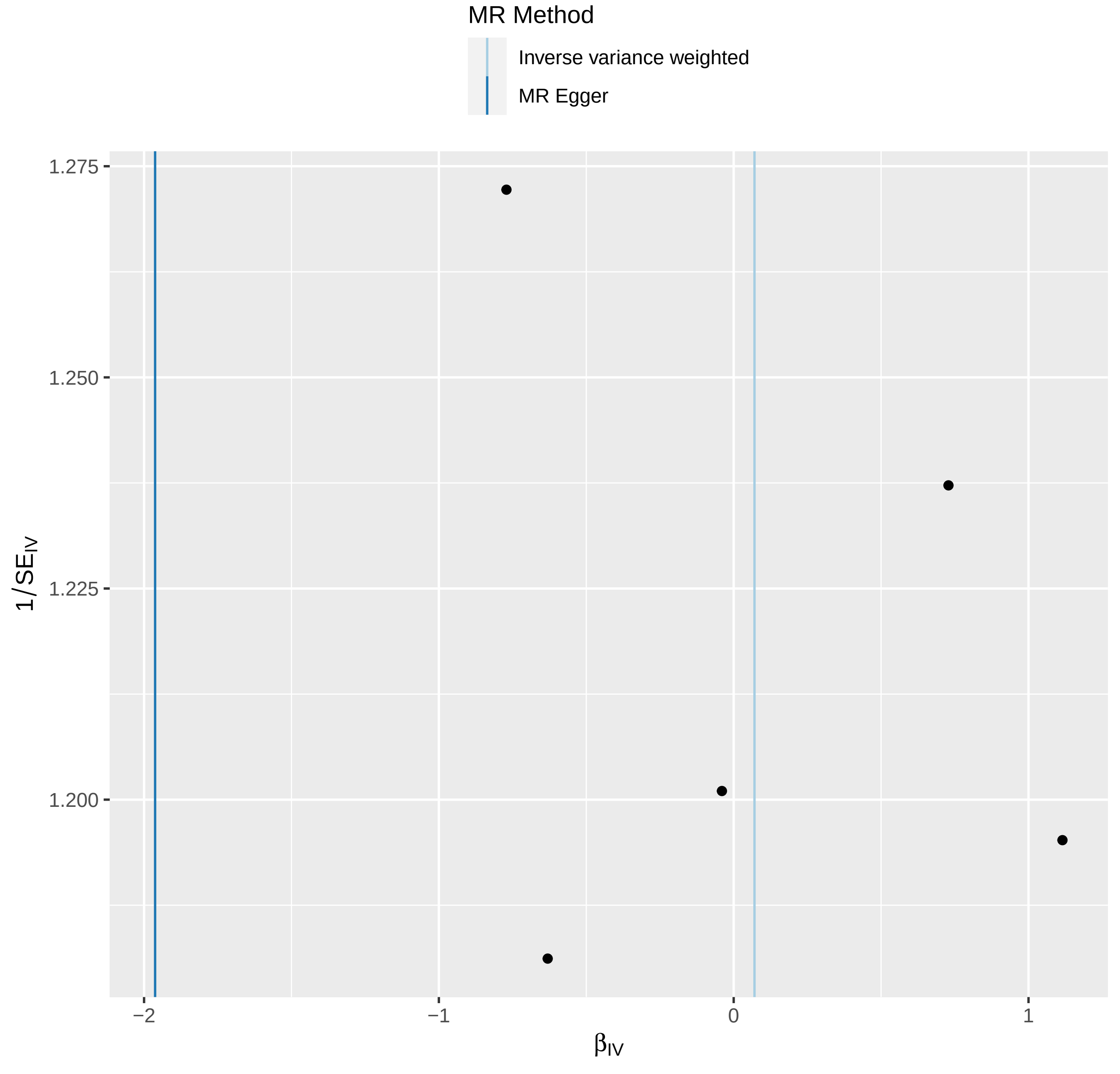

Batch 639 : Gut microbiota abundance (genus Anaerofilum id.2053) on Ulcerative colitis (strict) with PSC

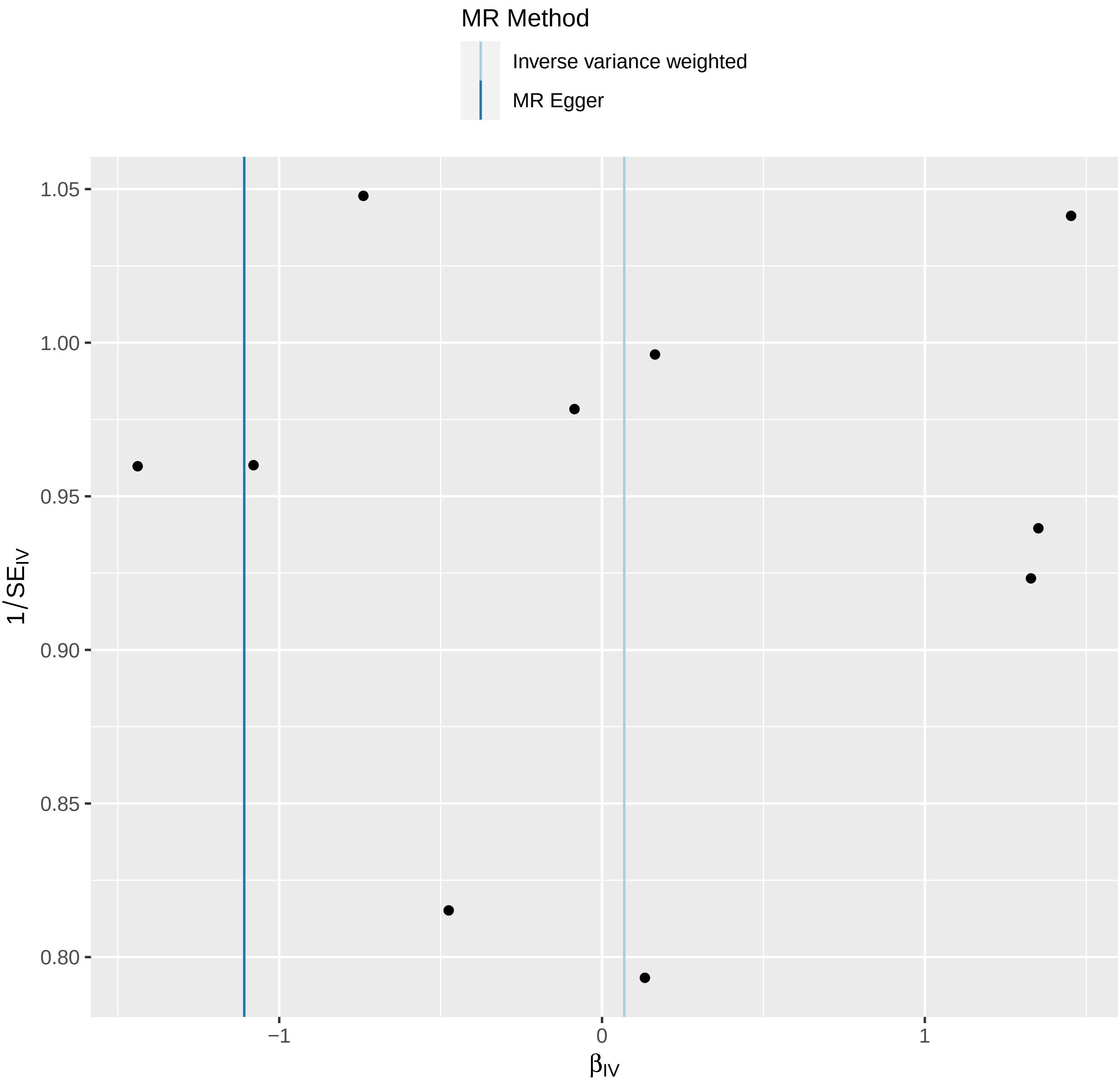

Batch 640 : Gut microbiota abundance (genus Anaerostipes id.1991) on Ulcerative colitis (strict) with PSC

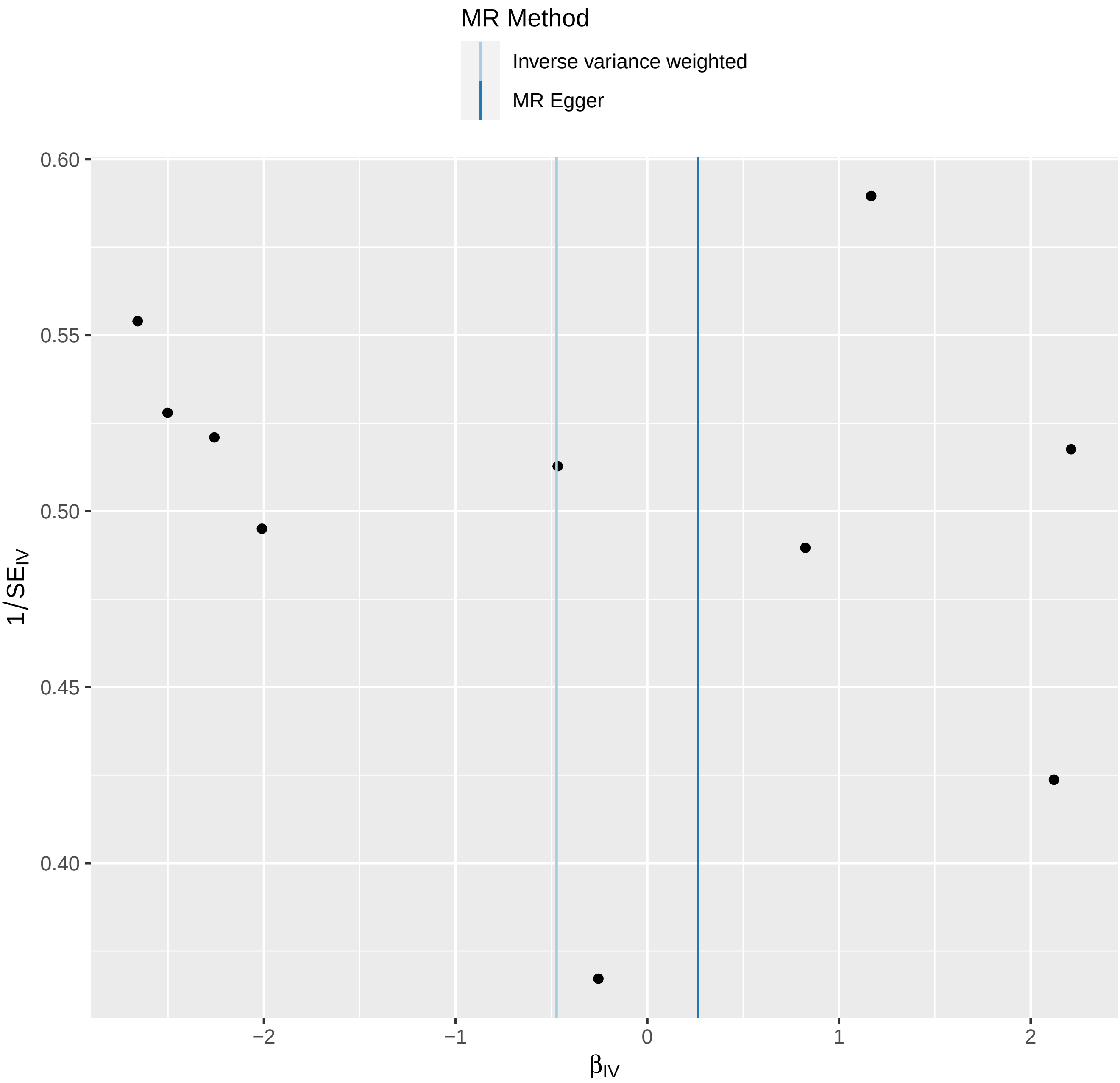

Batch 641 : Gut microbiota abundance (genus Anaerotruncus id.2054) on Ulcerative colitis (strict) with PSC

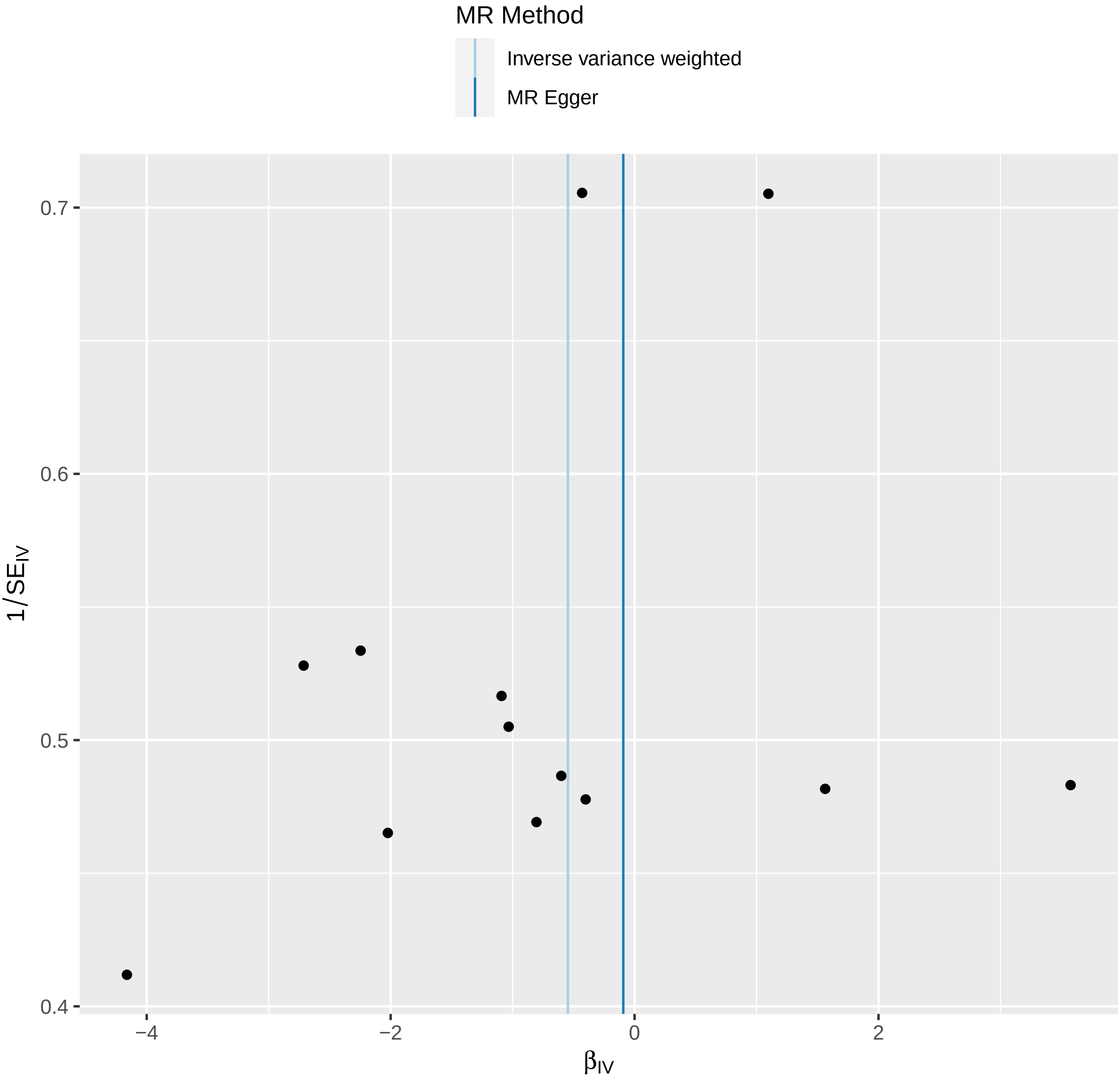

Batch 642 : Gut microbiota abundance (genus Bacteroides id.918) on Ulcerative colitis (strict) with PSC

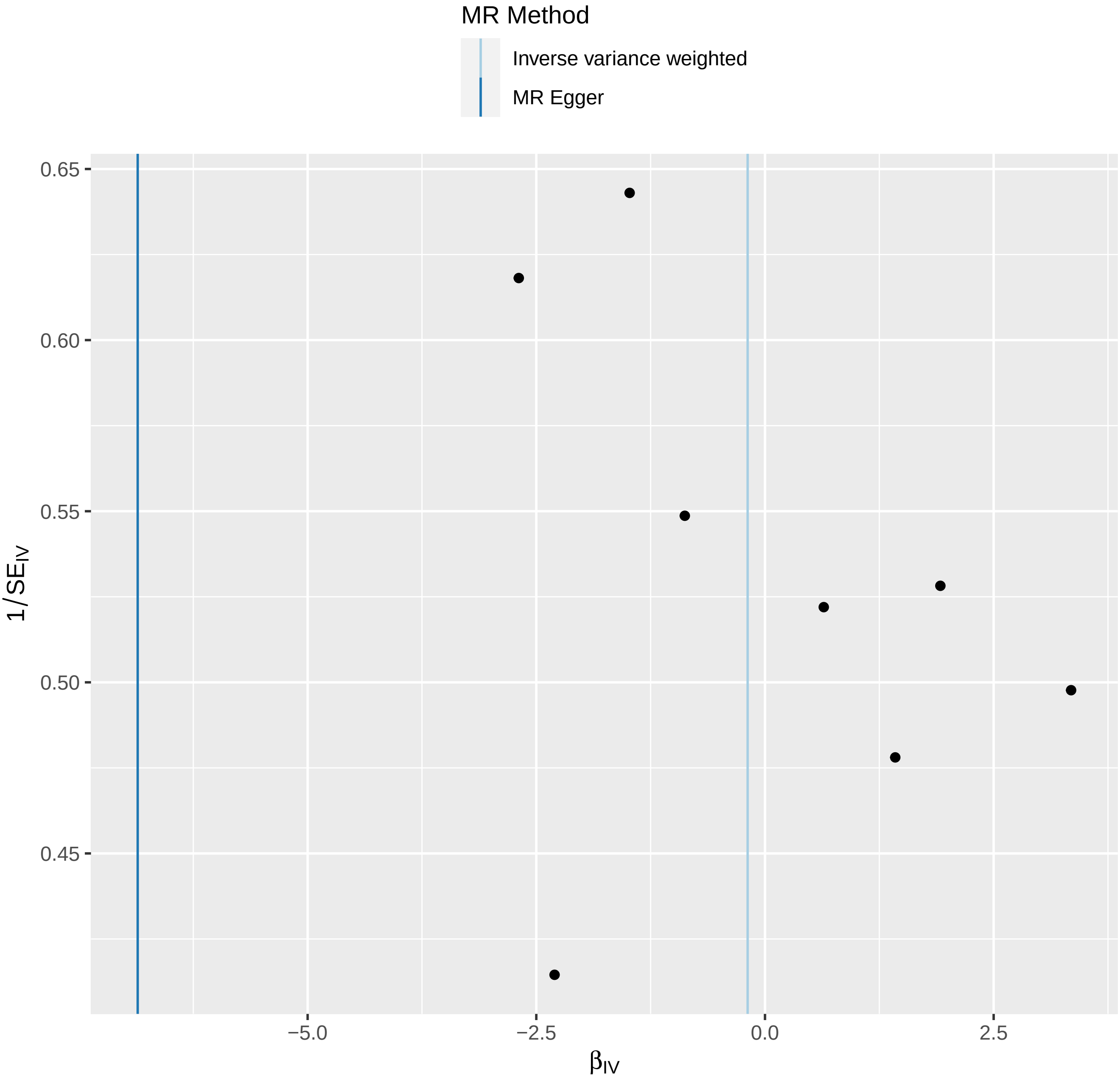

Batch 643 : Gut microbiota abundance (genus Barnesiella id.944) on Ulcerative colitis (strict) with PSC

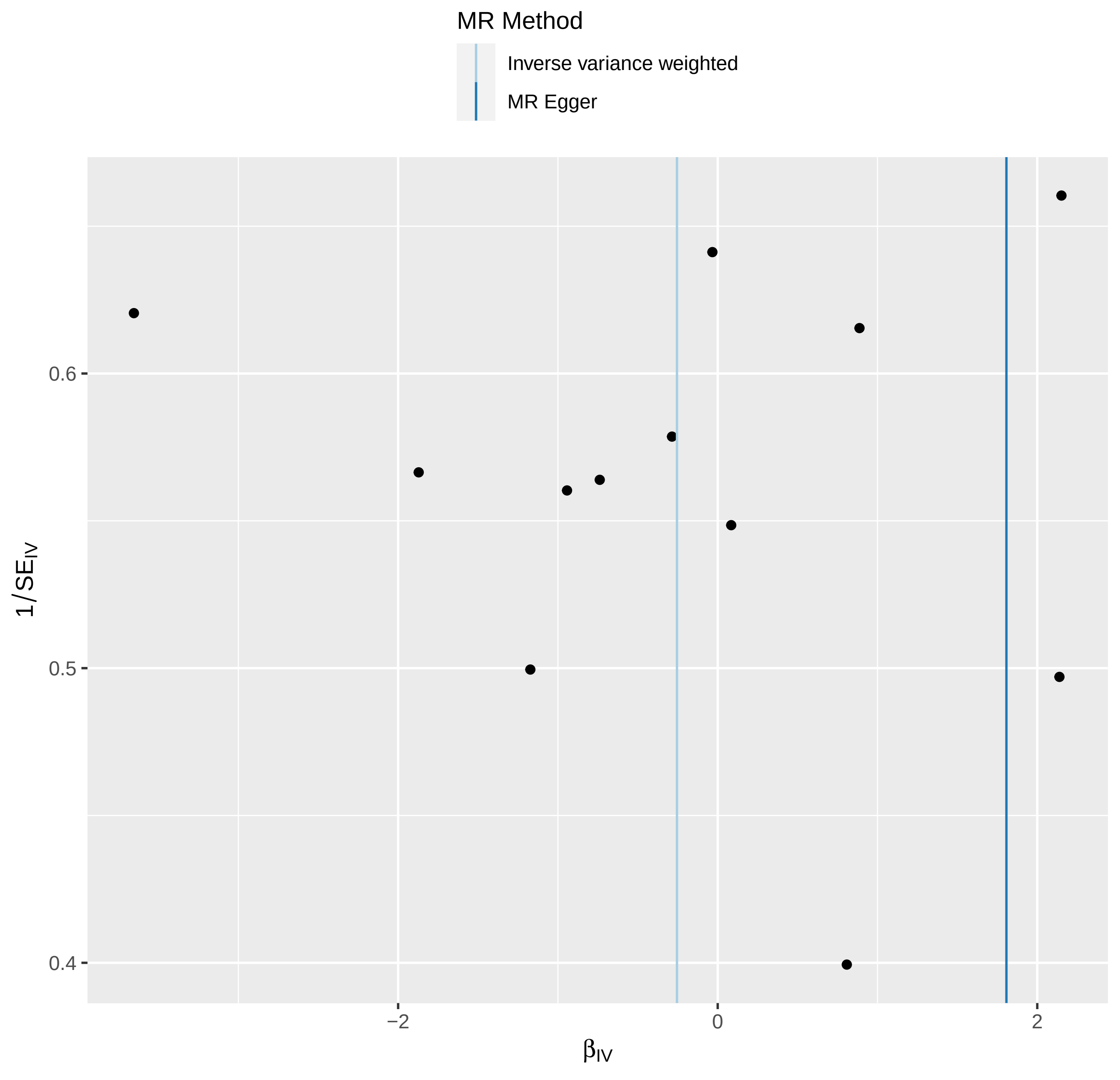

Batch 644 : Gut microbiota abundance (genus Bifidobacterium id.436) on Ulcerative colitis (strict) with PSC

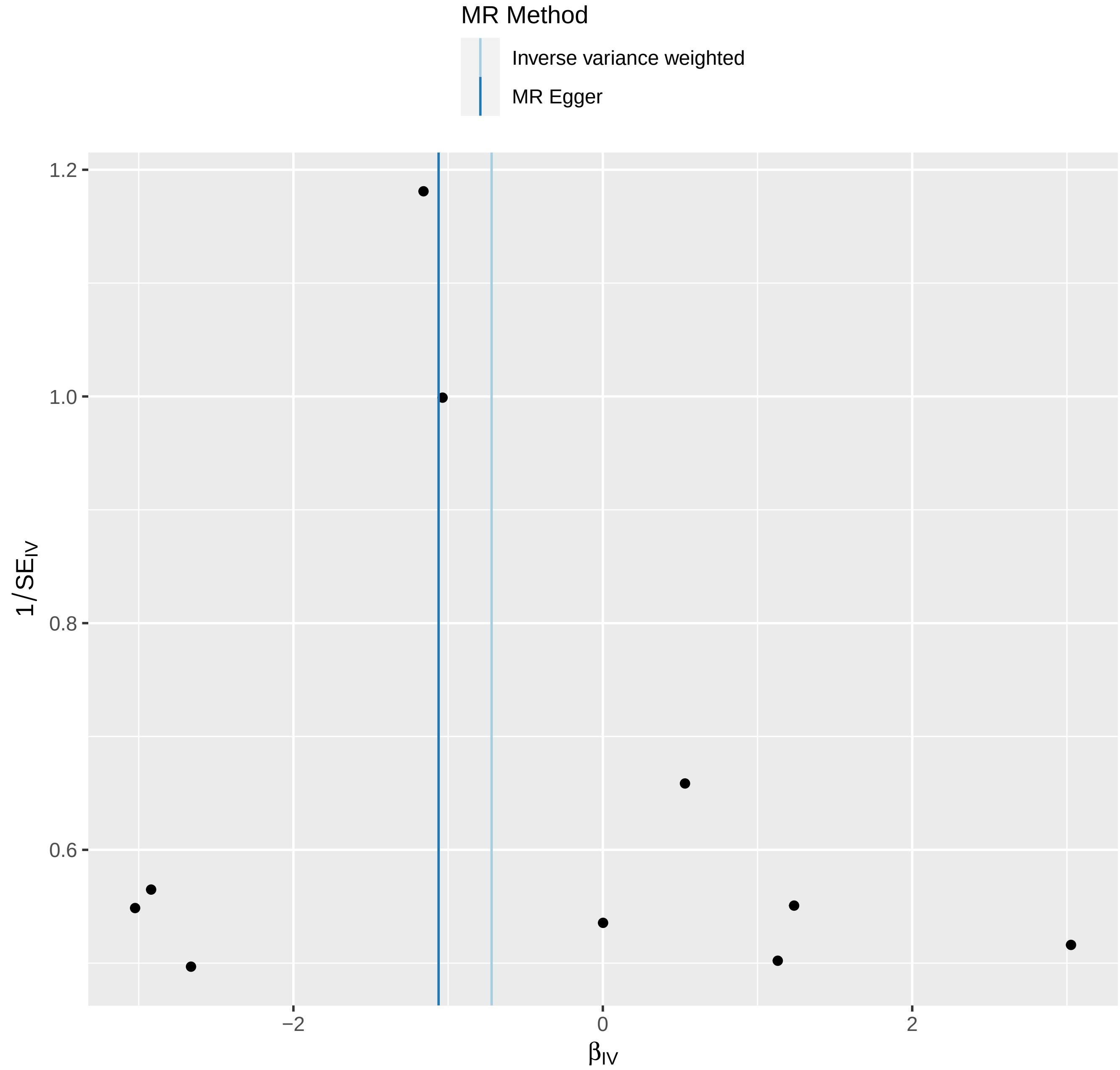

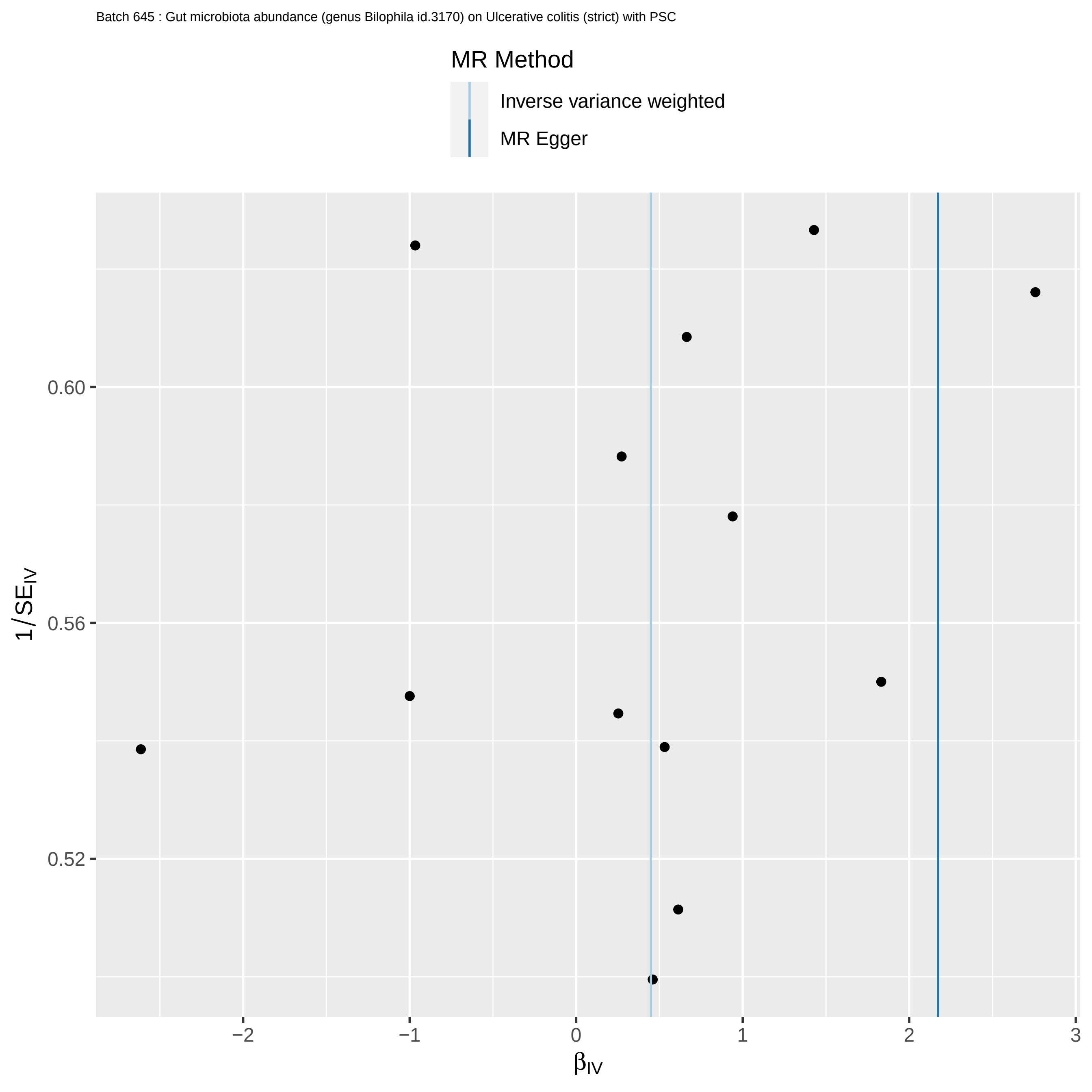

Batch 645 : Gut microbiota abundance (genus Bilophila id.3170) on Ulcerative colitis (strict) with PSC
MR Method
Inverse variance weighted
MR Egger
0.60
0.56
0.52
1/SE_IV
−2
−1
0
1
2
3
β_IV

Batch 646 : Gut microbiota abundance (genus Blautia id.1992) on Ulcerative colitis (strict) with PSC

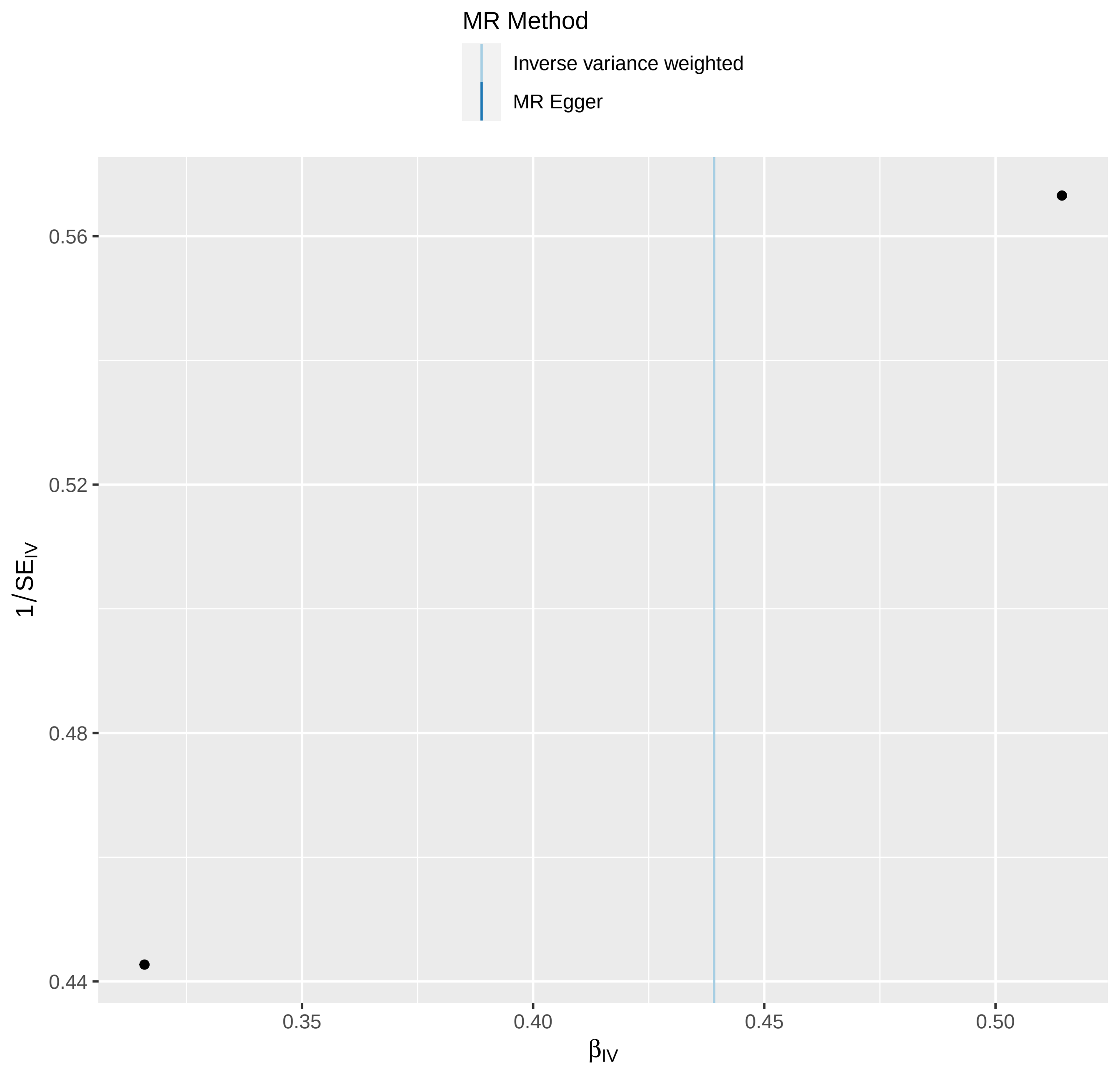

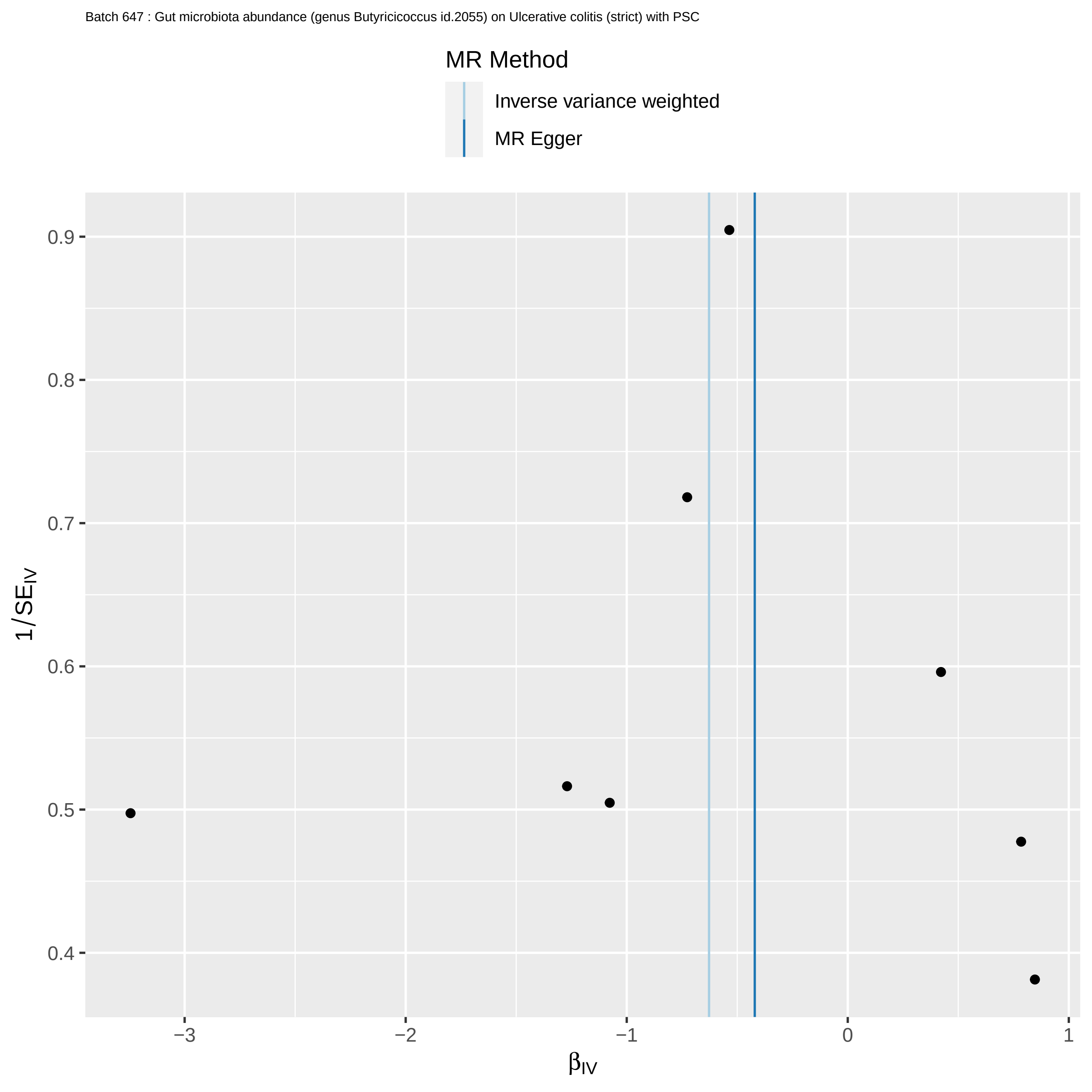
Batch 647 : Gut microbiota abundance (genus Butyricicoccus id.2055) on Ulcerative colitis (strict) with PSC
MR Method
Inverse variance weighted
MR Egger
0.9
0.8
0.7
0.6
0.5
0.4
1/SE_IV
−3
−2
−1
0
1
β_IV

Batch 648 : Gut microbiota abundance (genus Butyricimonas id.945) on Ulcerative colitis (strict) with PSC

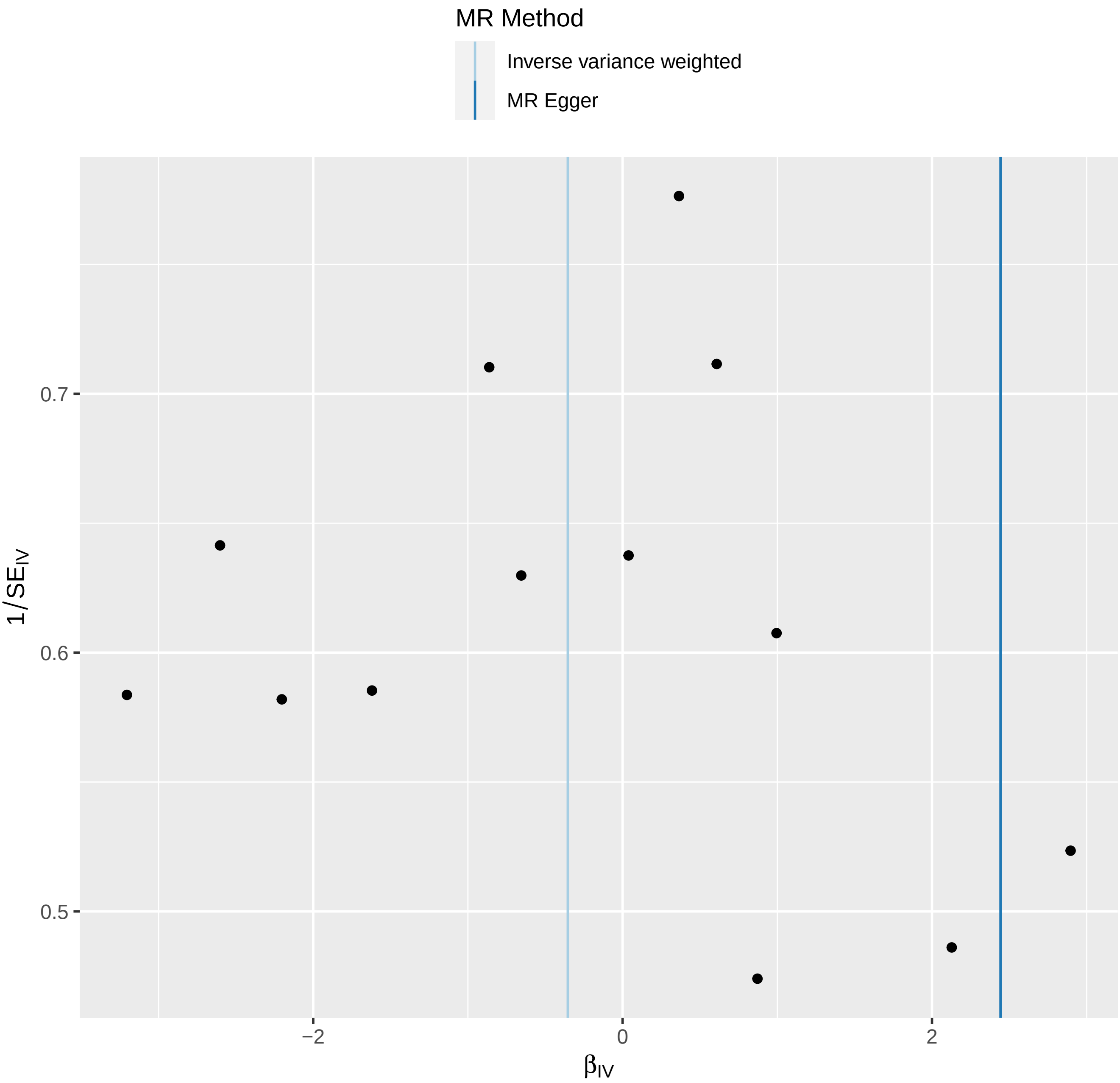

Batch 649 : Gut microbiota abundance (genus Butyrivibrio id.1993) on Ulcerative colitis (strict) with PSC

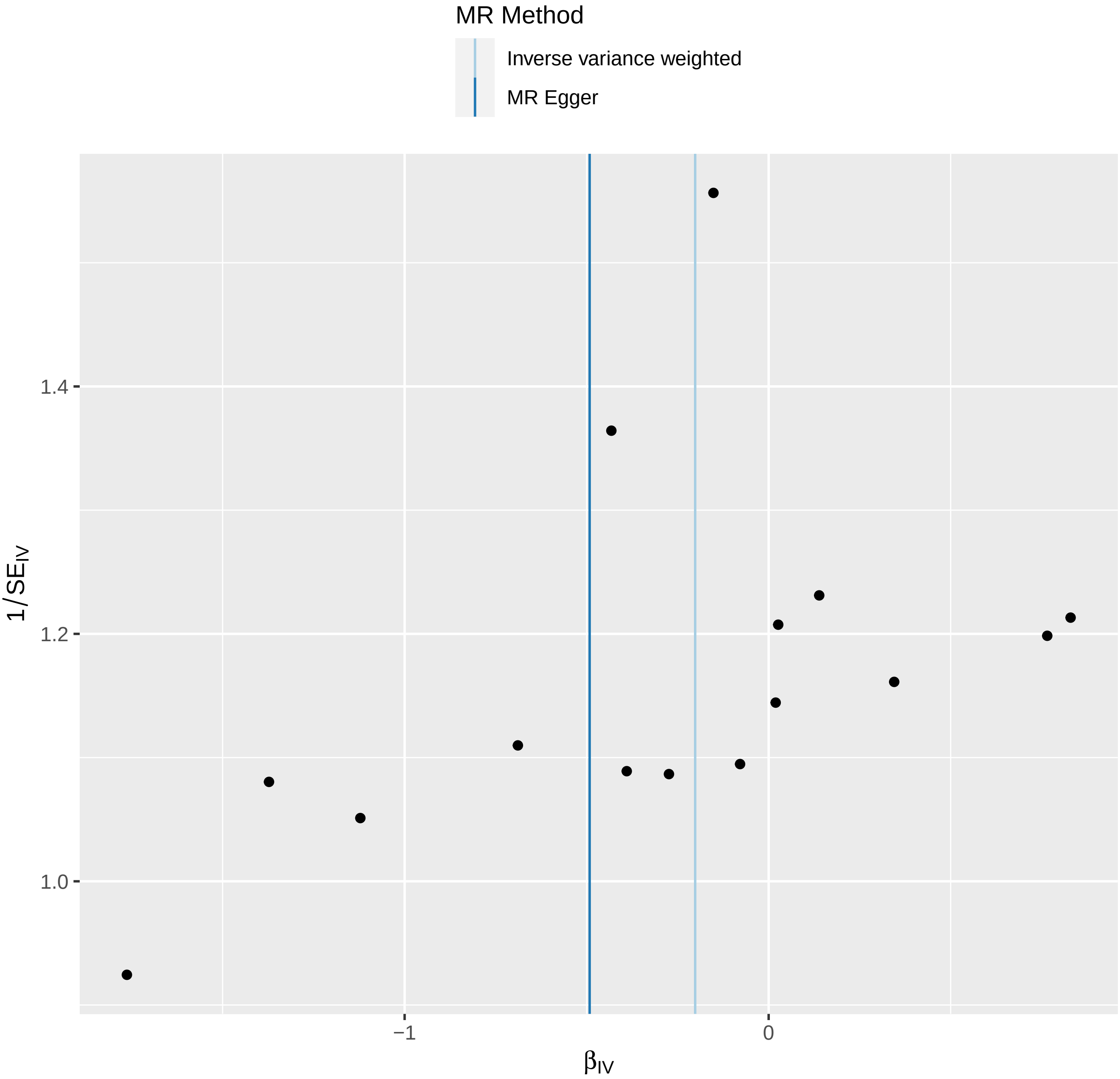

Batch 650 : Gut microbiota abundance (genus Candidatus Soleaferrea id.11350) on Ulcerative colitis (strict) with PSC

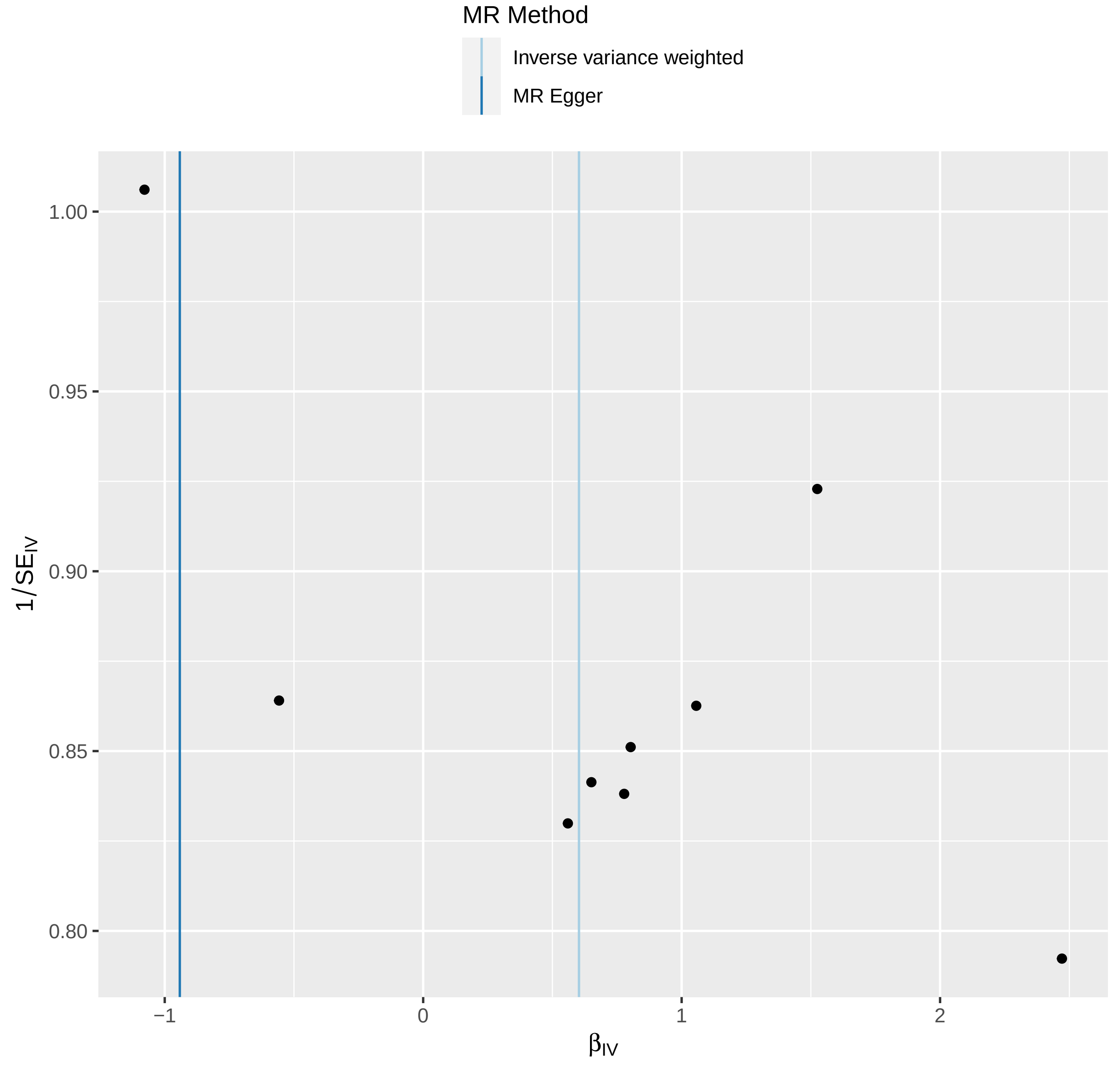

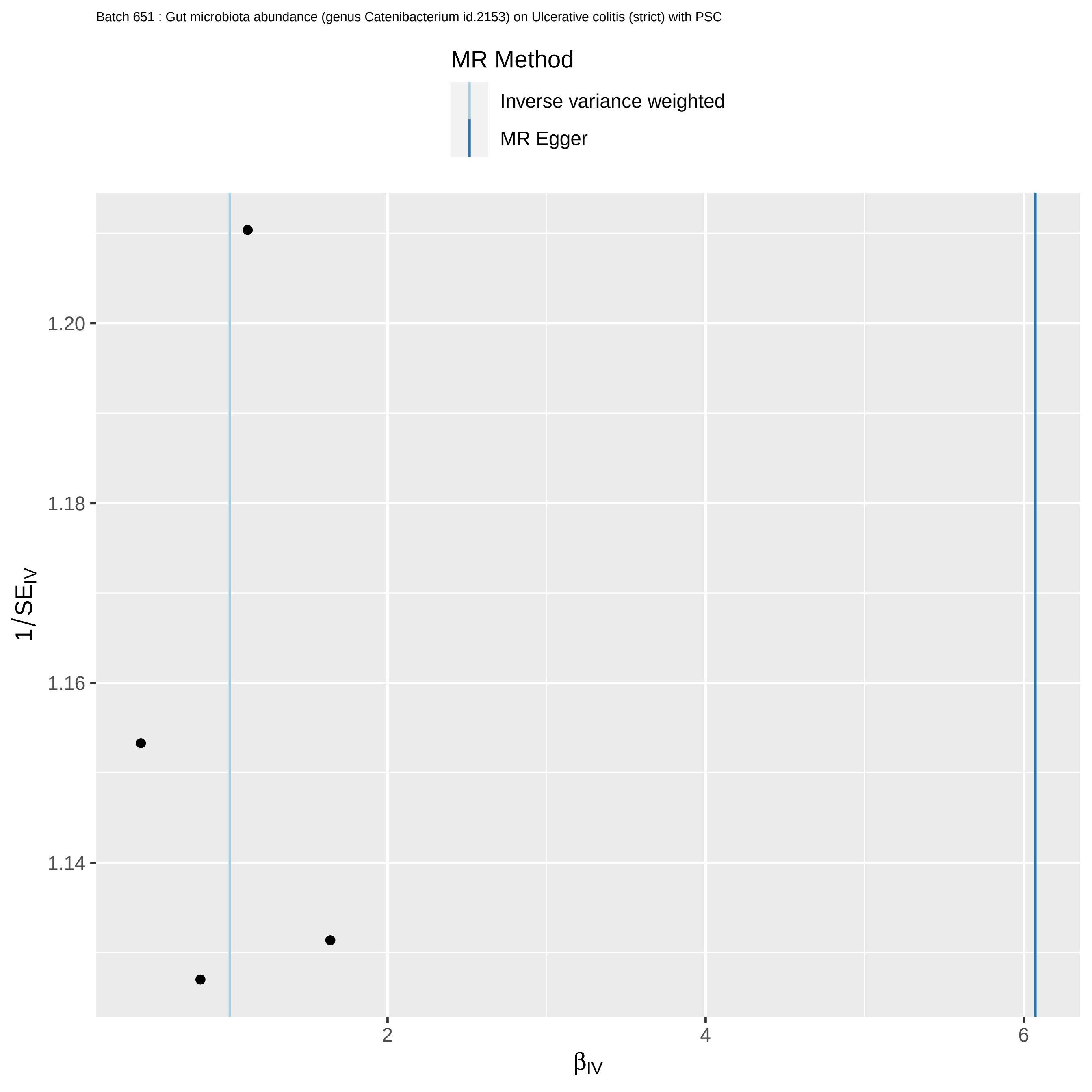

Batch 651 : Gut microbiota abundance (genus Catenibacterium id.2153) on Ulcerative colitis (strict) with PSC
MR Method
Inverse variance weighted
MR Egger
1.20
1.18
1.16
1.14
1/SE_IV
2
4
6
β_IV

Batch 652 : Gut microbiota abundance (genus Christensenellaceae R 7group id.11283) on Ulcerative colitis (strict) with PSC

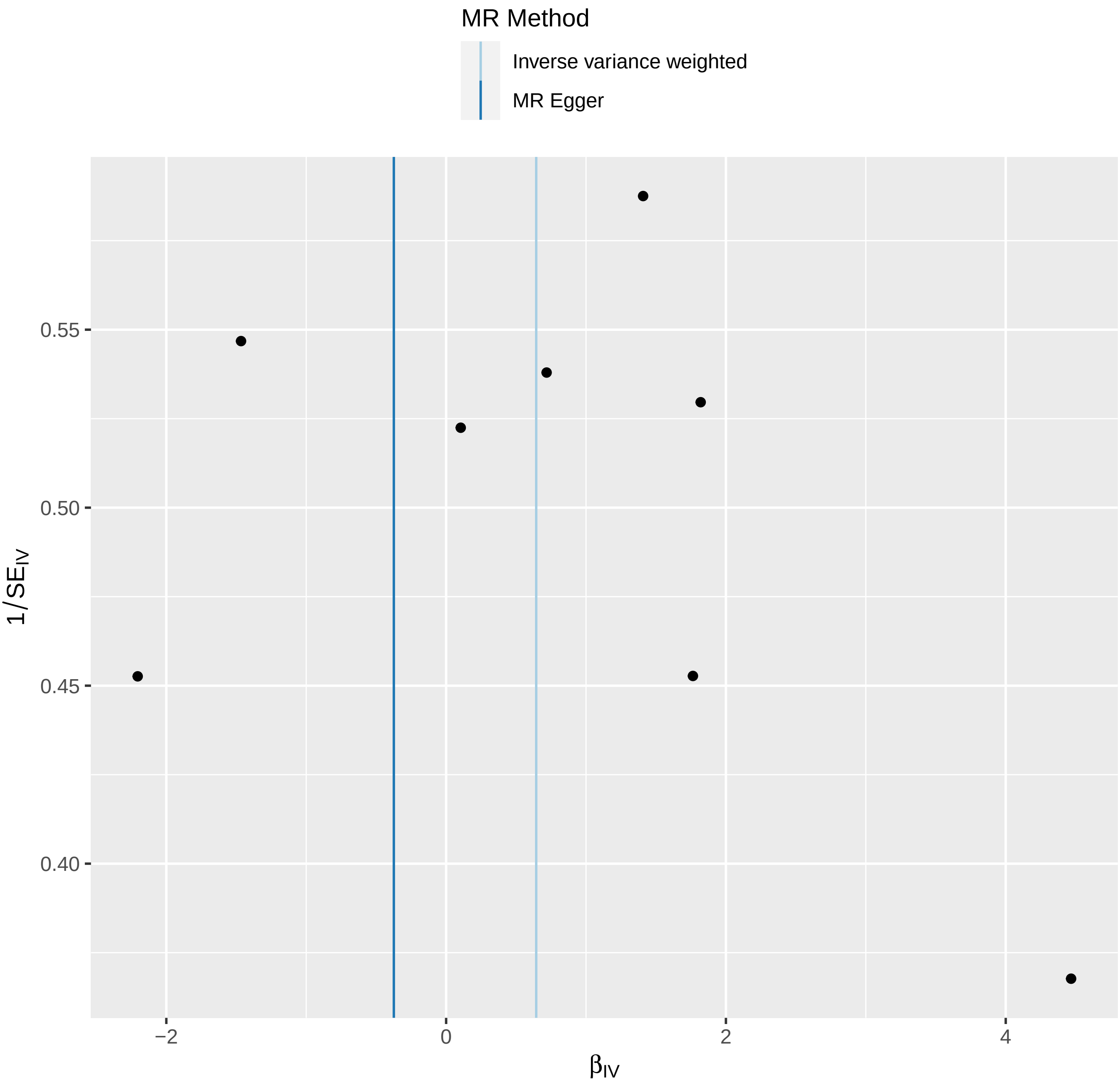

Batch 653 : Gut microbiota abundance (genus Clostridium innocuum group id.14397) on Ulcerative colitis (strict) with PSC

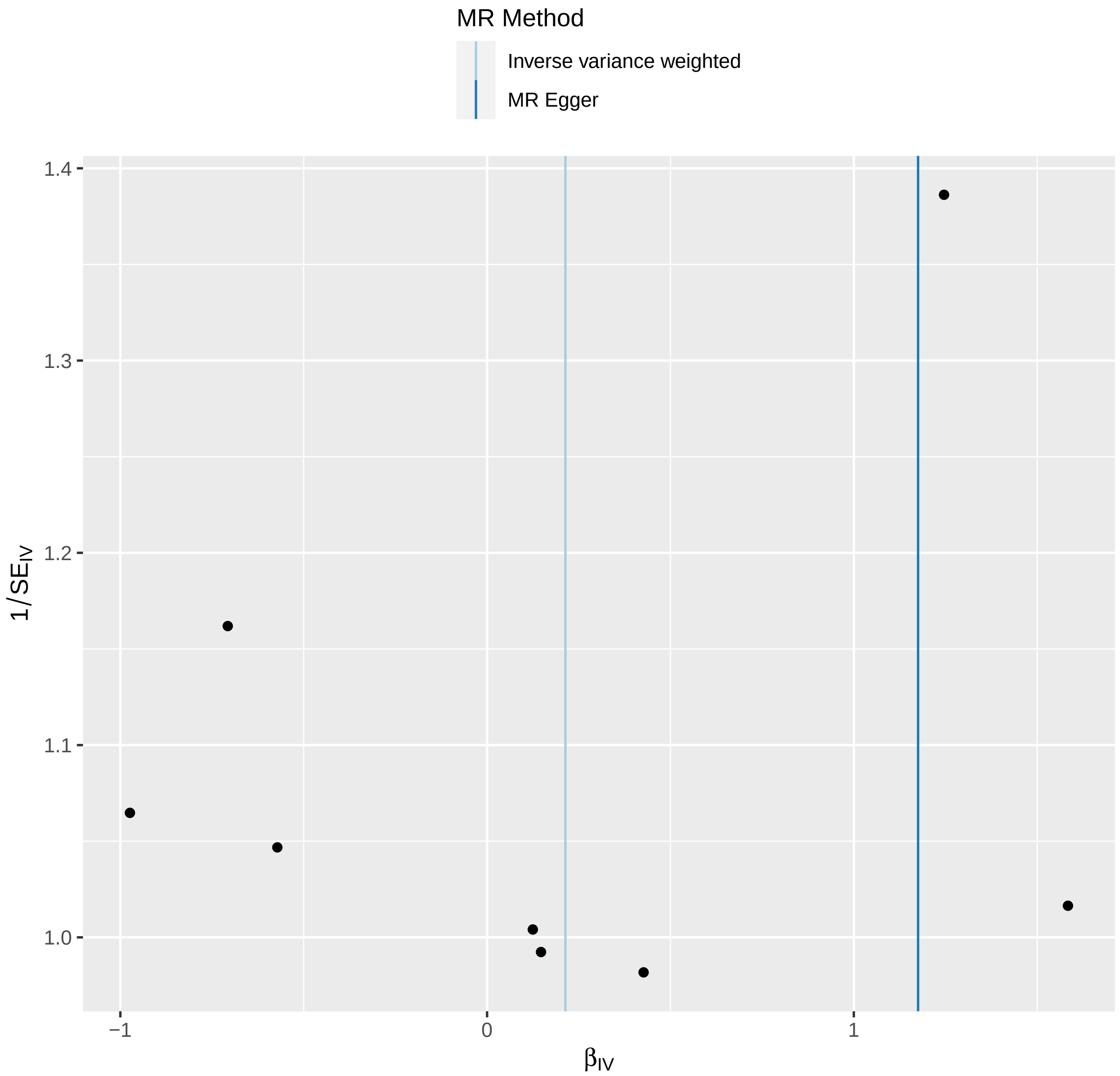

Batch 654 : Gut microbiota abundance (genus Clostridium sensustricto1 id.1873) on Ulcerative colitis (strict) with PSC

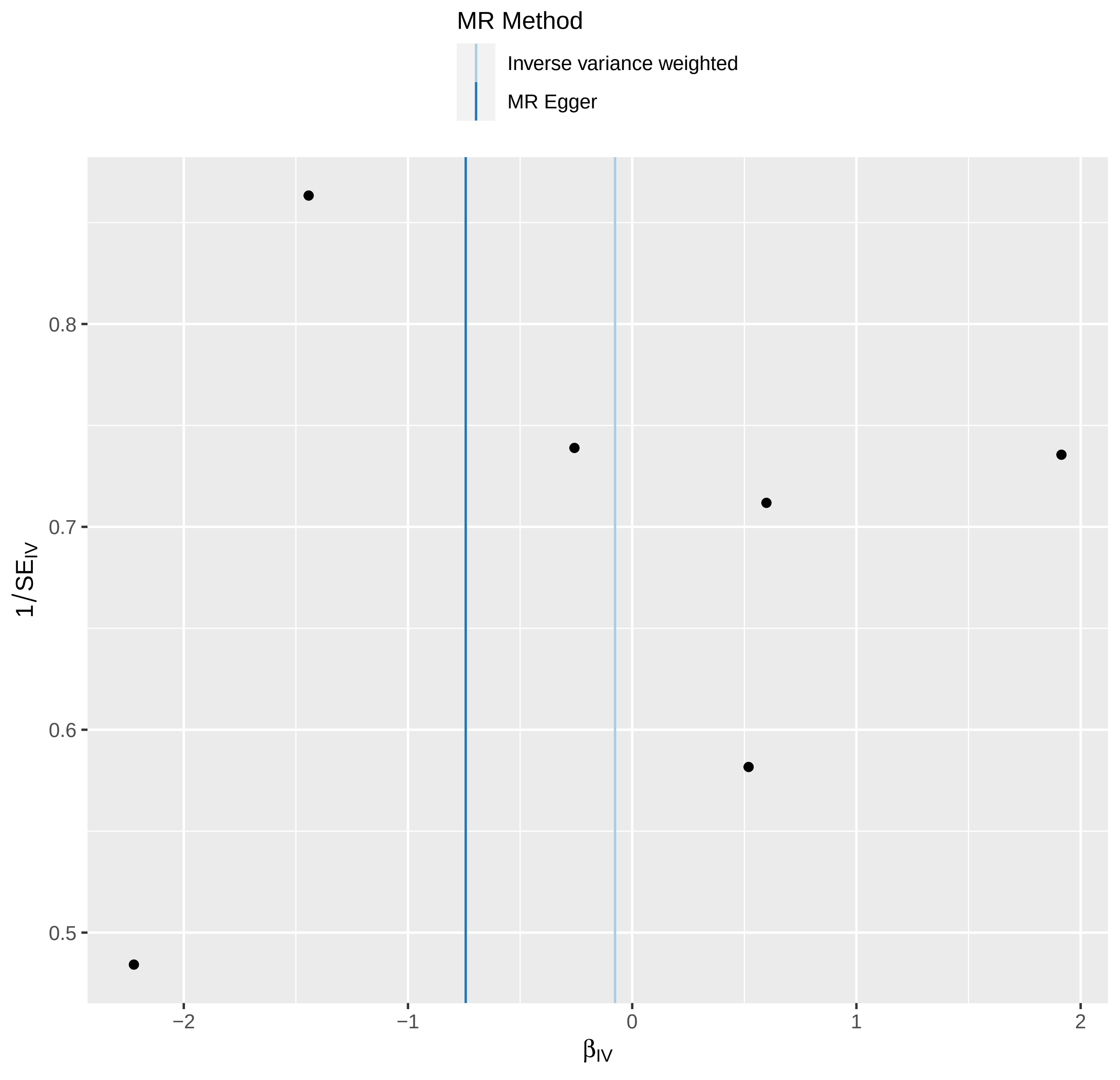

Batch 655 : Gut microbiota abundance (genus Collinsella id.815) on Ulcerative colitis (strict) with PSC

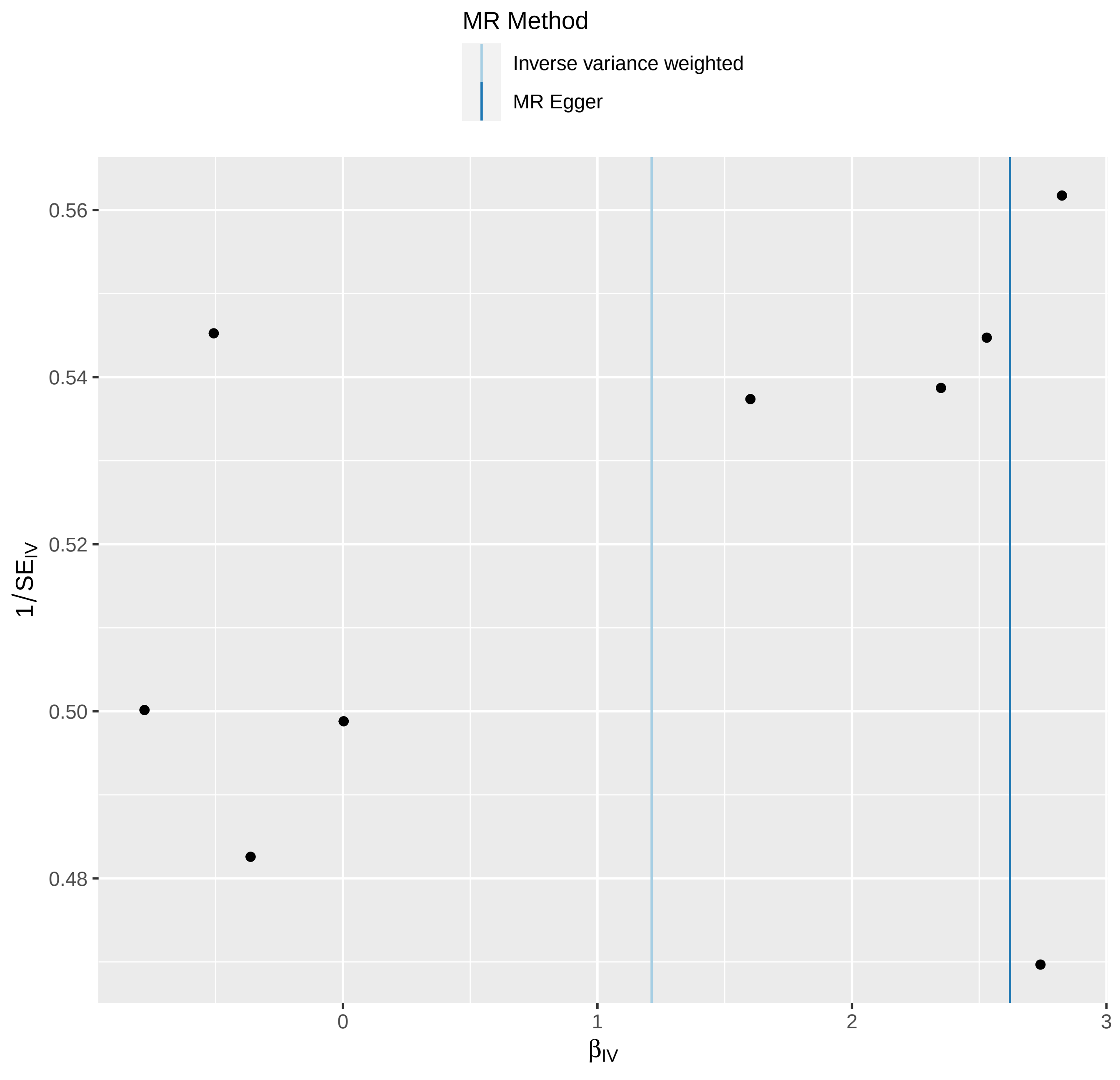

Batch 656 : Gut microbiota abundance (genus Coprobacter id.949) on Ulcerative colitis (strict) with PSC

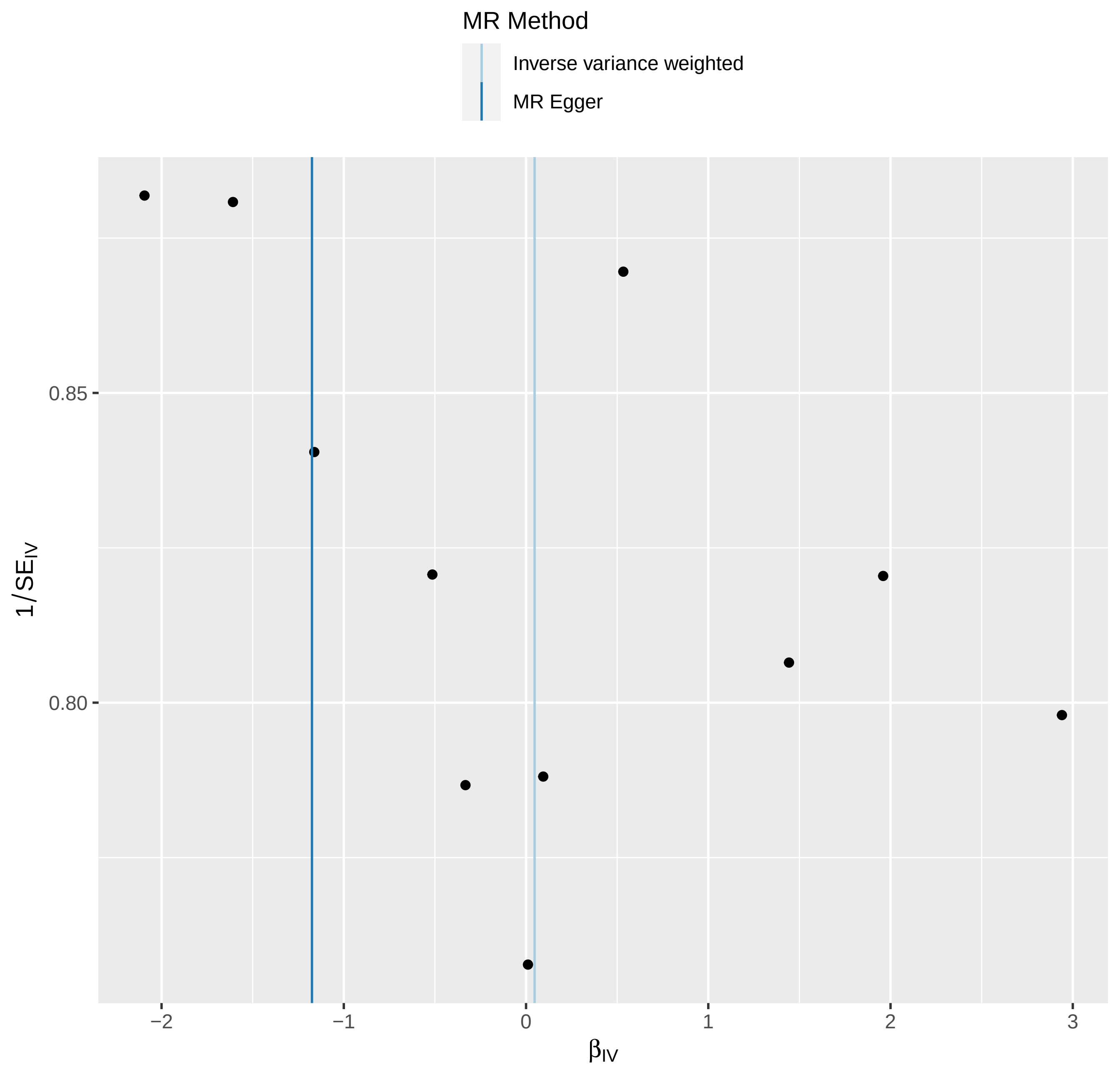

Batch 657 : Gut microbiota abundance (genus Coprococcus1 id.11301) on Ulcerative colitis (strict) with PSC

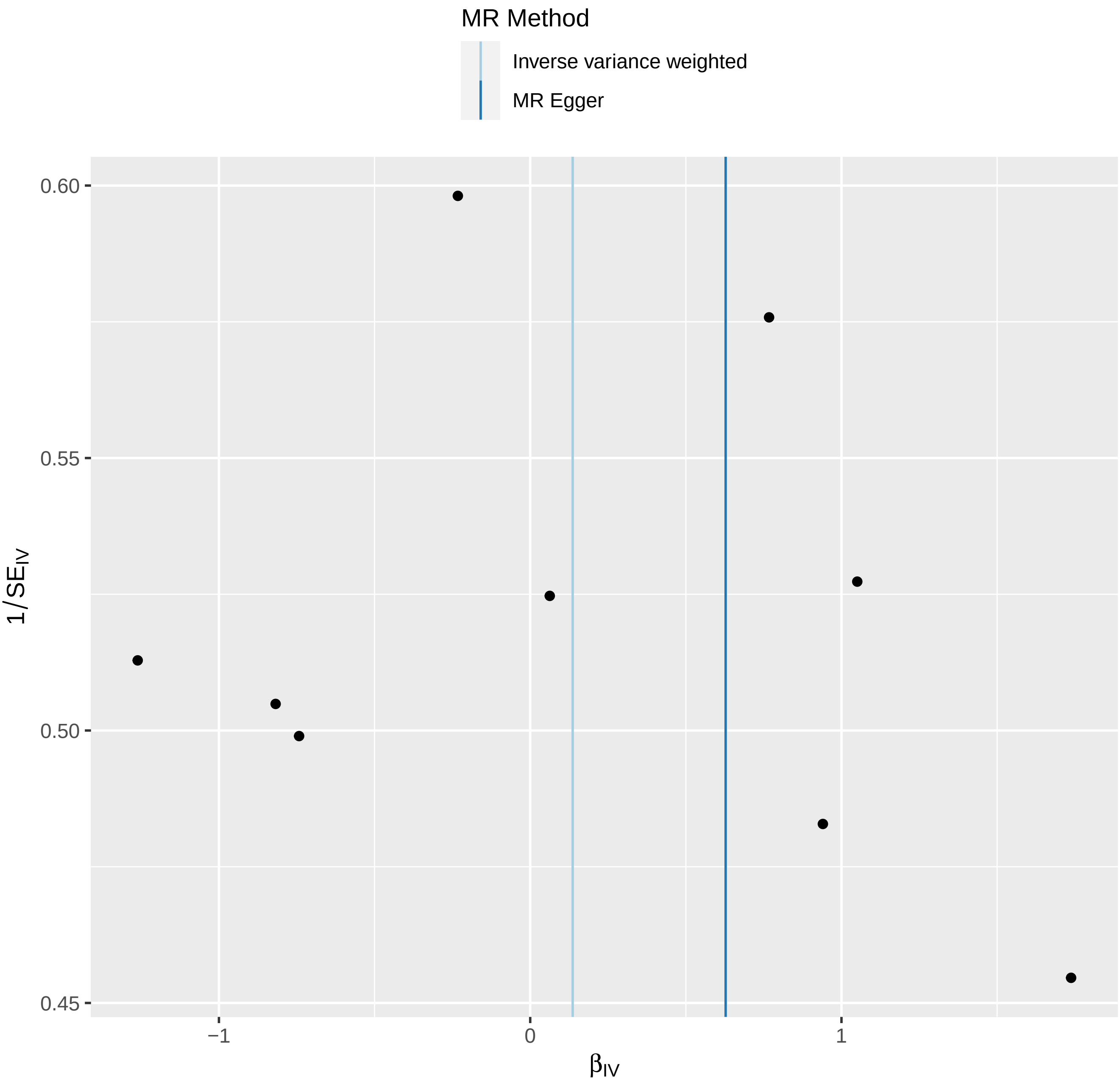

Batch 658 : Gut microbiota abundance (genus Coprococcus2 id.11302) on Ulcerative colitis (strict) with PSC

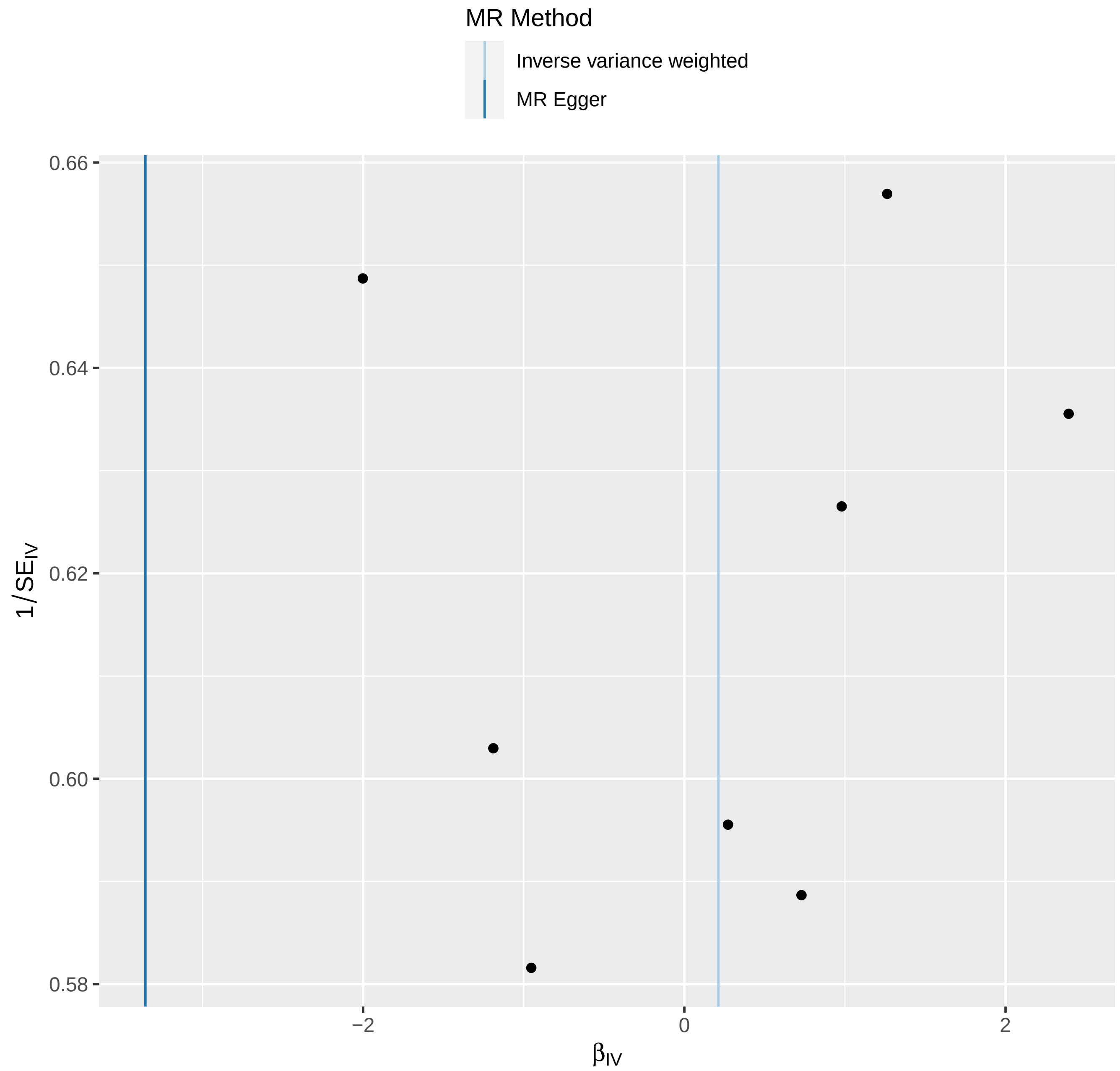

Batch 659 : Gut microbiota abundance (genus Coprococcus3 id.11303) on Ulcerative colitis (strict) with PSC

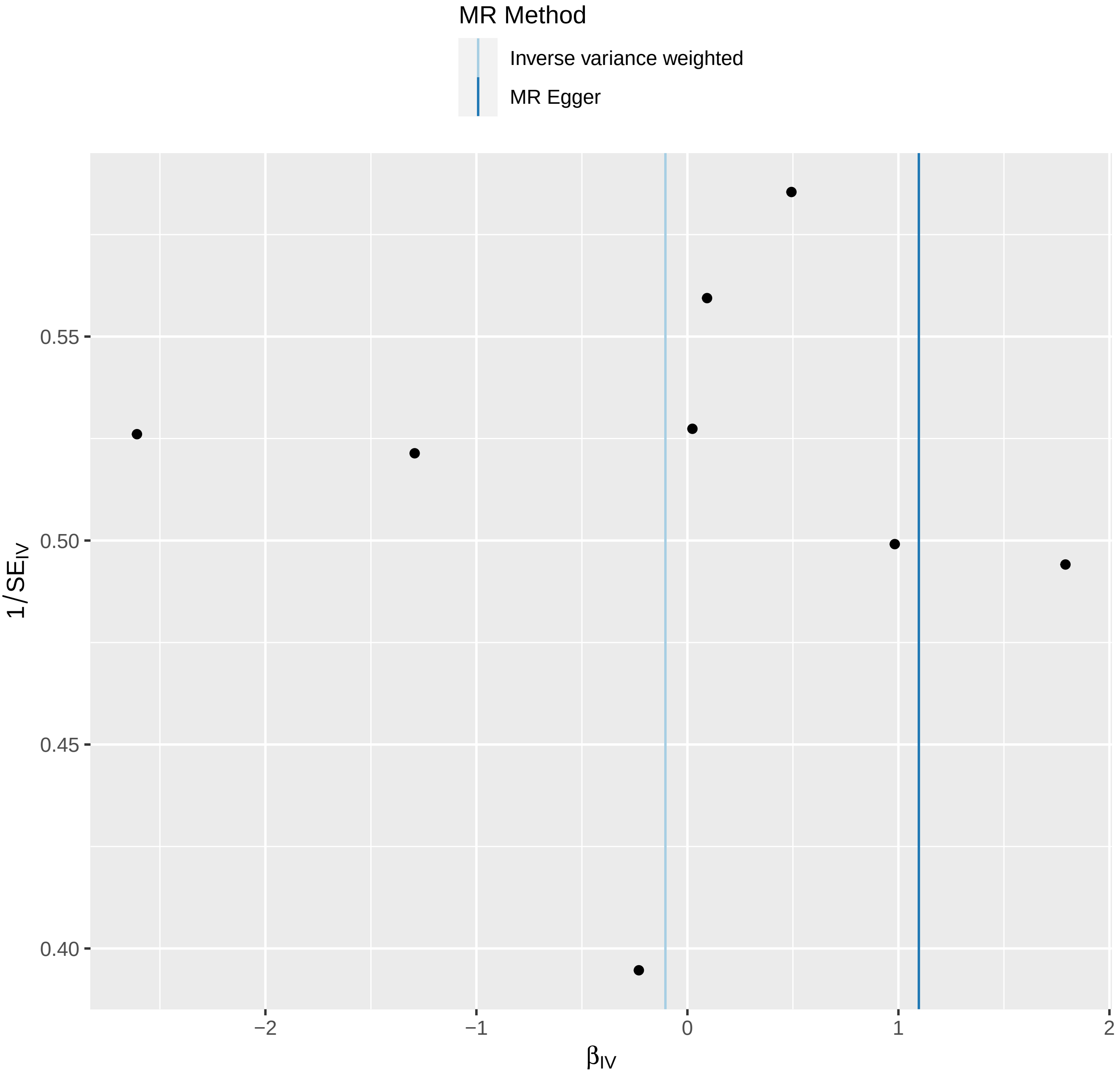

Batch 660 : Gut microbiota abundance (genus Defluviitaleaceae UCG011 id.11287) on Ulcerative colitis (strict) with PSC

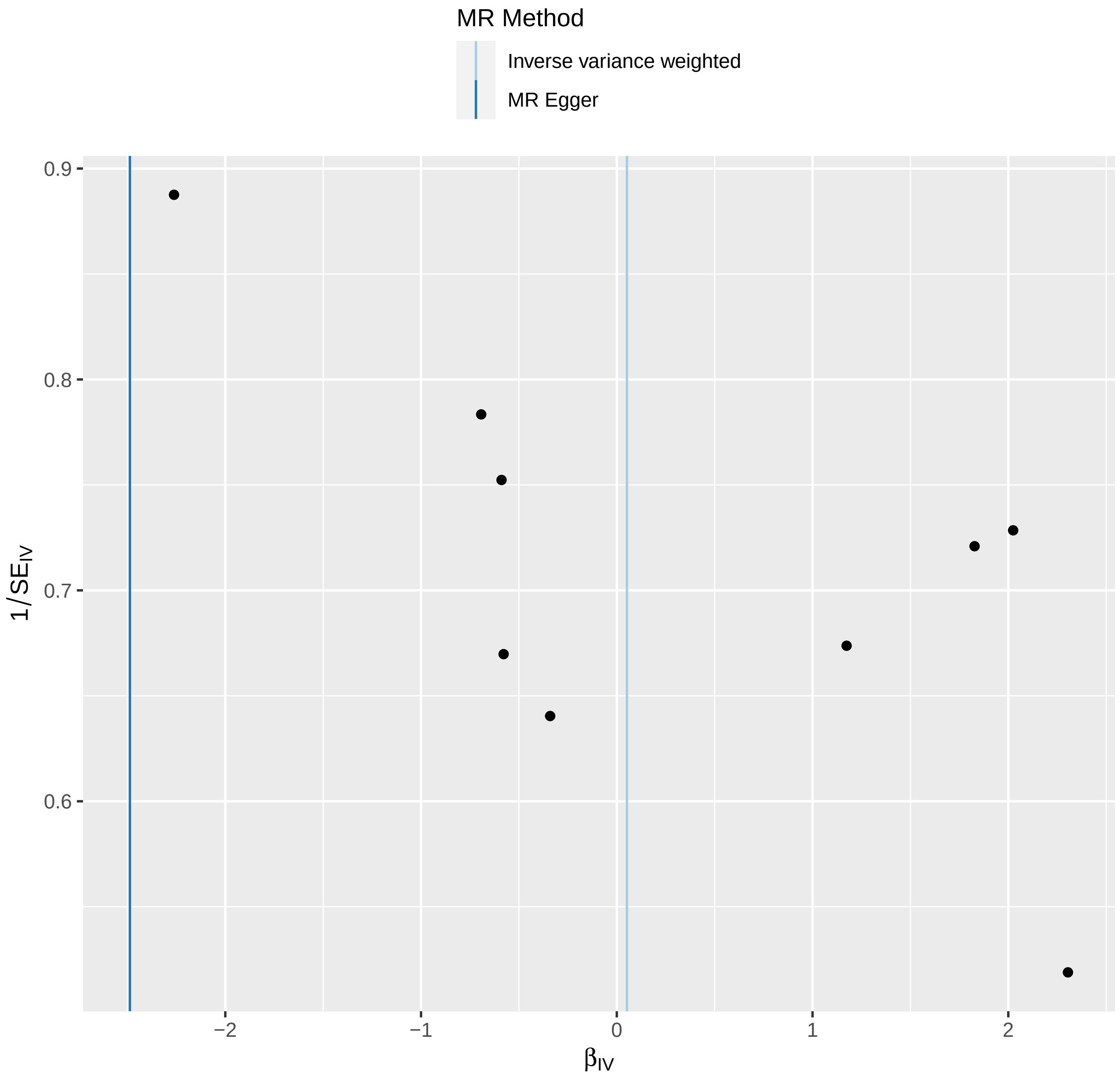

Batch 661 : Gut microbiota abundance (genus Desulfovibrio id.3173) on Ulcerative colitis (strict) with PSC

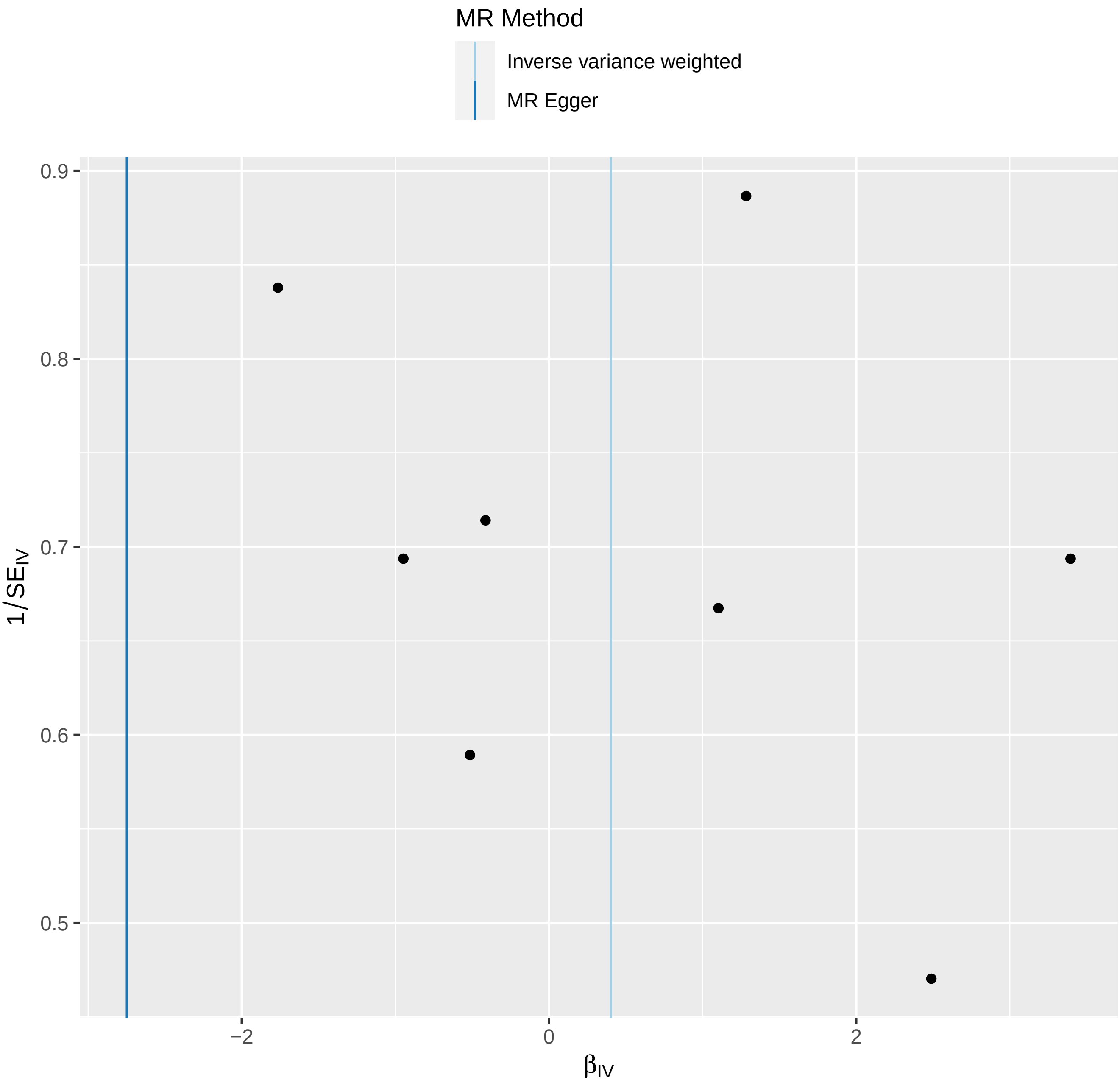

Batch 662 : Gut microbiota abundance (genus Dialister id.2183) on Ulcerative colitis (strict) with PSC

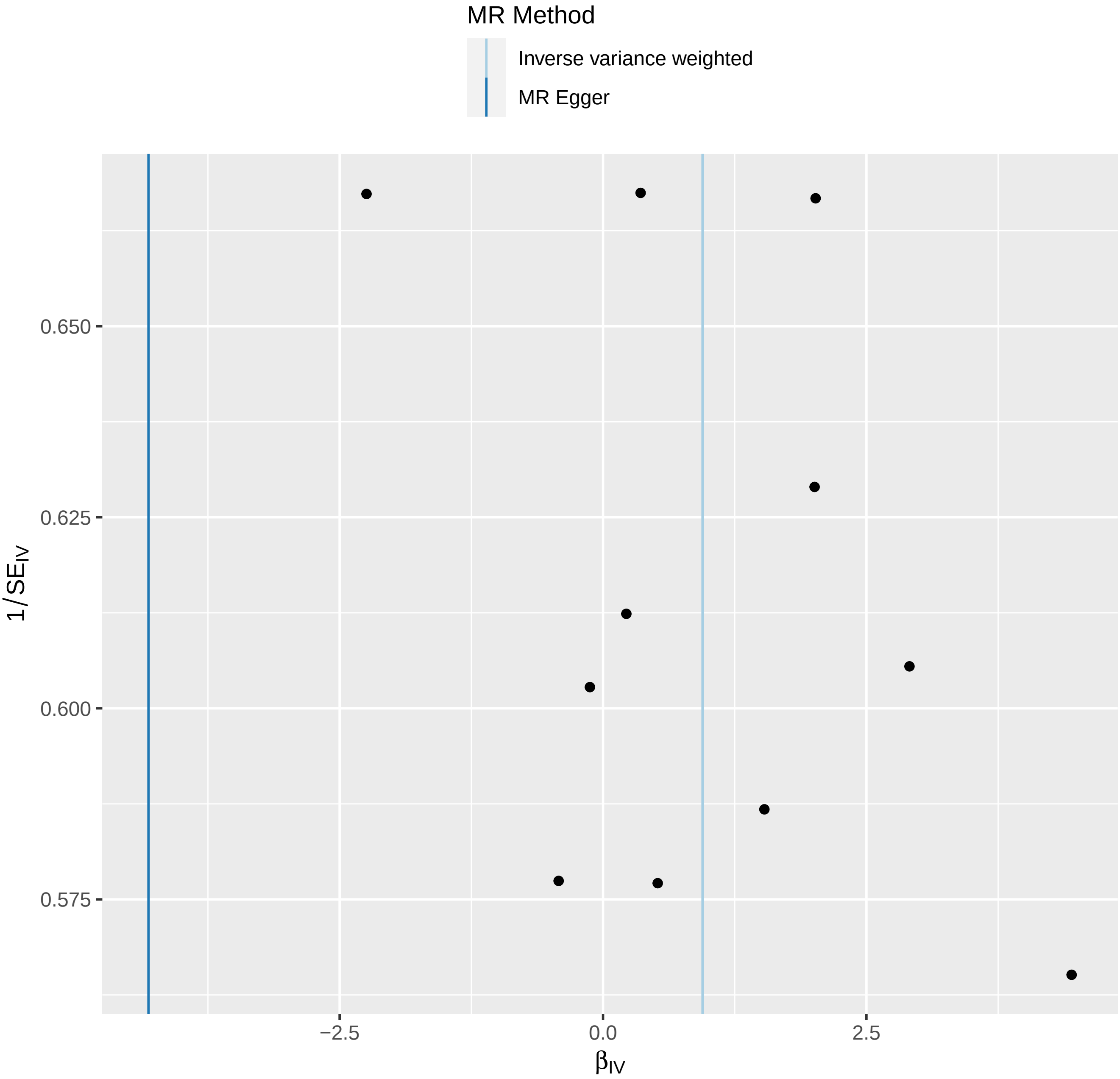

Batch 663 : Gut microbiota abundance (genus Dorea id.1997) on Ulcerative colitis (strict) with PSC

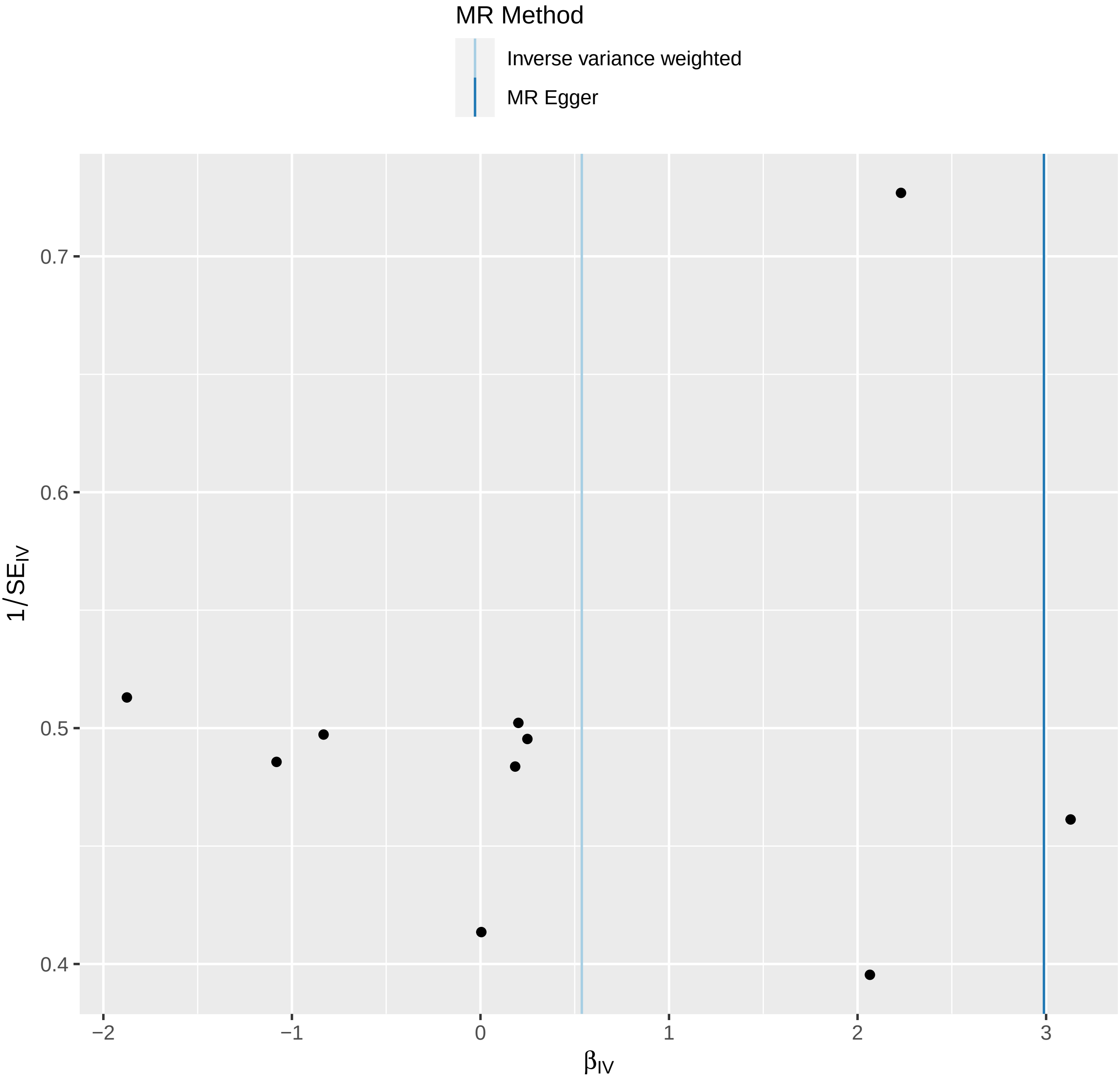

Batch 664 : Gut microbiota abundance (genus Eggerthella id.819) on Ulcerative colitis (strict) with PSC

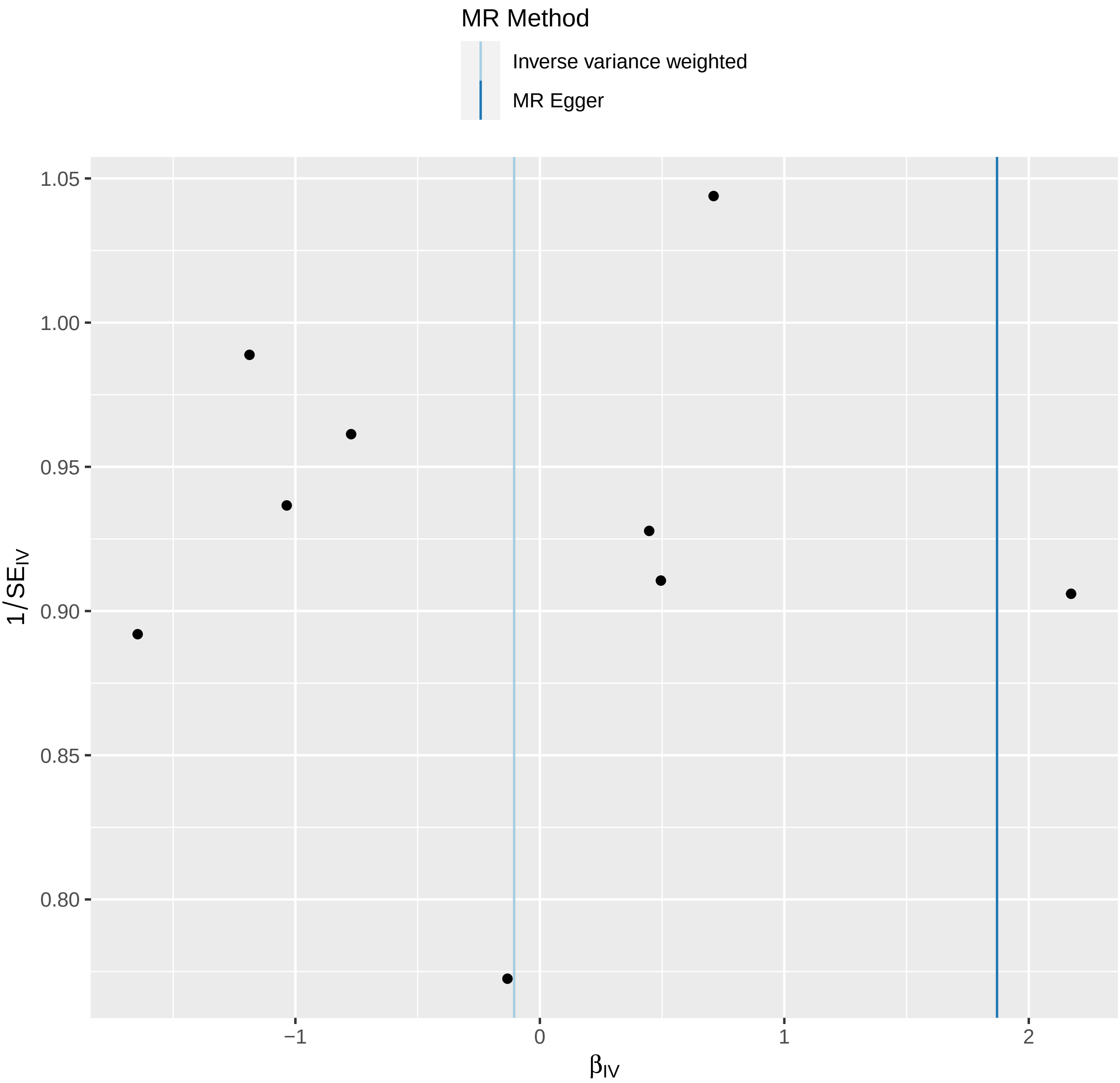

Batch 665 : Gut microbiota abundance (genus Eisenbergiella id.11304) on Ulcerative colitis (strict) with PSC

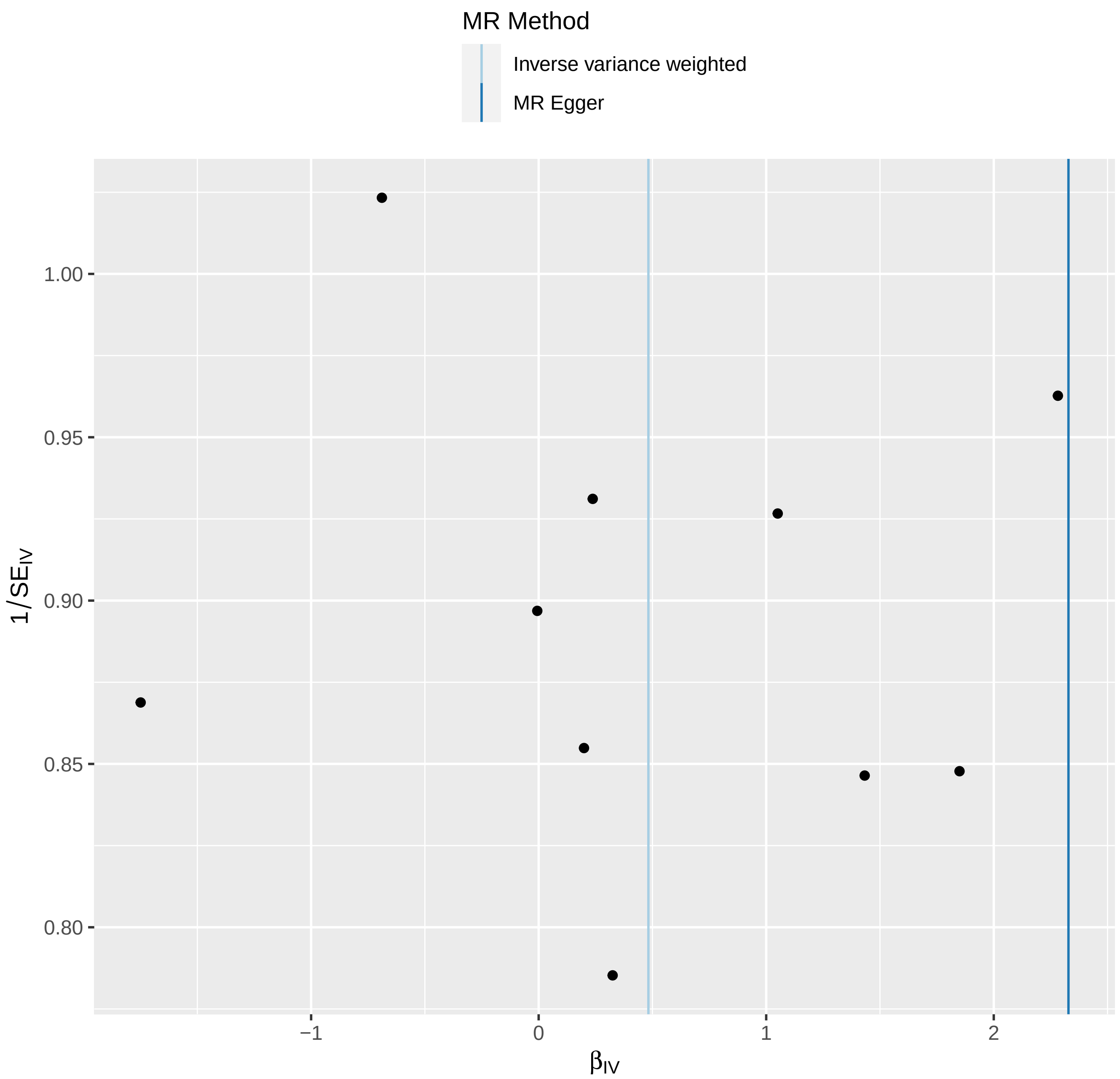

Batch 666 : Gut microbiota abundance (genus Enterorhabdus id.820) on Ulcerative colitis (strict) with PSC

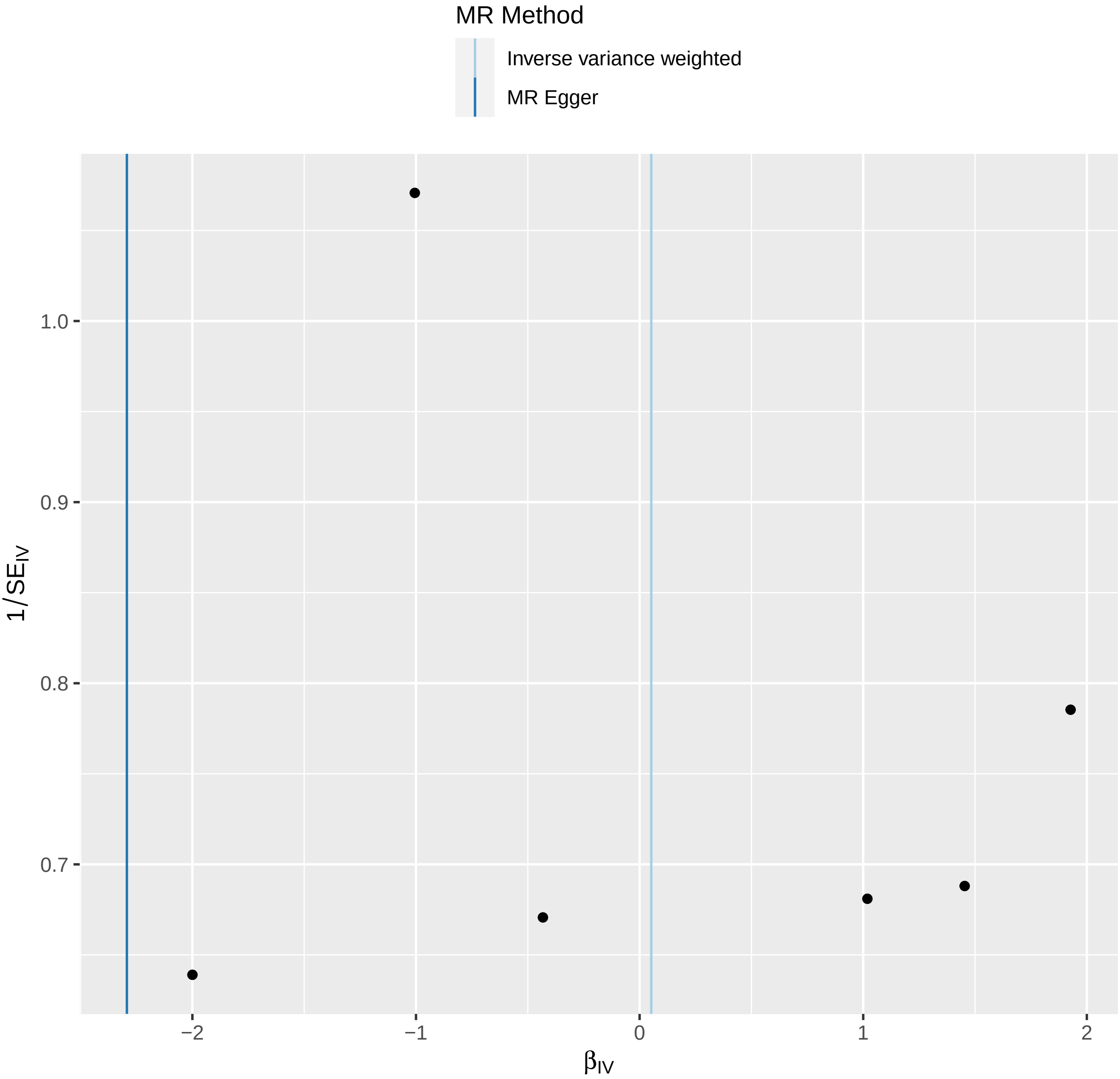

Batch 667 : Gut microbiota abundance (genus Erysipelatoclostridium id.11381) on Ulcerative colitis (strict) with PSC

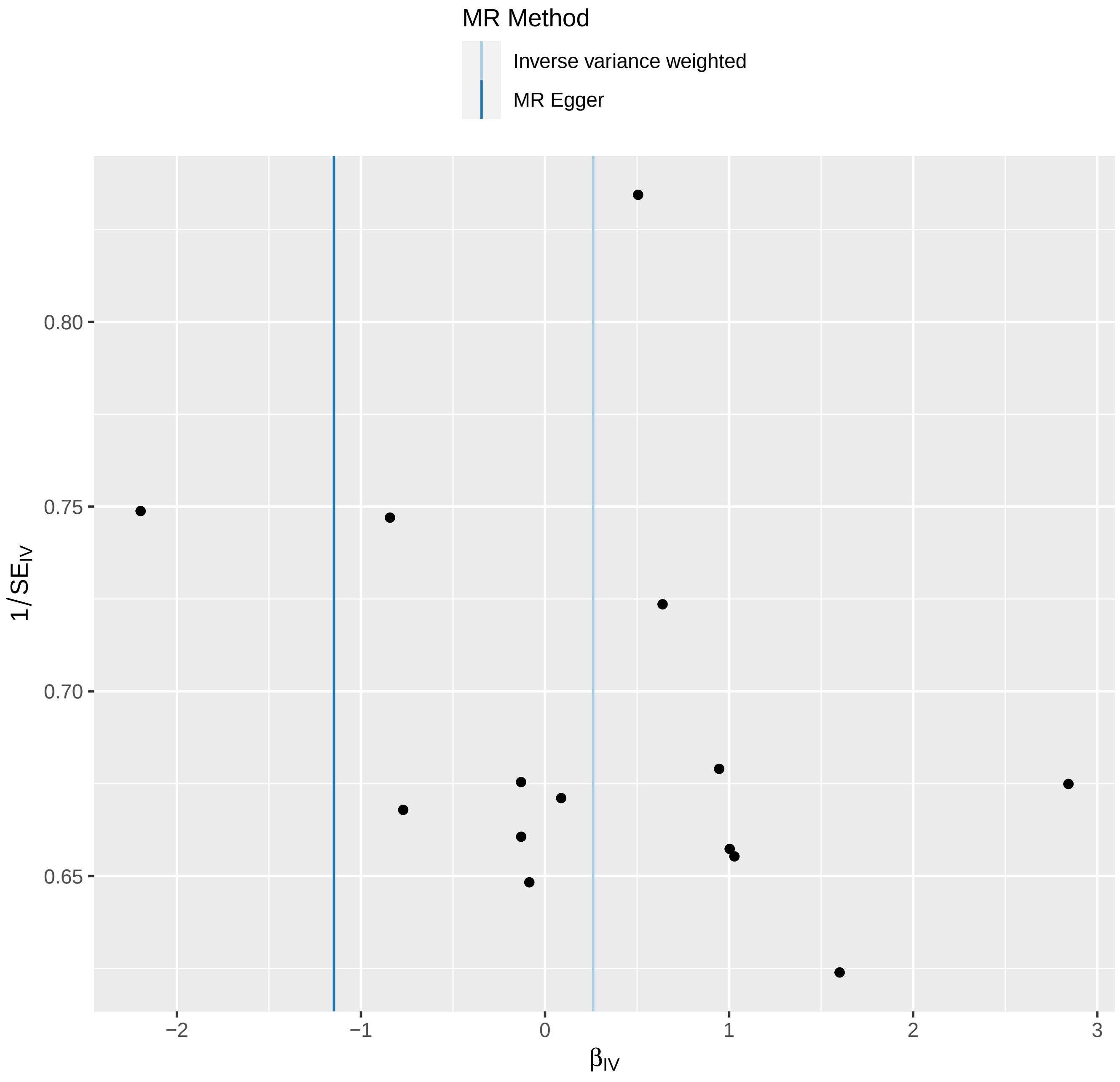

Insufficient number of SNPs

Batch 669 : Gut microbiota abundance (genus Escherichia Shigella id.3504) on Ulcerative colitis (strict) with PSC

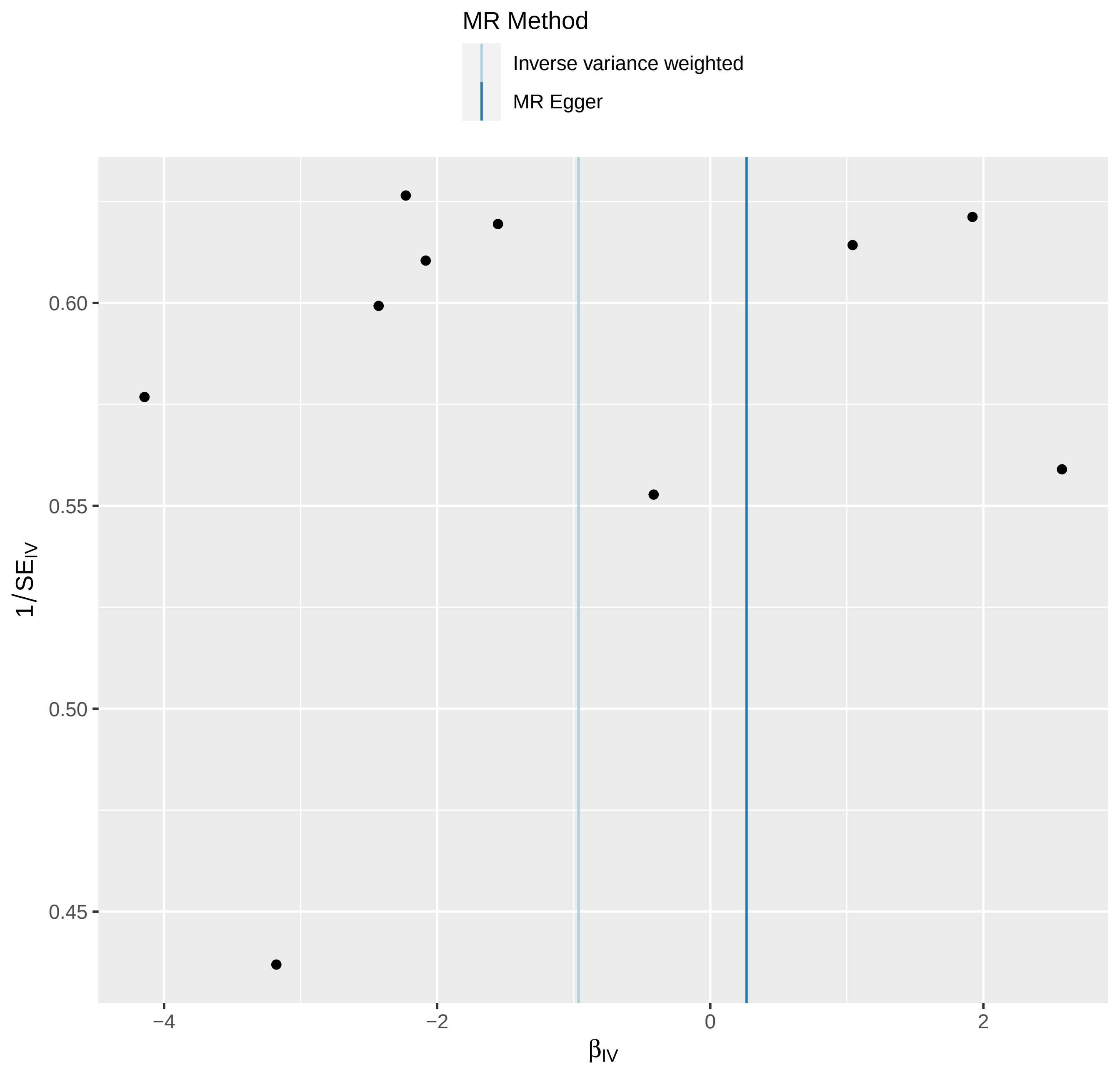

Batch 670 : Gut microbiota abundance (genus Eubacterium brachy group id.11296) on Ulcerative colitis (strict) with PSC

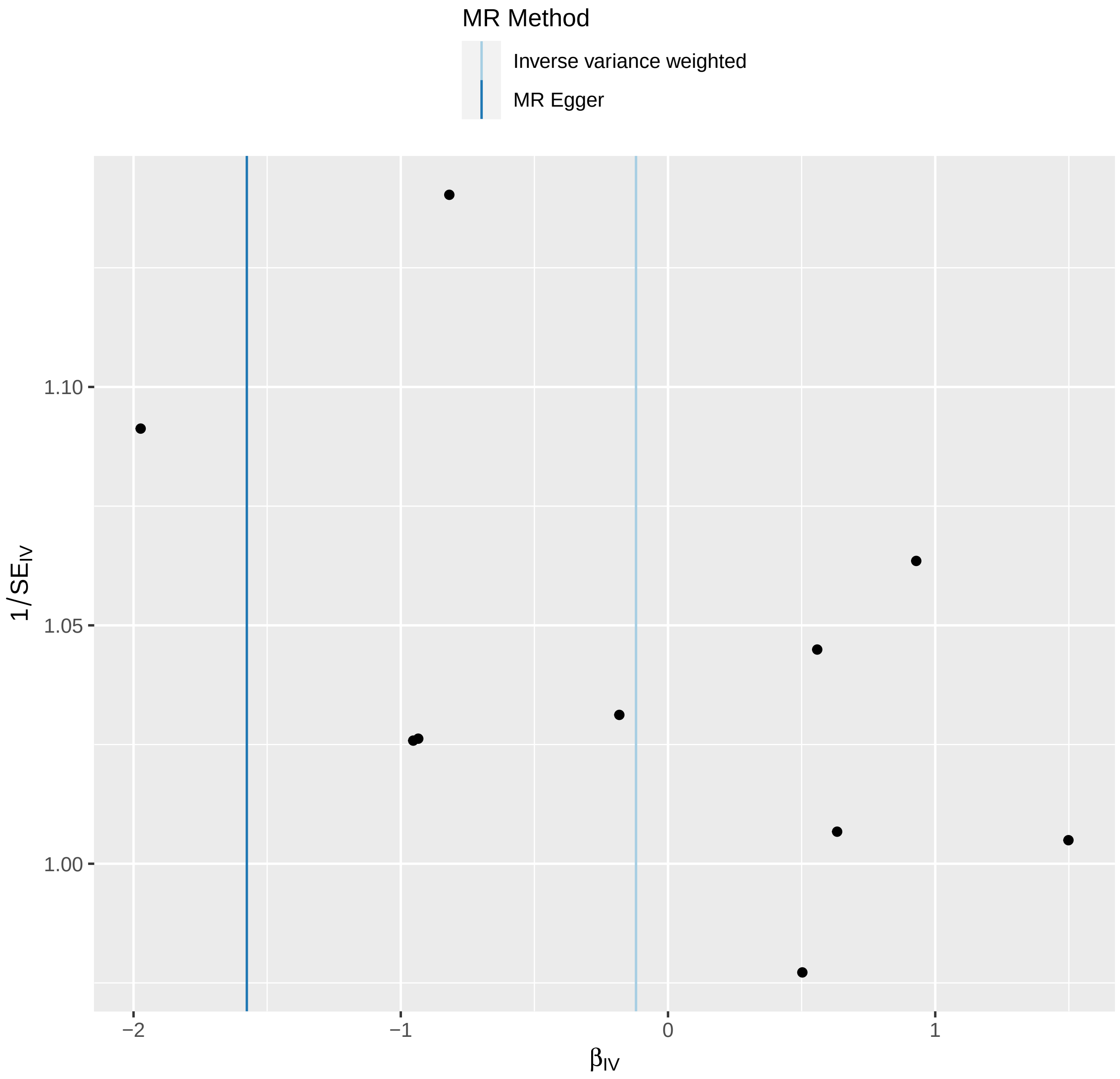

Batch 671 : Gut microbiota abundance (genus Eubacterium coprostanoligenes group id.11375) on Ulcerative colitis (strict) with PSC

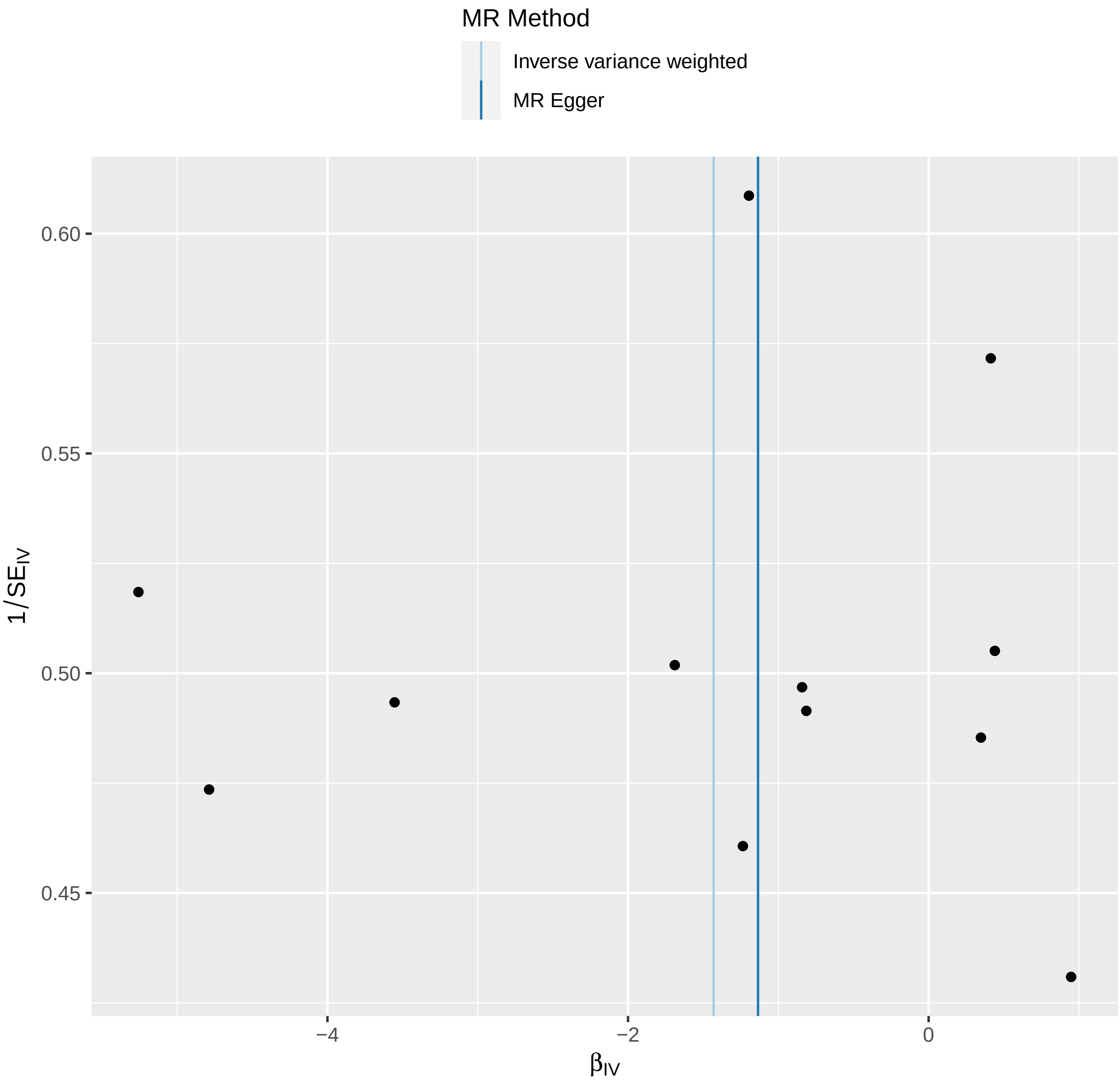

Batch 672 : Gut microbiota abundance (genus Eubacterium eligens group id.14372) on Ulcerative colitis (strict) with PSC

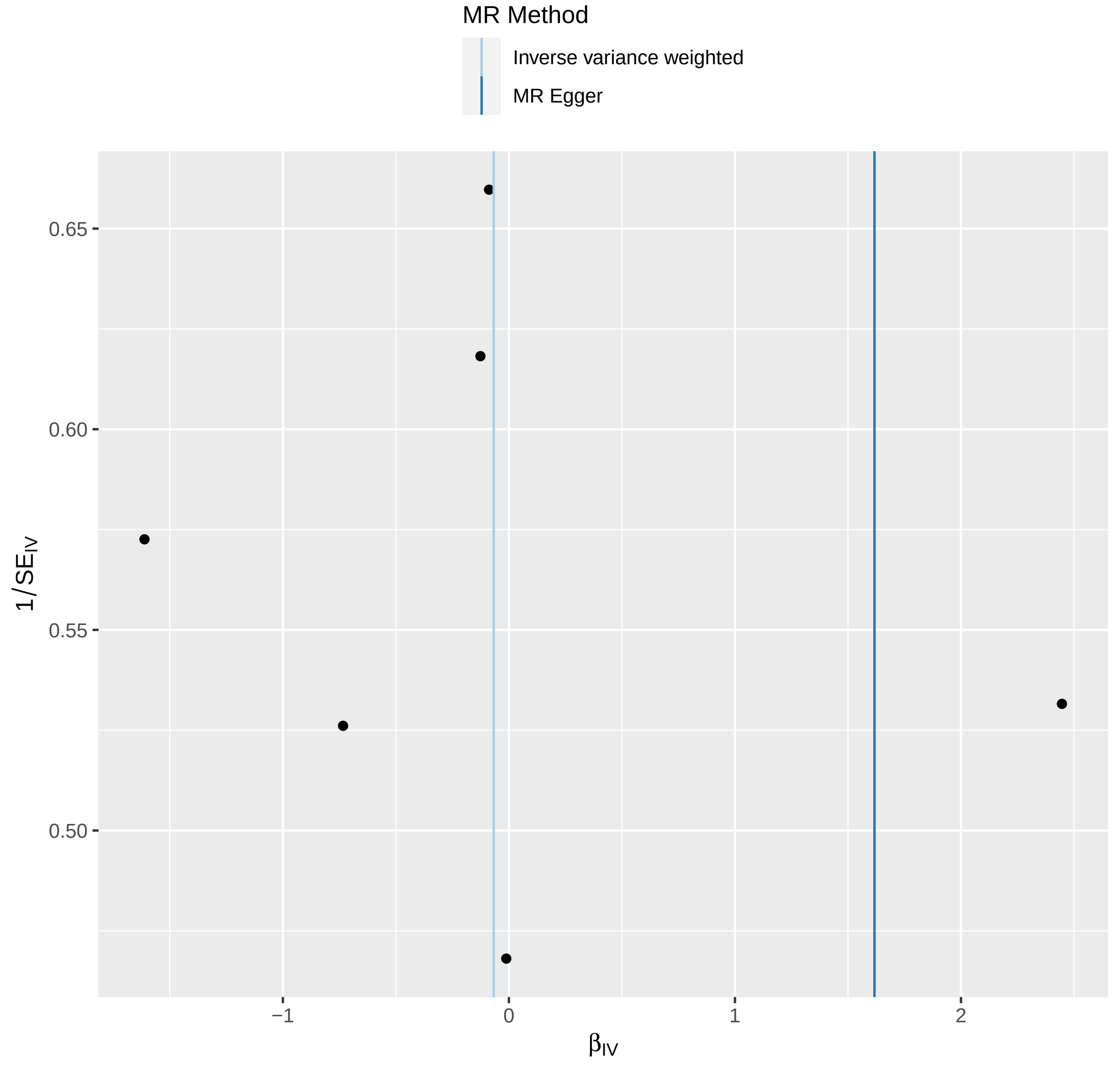

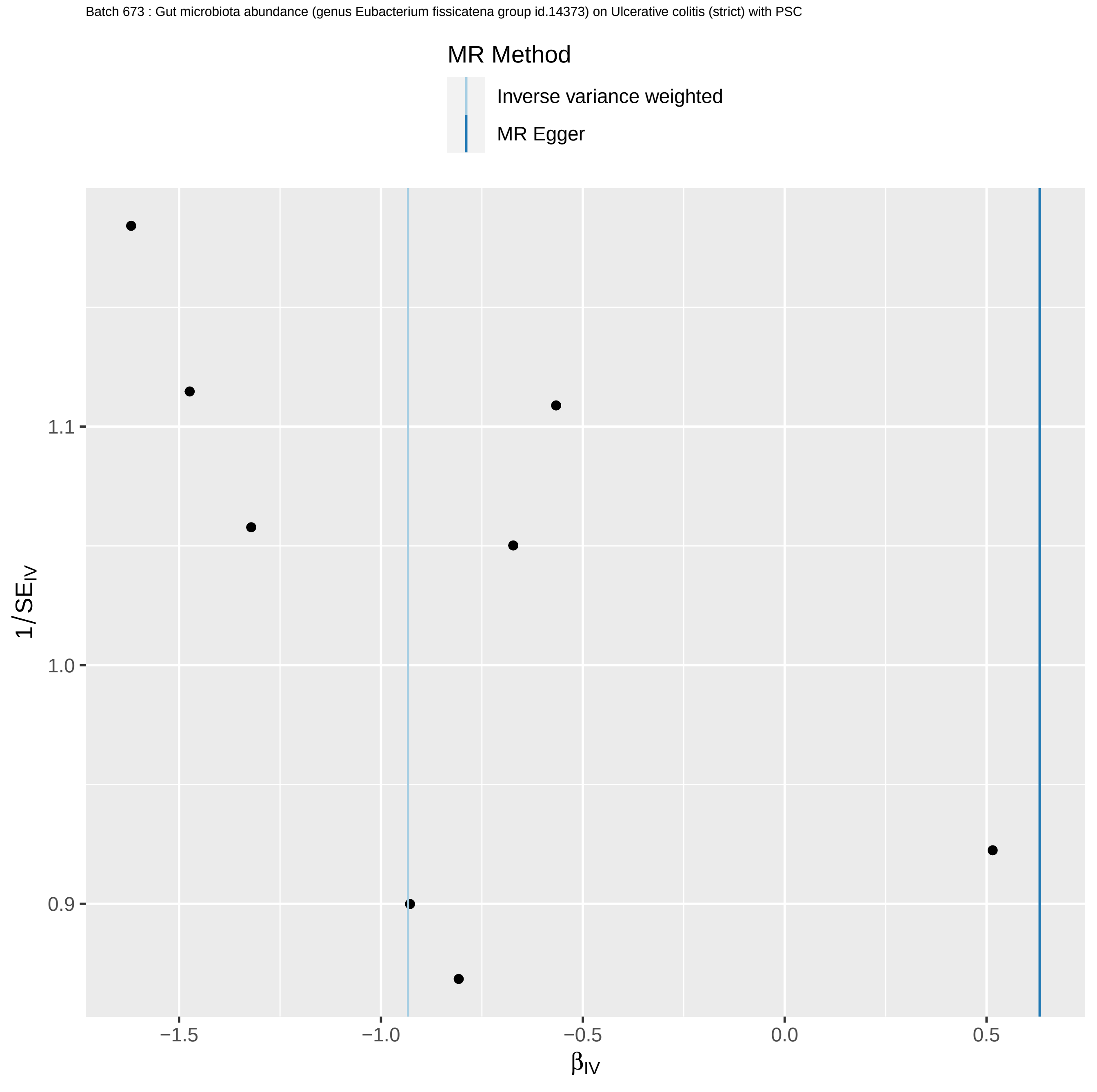

Batch 673 : Gut microbiota abundance (genus Eubacterium fissicatena group id.14373) on Ulcerative colitis (strict) with PSC
MR Method
Inverse variance weighted
MR Egger
1.1
1.0
0.9
1/SE$_{IV}$
−1.5
−1.0
−0.5
0.0
0.5
β_{IV}

Batch 674 : Gut microbiota abundance (genus Eubacterium hallii group id.11338) on Ulcerative colitis (strict) with PSC

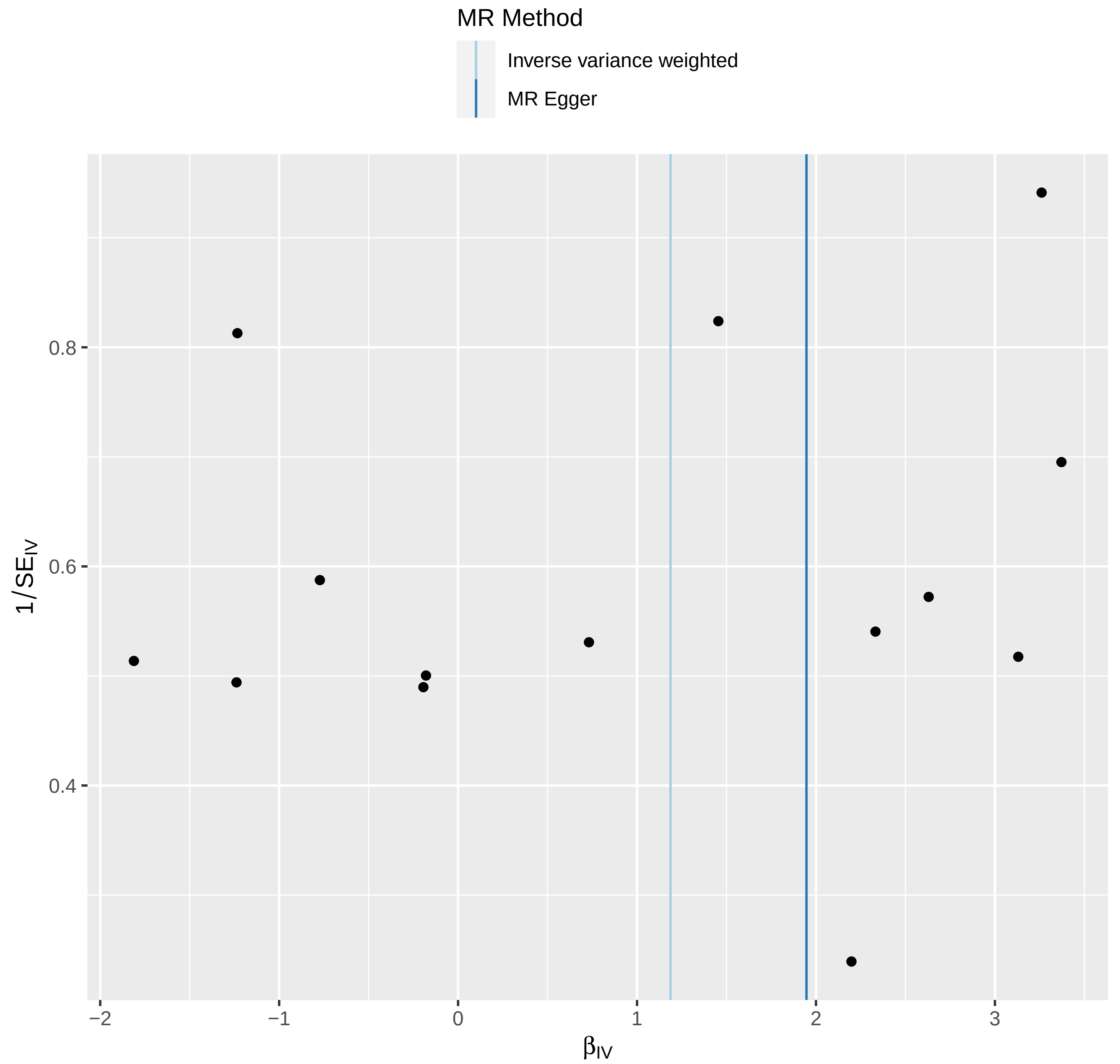

Batch 675 : Gut microbiota abundance (genus Eubacterium nodatum group id.11297) on Ulcerative colitis (strict) with PSC

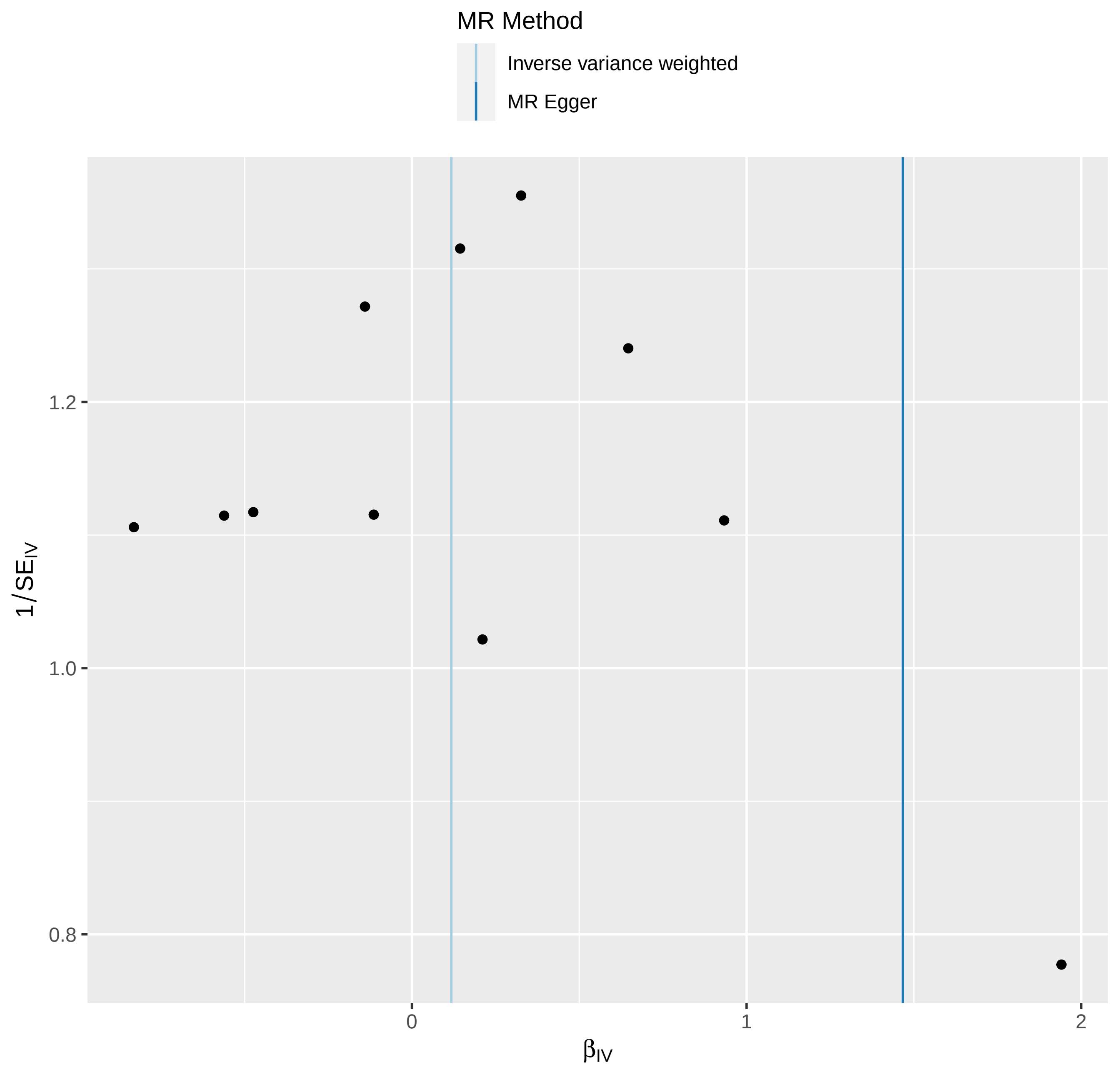

Batch 676 : Gut microbiota abundance (genus Eubacterium oxidoreducens group id.11339) on Ulcerative colitis (strict) with PSC

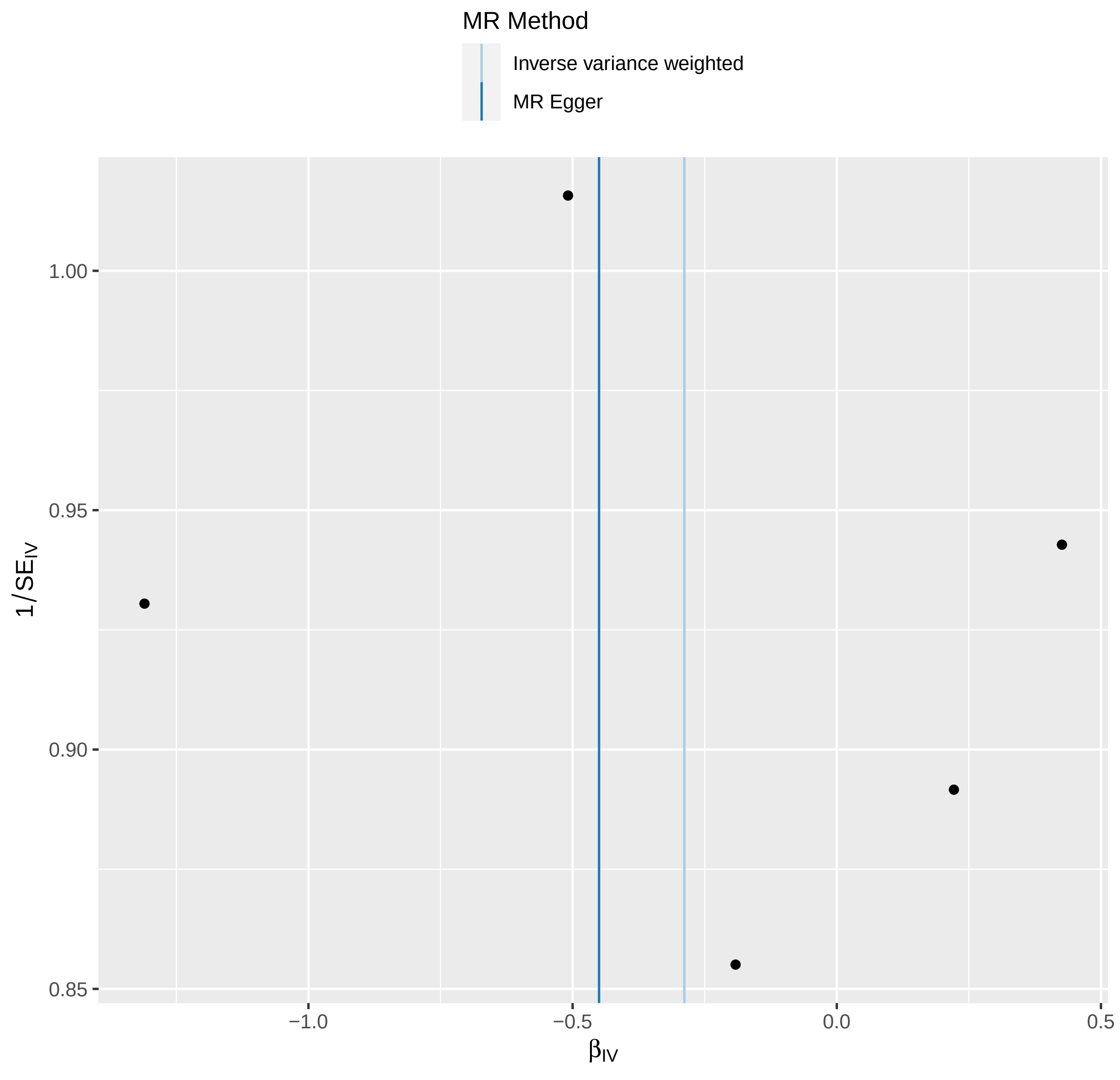

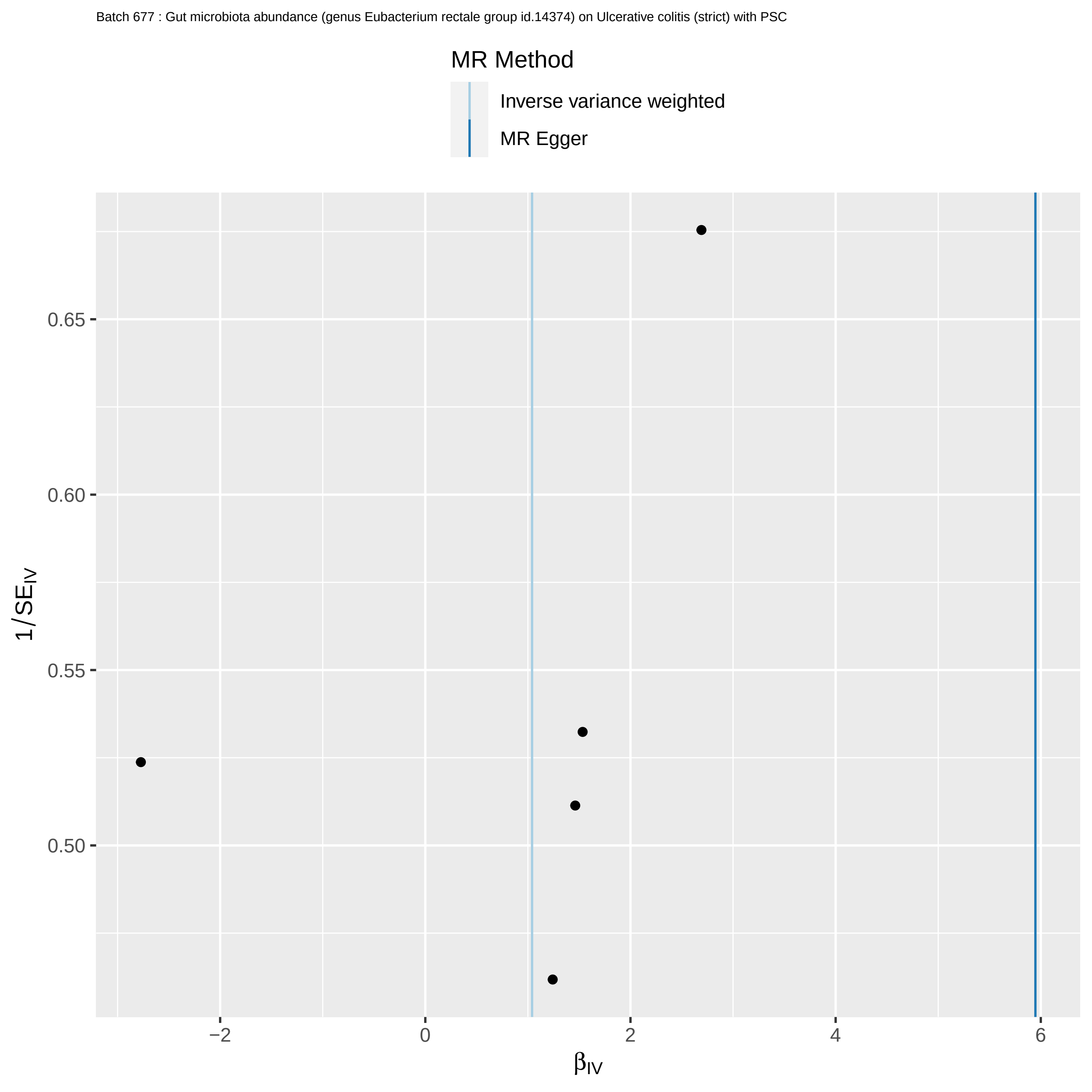
Batch 677 : Gut microbiota abundance (genus Eubacterium rectale group id.14374) on Ulcerative colitis (strict) with PSC
MR Method
Inverse variance weighted
MR Egger
0.65
0.60
0.55
0.50
1/SE_IV
−2
0
2
4
6
β_IV

Batch 678 : Gut microbiota abundance (genus Eubacterium ruminantium group id.11340) on Ulcerative colitis (strict) with PSC

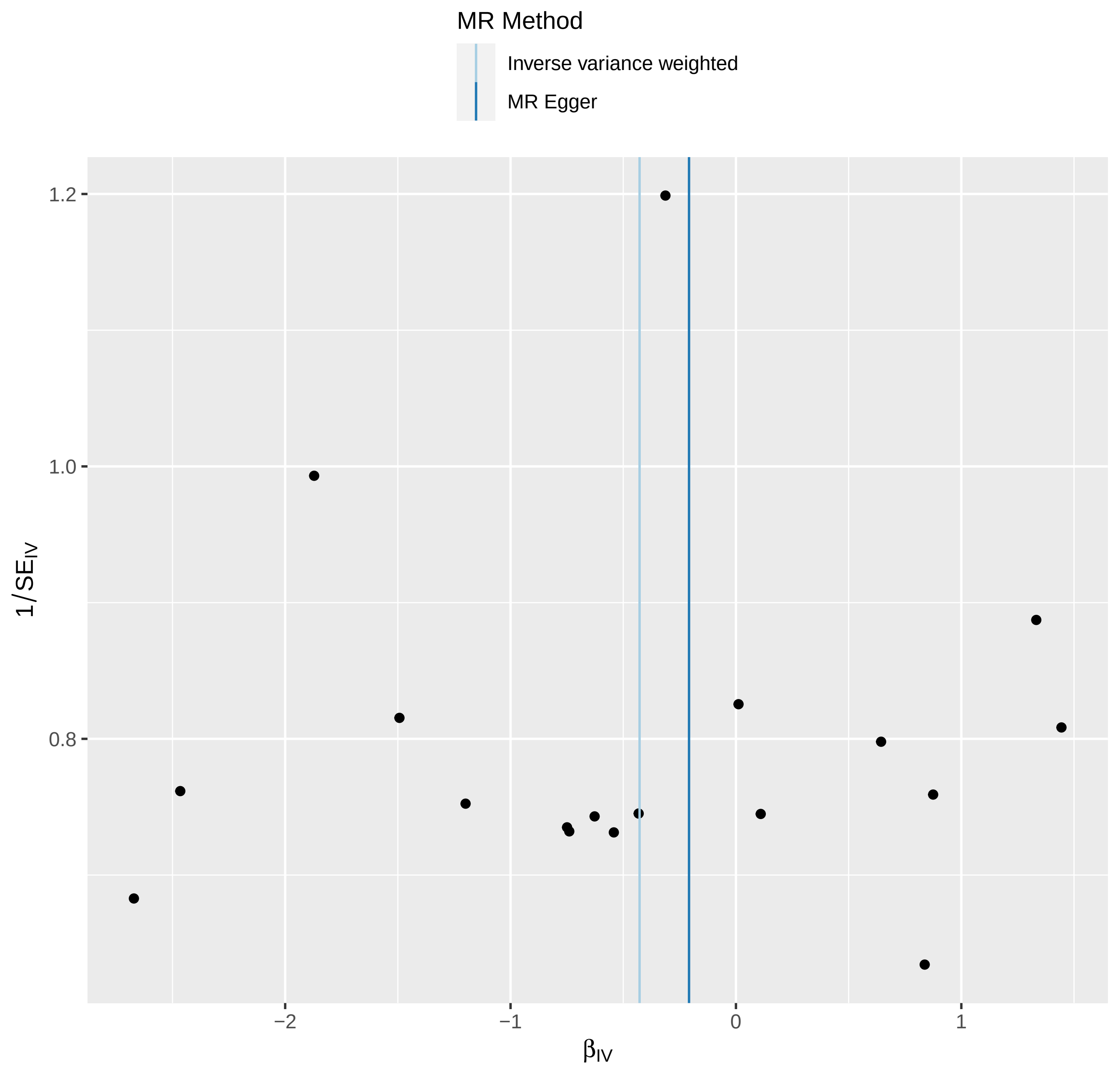

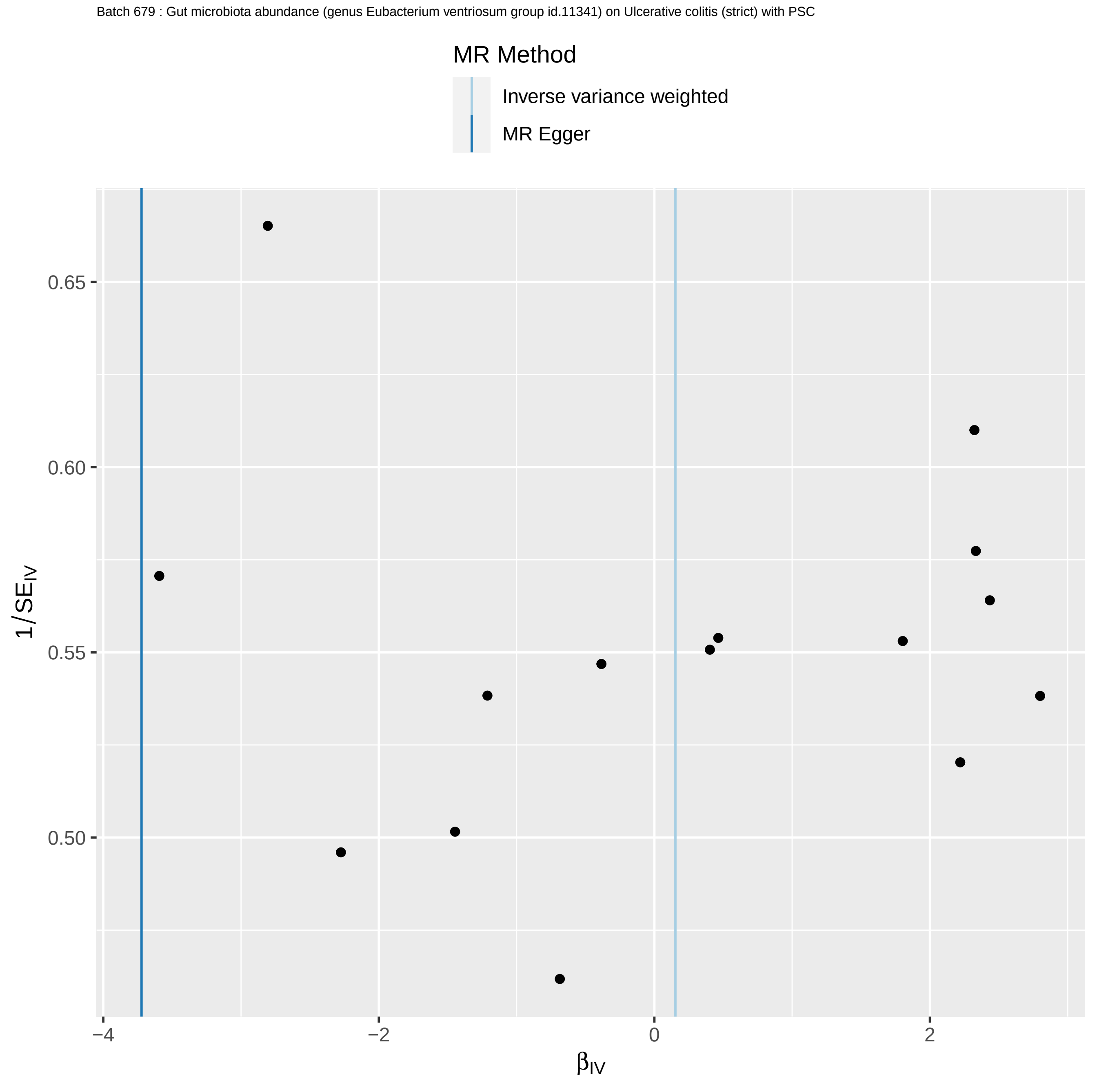

Batch 679 : Gut microbiota abundance (genus Eubacterium ventriosum group id.11341) on Ulcerative colitis (strict) with PSC
MR Method
Inverse variance weighted
MR Egger
0.65
0.60
0.55
0.50
1/SE_IV
−4
−2
0
2
β_IV

Batch 680 : Gut microbiota abundance (genus Eubacterium xylanophilum group id.14375) on Ulcerative colitis (strict) with PSC

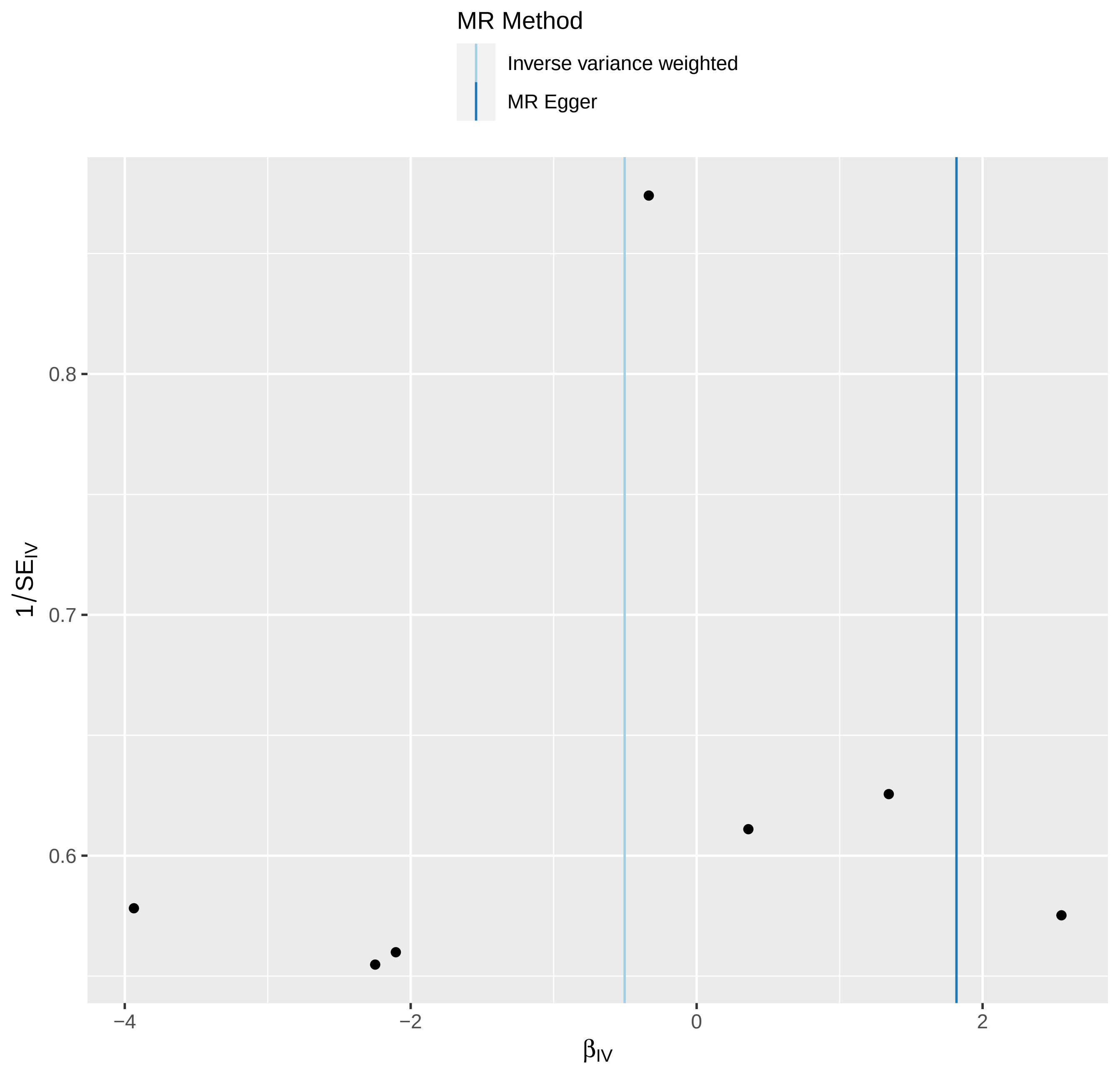

Batch 681 : Gut microbiota abundance (genus Faecalibacterium id.2057) on Ulcerative colitis (strict) with PSC

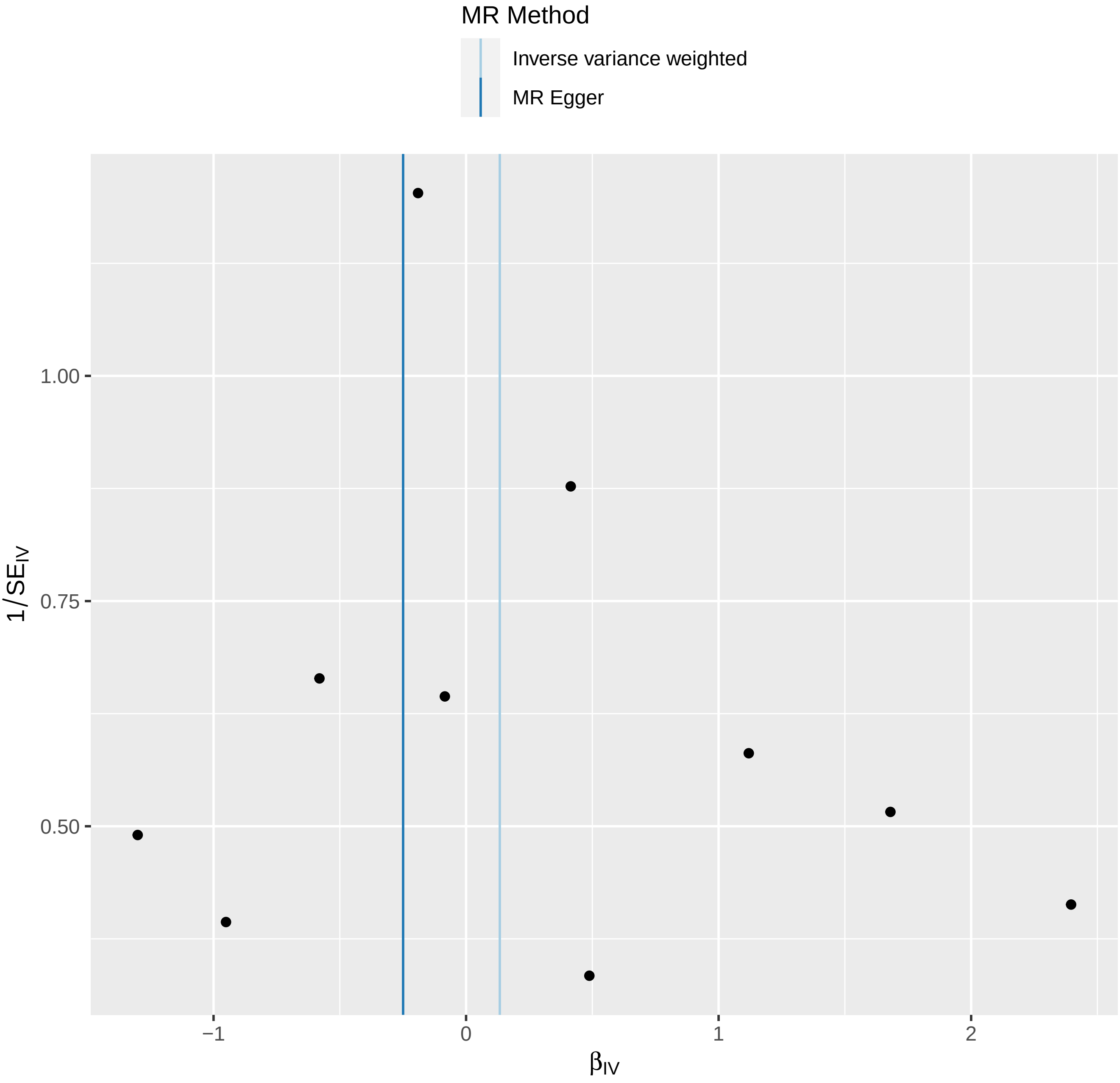

Batch 682 : Gut microbiota abundance (genus Family XIII AD3011 group id.11293) on Ulcerative colitis (strict) with PSC

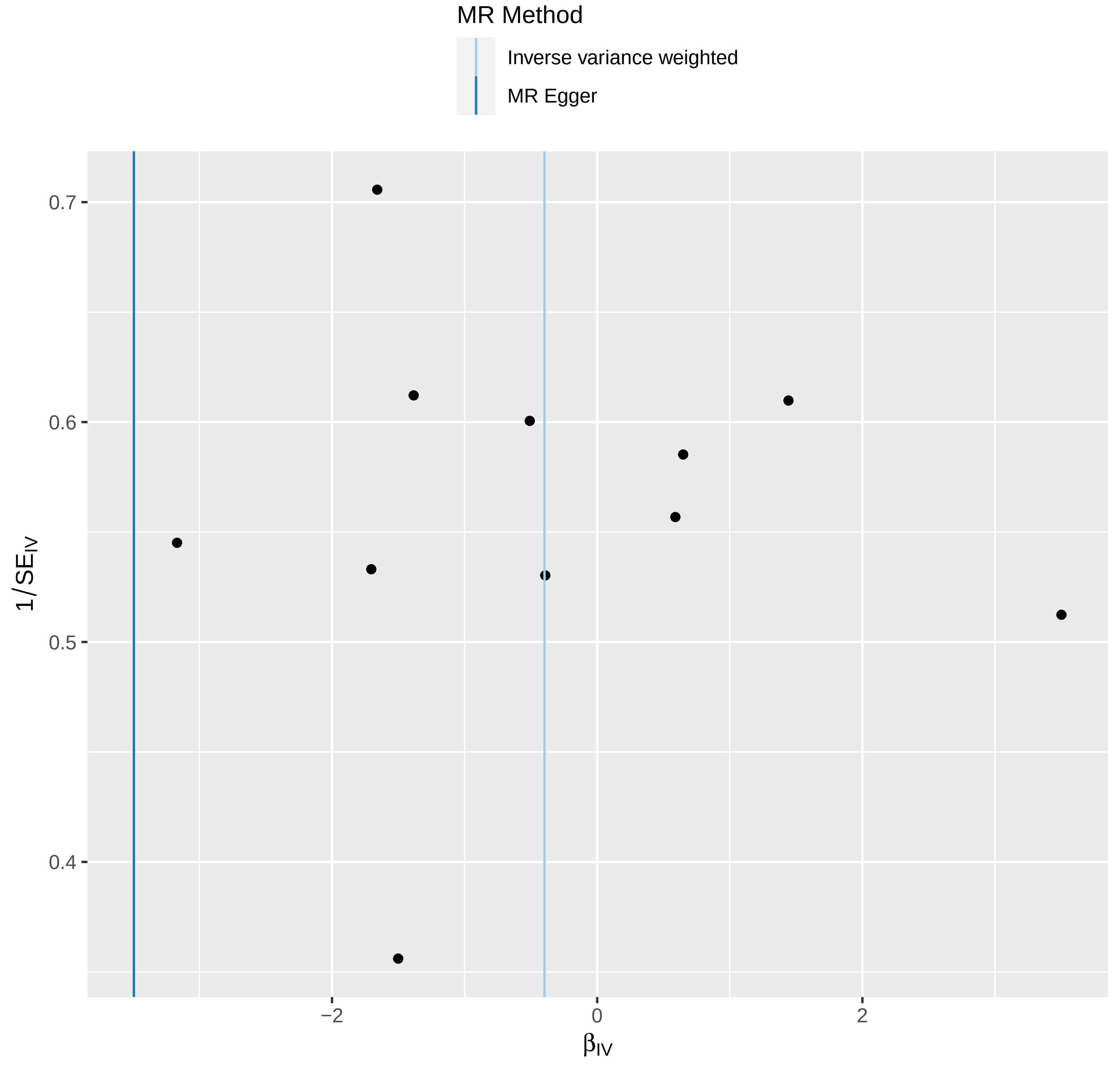

Batch 683 : Gut microbiota abundance (genus Family XIII UCG001 id.11294) on Ulcerative colitis (strict) with PSC

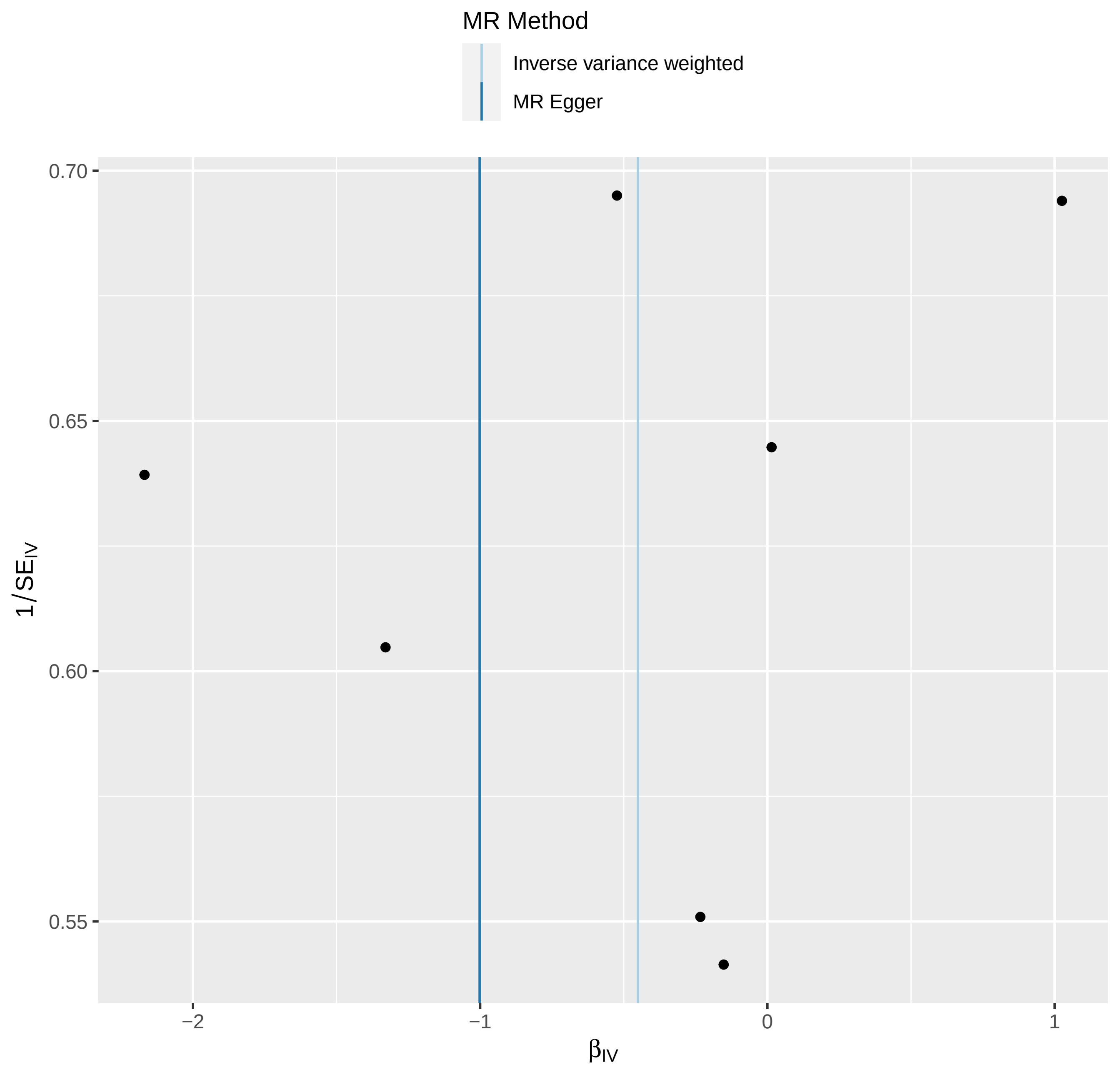

Batch 684 : Gut microbiota abundance (genus Flavonifractor id.2059) on Ulcerative colitis (strict) with PSC

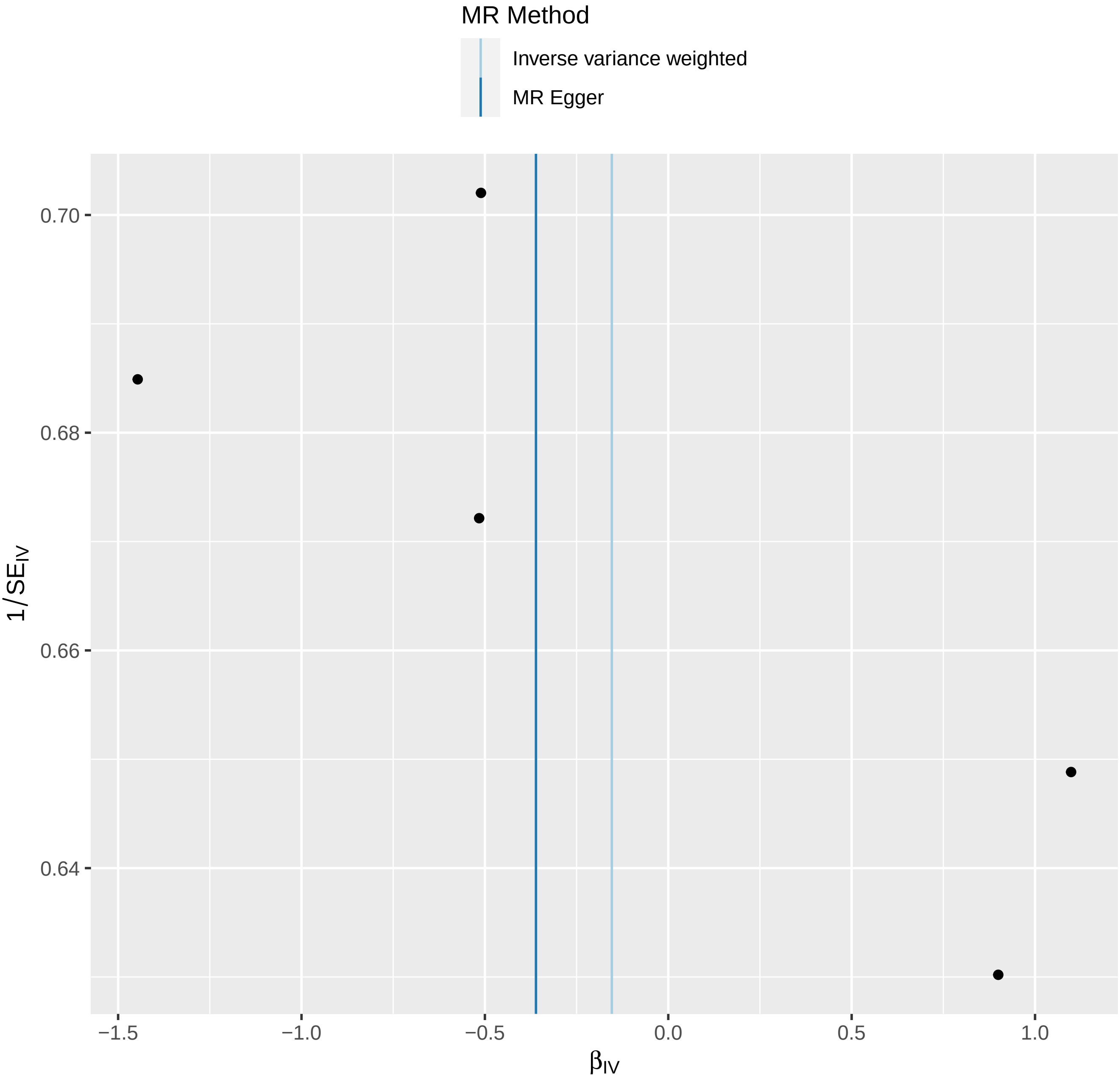

Batch 685 : Gut microbiota abundance (genus Fusicatenibacter id.11305) on Ulcerative colitis (strict) with PSC

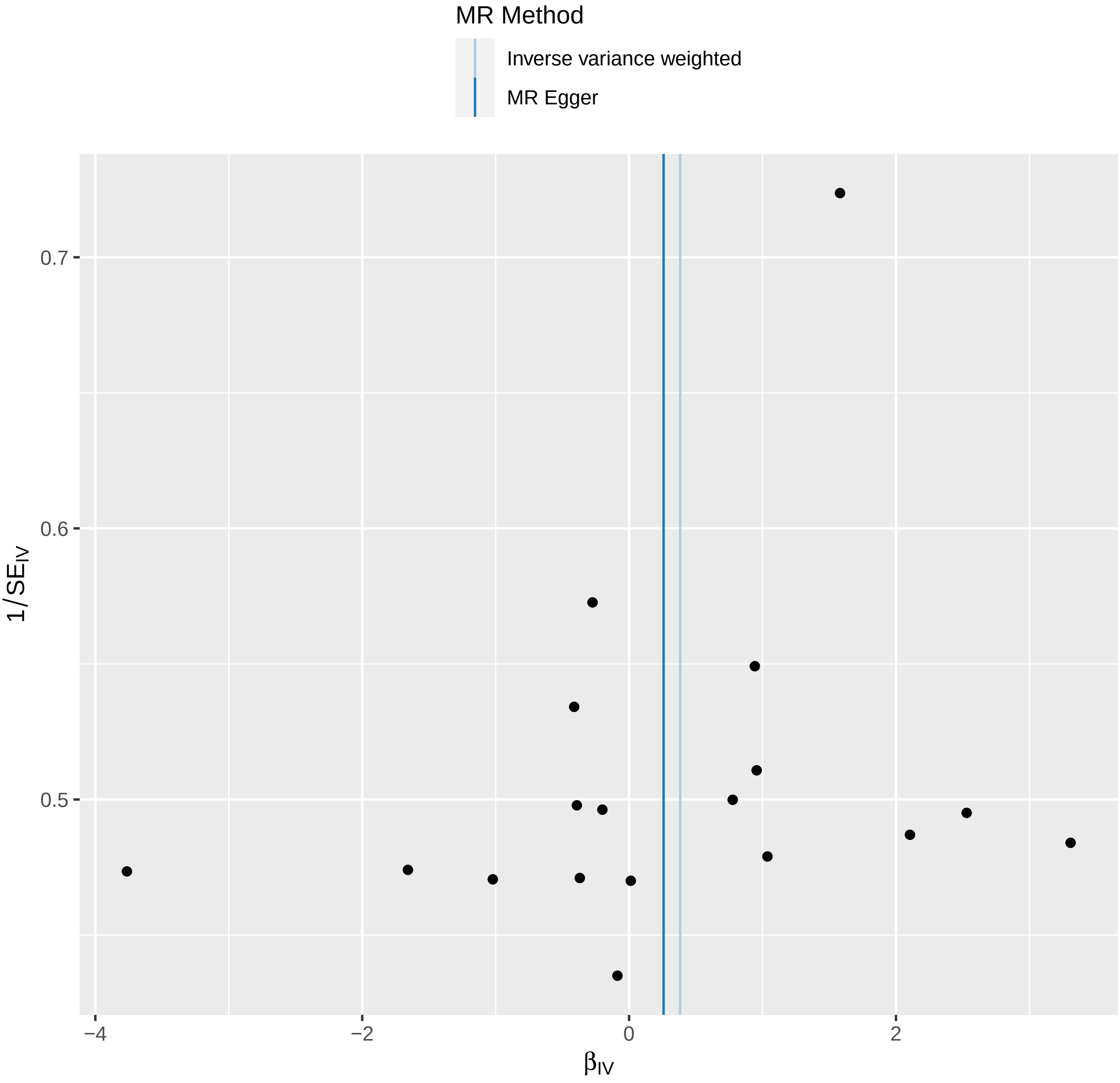

Batch 686 : Gut microbiota abundance (genus Gordonibacter id.821) on Ulcerative colitis (strict) with PSC

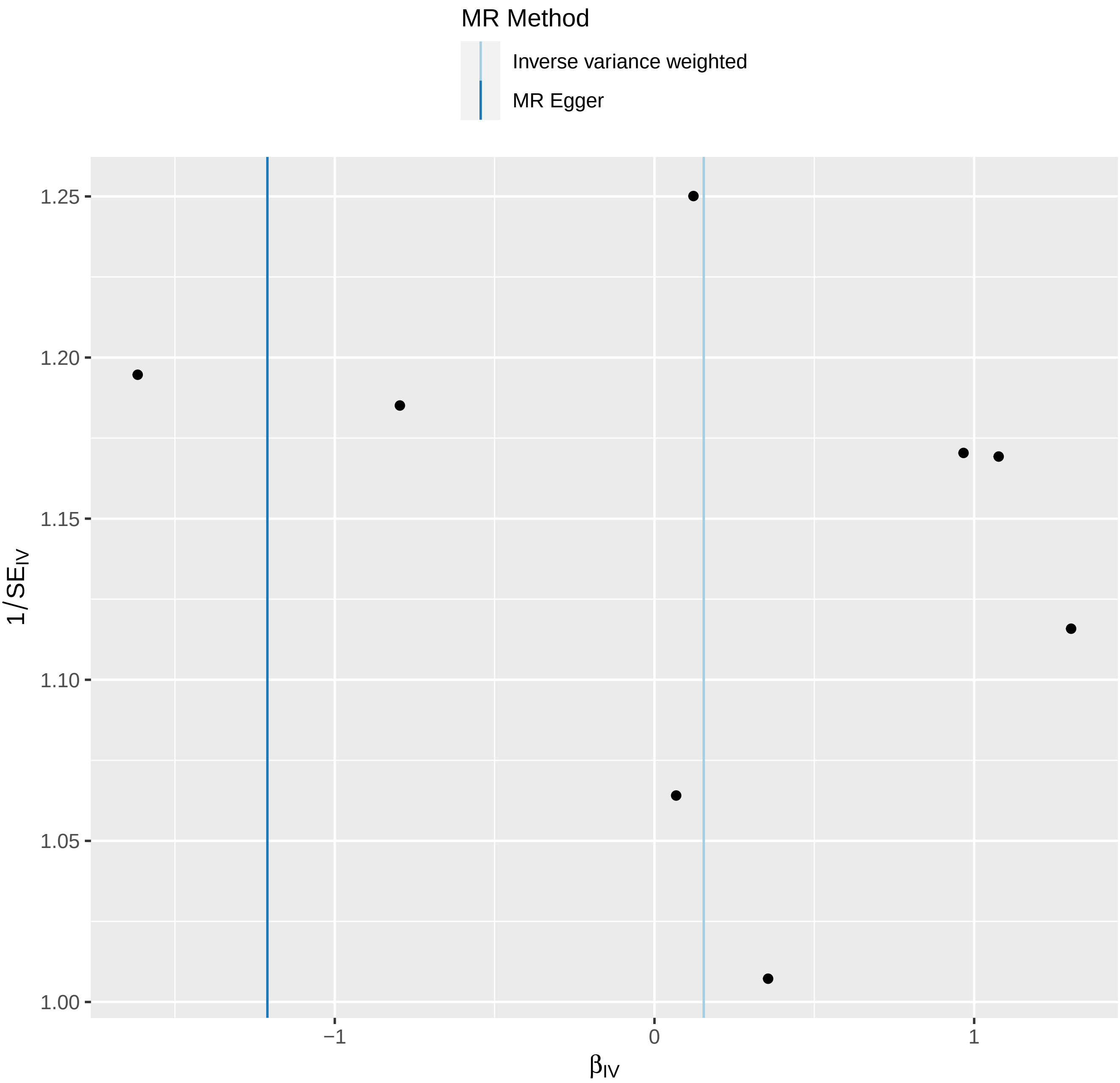

Batch 687 : Gut microbiota abundance (genus Haemophilus id.3698) on Ulcerative colitis (strict) with PSC

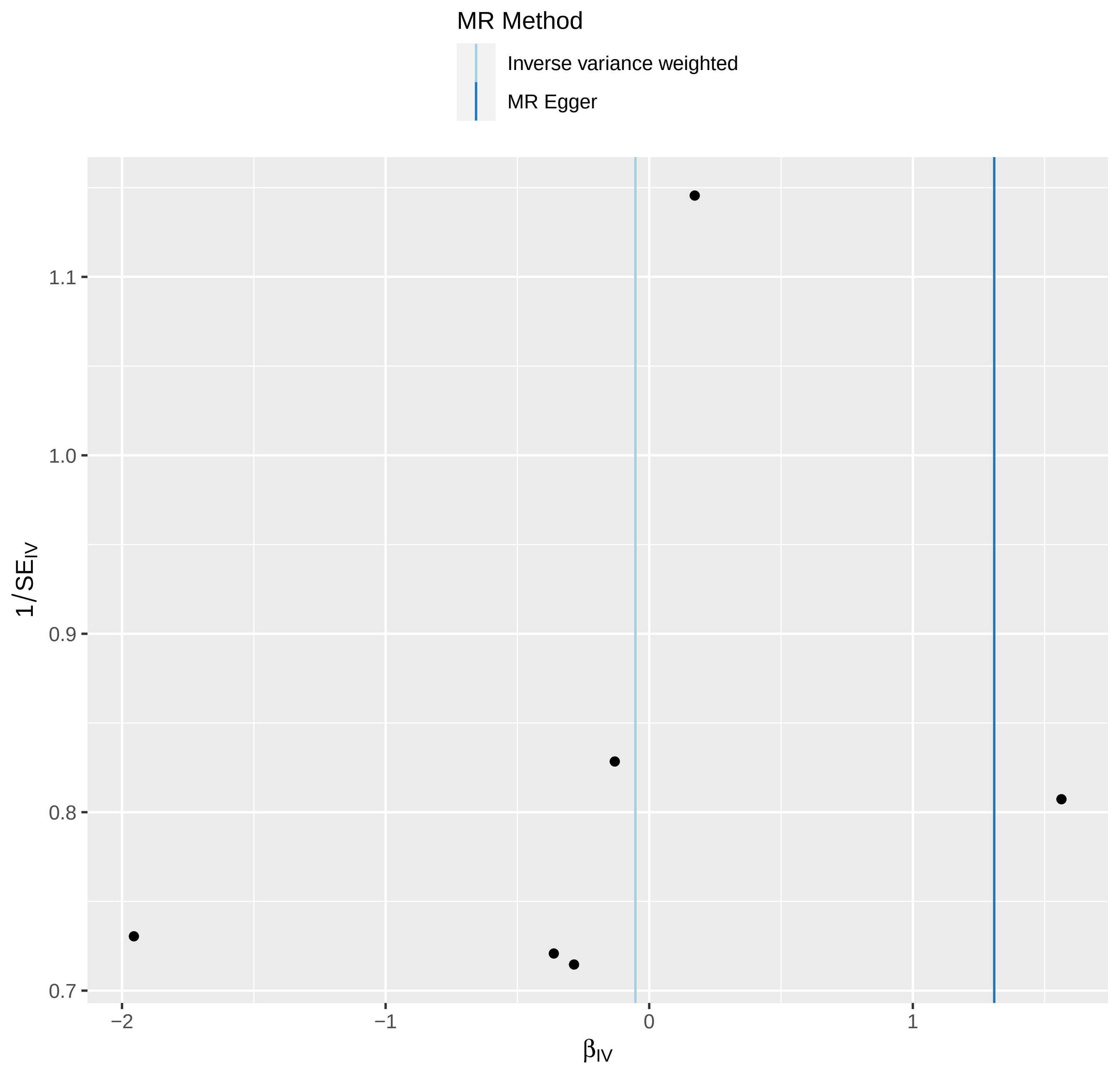

Batch 688 : Gut microbiota abundance (genus Holdemanella id.11393) on Ulcerative colitis (strict) with PSC

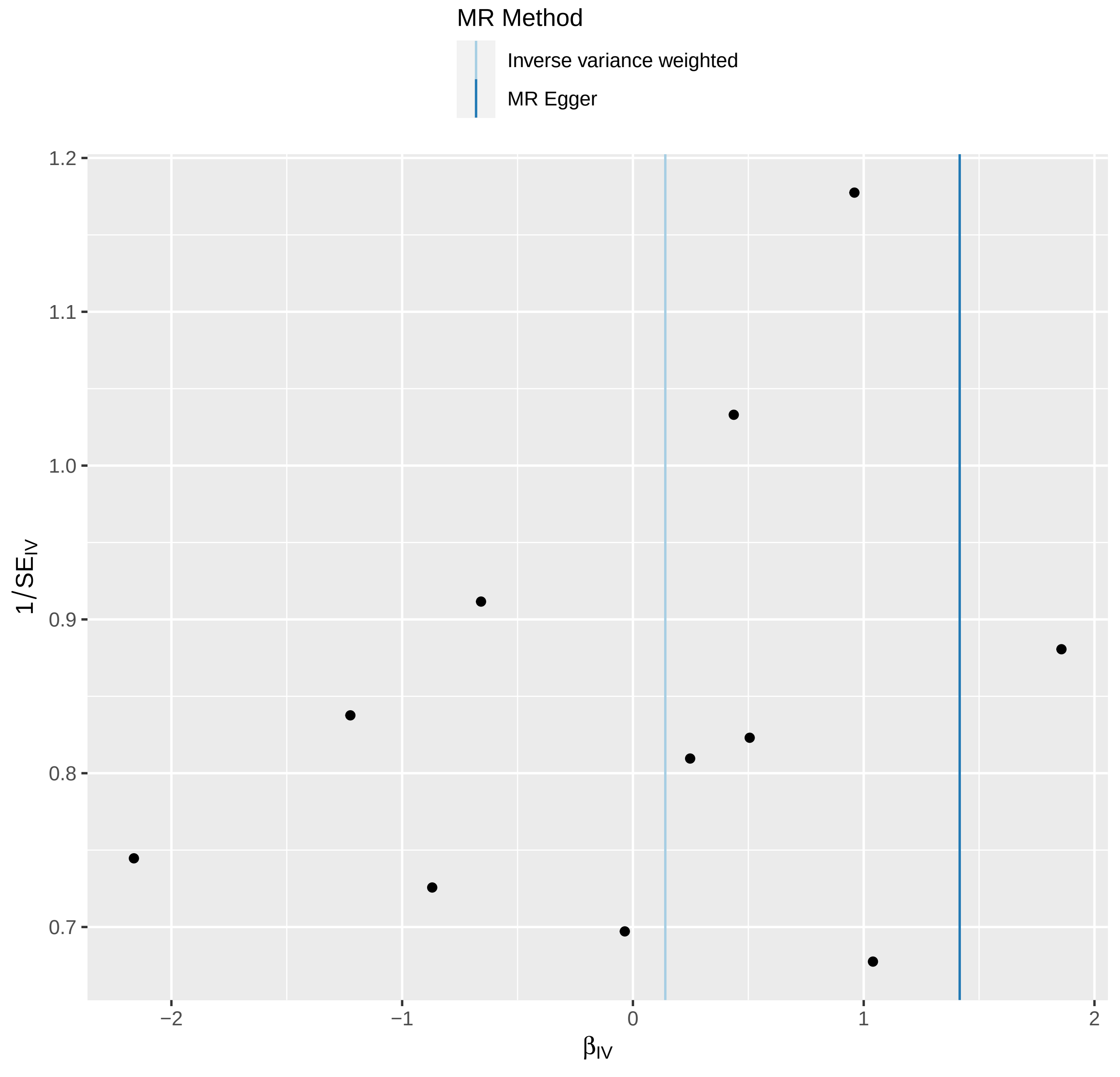

Batch 689 : Gut microbiota abundance (genus Holdemania id.2157) on Ulcerative colitis (strict) with PSC

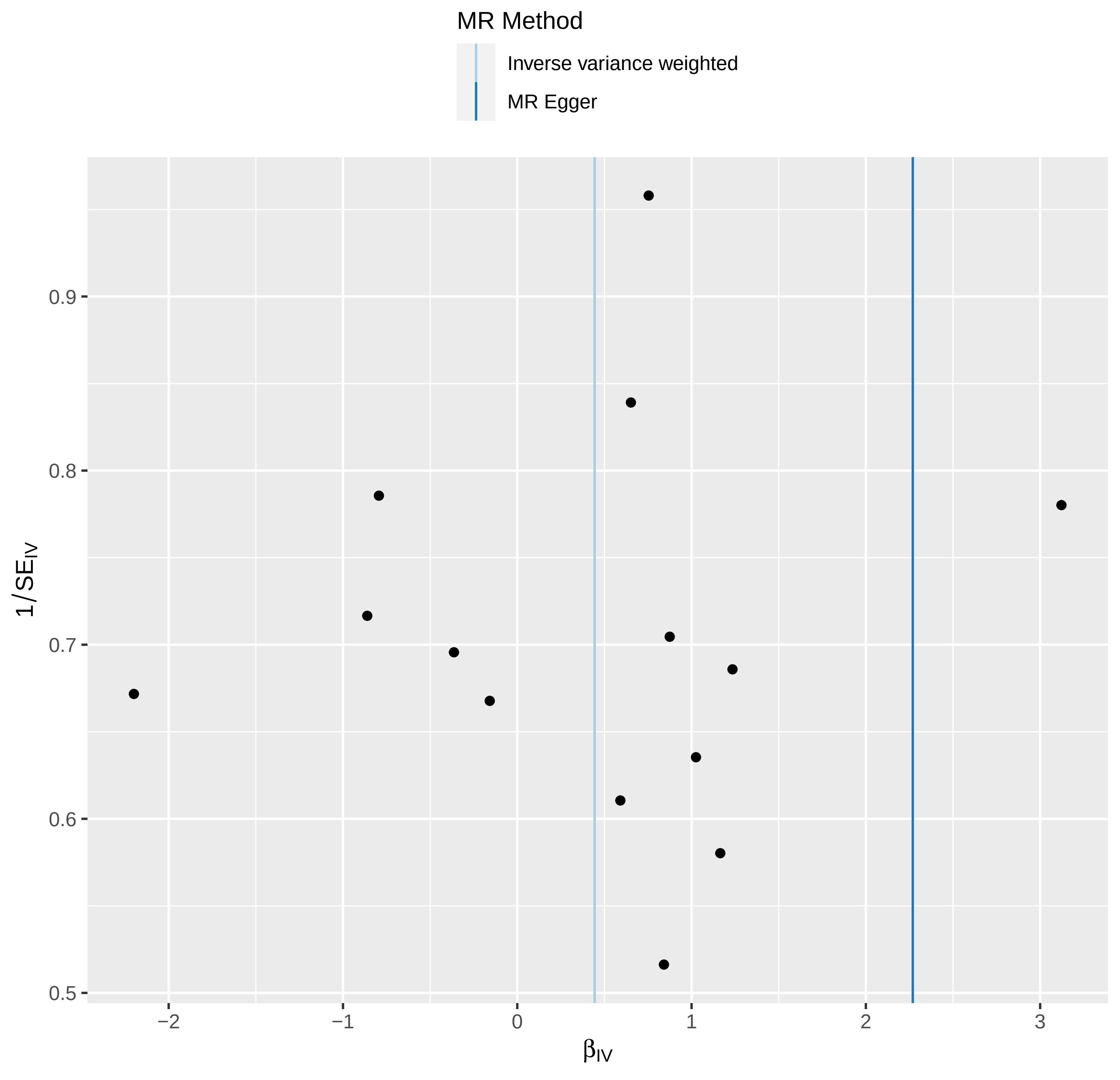

Batch 690 : Gut microbiota abundance (genus Howardella id.2000) on Ulcerative colitis (strict) with PSC

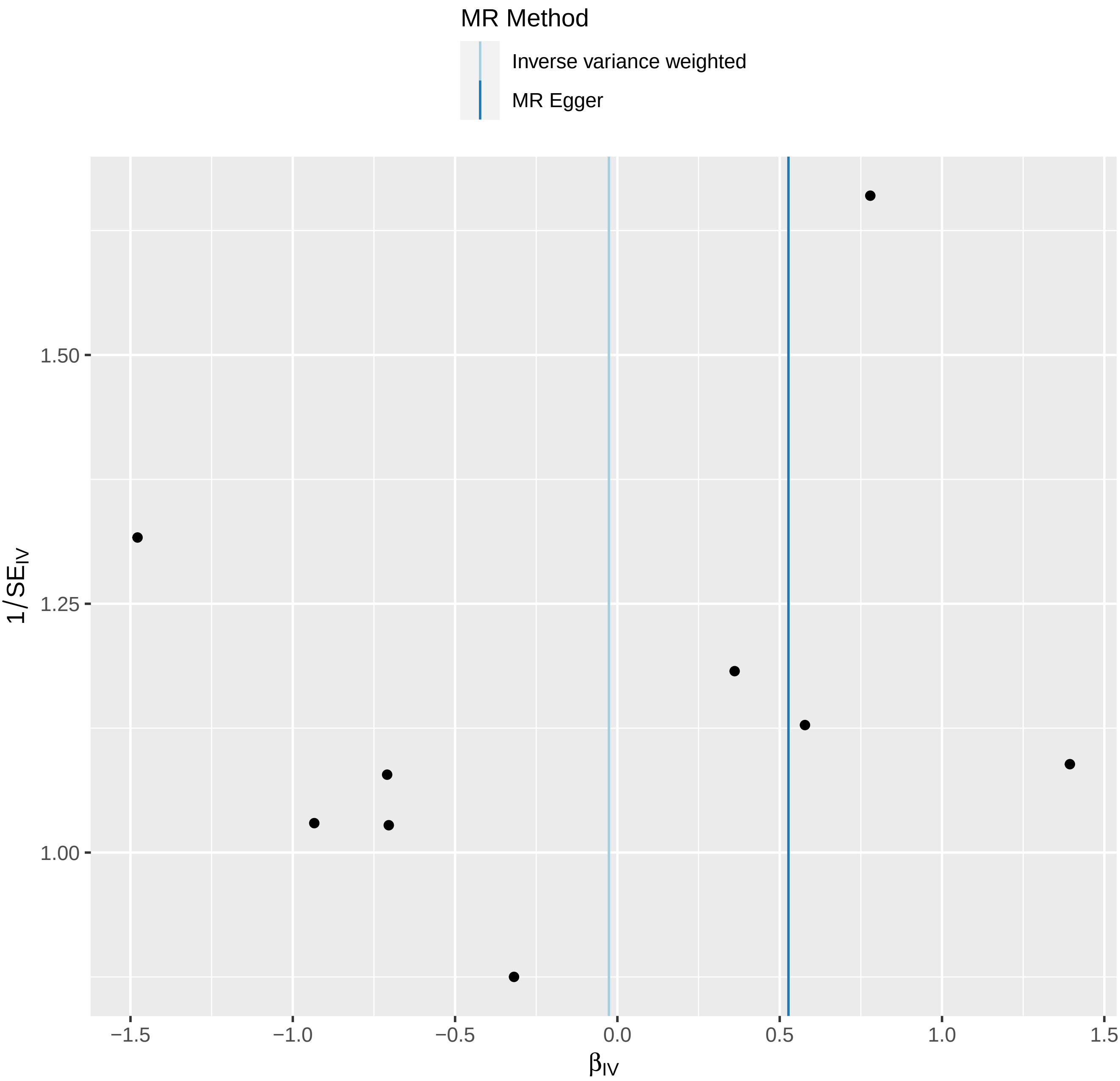

Batch 691 : Gut microbiota abundance (genus Hungatella id.11306) on Ulcerative colitis (strict) with PSC

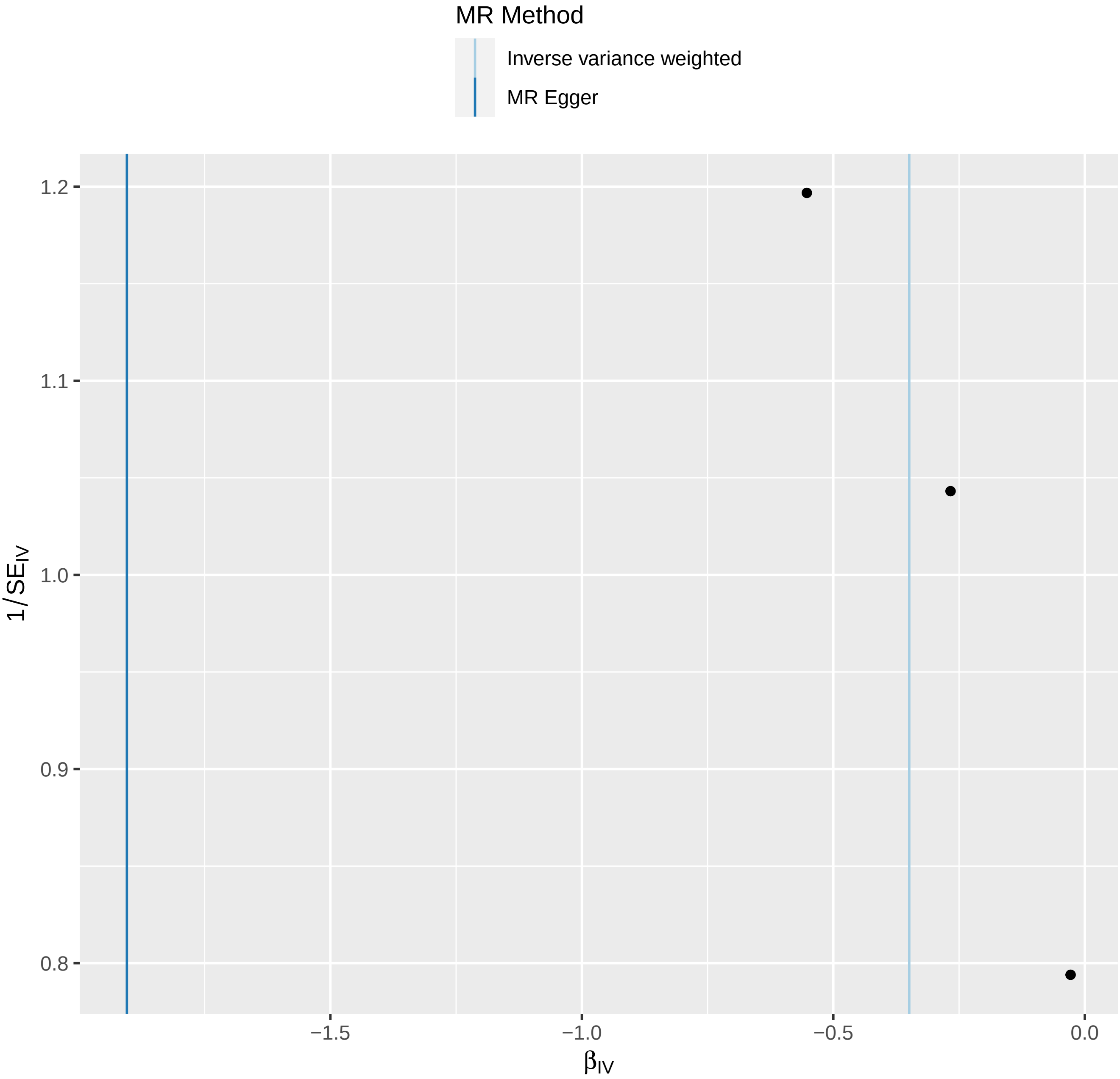

Batch 692 : Gut microbiota abundance (genus Intestinibacter id.11345) on Ulcerative colitis (strict) with PSC

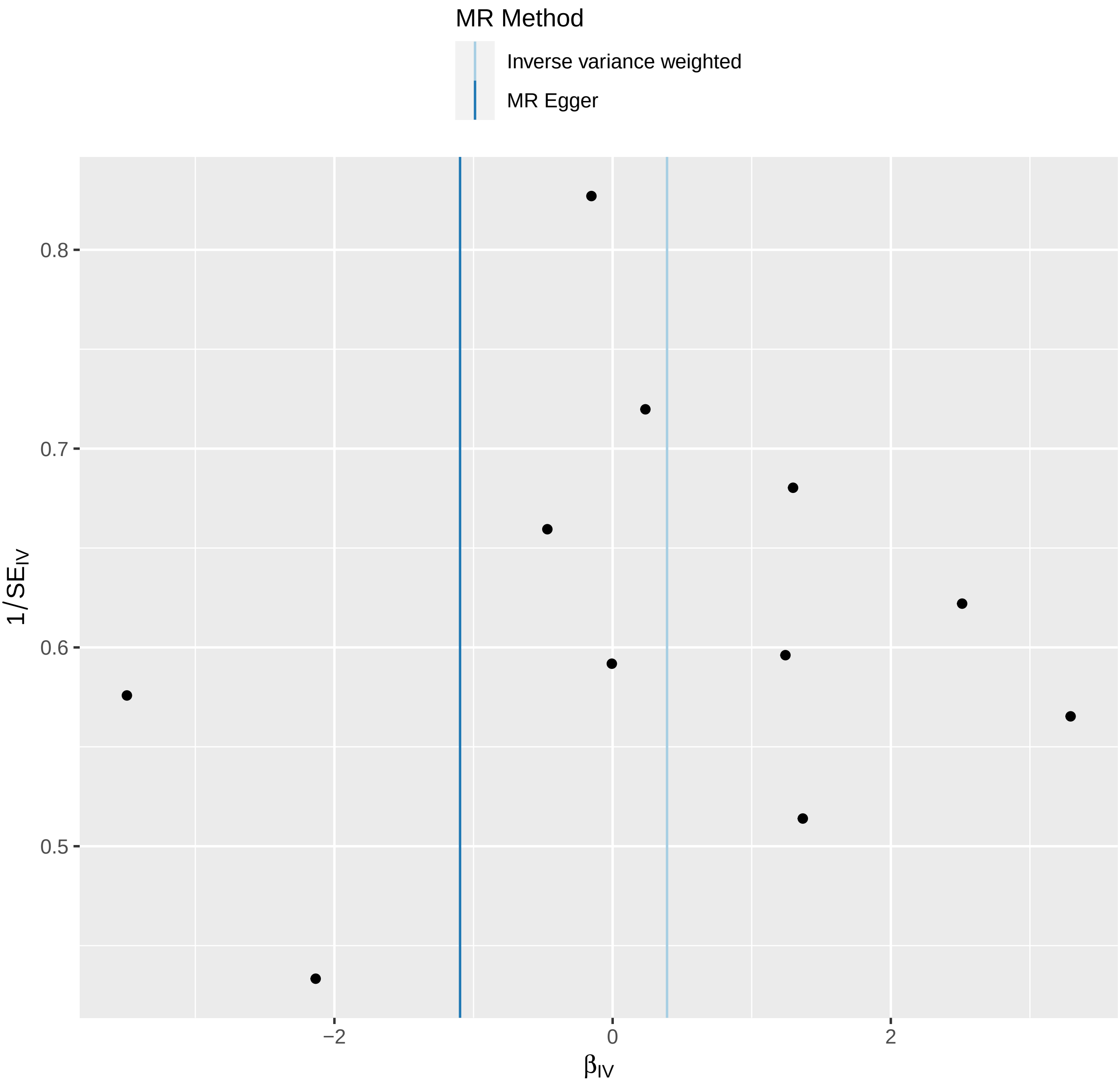

Batch 693 : Gut microbiota abundance (genus Intestinimonas id.2062) on Ulcerative colitis (strict) with PSC

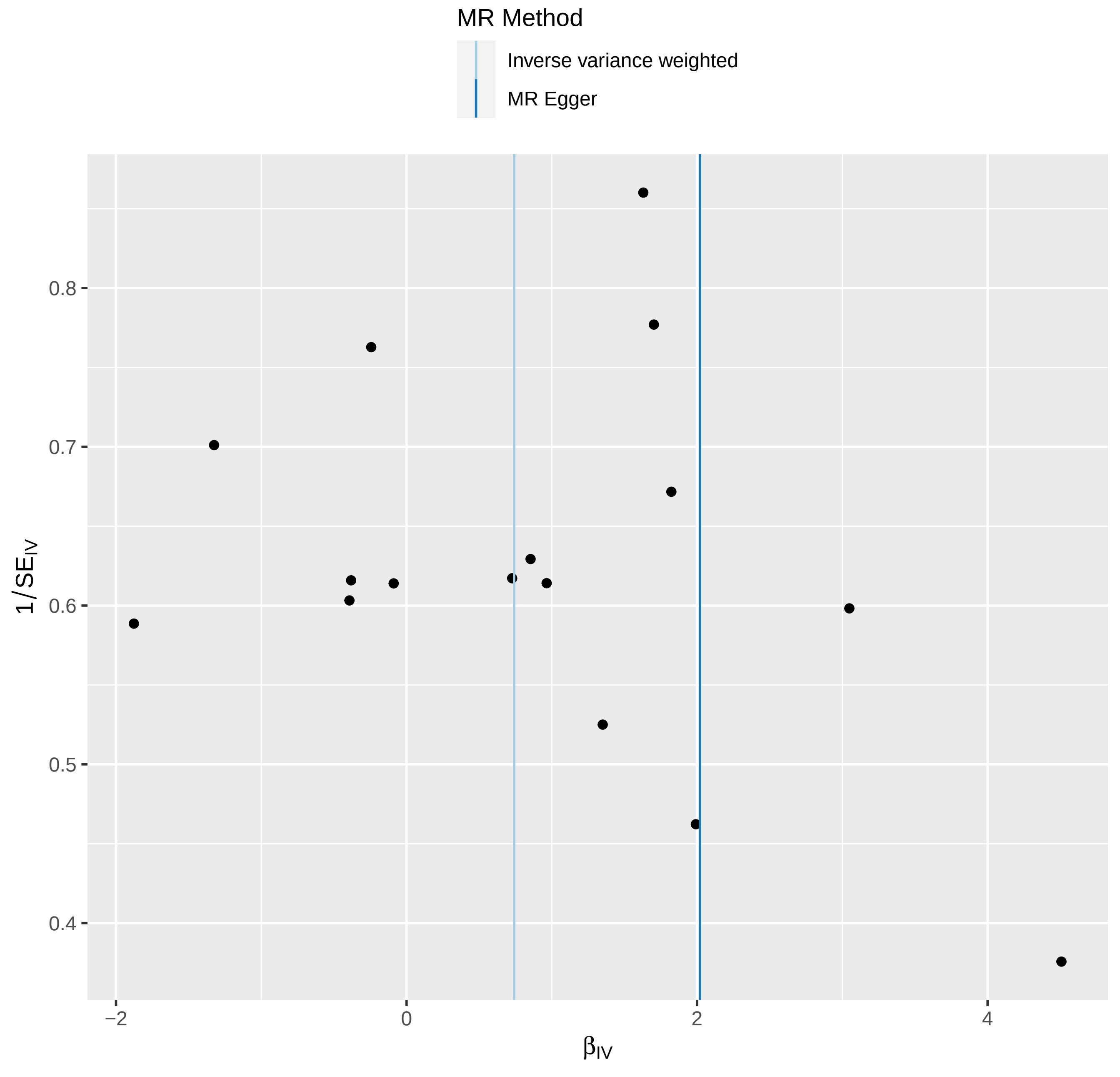

Batch 694 : Gut microbiota abundance (genus Lachnoclostridium id.11308) on Ulcerative colitis (strict) with PSC

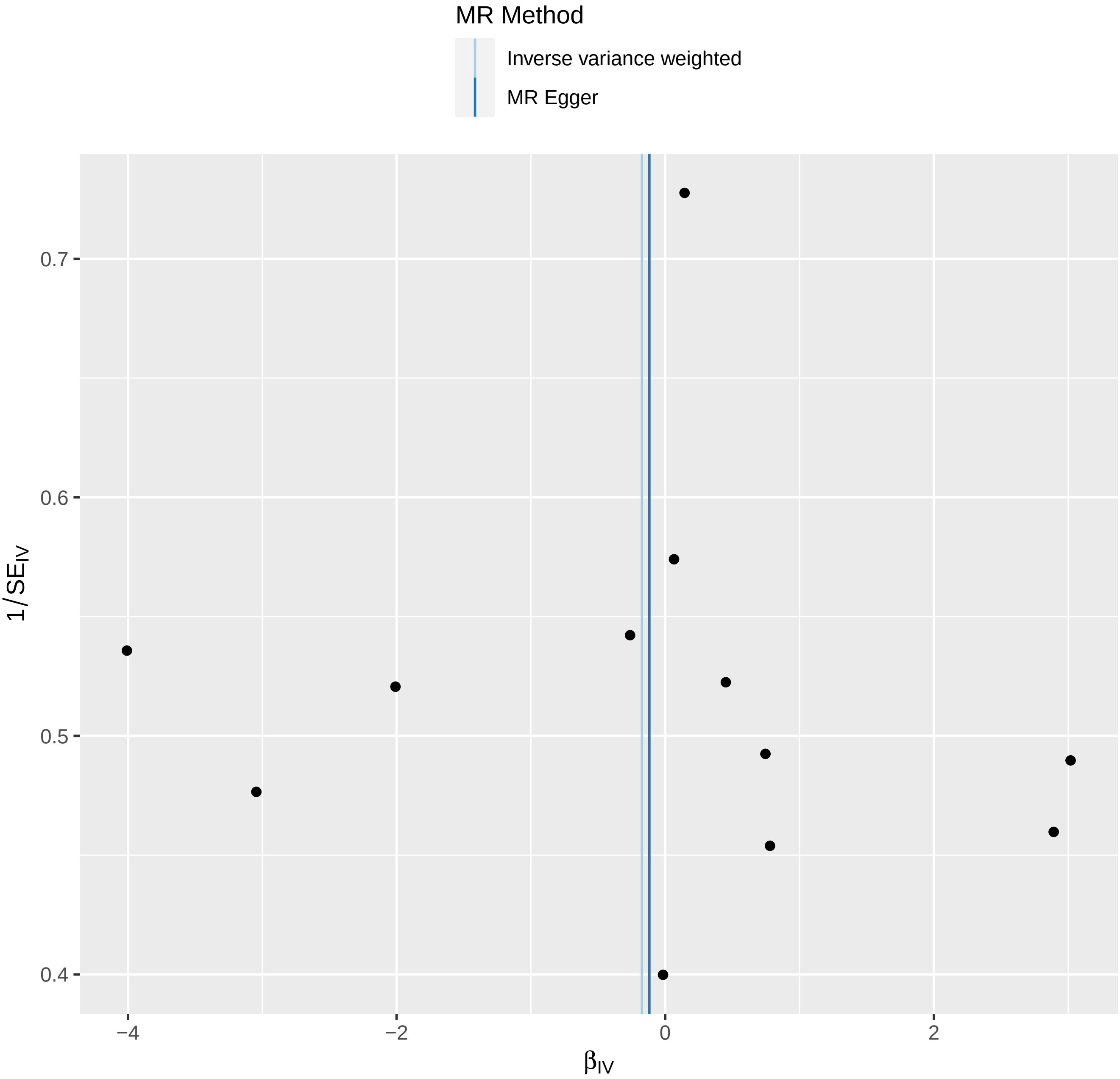

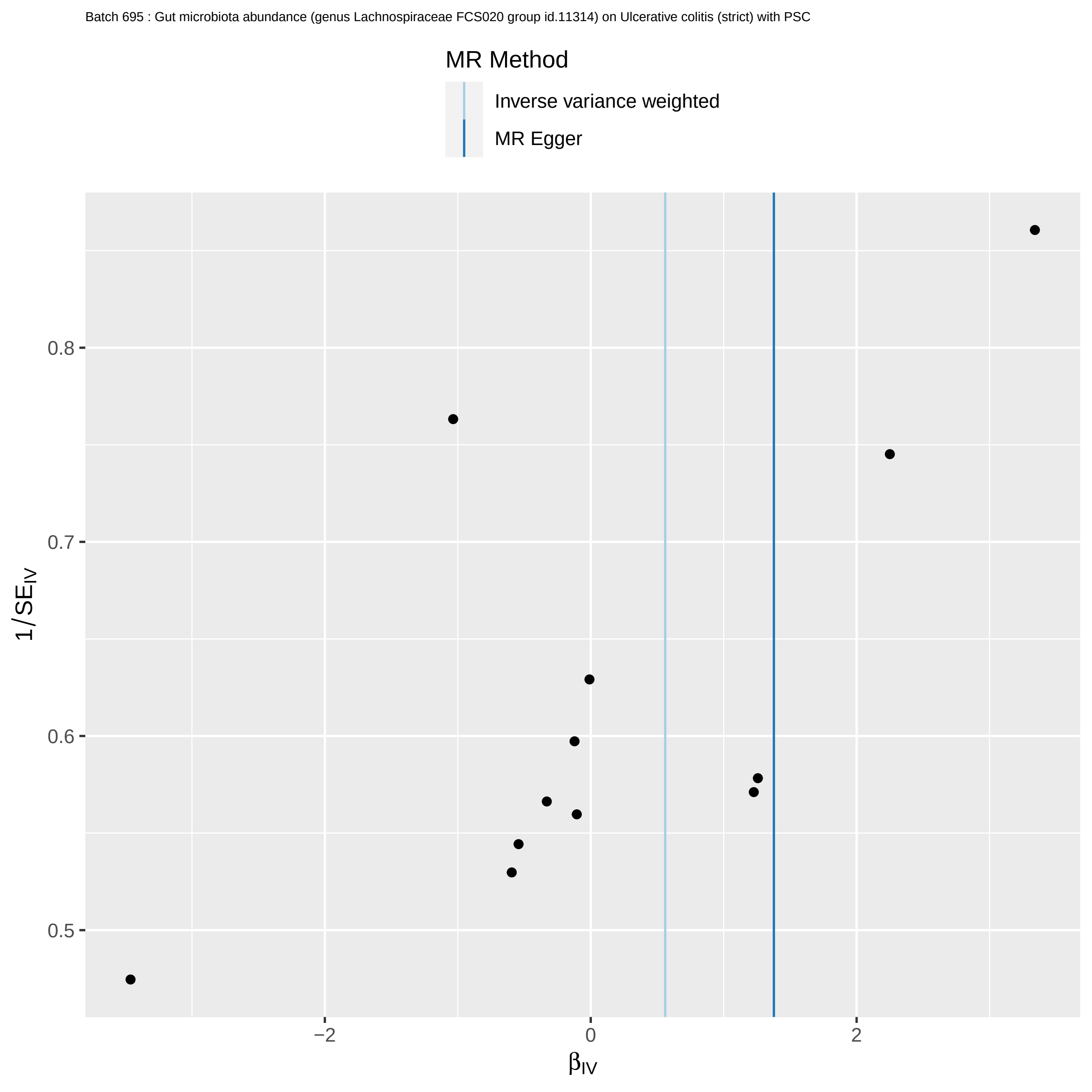
Batch 695 : Gut microbiota abundance (genus Lachnospiraceae FCS020 group id.11314) on Ulcerative colitis (strict) with PSC
MR Method
Inverse variance weighted
MR Egger
0.8
0.7
0.6
0.5
1/SE_IV
−2
0
2
β_IV

Batch 696 : Gut microbiota abundance (genus Lachnospiraceae NC2004 group id.11316) on Ulcerative colitis (strict) with PSC

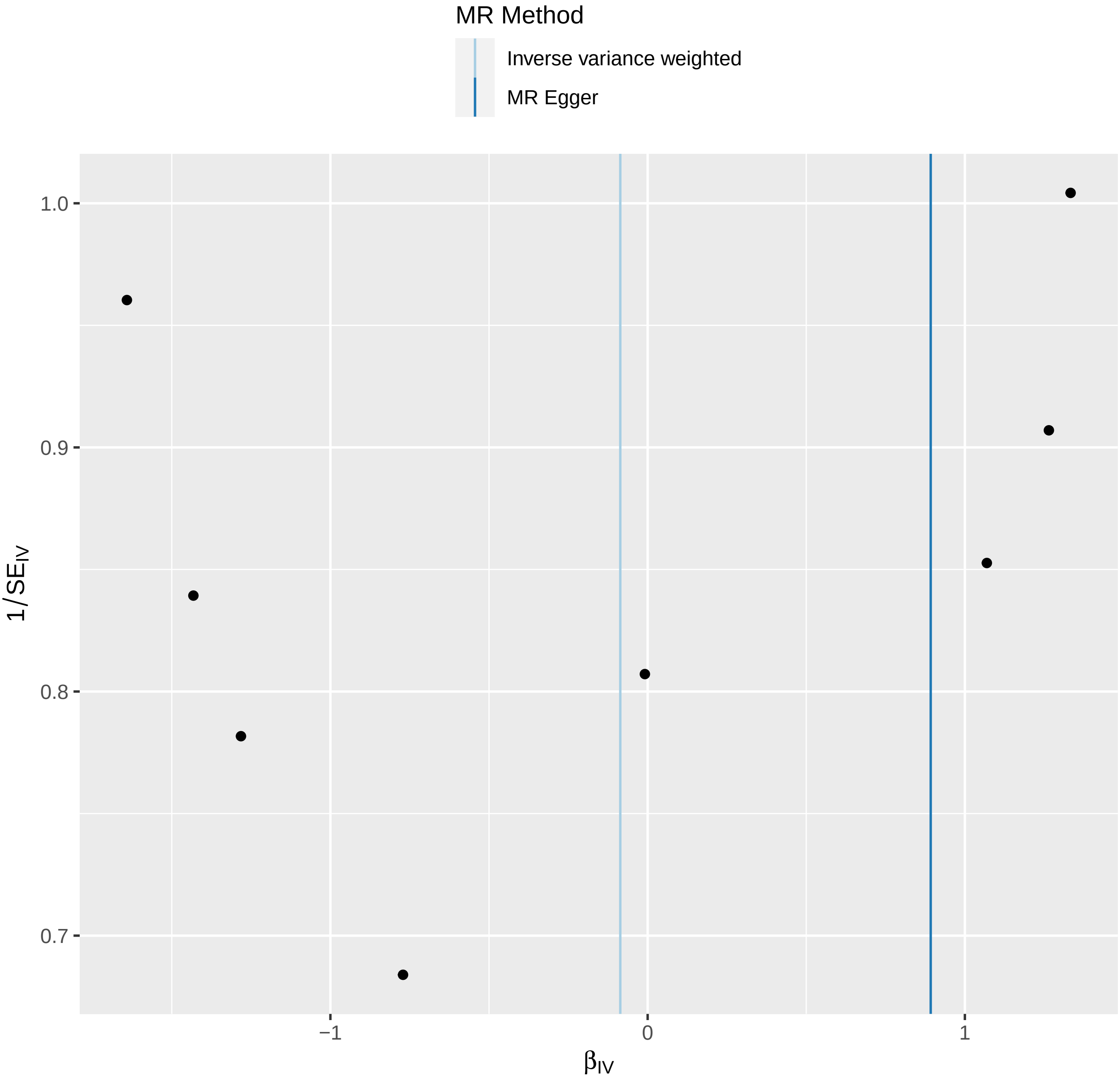

Batch 697 : Gut microbiota abundance (genus Lachnospiraceae ND3007 group id.11317) on Ulcerative colitis (strict) with PSC

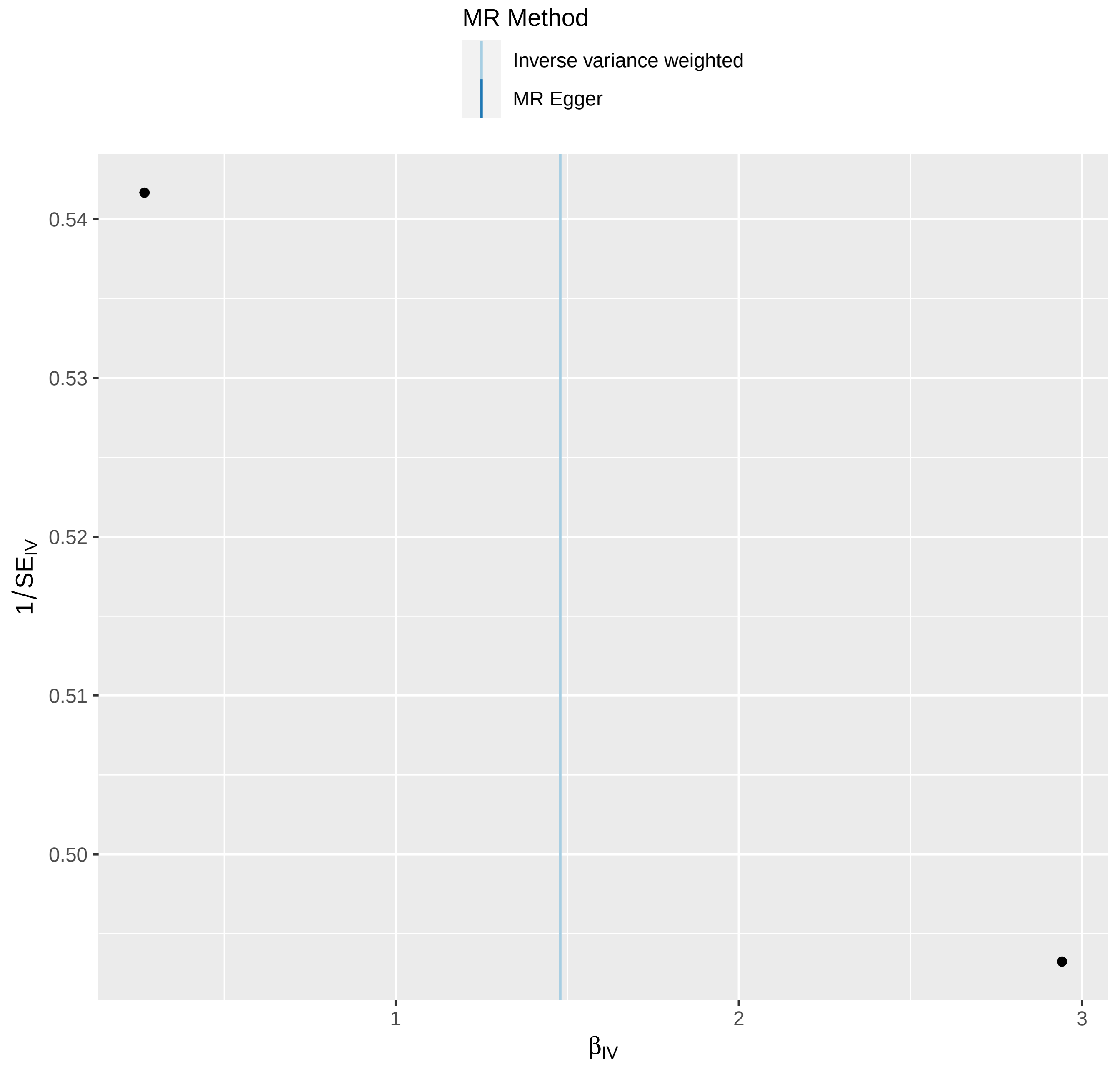

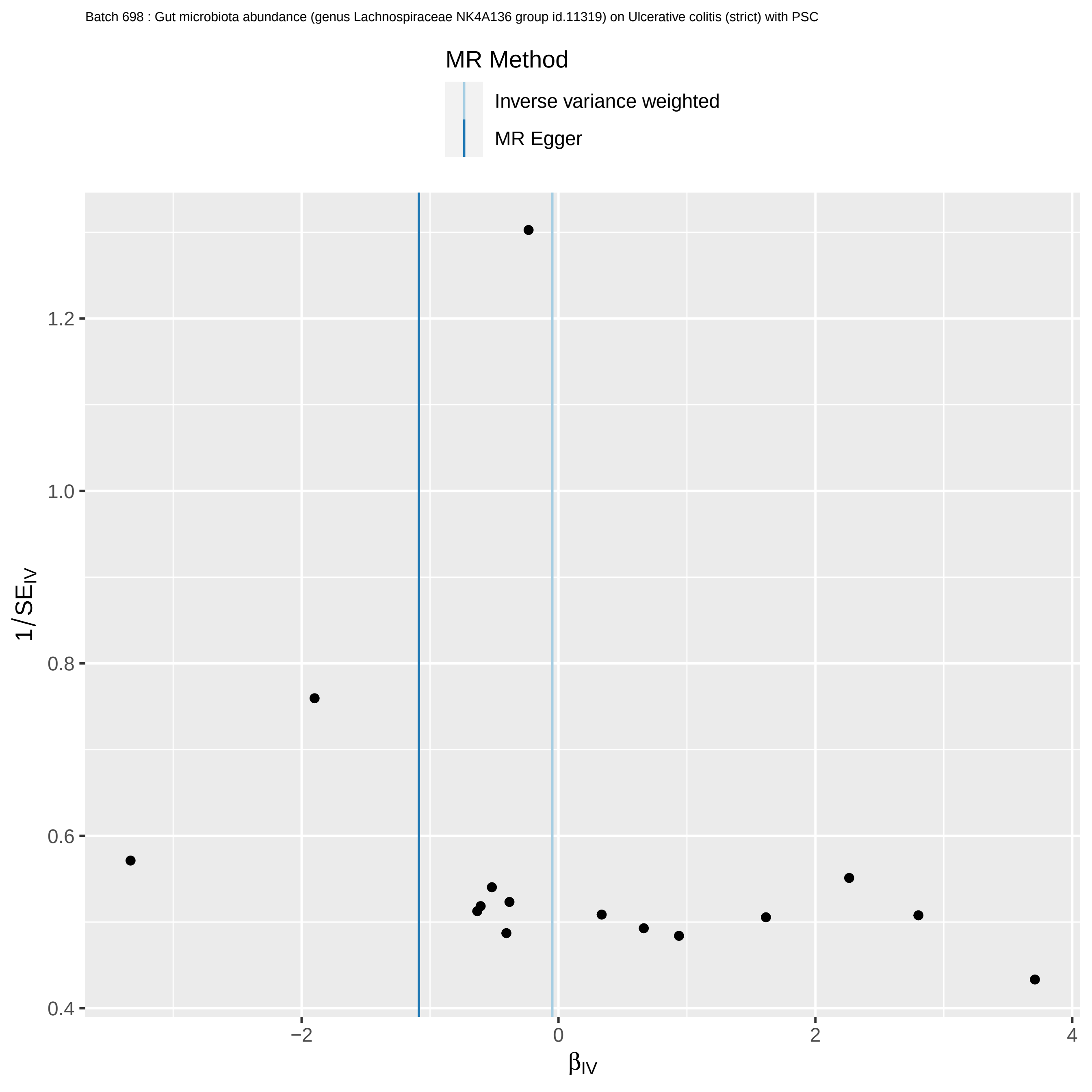

Batch 698 : Gut microbiota abundance (genus Lachnospiraceae NK4A136 group id.11319) on Ulcerative colitis (strict) with PSC
MR Method
Inverse variance weighted
MR Egger
1.2
1.0
0.8
0.6
0.4
1/SE_IV
−2
0
2
4
β_IV

Batch 699 : Gut microbiota abundance (genus Lachnospiraceae UCG001 id.11321) on Ulcerative colitis (strict) with PSC

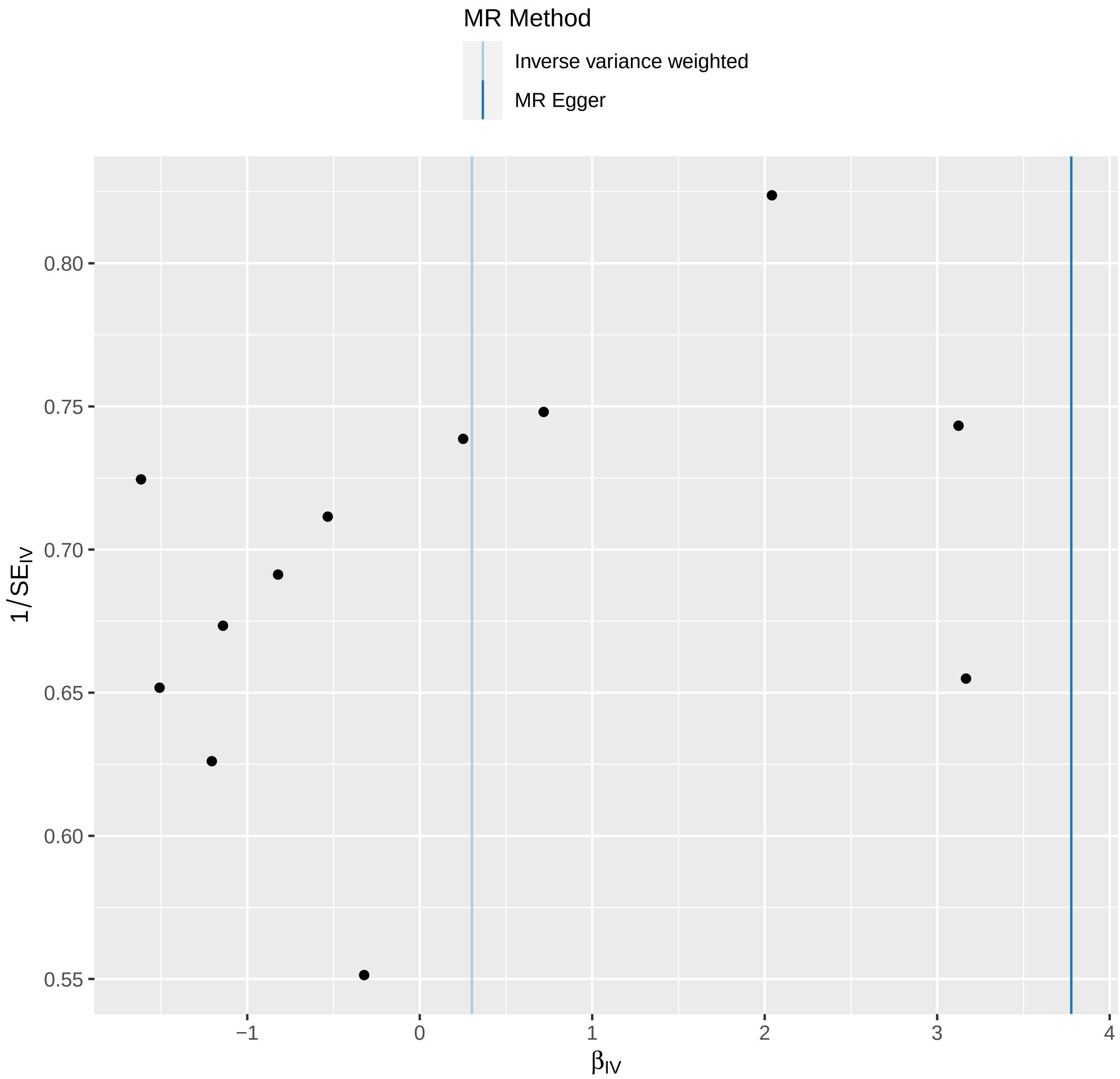

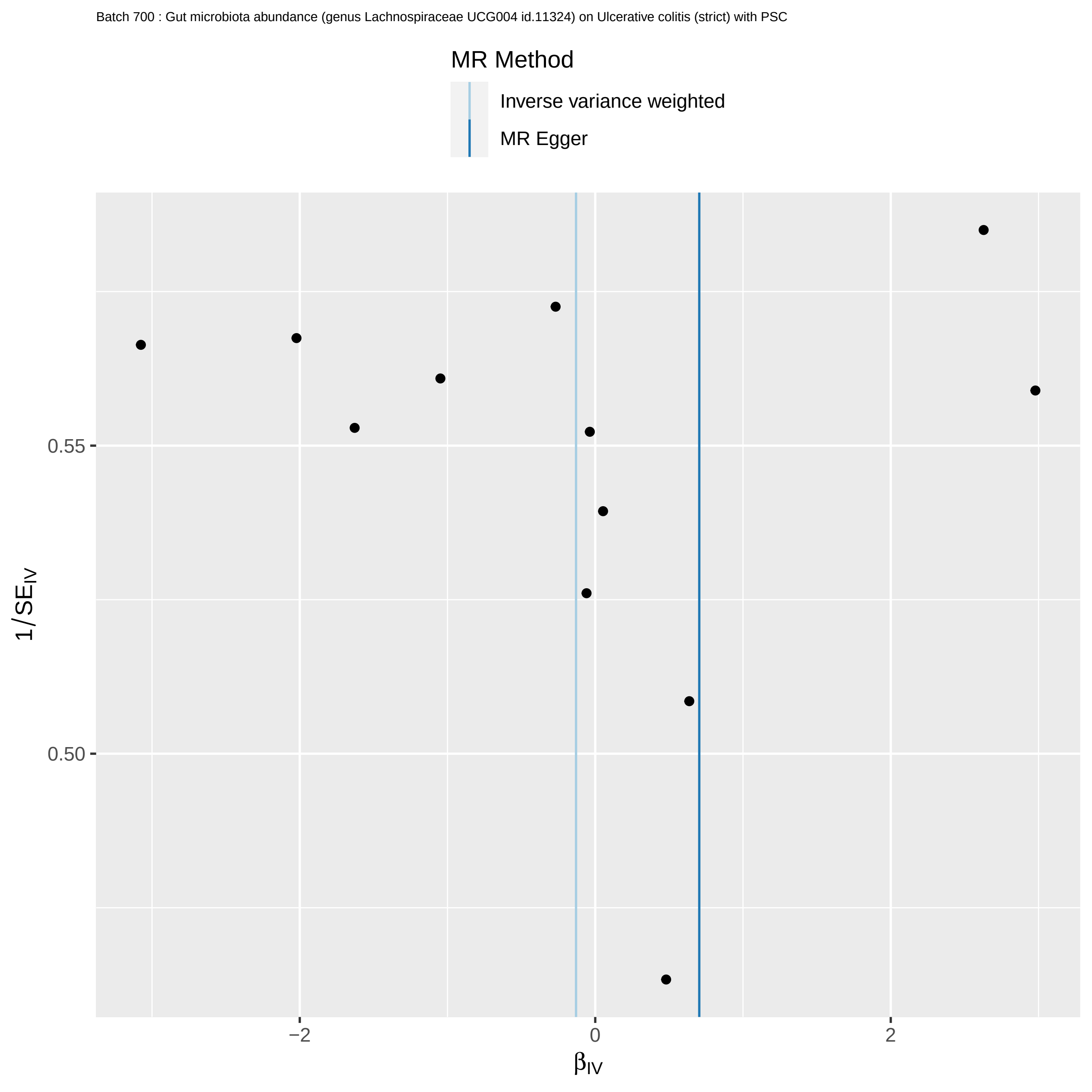
Batch 700 : Gut microbiota abundance (genus Lachnospiraceae UCG004 id.11324) on Ulcerative colitis (strict) with PSC
MR Method
Inverse variance weighted
MR Egger
0.55
0.50
1/SE$_{IV}$
−2
0
2
β_{IV}

Batch 701 : Gut microbiota abundance (genus Lachnospiraceae UCG008 id.11328) on Ulcerative colitis (strict) with PSC

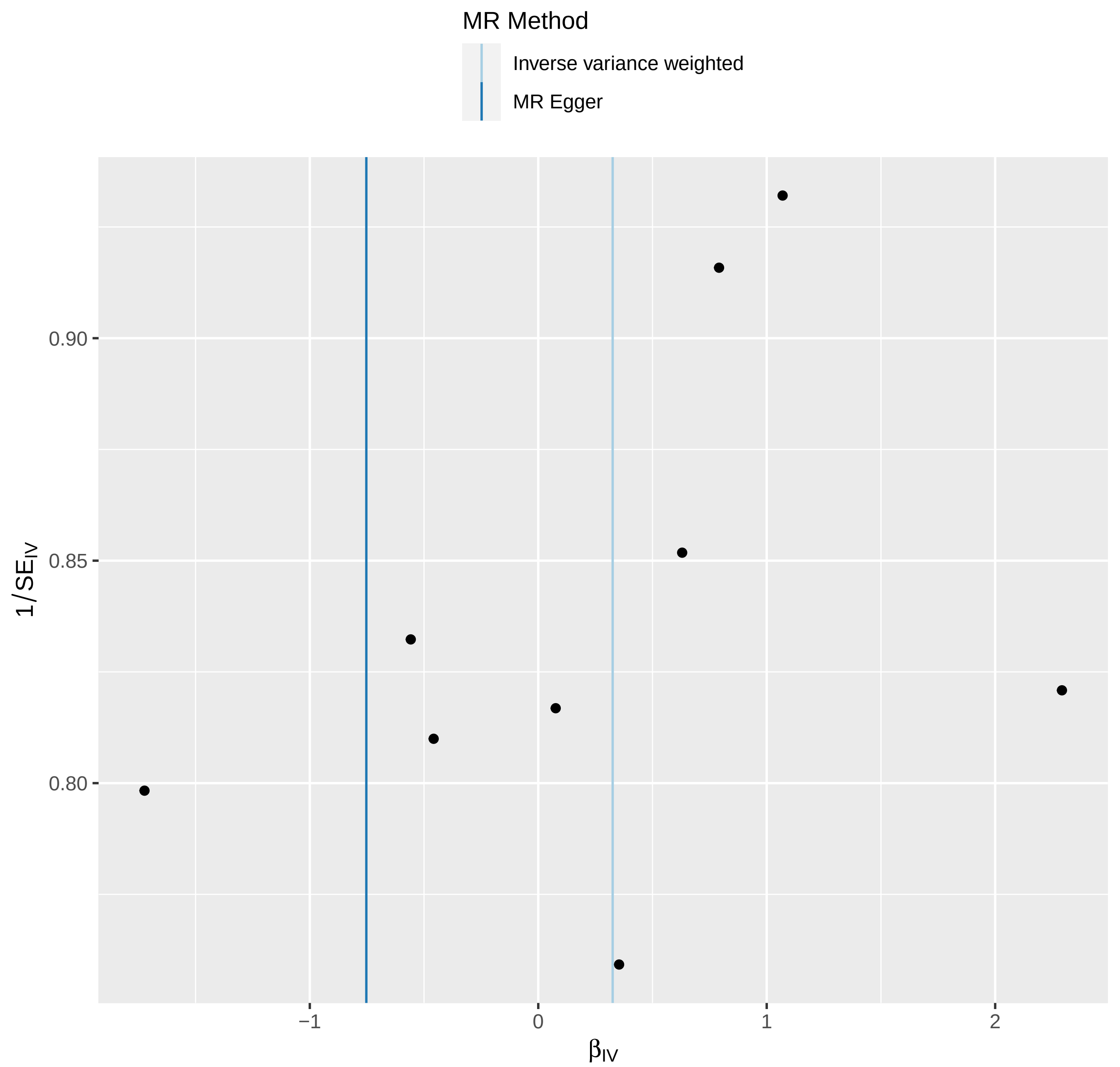

Batch 702 : Gut microbiota abundance (genus Lachnospiraceae UCG010 id.11330) on Ulcerative colitis (strict) with PSC

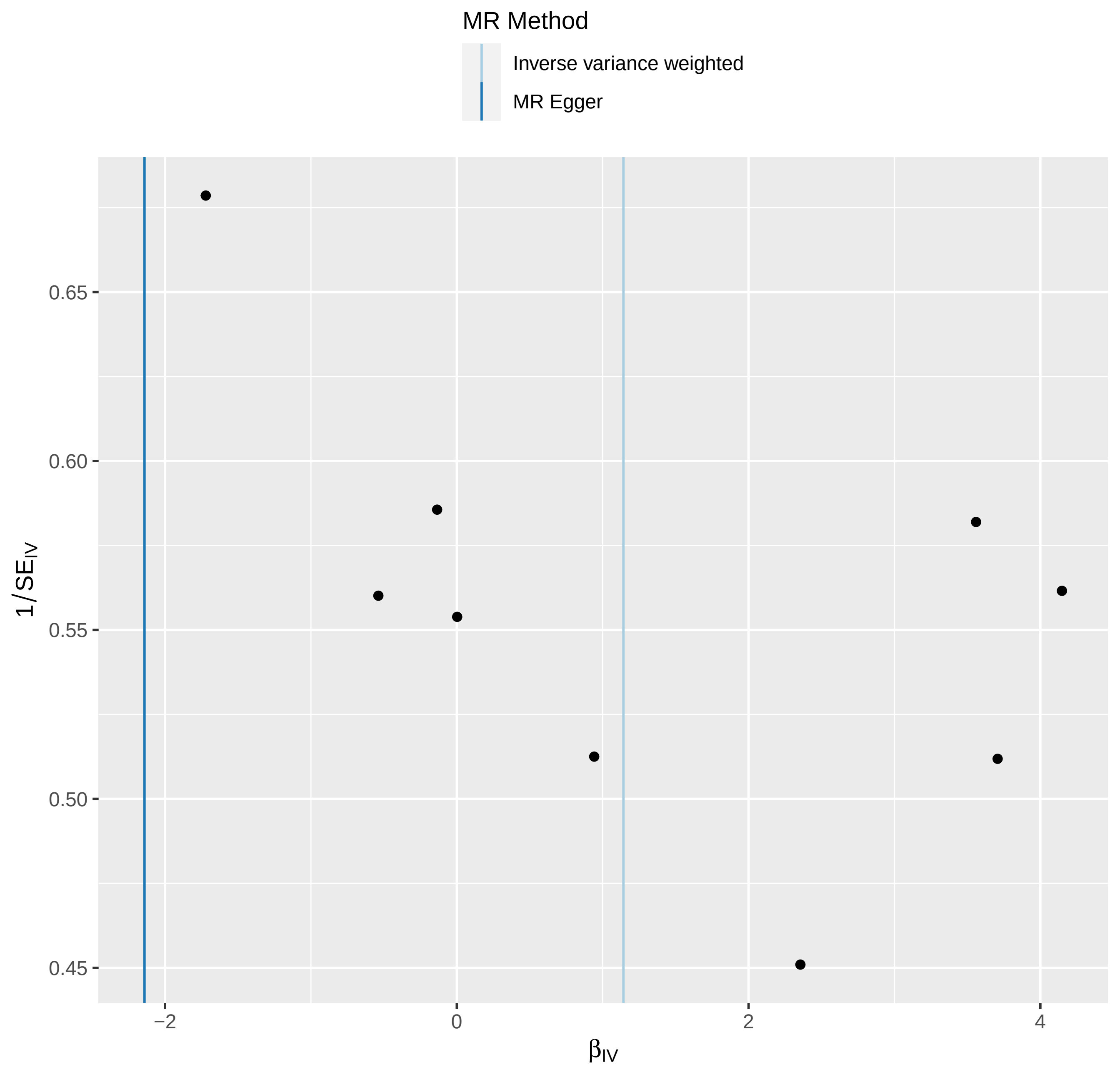

Insufficient number of SNPs

Batch 704 : Gut microbiota abundance (genus Lactobacillus id.1837) on Ulcerative colitis (strict) with PSC

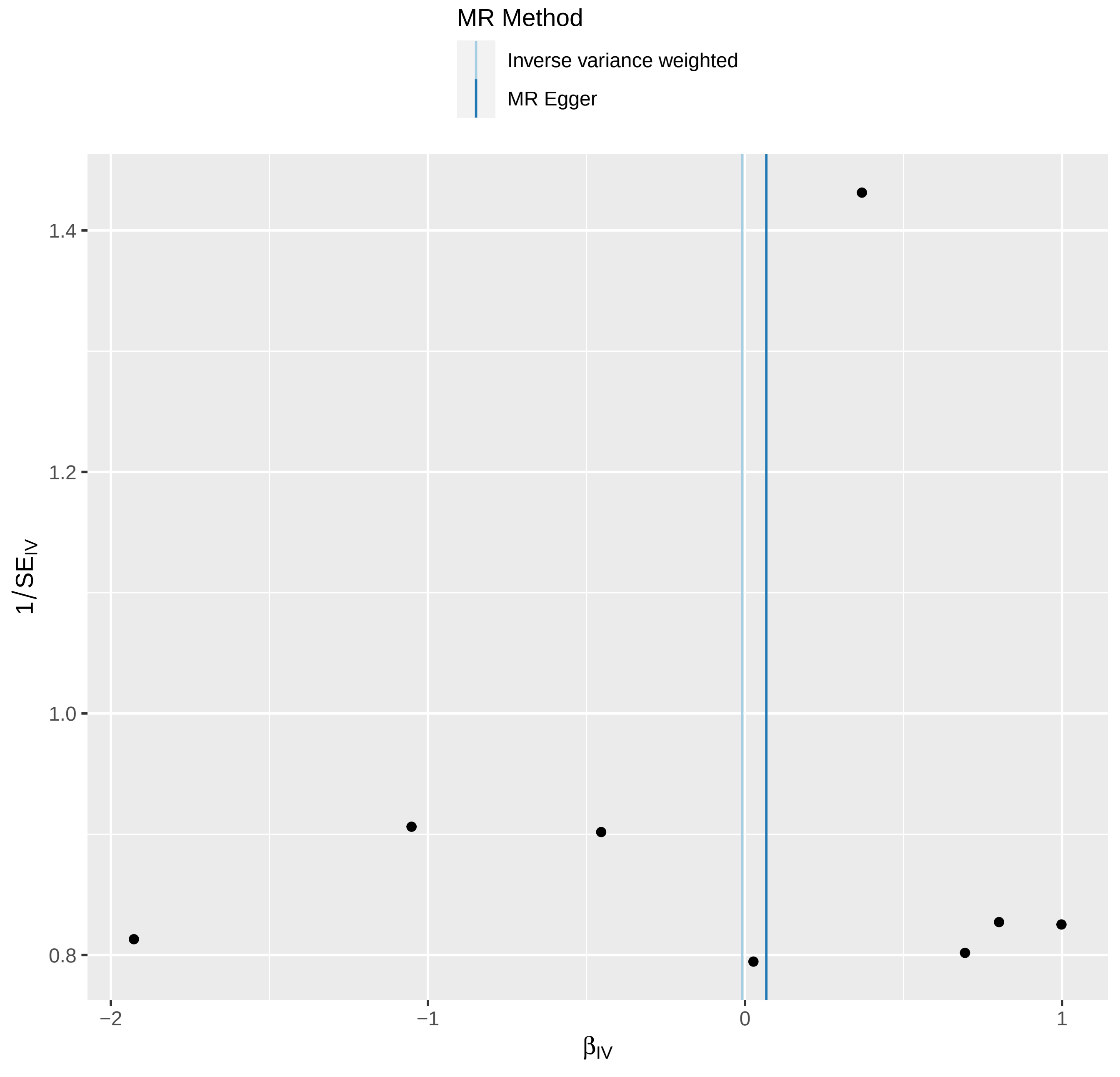

Batch 705 : Gut microbiota abundance (genus Lactococcus id.1851) on Ulcerative colitis (strict) with PSC

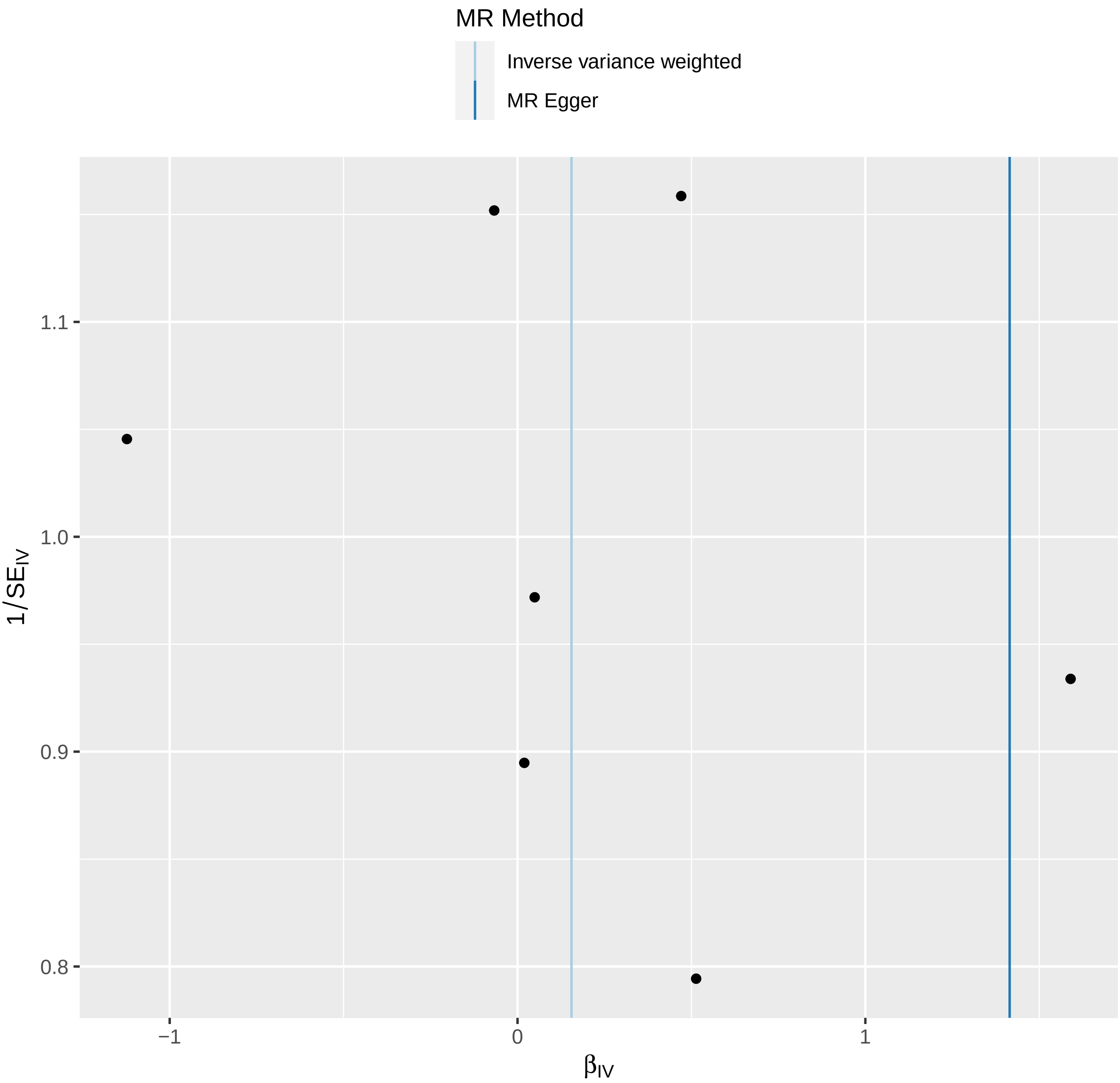

Batch 706 : Gut microbiota abundance (genus Marvinbryantia id.2005) on Ulcerative colitis (strict) with PSC

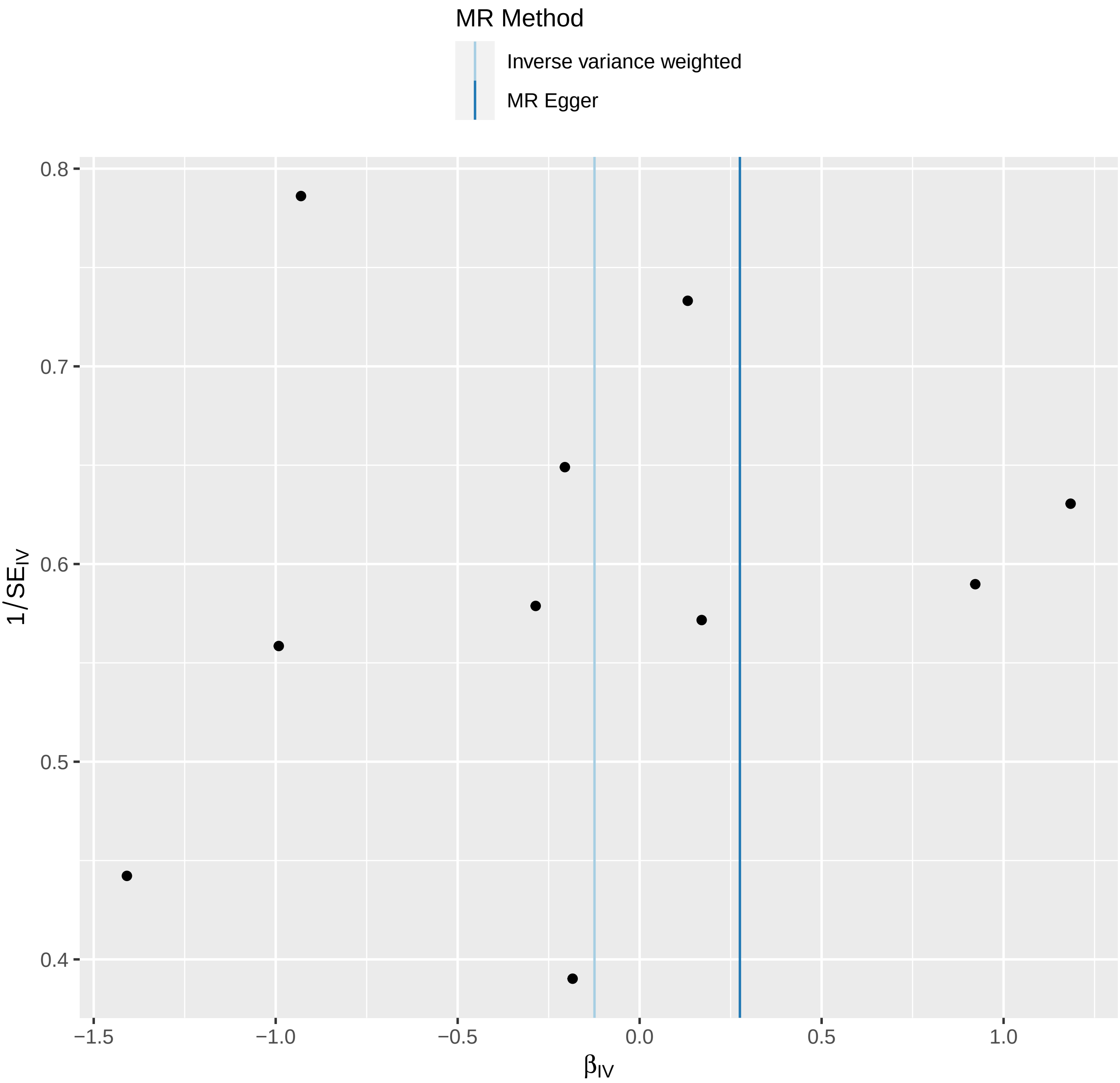

Batch 707 : Gut microbiota abundance (genus Methanobrevibacter id.123) on Ulcerative colitis (strict) with PSC

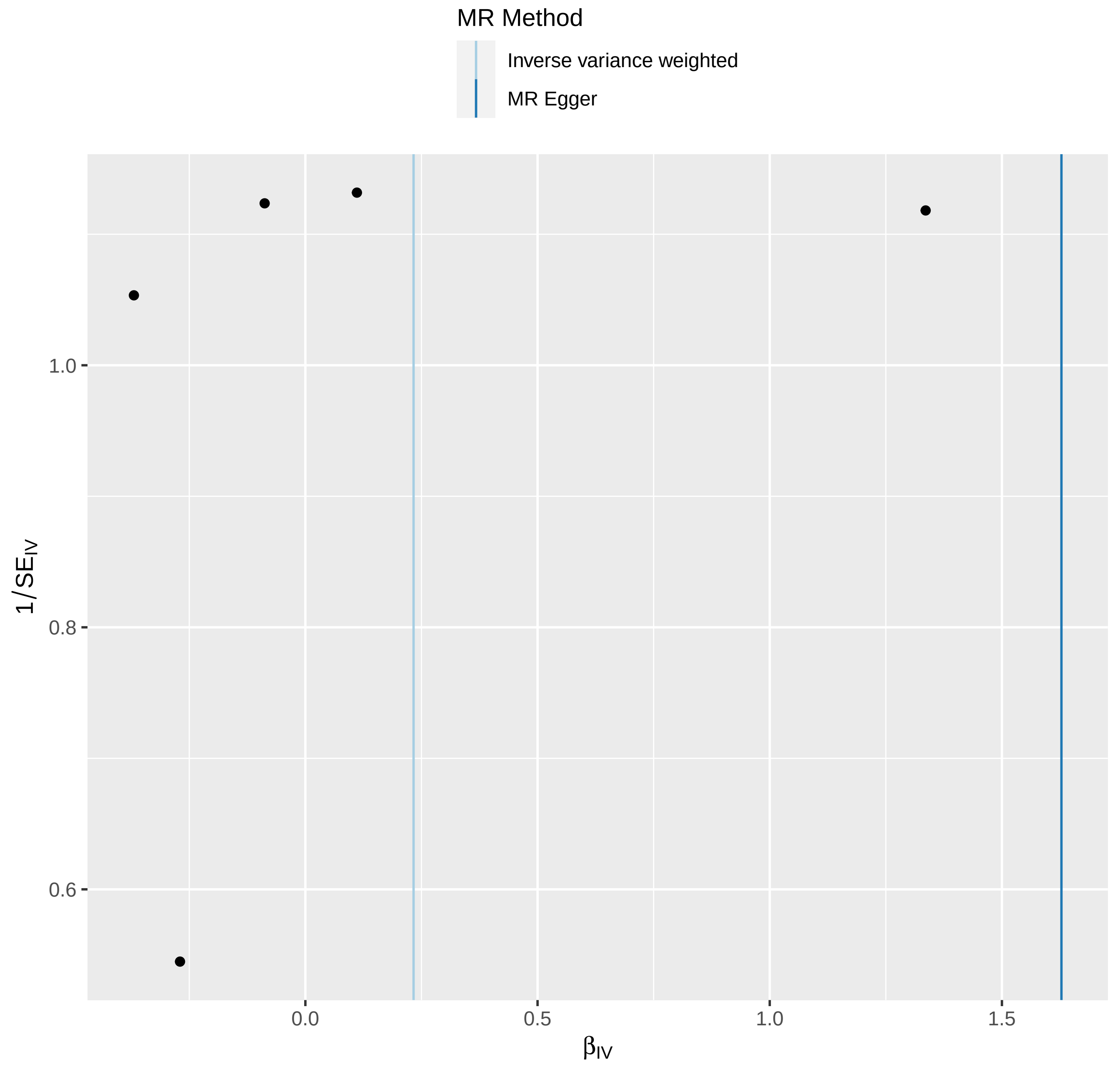

Batch 708 : Gut microbiota abundance (genus Odoribacter id.952) on Ulcerative colitis (strict) with PSC

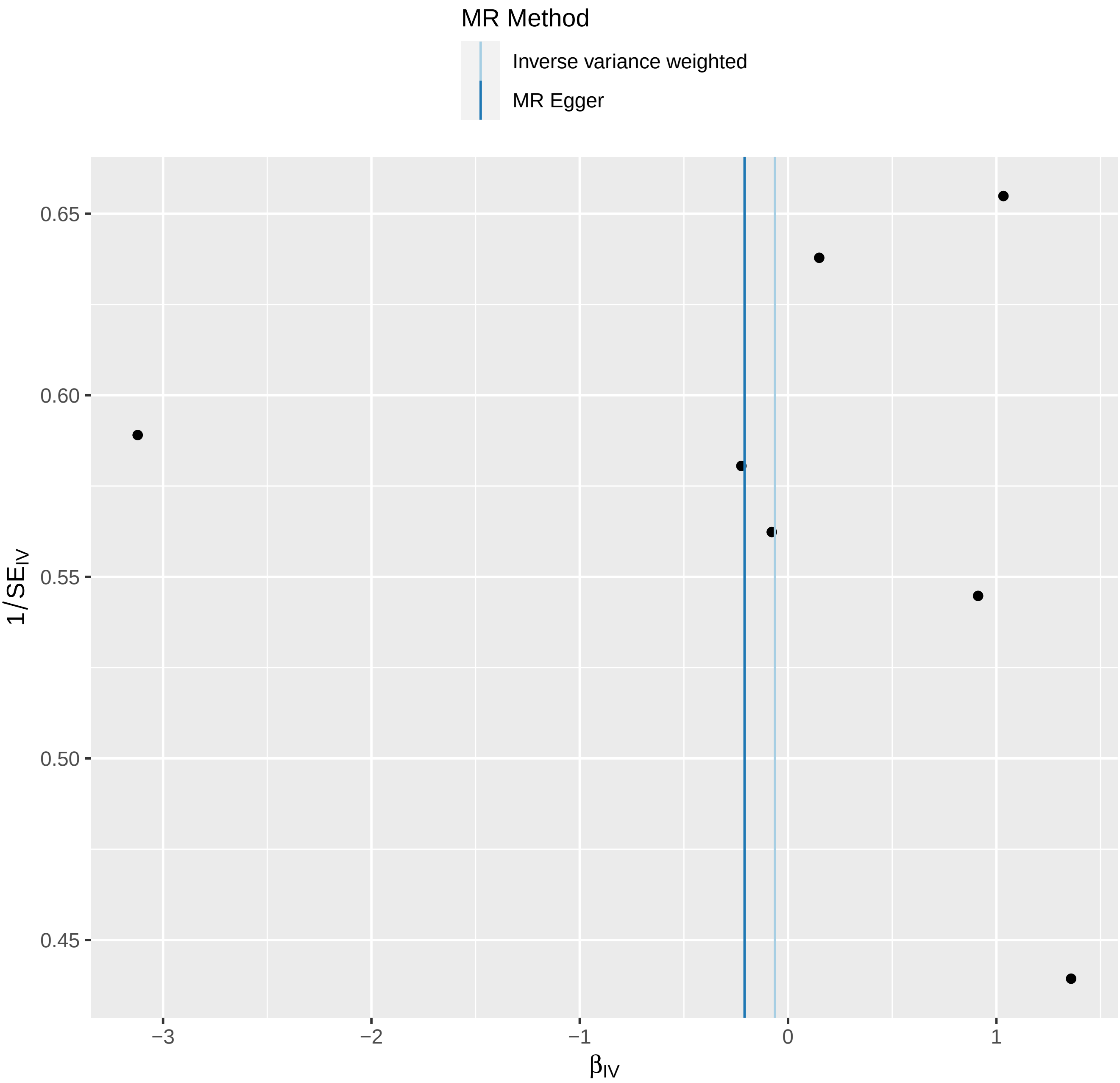

Batch 709 : Gut microbiota abundance (genus Olsenella id.822) on Ulcerative colitis (strict) with PSC

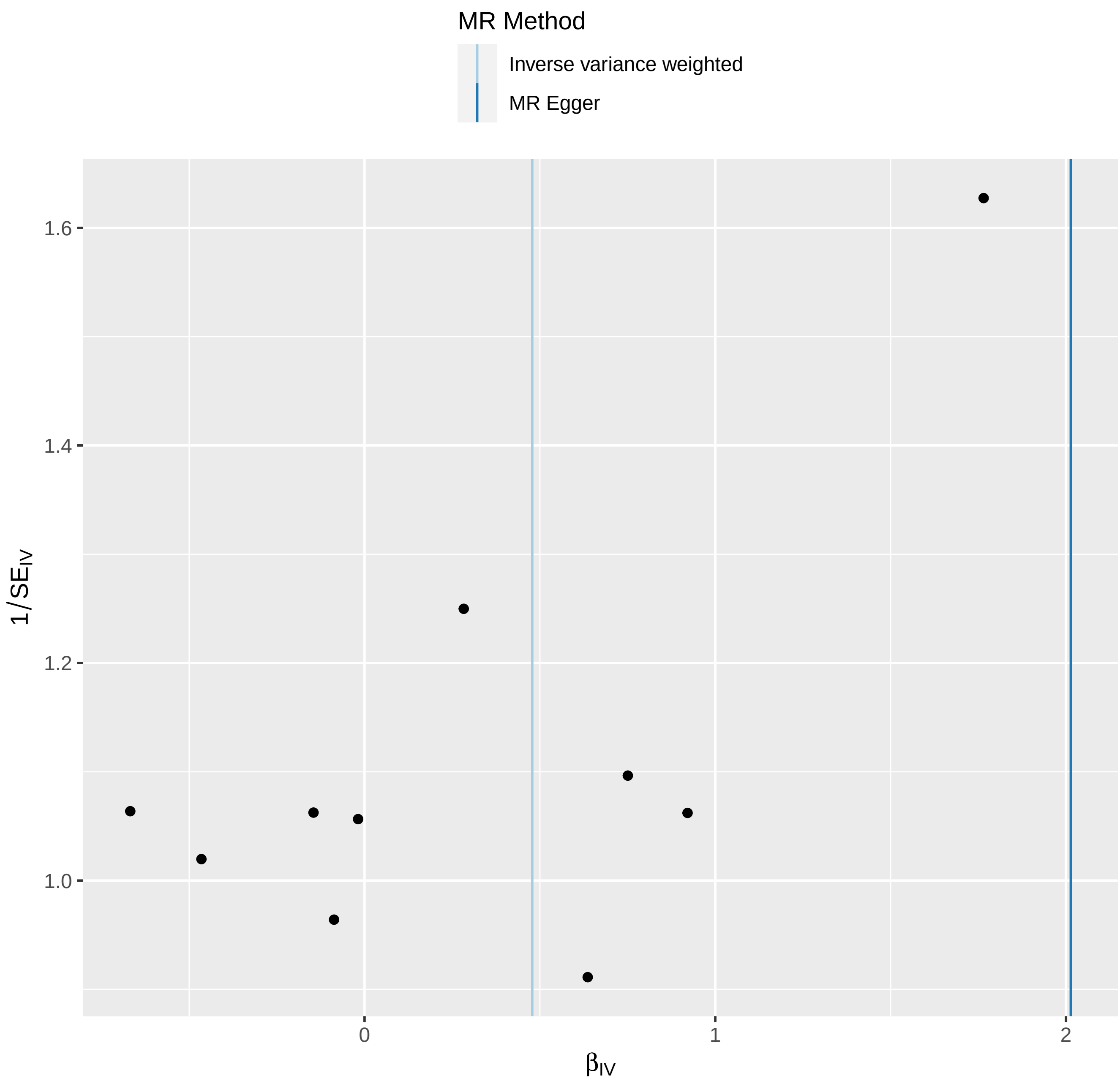

Batch 710 : Gut microbiota abundance (genus Oscillibacter id.2063) on Ulcerative colitis (strict) with PSC

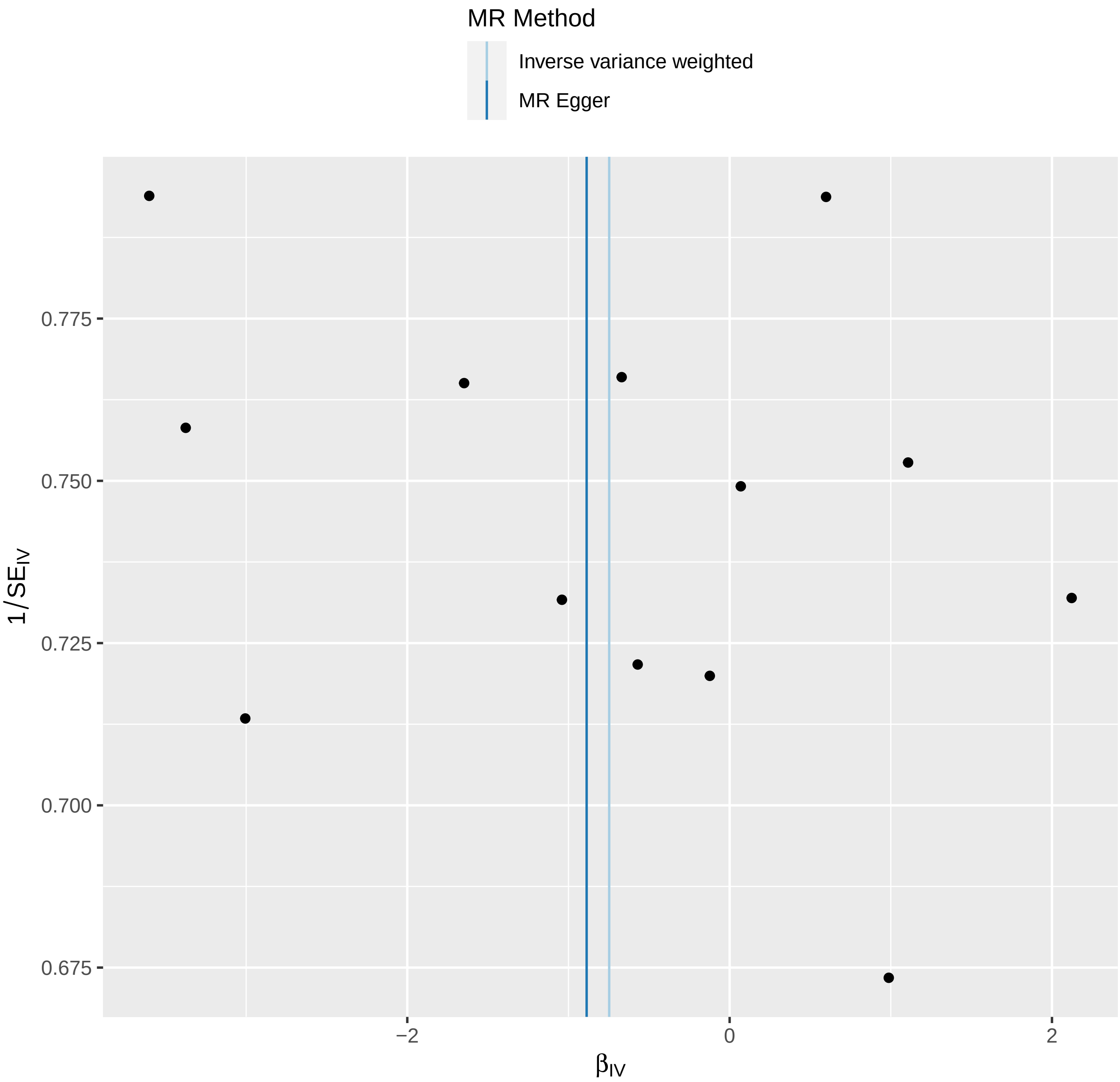

Batch 711 : Gut microbiota abundance (genus Oscillospira id.2064) on Ulcerative colitis (strict) with PSC

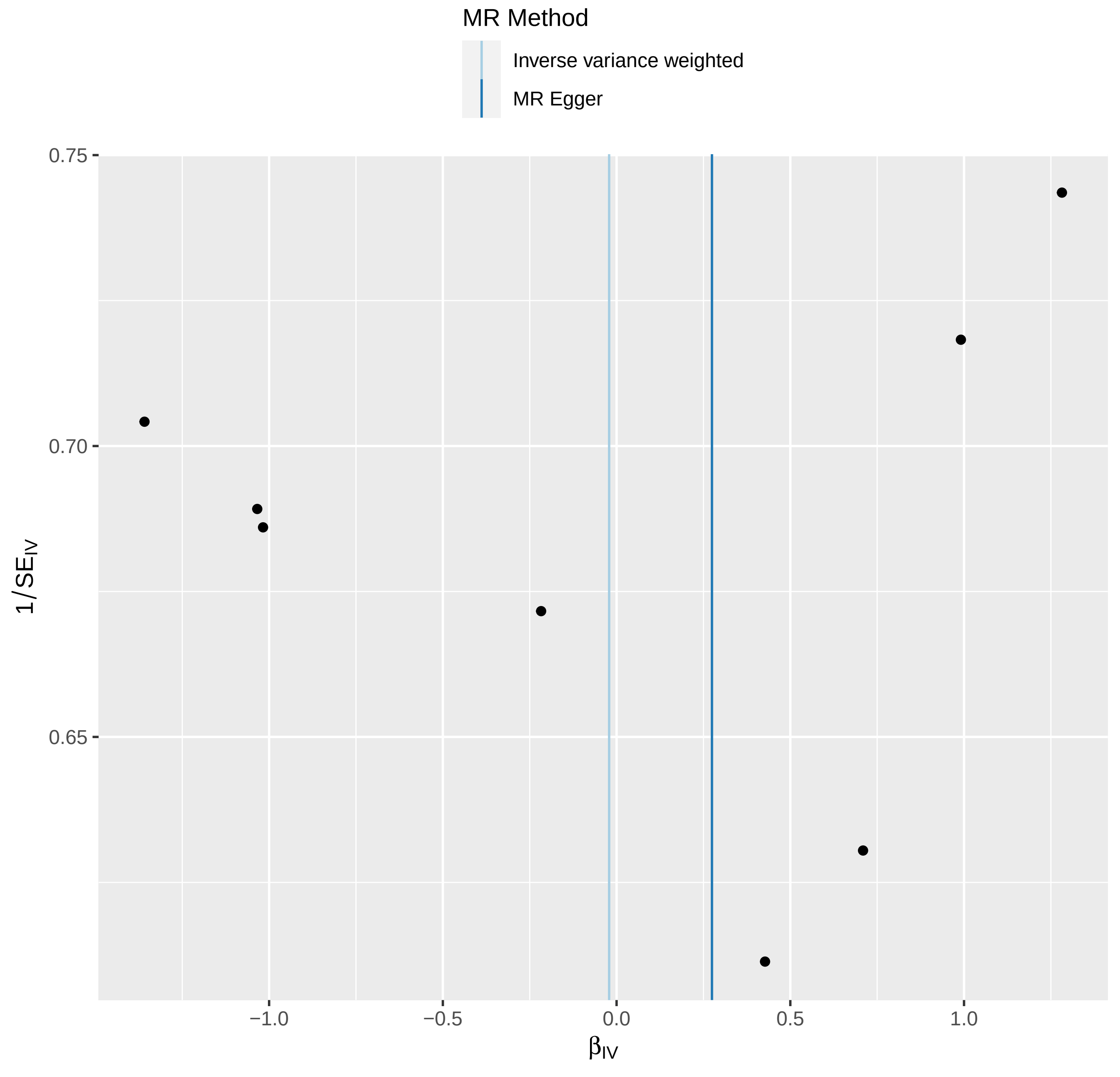

Batch 712 : Gut microbiota abundance (genus Oxalobacter id.2978) on Ulcerative colitis (strict) with PSC

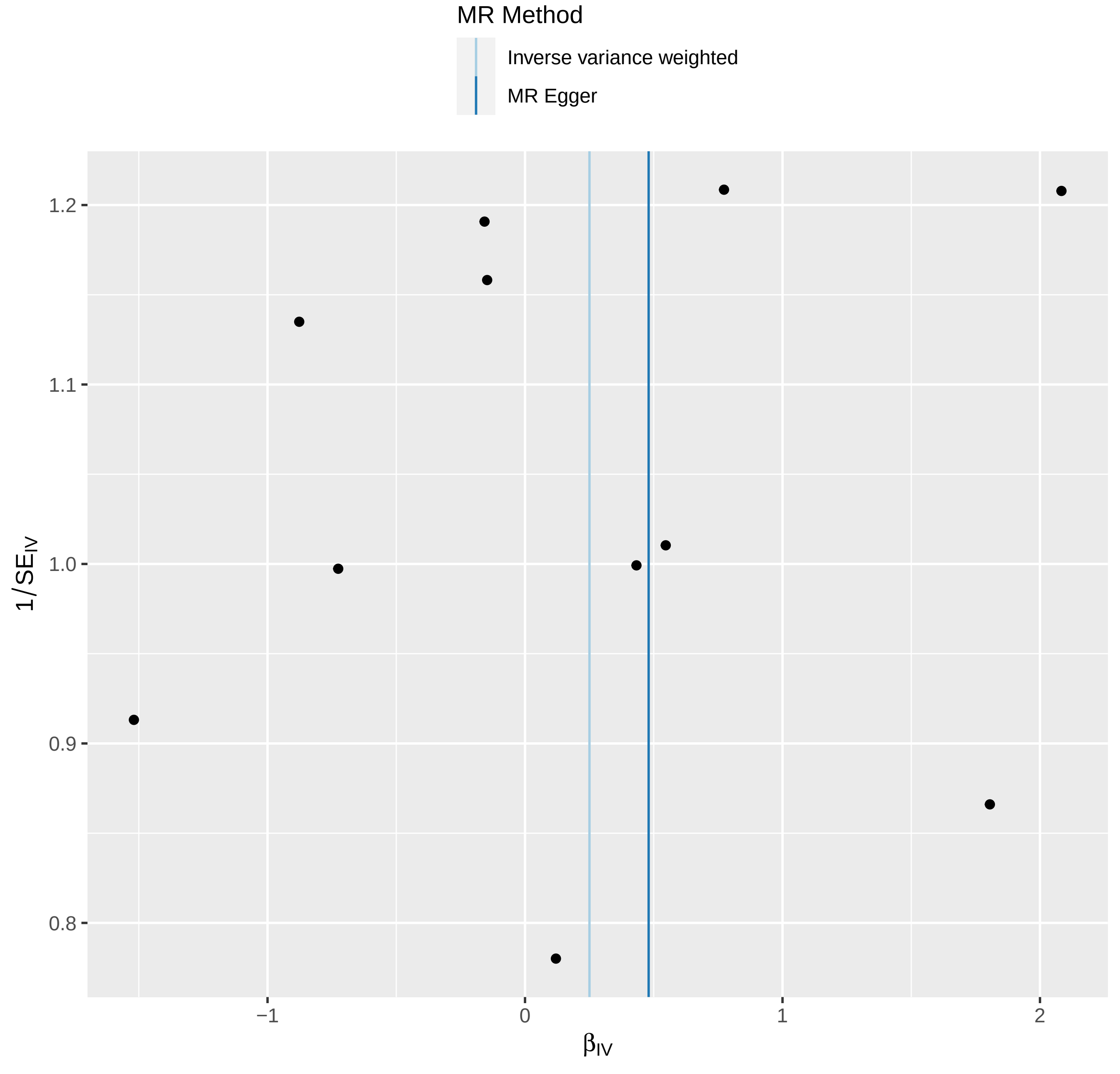

Batch 713 : Gut microbiota abundance (genus Parabacteroides id.954) on Ulcerative colitis (strict) with PSC

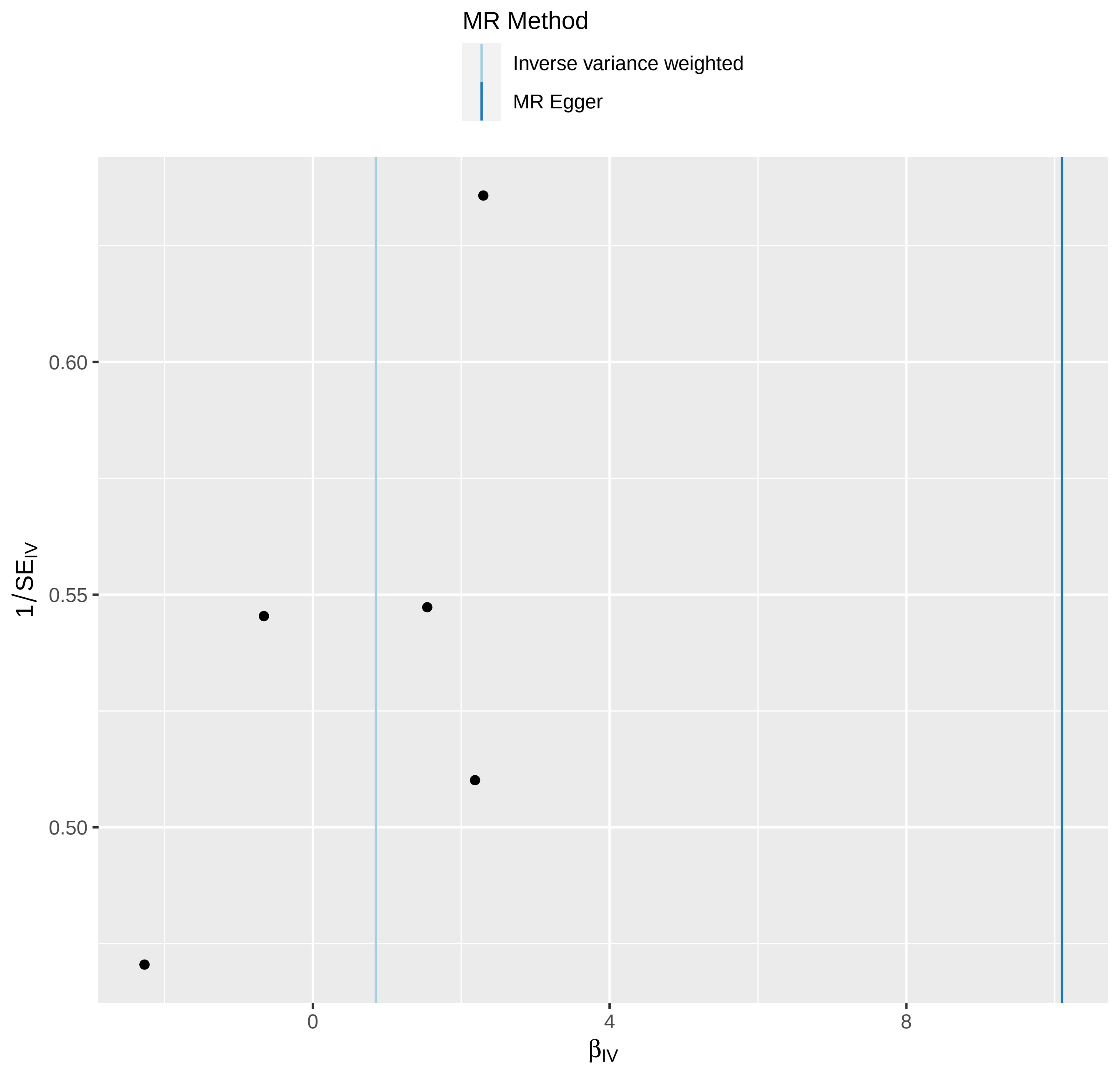

Batch 714 : Gut microbiota abundance (genus Paraprevotella id.962) on Ulcerative colitis (strict) with PSC

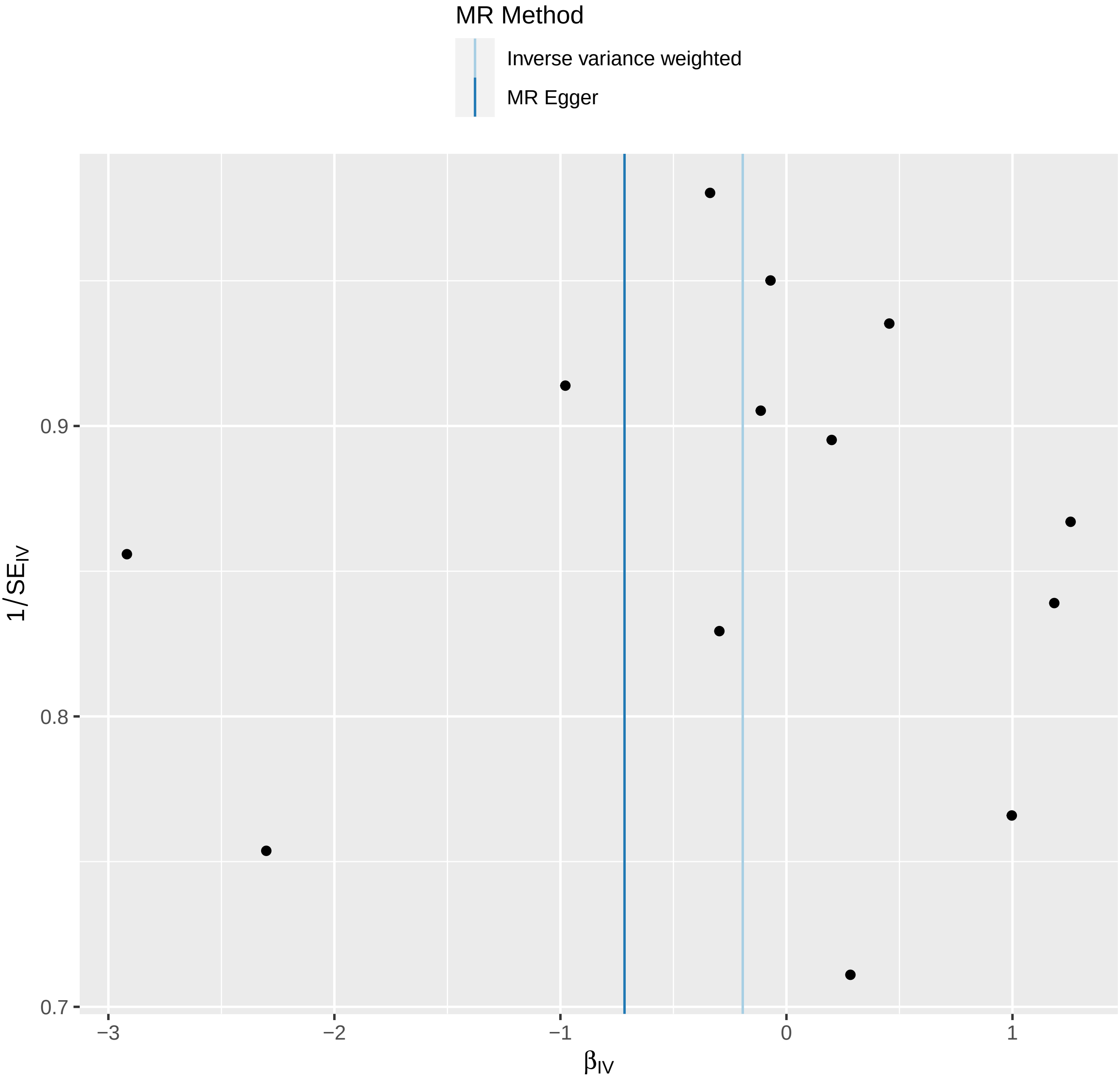

Batch 715 : Gut microbiota abundance (genus Parasutterella id.2892) on Ulcerative colitis (strict) with PSC

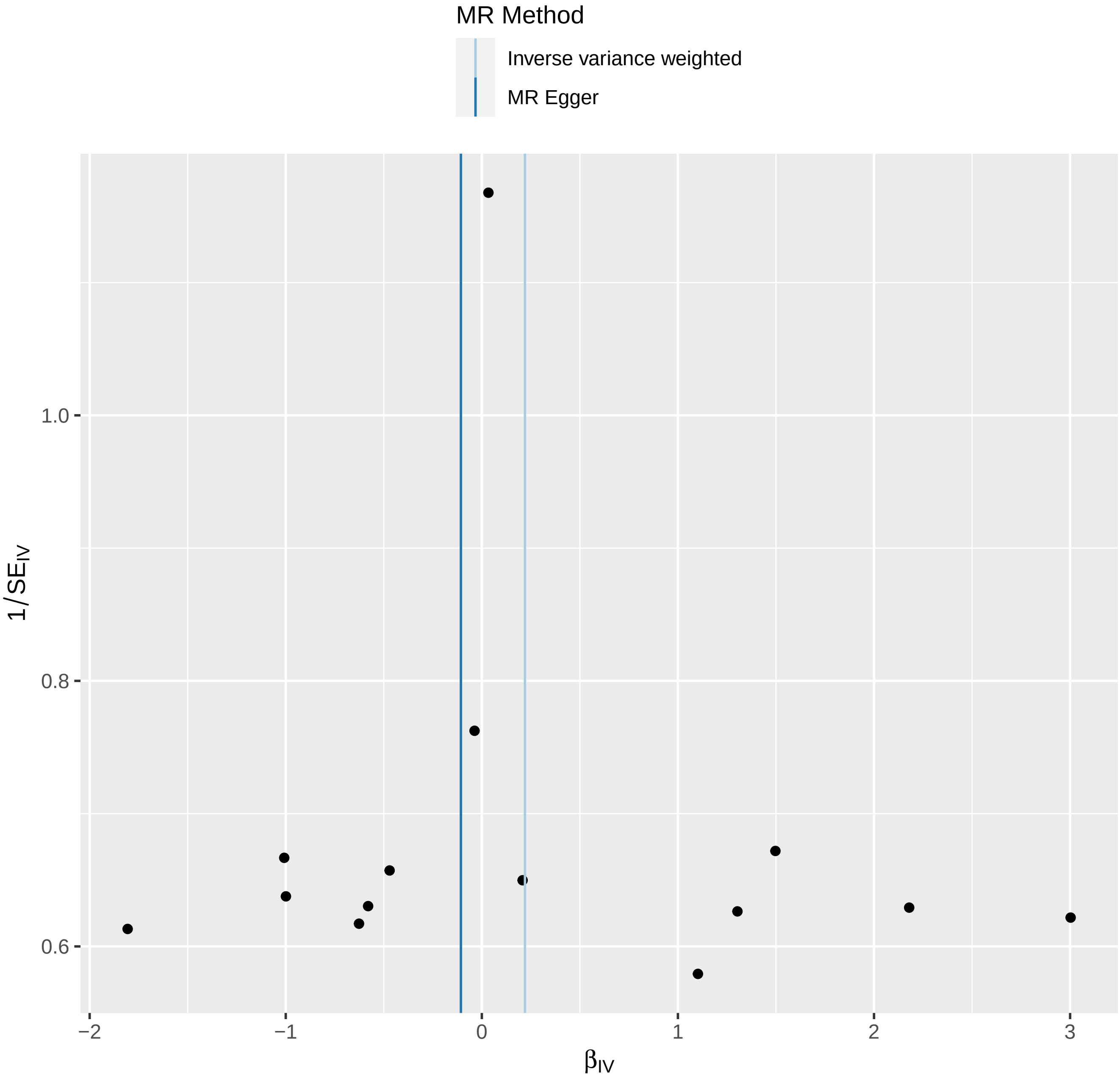

Batch 716 : Gut microbiota abundance (genus Peptococcus id.2037) on Ulcerative colitis (strict) with PSC

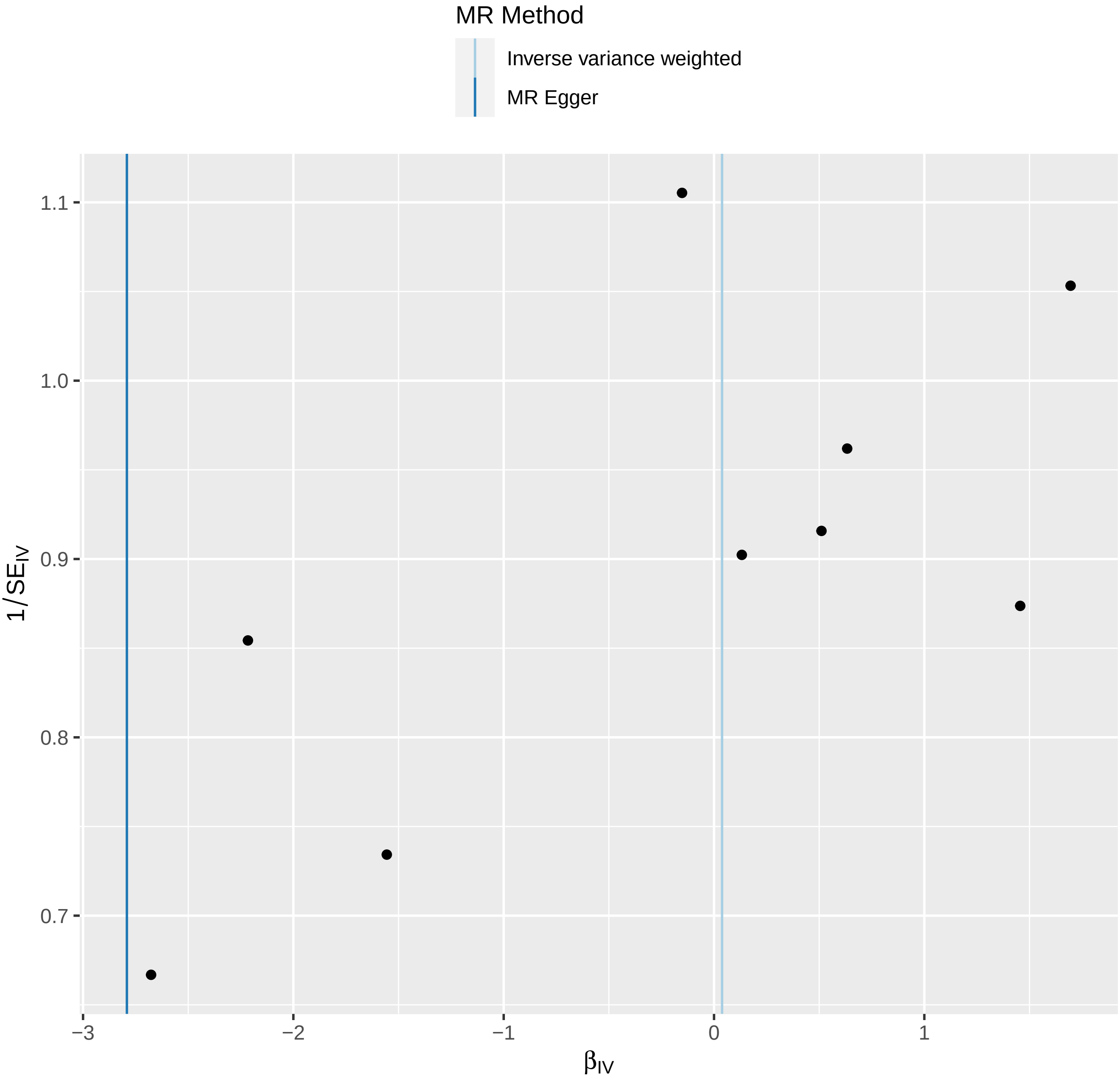

Batch 717 : Gut microbiota abundance (genus Phascolarctobacterium id.2168) on Ulcerative colitis (strict) with PSC

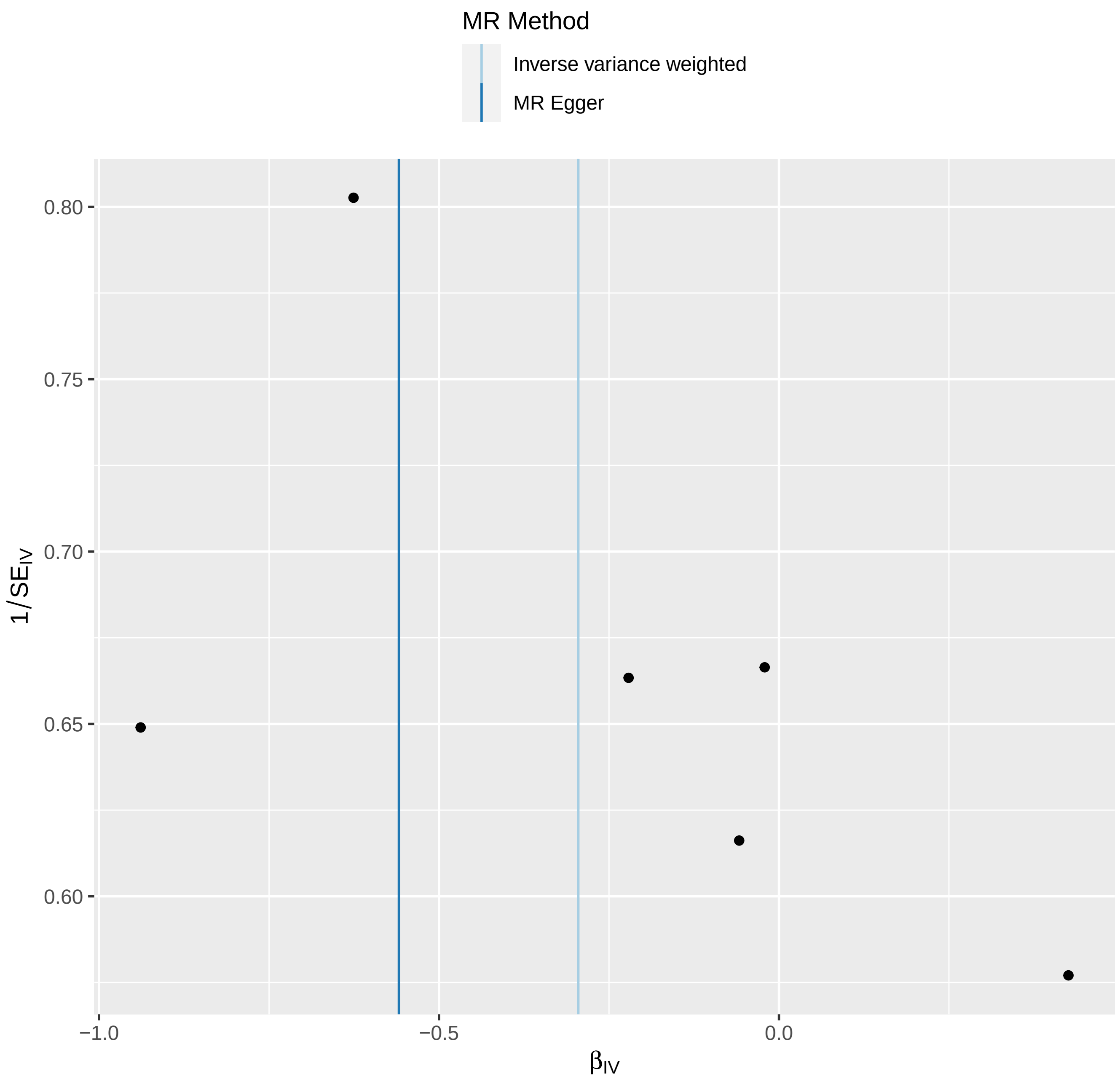

Batch 718 : Gut microbiota abundance (genus Prevotella7 id.11182) on Ulcerative colitis (strict) with PSC

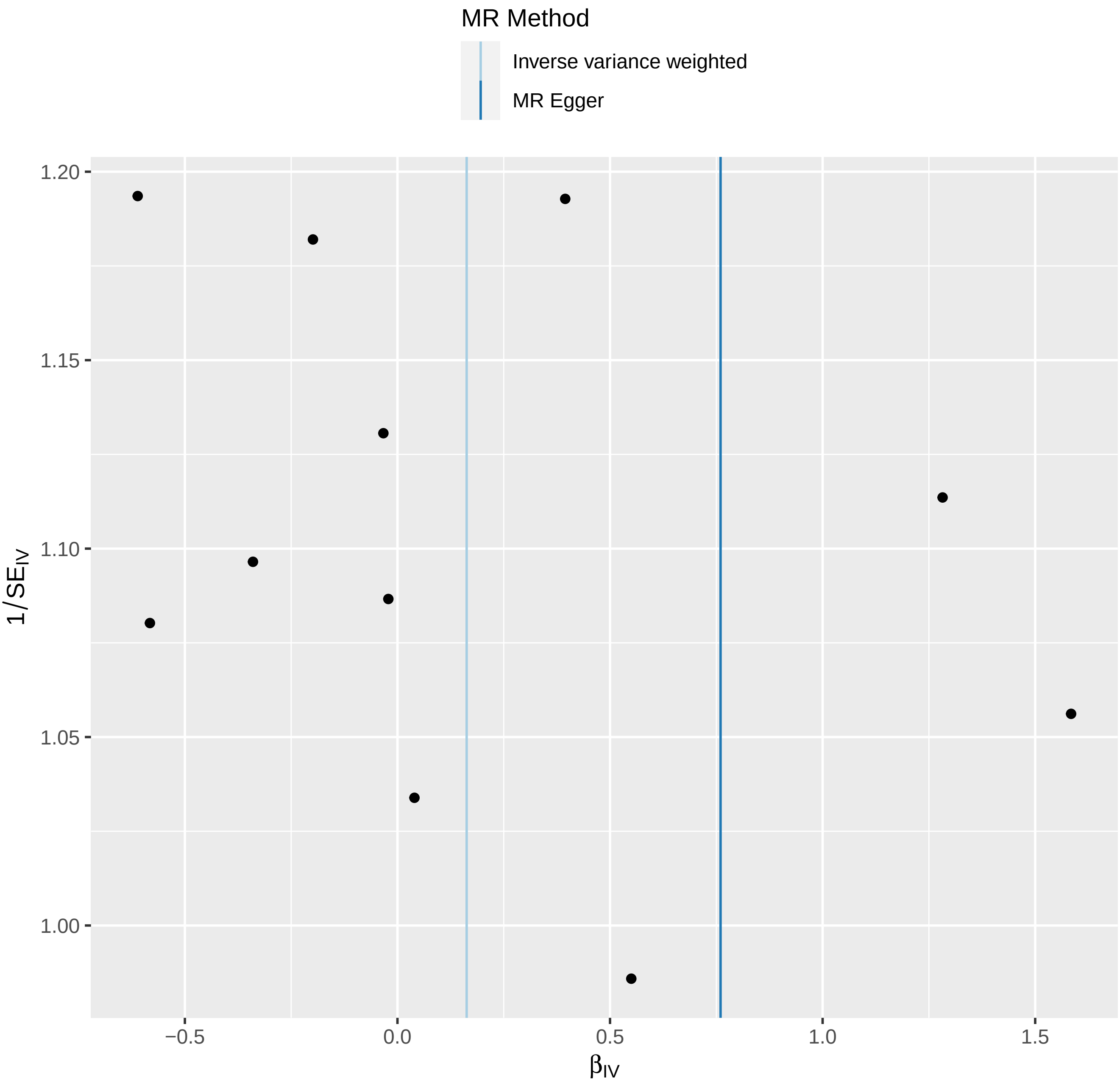

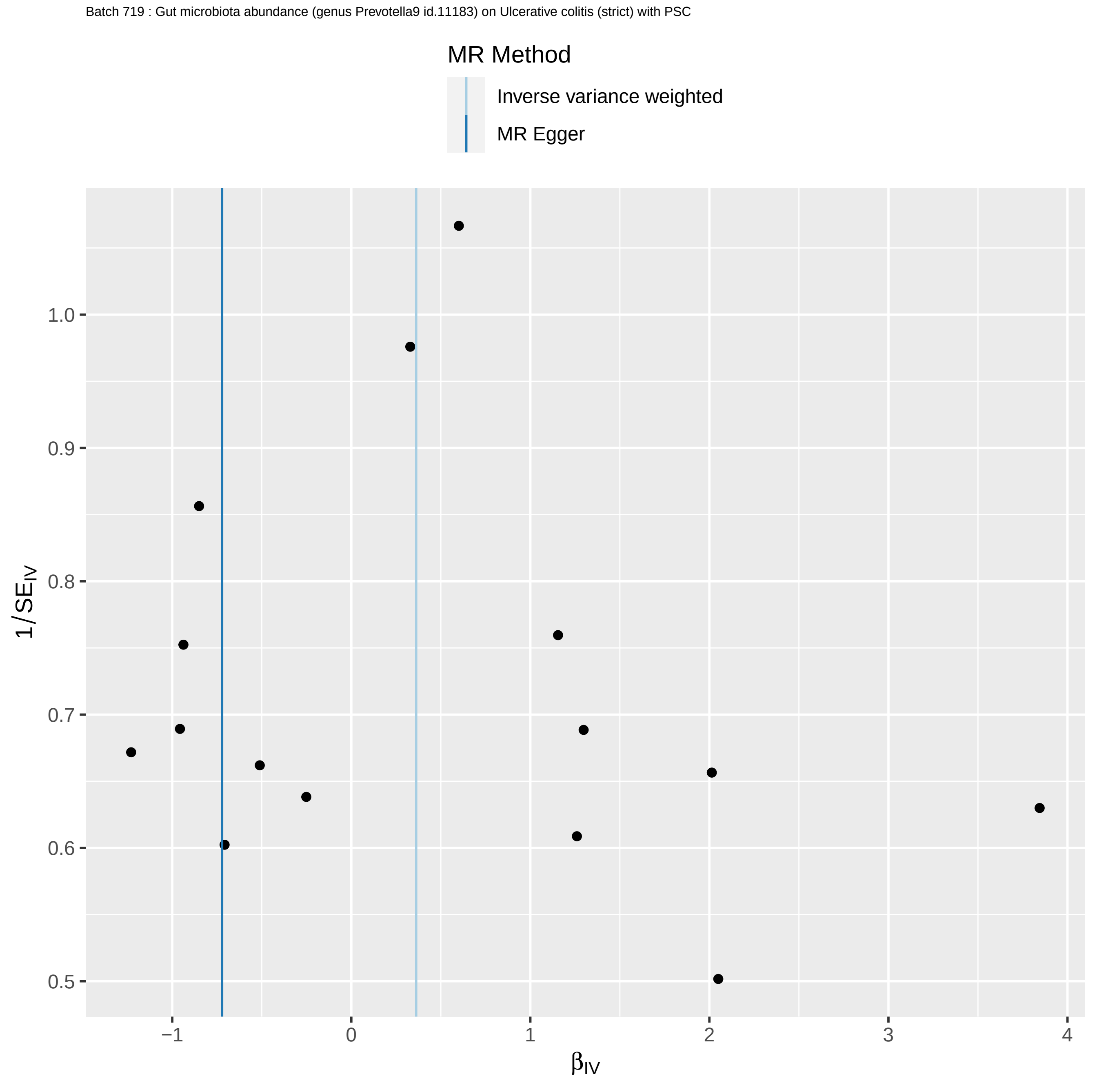
Batch 719 : Gut microbiota abundance (genus Prevotella9 id.11183) on Ulcerative colitis (strict) with PSC
MR Method
Inverse variance weighted
MR Egger
1/SE_IV
β_IV
1.0
0.9
0.8
0.7
0.6
0.5
−1
0
1
2
3
4

Batch 720 : Gut microbiota abundance (genus Rikenellaceae RC9 gut group id.11191) on Ulcerative colitis (strict) with PSC

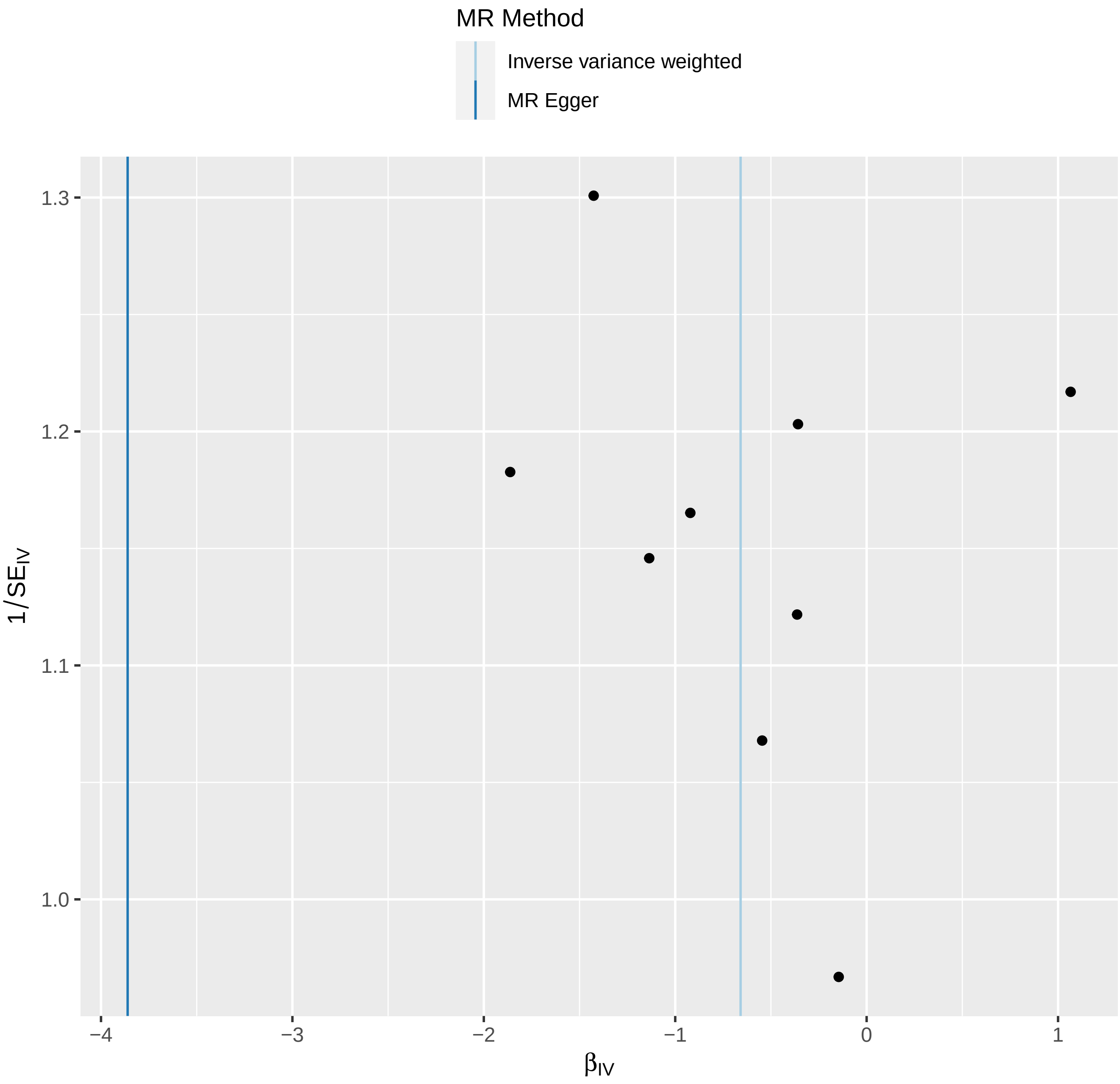

Batch 721 : Gut microbiota abundance (genus Romboutsia id.11347) on Ulcerative colitis (strict) with PSC

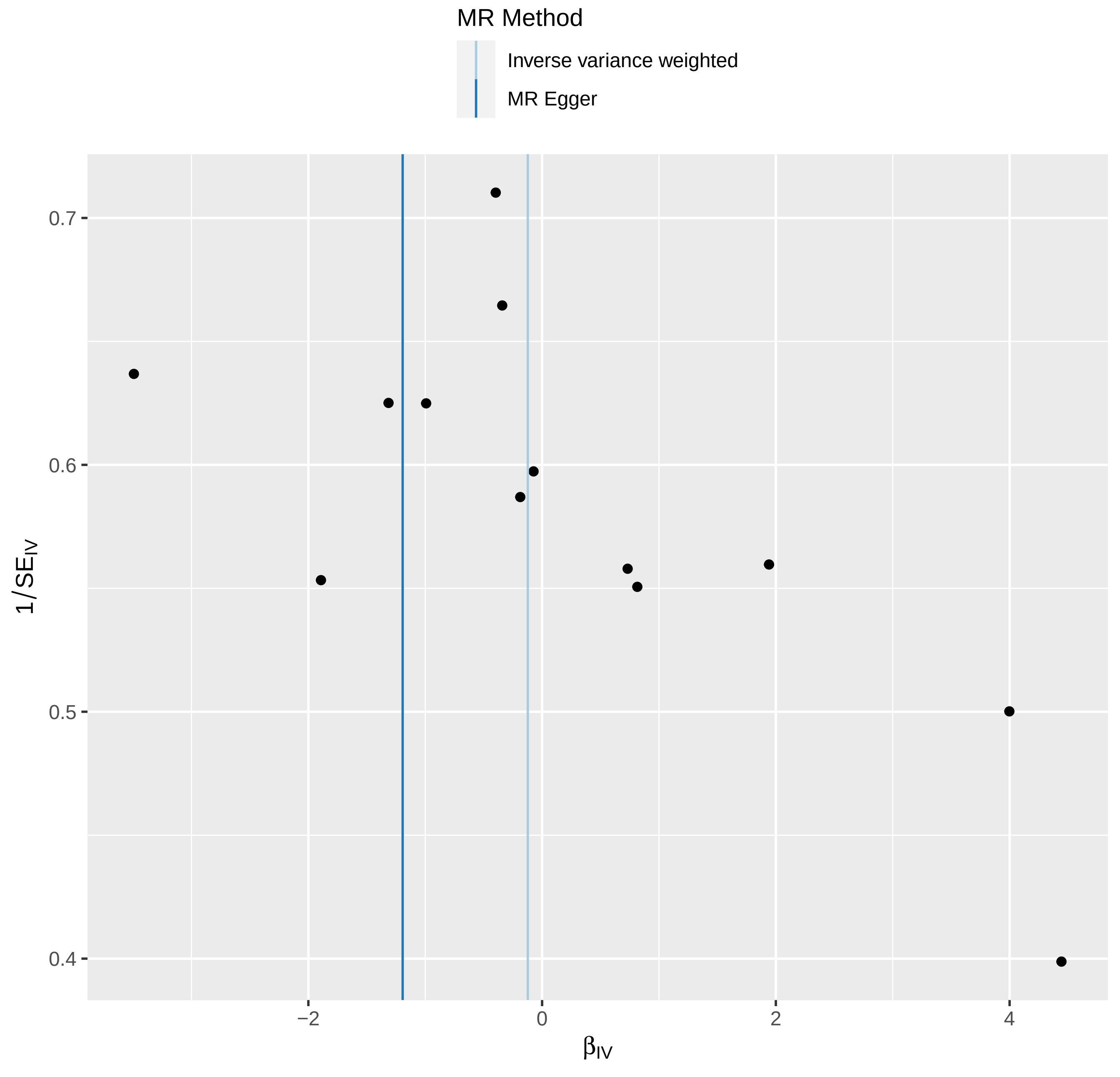

Batch 722 : Gut microbiota abundance (genus Roseburia id.2012) on Ulcerative colitis (strict) with PSC

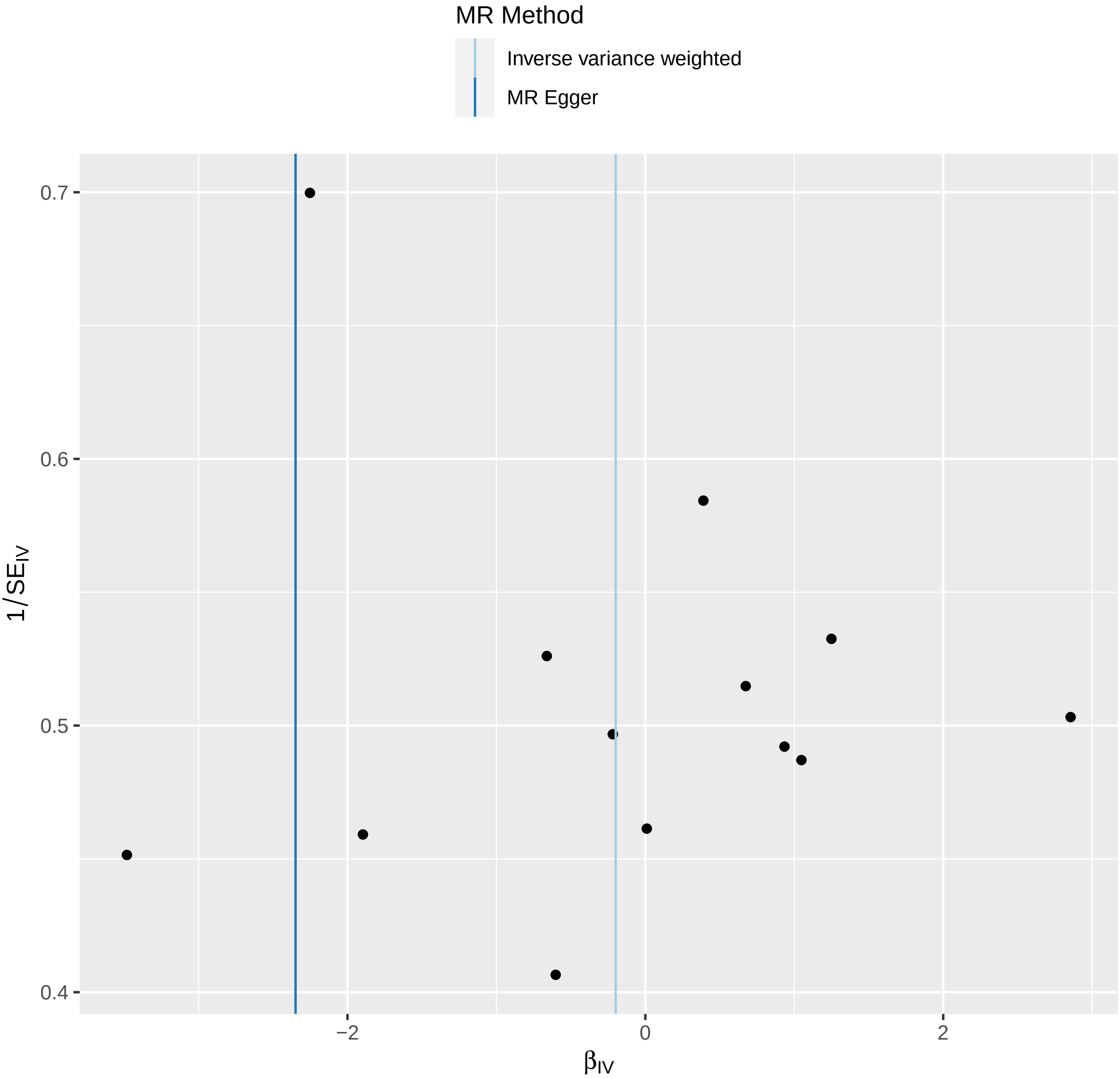

Batch 723 : Gut microbiota abundance (genus Ruminiclostridium5 id.11355) on Ulcerative colitis (strict) with PSC

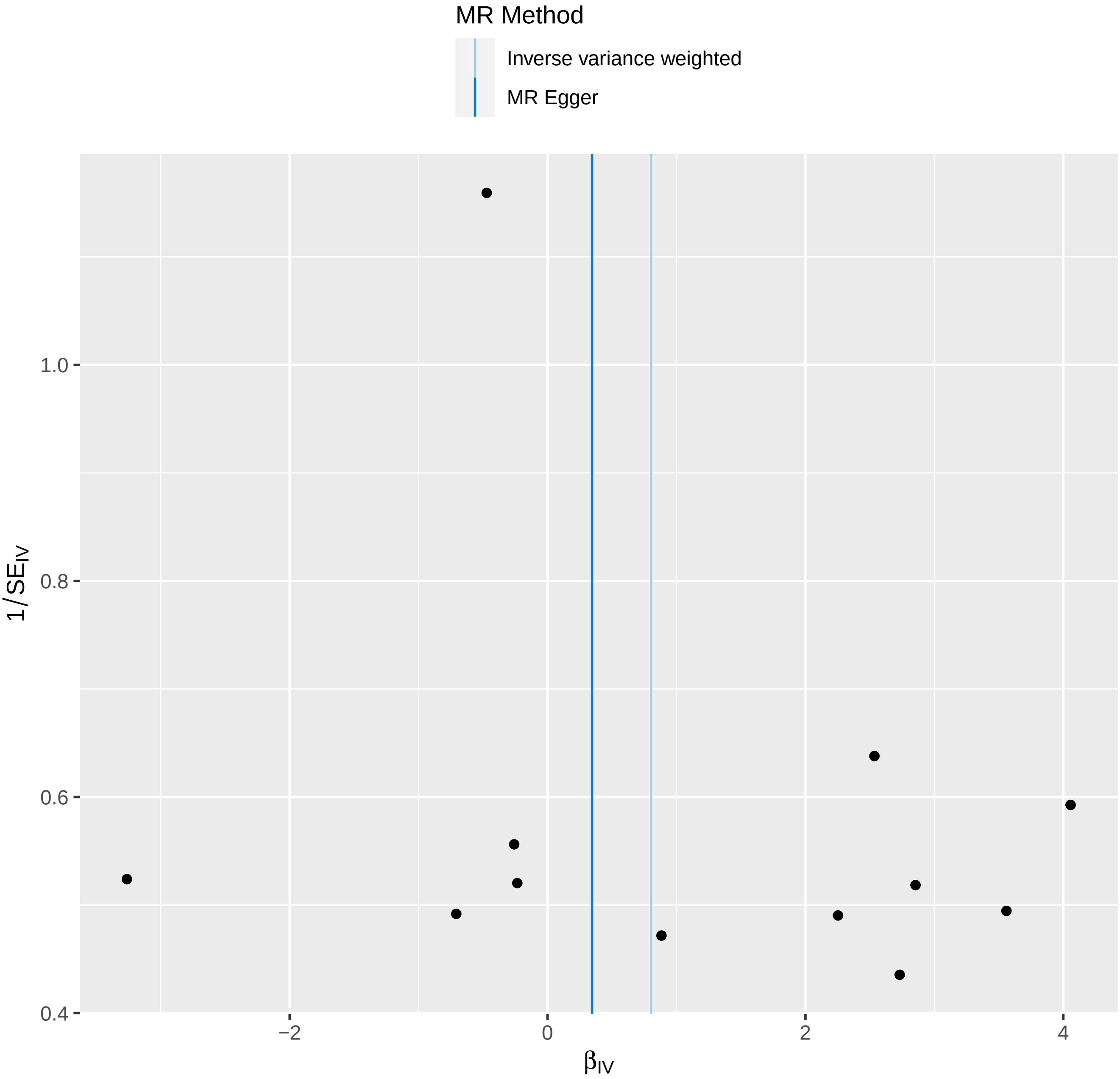

Batch 724 : Gut microbiota abundance (genus Ruminiclostridium6 id.11356) on Ulcerative colitis (strict) with PSC

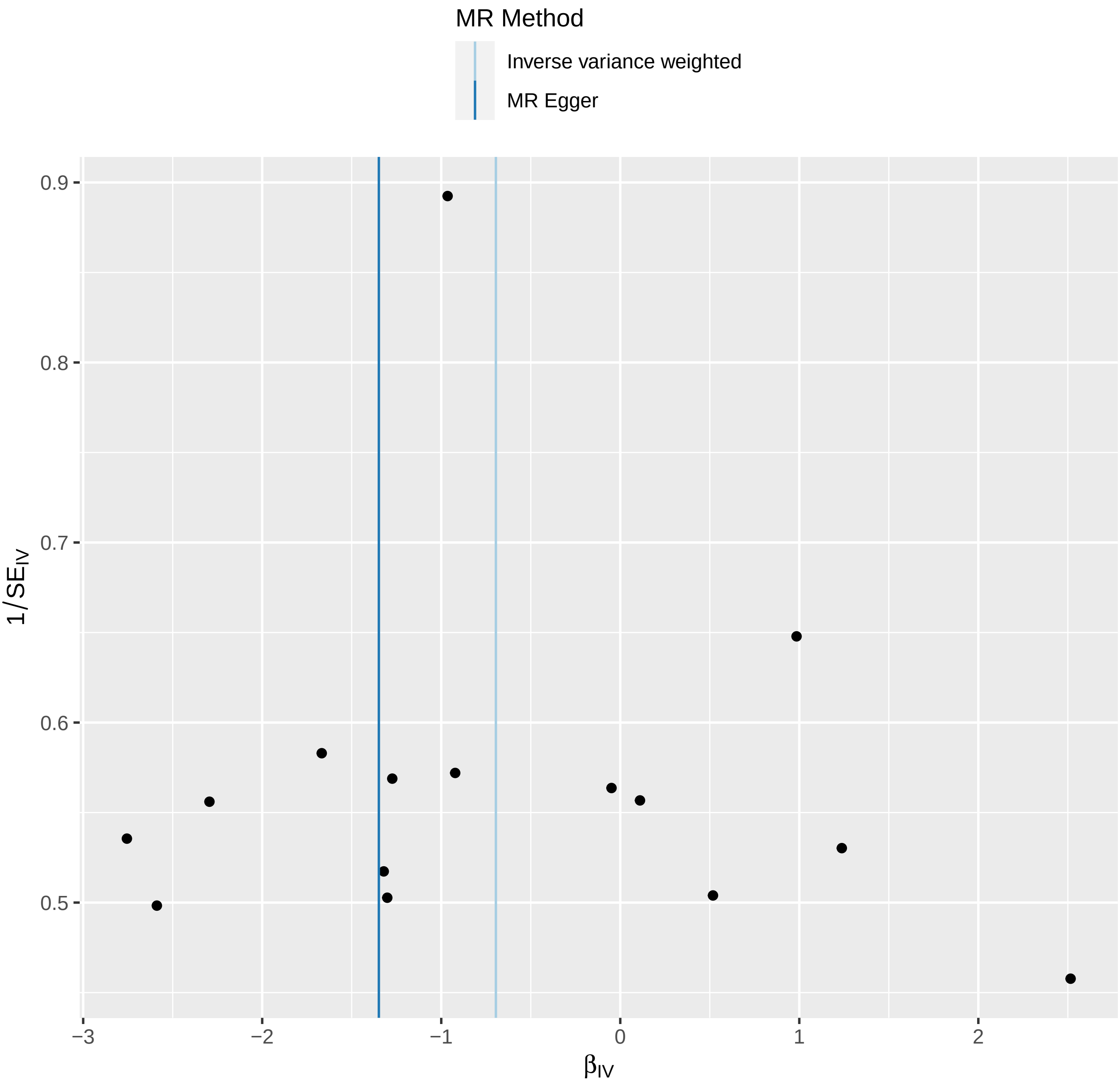

Batch 725 : Gut microbiota abundance (genus Ruminiclostridium9 id.11357) on Ulcerative colitis (strict) with PSC

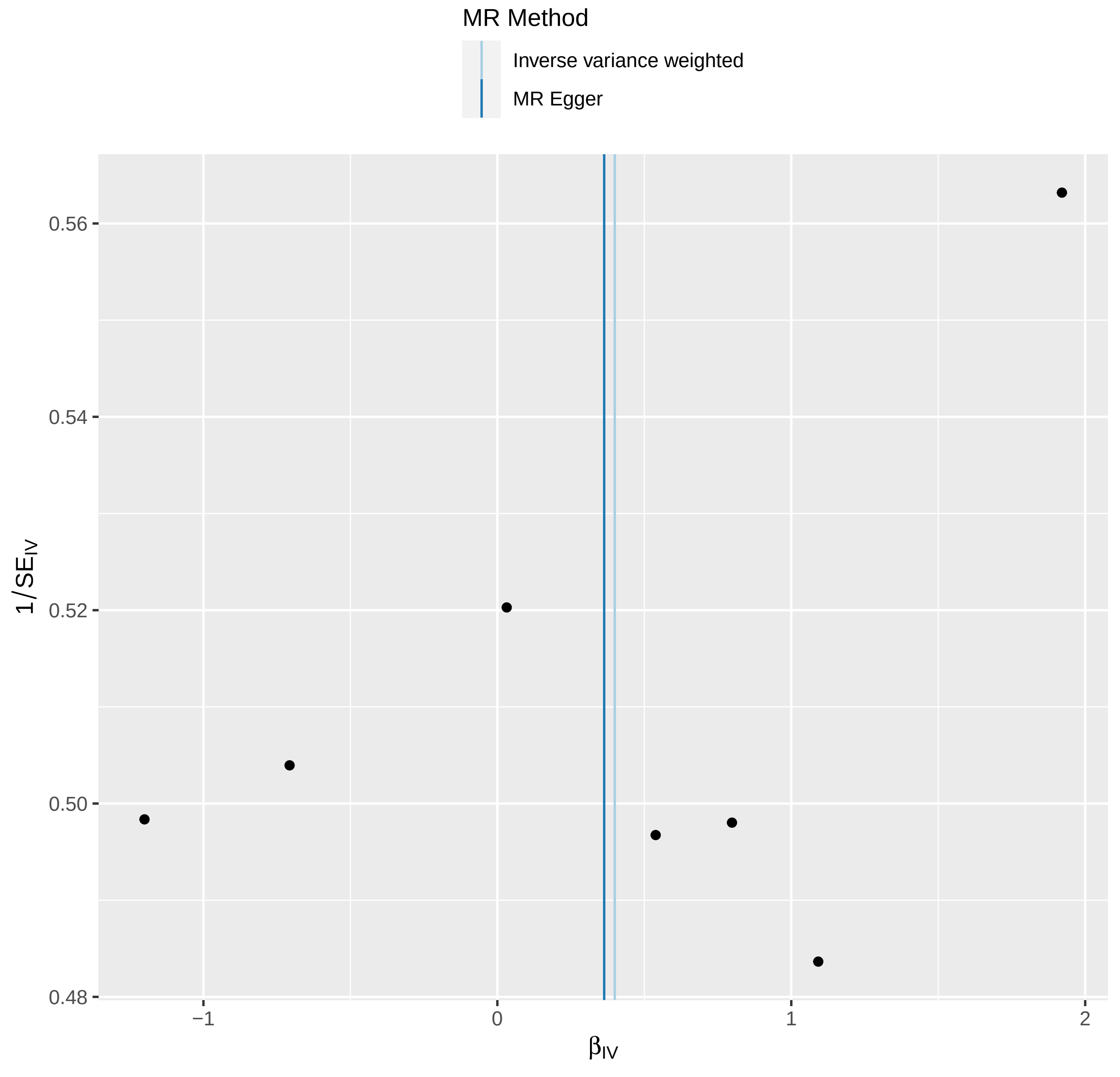

Batch 726 : Gut microbiota abundance (genus Ruminococcaceae NK4A214 group id.11358) on Ulcerative colitis (strict) with PSC

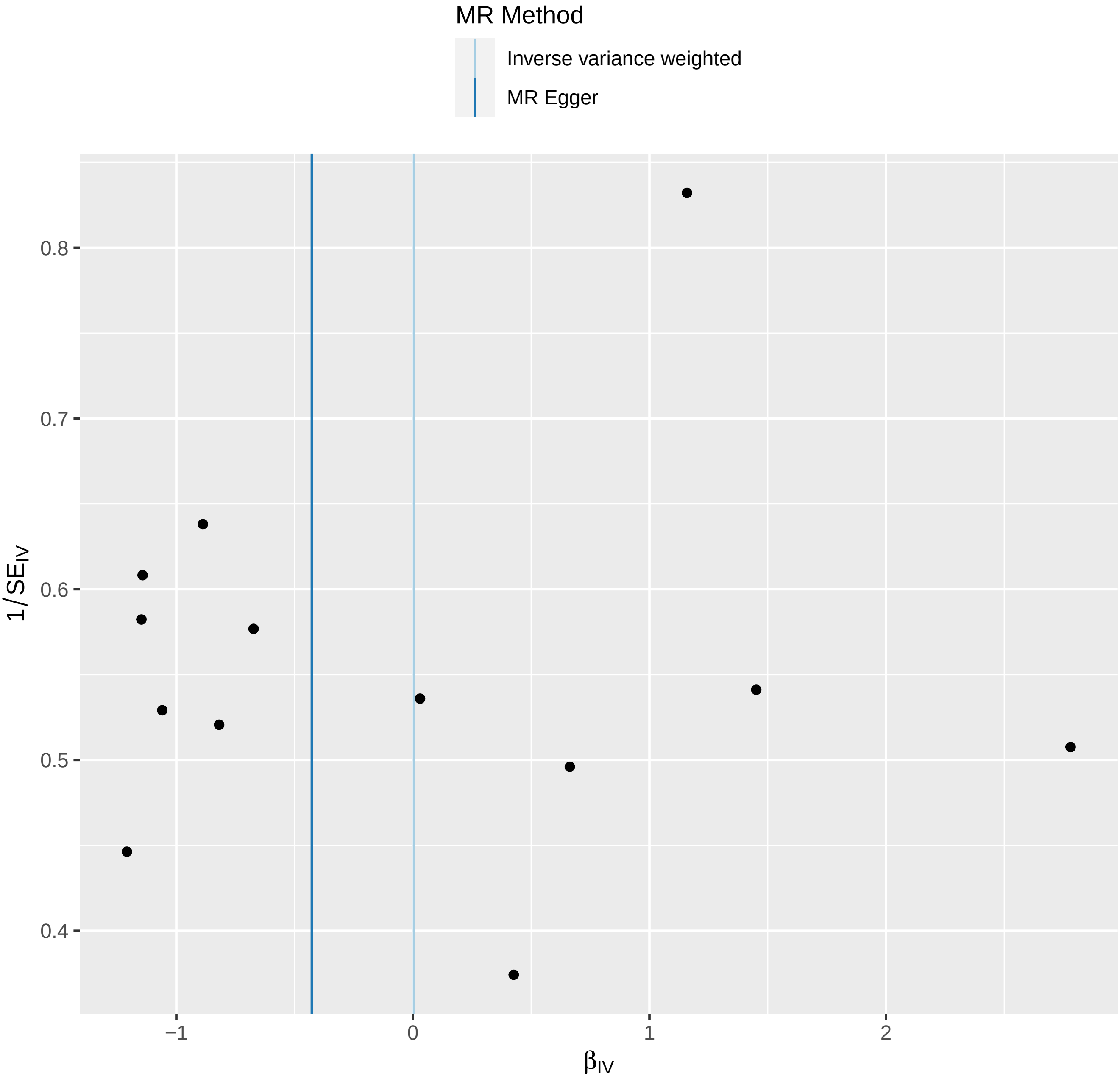

Batch 727 : Gut microbiota abundance (genus Ruminococcaceae UCG002 id.11360) on Ulcerative colitis (strict) with PSC

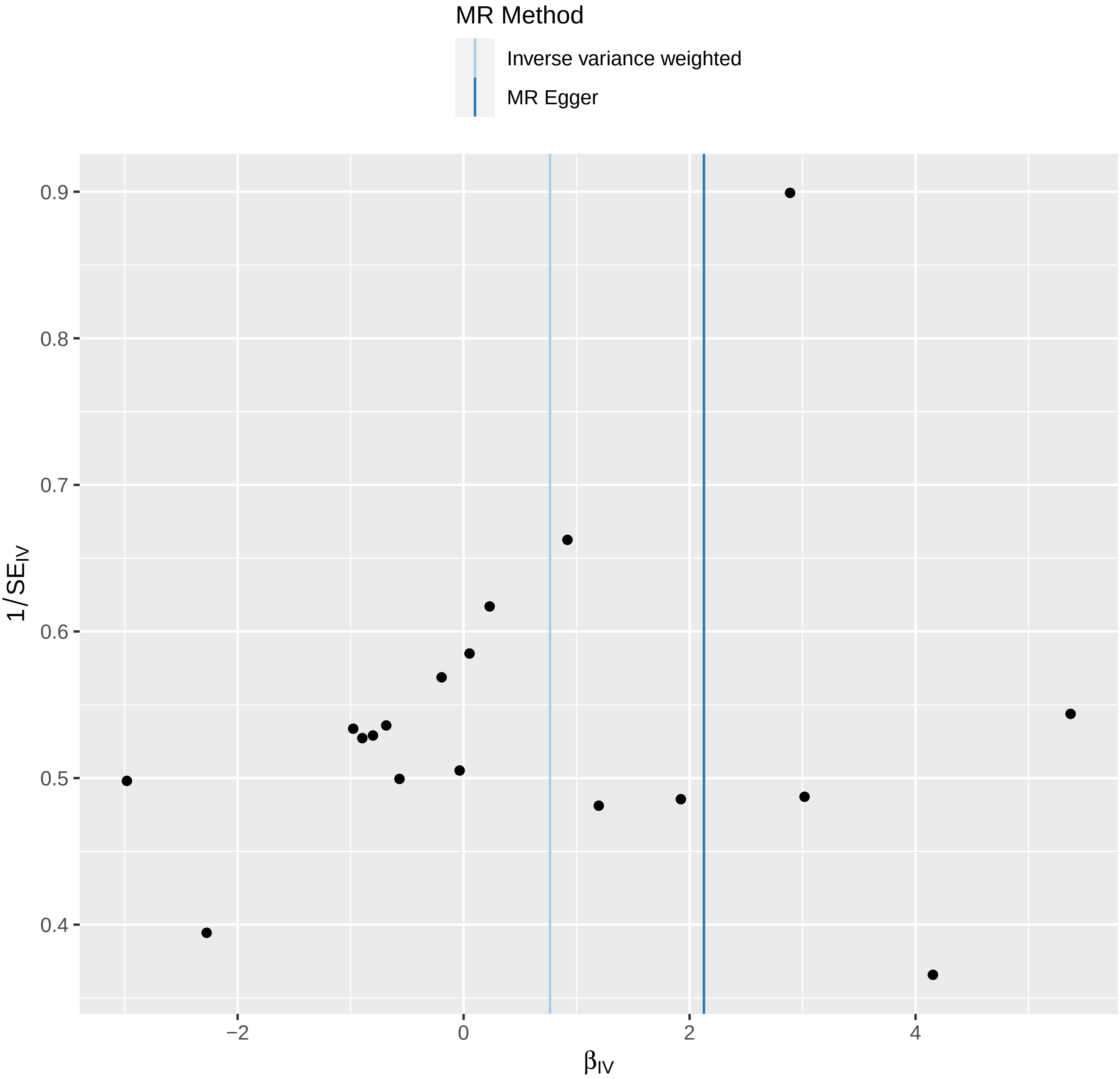

Batch 728 : Gut microbiota abundance (genus Ruminococcaceae UCG003 id.11361) on Ulcerative colitis (strict) with PSC

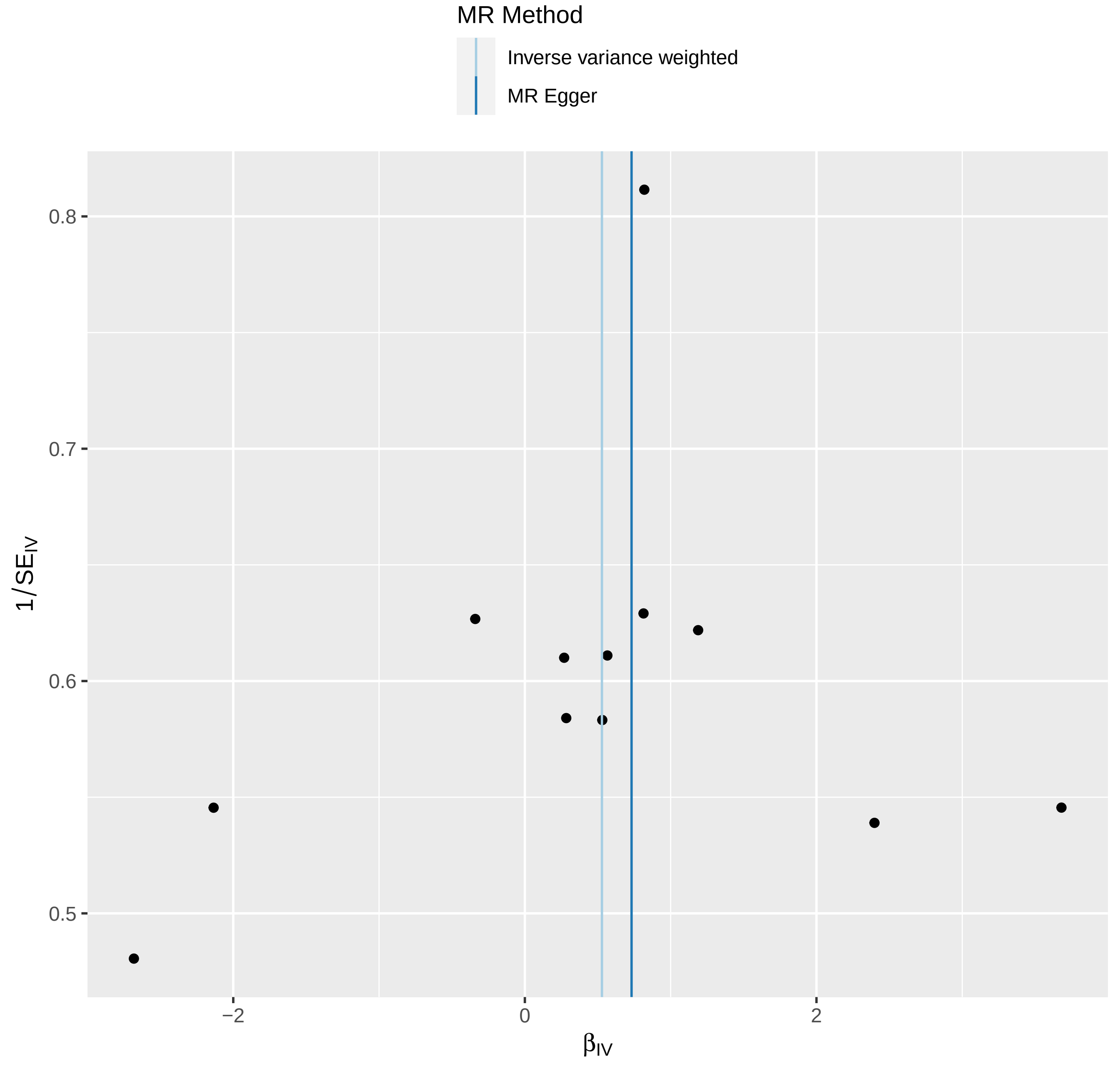

Batch 729 : Gut microbiota abundance (genus Ruminococcaceae UCG004 id.11362) on Ulcerative colitis (strict) with PSC

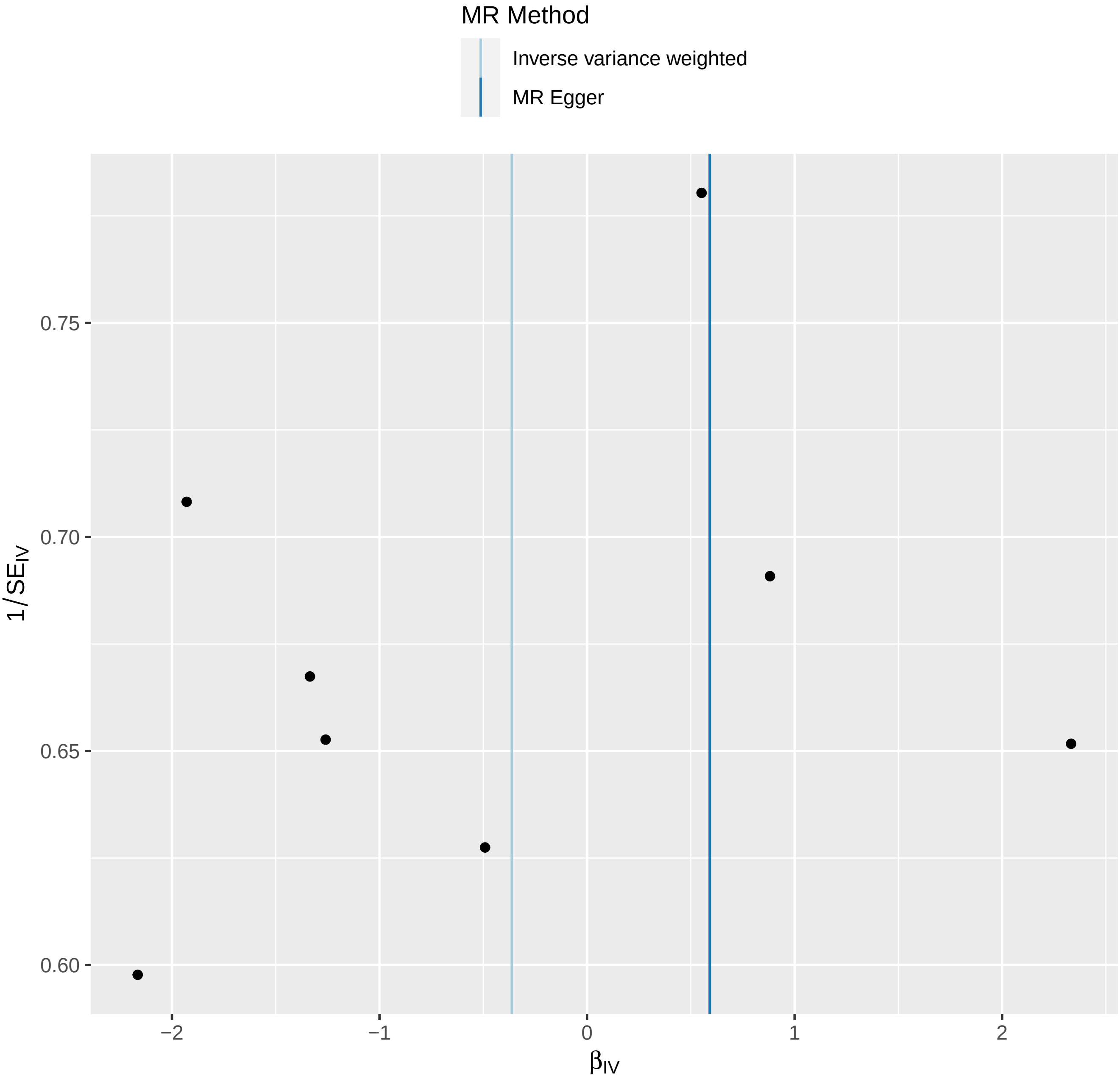

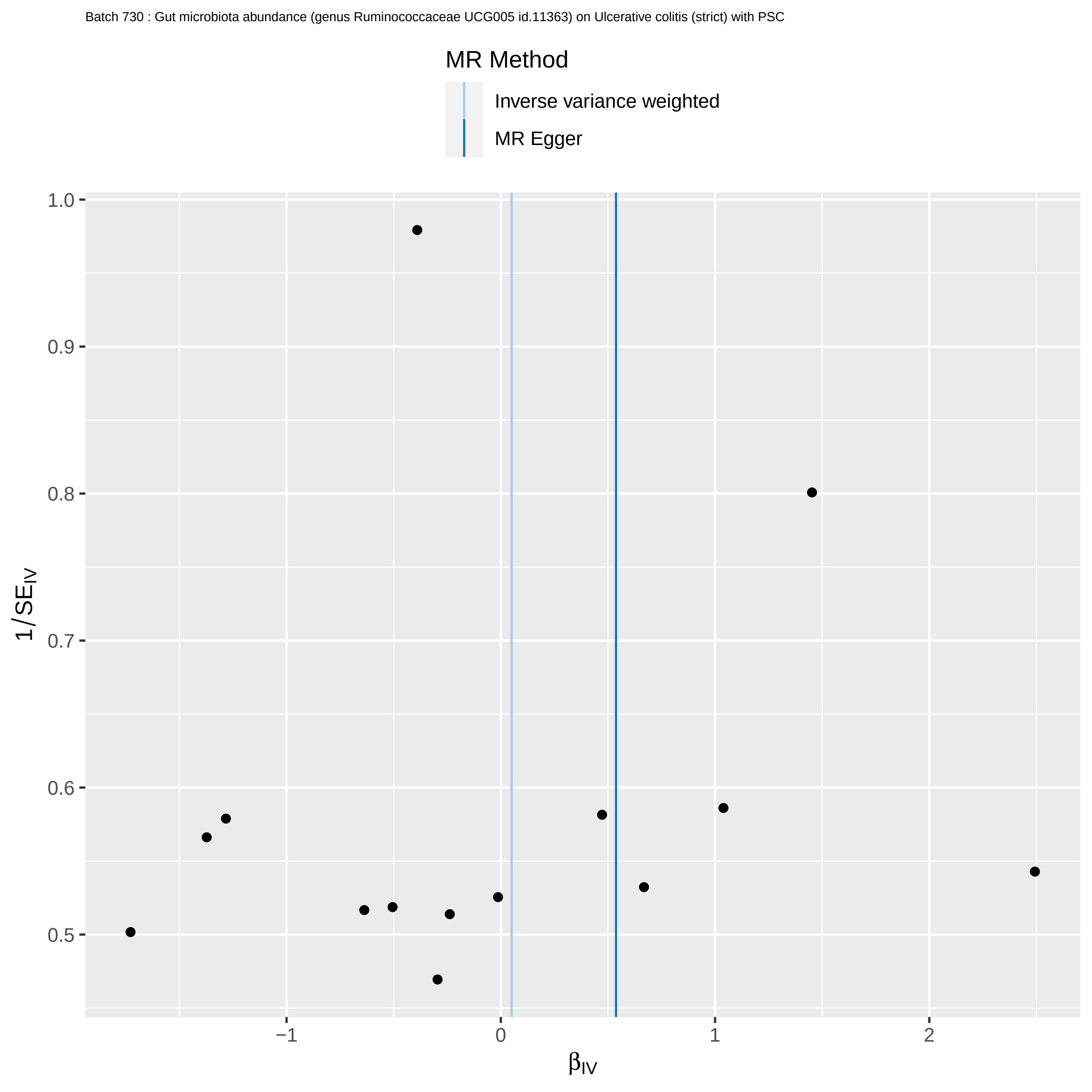

Batch 730 : Gut microbiota abundance (genus Ruminococcaceae UCG005 id.11363) on Ulcerative colitis (strict) with PSC
MR Method
Inverse variance weighted
MR Egger
1.0
0.9
0.8
0.7
0.6
0.5
$1/SE_{IV}$
−1
0
1
2
β_{IV}

Batch 731 : Gut microbiota abundance (genus Ruminococcaceae UCG009 id.11366) on Ulcerative colitis (strict) with PSC

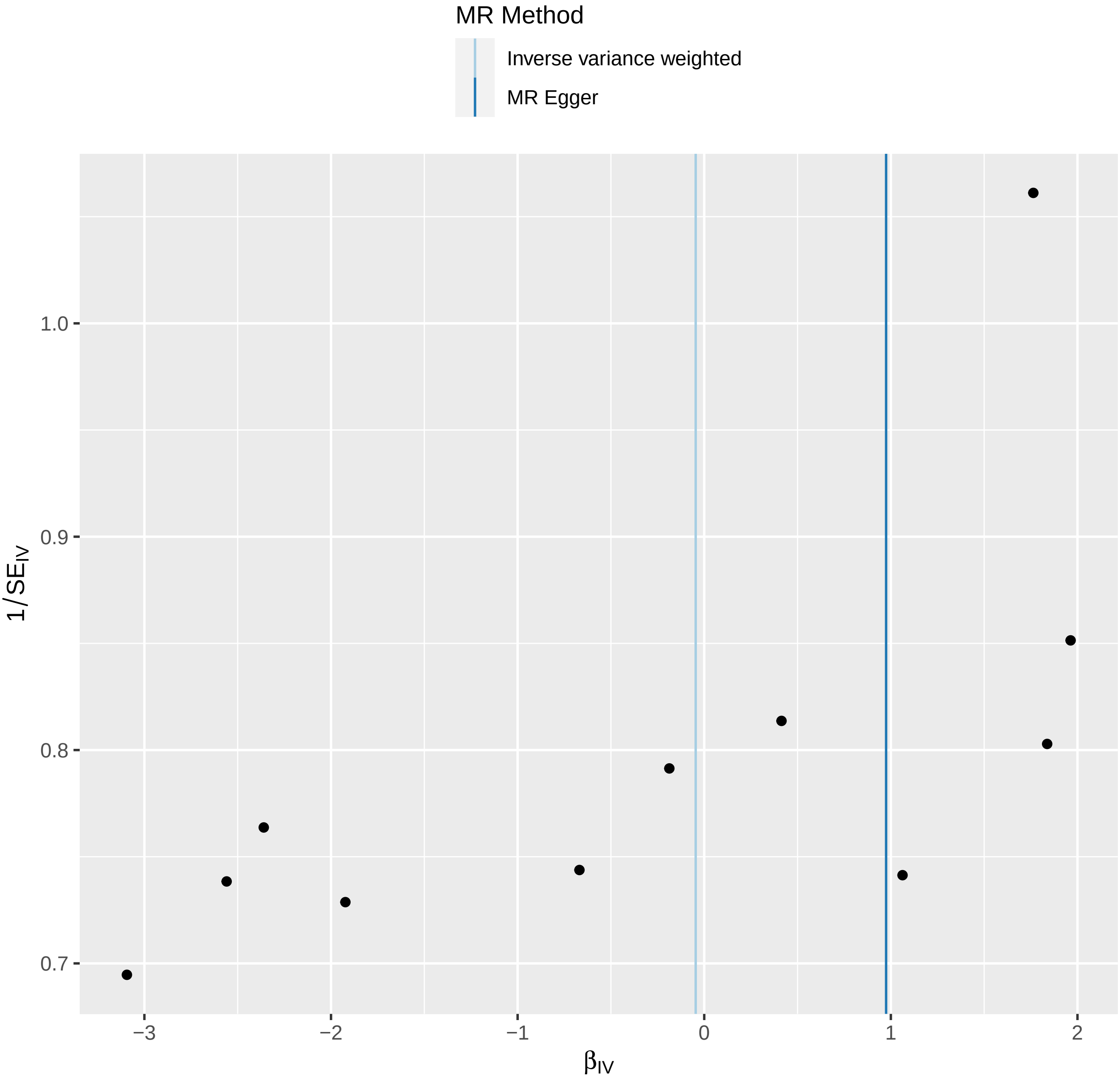

Batch 732 : Gut microbiota abundance (genus Ruminococcaceae UCG010 id.11367) on Ulcerative colitis (strict) with PSC

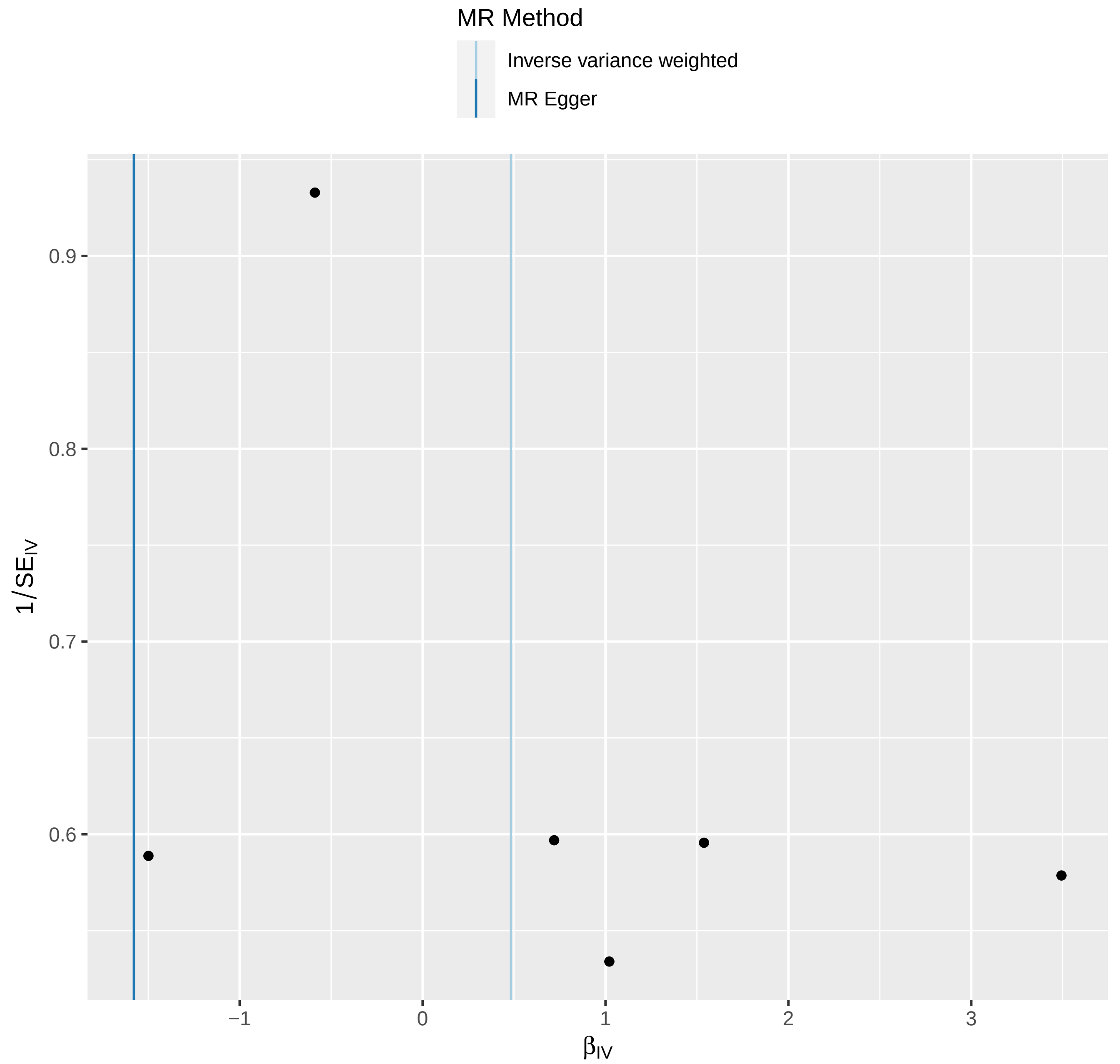

Batch 733 : Gut microbiota abundance (genus Ruminococcaceae UCG011 id.11368) on Ulcerative colitis (strict) with PSC

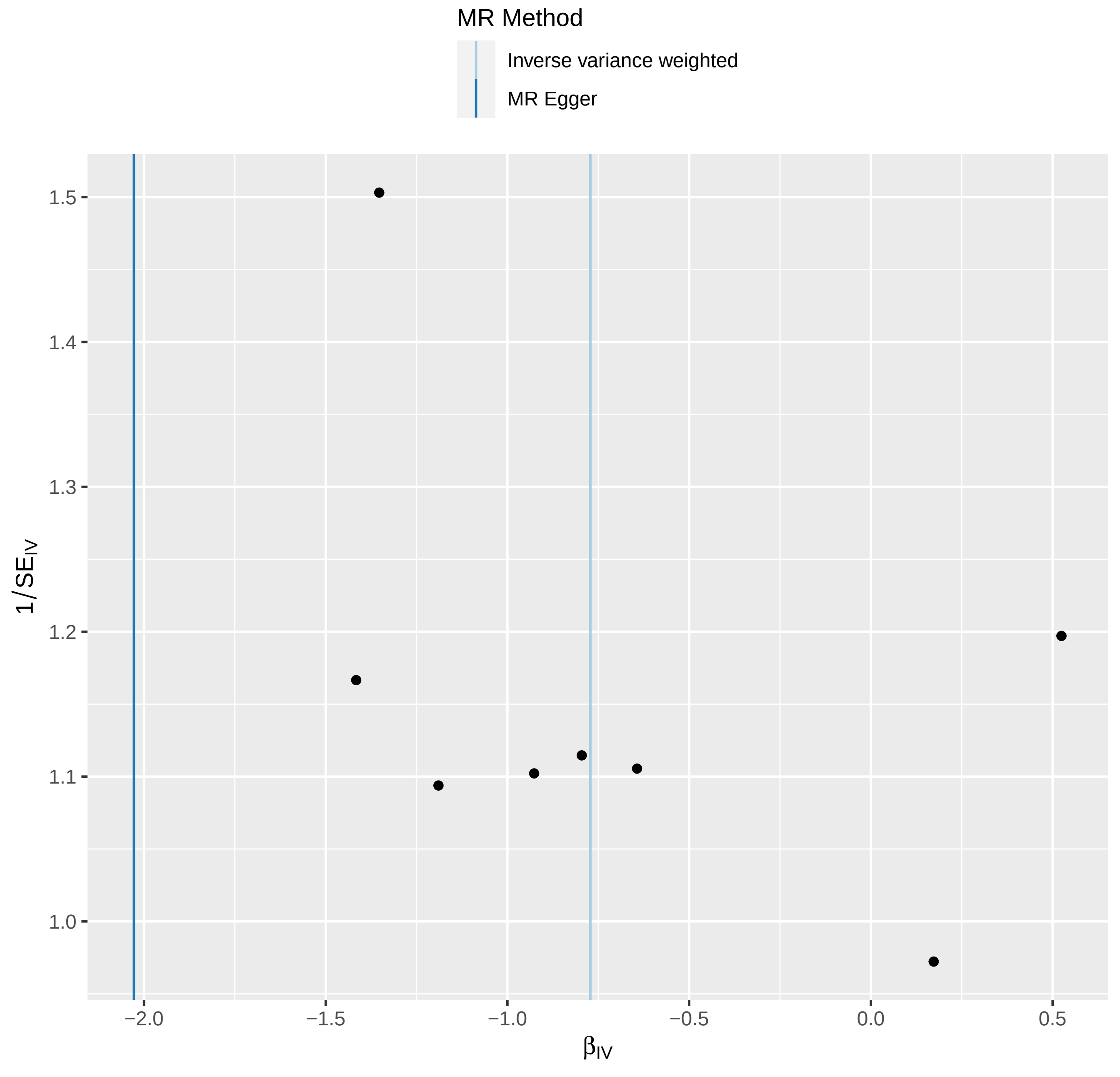

Batch 734 : Gut microbiota abundance (genus Ruminococcaceae UCG013 id.11370) on Ulcerative colitis (strict) with PSC

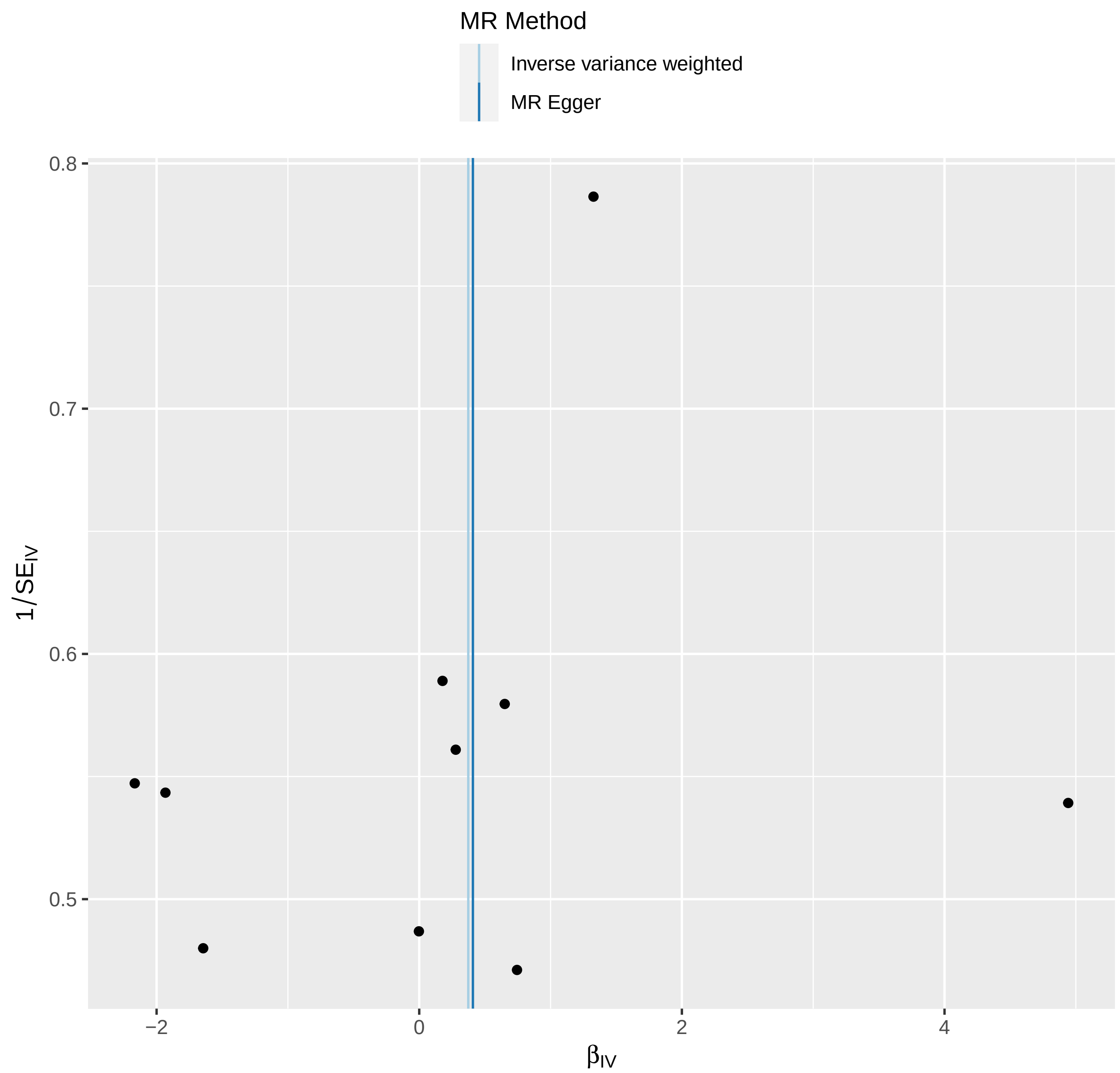

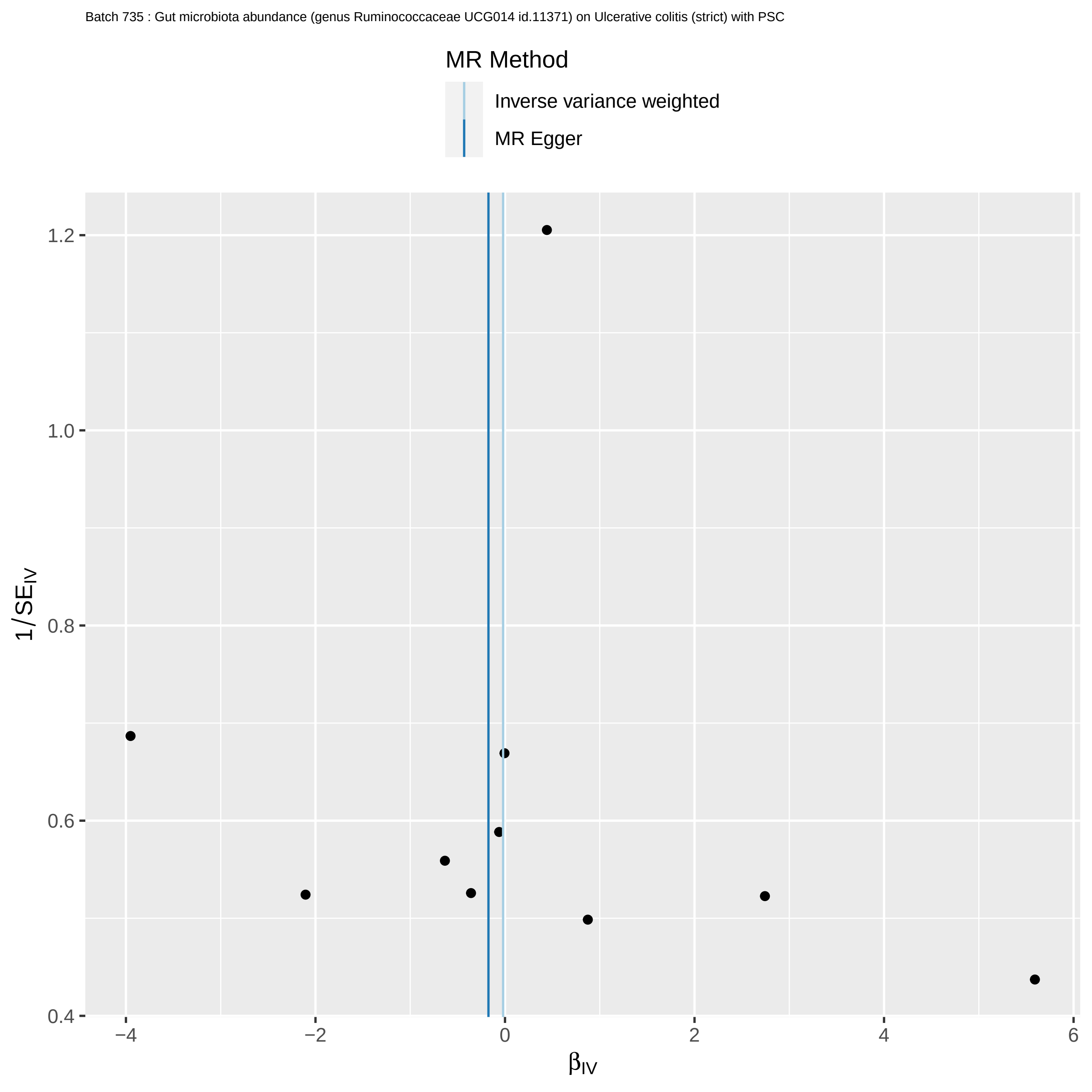

Batch 735 : Gut microbiota abundance (genus Ruminococcaceae UCG014 id.11371) on Ulcerative colitis (strict) with PSC
MR Method
Inverse variance weighted
MR Egger
1.2
1.0
0.8
0.6
0.4
1/SE_IV
−4
−2
0
2
4
6
β_IV

Batch 736 : Gut microbiota abundance (genus Ruminococcus1 id.11373) on Ulcerative colitis (strict) with PSC

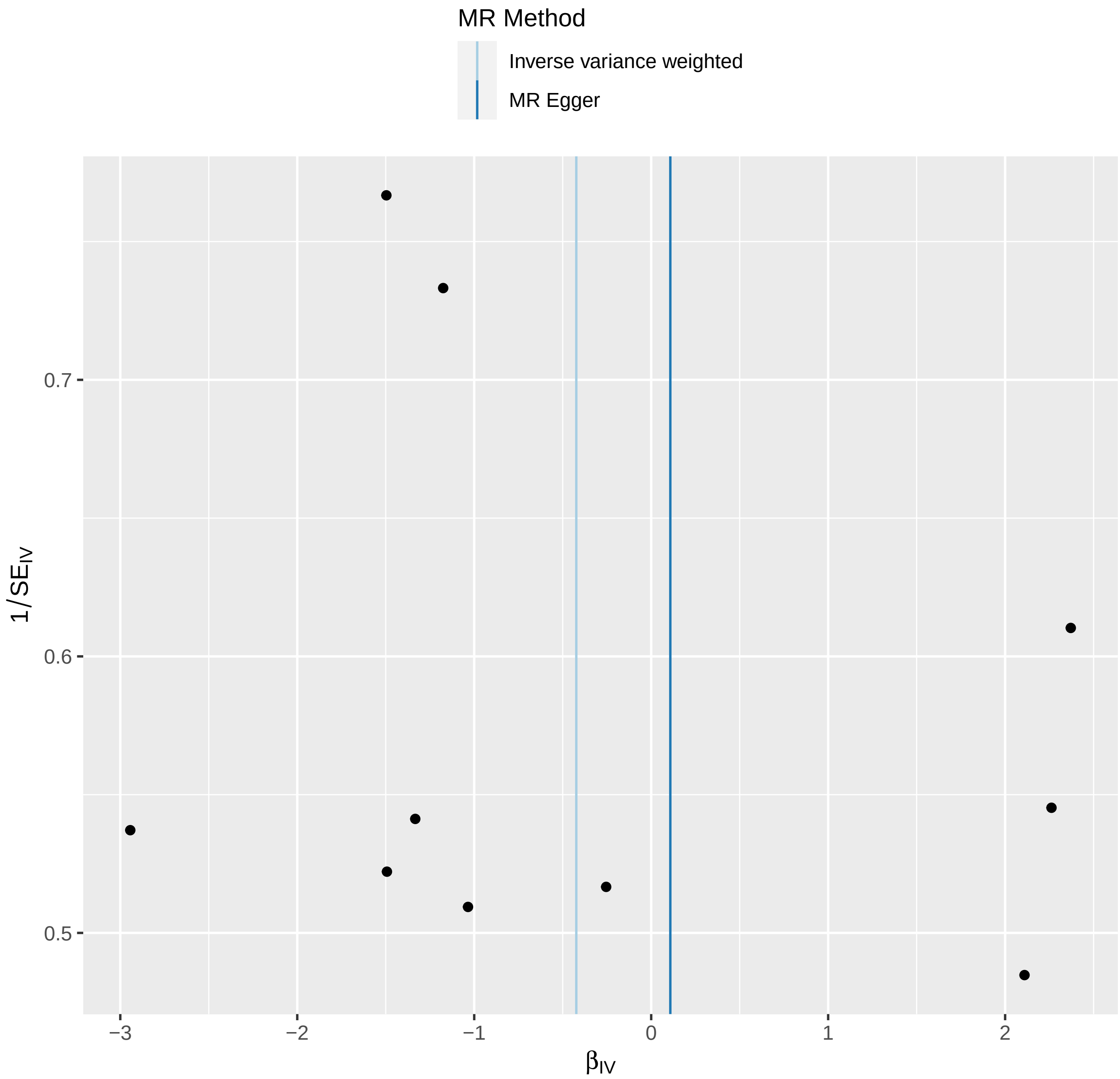

Batch 737 : Gut microbiota abundance (genus Ruminococcus2 id.11374) on Ulcerative colitis (strict) with PSC

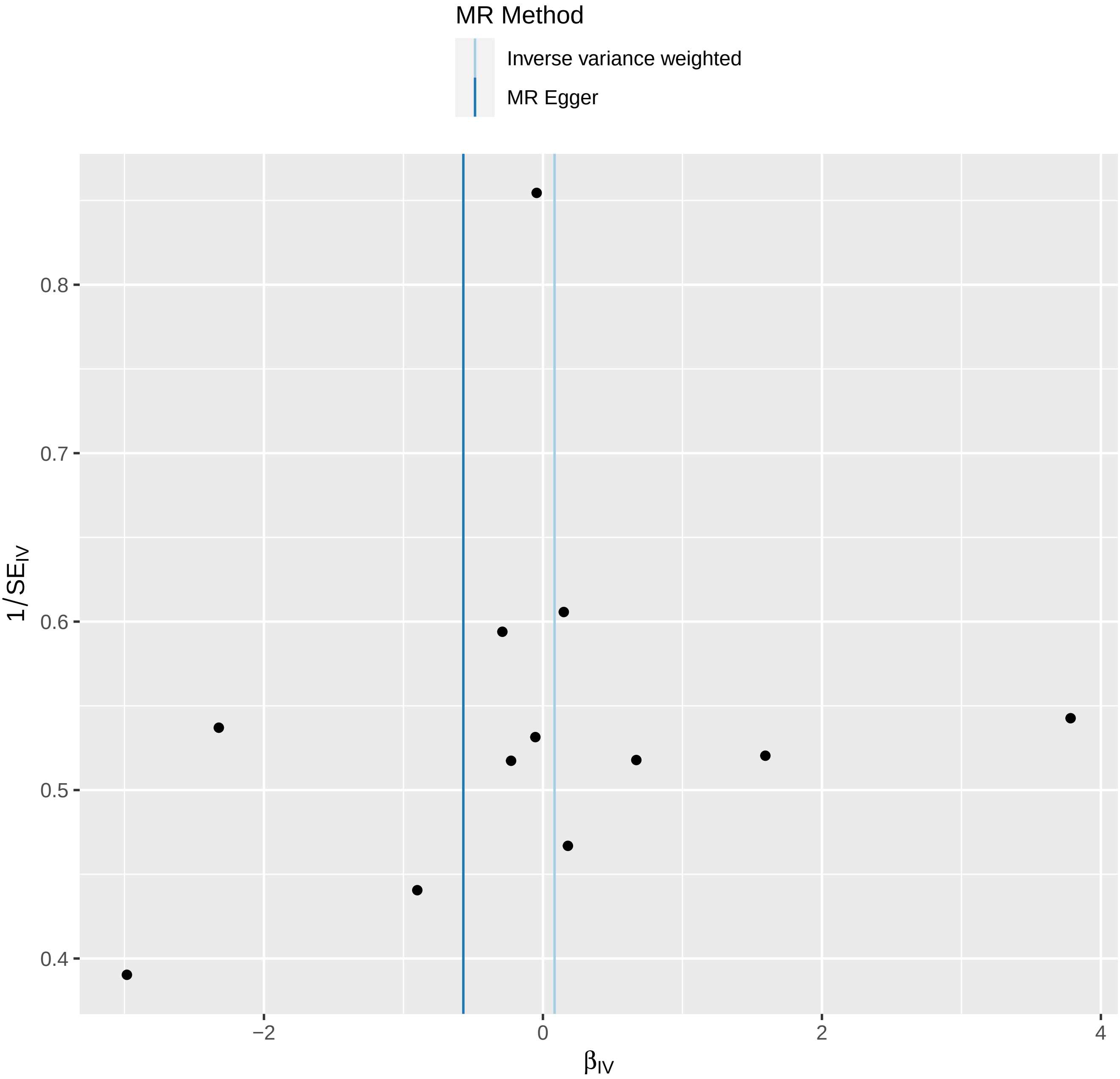

Batch 738 : Gut microbiota abundance (genus Ruminococcus gauvreauii group id.11342) on Ulcerative colitis (strict) with PSC

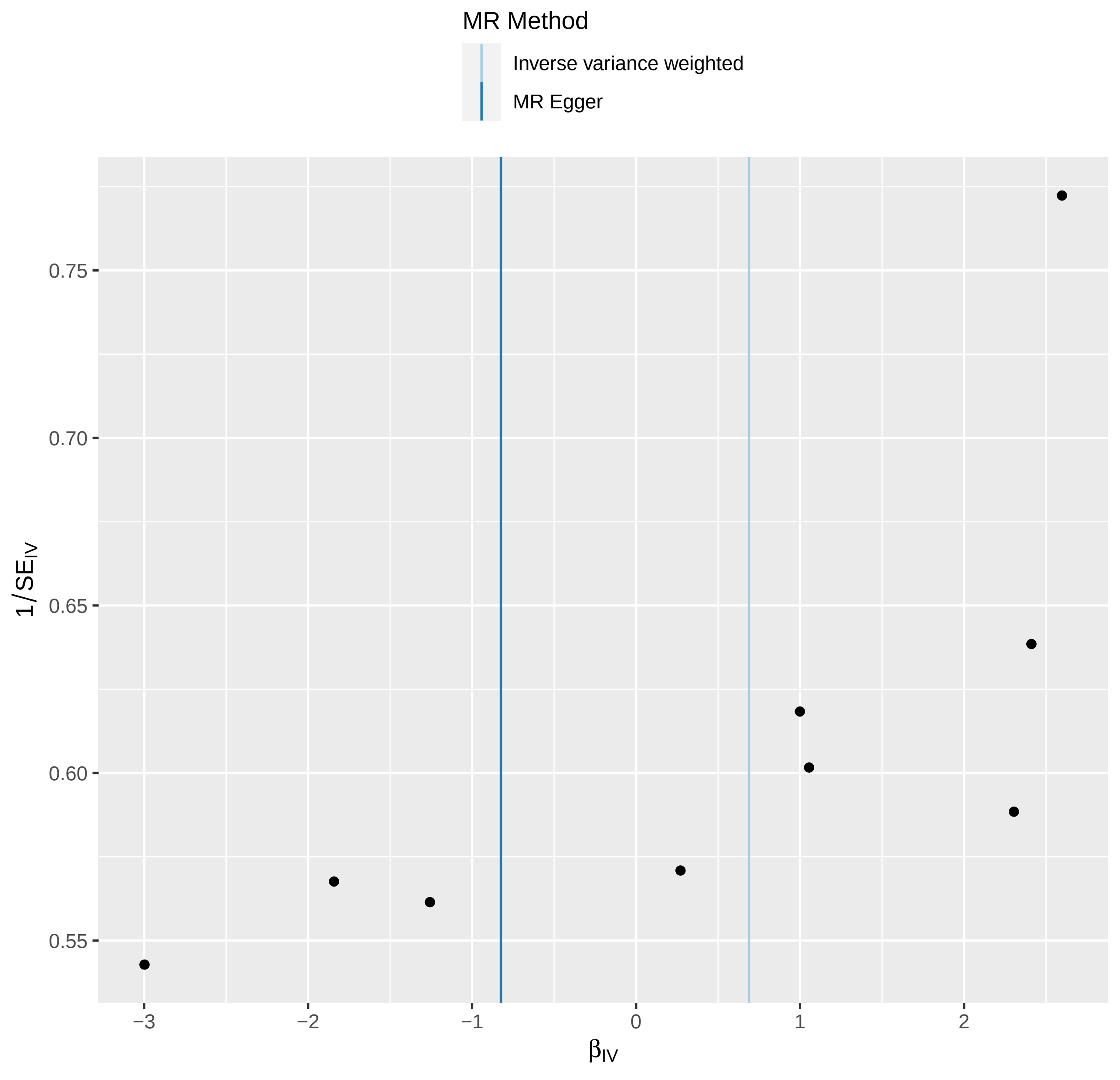

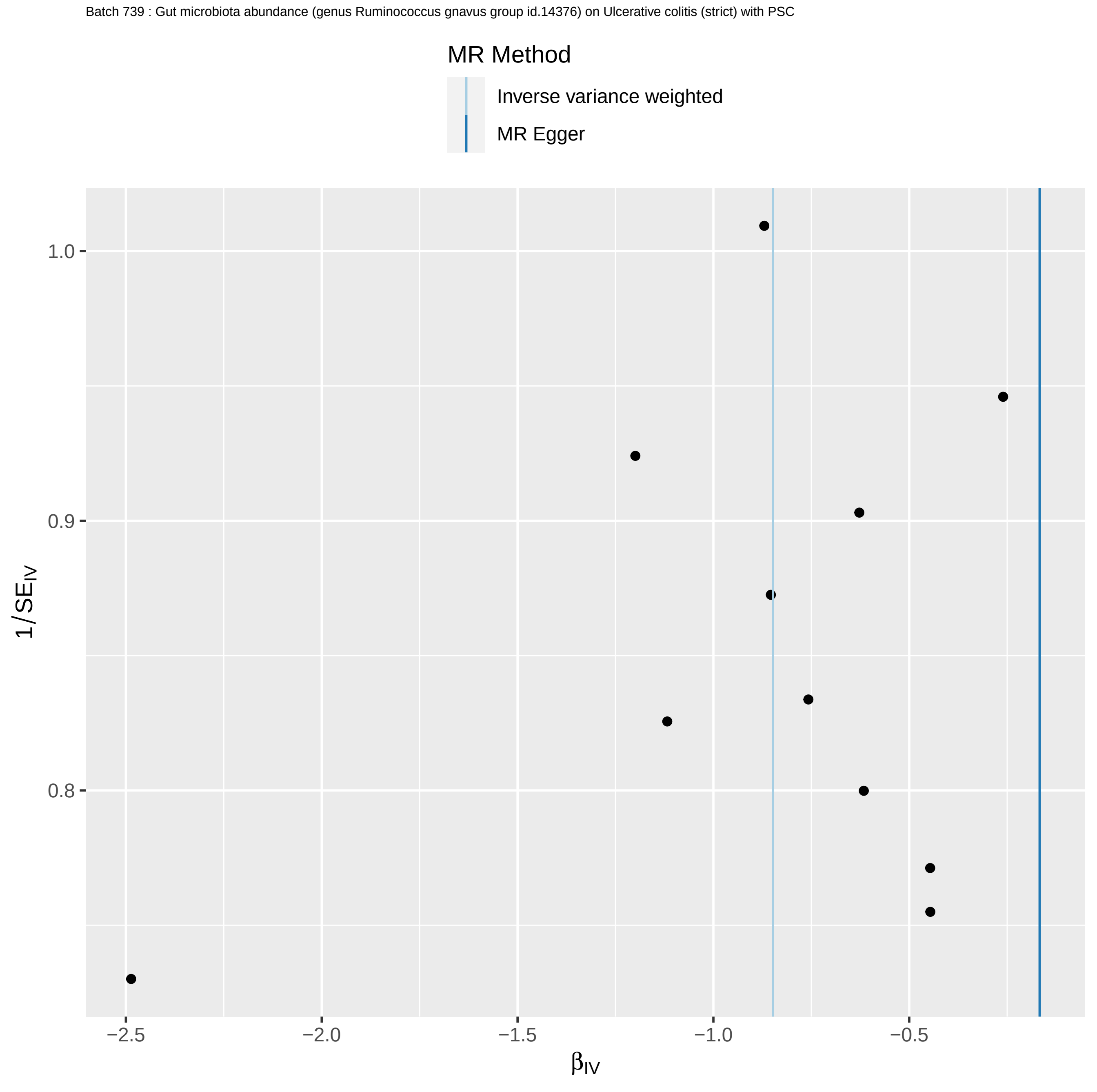

Batch 739 : Gut microbiota abundance (genus Ruminococcus gnavus group id.14376) on Ulcerative colitis (strict) with PSC
MR Method
Inverse variance weighted
MR Egger
1.0
0.9
0.8
1/SE_IV
−2.5
−2.0
−1.5
−1.0
−0.5
β_IV

Batch 740 : Gut microbiota abundance (genus Ruminococcus torques group id.14377) on Ulcerative colitis (strict) with PSC

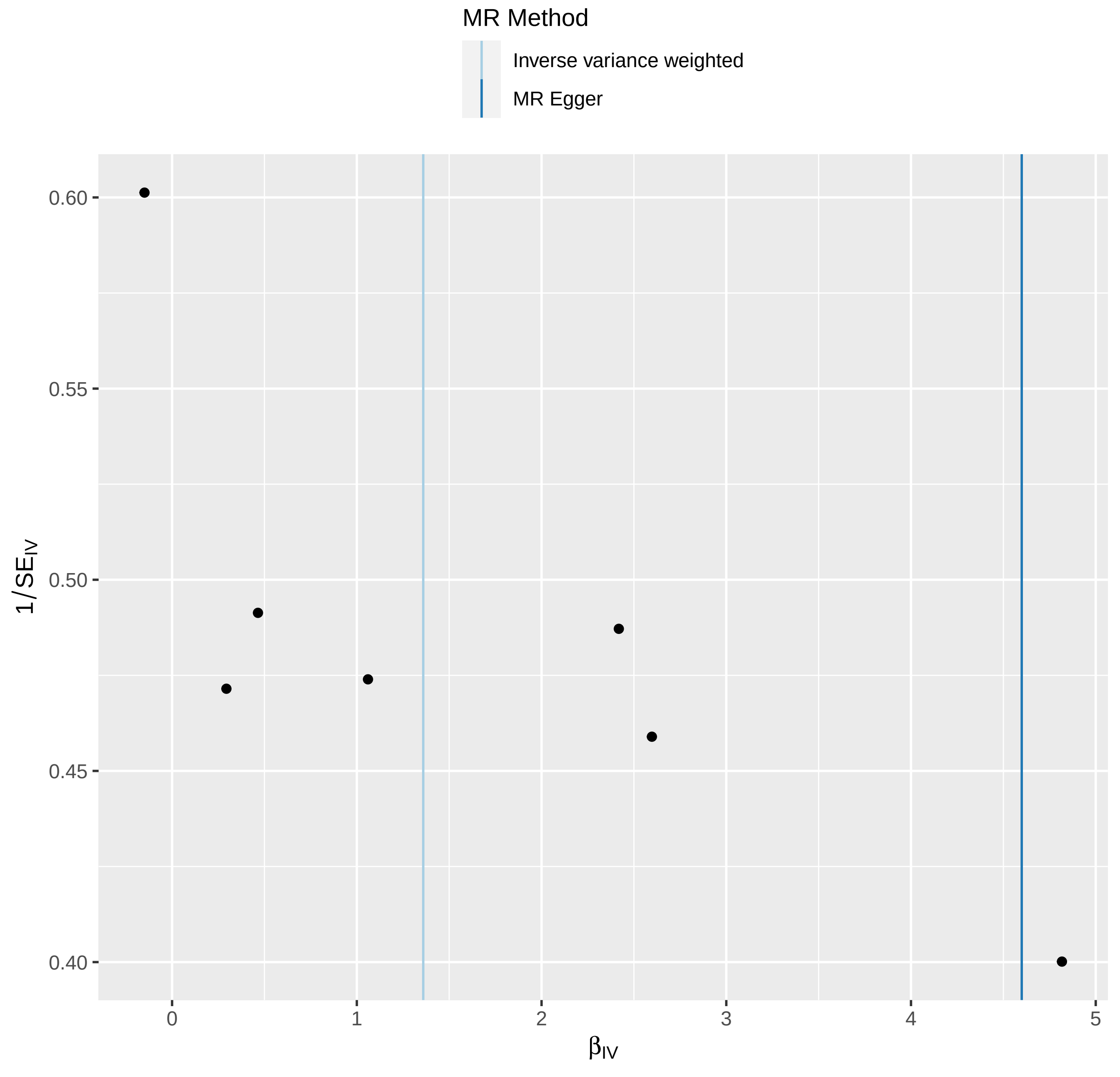

Batch 741 : Gut microbiota abundance (genus Sellimonas id.14369) on Ulcerative colitis (strict) with PSC

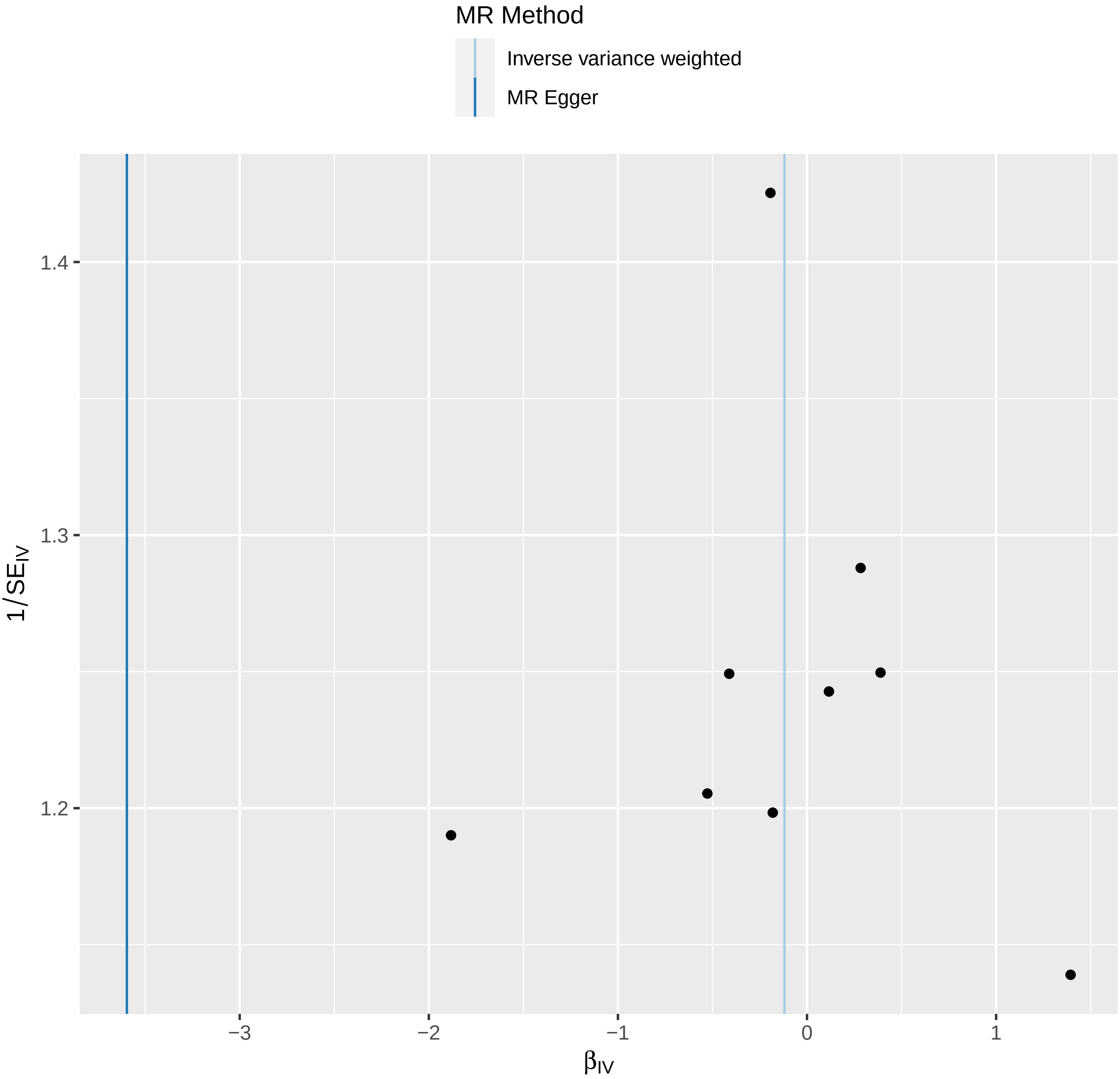

Batch 742 : Gut microbiota abundance (genus Senegalimassilia id.11160) on Ulcerative colitis (strict) with PSC

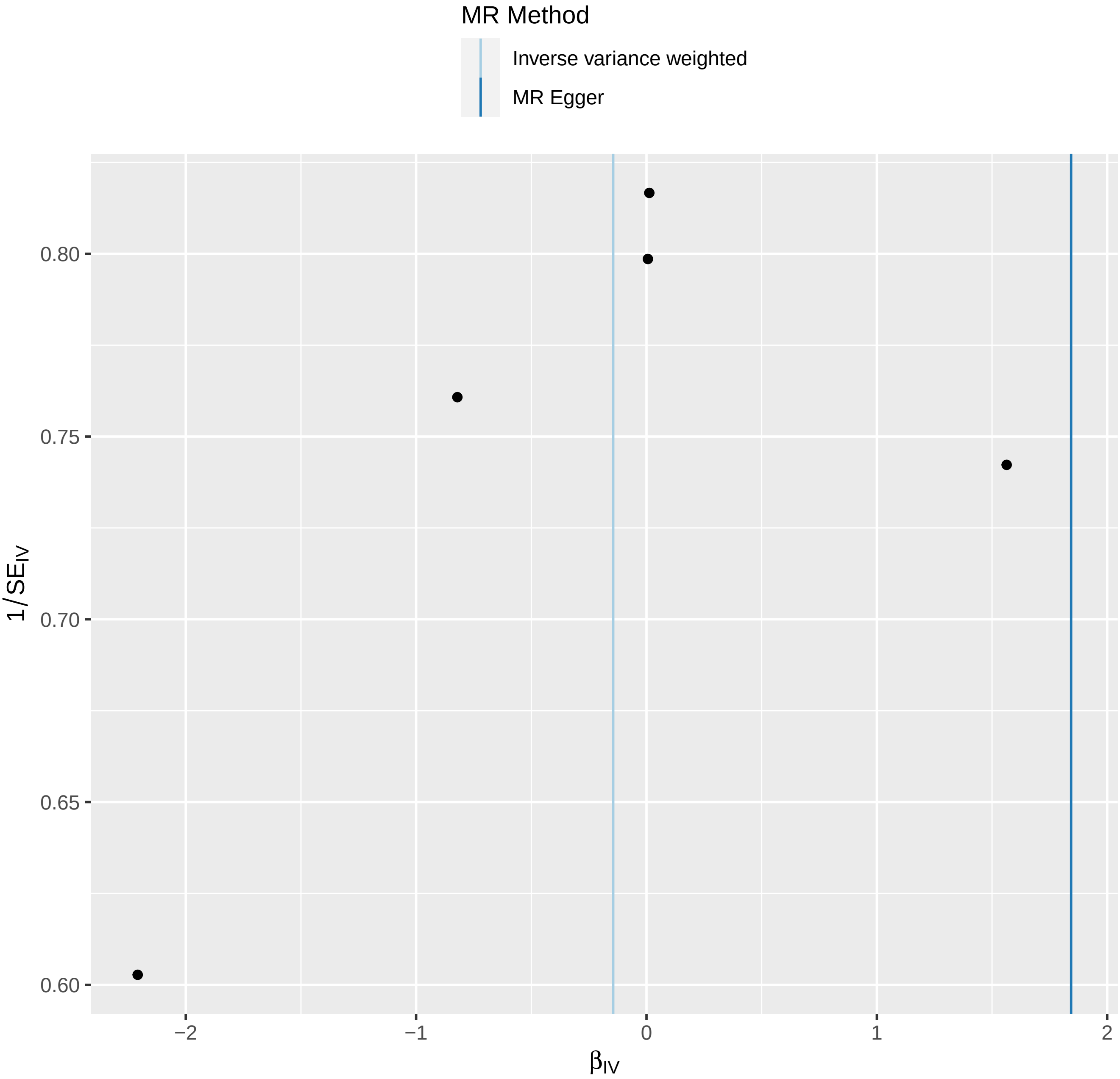

Batch 743 : Gut microbiota abundance (genus Slackia id.825) on Ulcerative colitis (strict) with PSC

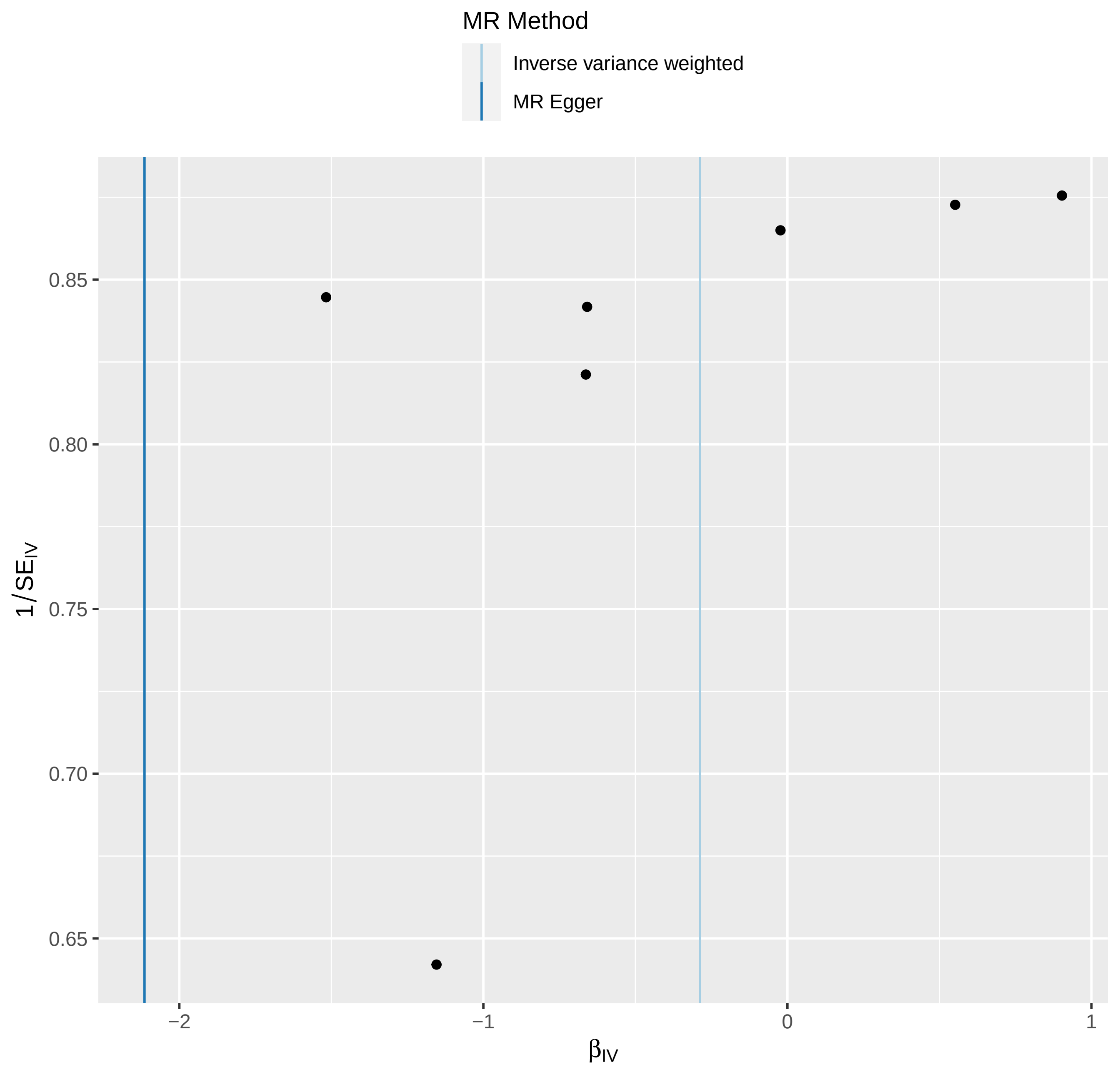

Batch 744 : Gut microbiota abundance (genus Streptococcus id.1853) on Ulcerative colitis (strict) with PSC

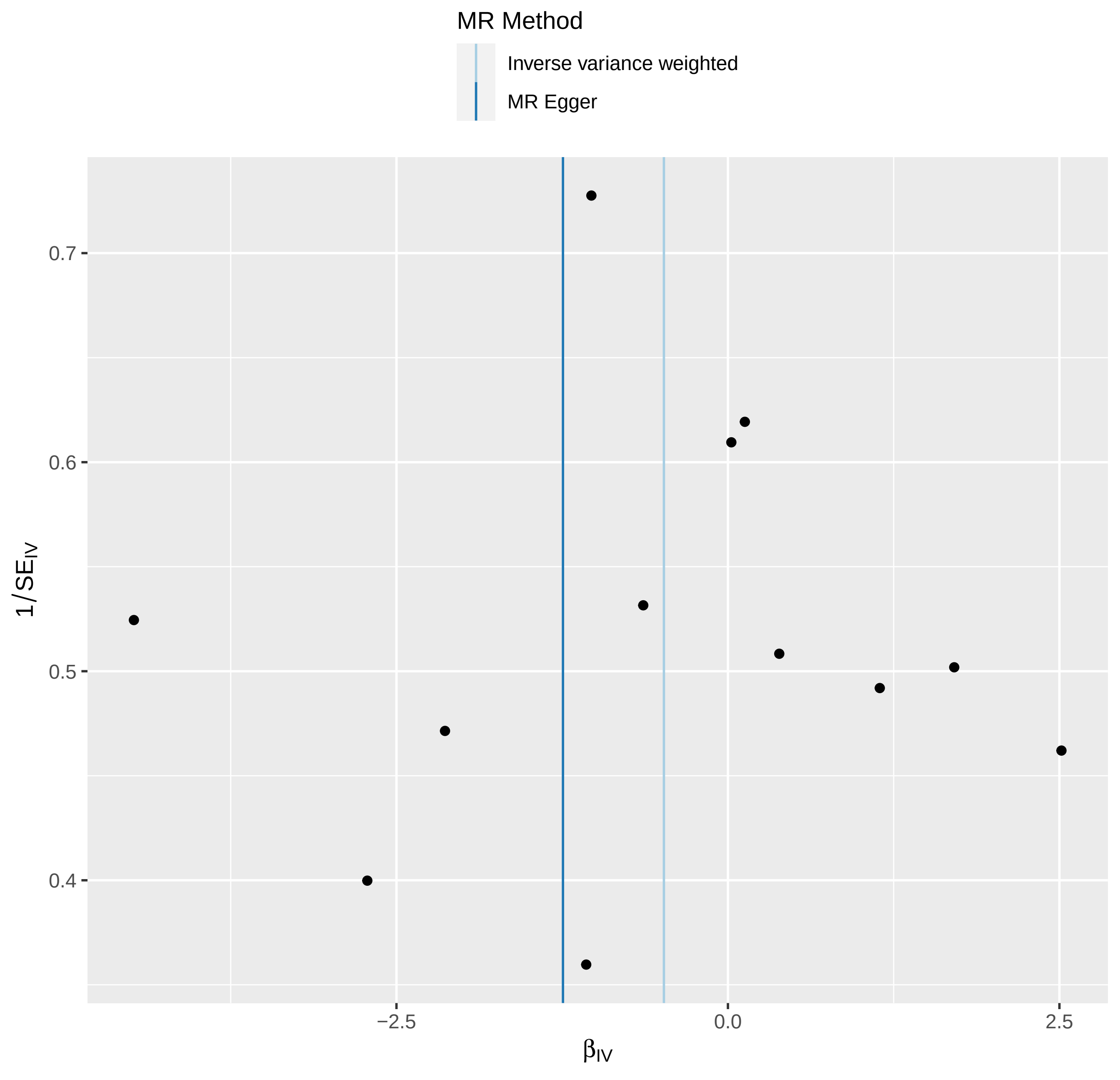

Batch 745 : Gut microbiota abundance (genus Subdoligranulum id.2070) on Ulcerative colitis (strict) with PSC

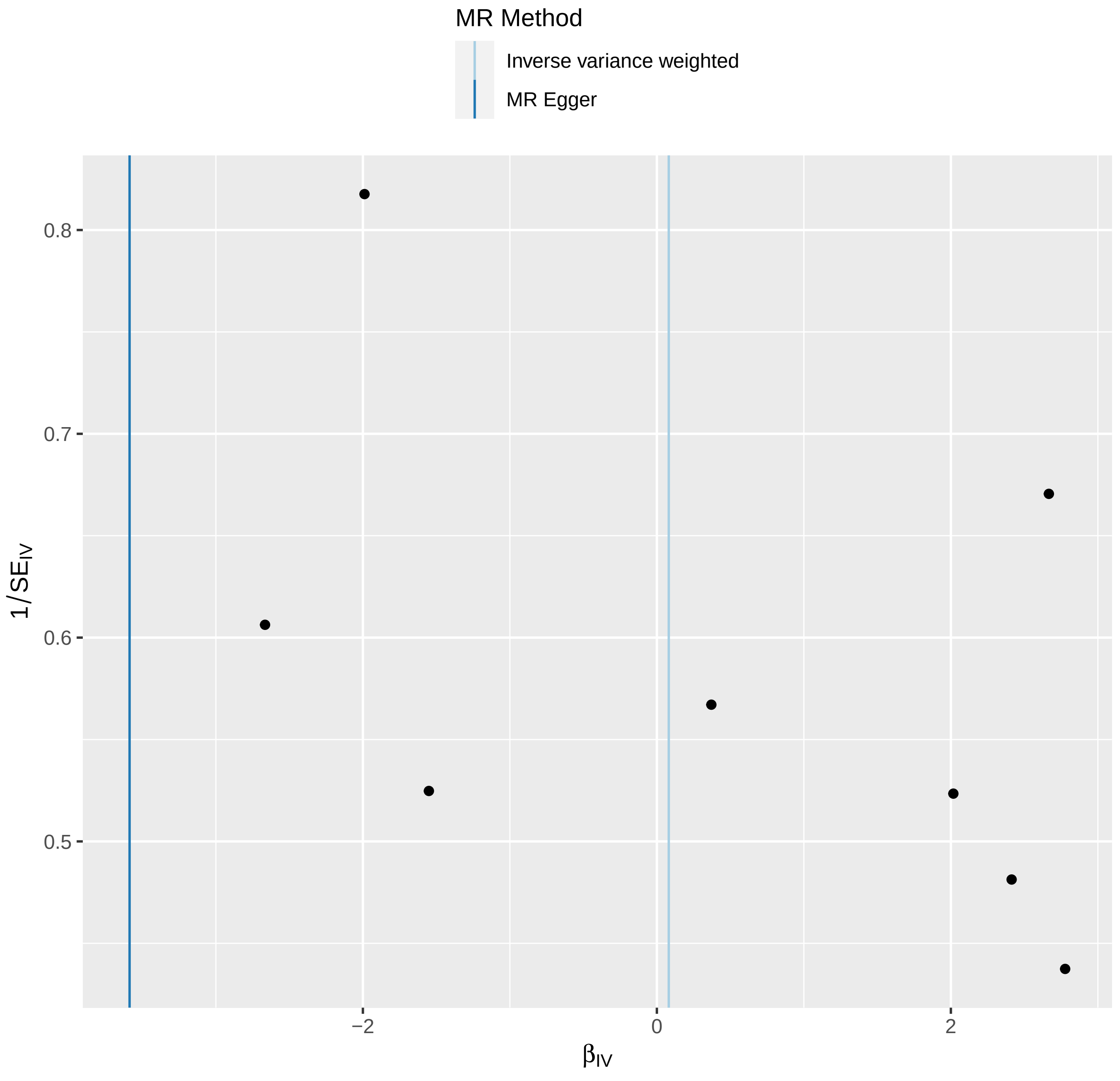

Batch 746 : Gut microbiota abundance (genus Sutterella id.2896) on Ulcerative colitis (strict) with PSC

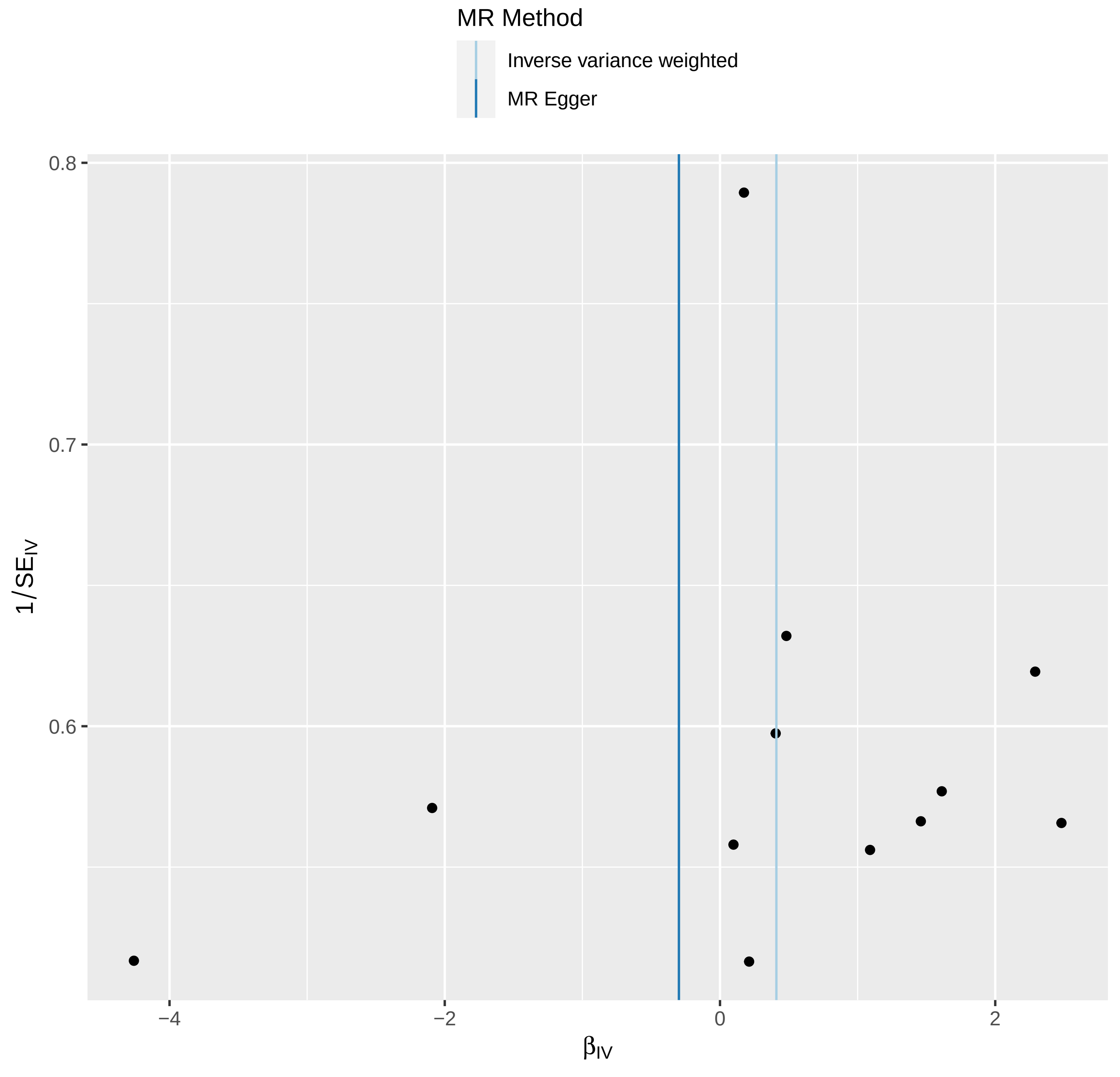

Batch 747 : Gut microbiota abundance (genus Terrisporobacter id.11348) on Ulcerative colitis (strict) with PSC

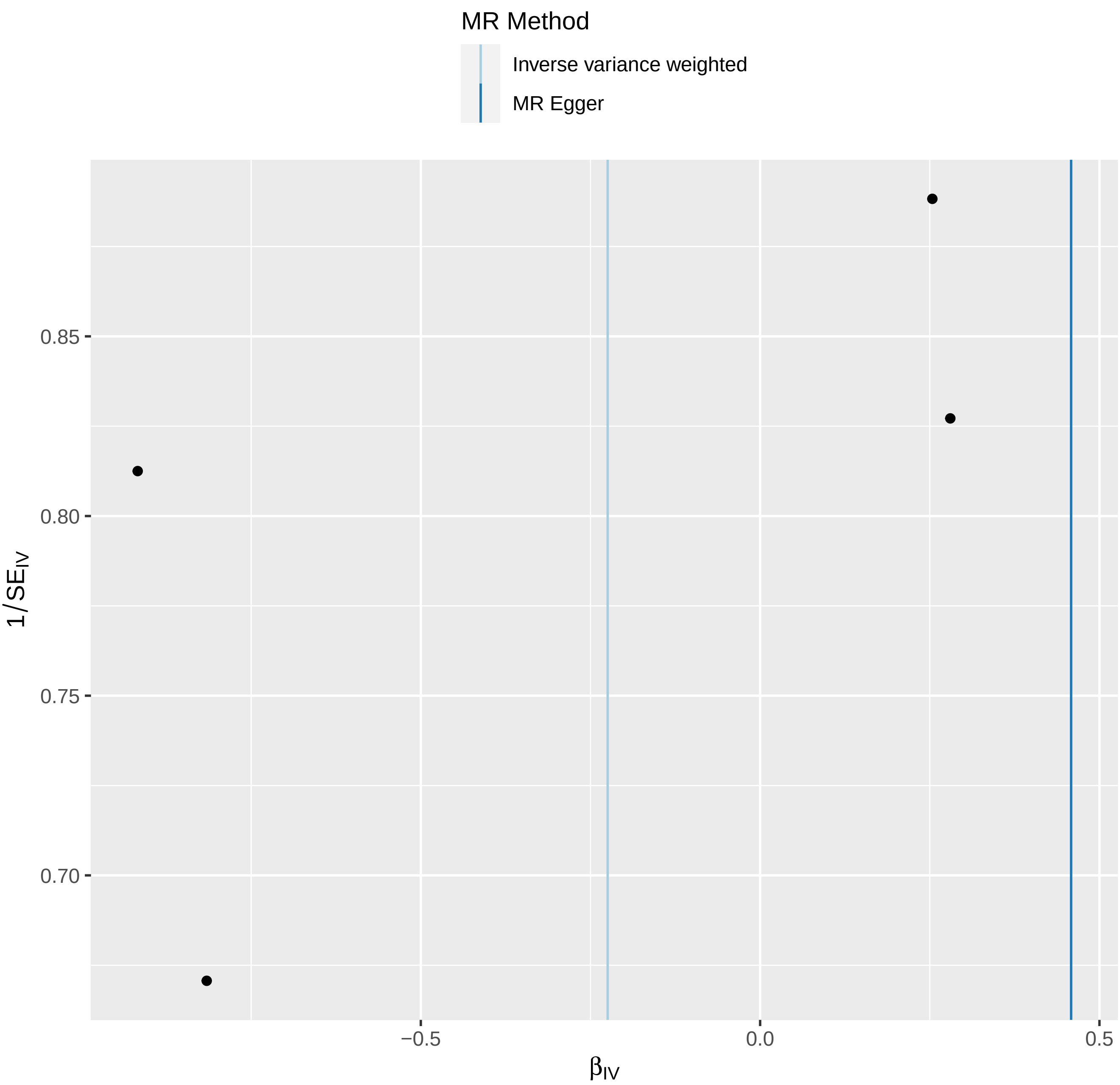

Batch 748 : Gut microbiota abundance (genus Turicibacter id.2162) on Ulcerative colitis (strict) with PSC

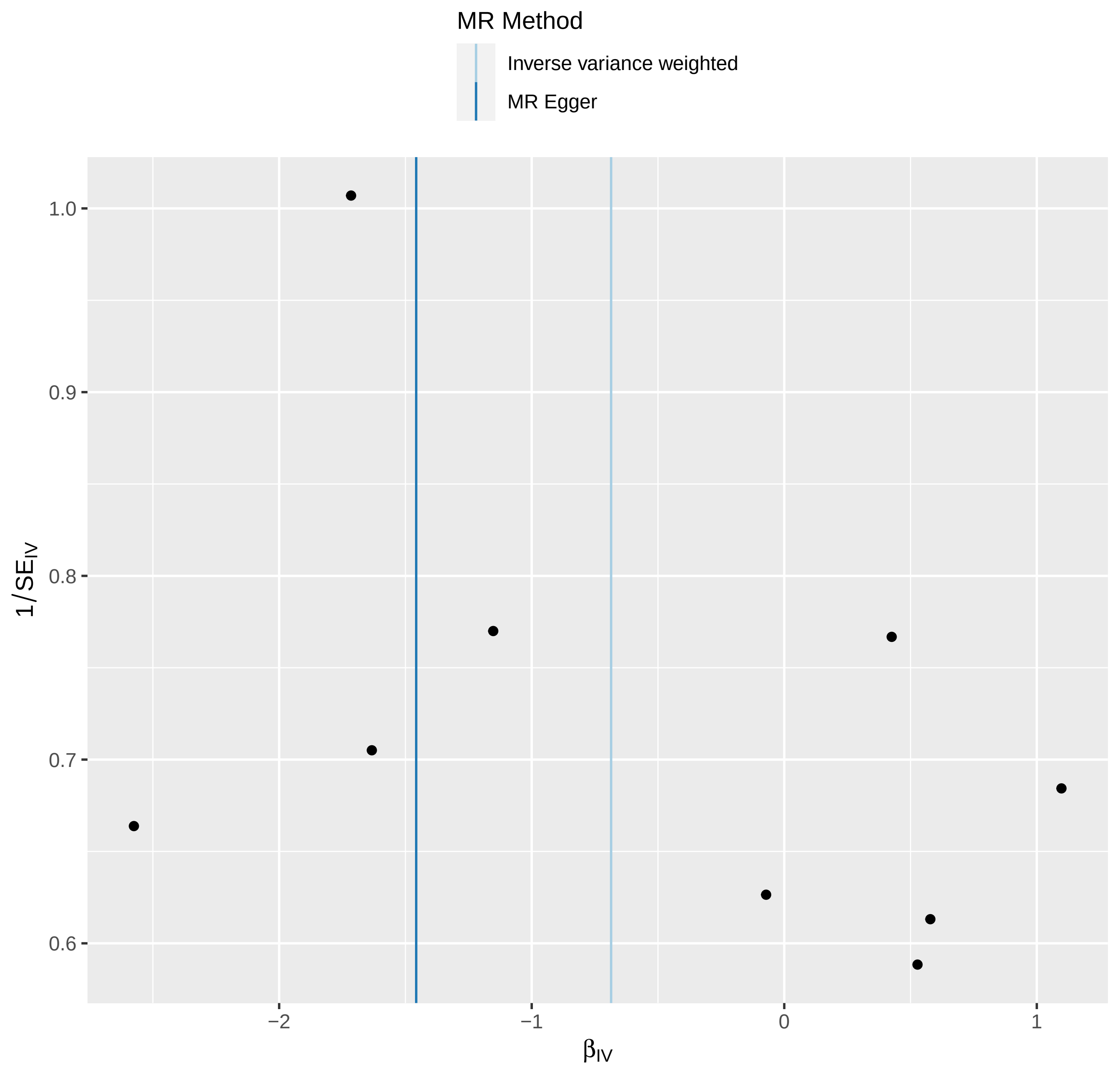

Batch 749 : Gut microbiota abundance (genus Tyzzerella3 id.11335) on Ulcerative colitis (strict) with PSC

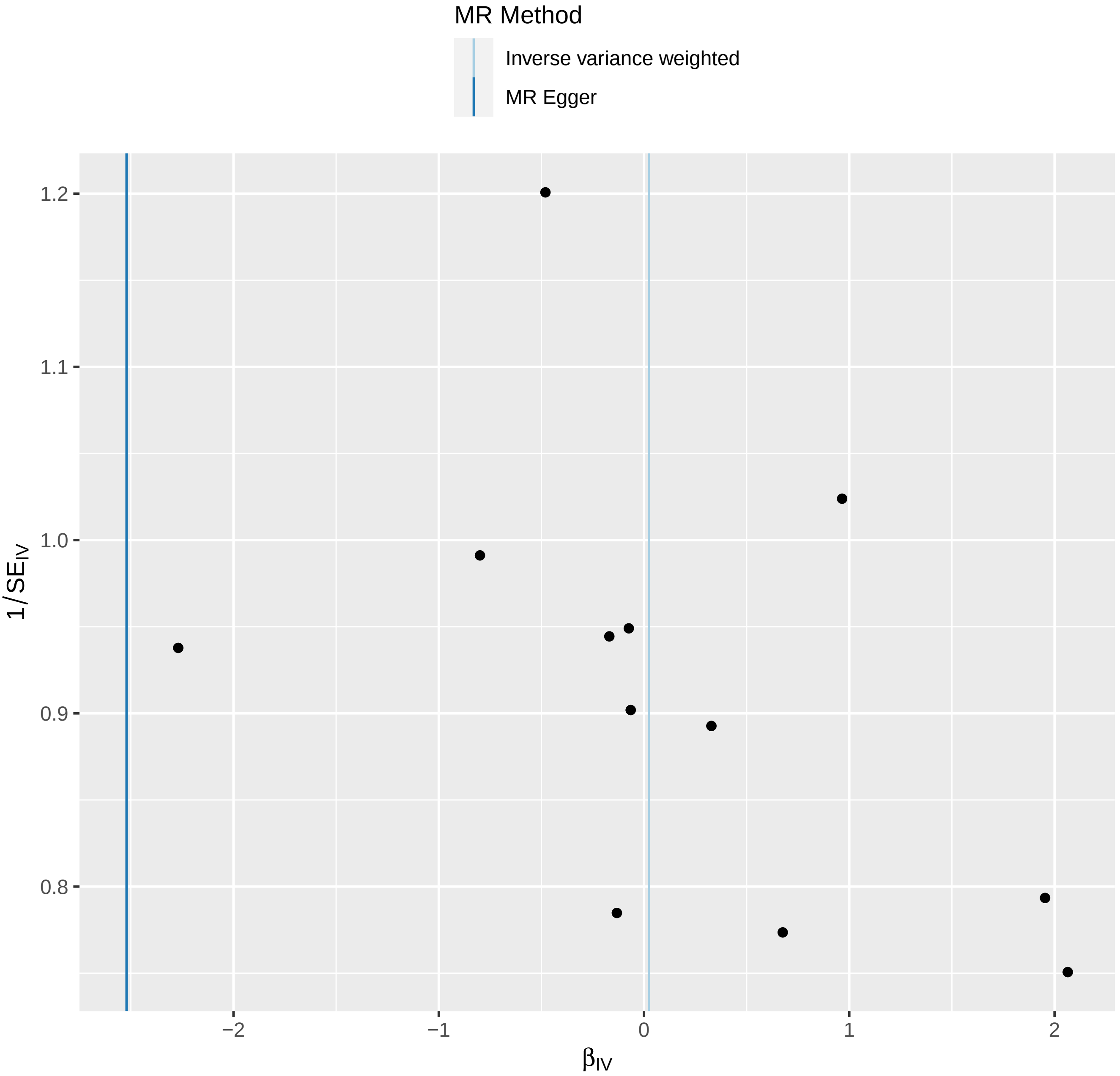

Batch 750 : Gut microbiota abundance (genus Veillonella id.2198) on Ulcerative colitis (strict) with PSC

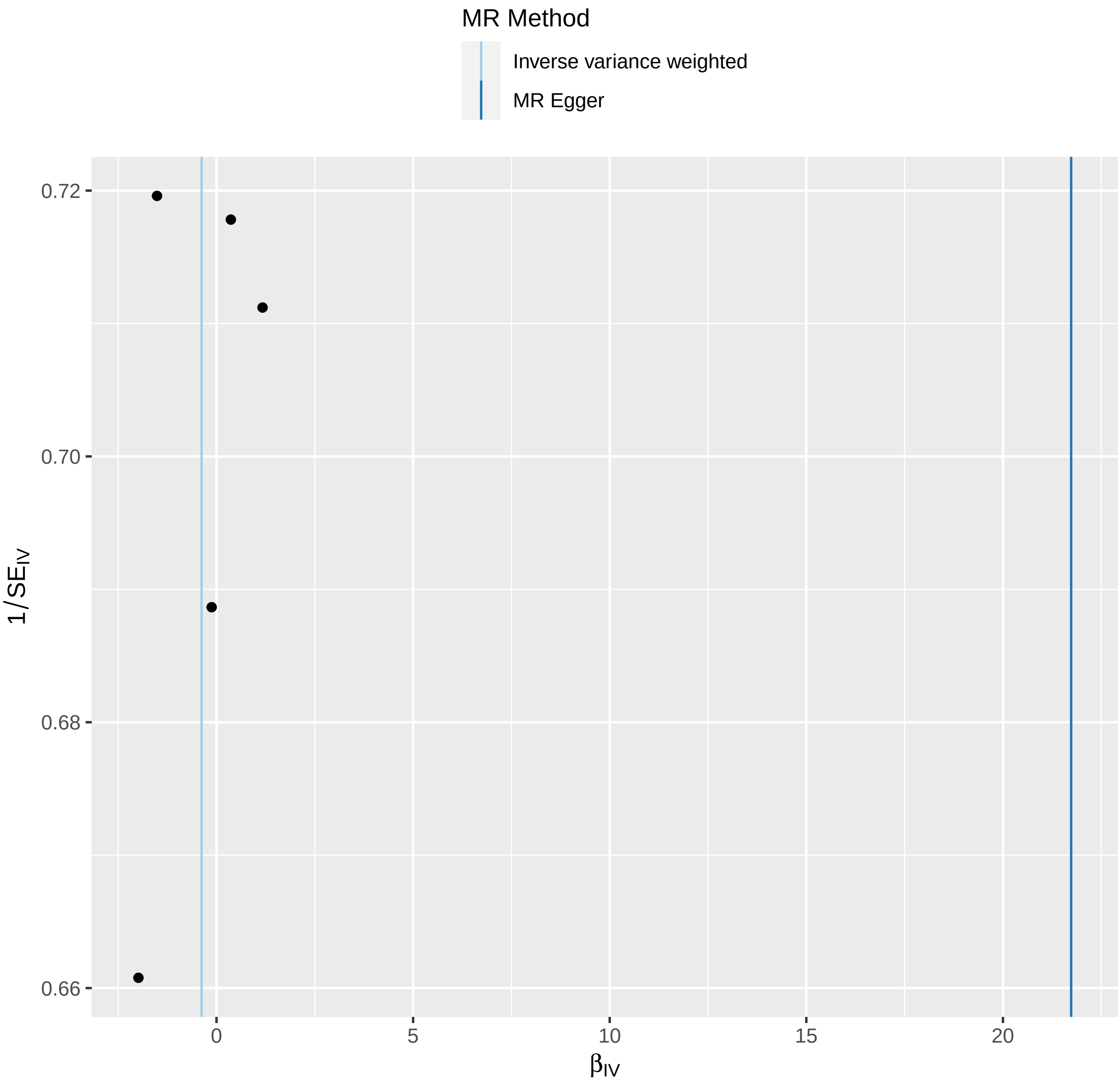

Batch 751 : Gut microbiota abundance (family Acidaminococcaceae id.2166) on Arthropathy in ulcerative colitis

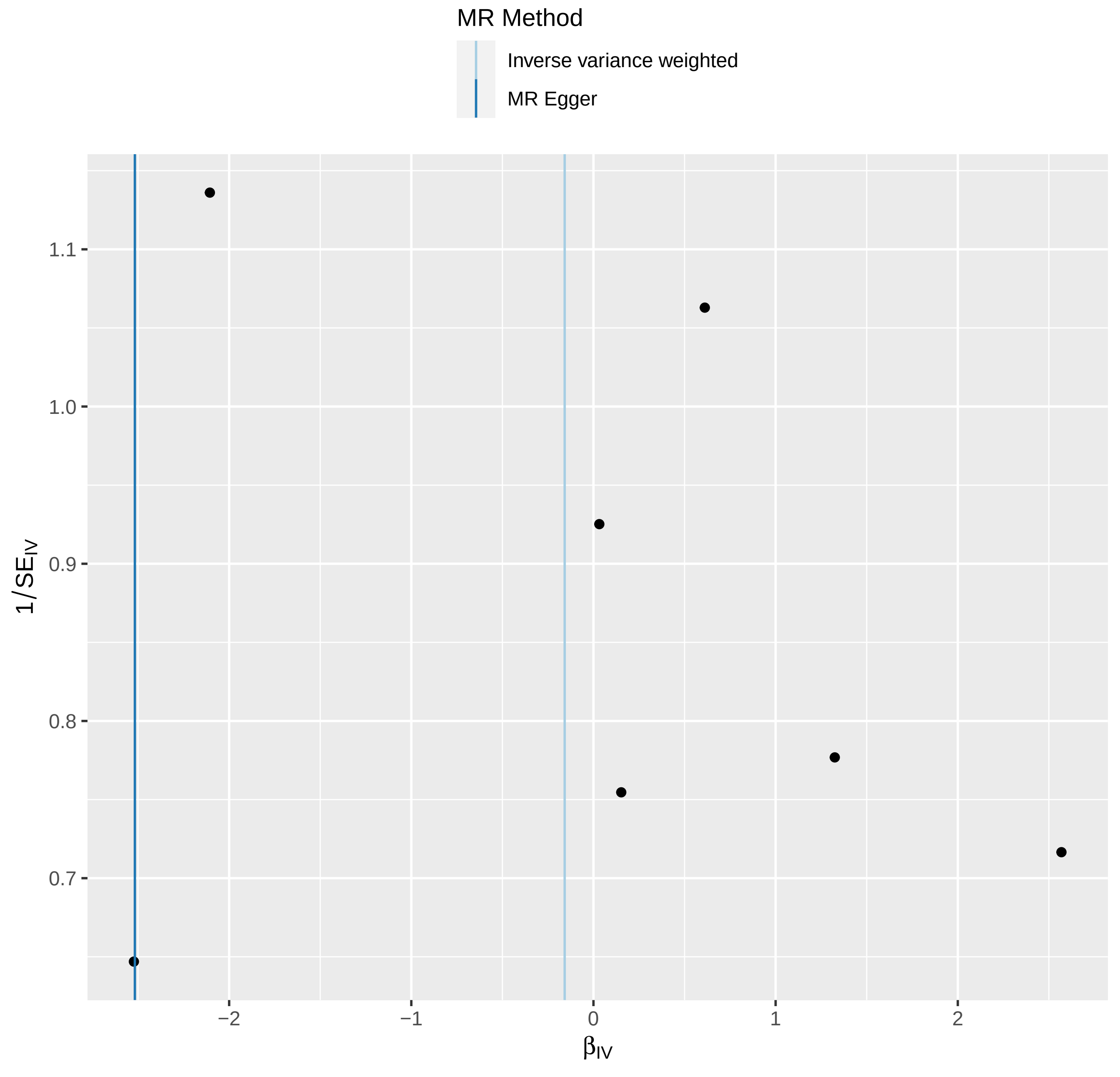

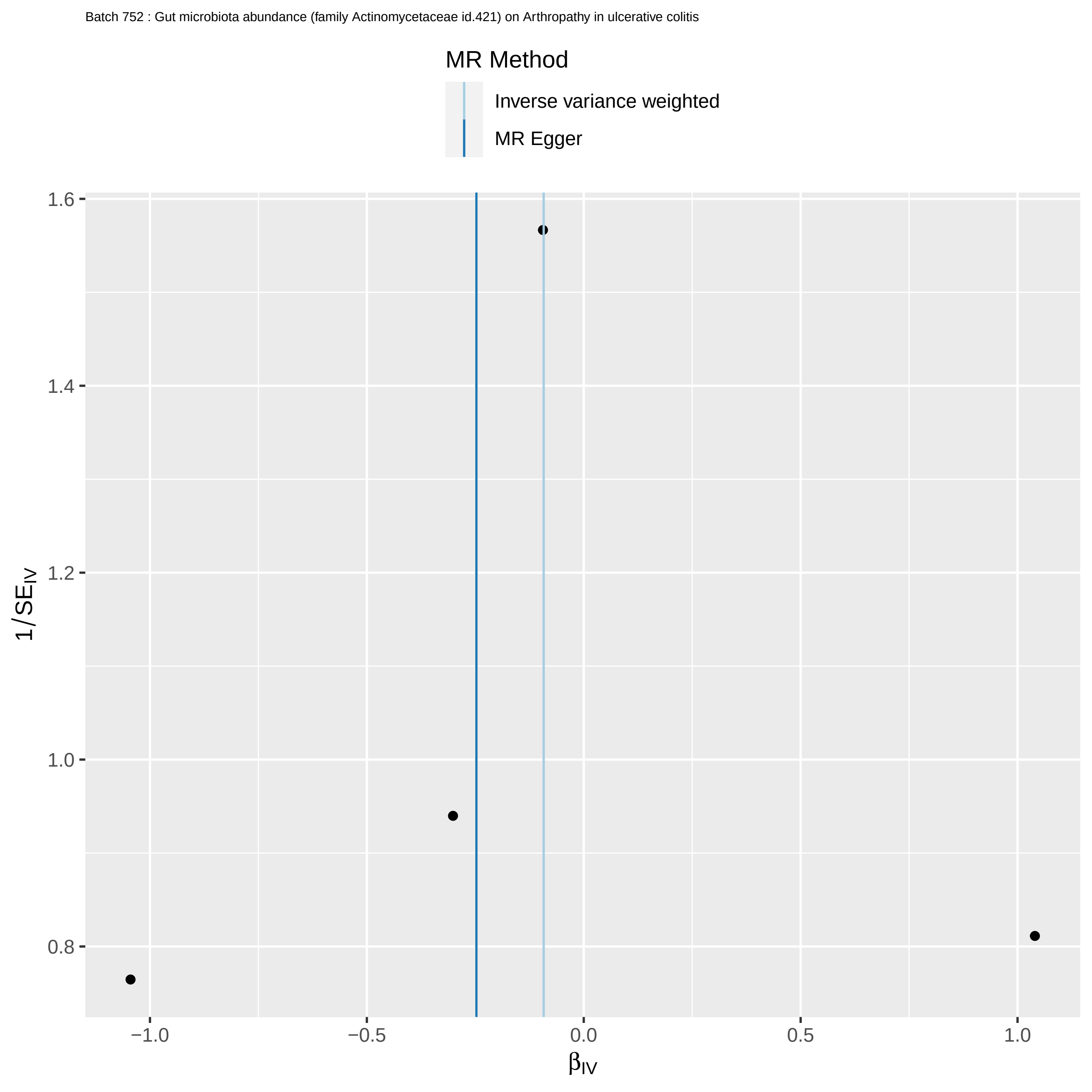

Batch 752 : Gut microbiota abundance (family Actinomycetaceae id.421) on Arthropathy in ulcerative colitis
MR Method
Inverse variance weighted
MR Egger
1.6
1.4
1.2
1.0
0.8
$1/SE_{IV}$
−1.0
−0.5
0.0
0.5
1.0
β_{IV}

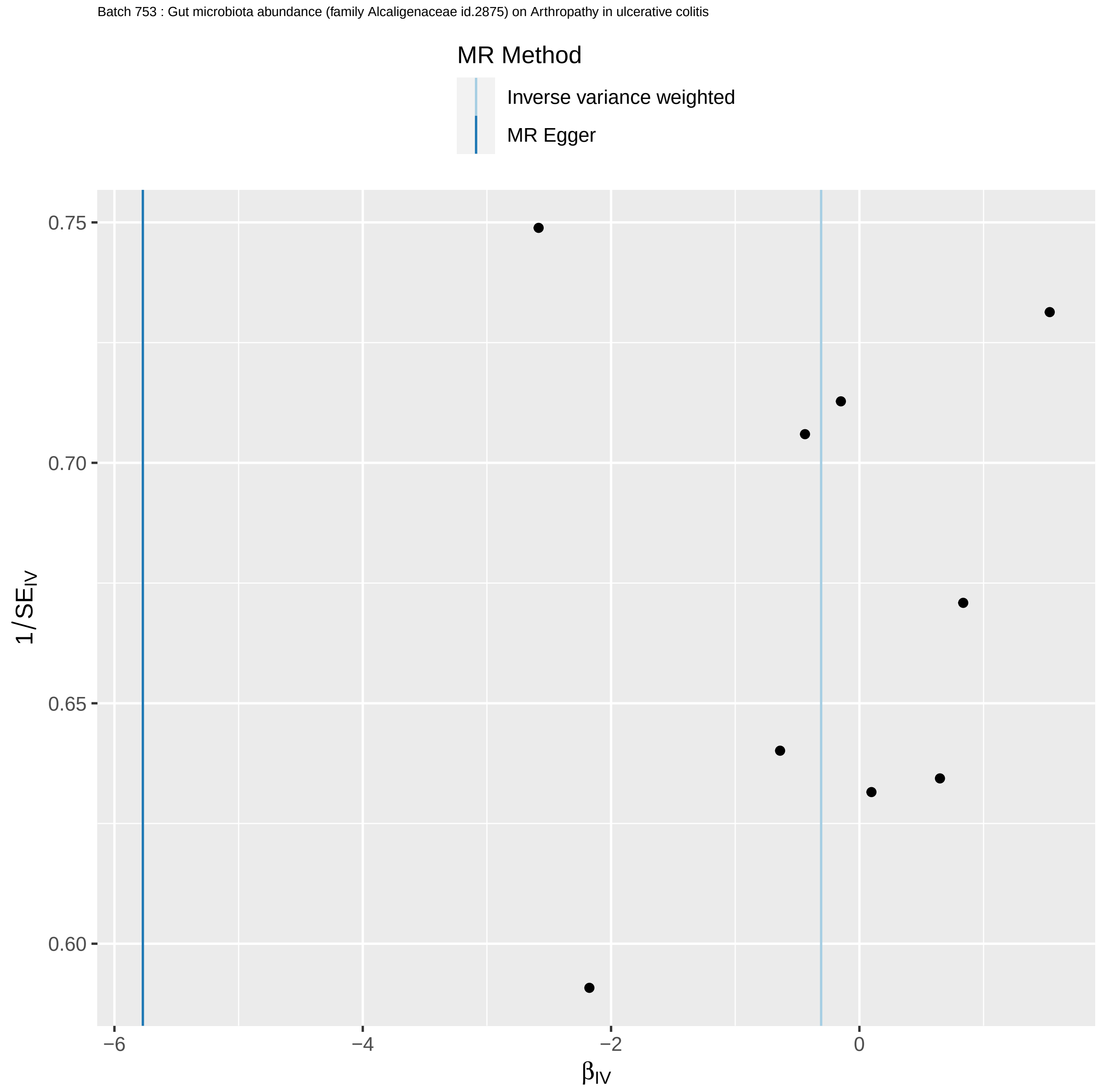
Batch 753 : Gut microbiota abundance (family Alcaligenaceae id.2875) on Arthropathy in ulcerative colitis
MR Method
Inverse variance weighted
MR Egger
0.75
0.70
0.65
0.60
$1/SE_{IV}$
−6
−4
−2
0
β_{IV}

Batch 754 : Gut microbiota abundance (family Bacteroidaceae id.917) on Arthropathy in ulcerative colitis

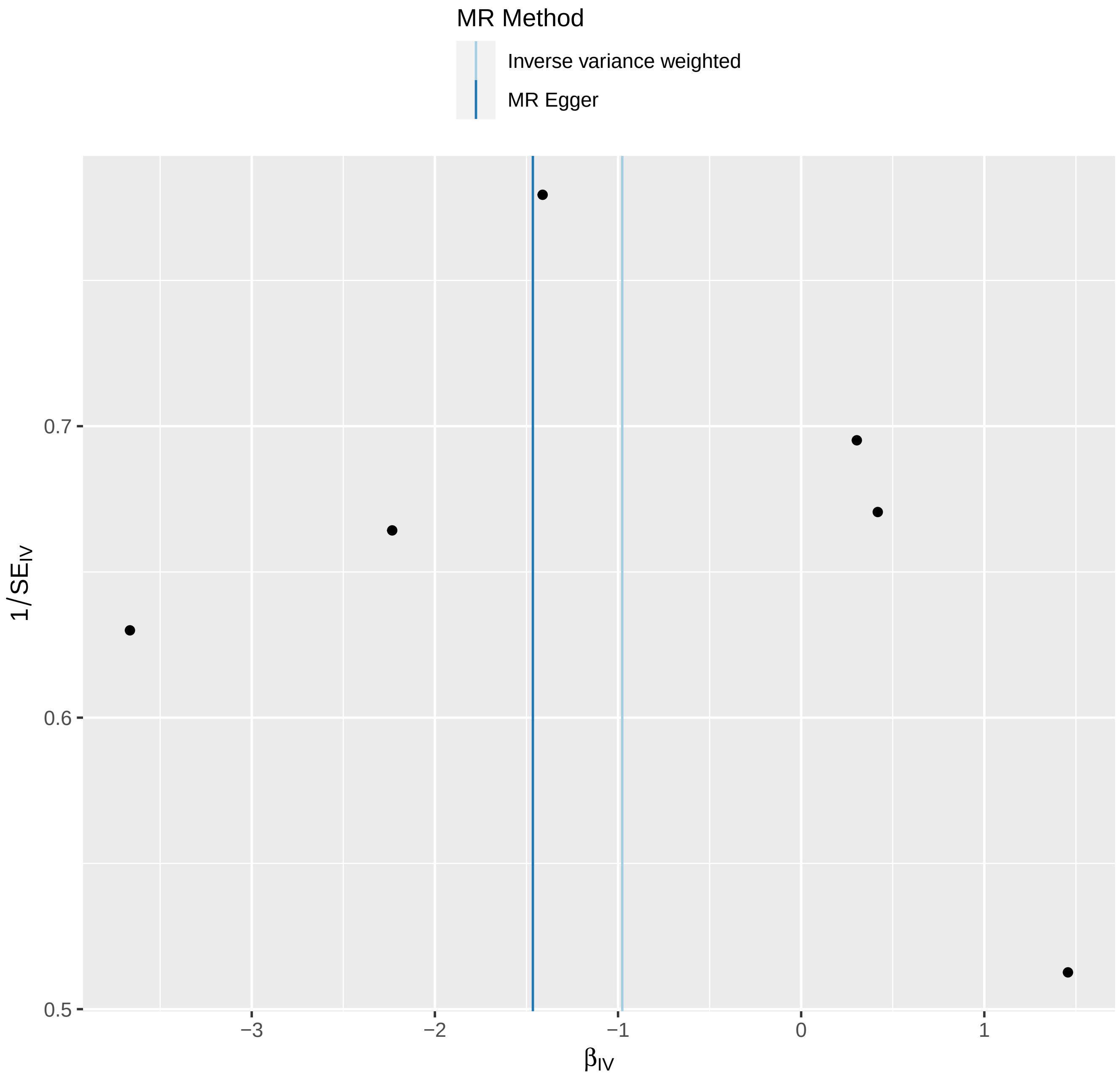

Batch 755 : Gut microbiota abundance (family Bacteroidales S24 7group id.11173) on Arthropathy in ulcerative colitis

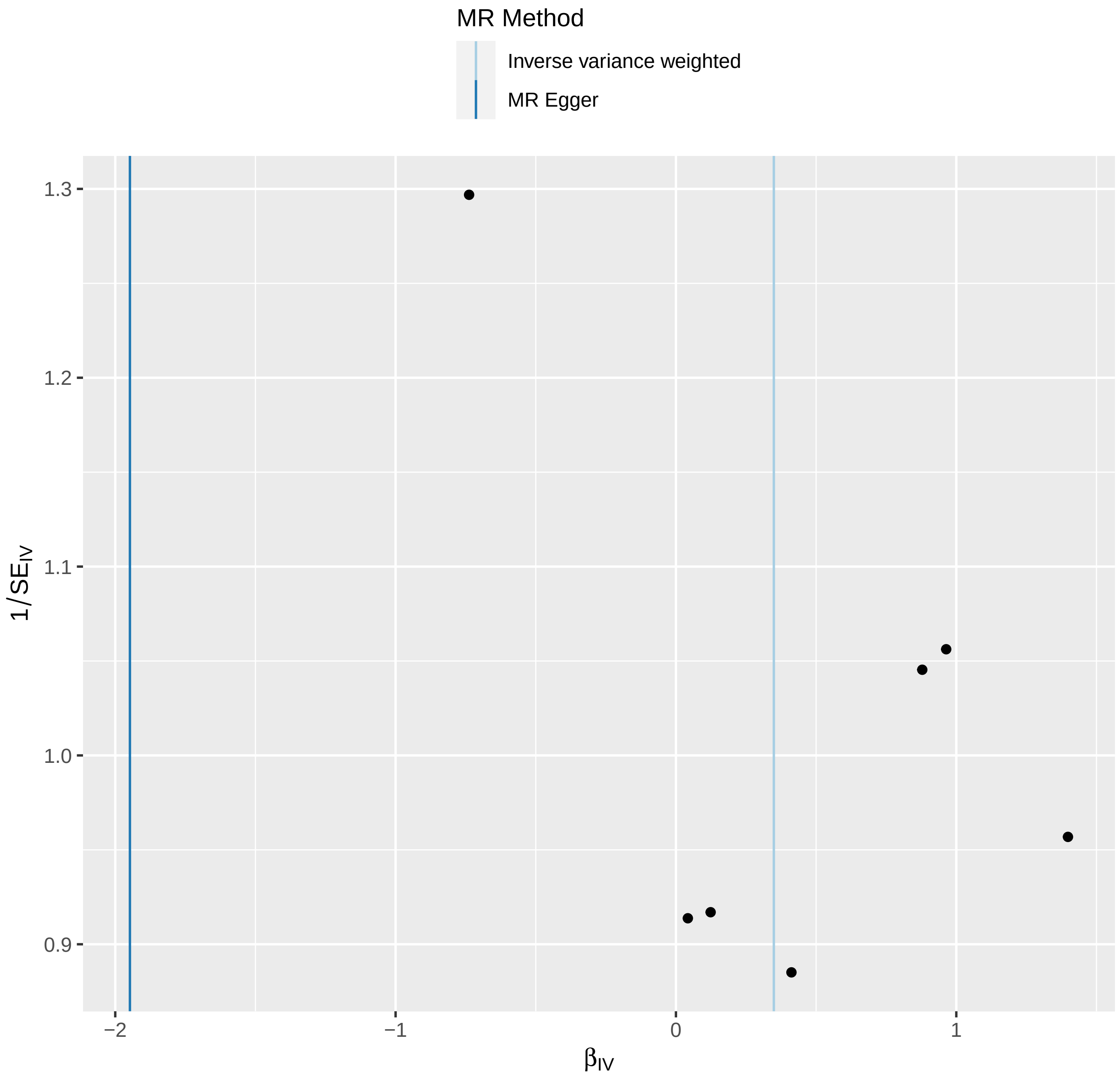

Batch 756 : Gut microbiota abundance (family Bifidobacteriaceae id.433) on Arthropathy in ulcerative colitis

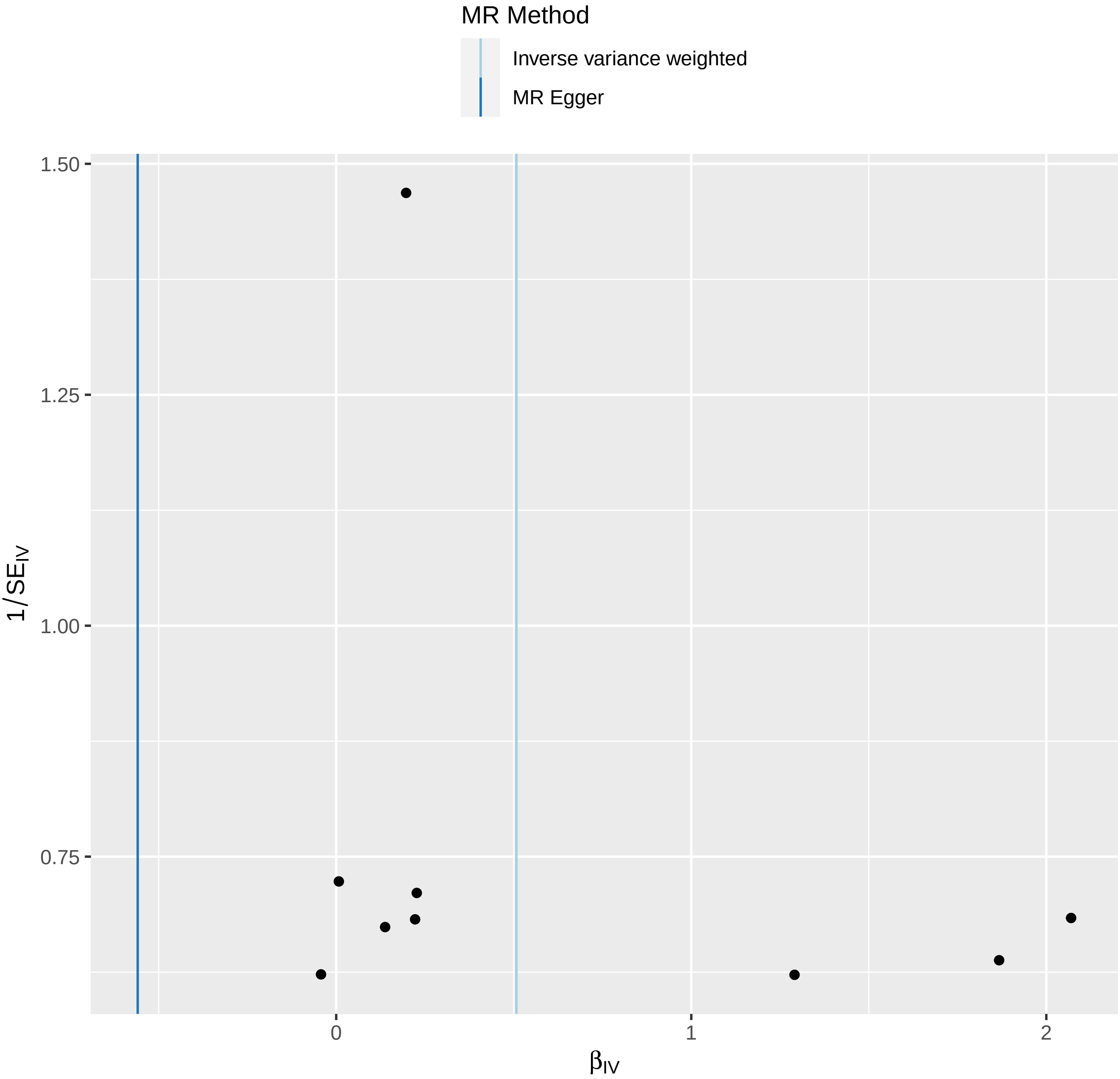

Batch 758 : Gut microbiota abundance (family Clostridiaceae1 id.1869) on Arthropathy in ulcerative colitis

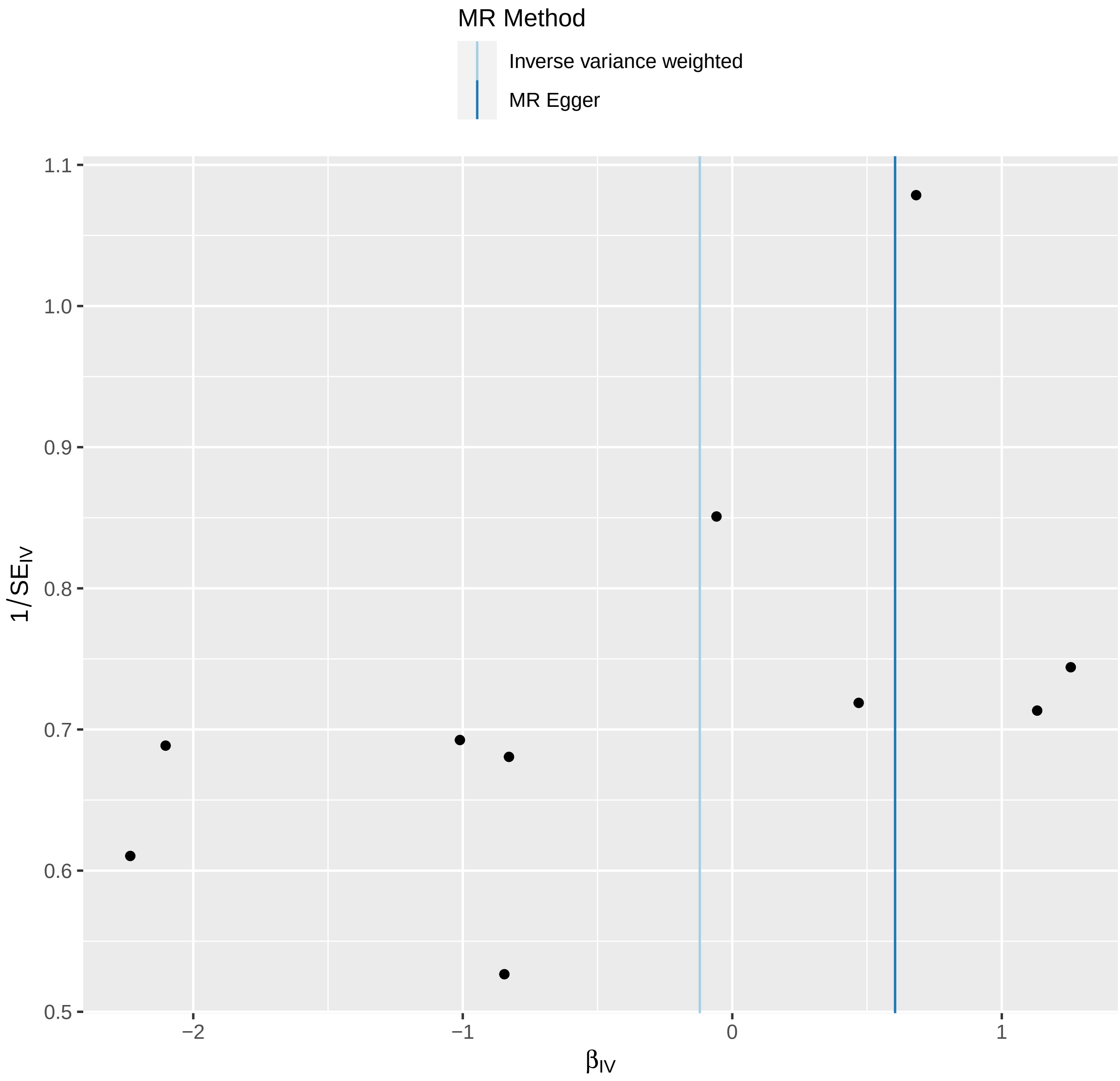

Batch 759 : Gut microbiota abundance (family Clostridiales vadin BB60 group id.11286) on Arthropathy in ulcerative colitis

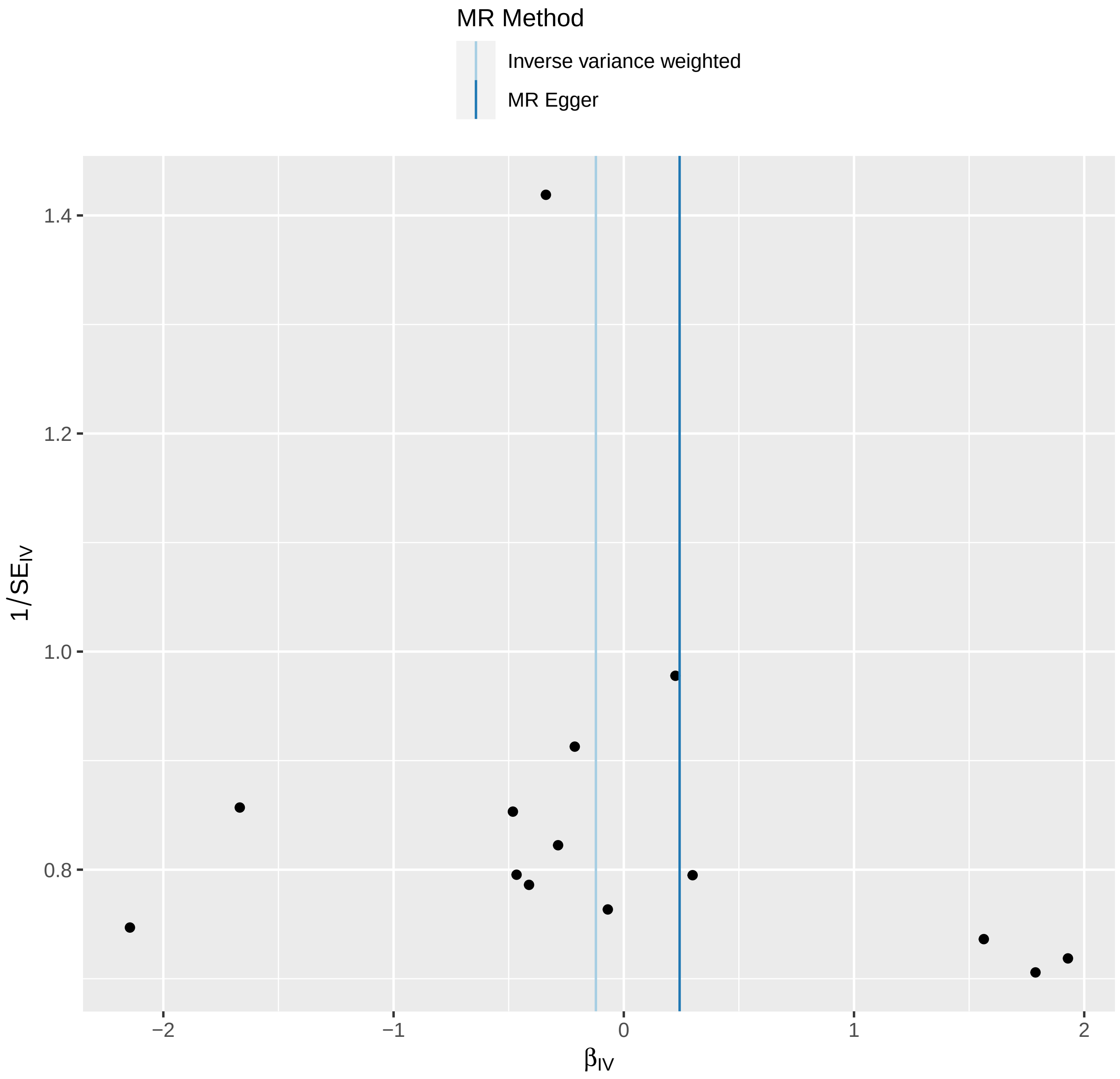

Batch 760 : Gut microbiota abundance (family Coriobacteriaceae id.811) on Arthropathy in ulcerative colitis

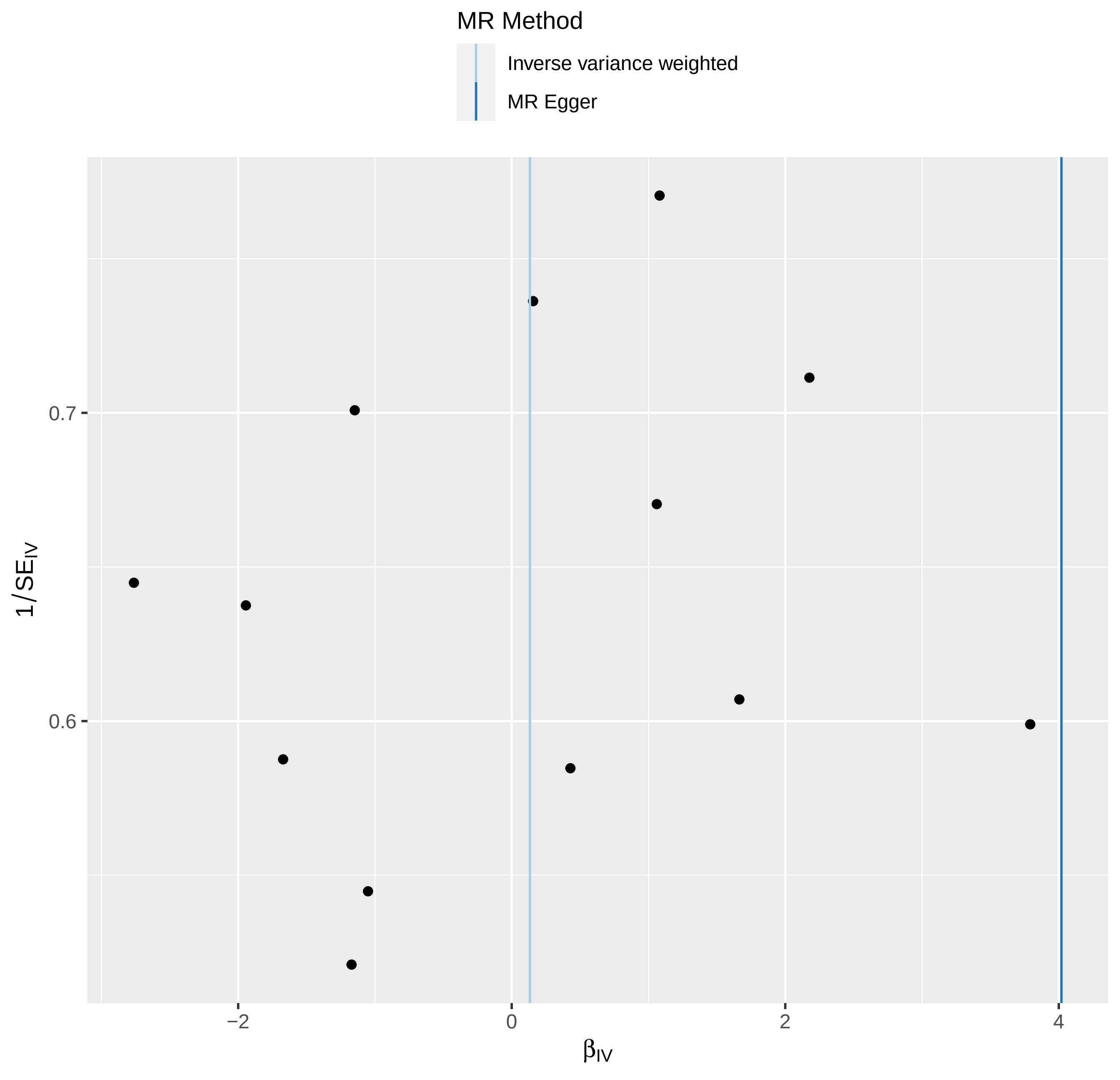

Batch 761 : Gut microbiota abundance (family Defluviitaleaceae id.1924) on Arthropathy in ulcerative colitis

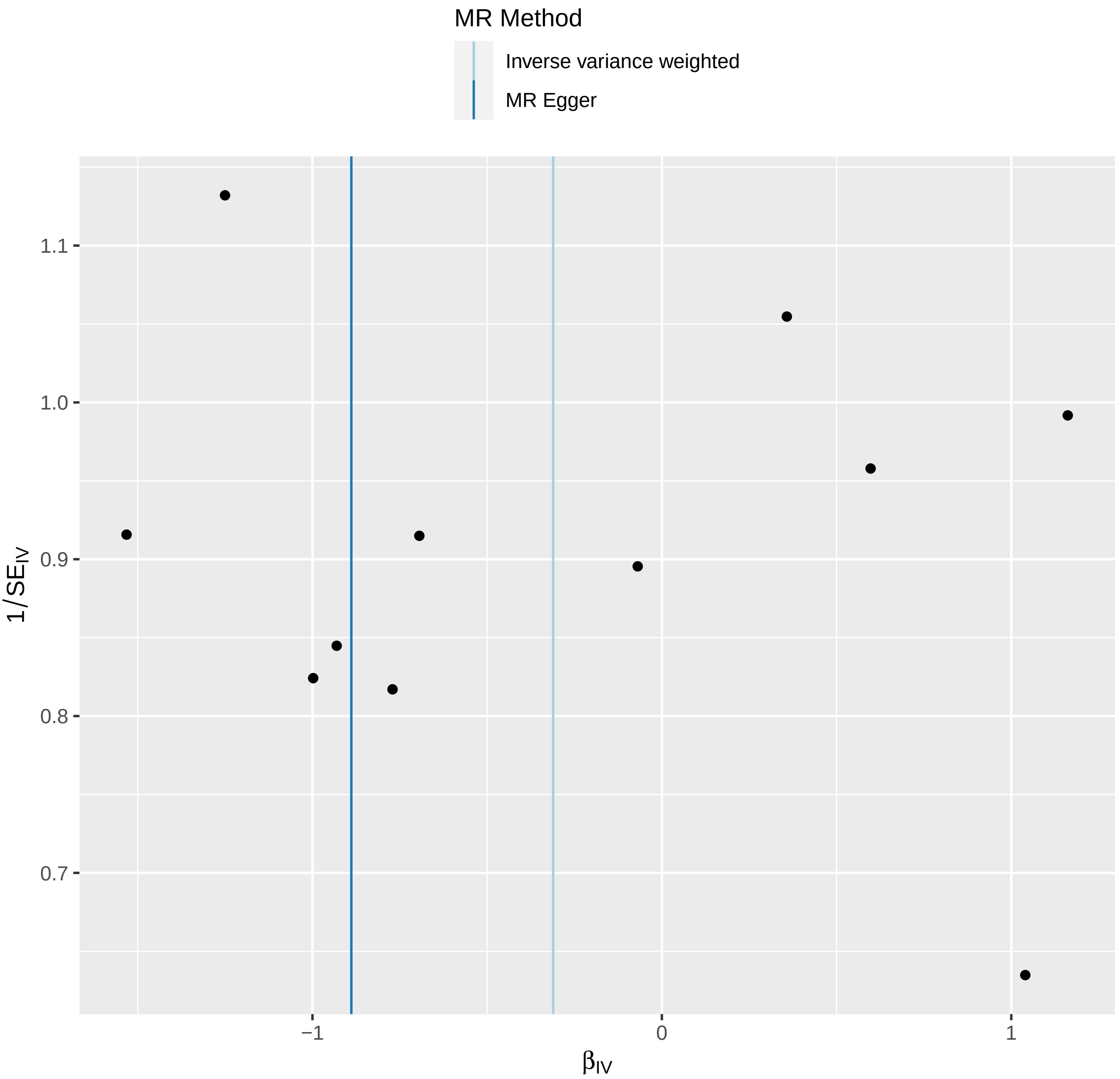

Batch 762 : Gut microbiota abundance (family Desulfovibrionaceae id.3169) on Arthropathy in ulcerative colitis

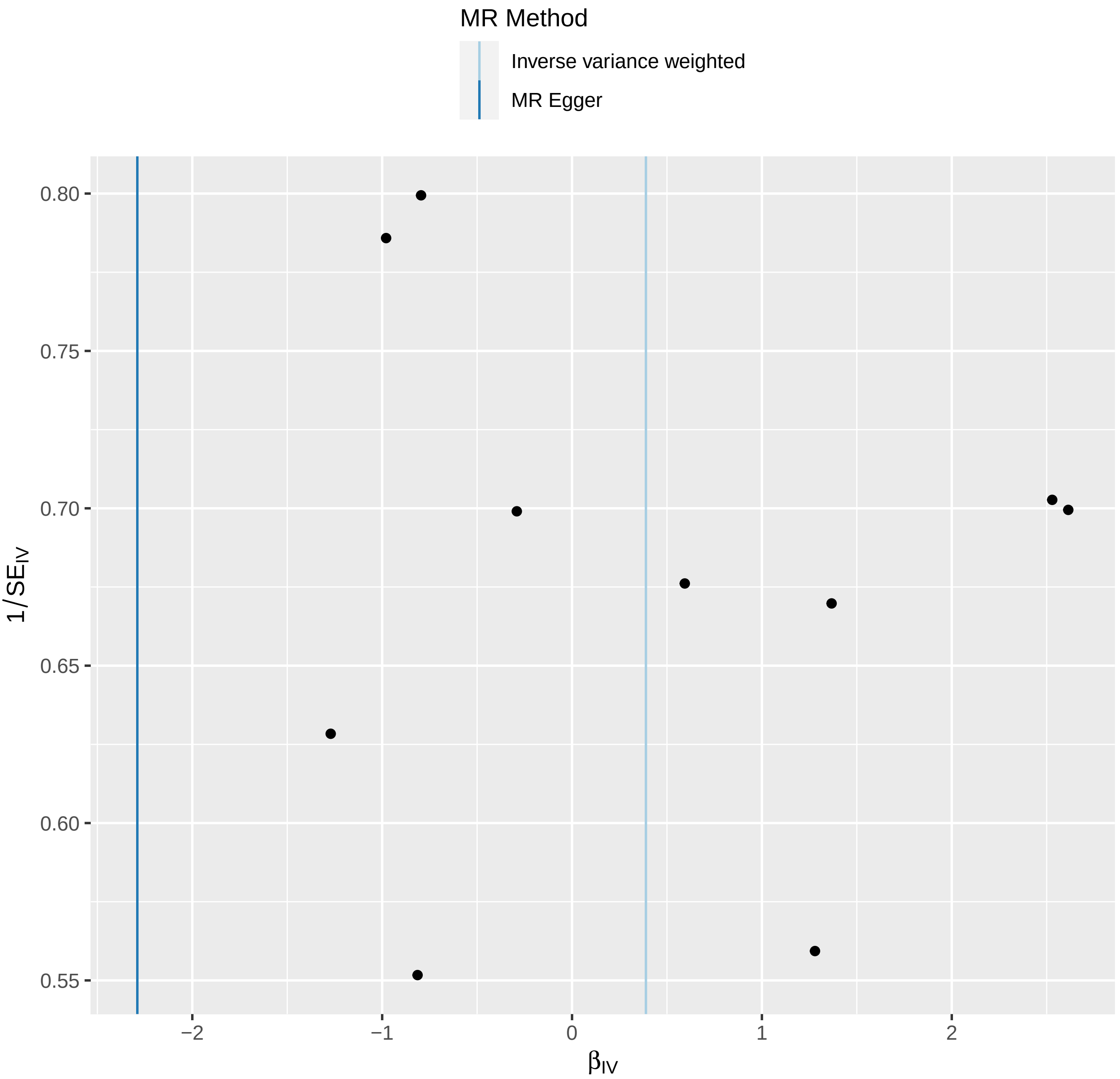

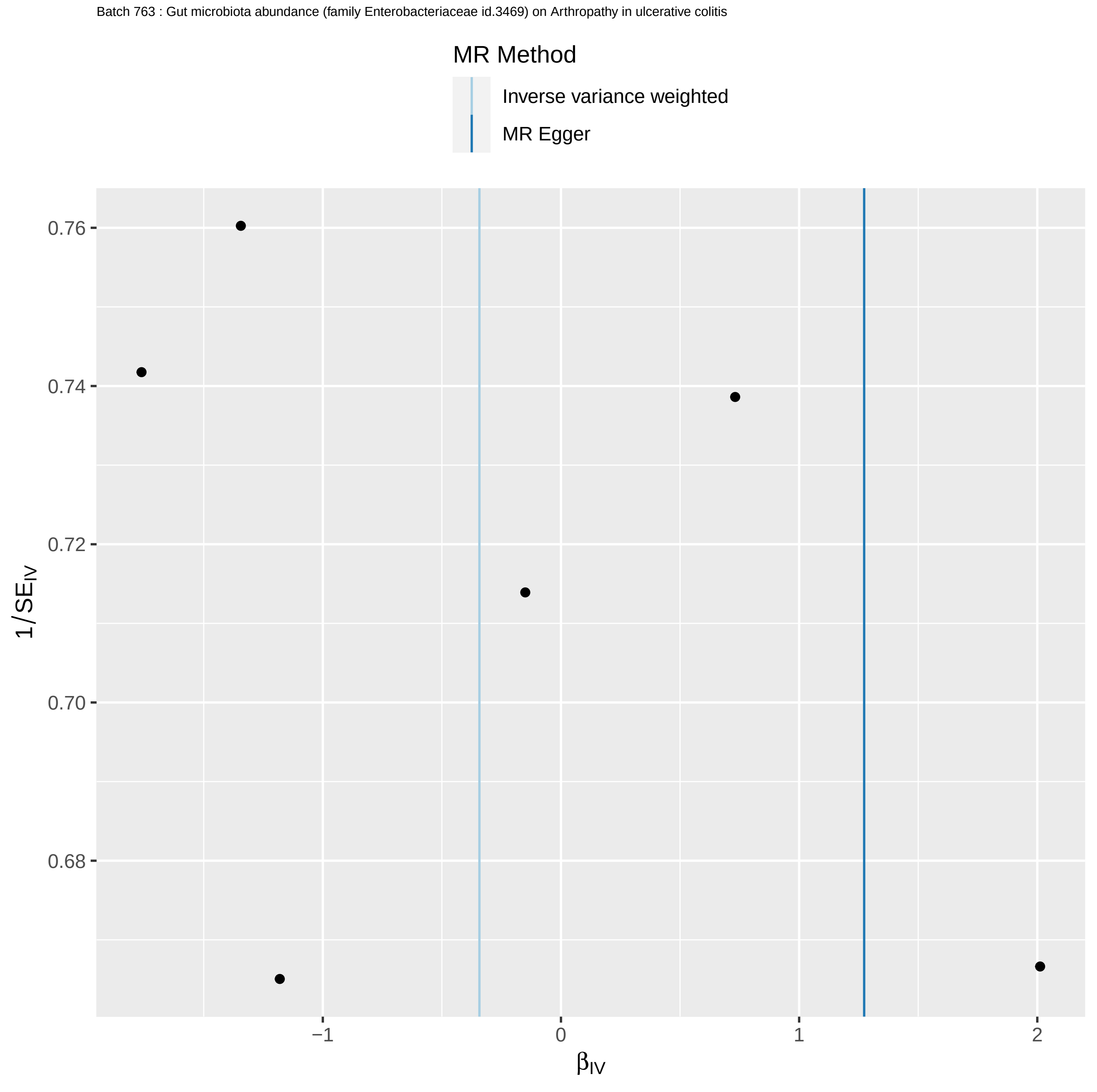
Batch 763 : Gut microbiota abundance (family Enterobacteriaceae id.3469) on Arthropathy in ulcerative colitis
MR Method
Inverse variance weighted
MR Egger
0.76
0.74
0.72
0.70
0.68
1/SE_IV
−1
0
1
2
β_IV

Batch 764 : Gut microbiota abundance (family Erysipelotrichaceae id.2149) on Arthropathy in ulcerative colitis

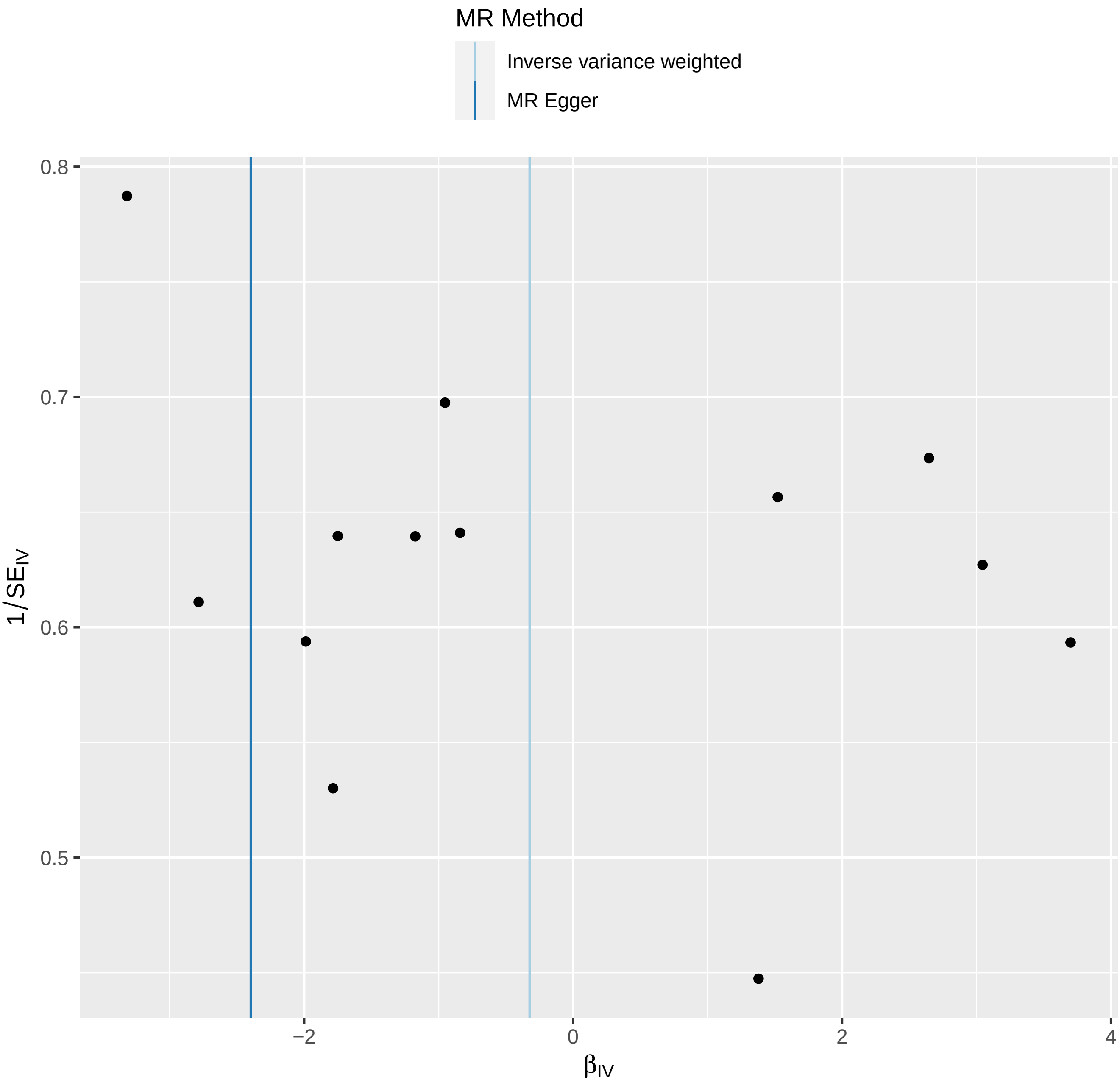

Batch 765 : Gut microbiota abundance (family Family XI id.1936) on Arthropathy in ulcerative colitis

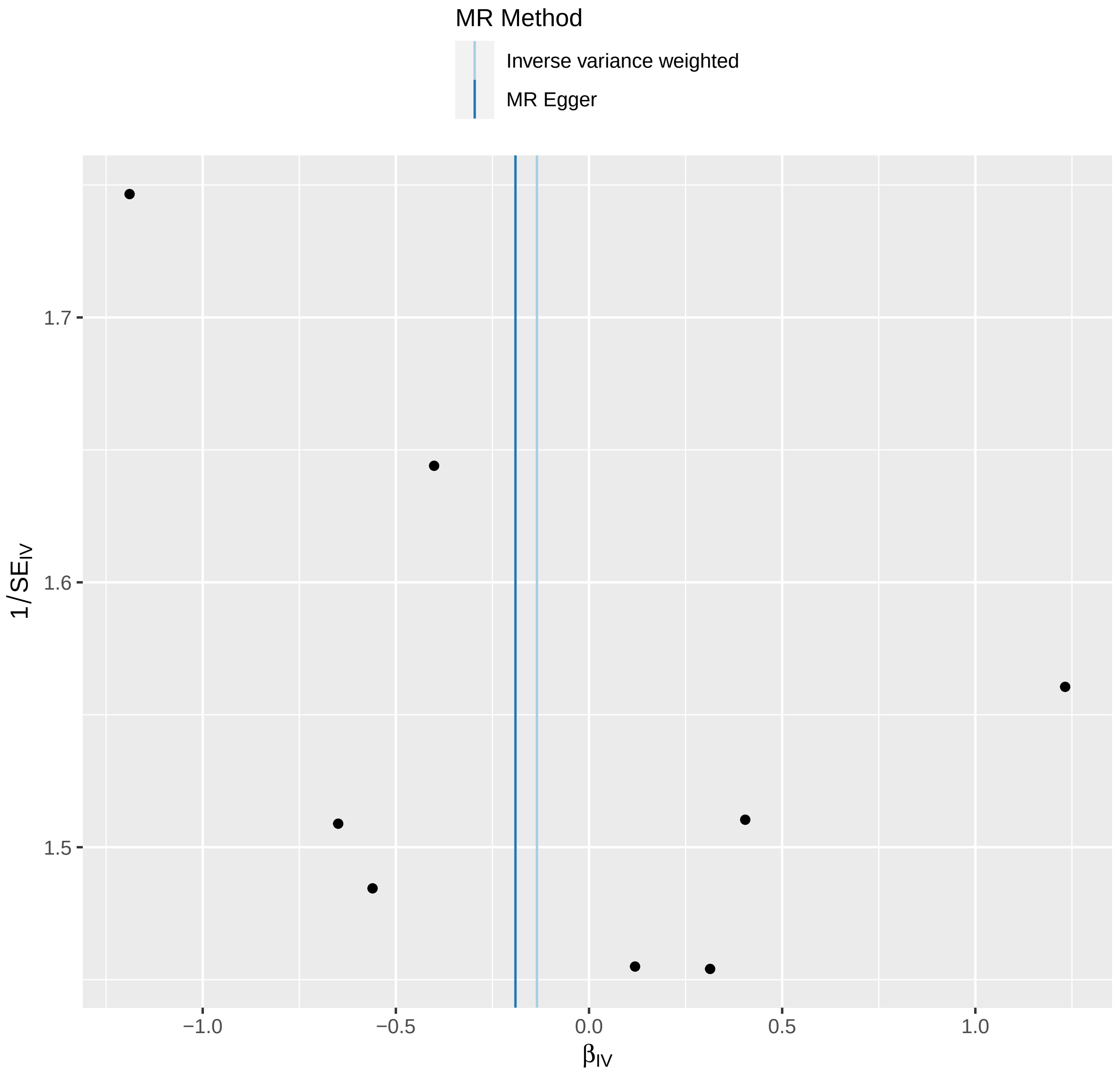

Batch 766 : Gut microbiota abundance (family Family XIII id.1957) on Arthropathy in ulcerative colitis

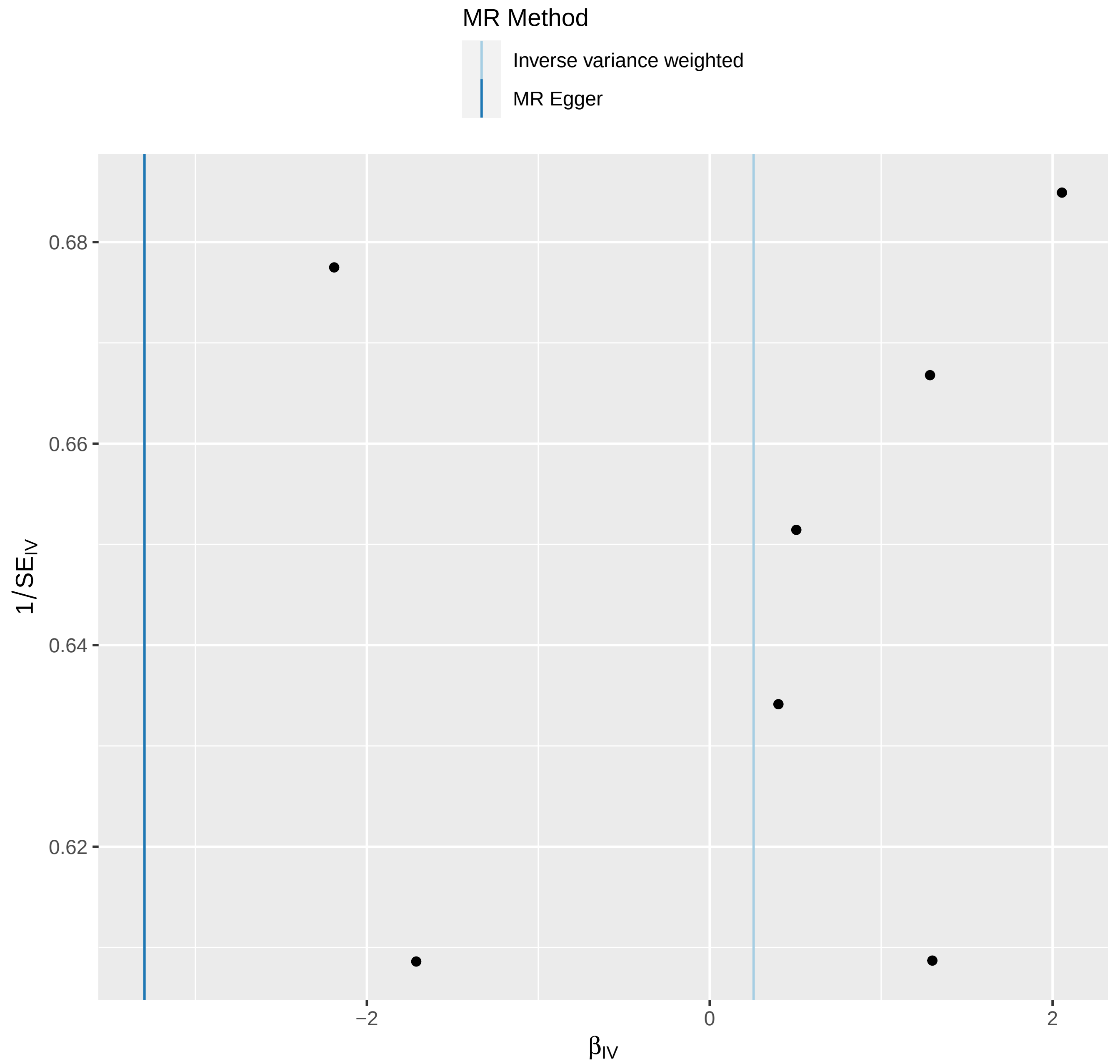

Batch 767 : Gut microbiota abundance (family Lachnospiraceae id.1987) on Arthropathy in ulcerative colitis

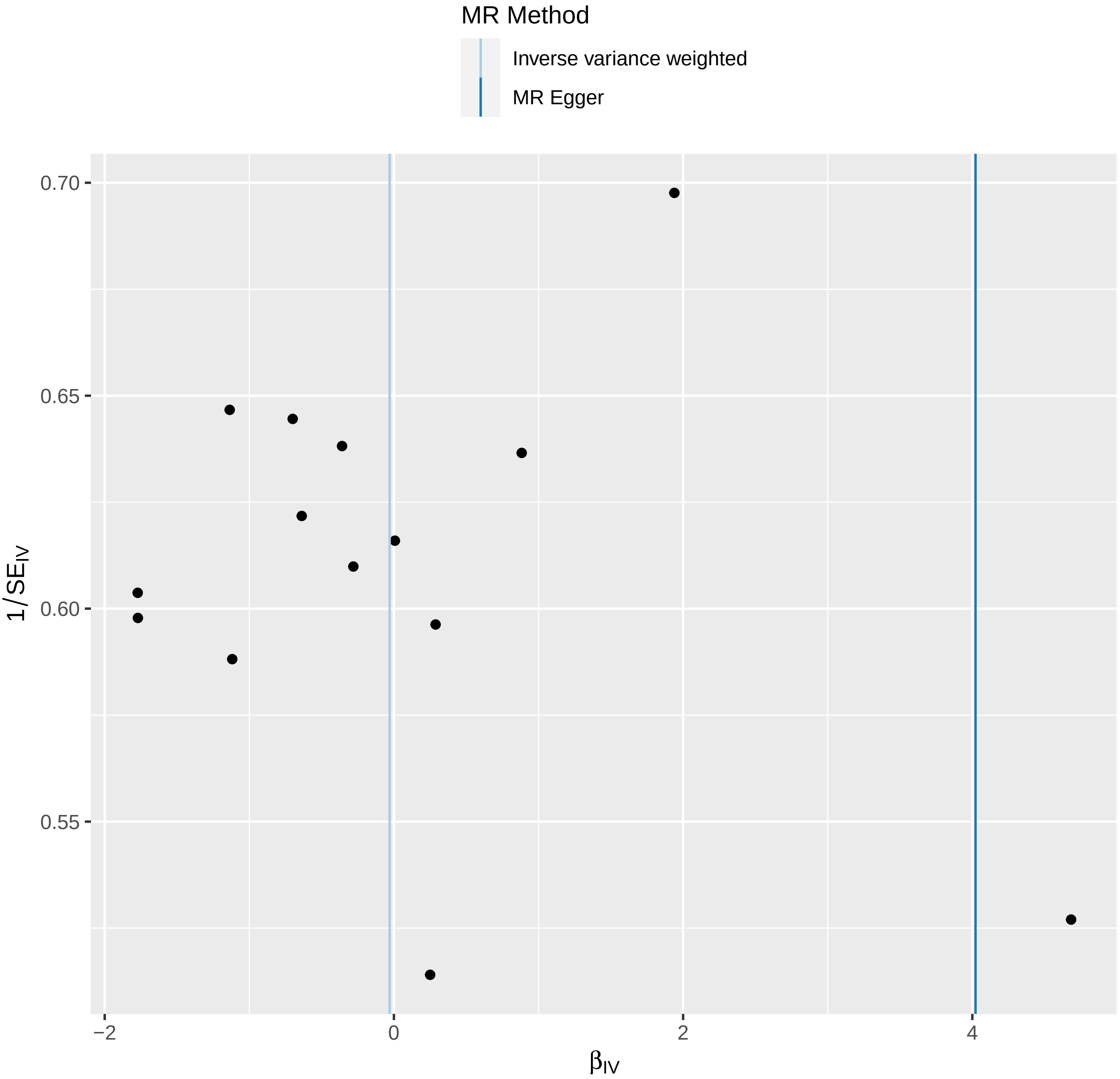

Batch 768 : Gut microbiota abundance (family Lactobacillaceae id.1836) on Arthropathy in ulcerative colitis

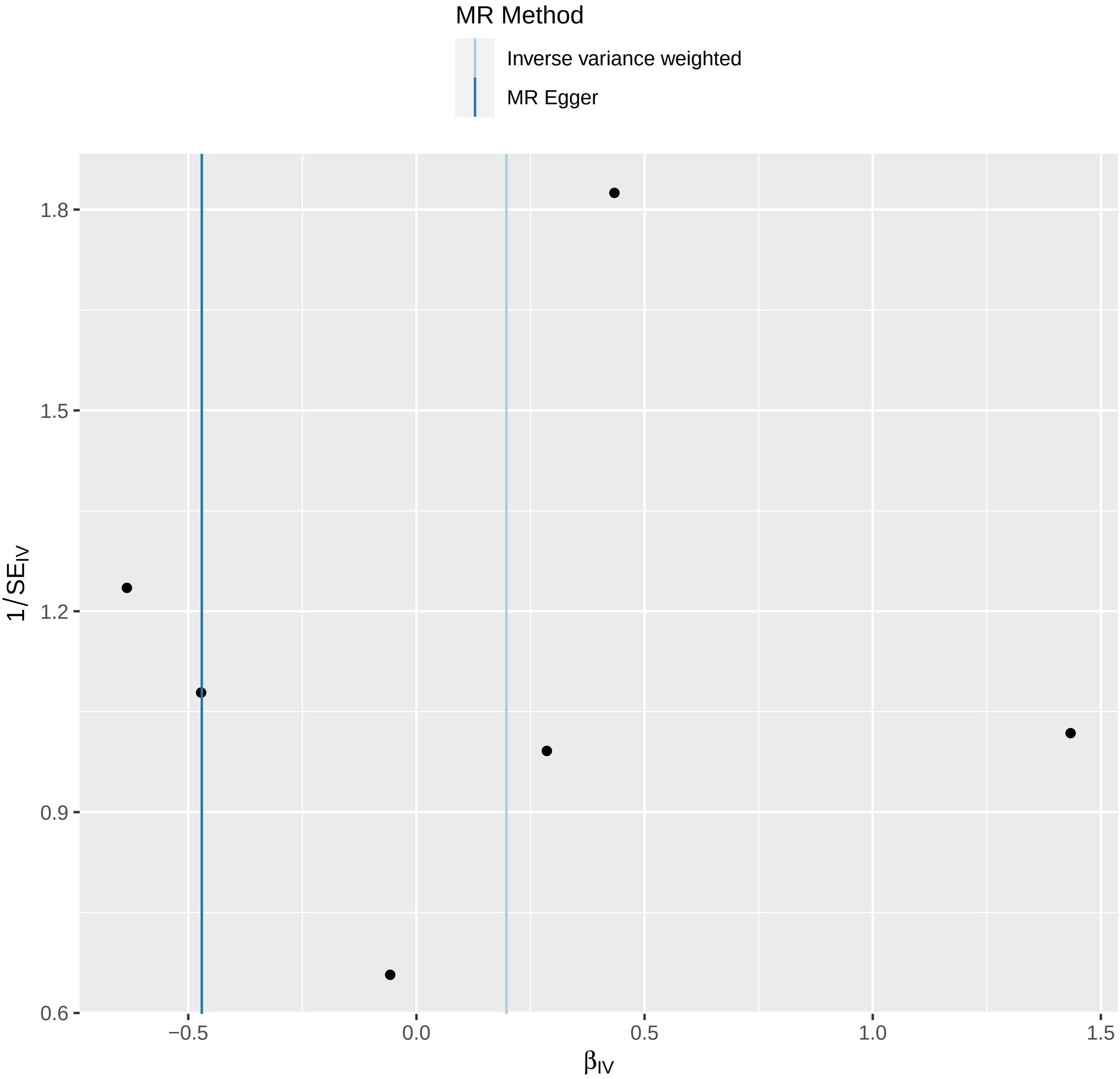

Batch 769 : Gut microbiota abundance (family Methanobacteriaceae id.121) on Arthropathy in ulcerative colitis

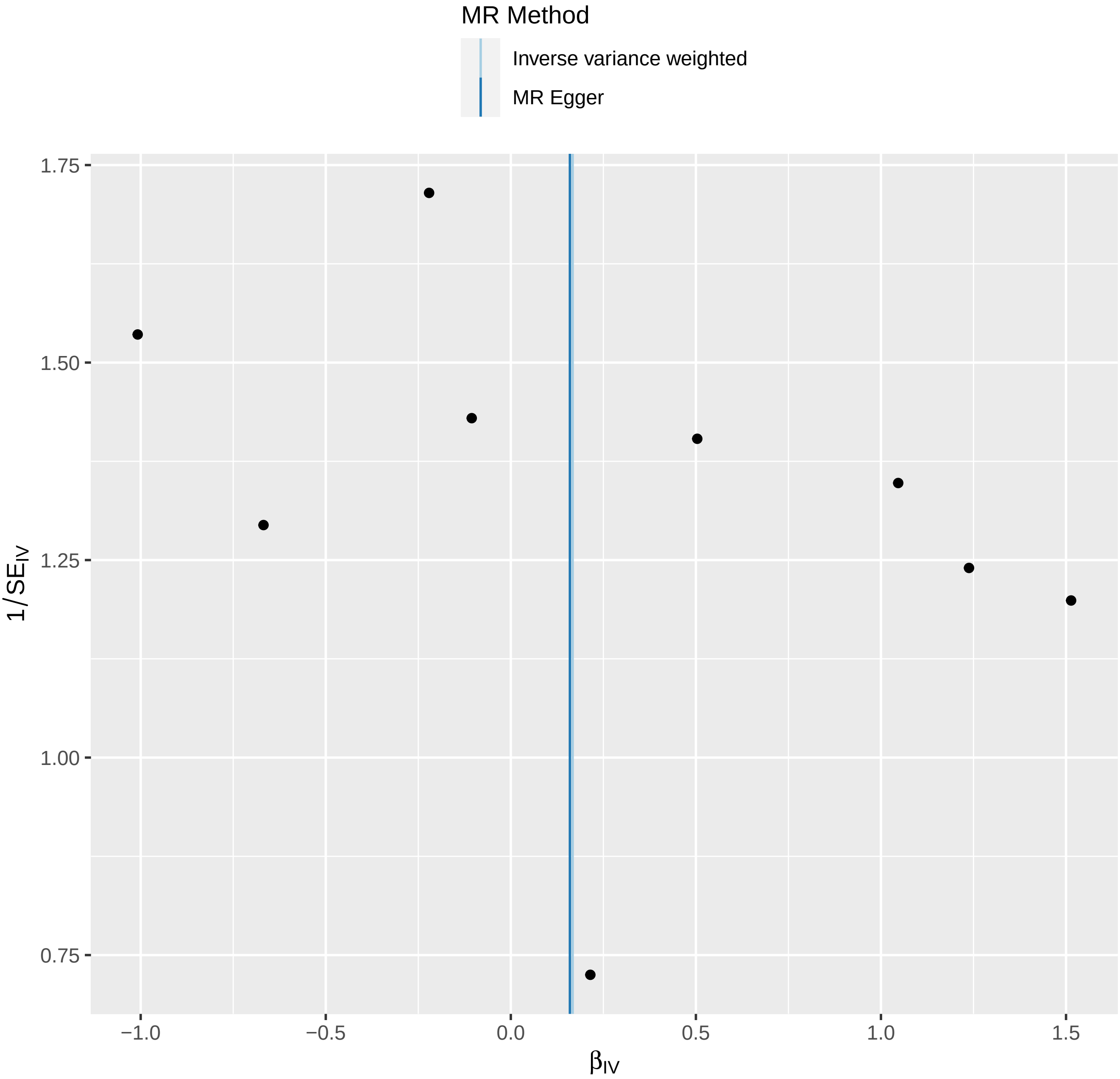

Batch 770 : Gut microbiota abundance (family Oxalobacteraceae id.2966) on Arthropathy in ulcerative colitis

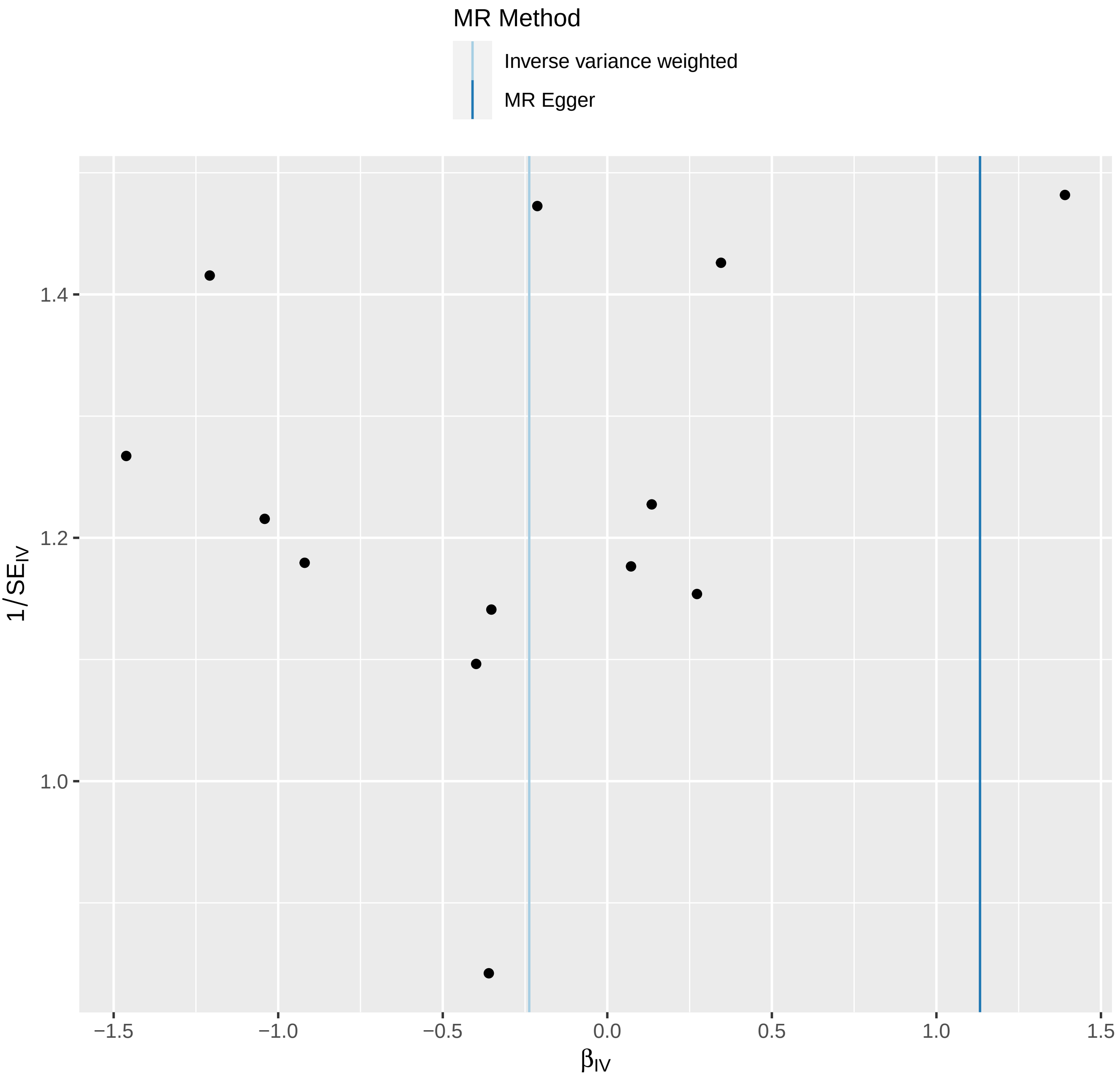

Batch 771 : Gut microbiota abundance (family Pasteurellaceae id.3689) on Arthropathy in ulcerative colitis

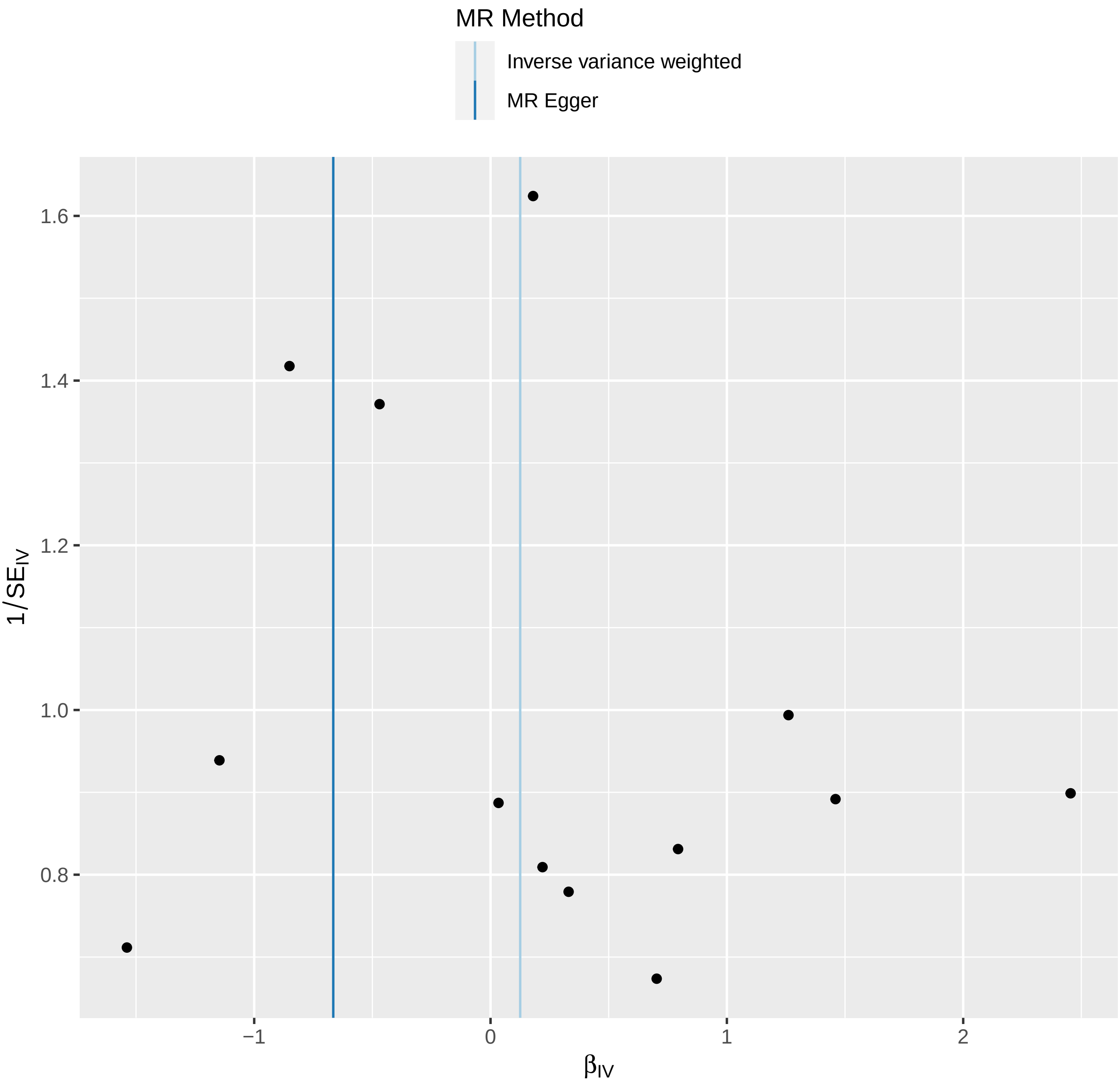

Batch 772 : Gut microbiota abundance (family Peptococcaceae id.2024) on Arthropathy in ulcerative colitis

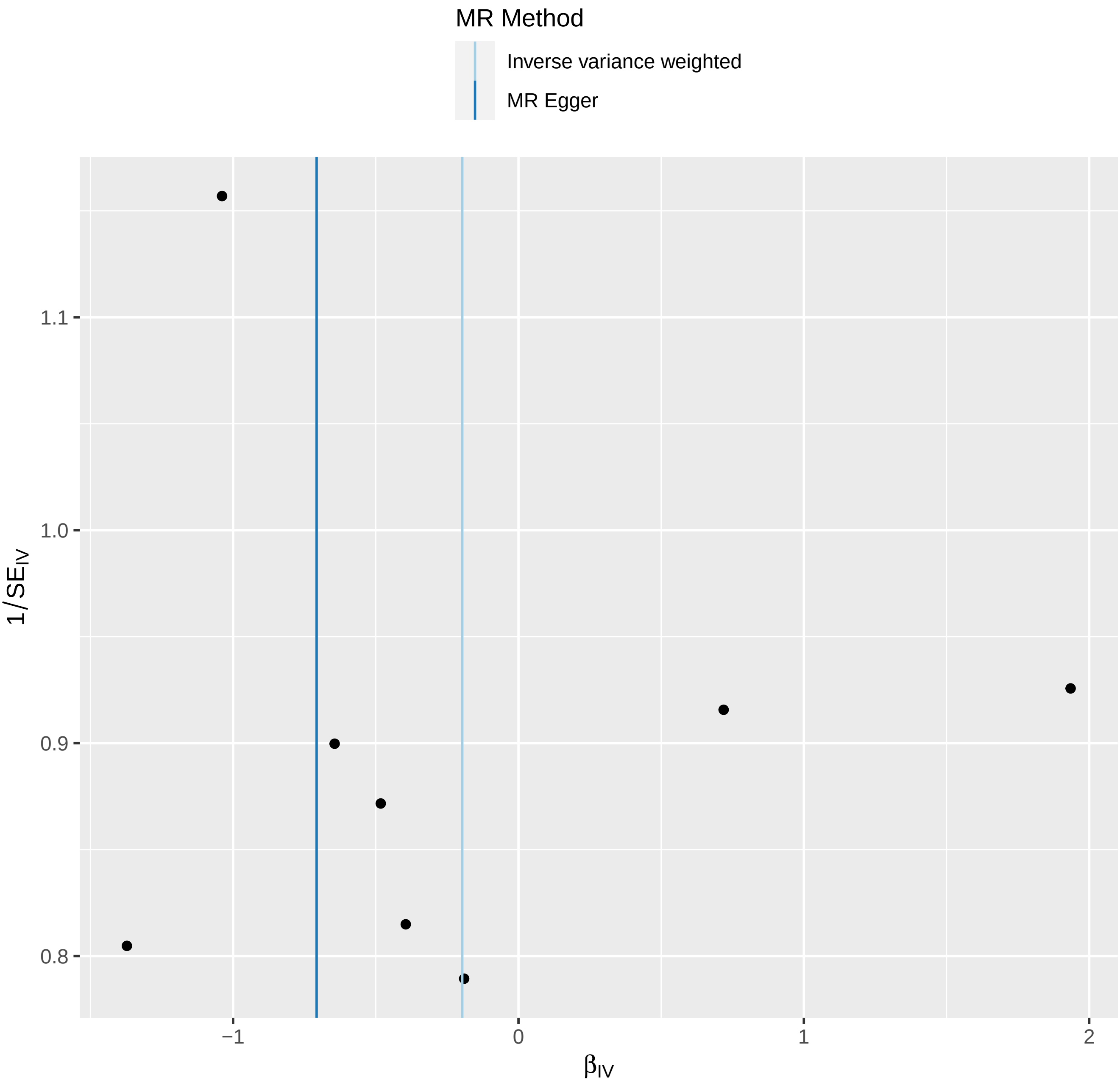

Batch 773 : Gut microbiota abundance (family Peptostreptococcaceae id.2042) on Arthropathy in ulcerative colitis

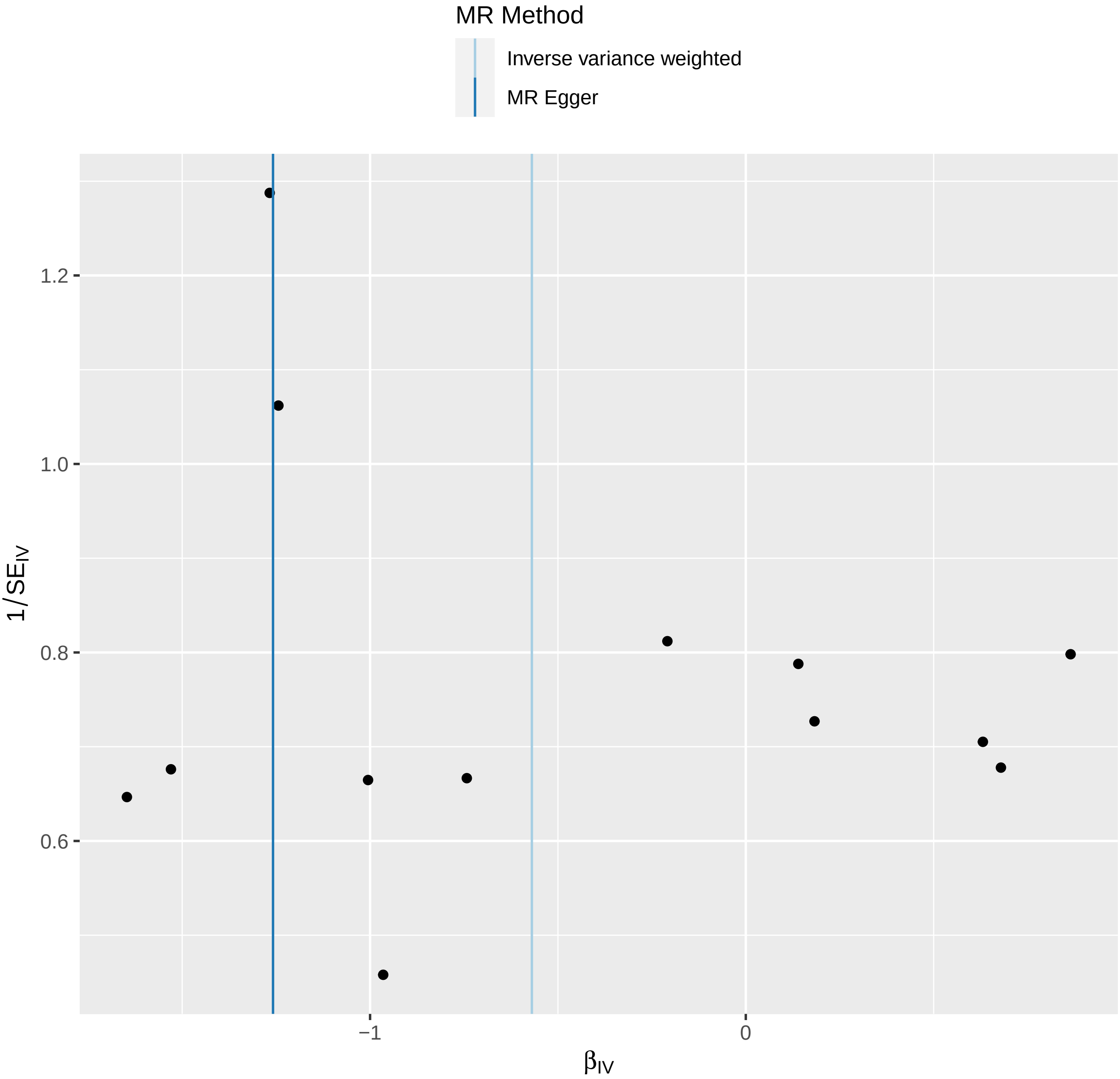

Batch 774 : Gut microbiota abundance (family Porphyromonadaceae id.943) on Arthropathy in ulcerative colitis

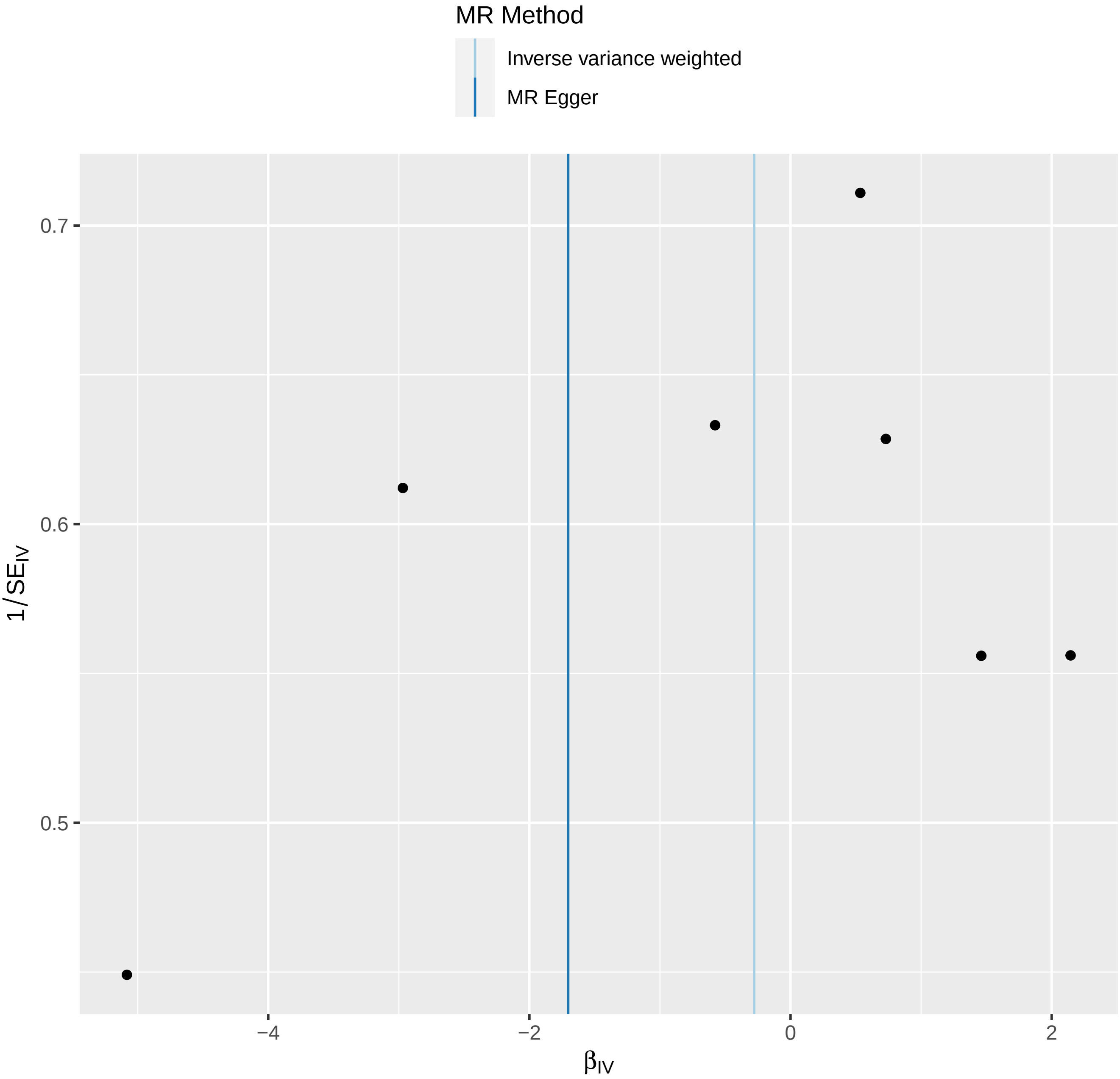

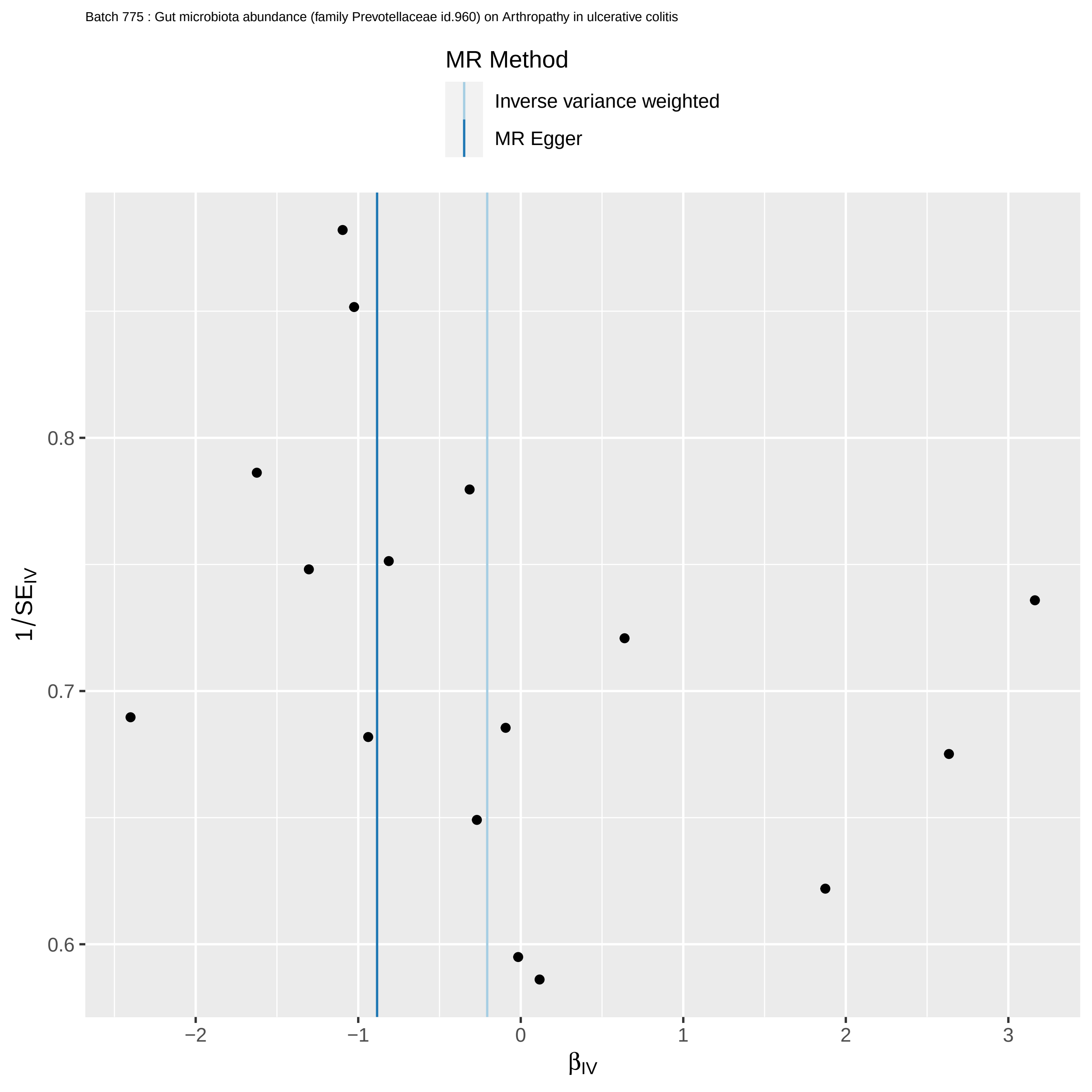

Batch 775 : Gut microbiota abundance (family Prevotellaceae id.960) on Arthropathy in ulcerative colitis
MR Method
Inverse variance weighted
MR Egger
0.8
0.7
0.6
1/SE_IV
−2
−1
0
1
2
3
β_IV

Batch 776 : Gut microbiota abundance (family Rhodospirillaceae id.2717) on Arthropathy in ulcerative colitis

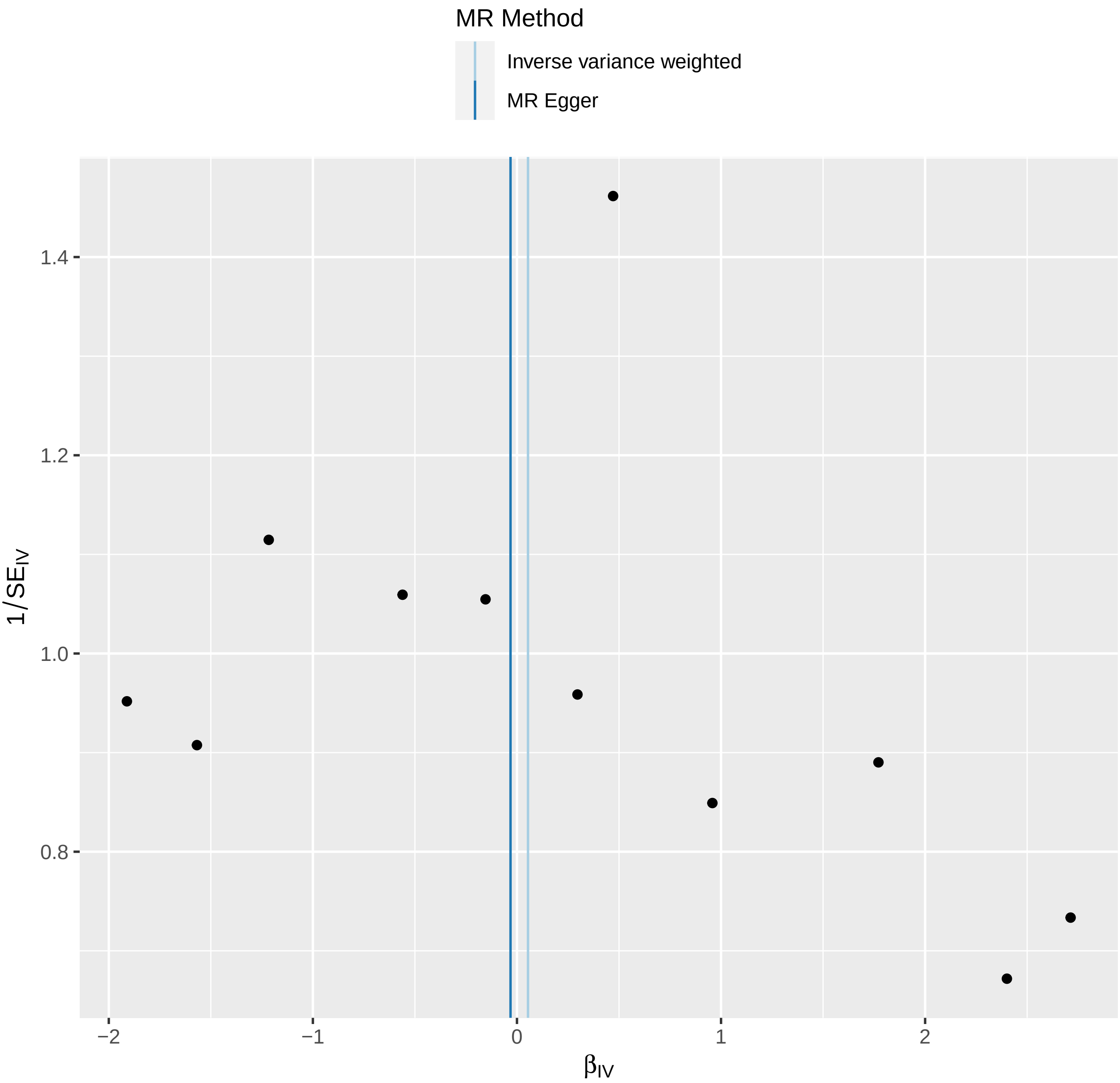

Batch 777 : Gut microbiota abundance (family Rikenellaceae id.967) on Arthropathy in ulcerative colitis

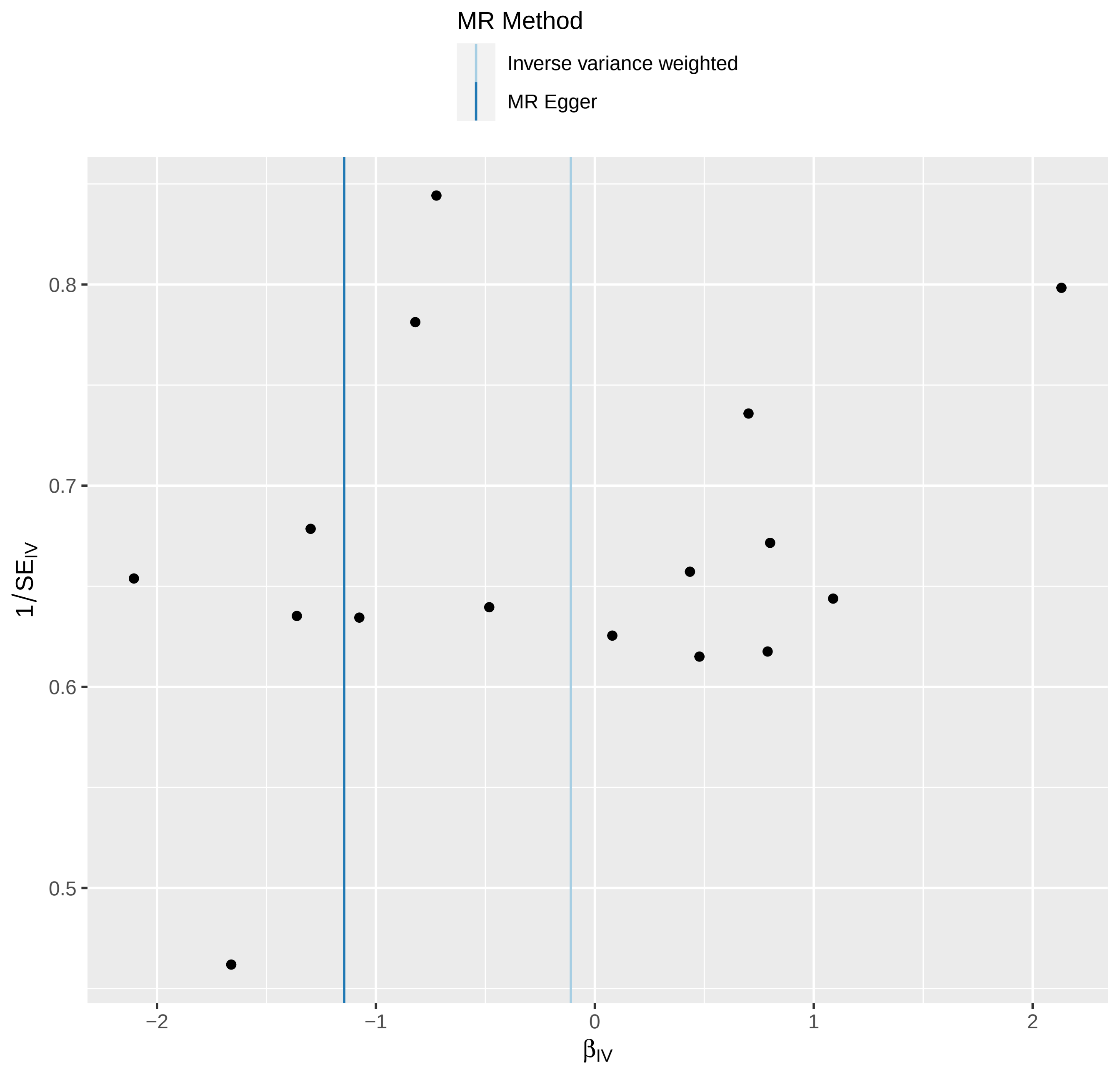

Batch 778 : Gut microbiota abundance (family Ruminococcaceae id.2050) on Arthropathy in ulcerative colitis

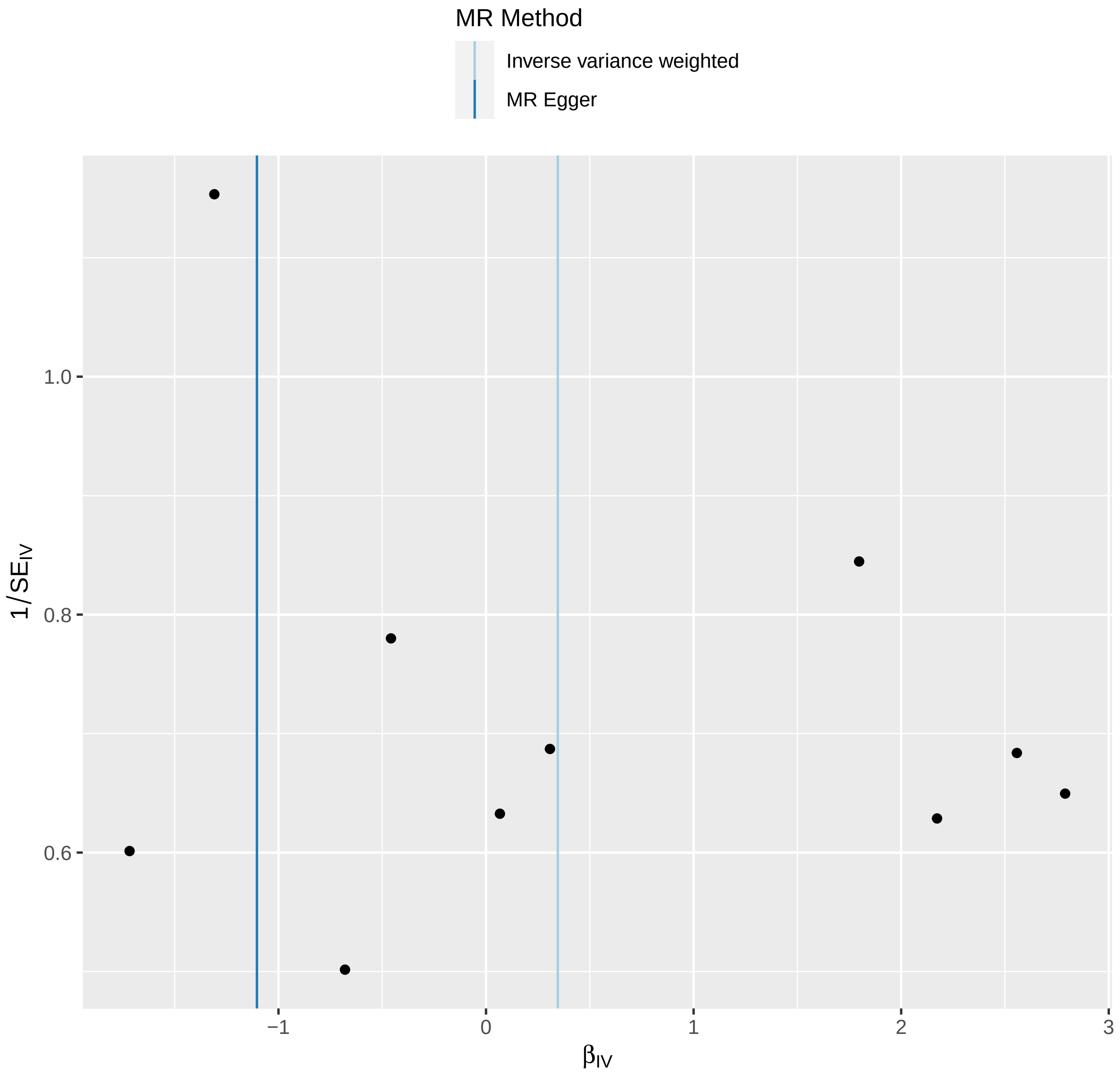

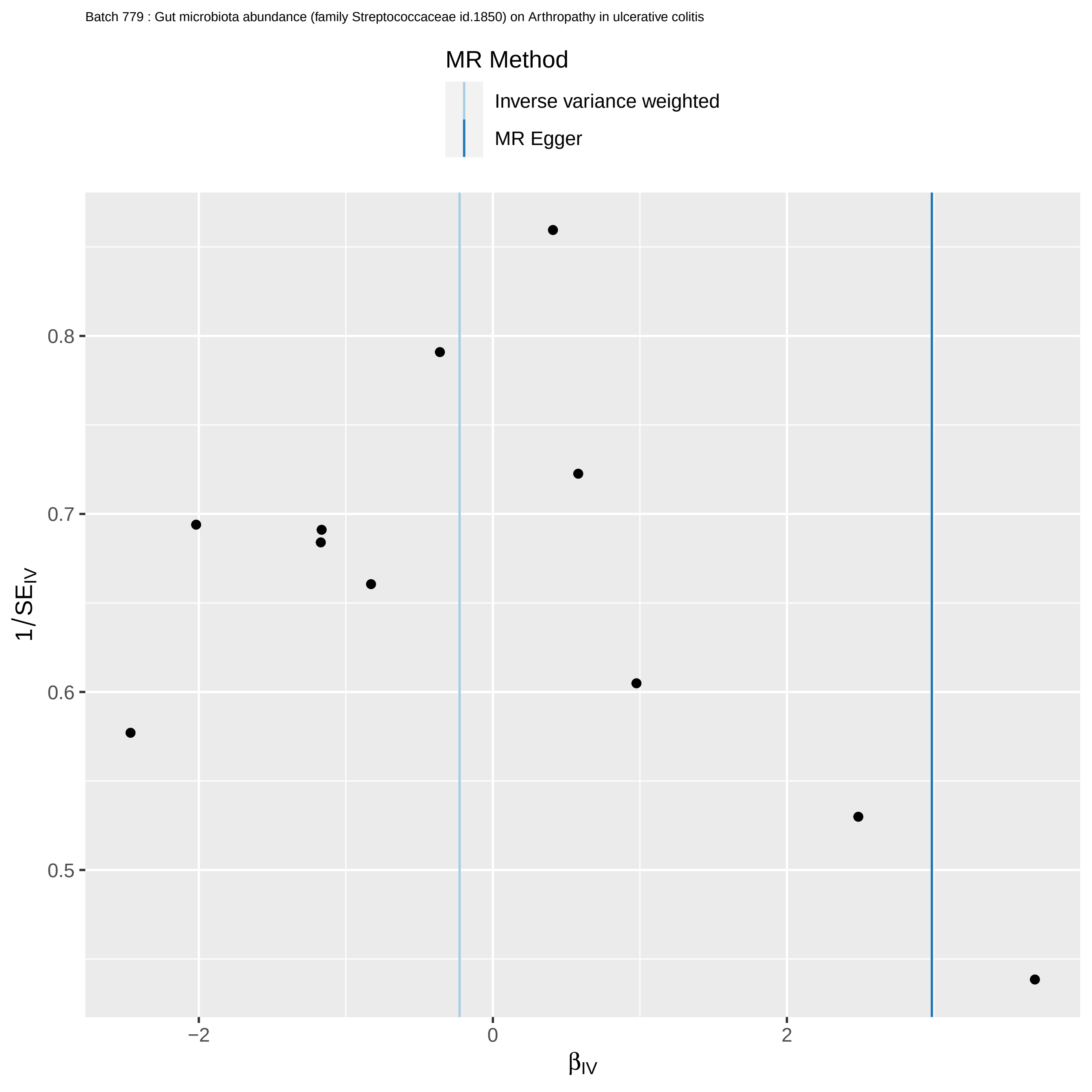

Batch 779 : Gut microbiota abundance (family Streptococcaceae id.1850) on Arthropathy in ulcerative colitis
MR Method
Inverse variance weighted
MR Egger
0.8
0.7
0.6
0.5
1/SE_IV
−2
0
2
β_IV

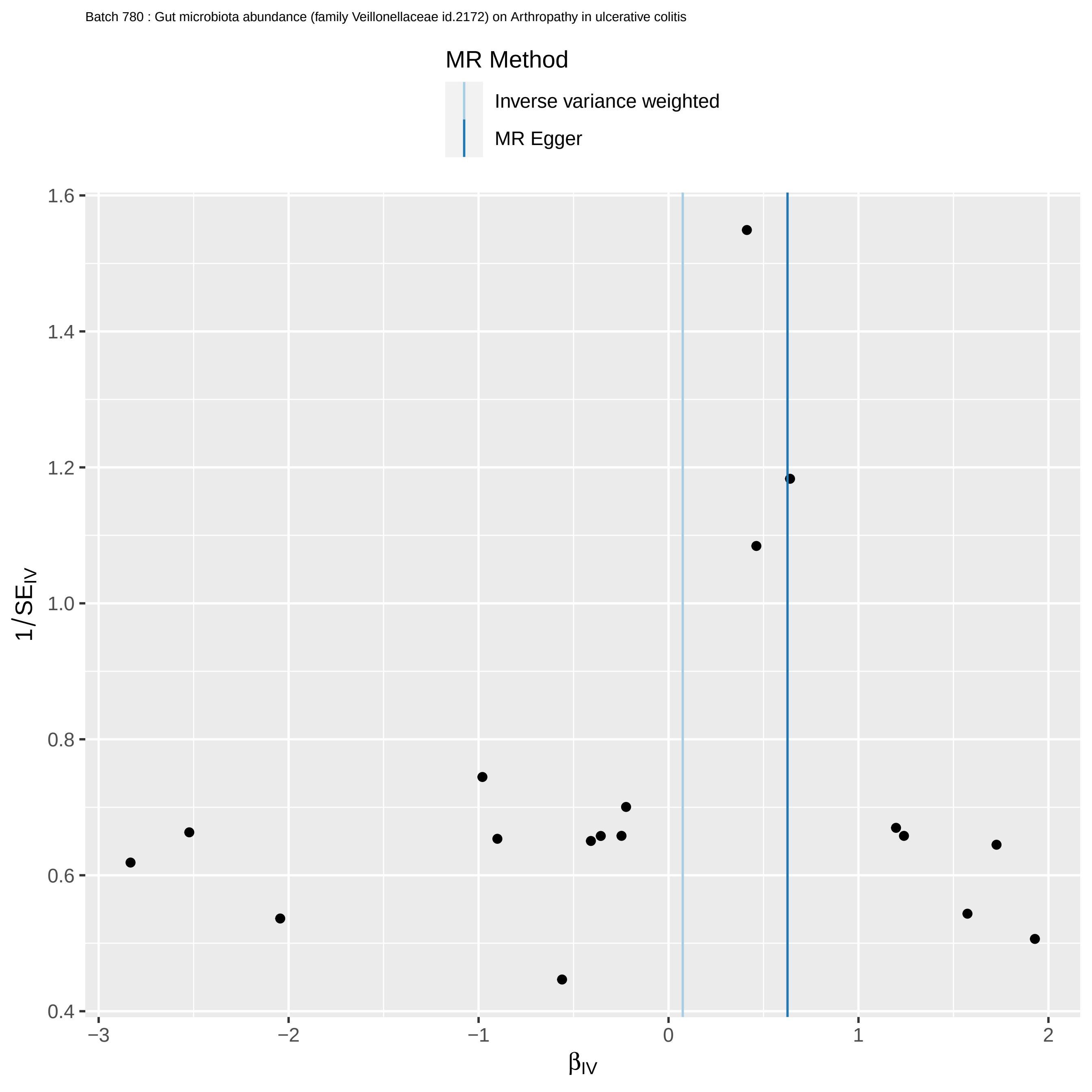
Batch 780 : Gut microbiota abundance (family Veillonellaceae id.2172) on Arthropathy in ulcerative colitis
MR Method
Inverse variance weighted
MR Egger
1.6
1.4
1.2
1.0
0.8
0.6
0.4
1/SE_IV
−3
−2
−1
0
1
2
β_IV

Batch 781 : Gut microbiota abundance (family Verrucomicrobiaceae id.4036) on Arthropathy in ulcerative colitis

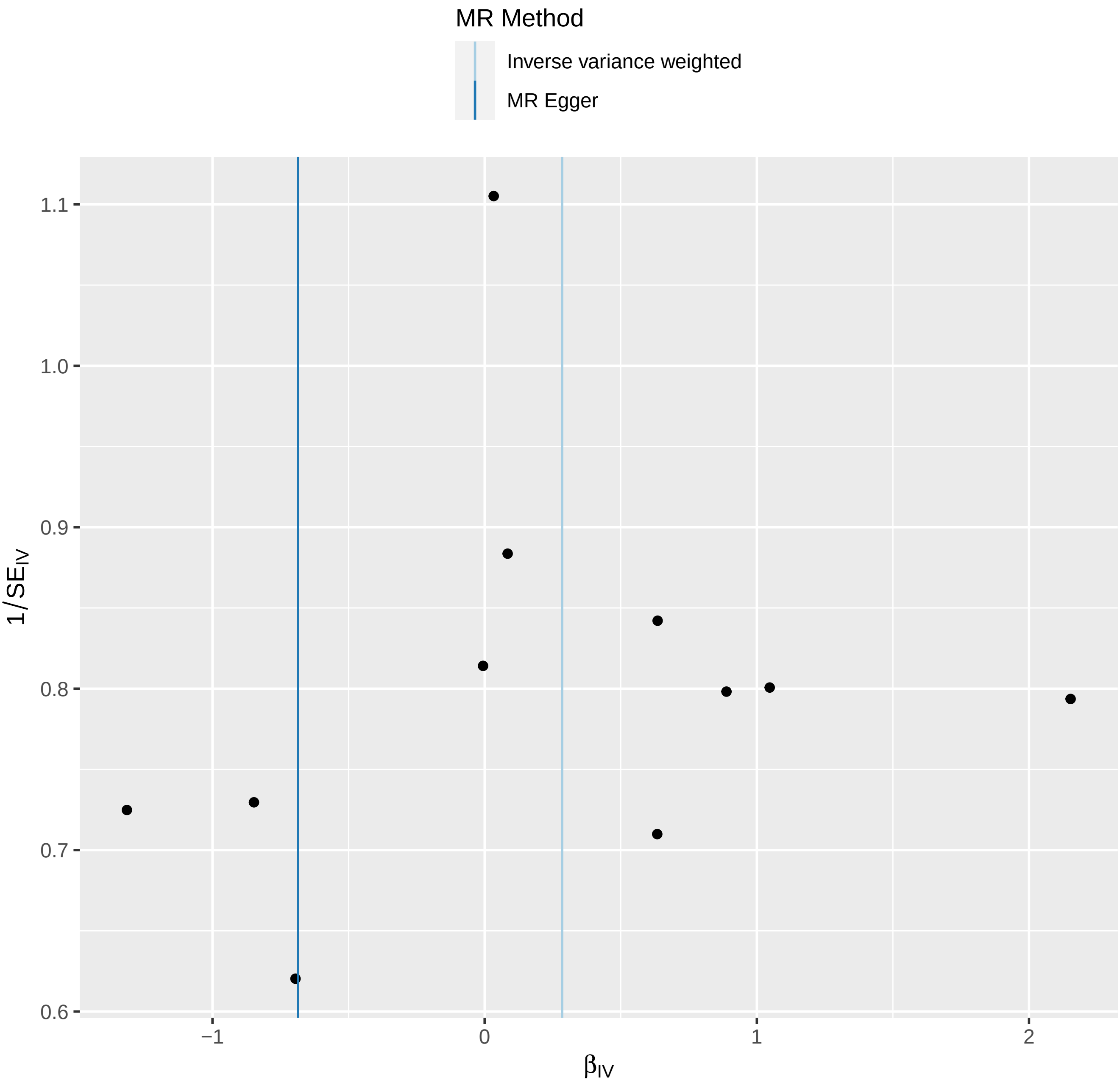

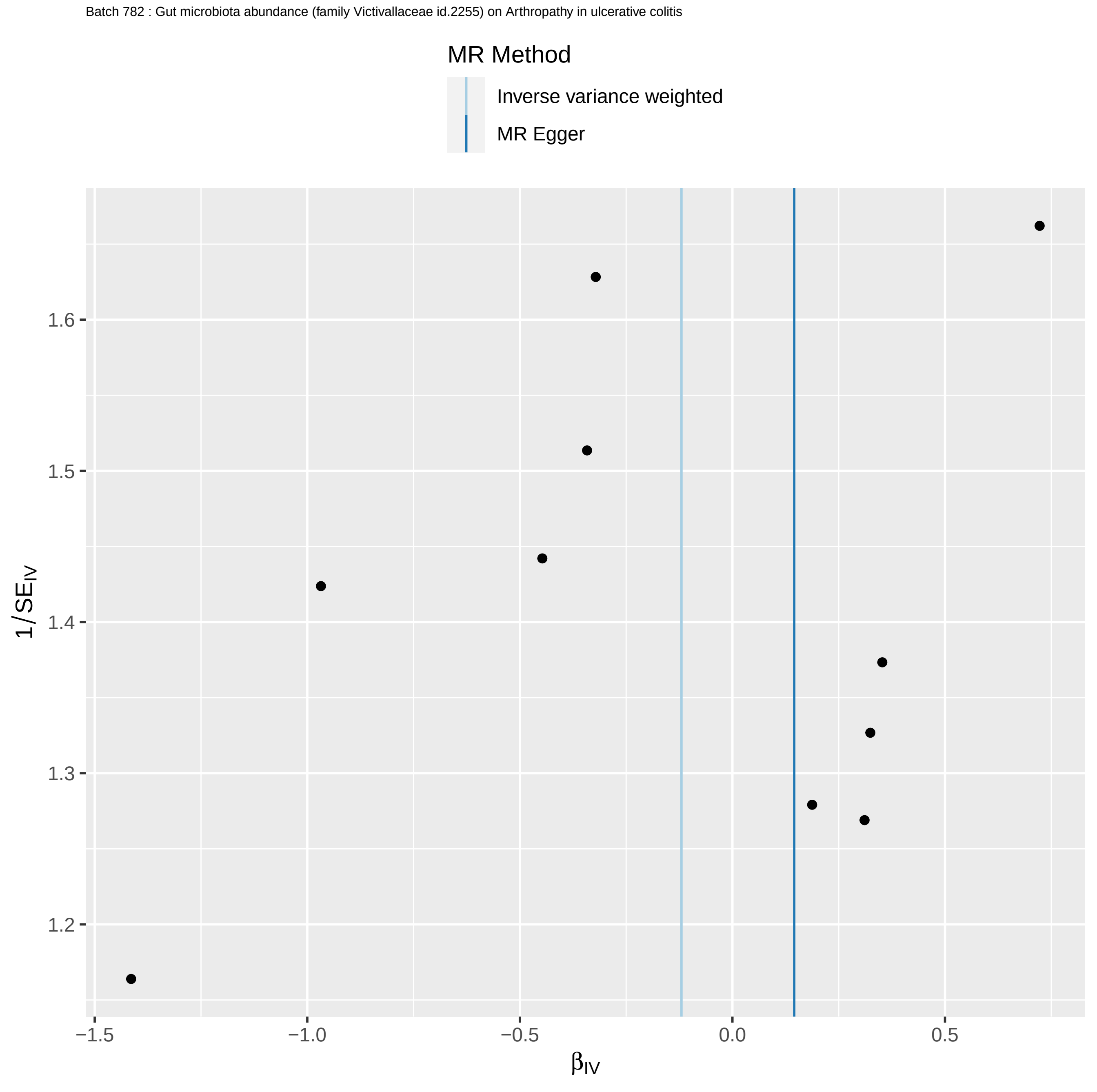
Batch 782 : Gut microbiota abundance (family Victivallaceae id.2255) on Arthropathy in ulcerative colitis
MR Method
Inverse variance weighted
MR Egger
1.6
1.5
1.4
1.3
1.2
1/SE$_{IV}$
−1.5
−1.0
−0.5
0.0
0.5
β$_{IV}$

Batch 783 : Gut microbiota abundance (genus Actinomyces id.423) on Arthropathy in ulcerative colitis

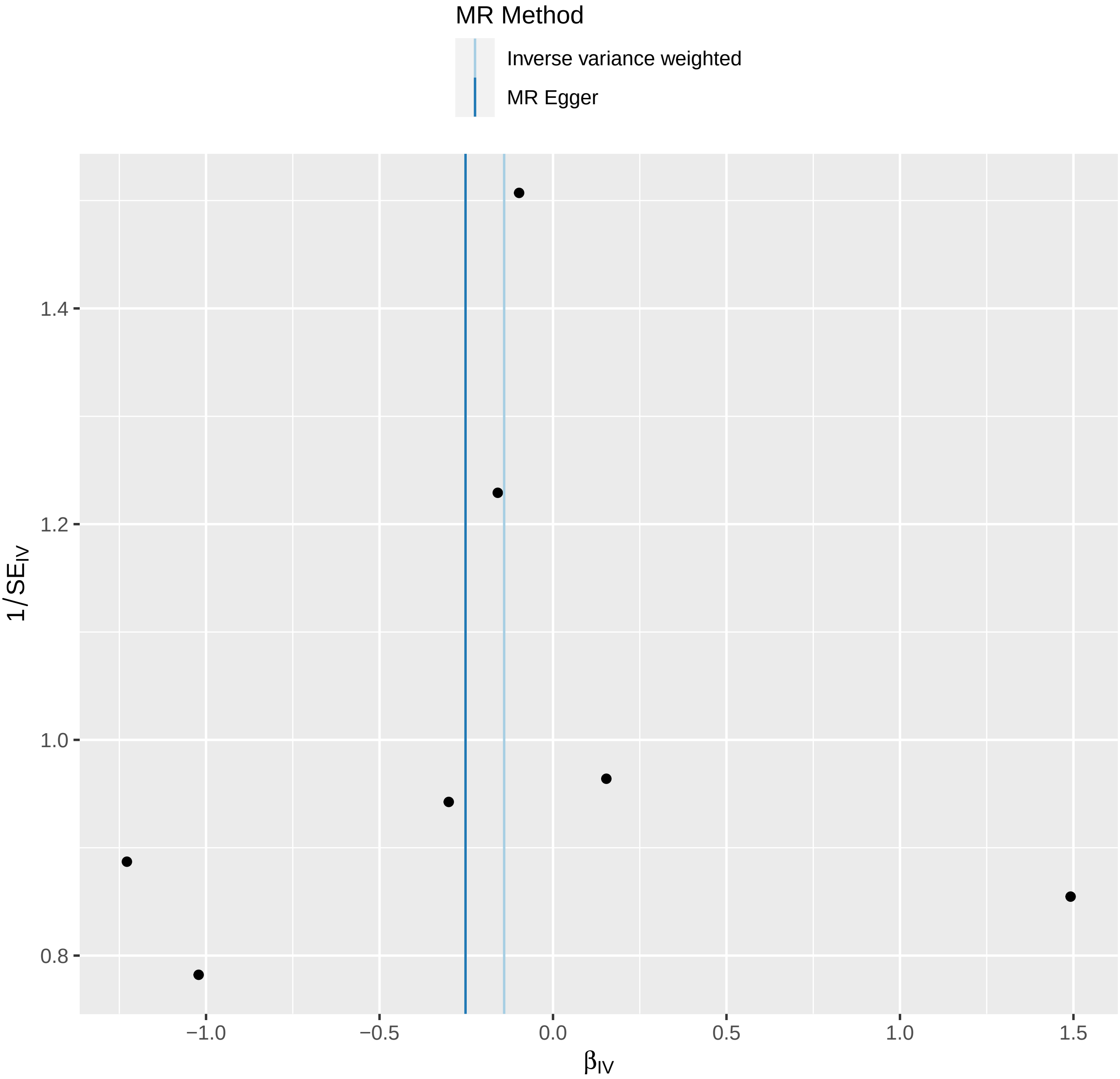

Batch 784 : Gut microbiota abundance (genus Adlercreutzia id.812) on Arthropathy in ulcerative colitis

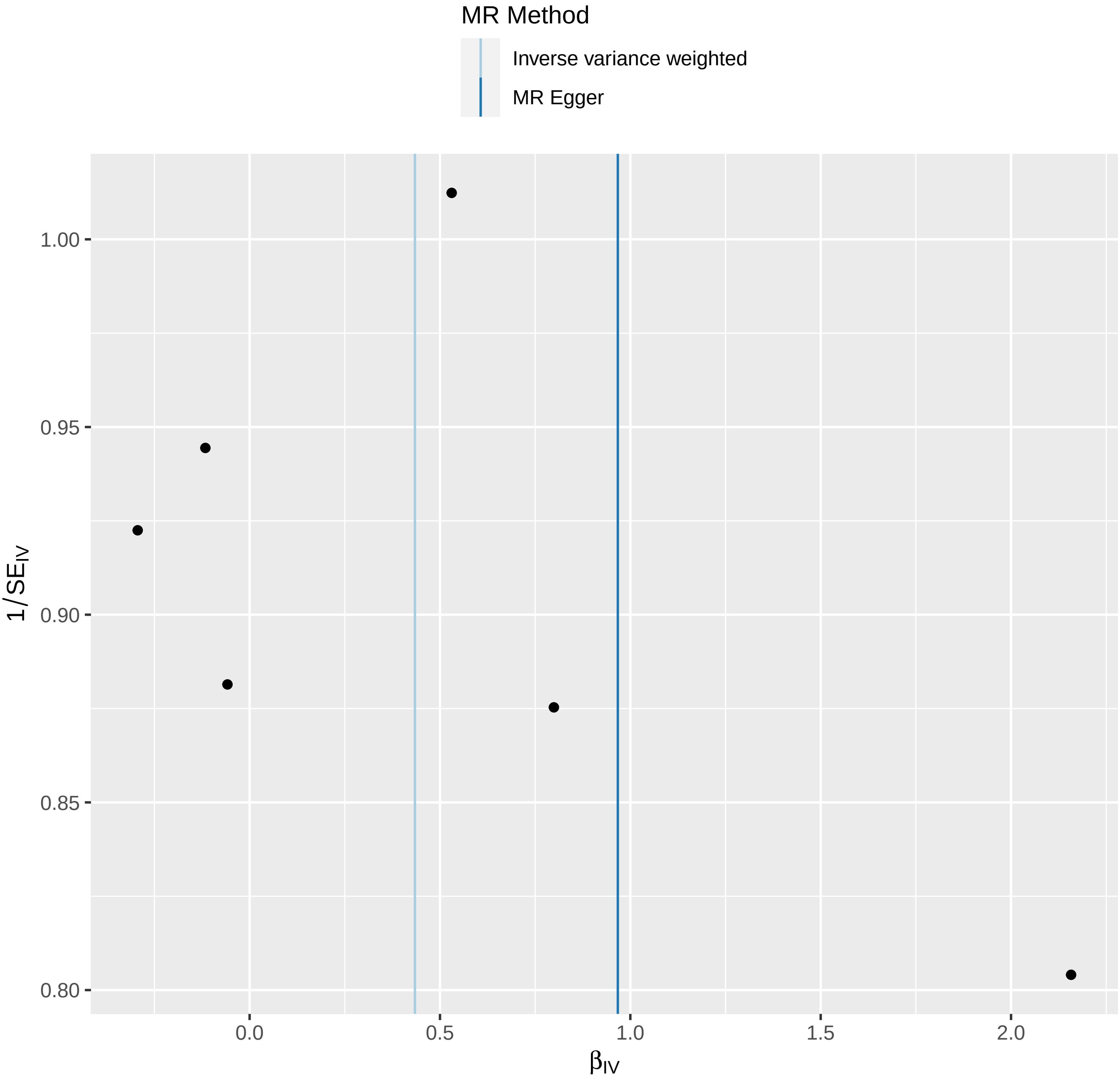

Batch 785 : Gut microbiota abundance (genus Akkermansia id.4037) on Arthropathy in ulcerative colitis

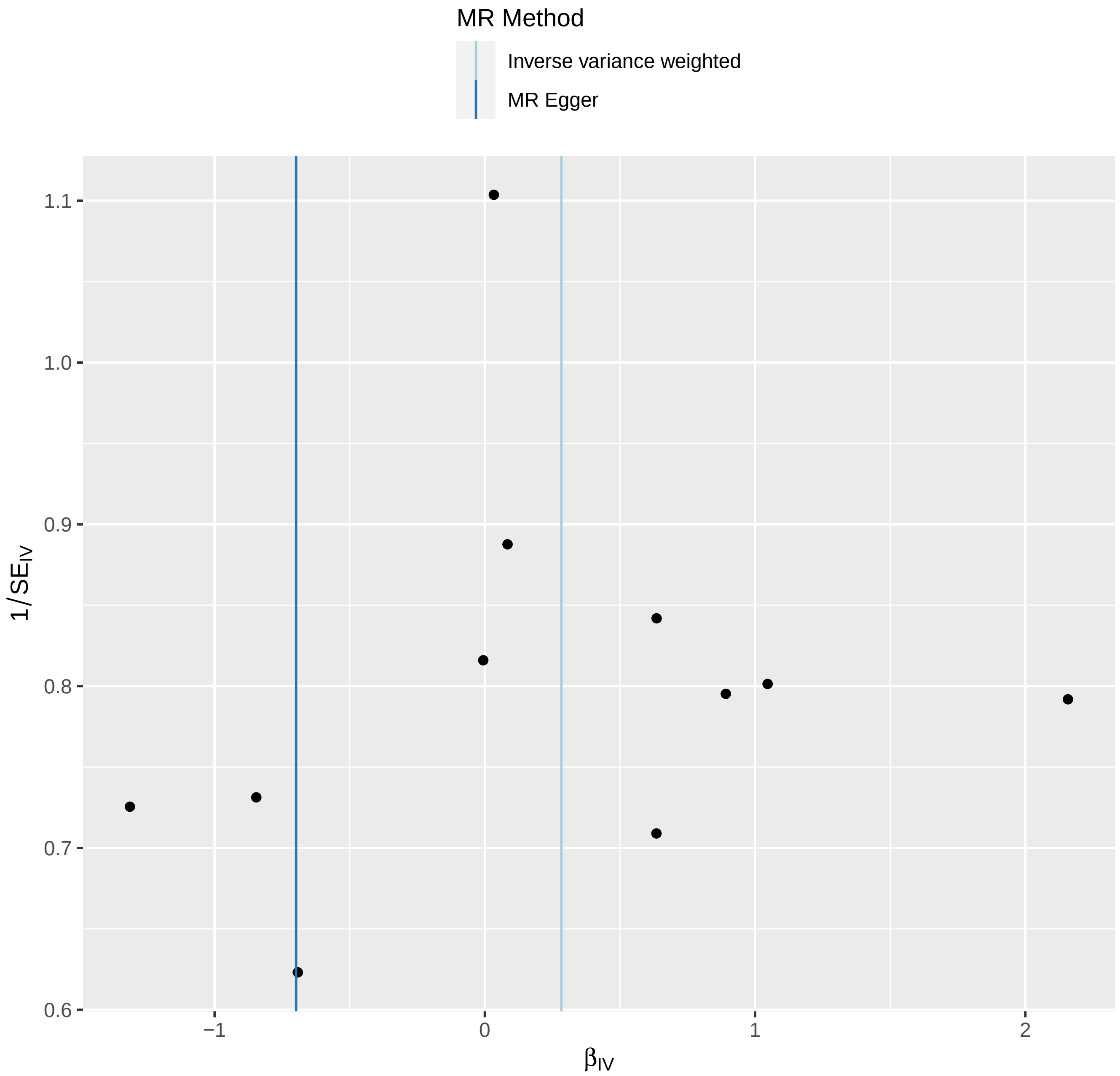

Batch 786 : Gut microbiota abundance (genus Alistipes id.968) on Arthropathy in ulcerative colitis

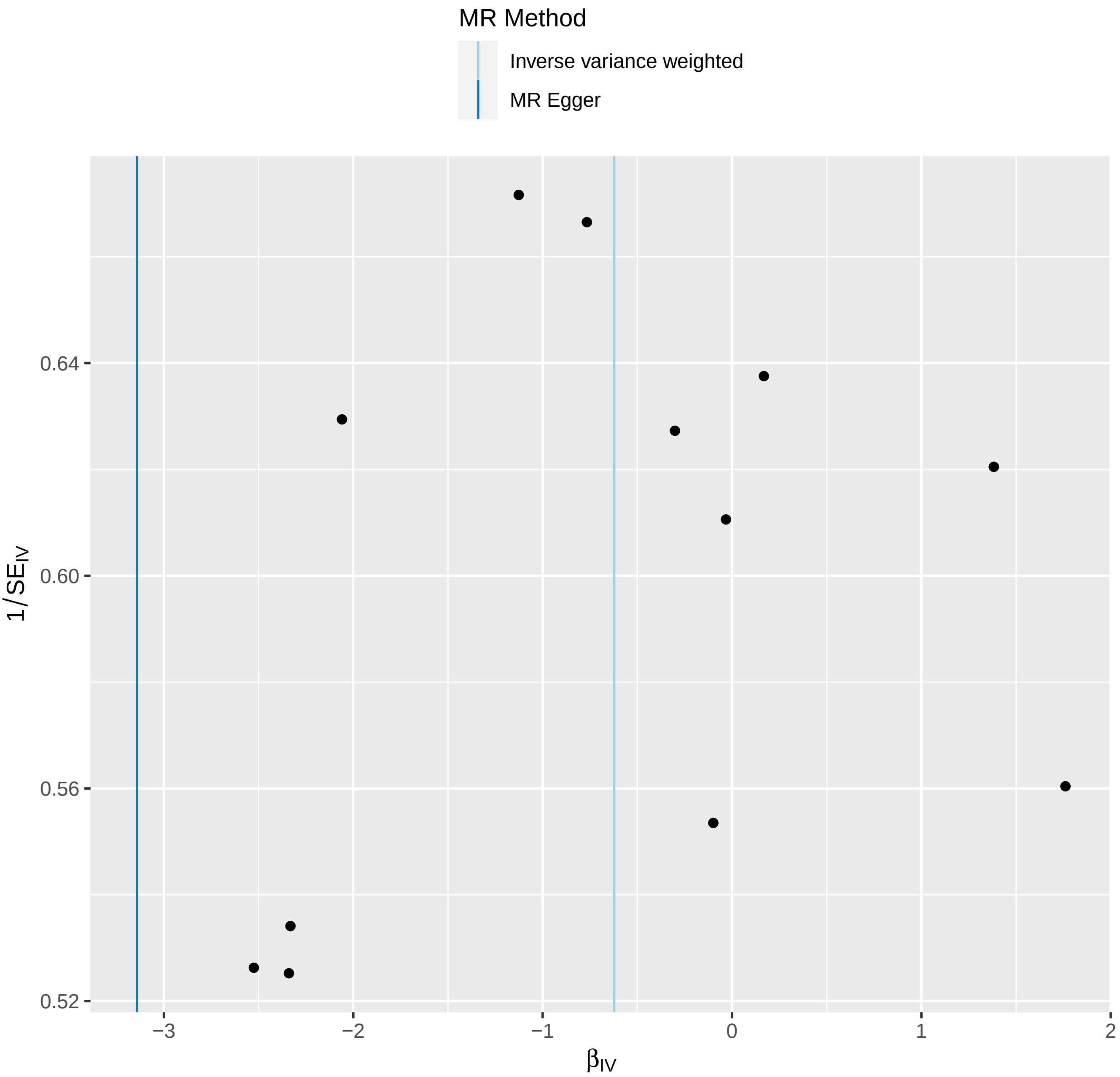

Batch 787 : Gut microbiota abundance (genus Allisonella id.2174) on Arthropathy in ulcerative colitis

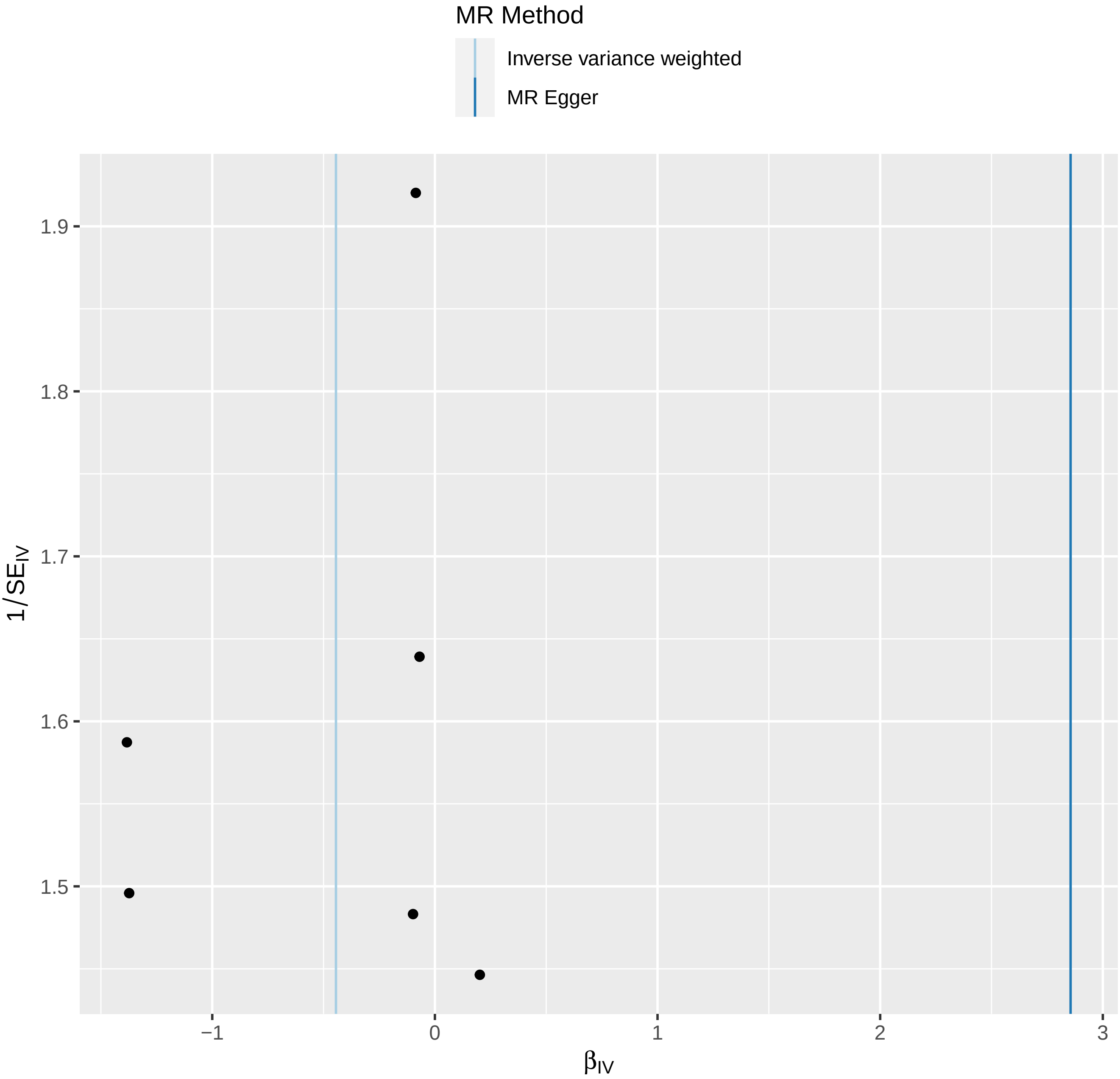

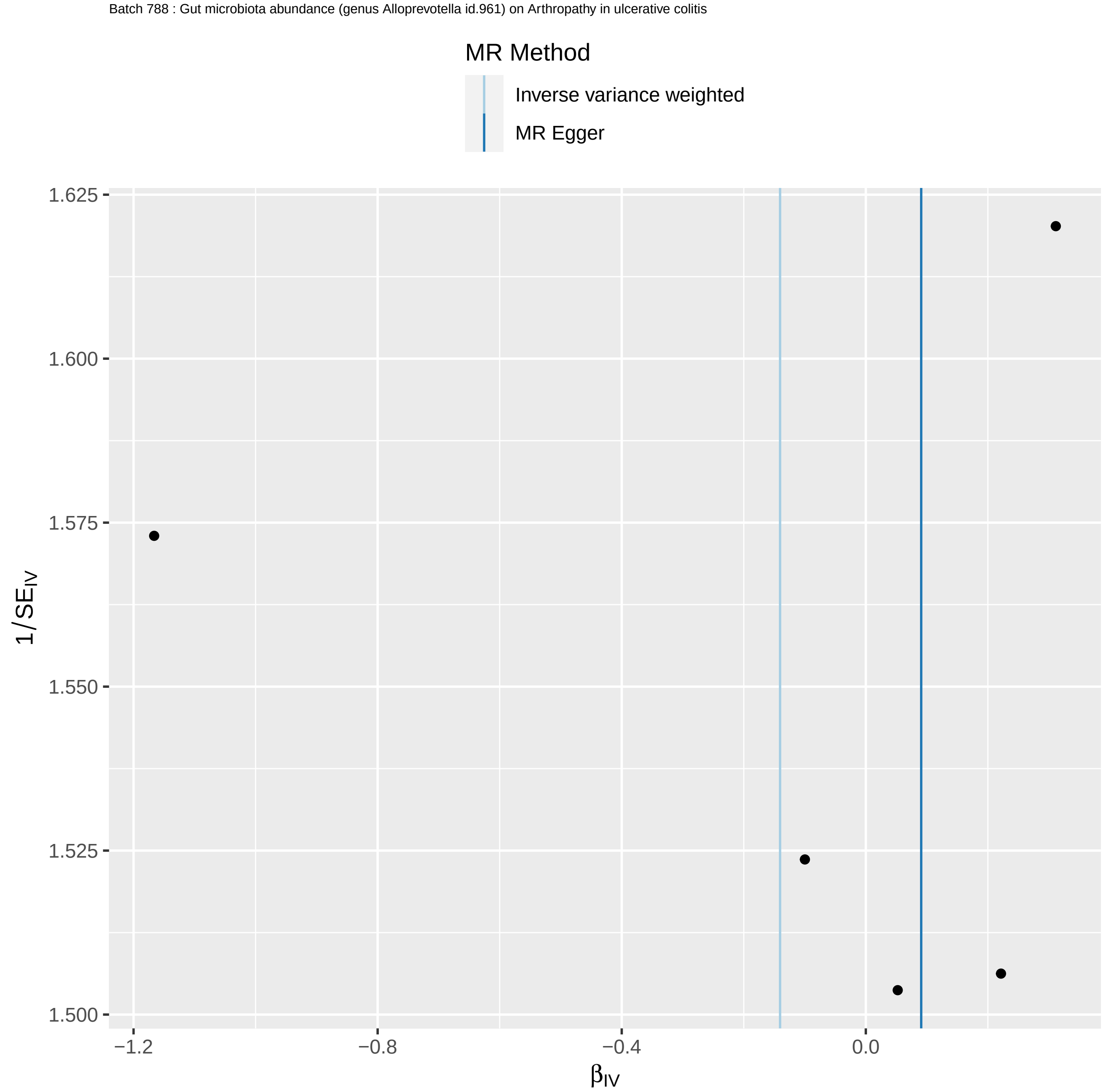
Batch 788 : Gut microbiota abundance (genus Alloprevotella id.961) on Arthropathy in ulcerative colitis
MR Method
Inverse variance weighted
MR Egger
1.625
1.600
1.575
1.550
1.525
1.500
1/SE_IV
−1.2
−0.8
−0.4
0.0
β_IV

Batch 789 : Gut microbiota abundance (genus Anaerofilum id.2053) on Arthropathy in ulcerative colitis

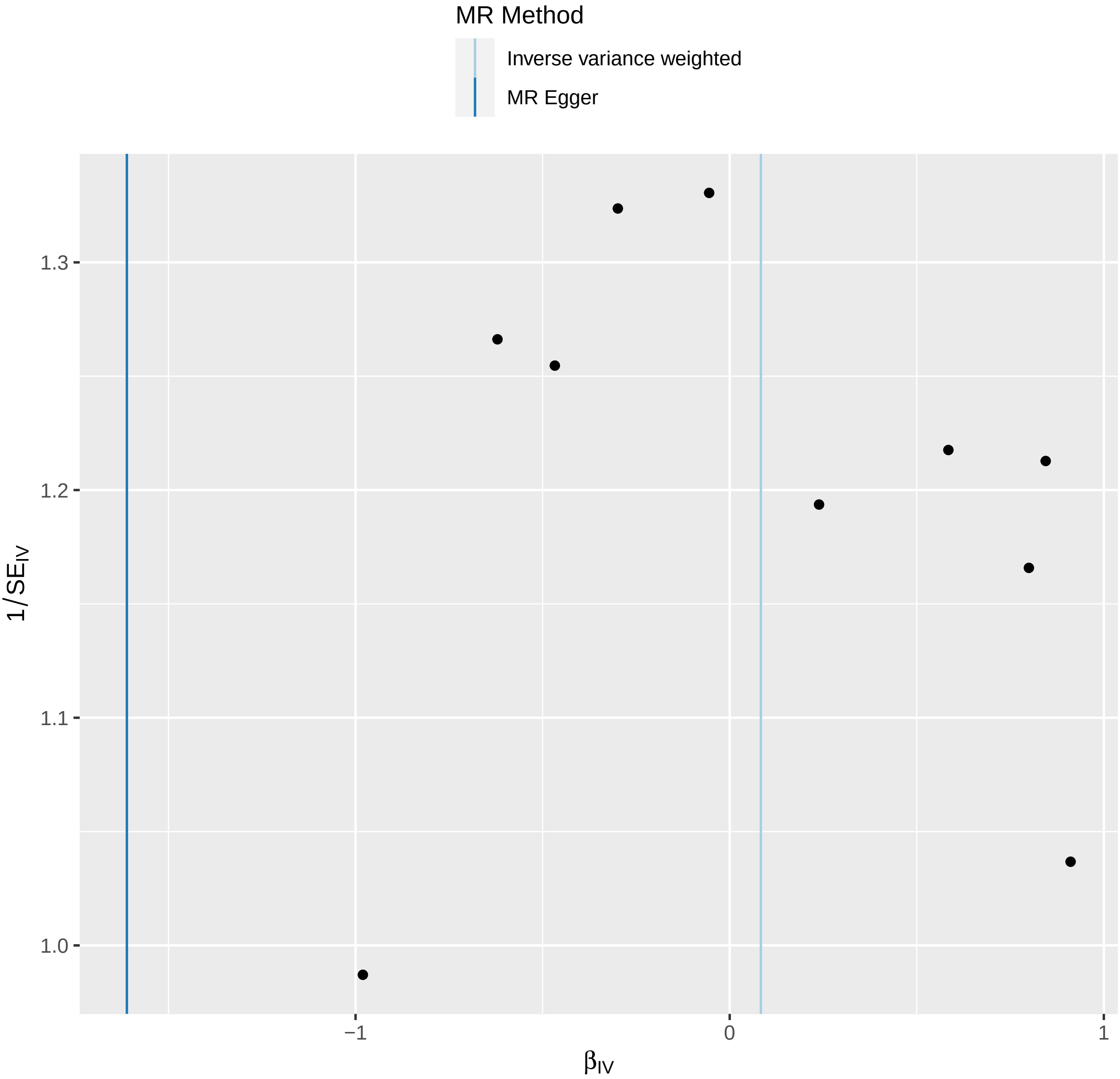

Batch 790 : Gut microbiota abundance (genus Anaerostipes id.1991) on Arthropathy in ulcerative colitis

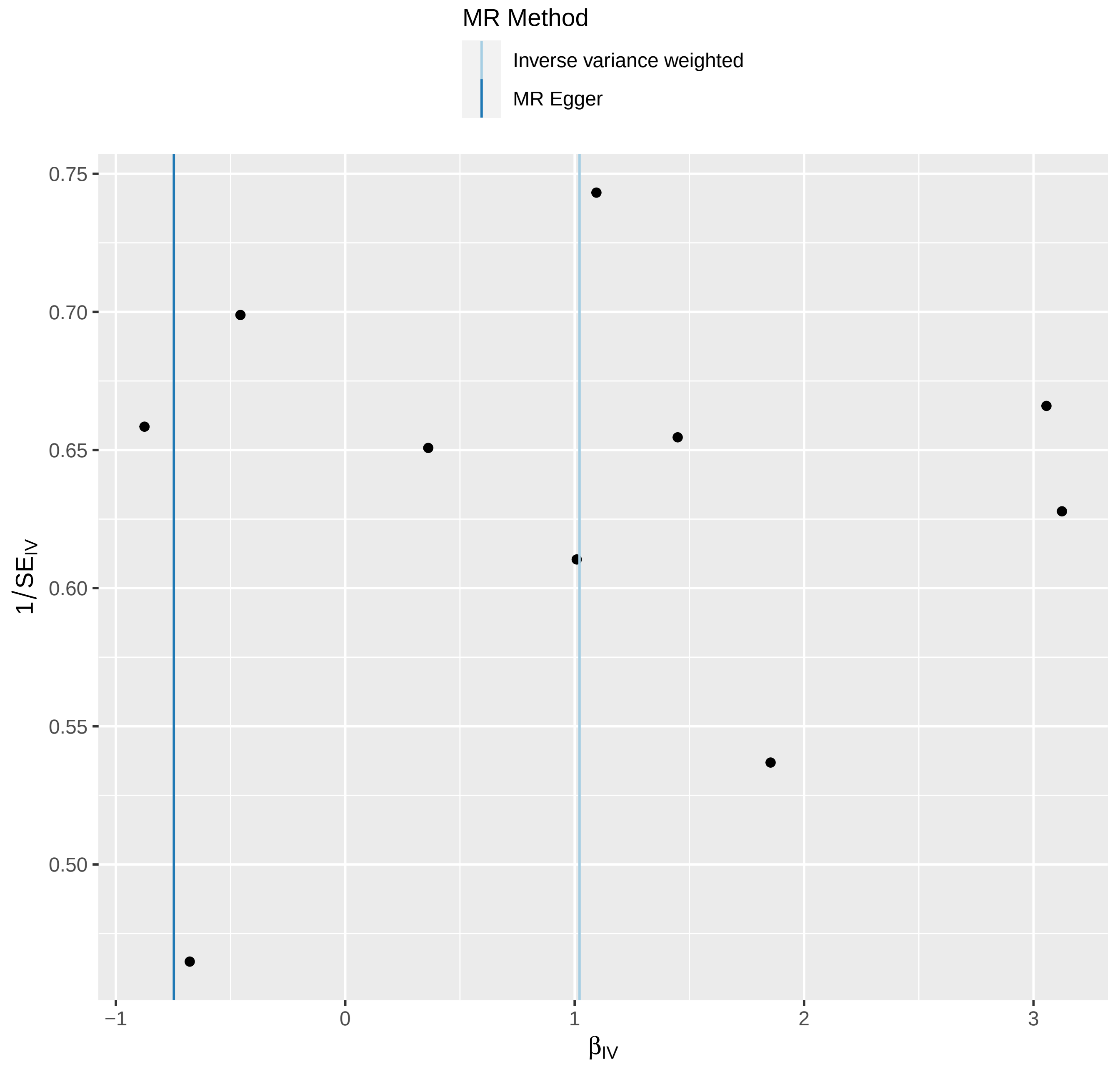

Batch 791 : Gut microbiota abundance (genus Anaerotruncus id.2054) on Arthropathy in ulcerative colitis

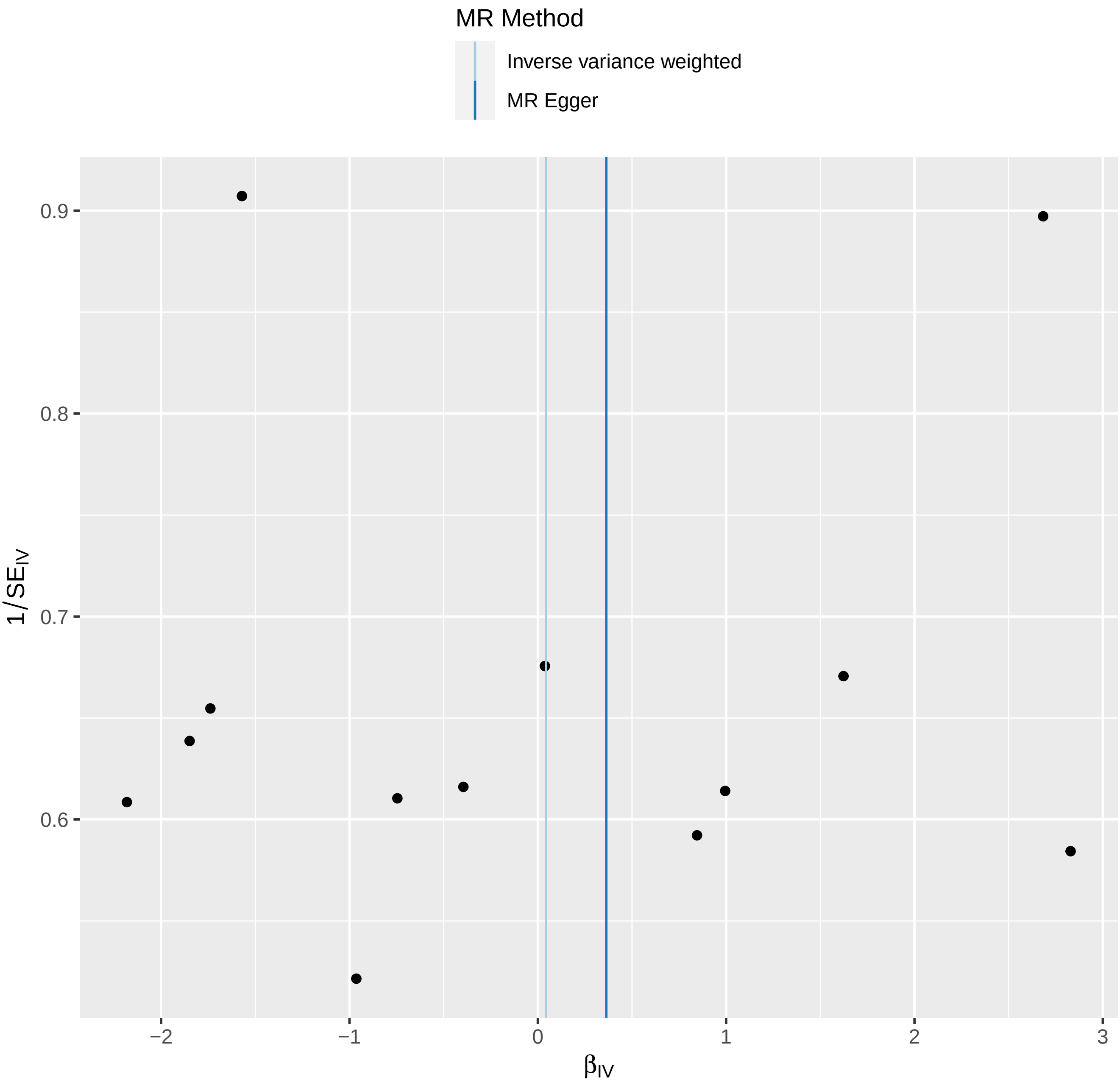

Batch 792 : Gut microbiota abundance (genus Bacteroides id.918) on Arthropathy in ulcerative colitis

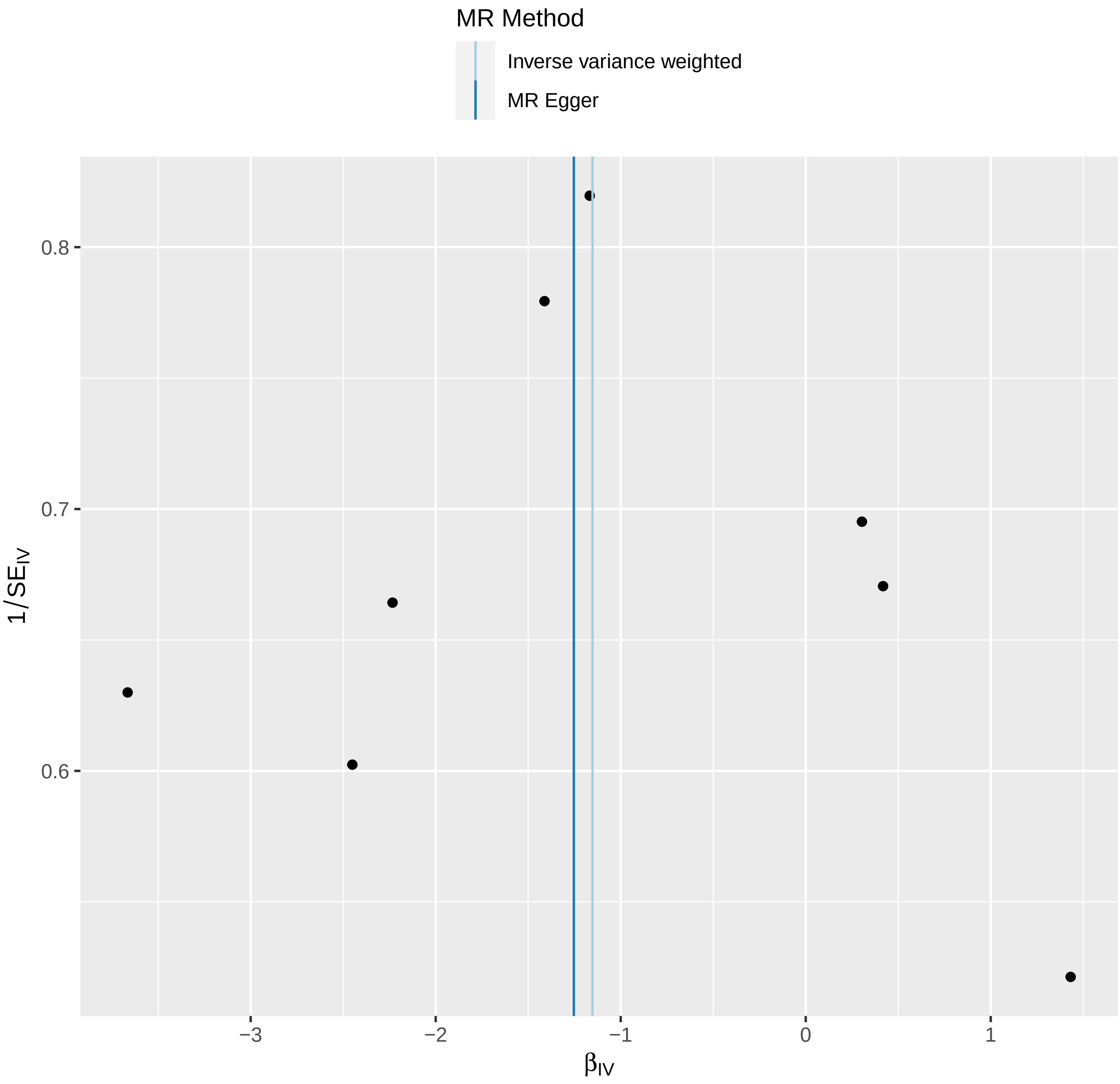

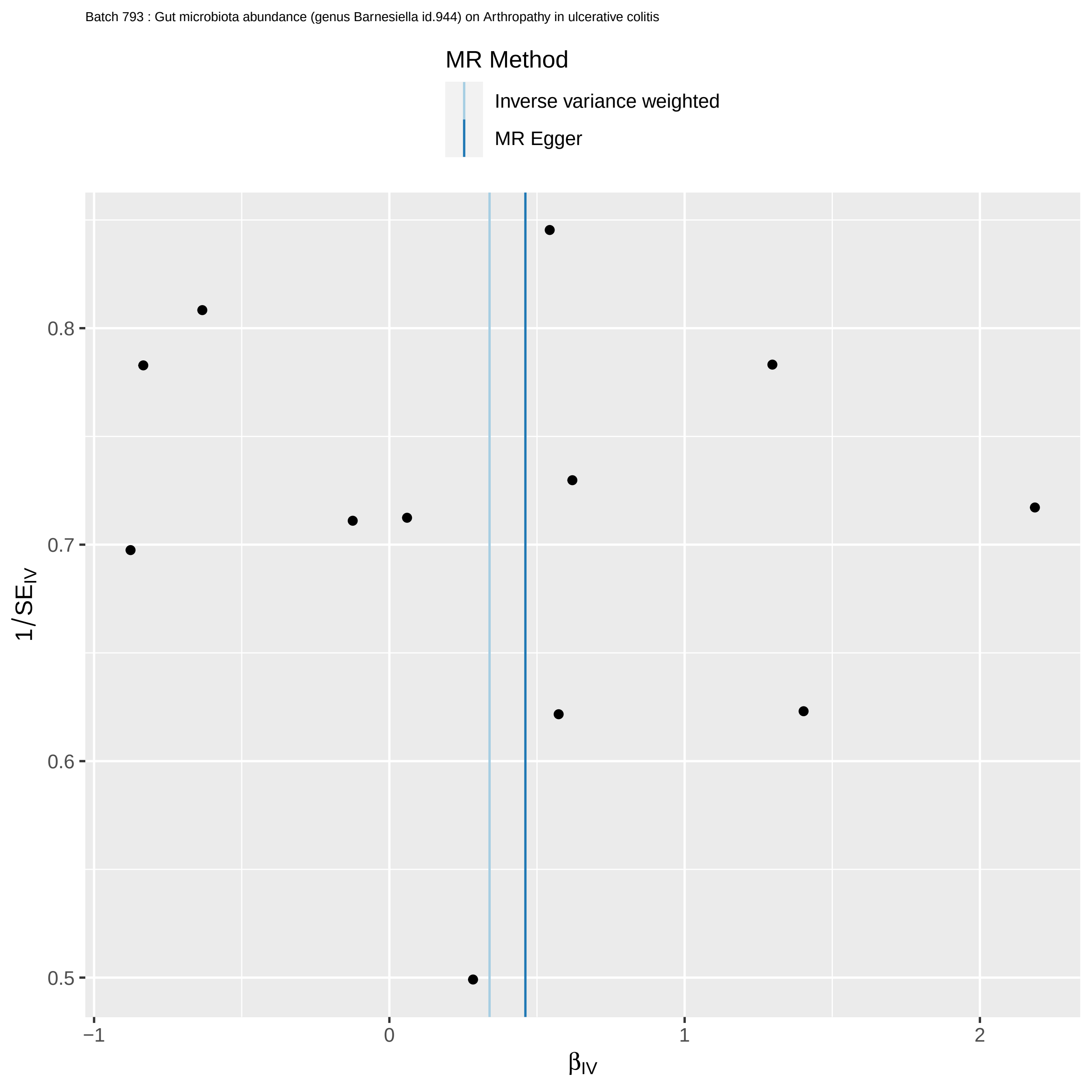

Batch 793 : Gut microbiota abundance (genus Barnesiella id.944) on Arthropathy in ulcerative colitis
MR Method
Inverse variance weighted
MR Egger
0.8
0.7
0.6
0.5
1/SE_IV
−1
0
1
2
β_IV

Batch 794 : Gut microbiota abundance (genus Bifidobacterium id.436) on Arthropathy in ulcerative colitis

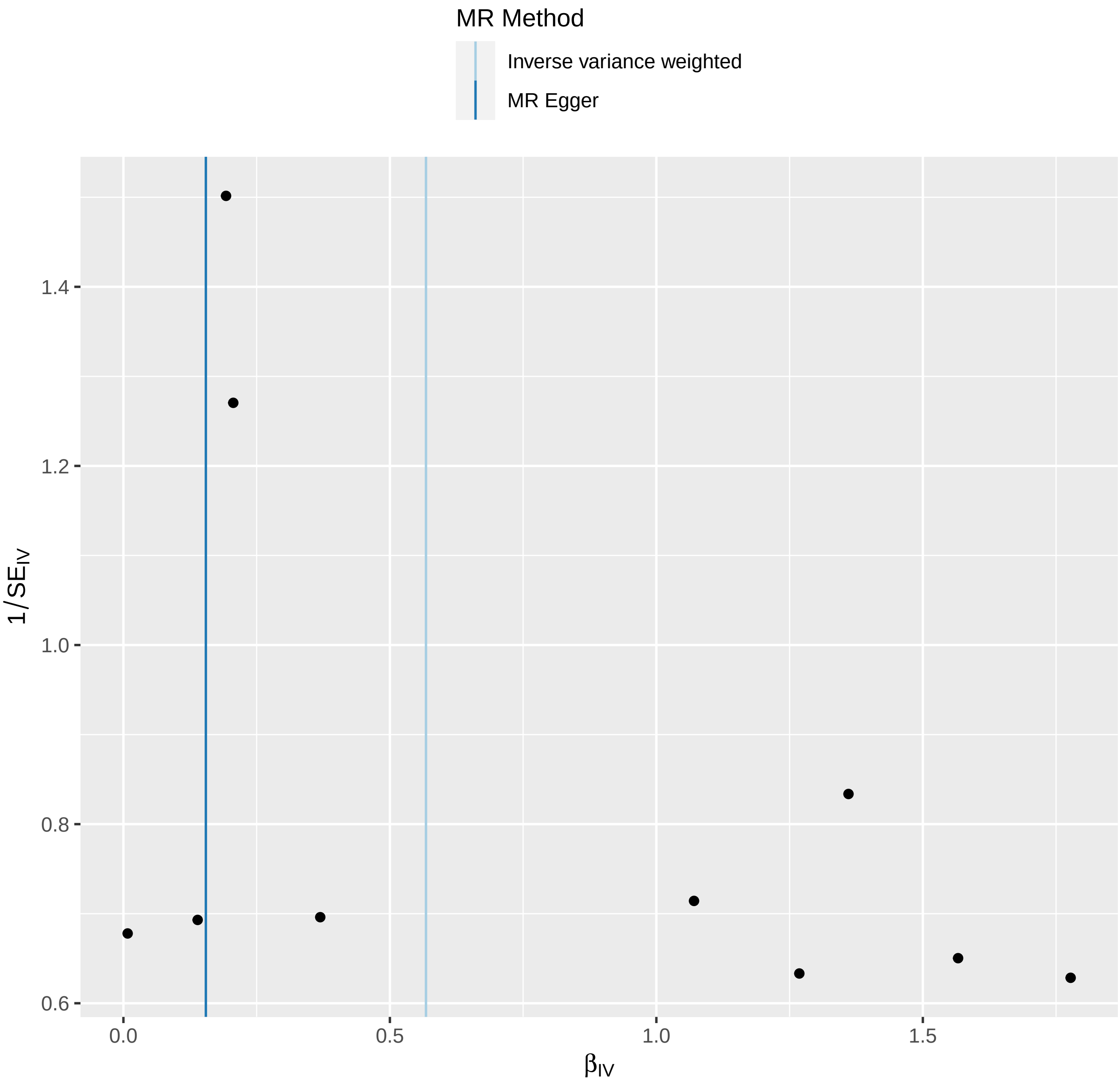

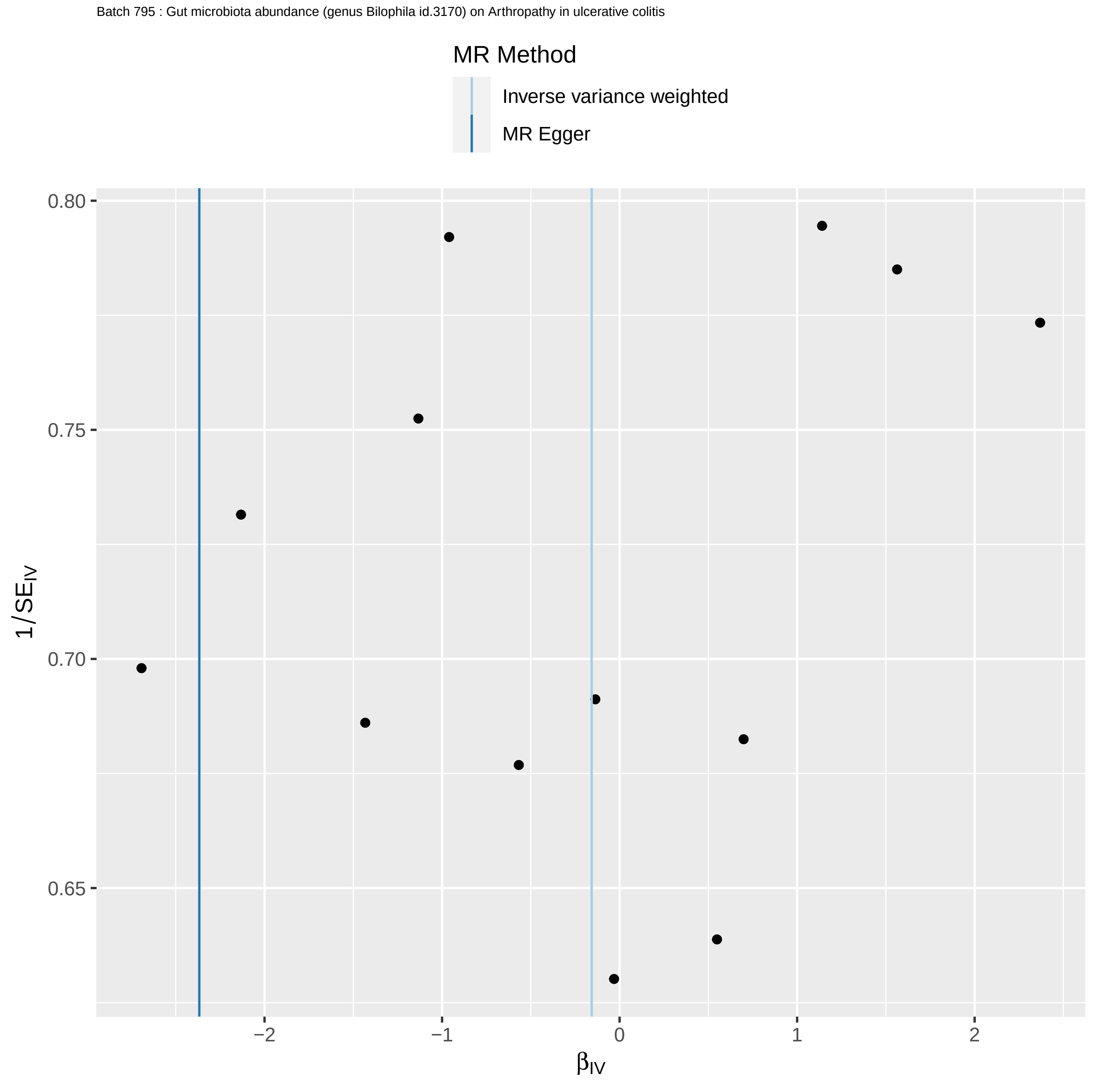

Batch 795 : Gut microbiota abundance (genus Bilophila id.3170) on Arthropathy in ulcerative colitis
MR Method
Inverse variance weighted
MR Egger
0.80
0.75
0.70
0.65
1/SE$_{IV}$
−2
−1
0
1
2
β_{IV}

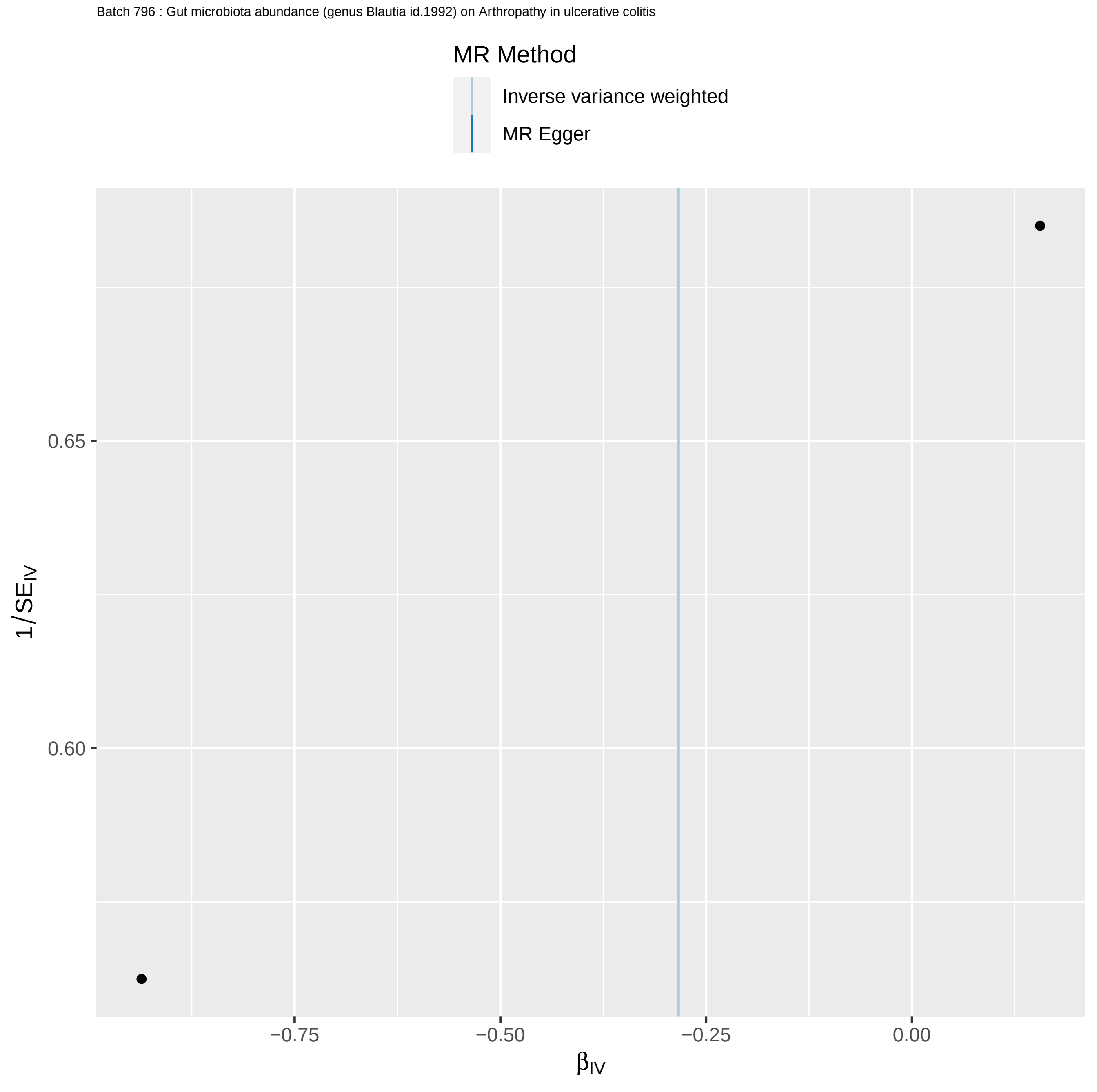
Batch 796 : Gut microbiota abundance (genus Blautia id.1992) on Arthropathy in ulcerative colitis
MR Method
Inverse variance weighted
MR Egger
0.65
0.60
1/SE_IV
−0.75
−0.50
−0.25
0.00
β_IV

Batch 797 : Gut microbiota abundance (genus Butyricicoccus id.2055) on Arthropathy in ulcerative colitis

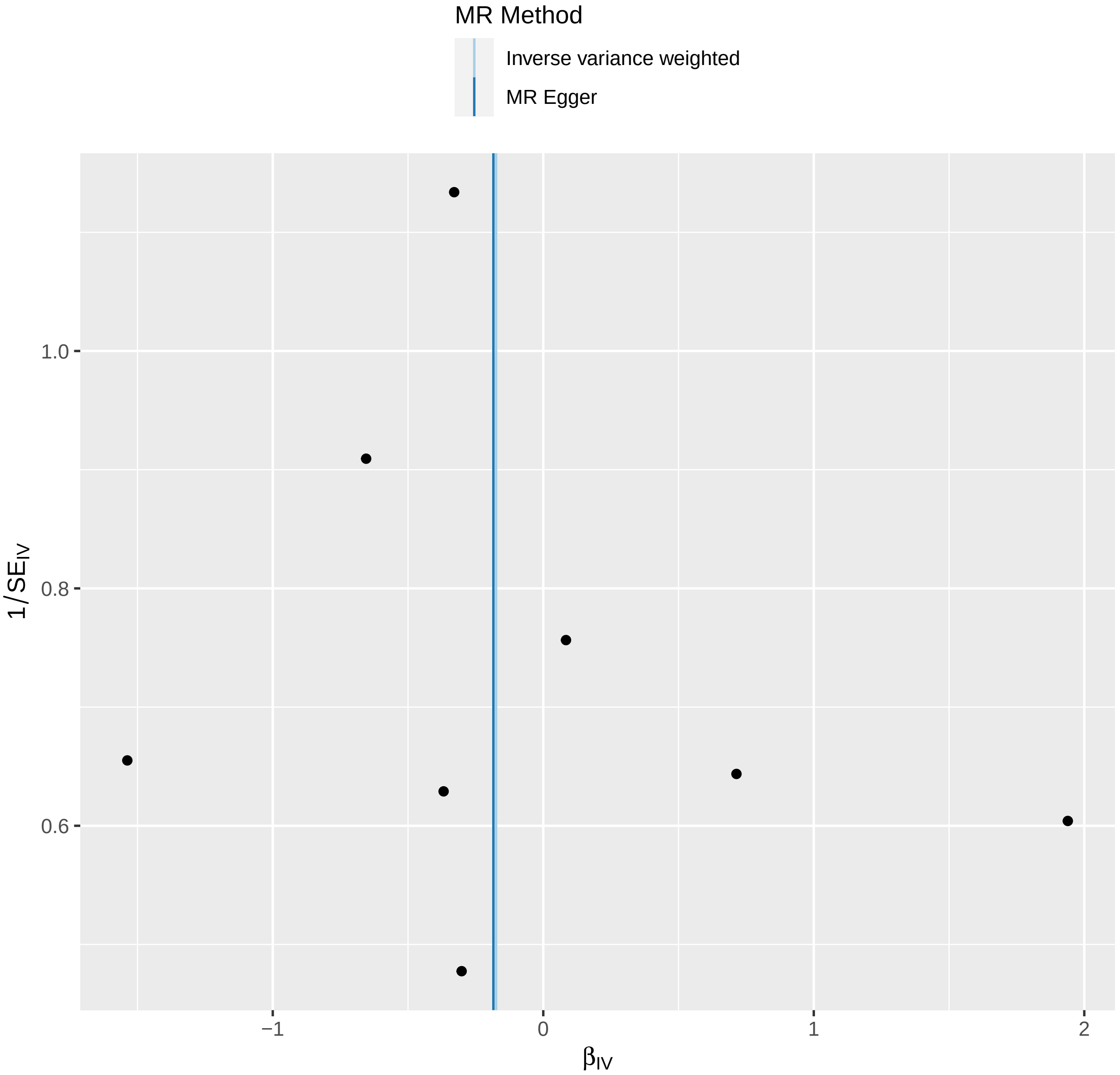

Batch 798 : Gut microbiota abundance (genus Butyricimonas id.945) on Arthropathy in ulcerative colitis

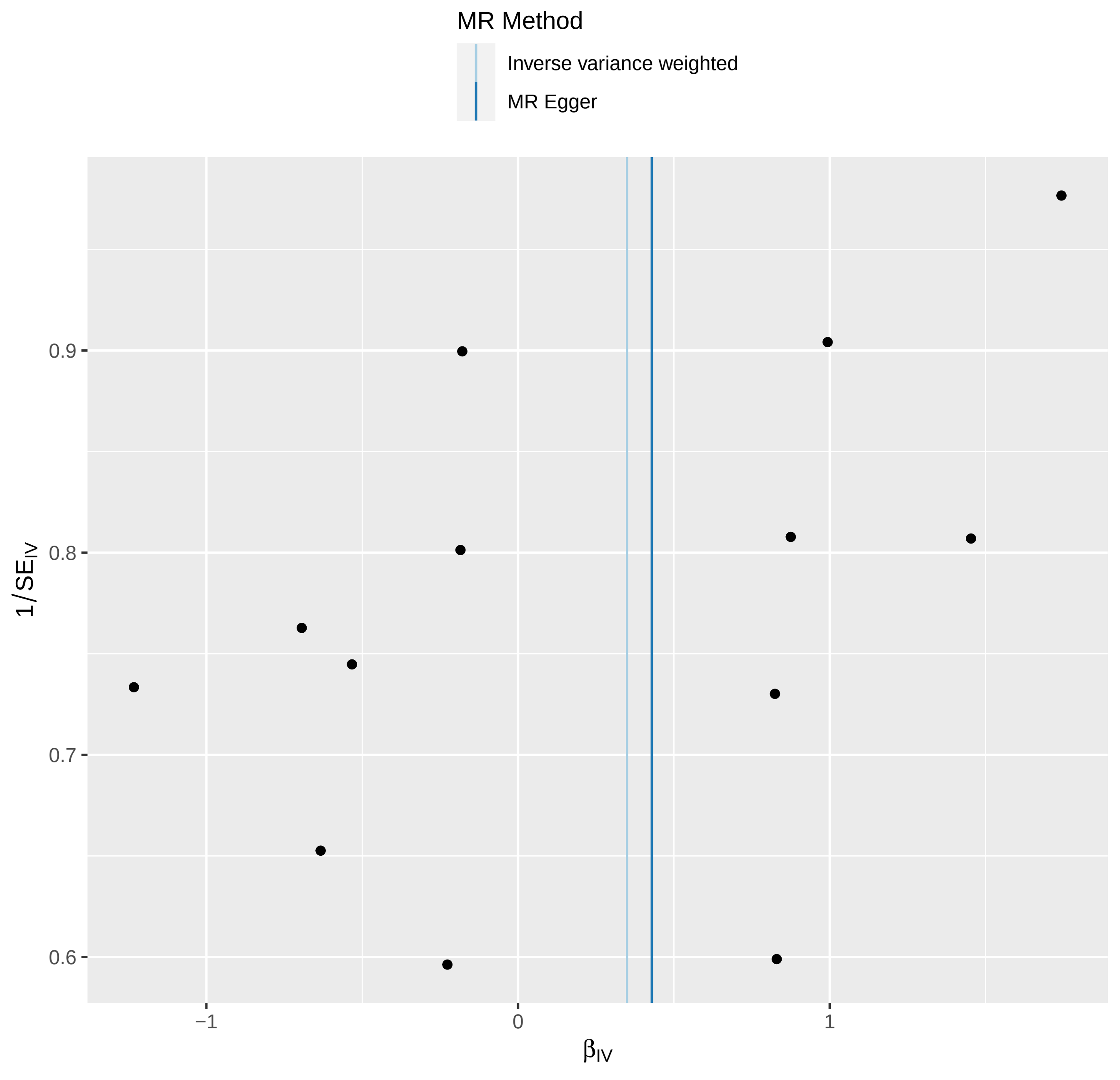

Batch 799 : Gut microbiota abundance (genus Butyrivibrio id.1993) on Arthropathy in ulcerative colitis

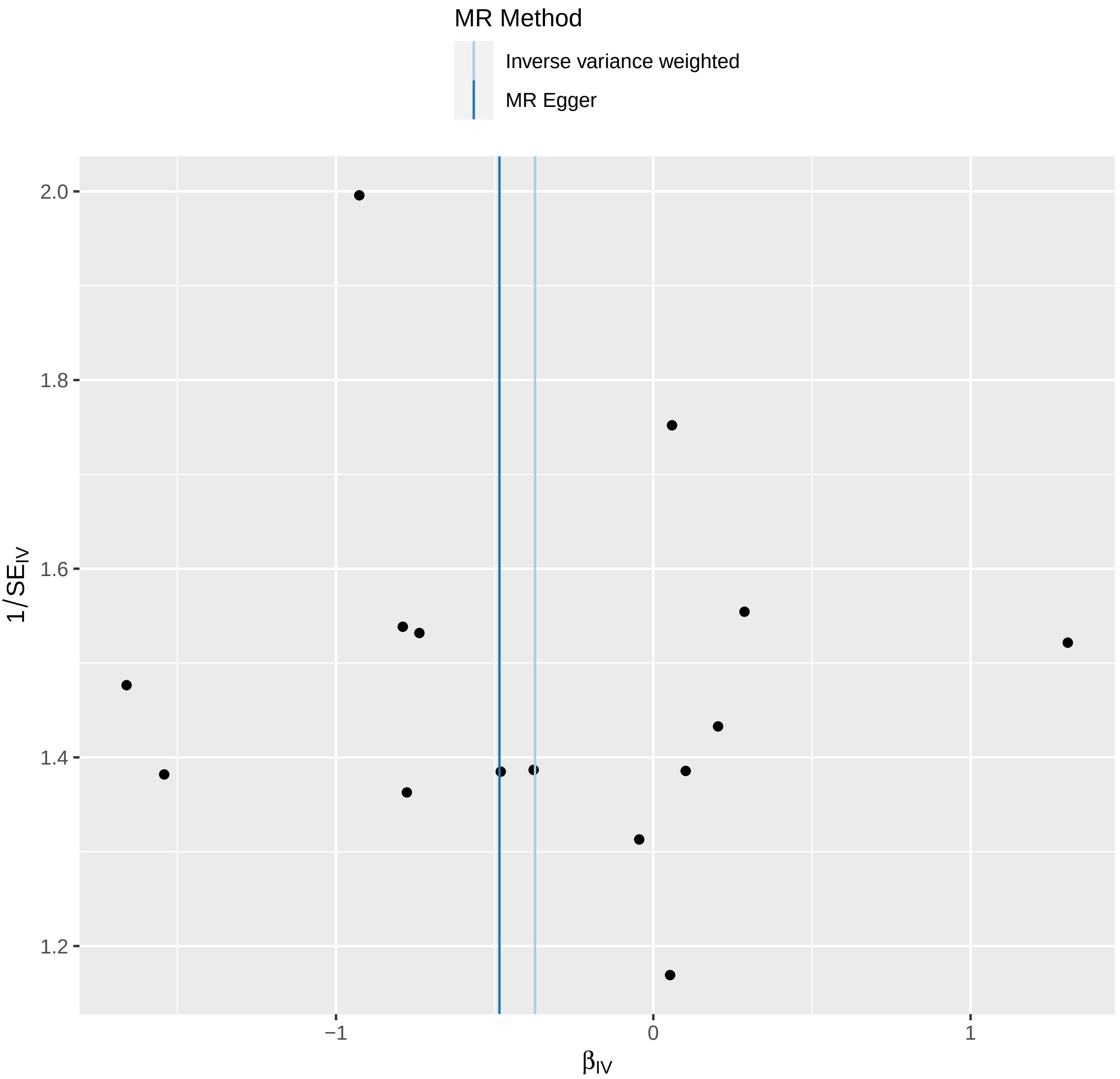

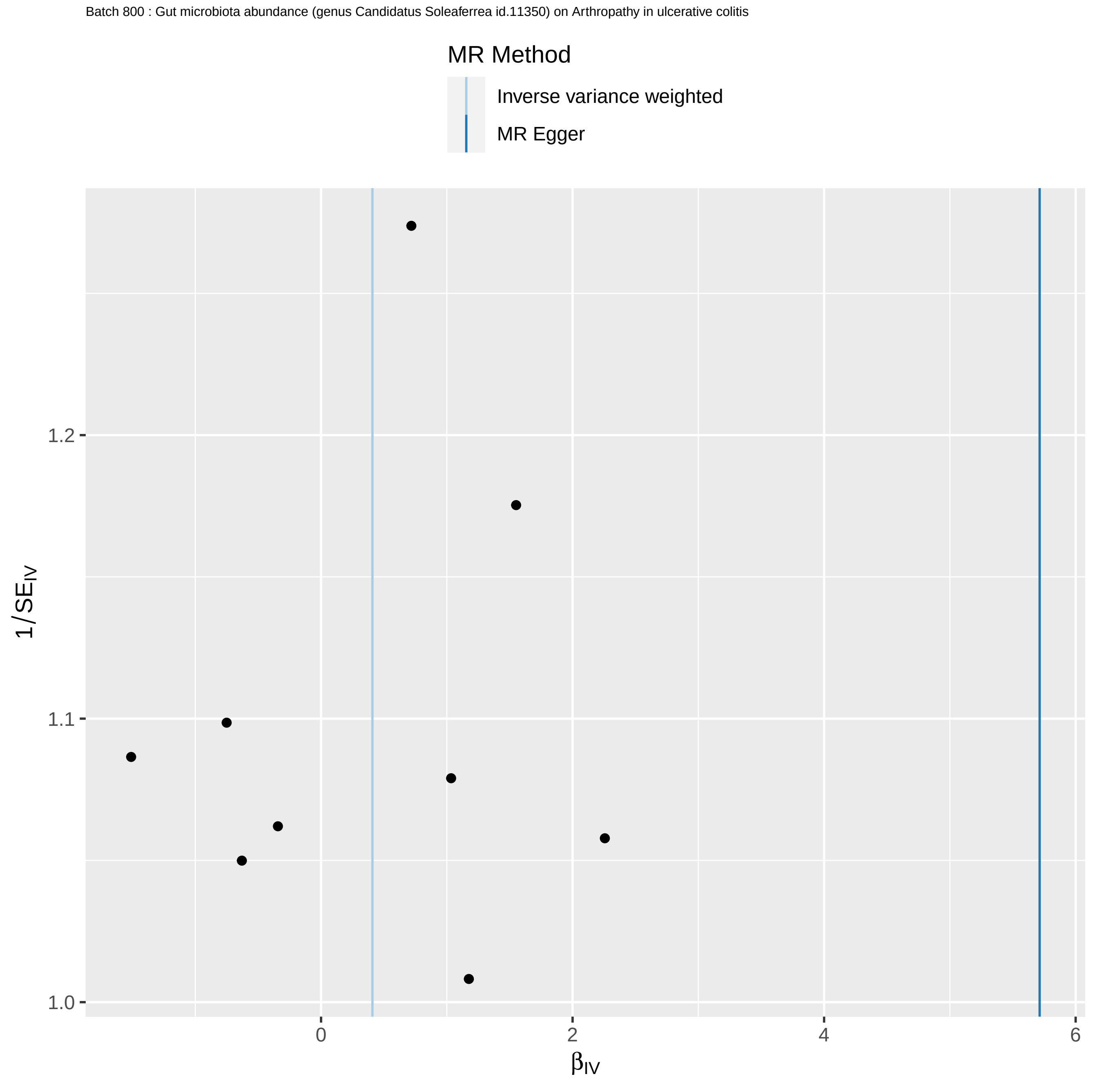

Batch 800 : Gut microbiota abundance (genus Candidatus Soleaferrea id.11350) on Arthropathy in ulcerative colitis
MR Method
Inverse variance weighted
MR Egger
1.2
1.1
1.0
0
2
4
6
1/SE_IV
β_IV

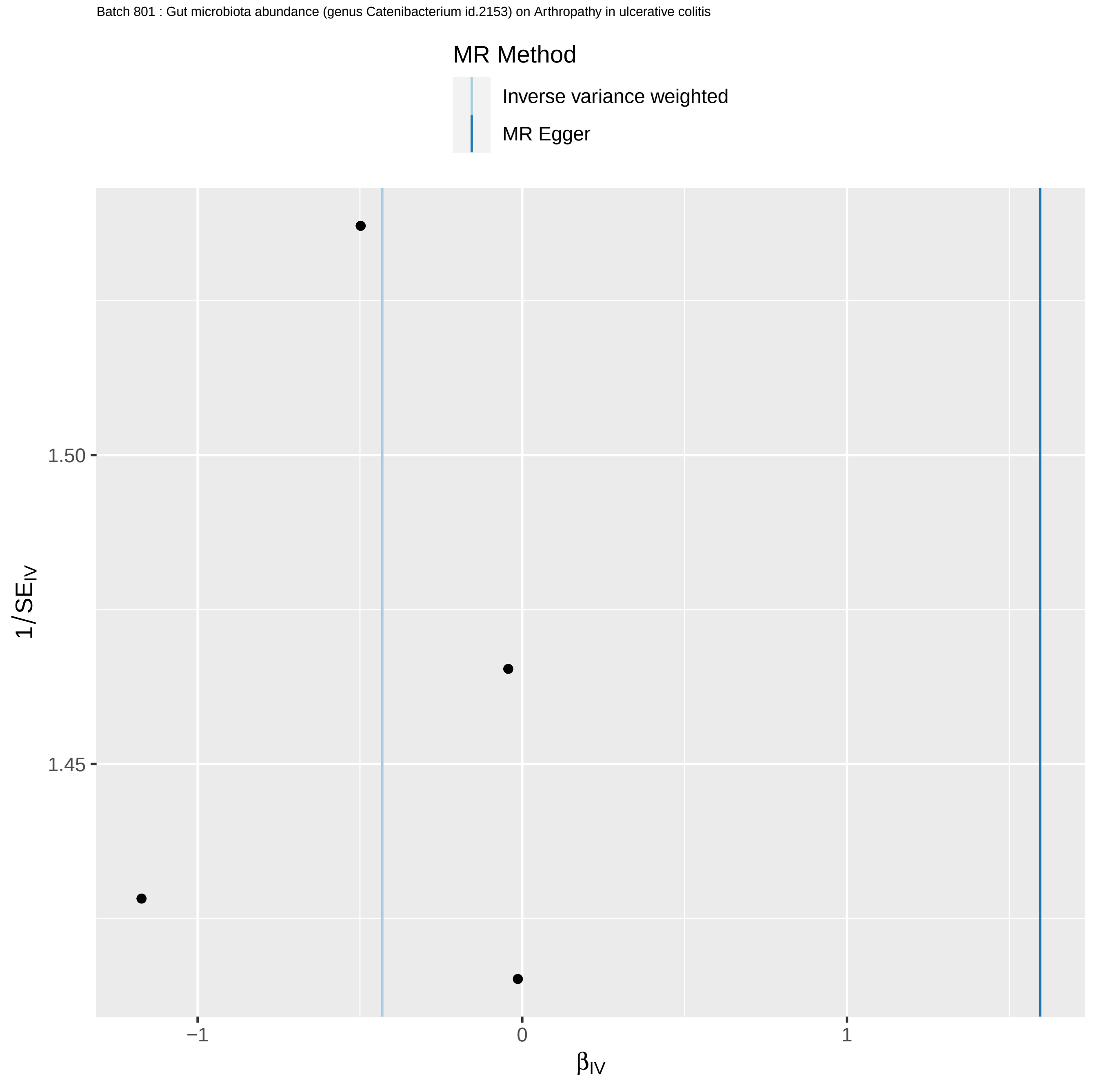

Batch 801 : Gut microbiota abundance (genus Catenibacterium id.2153) on Arthropathy in ulcerative colitis
MR Method
Inverse variance weighted
MR Egger
1.50
1.45
$1/SE_{IV}$
−1
0
1
β_{IV}

Batch 802 : Gut microbiota abundance (genus Christensenellaceae R 7group id.11283) on Arthropathy in ulcerative colitis

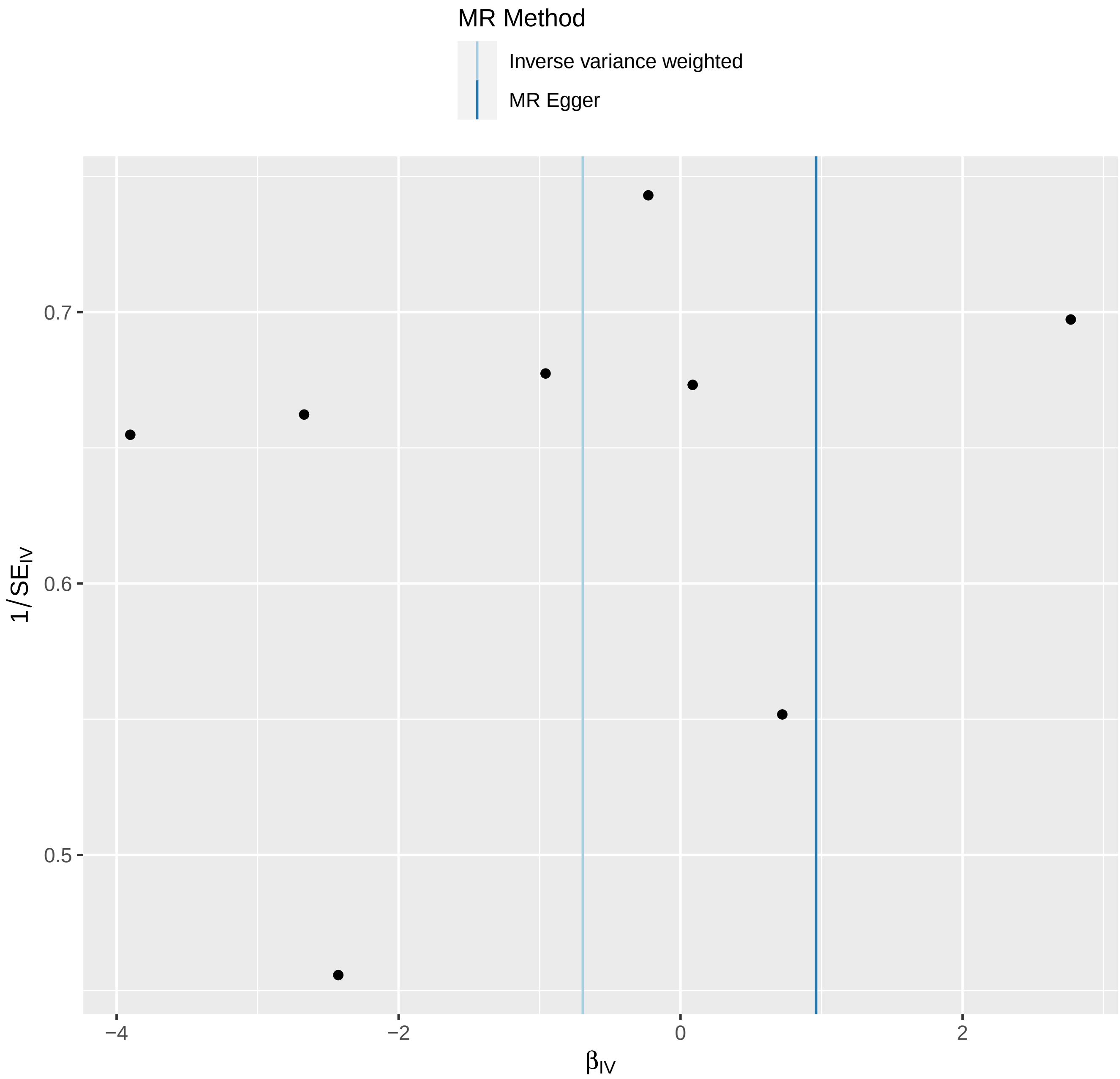

Batch 803 : Gut microbiota abundance (genus Clostridium innocuum group id.14397) on Arthropathy in ulcerative colitis

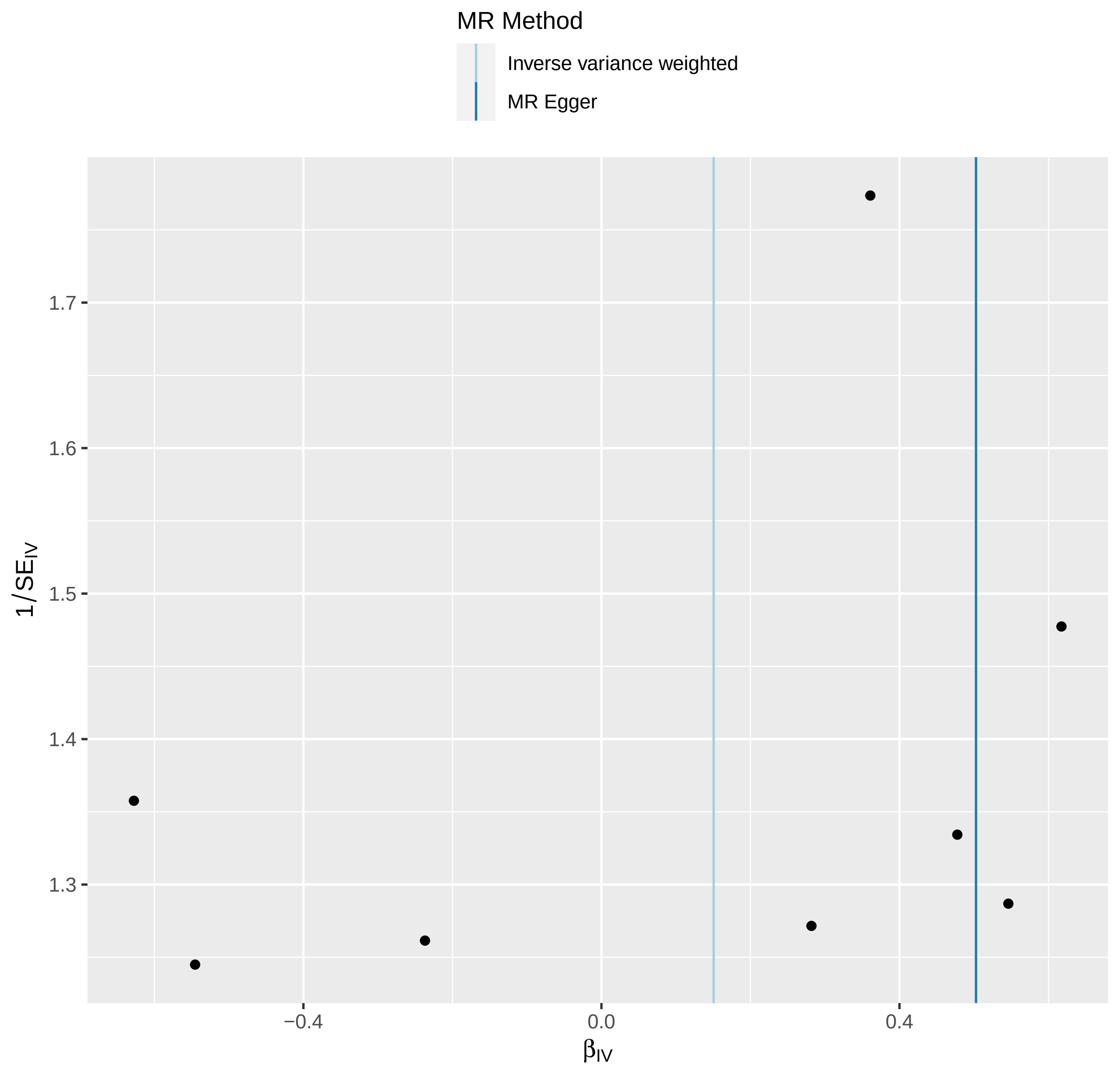

Batch 804 : Gut microbiota abundance (genus Clostridium sensustricto1 id.1873) on Arthropathy in ulcerative colitis

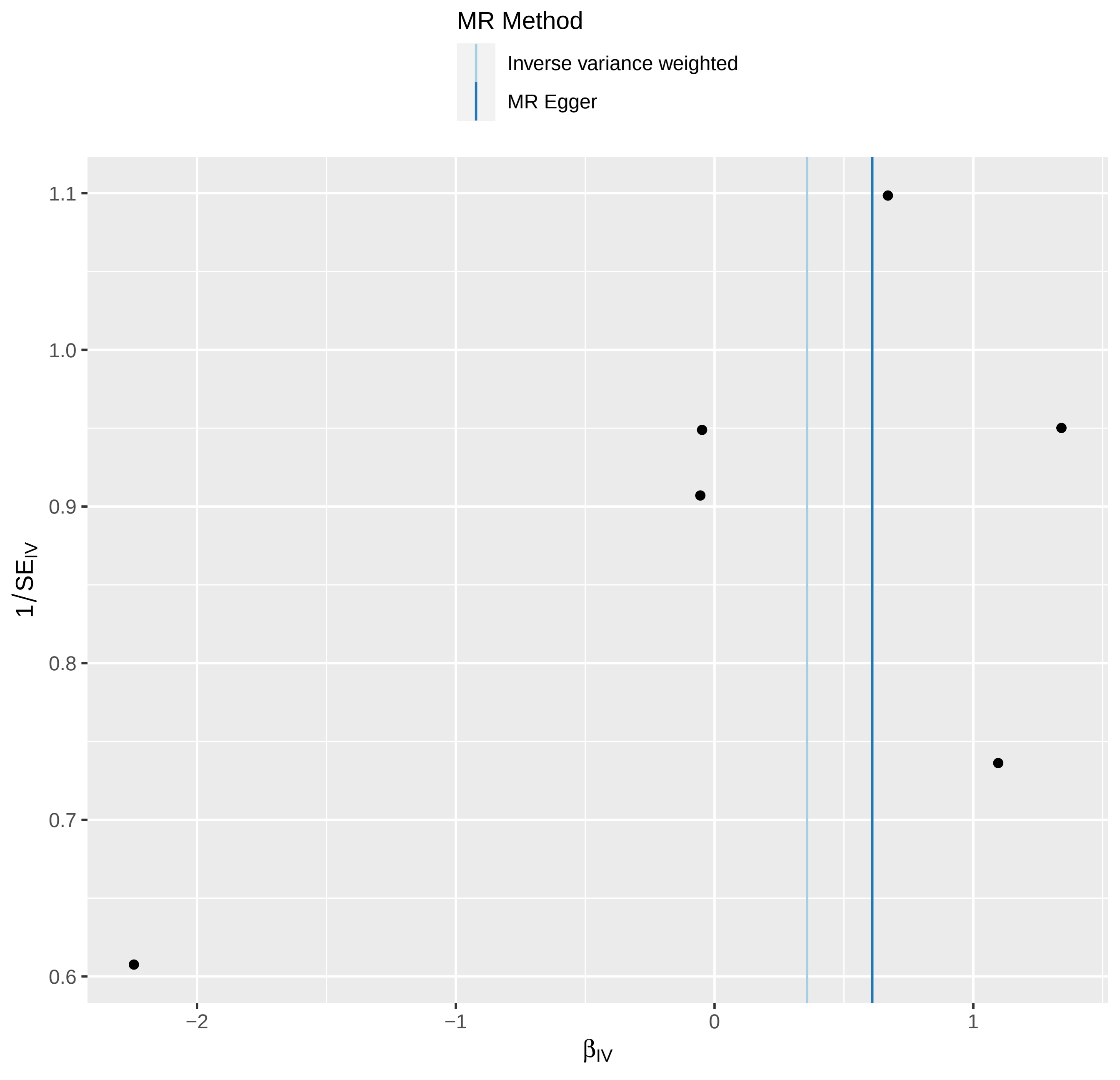

Batch 805 : Gut microbiota abundance (genus Collinsella id.815) on Arthropathy in ulcerative colitis

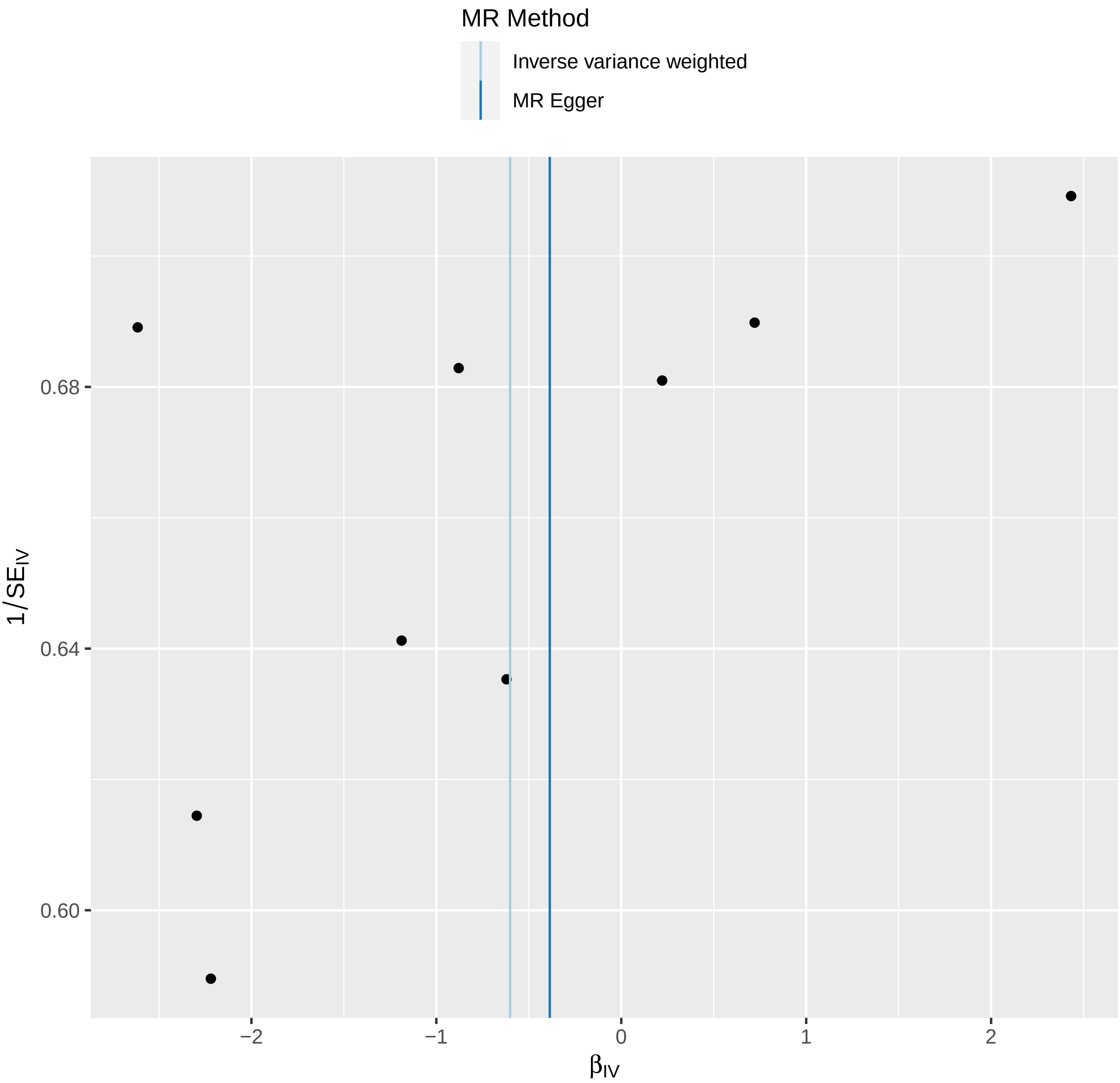

Batch 806 : Gut microbiota abundance (genus Coprobacter id.949) on Arthropathy in ulcerative colitis

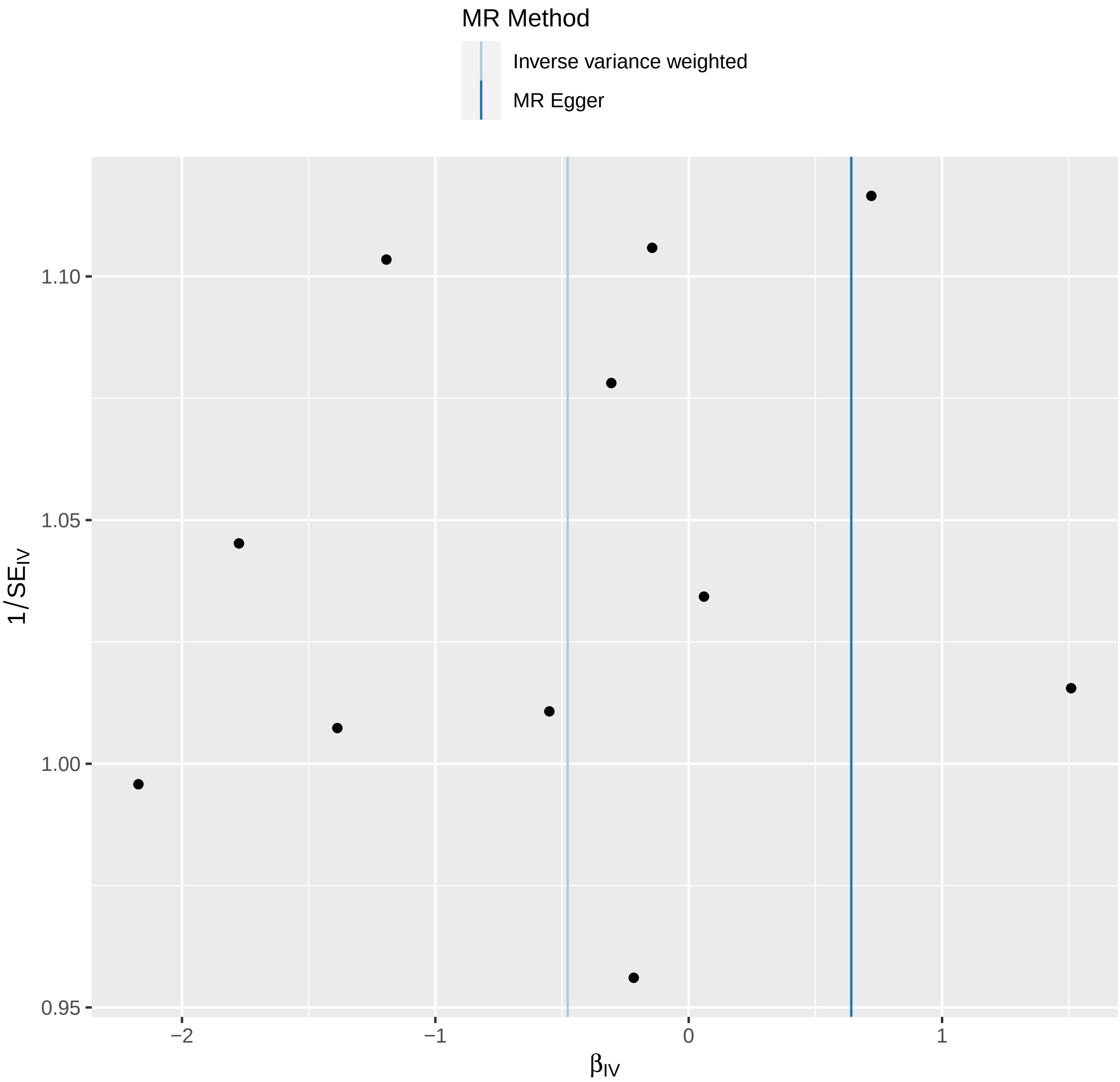

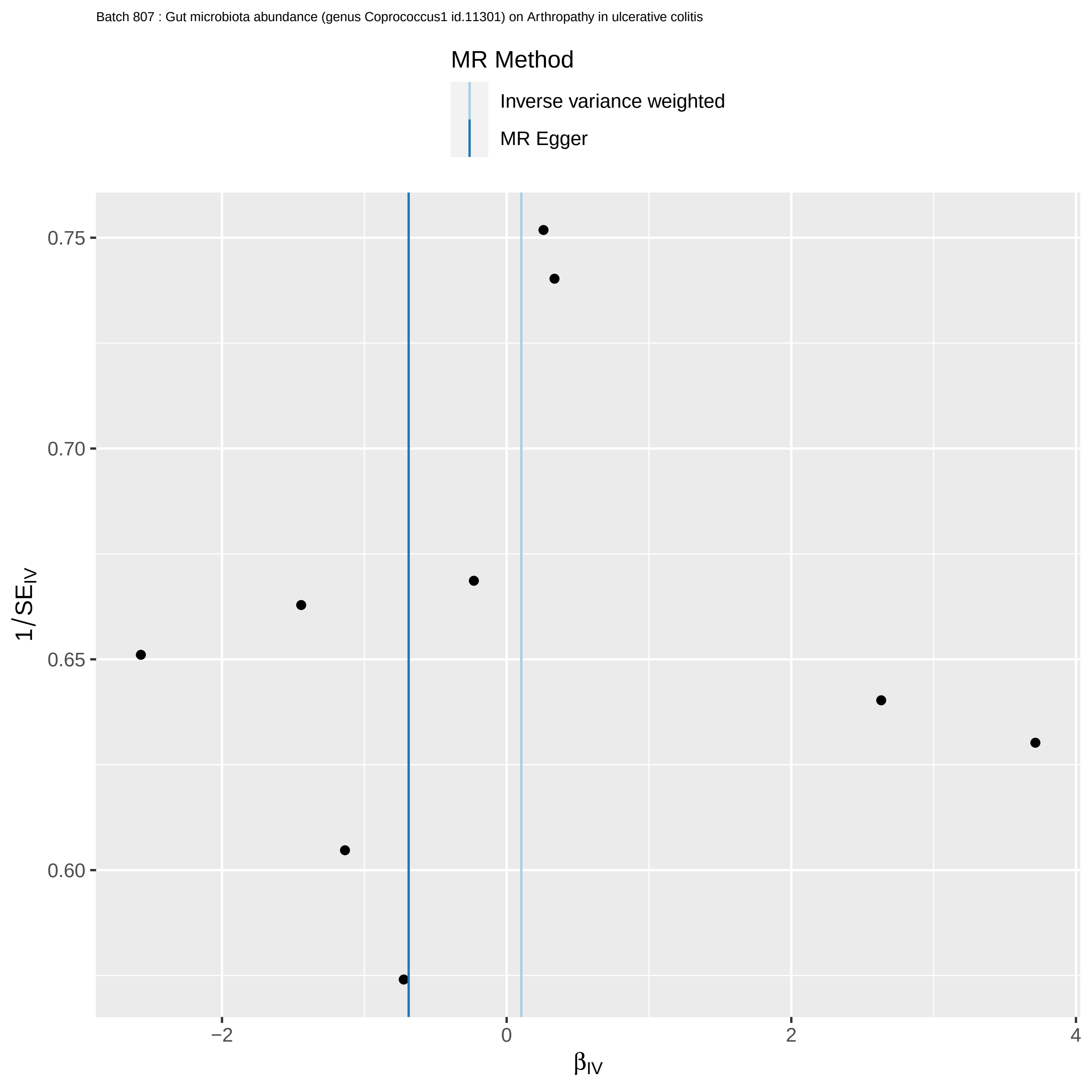
Batch 807 : Gut microbiota abundance (genus Coprococcus1 id.11301) on Arthropathy in ulcerative colitis
MR Method
Inverse variance weighted
MR Egger
0.75
0.70
0.65
0.60
1/SE_IV
−2
0
2
4
β_IV

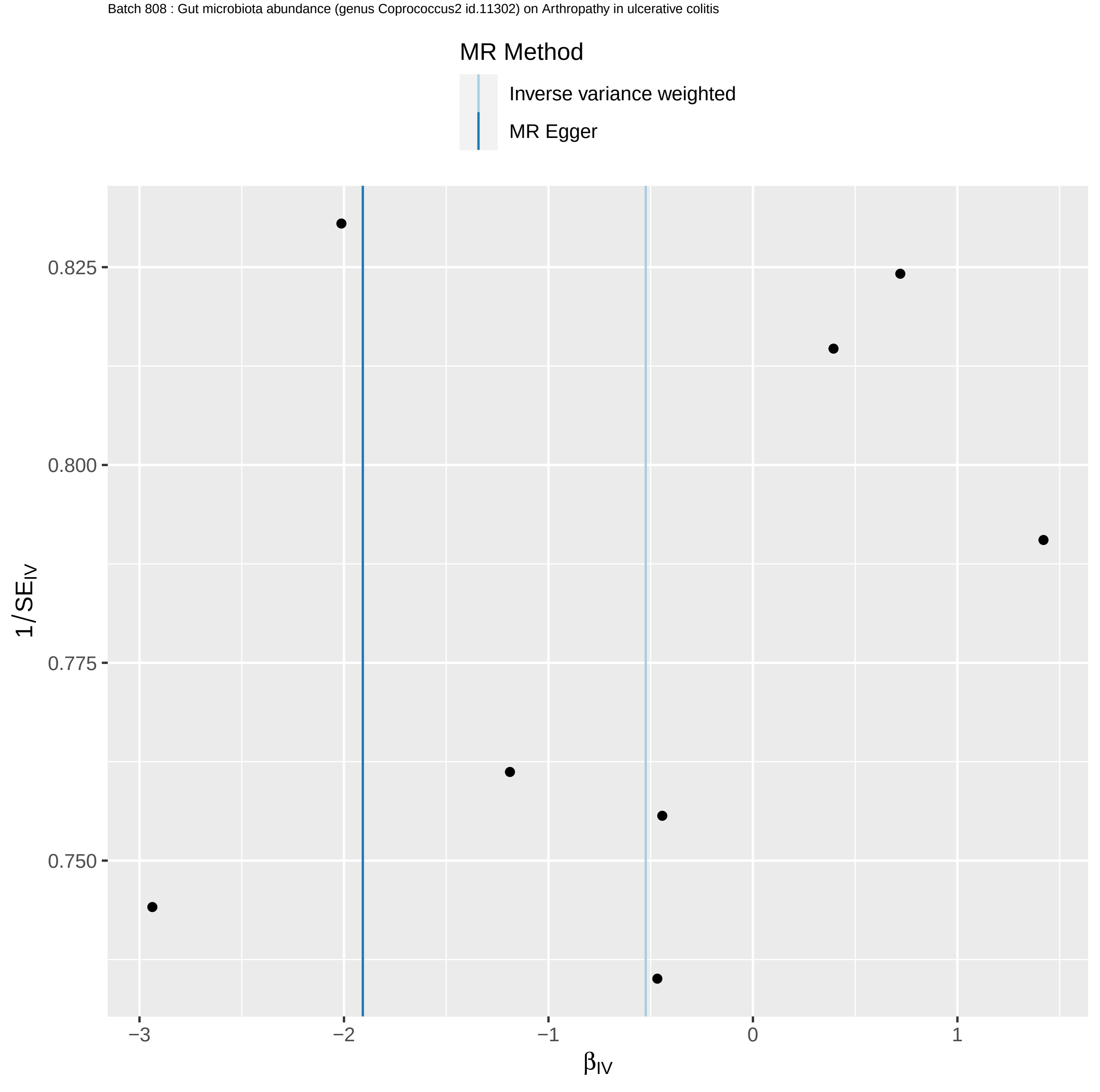
Batch 808 : Gut microbiota abundance (genus Coprococcus2 id.11302) on Arthropathy in ulcerative colitis
MR Method
Inverse variance weighted
MR Egger
0.825
0.800
0.775
0.750
1/SE$_{IV}$
−3
−2
−1
0
1
β$_{IV}$

Batch 809 : Gut microbiota abundance (genus Coprococcus3 id.11303) on Arthropathy in ulcerative colitis

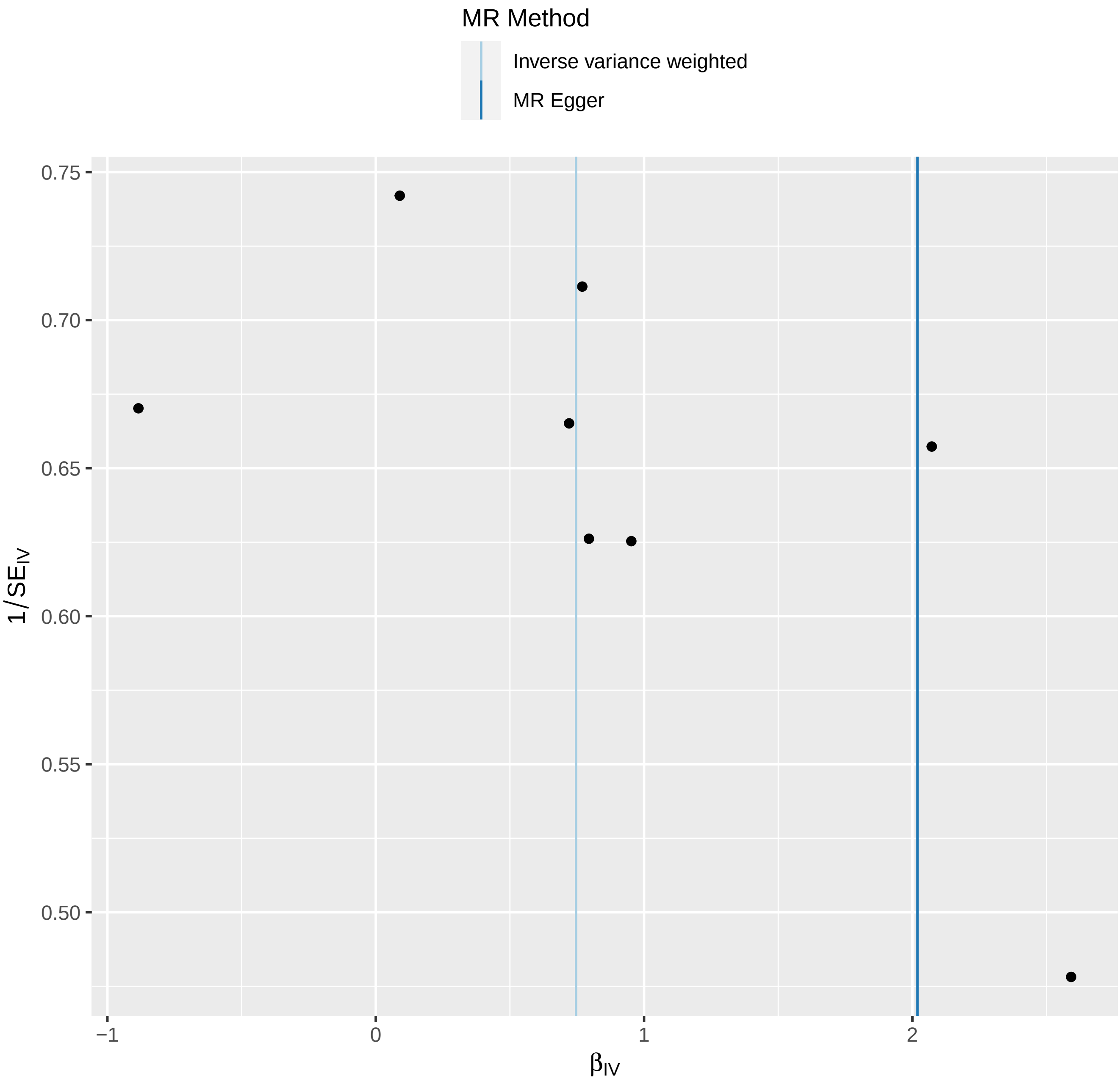

Batch 810 : Gut microbiota abundance (genus Defluviitaleaceae UCG011 id.11287) on Arthropathy in ulcerative colitis

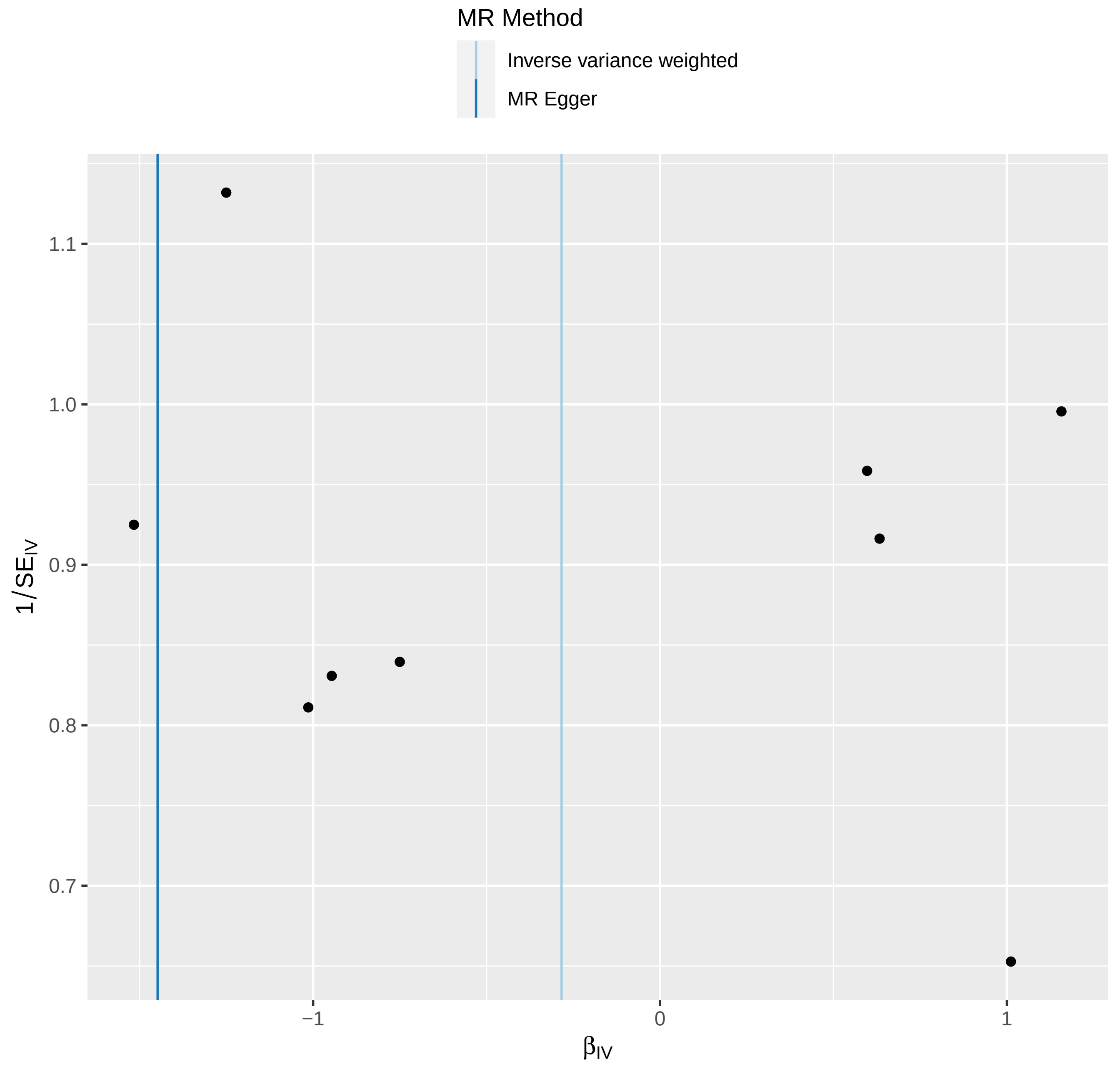

Batch 811 : Gut microbiota abundance (genus Desulfovibrio id.3173) on Arthropathy in ulcerative colitis

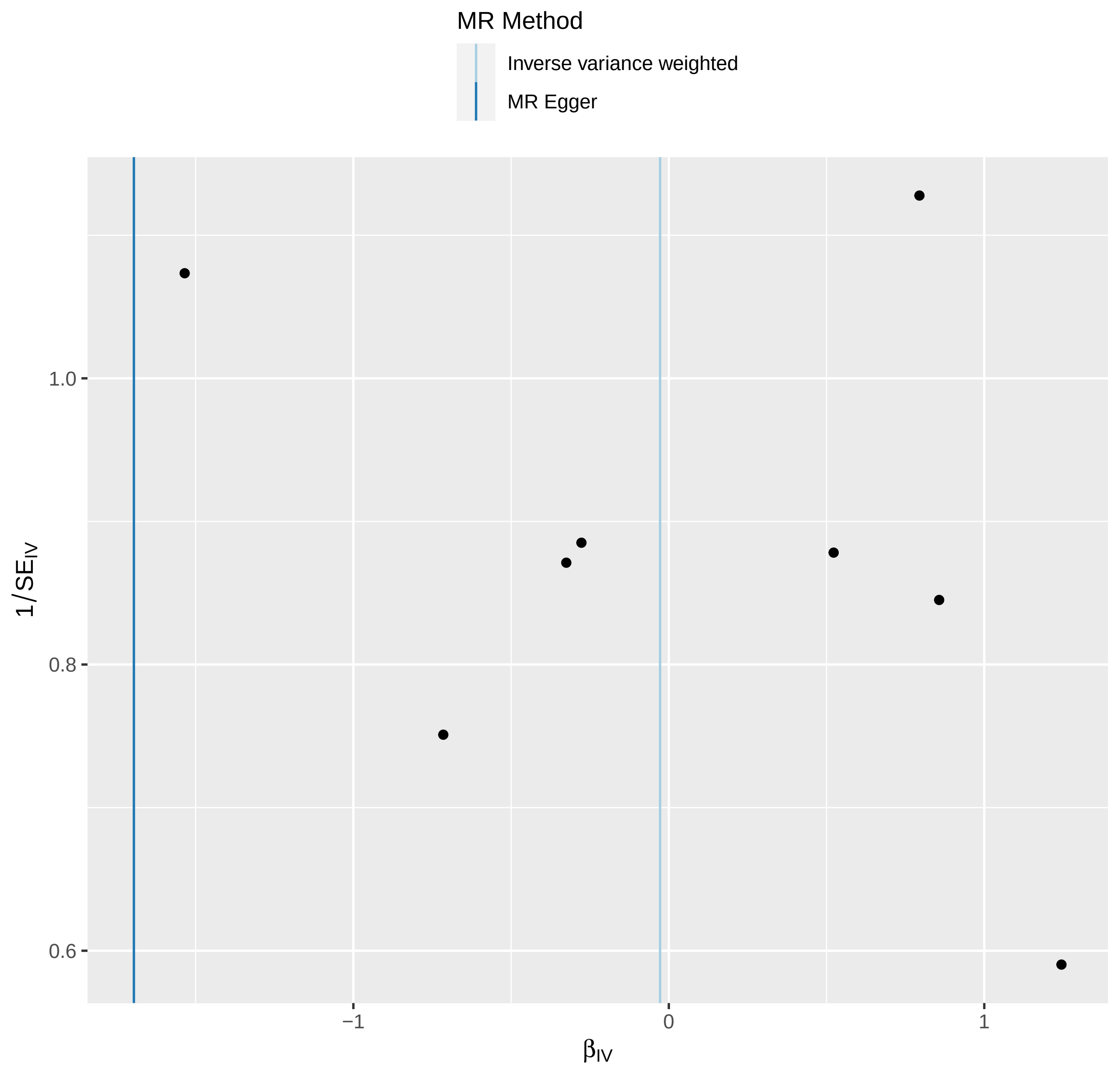

Batch 812 : Gut microbiota abundance (genus Dialister id.2183) on Arthropathy in ulcerative colitis

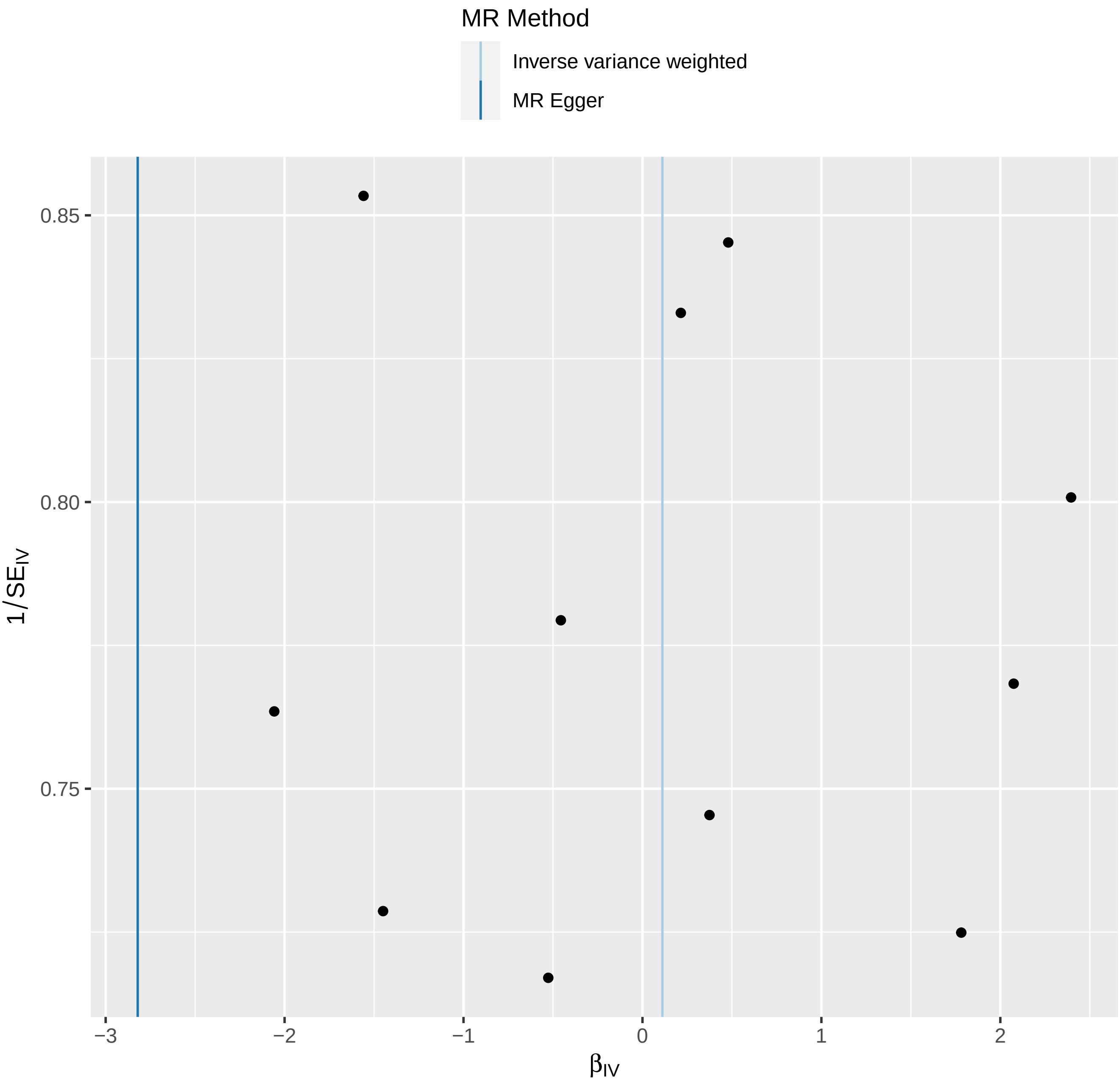

Batch 813 : Gut microbiota abundance (genus Dorea id.1997) on Arthropathy in ulcerative colitis

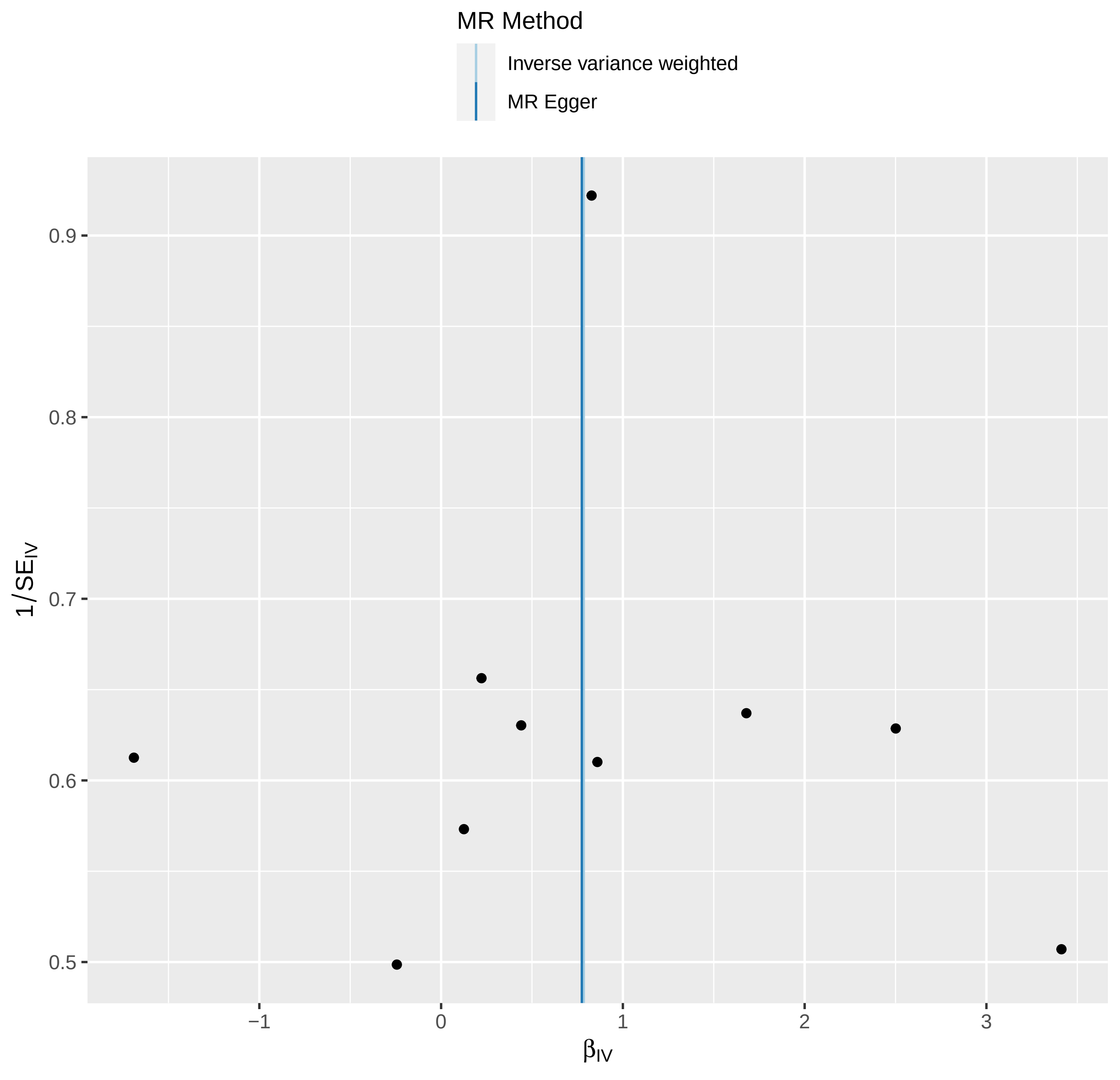

Batch 814 : Gut microbiota abundance (genus Eggerthella id.819) on Arthropathy in ulcerative colitis

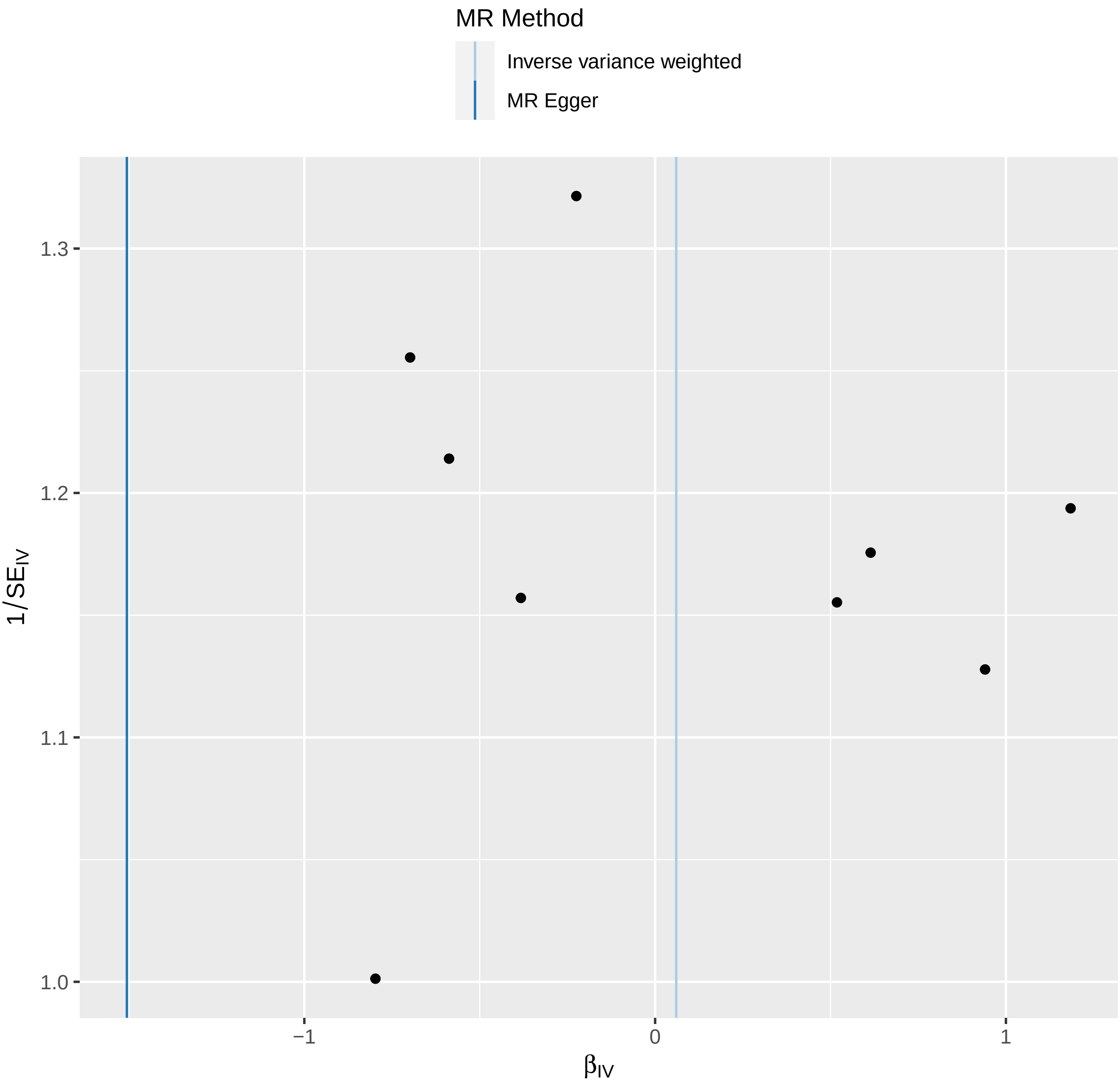

Batch 815 : Gut microbiota abundance (genus Eisenbergiella id.11304) on Arthropathy in ulcerative colitis

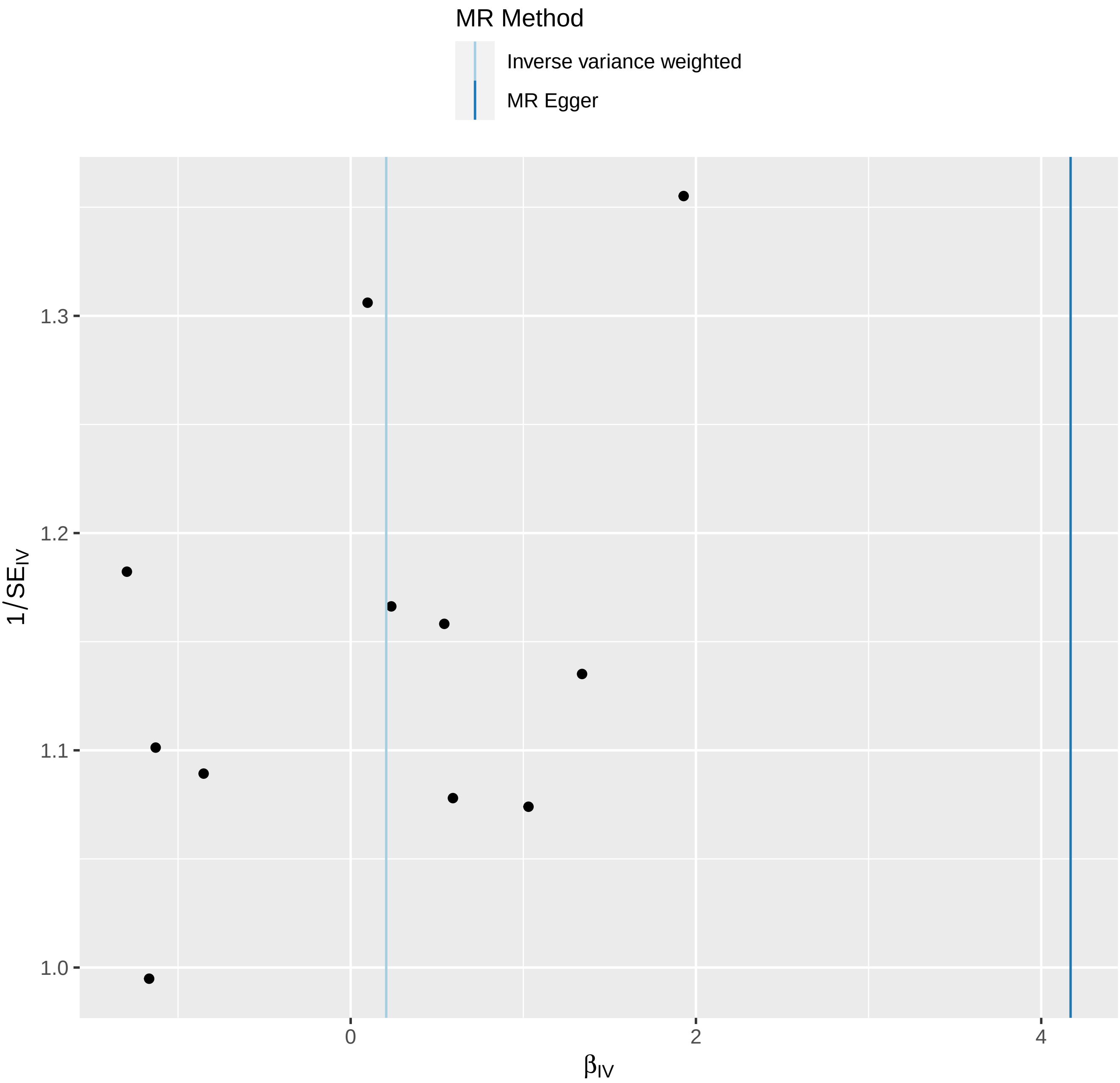

Batch 816 : Gut microbiota abundance (genus Enterorhabdus id.820) on Arthropathy in ulcerative colitis

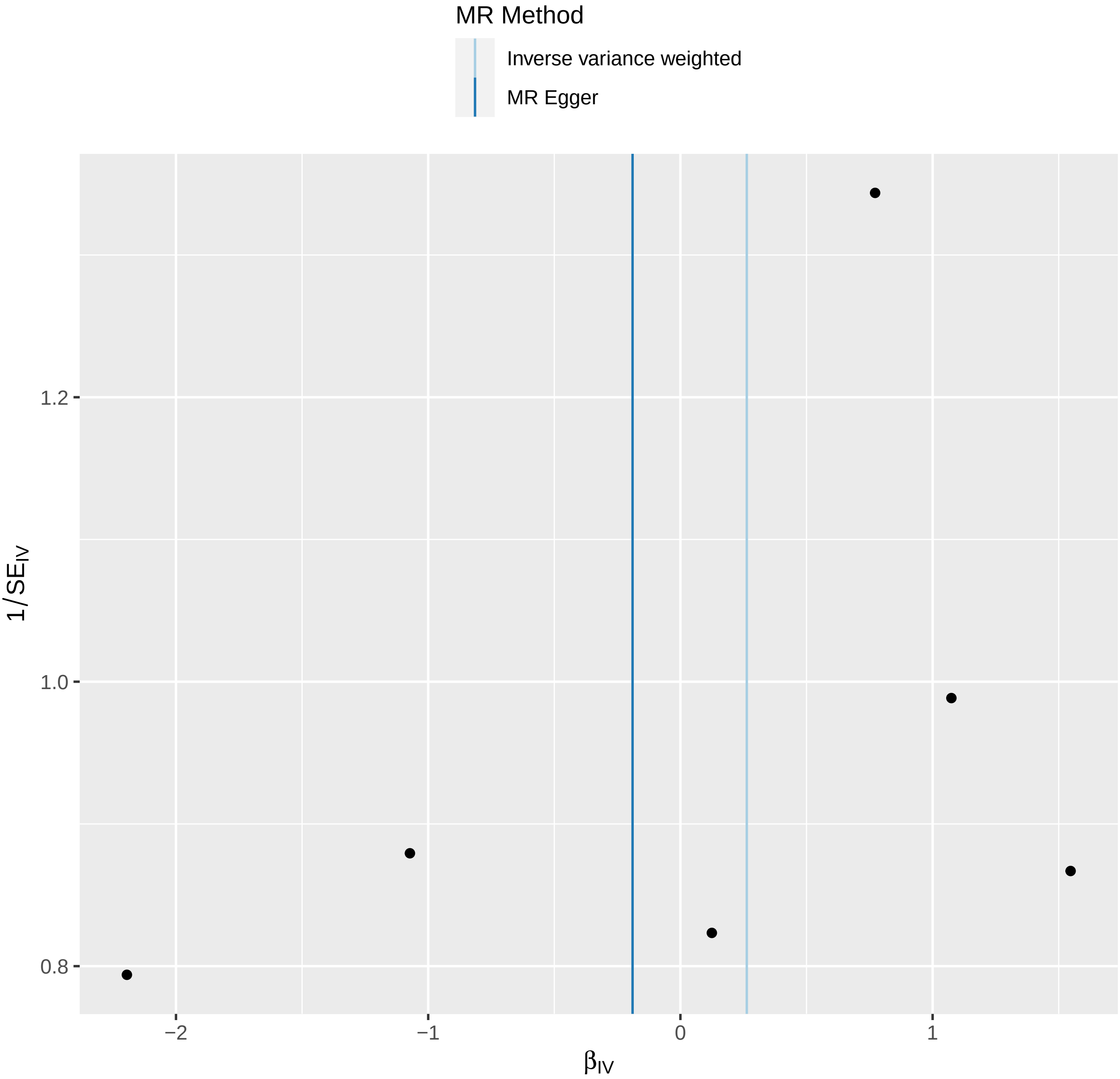

Batch 817 : Gut microbiota abundance (genus Erysipelatoclostridium id.11381) on Arthropathy in ulcerative colitis

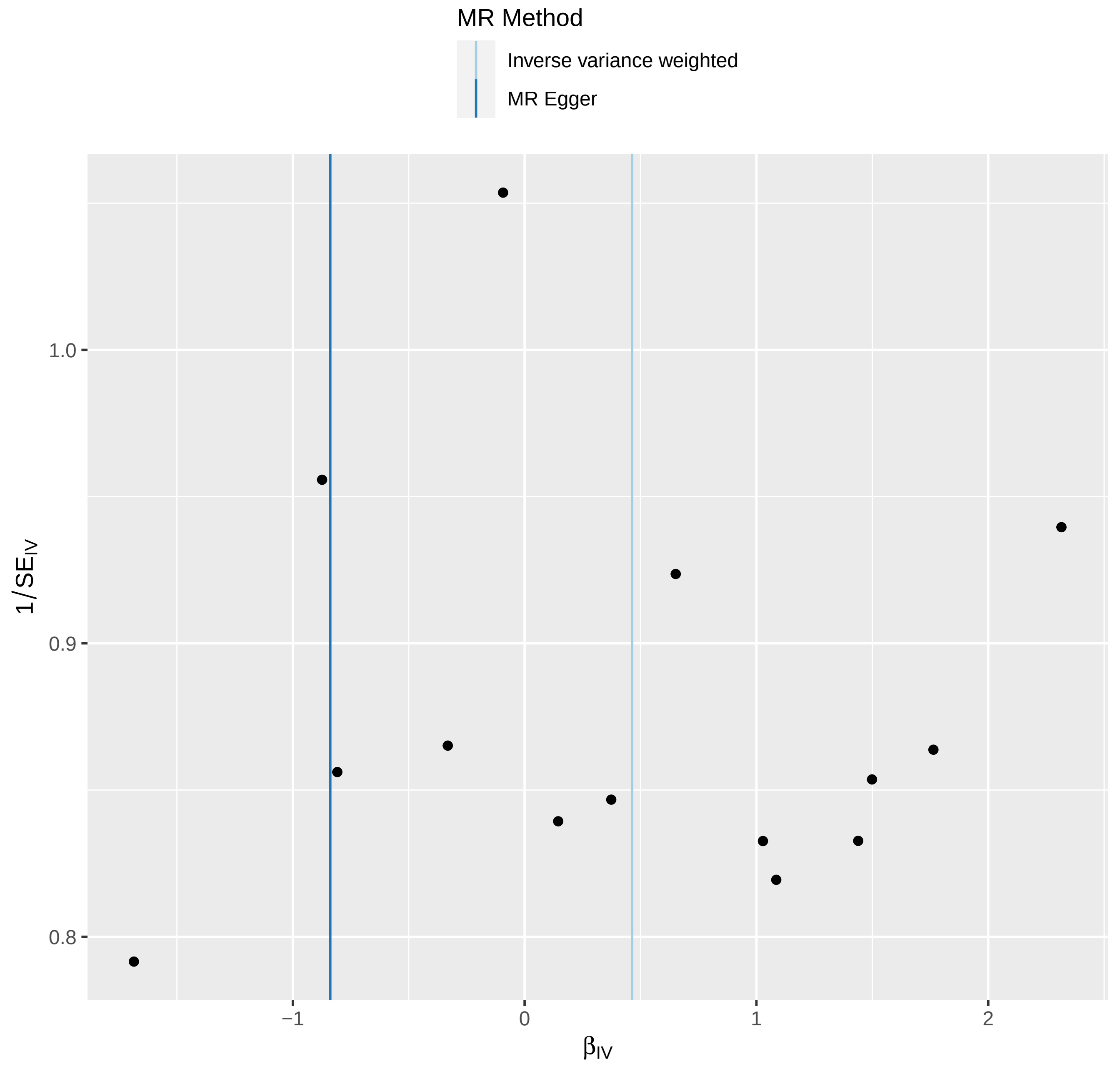

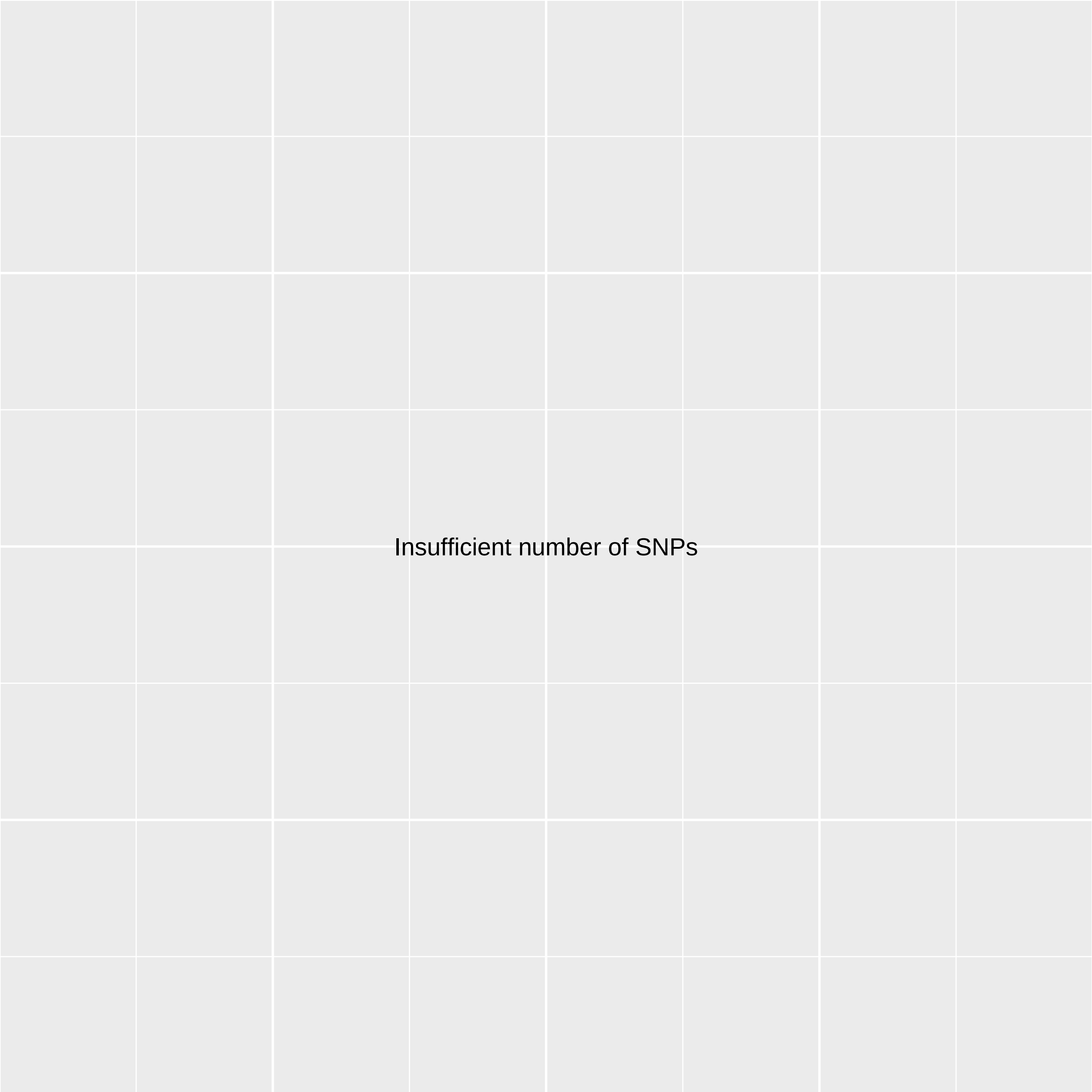
Insufficient number of SNPs

Batch 819 : Gut microbiota abundance (genus Escherichia Shigella id.3504) on Arthropathy in ulcerative colitis

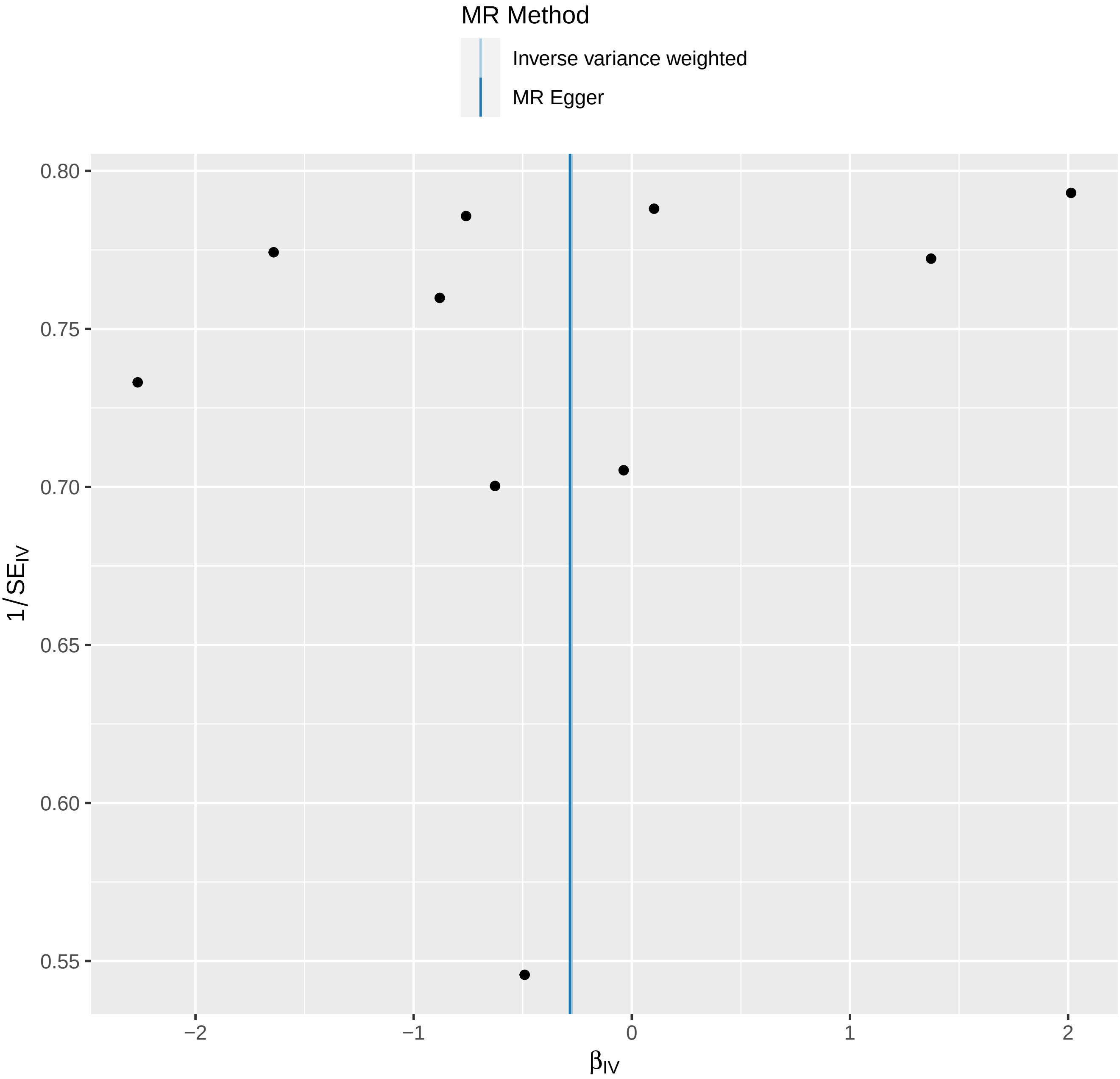

Batch 820 : Gut microbiota abundance (genus Eubacterium brachy group id.11296) on Arthropathy in ulcerative colitis

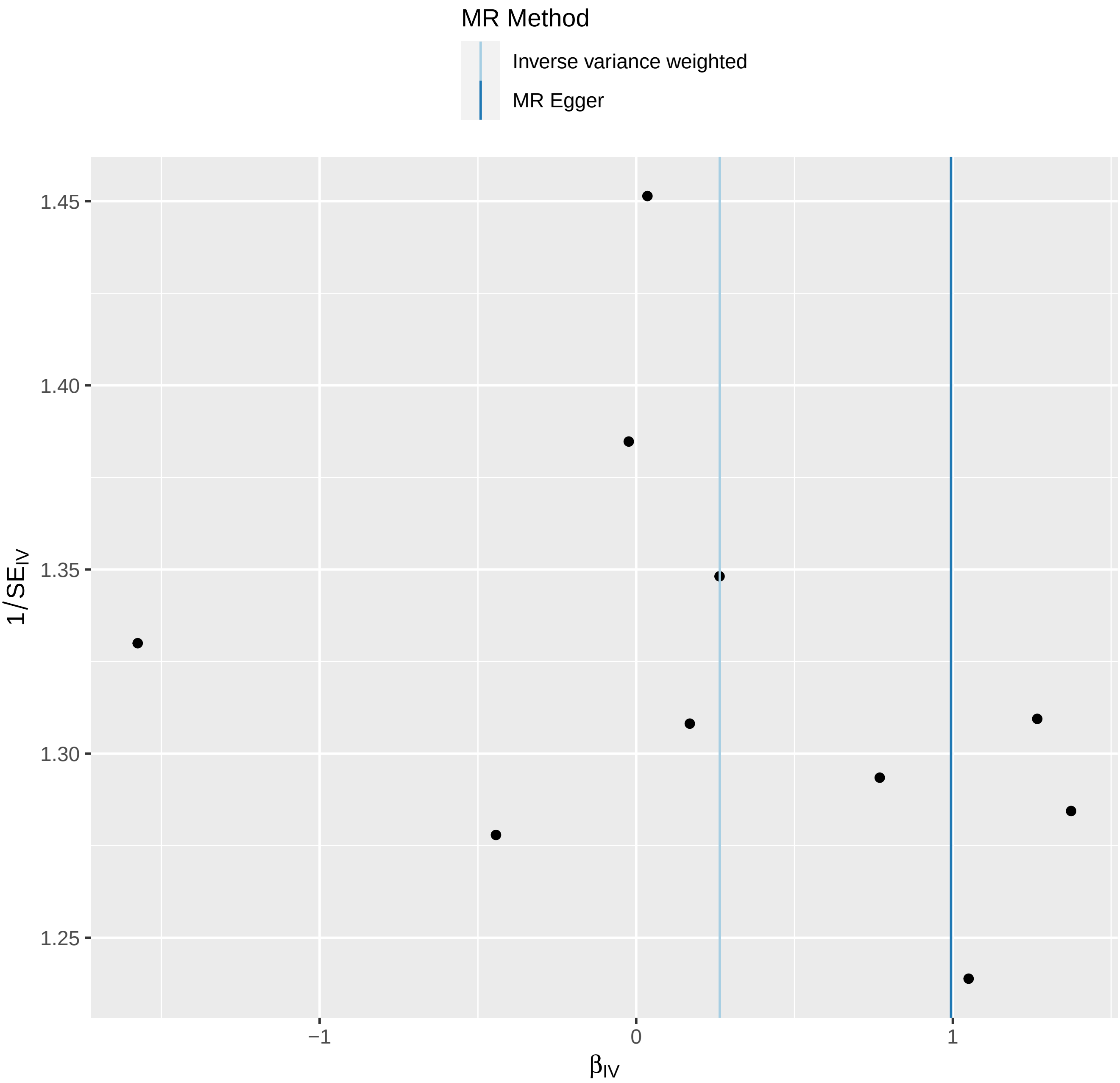

Batch 821 : Gut microbiota abundance (genus Eubacterium coprostanoligenes group id.11375) on Arthropathy in ulcerative colitis

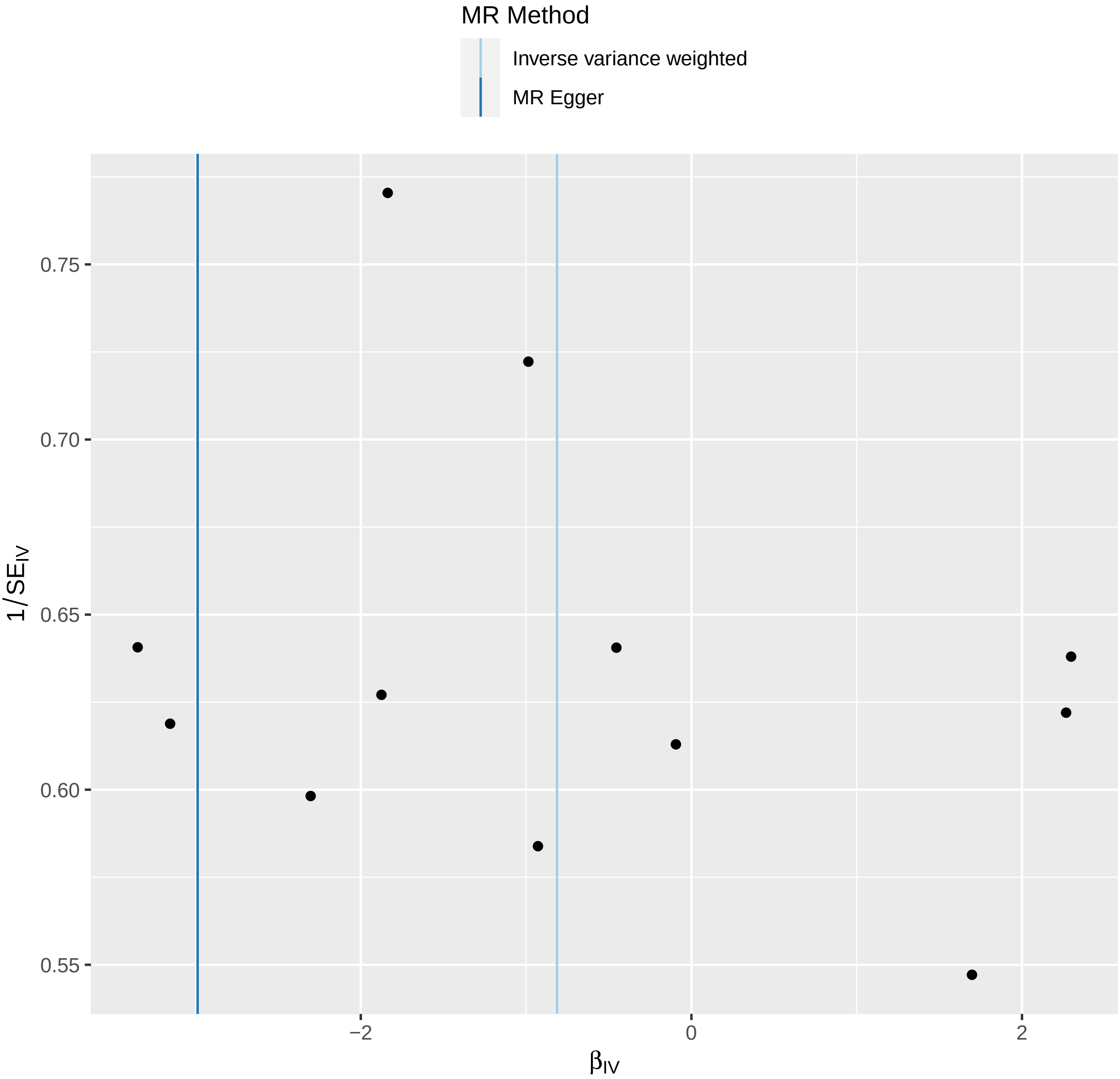

Batch 822 : Gut microbiota abundance (genus Eubacterium eligens group id.14372) on Arthropathy in ulcerative colitis

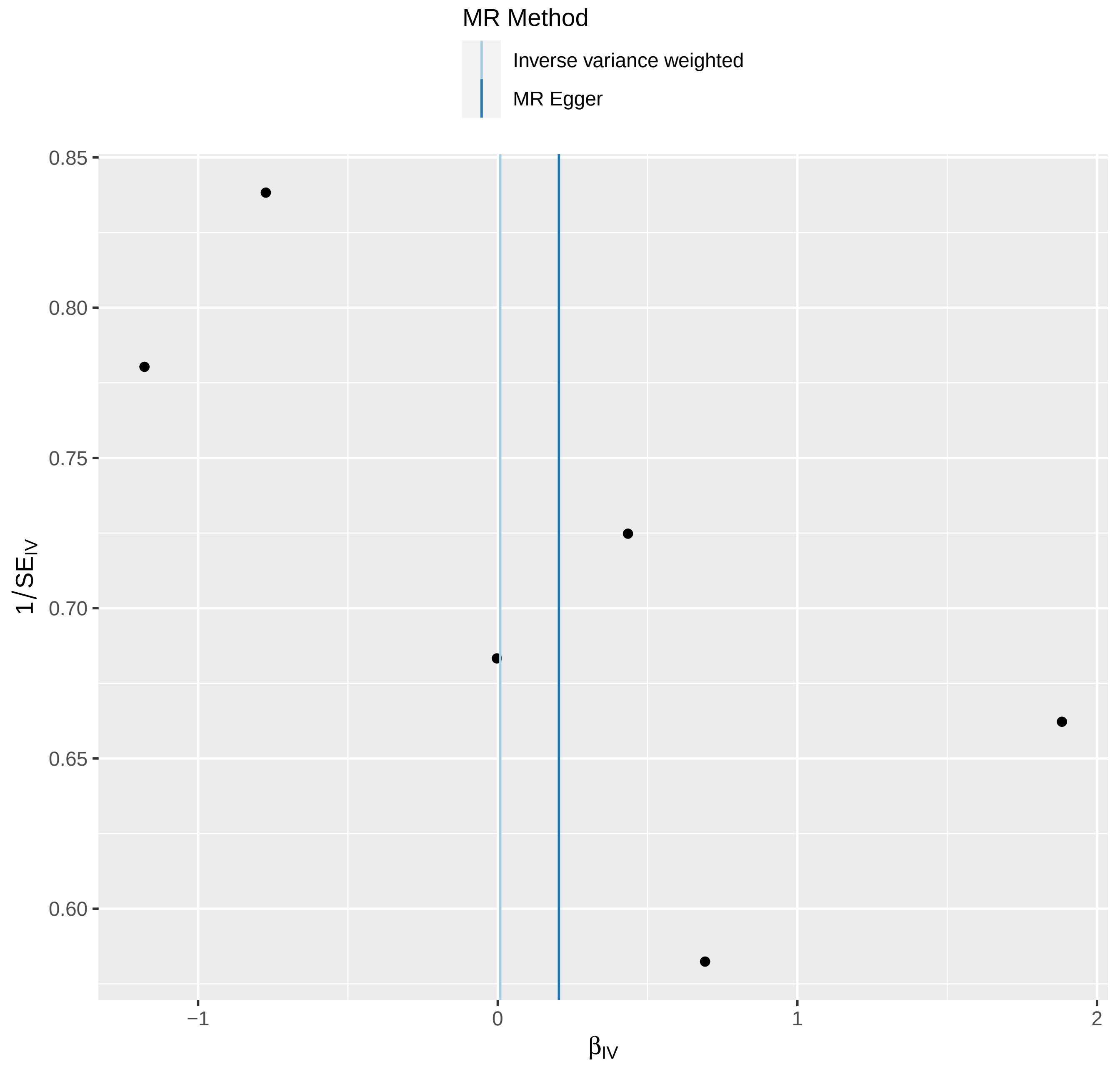

Batch 823 : Gut microbiota abundance (genus Eubacterium fissicatena group id.14373) on Arthropathy in ulcerative colitis

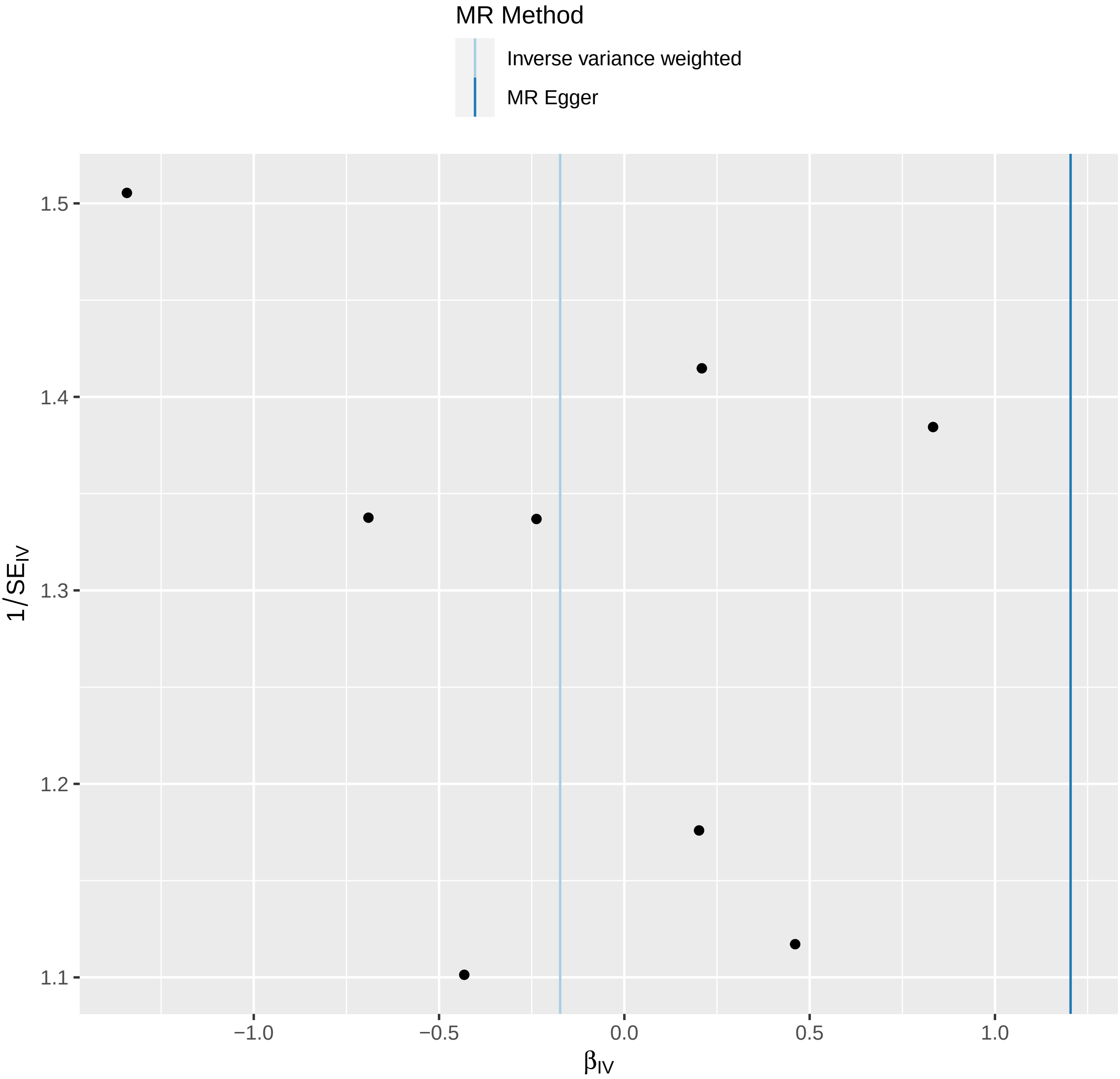

Batch 824 : Gut microbiota abundance (genus Eubacterium hallii group id.11338) on Arthropathy in ulcerative colitis

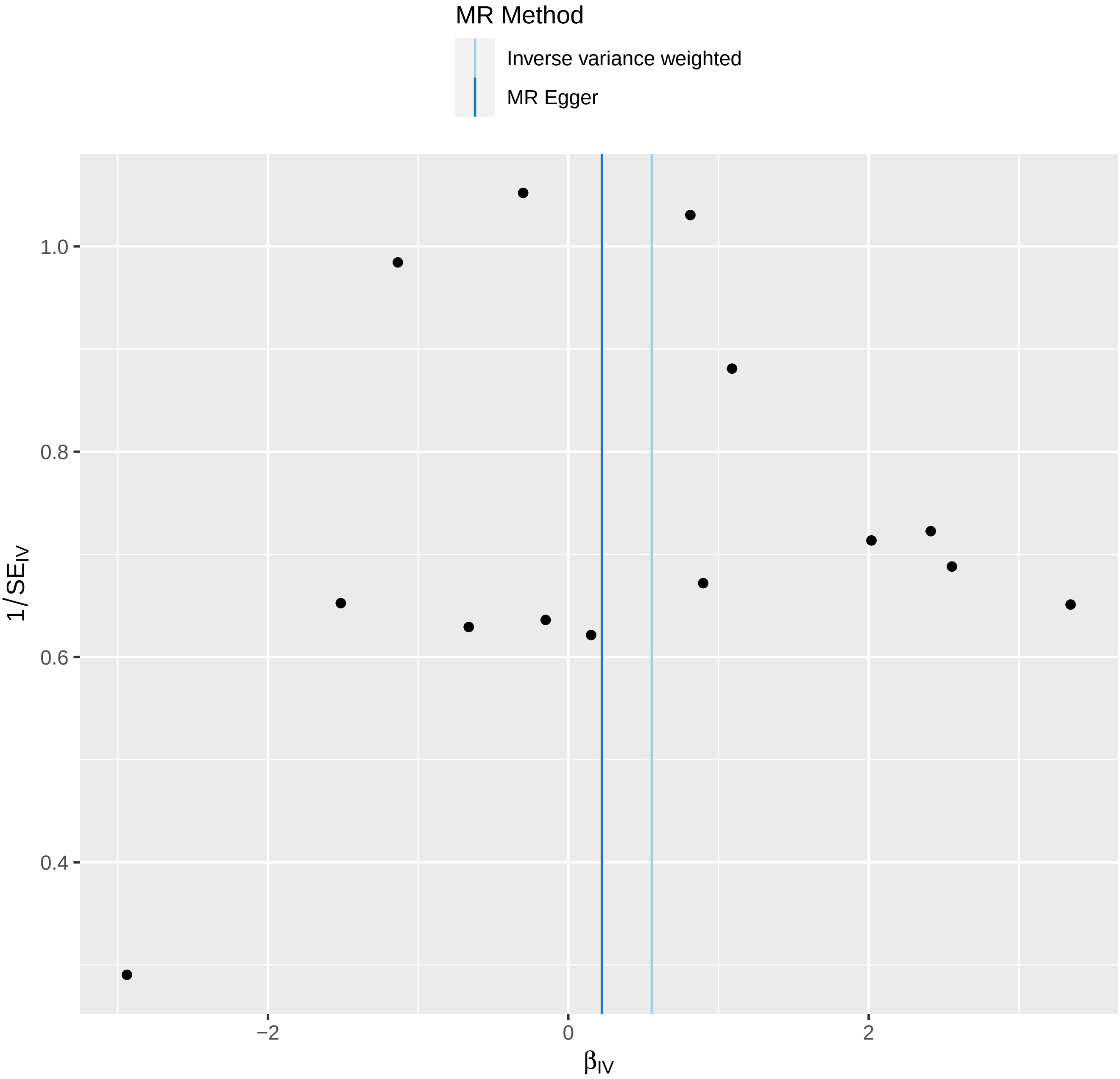

Batch 825 : Gut microbiota abundance (genus Eubacterium nodatum group id.11297) on Arthropathy in ulcerative colitis

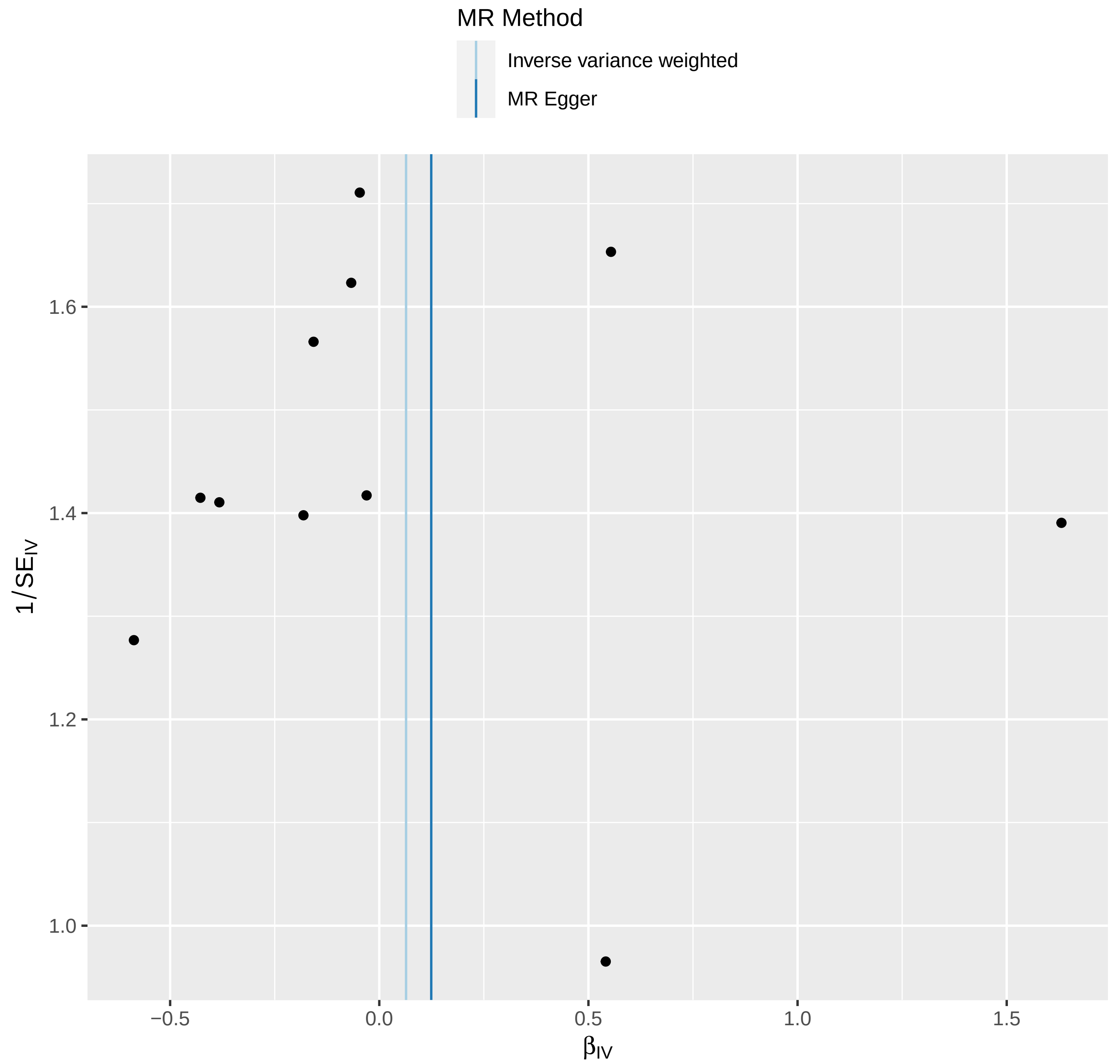

Batch 826 : Gut microbiota abundance (genus Eubacterium oxidoreducens group id.11339) on Arthropathy in ulcerative colitis

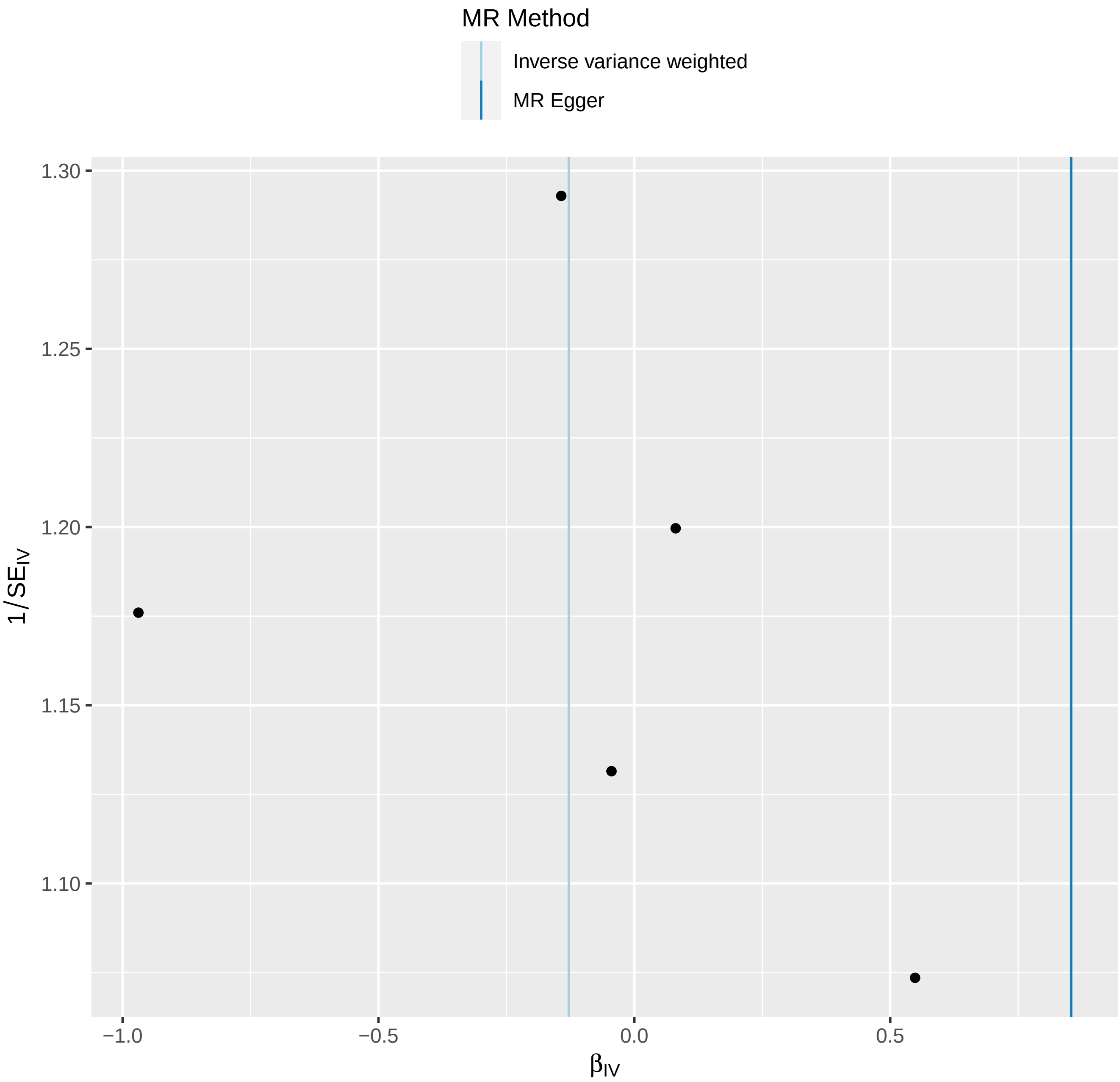

Batch 827 : Gut microbiota abundance (genus Eubacterium rectale group id.14374) on Arthropathy in ulcerative colitis

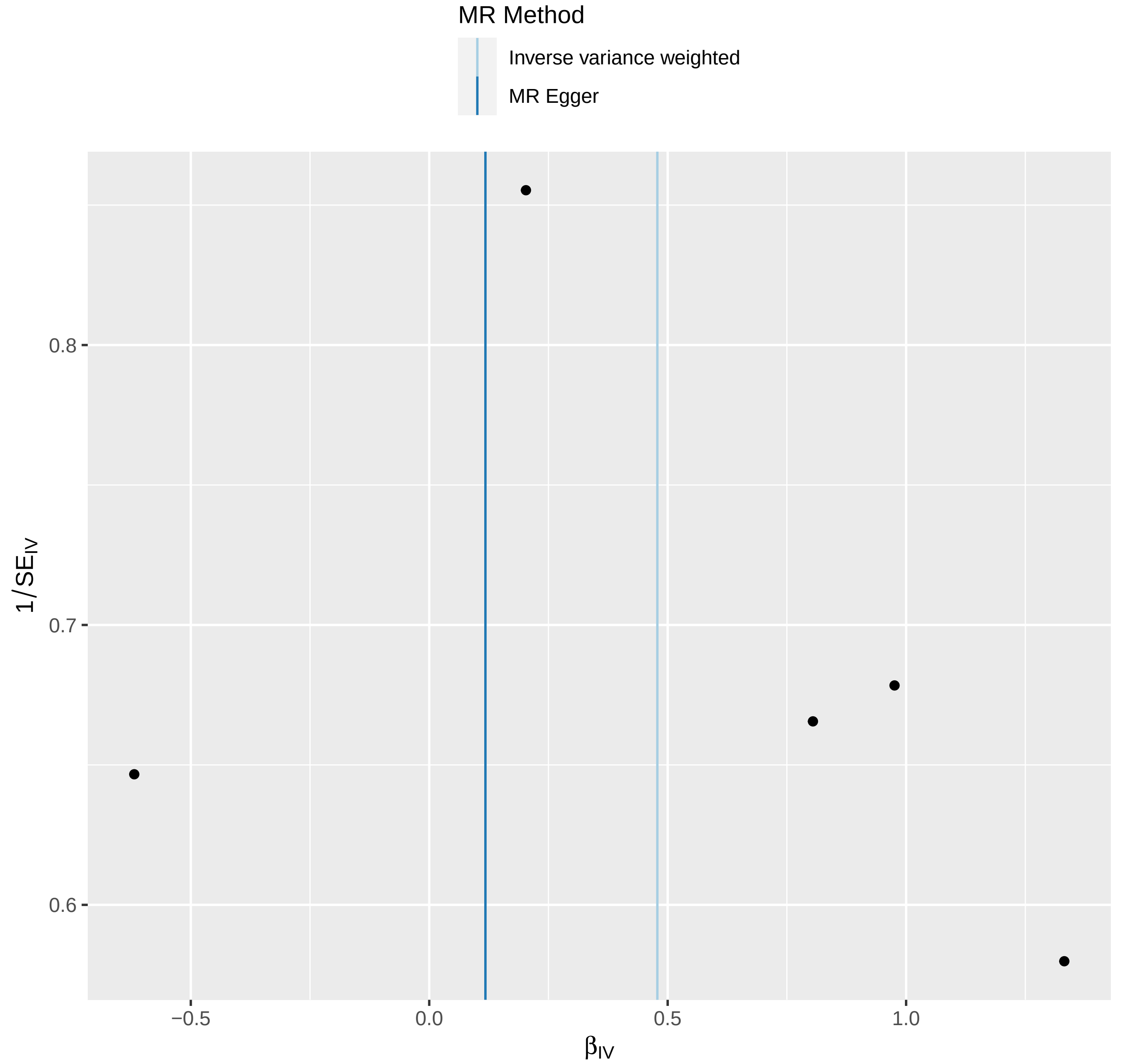

Batch 828 : Gut microbiota abundance (genus Eubacterium ruminantium group id.11340) on Arthropathy in ulcerative colitis

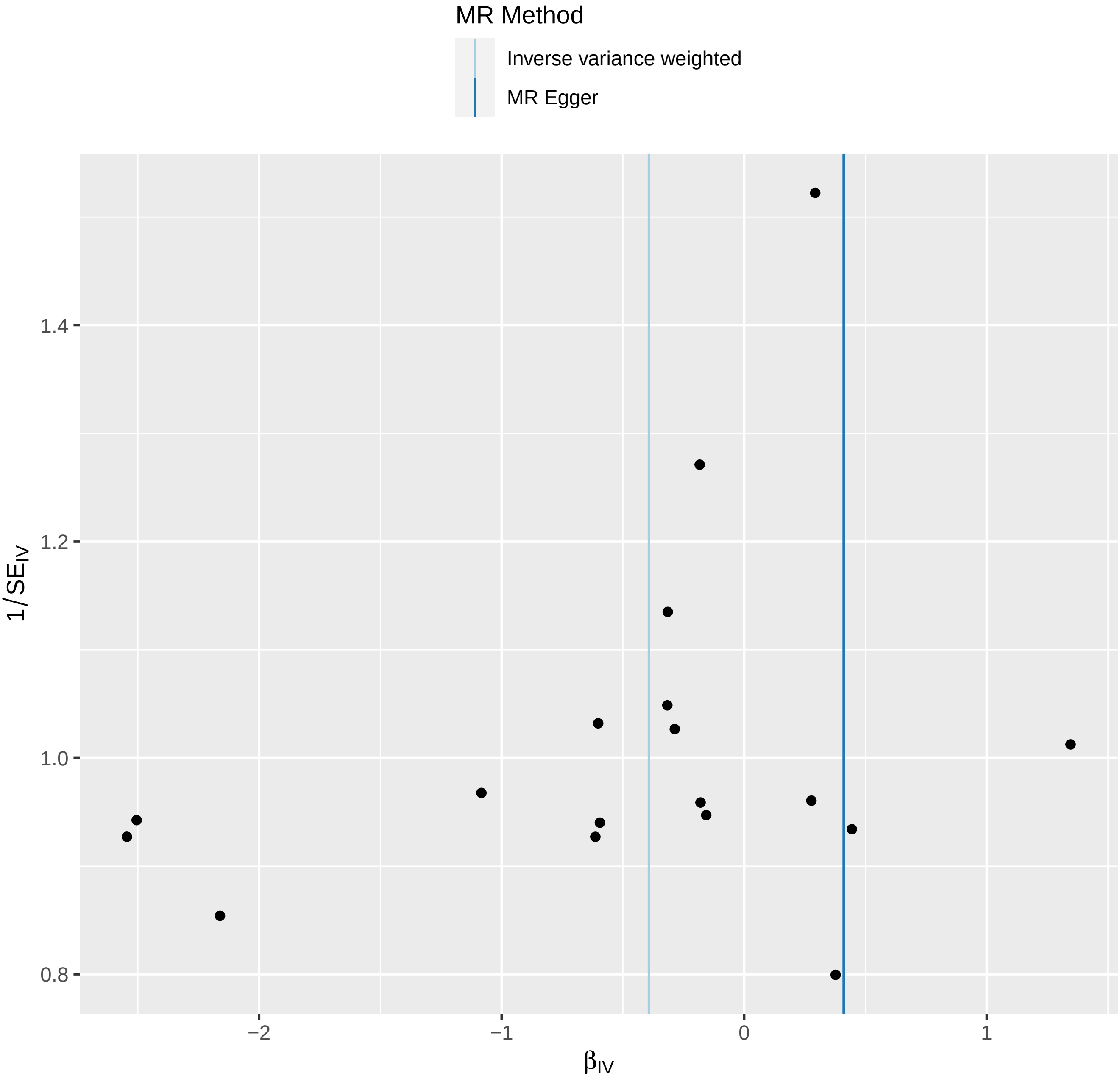

Batch 829 : Gut microbiota abundance (genus Eubacterium ventriosum group id.11341) on Arthropathy in ulcerative colitis

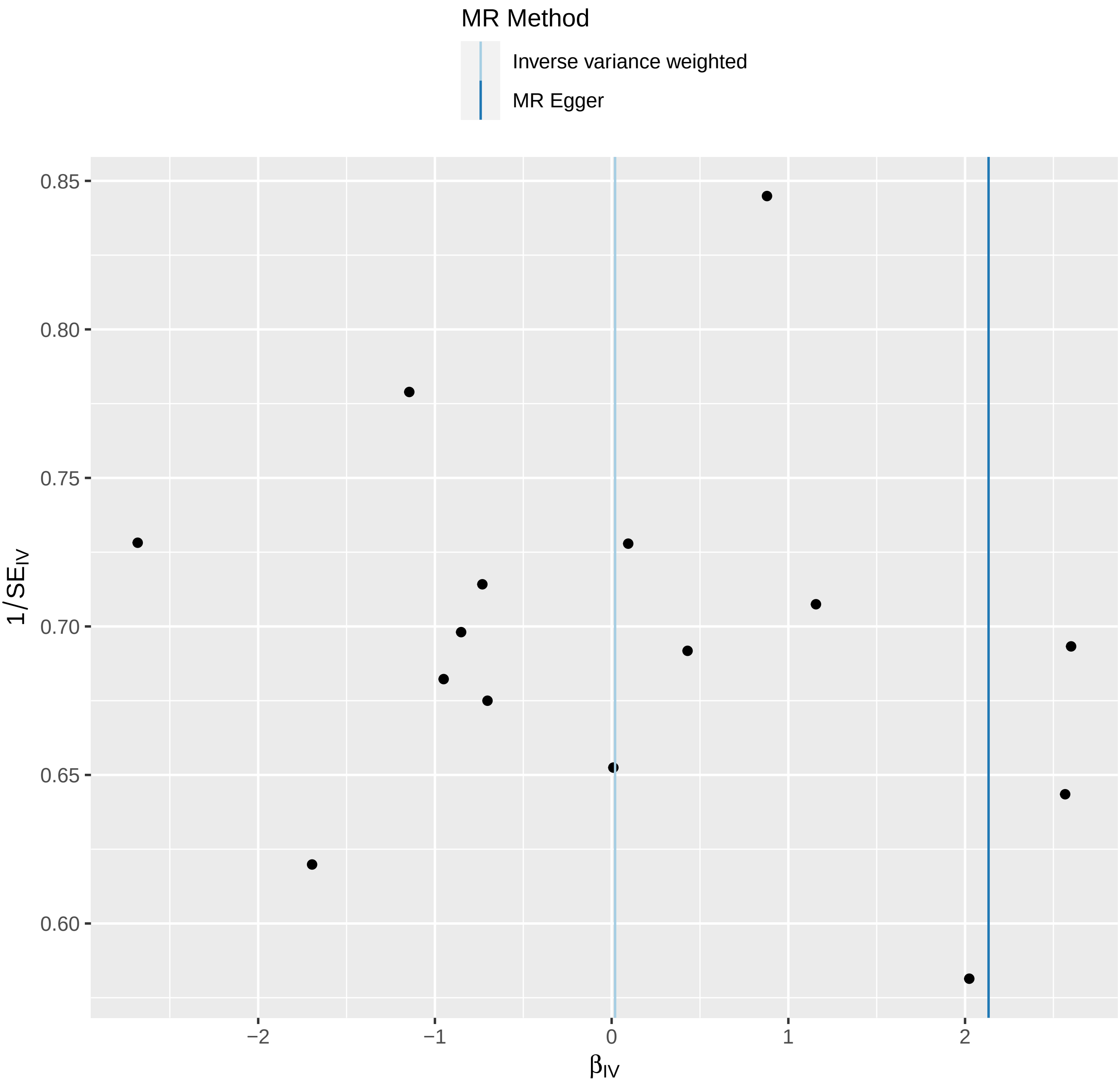

Batch 830 : Gut microbiota abundance (genus Eubacterium xylanophilum group id.14375) on Arthropathy in ulcerative colitis

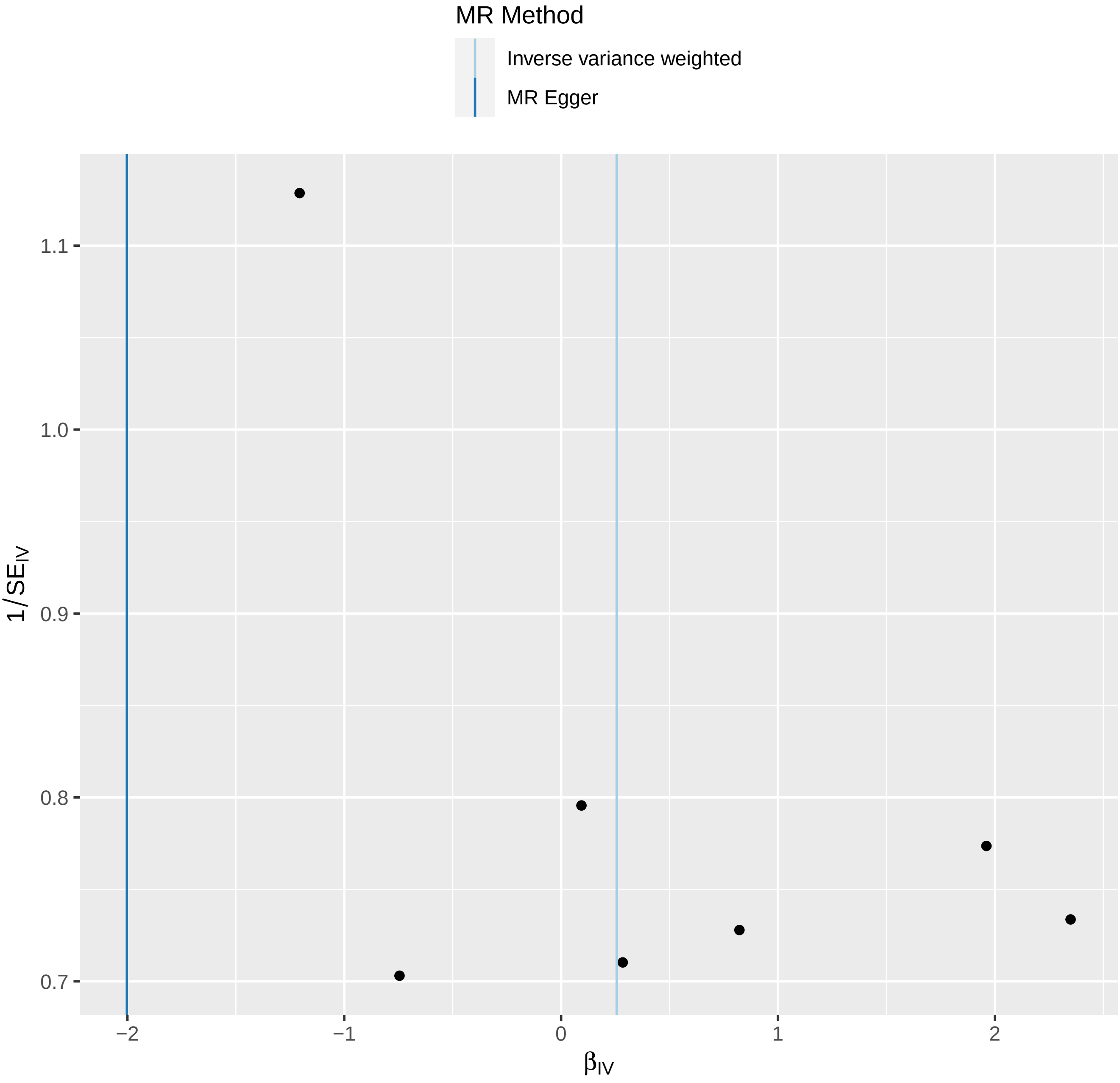

Batch 831 : Gut microbiota abundance (genus Faecalibacterium id.2057) on Arthropathy in ulcerative colitis

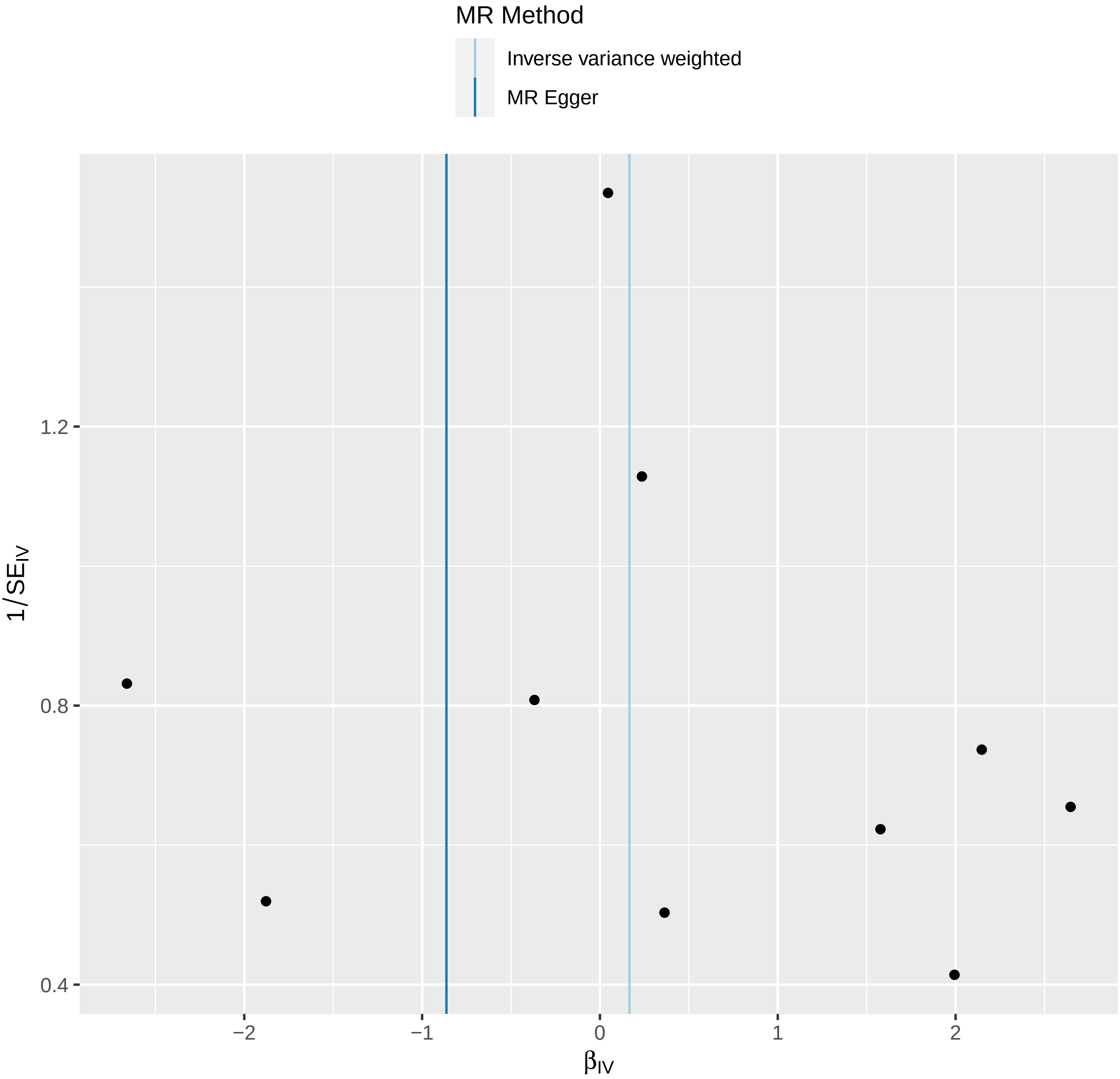

Batch 832 : Gut microbiota abundance (genus Family XIII AD3011 group id.11293) on Arthropathy in ulcerative colitis

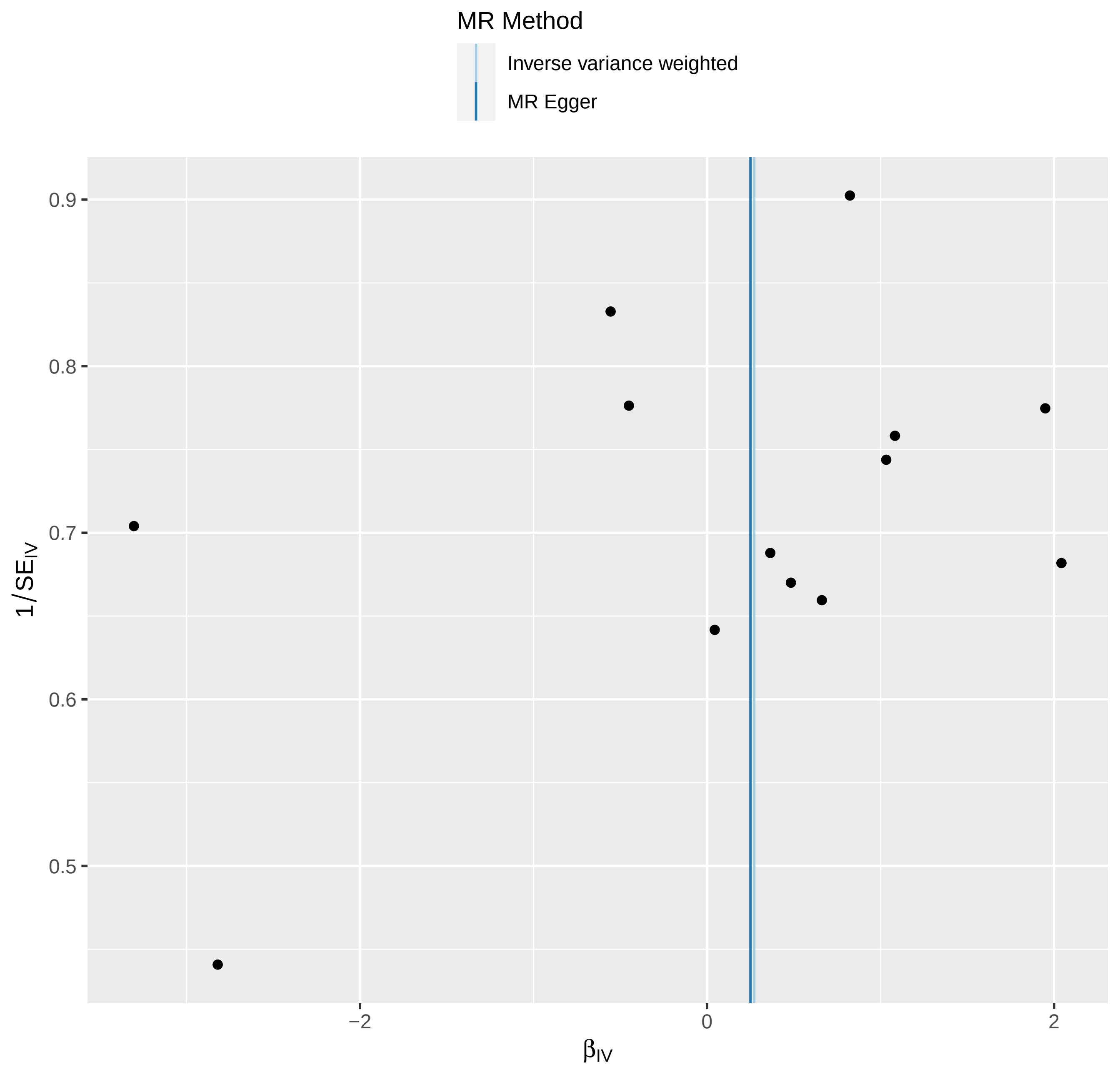

Batch 833 : Gut microbiota abundance (genus Family XIII UCG001 id.11294) on Arthropathy in ulcerative colitis

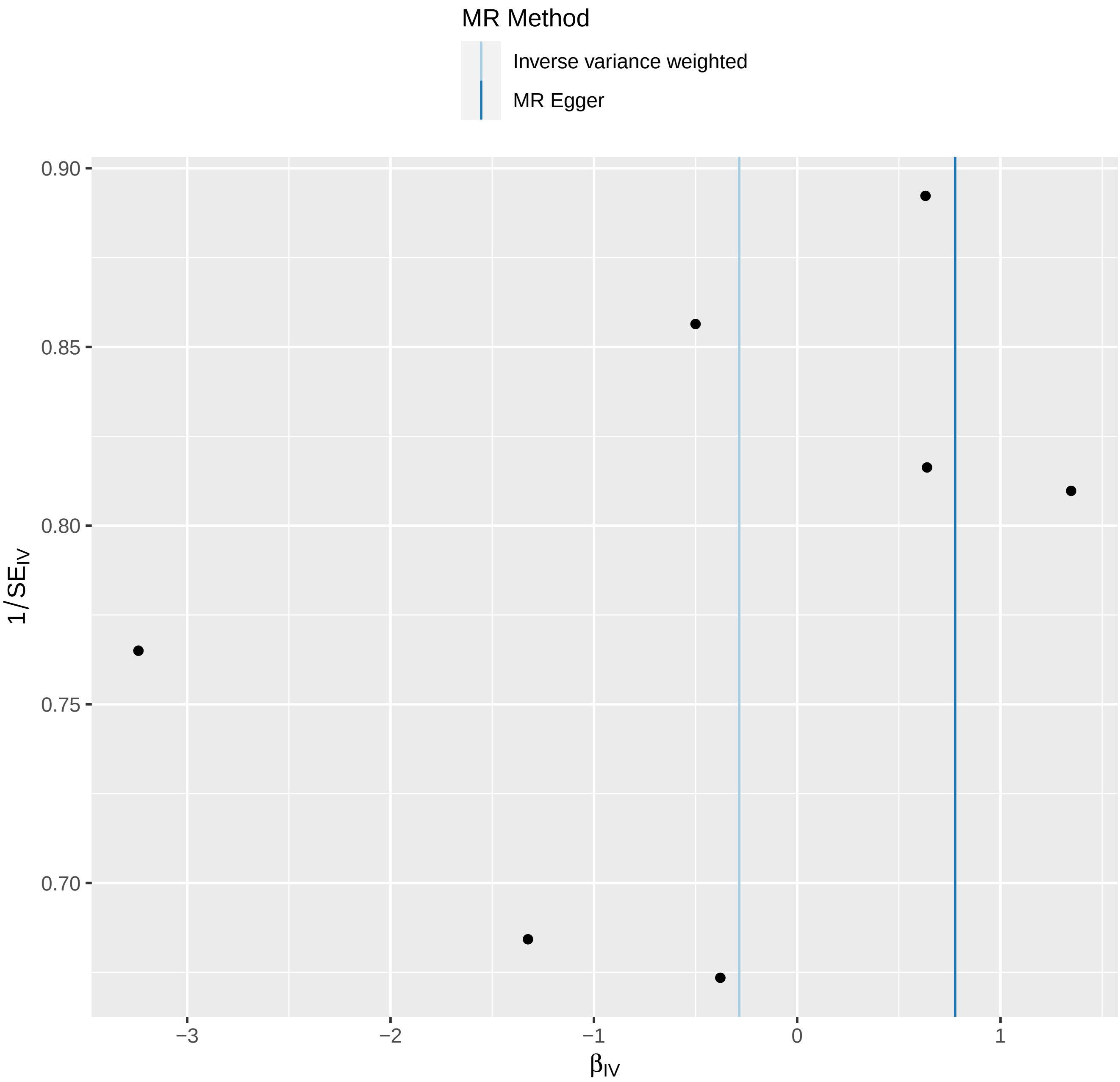

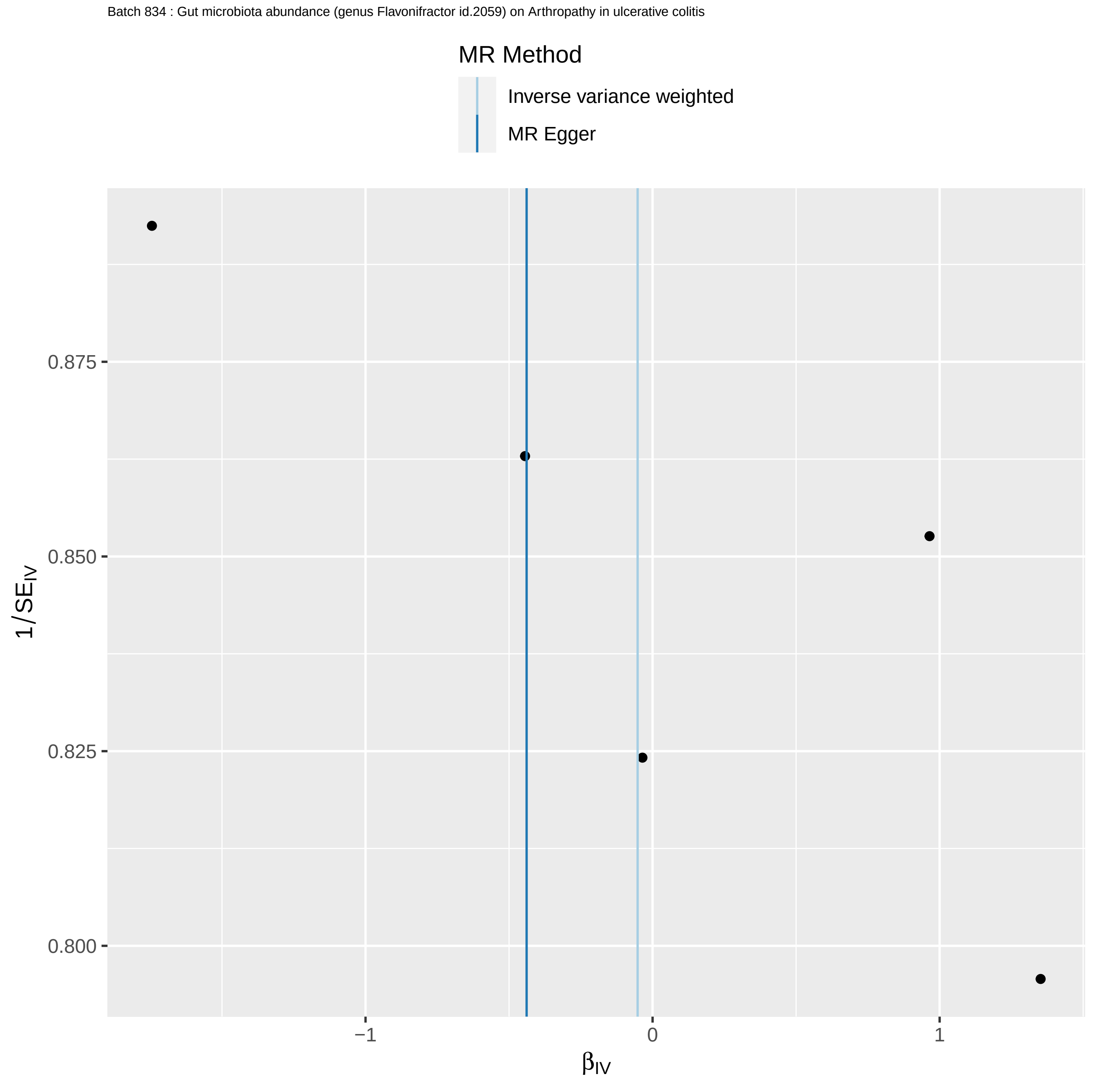
Batch 834 : Gut microbiota abundance (genus Flavonifractor id.2059) on Arthropathy in ulcerative colitis
MR Method
Inverse variance weighted
MR Egger
0.875
0.850
0.825
0.800
1/SE_IV
−1
0
1
β_IV

Batch 835 : Gut microbiota abundance (genus Fusicatenibacter id.11305) on Arthropathy in ulcerative colitis

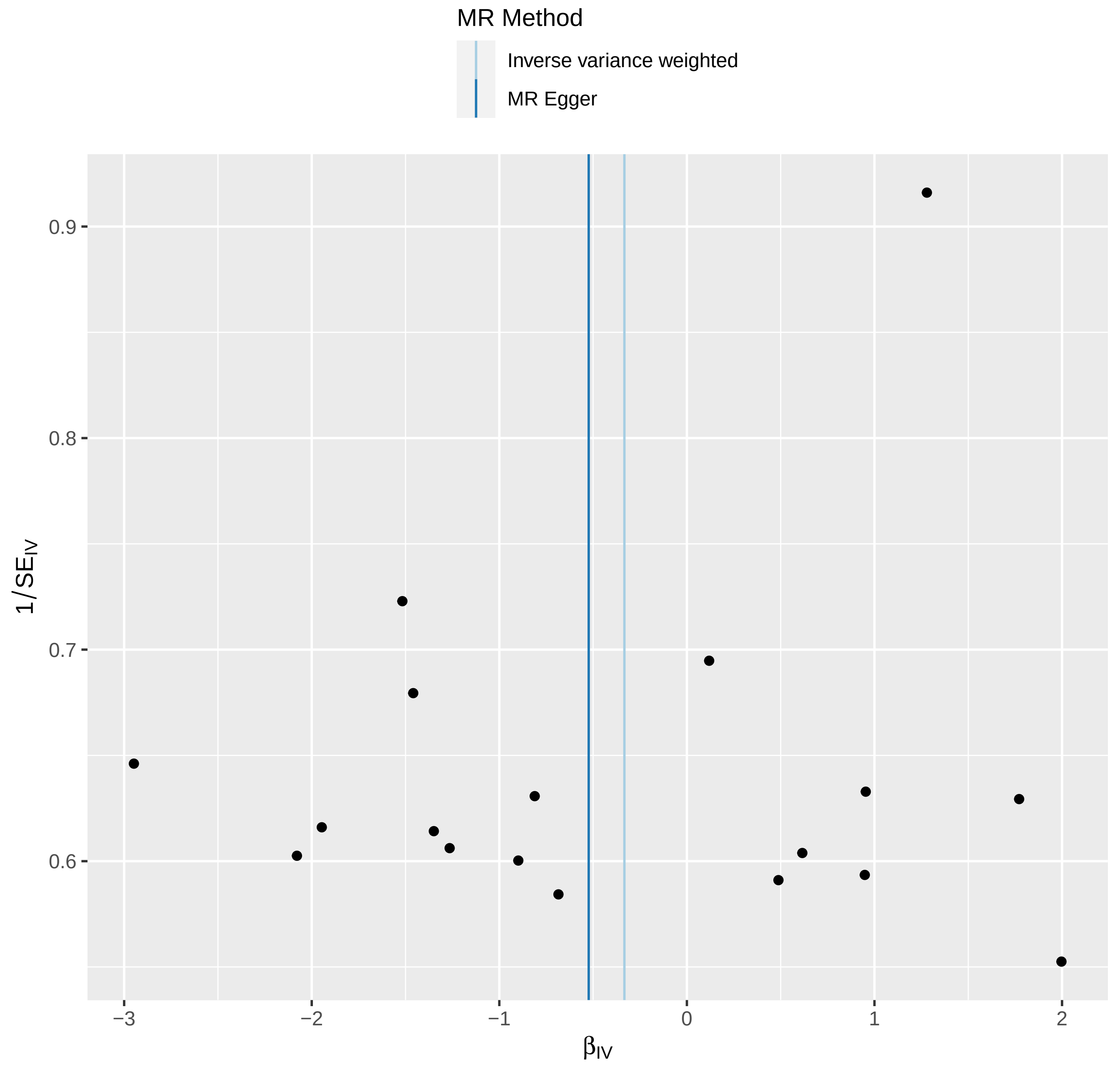

Batch 836 : Gut microbiota abundance (genus Gordonibacter id.821) on Arthropathy in ulcerative colitis

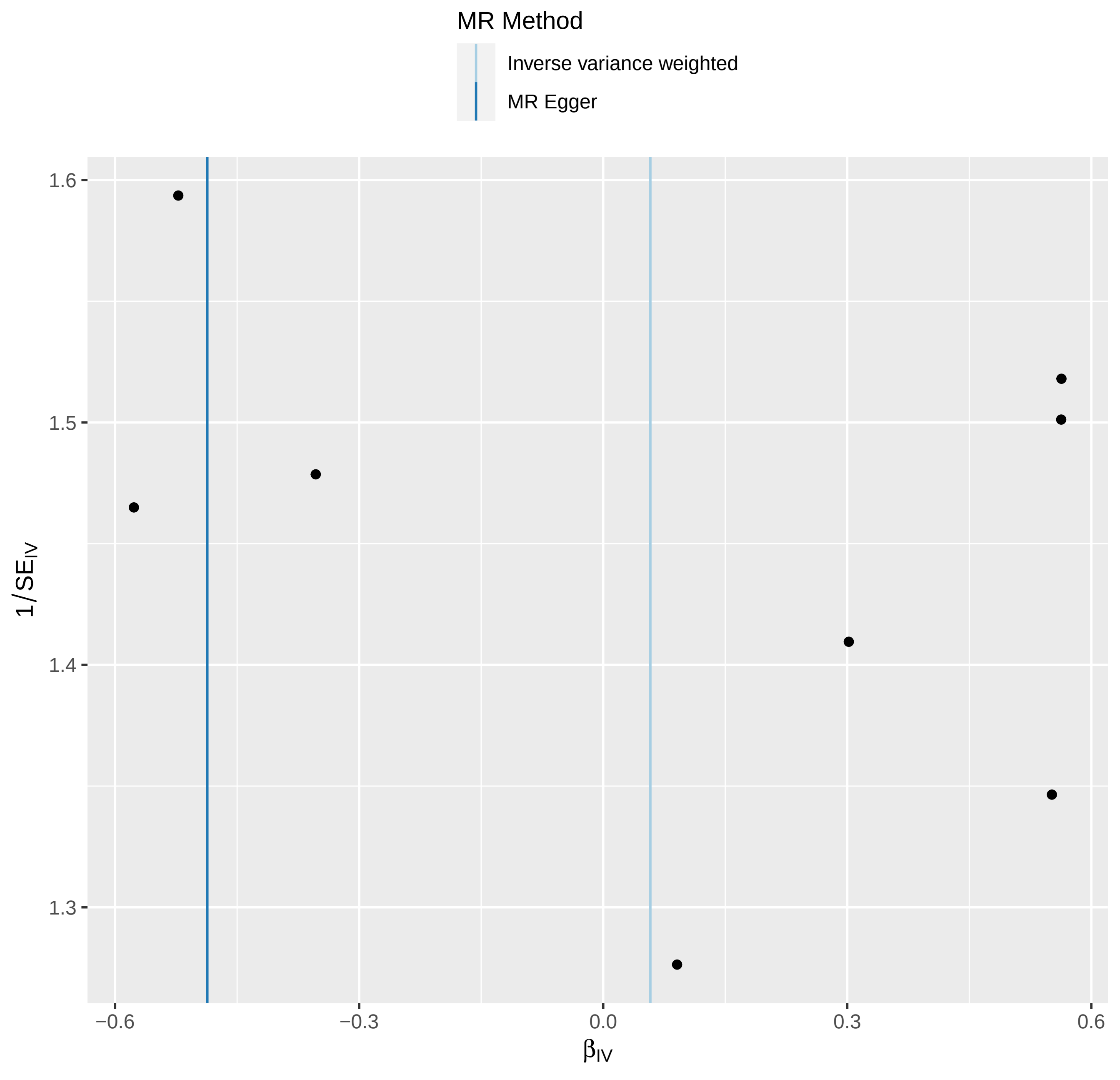

Batch 837 : Gut microbiota abundance (genus Haemophilus id.3698) on Arthropathy in ulcerative colitis

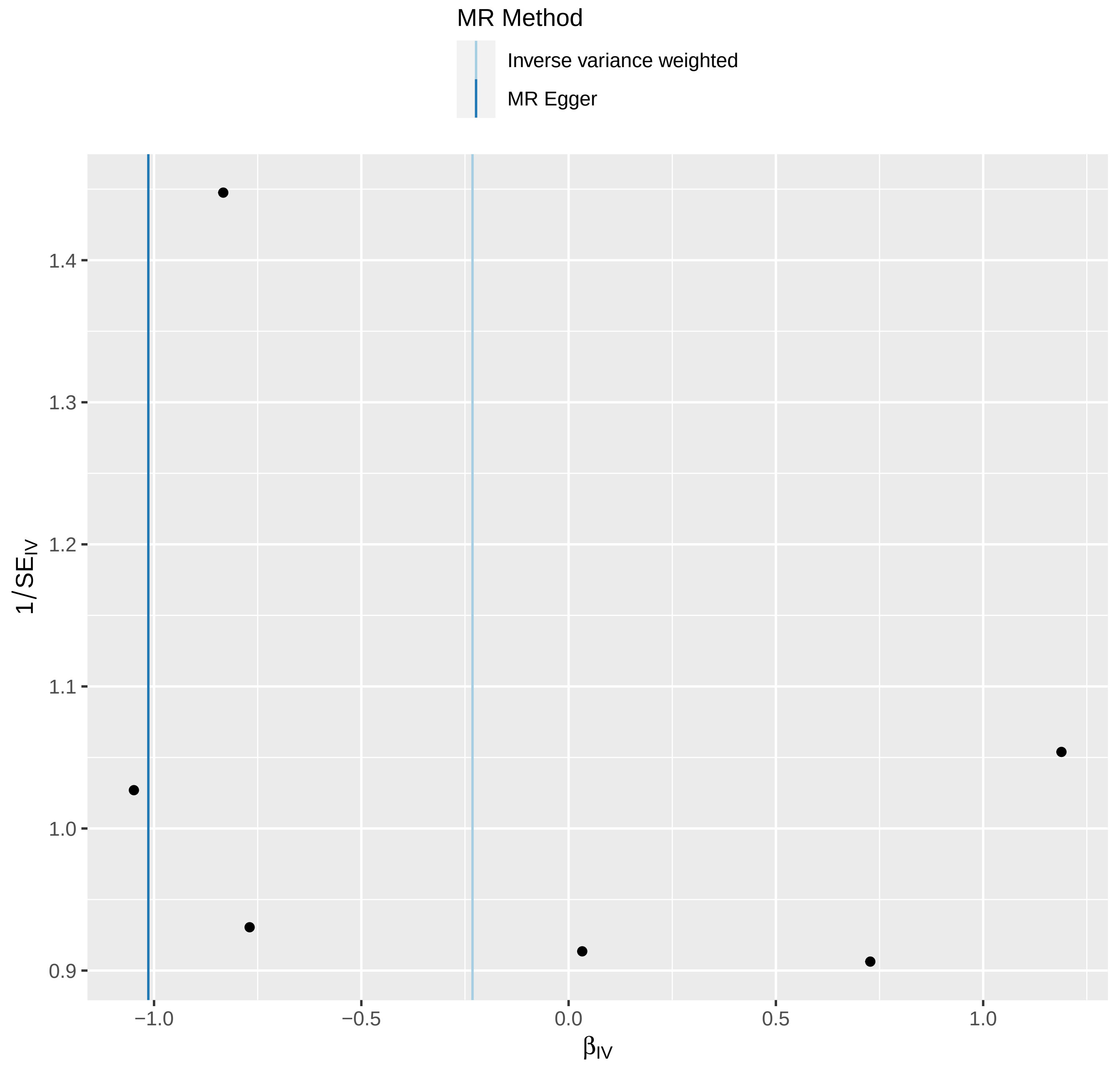

Batch 838 : Gut microbiota abundance (genus Holdemanella id.11393) on Arthropathy in ulcerative colitis

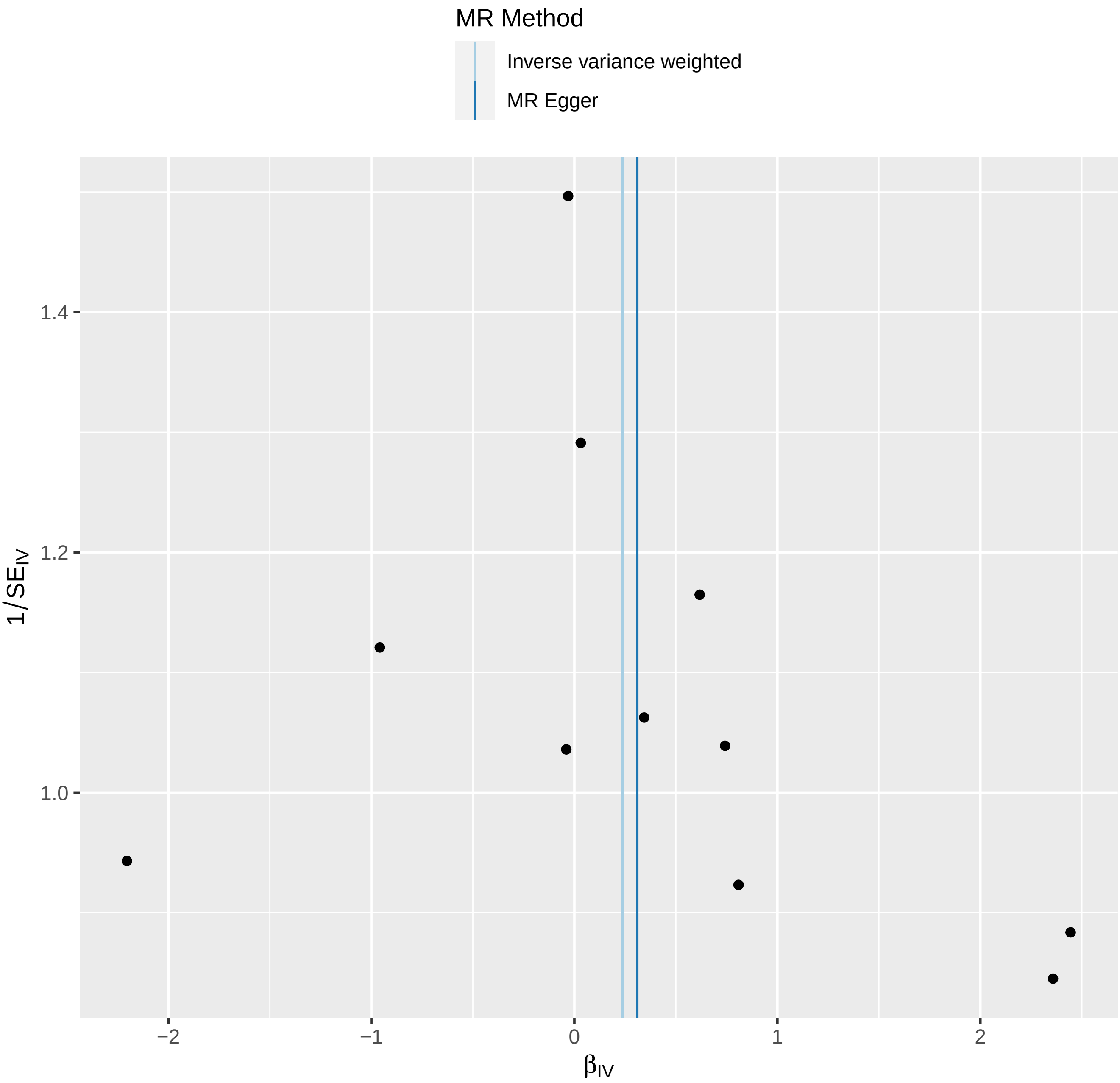

Batch 839 : Gut microbiota abundance (genus Holdemania id.2157) on Arthropathy in ulcerative colitis

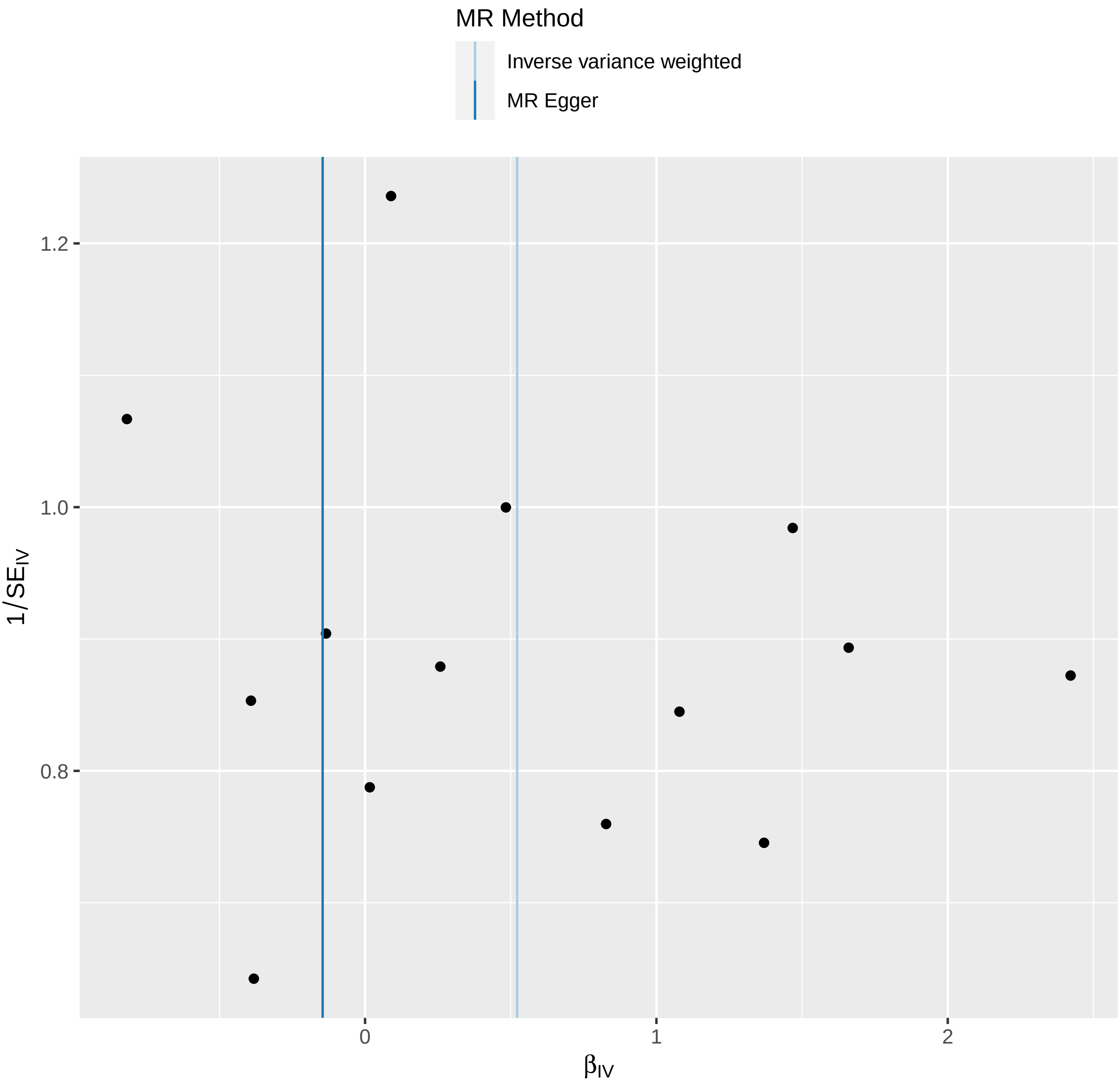

Batch 840 : Gut microbiota abundance (genus Howardella id.2000) on Arthropathy in ulcerative colitis

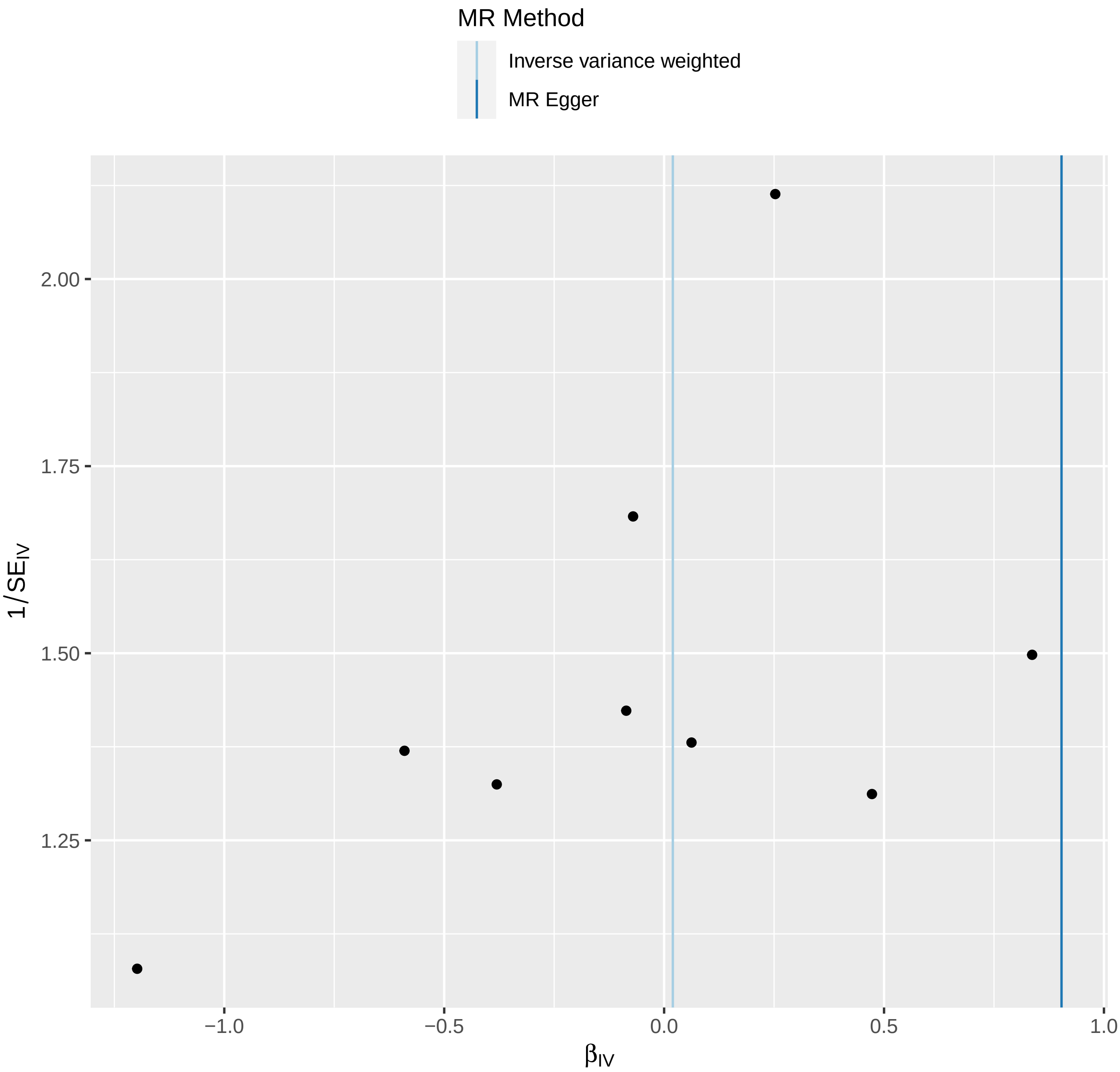

Batch 841 : Gut microbiota abundance (genus Hungatella id.11306) on Arthropathy in ulcerative colitis

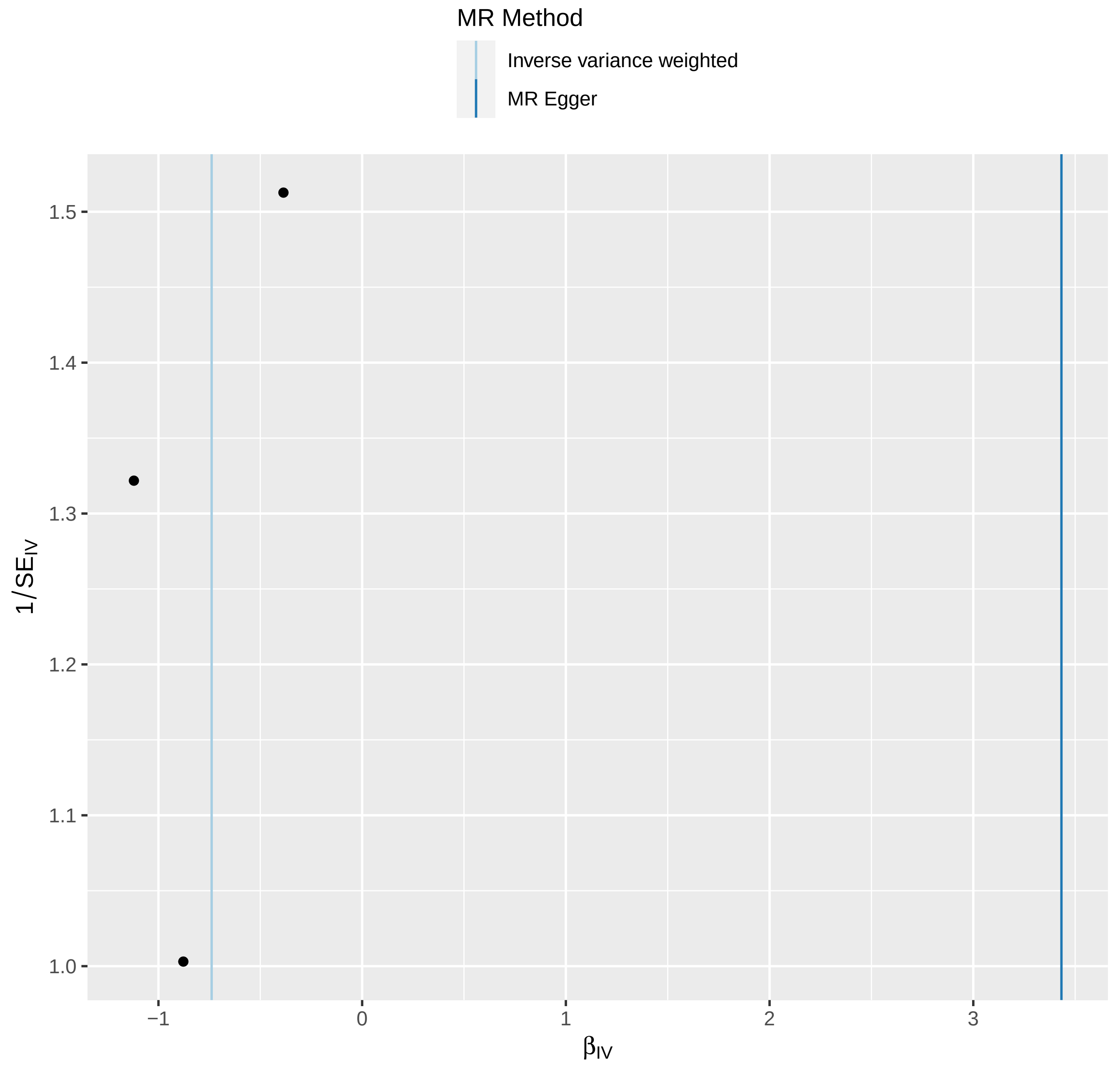

Batch 842 : Gut microbiota abundance (genus Intestinibacter id.11345) on Arthropathy in ulcerative colitis

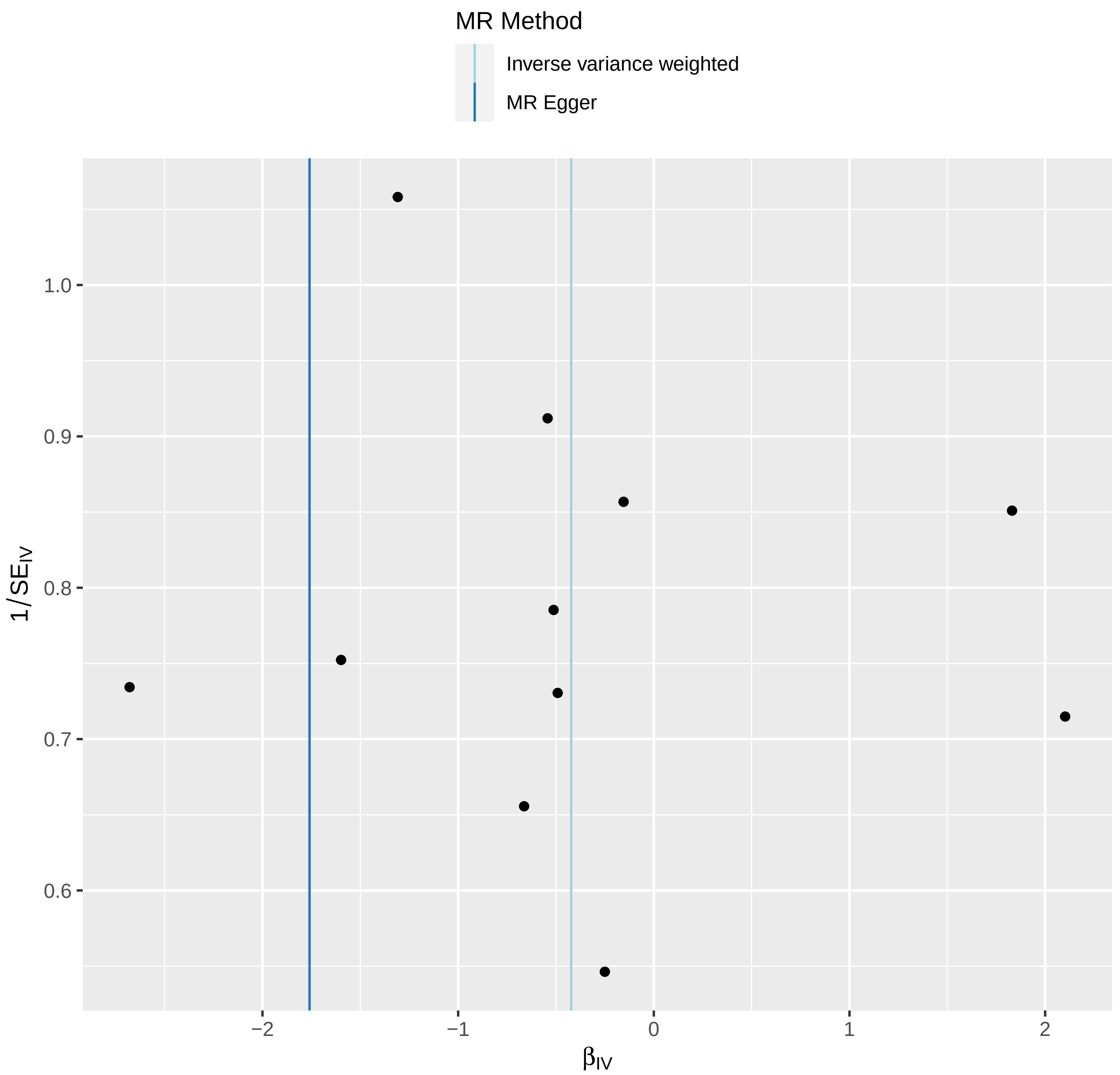

Batch 843 : Gut microbiota abundance (genus Intestinimonas id.2062) on Arthropathy in ulcerative colitis

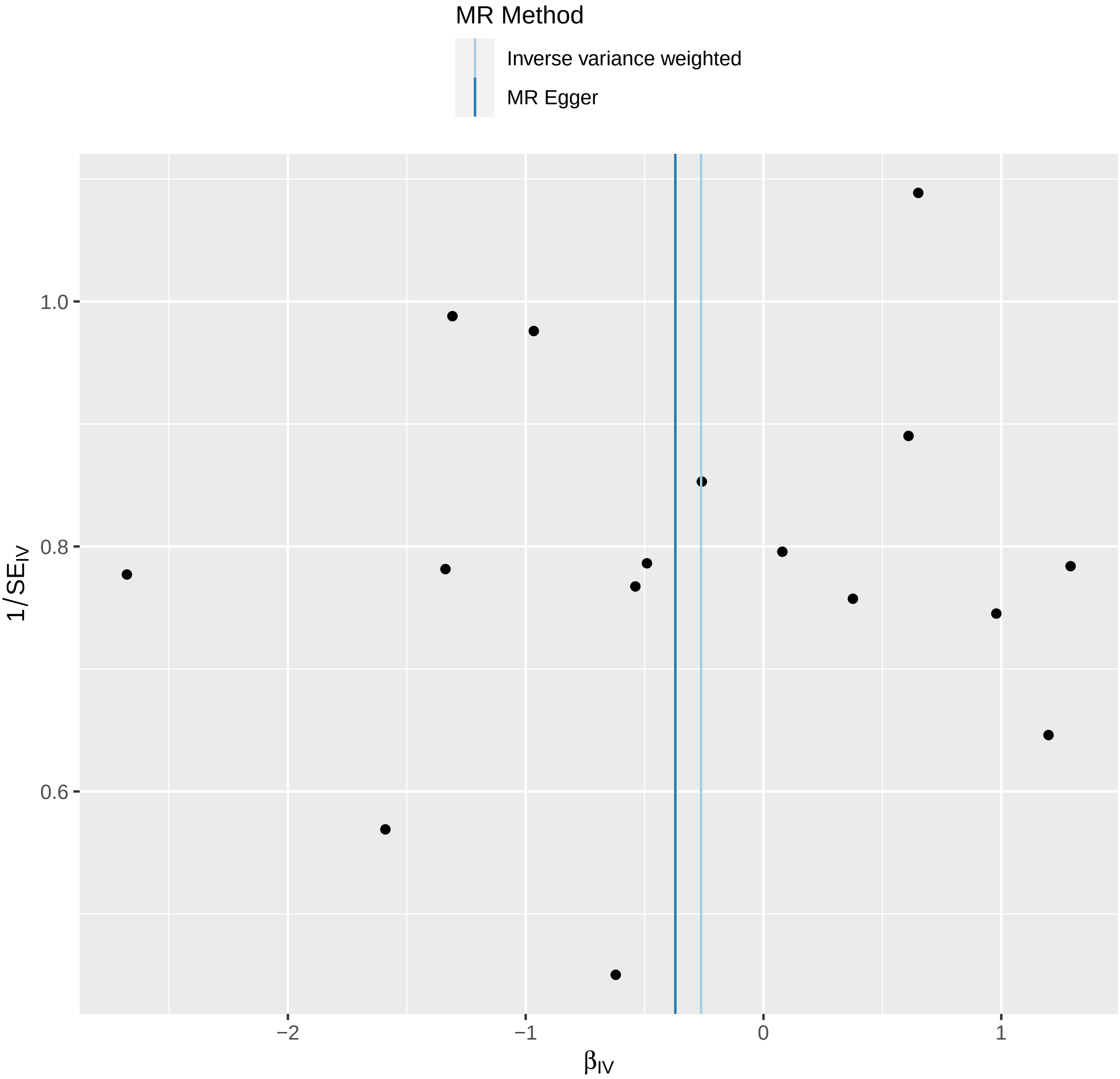

Batch 844 : Gut microbiota abundance (genus Lachnoclostridium id.11308) on Arthropathy in ulcerative colitis

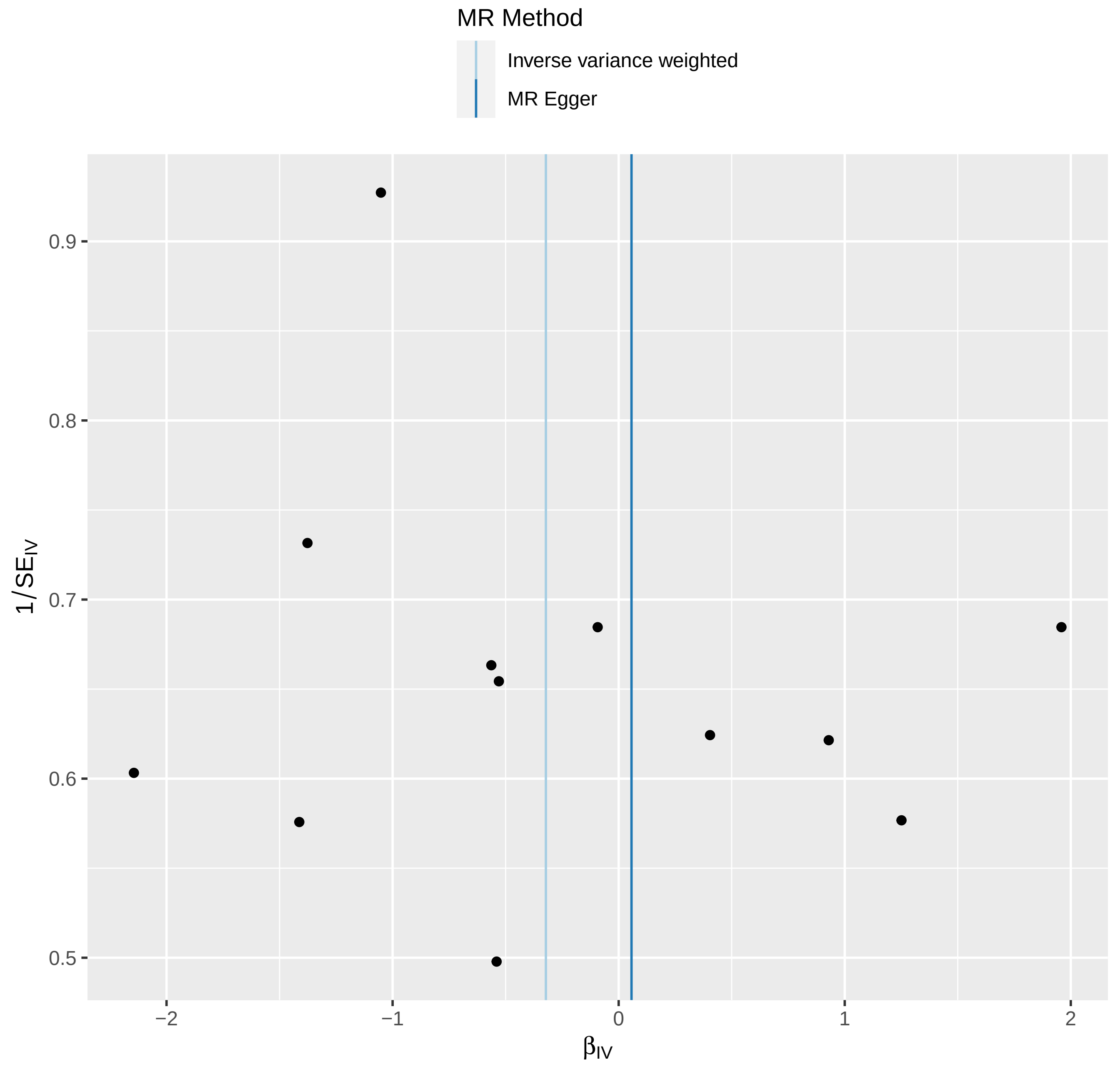

Batch 845 : Gut microbiota abundance (genus Lachnospiraceae FCS020 group id.11314) on Arthropathy in ulcerative colitis

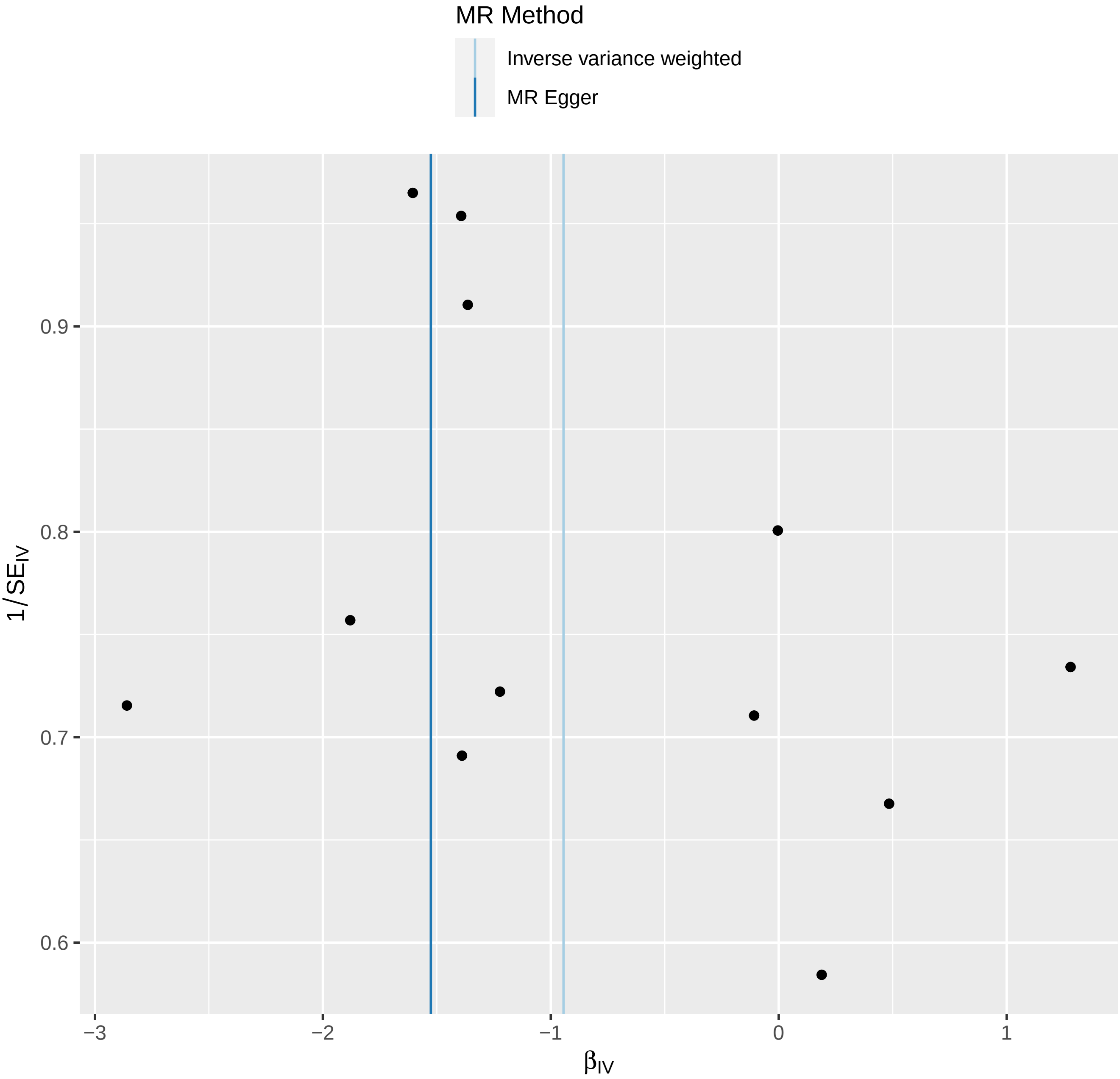

Batch 846 : Gut microbiota abundance (genus Lachnospiraceae NC2004 group id.11316) on Arthropathy in ulcerative colitis

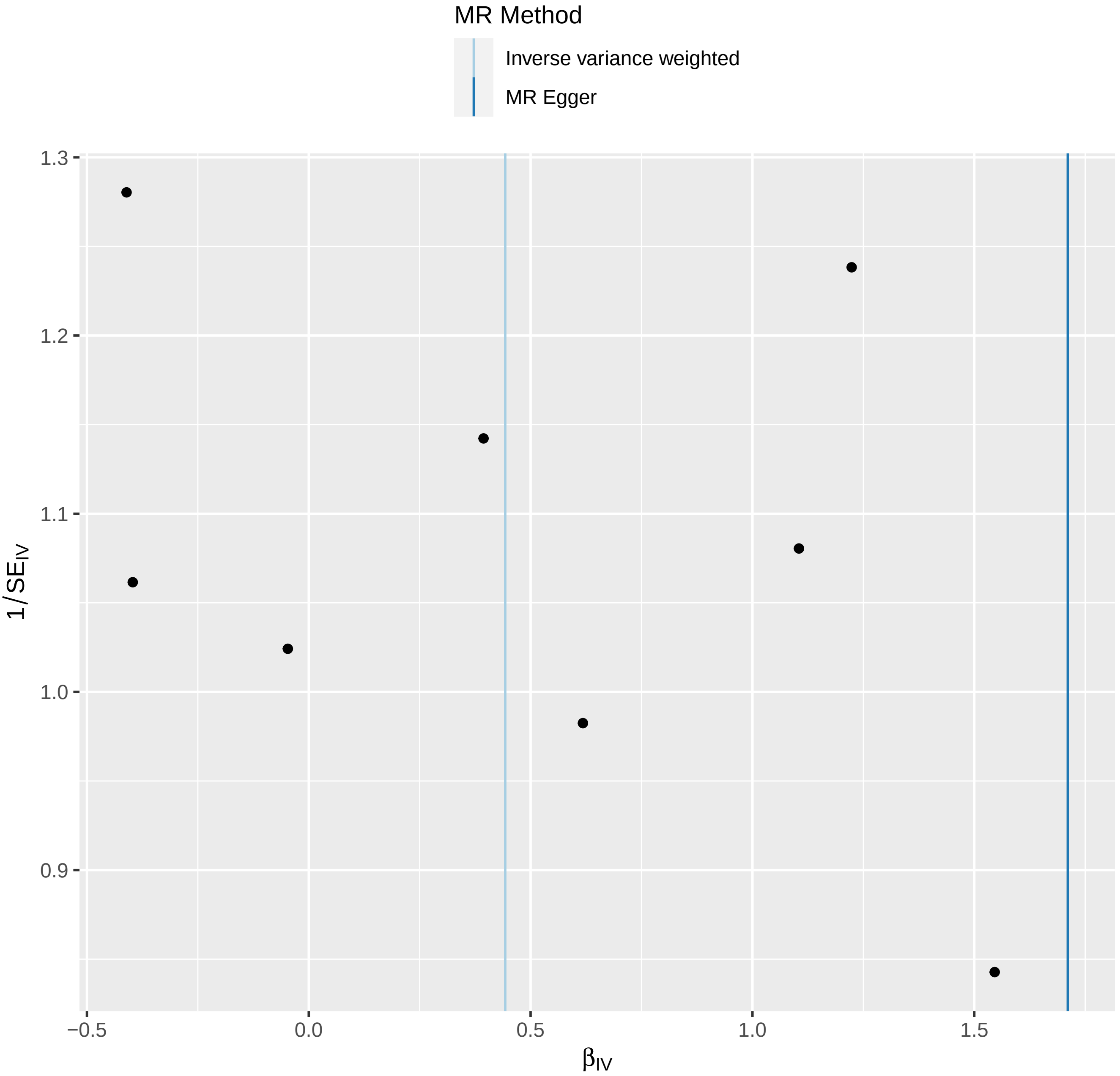

Batch 847 : Gut microbiota abundance (genus Lachnospiraceae ND3007 group id.11317) on Arthropathy in ulcerative colitis

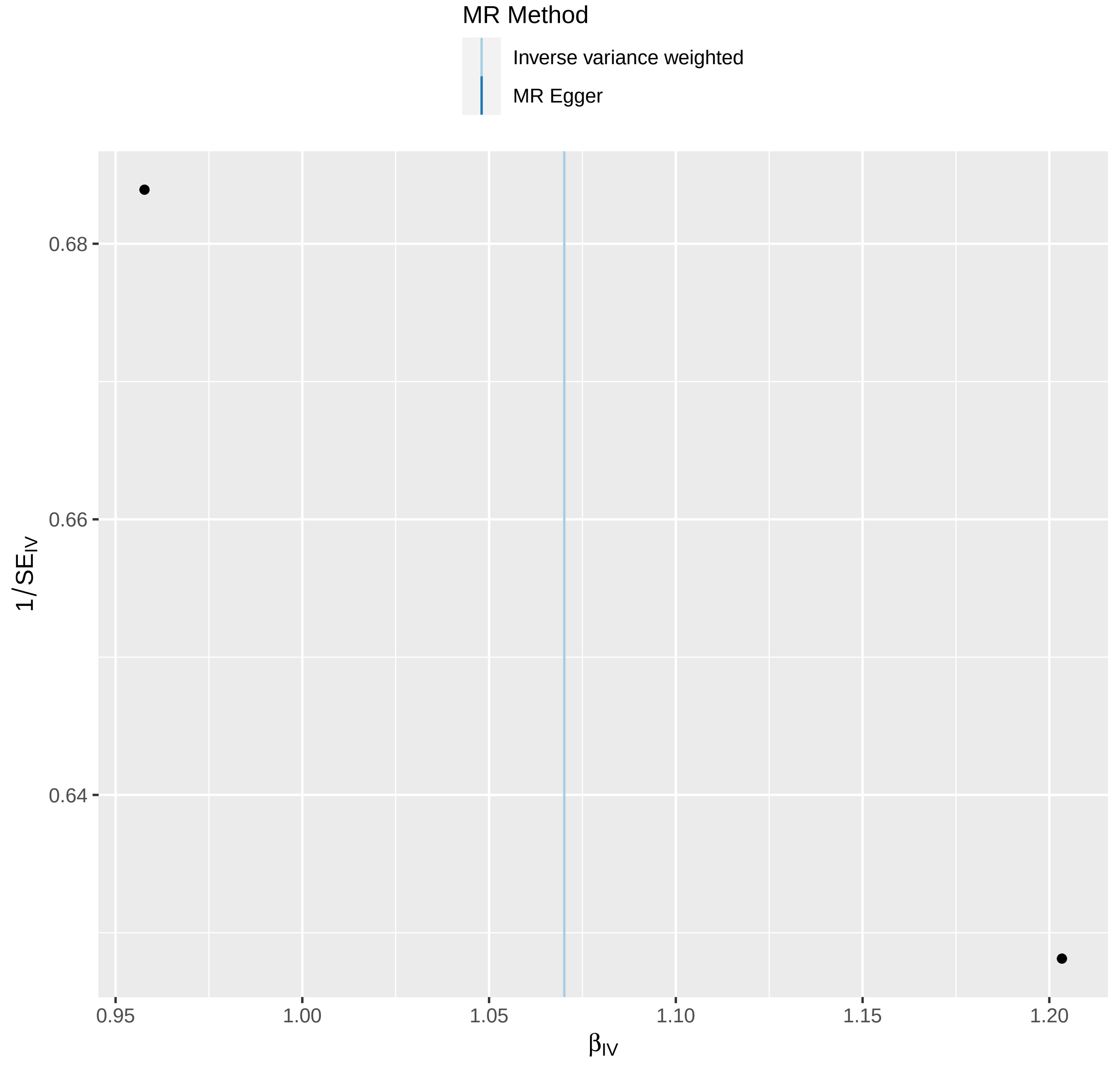

Batch 848 : Gut microbiota abundance (genus Lachnospiraceae NK4A136 group id.11319) on Arthropathy in ulcerative colitis

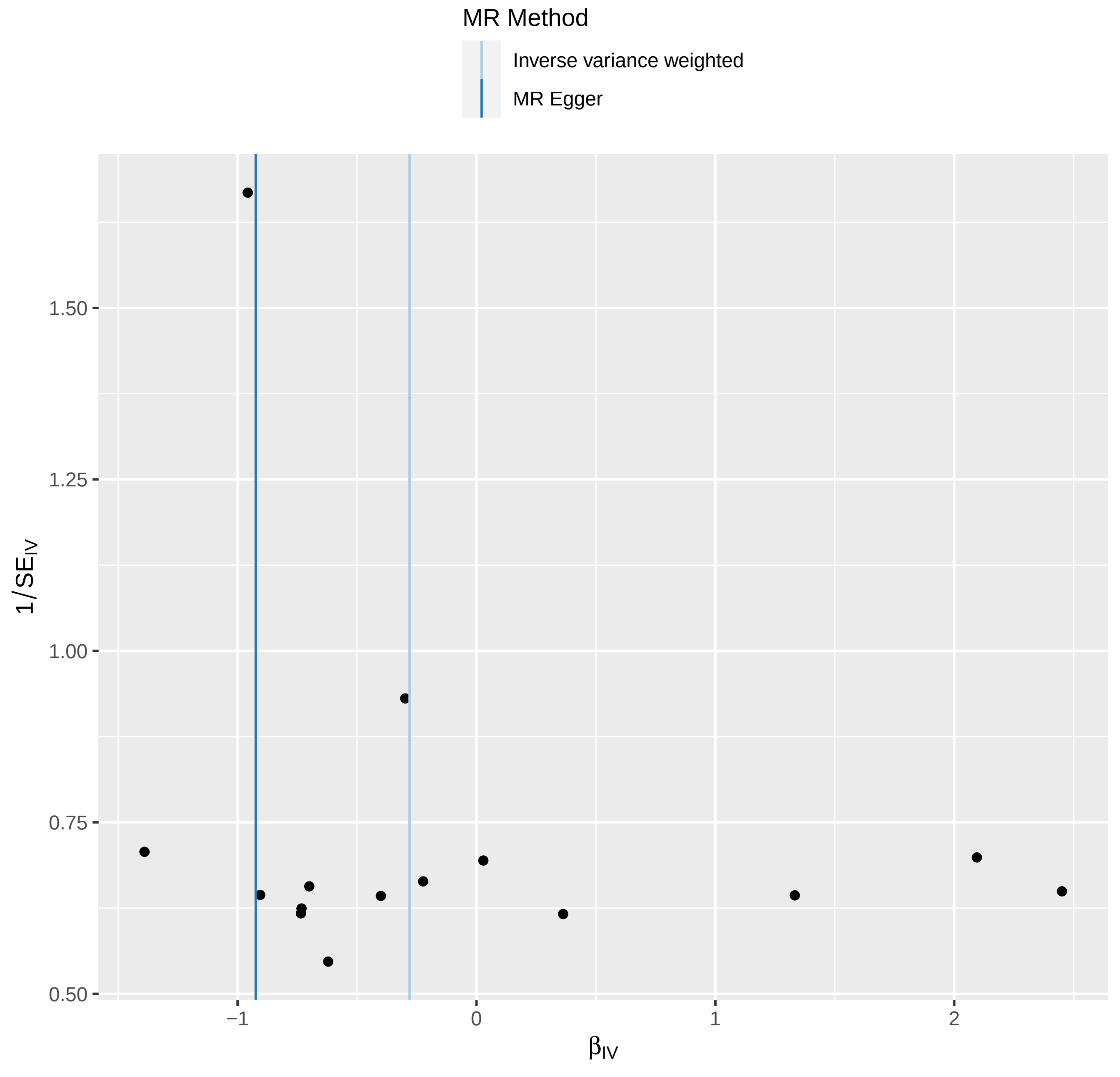

Batch 849 : Gut microbiota abundance (genus Lachnospiraceae UCG001 id.11321) on Arthropathy in ulcerative colitis

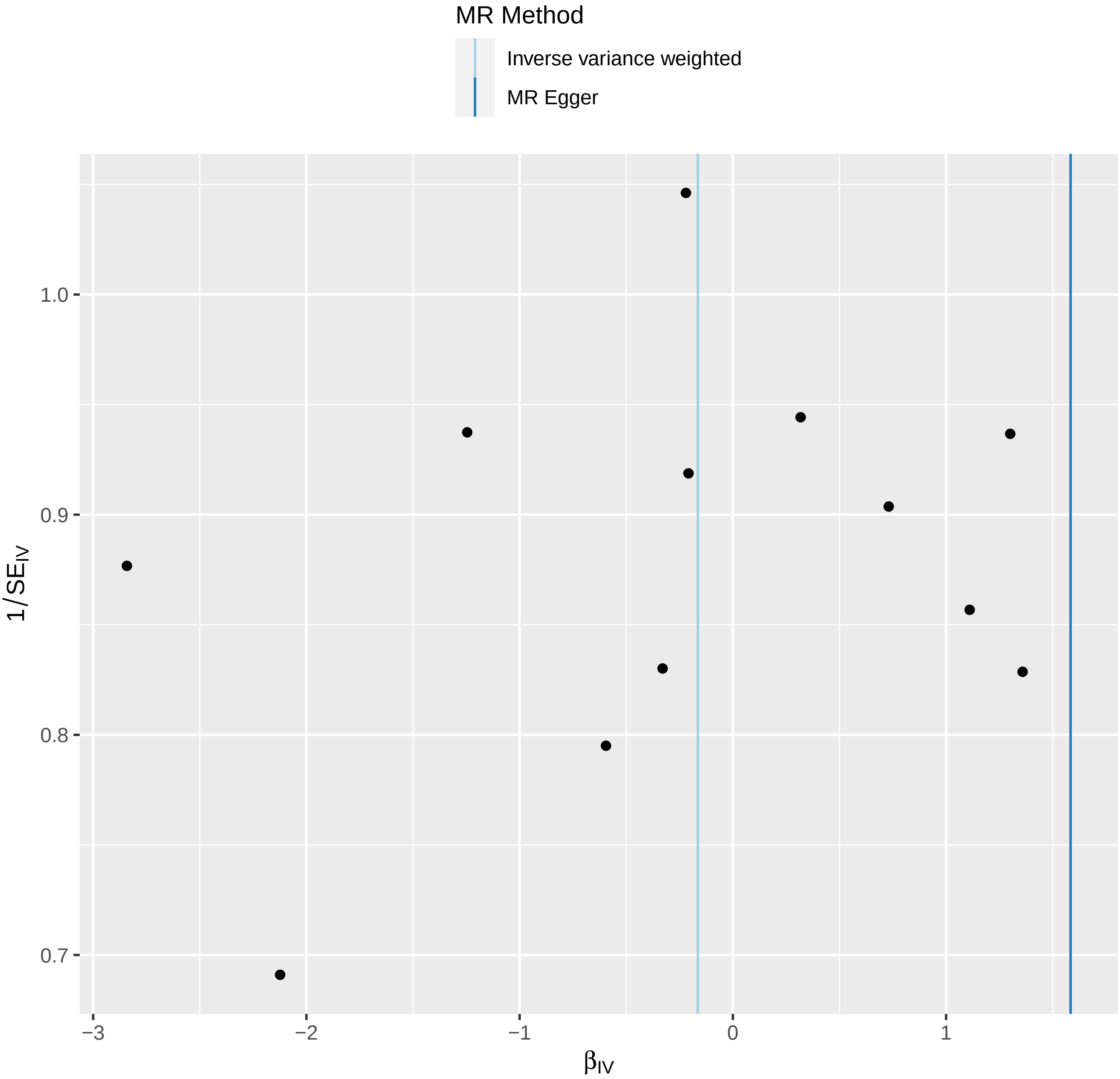

Batch 850 : Gut microbiota abundance (genus Lachnospiraceae UCG004 id.11324) on Arthropathy in ulcerative colitis

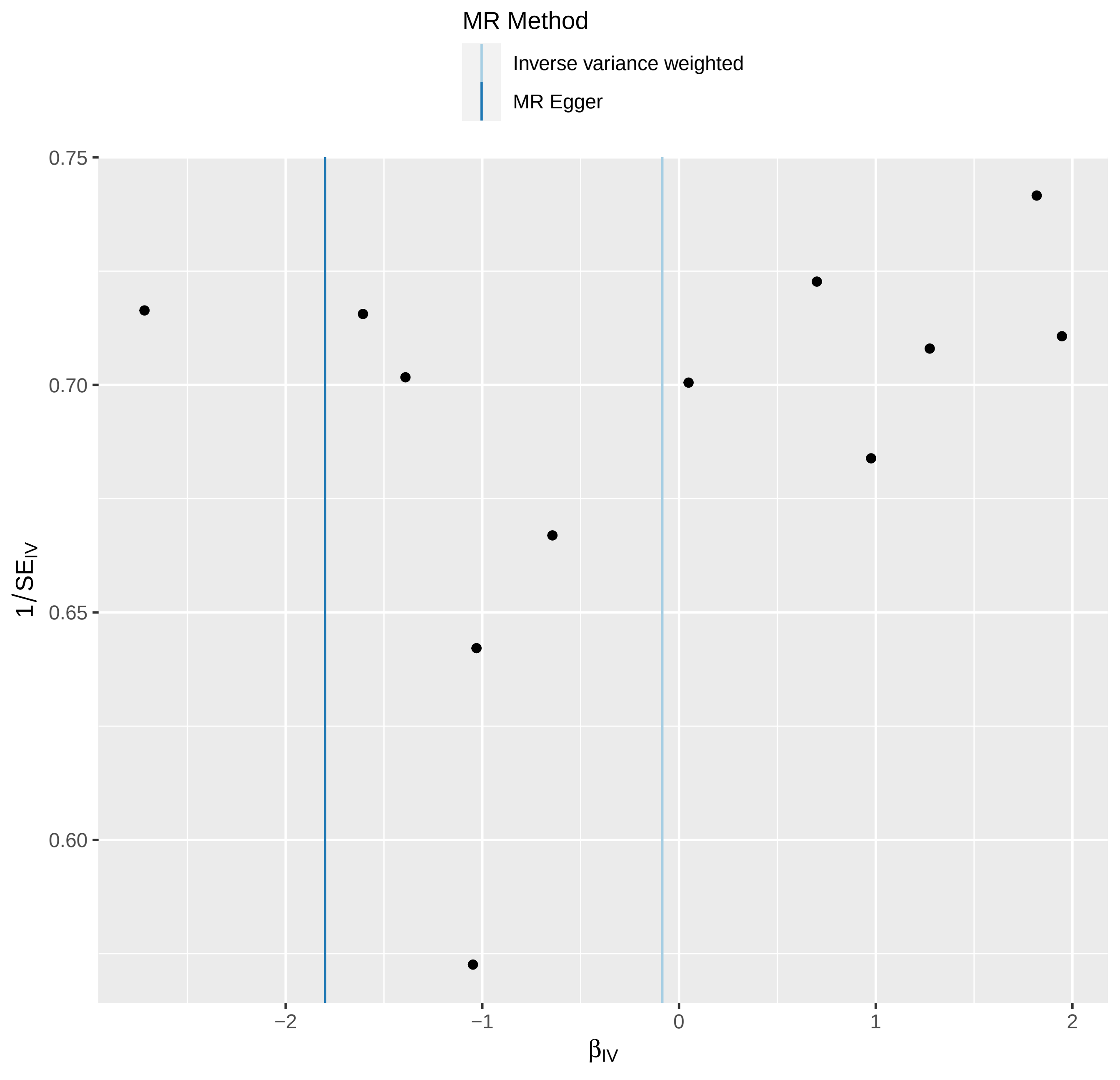

Batch 851 : Gut microbiota abundance (genus Lachnospiraceae UCG008 id.11328) on Arthropathy in ulcerative colitis

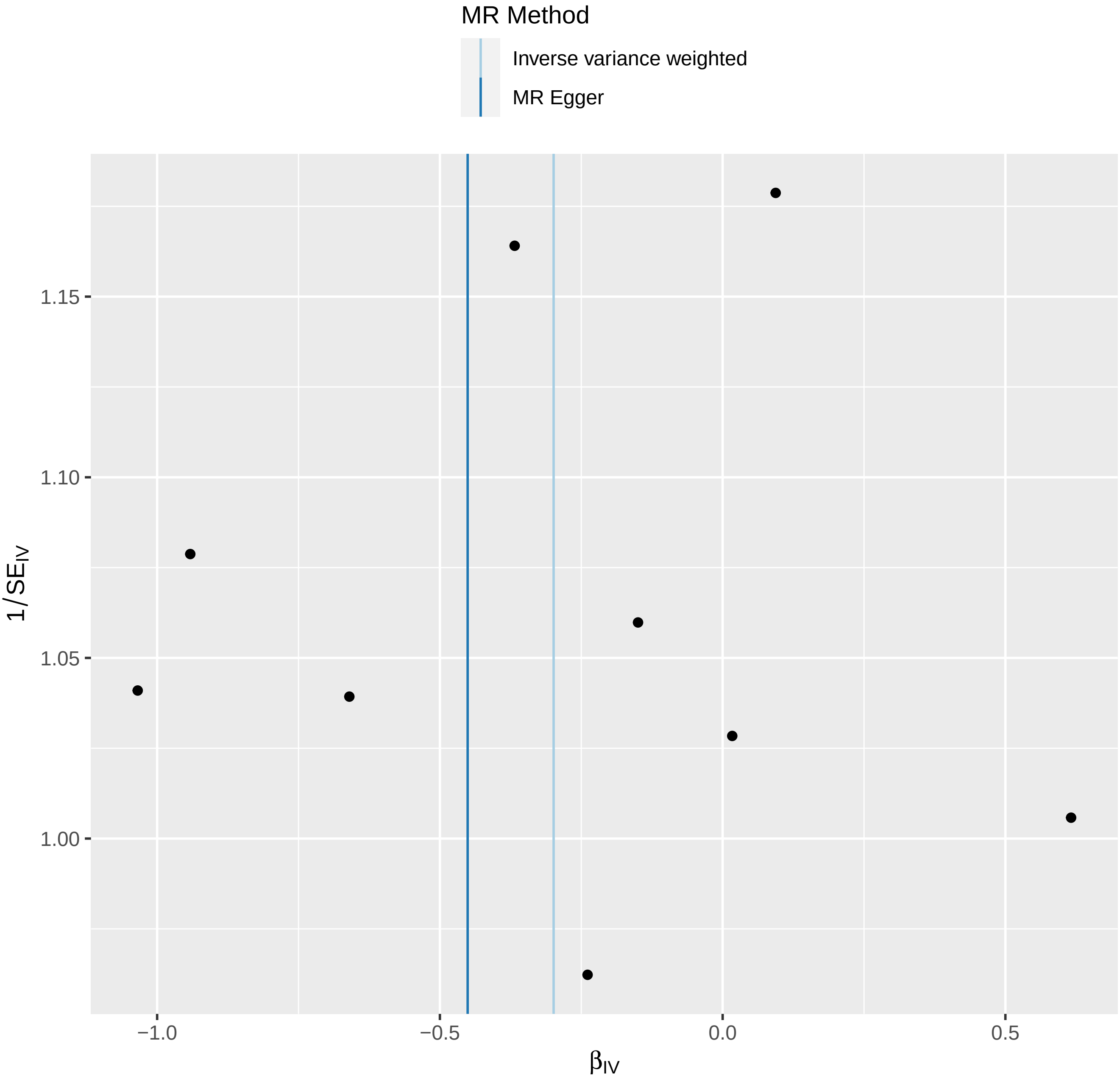

Batch 852 : Gut microbiota abundance (genus Lachnospiraceae UCG010 id.11330) on Arthropathy in ulcerative colitis

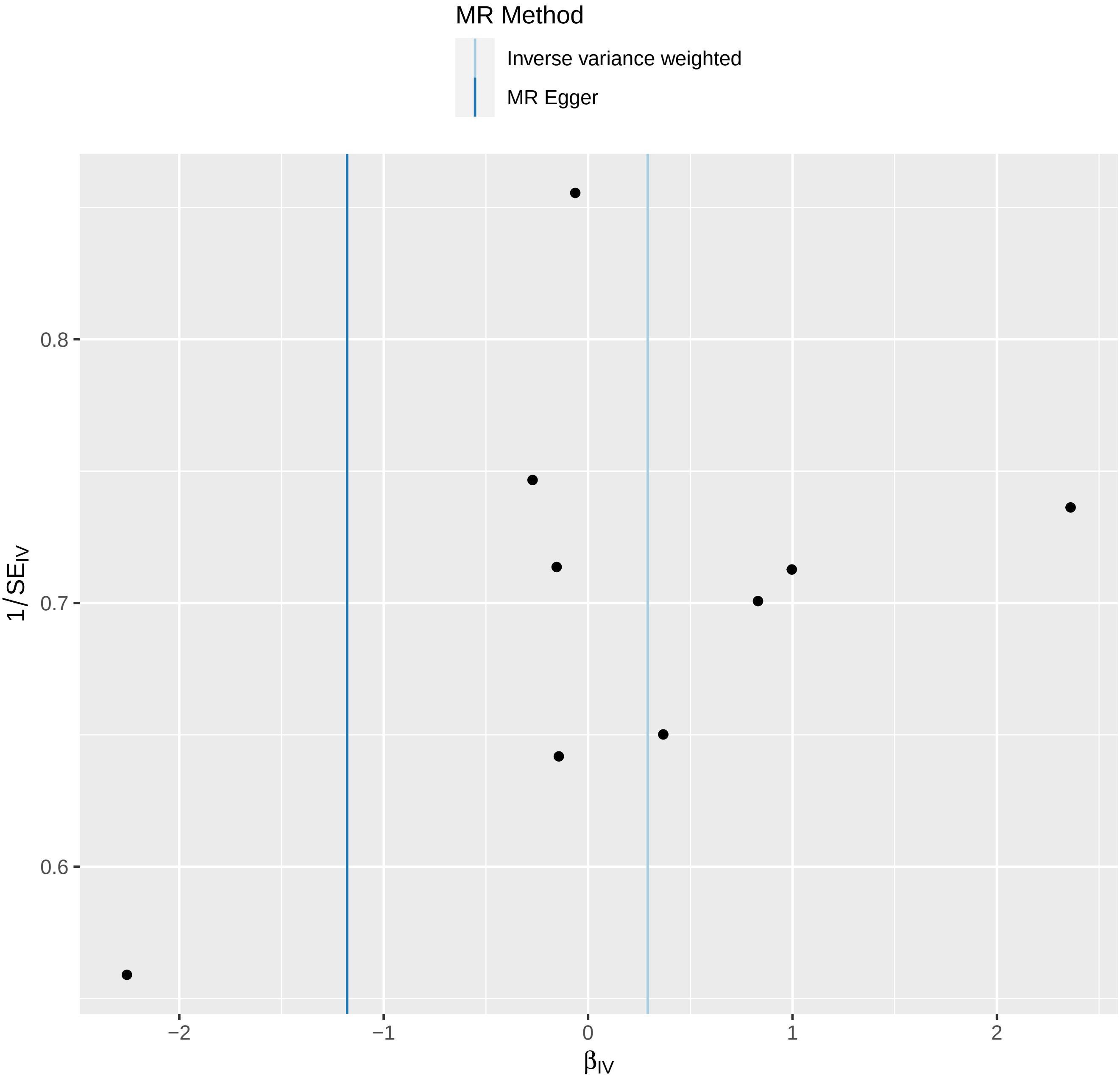

Insufficient number of SNPs

Batch 854 : Gut microbiota abundance (genus Lactobacillus id.1837) on Arthropathy in ulcerative colitis

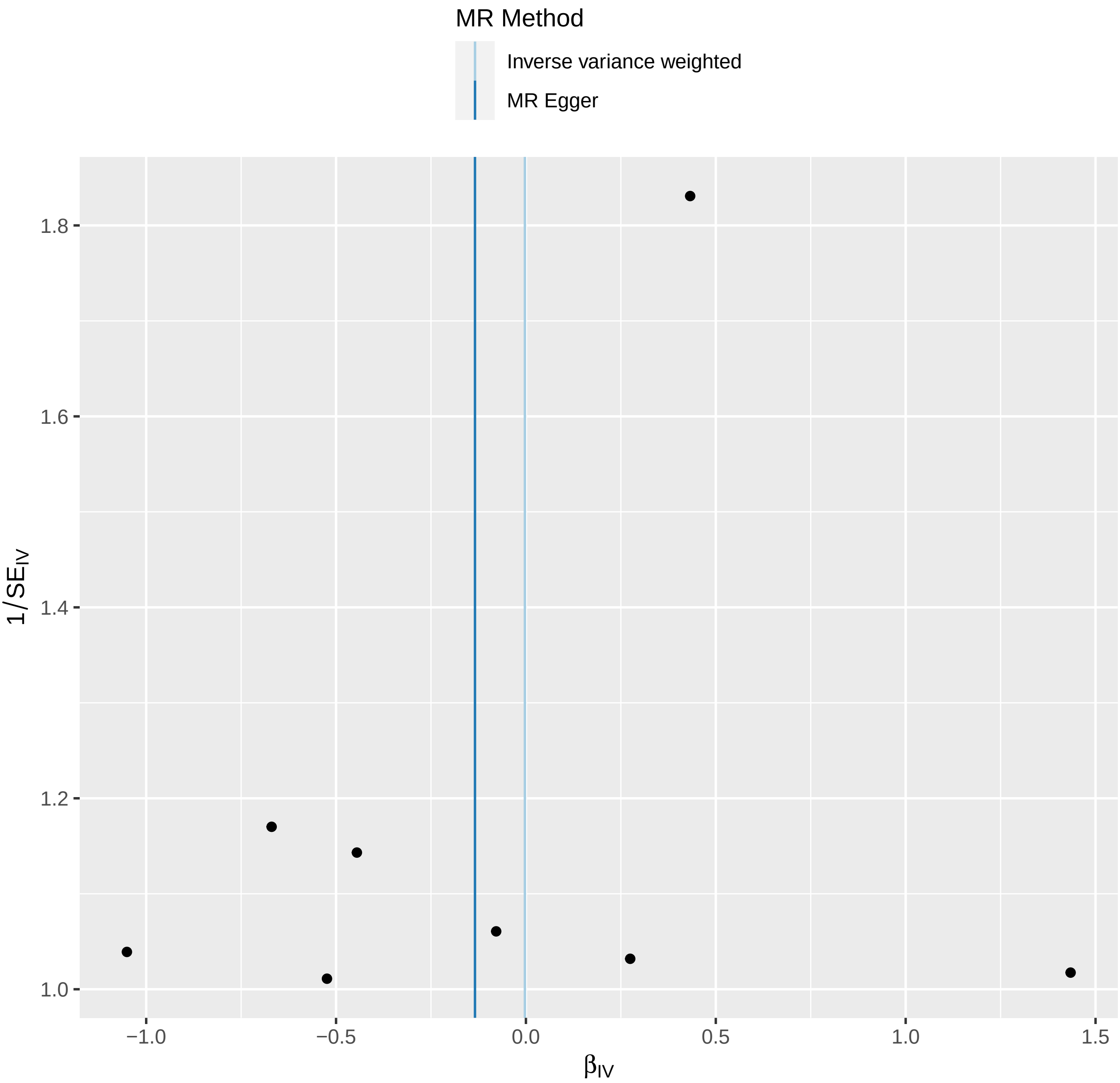

Batch 855 : Gut microbiota abundance (genus Lactococcus id.1851) on Arthropathy in ulcerative colitis

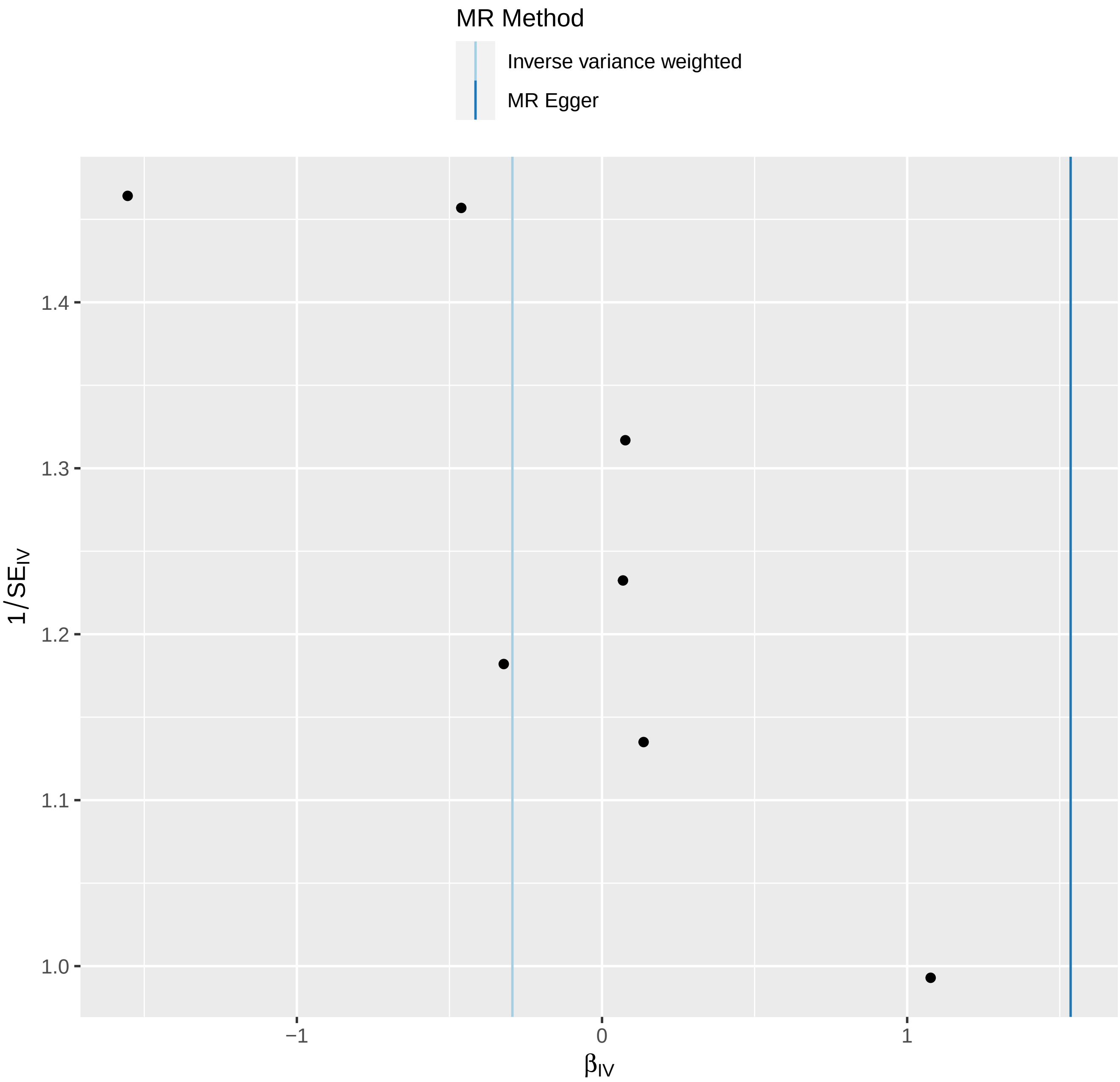

Batch 856 : Gut microbiota abundance (genus Marvinbryantia id.2005) on Arthropathy in ulcerative colitis

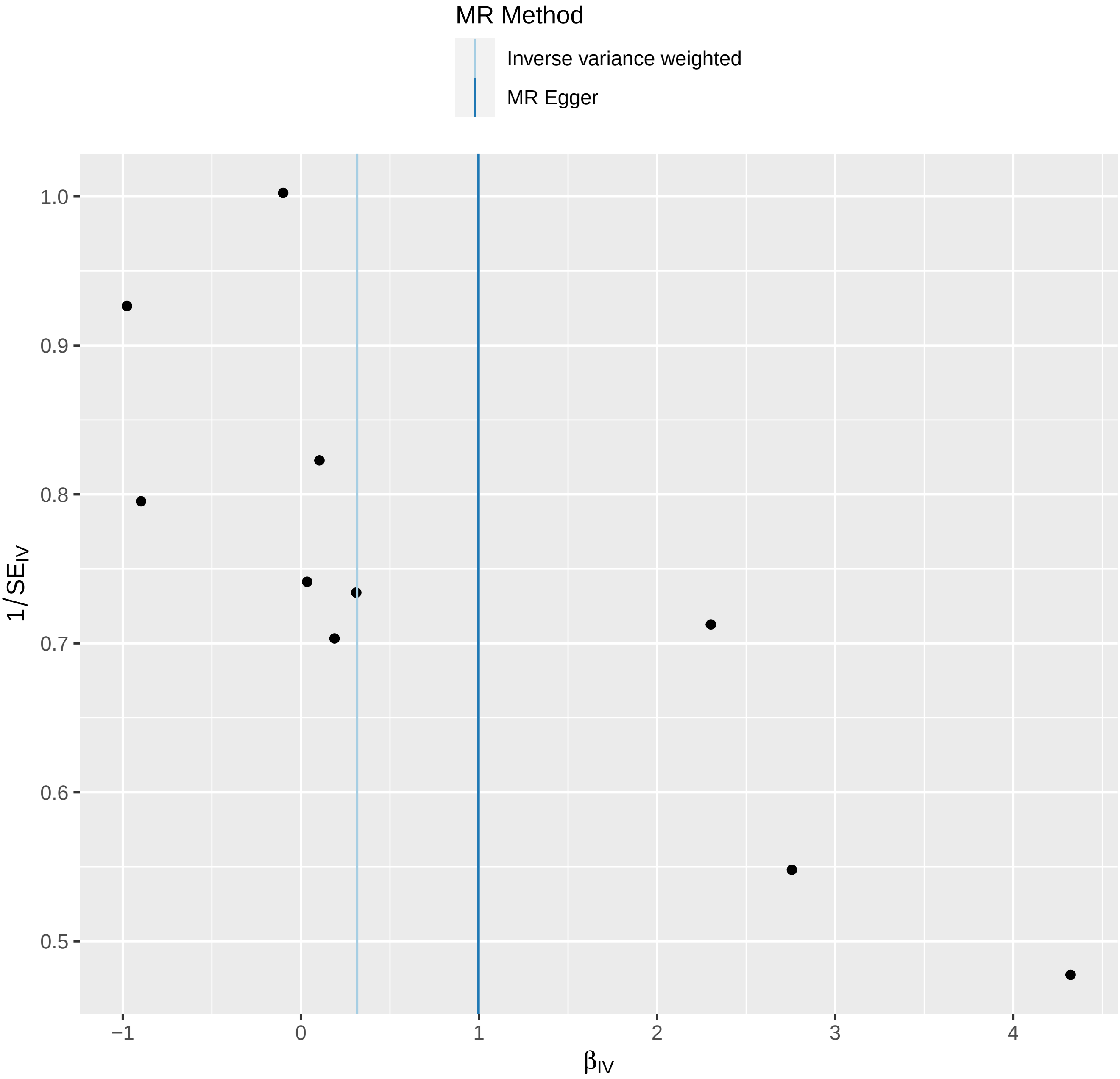

Batch 857 : Gut microbiota abundance (genus Methanobrevibacter id.123) on Arthropathy in ulcerative colitis

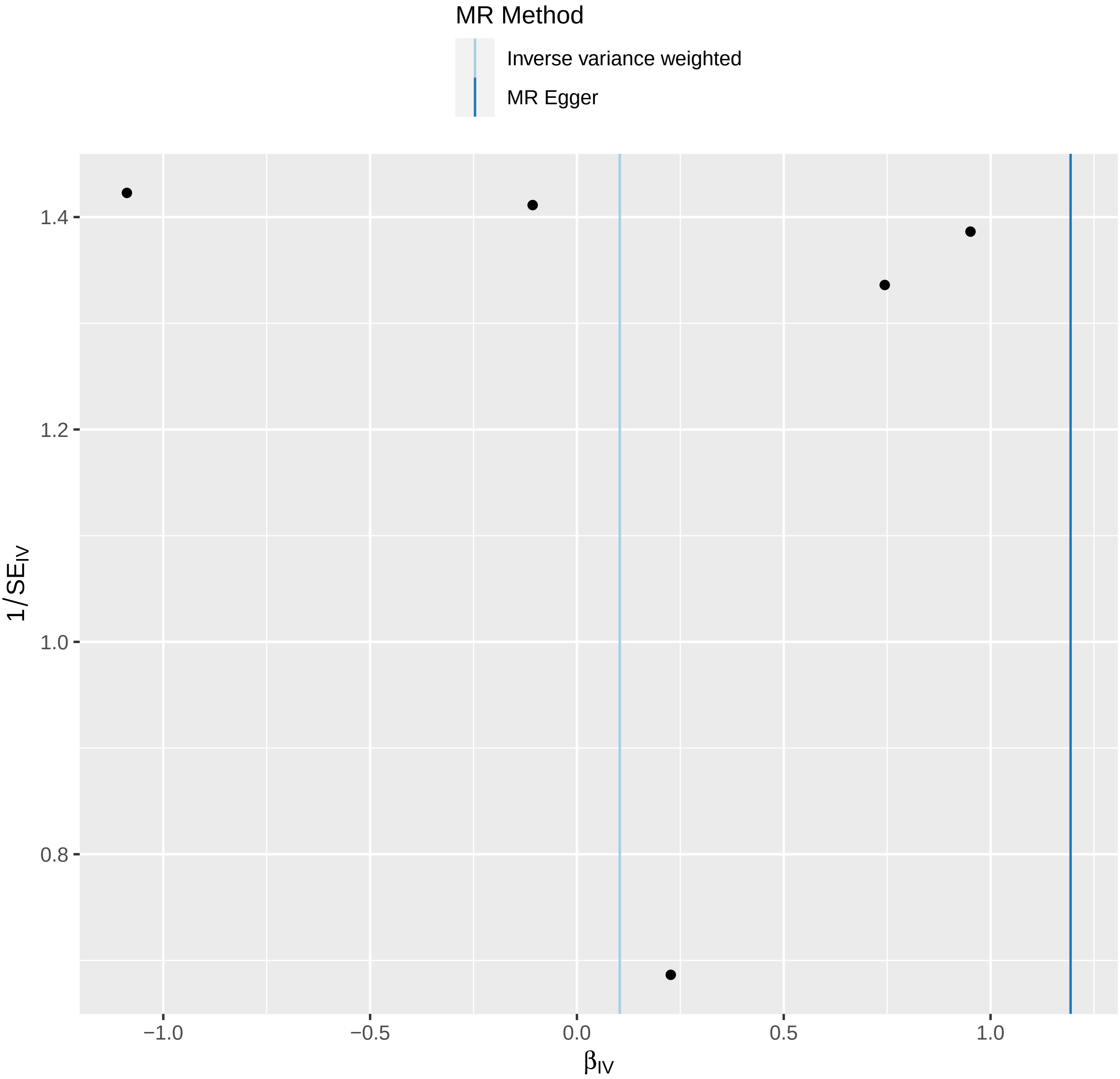

Batch 858 : Gut microbiota abundance (genus Odoribacter id.952) on Arthropathy in ulcerative colitis

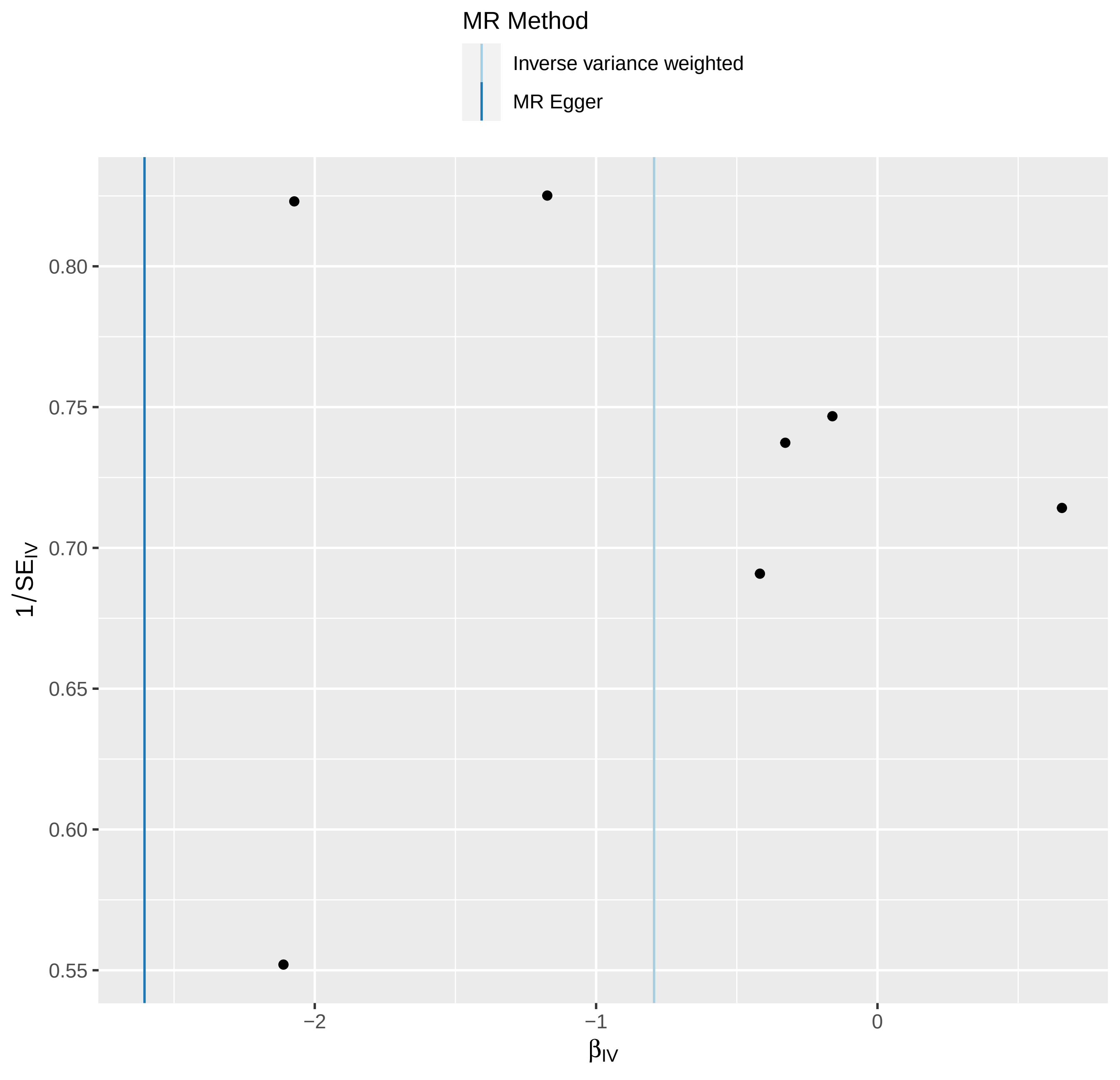

Batch 859 : Gut microbiota abundance (genus Olsenella id.822) on Arthropathy in ulcerative colitis

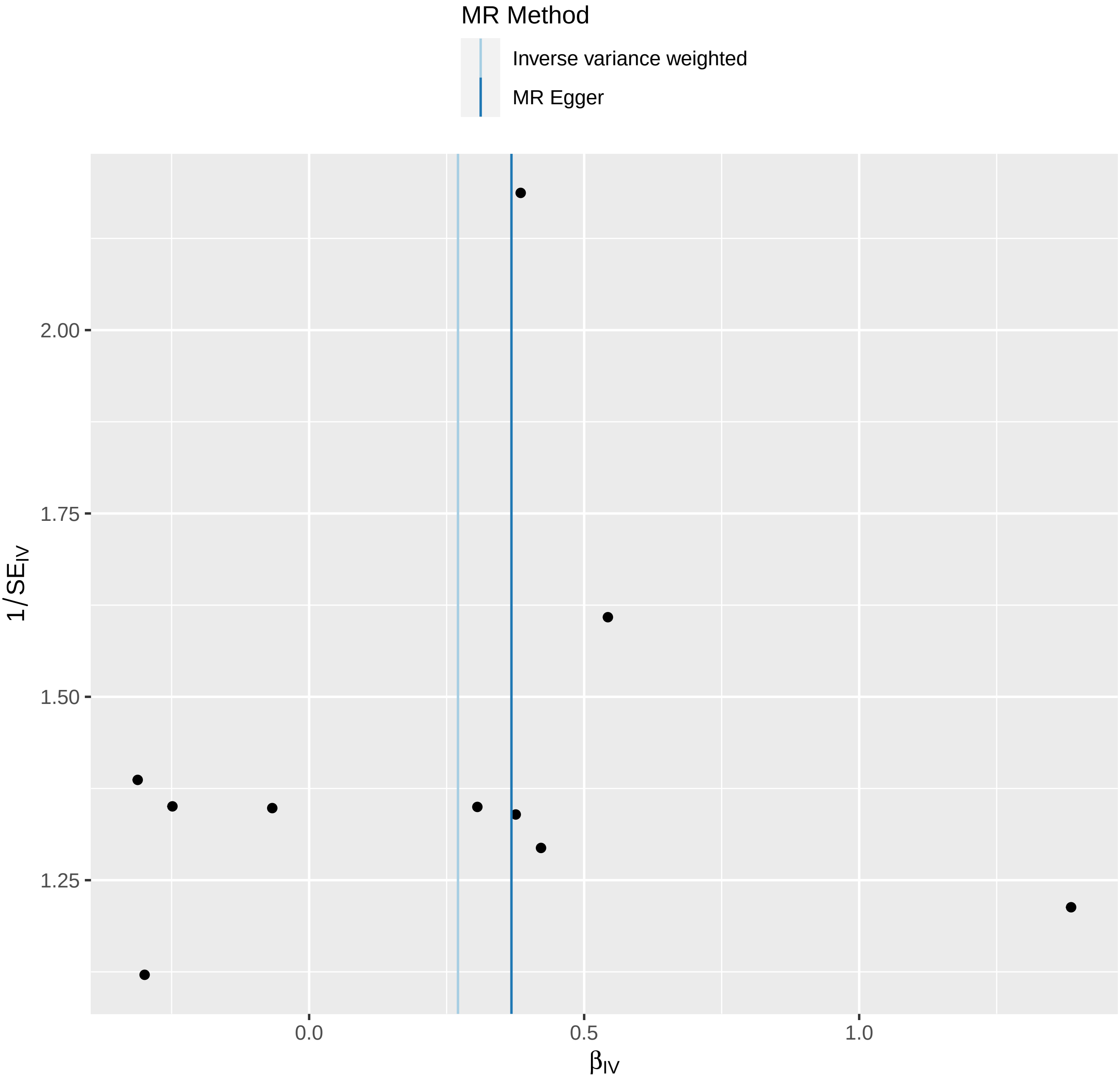

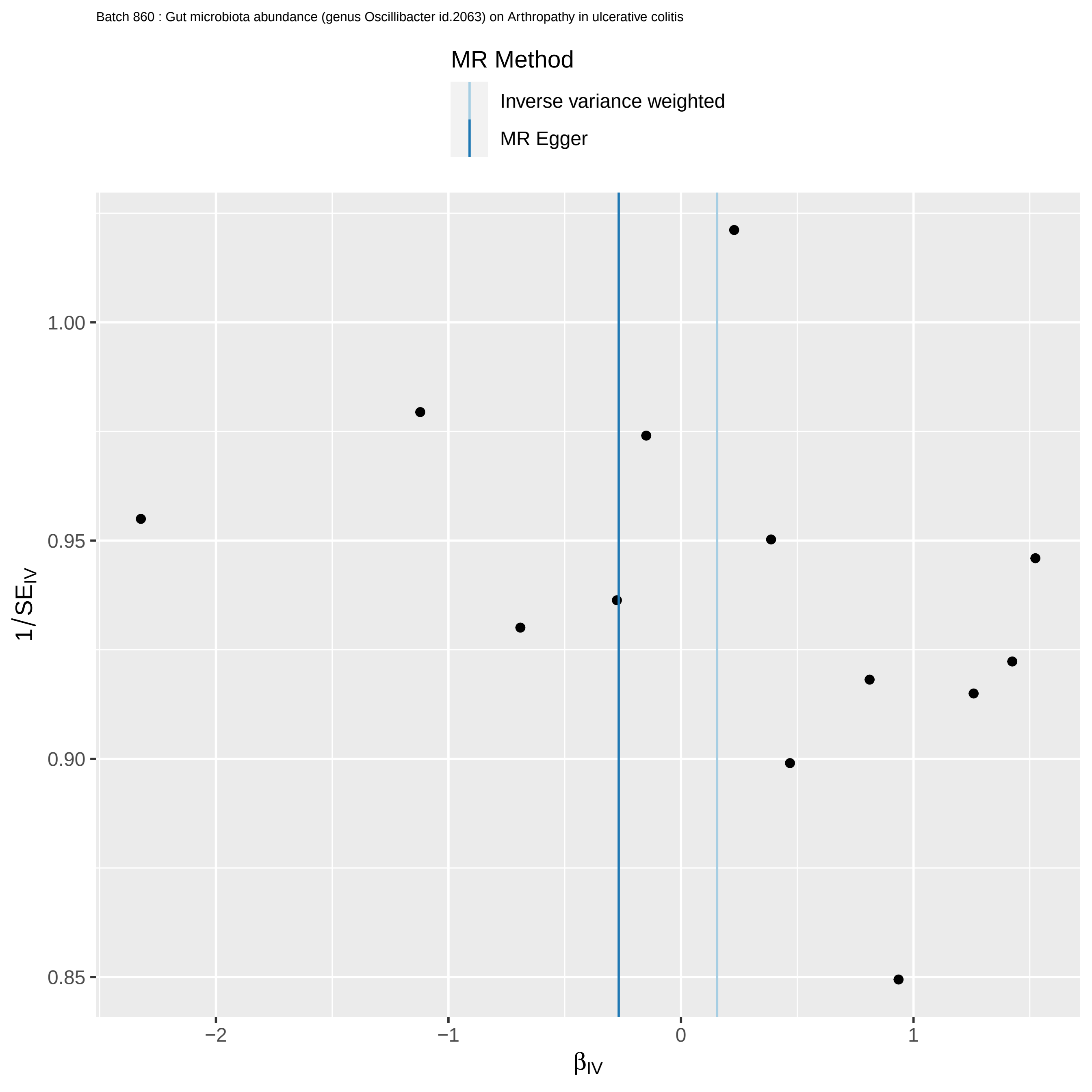
Batch 860 : Gut microbiota abundance (genus Oscillibacter id.2063) on Arthropathy in ulcerative colitis
MR Method
Inverse variance weighted
MR Egger
1.00
0.95
0.90
0.85
1/SE_IV
−2
−1
0
1
β_IV

Batch 861 : Gut microbiota abundance (genus Oscillospira id.2064) on Arthropathy in ulcerative colitis

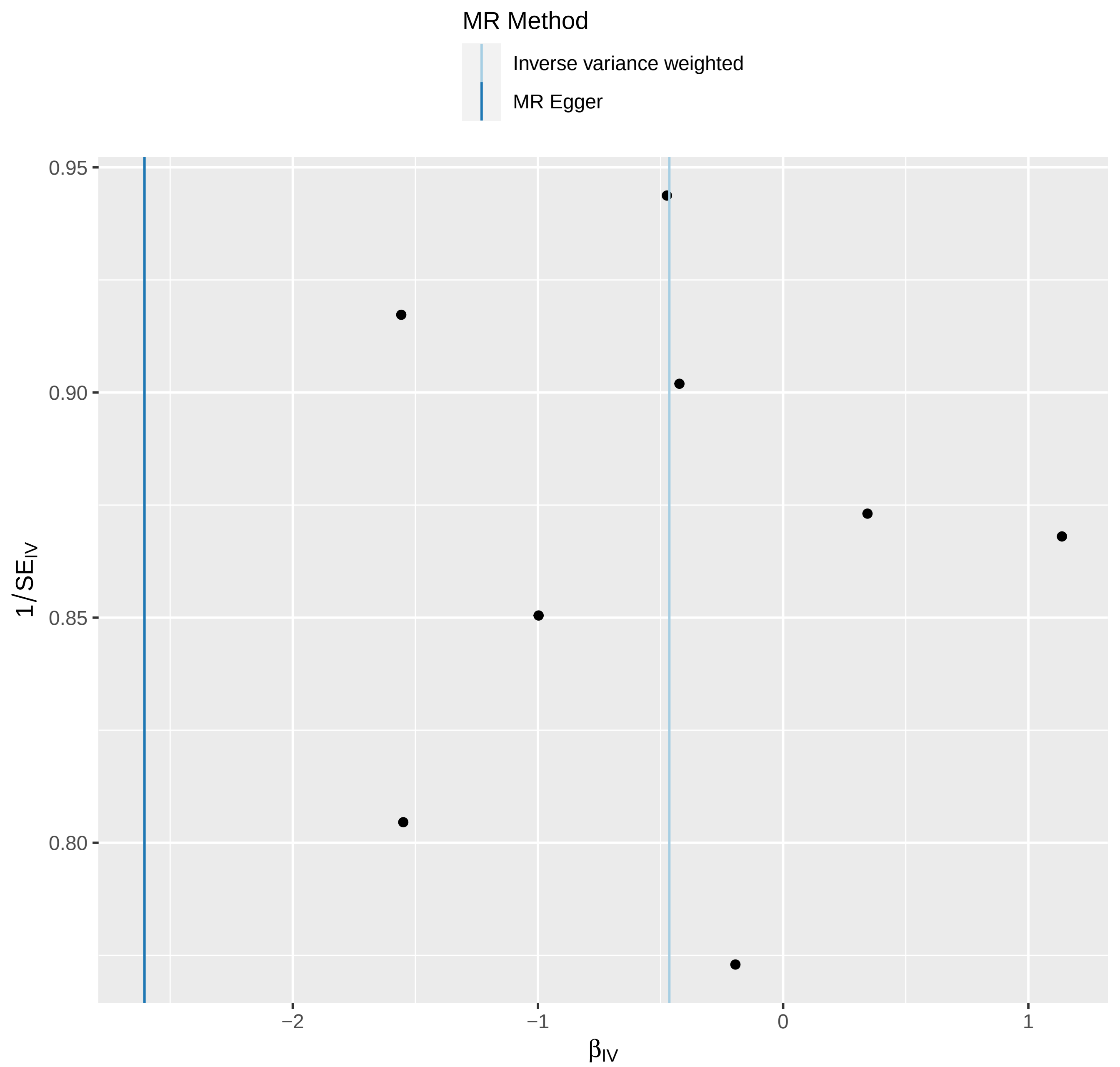

Batch 862 : Gut microbiota abundance (genus Oxalobacter id.2978) on Arthropathy in ulcerative colitis

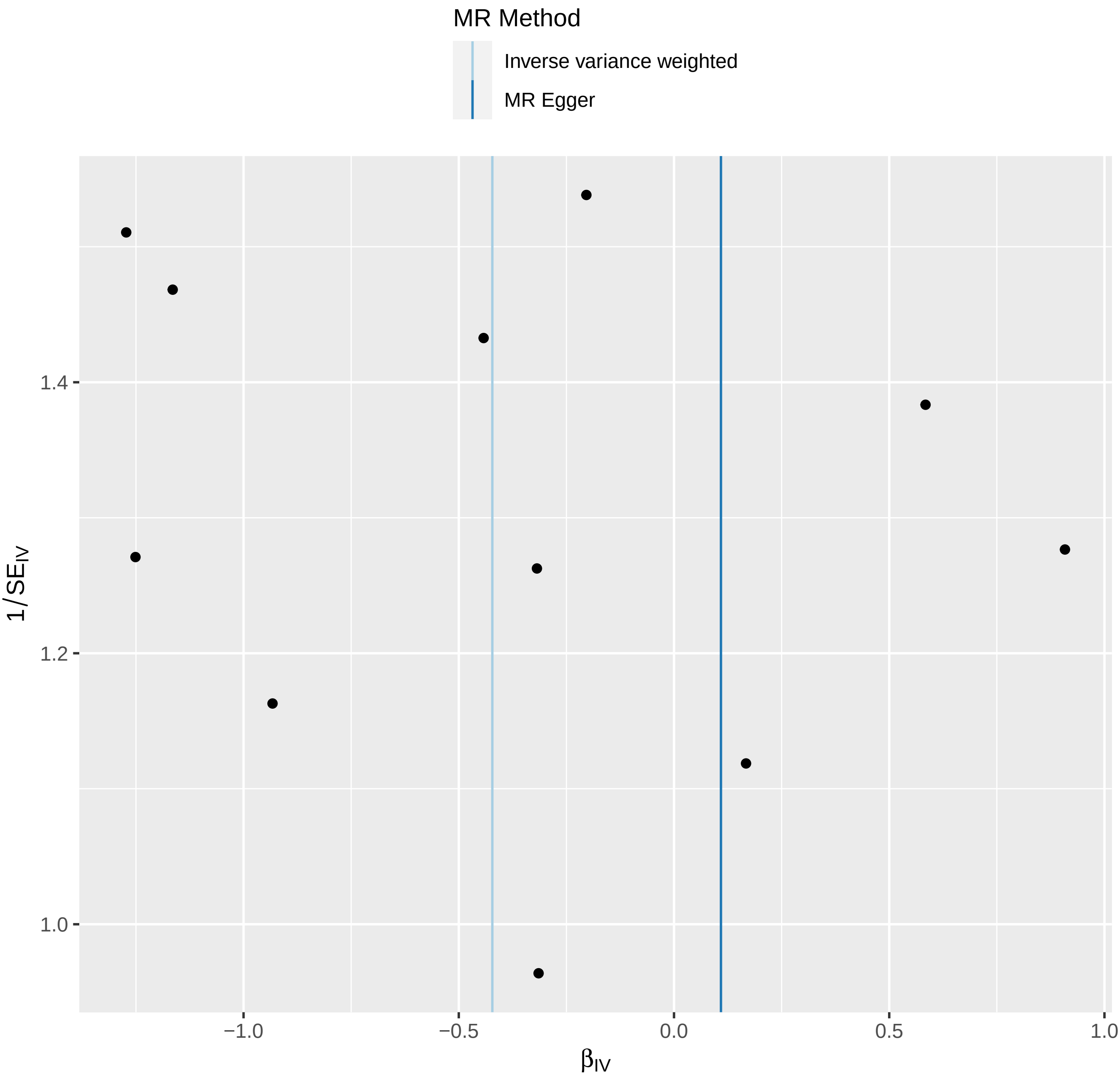

Batch 863 : Gut microbiota abundance (genus Parabacteroides id.954) on Arthropathy in ulcerative colitis

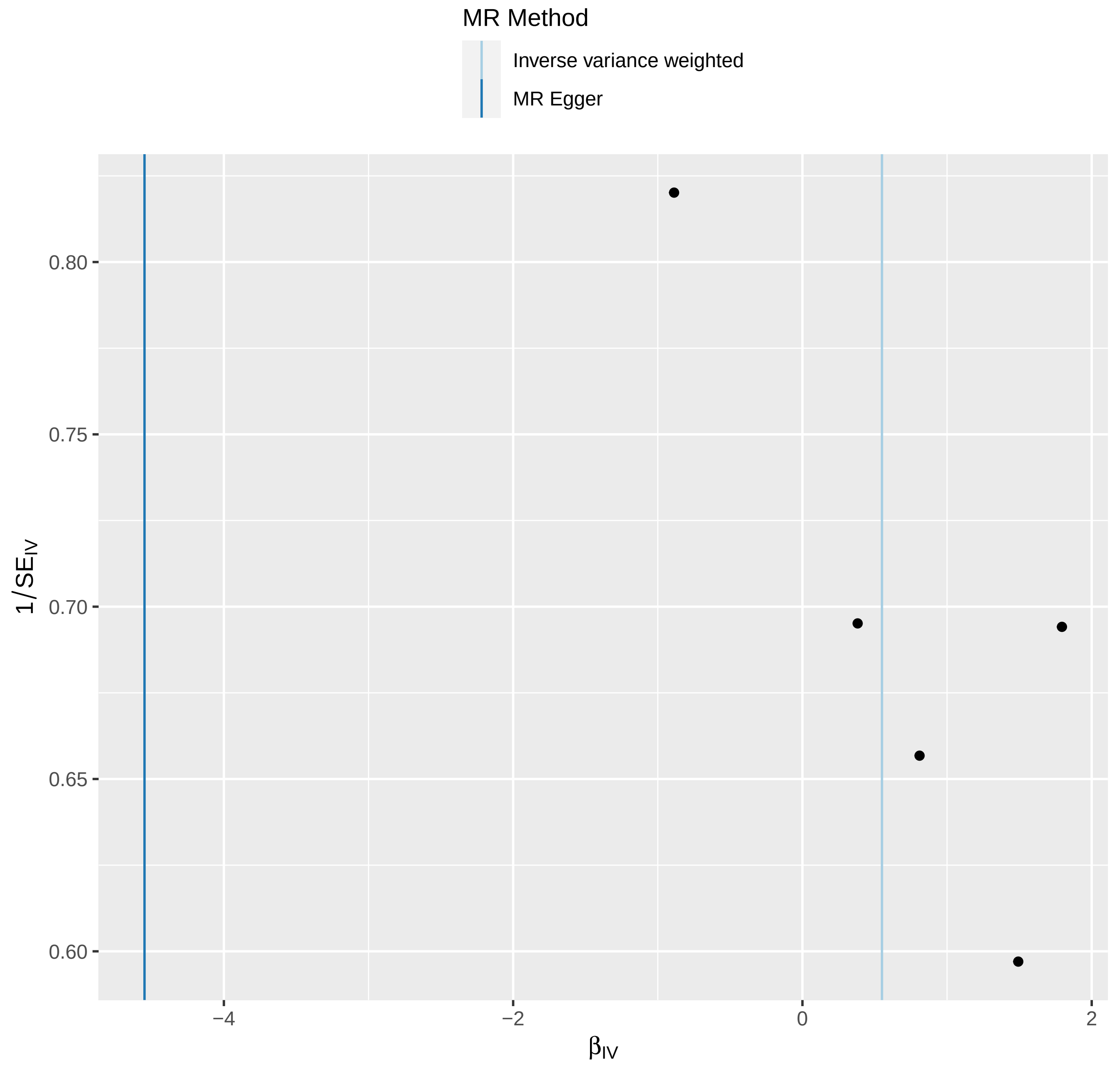

Batch 864 : Gut microbiota abundance (genus Paraprevotella id.962) on Arthropathy in ulcerative colitis

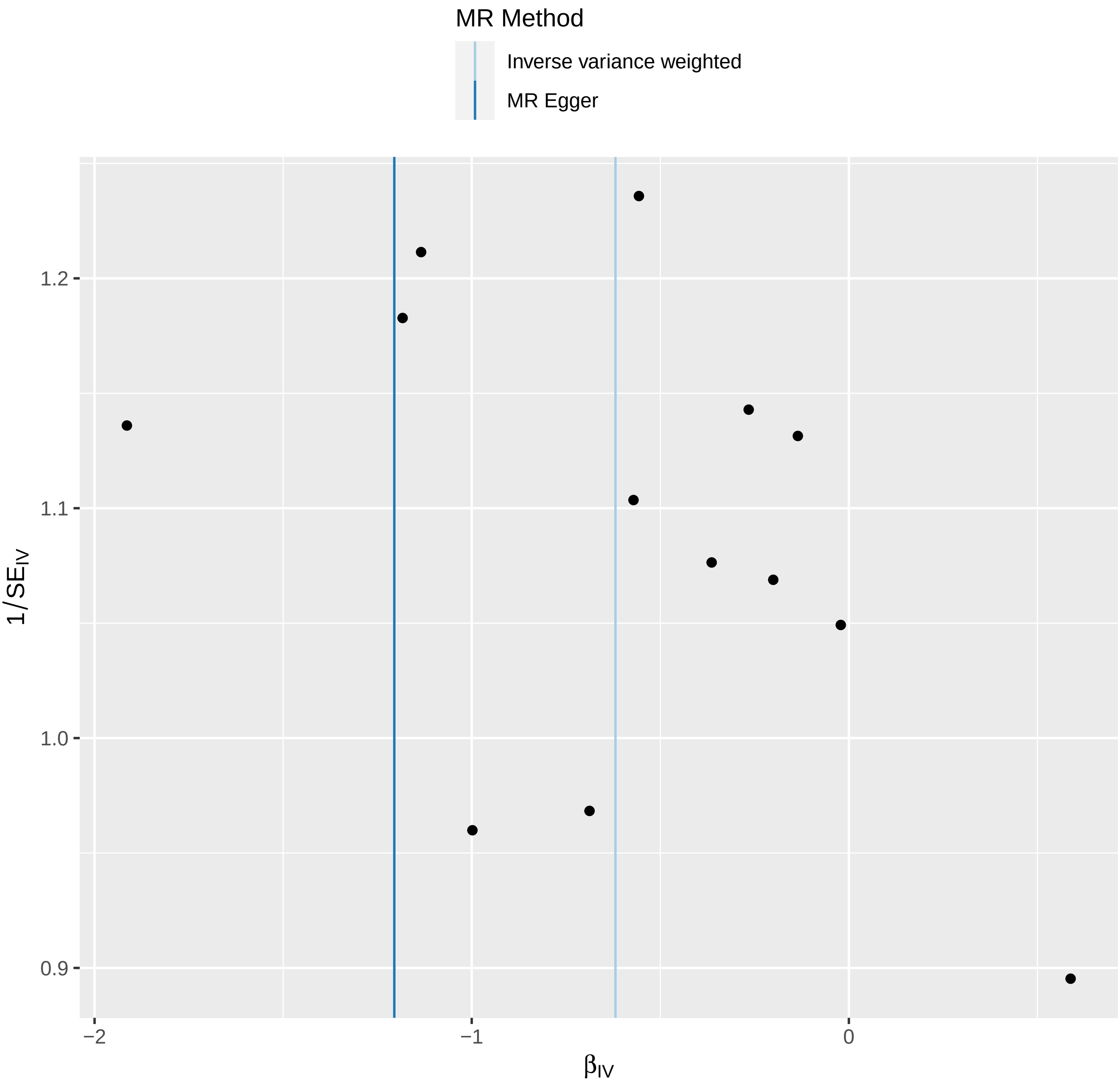

Batch 865 : Gut microbiota abundance (genus Parasutterella id.2892) on Arthropathy in ulcerative colitis

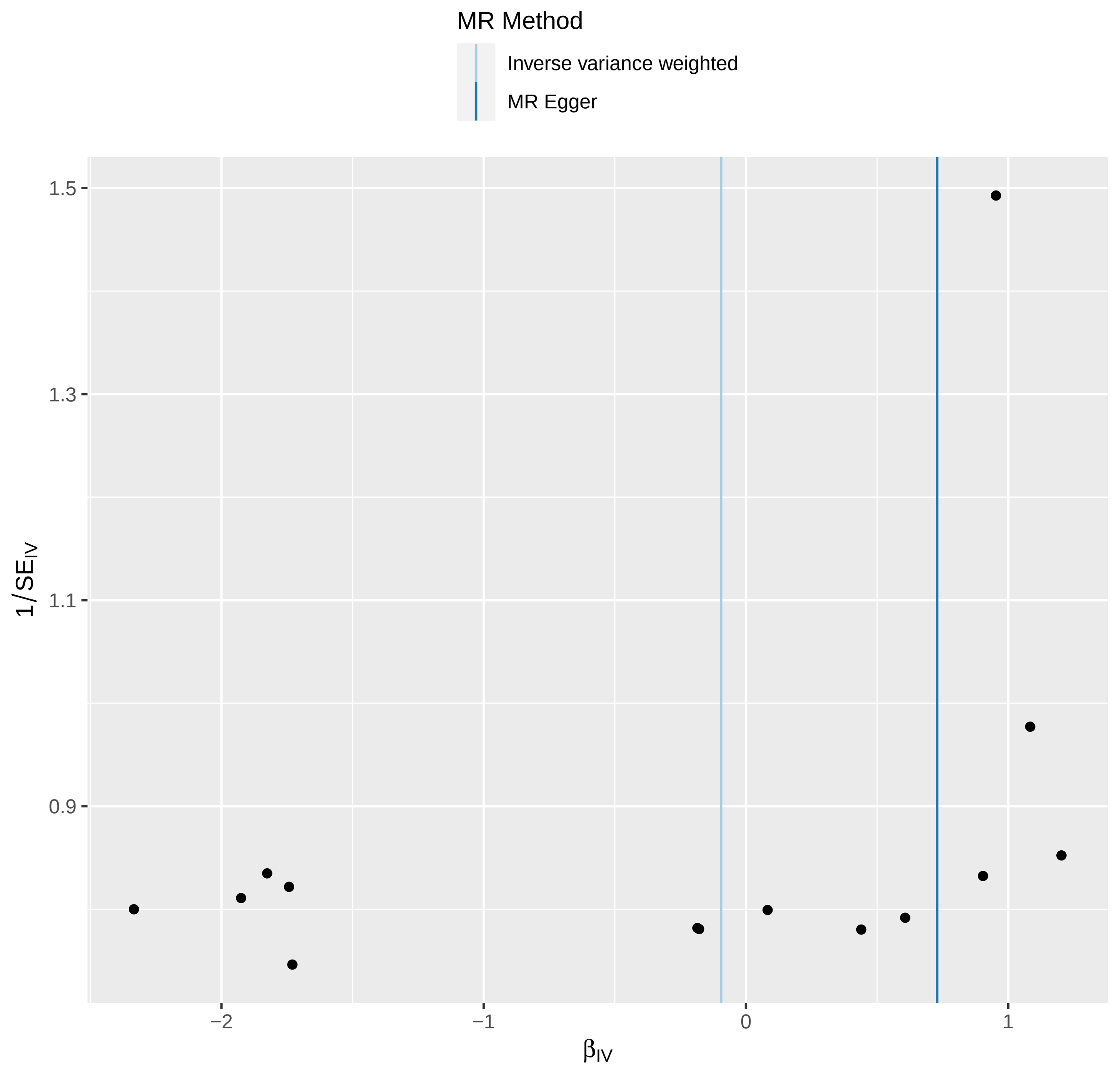

Batch 866 : Gut microbiota abundance (genus Peptococcus id.2037) on Arthropathy in ulcerative colitis

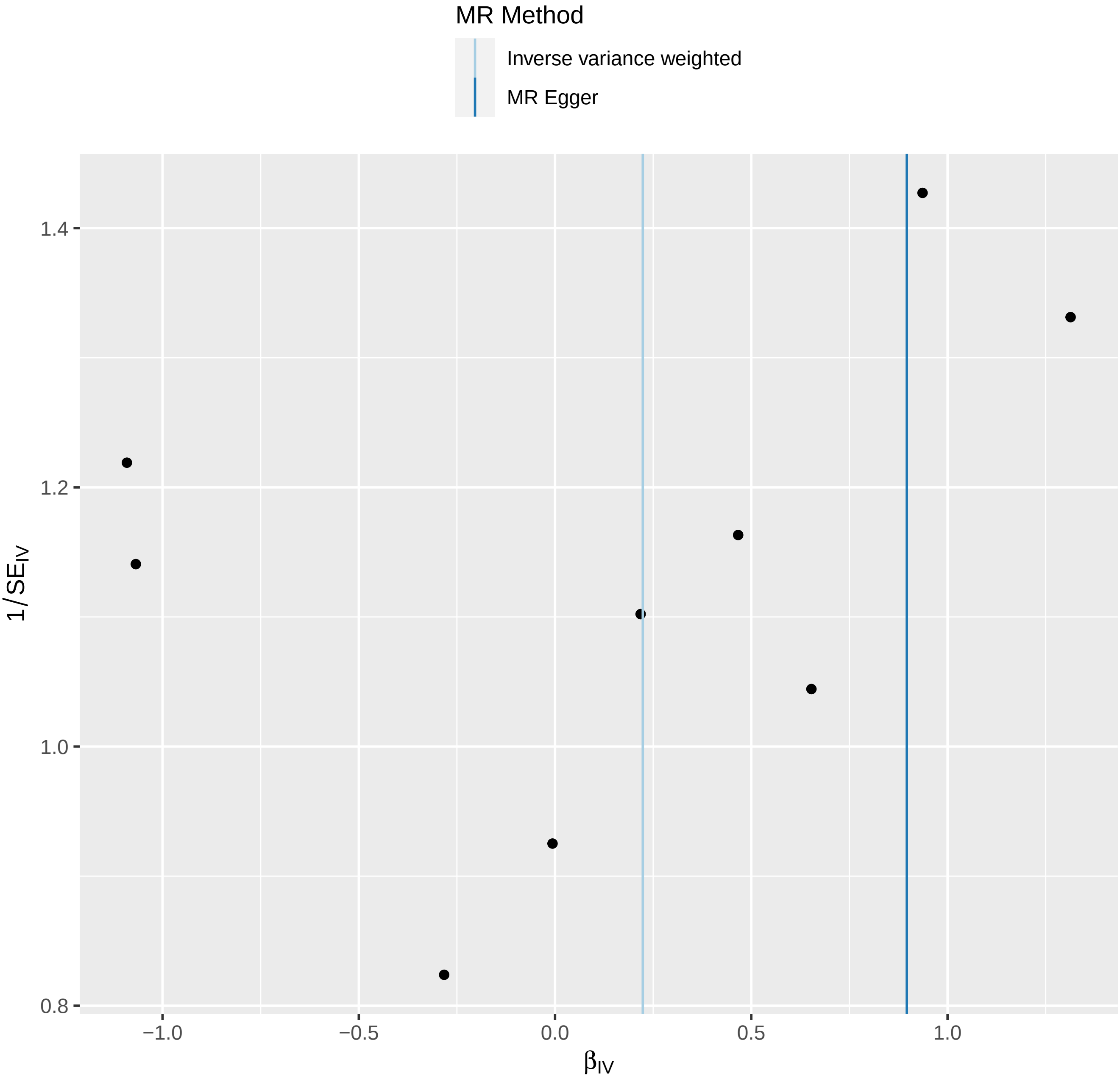

Batch 867 : Gut microbiota abundance (genus Phascolarctobacterium id.2168) on Arthropathy in ulcerative colitis

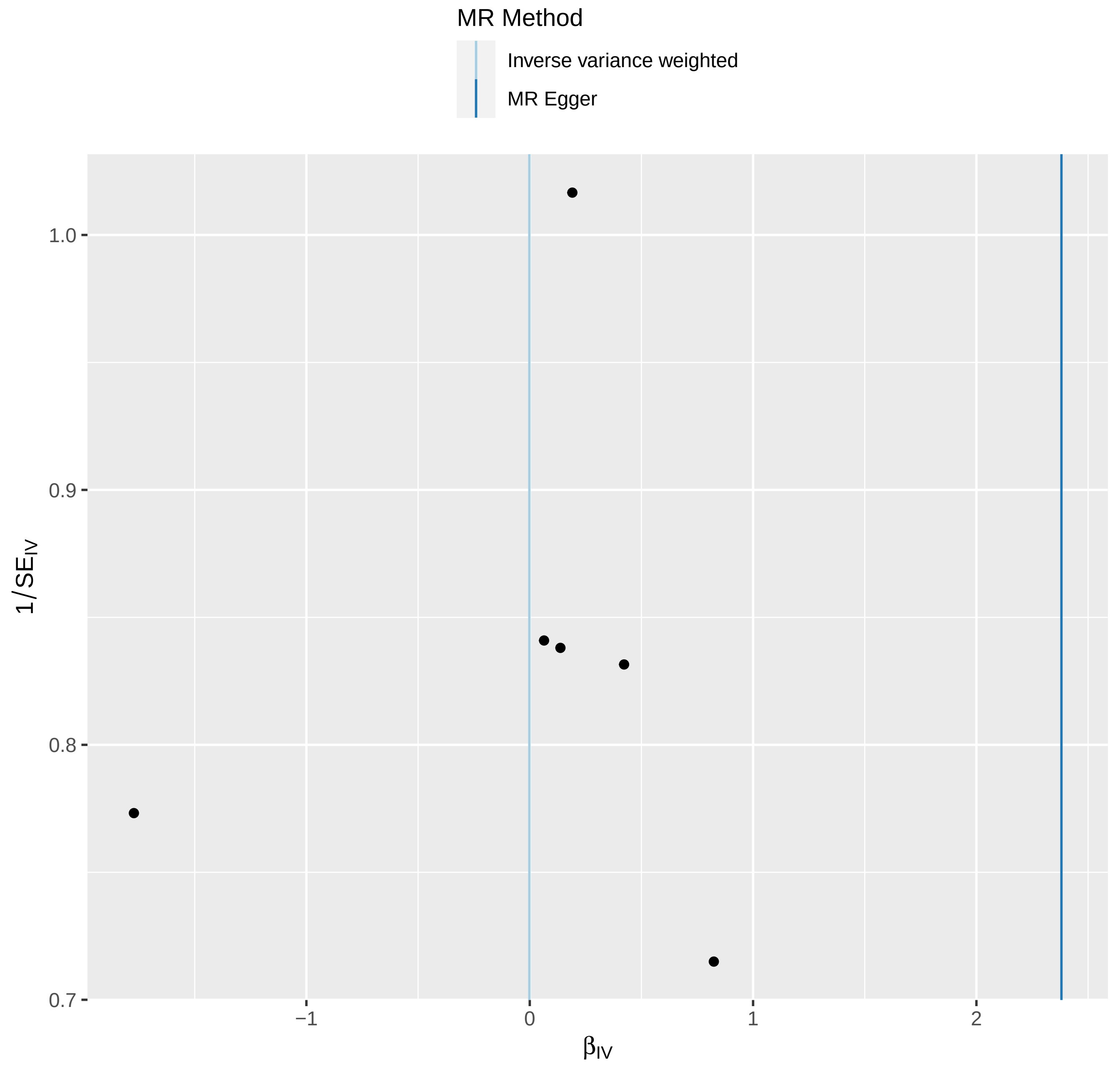

Batch 868 : Gut microbiota abundance (genus Prevotella7 id.11182) on Arthropathy in ulcerative colitis

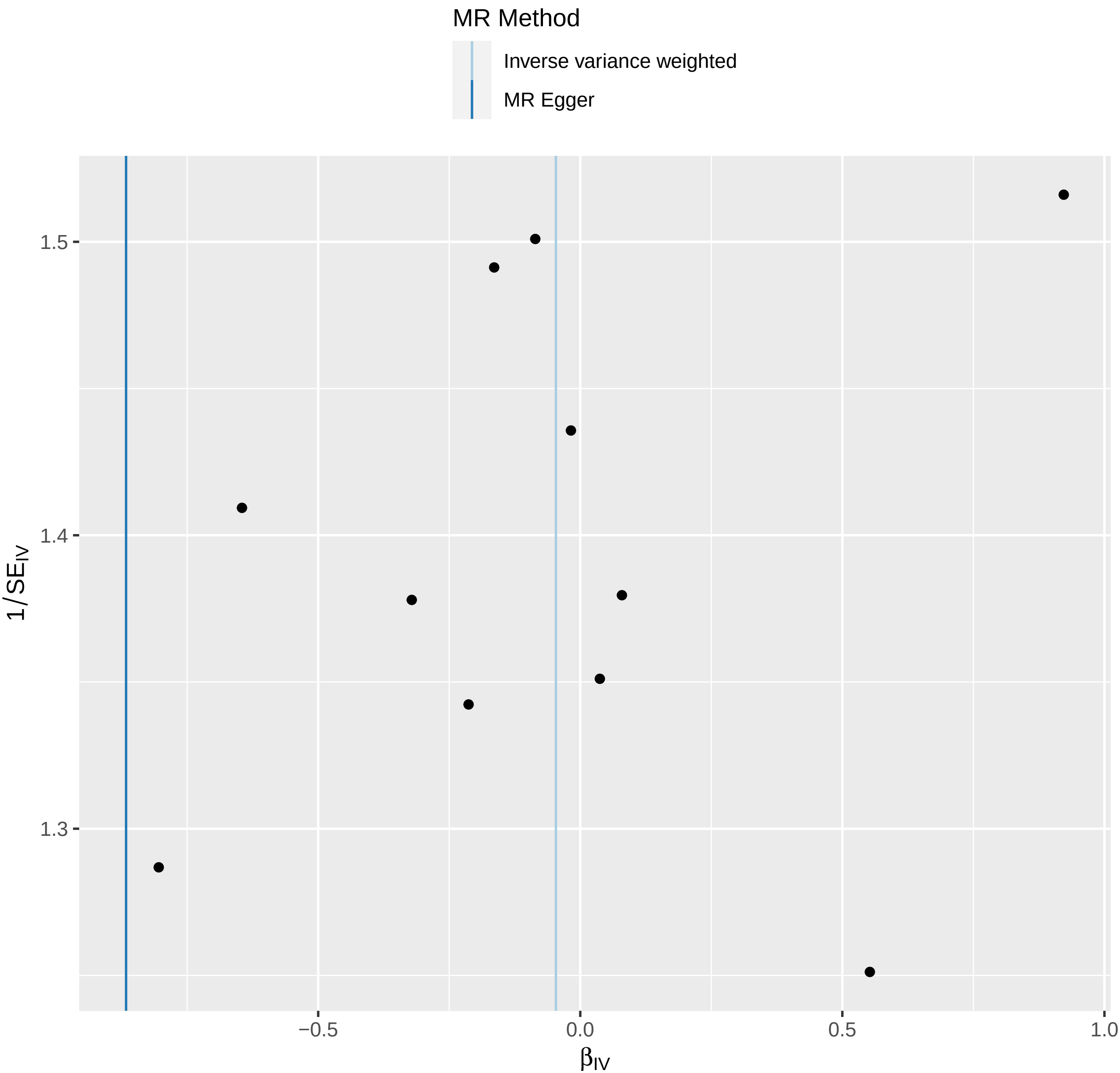

Batch 869 : Gut microbiota abundance (genus Prevotella9 id.11183) on Arthropathy in ulcerative colitis

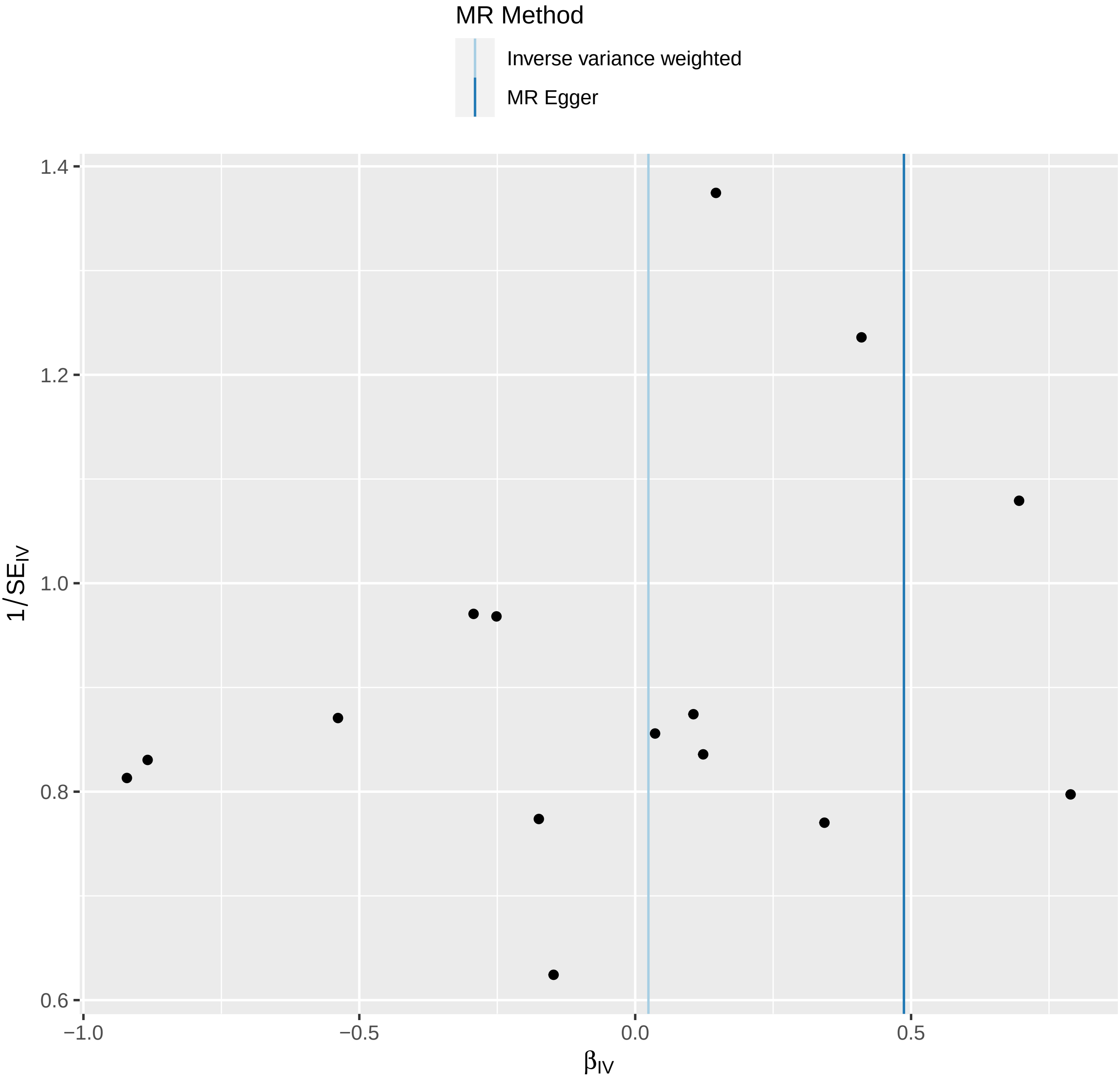

Batch 870 : Gut microbiota abundance (genus Rikenellaceae RC9 gut group id.11191) on Arthropathy in ulcerative colitis

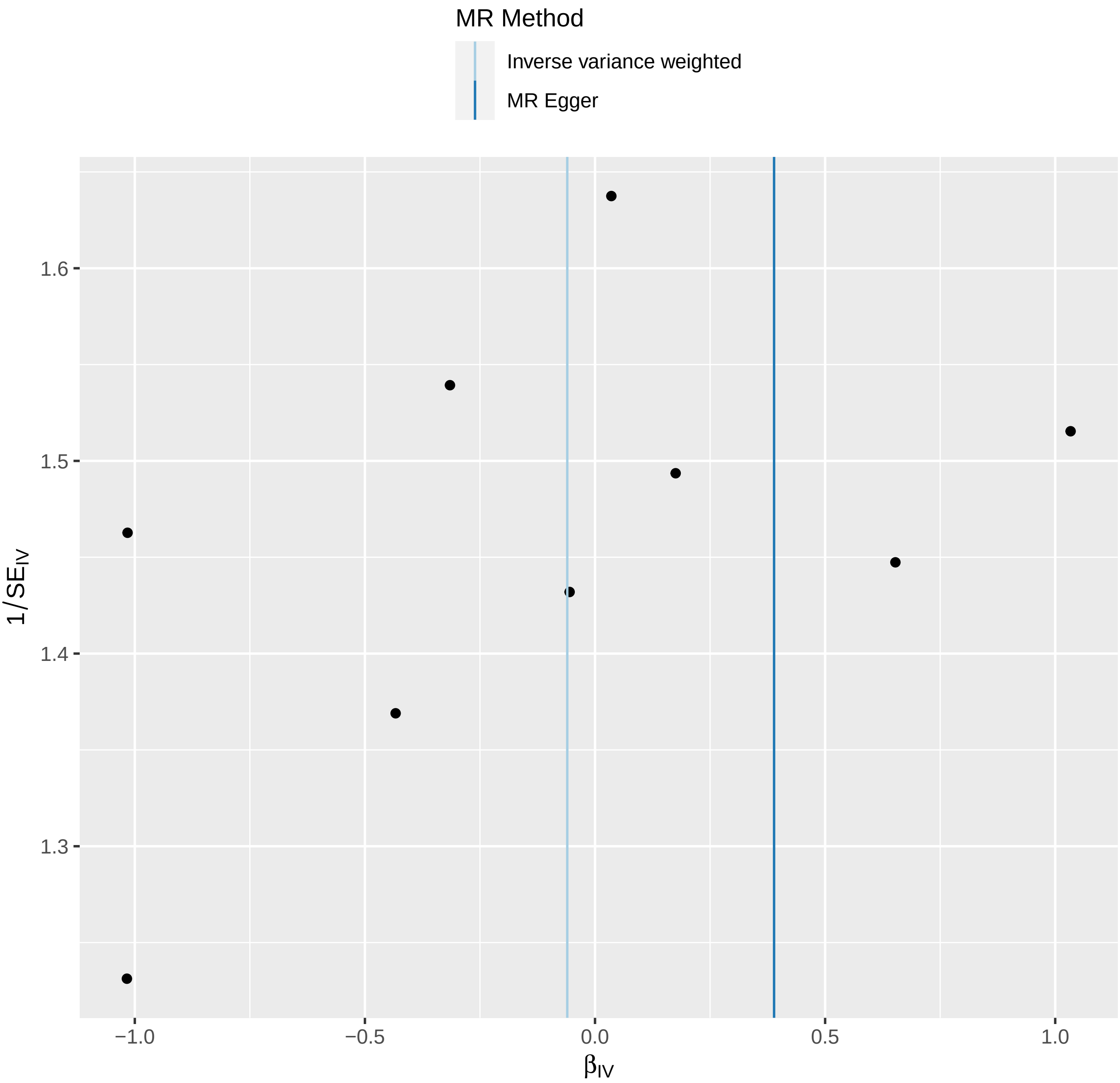

Batch 871 : Gut microbiota abundance (genus Romboutsia id.11347) on Arthropathy in ulcerative colitis

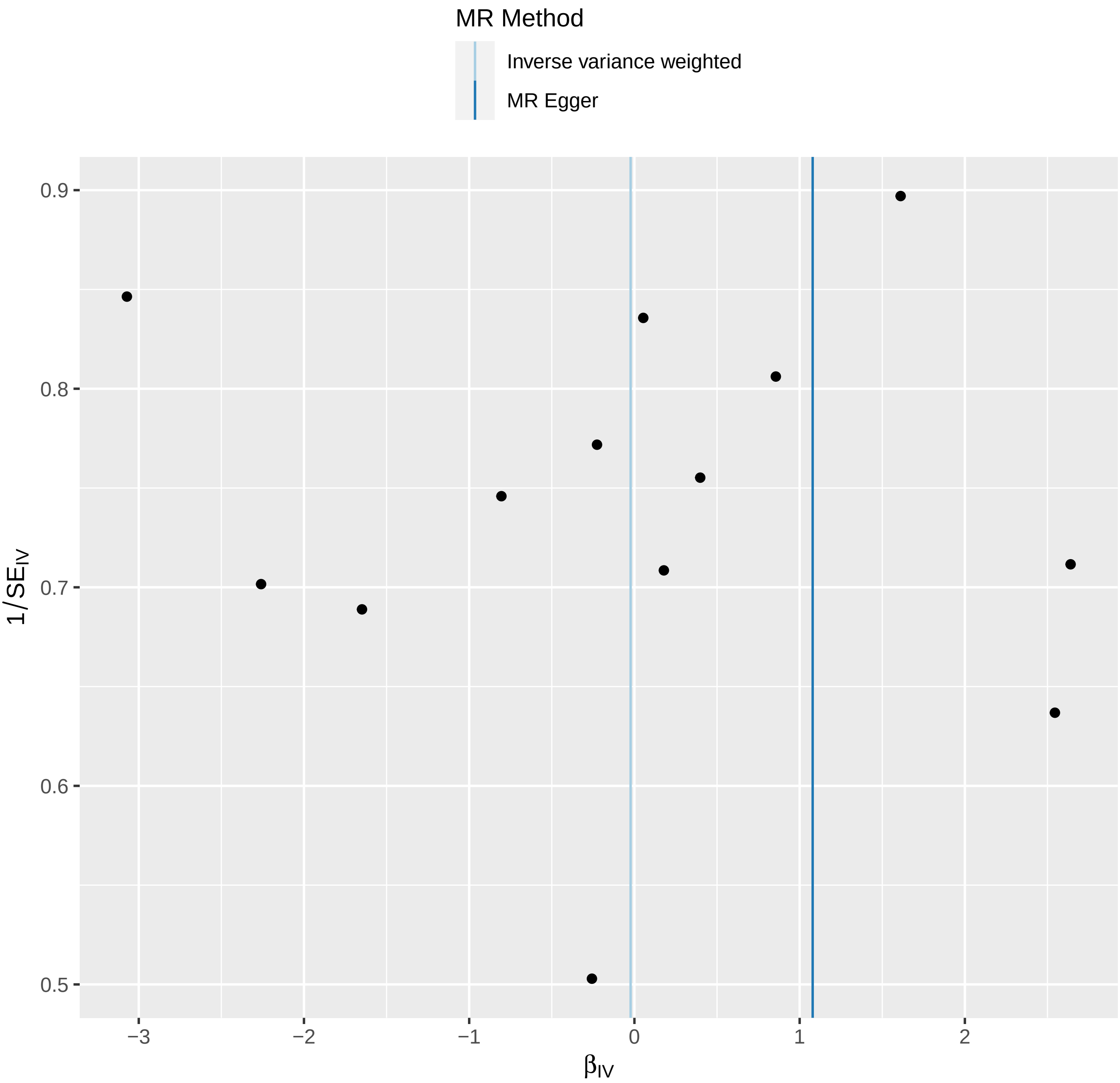

Batch 872 : Gut microbiota abundance (genus Roseburia id.2012) on Arthropathy in ulcerative colitis

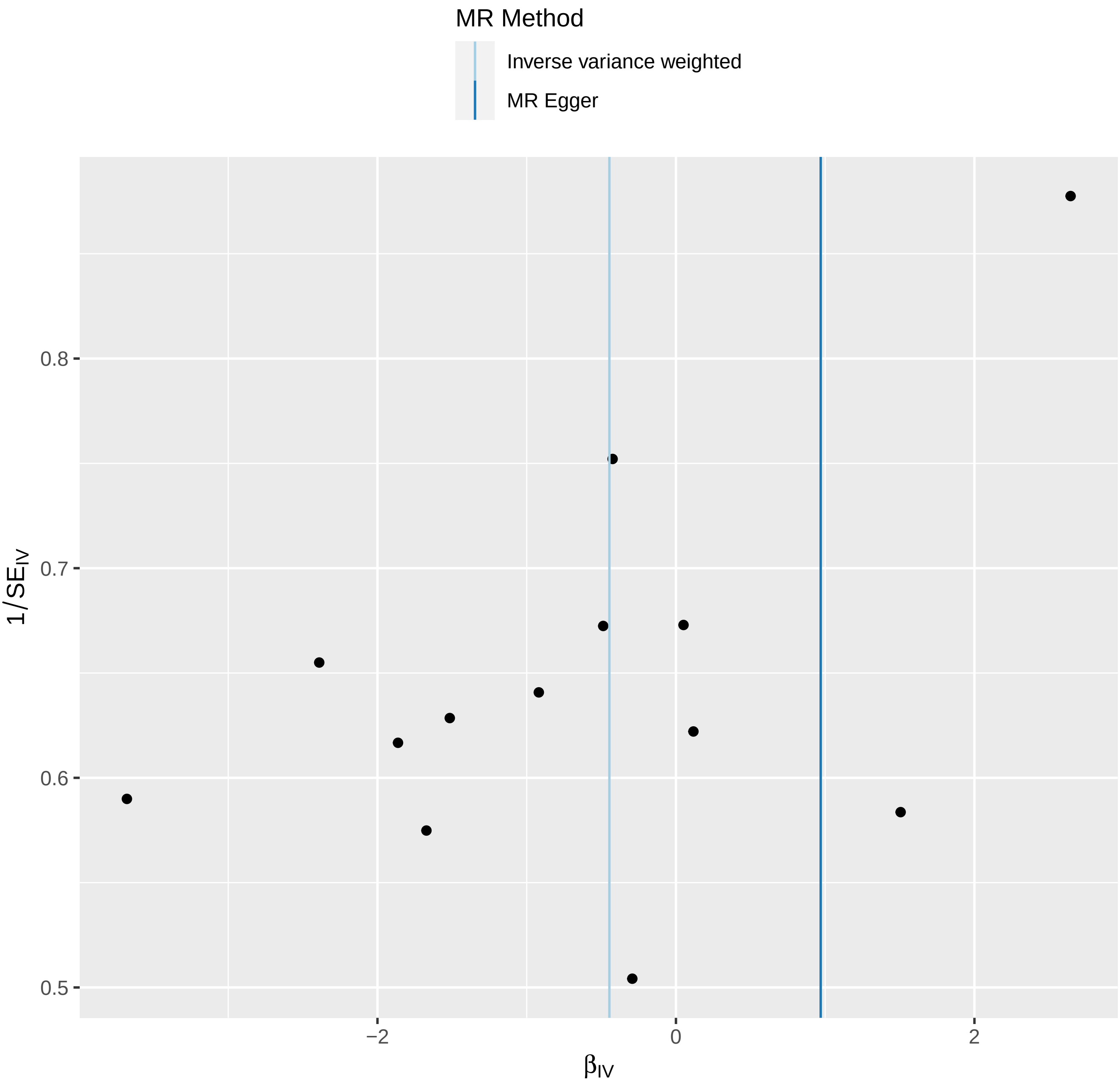

Batch 873 : Gut microbiota abundance (genus Ruminiclostridium5 id.11355) on Arthropathy in ulcerative colitis

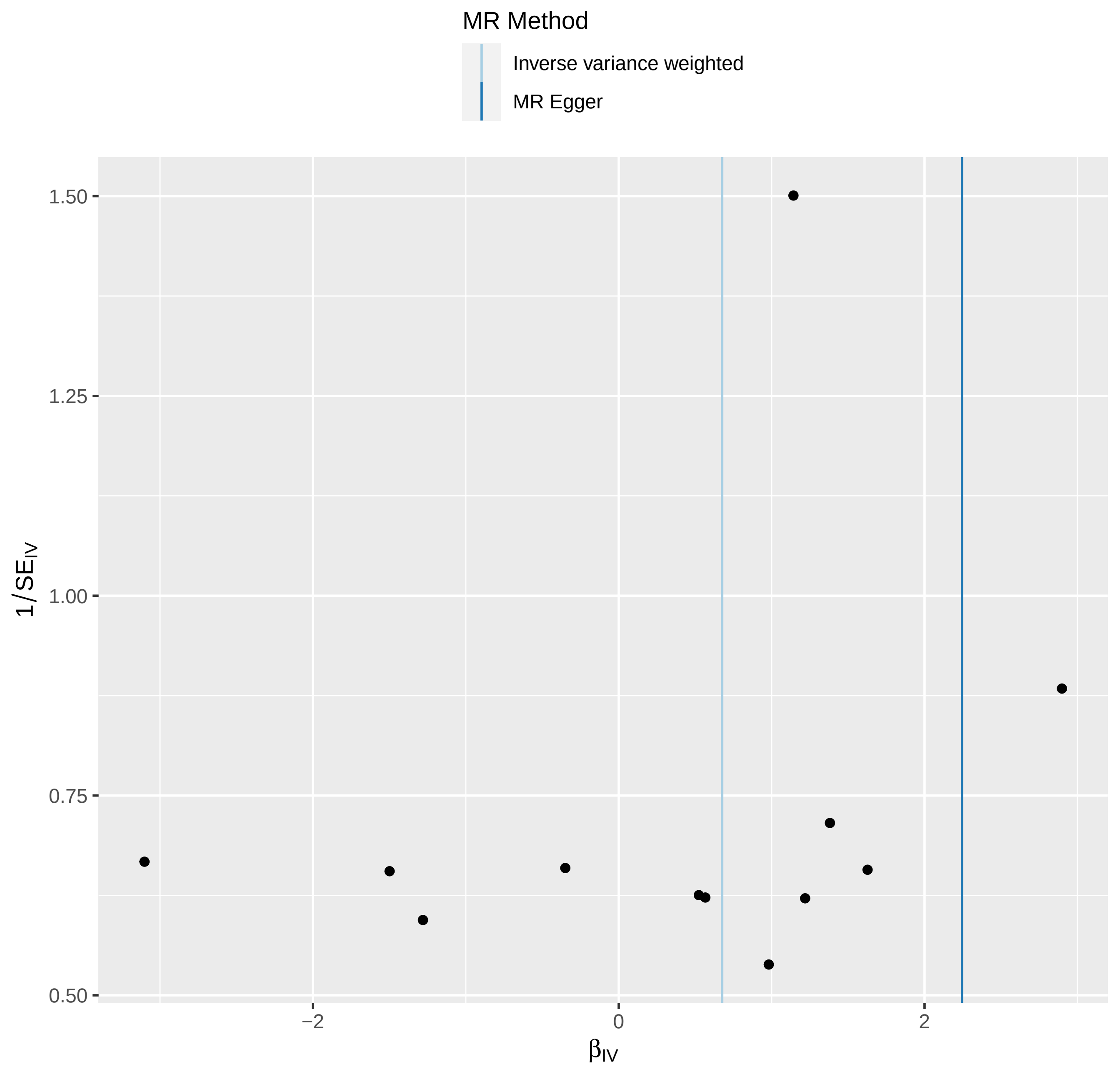

Batch 874 : Gut microbiota abundance (genus Ruminiclostridium6 id.11356) on Arthropathy in ulcerative colitis

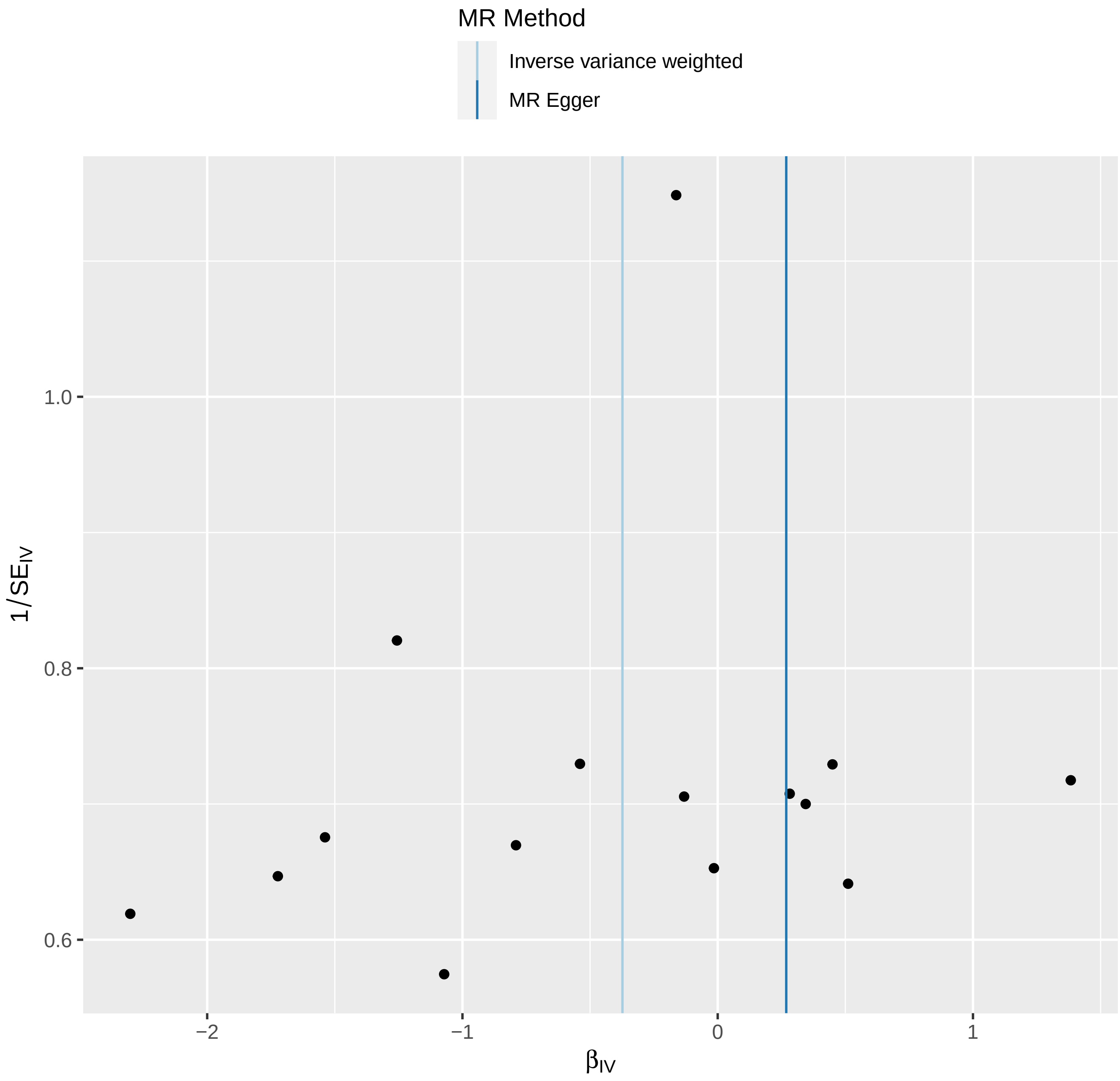

Batch 875 : Gut microbiota abundance (genus Ruminiclostridium9 id.11357) on Arthropathy in ulcerative colitis

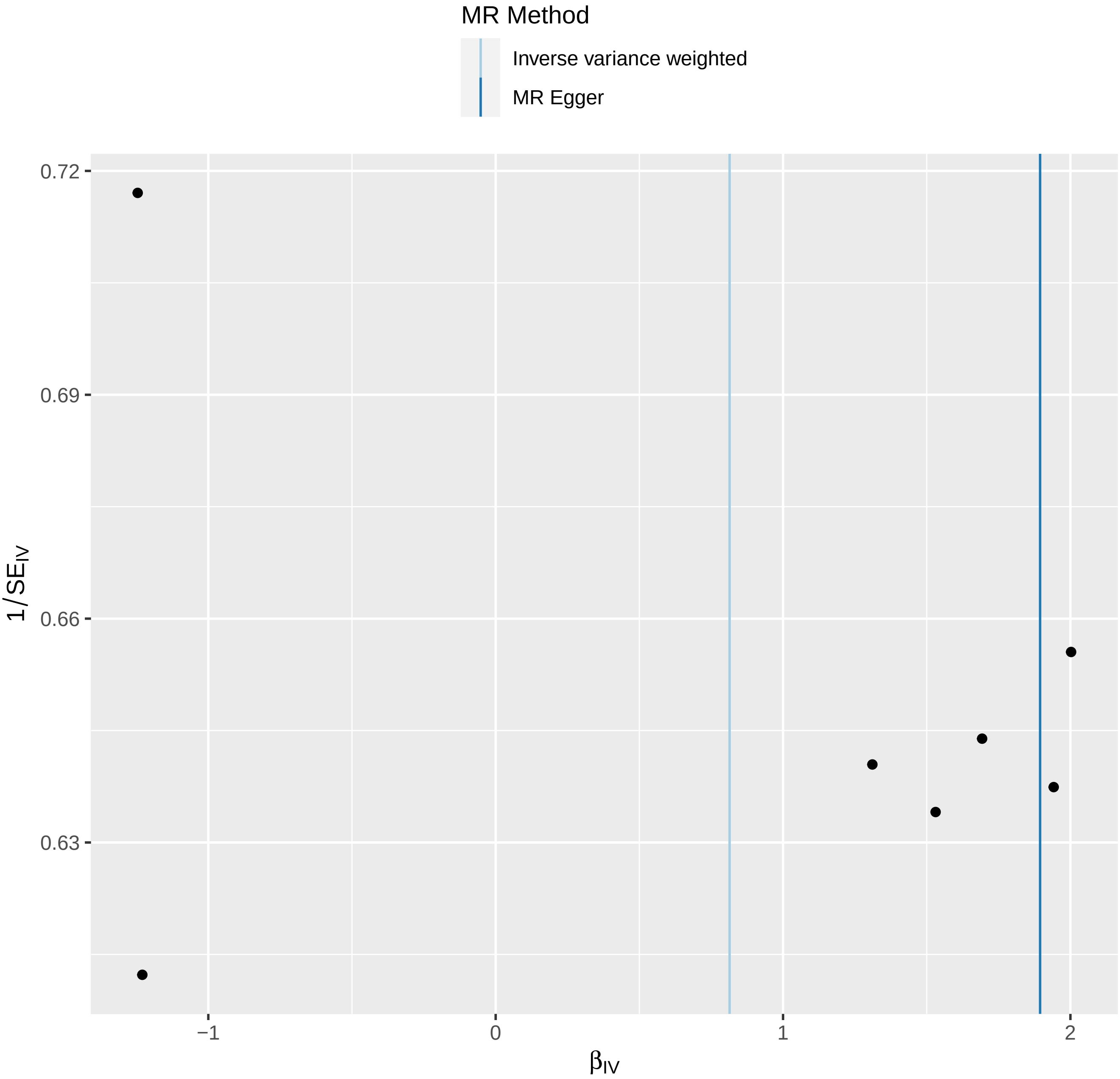

Batch 876 : Gut microbiota abundance (genus Ruminococcaceae NK4A214 group id.11358) on Arthropathy in ulcerative colitis

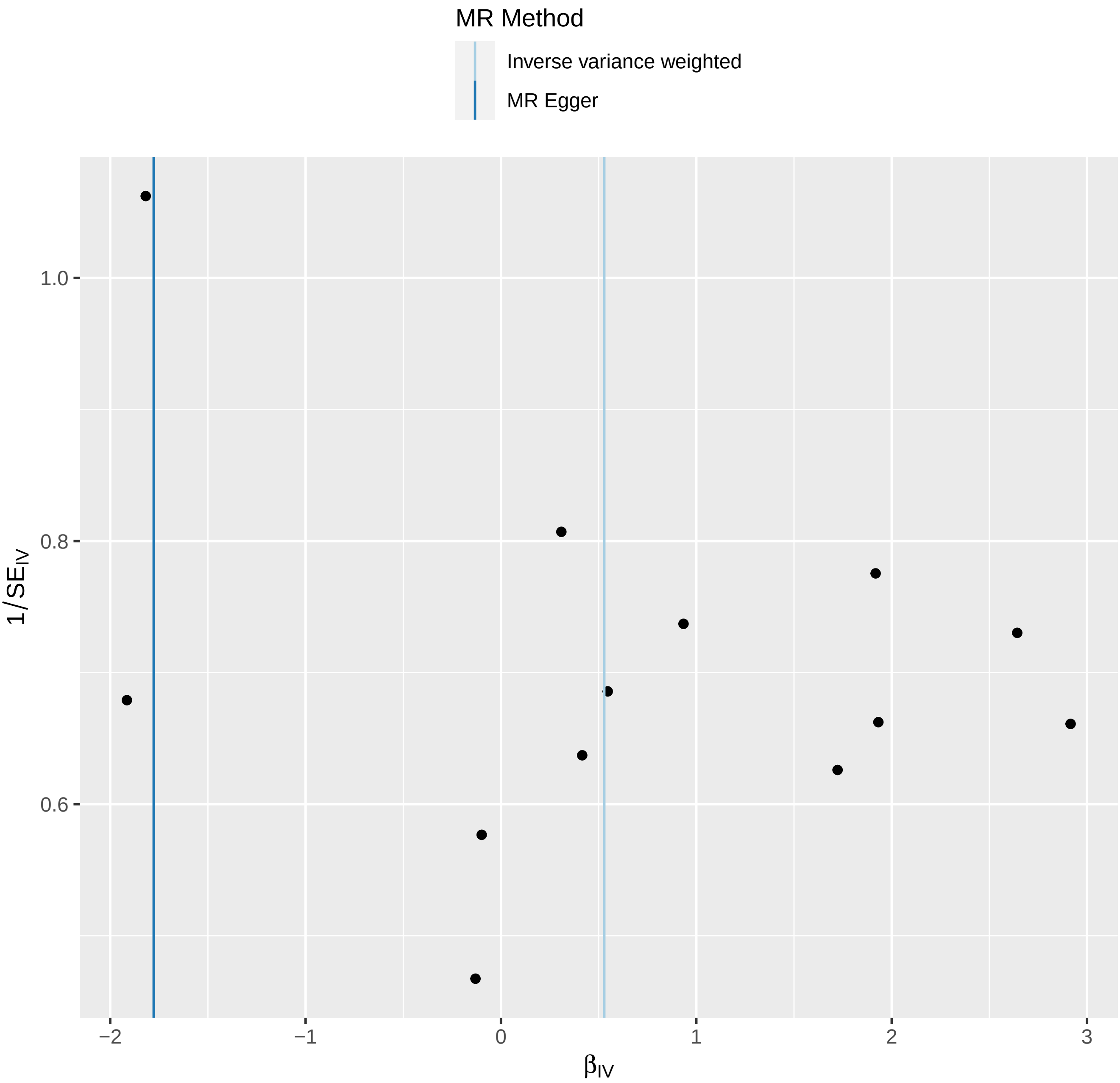

Batch 877 : Gut microbiota abundance (genus Ruminococcaceae UCG002 id.11360) on Arthropathy in ulcerative colitis

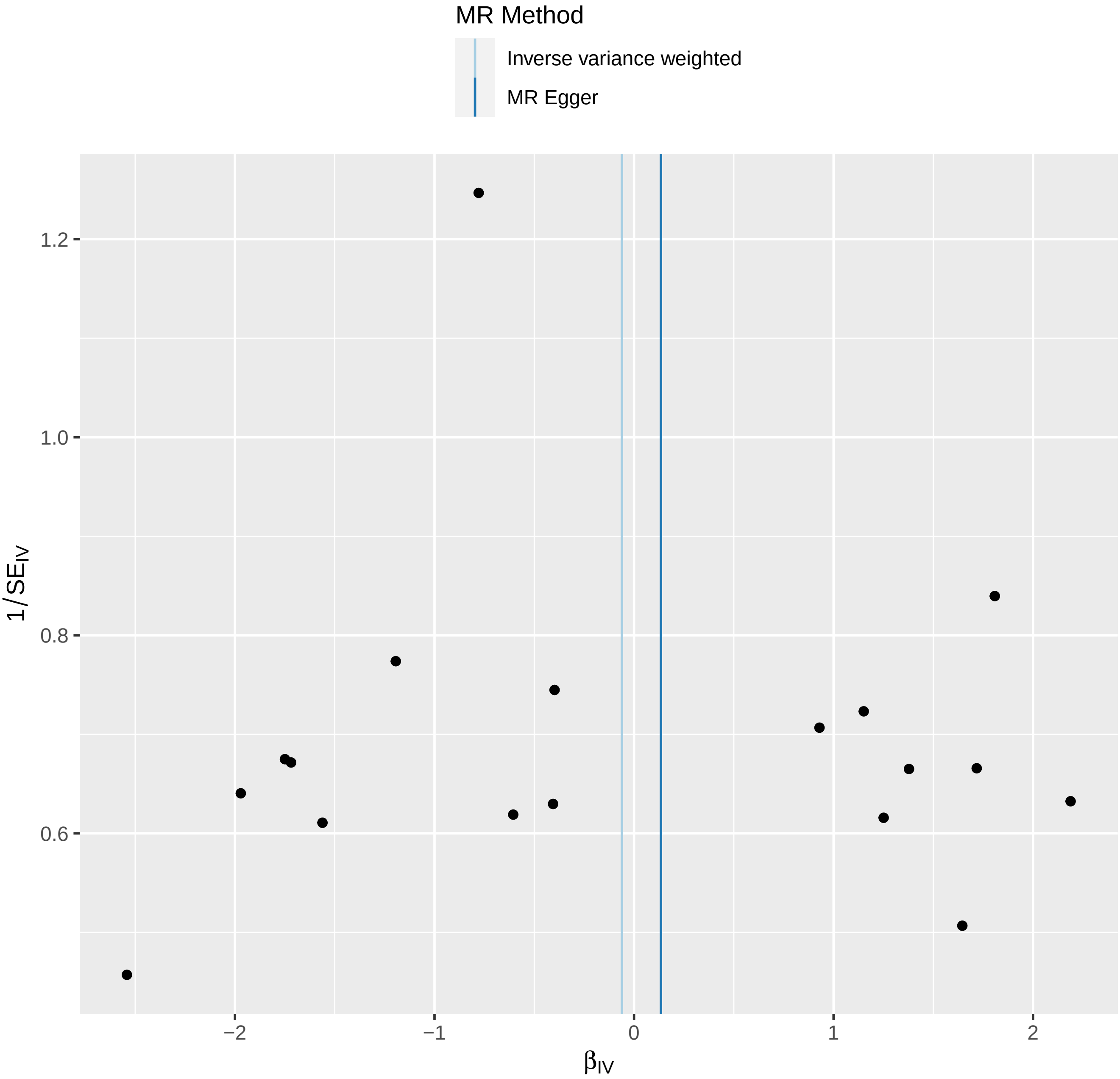

Batch 878 : Gut microbiota abundance (genus Ruminococcaceae UCG003 id.11361) on Arthropathy in ulcerative colitis

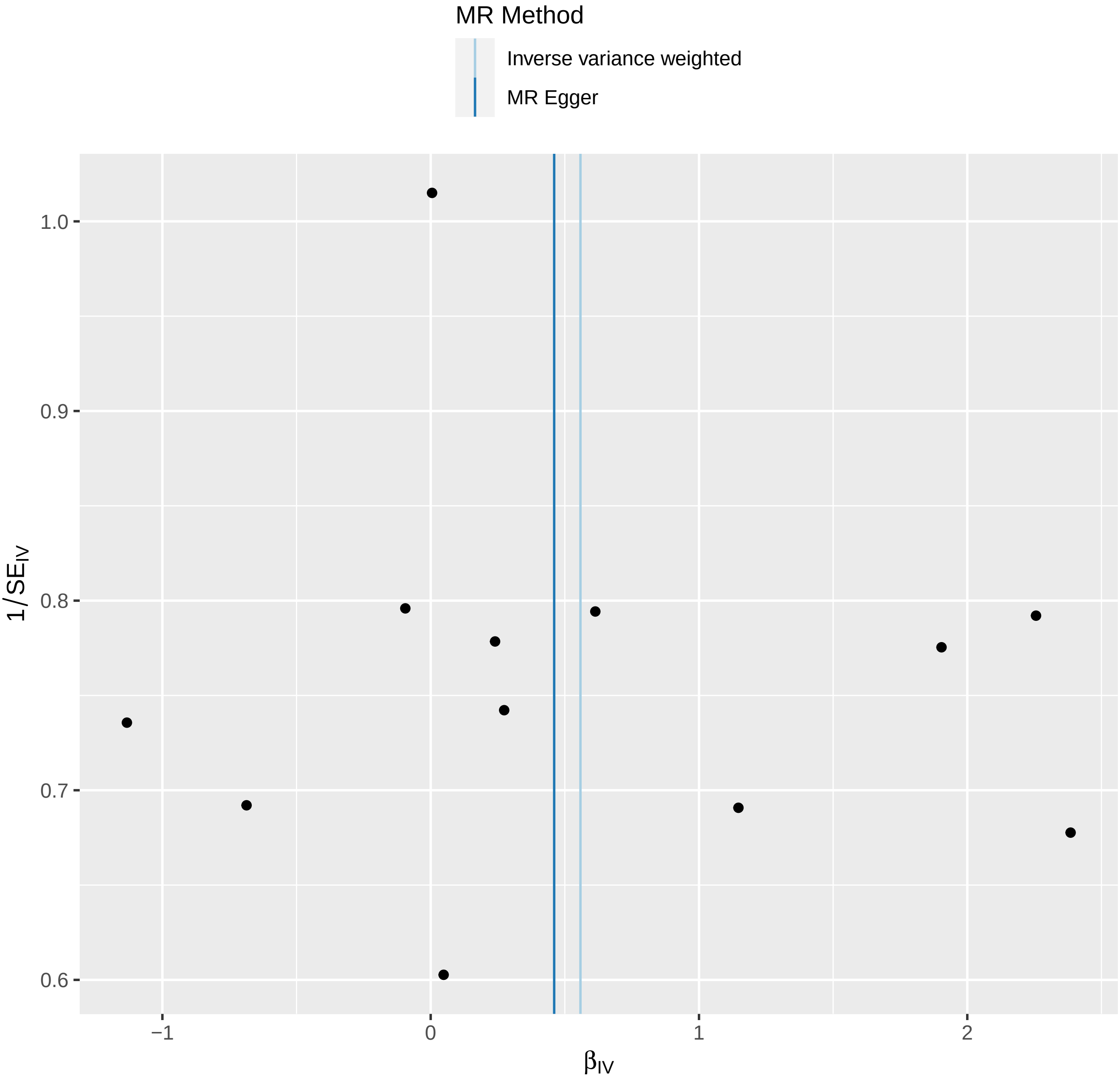

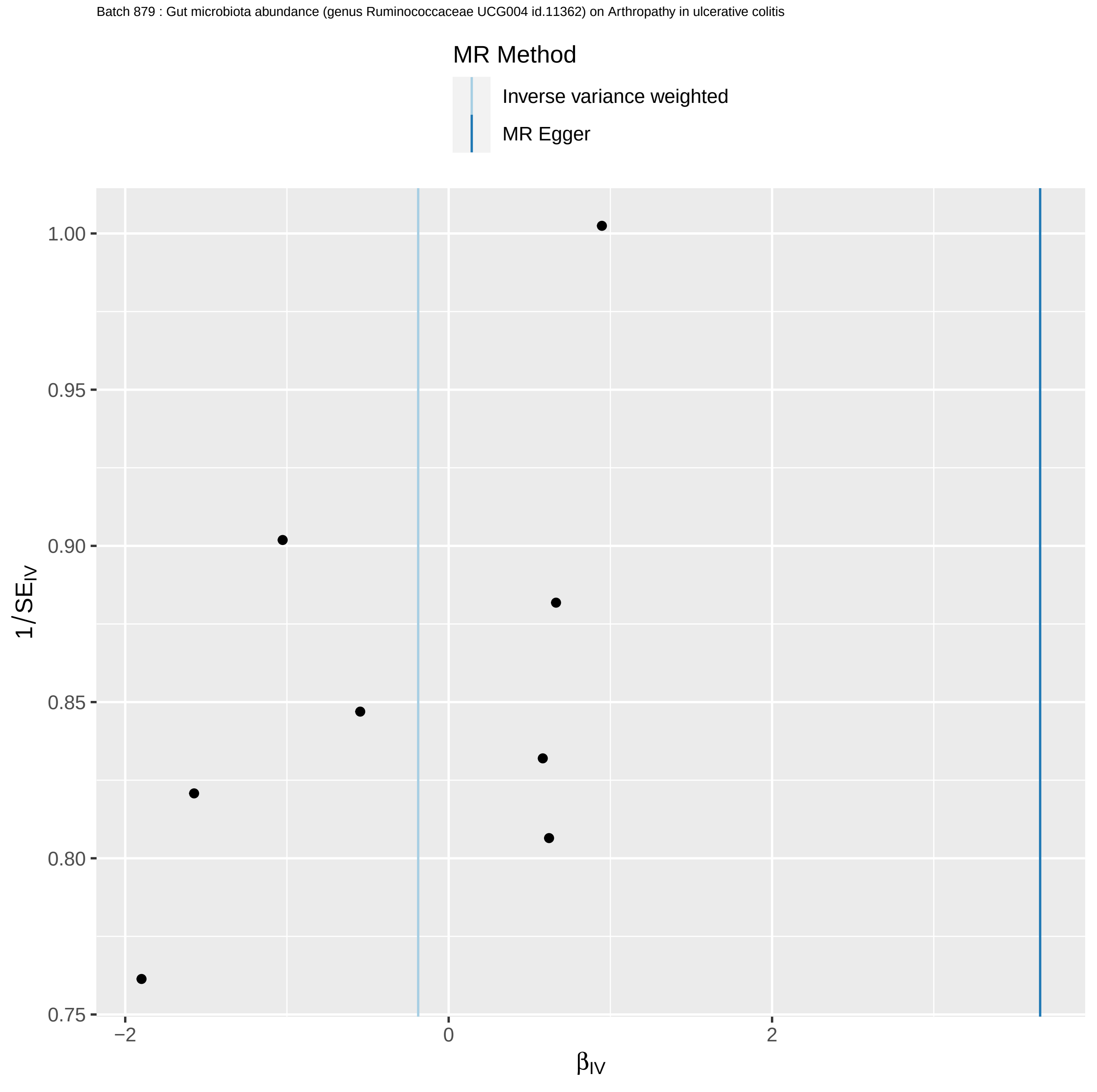
Batch 879 : Gut microbiota abundance (genus Ruminococcaceae UCG004 id.11362) on Arthropathy in ulcerative colitis
MR Method
Inverse variance weighted
MR Egger
1.00
0.95
0.90
0.85
0.80
0.75
1/SE_IV
−2
0
2
β_IV

Batch 880 : Gut microbiota abundance (genus Ruminococcaceae UCG005 id.11363) on Arthropathy in ulcerative colitis

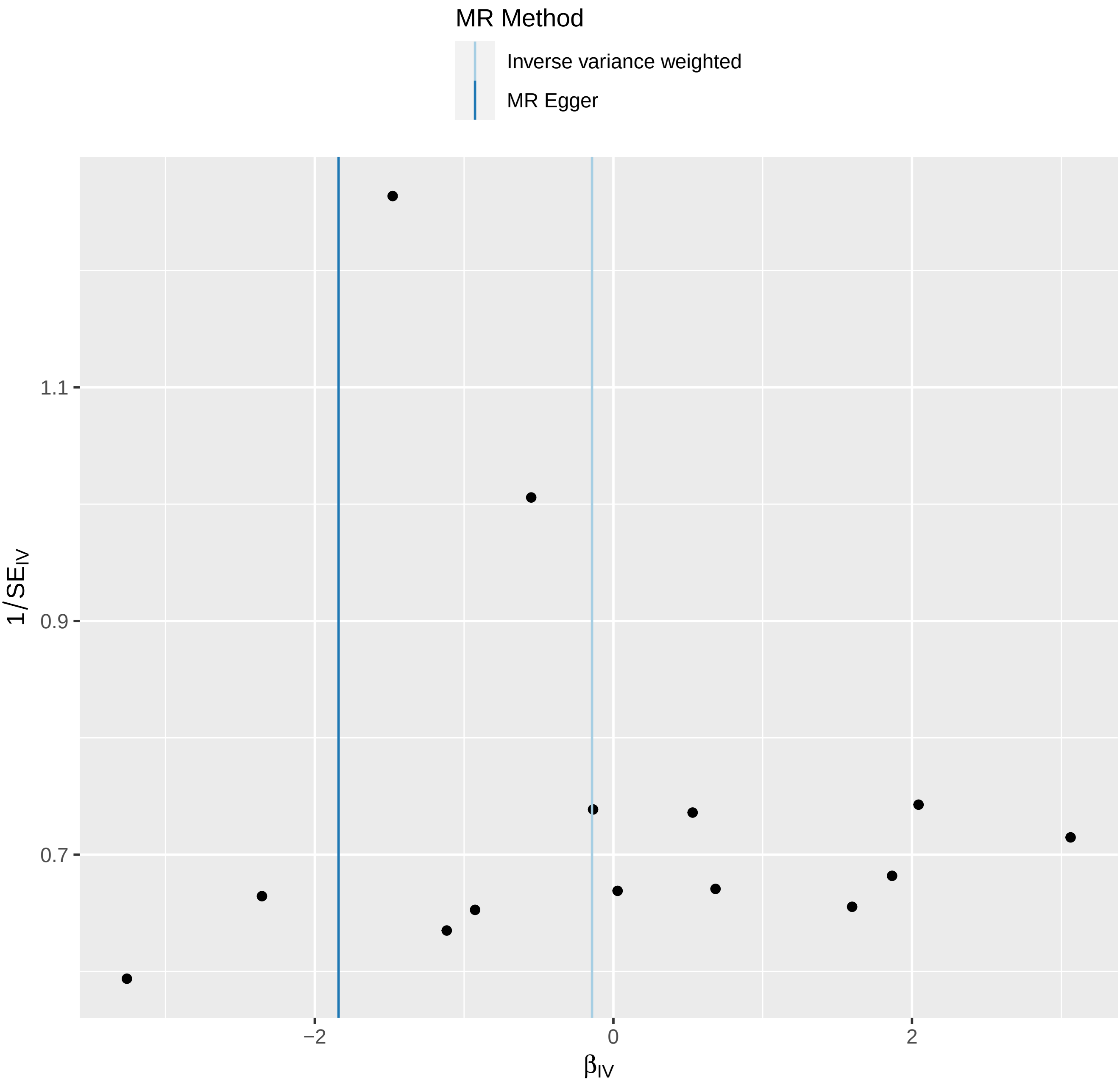

Batch 881 : Gut microbiota abundance (genus Ruminococcaceae UCG009 id.11366) on Arthropathy in ulcerative colitis

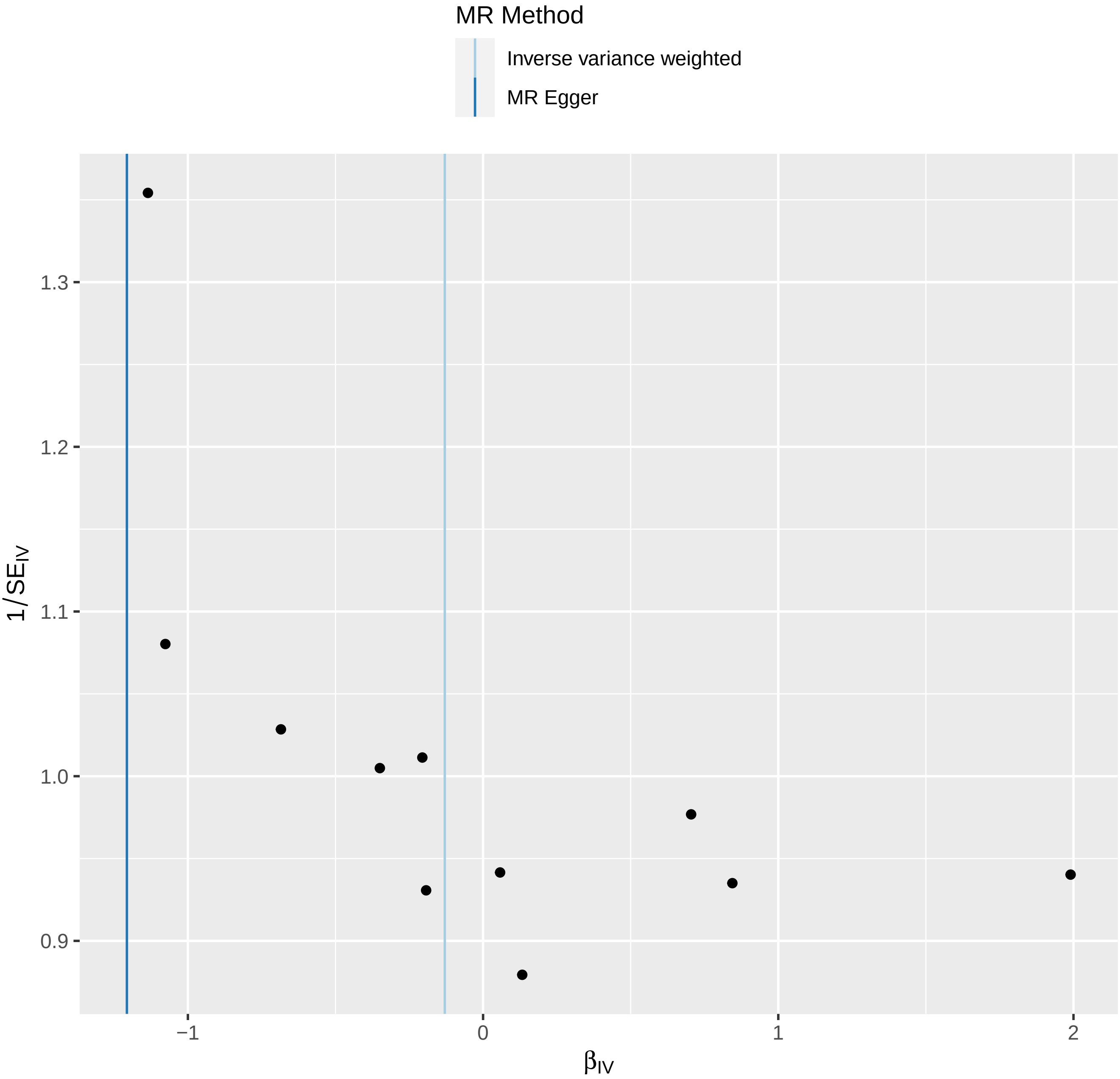

Batch 882 : Gut microbiota abundance (genus Ruminococcaceae UCG010 id.11367) on Arthropathy in ulcerative colitis

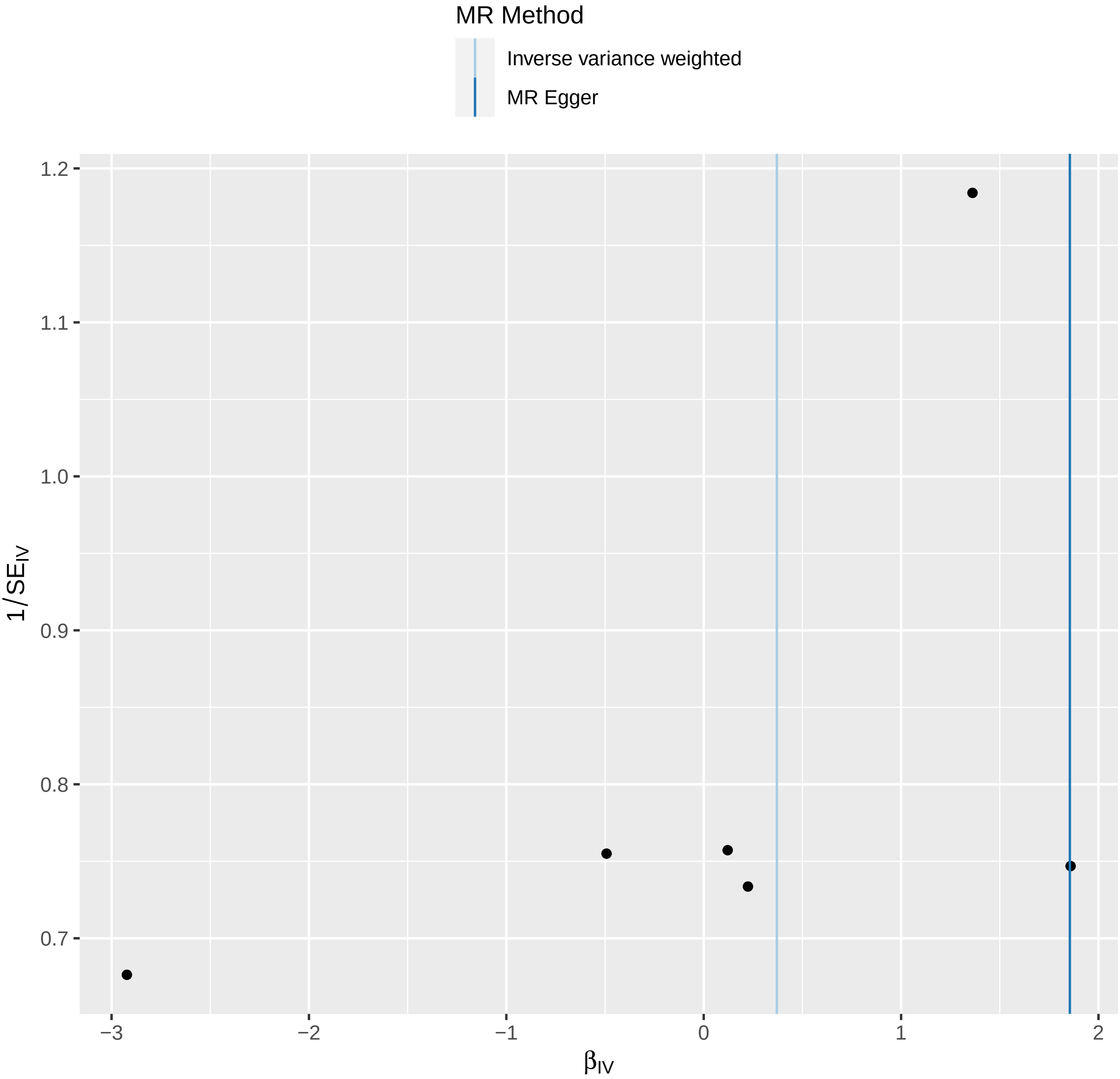

Batch 883 : Gut microbiota abundance (genus Ruminococcaceae UCG011 id.11368) on Arthropathy in ulcerative colitis

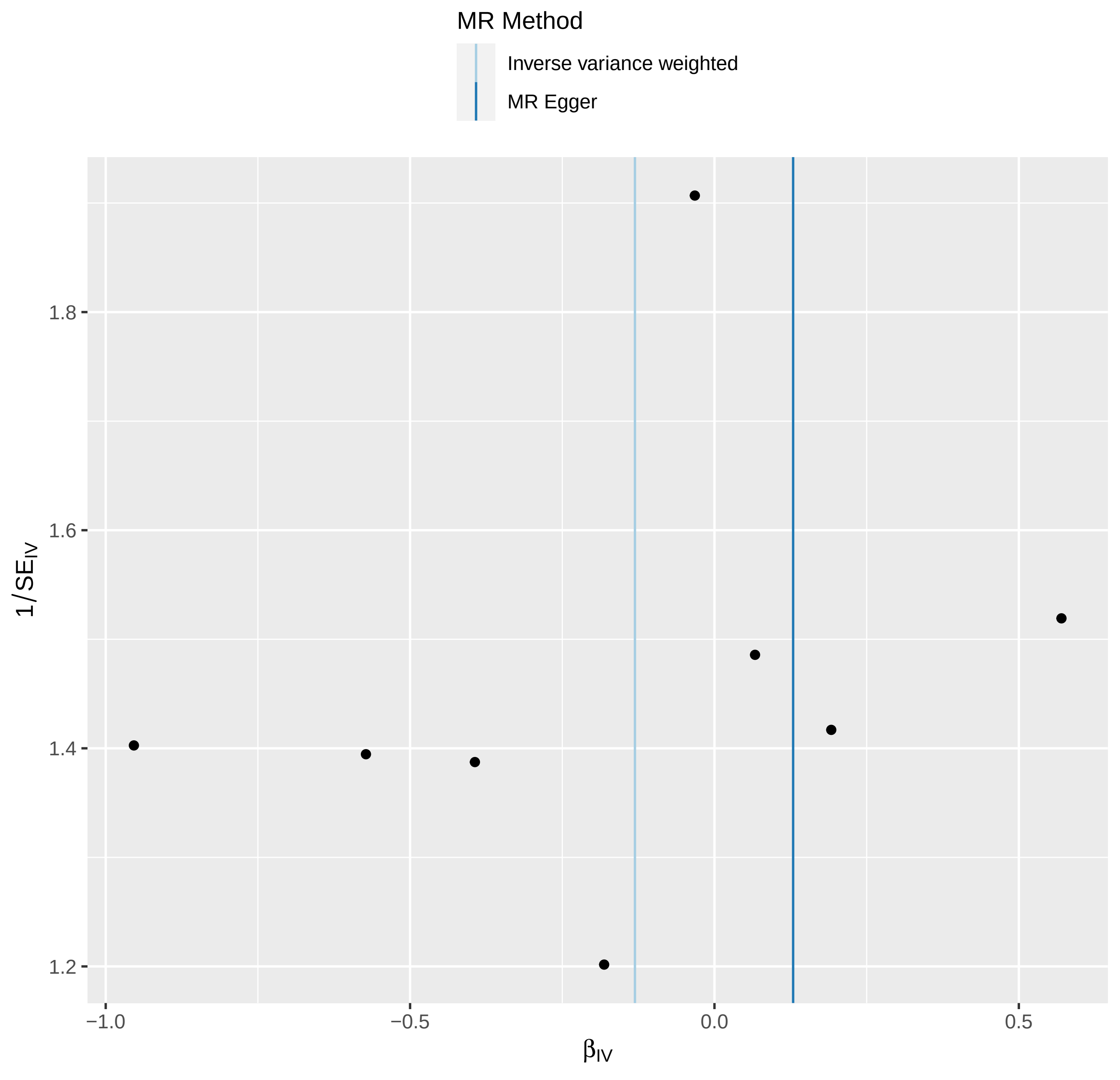

Batch 884 : Gut microbiota abundance (genus Ruminococcaceae UCG013 id.11370) on Arthropathy in ulcerative colitis

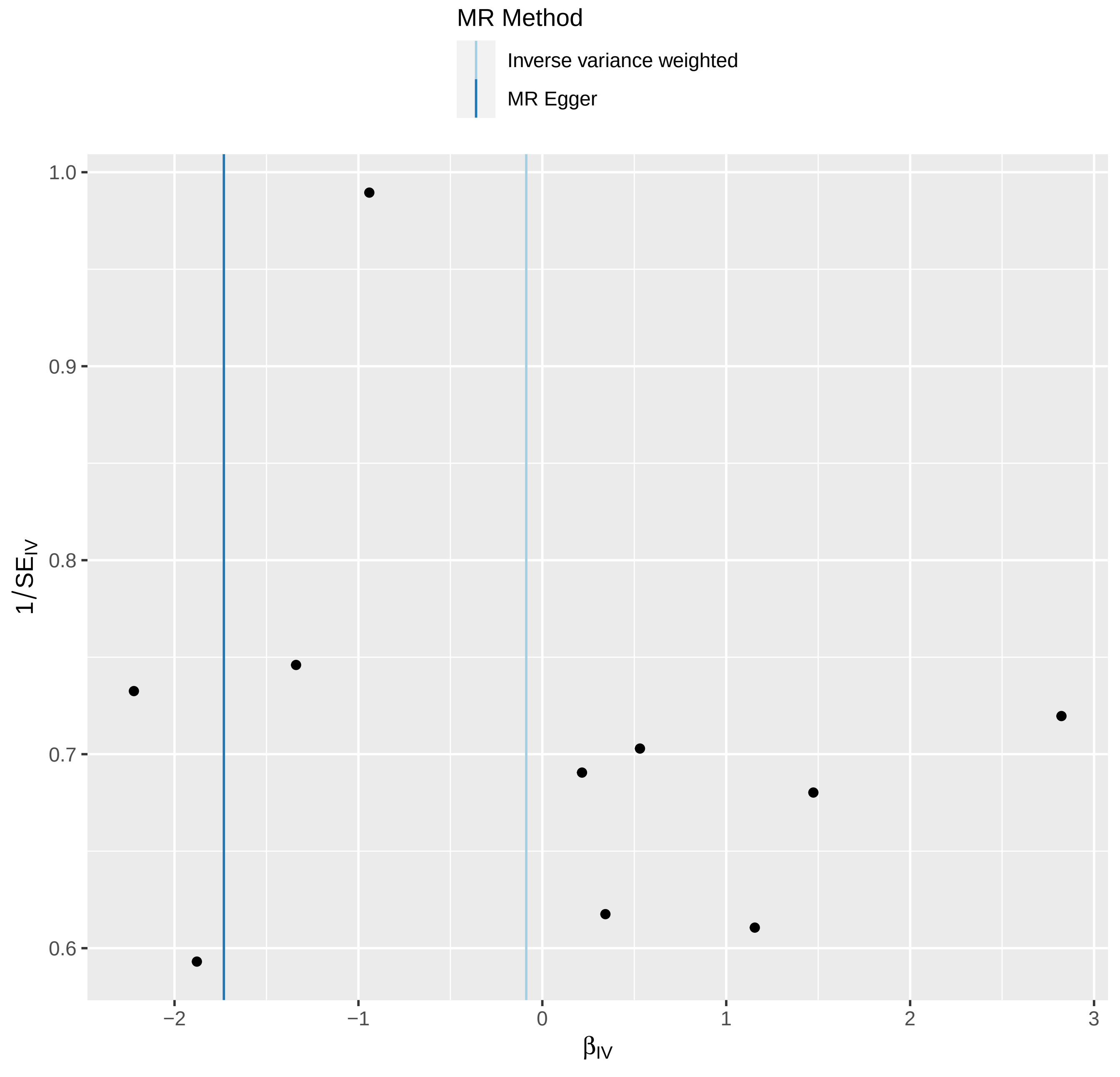

Batch 885 : Gut microbiota abundance (genus Ruminococcaceae UCG014 id.11371) on Arthropathy in ulcerative colitis

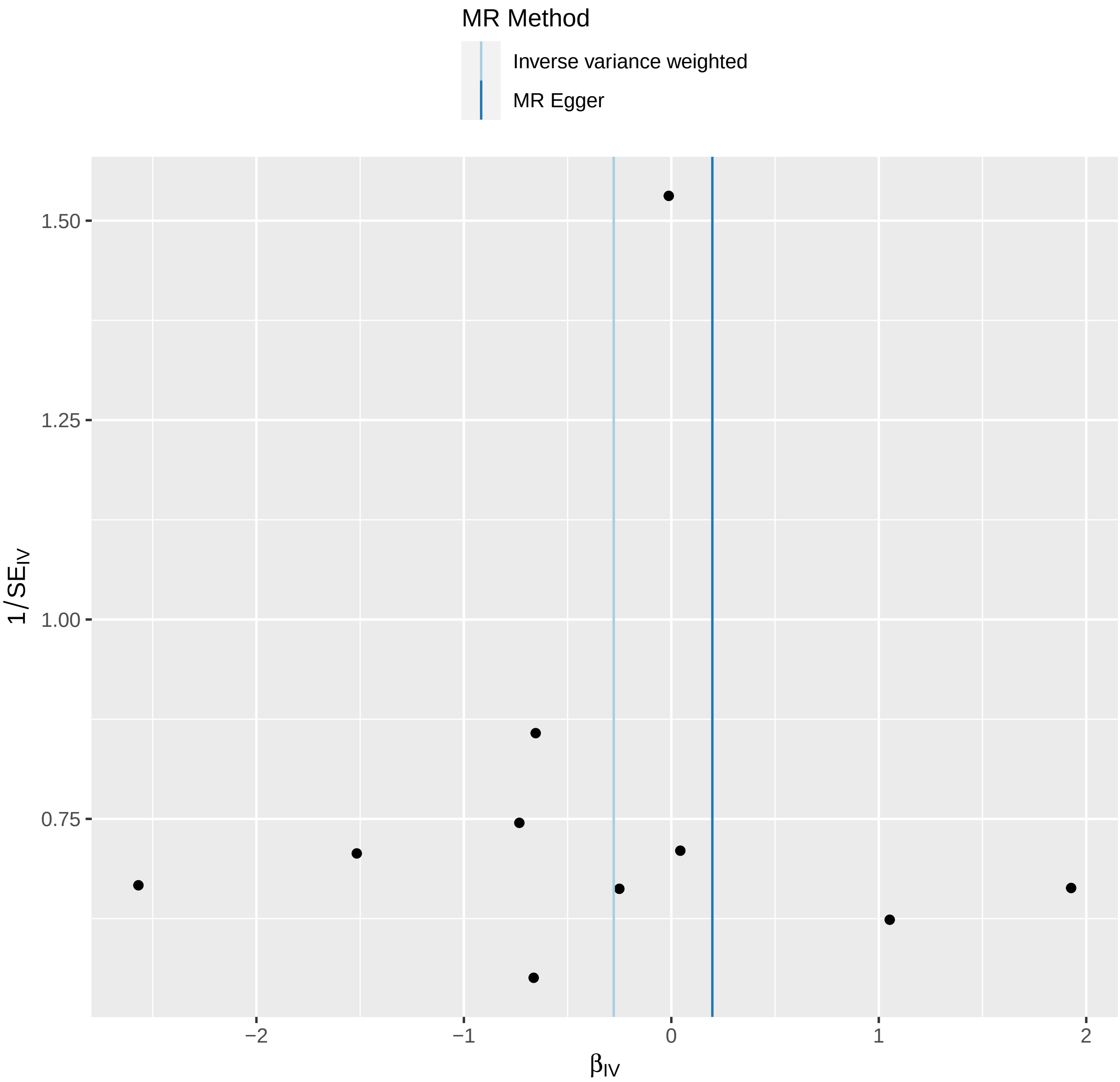

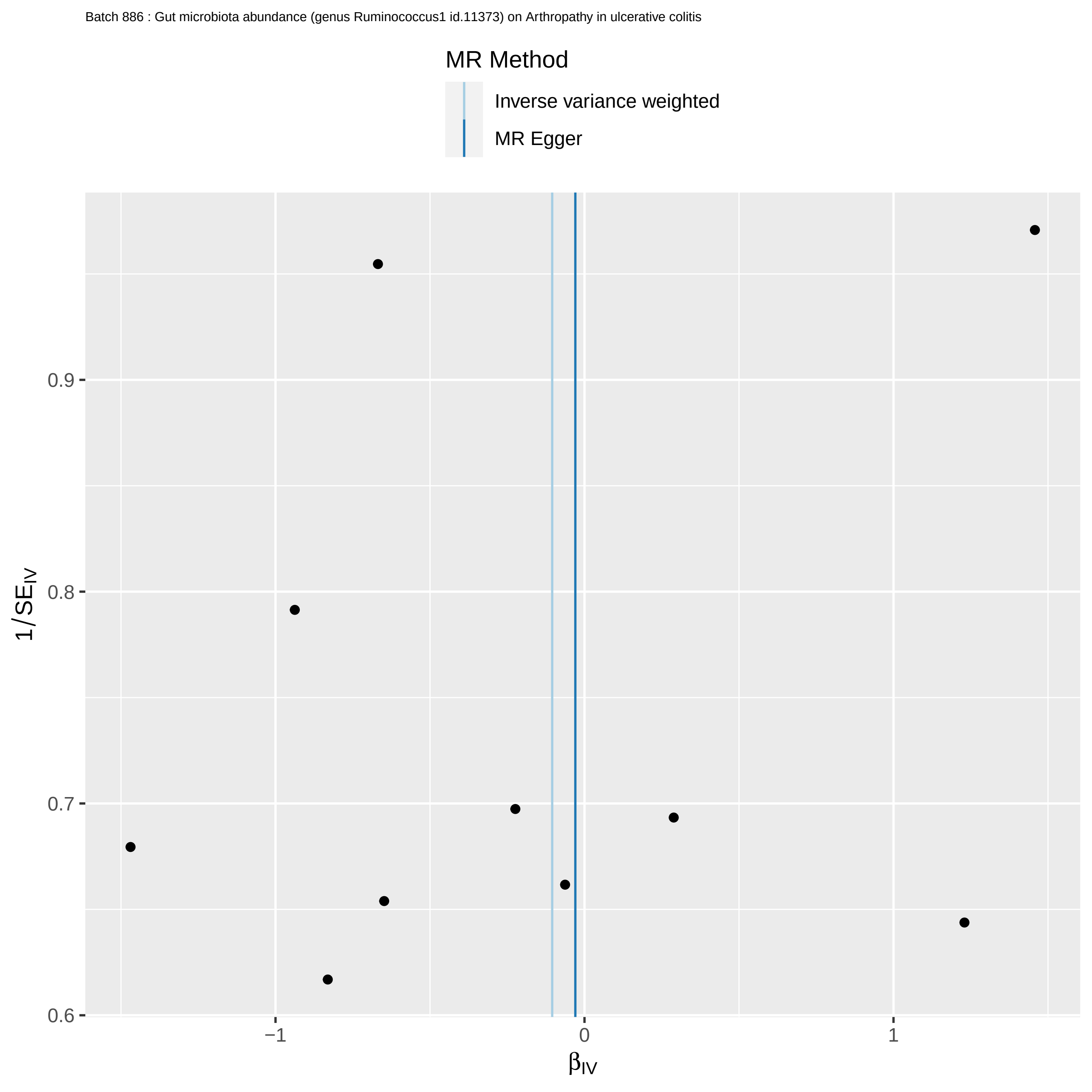

Batch 886 : Gut microbiota abundance (genus Ruminococcus1 id.11373) on Arthropathy in ulcerative colitis
MR Method
Inverse variance weighted
MR Egger
0.9
0.8
0.7
0.6
1/SE_IV
−1
0
1
β_IV

Batch 887 : Gut microbiota abundance (genus Ruminococcus2 id.11374) on Arthropathy in ulcerative colitis

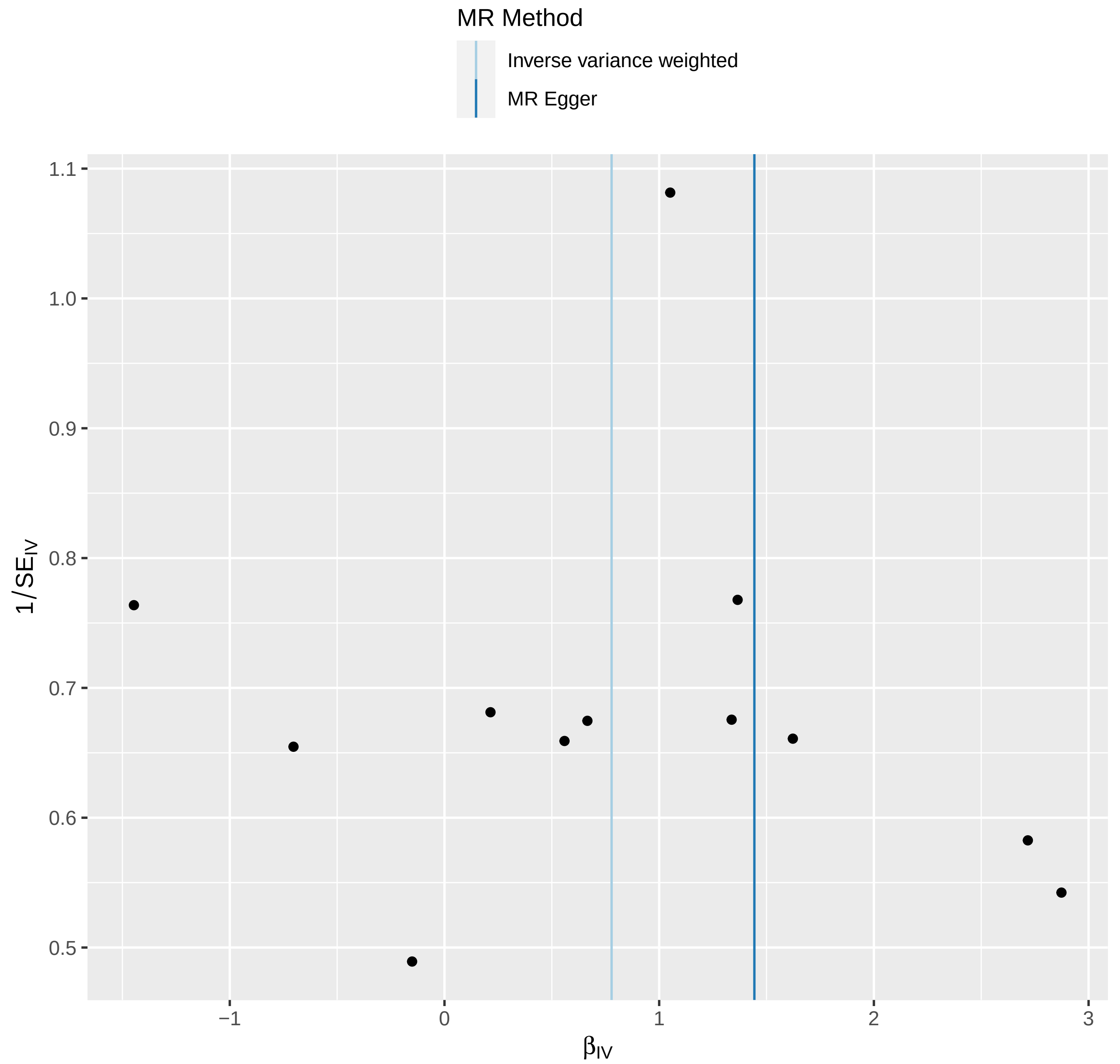

Batch 888 : Gut microbiota abundance (genus Ruminococcus gauvreauii group id.11342) on Arthropathy in ulcerative colitis

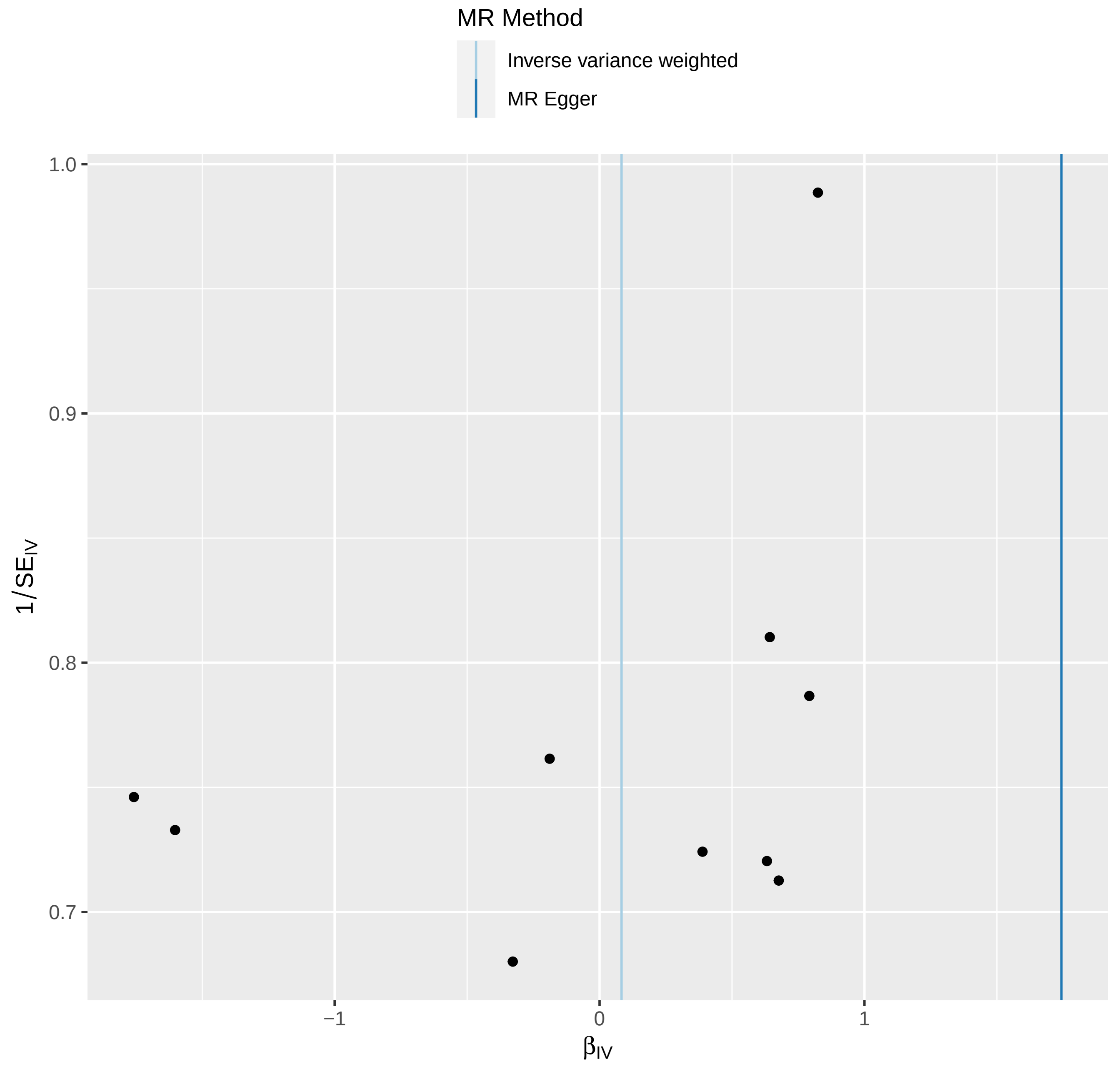

Batch 889 : Gut microbiota abundance (genus Ruminococcus gnavus group id.14376) on Arthropathy in ulcerative colitis

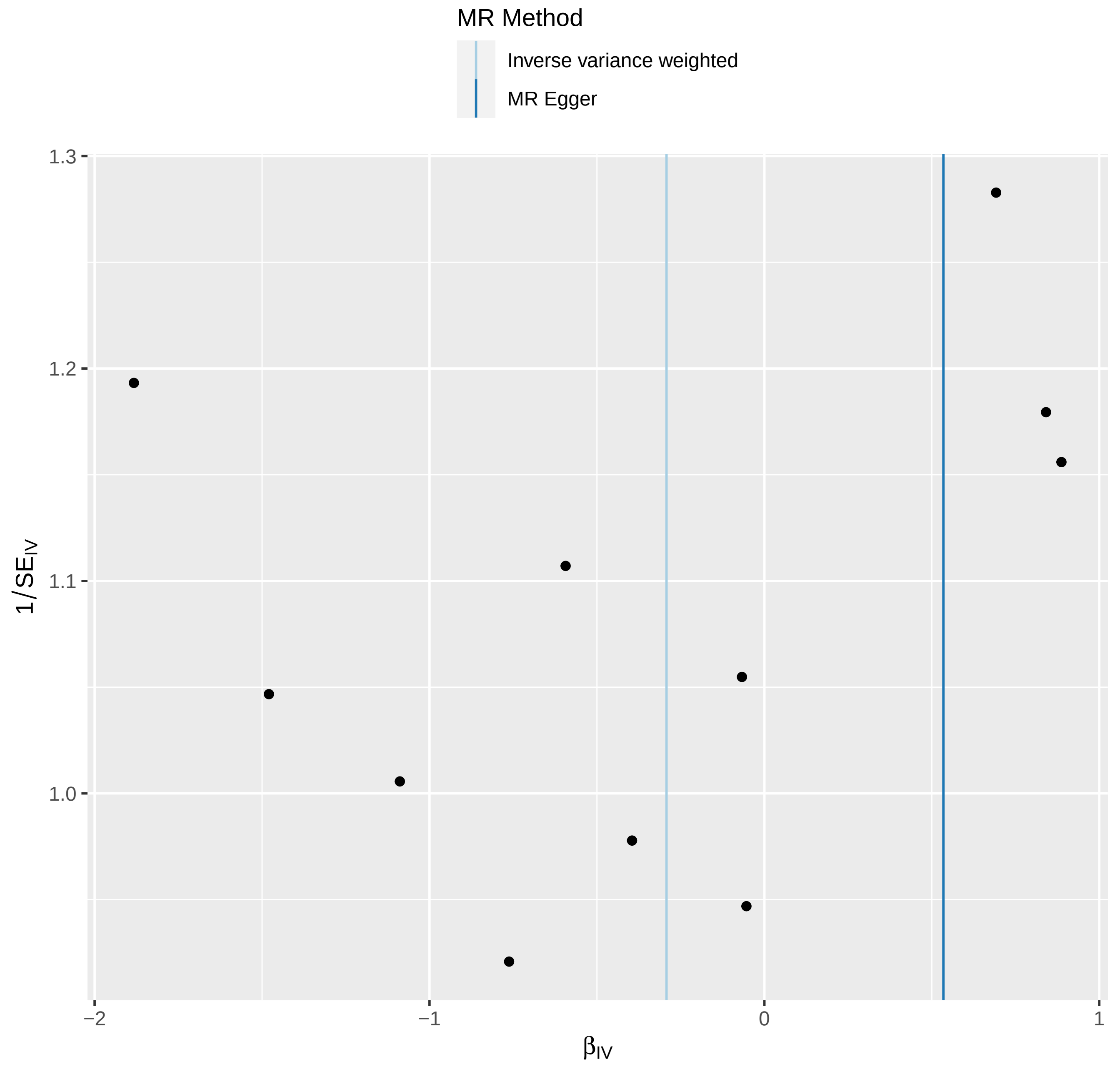

Batch 890 : Gut microbiota abundance (genus Ruminococcus torques group id.14377) on Arthropathy in ulcerative colitis

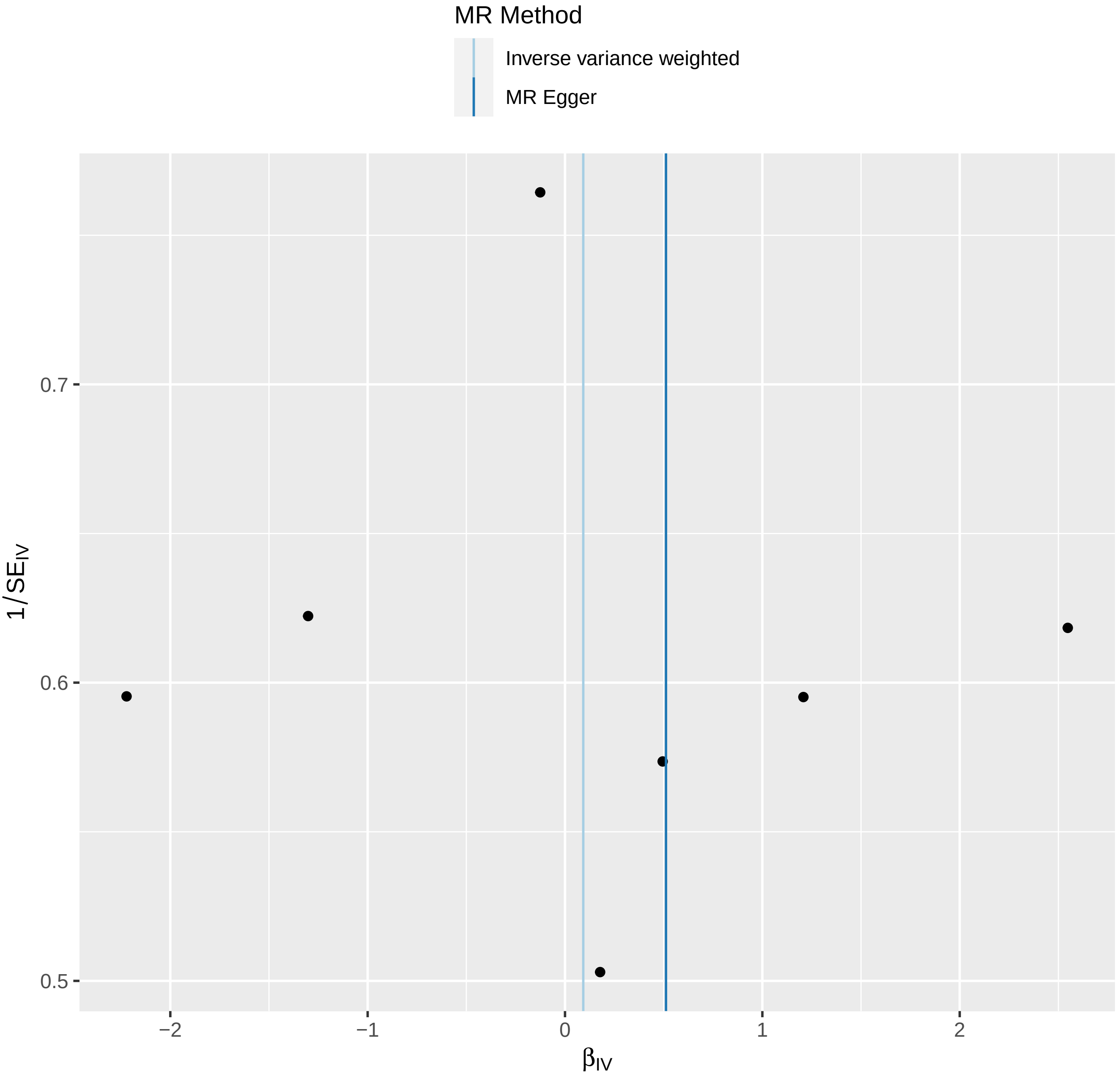

Batch 891 : Gut microbiota abundance (genus Sellimonas id.14369) on Arthropathy in ulcerative colitis

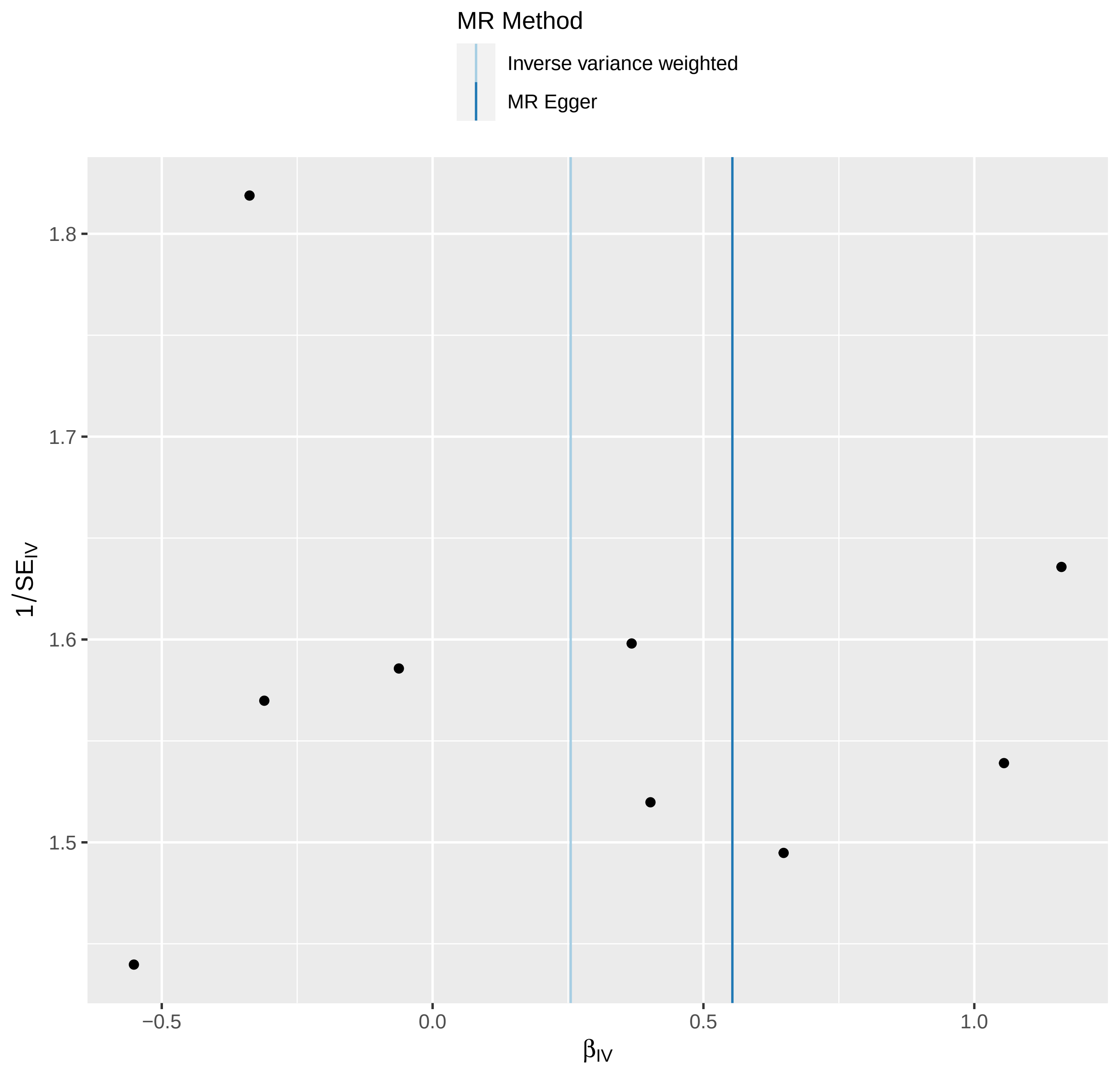

Batch 892 : Gut microbiota abundance (genus Senegalimassilia id.11160) on Arthropathy in ulcerative colitis

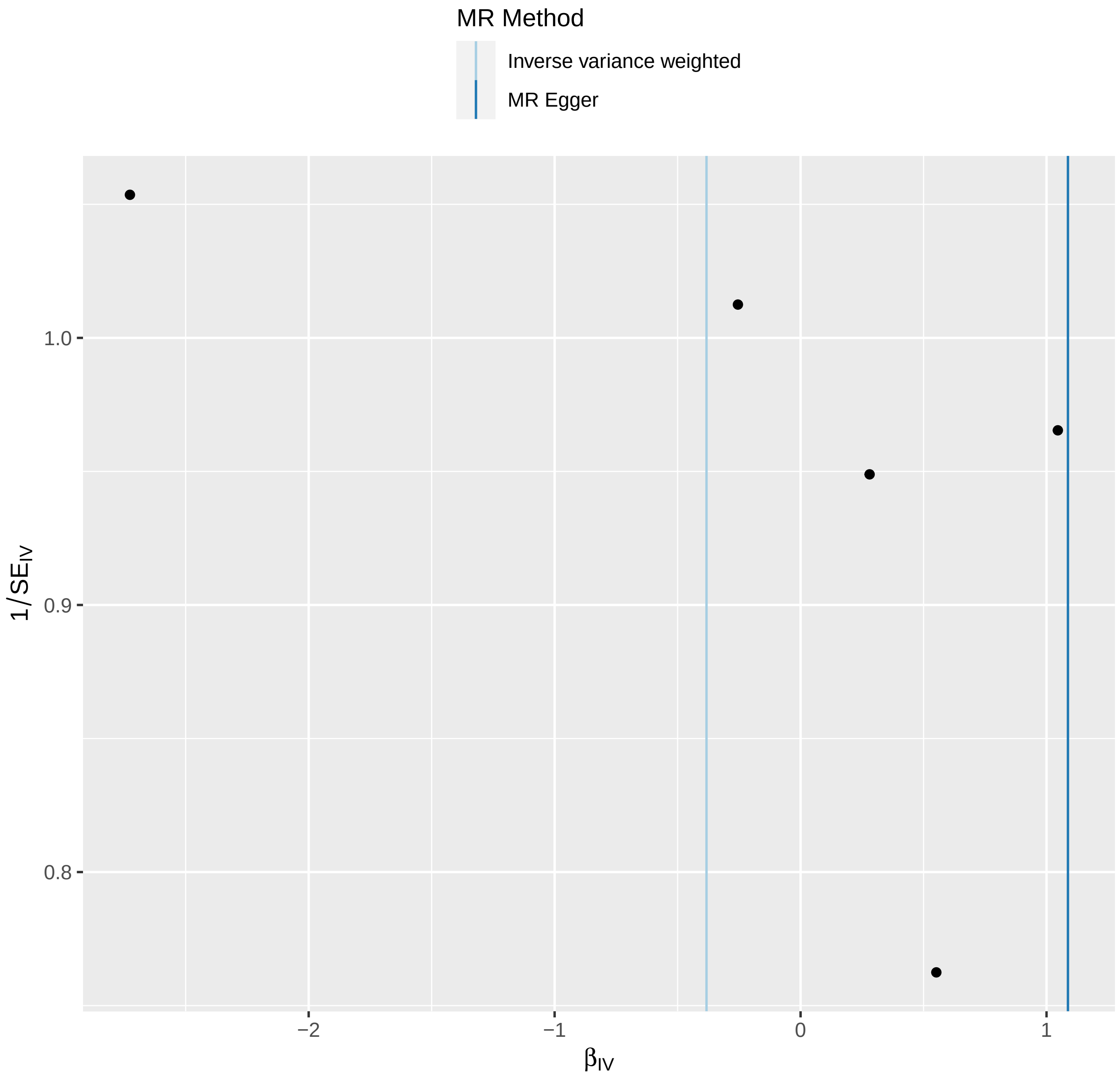

Batch 893 : Gut microbiota abundance (genus Slackia id.825) on Arthropathy in ulcerative colitis

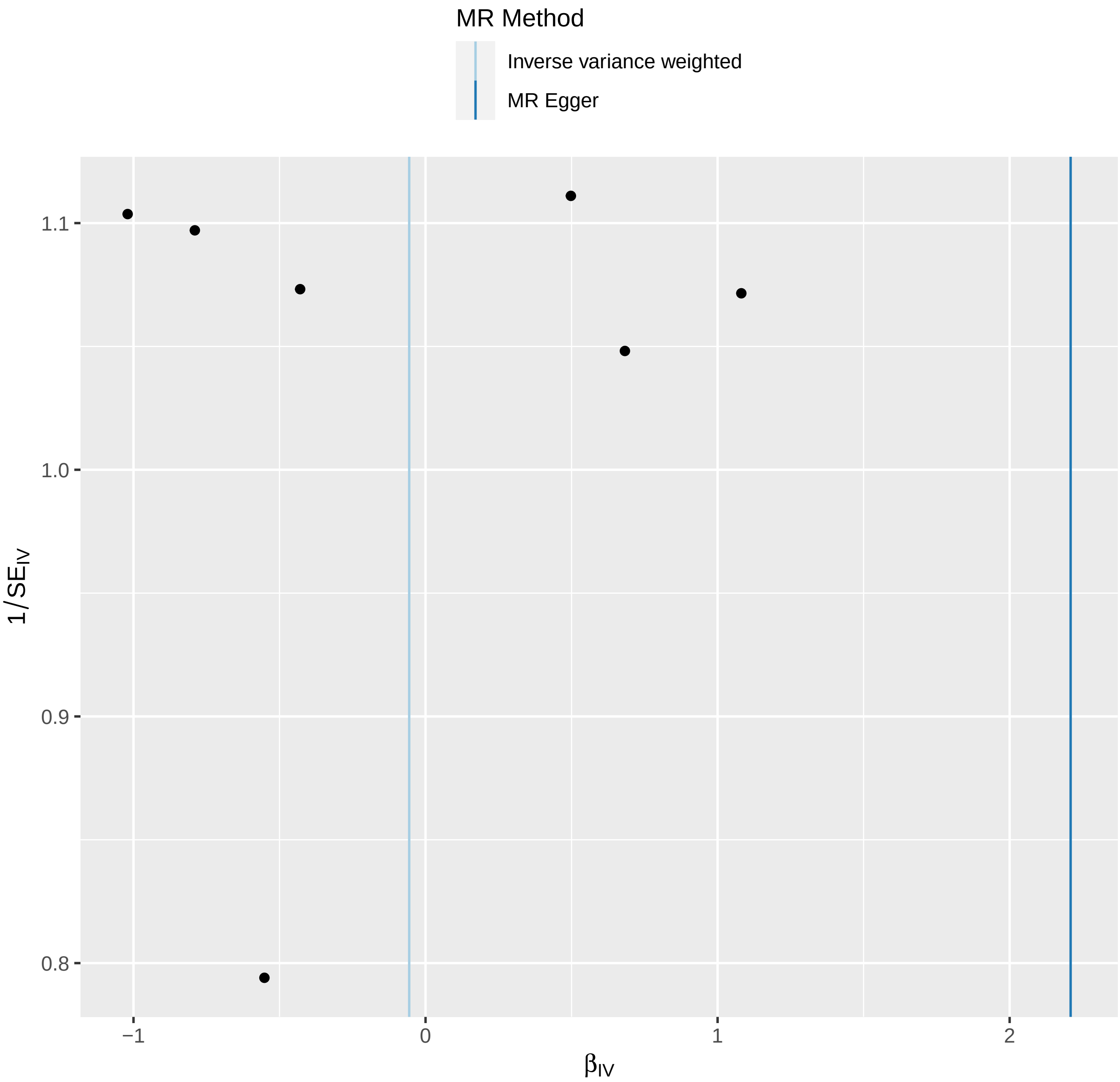

Batch 894 : Gut microbiota abundance (genus Streptococcus id.1853) on Arthropathy in ulcerative colitis

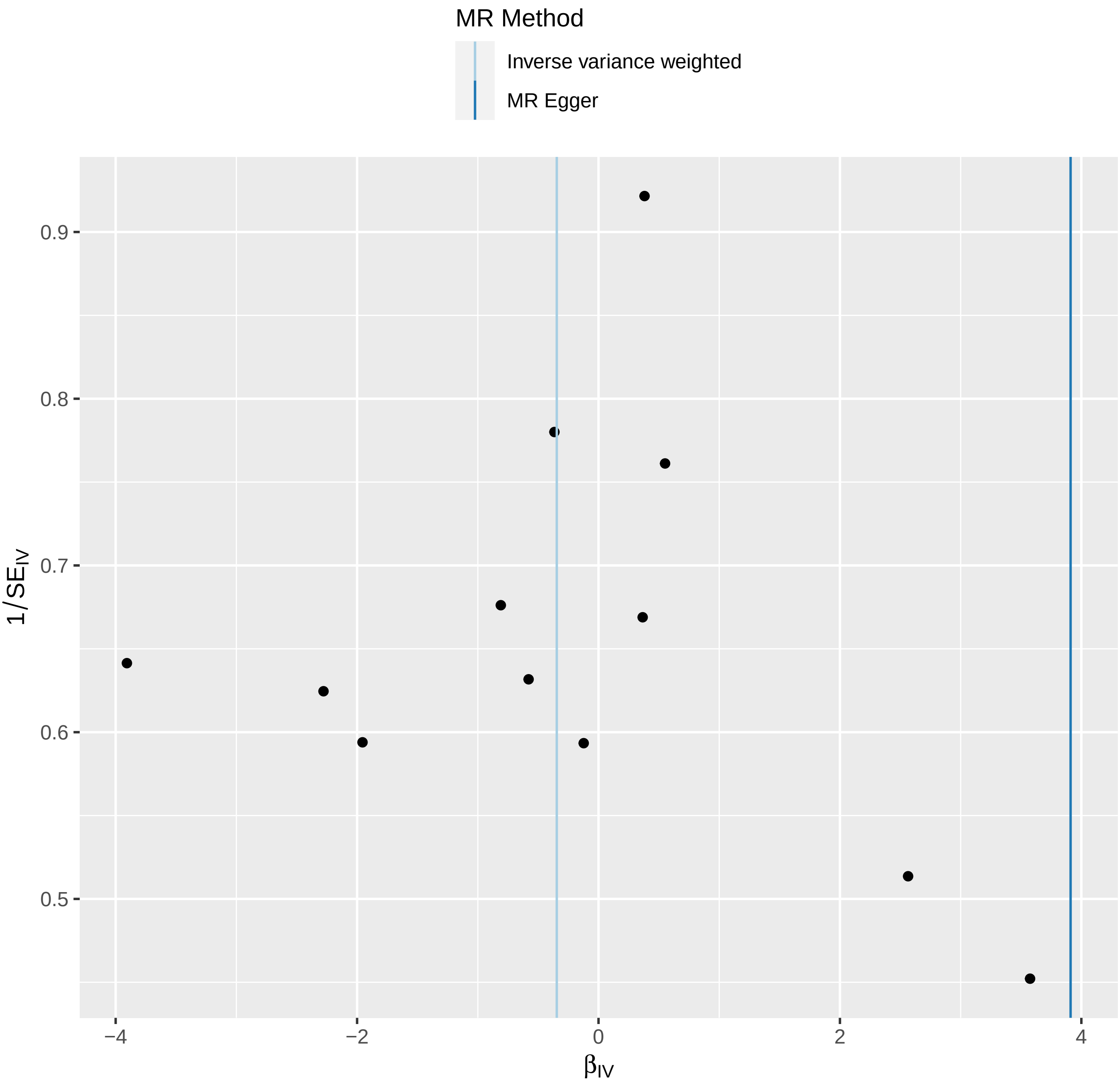

Batch 895 : Gut microbiota abundance (genus Subdoligranulum id.2070) on Arthropathy in ulcerative colitis

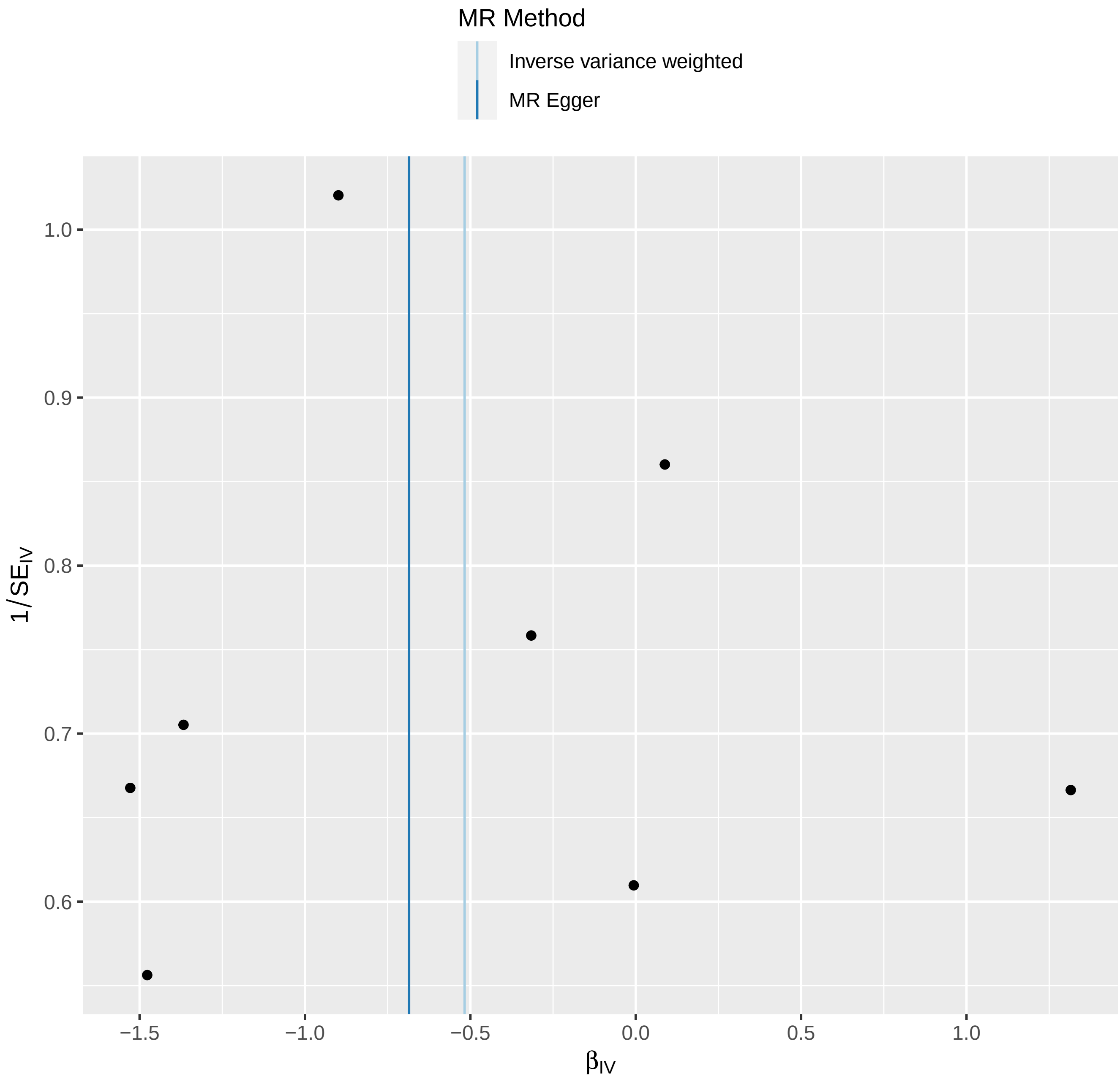

Batch 896 : Gut microbiota abundance (genus Sutterella id.2896) on Arthropathy in ulcerative colitis

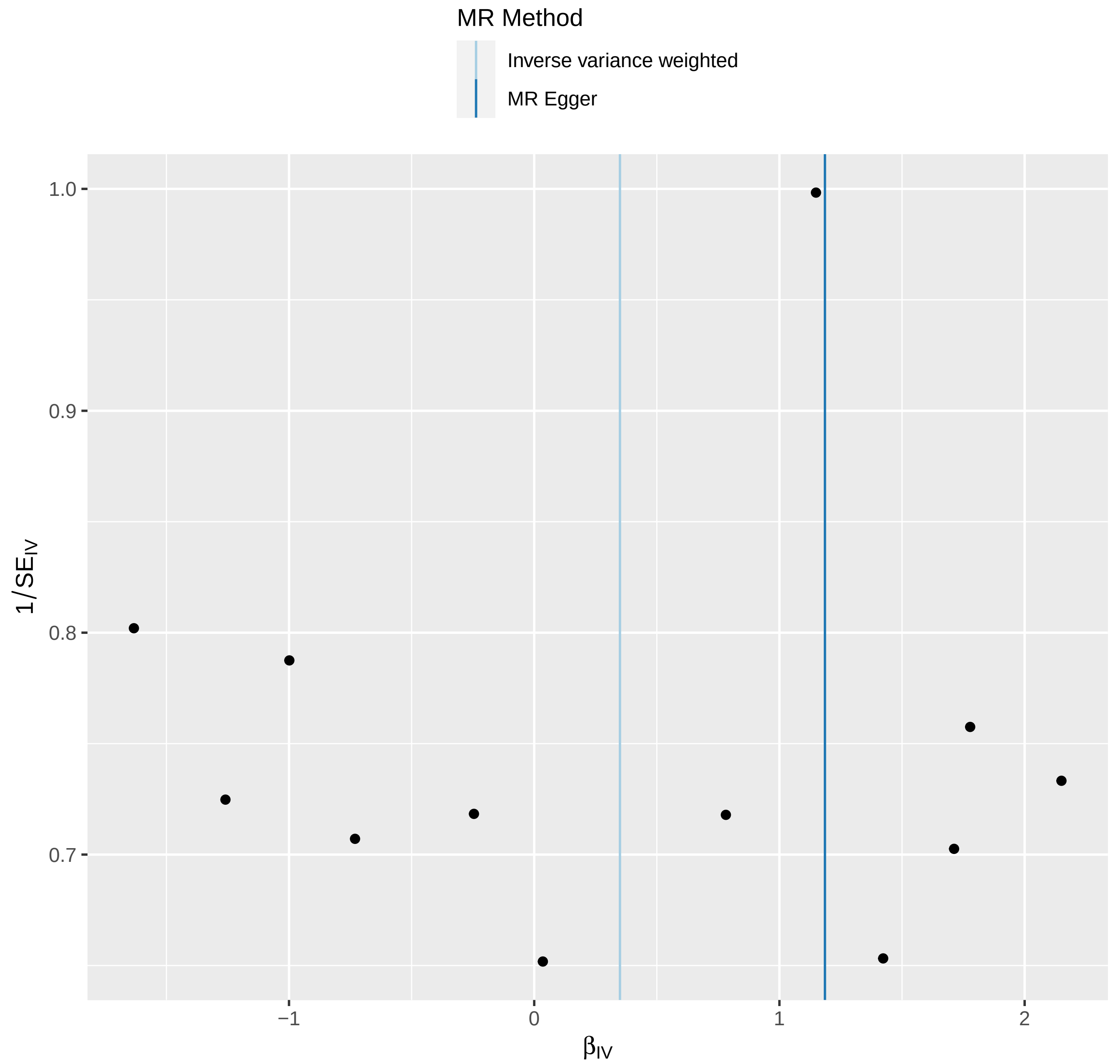

Batch 897 : Gut microbiota abundance (genus Terrisporobacter id.11348) on Arthropathy in ulcerative colitis

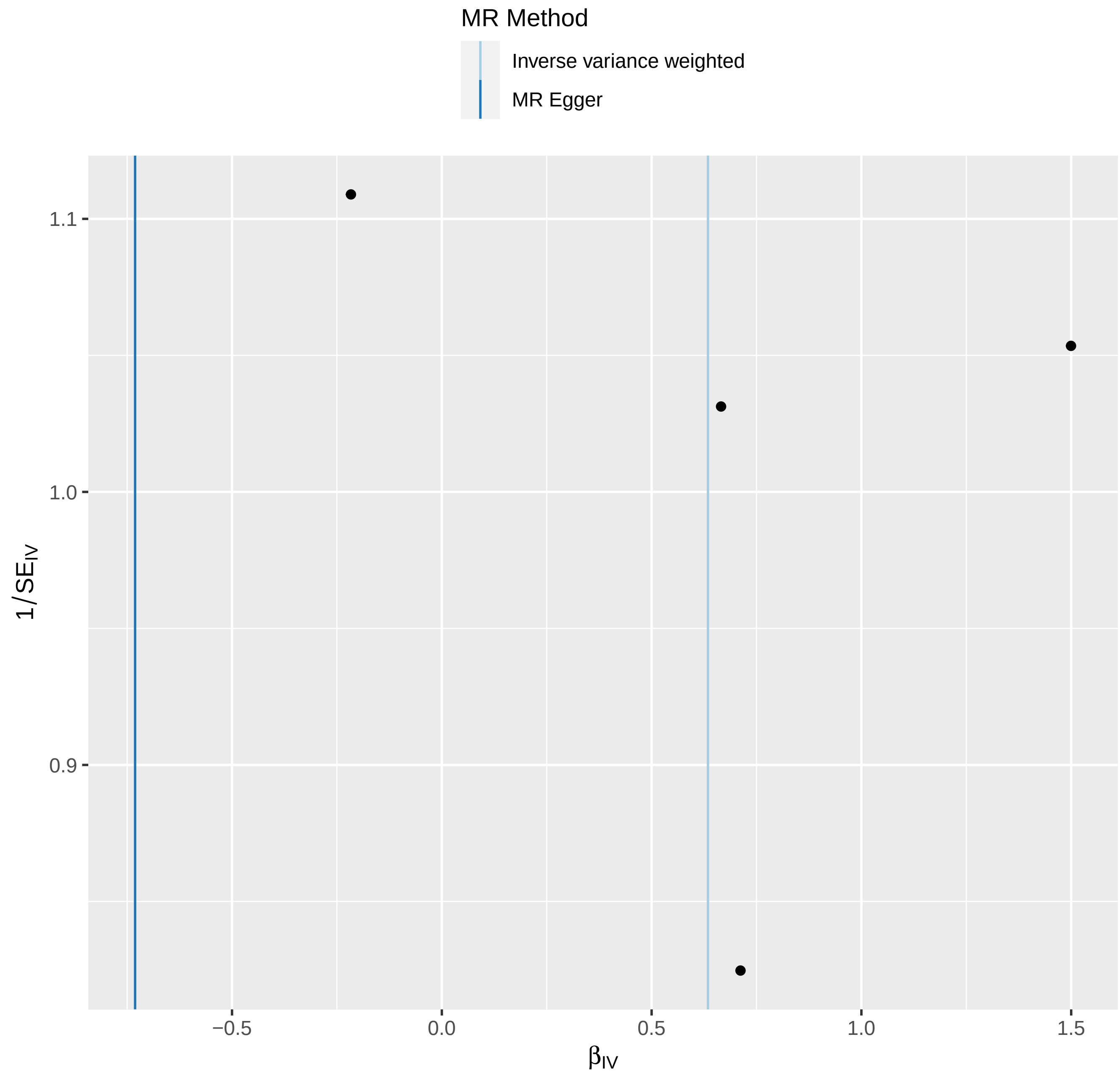

Batch 898 : Gut microbiota abundance (genus Turicibacter id.2162) on Arthropathy in ulcerative colitis

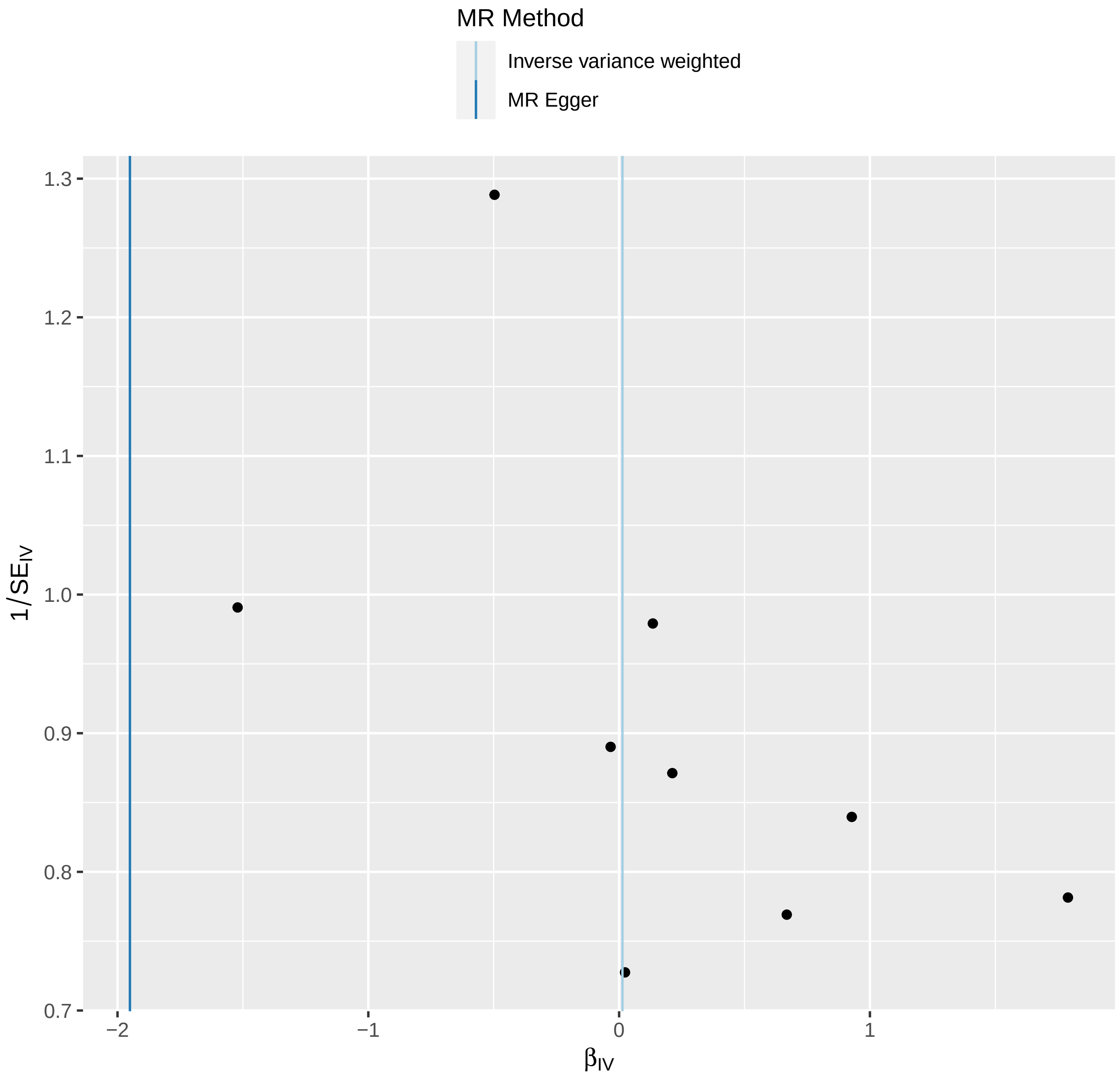

Batch 899 : Gut microbiota abundance (genus Tyzzerella3 id.11335) on Arthropathy in ulcerative colitis

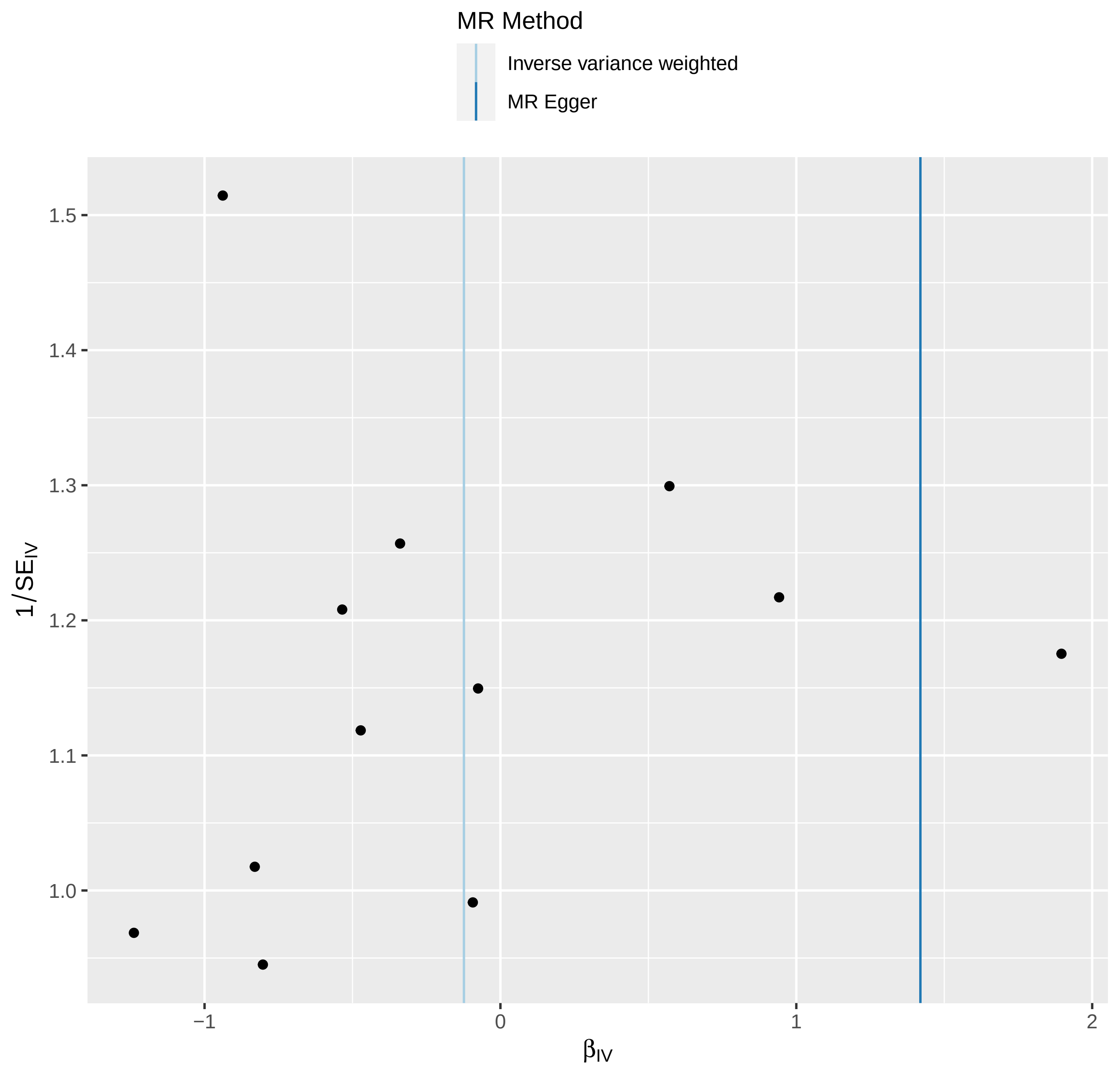

Batch 900 : Gut microbiota abundance (genus Veillonella id.2198) on Arthropathy in ulcerative colitis

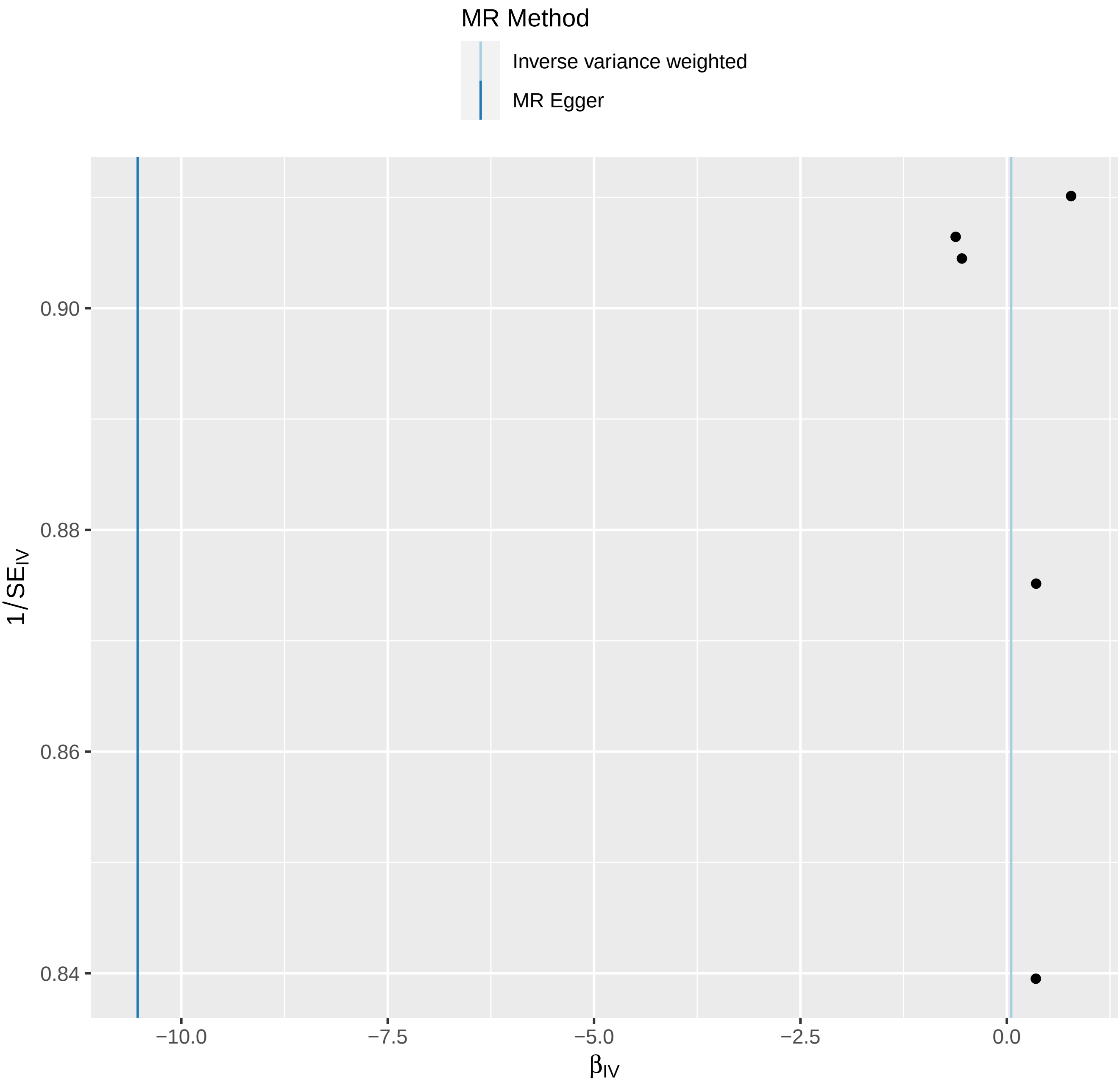

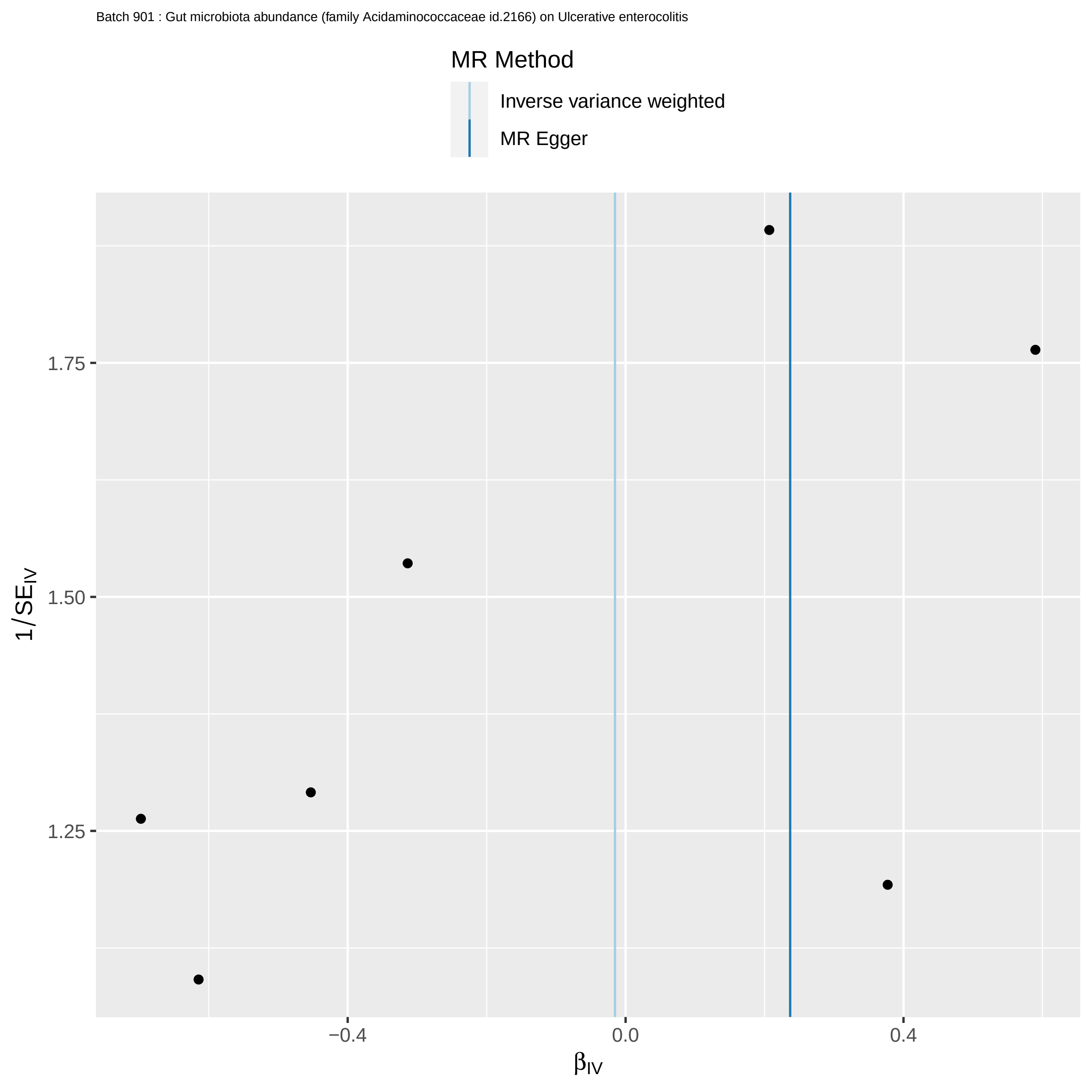
Batch 901 : Gut microbiota abundance (family Acidaminococcaceae id.2166) on Ulcerative enterocolitis
MR Method
Inverse variance weighted
MR Egger
1.75
1.50
1.25
1/SE_IV
−0.4
0.0
0.4
β_IV

Batch 902 : Gut microbiota abundance (family Actinomycetaceae id.421) on Ulcerative enterocolitis

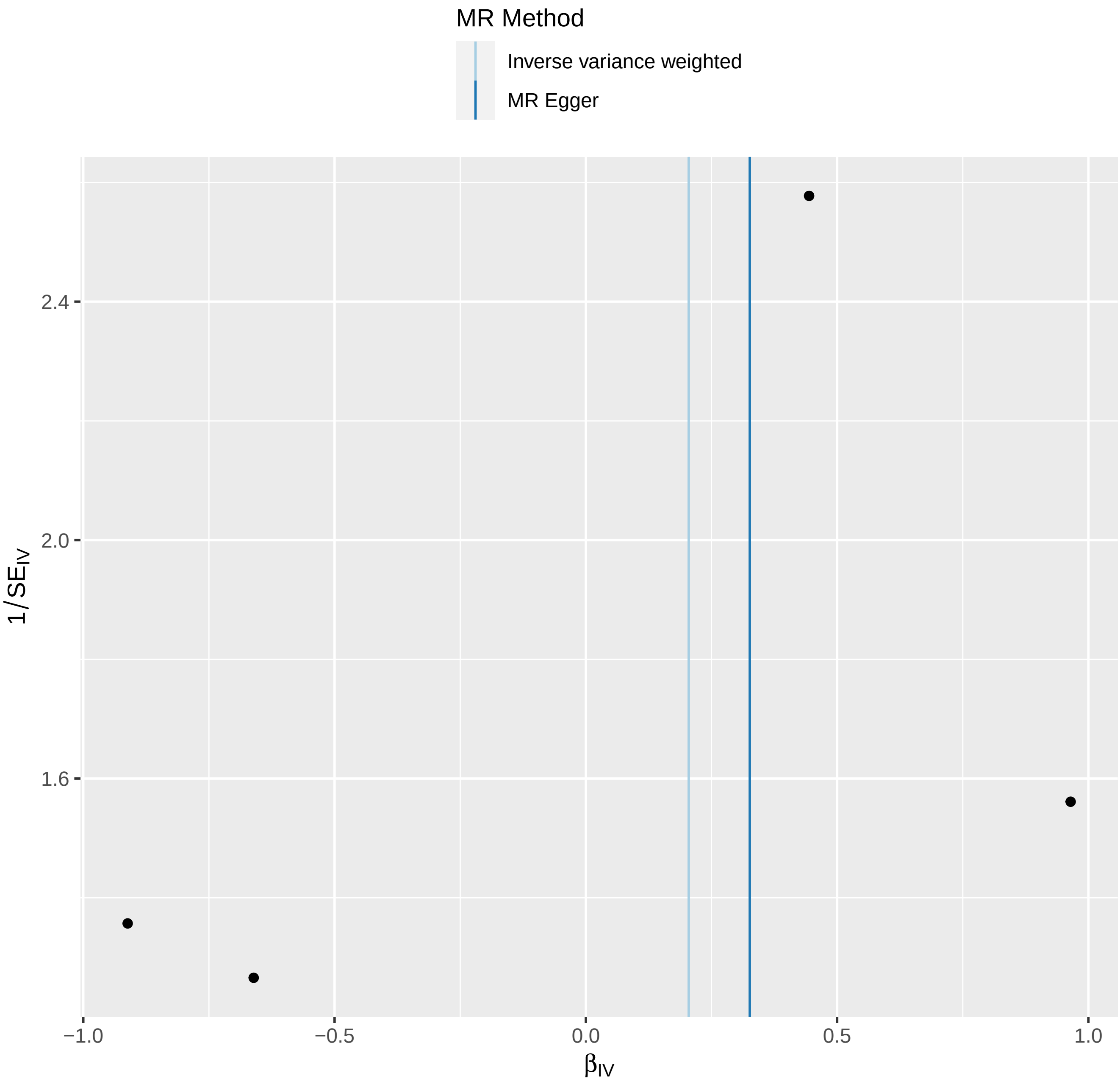

Batch 903 : Gut microbiota abundance (family Alcaligenaceae id.2875) on Ulcerative enterocolitis

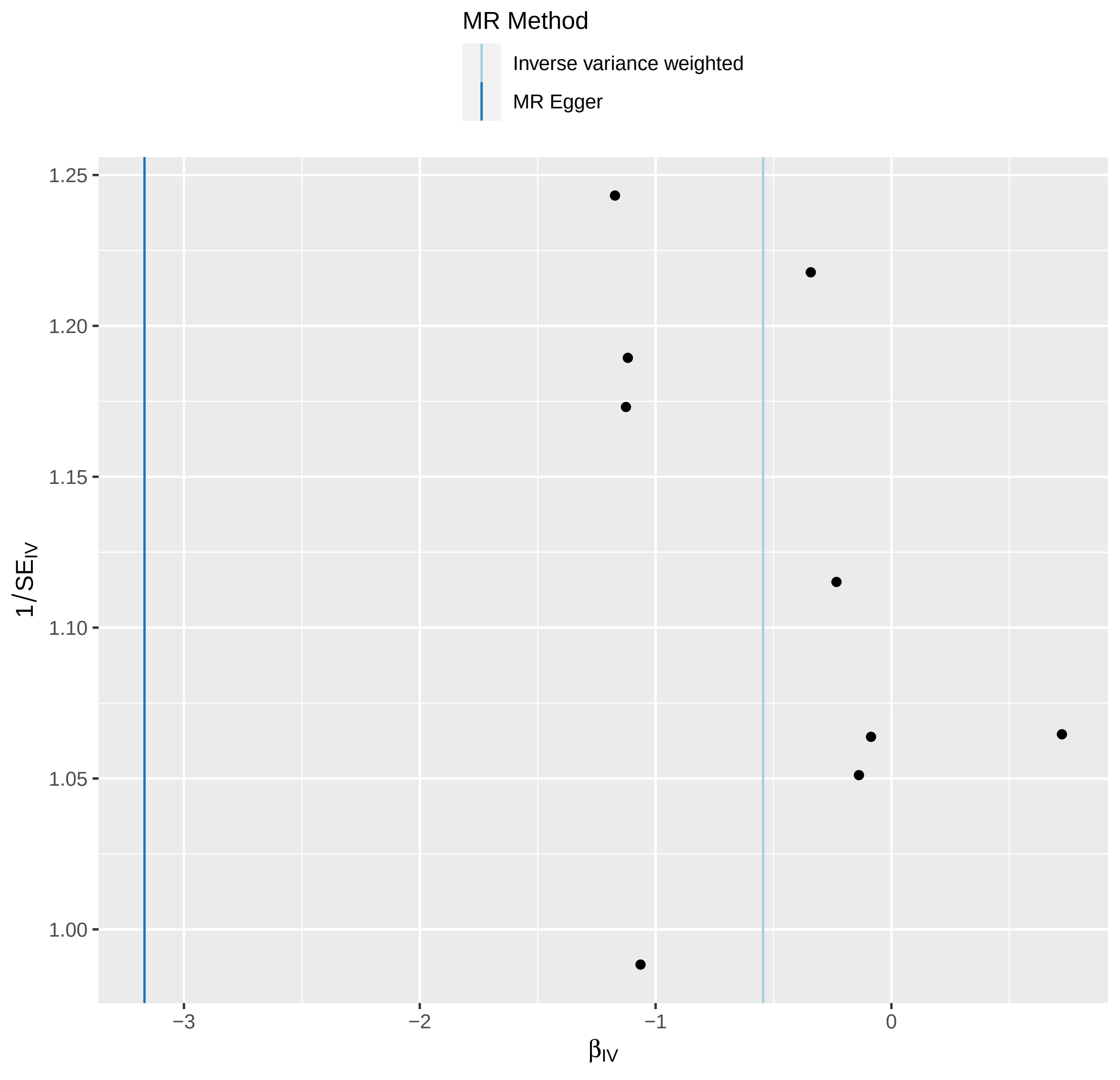

Batch 904 : Gut microbiota abundance (family Bacteroidaceae id.917) on Ulcerative enterocolitis

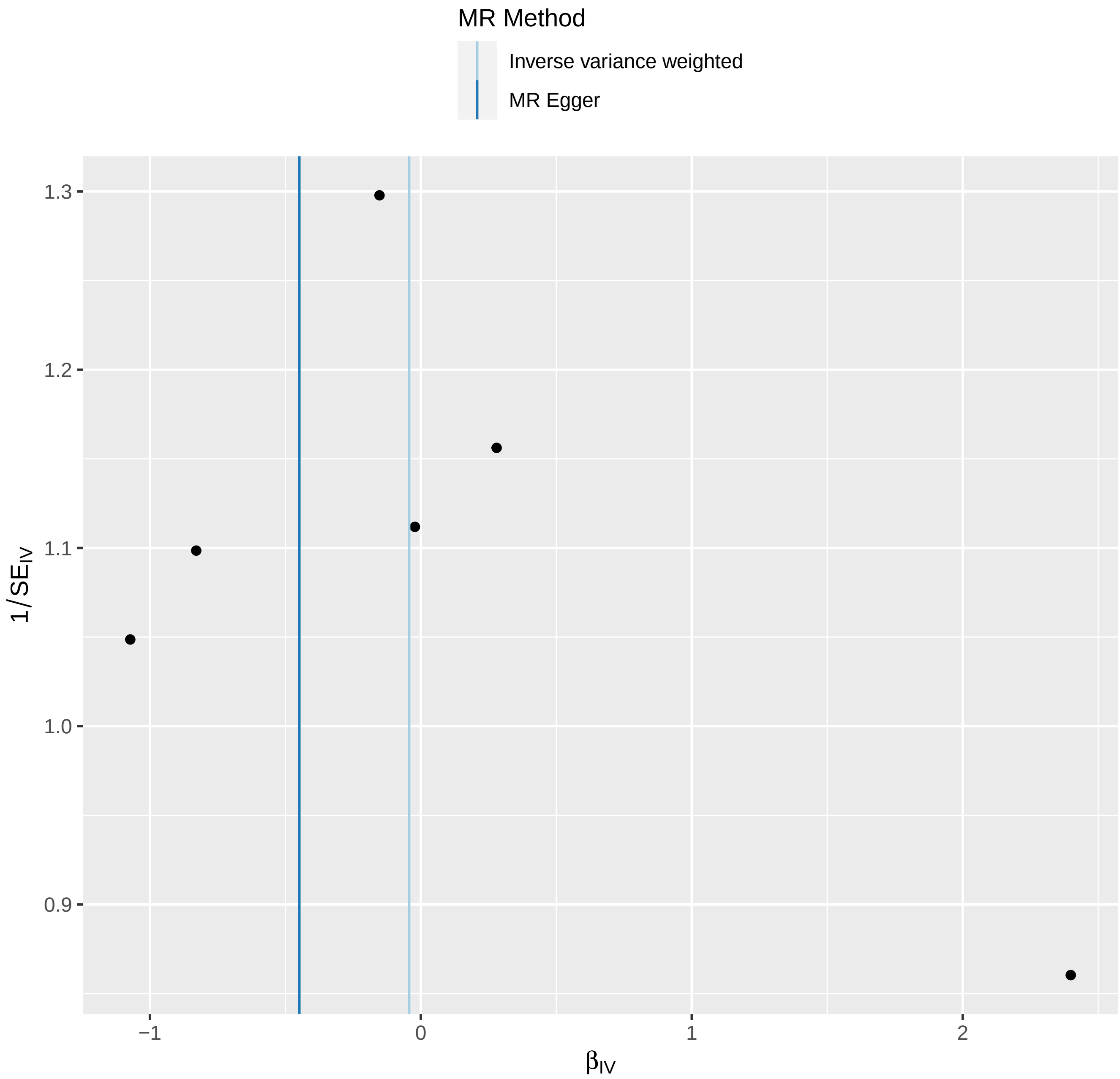

Batch 905 : Gut microbiota abundance (family Bacteroidales S24 7group id.11173) on Ulcerative enterocolitis

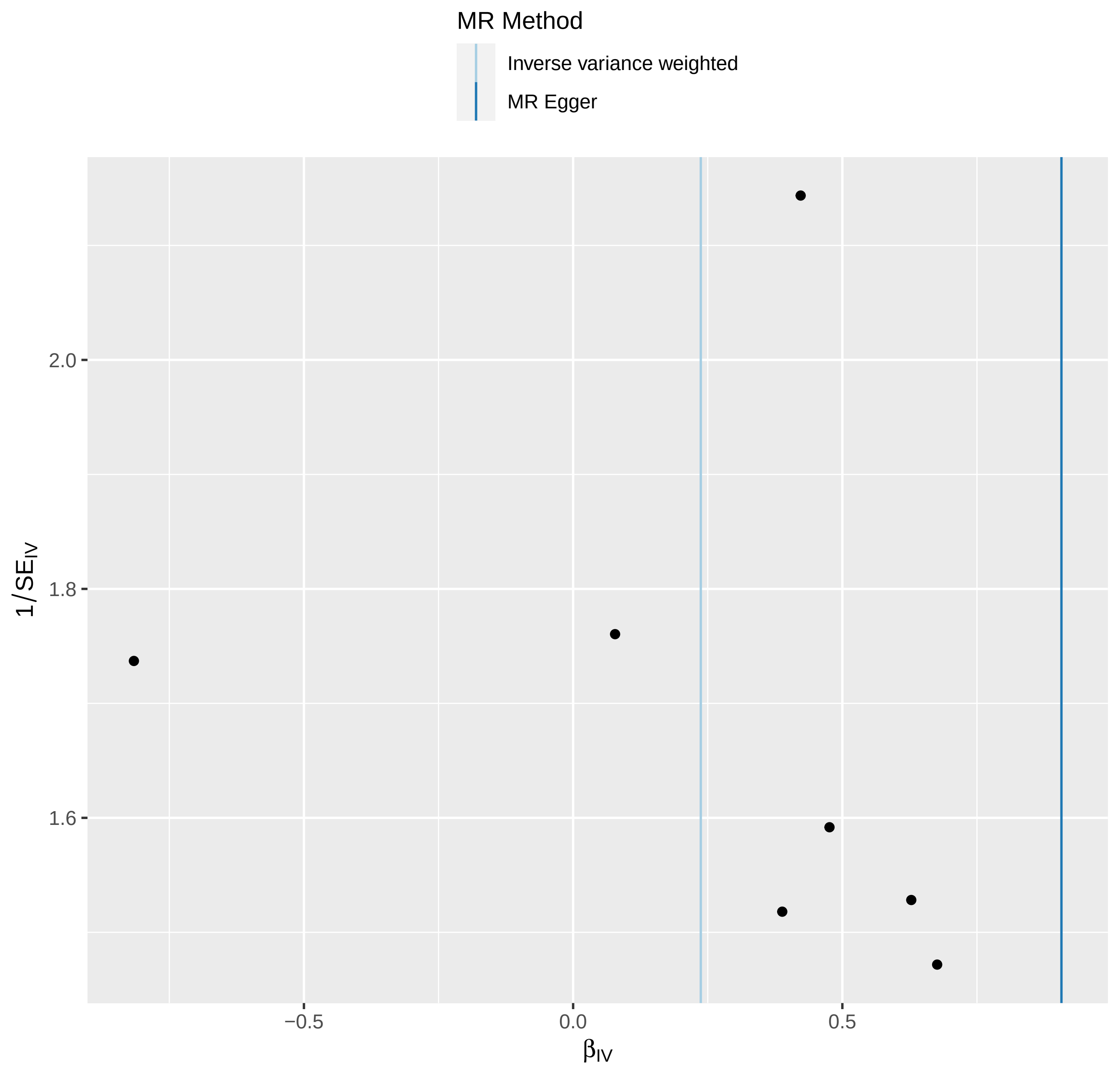

Batch 906 : Gut microbiota abundance (family Bifidobacteriaceae id.433) on Ulcerative enterocolitis

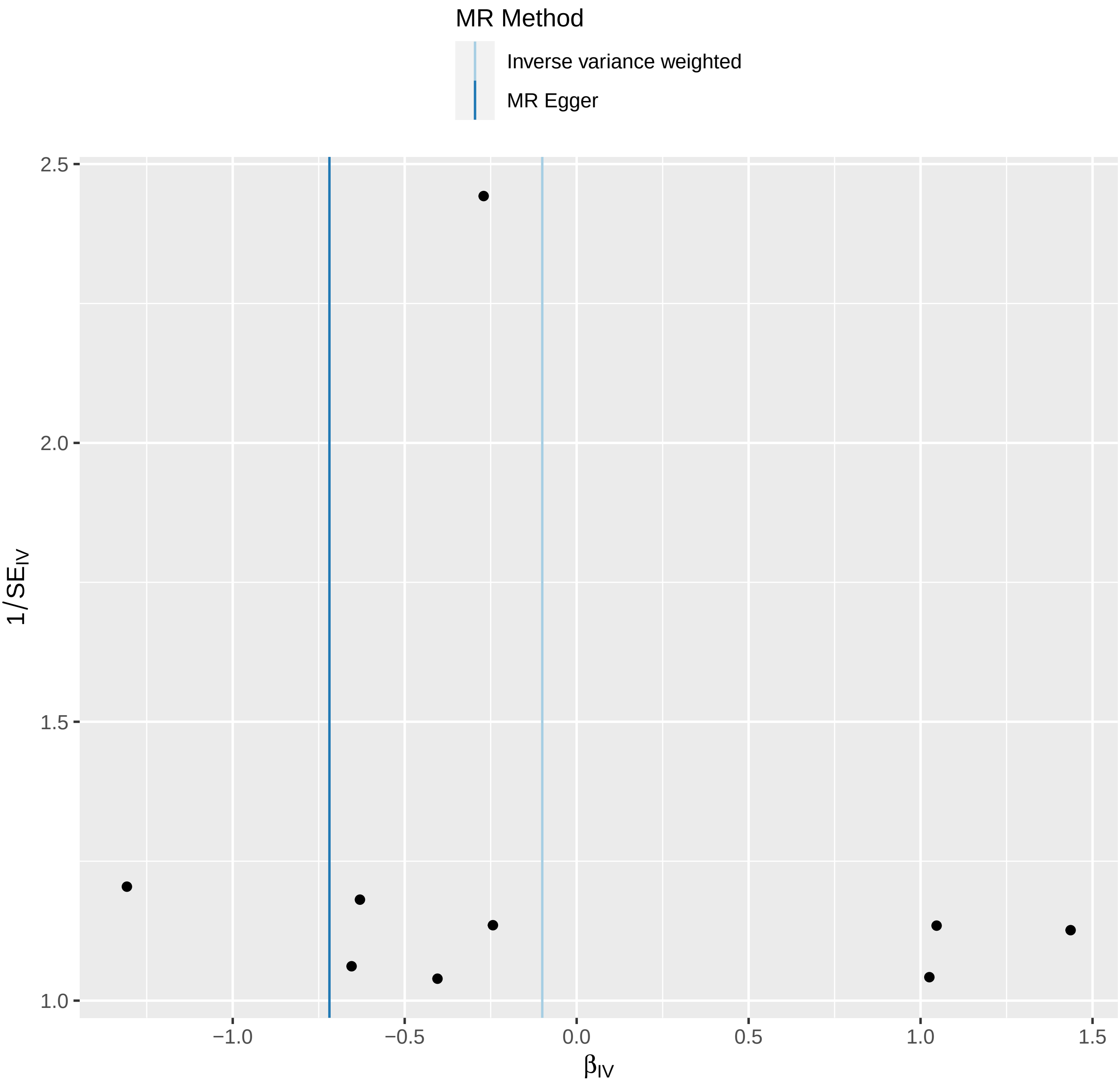

Batch 908 : Gut microbiota abundance (family Clostridiaceae1 id.1869) on Ulcerative enterocolitis

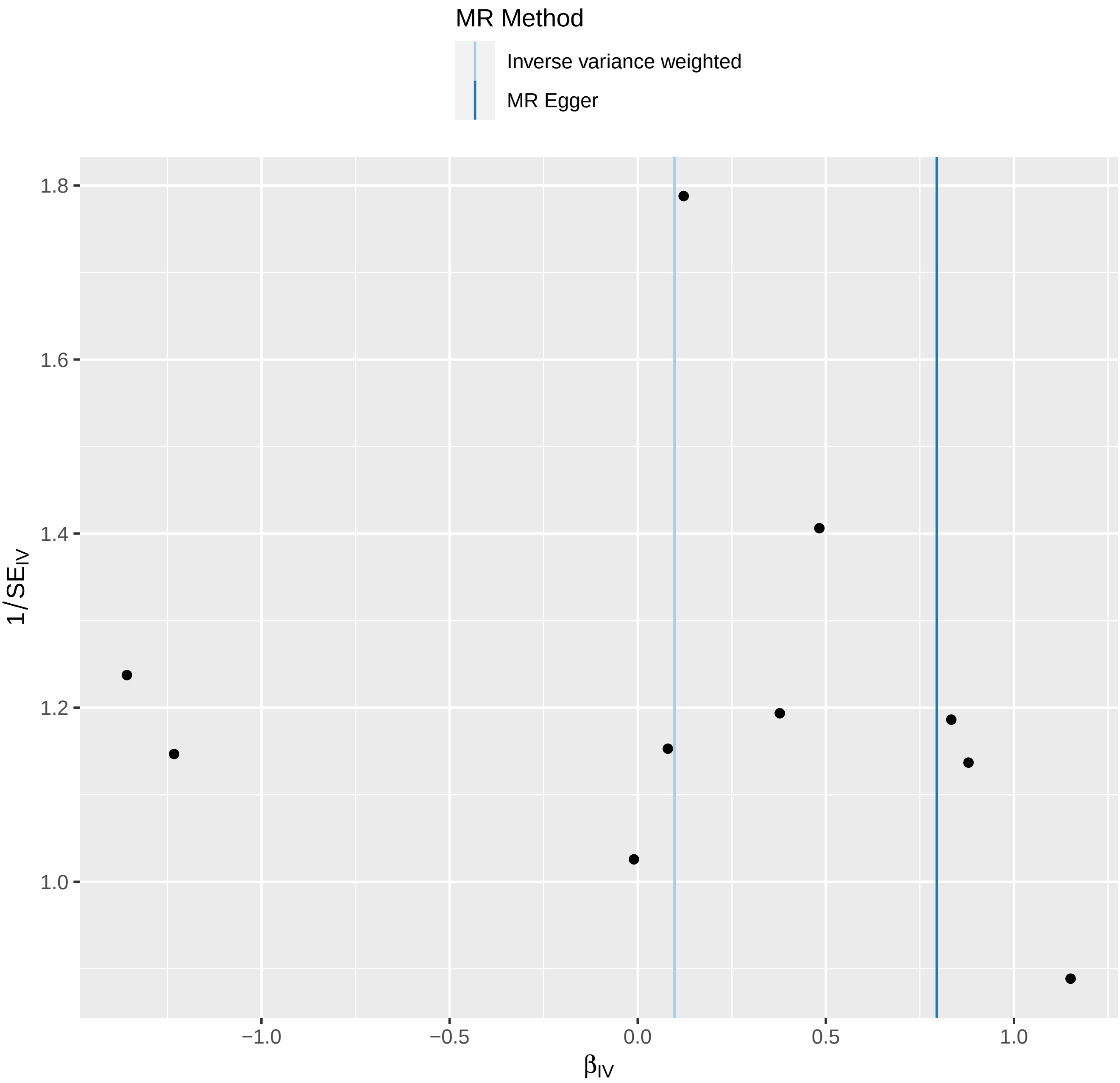

Batch 909 : Gut microbiota abundance (family Clostridiales vadin BB60 group id.11286) on Ulcerative enterocolitis

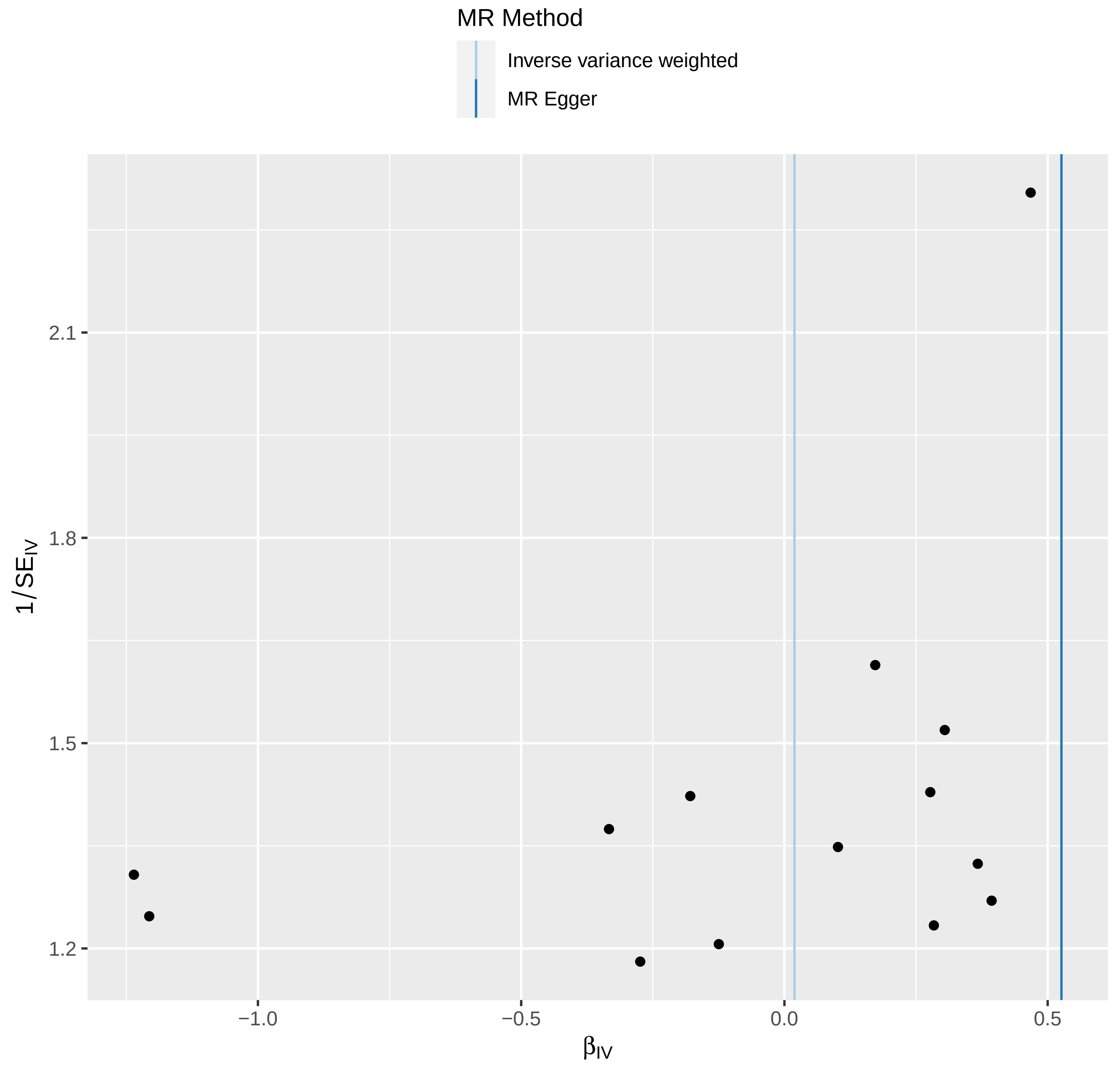

Batch 910 : Gut microbiota abundance (family Coriobacteriaceae id.811) on Ulcerative enterocolitis

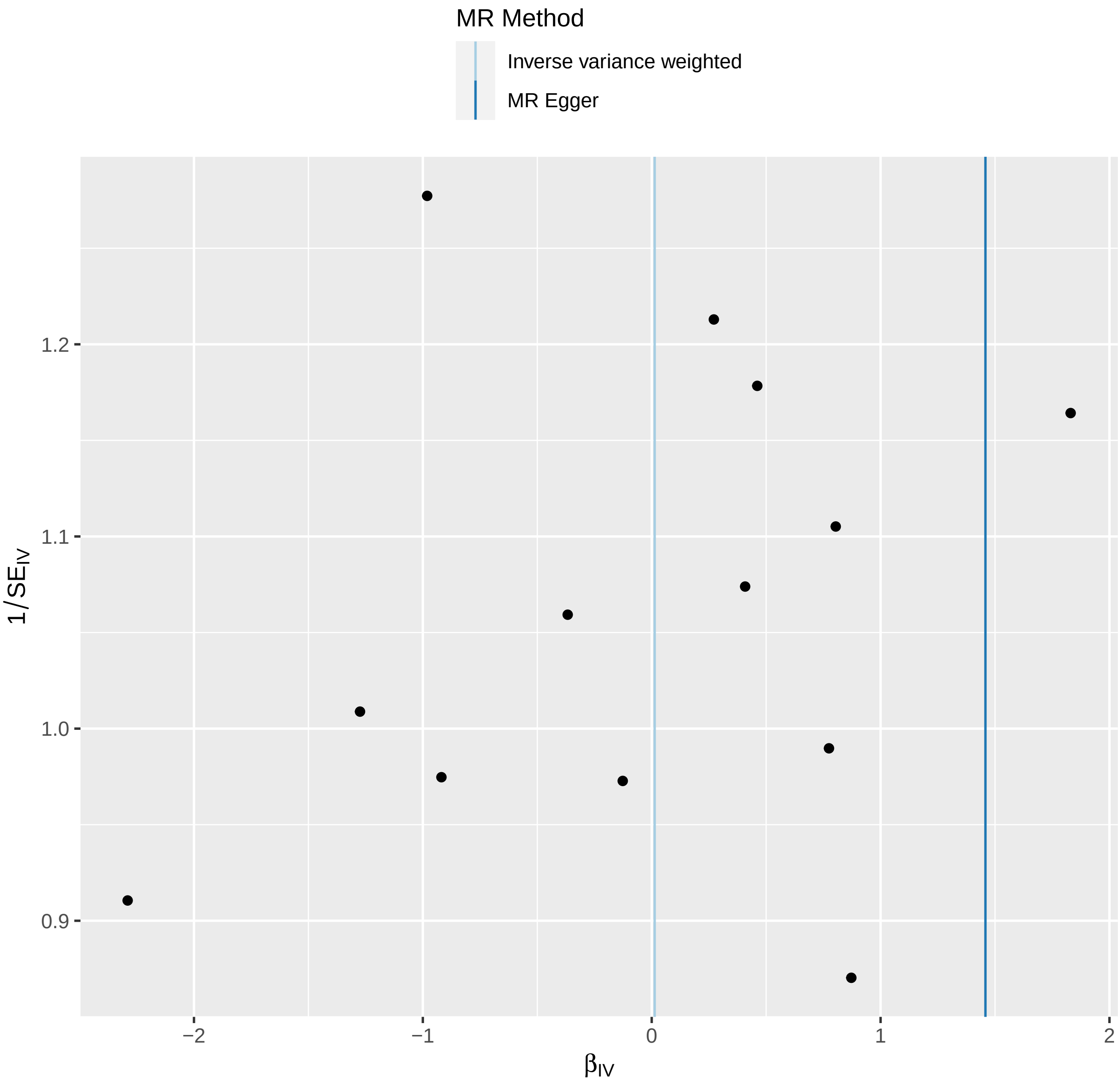

Batch 911 : Gut microbiota abundance (family Defluviitaleaceae id.1924) on Ulcerative enterocolitis

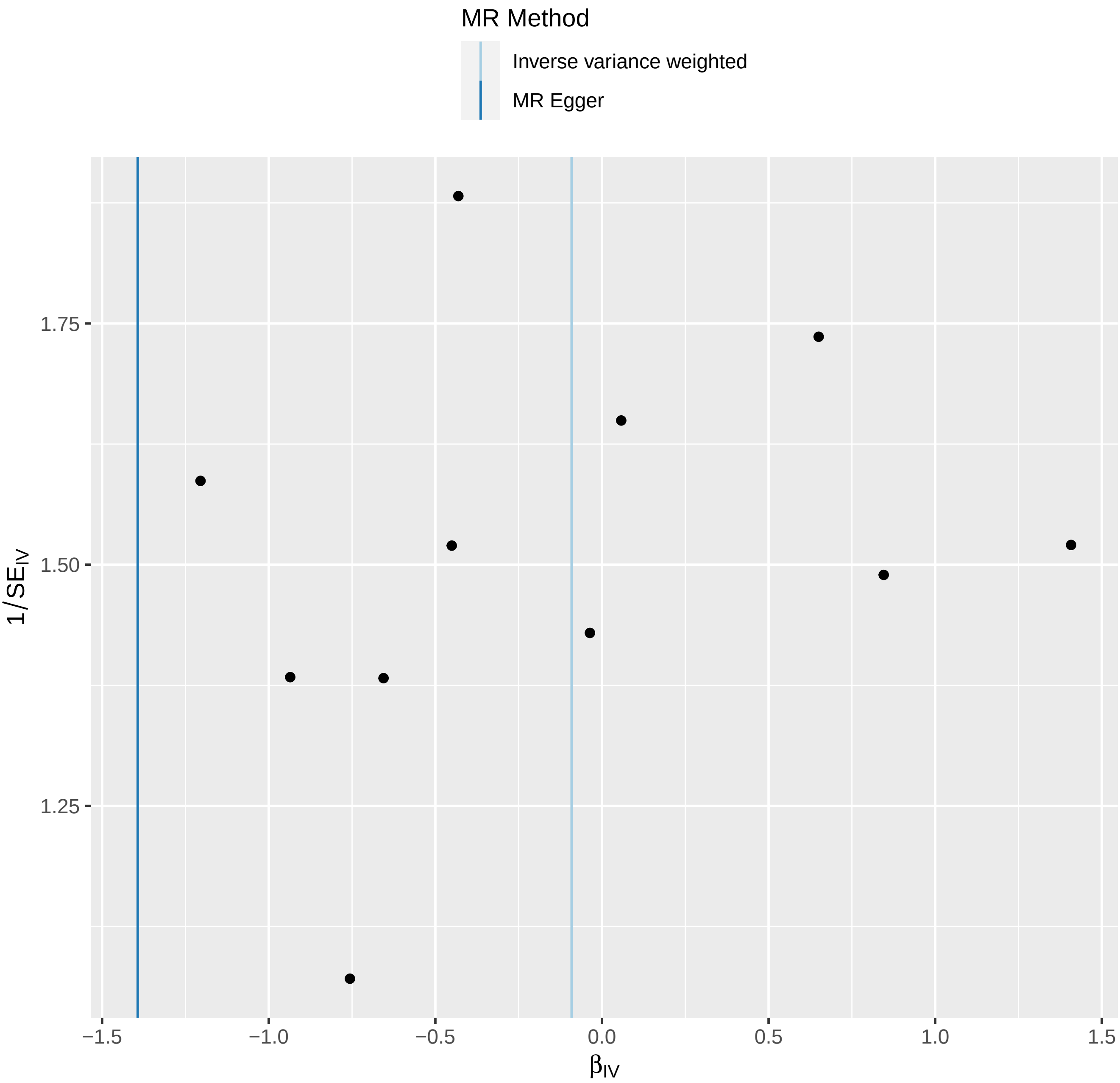

Batch 912 : Gut microbiota abundance (family Desulfovibrionaceae id.3169) on Ulcerative enterocolitis

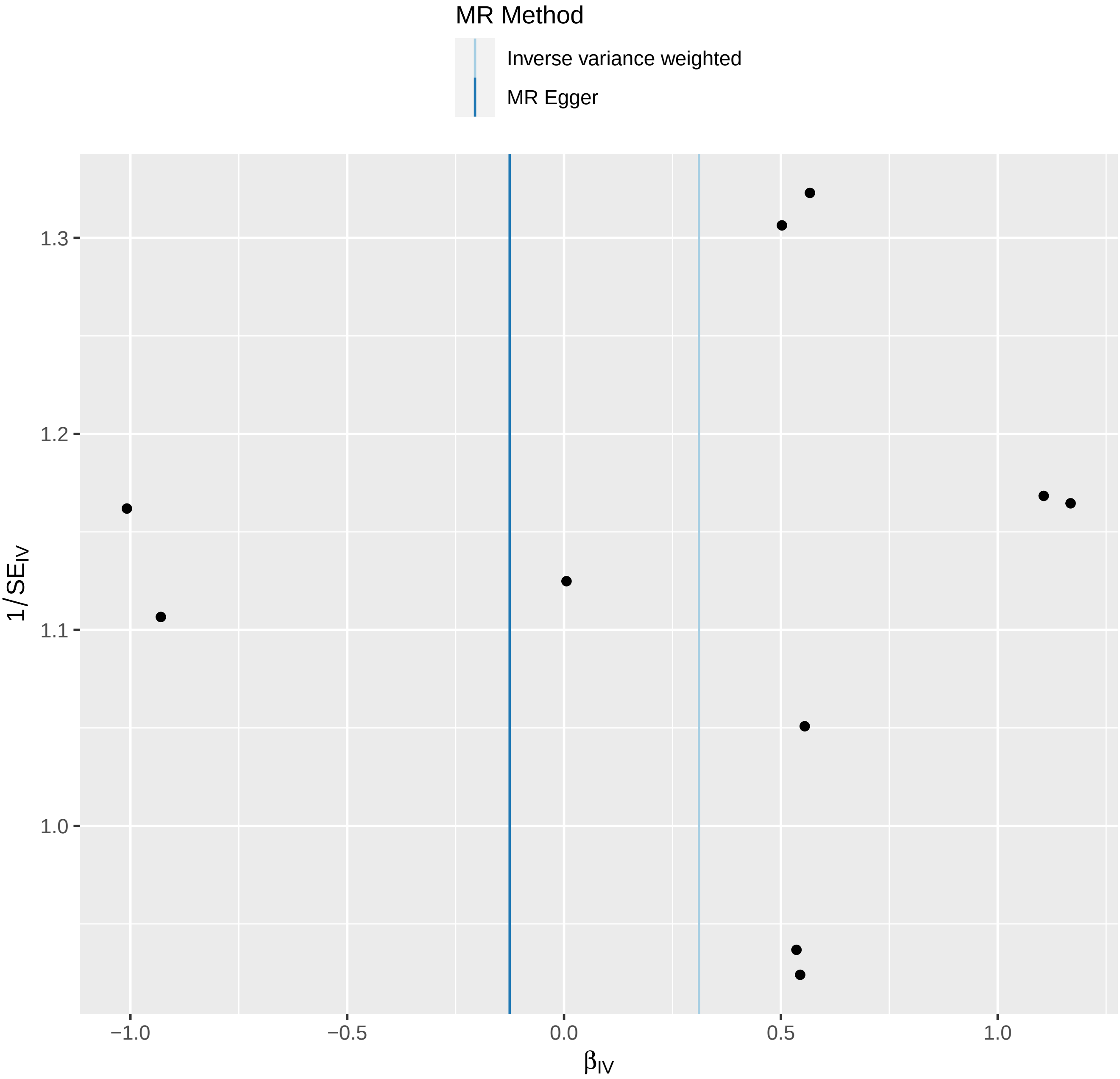

Batch 913 : Gut microbiota abundance (family Enterobacteriaceae id.3469) on Ulcerative enterocolitis

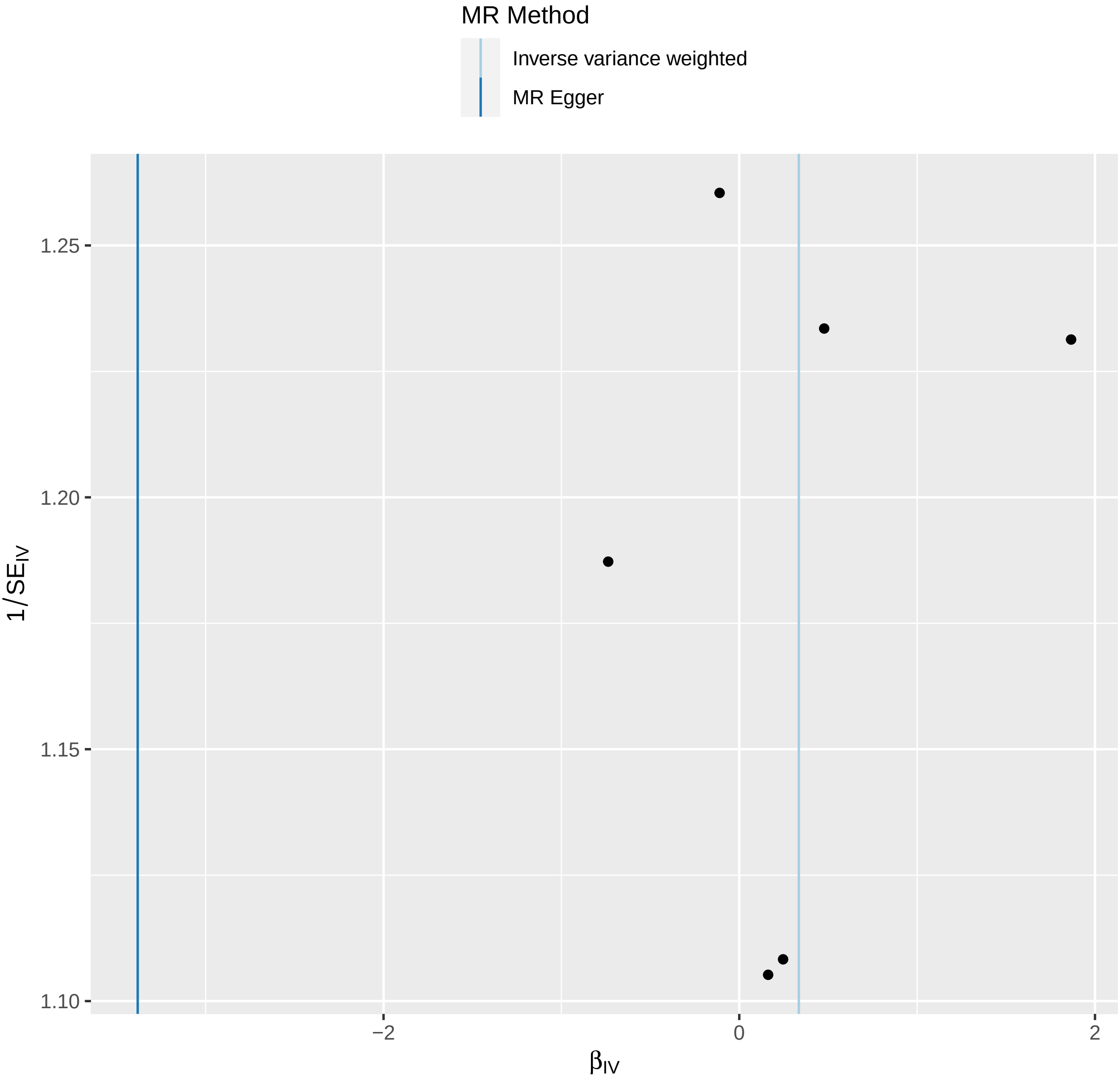

Batch 914 : Gut microbiota abundance (family Erysipelotrichaceae id.2149) on Ulcerative enterocolitis

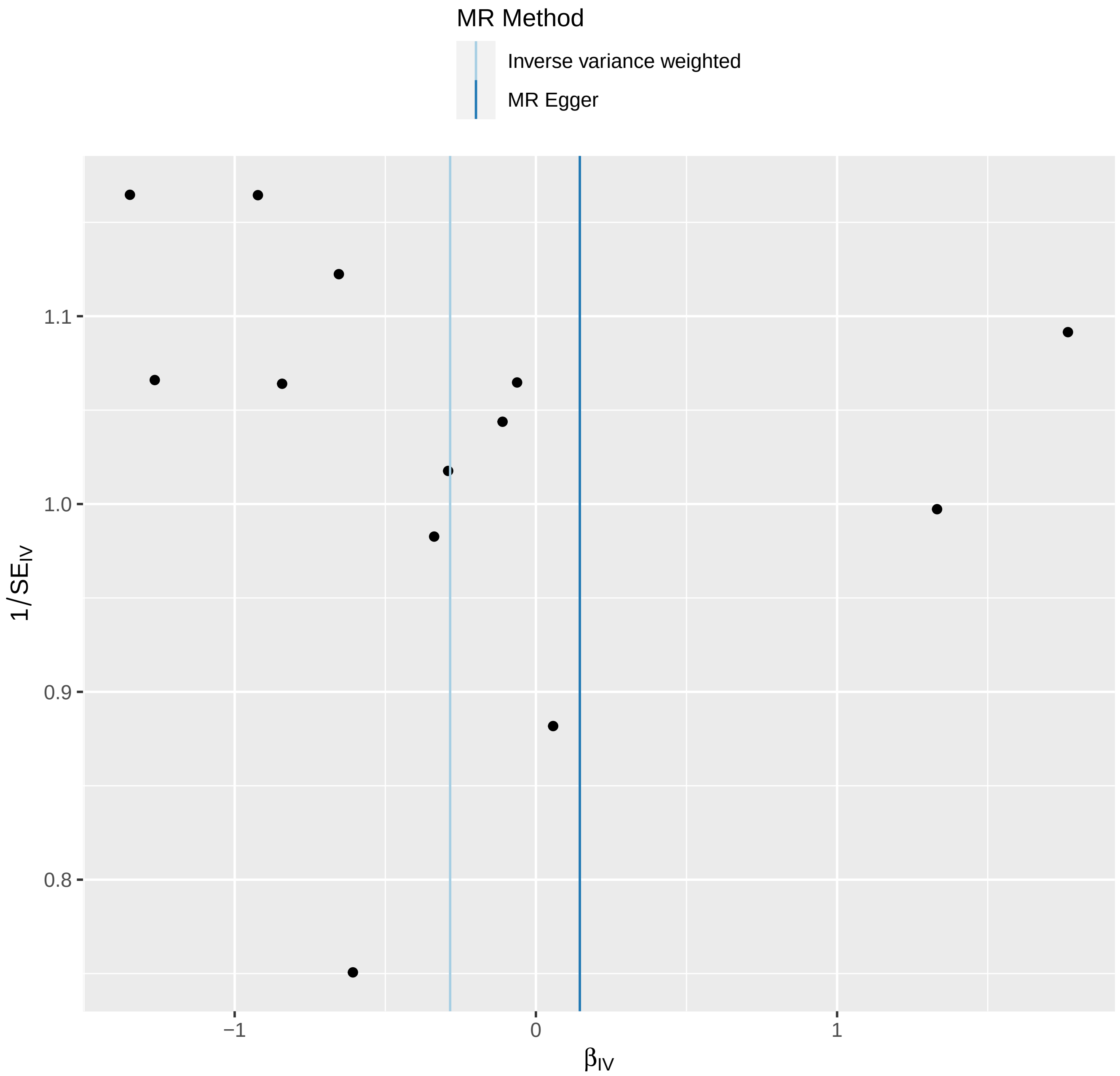

Batch 915 : Gut microbiota abundance (family Family XI id.1936) on Ulcerative enterocolitis

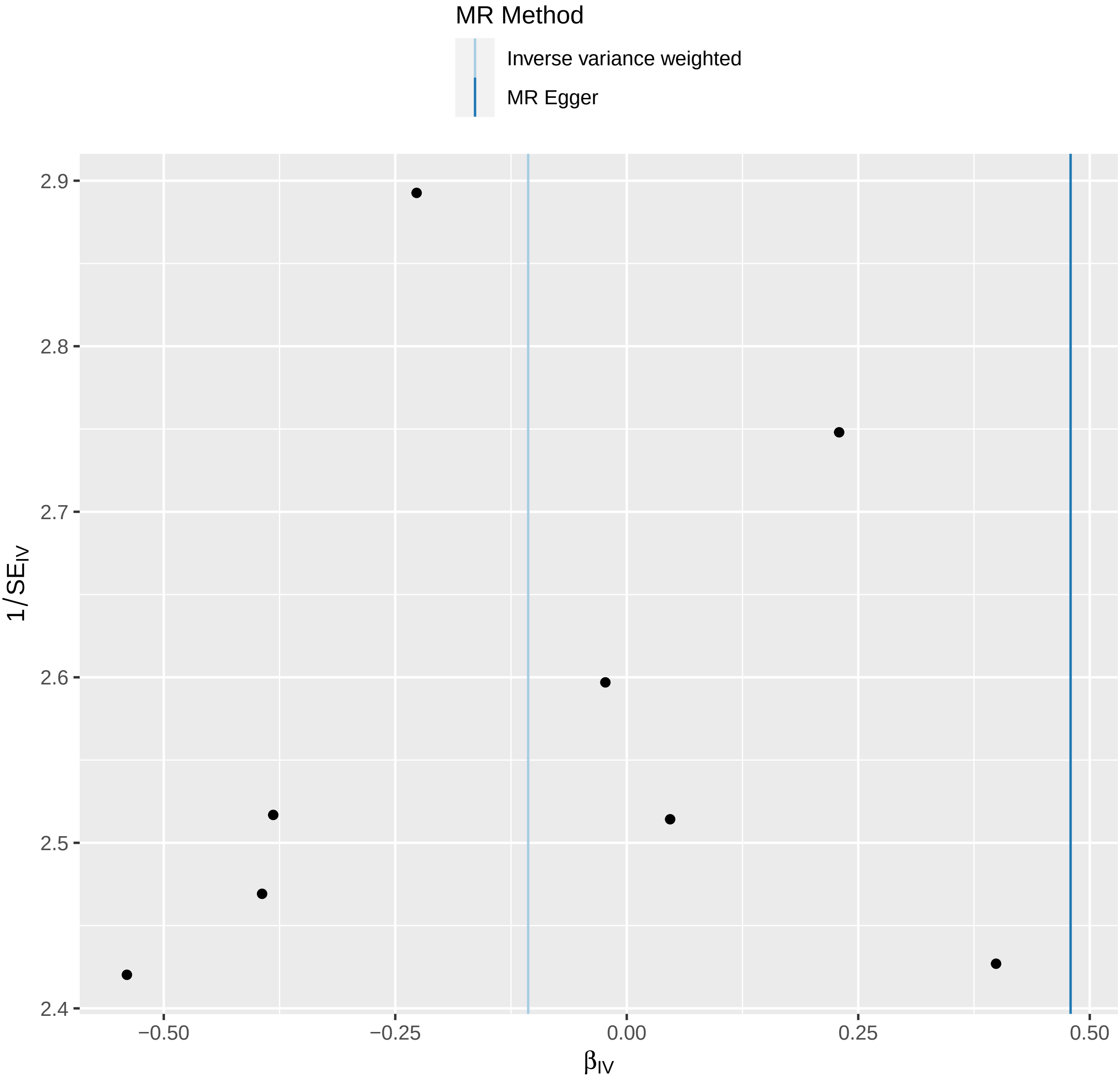

Batch 916 : Gut microbiota abundance (family Family XIII id.1957) on Ulcerative enterocolitis

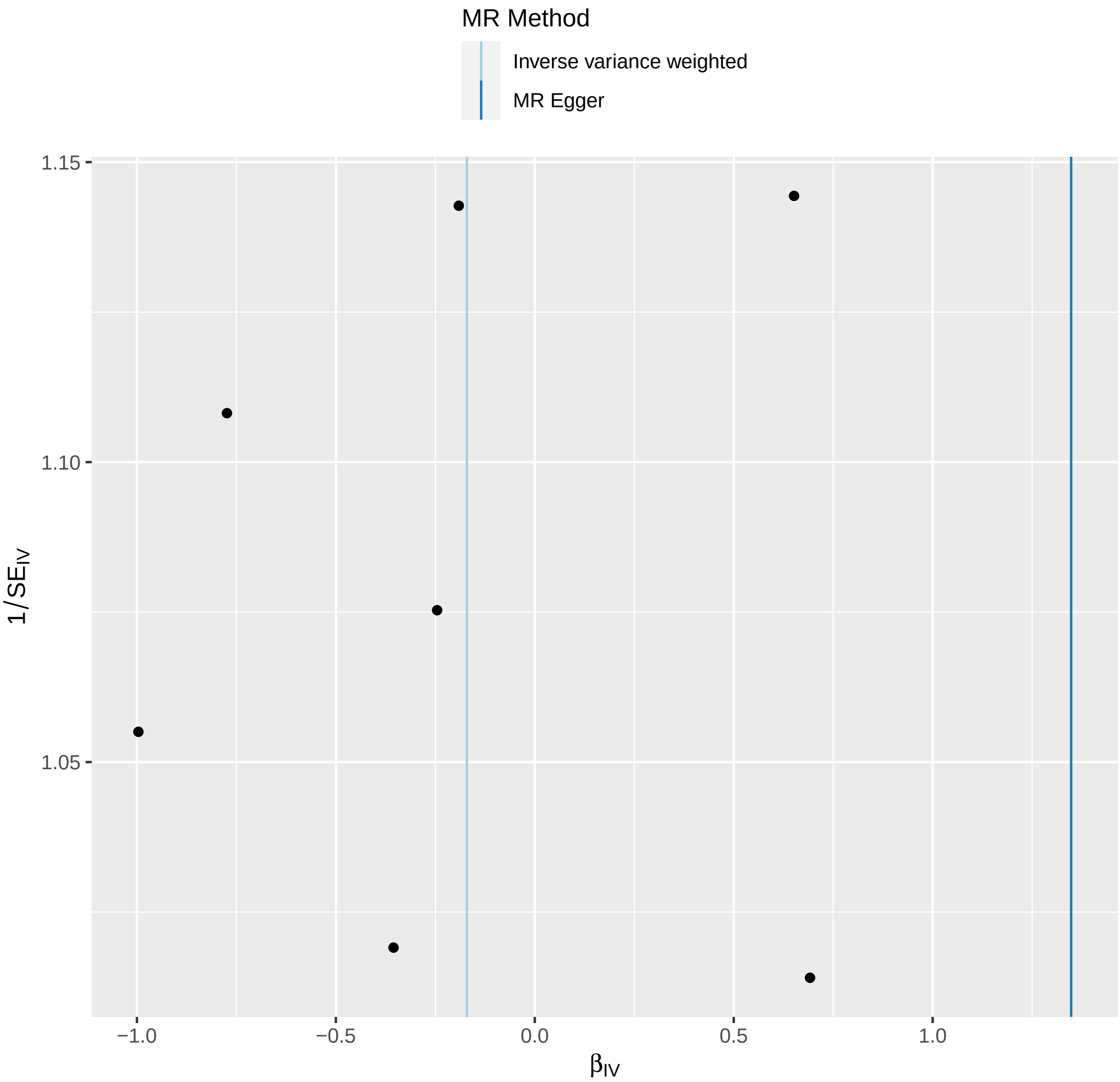

Batch 917 : Gut microbiota abundance (family Lachnospiraceae id.1987) on Ulcerative enterocolitis

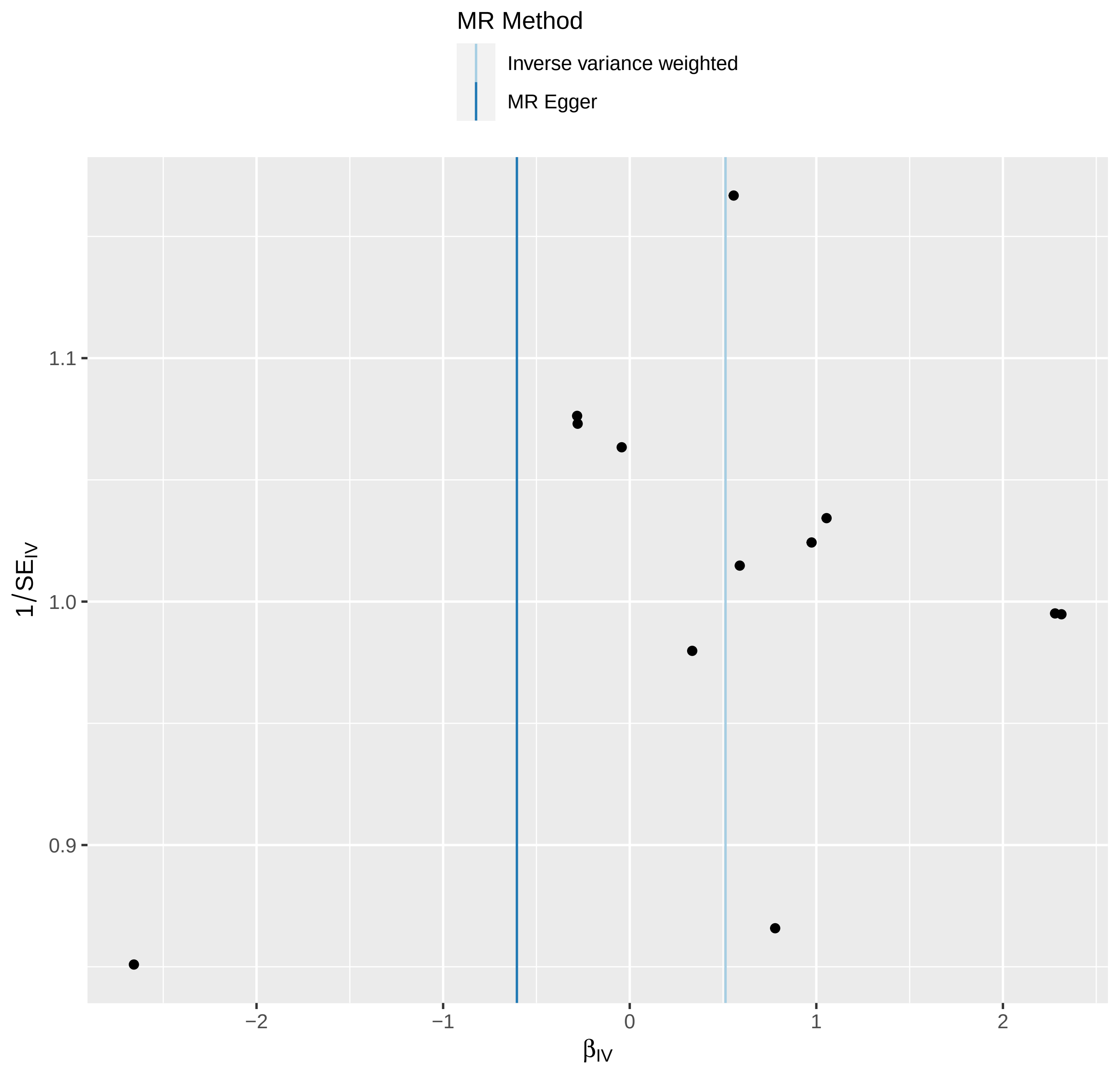

Batch 918 : Gut microbiota abundance (family Lactobacillaceae id.1836) on Ulcerative enterocolitis

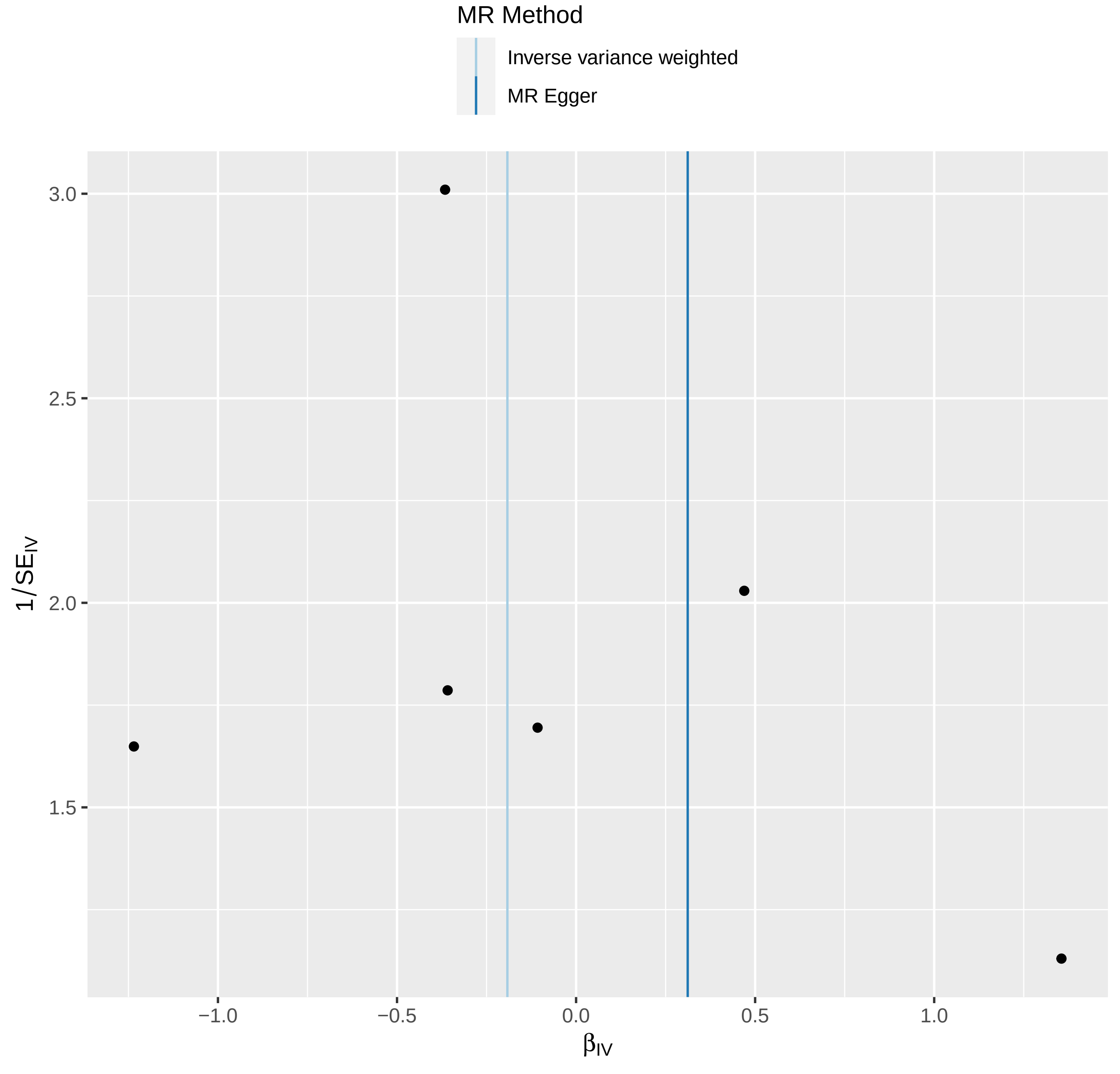

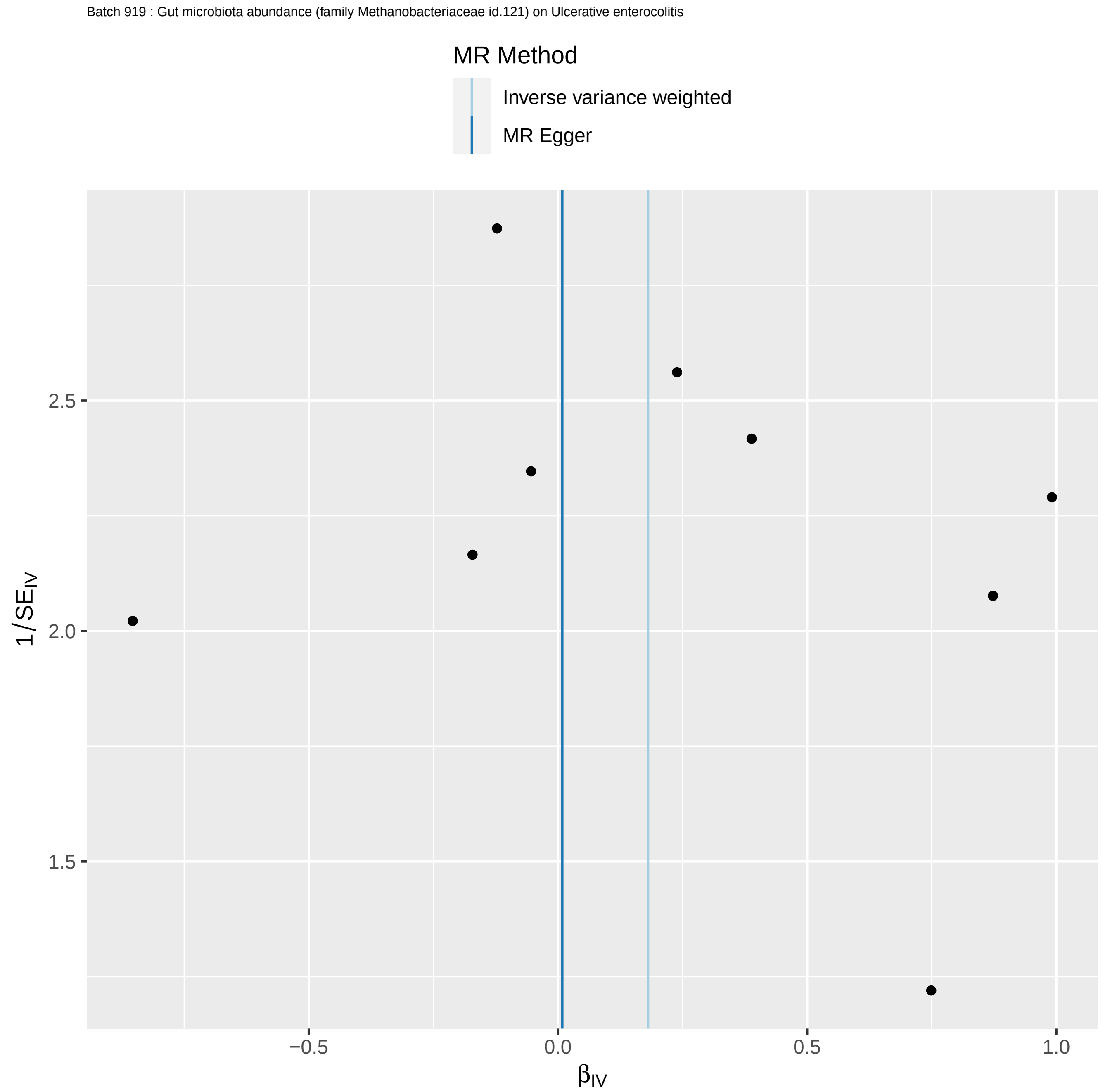

Batch 919 : Gut microbiota abundance (family Methanobacteriaceae id.121) on Ulcerative enterocolitis
MR Method
Inverse variance weighted
MR Egger
2.5
2.0
1.5
1/SE_IV
−0.5
0.0
0.5
1.0
β_IV

Batch 920 : Gut microbiota abundance (family Oxalobacteraceae id.2966) on Ulcerative enterocolitis

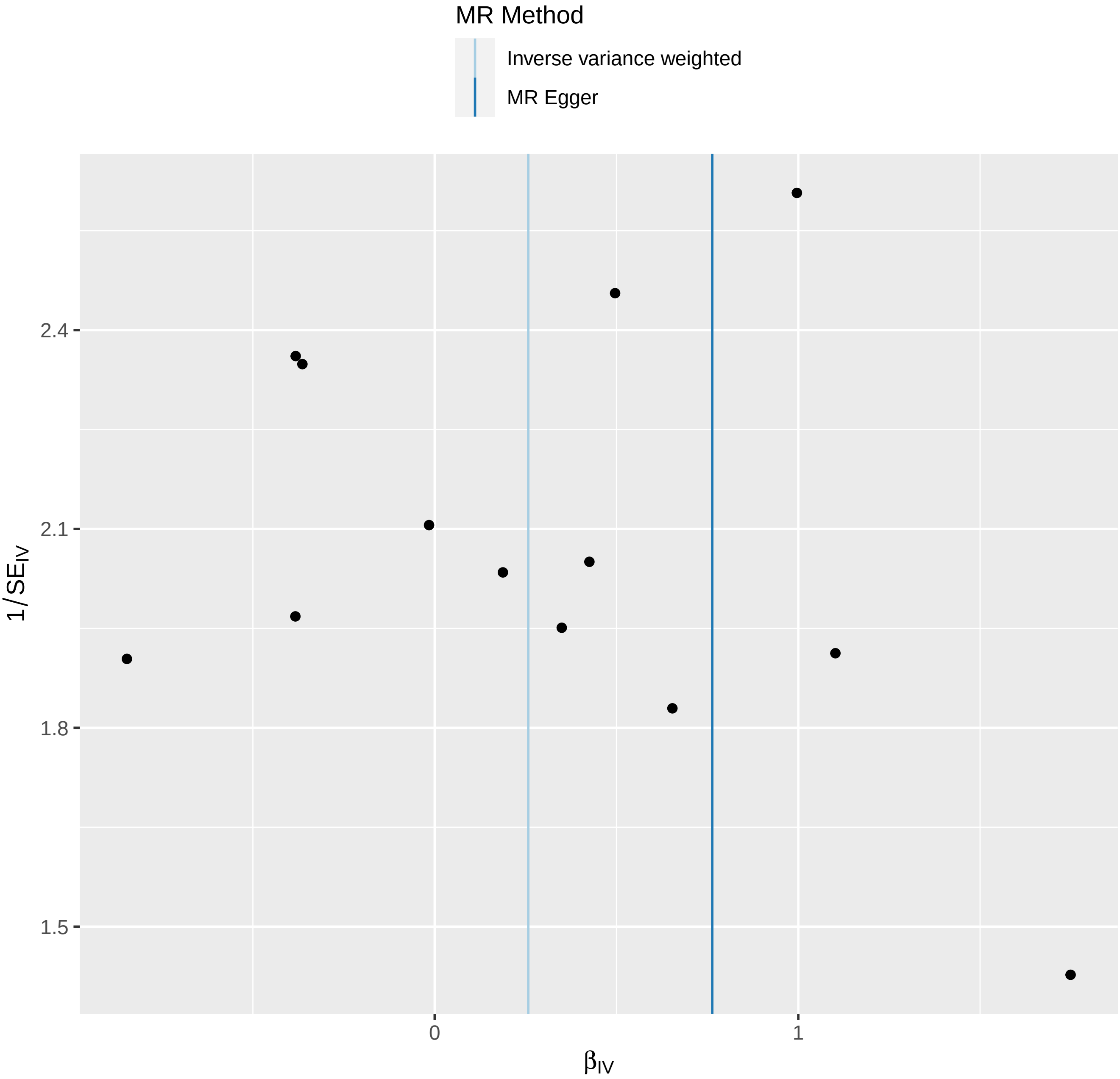

Batch 921 : Gut microbiota abundance (family Pasteurellaceae id.3689) on Ulcerative enterocolitis

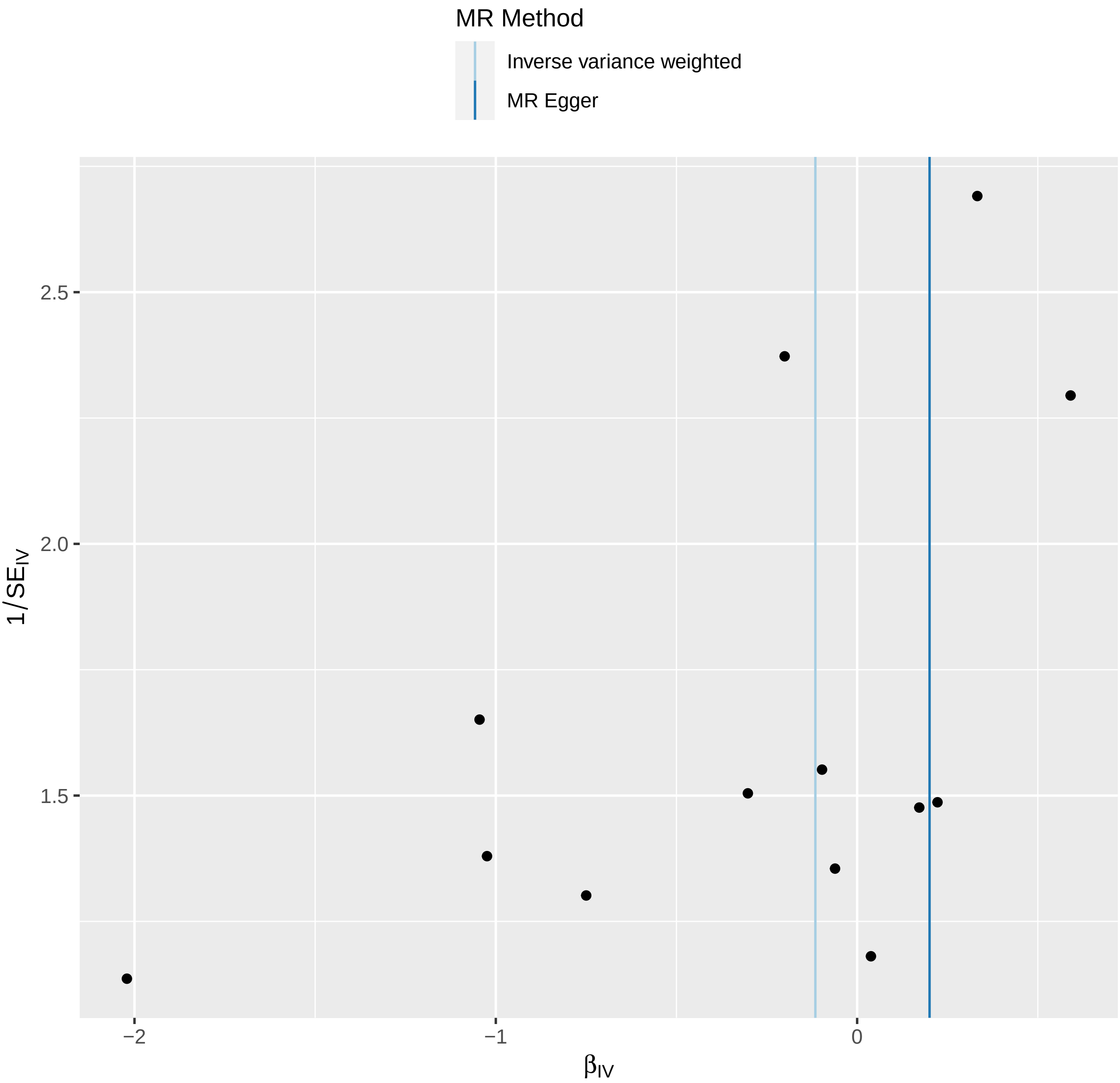

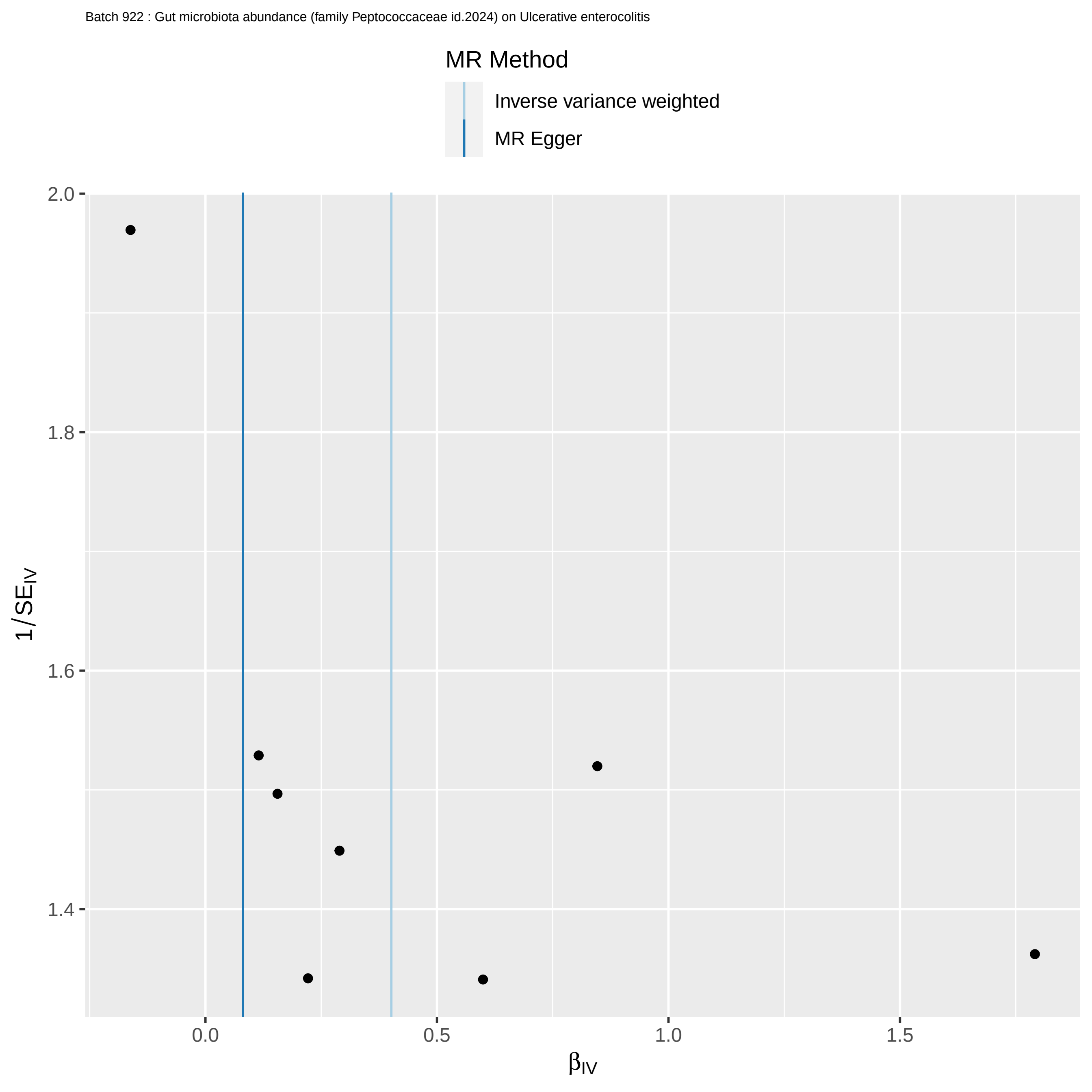
Batch 922 : Gut microbiota abundance (family Peptococcaceae id.2024) on Ulcerative enterocolitis
MR Method
Inverse variance weighted
MR Egger
2.0
1.8
1.6
1.4
$1/SE_{IV}$
0.0
0.5
1.0
1.5
β_{IV}

Batch 923 : Gut microbiota abundance (family Peptostreptococcaceae id.2042) on Ulcerative enterocolitis

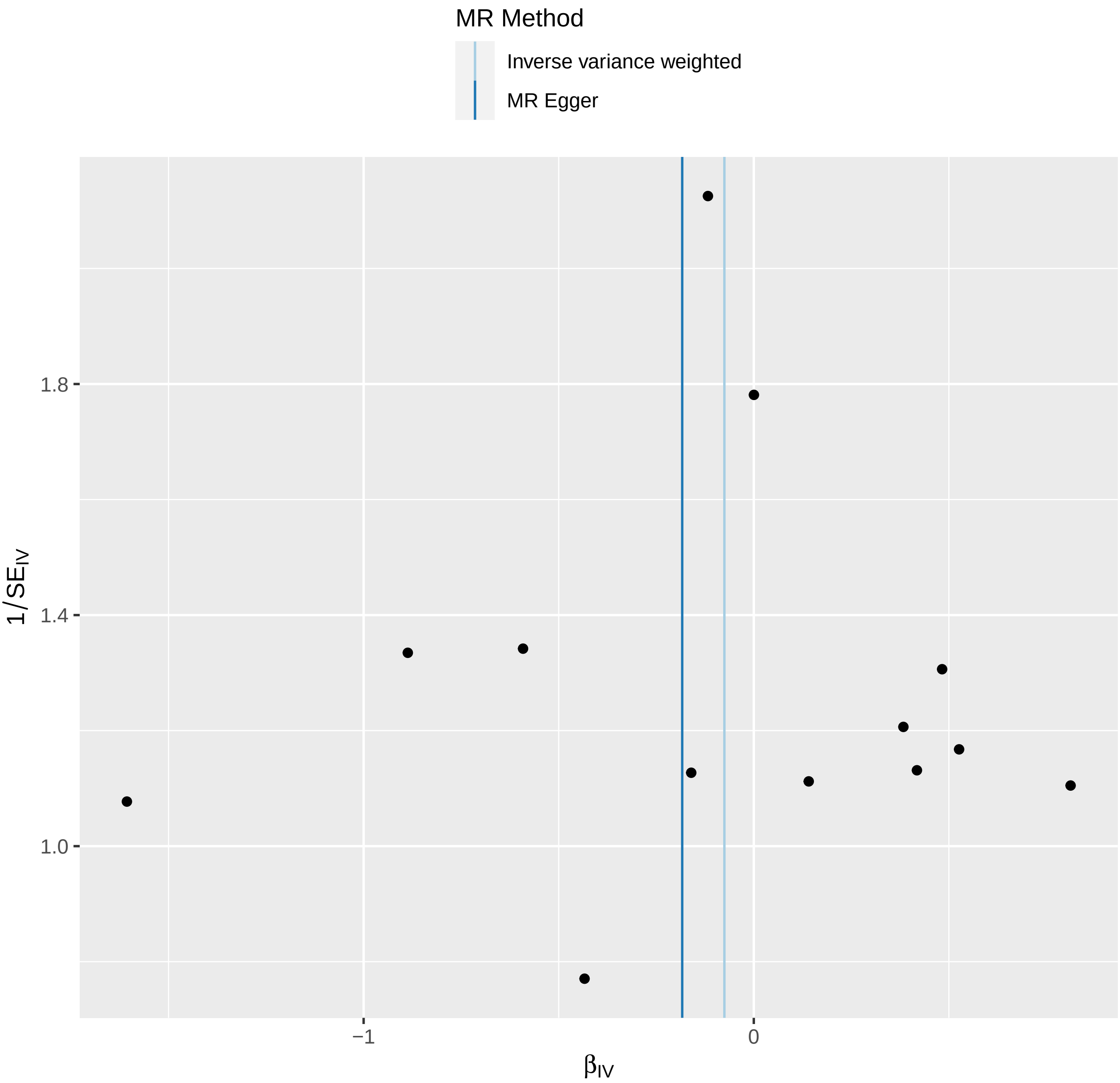

Batch 924 : Gut microbiota abundance (family Porphyromonadaceae id.943) on Ulcerative enterocolitis

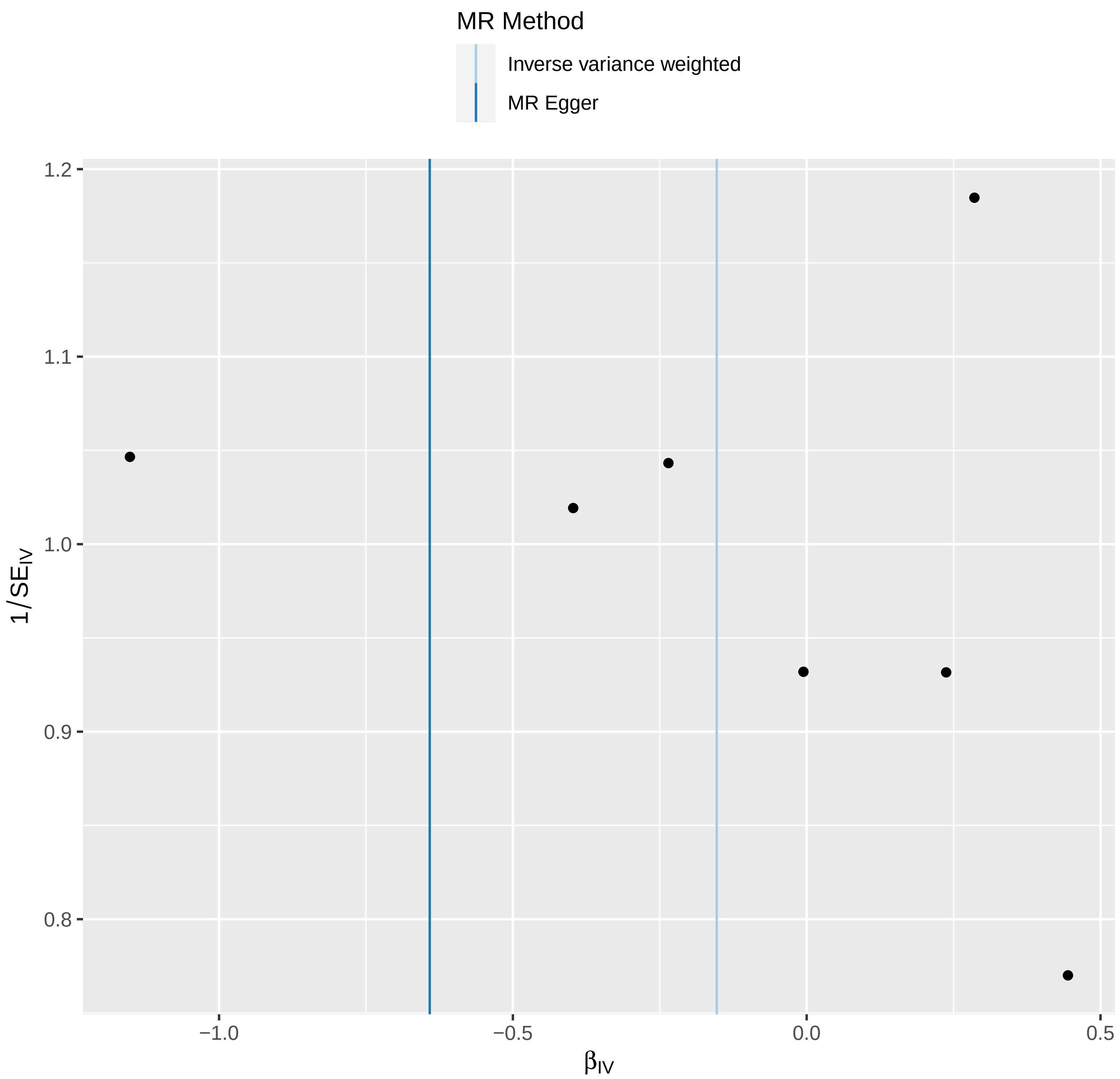

Batch 925 : Gut microbiota abundance (family Prevotellaceae id.960) on Ulcerative enterocolitis

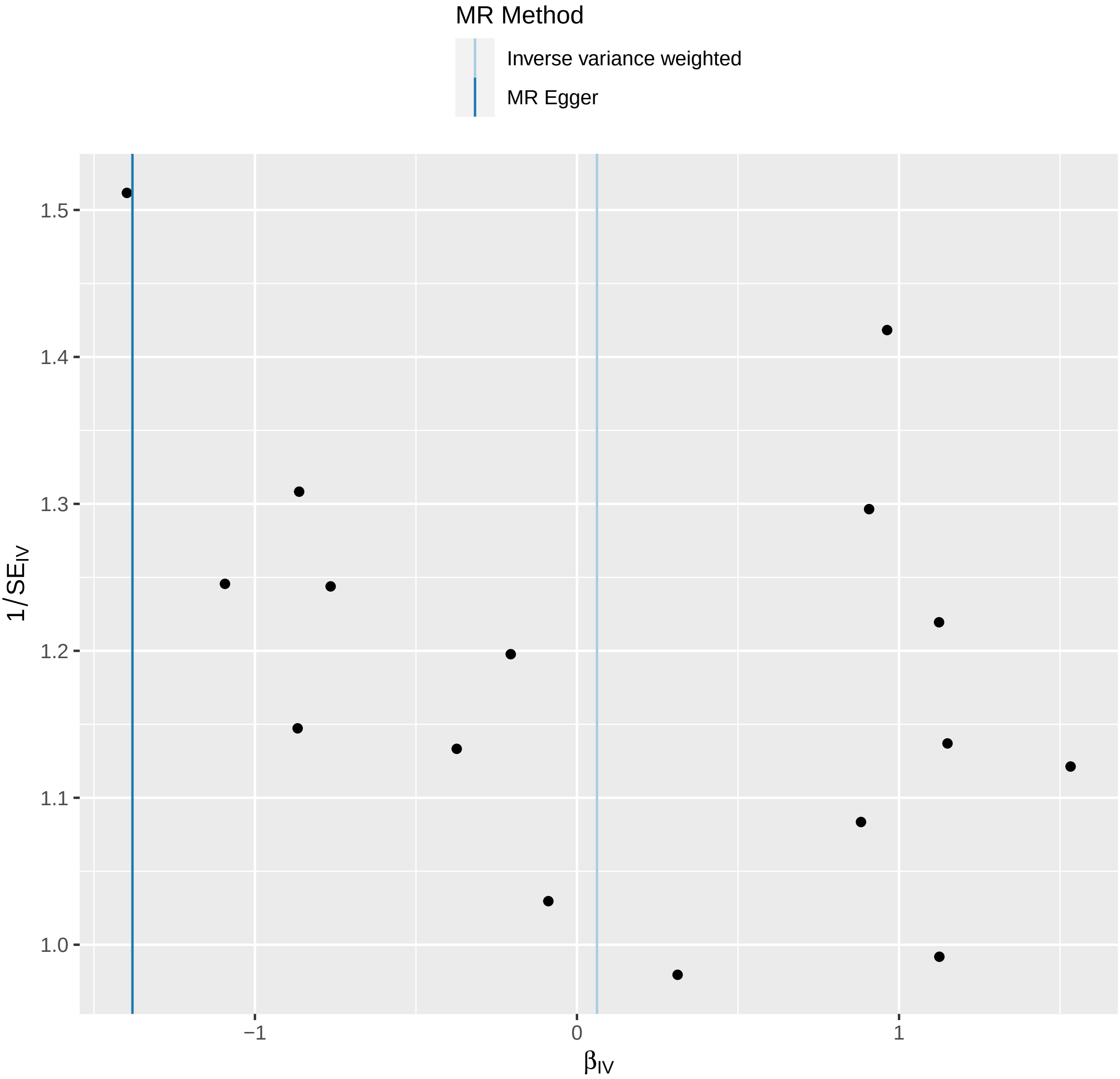

Batch 926 : Gut microbiota abundance (family Rhodospirillaceae id.2717) on Ulcerative enterocolitis

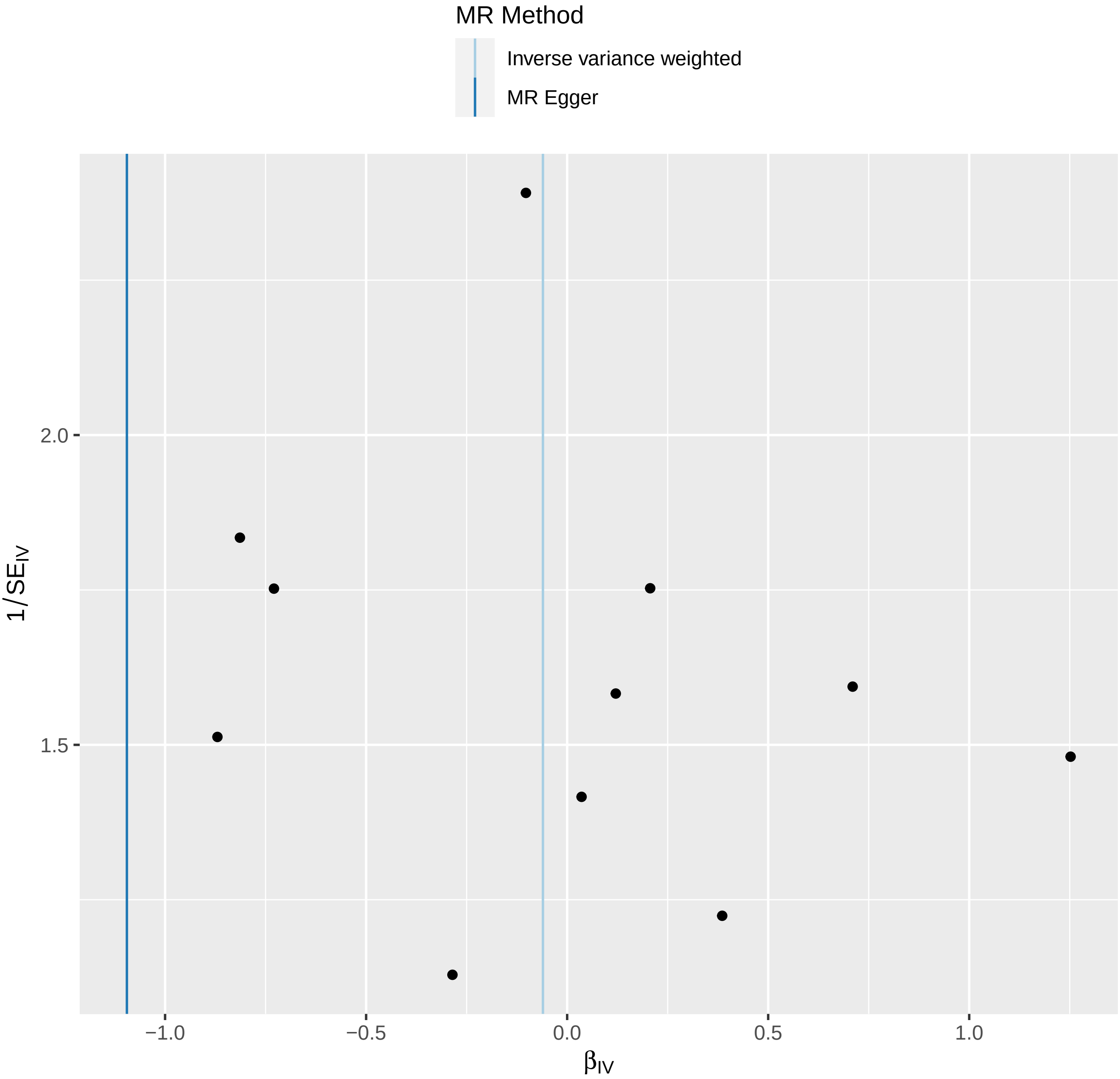

Batch 927 : Gut microbiota abundance (family Rikenellaceae id.967) on Ulcerative enterocolitis

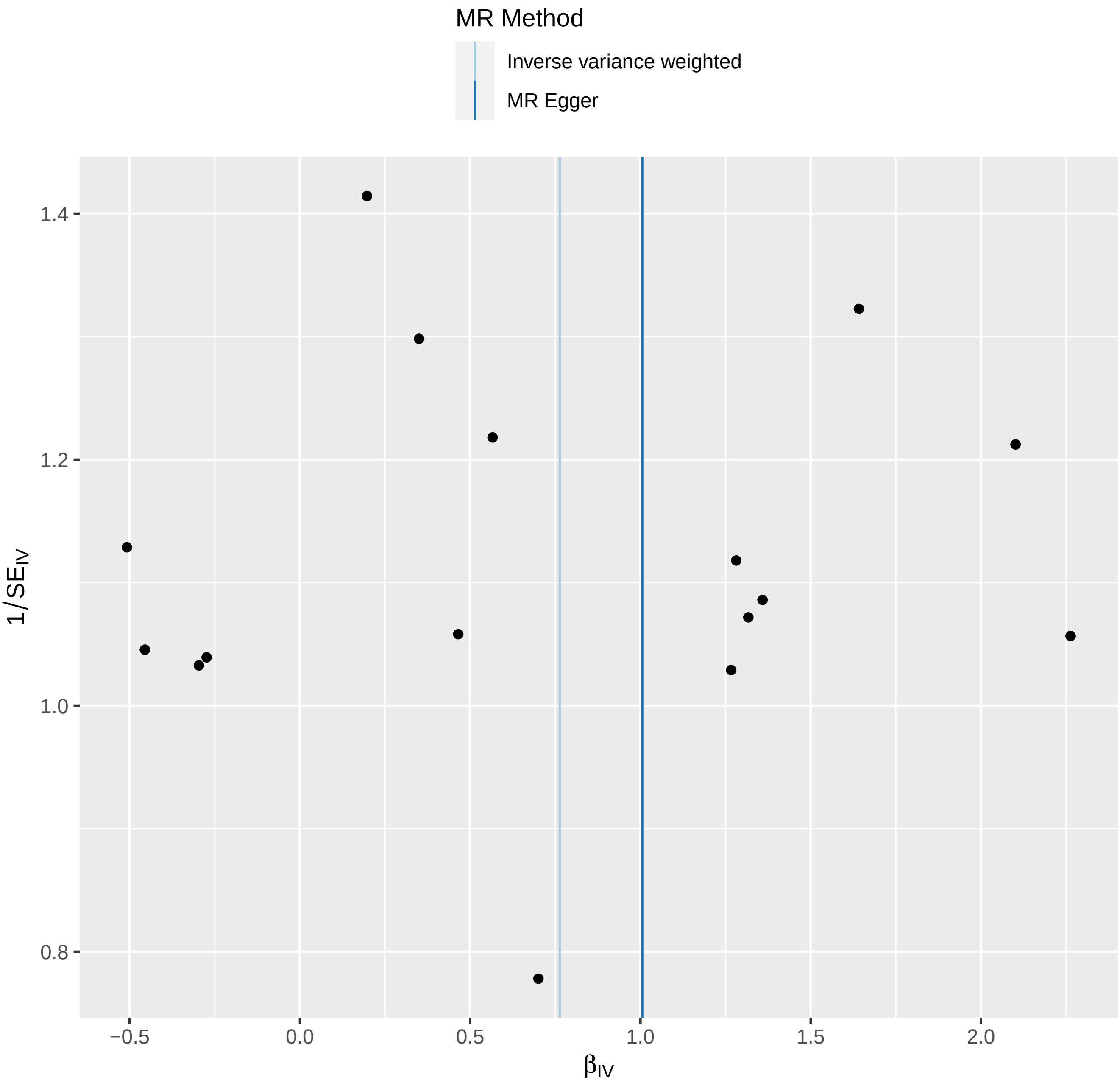

Batch 928 : Gut microbiota abundance (family Ruminococcaceae id.2050) on Ulcerative enterocolitis

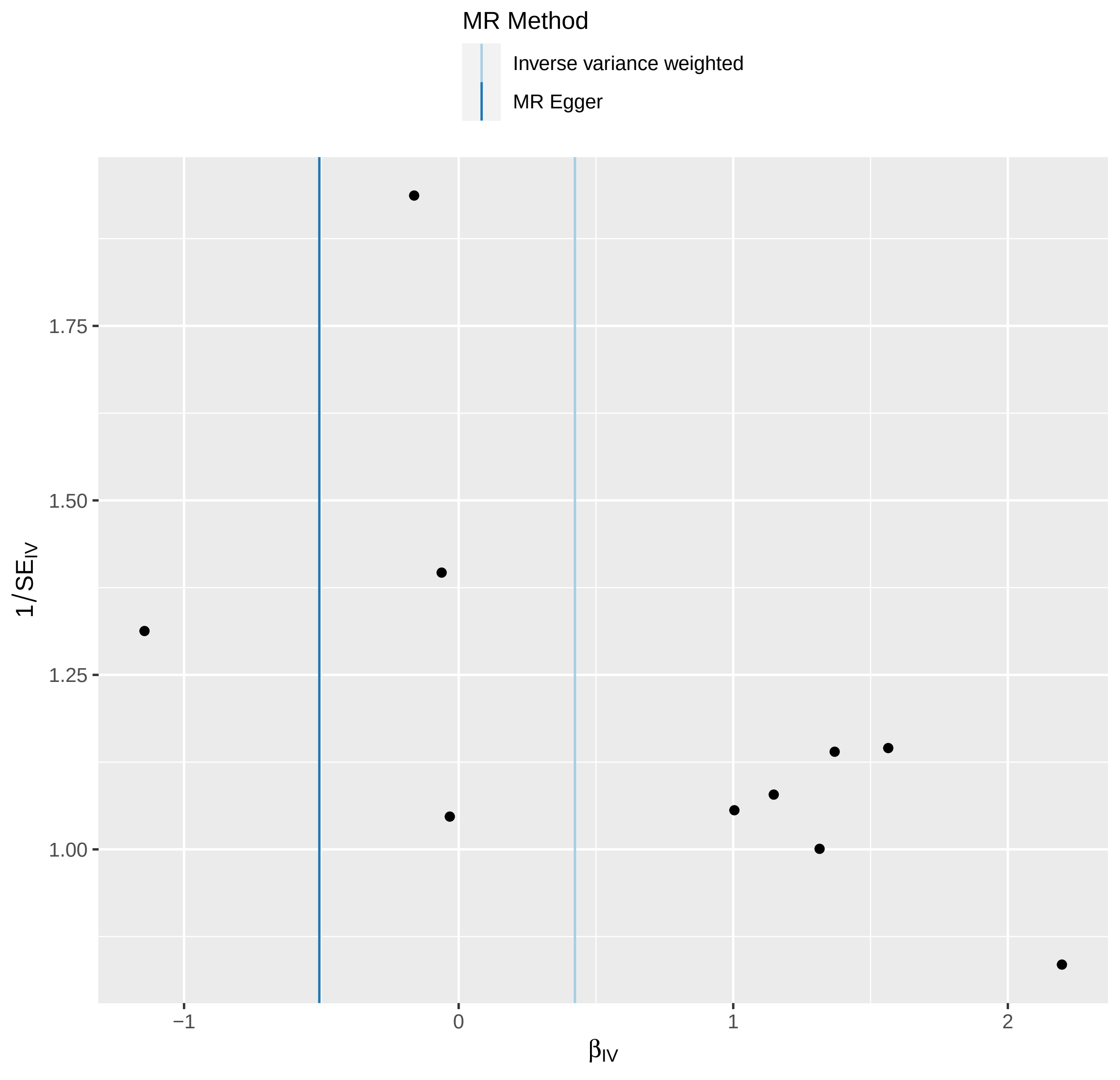

Batch 929 : Gut microbiota abundance (family Streptococcaceae id.1850) on Ulcerative enterocolitis

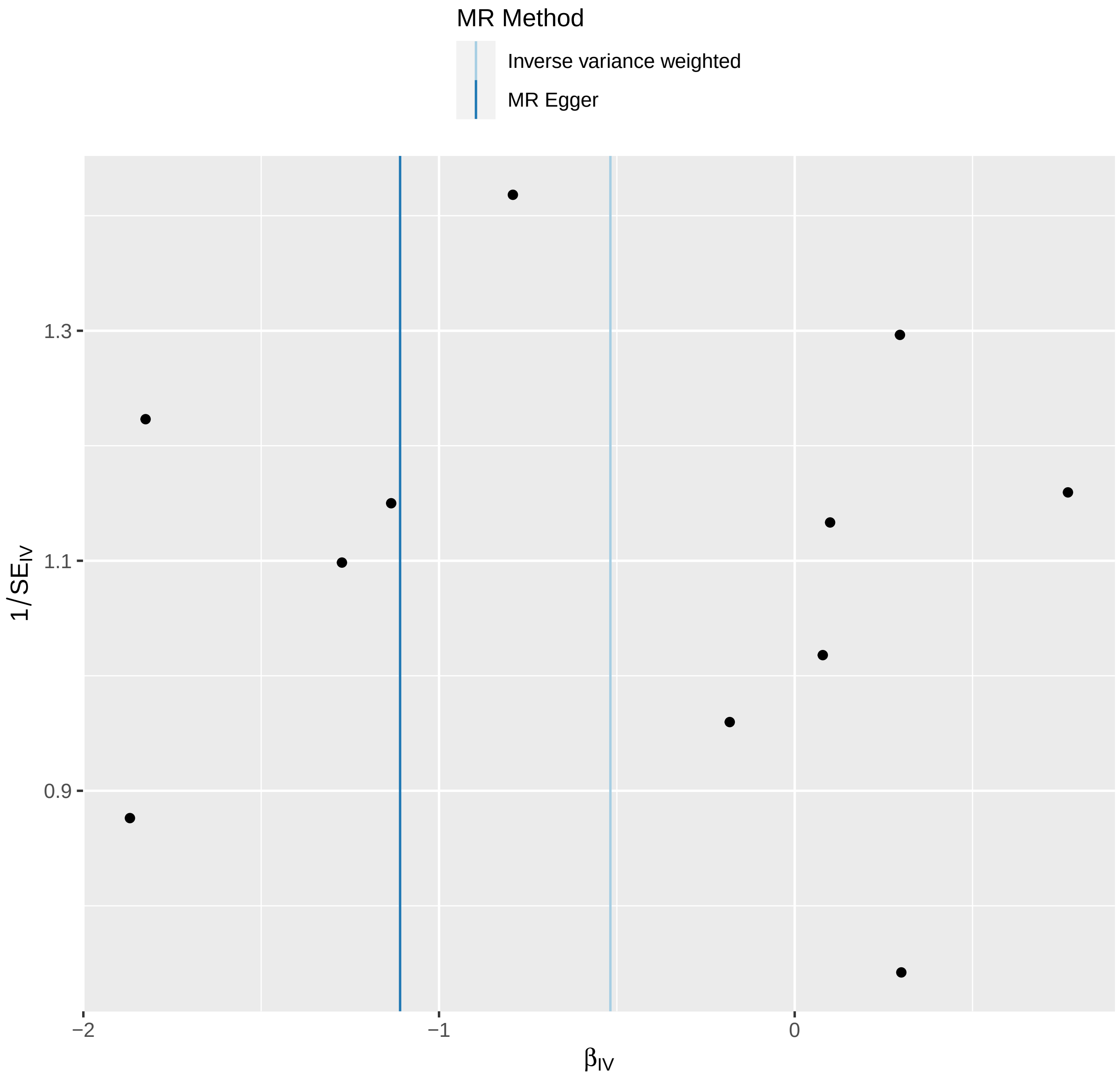

Batch 930 : Gut microbiota abundance (family Veillonellaceae id.2172) on Ulcerative enterocolitis

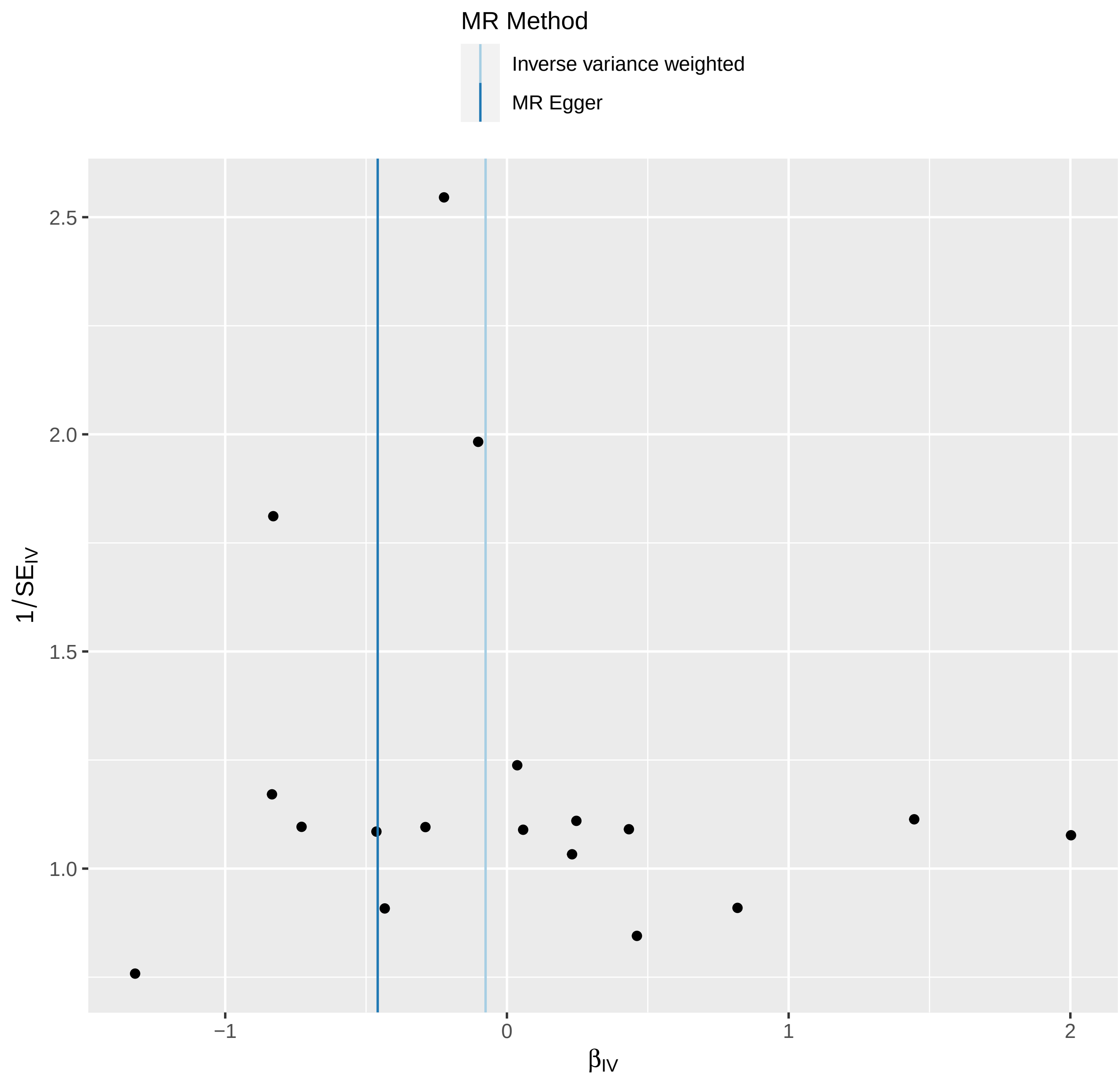

Batch 931 : Gut microbiota abundance (family Verrucomicrobiaceae id.4036) on Ulcerative enterocolitis

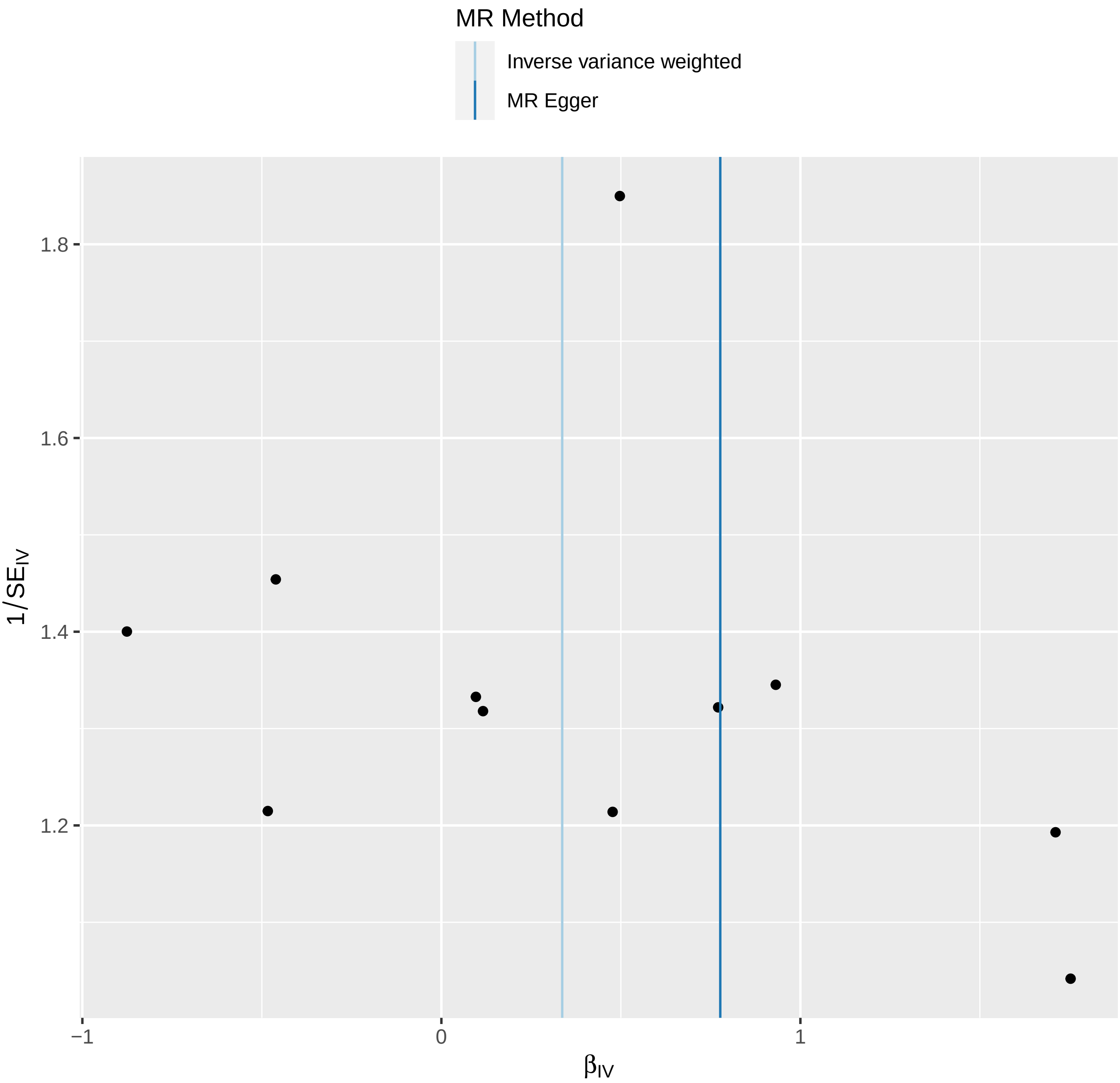

Batch 932 : Gut microbiota abundance (family Victivallaceae id.2255) on Ulcerative enterocolitis

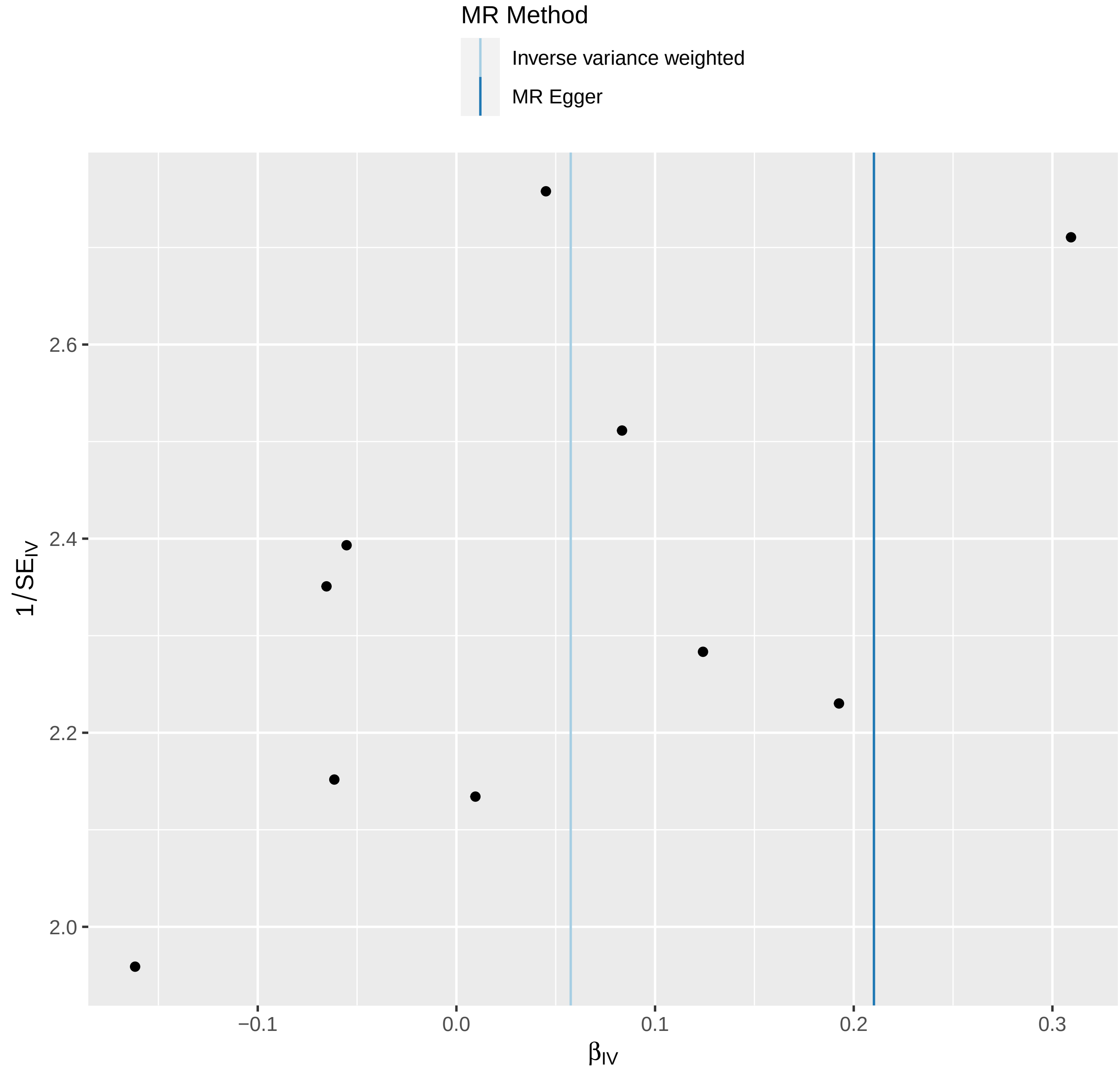

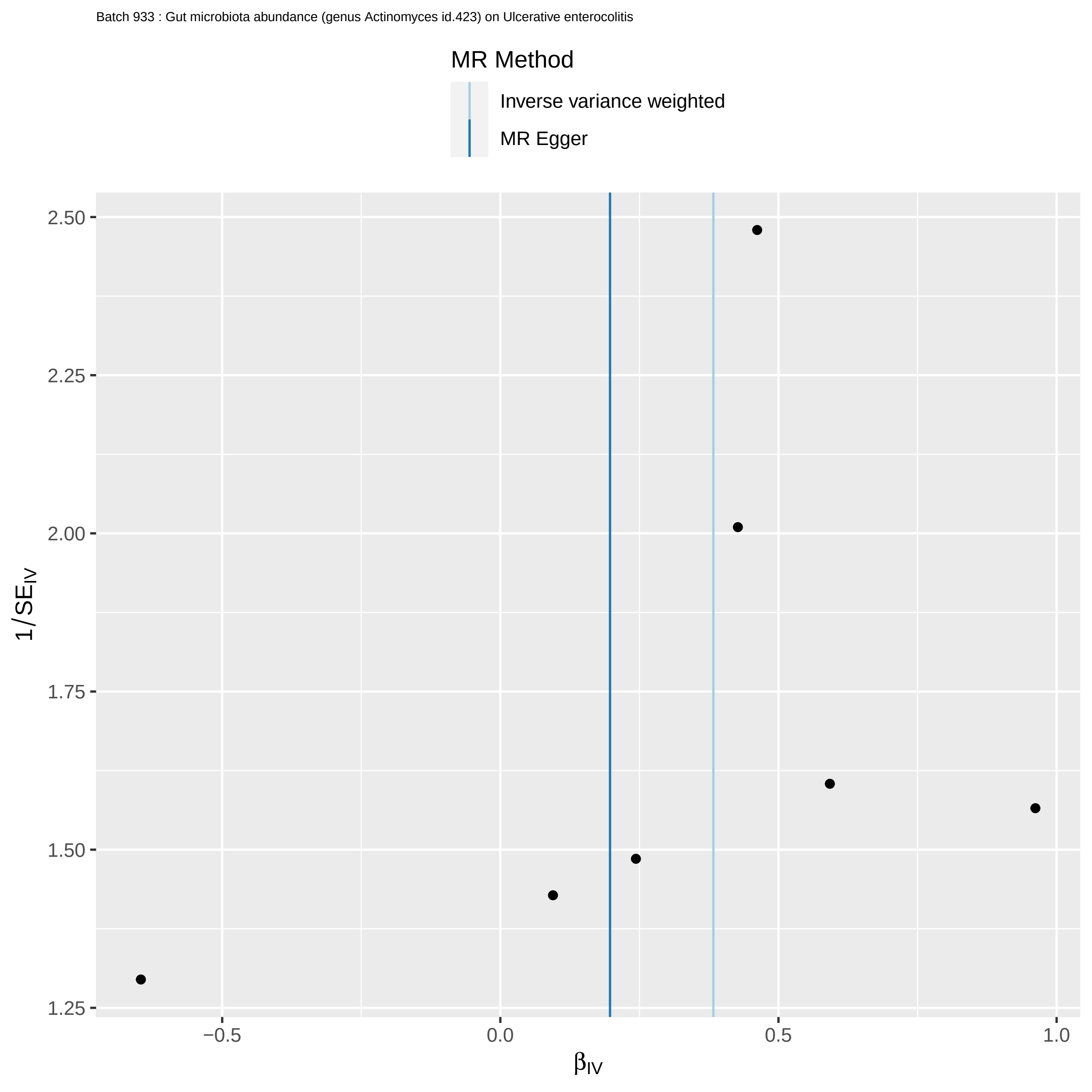

Batch 933 : Gut microbiota abundance (genus Actinomyces id.423) on Ulcerative enterocolitis
MR Method
Inverse variance weighted
MR Egger
2.50
2.25
2.00
1.75
1.50
1.25
1/SE_IV
−0.5
0.0
0.5
1.0
β_IV

Batch 934 : Gut microbiota abundance (genus Adlercreutzia id.812) on Ulcerative enterocolitis

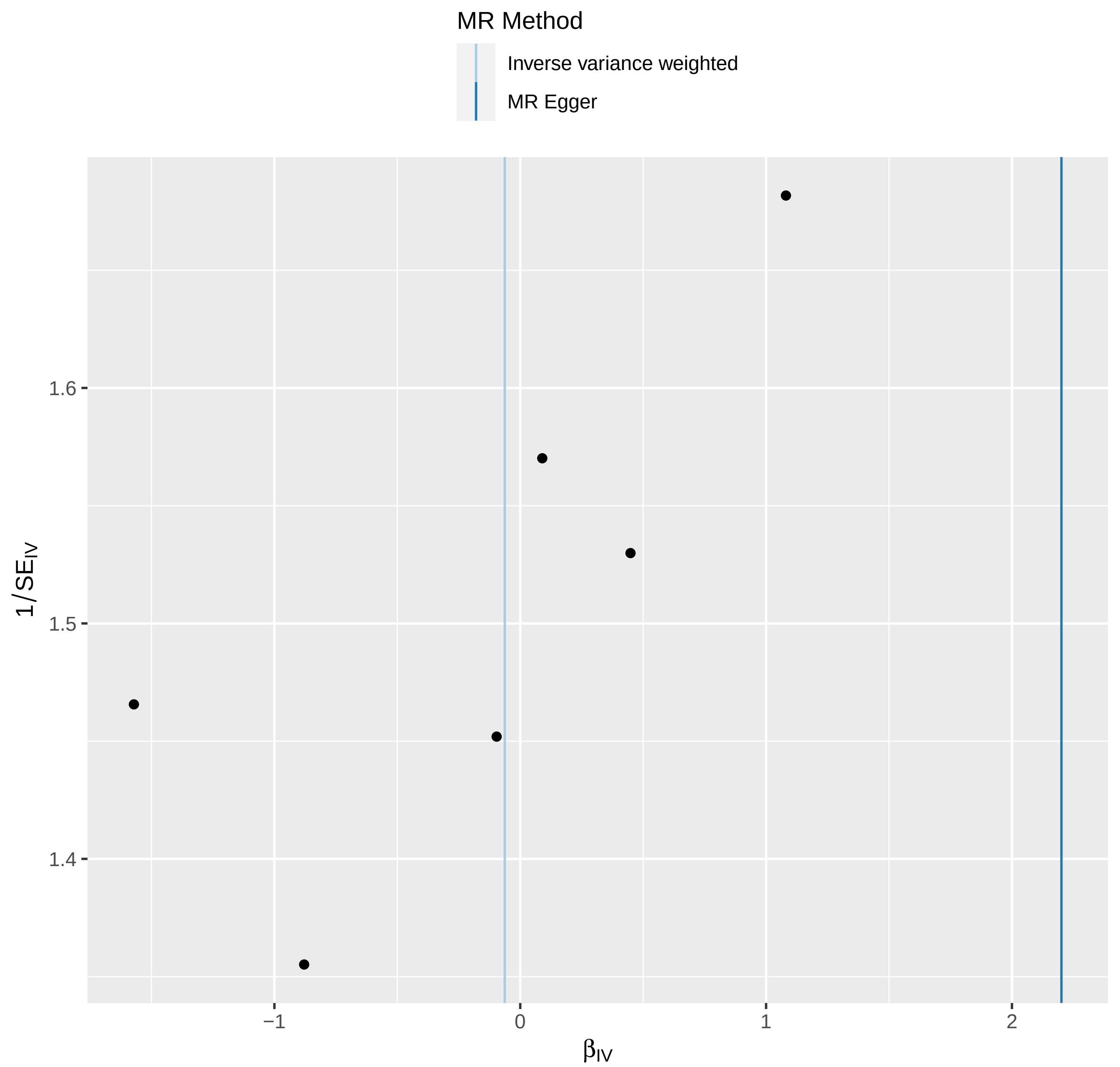

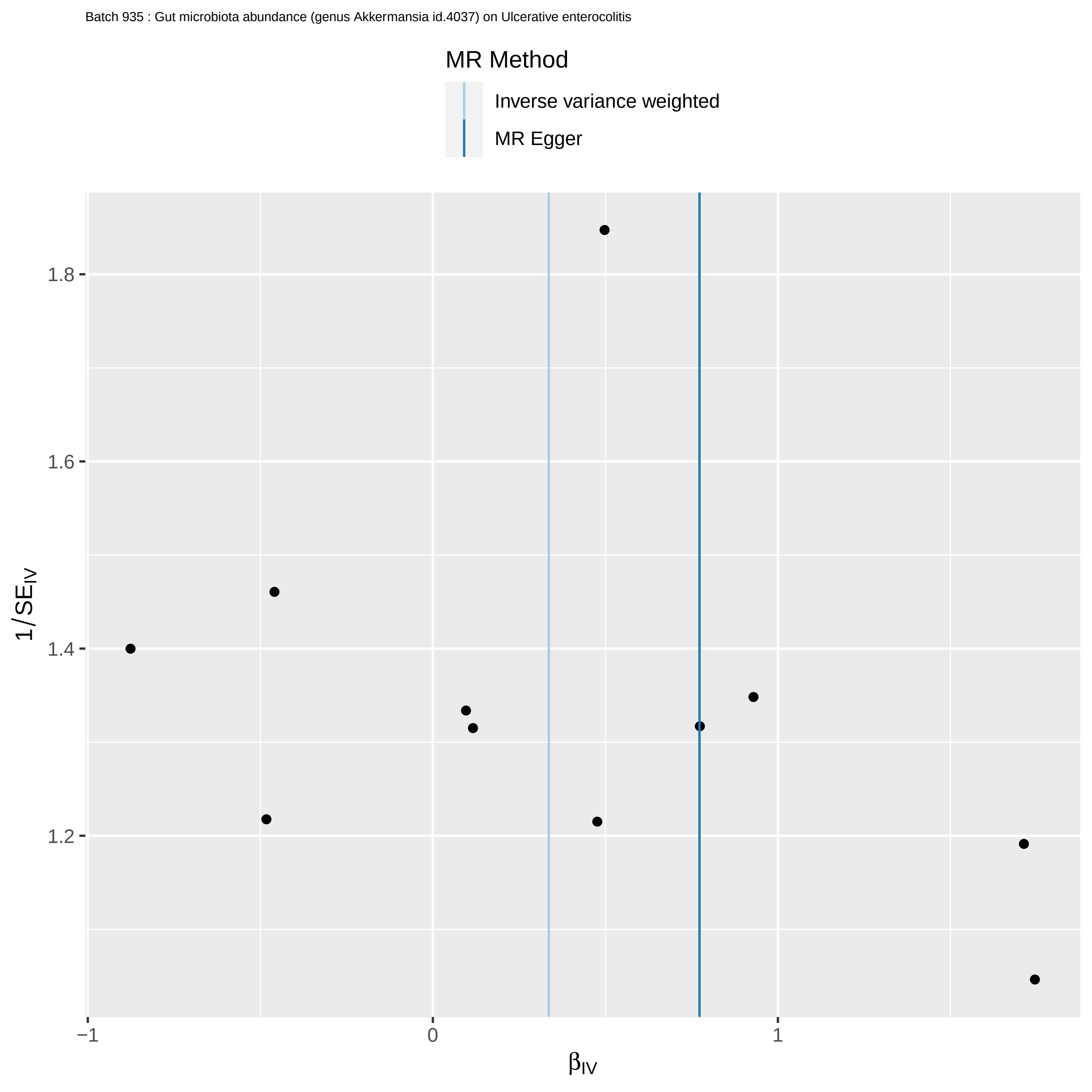
Batch 935 : Gut microbiota abundance (genus Akkermansia id.4037) on Ulcerative enterocolitis
MR Method
Inverse variance weighted
MR Egger
1.8
1.6
1.4
1.2
1/SE_IV
−1
0
1
β_IV

Batch 936 : Gut microbiota abundance (genus Alistipes id.968) on Ulcerative enterocolitis

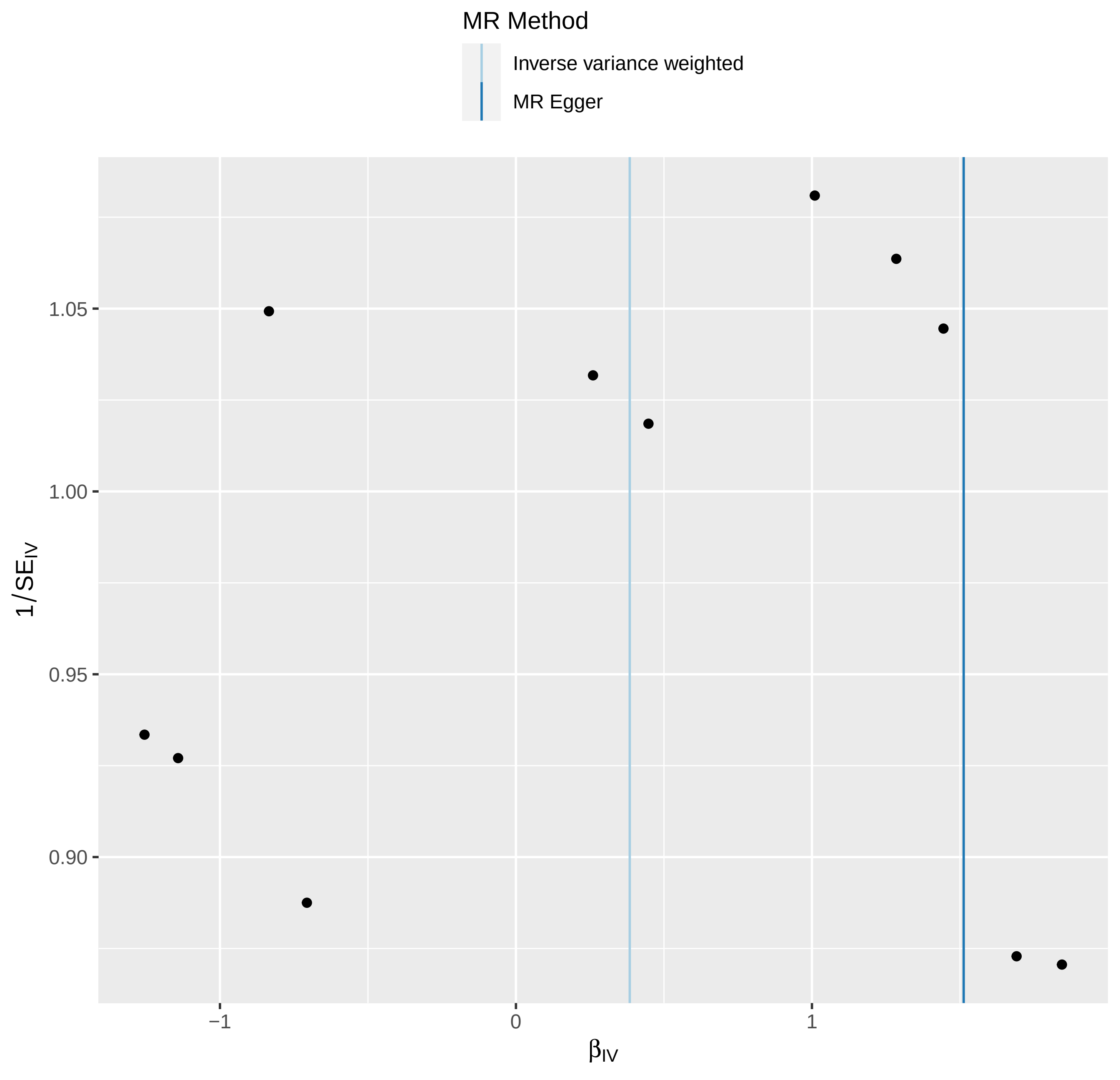

Batch 937 : Gut microbiota abundance (genus Allisonella id.2174) on Ulcerative enterocolitis

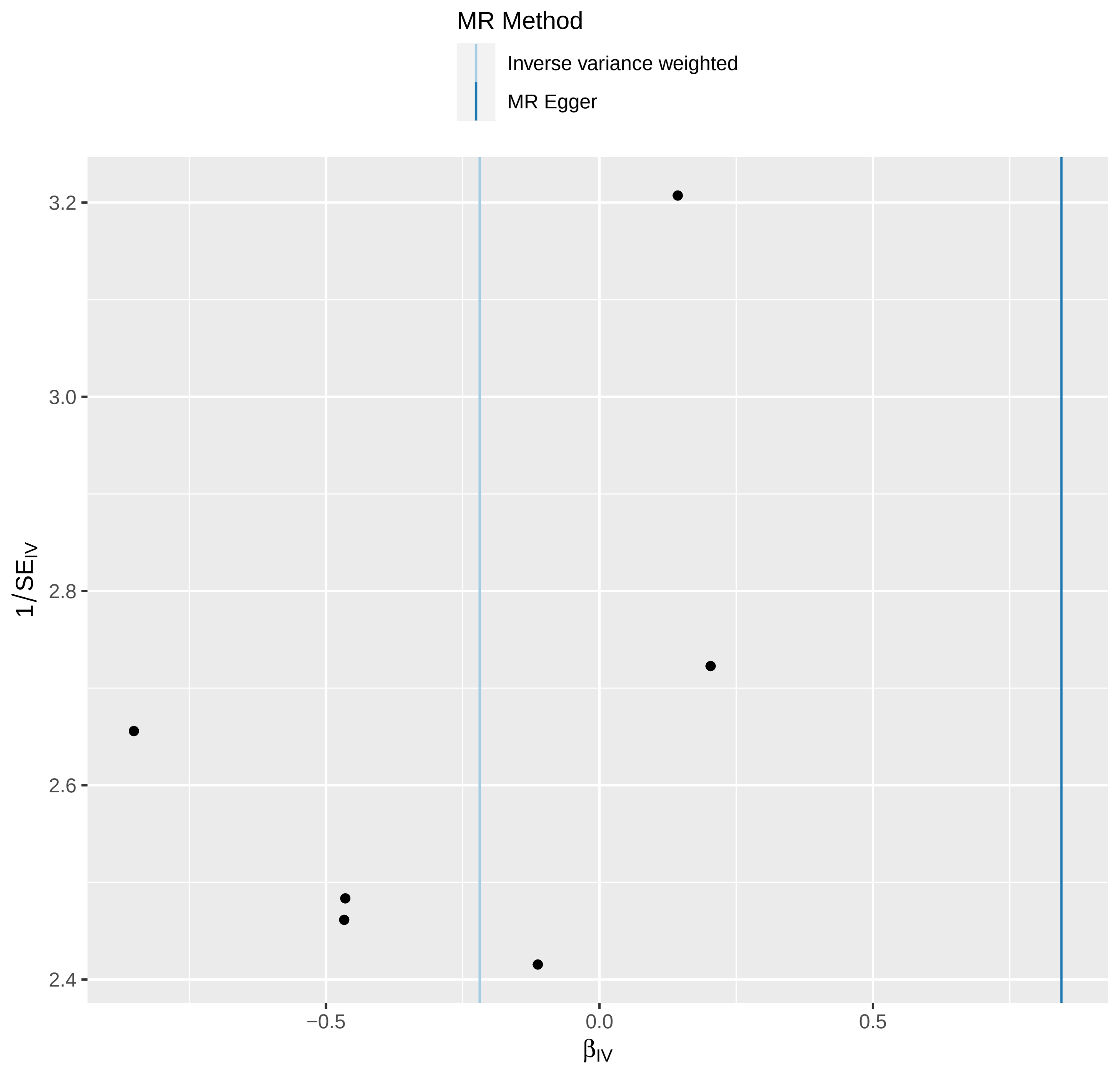

Batch 938 : Gut microbiota abundance (genus Alloprevotella id.961) on Ulcerative enterocolitis

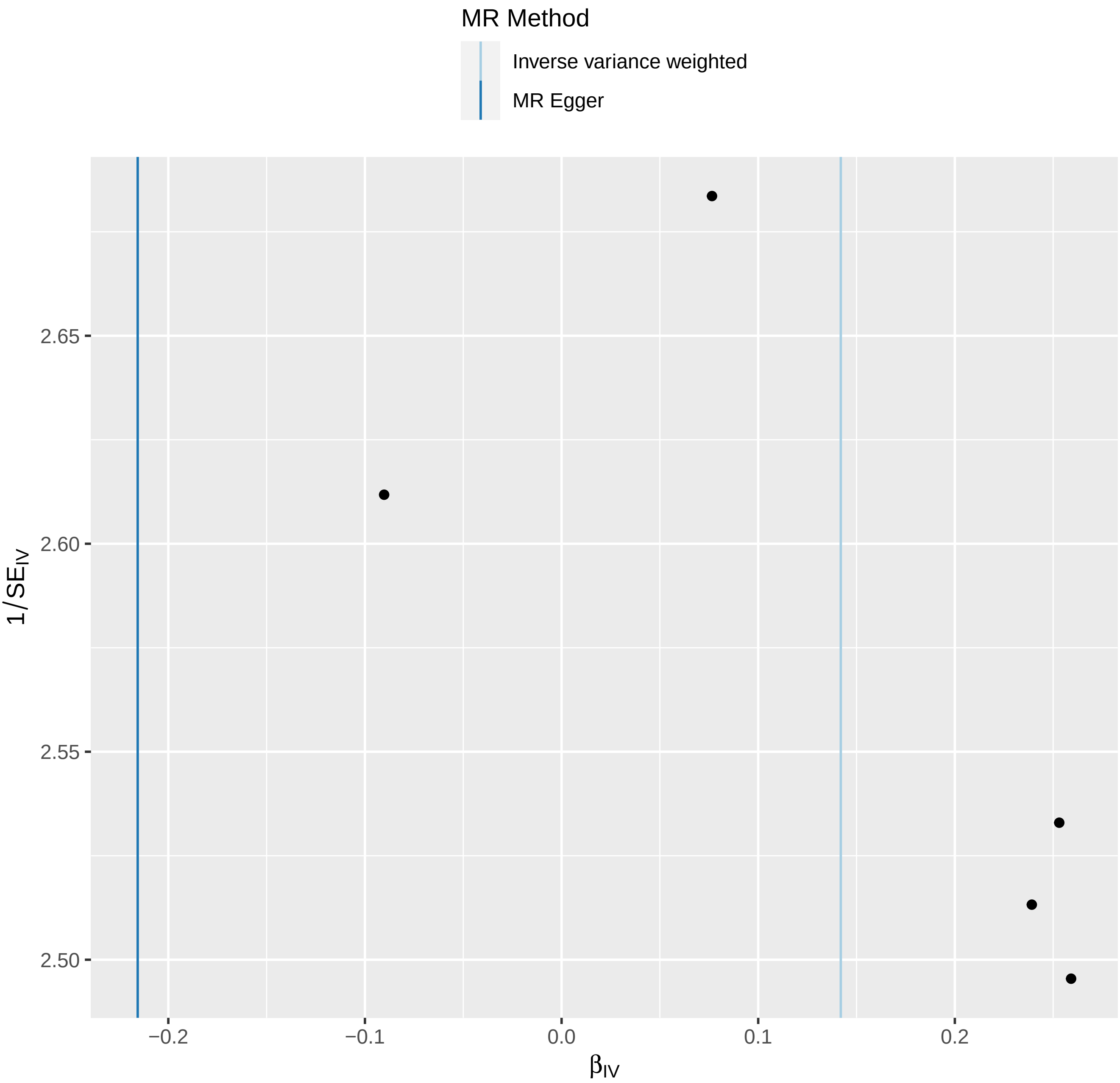

Batch 939 : Gut microbiota abundance (genus Anaerofilum id.2053) on Ulcerative enterocolitis

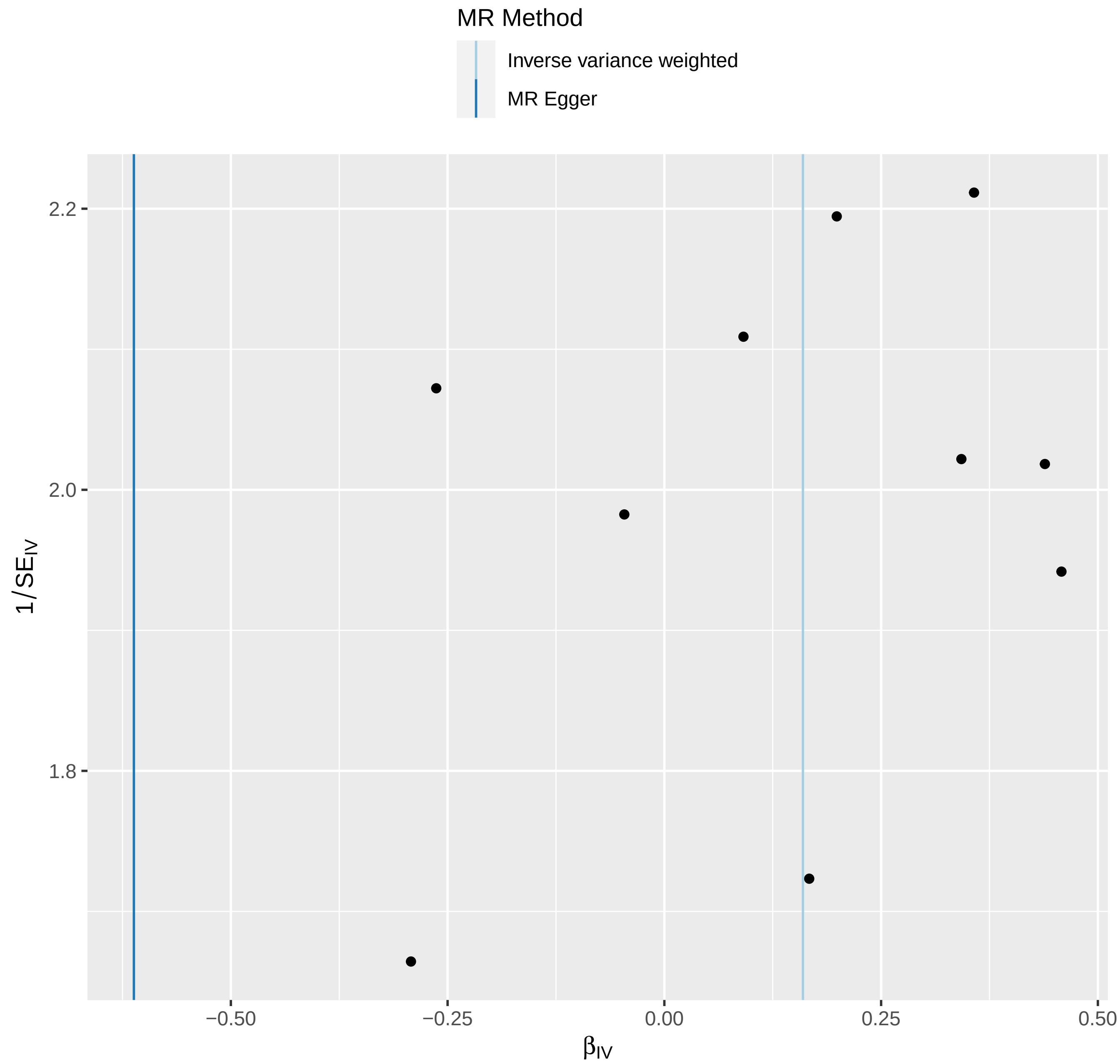

Batch 940 : Gut microbiota abundance (genus Anaerostipes id.1991) on Ulcerative enterocolitis

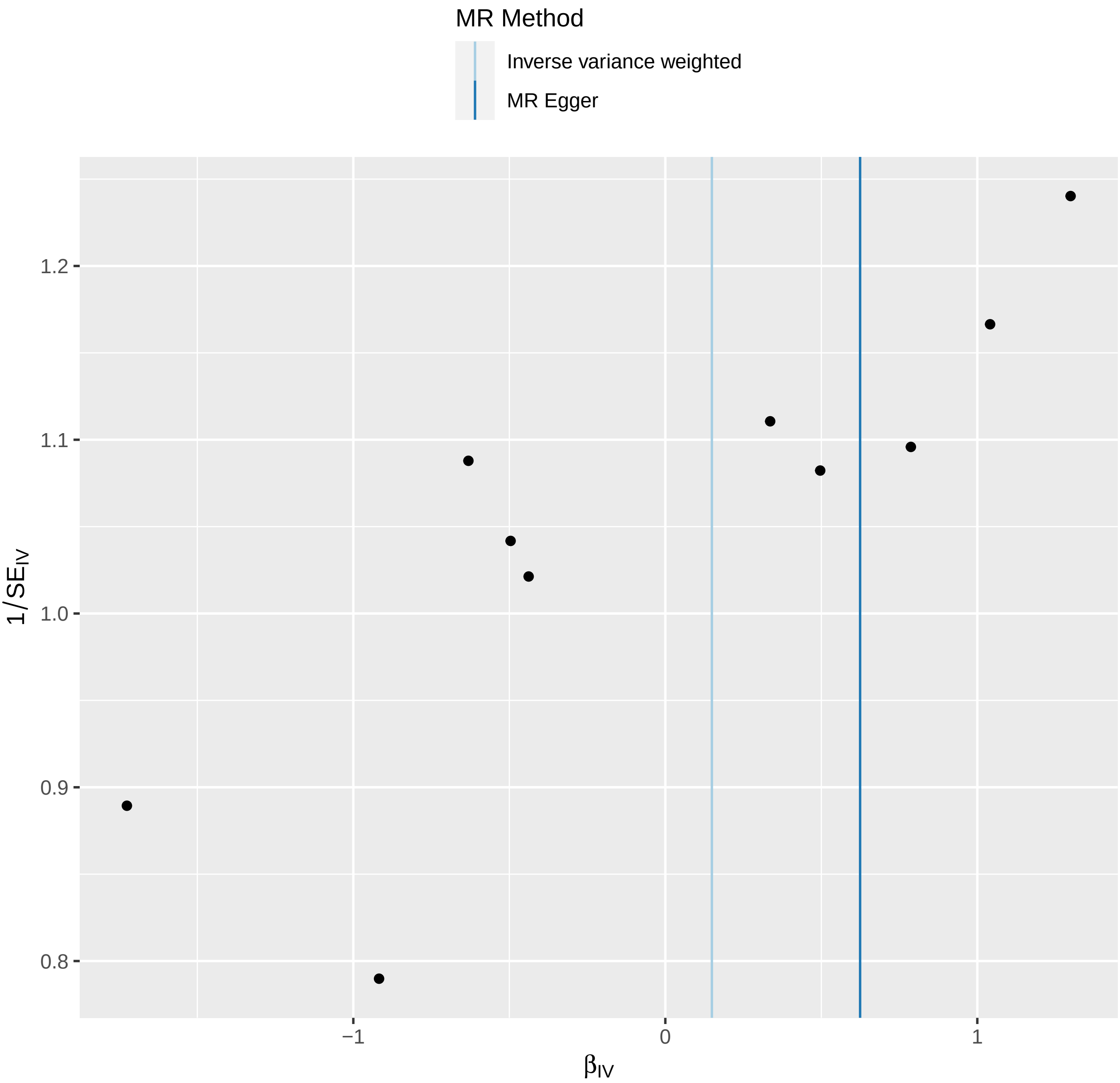

Batch 941 : Gut microbiota abundance (genus Anaerotruncus id.2054) on Ulcerative enterocolitis

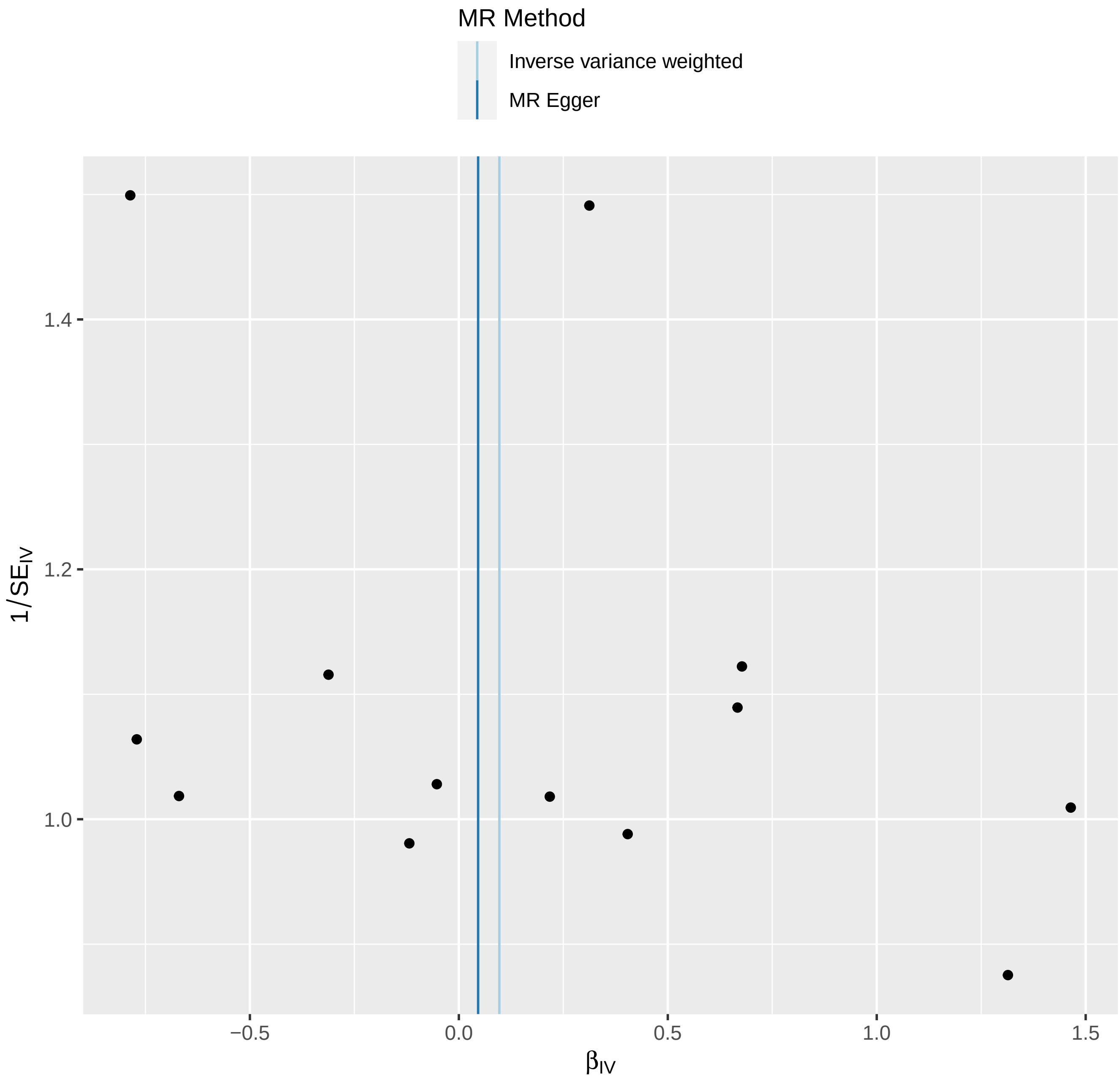

Batch 942 : Gut microbiota abundance (genus Bacteroides id.918) on Ulcerative enterocolitis

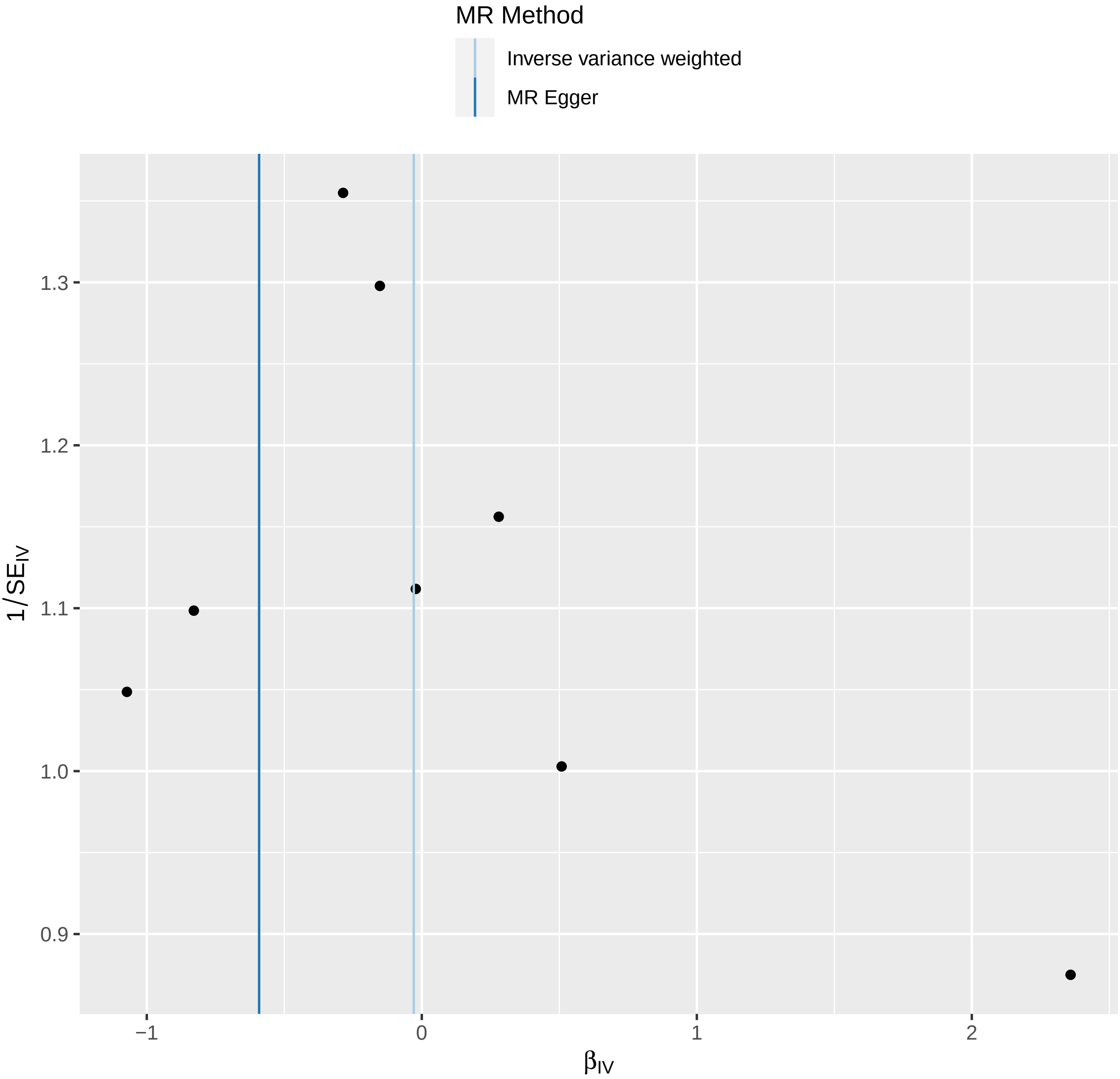

Batch 943 : Gut microbiota abundance (genus Barnesiella id.944) on Ulcerative enterocolitis

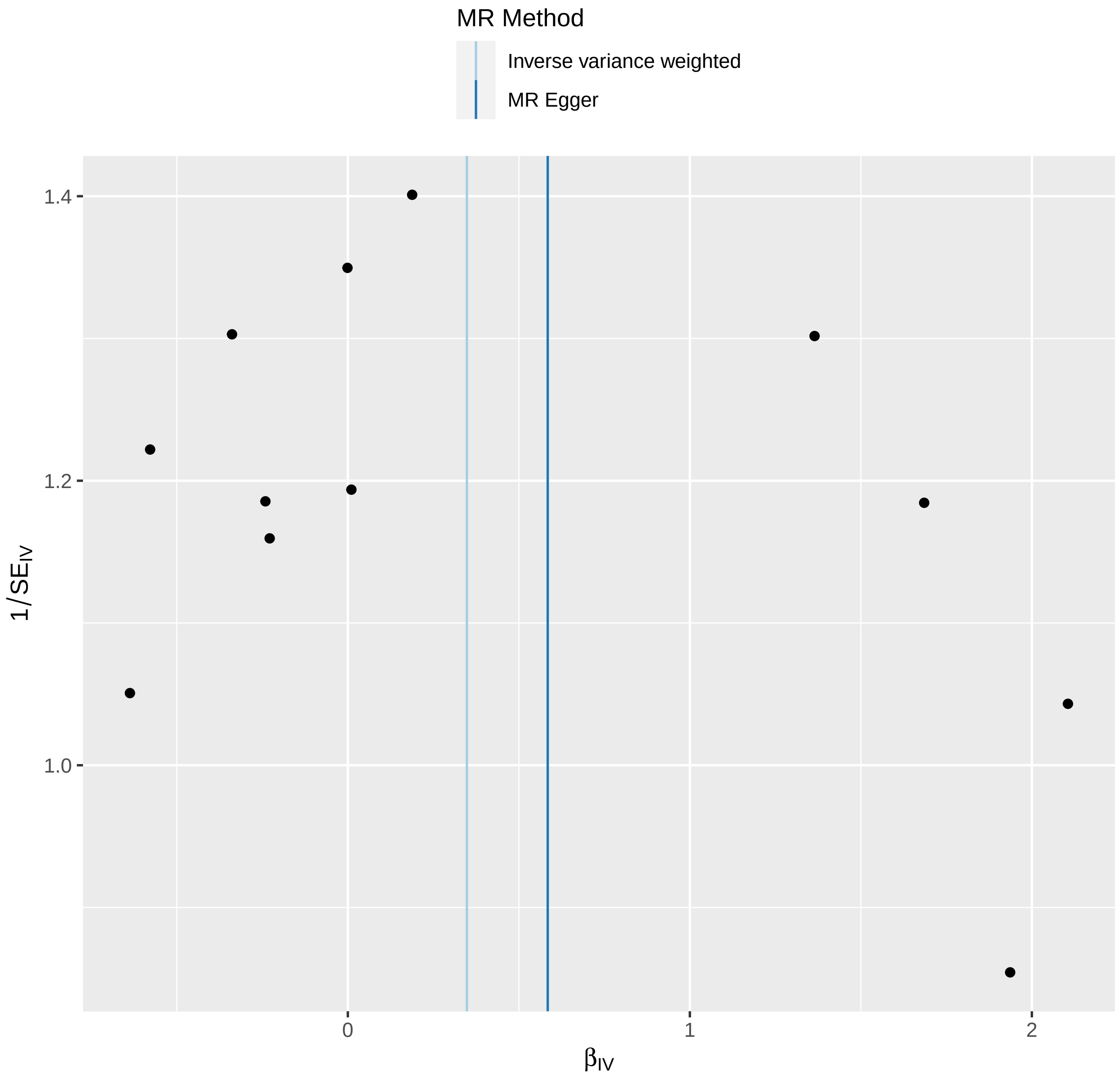

Batch 944 : Gut microbiota abundance (genus Bifidobacterium id.436) on Ulcerative enterocolitis

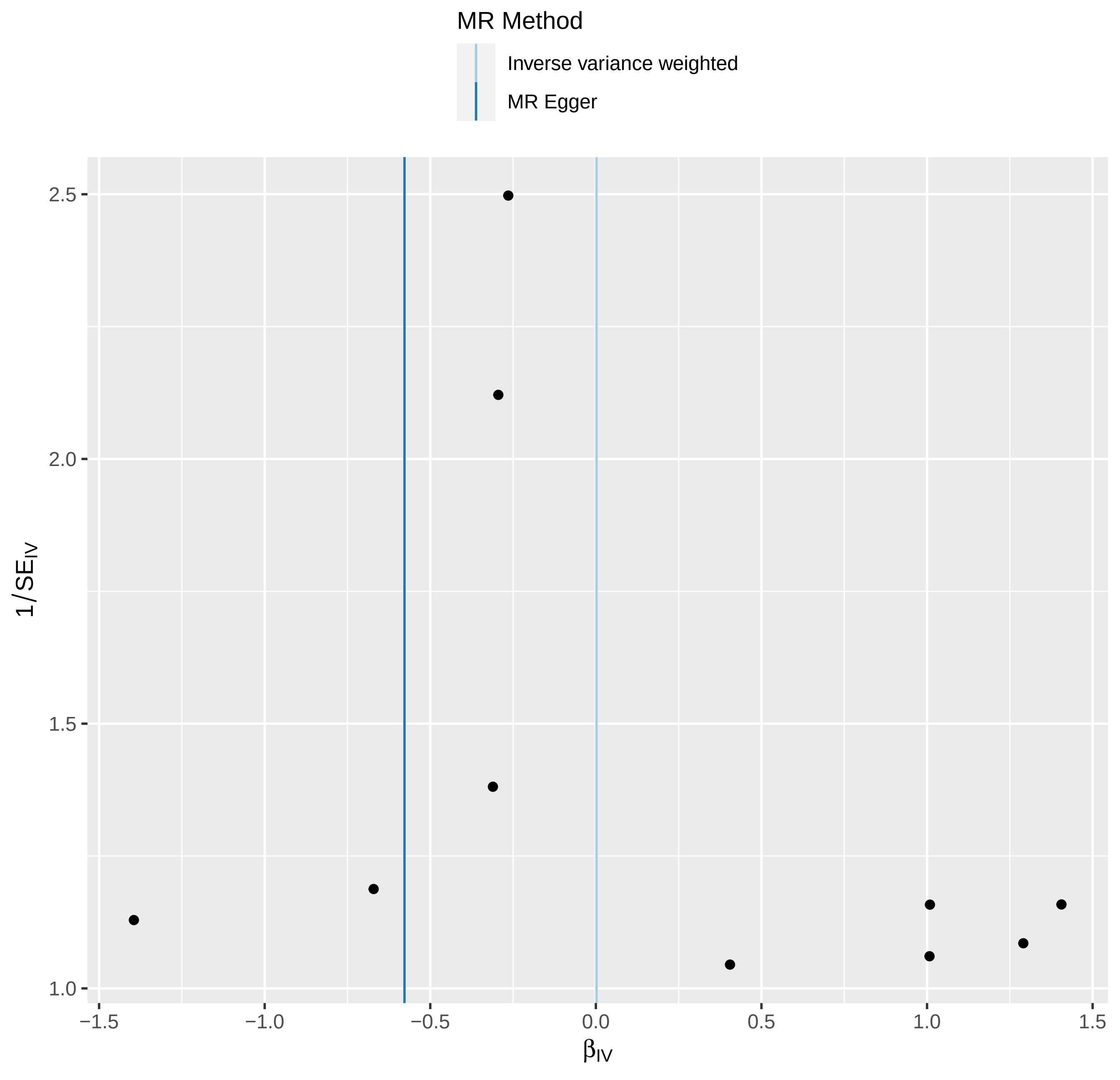

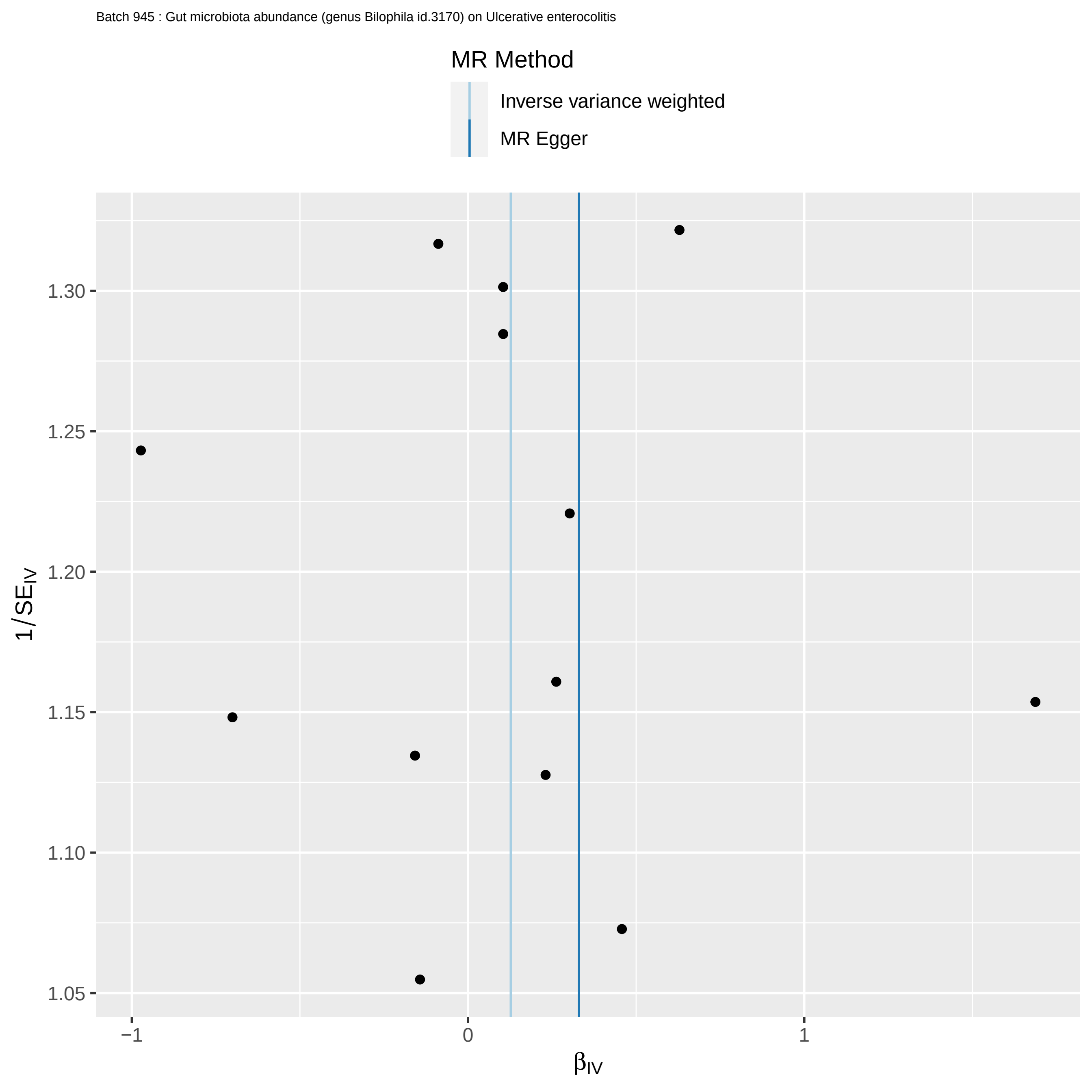
Batch 945 : Gut microbiota abundance (genus Bilophila id.3170) on Ulcerative enterocolitis
MR Method
Inverse variance weighted
MR Egger
1.30
1.25
1.20
1.15
1.10
1.05
1/SE_IV
−1
0
1
β_IV

Batch 946 : Gut microbiota abundance (genus Blautia id.1992) on Ulcerative enterocolitis

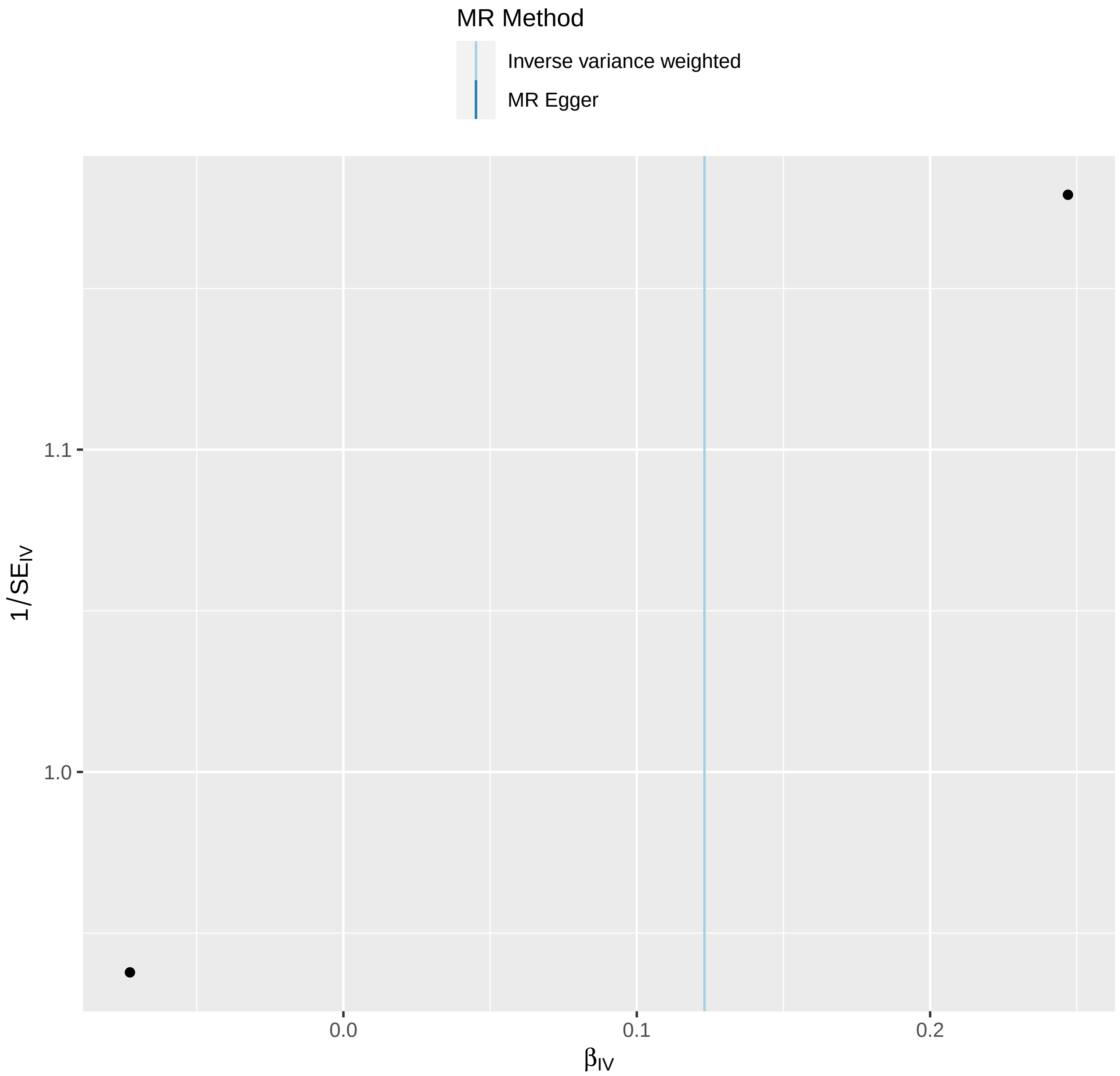

Batch 947 : Gut microbiota abundance (genus Butyricicoccus id.2055) on Ulcerative enterocolitis

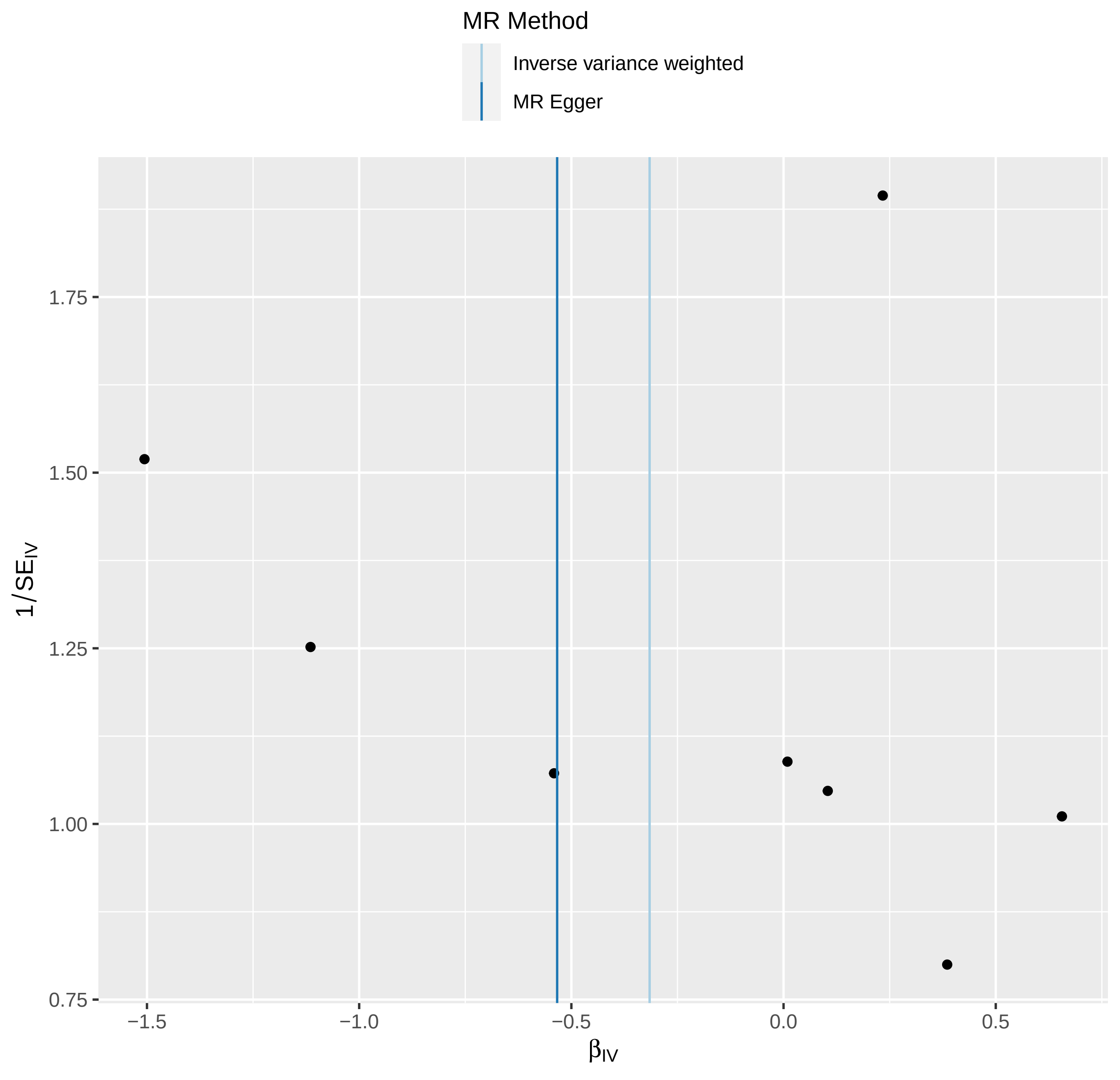

Batch 948 : Gut microbiota abundance (genus Butyricimonas id.945) on Ulcerative enterocolitis

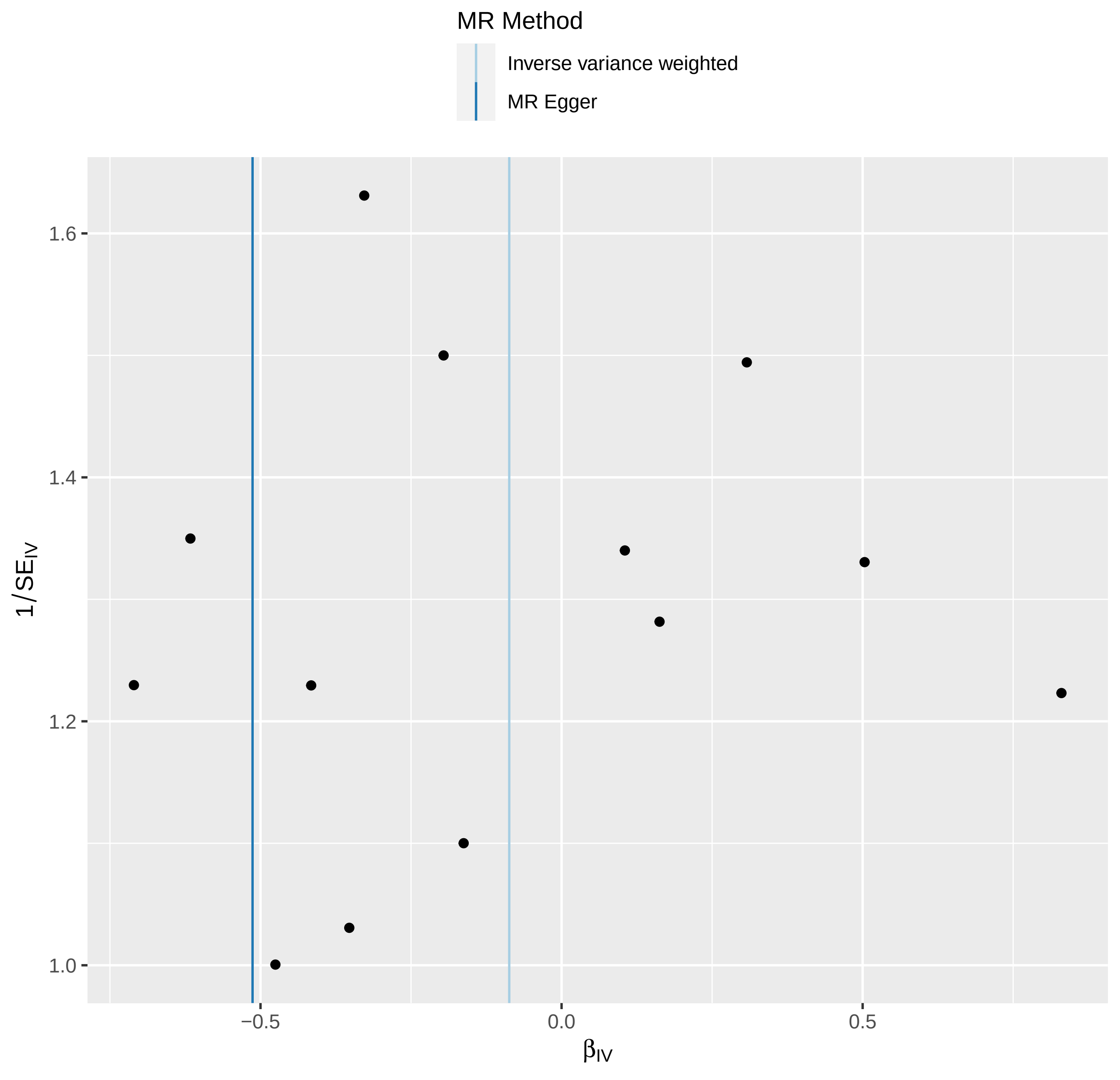

Batch 949 : Gut microbiota abundance (genus Butyrivibrio id.1993) on Ulcerative enterocolitis

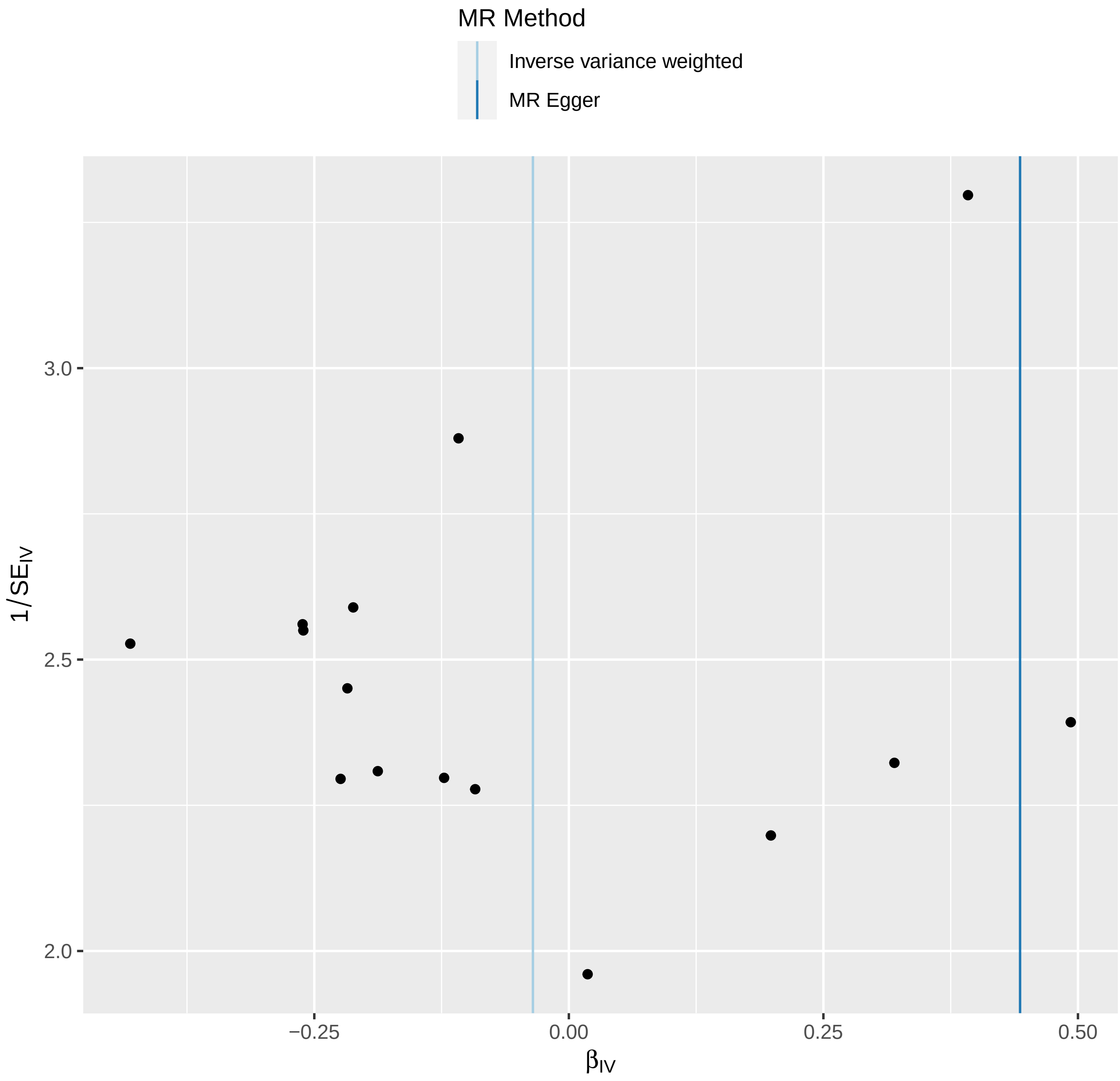

Batch 950 : Gut microbiota abundance (genus Candidatus Soleaferrea id.11350) on Ulcerative enterocolitis

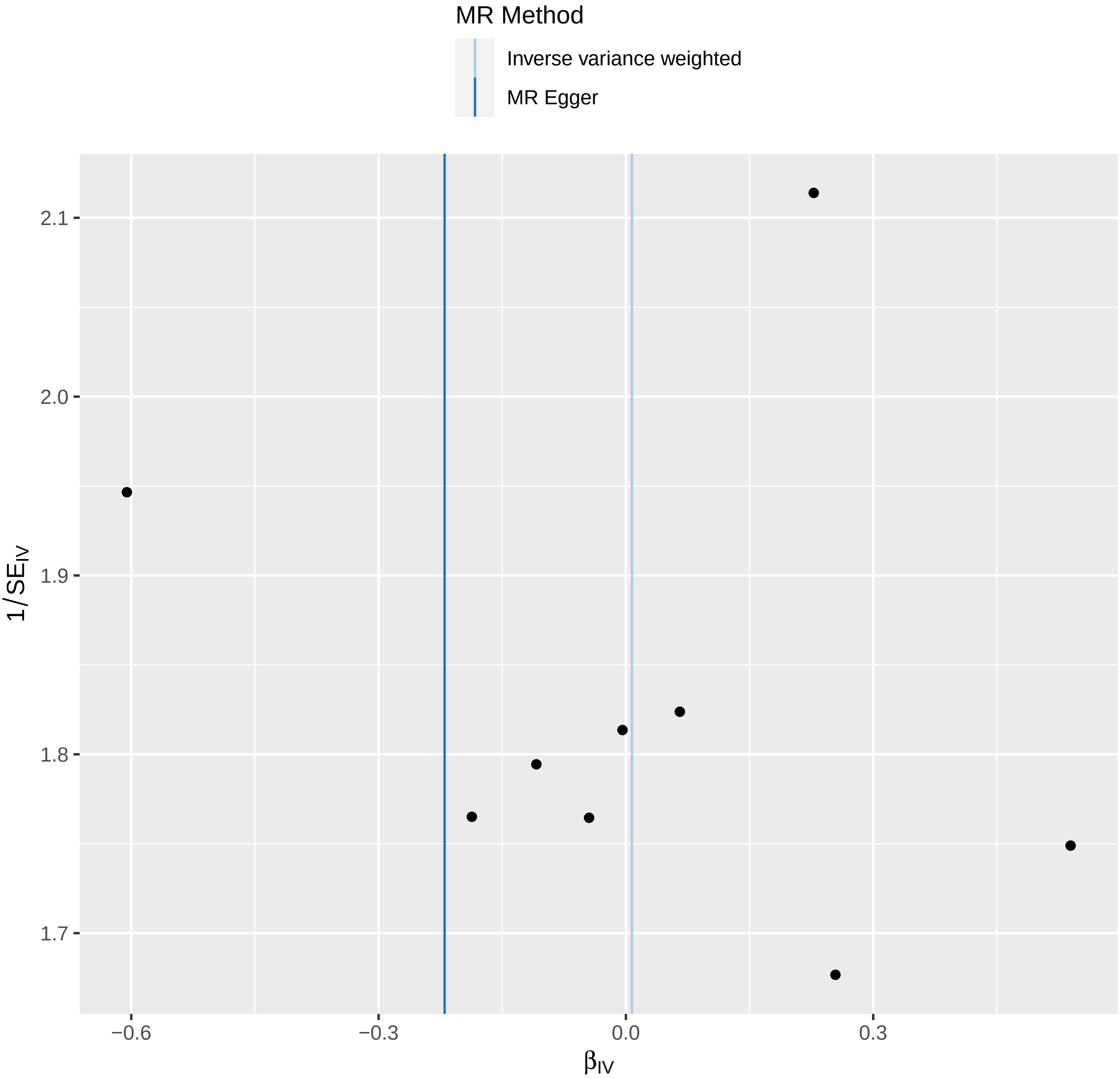

Batch 951 : Gut microbiota abundance (genus Catenibacterium id.2153) on Ulcerative enterocolitis

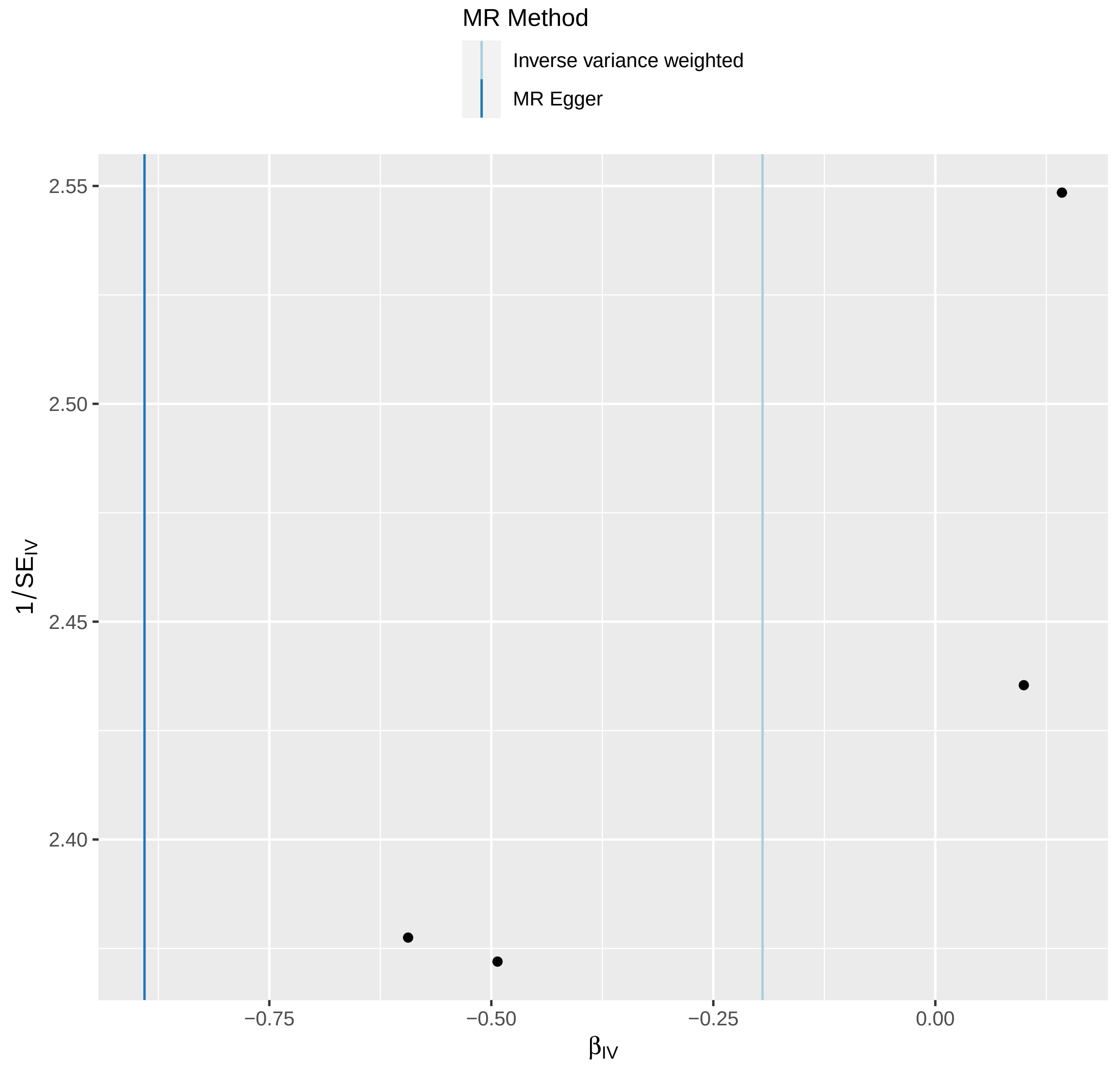

Batch 952 : Gut microbiota abundance (genus Christensenellaceae R 7group id.11283) on Ulcerative enterocolitis

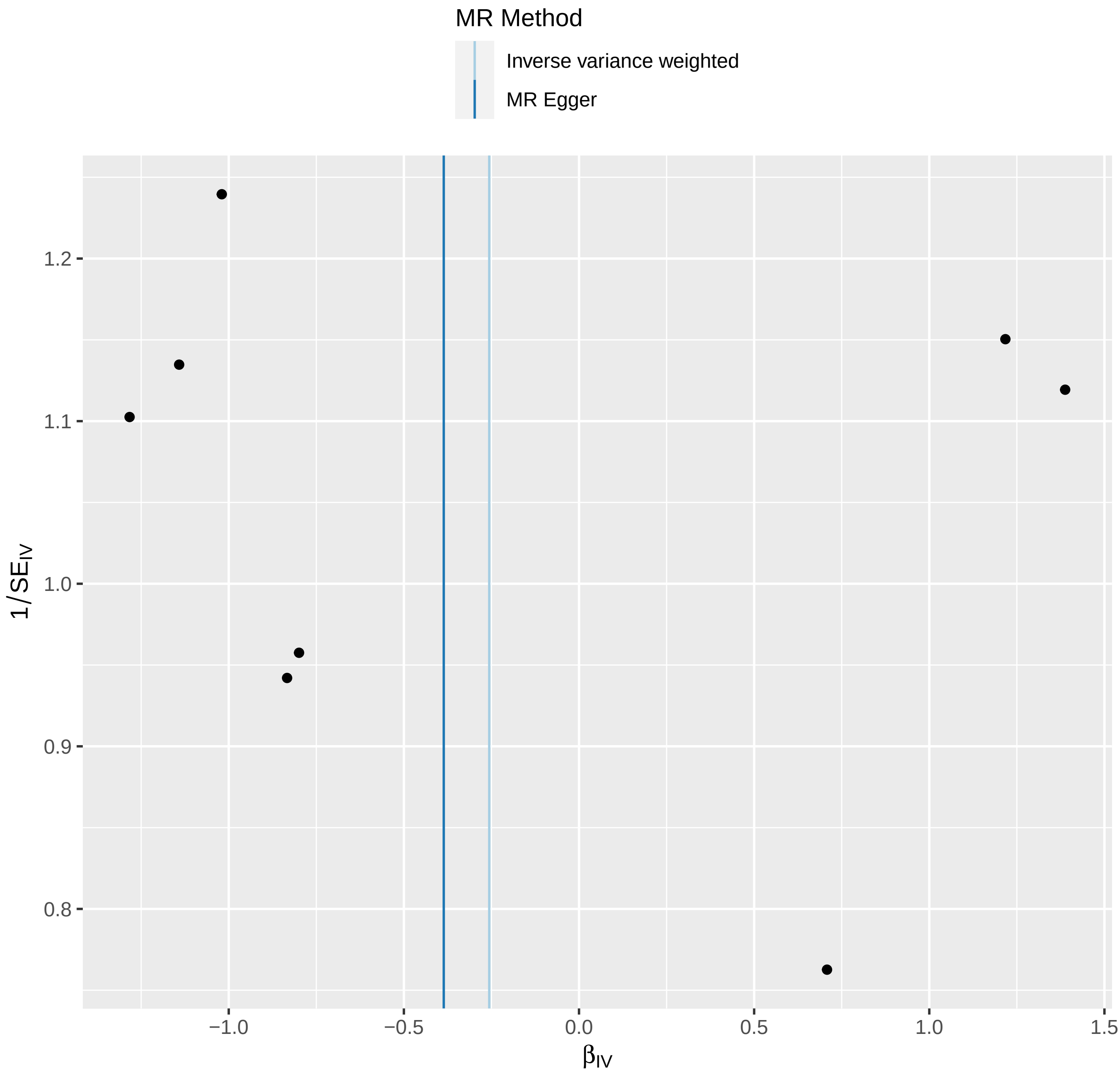

Batch 953 : Gut microbiota abundance (genus Clostridium innocuum group id.14397) on Ulcerative enterocolitis

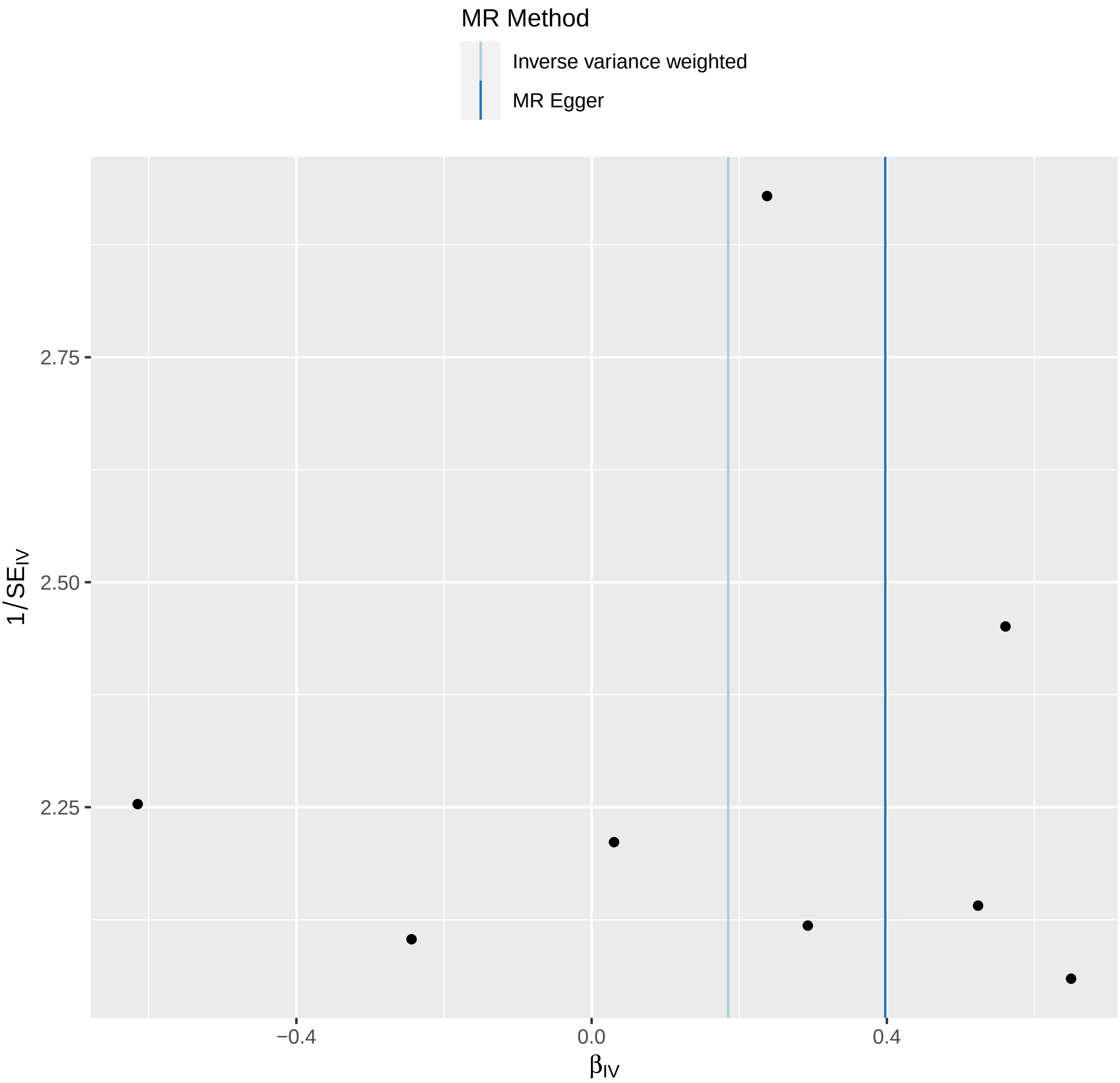

Batch 954 : Gut microbiota abundance (genus Clostridium sensustricto1 id.1873) on Ulcerative enterocolitis

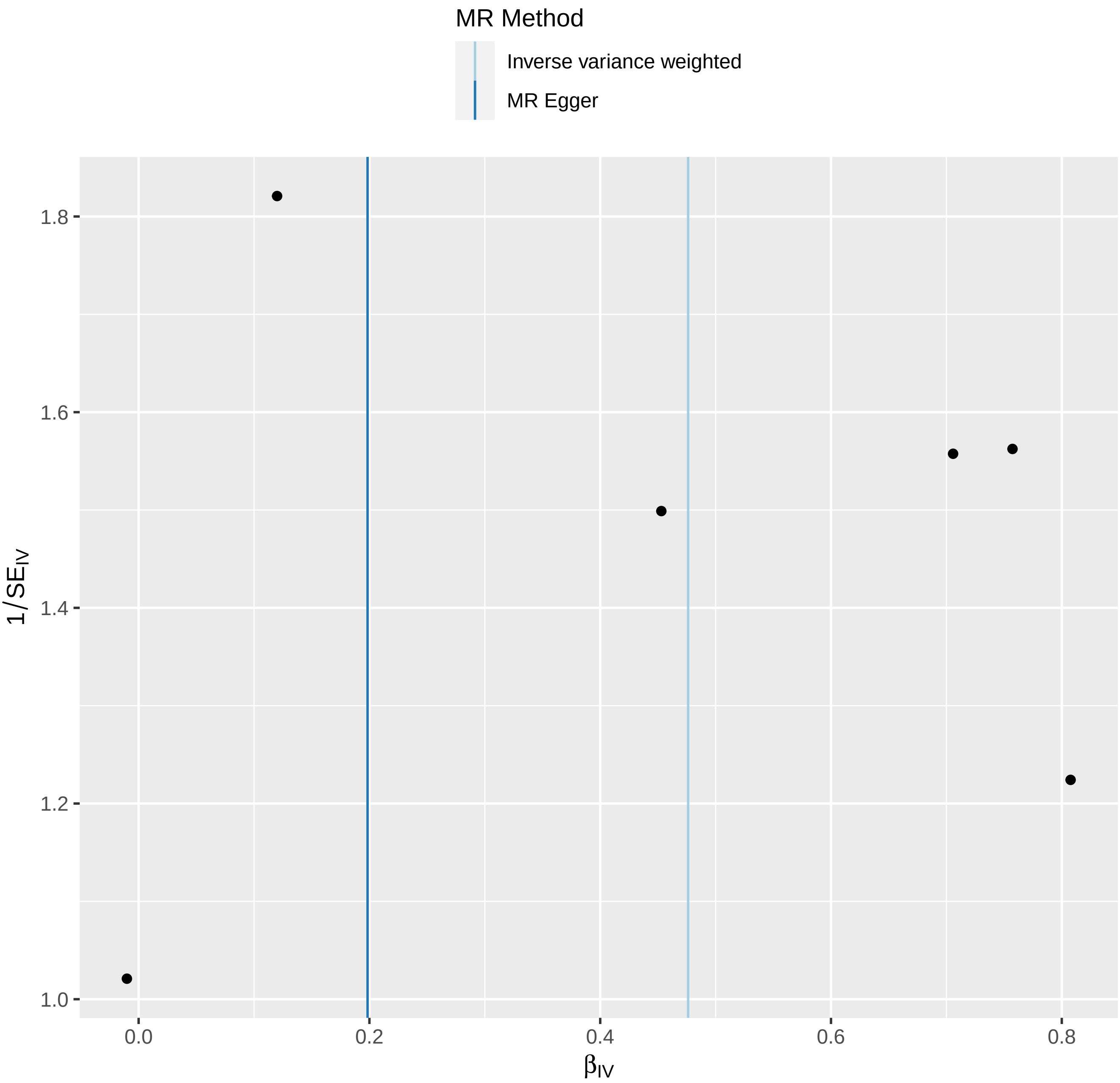

Batch 955 : Gut microbiota abundance (genus Collinsella id.815) on Ulcerative enterocolitis

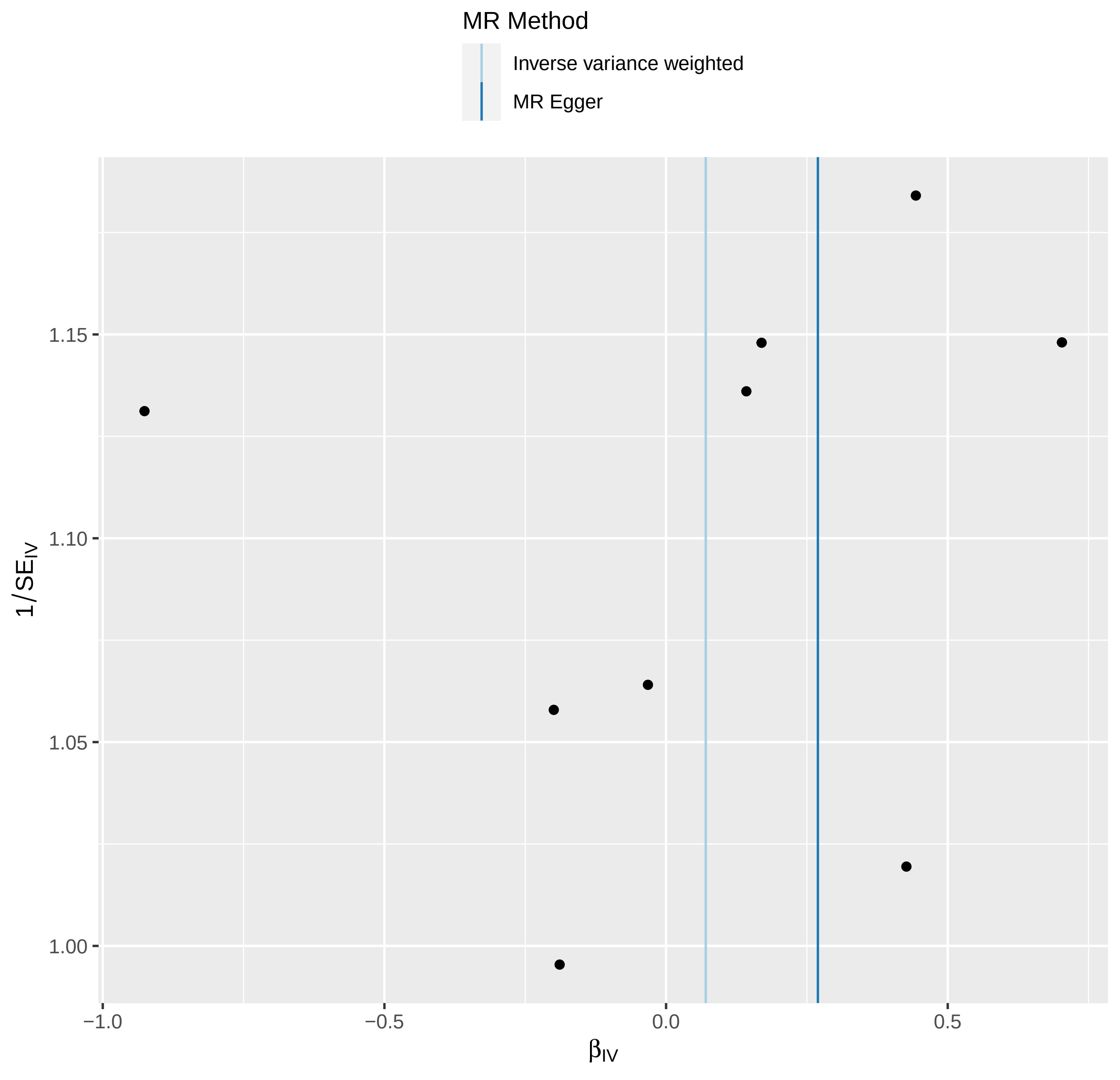

Batch 956 : Gut microbiota abundance (genus Coprobacter id.949) on Ulcerative enterocolitis

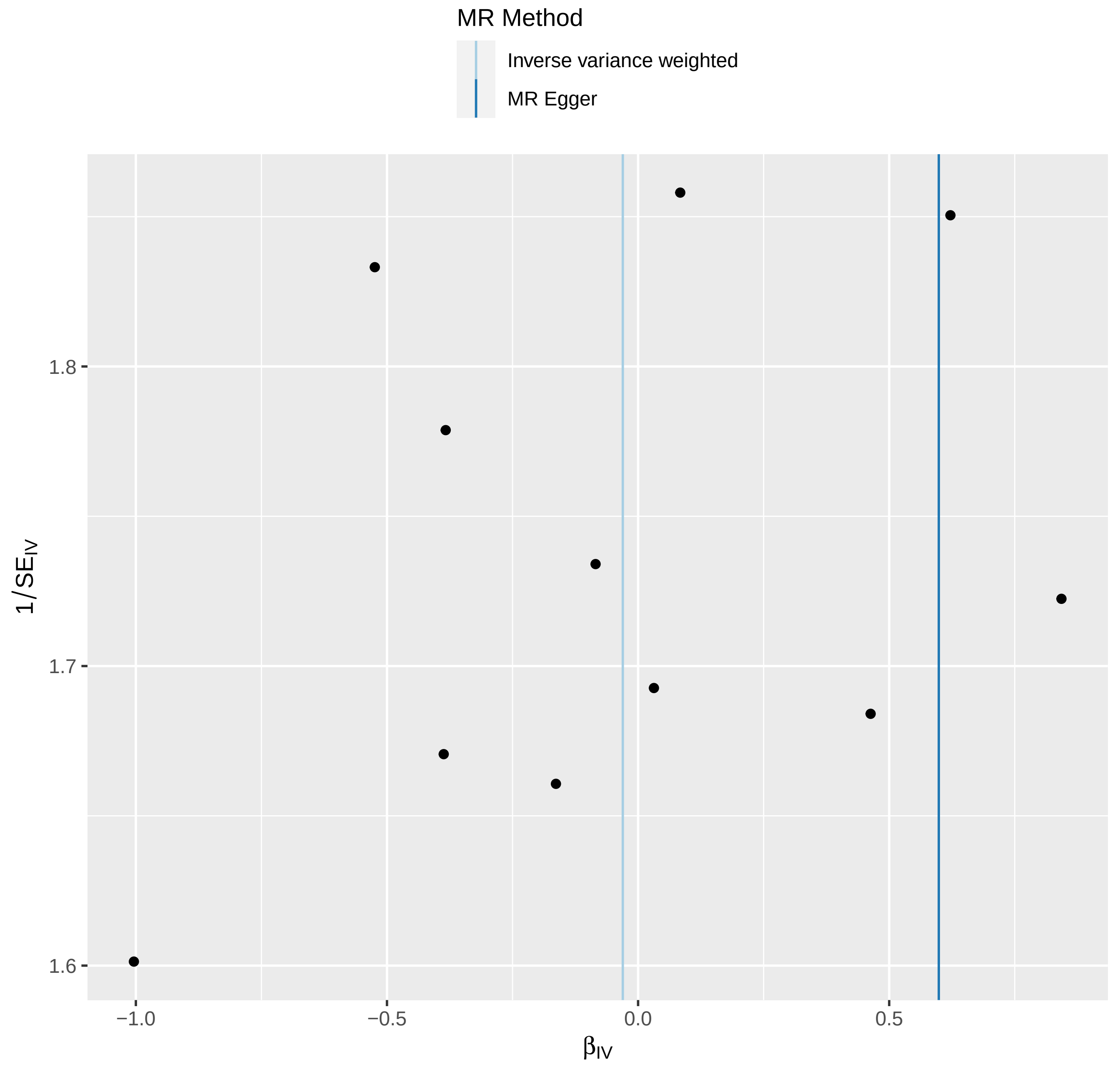

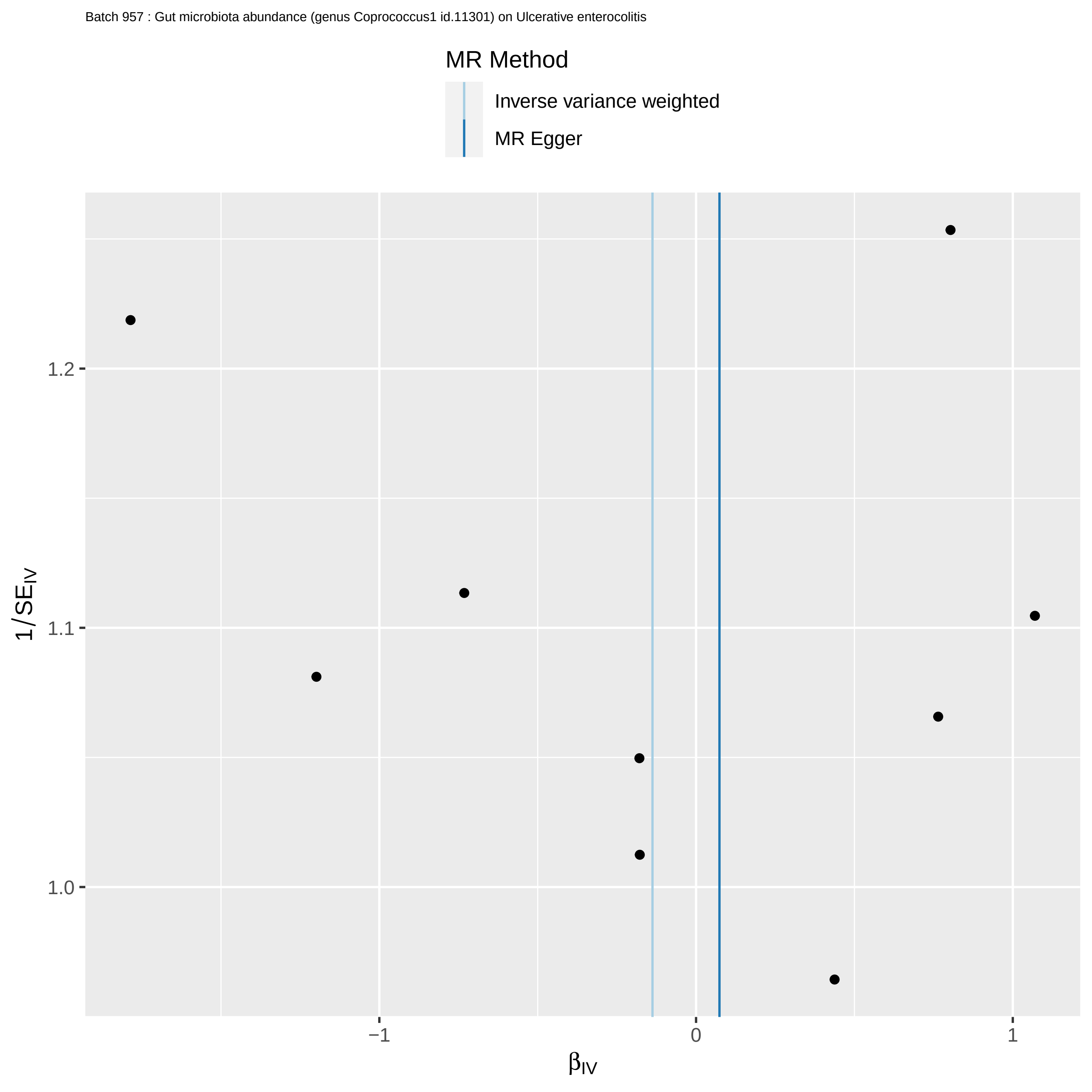

Batch 957 : Gut microbiota abundance (genus Coprococcus1 id.11301) on Ulcerative enterocolitis
MR Method
Inverse variance weighted
MR Egger
1.2
1.1
1.0
−1
0
1
1/SE_IV
β_IV

Batch 958 : Gut microbiota abundance (genus Coprococcus2 id.11302) on Ulcerative enterocolitis

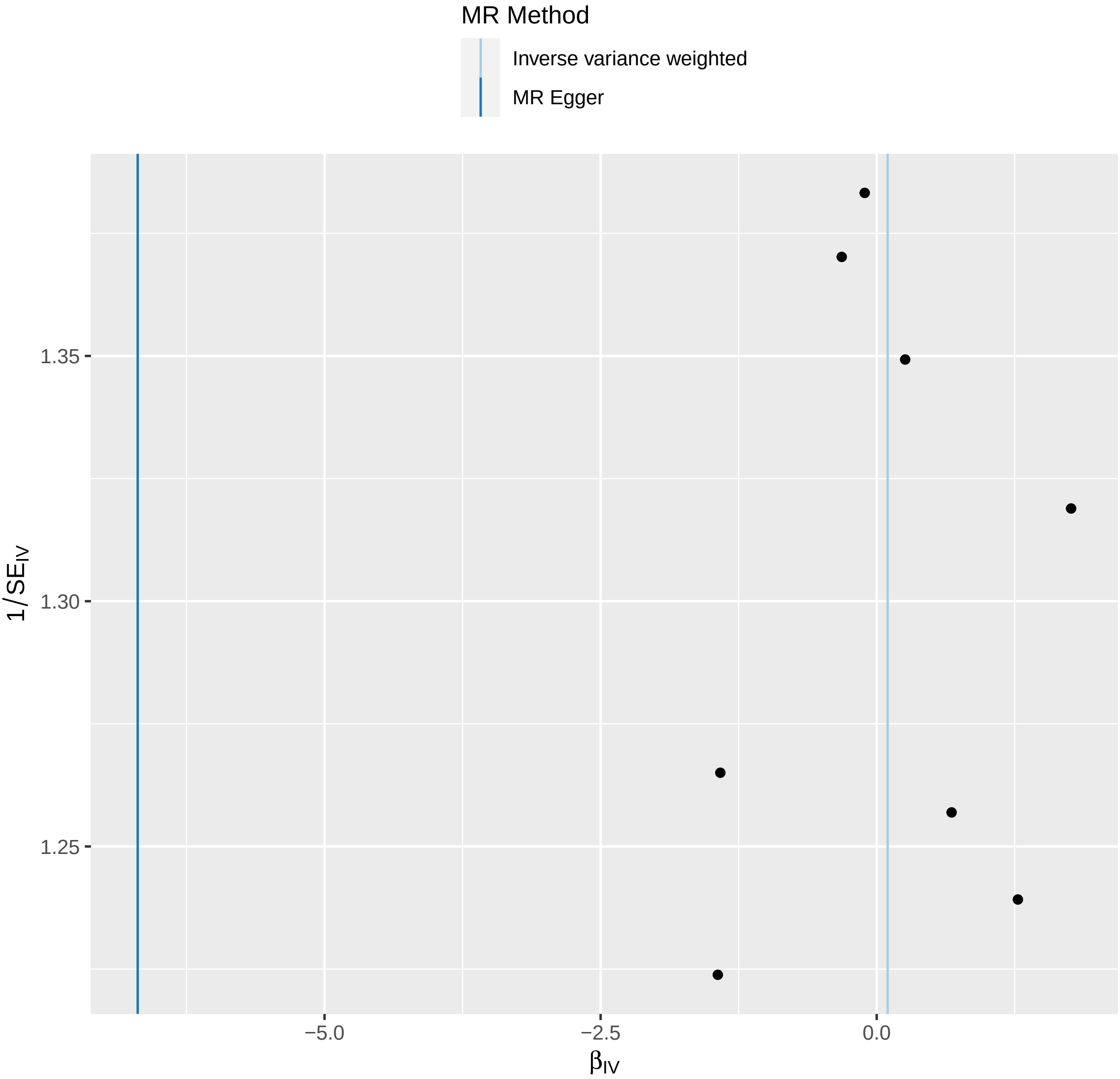

Batch 959 : Gut microbiota abundance (genus Coprococcus3 id.11303) on Ulcerative enterocolitis

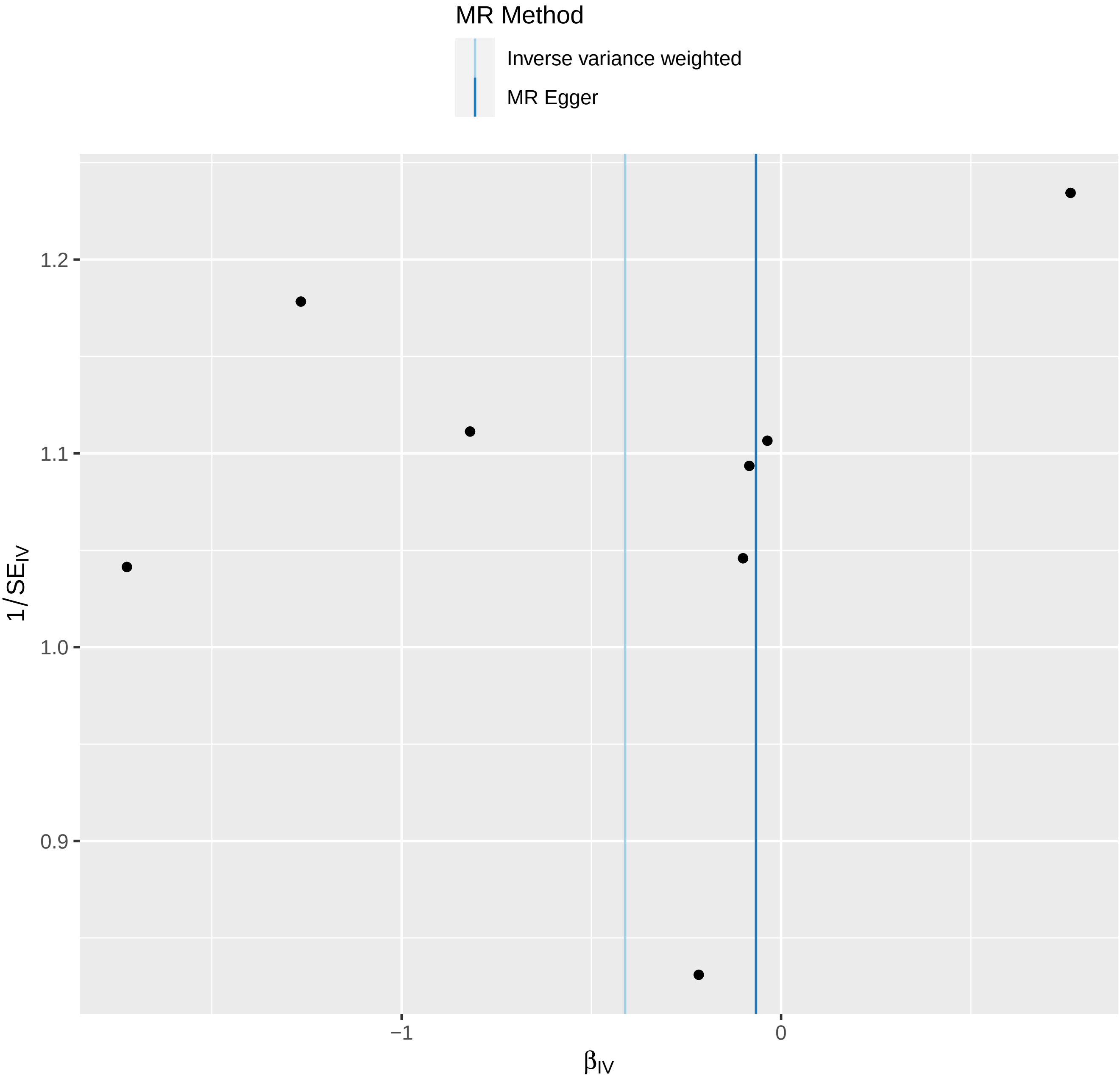

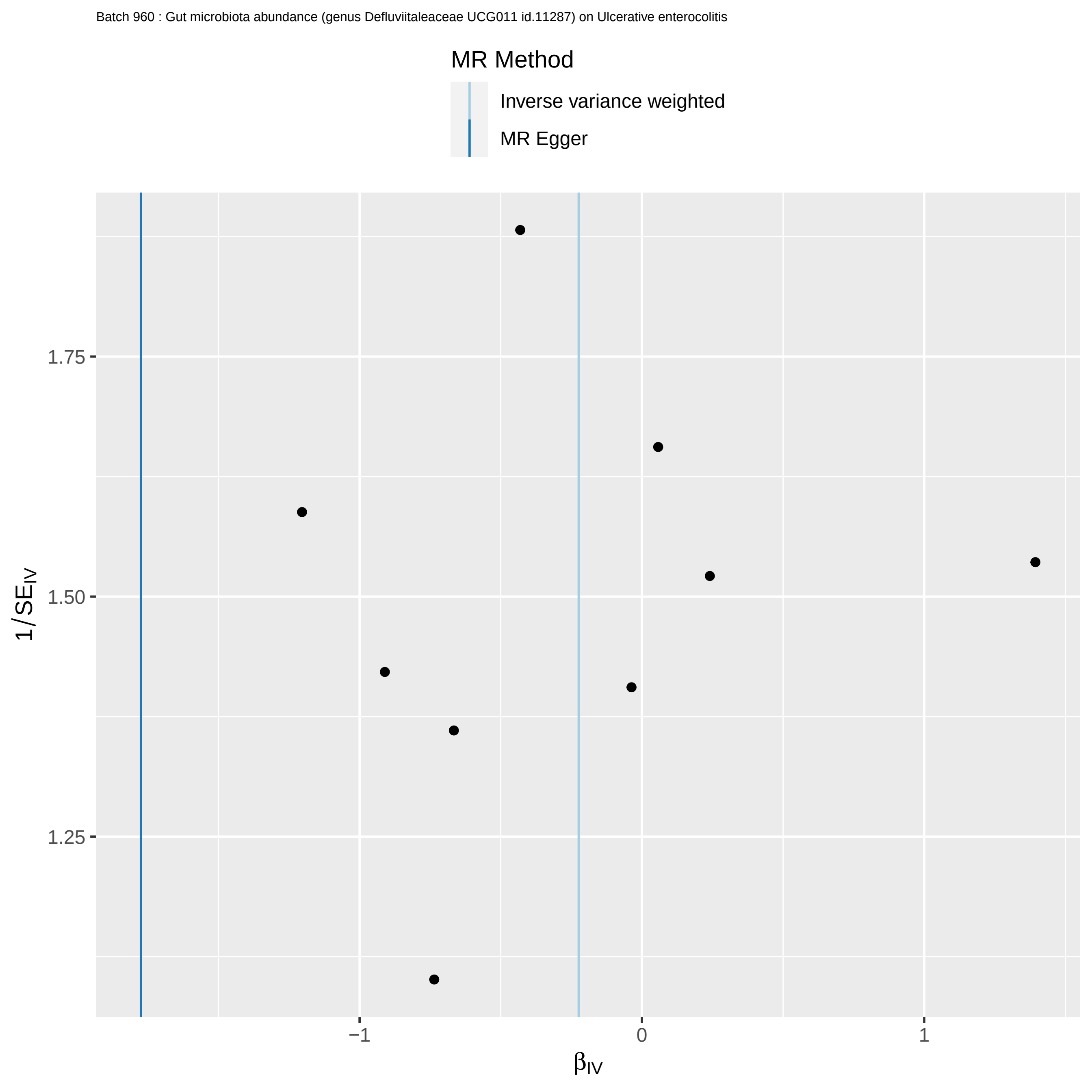

Batch 960 : Gut microbiota abundance (genus Defluviitaleaceae UCG011 id.11287) on Ulcerative enterocolitis
MR Method
Inverse variance weighted
MR Egger
1.75
1.50
1.25
$1/SE_{IV}$
−1
0
1
β_{IV}

Batch 961 : Gut microbiota abundance (genus Desulfovibrio id.3173) on Ulcerative enterocolitis

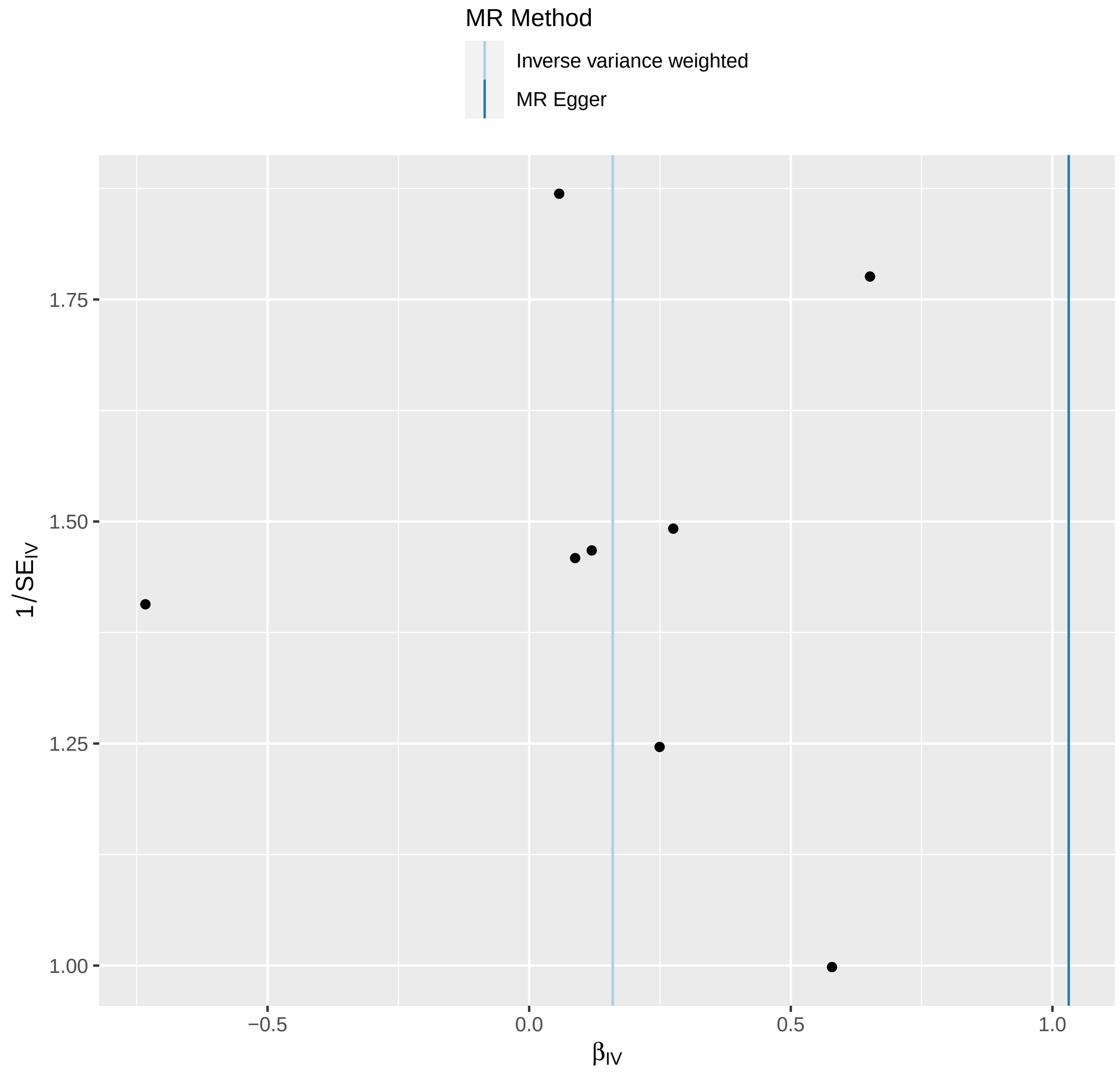

Batch 962 : Gut microbiota abundance (genus Dialister id.2183) on Ulcerative enterocolitis

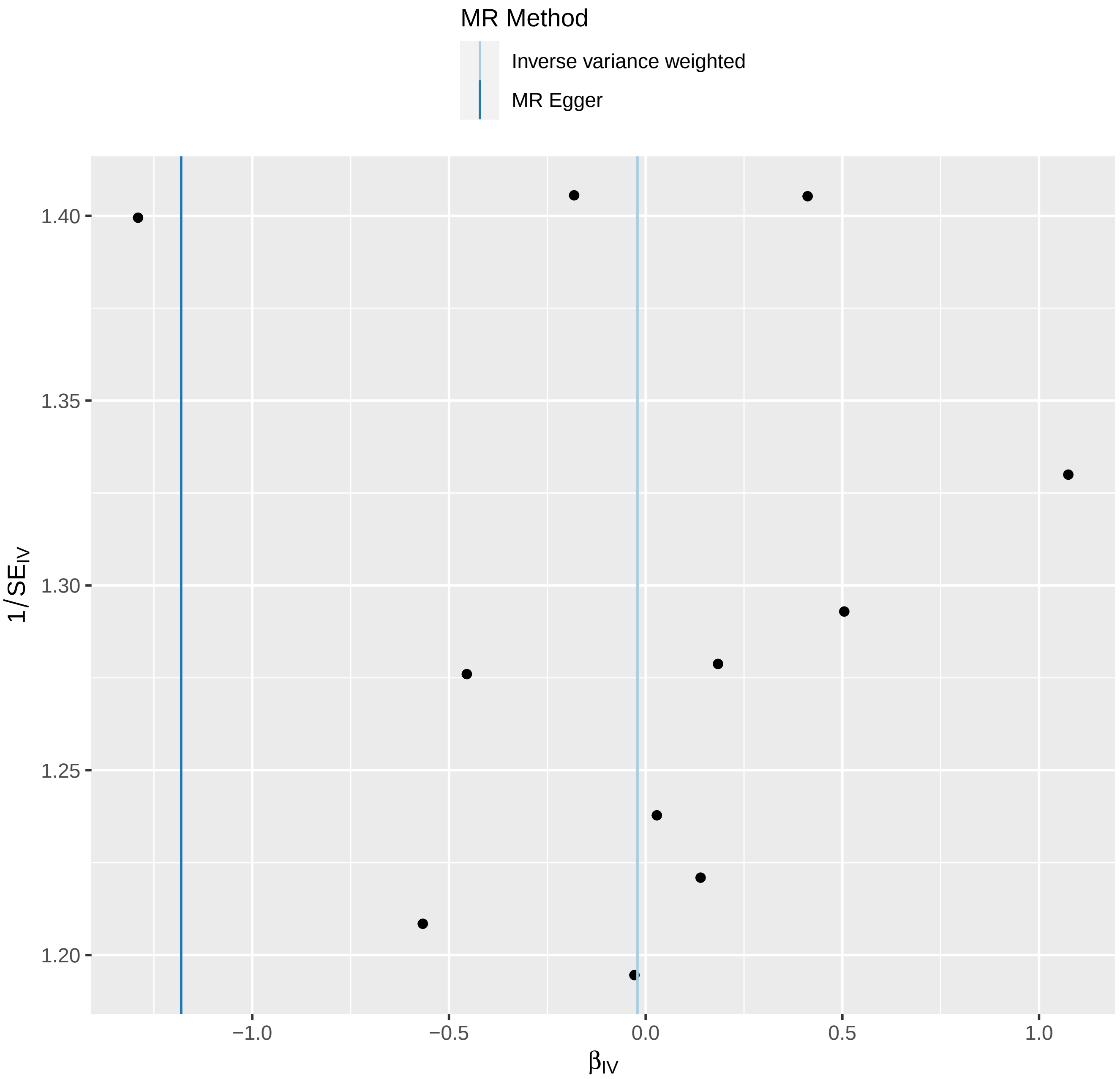

Batch 963 : Gut microbiota abundance (genus Dorea id.1997) on Ulcerative enterocolitis

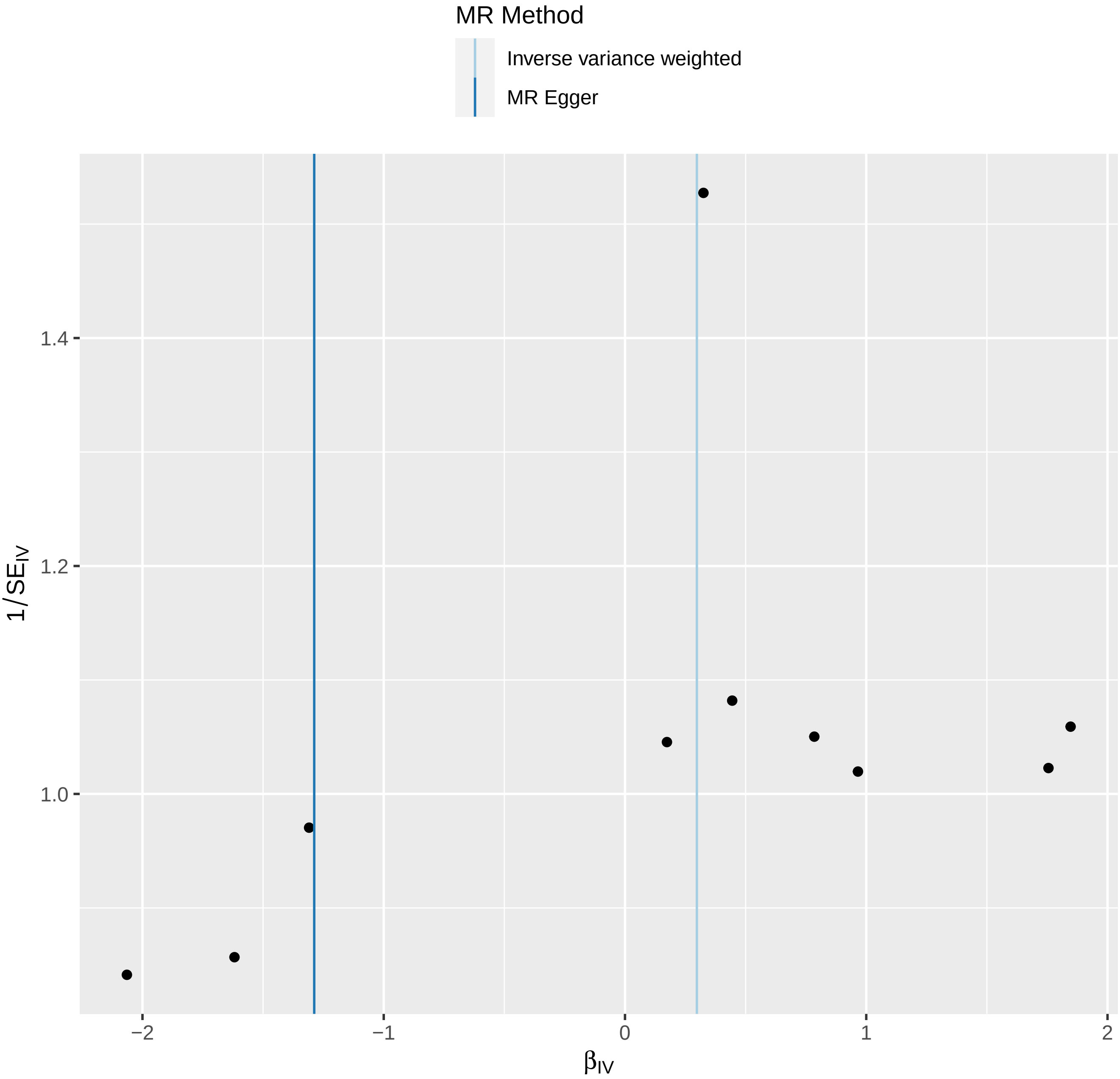

Batch 964 : Gut microbiota abundance (genus Eggerthella id.819) on Ulcerative enterocolitis

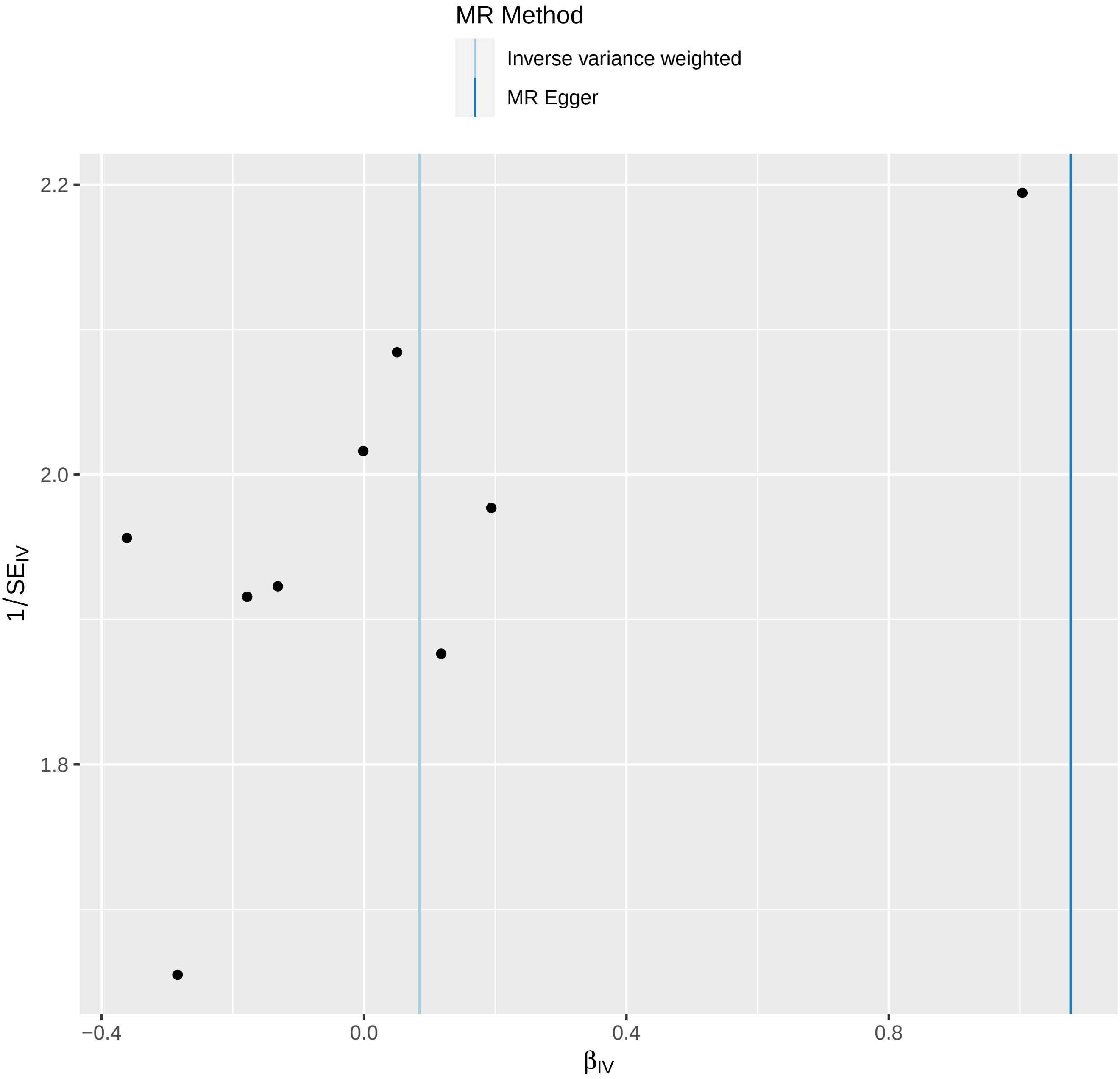

Batch 965 : Gut microbiota abundance (genus Eisenbergiella id.11304) on Ulcerative enterocolitis

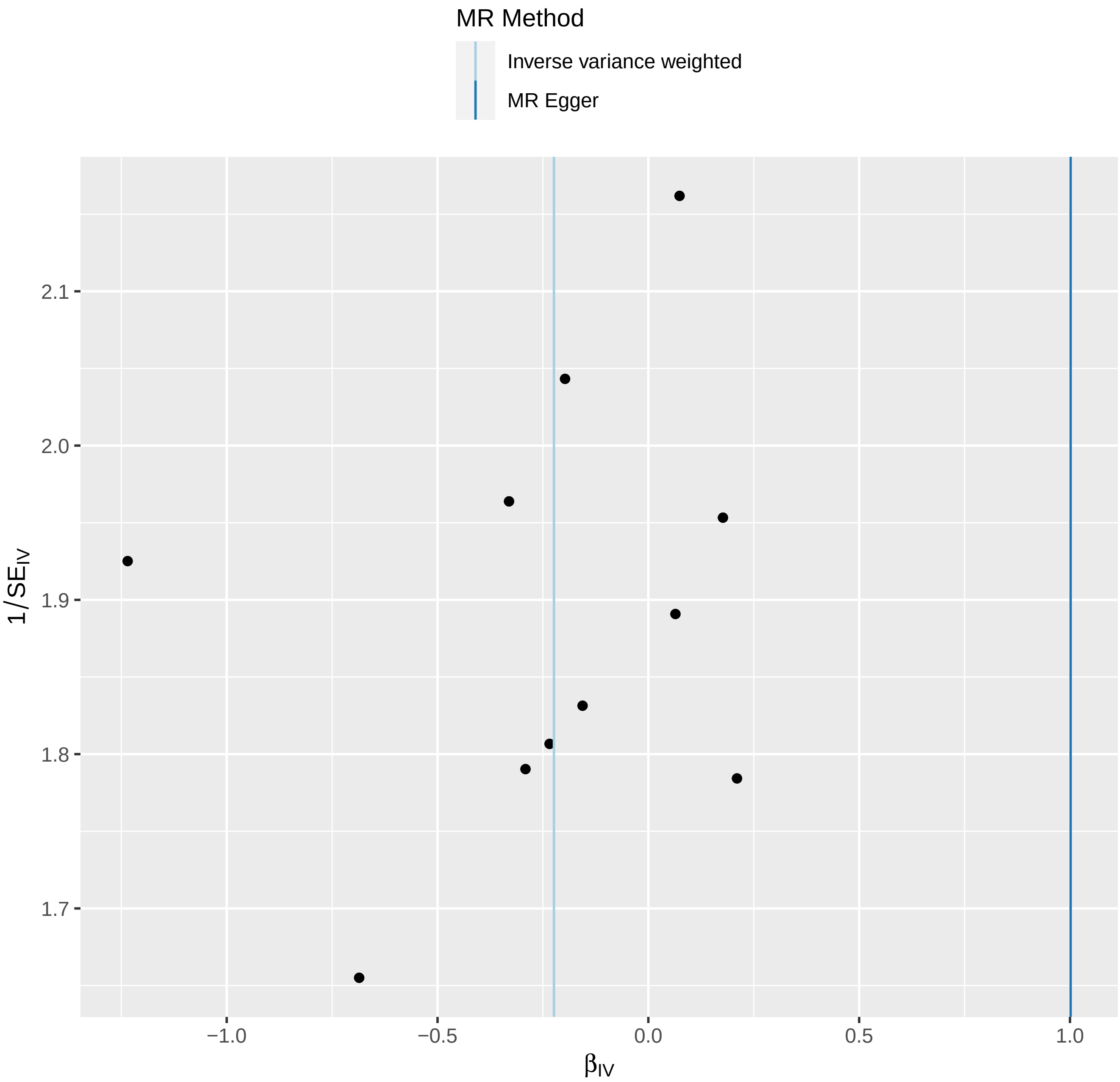

Batch 966 : Gut microbiota abundance (genus Enterorhabdus id.820) on Ulcerative enterocolitis

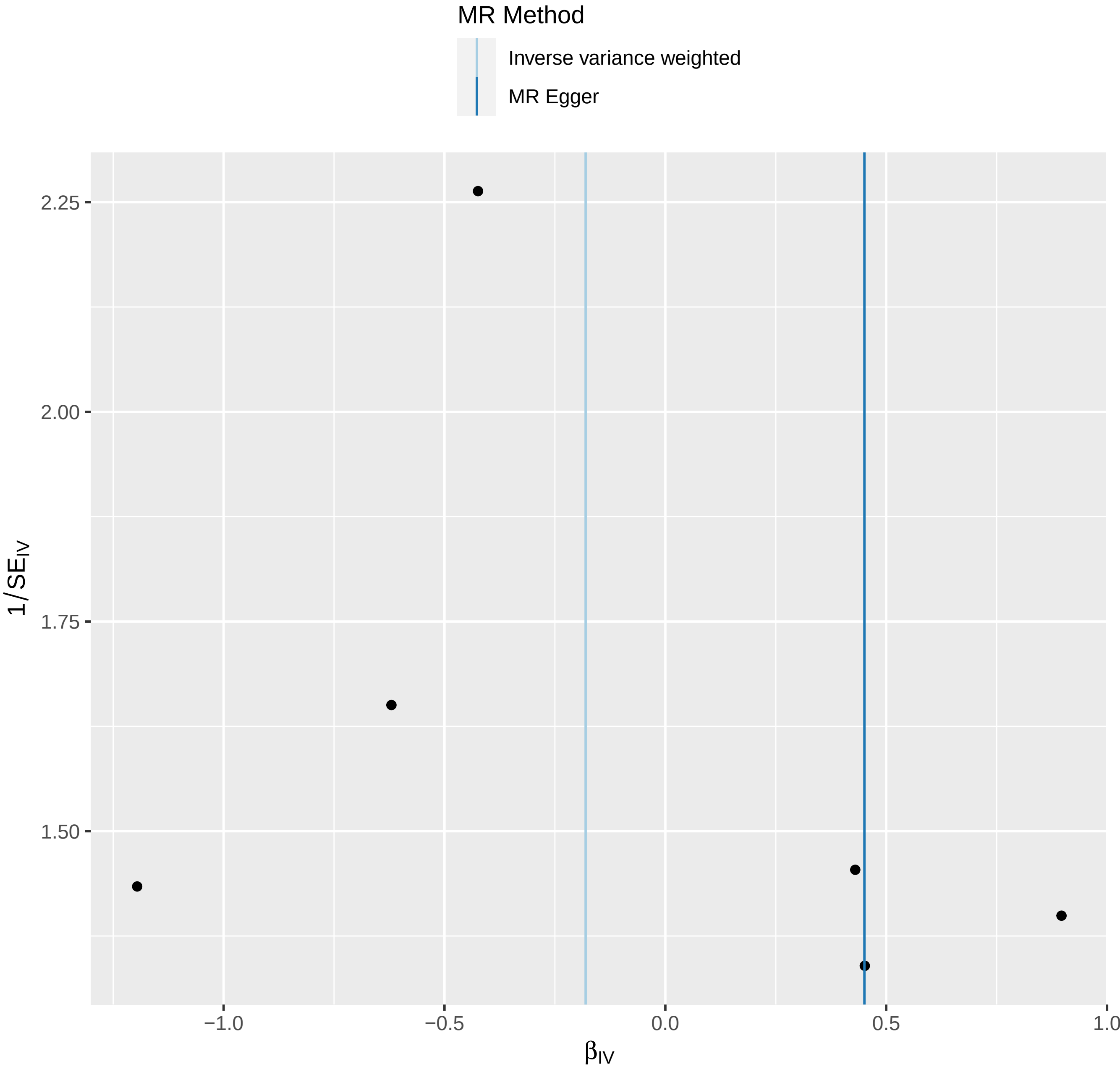

Batch 967 : Gut microbiota abundance (genus Erysipelatoclostridium id.11381) on Ulcerative enterocolitis

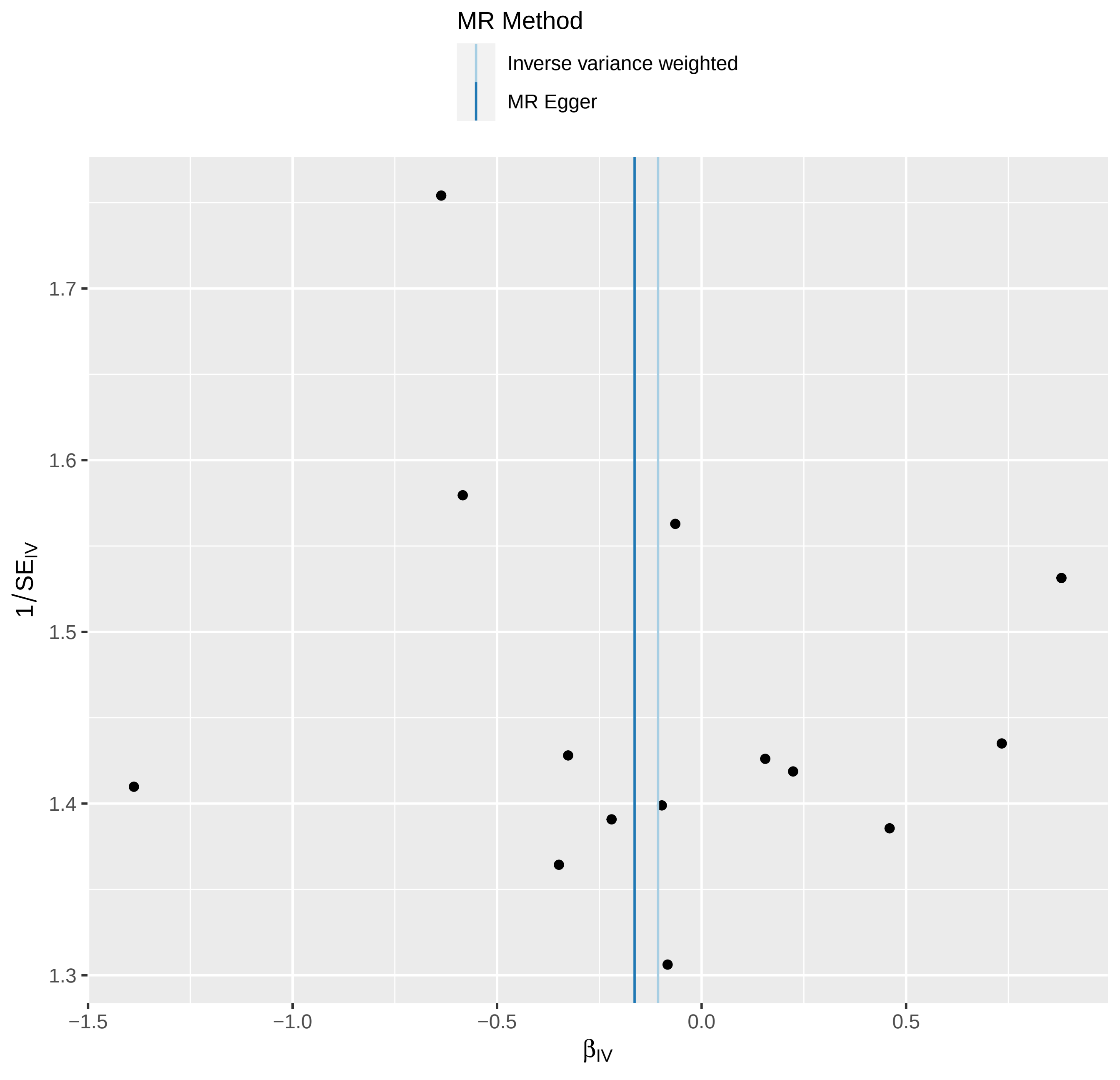

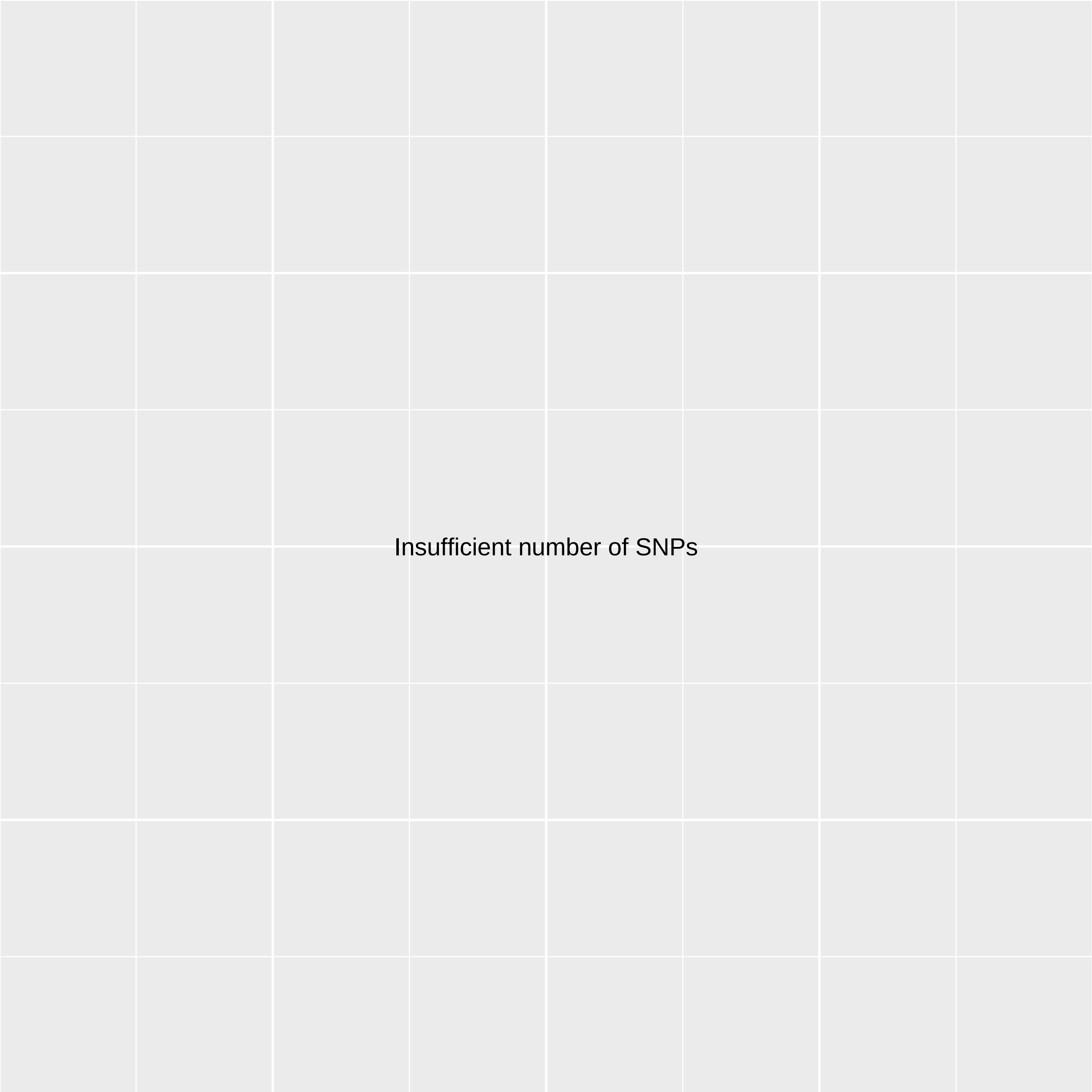
Insufficient number of SNPs

Batch 969 : Gut microbiota abundance (genus Escherichia Shigella id.3504) on Ulcerative enterocolitis

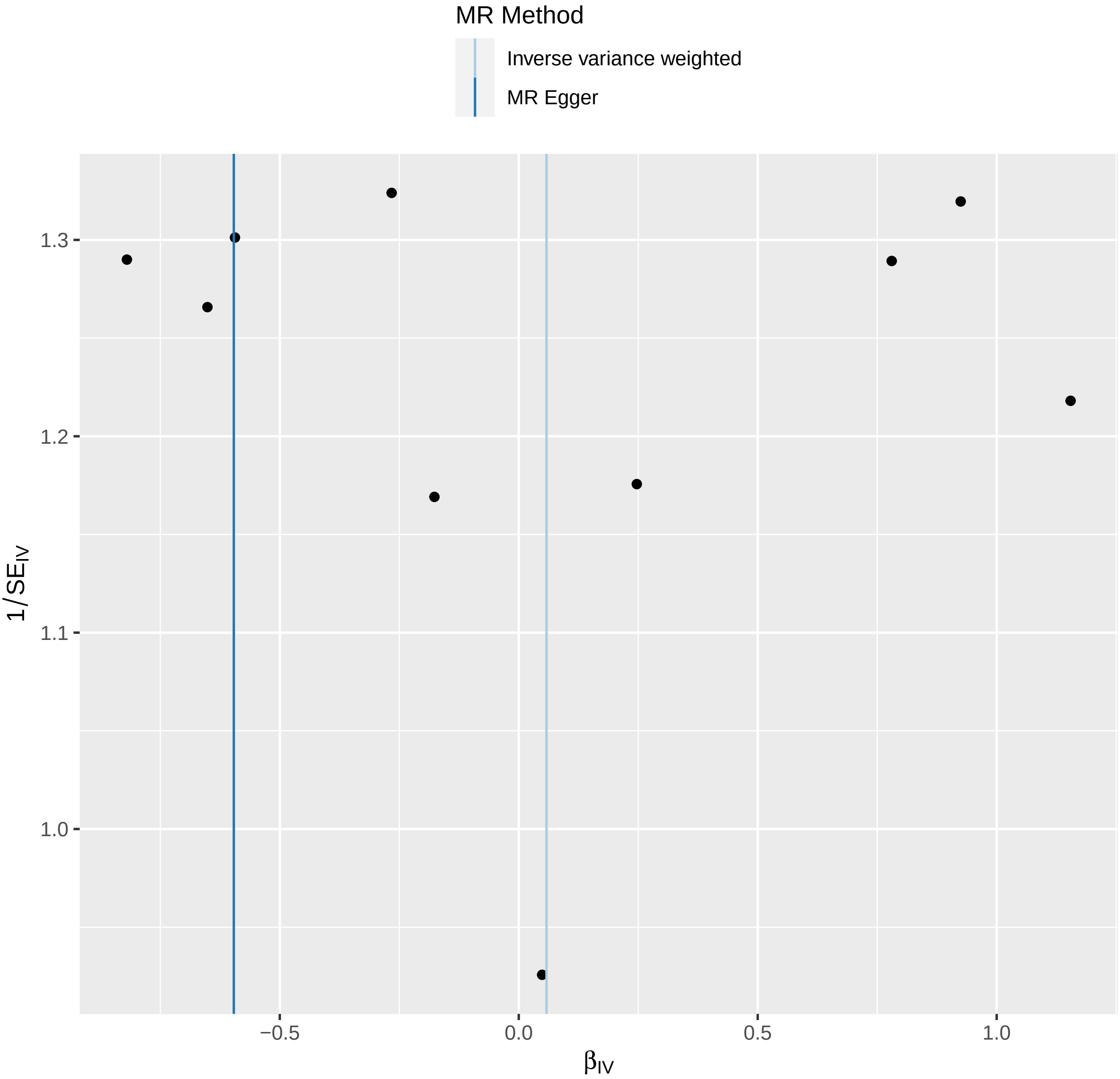

Batch 970 : Gut microbiota abundance (genus Eubacterium brachy group id.11296) on Ulcerative enterocolitis

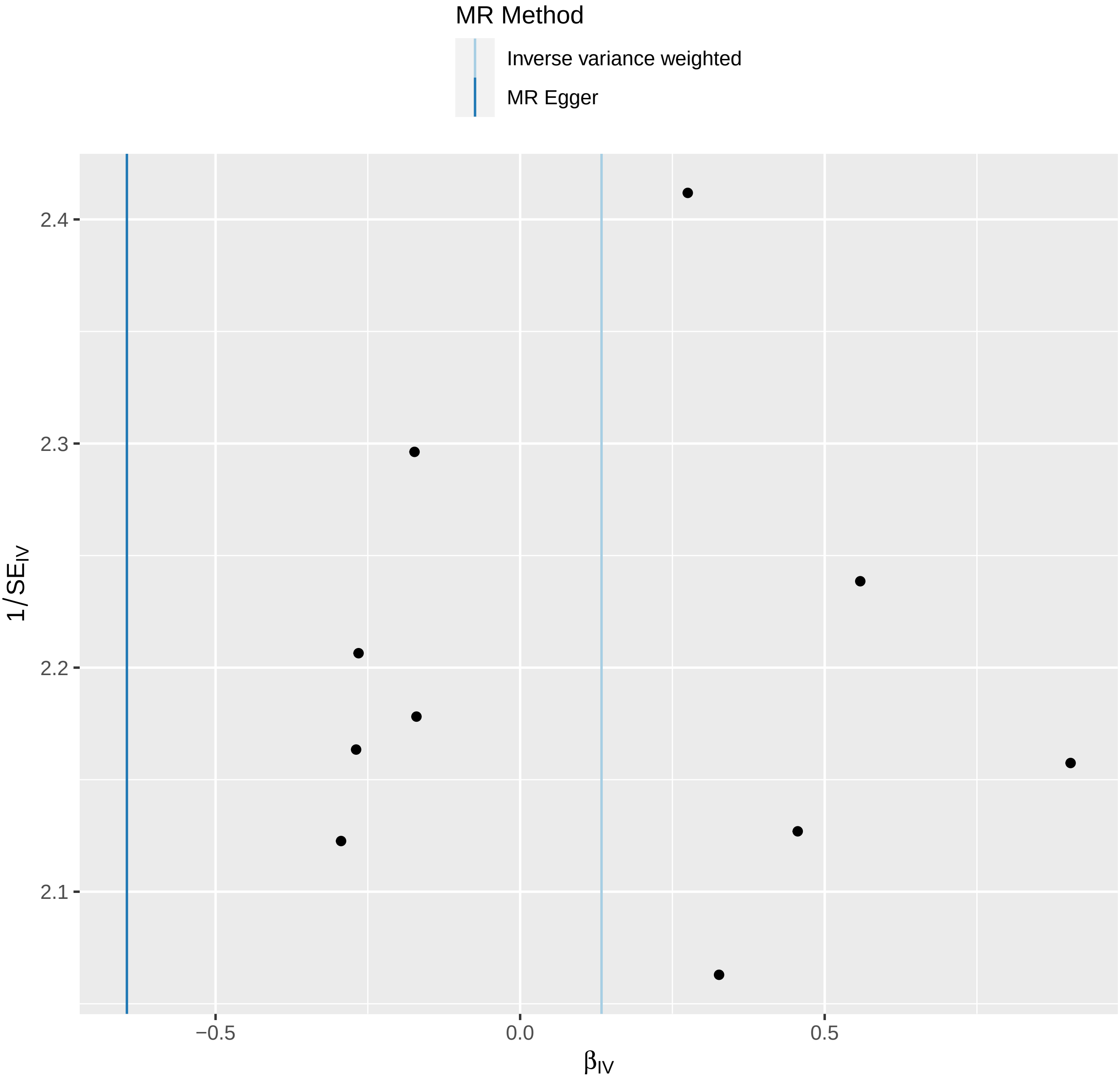

Batch 971 : Gut microbiota abundance (genus Eubacterium coprostanoligenes group id.11375) on Ulcerative enterocolitis

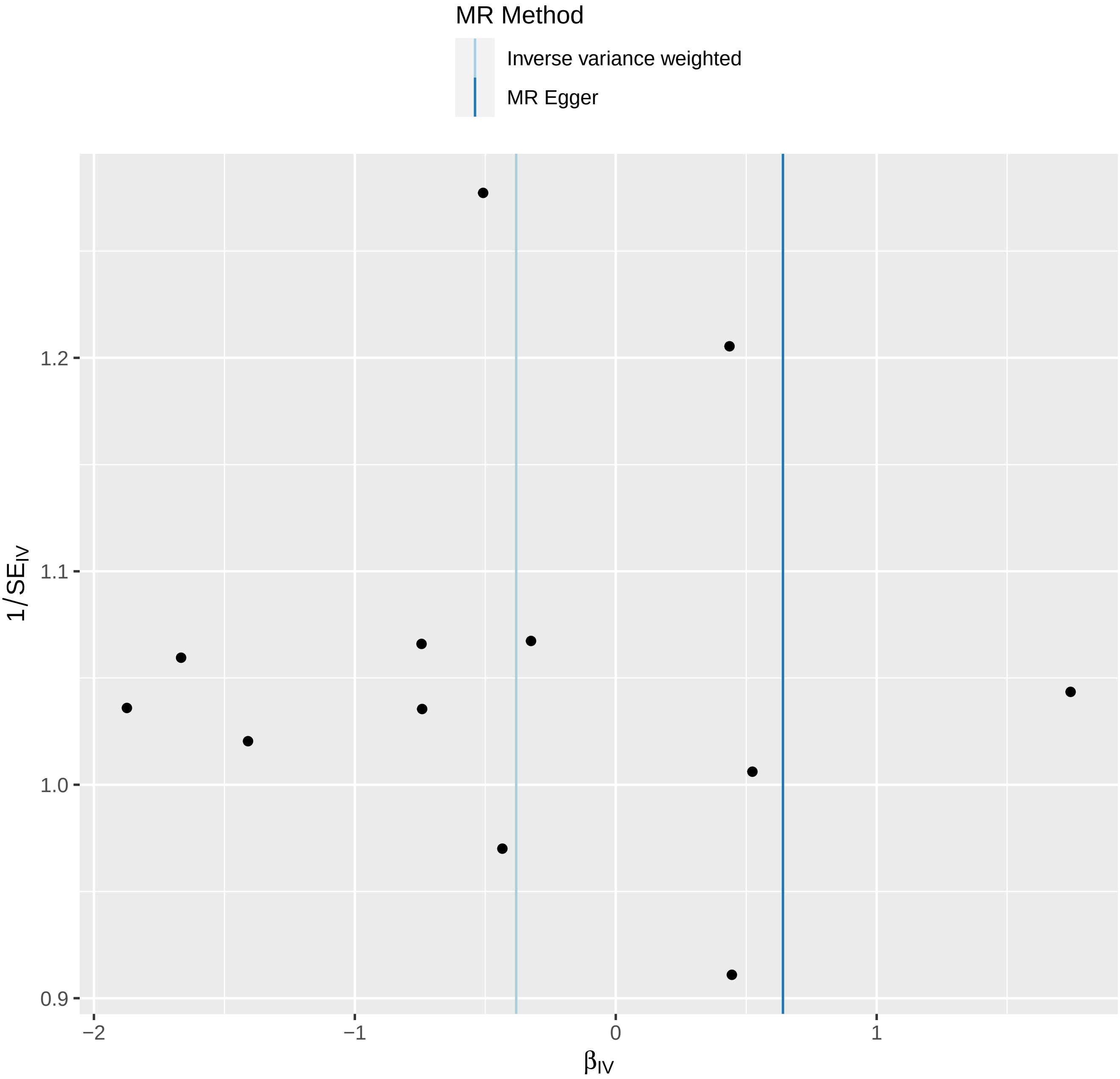

Batch 972 : Gut microbiota abundance (genus Eubacterium eligens group id.14372) on Ulcerative enterocolitis

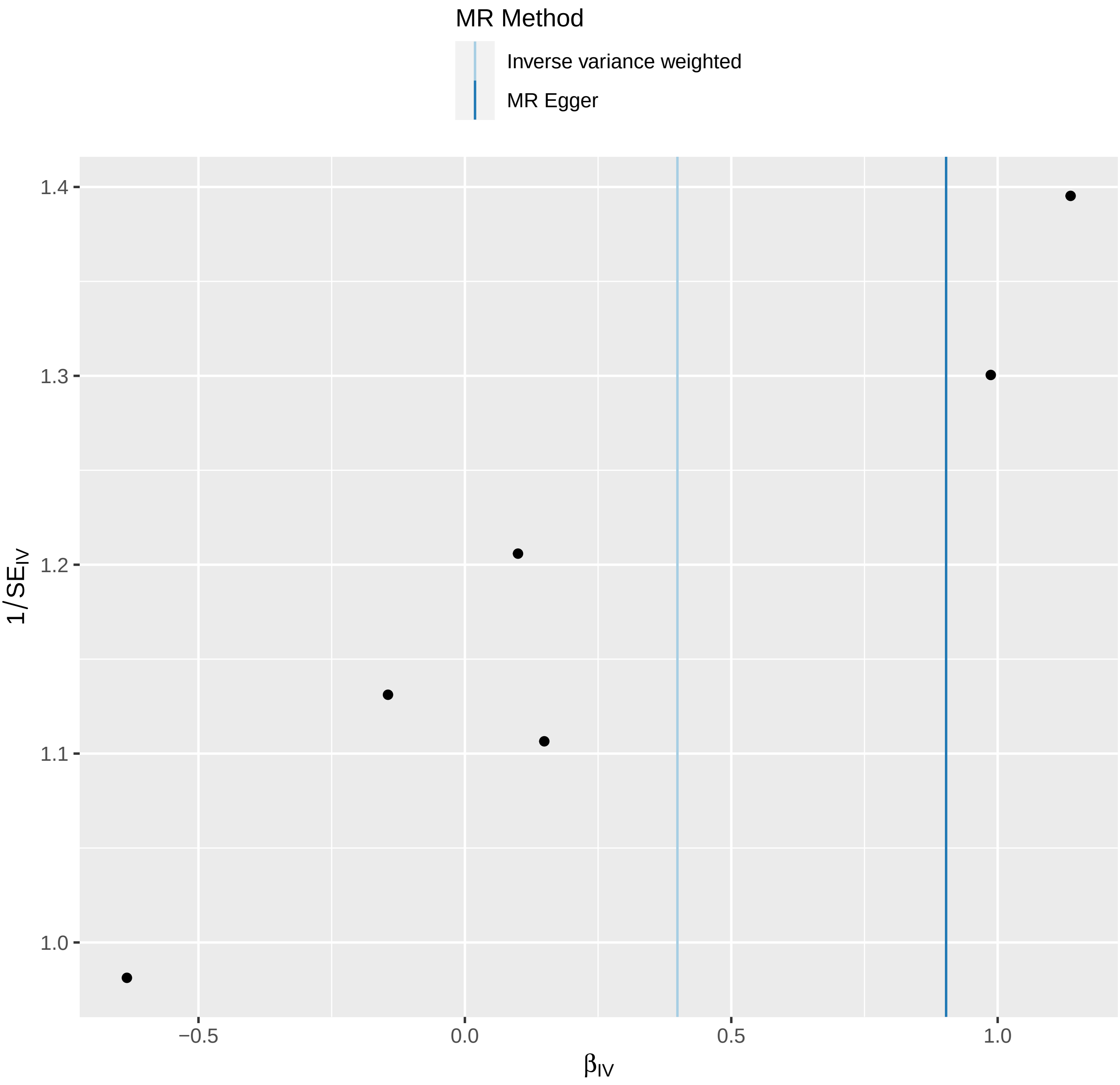

Batch 973 : Gut microbiota abundance (genus Eubacterium fissicatena group id.14373) on Ulcerative enterocolitis

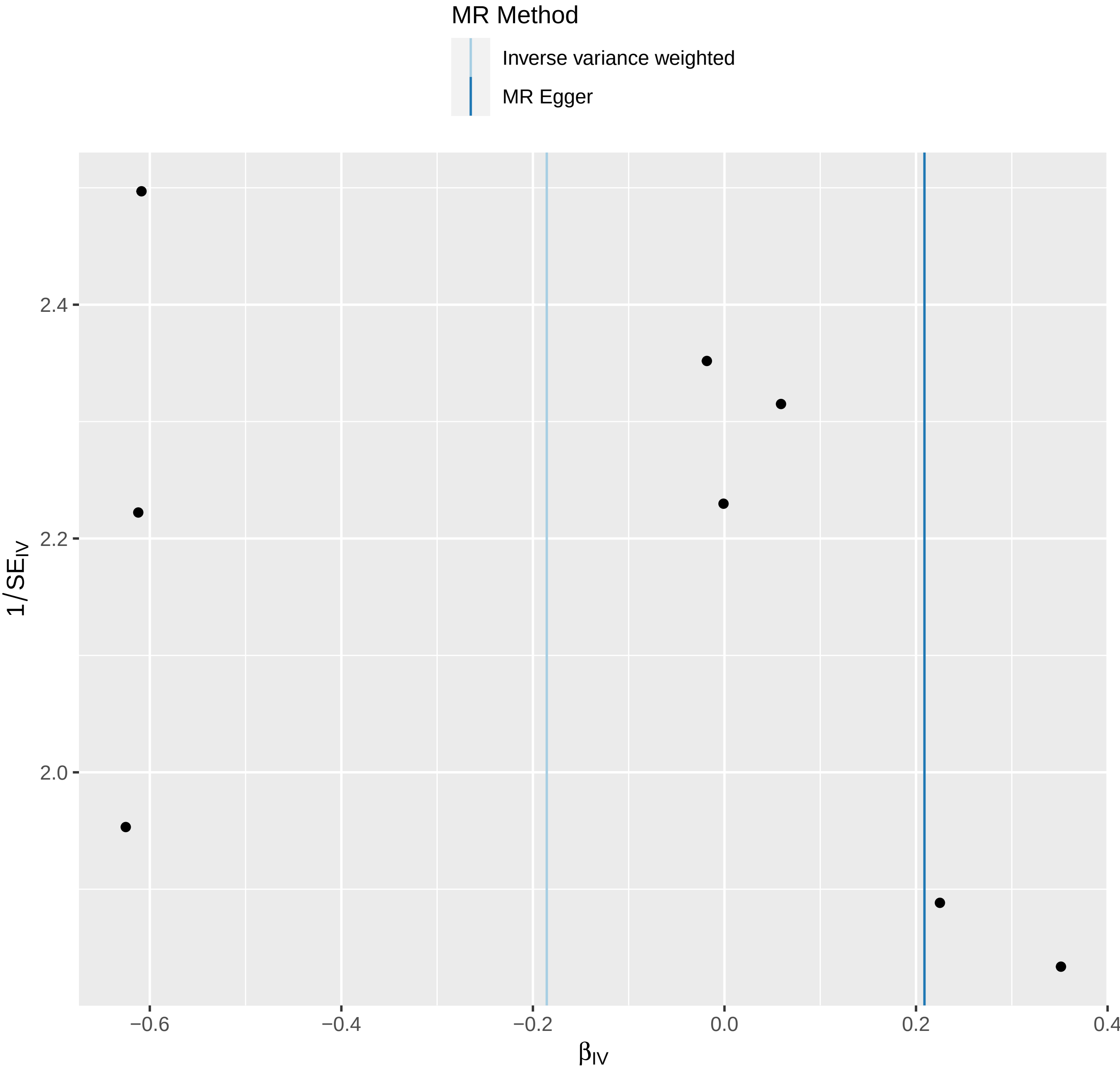

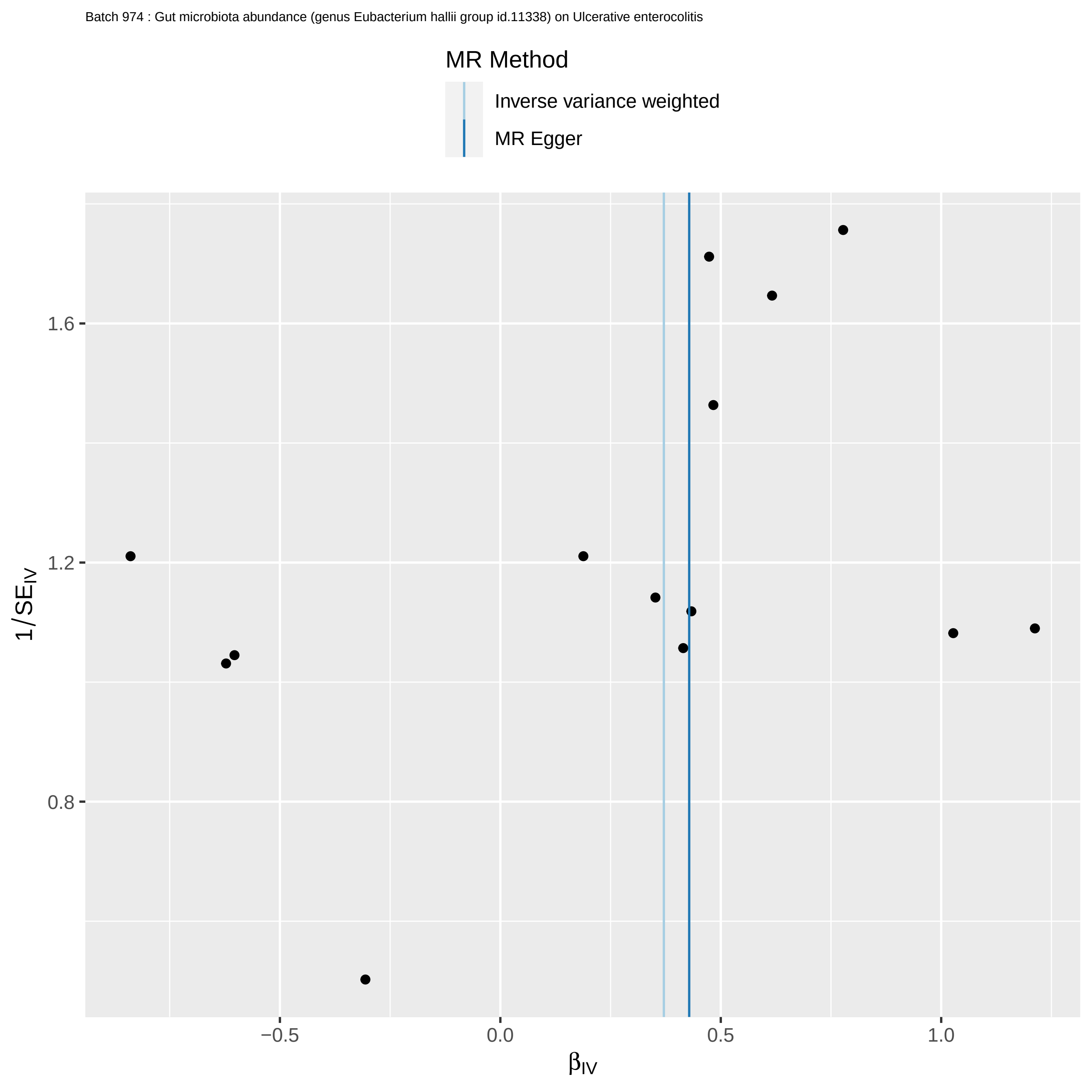

Batch 974 : Gut microbiota abundance (genus Eubacterium hallii group id.11338) on Ulcerative enterocolitis
MR Method
Inverse variance weighted
MR Egger
1.6
1.2
0.8
1/SE_IV
−0.5
0.0
0.5
1.0
β_IV

Batch 975 : Gut microbiota abundance (genus Eubacterium nodatum group id.11297) on Ulcerative enterocolitis

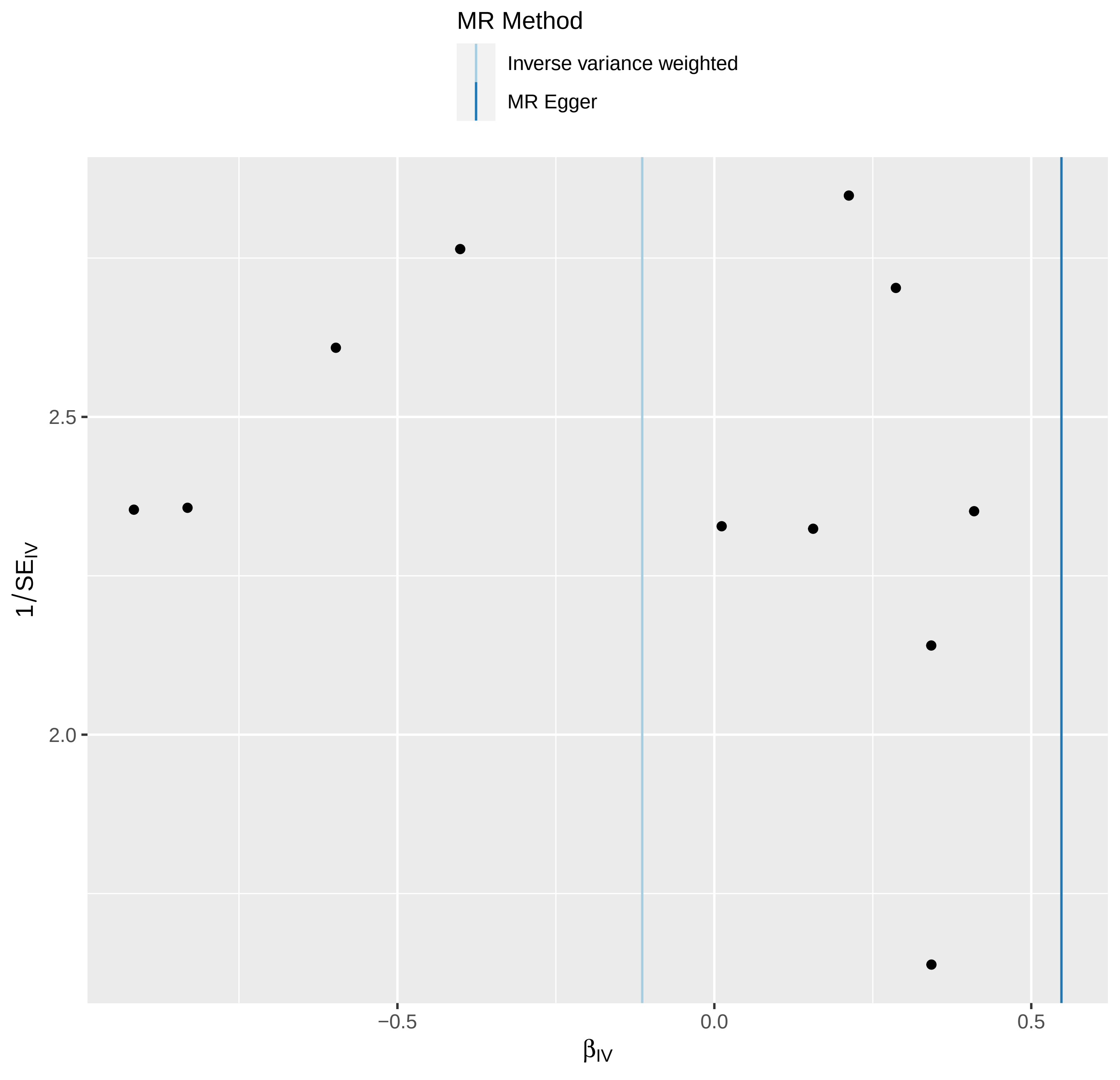

Batch 976 : Gut microbiota abundance (genus Eubacterium oxidoreducens group id.11339) on Ulcerative enterocolitis

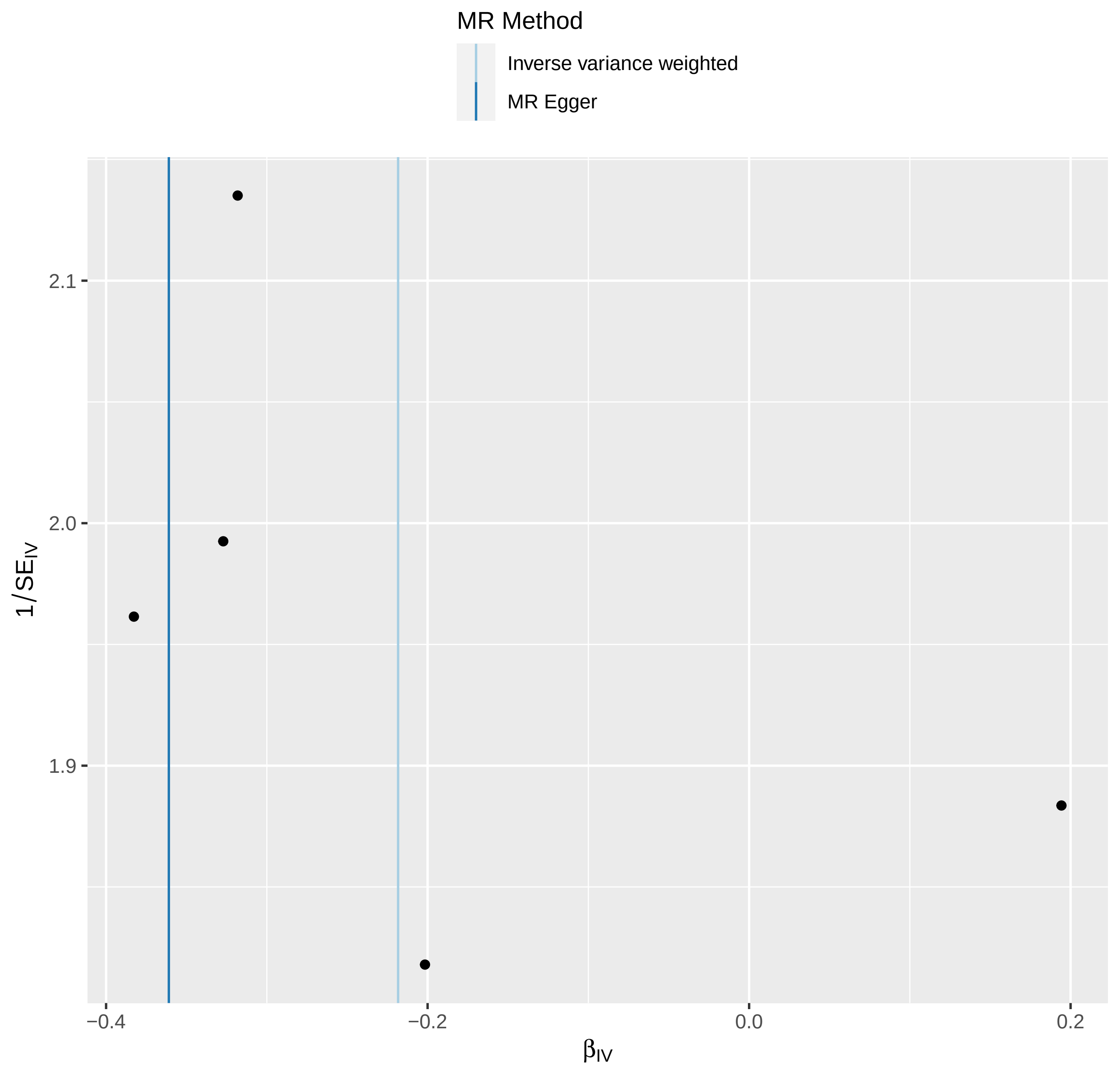

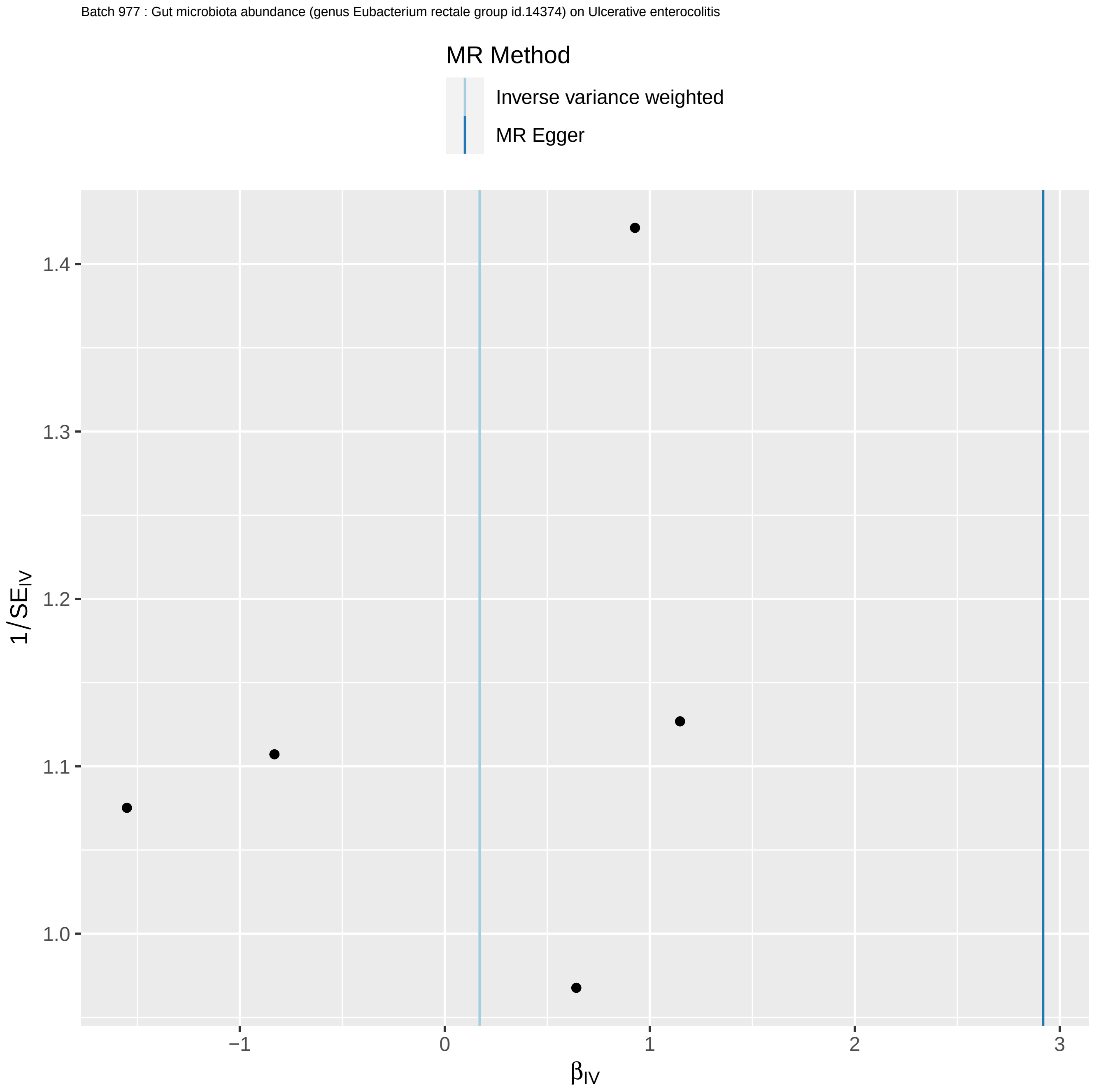
Batch 977 : Gut microbiota abundance (genus Eubacterium rectale group id.14374) on Ulcerative enterocolitis
MR Method
Inverse variance weighted
MR Egger
1.4
1.3
1.2
1.1
1.0
$1/SE_{IV}$
−1
0
1
2
3
β_{IV}

Batch 978 : Gut microbiota abundance (genus Eubacterium ruminantium group id.11340) on Ulcerative enterocolitis

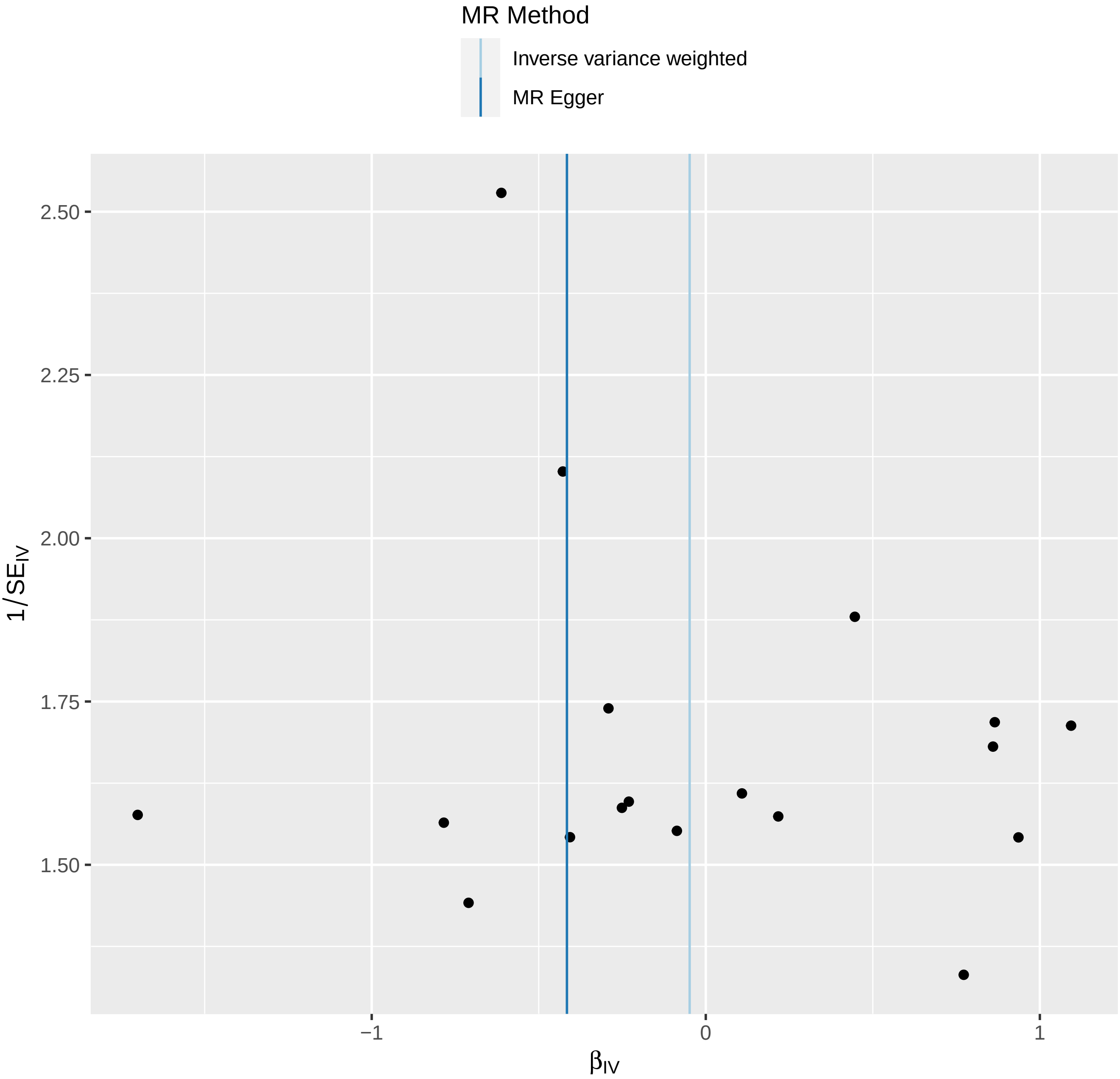

Batch 979 : Gut microbiota abundance (genus Eubacterium ventriosum group id.11341) on Ulcerative enterocolitis

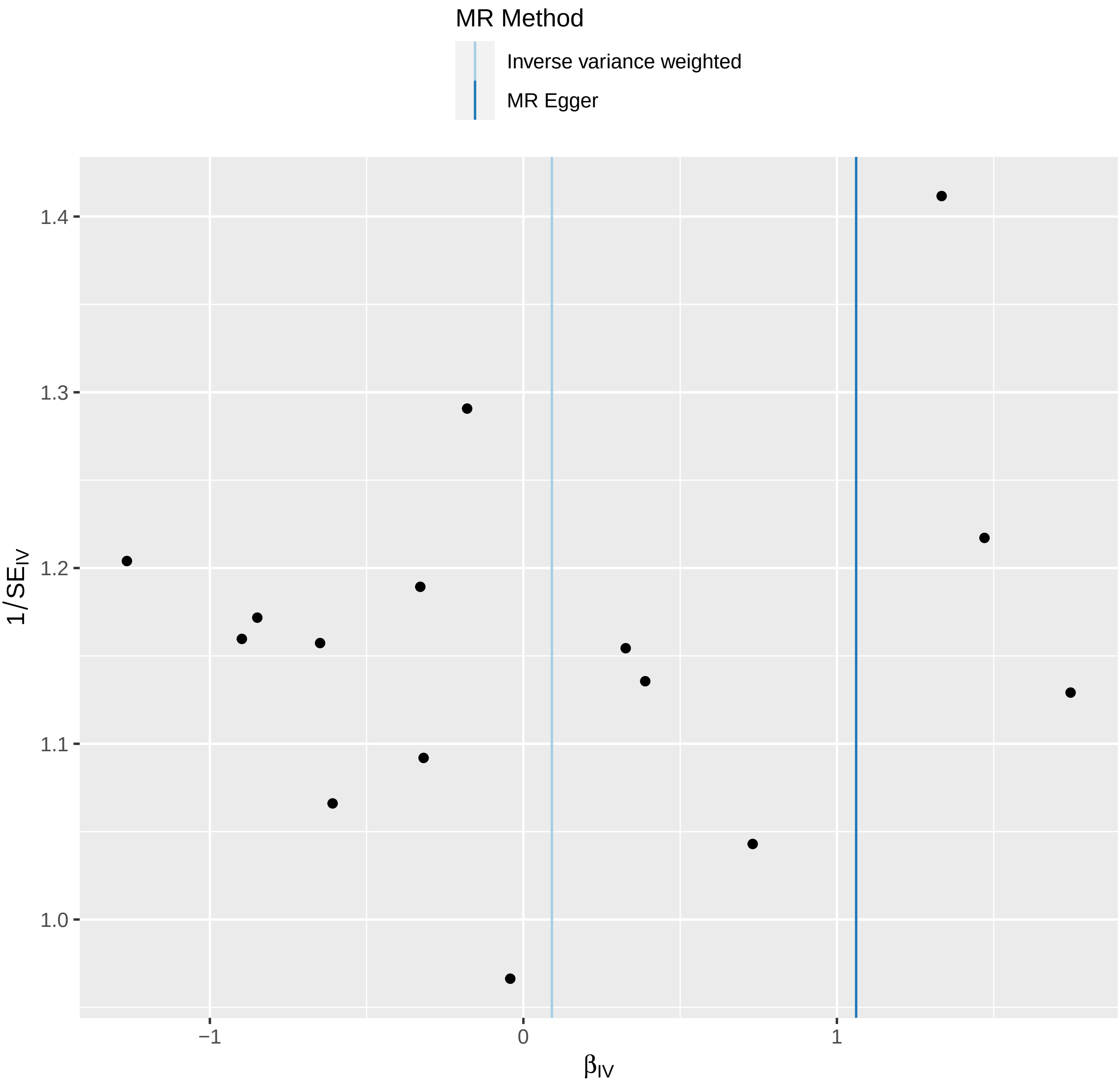

Batch 980 : Gut microbiota abundance (genus Eubacterium xylanophilum group id.14375) on Ulcerative enterocolitis

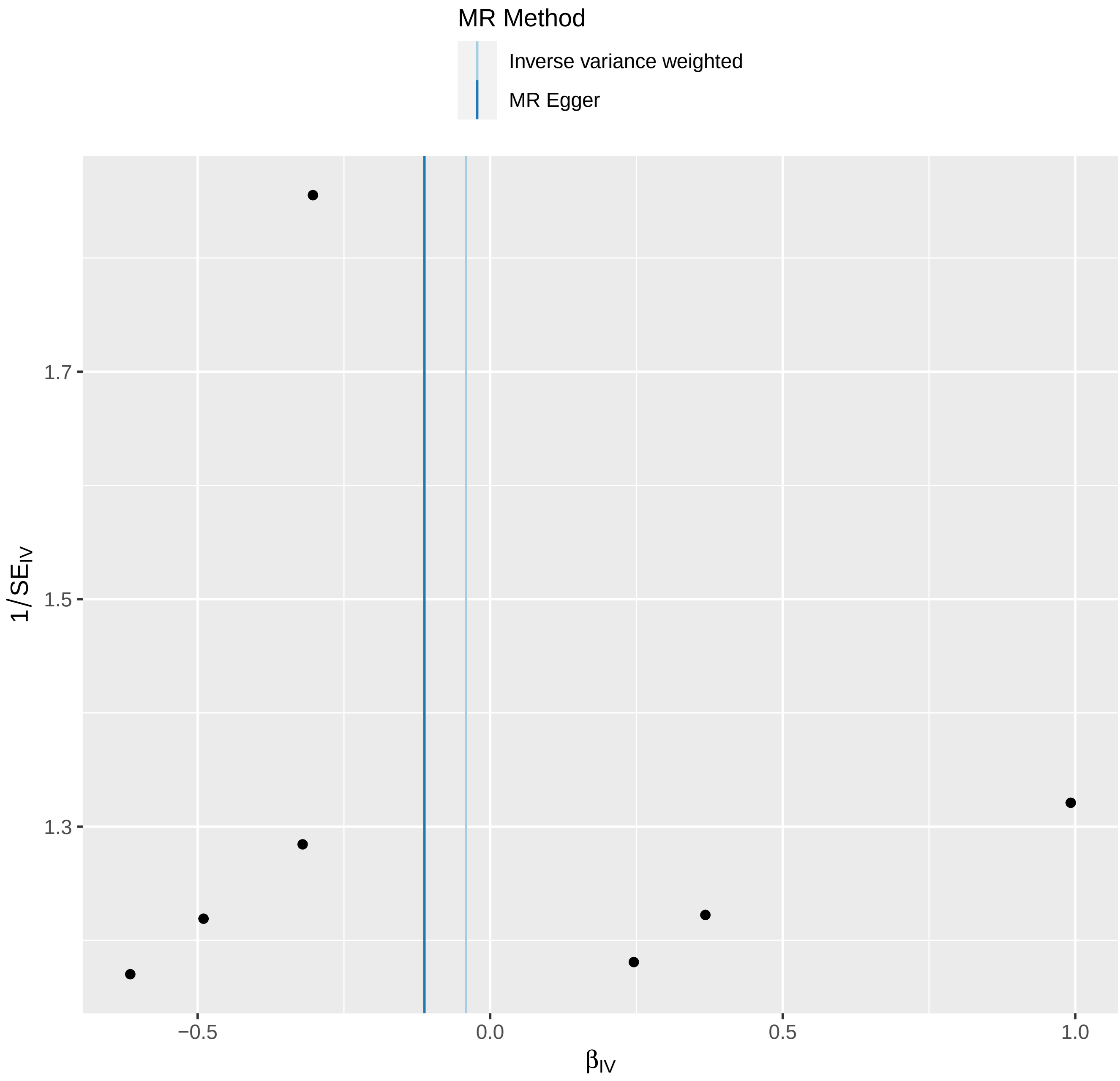

Batch 981 : Gut microbiota abundance (genus Faecalibacterium id.2057) on Ulcerative enterocolitis

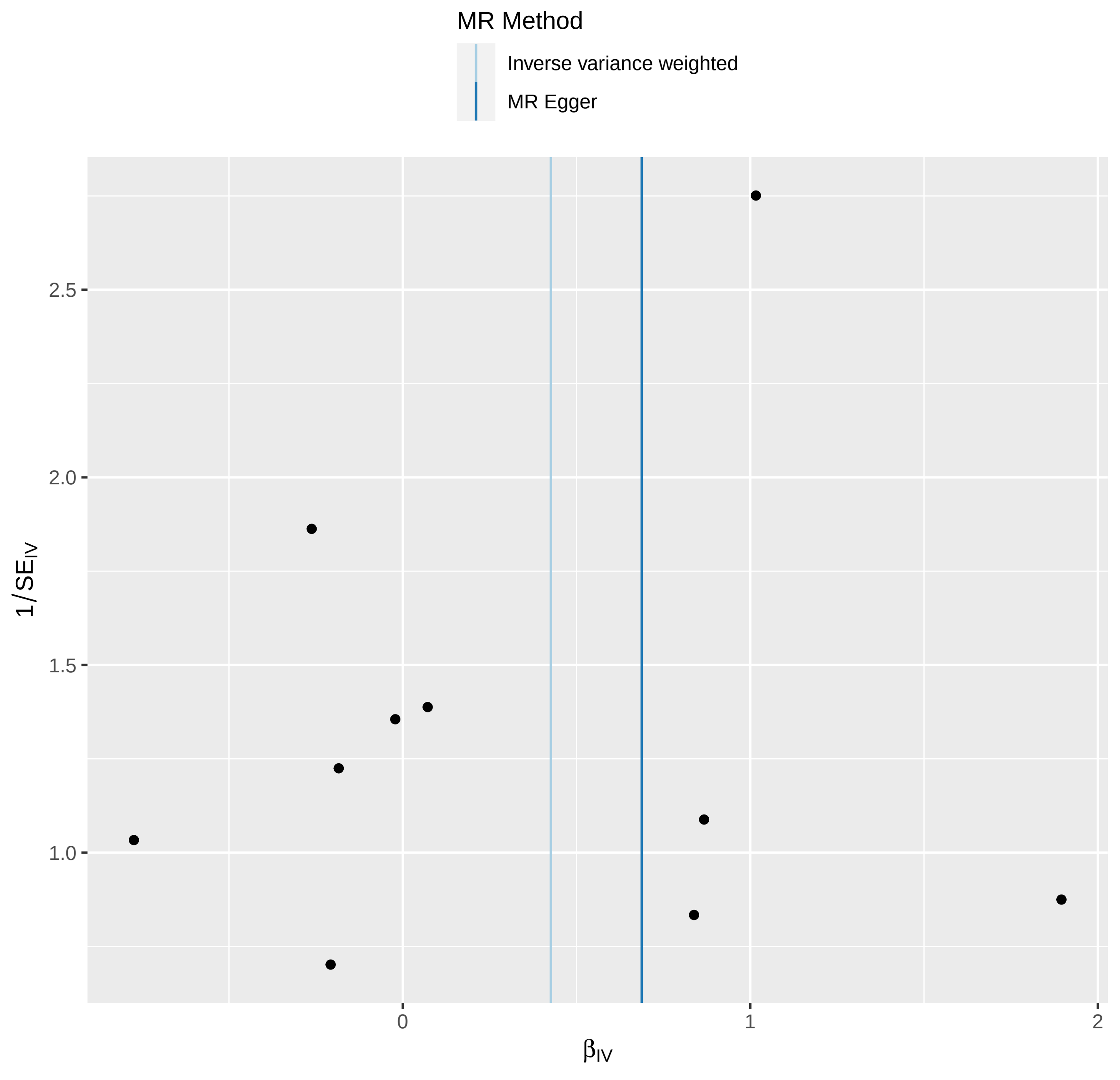

Batch 982 : Gut microbiota abundance (genus Family XIII AD3011 group id.11293) on Ulcerative enterocolitis

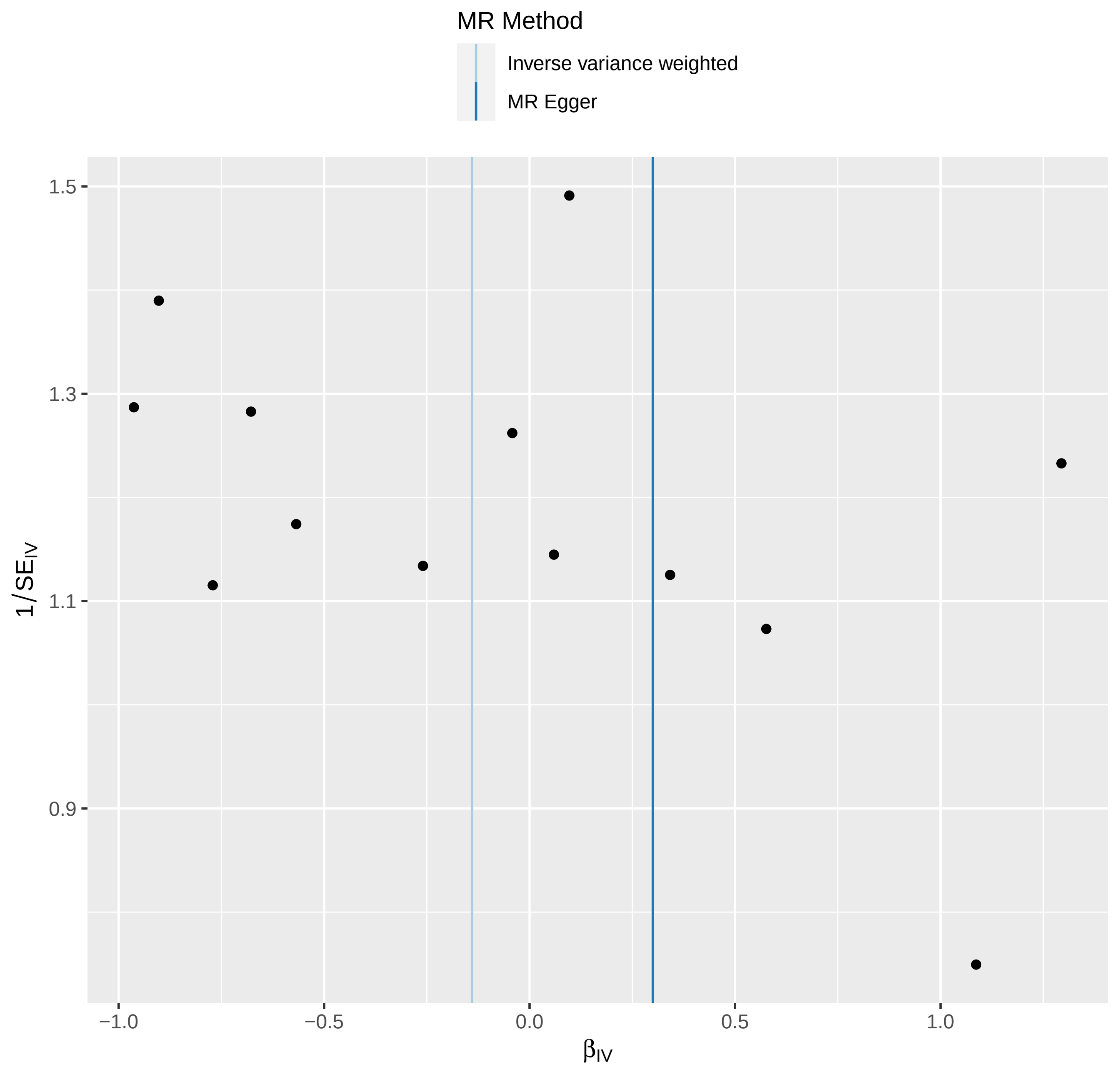

Batch 983 : Gut microbiota abundance (genus Family XIII UCG001 id.11294) on Ulcerative enterocolitis

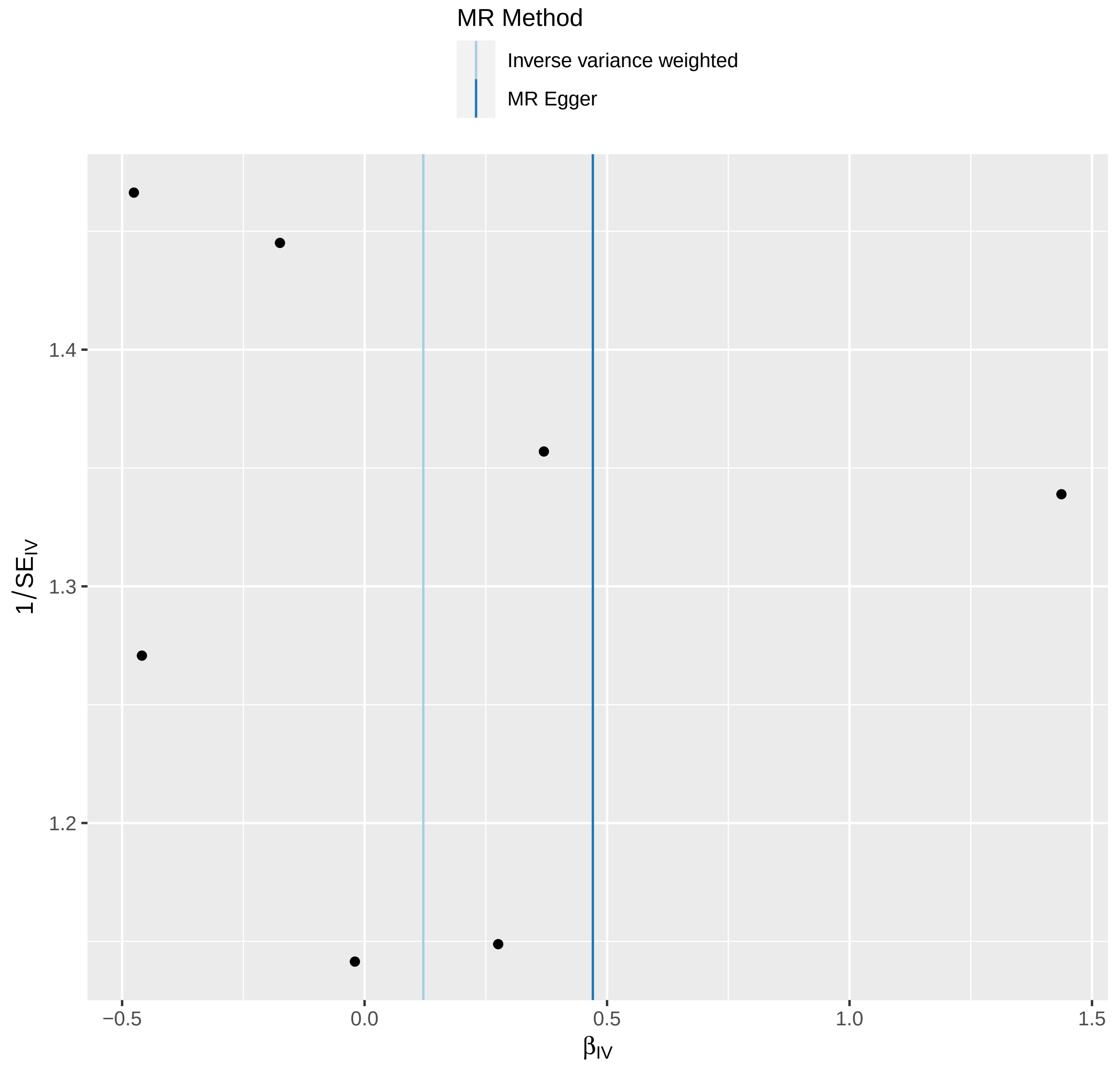

Batch 984 : Gut microbiota abundance (genus Flavonifractor id.2059) on Ulcerative enterocolitis

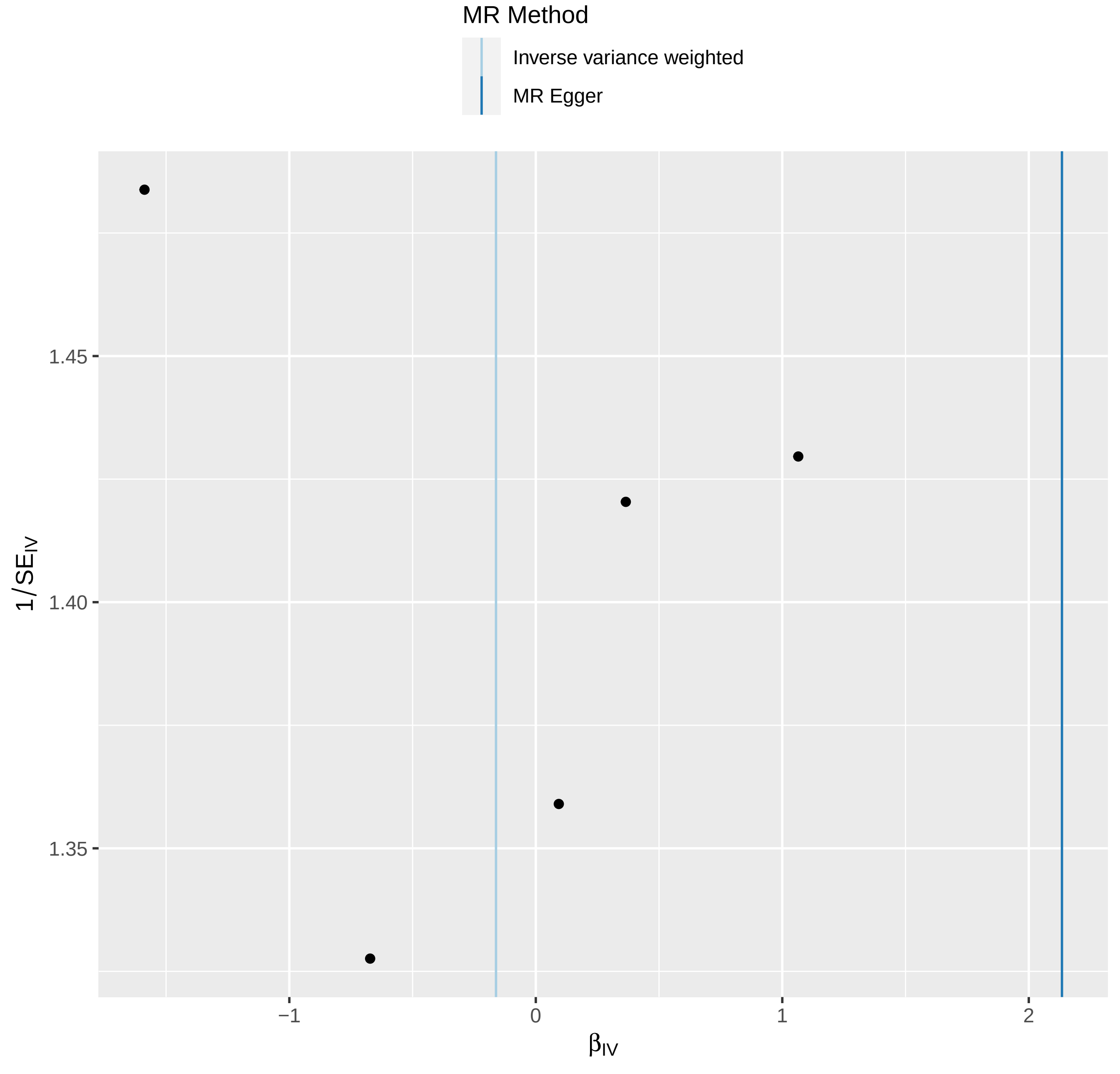

Batch 985 : Gut microbiota abundance (genus Fusicatenibacter id.11305) on Ulcerative enterocolitis

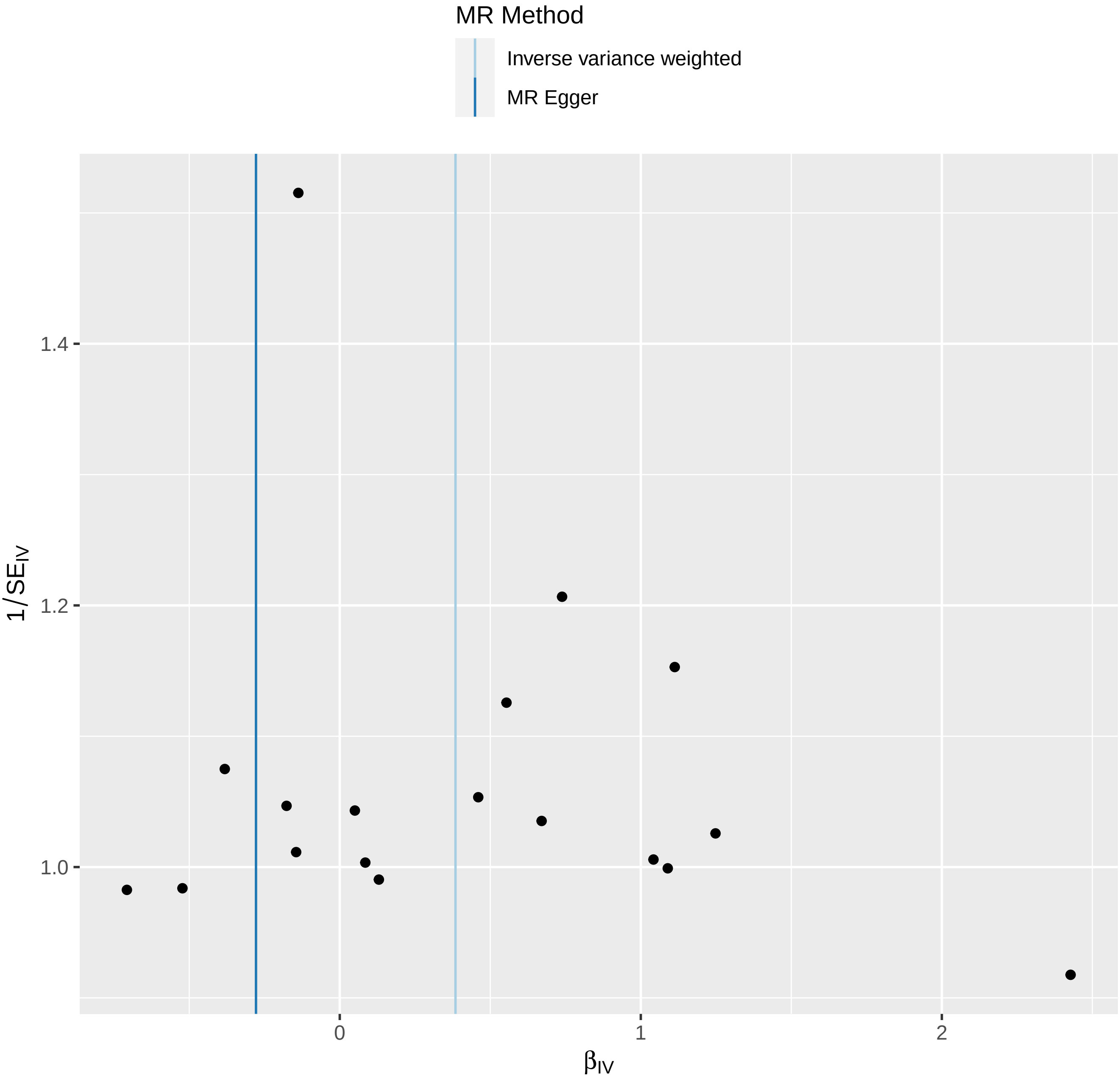

Batch 986 : Gut microbiota abundance (genus Gordonibacter id.821) on Ulcerative enterocolitis

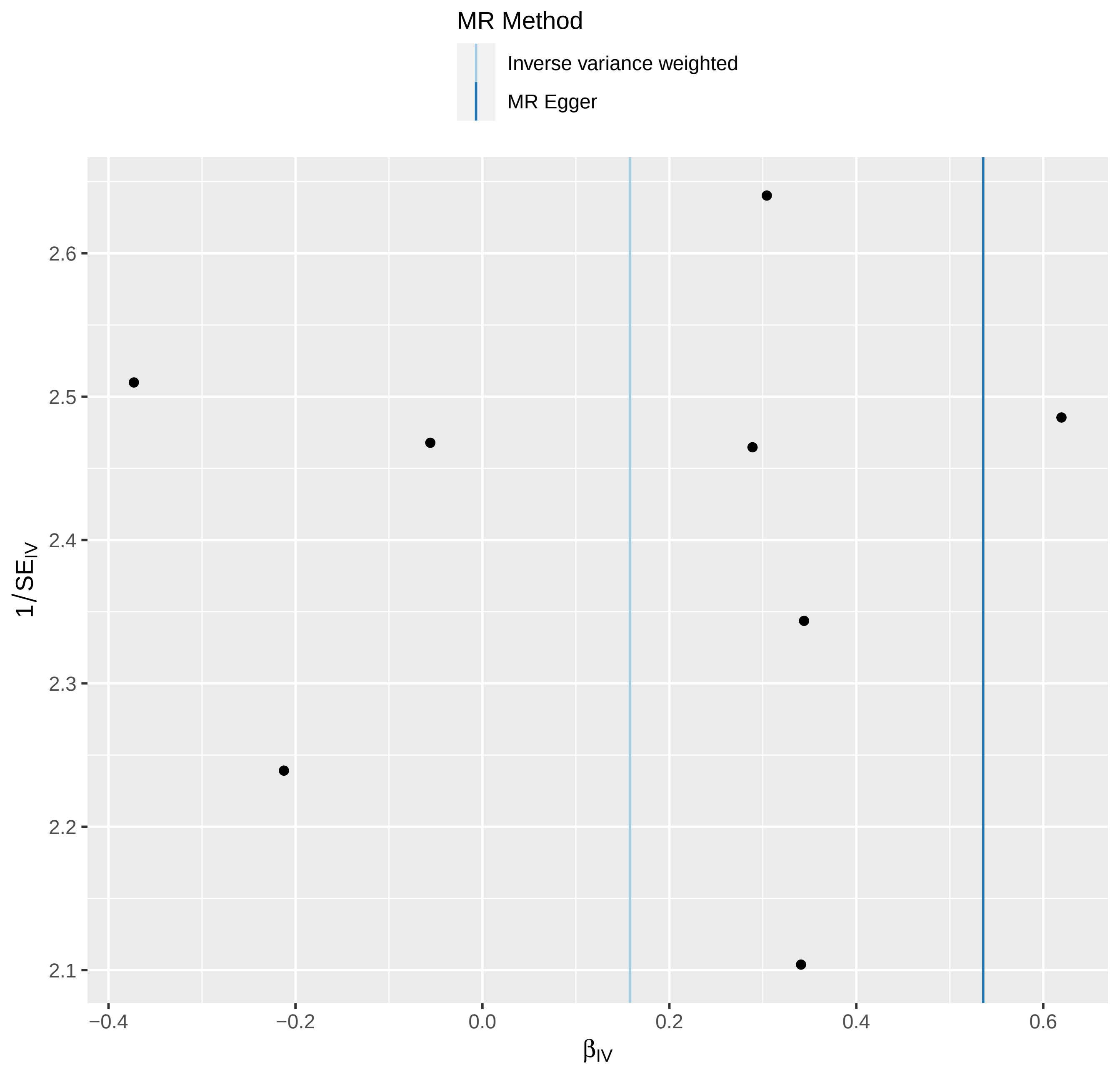

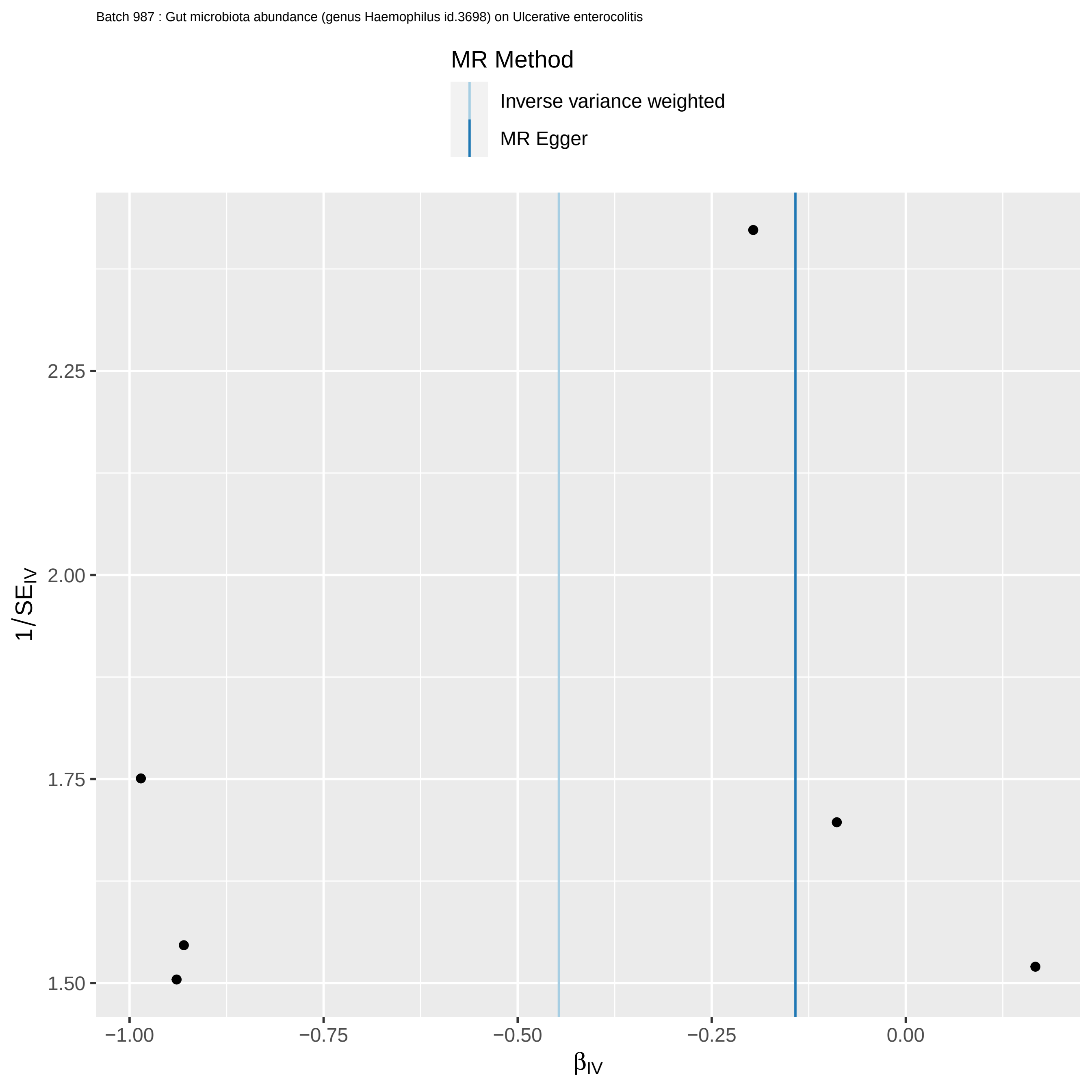
Batch 987 : Gut microbiota abundance (genus Haemophilus id.3698) on Ulcerative enterocolitis
MR Method
Inverse variance weighted
MR Egger
1/SE_IV
2.25
2.00
1.75
1.50
−1.00
−0.75
−0.50
−0.25
0.00
β_IV

Batch 988 : Gut microbiota abundance (genus Holdemanella id.11393) on Ulcerative enterocolitis

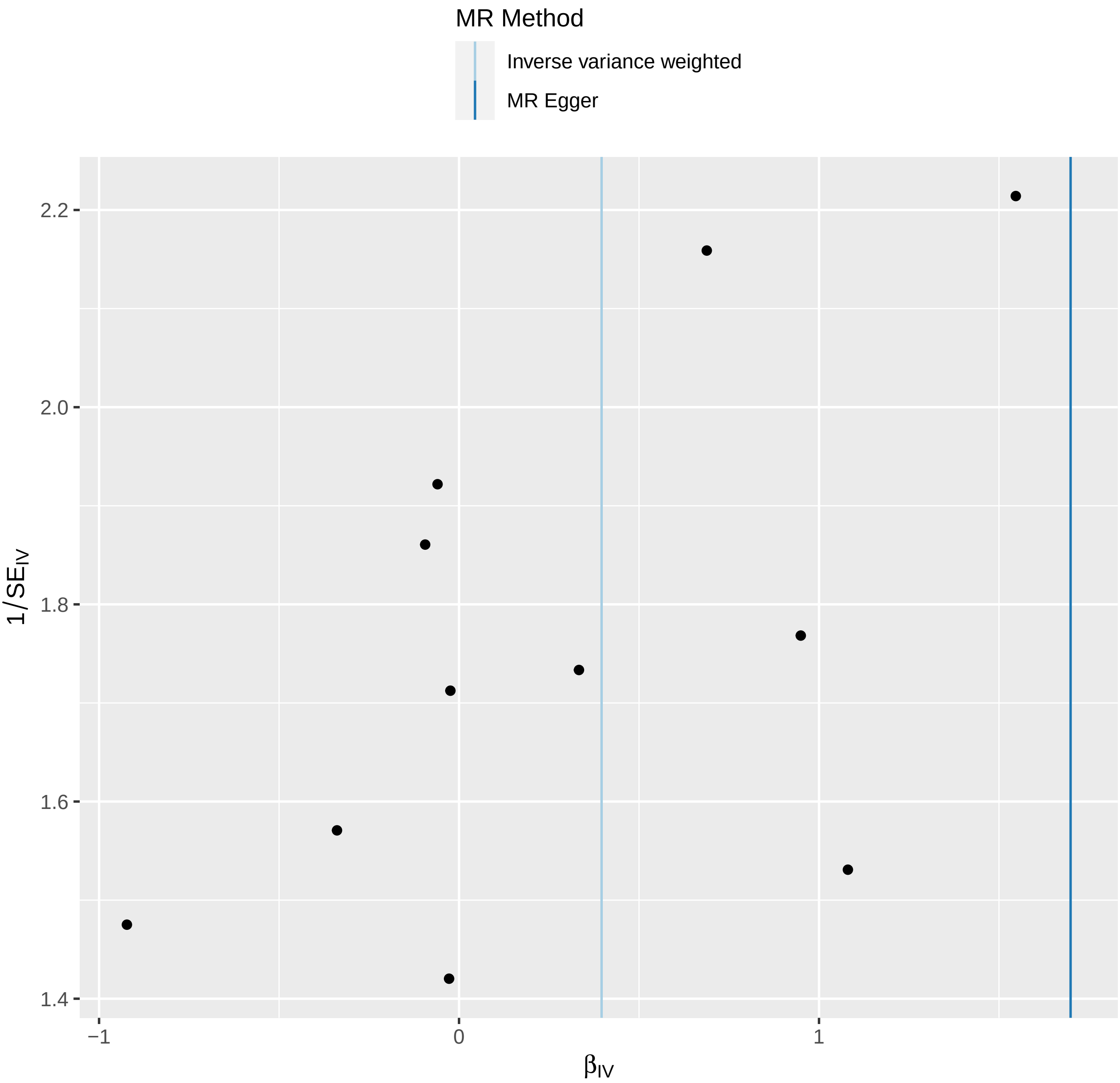

Batch 989 : Gut microbiota abundance (genus Holdemania id.2157) on Ulcerative enterocolitis

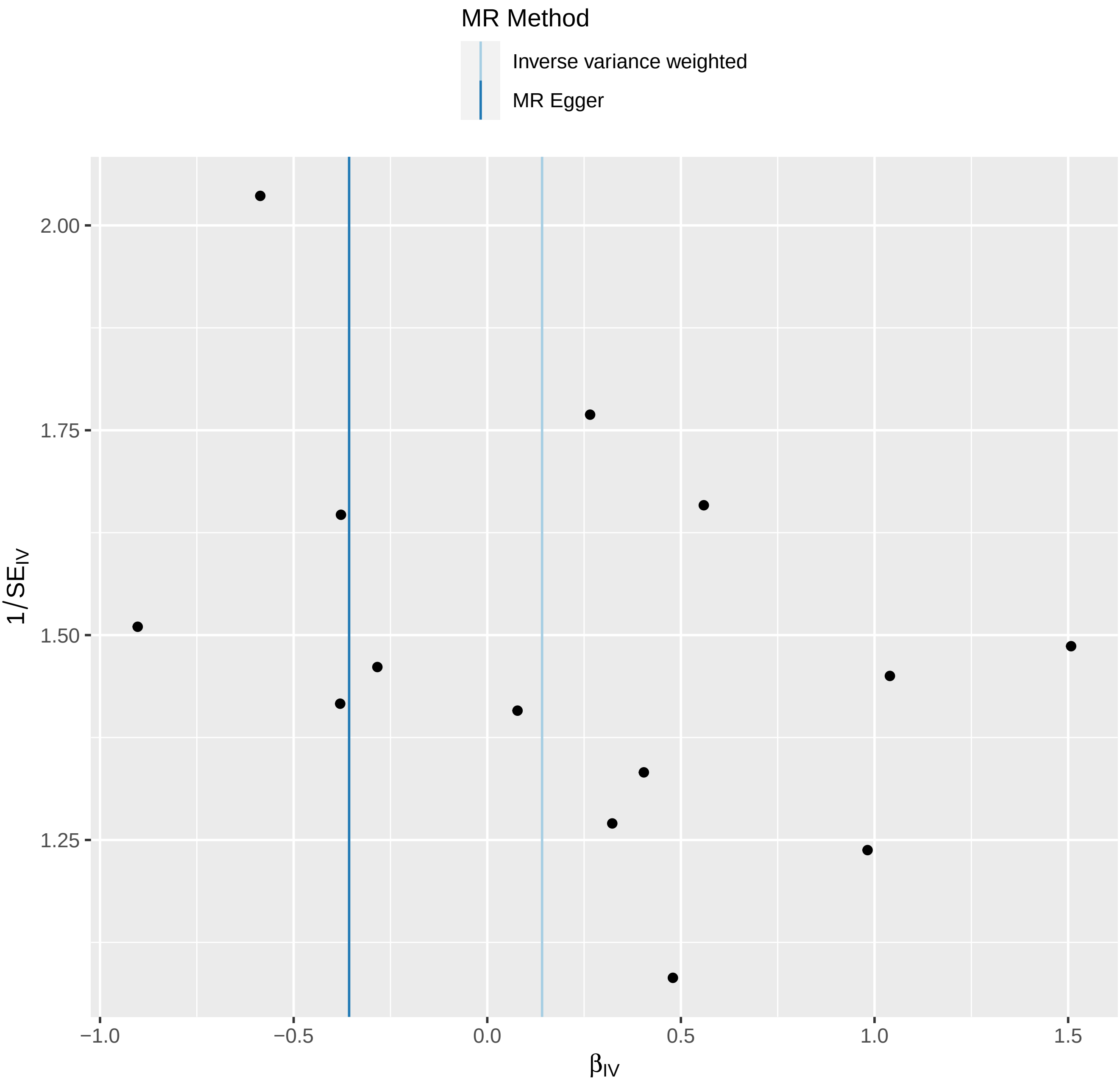

Batch 990 : Gut microbiota abundance (genus Howardella id.2000) on Ulcerative enterocolitis

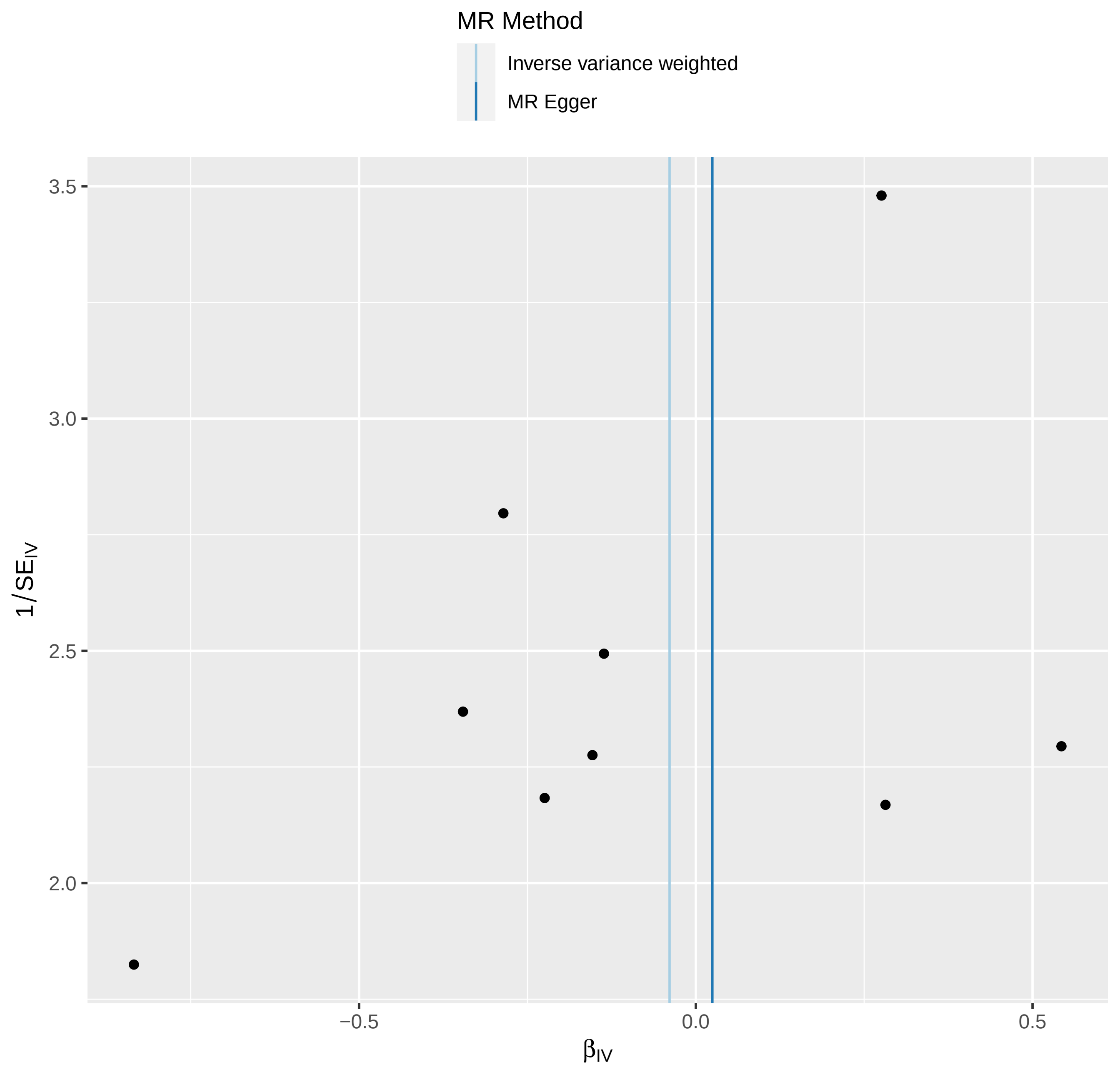

Batch 991 : Gut microbiota abundance (genus Hungatella id.11306) on Ulcerative enterocolitis

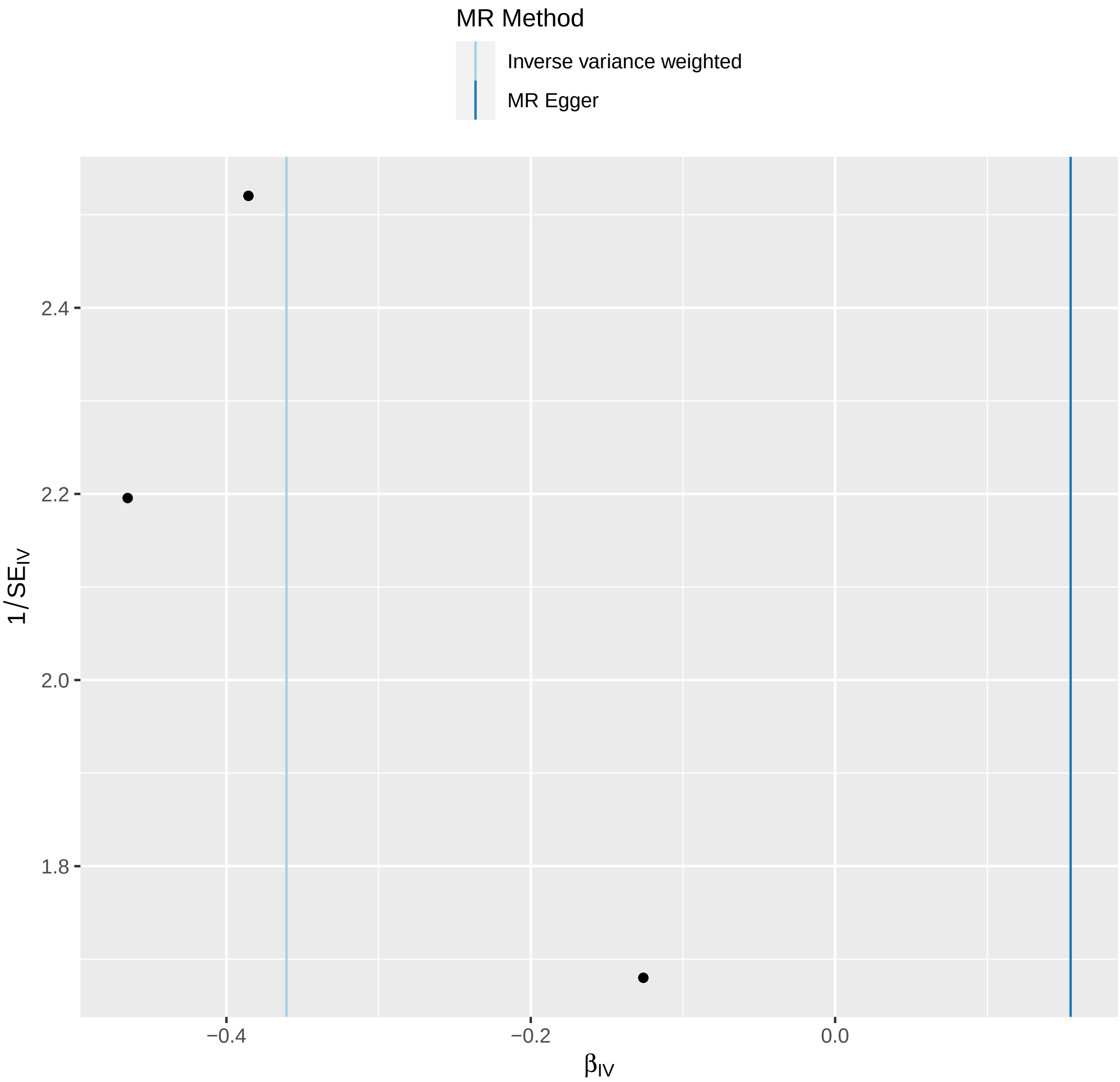

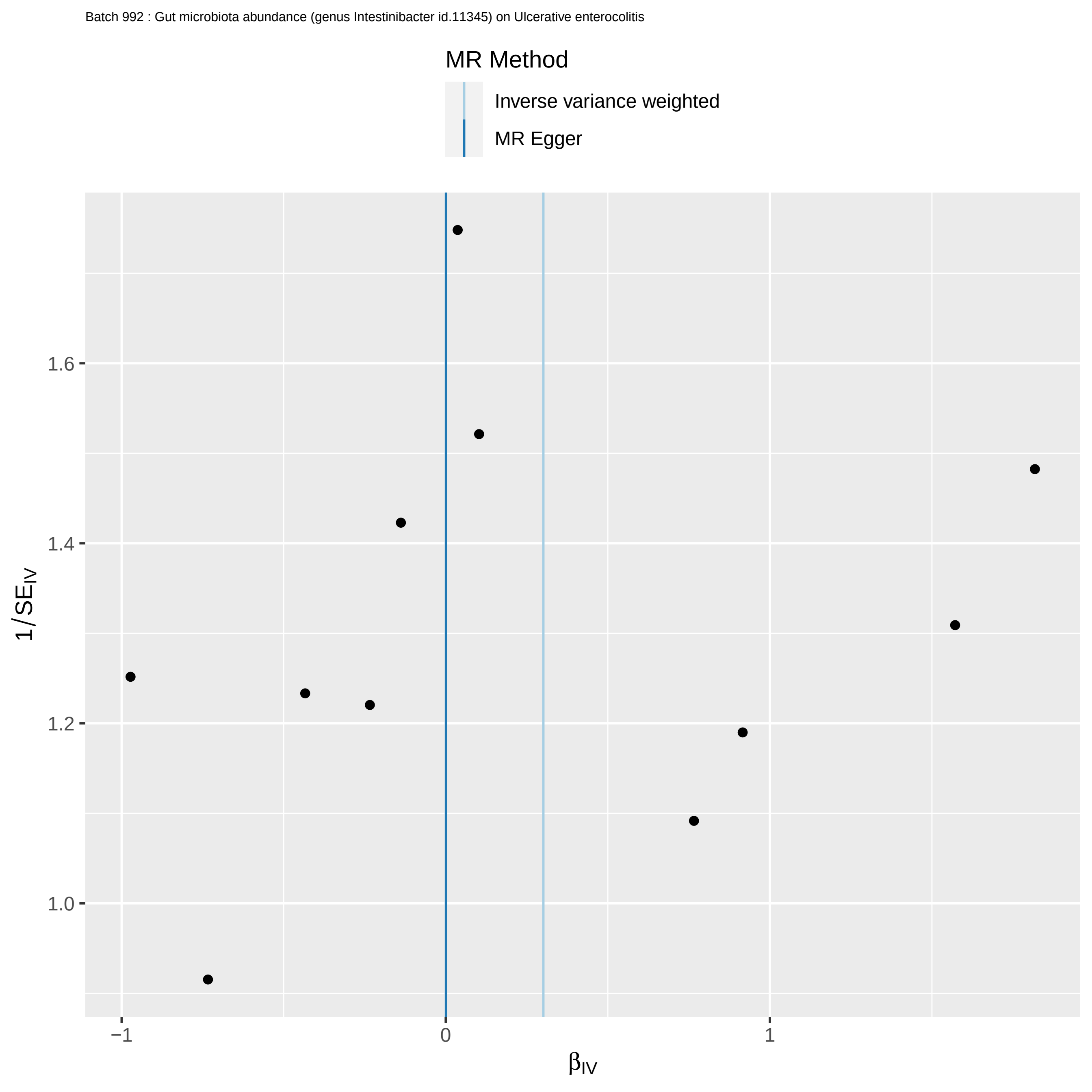

Batch 992 : Gut microbiota abundance (genus Intestinibacter id.11345) on Ulcerative enterocolitis
MR Method
Inverse variance weighted
MR Egger
1.6
1.4
1.2
1.0
1/SE_IV
−1
0
1
β_IV

Batch 993 : Gut microbiota abundance (genus Intestinimonas id.2062) on Ulcerative enterocolitis

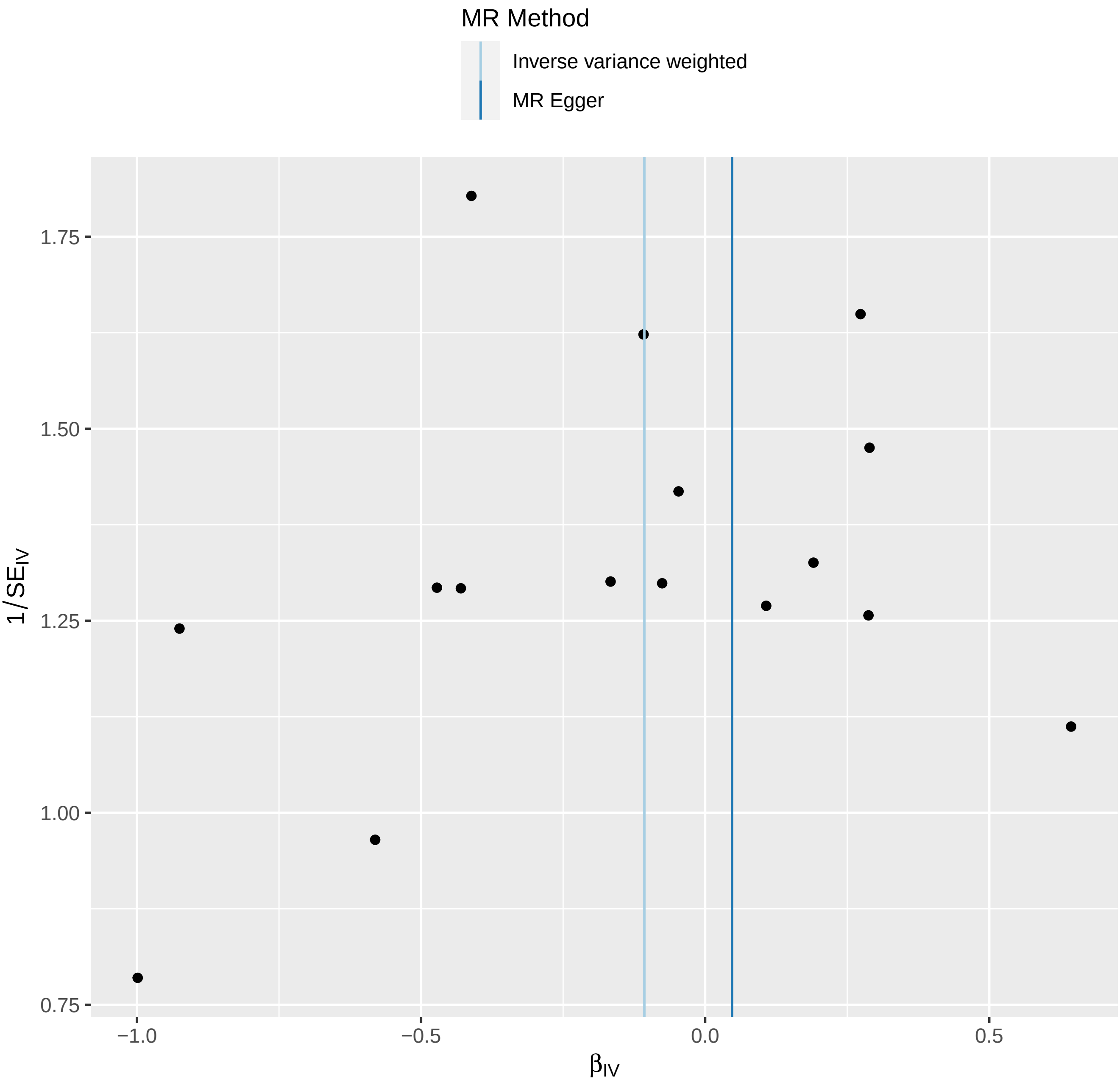

Batch 994 : Gut microbiota abundance (genus Lachnoclostridium id.11308) on Ulcerative enterocolitis

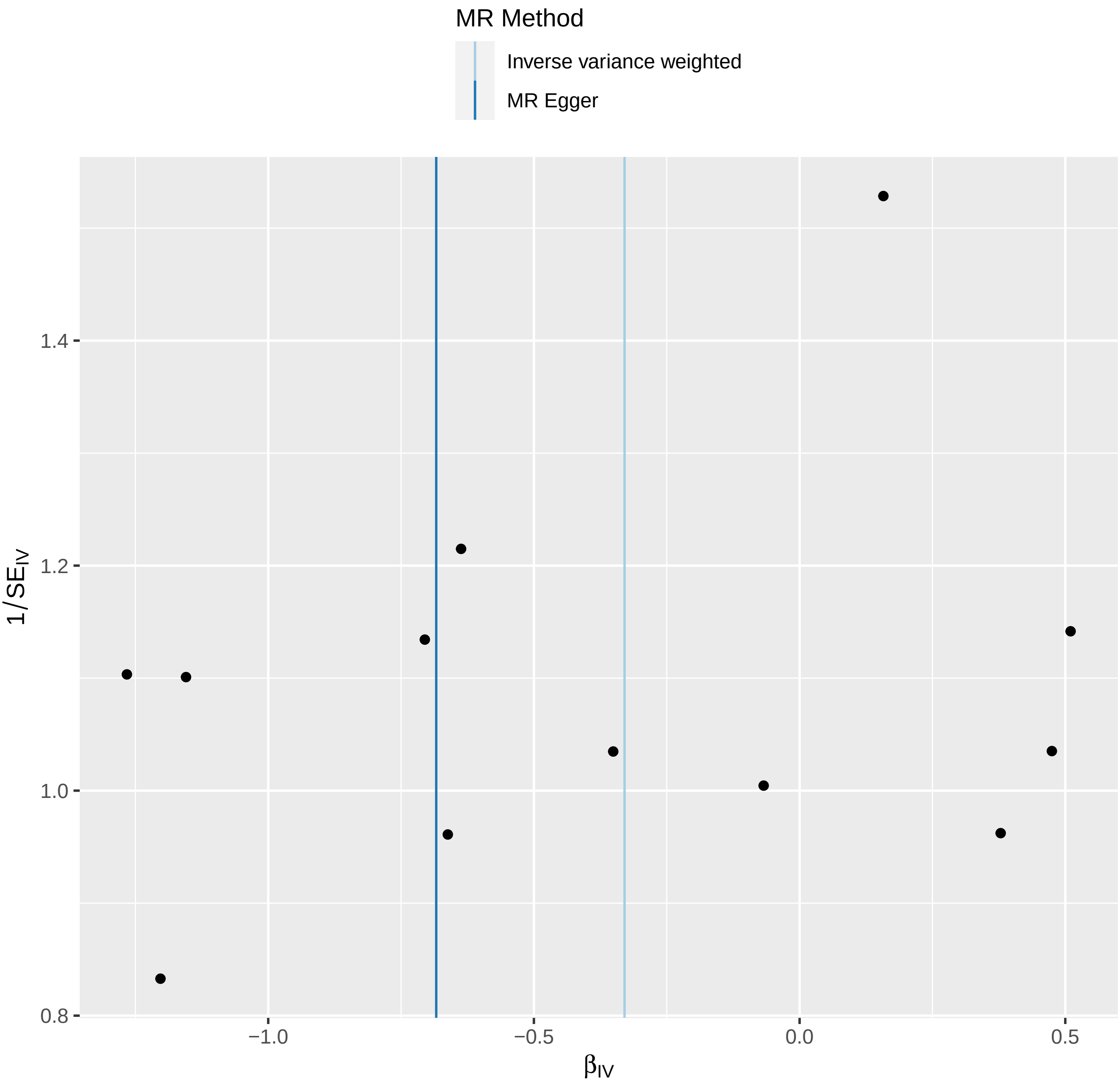

Batch 995 : Gut microbiota abundance (genus Lachnospiraceae FCS020 group id.11314) on Ulcerative enterocolitis

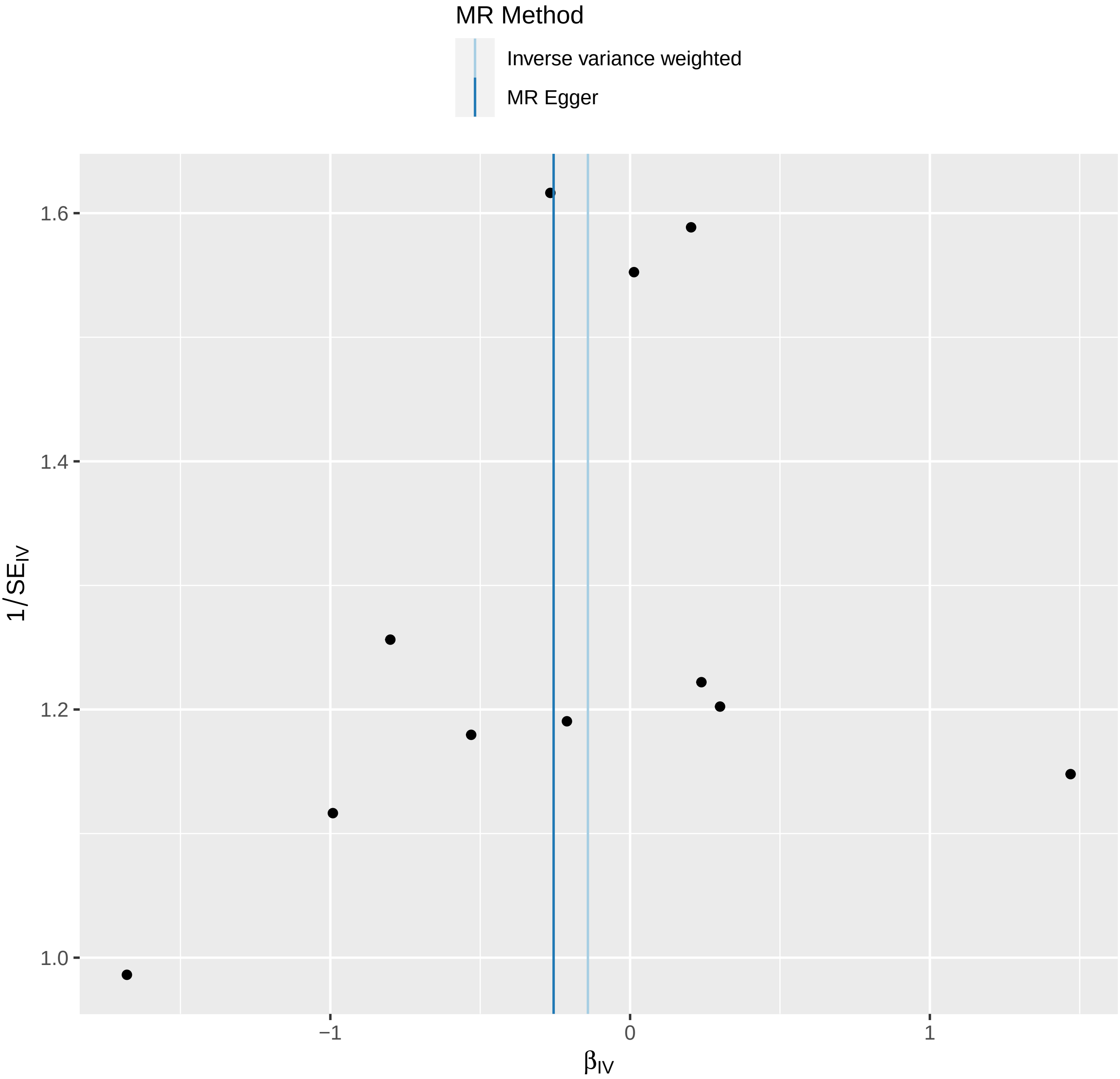

Batch 996 : Gut microbiota abundance (genus Lachnospiraceae NC2004 group id.11316) on Ulcerative enterocolitis

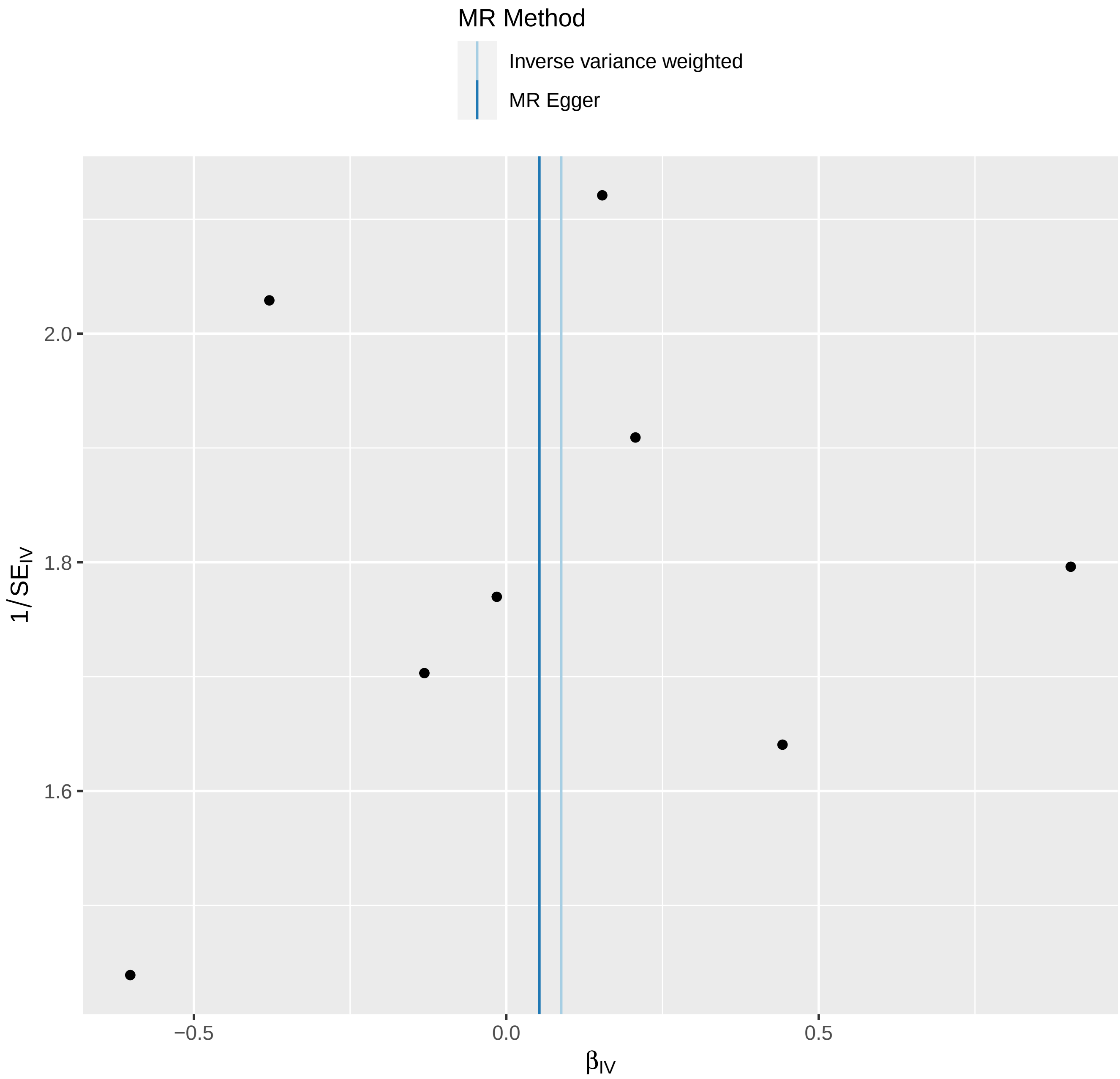

Batch 997 : Gut microbiota abundance (genus Lachnospiraceae ND3007 group id.11317) on Ulcerative enterocolitis

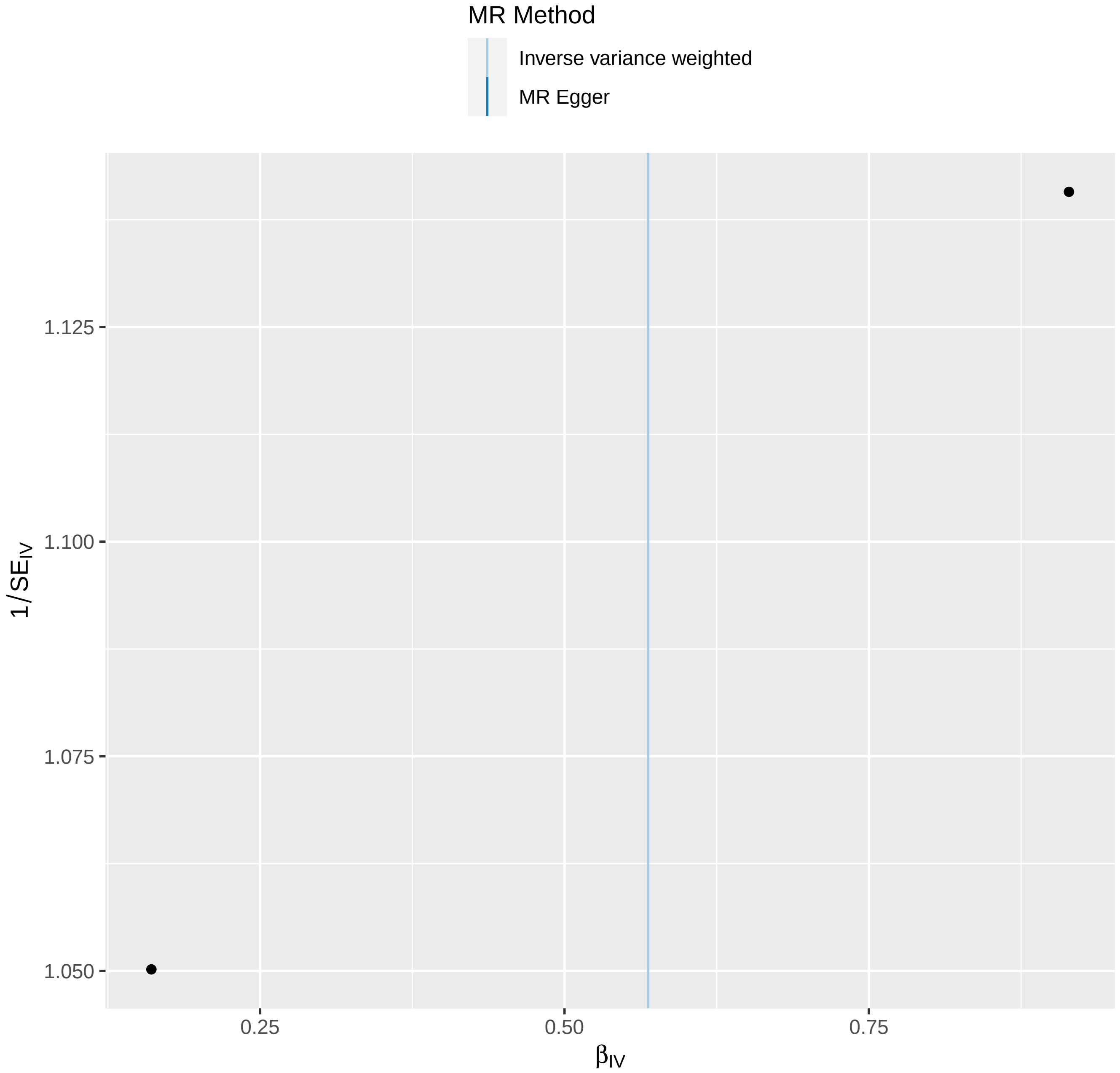

Batch 998 : Gut microbiota abundance (genus Lachnospiraceae NK4A136 group id.11319) on Ulcerative enterocolitis

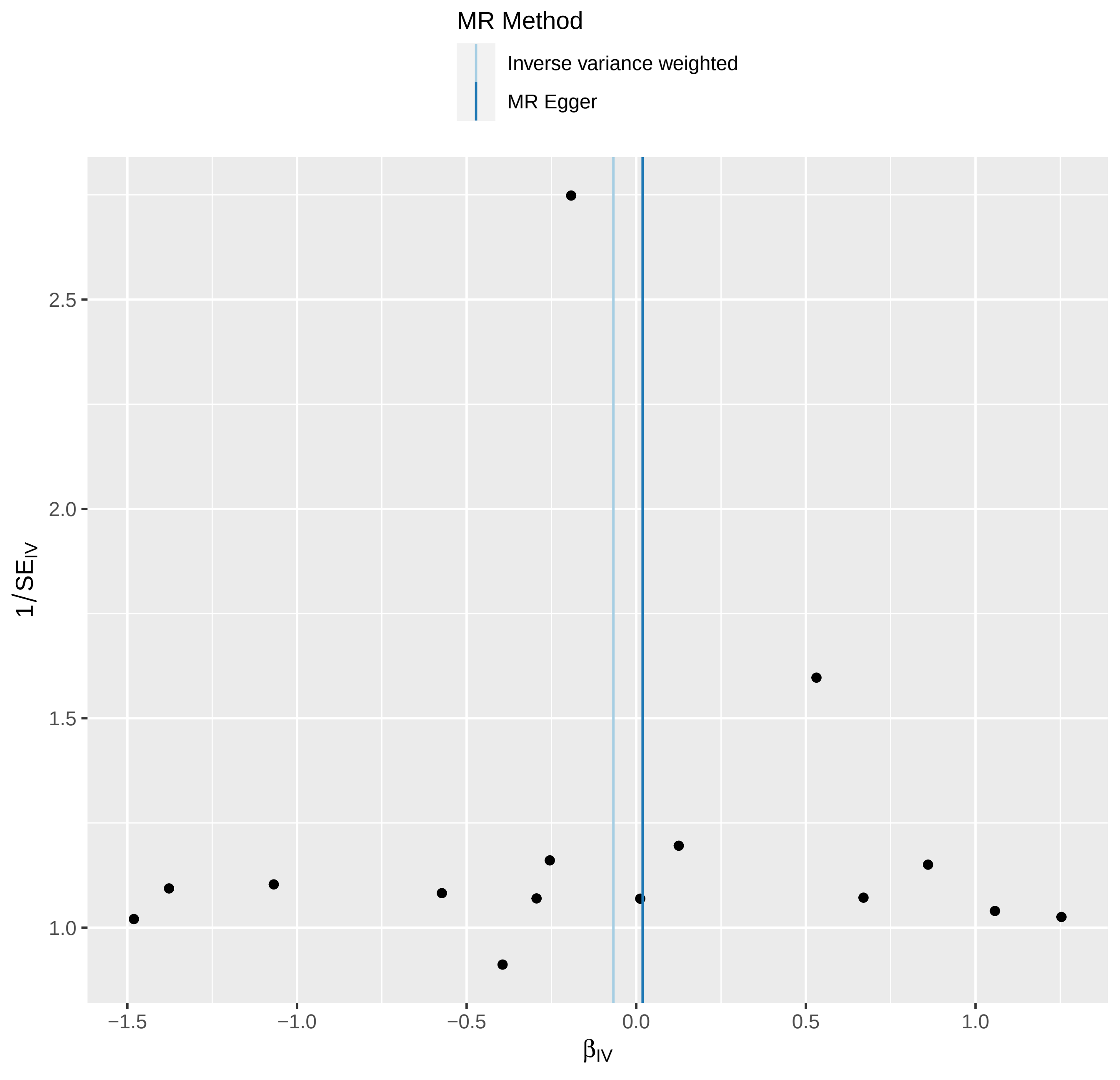

Batch 999 : Gut microbiota abundance (genus Lachnospiraceae UCG001 id.11321) on Ulcerative enterocolitis

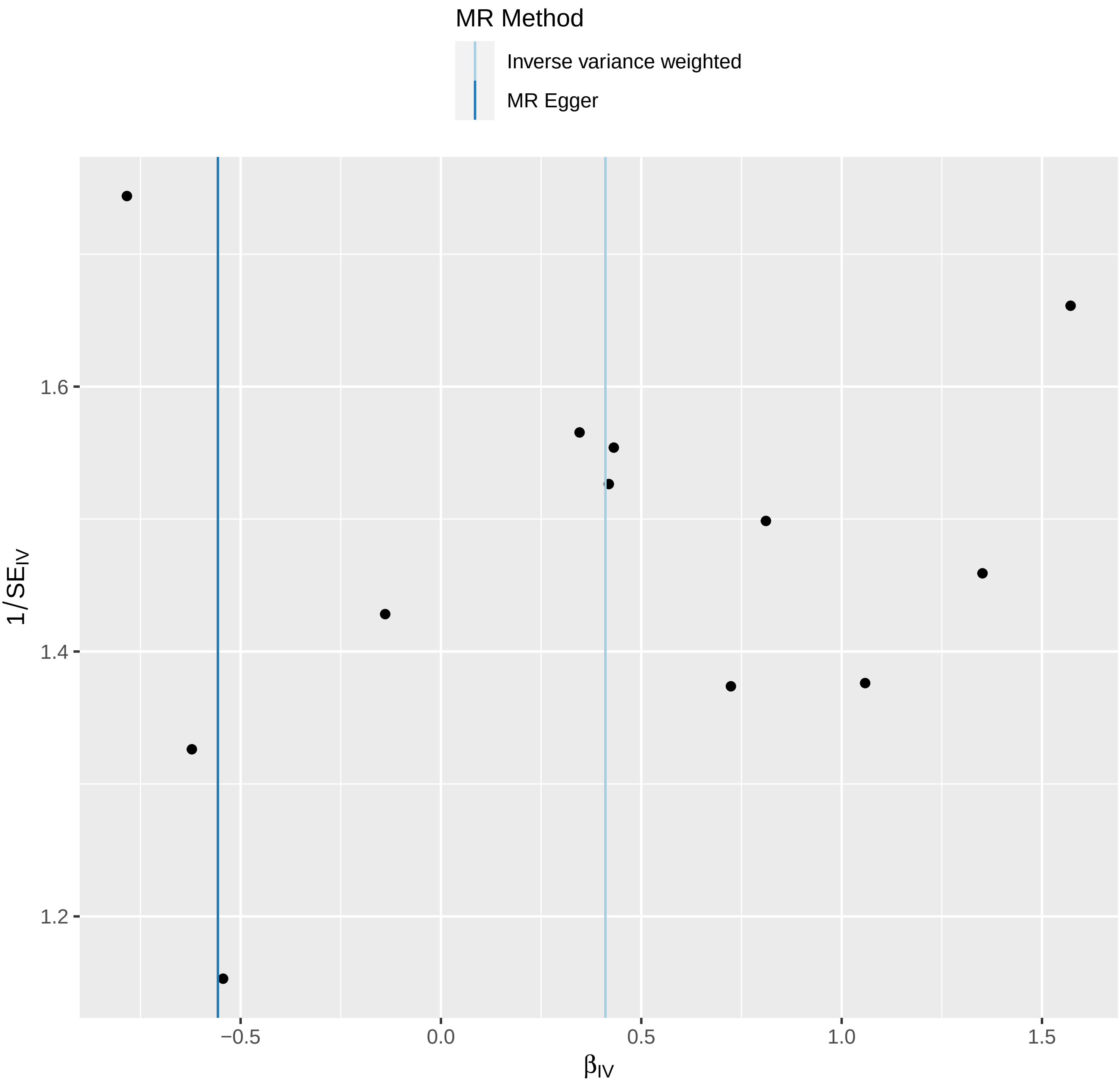

Batch 1000 : Gut microbiota abundance (genus Lachnospiraceae UCG004 id.11324) on Ulcerative enterocolitis

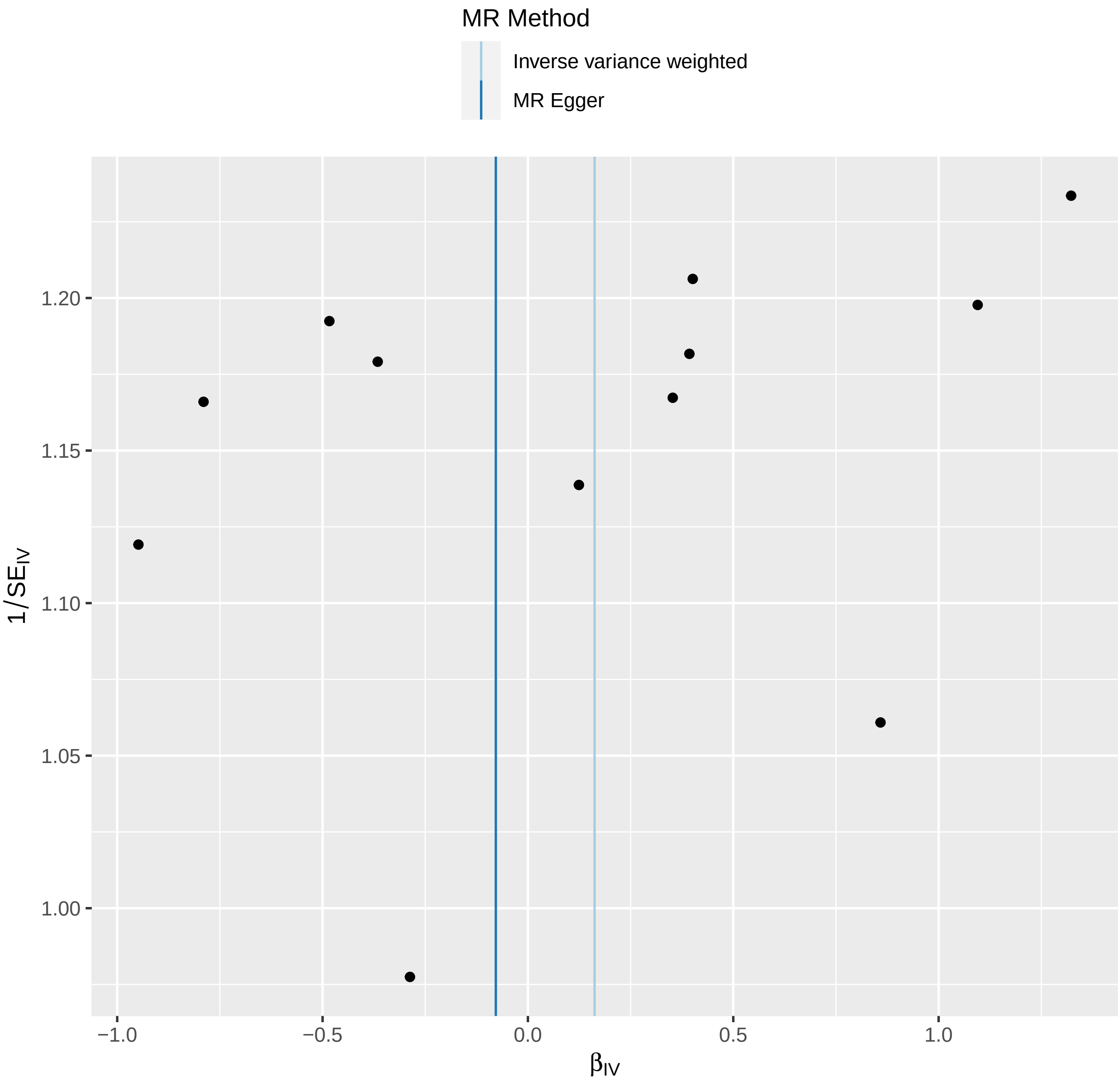

Batch 1001 : Gut microbiota abundance (genus Lachnospiraceae UCG008 id.11328) on Ulcerative enterocolitis

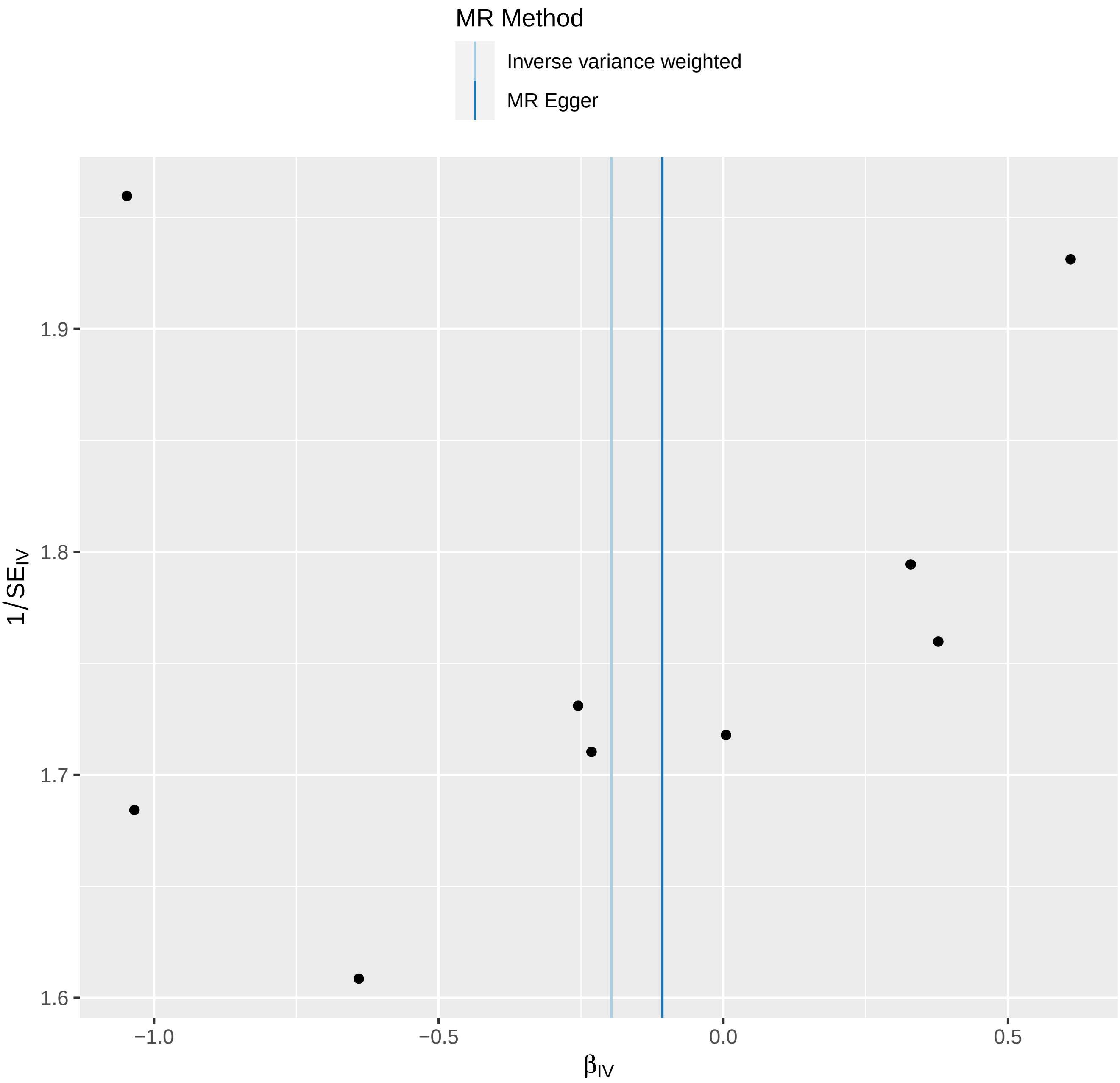

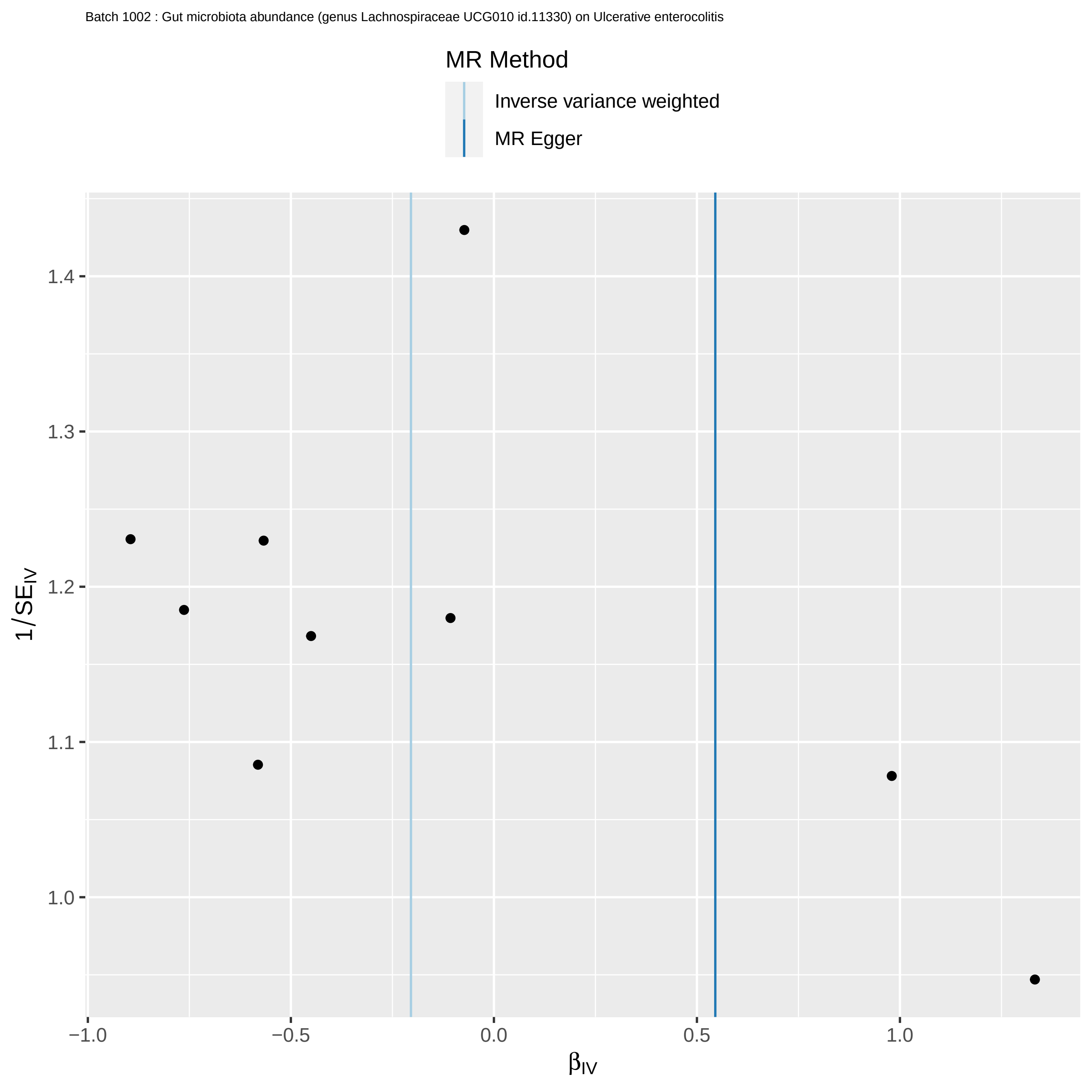
Batch 1002 : Gut microbiota abundance (genus Lachnospiraceae UCG010 id.11330) on Ulcerative enterocolitis
MR Method
Inverse variance weighted
MR Egger
1.4
1.3
1.2
1.1
1.0
1/SE_IV
−1.0
−0.5
0.0
0.5
1.0
β_IV

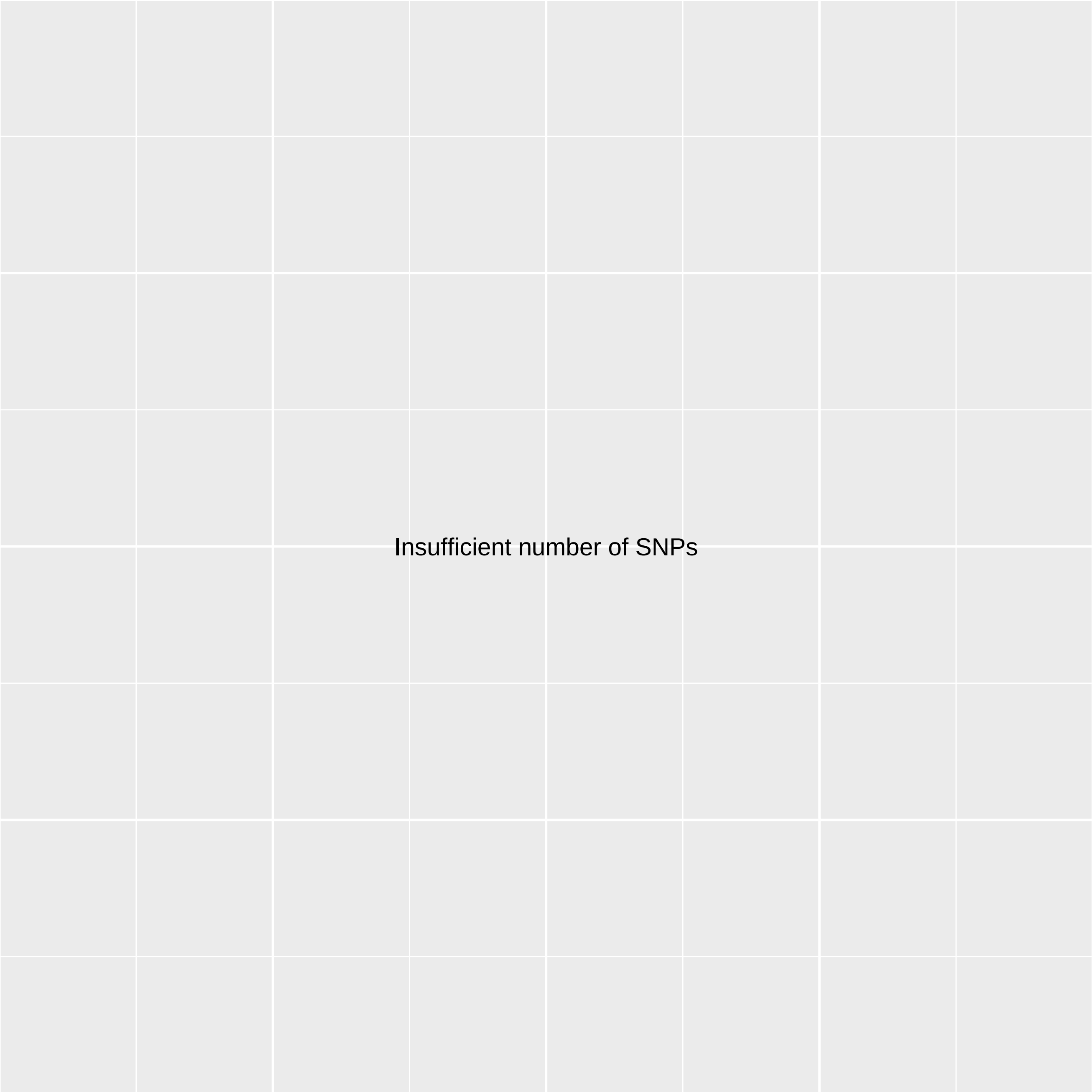
Insufficient number of SNPs

Batch 1004 : Gut microbiota abundance (genus Lactobacillus id.1837) on Ulcerative enterocolitis

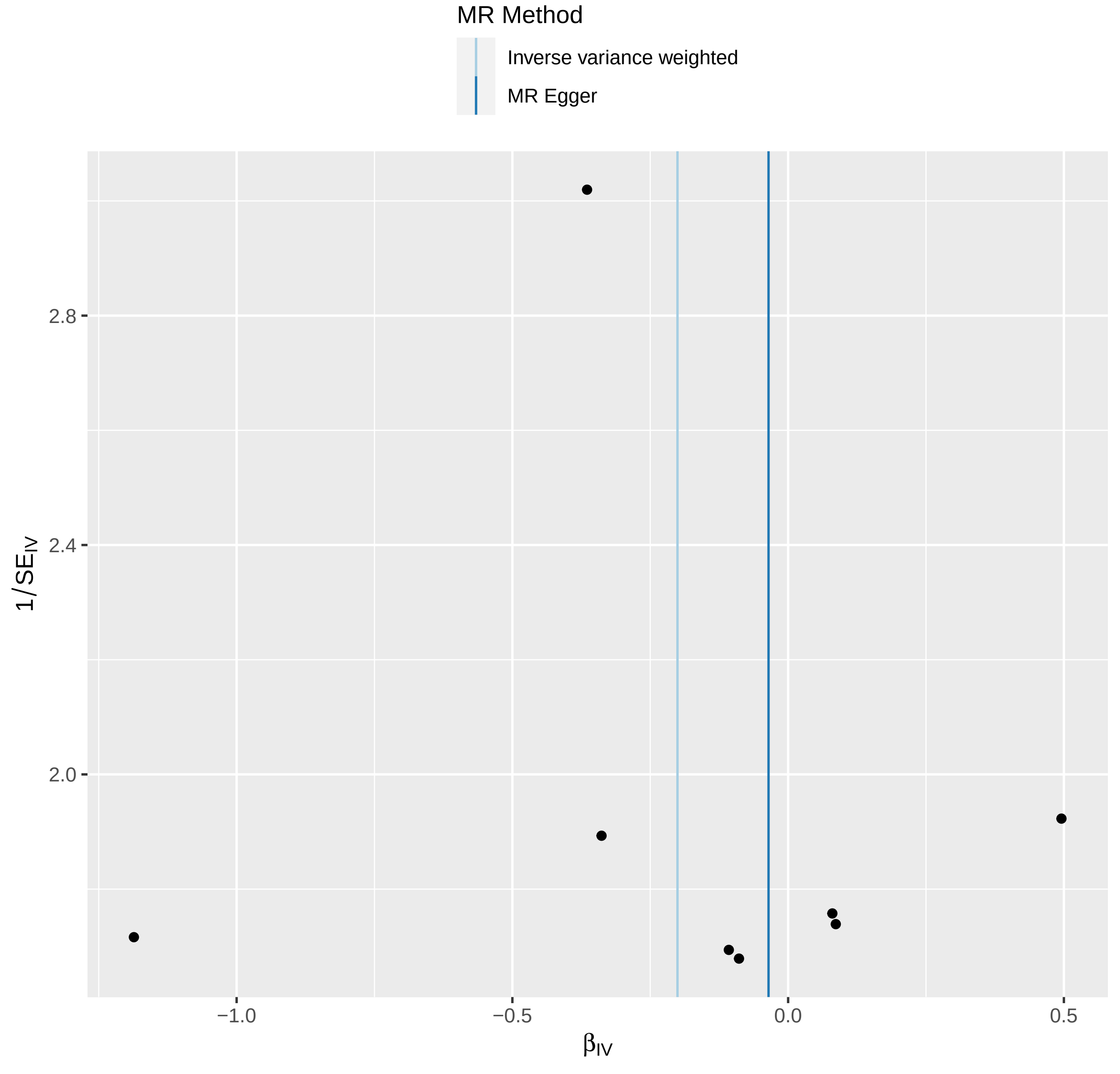

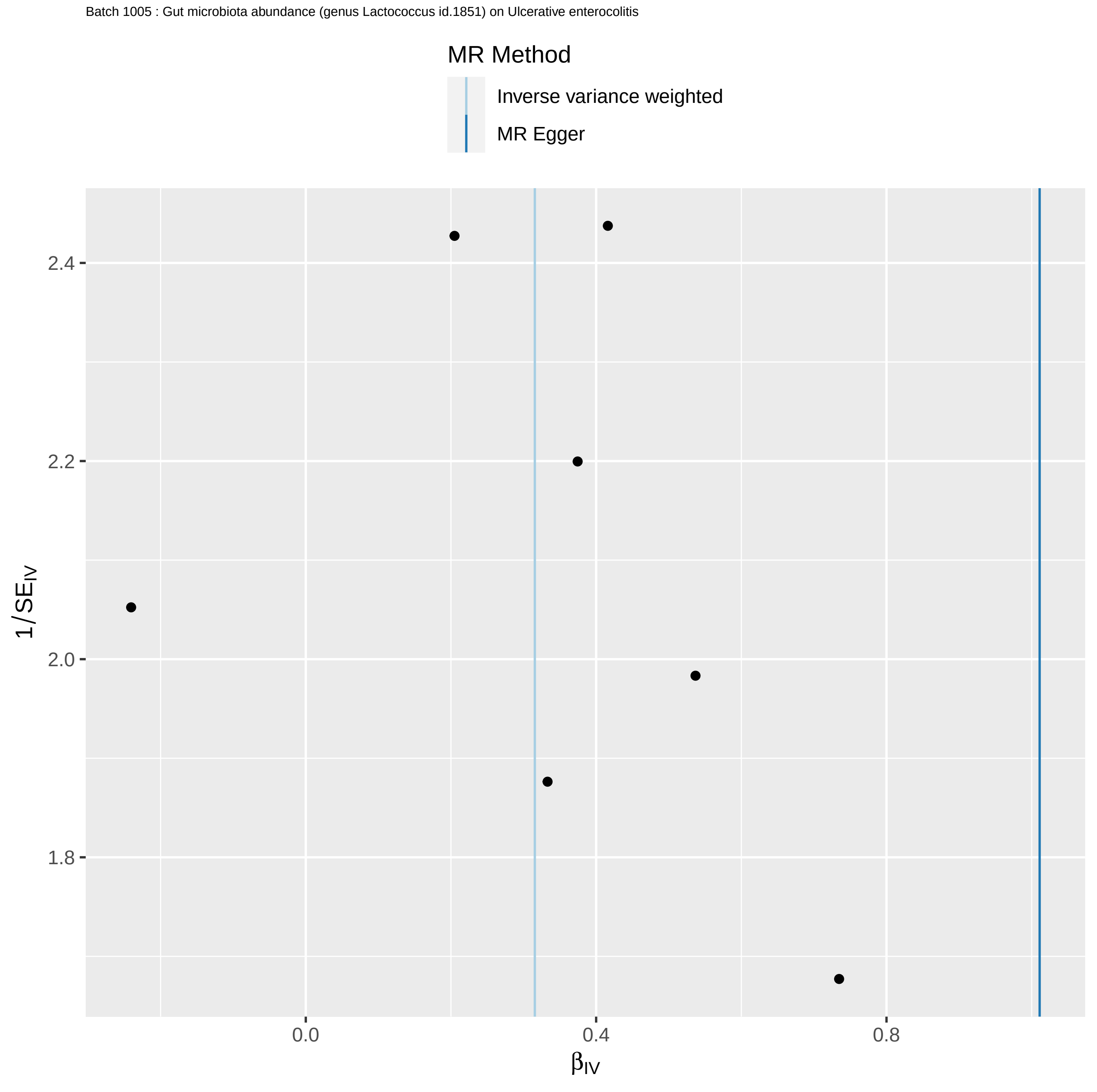
Batch 1005 : Gut microbiota abundance (genus Lactococcus id.1851) on Ulcerative enterocolitis
MR Method
Inverse variance weighted
MR Egger
2.4
2.2
2.0
1.8
1/SE_IV
0.0
0.4
0.8
β_IV

Batch 1006 : Gut microbiota abundance (genus Marvinbryantia id.2005) on Ulcerative enterocolitis

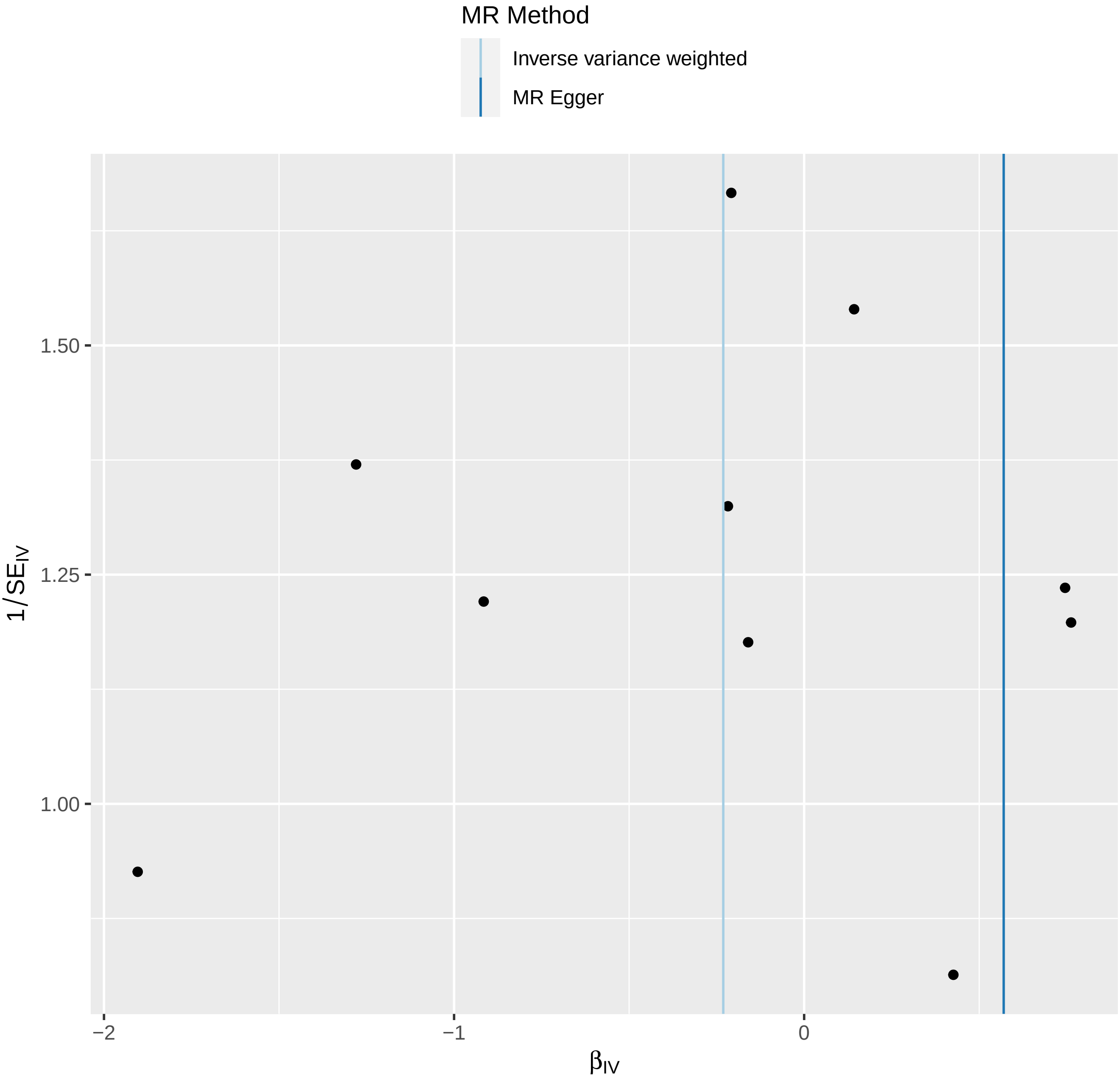

Batch 1007 : Gut microbiota abundance (genus Methanobrevibacter id.123) on Ulcerative enterocolitis

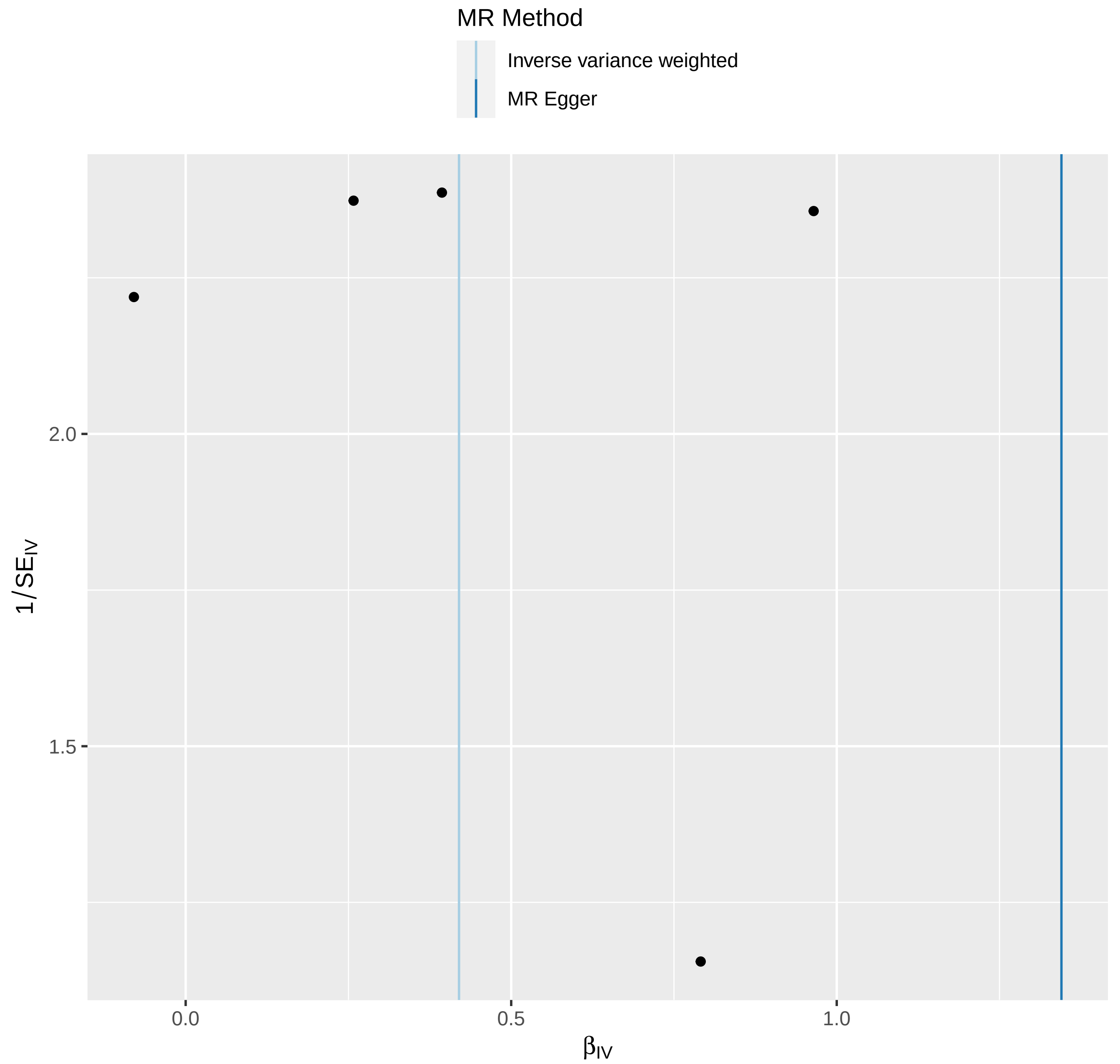

Batch 1008 : Gut microbiota abundance (genus Odoribacter id.952) on Ulcerative enterocolitis

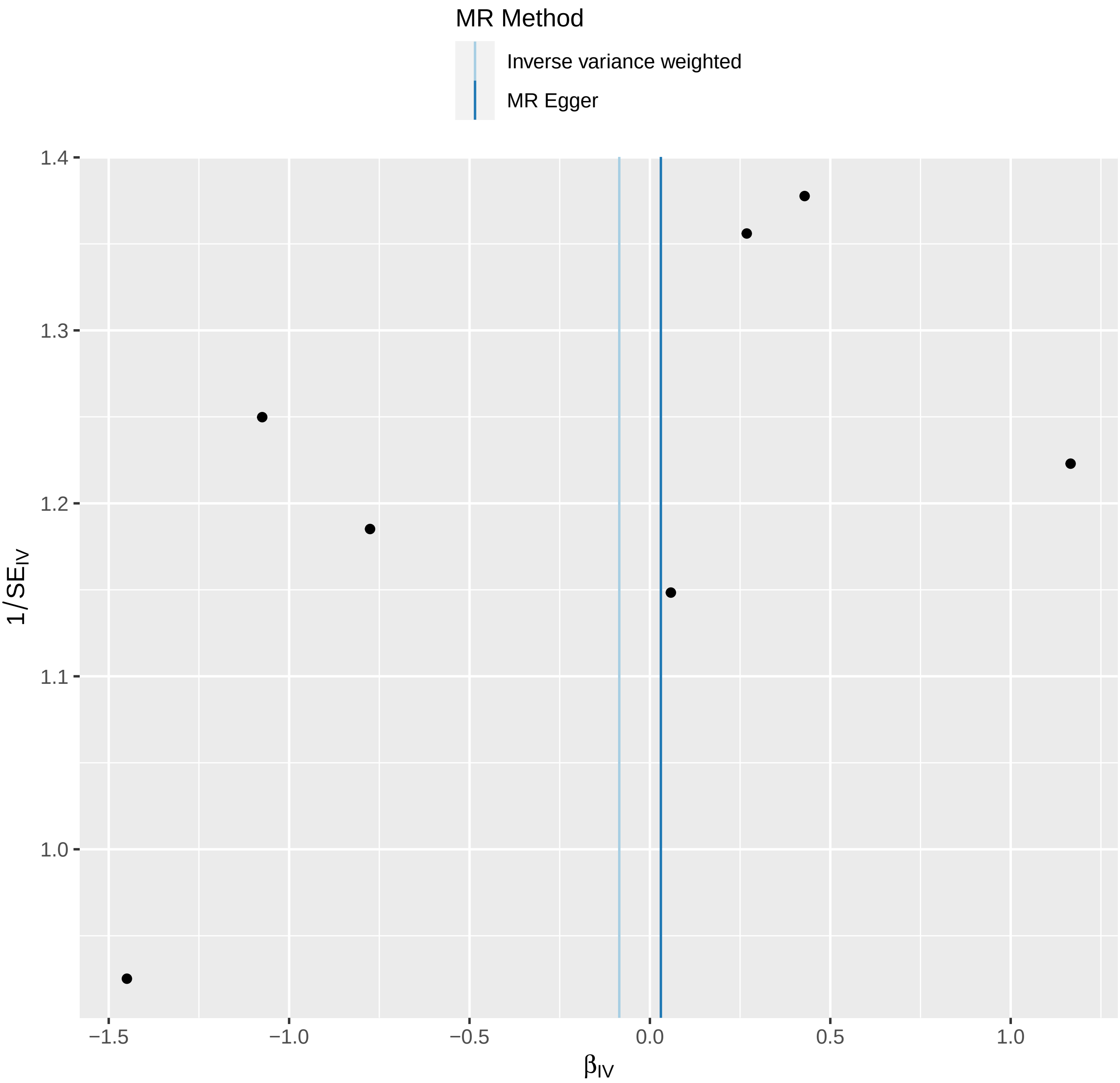

Batch 1009 : Gut microbiota abundance (genus Olsenella id.822) on Ulcerative enterocolitis

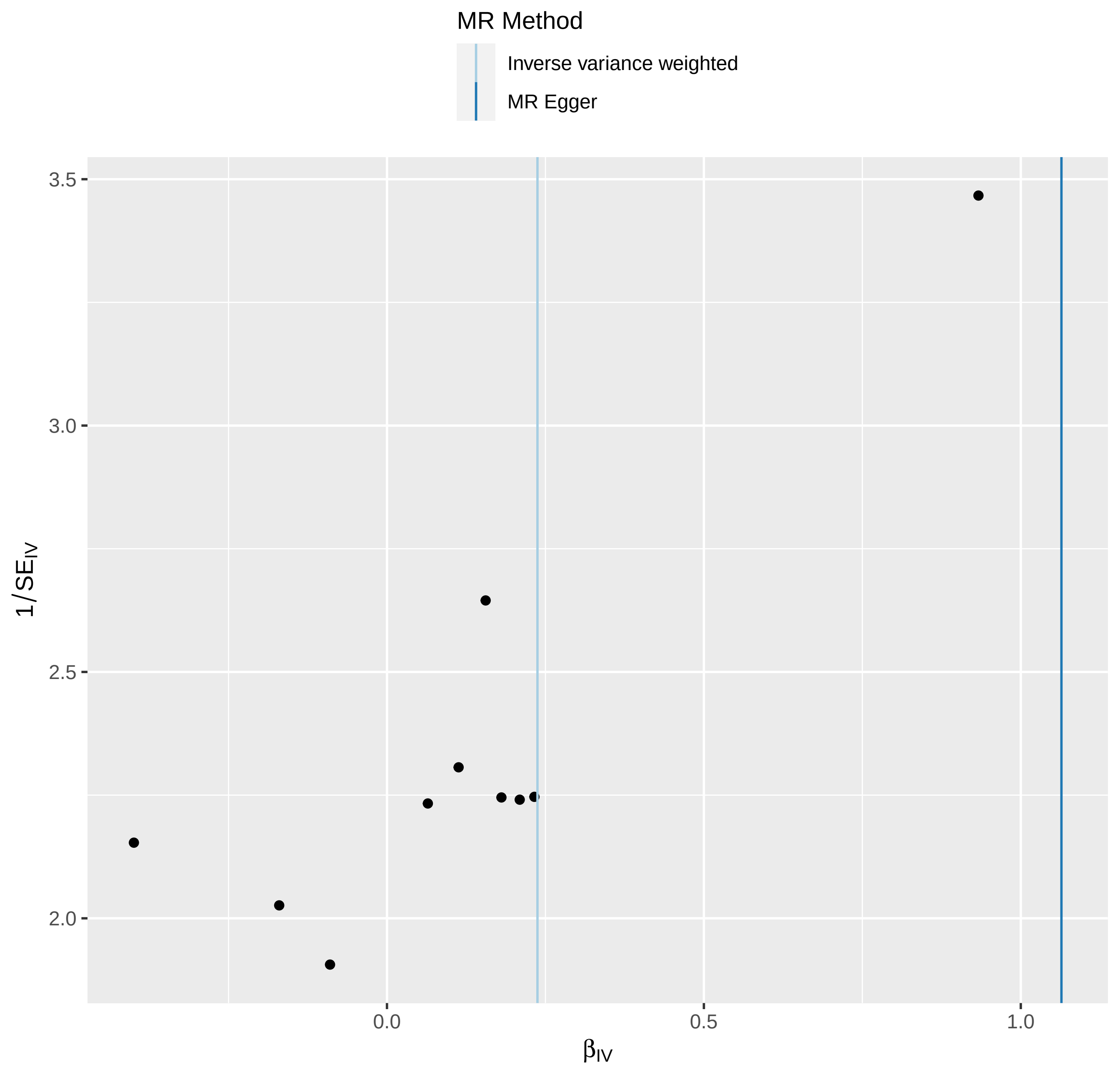

Batch 1010 : Gut microbiota abundance (genus Oscillibacter id.2063) on Ulcerative enterocolitis

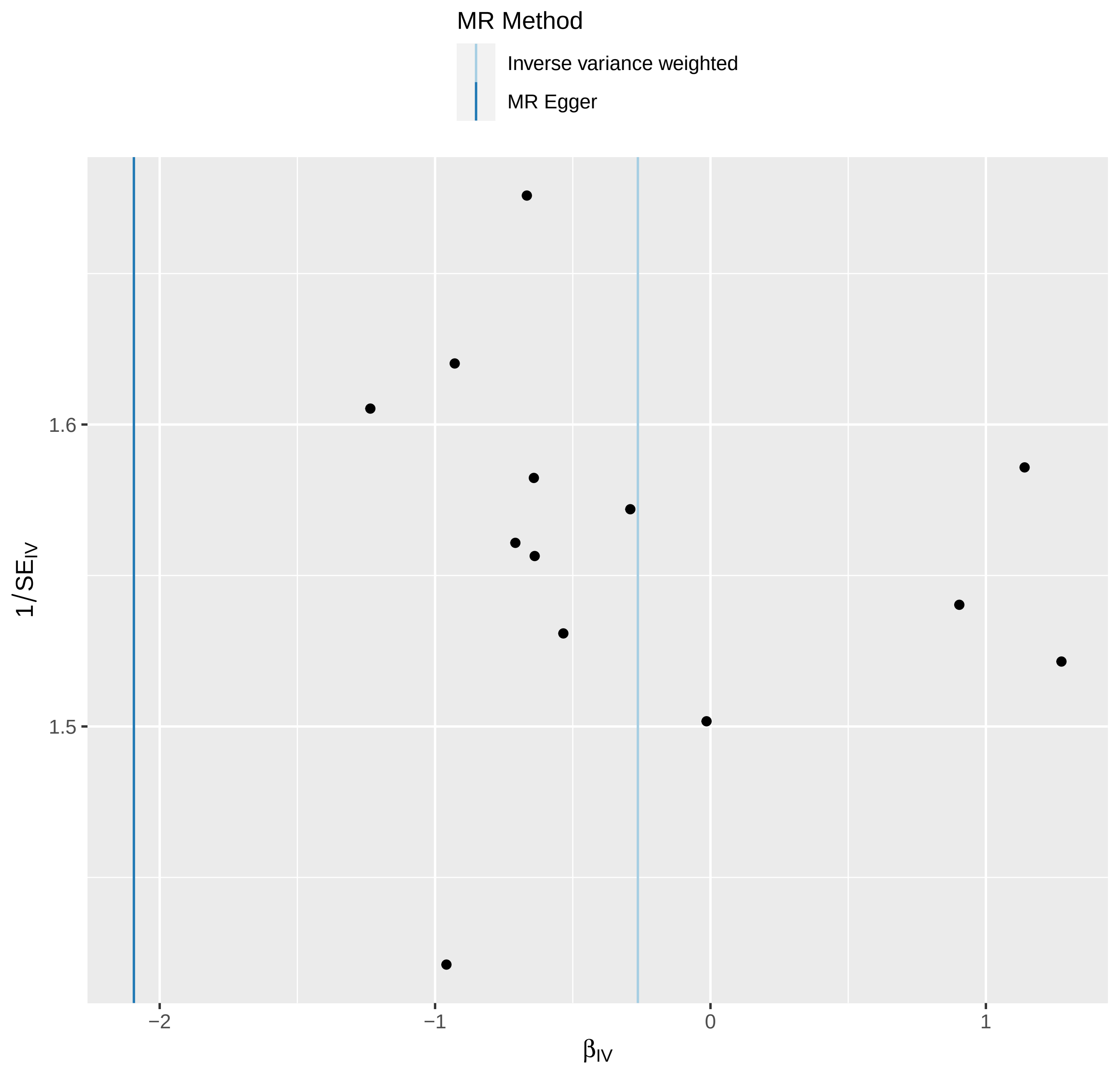

Batch 1011 : Gut microbiota abundance (genus Oscillospira id.2064) on Ulcerative enterocolitis

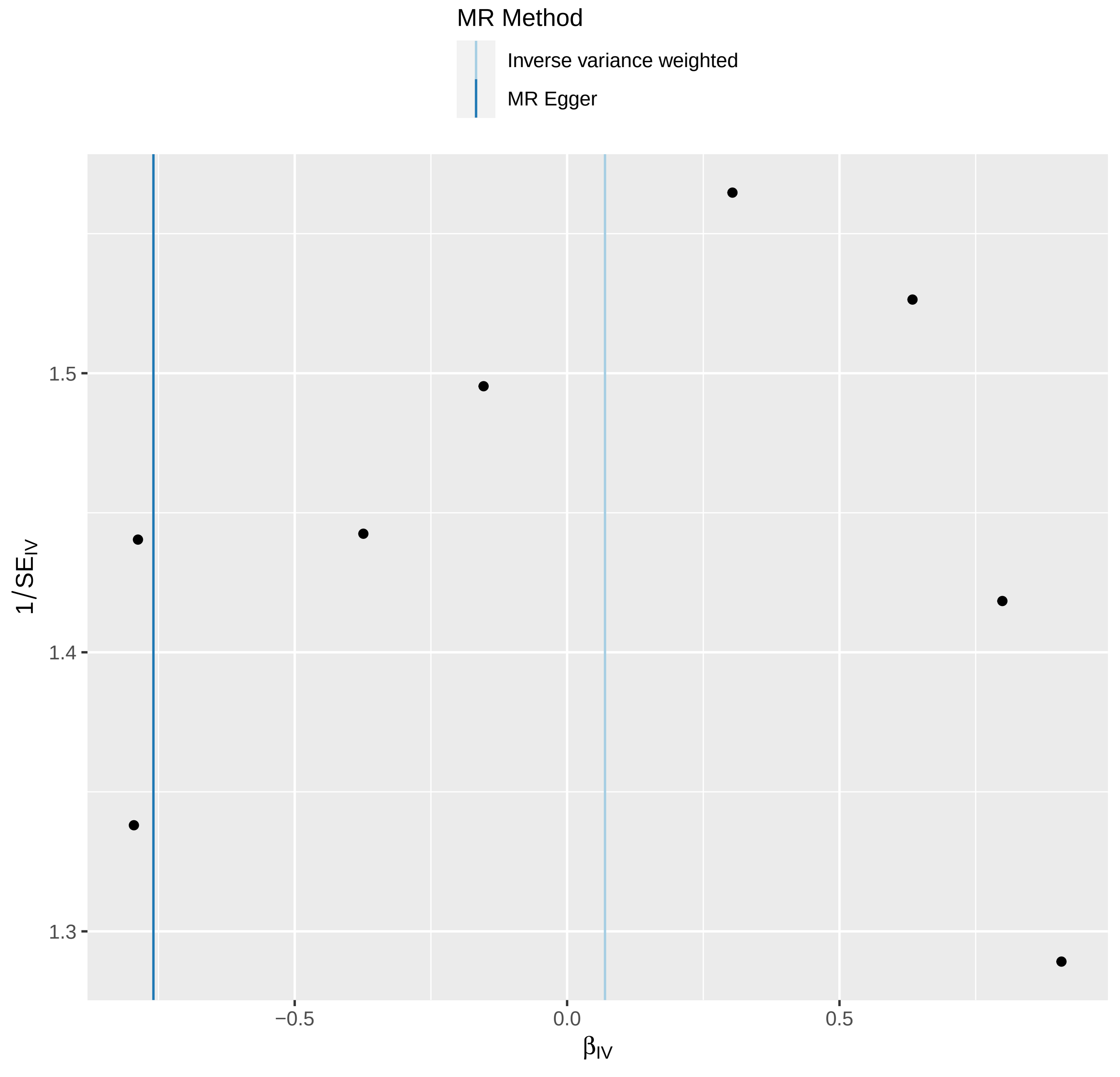

Batch 1012 : Gut microbiota abundance (genus Oxalobacter id.2978) on Ulcerative enterocolitis

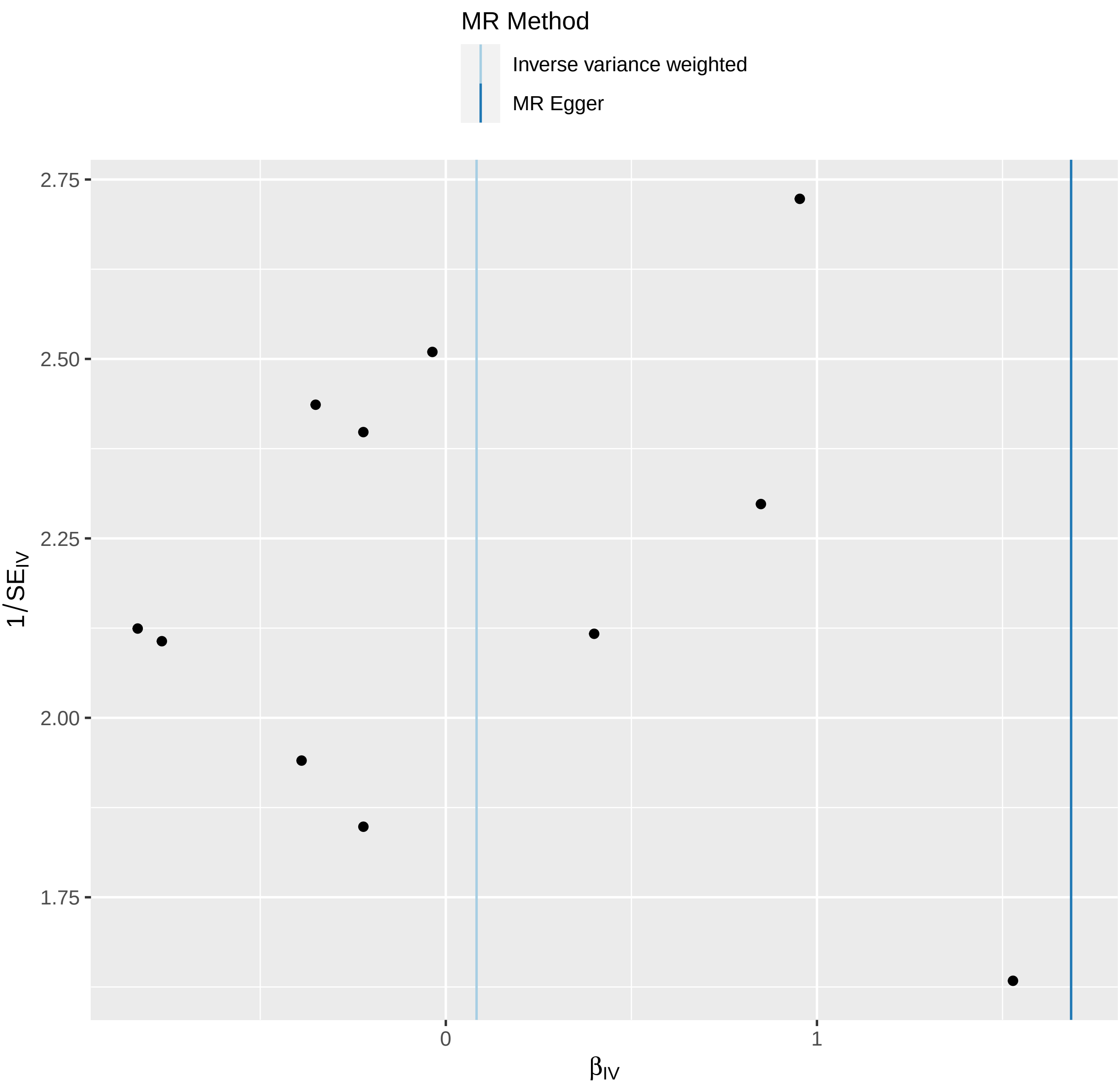

Batch 1013 : Gut microbiota abundance (genus Parabacteroides id.954) on Ulcerative enterocolitis

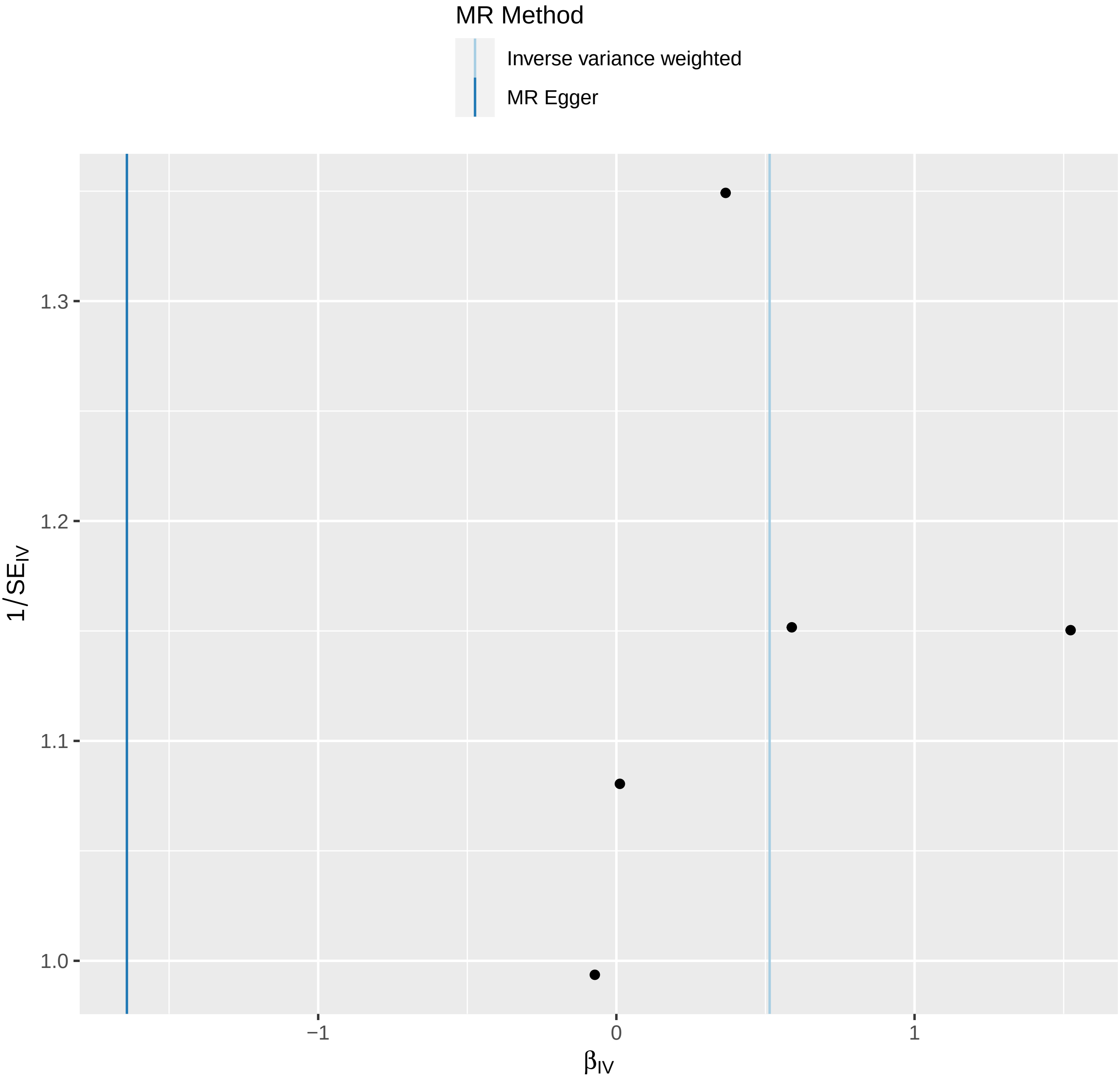

Batch 1014 : Gut microbiota abundance (genus Paraprevotella id.962) on Ulcerative enterocolitis

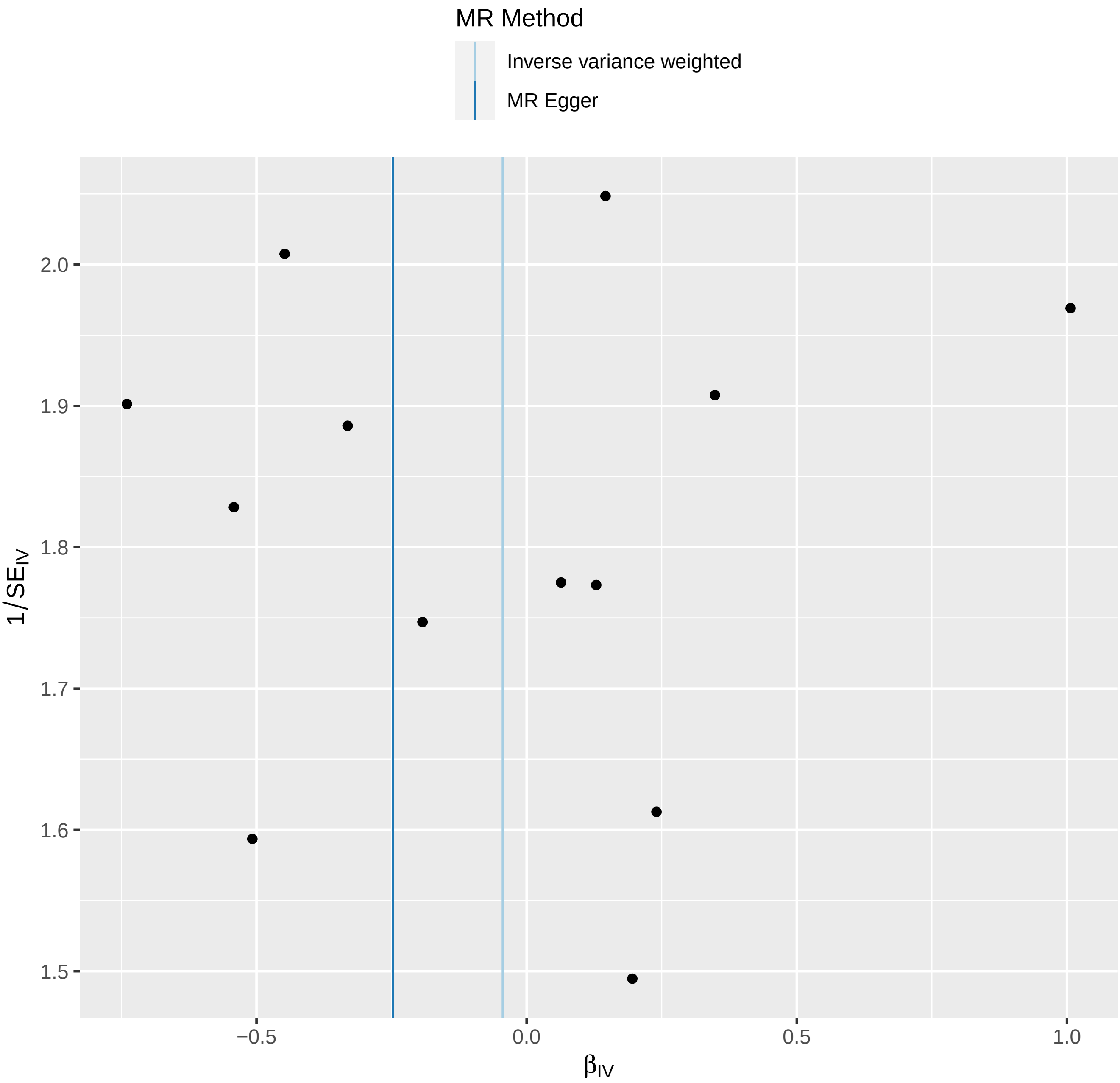

Batch 1015 : Gut microbiota abundance (genus Parasutterella id.2892) on Ulcerative enterocolitis

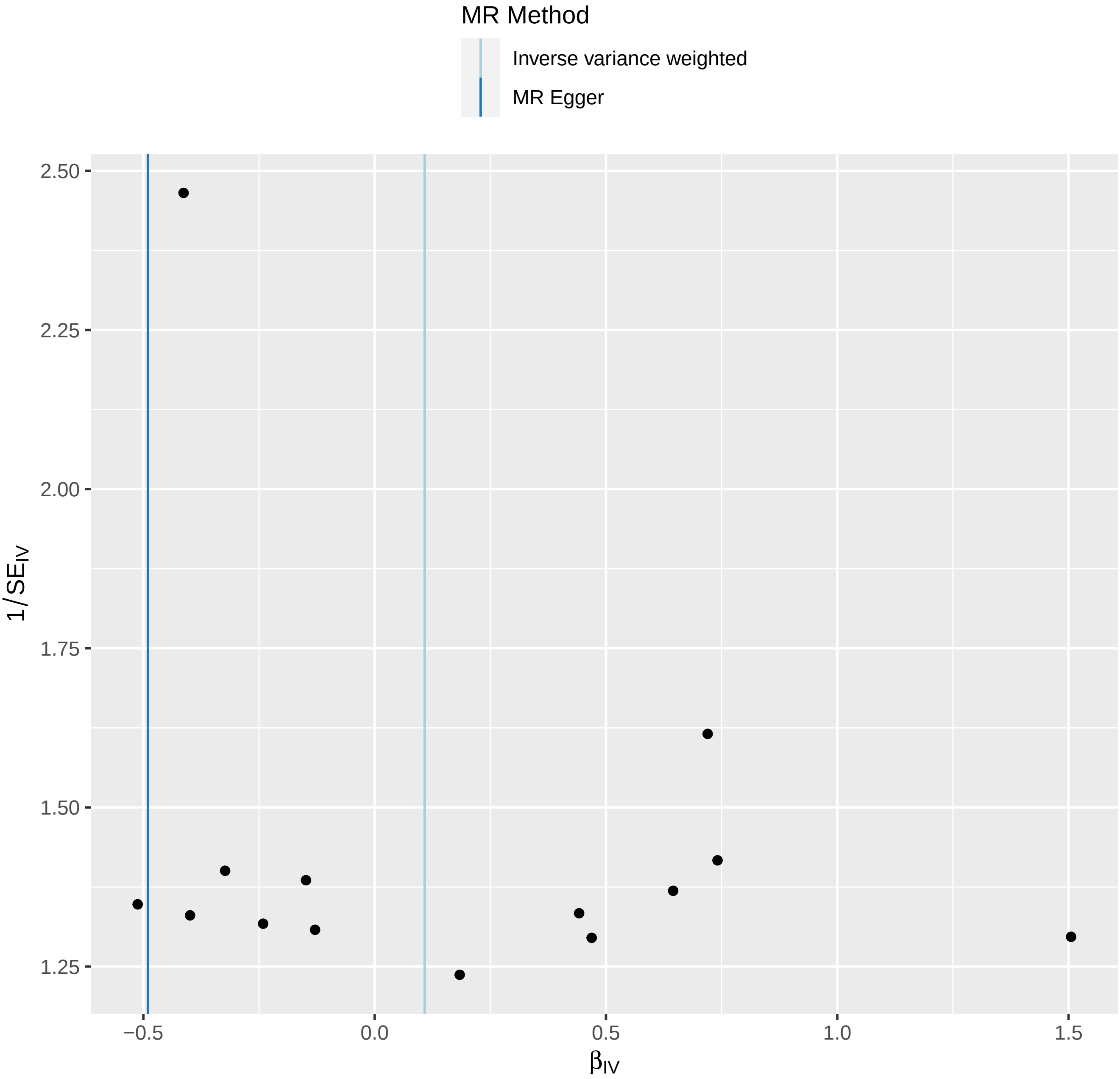

Batch 1016 : Gut microbiota abundance (genus Peptococcus id.2037) on Ulcerative enterocolitis

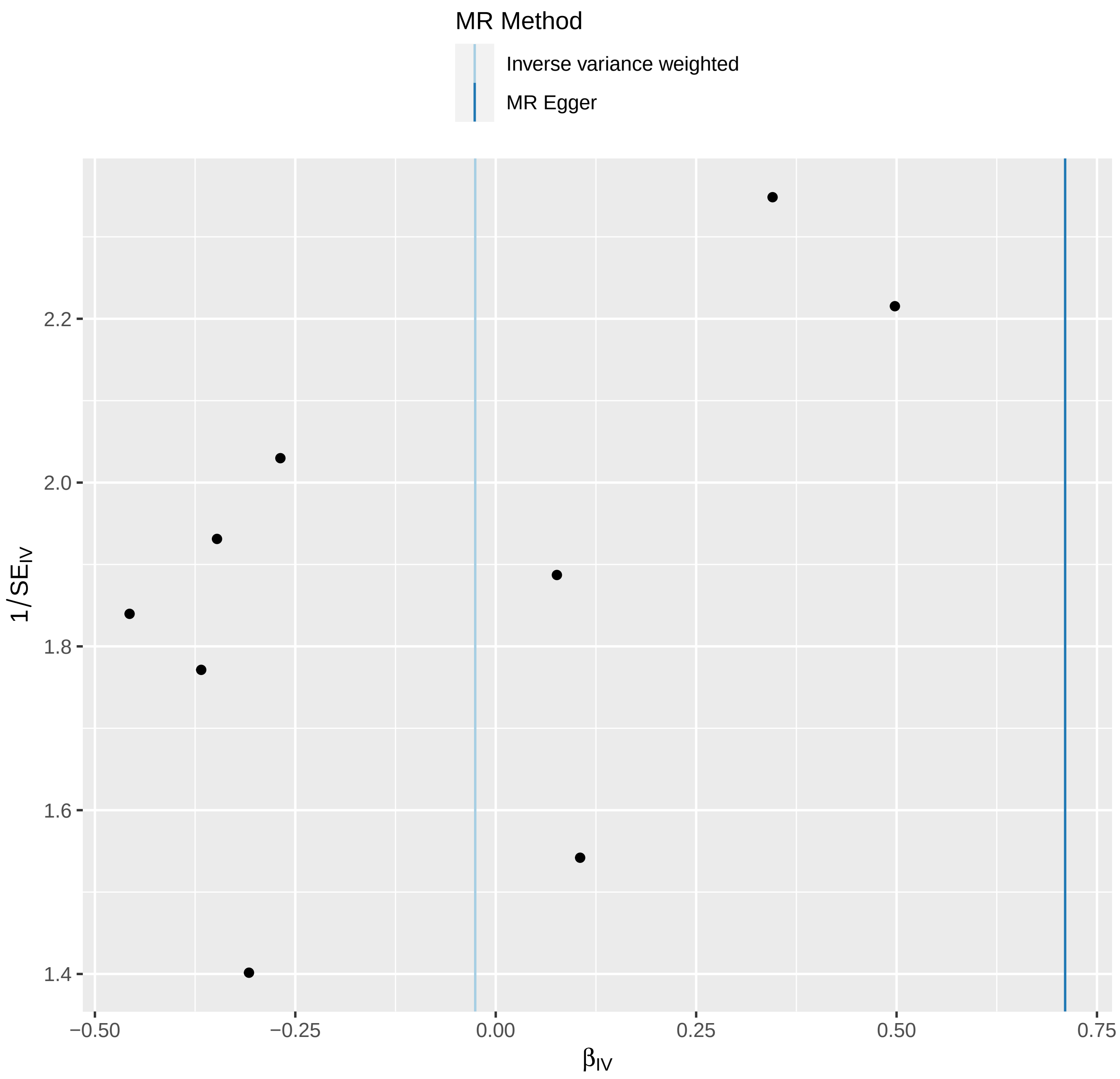

Batch 1017 : Gut microbiota abundance (genus Phascolarctobacterium id.2168) on Ulcerative enterocolitis

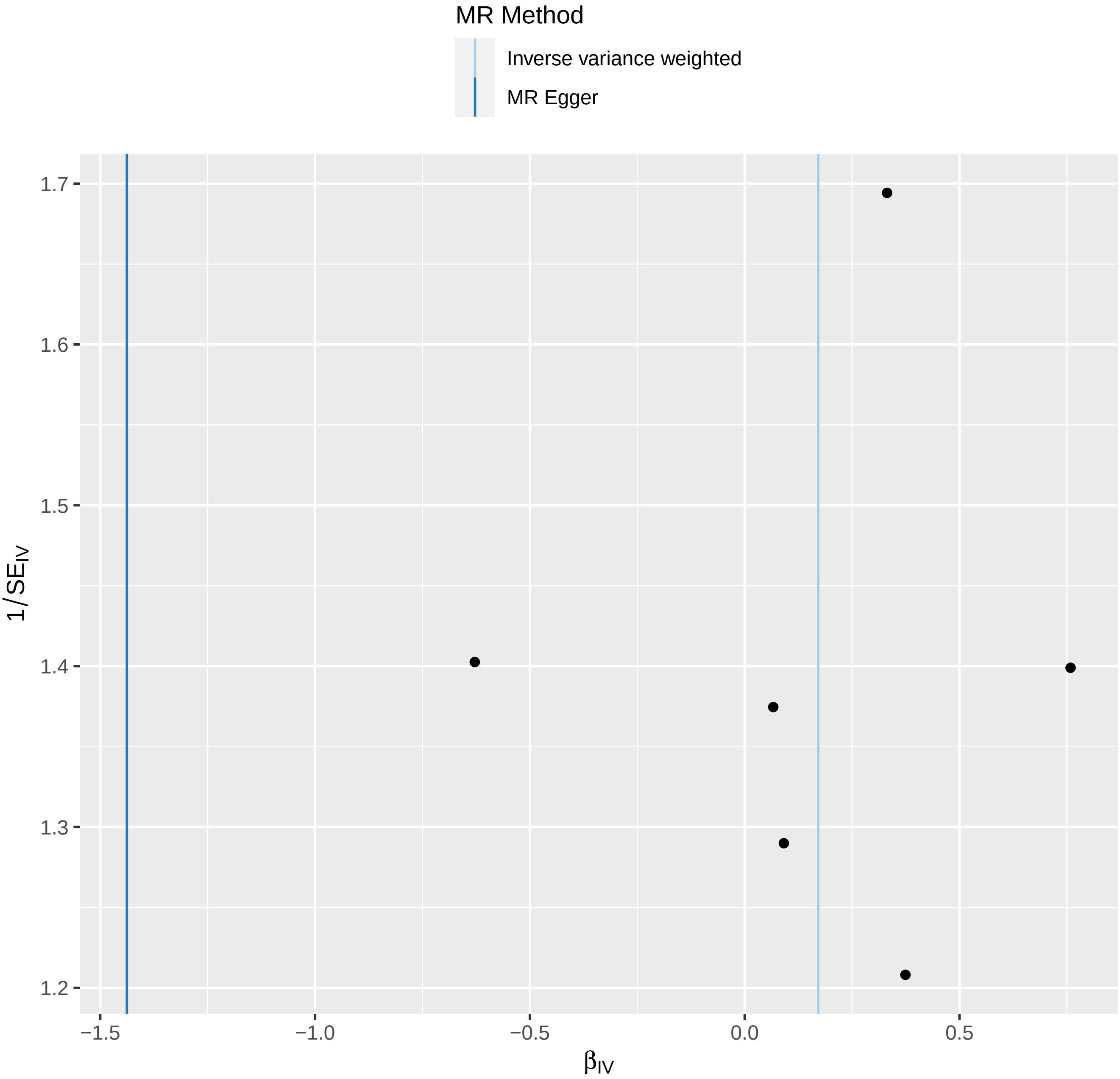

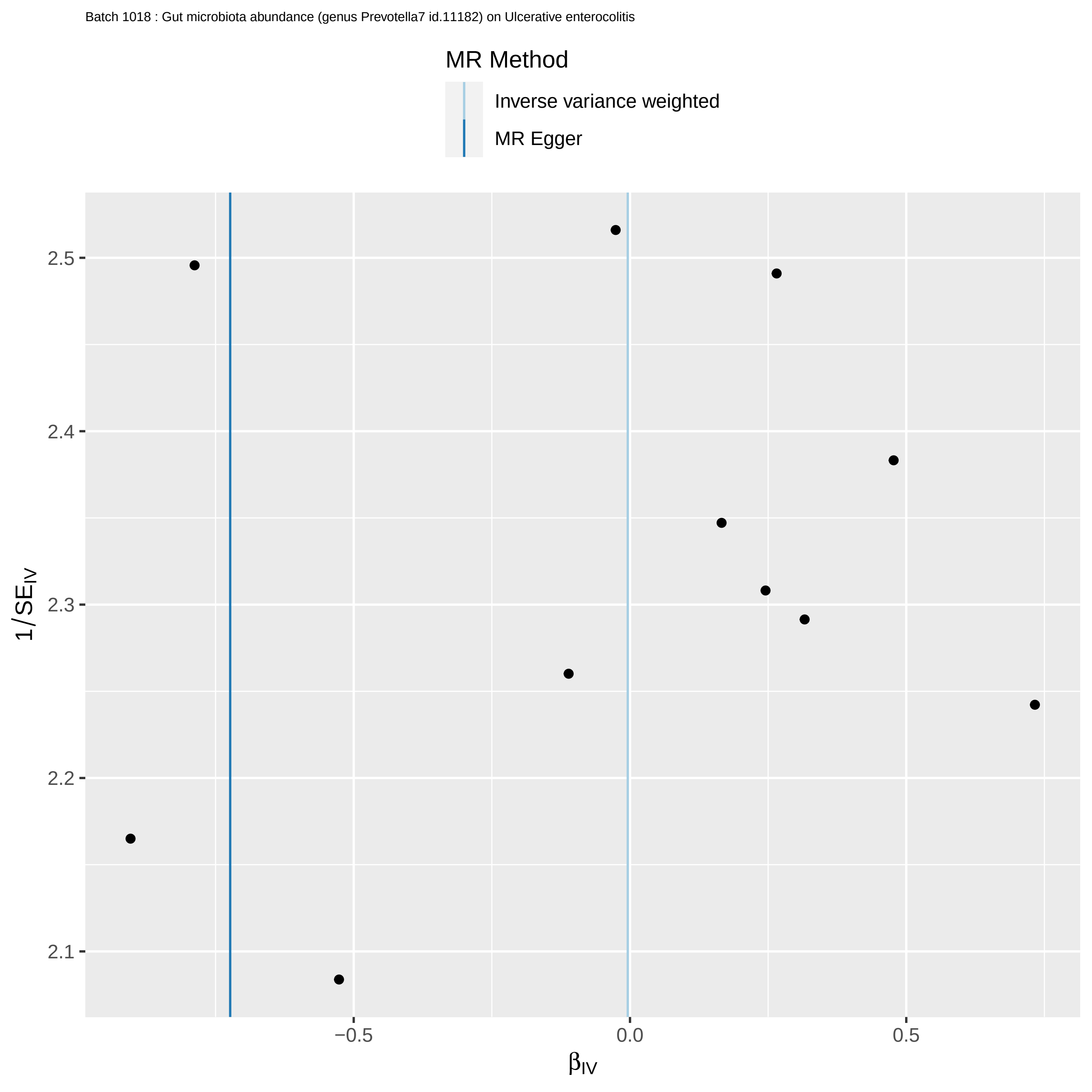
Batch 1018 : Gut microbiota abundance (genus Prevotella7 id.11182) on Ulcerative enterocolitis
MR Method
Inverse variance weighted
MR Egger
2.5
2.4
2.3
2.2
2.1
1/SE_IV
−0.5
0.0
0.5
β_IV

Batch 1019 : Gut microbiota abundance (genus Prevotella9 id.11183) on Ulcerative enterocolitis

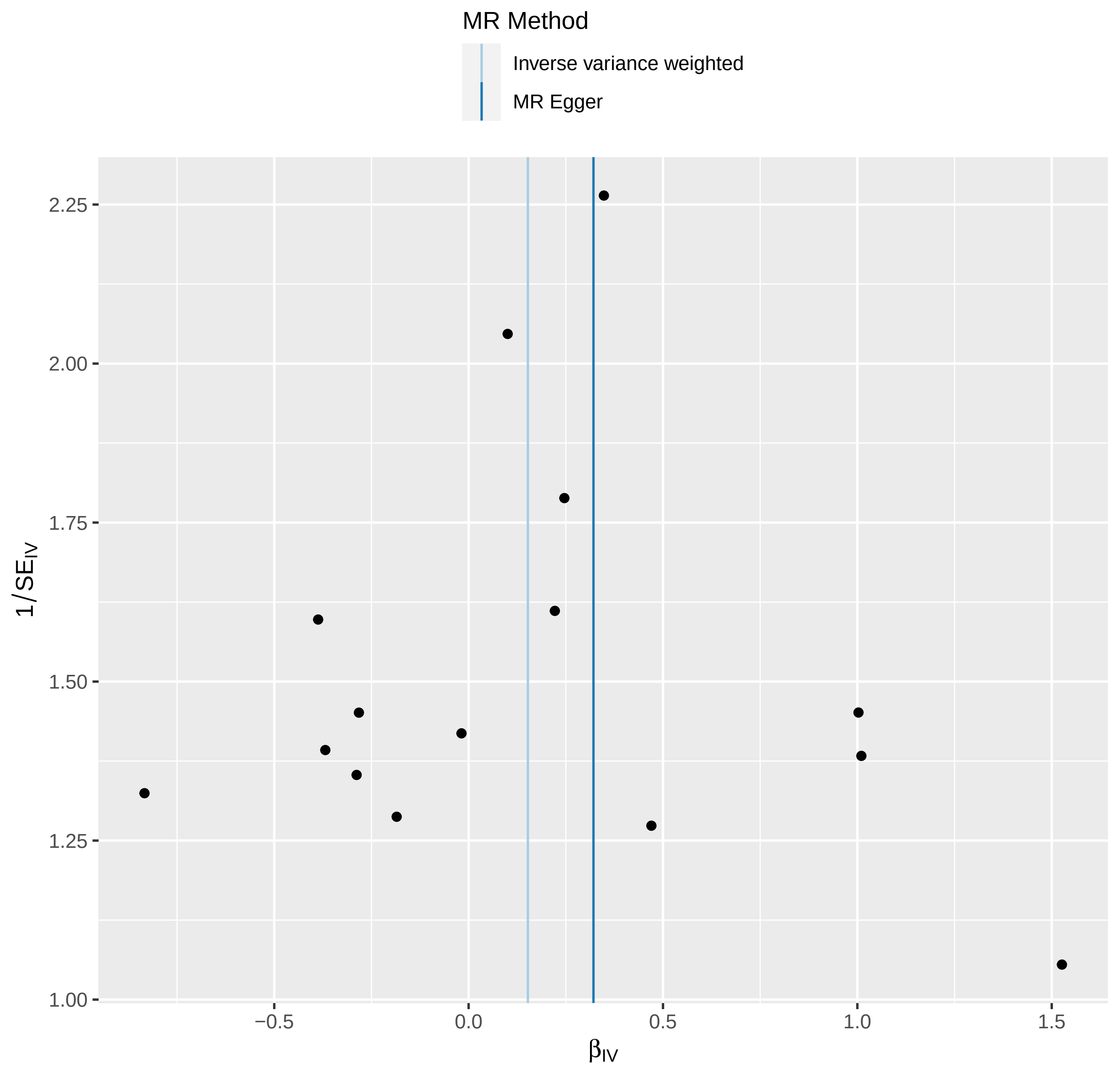

Batch 1020 : Gut microbiota abundance (genus Rikenellaceae RC9 gut group id.11191) on Ulcerative enterocolitis

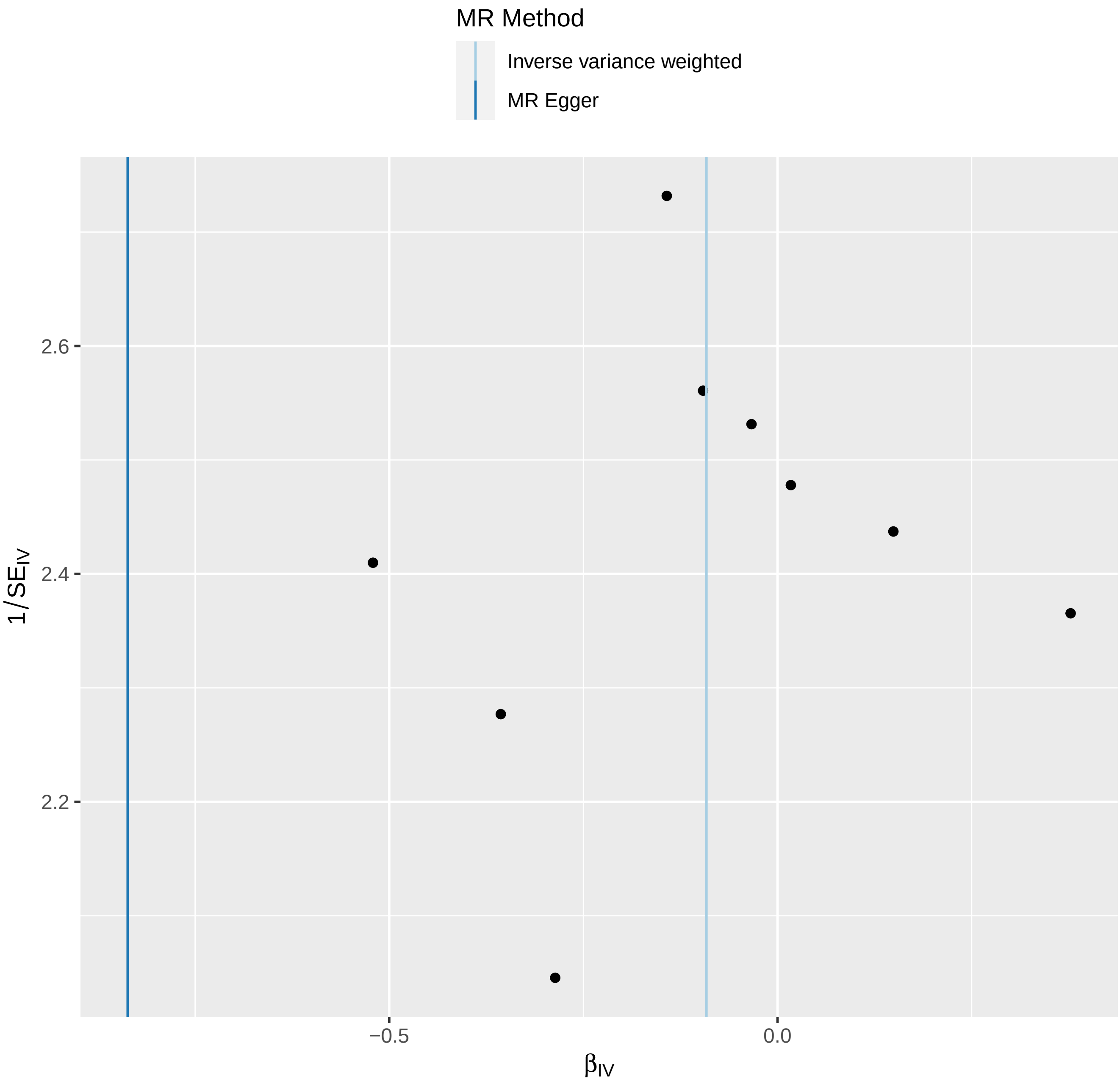

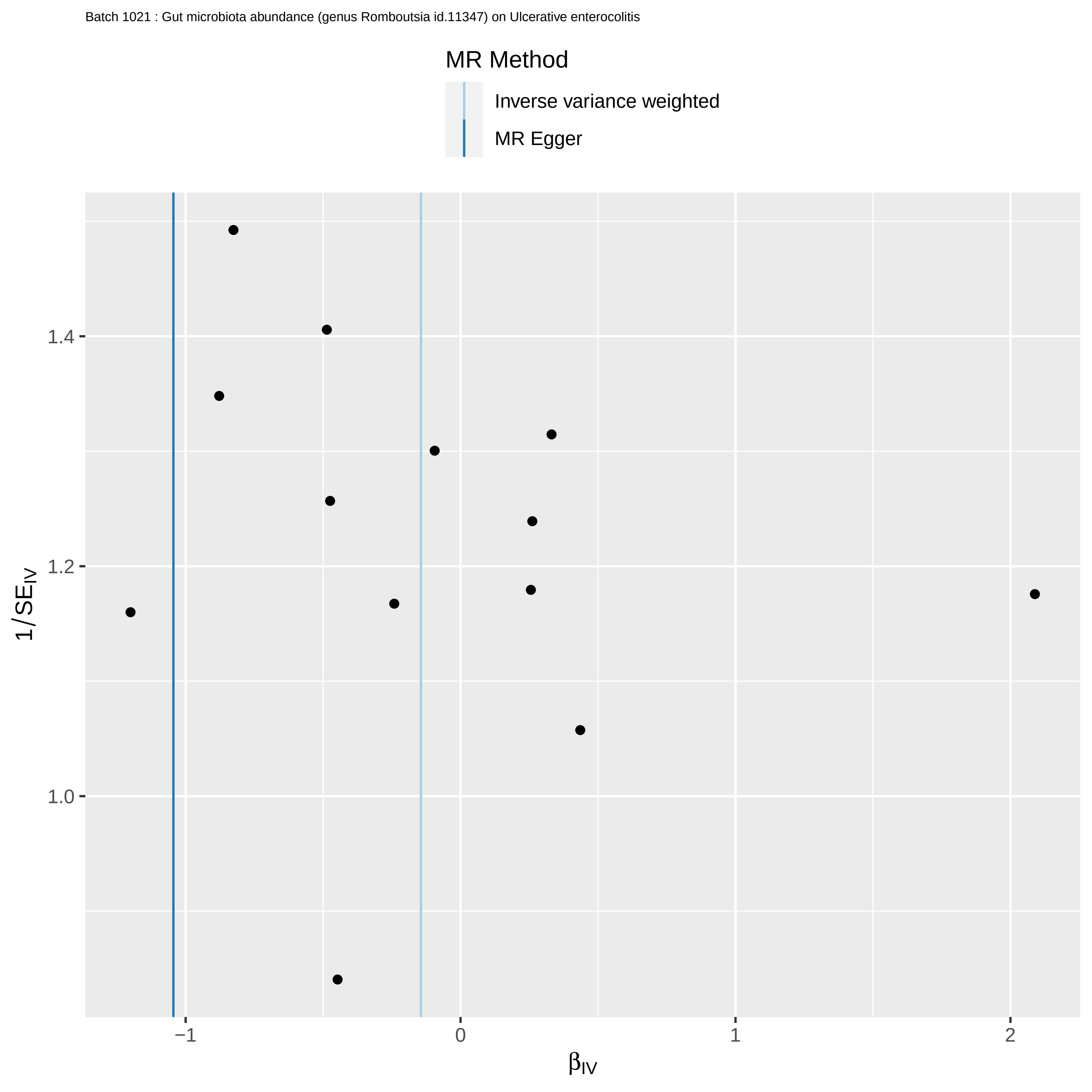

Batch 1021 : Gut microbiota abundance (genus Romboutsia id.11347) on Ulcerative enterocolitis
MR Method
Inverse variance weighted
MR Egger
1.4
1.2
1.0
1/SE_IV
−1
0
1
2
β_IV

Batch 1022 : Gut microbiota abundance (genus Roseburia id.2012) on Ulcerative enterocolitis

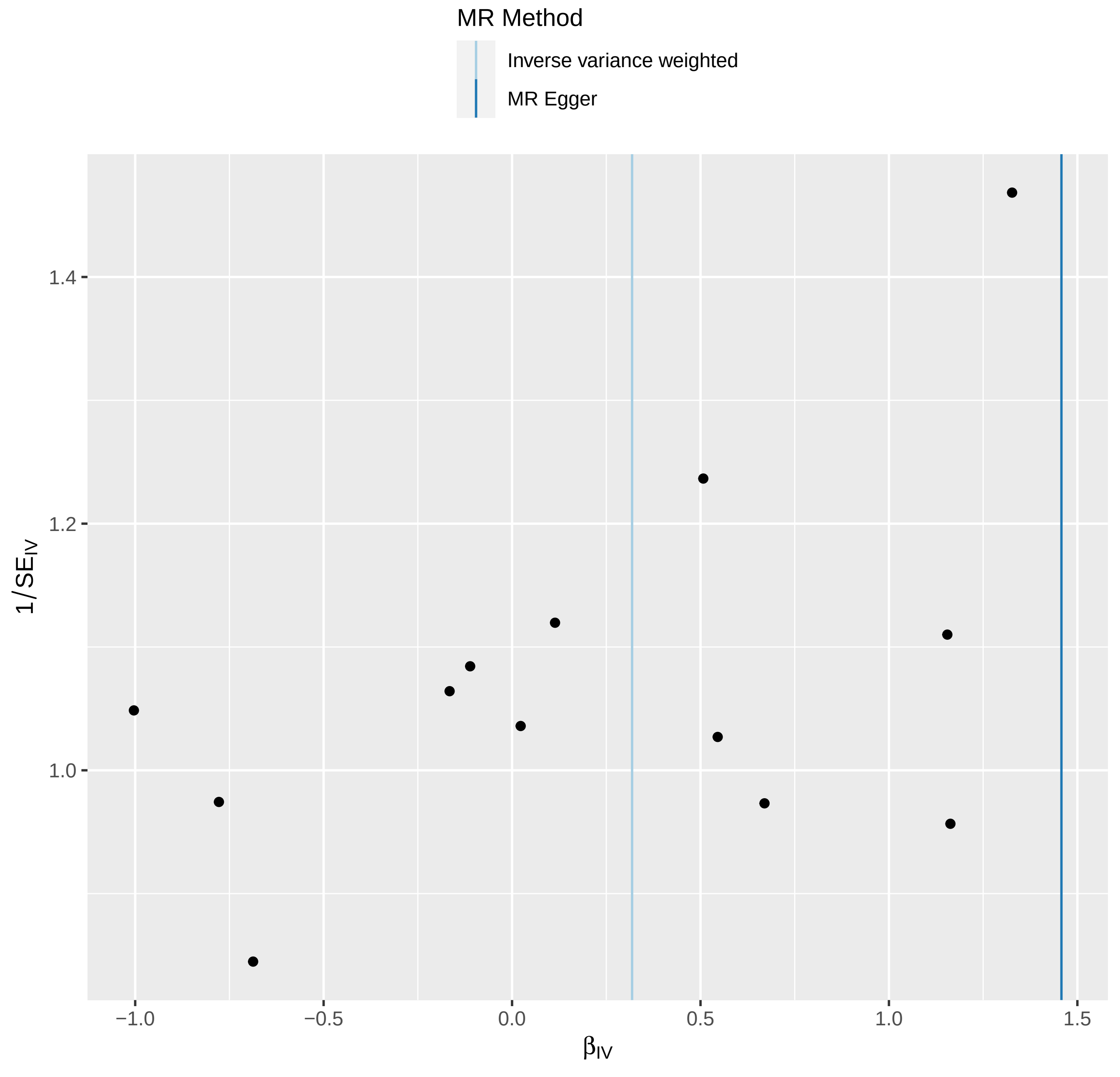

Batch 1023 : Gut microbiota abundance (genus Ruminiclostridium5 id.11355) on Ulcerative enterocolitis

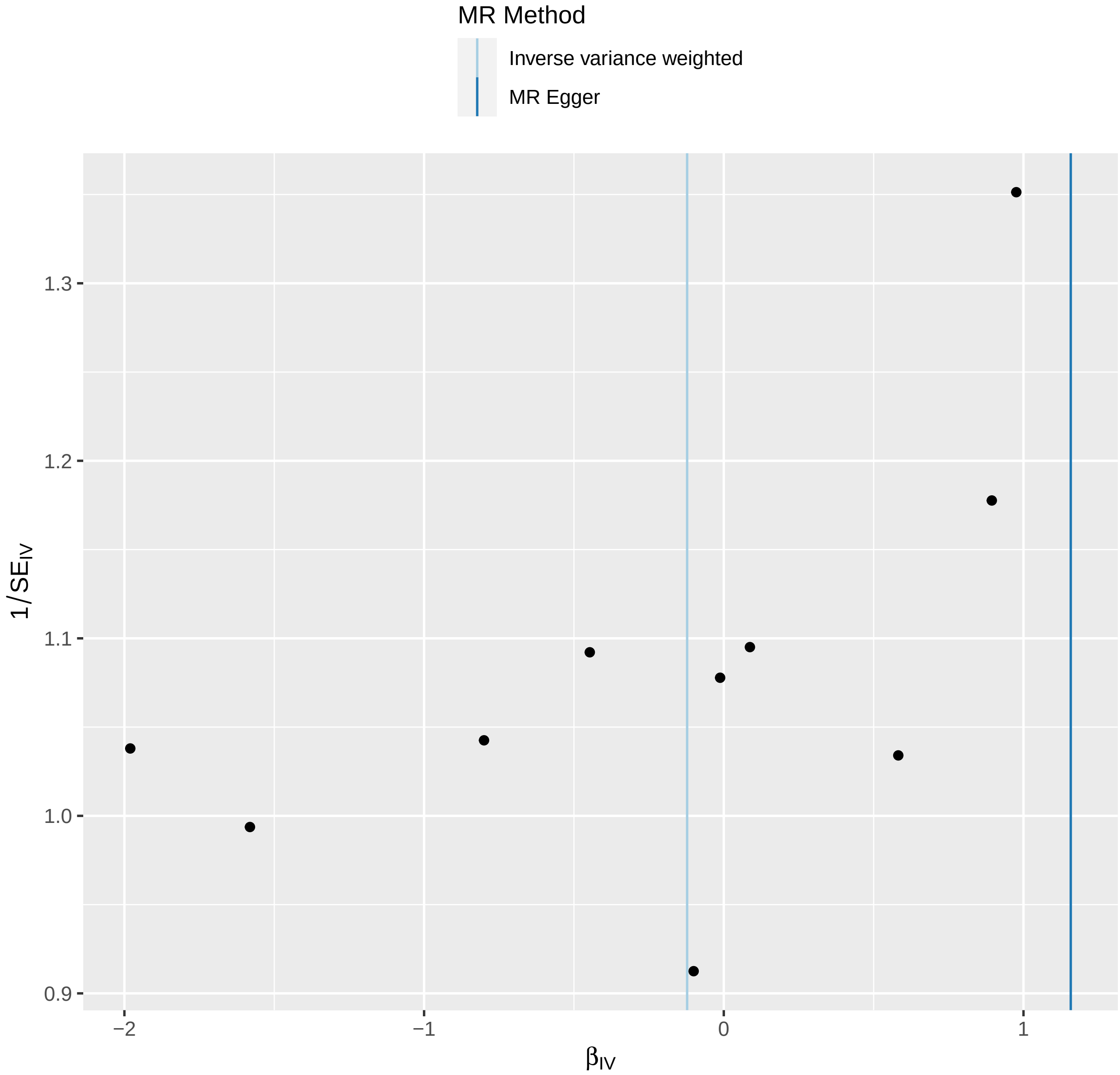

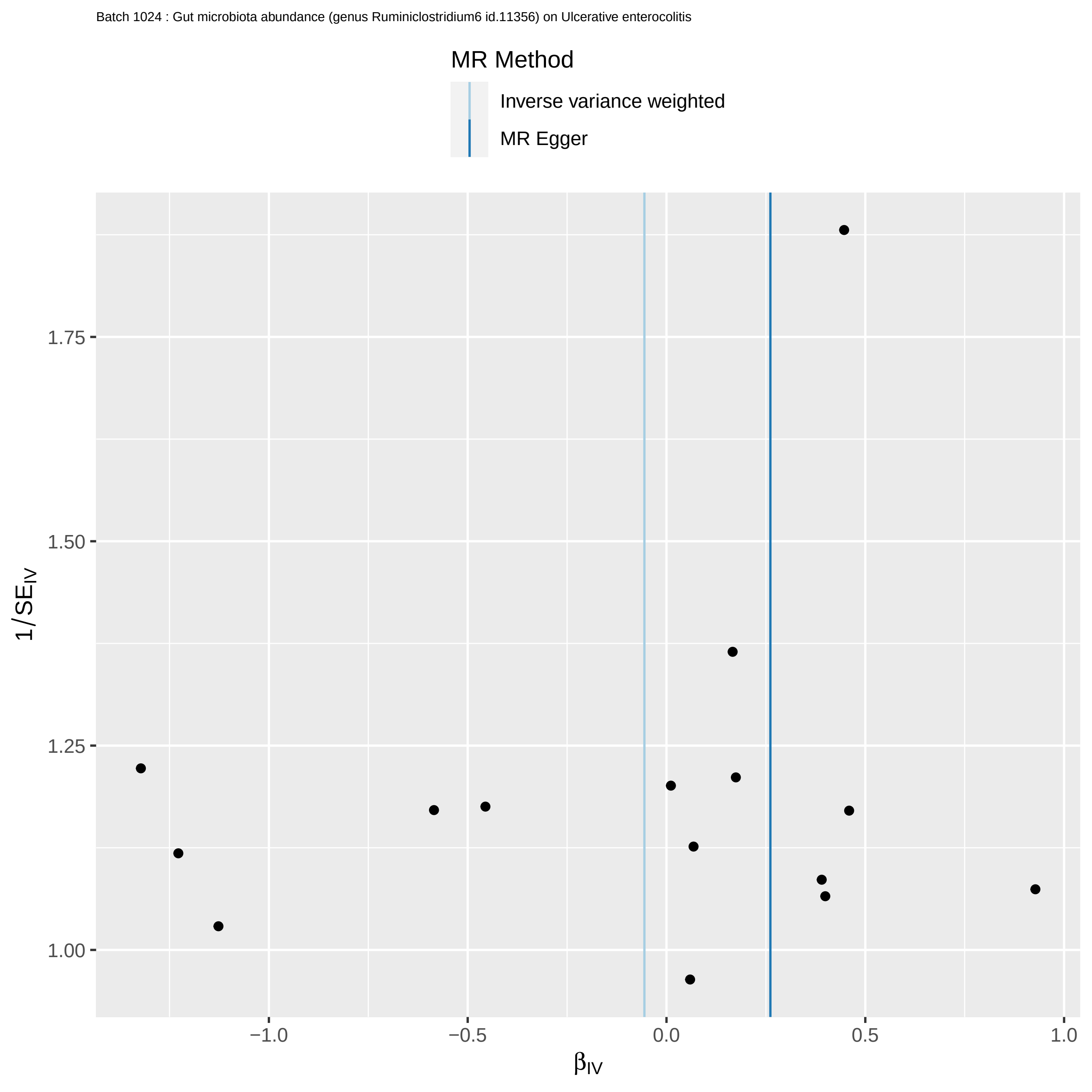
Batch 1024 : Gut microbiota abundance (genus Ruminiclostridium6 id.11356) on Ulcerative enterocolitis
MR Method
Inverse variance weighted
MR Egger
1.75
1.50
1.25
1.00
1/SE$_{IV}$
−1.0
−0.5
0.0
0.5
1.0
β$_{IV}$

Batch 1025 : Gut microbiota abundance (genus Ruminiclostridium9 id.11357) on Ulcerative enterocolitis

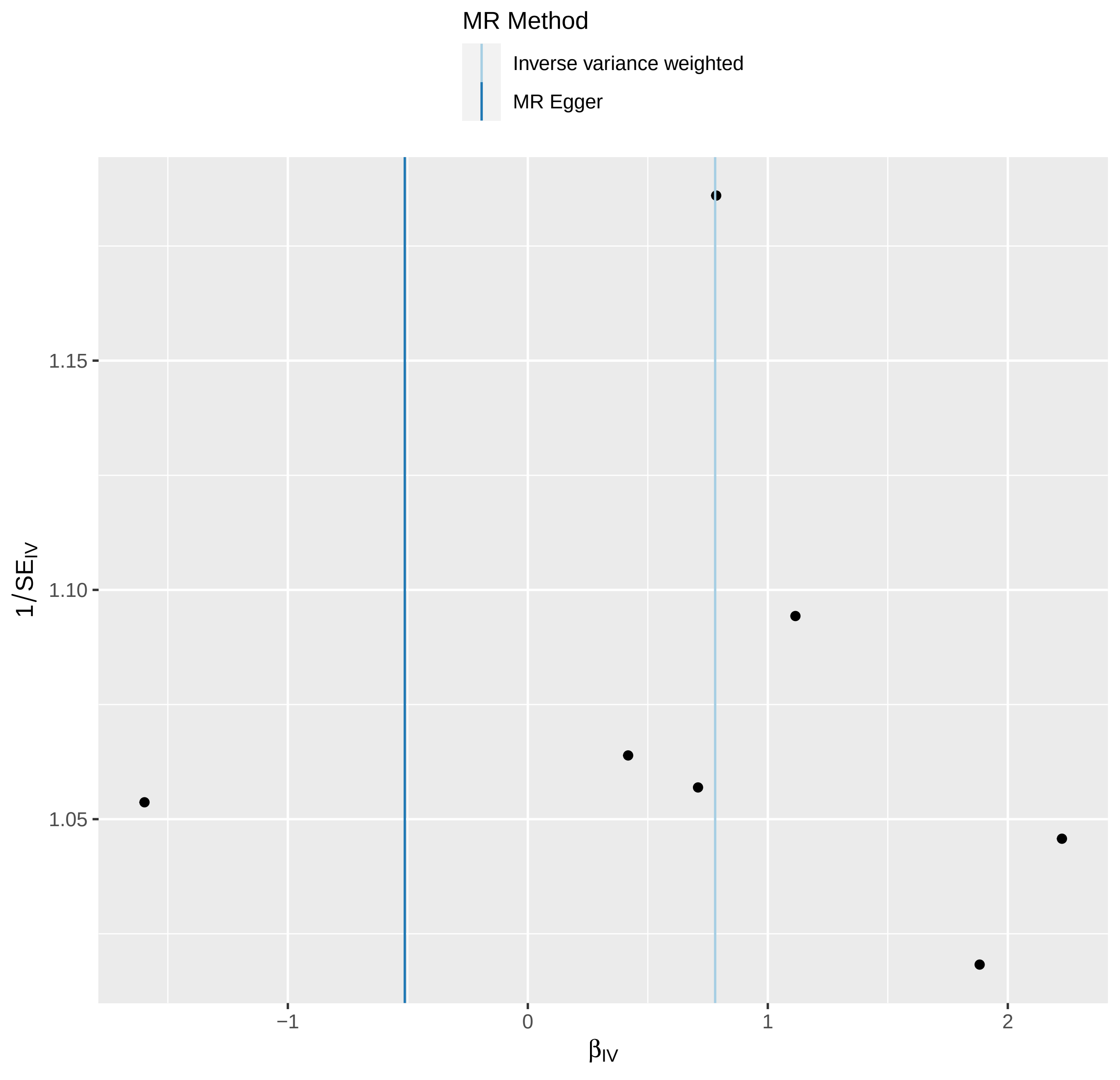

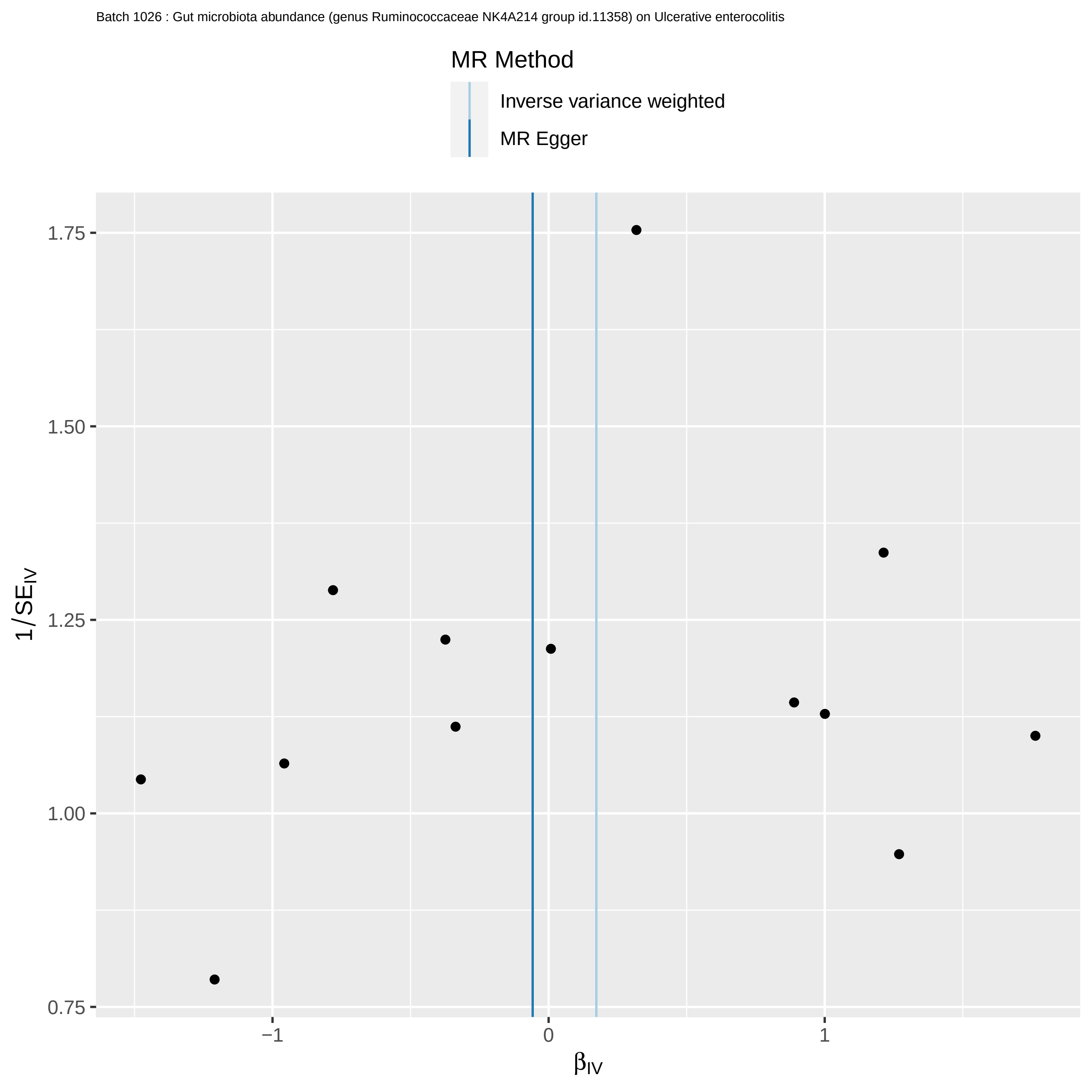

Batch 1026 : Gut microbiota abundance (genus Ruminococcaceae NK4A214 group id.11358) on Ulcerative enterocolitis
MR Method
Inverse variance weighted
MR Egger
1.75
1.50
1.25
1.00
0.75
1/SE_IV
−1
0
1
β_IV

Batch 1027 : Gut microbiota abundance (genus Ruminococcaceae UCG002 id.11360) on Ulcerative enterocolitis

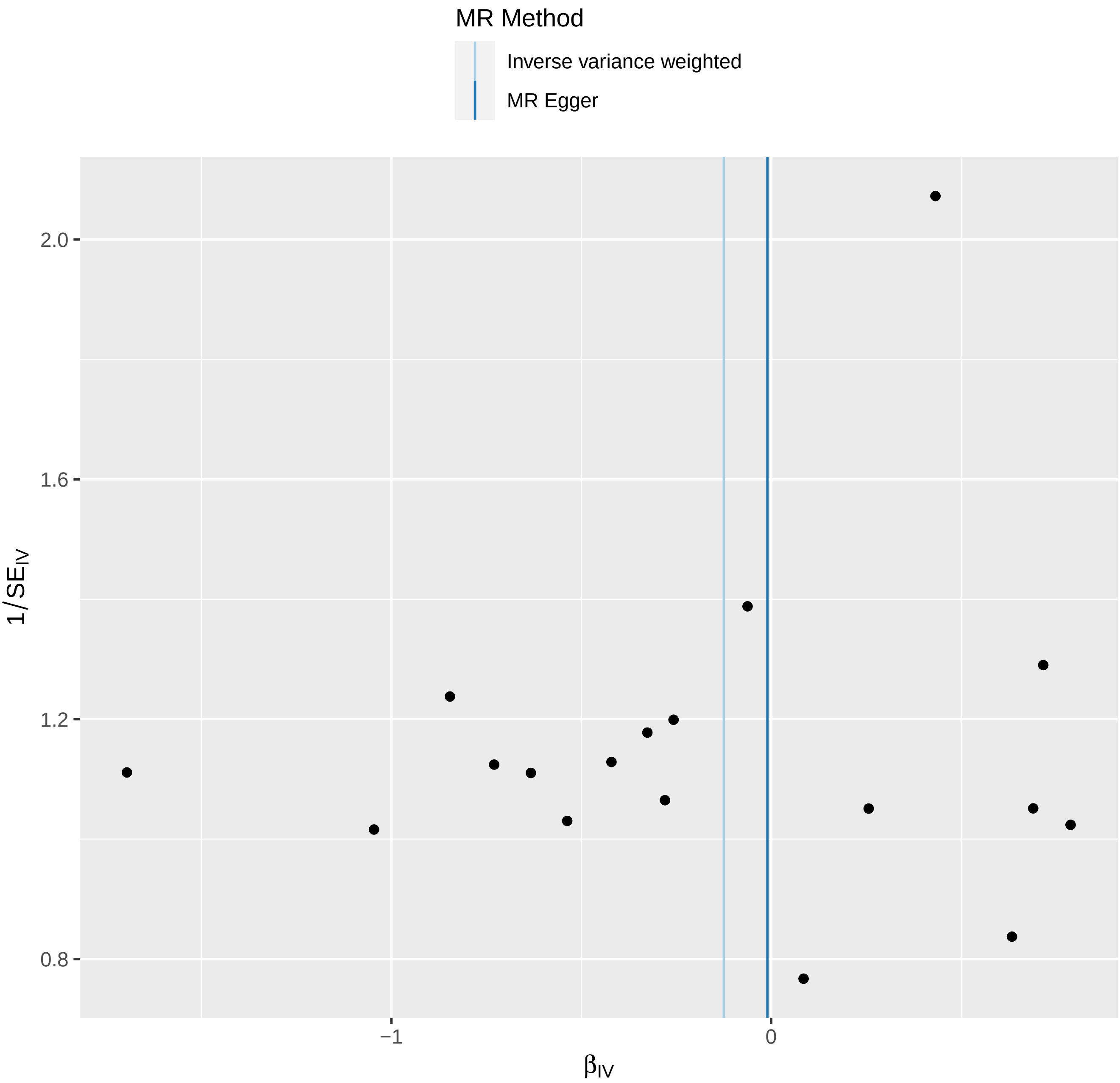

Batch 1028 : Gut microbiota abundance (genus Ruminococcaceae UCG003 id.11361) on Ulcerative enterocolitis

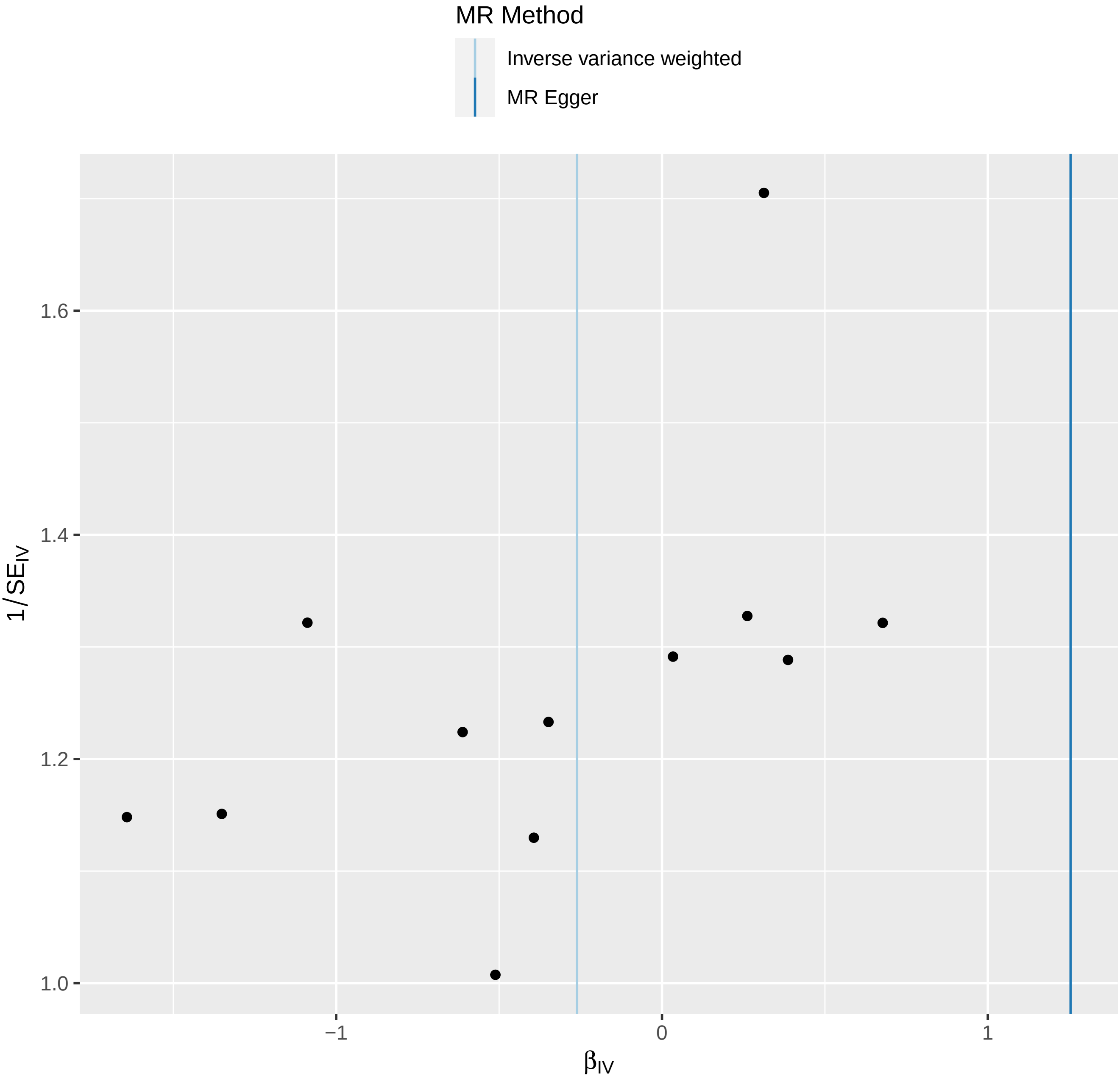

Batch 1029 : Gut microbiota abundance (genus Ruminococcaceae UCG004 id.11362) on Ulcerative enterocolitis

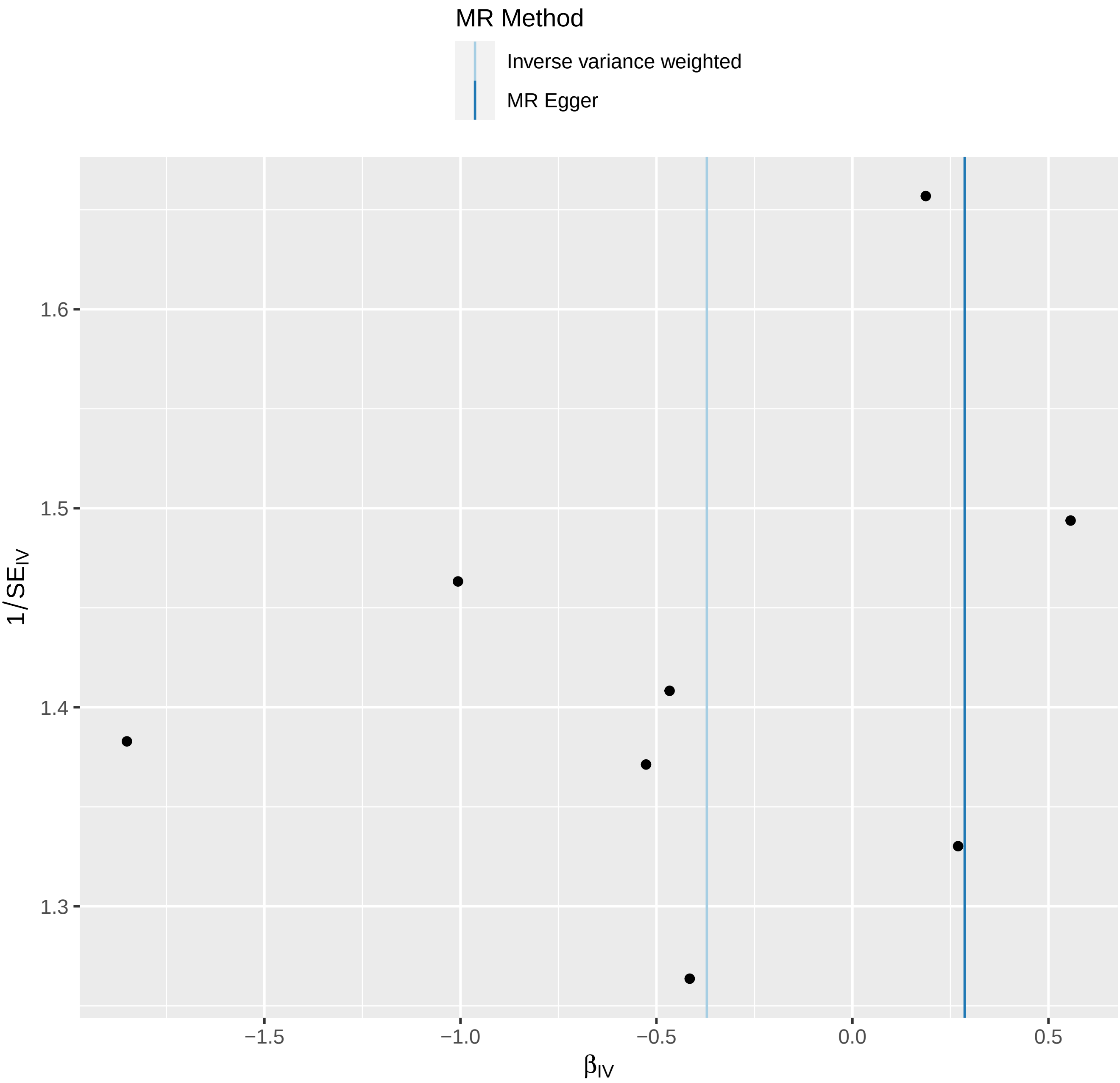

Batch 1030 : Gut microbiota abundance (genus Ruminococcaceae UCG005 id.11363) on Ulcerative enterocolitis

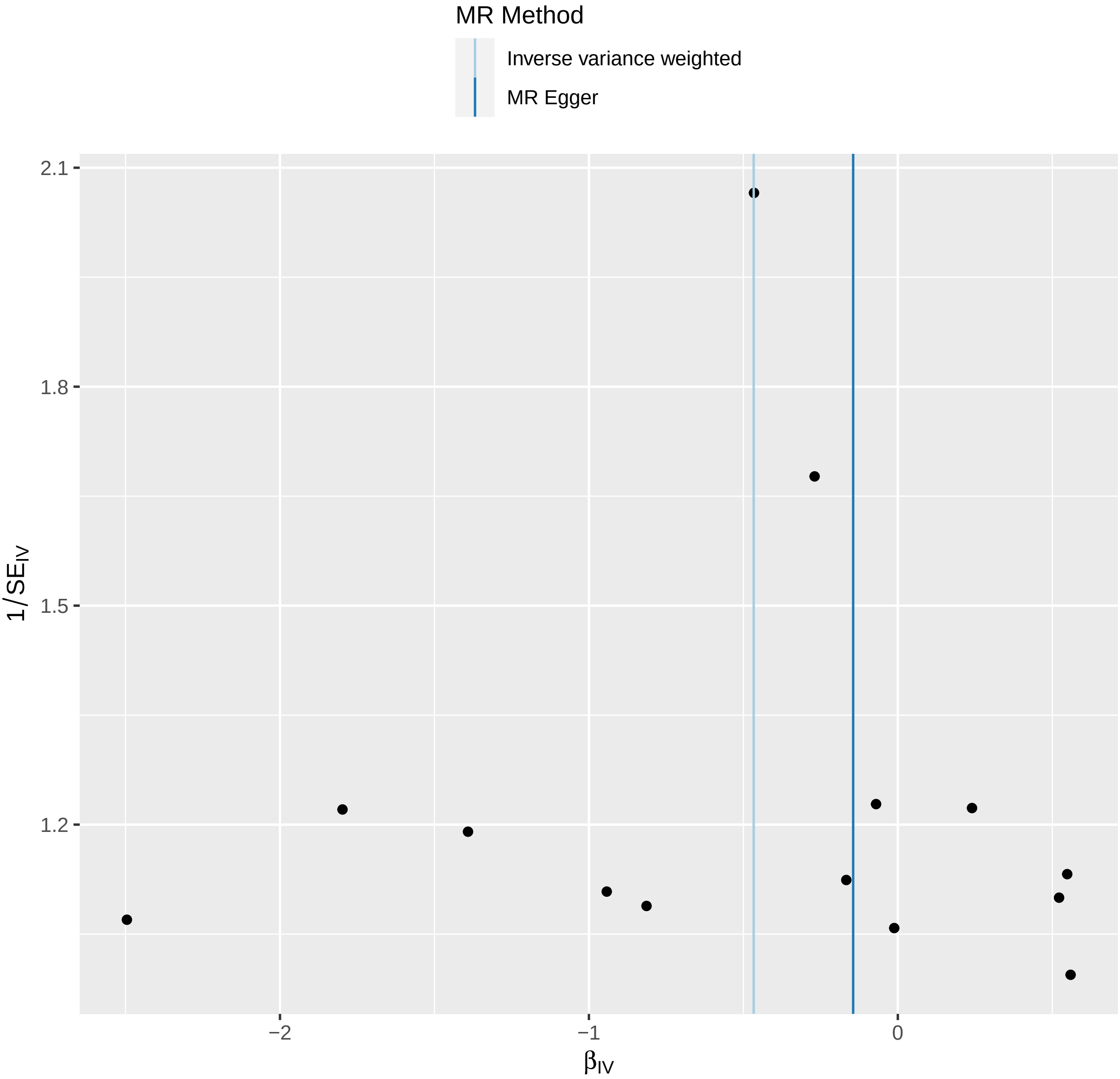

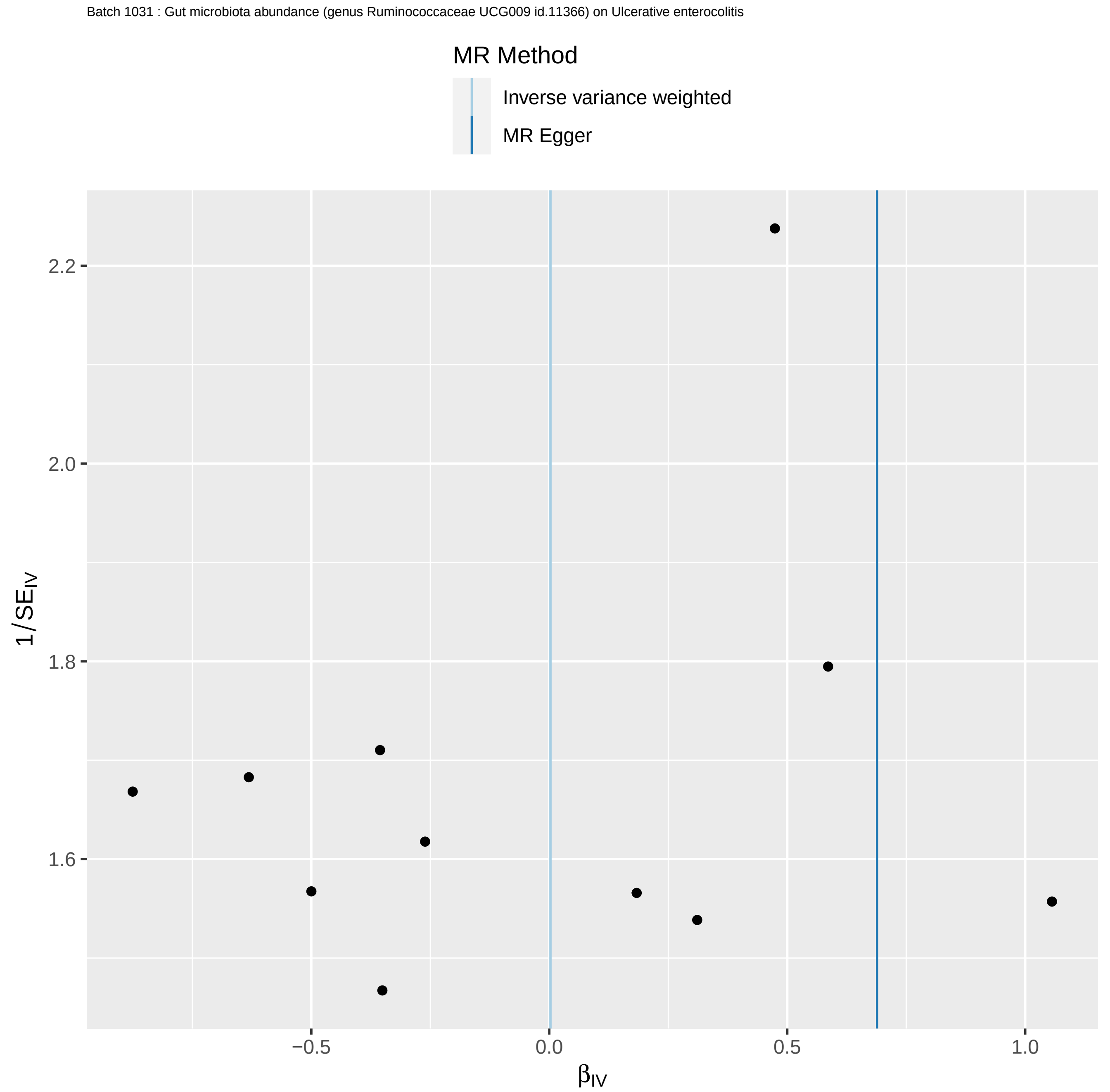
Batch 1031 : Gut microbiota abundance (genus Ruminococcaceae UCG009 id.11366) on Ulcerative enterocolitis
MR Method
Inverse variance weighted
MR Egger
2.2
2.0
1.8
1.6
$1/SE_{IV}$
−0.5
0.0
0.5
1.0
β_{IV}

Batch 1032 : Gut microbiota abundance (genus Ruminococcaceae UCG010 id.11367) on Ulcerative enterocolitis

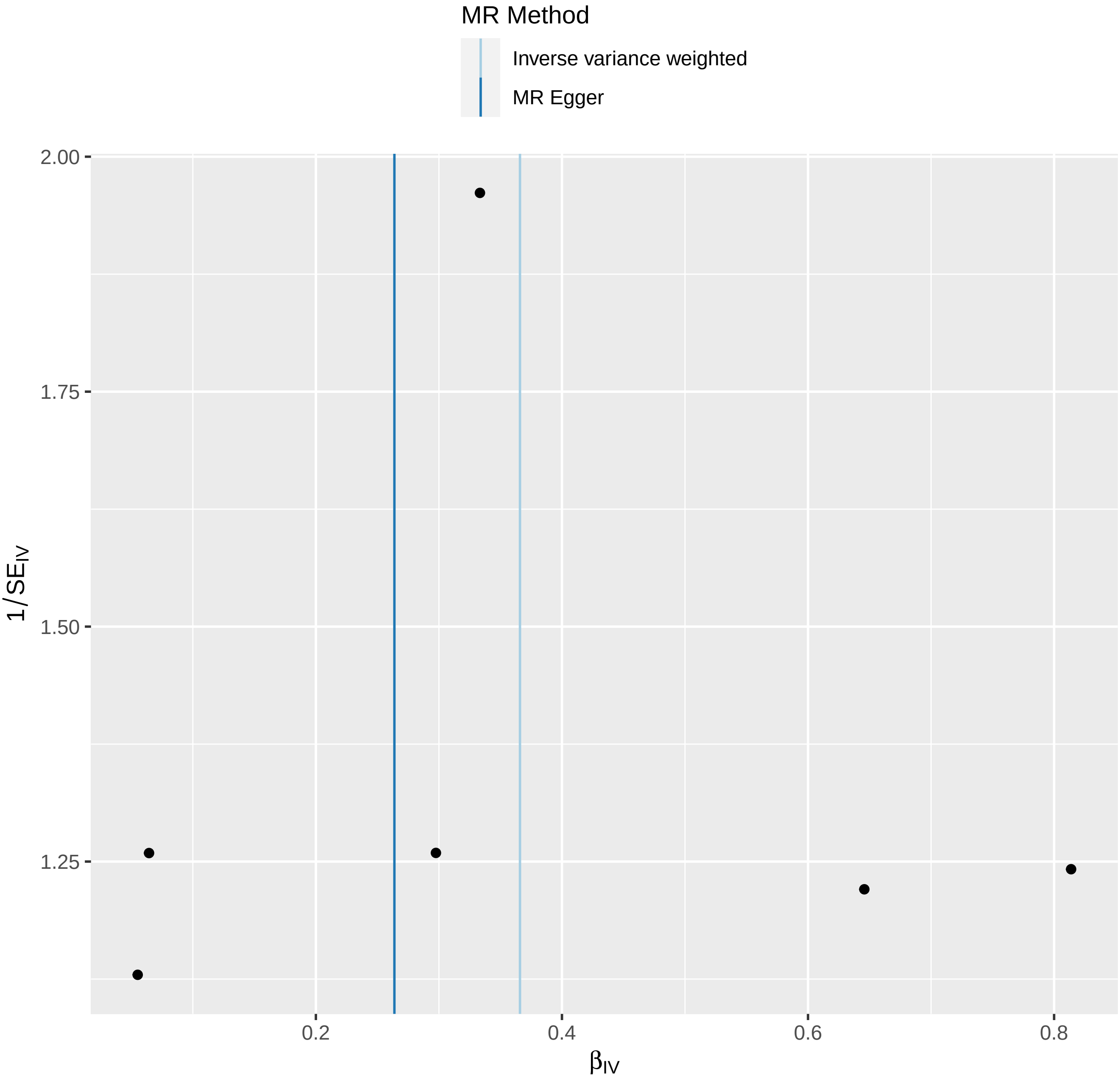

Batch 1033 : Gut microbiota abundance (genus Ruminococcaceae UCG011 id.11368) on Ulcerative enterocolitis

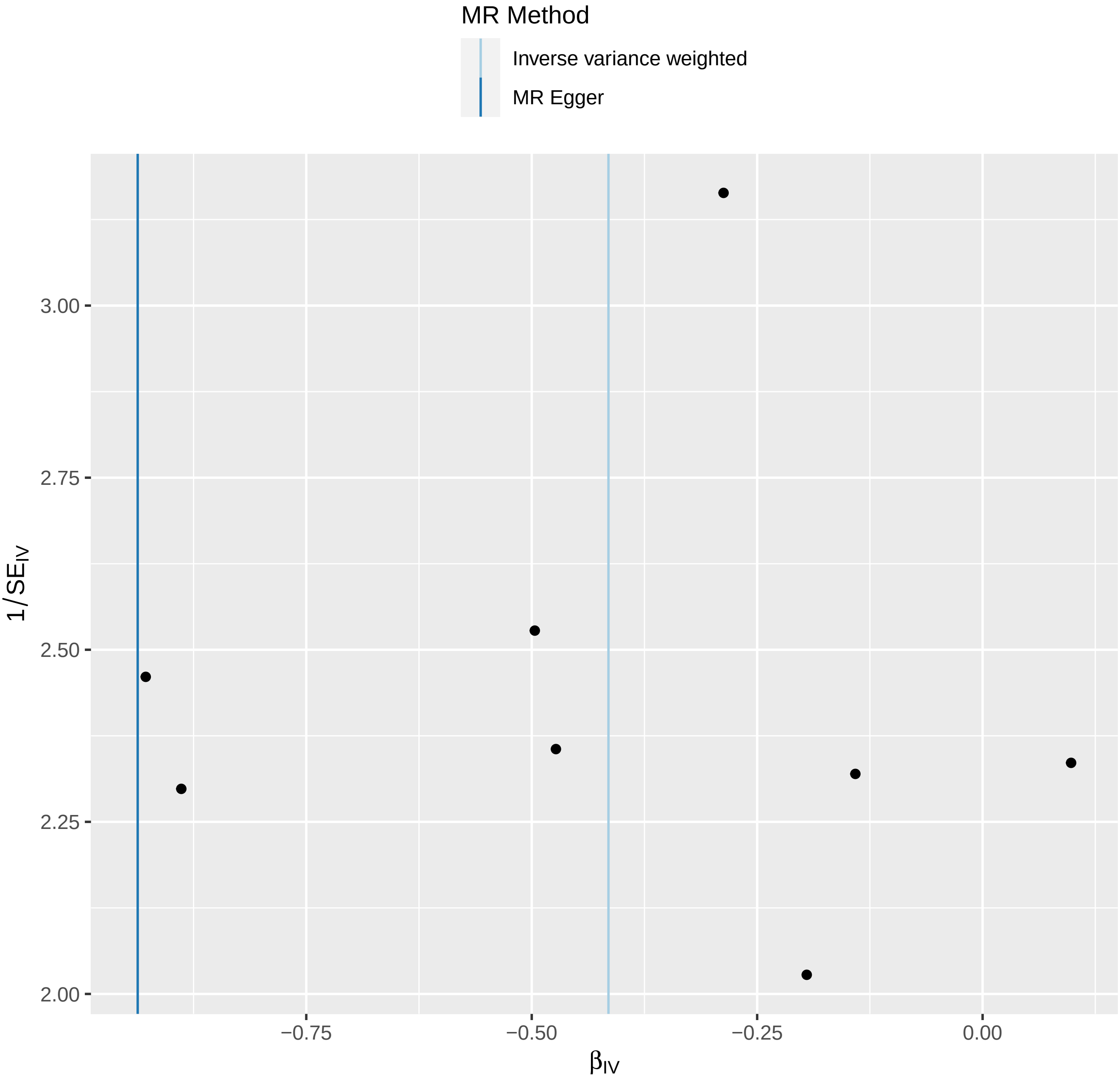

Batch 1034 : Gut microbiota abundance (genus Ruminococcaceae UCG013 id.11370) on Ulcerative enterocolitis

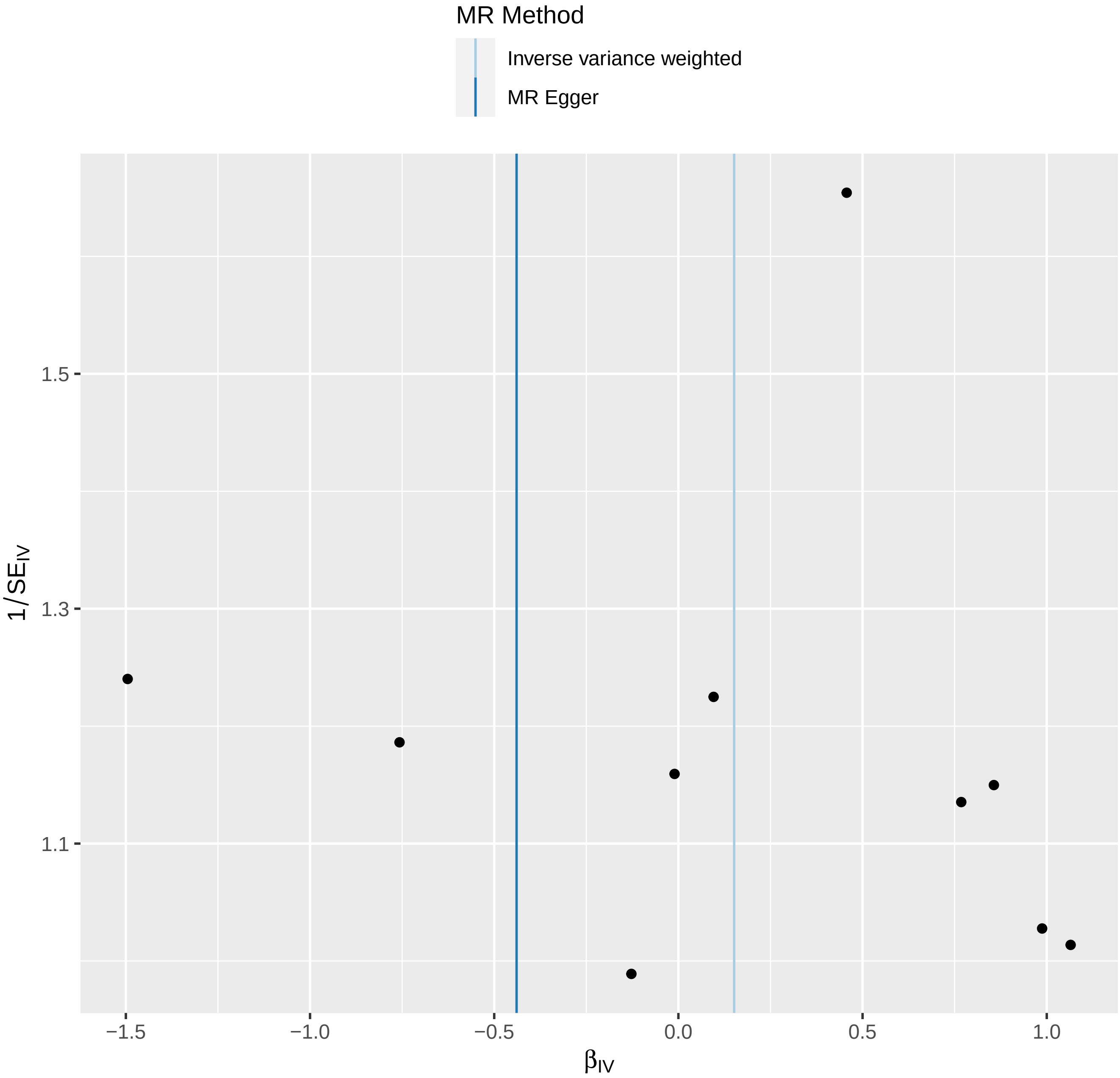

Batch 1035 : Gut microbiota abundance (genus Ruminococcaceae UCG014 id.11371) on Ulcerative enterocolitis

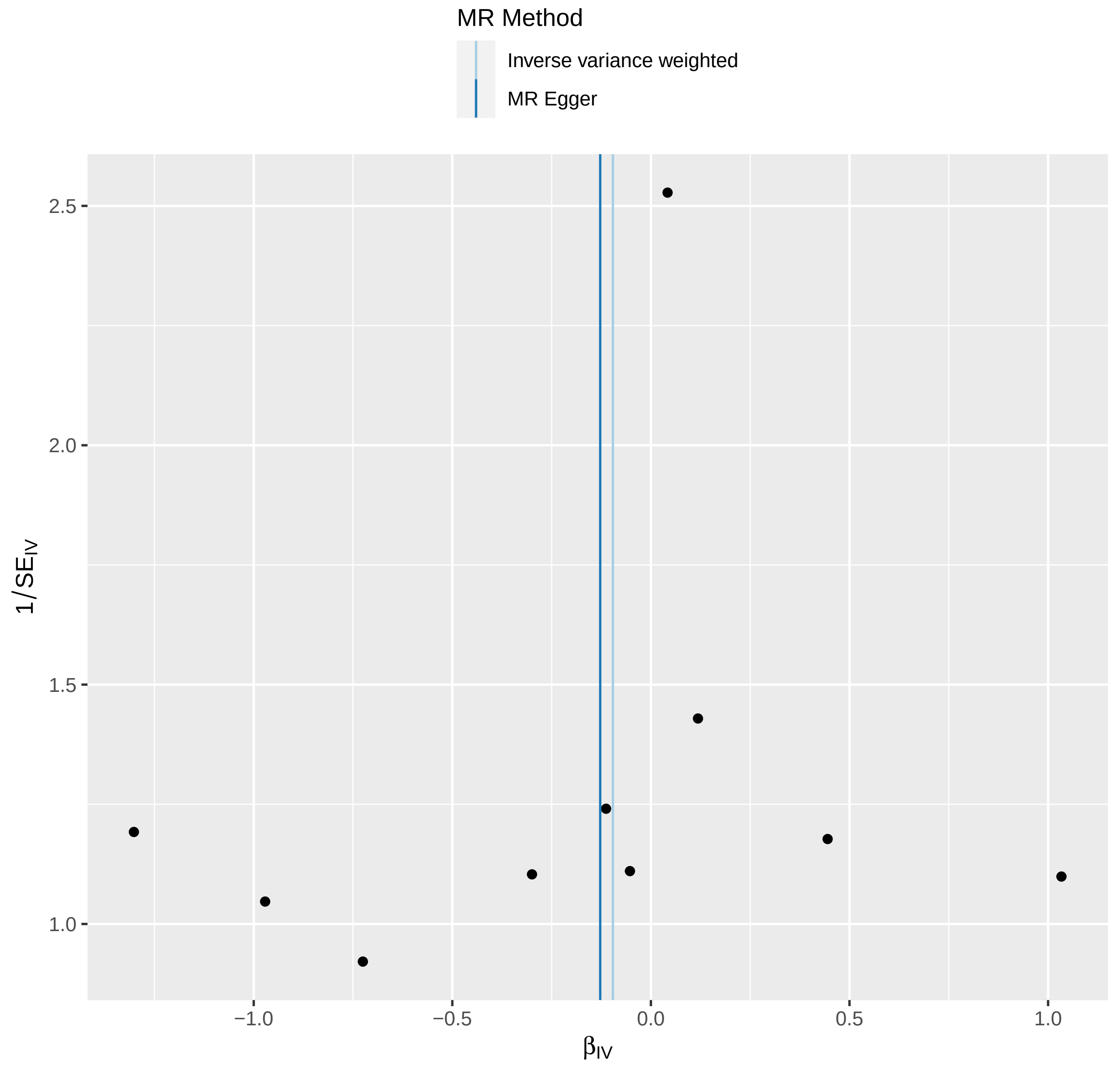

Batch 1036 : Gut microbiota abundance (genus Ruminococcus1 id.11373) on Ulcerative enterocolitis

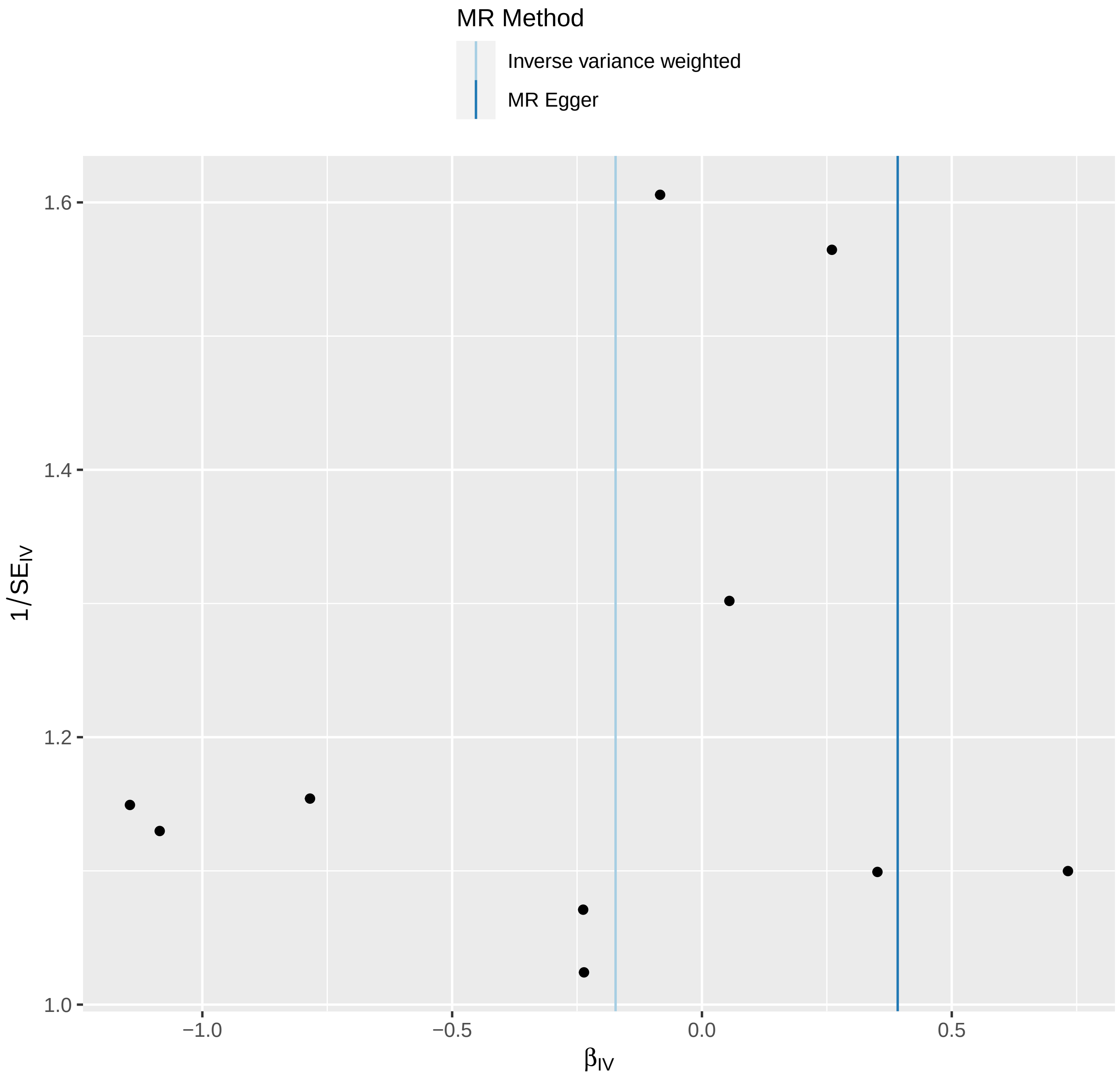

Batch 1037 : Gut microbiota abundance (genus Ruminococcus2 id.11374) on Ulcerative enterocolitis

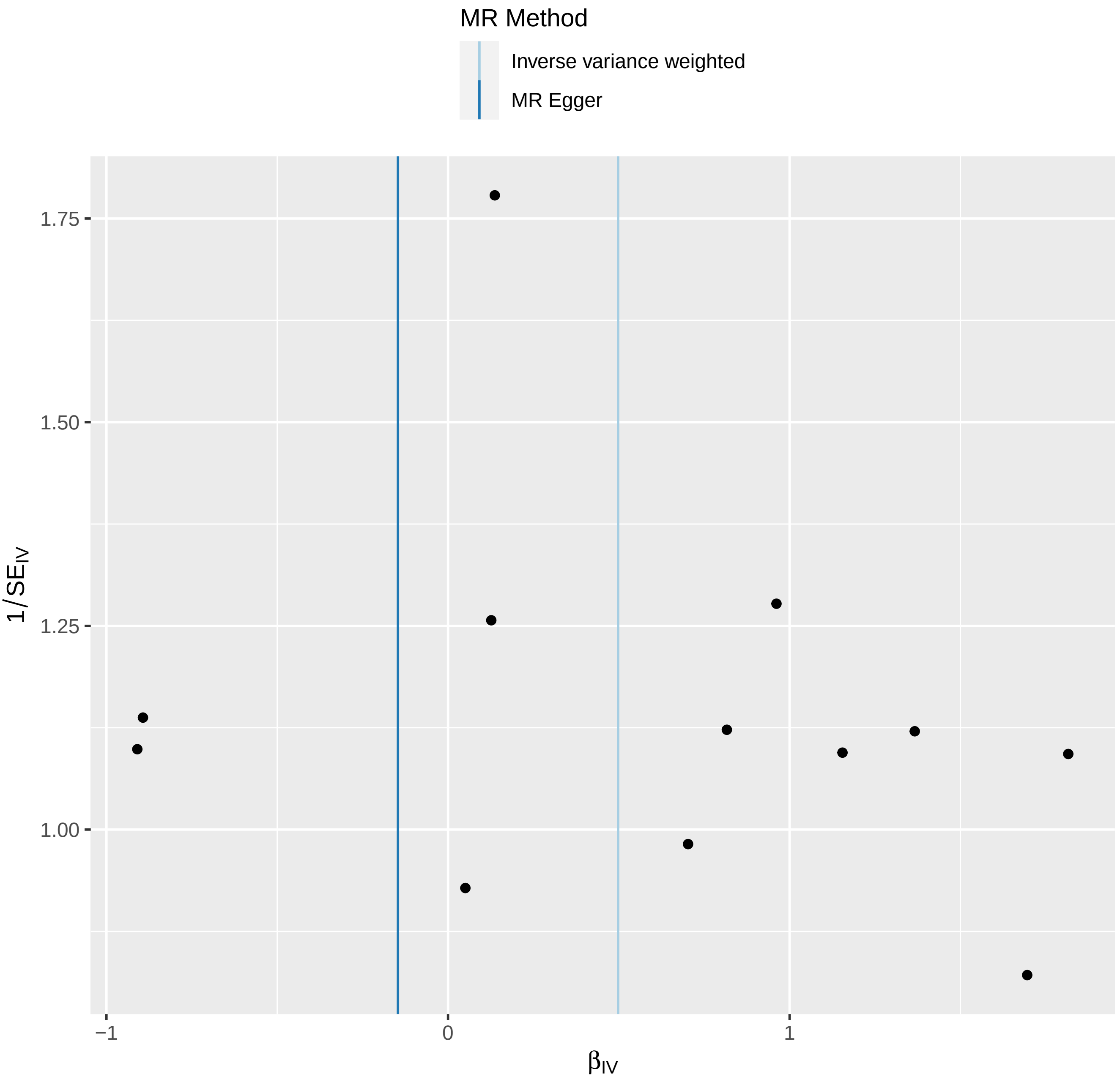

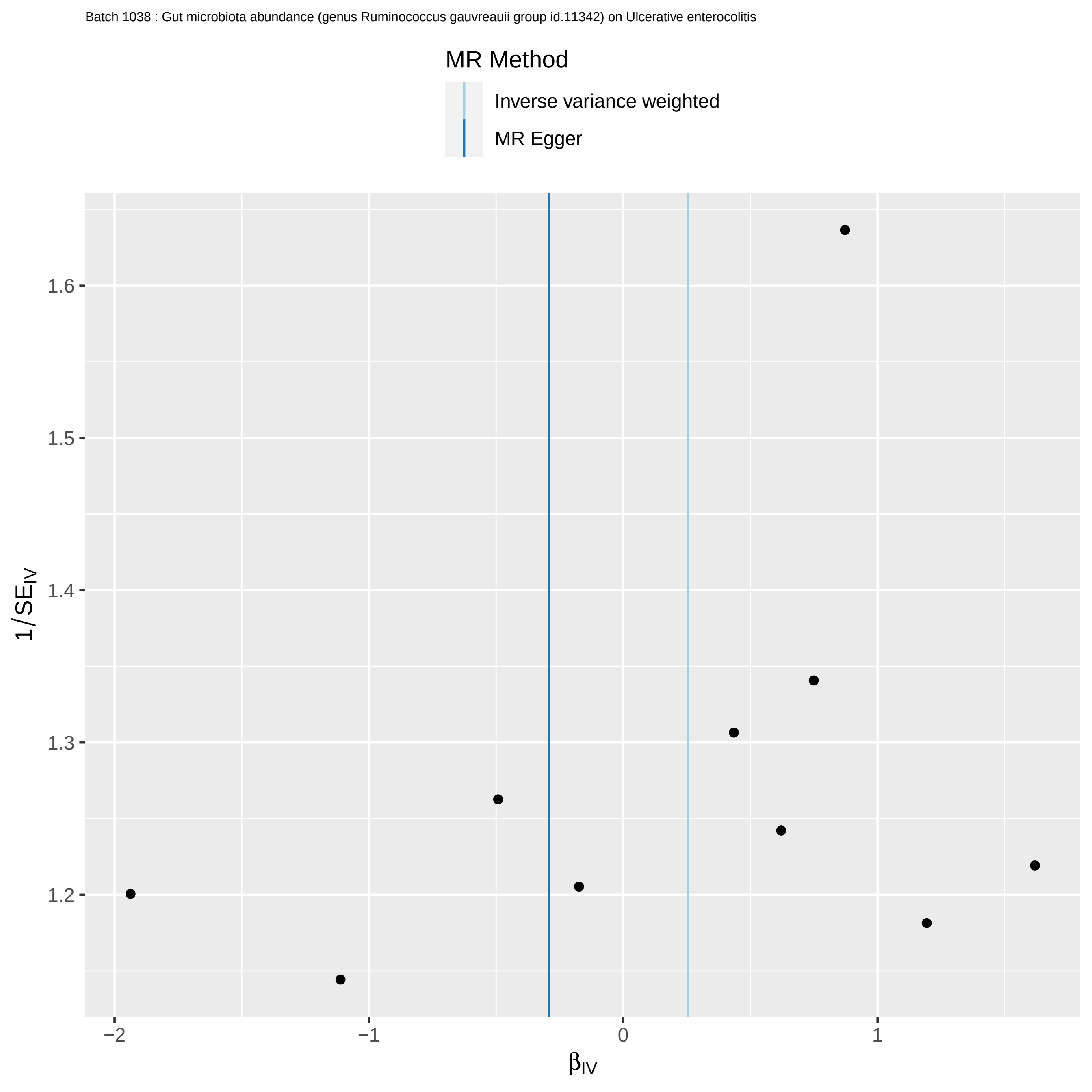

Batch 1038 : Gut microbiota abundance (genus Ruminococcus gauvreauii group id.11342) on Ulcerative enterocolitis
MR Method
Inverse variance weighted
MR Egger
1.6
1.5
1.4
1.3
1.2
1/SE$_{IV}$
−2
−1
0
1
β$_{IV}$

Batch 1039 : Gut microbiota abundance (genus Ruminococcus gnavus group id.14376) on Ulcerative enterocolitis

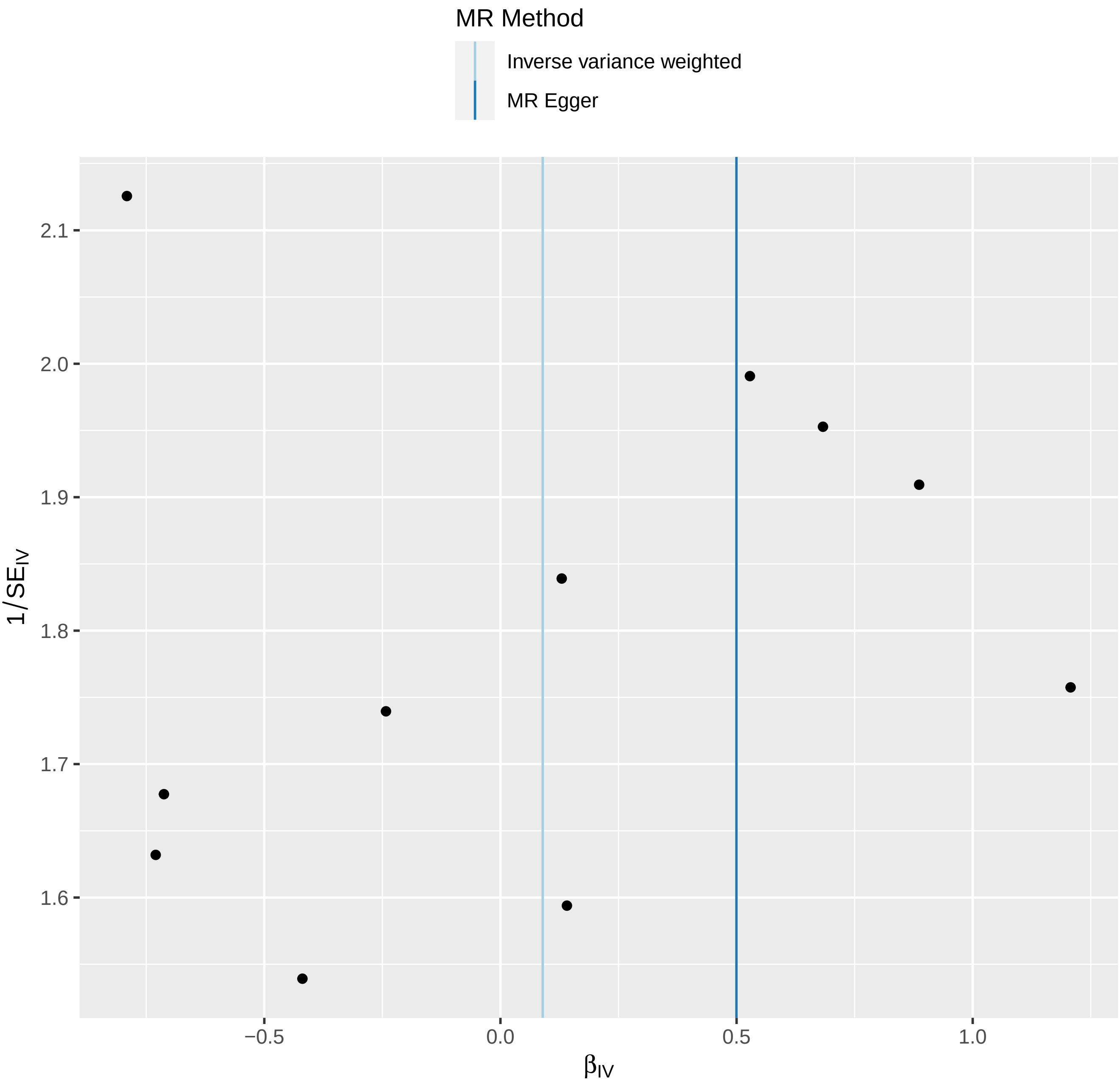

Batch 1040 : Gut microbiota abundance (genus Ruminococcus torques group id.14377) on Ulcerative enterocolitis

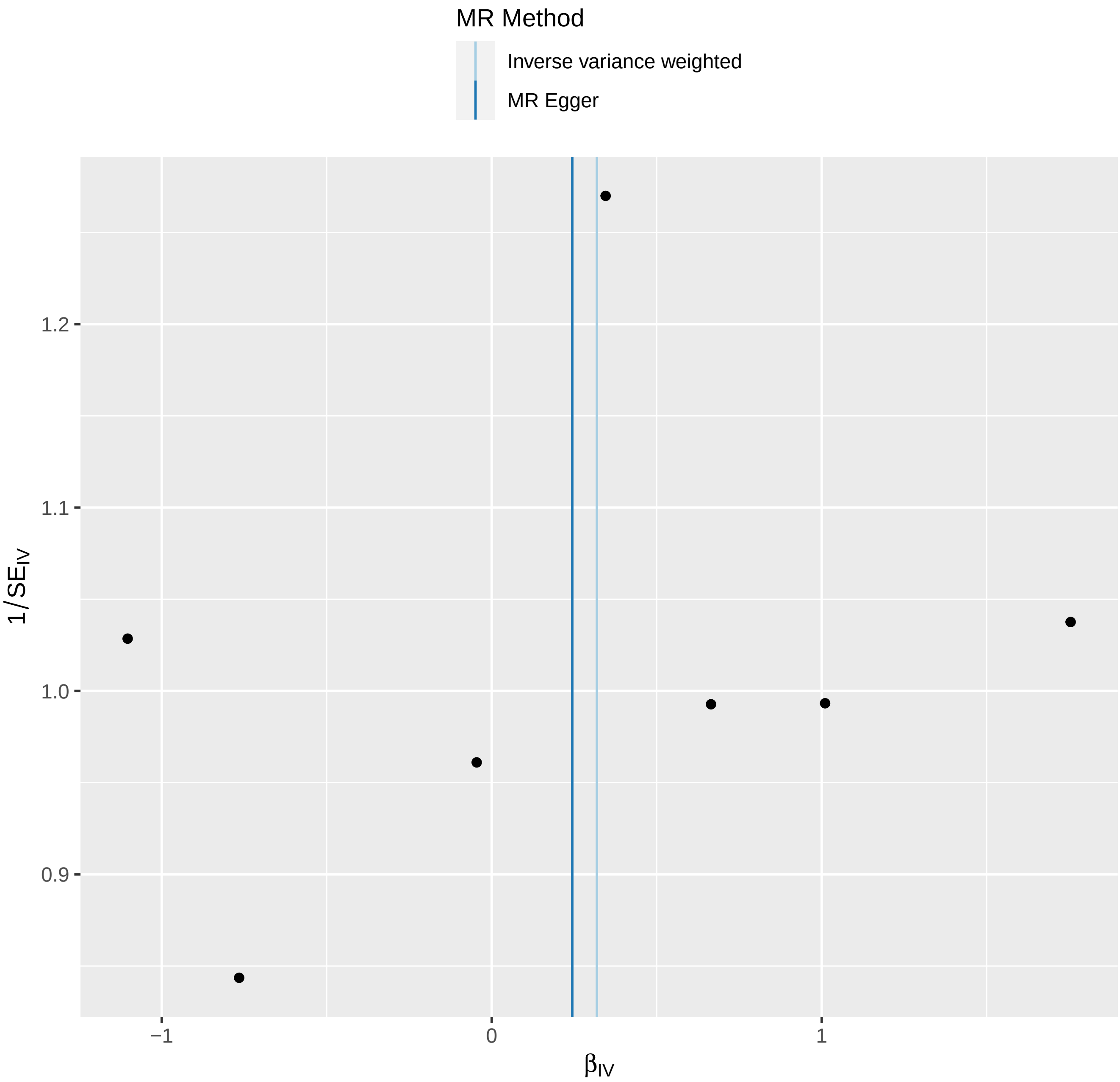

Batch 1041 : Gut microbiota abundance (genus Sellimonas id.14369) on Ulcerative enterocolitis

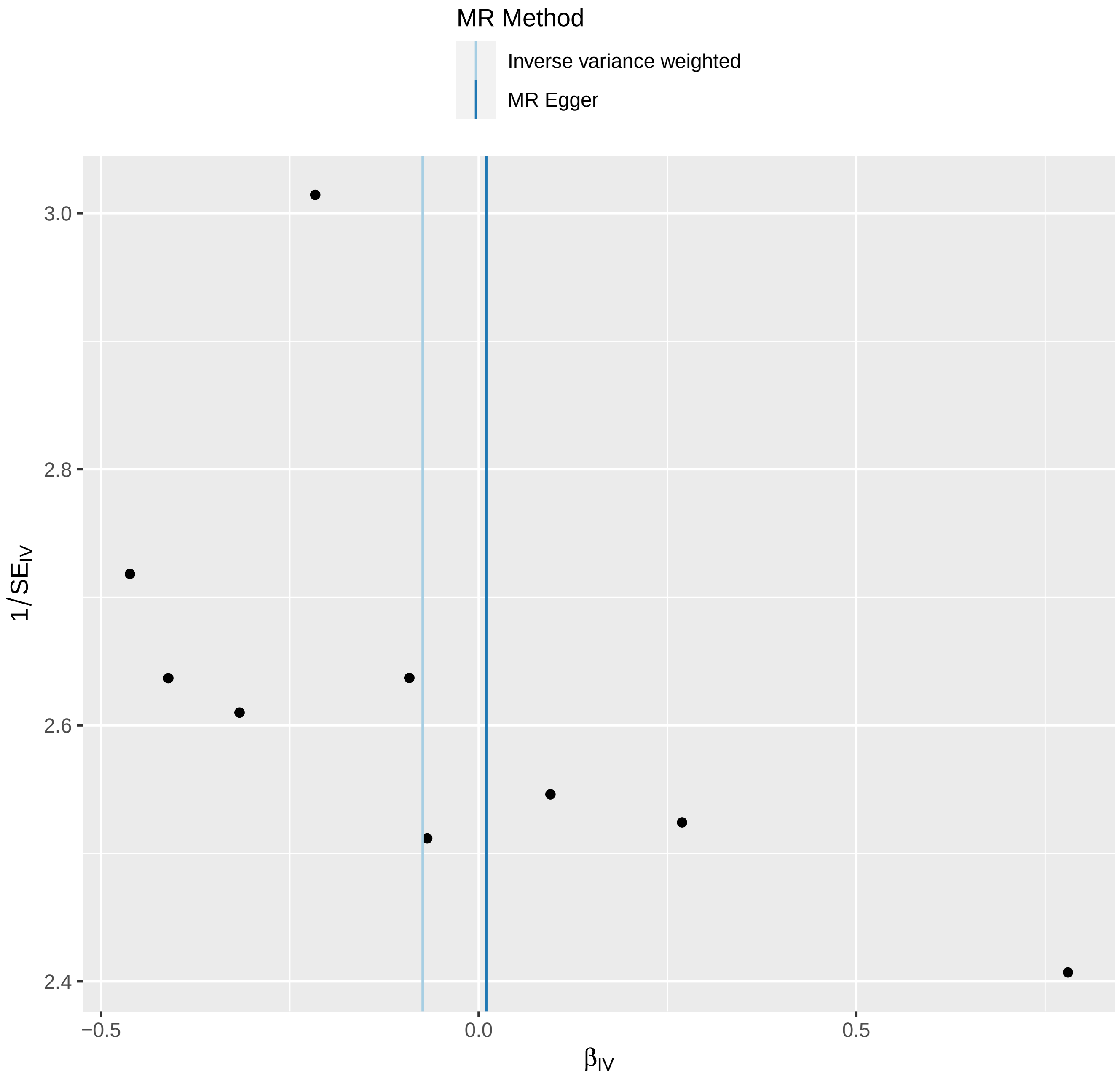

Batch 1042 : Gut microbiota abundance (genus Senegalimassilia id.11160) on Ulcerative enterocolitis

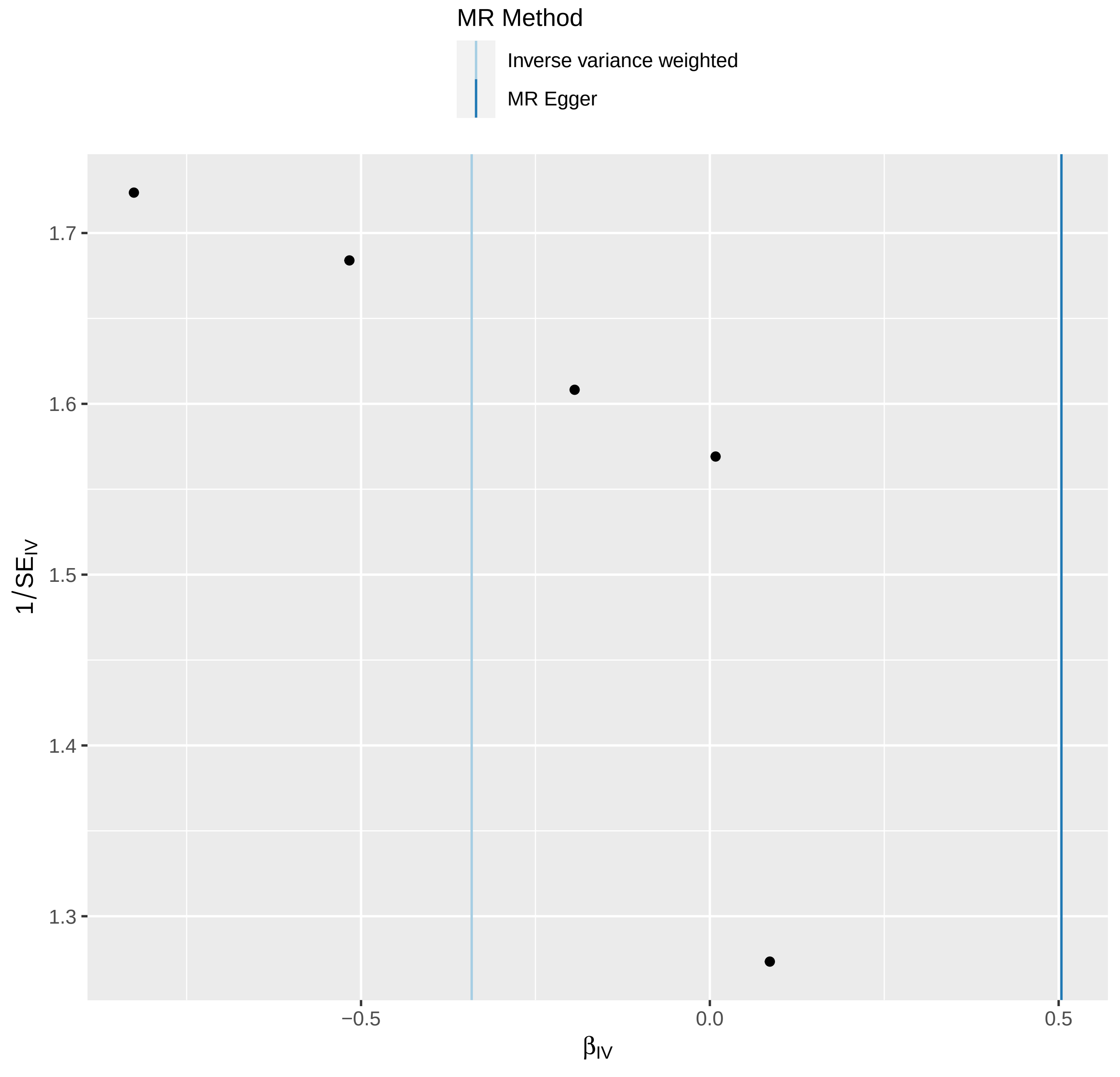

Batch 1043 : Gut microbiota abundance (genus Slackia id.825) on Ulcerative enterocolitis

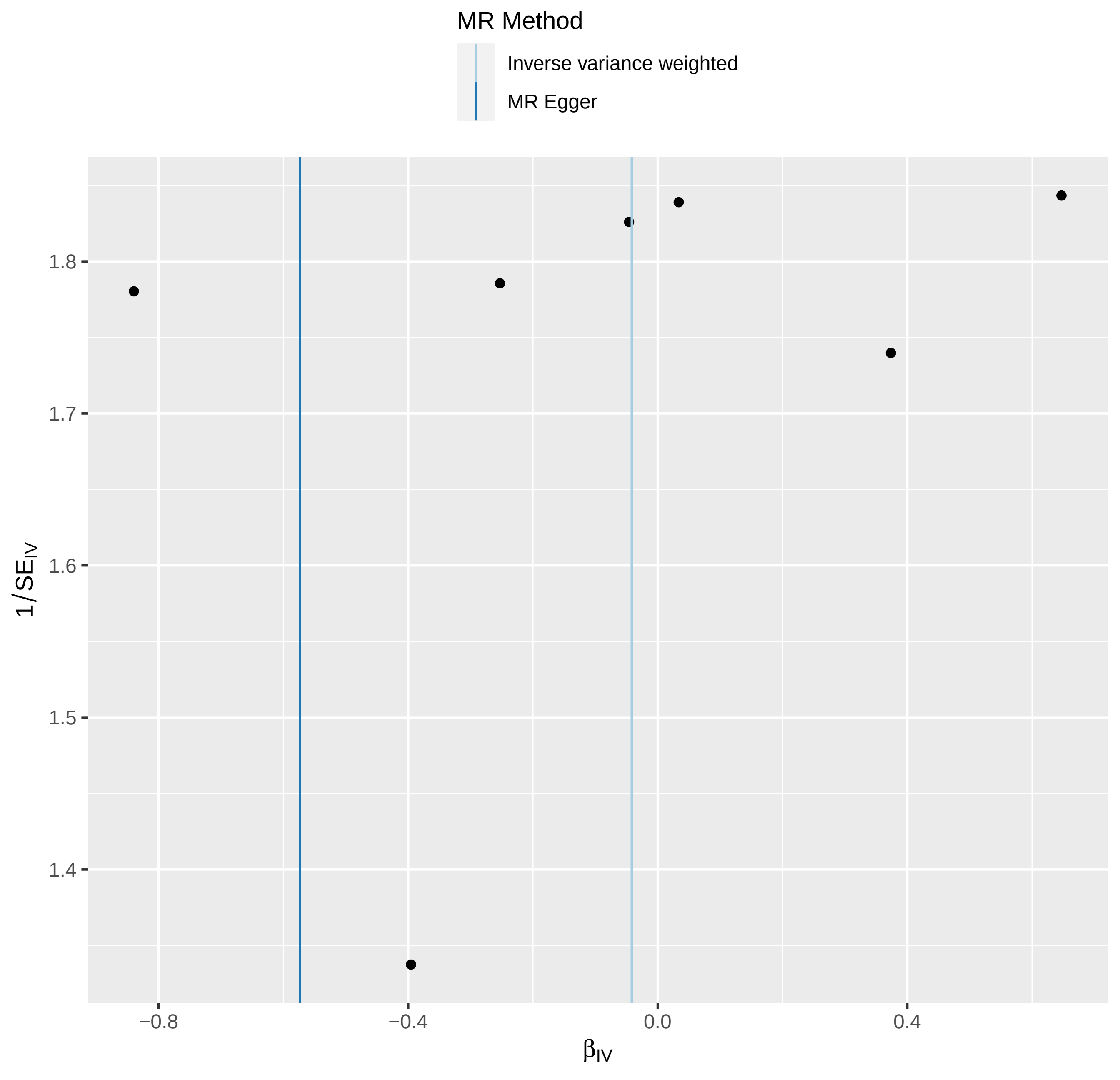

Batch 1044 : Gut microbiota abundance (genus Streptococcus id.1853) on Ulcerative enterocolitis

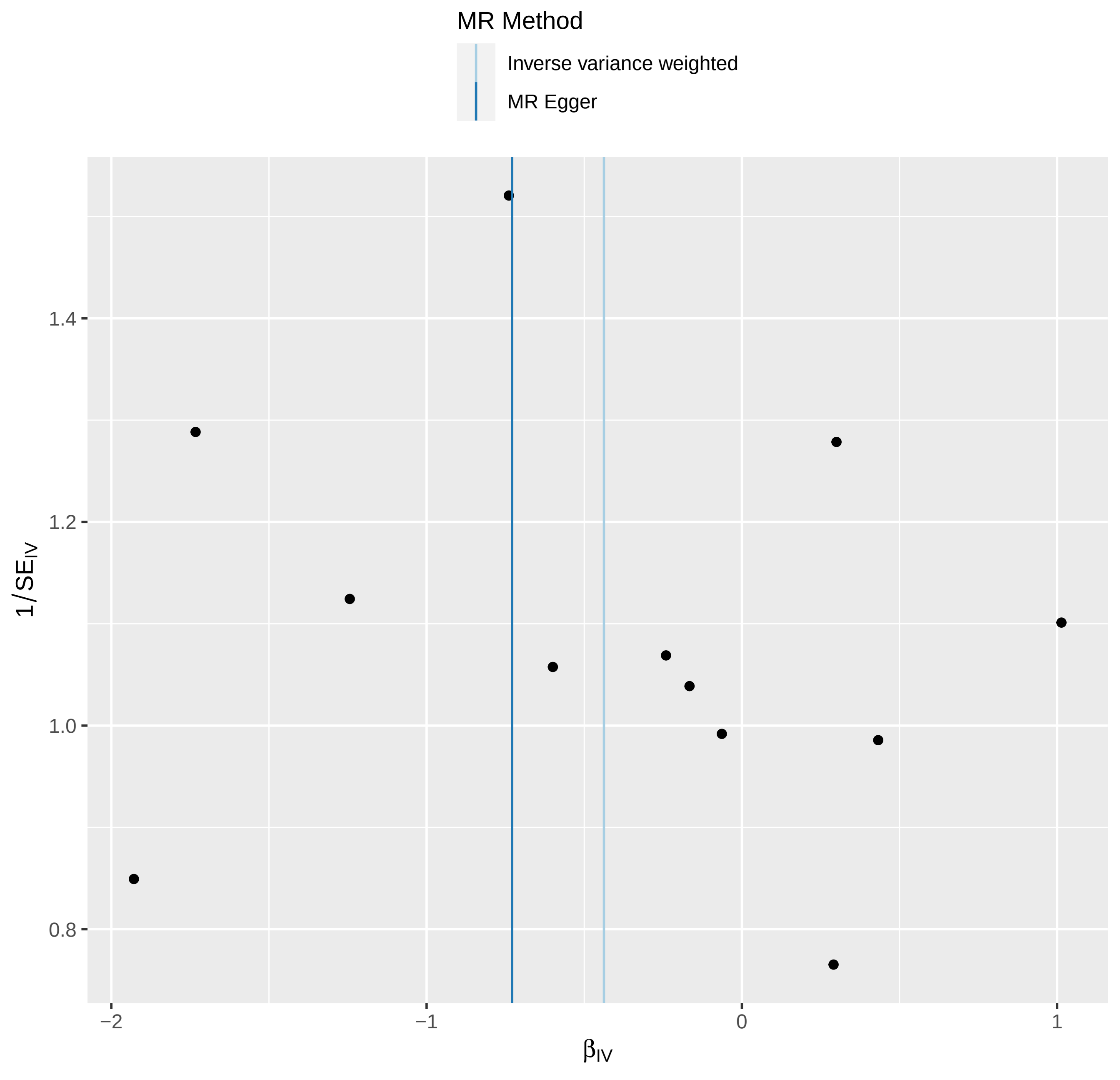

Batch 1045 : Gut microbiota abundance (genus Subdoligranulum id.2070) on Ulcerative enterocolitis

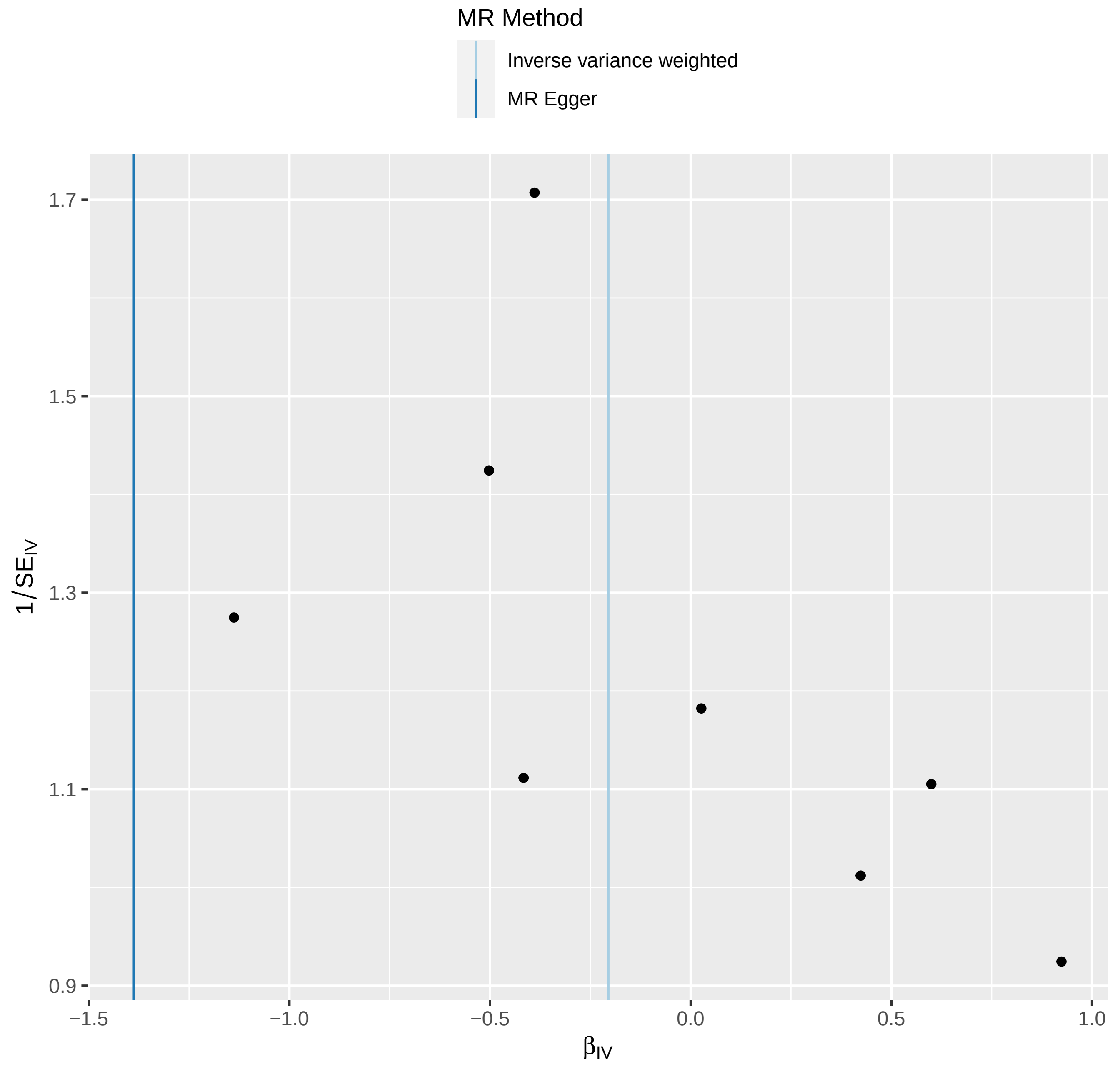

Batch 1046 : Gut microbiota abundance (genus Sutterella id.2896) on Ulcerative enterocolitis

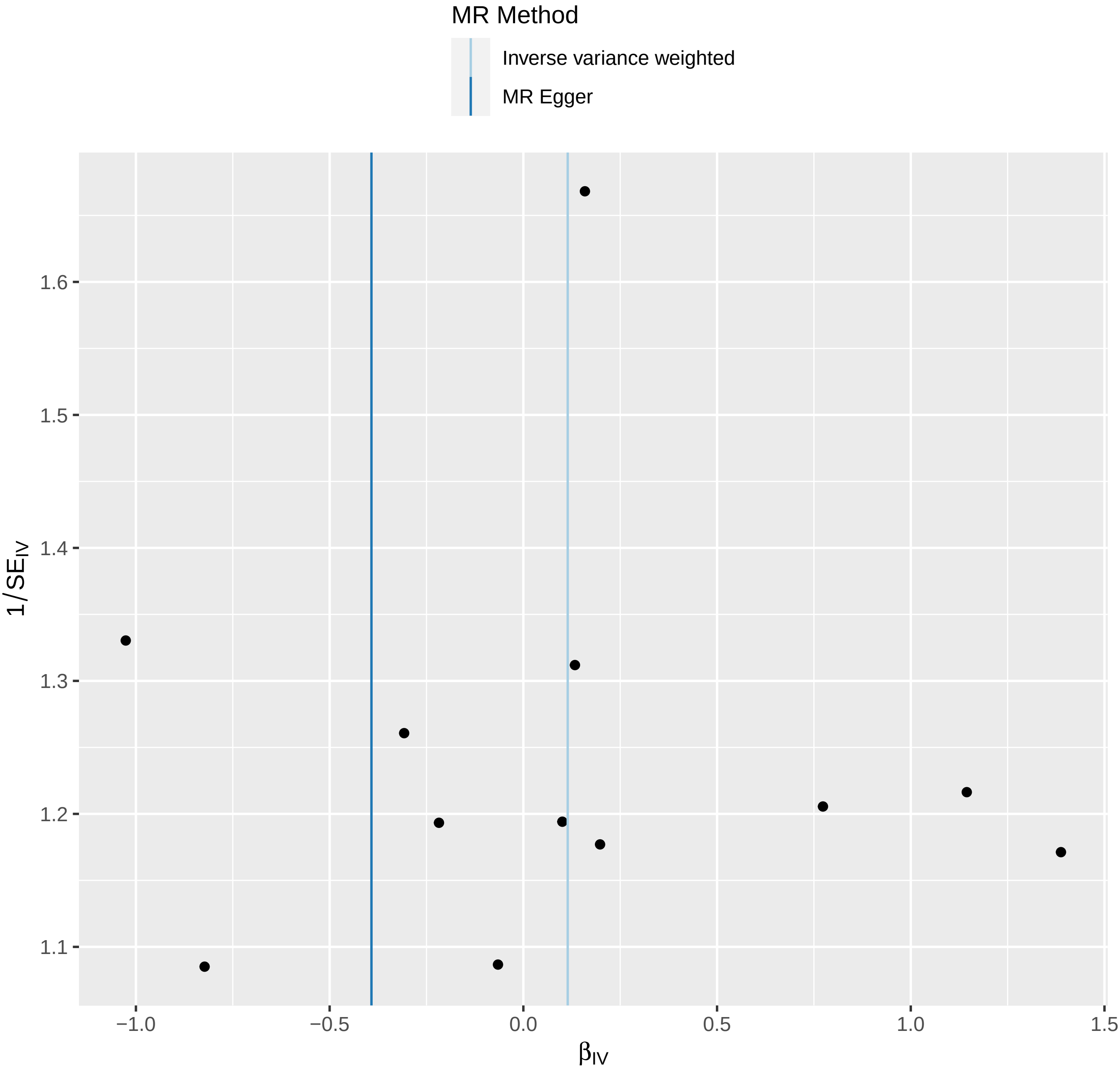

Batch 1047 : Gut microbiota abundance (genus Terrisporobacter id.11348) on Ulcerative enterocolitis

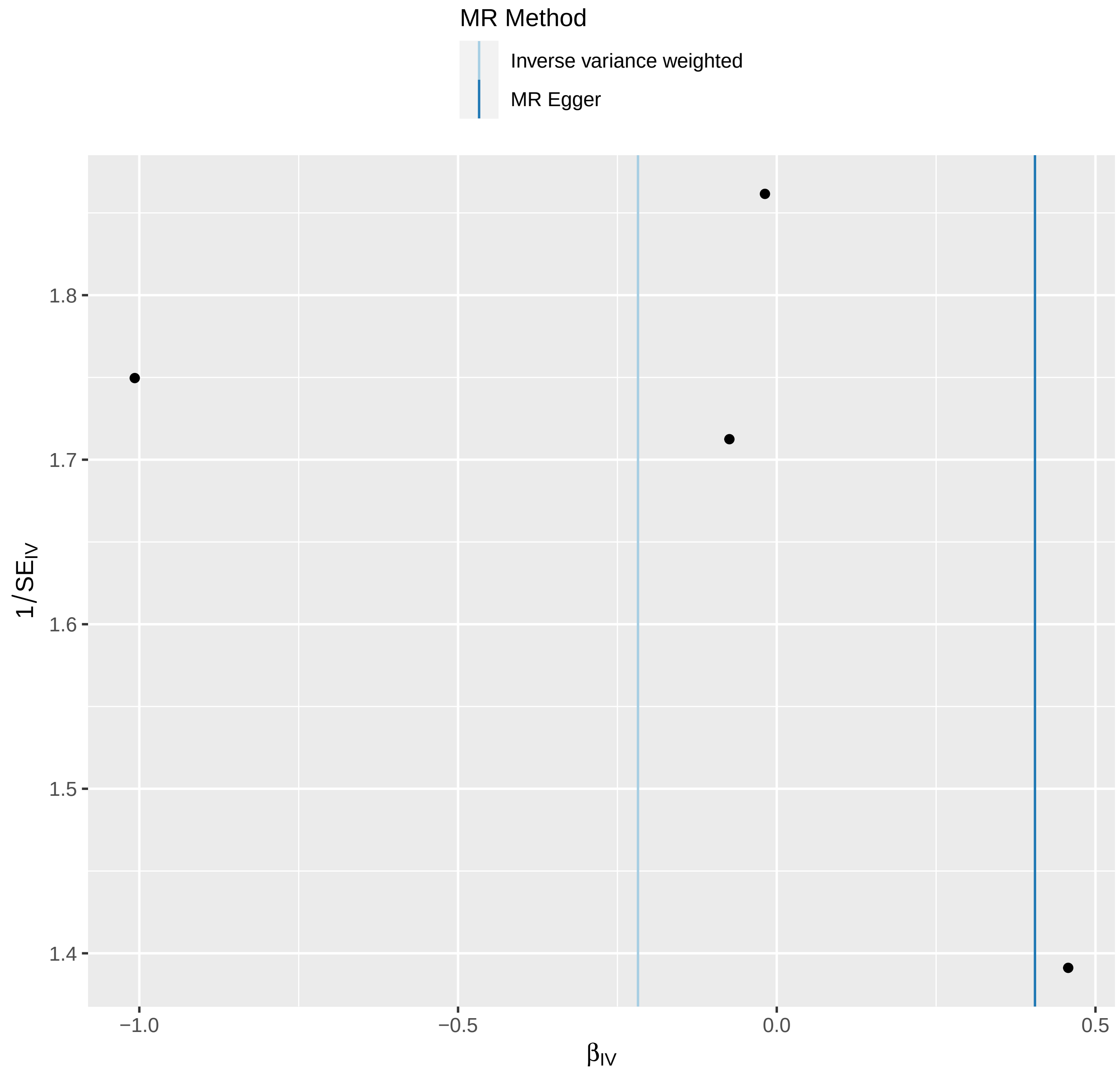

Batch 1048 : Gut microbiota abundance (genus Turicibacter id.2162) on Ulcerative enterocolitis

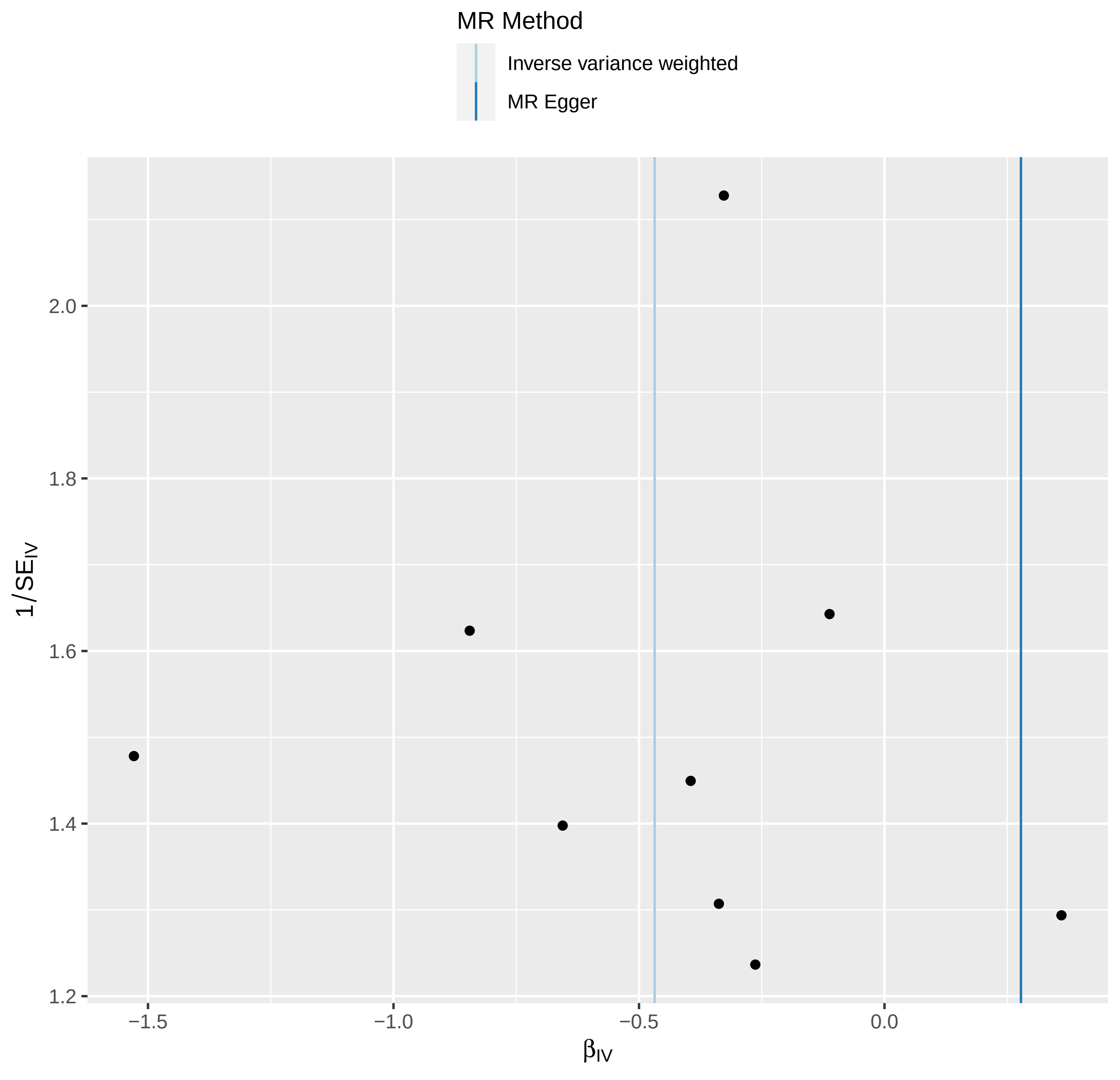

Batch 1049 : Gut microbiota abundance (genus Tyzzerella3 id.11335) on Ulcerative enterocolitis

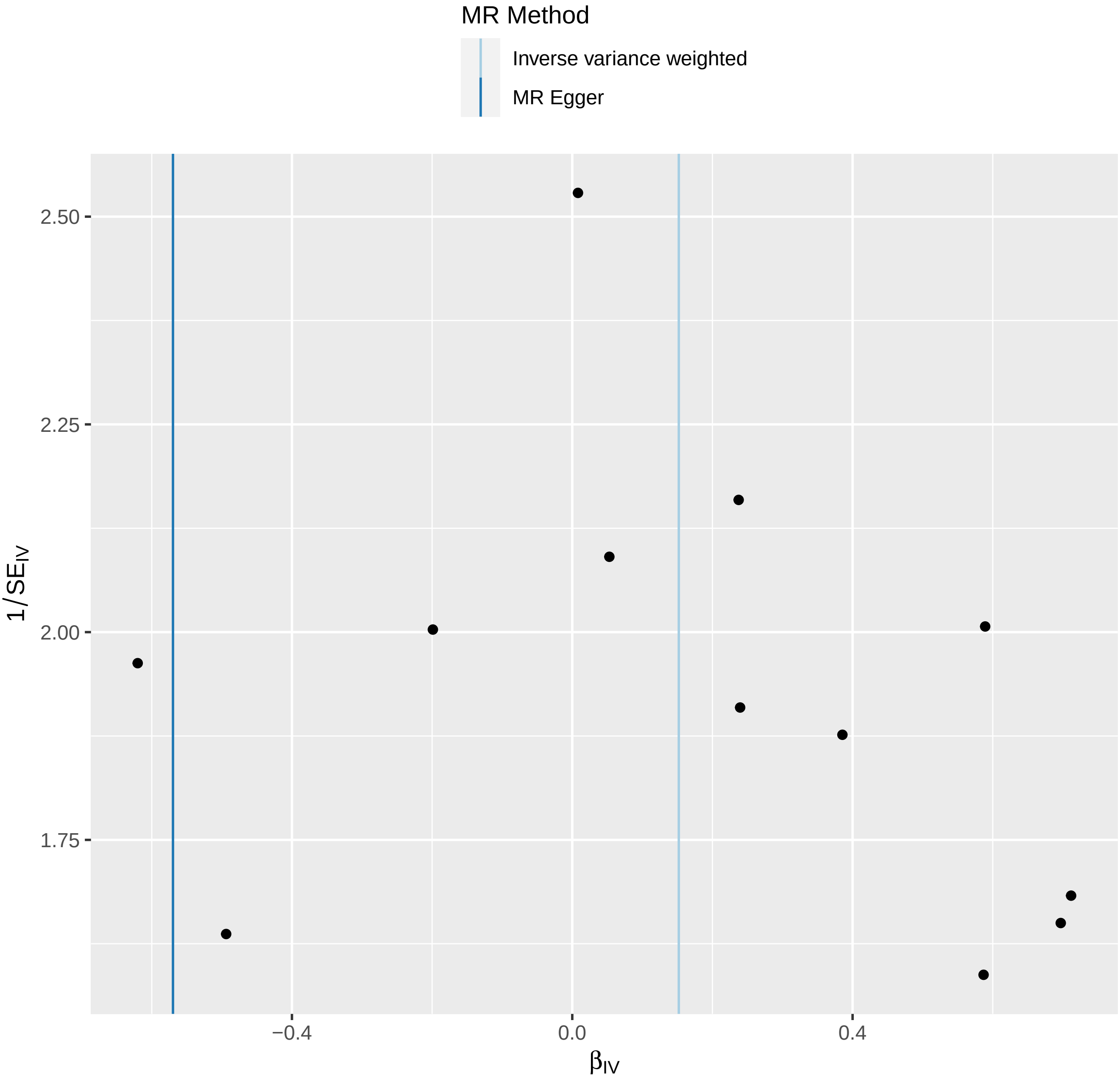

Batch 1050 : Gut microbiota abundance (genus Veillonella id.2198) on Ulcerative enterocolitis

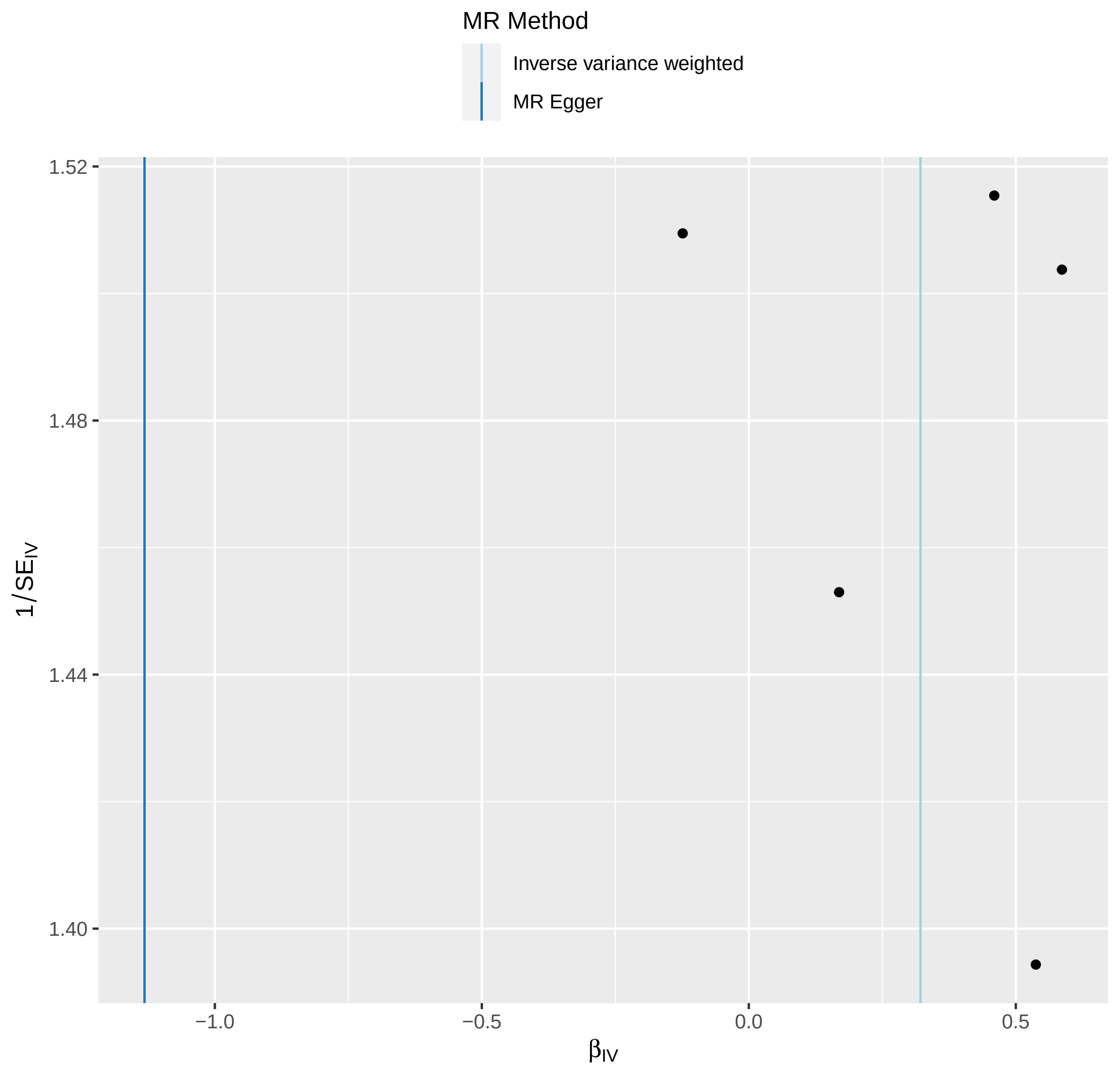

Batch 1051 : Gut microbiota abundance (family Acidaminococcaceae id.2166) on Ulcerative ileocolitis

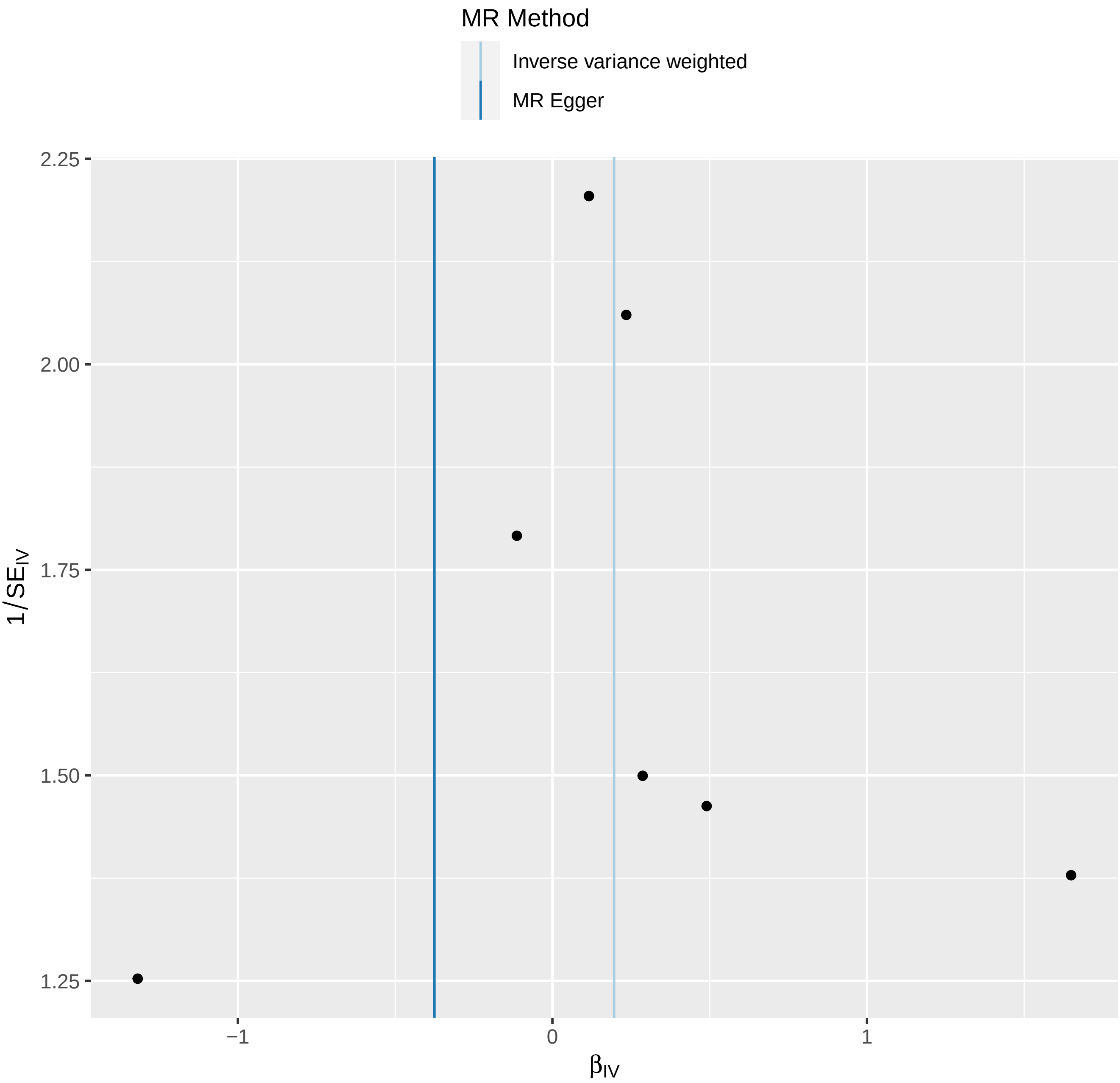

Batch 1052 : Gut microbiota abundance (family Actinomycetaceae id.421) on Ulcerative ileocolitis

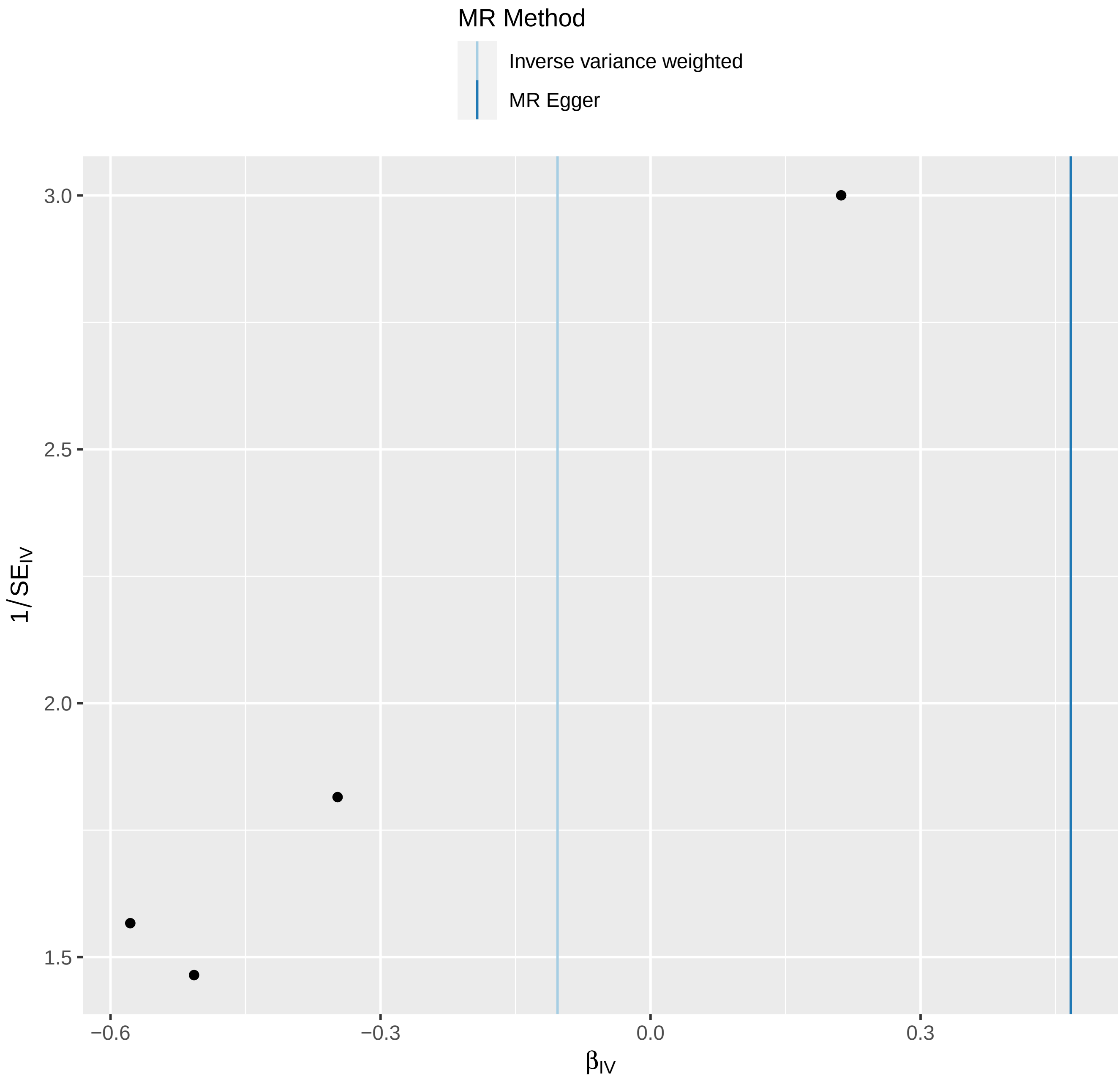

Batch 1053 : Gut microbiota abundance (family Alcaligenaceae id.2875) on Ulcerative ileocolitis

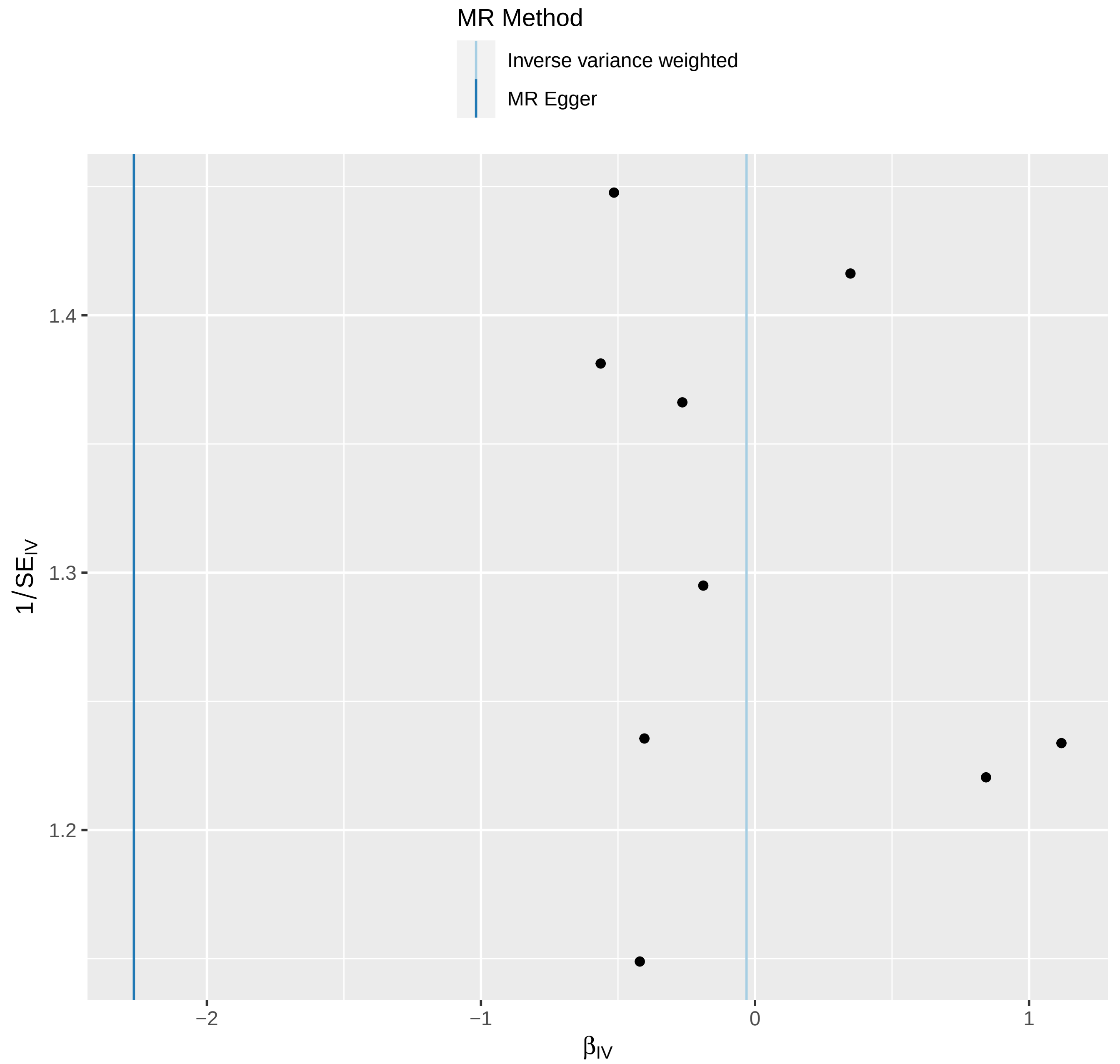

Batch 1054 : Gut microbiota abundance (family Bacteroidaceae id.917) on Ulcerative ileocolitis

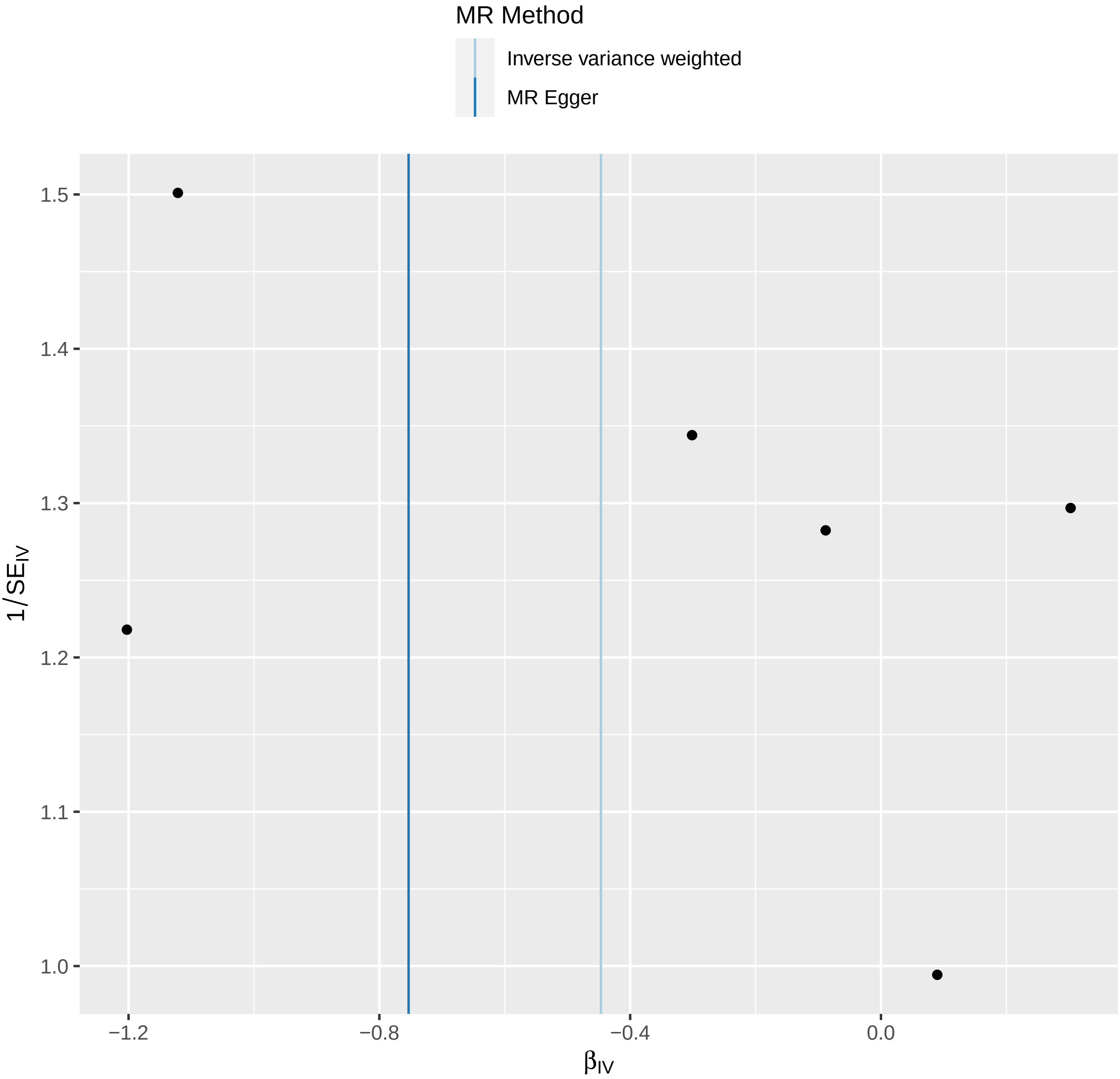

Batch 1055 : Gut microbiota abundance (family Bacteroidales S24 7group id.11173) on Ulcerative ileocolitis

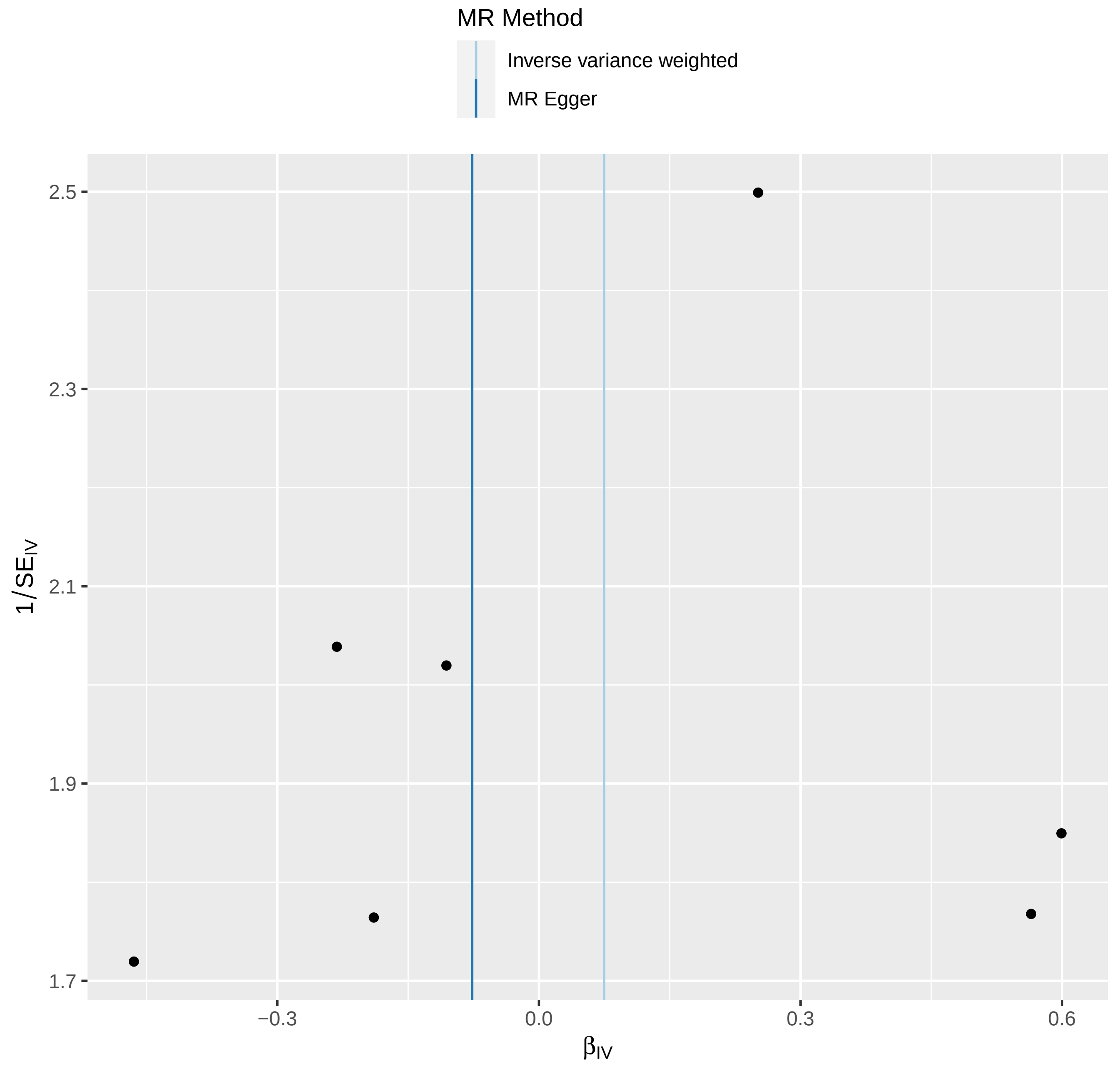

Batch 1056 : Gut microbiota abundance (family Bifidobacteriaceae id.433) on Ulcerative ileocolitis

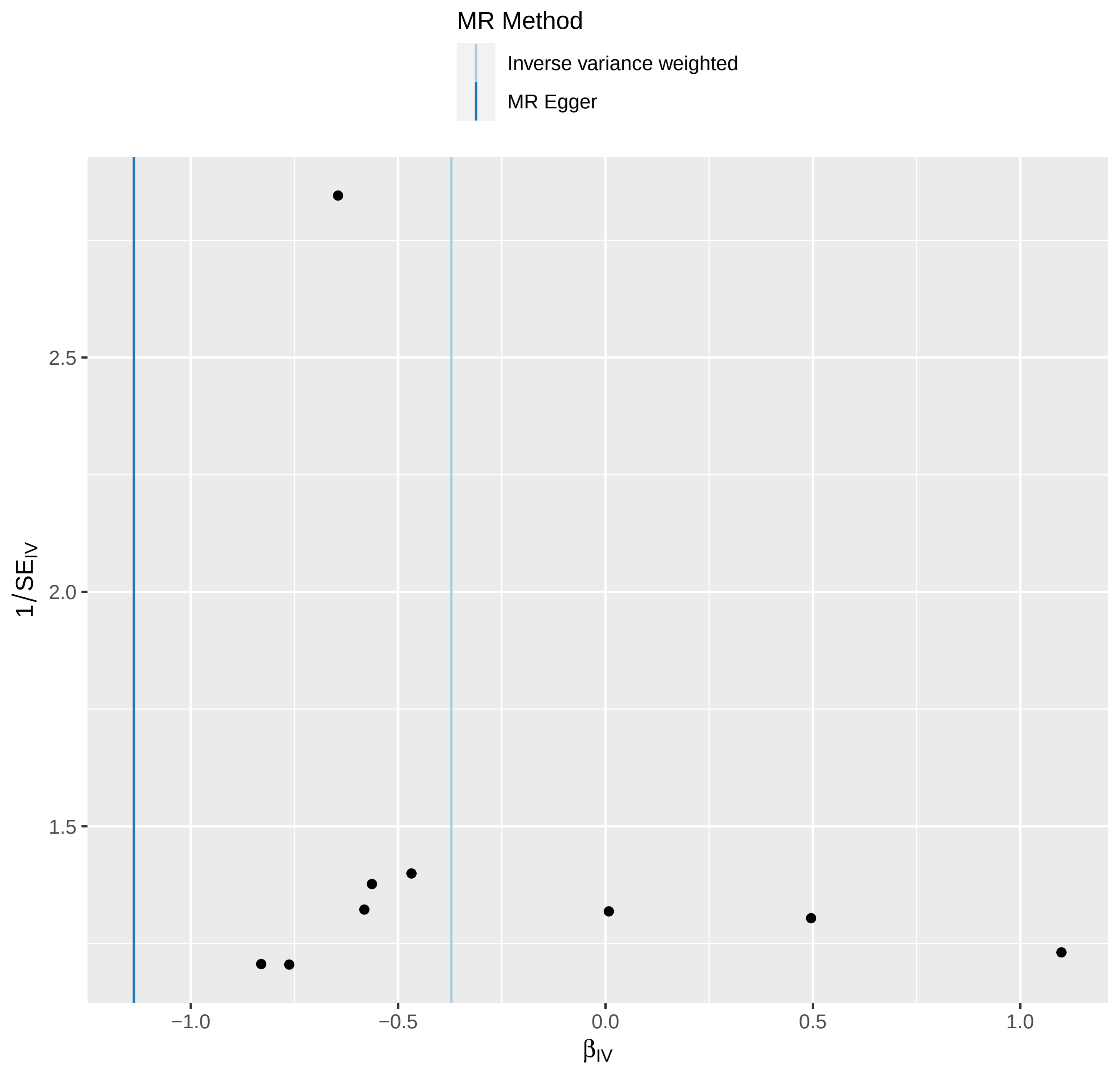

Batch 1058 : Gut microbiota abundance (family Clostridiaceae1 id.1869) on Ulcerative ileocolitis

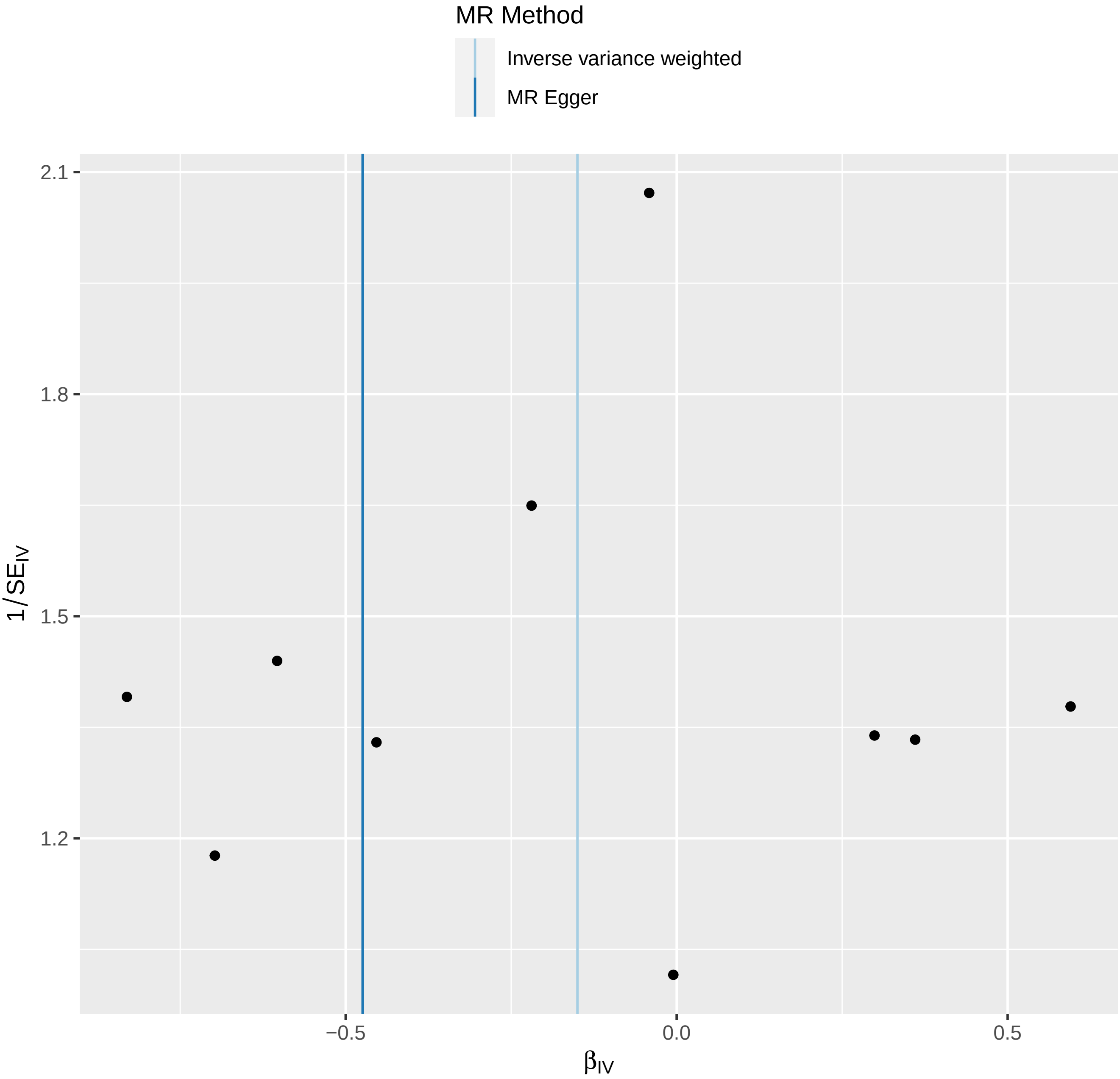

Batch 1059 : Gut microbiota abundance (family Clostridiales vadin BB60 group id.11286) on Ulcerative ileocolitis

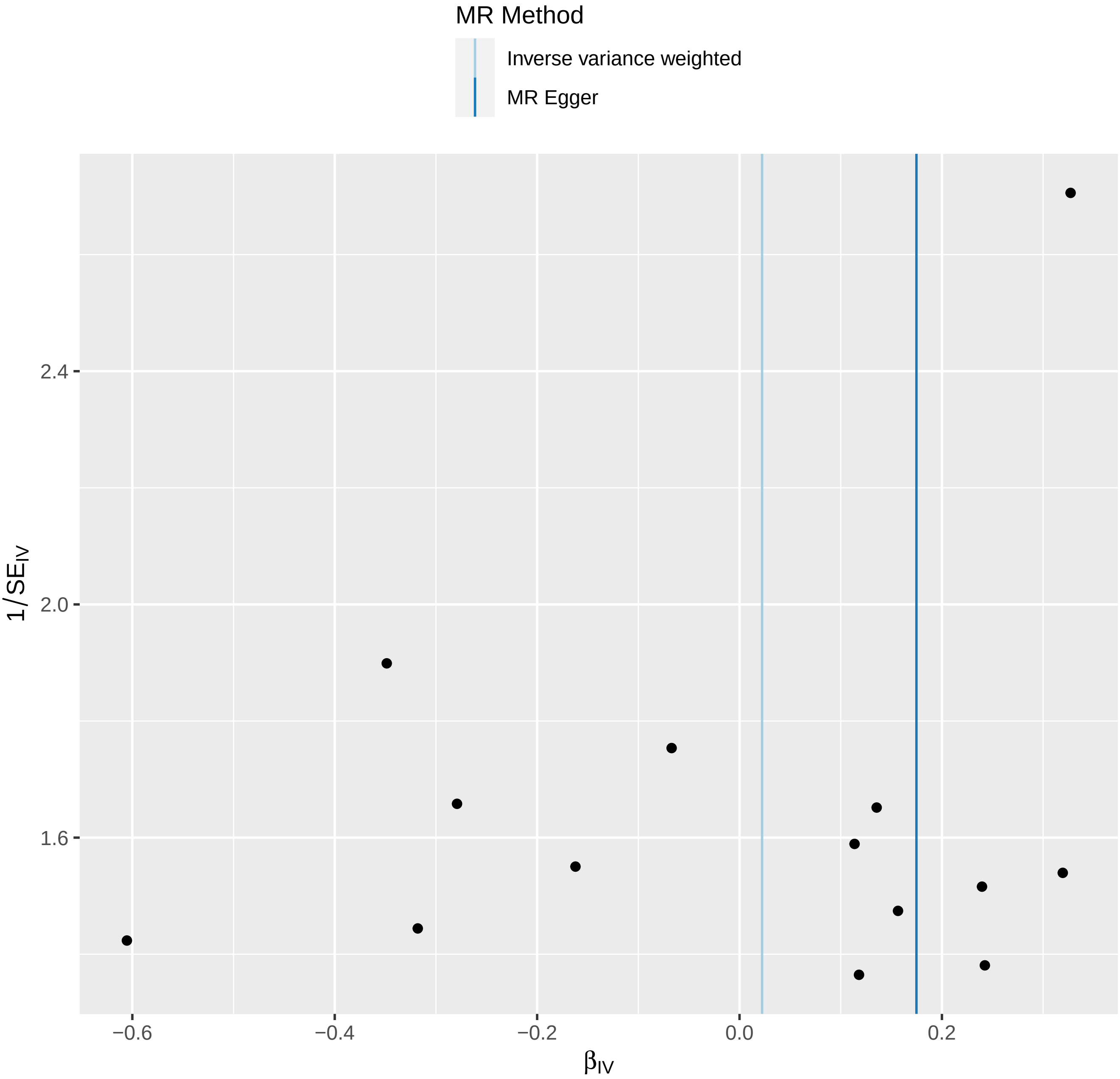

Batch 1060 : Gut microbiota abundance (family Coriobacteriaceae id.811) on Ulcerative ileocolitis

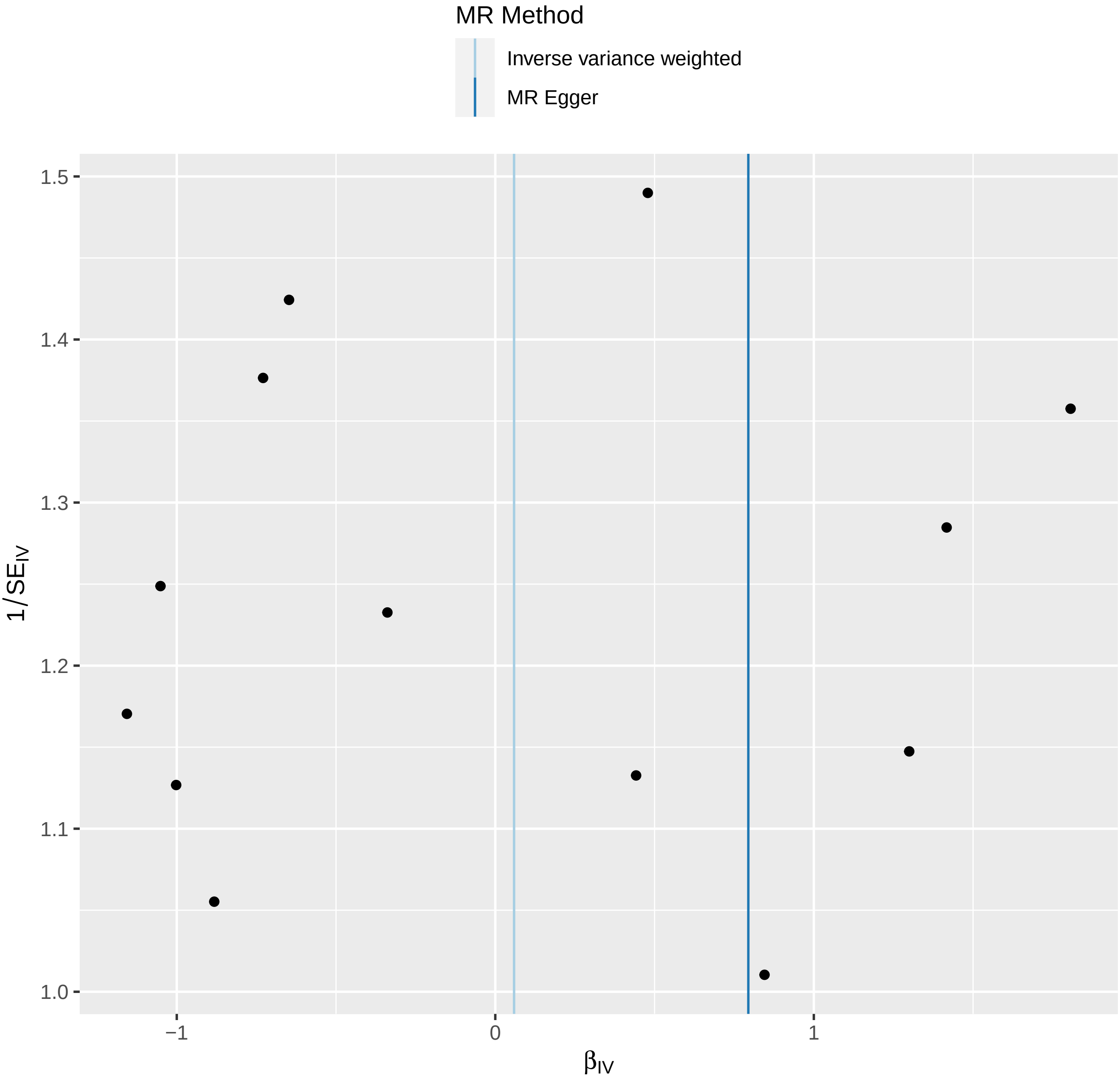

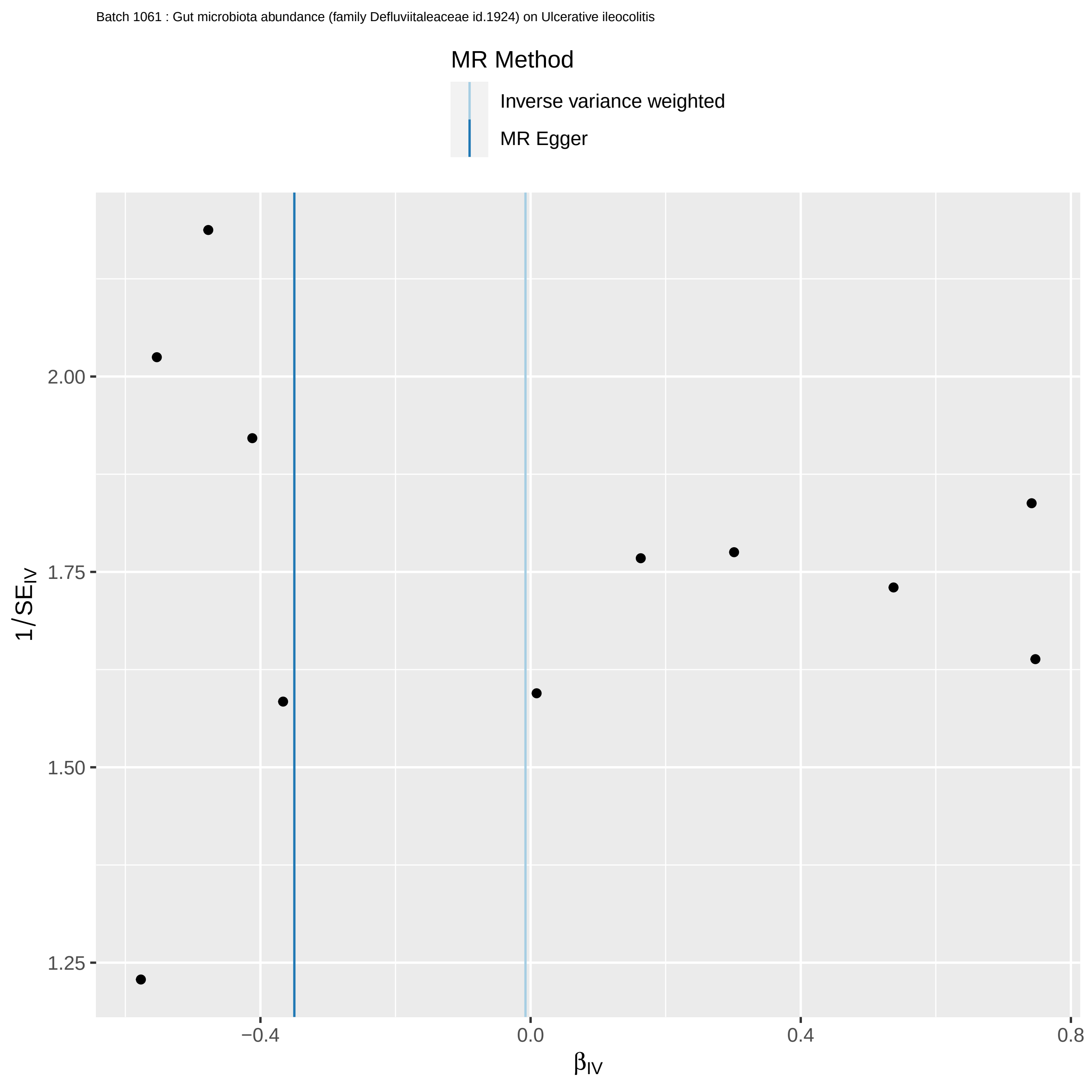
Batch 1061 : Gut microbiota abundance (family Defluviitaleaceae id.1924) on Ulcerative ileocolitis
MR Method
Inverse variance weighted
MR Egger
2.00
1.75
1.50
1.25
1/SE_IV
−0.4
0.0
0.4
0.8
β_IV

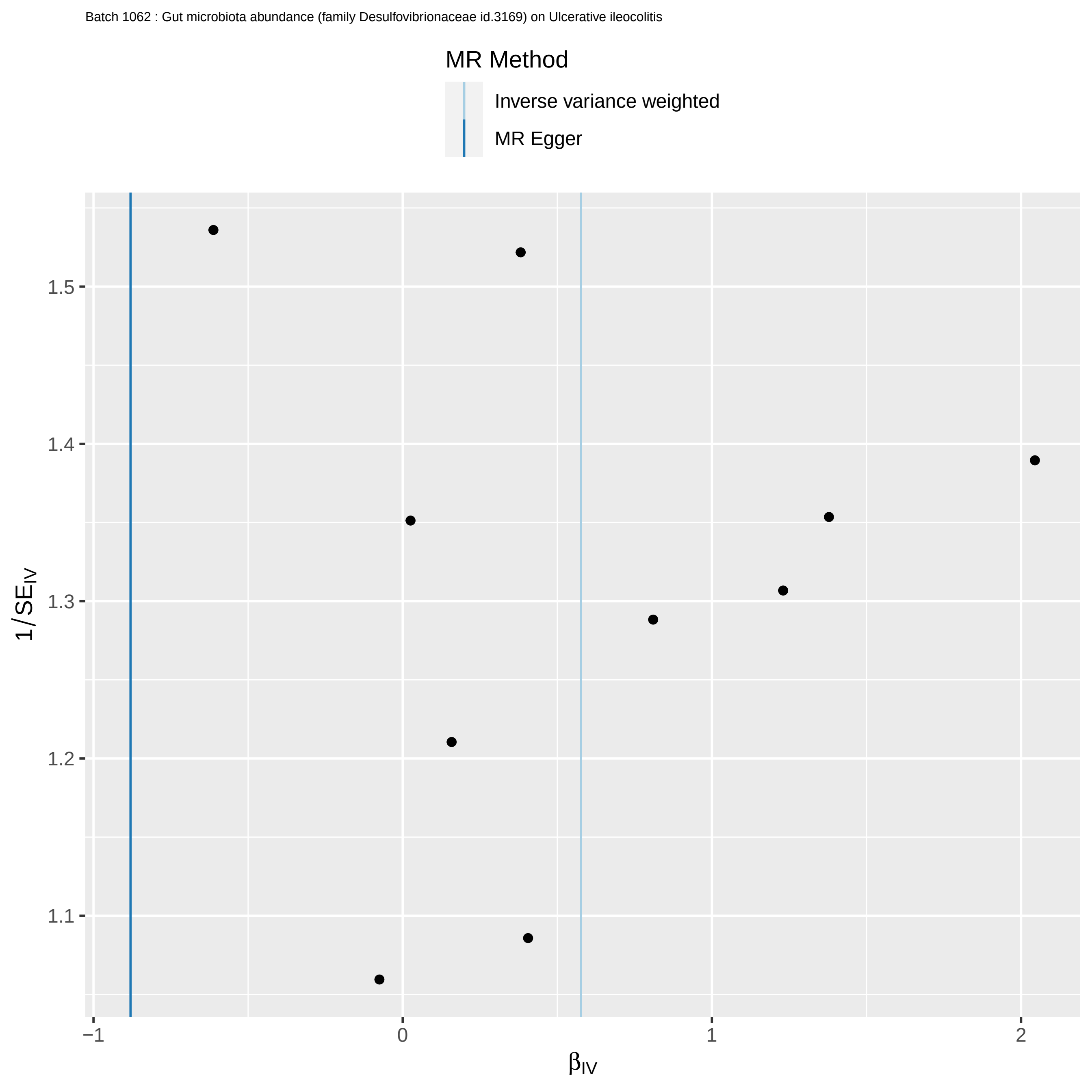
Batch 1062 : Gut microbiota abundance (family Desulfovibrionaceae id.3169) on Ulcerative ileocolitis
MR Method
Inverse variance weighted
MR Egger
1.5
1.4
1.3
1.2
1.1
$1/SE_{IV}$
−1
0
1
2
β_{IV}

Batch 1063 : Gut microbiota abundance (family Enterobacteriaceae id.3469) on Ulcerative ileocolitis

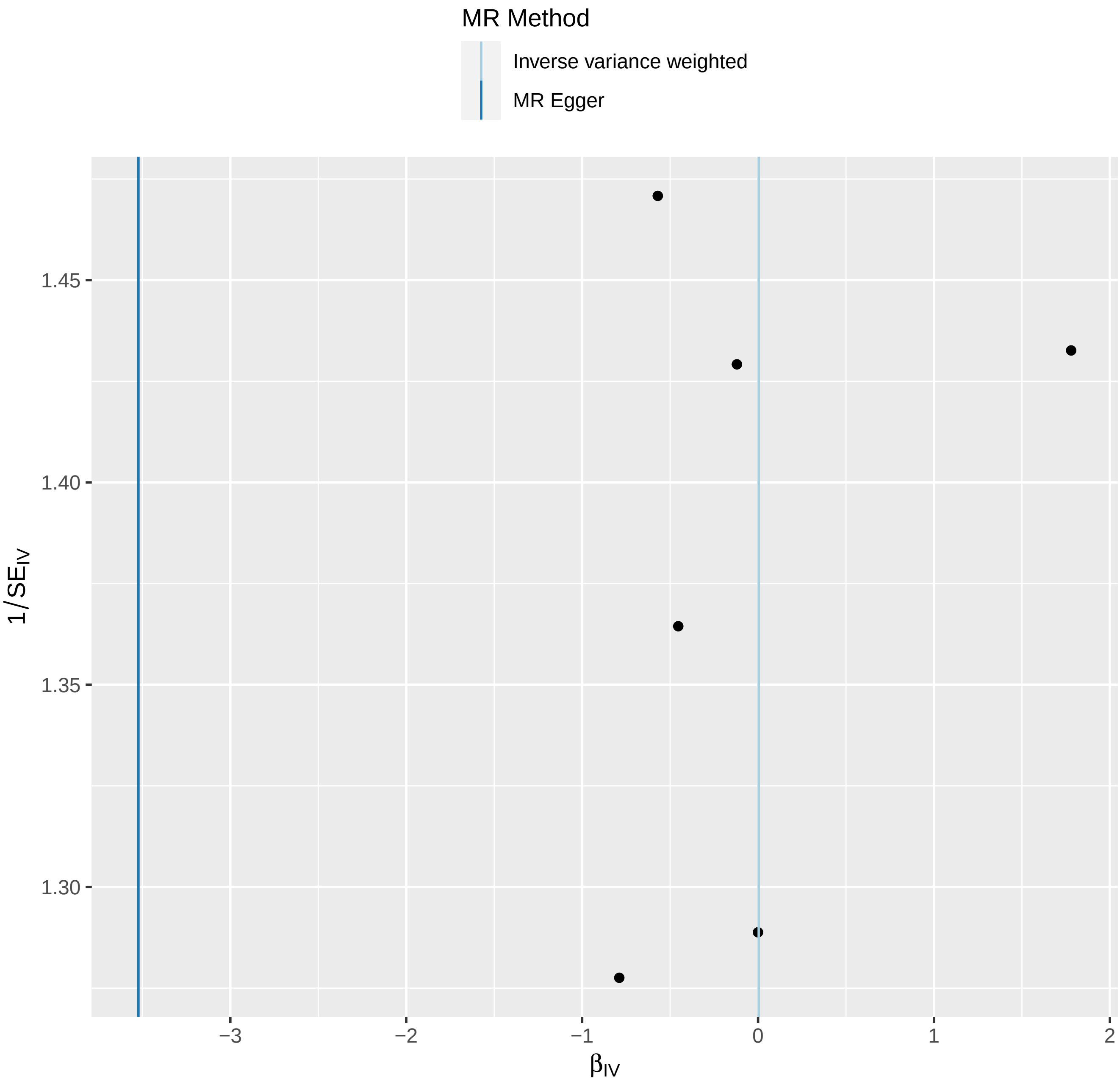

Batch 1064 : Gut microbiota abundance (family Erysipelotrichaceae id.2149) on Ulcerative ileocolitis

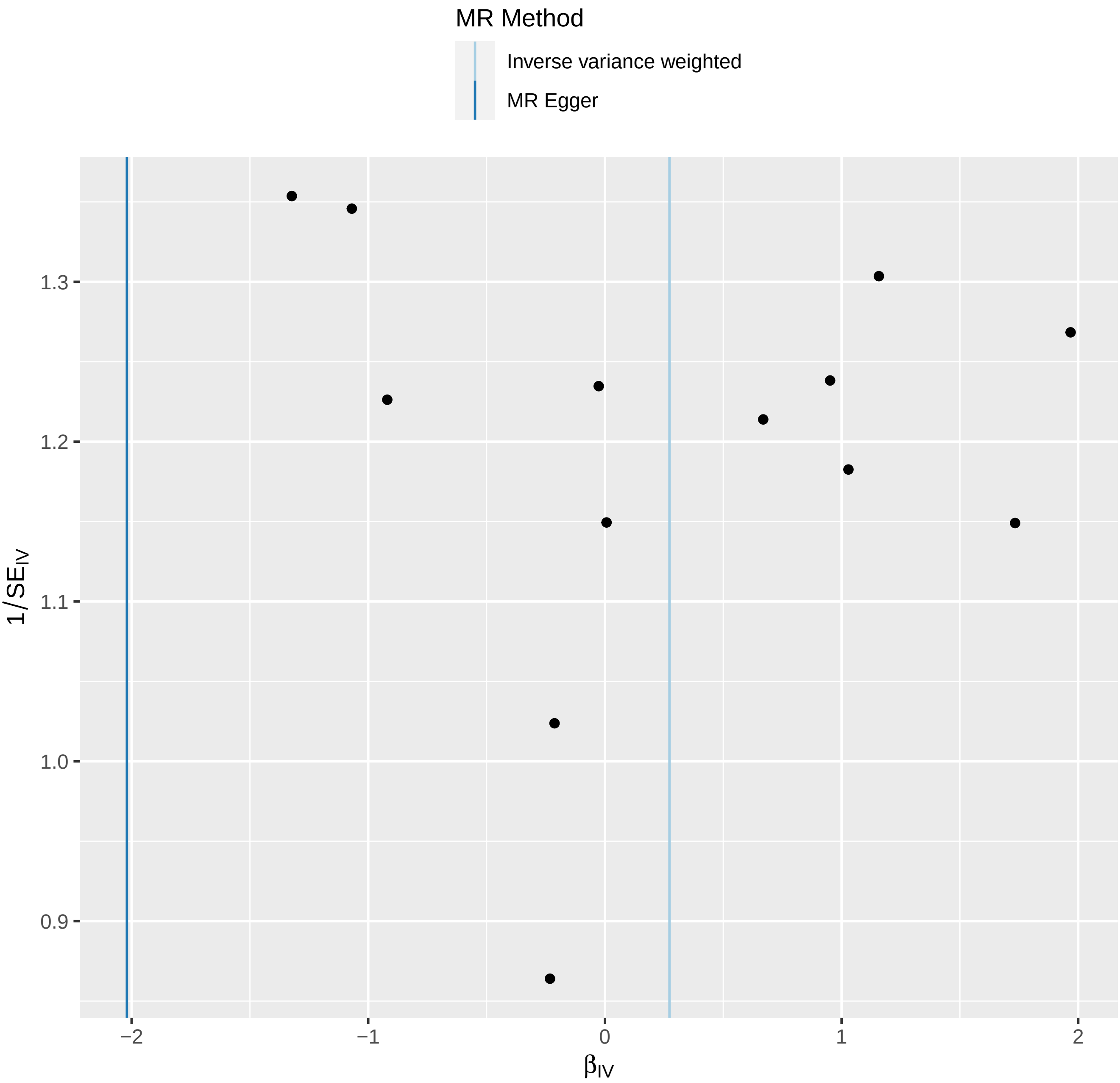

Batch 1065 : Gut microbiota abundance (family Family XI id.1936) on Ulcerative ileocolitis

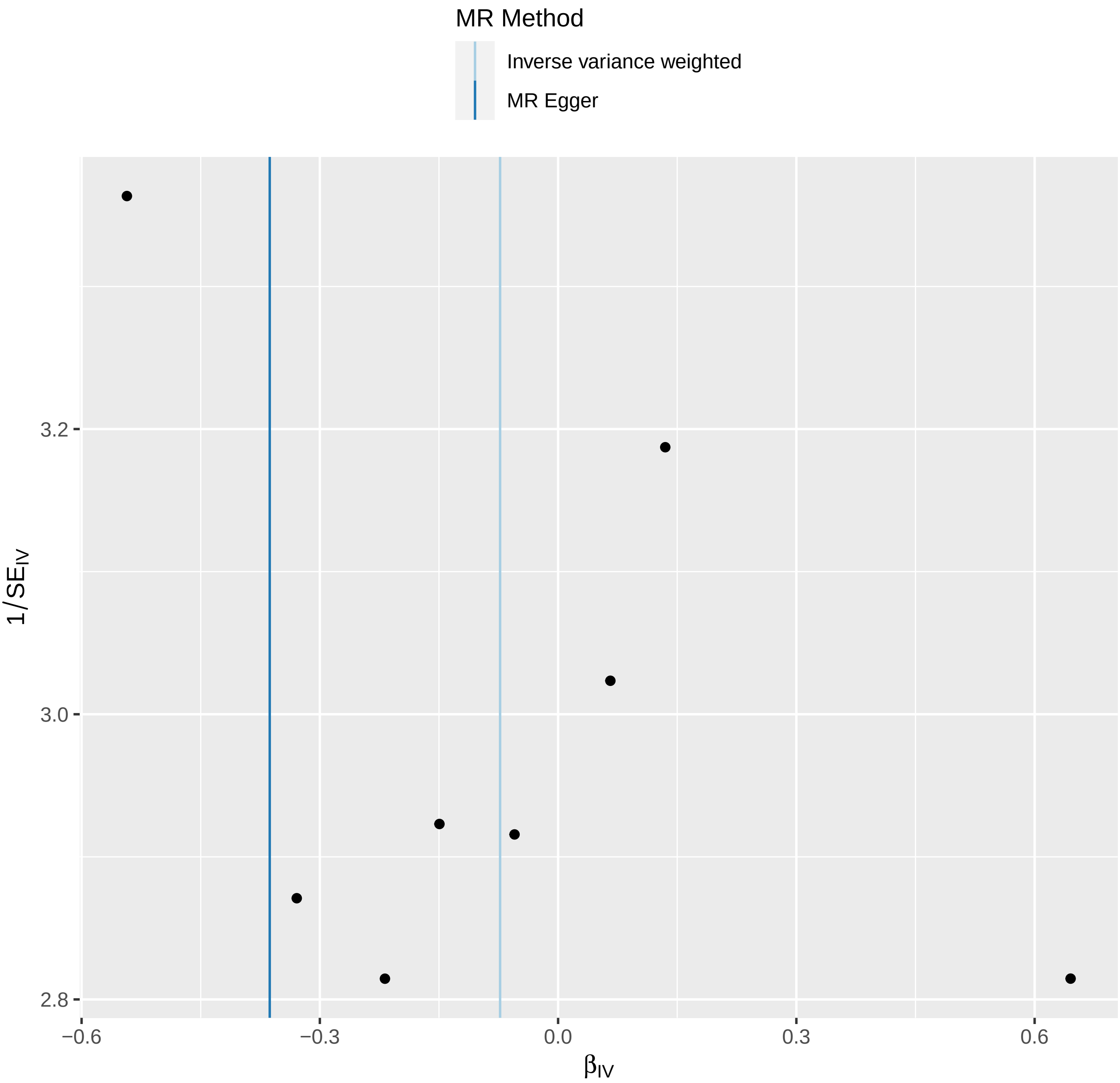

Batch 1066 : Gut microbiota abundance (family Family XIII id.1957) on Ulcerative ileocolitis

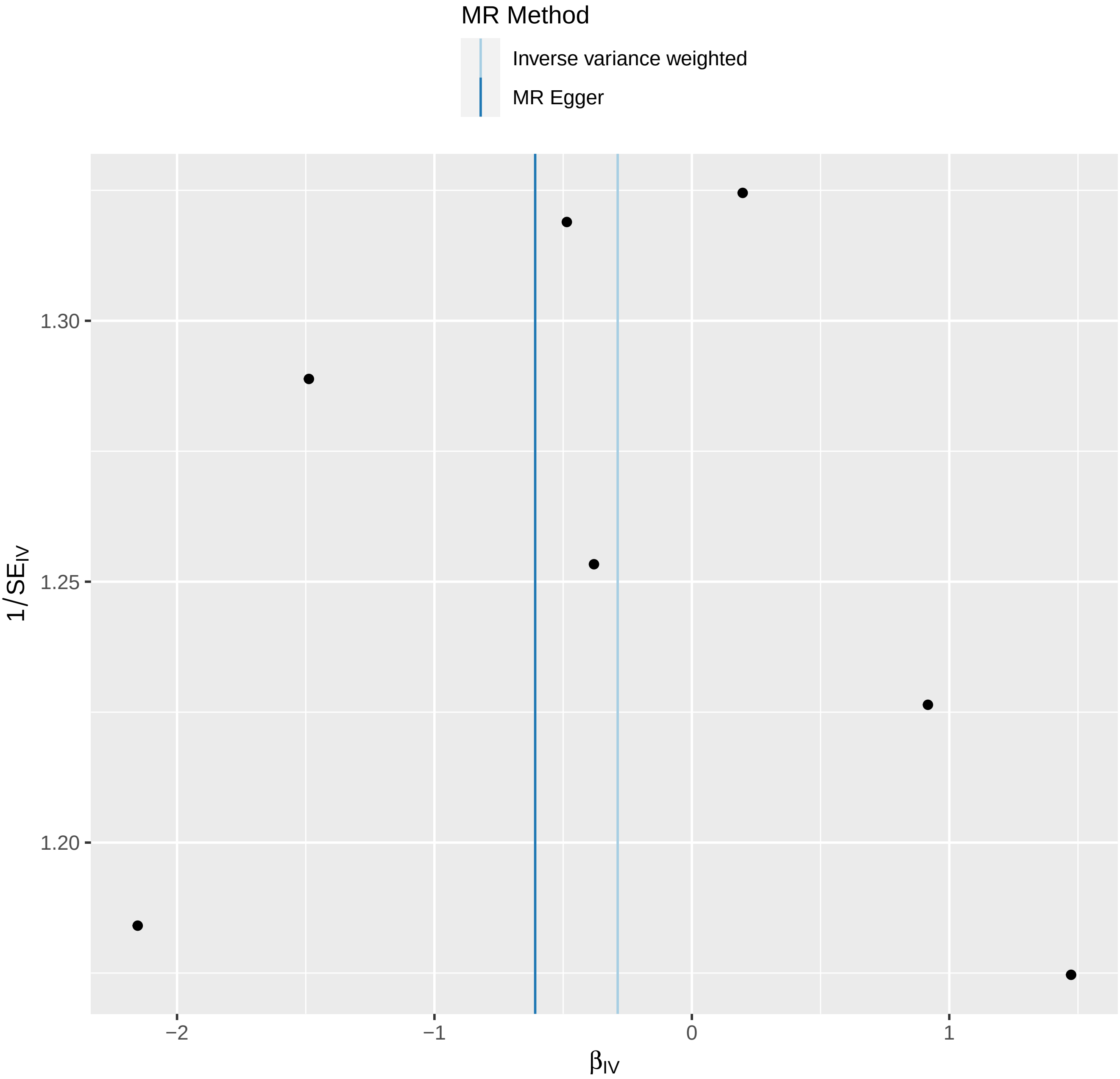

Batch 1067 : Gut microbiota abundance (family Lachnospiraceae id.1987) on Ulcerative ileocolitis

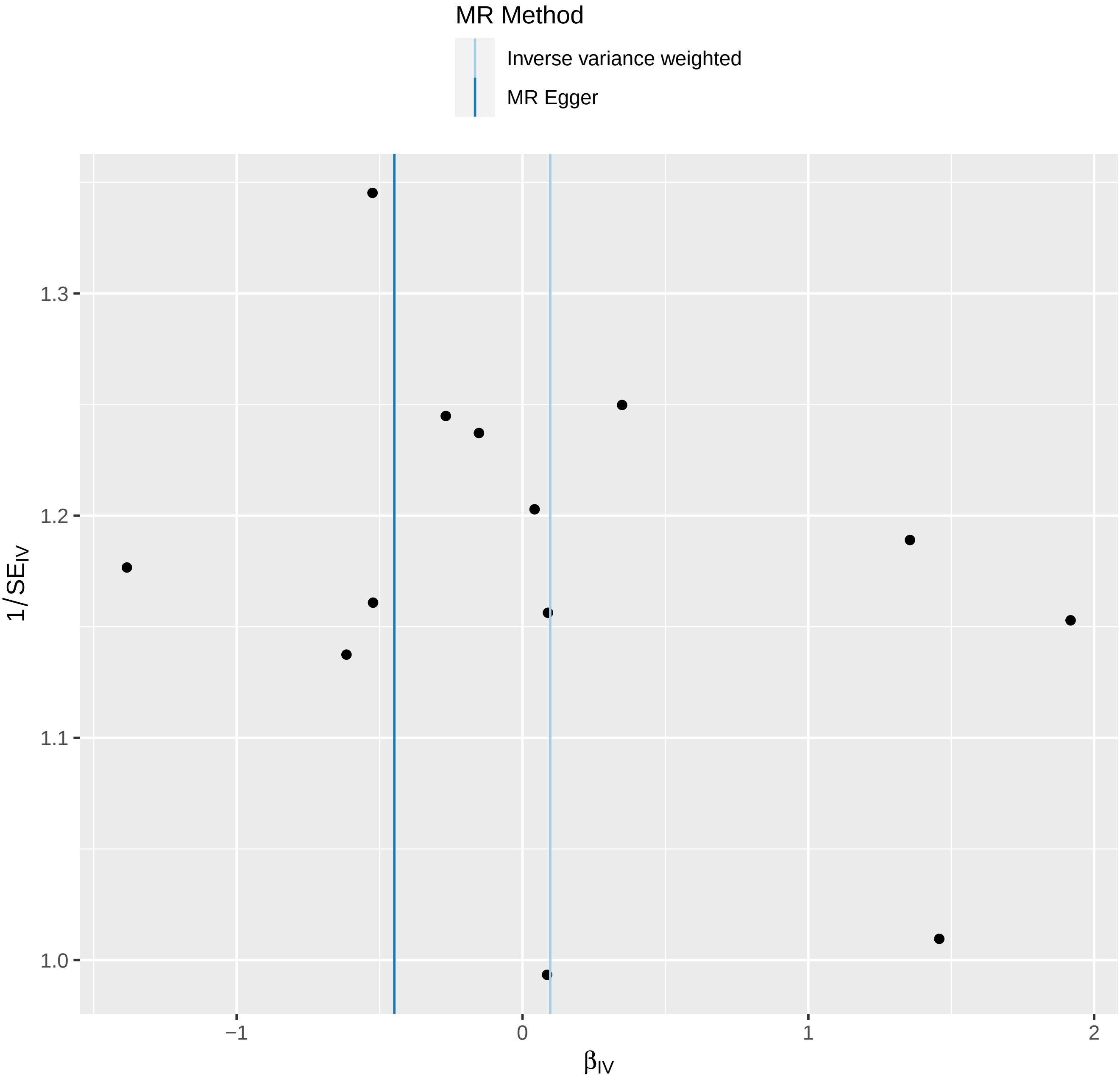

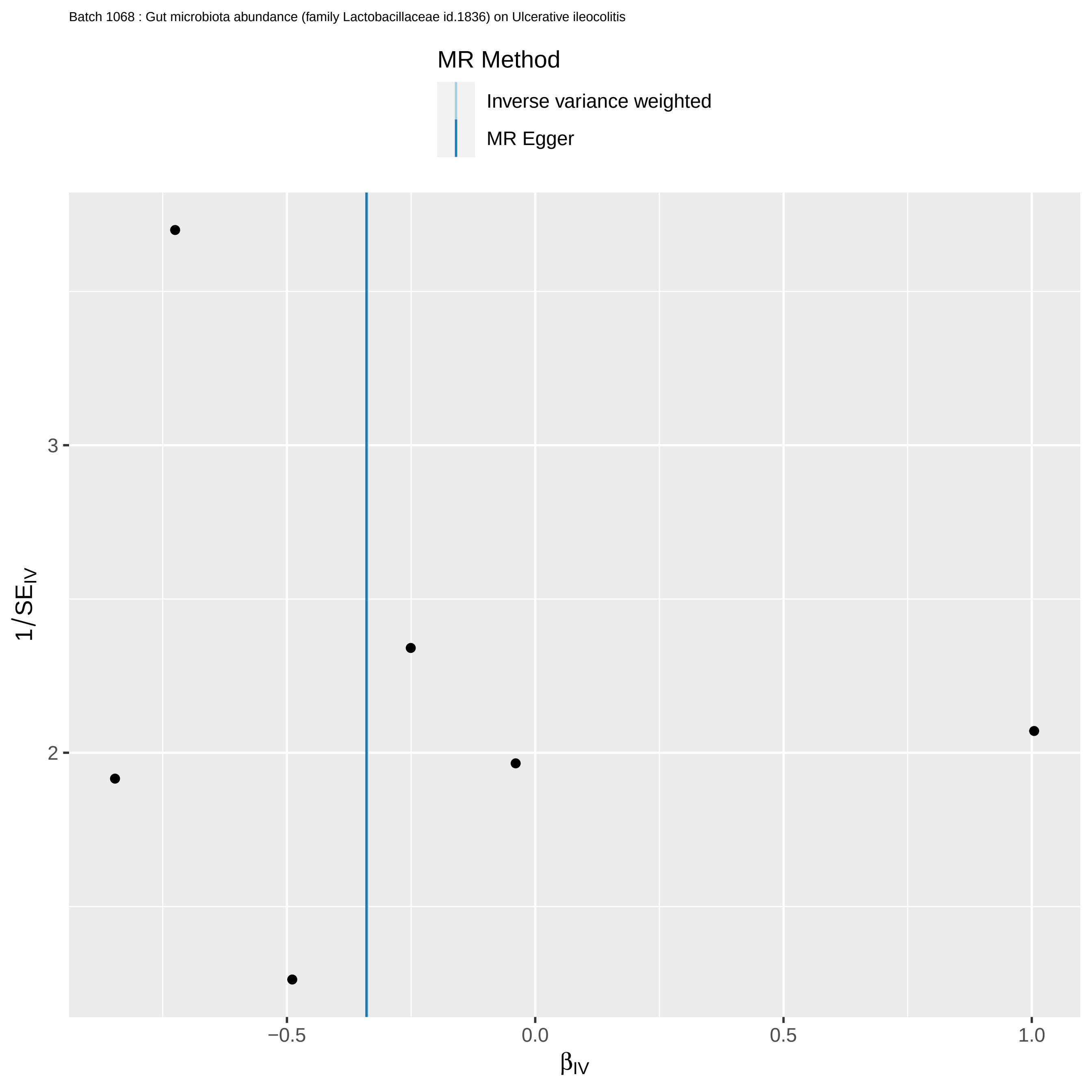
Batch 1068 : Gut microbiota abundance (family Lactobacillaceae id.1836) on Ulcerative ileocolitis
MR Method
Inverse variance weighted
MR Egger
3
2
1/SE_IV
−0.5
0.0
0.5
1.0
β_IV

Batch 1069 : Gut microbiota abundance (family Methanobacteriaceae id.121) on Ulcerative ileocolitis

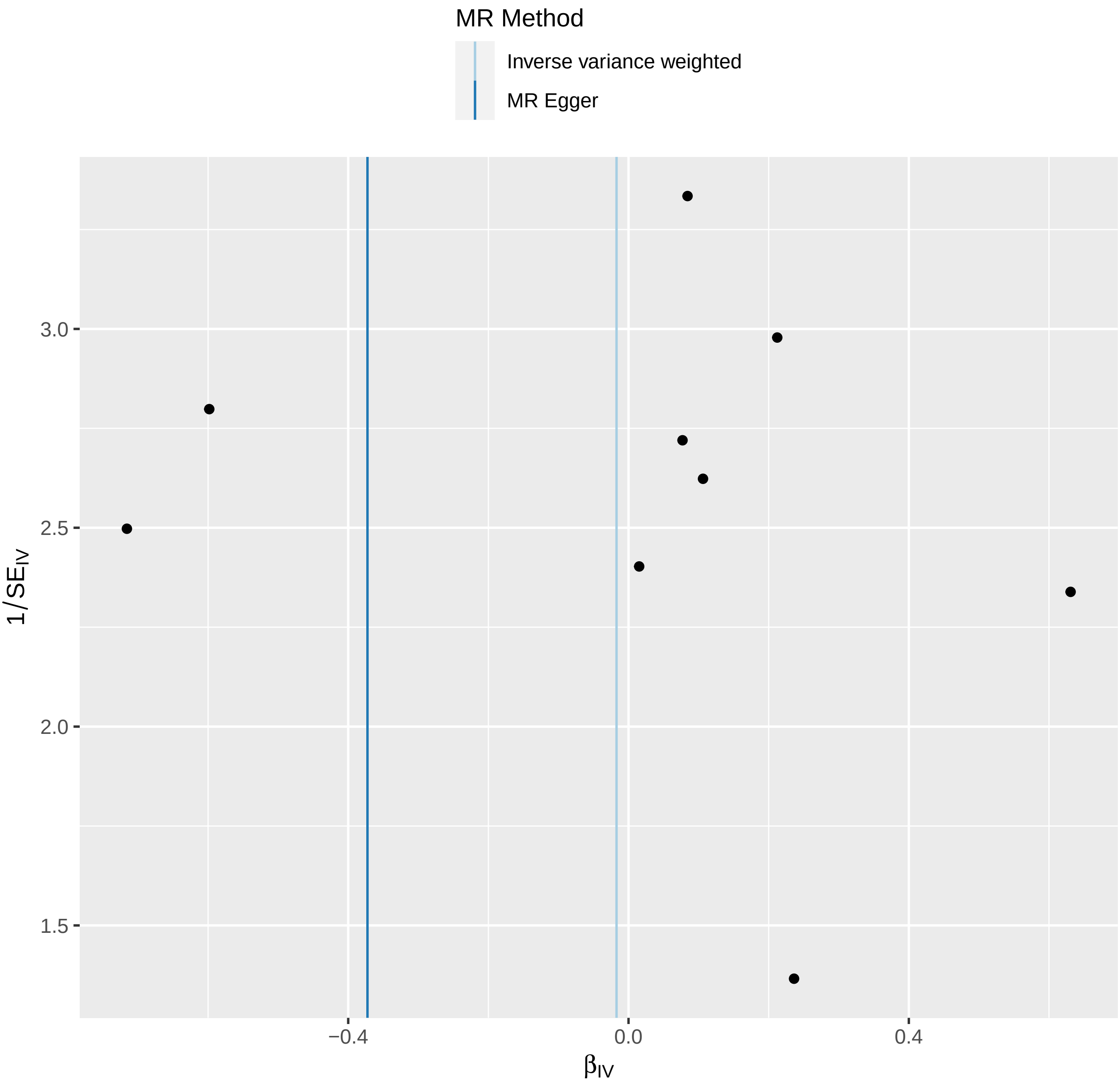

Batch 1070 : Gut microbiota abundance (family Oxalobacteraceae id.2966) on Ulcerative ileocolitis

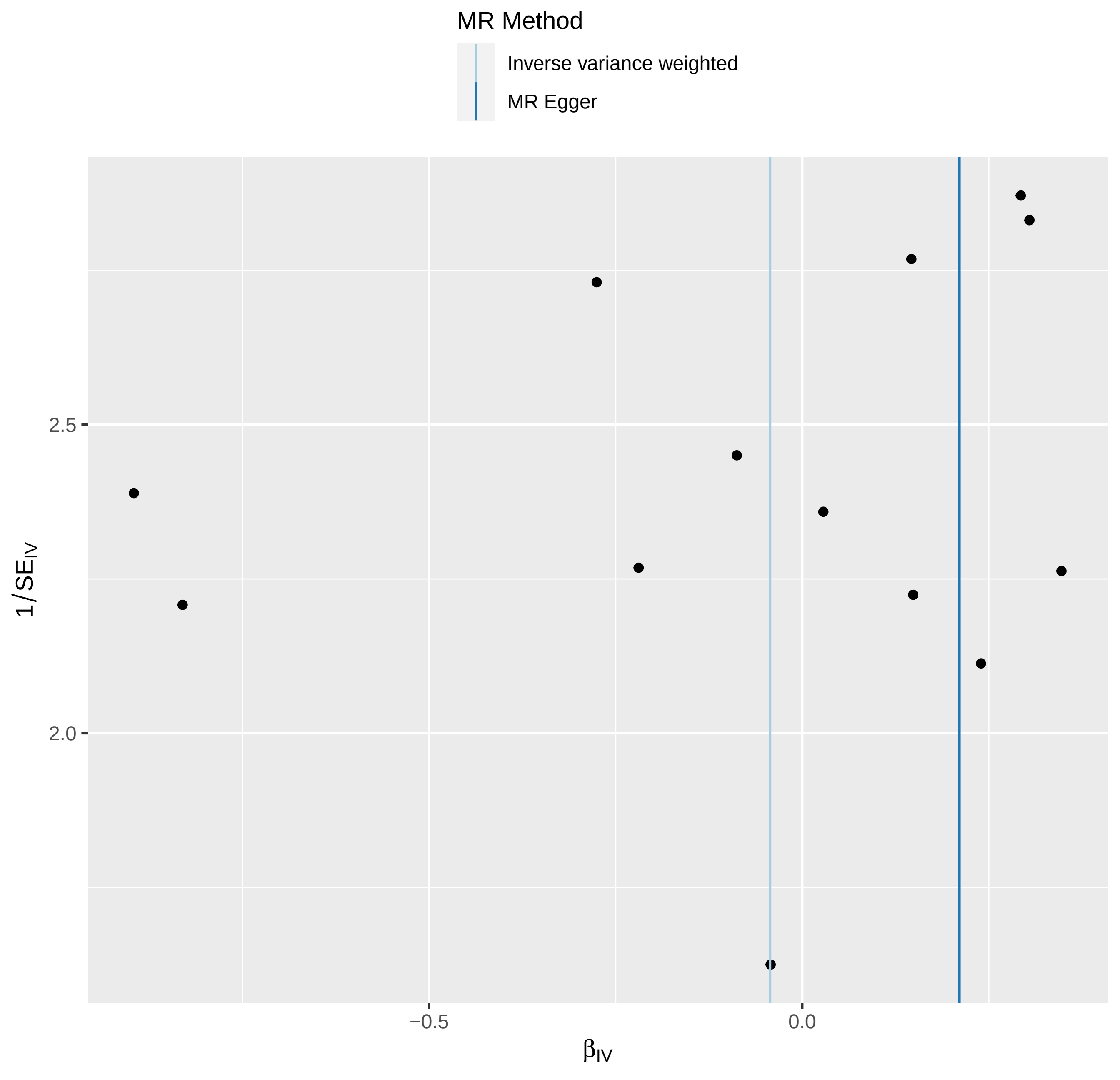

Batch 1071 : Gut microbiota abundance (family Pasteurellaceae id.3689) on Ulcerative ileocolitis

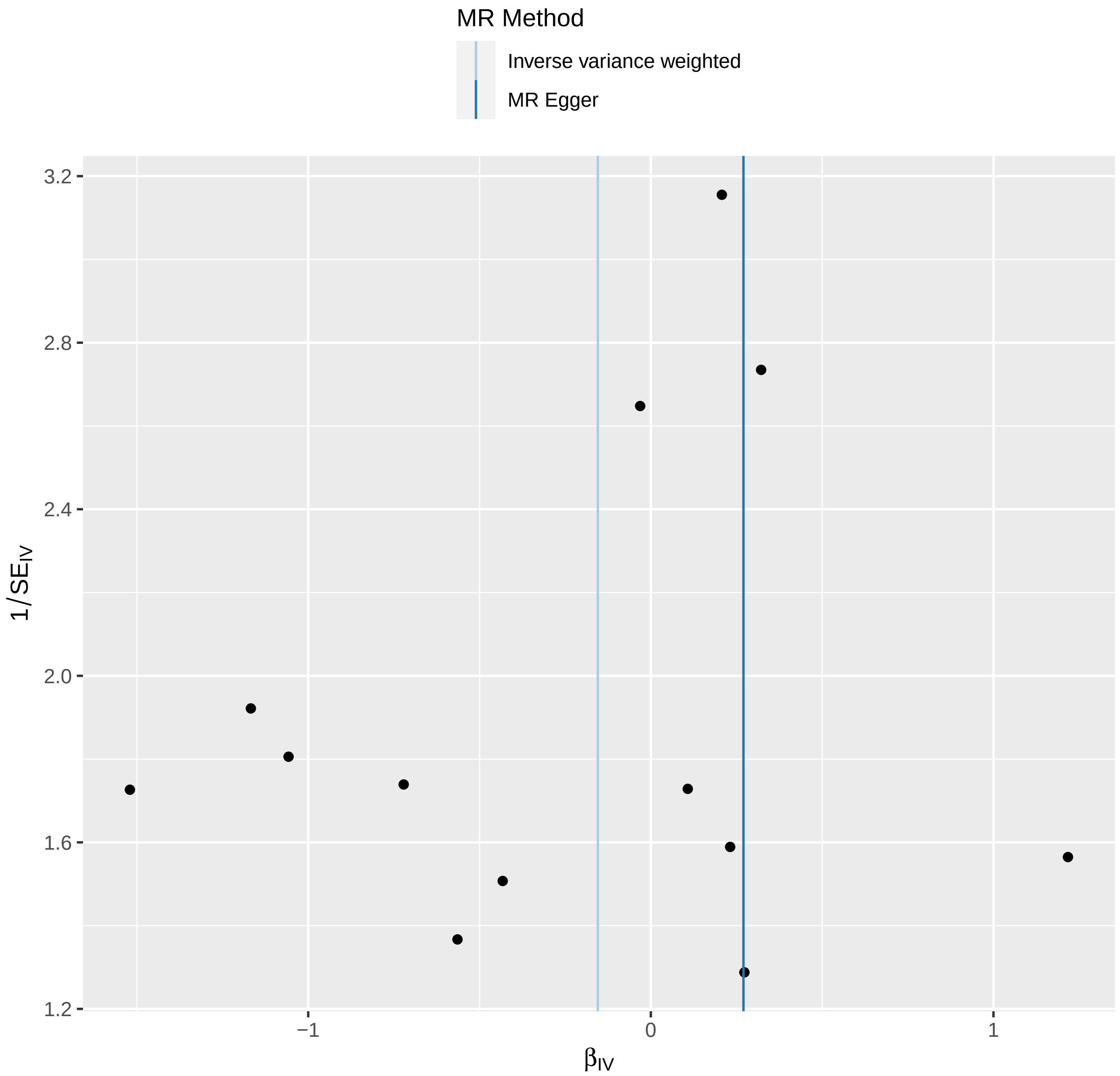

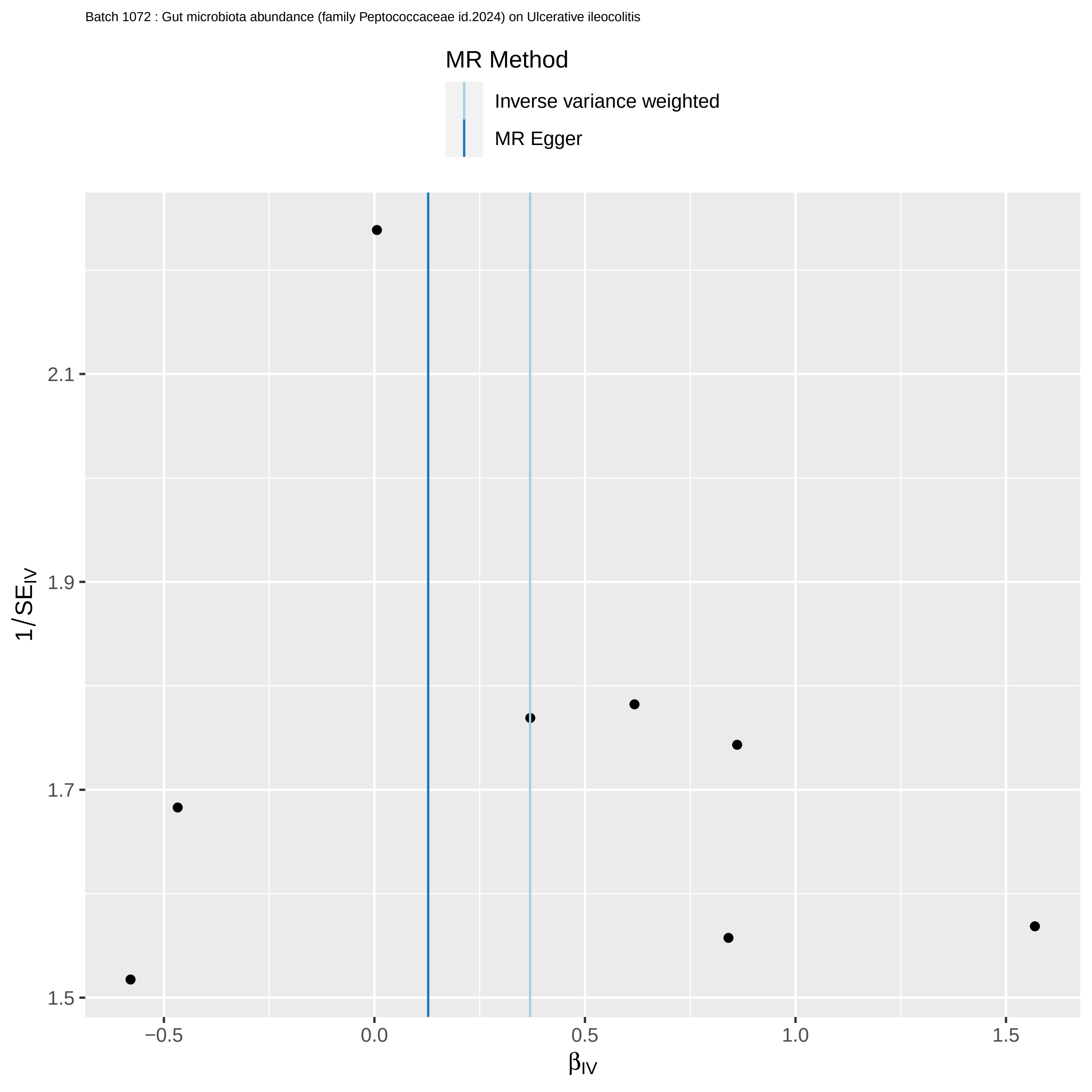
Batch 1072 : Gut microbiota abundance (family Peptococcaceae id.2024) on Ulcerative ileocolitis
MR Method
Inverse variance weighted
MR Egger
1/SE_IV
2.1
1.9
1.7
1.5
−0.5
0.0
0.5
1.0
1.5
β_IV

Batch 1073 : Gut microbiota abundance (family Peptostreptococcaceae id.2042) on Ulcerative ileocolitis

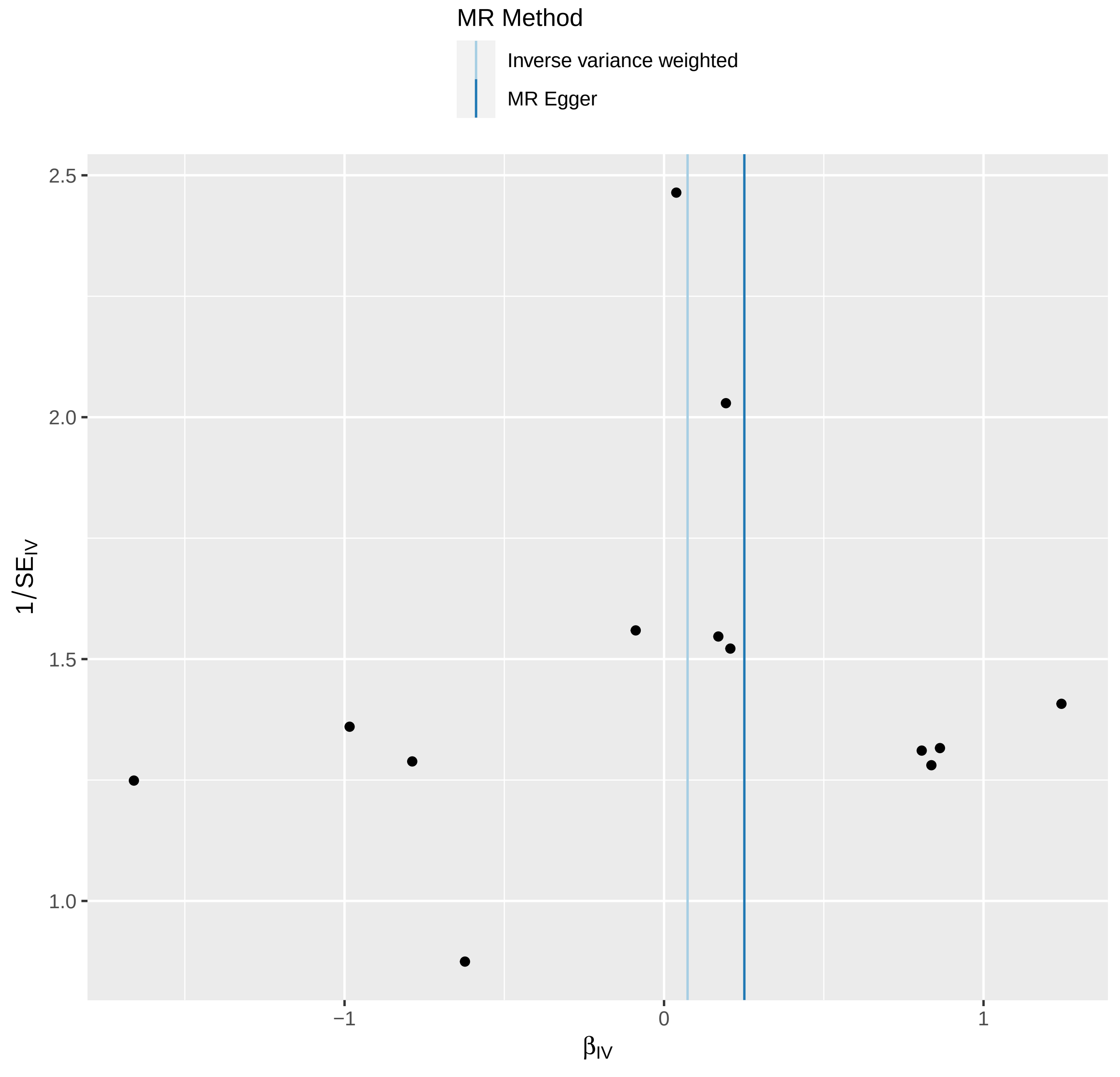

Batch 1074 : Gut microbiota abundance (family Porphyromonadaceae id.943) on Ulcerative ileocolitis

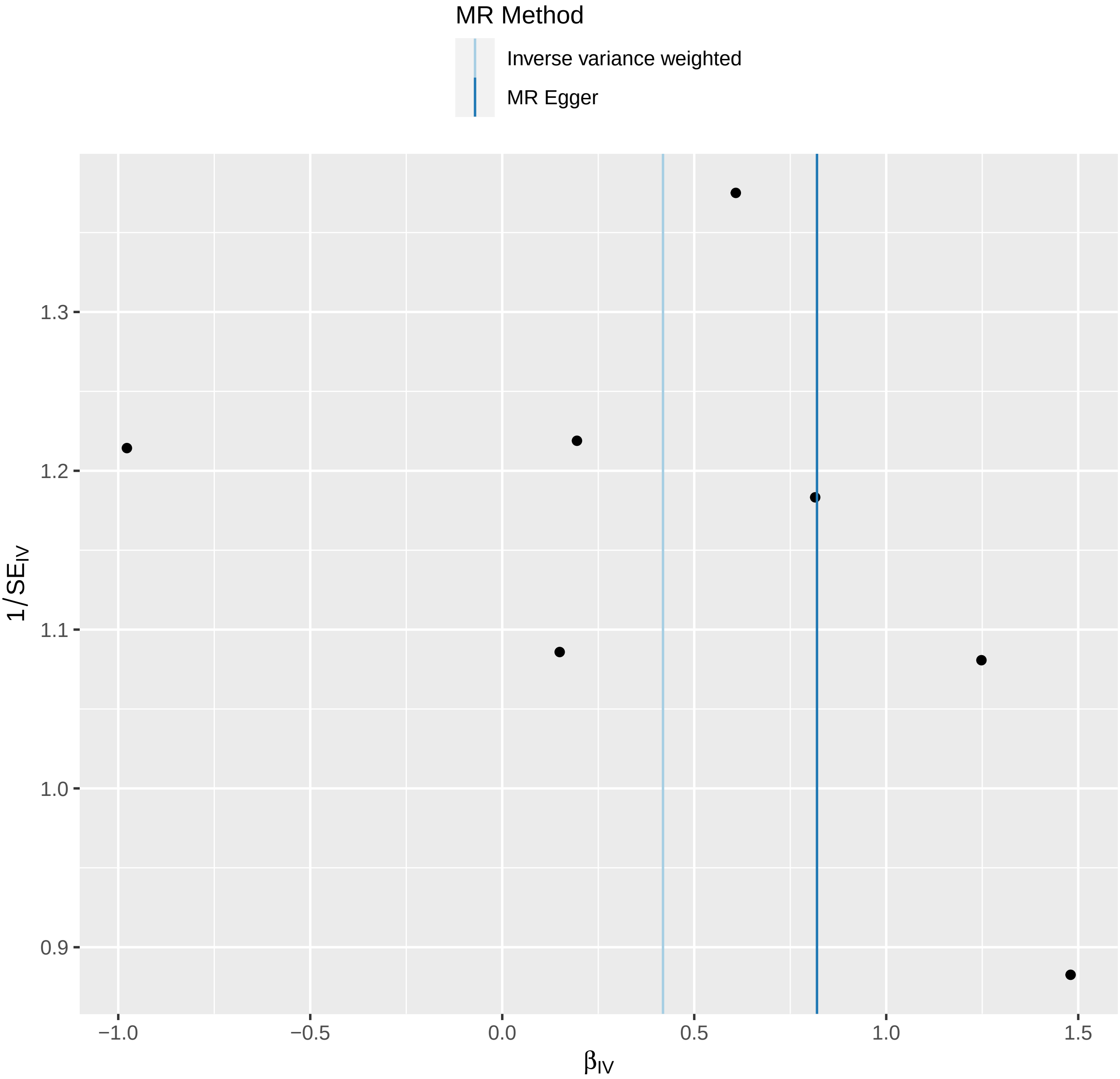

Batch 1075 : Gut microbiota abundance (family Prevotellaceae id.960) on Ulcerative ileocolitis

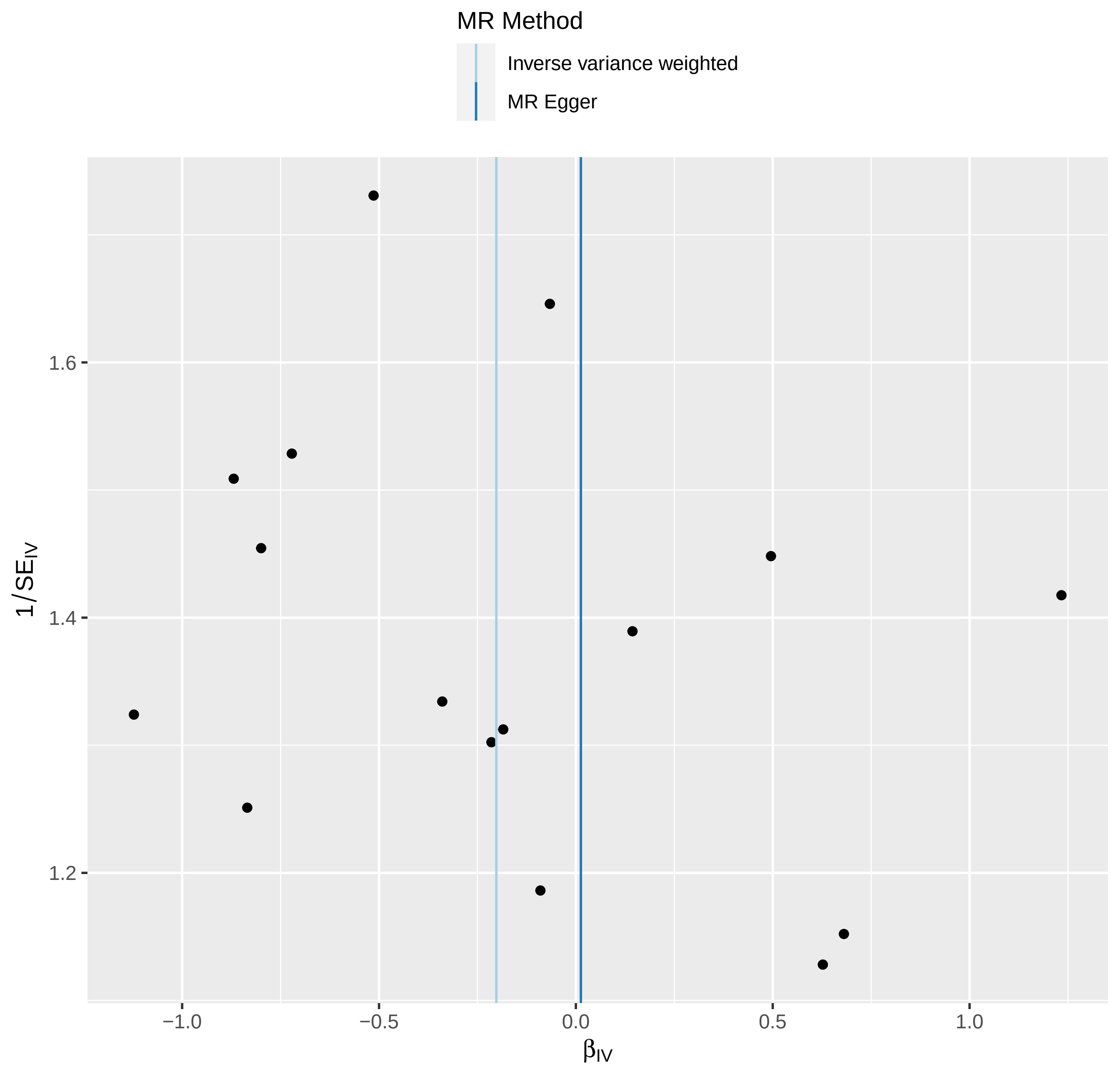

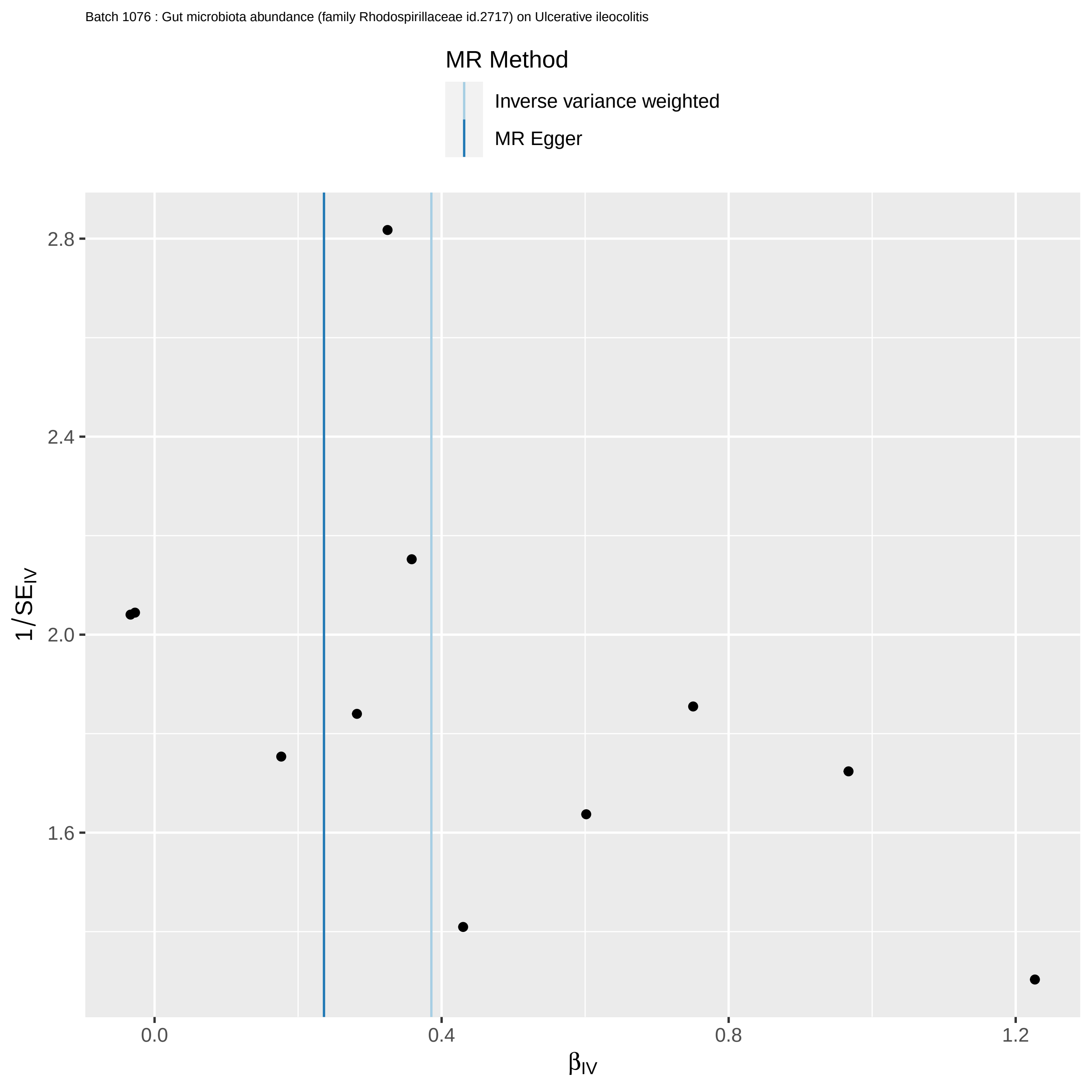
Batch 1076 : Gut microbiota abundance (family Rhodospirillaceae id.2717) on Ulcerative ileocolitis
MR Method
Inverse variance weighted
MR Egger
2.8
2.4
2.0
1.6
1/SE_IV
0.0
0.4
0.8
1.2
β_IV

Batch 1077 : Gut microbiota abundance (family Rikenellaceae id.967) on Ulcerative ileocolitis

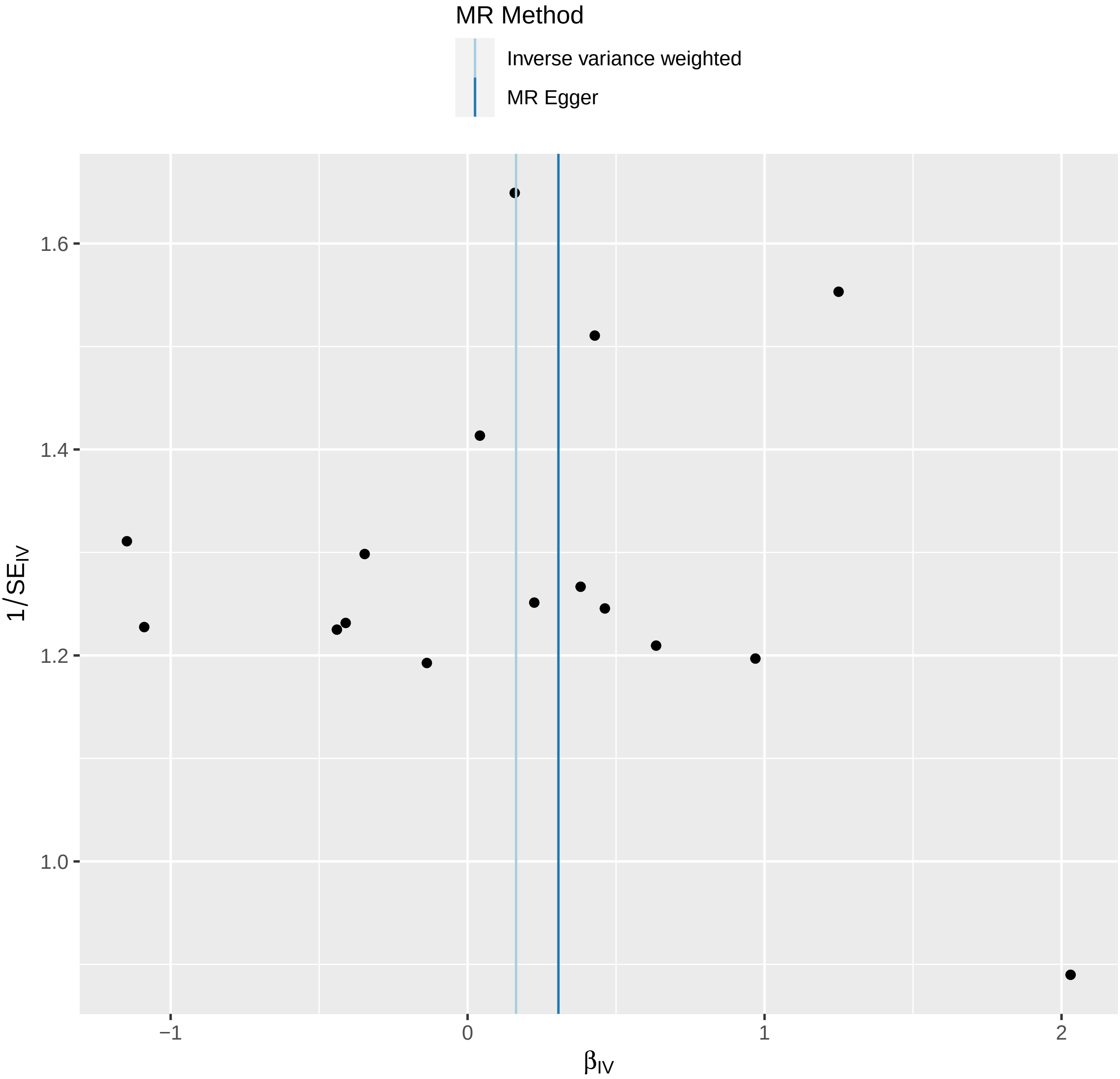

Batch 1078 : Gut microbiota abundance (family Ruminococcaceae id.2050) on Ulcerative ileocolitis

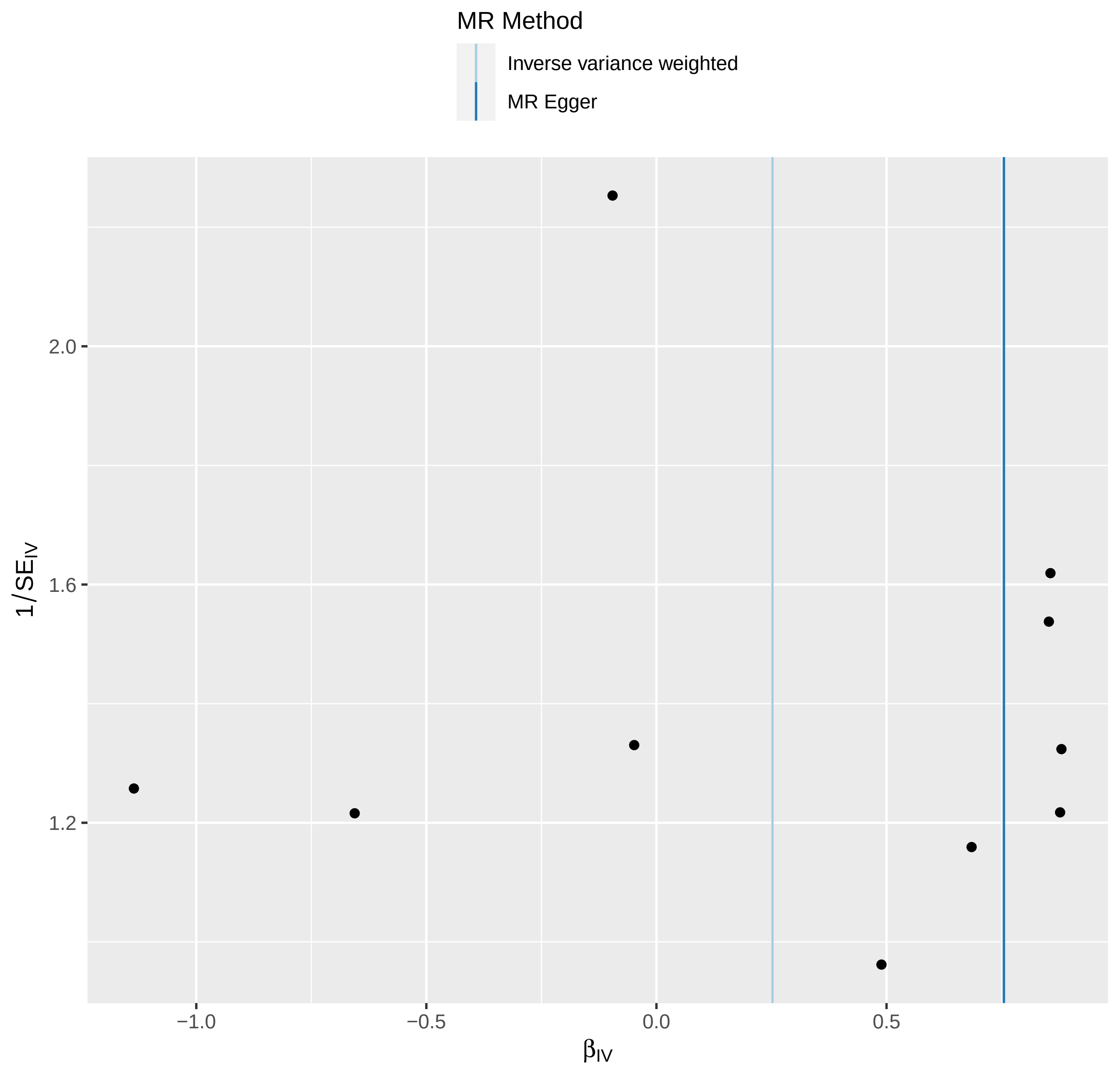

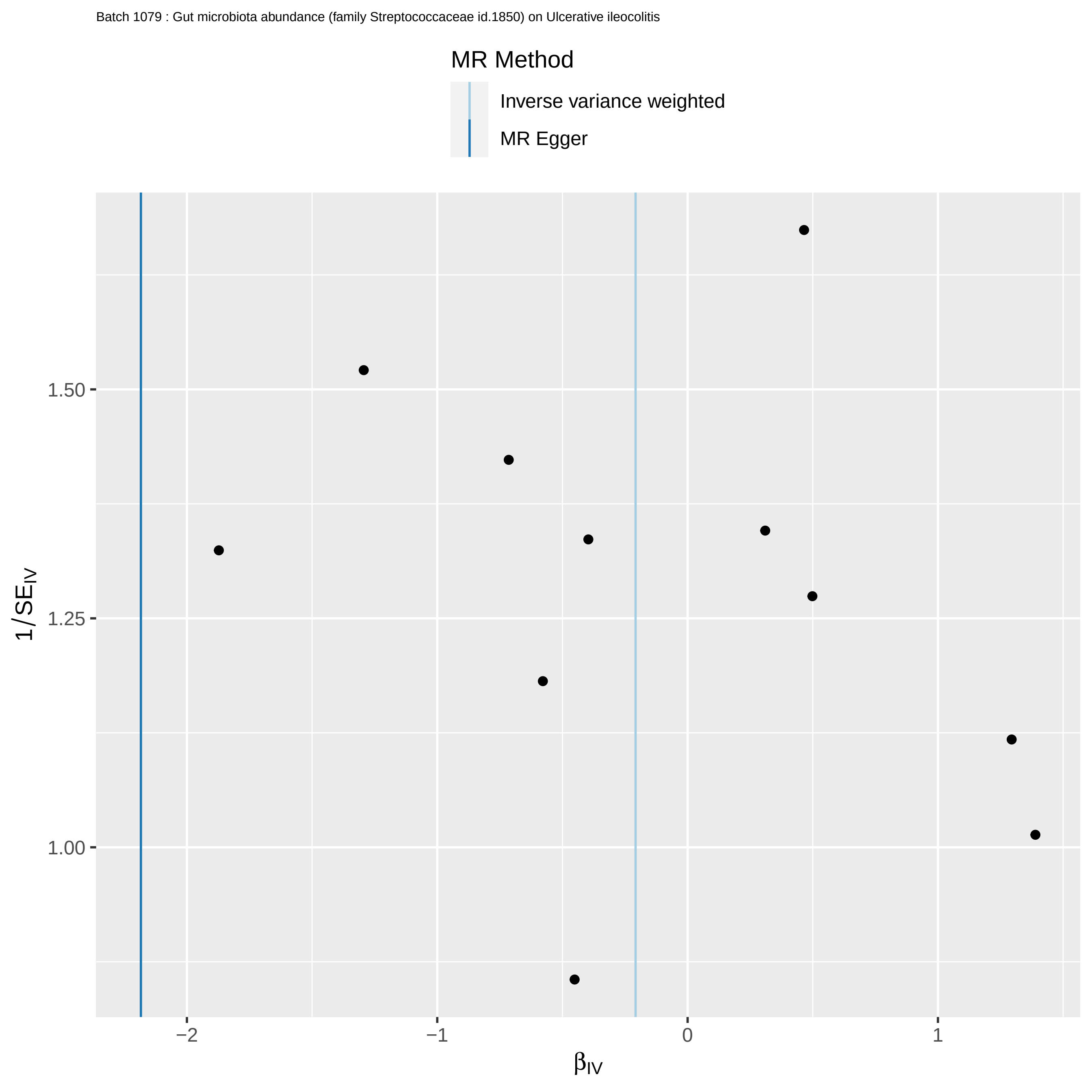

Batch 1079 : Gut microbiota abundance (family Streptococcaceae id.1850) on Ulcerative ileocolitis
MR Method
Inverse variance weighted
MR Egger
1.50
1.25
1.00
1/SE_IV
−2
−1
0
1
β_IV

Batch 1080 : Gut microbiota abundance (family Veillonellaceae id.2172) on Ulcerative ileocolitis

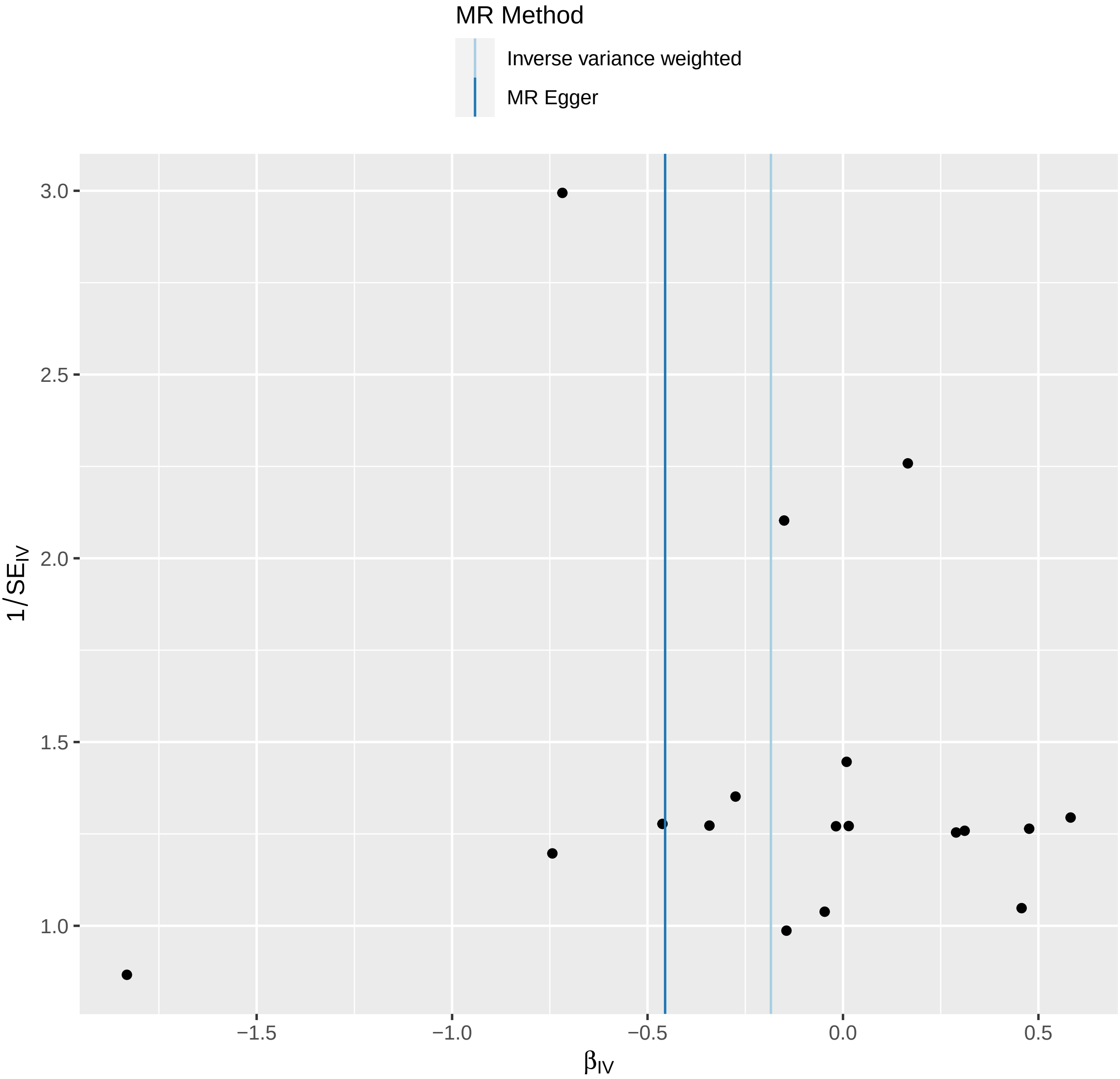

Batch 1081 : Gut microbiota abundance (family Verrucomicrobiaceae id.4036) on Ulcerative ileocolitis

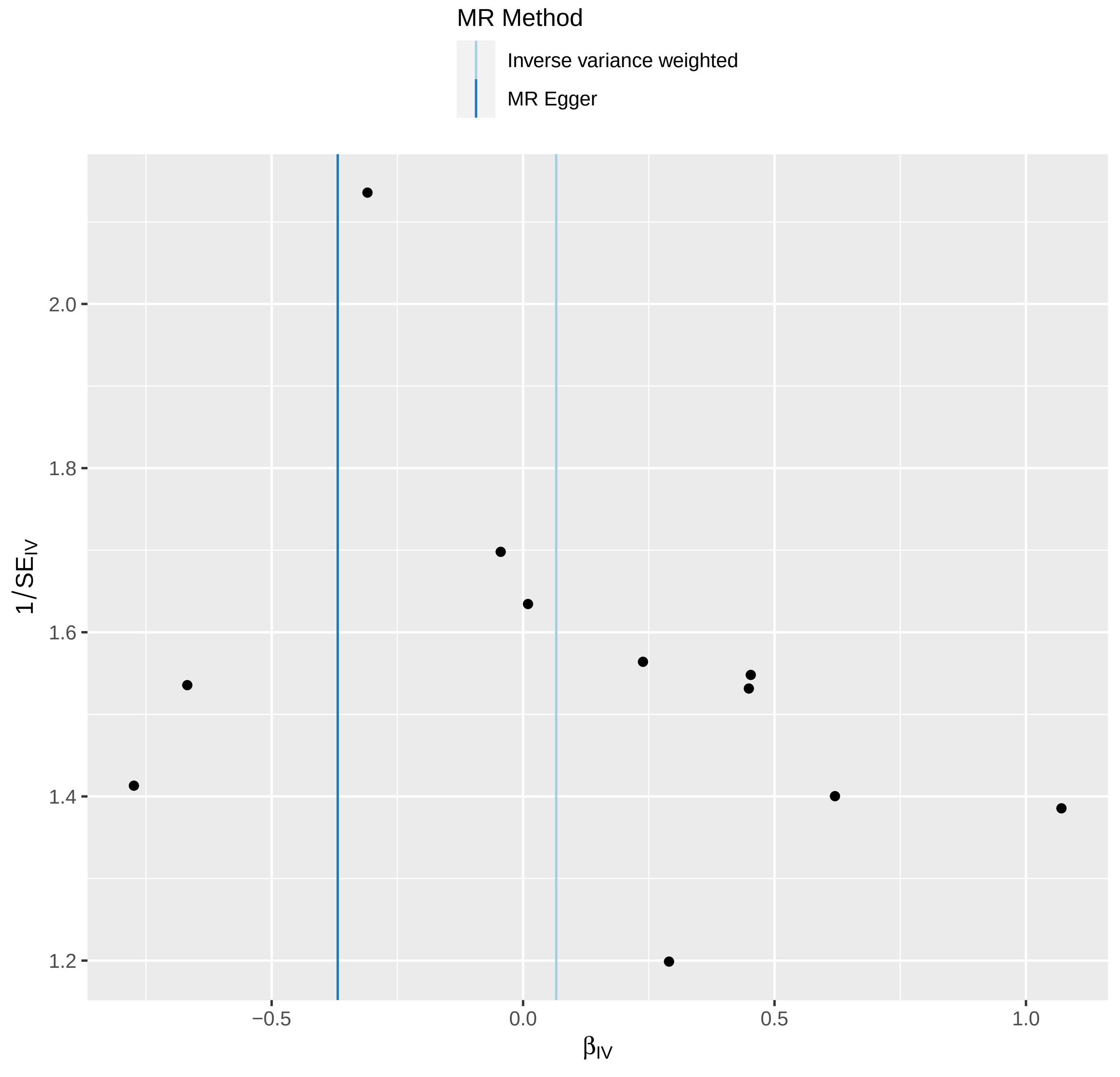

Batch 1082 : Gut microbiota abundance (family Victivallaceae id.2255) on Ulcerative ileocolitis

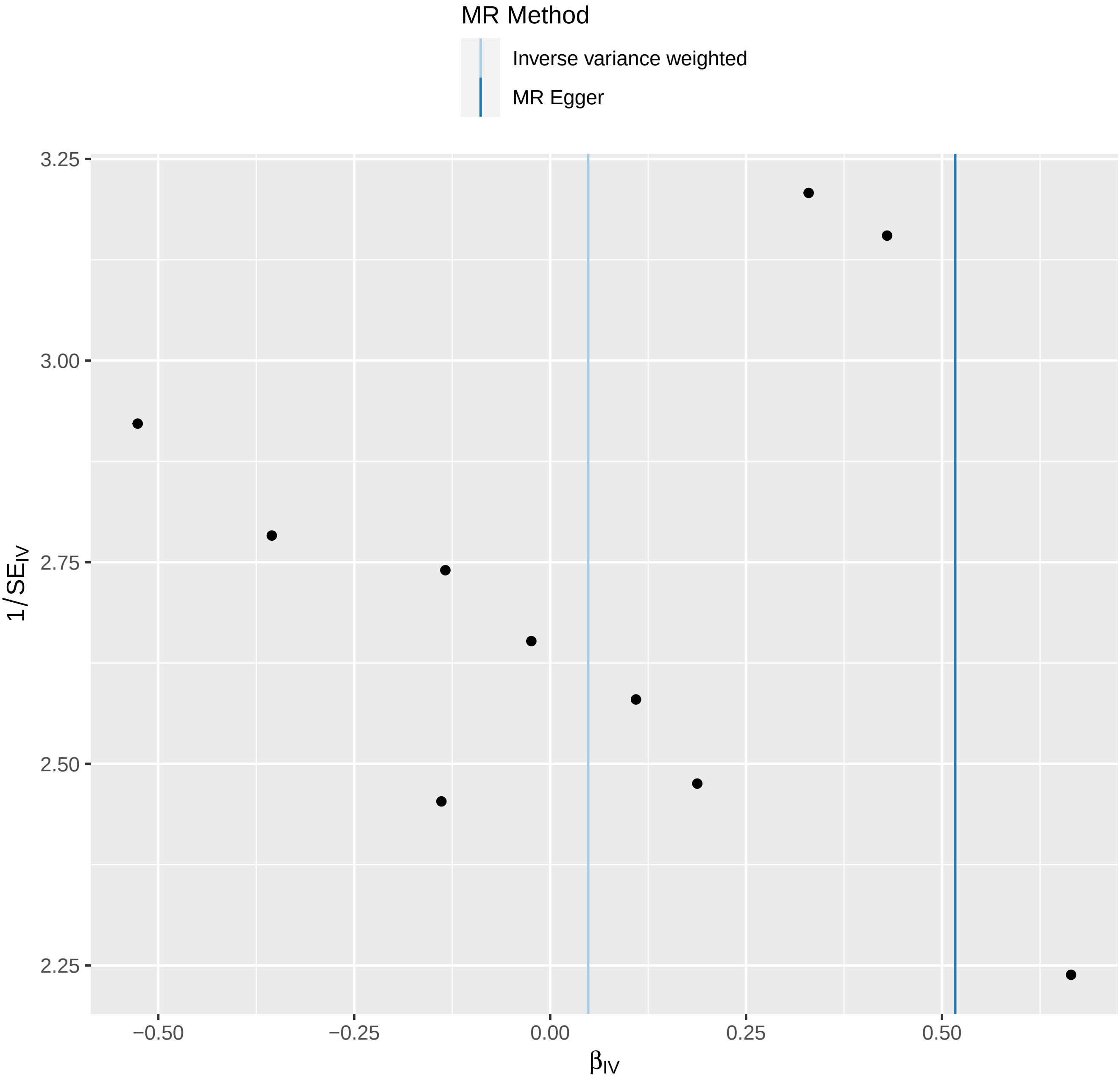

Batch 1083 : Gut microbiota abundance (genus Actinomyces id.423) on Ulcerative ileocolitis

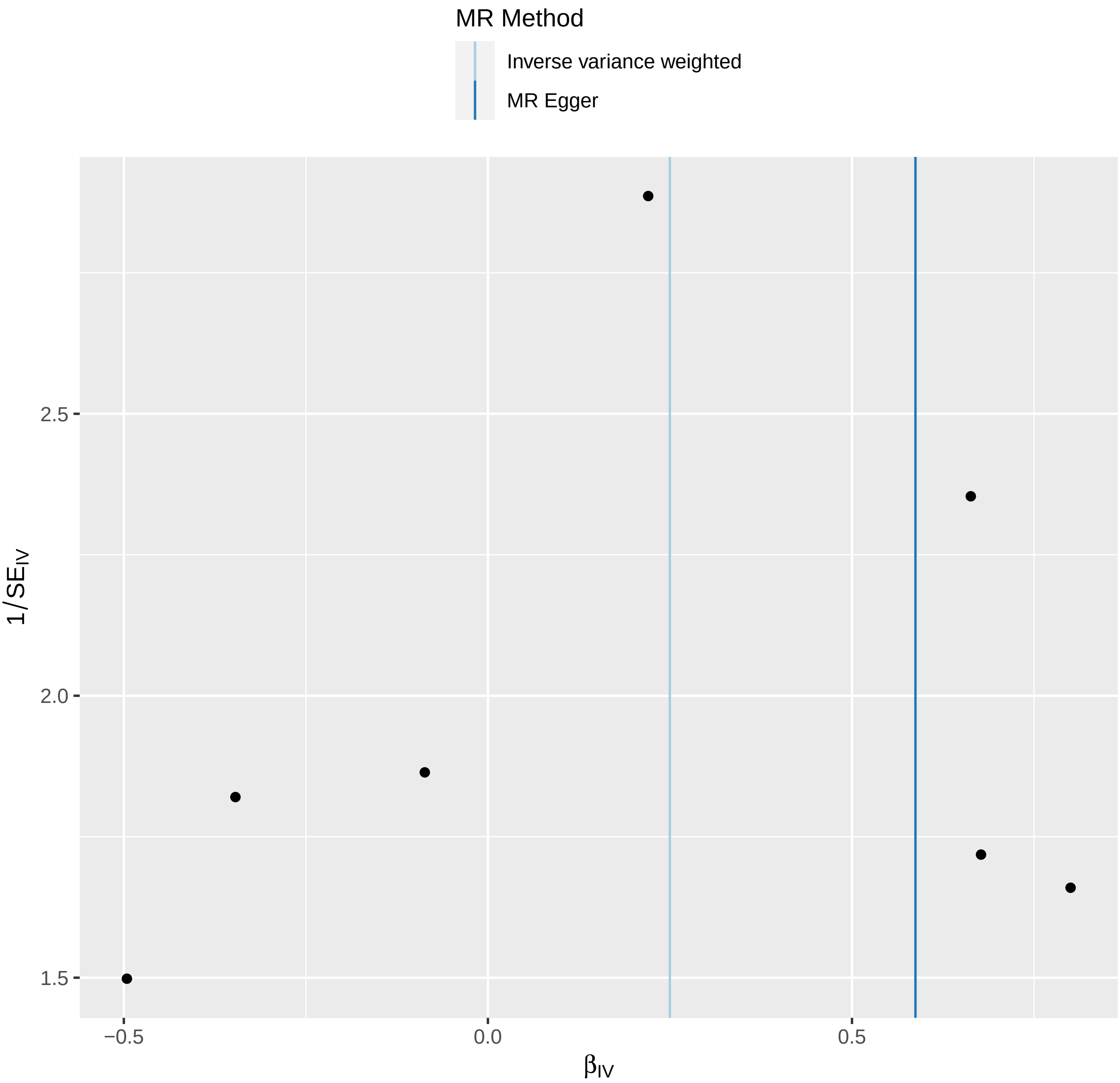

Batch 1084 : Gut microbiota abundance (genus Adlercreutzia id.812) on Ulcerative ileocolitis

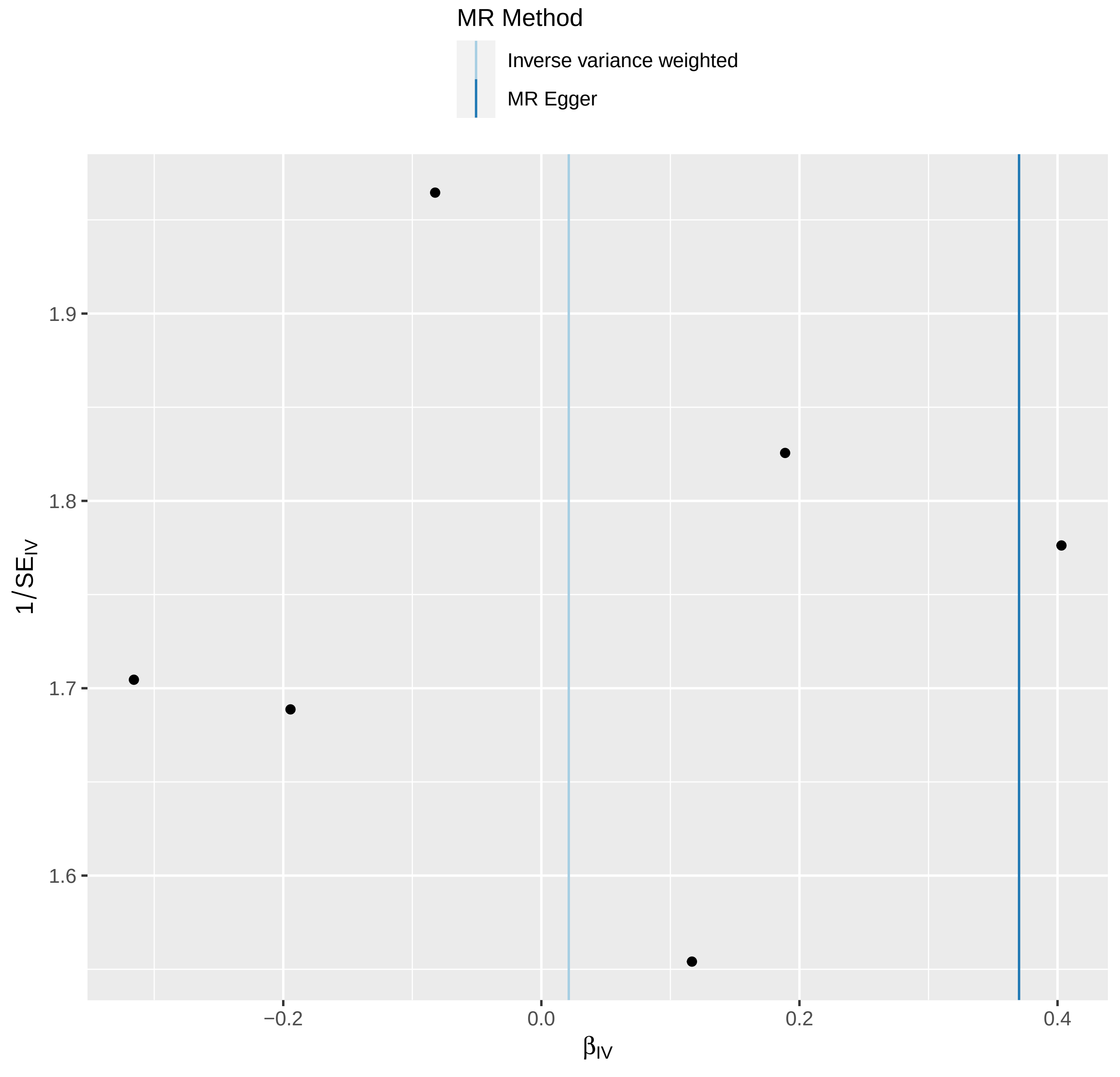

Batch 1085 : Gut microbiota abundance (genus Akkermansia id.4037) on Ulcerative ileocolitis

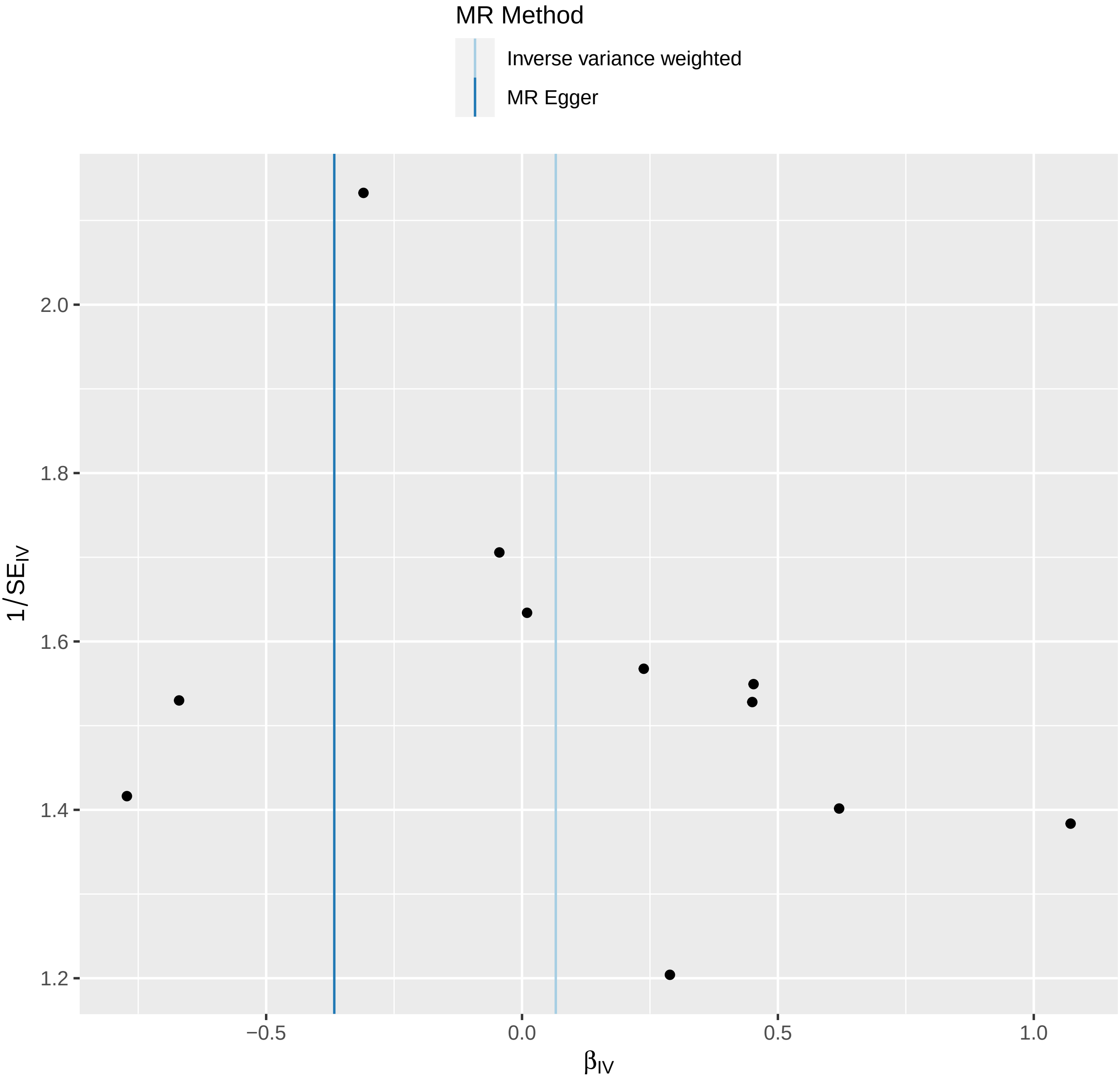

Batch 1086 : Gut microbiota abundance (genus Alistipes id.968) on Ulcerative ileocolitis

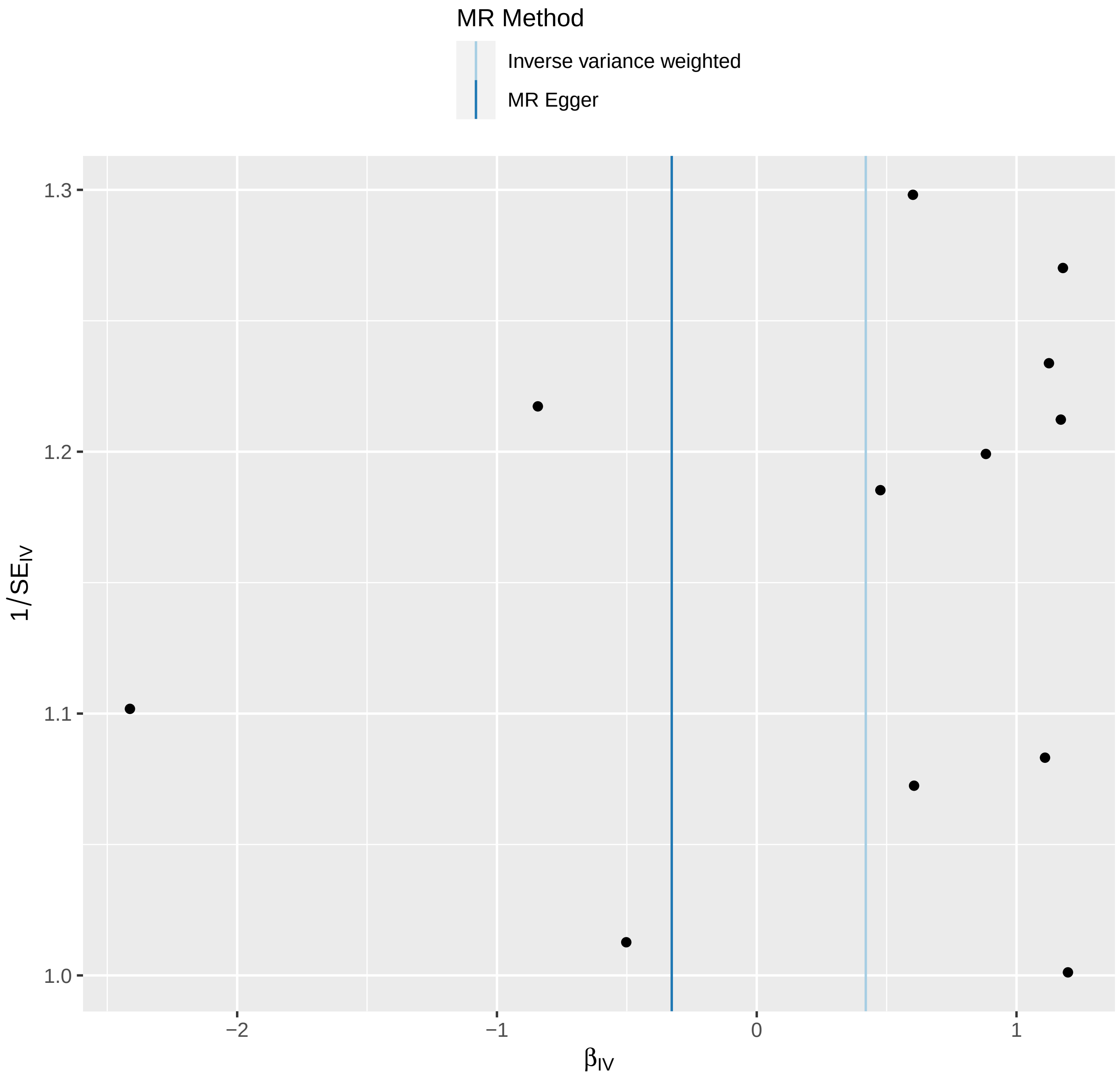

Batch 1087 : Gut microbiota abundance (genus Allisonella id.2174) on Ulcerative ileocolitis

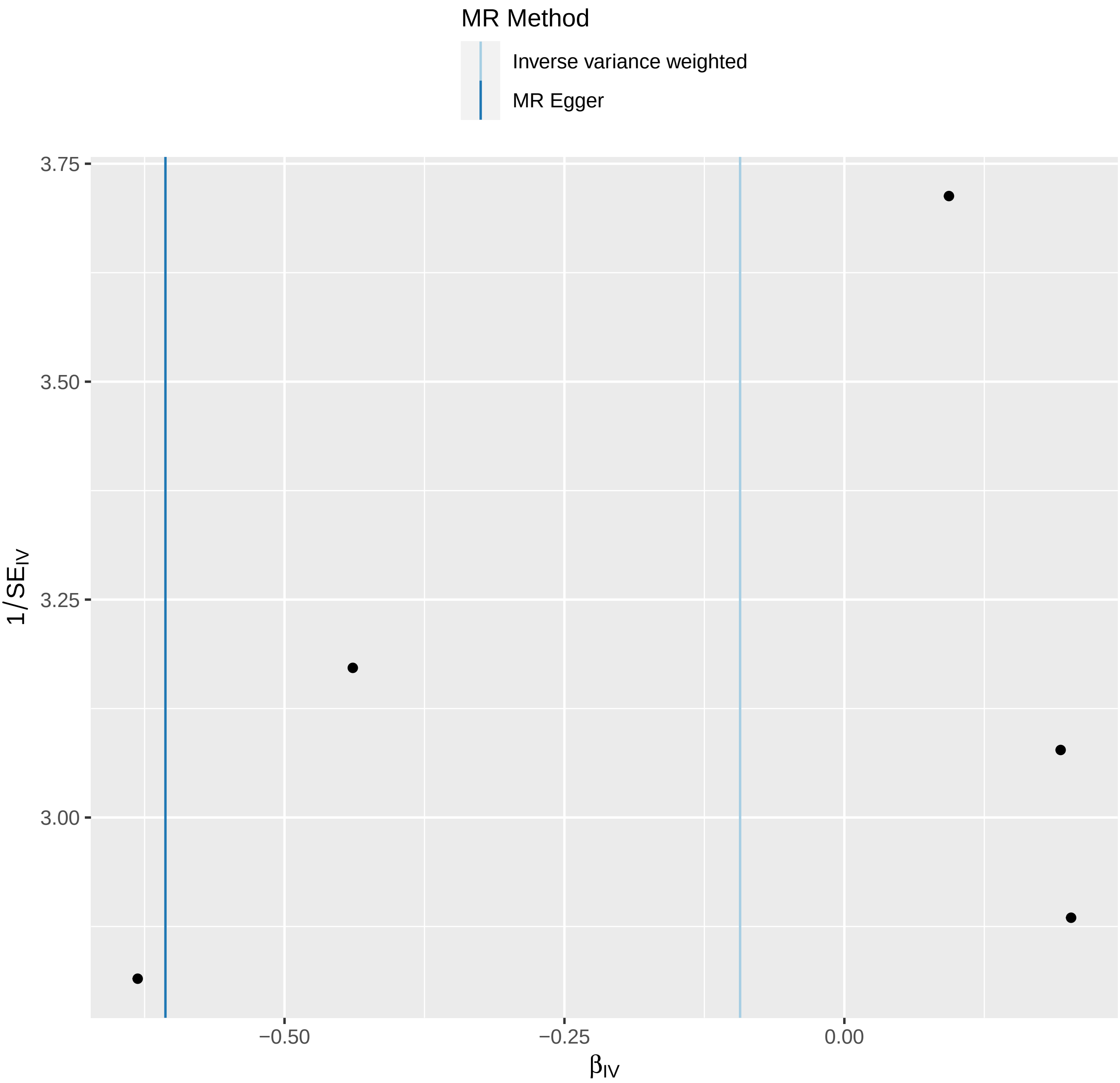

Batch 1088 : Gut microbiota abundance (genus Alloprevotella id.961) on Ulcerative ileocolitis

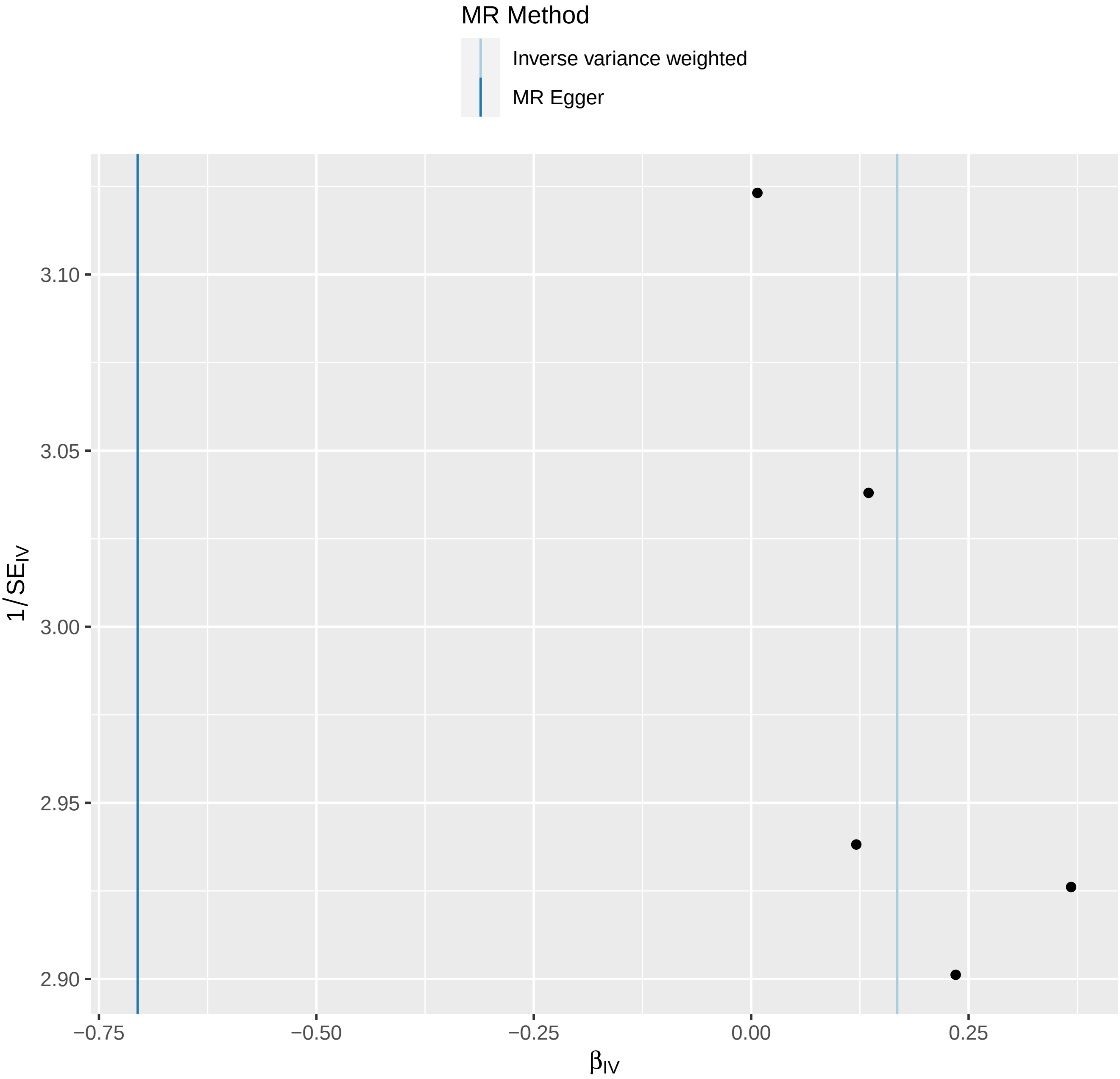

Batch 1089 : Gut microbiota abundance (genus Anaerofilum id.2053) on Ulcerative ileocolitis

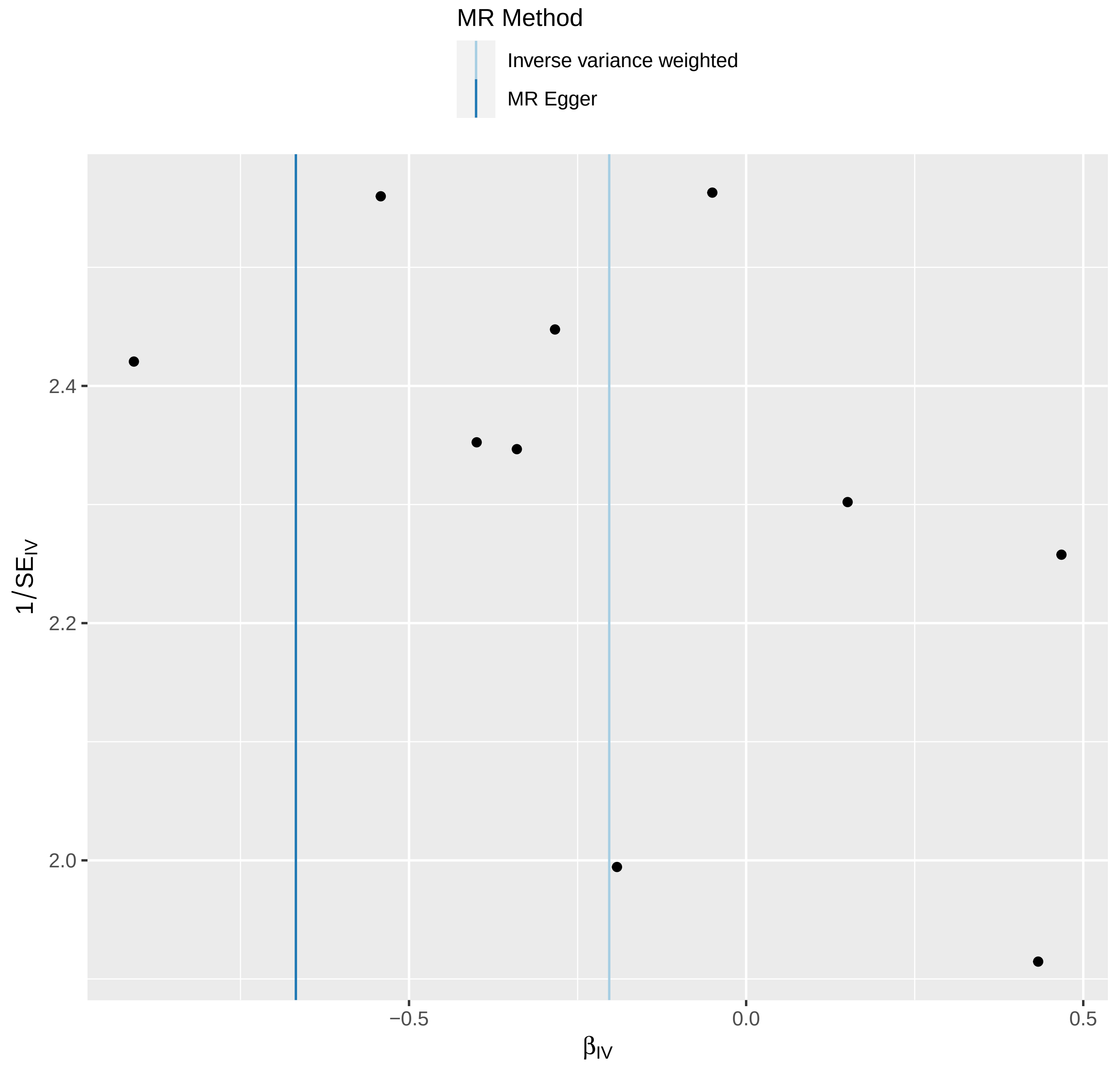

Batch 1090 : Gut microbiota abundance (genus Anaerostipes id.1991) on Ulcerative ileocolitis

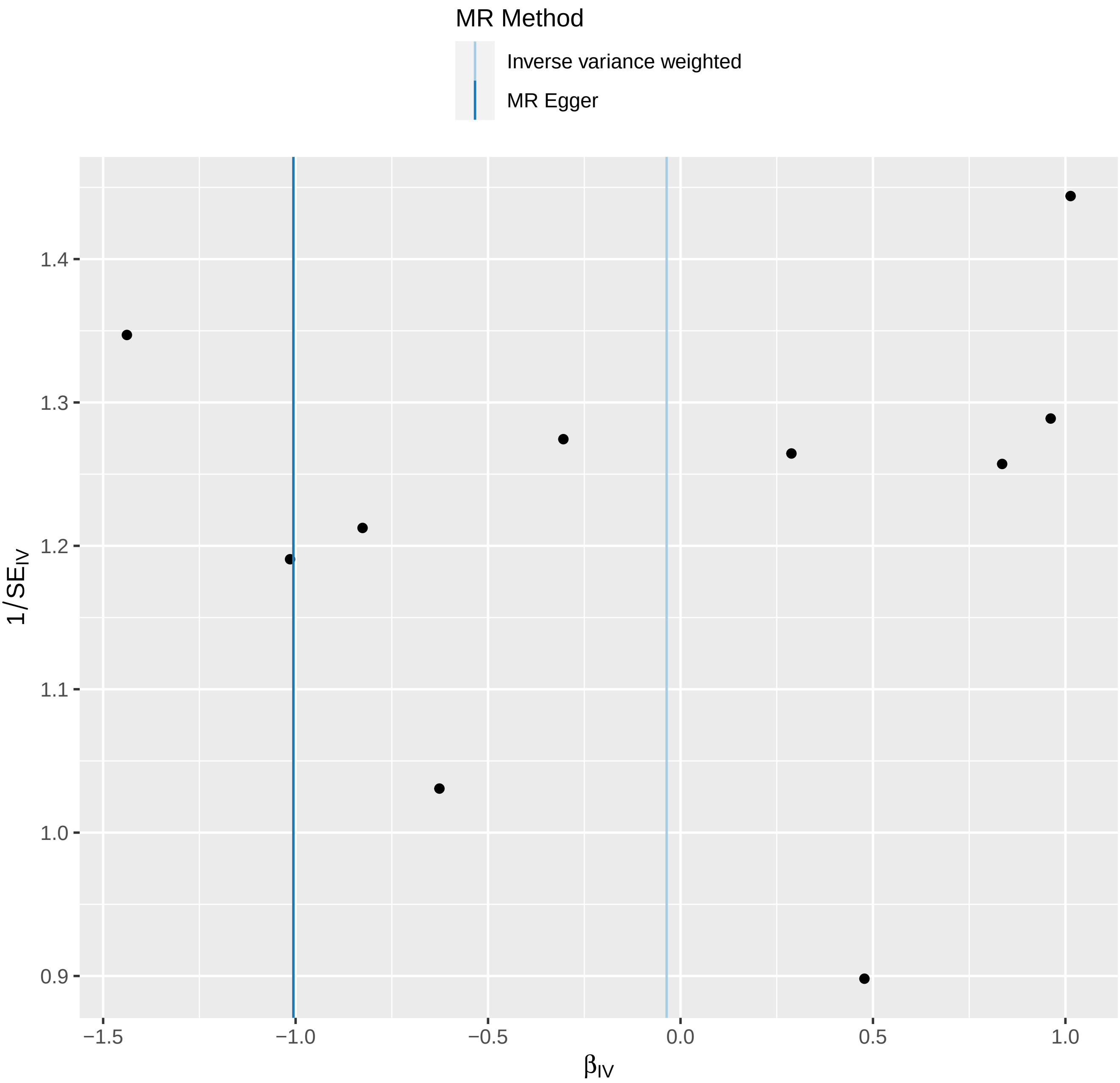

Batch 1091 : Gut microbiota abundance (genus Anaerotruncus id.2054) on Ulcerative ileocolitis

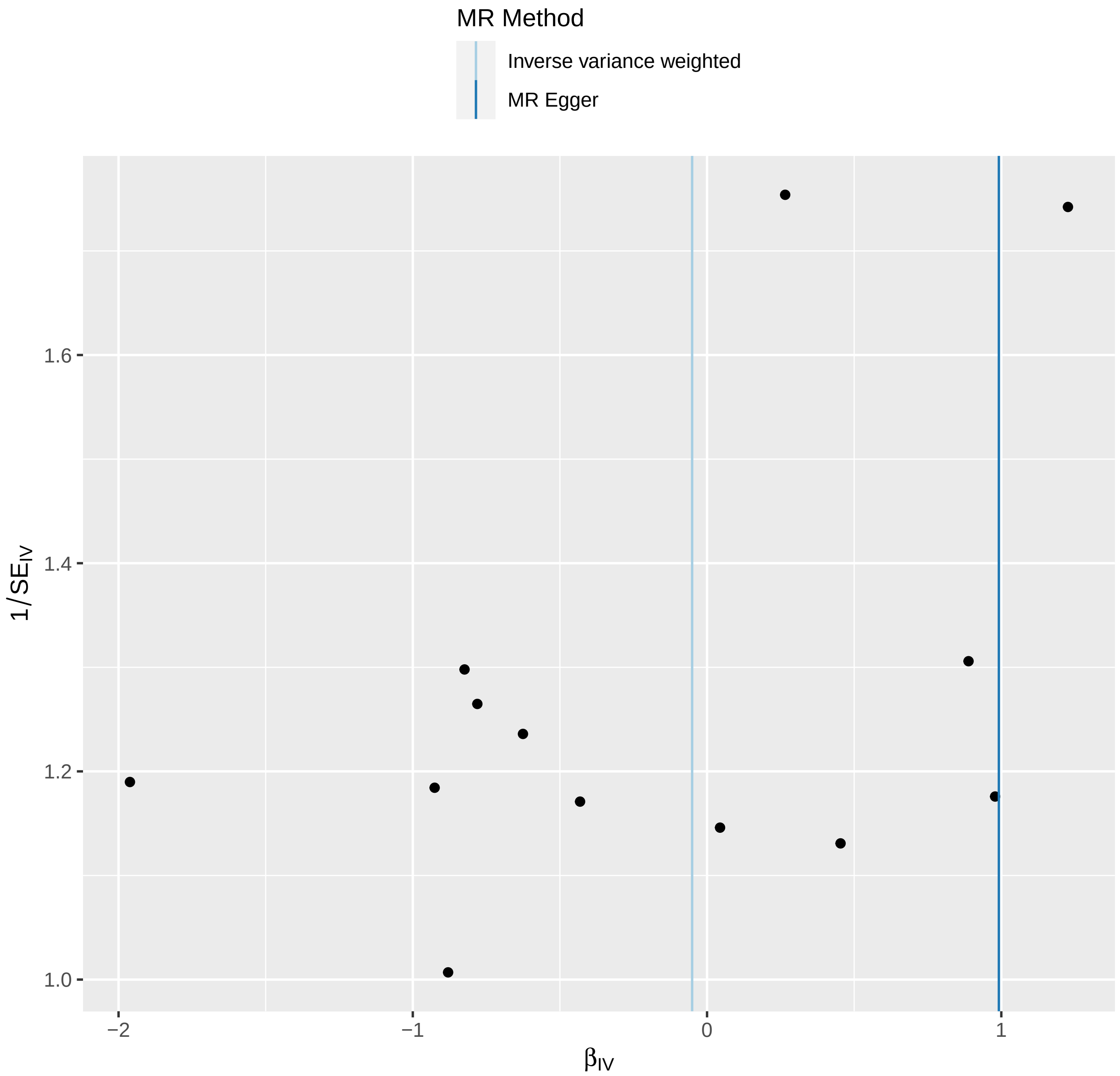

Batch 1092 : Gut microbiota abundance (genus Bacteroides id.918) on Ulcerative ileocolitis

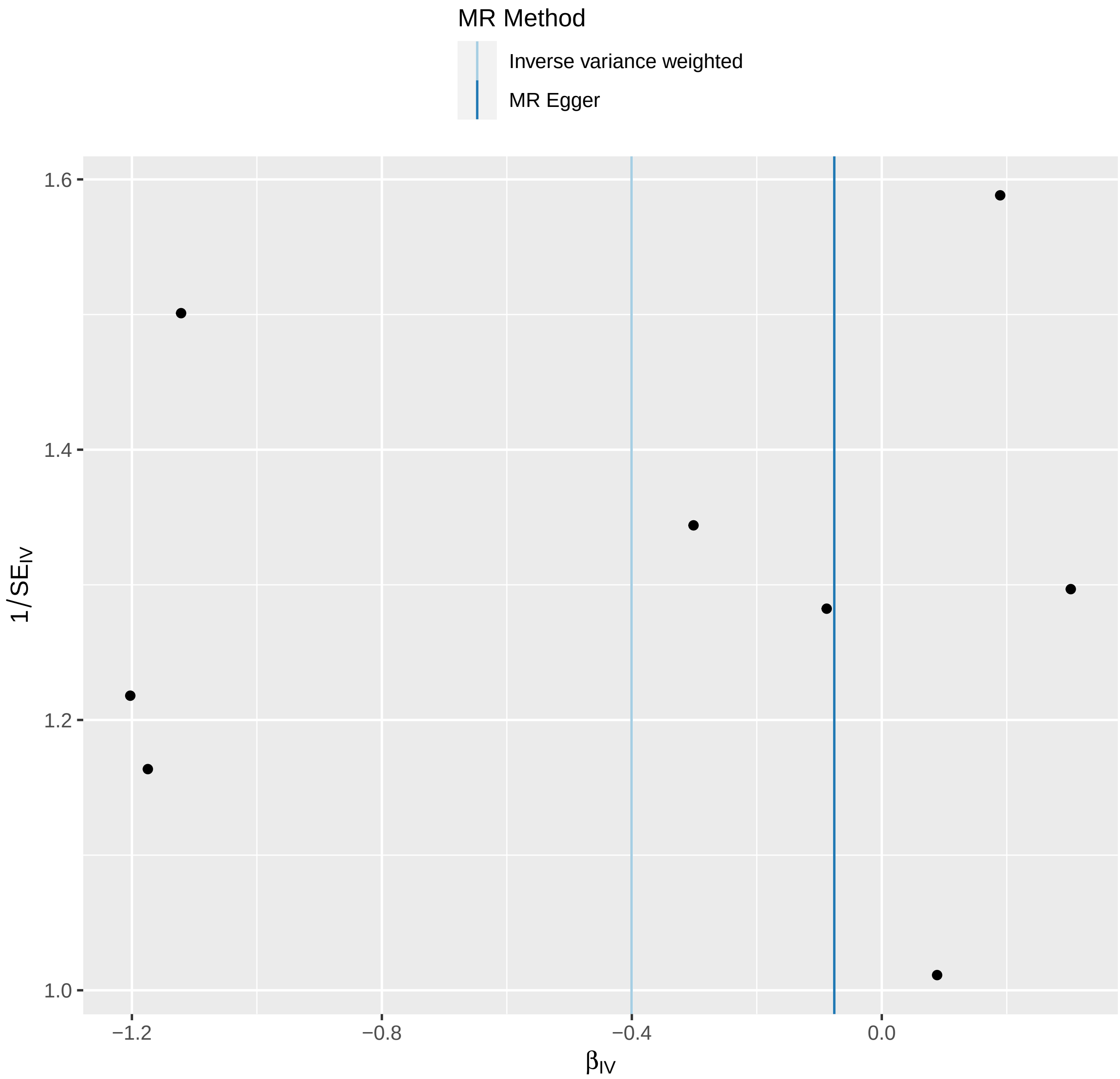

Batch 1093 : Gut microbiota abundance (genus Barnesiella id.944) on Ulcerative ileocolitis

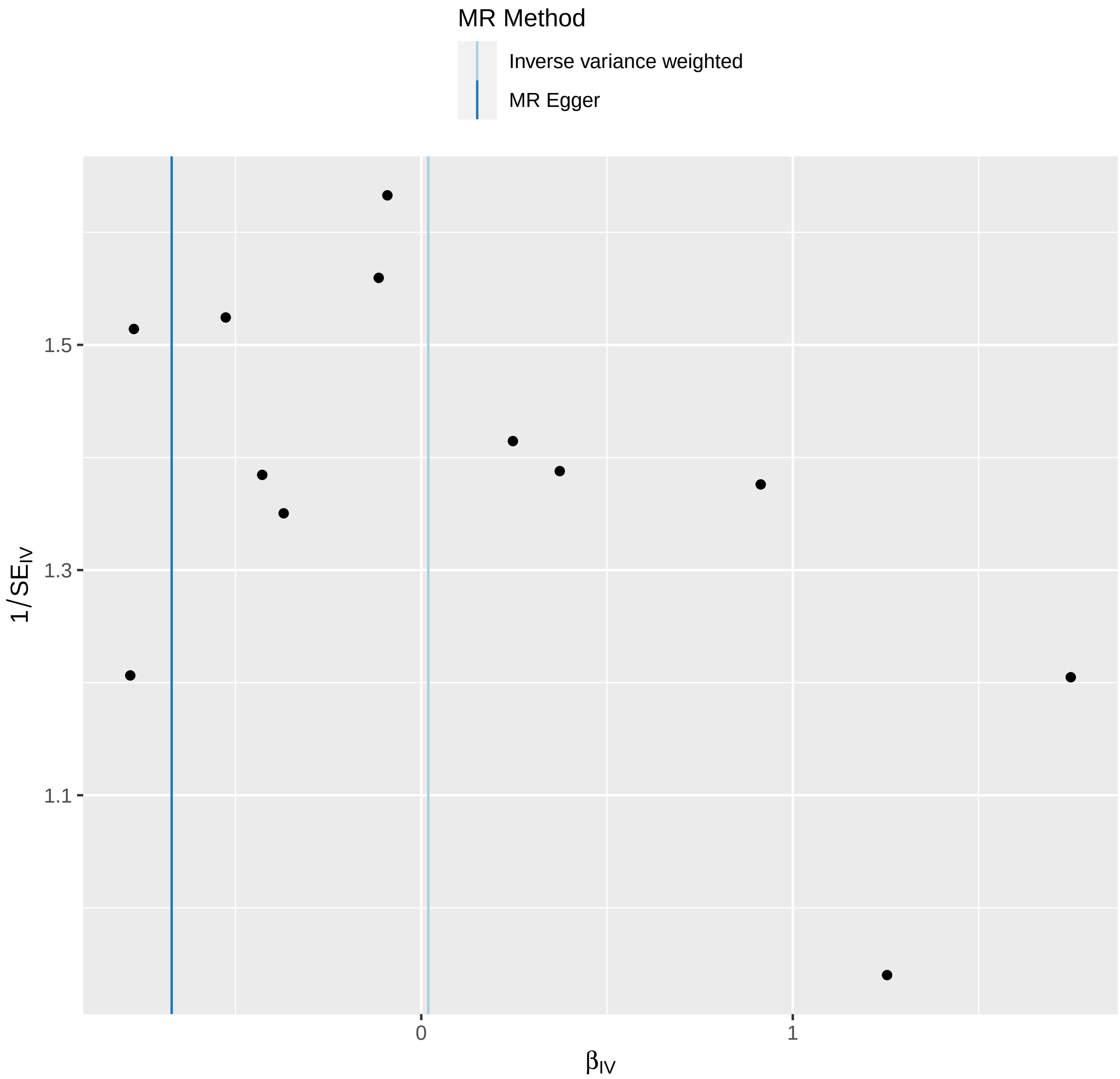

Batch 1094 : Gut microbiota abundance (genus Bifidobacterium id.436) on Ulcerative ileocolitis

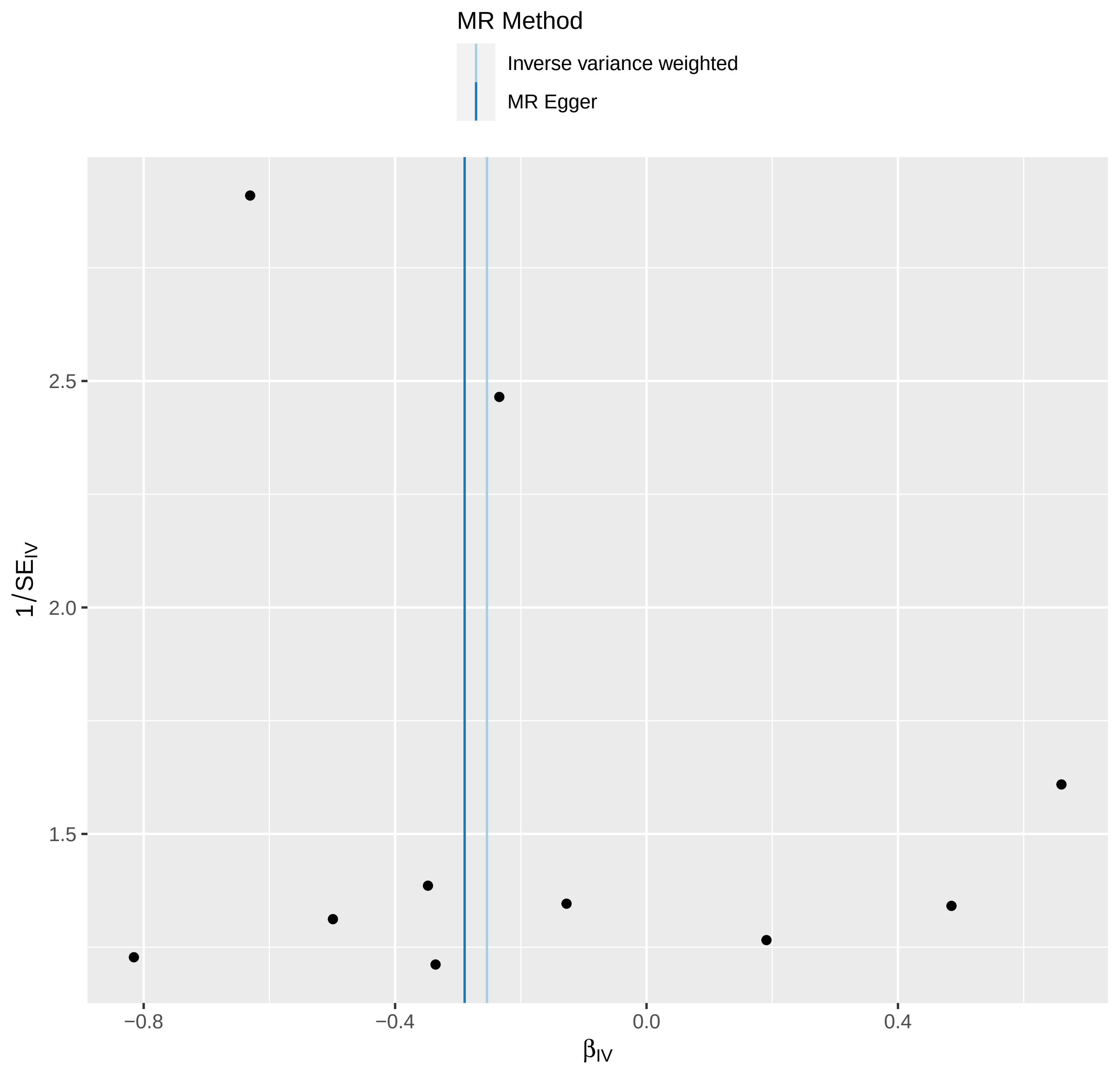

Batch 1095 : Gut microbiota abundance (genus Bilophila id.3170) on Ulcerative ileocolitis

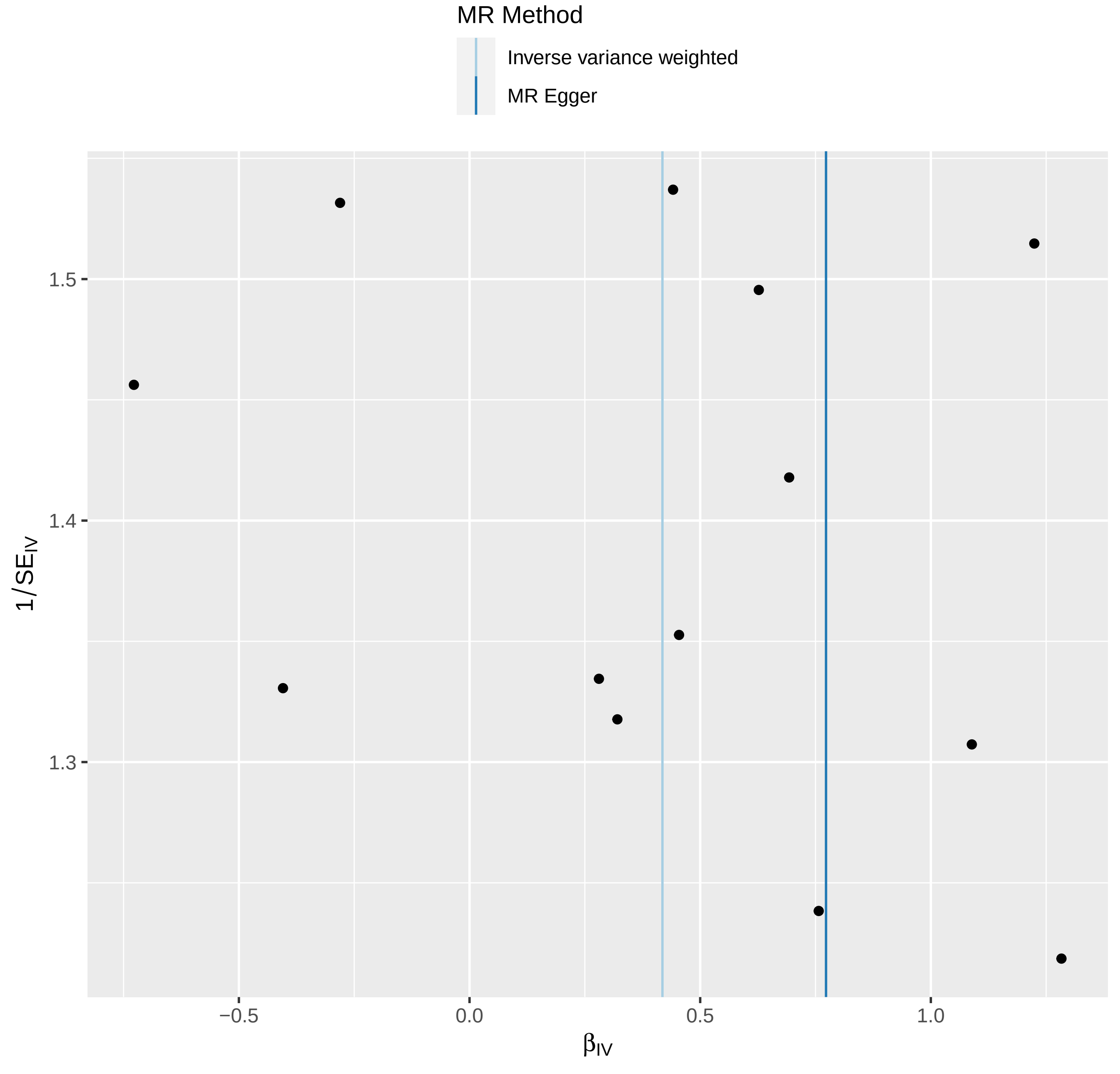

Batch 1096 : Gut microbiota abundance (genus Blautia id.1992) on Ulcerative ileocolitis

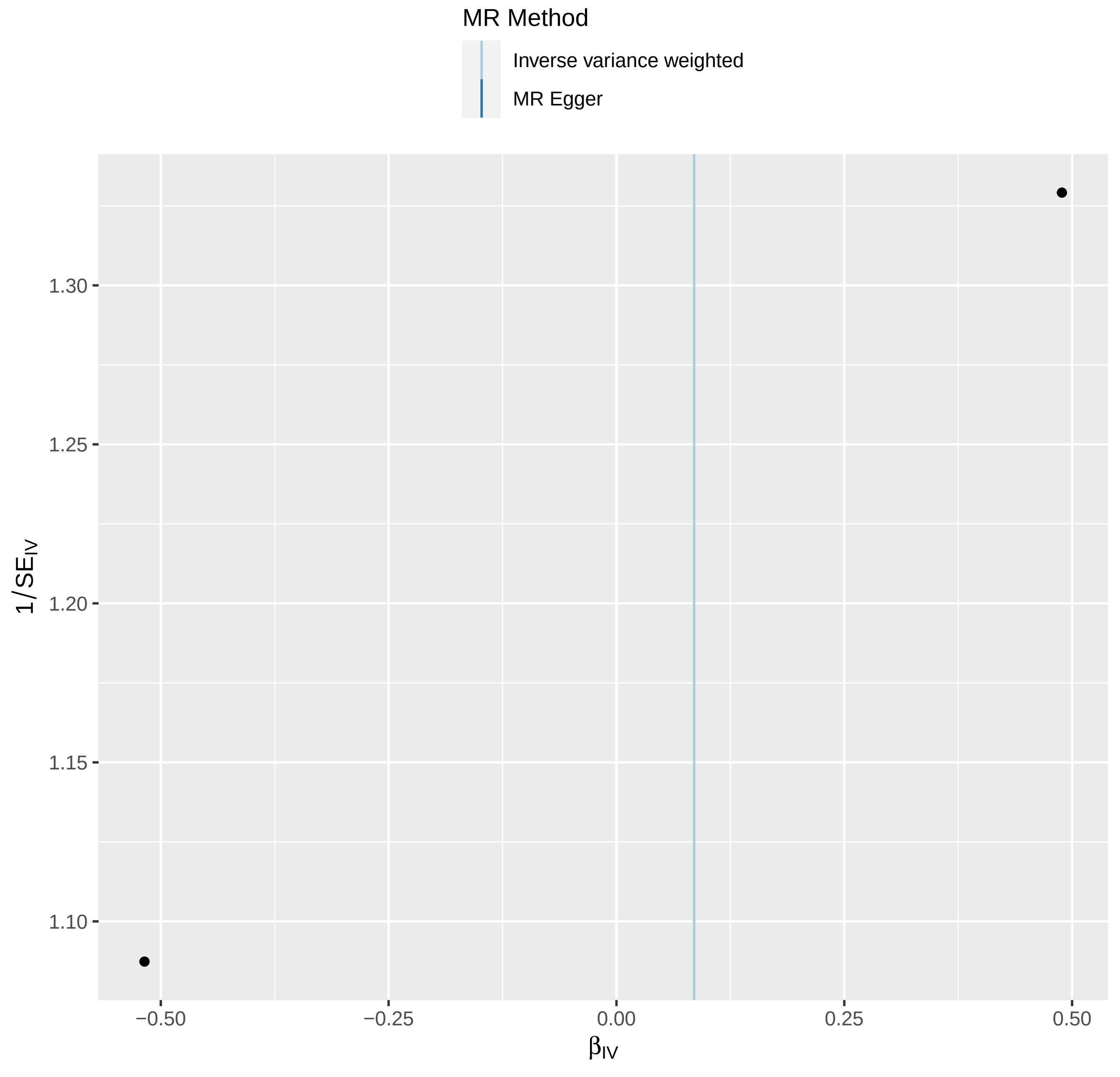

Batch 1097 : Gut microbiota abundance (genus Butyricicoccus id.2055) on Ulcerative ileocolitis

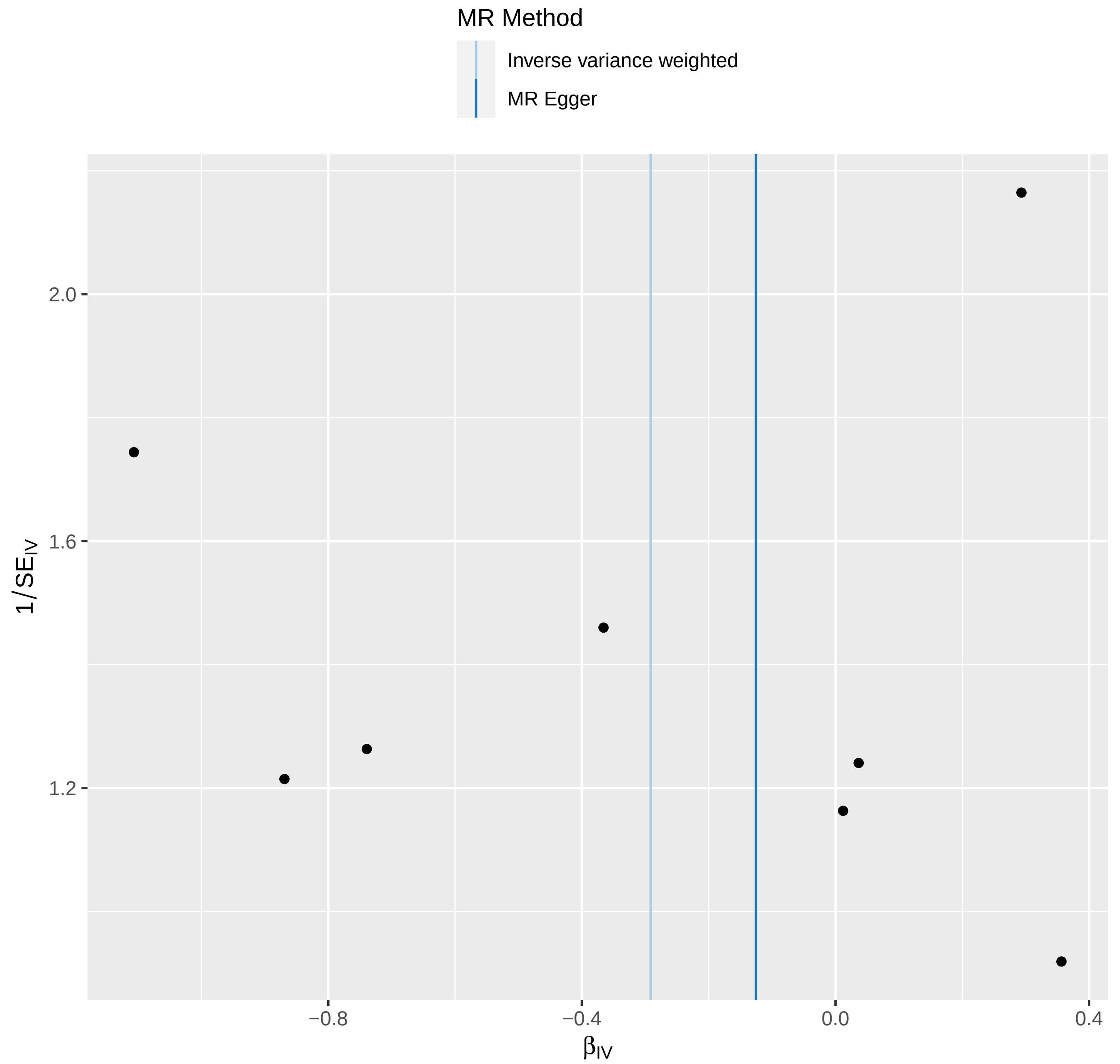

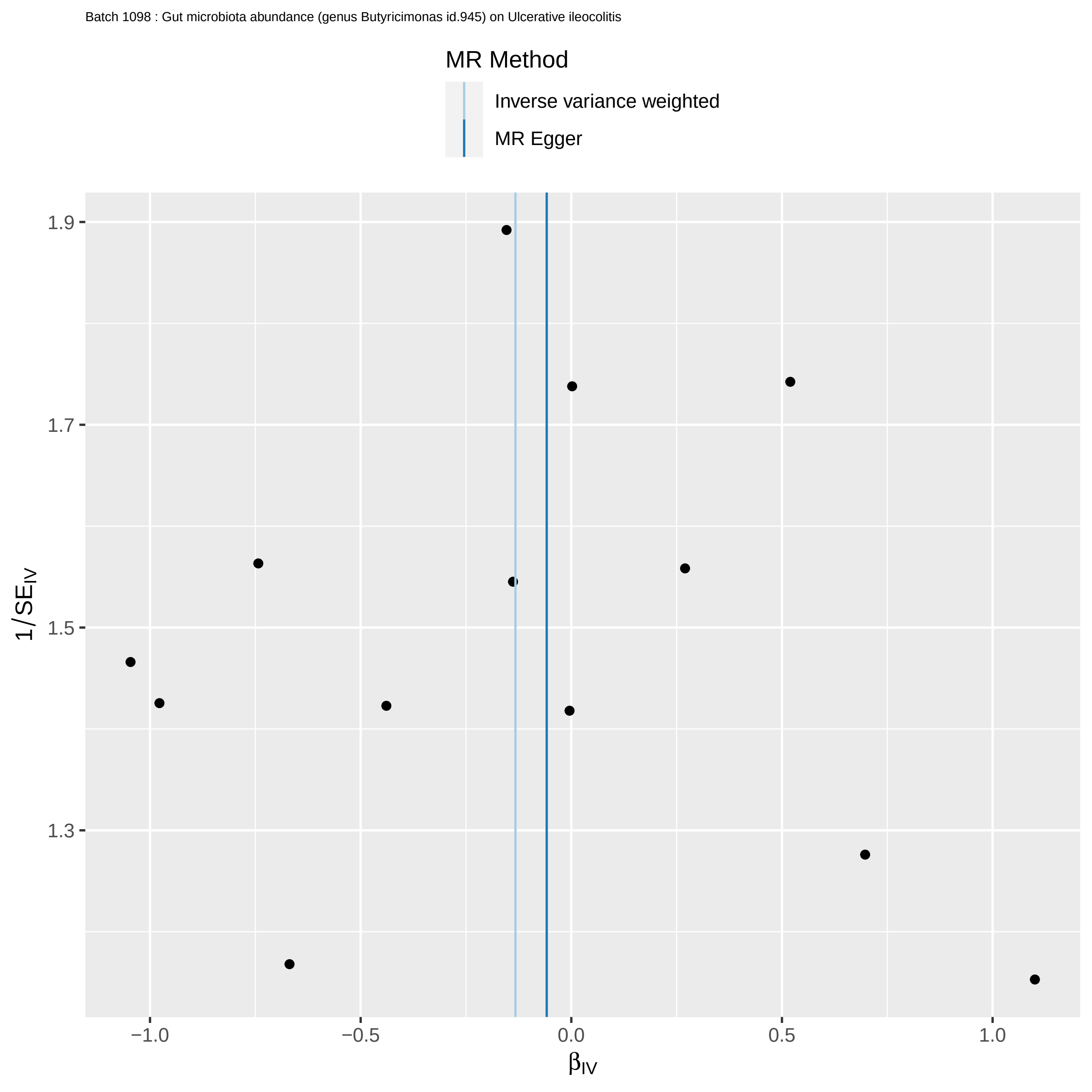

Batch 1098 : Gut microbiota abundance (genus Butyricimonas id.945) on Ulcerative ileocolitis
MR Method
Inverse variance weighted
MR Egger
1.9
1.7
1.5
1.3
1/SE_IV
−1.0
−0.5
0.0
0.5
1.0
β_IV

Batch 1099 : Gut microbiota abundance (genus Butyrivibrio id.1993) on Ulcerative ileocolitis

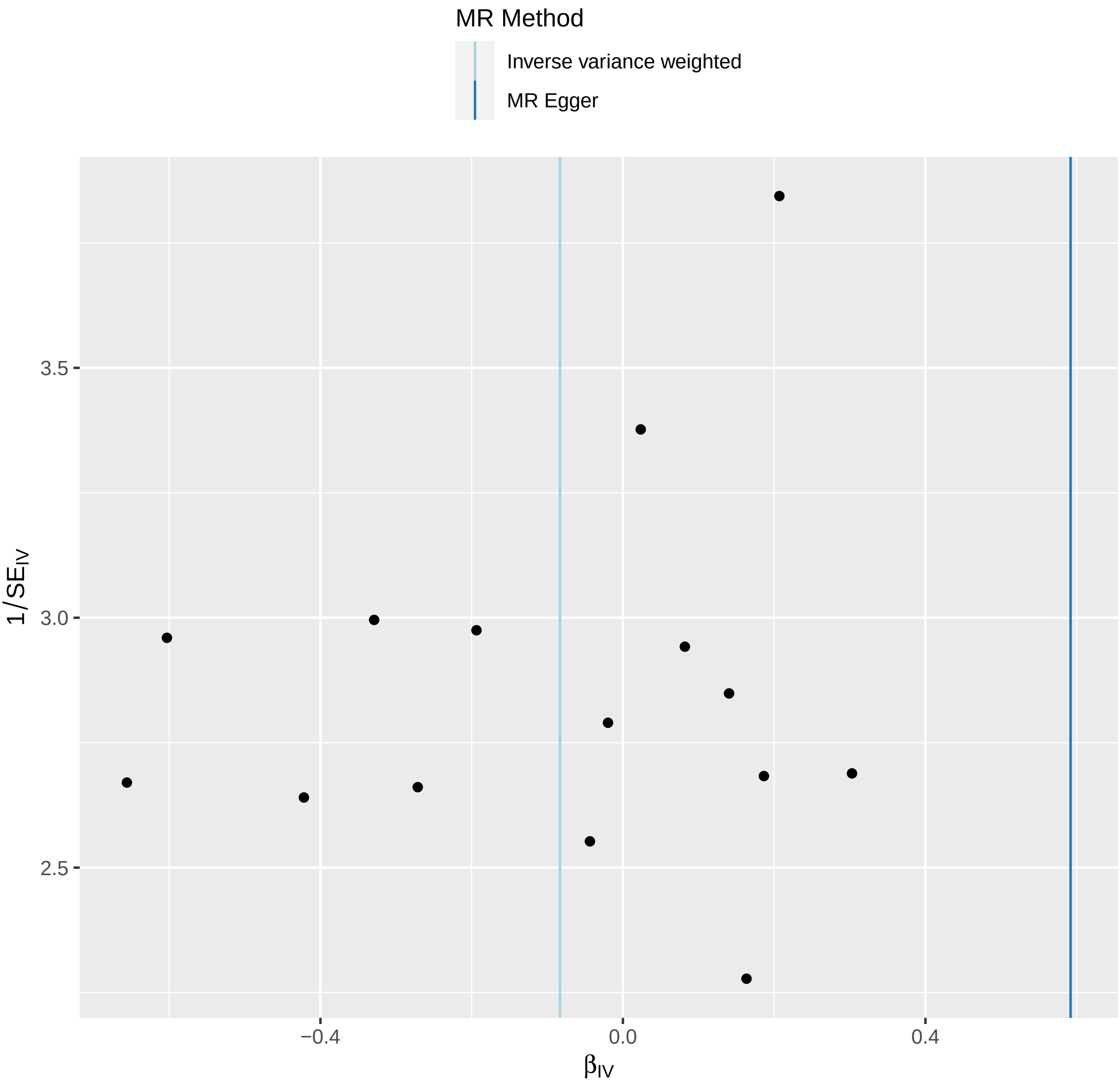

Batch 1100 : Gut microbiota abundance (genus Candidatus Soleaferrea id.11350) on Ulcerative ileocolitis

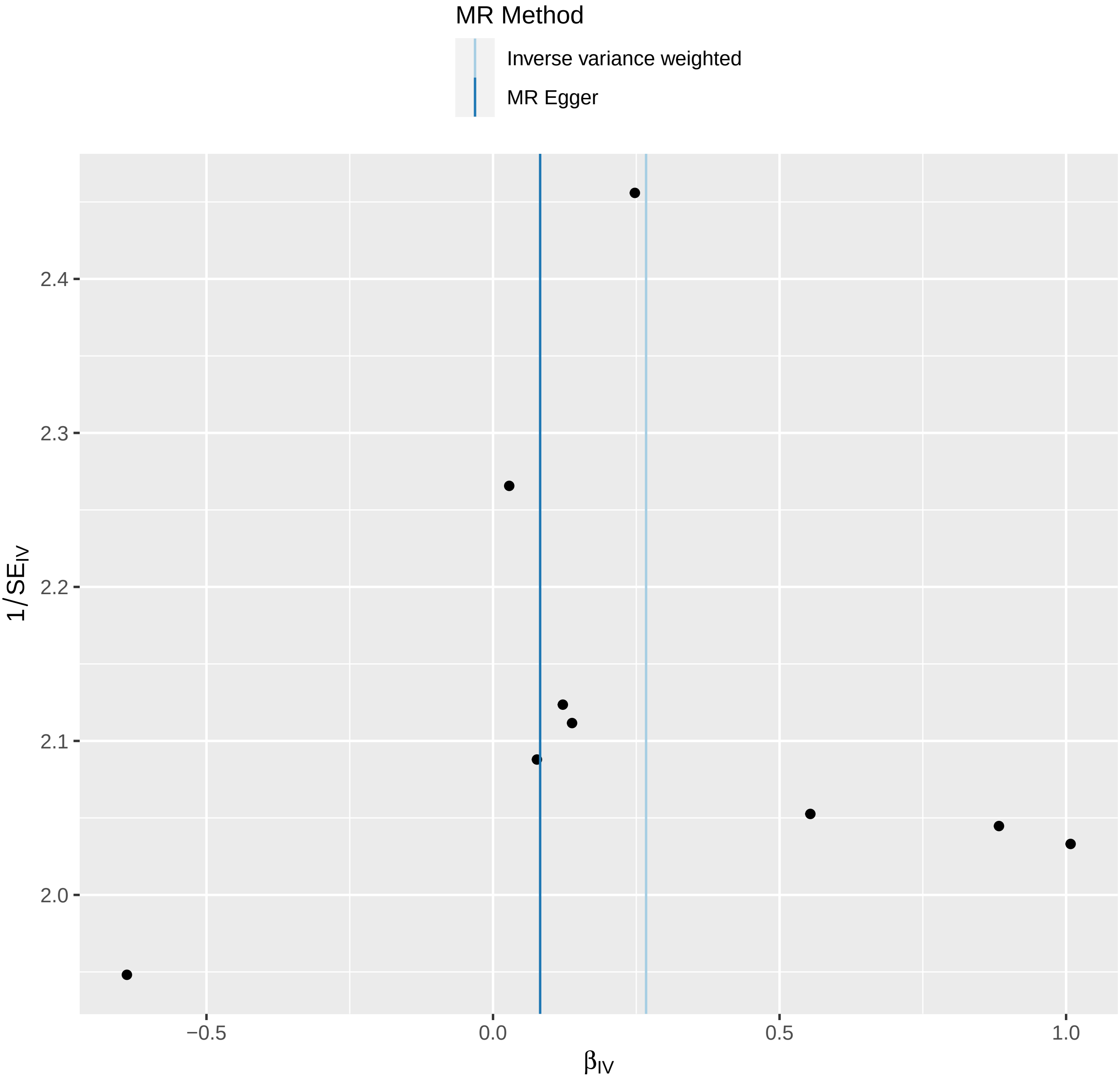

Batch 1101 : Gut microbiota abundance (genus Catenibacterium id.2153) on Ulcerative ileocolitis

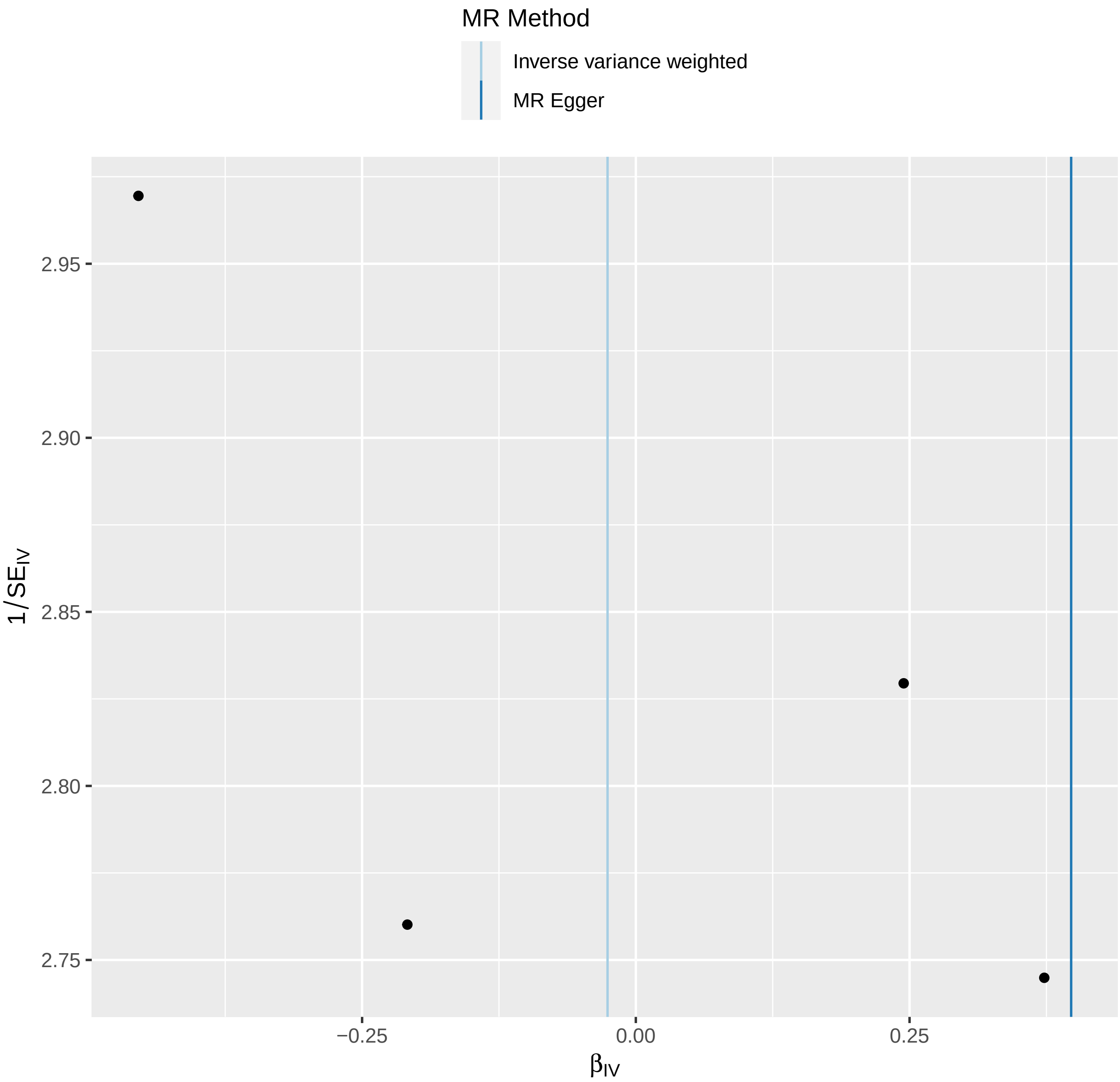

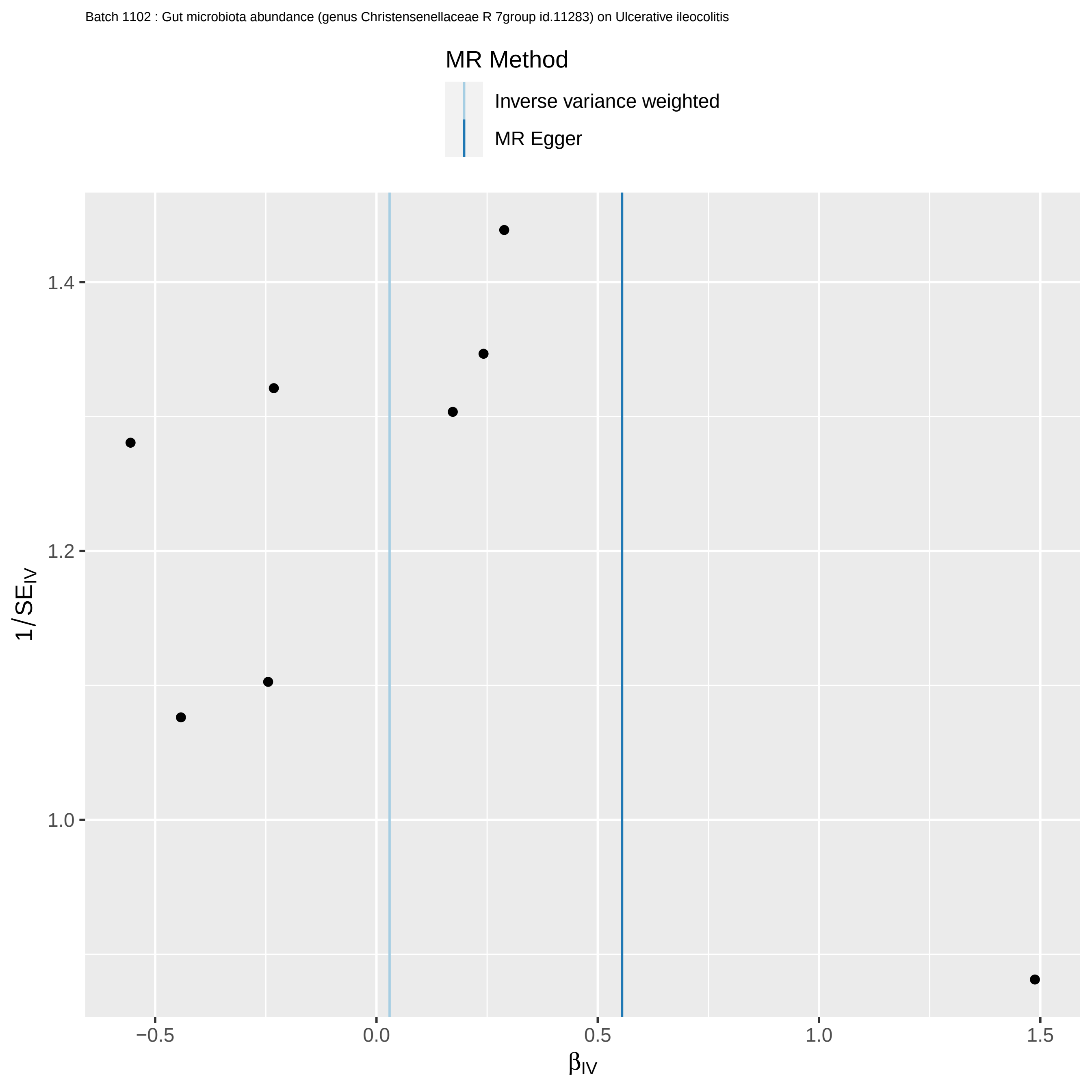

Batch 1102 : Gut microbiota abundance (genus Christensenellaceae R 7group id.11283) on Ulcerative ileocolitis
MR Method
Inverse variance weighted
MR Egger
1.4
1.2
1.0
1/SE_IV
−0.5
0.0
0.5
1.0
1.5
β_IV

Batch 1103 : Gut microbiota abundance (genus Clostridium innocuum group id.14397) on Ulcerative ileocolitis

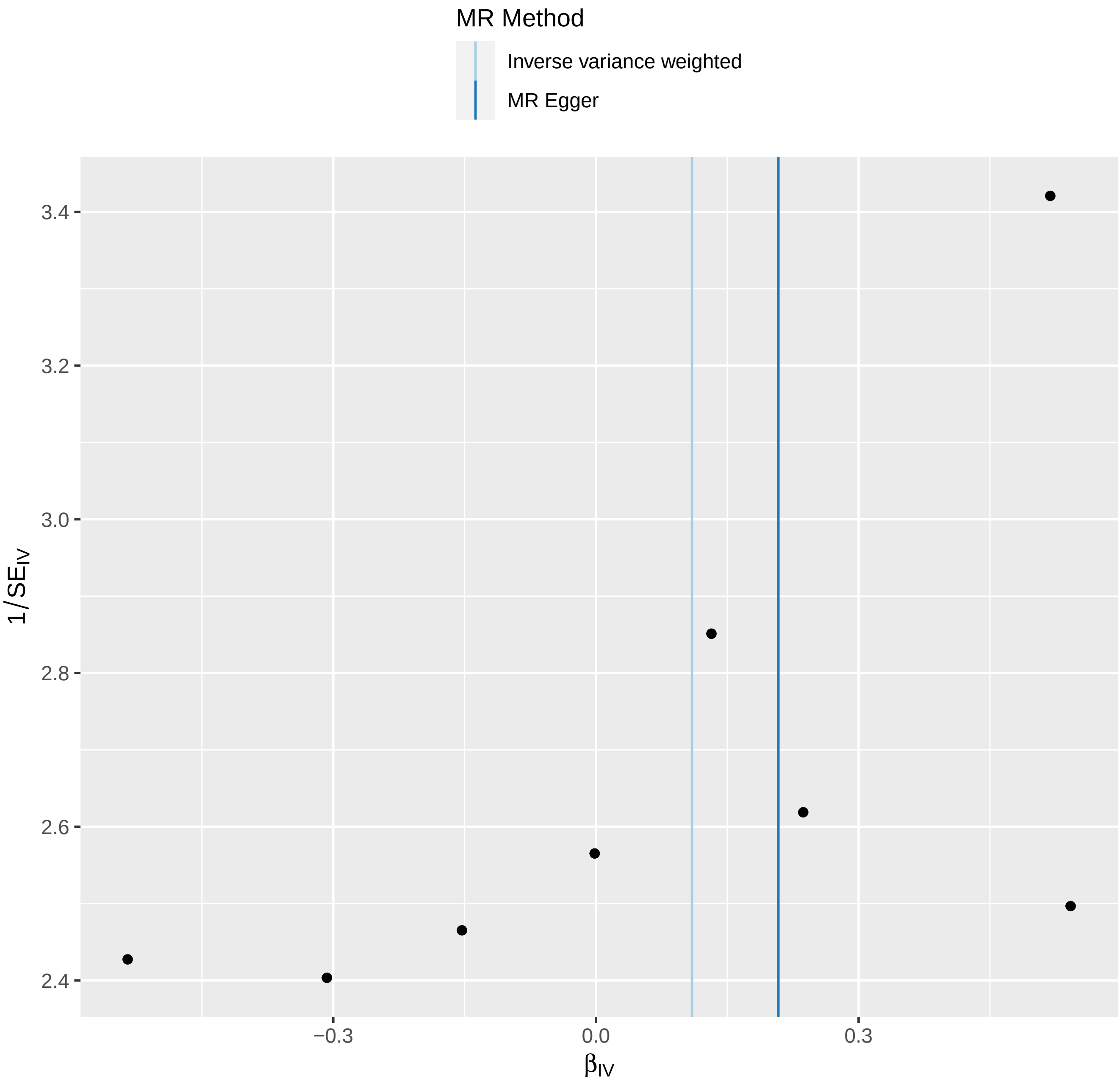

Batch 1104 : Gut microbiota abundance (genus Clostridium sensustricto1 id.1873) on Ulcerative ileocolitis

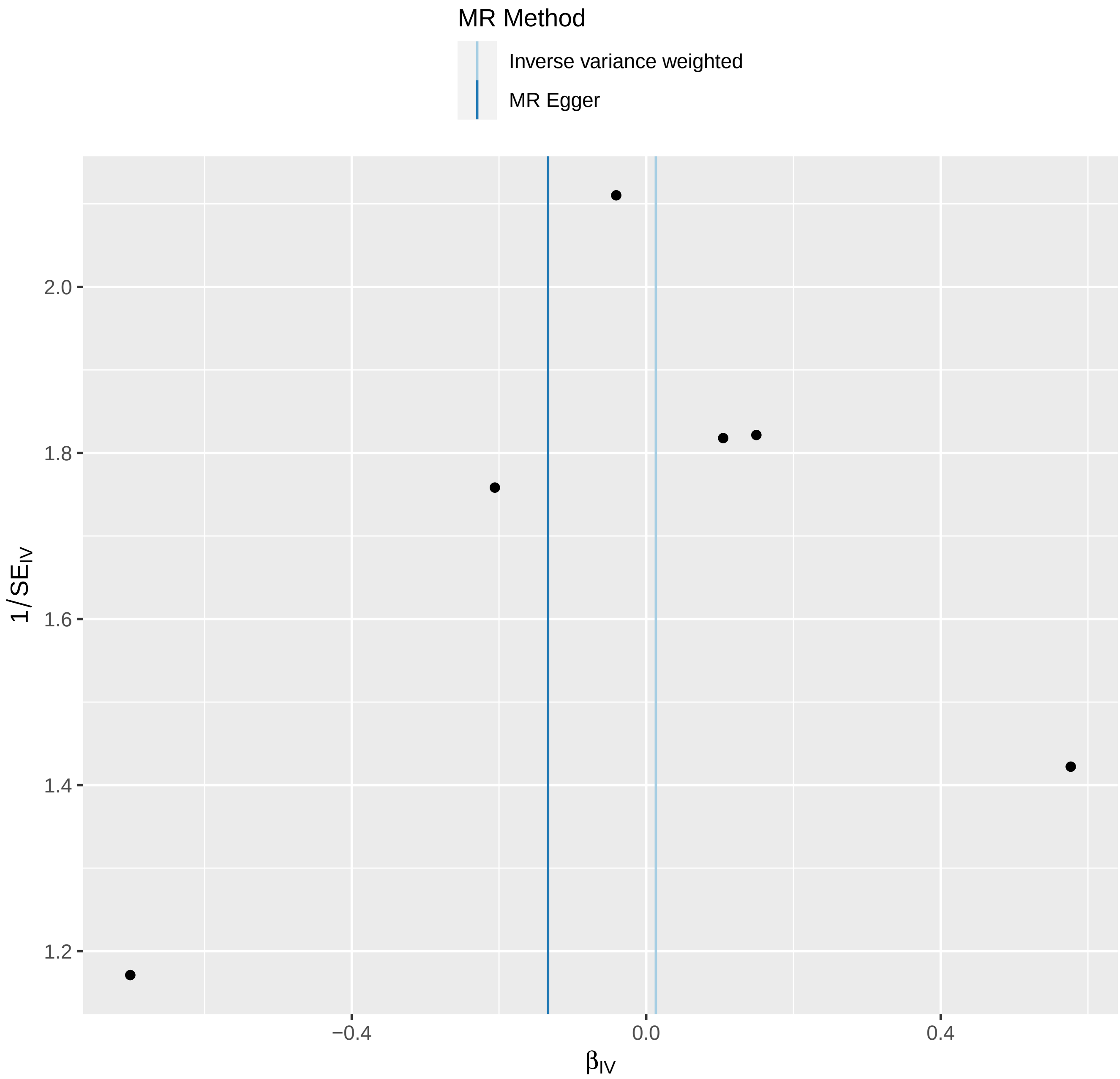

Batch 1105 : Gut microbiota abundance (genus Collinsella id.815) on Ulcerative ileocolitis

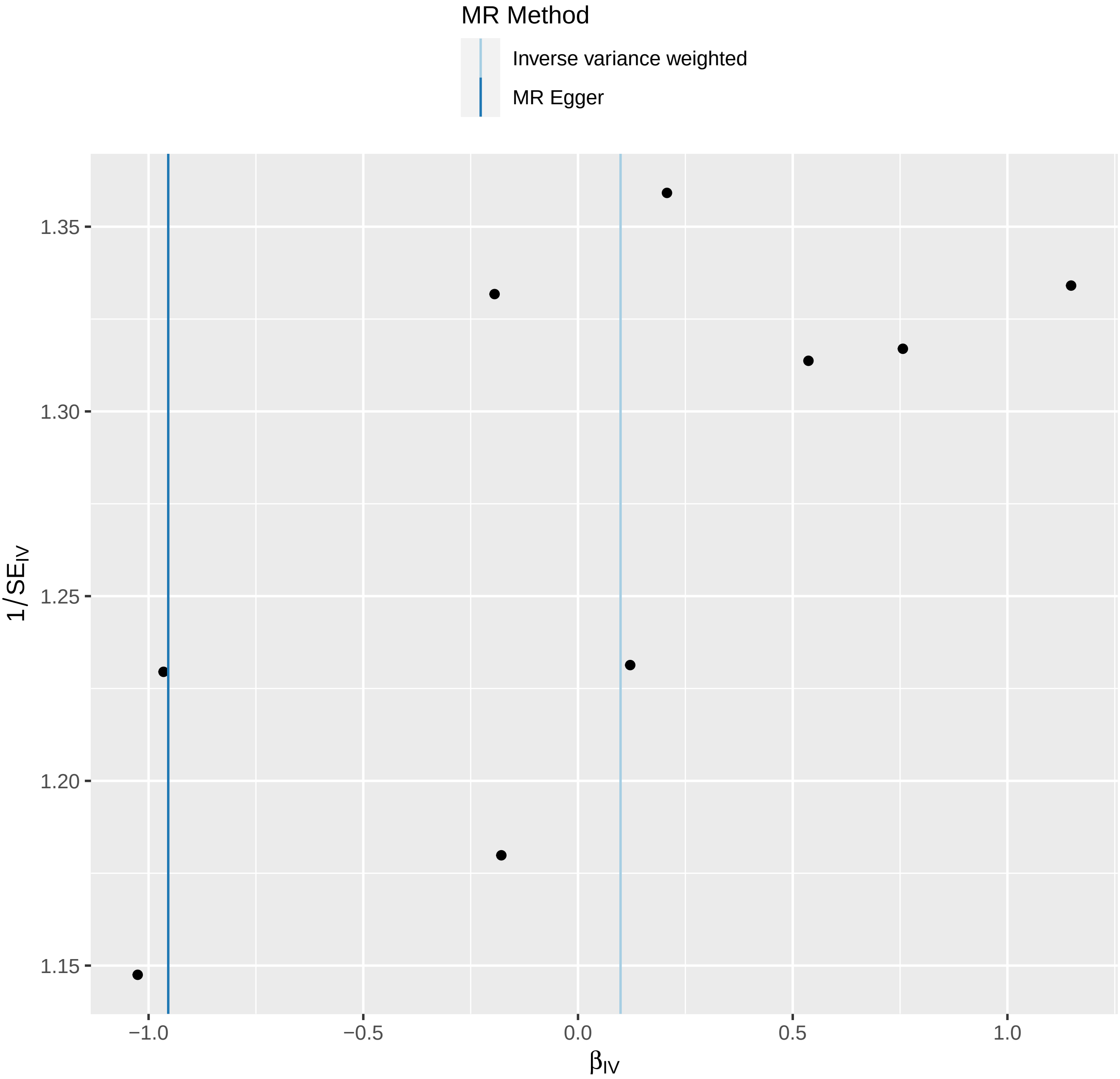

Batch 1106 : Gut microbiota abundance (genus Coprobacter id.949) on Ulcerative ileocolitis

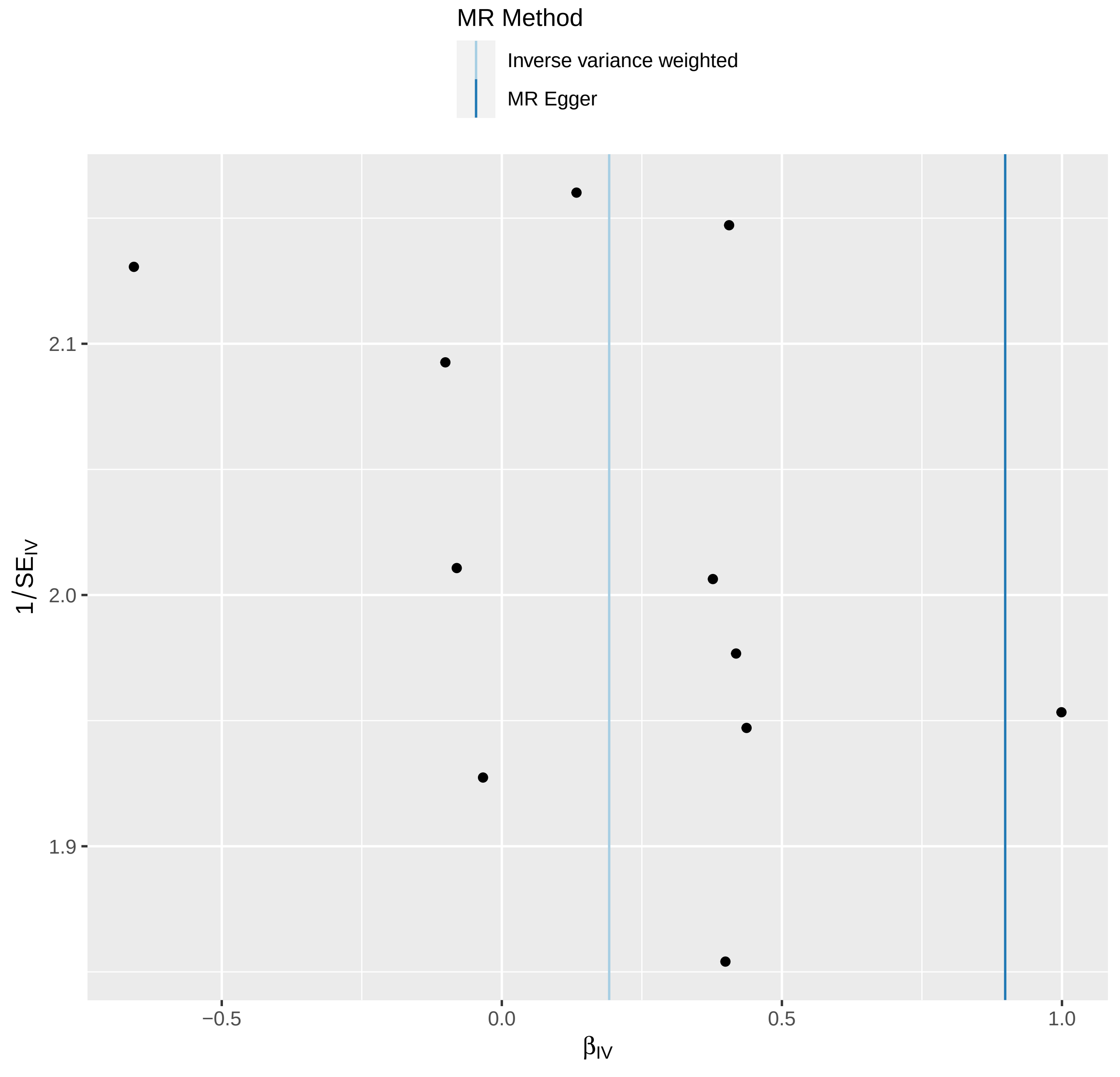

Batch 1107 : Gut microbiota abundance (genus Coprococcus1 id.11301) on Ulcerative ileocolitis

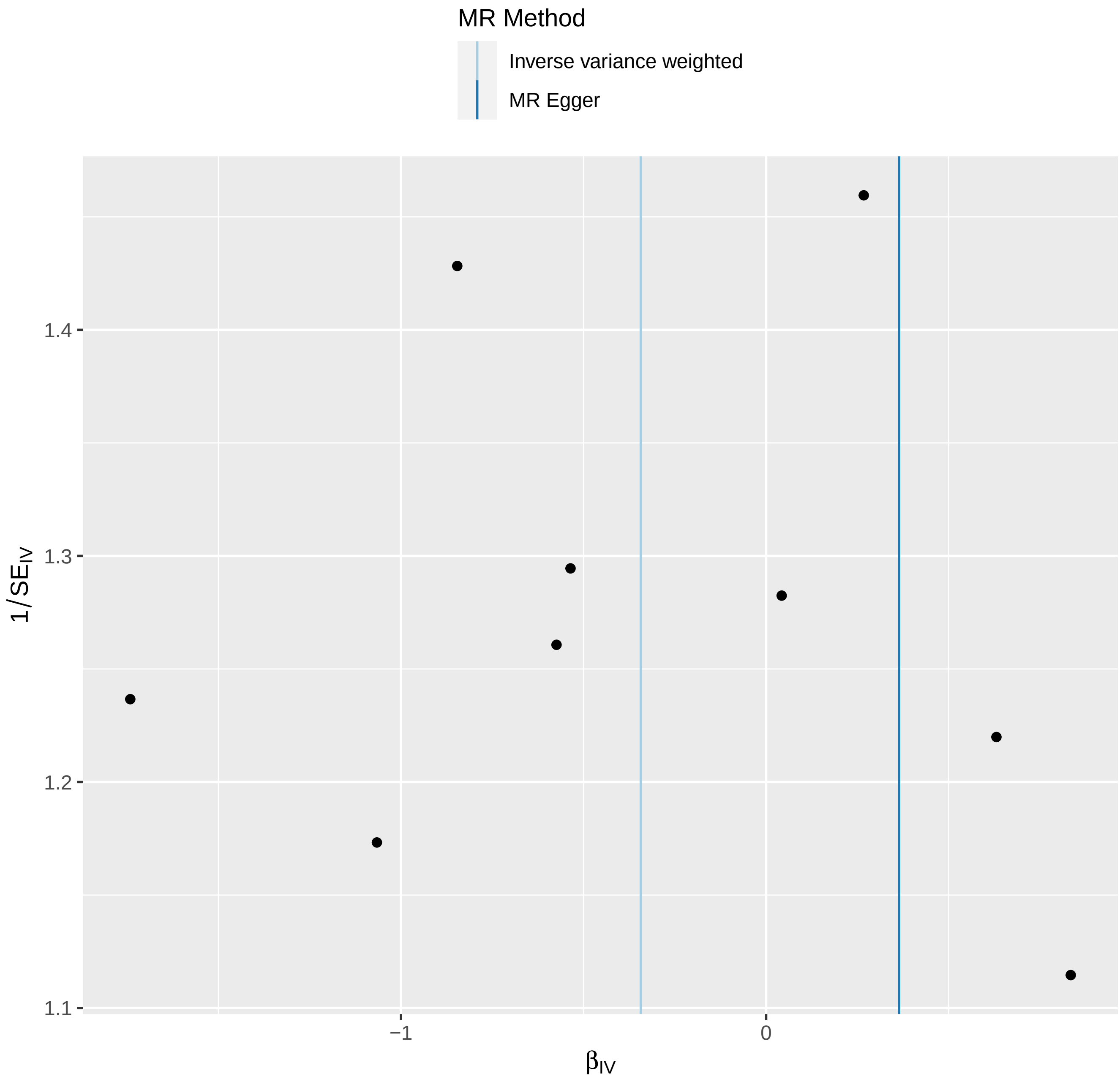

Batch 1108 : Gut microbiota abundance (genus Coprococcus2 id.11302) on Ulcerative ileocolitis

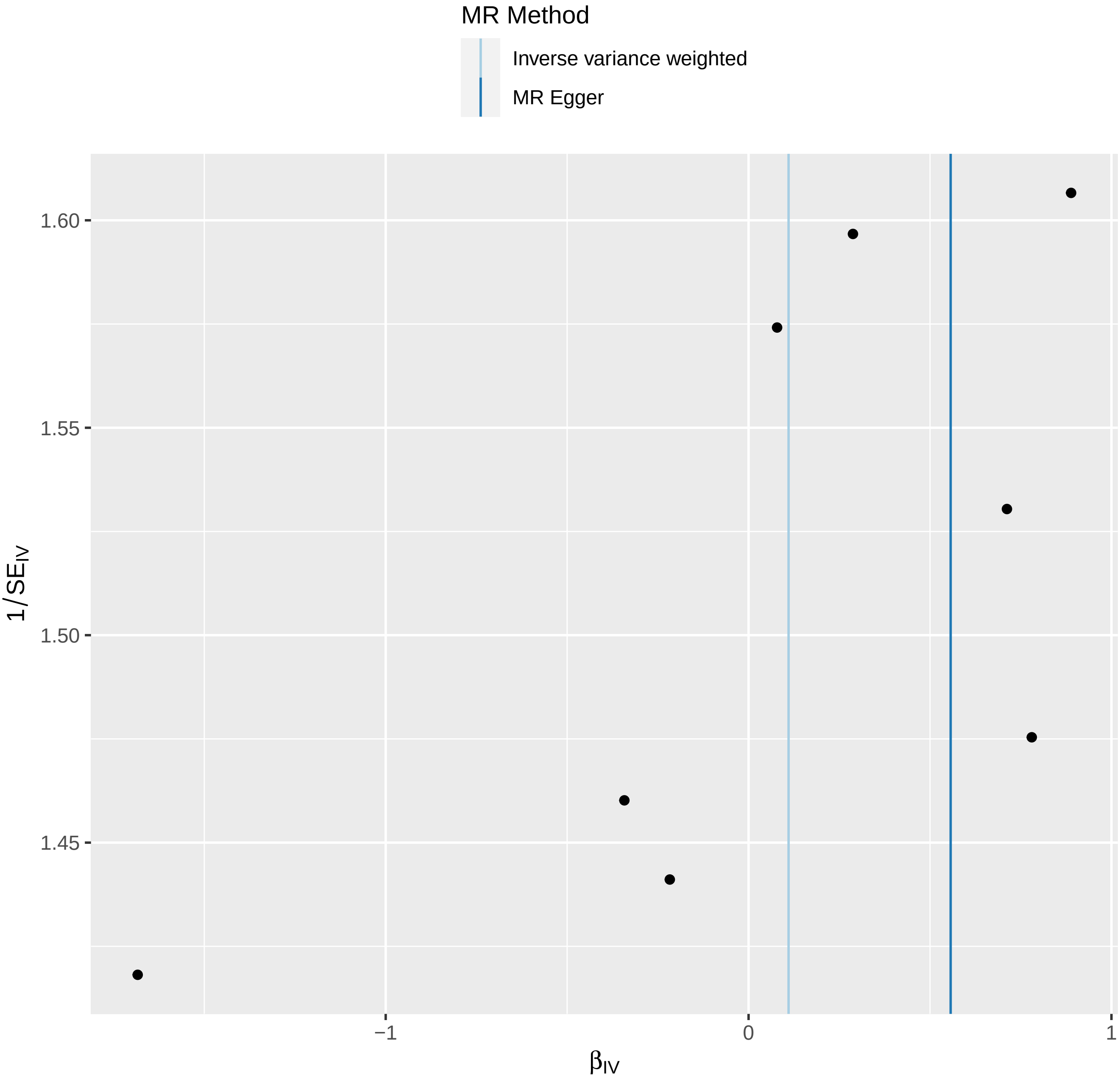

Batch 1109 : Gut microbiota abundance (genus Coprococcus3 id.11303) on Ulcerative ileocolitis

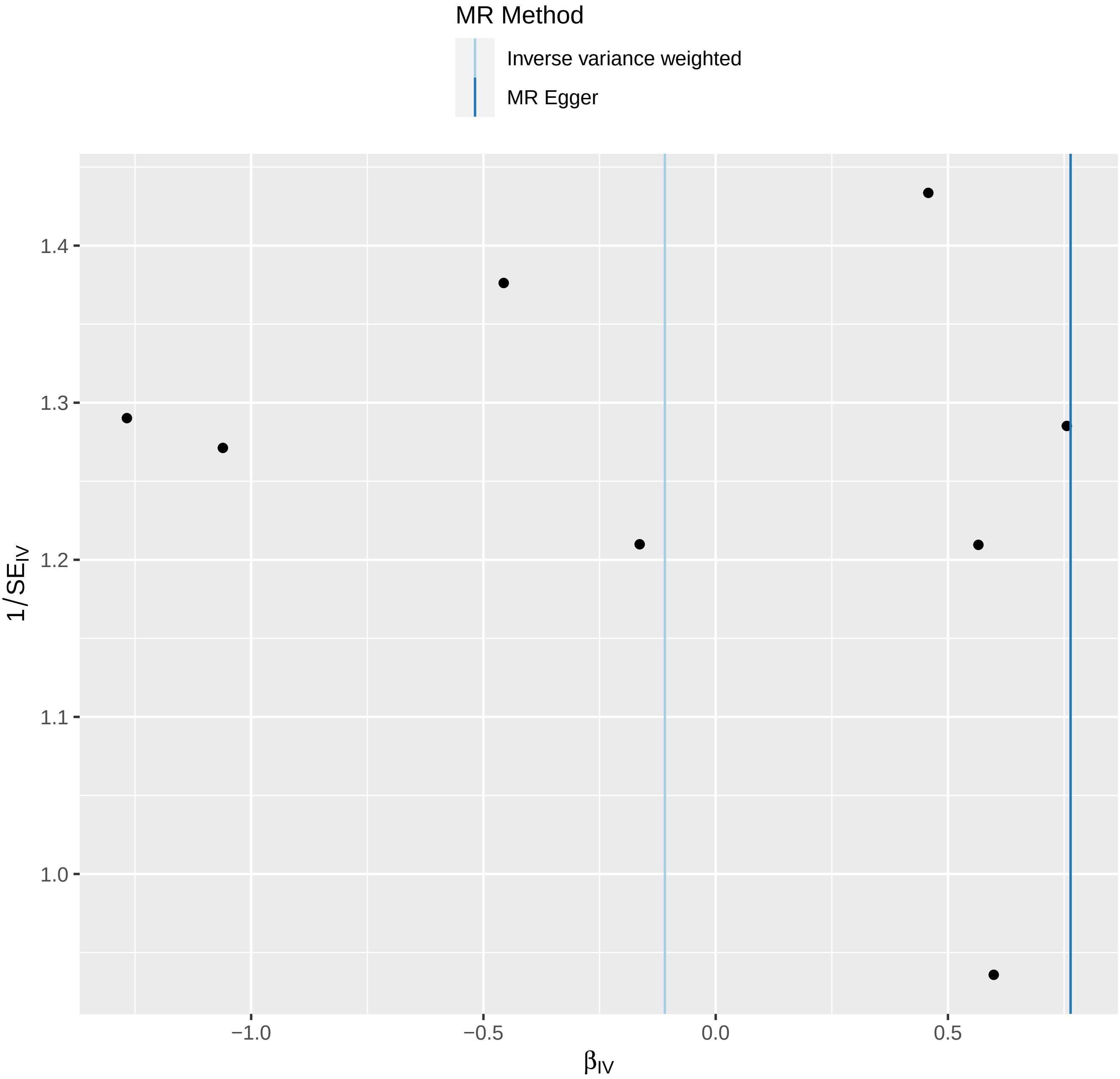

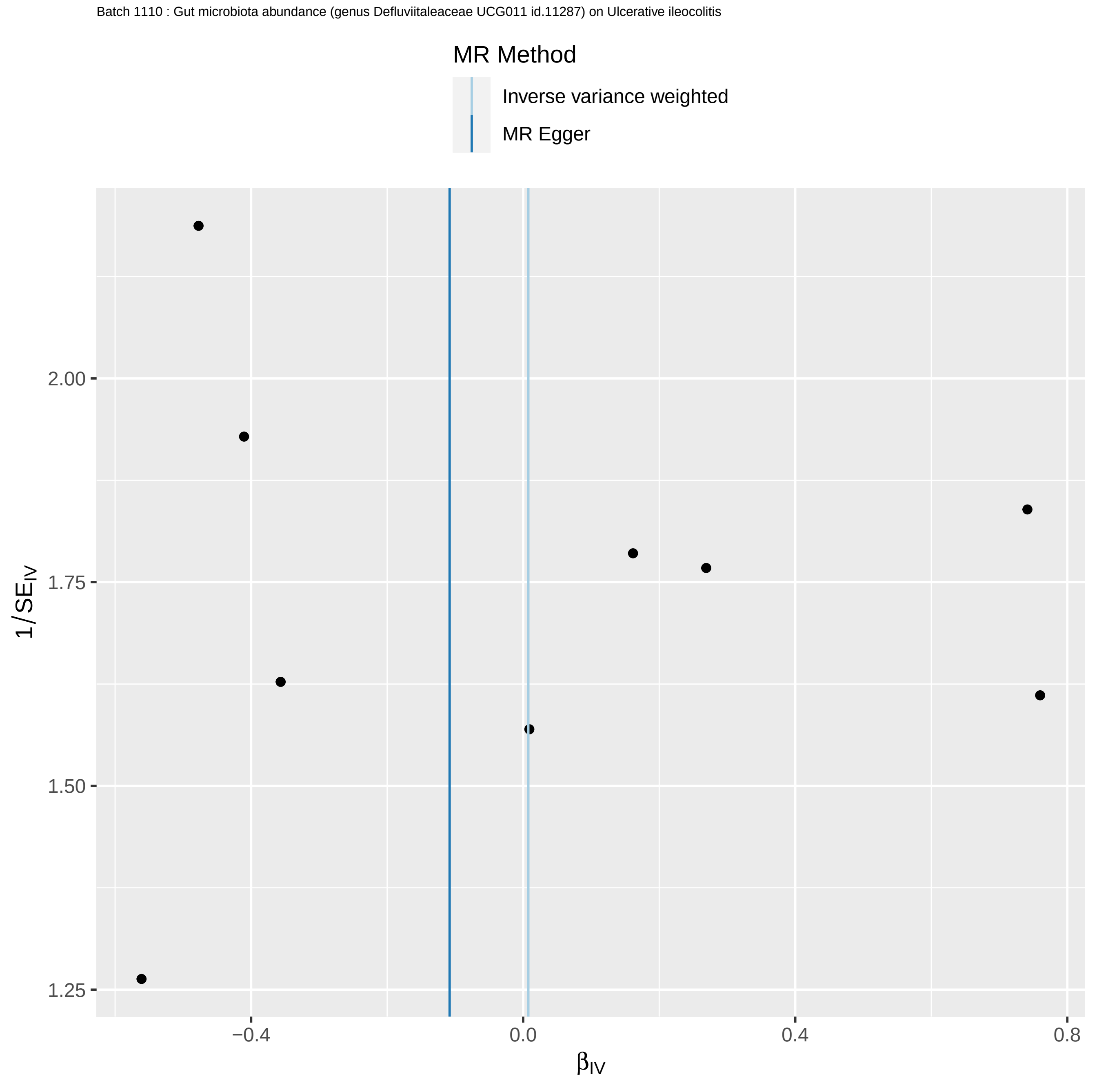

Batch 1110 : Gut microbiota abundance (genus Defluviitaleaceae UCG011 id.11287) on Ulcerative ileocolitis
MR Method
Inverse variance weighted
MR Egger
2.00
1.75
1.50
1.25
1/SE_IV
−0.4
0.0
0.4
0.8
β_IV

Batch 1111 : Gut microbiota abundance (genus Desulfovibrio id.3173) on Ulcerative ileocolitis

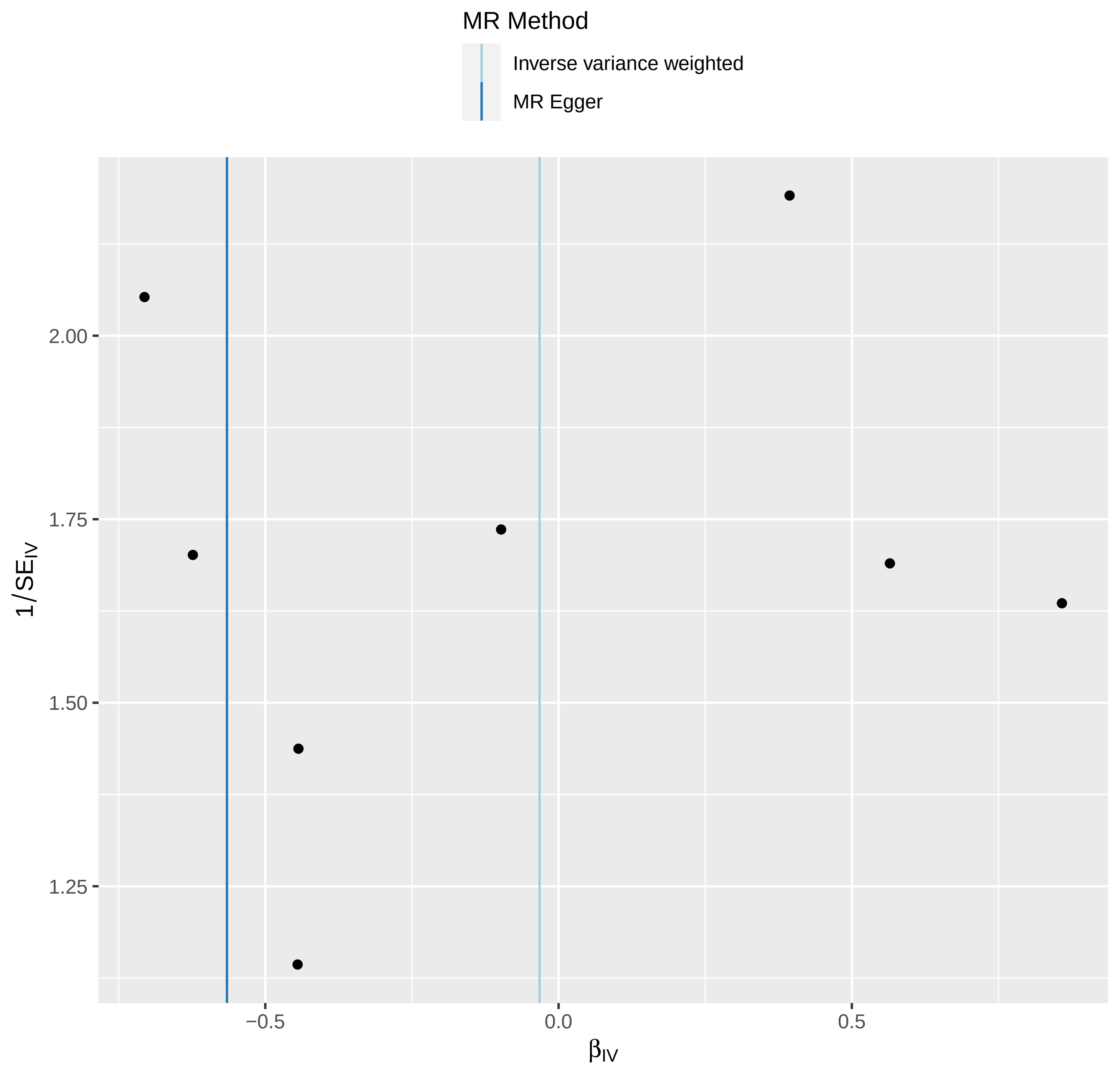

Batch 1112 : Gut microbiota abundance (genus Dialister id.2183) on Ulcerative ileocolitis

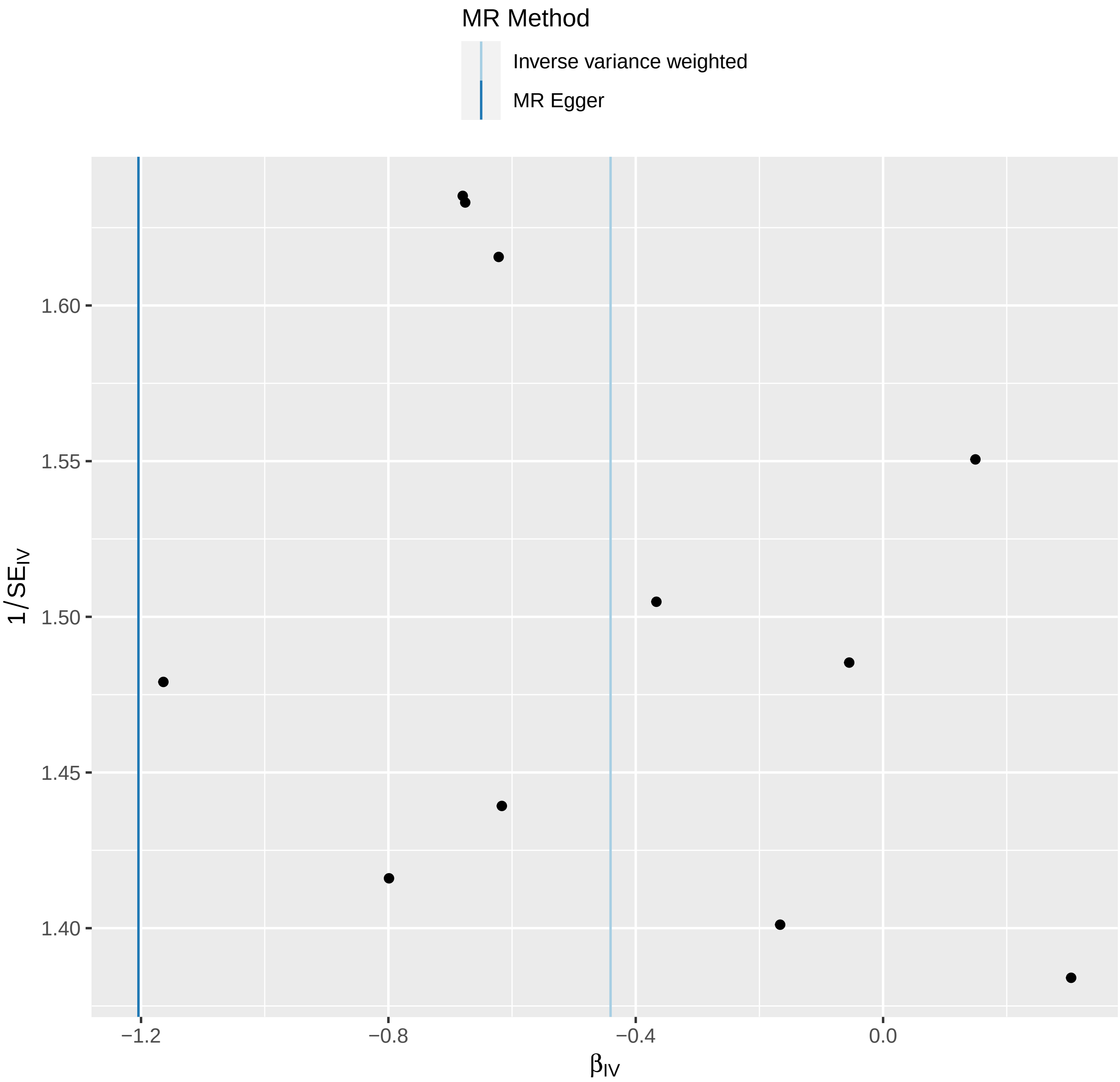

Batch 1113 : Gut microbiota abundance (genus Dorea id.1997) on Ulcerative ileocolitis

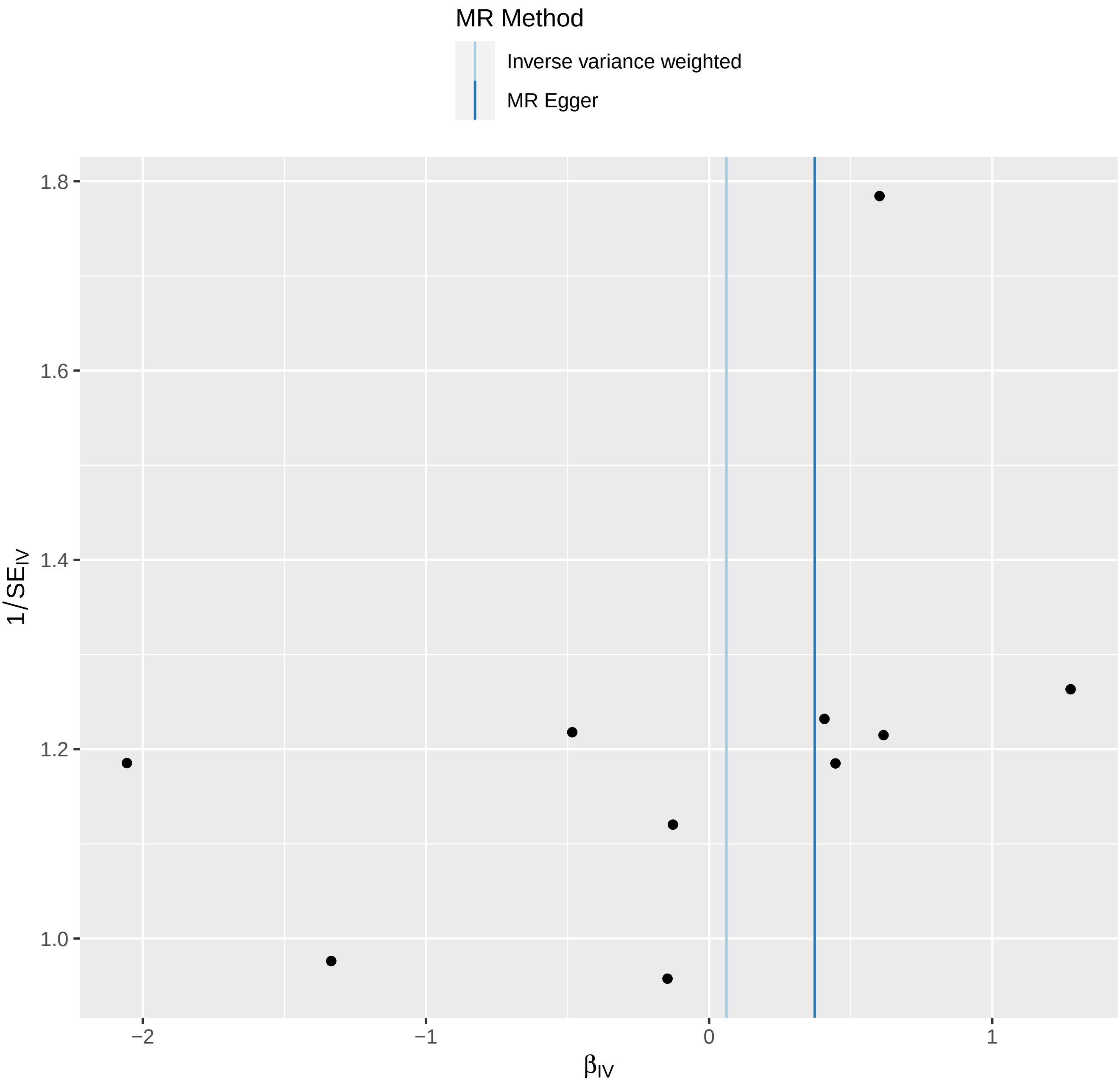

Batch 1114 : Gut microbiota abundance (genus Eggerthella id.819) on Ulcerative ileocolitis

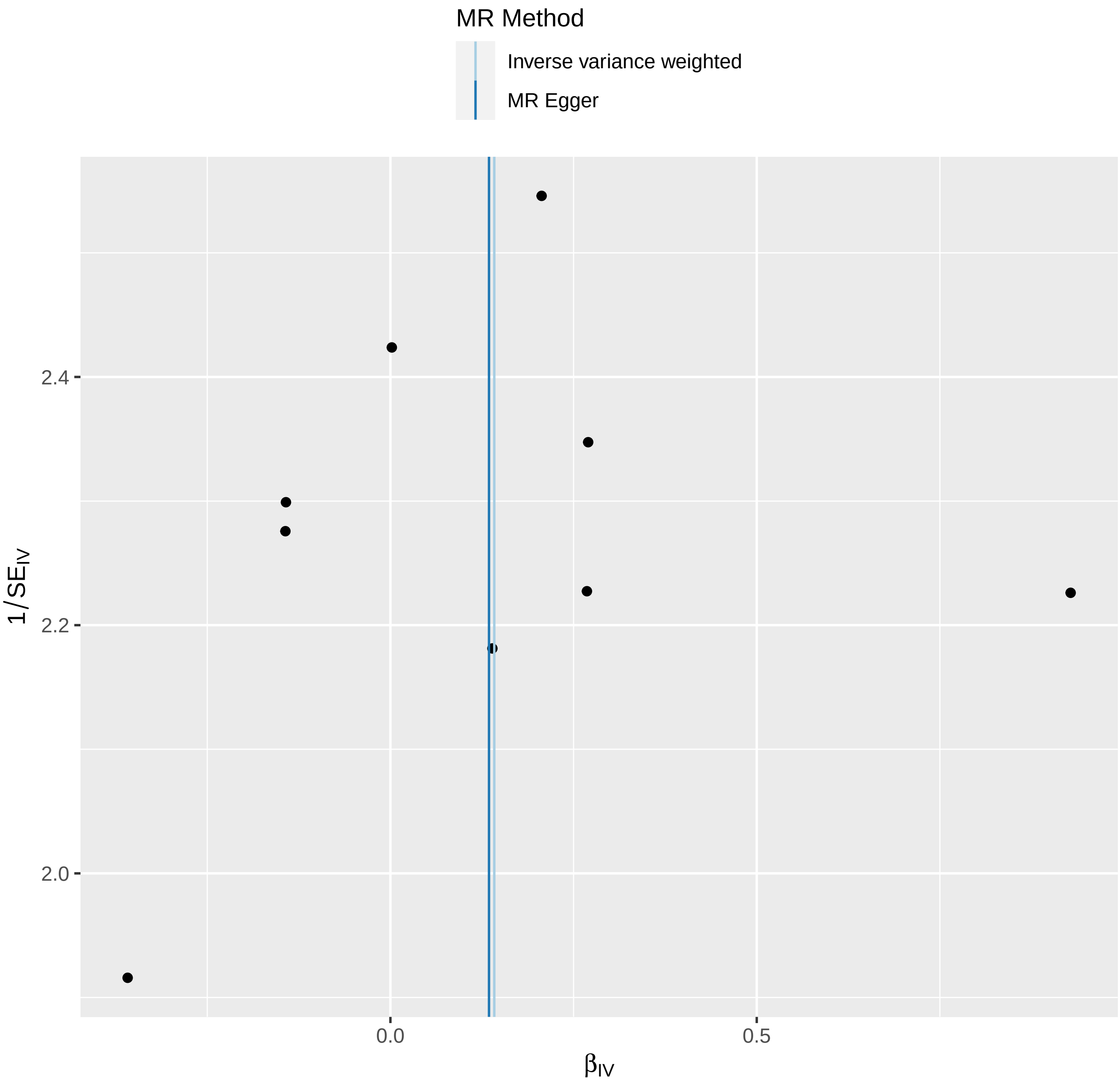

Batch 1115 : Gut microbiota abundance (genus Eisenbergiella id.11304) on Ulcerative ileocolitis

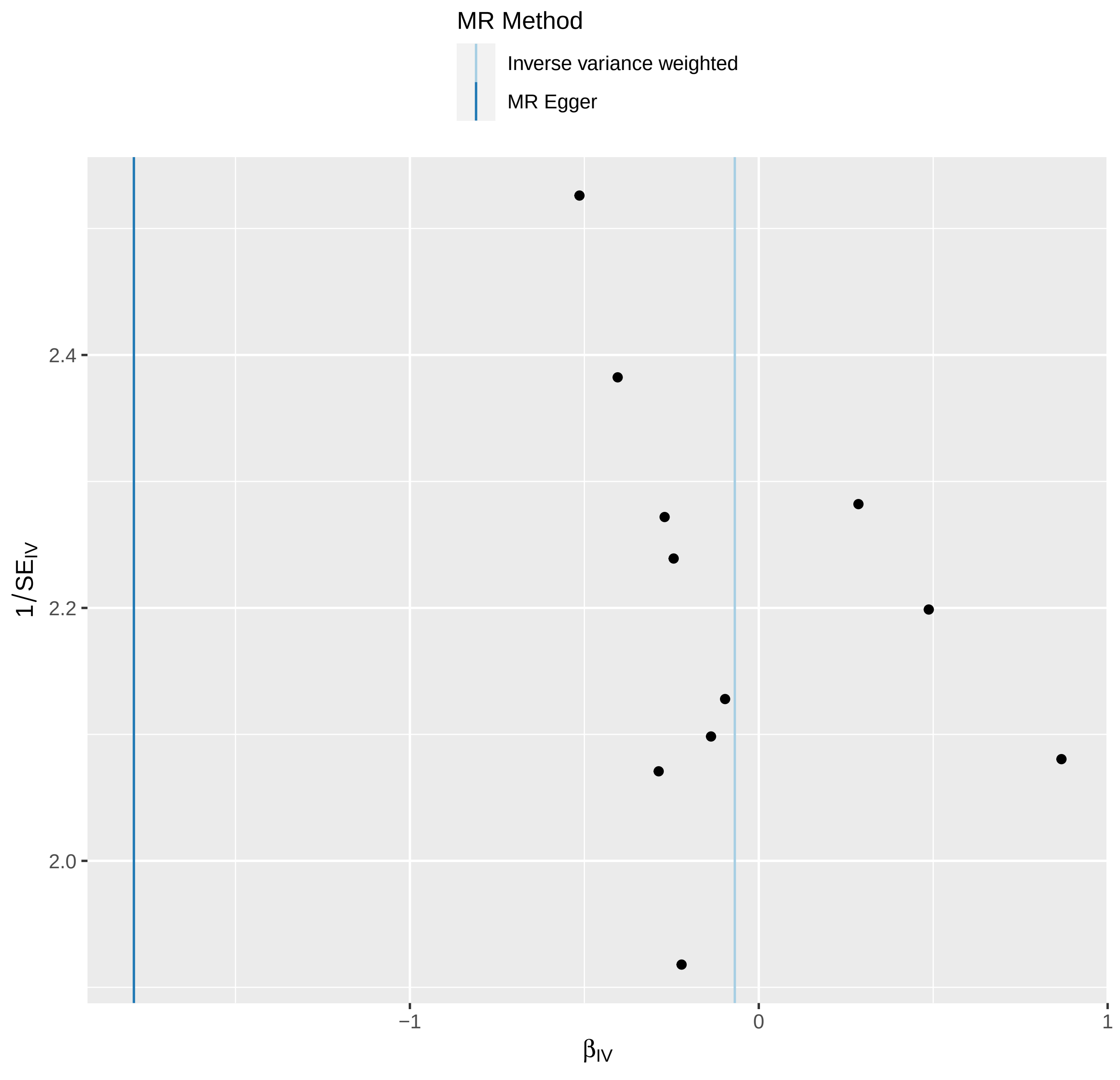

Batch 1116 : Gut microbiota abundance (genus Enterorhabdus id.820) on Ulcerative ileocolitis

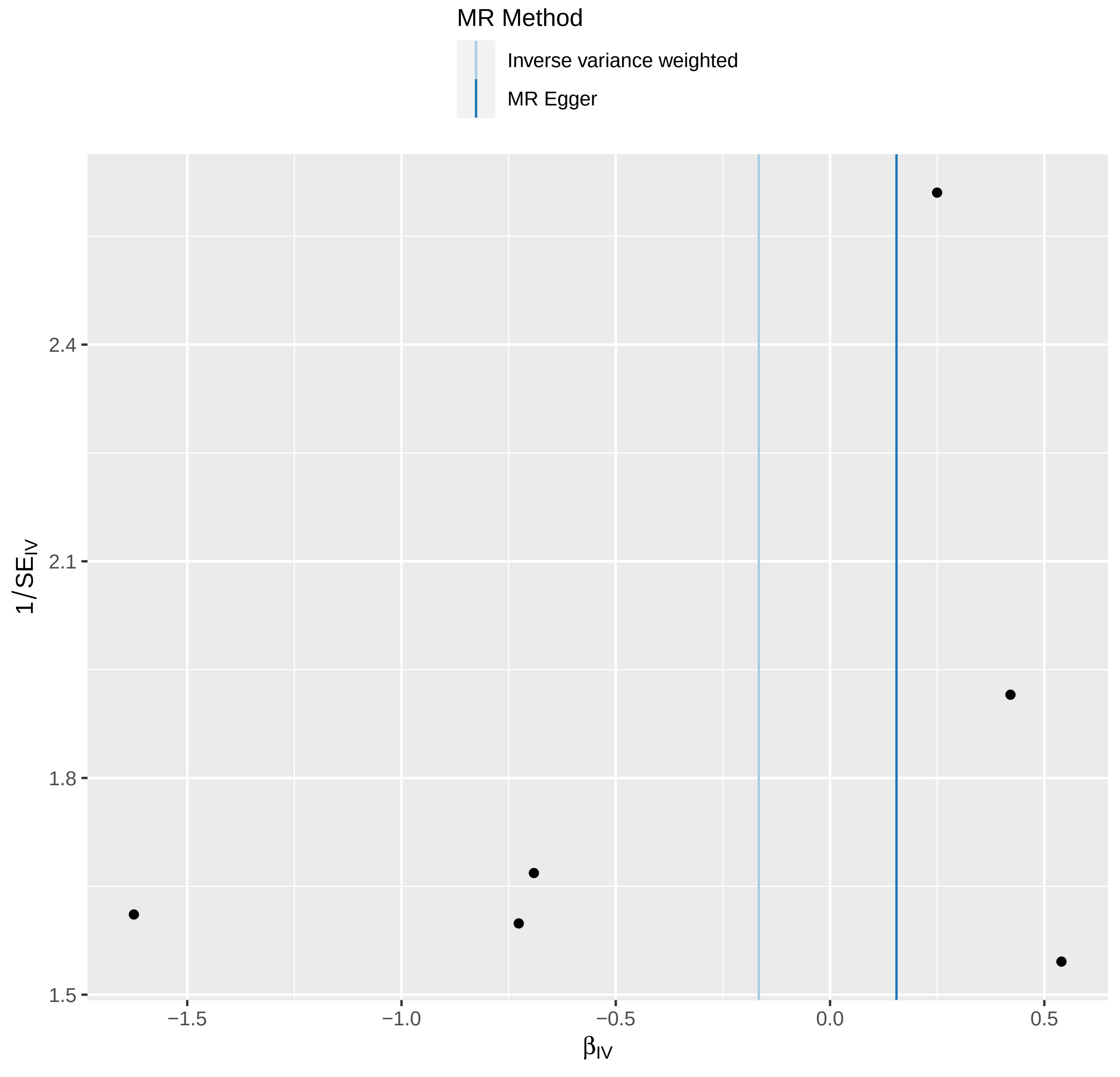

Batch 1117 : Gut microbiota abundance (genus Erysipelatoclostridium id.11381) on Ulcerative ileocolitis

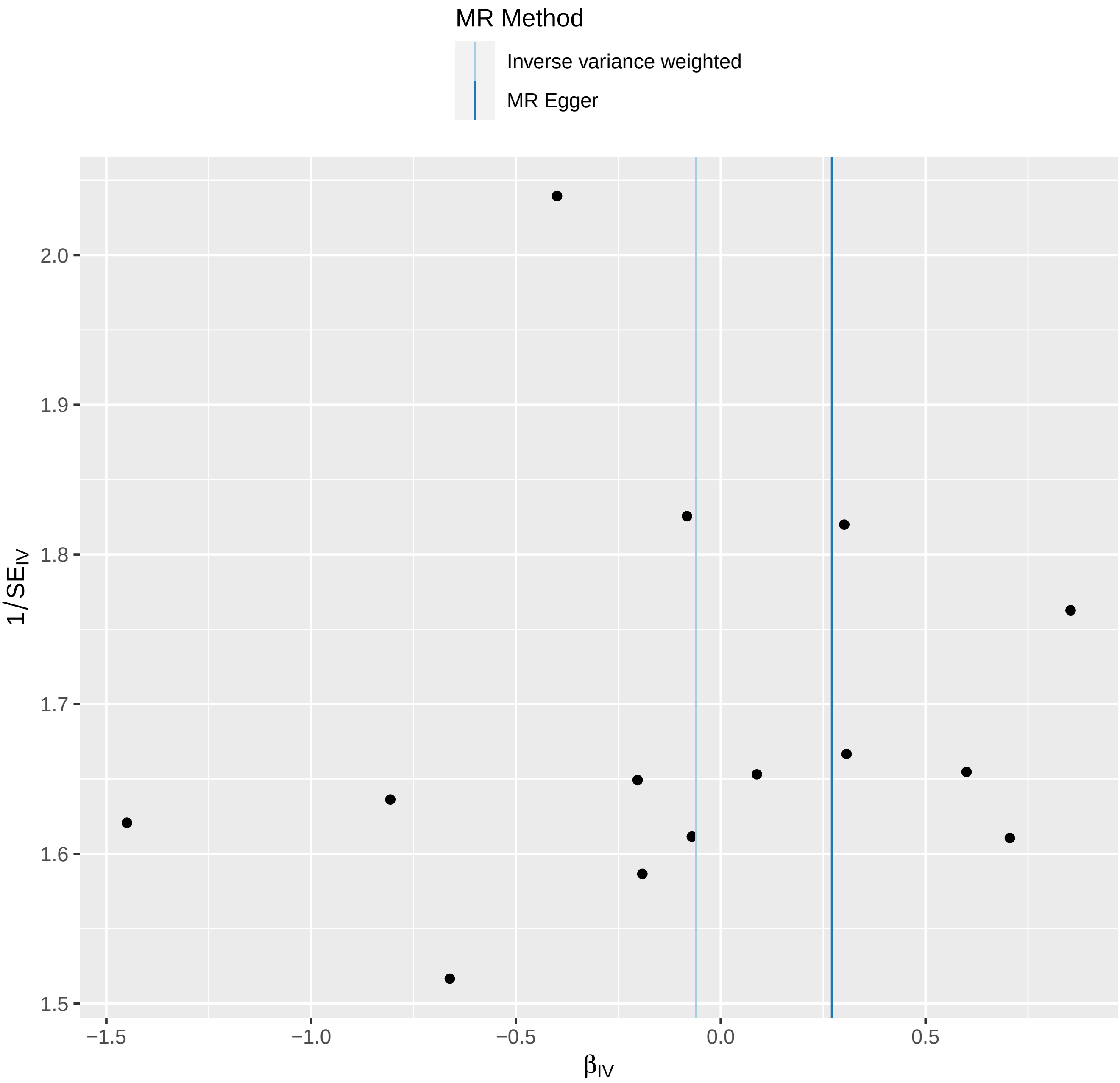

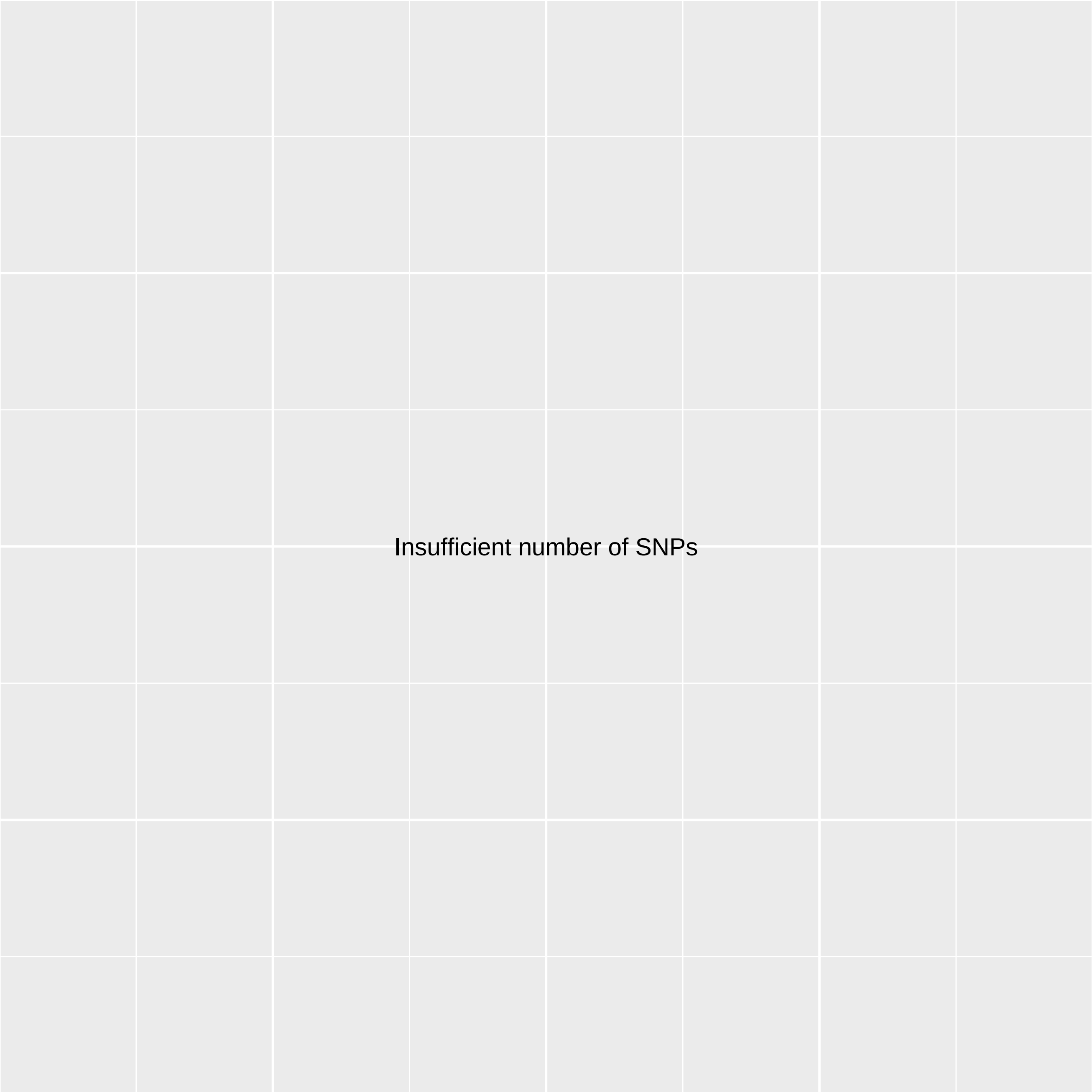
Insufficient number of SNPs

Batch 1119 : Gut microbiota abundance (genus Escherichia Shigella id.3504) on Ulcerative ileocolitis

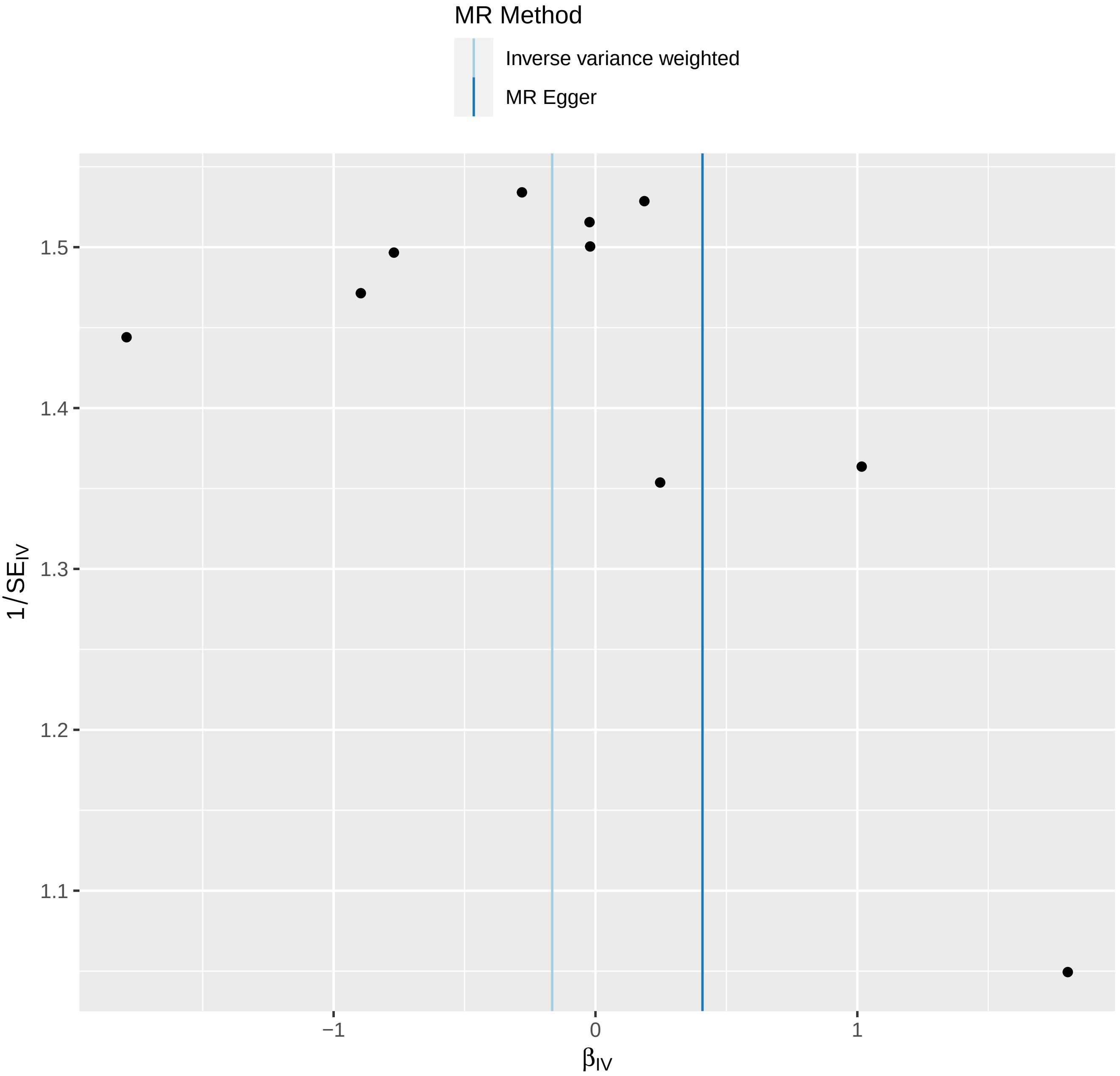

Batch 1120 : Gut microbiota abundance (genus Eubacterium brachy group id.11296) on Ulcerative ileocolitis

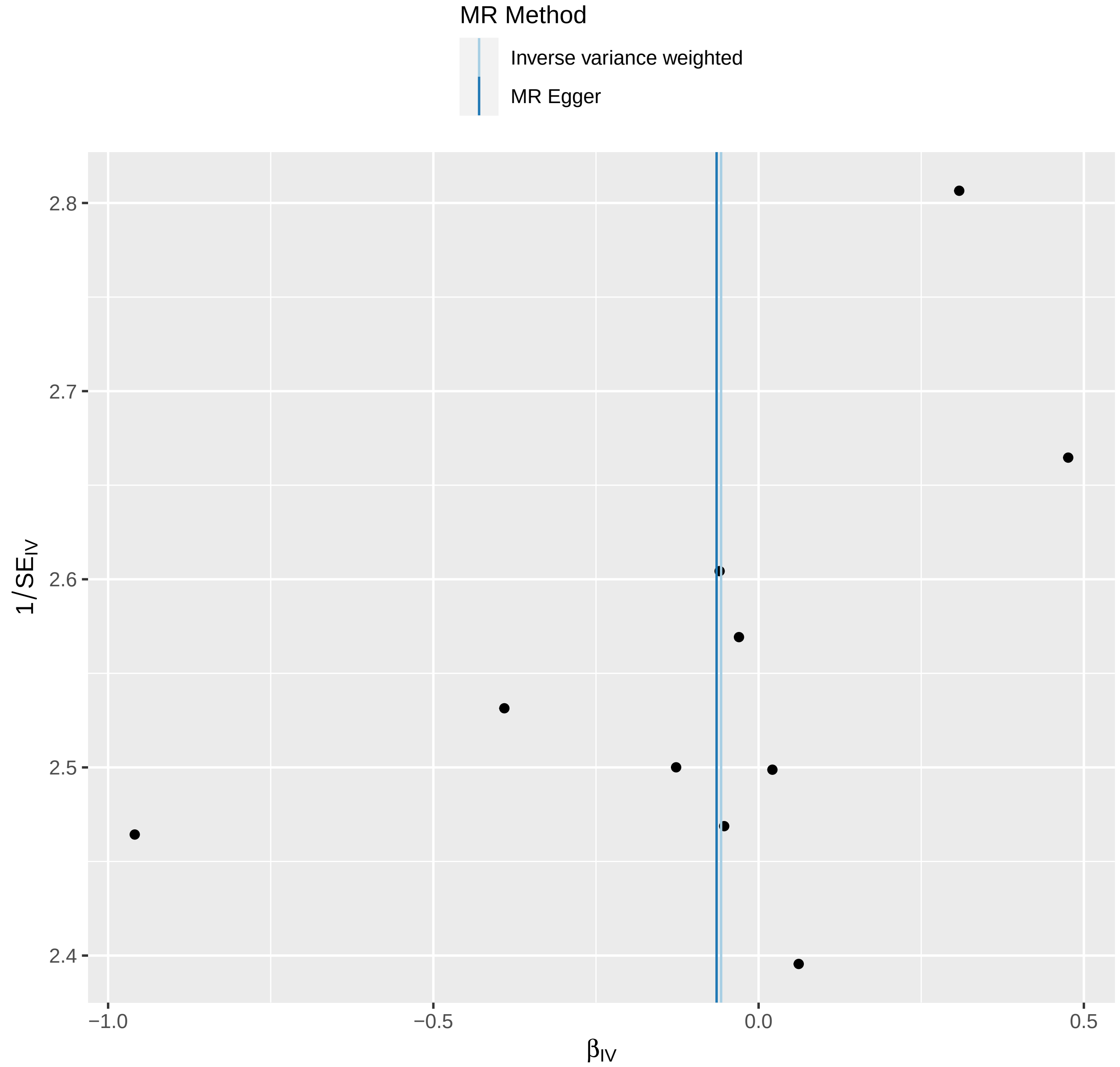

Batch 1121 : Gut microbiota abundance (genus Eubacterium coprostanoligenes group id.11375) on Ulcerative ileocolitis

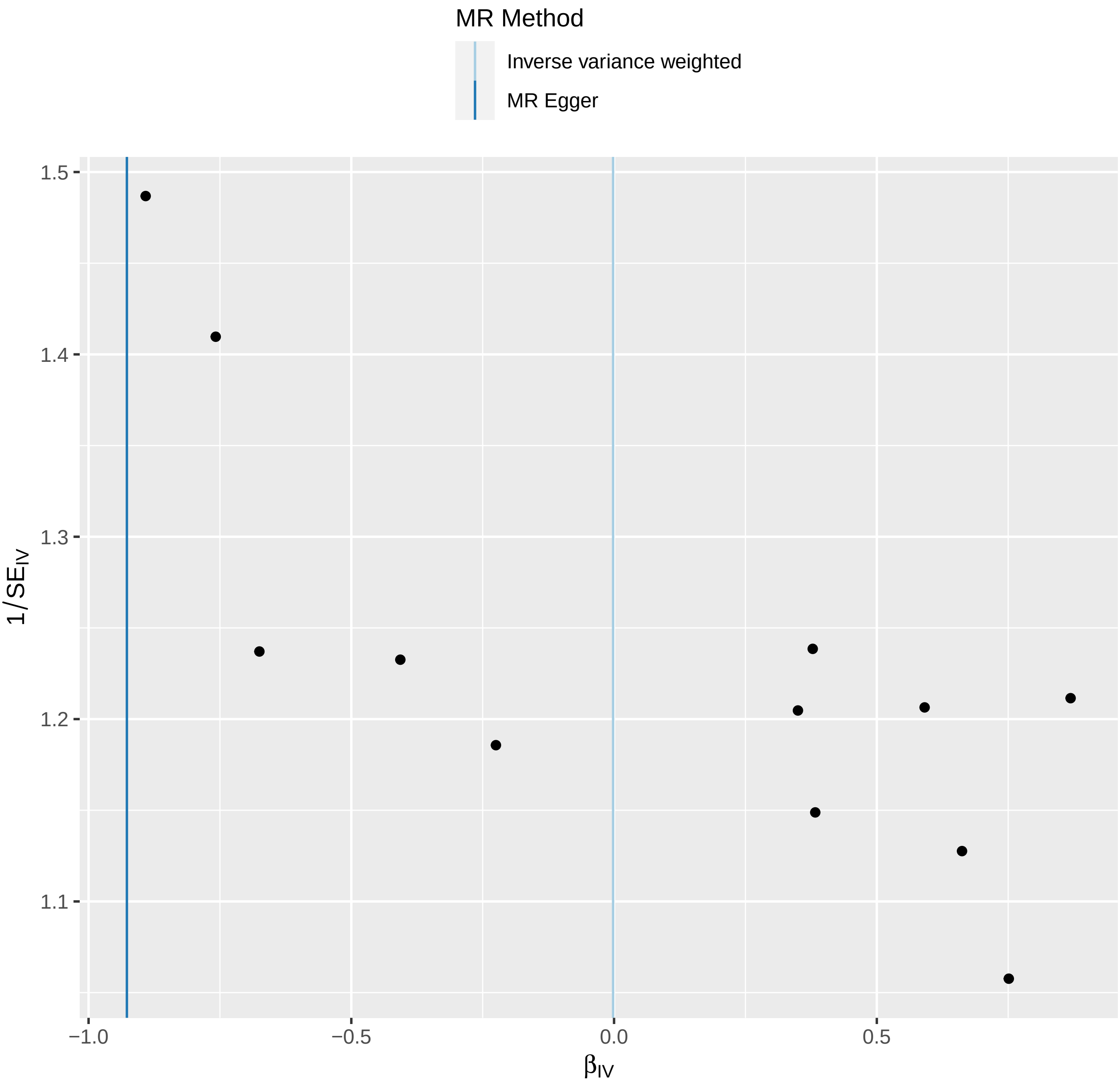

Batch 1122 : Gut microbiota abundance (genus Eubacterium eligens group id.14372) on Ulcerative ileocolitis

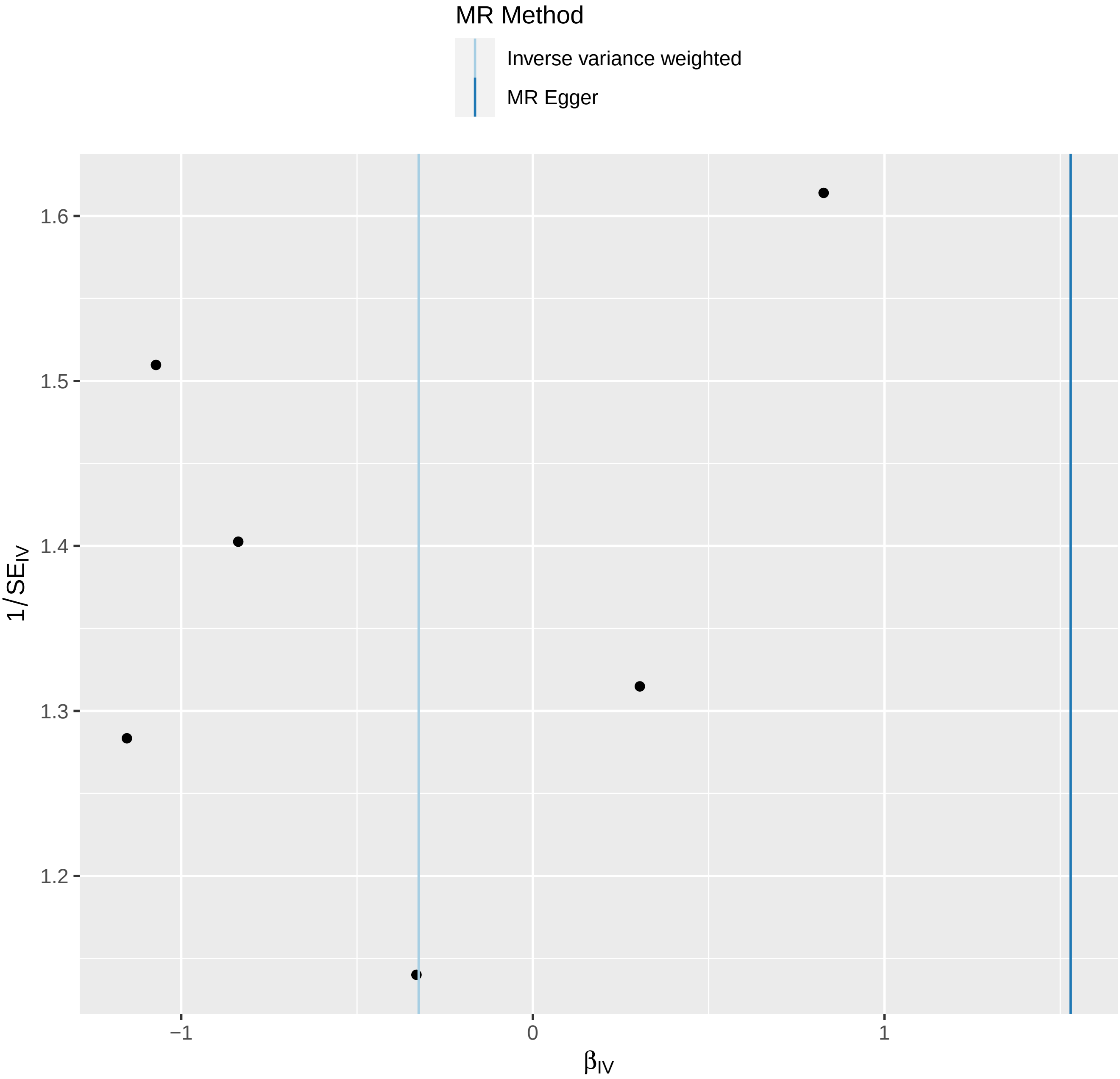

Batch 1123 : Gut microbiota abundance (genus Eubacterium fissicatena group id.14373) on Ulcerative ileocolitis

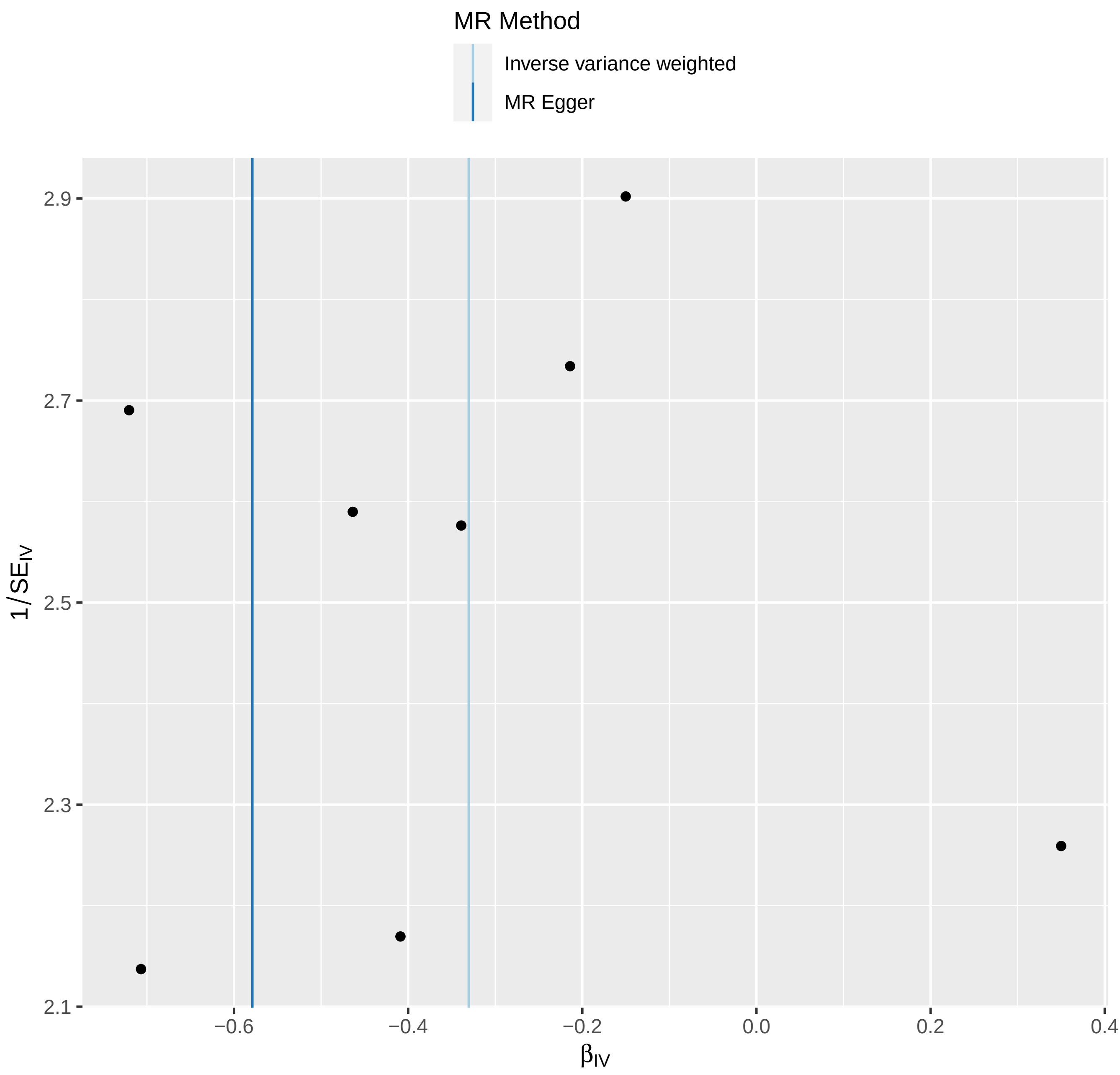

Batch 1124 : Gut microbiota abundance (genus Eubacterium hallii group id.11338) on Ulcerative ileocolitis

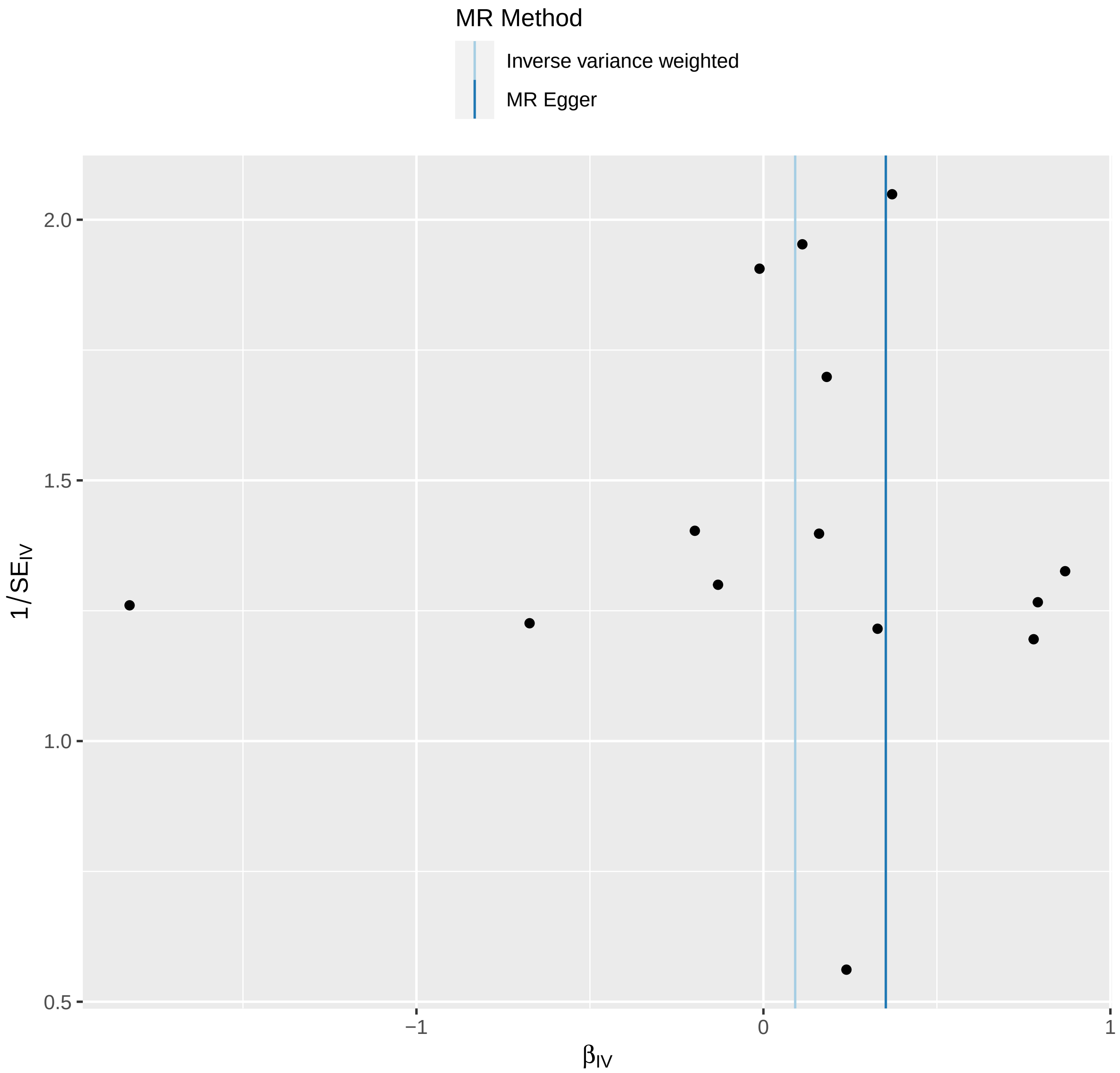

Batch 1125 : Gut microbiota abundance (genus Eubacterium nodatum group id.11297) on Ulcerative ileocolitis

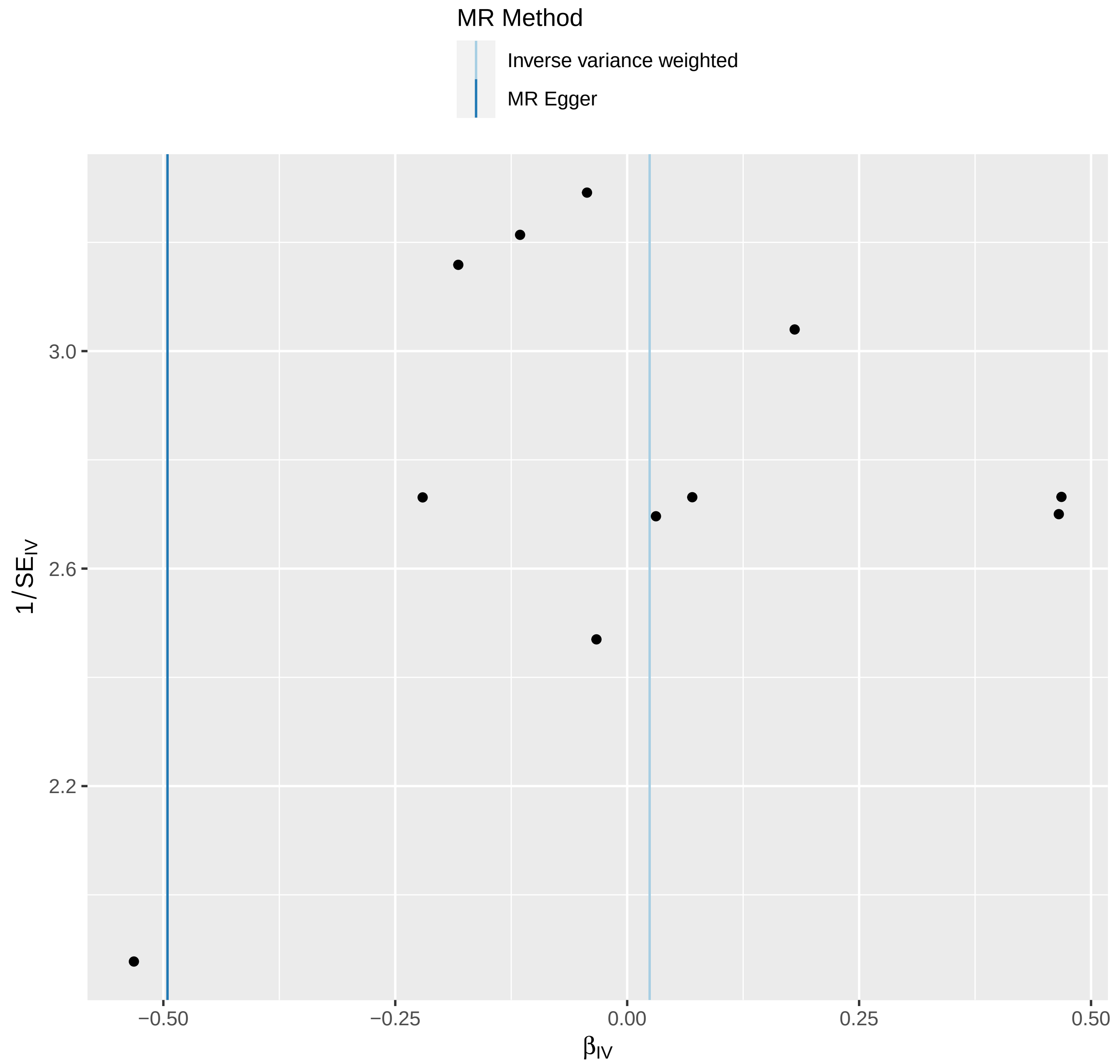

Batch 1126 : Gut microbiota abundance (genus Eubacterium oxidoreducens group id.11339) on Ulcerative ileocolitis

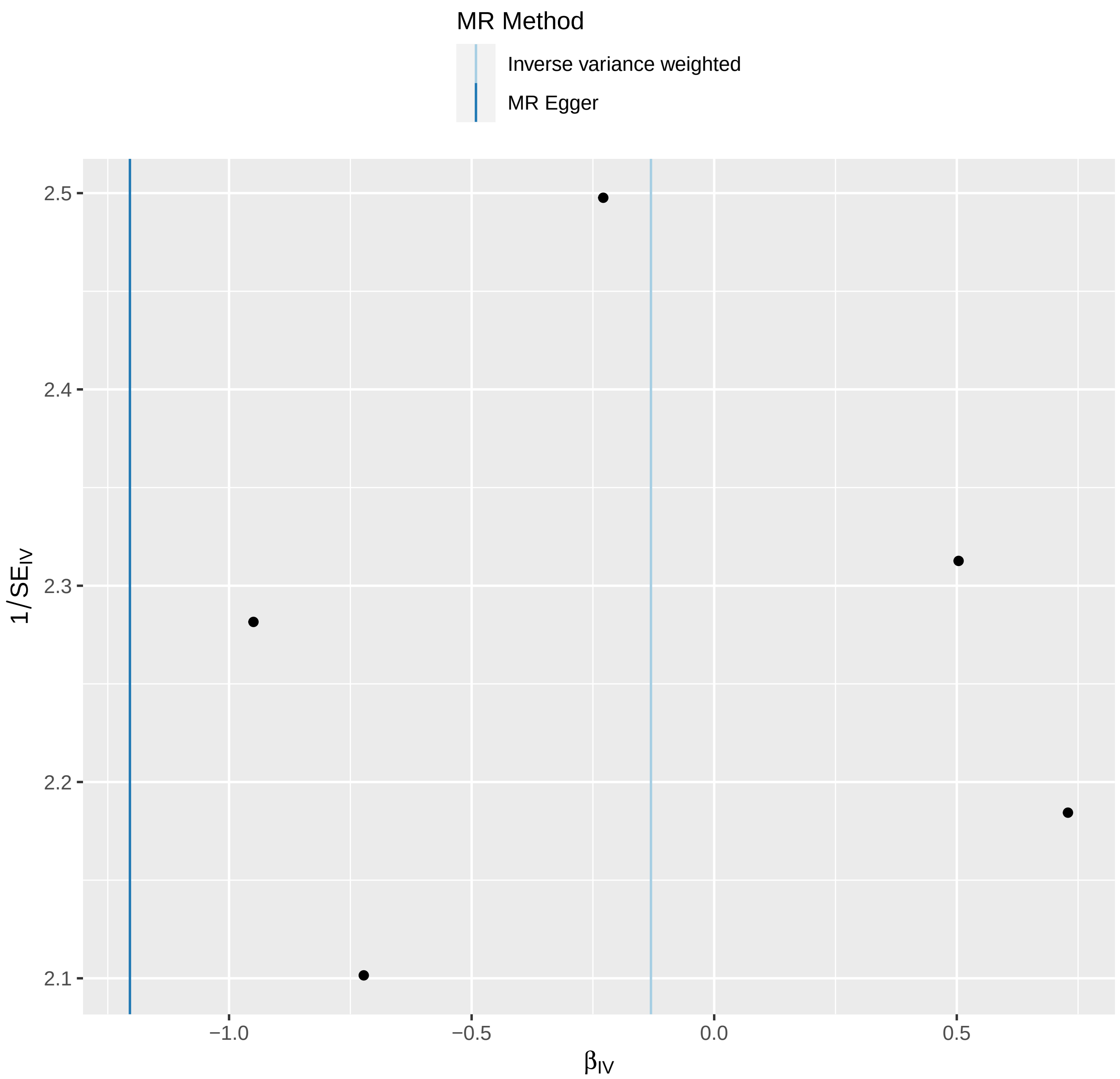

Batch 1127 : Gut microbiota abundance (genus Eubacterium rectale group id.14374) on Ulcerative ileocolitis

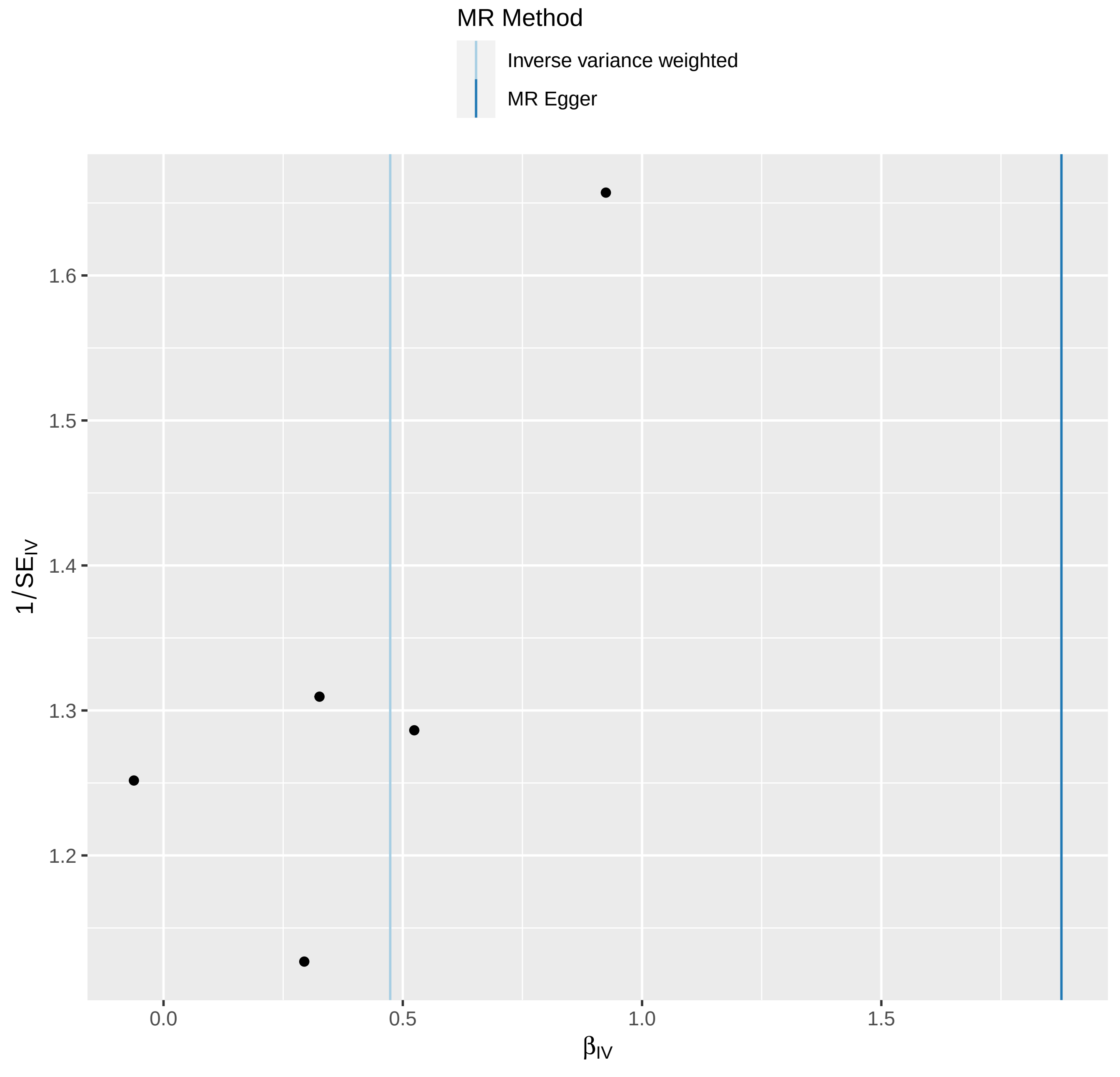

Batch 1128 : Gut microbiota abundance (genus Eubacterium ruminantium group id.11340) on Ulcerative ileocolitis

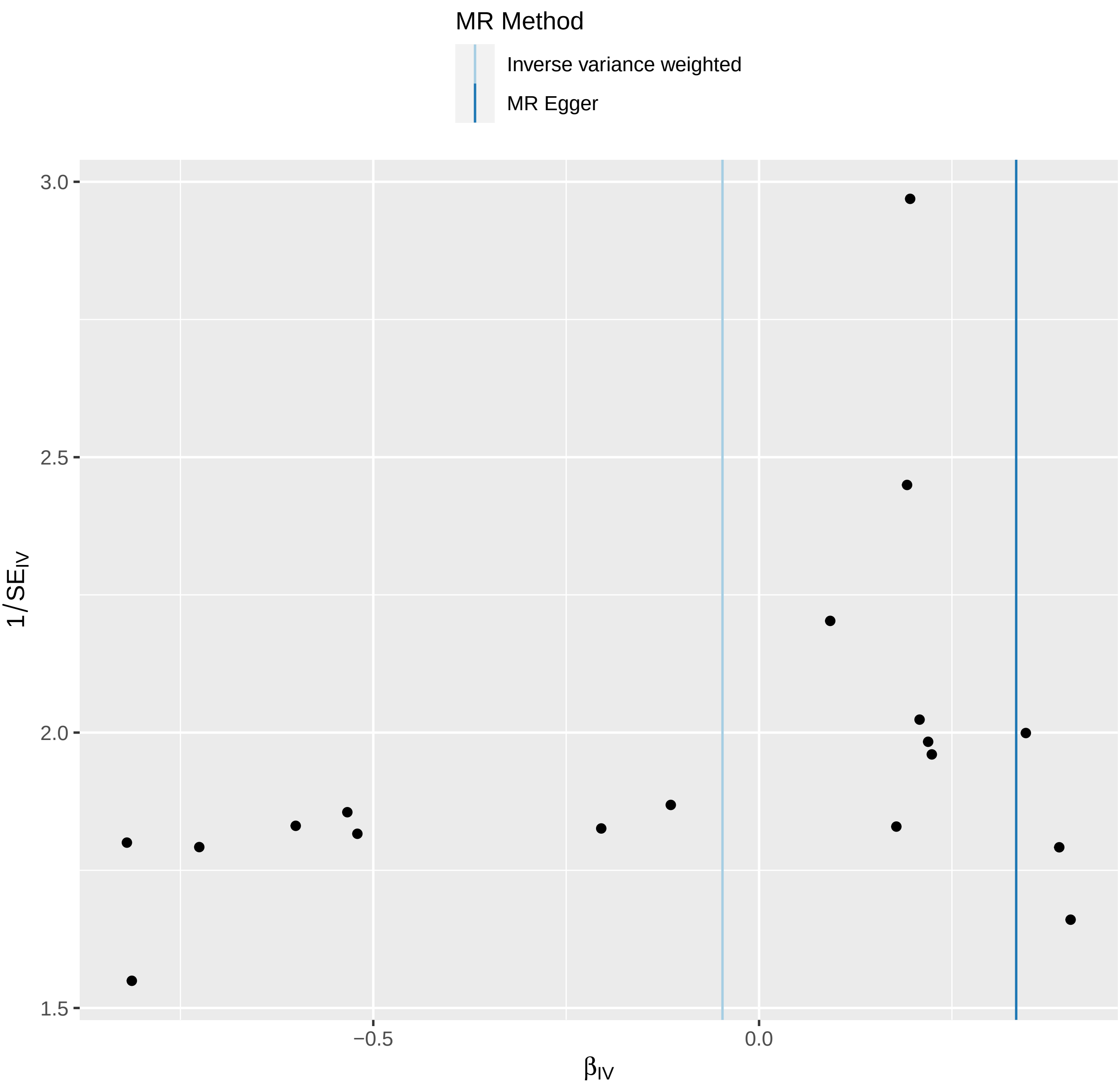

Batch 1129 : Gut microbiota abundance (genus Eubacterium ventriosum group id.11341) on Ulcerative ileocolitis

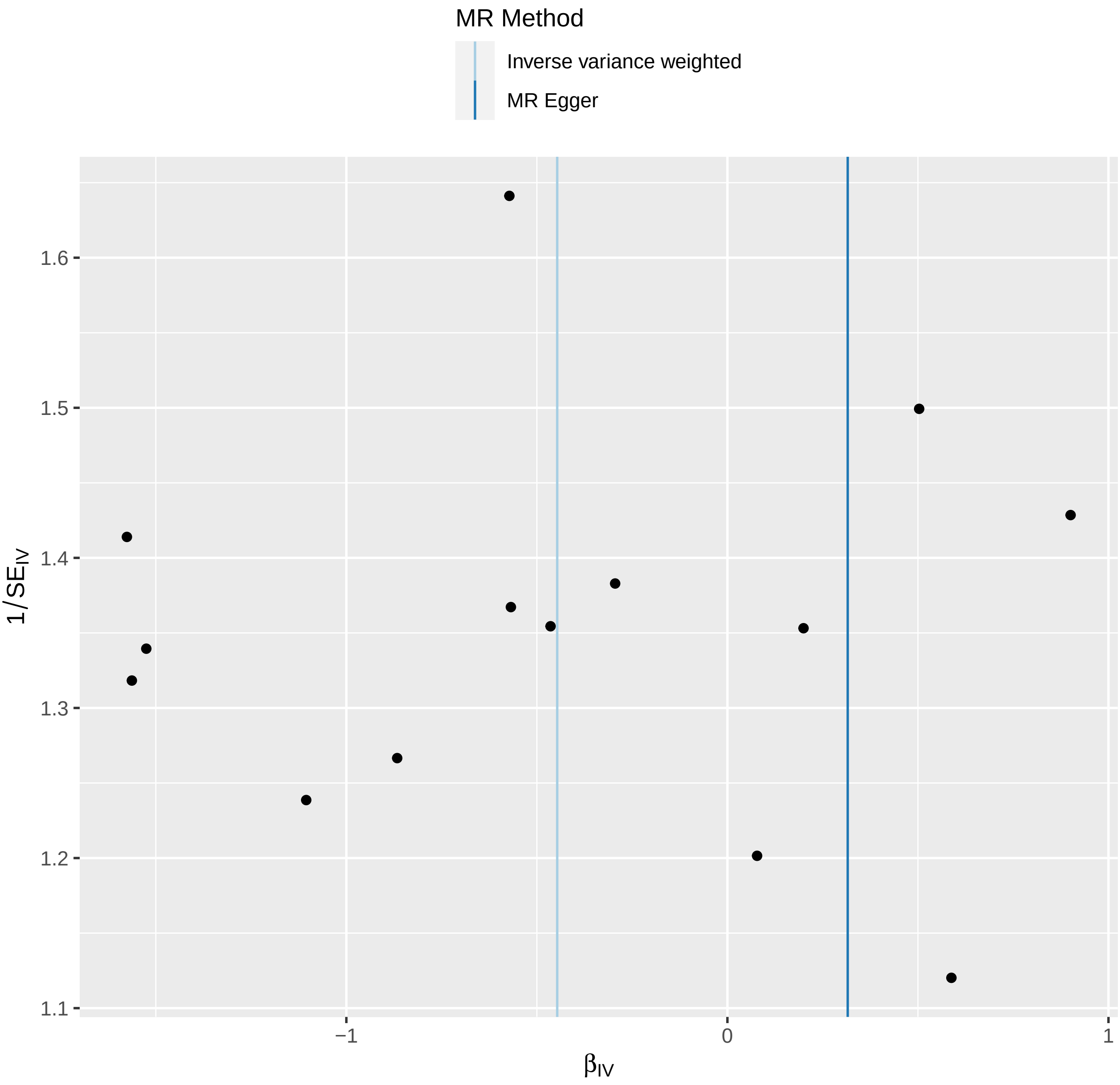

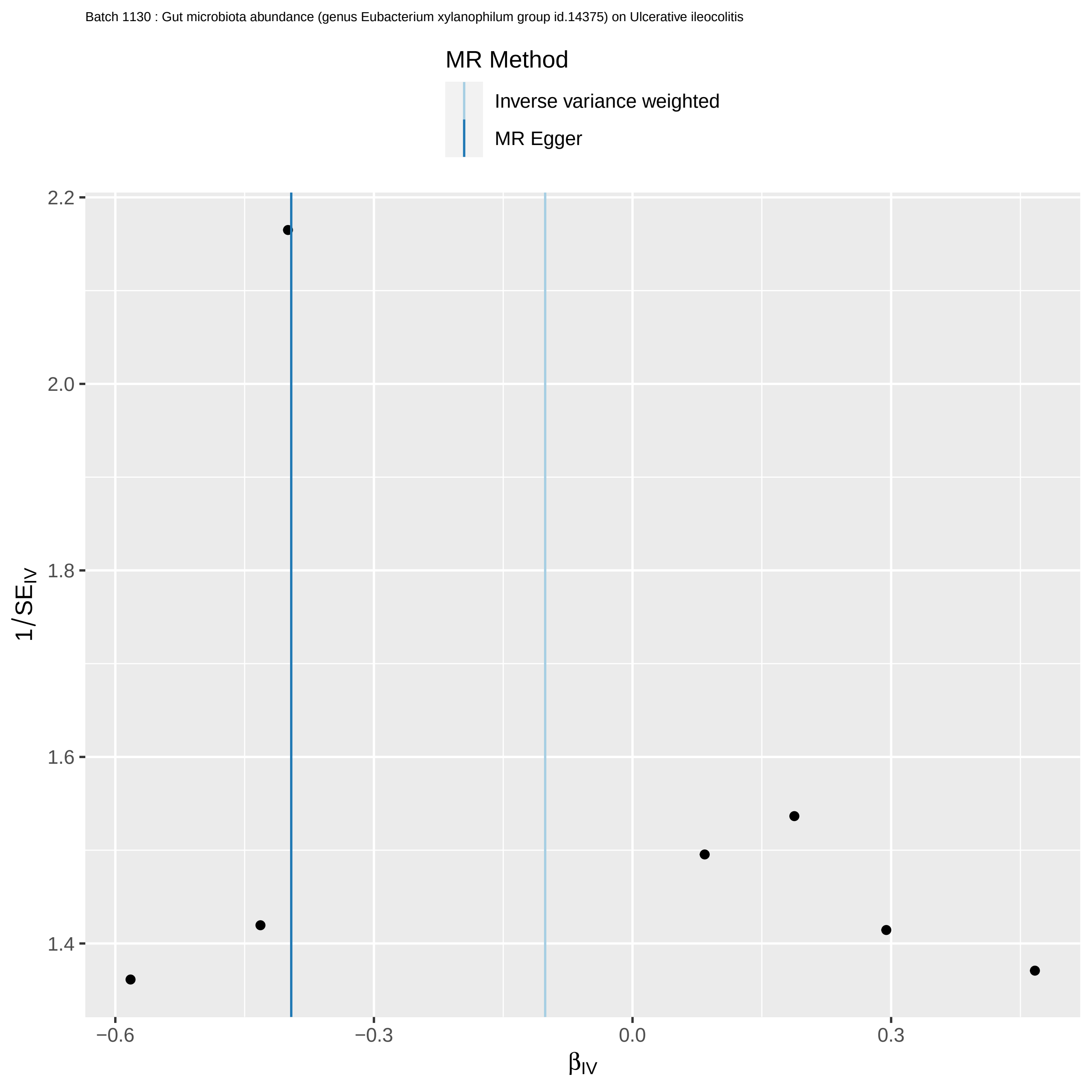
Batch 1130 : Gut microbiota abundance (genus Eubacterium xylanophilum group id.14375) on Ulcerative ileocolitis
MR Method
Inverse variance weighted
MR Egger
2.2
2.0
1.8
1.6
1.4
1/SE_IV
−0.6
−0.3
0.0
0.3
β_IV

Batch 1131 : Gut microbiota abundance (genus Faecalibacterium id.2057) on Ulcerative ileocolitis

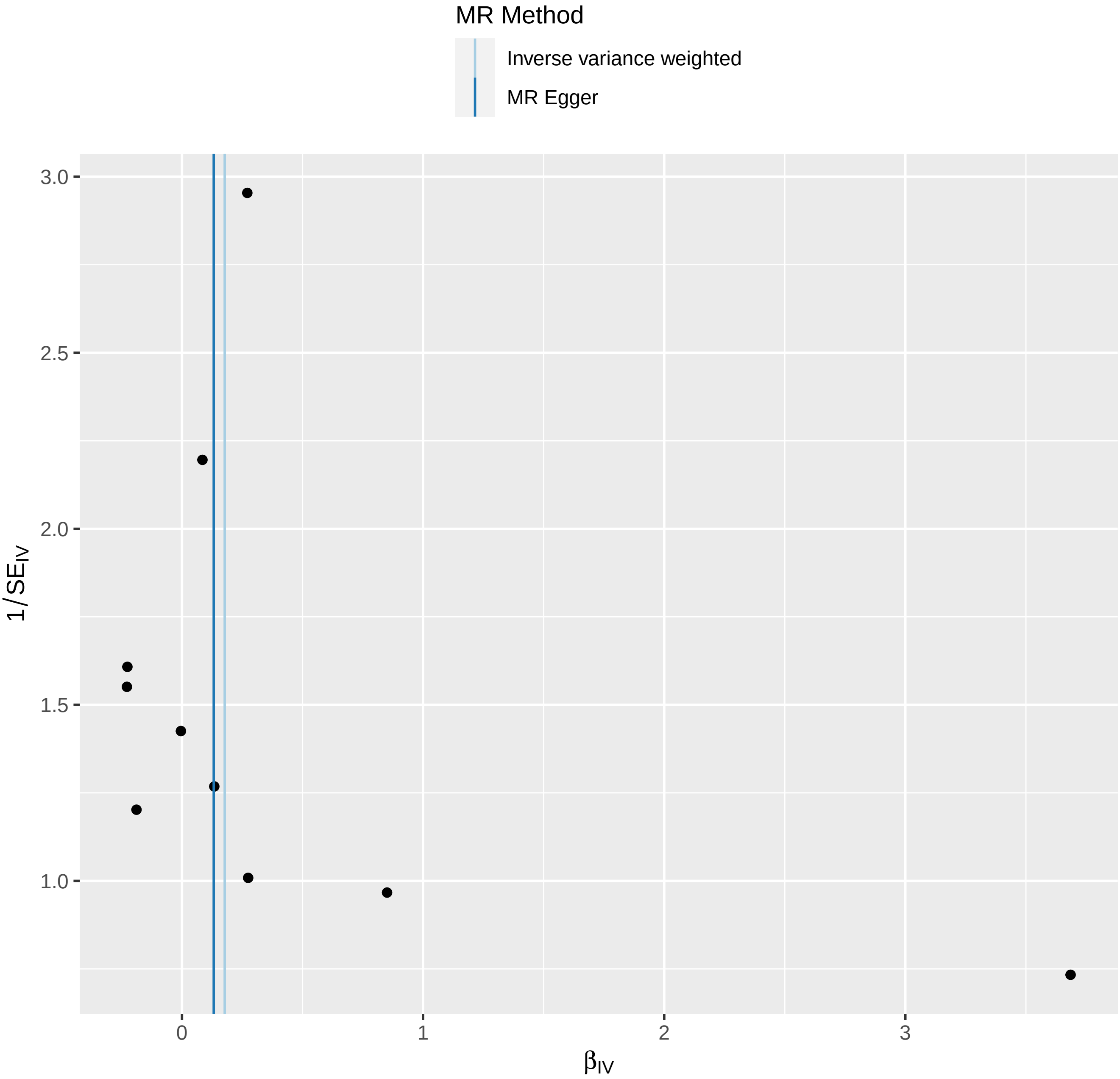

Batch 1132 : Gut microbiota abundance (genus Family XIII AD3011 group id.11293) on Ulcerative ileocolitis

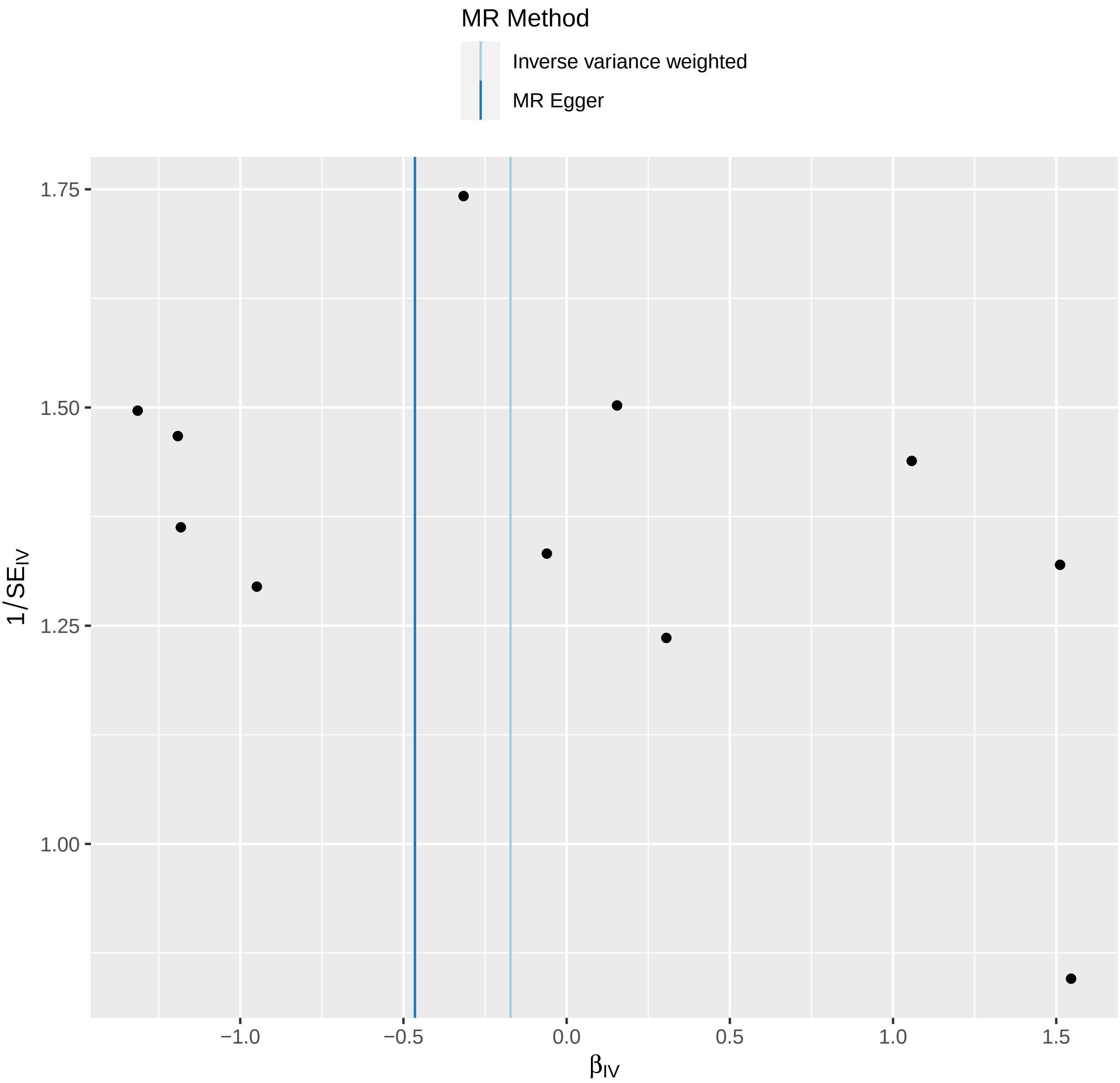

Batch 1133 : Gut microbiota abundance (genus Family XIII UCG001 id.11294) on Ulcerative ileocolitis

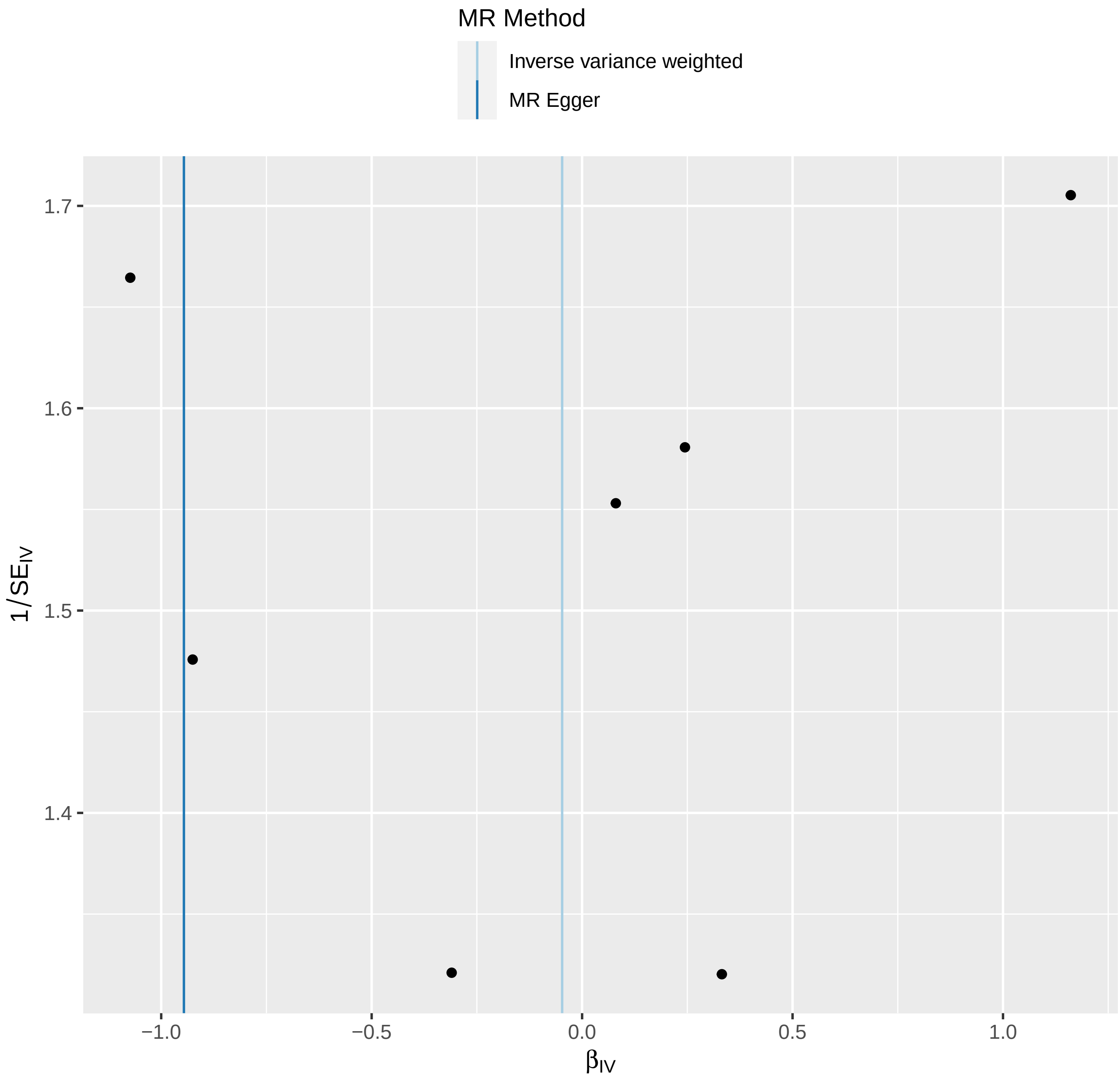

Batch 1134 : Gut microbiota abundance (genus Flavonifractor id.2059) on Ulcerative ileocolitis

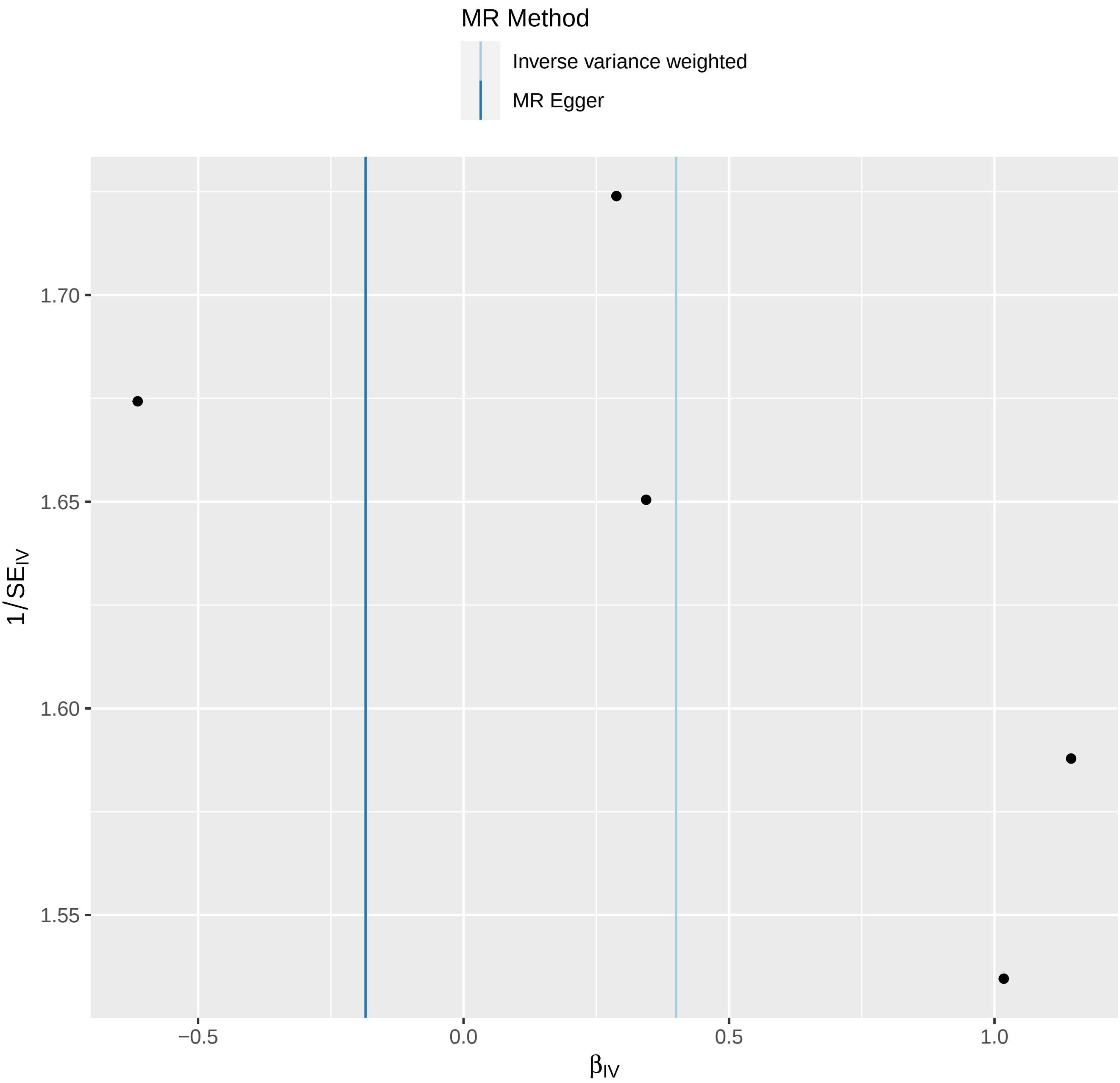

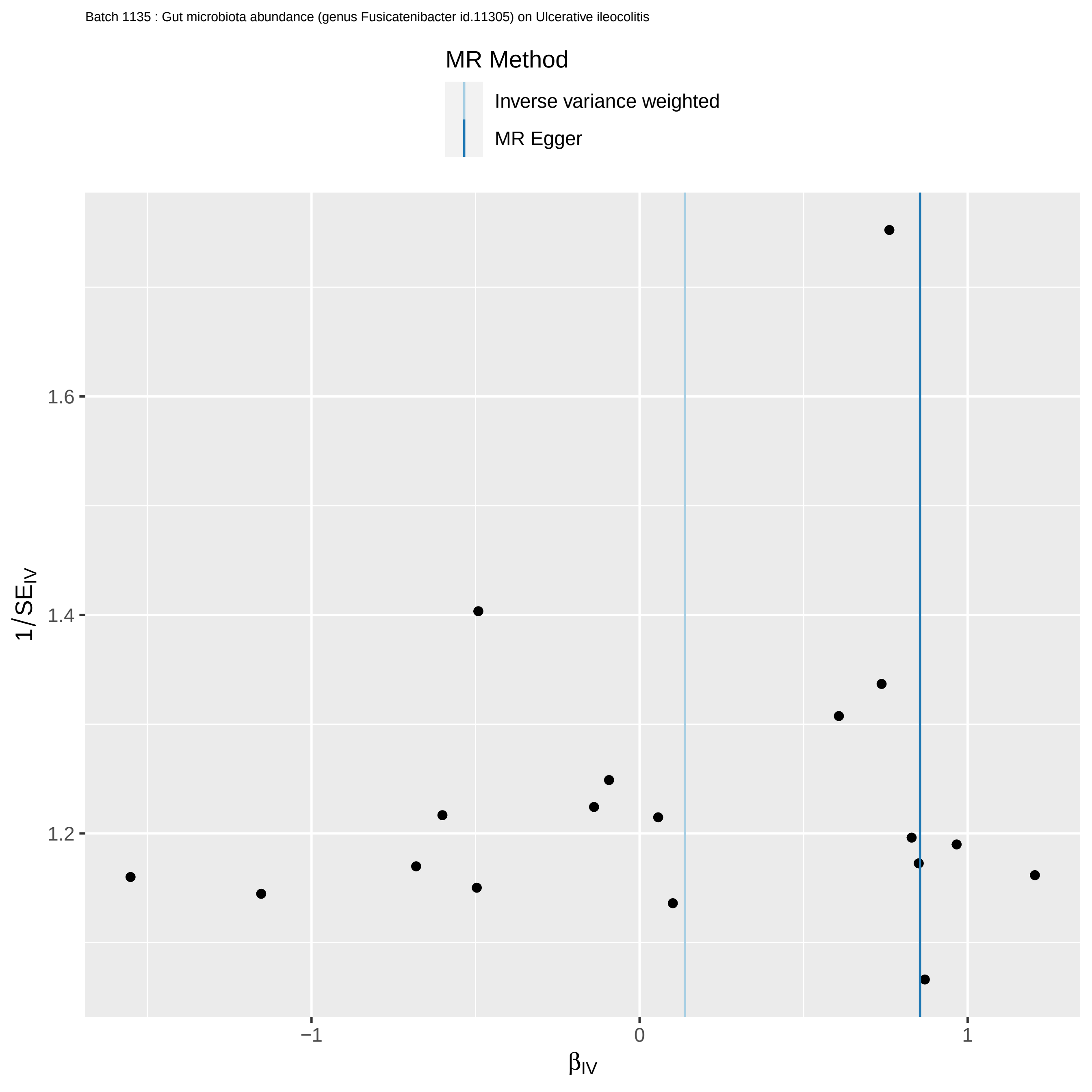
Batch 1135 : Gut microbiota abundance (genus Fusicatenibacter id.11305) on Ulcerative ileocolitis
MR Method
Inverse variance weighted
MR Egger
1.6
1.4
1.2
1/SE_IV
−1
0
1
β_IV

Batch 1136 : Gut microbiota abundance (genus Gordonibacter id.821) on Ulcerative ileocolitis

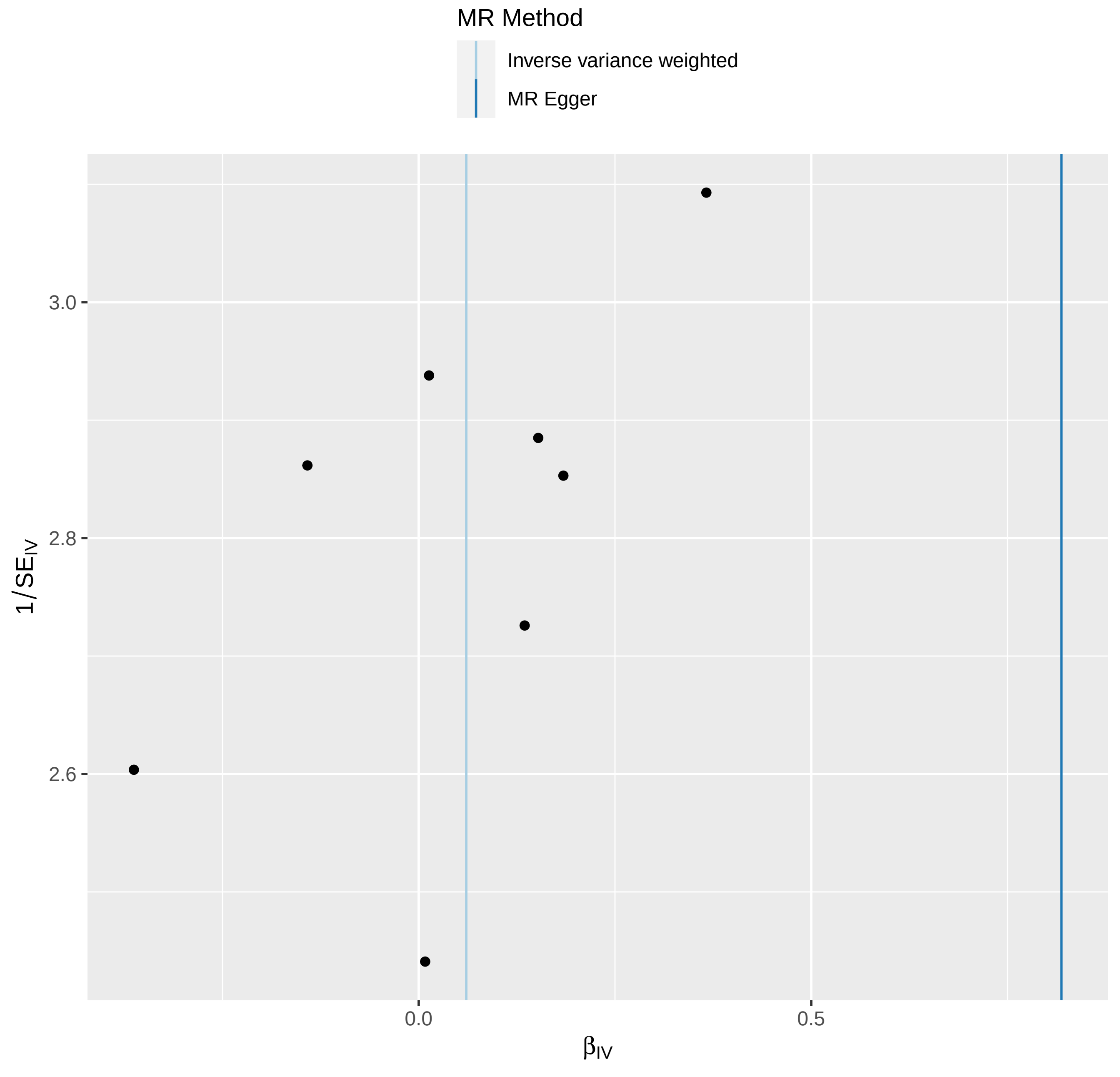

Batch 1137 : Gut microbiota abundance (genus Haemophilus id.3698) on Ulcerative ileocolitis

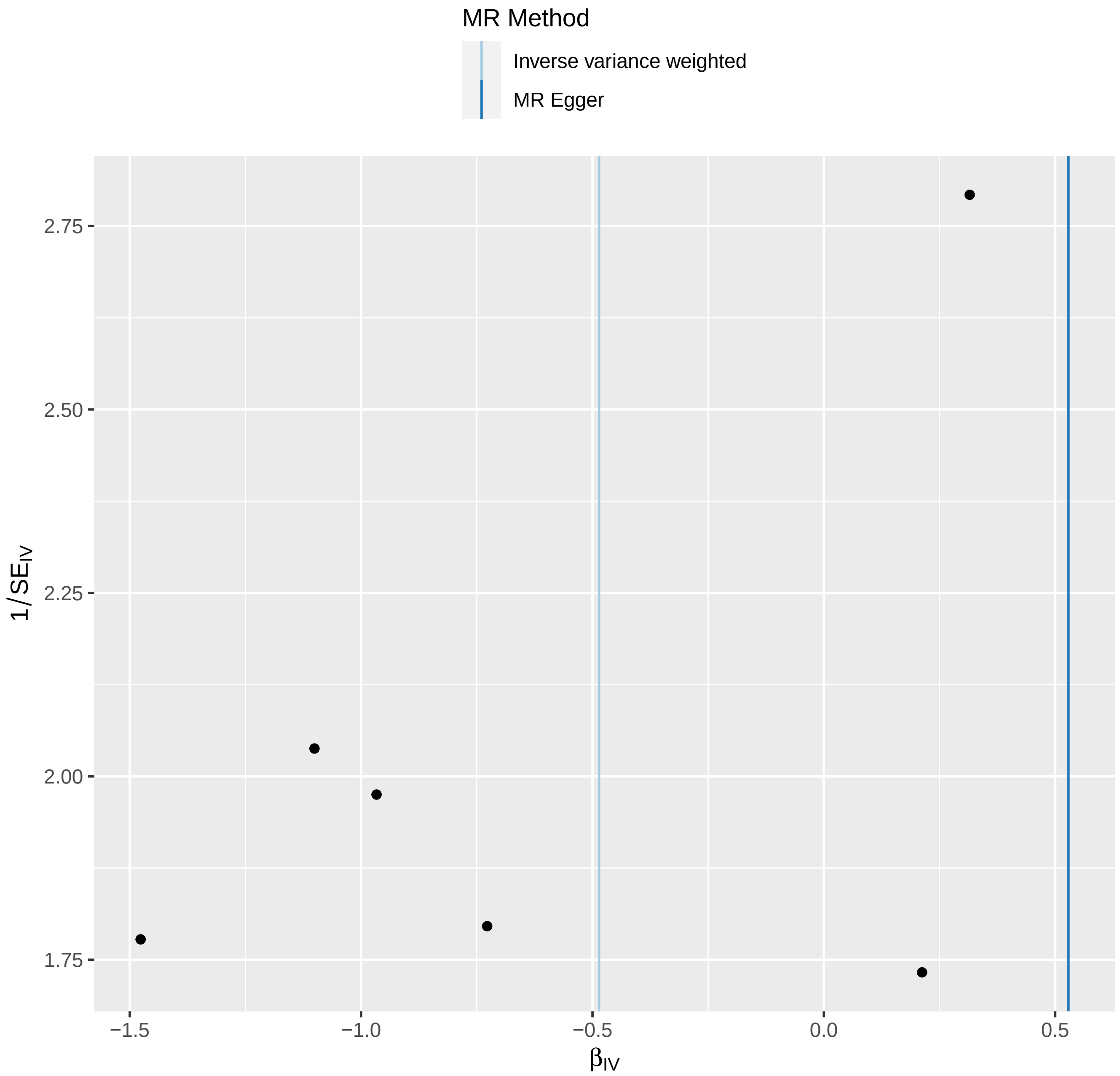

Batch 1138 : Gut microbiota abundance (genus Holdemanella id.11393) on Ulcerative ileocolitis

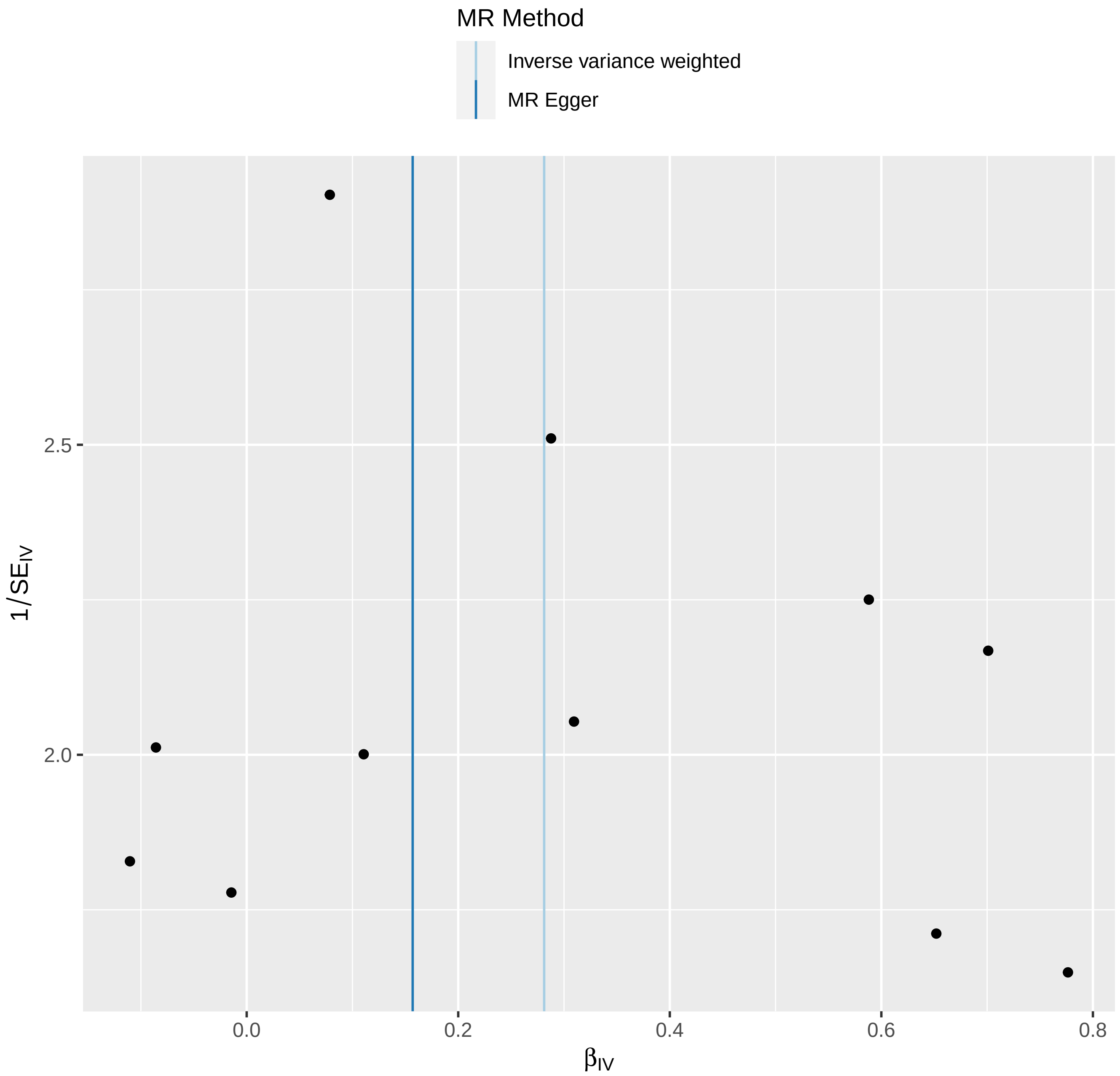

Batch 1139 : Gut microbiota abundance (genus Holdemania id.2157) on Ulcerative ileocolitis

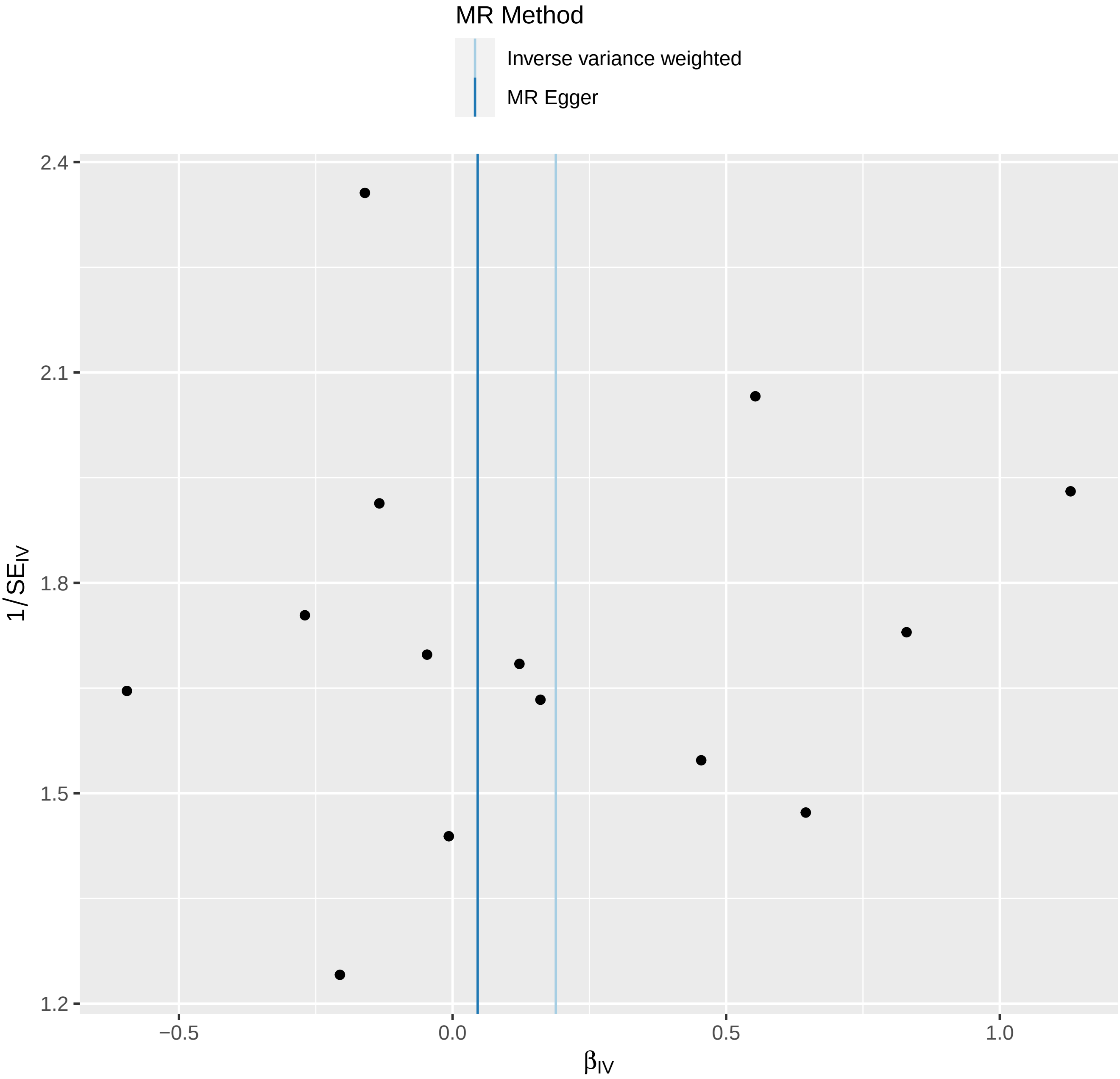

Batch 1140 : Gut microbiota abundance (genus Howardella id.2000) on Ulcerative ileocolitis

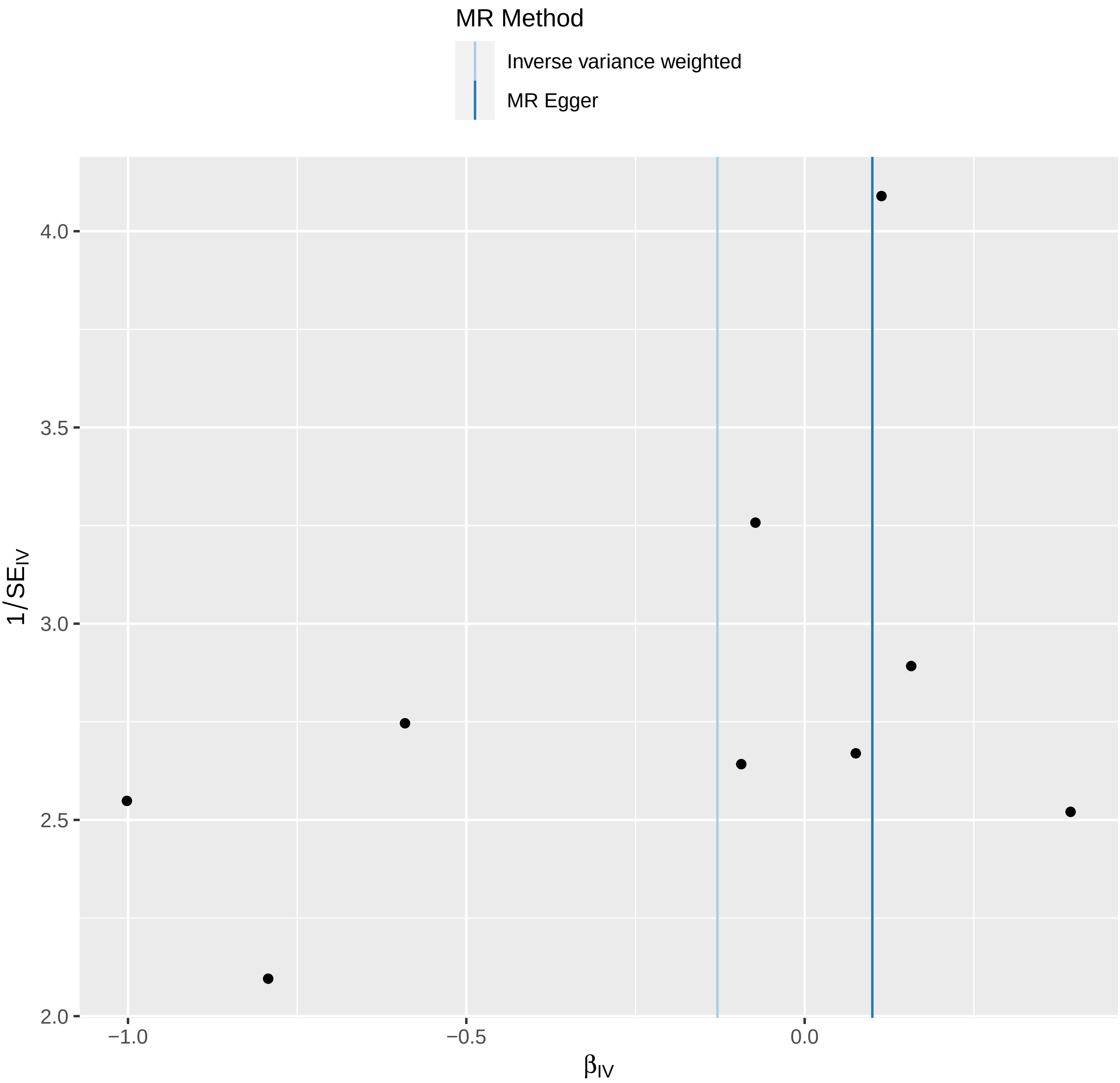

Batch 1141 : Gut microbiota abundance (genus Hungatella id.11306) on Ulcerative ileocolitis

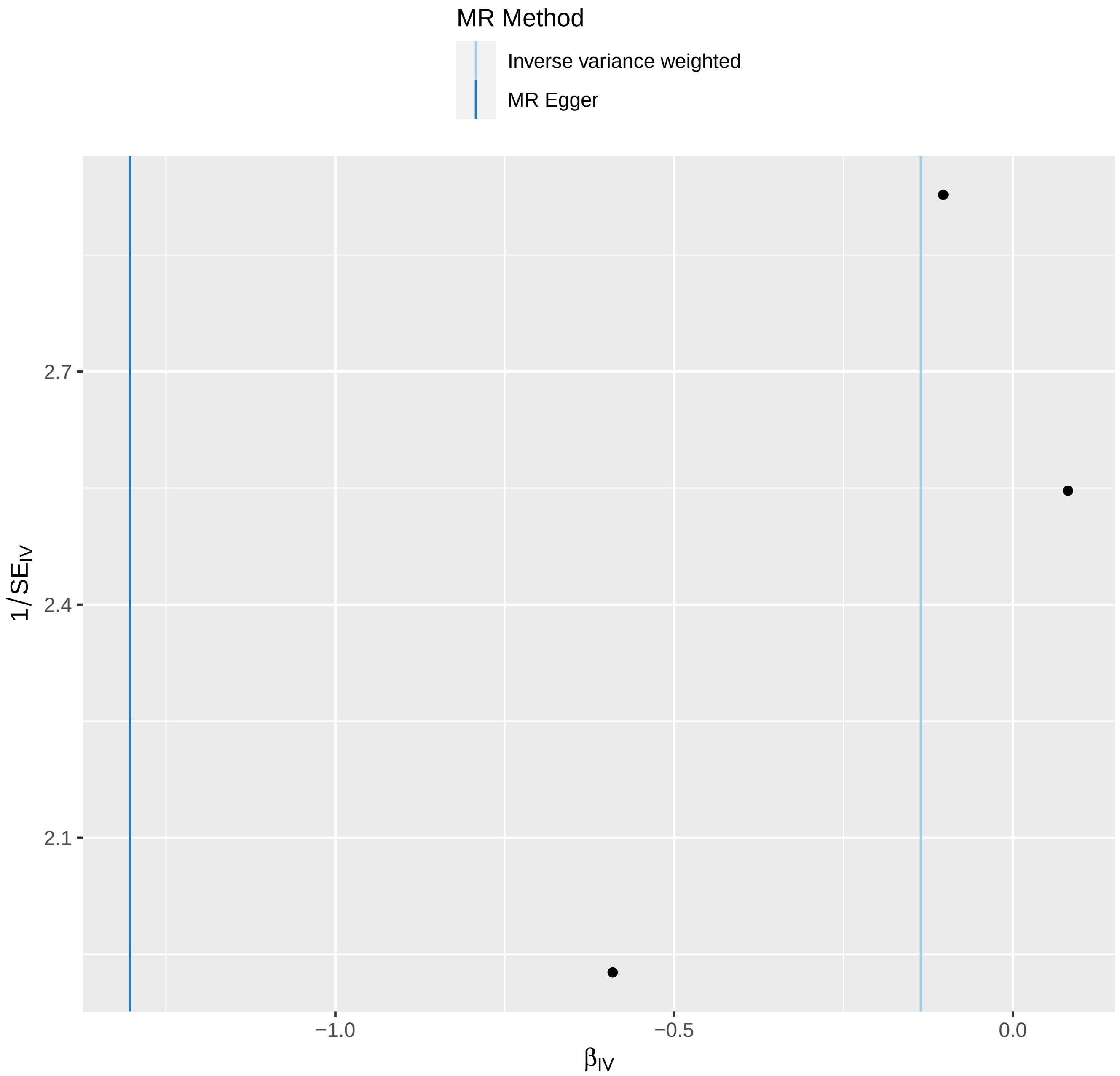

Batch 1142 : Gut microbiota abundance (genus Intestinibacter id.11345) on Ulcerative ileocolitis

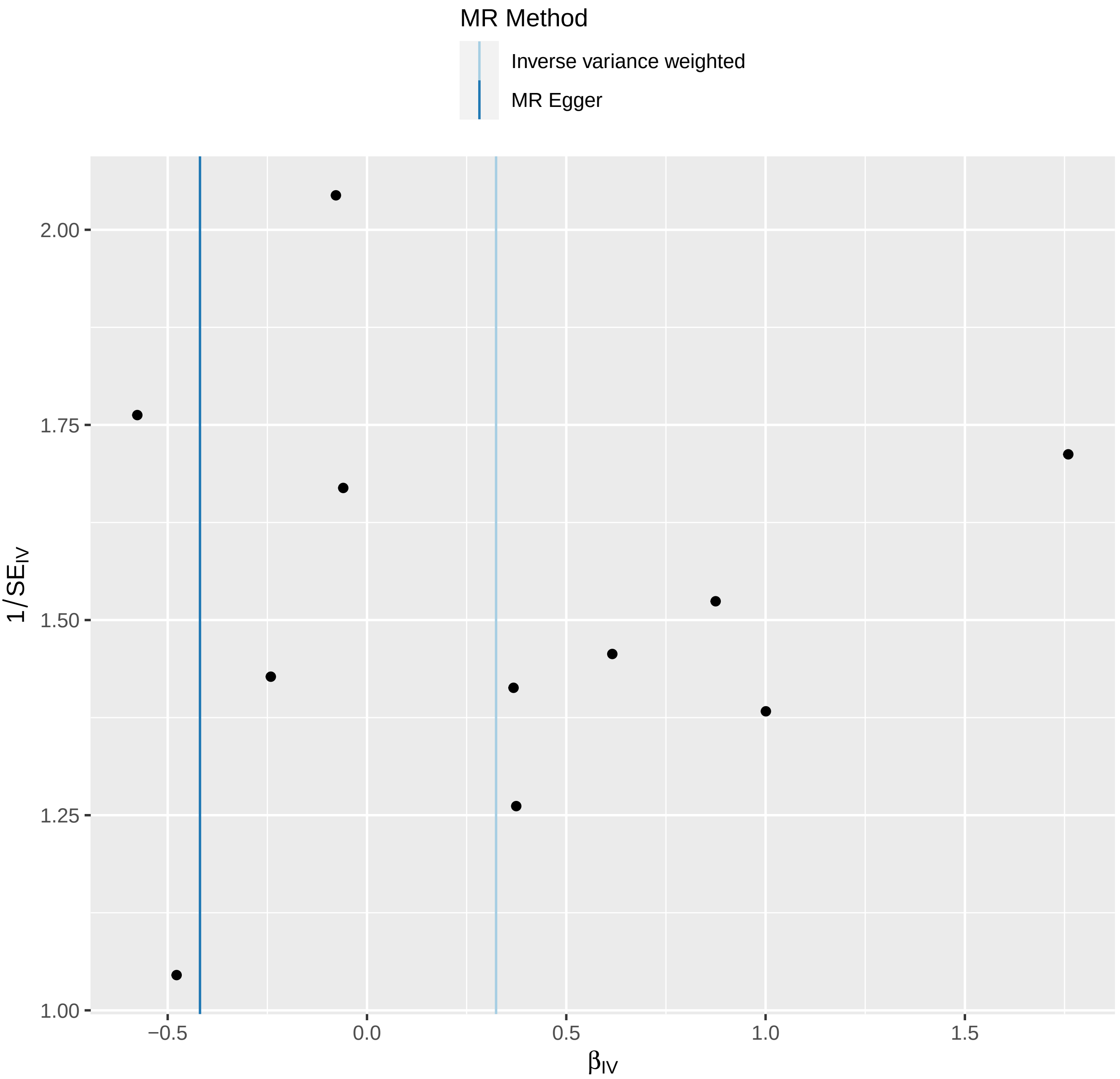

Batch 1143 : Gut microbiota abundance (genus Intestinimonas id.2062) on Ulcerative ileocolitis

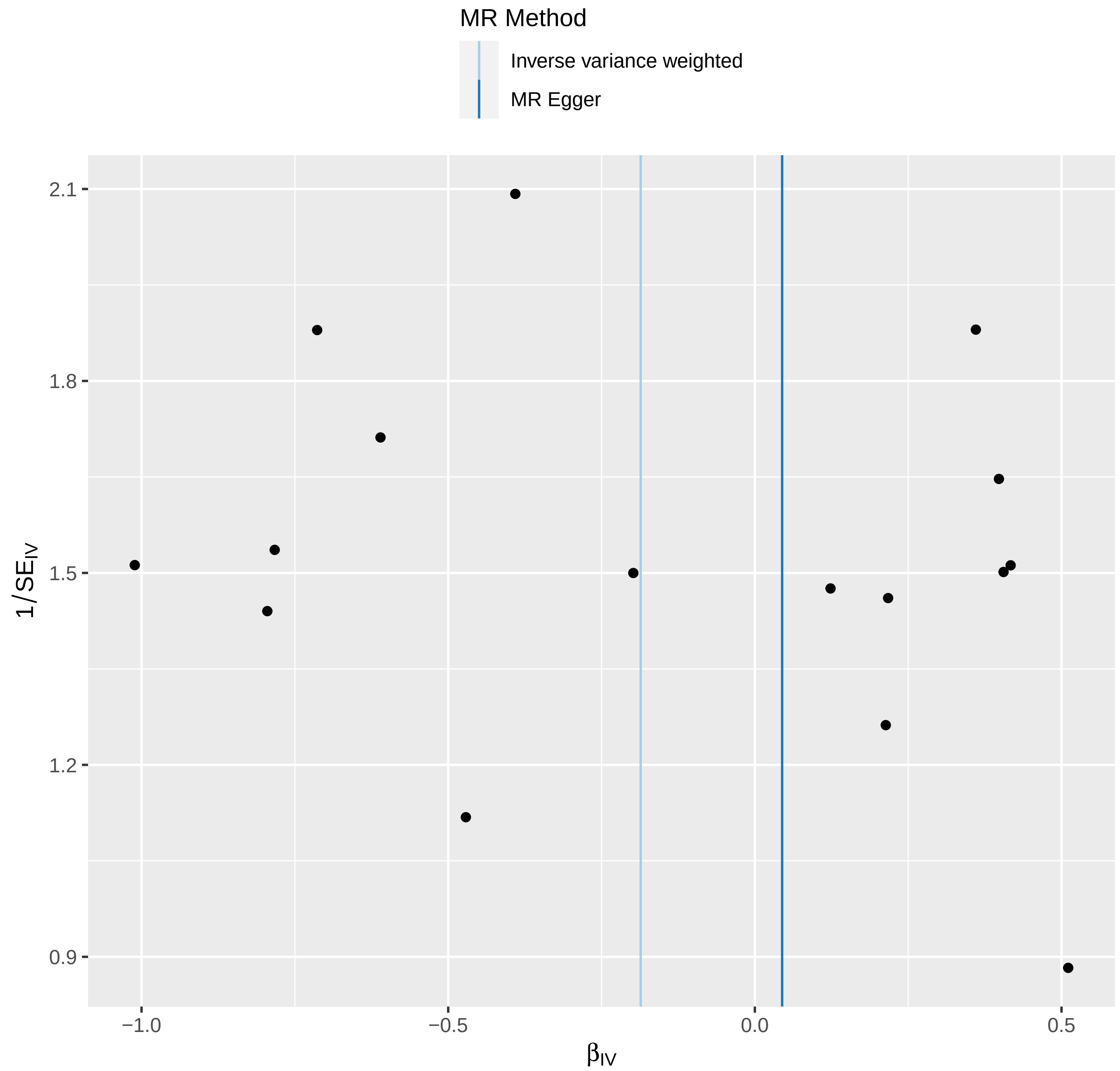

Batch 1144 : Gut microbiota abundance (genus Lachnoclostridium id.11308) on Ulcerative ileocolitis

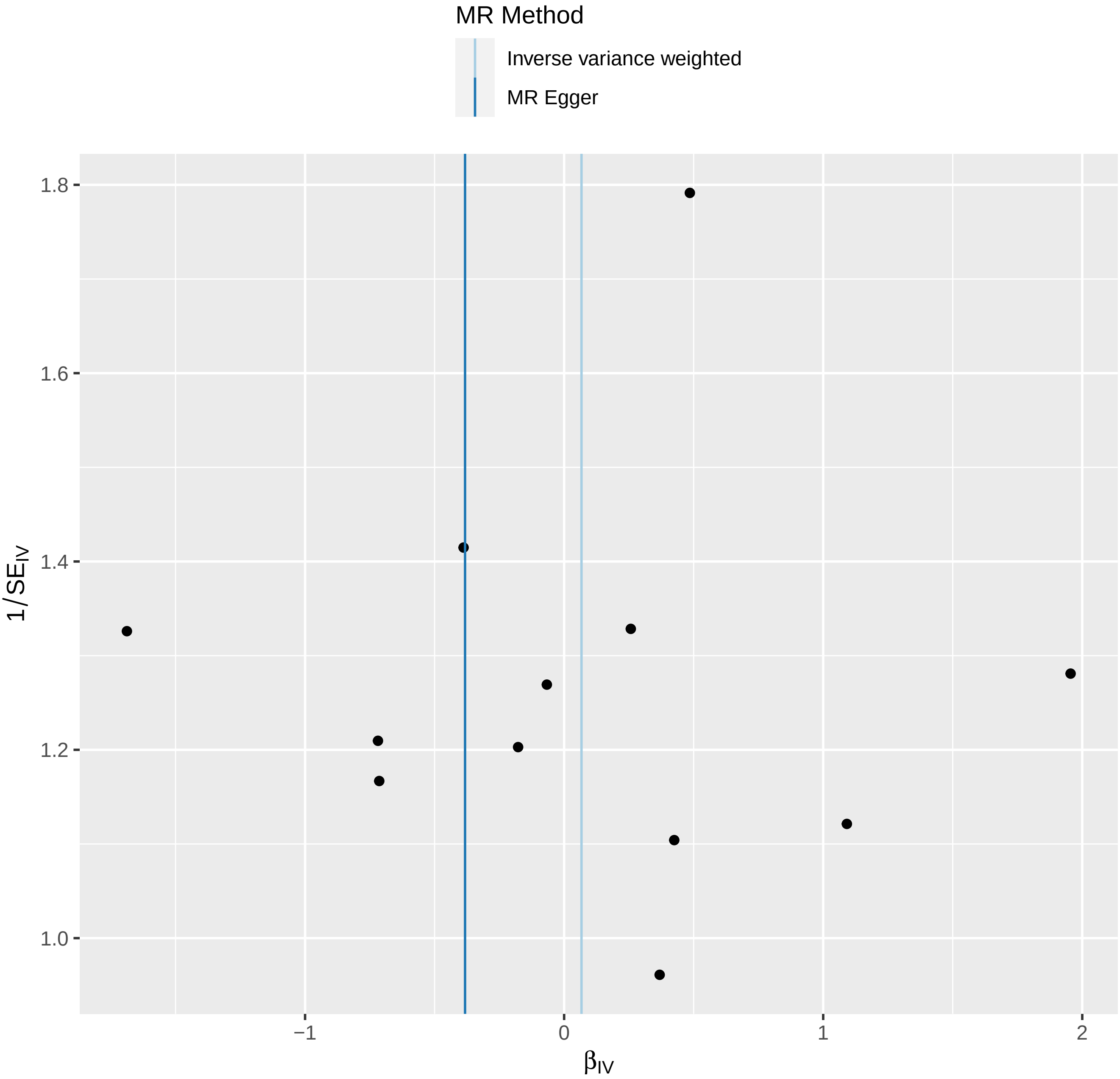

Batch 1145 : Gut microbiota abundance (genus Lachnospiraceae FCS020 group id.11314) on Ulcerative ileocolitis

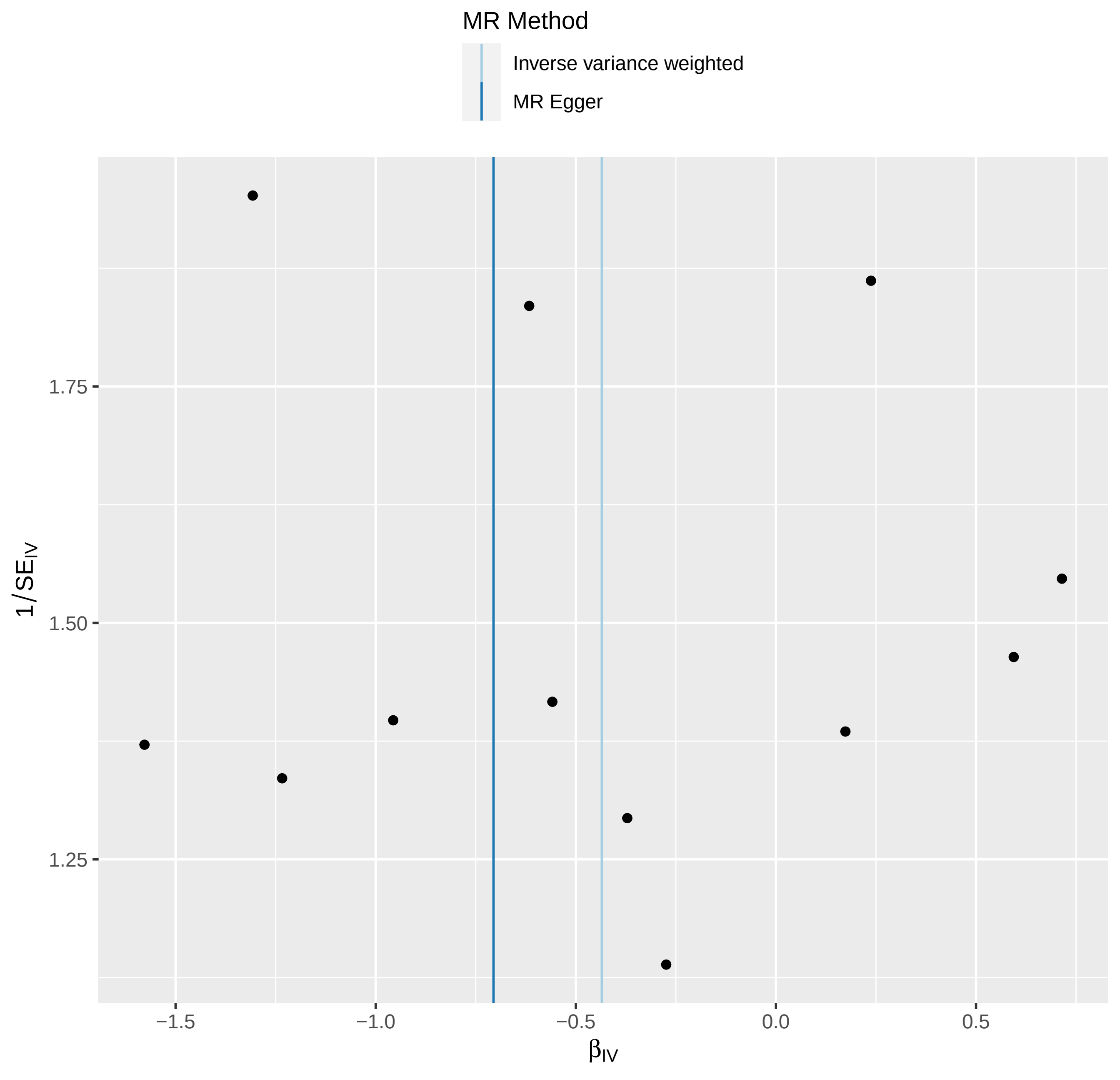

Batch 1146 : Gut microbiota abundance (genus Lachnospiraceae NC2004 group id.11316) on Ulcerative ileocolitis

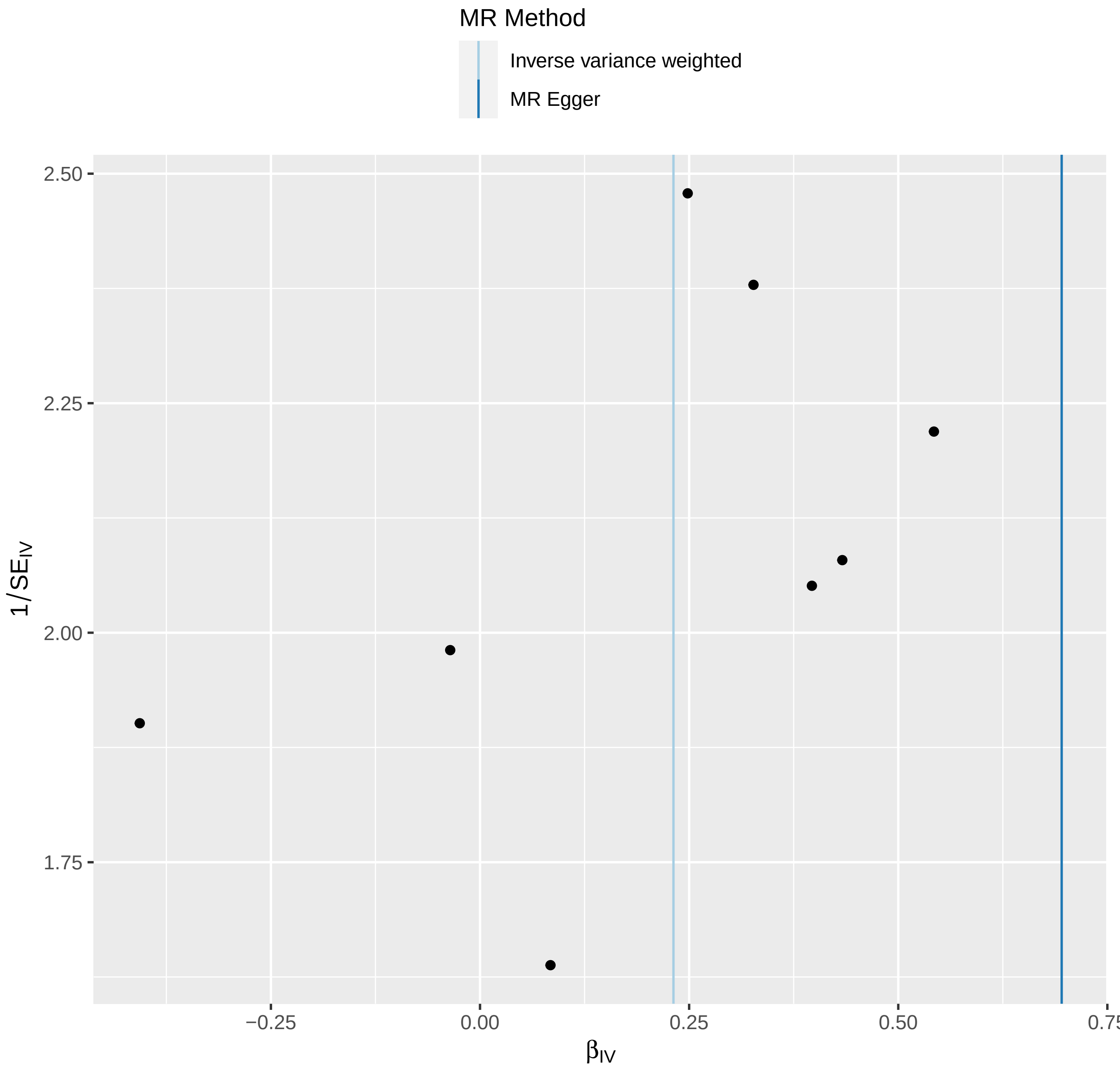

Batch 1147 : Gut microbiota abundance (genus Lachnospiraceae ND3007 group id.11317) on Ulcerative ileocolitis

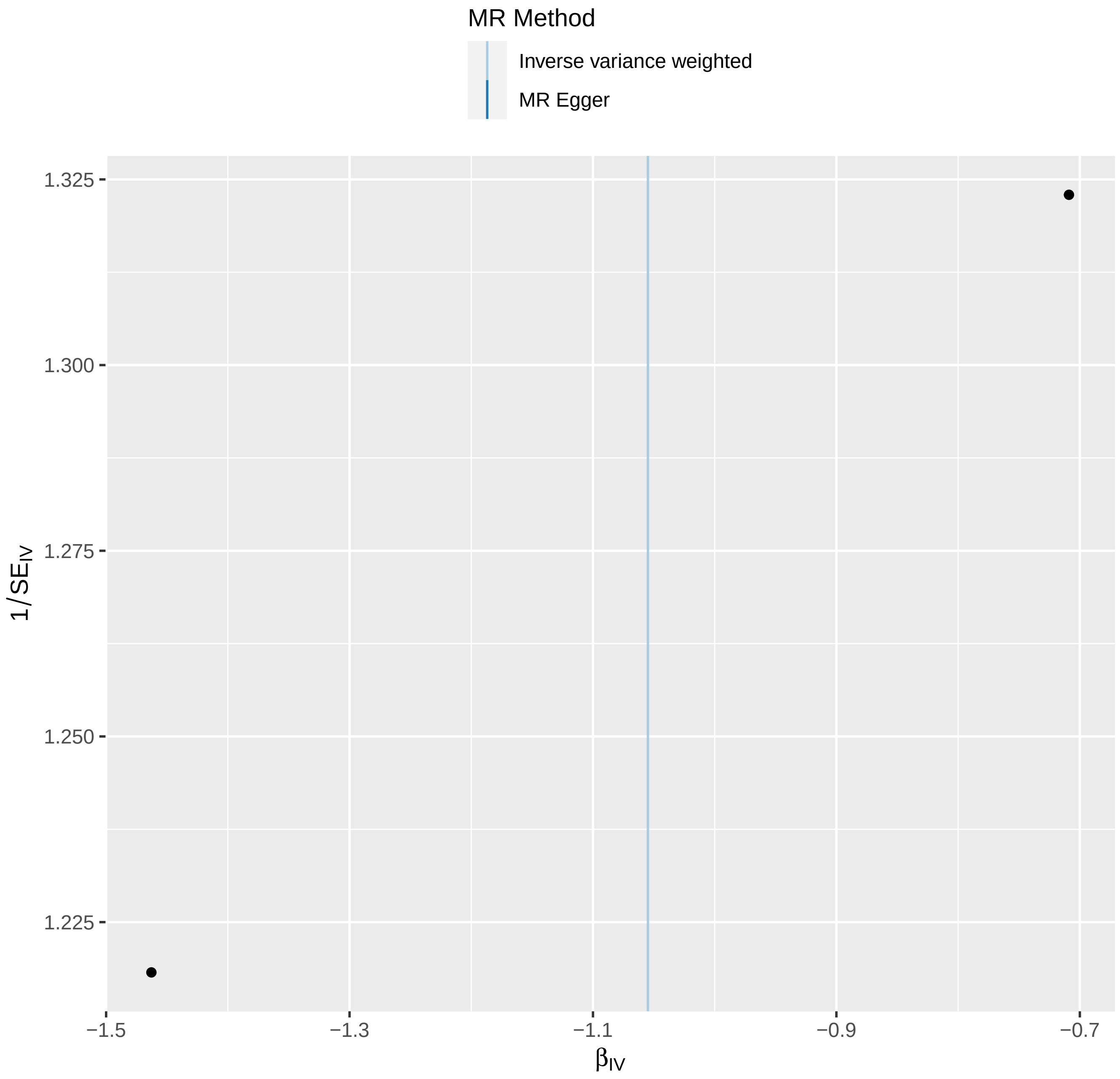

Batch 1148 : Gut microbiota abundance (genus Lachnospiraceae NK4A136 group id.11319) on Ulcerative ileocolitis

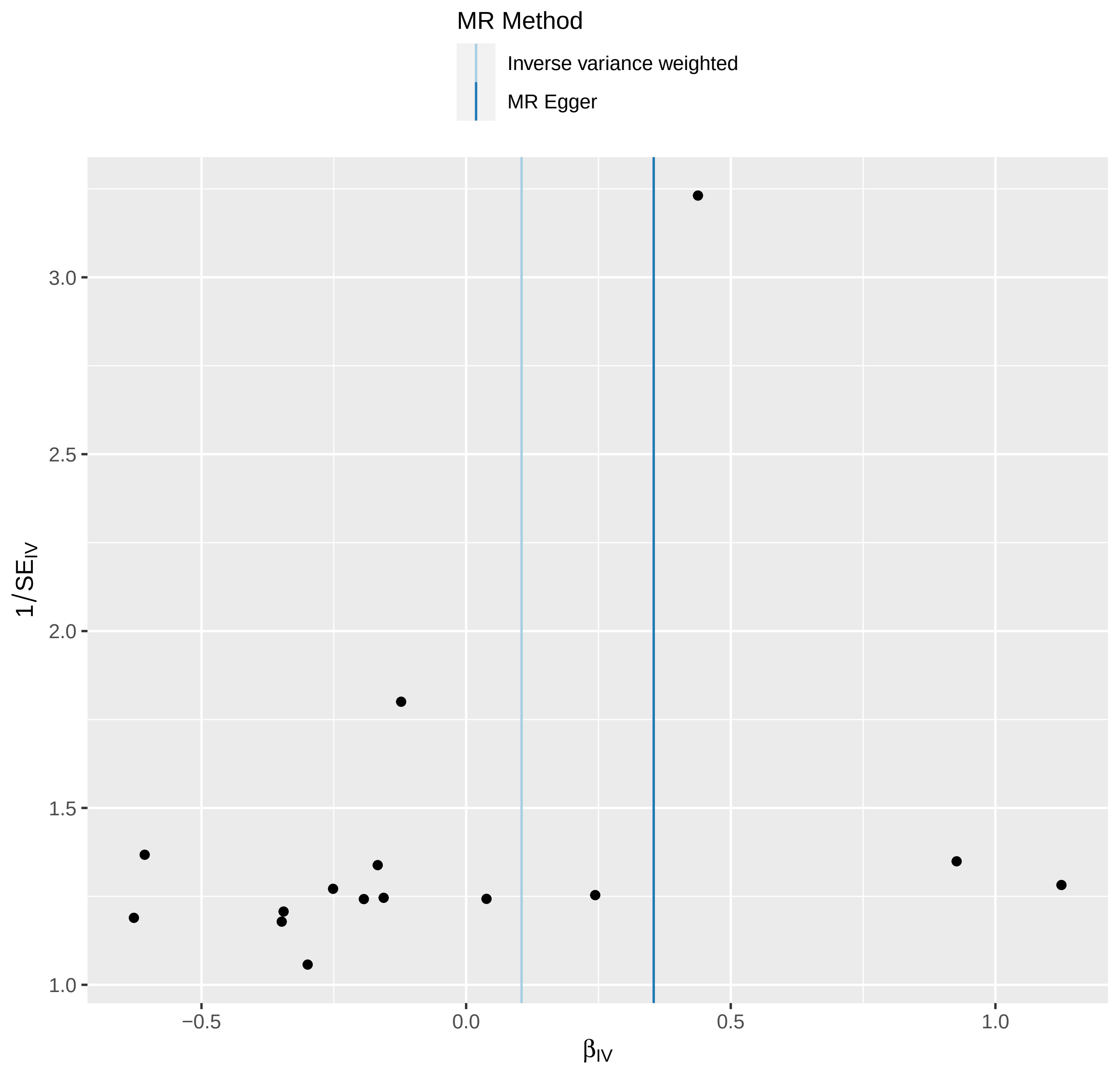

Batch 1149 : Gut microbiota abundance (genus Lachnospiraceae UCG001 id.11321) on Ulcerative ileocolitis

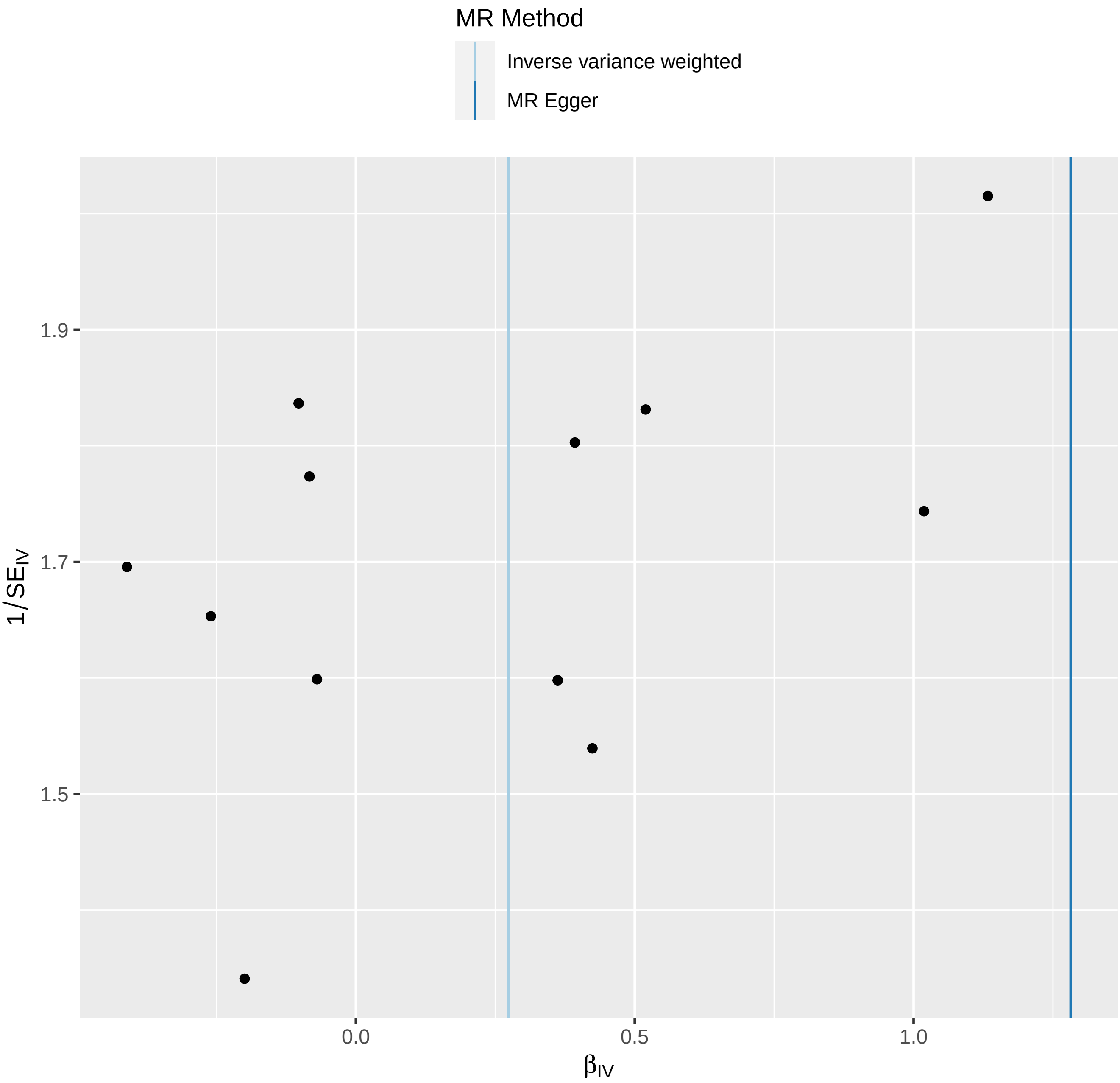

Batch 1150 : Gut microbiota abundance (genus Lachnospiraceae UCG004 id.11324) on Ulcerative ileocolitis

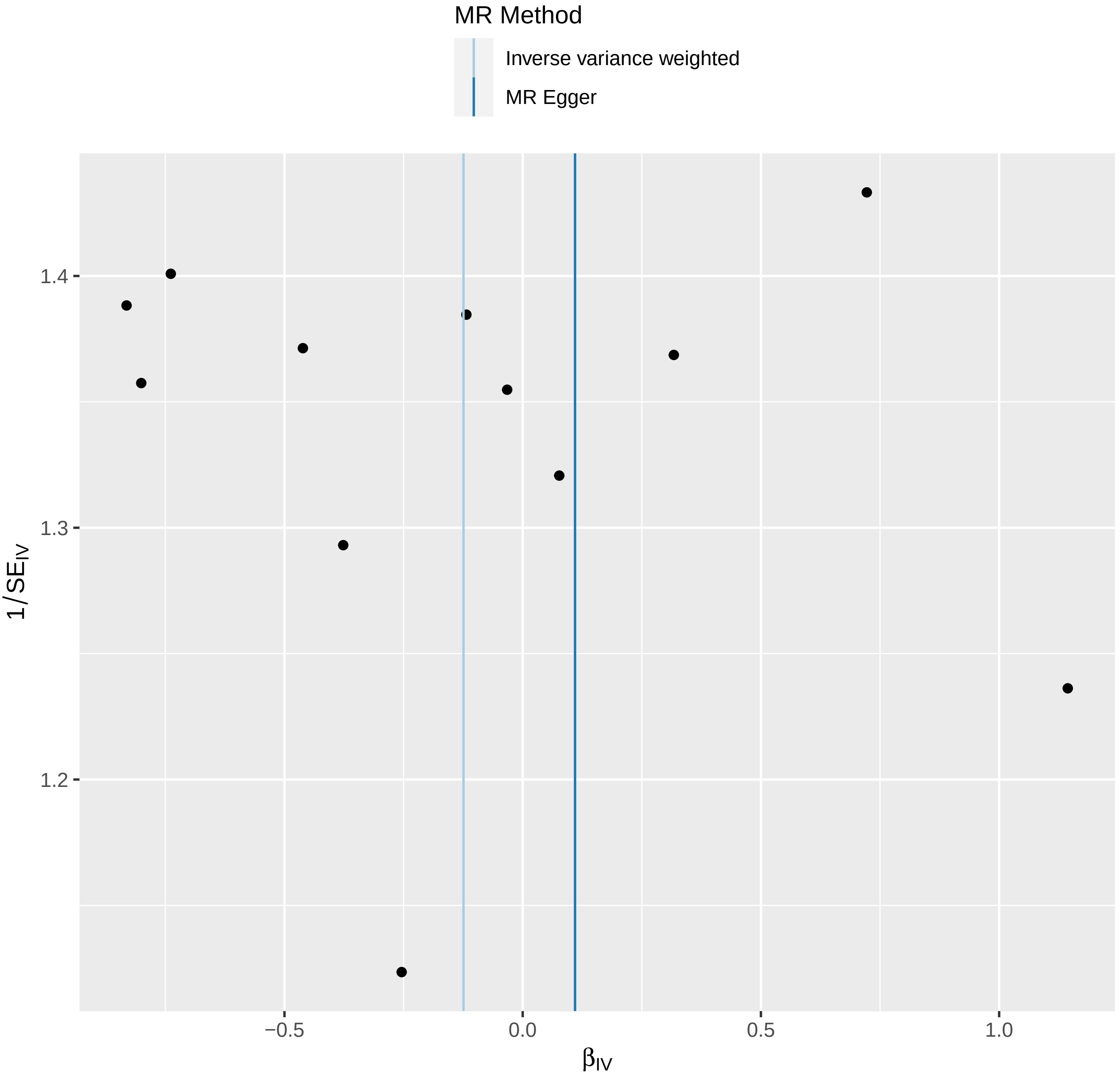

Batch 1151 : Gut microbiota abundance (genus Lachnospiraceae UCG008 id.11328) on Ulcerative ileocolitis

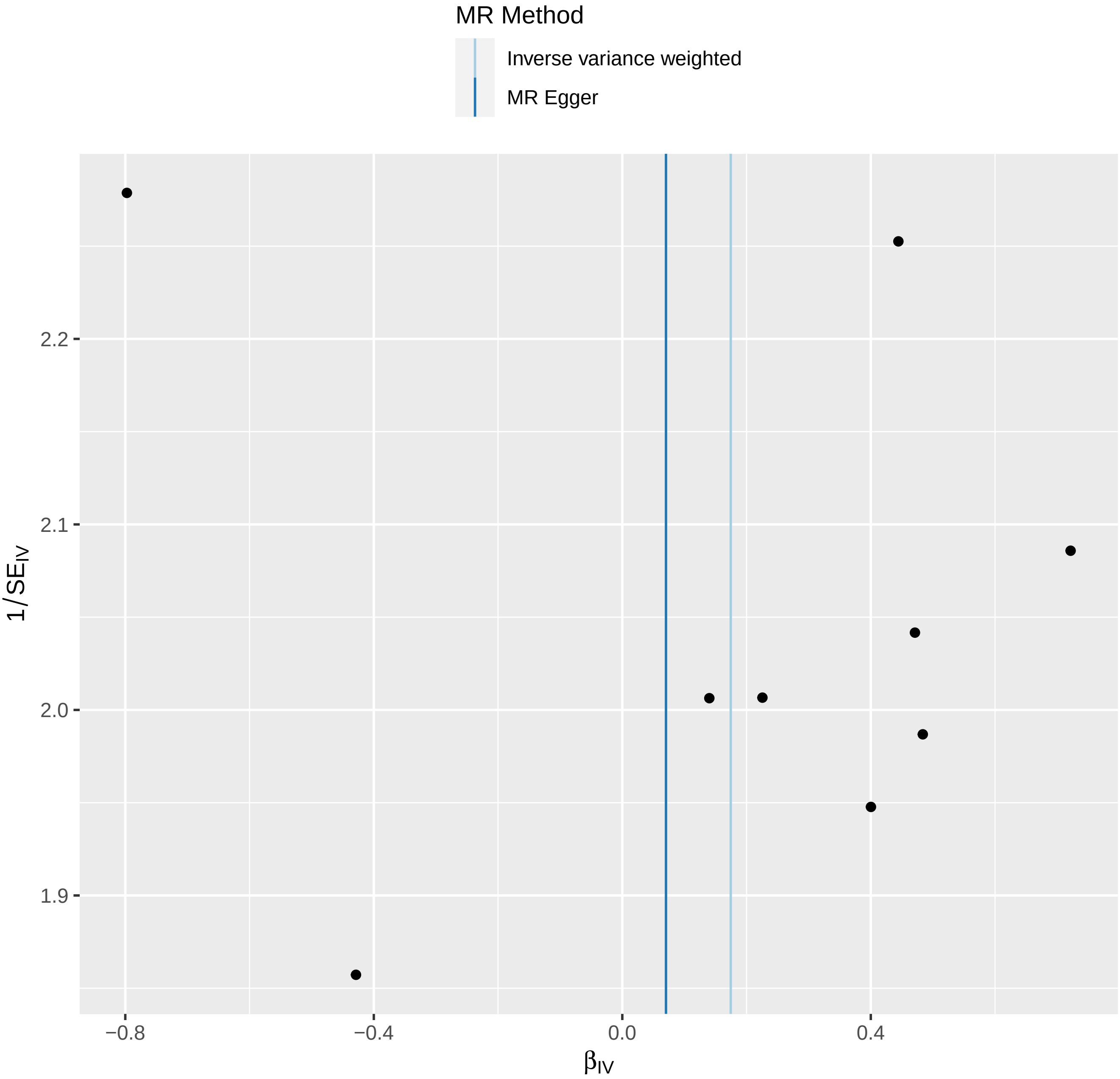

Batch 1152 : Gut microbiota abundance (genus Lachnospiraceae UCG010 id.11330) on Ulcerative ileocolitis

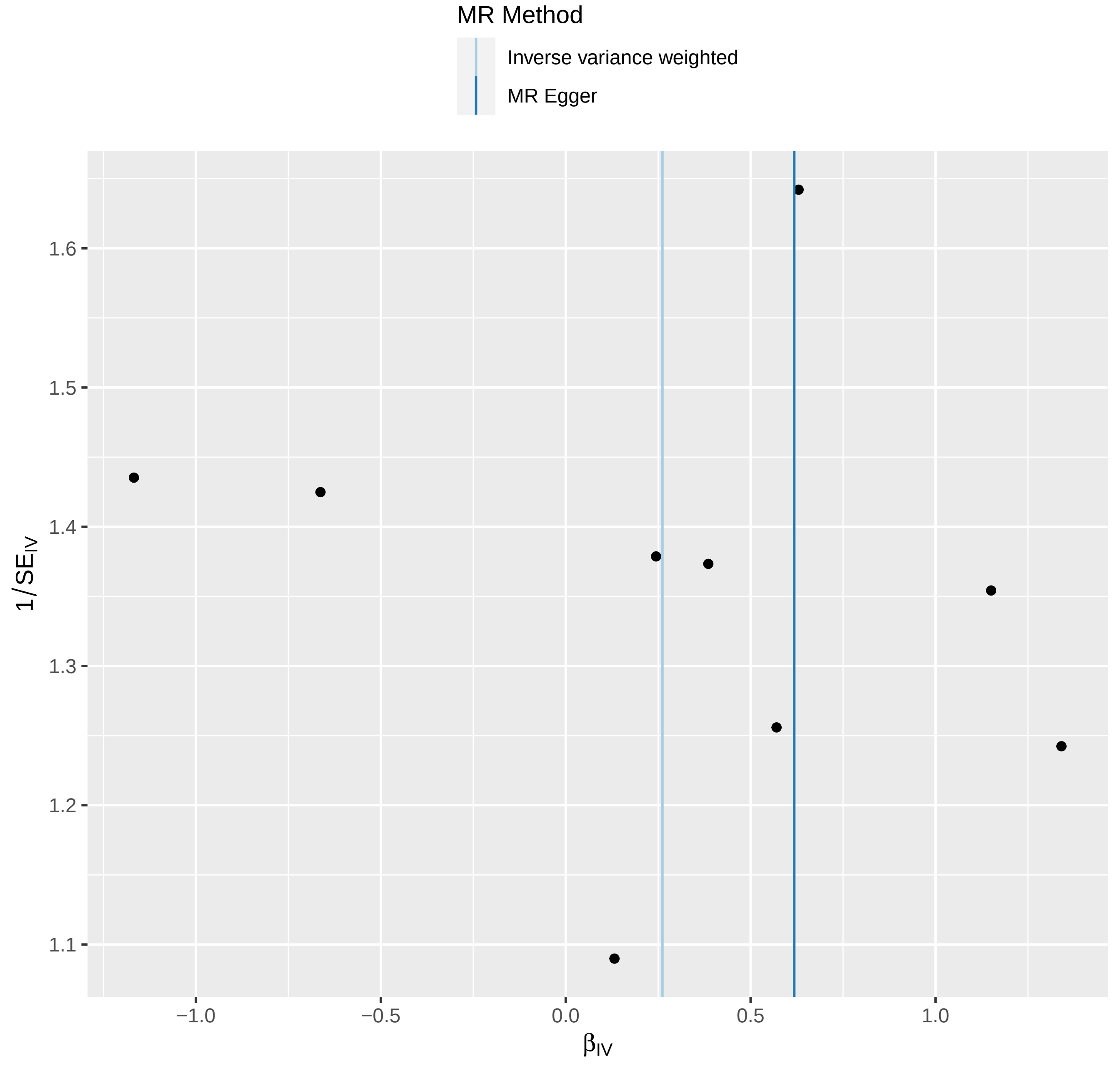

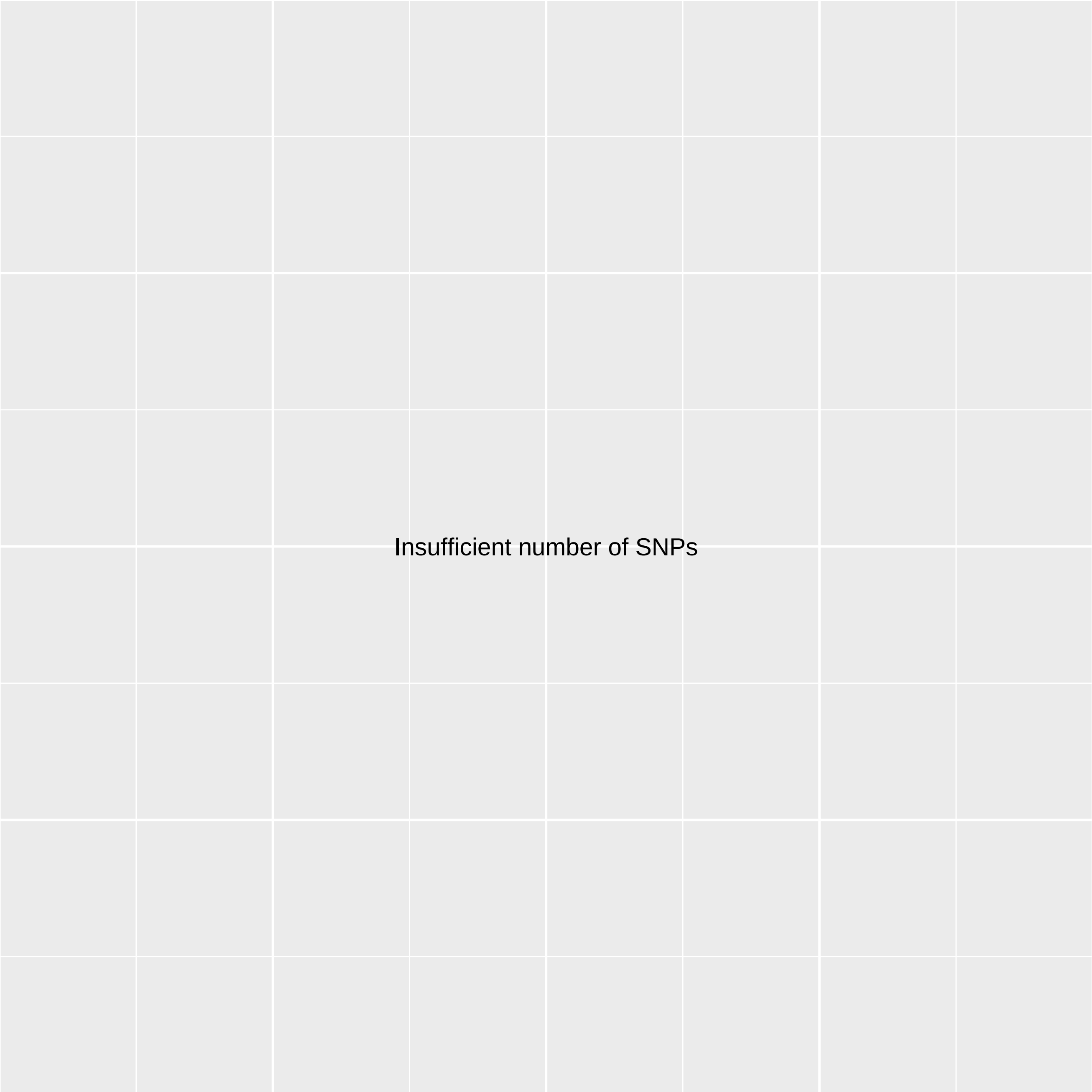
Insufficient number of SNPs

Batch 1154 : Gut microbiota abundance (genus Lactobacillus id.1837) on Ulcerative ileocolitis

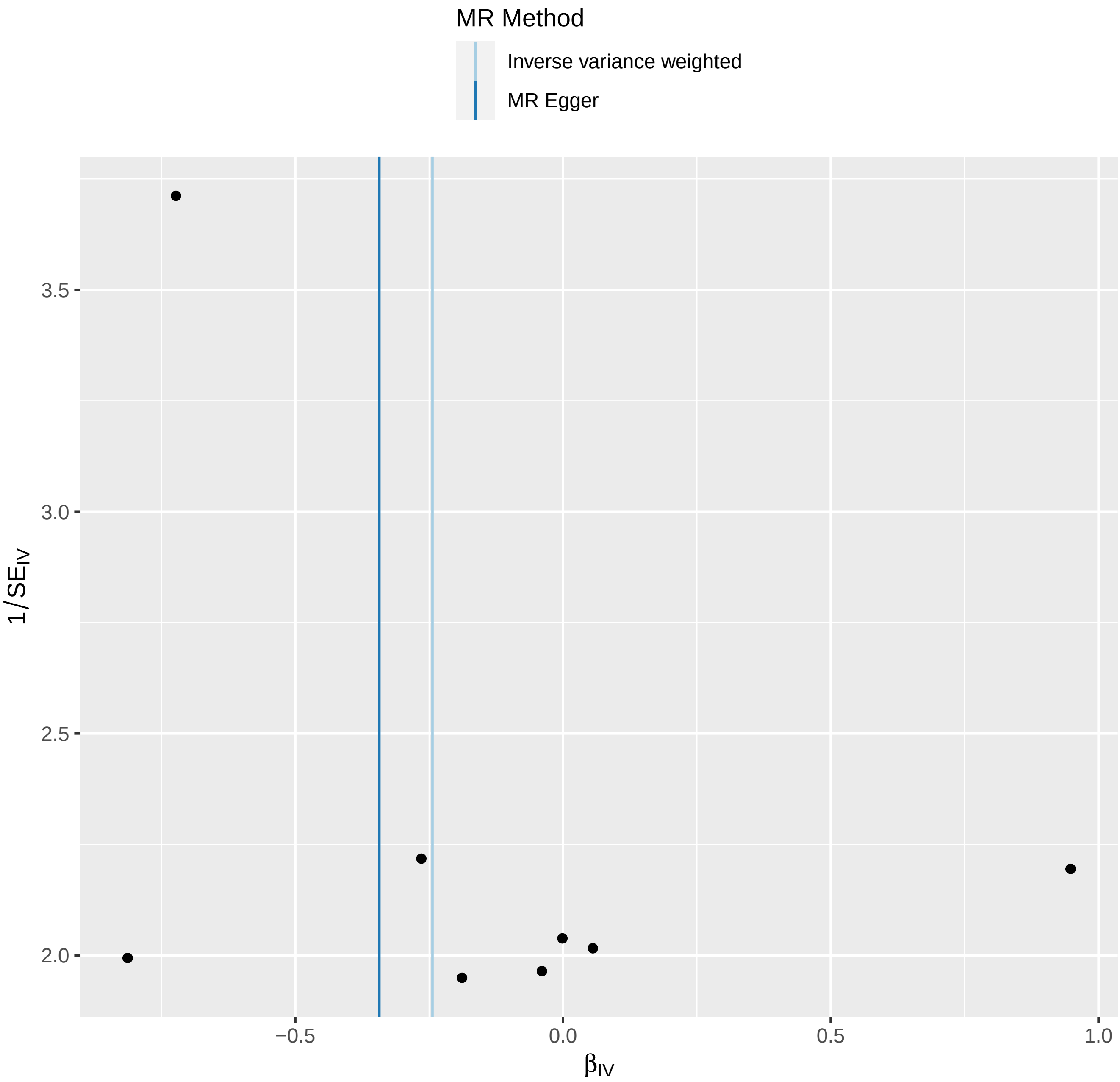

Batch 1155 : Gut microbiota abundance (genus Lactococcus id.1851) on Ulcerative ileocolitis

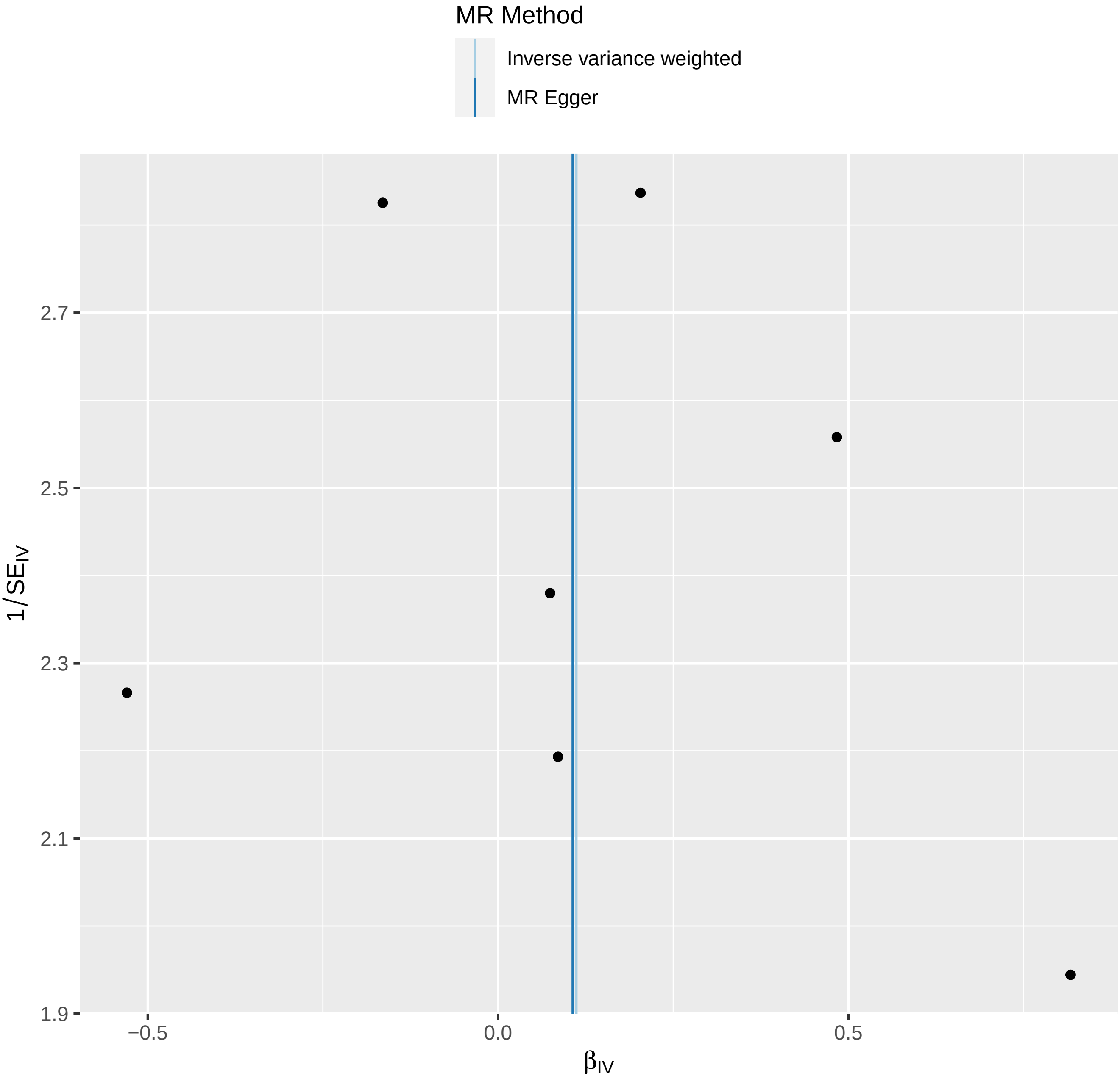

Batch 1156 : Gut microbiota abundance (genus Marvinbryantia id.2005) on Ulcerative ileocolitis

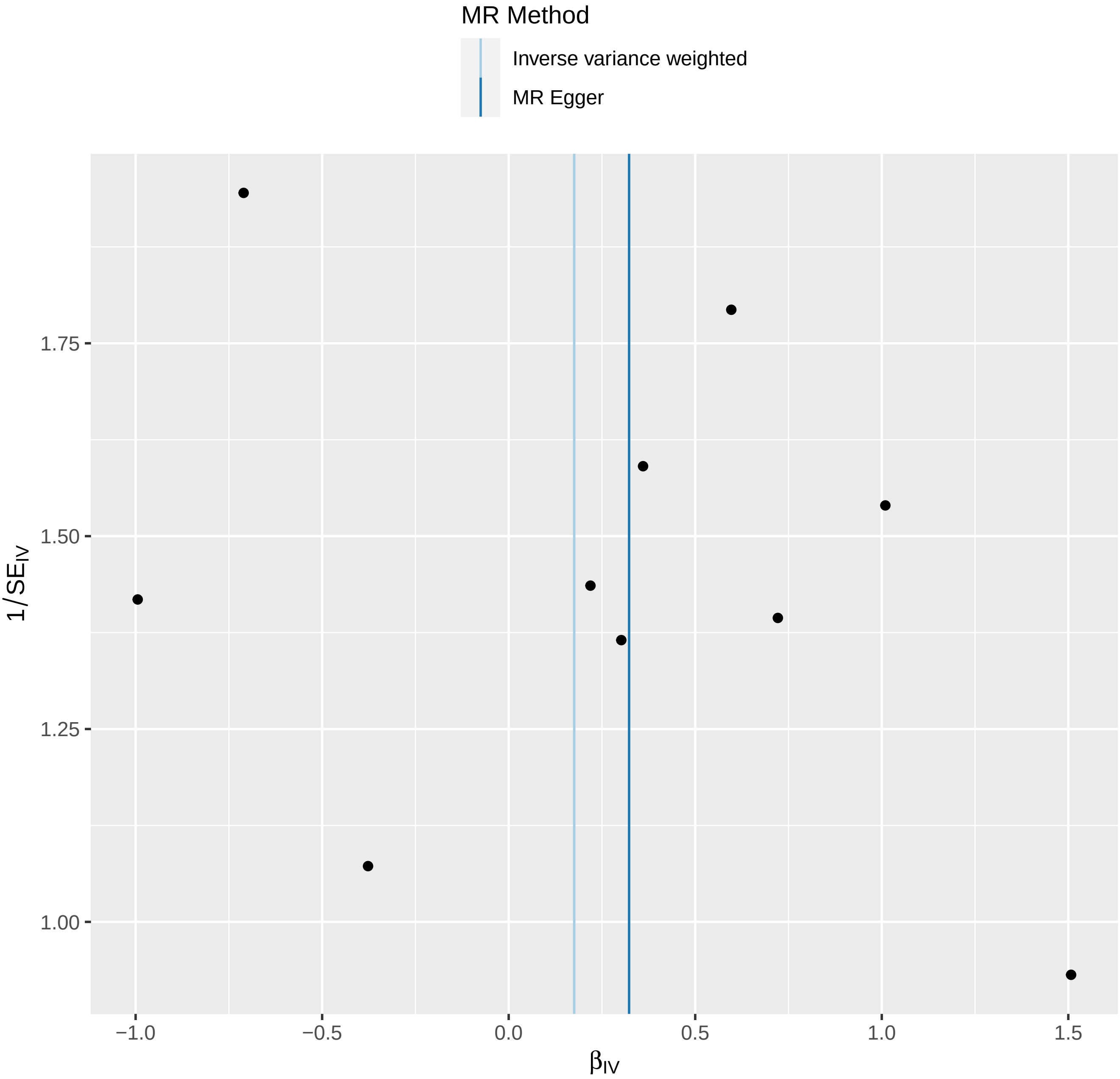

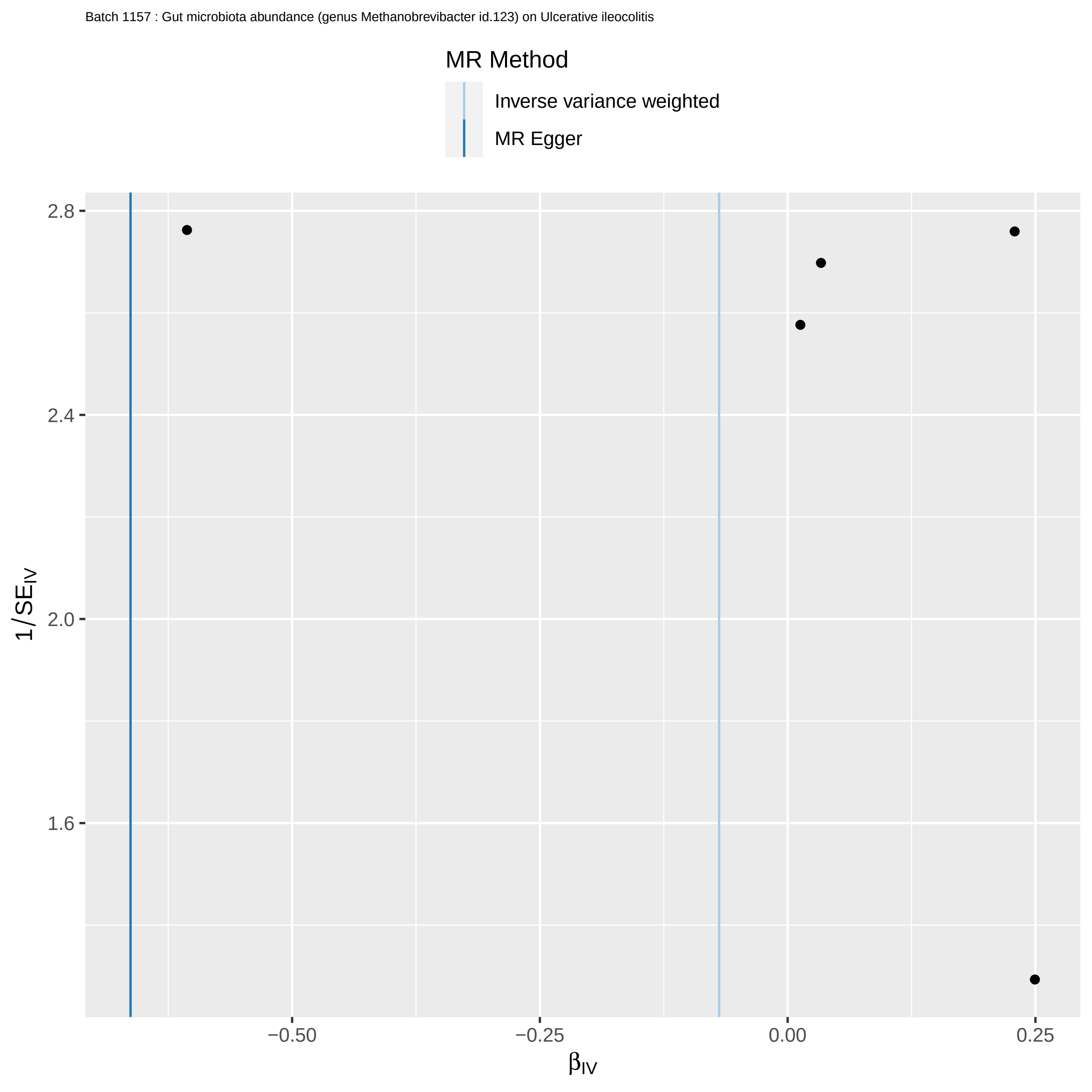

Batch 1157 : Gut microbiota abundance (genus Methanobrevibacter id.123) on Ulcerative ileocolitis
MR Method
Inverse variance weighted
MR Egger
2.8
2.4
2.0
1.6
1/SE$_{IV}$
−0.50
−0.25
0.00
0.25
β$_{IV}$

Batch 1158 : Gut microbiota abundance (genus Odoribacter id.952) on Ulcerative ileocolitis

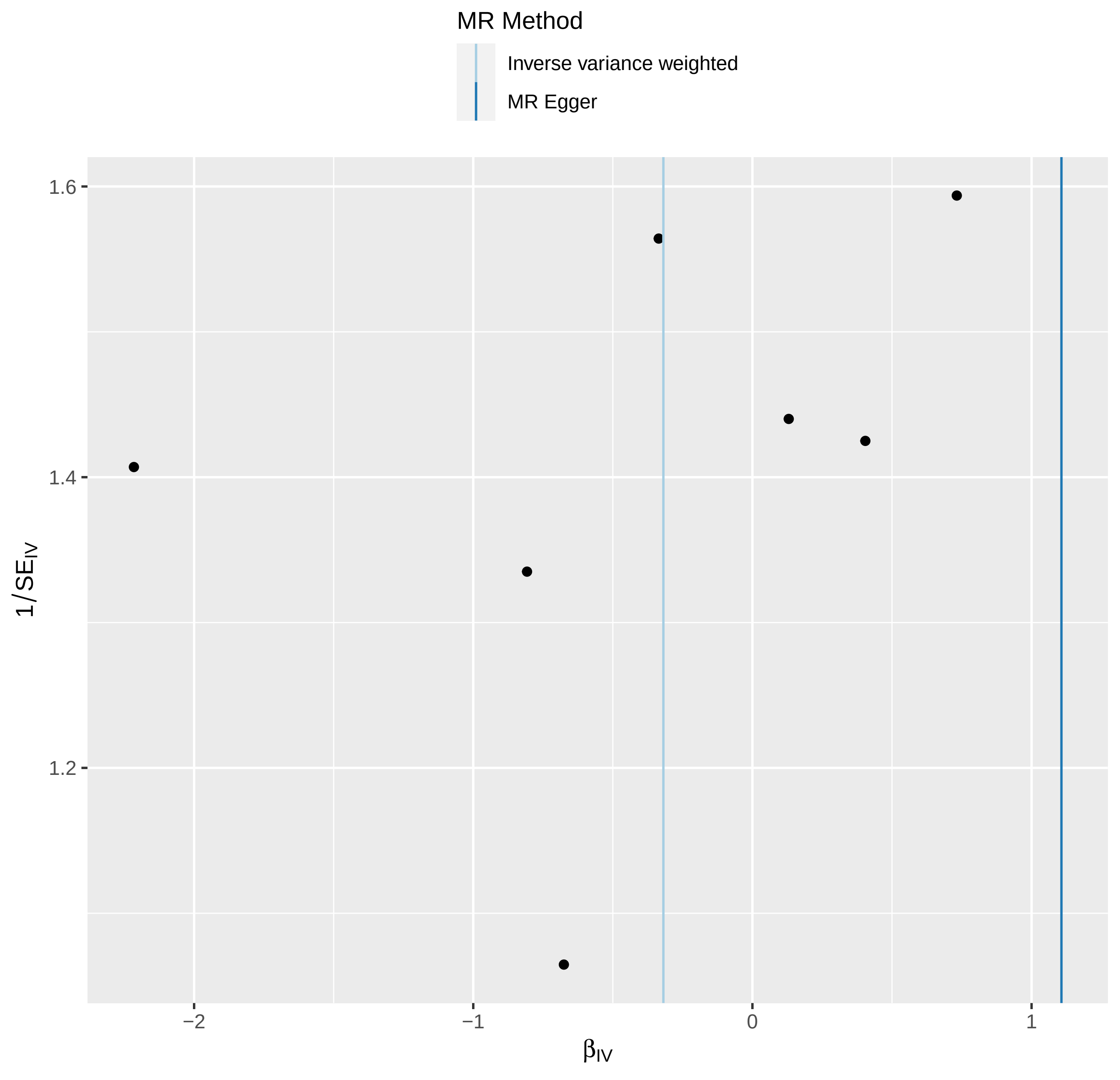

Batch 1159 : Gut microbiota abundance (genus Olsenella id.822) on Ulcerative ileocolitis

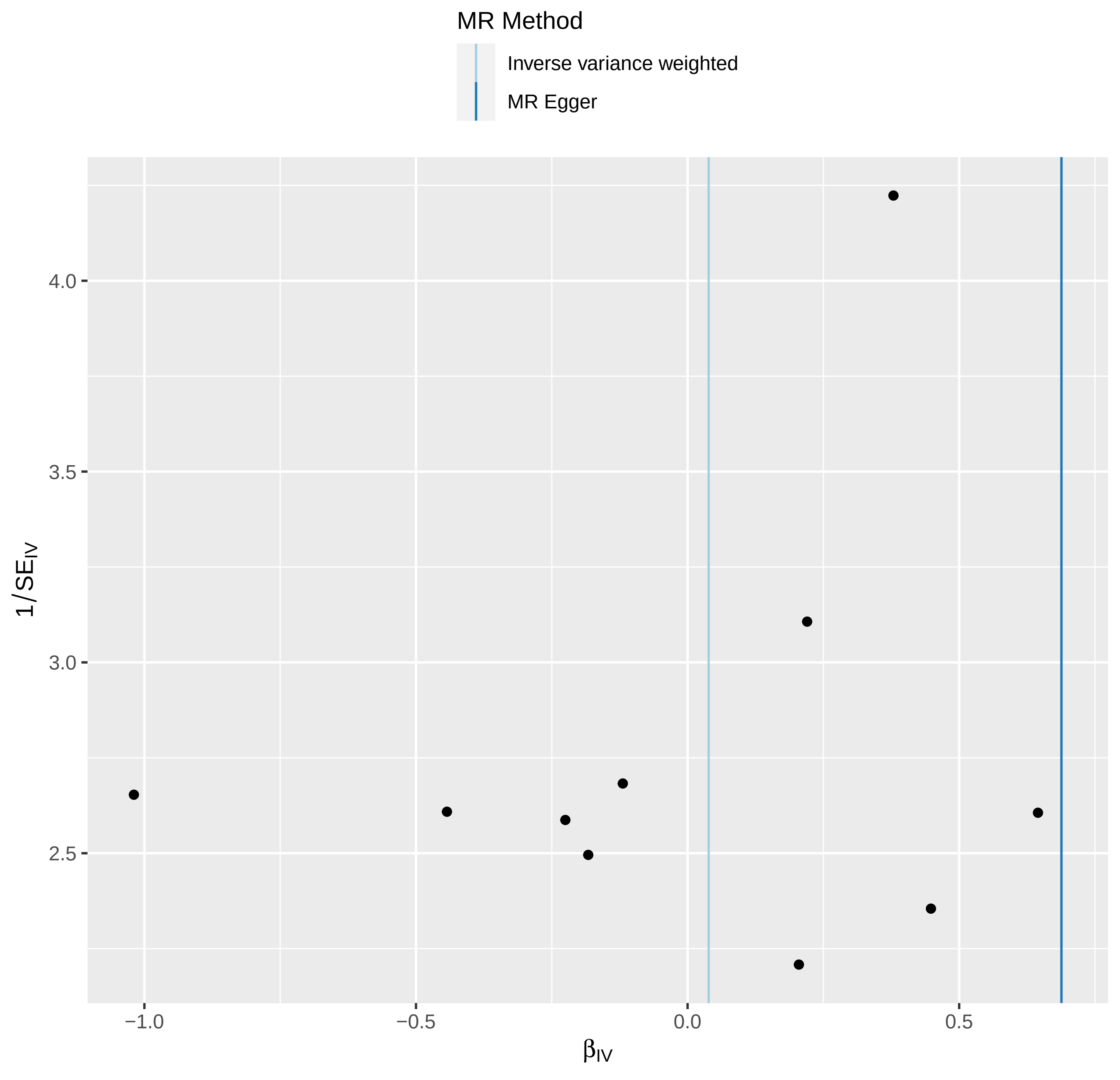

Batch 1160 : Gut microbiota abundance (genus Oscillibacter id.2063) on Ulcerative ileocolitis

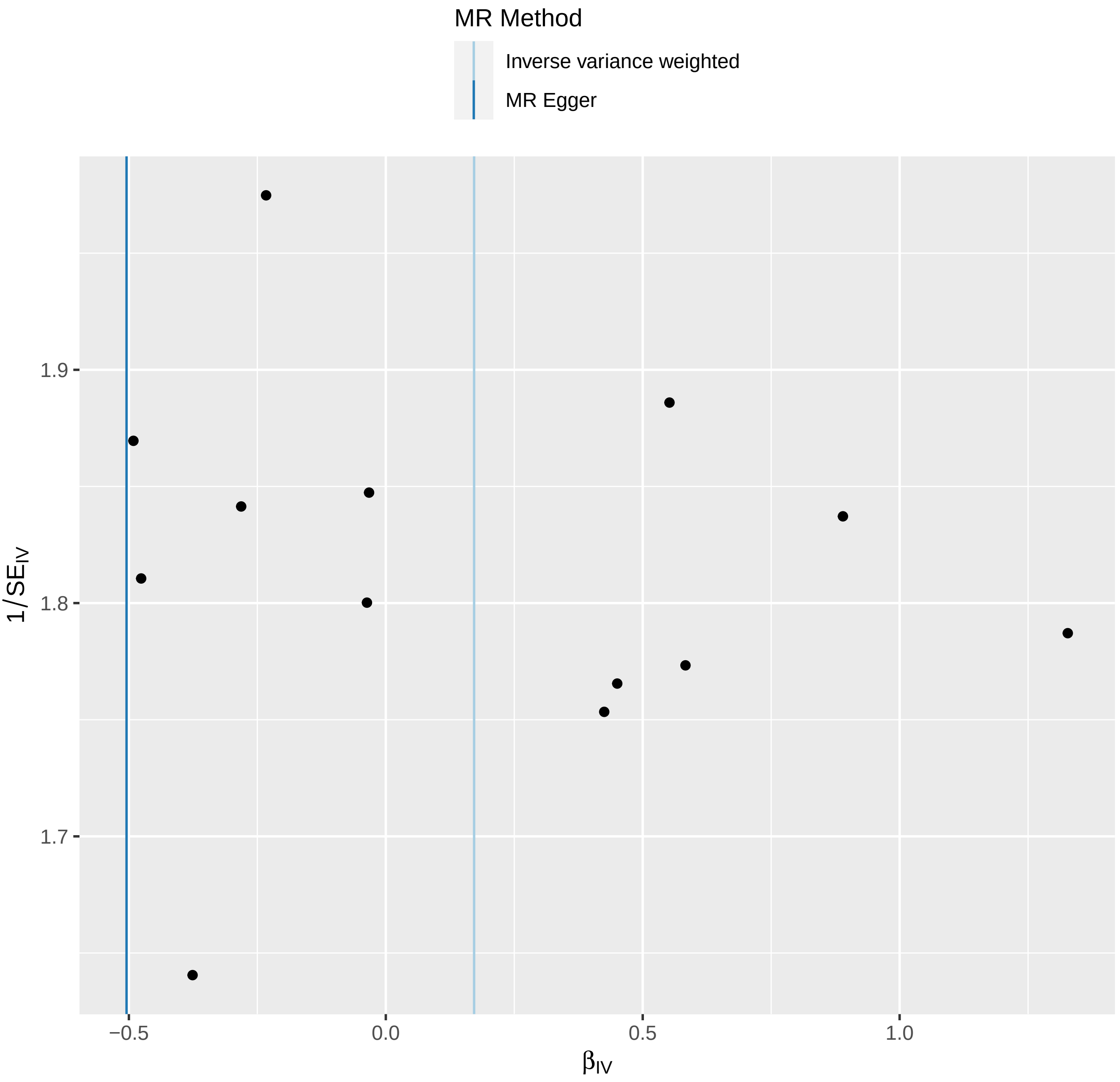

Batch 1161 : Gut microbiota abundance (genus Oscillospira id.2064) on Ulcerative ileocolitis

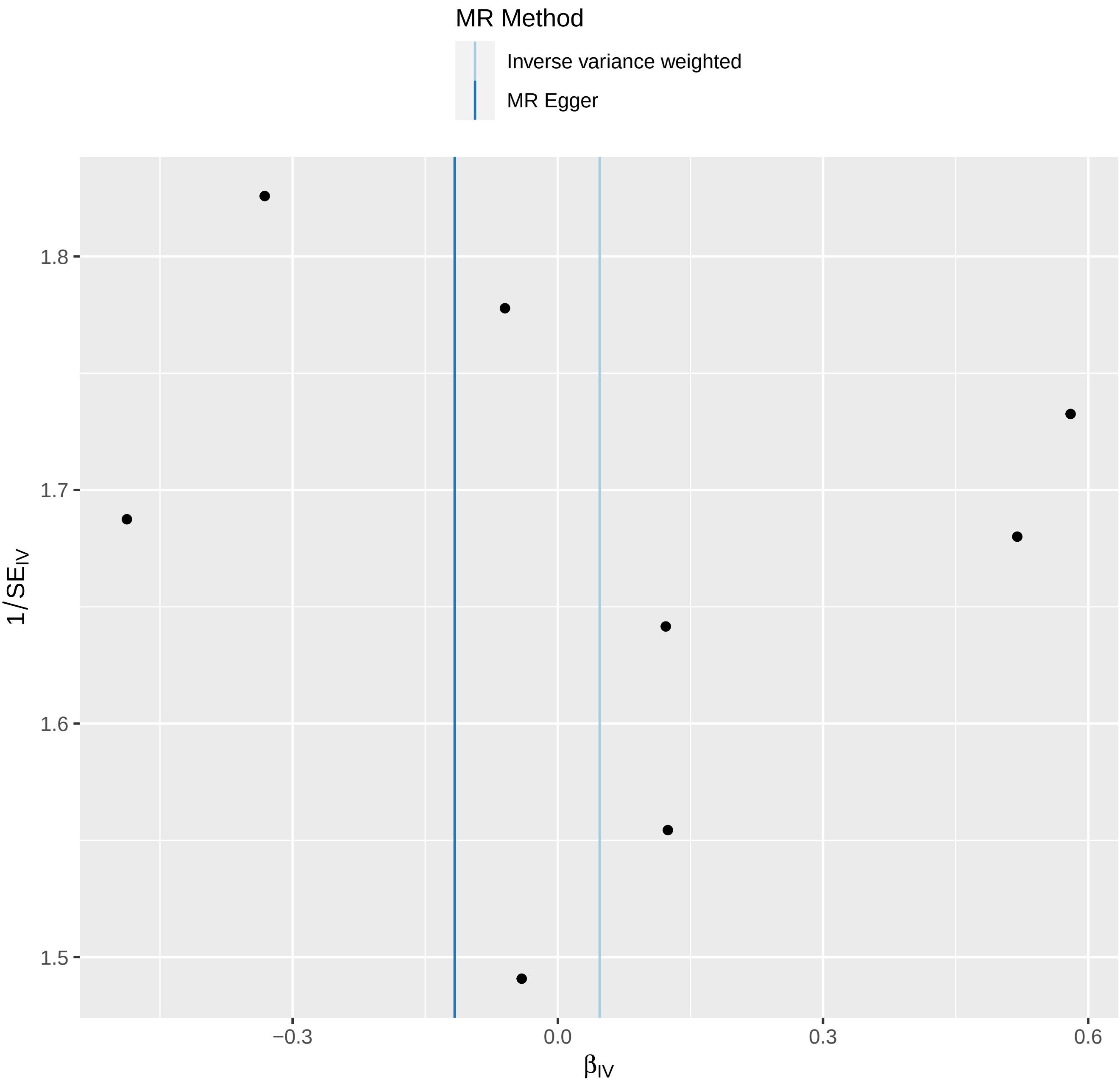

Batch 1162 : Gut microbiota abundance (genus Oxalobacter id.2978) on Ulcerative ileocolitis

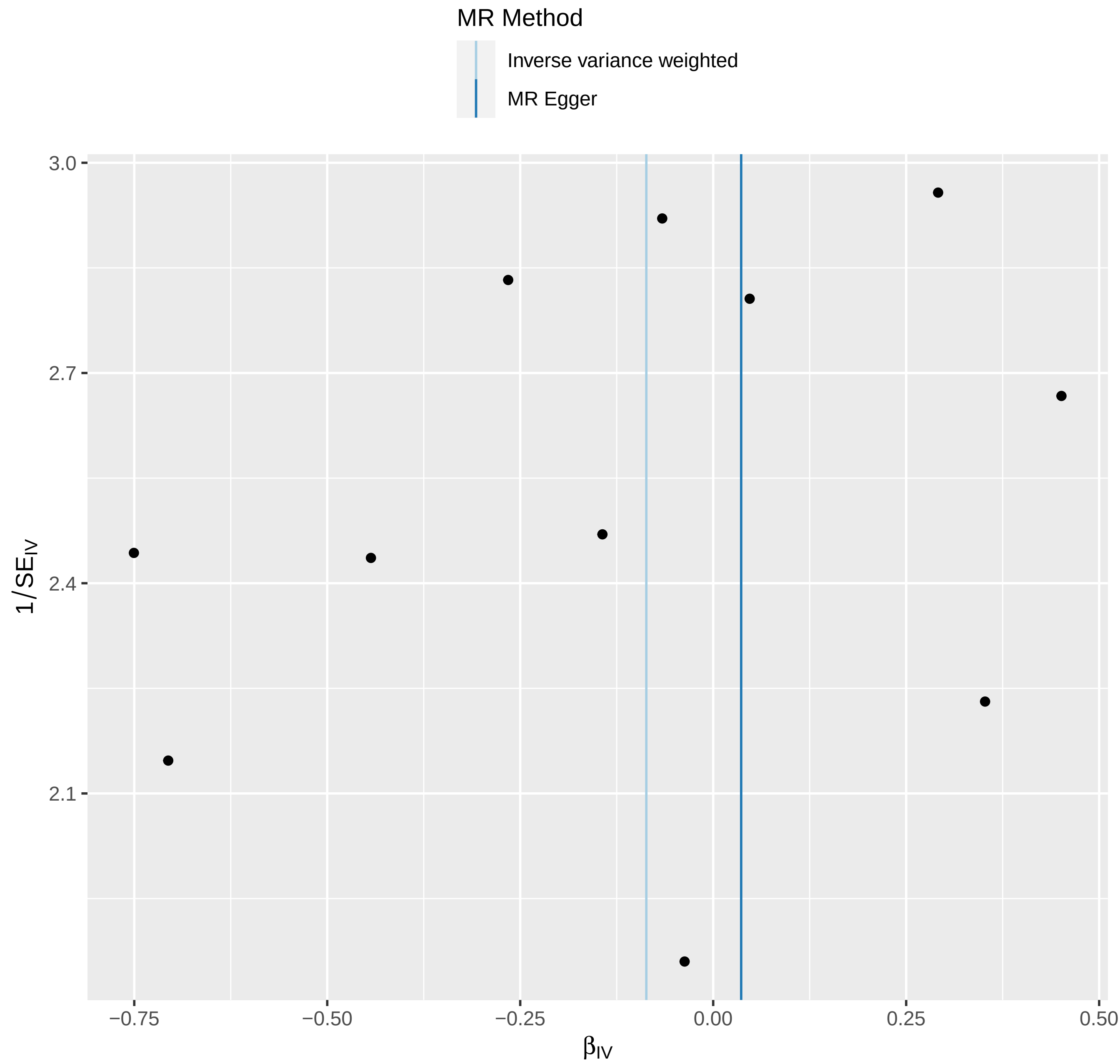

Batch 1163 : Gut microbiota abundance (genus Parabacteroides id.954) on Ulcerative ileocolitis

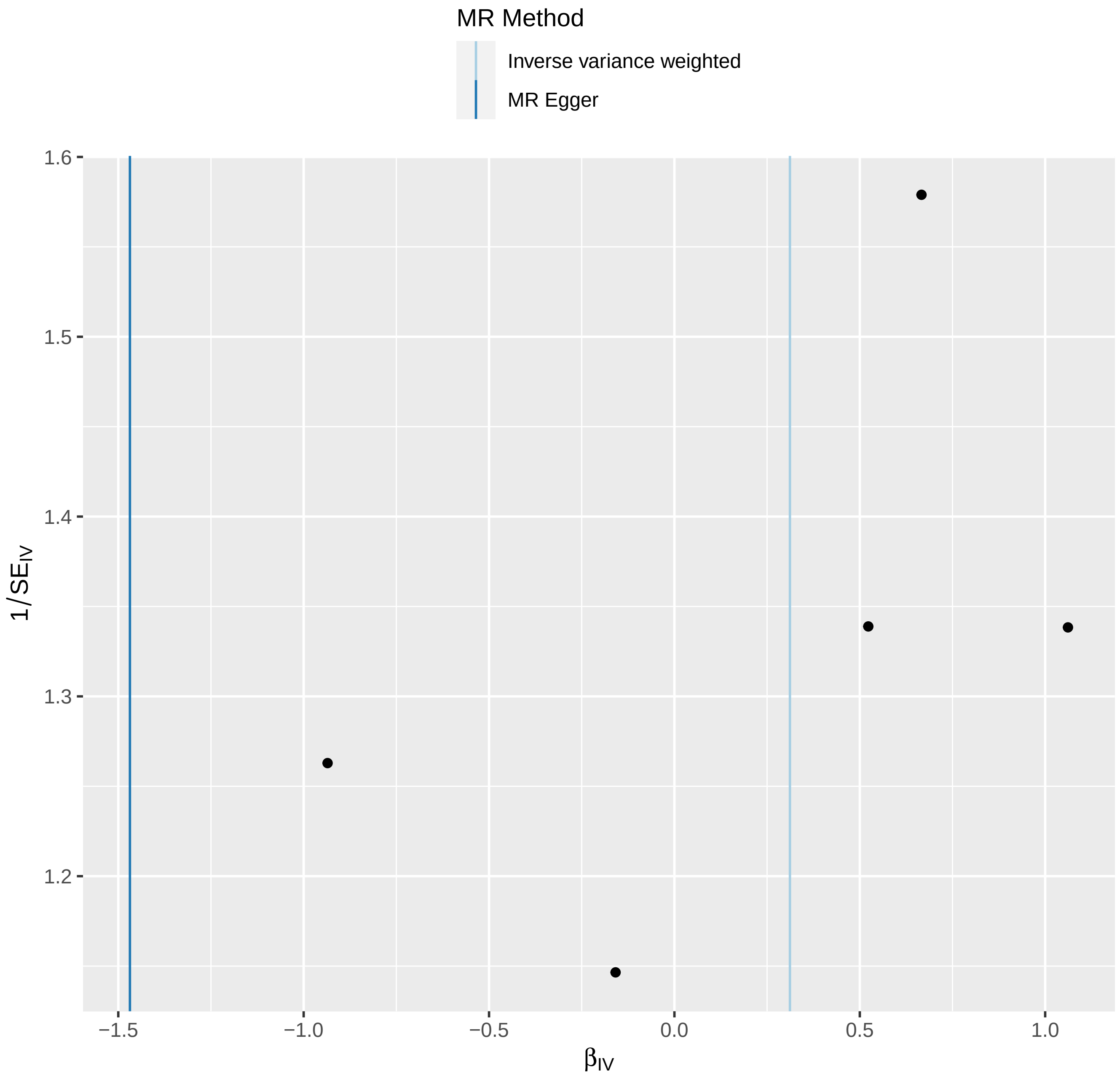

Batch 1164 : Gut microbiota abundance (genus Paraprevotella id.962) on Ulcerative ileocolitis

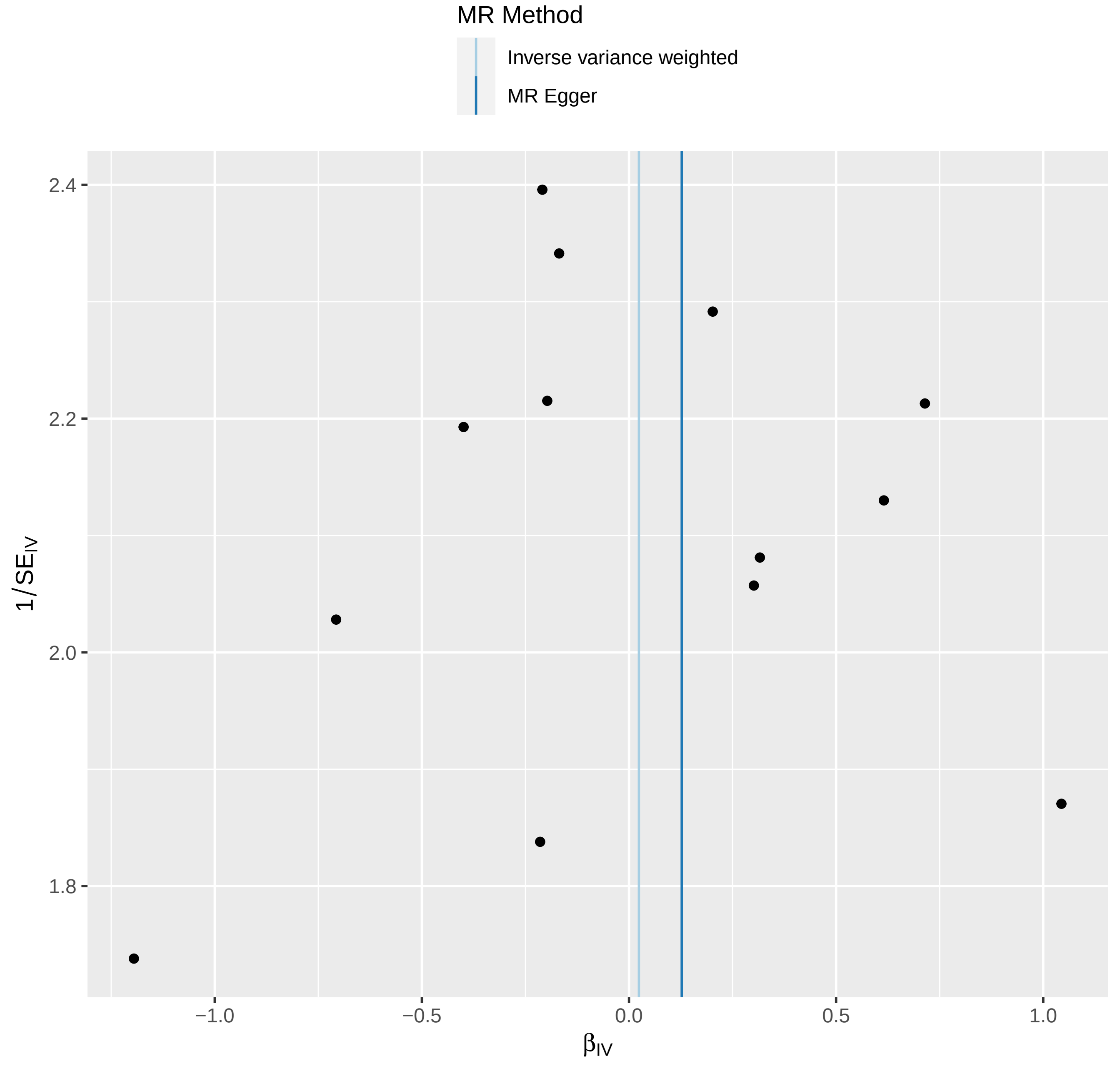

Batch 1165 : Gut microbiota abundance (genus Parasutterella id.2892) on Ulcerative ileocolitis

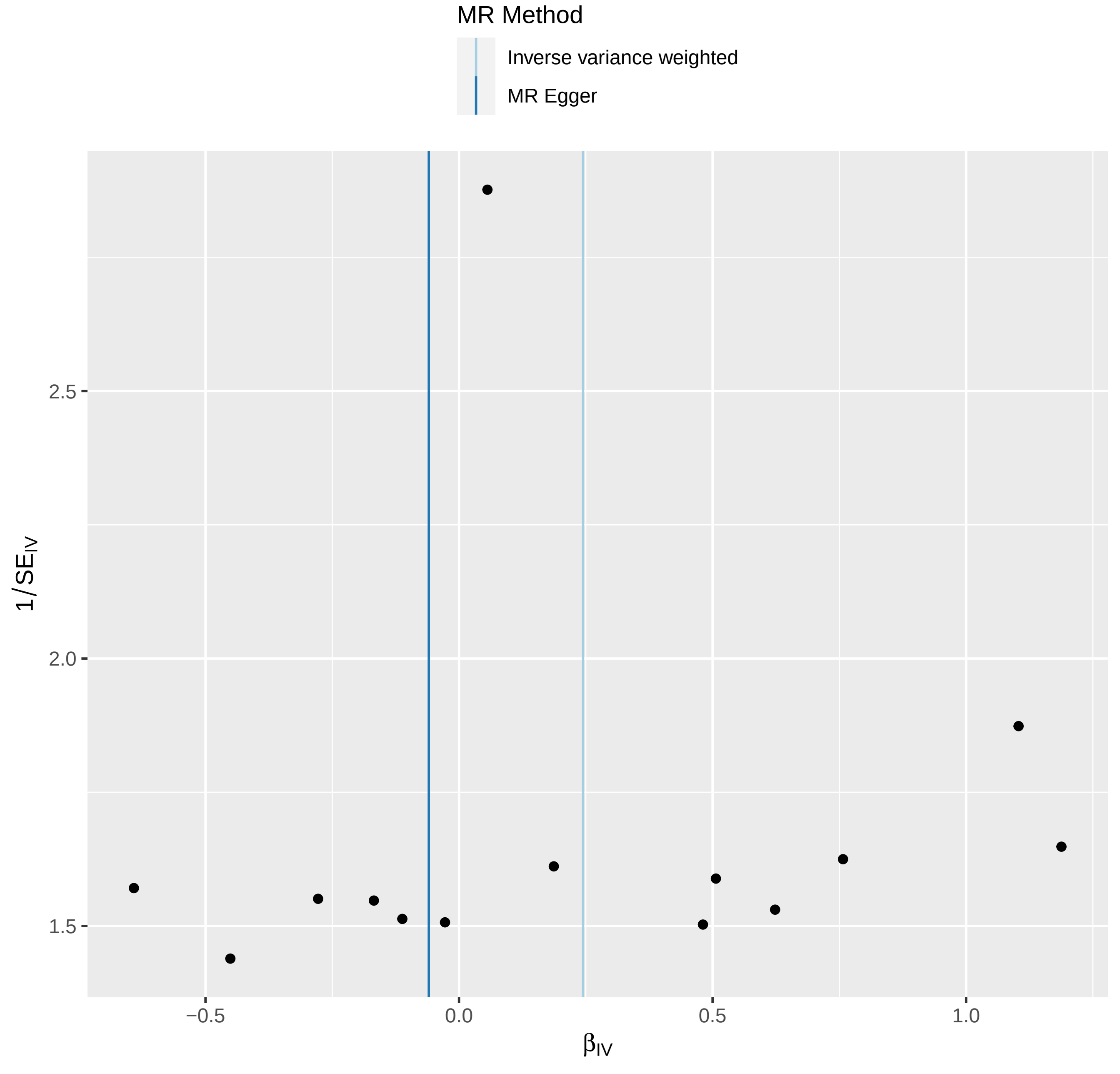

Batch 1166 : Gut microbiota abundance (genus Peptococcus id.2037) on Ulcerative ileocolitis

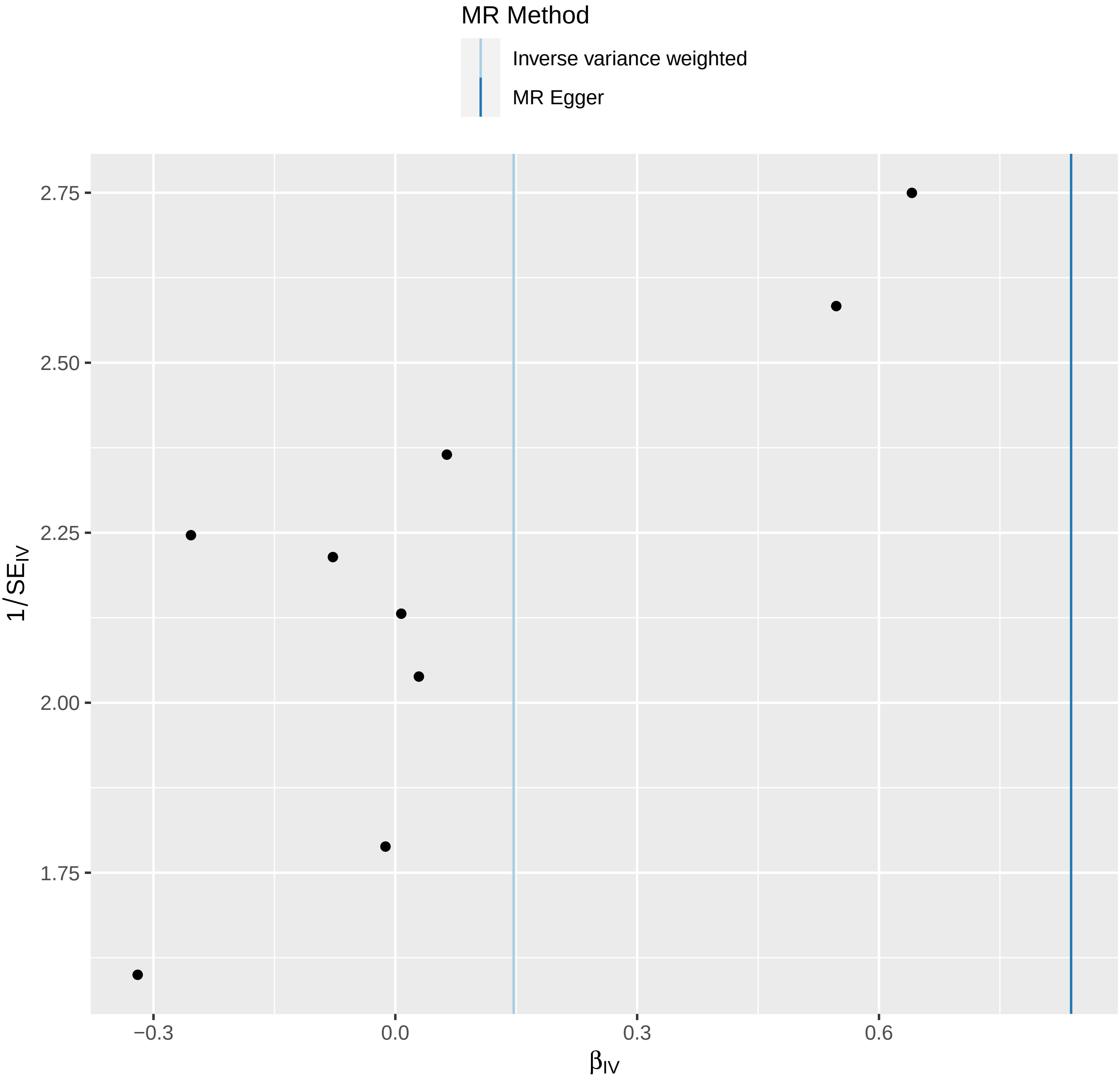

Batch 1167 : Gut microbiota abundance (genus Phascolarctobacterium id.2168) on Ulcerative ileocolitis

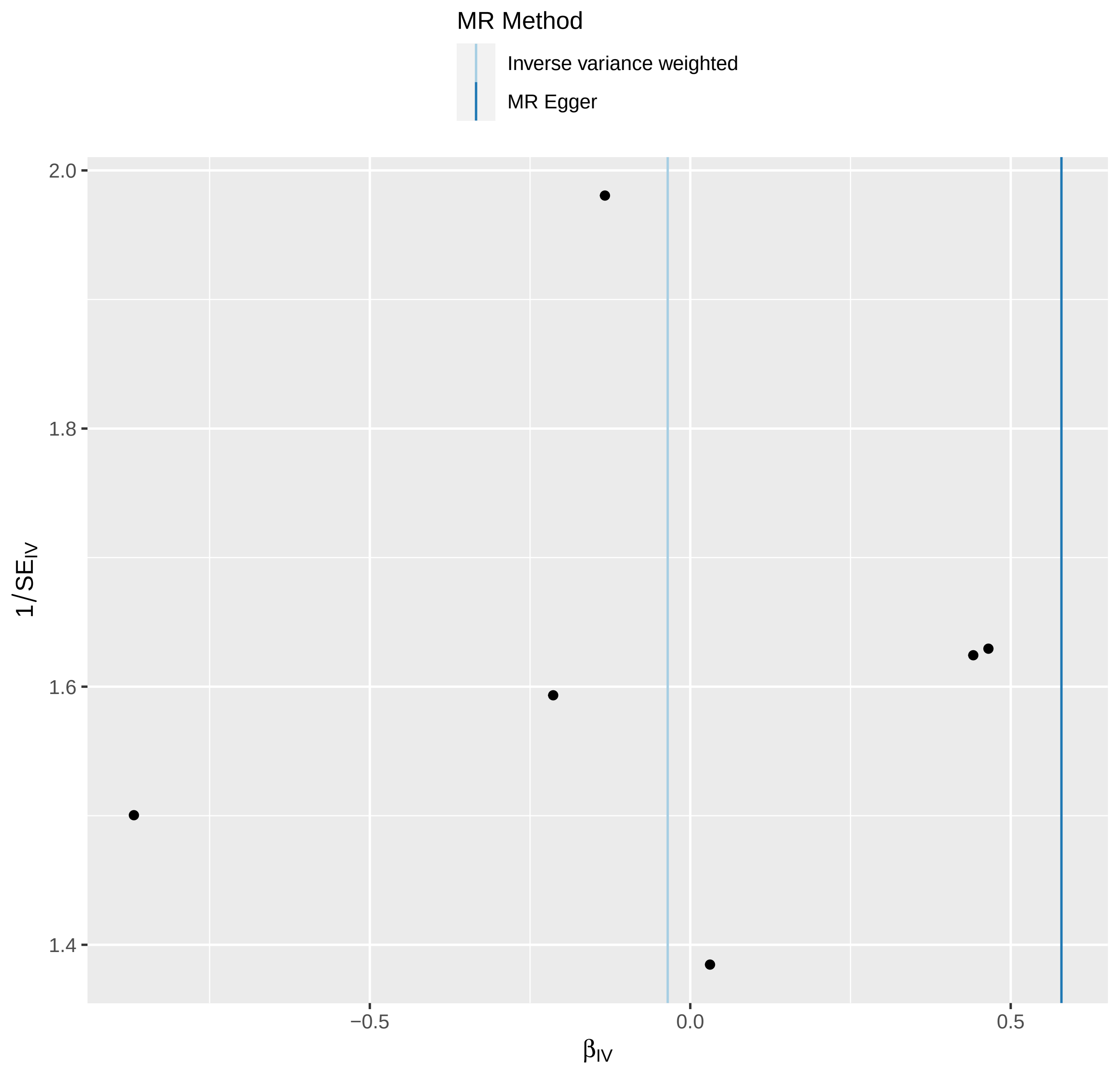

Batch 1168 : Gut microbiota abundance (genus Prevotella7 id.11182) on Ulcerative ileocolitis

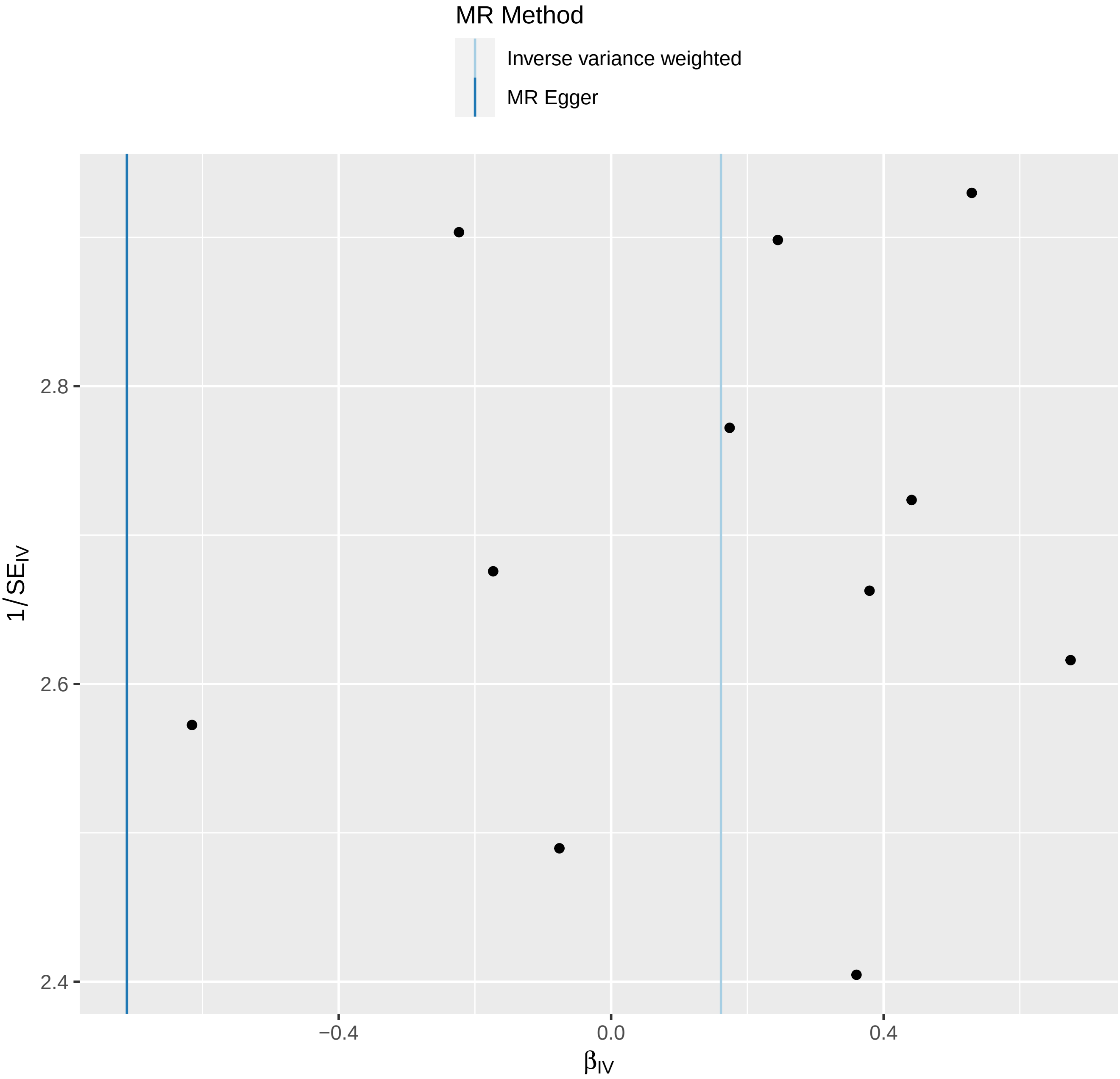

Batch 1169 : Gut microbiota abundance (genus Prevotella9 id.11183) on Ulcerative ileocolitis

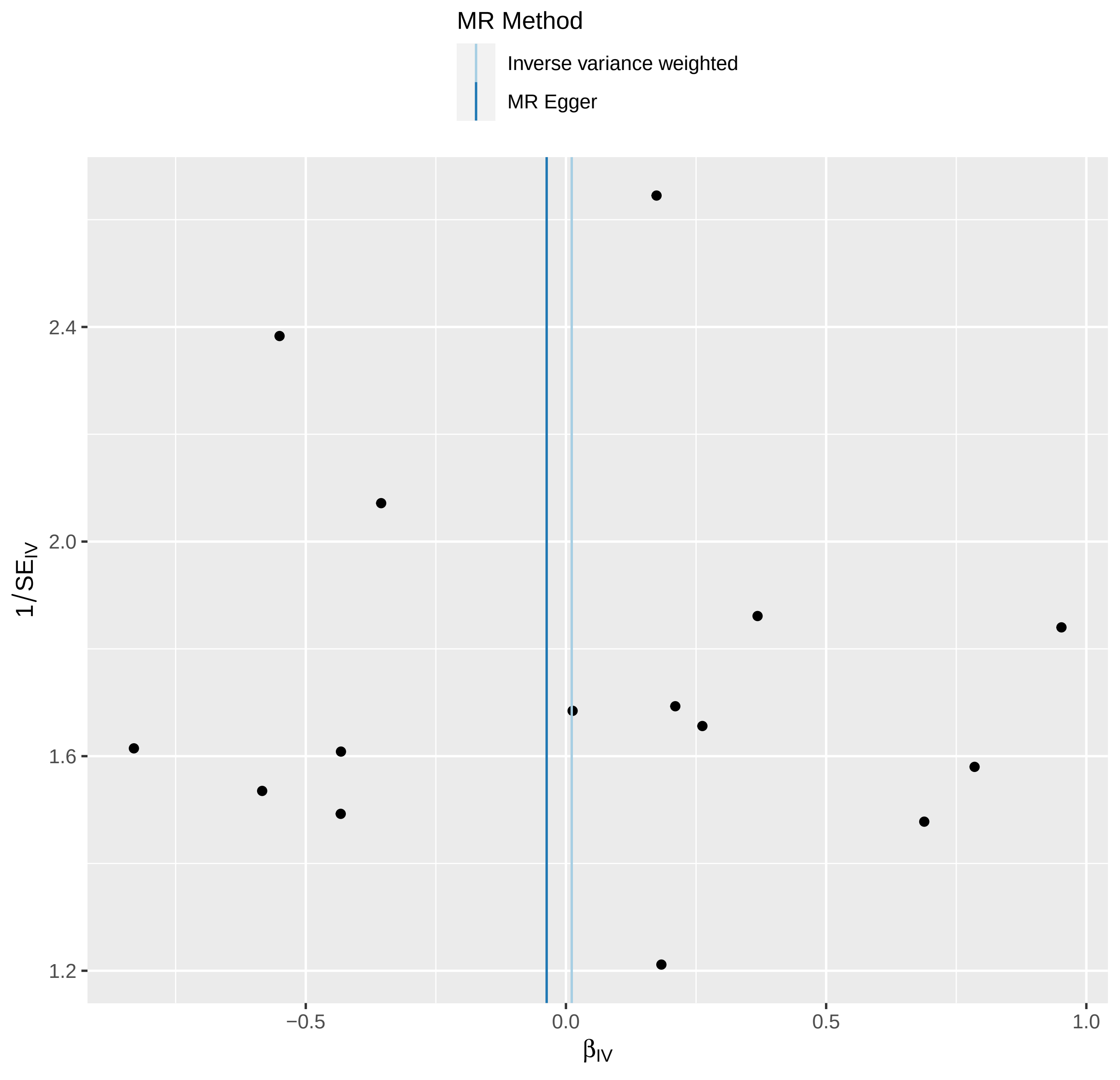

Batch 1170 : Gut microbiota abundance (genus Rikenellaceae RC9 gut group id.11191) on Ulcerative ileocolitis

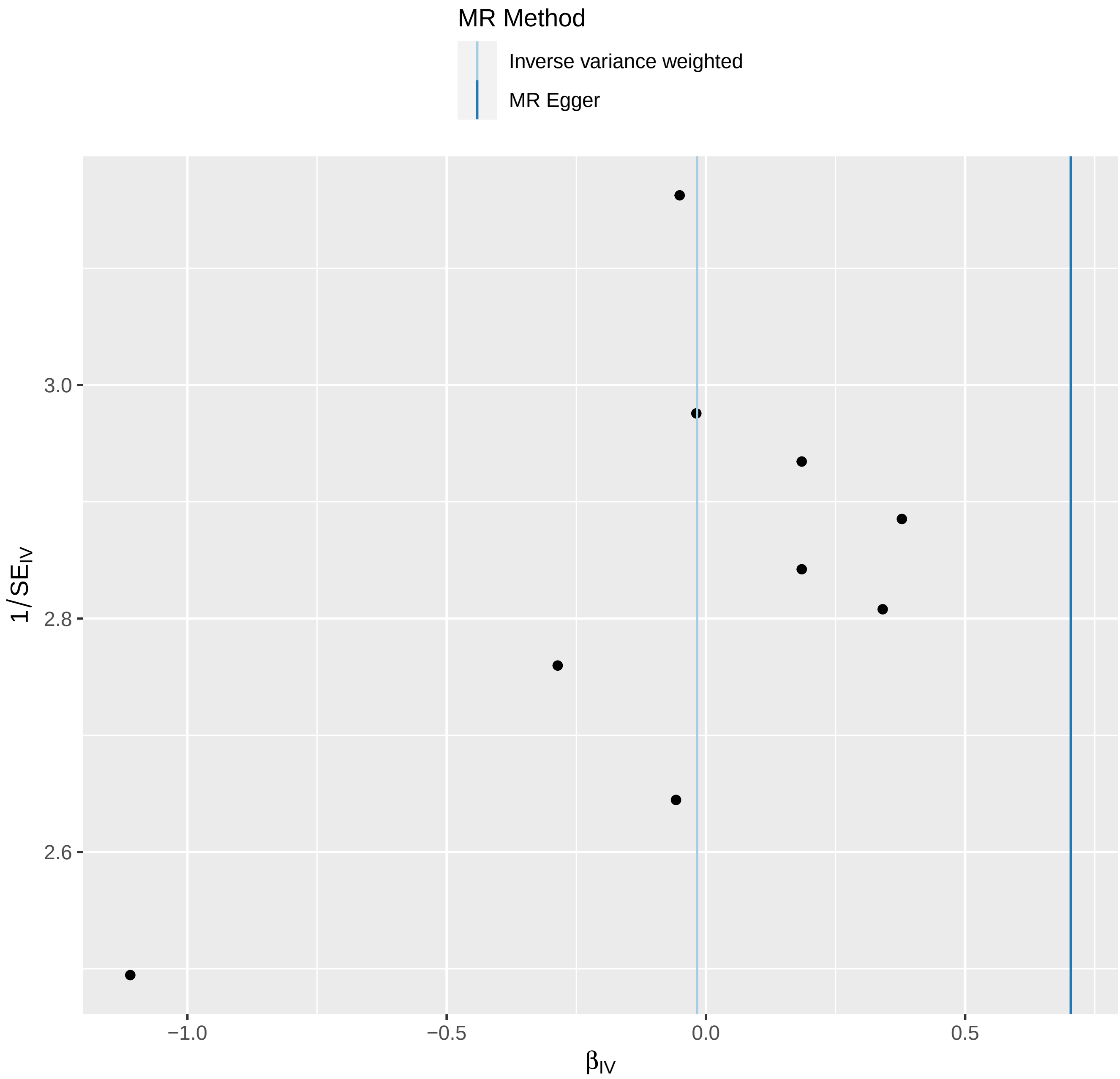

Batch 1171 : Gut microbiota abundance (genus Romboutsia id.11347) on Ulcerative ileocolitis

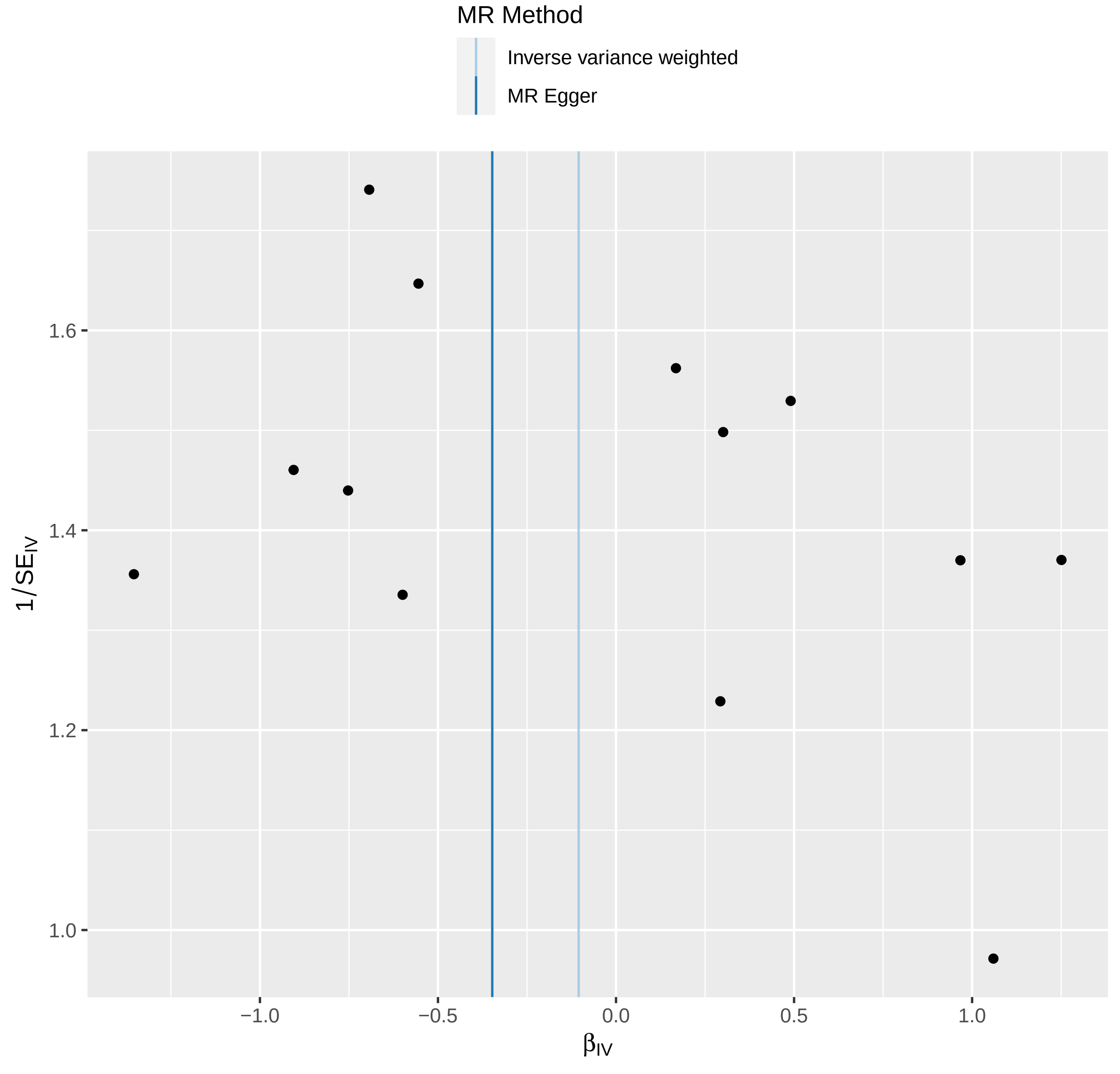

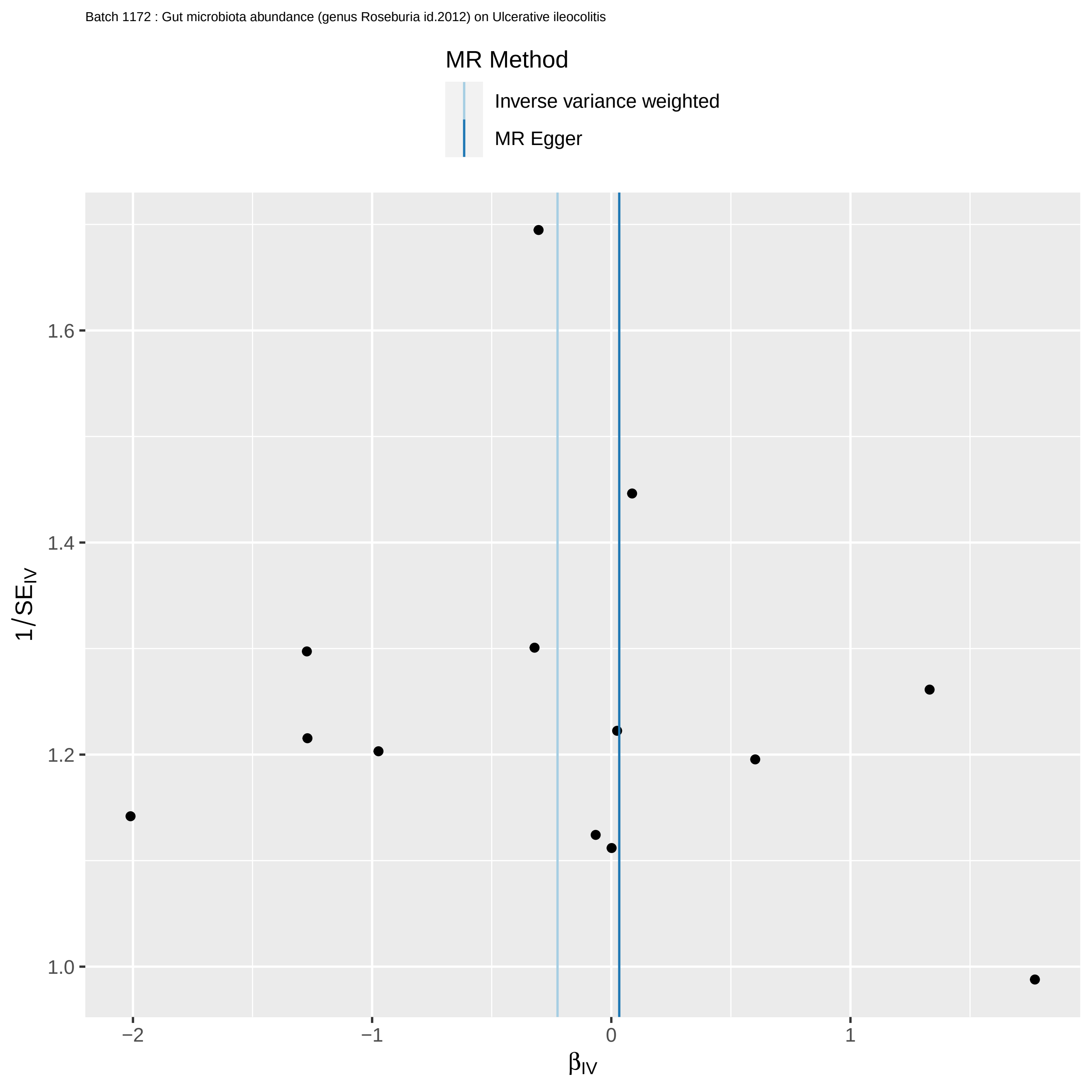

Batch 1172 : Gut microbiota abundance (genus Roseburia id.2012) on Ulcerative ileocolitis
MR Method
Inverse variance weighted
MR Egger
1.6
1.4
1.2
1.0
$1/SE_{IV}$
−2
−1
0
1
β_{IV}

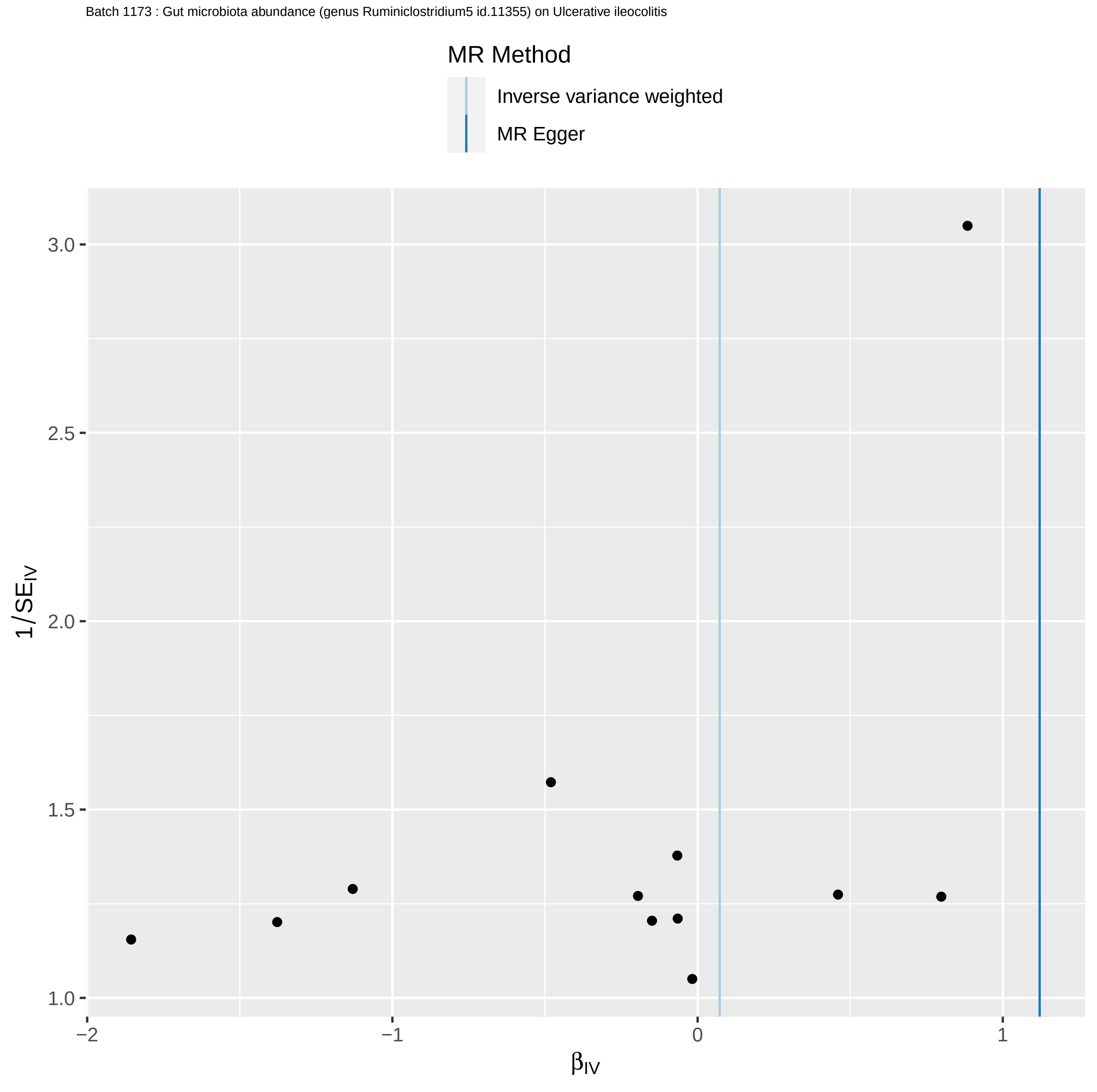
Batch 1173 : Gut microbiota abundance (genus Ruminiclostridium5 id.11355) on Ulcerative ileocolitis
MR Method
Inverse variance weighted
MR Egger
3.0
2.5
2.0
1.5
1.0
−2
−1
0
1
1/SE$_{IV}$
β$_{IV}$

Batch 1174 : Gut microbiota abundance (genus Ruminiclostridium6 id.11356) on Ulcerative ileocolitis

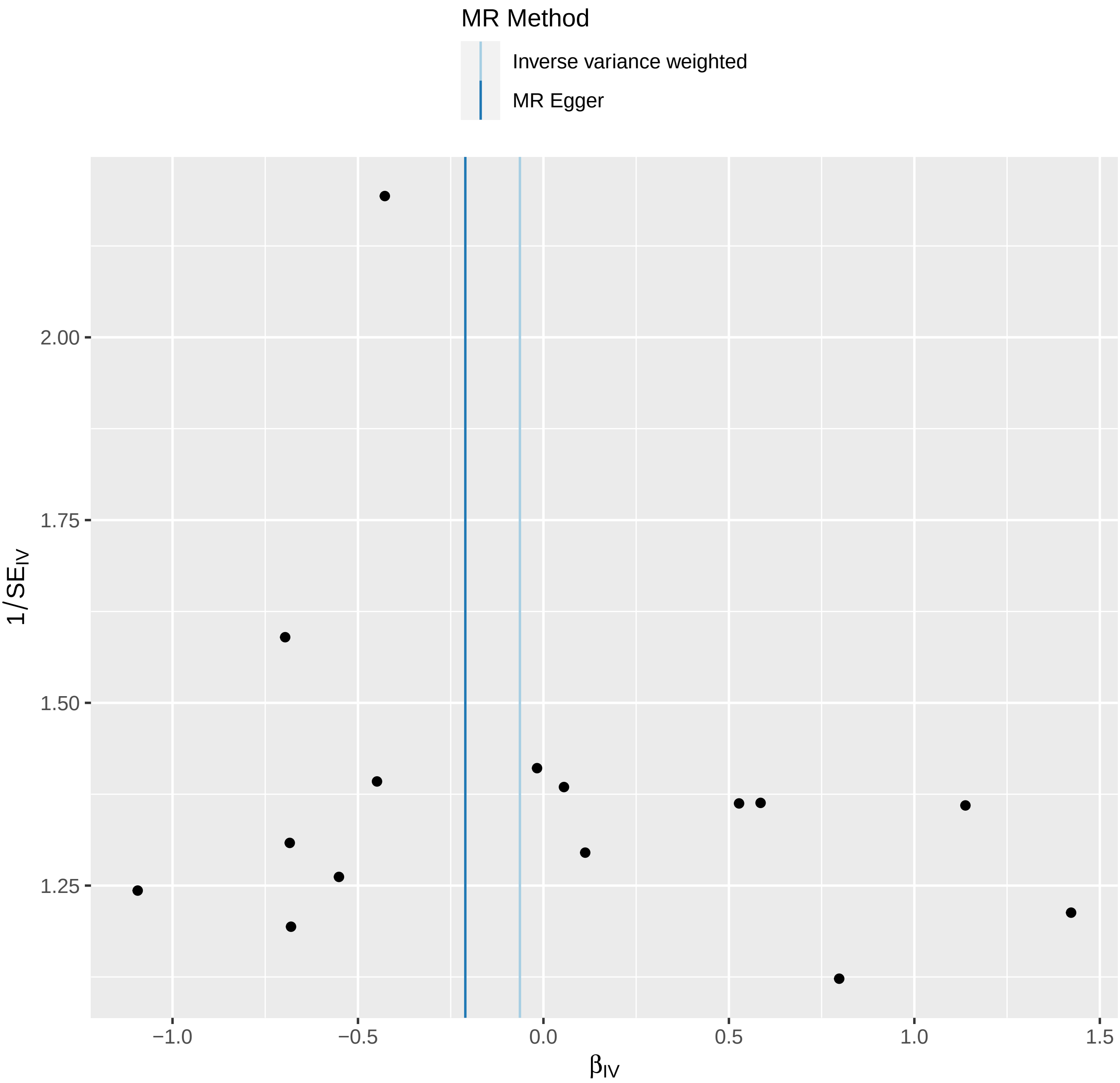

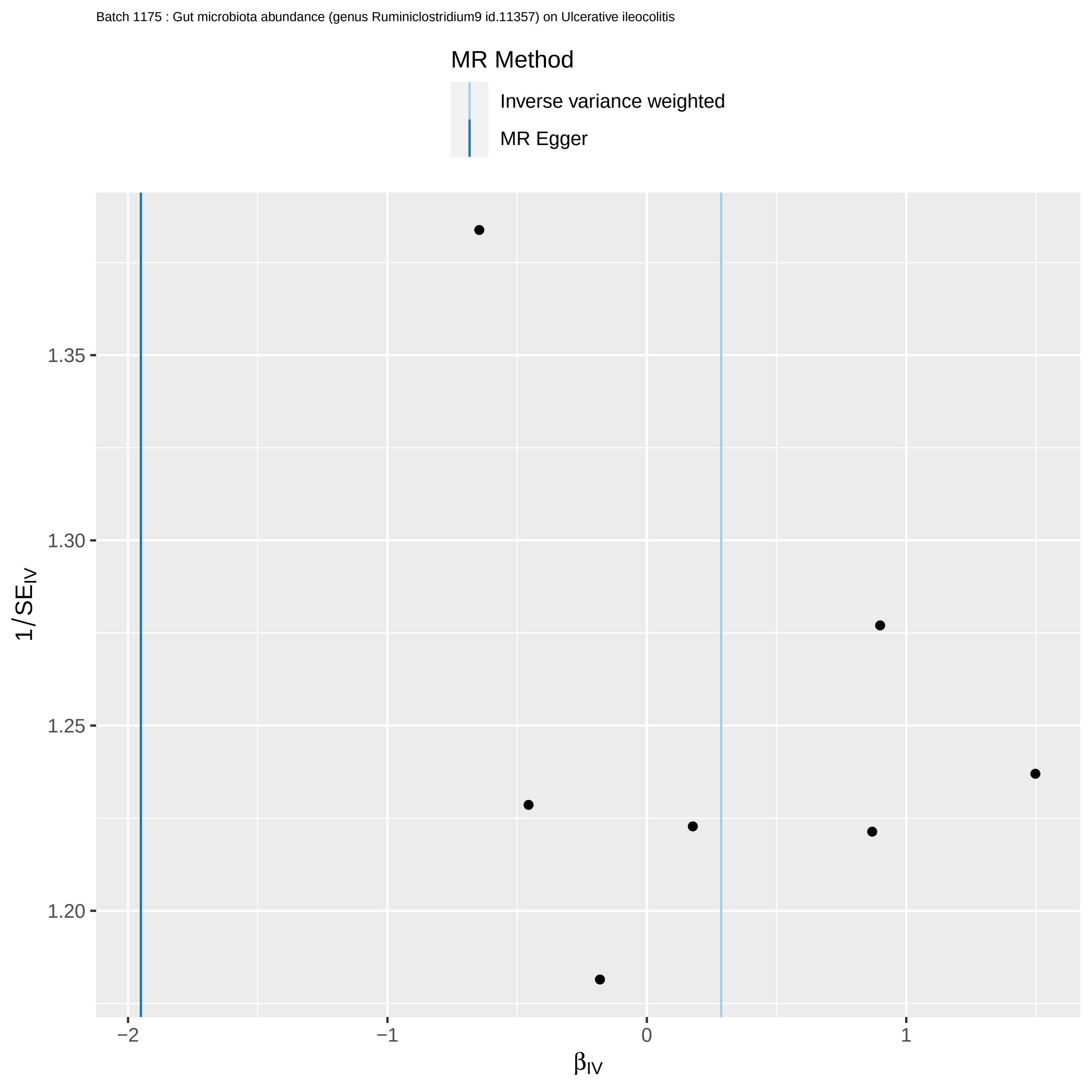
Batch 1175 : Gut microbiota abundance (genus Ruminiclostridium9 id.11357) on Ulcerative ileocolitis
MR Method
Inverse variance weighted
MR Egger
1.35
1.30
1.25
1.20
1/SE_IV
−2
−1
0
1
β_IV

Batch 1176 : Gut microbiota abundance (genus Ruminococcaceae NK4A214 group id.11358) on Ulcerative ileocolitis

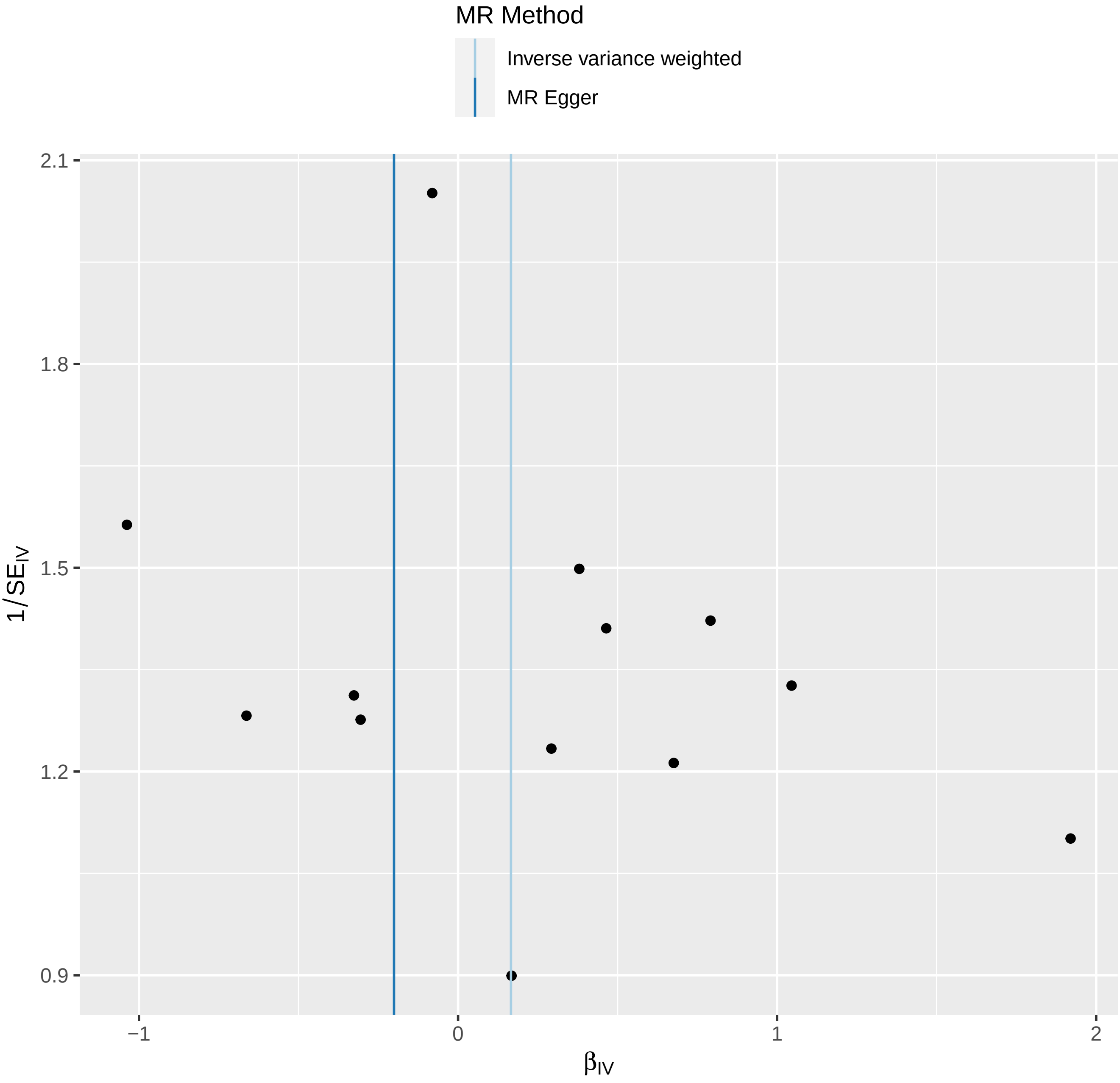

Batch 1177 : Gut microbiota abundance (genus Ruminococcaceae UCG002 id.11360) on Ulcerative ileocolitis

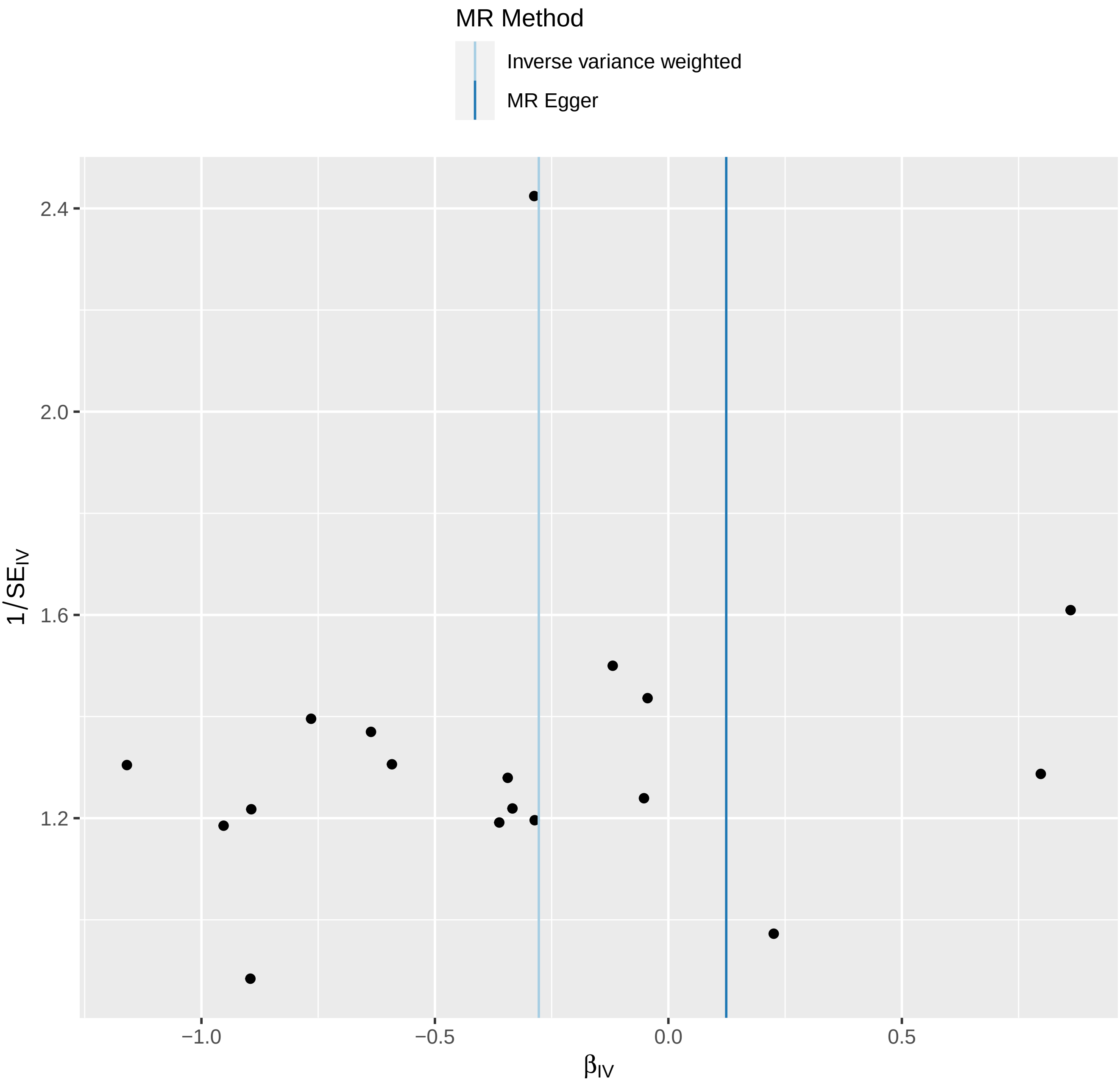

Batch 1178 : Gut microbiota abundance (genus Ruminococcaceae UCG003 id.11361) on Ulcerative ileocolitis

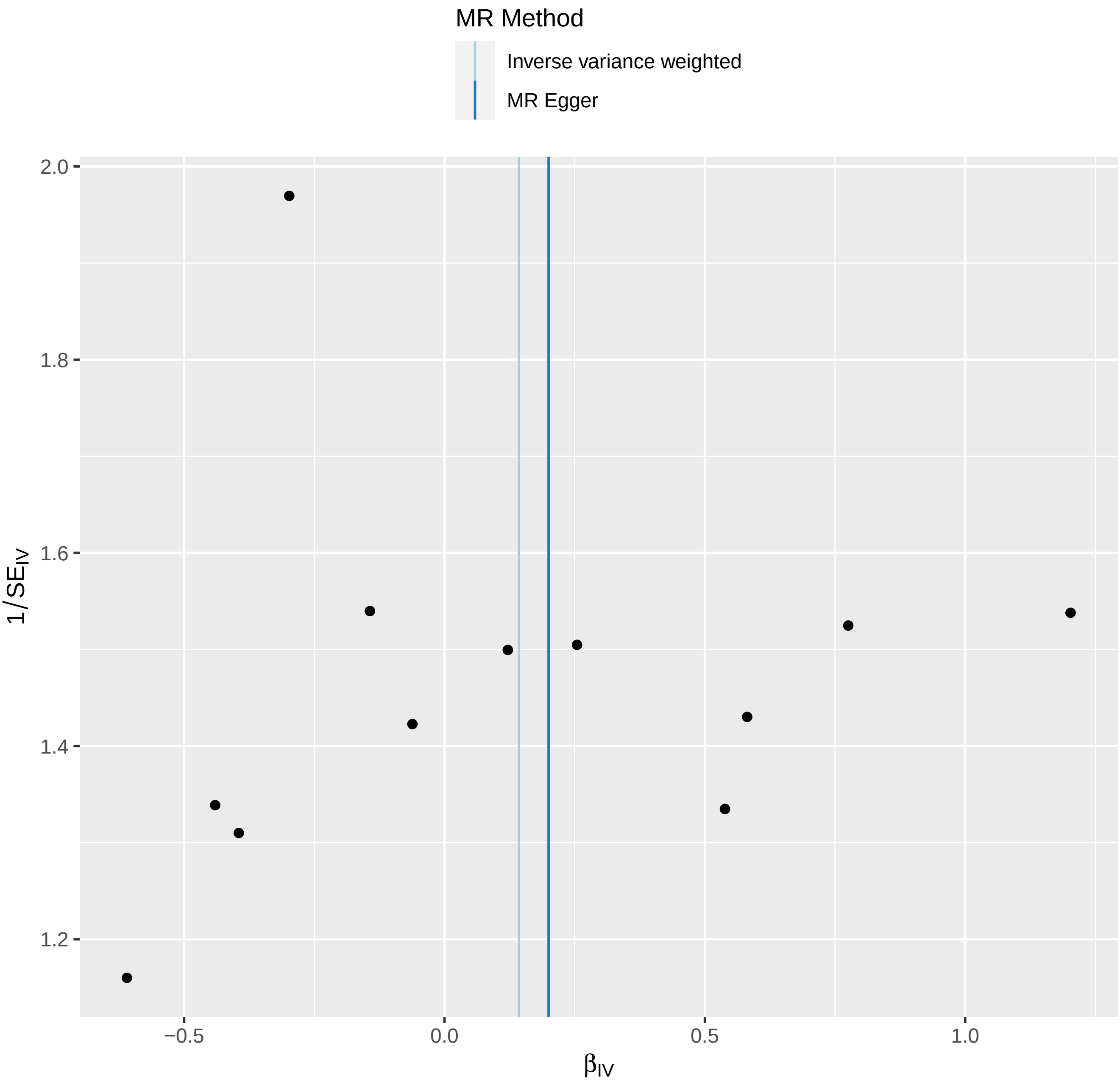

Batch 1179 : Gut microbiota abundance (genus Ruminococcaceae UCG004 id.11362) on Ulcerative ileocolitis

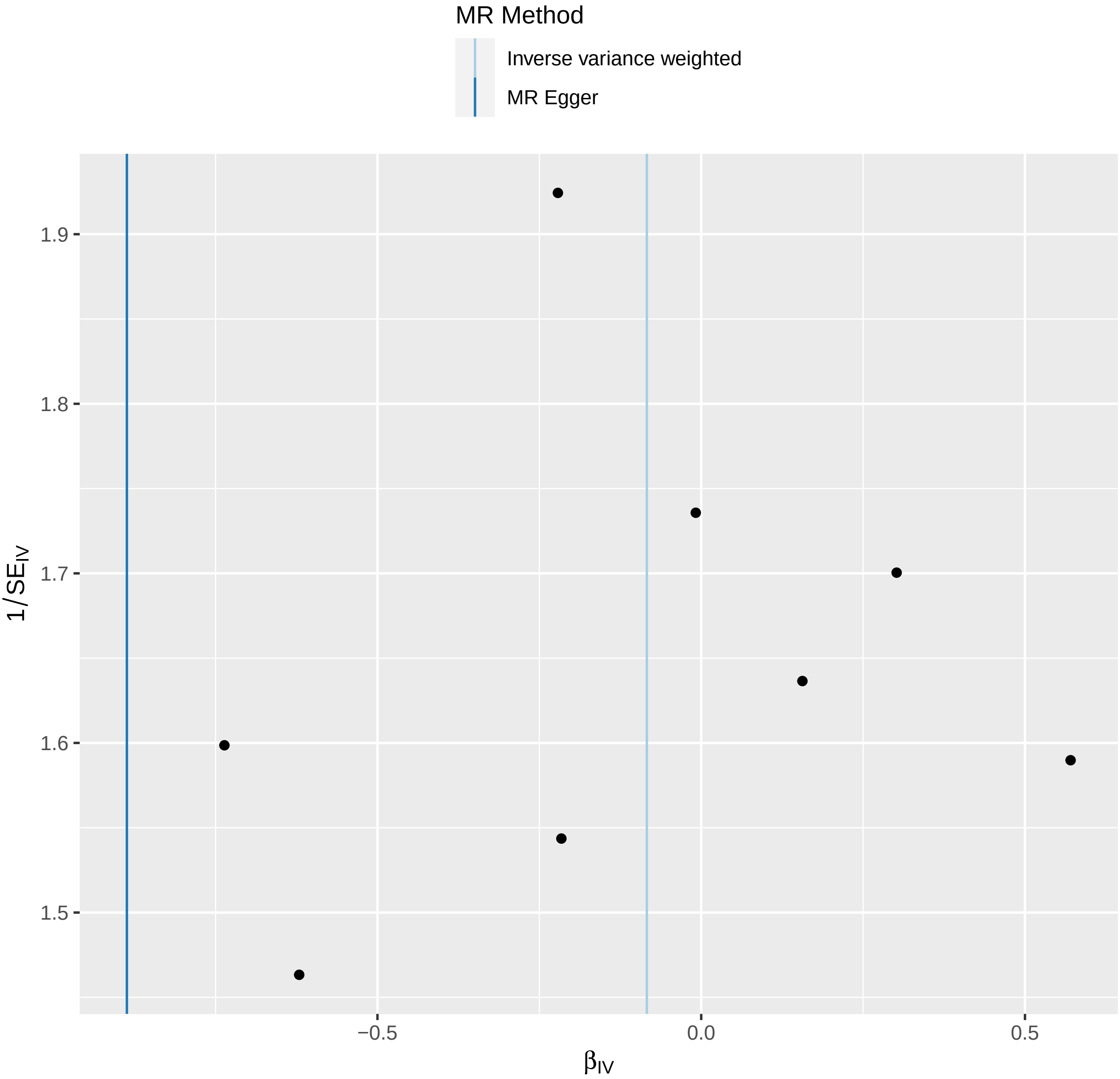

Batch 1180 : Gut microbiota abundance (genus Ruminococcaceae UCG005 id.11363) on Ulcerative ileocolitis

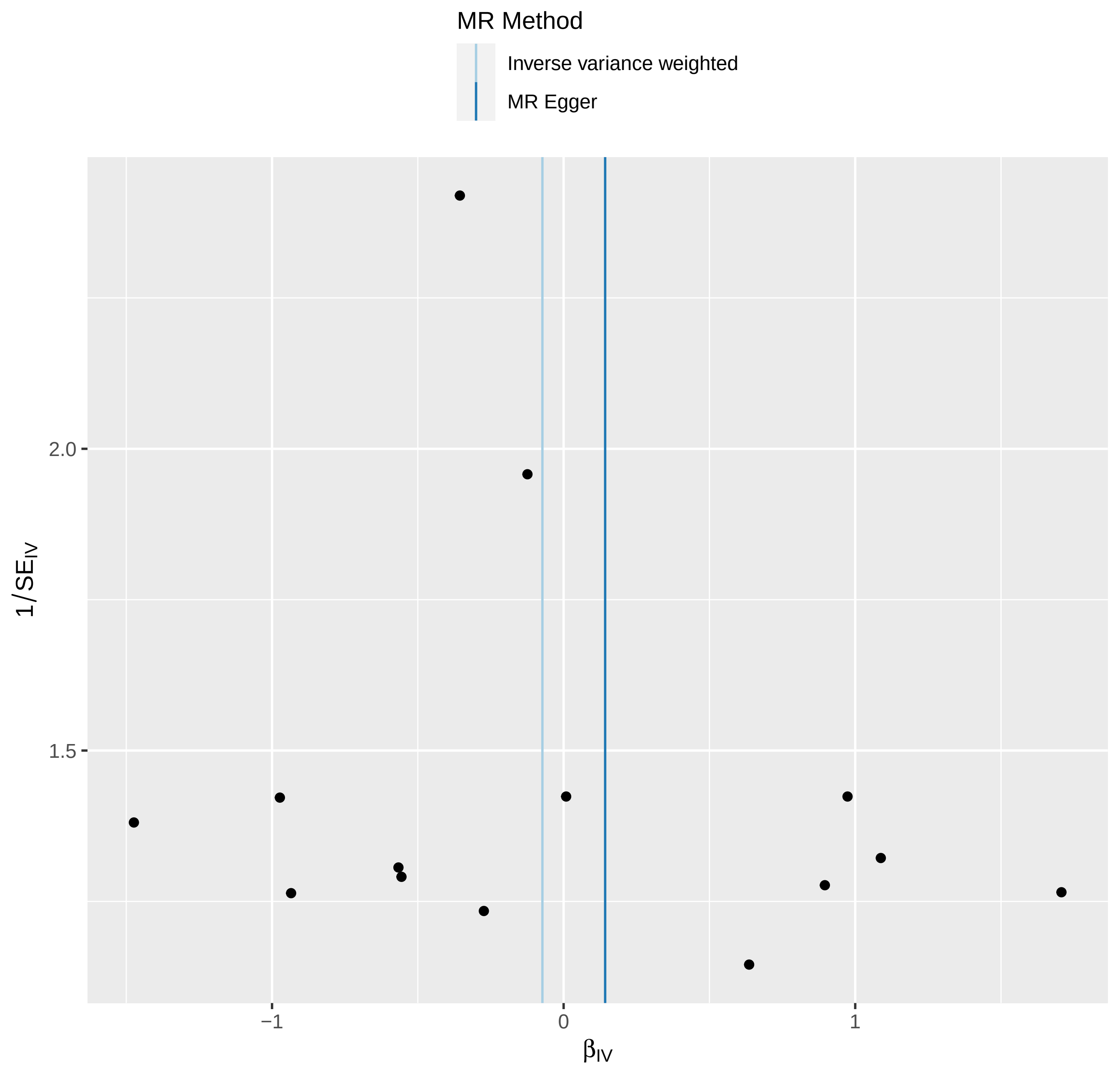

Batch 1181 : Gut microbiota abundance (genus Ruminococcaceae UCG009 id.11366) on Ulcerative ileocolitis

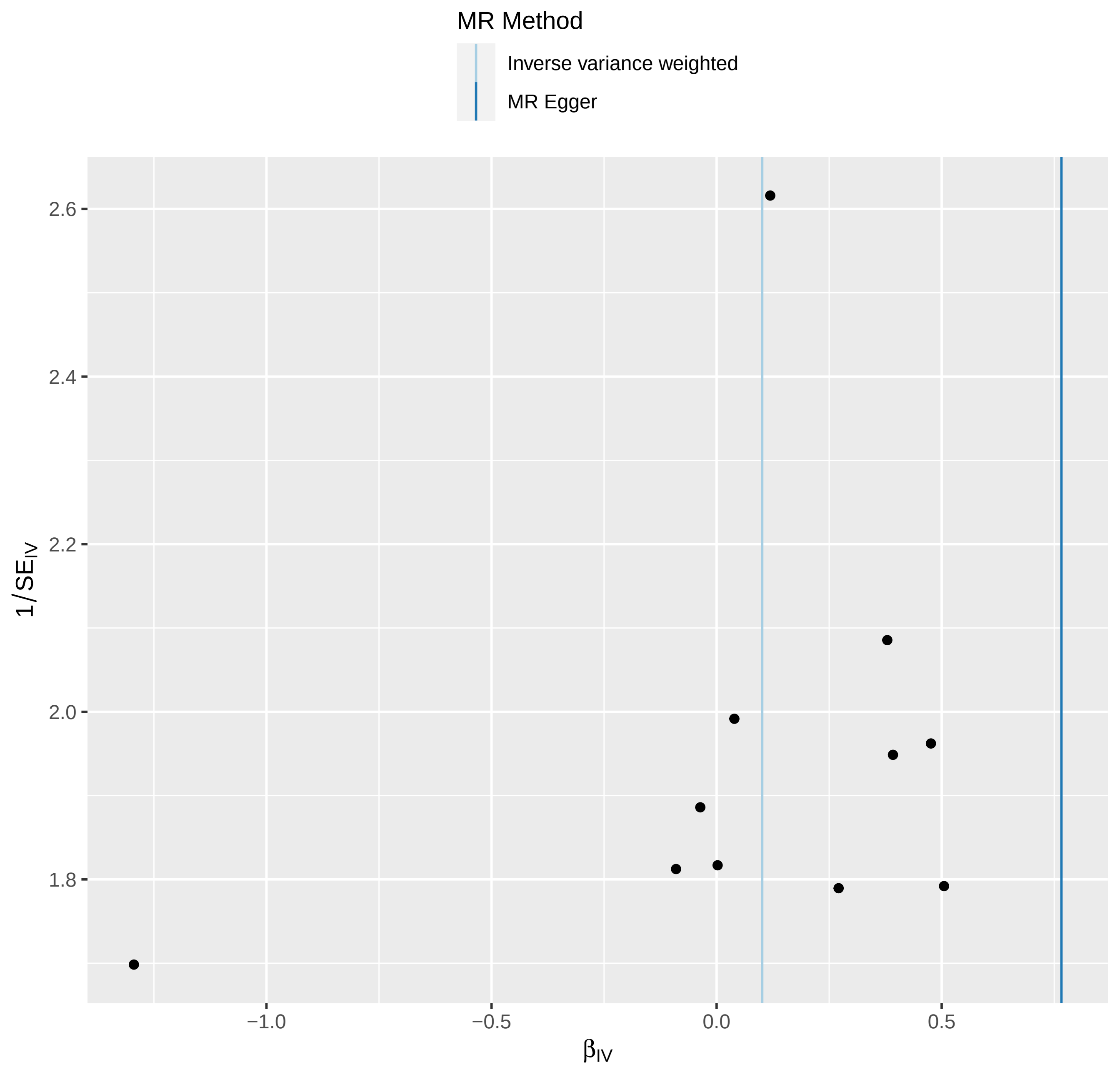

Batch 1182 : Gut microbiota abundance (genus Ruminococcaceae UCG010 id.11367) on Ulcerative ileocolitis

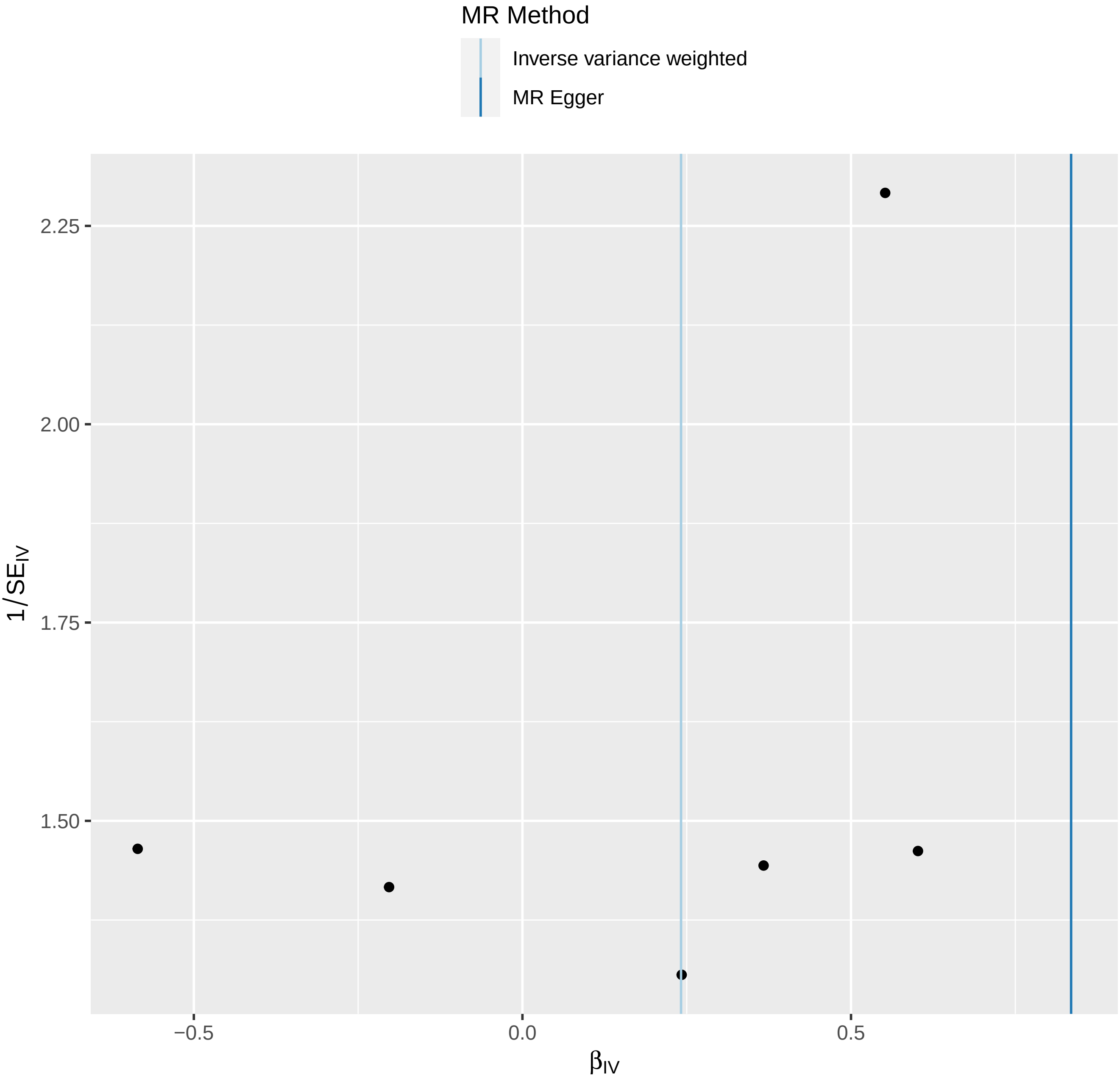

Batch 1183 : Gut microbiota abundance (genus Ruminococcaceae UCG011 id.11368) on Ulcerative ileocolitis

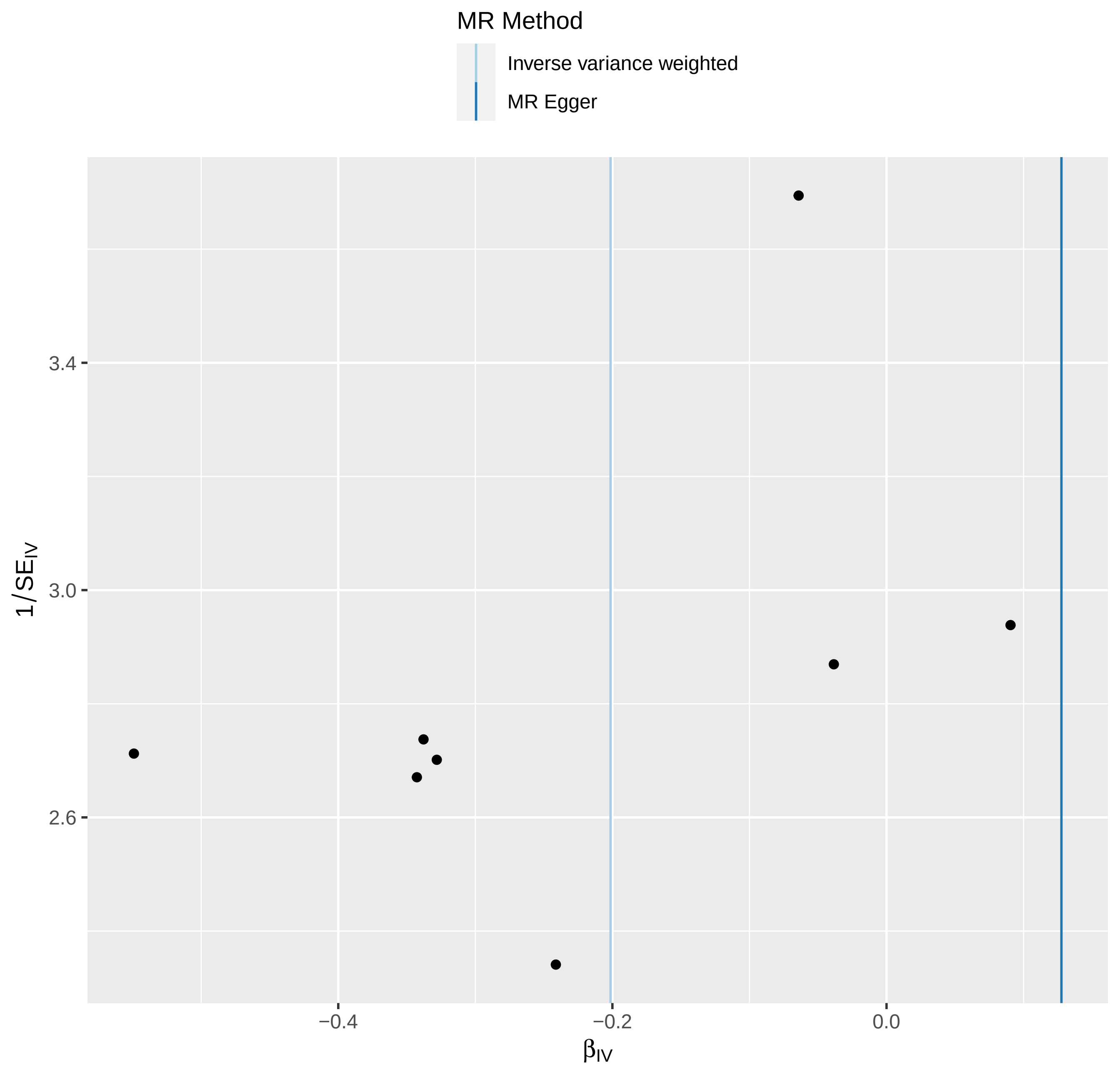

Batch 1184 : Gut microbiota abundance (genus Ruminococcaceae UCG013 id.11370) on Ulcerative ileocolitis

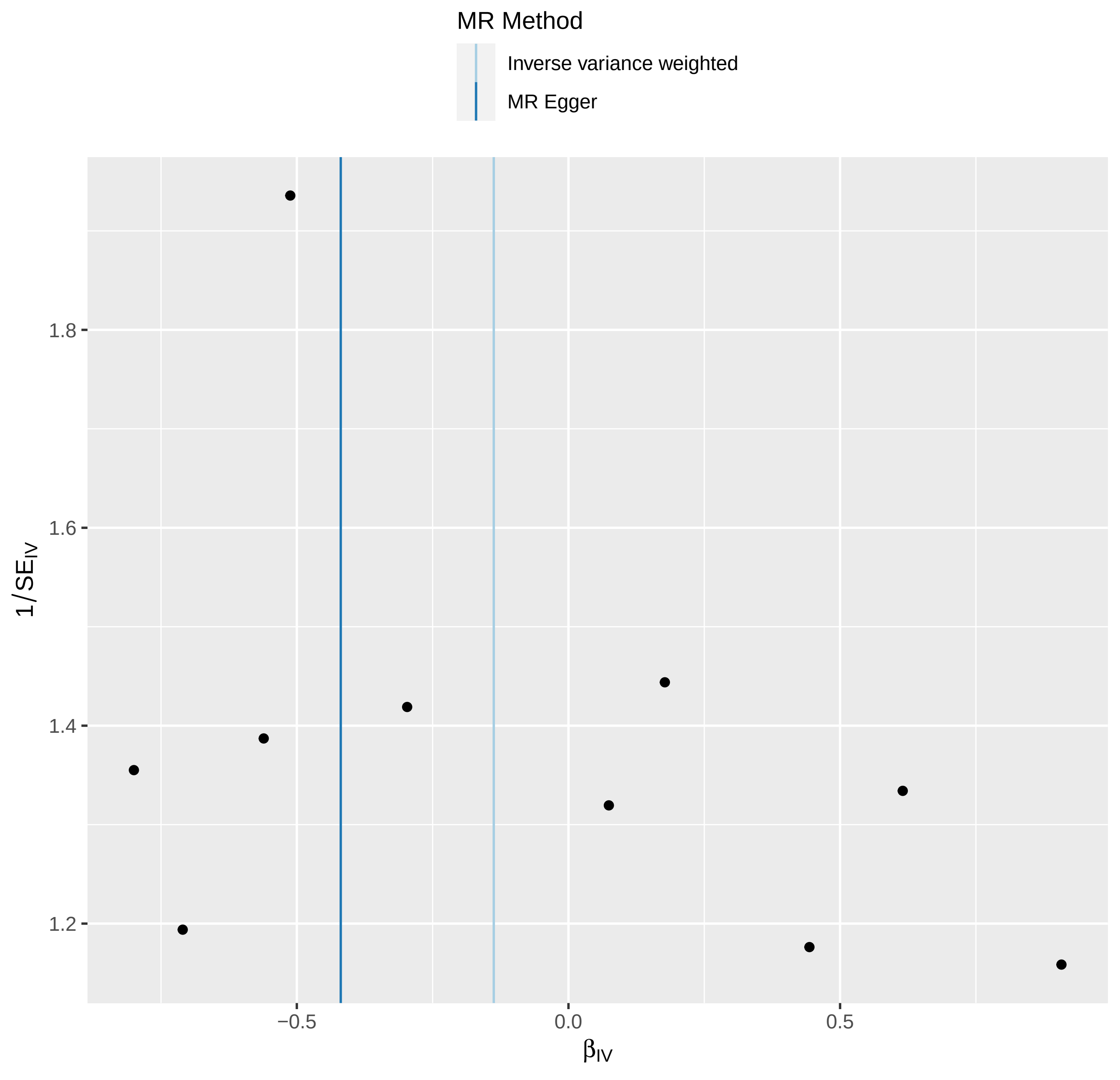

Batch 1185 : Gut microbiota abundance (genus Ruminococcaceae UCG014 id.11371) on Ulcerative ileocolitis

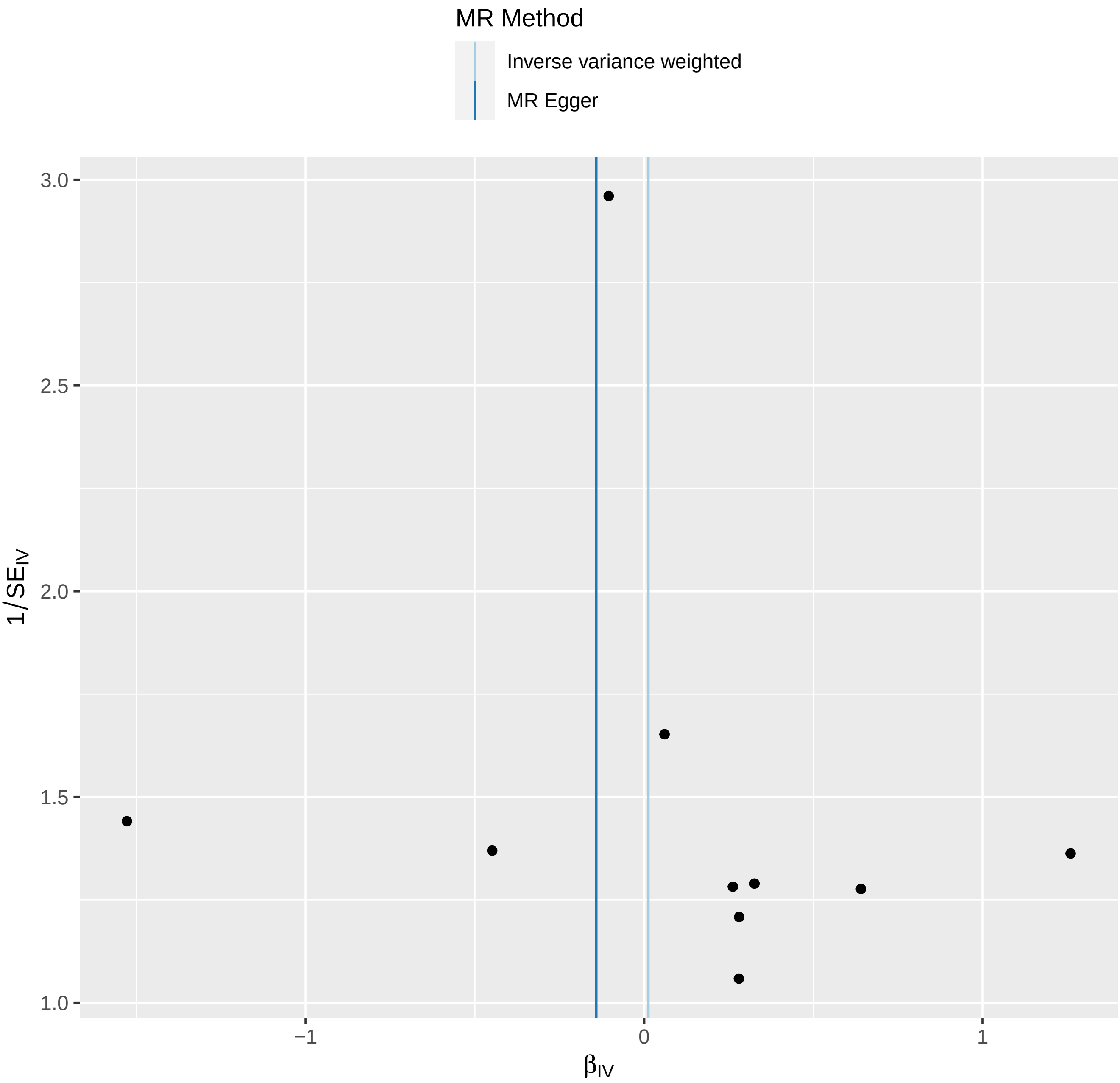

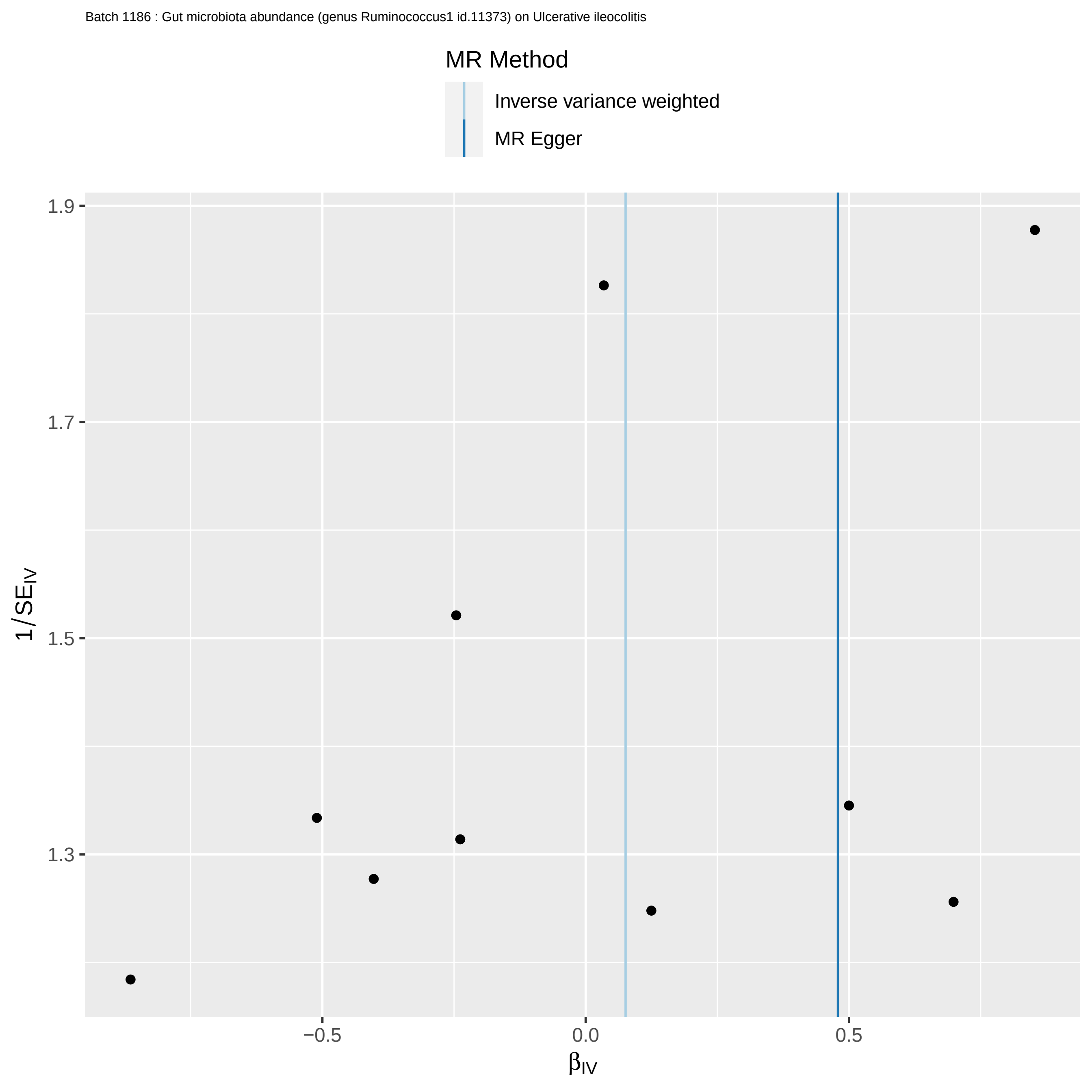

Batch 1186 : Gut microbiota abundance (genus Ruminococcus1 id.11373) on Ulcerative ileocolitis
MR Method
Inverse variance weighted
MR Egger
1.9
1.7
1.5
1.3
1/SE$_{IV}$
−0.5
0.0
0.5
β$_{IV}$

Batch 1187 : Gut microbiota abundance (genus Ruminococcus2 id.11374) on Ulcerative ileocolitis

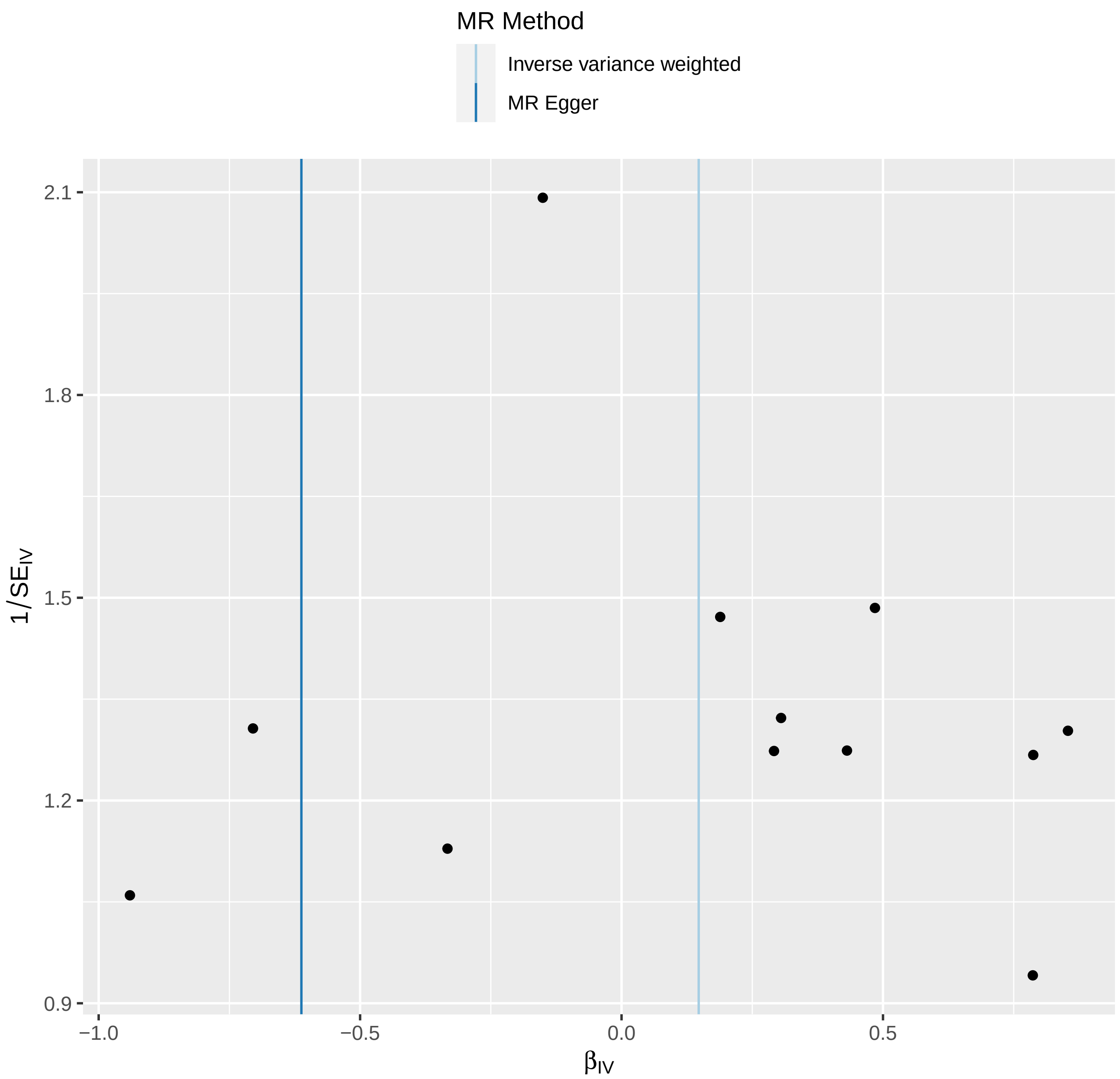

Batch 1188 : Gut microbiota abundance (genus Ruminococcus gauvreauii group id.11342) on Ulcerative ileocolitis

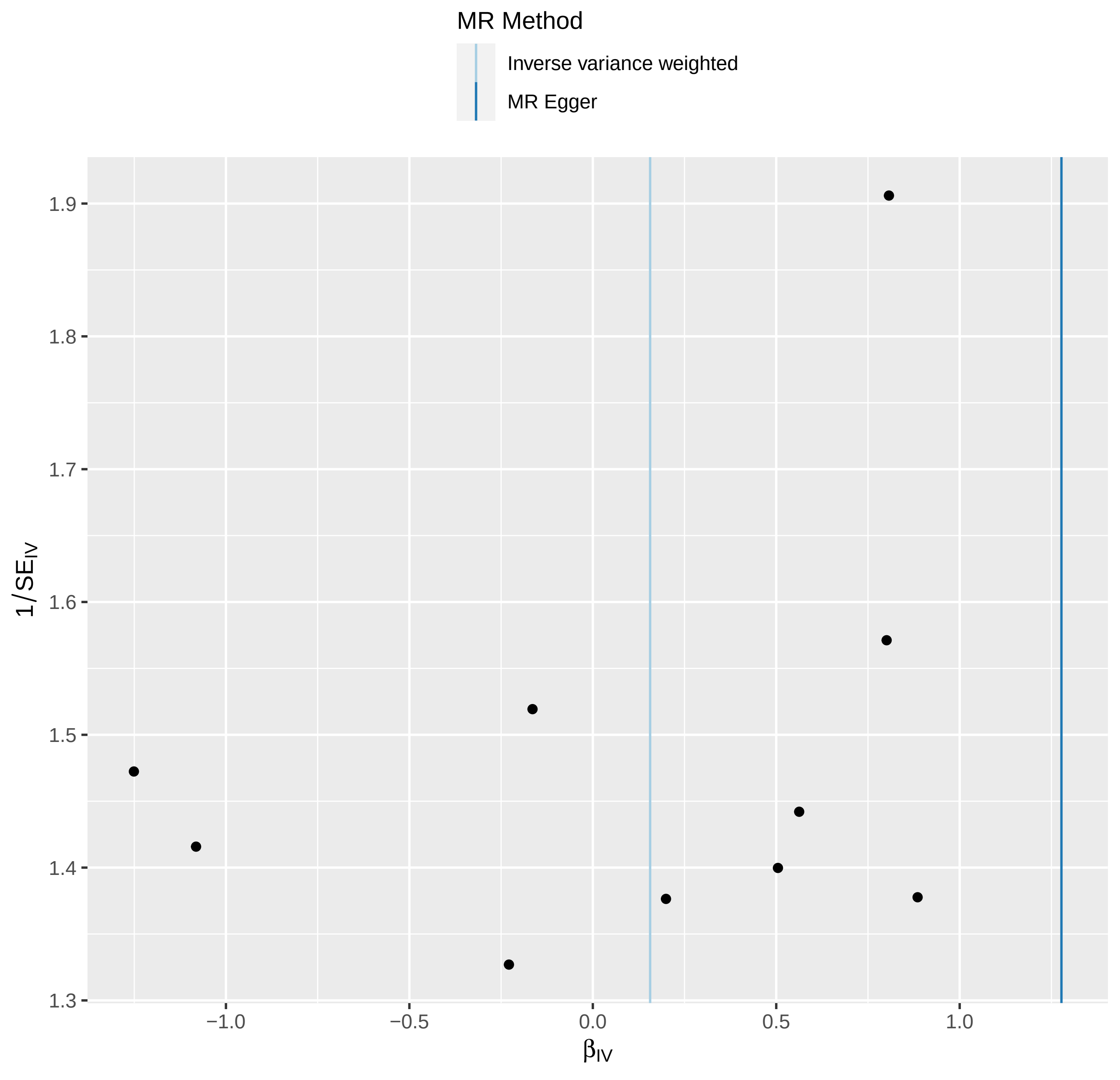

Batch 1189 : Gut microbiota abundance (genus Ruminococcus gnavus group id.14376) on Ulcerative ileocolitis

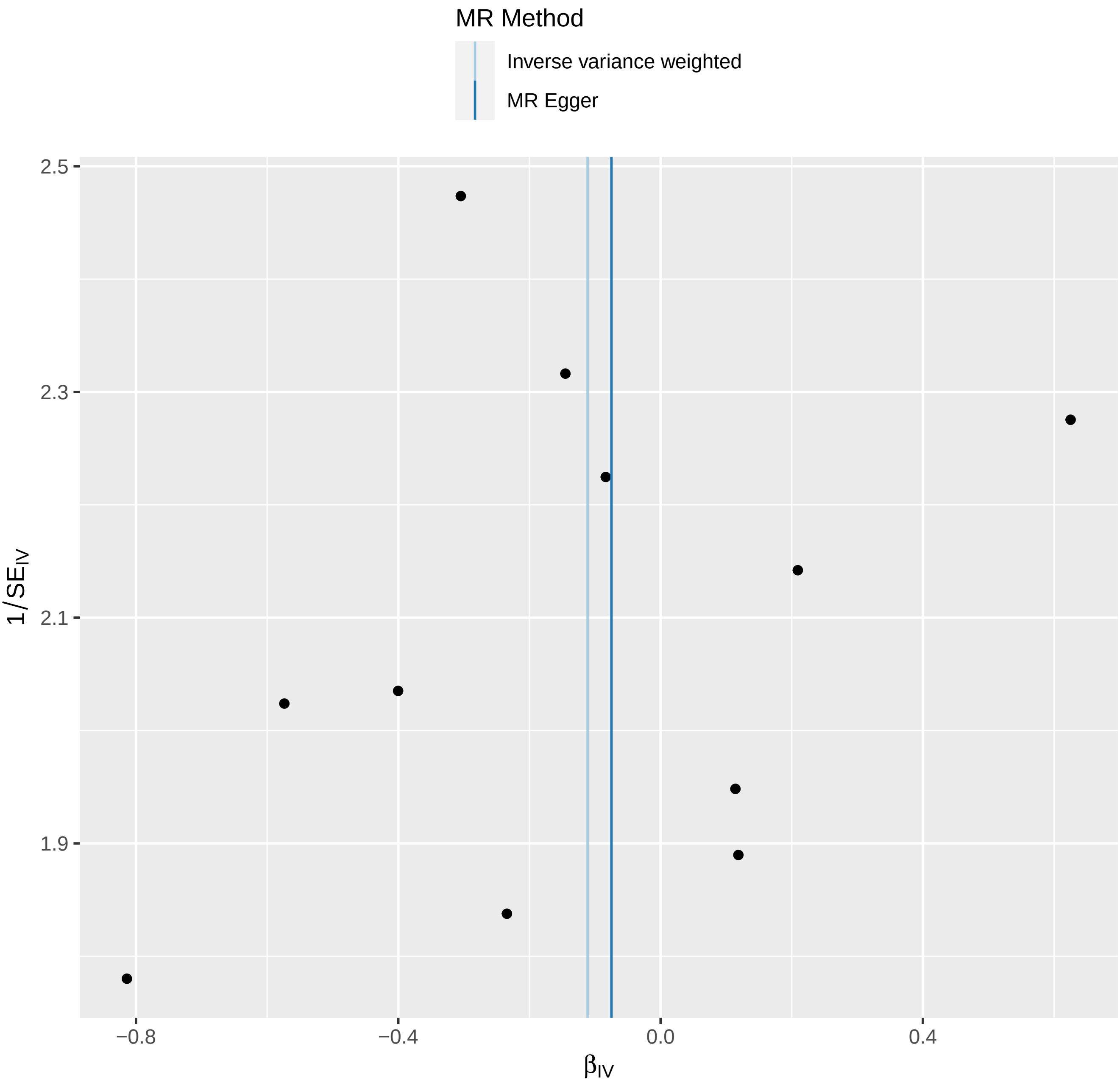

Batch 1190 : Gut microbiota abundance (genus Ruminococcus torques group id.14377) on Ulcerative ileocolitis

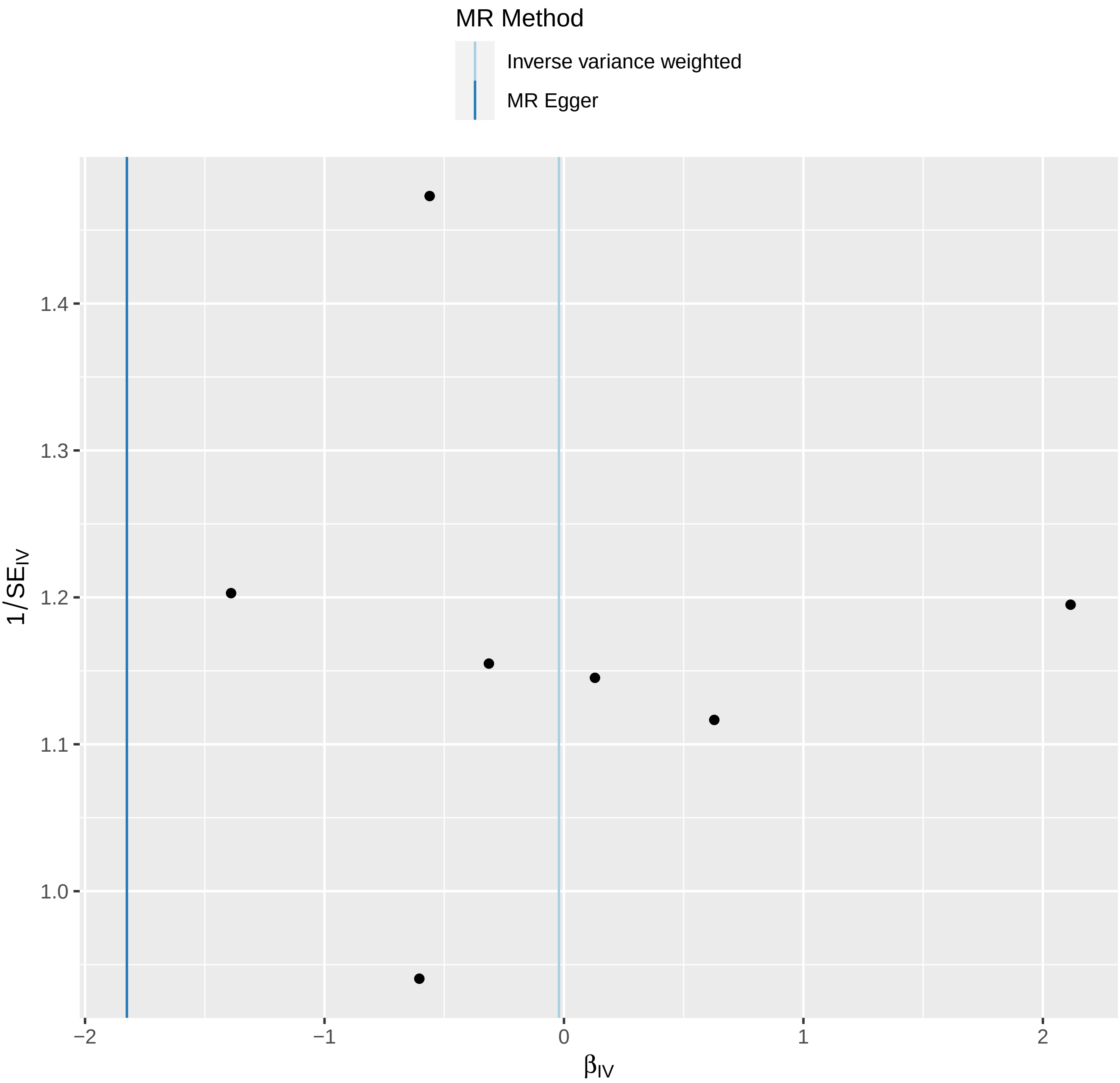

Batch 1191 : Gut microbiota abundance (genus Sellimonas id.14369) on Ulcerative ileocolitis

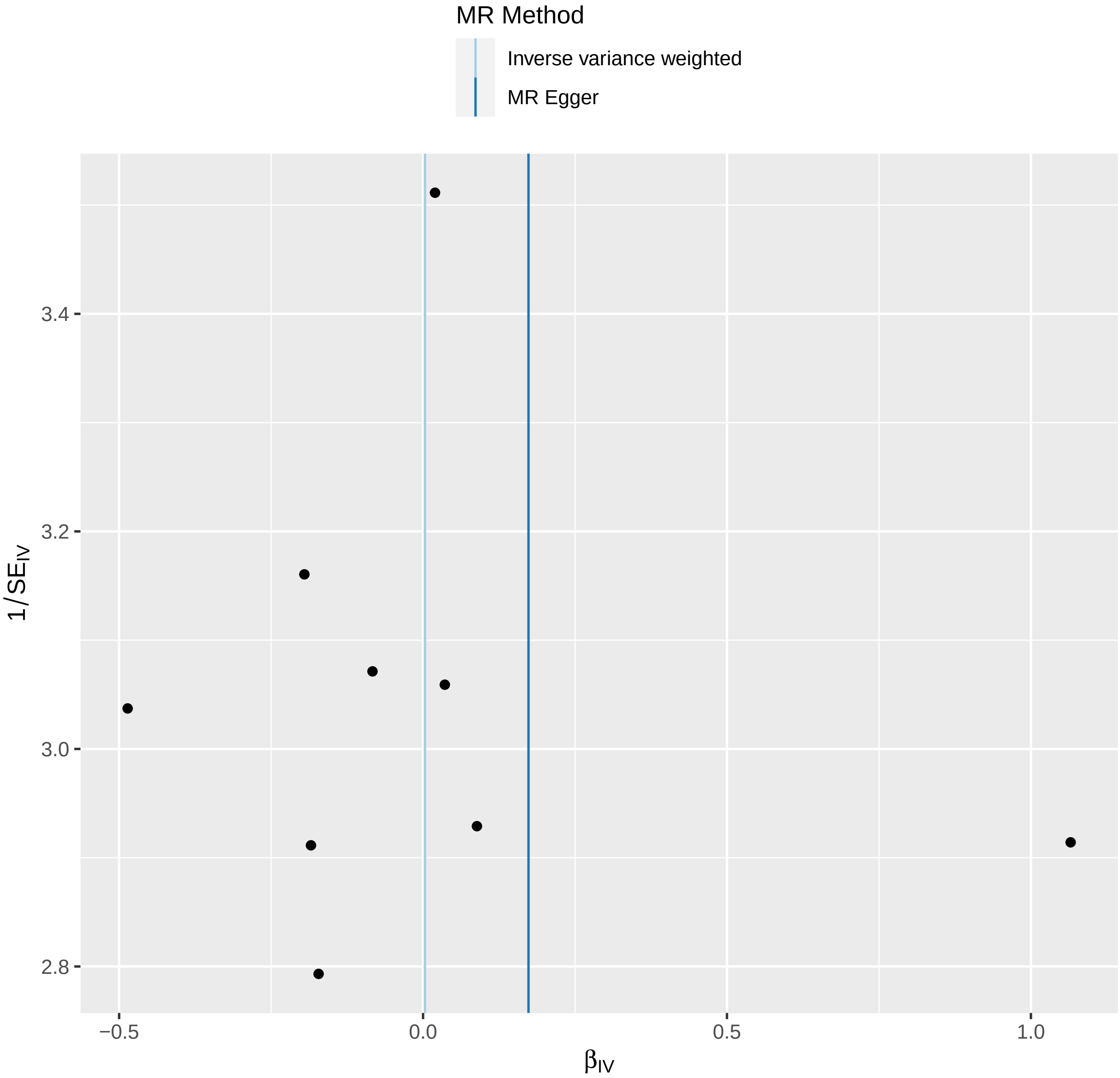

Batch 1192 : Gut microbiota abundance (genus Senegalimassilia id.11160) on Ulcerative ileocolitis

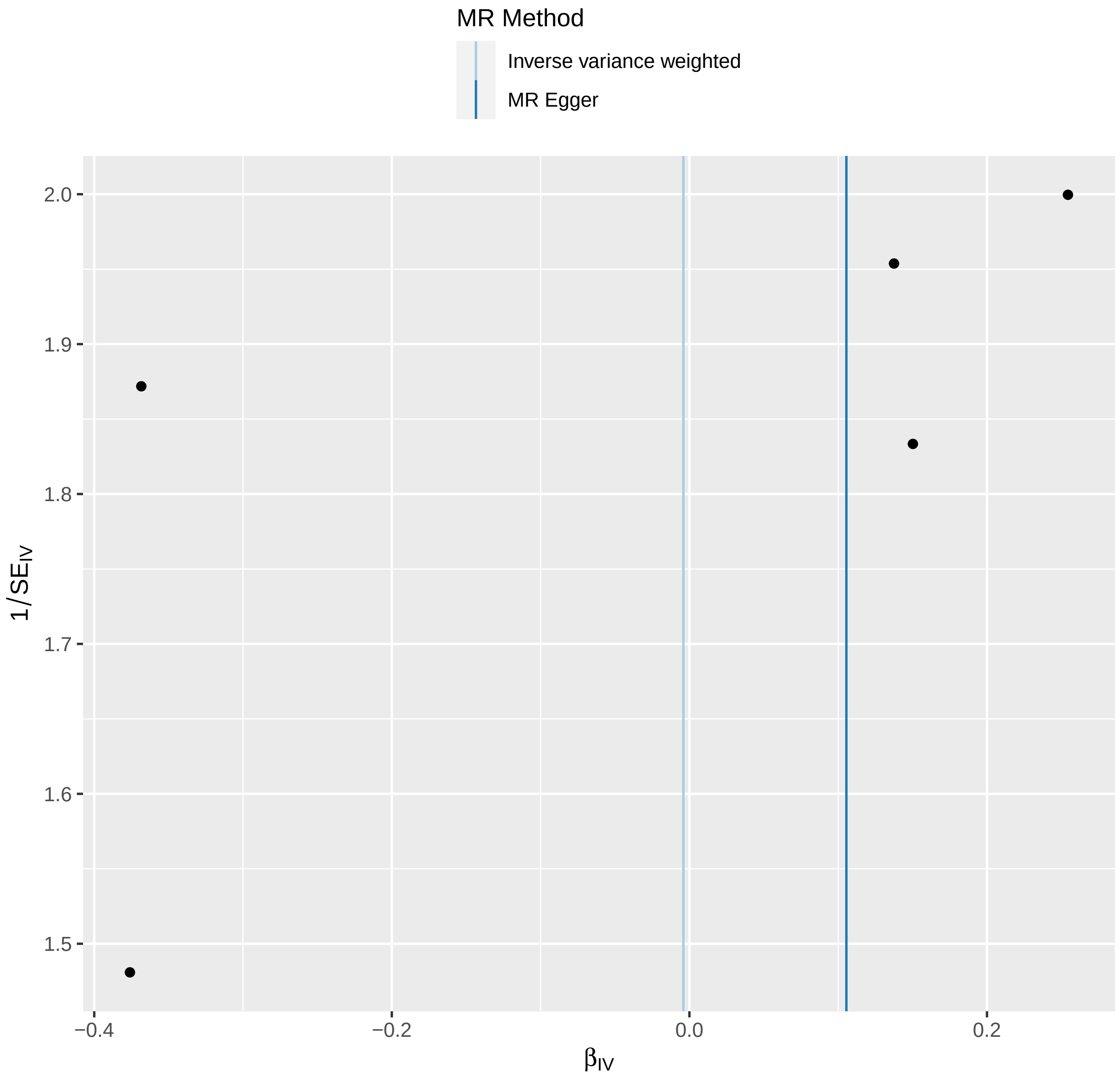

Batch 1193 : Gut microbiota abundance (genus Slackia id.825) on Ulcerative ileocolitis

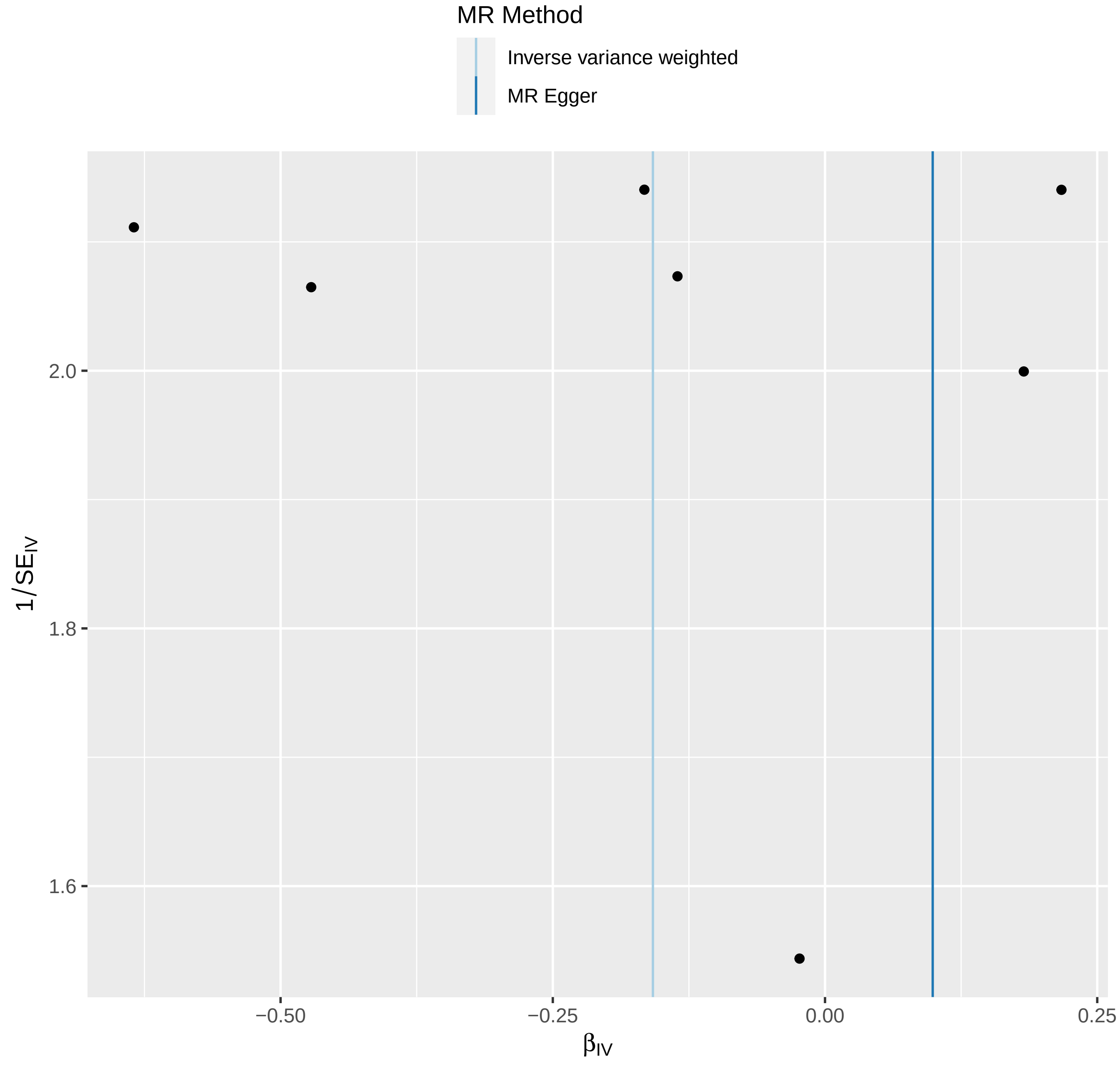

Batch 1194 : Gut microbiota abundance (genus Streptococcus id.1853) on Ulcerative ileocolitis

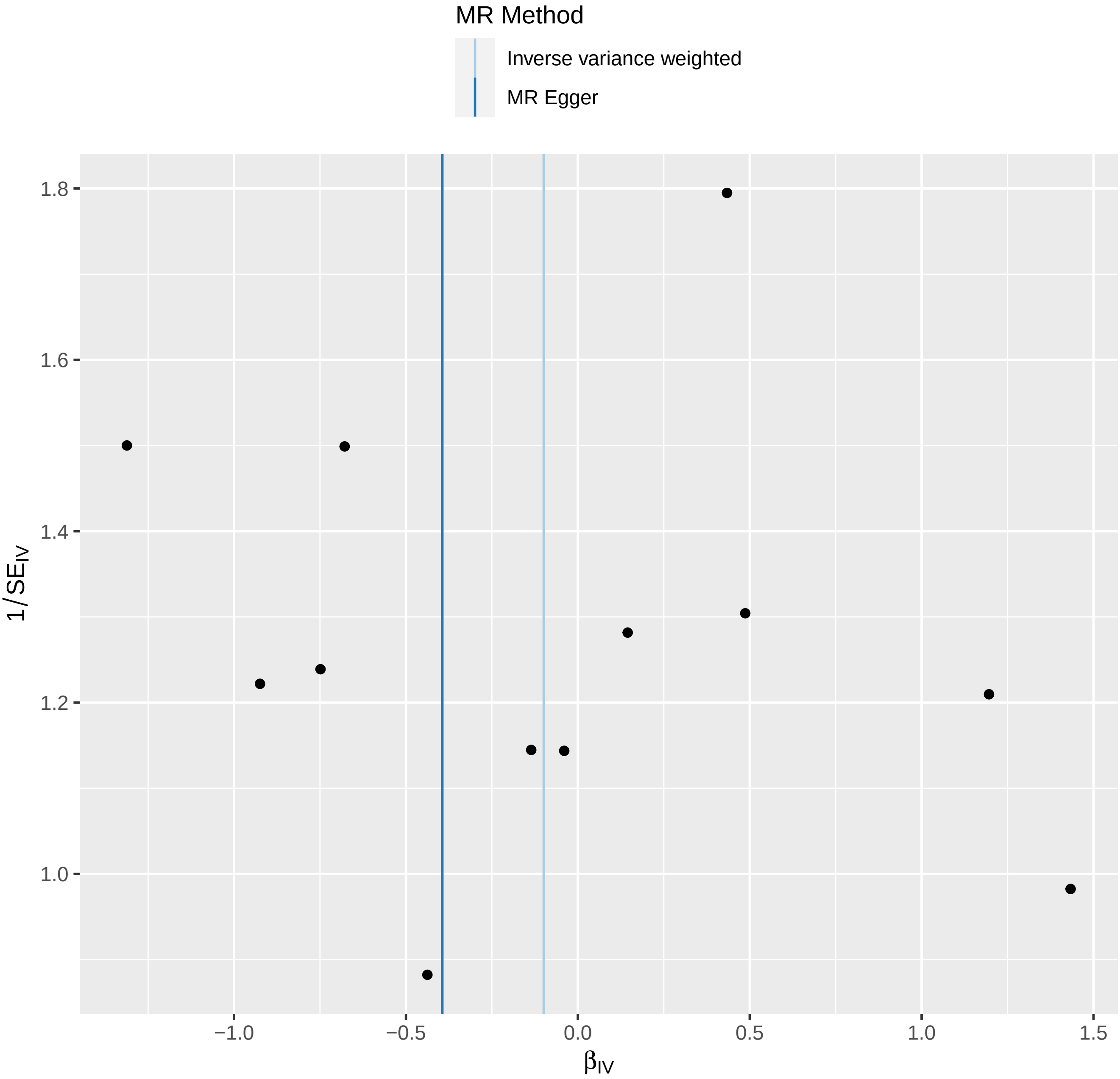

Batch 1195 : Gut microbiota abundance (genus Subdoligranulum id.2070) on Ulcerative ileocolitis

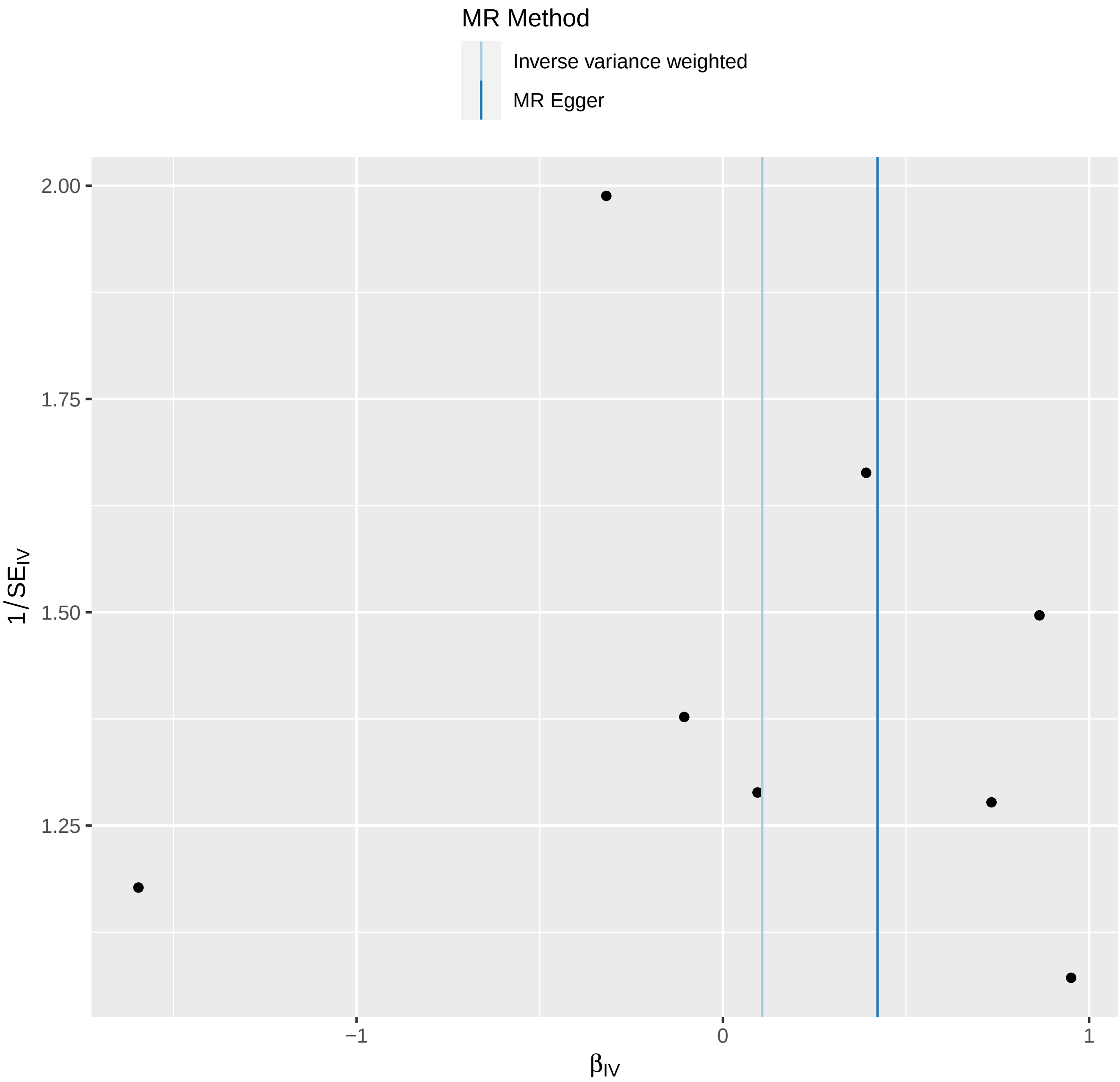

Batch 1196 : Gut microbiota abundance (genus Sutterella id.2896) on Ulcerative ileocolitis

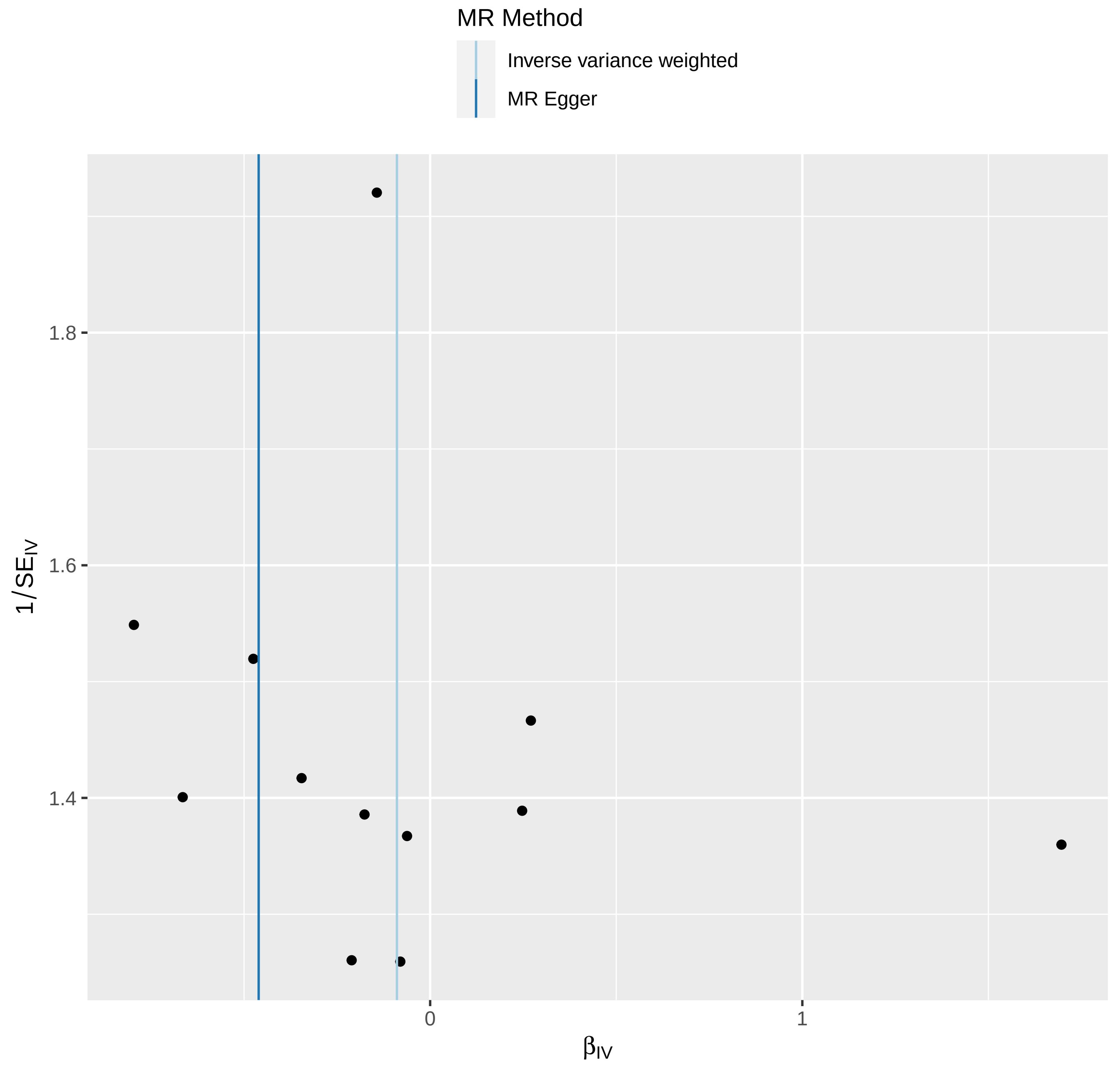

Batch 1197 : Gut microbiota abundance (genus Terrisporobacter id.11348) on Ulcerative ileocolitis

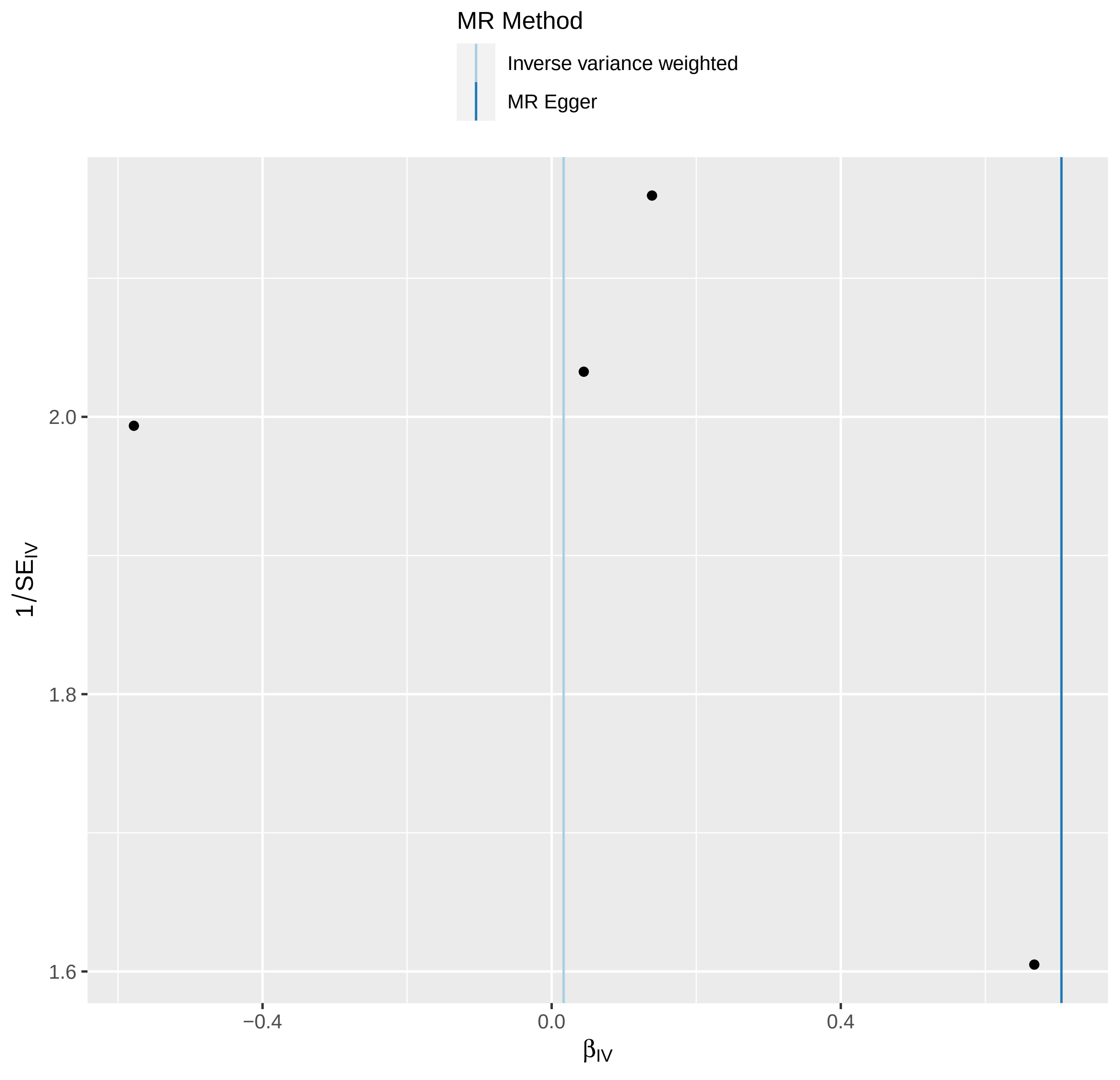

Batch 1198 : Gut microbiota abundance (genus Turicibacter id.2162) on Ulcerative ileocolitis

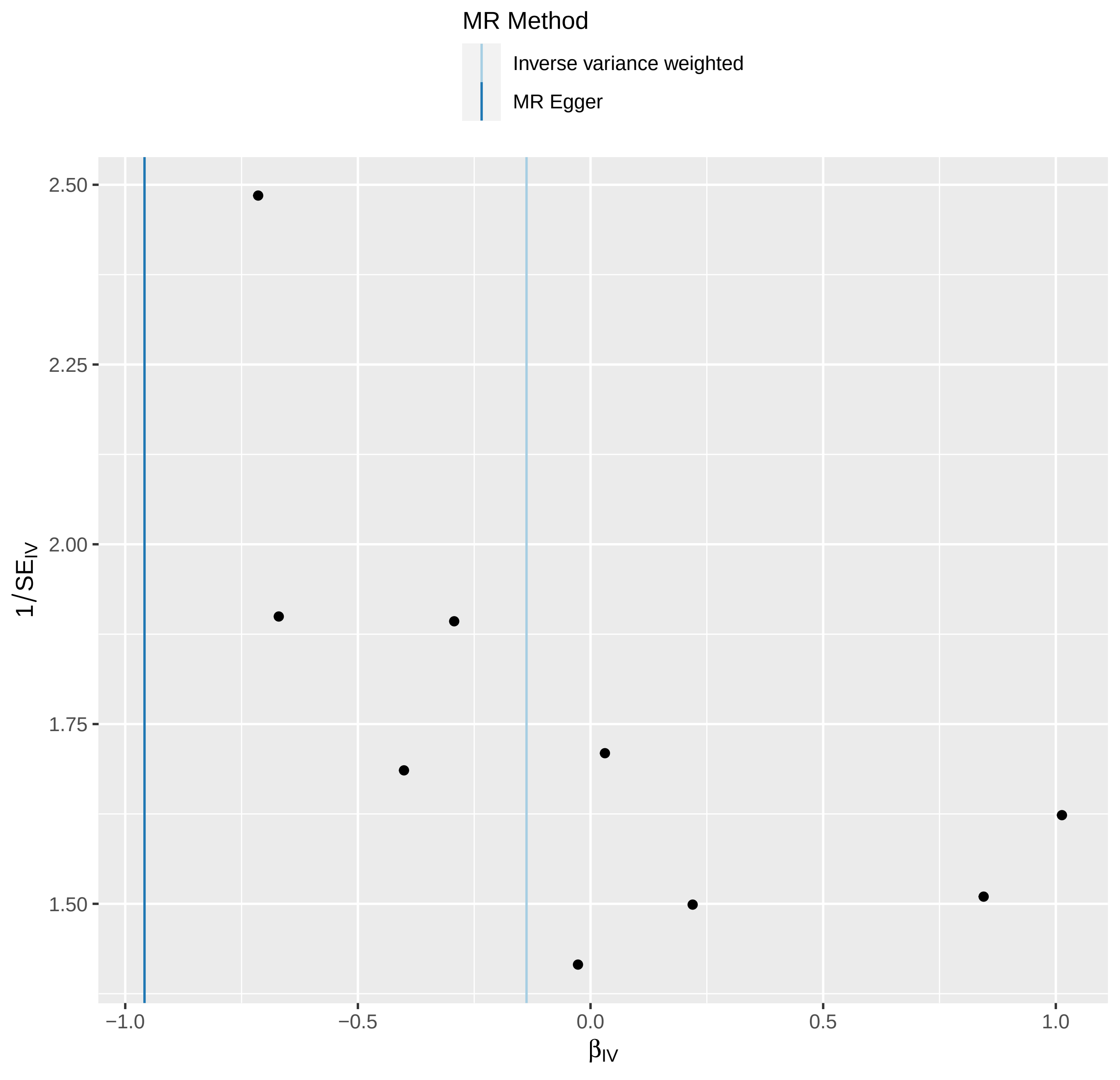

Batch 1199 : Gut microbiota abundance (genus Tyzzerella3 id.11335) on Ulcerative ileocolitis

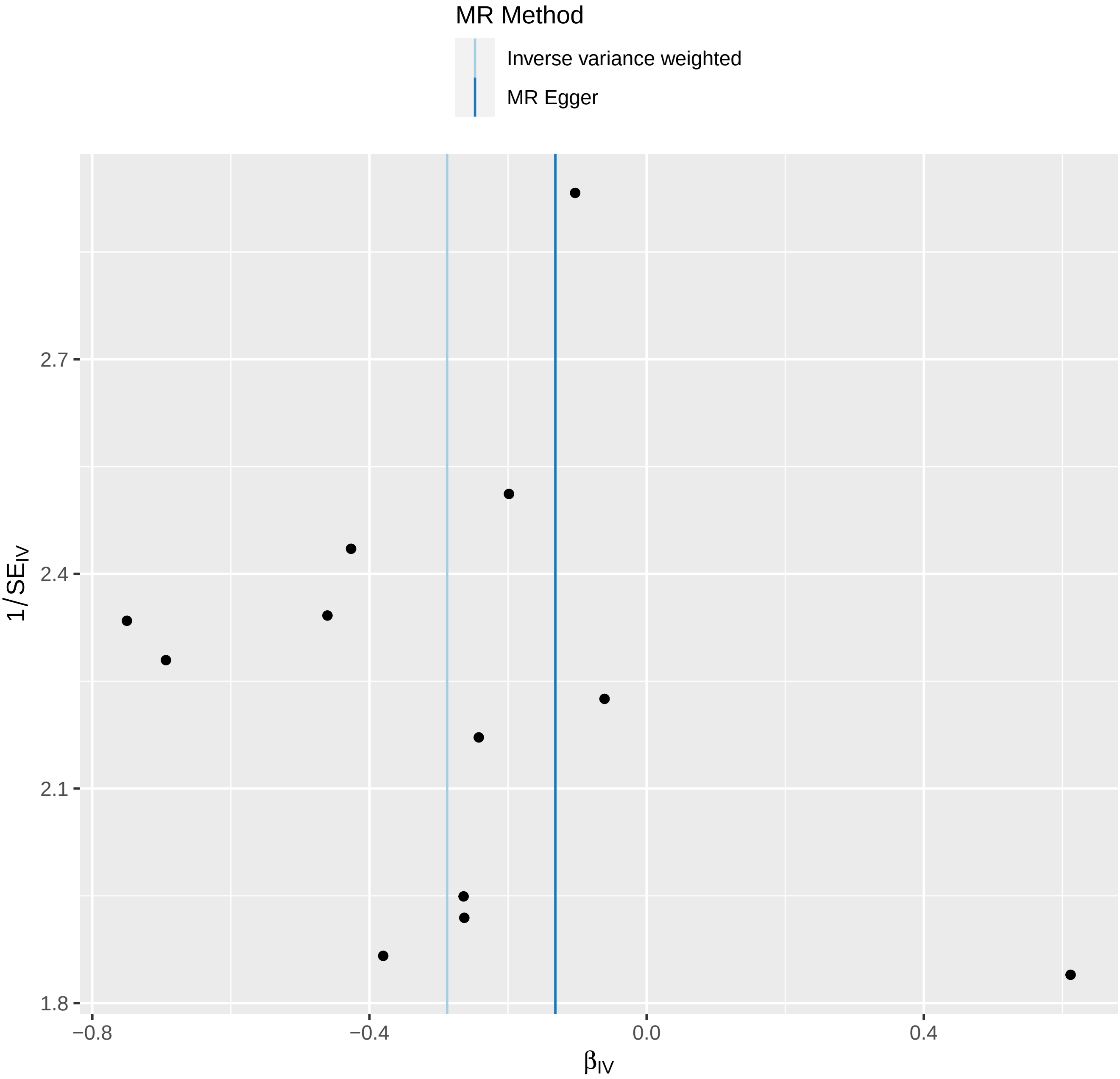

Batch 1200 : Gut microbiota abundance (genus Veillonella id.2198) on Ulcerative ileocolitis

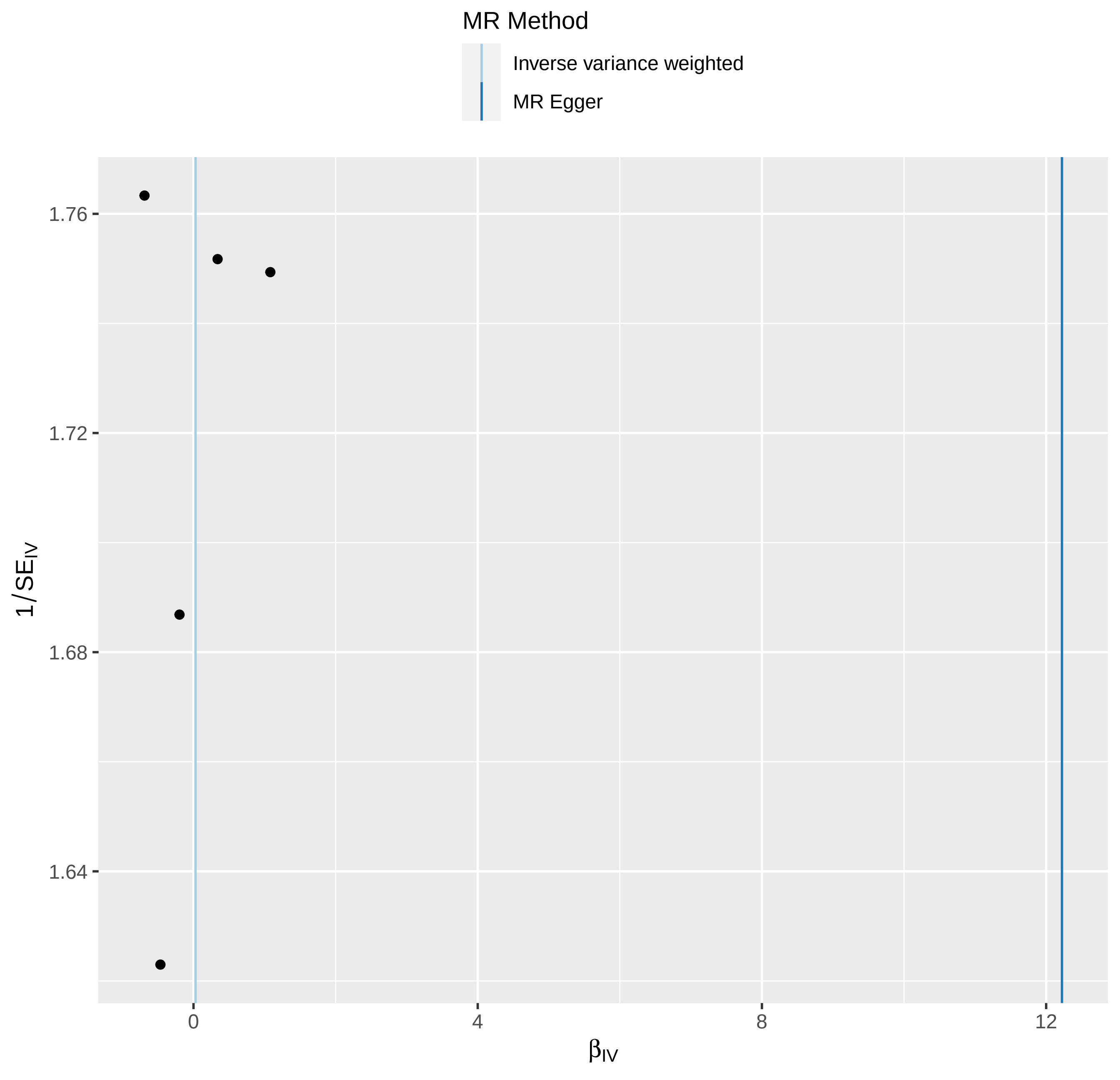

Batch 1201 : Gut microbiota abundance (family Acidaminococcaceae id.2166) on Other ulcerative colitis

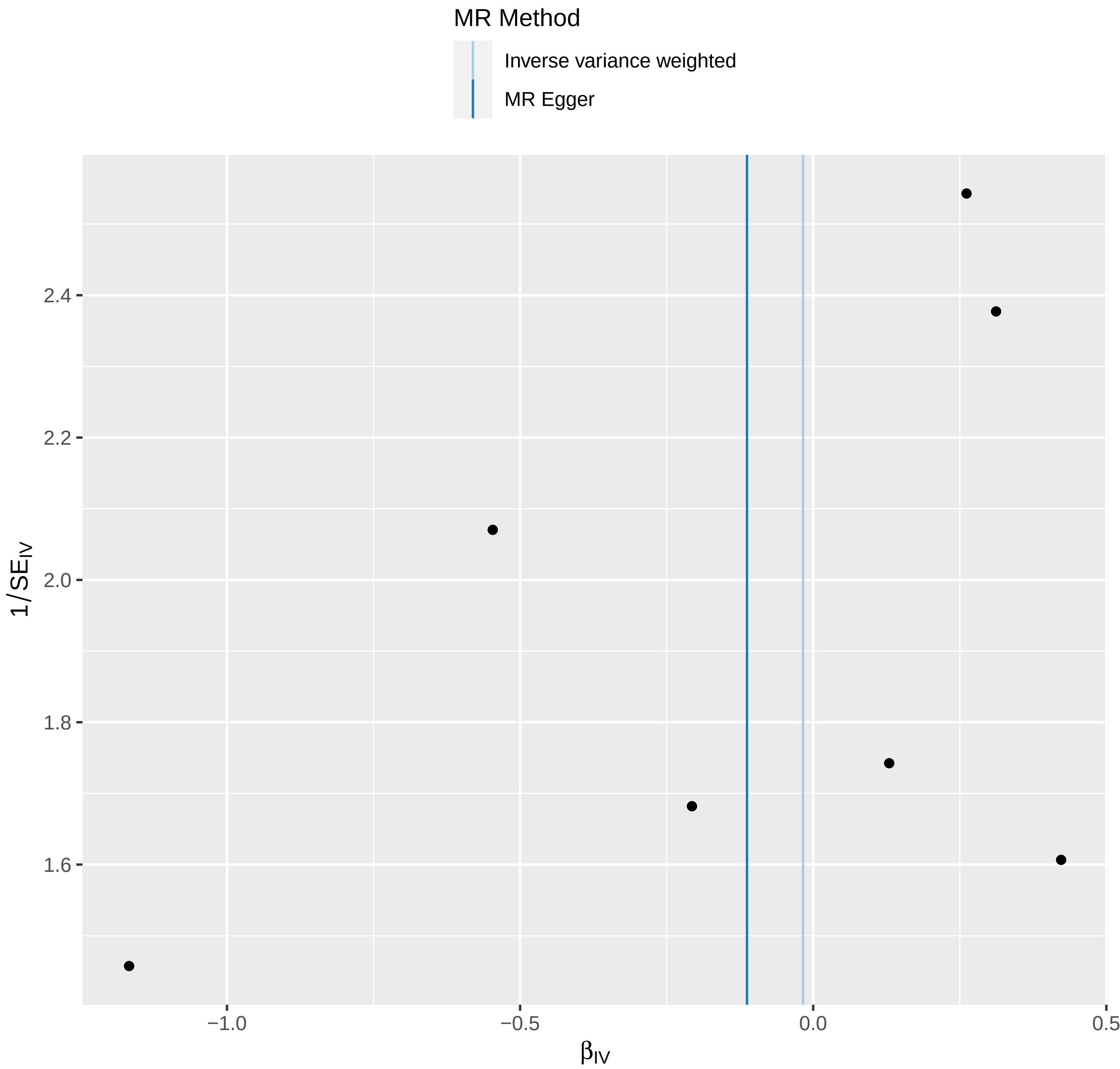

Batch 1202 : Gut microbiota abundance (family Actinomycetaceae id.421) on Other ulcerative colitis

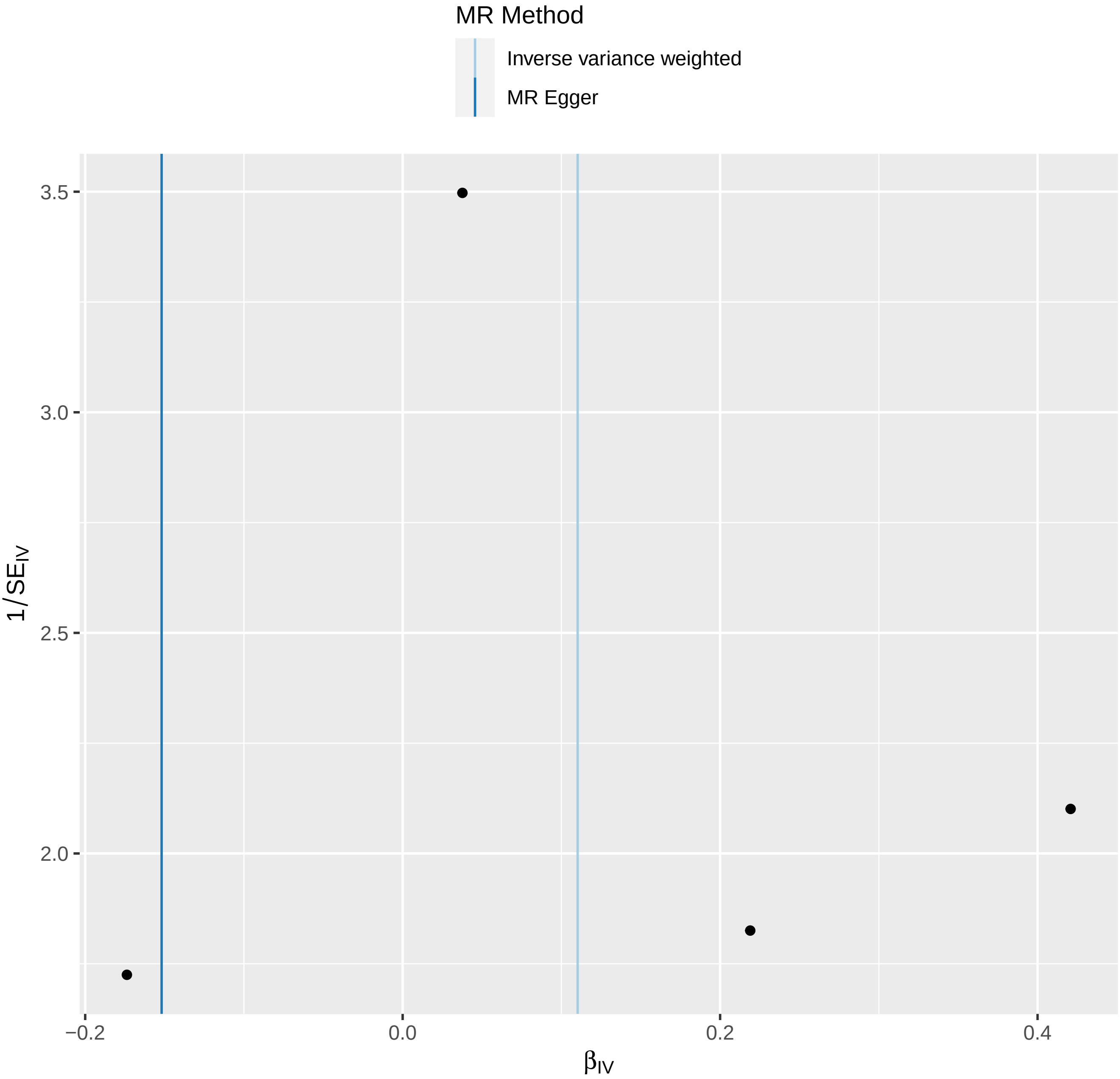

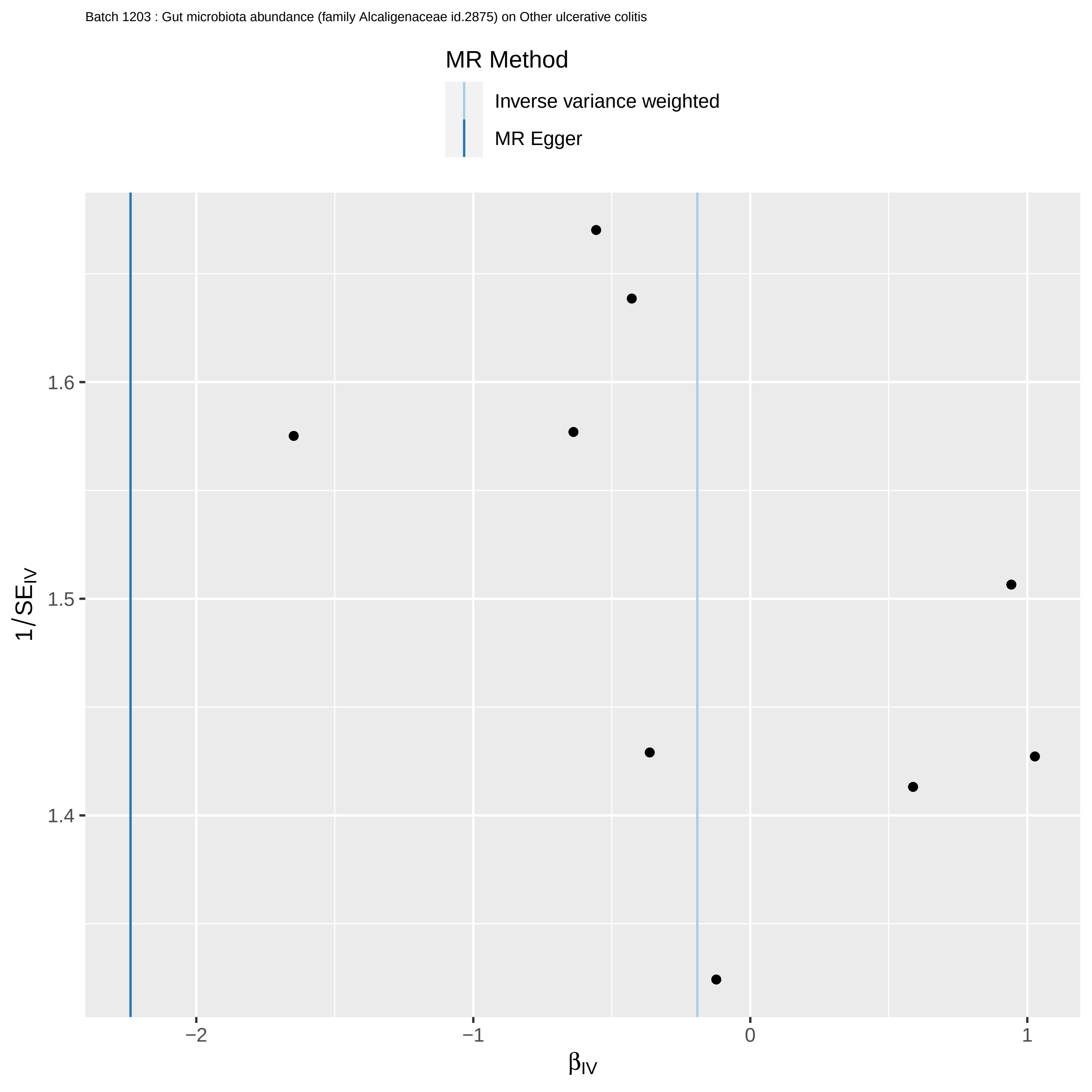

Batch 1203 : Gut microbiota abundance (family Alcaligenaceae id.2875) on Other ulcerative colitis
MR Method
Inverse variance weighted
MR Egger
1.6
1.5
1.4
−2
−1
0
1
1/SE_IV
β_IV

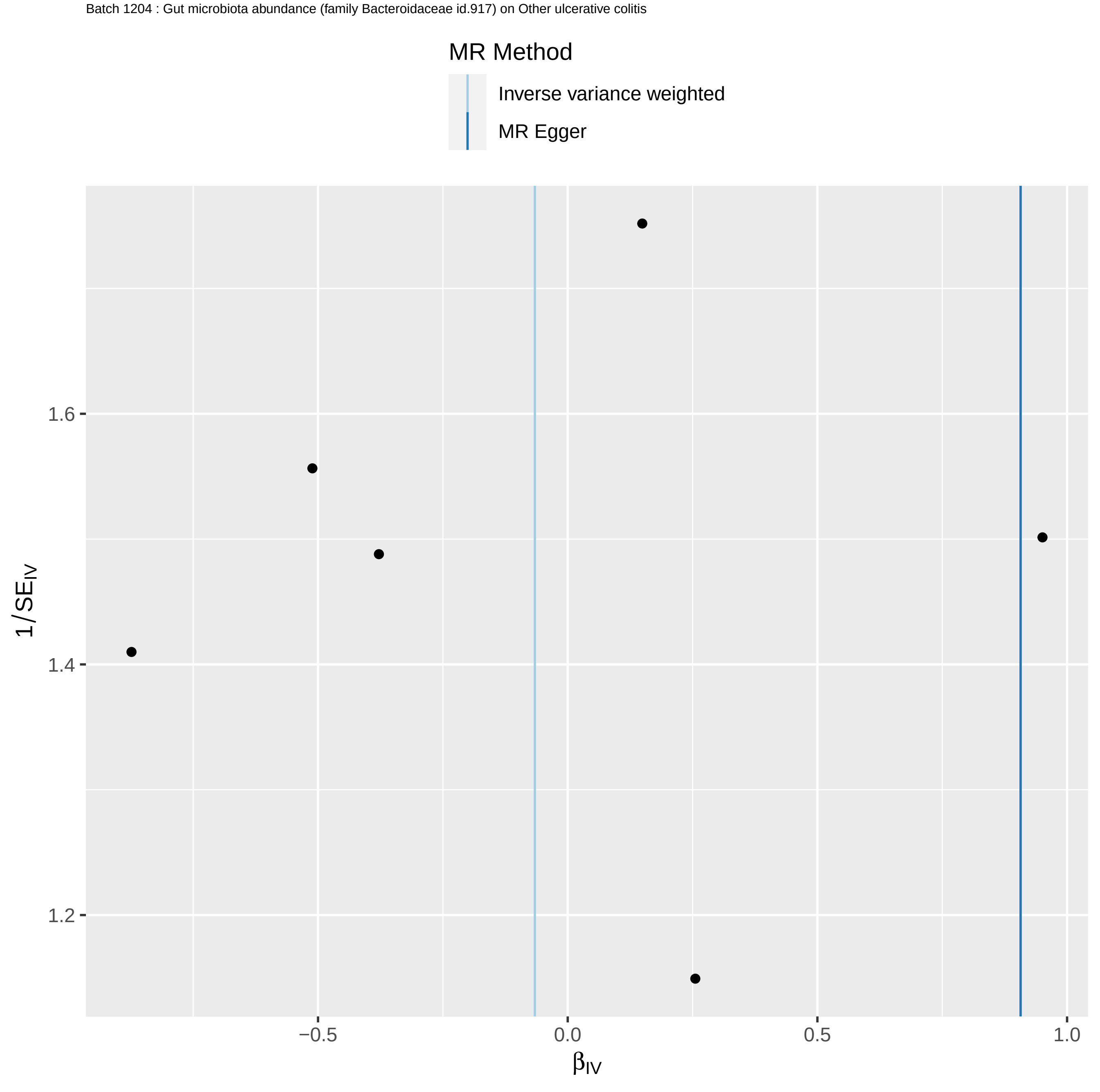

Batch 1204 : Gut microbiota abundance (family Bacteroidaceae id.917) on Other ulcerative colitis
MR Method
Inverse variance weighted
MR Egger
1.6
1.4
1.2
1/SE_IV
−0.5
0.0
0.5
1.0
β_IV

Batch 1205 : Gut microbiota abundance (family Bacteroidales S24 7group id.11173) on Other ulcerative colitis

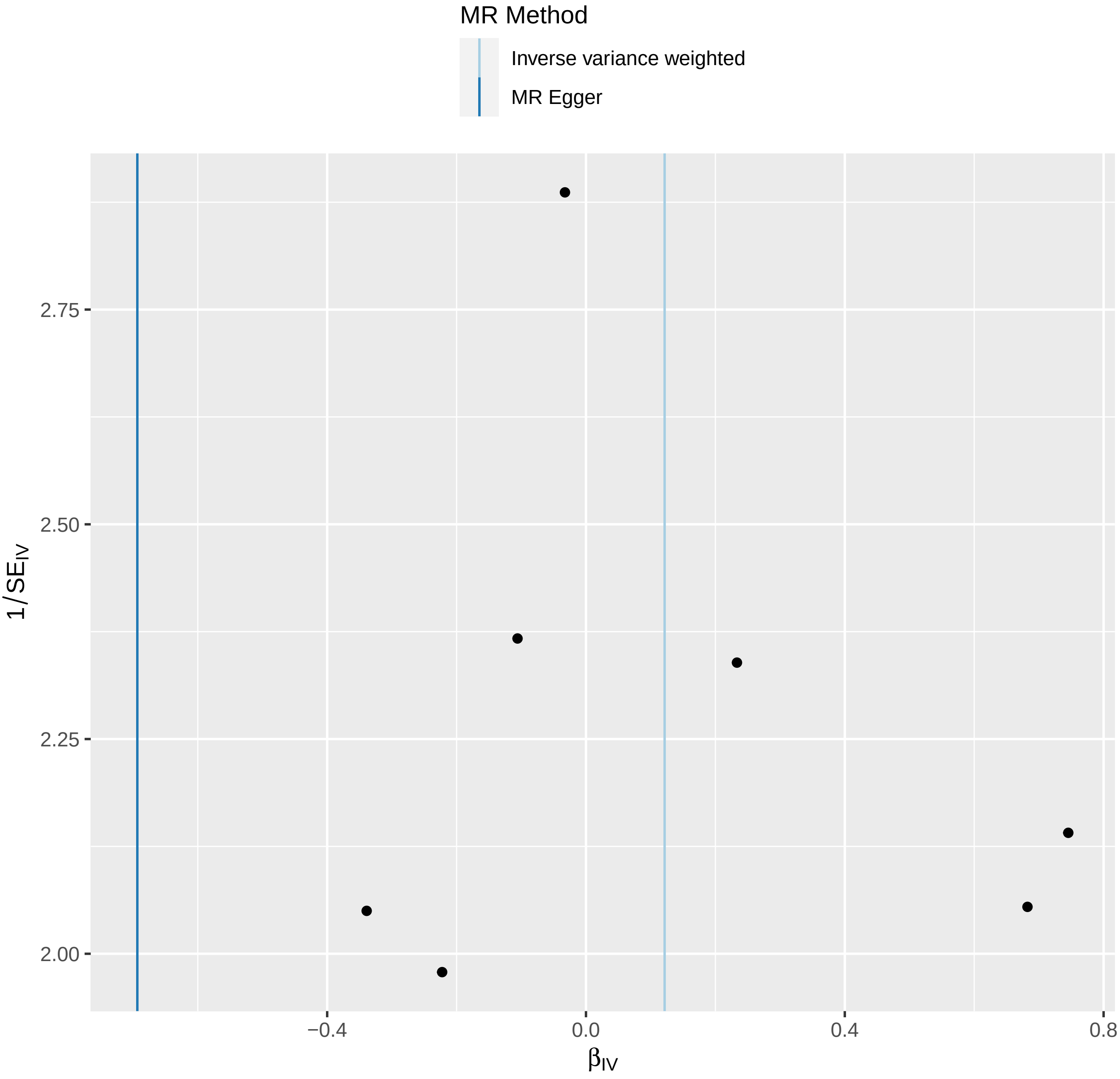

Batch 1206 : Gut microbiota abundance (family Bifidobacteriaceae id.433) on Other ulcerative colitis

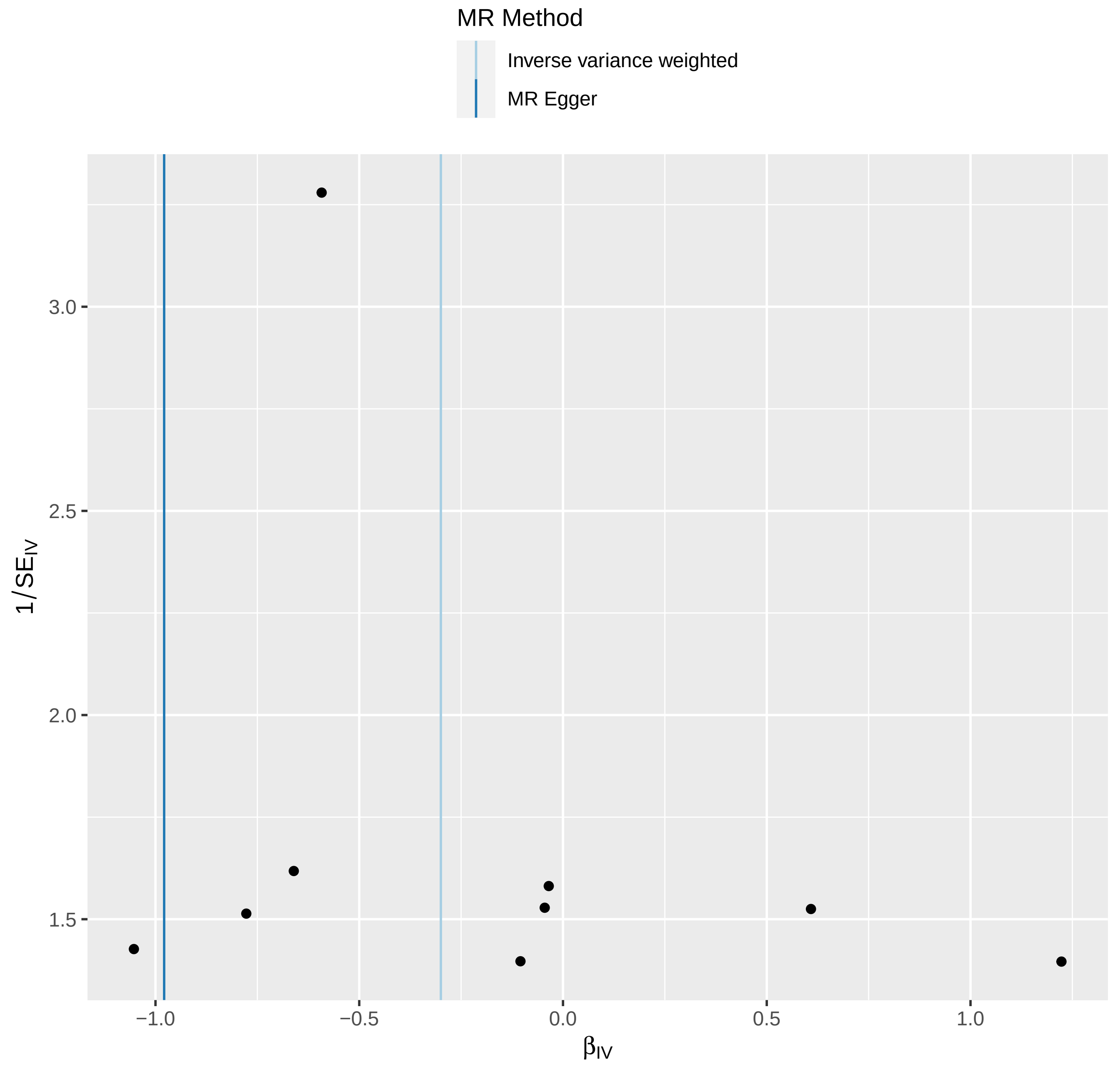

Batch 1208 : Gut microbiota abundance (family Clostridiaceae1 id.1869) on Other ulcerative colitis

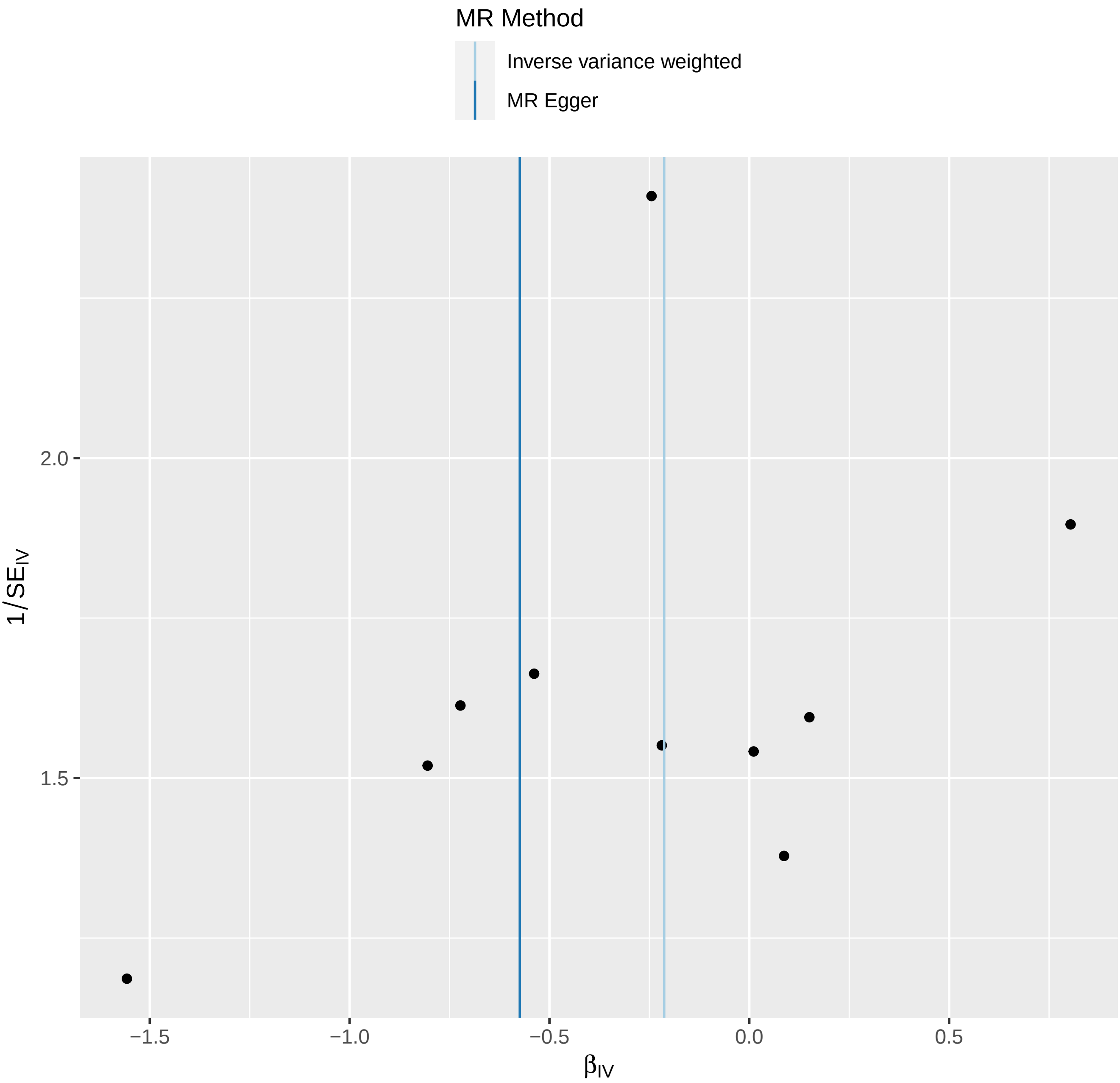

Batch 1209 : Gut microbiota abundance (family Clostridiales vadin BB60 group id.11286) on Other ulcerative colitis

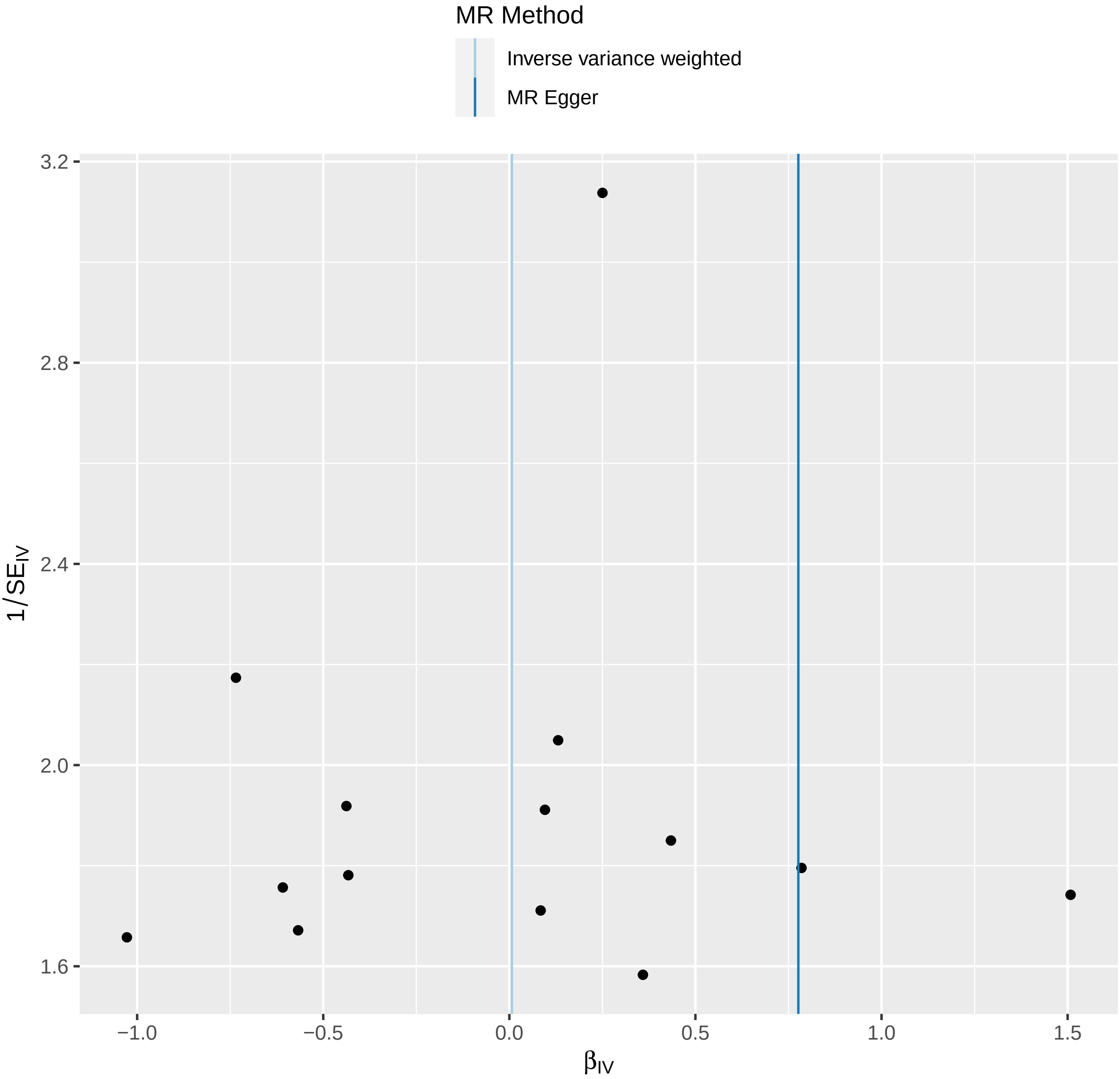

Batch 1210 : Gut microbiota abundance (family Coriobacteriaceae id.811) on Other ulcerative colitis

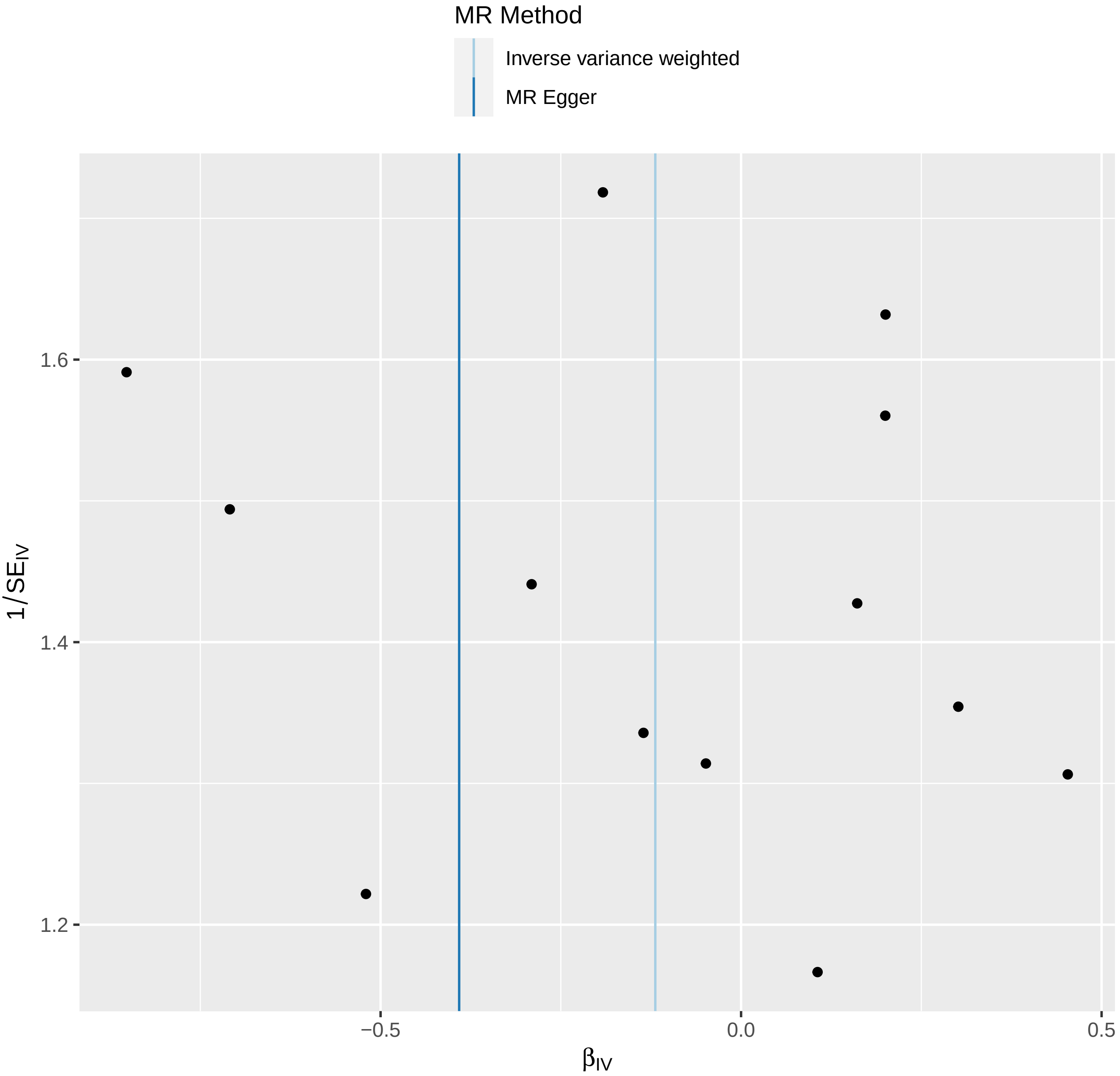

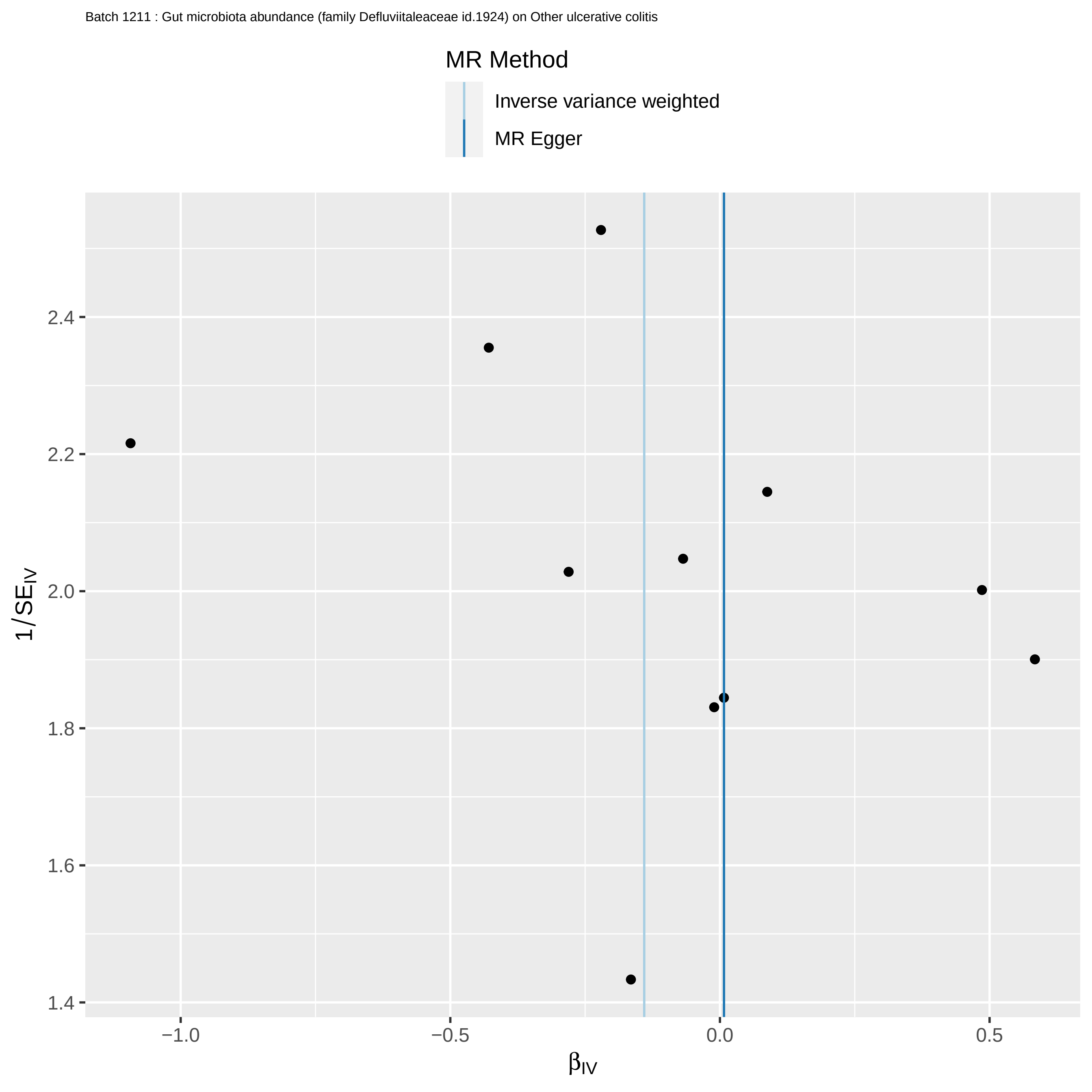

Batch 1211 : Gut microbiota abundance (family Defluviitaleaceae id.1924) on Other ulcerative colitis
MR Method
Inverse variance weighted
MR Egger
2.4
2.2
2.0
1.8
1.6
1.4
1/SE_IV
−1.0
−0.5
0.0
0.5
β_IV

Batch 1212 : Gut microbiota abundance (family Desulfovibrionaceae id.3169) on Other ulcerative colitis

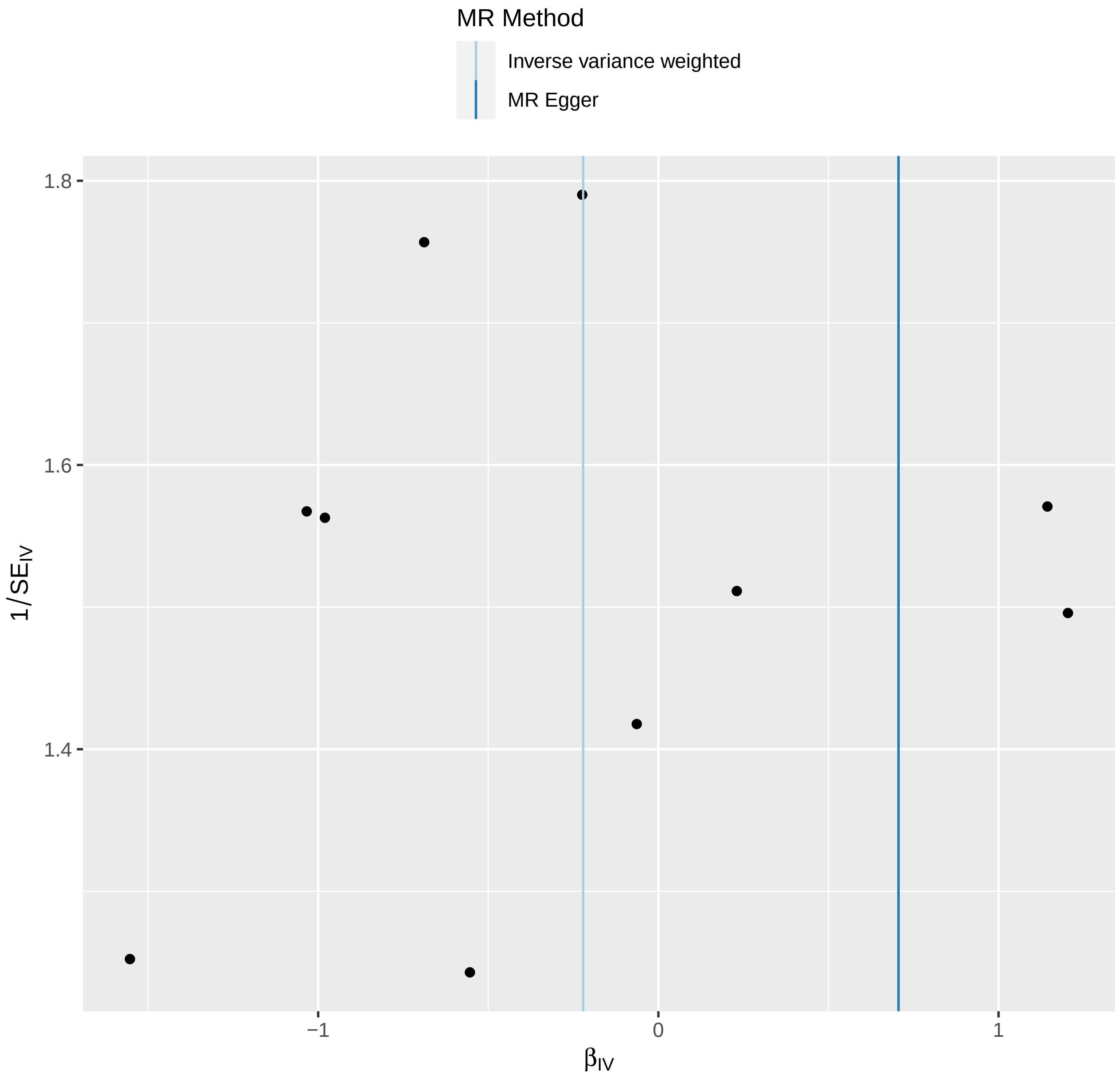

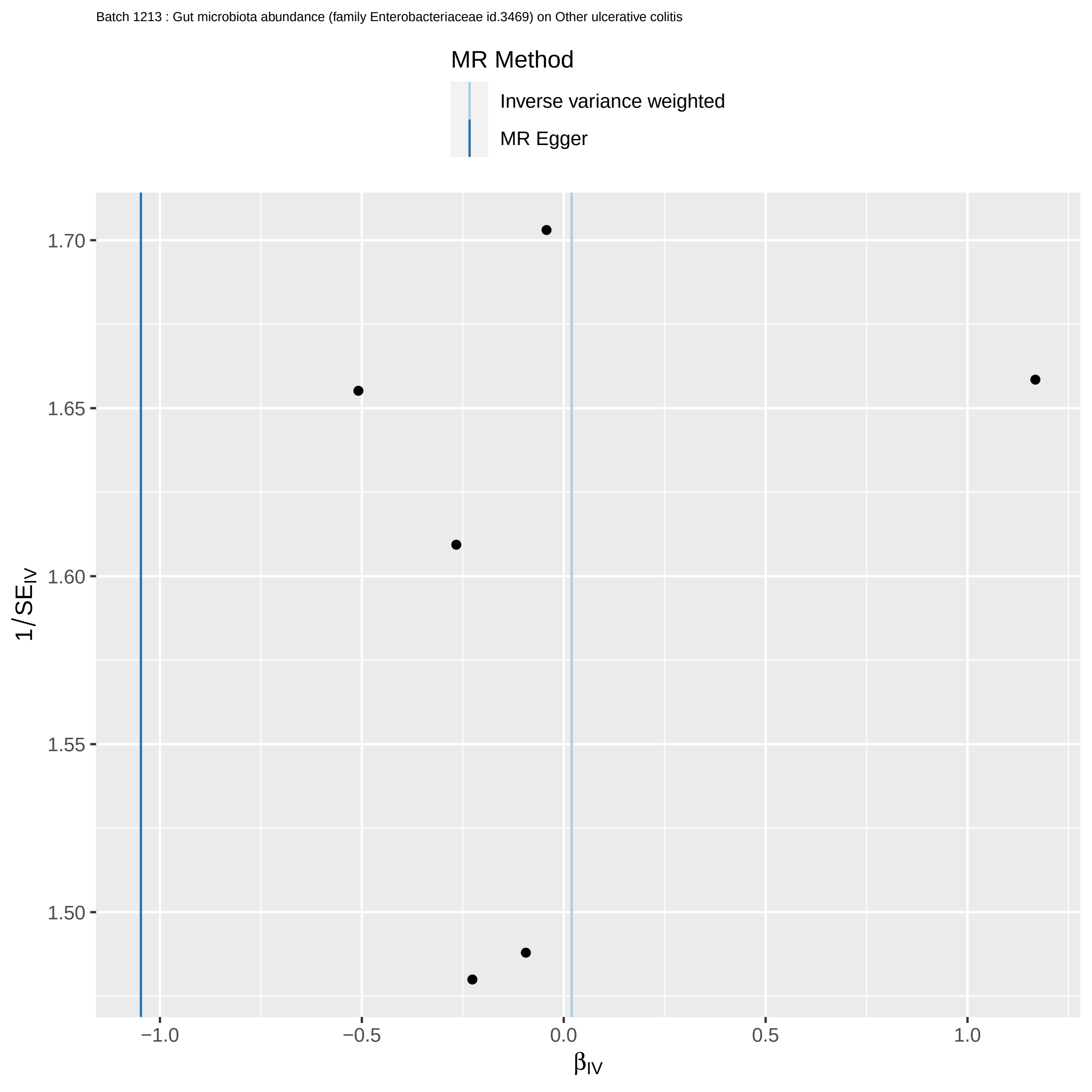

Batch 1213 : Gut microbiota abundance (family Enterobacteriaceae id.3469) on Other ulcerative colitis
MR Method
Inverse variance weighted
MR Egger
1.70
1.65
1.60
1.55
1.50
1/SE_IV
−1.0
−0.5
0.0
0.5
1.0
β_IV

Batch 1214 : Gut microbiota abundance (family Erysipelotrichaceae id.2149) on Other ulcerative colitis

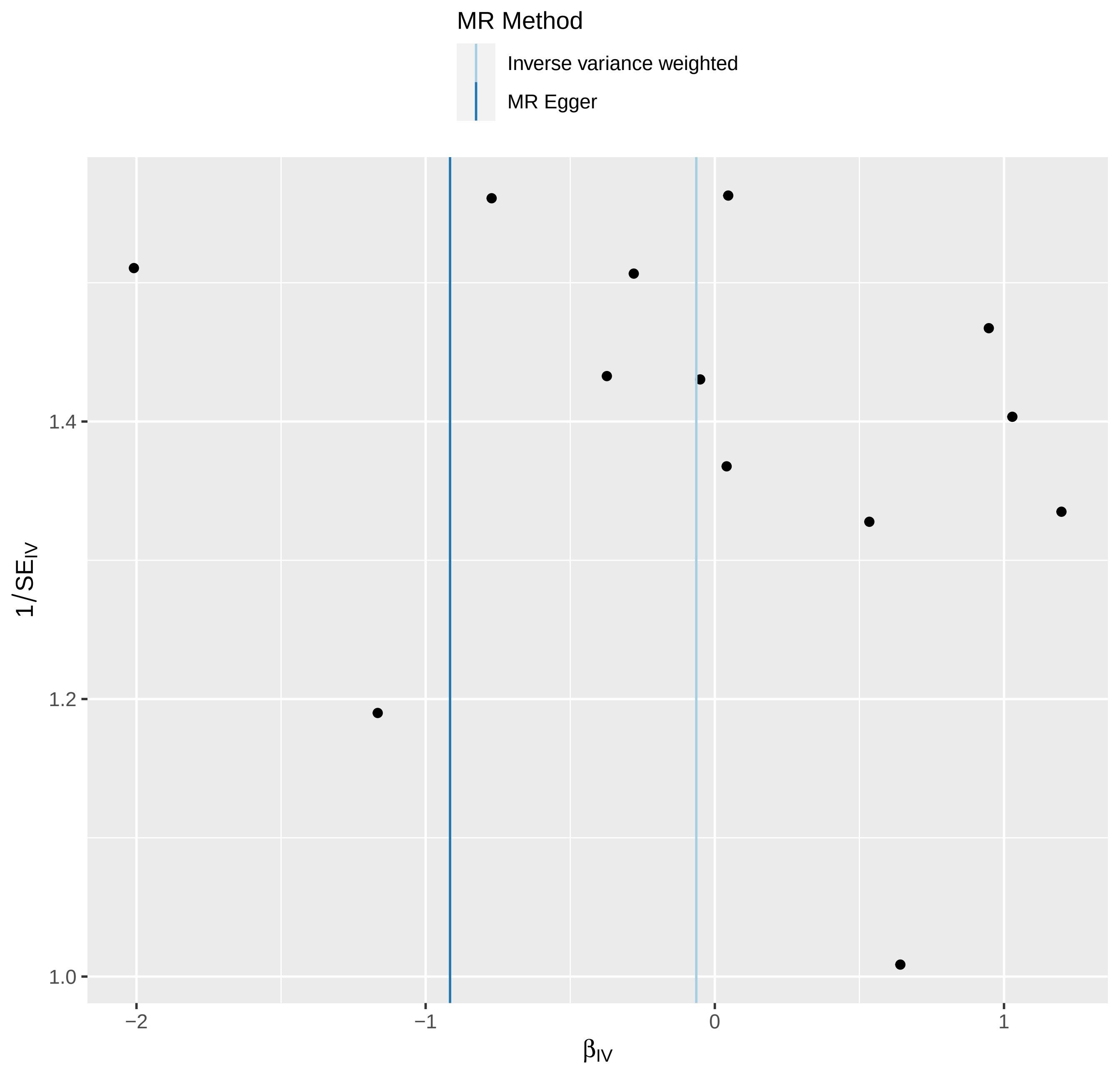

Batch 1215 : Gut microbiota abundance (family Family XI id.1936) on Other ulcerative colitis

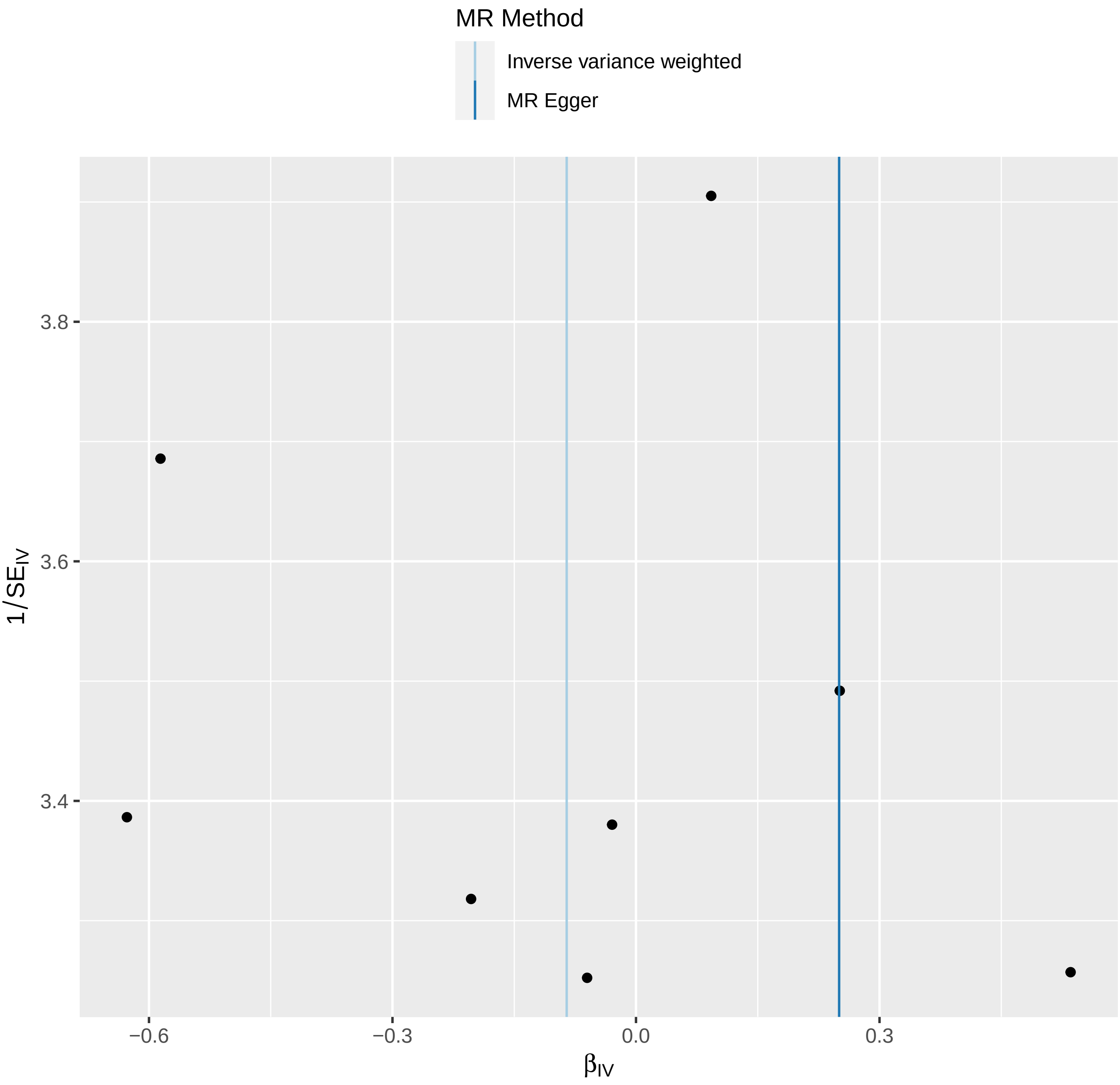

Batch 1216 : Gut microbiota abundance (family Family XIII id.1957) on Other ulcerative colitis

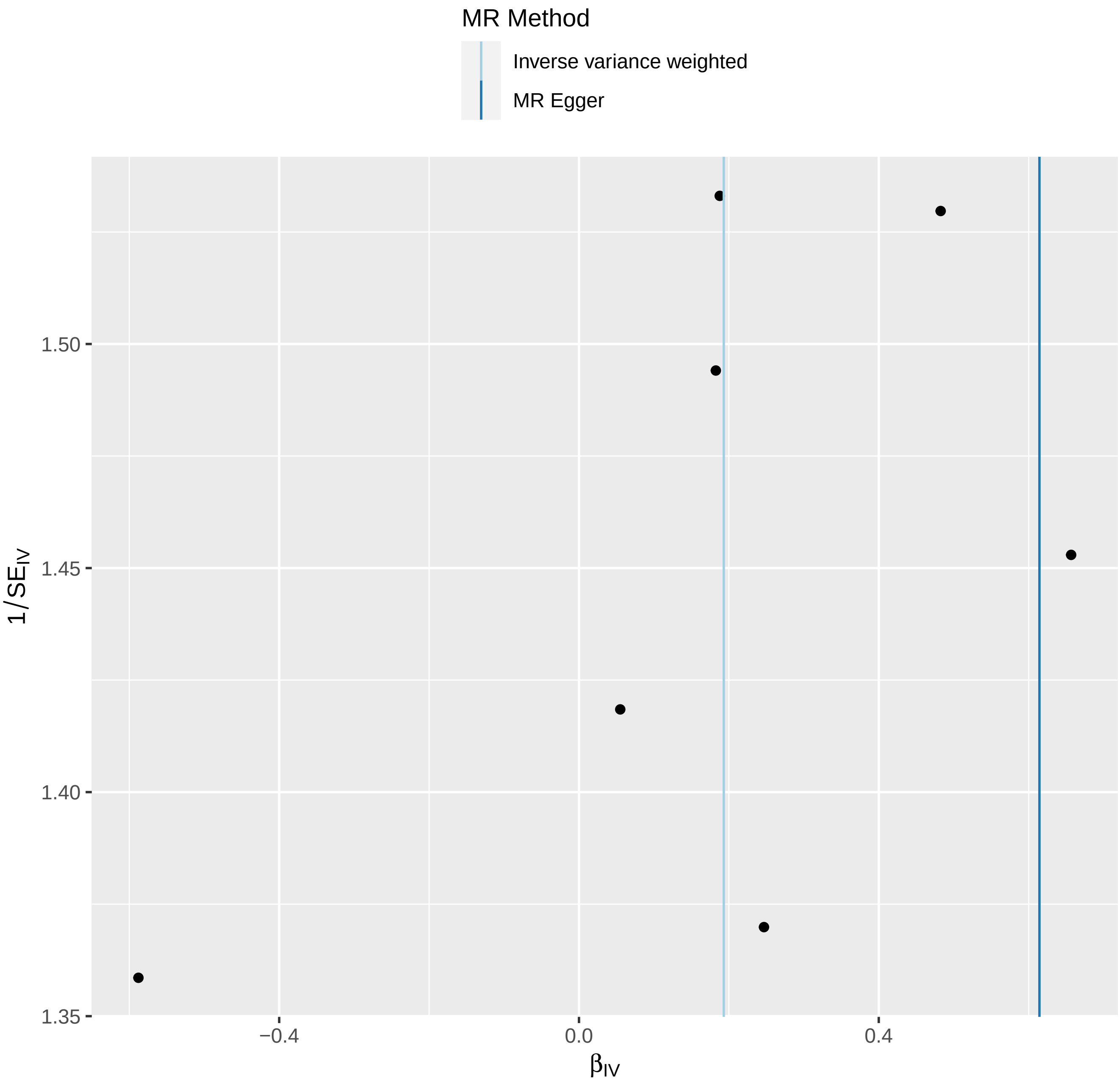

Batch 1217 : Gut microbiota abundance (family Lachnospiraceae id.1987) on Other ulcerative colitis

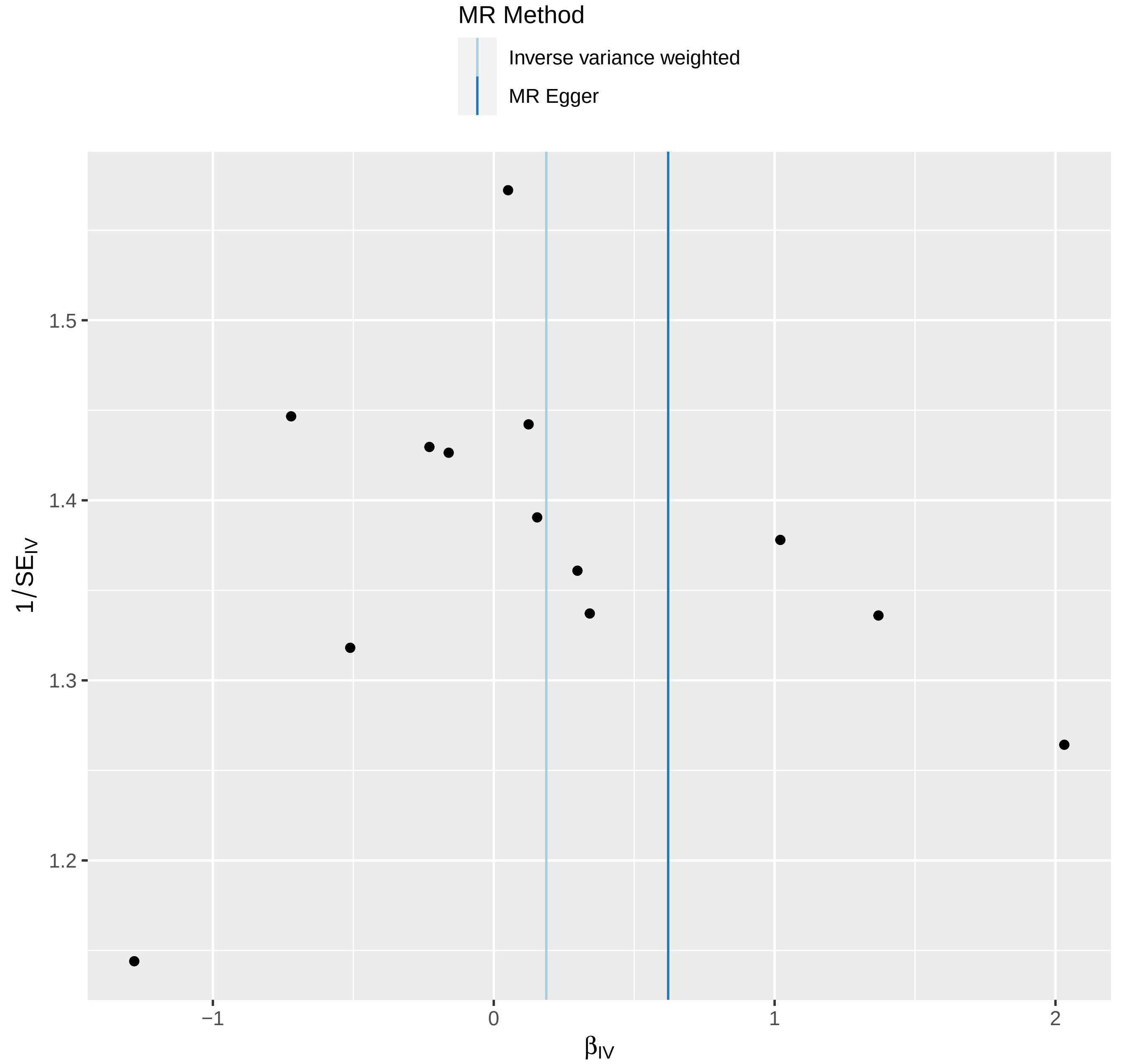

Batch 1218 : Gut microbiota abundance (family Lactobacillaceae id.1836) on Other ulcerative colitis

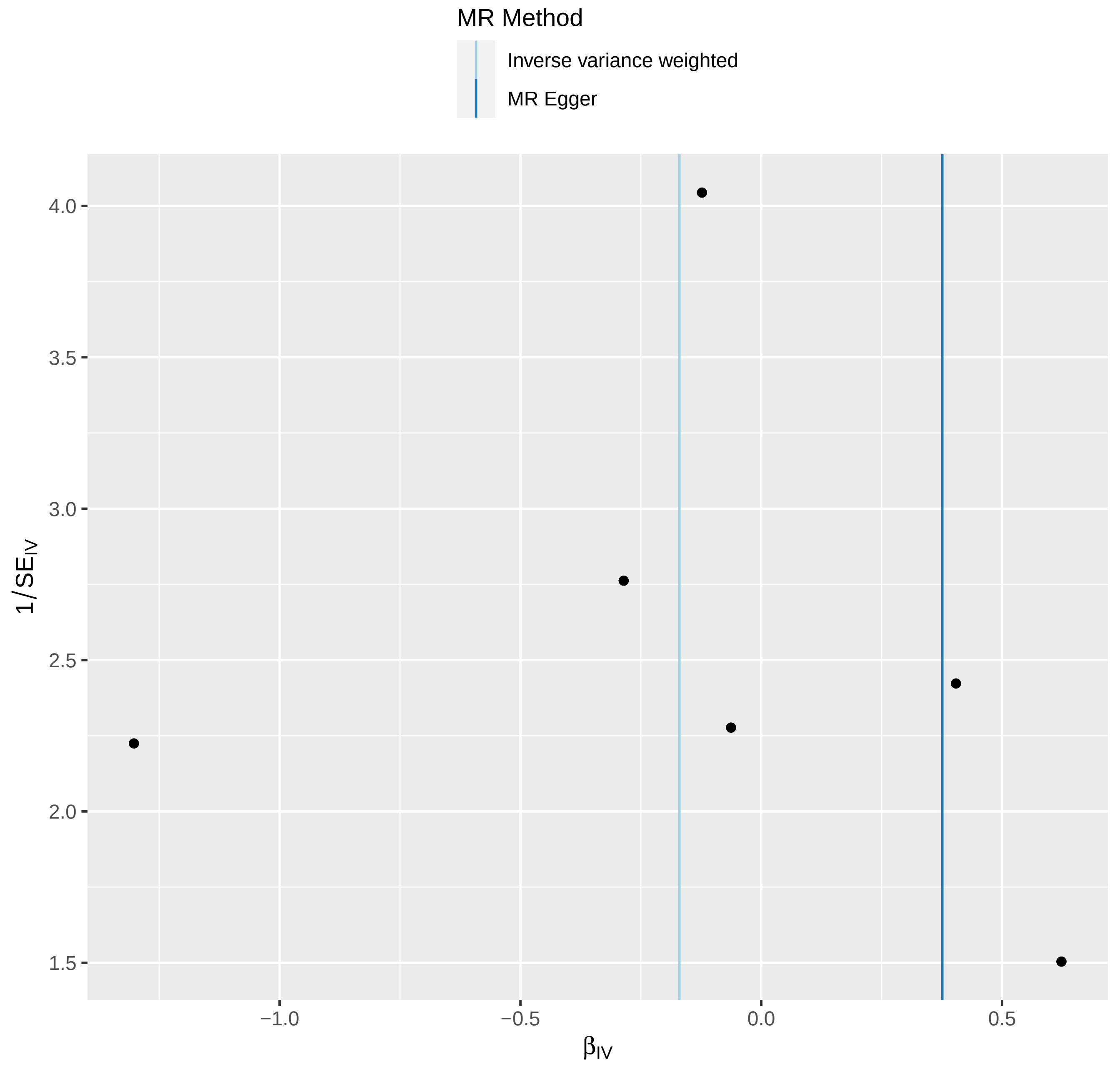

Batch 1219 : Gut microbiota abundance (family Methanobacteriaceae id.121) on Other ulcerative colitis

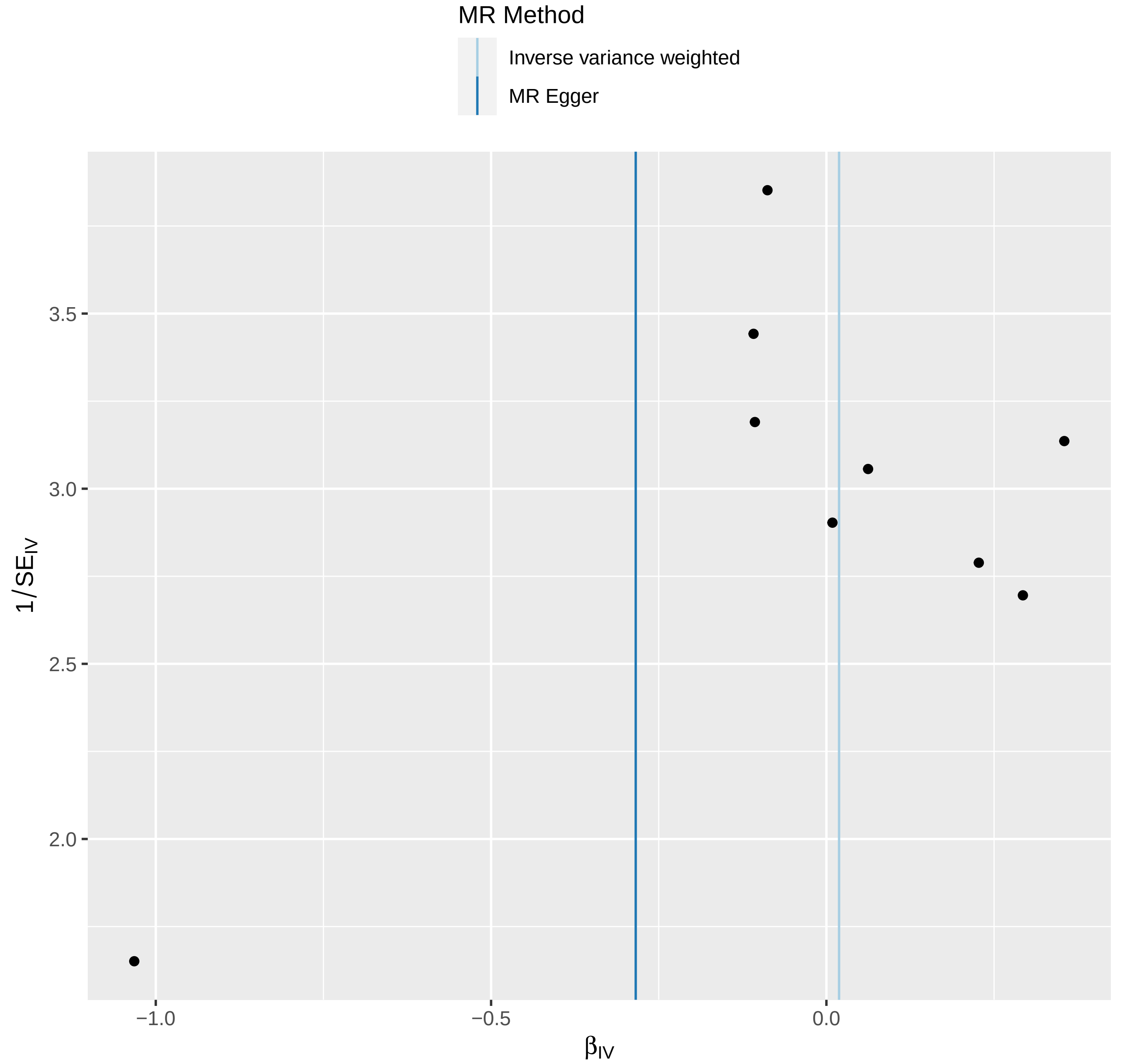

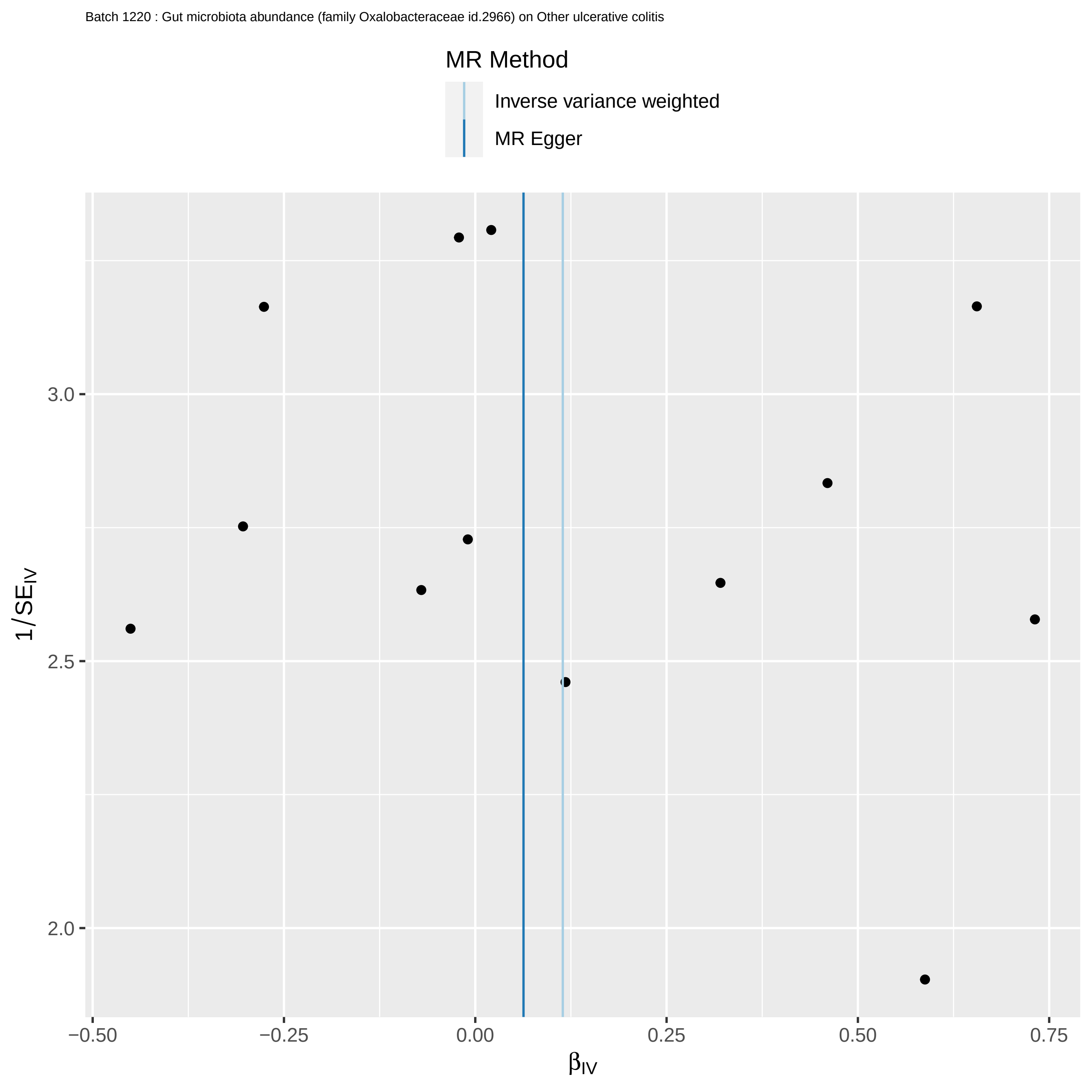

Batch 1220 : Gut microbiota abundance (family Oxalobacteraceae id.2966) on Other ulcerative colitis
MR Method
Inverse variance weighted
MR Egger
3.0
2.5
2.0
1/SE_IV
−0.50
−0.25
0.00
0.25
0.50
0.75
β_IV

Batch 1221 : Gut microbiota abundance (family Pasteurellaceae id.3689) on Other ulcerative colitis

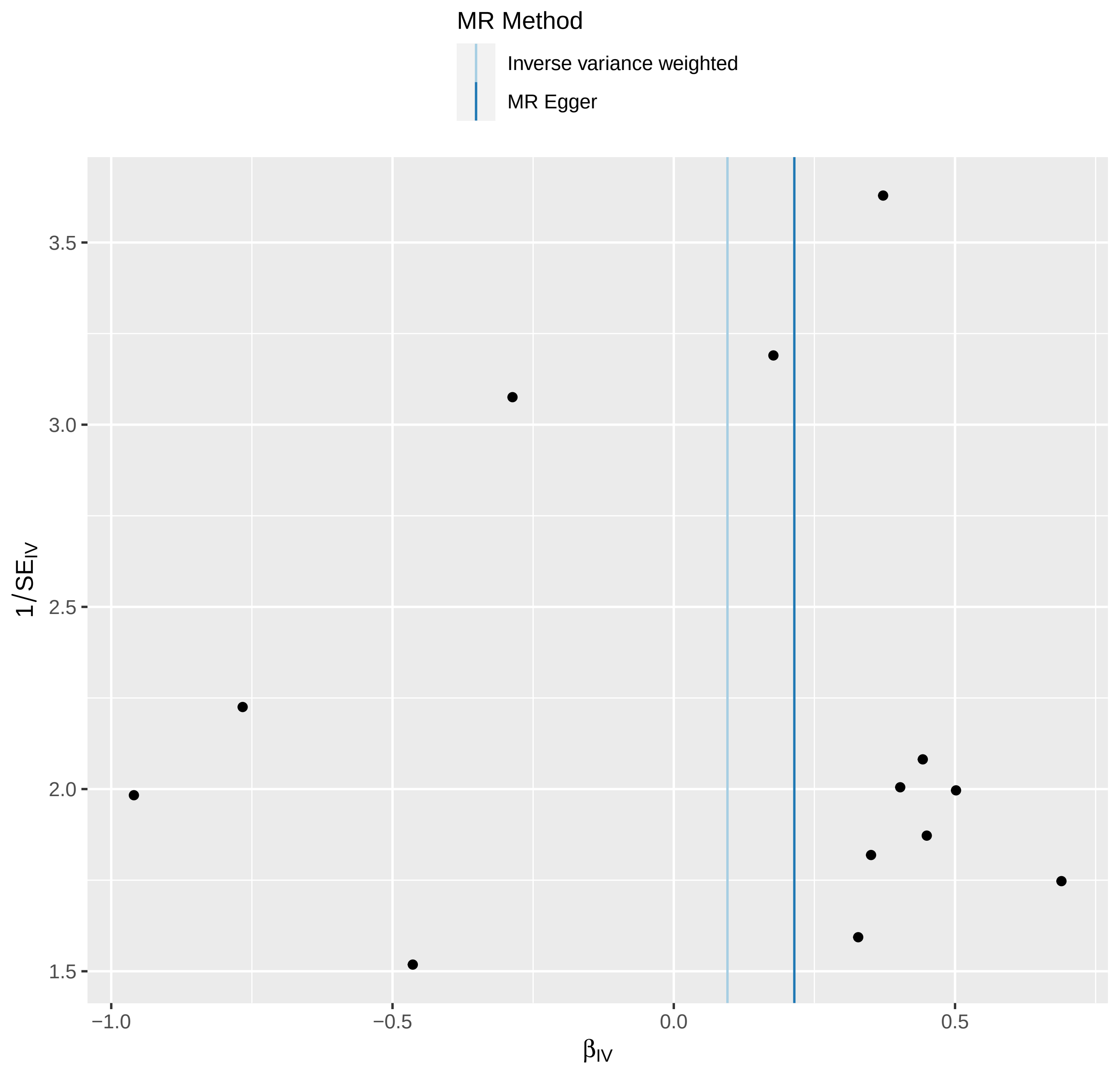

Batch 1222 : Gut microbiota abundance (family Peptococcaceae id.2024) on Other ulcerative colitis

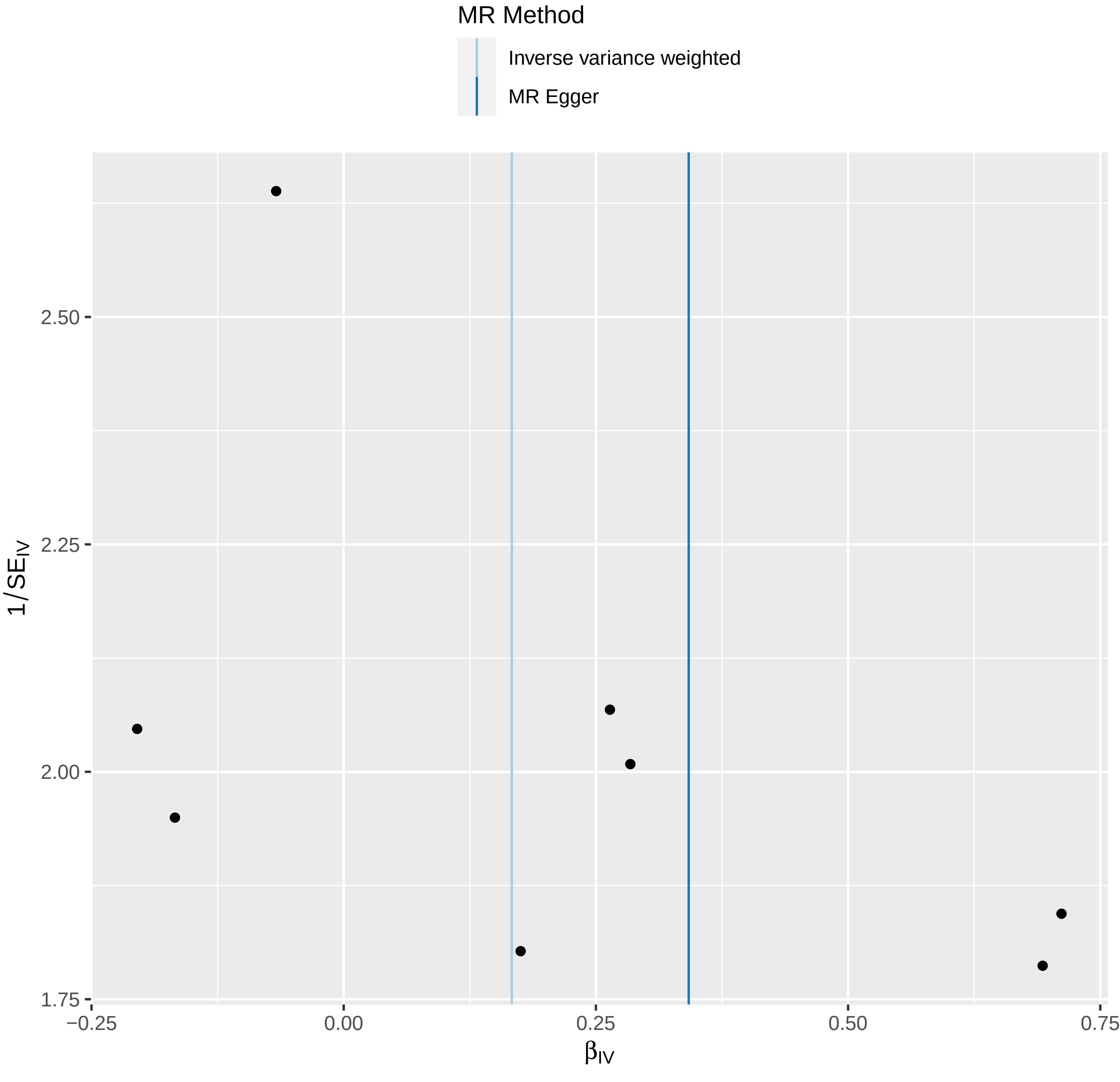

Batch 1223 : Gut microbiota abundance (family Peptostreptococcaceae id.2042) on Other ulcerative colitis

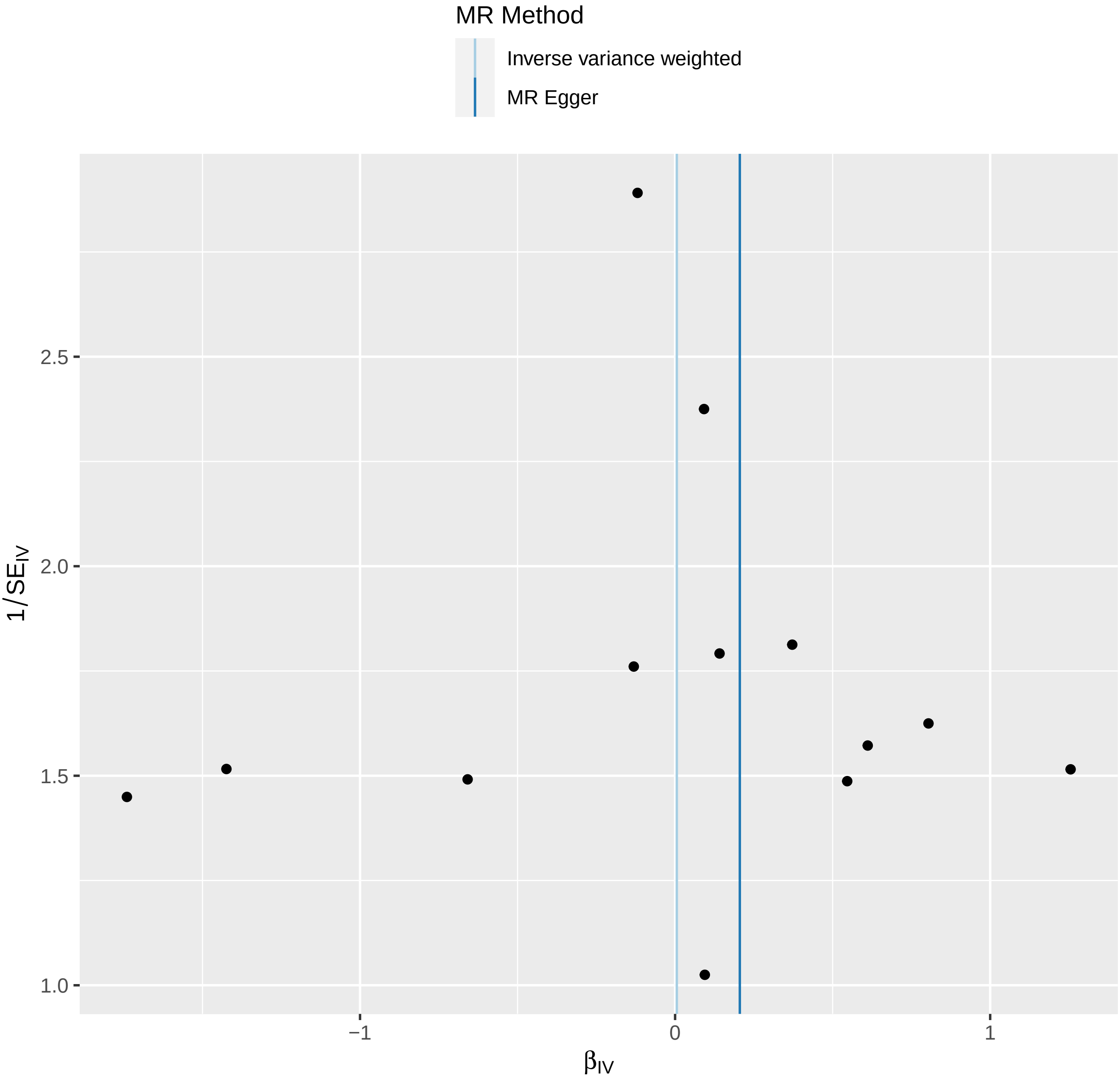

Batch 1224 : Gut microbiota abundance (family Porphyromonadaceae id.943) on Other ulcerative colitis

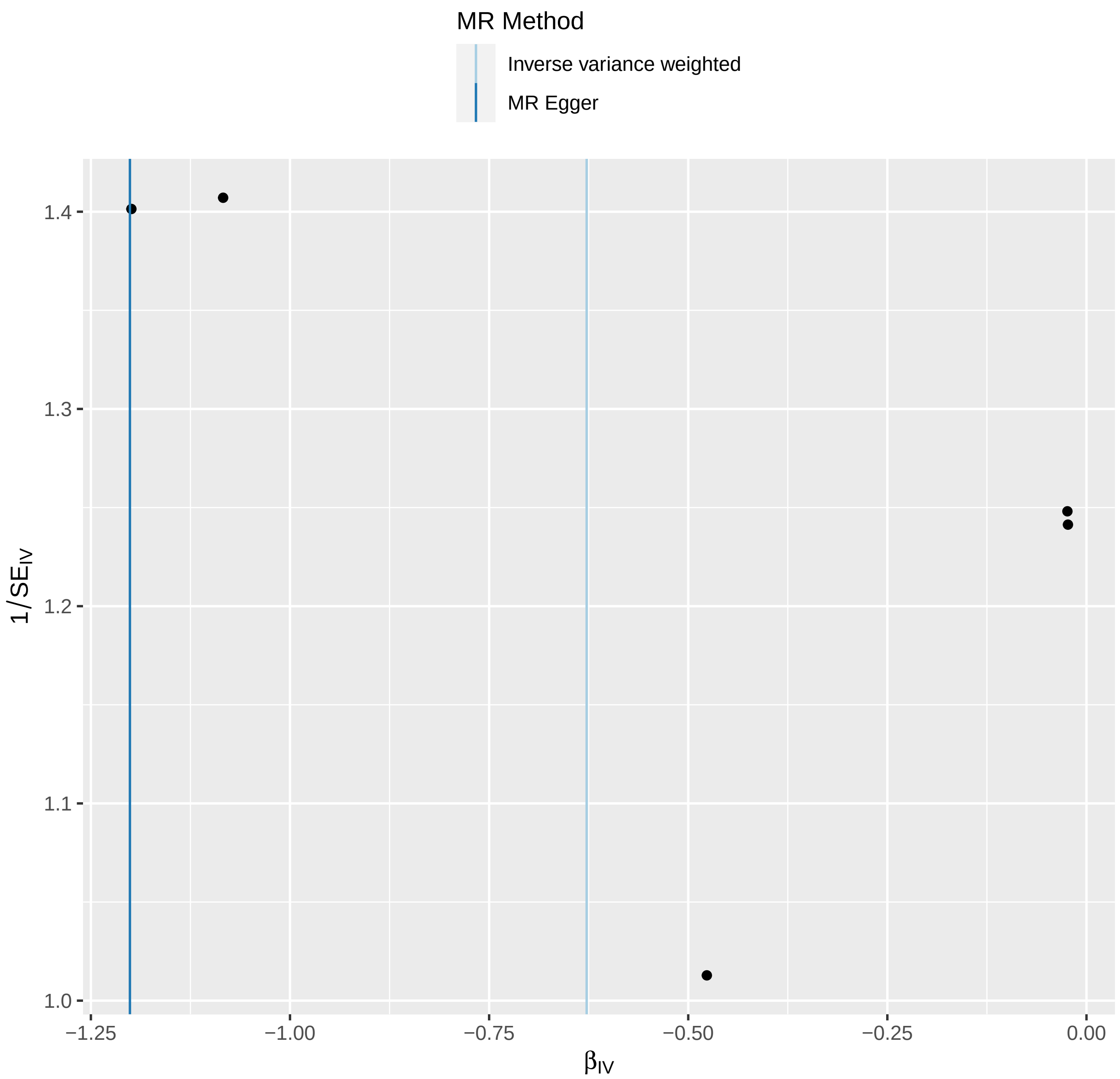

Batch 1225 : Gut microbiota abundance (family Prevotellaceae id.960) on Other ulcerative colitis

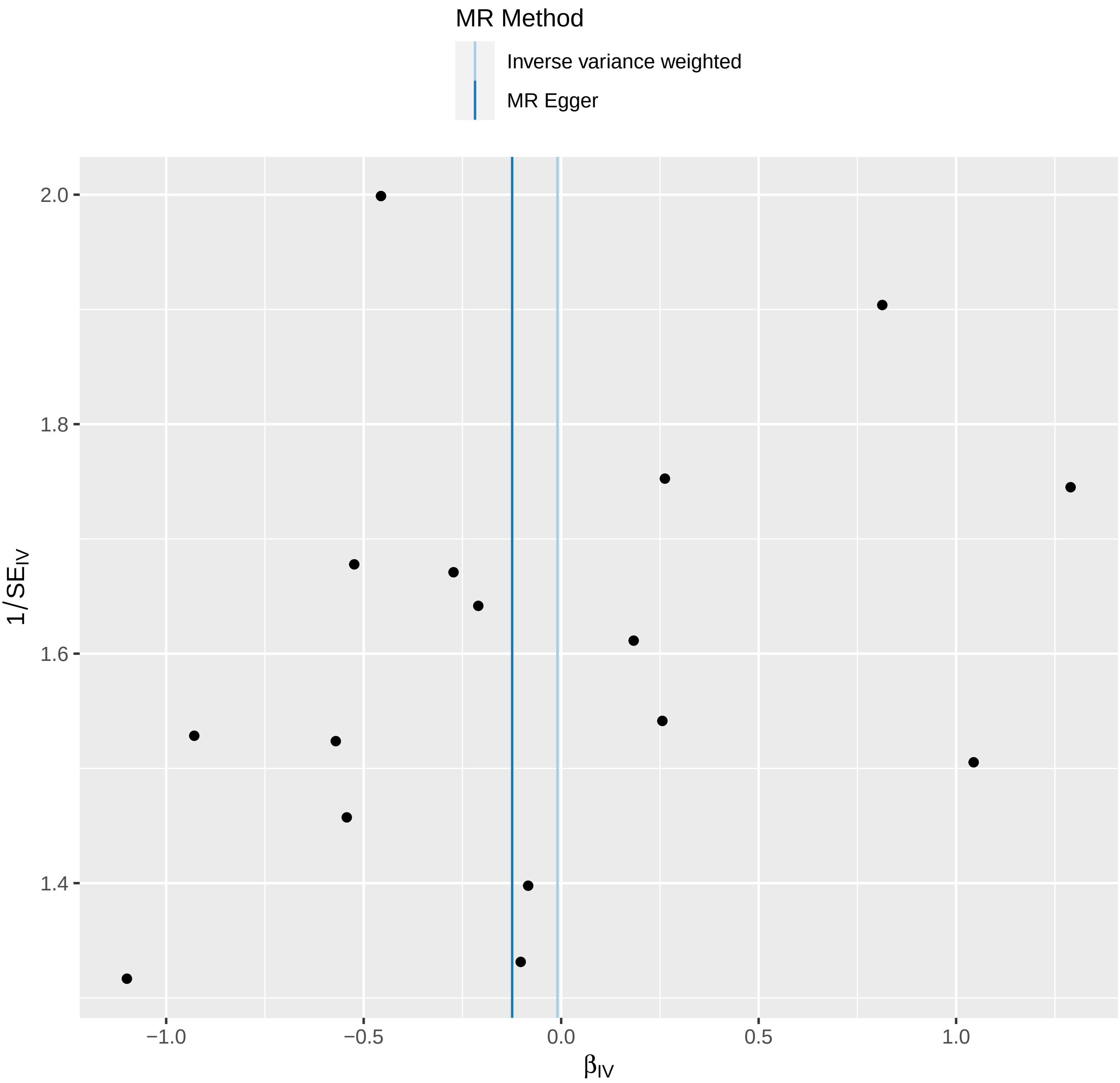

Batch 1226 : Gut microbiota abundance (family Rhodospirillaceae id.2717) on Other ulcerative colitis

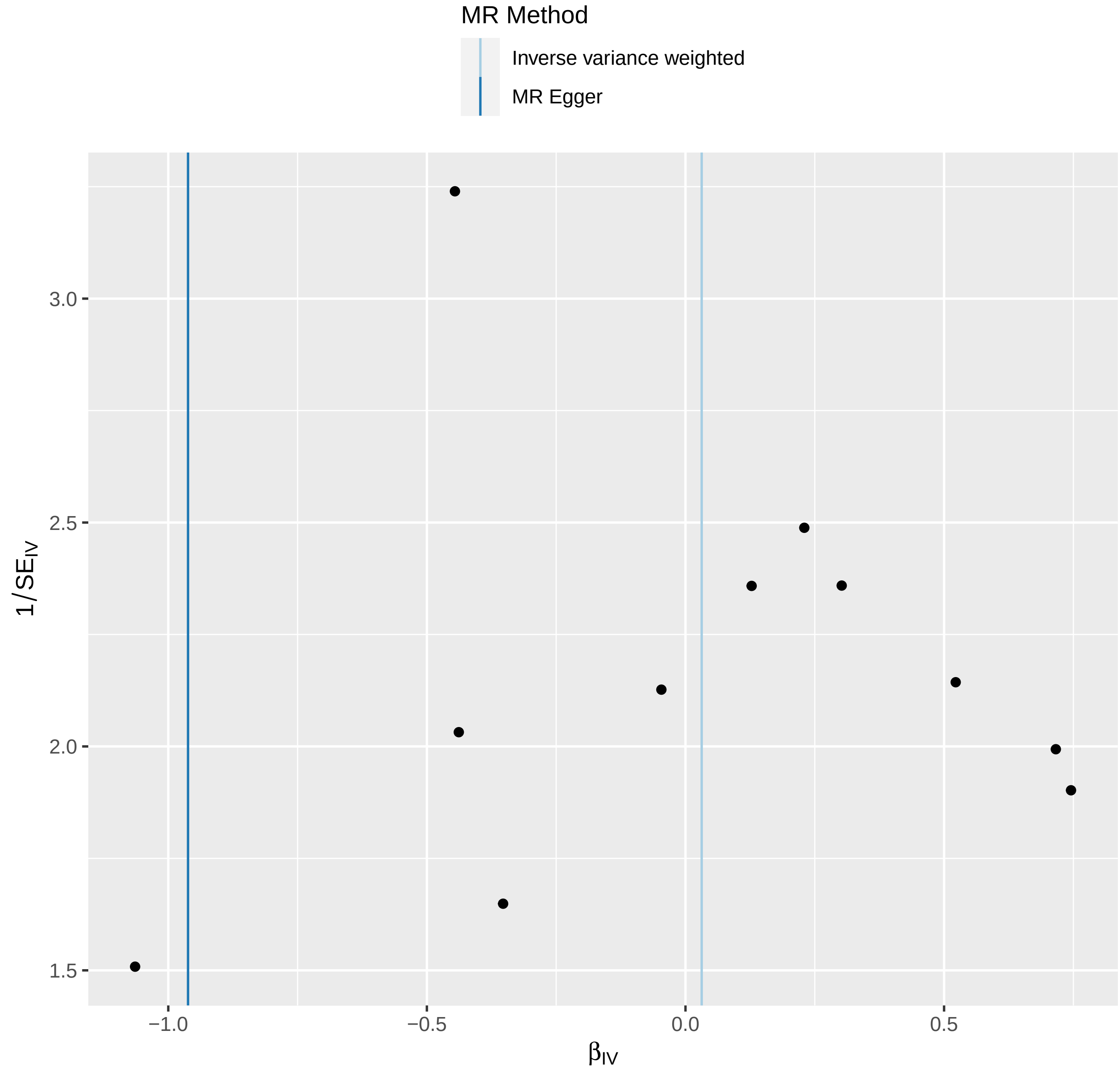

Batch 1227 : Gut microbiota abundance (family Rikenellaceae id.967) on Other ulcerative colitis

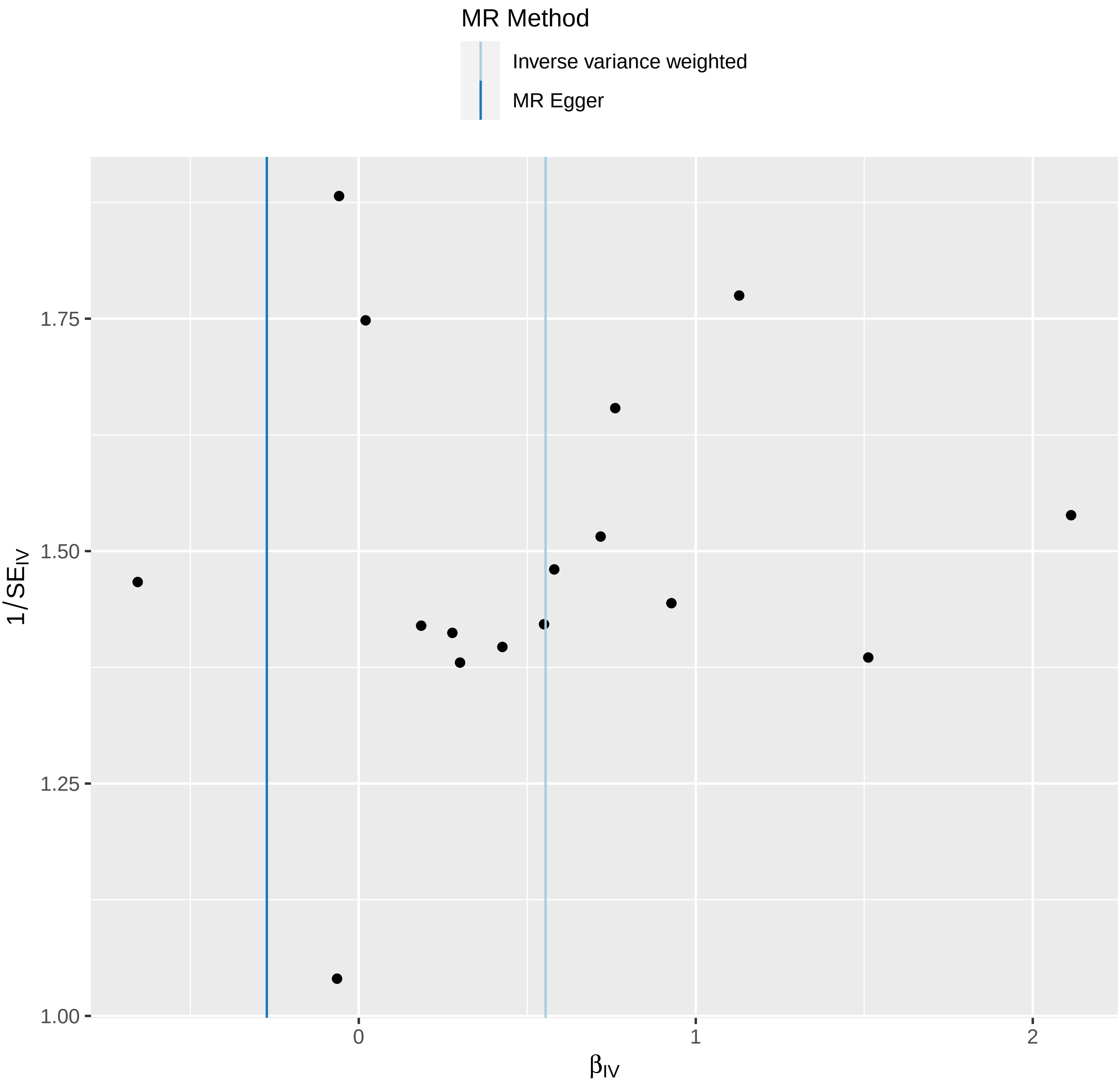

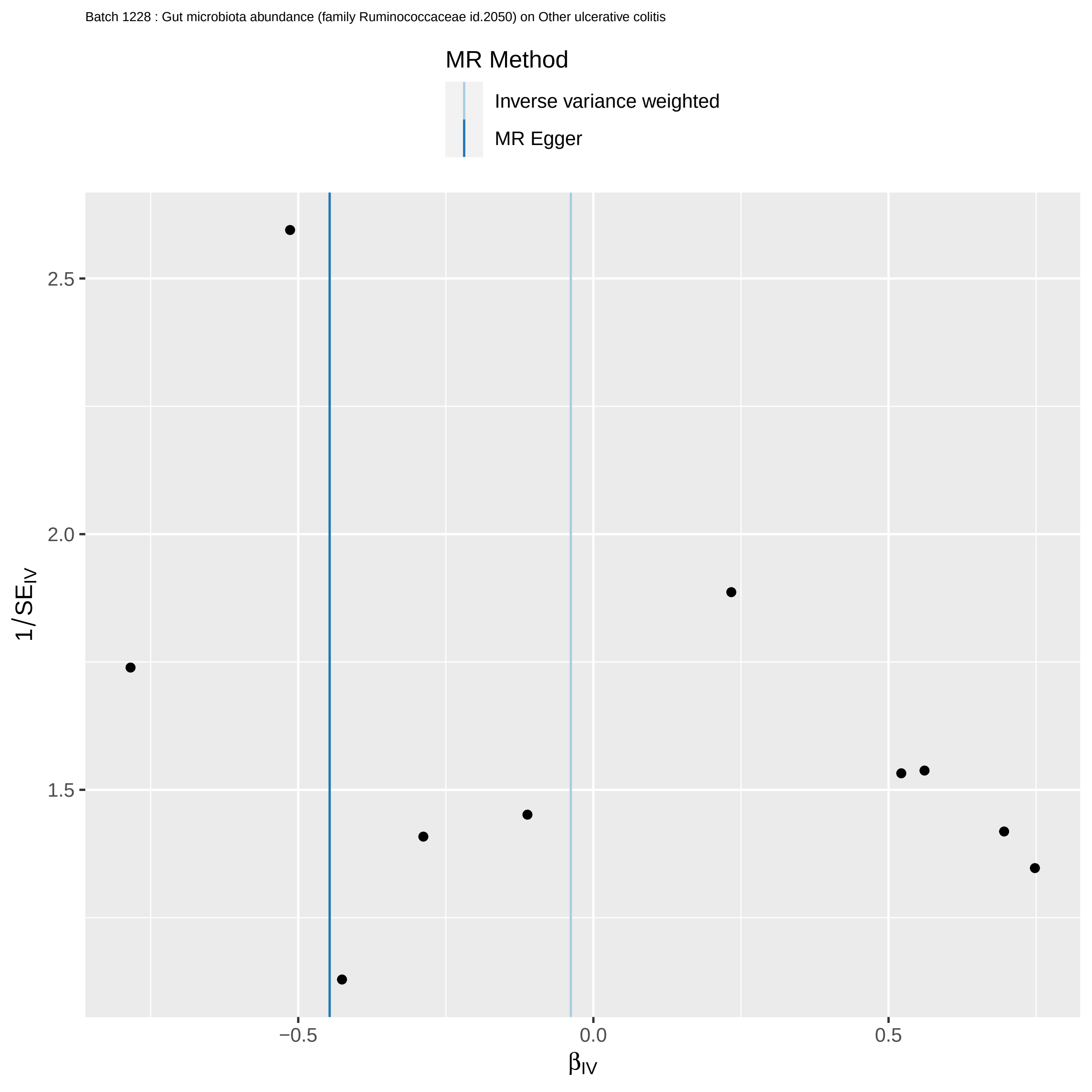
Batch 1228 : Gut microbiota abundance (family Ruminococcaceae id.2050) on Other ulcerative colitis
MR Method
Inverse variance weighted
MR Egger
2.5
2.0
1.5
1/SE_IV
−0.5
0.0
0.5
β_IV

Batch 1229 : Gut microbiota abundance (family Streptococcaceae id.1850) on Other ulcerative colitis

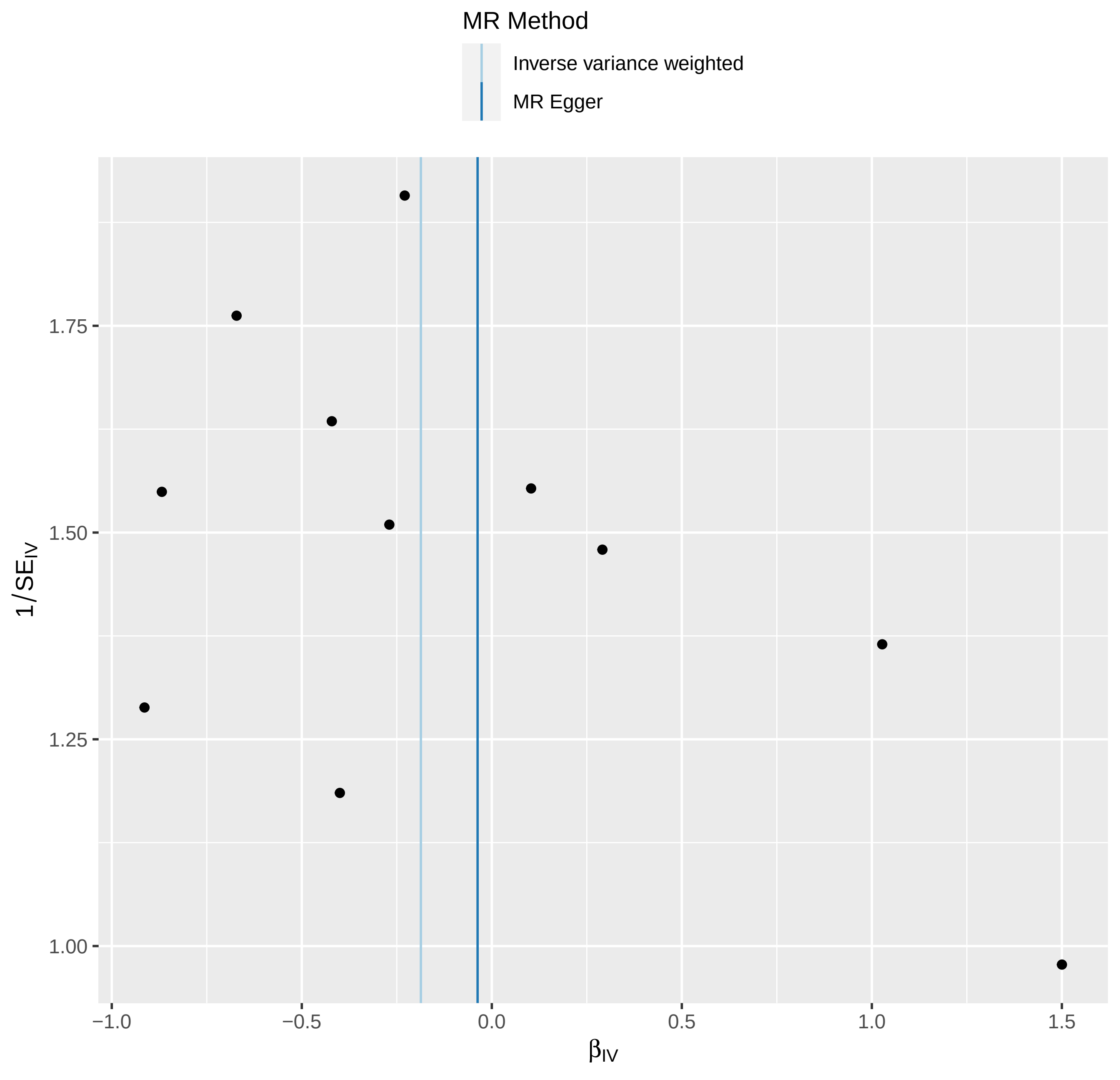

Batch 1230 : Gut microbiota abundance (family Veillonellaceae id.2172) on Other ulcerative colitis

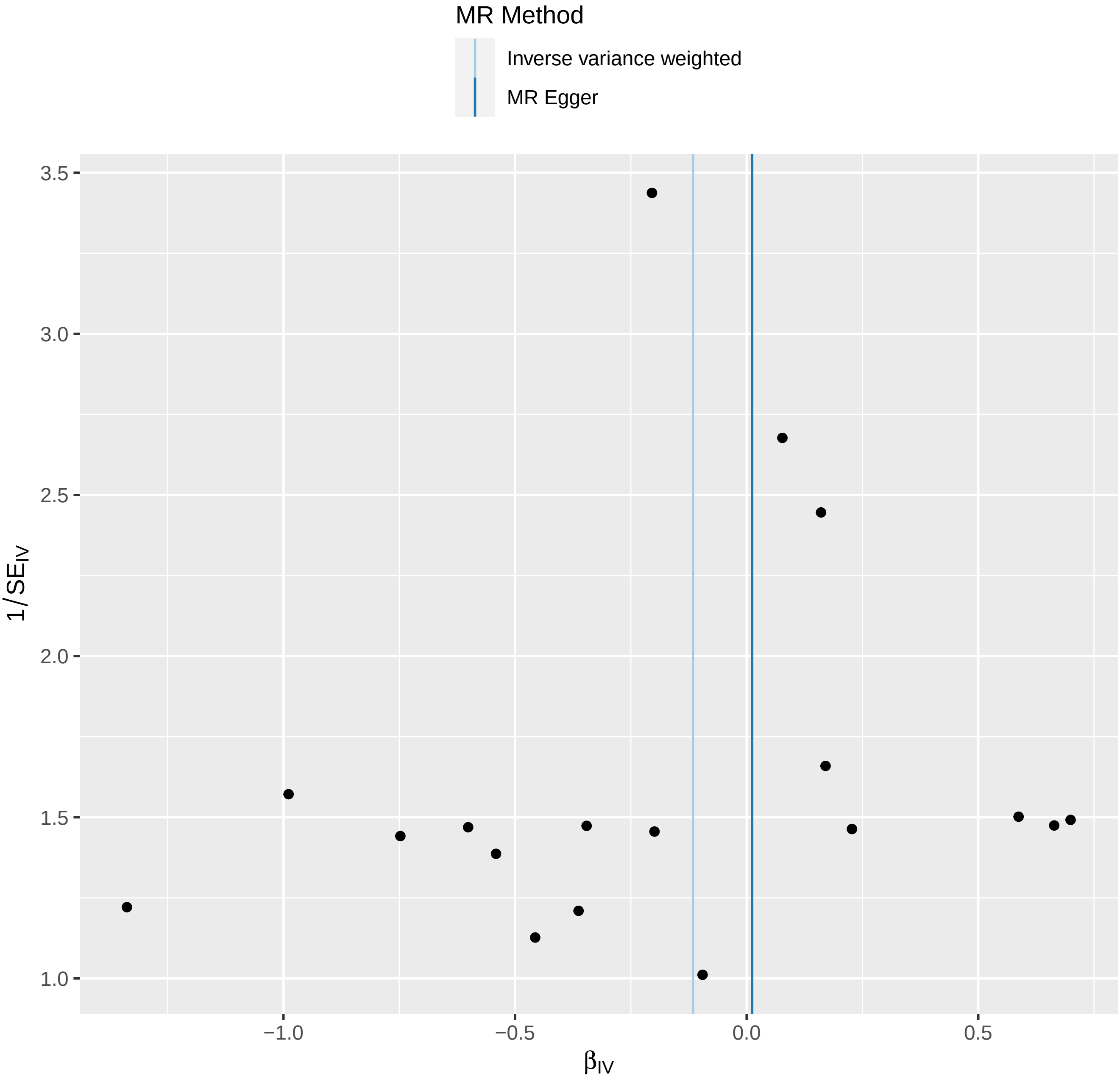

Batch 1231 : Gut microbiota abundance (family Verrucomicrobiaceae id.4036) on Other ulcerative colitis

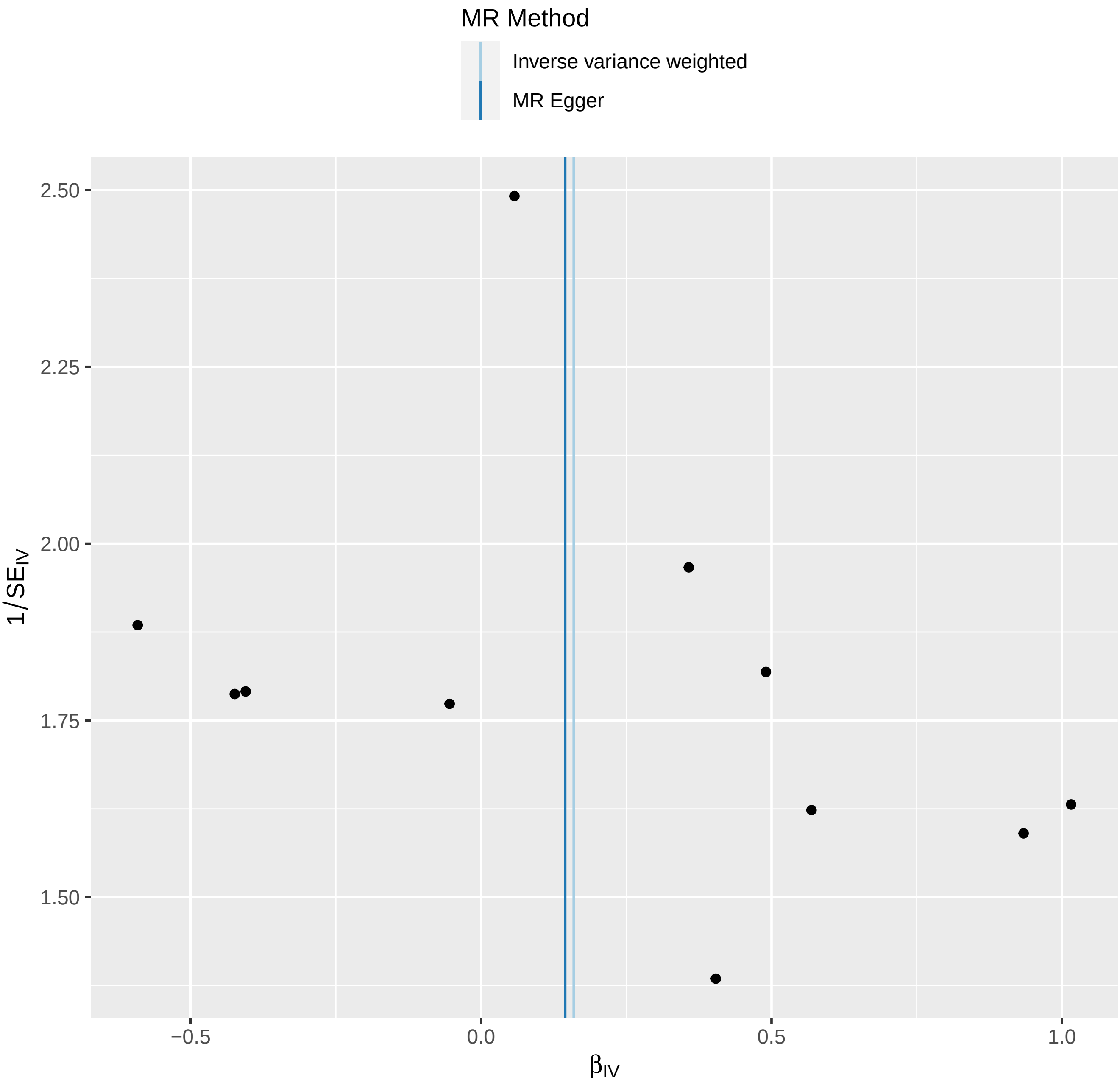

Batch 1232 : Gut microbiota abundance (family Victivallaceae id.2255) on Other ulcerative colitis

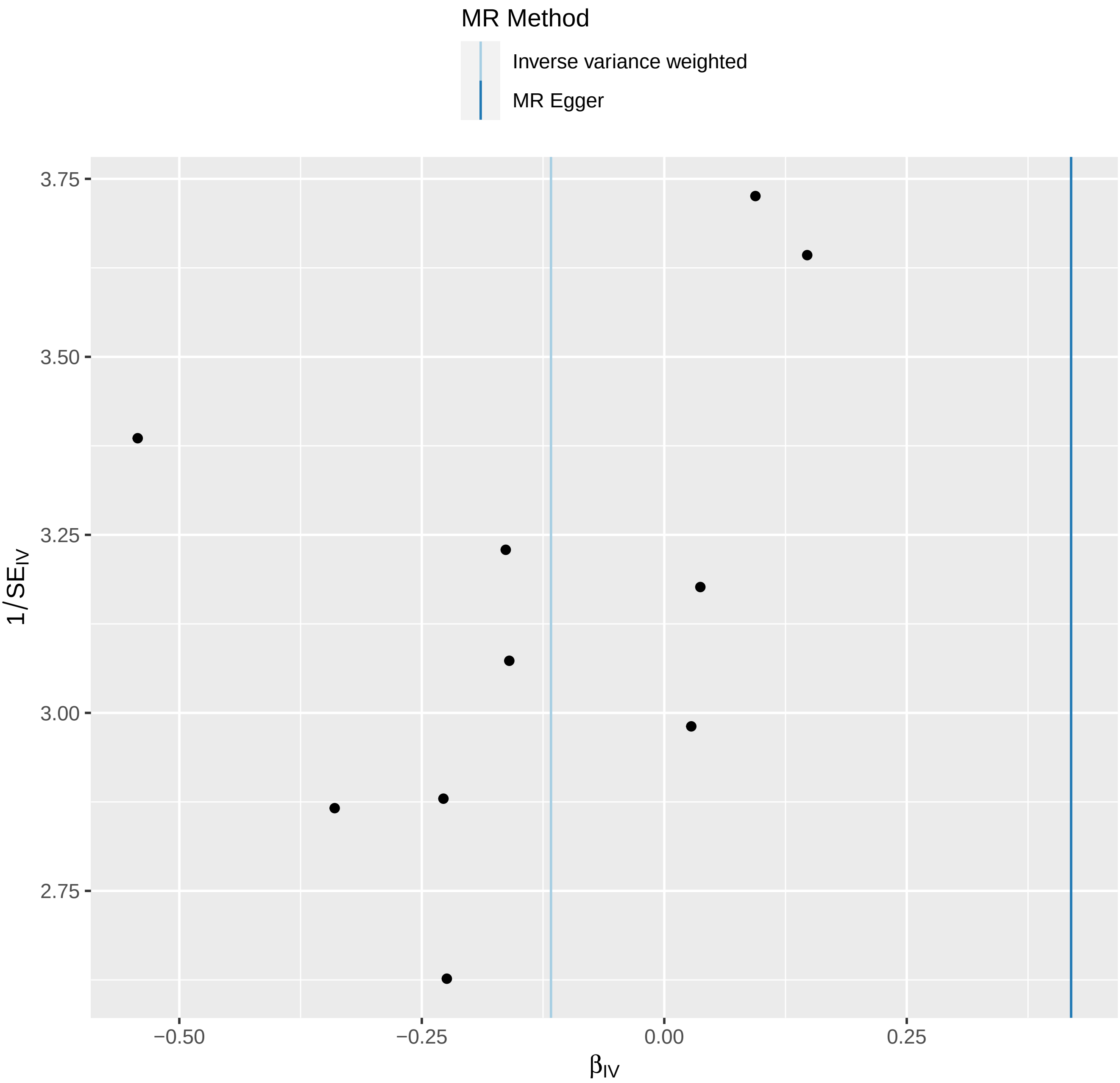

Batch 1233 : Gut microbiota abundance (genus Actinomyces id.423) on Other ulcerative colitis

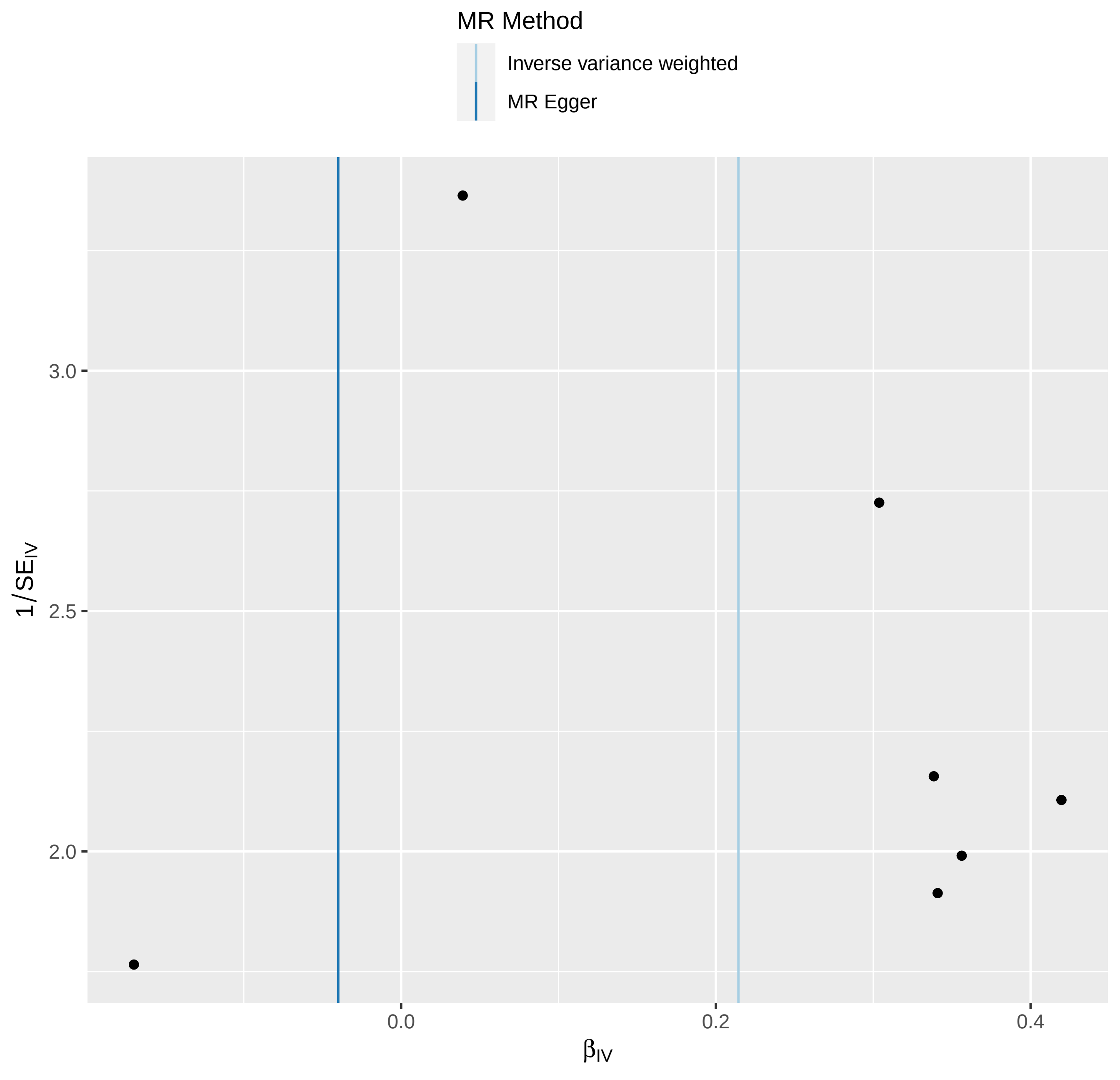

Batch 1234 : Gut microbiota abundance (genus Adlercreutzia id.812) on Other ulcerative colitis

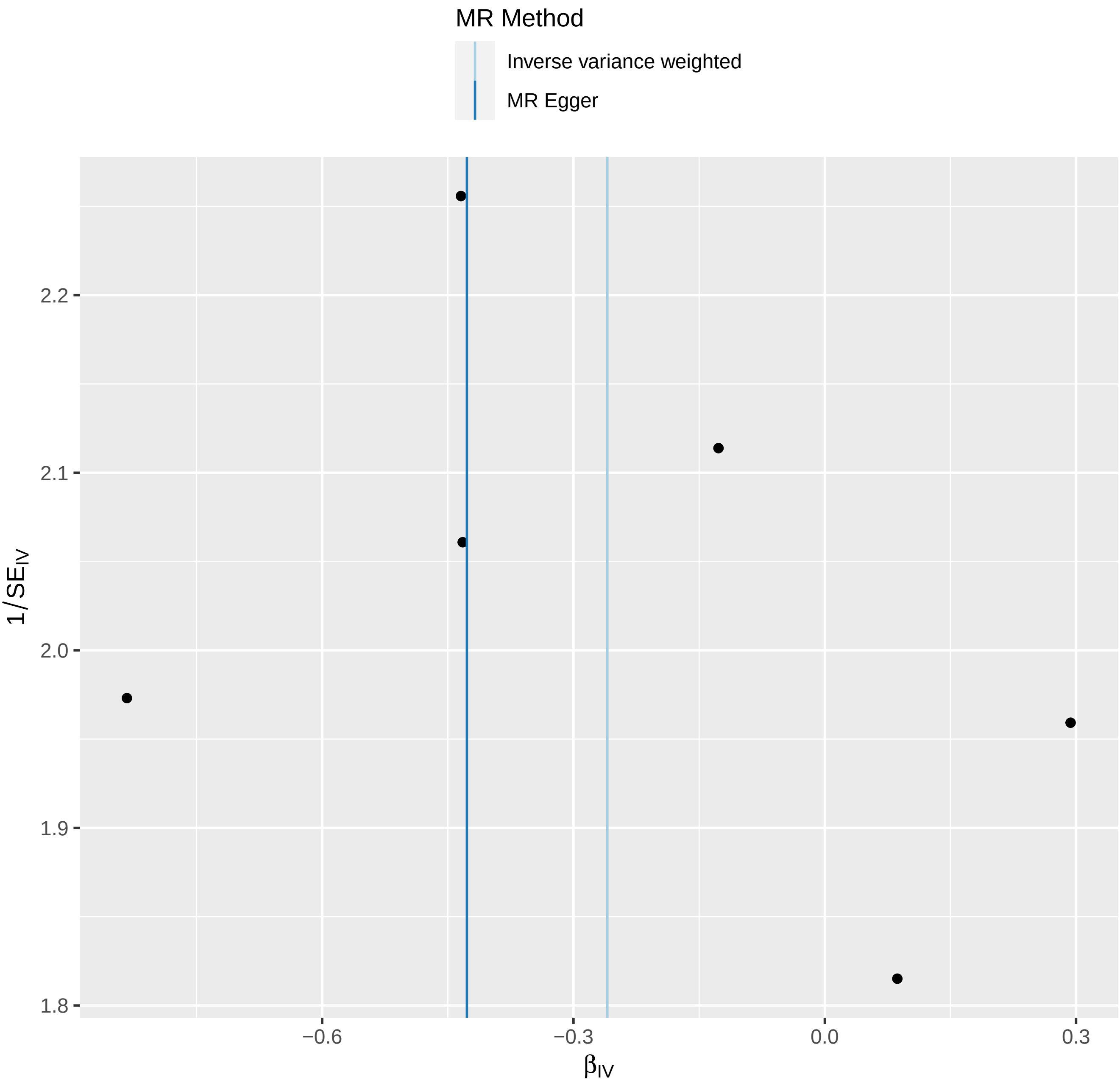

Batch 1235 : Gut microbiota abundance (genus Akkermansia id.4037) on Other ulcerative colitis

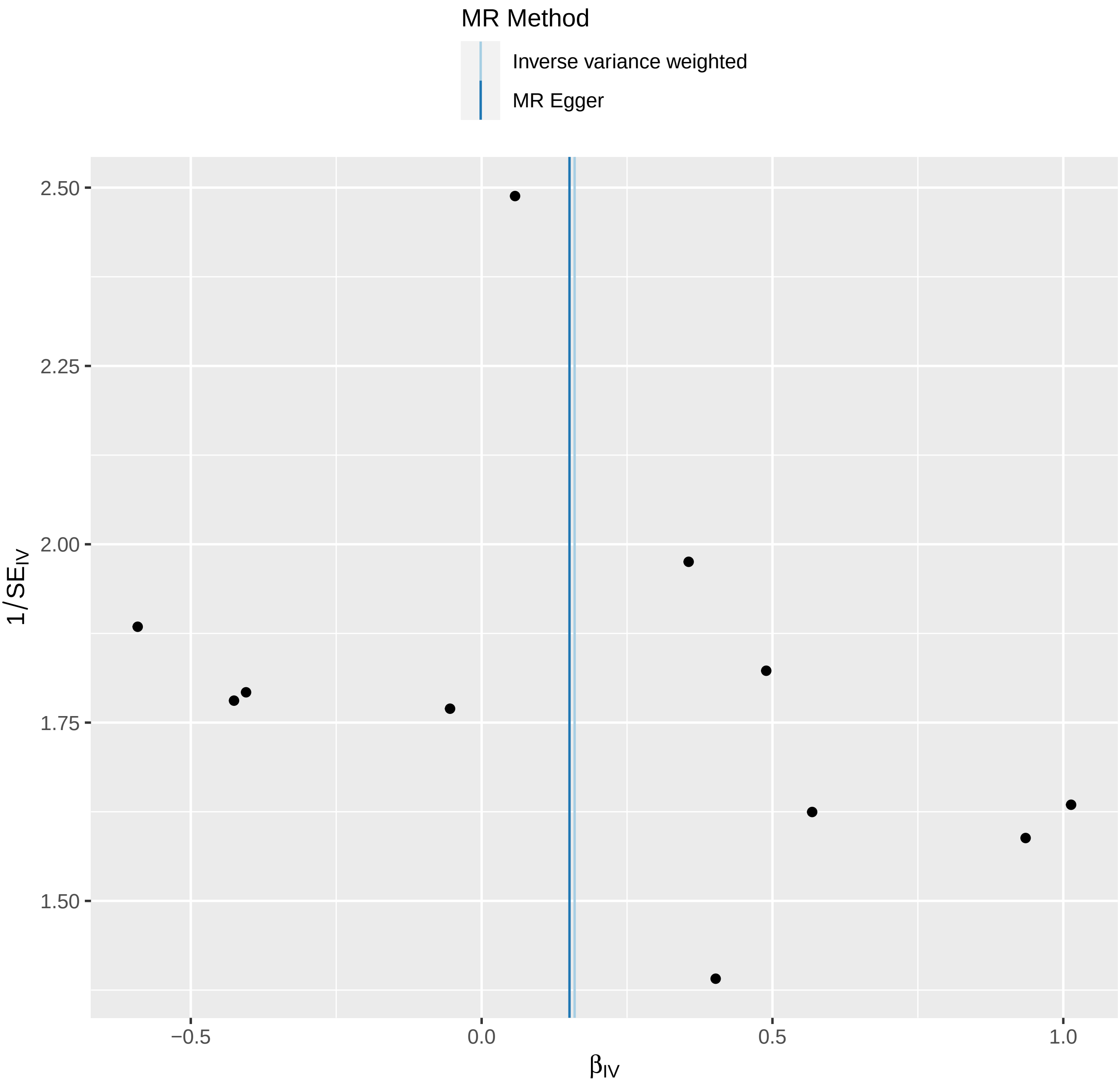

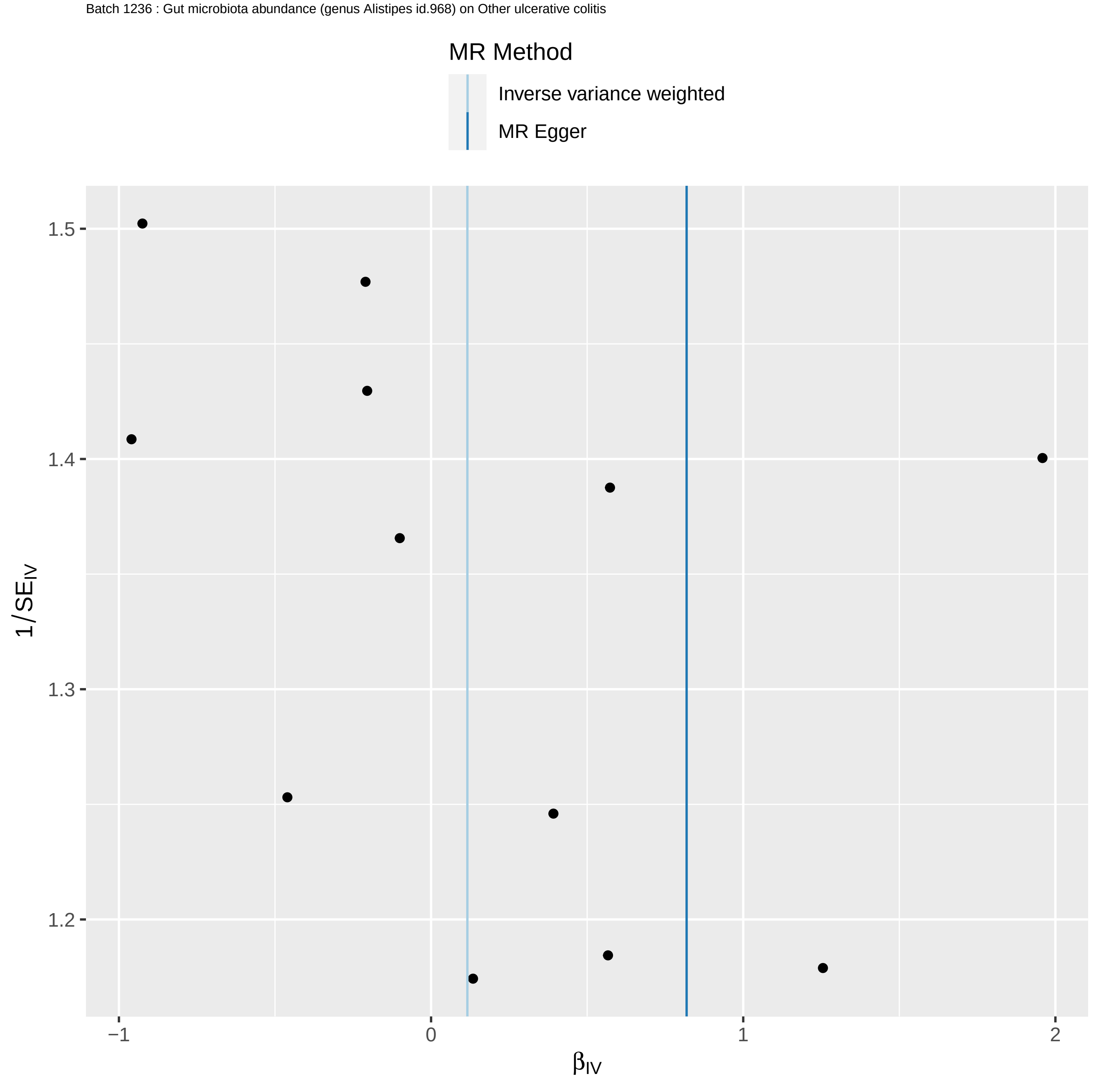

Batch 1236 : Gut microbiota abundance (genus Alistipes id.968) on Other ulcerative colitis
MR Method
Inverse variance weighted
MR Egger
1.5
1.4
1.3
1.2
1/SE_IV
−1
0
1
2
β_IV

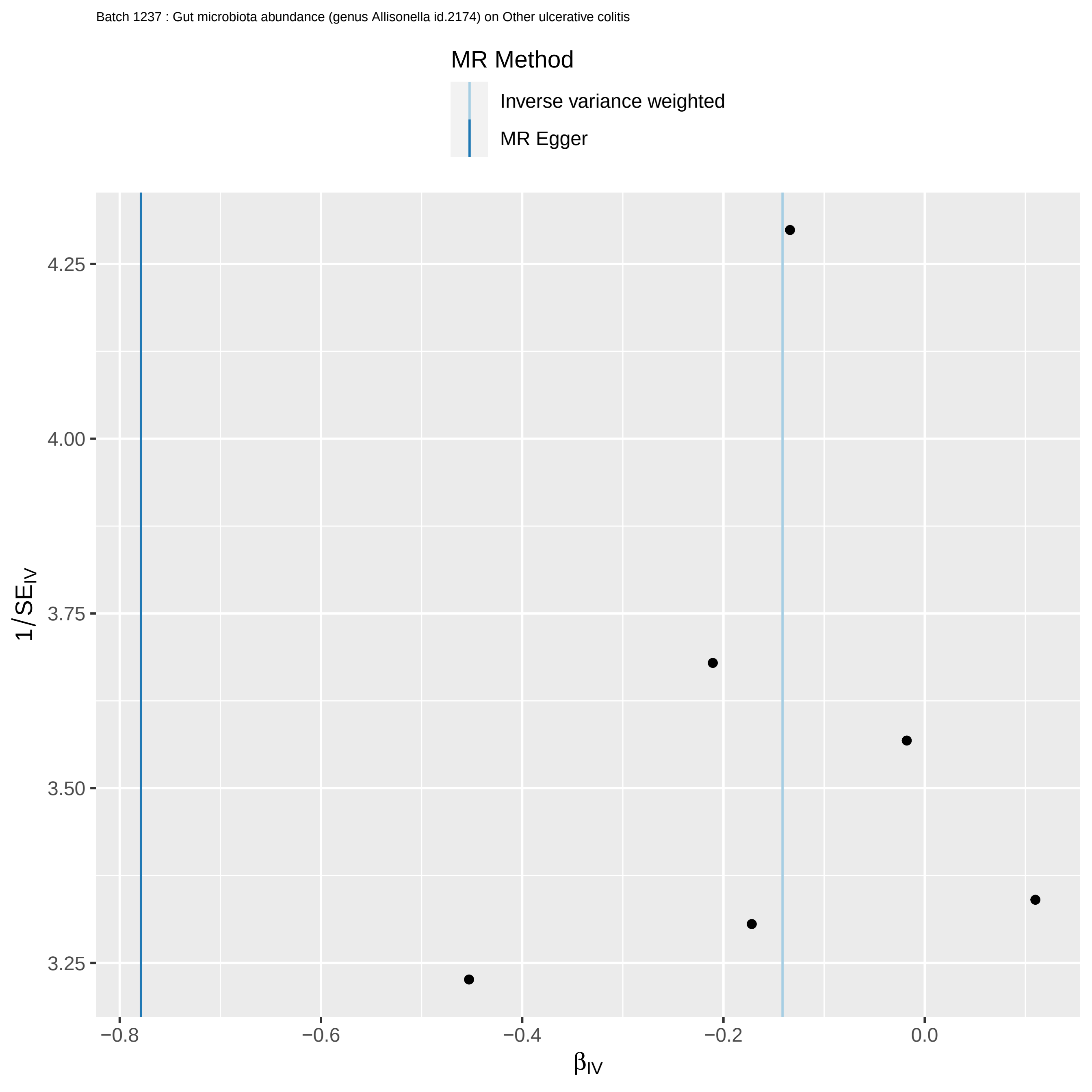
Batch 1237 : Gut microbiota abundance (genus Allisonella id.2174) on Other ulcerative colitis
MR Method
Inverse variance weighted
MR Egger
1/SE_IV
4.25
4.00
3.75
3.50
3.25
−0.8
−0.6
−0.4
−0.2
0.0
β_IV

Batch 1238 : Gut microbiota abundance (genus Alloprevotella id.961) on Other ulcerative colitis

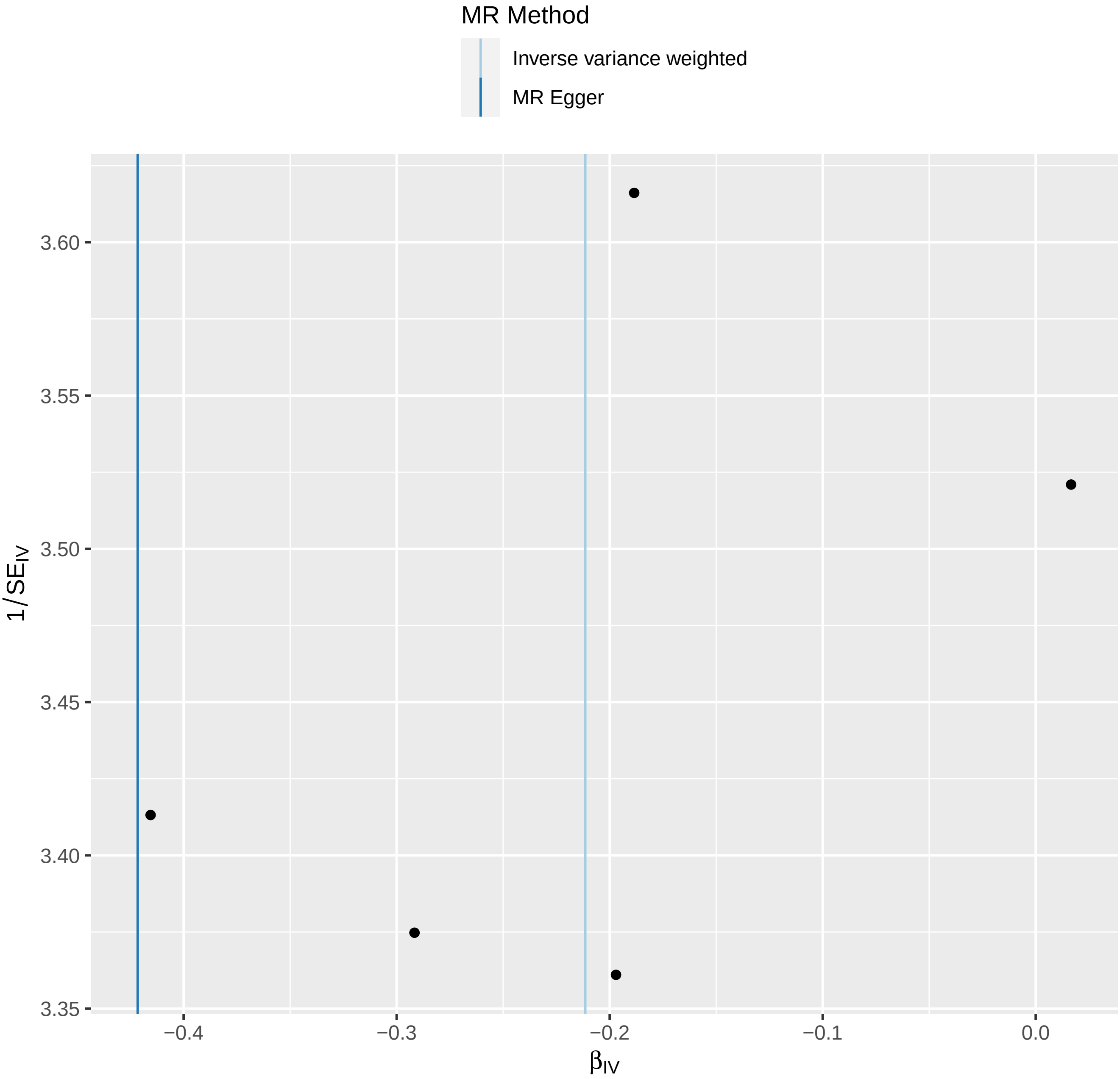

Batch 1239 : Gut microbiota abundance (genus Anaerofilum id.2053) on Other ulcerative colitis

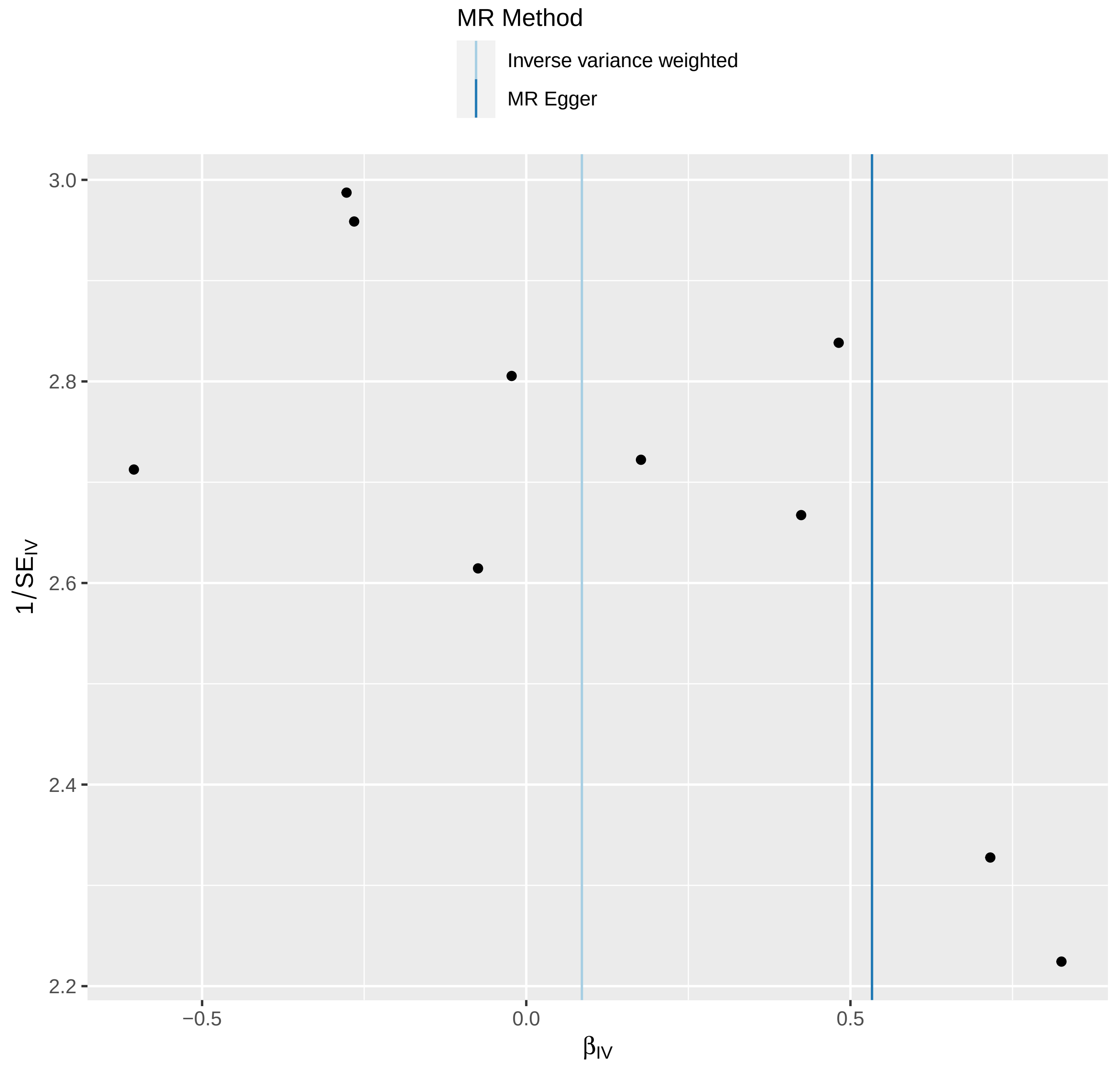

Batch 1240 : Gut microbiota abundance (genus Anaerostipes id.1991) on Other ulcerative colitis

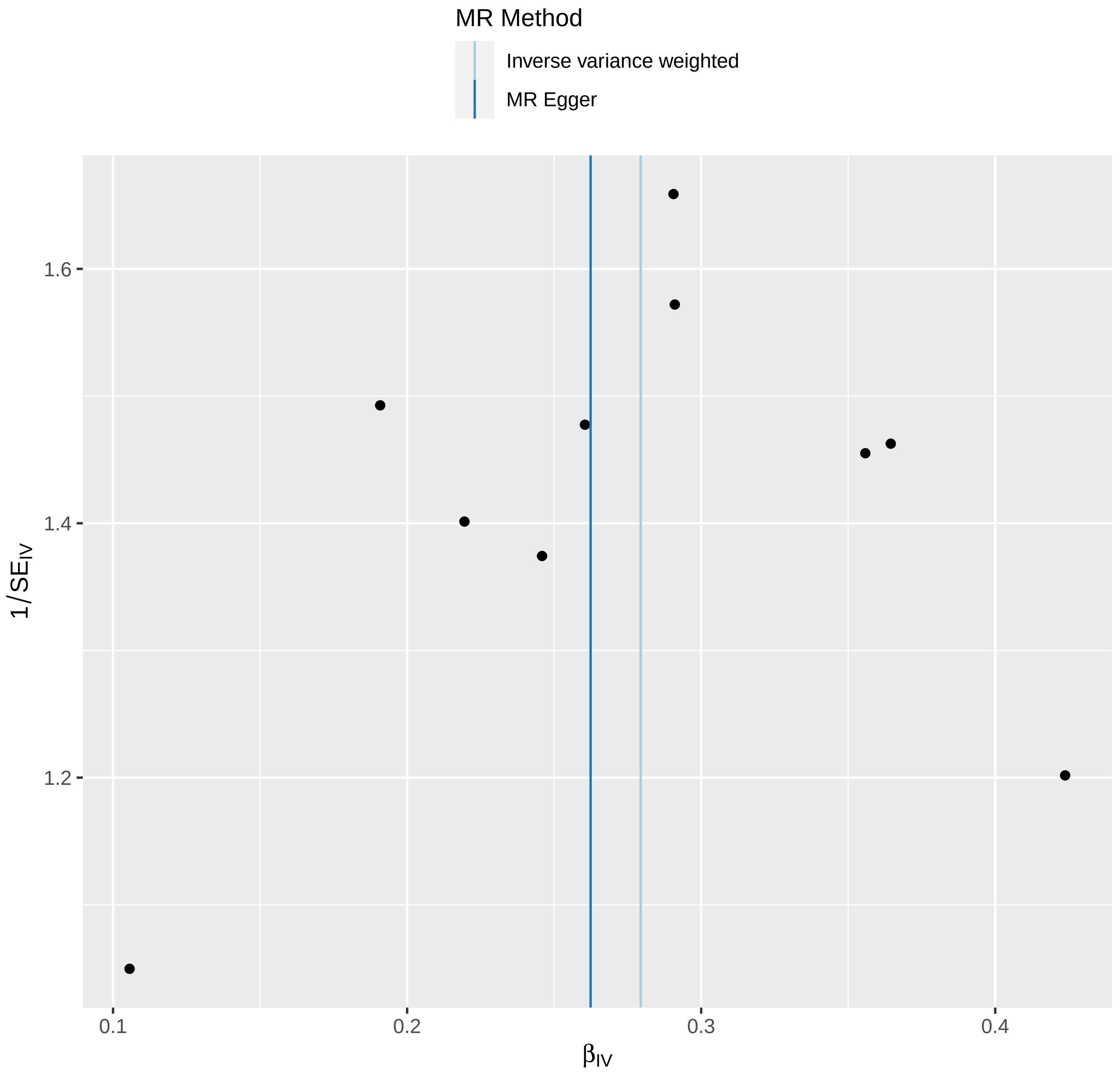

Batch 1241 : Gut microbiota abundance (genus Anaerotruncus id.2054) on Other ulcerative colitis

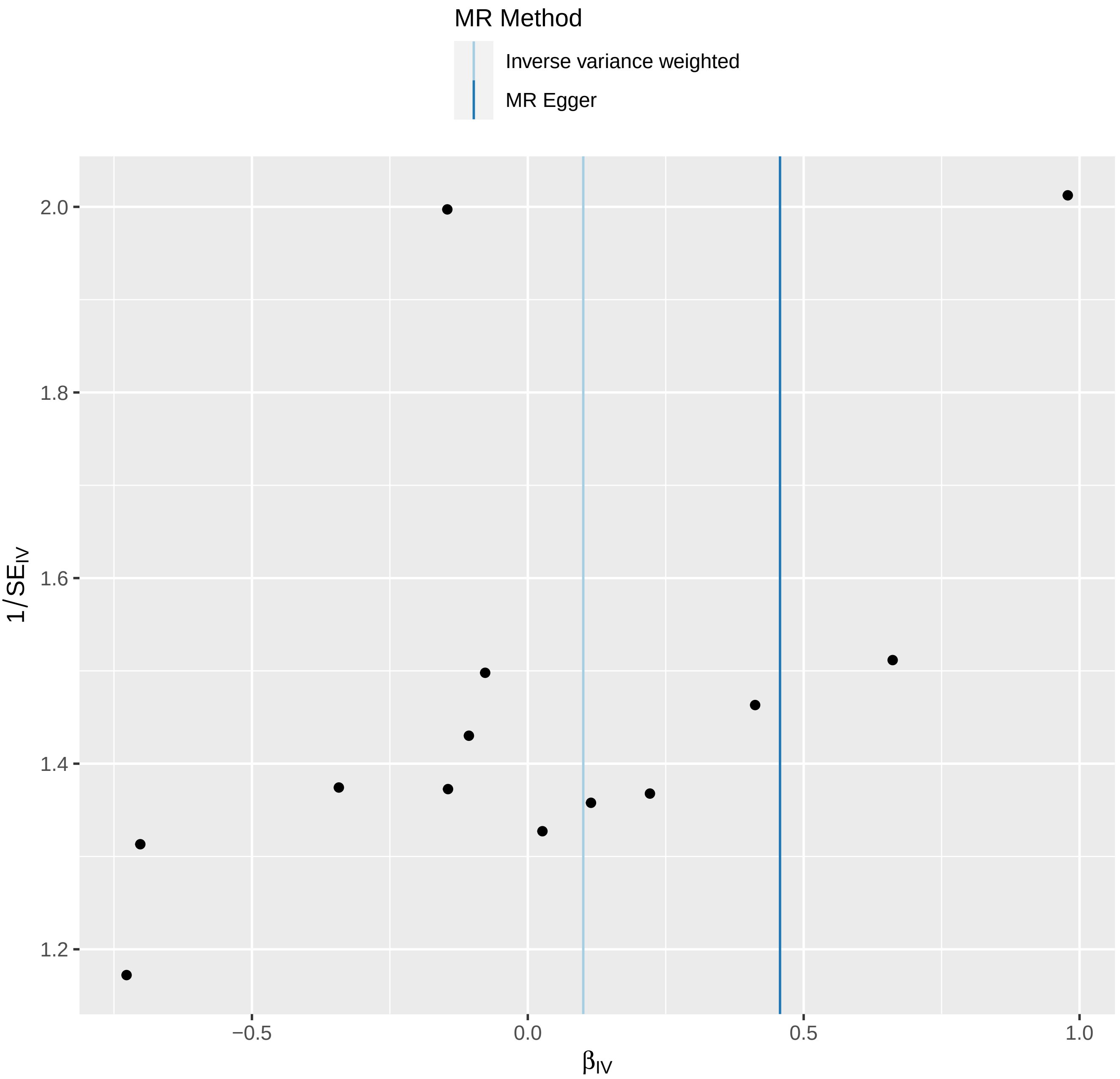

Batch 1242 : Gut microbiota abundance (genus Bacteroides id.918) on Other ulcerative colitis

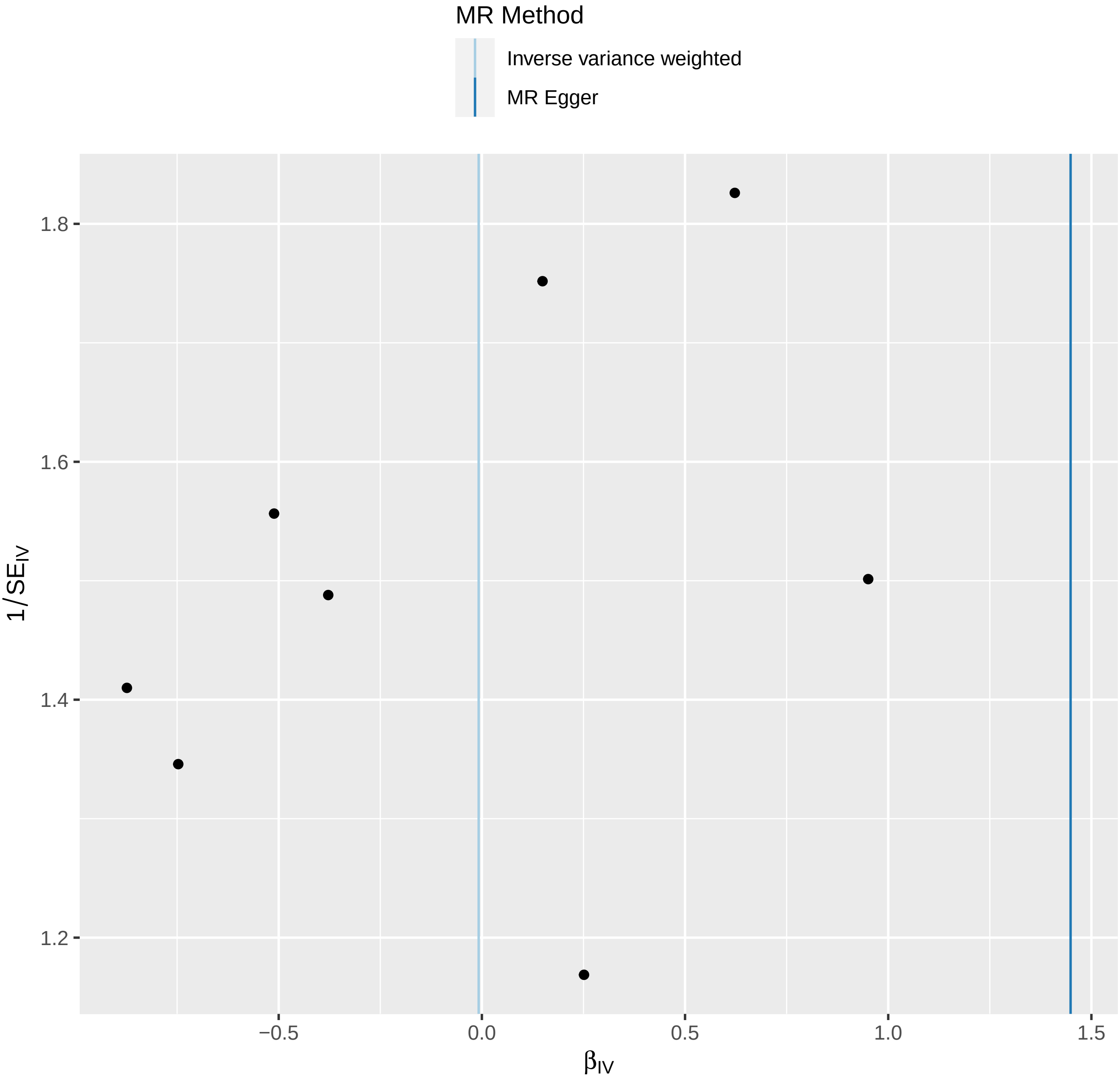

Batch 1243 : Gut microbiota abundance (genus Barnesiella id.944) on Other ulcerative colitis

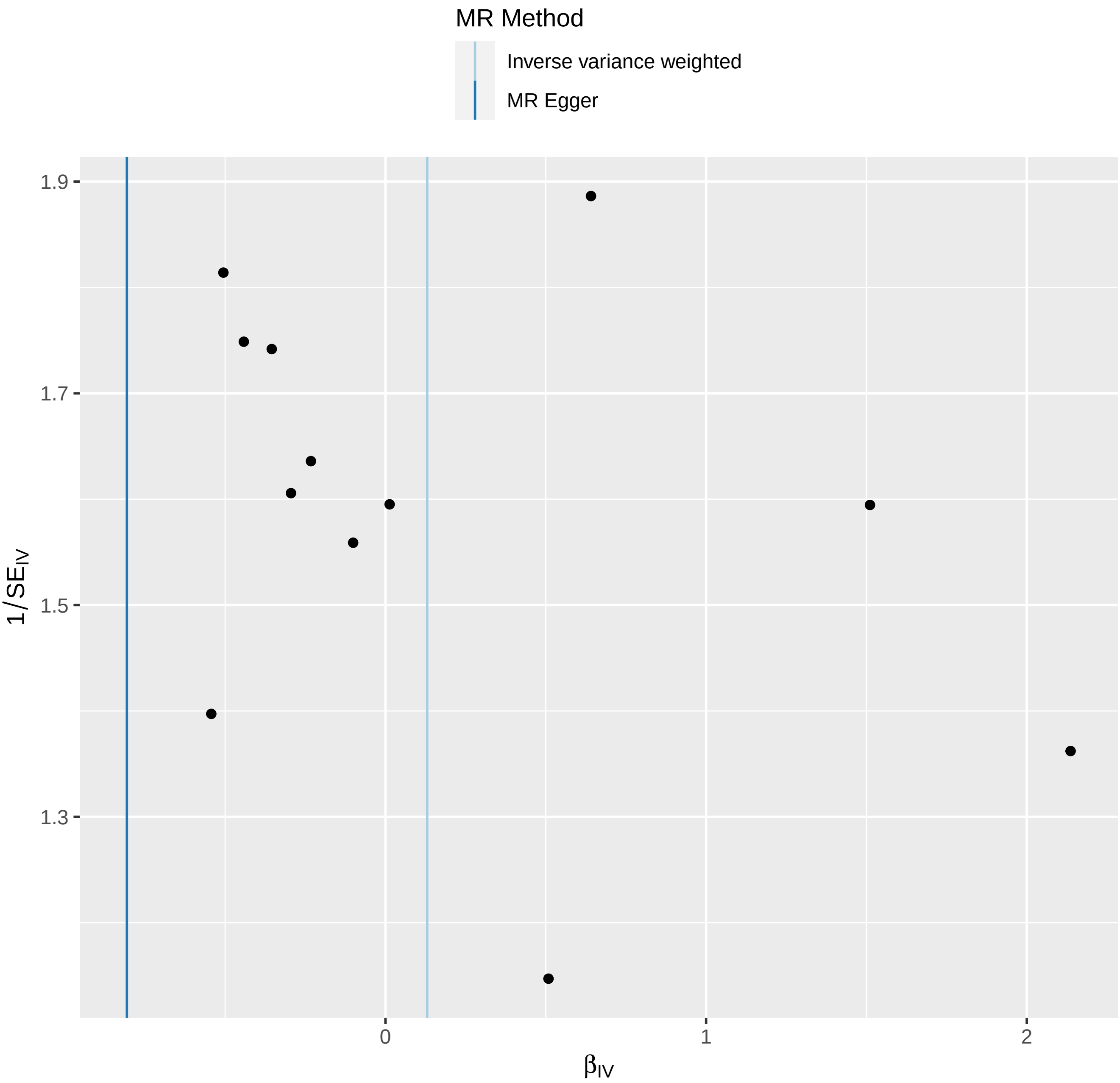

Batch 1244 : Gut microbiota abundance (genus Bifidobacterium id.436) on Other ulcerative colitis

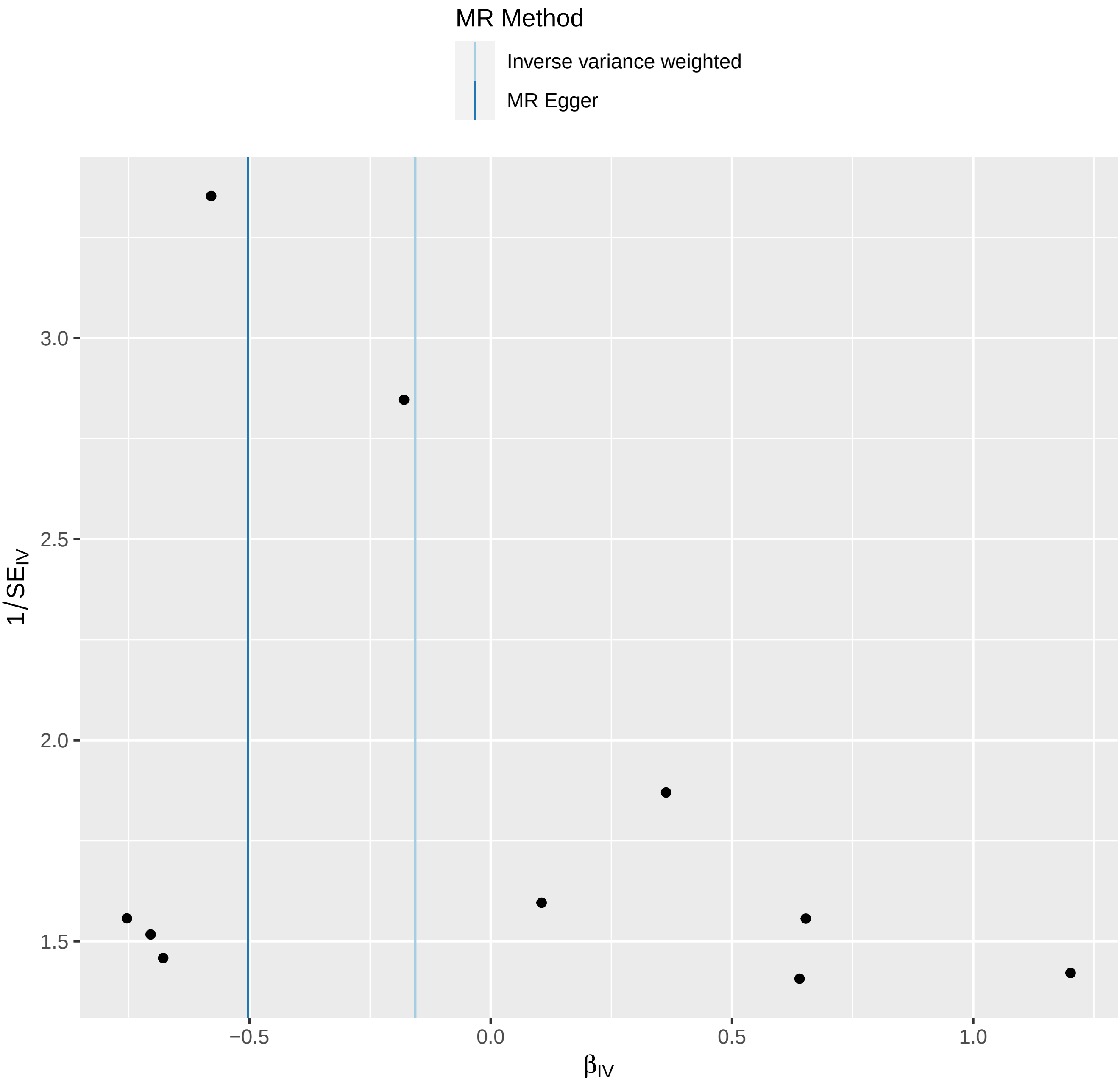

Batch 1245 : Gut microbiota abundance (genus Bilophila id.3170) on Other ulcerative colitis

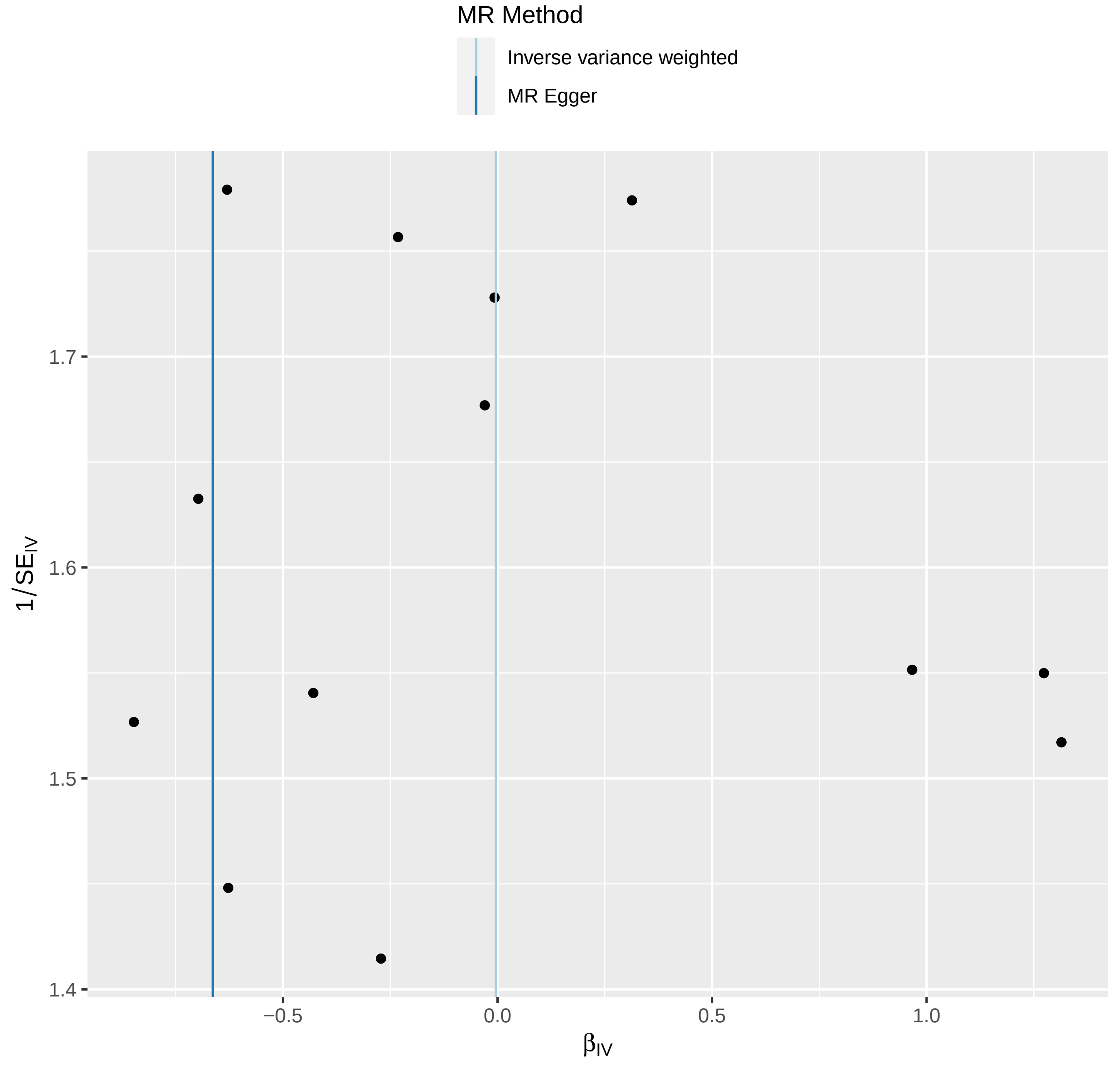

Batch 1246 : Gut microbiota abundance (genus Blautia id.1992) on Other ulcerative colitis

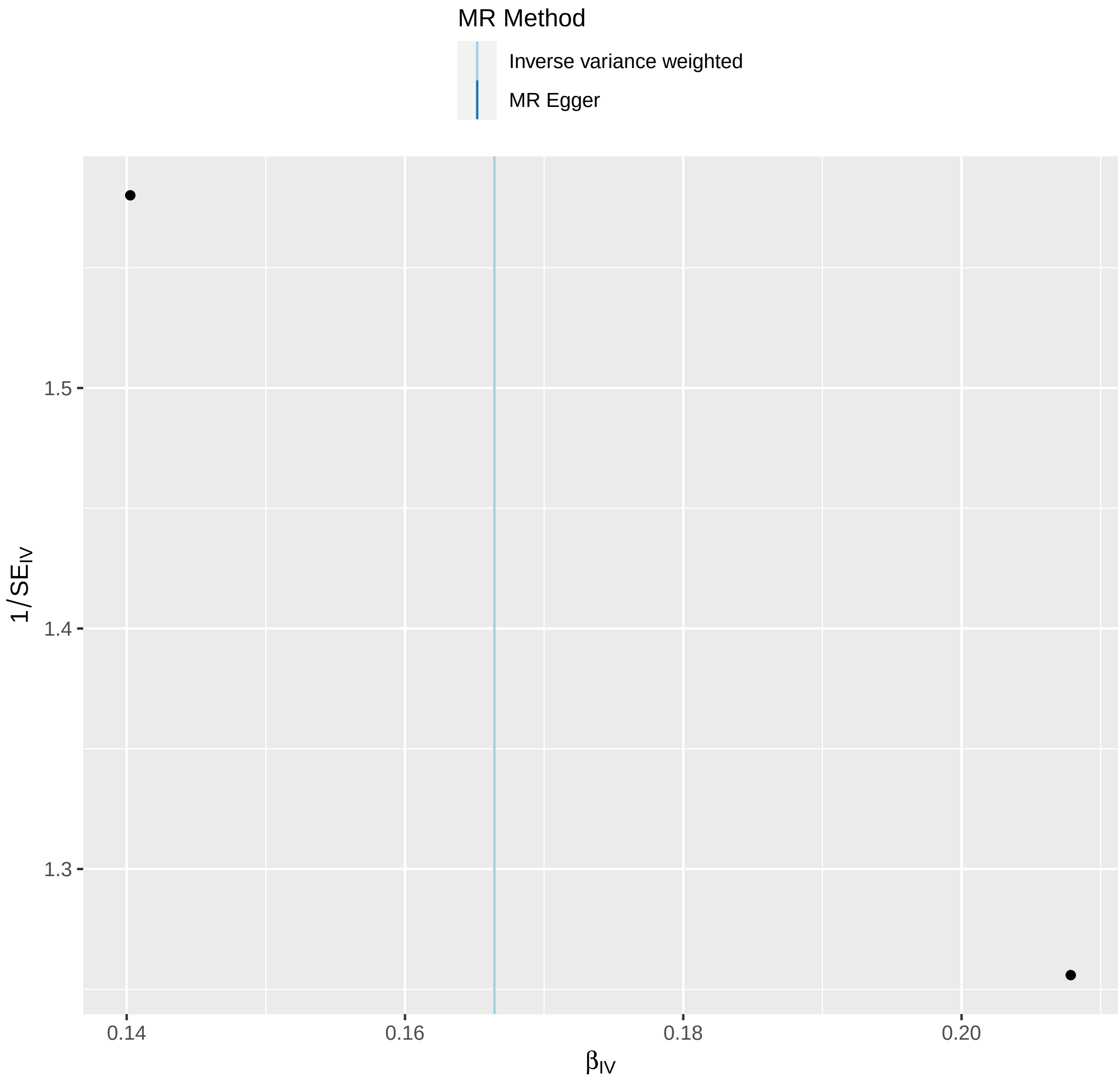

Batch 1247 : Gut microbiota abundance (genus Butyricicoccus id.2055) on Other ulcerative colitis

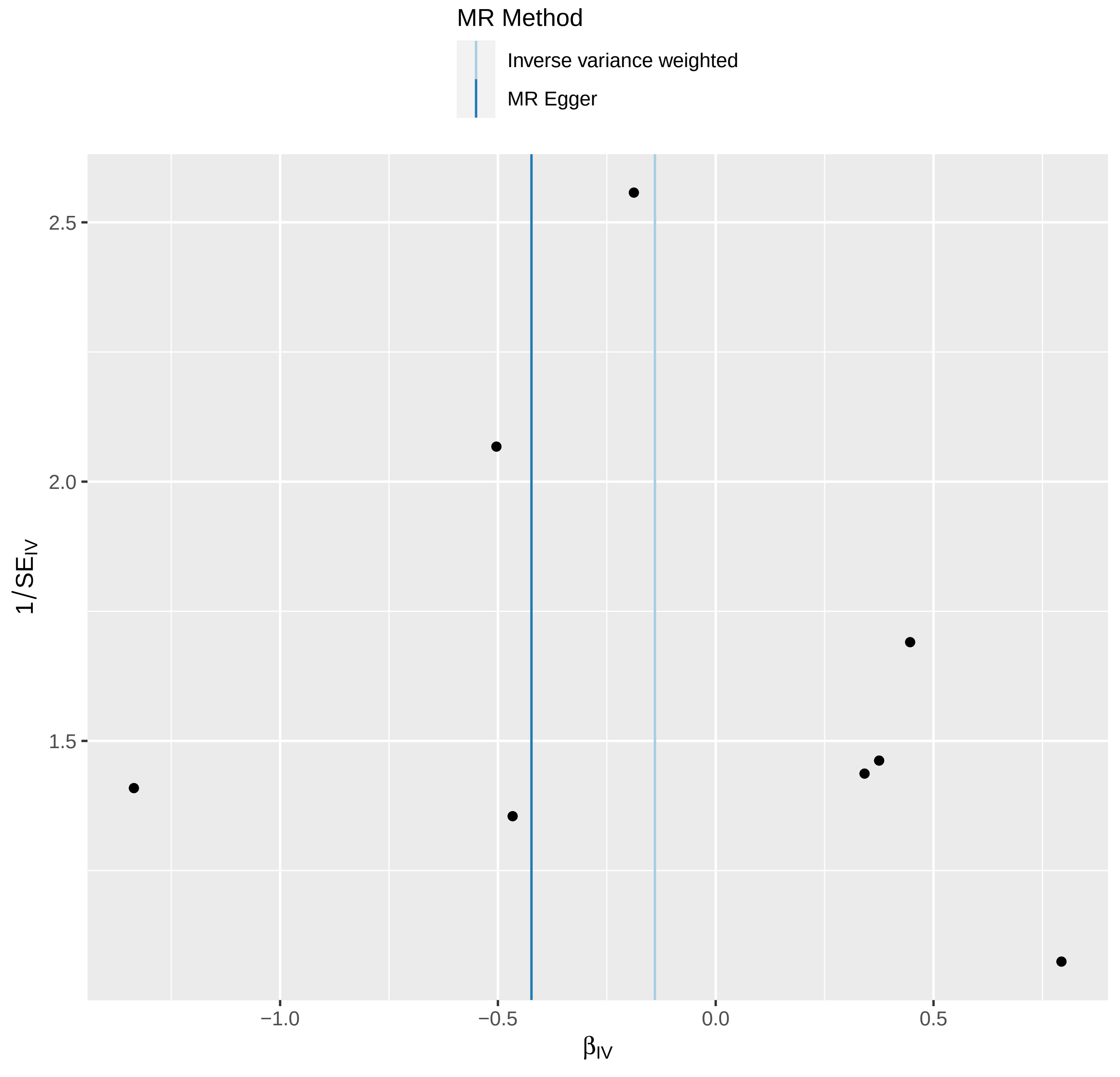

Batch 1248 : Gut microbiota abundance (genus Butyricimonas id.945) on Other ulcerative colitis

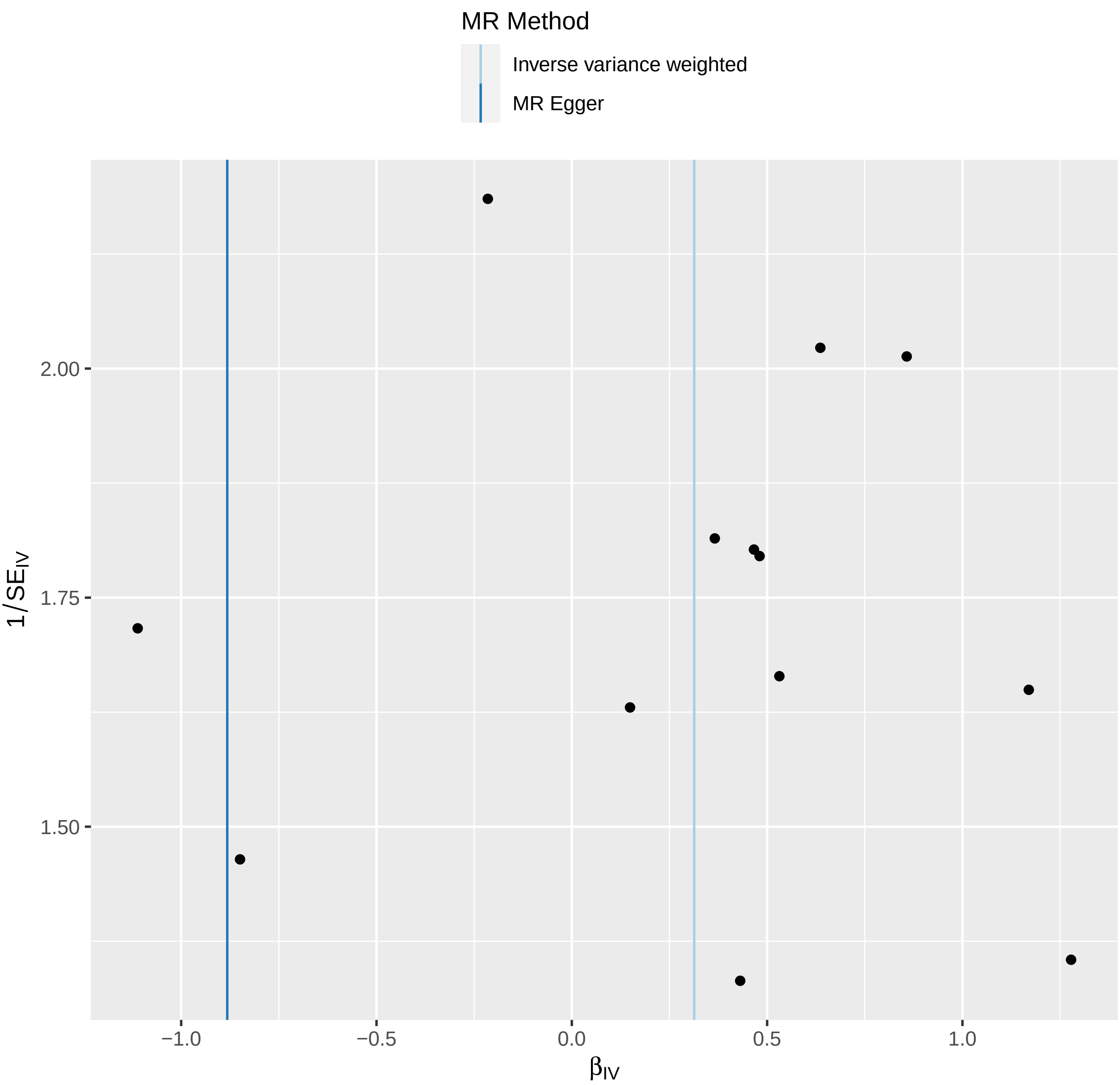

Batch 1249 : Gut microbiota abundance (genus Butyrivibrio id.1993) on Other ulcerative colitis

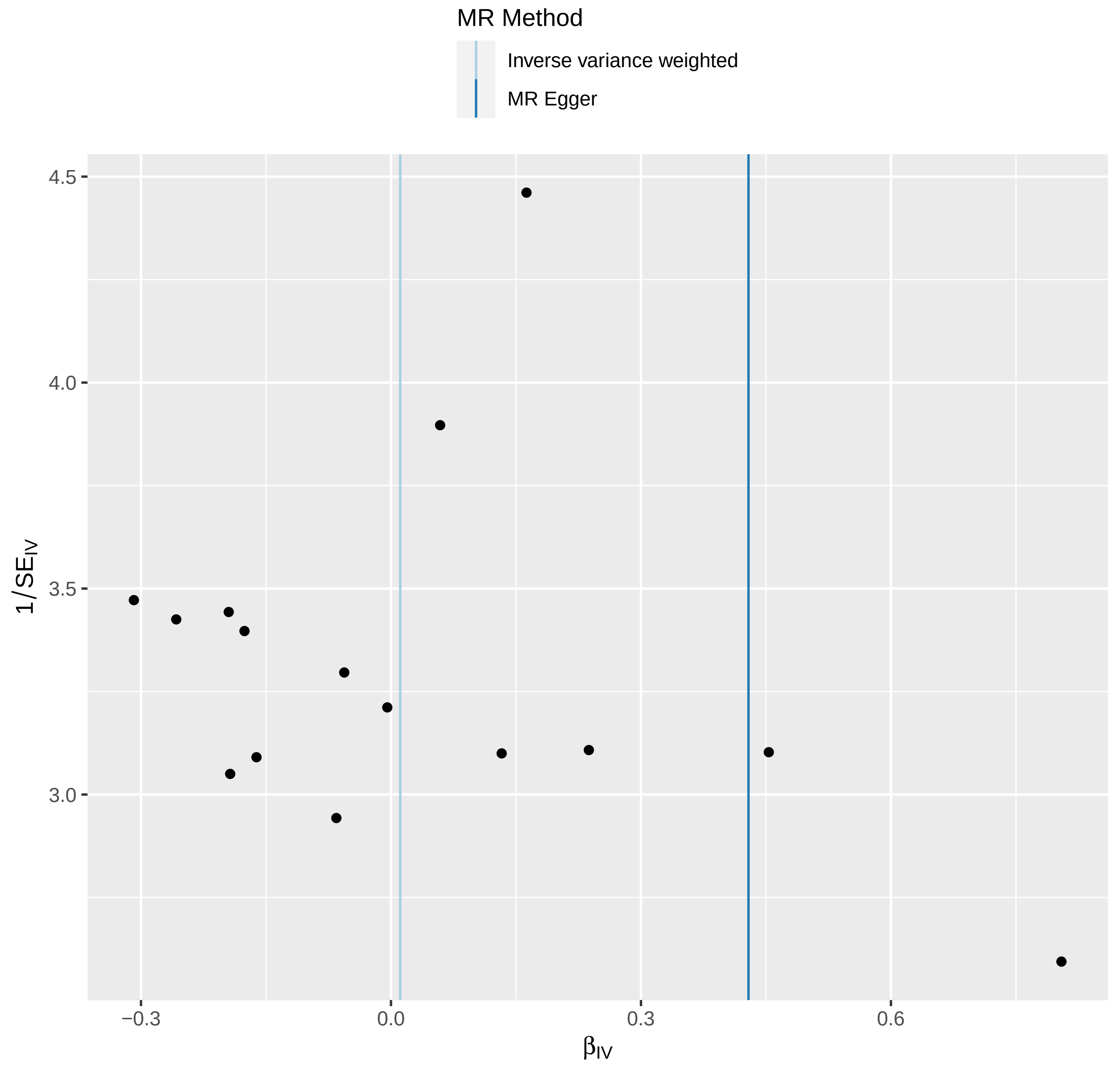

Batch 1250 : Gut microbiota abundance (genus Candidatus Soleaferrea id.11350) on Other ulcerative colitis

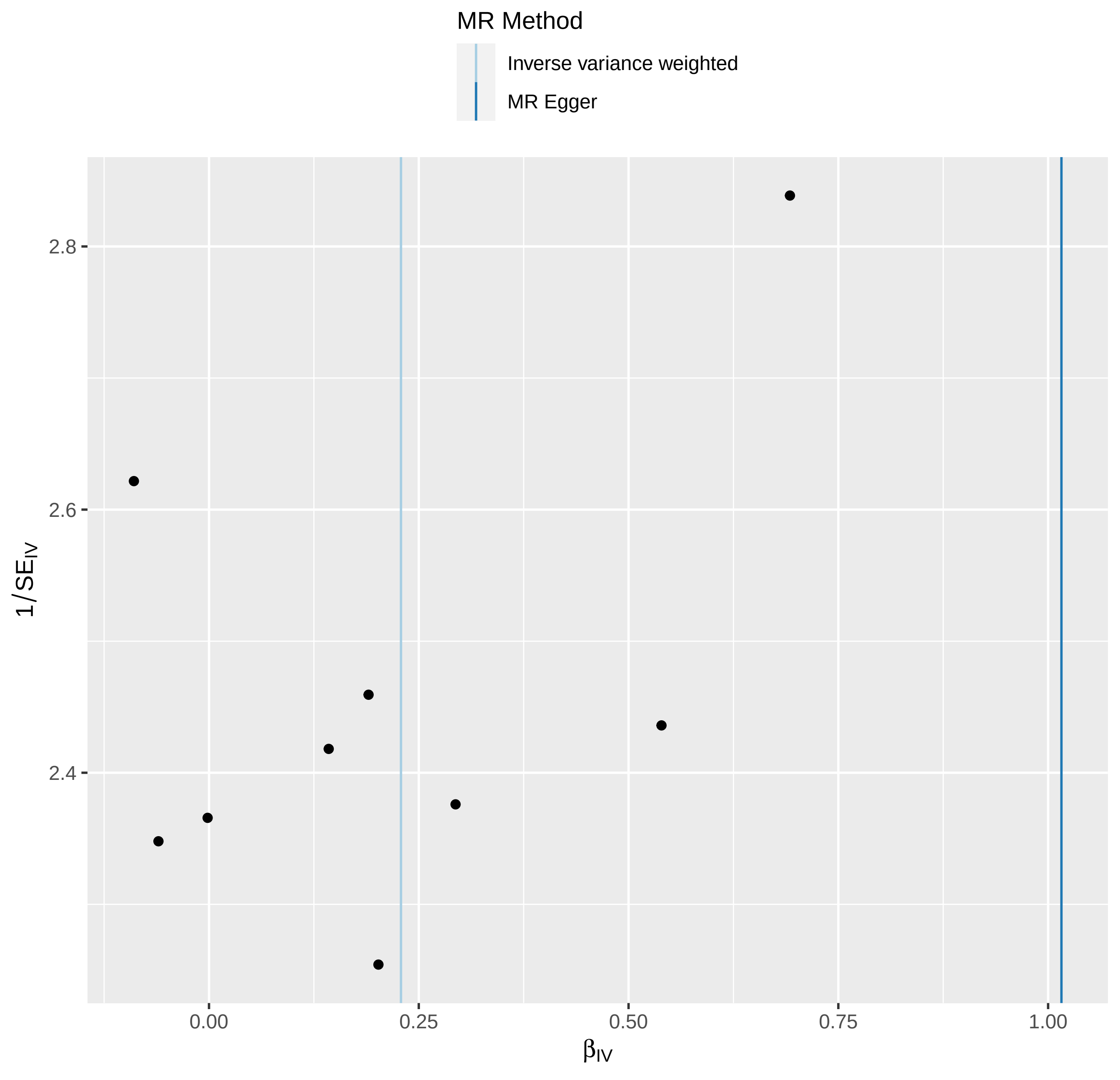

Batch 1251 : Gut microbiota abundance (genus Catenibacterium id.2153) on Other ulcerative colitis

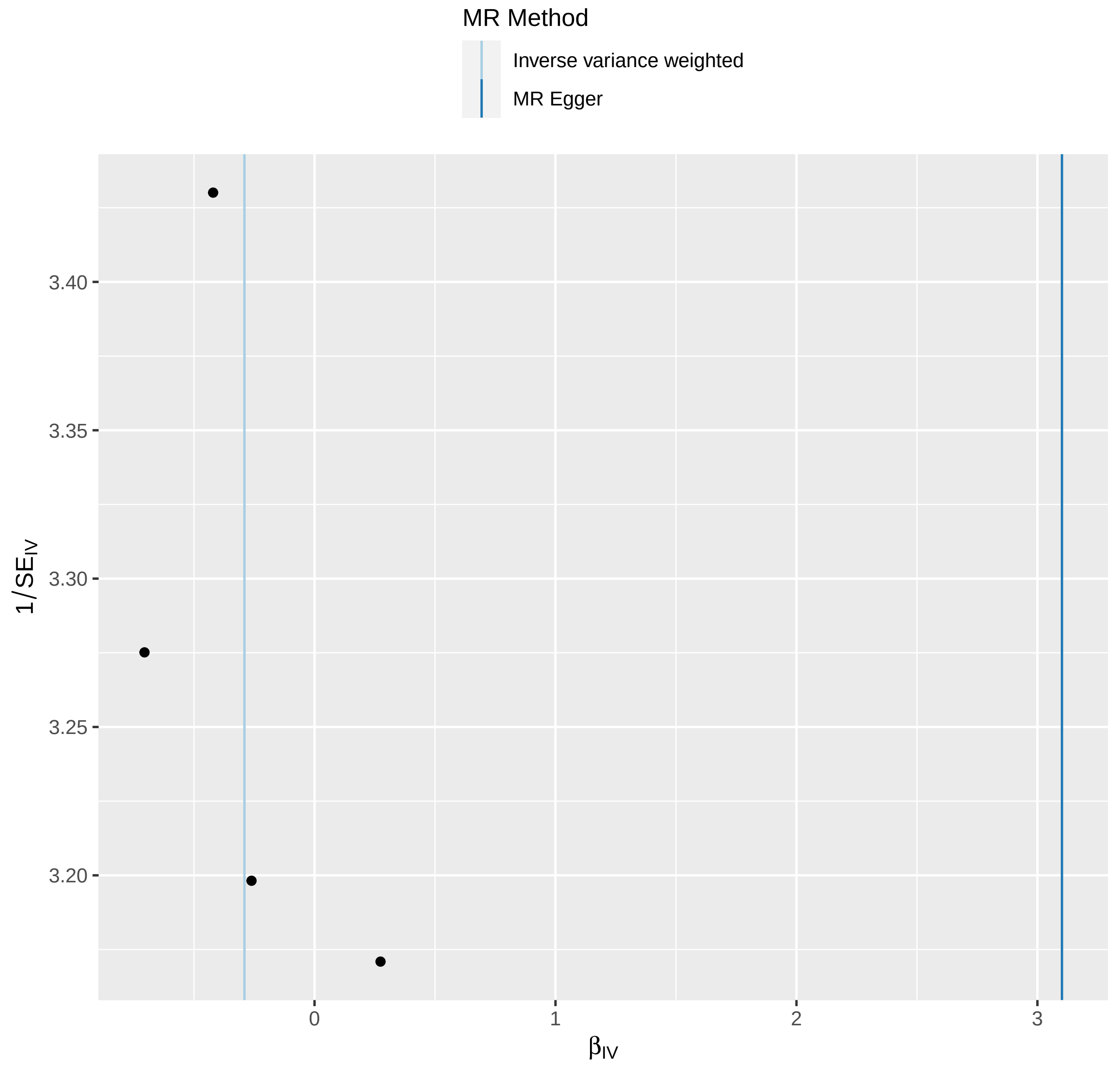

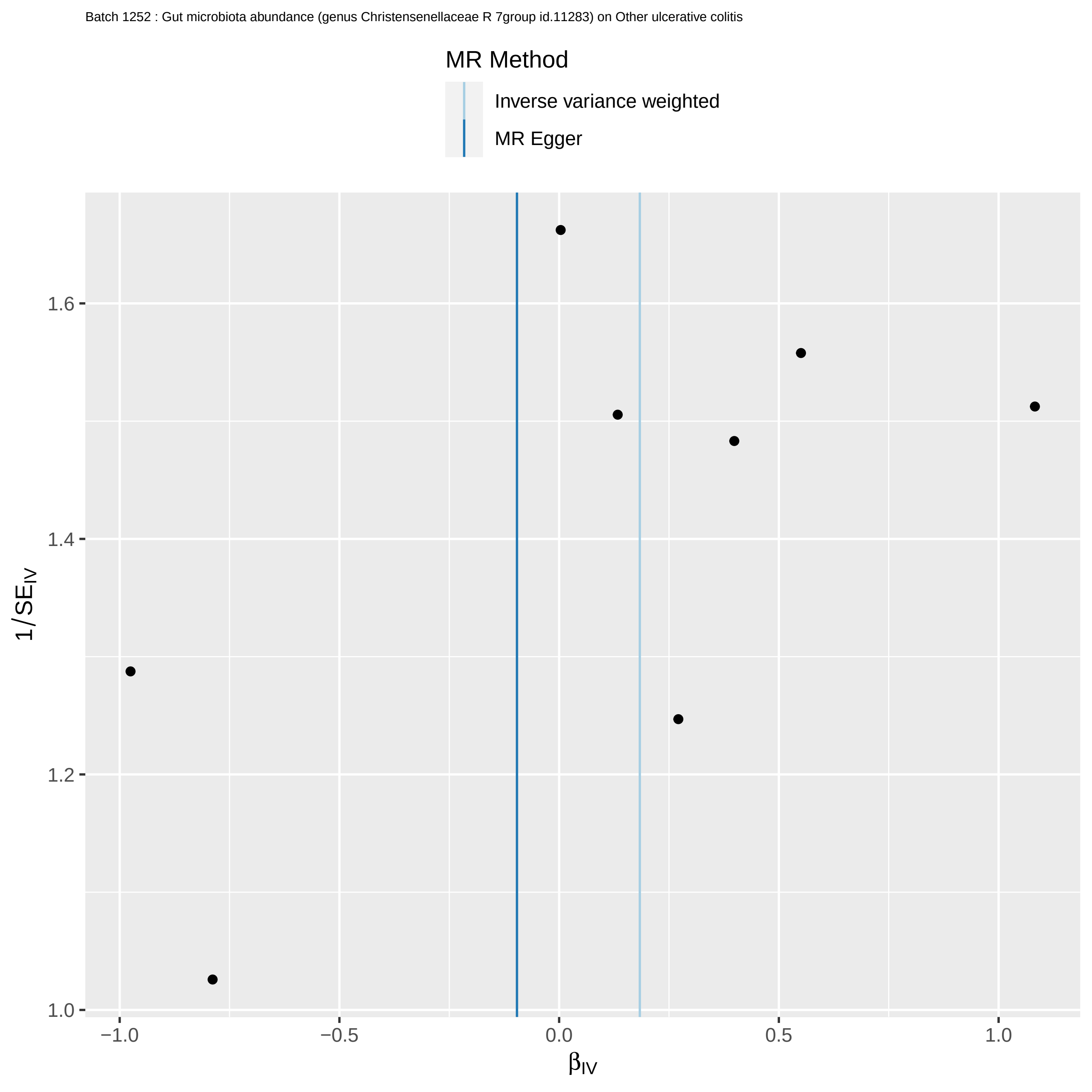
Batch 1252 : Gut microbiota abundance (genus Christensenellaceae R 7group id.11283) on Other ulcerative colitis
MR Method
Inverse variance weighted
MR Egger
1.6
1.4
1.2
1.0
1/SE_IV
−1.0
−0.5
0.0
0.5
1.0
β_IV

Batch 1253 : Gut microbiota abundance (genus Clostridium innocuum group id.14397) on Other ulcerative colitis

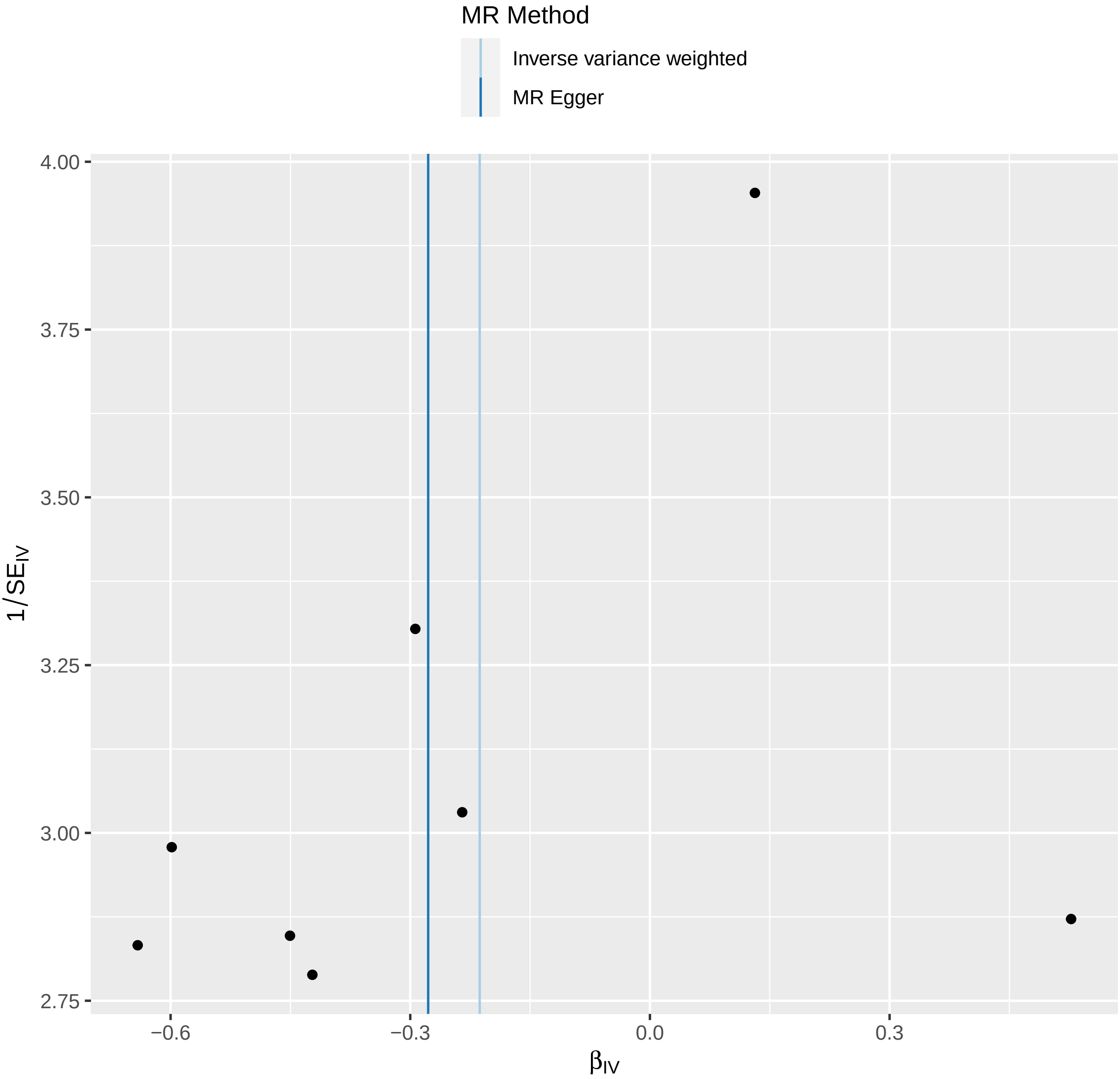

Batch 1254 : Gut microbiota abundance (genus Clostridium sensustricto1 id.1873) on Other ulcerative colitis

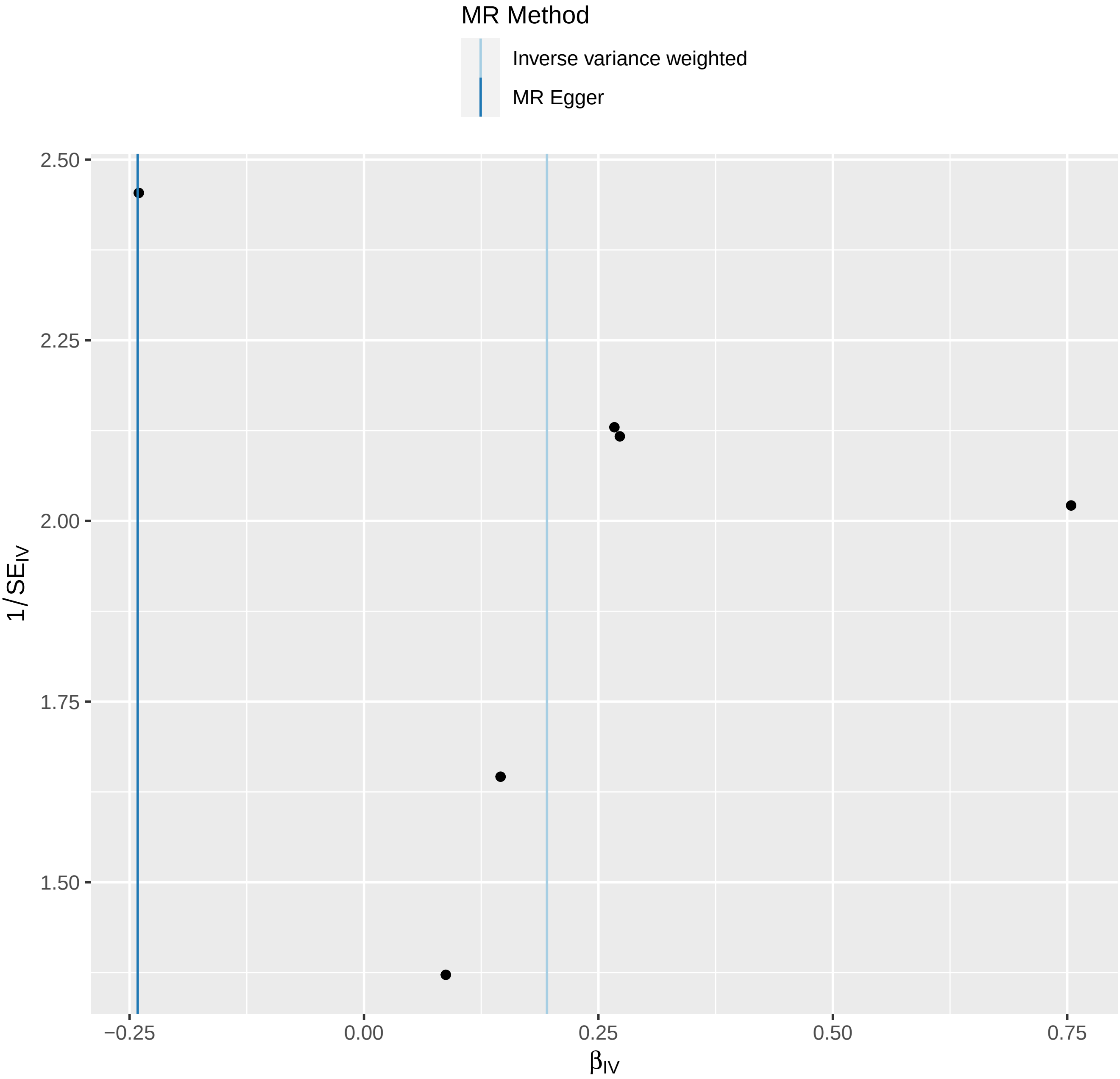

Batch 1255 : Gut microbiota abundance (genus Collinsella id.815) on Other ulcerative colitis

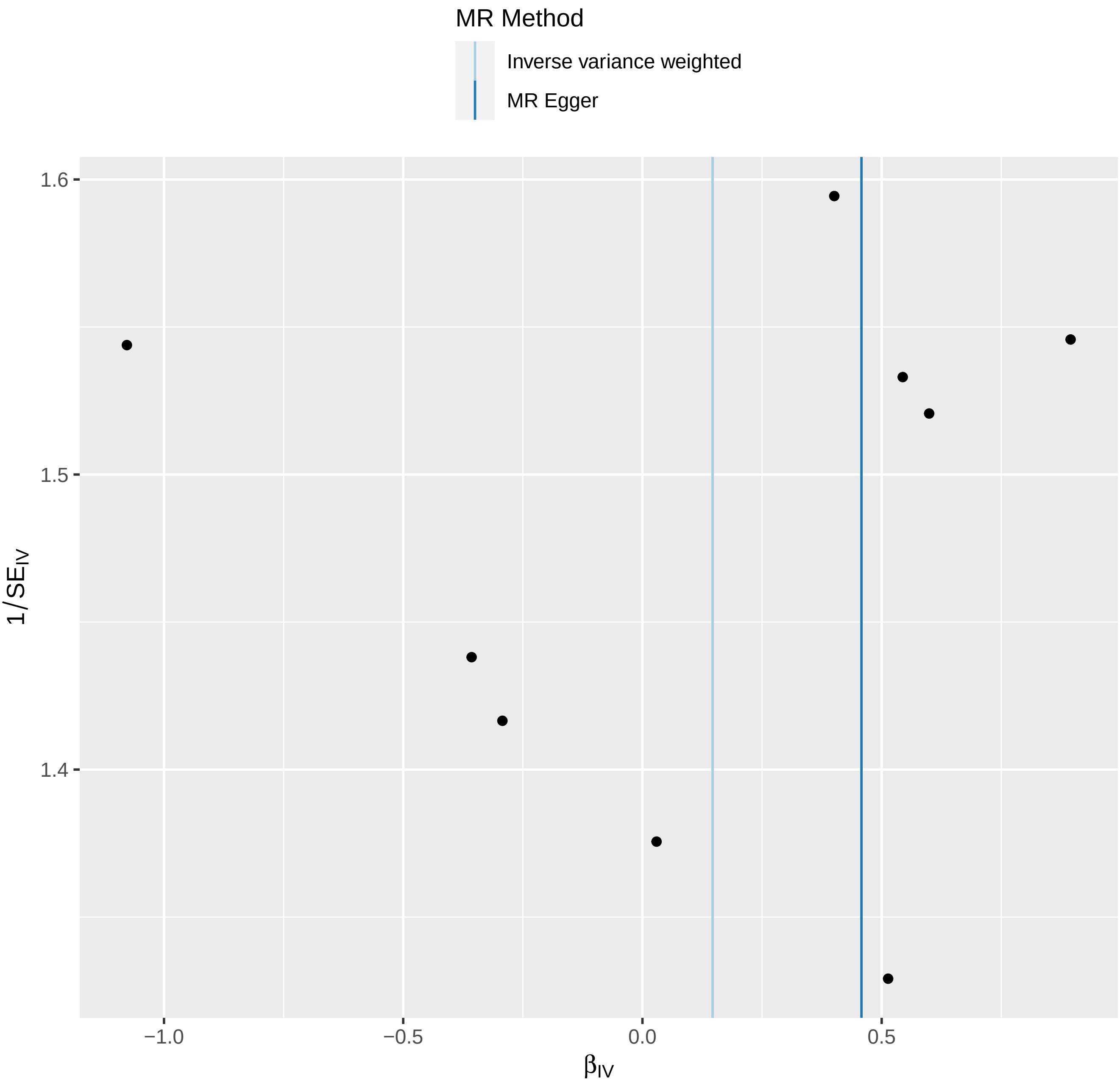

Batch 1256 : Gut microbiota abundance (genus Coprobacter id.949) on Other ulcerative colitis

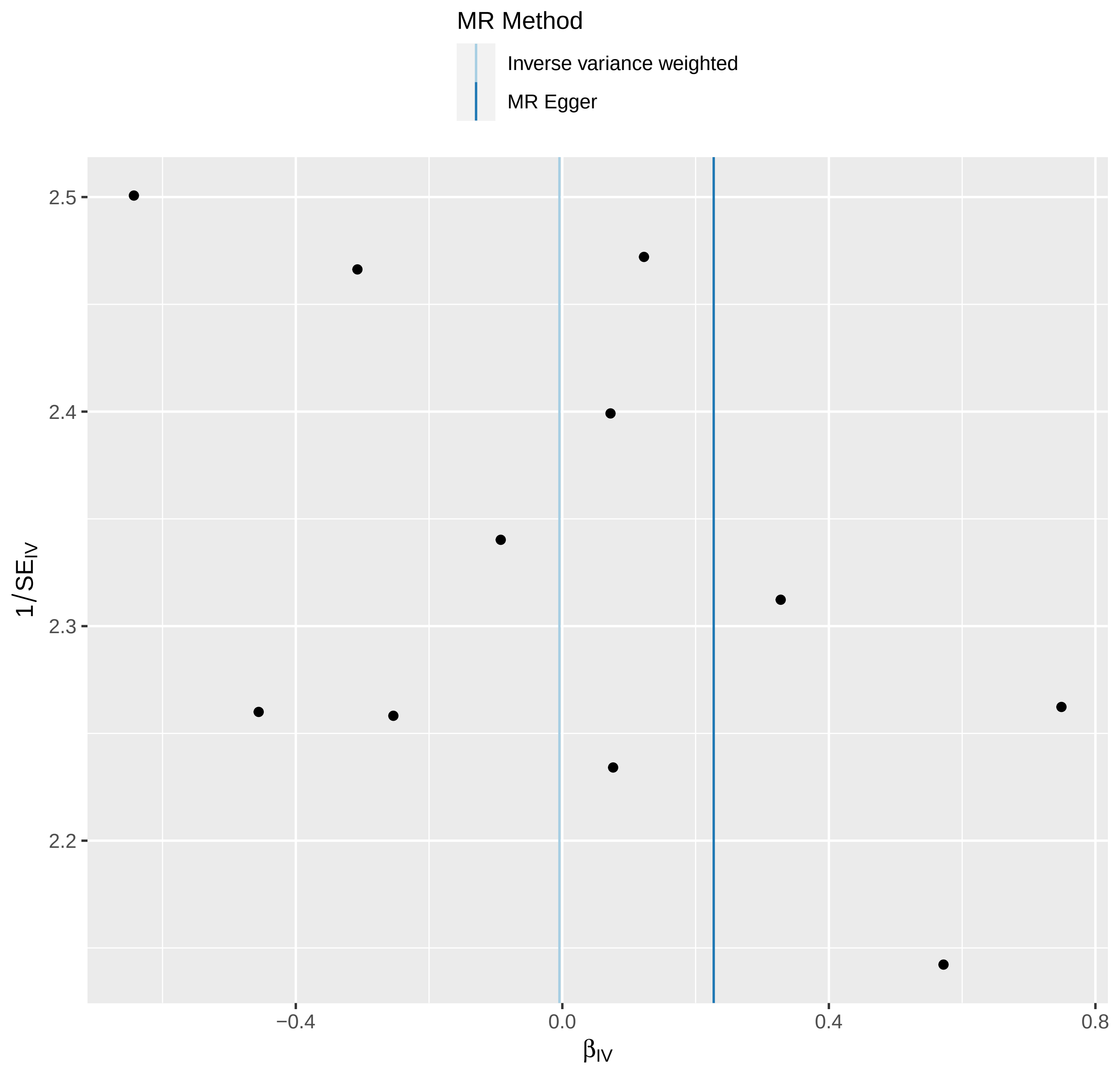

Batch 1257 : Gut microbiota abundance (genus Coprococcus1 id.11301) on Other ulcerative colitis

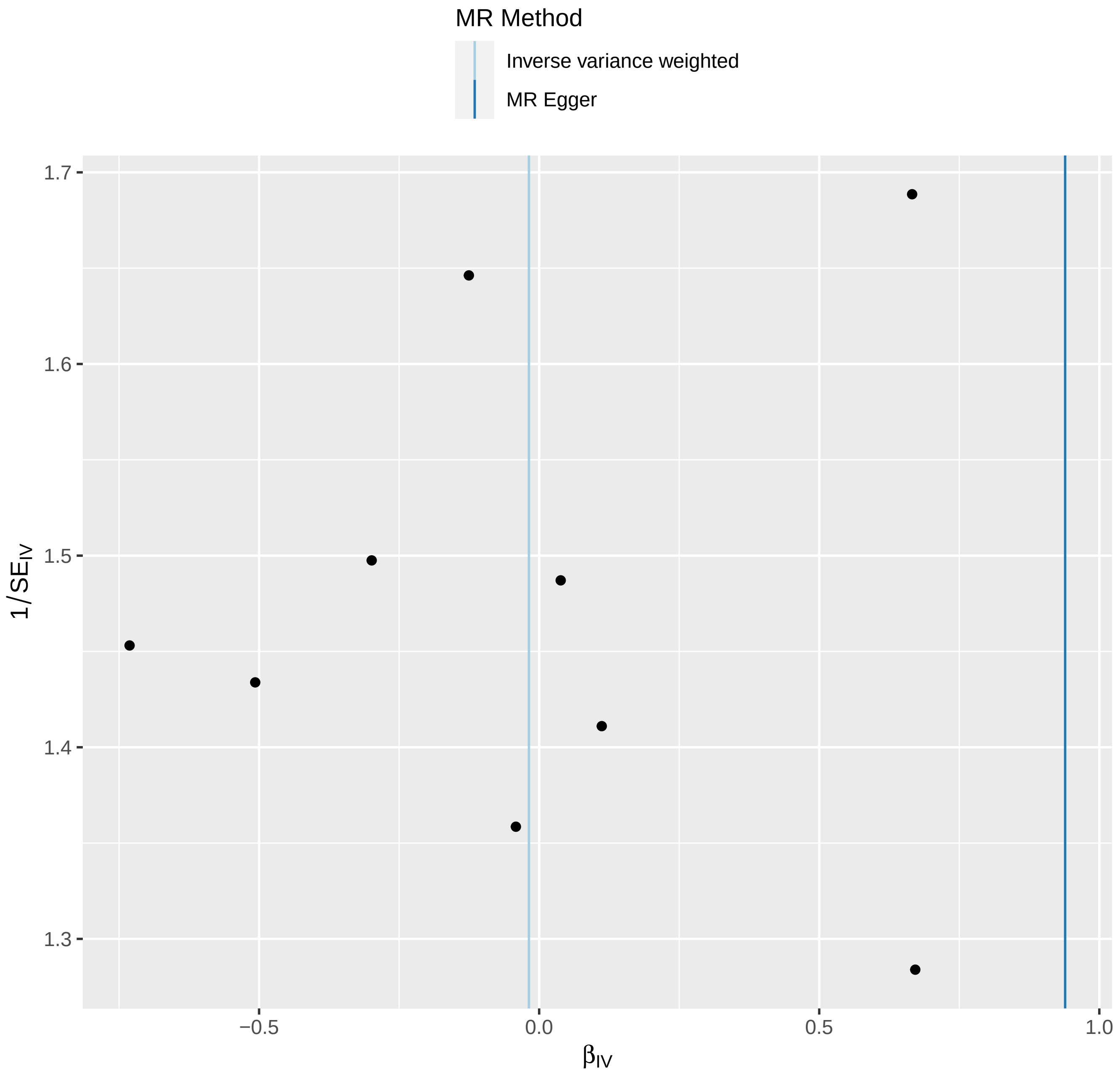

Batch 1258 : Gut microbiota abundance (genus Coprococcus2 id.11302) on Other ulcerative colitis

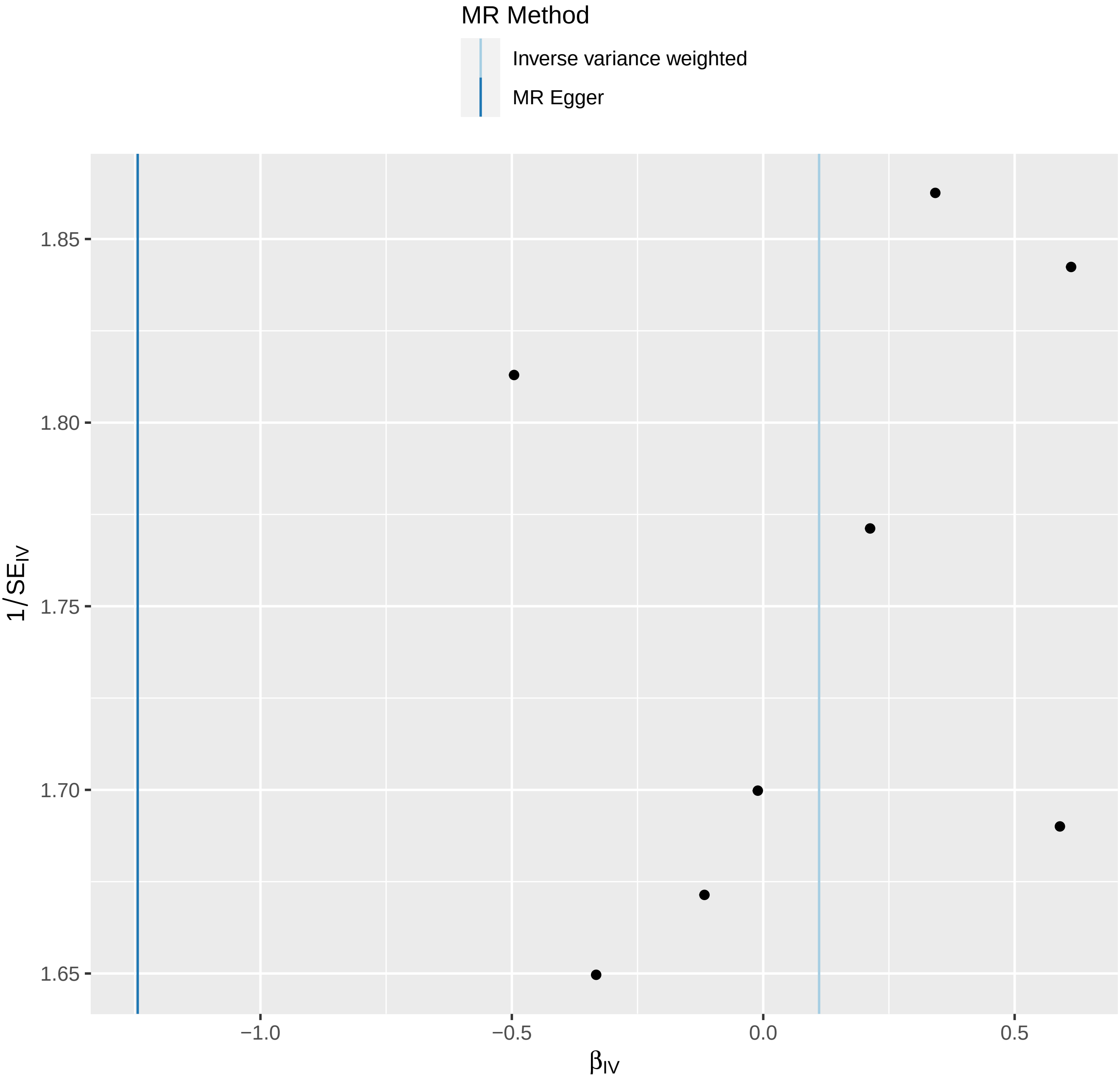

Batch 1259 : Gut microbiota abundance (genus Coprococcus3 id.11303) on Other ulcerative colitis

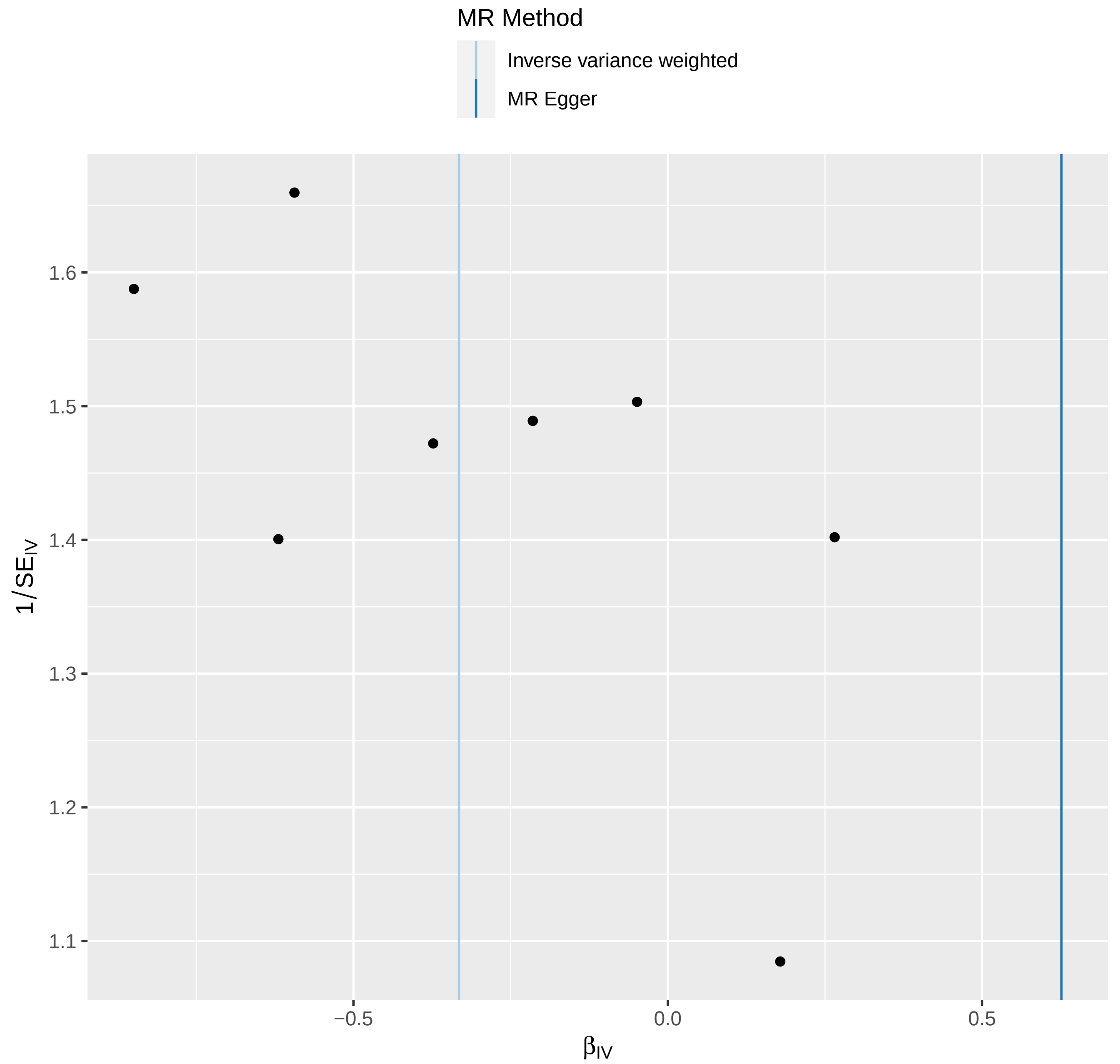

Batch 1260 : Gut microbiota abundance (genus Defluviitaleaceae UCG011 id.11287) on Other ulcerative colitis

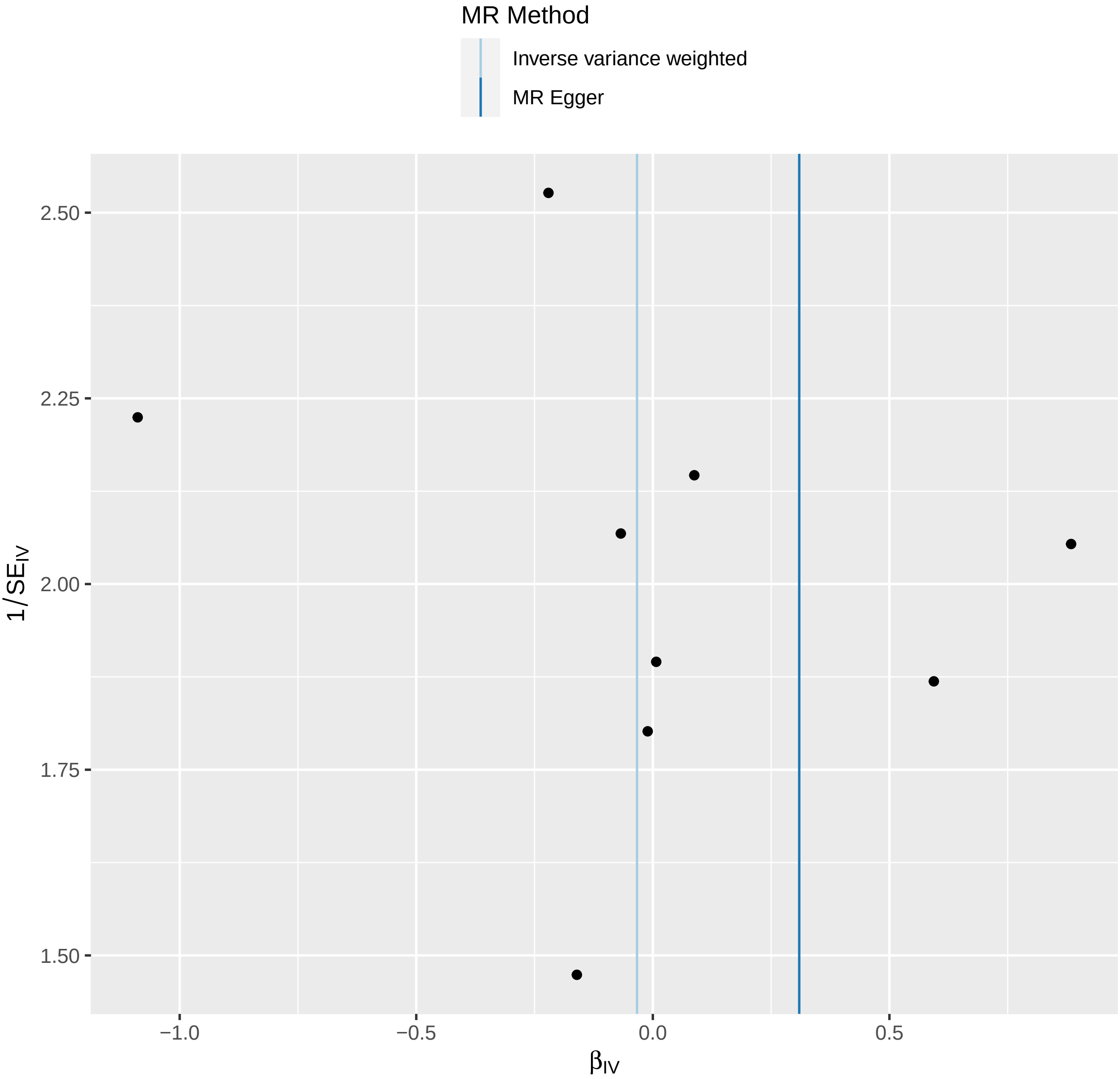

Batch 1261 : Gut microbiota abundance (genus Desulfovibrio id.3173) on Other ulcerative colitis

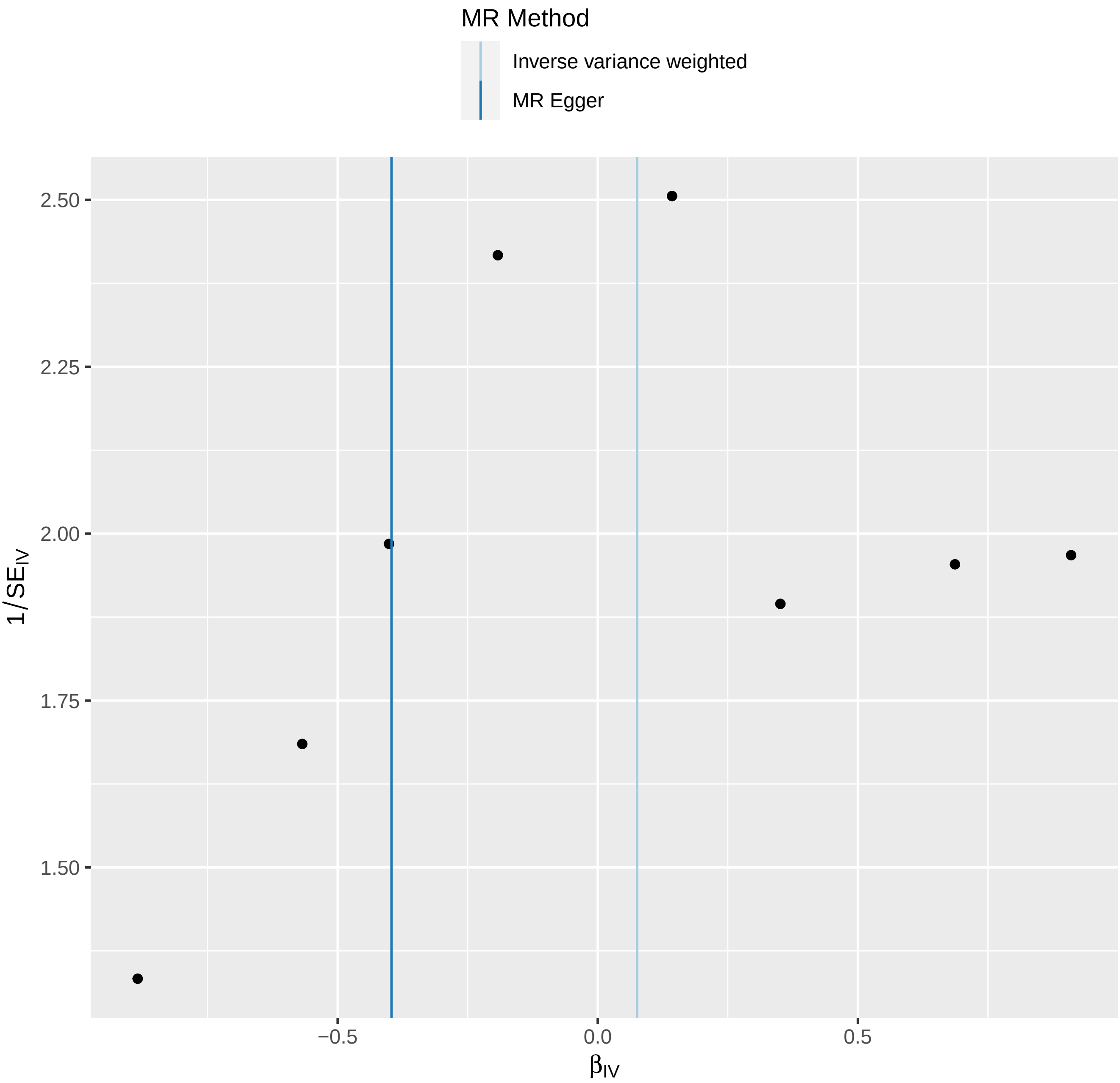

Batch 1262 : Gut microbiota abundance (genus Dialister id.2183) on Other ulcerative colitis

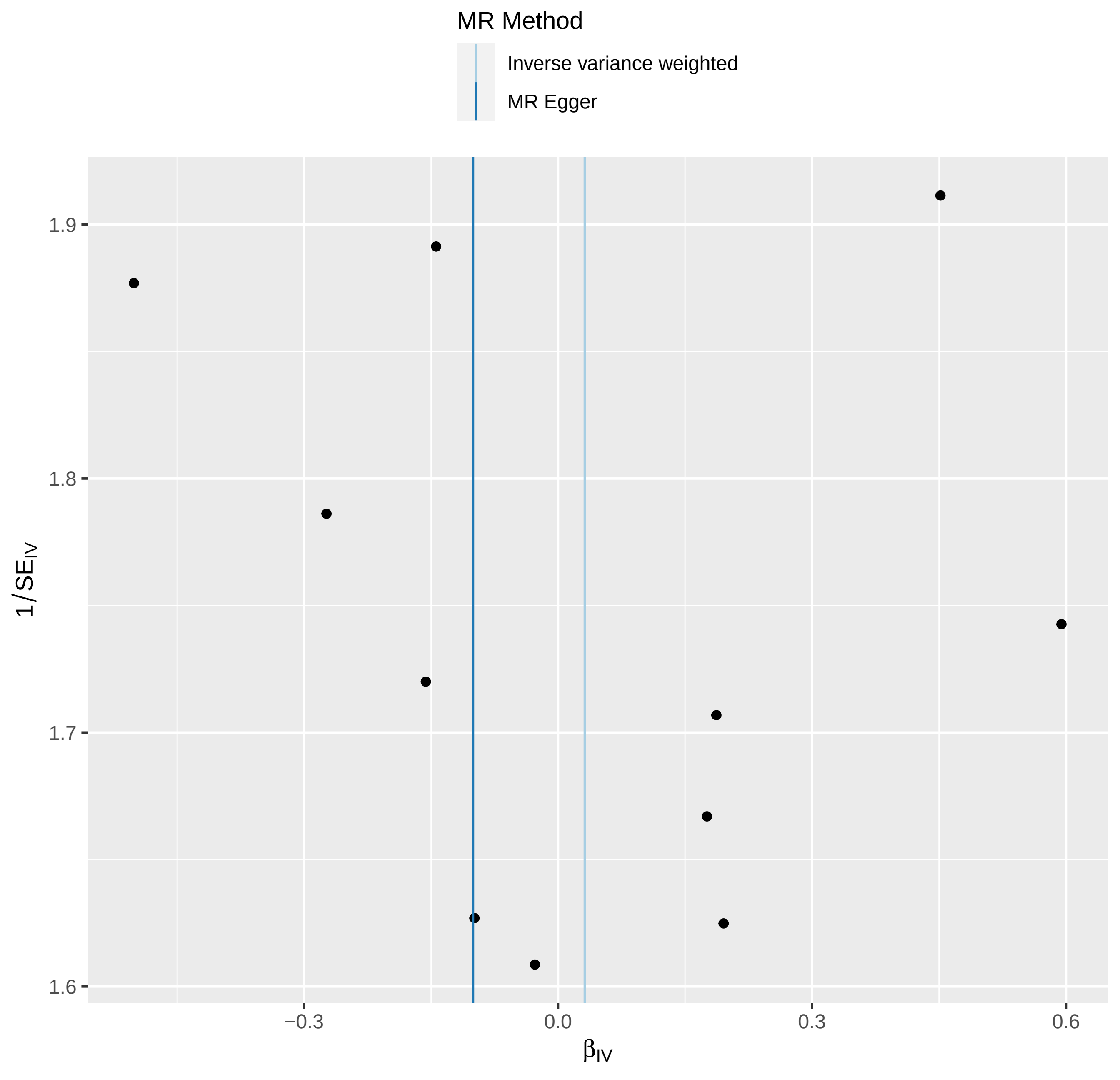

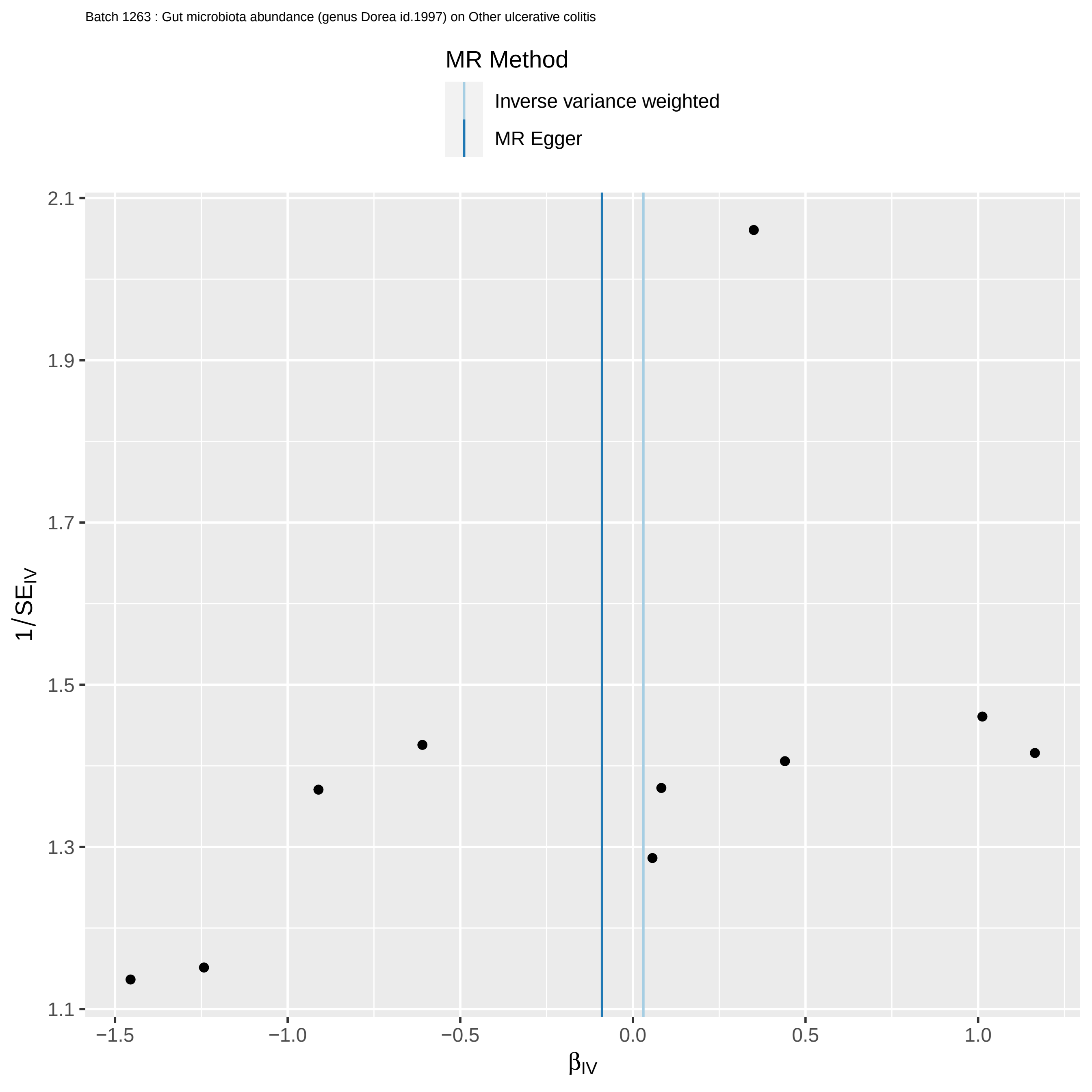

Batch 1263 : Gut microbiota abundance (genus Dorea id.1997) on Other ulcerative colitis
MR Method
Inverse variance weighted
MR Egger
2.1
1.9
1.7
1.5
1.3
1.1
$1/SE_{IV}$
−1.5
−1.0
−0.5
0.0
0.5
1.0
β_{IV}

Batch 1264 : Gut microbiota abundance (genus Eggerthella id.819) on Other ulcerative colitis

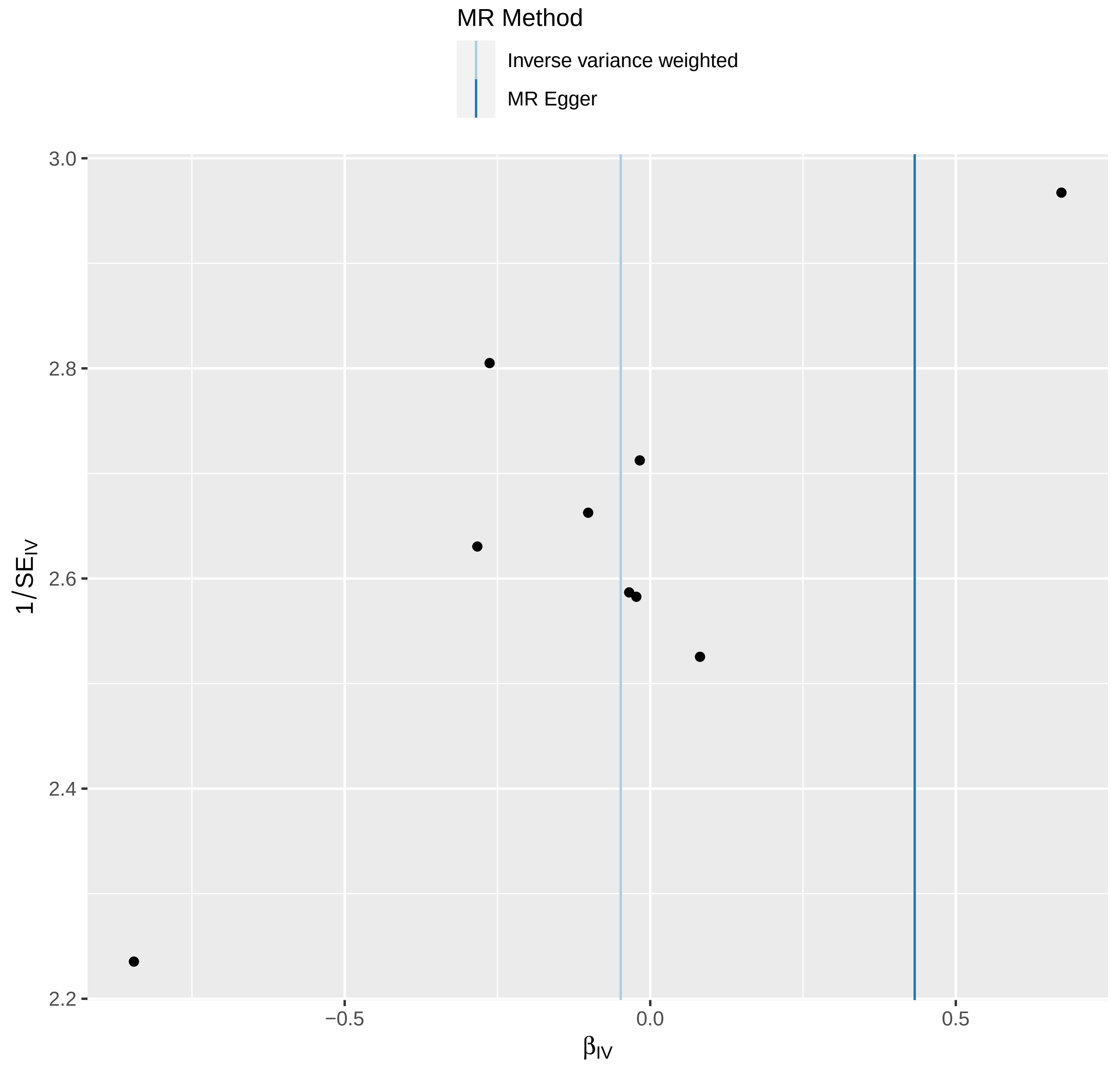

Batch 1265 : Gut microbiota abundance (genus Eisenbergiella id.11304) on Other ulcerative colitis

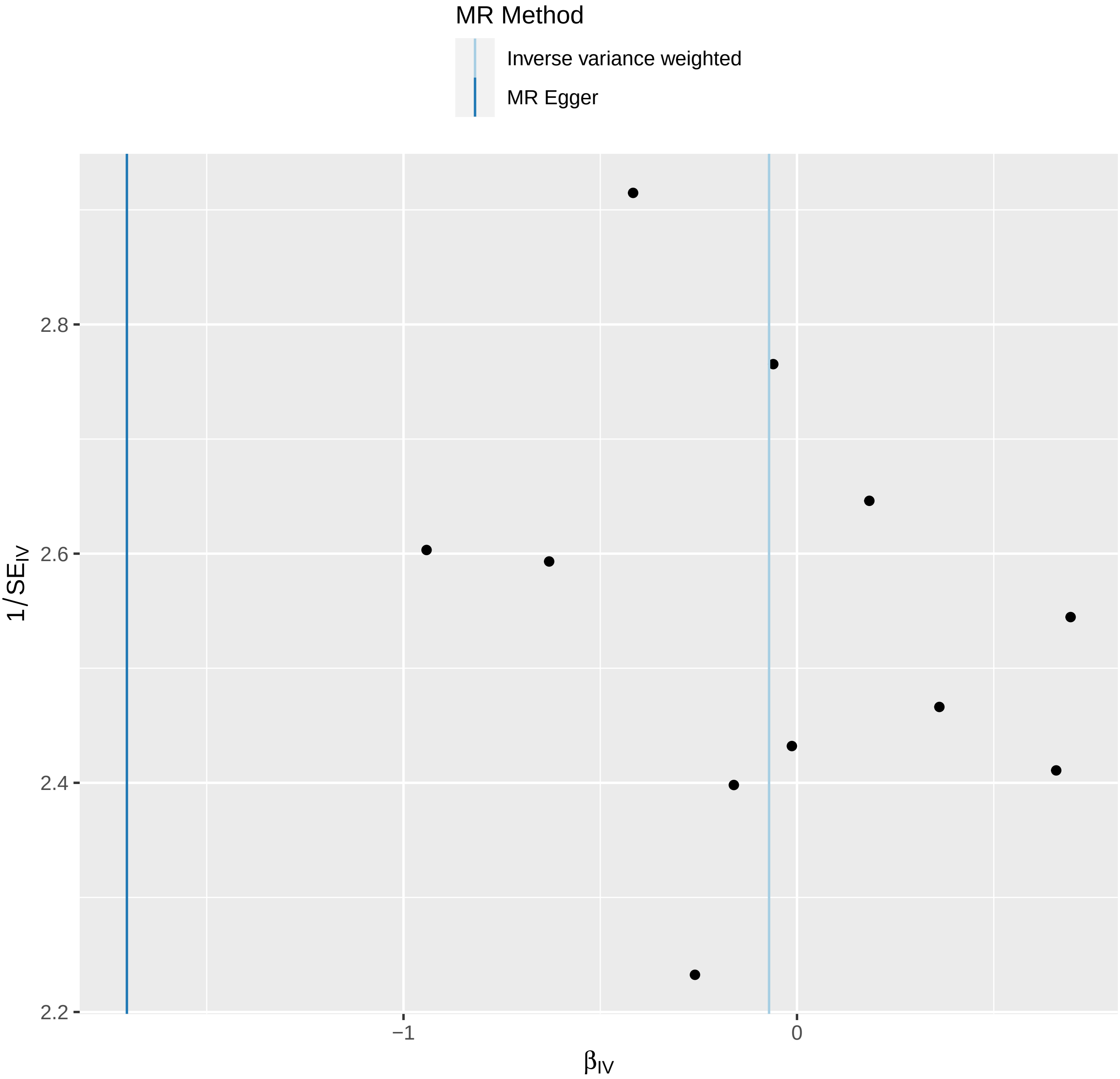

Batch 1266 : Gut microbiota abundance (genus Enterorhabdus id.820) on Other ulcerative colitis

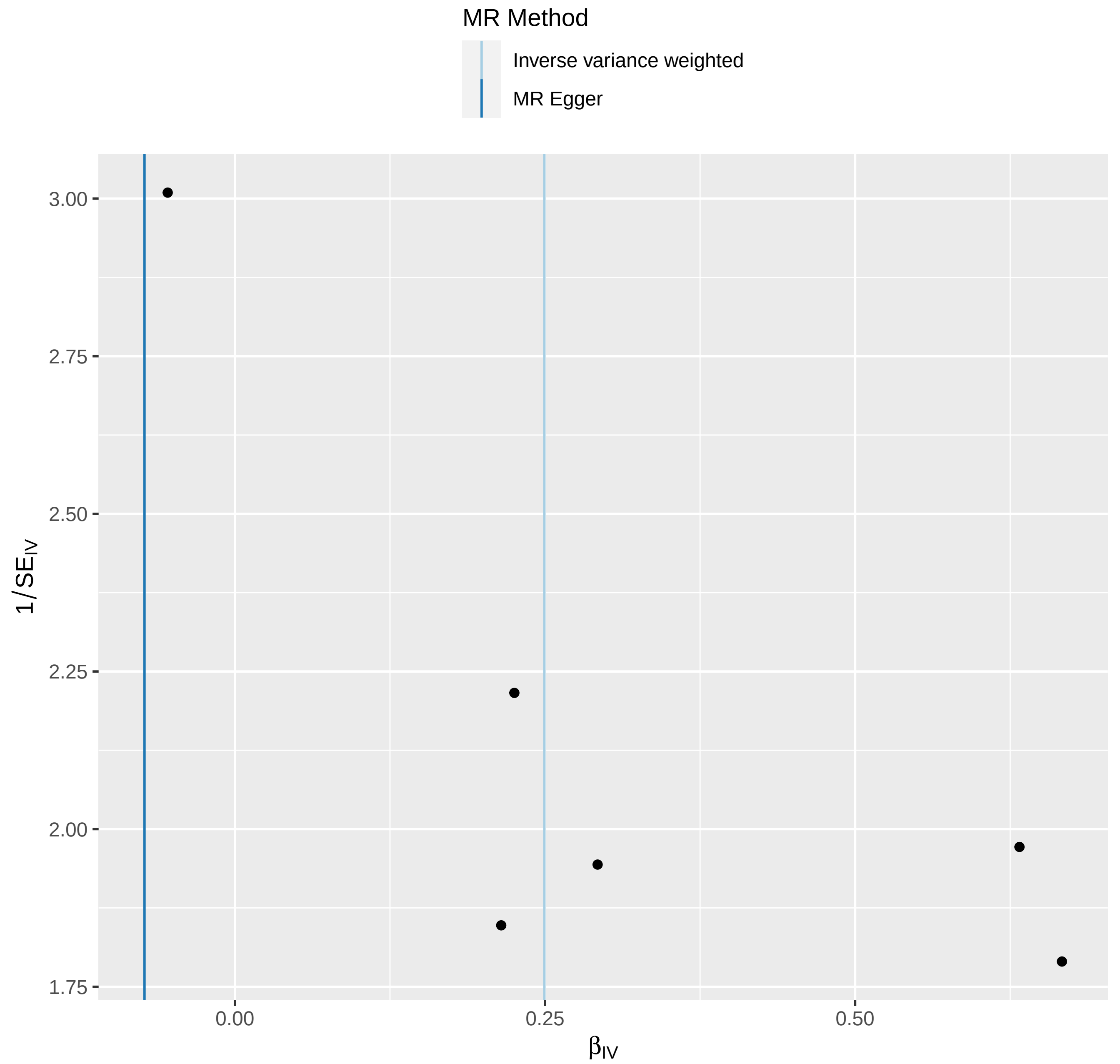

Batch 1267 : Gut microbiota abundance (genus Erysipelatoclostridium id.11381) on Other ulcerative colitis

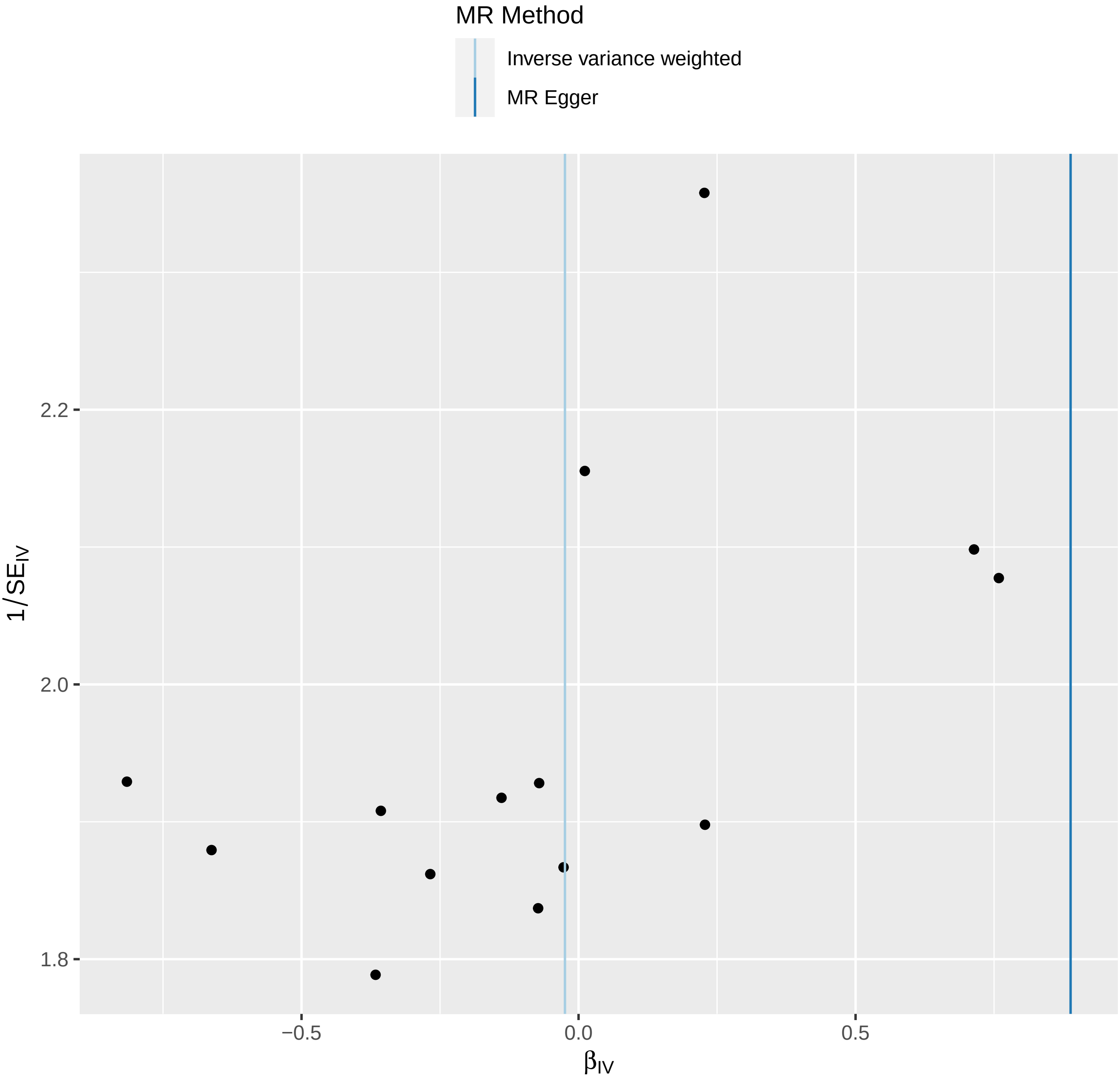

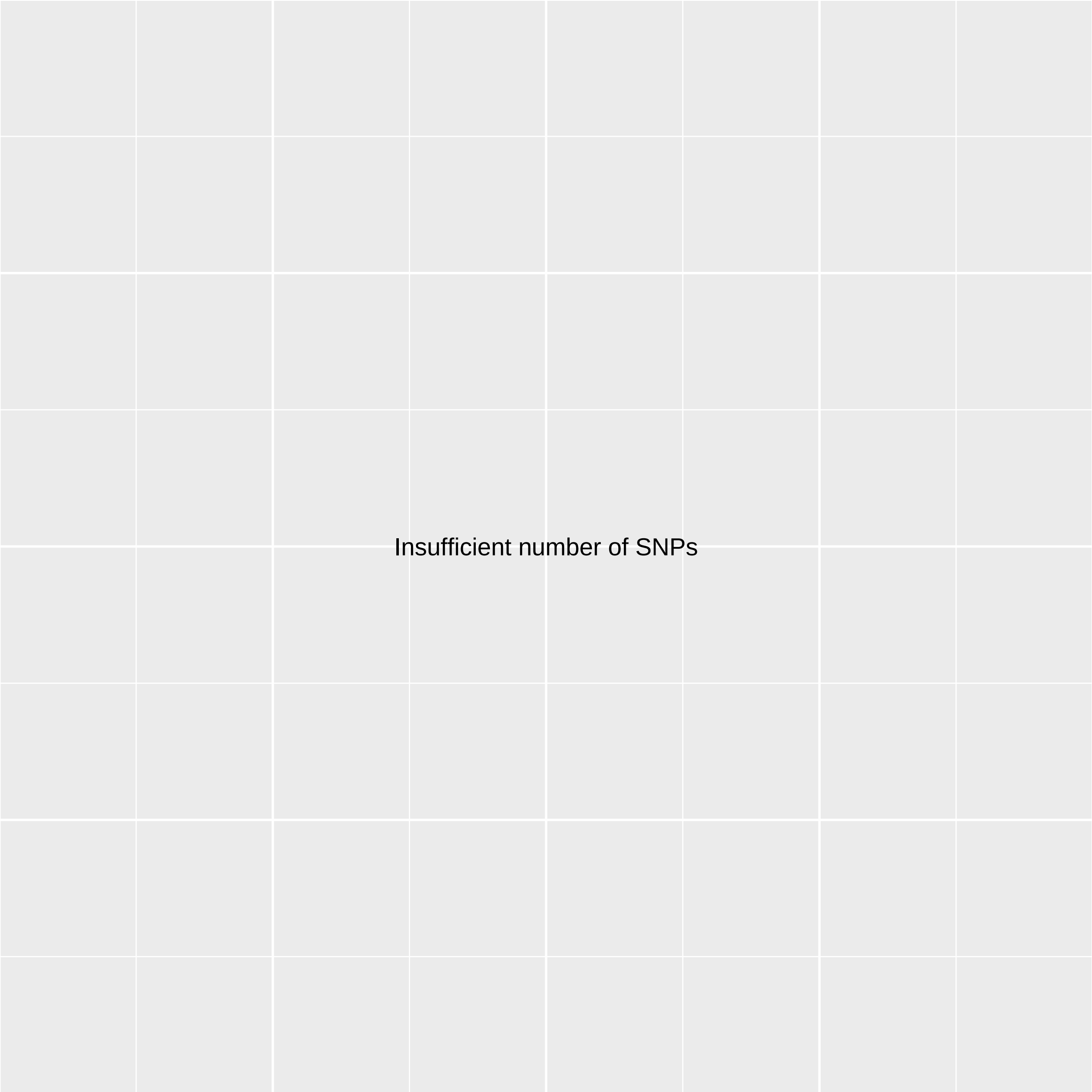
Insufficient number of SNPs

Batch 1269 : Gut microbiota abundance (genus Escherichia Shigella id.3504) on Other ulcerative colitis

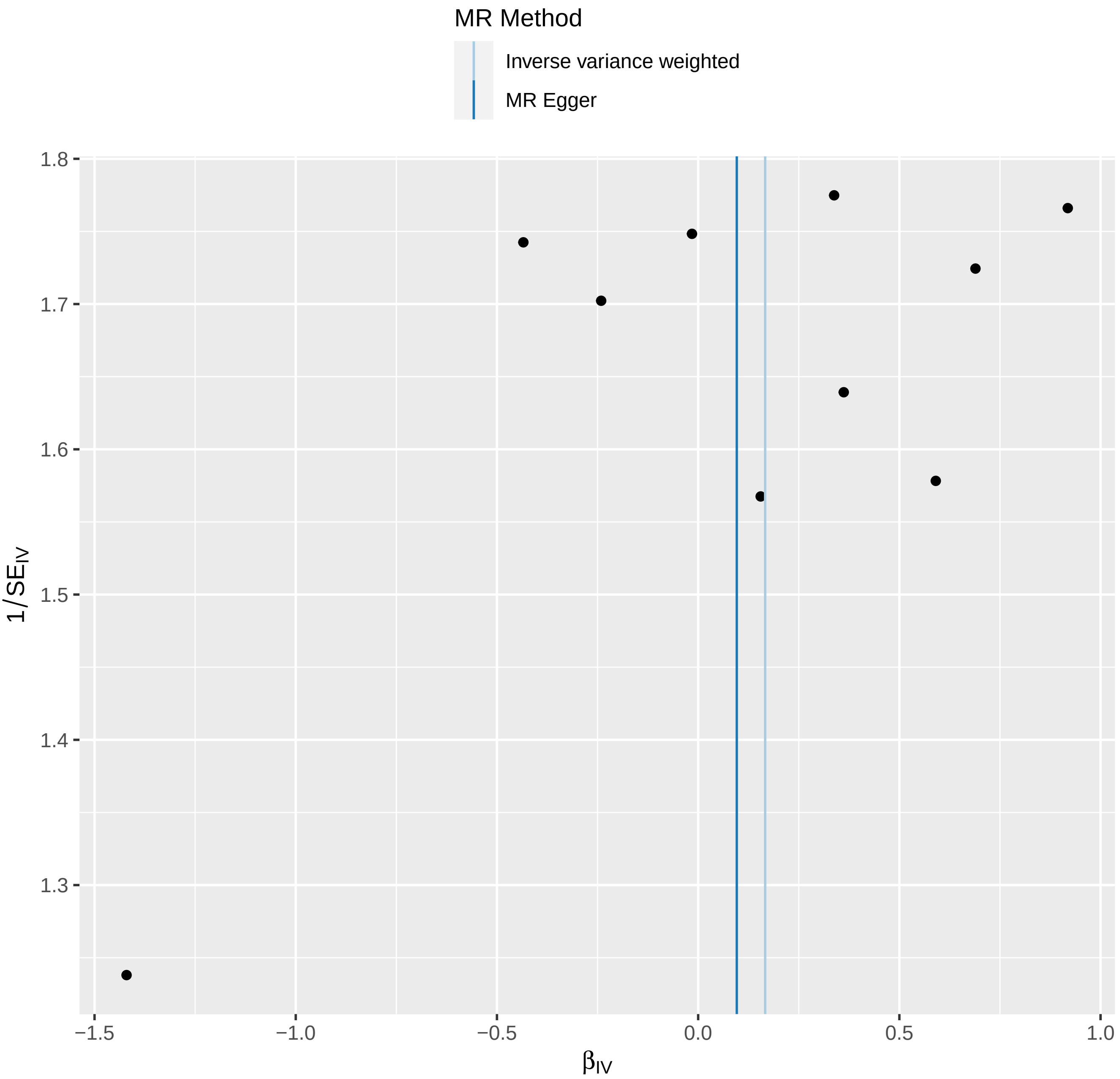

Batch 1270 : Gut microbiota abundance (genus Eubacterium brachy group id.11296) on Other ulcerative colitis

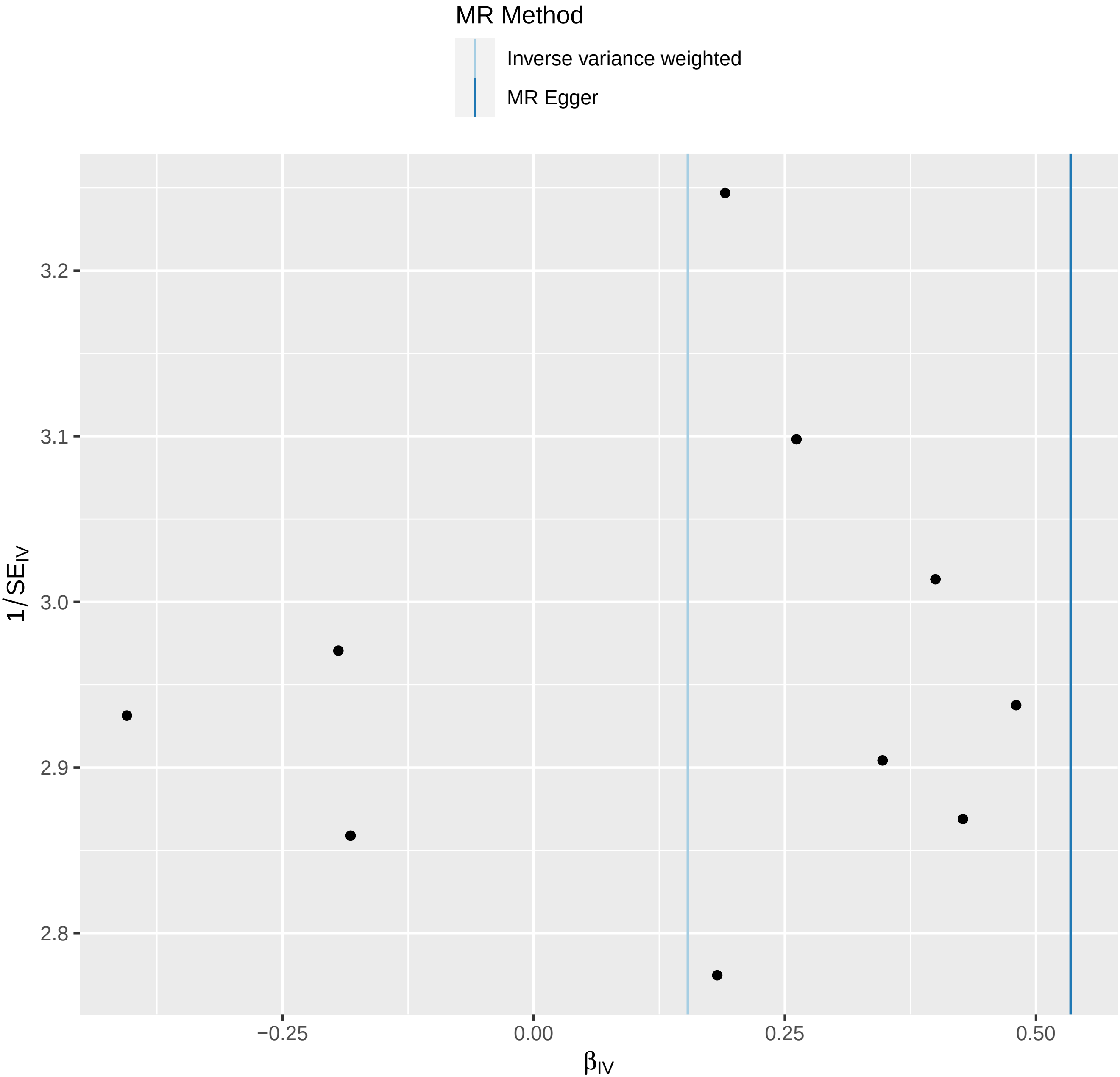

Batch 1271 : Gut microbiota abundance (genus Eubacterium coprostanoligenes group id.11375) on Other ulcerative colitis

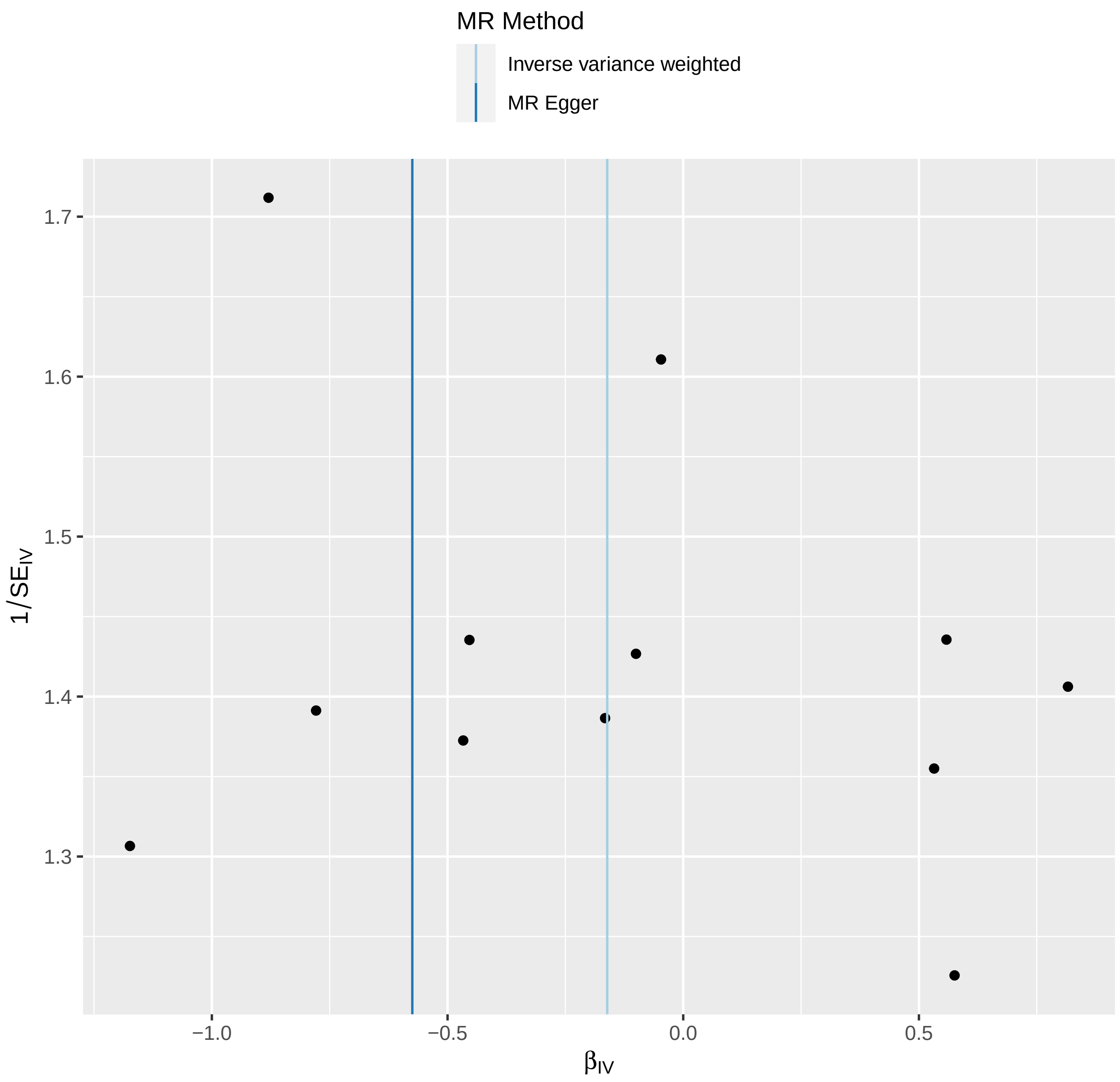

Batch 1272 : Gut microbiota abundance (genus Eubacterium eligens group id.14372) on Other ulcerative colitis

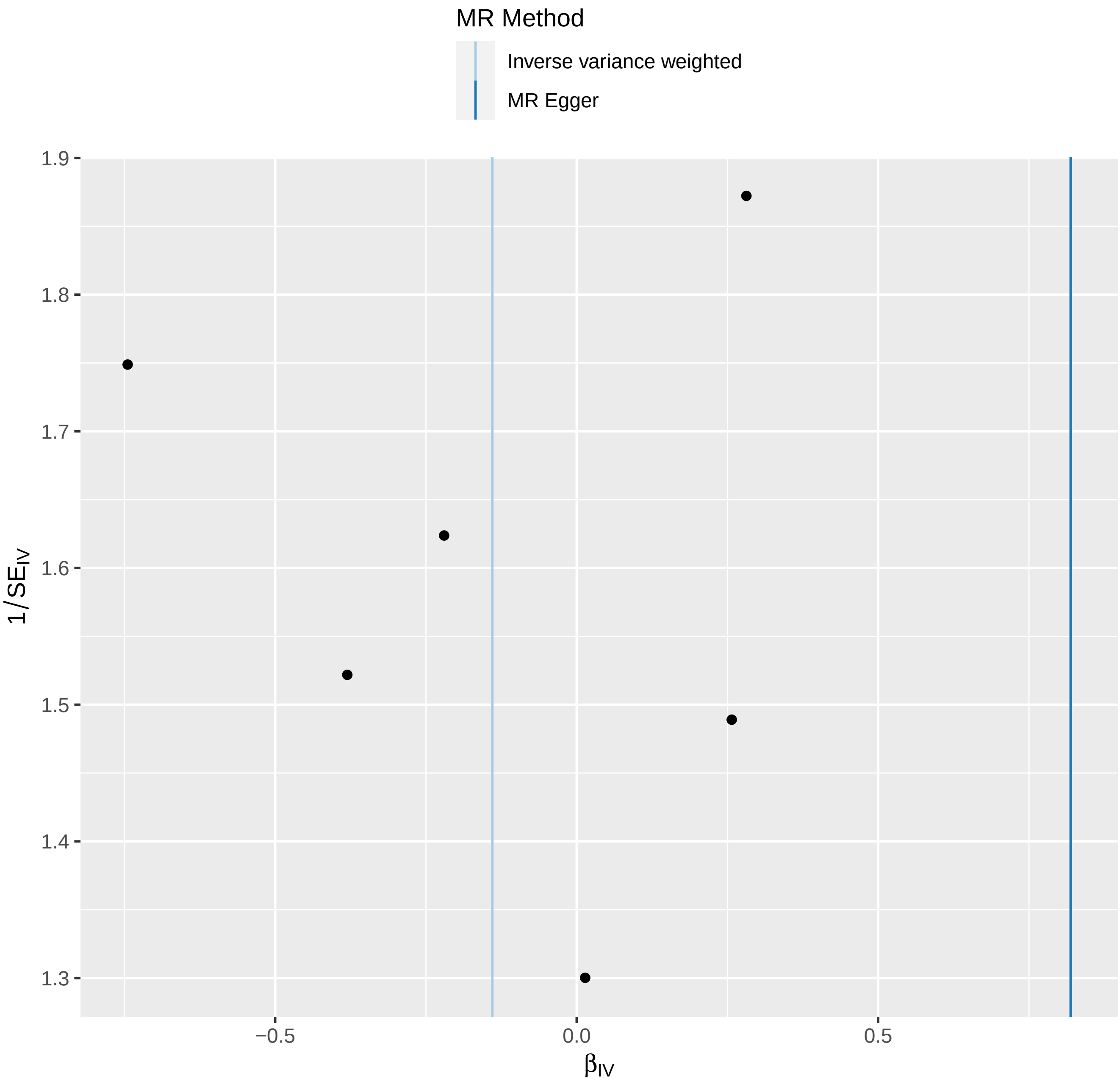

Batch 1273 : Gut microbiota abundance (genus Eubacterium fissicatena group id.14373) on Other ulcerative colitis

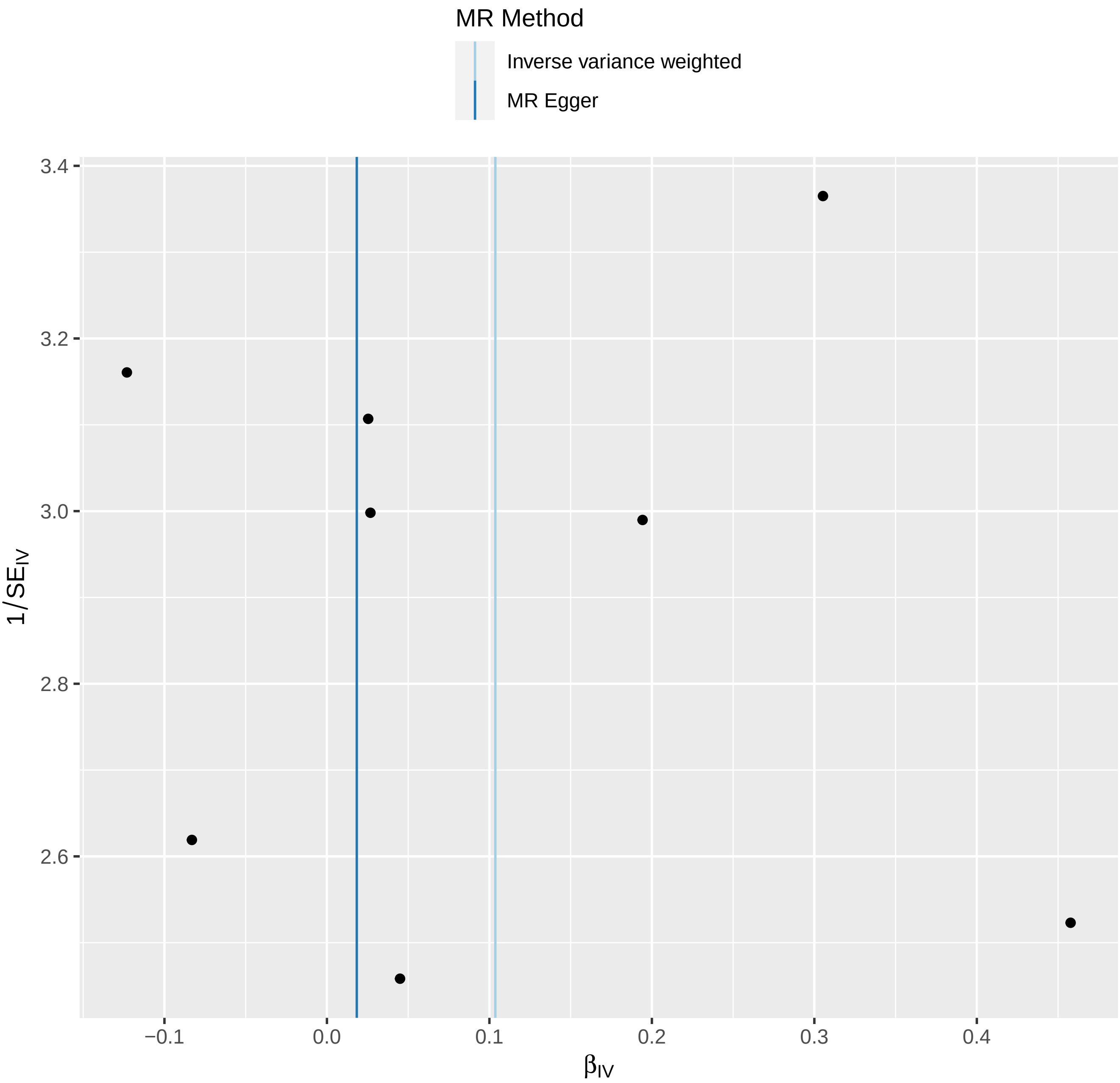

Batch 1274 : Gut microbiota abundance (genus Eubacterium hallii group id.11338) on Other ulcerative colitis

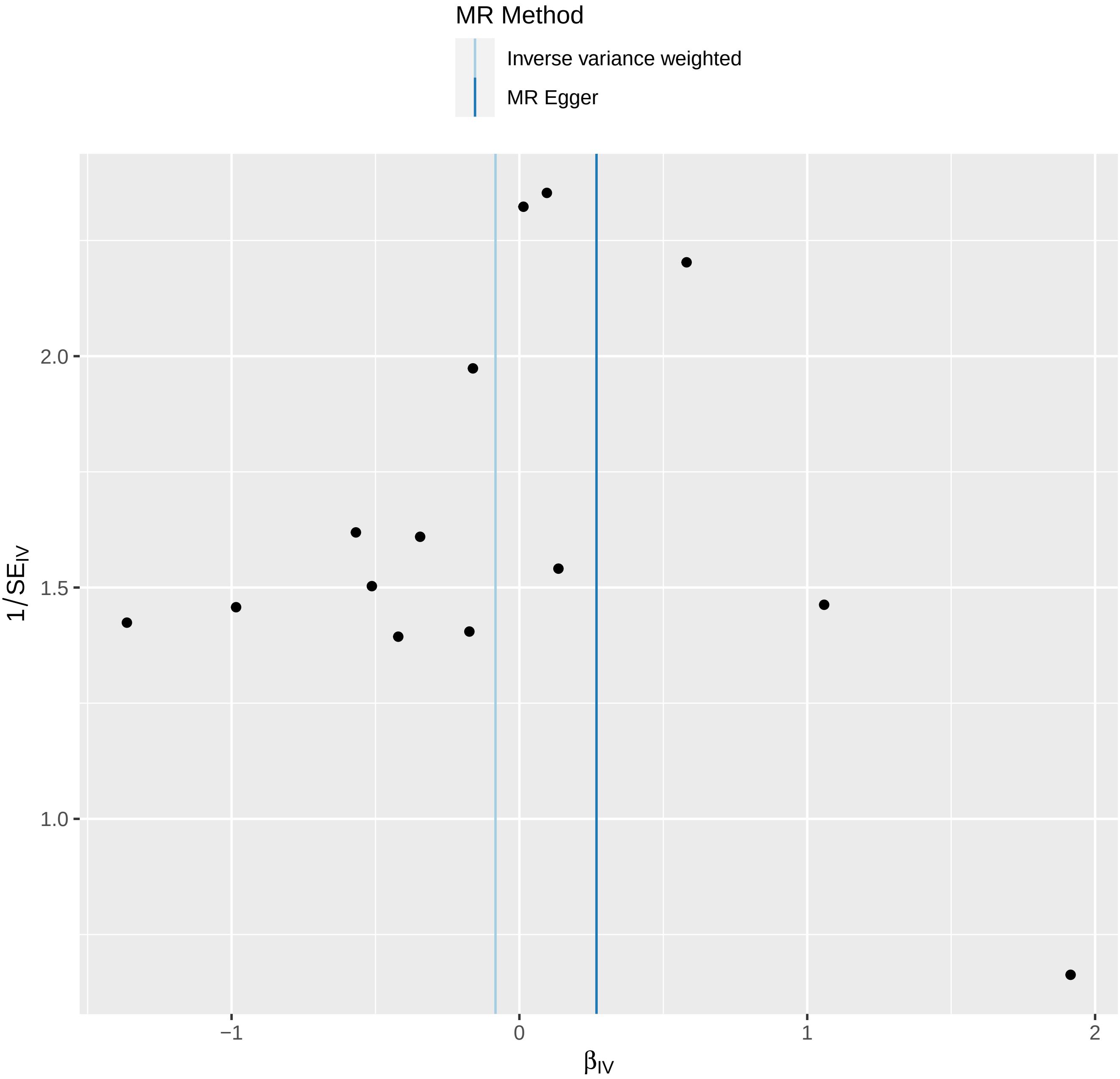

Batch 1275 : Gut microbiota abundance (genus Eubacterium nodatum group id.11297) on Other ulcerative colitis

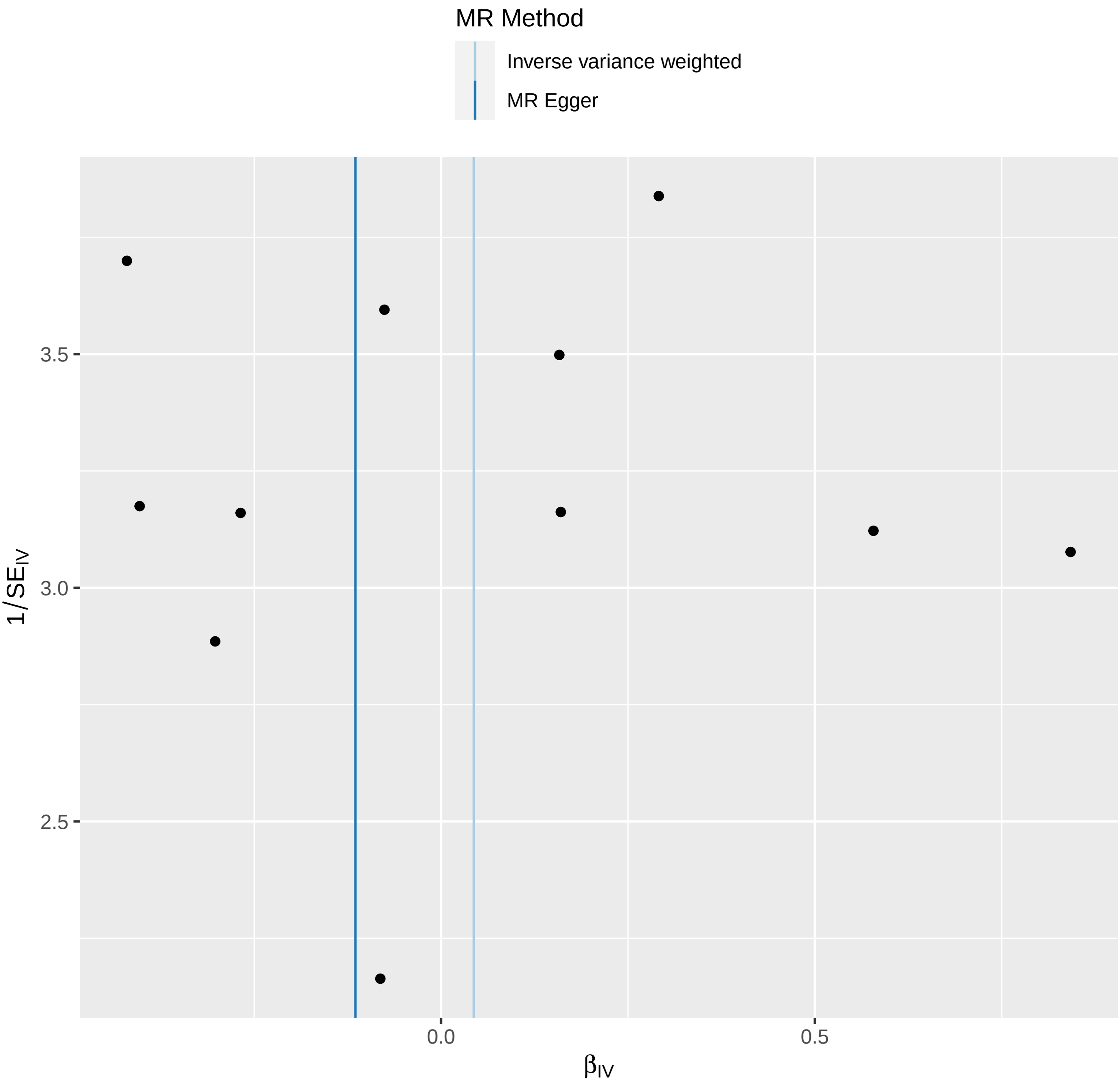

Batch 1276 : Gut microbiota abundance (genus Eubacterium oxidoreducens group id.11339) on Other ulcerative colitis

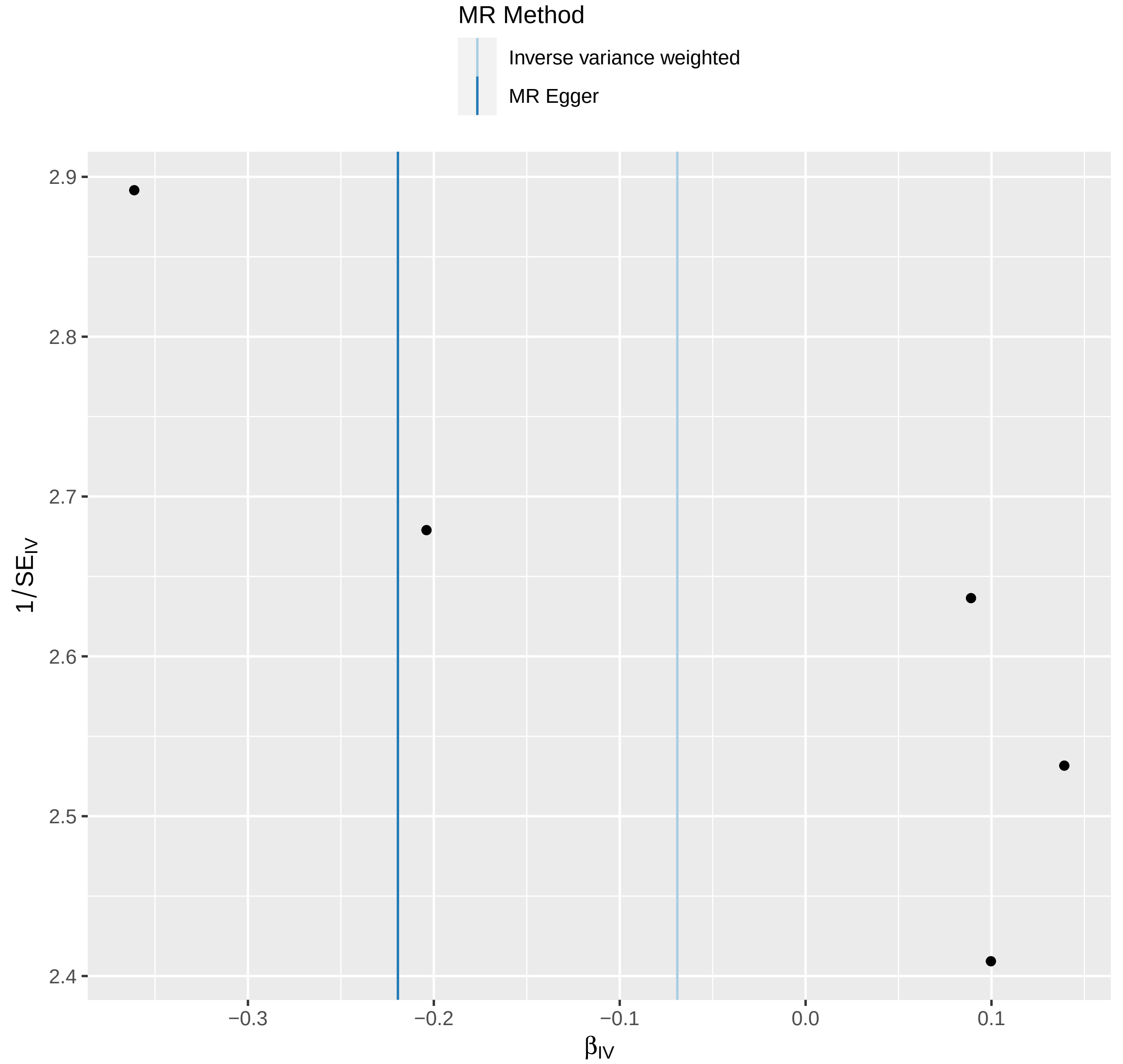

Batch 1277 : Gut microbiota abundance (genus Eubacterium rectale group id.14374) on Other ulcerative colitis

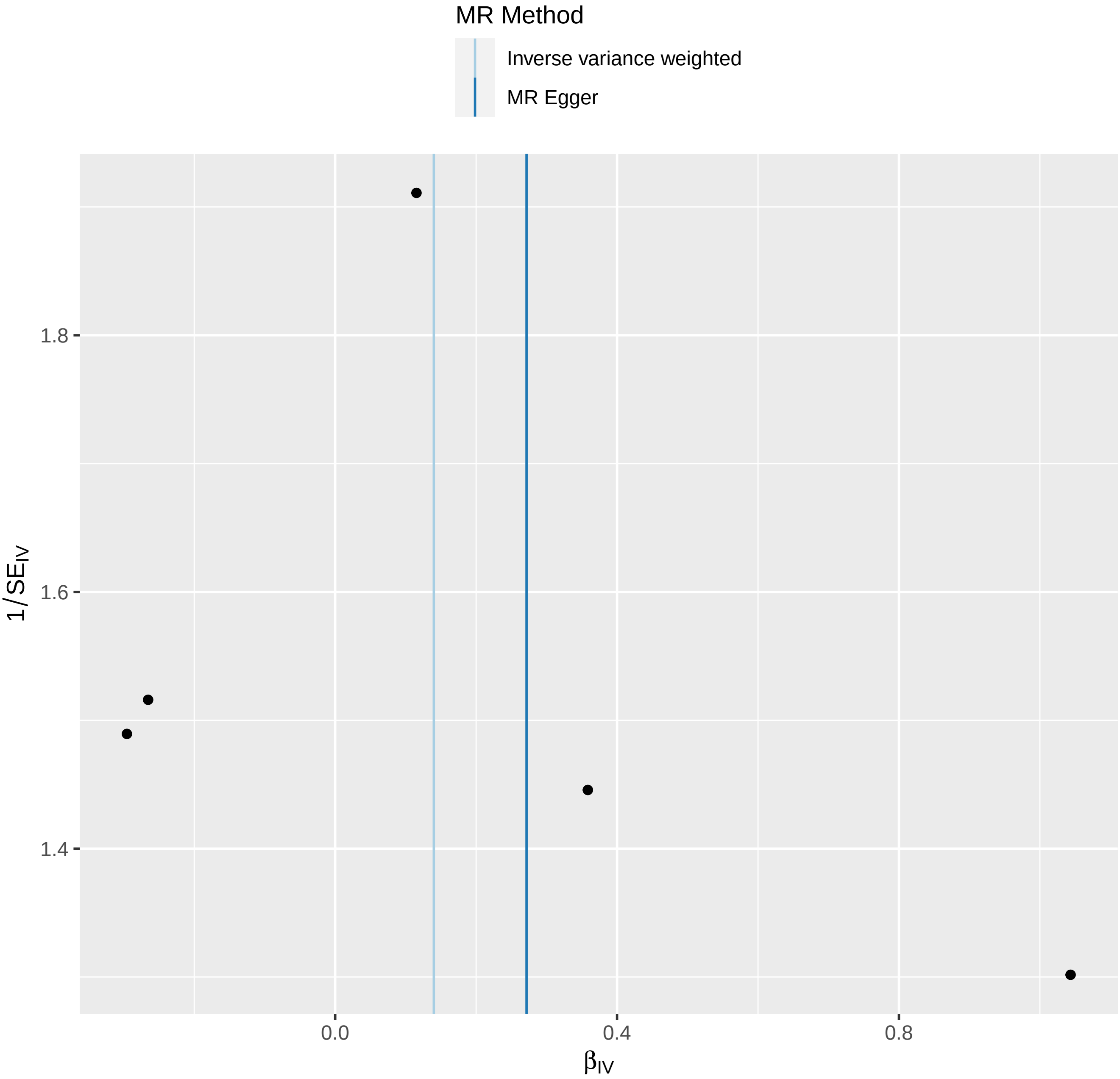

Batch 1278 : Gut microbiota abundance (genus Eubacterium ruminantium group id.11340) on Other ulcerative colitis

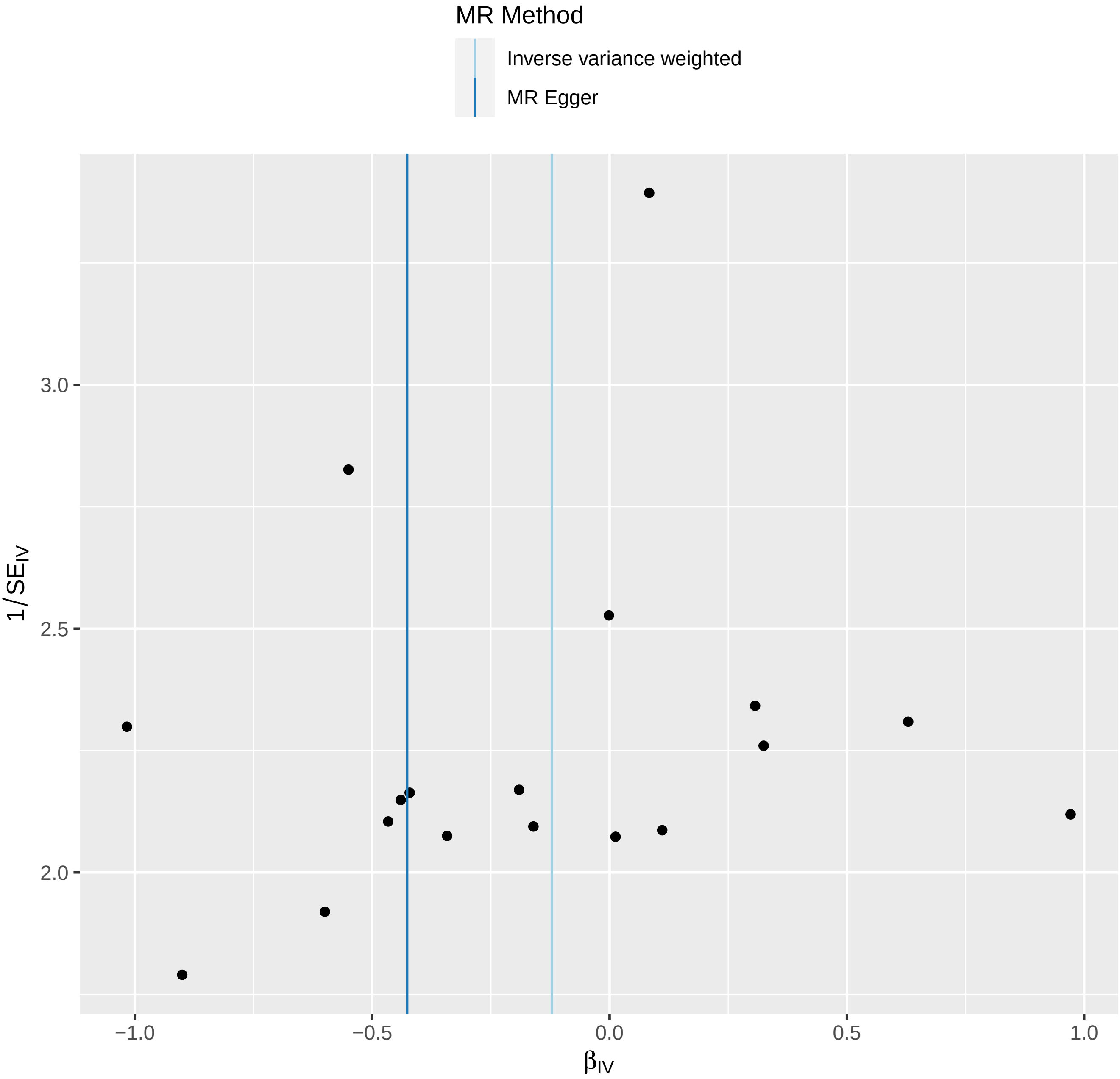

Batch 1279 : Gut microbiota abundance (genus Eubacterium ventriosum group id.11341) on Other ulcerative colitis

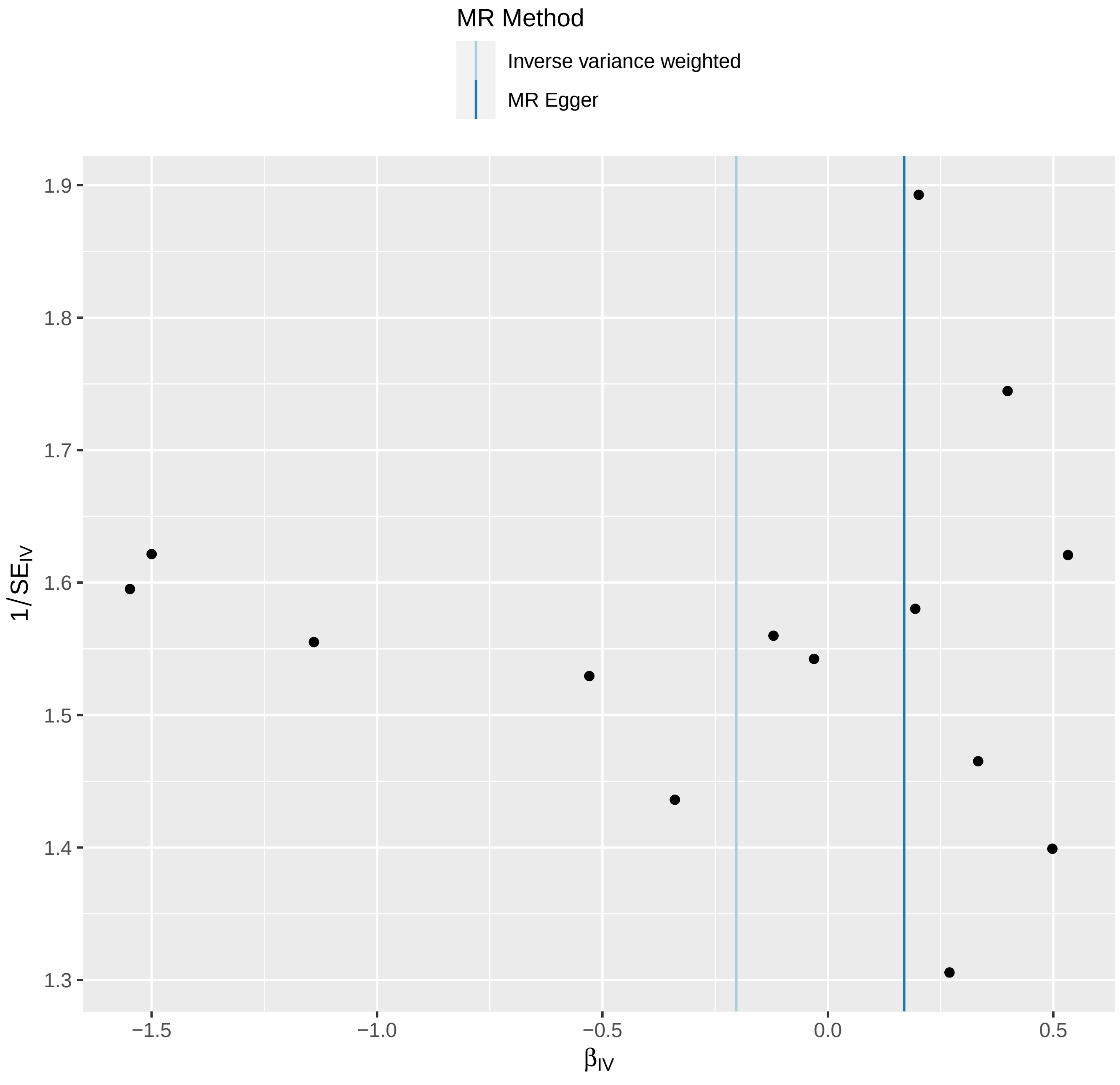

Batch 1280 : Gut microbiota abundance (genus Eubacterium xylanophilum group id.14375) on Other ulcerative colitis

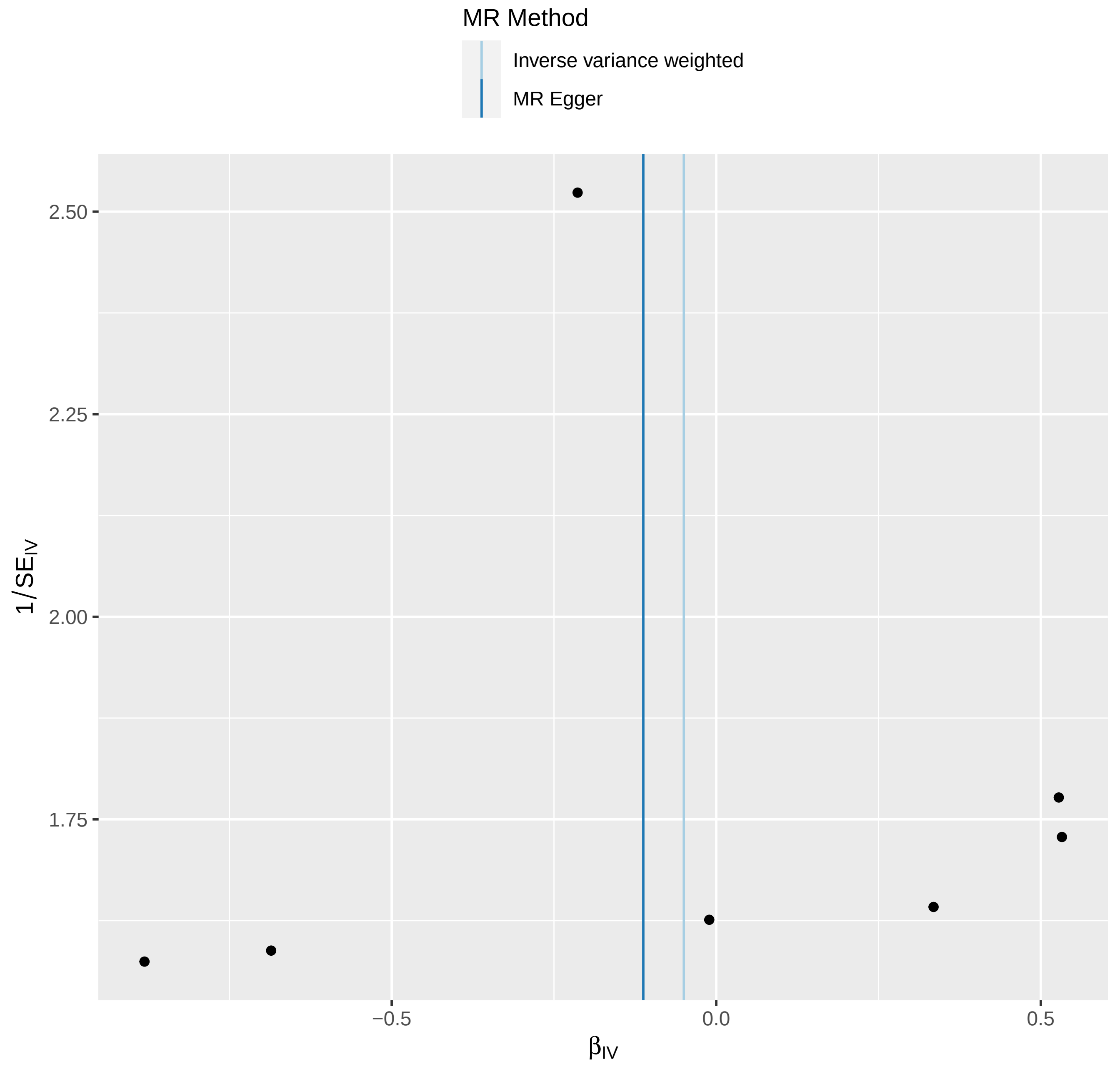

Batch 1281 : Gut microbiota abundance (genus Faecalibacterium id.2057) on Other ulcerative colitis

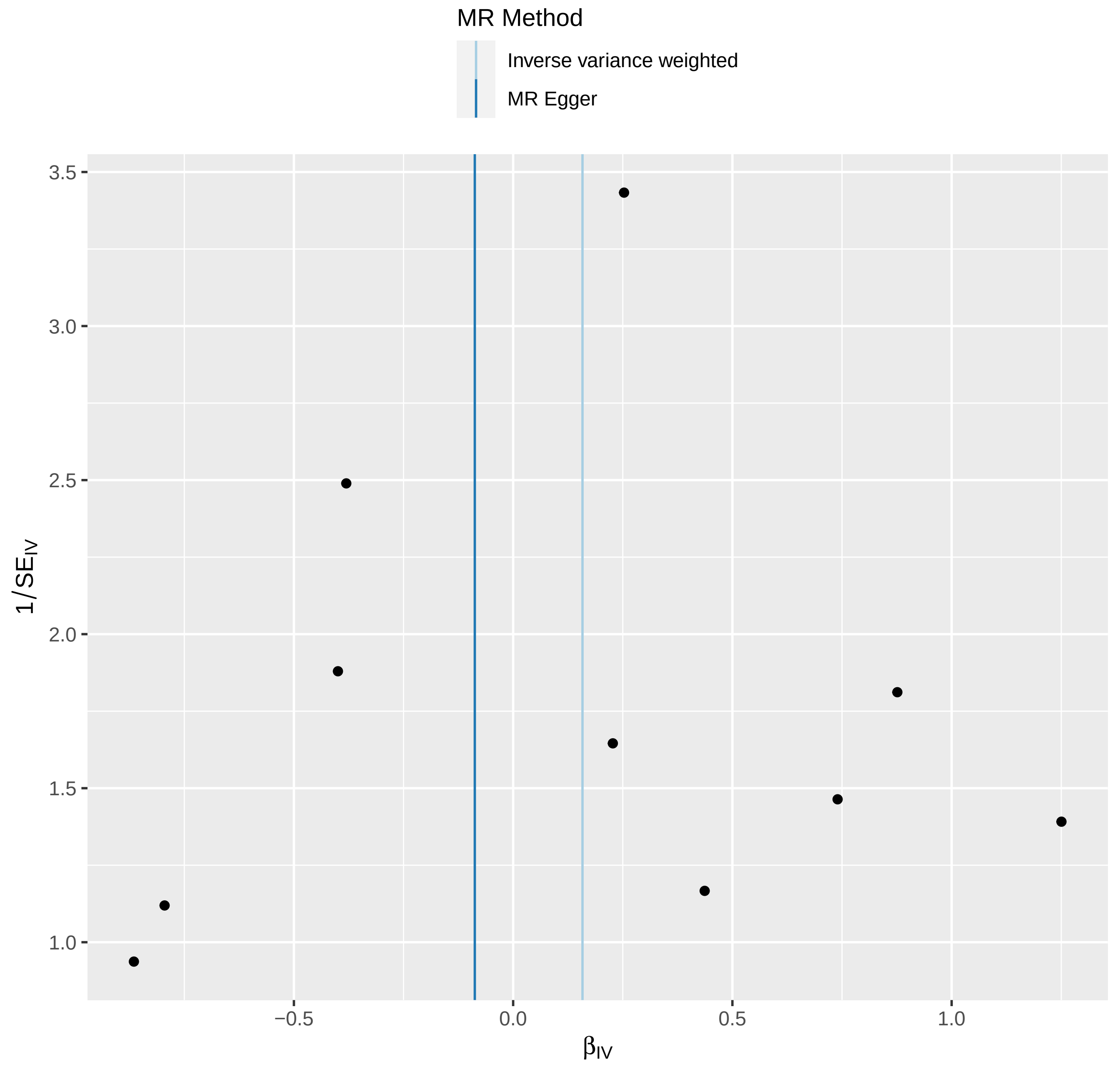

Batch 1282 : Gut microbiota abundance (genus Family XIII AD3011 group id.11293) on Other ulcerative colitis

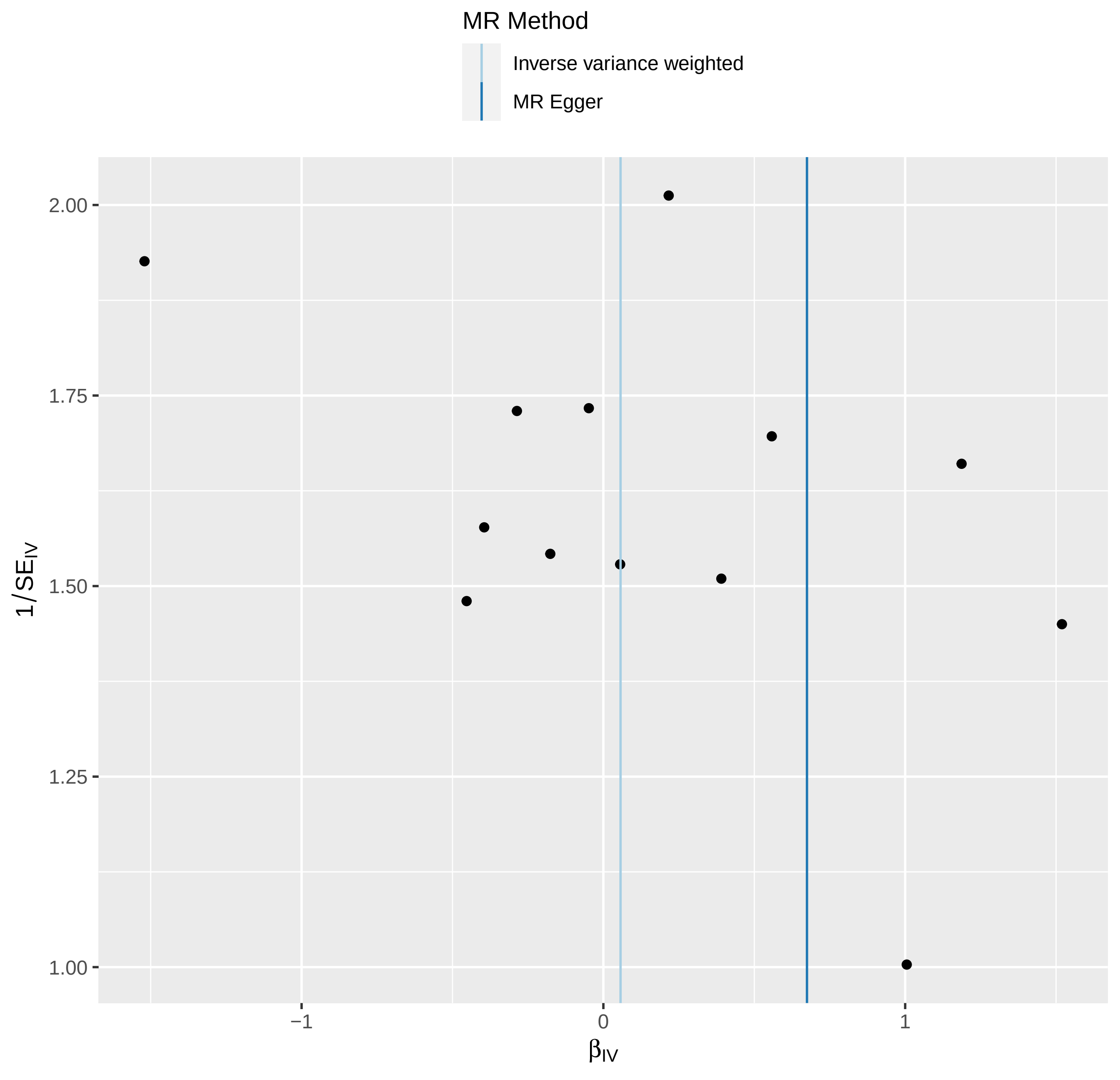

Batch 1283 : Gut microbiota abundance (genus Family XIII UCG001 id.11294) on Other ulcerative colitis

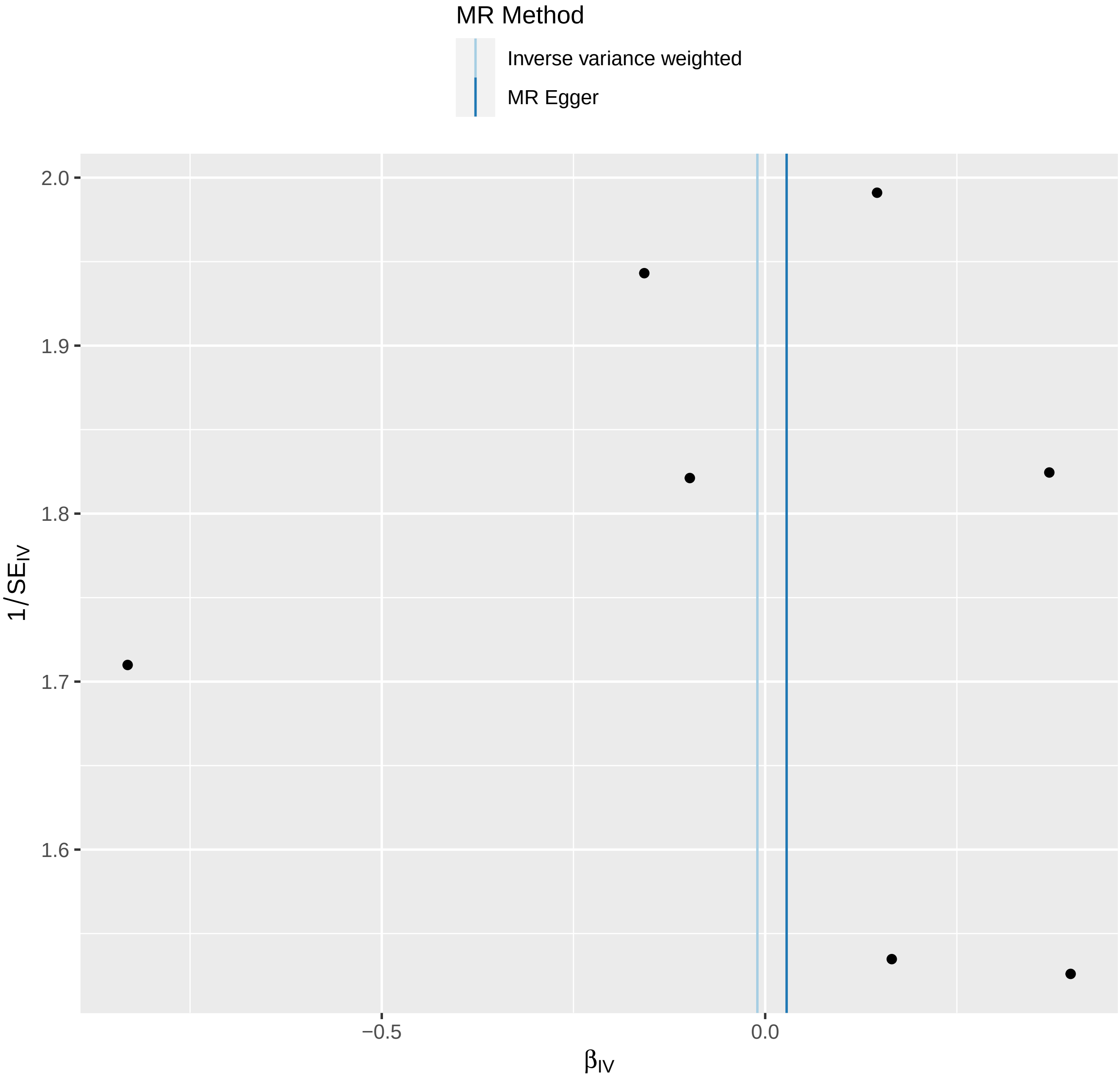

Batch 1284 : Gut microbiota abundance (genus Flavonifractor id.2059) on Other ulcerative colitis

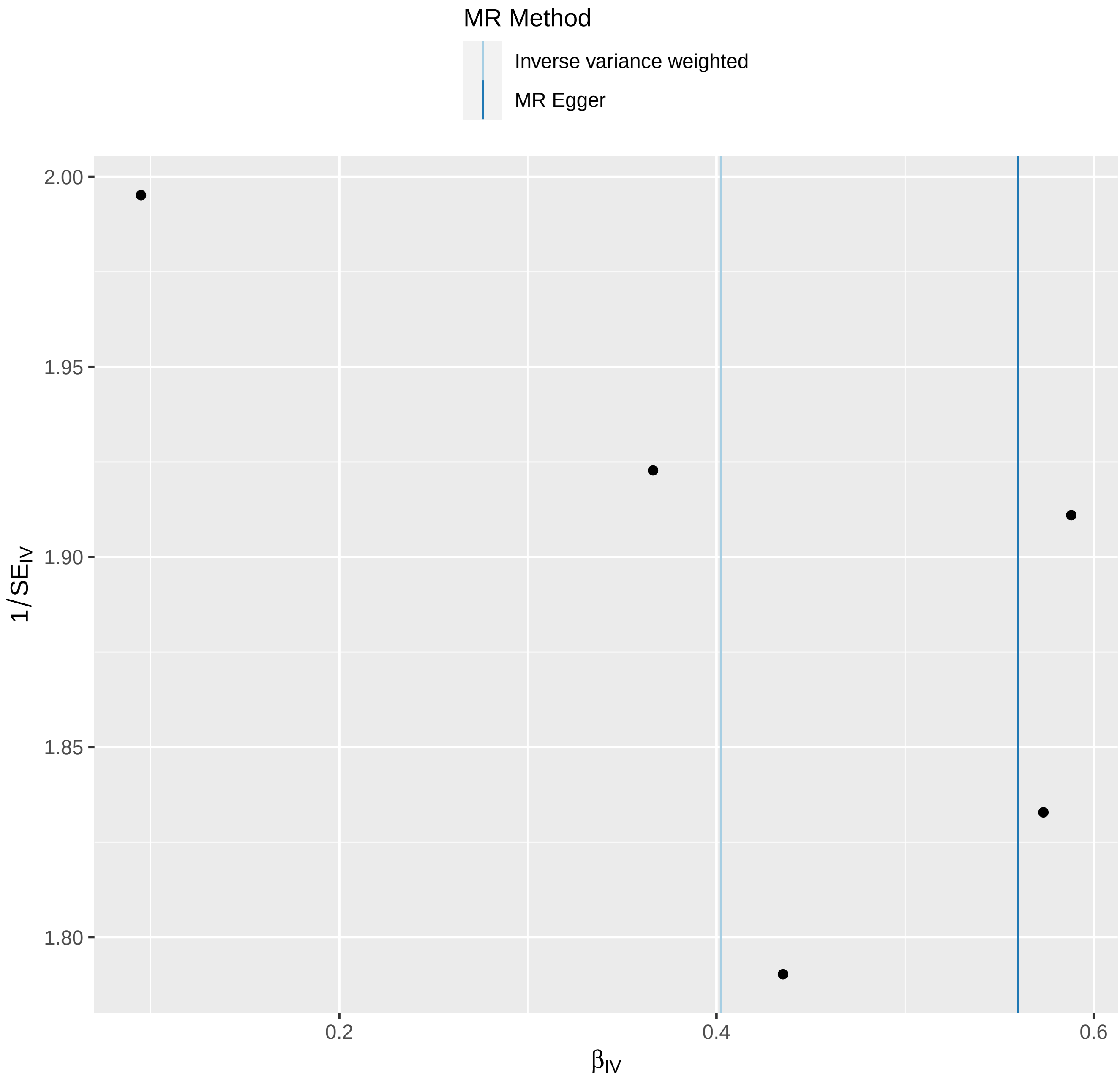

Batch 1285 : Gut microbiota abundance (genus Fusicatenibacter id.11305) on Other ulcerative colitis

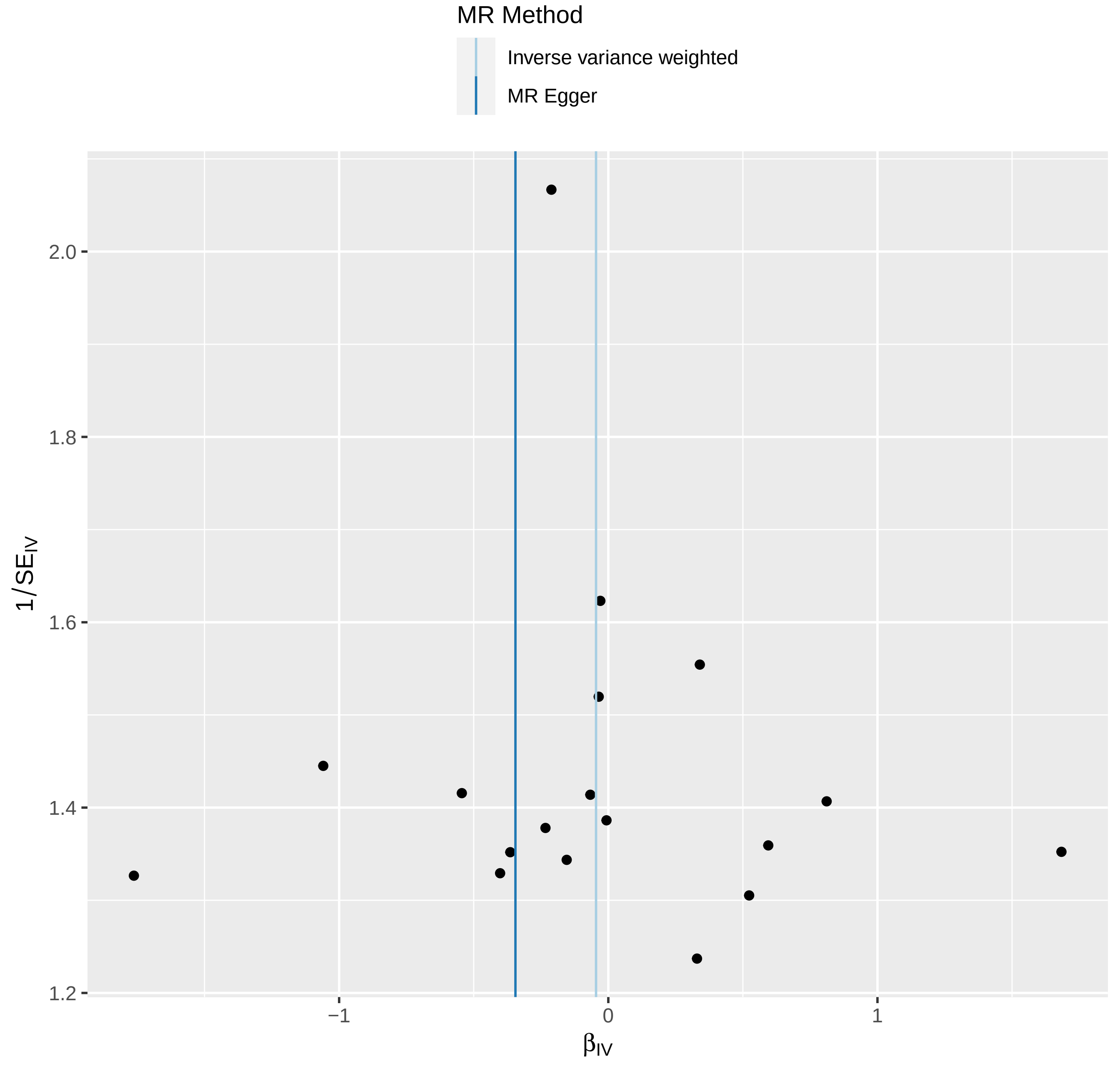

Batch 1286 : Gut microbiota abundance (genus Gordonibacter id.821) on Other ulcerative colitis

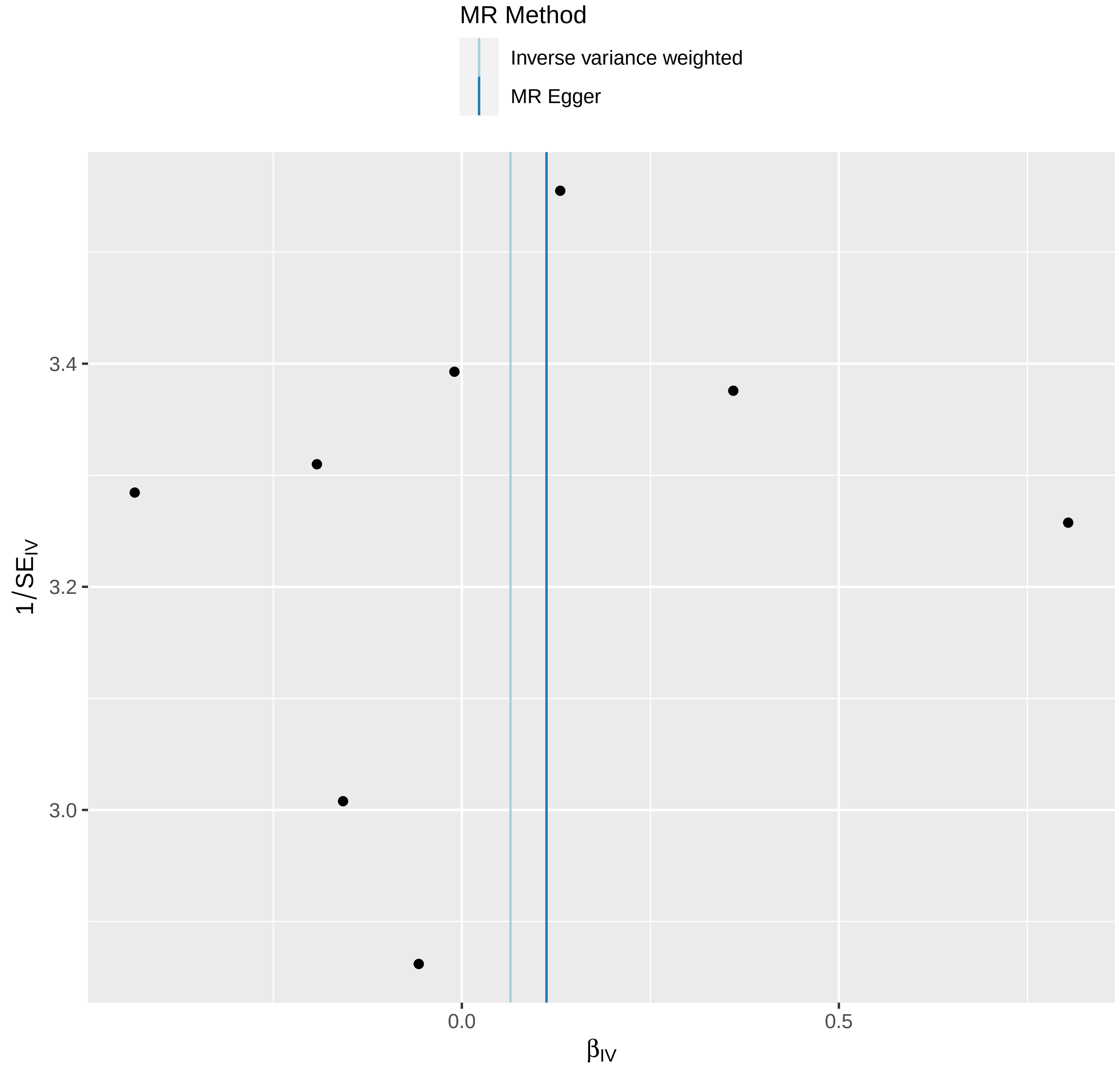

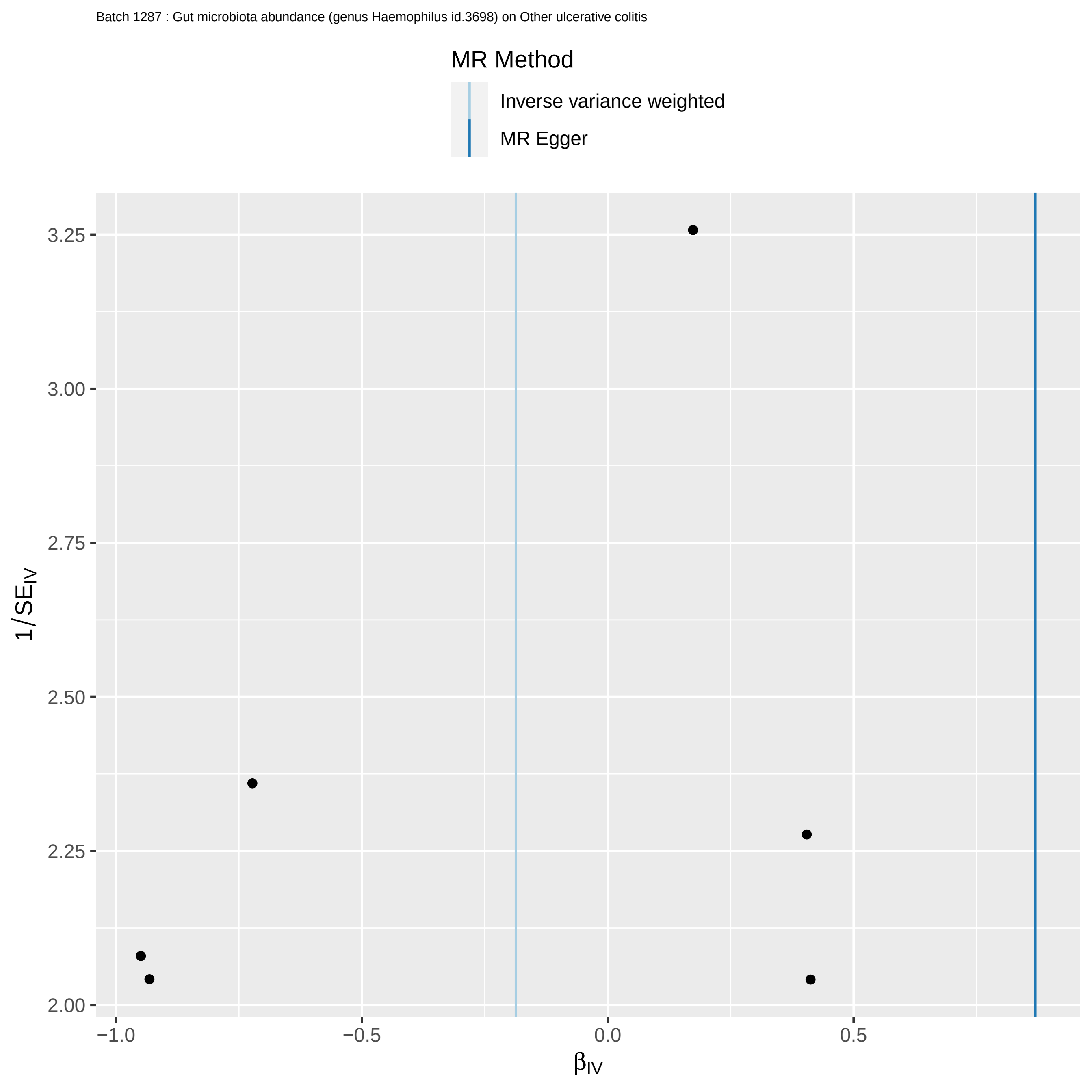
Batch 1287 : Gut microbiota abundance (genus Haemophilus id.3698) on Other ulcerative colitis
MR Method
Inverse variance weighted
MR Egger
3.25
3.00
2.75
2.50
2.25
2.00
1/SE_IV
−1.0
−0.5
0.0
0.5
β_IV

Batch 1288 : Gut microbiota abundance (genus Holdemanella id.11393) on Other ulcerative colitis

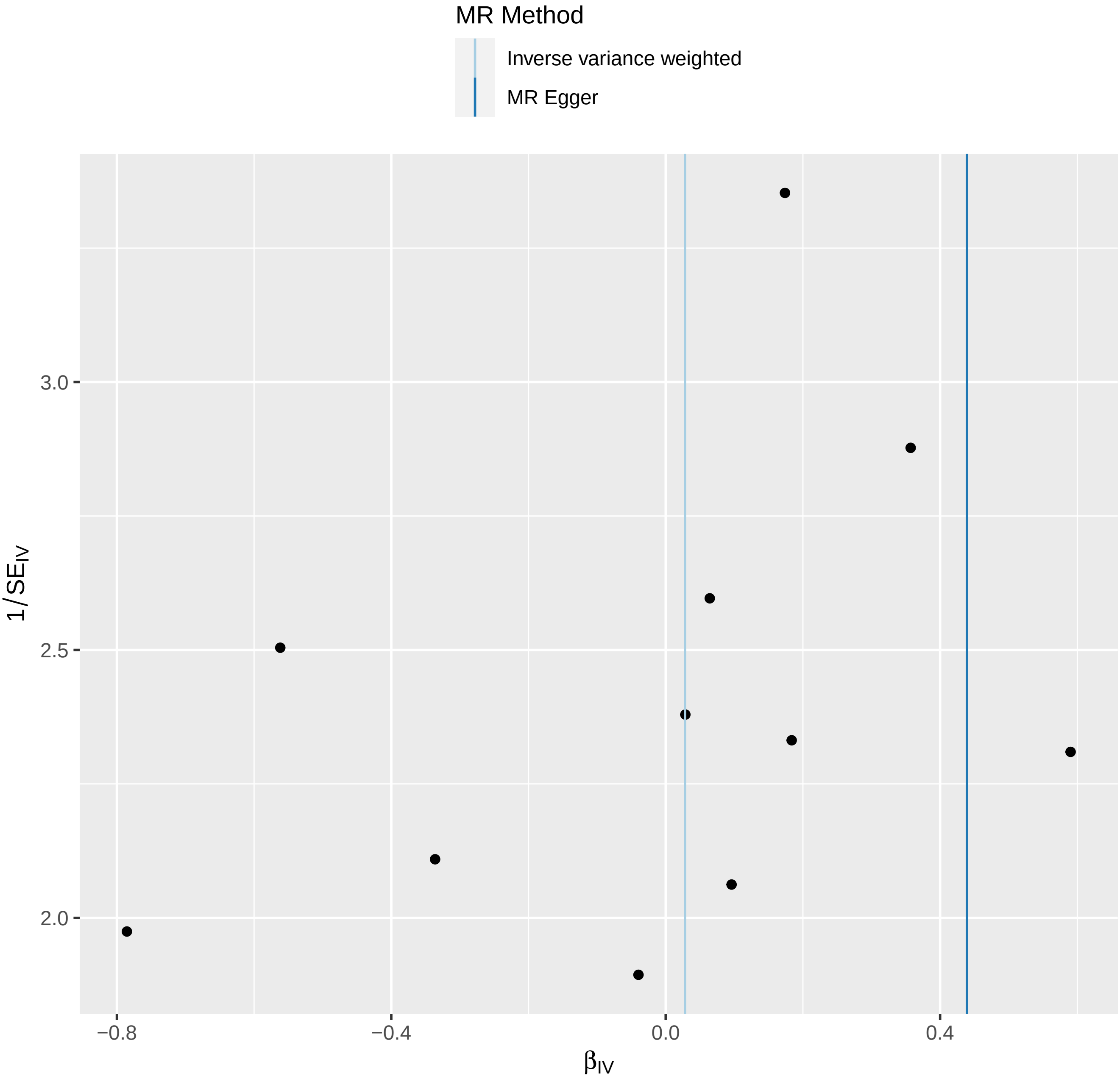

Batch 1289 : Gut microbiota abundance (genus Holdemania id.2157) on Other ulcerative colitis

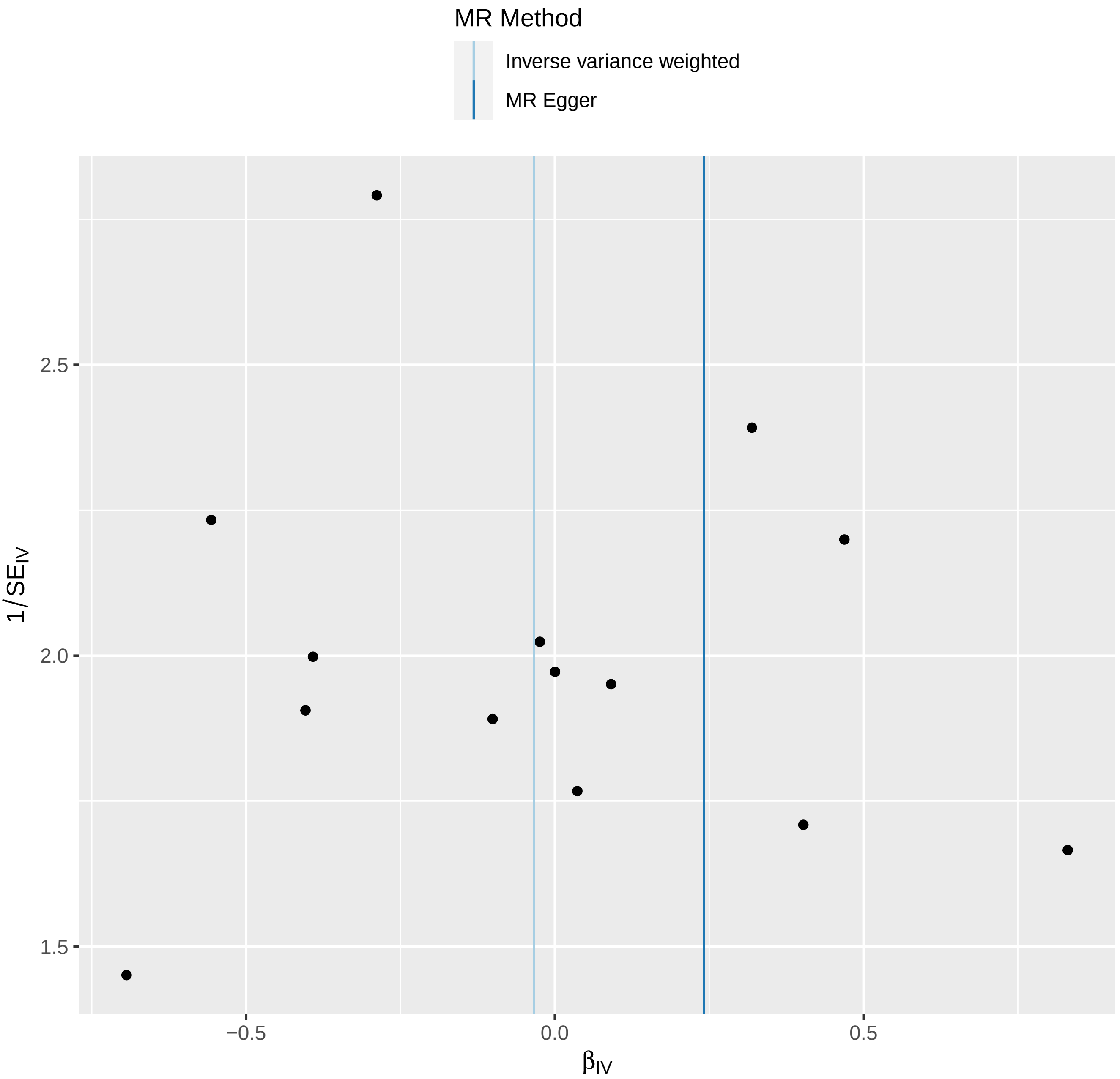

Batch 1290 : Gut microbiota abundance (genus Howardella id.2000) on Other ulcerative colitis

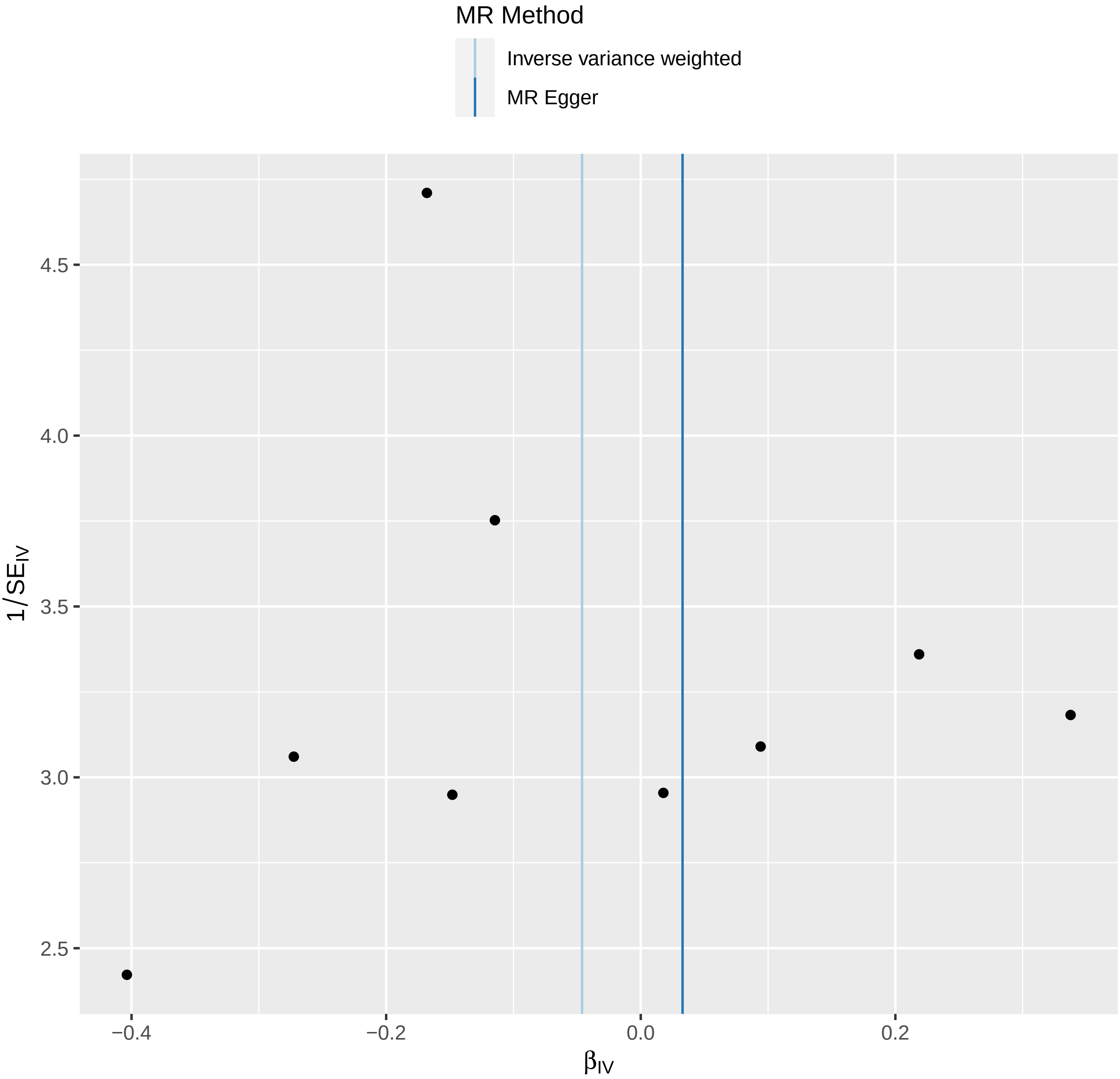

Batch 1291 : Gut microbiota abundance (genus Hungatella id.11306) on Other ulcerative colitis

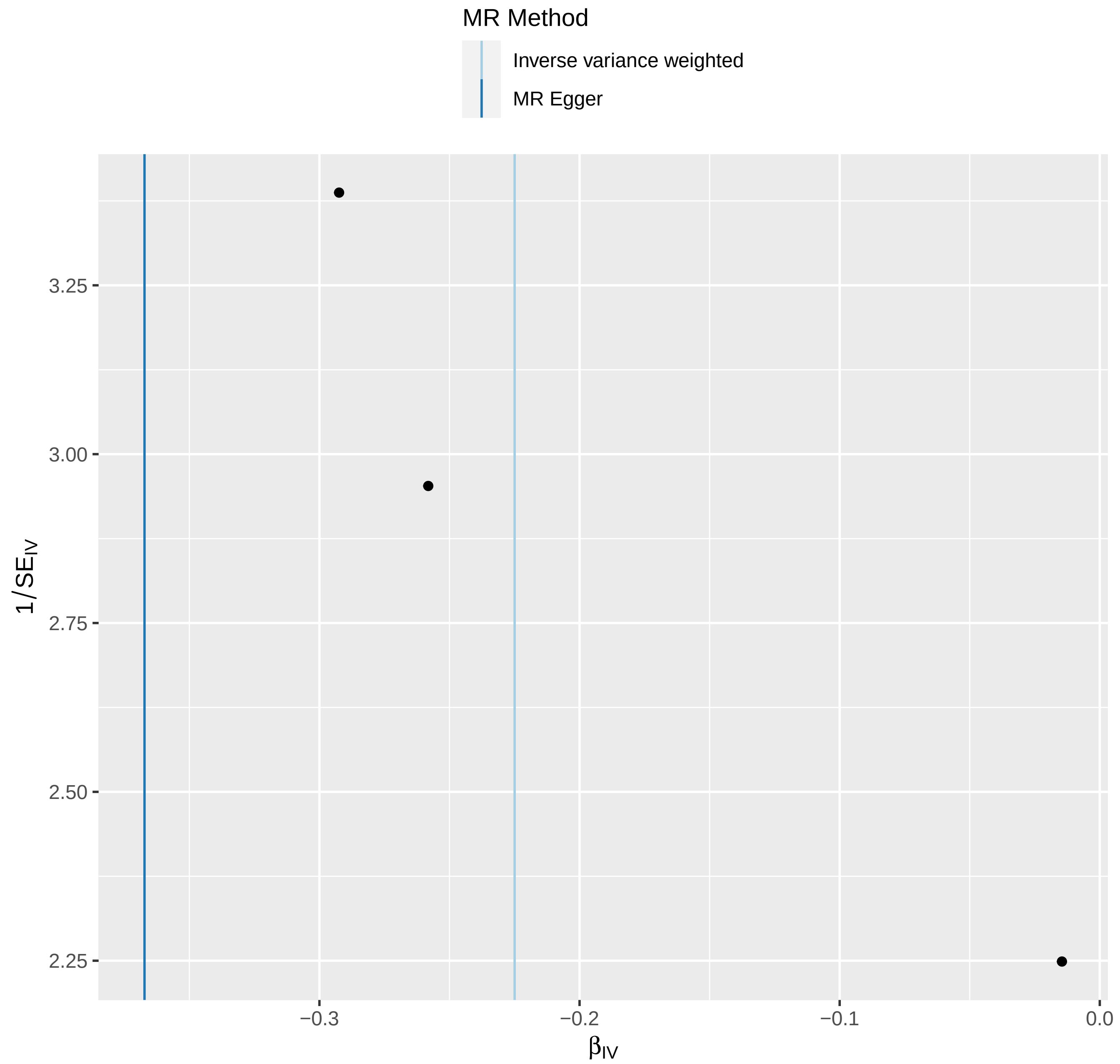

Batch 1292 : Gut microbiota abundance (genus Intestinibacter id.11345) on Other ulcerative colitis

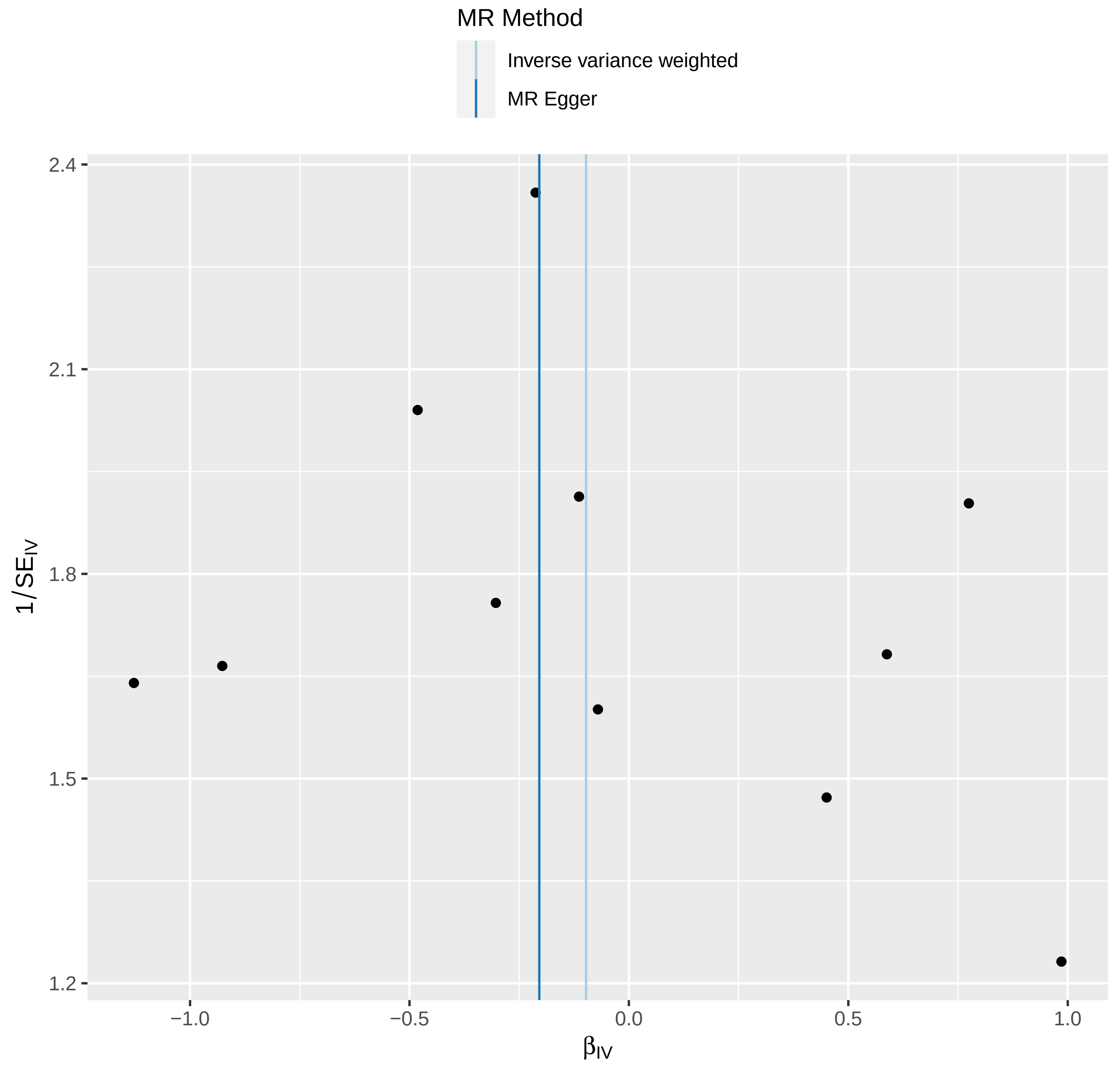

Batch 1293 : Gut microbiota abundance (genus Intestinimonas id.2062) on Other ulcerative colitis

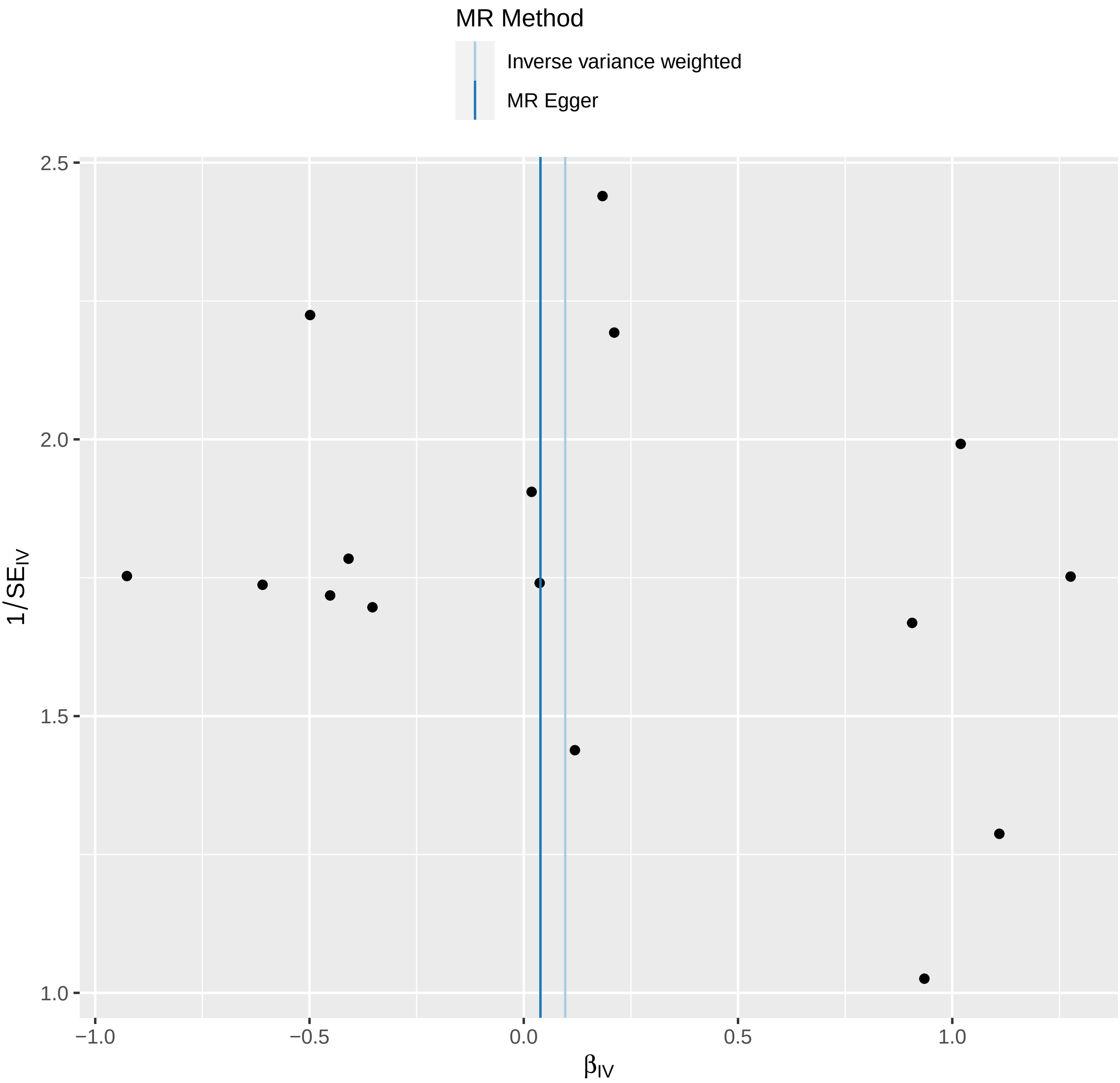

Batch 1294 : Gut microbiota abundance (genus Lachnoclostridium id.11308) on Other ulcerative colitis

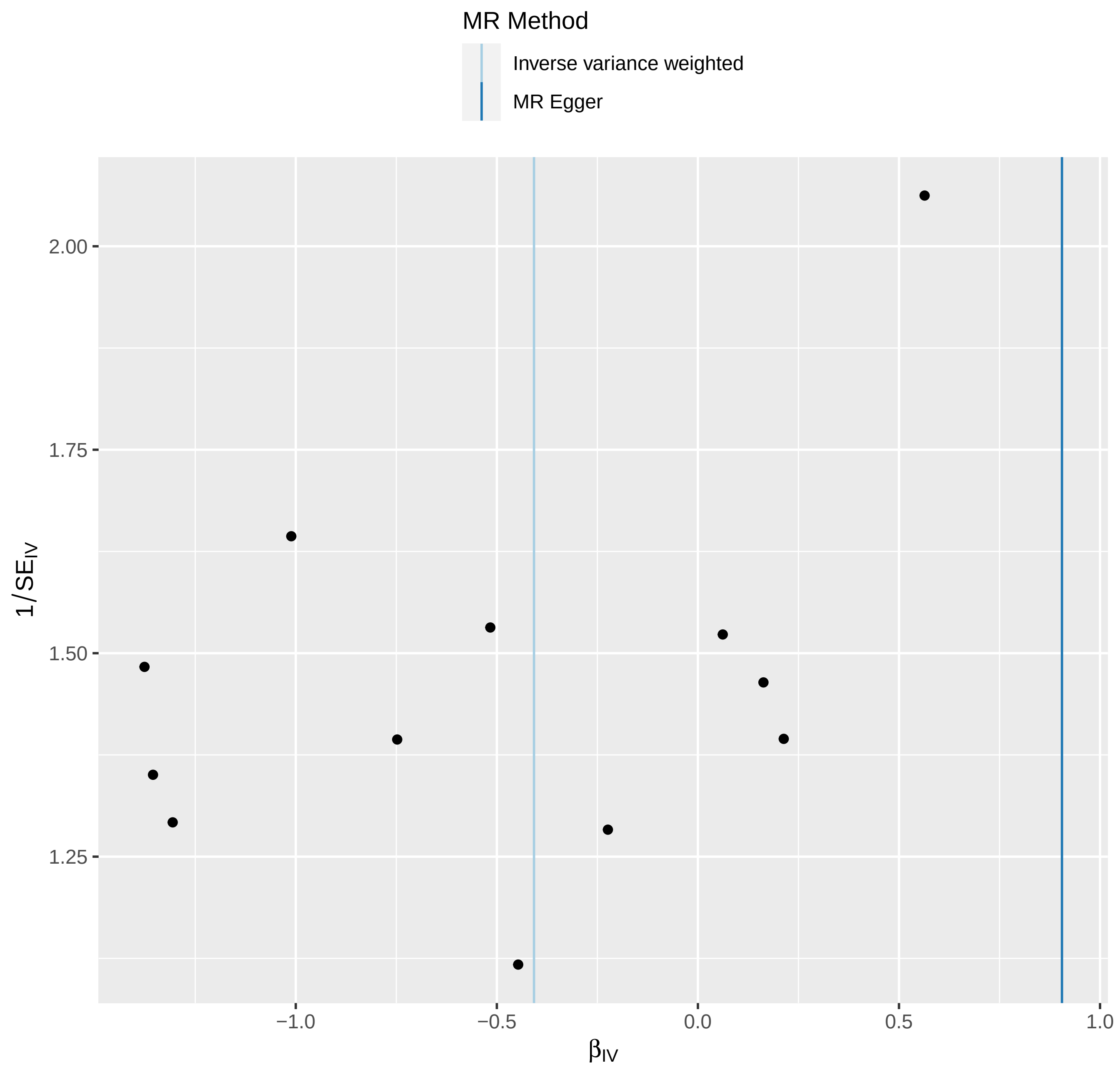

Batch 1295 : Gut microbiota abundance (genus Lachnospiraceae FCS020 group id.11314) on Other ulcerative colitis

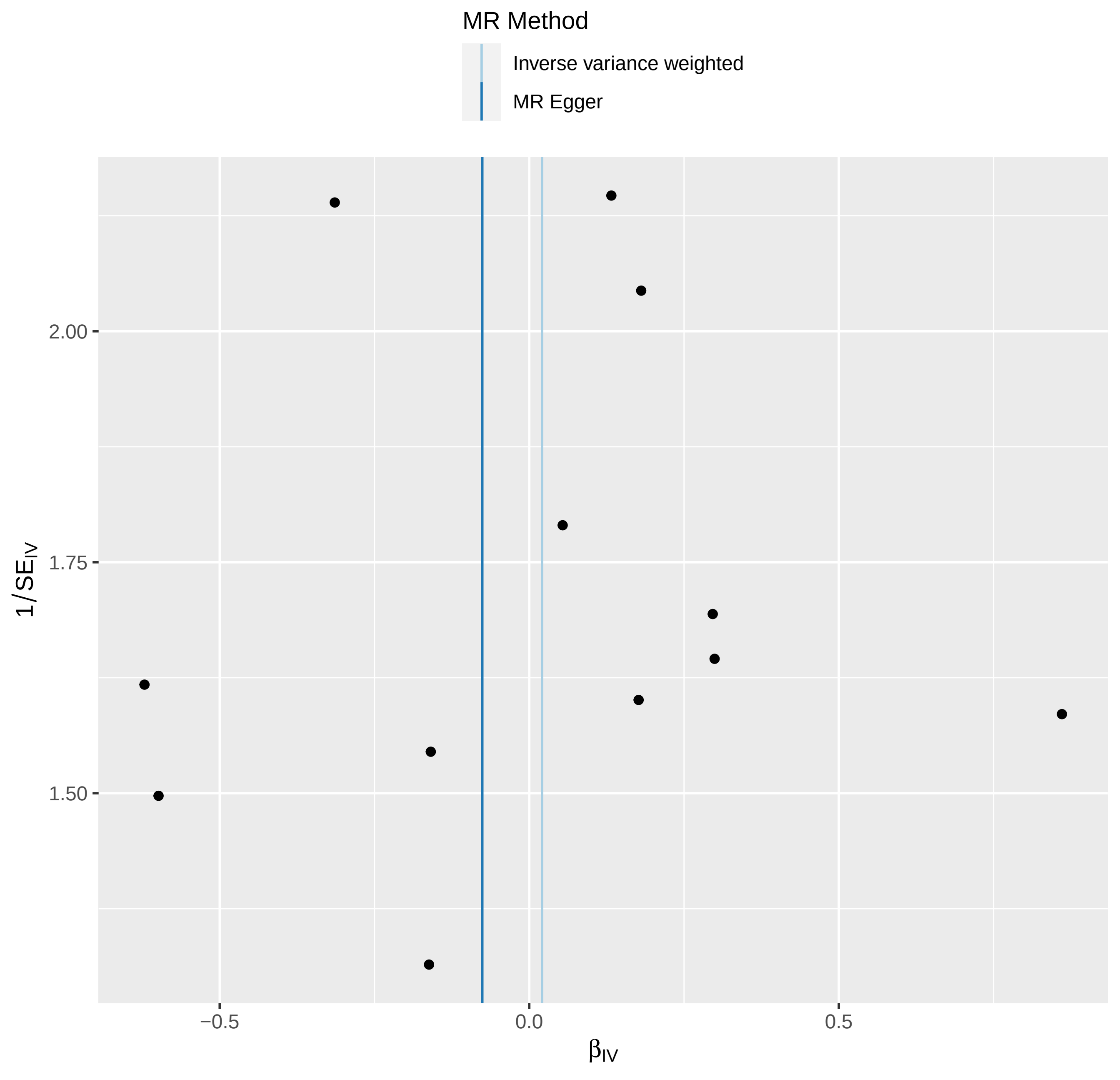

Batch 1296 : Gut microbiota abundance (genus Lachnospiraceae NC2004 group id.11316) on Other ulcerative colitis

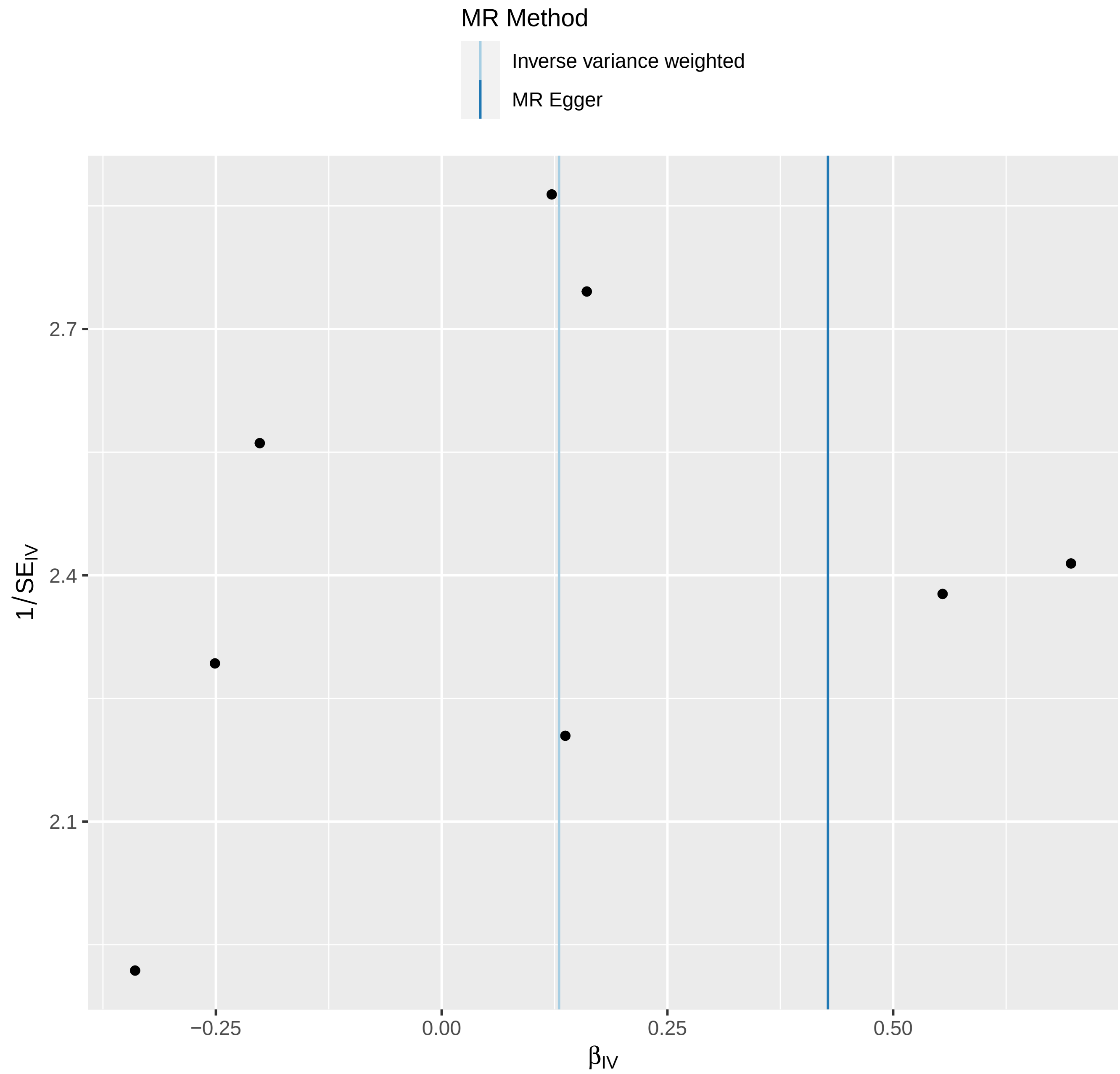

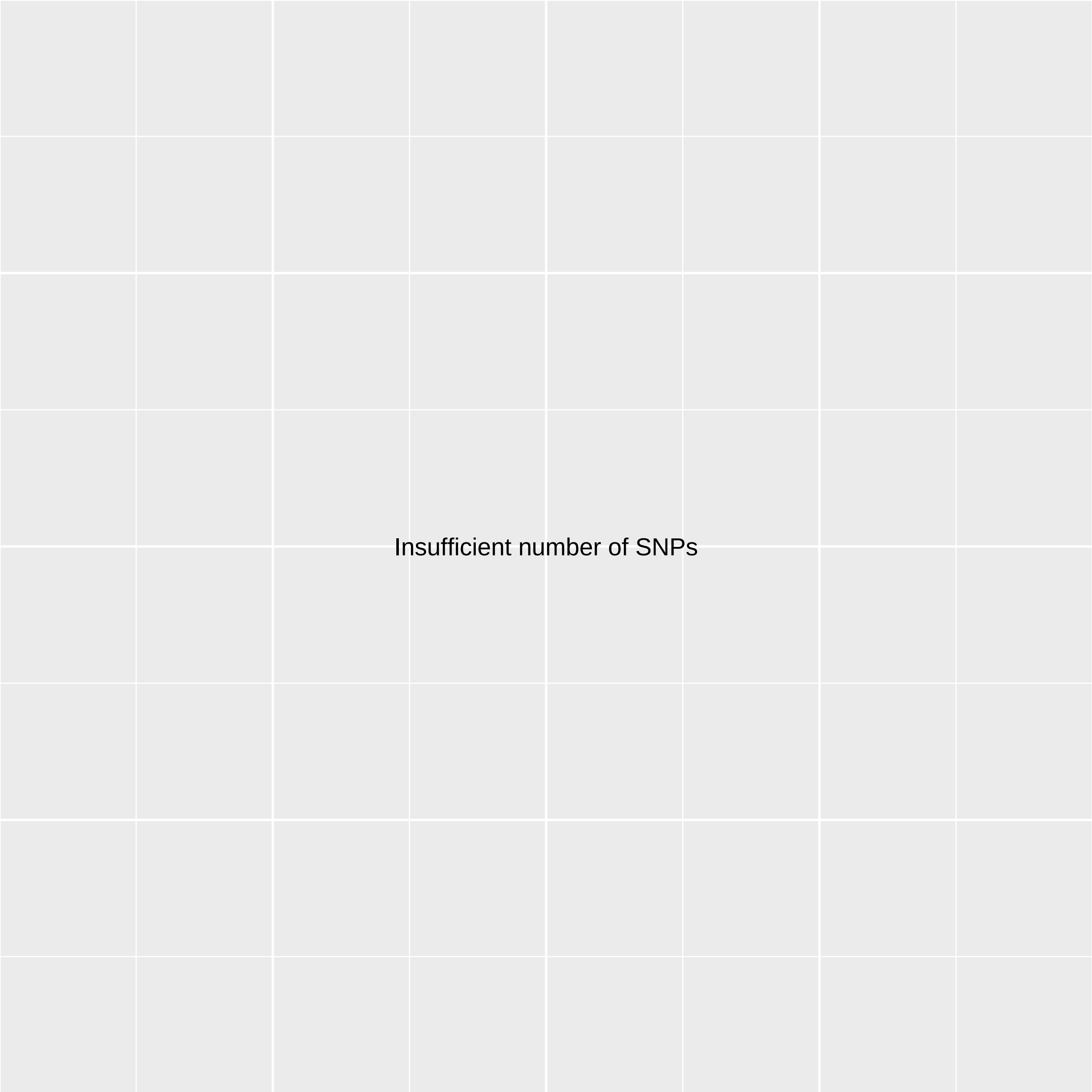
Insufficient number of SNPs

Batch 1298 : Gut microbiota abundance (genus Lachnospiraceae NK4A136 group id.11319) on Other ulcerative colitis

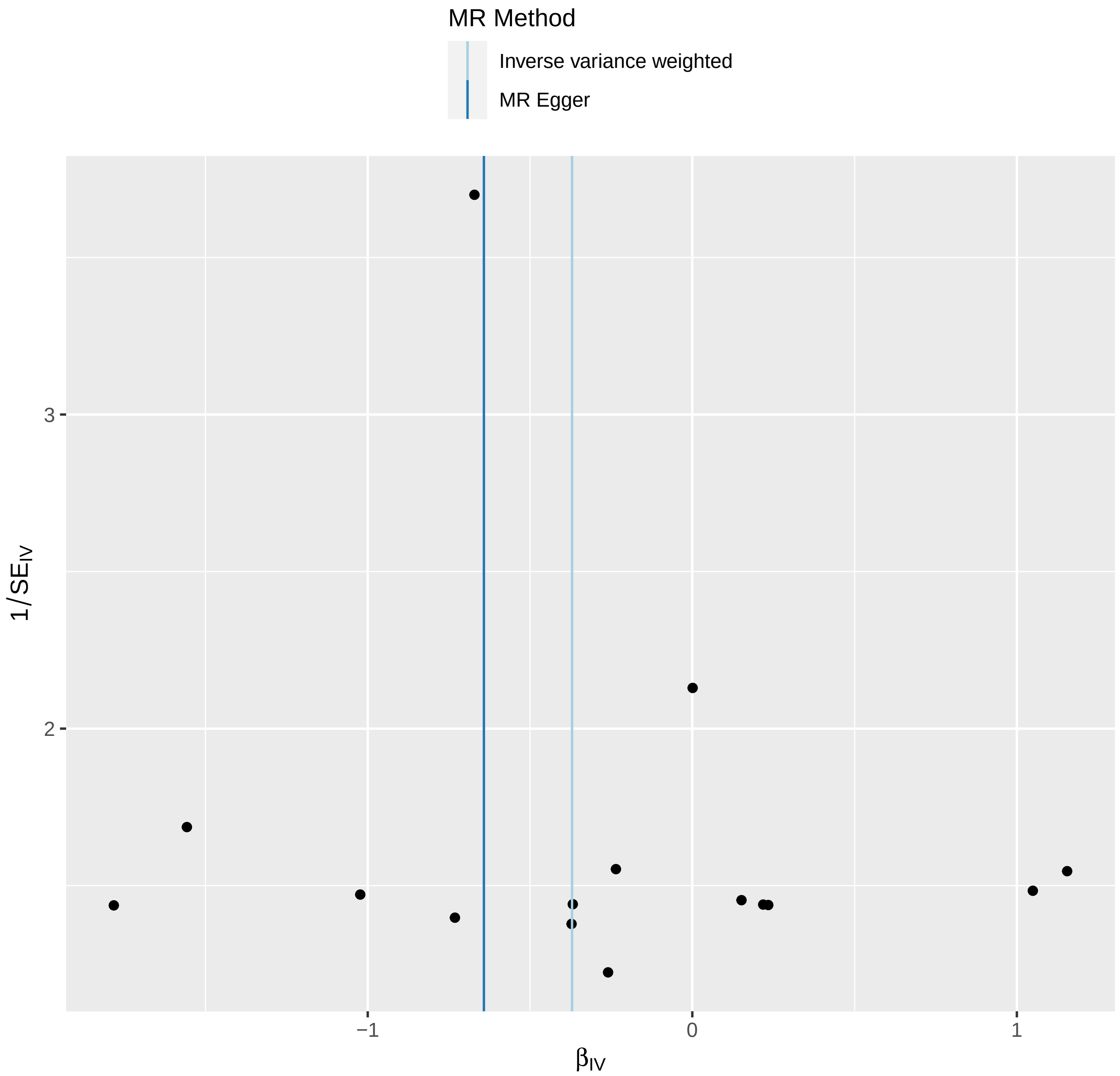

Batch 1299 : Gut microbiota abundance (genus Lachnospiraceae UCG001 id.11321) on Other ulcerative colitis

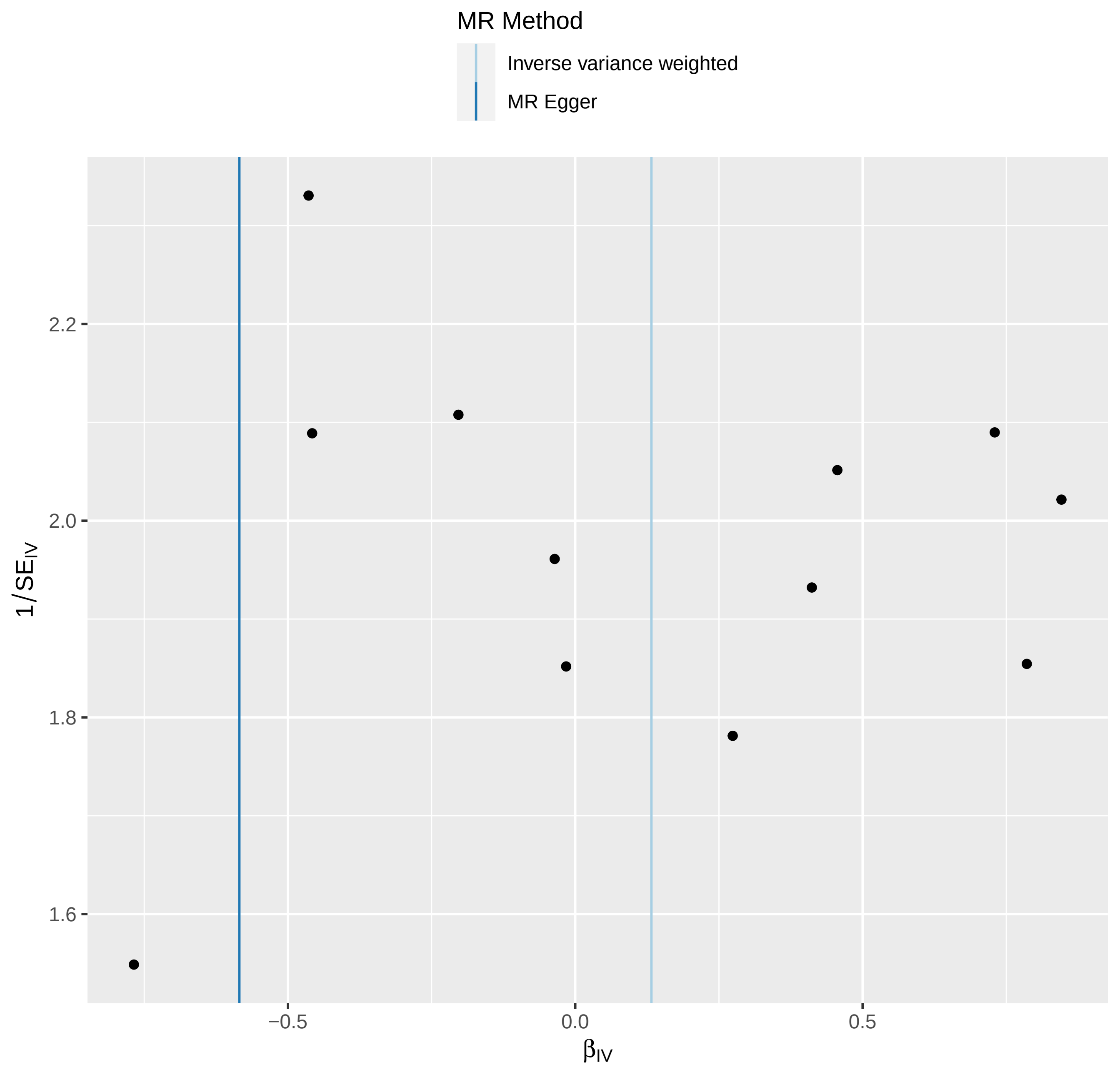

Batch 1300 : Gut microbiota abundance (genus Lachnospiraceae UCG004 id.11324) on Other ulcerative colitis

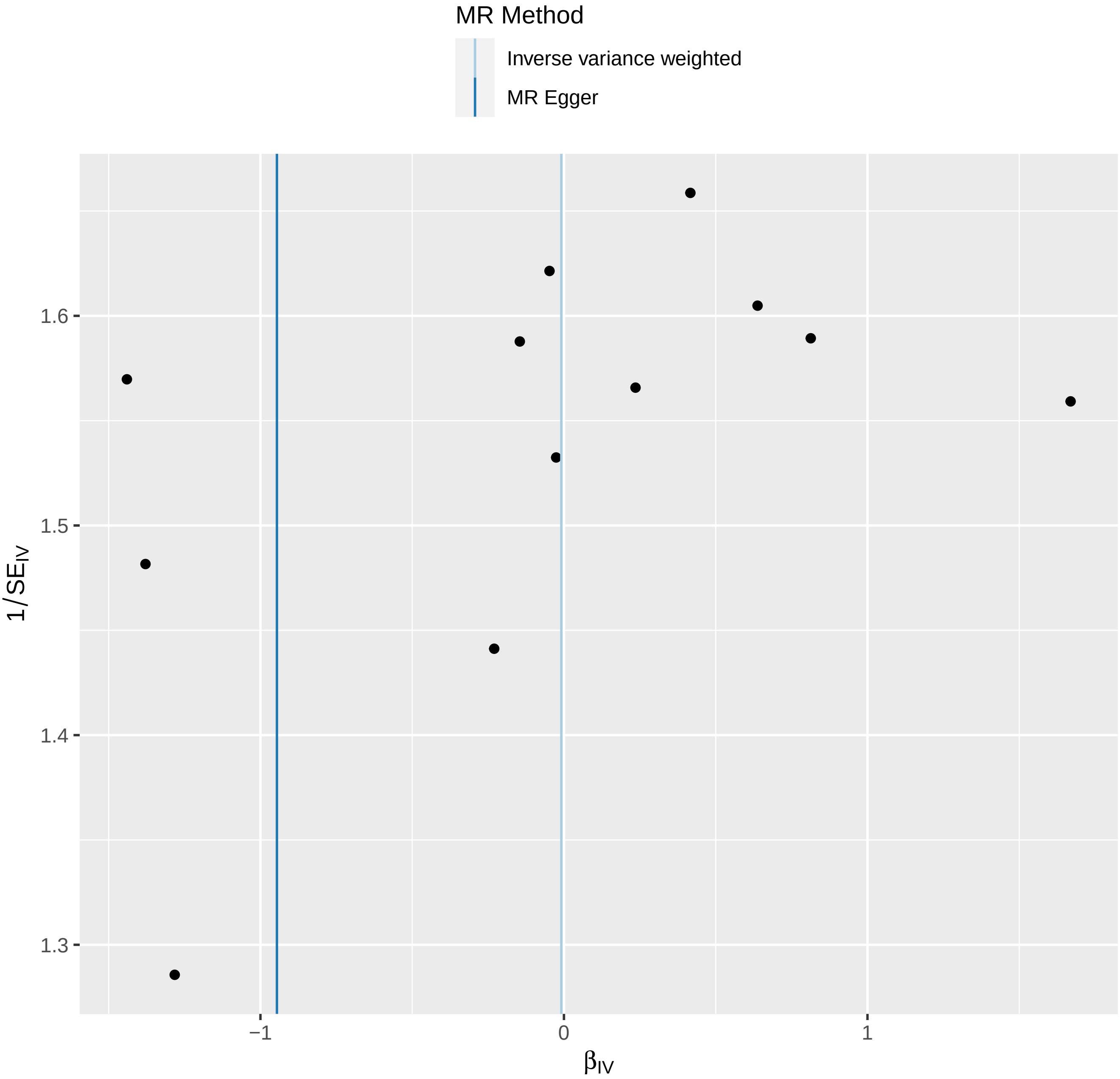

Batch 1301 : Gut microbiota abundance (genus Lachnospiraceae UCG008 id.11328) on Other ulcerative colitis

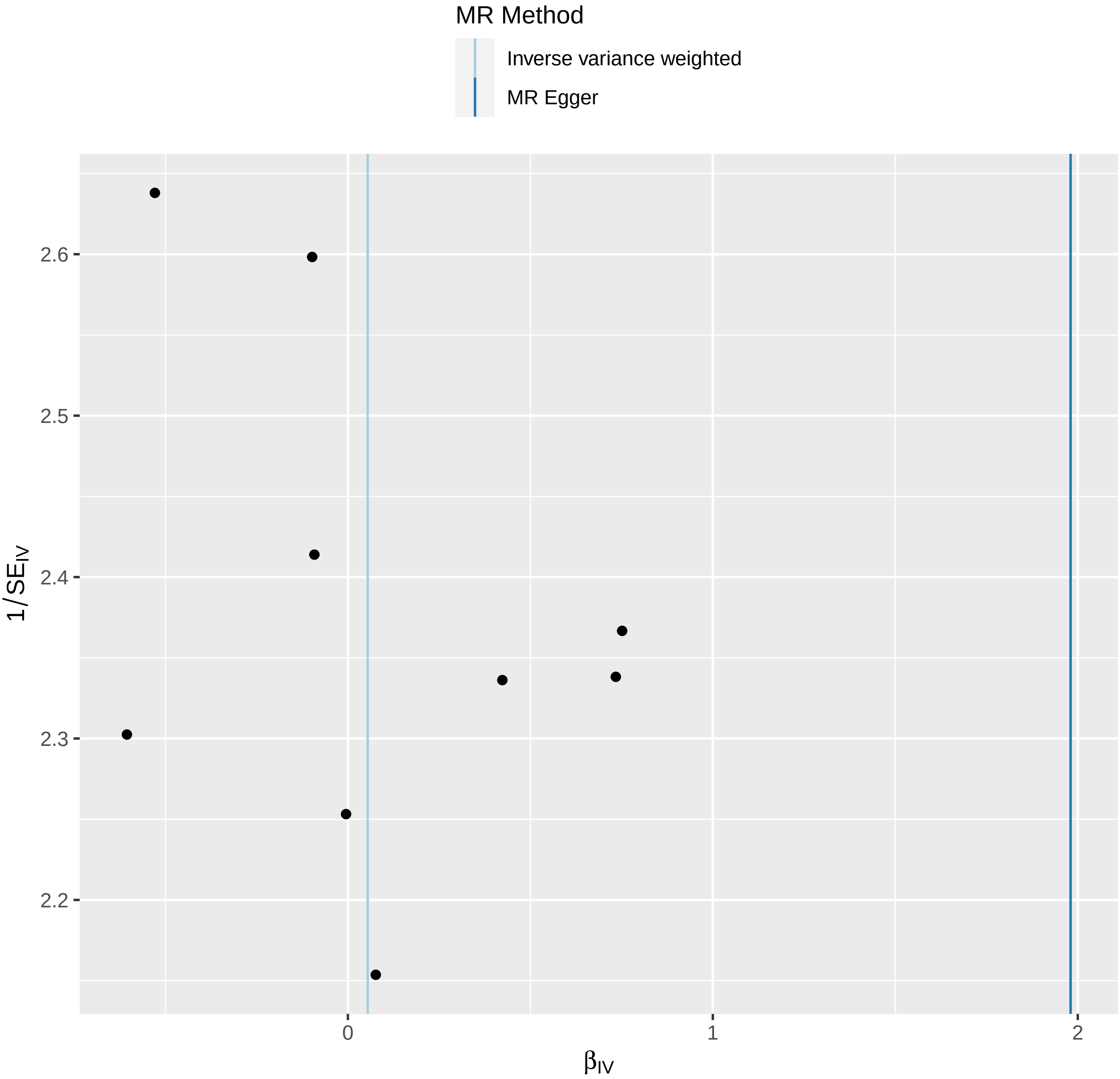

Batch 1302 : Gut microbiota abundance (genus Lachnospiraceae UCG010 id.11330) on Other ulcerative colitis

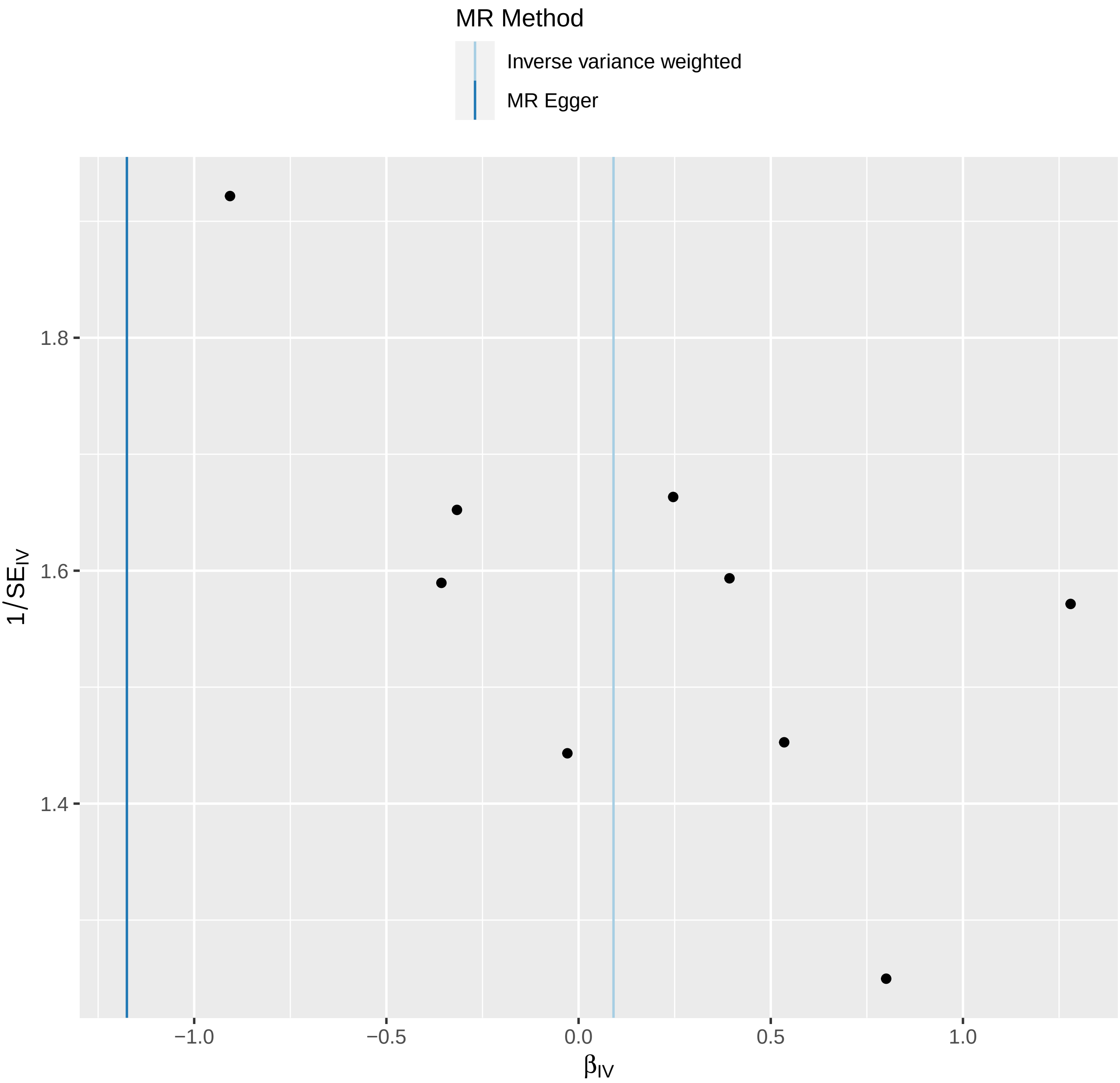

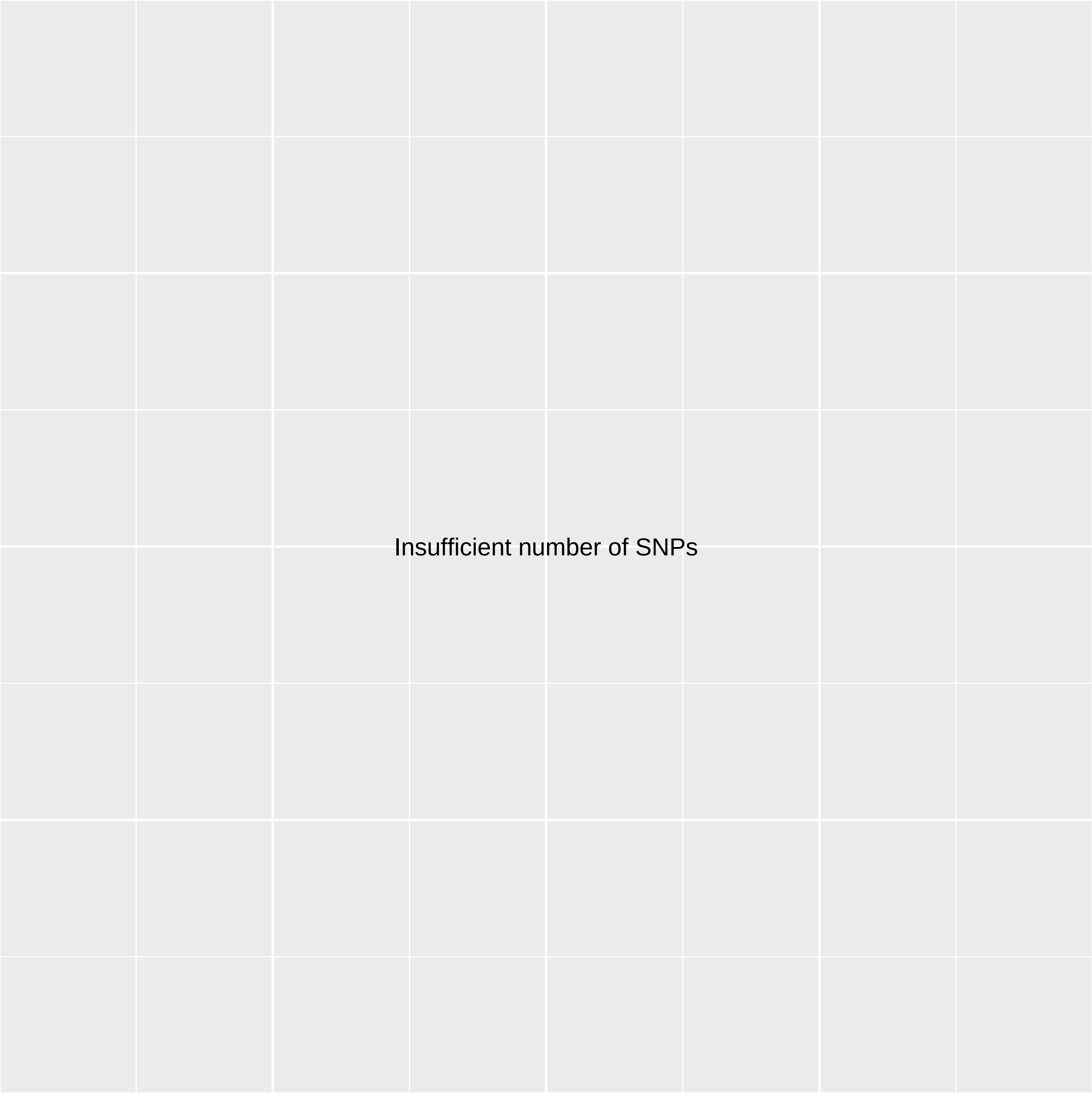
Insufficient number of SNPs

Batch 1304 : Gut microbiota abundance (genus Lactobacillus id.1837) on Other ulcerative colitis

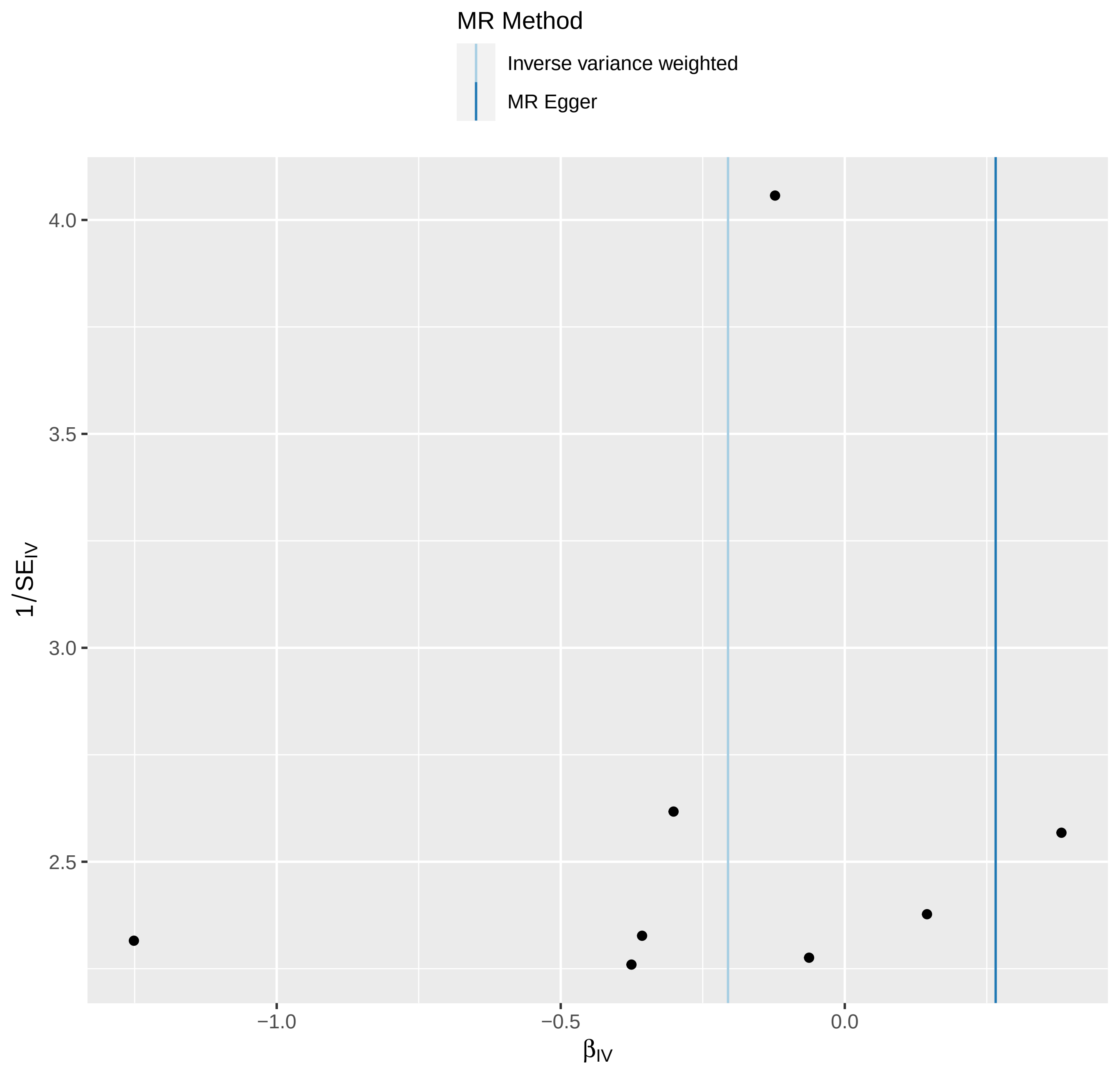

Batch 1305 : Gut microbiota abundance (genus Lactococcus id.1851) on Other ulcerative colitis

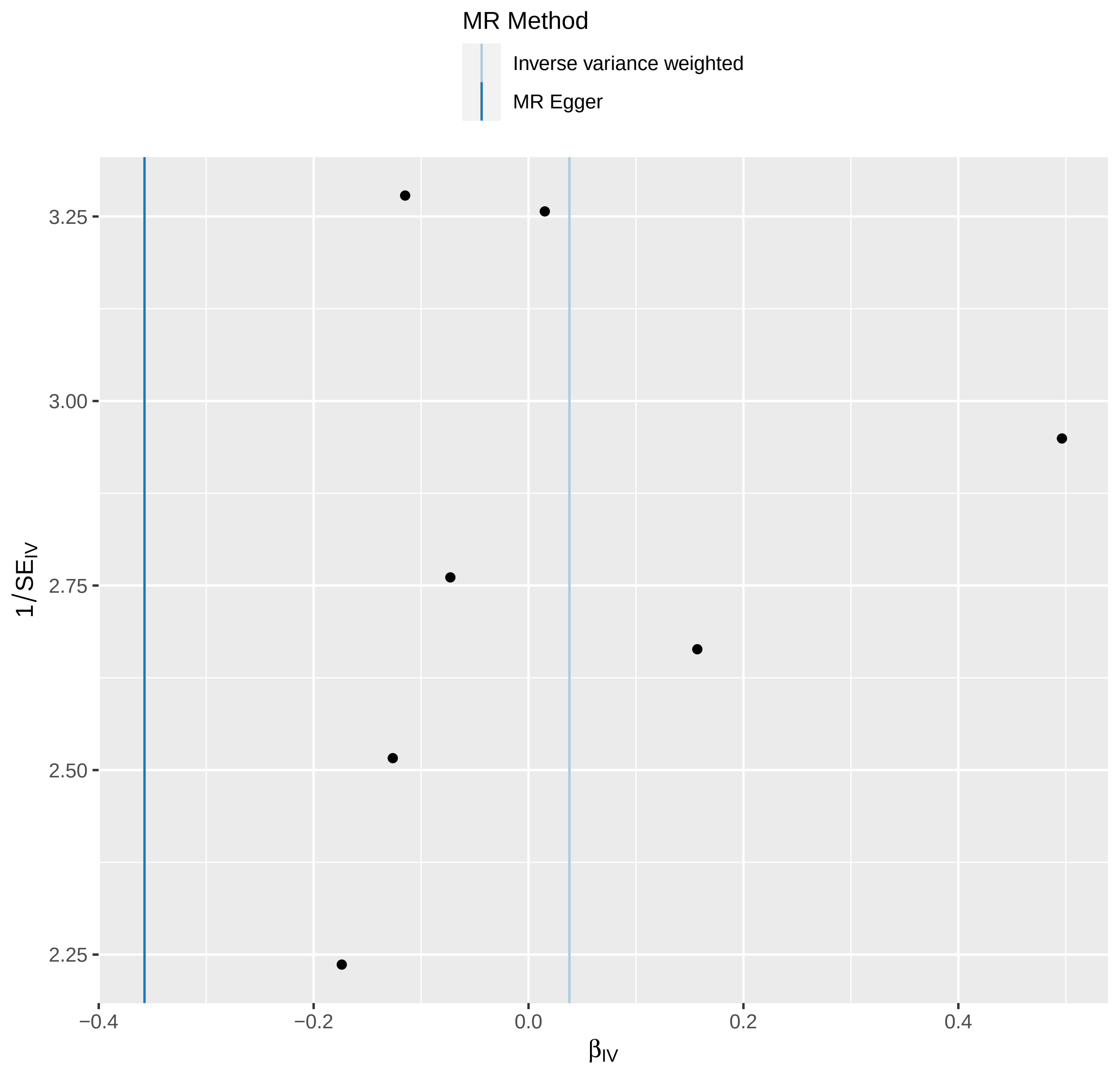

Batch 1306 : Gut microbiota abundance (genus Marvinbryantia id.2005) on Other ulcerative colitis

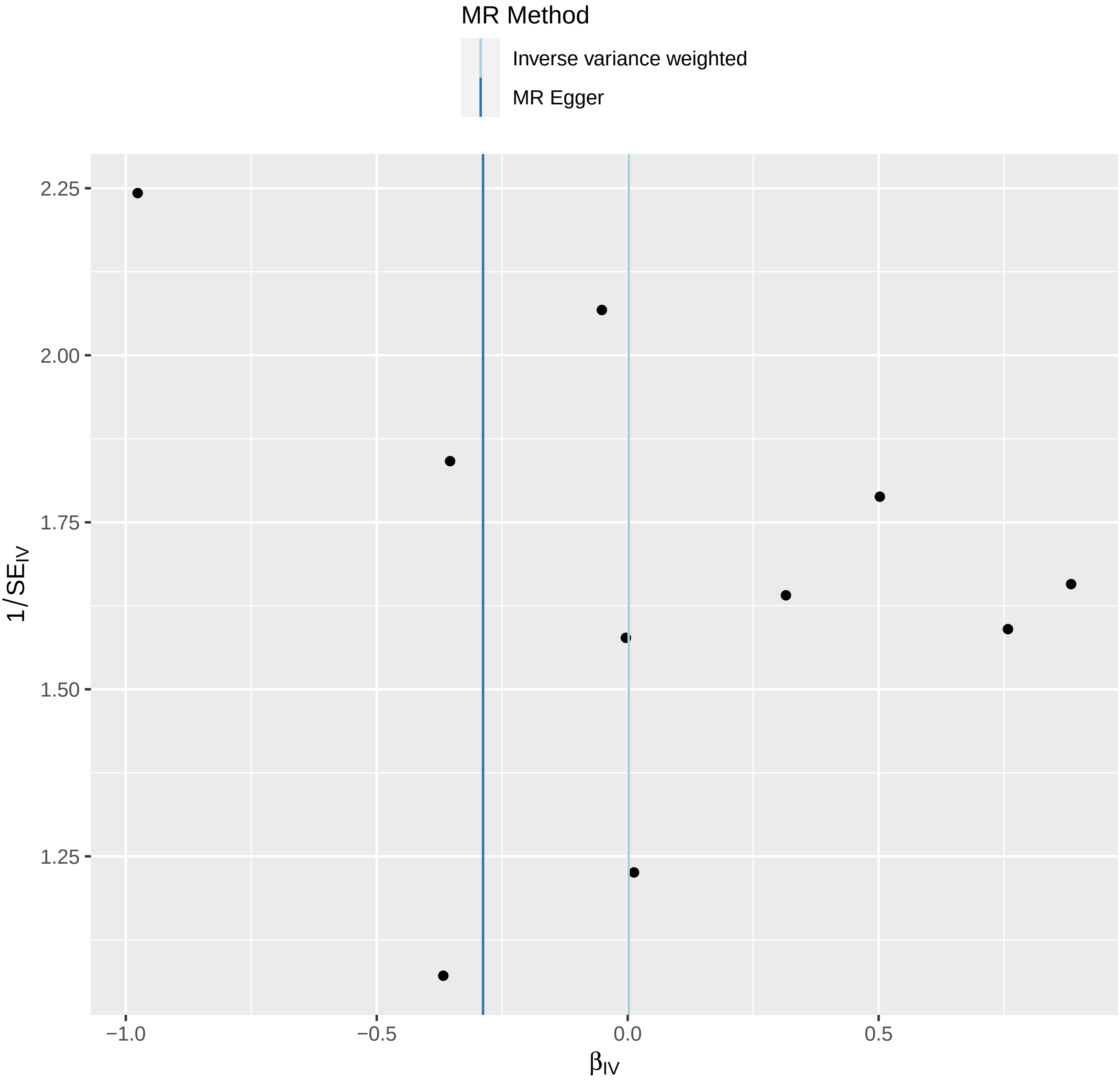

Batch 1307 : Gut microbiota abundance (genus Methanobrevibacter id.123) on Other ulcerative colitis

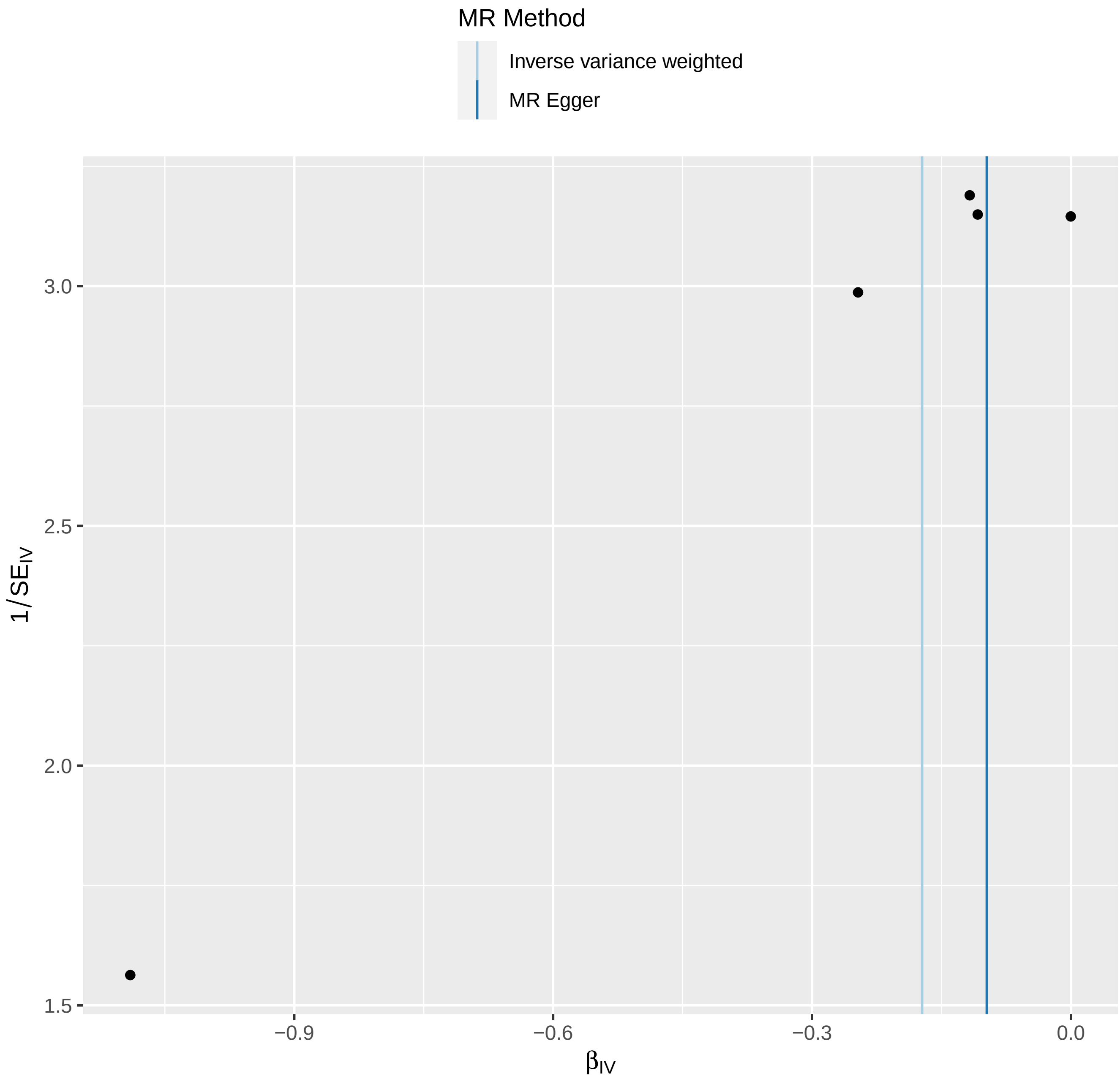

Batch 1308 : Gut microbiota abundance (genus Odoribacter id.952) on Other ulcerative colitis

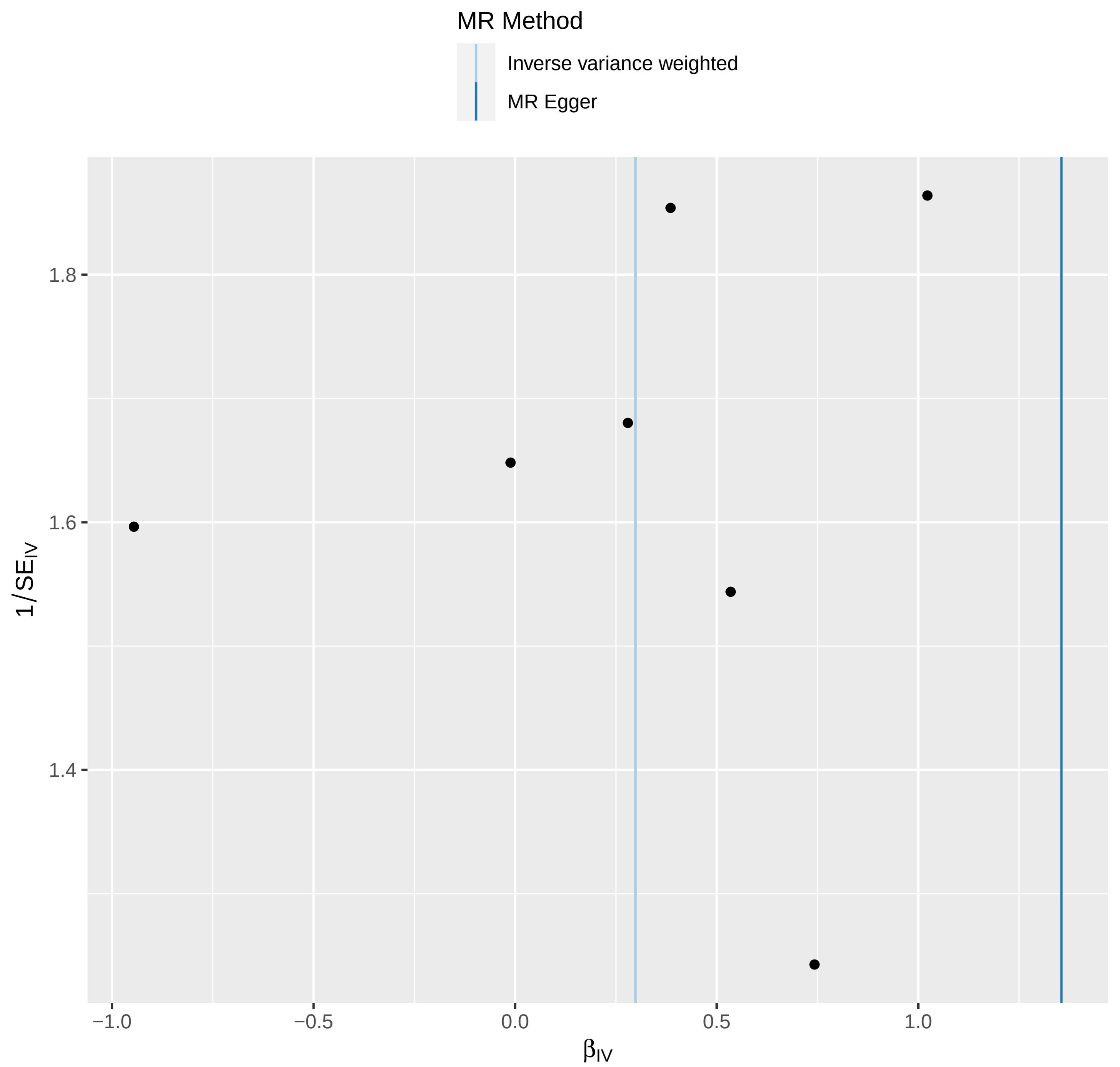

Batch 1309 : Gut microbiota abundance (genus Olsenella id.822) on Other ulcerative colitis

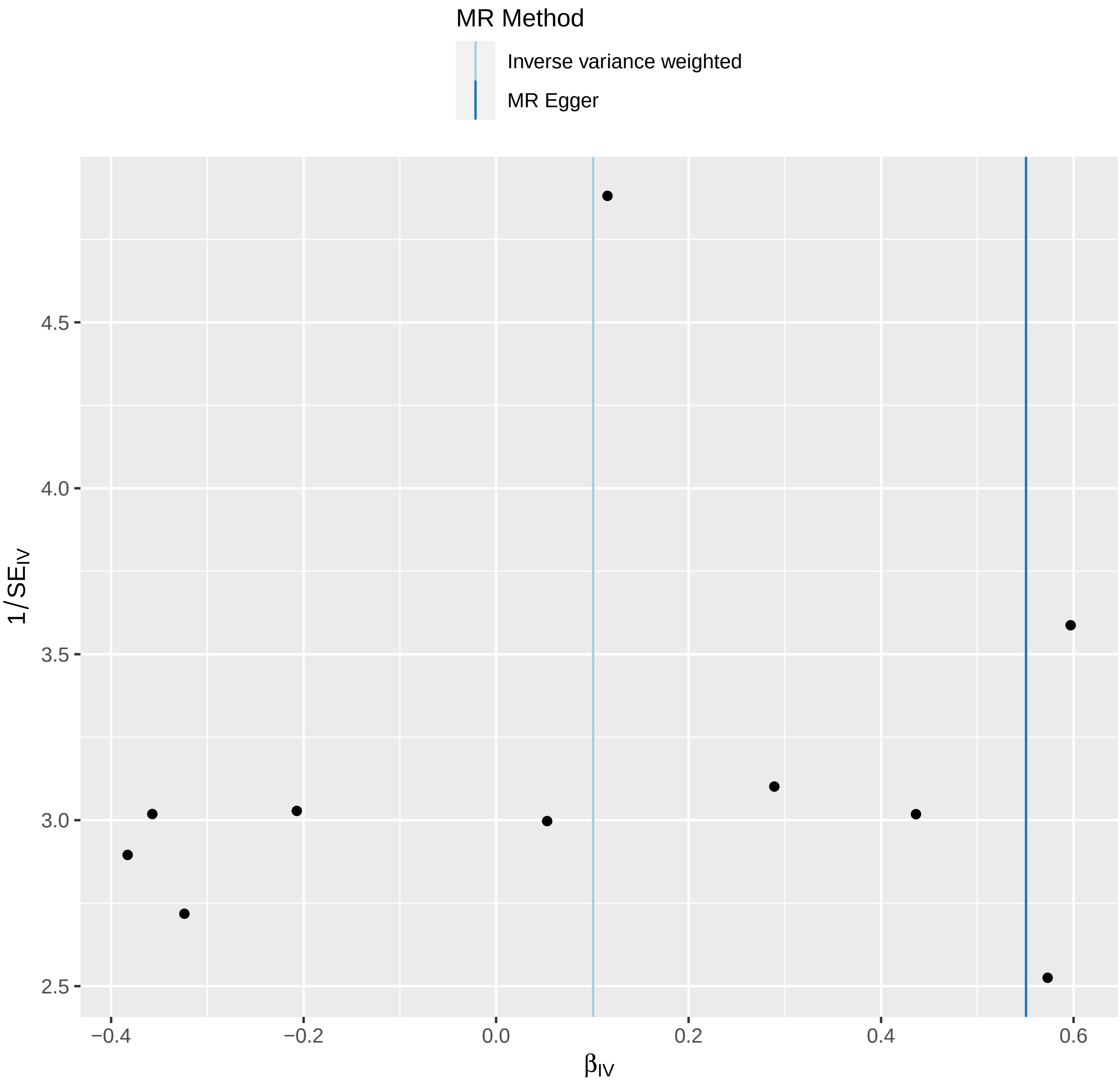

Batch 1310 : Gut microbiota abundance (genus Oscillibacter id.2063) on Other ulcerative colitis

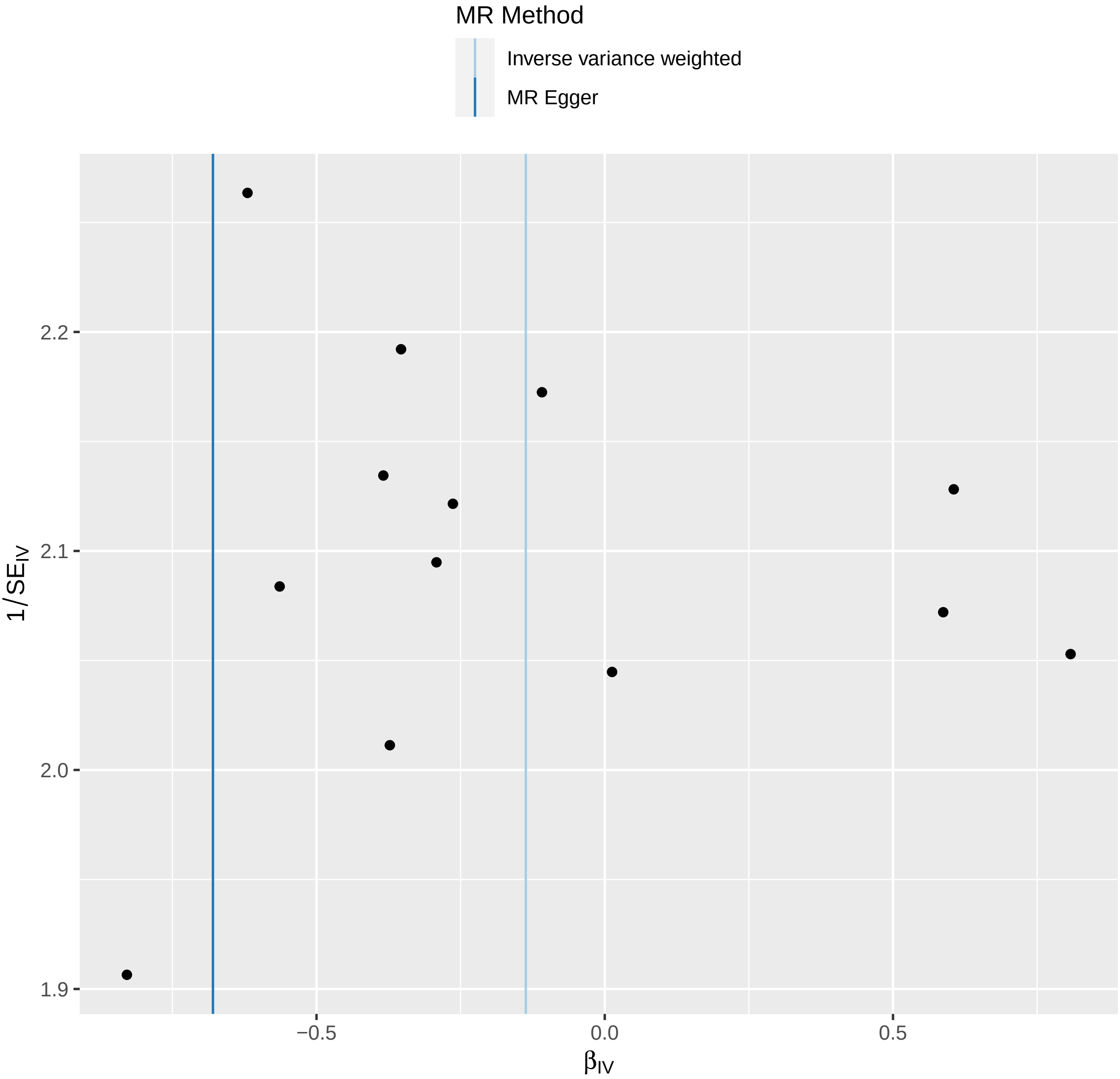

Batch 1311 : Gut microbiota abundance (genus Oscillospira id.2064) on Other ulcerative colitis

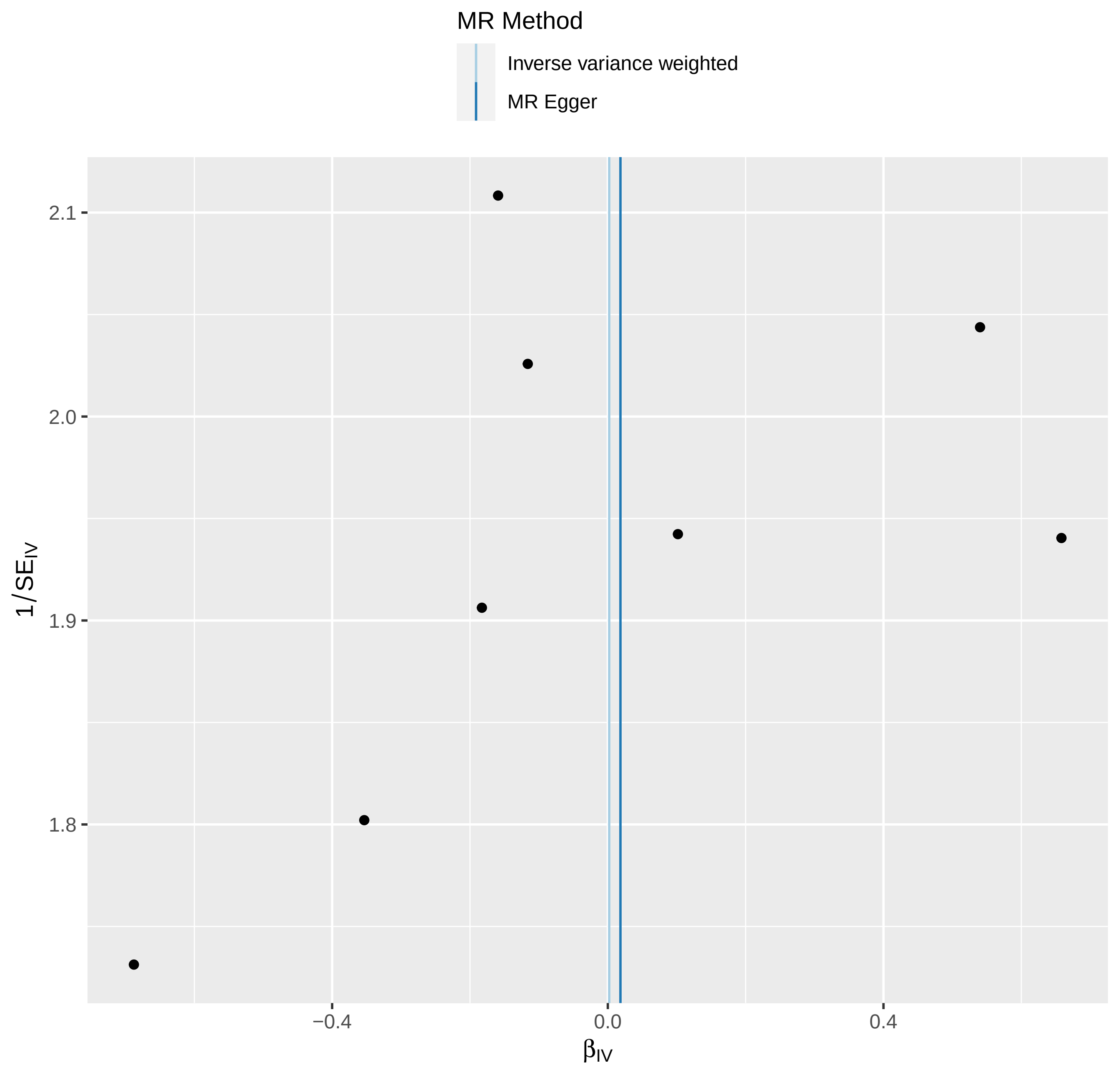

Batch 1312 : Gut microbiota abundance (genus Oxalobacter id.2978) on Other ulcerative colitis

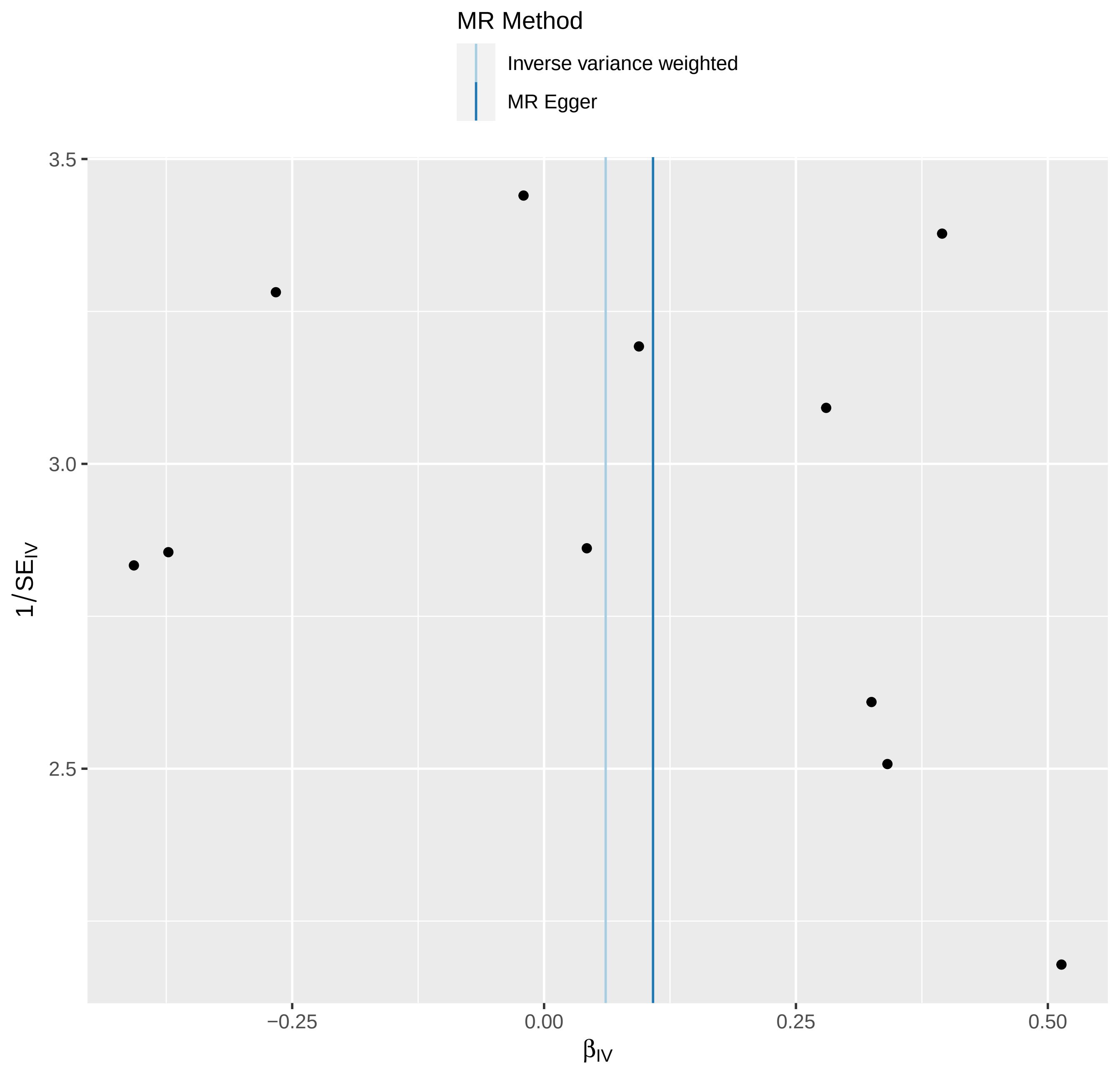

Batch 1313 : Gut microbiota abundance (genus Parabacteroides id.954) on Other ulcerative colitis

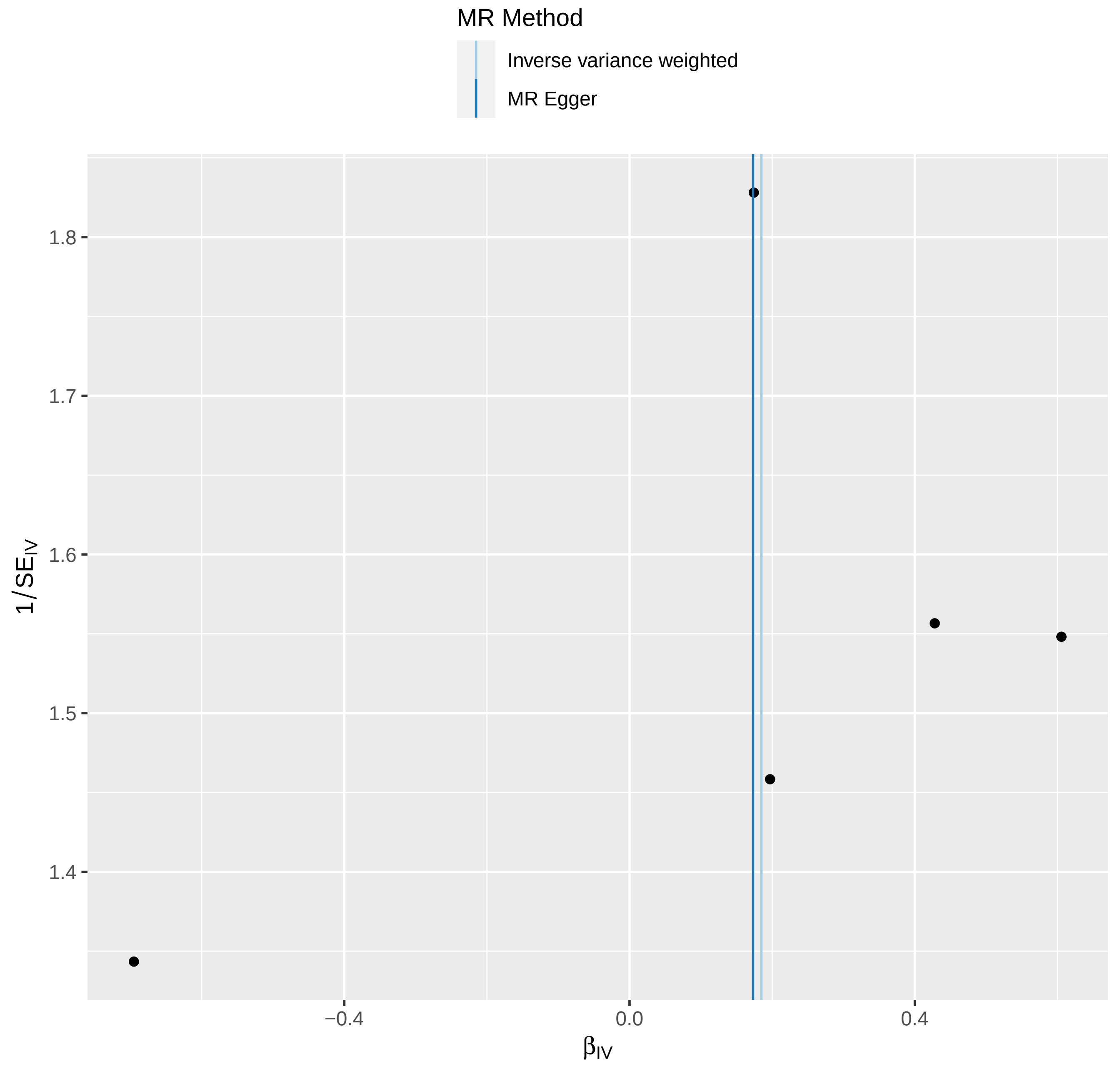

Batch 1314 : Gut microbiota abundance (genus Paraprevotella id.962) on Other ulcerative colitis

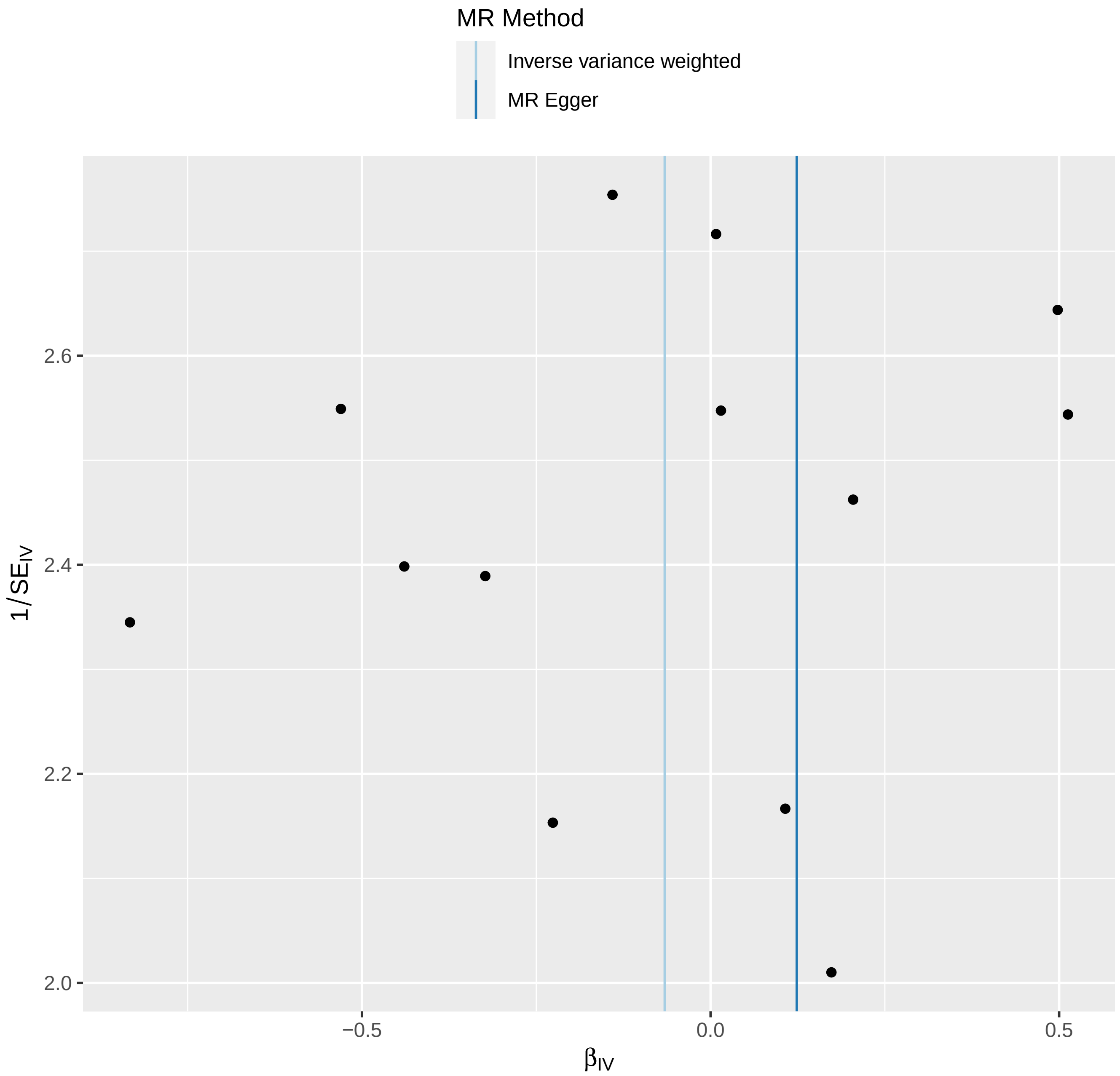

Batch 1315 : Gut microbiota abundance (genus Parasutterella id.2892) on Other ulcerative colitis

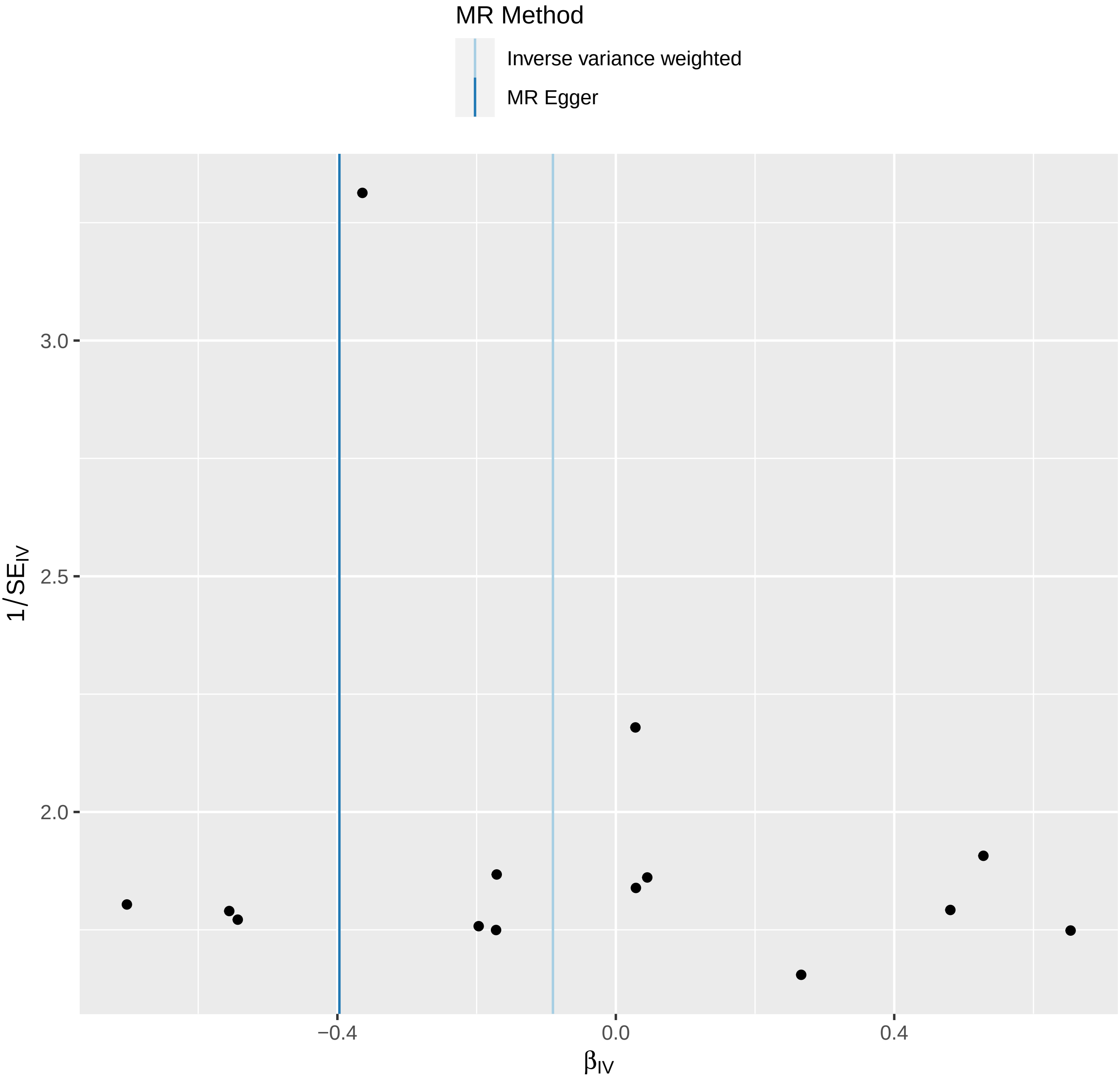

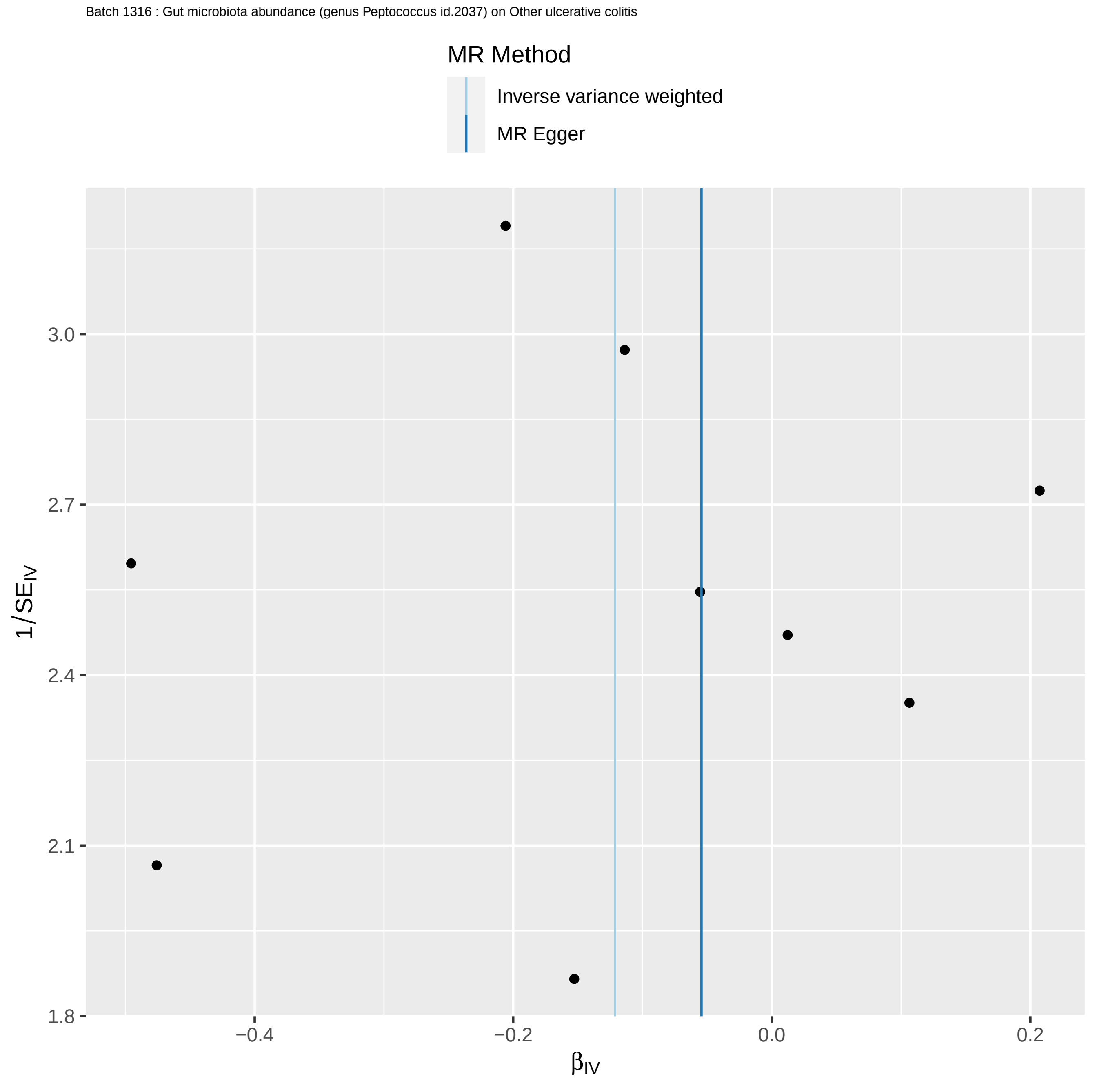
Batch 1316 : Gut microbiota abundance (genus Peptococcus id.2037) on Other ulcerative colitis
MR Method
Inverse variance weighted
MR Egger
3.0
2.7
2.4
2.1
1.8
1/SE_IV
−0.4
−0.2
0.0
0.2
β_IV

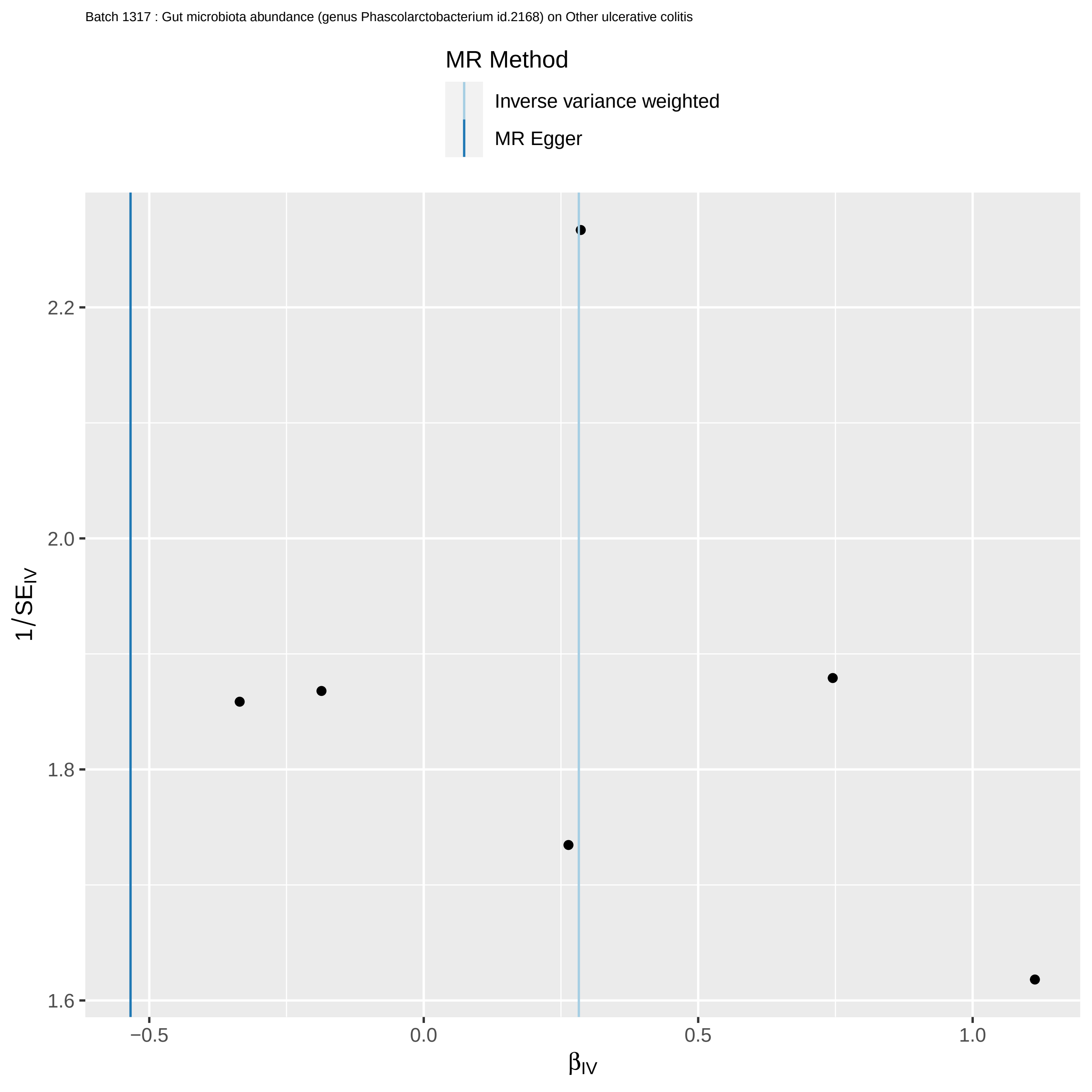
Batch 1317 : Gut microbiota abundance (genus Phascolarctobacterium id.2168) on Other ulcerative colitis
MR Method
Inverse variance weighted
MR Egger
2.2
2.0
1.8
1.6
1/SE_IV
−0.5
0.0
0.5
1.0
β_IV

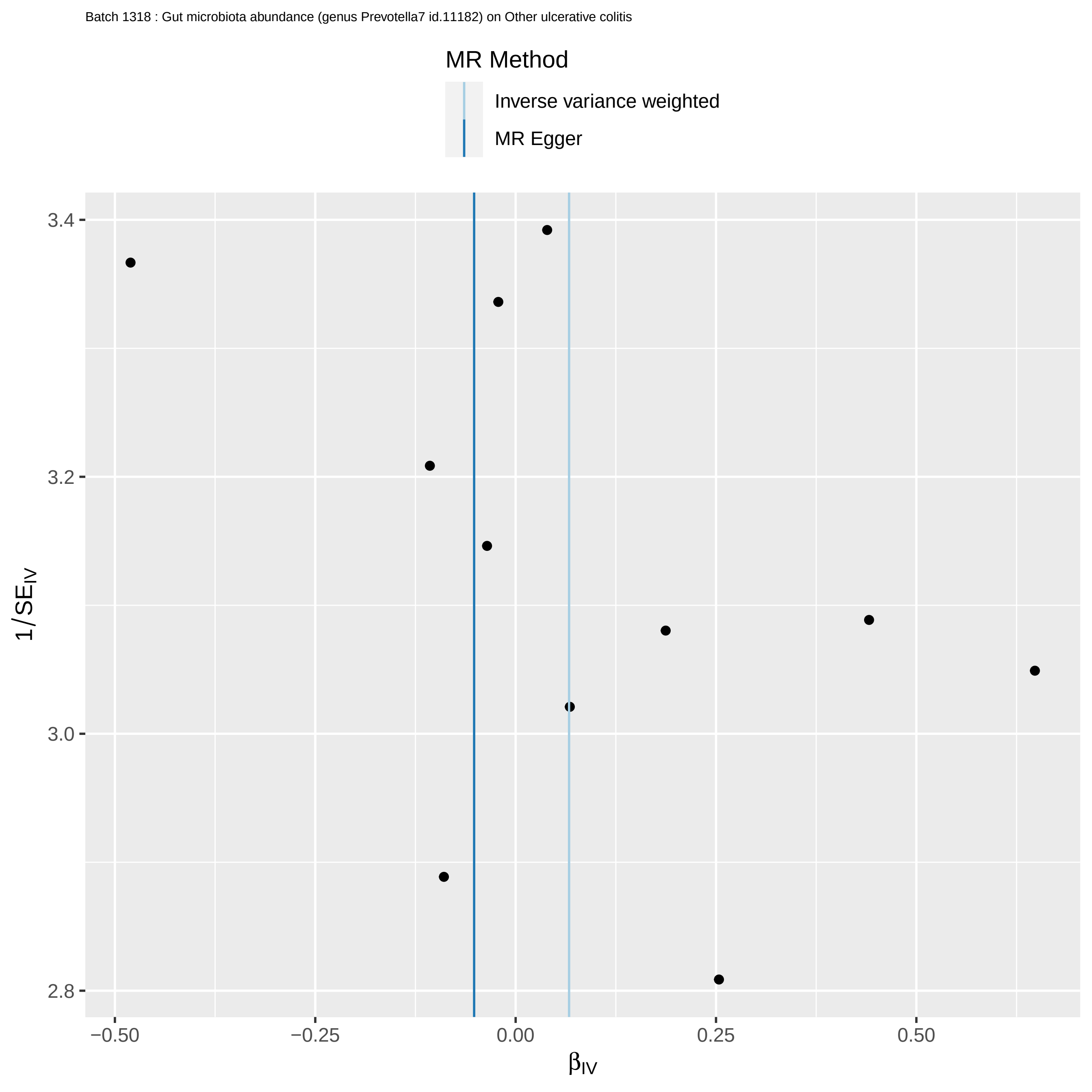

Batch 1318 : Gut microbiota abundance (genus Prevotella7 id.11182) on Other ulcerative colitis
MR Method
Inverse variance weighted
MR Egger
3.4
3.2
3.0
2.8
1/SE_IV
−0.50
−0.25
0.00
0.25
0.50
β_IV

Batch 1319 : Gut microbiota abundance (genus Prevotella9 id.11183) on Other ulcerative colitis

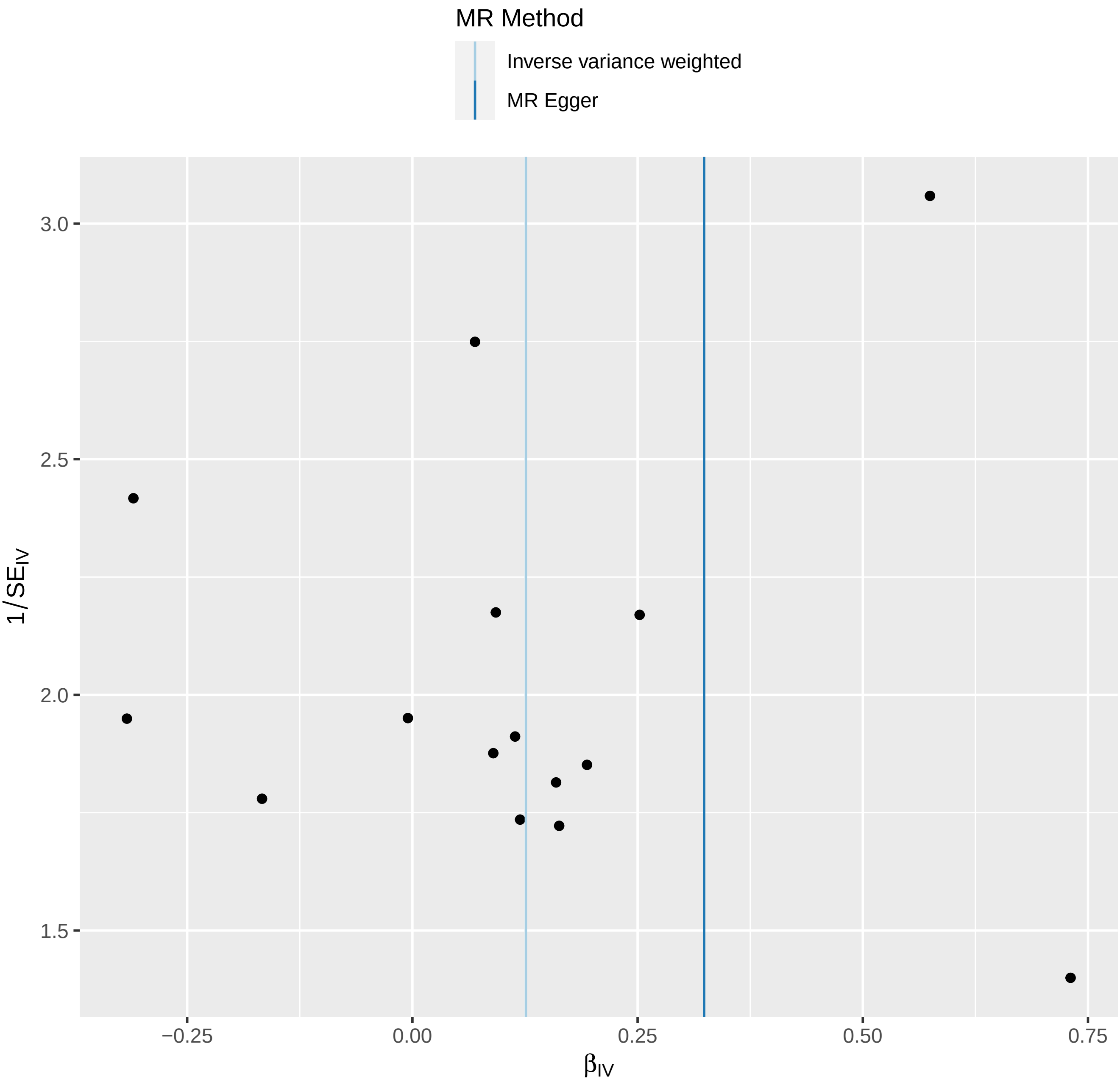

Batch 1320 : Gut microbiota abundance (genus Rikenellaceae RC9 gut group id.11191) on Other ulcerative colitis

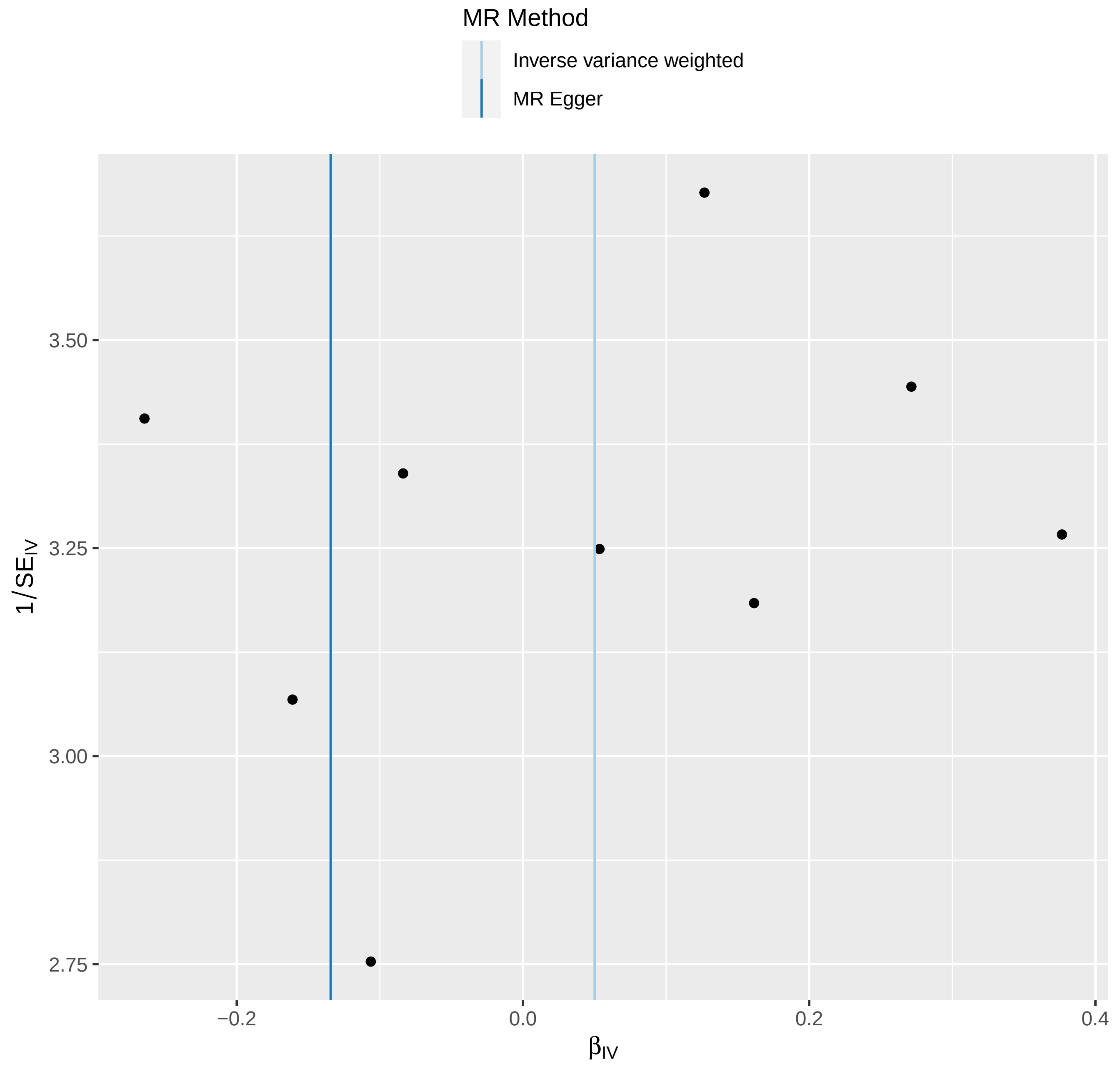

Batch 1321 : Gut microbiota abundance (genus Romboutsia id.11347) on Other ulcerative colitis

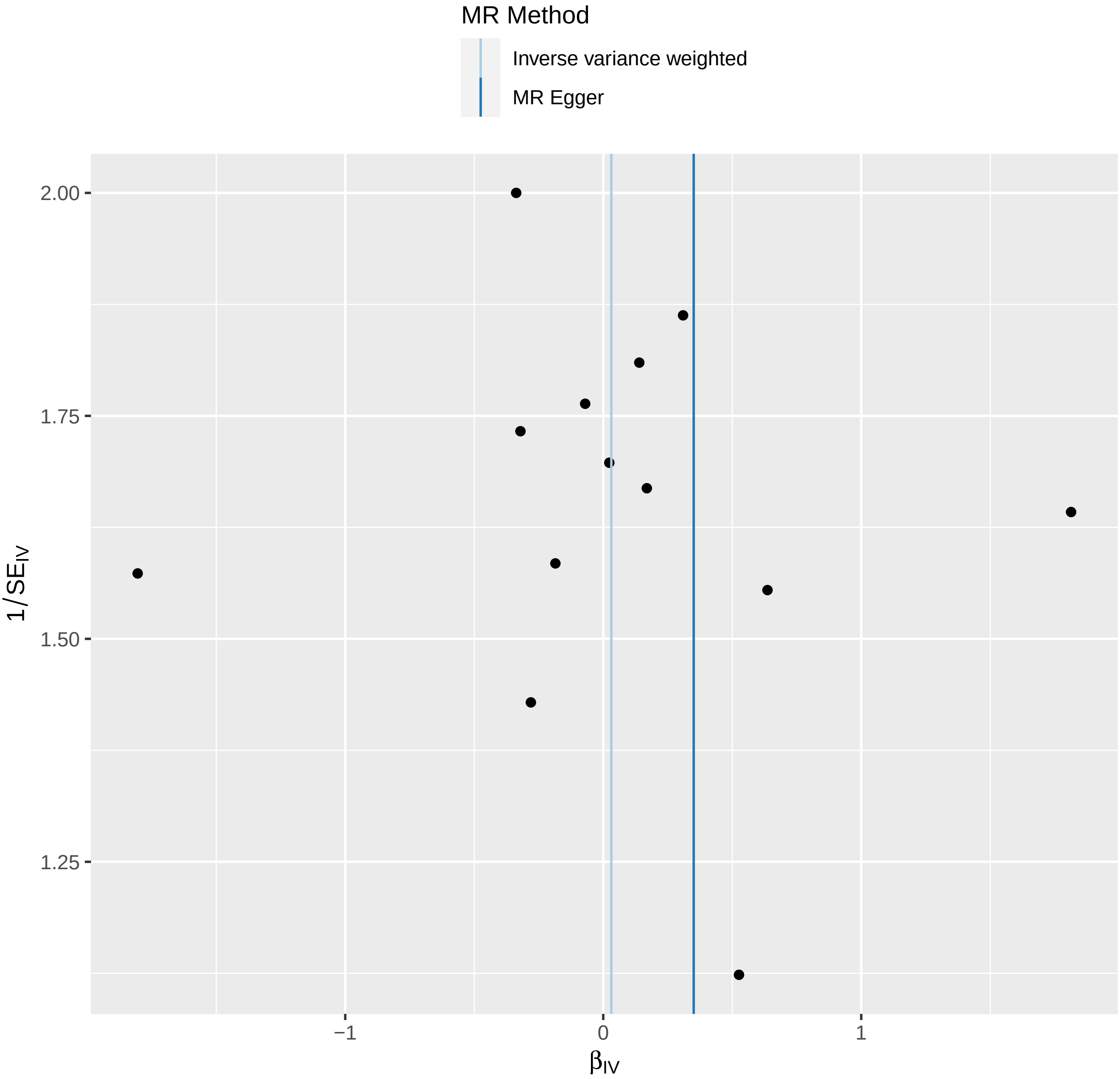

Batch 1322 : Gut microbiota abundance (genus Roseburia id.2012) on Other ulcerative colitis

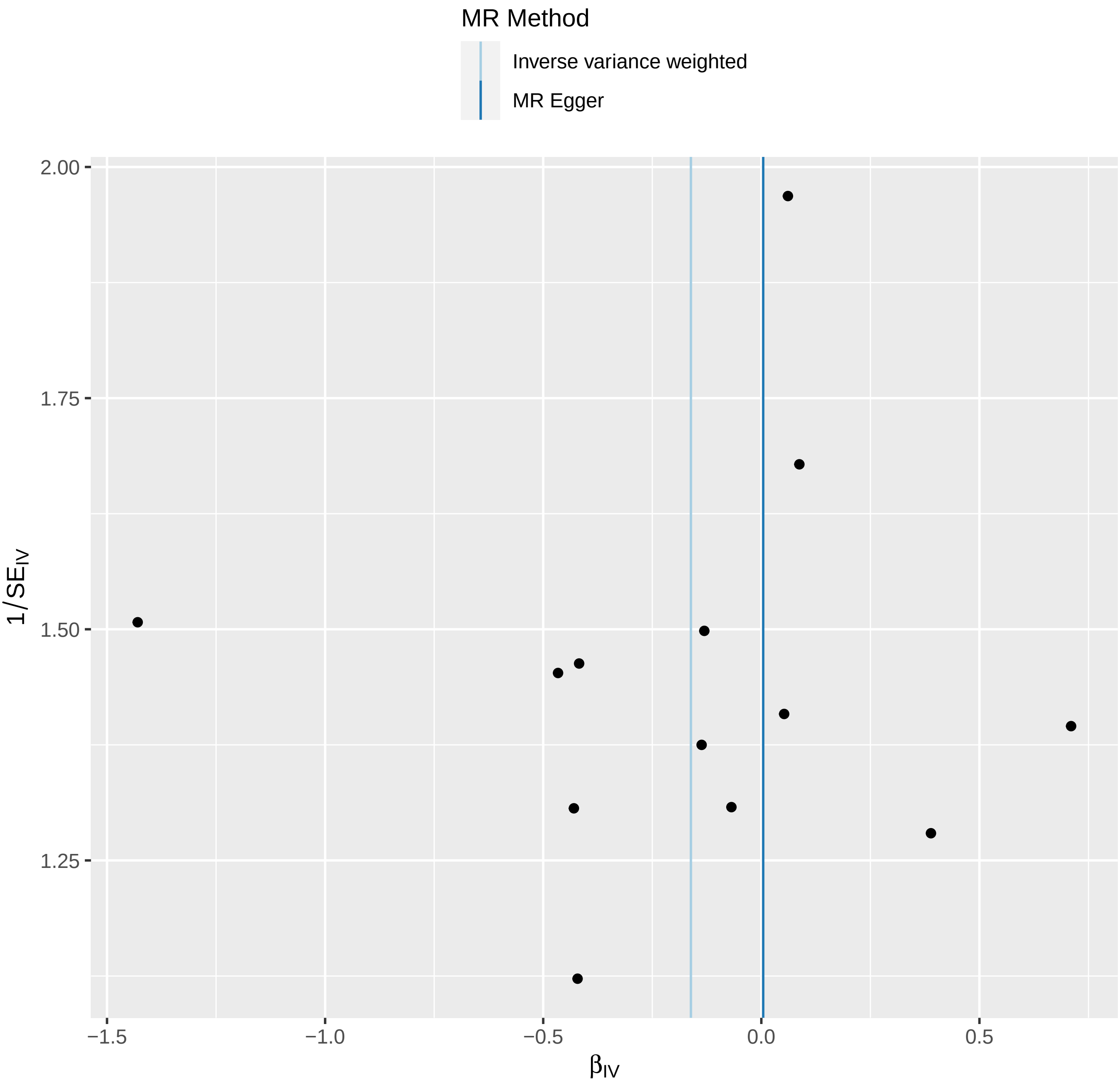

Batch 1323 : Gut microbiota abundance (genus Ruminiclostridium5 id.11355) on Other ulcerative colitis

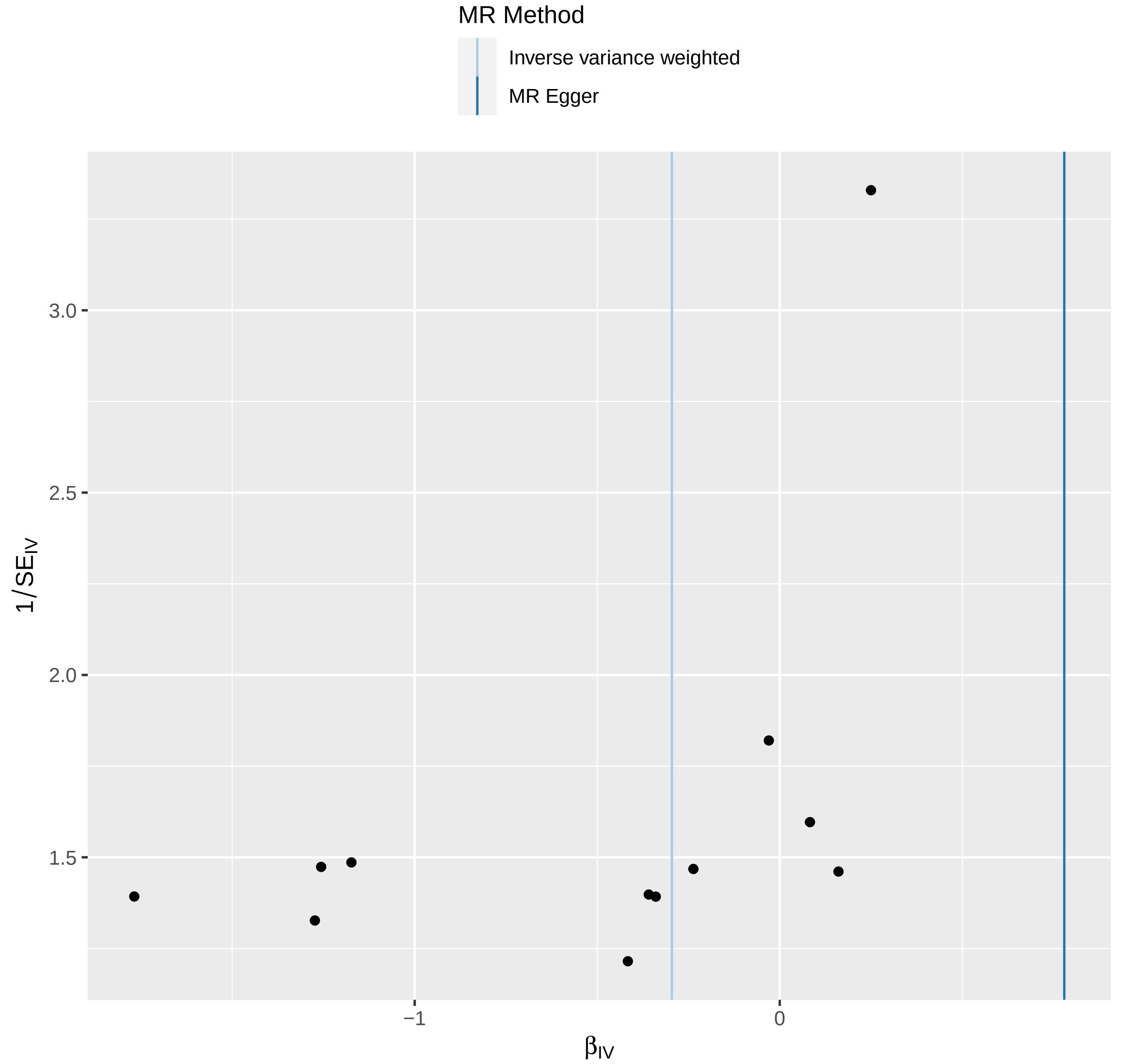

Batch 1324 : Gut microbiota abundance (genus Ruminiclostridium6 id.11356) on Other ulcerative colitis

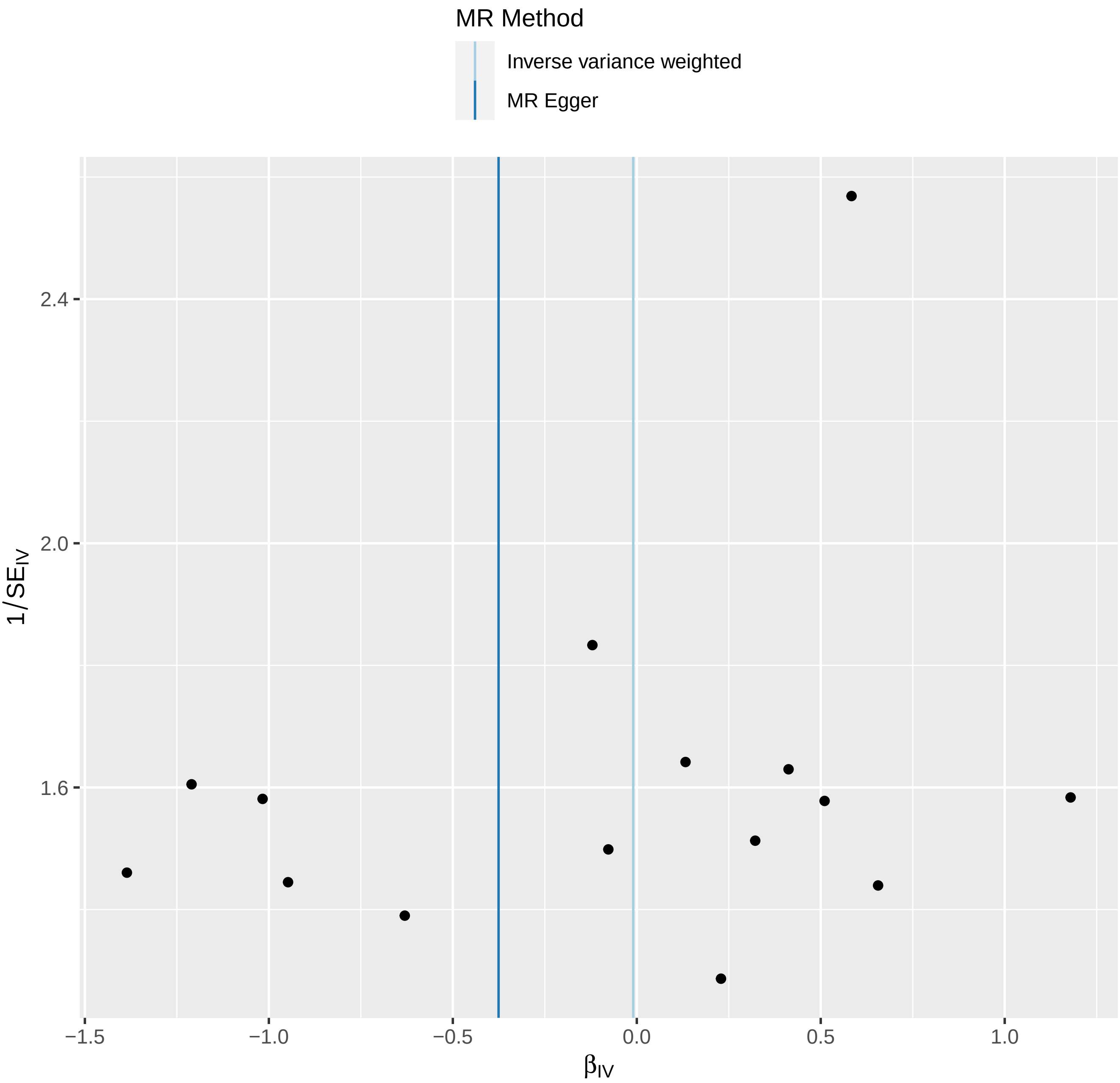

Batch 1325 : Gut microbiota abundance (genus Ruminiclostridium9 id.11357) on Other ulcerative colitis

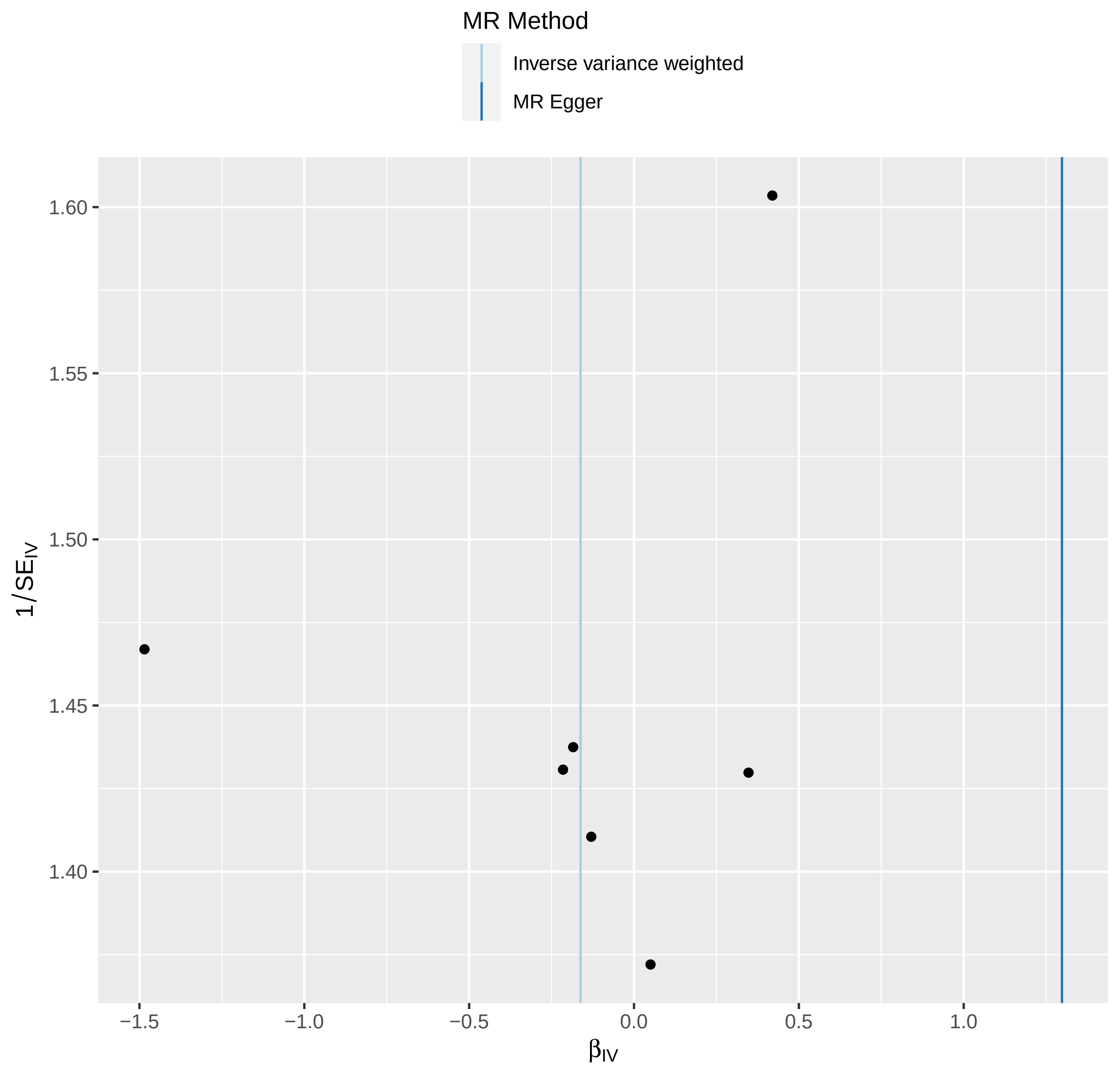

Batch 1326 : Gut microbiota abundance (genus Ruminococcaceae NK4A214 group id.11358) on Other ulcerative colitis

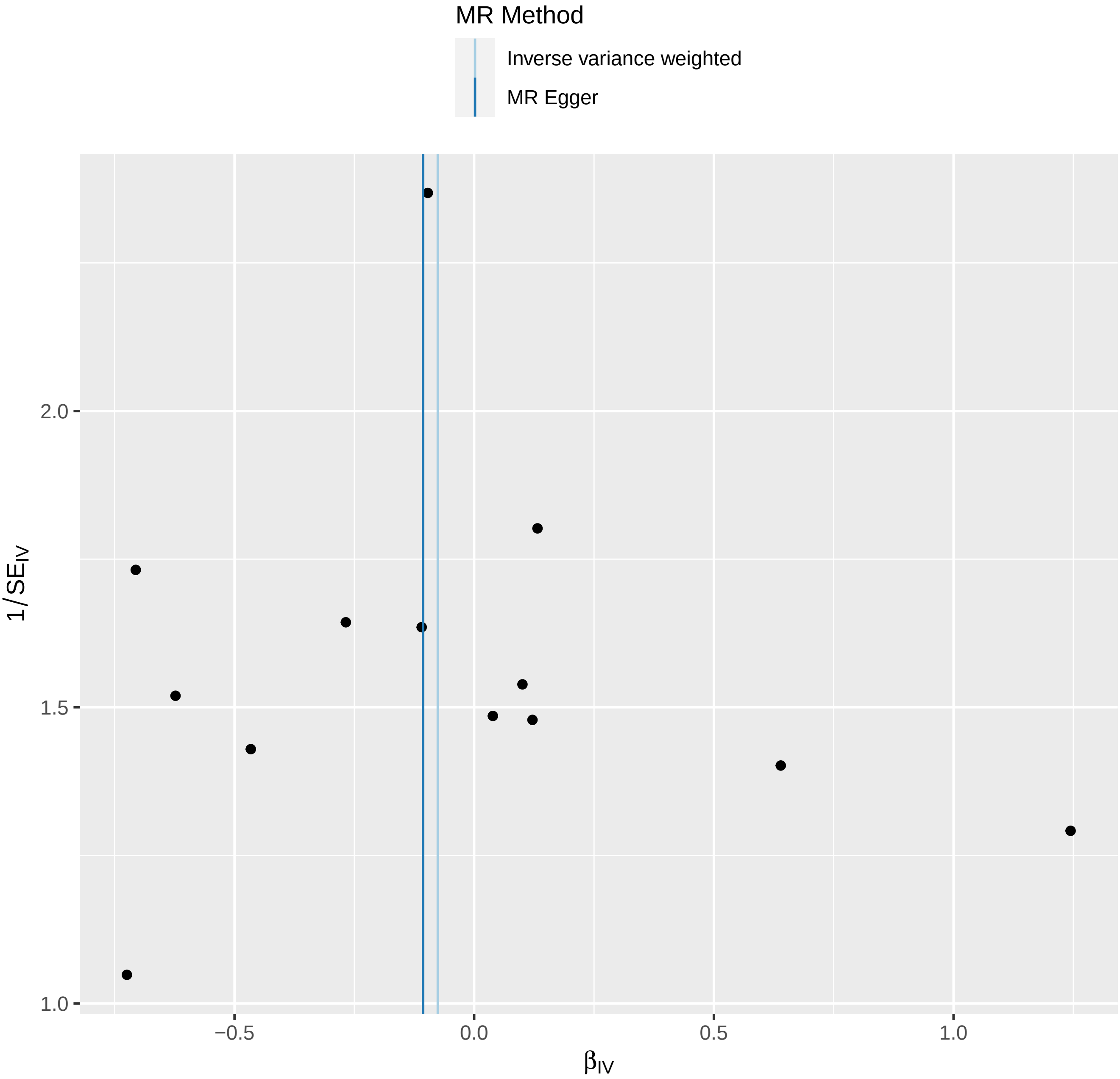

Batch 1327 : Gut microbiota abundance (genus Ruminococcaceae UCG002 id.11360) on Other ulcerative colitis

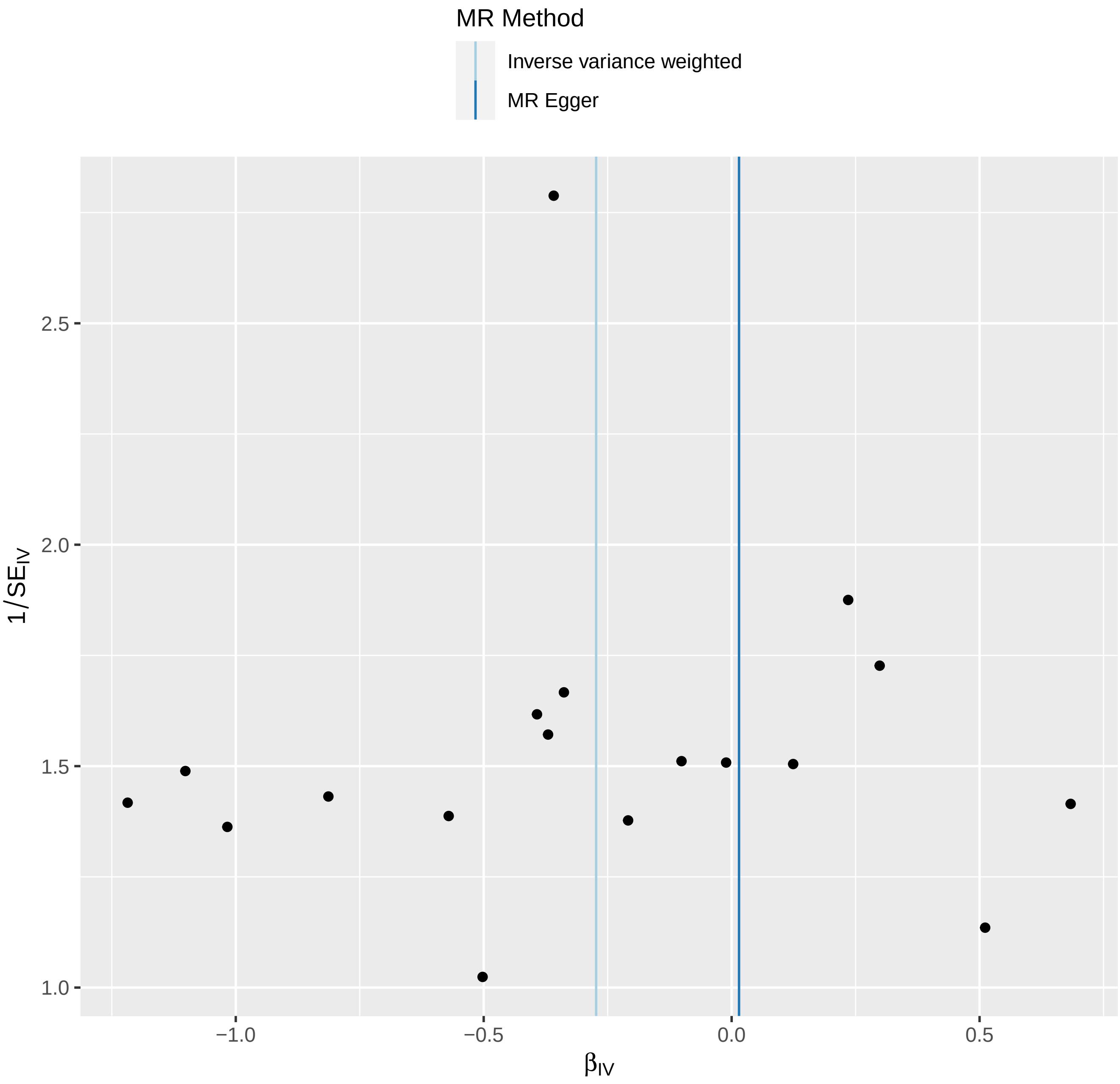

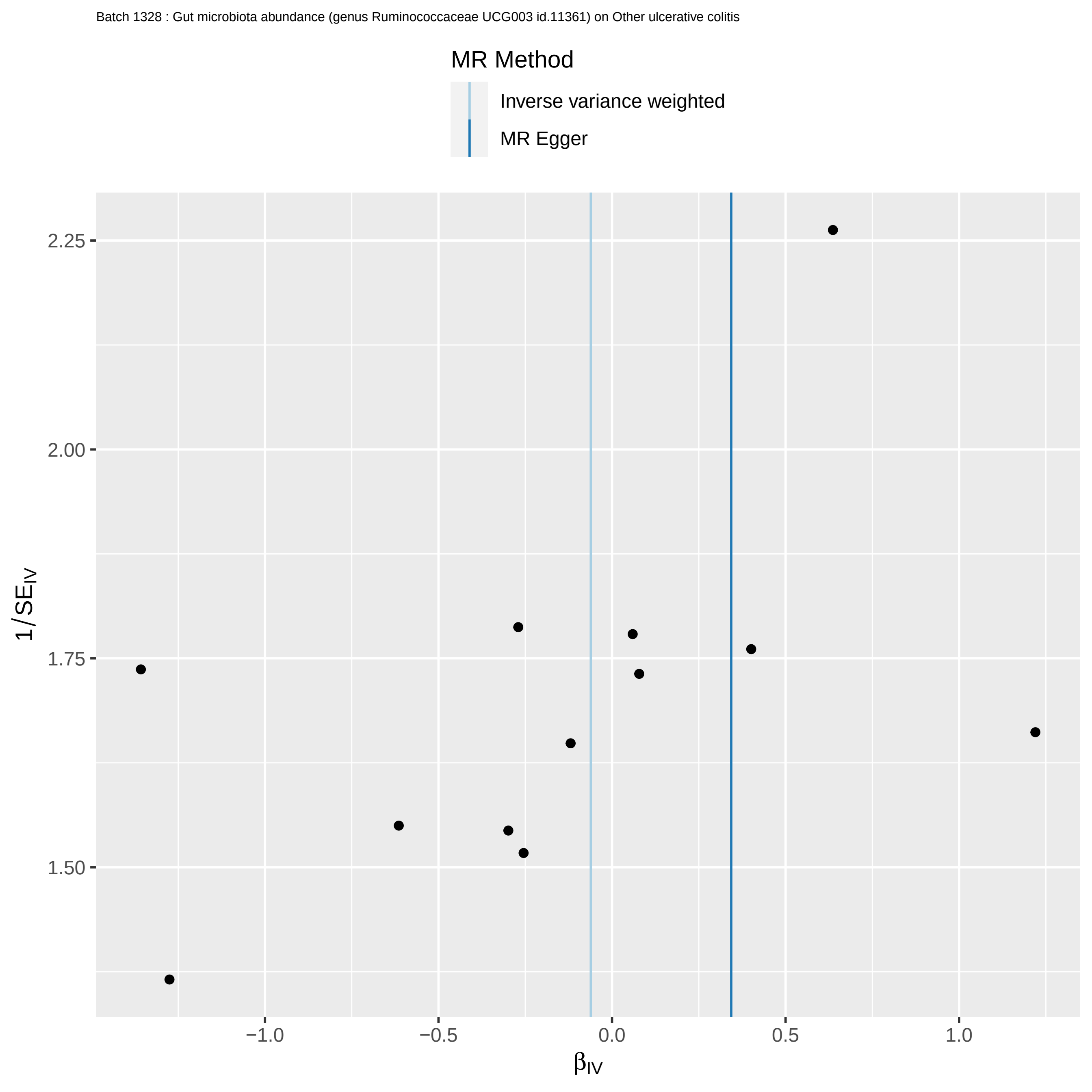

Batch 1328 : Gut microbiota abundance (genus Ruminococcaceae UCG003 id.11361) on Other ulcerative colitis
MR Method
Inverse variance weighted
MR Egger
2.25
2.00
1.75
1.50
1/SE_IV
−1.0
−0.5
0.0
0.5
1.0
β_IV

Batch 1329 : Gut microbiota abundance (genus Ruminococcaceae UCG004 id.11362) on Other ulcerative colitis

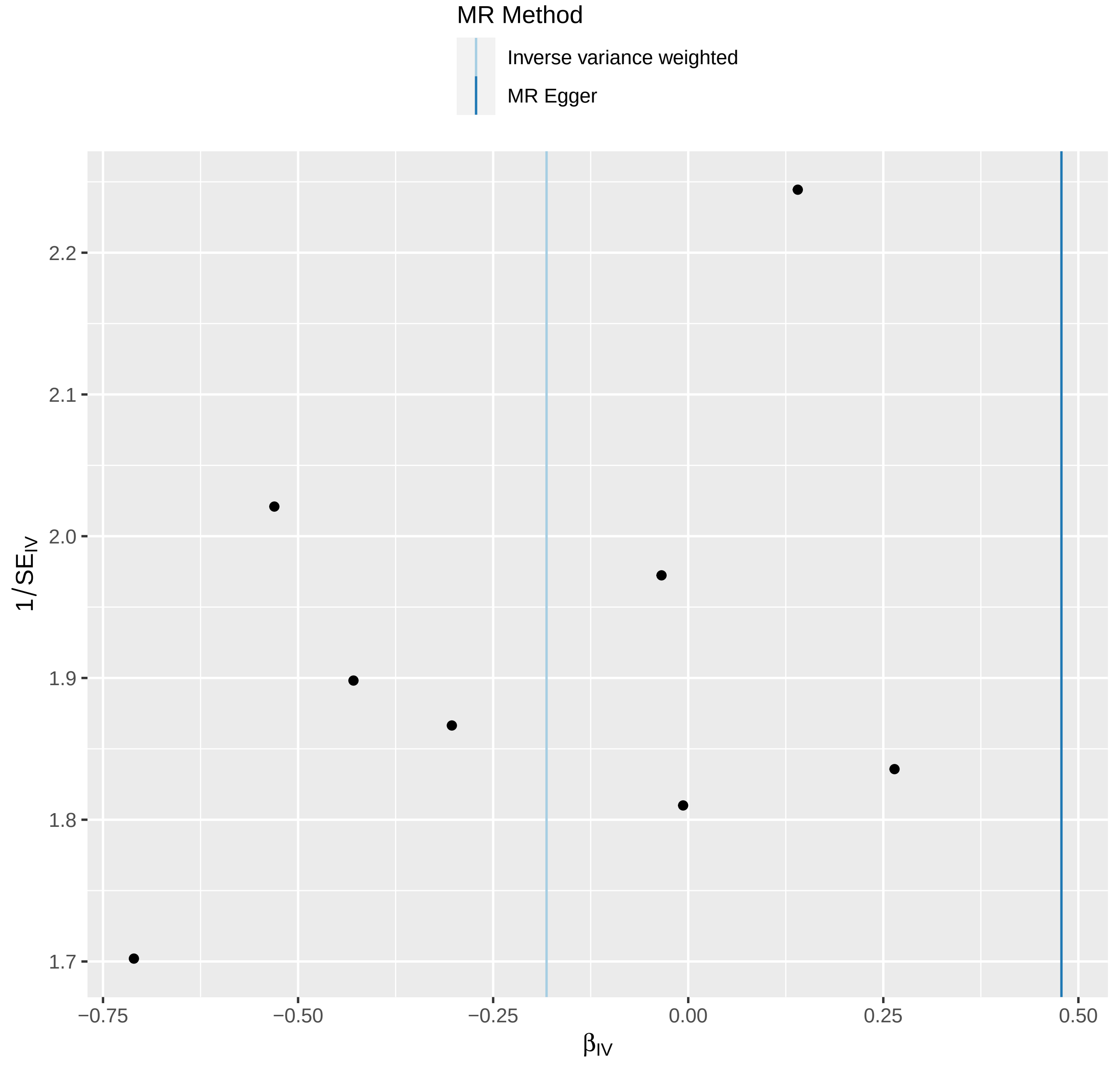

Batch 1330 : Gut microbiota abundance (genus Ruminococcaceae UCG005 id.11363) on Other ulcerative colitis

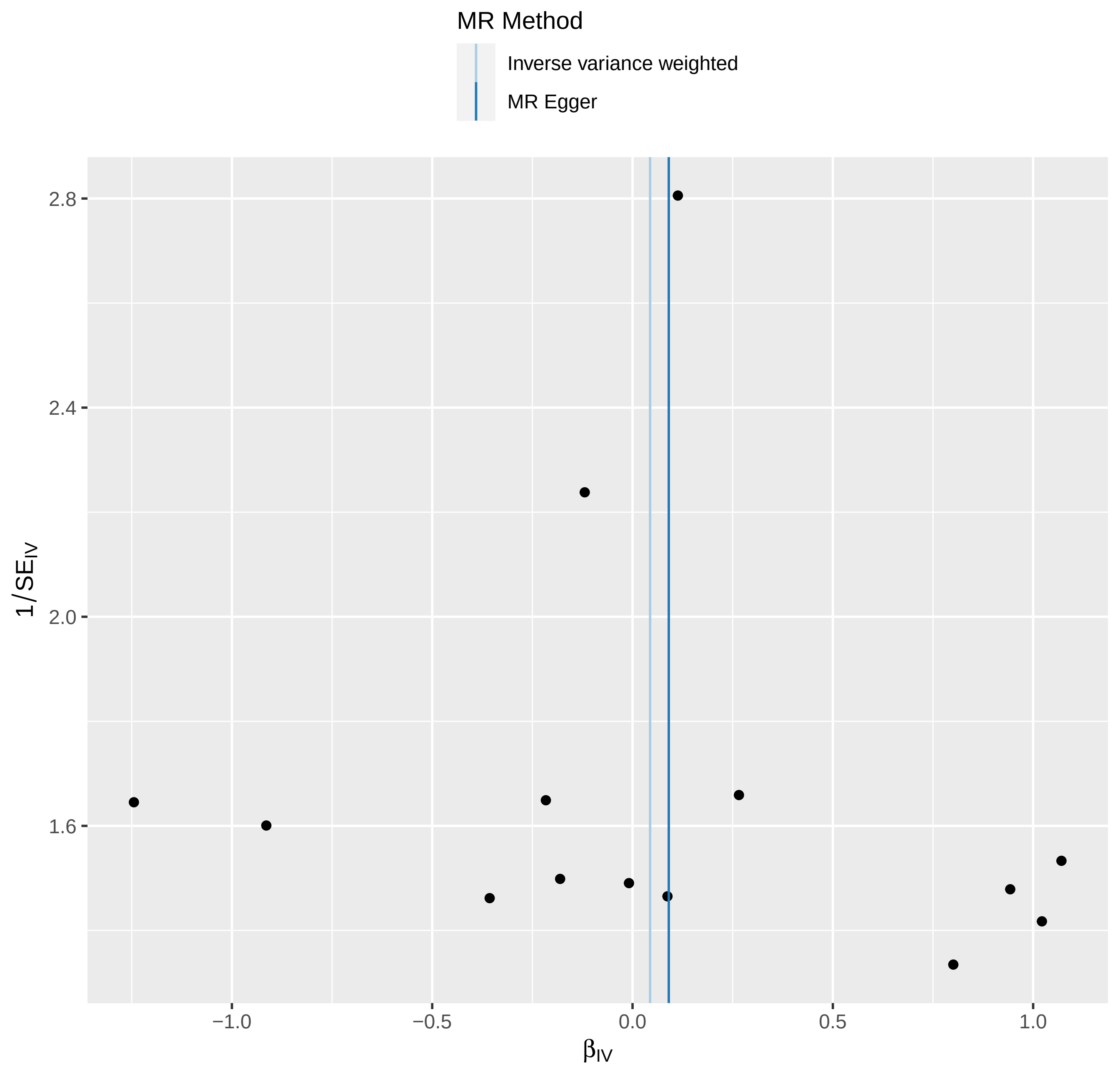

Batch 1331 : Gut microbiota abundance (genus Ruminococcaceae UCG009 id.11366) on Other ulcerative colitis

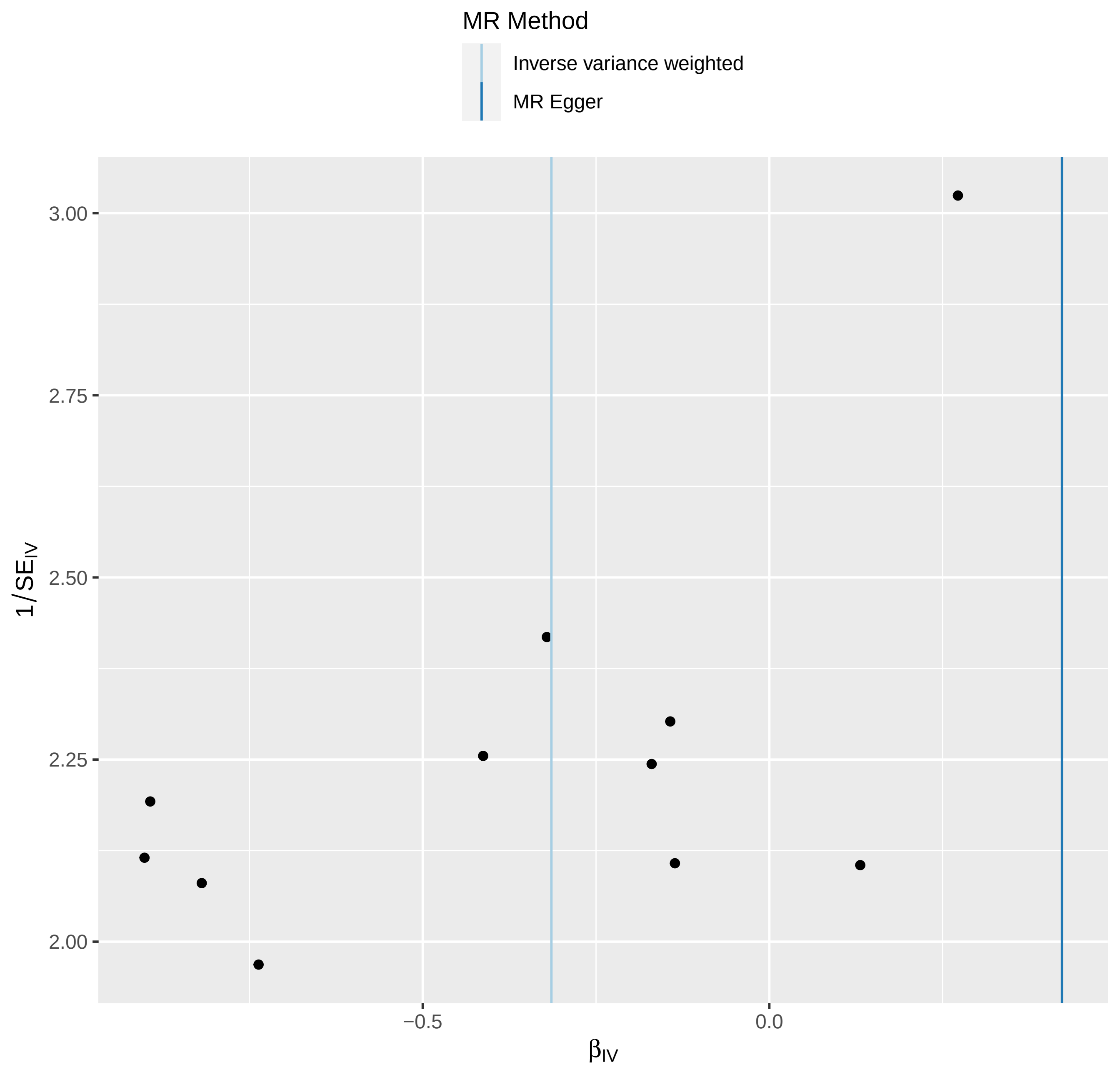

Batch 1332 : Gut microbiota abundance (genus Ruminococcaceae UCG010 id.11367) on Other ulcerative colitis

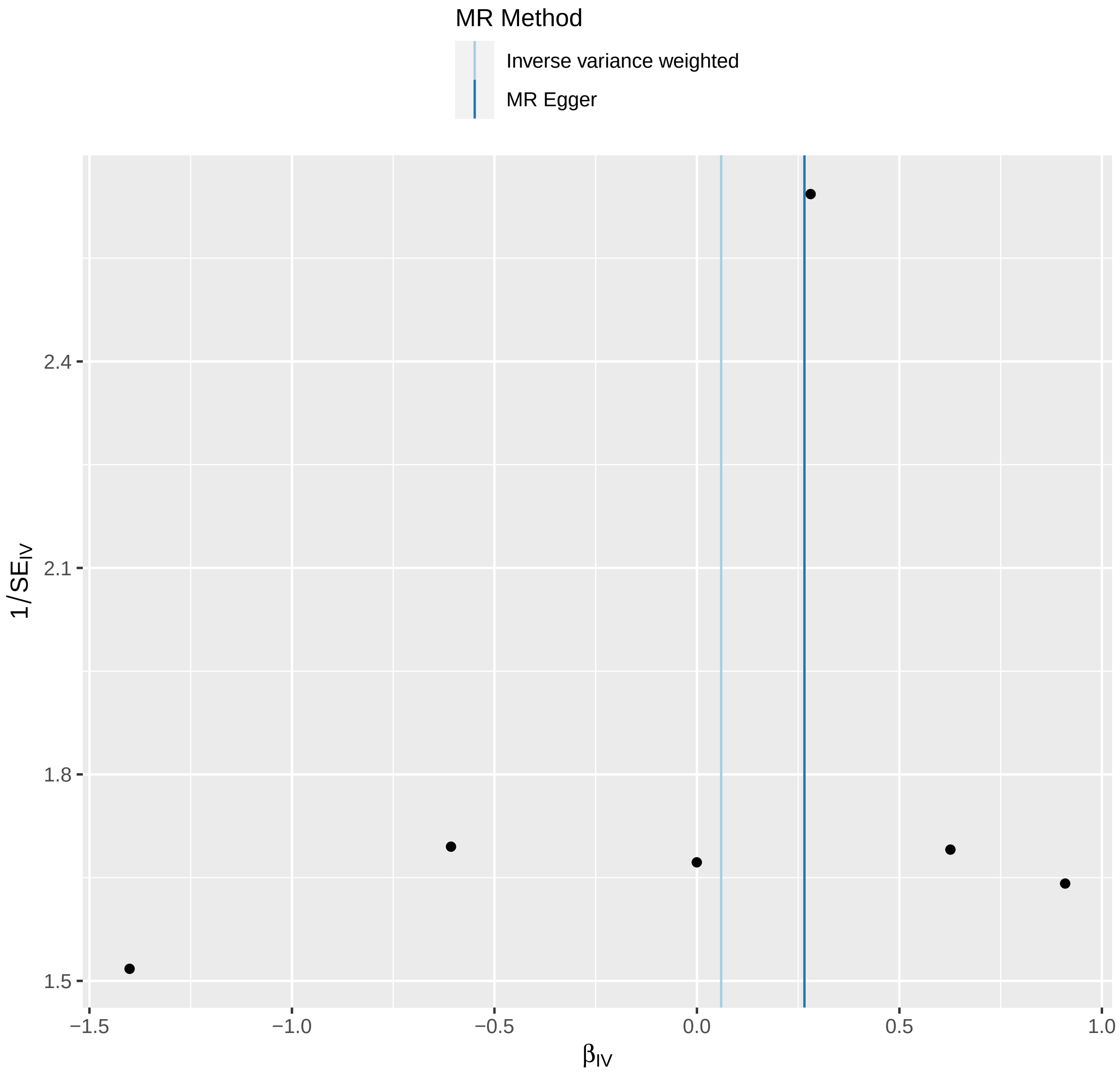

Batch 1333 : Gut microbiota abundance (genus Ruminococcaceae UCG011 id.11368) on Other ulcerative colitis

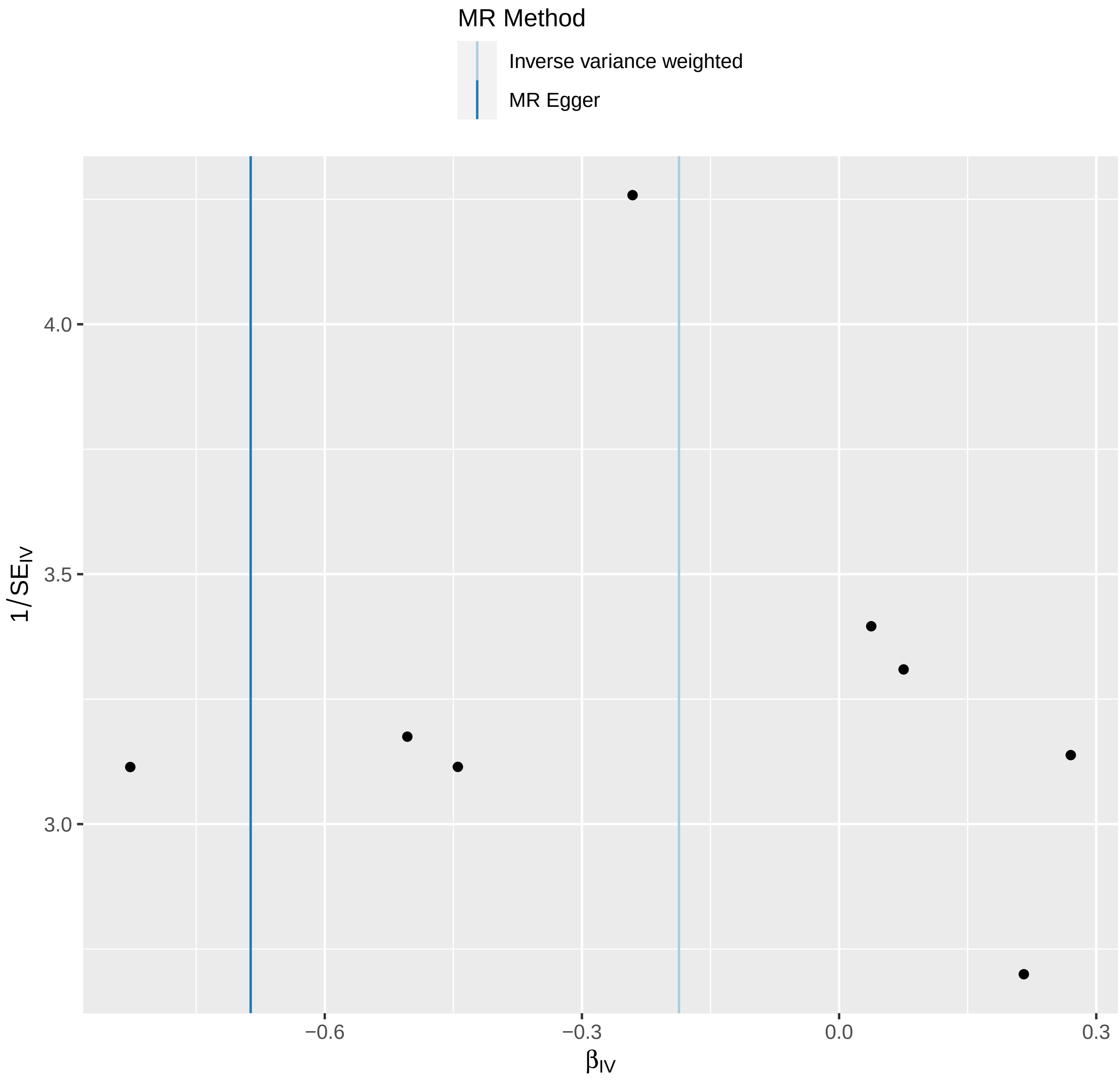

Batch 1334 : Gut microbiota abundance (genus Ruminococcaceae UCG013 id.11370) on Other ulcerative colitis

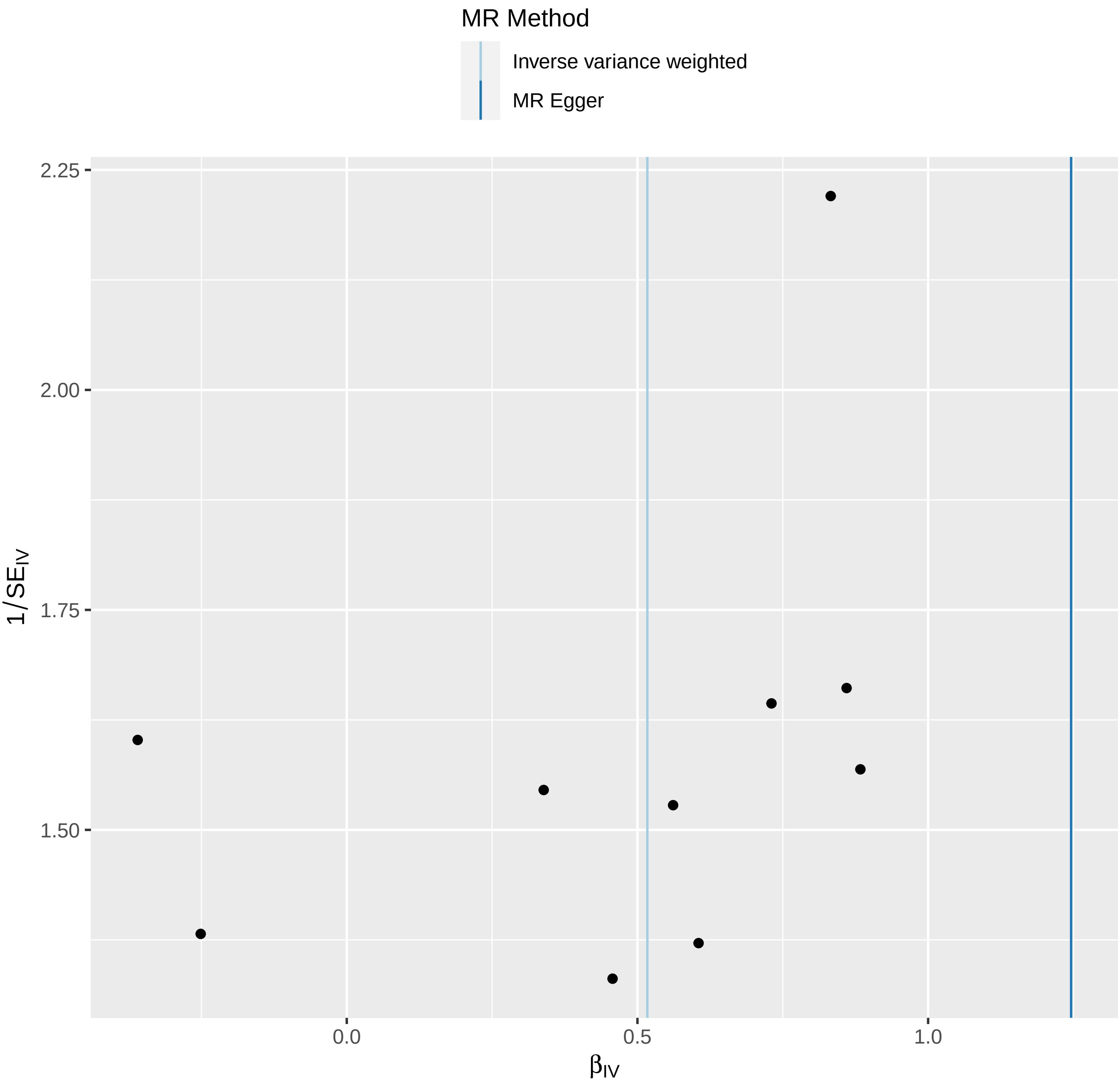

Batch 1335 : Gut microbiota abundance (genus Ruminococcaceae UCG014 id.11371) on Other ulcerative colitis

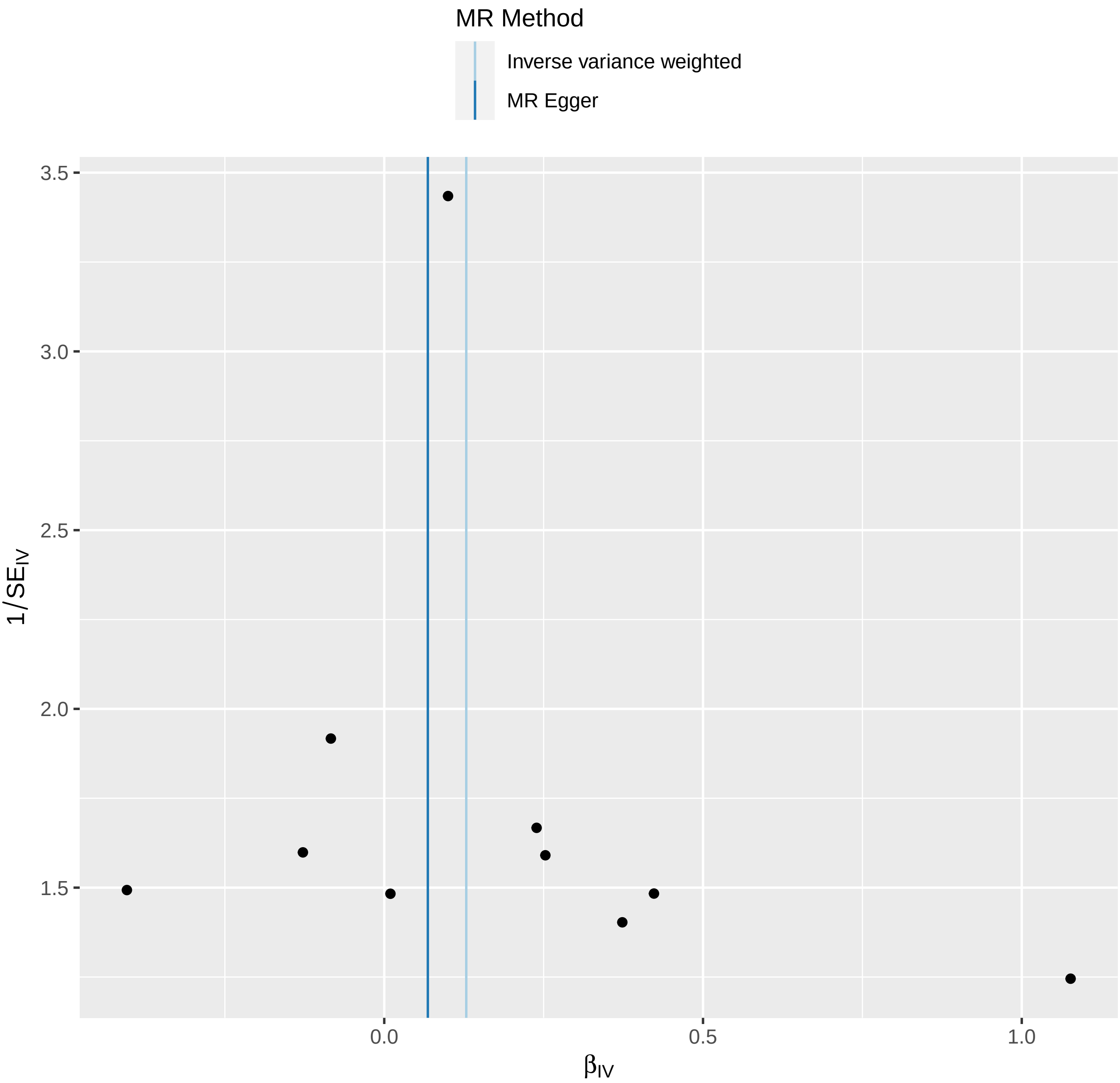

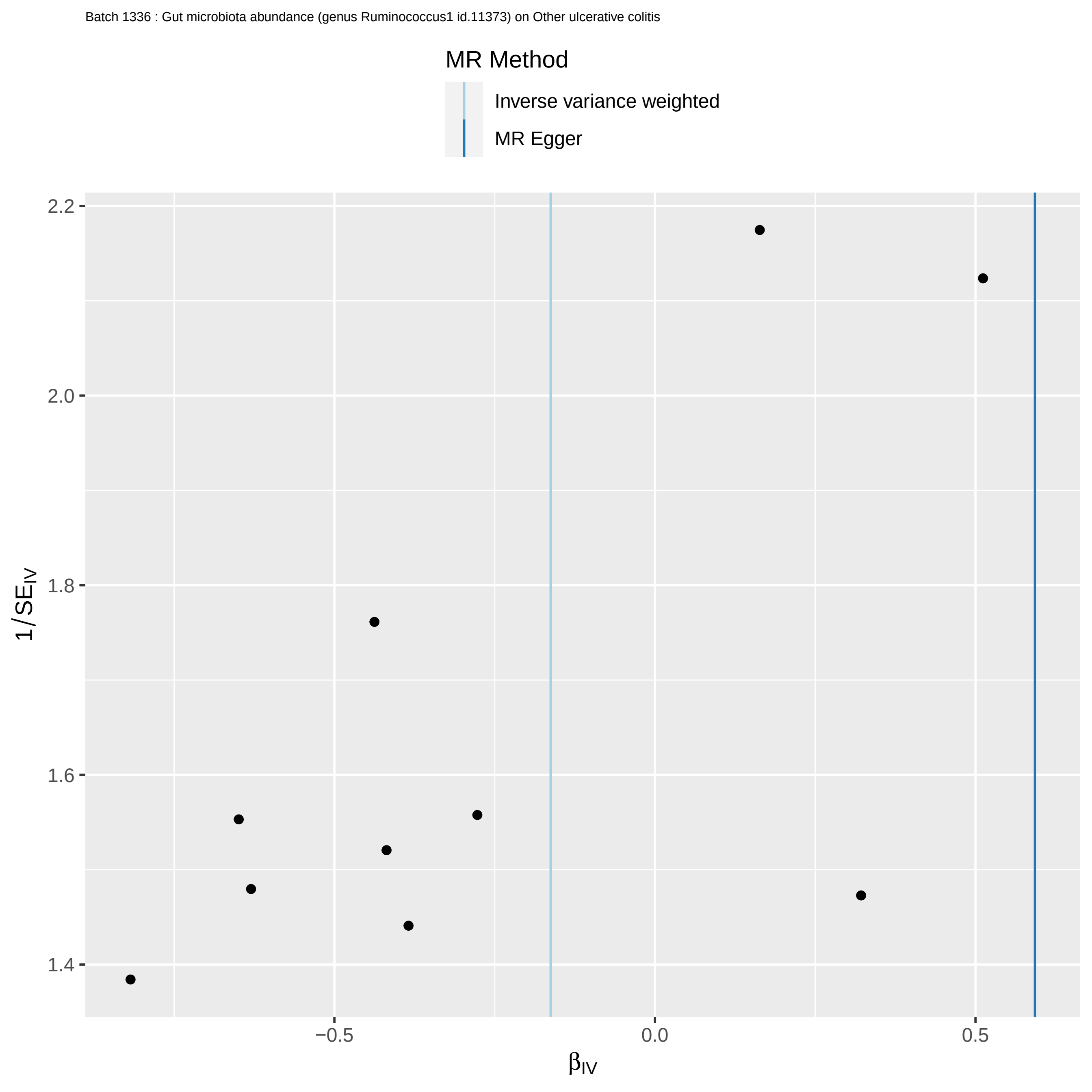
Batch 1336 : Gut microbiota abundance (genus Ruminococcus1 id.11373) on Other ulcerative colitis
MR Method
Inverse variance weighted
MR Egger
2.2
2.0
1.8
1.6
1.4
1/SE_IV
−0.5
0.0
0.5
β_IV

Batch 1337 : Gut microbiota abundance (genus Ruminococcus2 id.11374) on Other ulcerative colitis

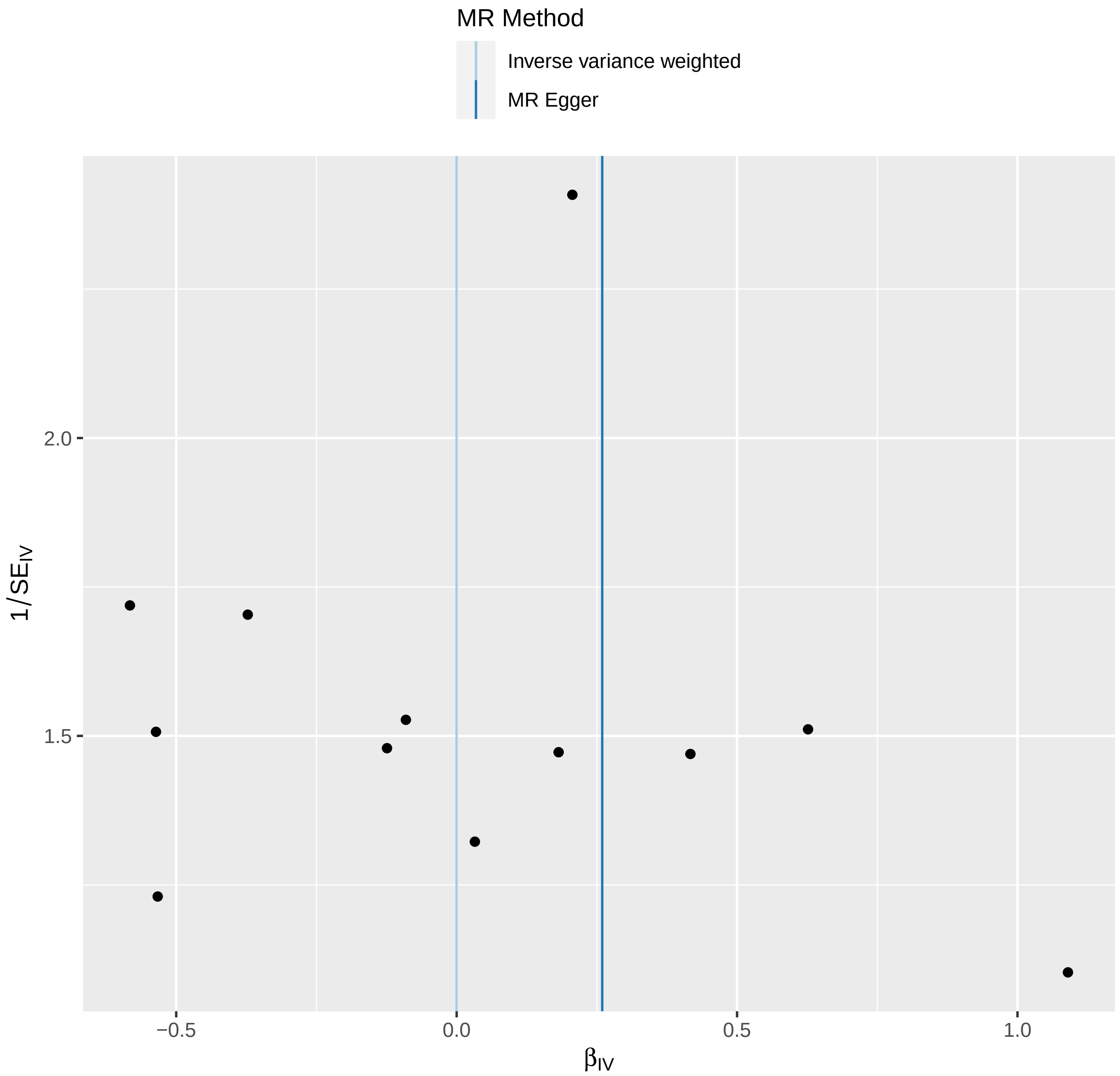

Batch 1338 : Gut microbiota abundance (genus Ruminococcus gauvreauii group id.11342) on Other ulcerative colitis

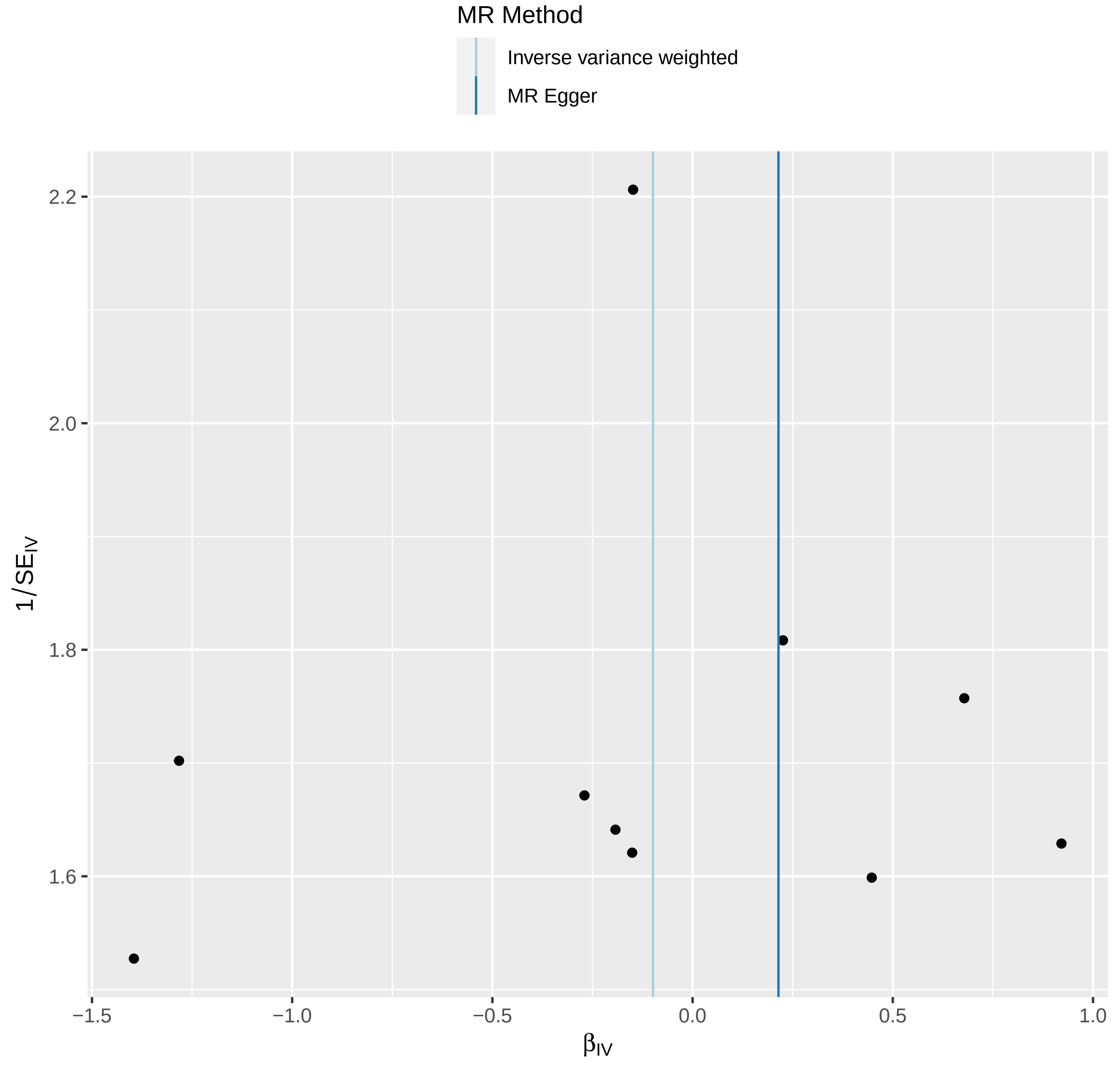

Batch 1339 : Gut microbiota abundance (genus Ruminococcus gnavus group id.14376) on Other ulcerative colitis

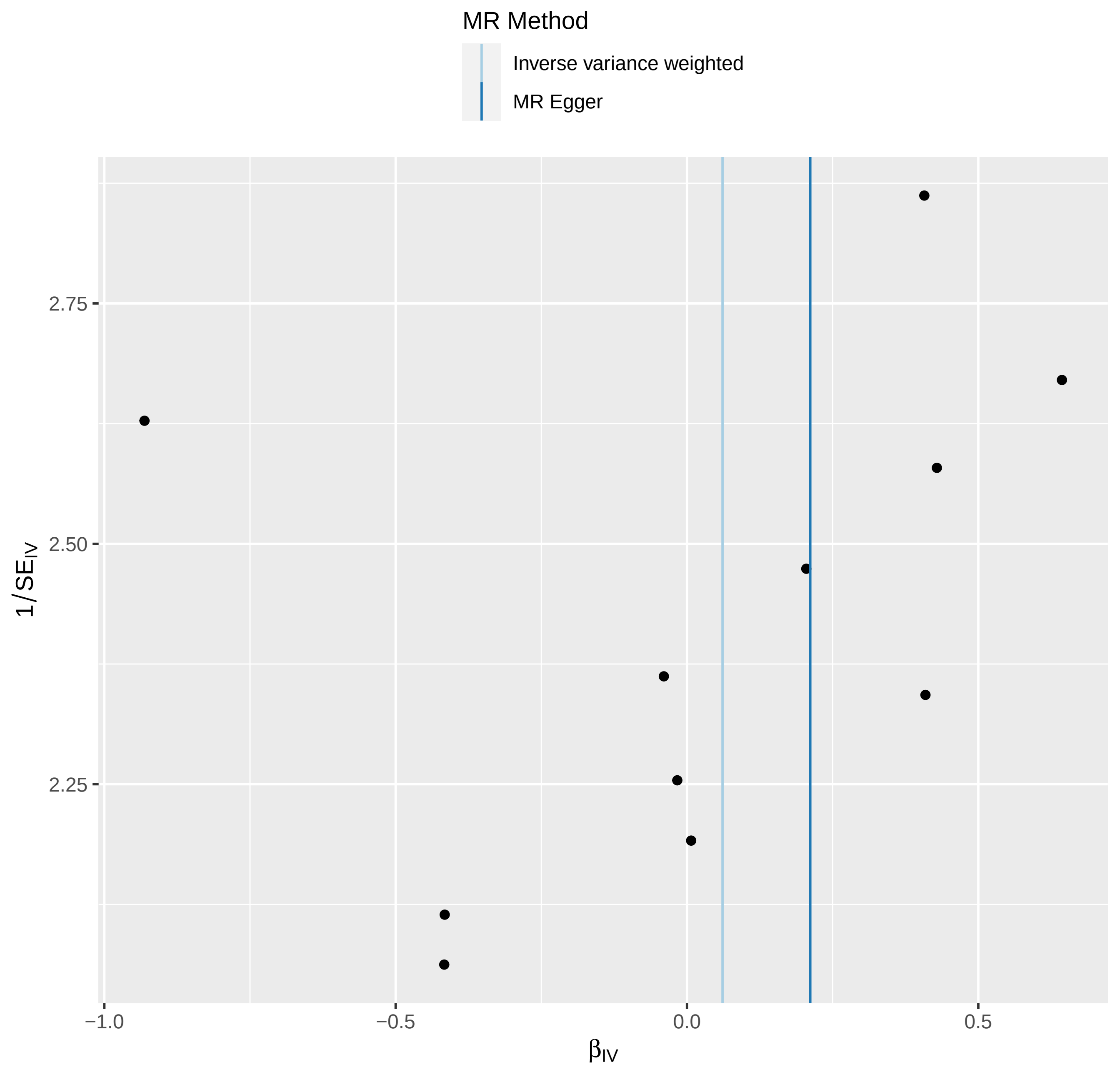

Batch 1340 : Gut microbiota abundance (genus Ruminococcus torques group id.14377) on Other ulcerative colitis

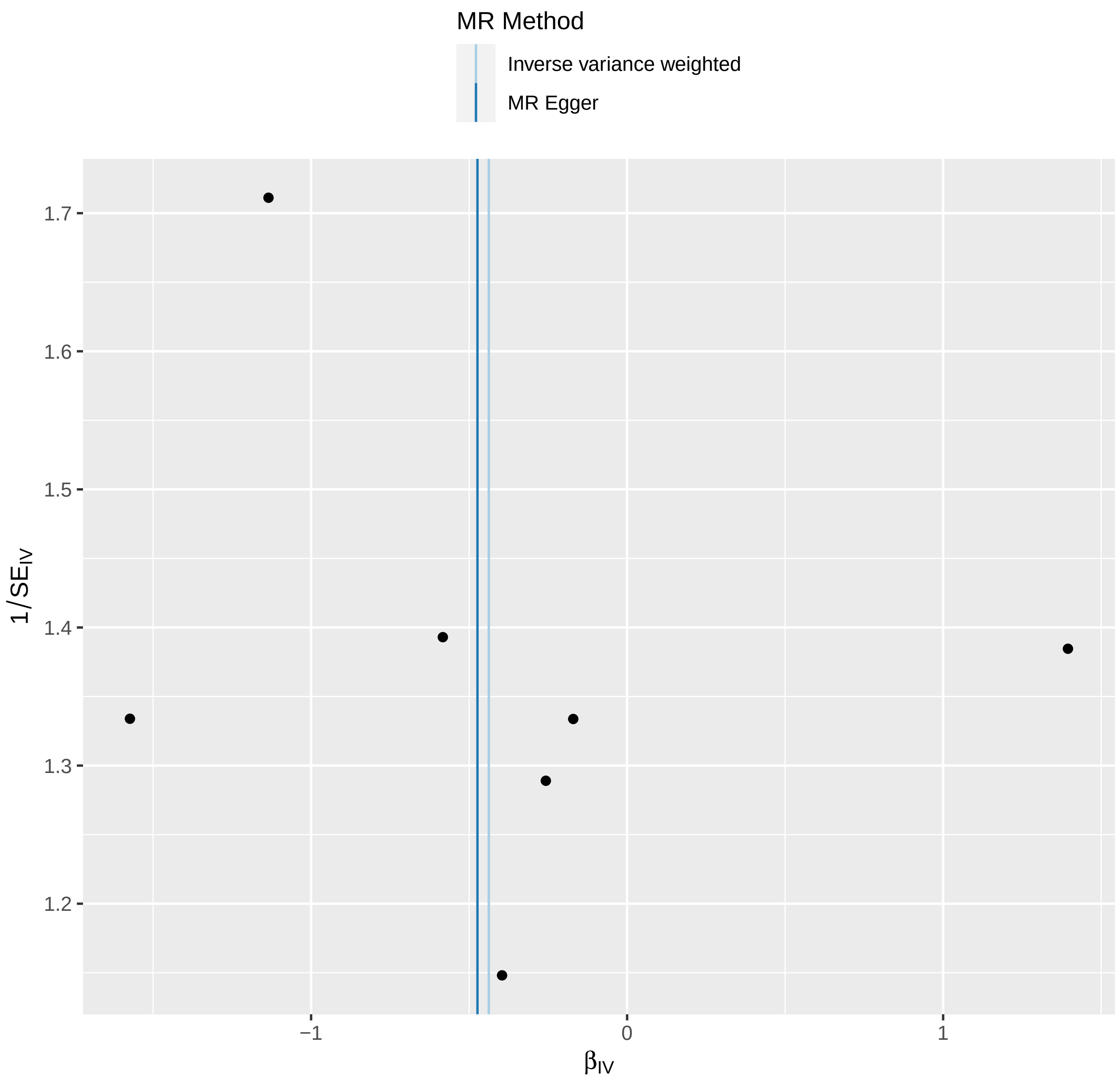

Batch 1341 : Gut microbiota abundance (genus Sellimonas id.14369) on Other ulcerative colitis

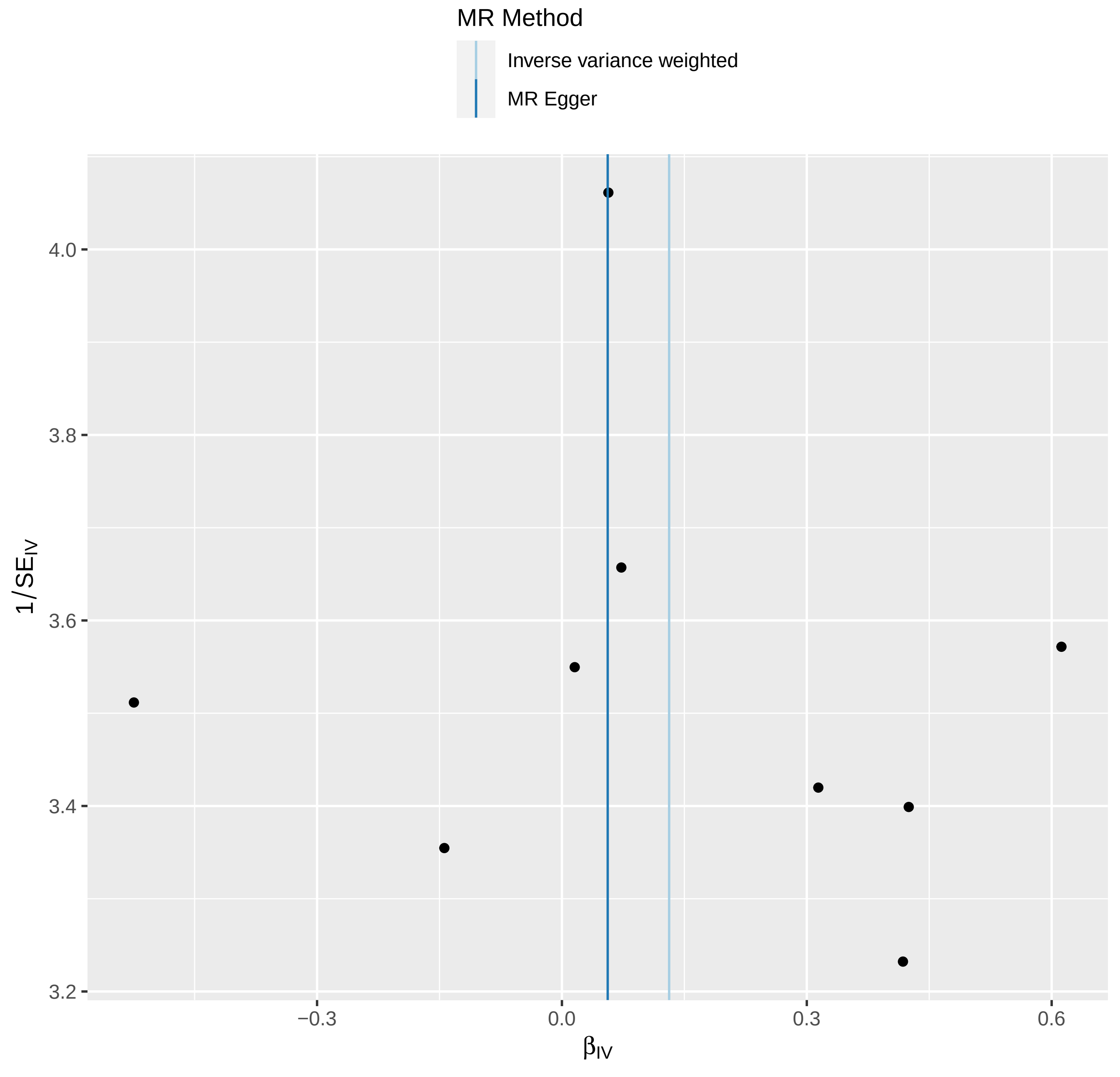

Batch 1342 : Gut microbiota abundance (genus Senegalimassilia id.11160) on Other ulcerative colitis

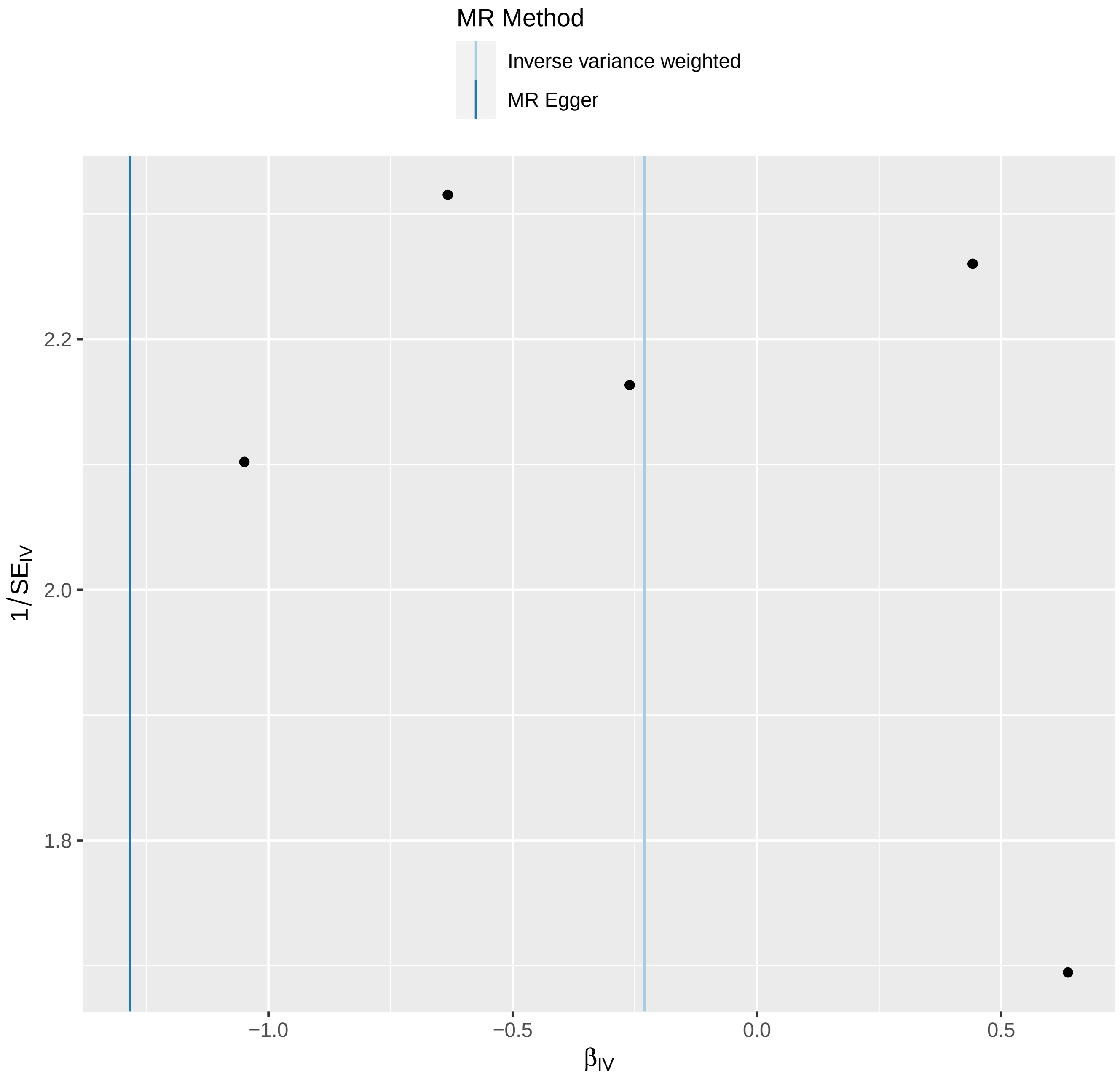

Batch 1343 : Gut microbiota abundance (genus Slackia id.825) on Other ulcerative colitis

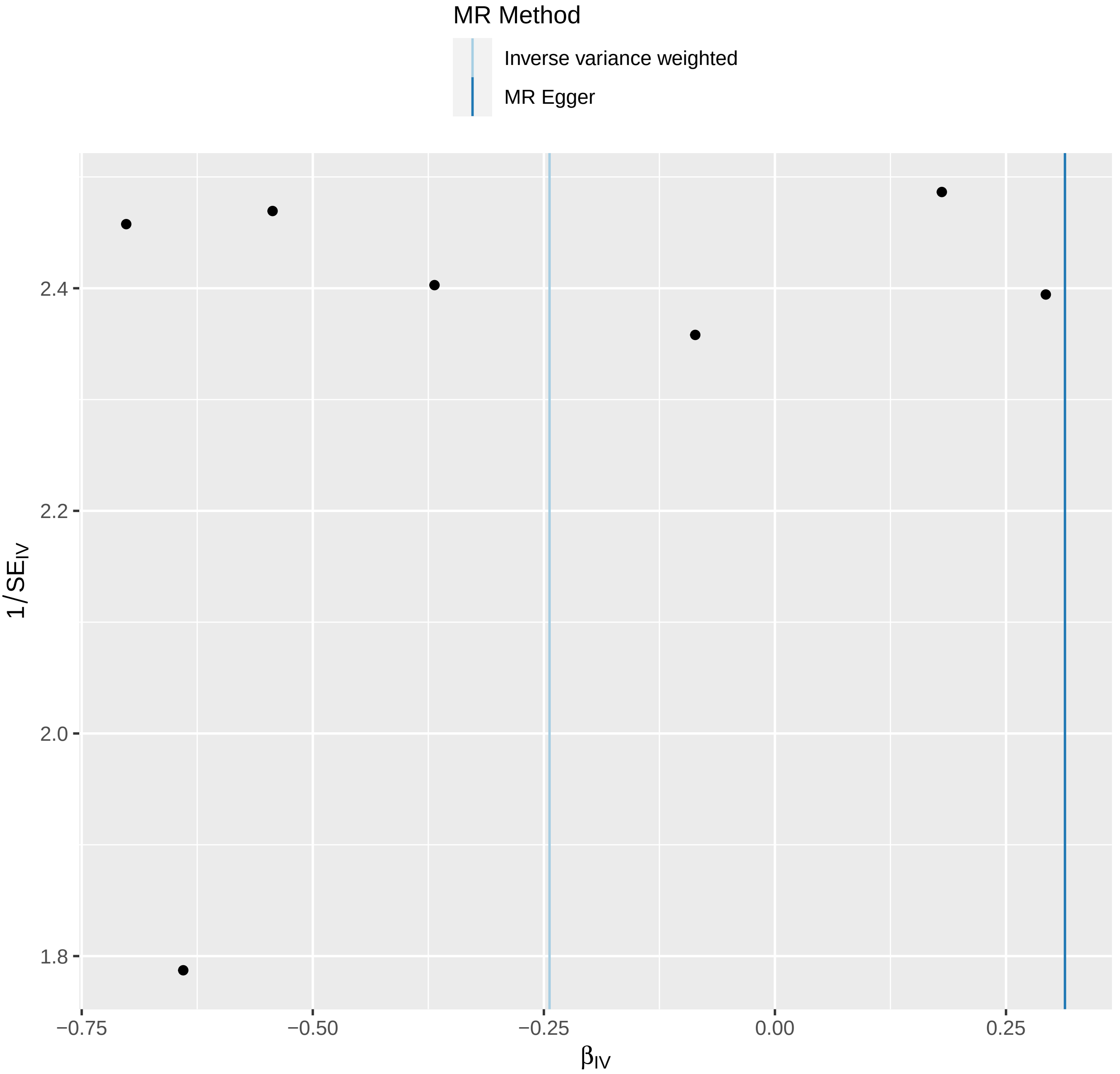

Batch 1344 : Gut microbiota abundance (genus Streptococcus id.1853) on Other ulcerative colitis

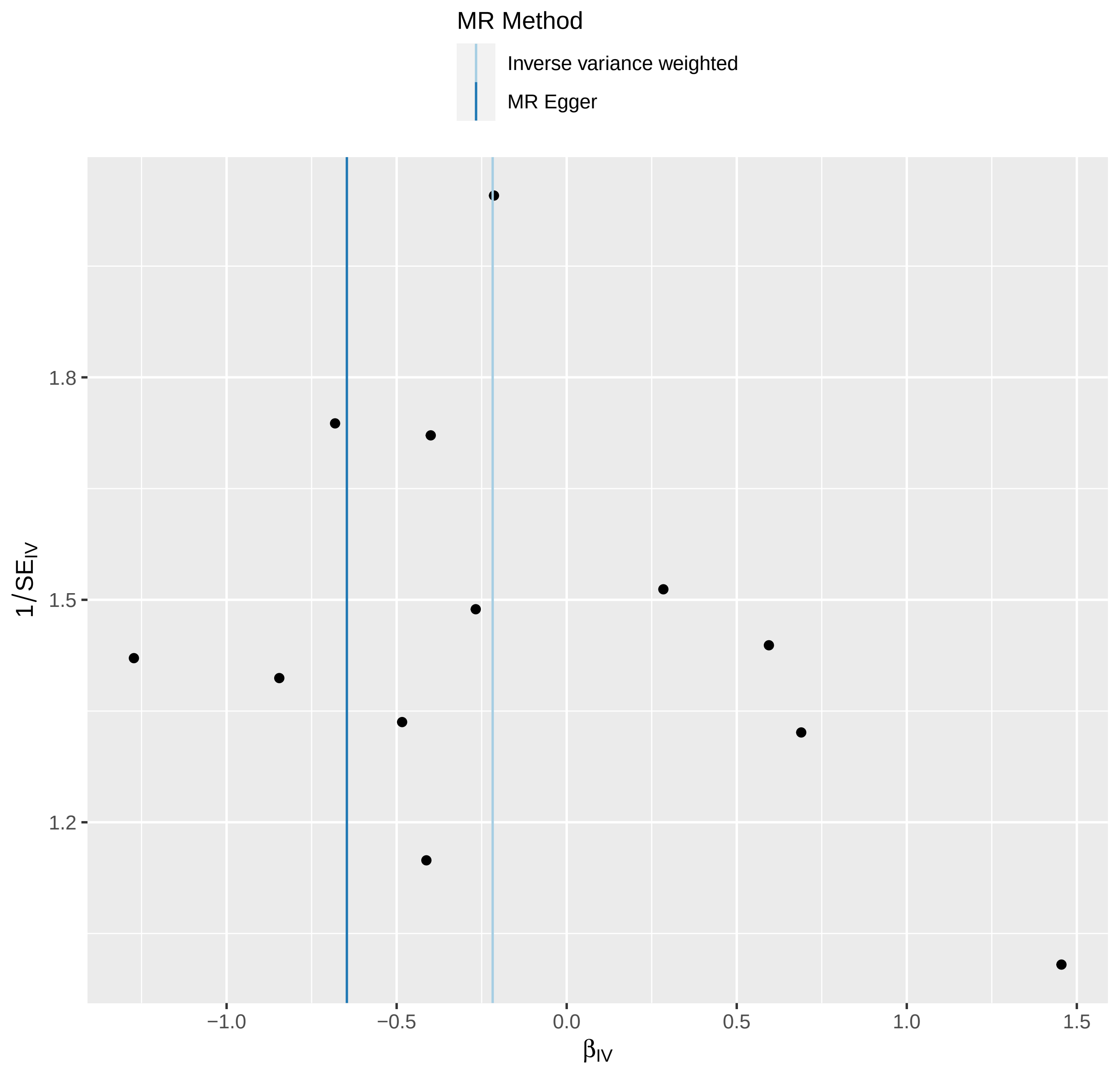

Batch 1345 : Gut microbiota abundance (genus Subdoligranulum id.2070) on Other ulcerative colitis

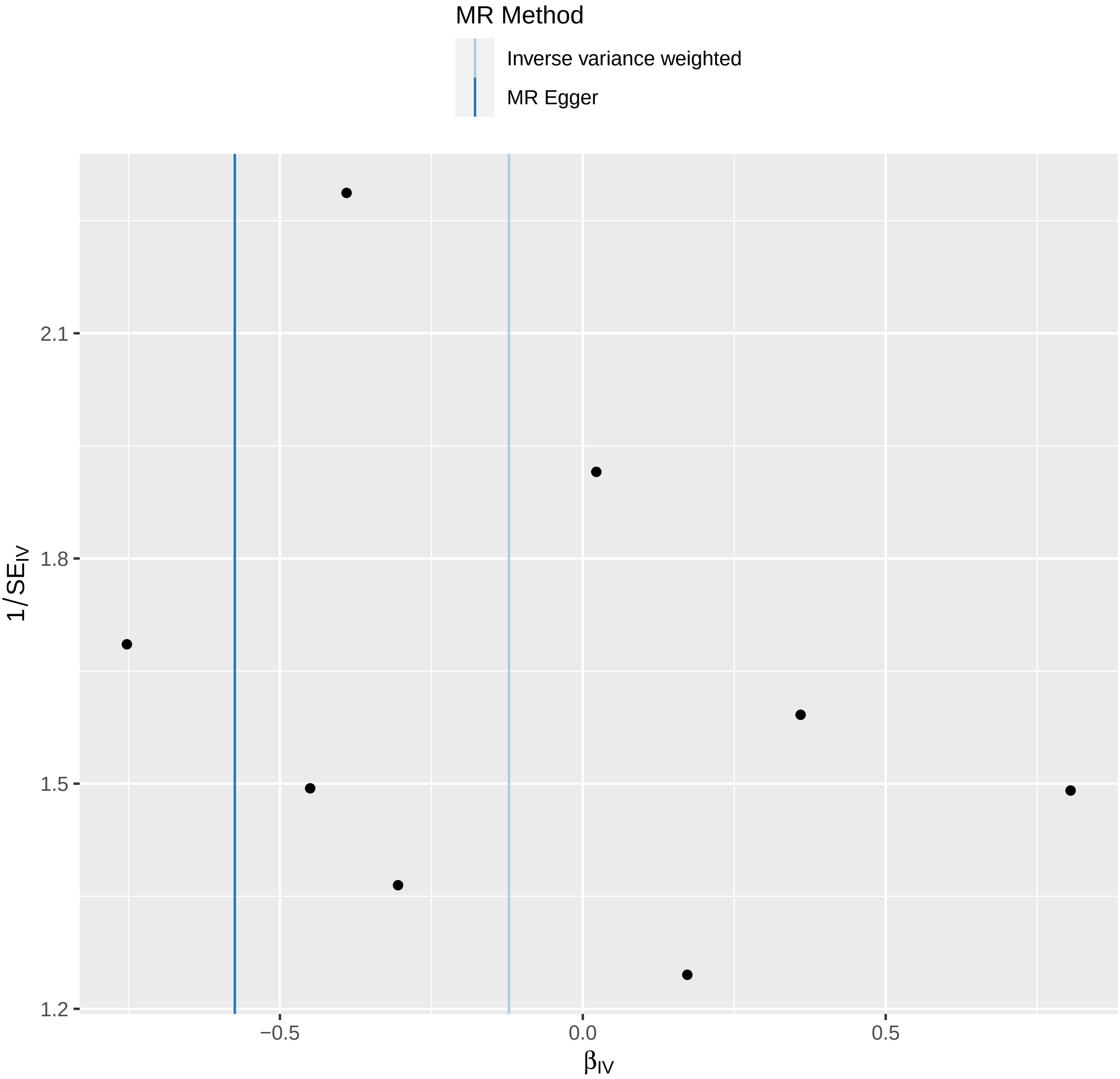

Batch 1346 : Gut microbiota abundance (genus Sutterella id.2896) on Other ulcerative colitis

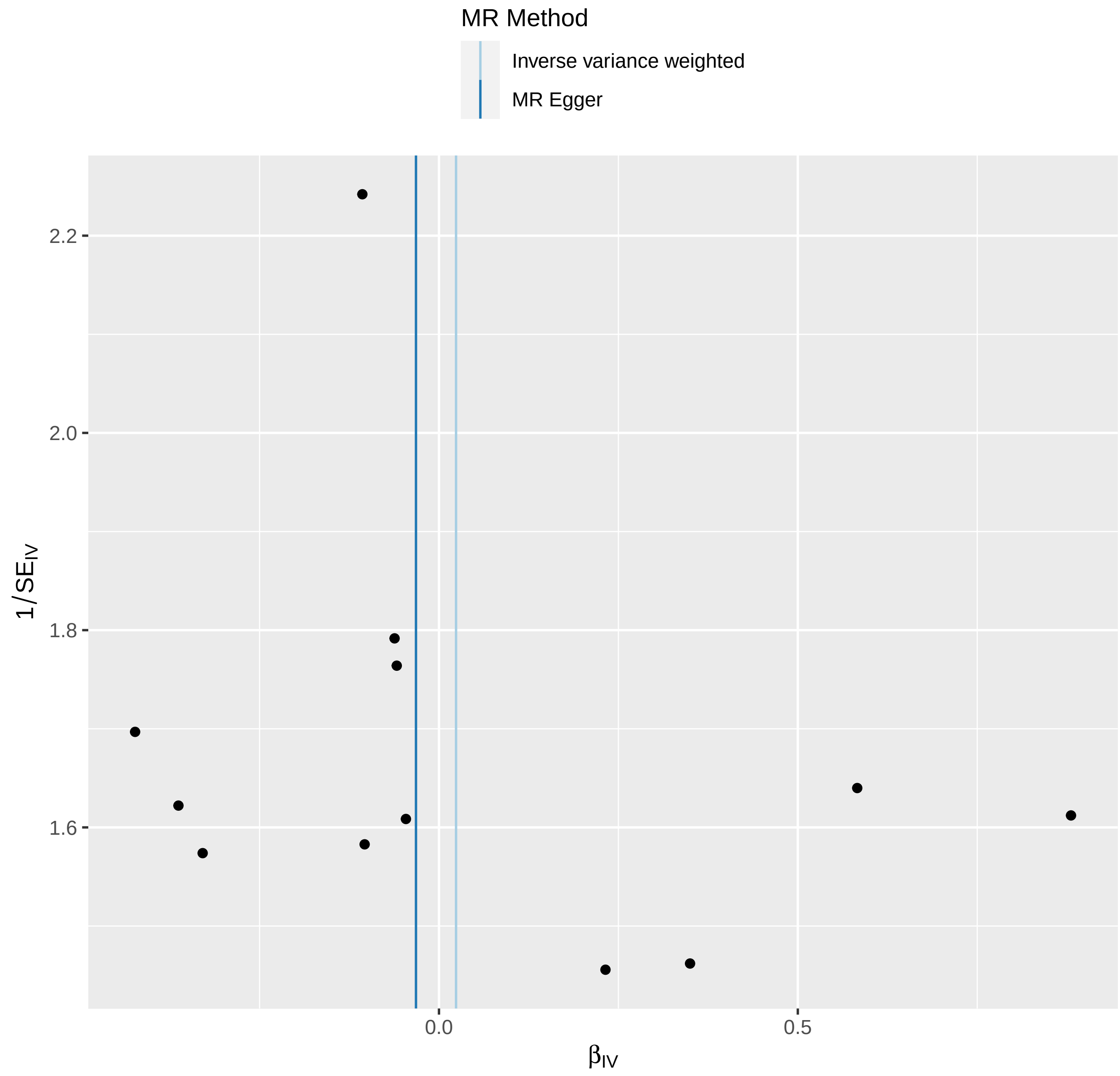

Batch 1347 : Gut microbiota abundance (genus Terrisporobacter id.11348) on Other ulcerative colitis

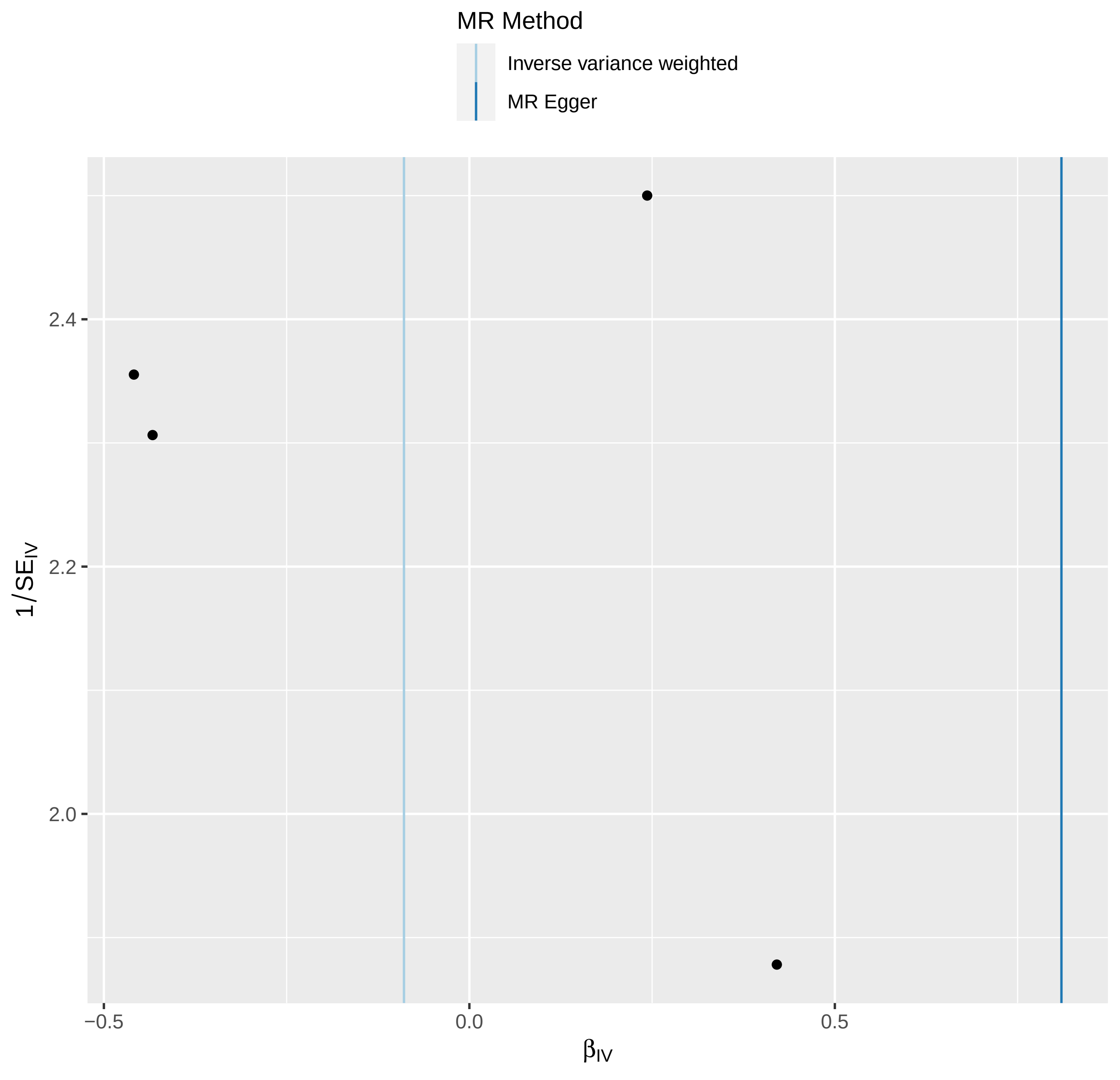

Batch 1348 : Gut microbiota abundance (genus Turicibacter id.2162) on Other ulcerative colitis

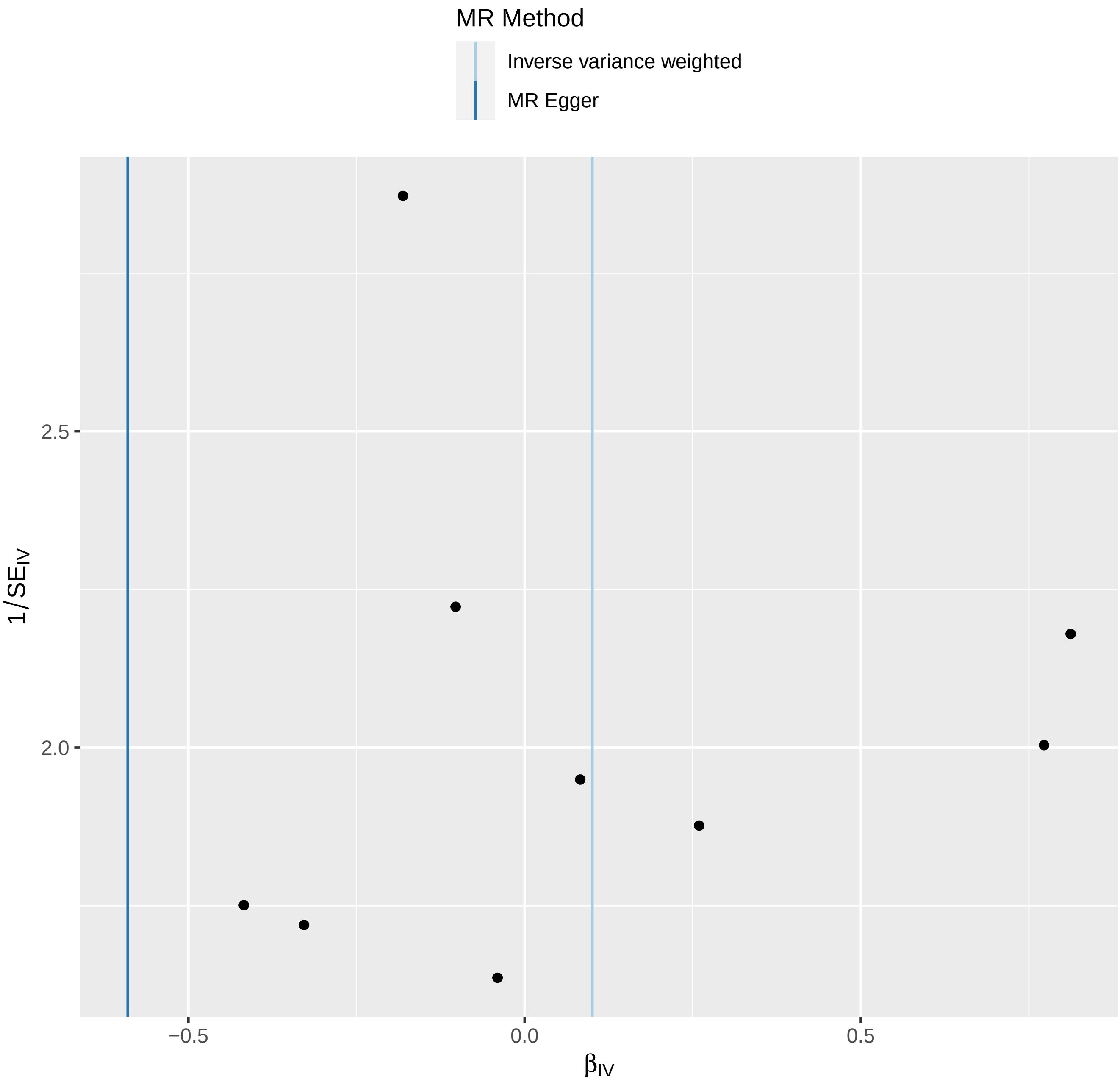

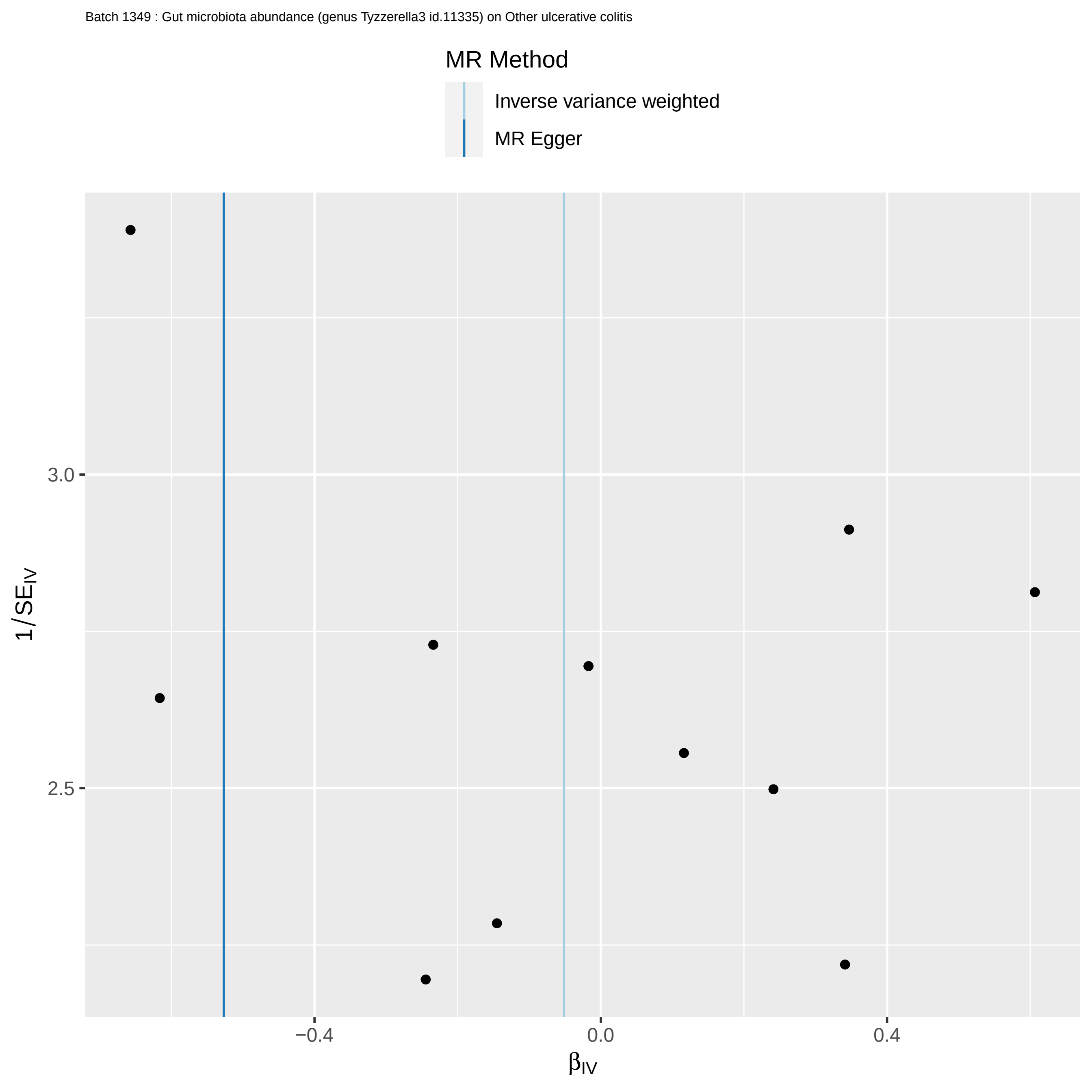

Batch 1349 : Gut microbiota abundance (genus Tyzzerella3 id.11335) on Other ulcerative colitis
MR Method
Inverse variance weighted
MR Egger
3.0
2.5
1/SE$_{IV}$
−0.4
0.0
0.4
β$_{IV}$

Batch 1350 : Gut microbiota abundance (genus Veillonella id.2198) on Other ulcerative colitis

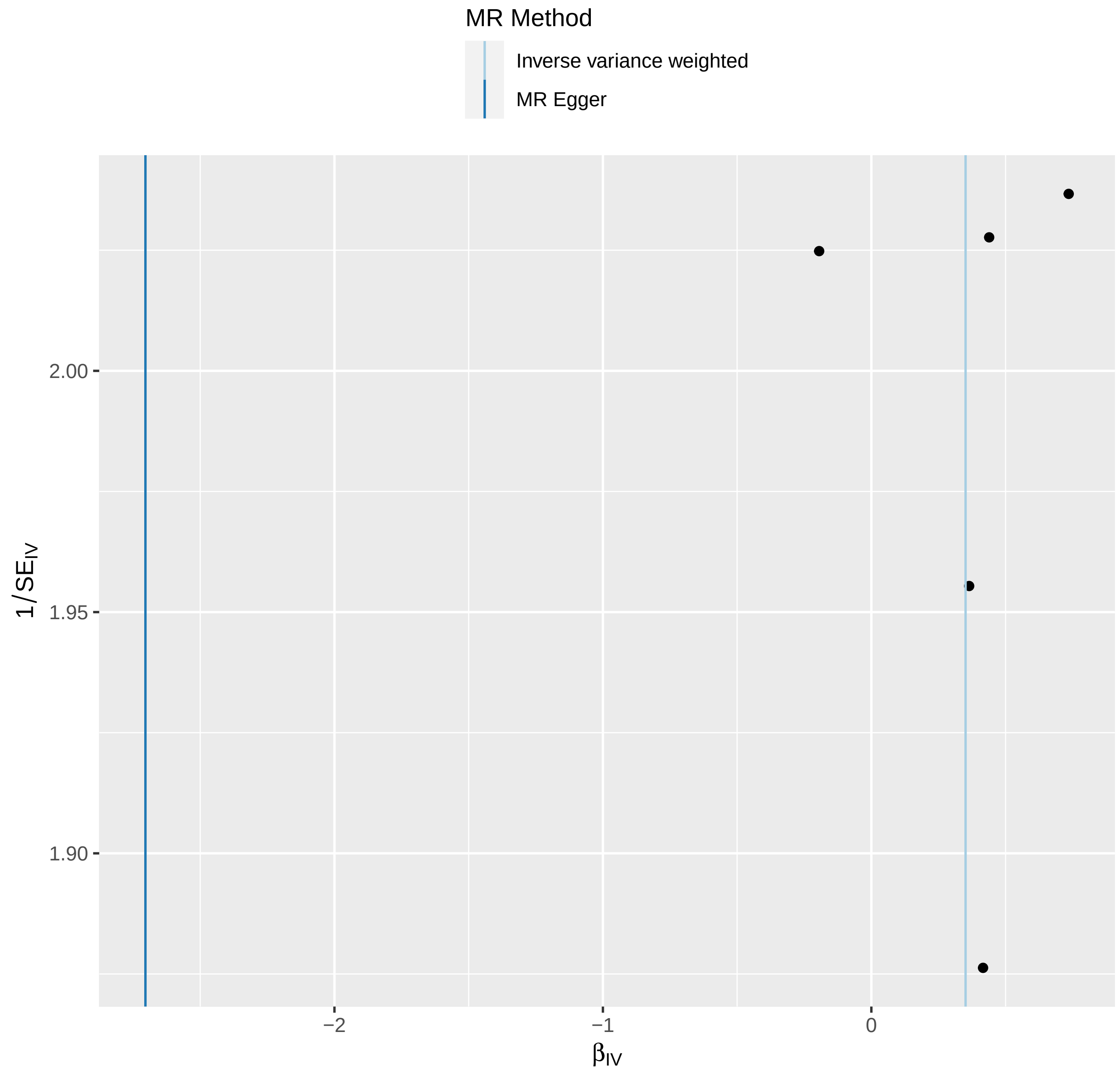

Batch 1351 : Gut microbiota abundance (family Acidaminococcaceae id.2166) on Ulcerative proctitis

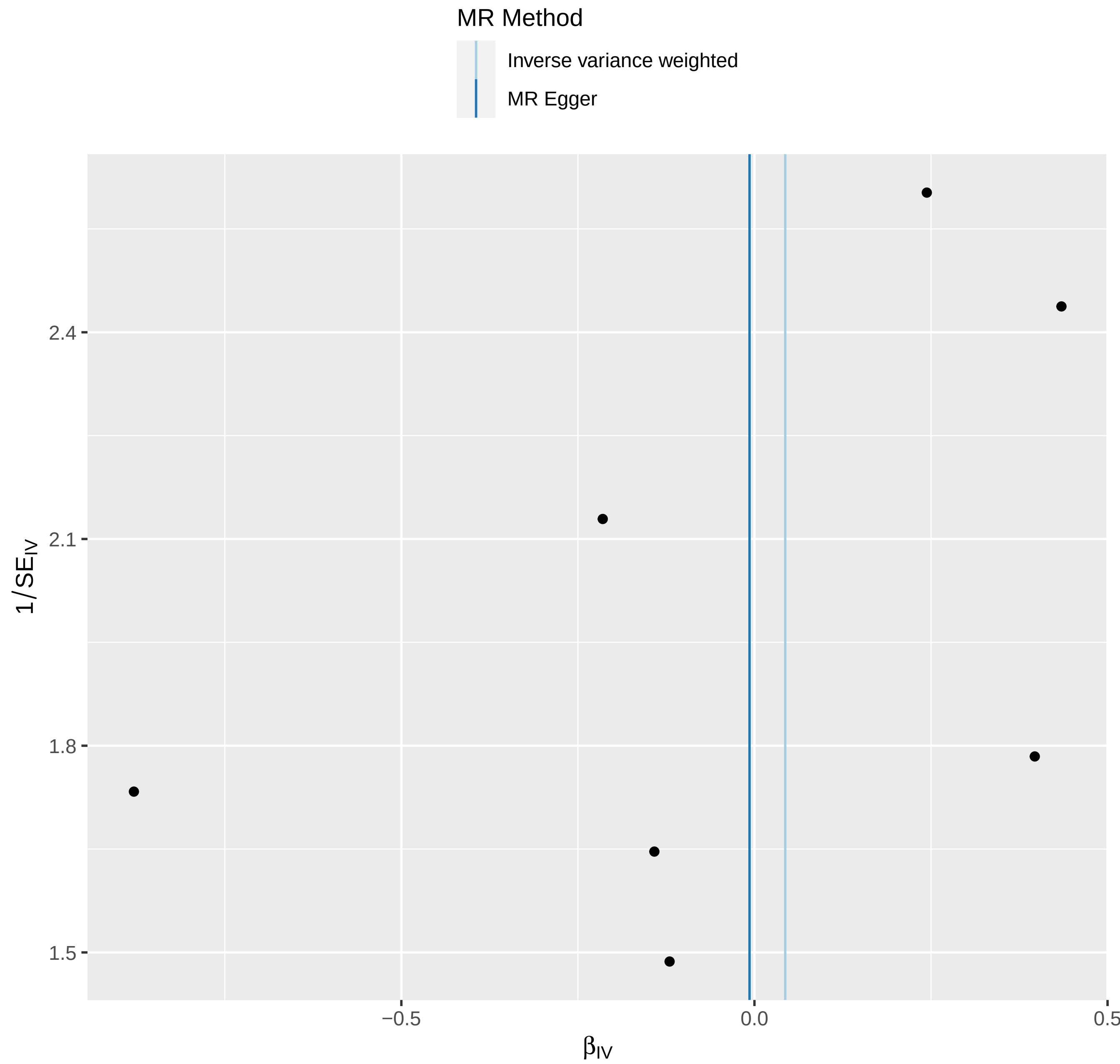

Batch 1352 : Gut microbiota abundance (family Actinomycetaceae id.421) on Ulcerative proctitis

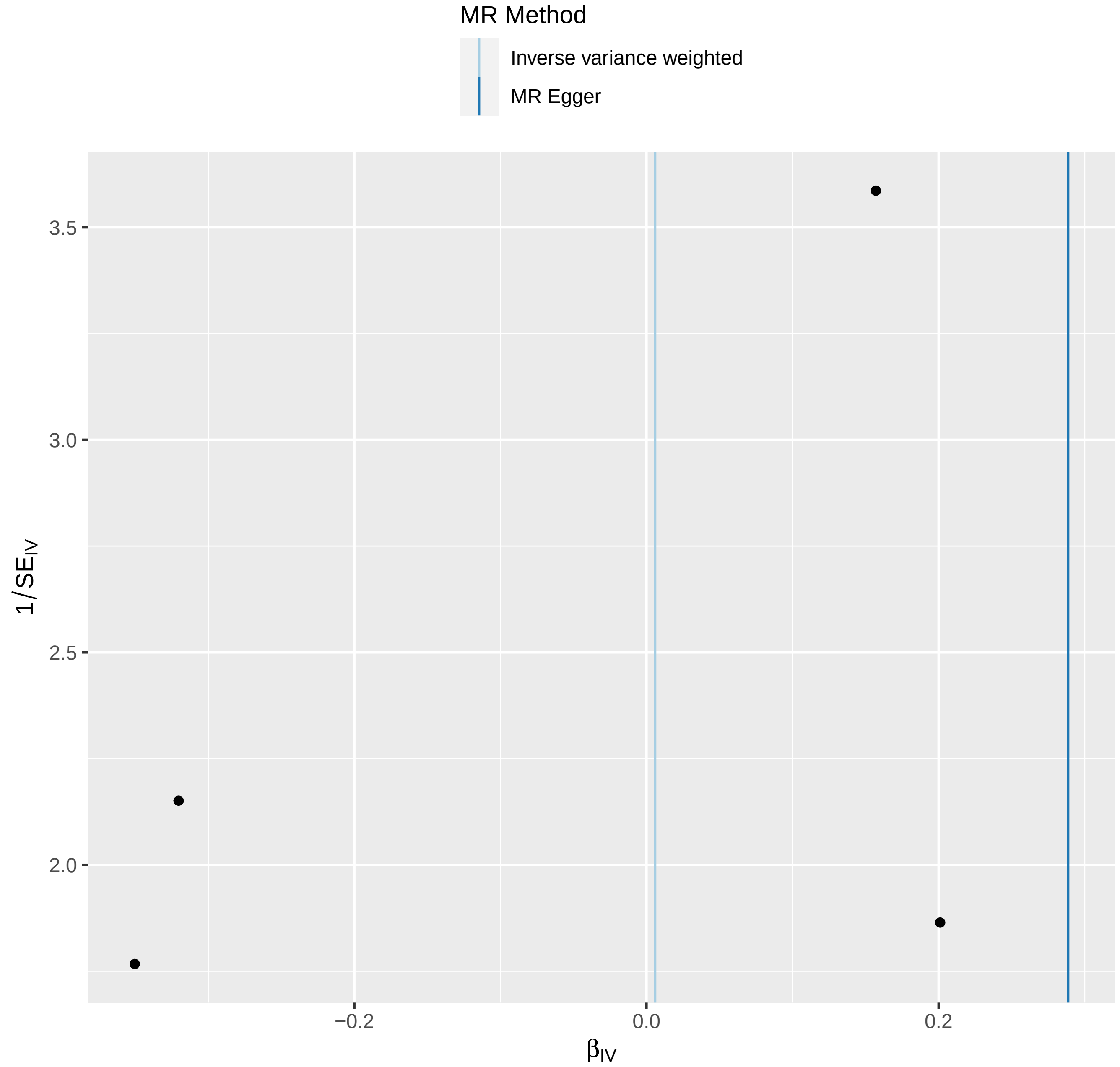

Batch 1353 : Gut microbiota abundance (family Alcaligenaceae id.2875) on Ulcerative proctitis

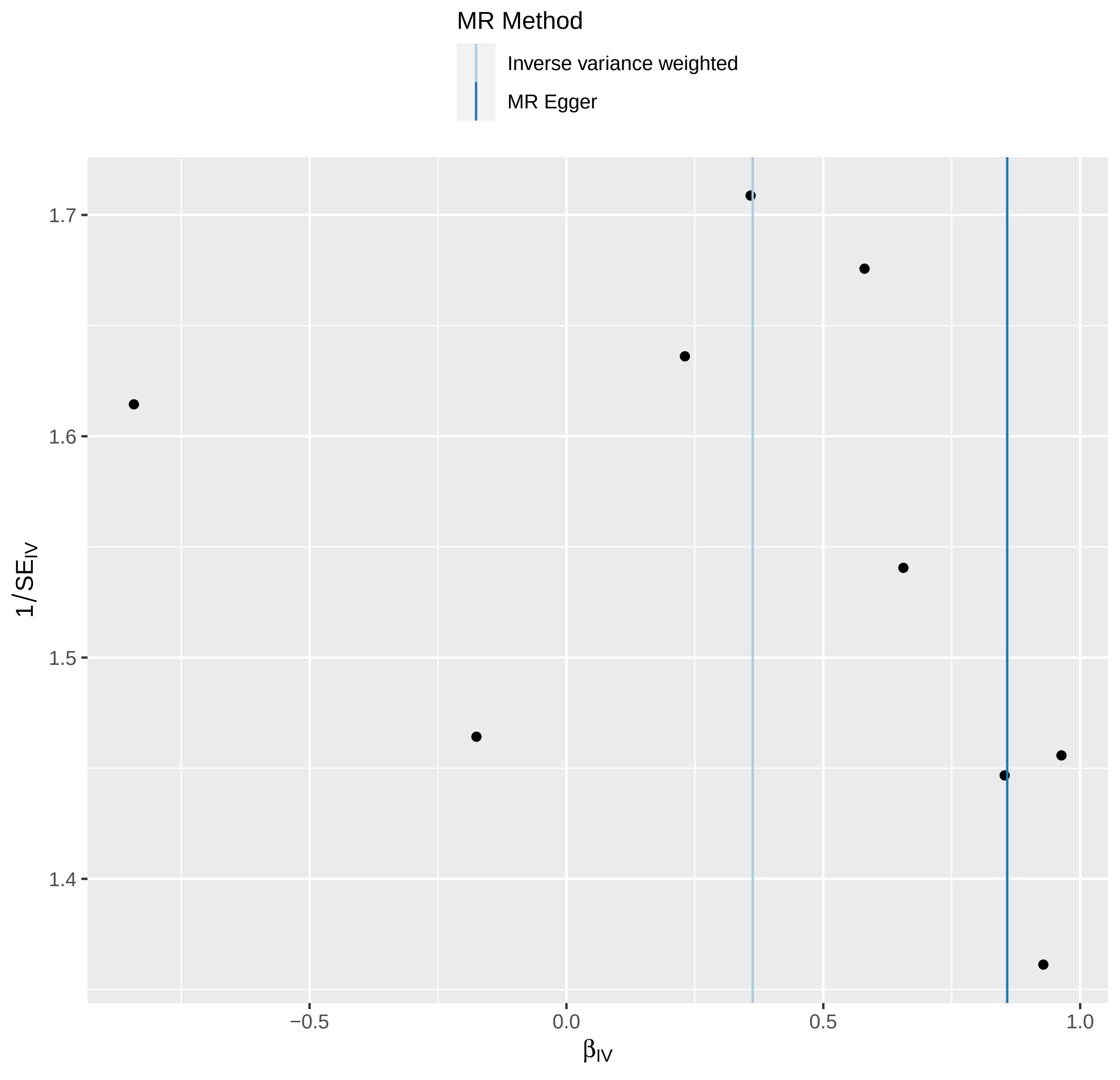

Batch 1354 : Gut microbiota abundance (family Bacteroidaceae id.917) on Ulcerative proctitis

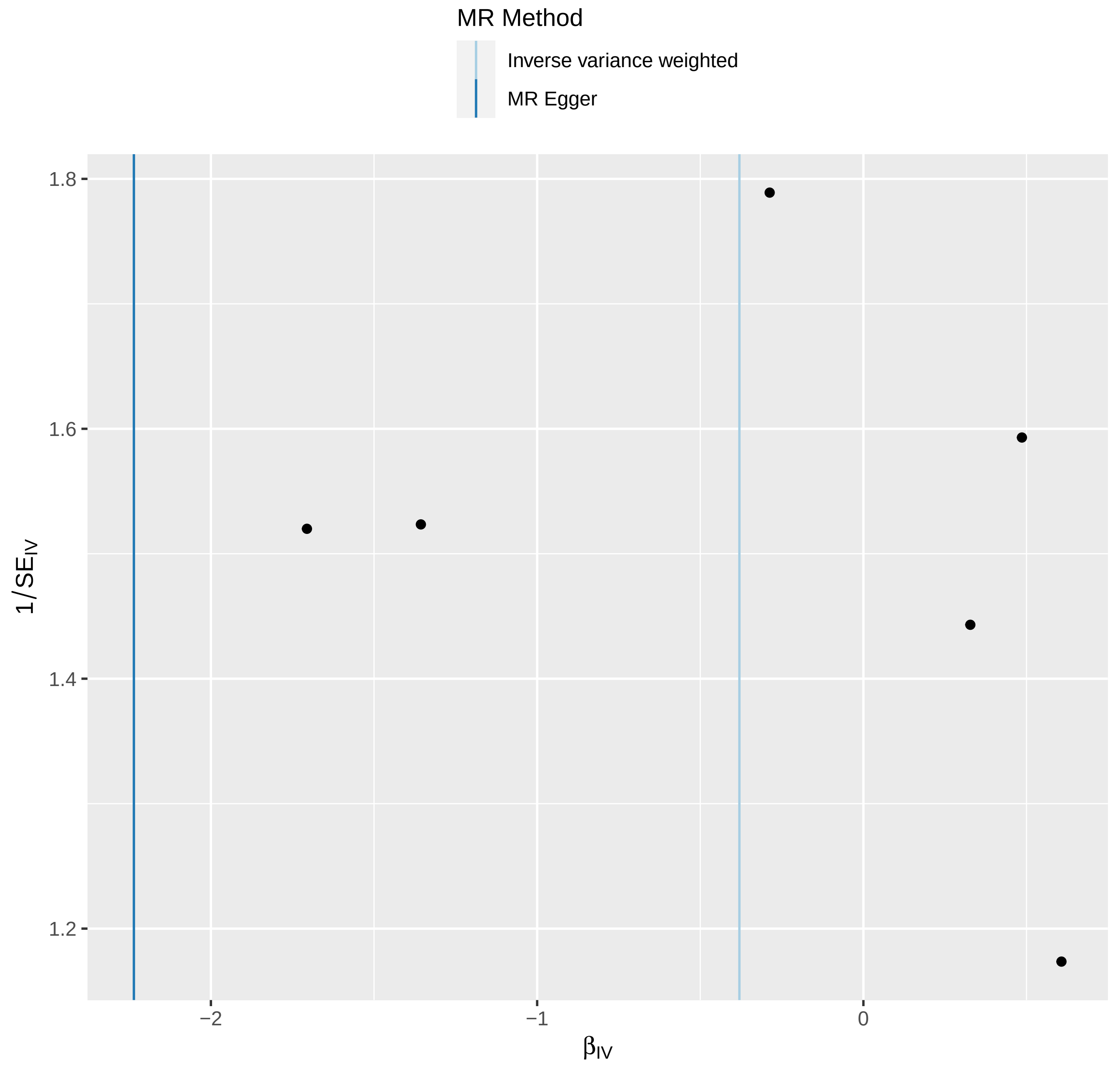

Batch 1355 : Gut microbiota abundance (family Bacteroidales S24 7group id.11173) on Ulcerative proctitis

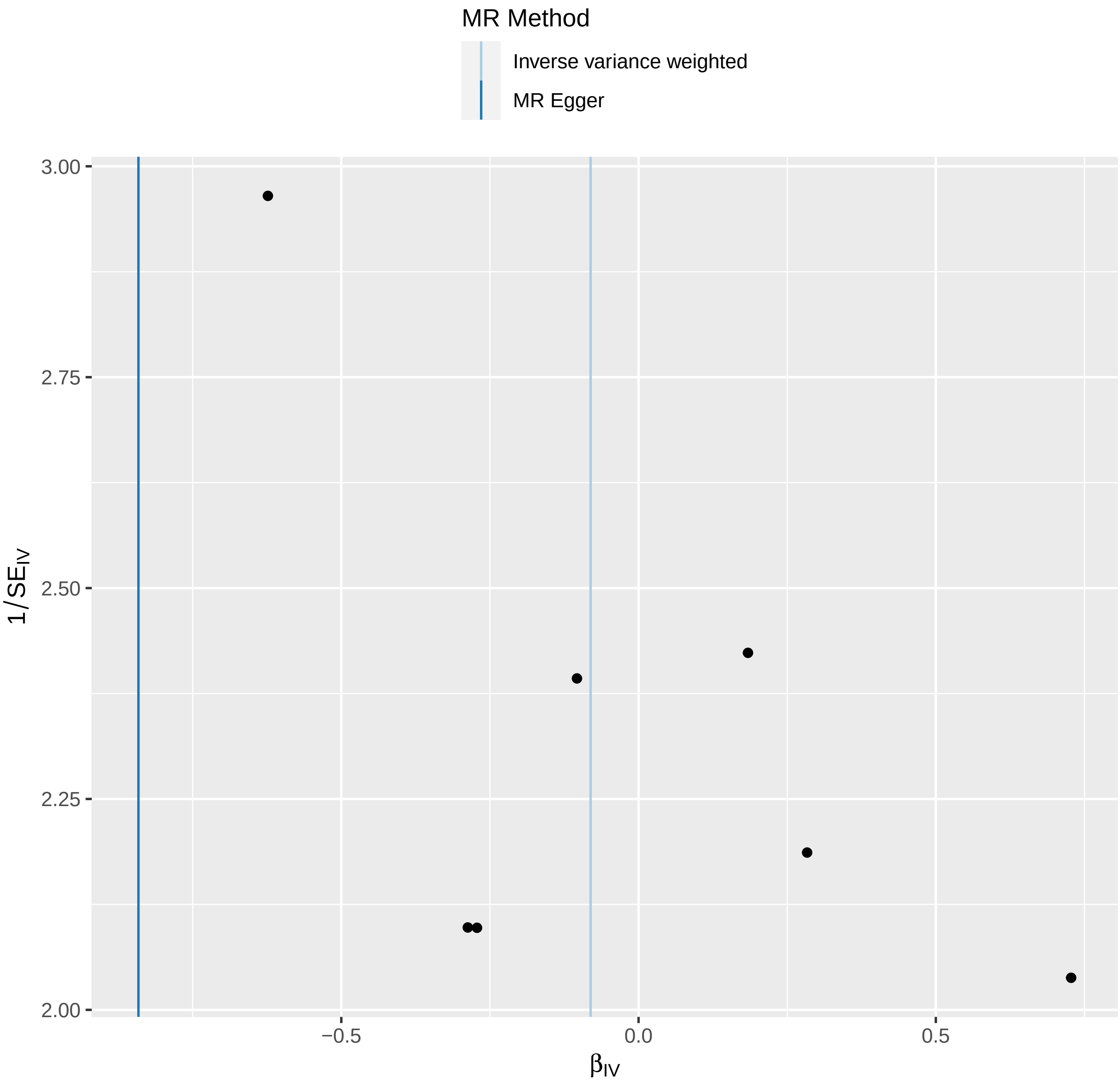

Batch 1356 : Gut microbiota abundance (family Bifidobacteriaceae id.433) on Ulcerative proctitis

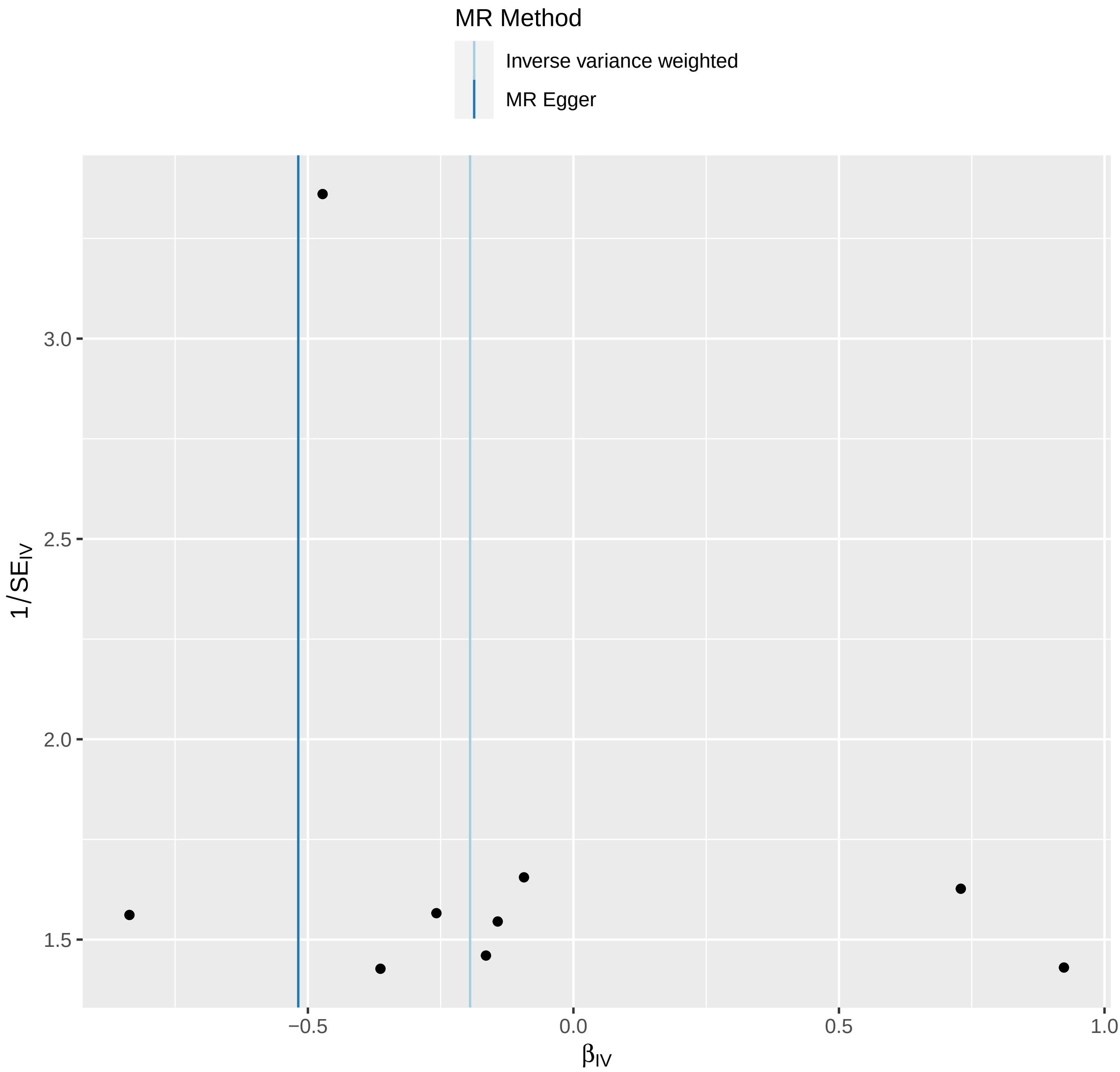

Batch 1358 : Gut microbiota abundance (family Clostridiaceae1 id.1869) on Ulcerative proctitis

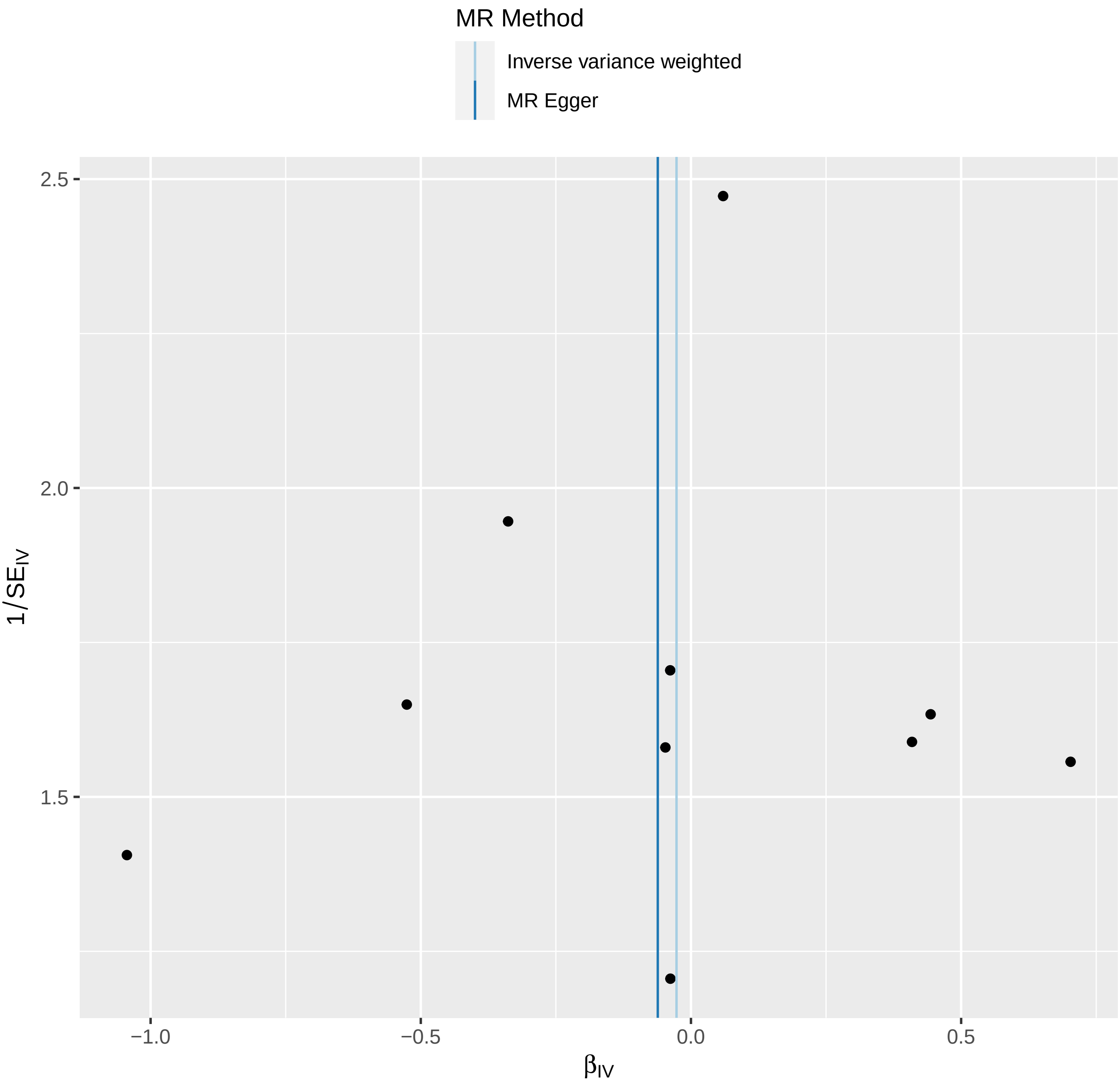

Batch 1359 : Gut microbiota abundance (family Clostridiales vadin BB60 group id.11286) on Ulcerative proctitis

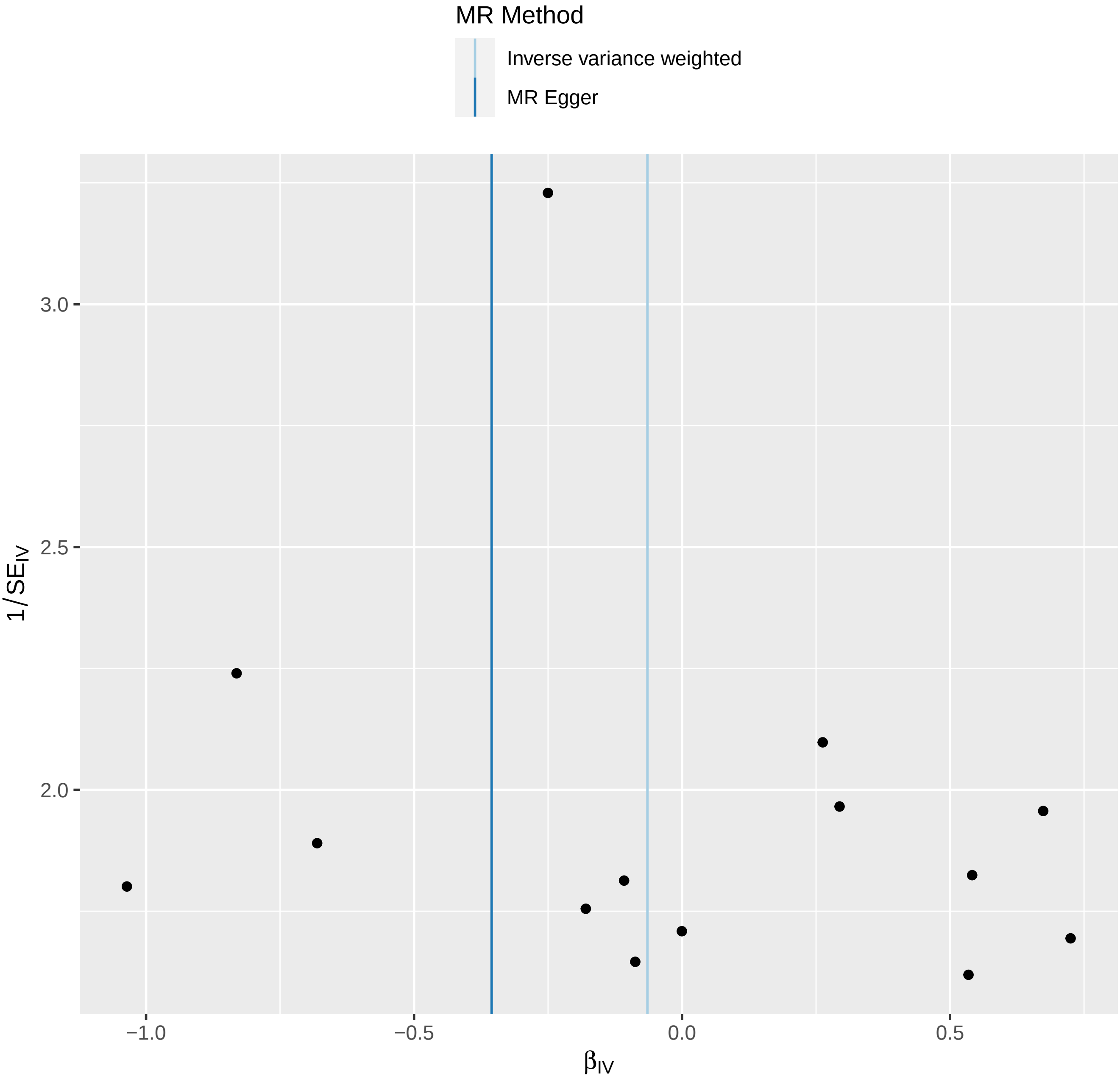

Batch 1360 : Gut microbiota abundance (family Coriobacteriaceae id.811) on Ulcerative proctitis

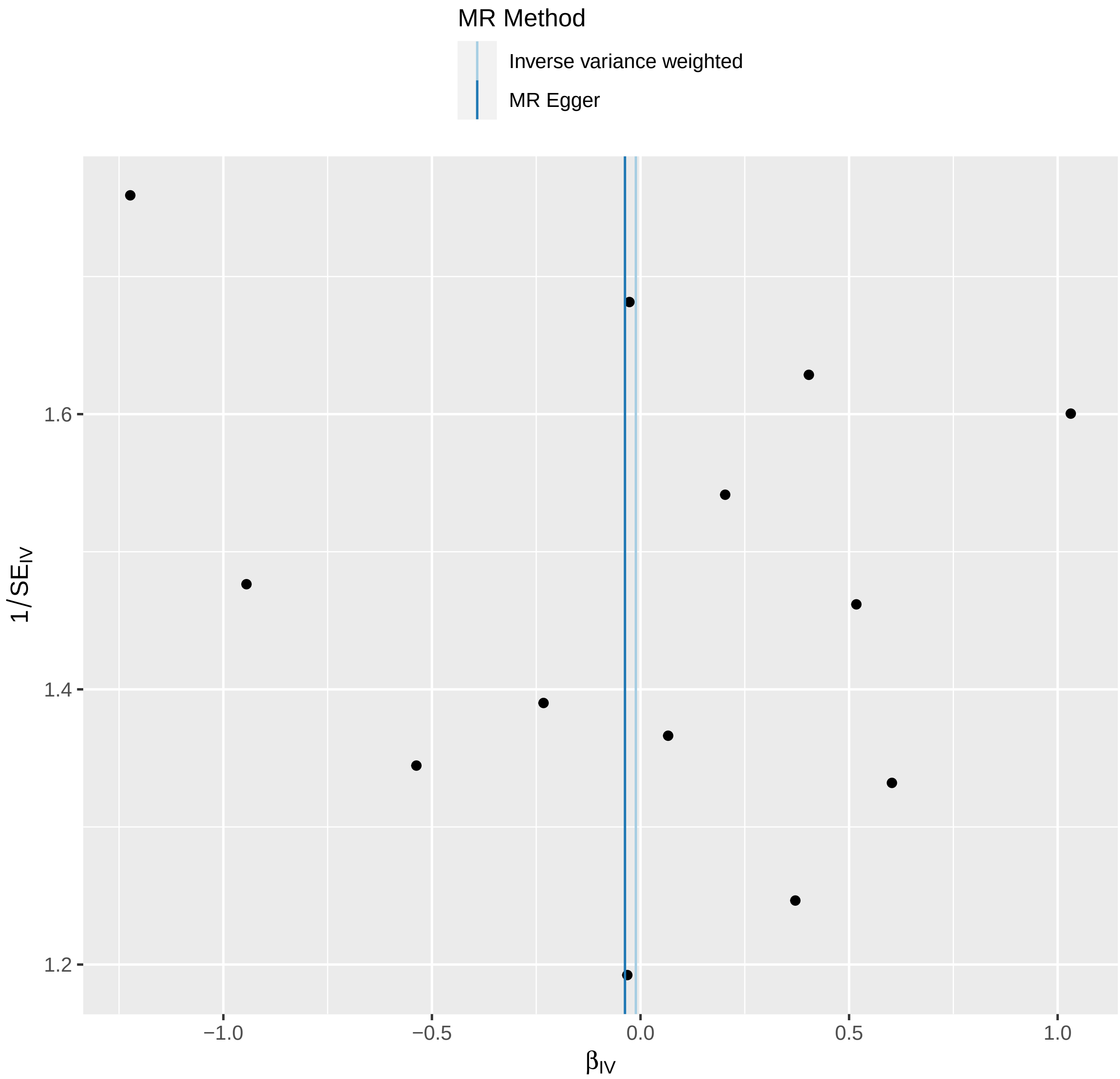

Batch 1361 : Gut microbiota abundance (family Defluviitaleaceae id.1924) on Ulcerative proctitis

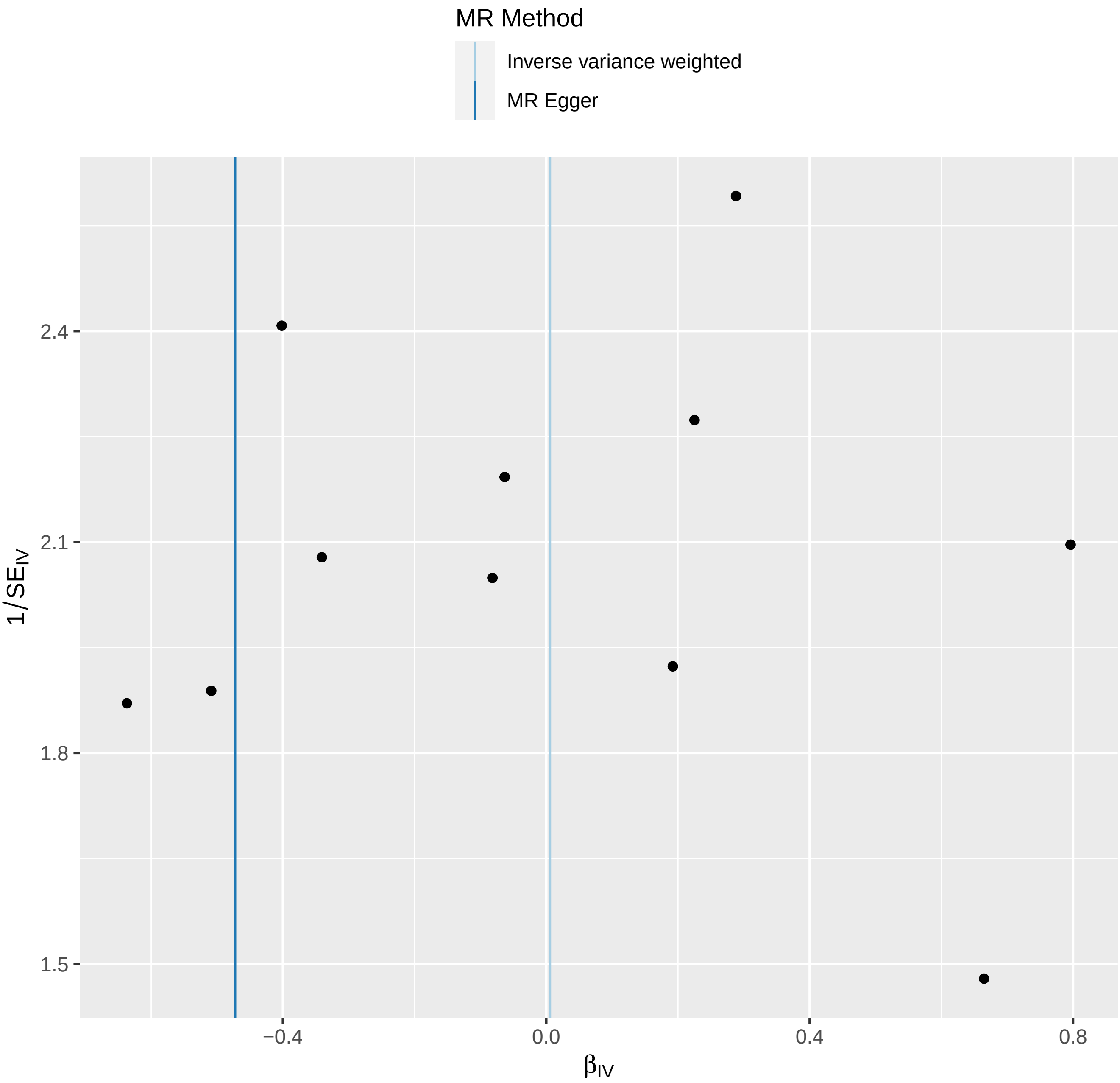

Batch 1362 : Gut microbiota abundance (family Desulfovibrionaceae id.3169) on Ulcerative proctitis

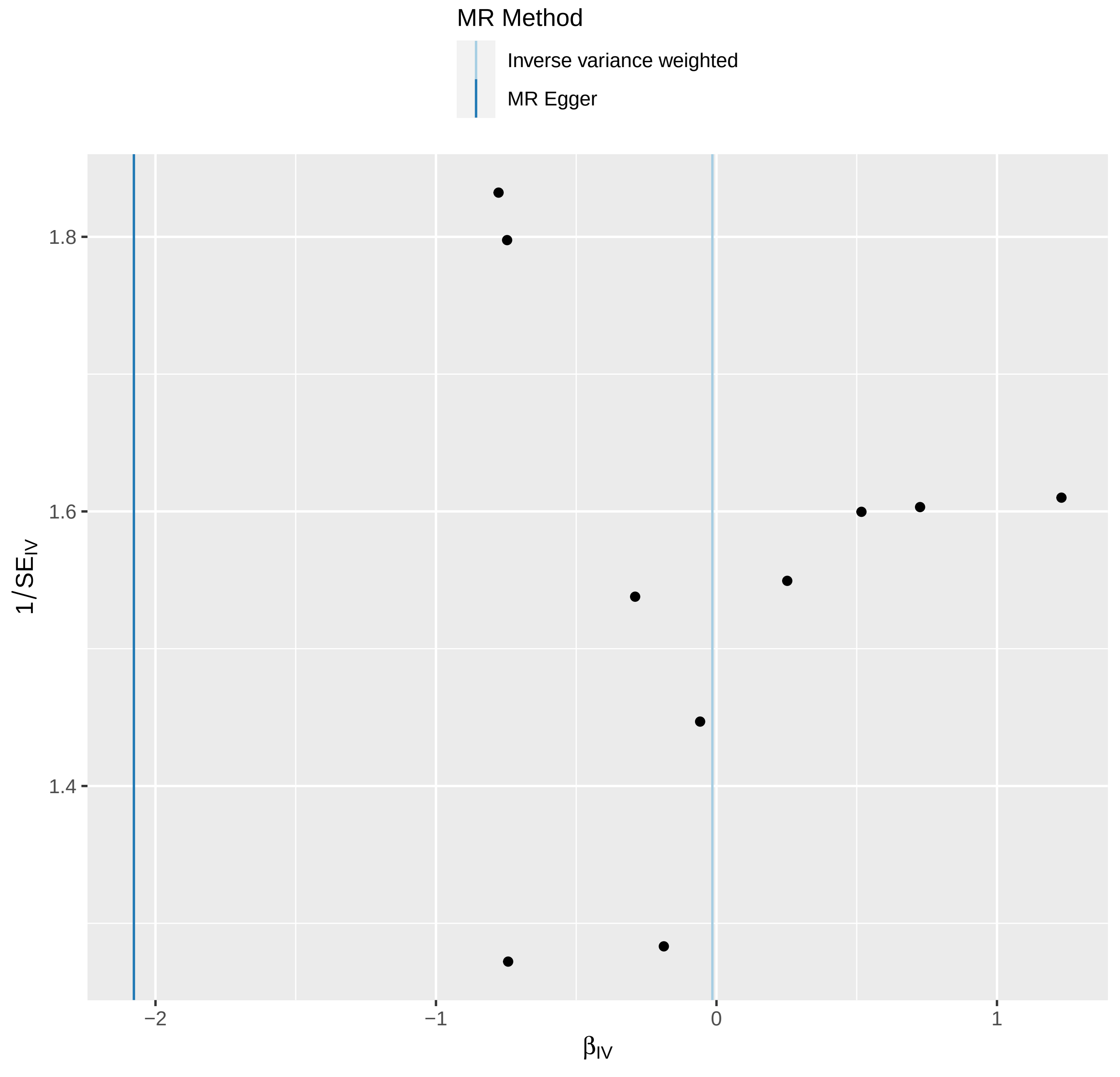

Batch 1363 : Gut microbiota abundance (family Enterobacteriaceae id.3469) on Ulcerative proctitis

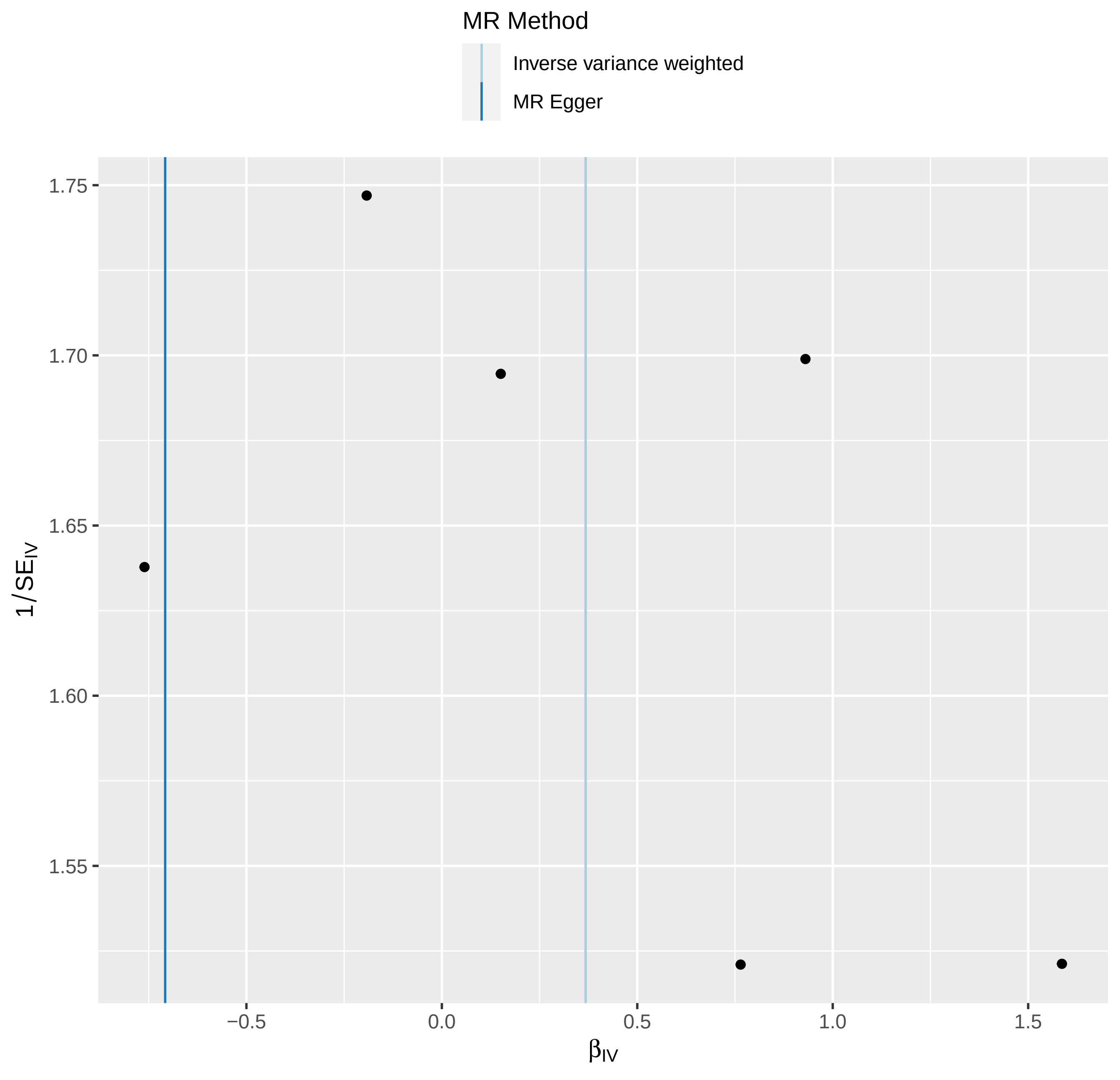

Batch 1364 : Gut microbiota abundance (family Erysipelotrichaceae id.2149) on Ulcerative proctitis

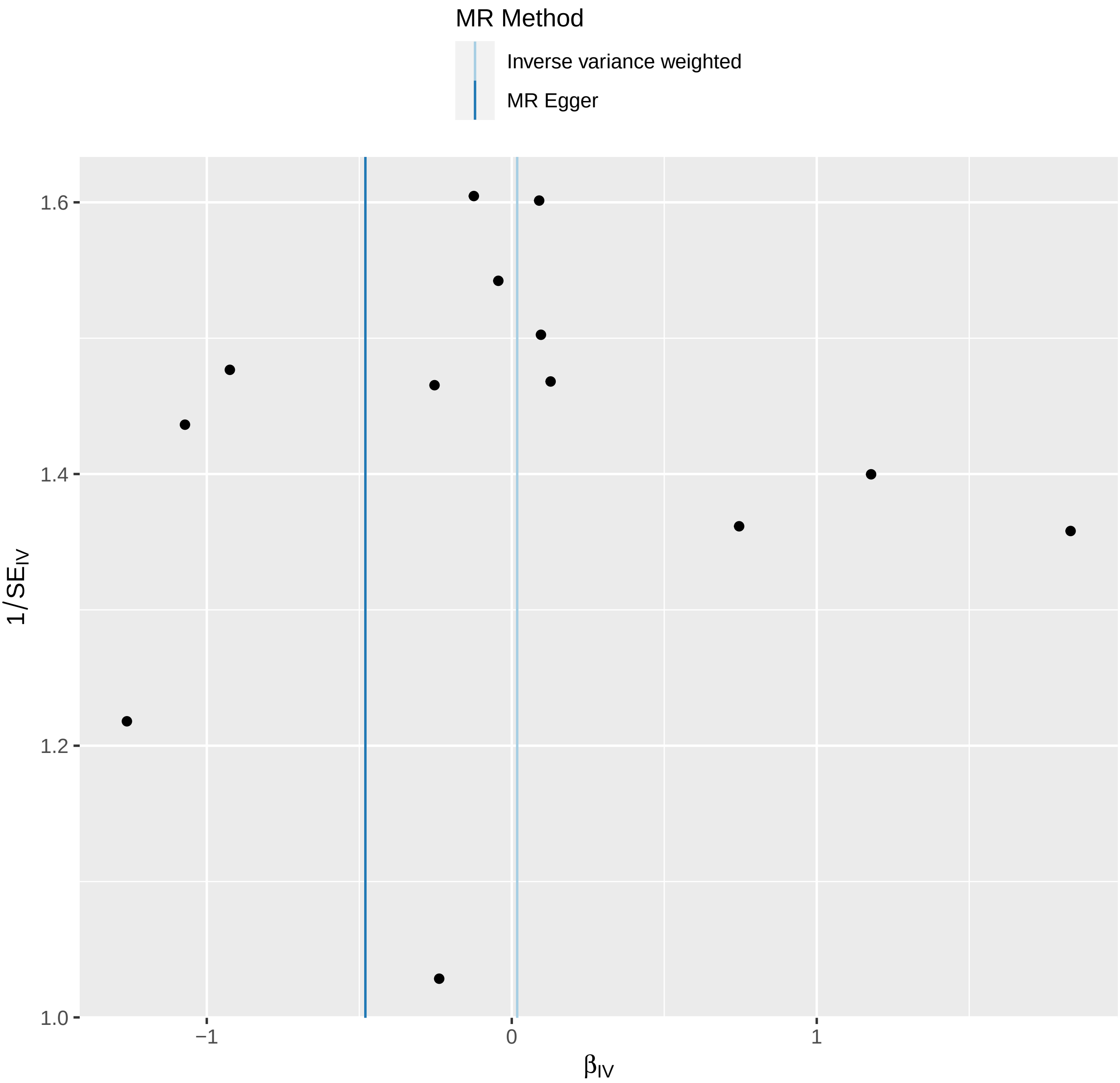

Batch 1365 : Gut microbiota abundance (family Family XI id.1936) on Ulcerative proctitis

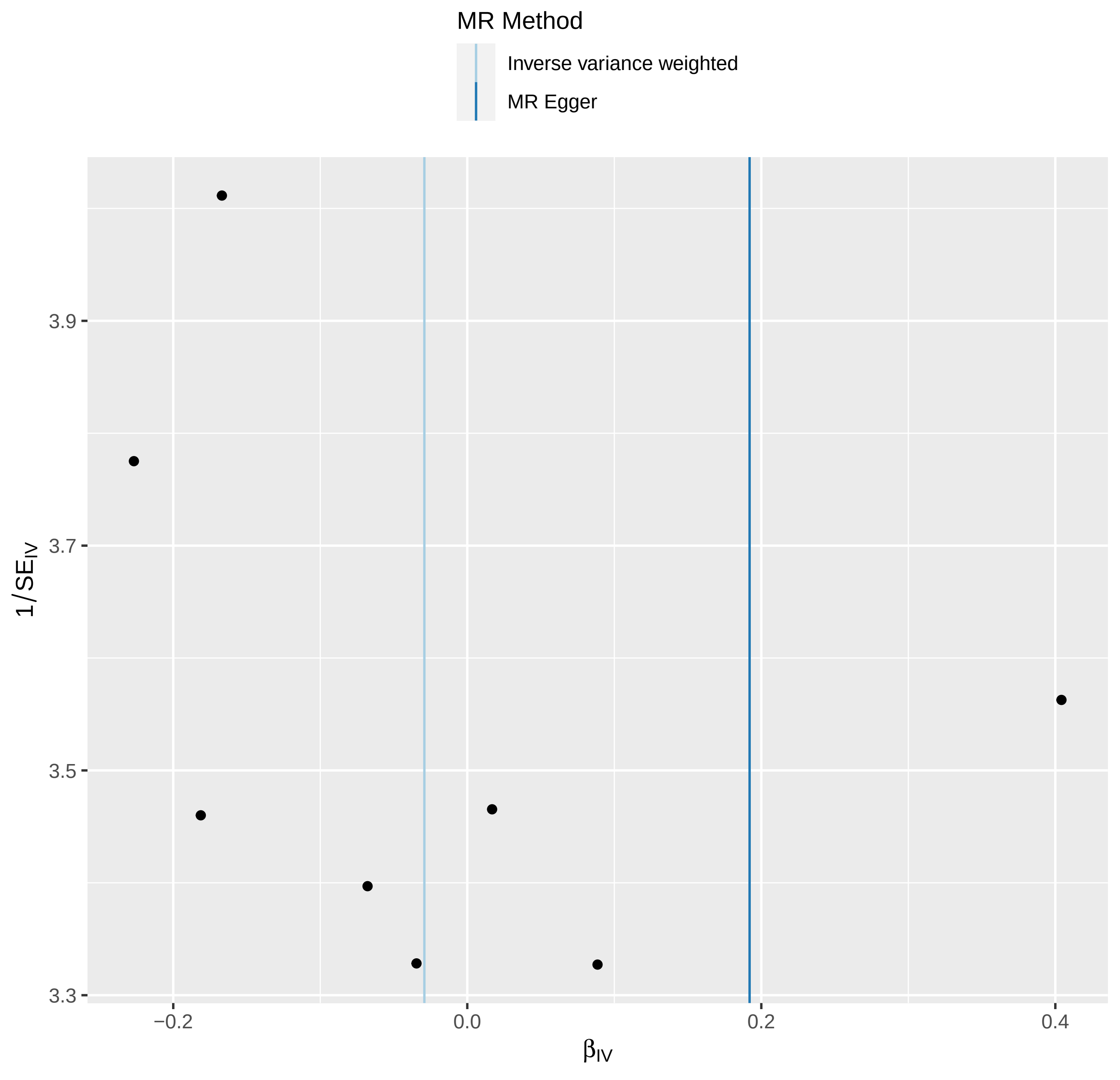

Batch 1366 : Gut microbiota abundance (family Family XIII id.1957) on Ulcerative proctitis

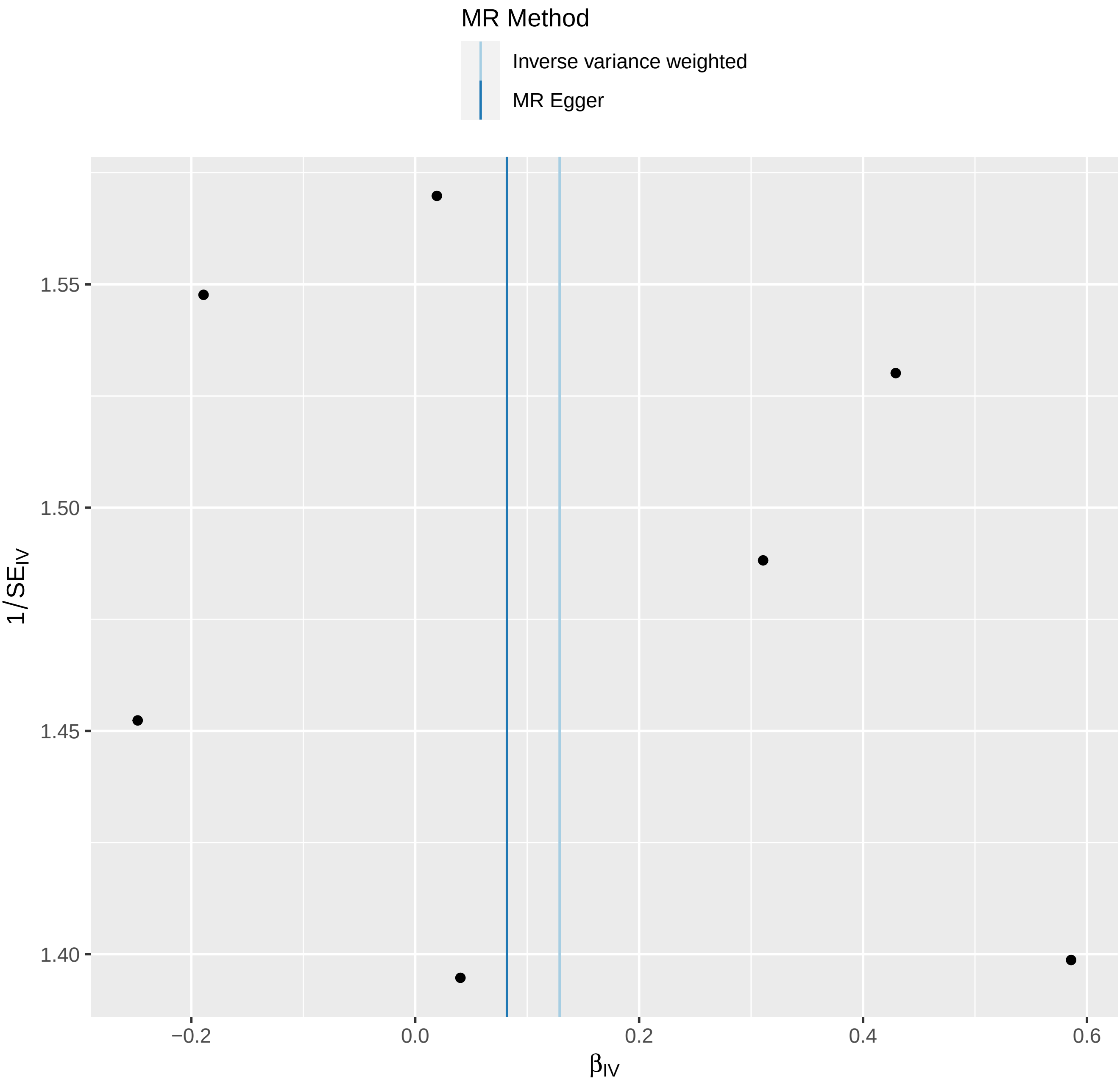

Batch 1367 : Gut microbiota abundance (family Lachnospiraceae id.1987) on Ulcerative proctitis

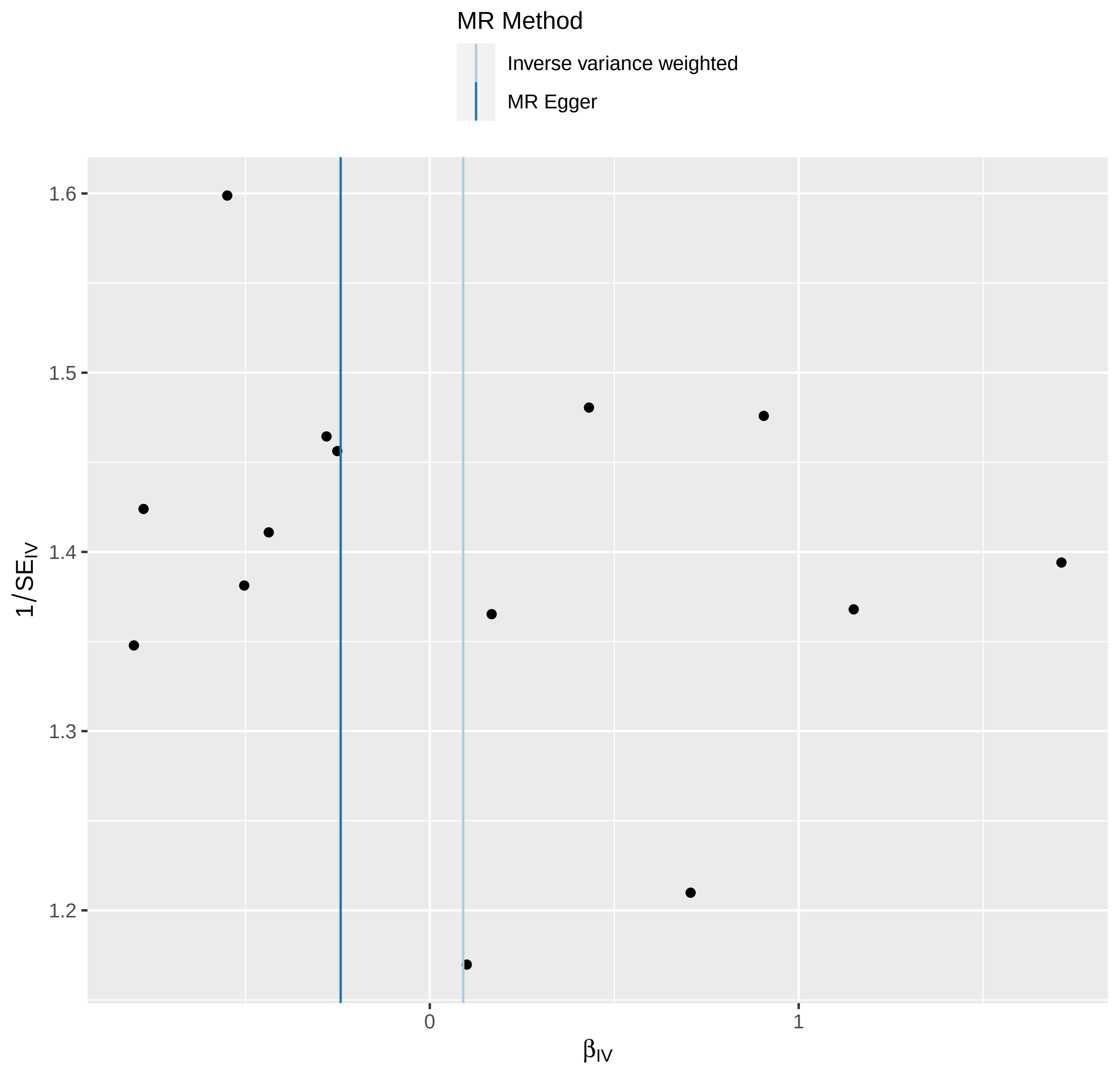

Batch 1368 : Gut microbiota abundance (family Lactobacillaceae id.1836) on Ulcerative proctitis

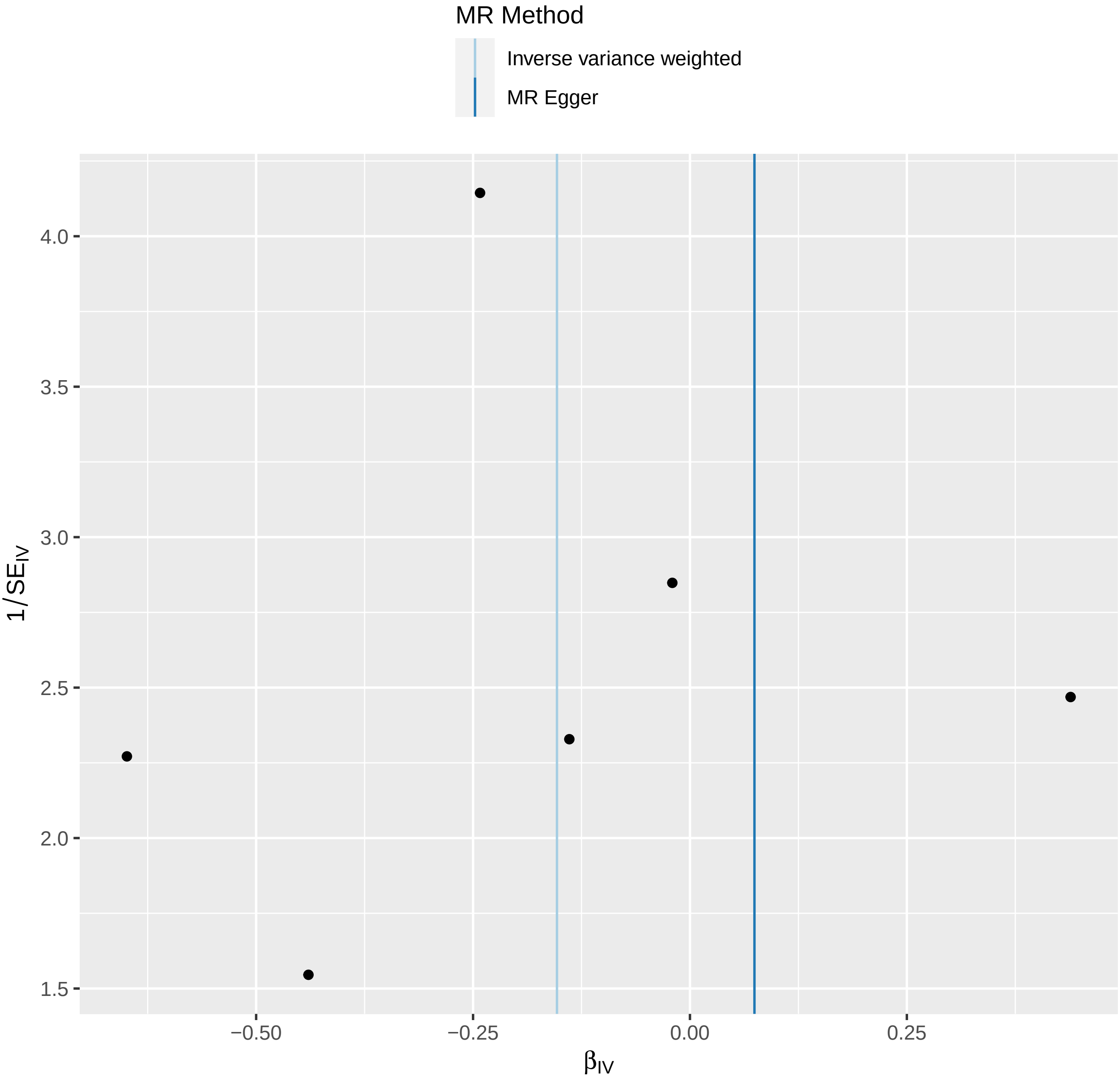

Batch 1369 : Gut microbiota abundance (family Methanobacteriaceae id.121) on Ulcerative proctitis

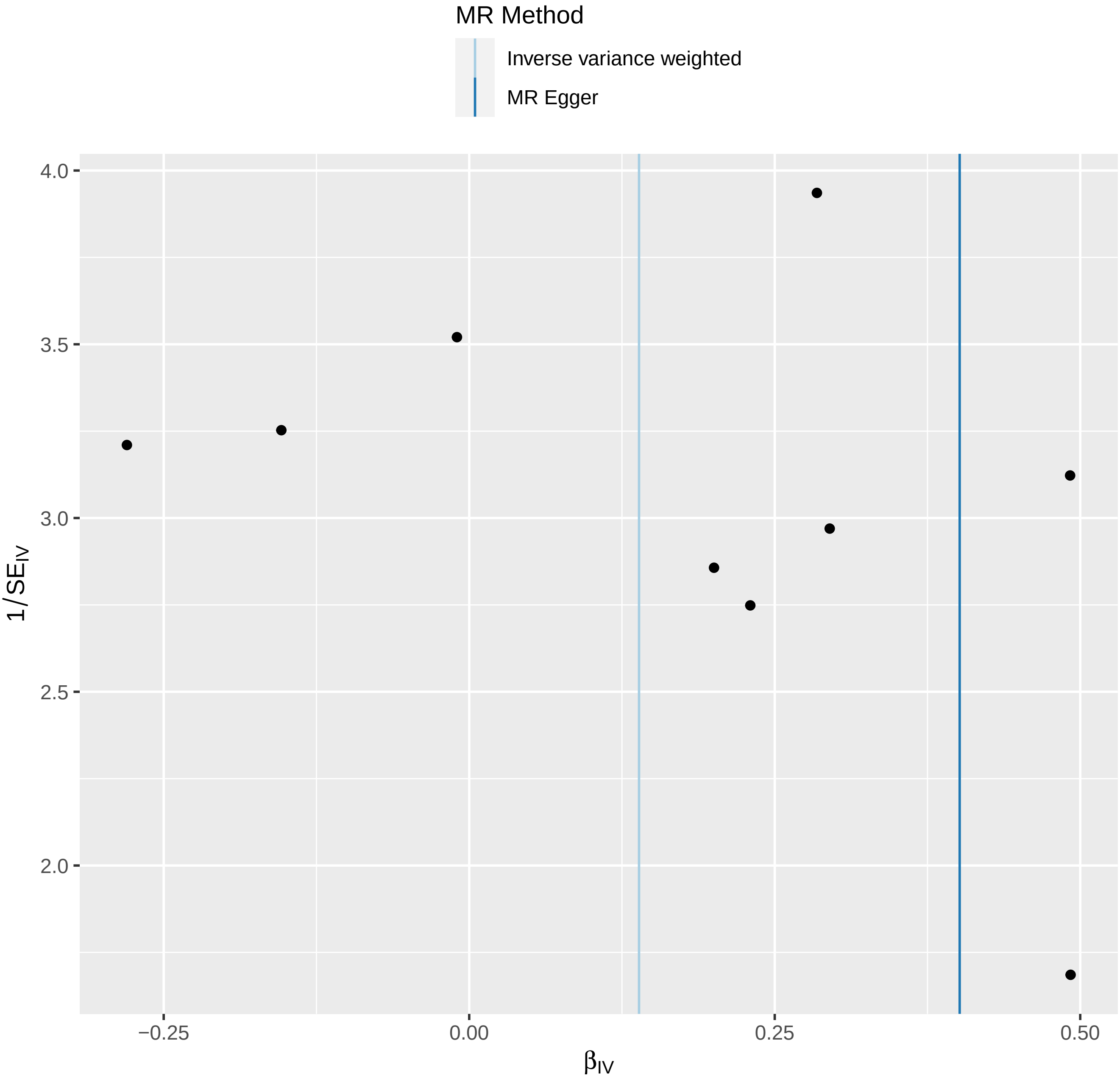

Batch 1370 : Gut microbiota abundance (family Oxalobacteraceae id.2966) on Ulcerative proctitis

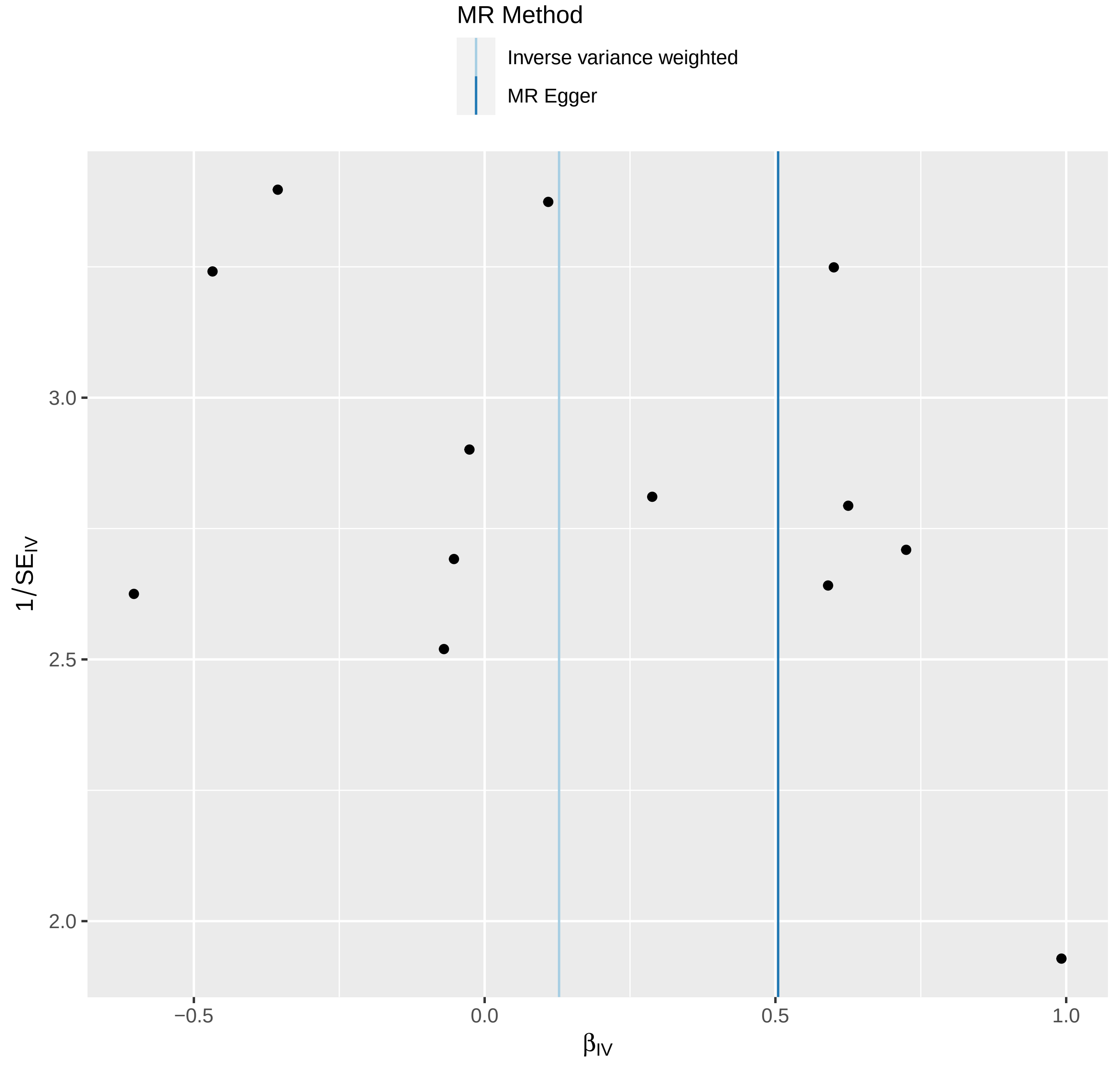

Batch 1371 : Gut microbiota abundance (family Pasteurellaceae id.3689) on Ulcerative proctitis

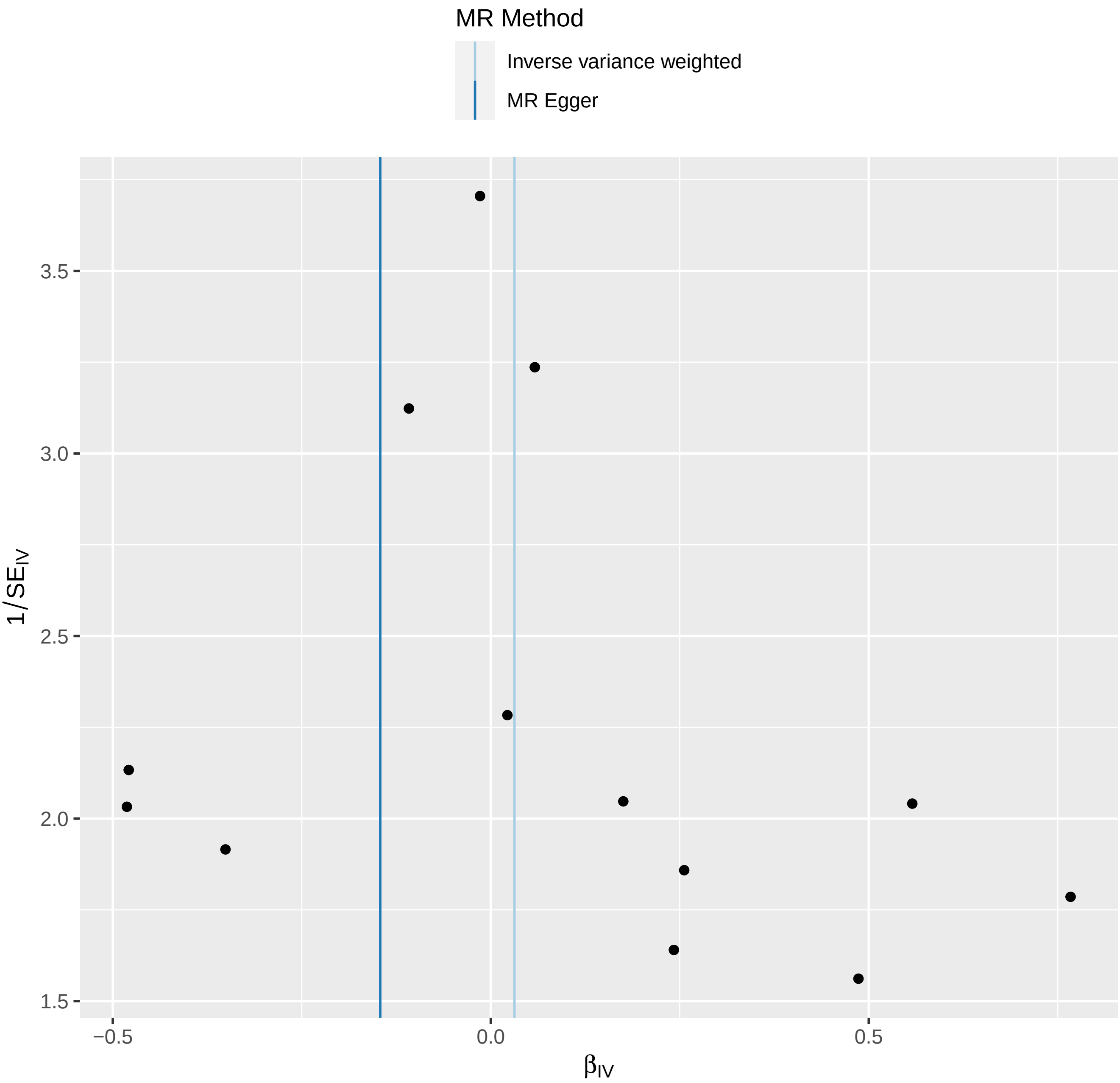

Batch 1372 : Gut microbiota abundance (family Peptococcaceae id.2024) on Ulcerative proctitis

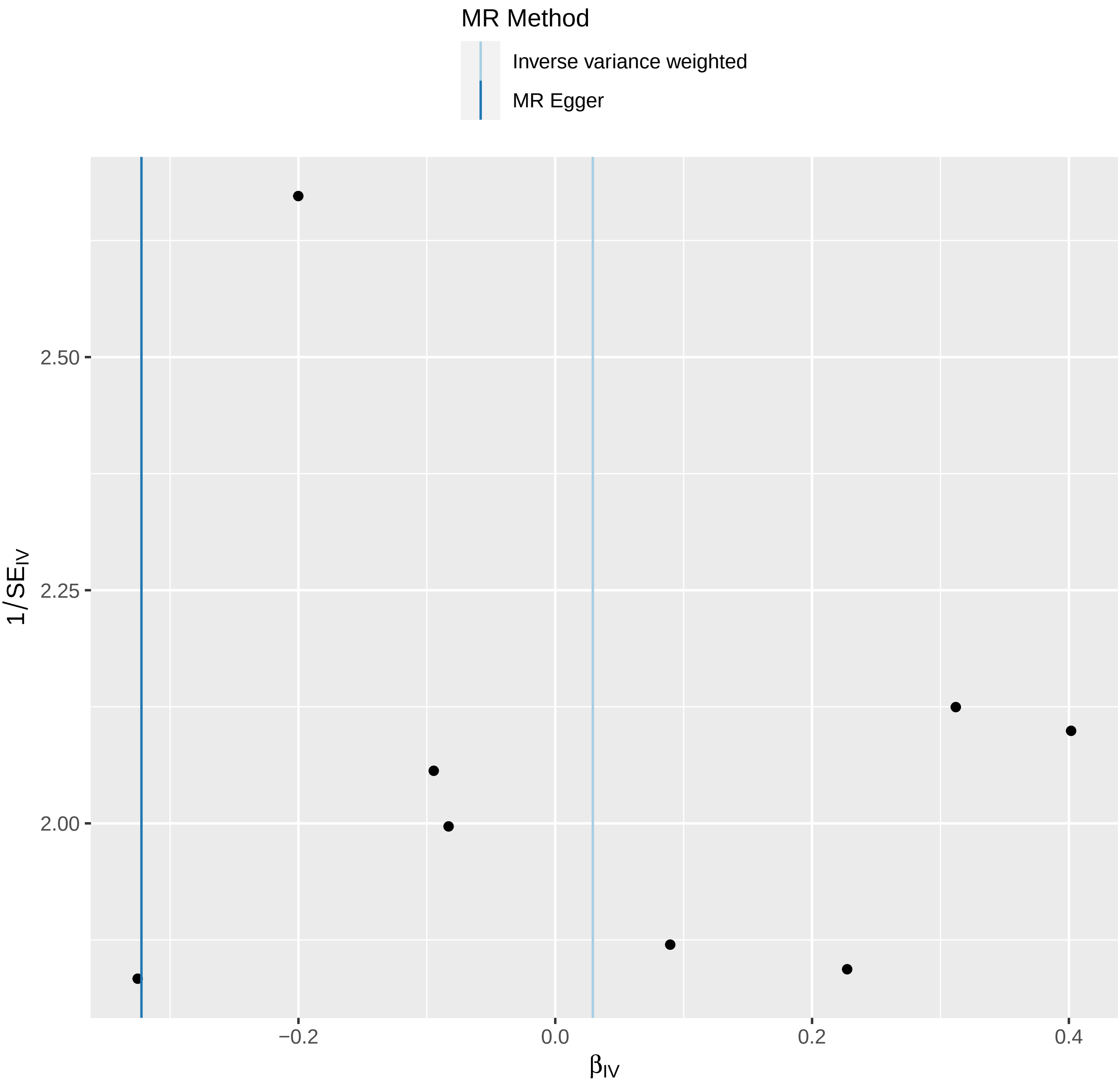

Batch 1373 : Gut microbiota abundance (family Peptostreptococcaceae id.2042) on Ulcerative proctitis

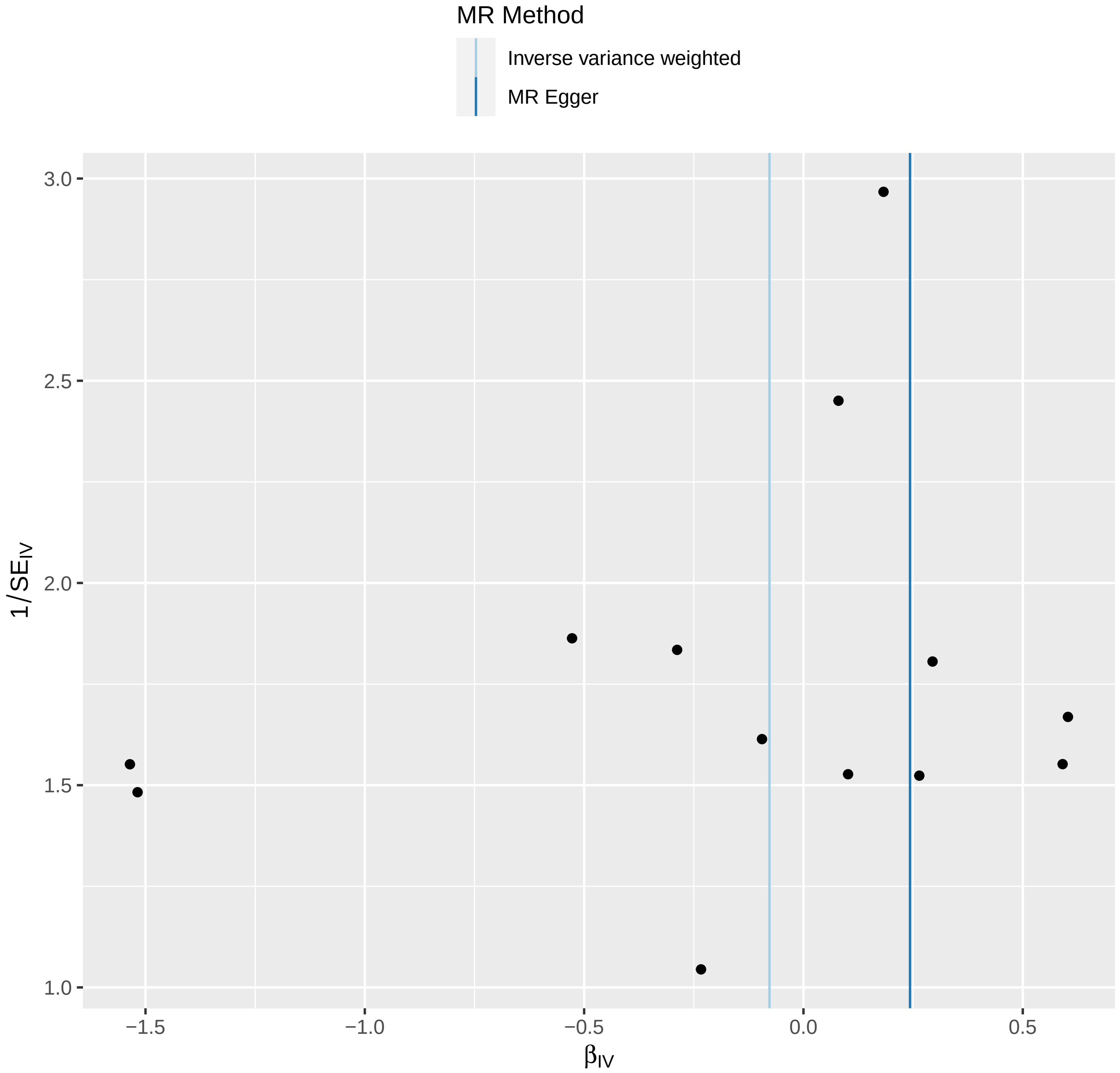

Batch 1374 : Gut microbiota abundance (family Porphyromonadaceae id.943) on Ulcerative proctitis

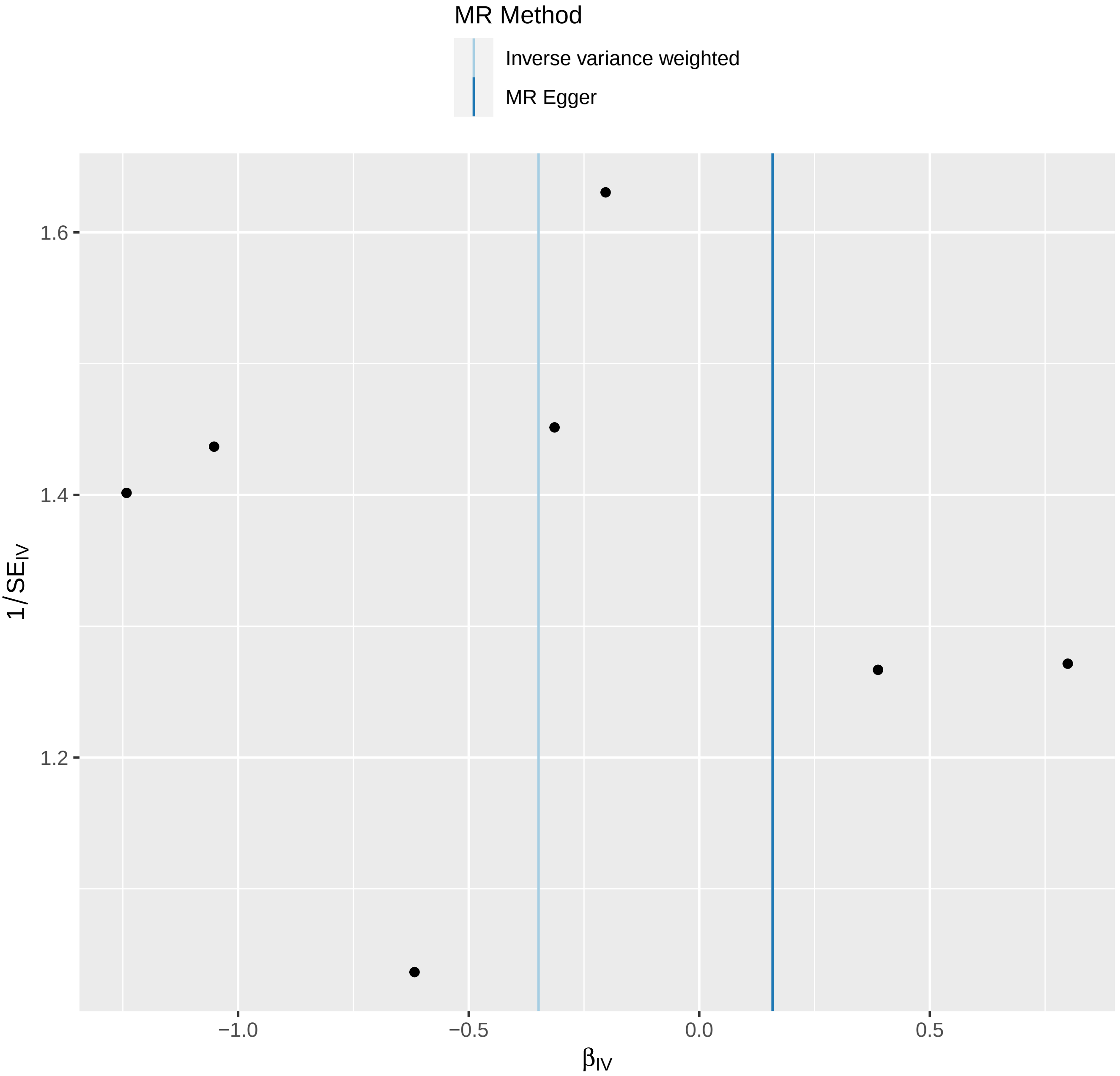

Batch 1375 : Gut microbiota abundance (family Prevotellaceae id.960) on Ulcerative proctitis

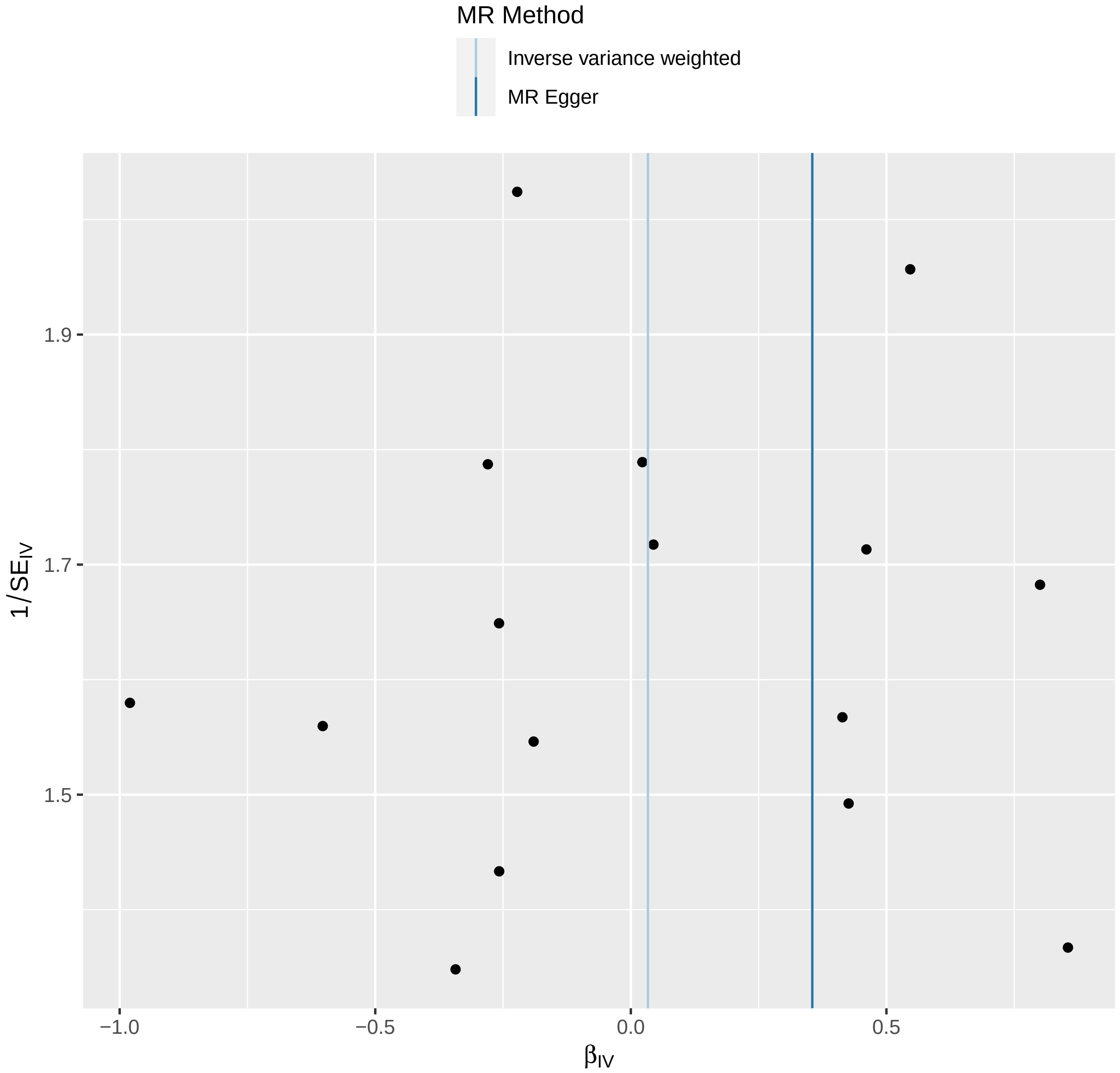

Batch 1376 : Gut microbiota abundance (family Rhodospirillaceae id.2717) on Ulcerative proctitis

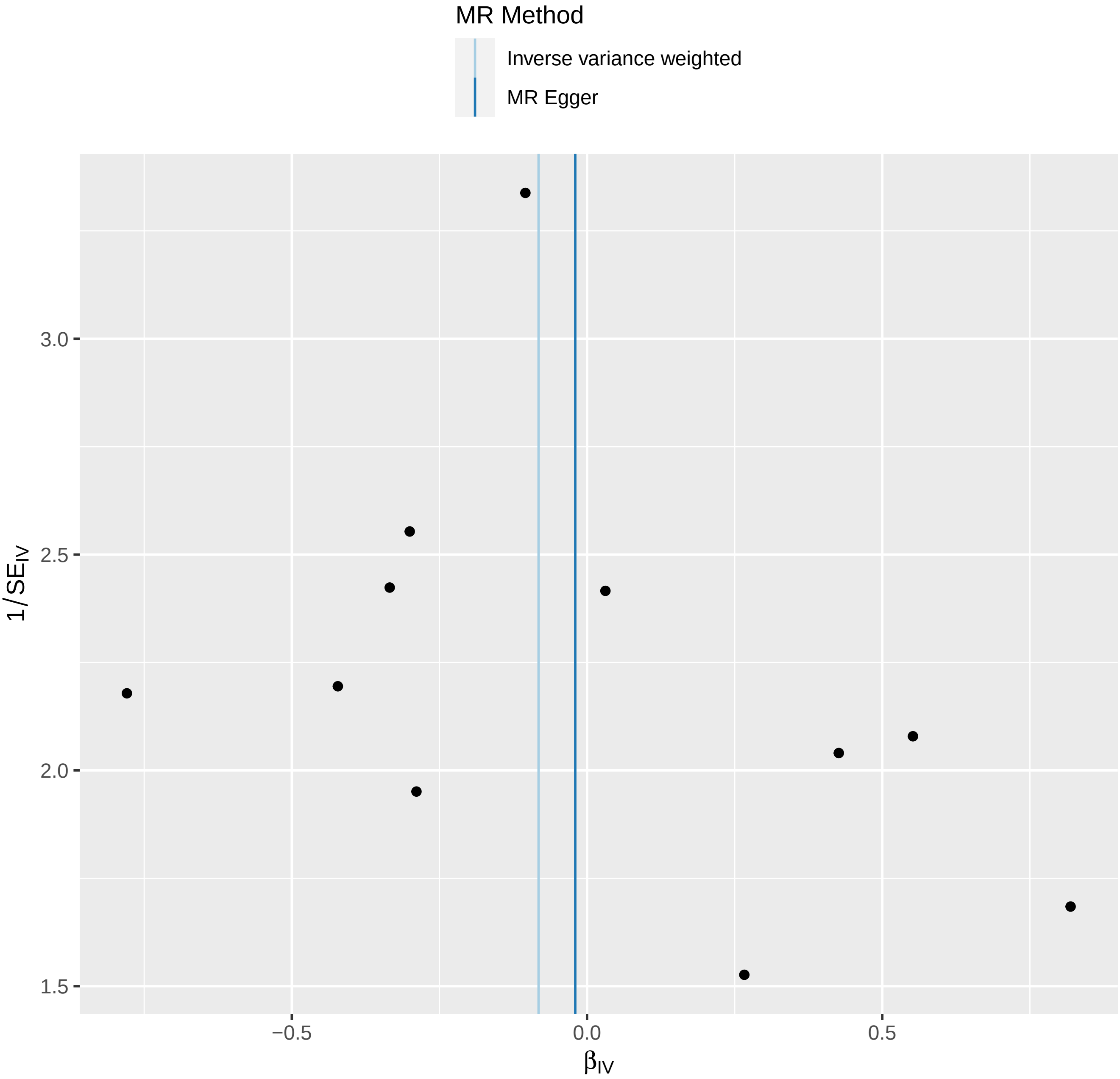

Batch 1377 : Gut microbiota abundance (family Rikenellaceae id.967) on Ulcerative proctitis

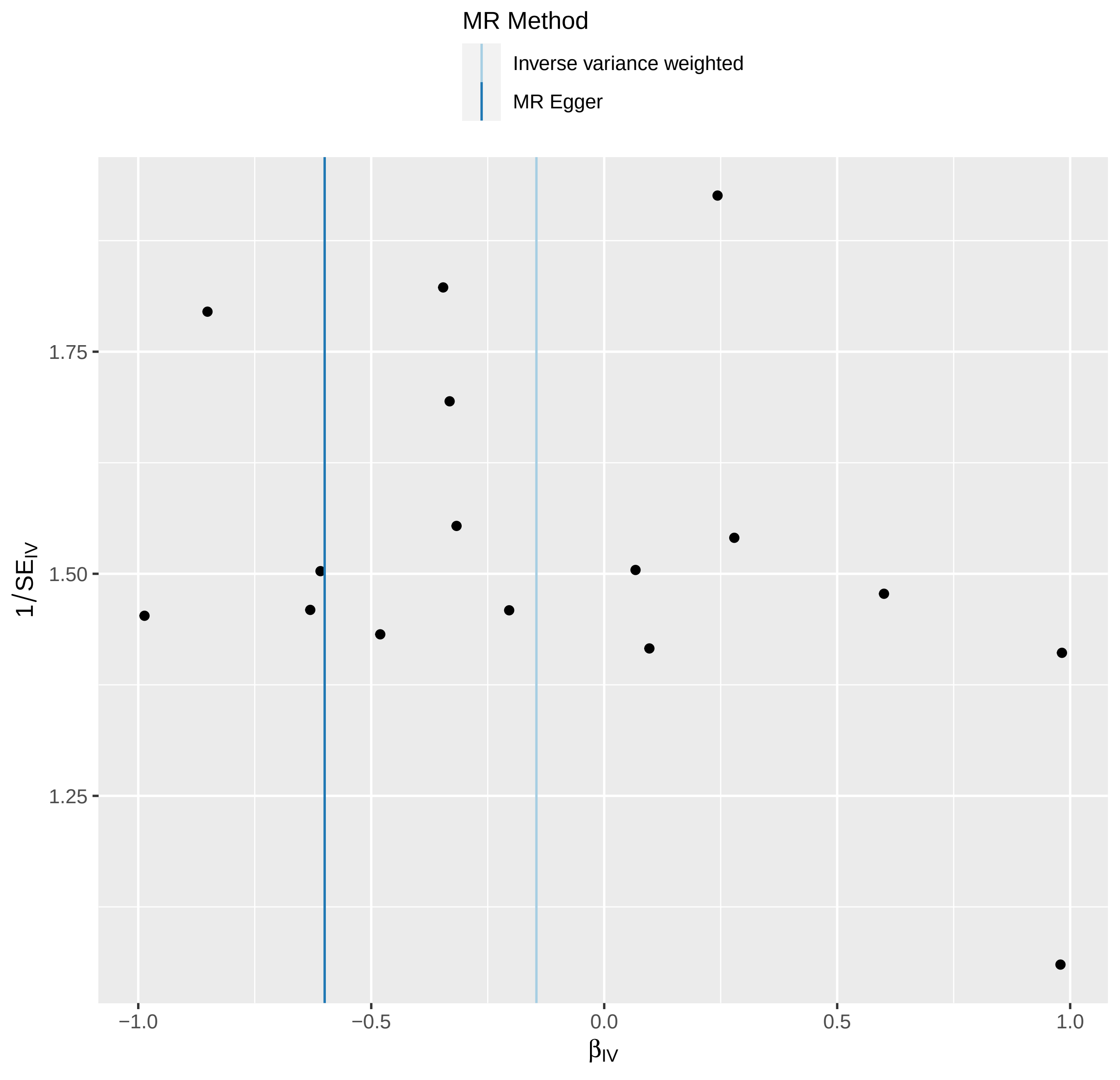

Batch 1378 : Gut microbiota abundance (family Ruminococcaceae id.2050) on Ulcerative proctitis

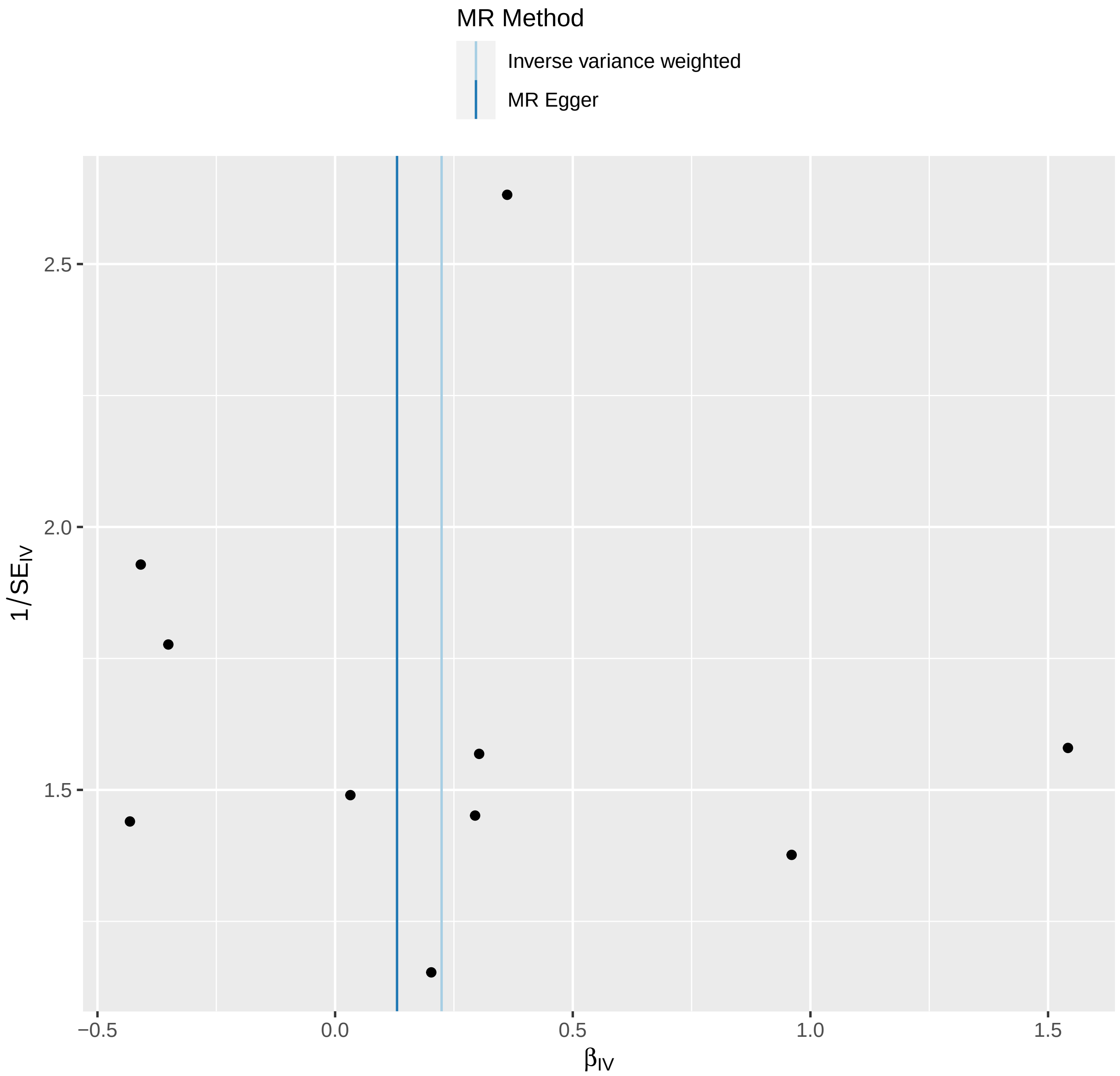

Batch 1379 : Gut microbiota abundance (family Streptococcaceae id.1850) on Ulcerative proctitis

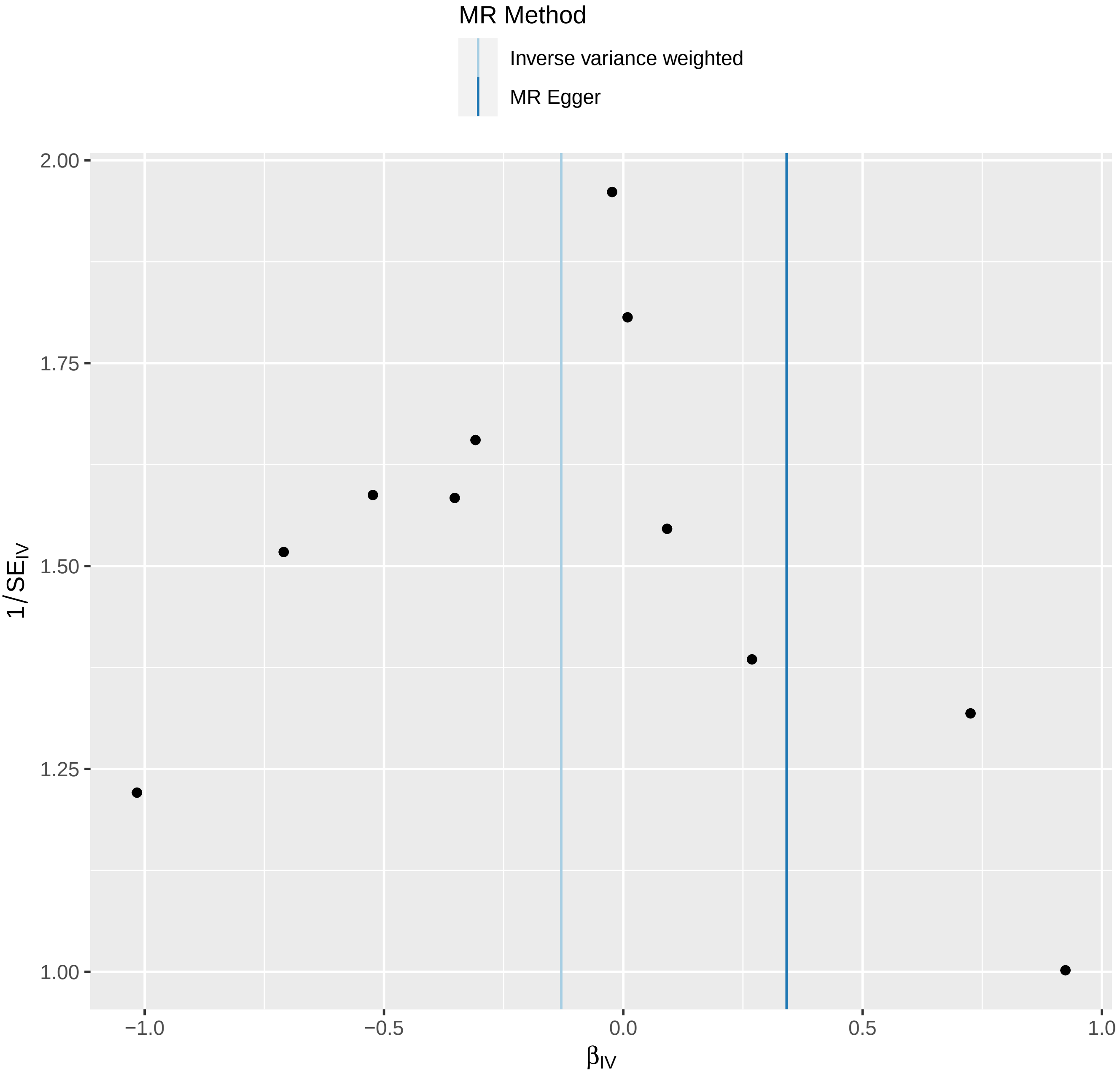

Batch 1380 : Gut microbiota abundance (family Veillonellaceae id.2172) on Ulcerative proctitis

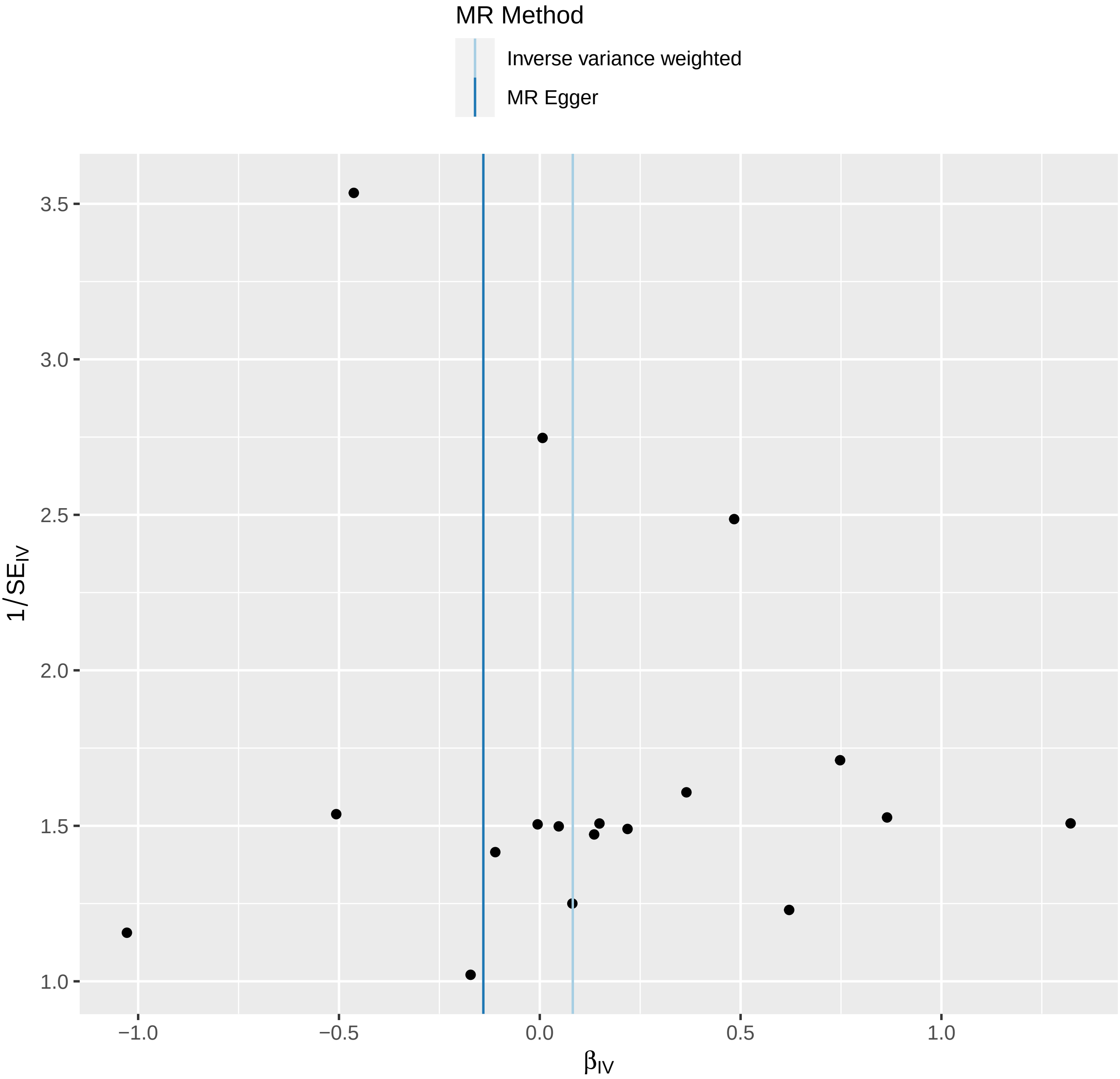

Batch 1381 : Gut microbiota abundance (family Verrucomicrobiaceae id.4036) on Ulcerative proctitis

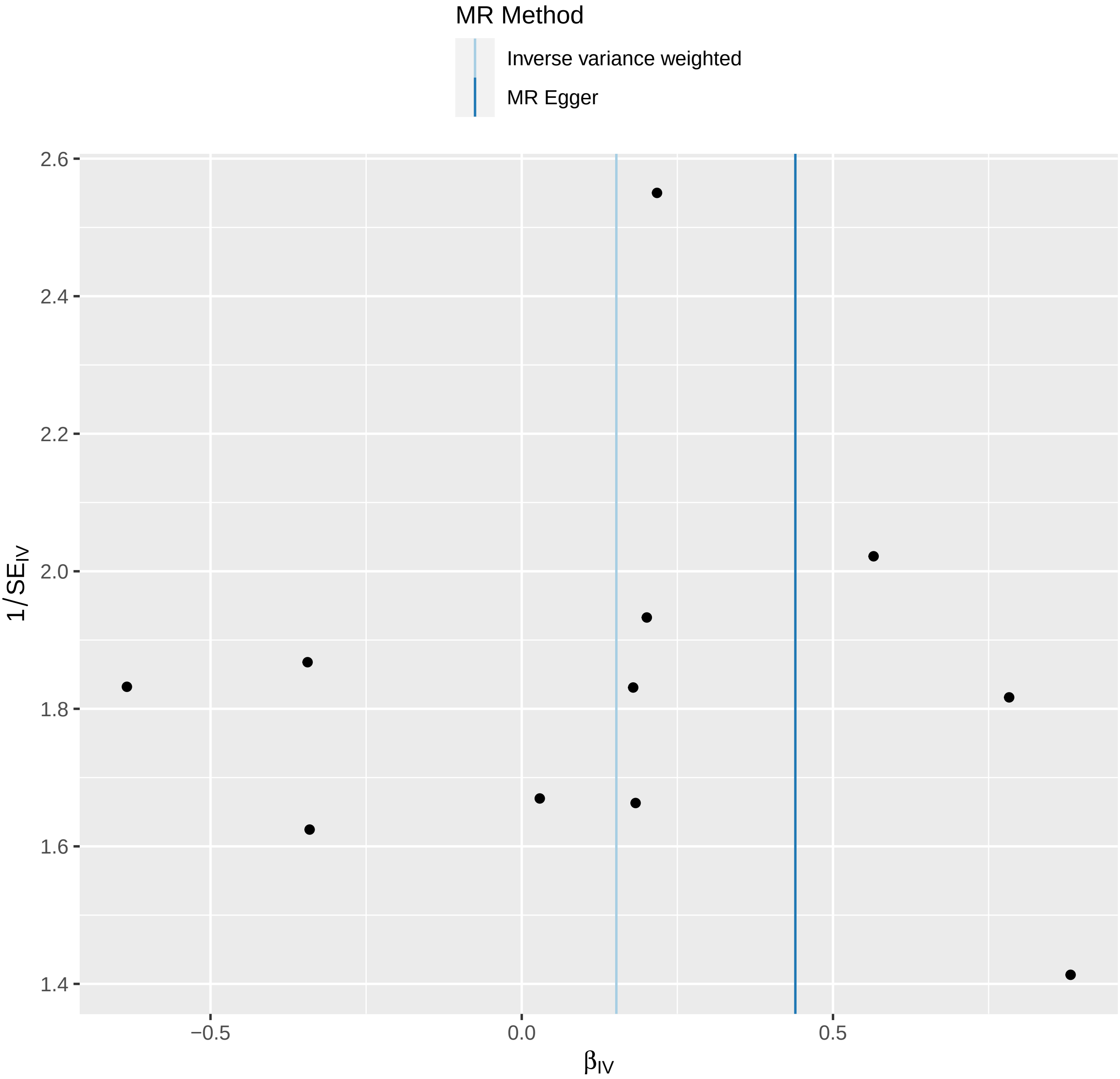

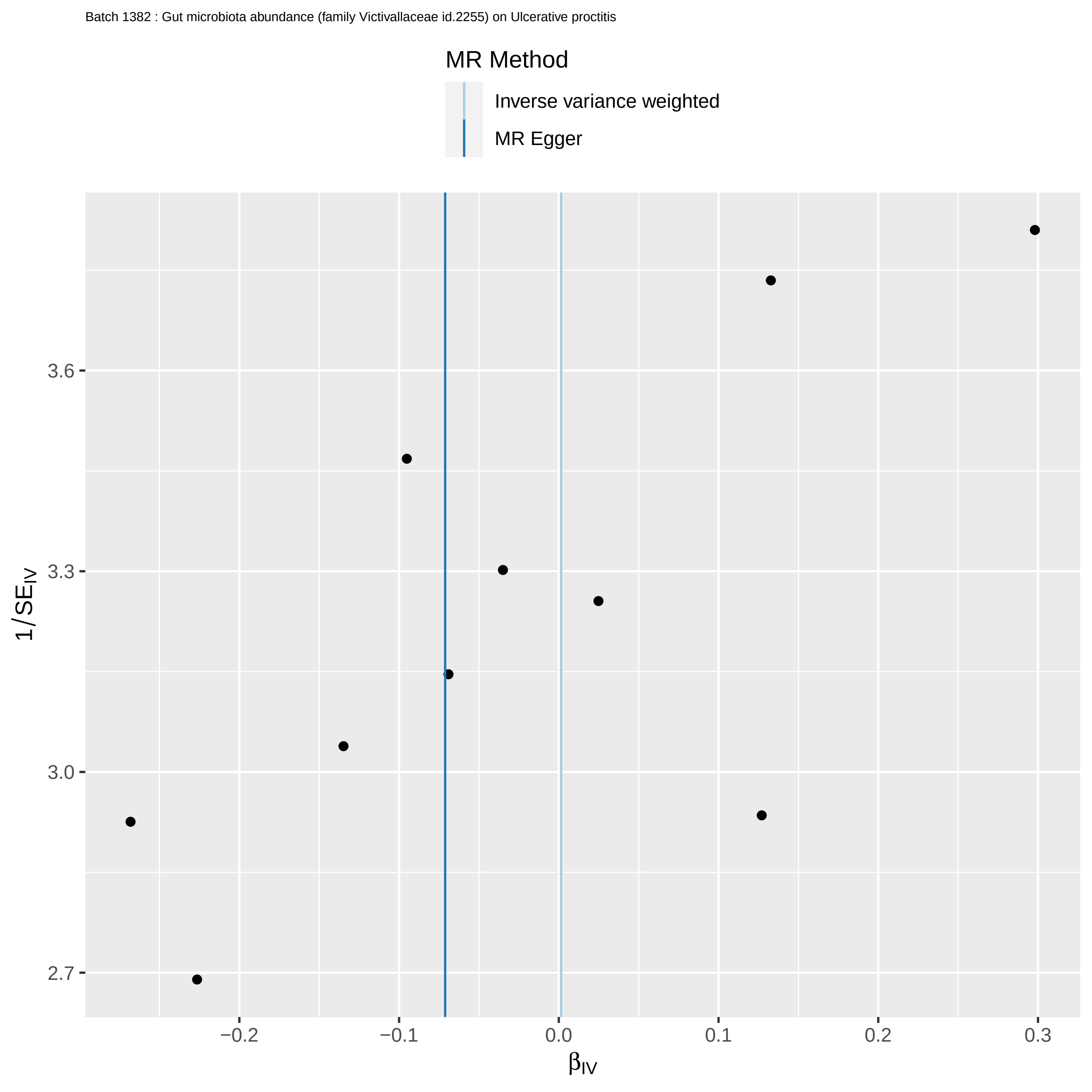
Batch 1382 : Gut microbiota abundance (family Victivallaceae id.2255) on Ulcerative proctitis
MR Method
Inverse variance weighted
MR Egger
3.6
3.3
3.0
2.7
1/SE_IV
−0.2
−0.1
0.0
0.1
0.2
0.3
β_IV

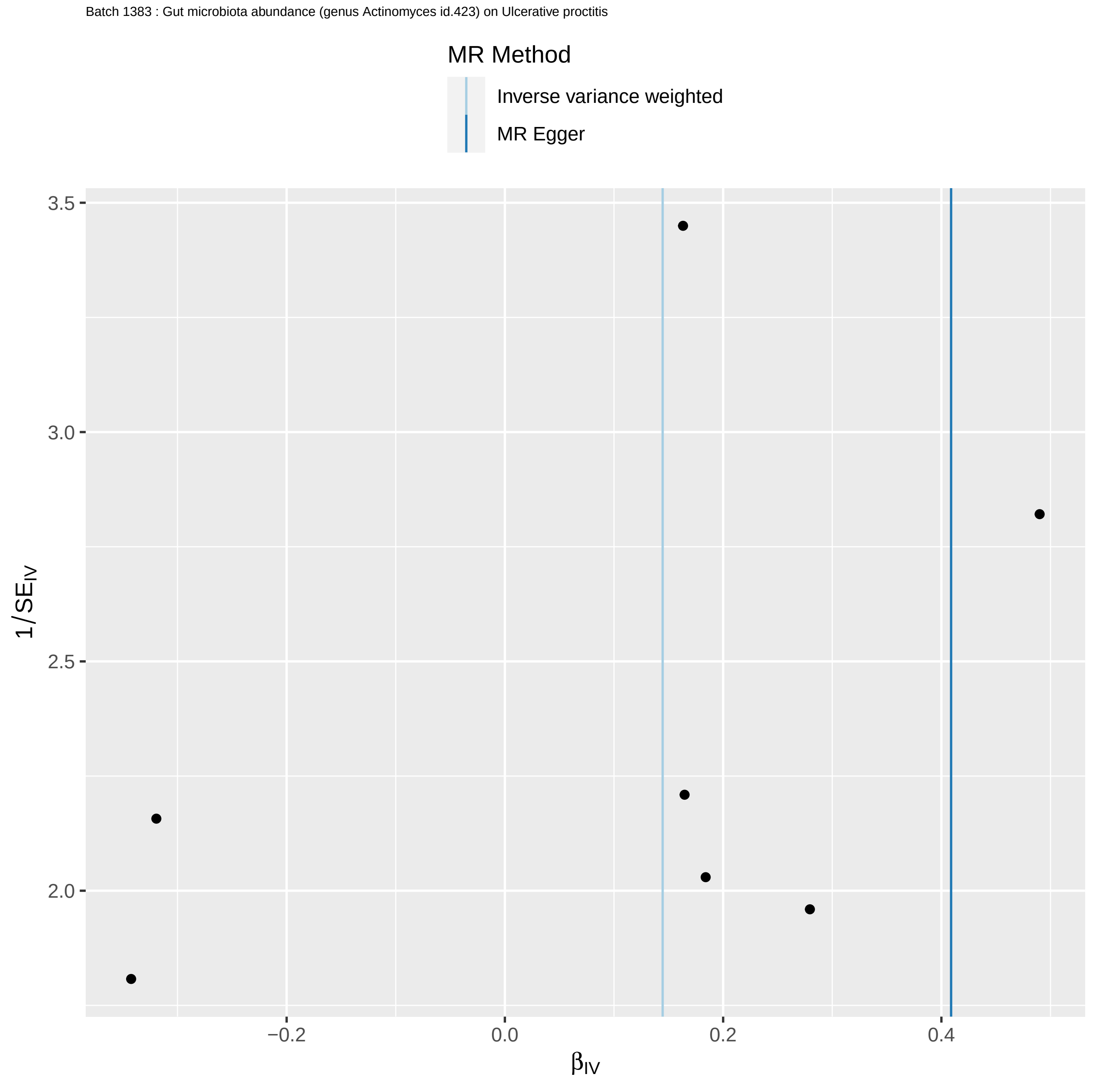

Batch 1383 : Gut microbiota abundance (genus Actinomyces id.423) on Ulcerative proctitis
MR Method
Inverse variance weighted
MR Egger
3.5
3.0
2.5
2.0
1/SE_IV
−0.2
0.0
0.2
0.4
β_IV

Batch 1384 : Gut microbiota abundance (genus Adlercreutzia id.812) on Ulcerative proctitis

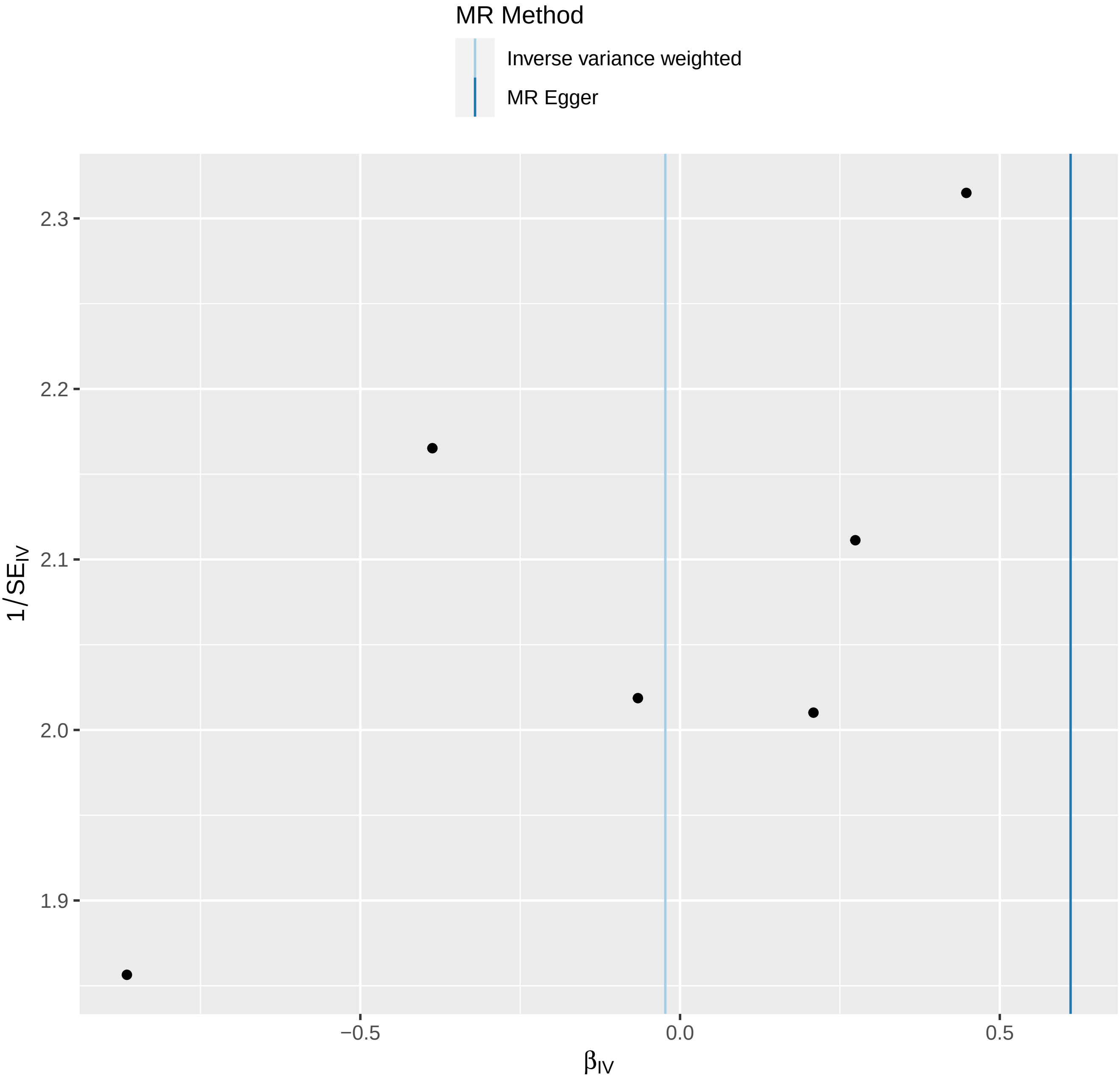

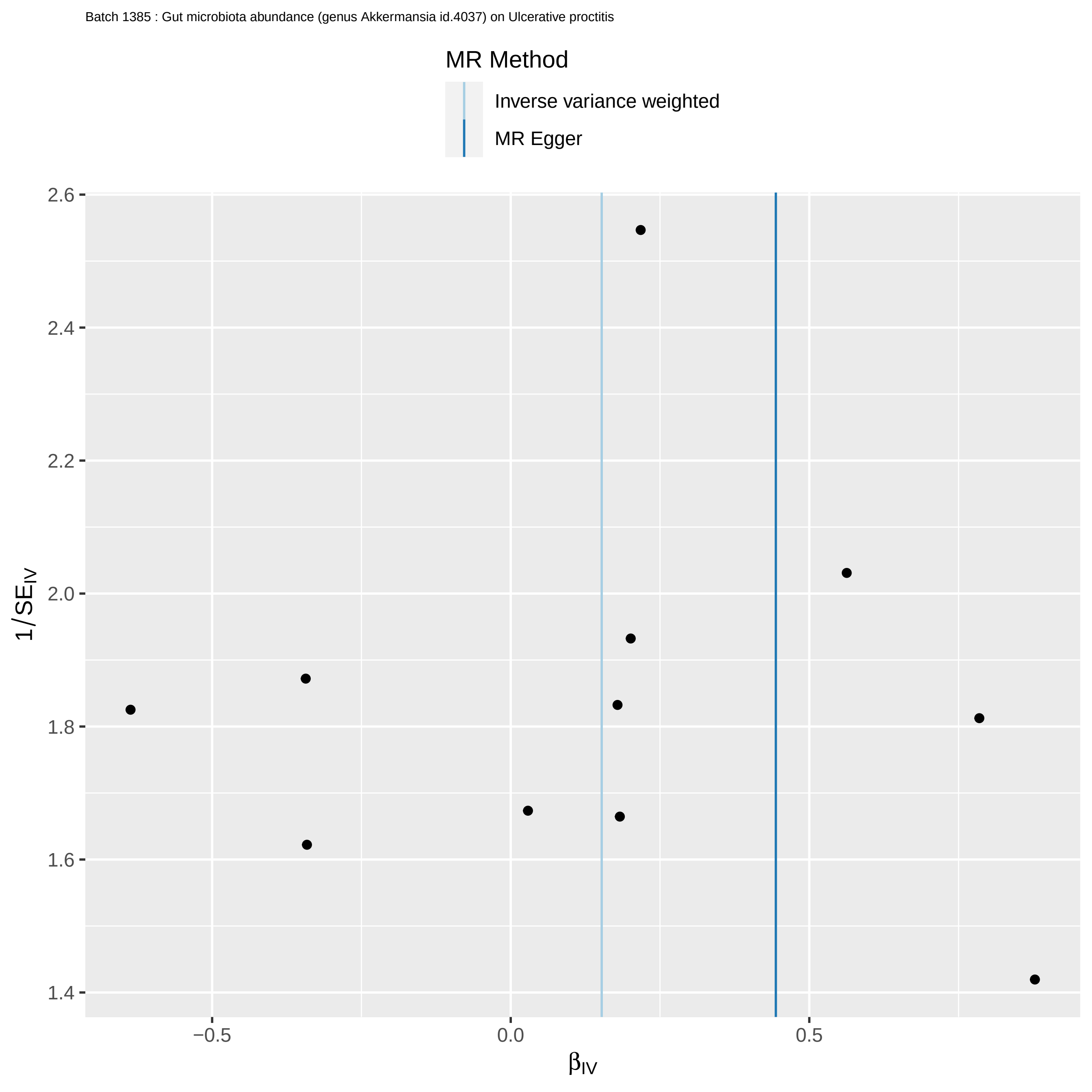
Batch 1385 : Gut microbiota abundance (genus Akkermansia id.4037) on Ulcerative proctitis
MR Method
Inverse variance weighted
MR Egger
2.6
2.4
2.2
2.0
1.8
1.6
1.4
$1/SE_{IV}$
−0.5
0.0
0.5
β_{IV}

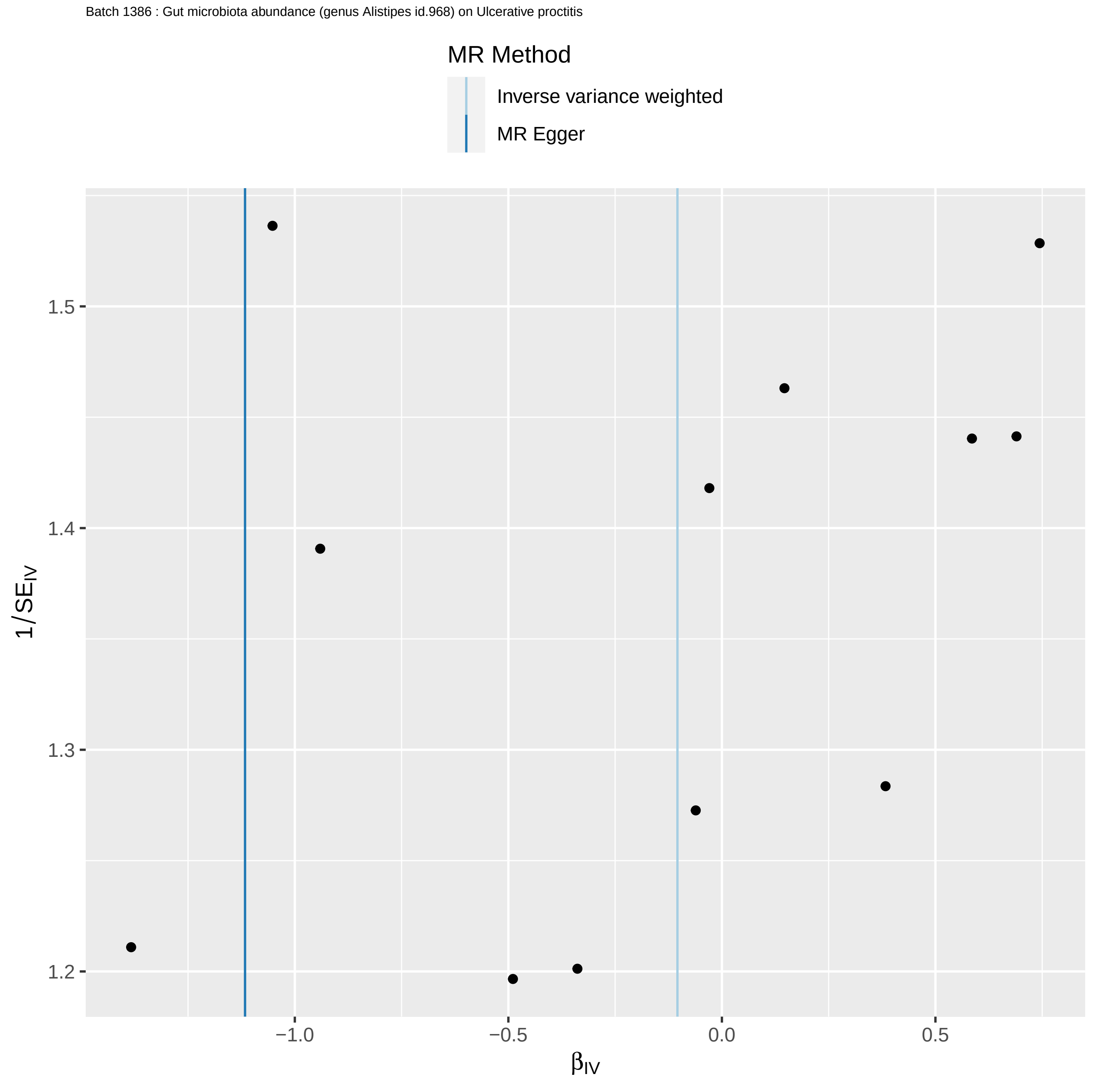
Batch 1386 : Gut microbiota abundance (genus Alistipes id.968) on Ulcerative proctitis
MR Method
Inverse variance weighted
MR Egger
1.5
1.4
1.3
1.2
1/SE_IV
−1.0
−0.5
0.0
0.5
β_IV

Batch 1387 : Gut microbiota abundance (genus Allisonella id.2174) on Ulcerative proctitis

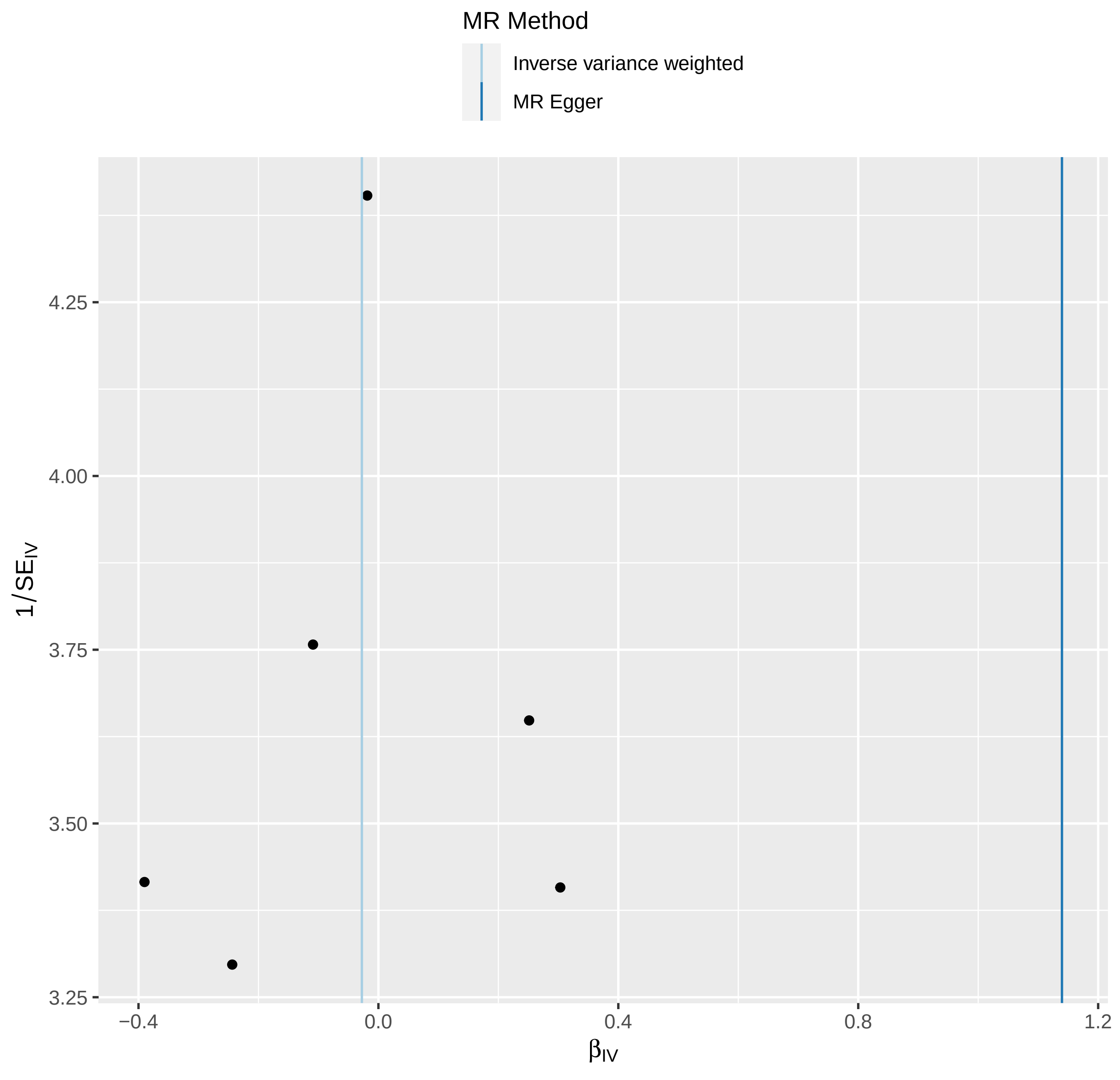

Batch 1388 : Gut microbiota abundance (genus Alloprevotella id.961) on Ulcerative proctitis

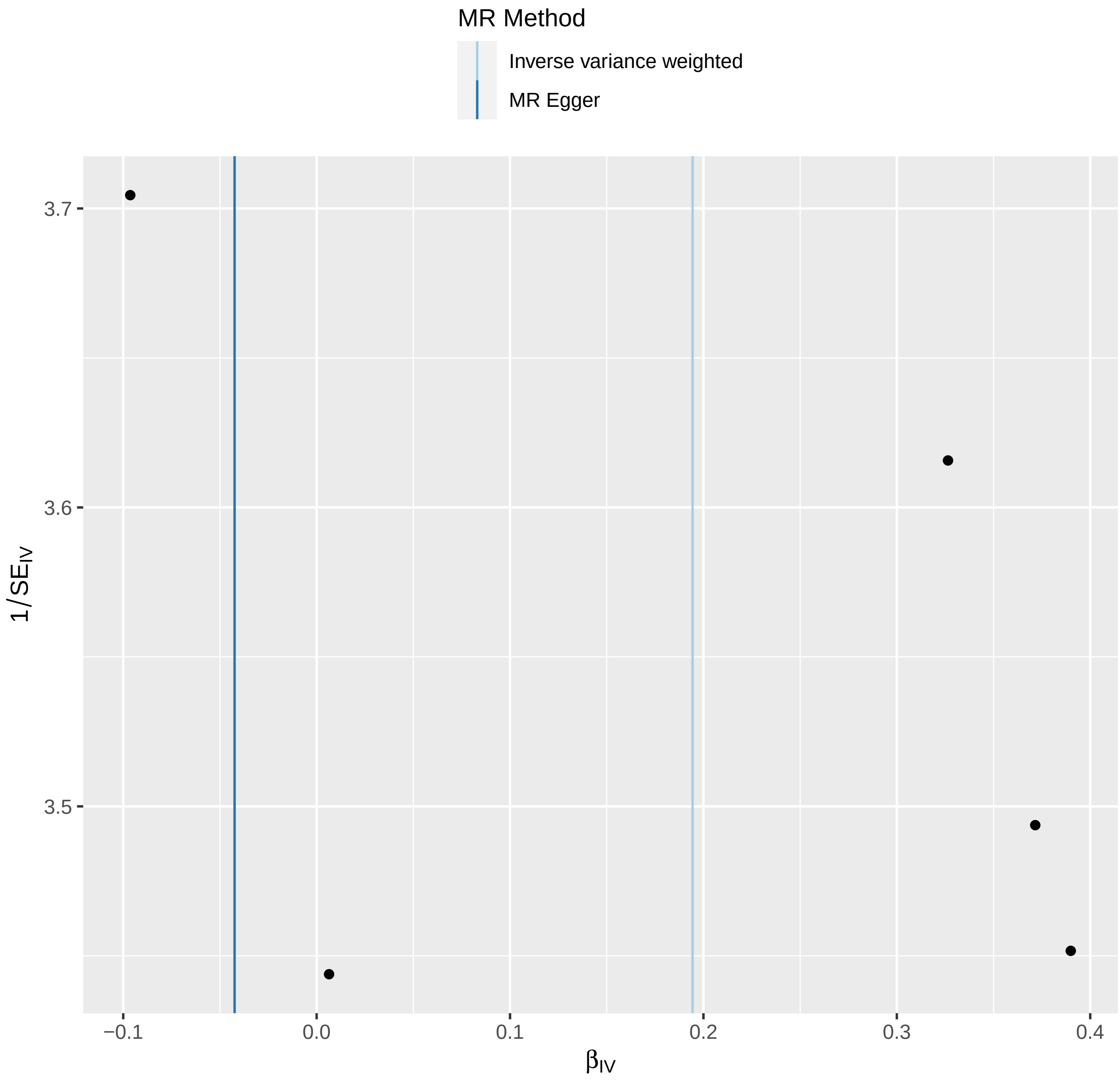

Batch 1389 : Gut microbiota abundance (genus Anaerofilum id.2053) on Ulcerative proctitis

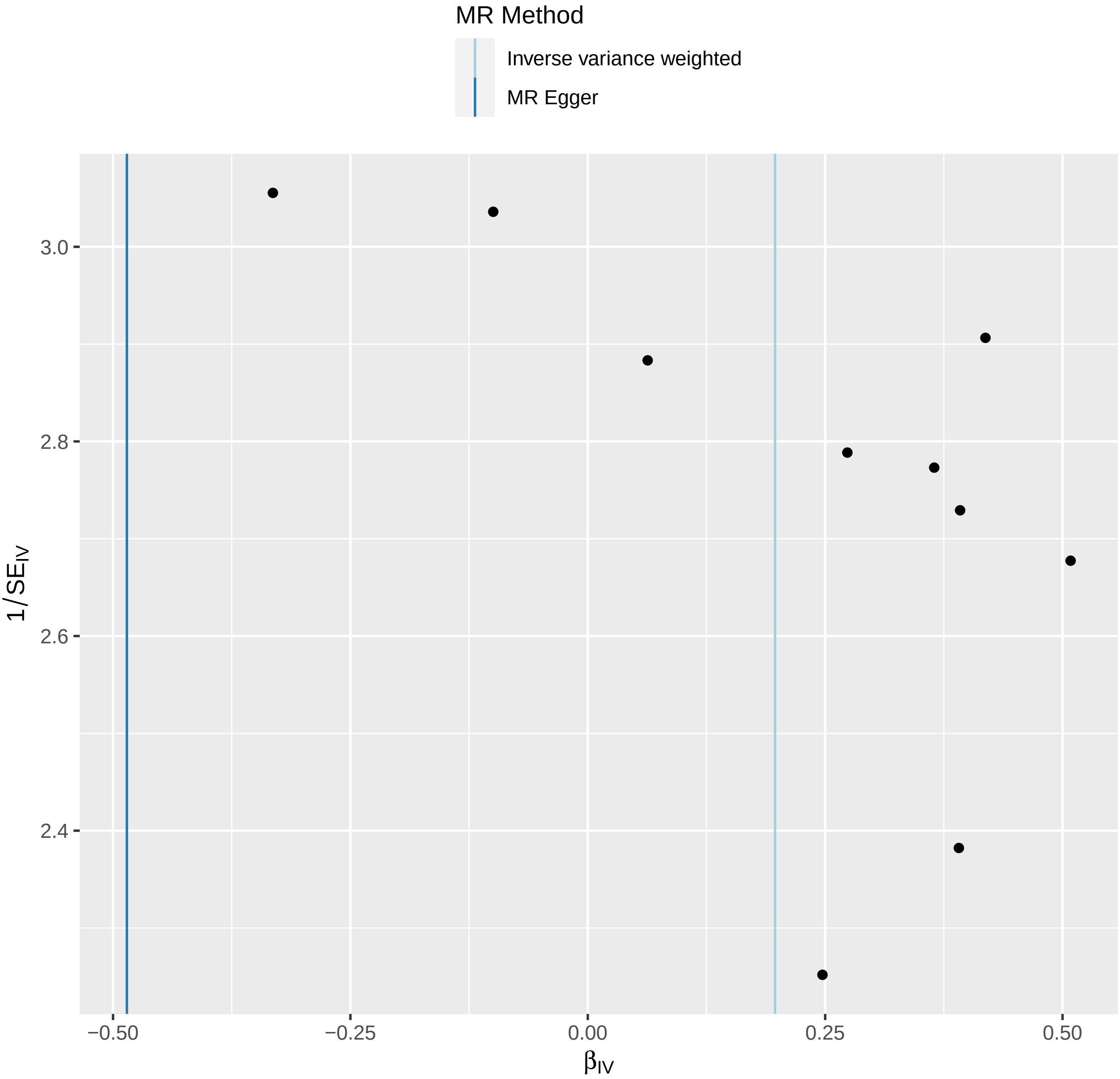

Batch 1390 : Gut microbiota abundance (genus Anaerostipes id.1991) on Ulcerative proctitis

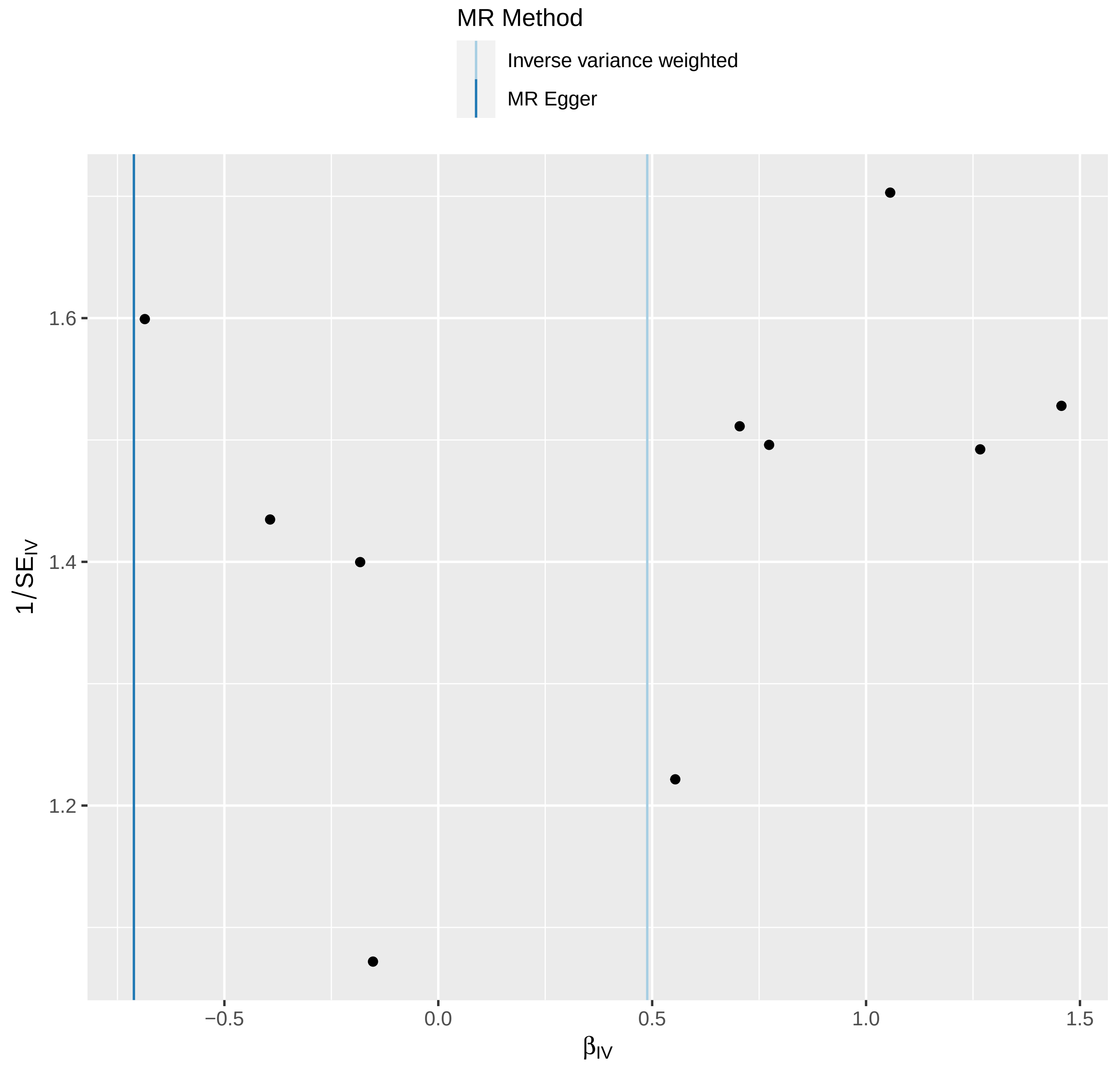

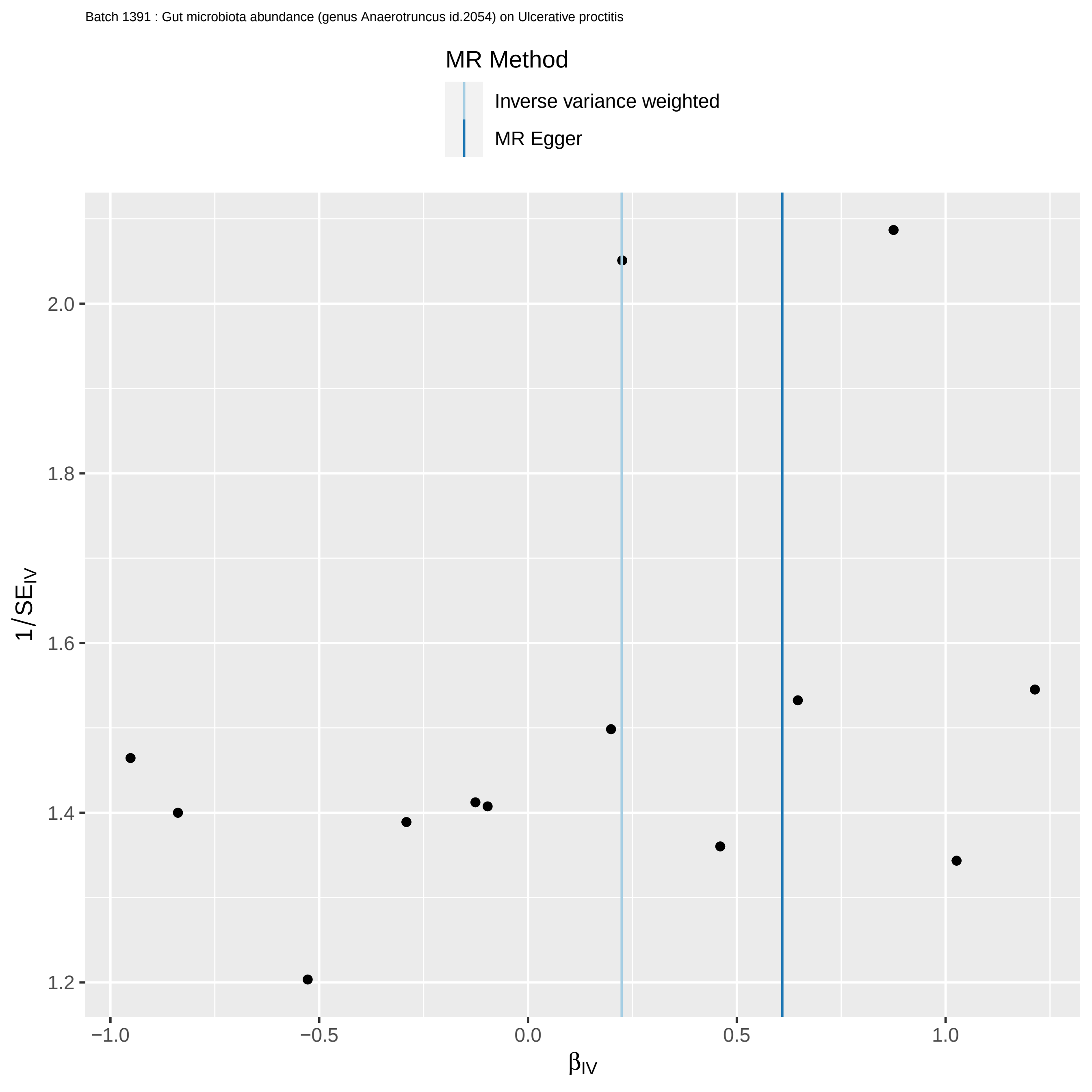
Batch 1391 : Gut microbiota abundance (genus Anaerotruncus id.2054) on Ulcerative proctitis
MR Method
Inverse variance weighted
MR Egger
2.0
1.8
1.6
1.4
1.2
1/SE_IV
−1.0
−0.5
0.0
0.5
1.0
β_IV

Batch 1392 : Gut microbiota abundance (genus Bacteroides id.918) on Ulcerative proctitis

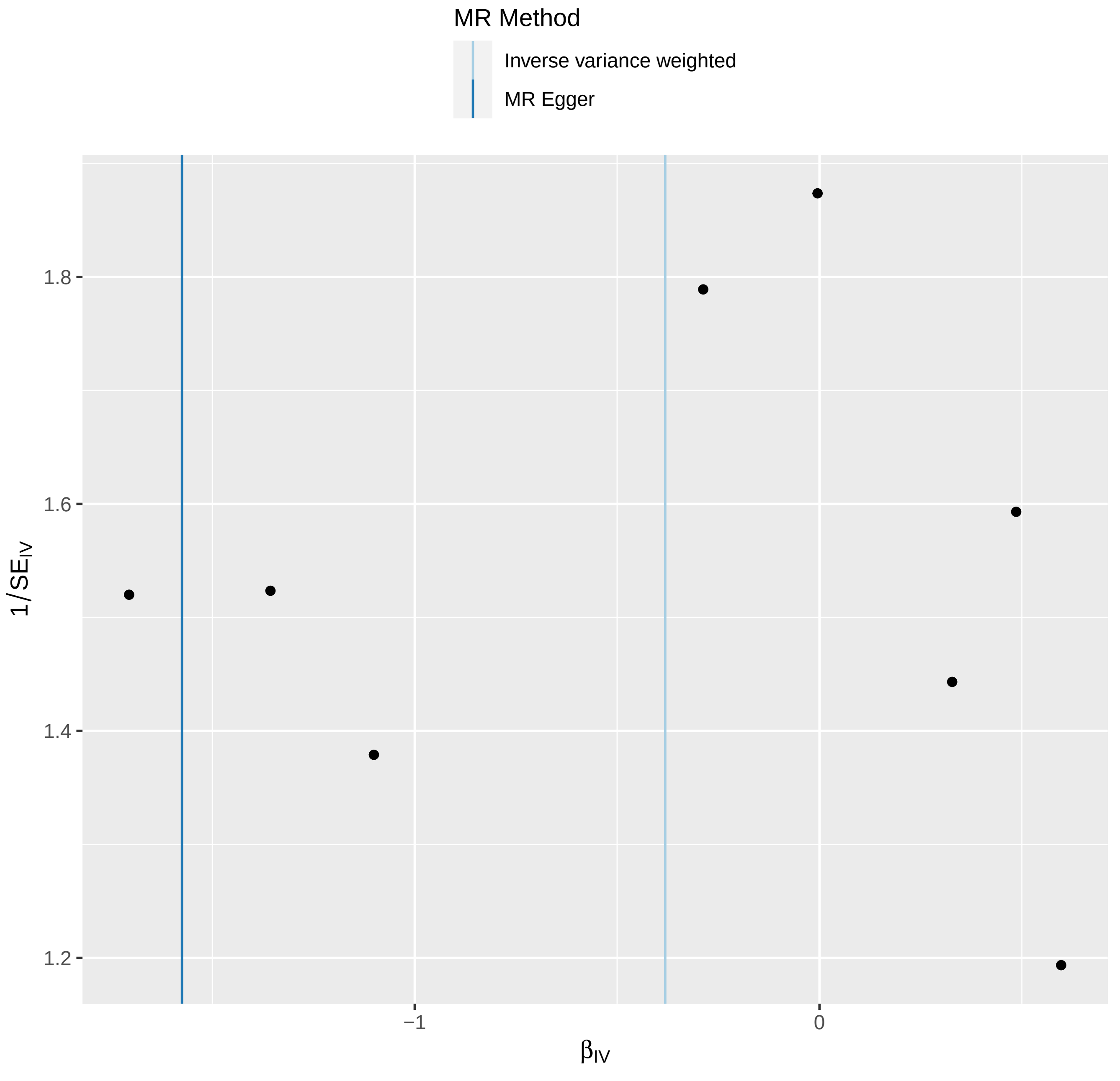

Batch 1393 : Gut microbiota abundance (genus Barnesiella id.944) on Ulcerative proctitis

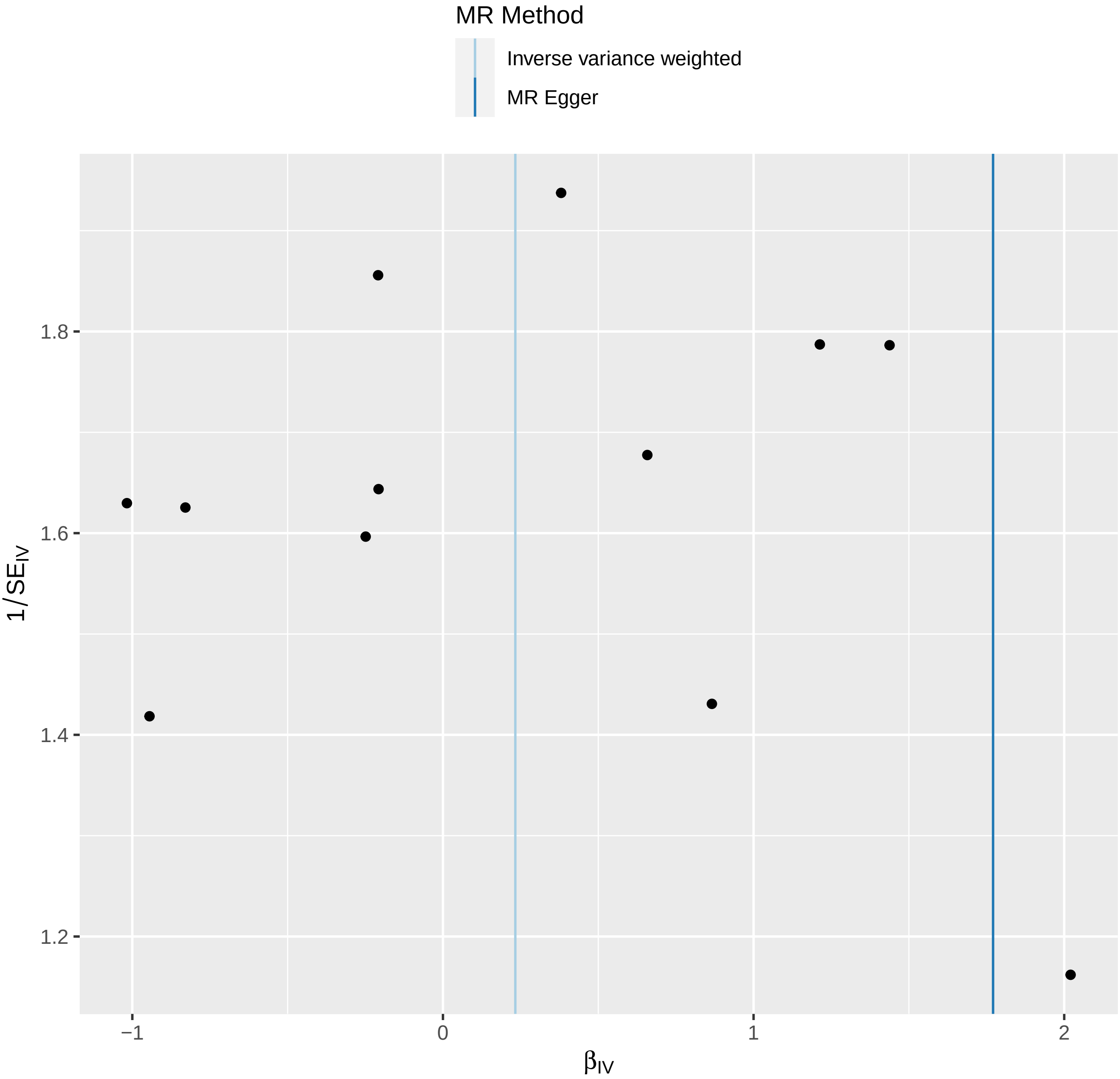

Batch 1394 : Gut microbiota abundance (genus Bifidobacterium id.436) on Ulcerative proctitis

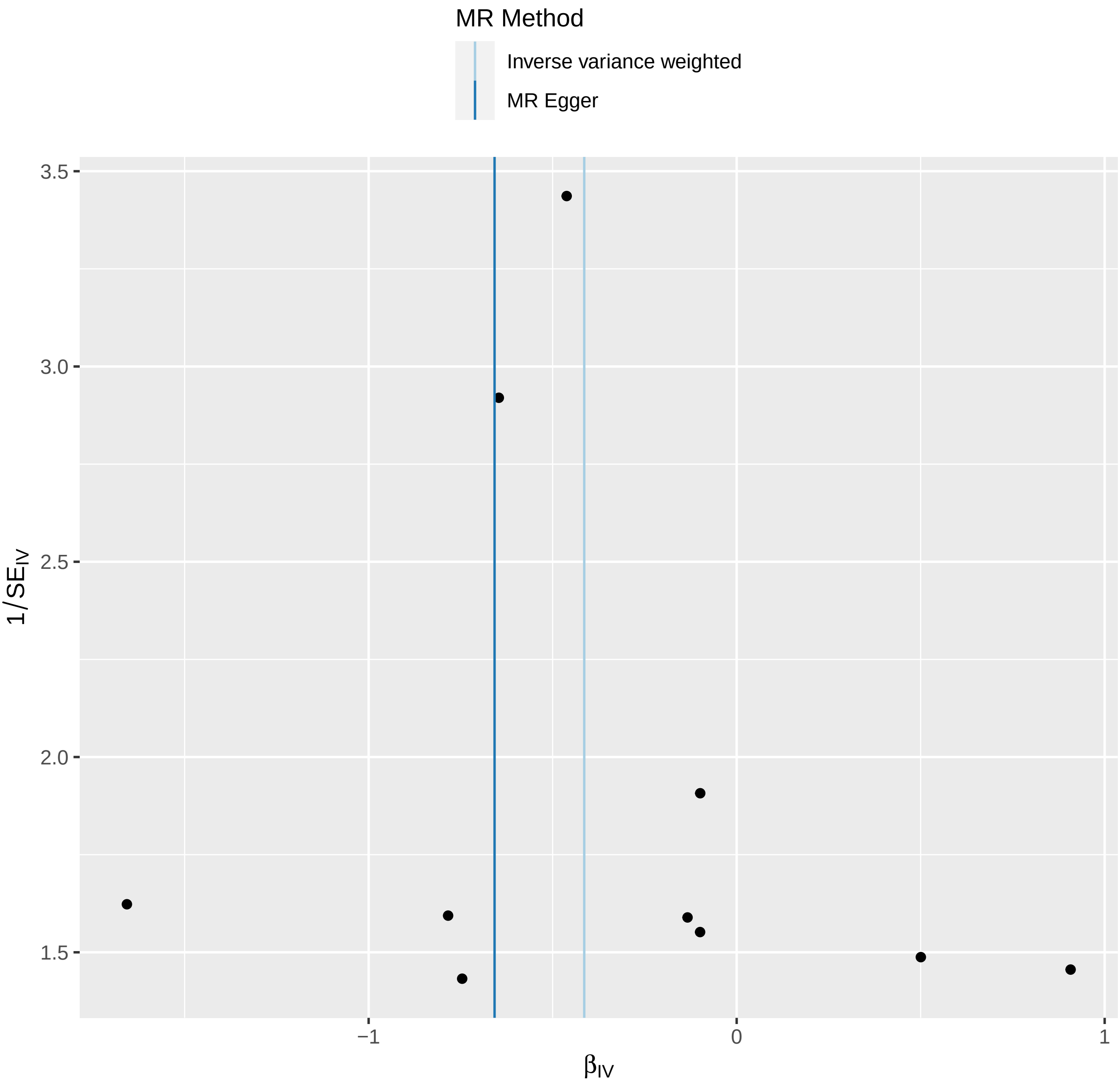

Batch 1395 : Gut microbiota abundance (genus Bilophila id.3170) on Ulcerative proctitis

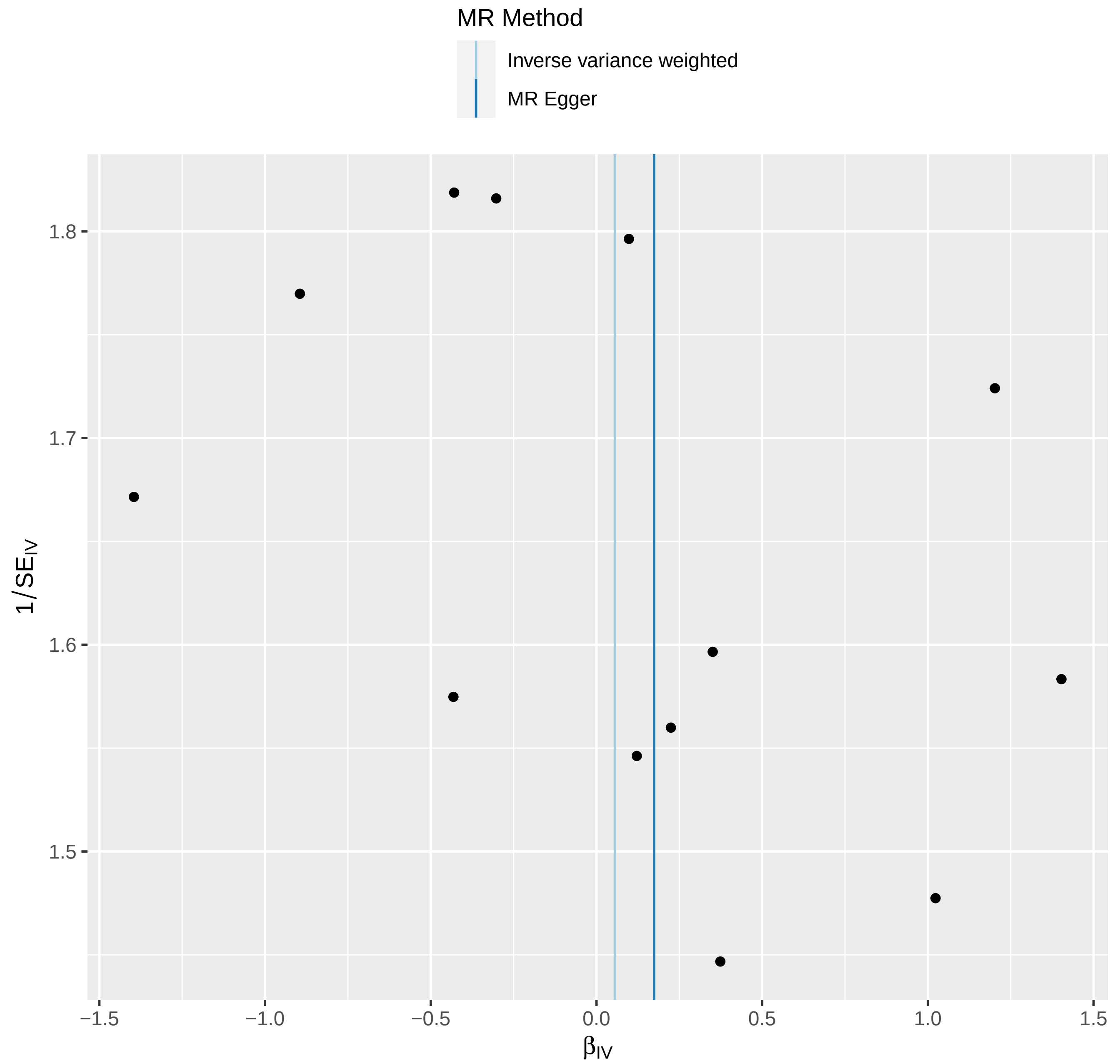

Batch 1396 : Gut microbiota abundance (genus Blautia id.1992) on Ulcerative proctitis

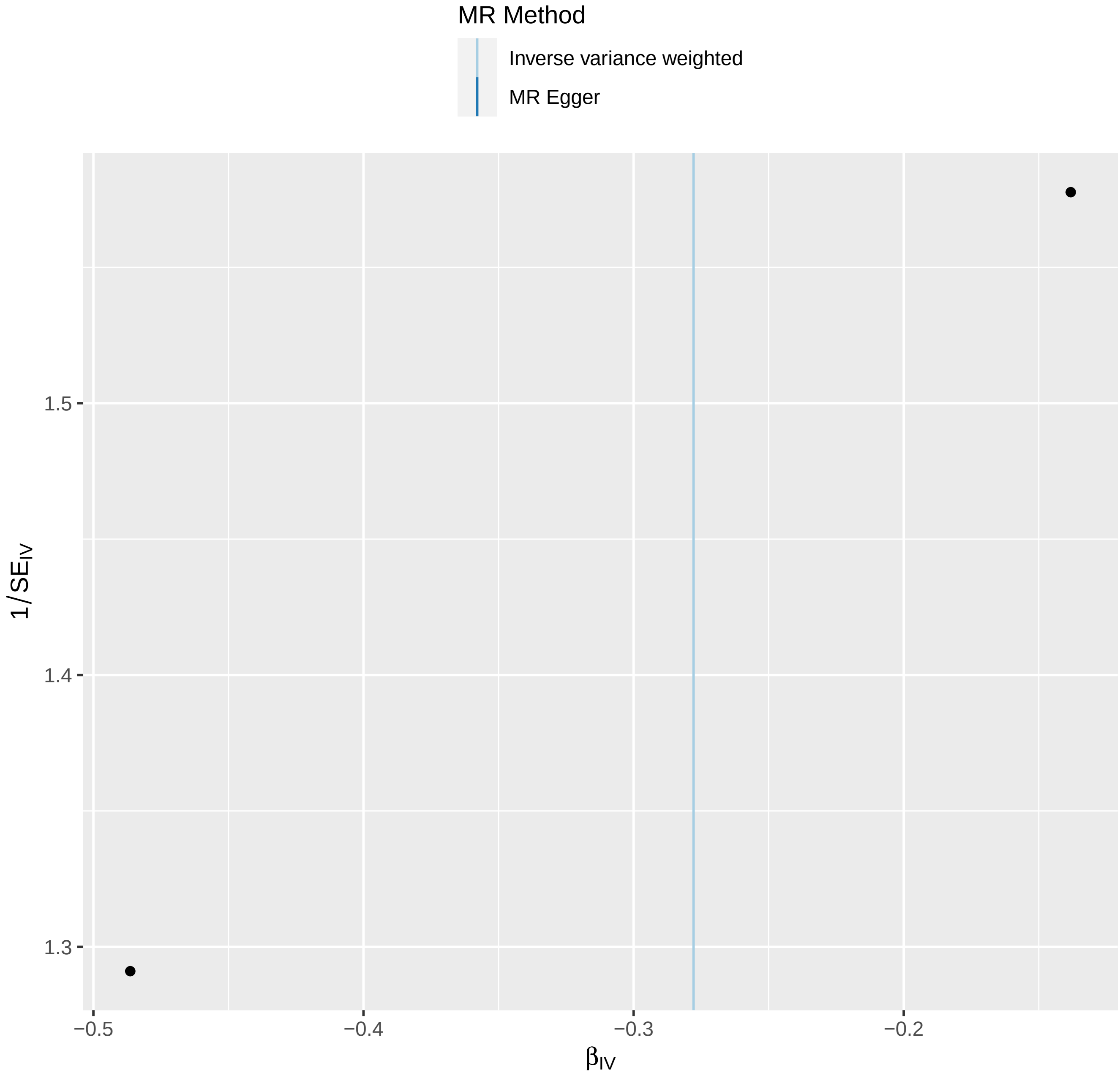

Batch 1397 : Gut microbiota abundance (genus Butyricicoccus id.2055) on Ulcerative proctitis

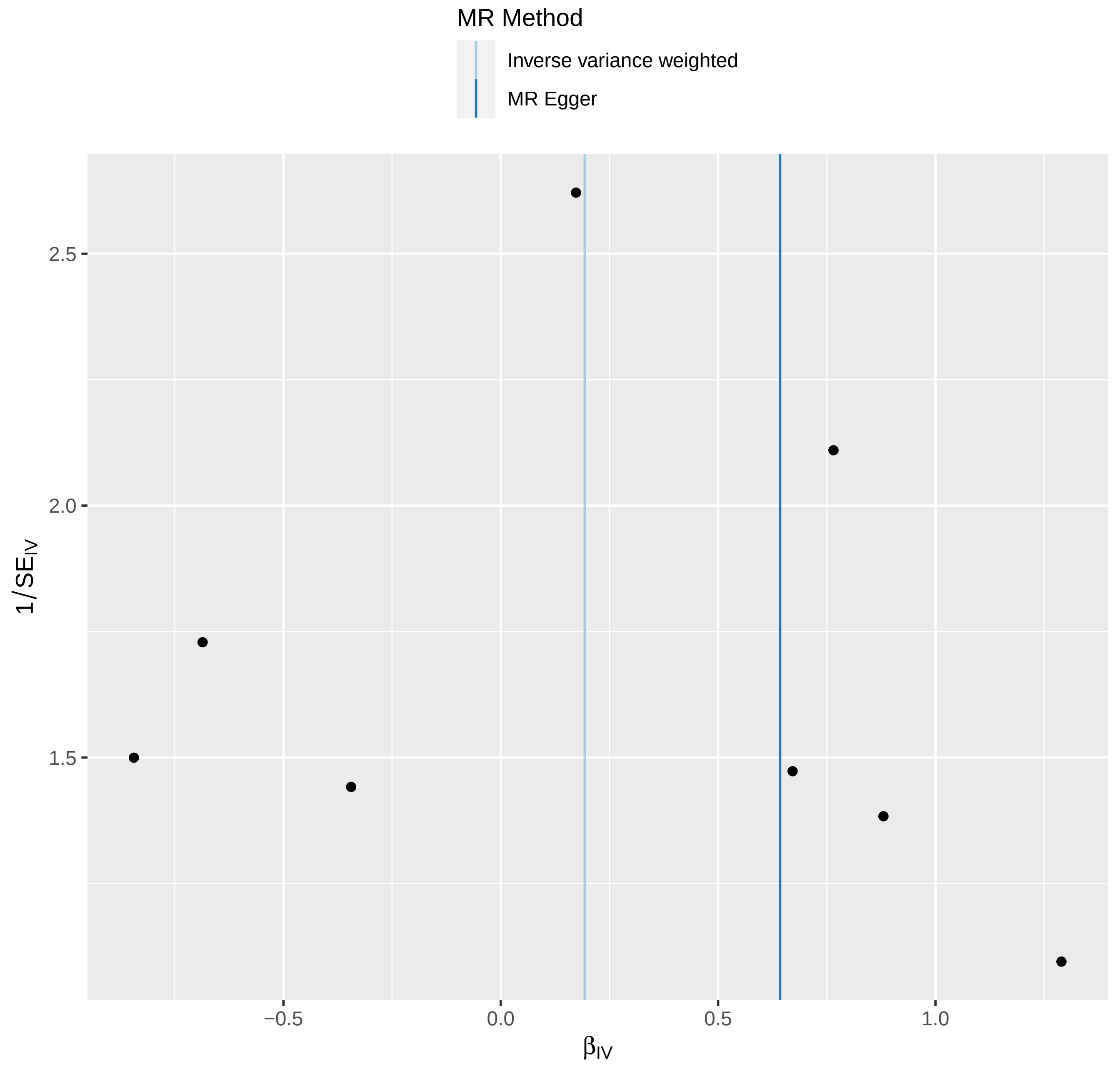

Batch 1398 : Gut microbiota abundance (genus Butyricimonas id.945) on Ulcerative proctitis

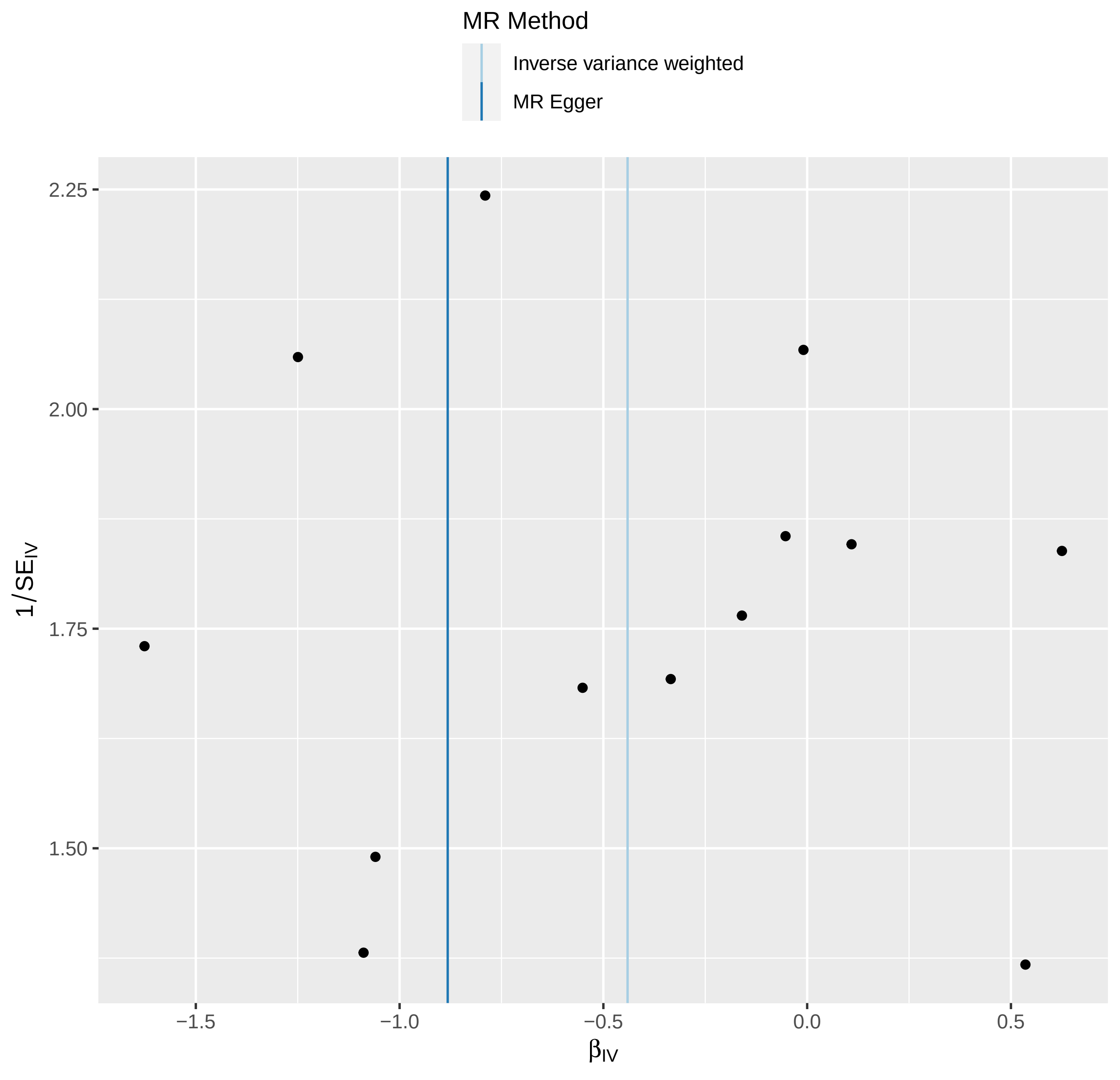

Batch 1399 : Gut microbiota abundance (genus Butyrivibrio id.1993) on Ulcerative proctitis

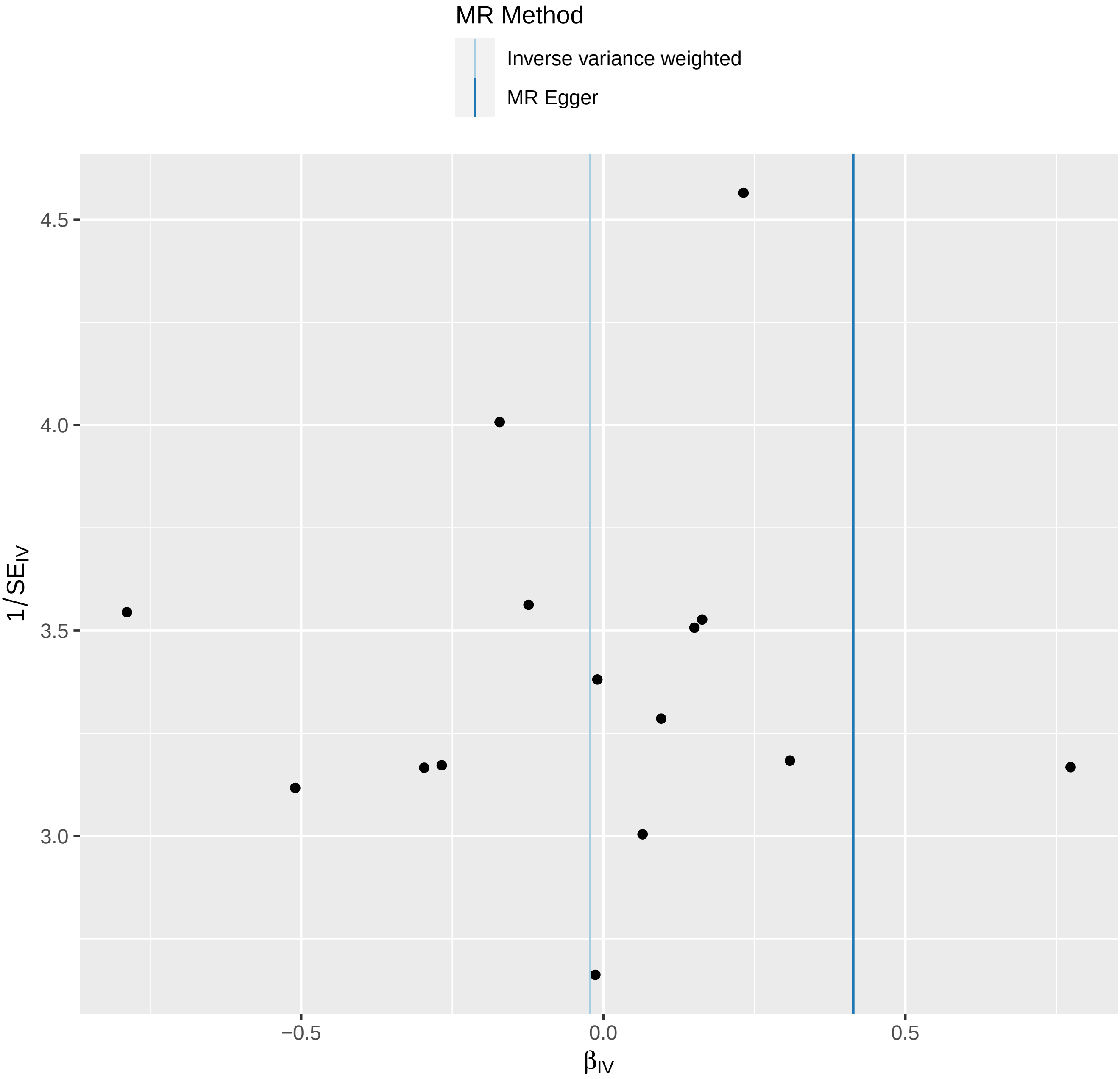

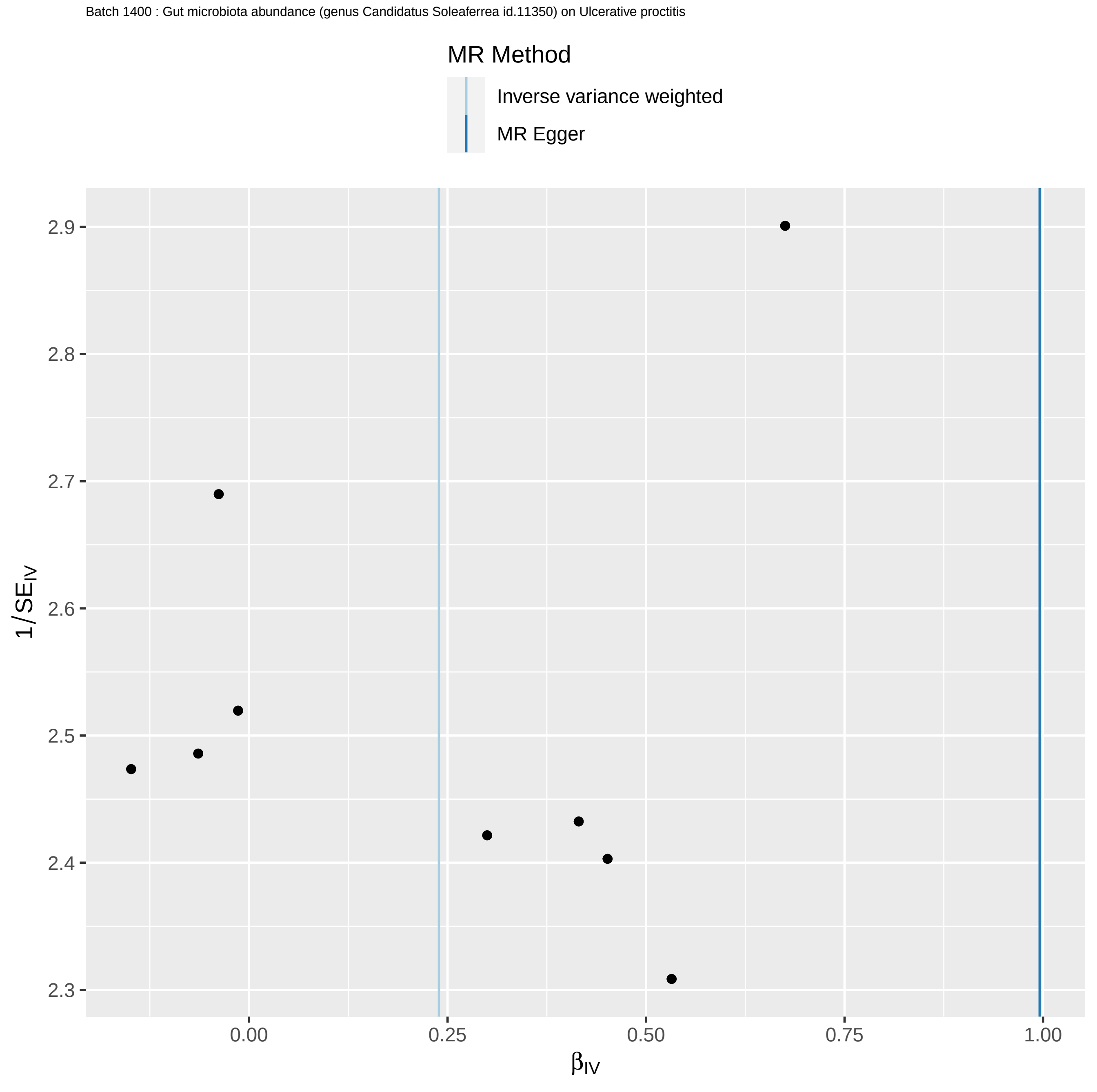
Batch 1400 : Gut microbiota abundance (genus Candidatus Soleaferrea id.11350) on Ulcerative proctitis
MR Method
Inverse variance weighted
MR Egger
1/SE_IV
2.9
2.8
2.7
2.6
2.5
2.4
2.3
0.00
0.25
0.50
0.75
1.00
β_IV

Batch 1401 : Gut microbiota abundance (genus Catenibacterium id.2153) on Ulcerative proctitis

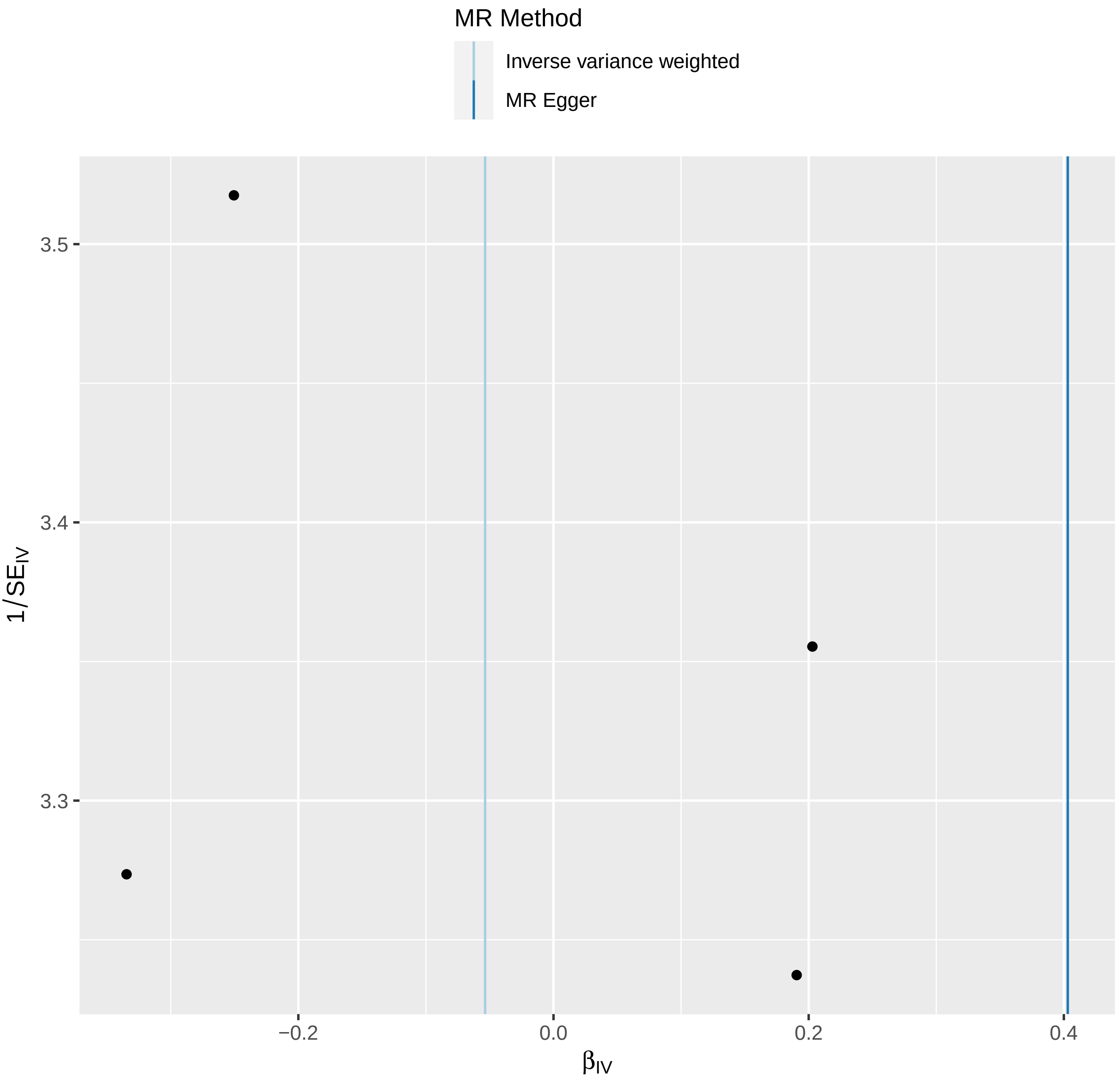

Batch 1402 : Gut microbiota abundance (genus Christensenellaceae R 7group id.11283) on Ulcerative proctitis

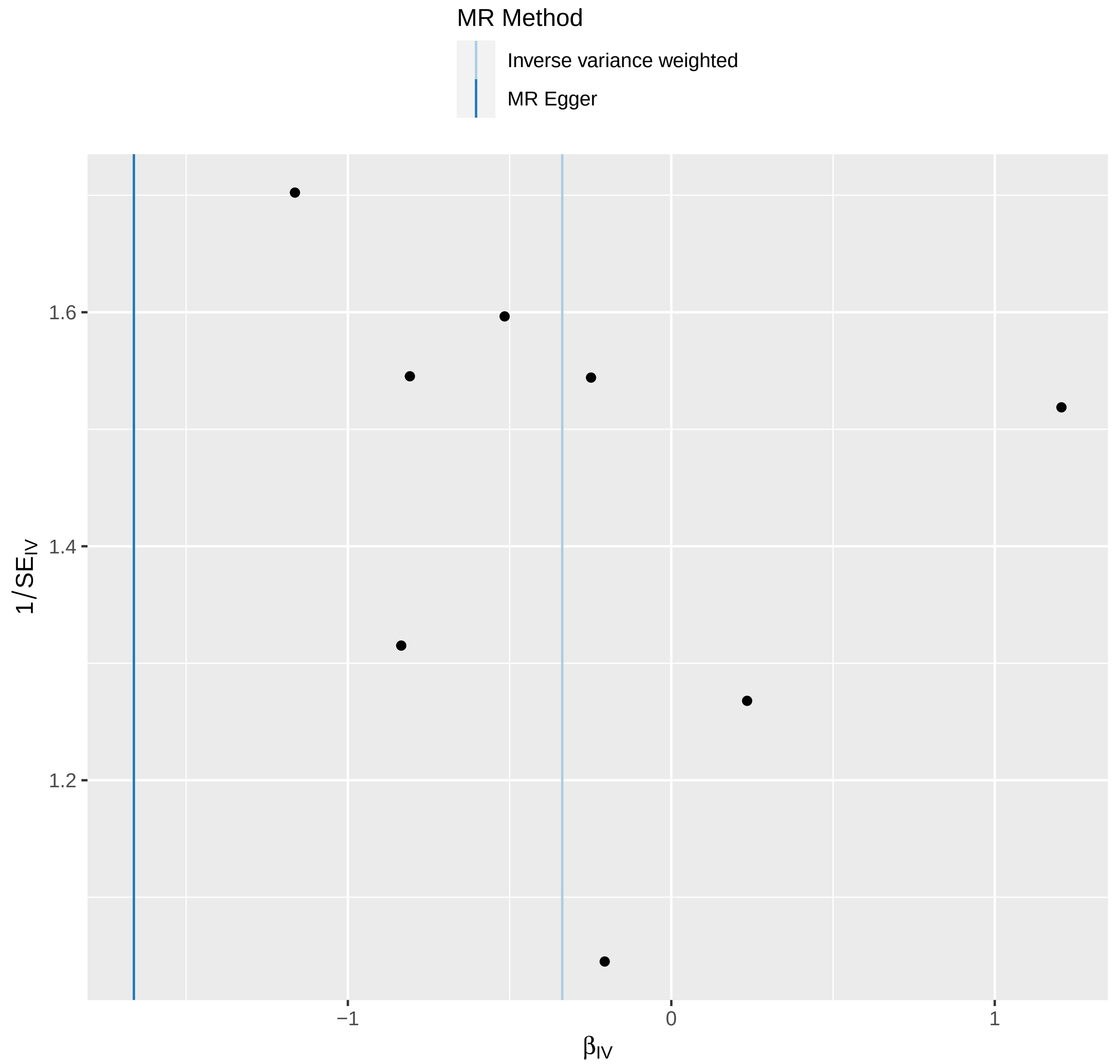

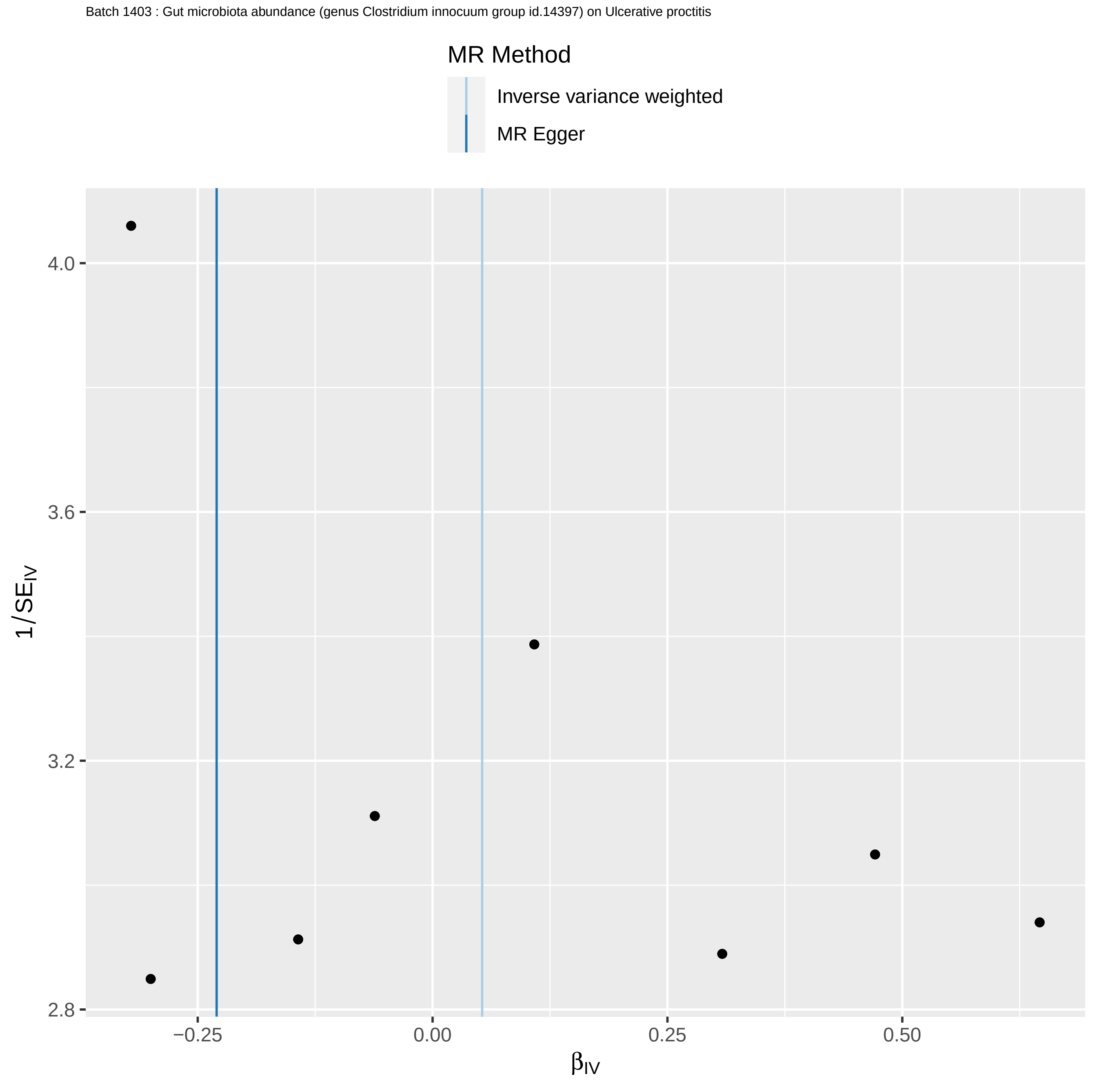
Batch 1403 : Gut microbiota abundance (genus Clostridium innocuum group id.14397) on Ulcerative proctitis
MR Method
Inverse variance weighted
MR Egger
4.0
3.6
3.2
2.8
1/SE$_{IV}$
−0.25
0.00
0.25
0.50
β$_{IV}$

Batch 1404 : Gut microbiota abundance (genus Clostridium sensustricto1 id.1873) on Ulcerative proctitis

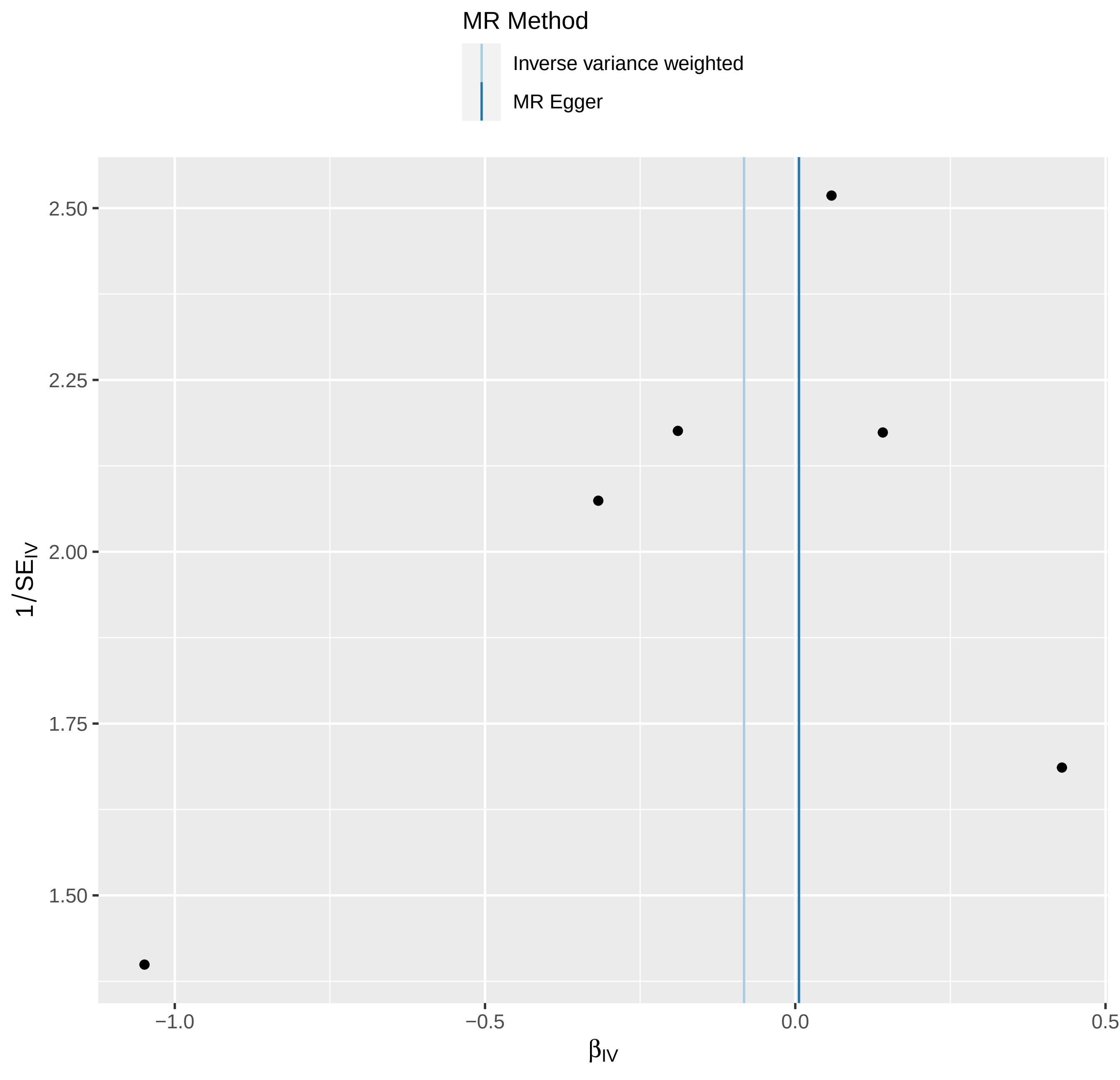

Batch 1405 : Gut microbiota abundance (genus Collinsella id.815) on Ulcerative proctitis

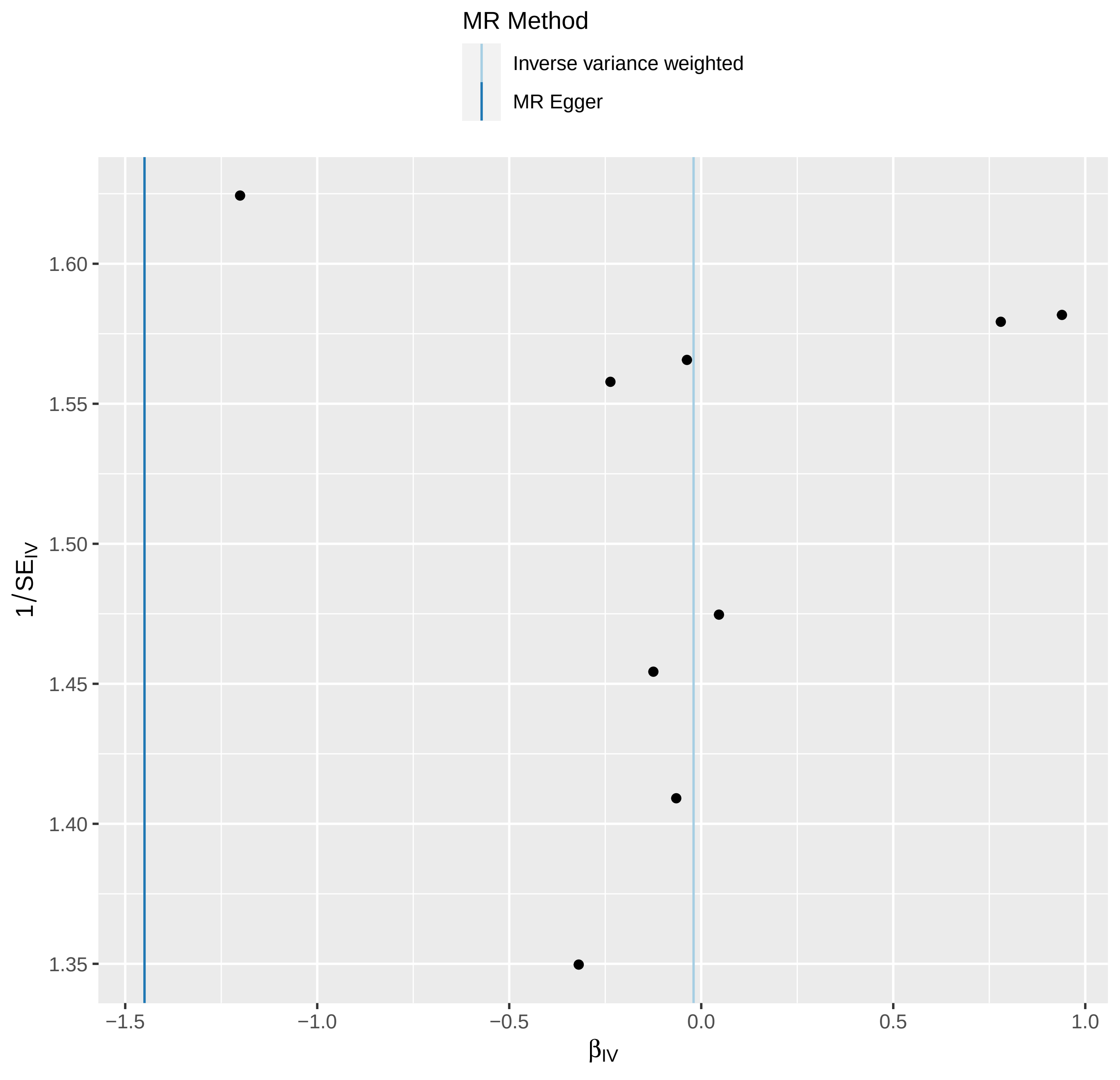

Batch 1406 : Gut microbiota abundance (genus Coprobacter id.949) on Ulcerative proctitis

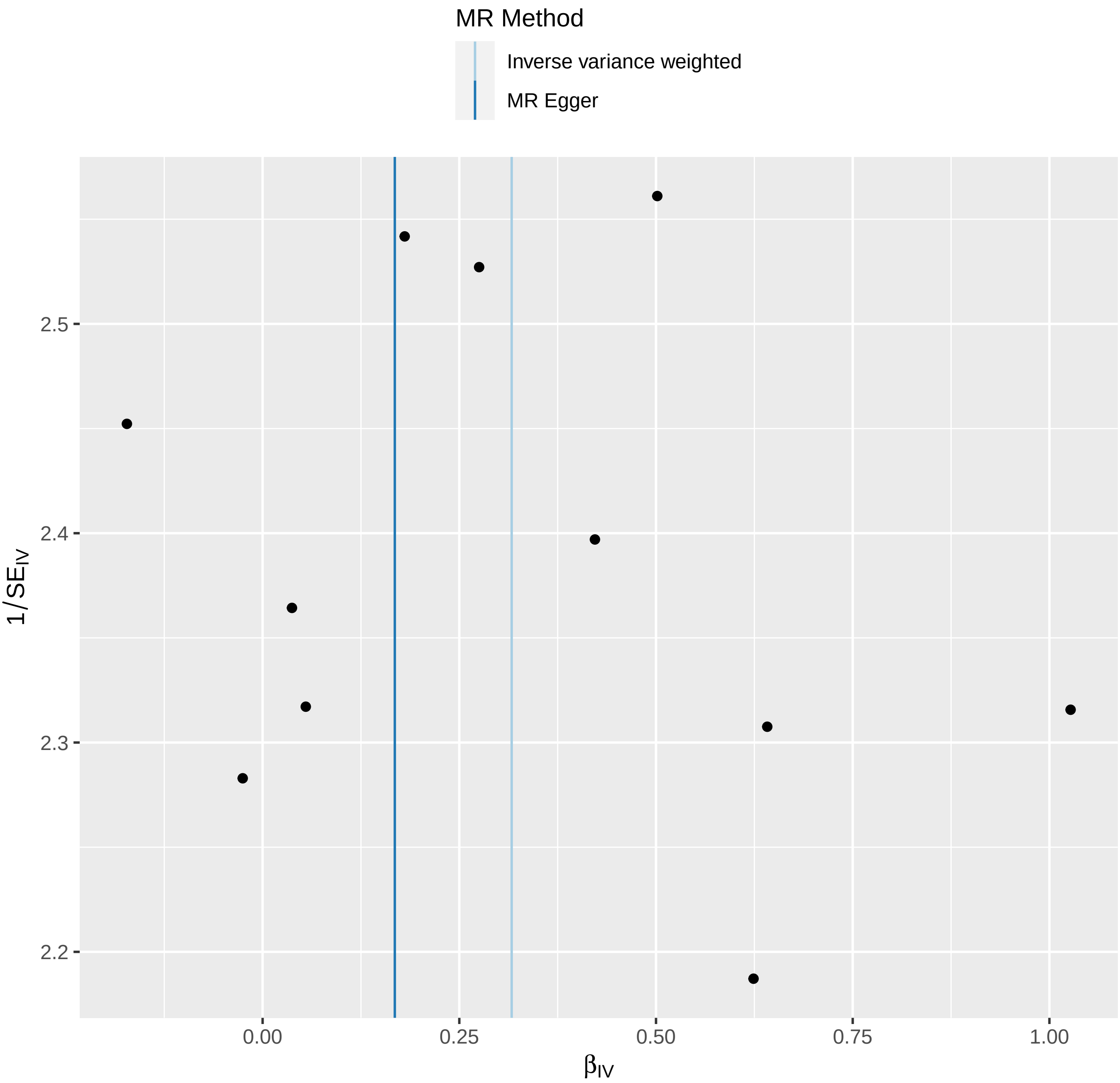

Batch 1407 : Gut microbiota abundance (genus Coprococcus1 id.11301) on Ulcerative proctitis

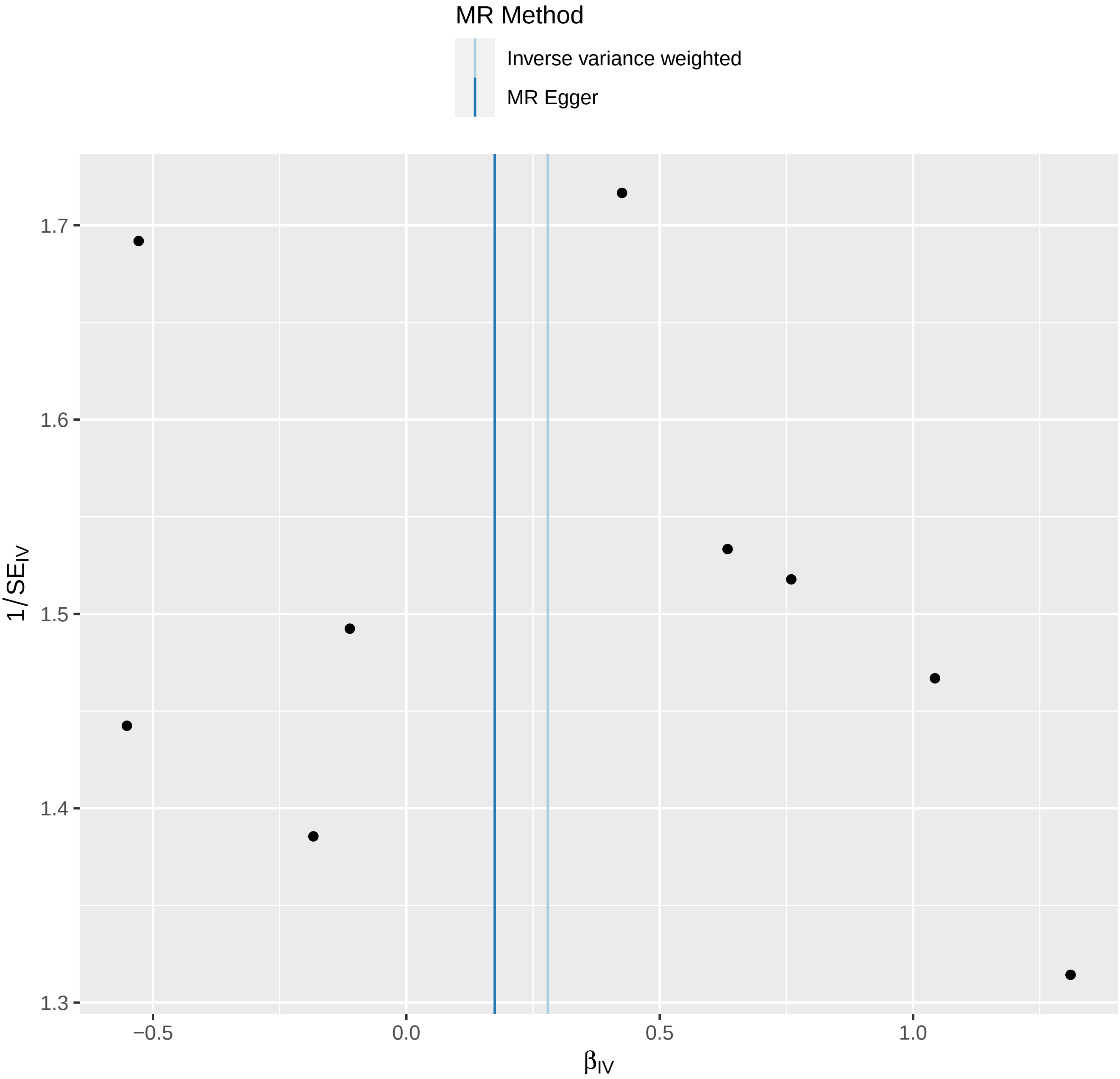

Batch 1408 : Gut microbiota abundance (genus Coprococcus2 id.11302) on Ulcerative proctitis

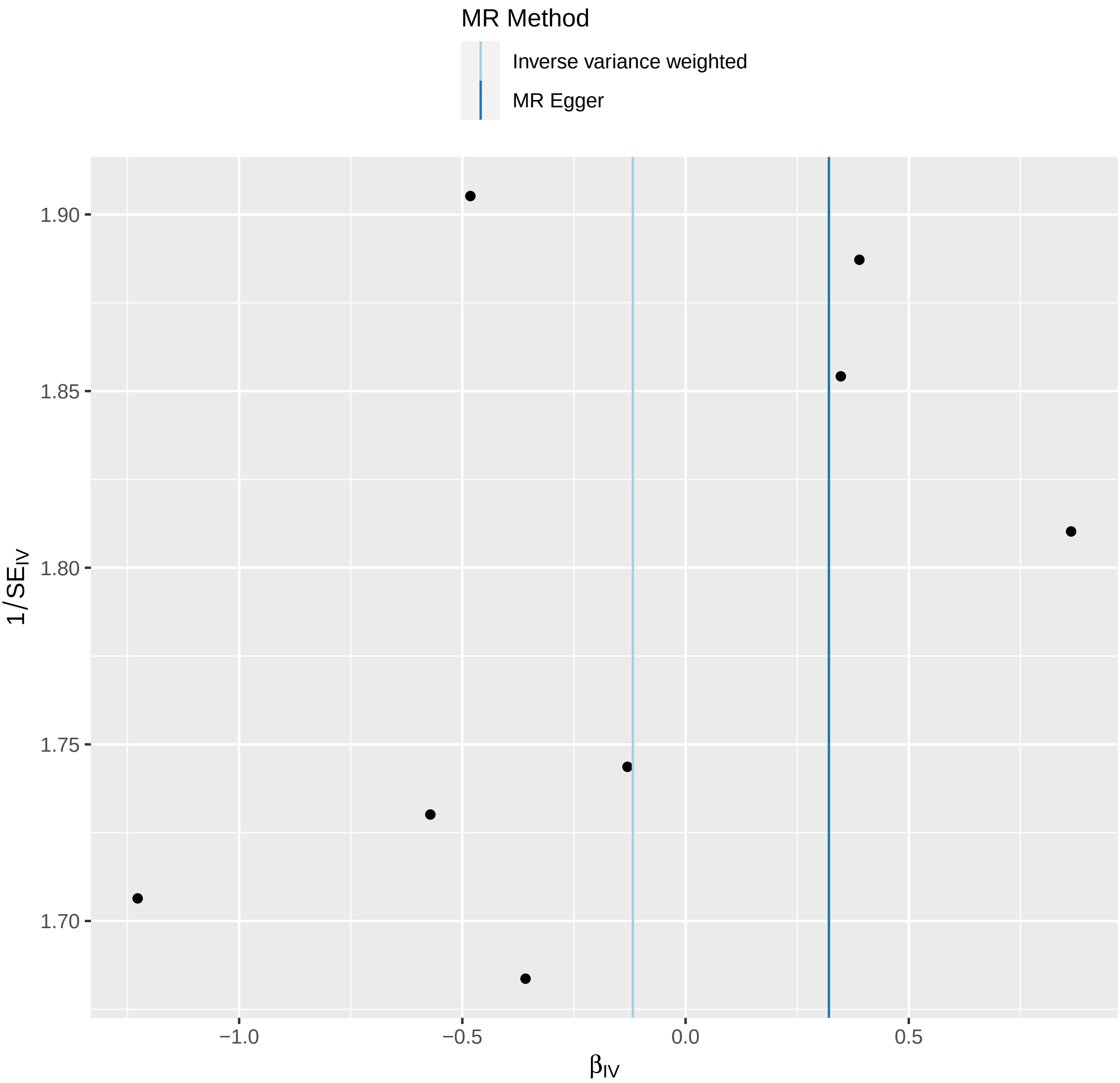

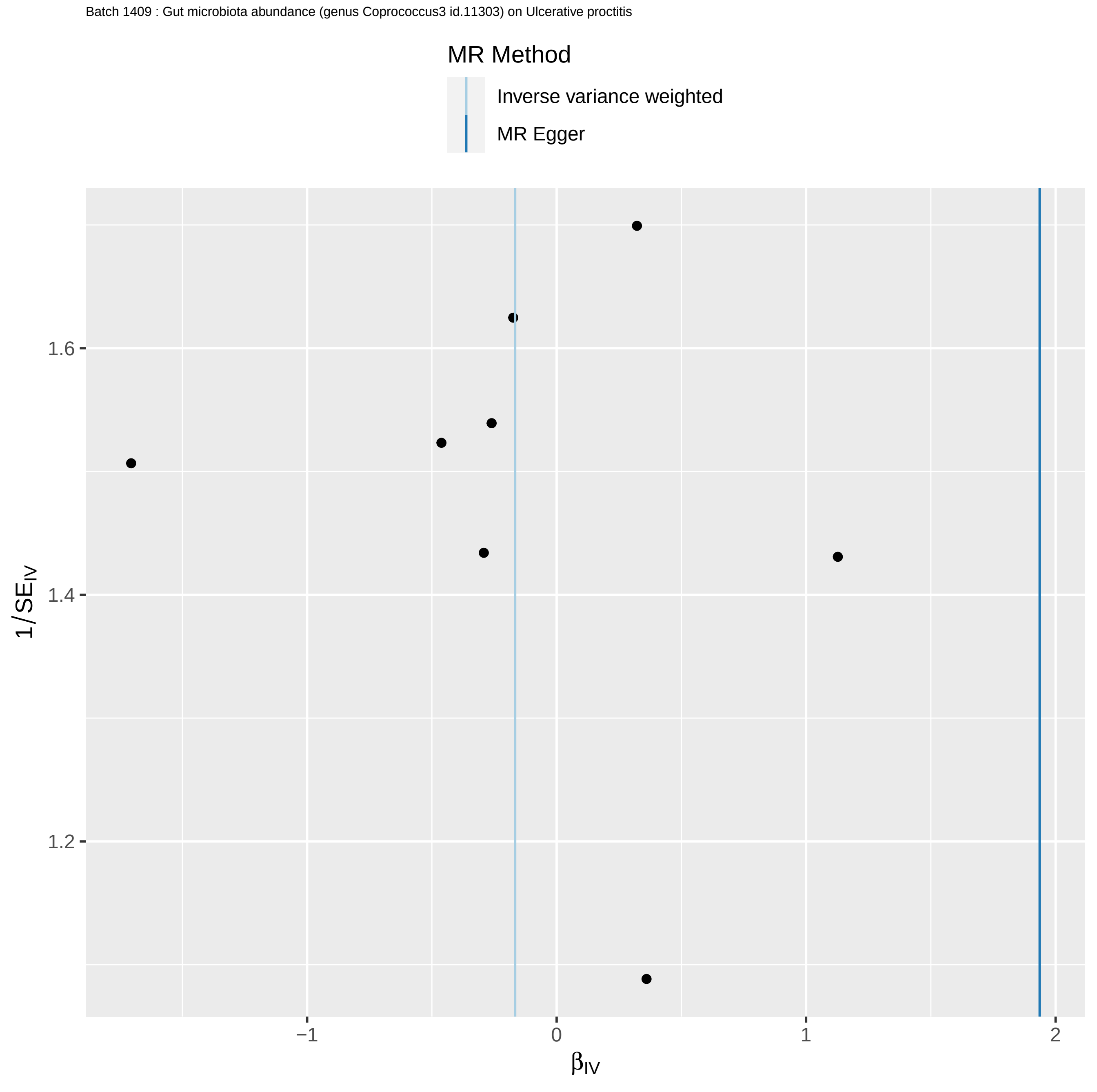

Batch 1409 : Gut microbiota abundance (genus Coprococcus3 id.11303) on Ulcerative proctitis
MR Method
Inverse variance weighted
MR Egger
1.6
1.4
1.2
1/SE_IV
−1
0
1
2
β_IV

Batch 1410 : Gut microbiota abundance (genus Defluviitaleaceae UCG011 id.11287) on Ulcerative proctitis

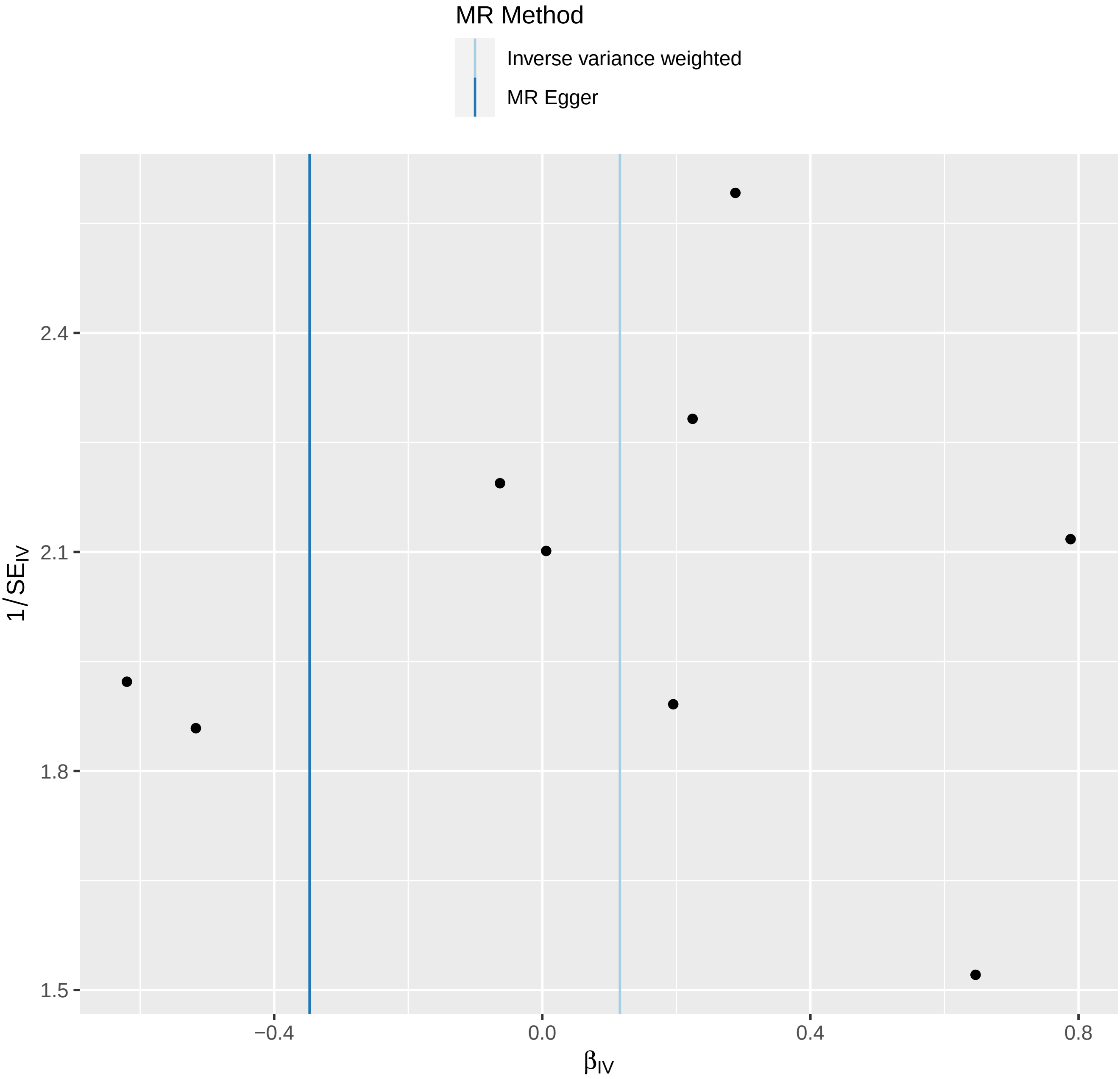

Batch 1411 : Gut microbiota abundance (genus Desulfovibrio id.3173) on Ulcerative proctitis

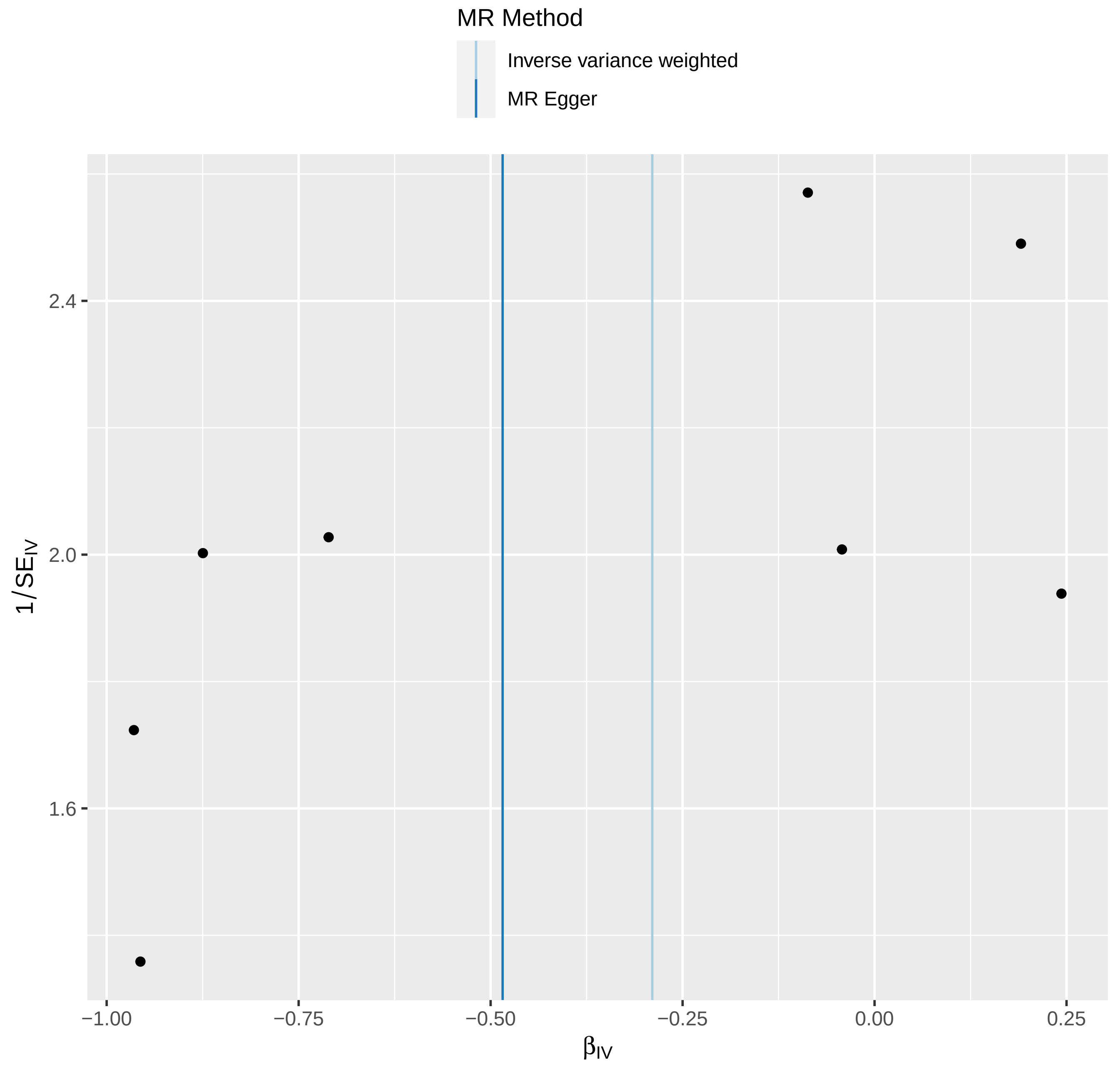

Batch 1412 : Gut microbiota abundance (genus Dialister id.2183) on Ulcerative proctitis

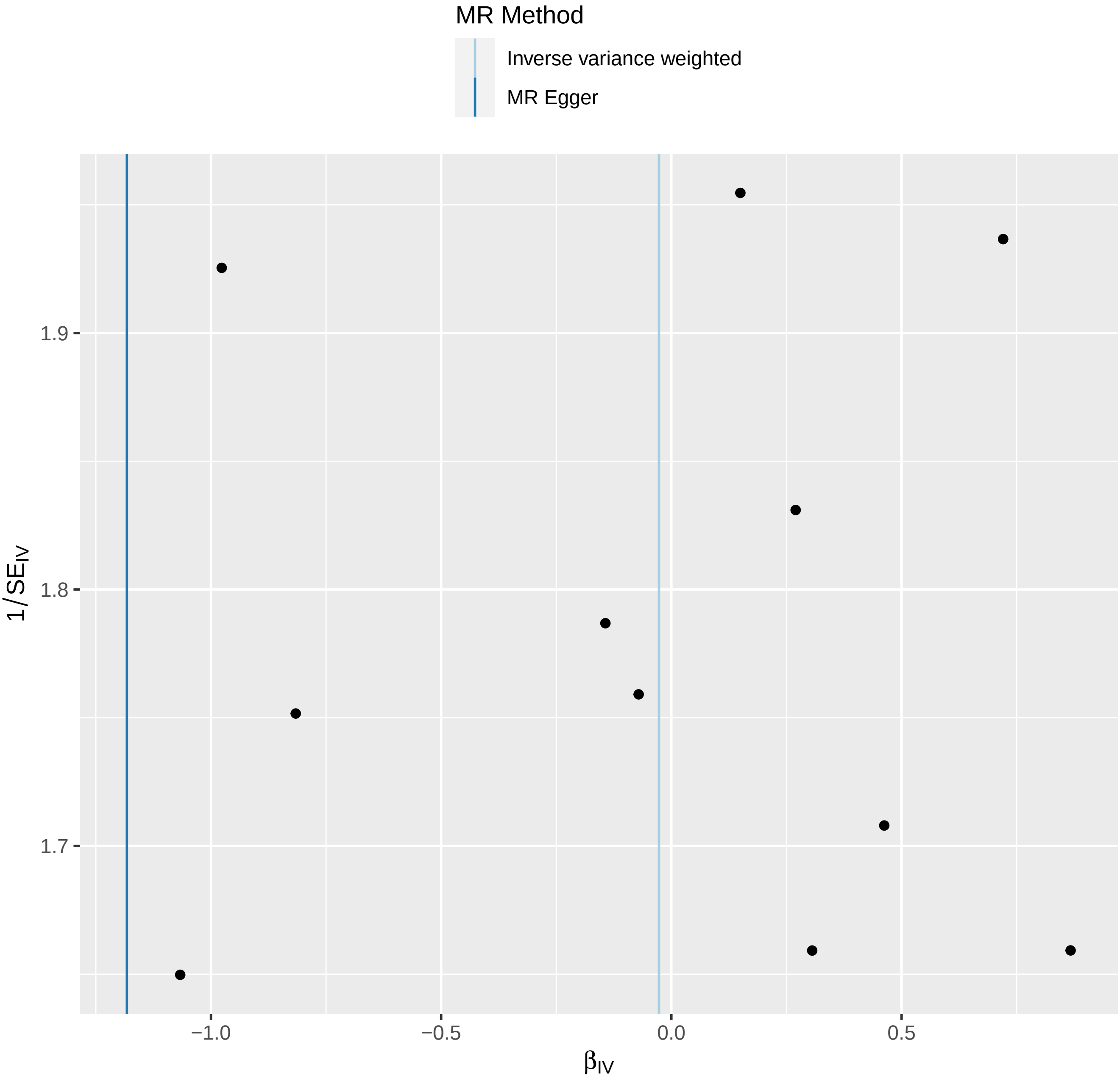

Batch 1413 : Gut microbiota abundance (genus Dorea id.1997) on Ulcerative proctitis

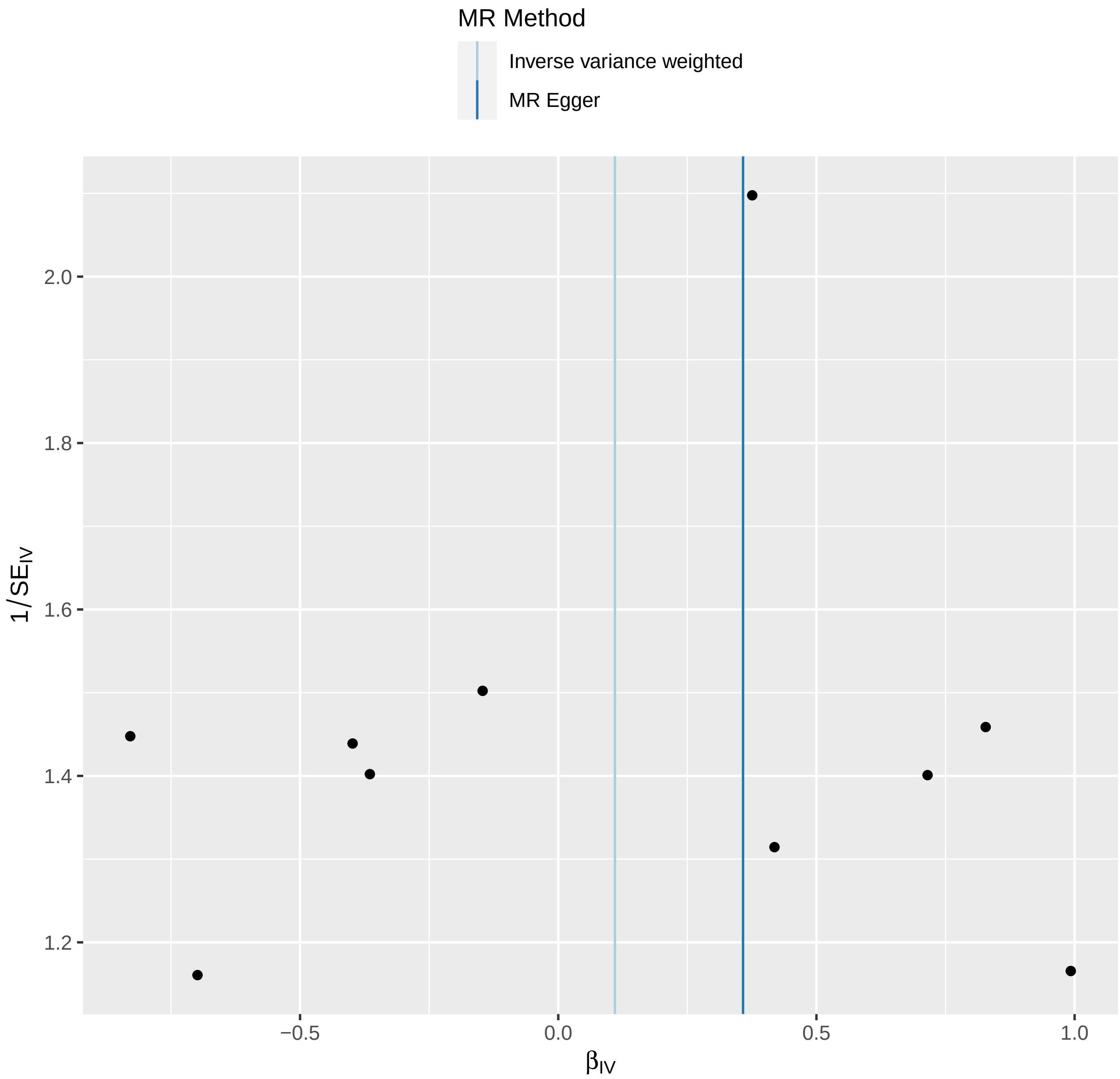

Batch 1414 : Gut microbiota abundance (genus Eggerthella id.819) on Ulcerative proctitis

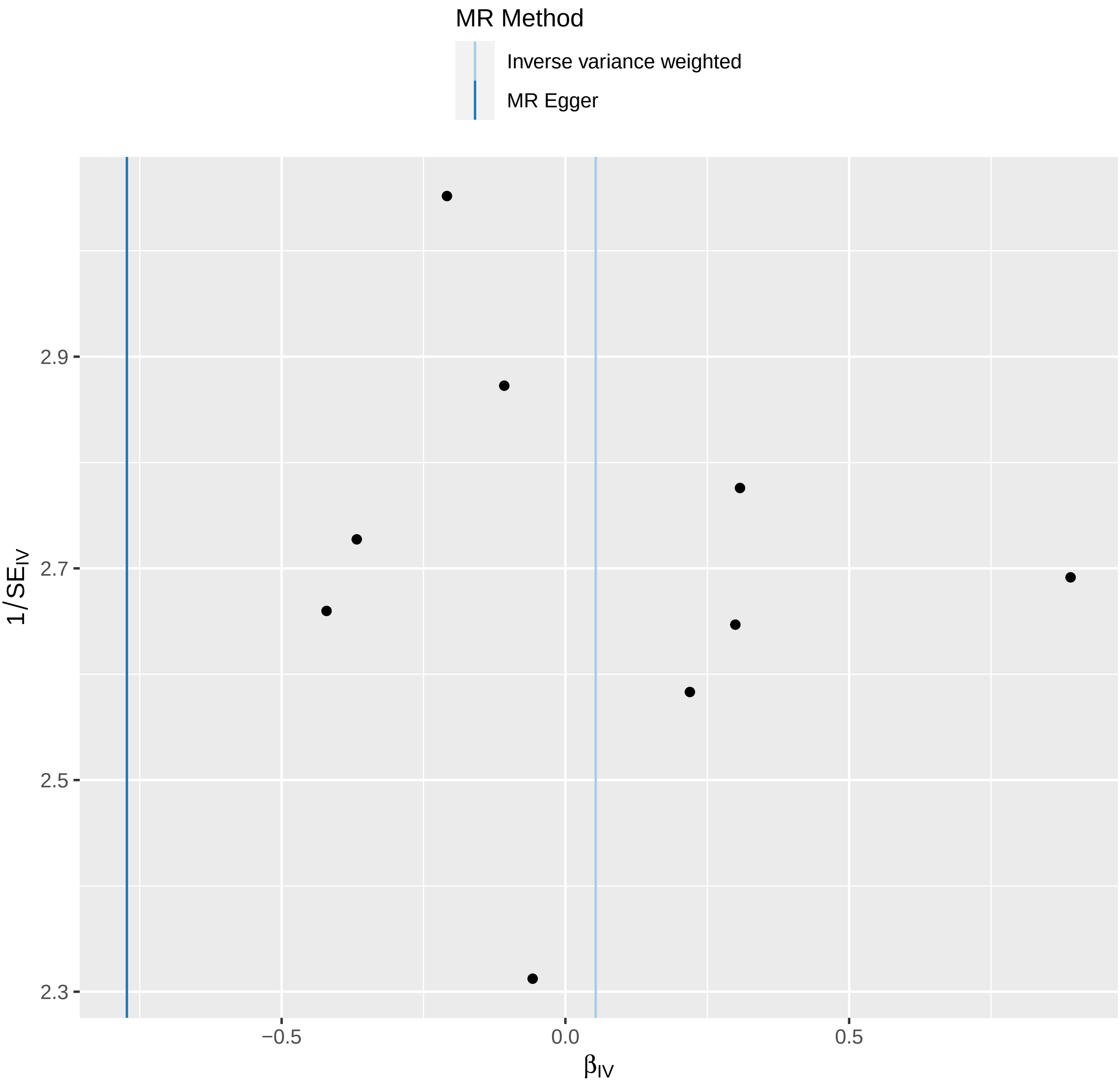

Batch 1415 : Gut microbiota abundance (genus Eisenbergiella id.11304) on Ulcerative proctitis

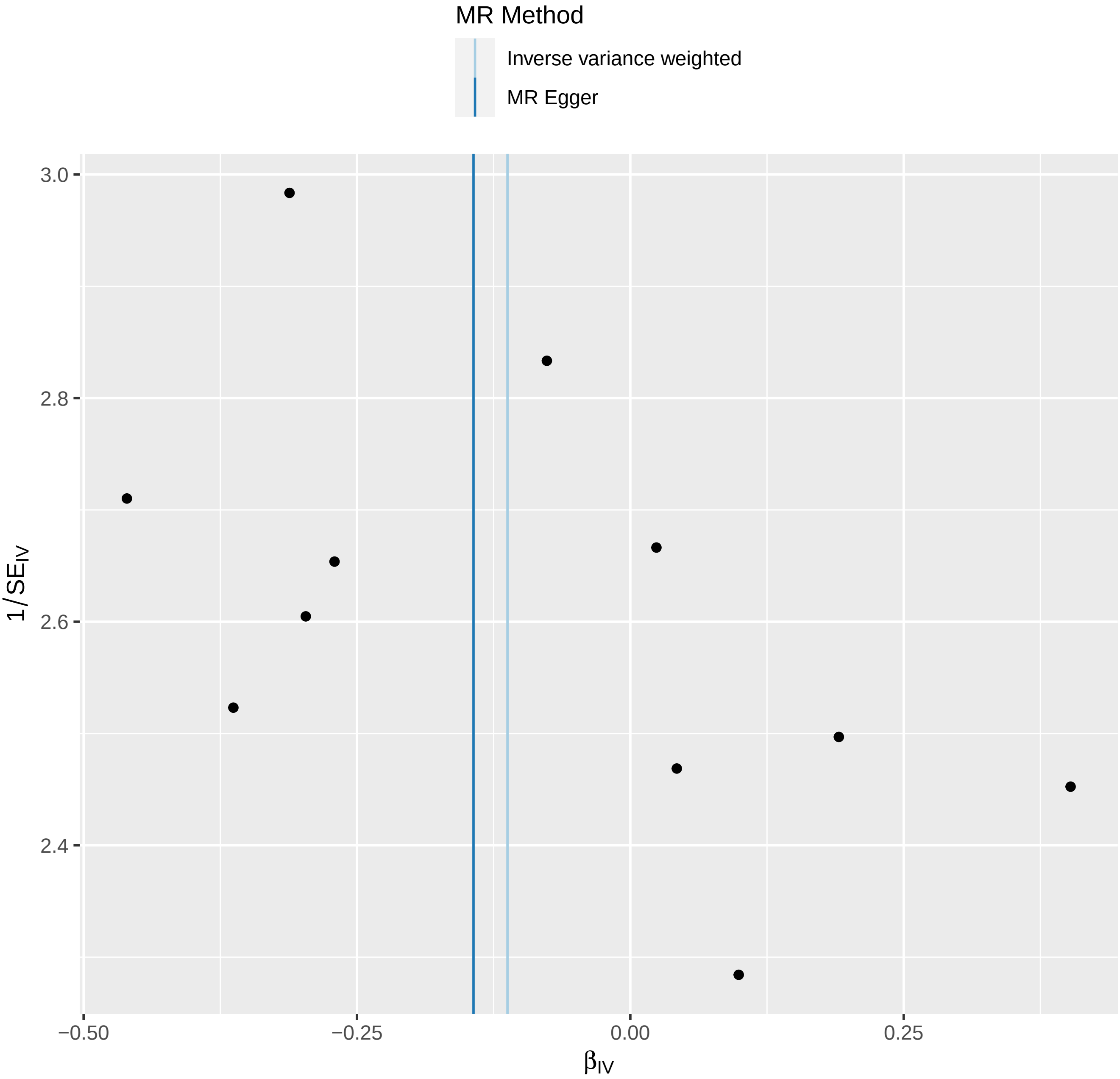

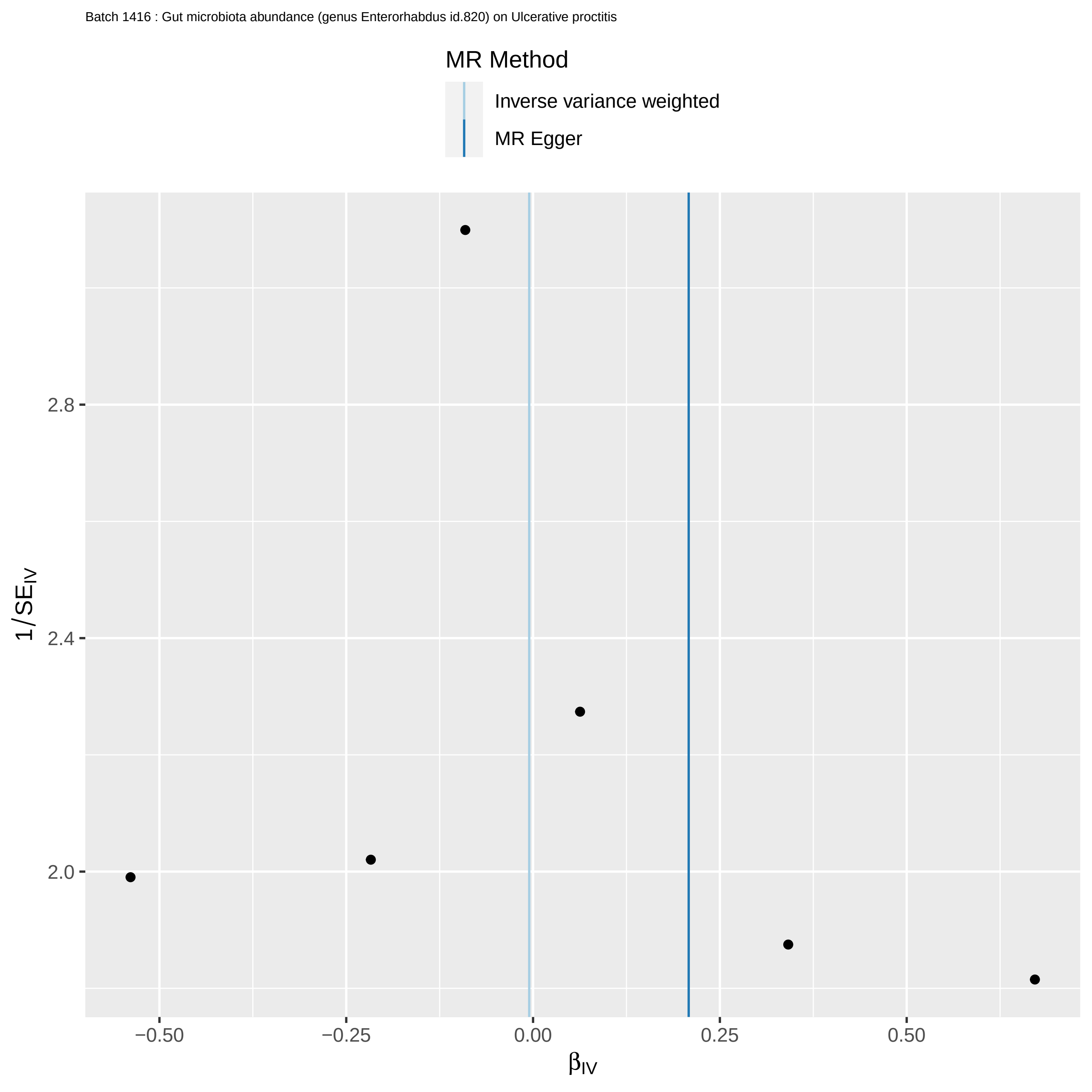

Batch 1416 : Gut microbiota abundance (genus Enterorhabdus id.820) on Ulcerative proctitis
MR Method
Inverse variance weighted
MR Egger
2.8
2.4
2.0
1/SE_IV
−0.50
−0.25
0.00
0.25
0.50
β_IV

Batch 1417 : Gut microbiota abundance (genus Erysipelatoclostridium id.11381) on Ulcerative proctitis

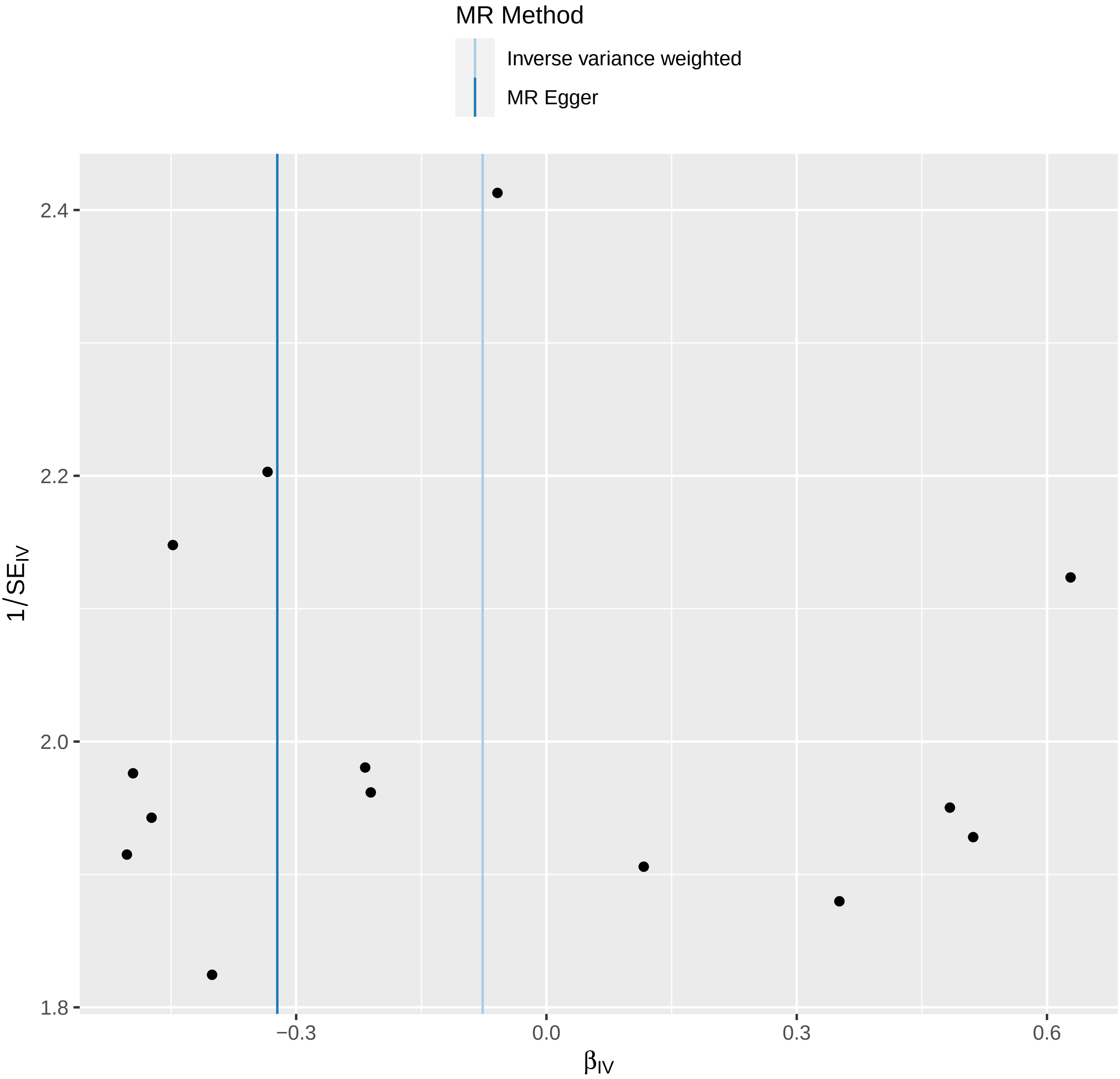

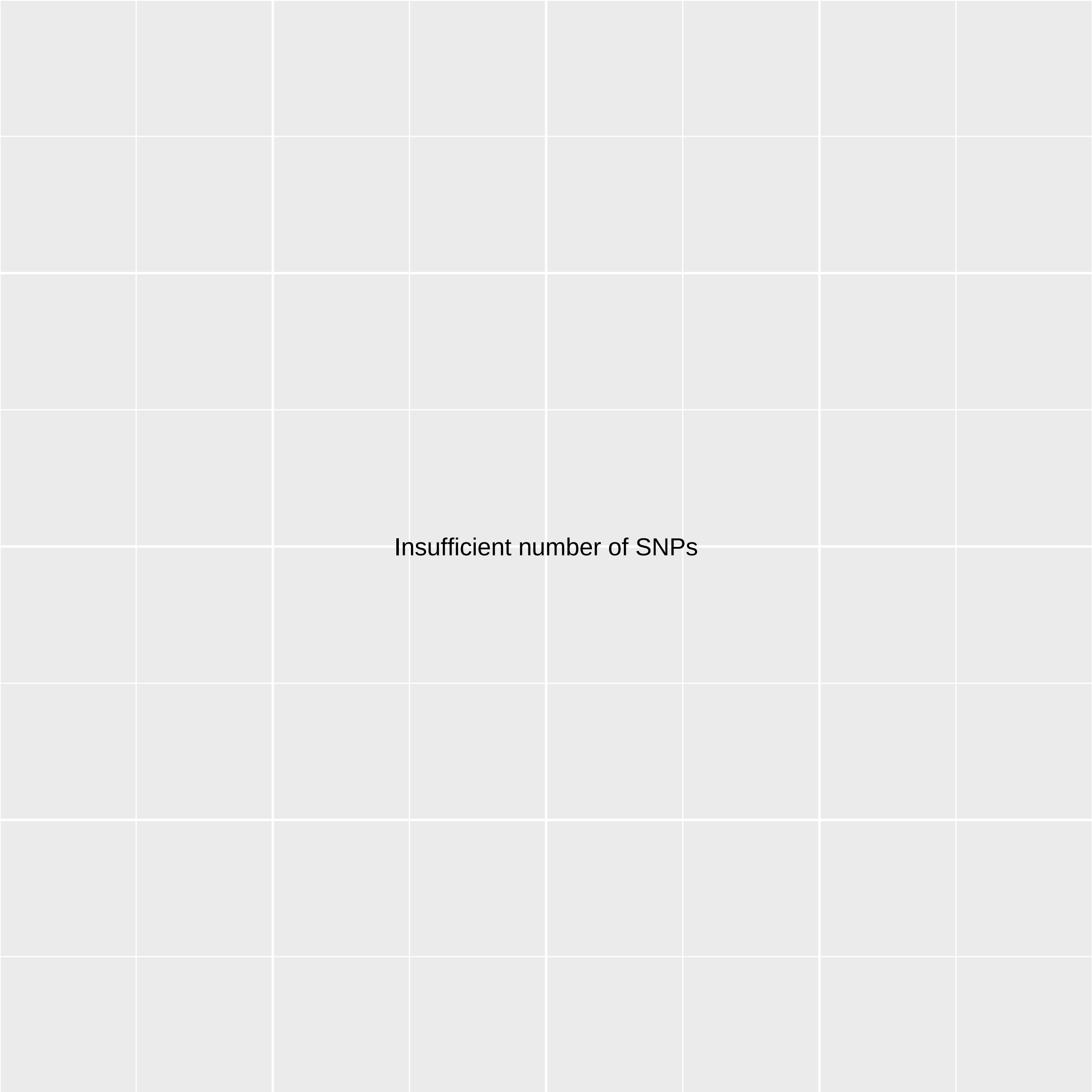
Insufficient number of SNPs

Batch 1419 : Gut microbiota abundance (genus Escherichia Shigella id.3504) on Ulcerative proctitis

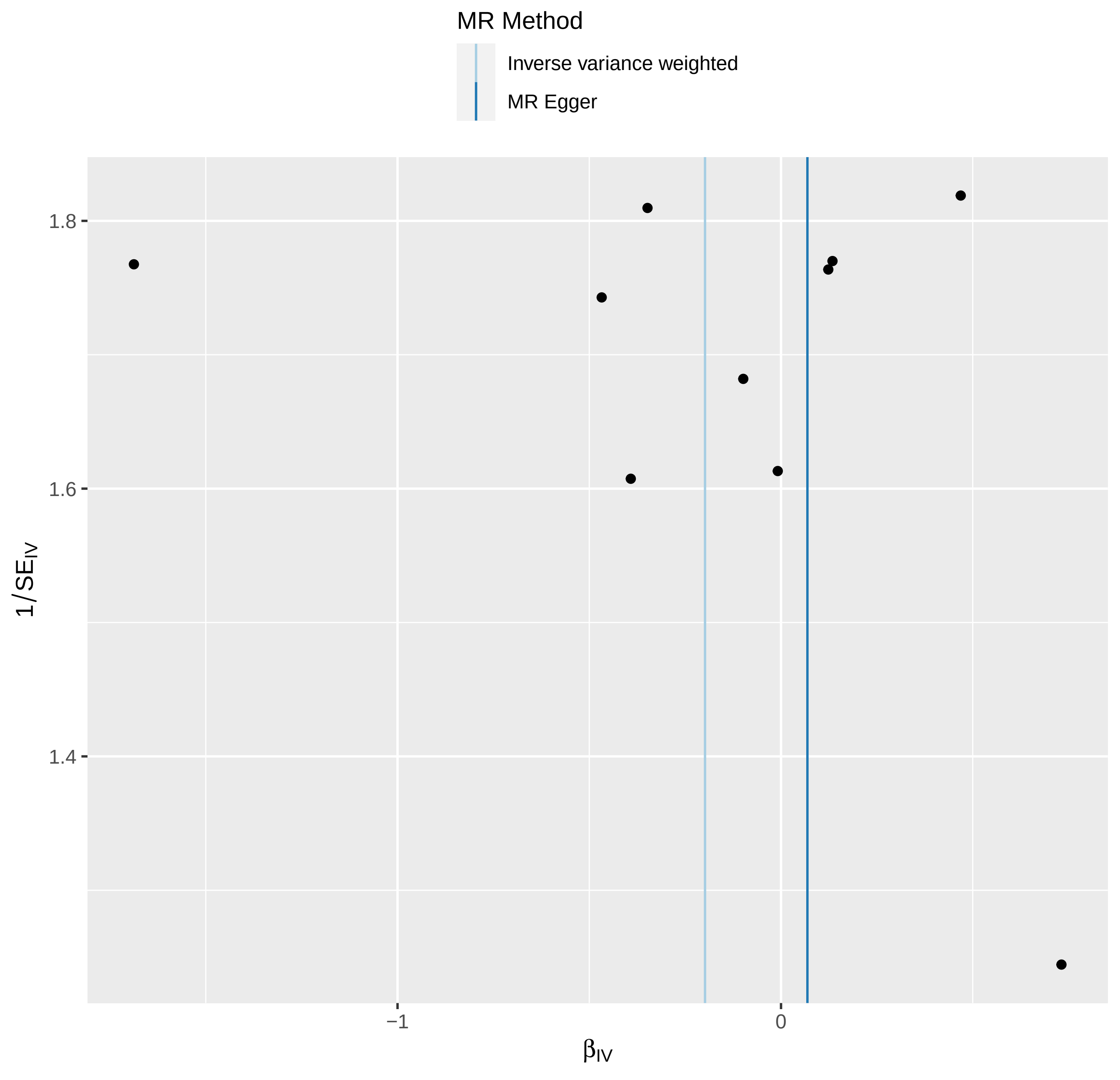

Batch 1420 : Gut microbiota abundance (genus Eubacterium brachy group id.11296) on Ulcerative proctitis

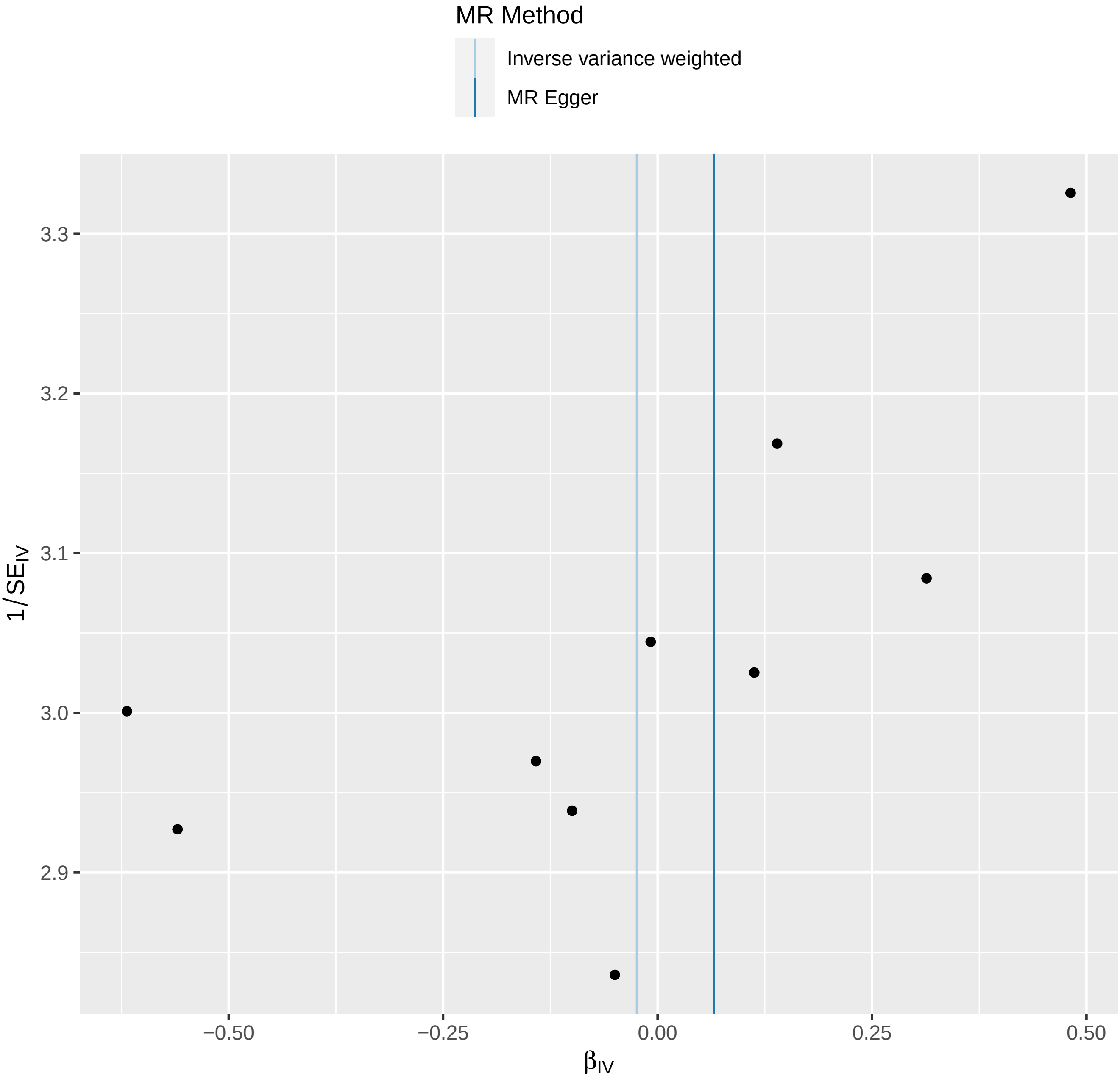

Batch 1421 : Gut microbiota abundance (genus Eubacterium coprostanoligenes group id.11375) on Ulcerative proctitis

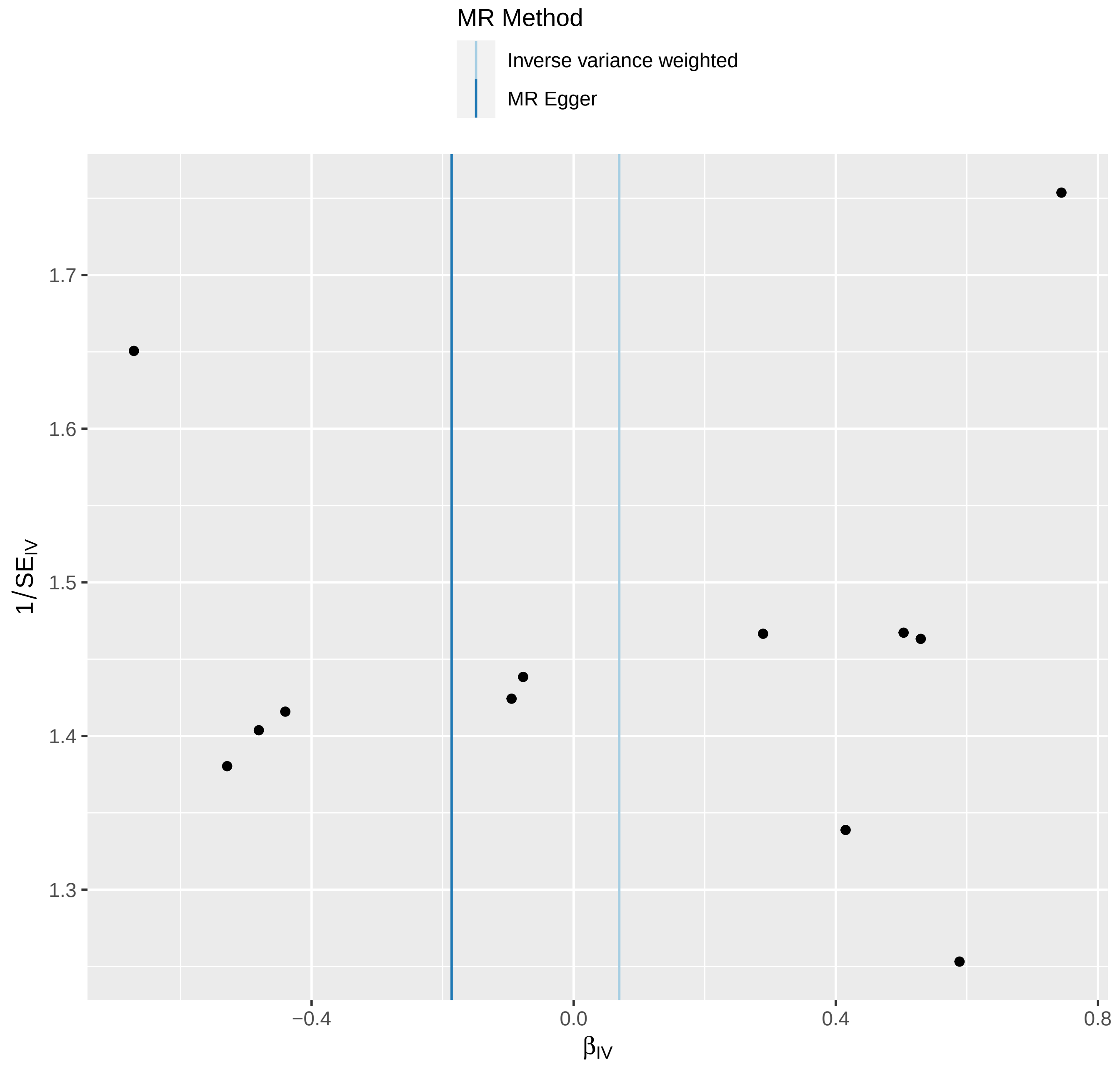

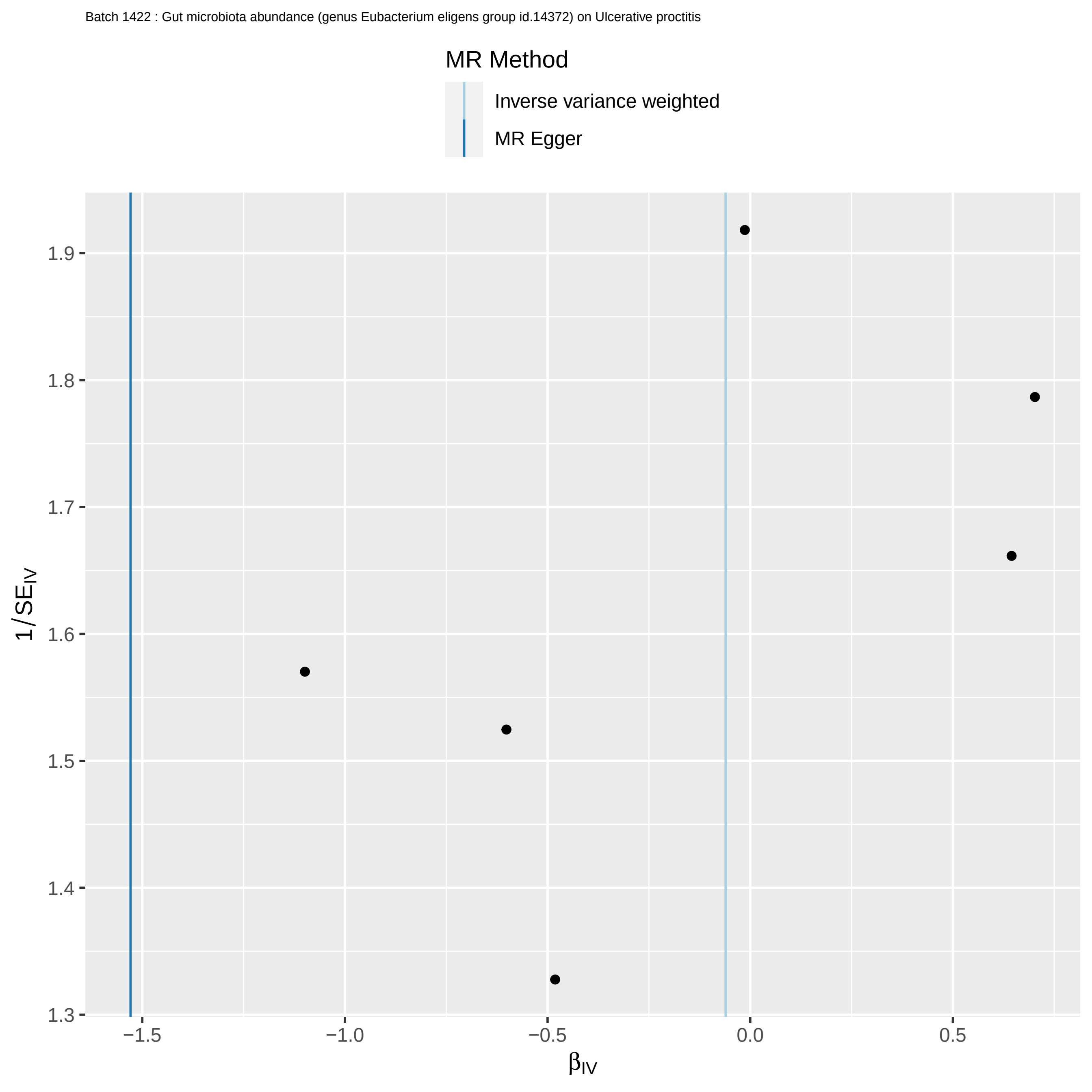
Batch 1422 : Gut microbiota abundance (genus Eubacterium eligens group id.14372) on Ulcerative proctitis
MR Method
Inverse variance weighted
MR Egger
1.9
1.8
1.7
1.6
1.5
1.4
1.3
1/SE_IV
−1.5
−1.0
−0.5
0.0
0.5
β_IV

Batch 1423 : Gut microbiota abundance (genus Eubacterium fissicatena group id.14373) on Ulcerative proctitis

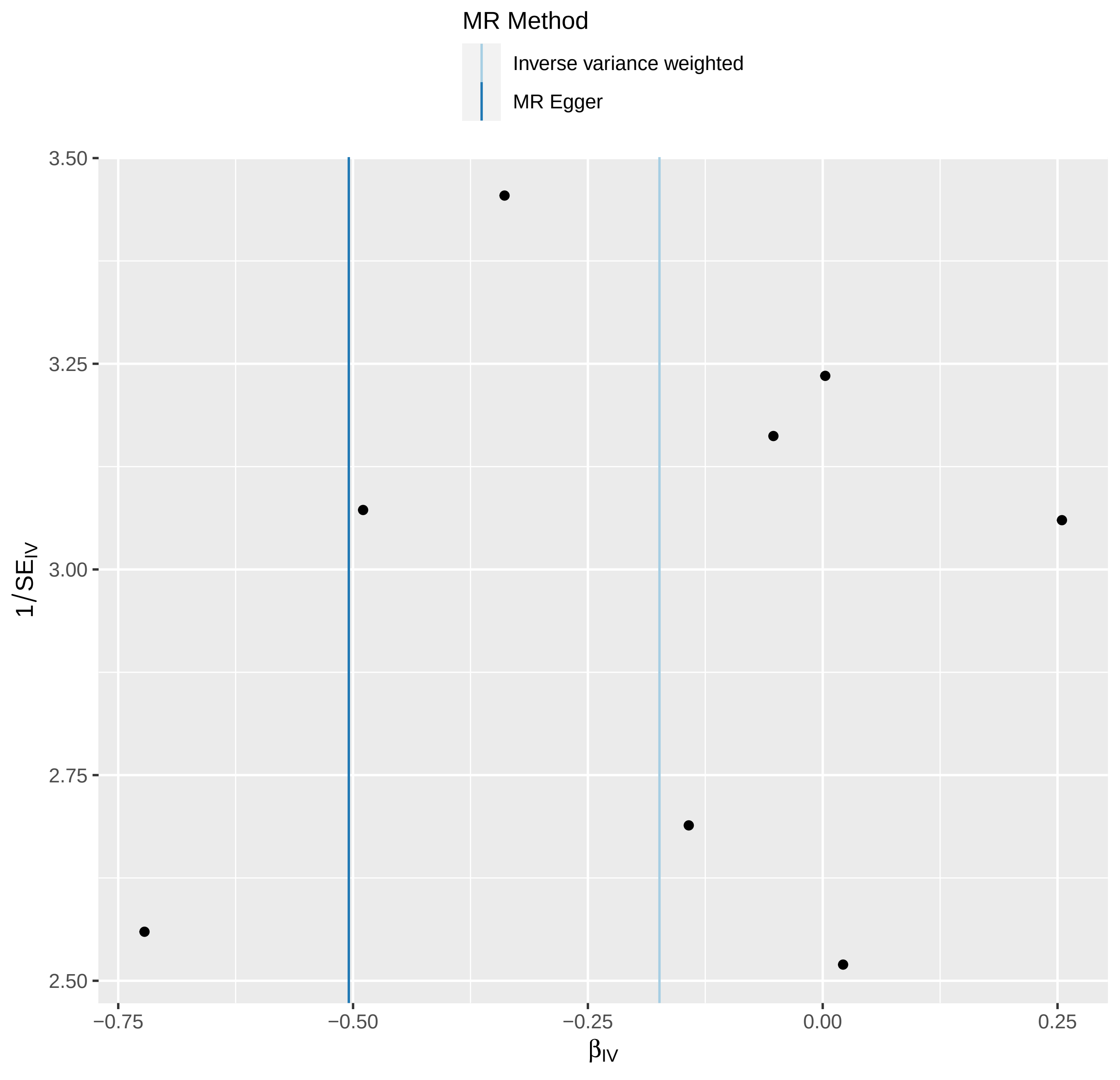

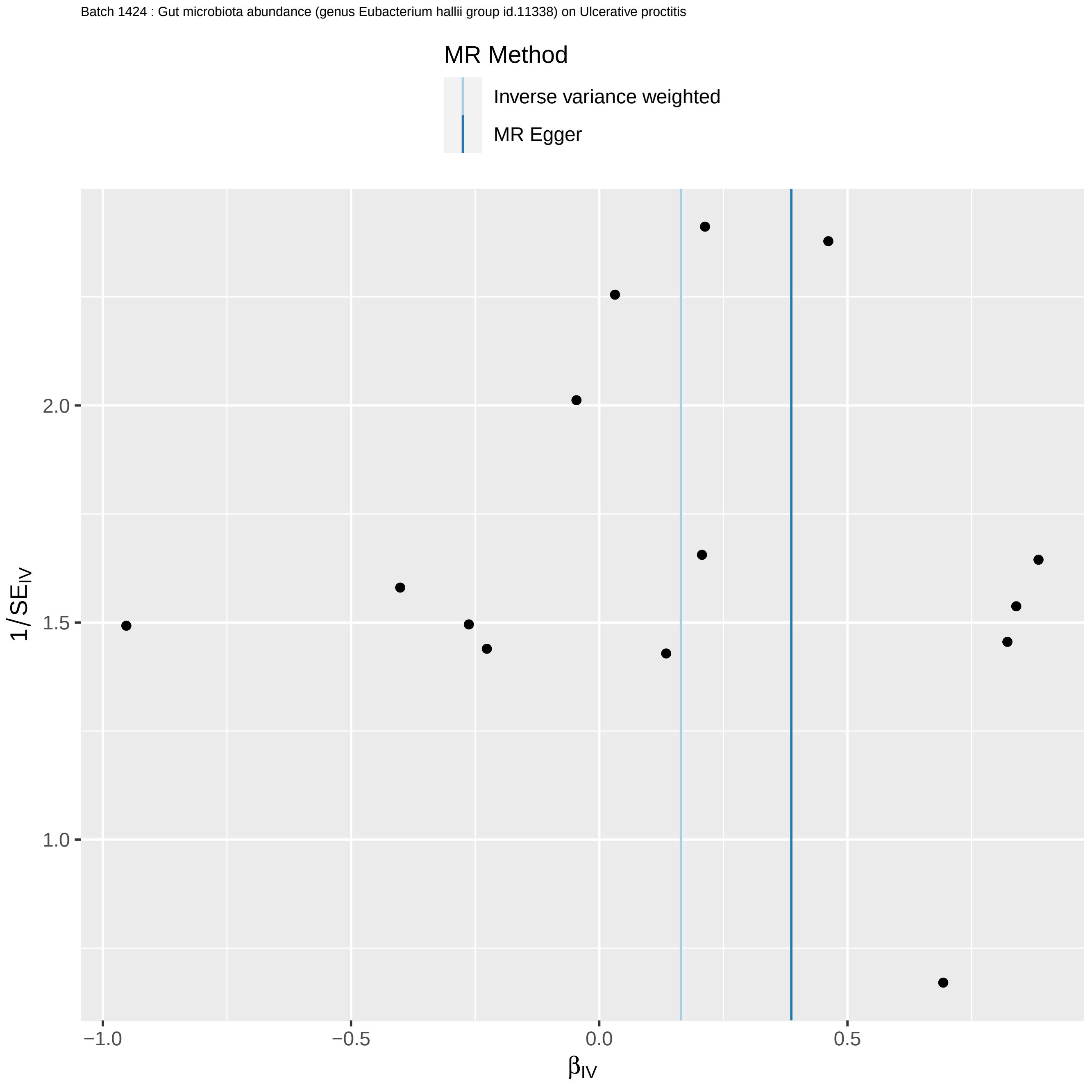
Batch 1424 : Gut microbiota abundance (genus Eubacterium hallii group id.11338) on Ulcerative proctitis
MR Method
Inverse variance weighted
MR Egger
2.0
1.5
1.0
1/SE_IV
−1.0
−0.5
0.0
0.5
β_IV

Batch 1425 : Gut microbiota abundance (genus Eubacterium nodatum group id.11297) on Ulcerative proctitis

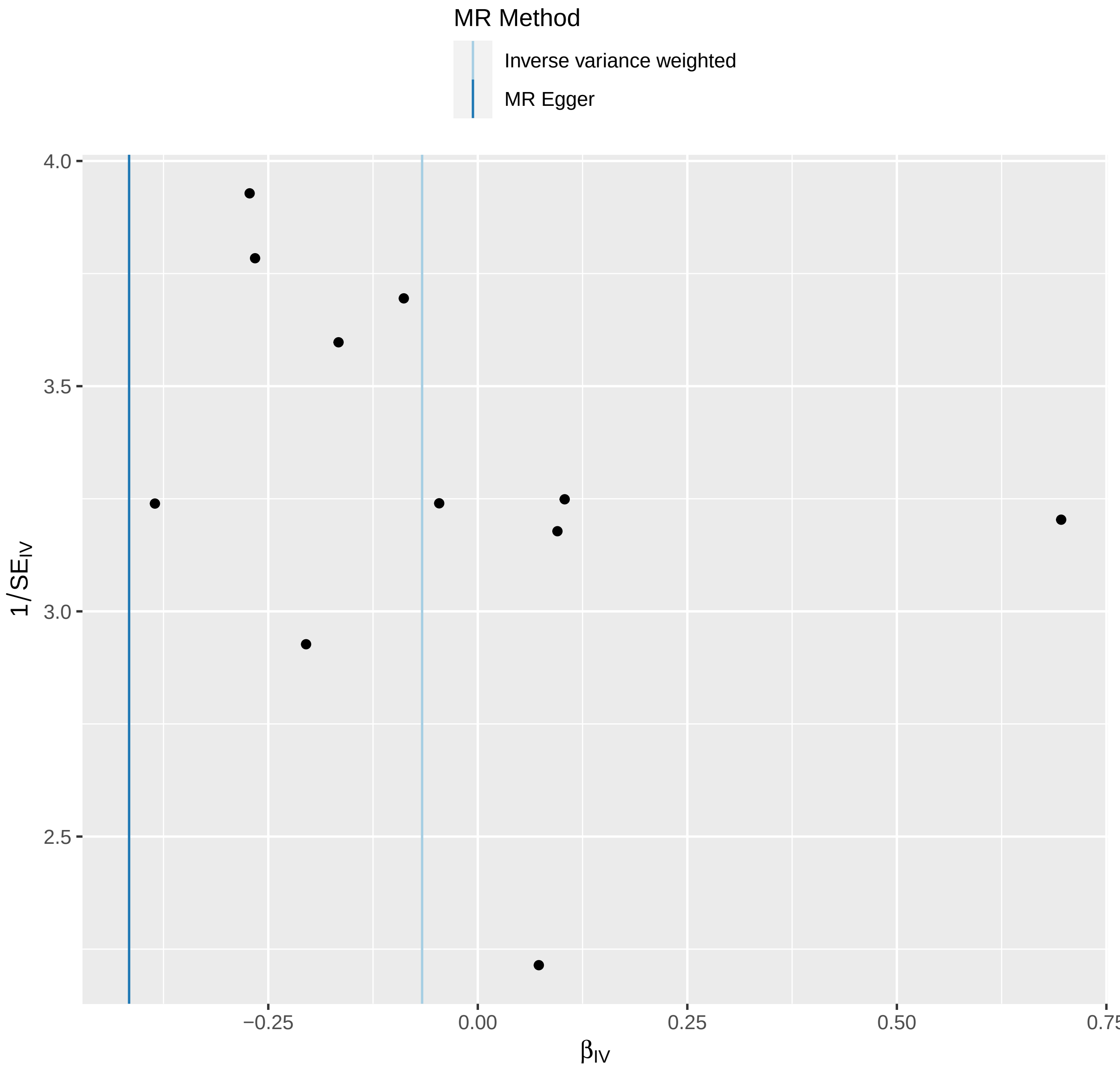

Batch 1426 : Gut microbiota abundance (genus Eubacterium oxidoreducens group id.11339) on Ulcerative proctitis

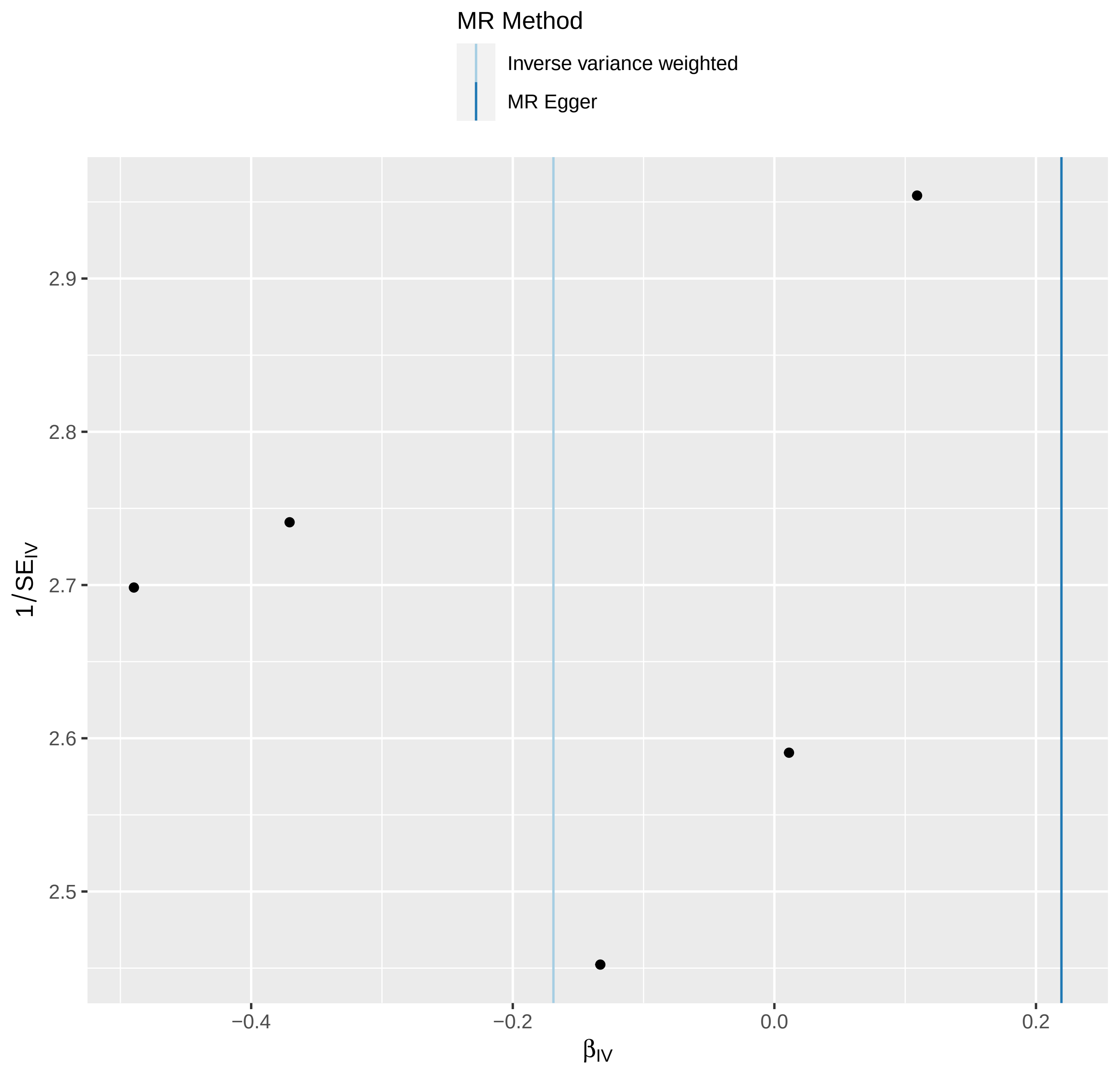

Batch 1427 : Gut microbiota abundance (genus Eubacterium rectale group id.14374) on Ulcerative proctitis

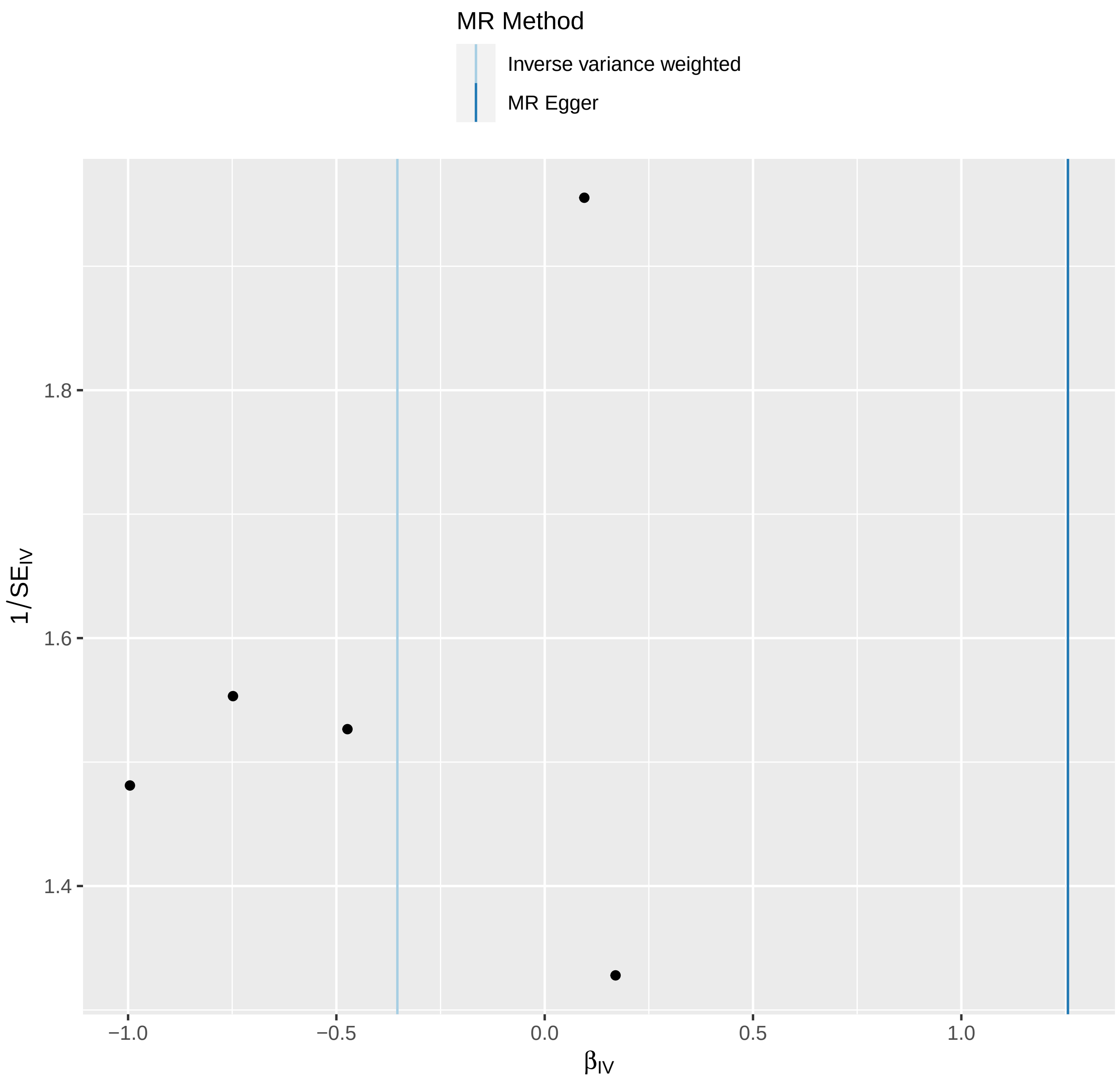

Batch 1428 : Gut microbiota abundance (genus Eubacterium ruminantium group id.11340) on Ulcerative proctitis

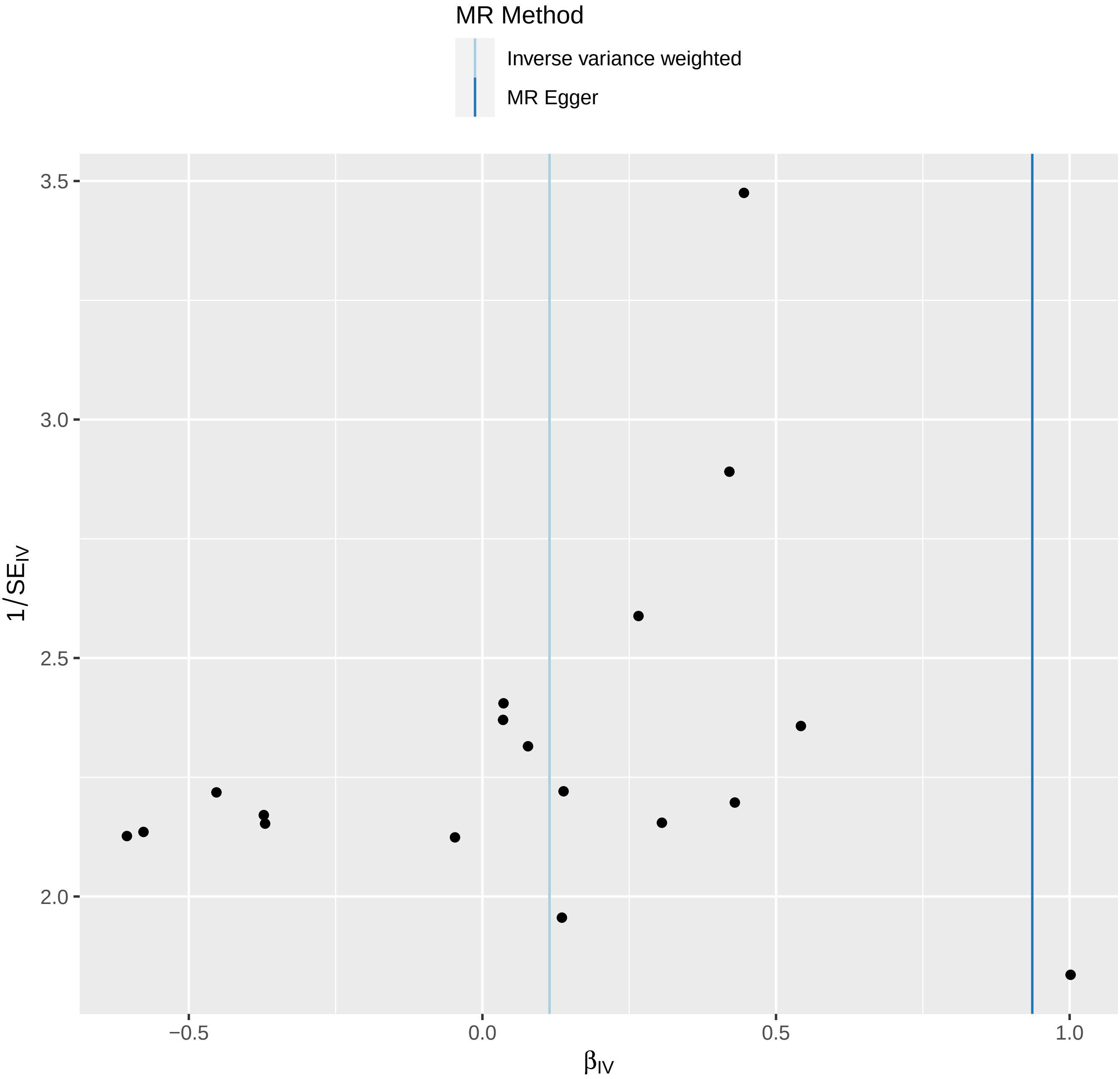

Batch 1429 : Gut microbiota abundance (genus Eubacterium ventriosum group id.11341) on Ulcerative proctitis

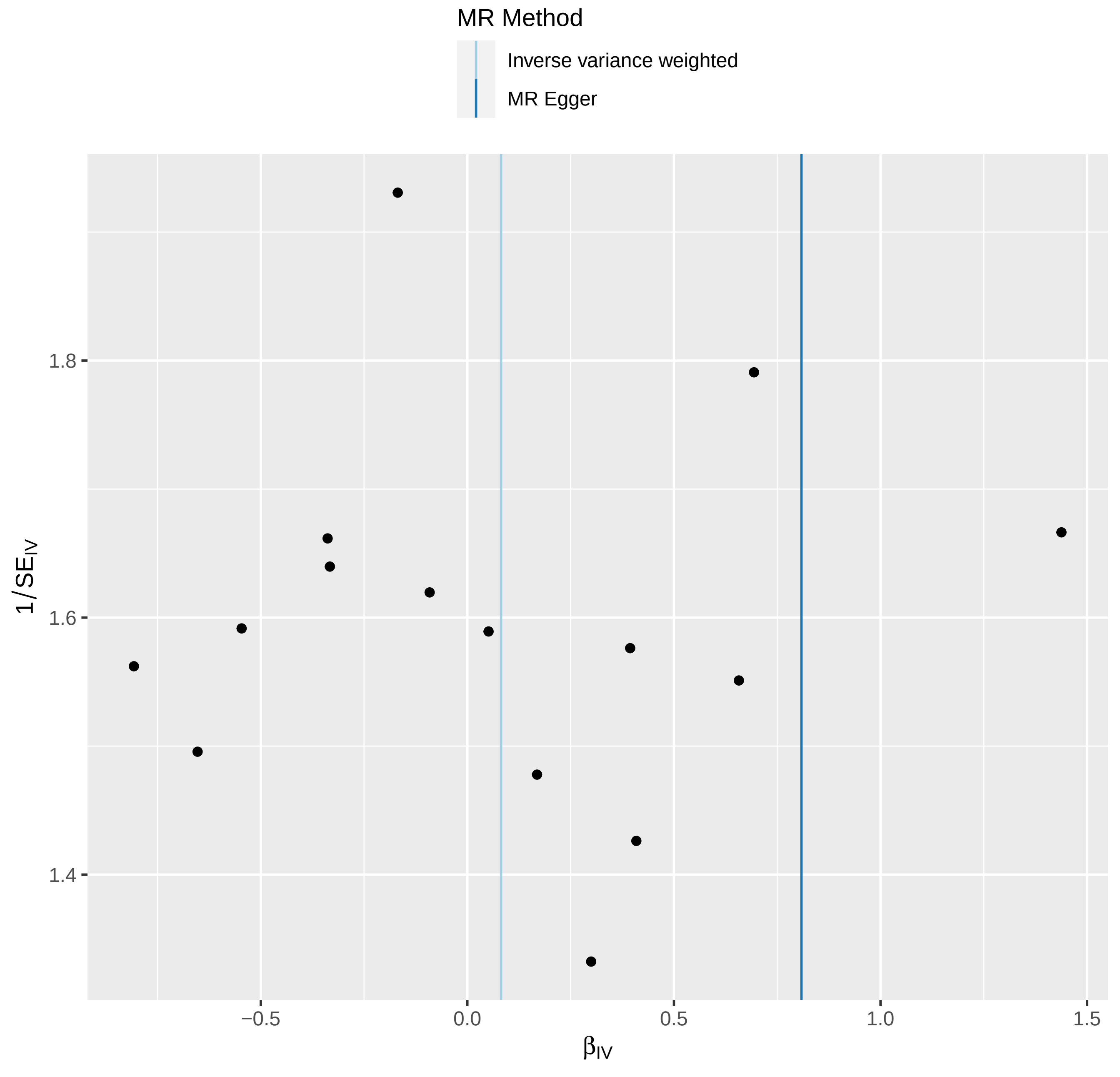

Batch 1430 : Gut microbiota abundance (genus Eubacterium xylanophilum group id.14375) on Ulcerative proctitis

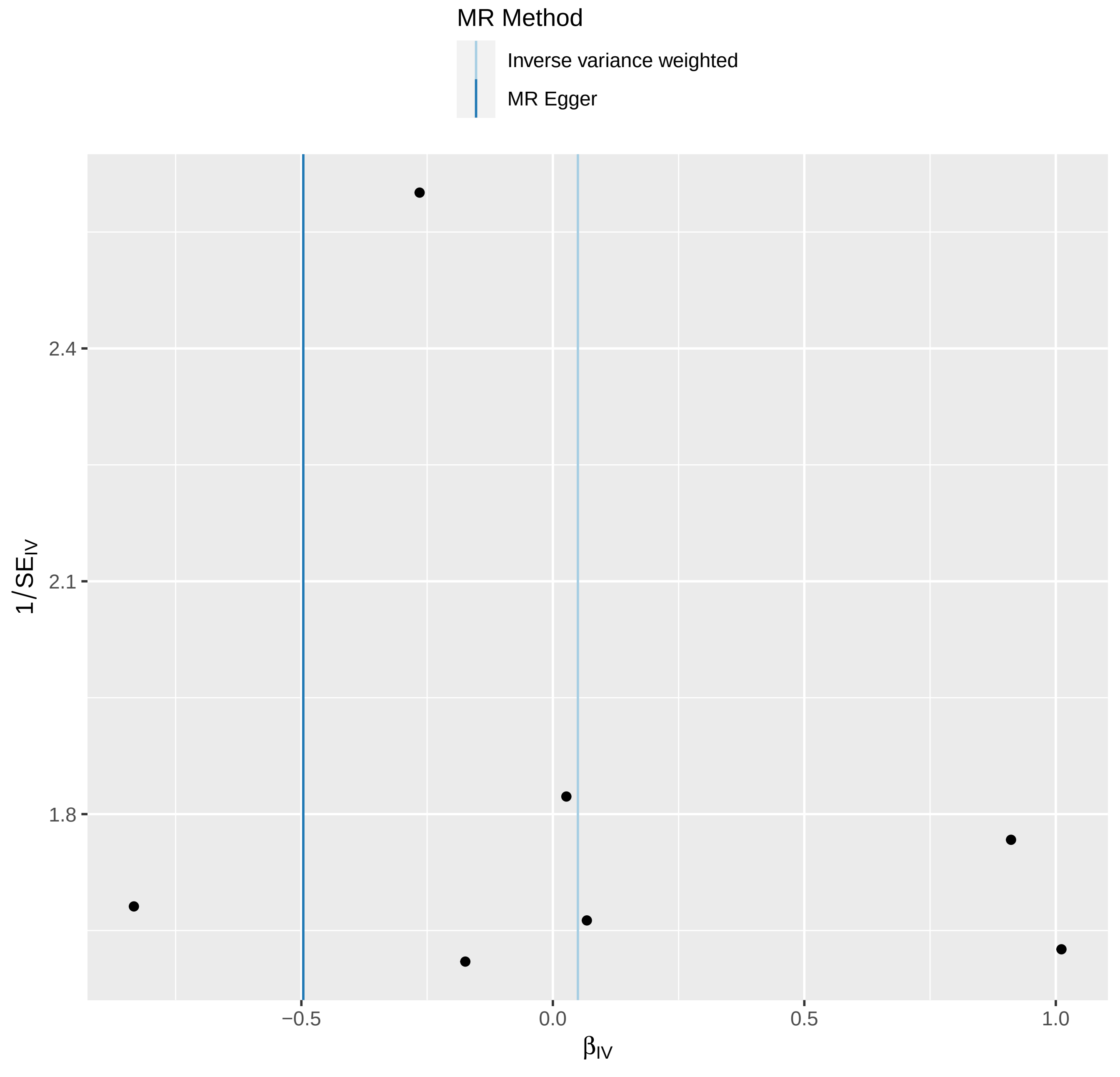

Batch 1431 : Gut microbiota abundance (genus Faecalibacterium id.2057) on Ulcerative proctitis

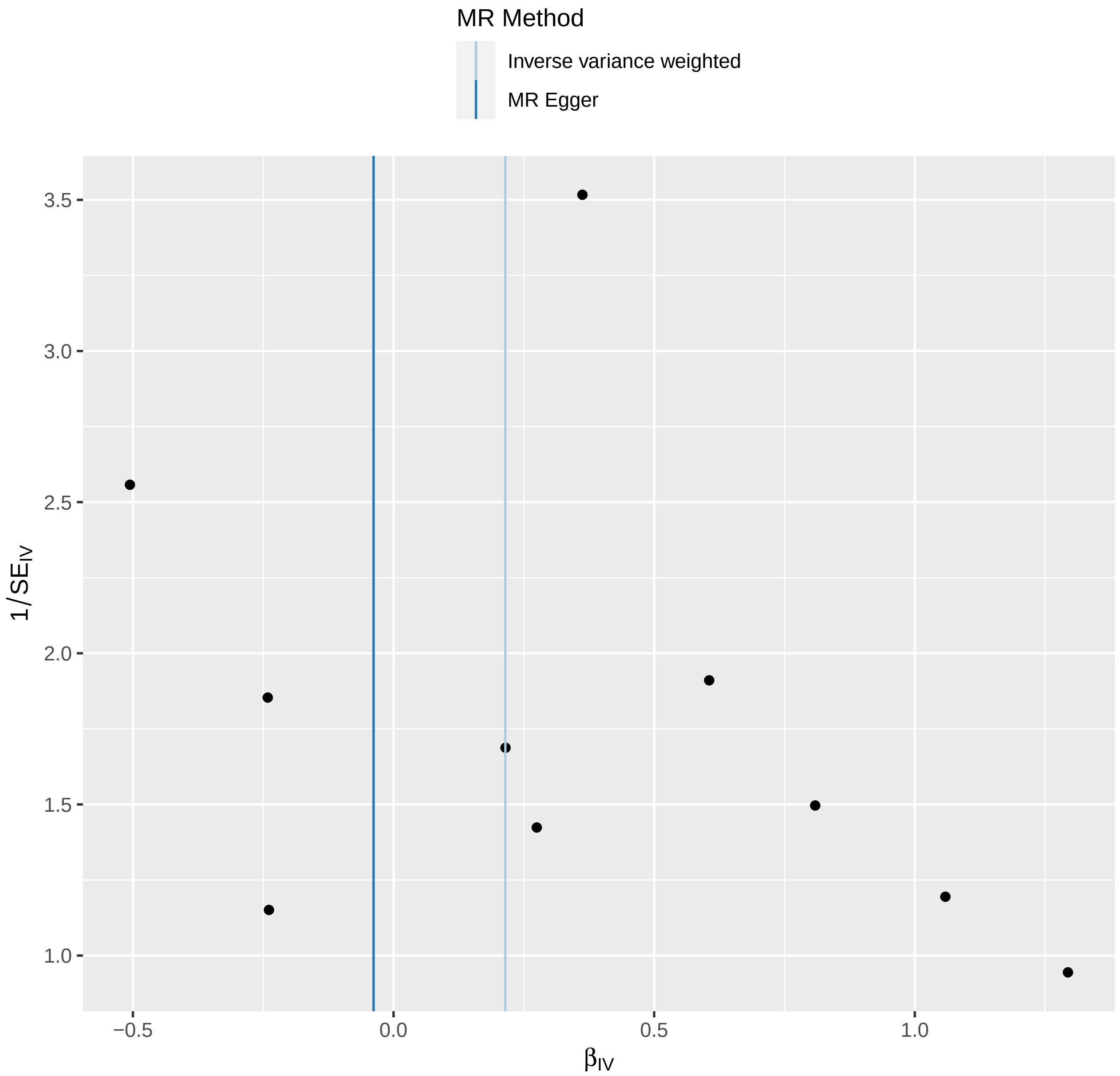

Batch 1432 : Gut microbiota abundance (genus Family XIII AD3011 group id.11293) on Ulcerative proctitis

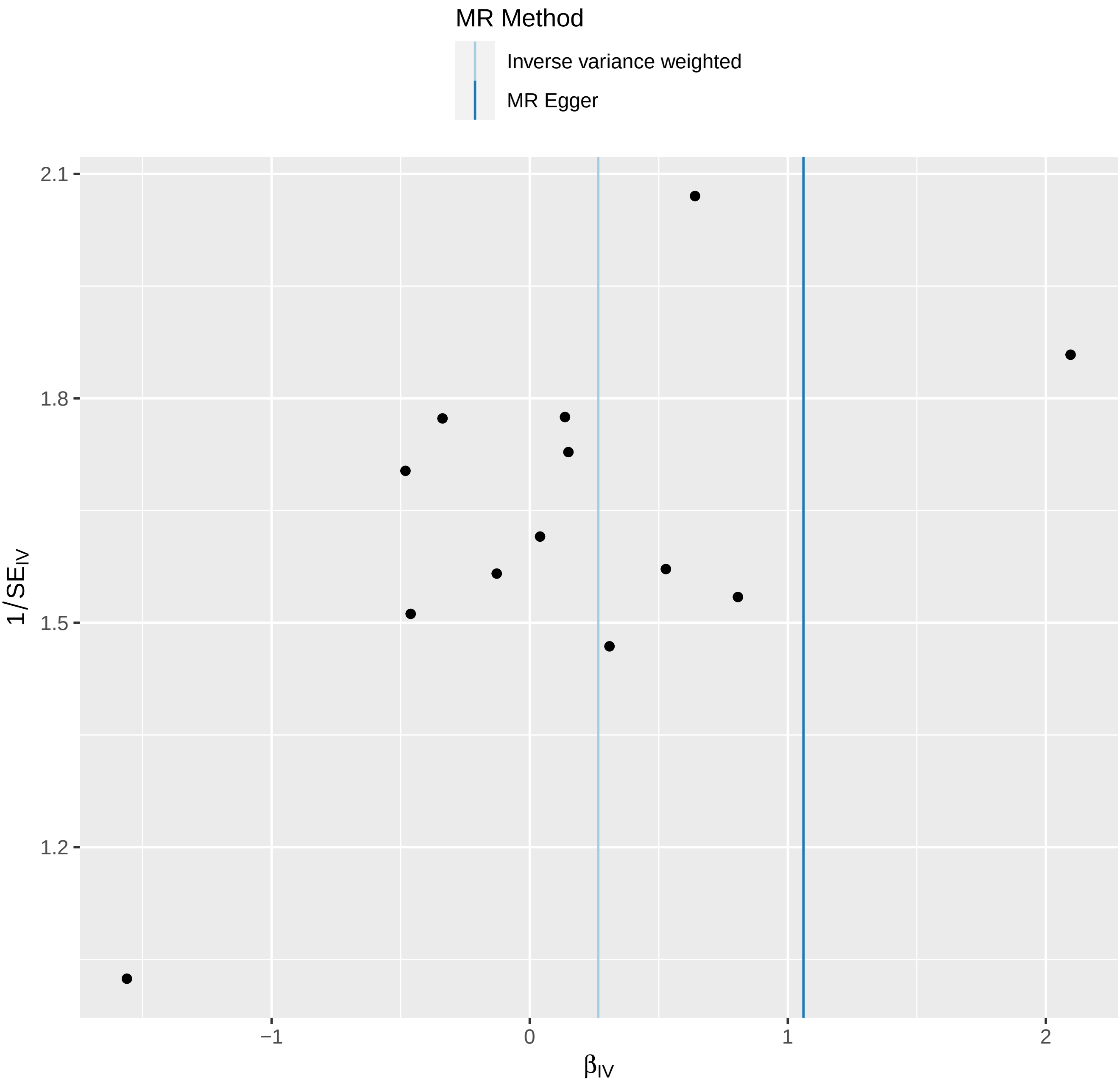

Batch 1433 : Gut microbiota abundance (genus Family XIII UCG001 id.11294) on Ulcerative proctitis

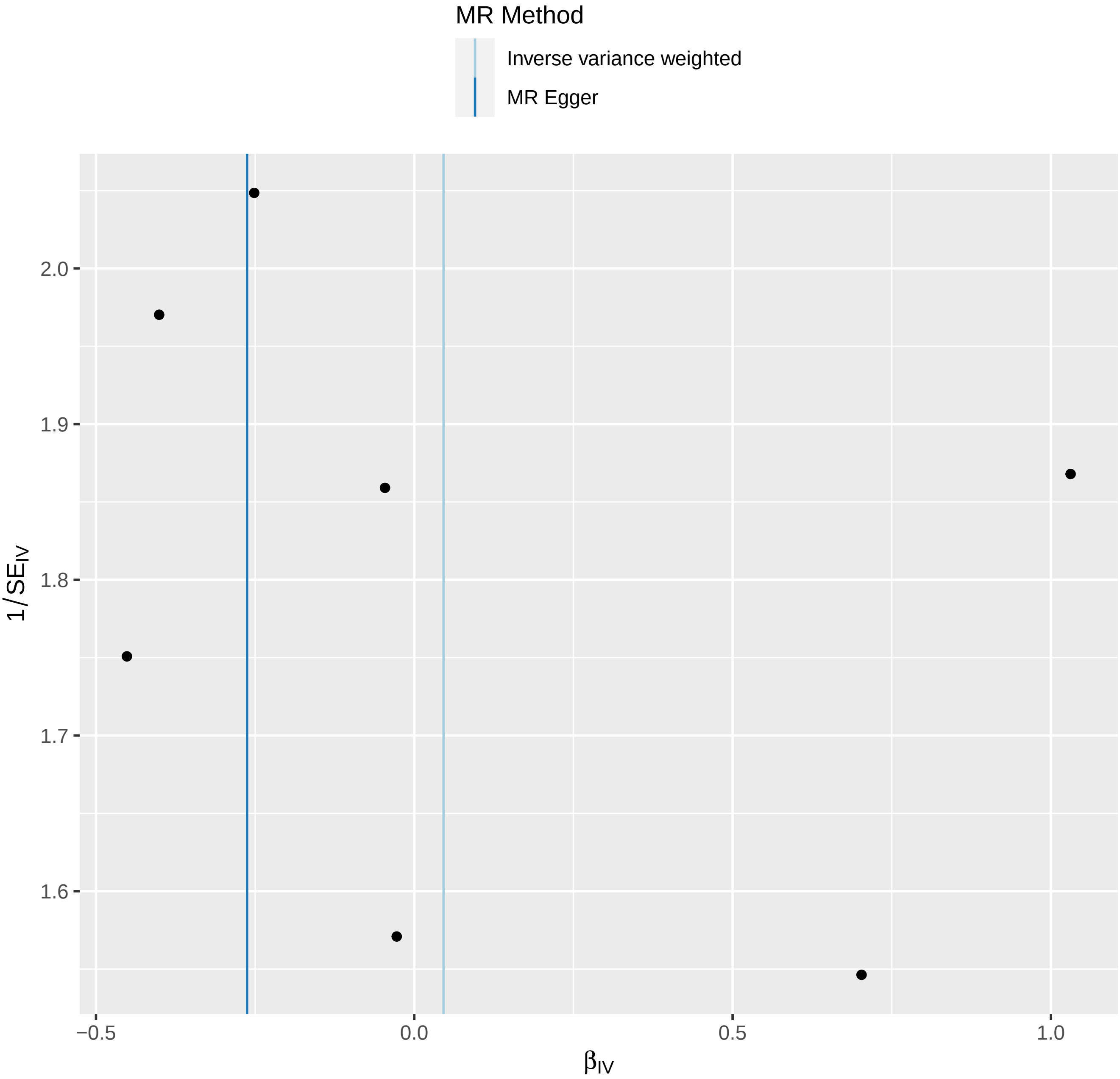

Batch 1434 : Gut microbiota abundance (genus Flavonifractor id.2059) on Ulcerative proctitis

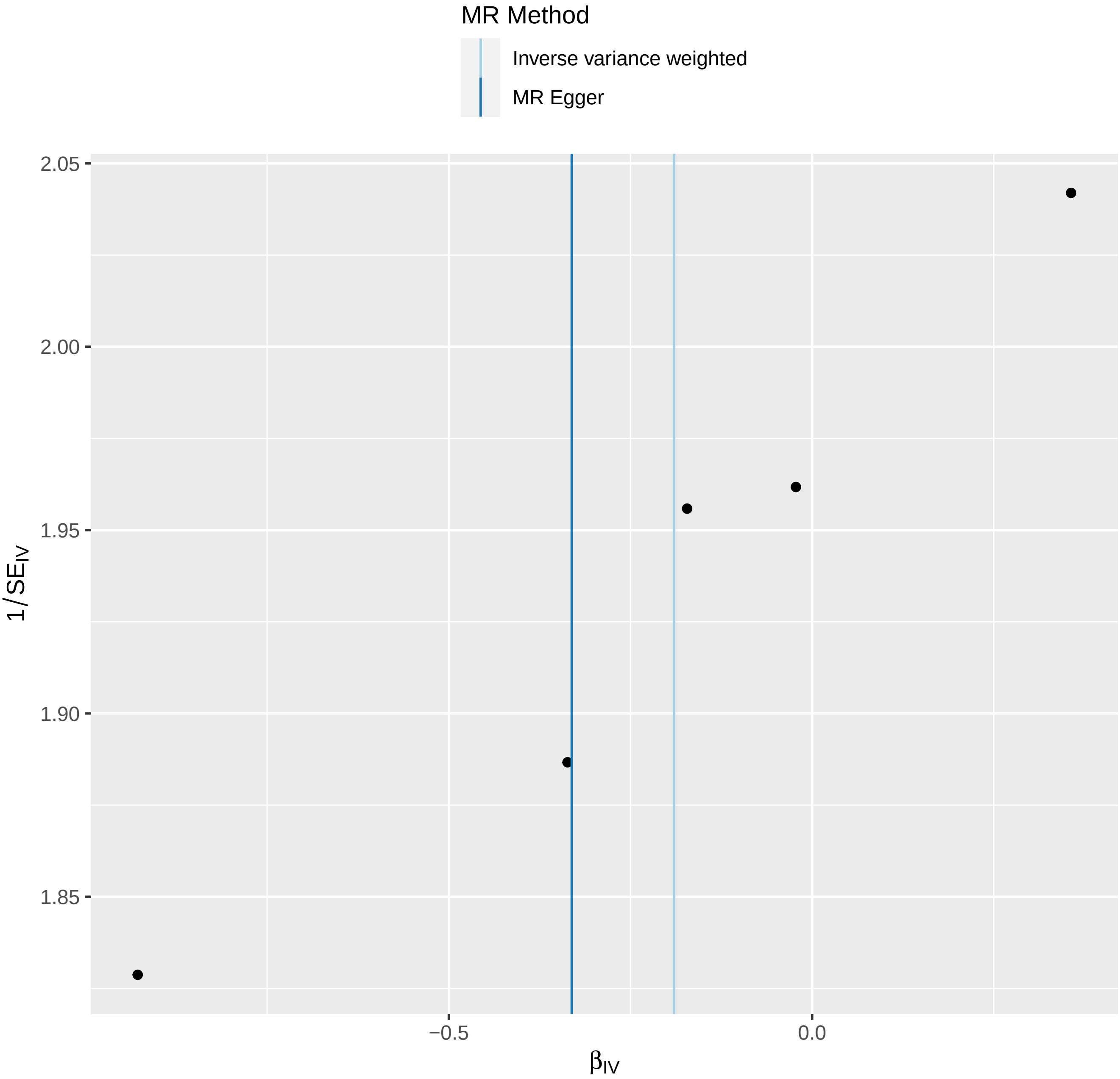

Batch 1435 : Gut microbiota abundance (genus Fusicatenibacter id.11305) on Ulcerative proctitis

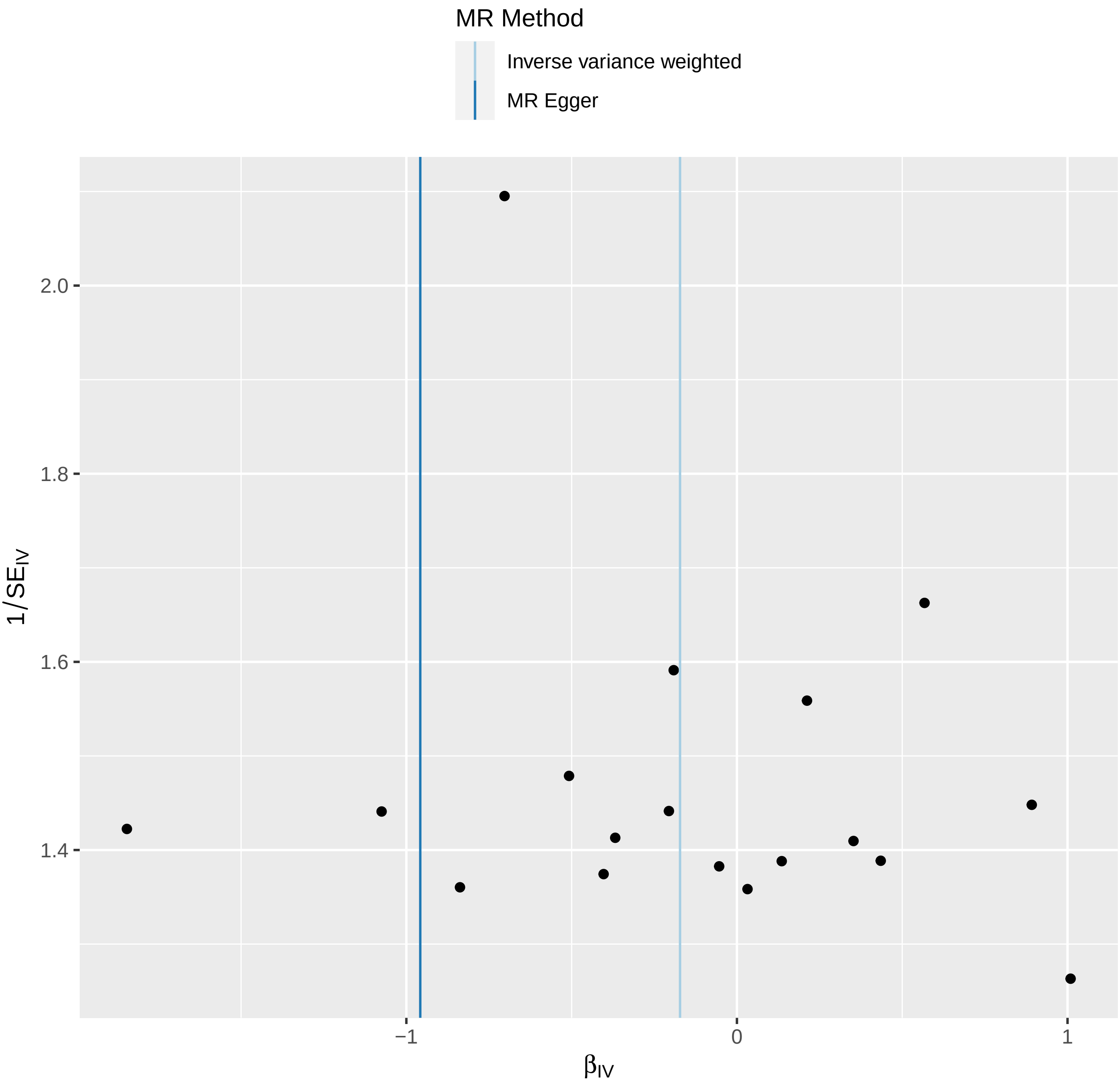

Batch 1436 : Gut microbiota abundance (genus Gordonibacter id.821) on Ulcerative proctitis

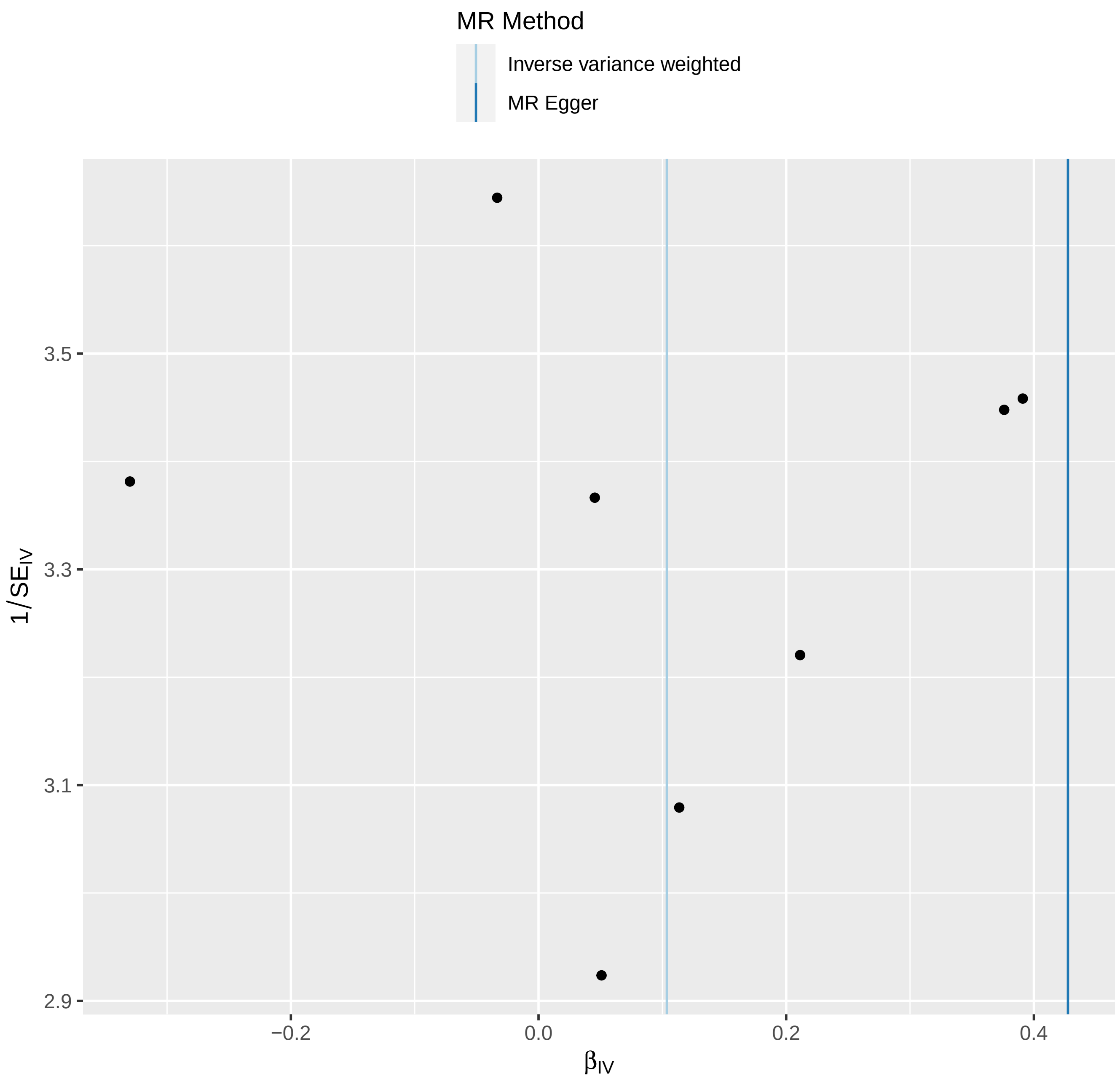

Batch 1437 : Gut microbiota abundance (genus Haemophilus id.3698) on Ulcerative proctitis

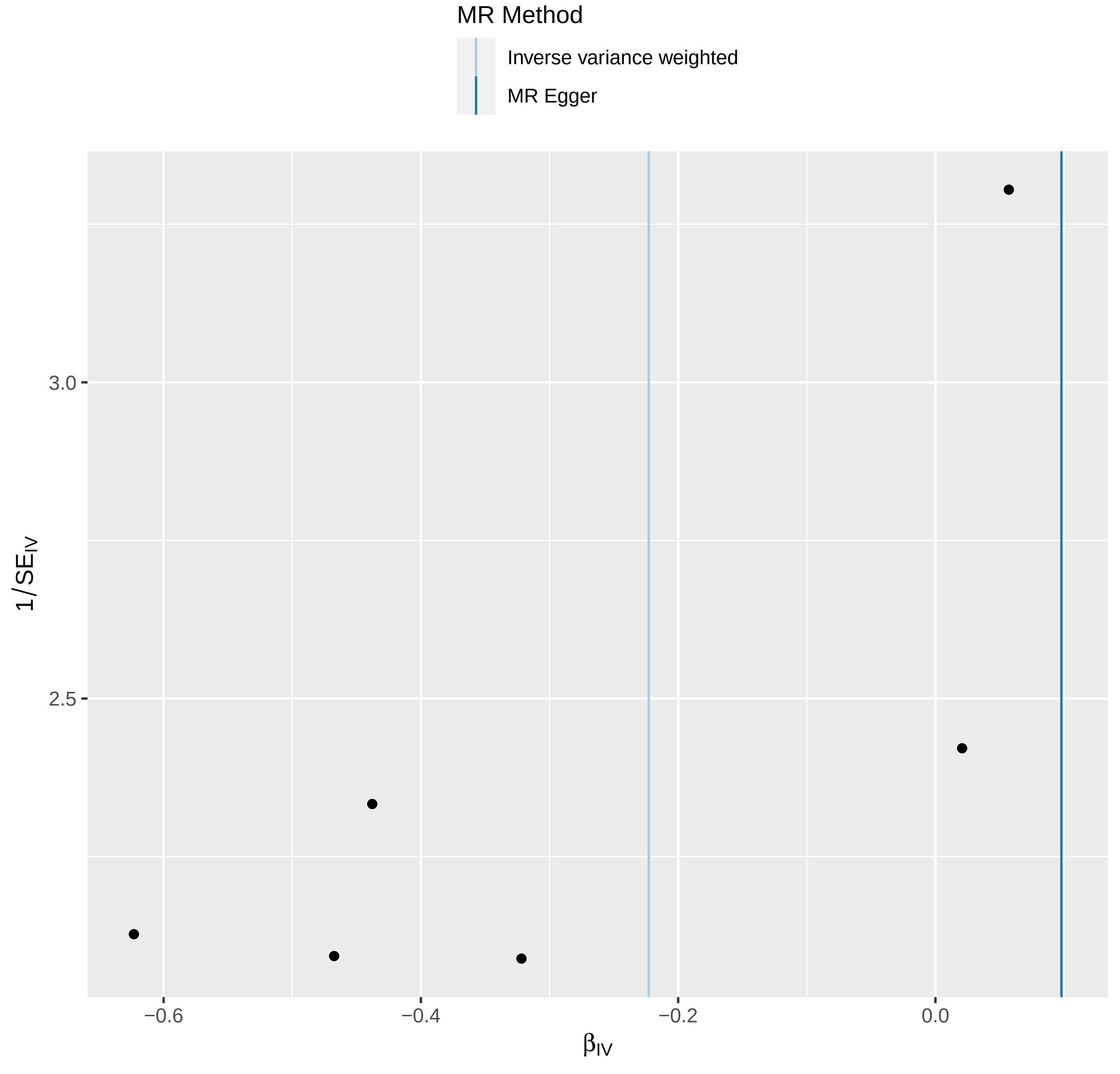

Batch 1438 : Gut microbiota abundance (genus Holdemanella id.11393) on Ulcerative proctitis

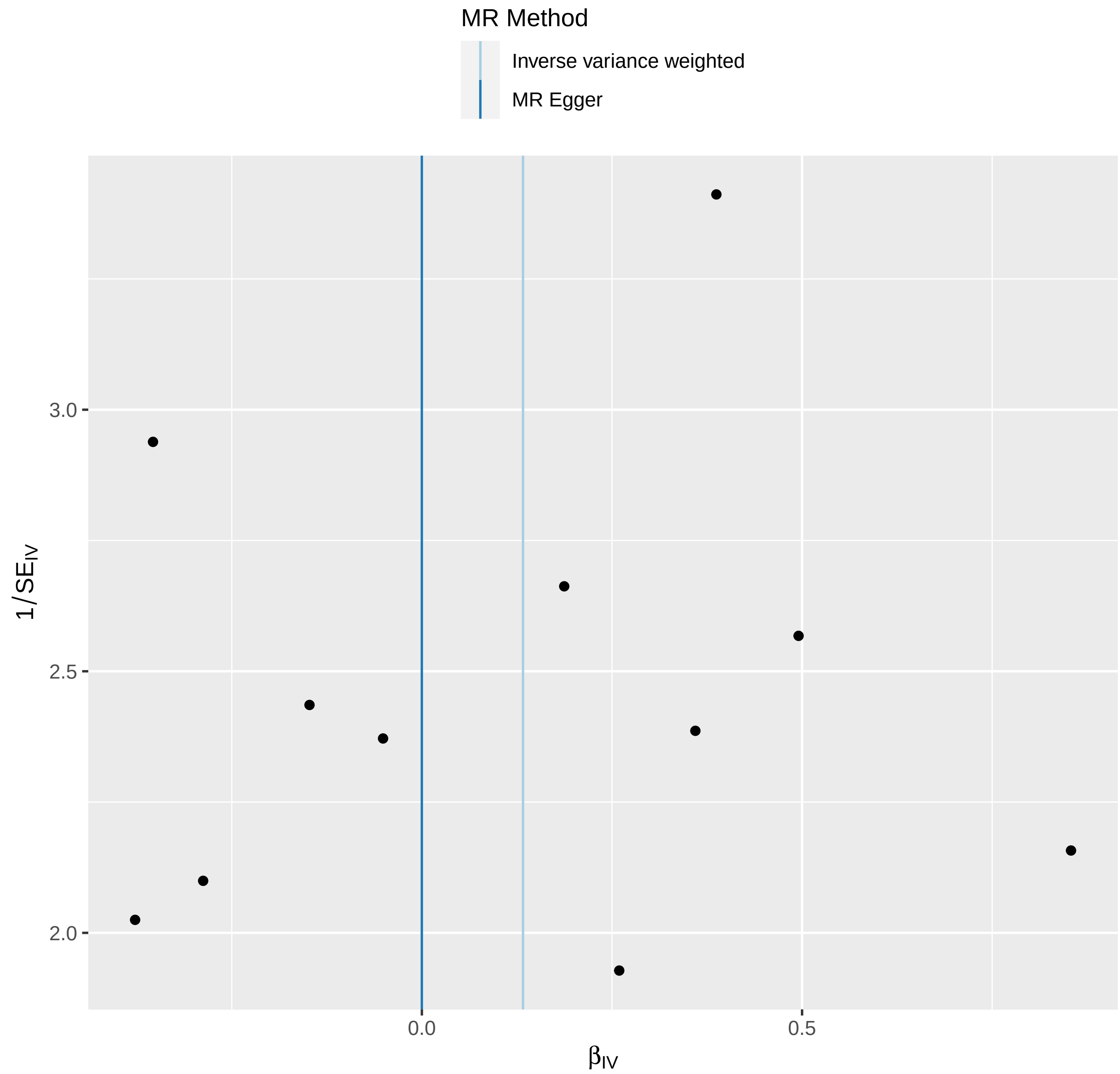

Batch 1439 : Gut microbiota abundance (genus Holdemania id.2157) on Ulcerative proctitis

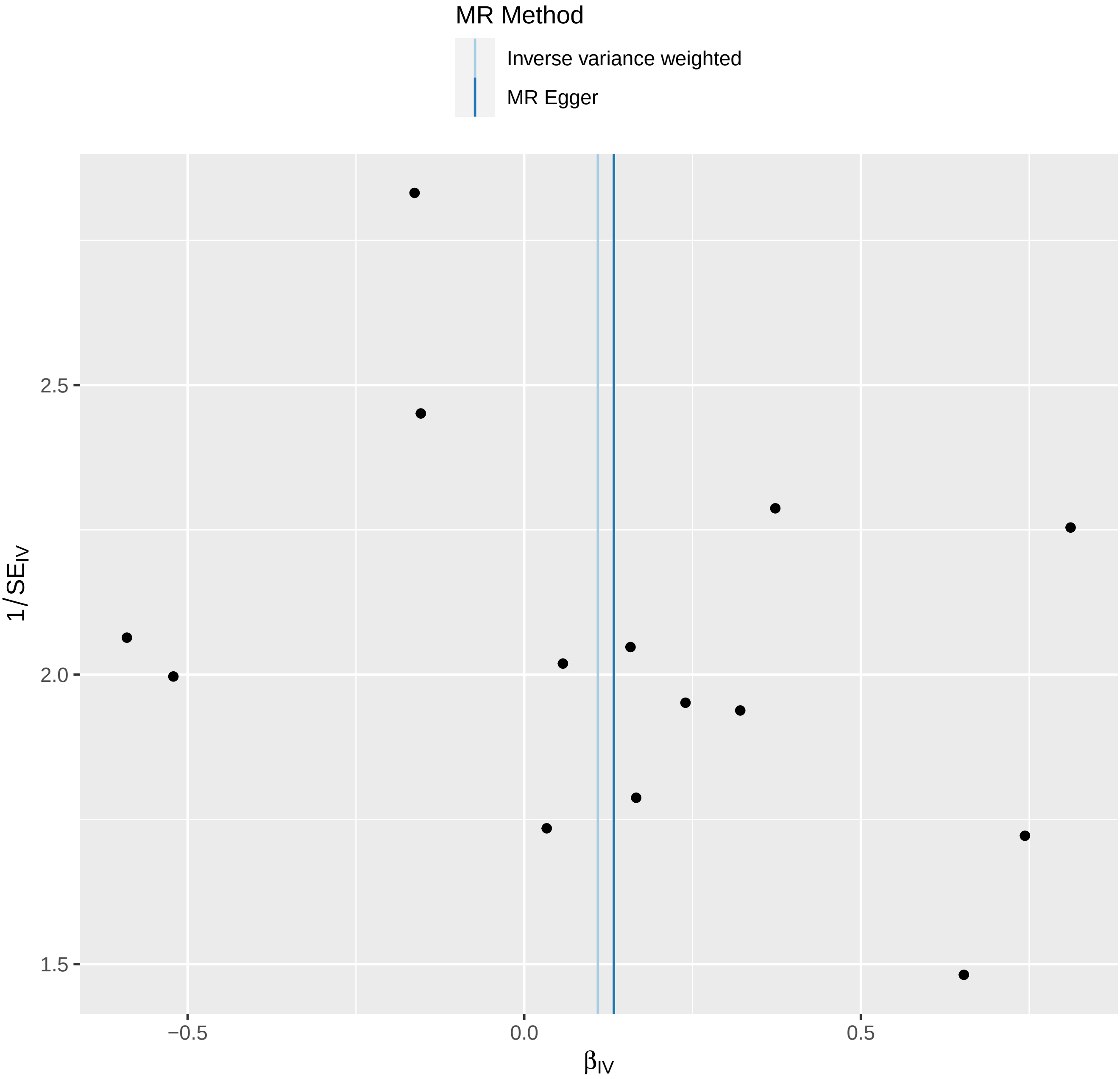

Batch 1440 : Gut microbiota abundance (genus Howardella id.2000) on Ulcerative proctitis

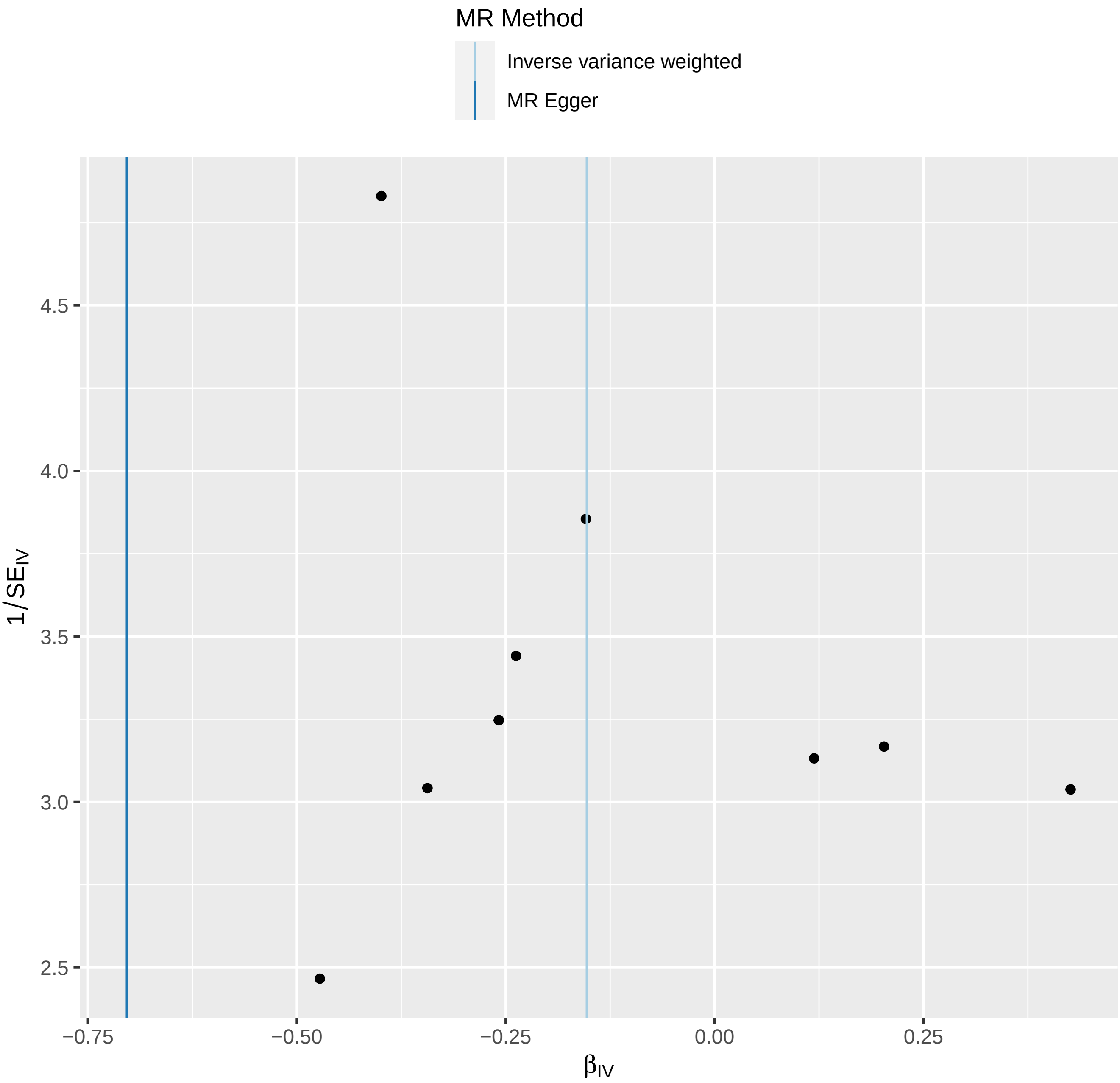

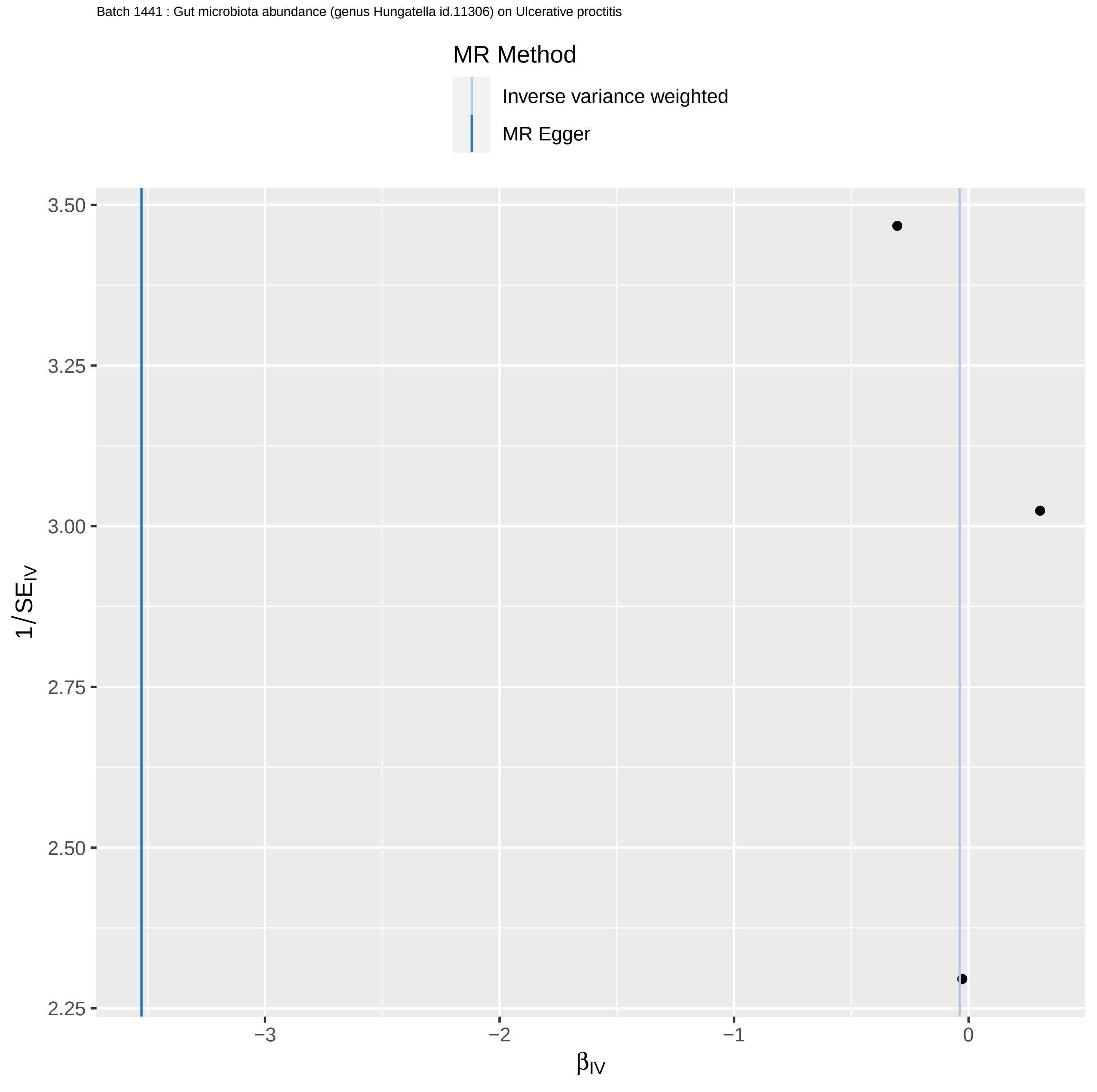
Batch 1441 : Gut microbiota abundance (genus Hungatella id.11306) on Ulcerative proctitis
MR Method
Inverse variance weighted
MR Egger
3.50
3.25
3.00
2.75
2.50
2.25
1/SE_IV
−3
−2
−1
0
β_IV

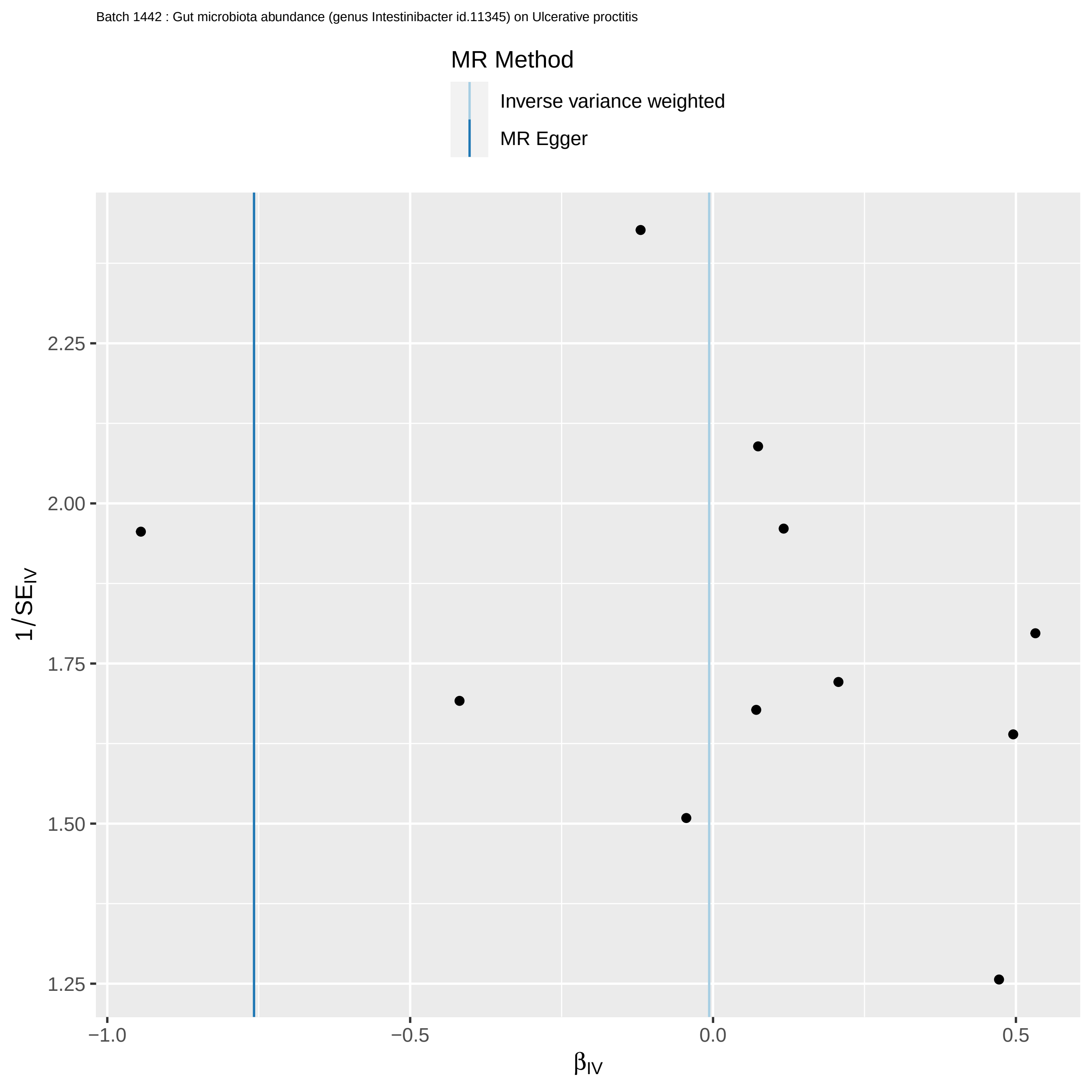

Batch 1442 : Gut microbiota abundance (genus Intestinibacter id.11345) on Ulcerative proctitis
MR Method
Inverse variance weighted
MR Egger
1/SE_IV
2.25
2.00
1.75
1.50
1.25
−1.0
−0.5
0.0
0.5
β_IV

Batch 1443 : Gut microbiota abundance (genus Intestinimonas id.2062) on Ulcerative proctitis

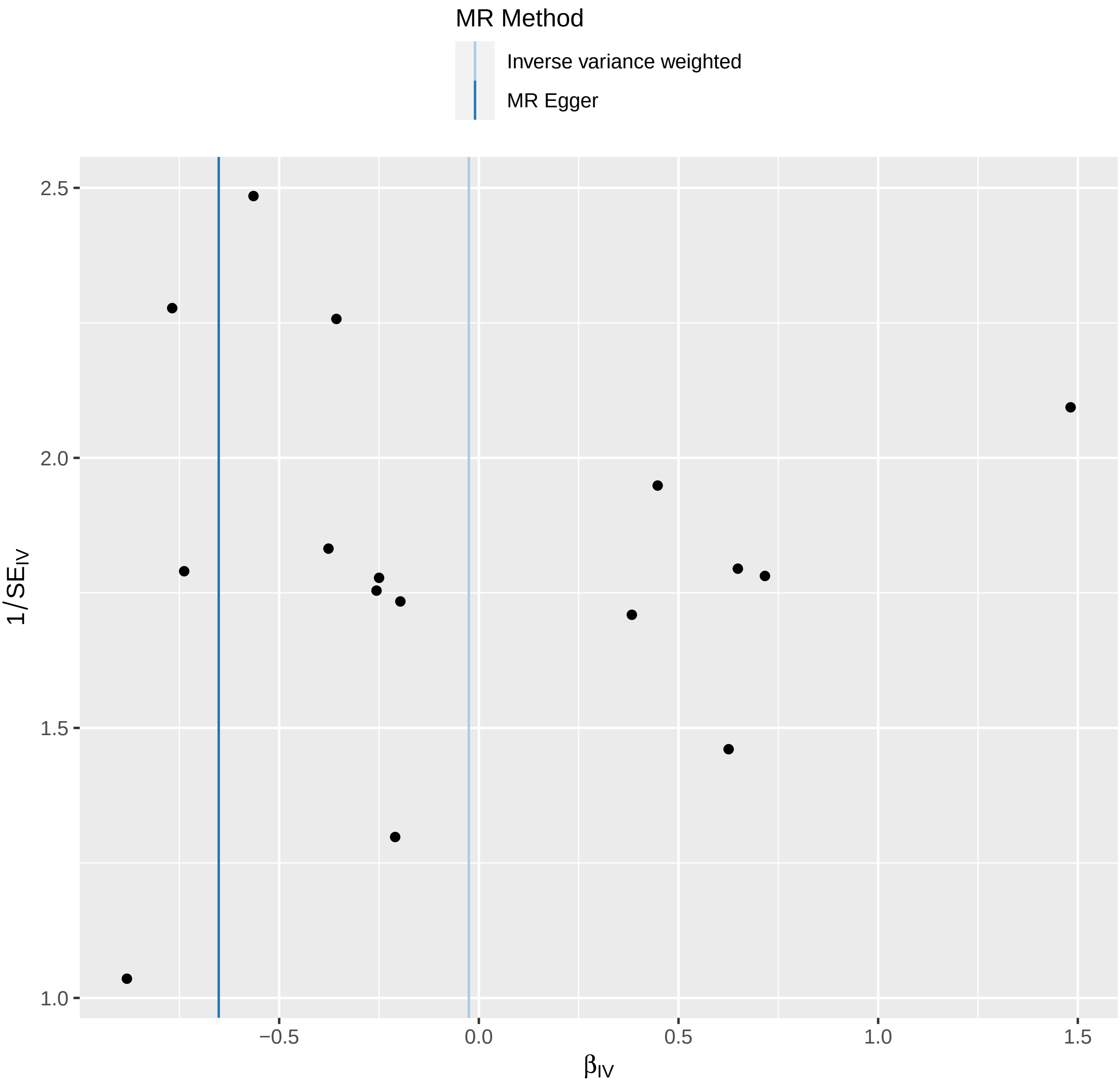

Batch 1444 : Gut microbiota abundance (genus Lachnoclostridium id.11308) on Ulcerative proctitis

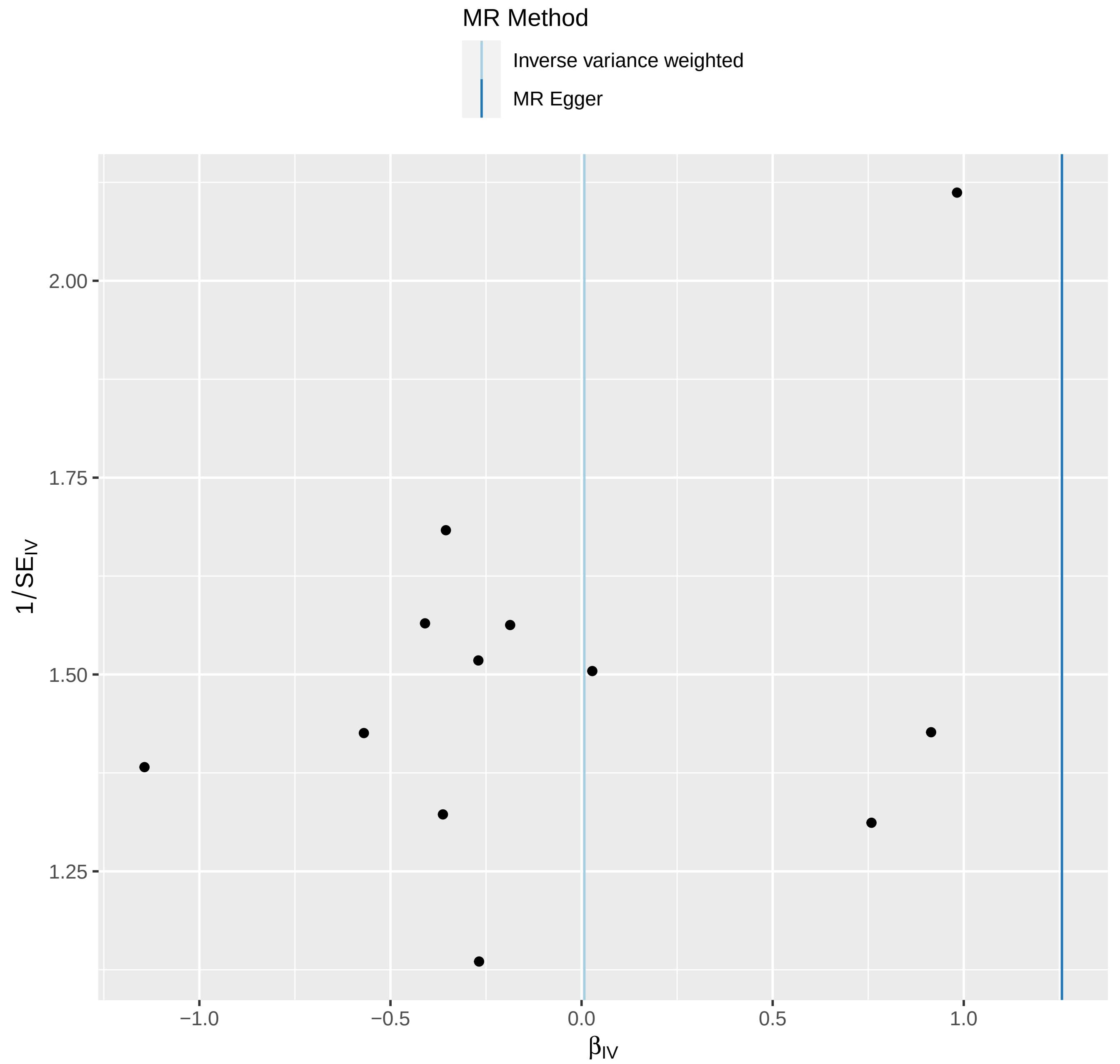

Batch 1445 : Gut microbiota abundance (genus Lachnospiraceae FCS020 group id.11314) on Ulcerative proctitis

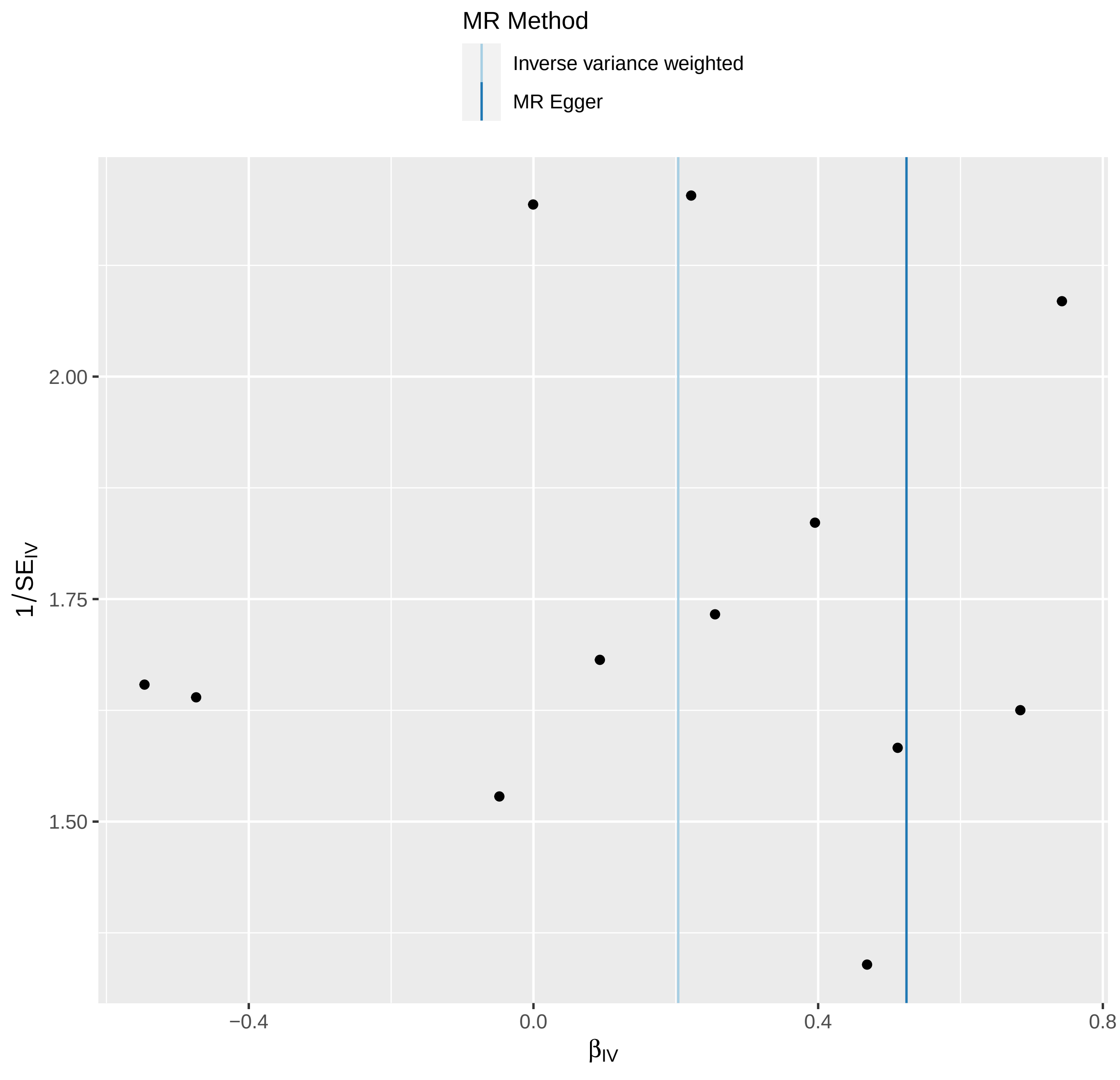

Batch 1446 : Gut microbiota abundance (genus Lachnospiraceae NC2004 group id.11316) on Ulcerative proctitis

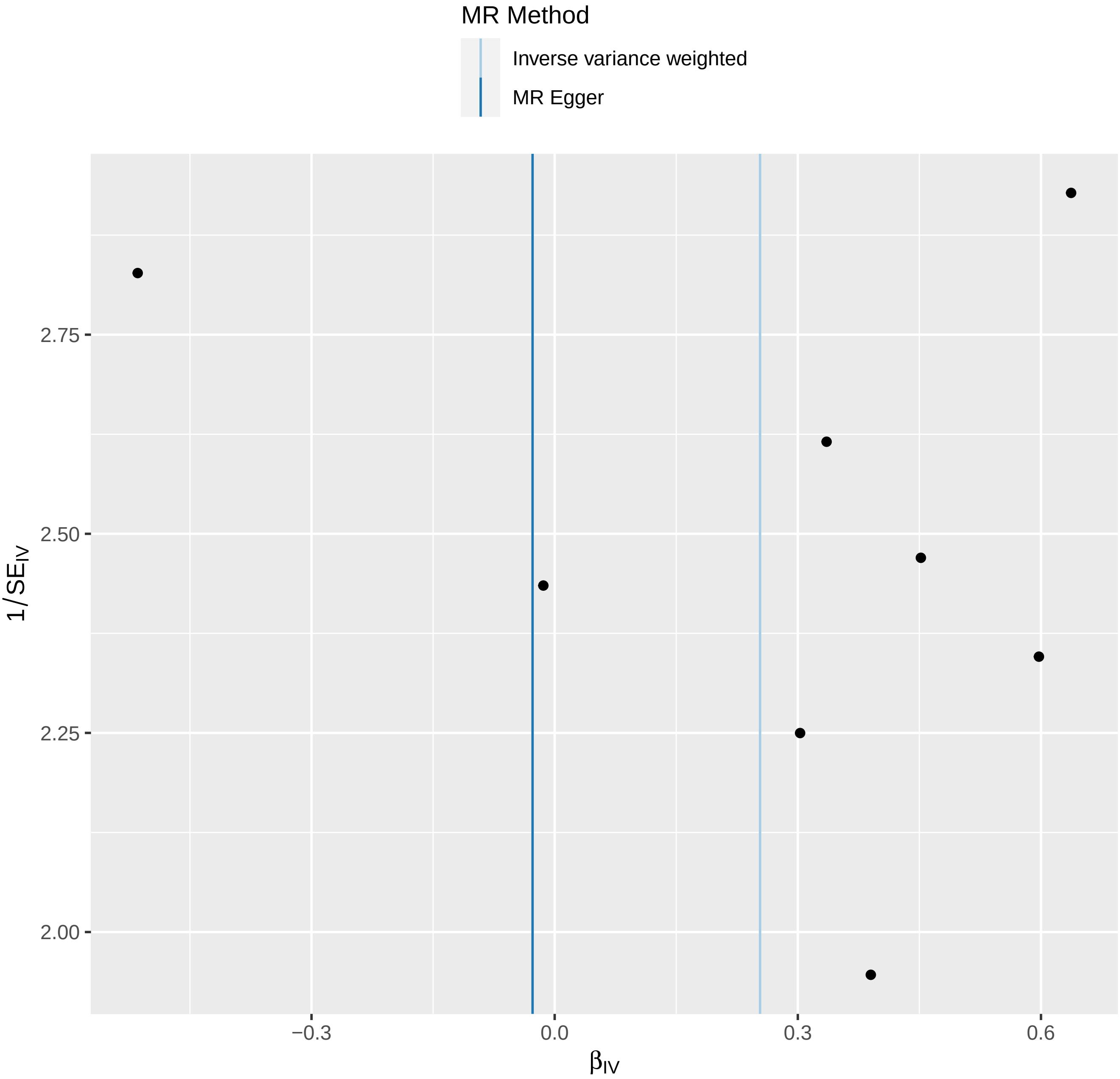

Batch 1447 : Gut microbiota abundance (genus Lachnospiraceae ND3007 group id.11317) on Ulcerative proctitis

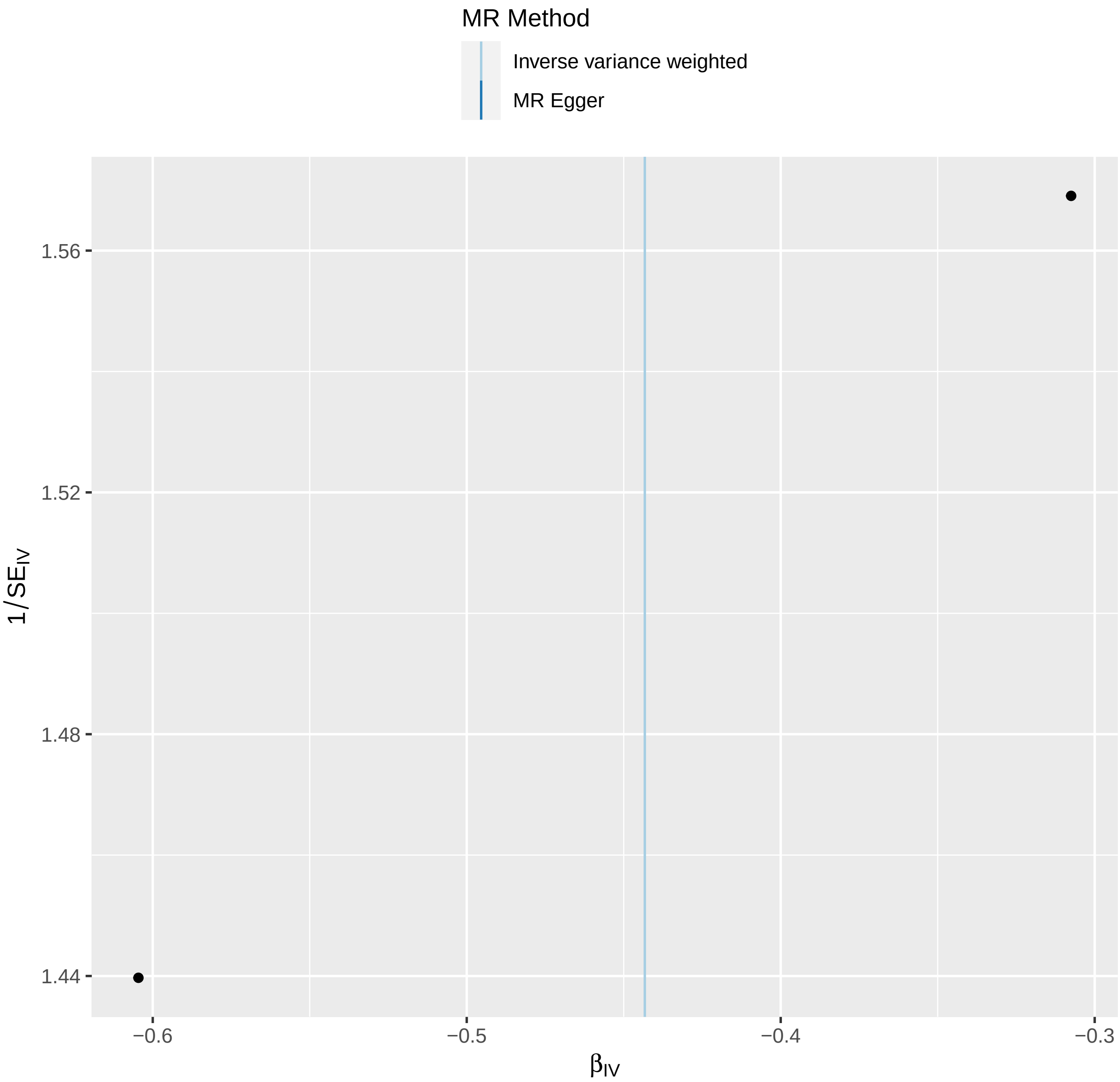

Batch 1448 : Gut microbiota abundance (genus Lachnospiraceae NK4A136 group id.11319) on Ulcerative proctitis

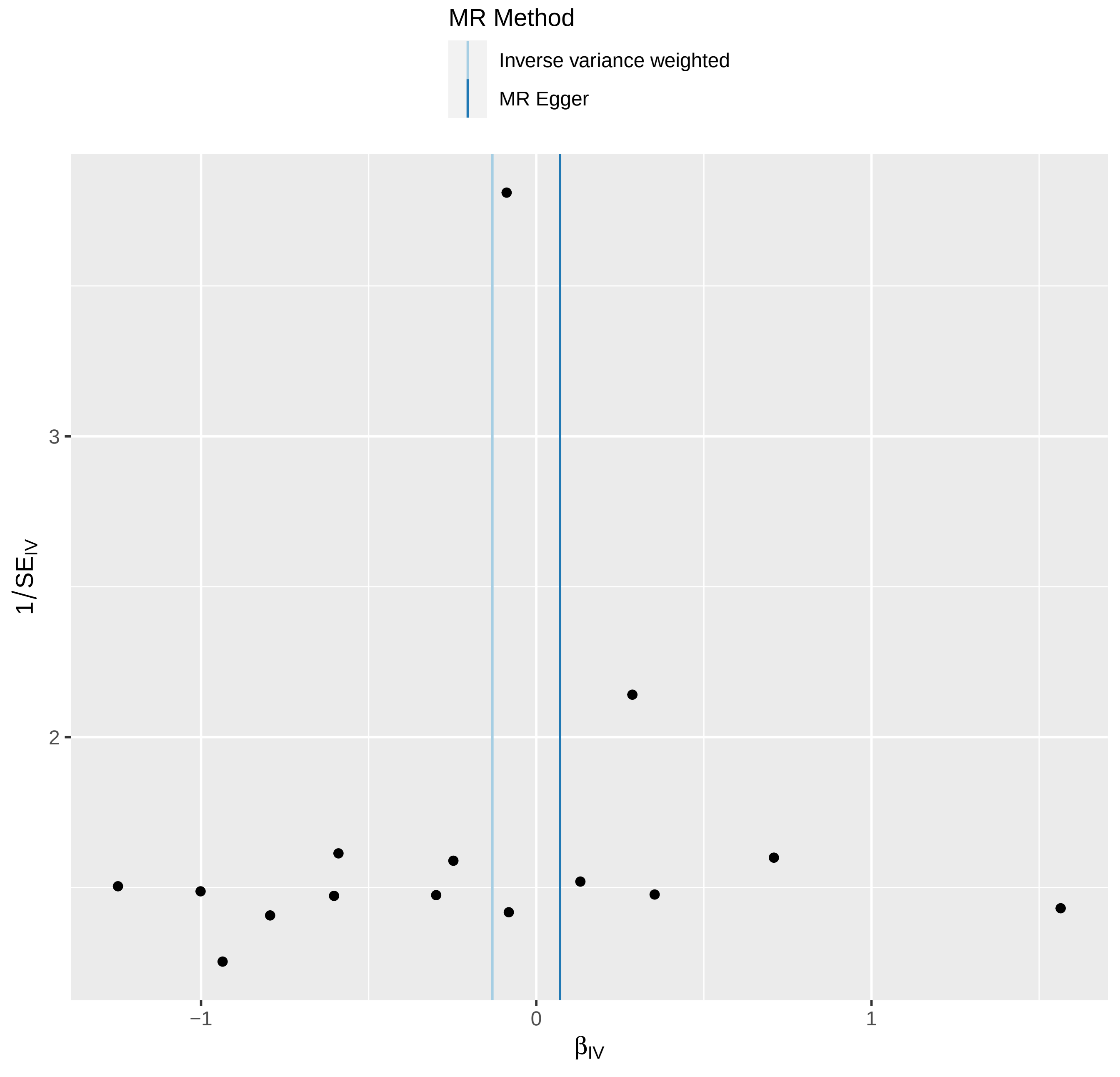

Batch 1449 : Gut microbiota abundance (genus Lachnospiraceae UCG001 id.11321) on Ulcerative proctitis

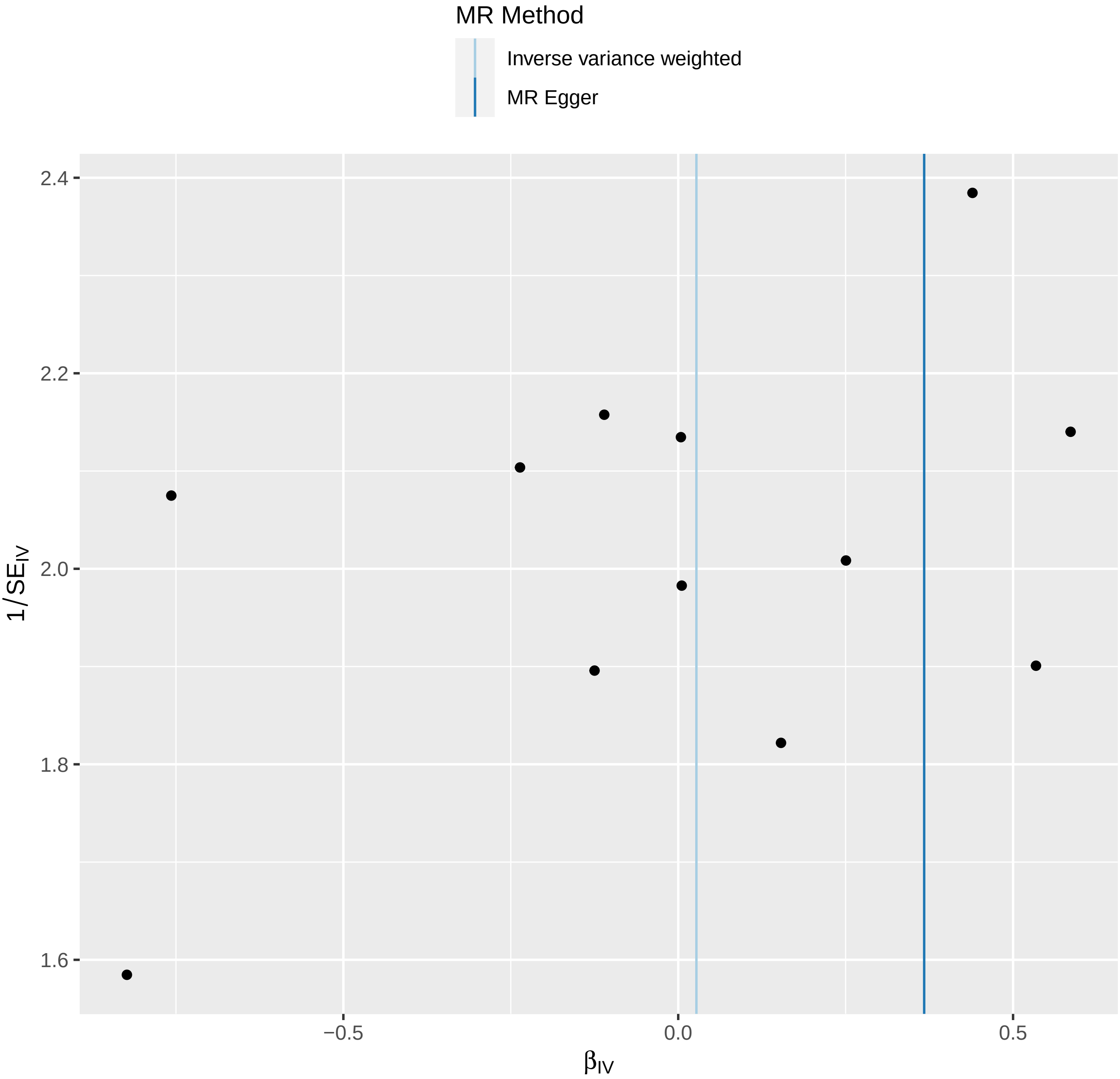

Batch 1450 : Gut microbiota abundance (genus Lachnospiraceae UCG004 id.11324) on Ulcerative proctitis

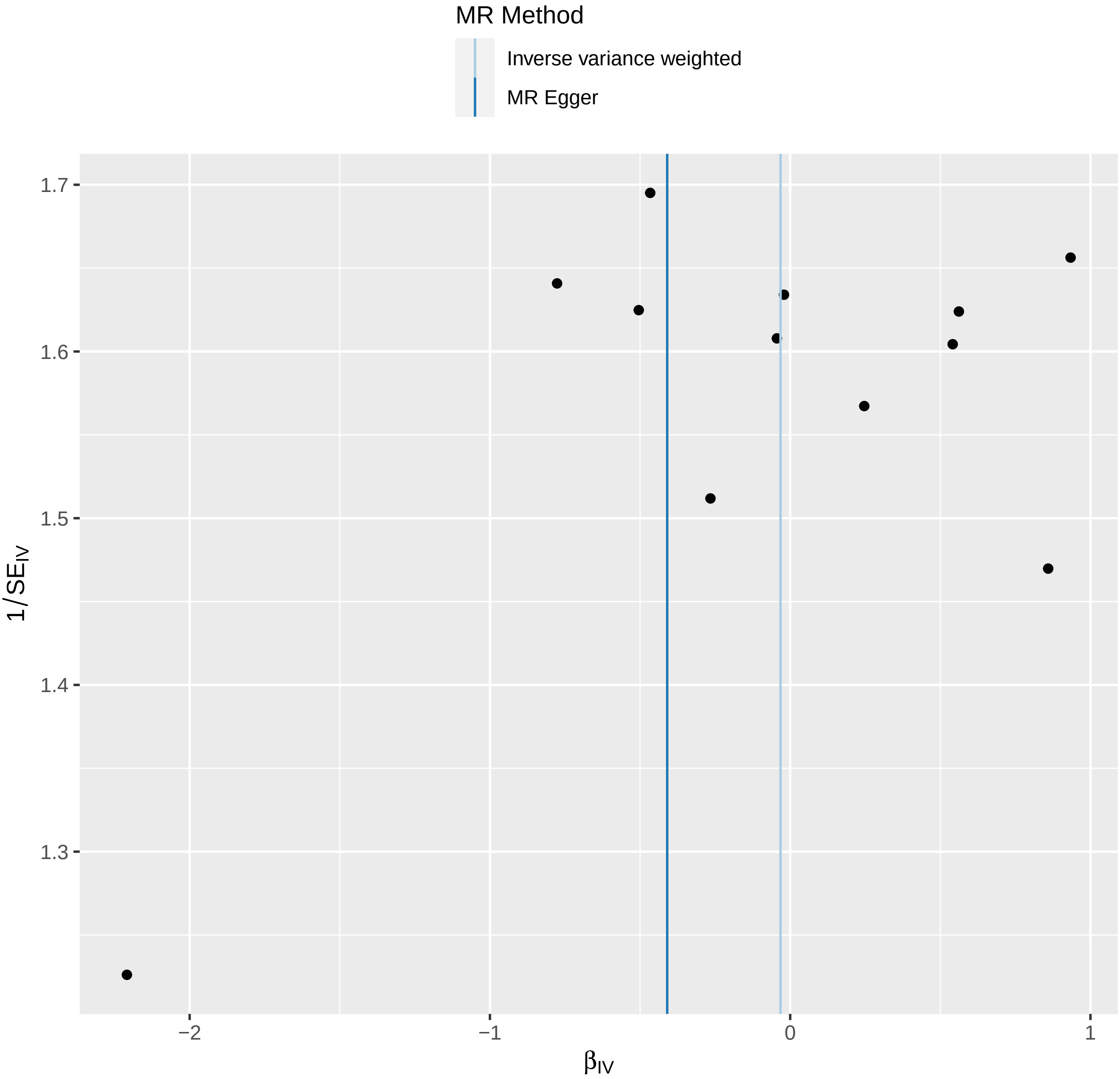

Batch 1451 : Gut microbiota abundance (genus Lachnospiraceae UCG008 id.11328) on Ulcerative proctitis

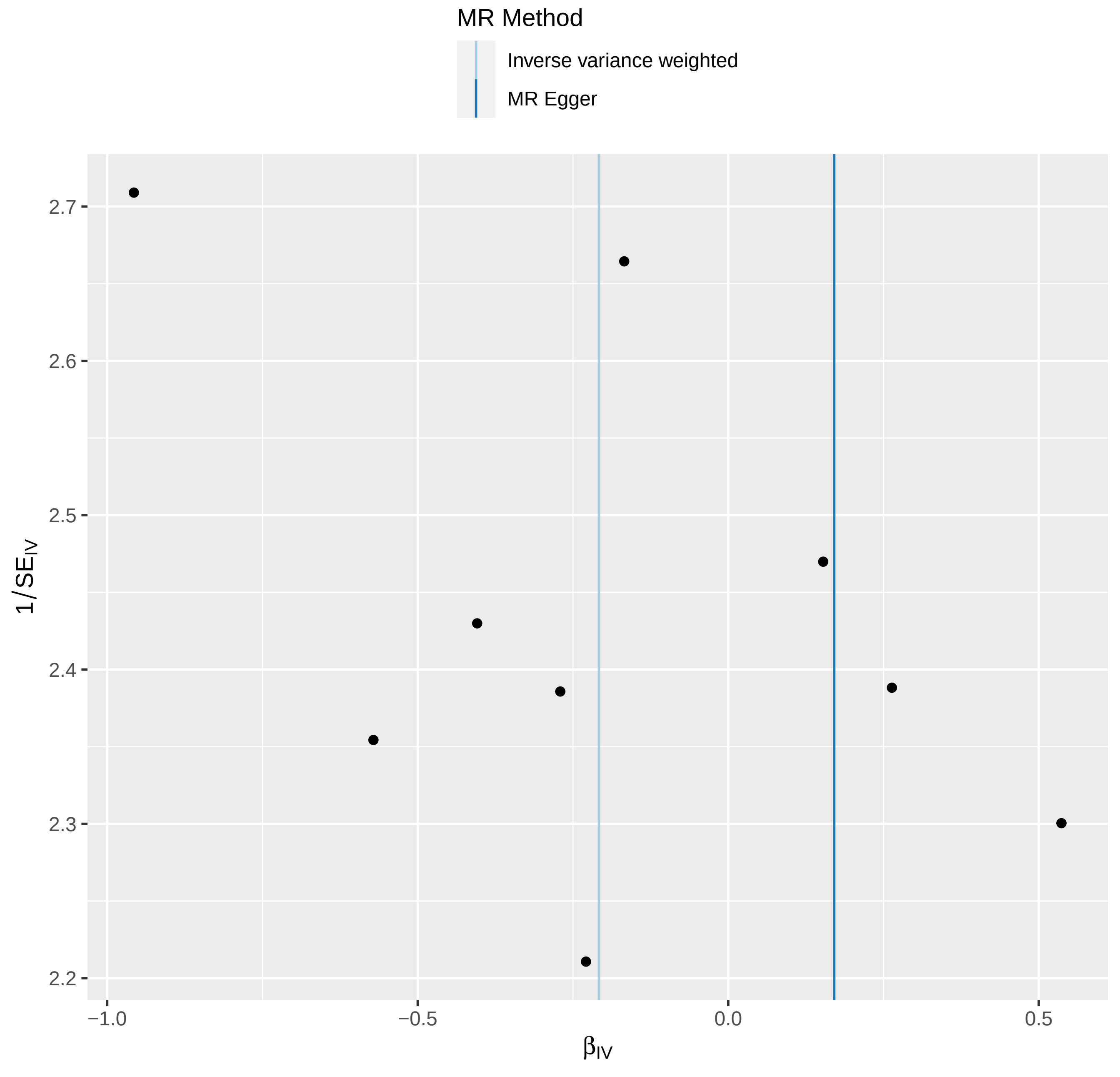

Batch 1452 : Gut microbiota abundance (genus Lachnospiraceae UCG010 id.11330) on Ulcerative proctitis

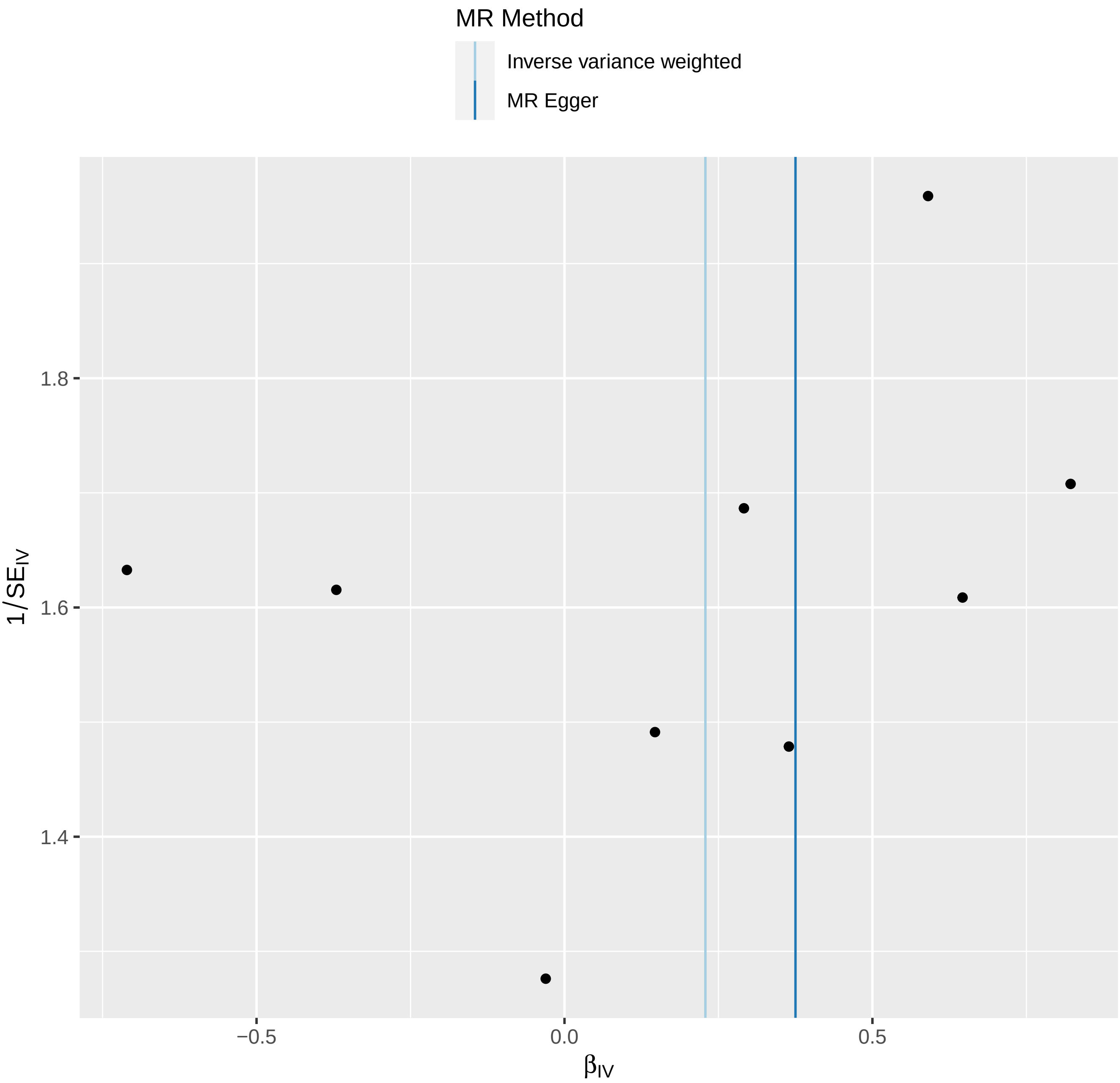

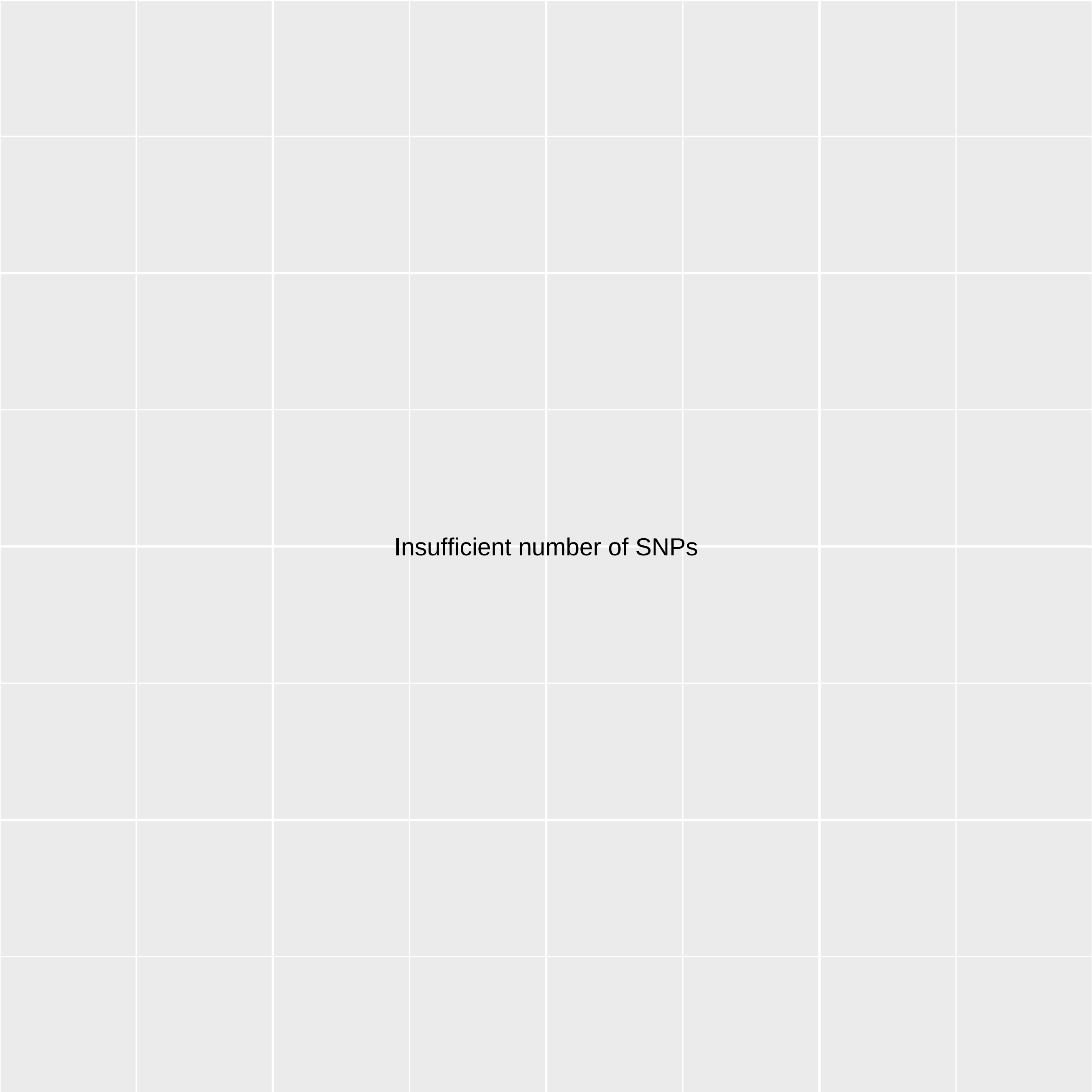
Insufficient number of SNPs

Batch 1454 : Gut microbiota abundance (genus Lactobacillus id.1837) on Ulcerative proctitis

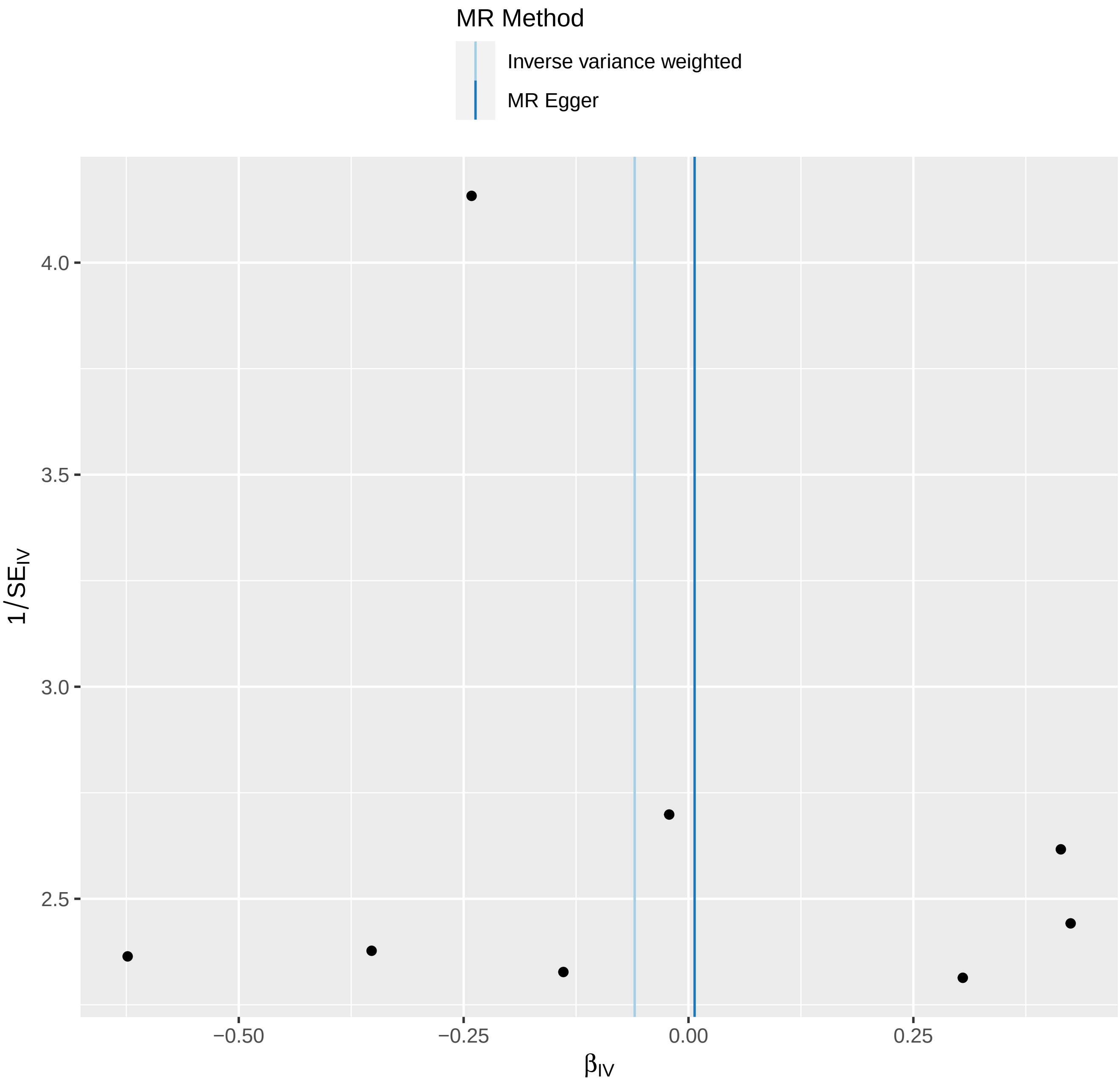

Batch 1455 : Gut microbiota abundance (genus Lactococcus id.1851) on Ulcerative proctitis

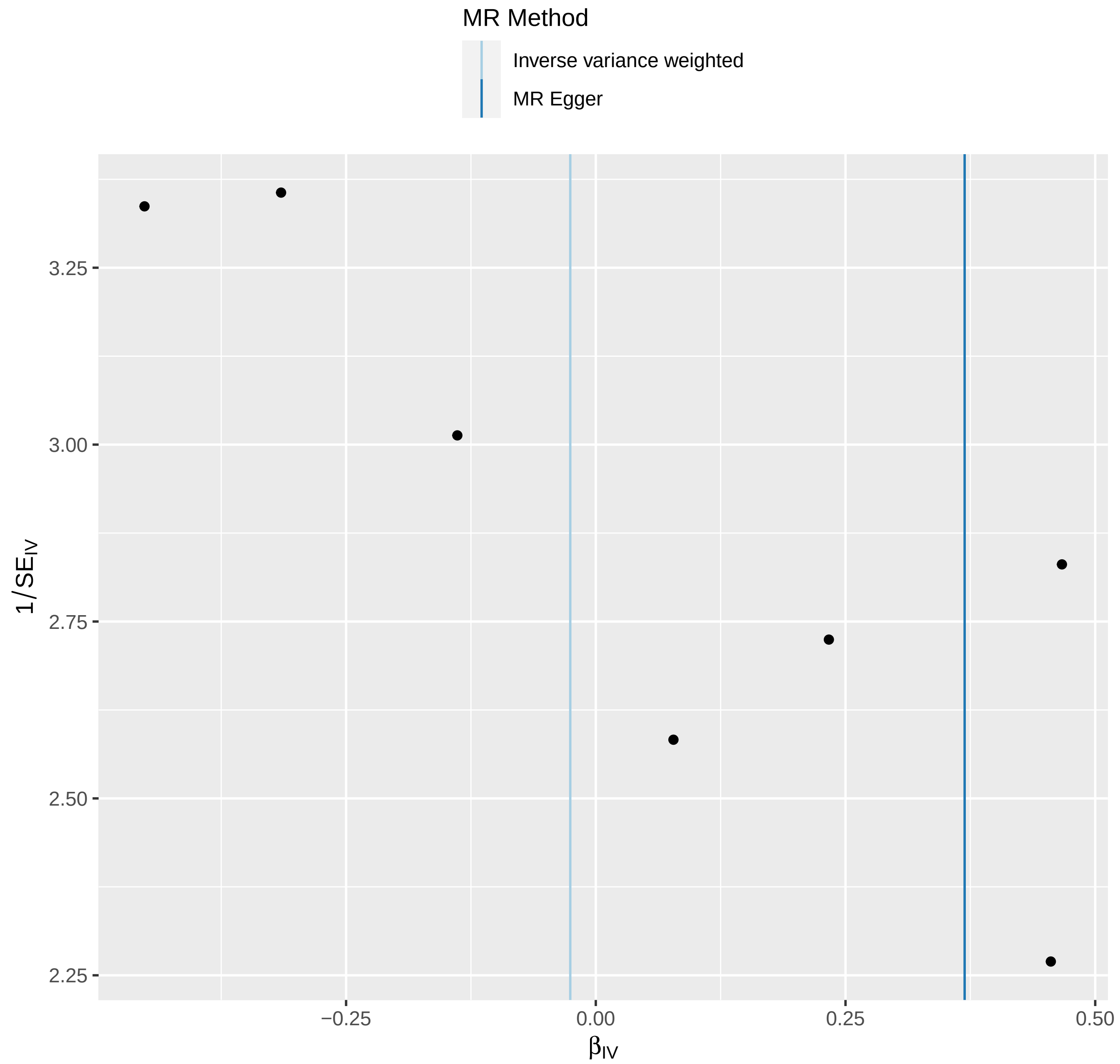

Batch 1456 : Gut microbiota abundance (genus Marvinbryantia id.2005) on Ulcerative proctitis

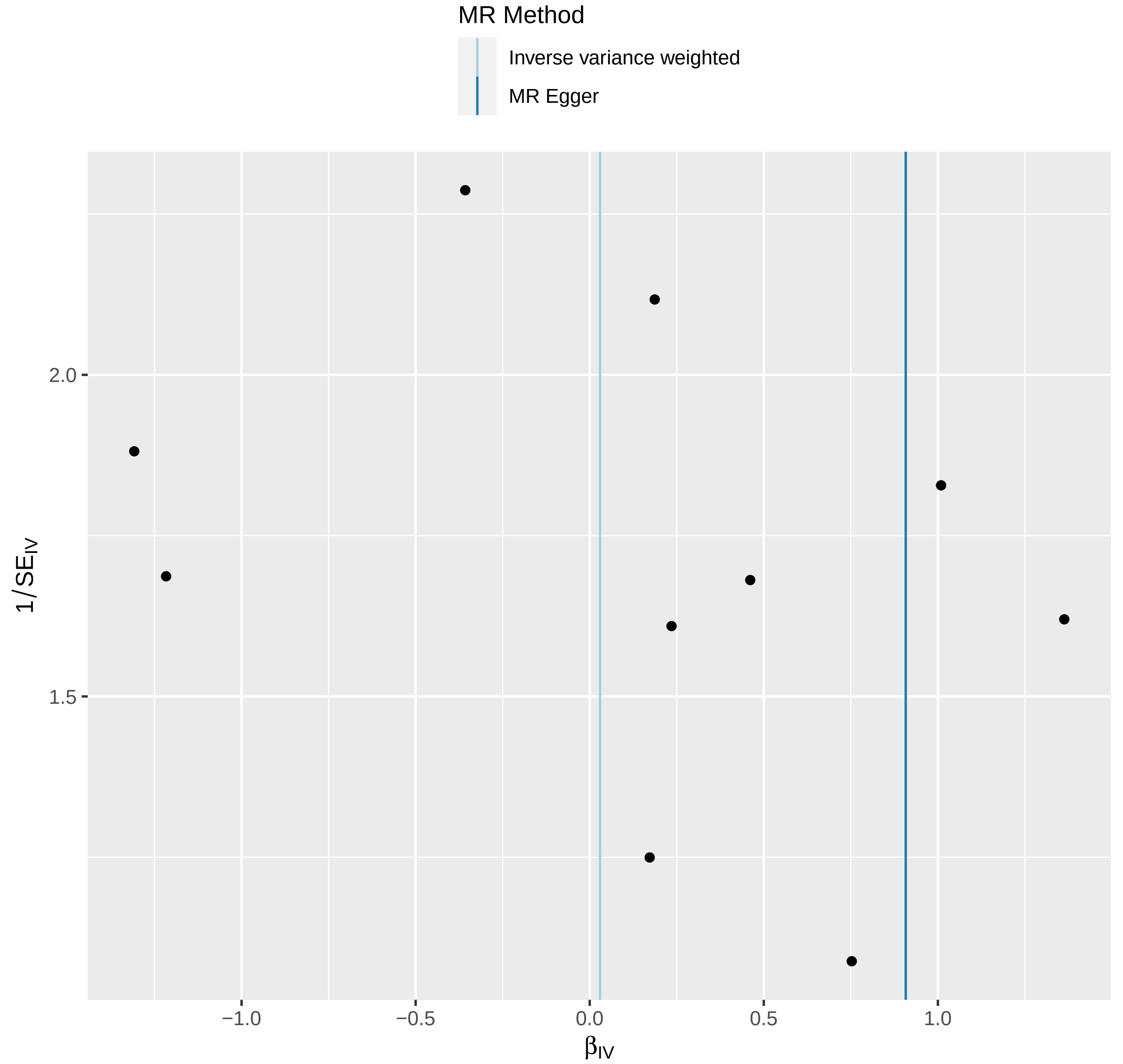

Batch 1457 : Gut microbiota abundance (genus Methanobrevibacter id.123) on Ulcerative proctitis

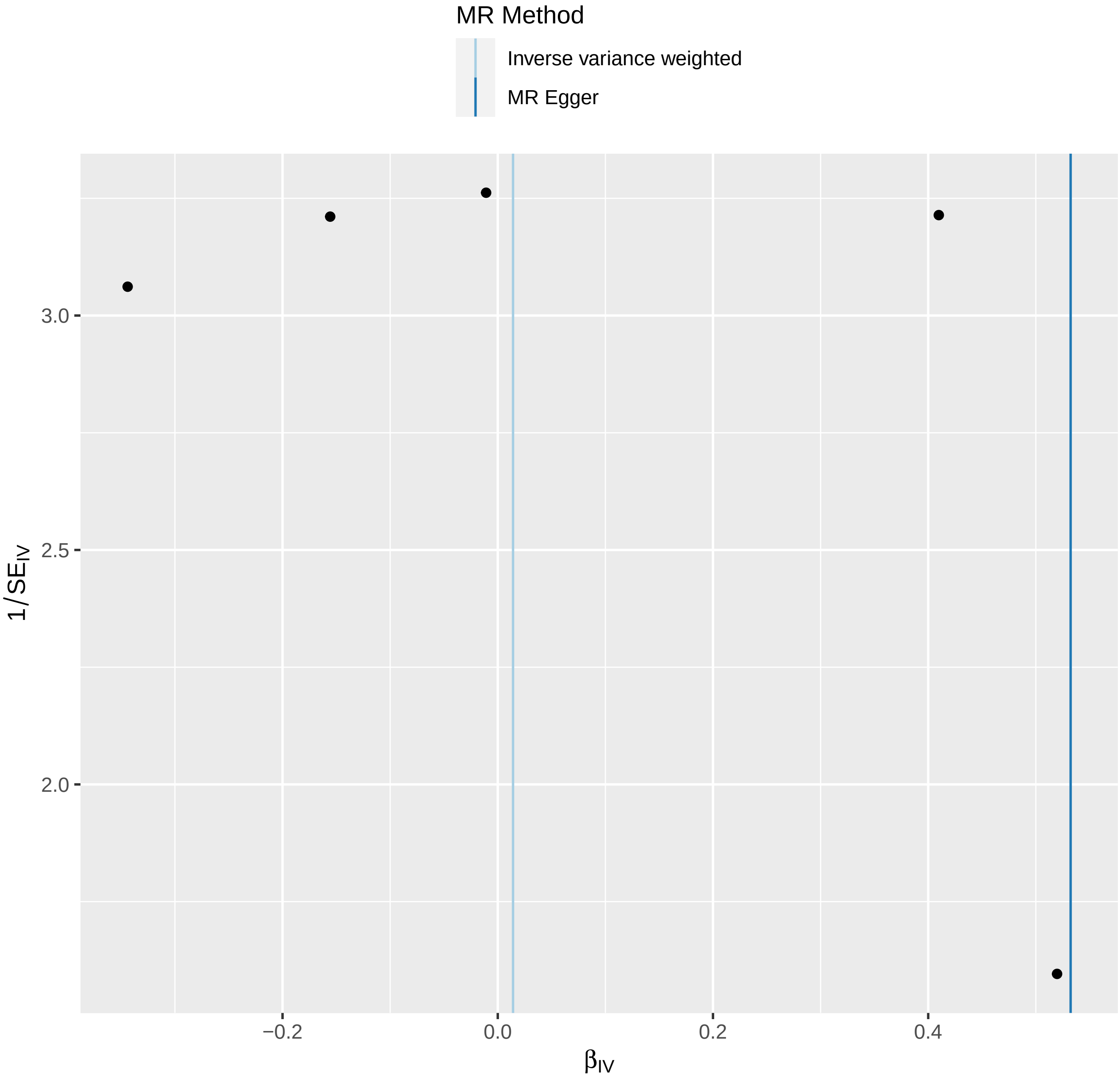

Batch 1458 : Gut microbiota abundance (genus Odoribacter id.952) on Ulcerative proctitis

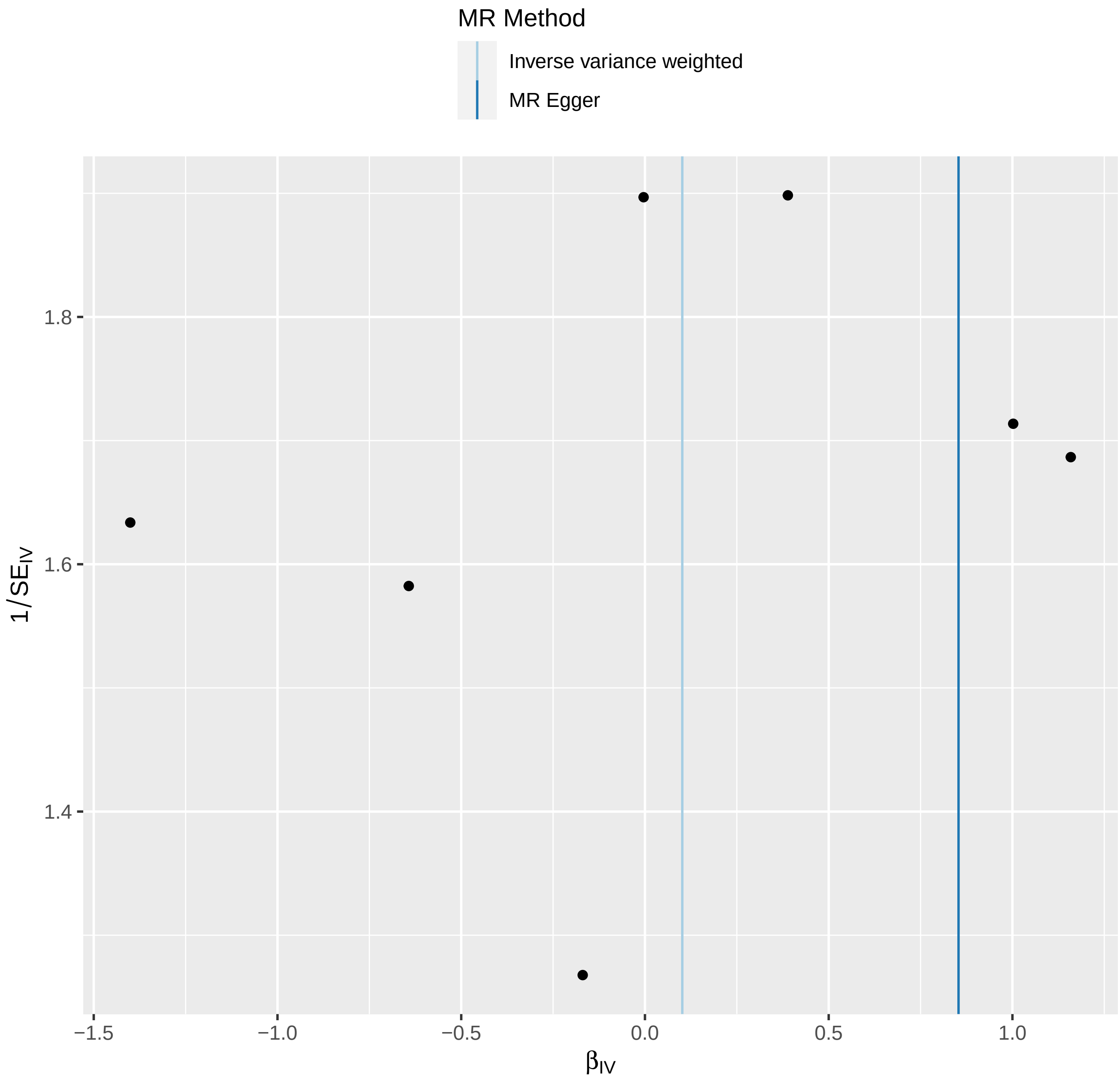

Batch 1459 : Gut microbiota abundance (genus Olsenella id.822) on Ulcerative proctitis

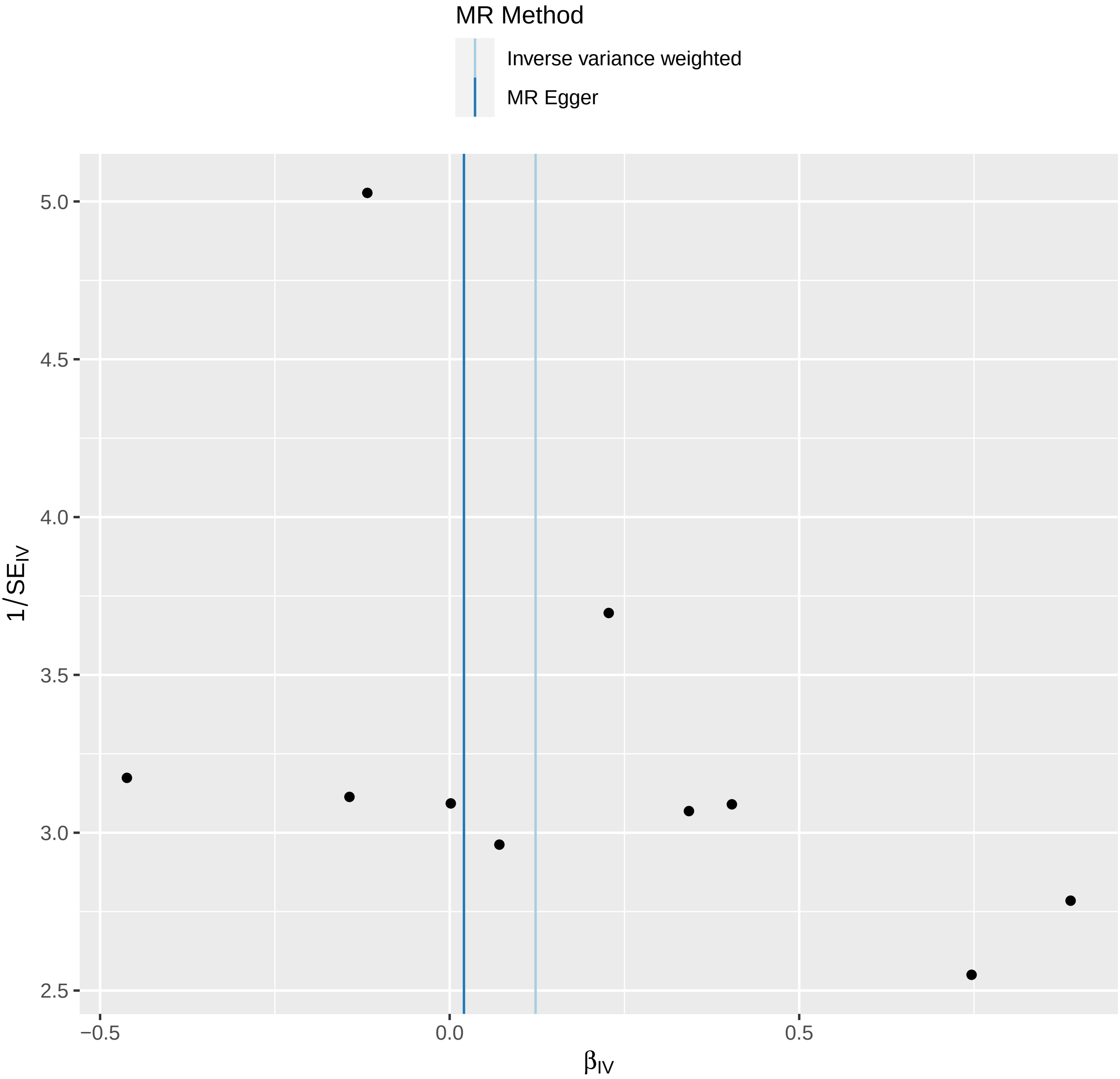

Batch 1460 : Gut microbiota abundance (genus Oscillibacter id.2063) on Ulcerative proctitis

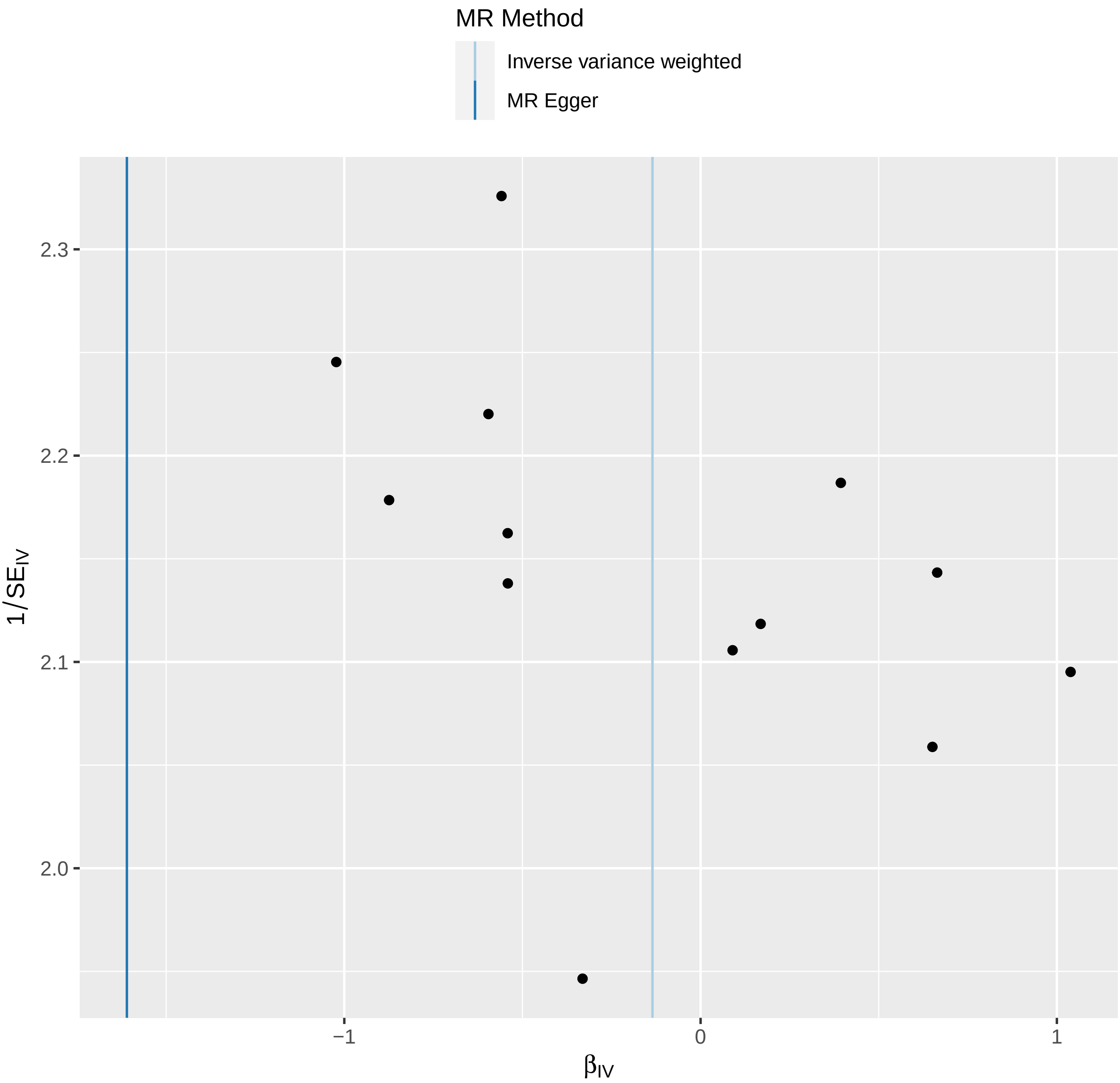

Batch 1461 : Gut microbiota abundance (genus Oscillospira id.2064) on Ulcerative proctitis

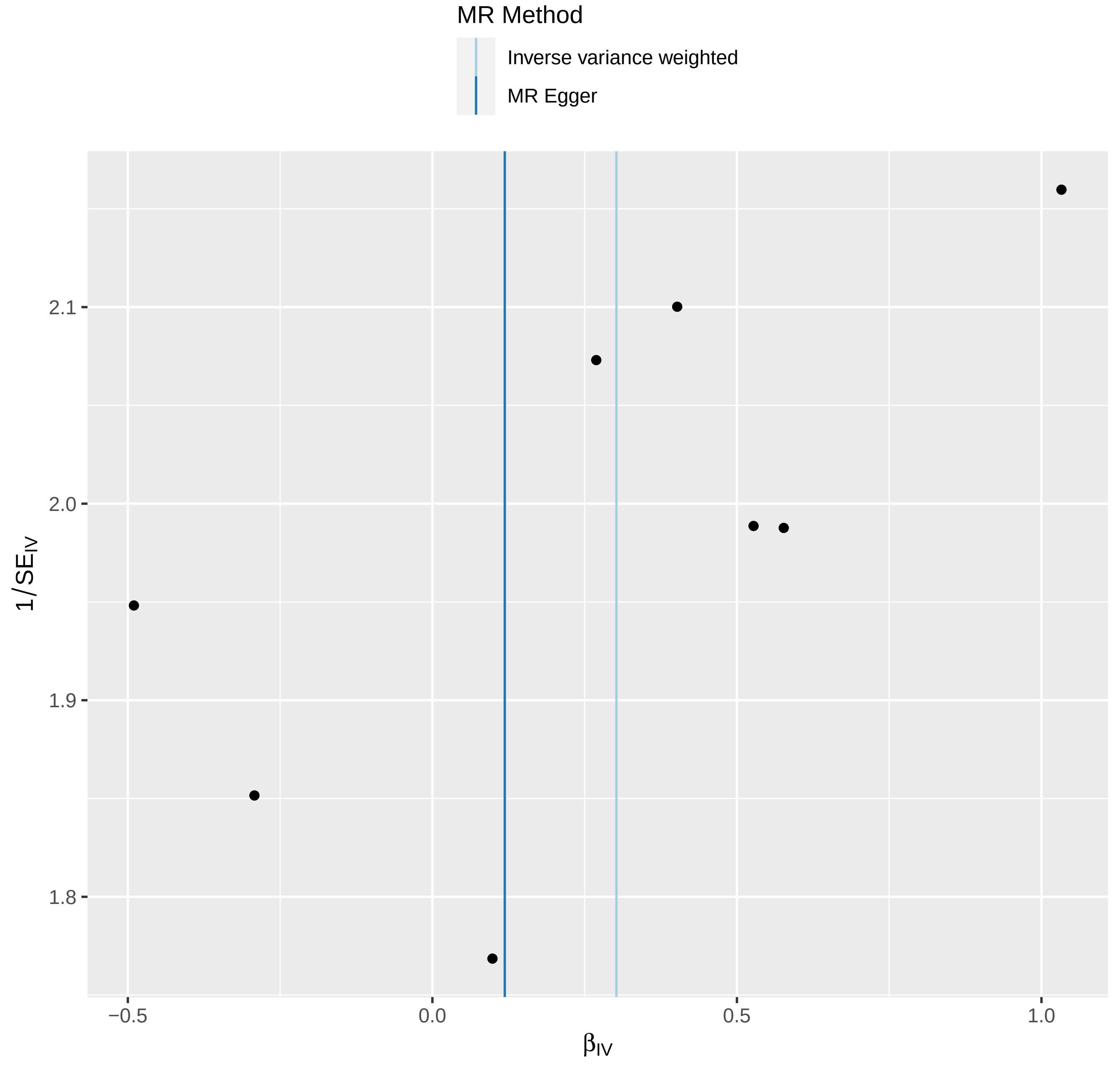

Batch 1462 : Gut microbiota abundance (genus Oxalobacter id.2978) on Ulcerative proctitis

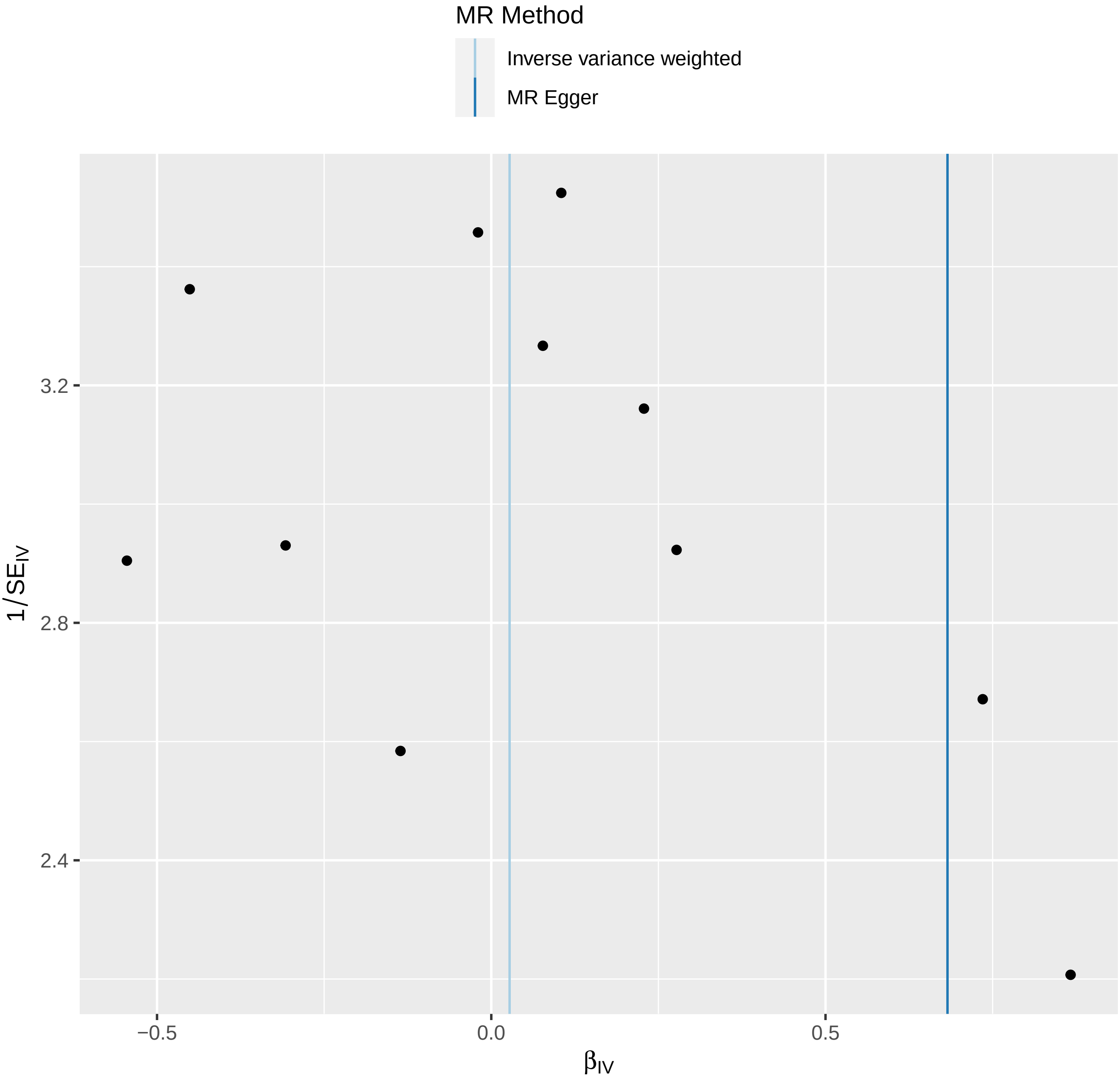

Batch 1463 : Gut microbiota abundance (genus Parabacteroides id.954) on Ulcerative proctitis

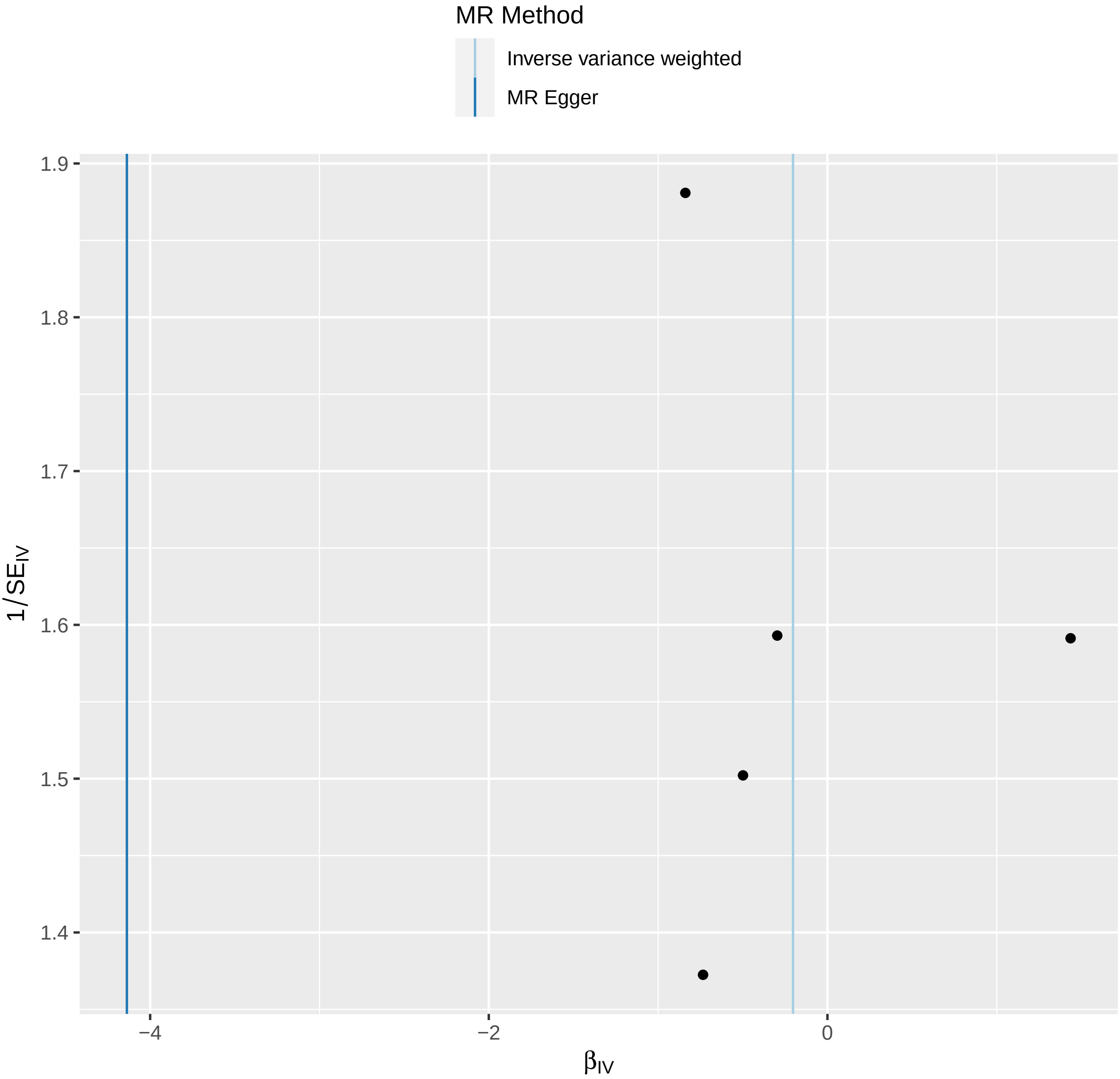

Batch 1464 : Gut microbiota abundance (genus Paraprevotella id.962) on Ulcerative proctitis

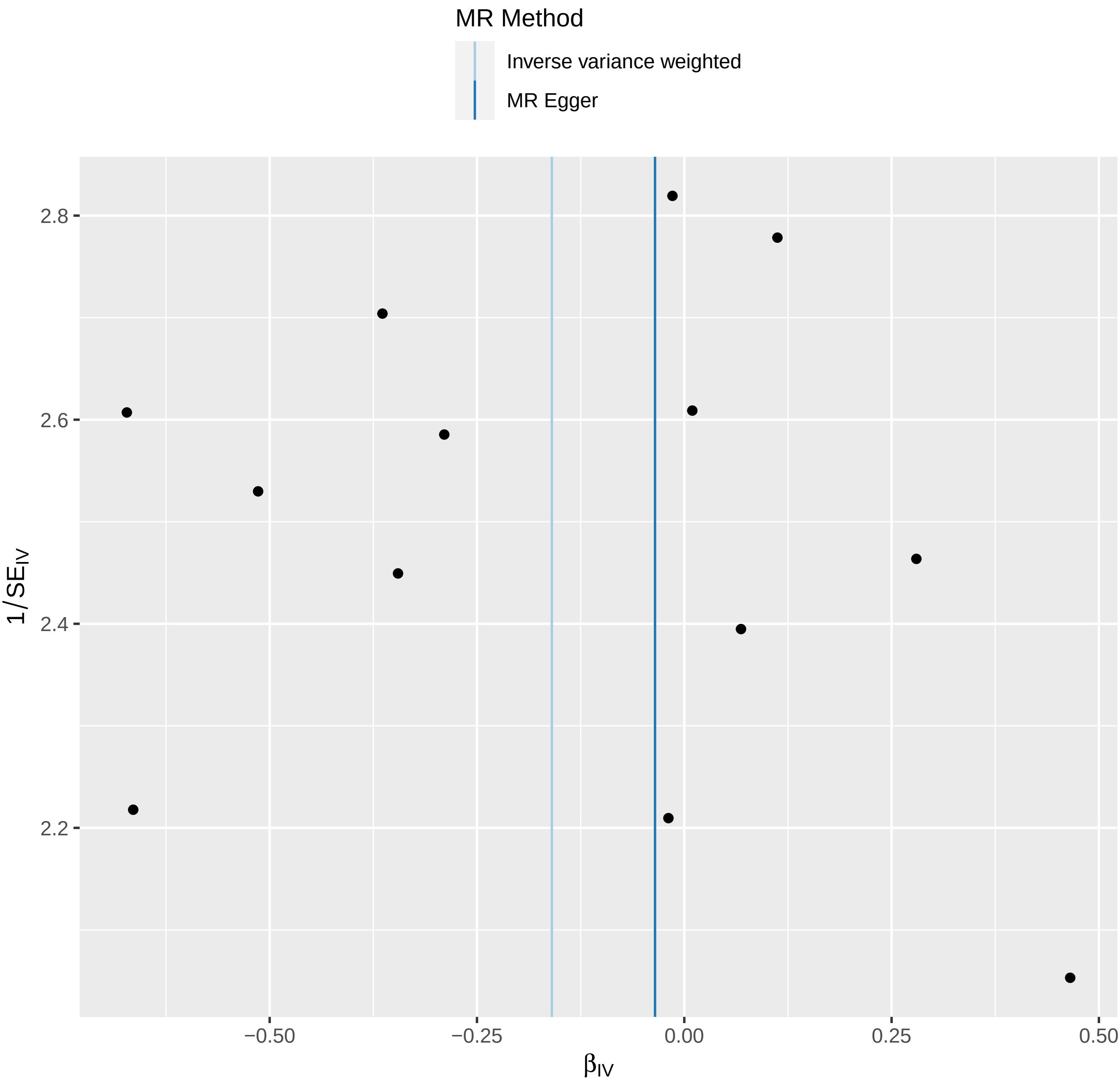

Batch 1465 : Gut microbiota abundance (genus Parasutterella id.2892) on Ulcerative proctitis

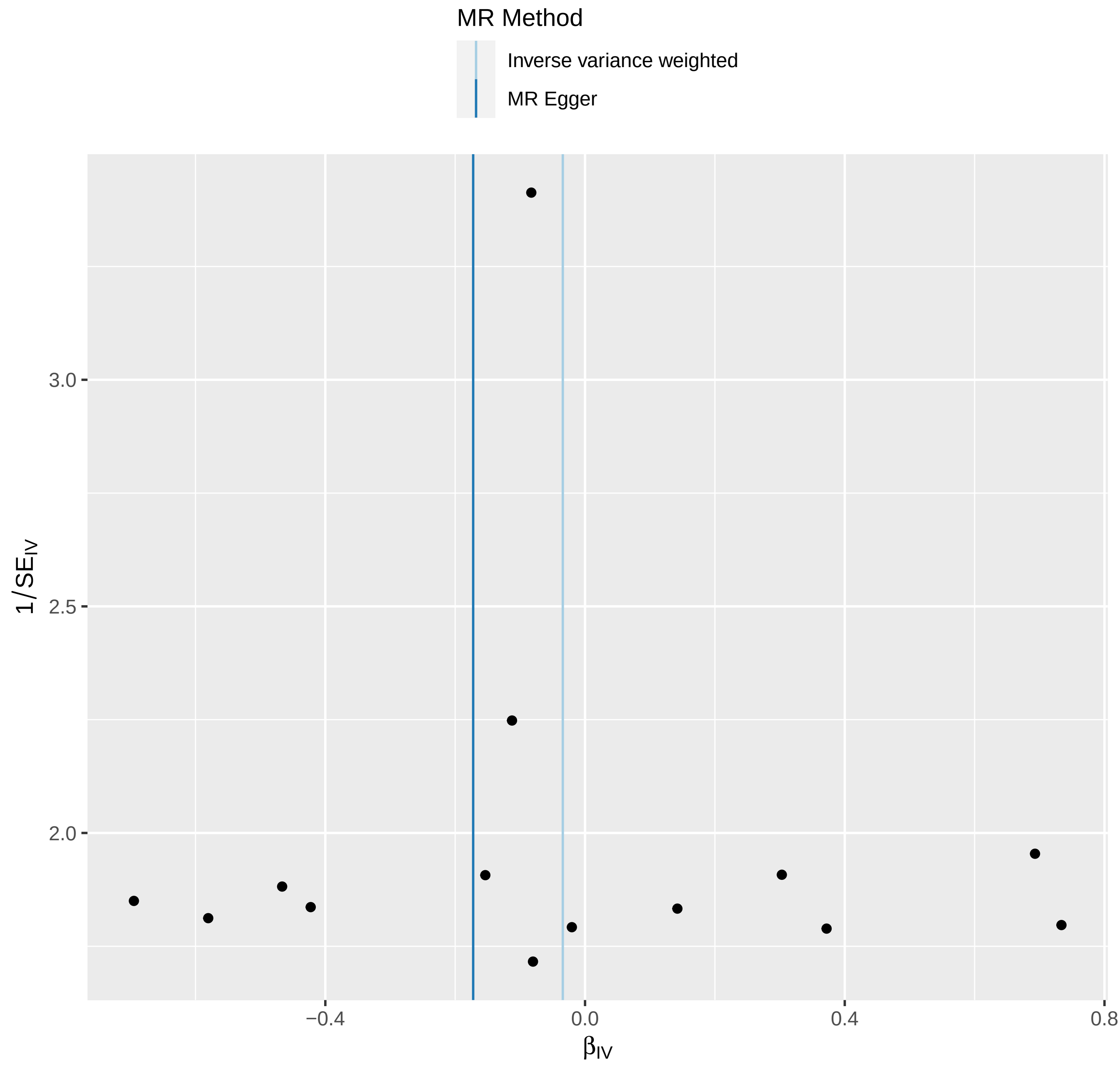

Batch 1466 : Gut microbiota abundance (genus Peptococcus id.2037) on Ulcerative proctitis

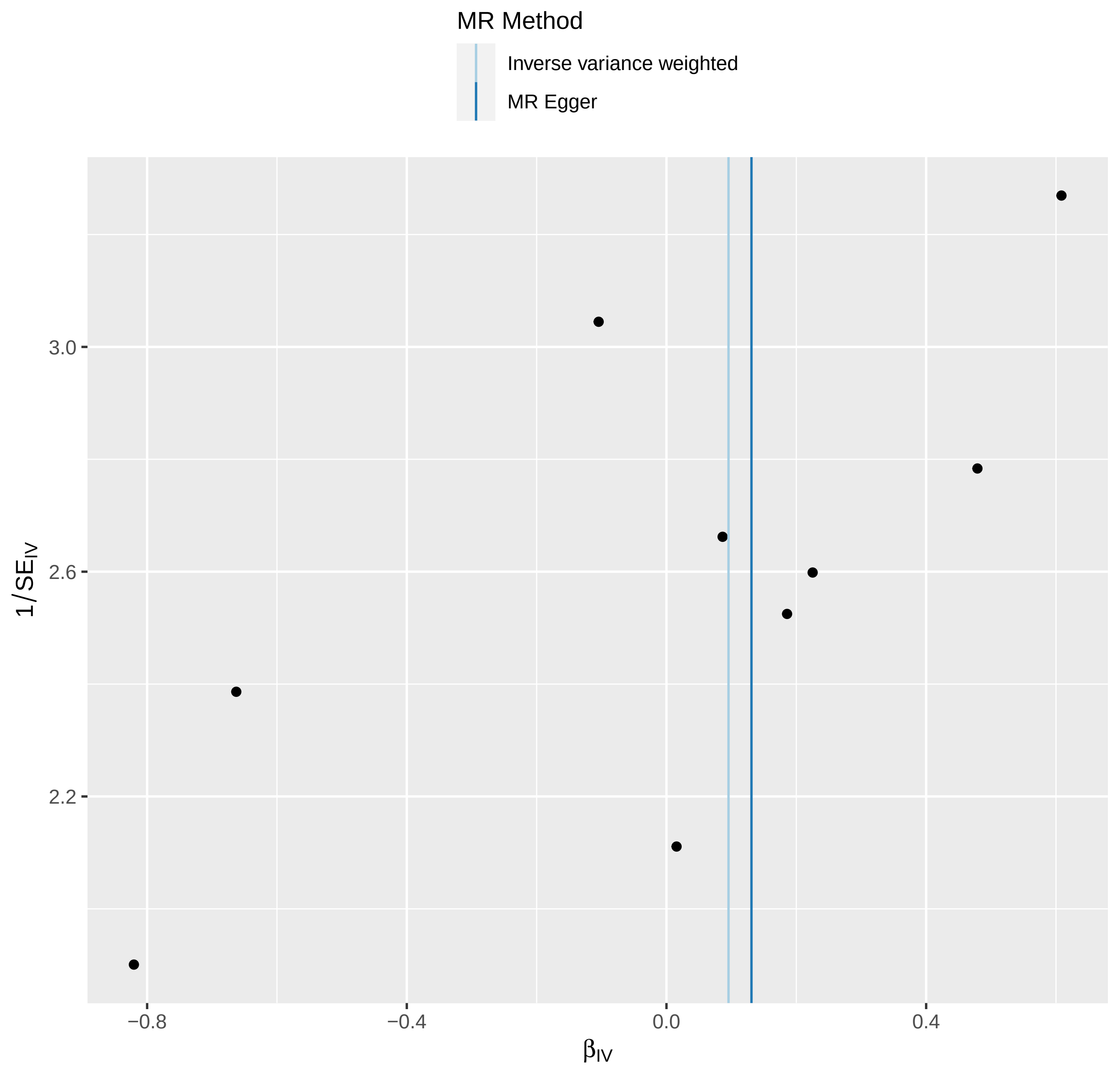

Batch 1467 : Gut microbiota abundance (genus Phascolarctobacterium id.2168) on Ulcerative proctitis

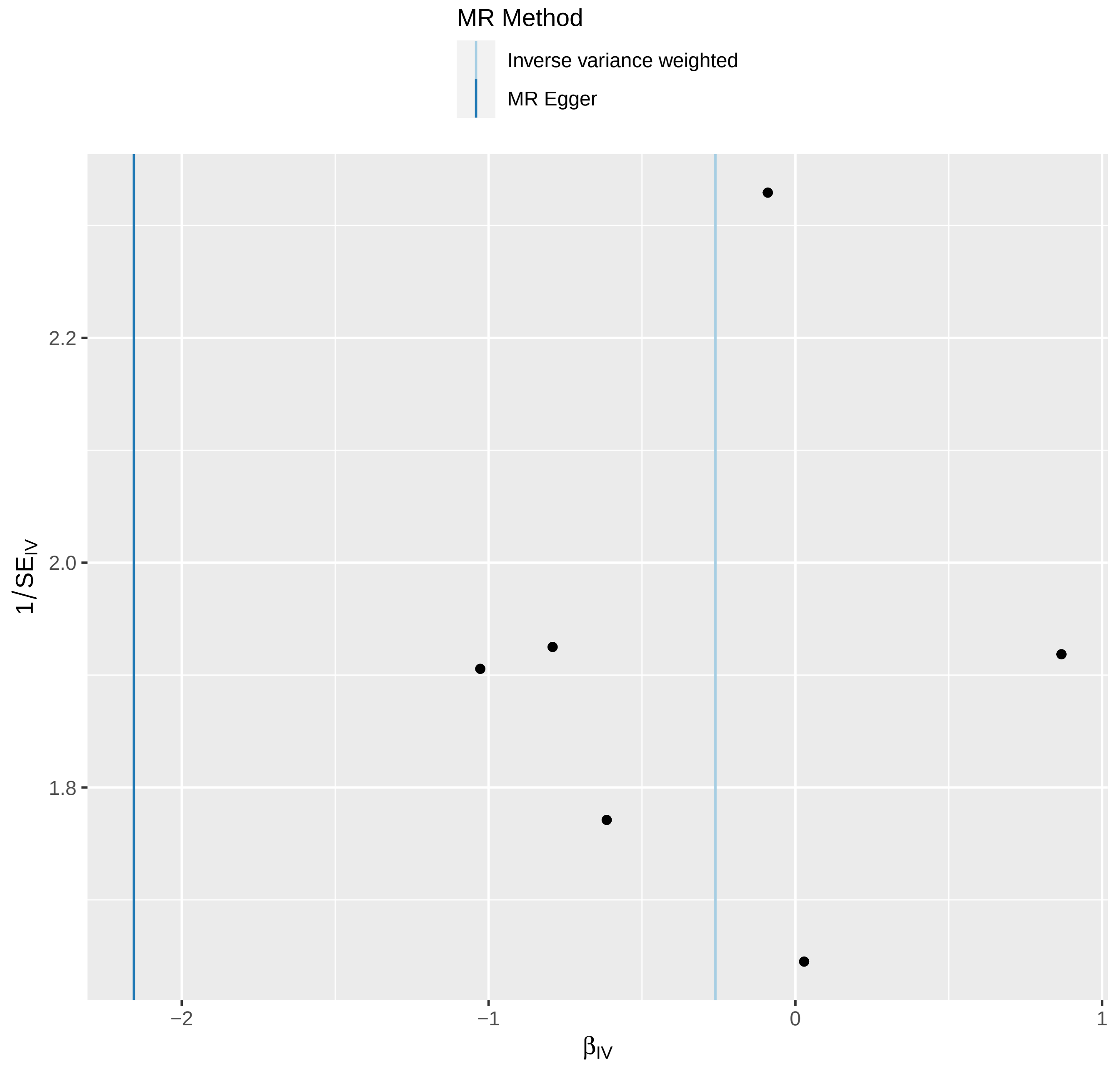

Batch 1468 : Gut microbiota abundance (genus Prevotella7 id.11182) on Ulcerative proctitis

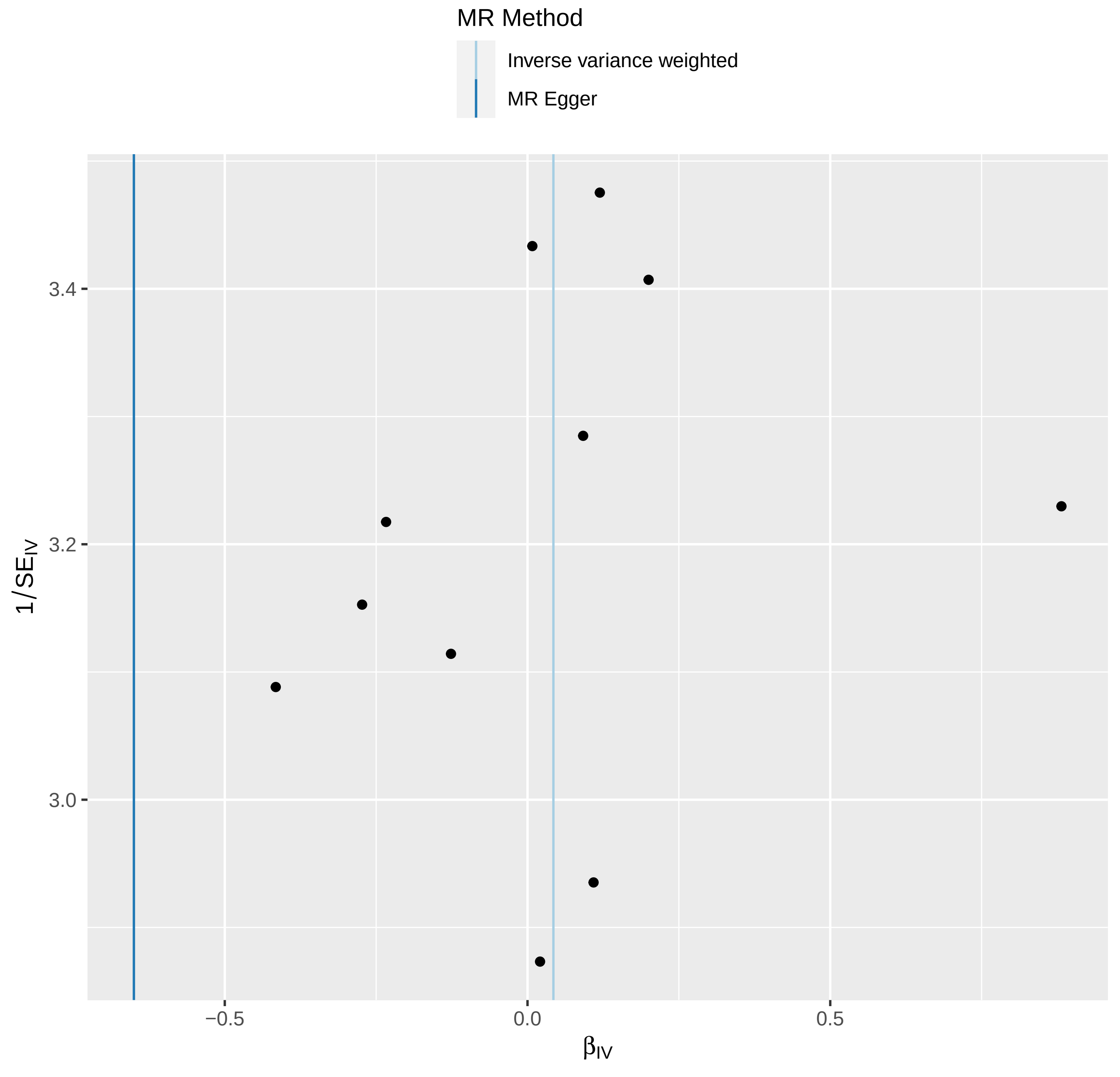

Batch 1469 : Gut microbiota abundance (genus Prevotella9 id.11183) on Ulcerative proctitis

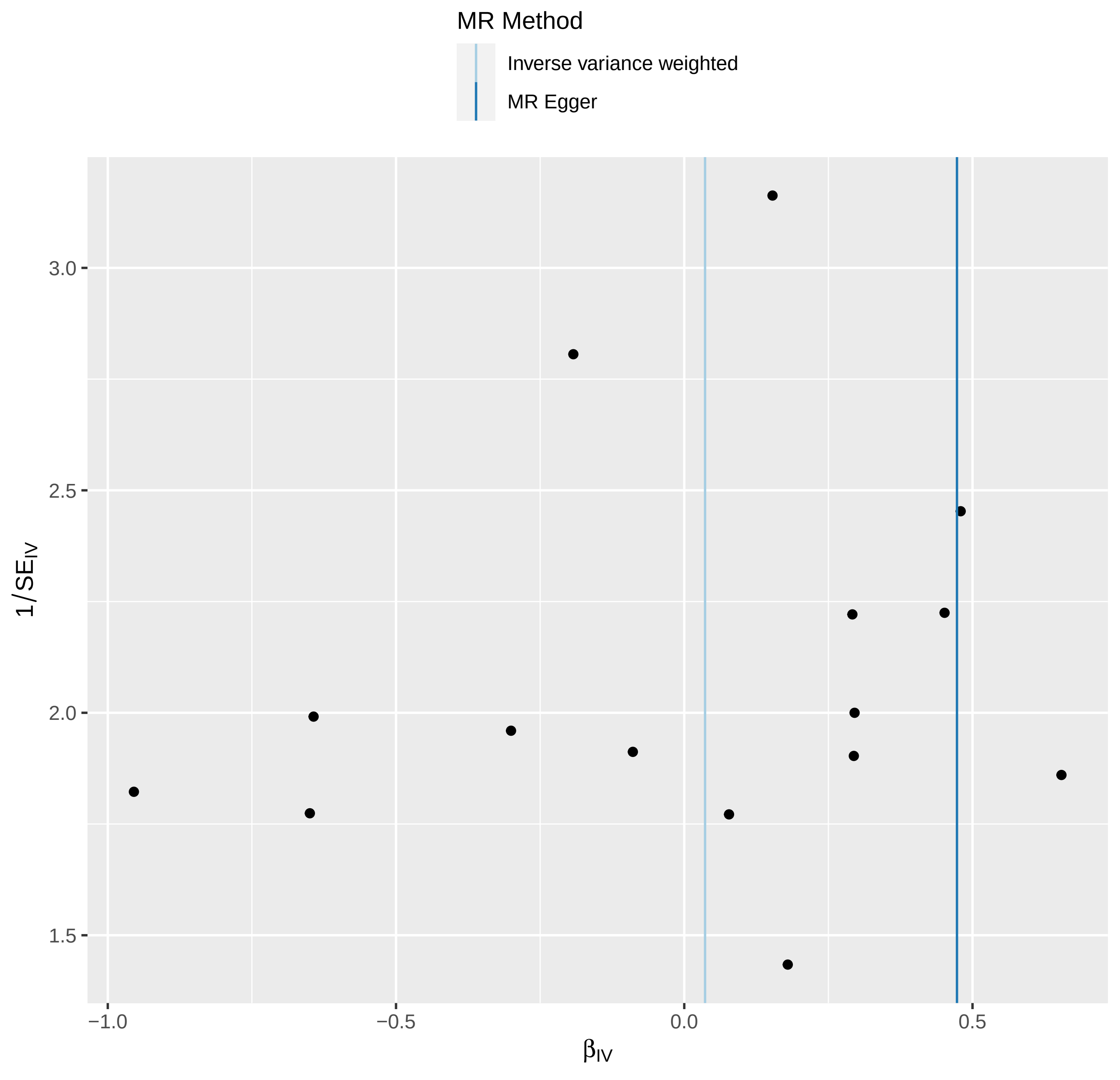

Batch 1470 : Gut microbiota abundance (genus Rikenellaceae RC9 gut group id.11191) on Ulcerative proctitis

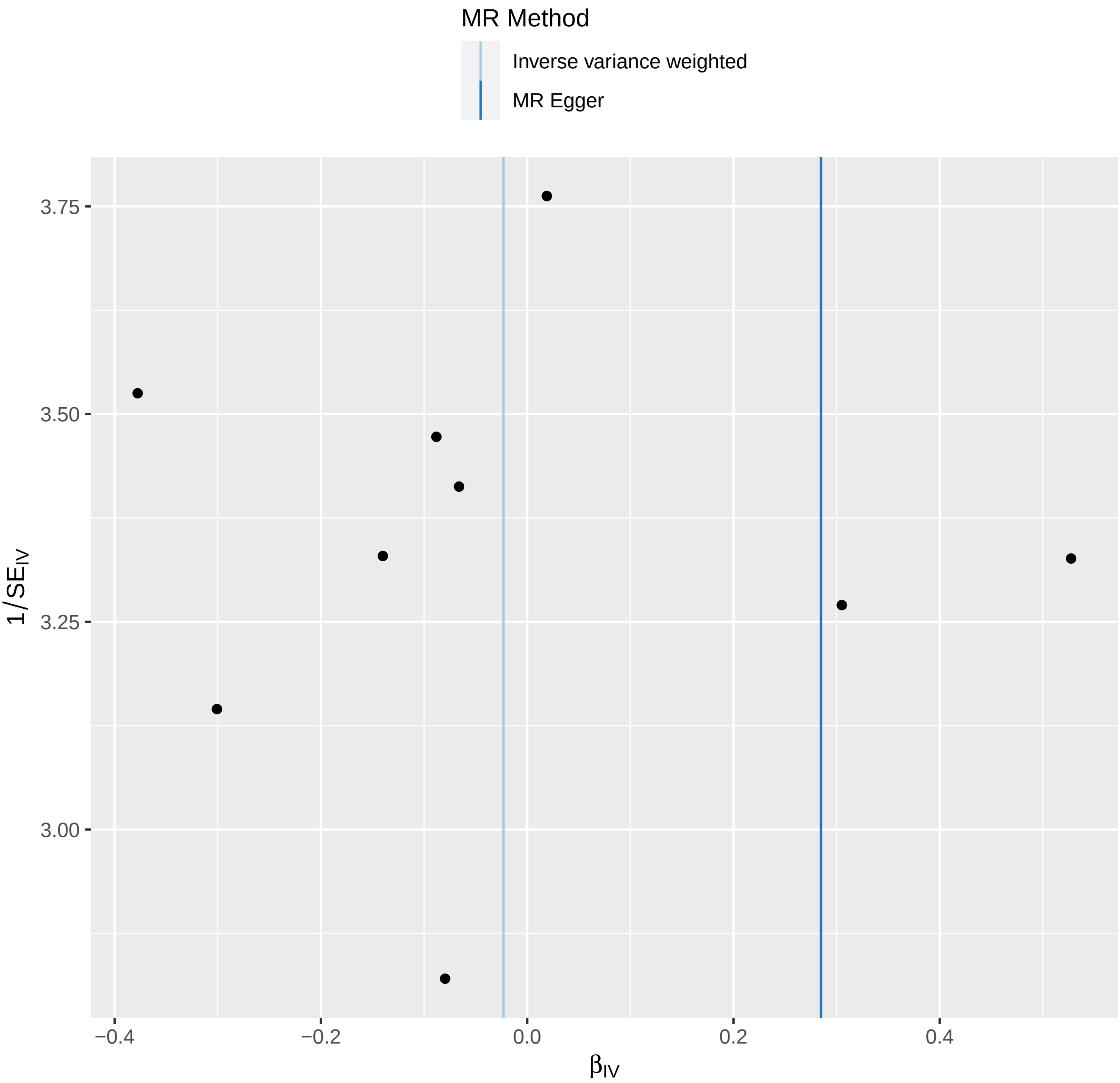

Batch 1471 : Gut microbiota abundance (genus Romboutsia id.11347) on Ulcerative proctitis

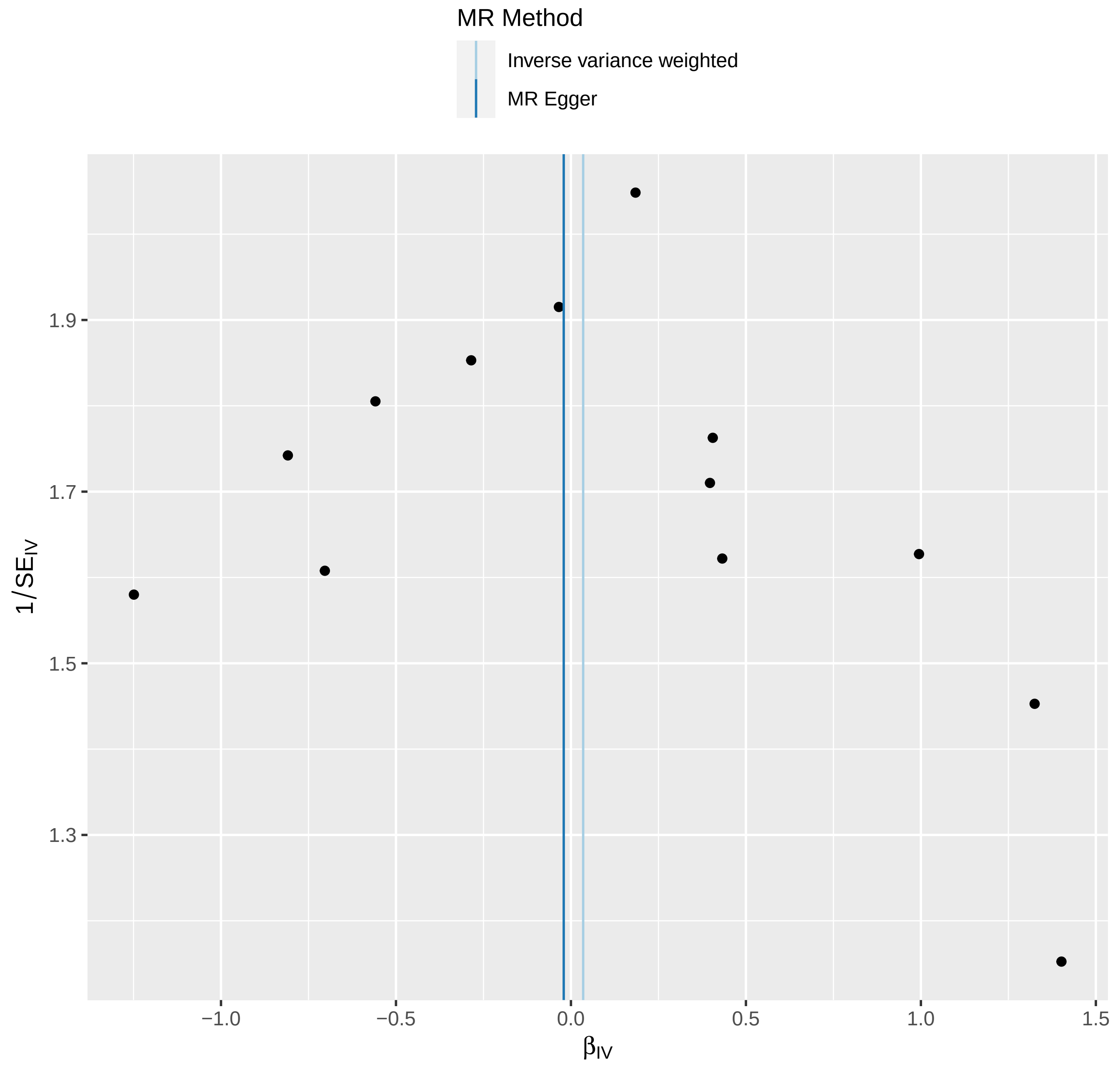

Batch 1472 : Gut microbiota abundance (genus Roseburia id.2012) on Ulcerative proctitis

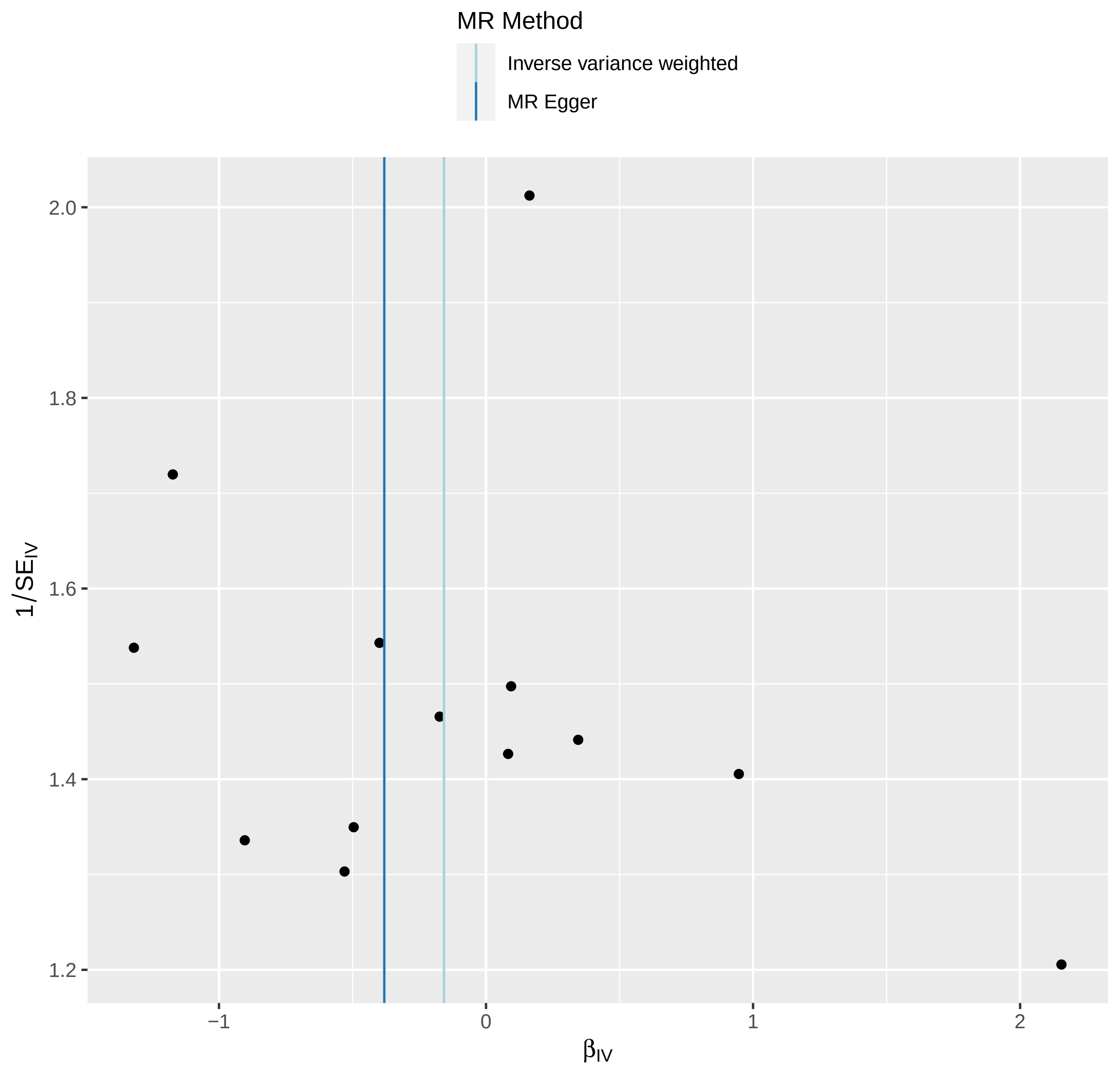

Batch 1473 : Gut microbiota abundance (genus Ruminiclostridium5 id.11355) on Ulcerative proctitis

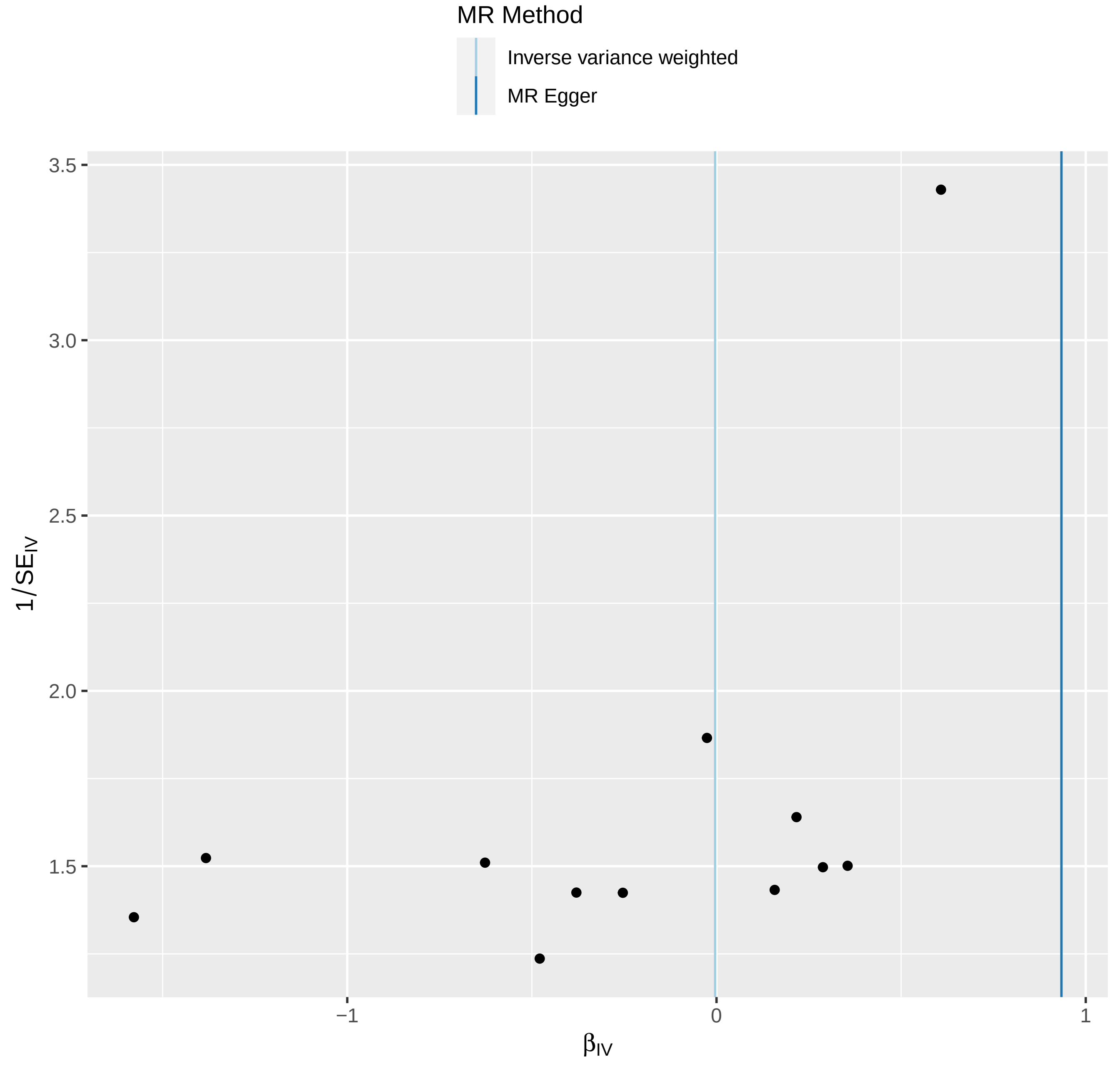

Batch 1474 : Gut microbiota abundance (genus Ruminiclostridium6 id.11356) on Ulcerative proctitis

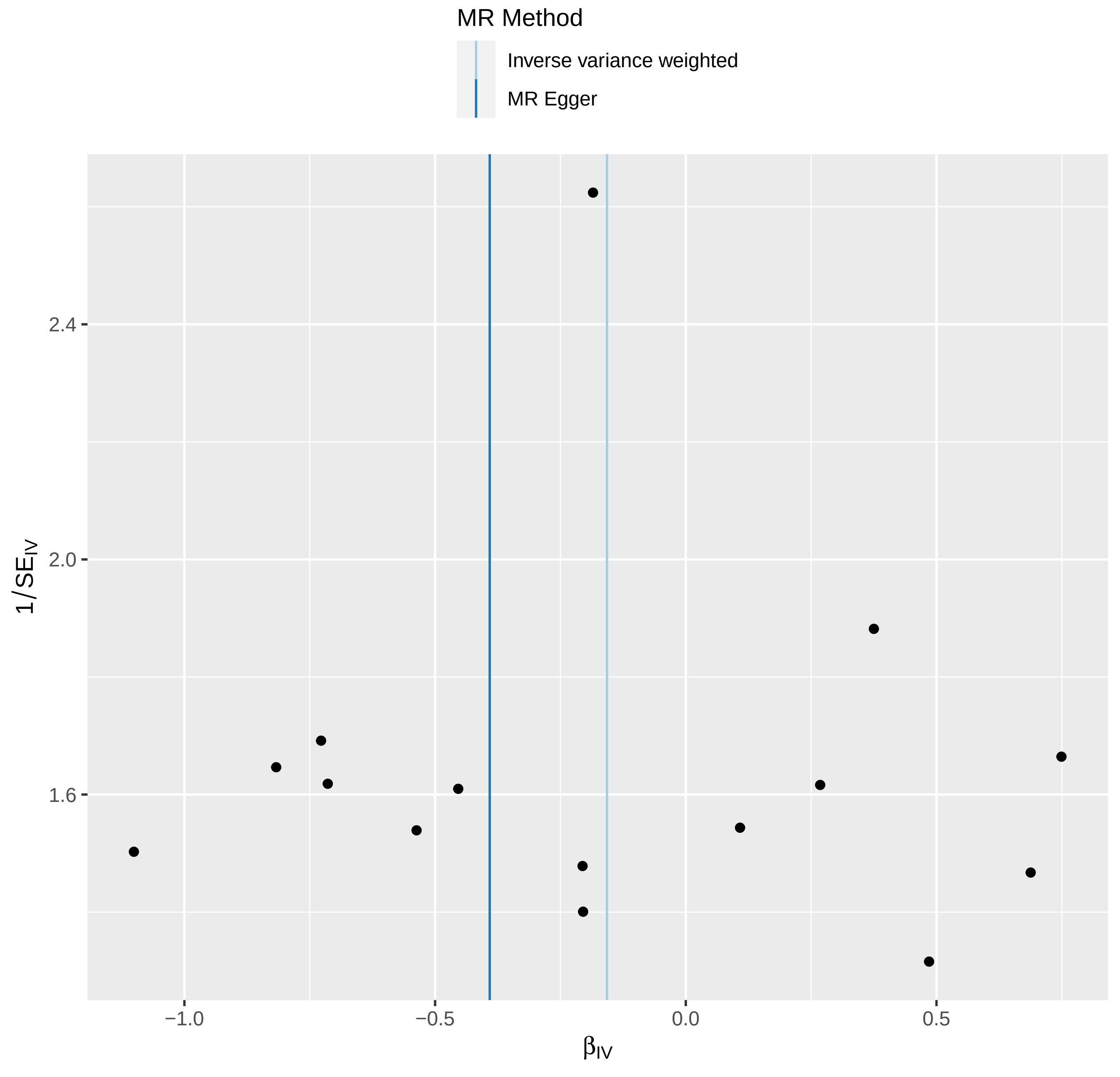

Batch 1475 : Gut microbiota abundance (genus Ruminiclostridium9 id.11357) on Ulcerative proctitis

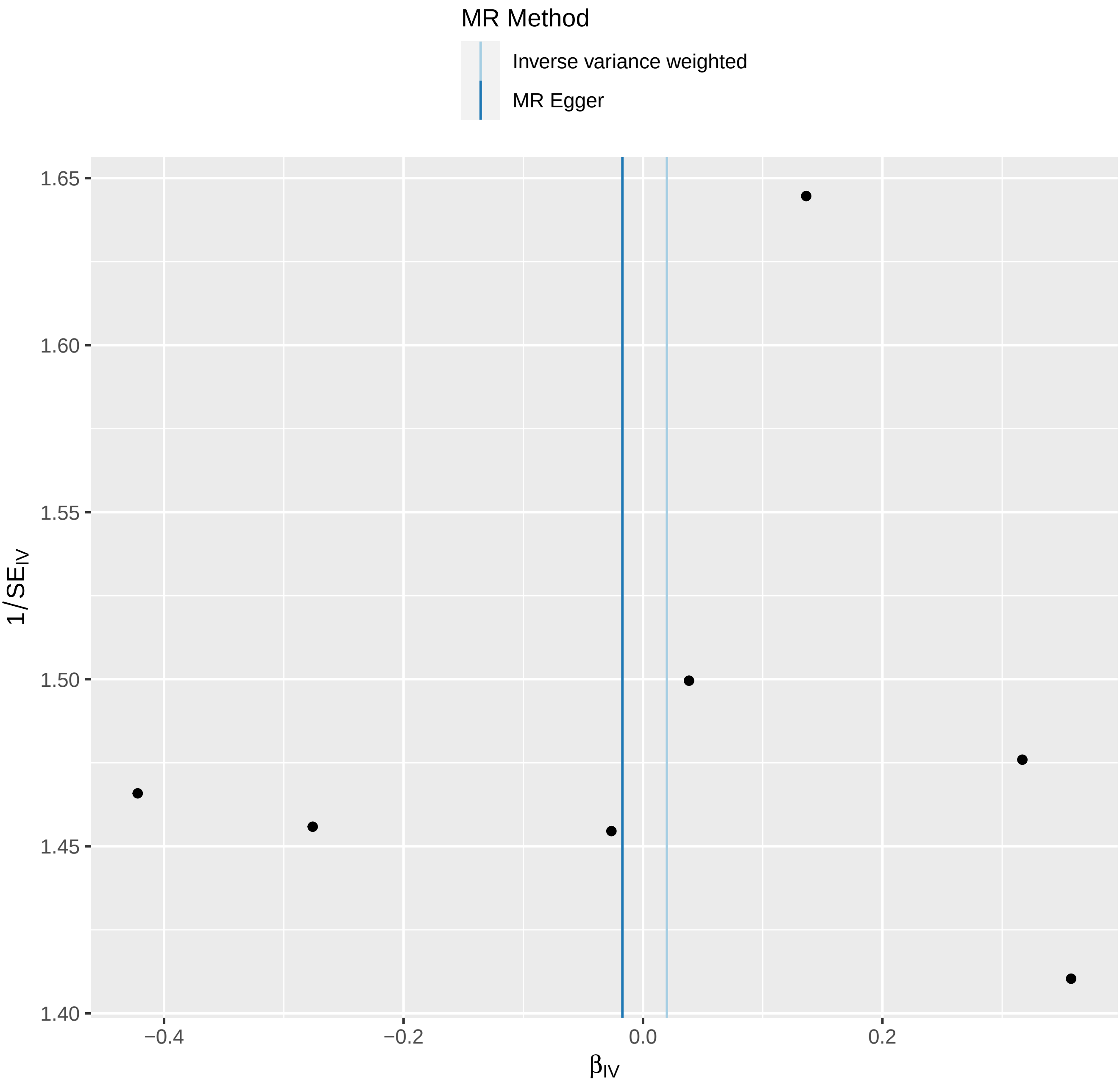

Batch 1476 : Gut microbiota abundance (genus Ruminococcaceae NK4A214 group id.11358) on Ulcerative proctitis

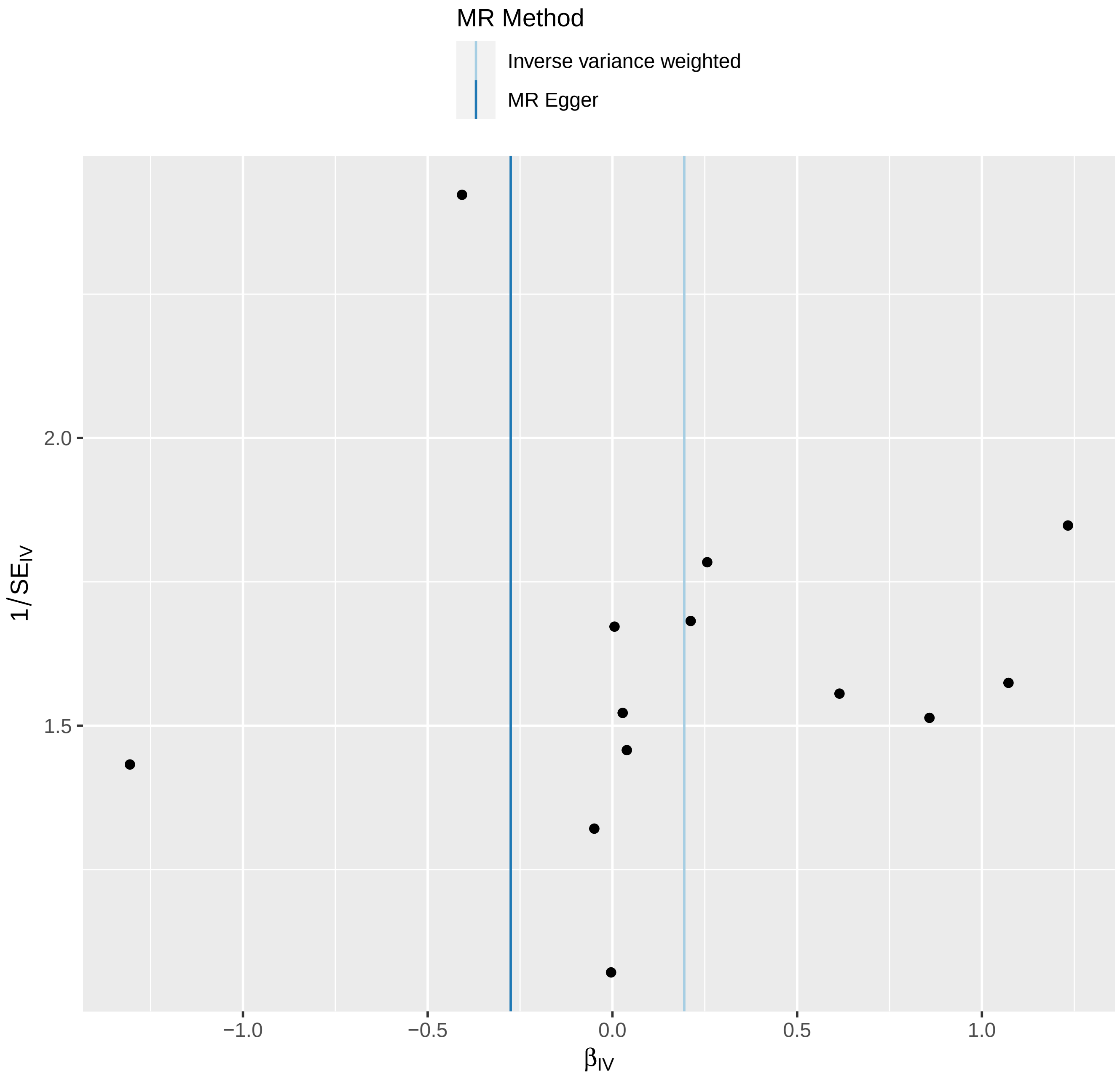

Batch 1477 : Gut microbiota abundance (genus Ruminococcaceae UCG002 id.11360) on Ulcerative proctitis

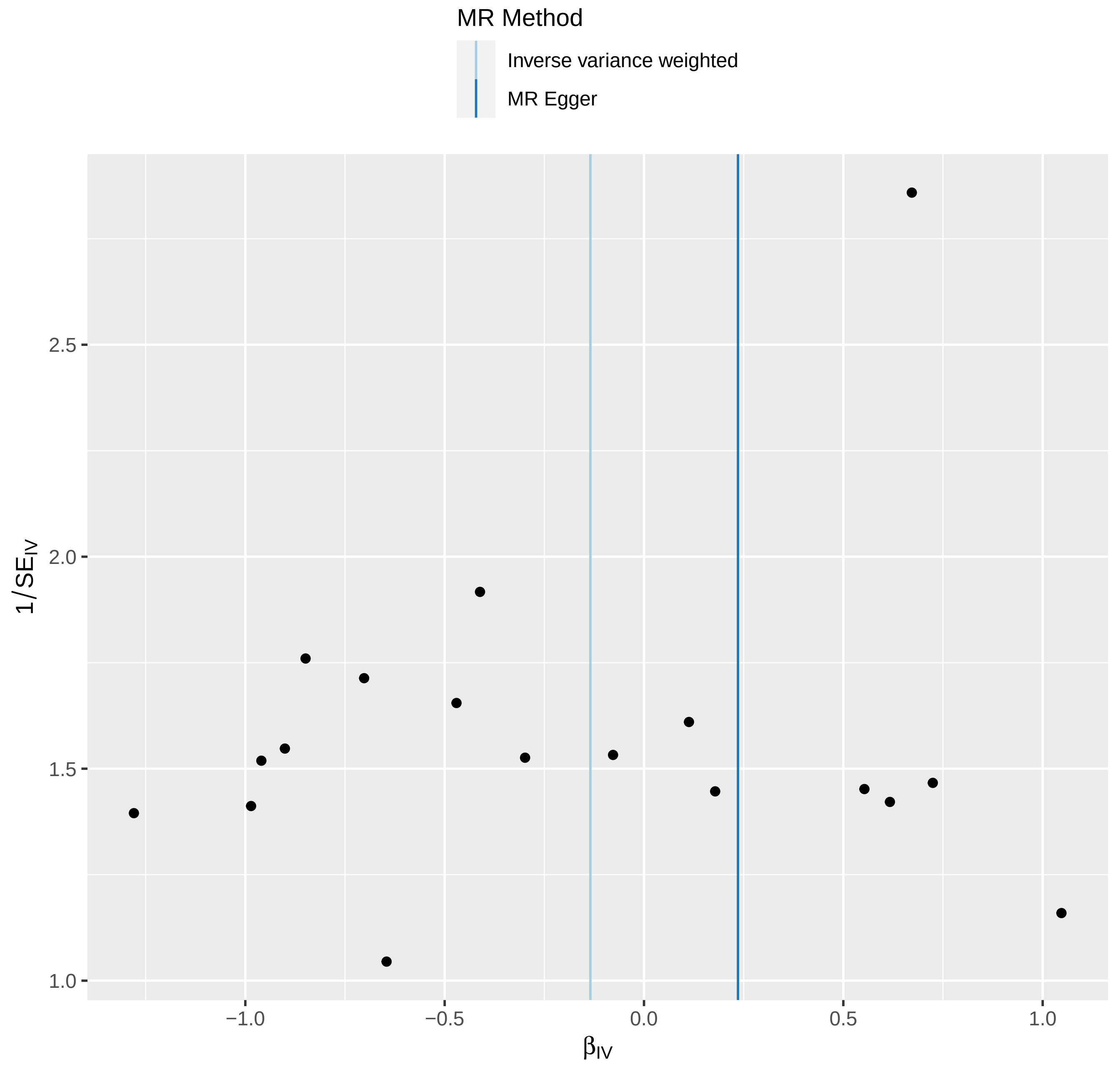

Batch 1478 : Gut microbiota abundance (genus Ruminococcaceae UCG003 id.11361) on Ulcerative proctitis

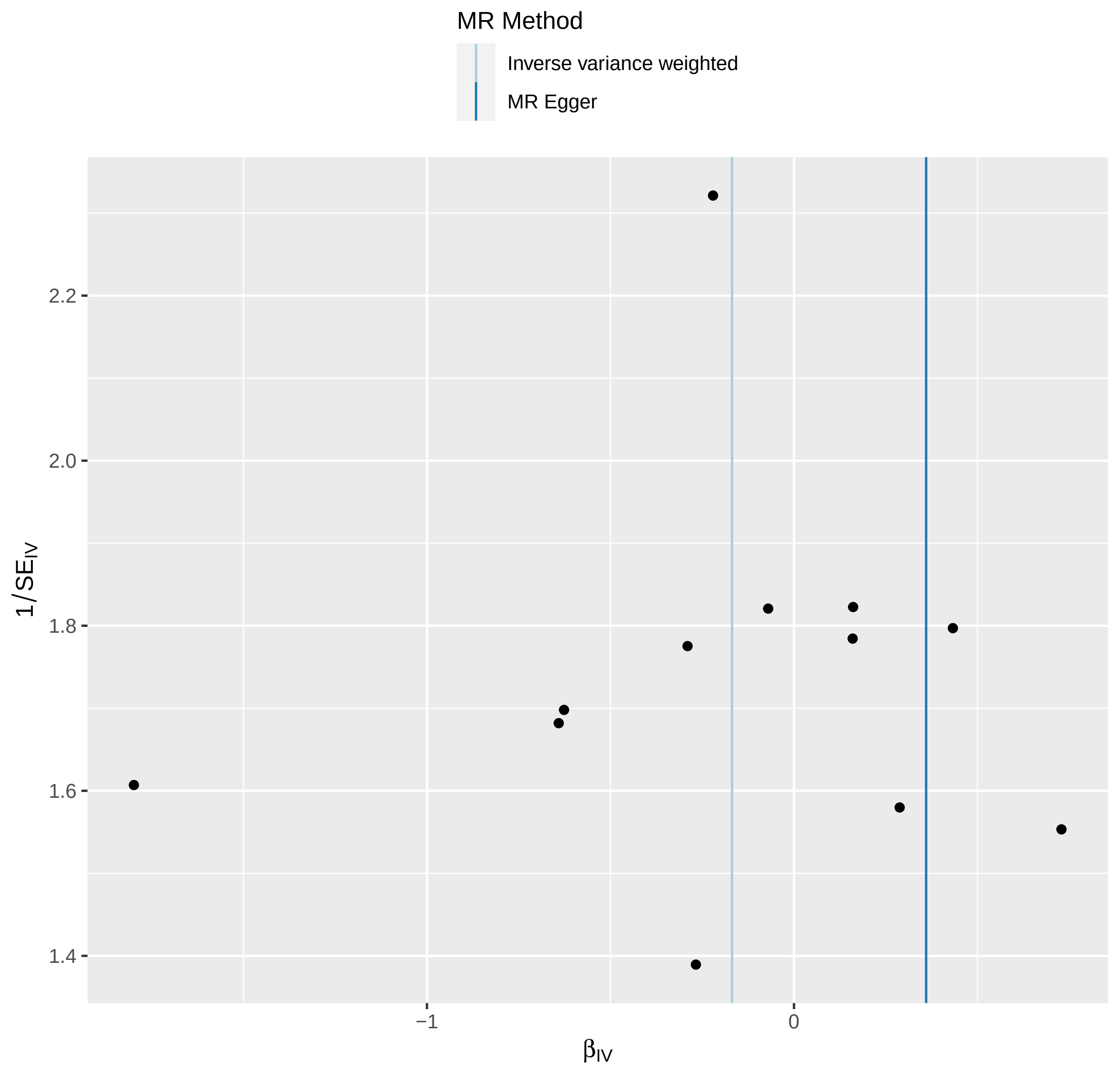

Batch 1479 : Gut microbiota abundance (genus Ruminococcaceae UCG004 id.11362) on Ulcerative proctitis

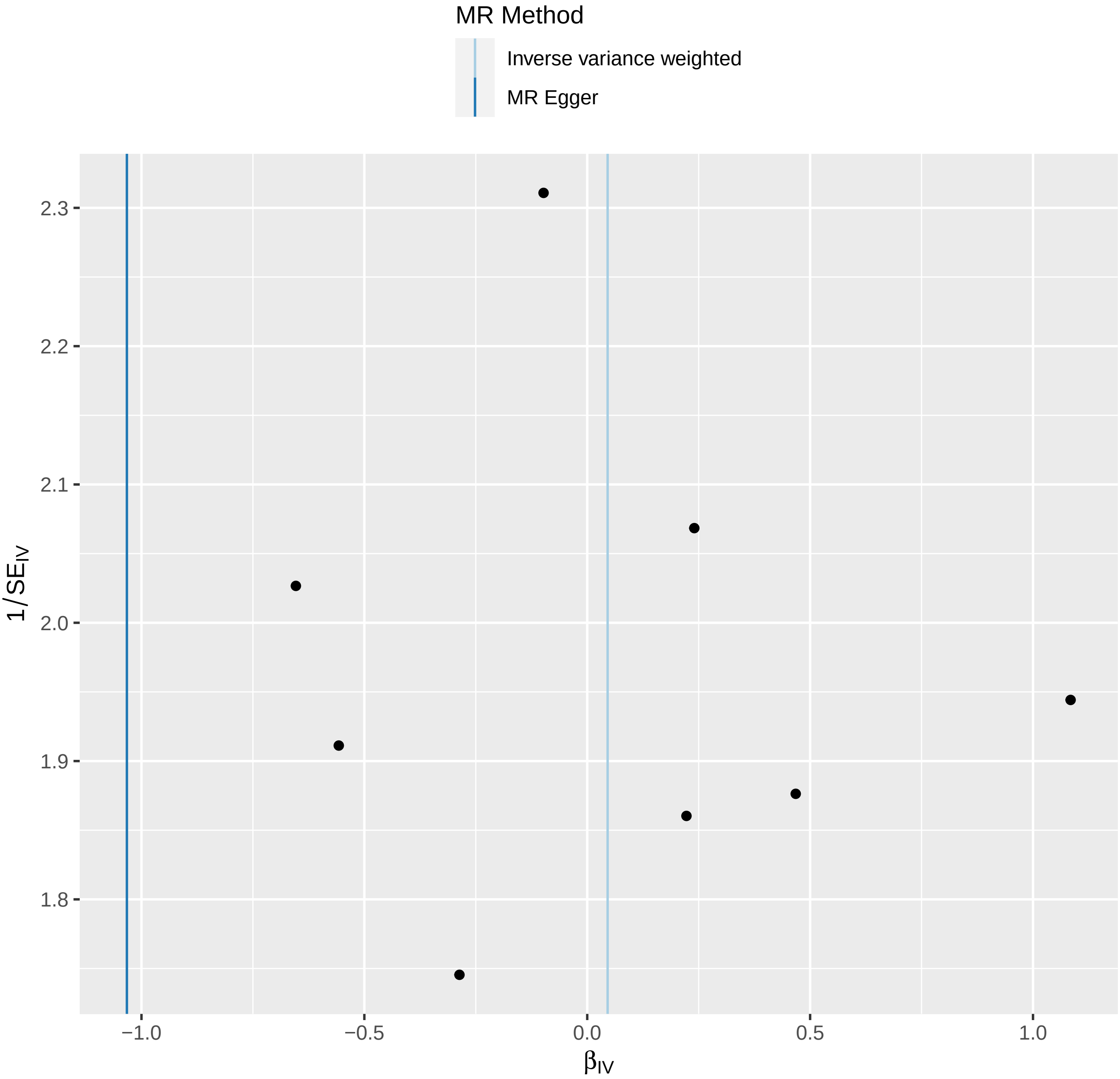

Batch 1480 : Gut microbiota abundance (genus Ruminococcaceae UCG005 id.11363) on Ulcerative proctitis

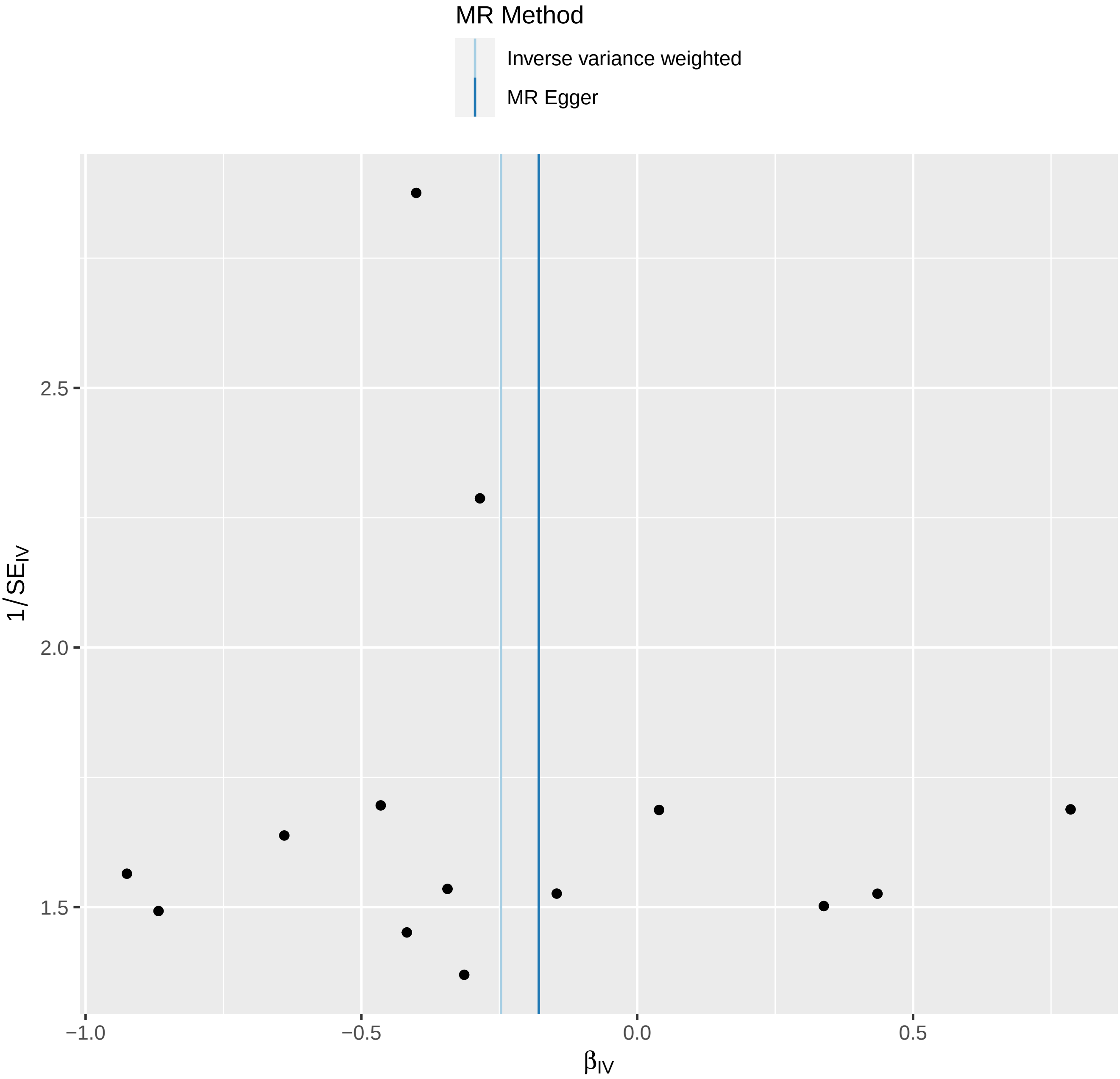

Batch 1481 : Gut microbiota abundance (genus Ruminococcaceae UCG009 id.11366) on Ulcerative proctitis

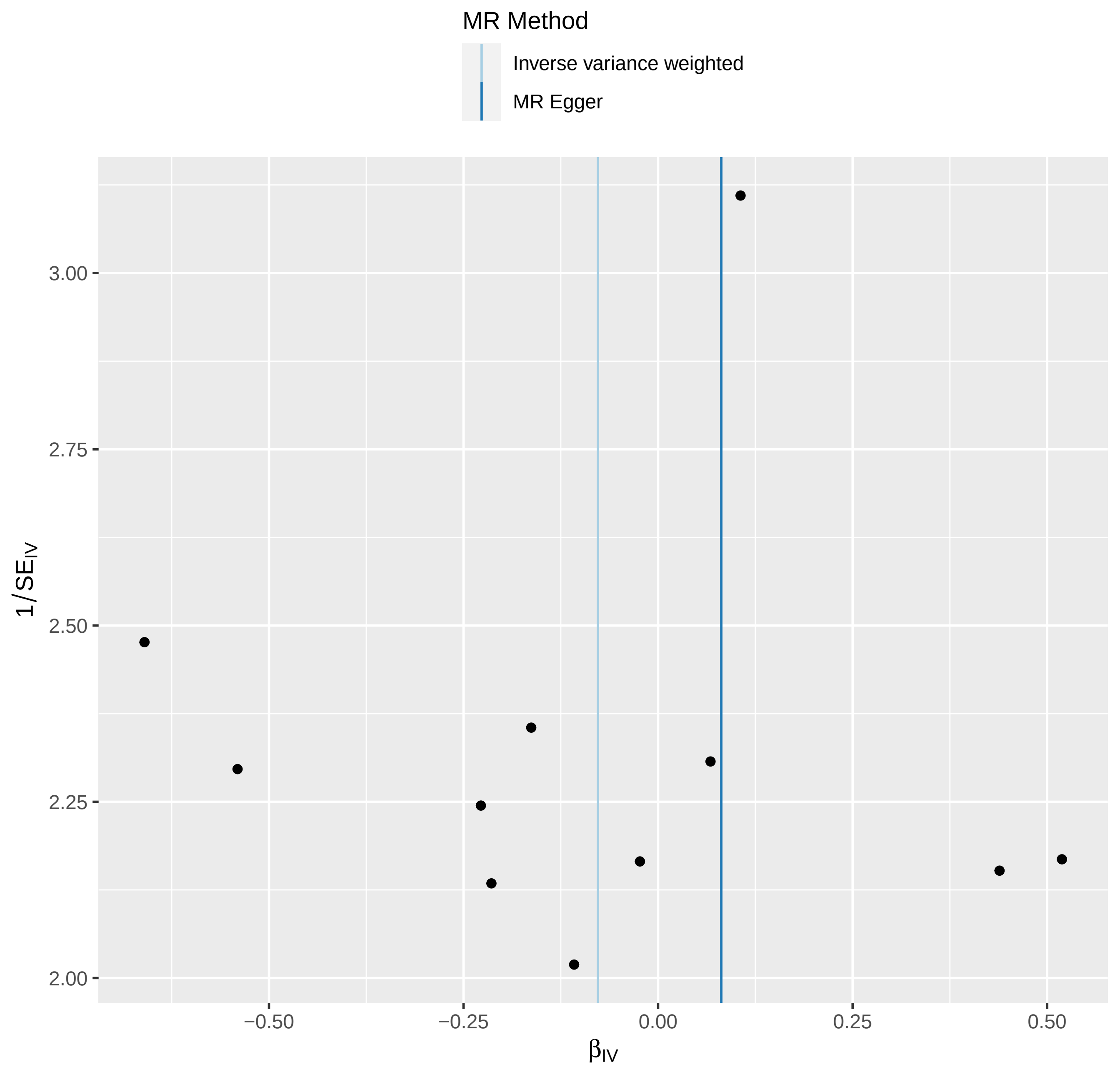

Batch 1482 : Gut microbiota abundance (genus Ruminococcaceae UCG010 id.11367) on Ulcerative proctitis

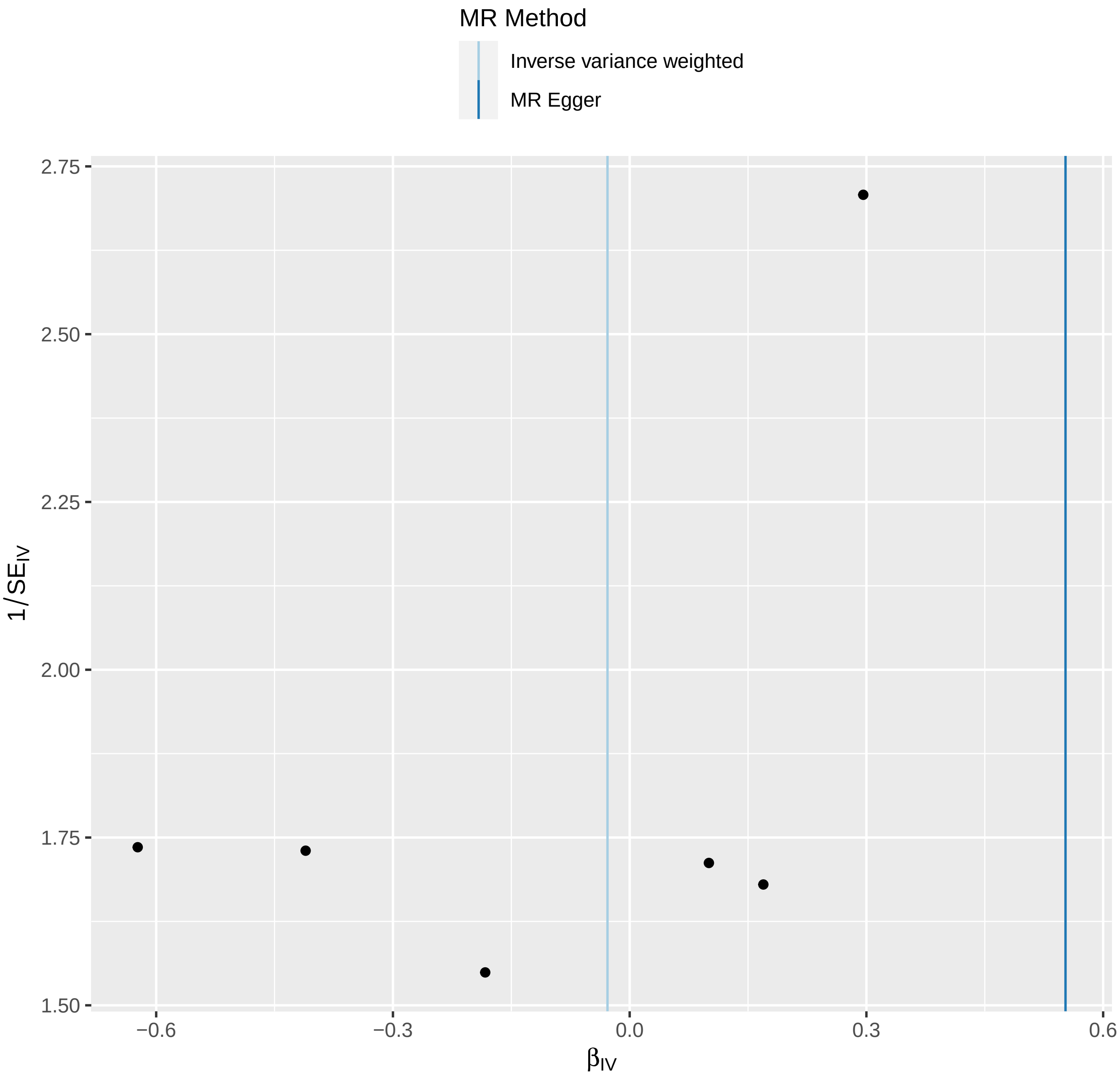

Batch 1483 : Gut microbiota abundance (genus Ruminococcaceae UCG011 id.11368) on Ulcerative proctitis

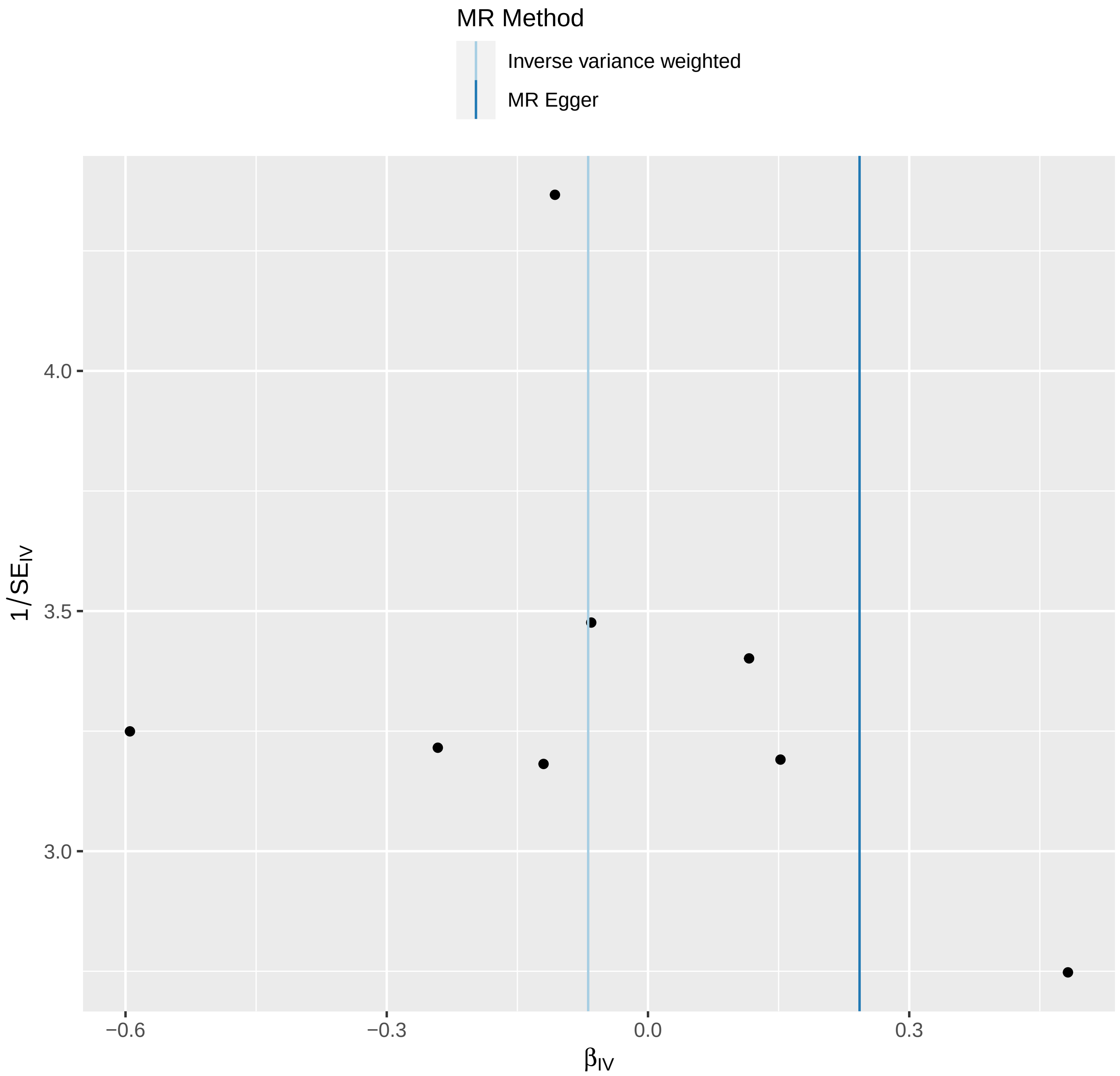

Batch 1484 : Gut microbiota abundance (genus Ruminococcaceae UCG013 id.11370) on Ulcerative proctitis

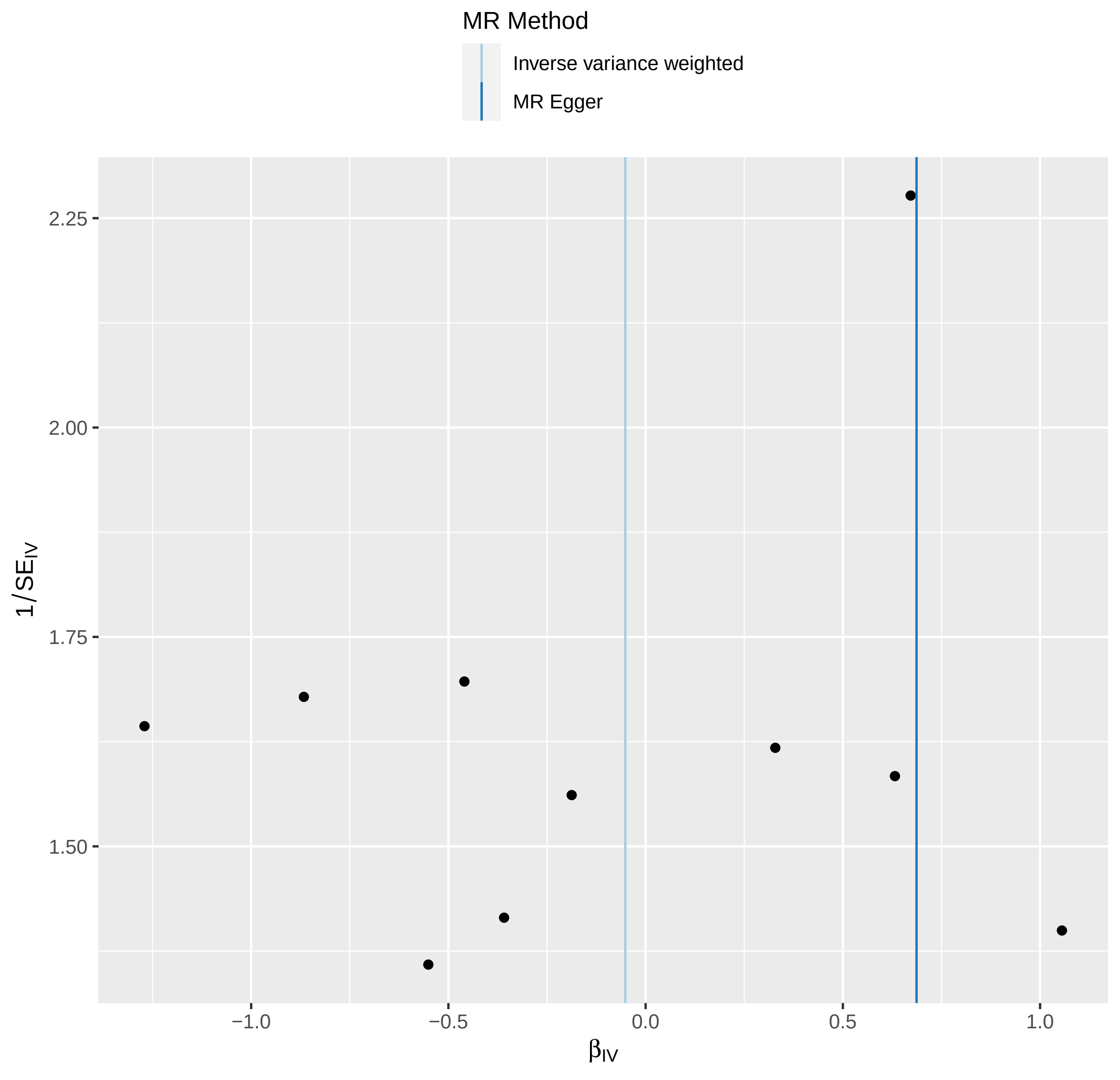

Batch 1485 : Gut microbiota abundance (genus Ruminococcaceae UCG014 id.11371) on Ulcerative proctitis

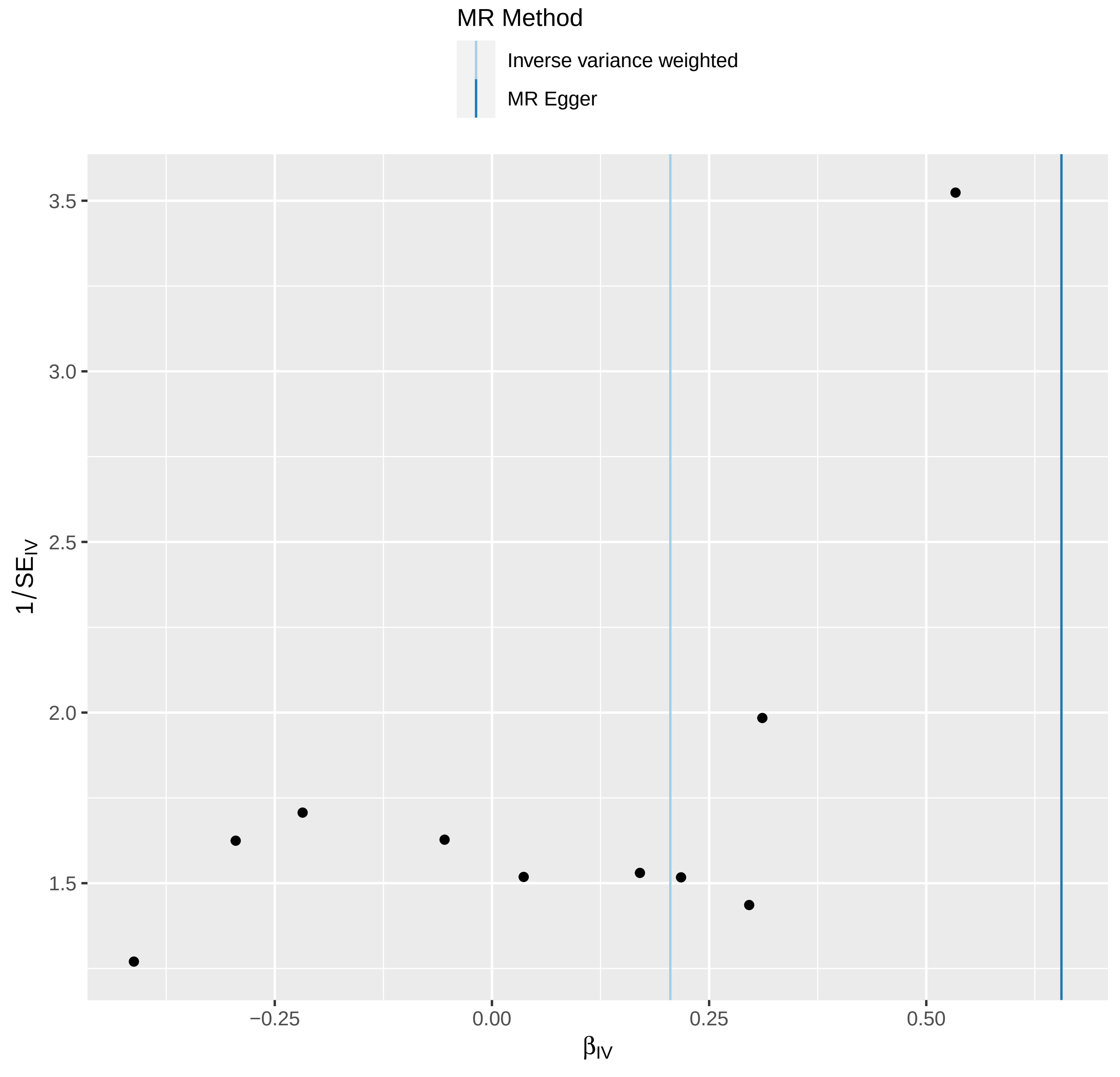

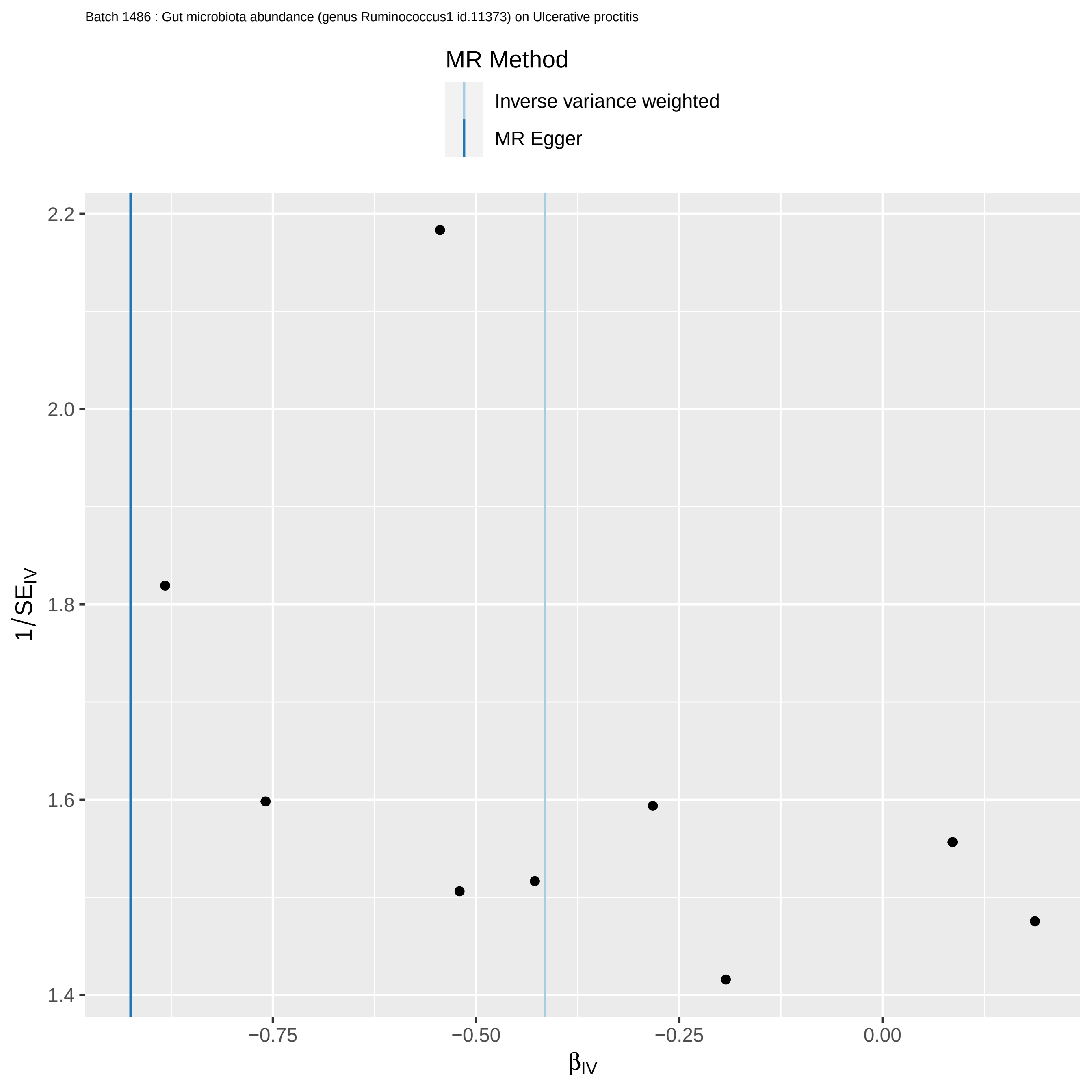
Batch 1486 : Gut microbiota abundance (genus Ruminococcus1 id.11373) on Ulcerative proctitis
MR Method
Inverse variance weighted
MR Egger
2.2
2.0
1.8
1.6
1.4
1/SE$_{IV}$
−0.75
−0.50
−0.25
0.00
β$_{IV}$

Batch 1487 : Gut microbiota abundance (genus Ruminococcus2 id.11374) on Ulcerative proctitis

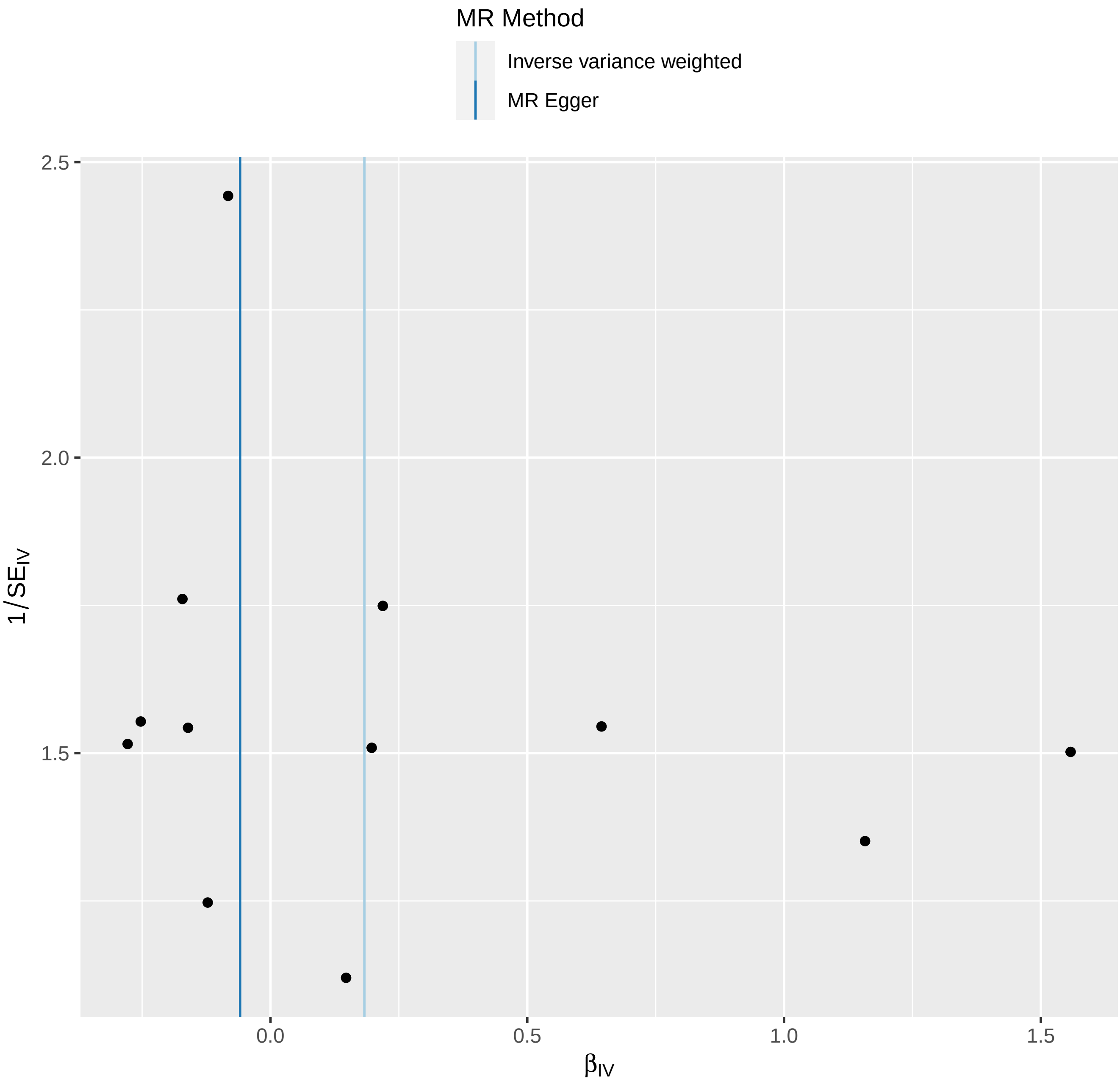

Batch 1488 : Gut microbiota abundance (genus Ruminococcus gauvreauii group id.11342) on Ulcerative proctitis

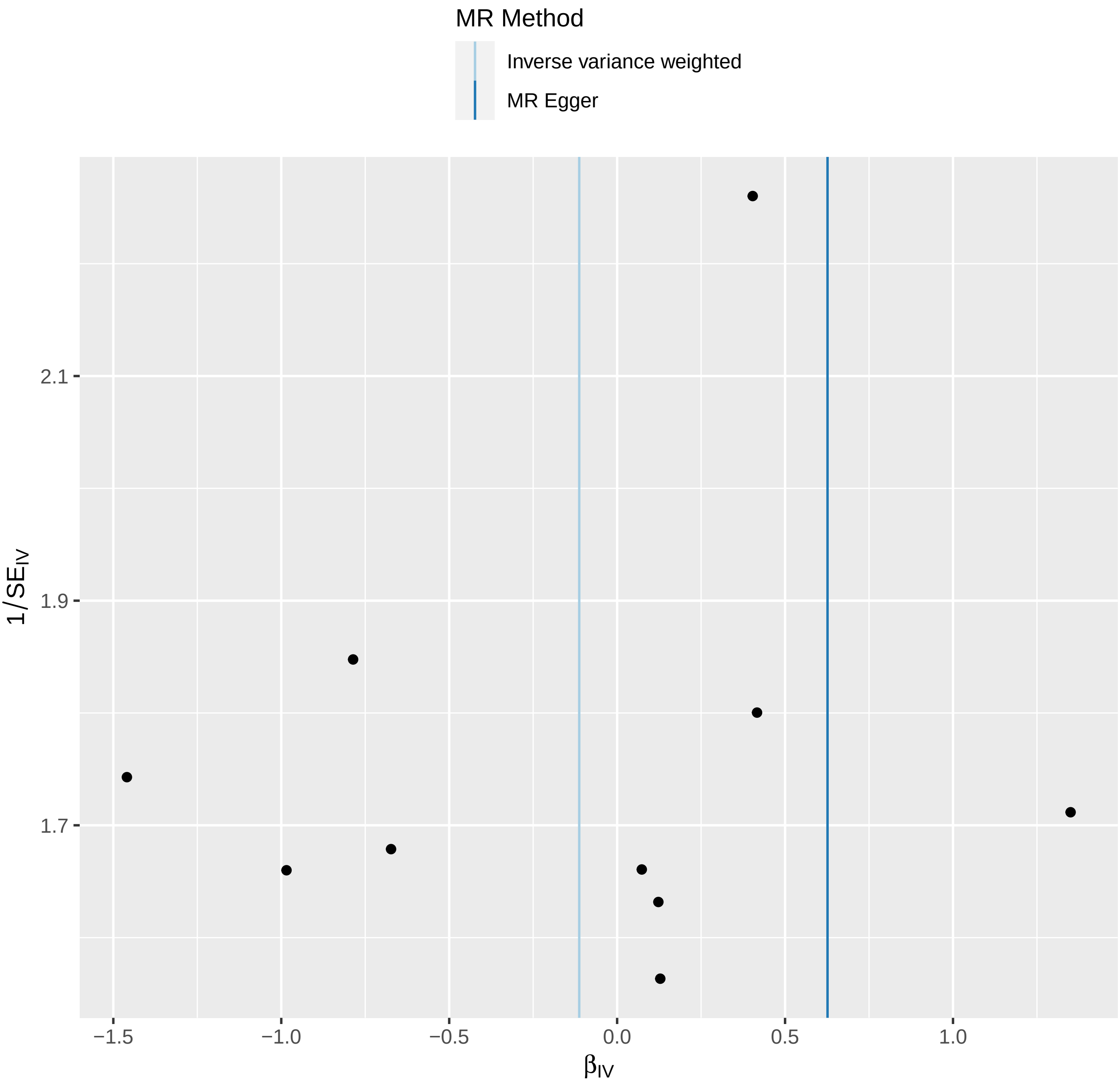

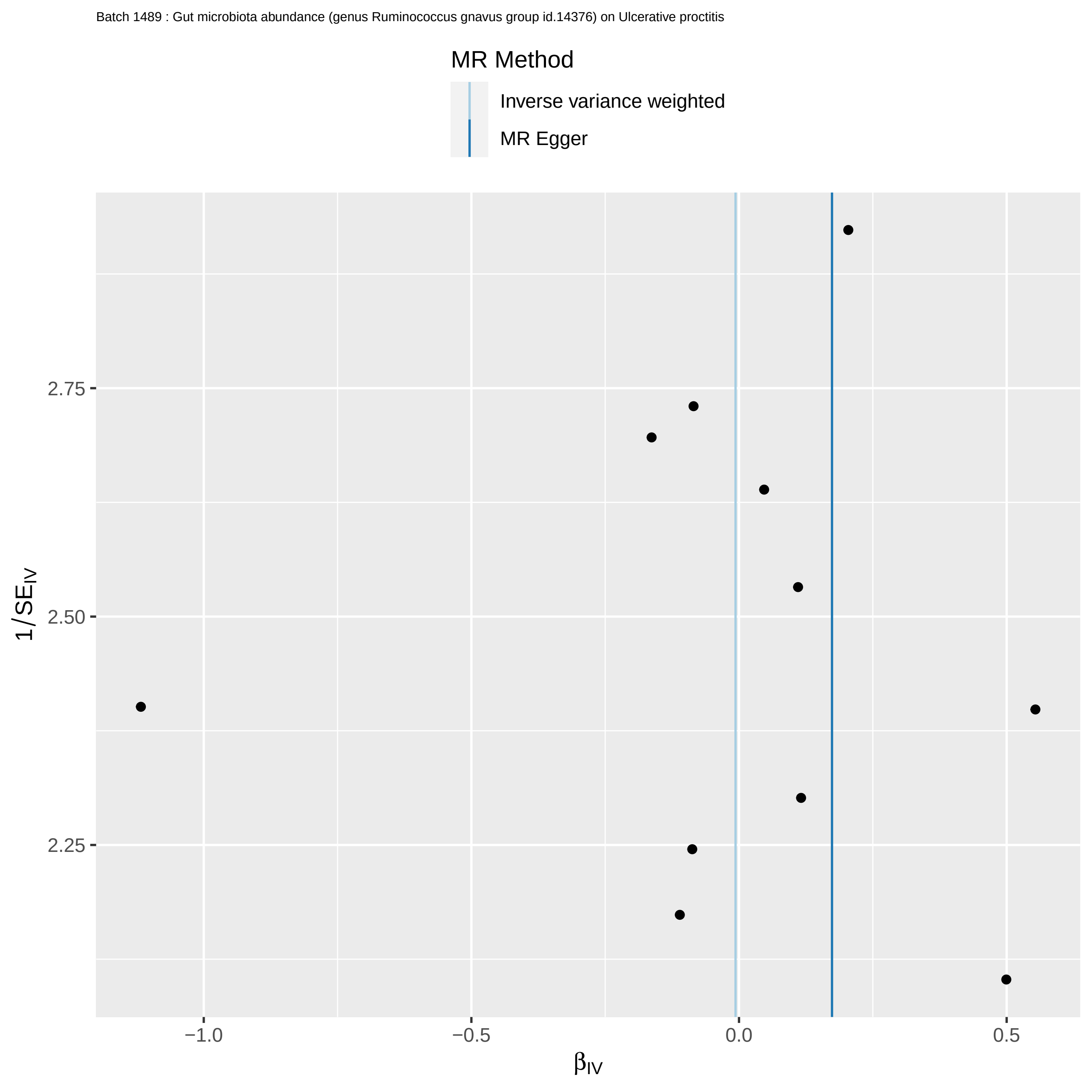
Batch 1489 : Gut microbiota abundance (genus Ruminococcus gnavus group id.14376) on Ulcerative proctitis
MR Method
Inverse variance weighted
MR Egger
2.75
2.50
2.25
1/SE_IV
−1.0
−0.5
0.0
0.5
β_IV

Batch 1490 : Gut microbiota abundance (genus Ruminococcus torques group id.14377) on Ulcerative proctitis

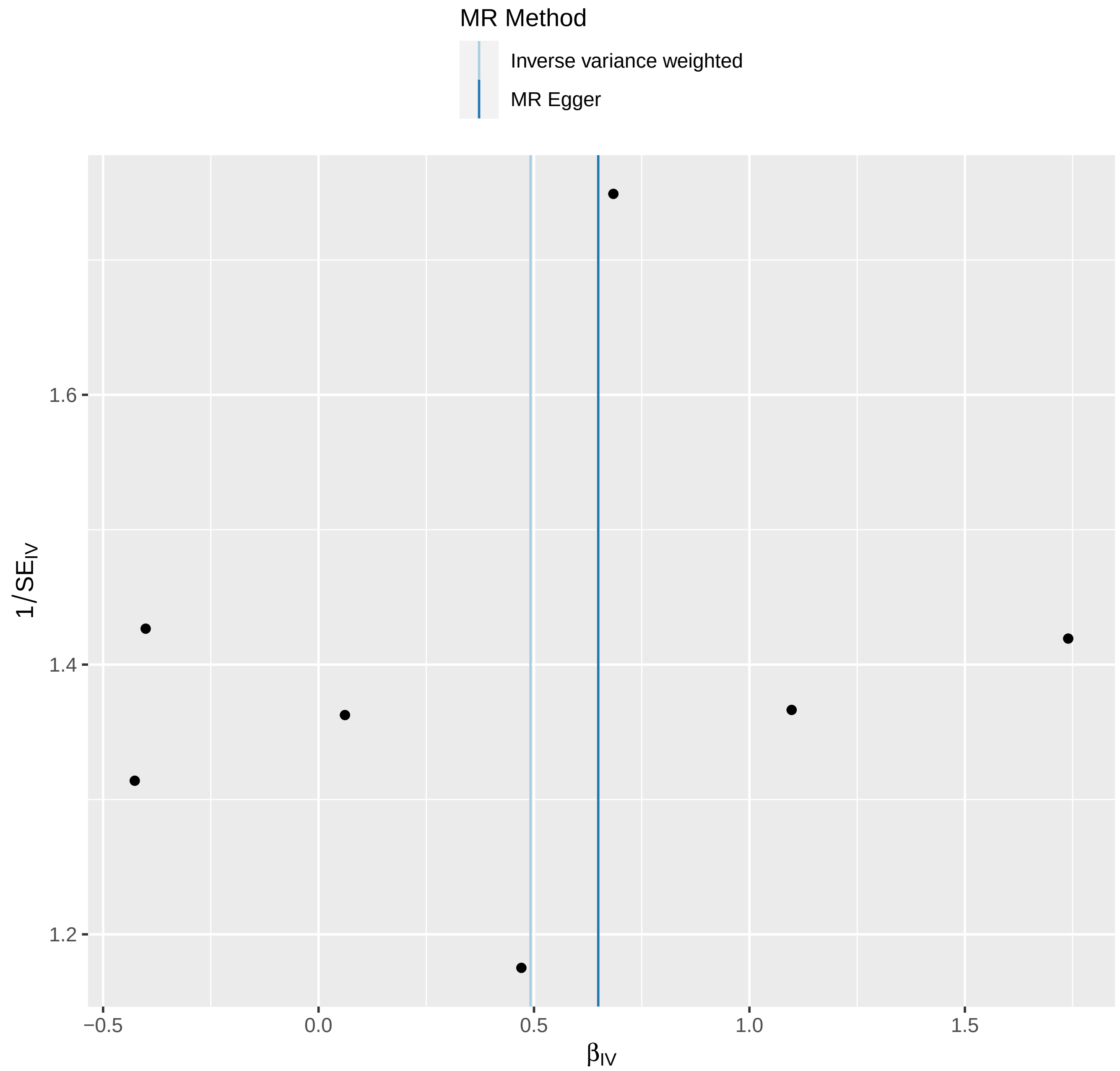

Batch 1491 : Gut microbiota abundance (genus Sellimonas id.14369) on Ulcerative proctitis

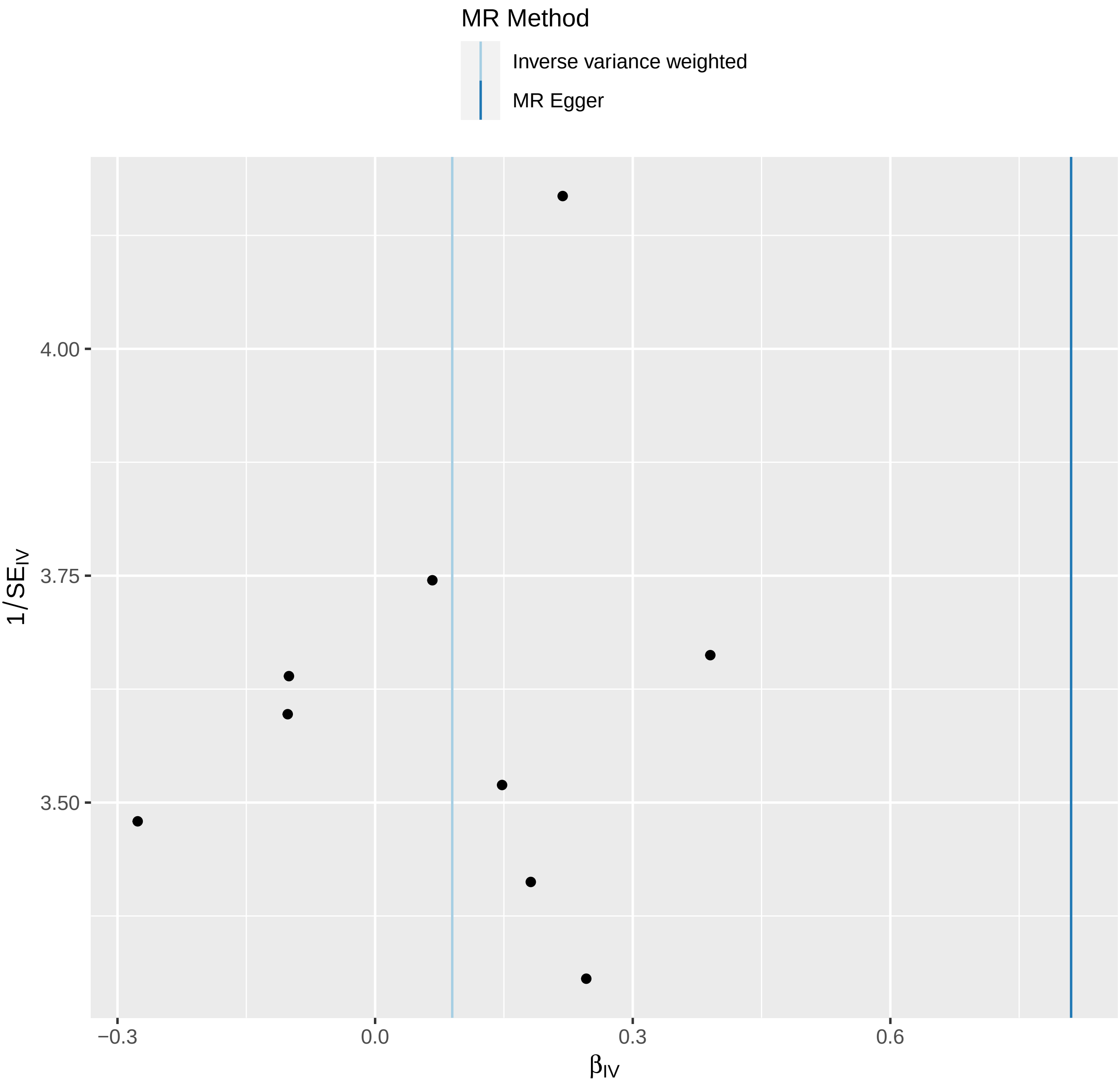

Batch 1492 : Gut microbiota abundance (genus Senegalimassilia id.11160) on Ulcerative proctitis

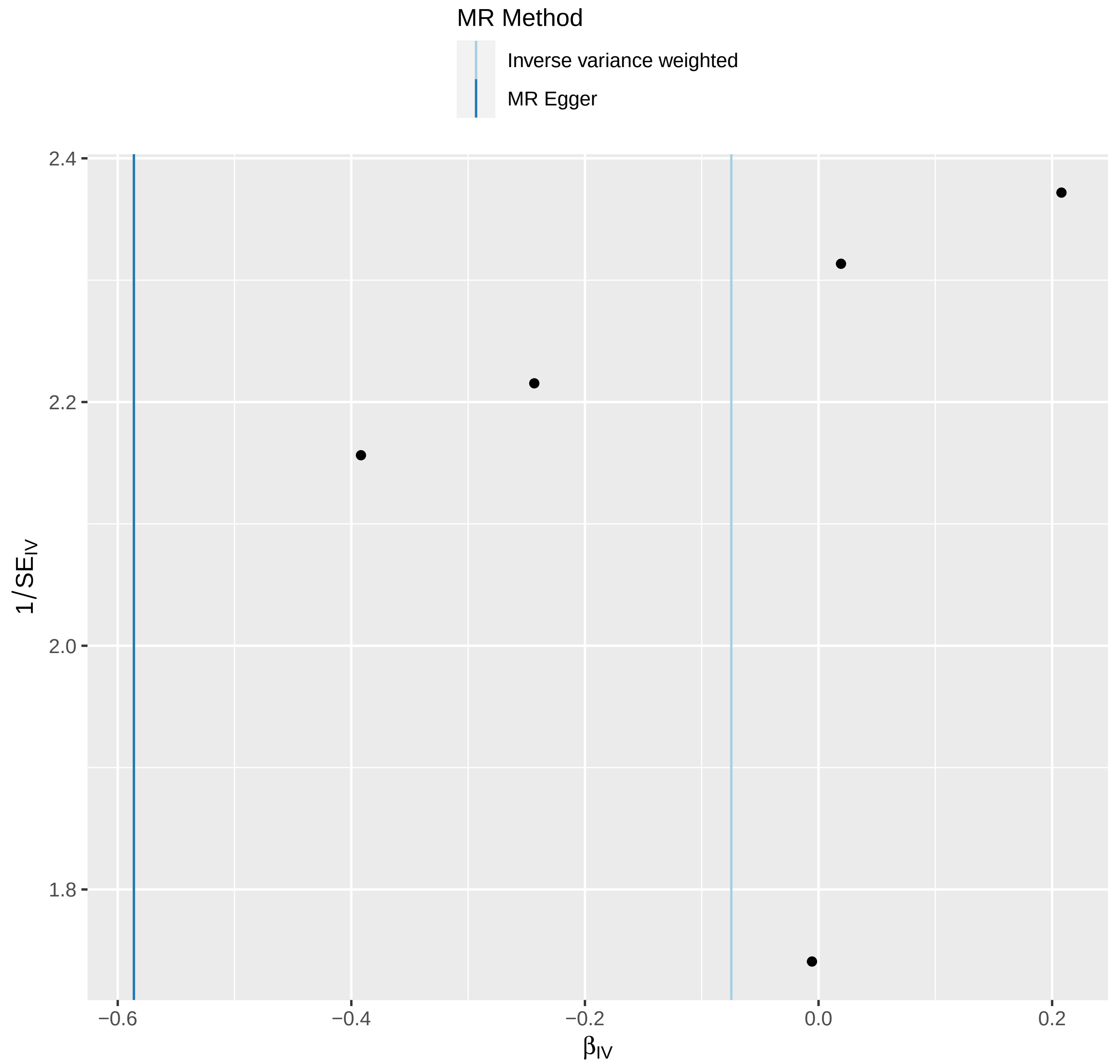

Batch 1493 : Gut microbiota abundance (genus Slackia id.825) on Ulcerative proctitis

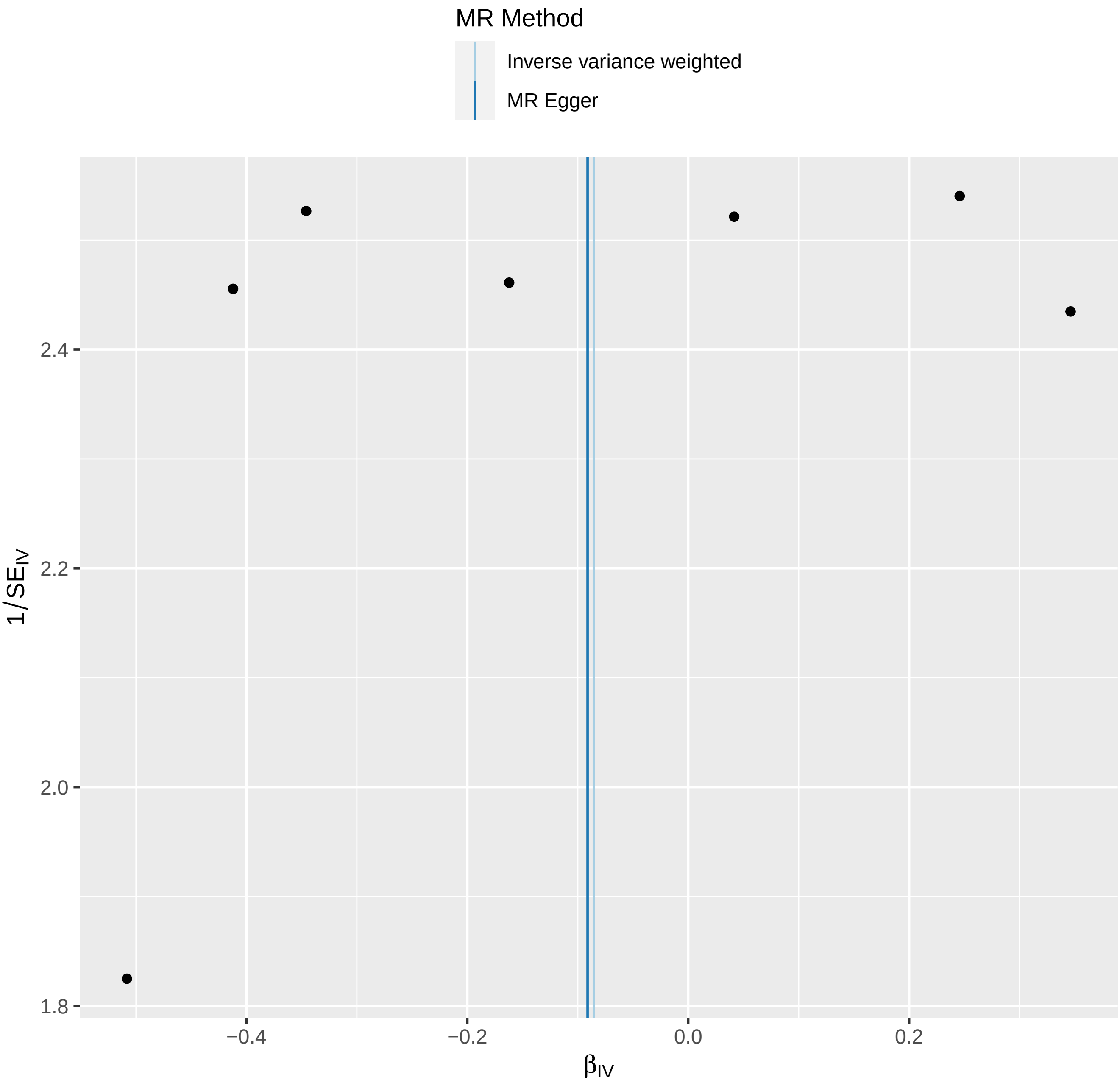

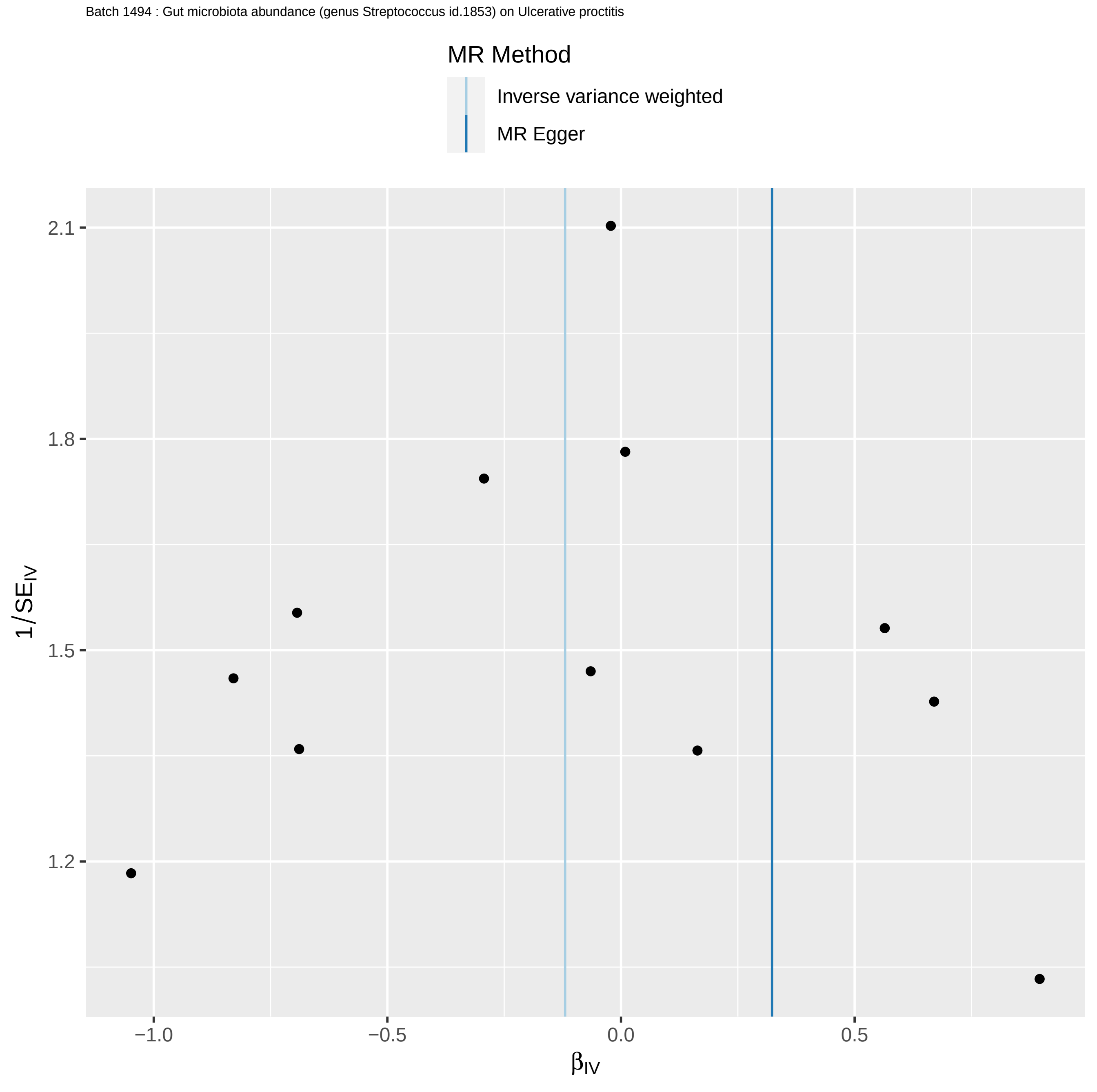
Batch 1494 : Gut microbiota abundance (genus Streptococcus id.1853) on Ulcerative proctitis
MR Method
Inverse variance weighted
MR Egger
2.1
1.8
1.5
1.2
$1/SE_{IV}$
−1.0
−0.5
0.0
0.5
β_{IV}

Batch 1495 : Gut microbiota abundance (genus Subdoligranulum id.2070) on Ulcerative proctitis

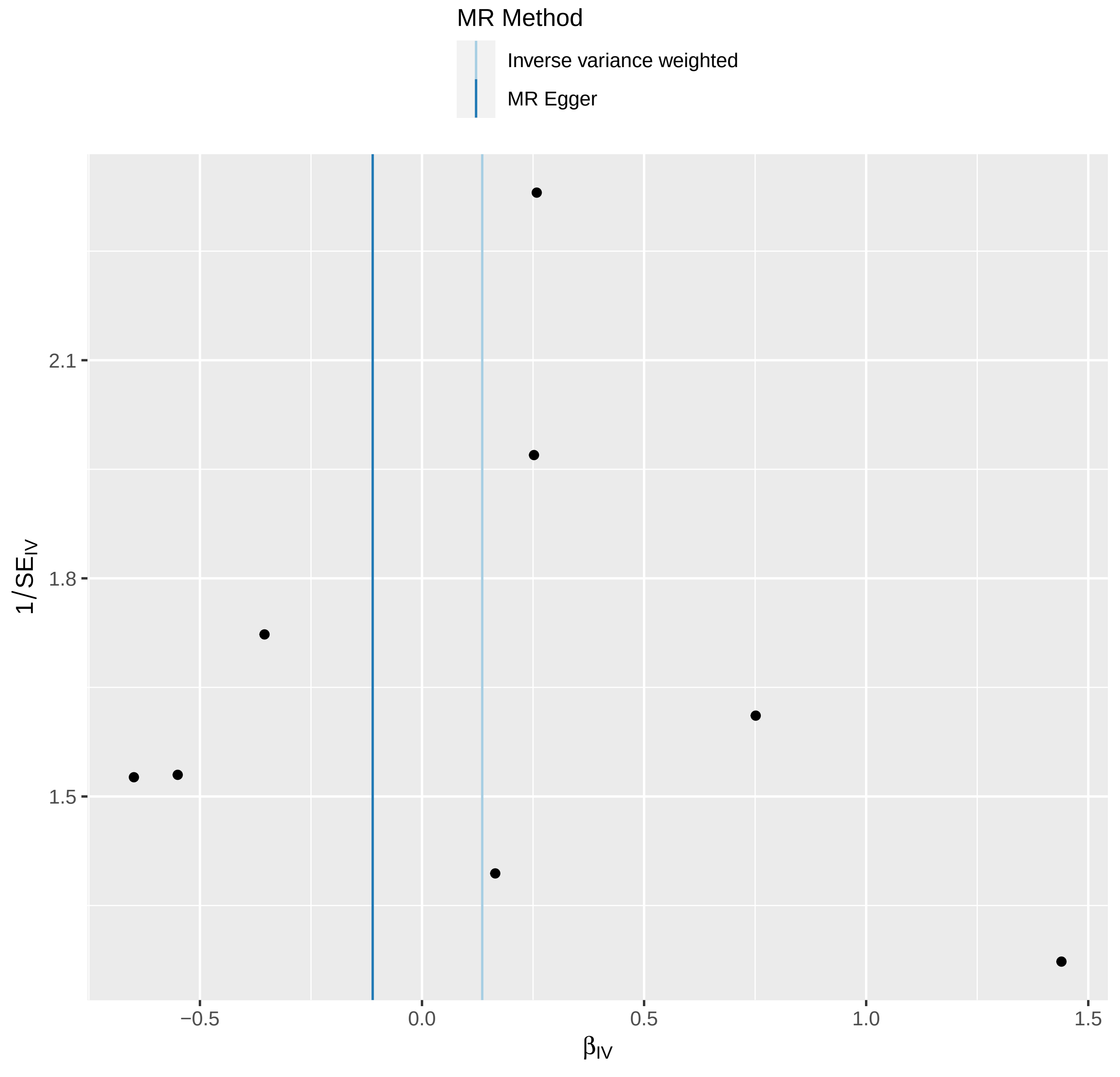

Batch 1496 : Gut microbiota abundance (genus Sutterella id.2896) on Ulcerative proctitis

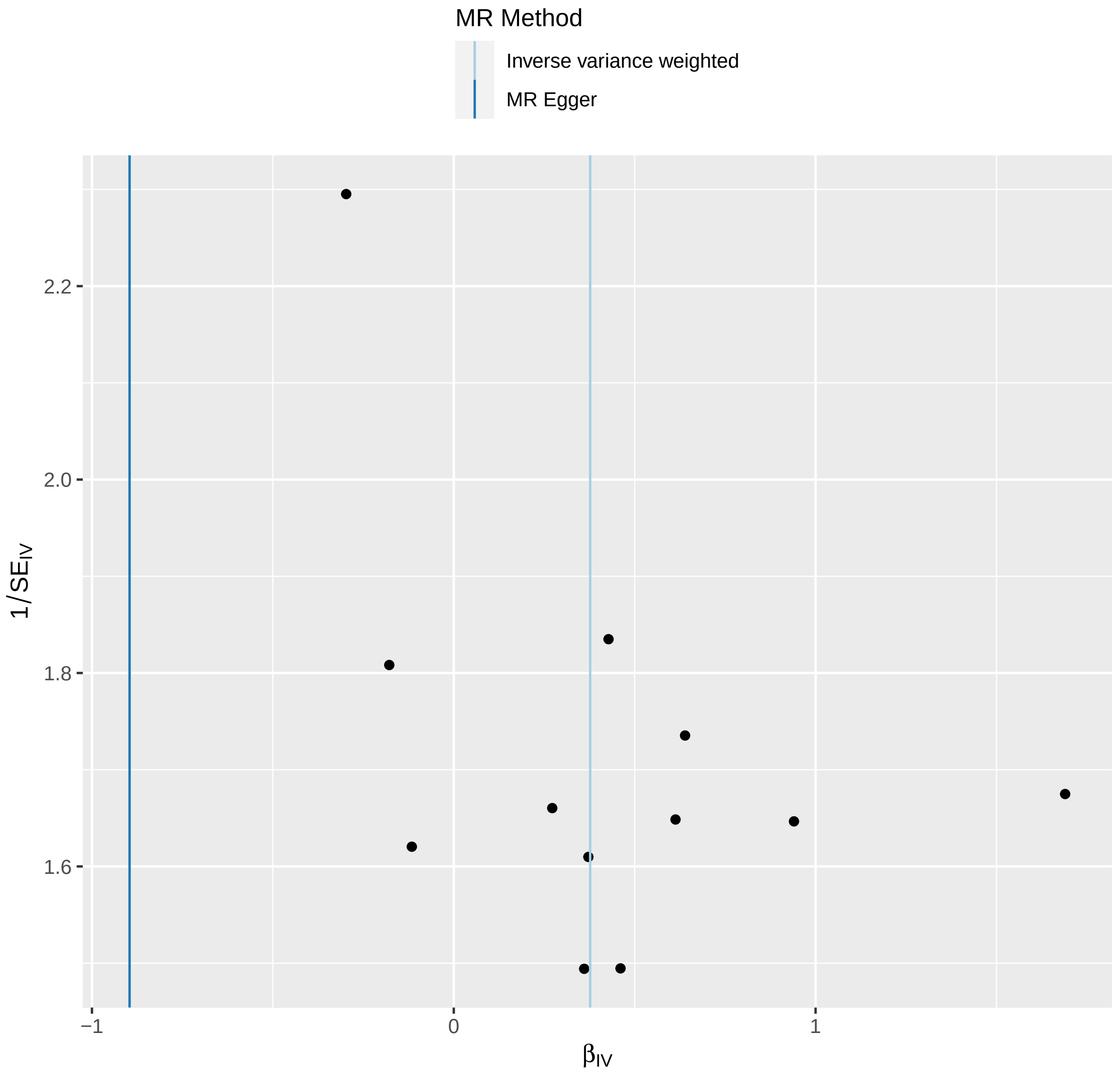

Batch 1497 : Gut microbiota abundance (genus Terrisporobacter id.11348) on Ulcerative proctitis

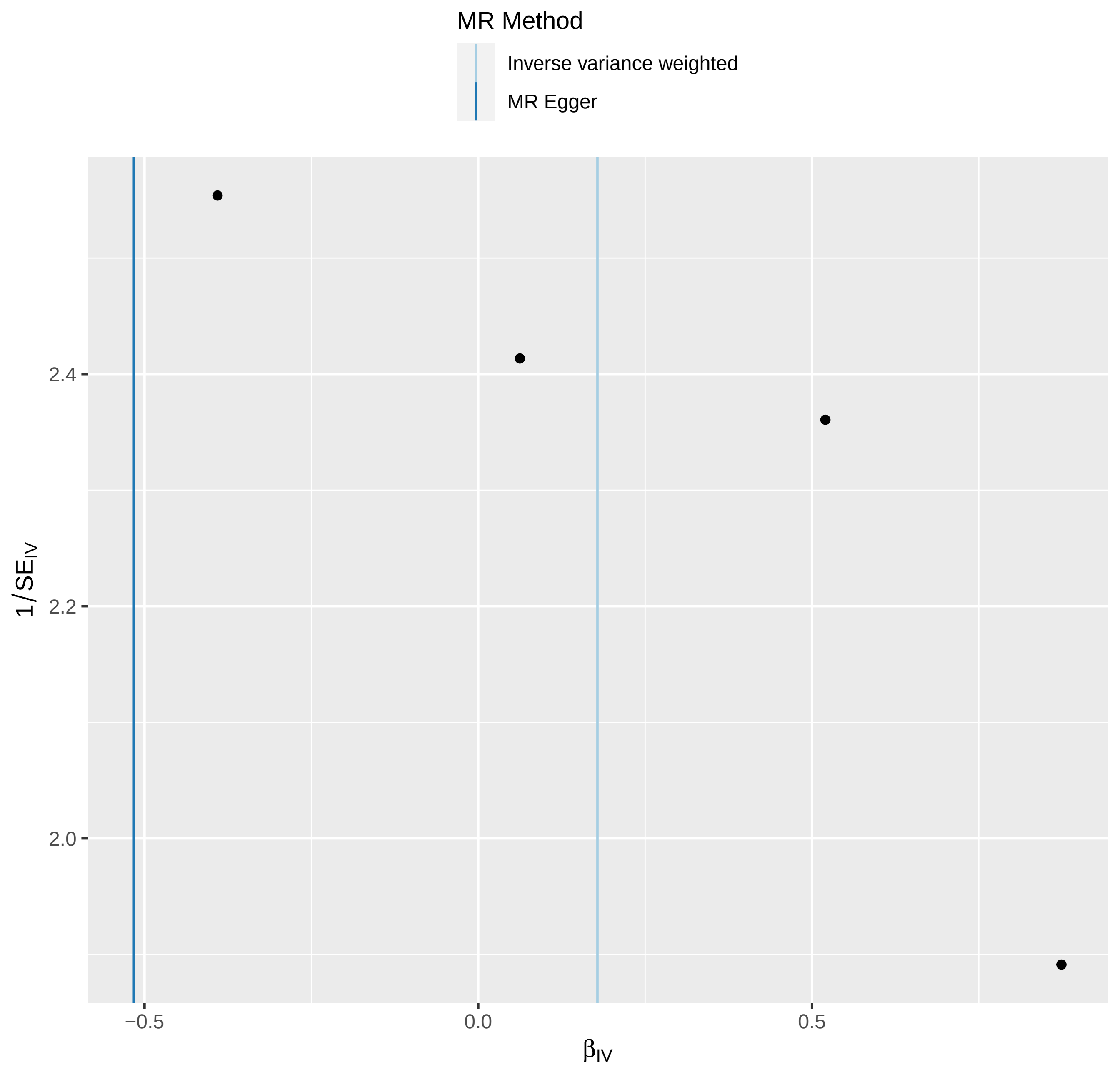

Batch 1498 : Gut microbiota abundance (genus Turicibacter id.2162) on Ulcerative proctitis

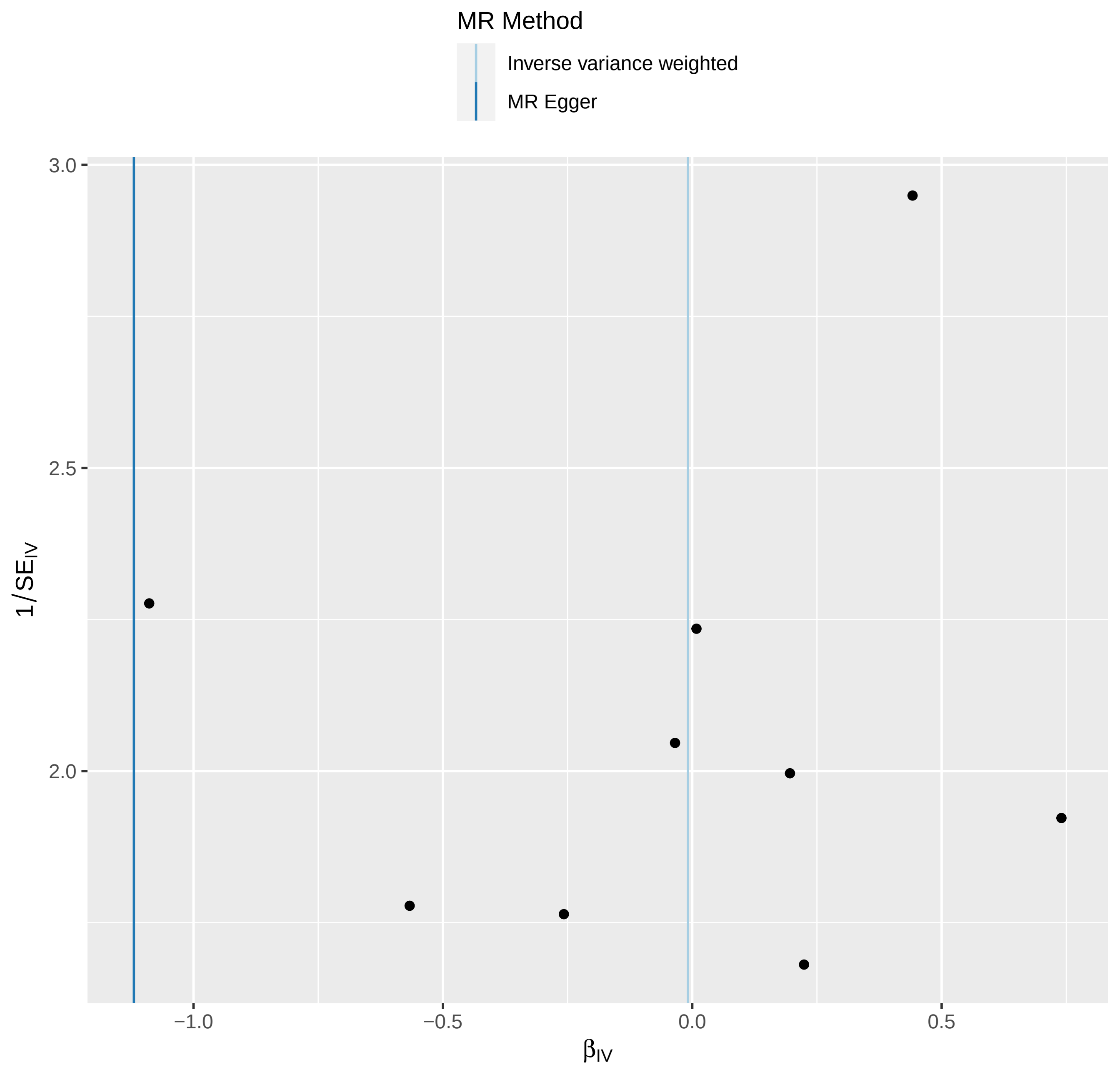

Batch 1499 : Gut microbiota abundance (genus Tyzzerella3 id.11335) on Ulcerative proctitis

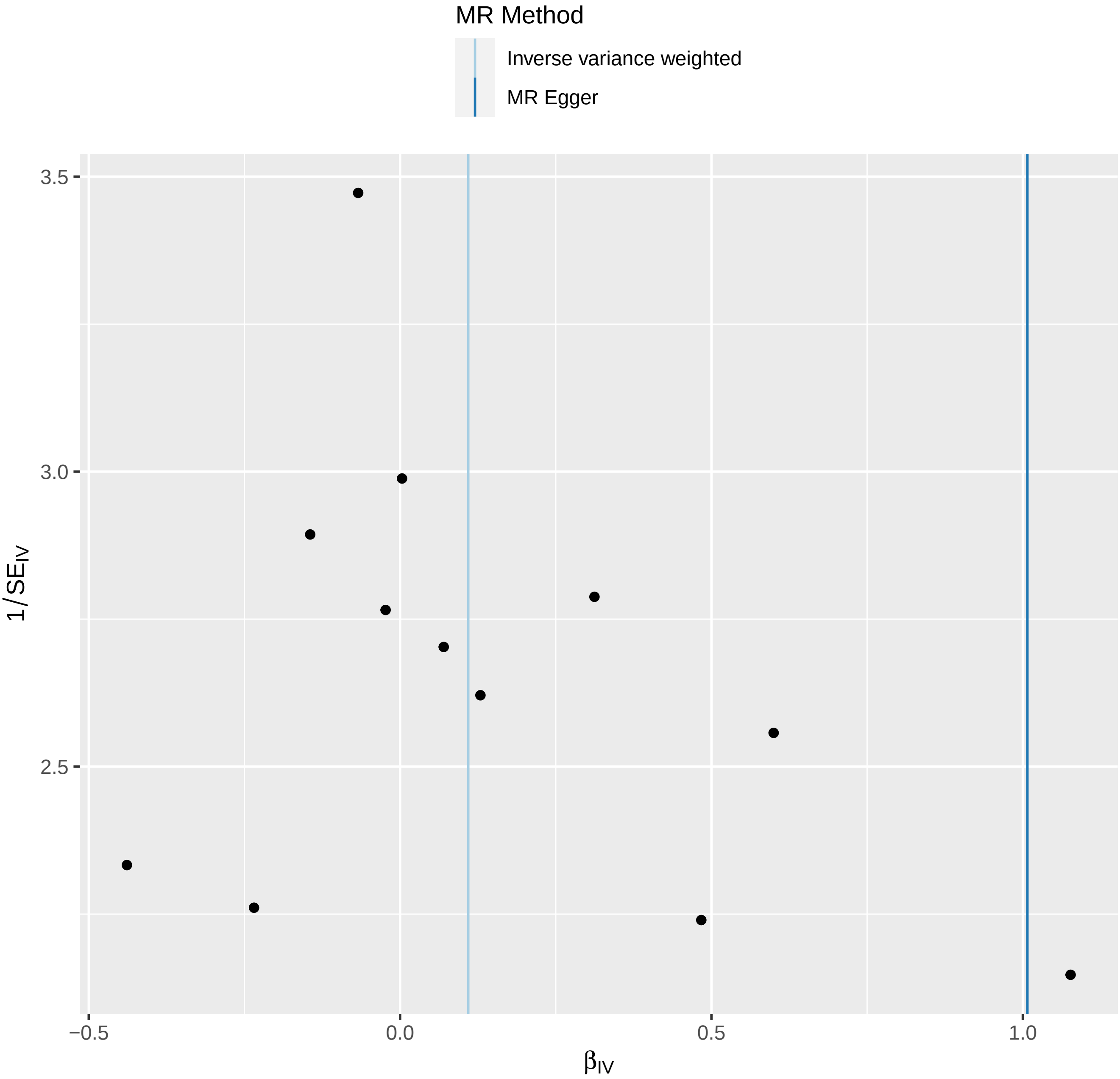

Batch 1500 : Gut microbiota abundance (genus Veillonella id.2198) on Ulcerative proctitis

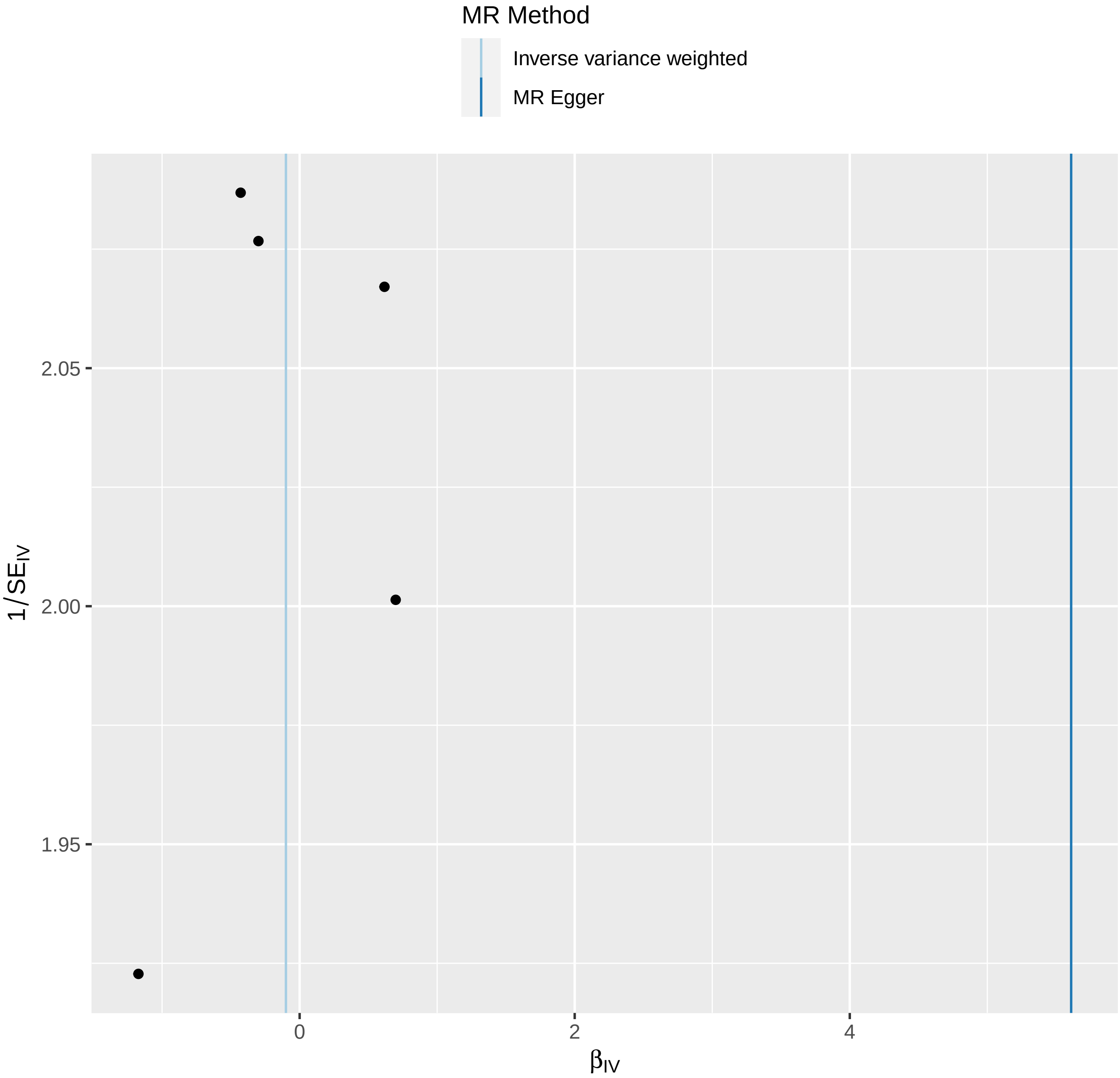

Batch 1501 : Gut microbiota abundance (family Acidaminococcaceae id.2166) on Ulcerative rectosigmoiditis

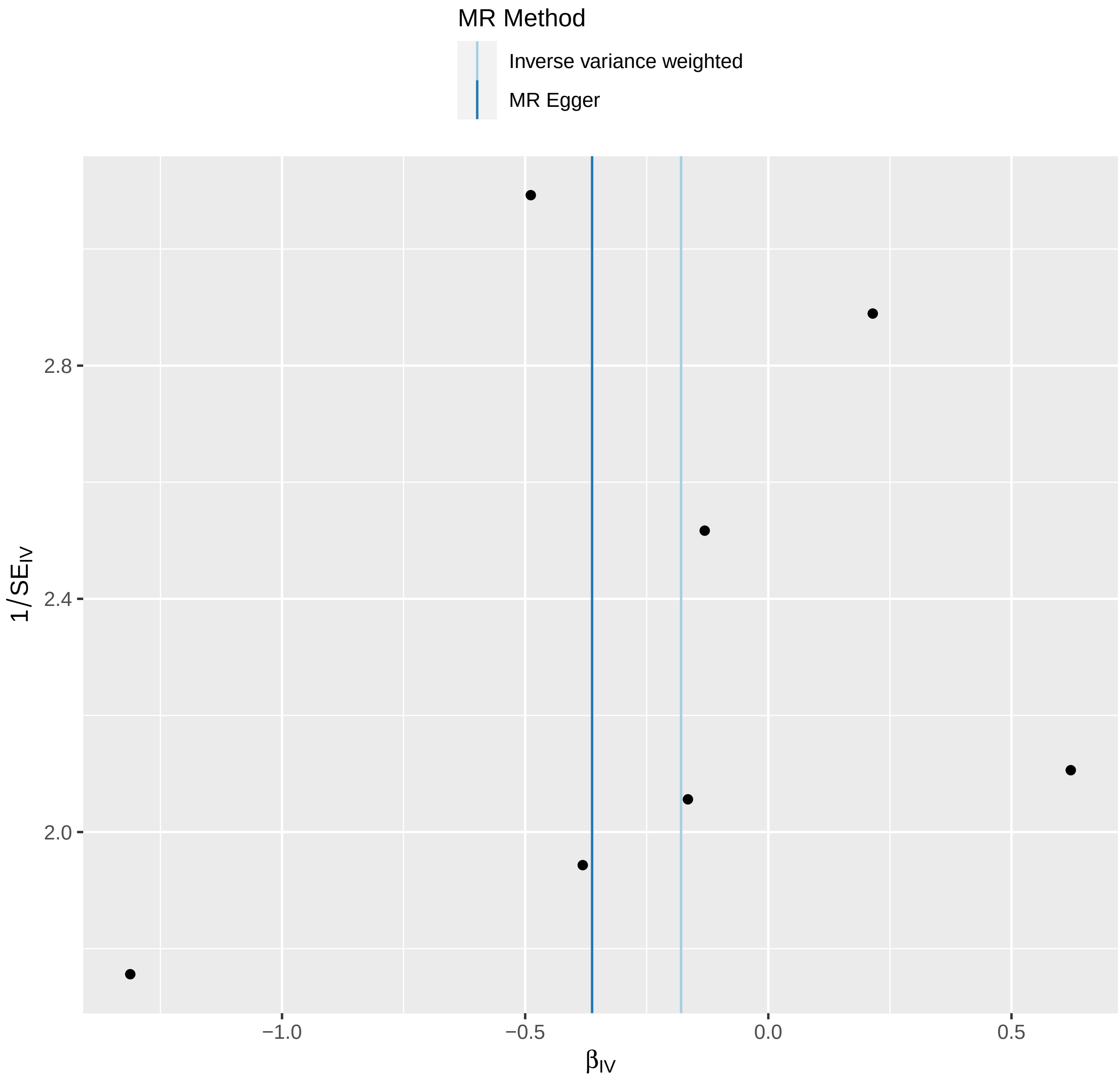

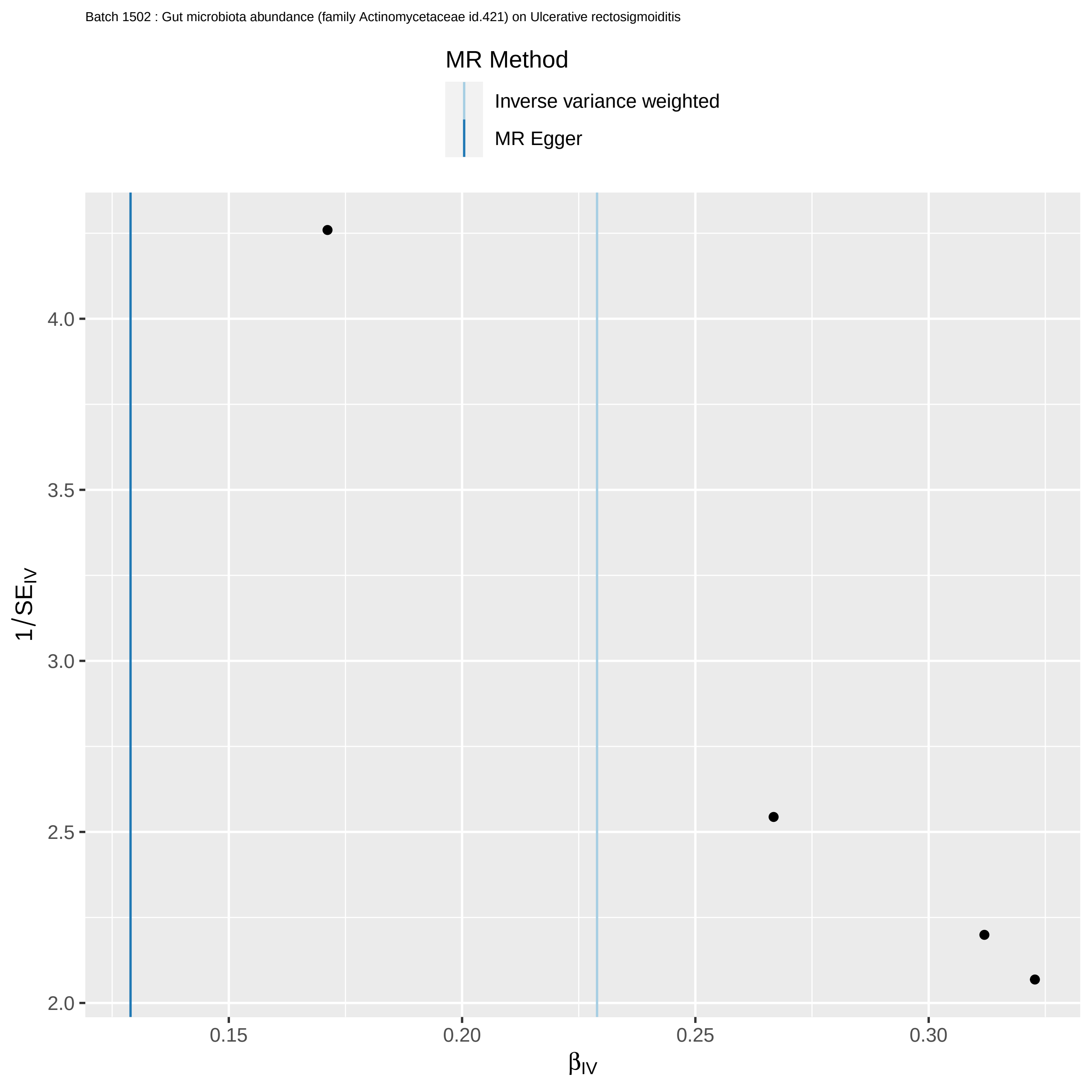

Batch 1502 : Gut microbiota abundance (family Actinomycetaceae id.421) on Ulcerative rectosigmoiditis
MR Method
Inverse variance weighted
MR Egger
4.0
3.5
3.0
2.5
2.0
0.15
0.20
0.25
0.30
$1/SE_{IV}$
β_{IV}

Batch 1503 : Gut microbiota abundance (family Alcaligenaceae id.2875) on Ulcerative rectosigmoiditis

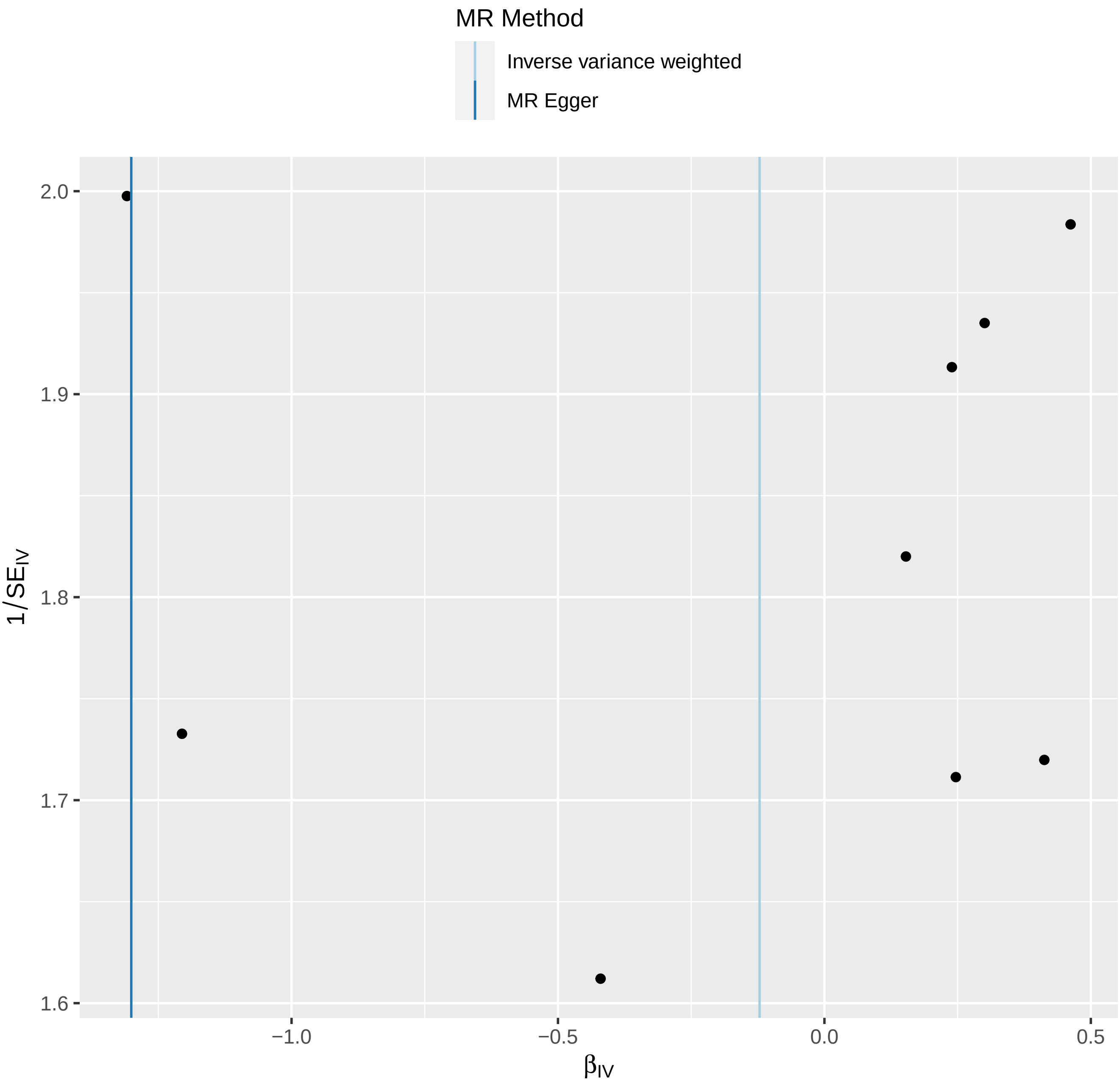

Batch 1504 : Gut microbiota abundance (family Bacteroidaceae id.917) on Ulcerative rectosigmoiditis

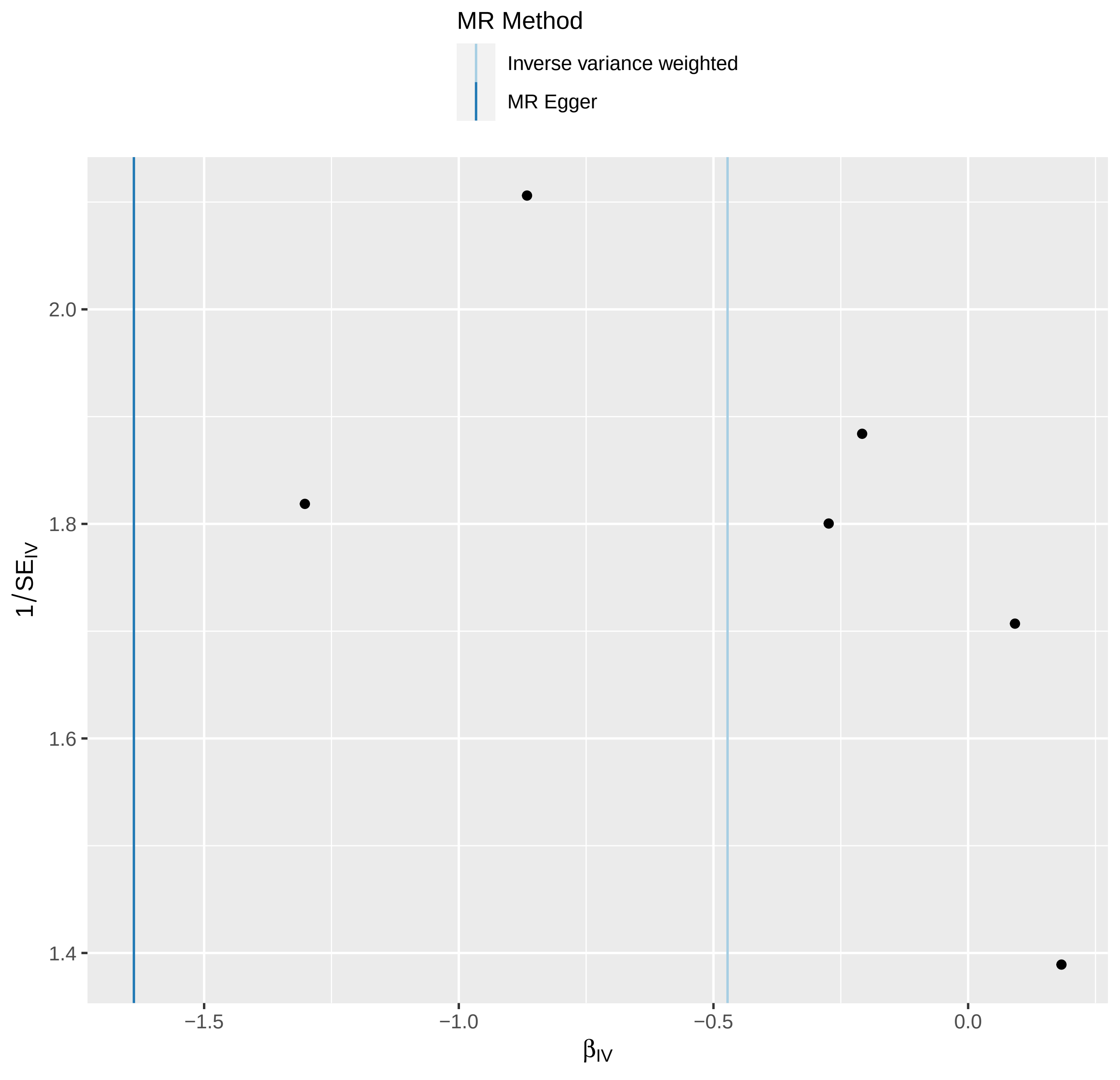

Batch 1505 : Gut microbiota abundance (family Bacteroidales S24 7group id.11173) on Ulcerative rectosigmoiditis

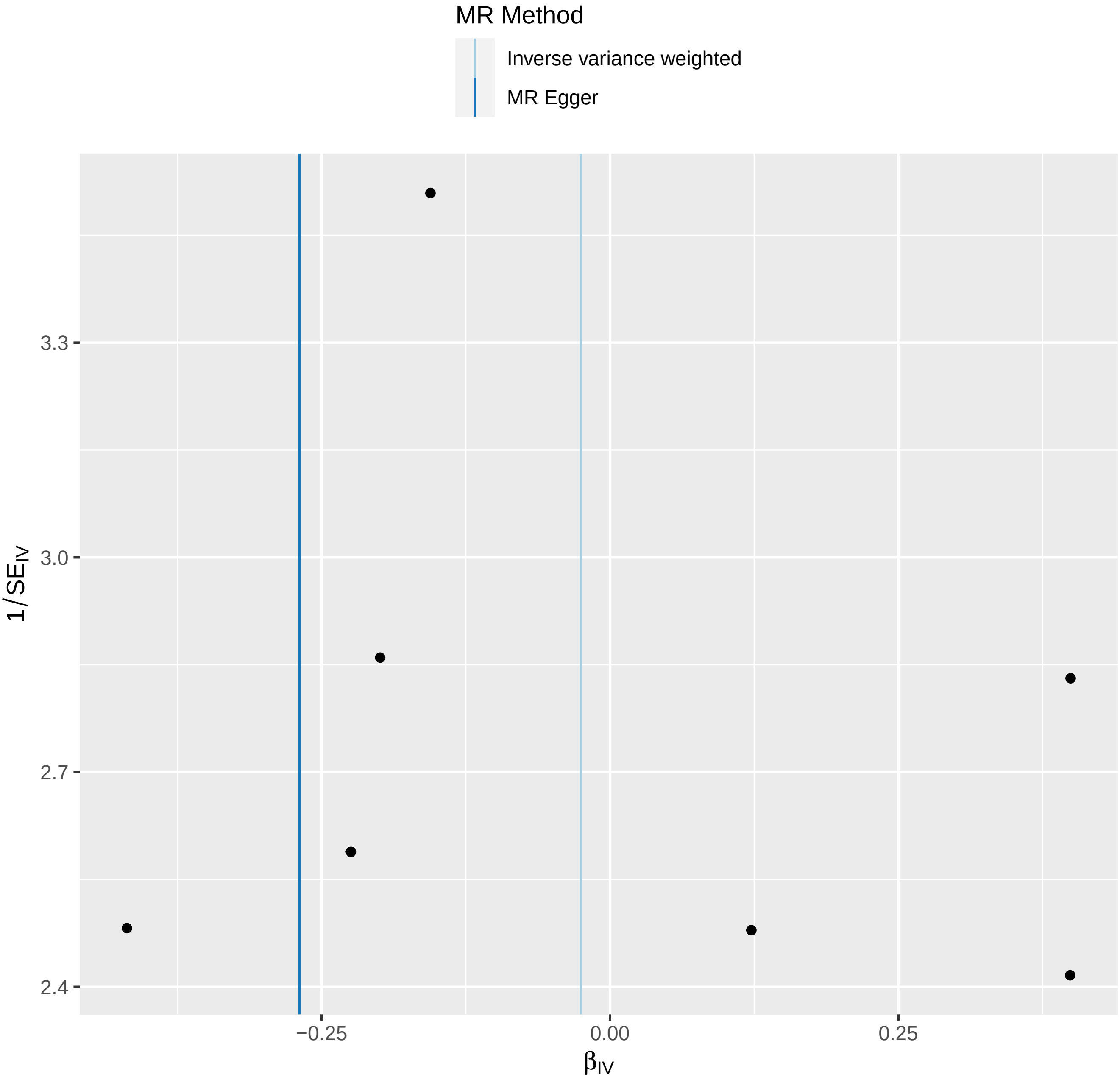

Batch 1506 : Gut microbiota abundance (family Bifidobacteriaceae id.433) on Ulcerative rectosigmoiditis

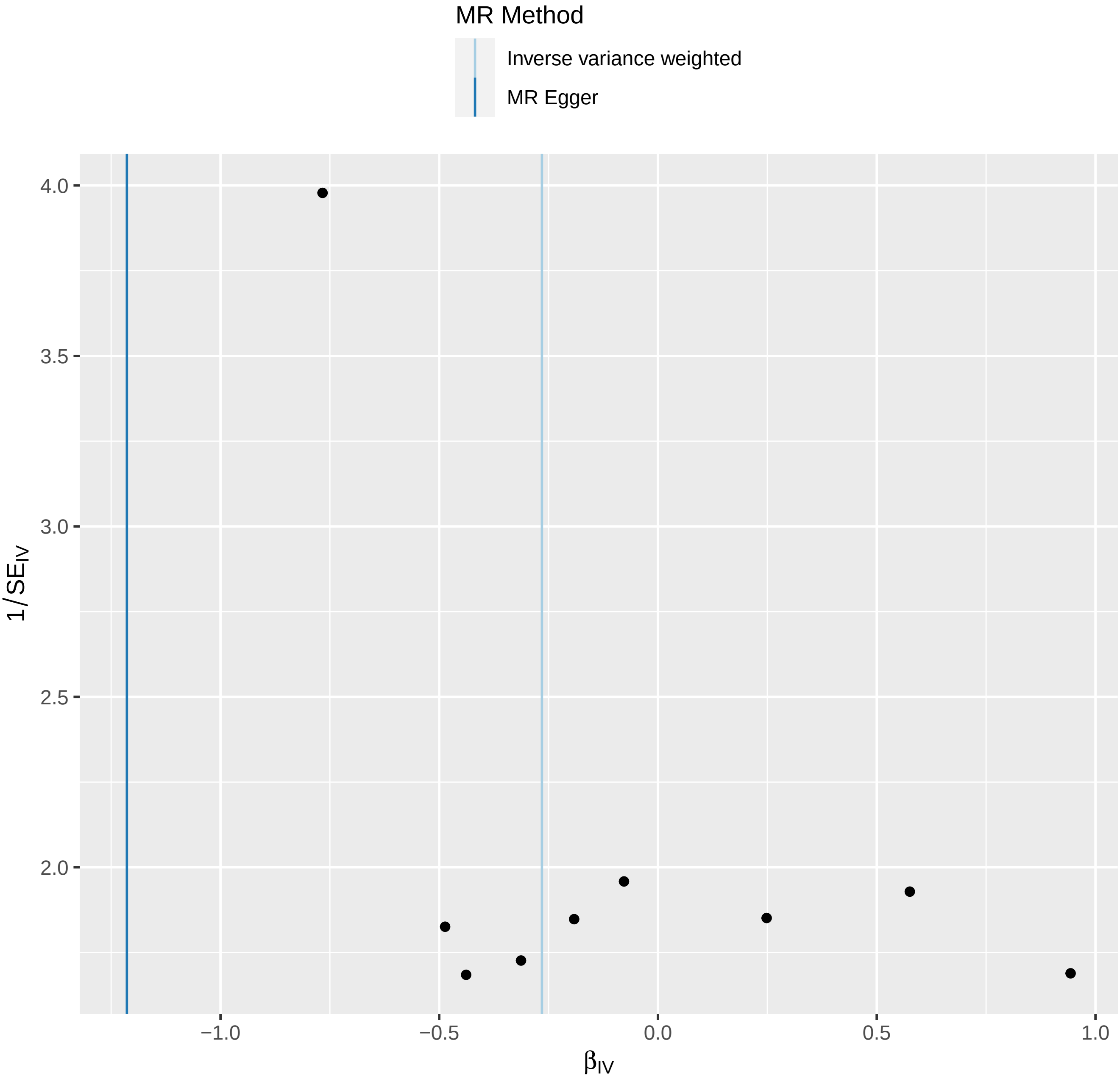

Batch 1508 : Gut microbiota abundance (family Clostridiaceae1 id.1869) on Ulcerative rectosigmoiditis

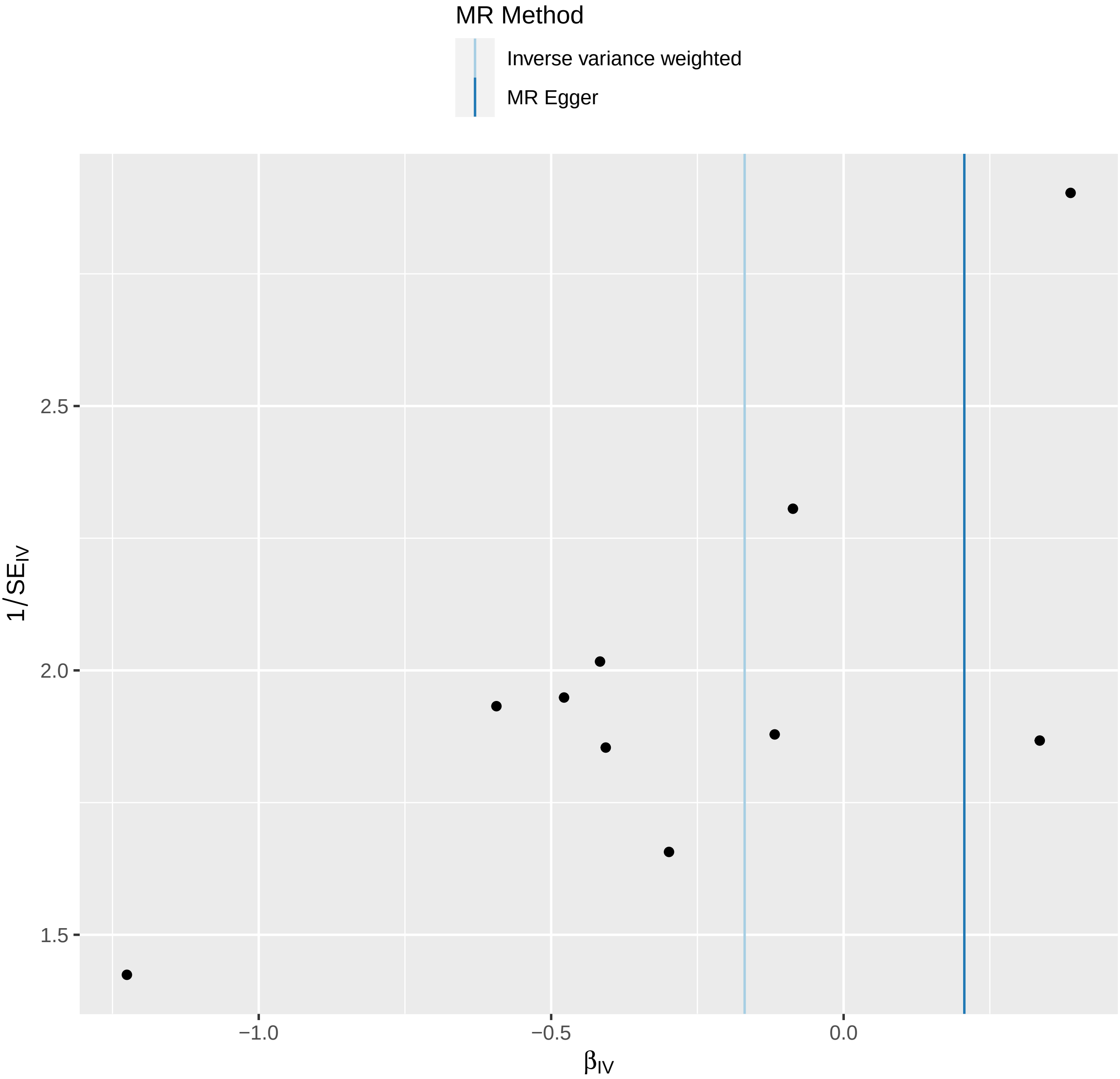

Batch 1509 : Gut microbiota abundance (family Clostridiales vadin BB60 group id.11286) on Ulcerative rectosigmoiditis

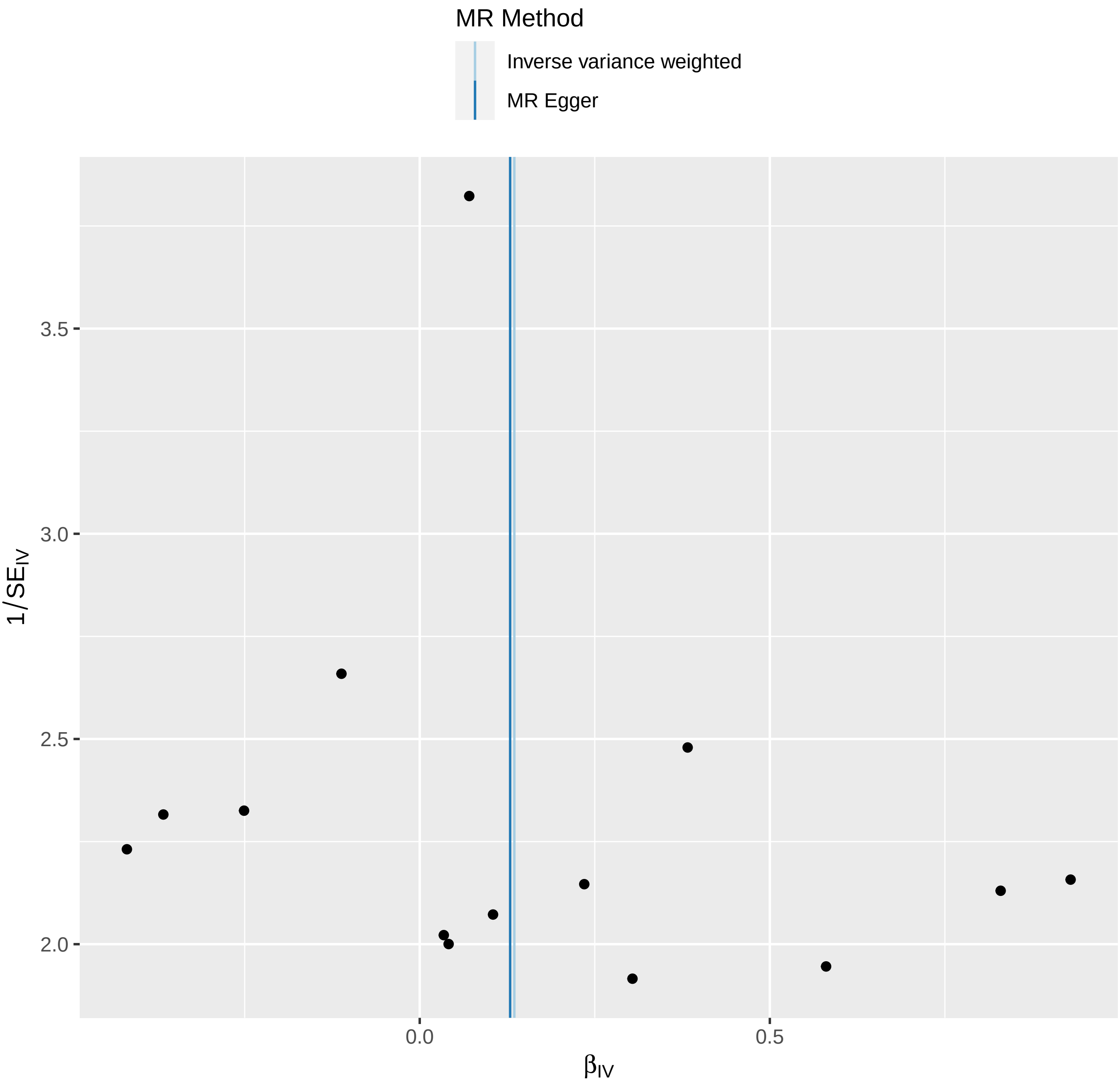

Batch 1510 : Gut microbiota abundance (family Coriobacteriaceae id.811) on Ulcerative rectosigmoiditis

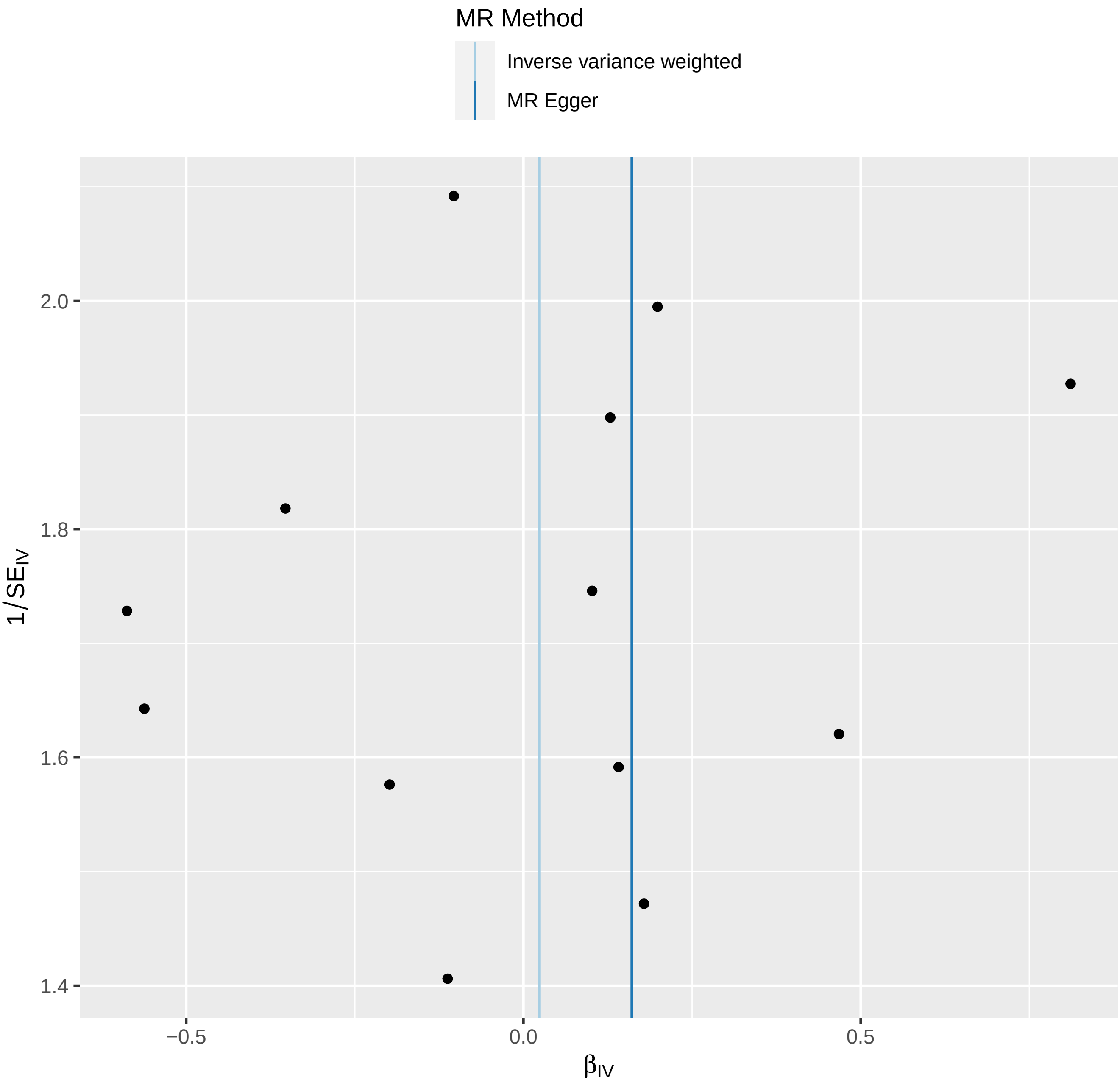

Batch 1511 : Gut microbiota abundance (family Defluviitaleaceae id.1924) on Ulcerative rectosigmoiditis

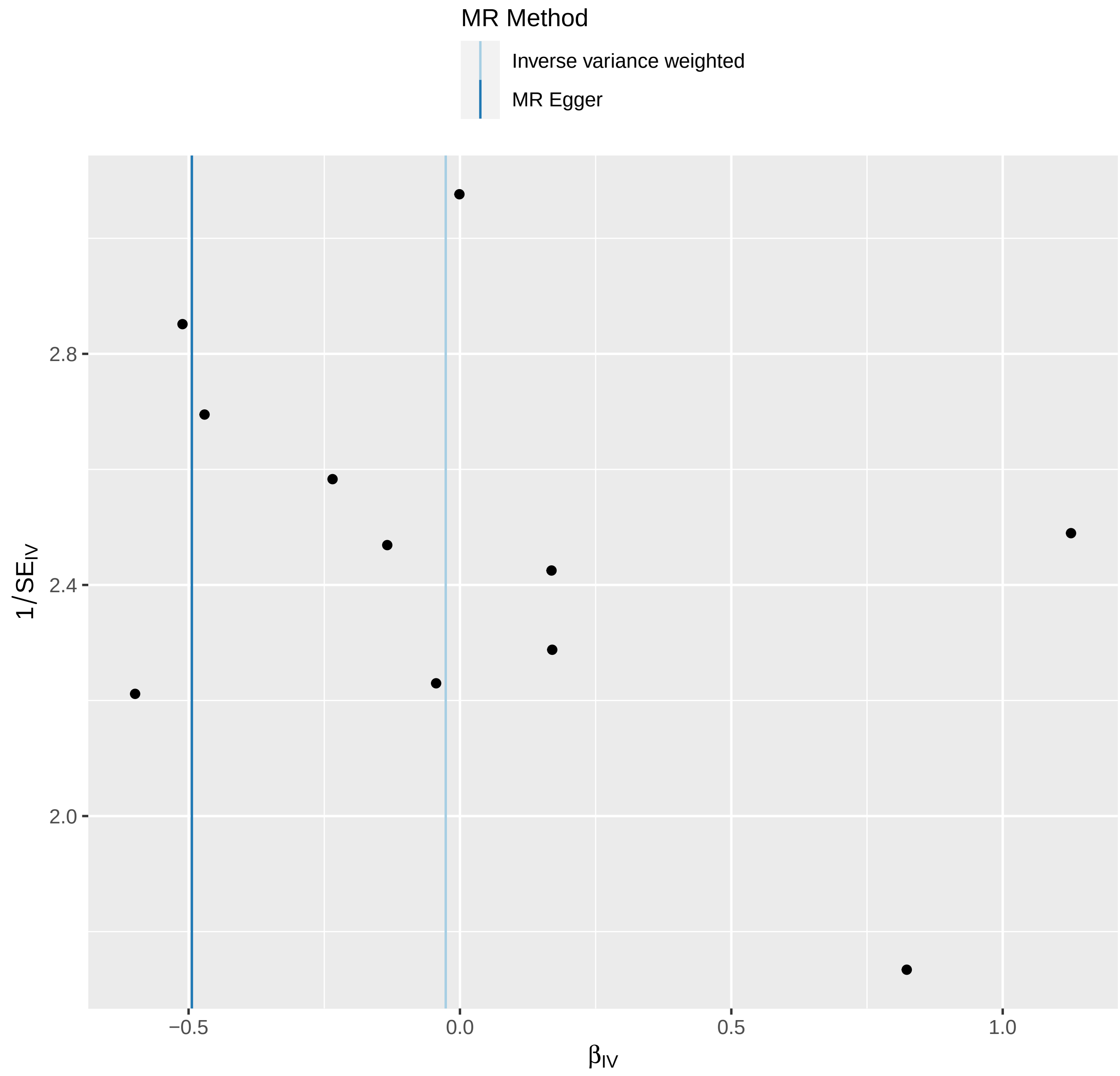

Batch 1512 : Gut microbiota abundance (family Desulfovibrionaceae id.3169) on Ulcerative rectosigmoiditis

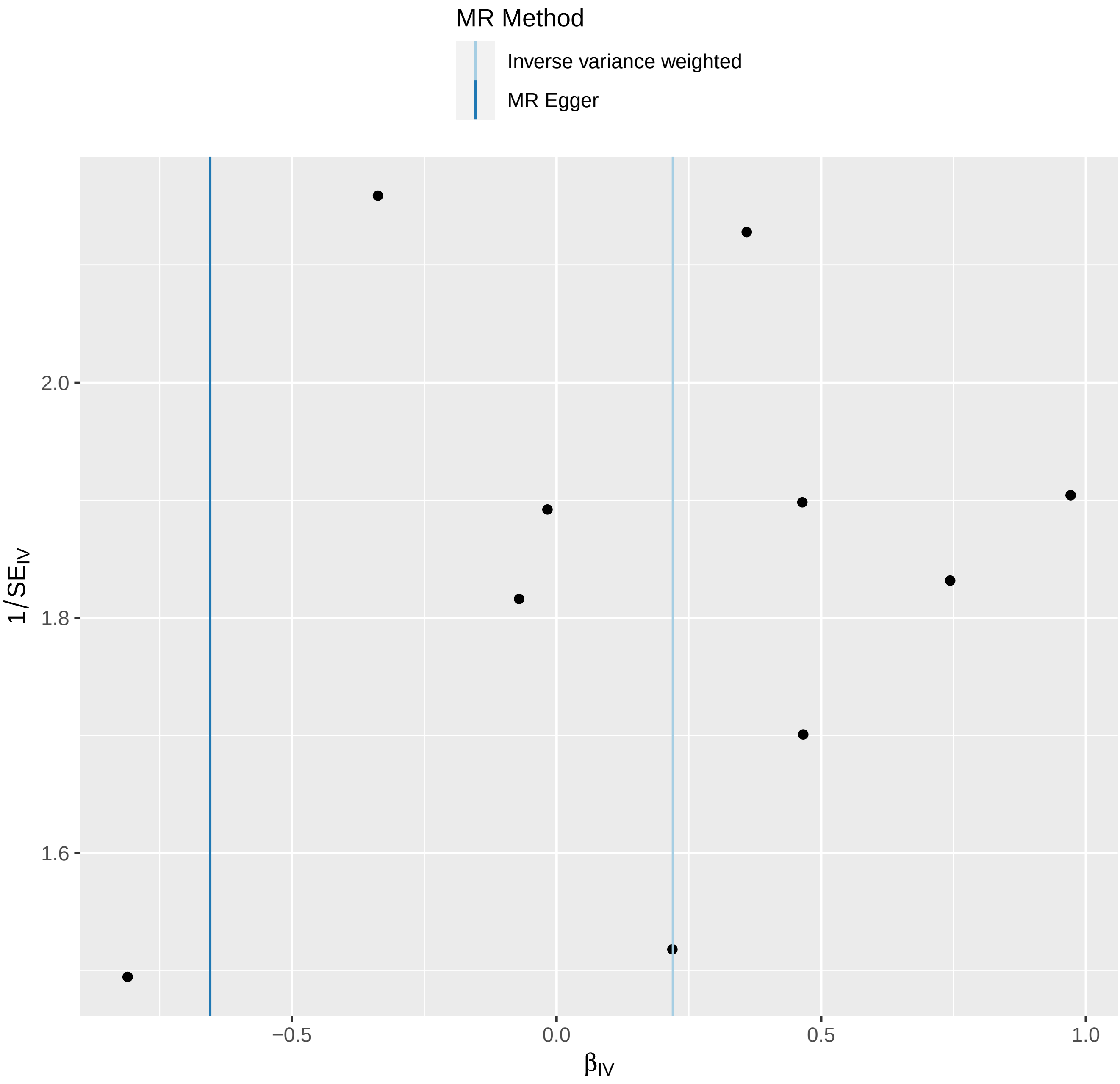

Batch 1513 : Gut microbiota abundance (family Enterobacteriaceae id.3469) on Ulcerative rectosigmoiditis

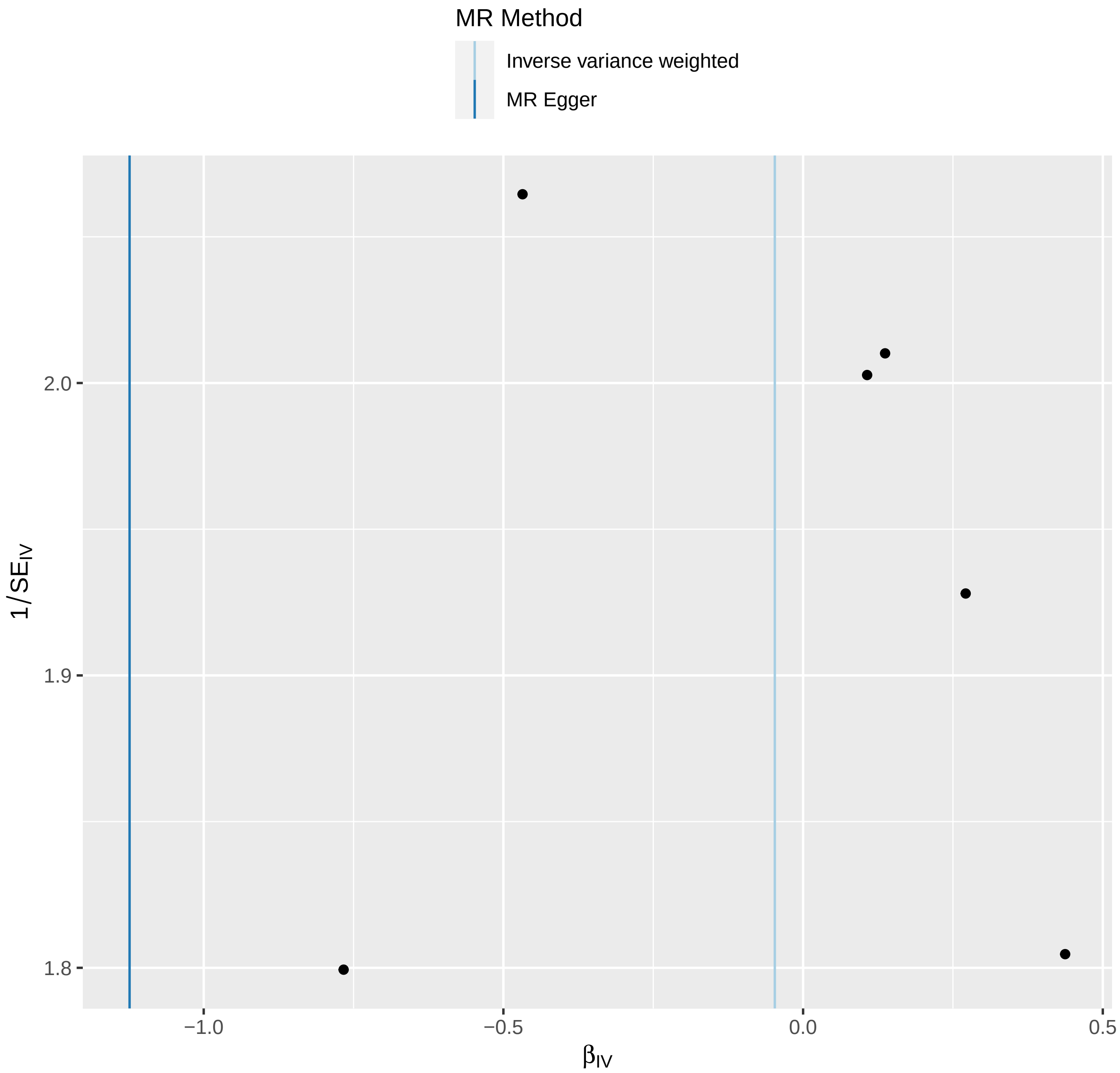

Batch 1514 : Gut microbiota abundance (family Erysipelotrichaceae id.2149) on Ulcerative rectosigmoiditis

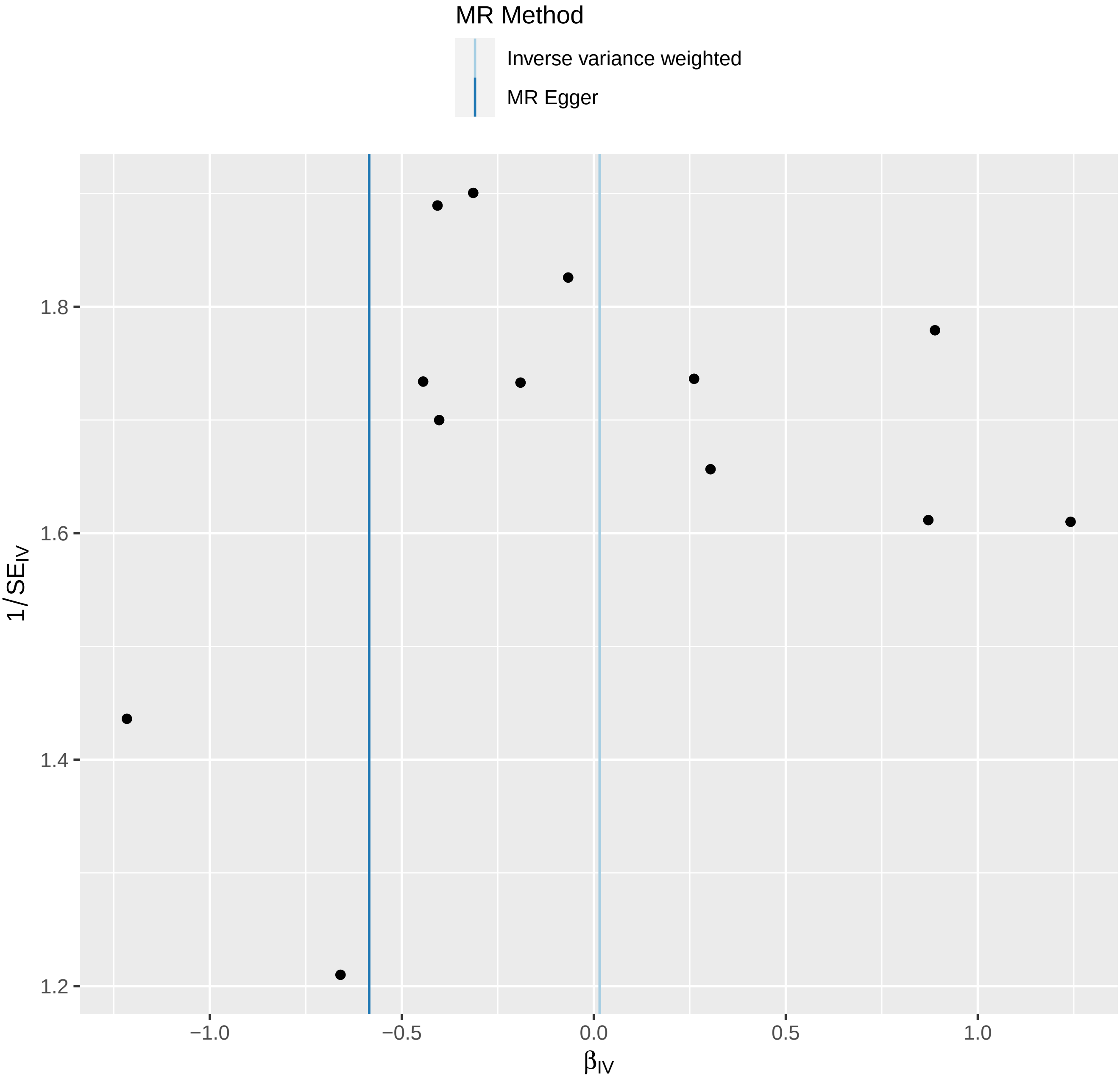

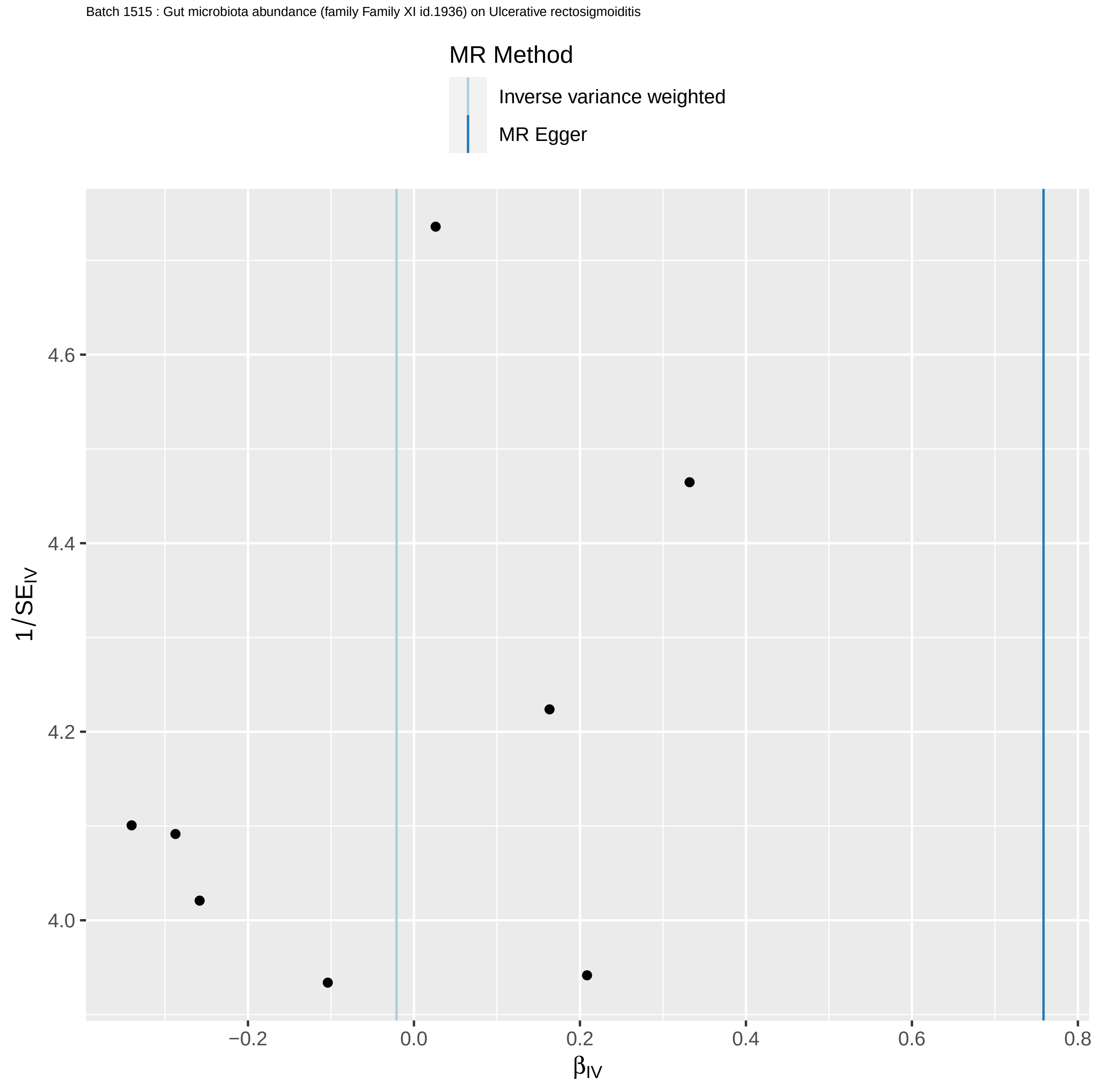
Batch 1515 : Gut microbiota abundance (family Family XI id.1936) on Ulcerative rectosigmoiditis
MR Method
Inverse variance weighted
MR Egger
4.6
4.4
4.2
4.0
1/SE_IV
−0.2
0.0
0.2
0.4
0.6
0.8
β_IV

Batch 1516 : Gut microbiota abundance (family Family XIII id.1957) on Ulcerative rectosigmoiditis

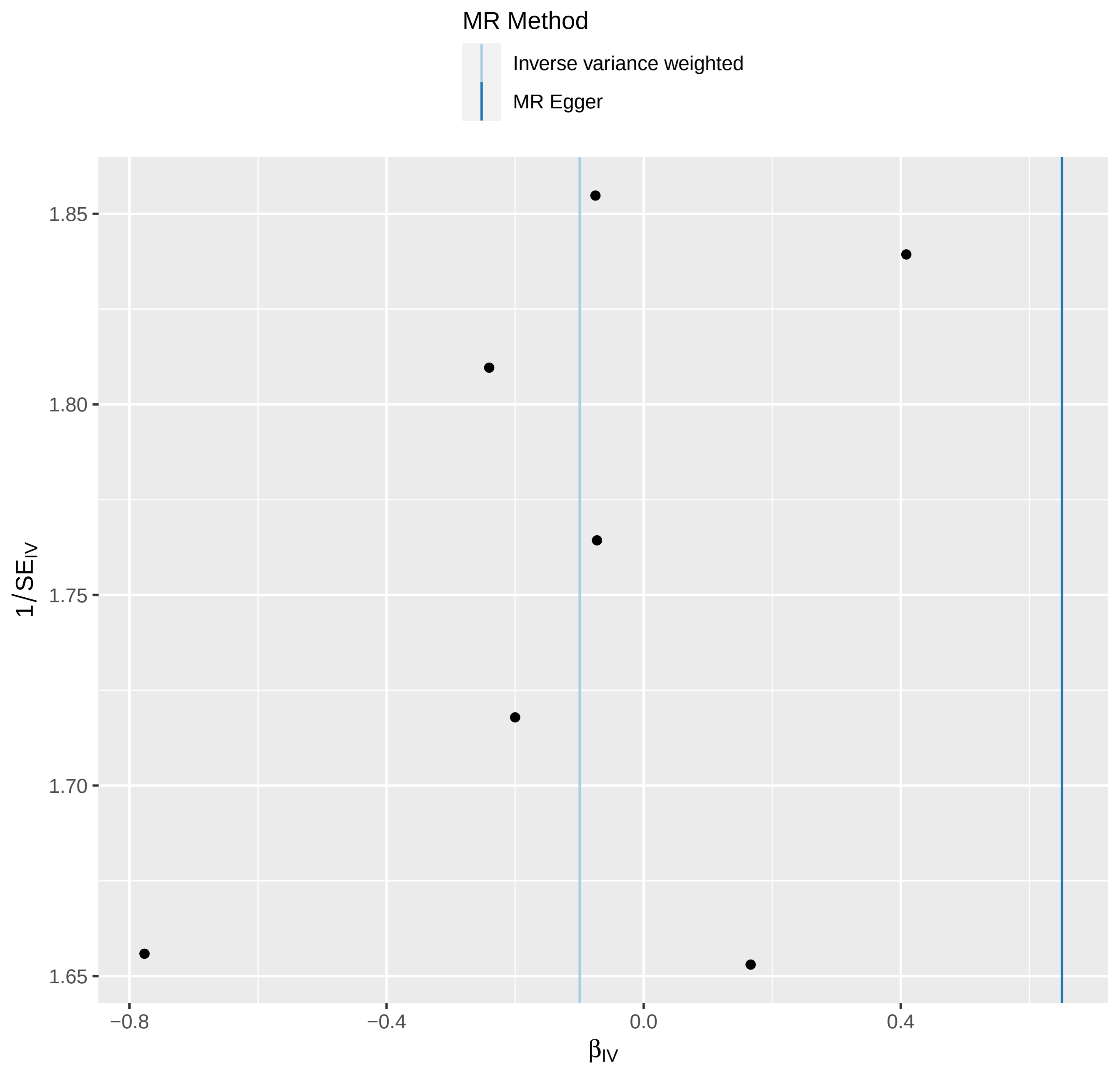

Batch 1517 : Gut microbiota abundance (family Lachnospiraceae id.1987) on Ulcerative rectosigmoiditis

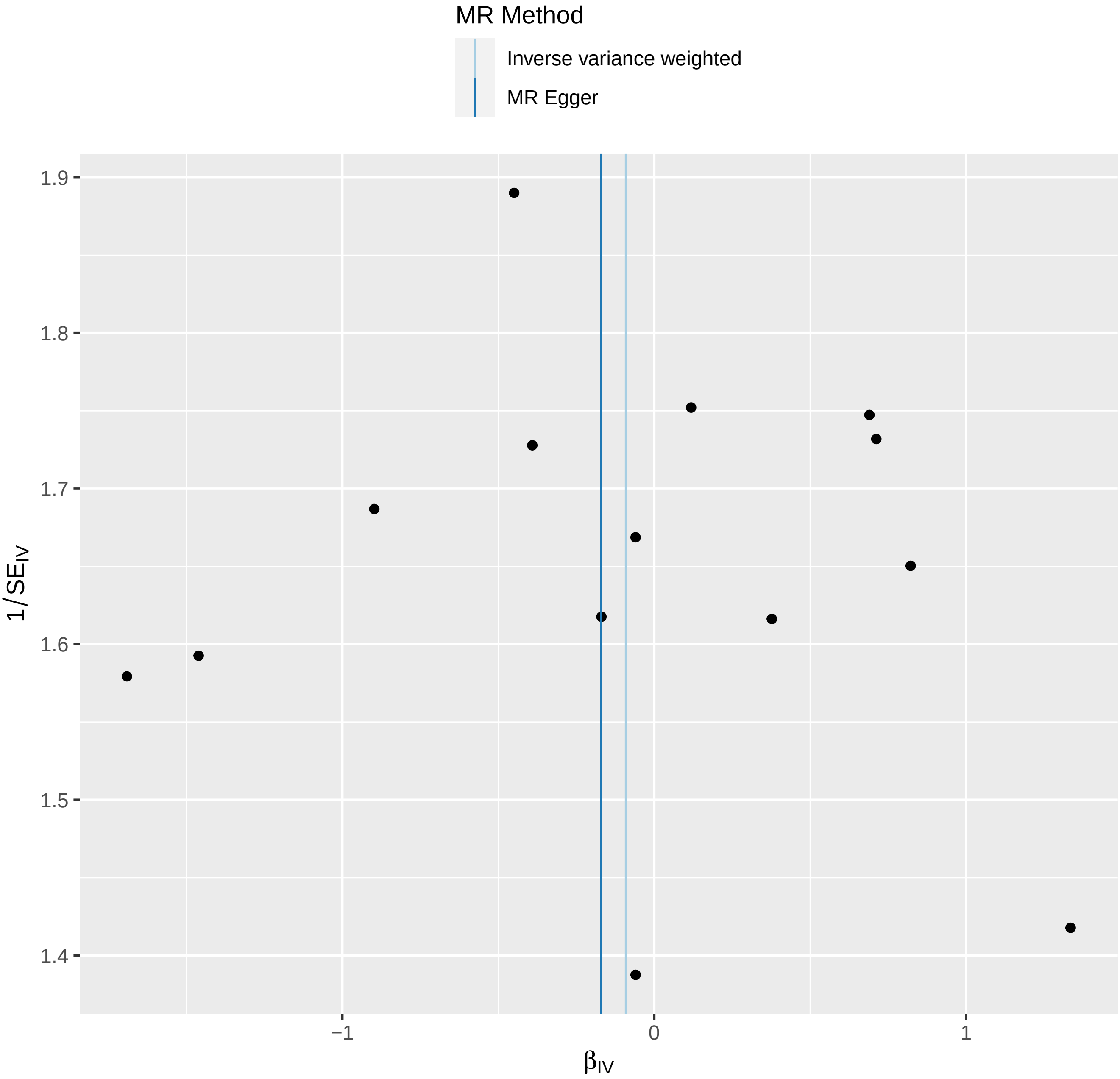

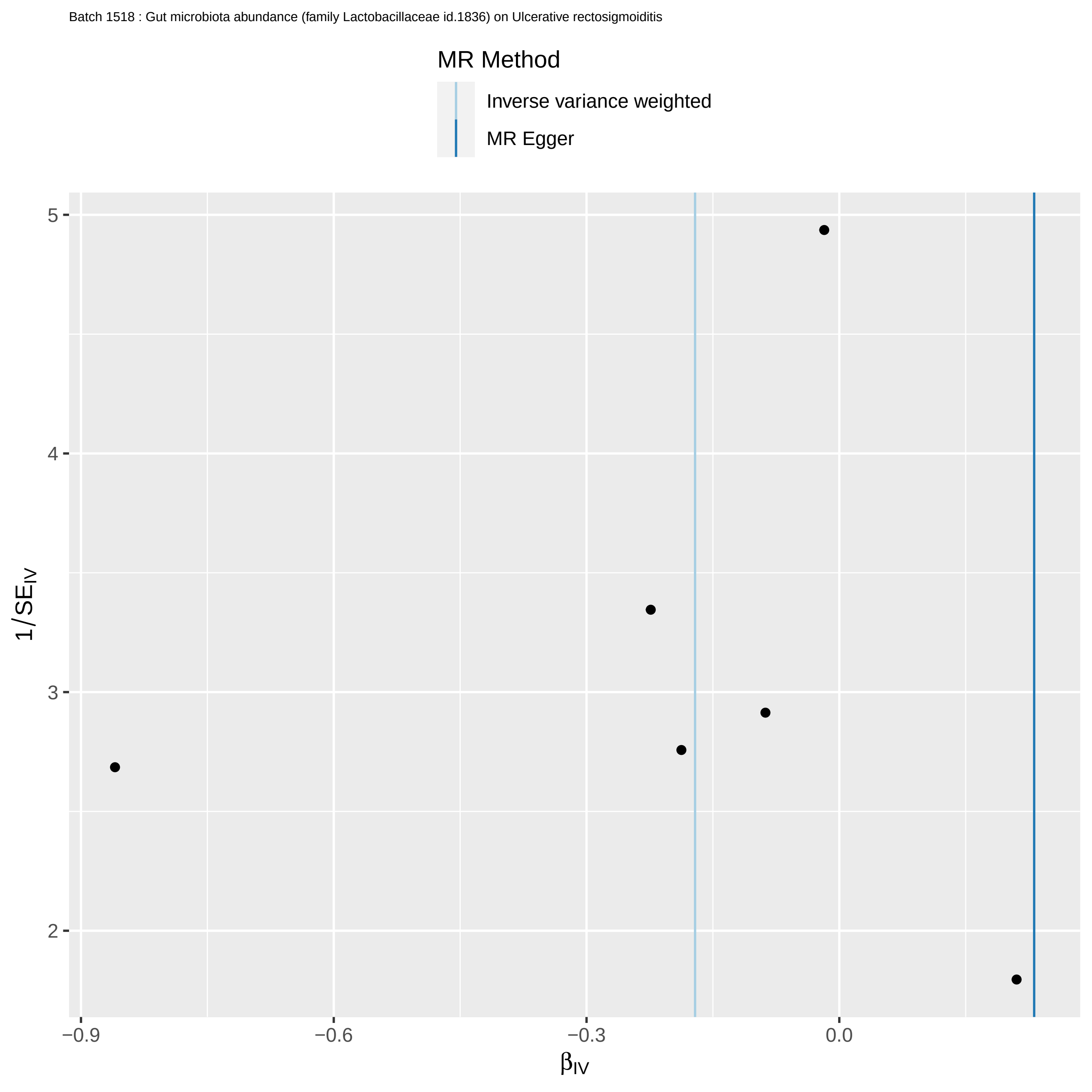
Batch 1518 : Gut microbiota abundance (family Lactobacillaceae id.1836) on Ulcerative rectosigmoiditis
MR Method
Inverse variance weighted
MR Egger
5
4
3
2
1/SE_IV
−0.9
−0.6
−0.3
0.0
β_IV

Batch 1519 : Gut microbiota abundance (family Methanobacteriaceae id.121) on Ulcerative rectosigmoiditis

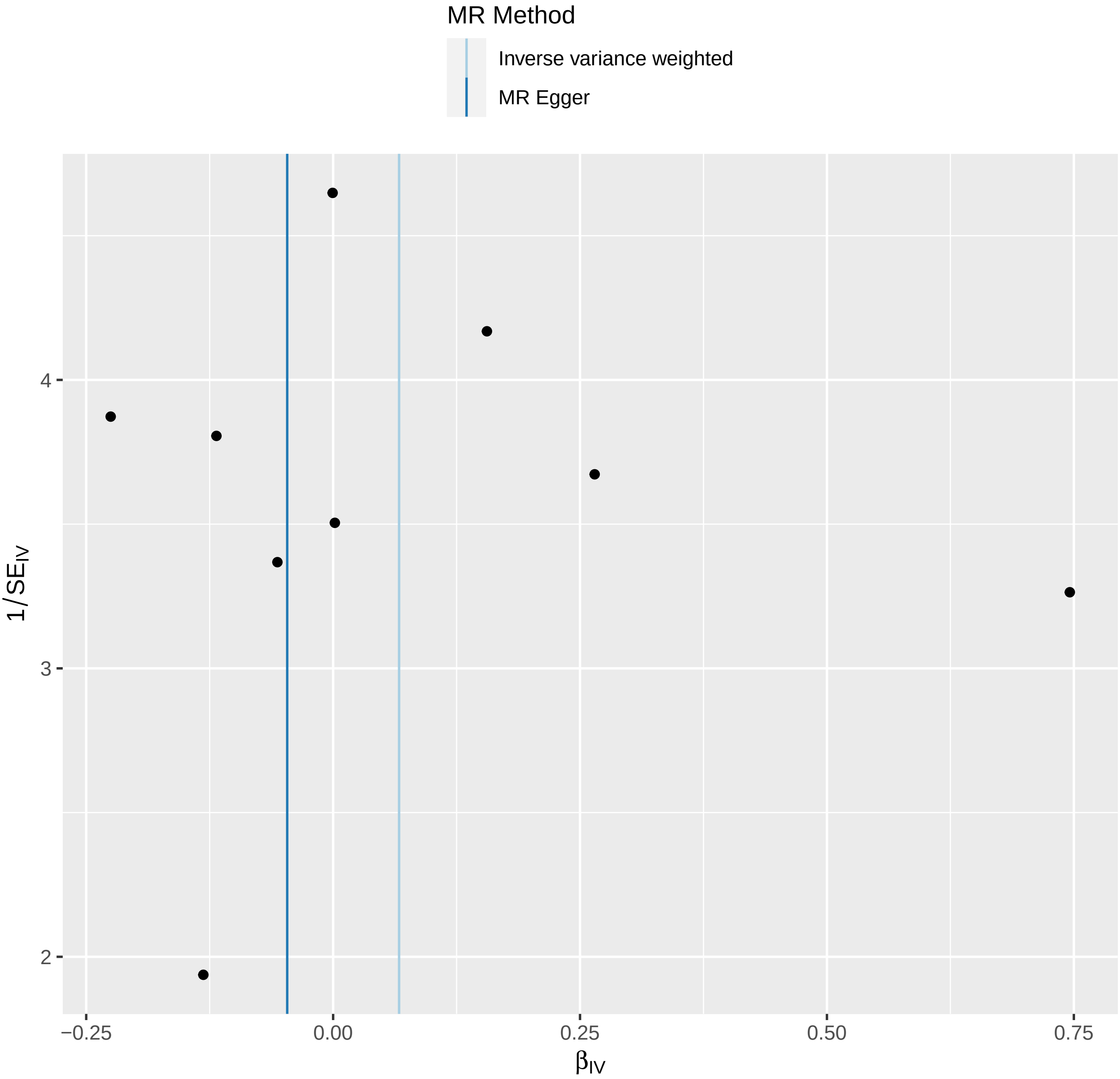

Batch 1520 : Gut microbiota abundance (family Oxalobacteraceae id.2966) on Ulcerative rectosigmoiditis

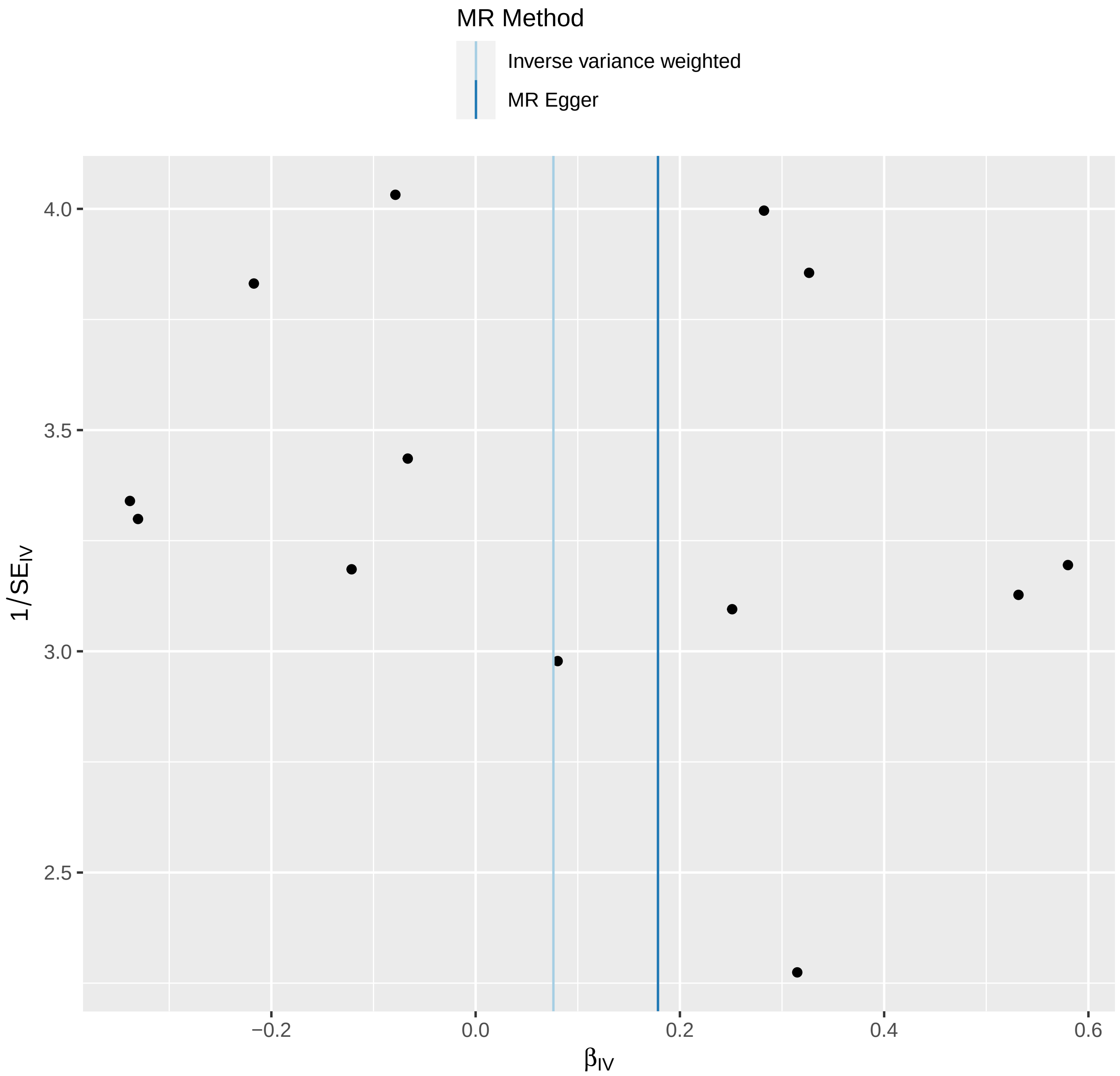

Batch 1521 : Gut microbiota abundance (family Pasteurellaceae id.3689) on Ulcerative rectosigmoiditis

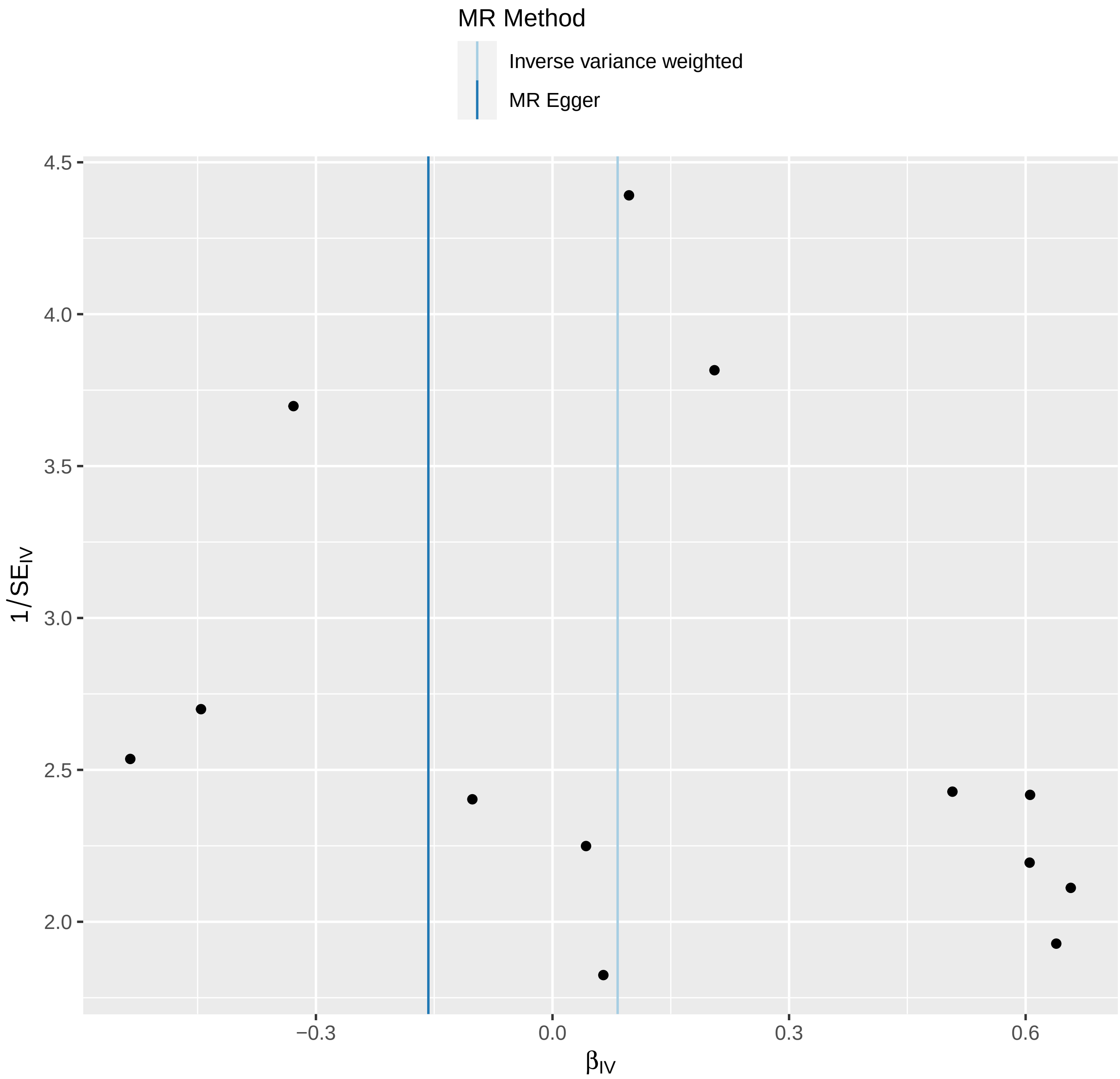

Batch 1522 : Gut microbiota abundance (family Peptococcaceae id.2024) on Ulcerative rectosigmoiditis

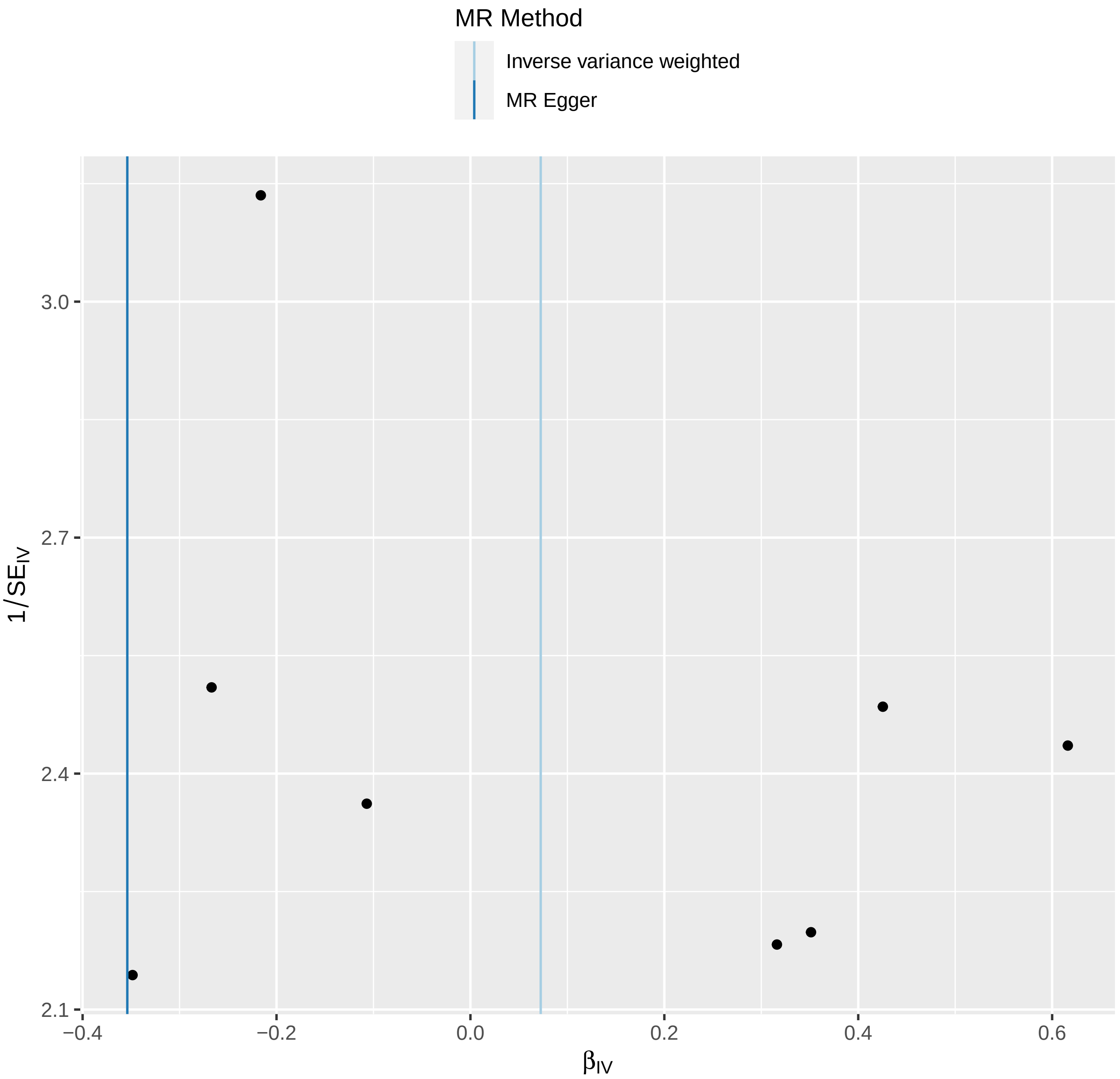

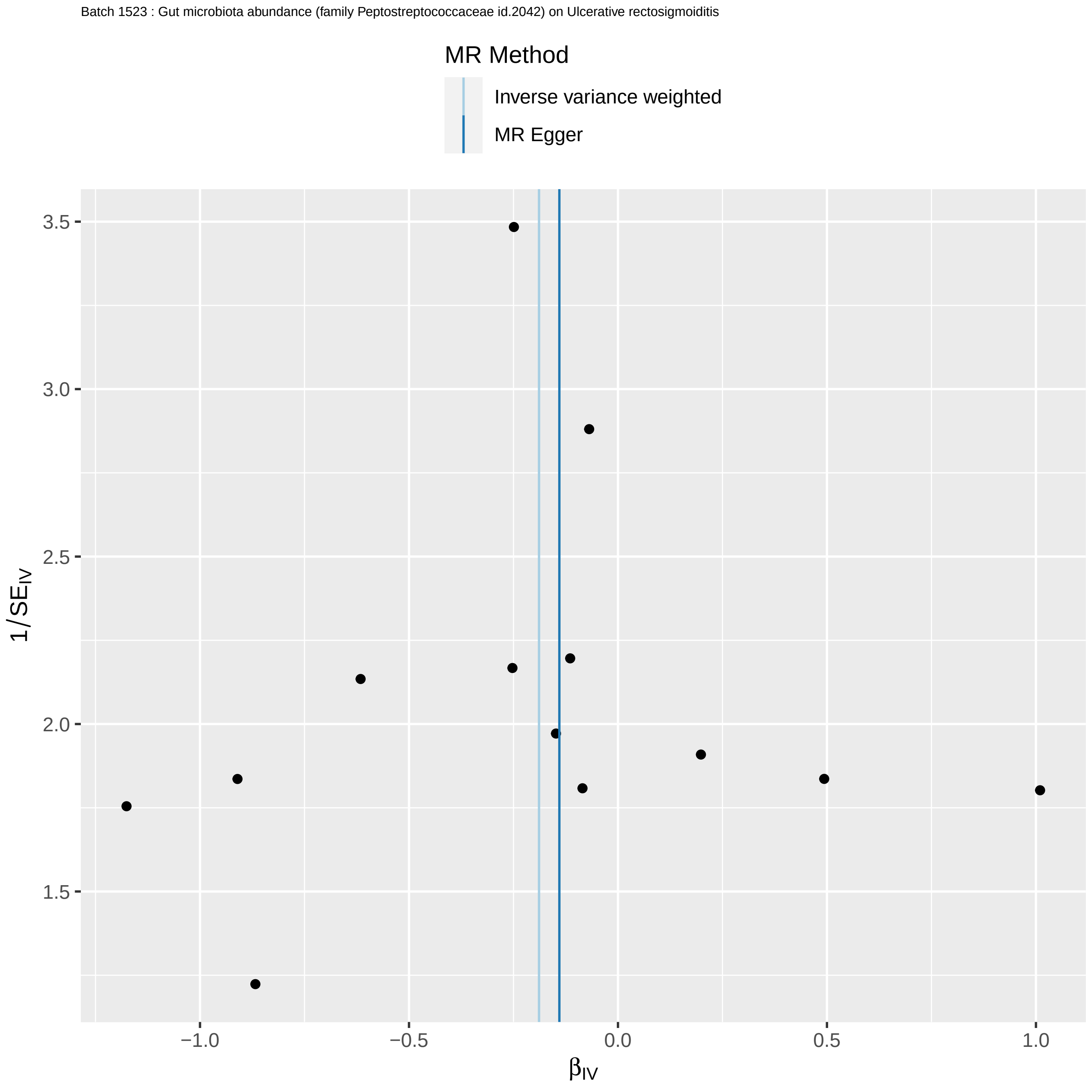

Batch 1523 : Gut microbiota abundance (family Peptostreptococcaceae id.2042) on Ulcerative rectosigmoiditis
MR Method
Inverse variance weighted
MR Egger
3.5
3.0
2.5
2.0
1.5
1/SE_IV
−1.0
−0.5
0.0
0.5
1.0
β_IV

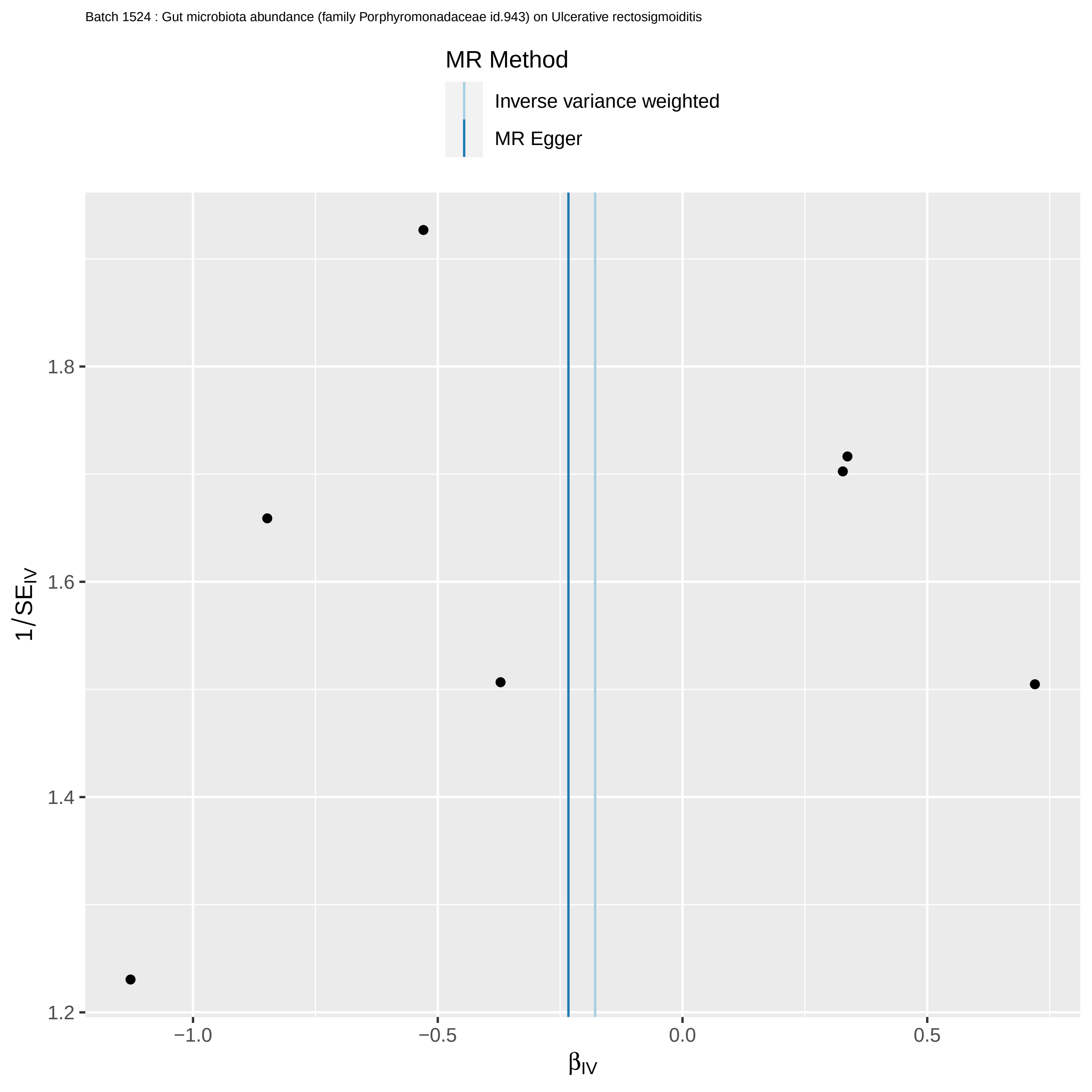
Batch 1524 : Gut microbiota abundance (family Porphyromonadaceae id.943) on Ulcerative rectosigmoiditis
MR Method
Inverse variance weighted
MR Egger
1.8
1.6
1.4
1.2
1/SE$_{IV}$
−1.0
−0.5
0.0
0.5
β_{IV}

Batch 1525 : Gut microbiota abundance (family Prevotellaceae id.960) on Ulcerative rectosigmoiditis

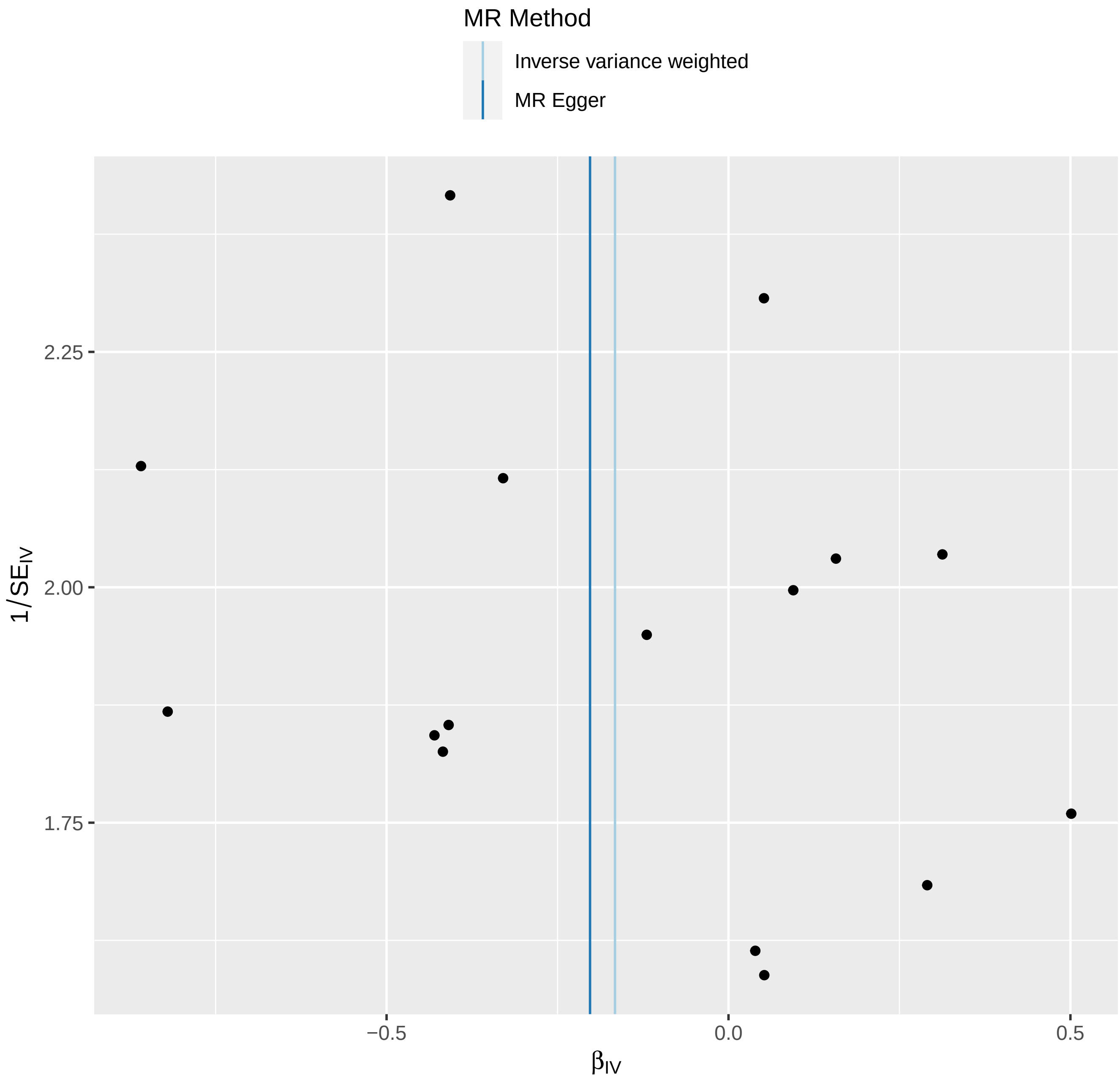

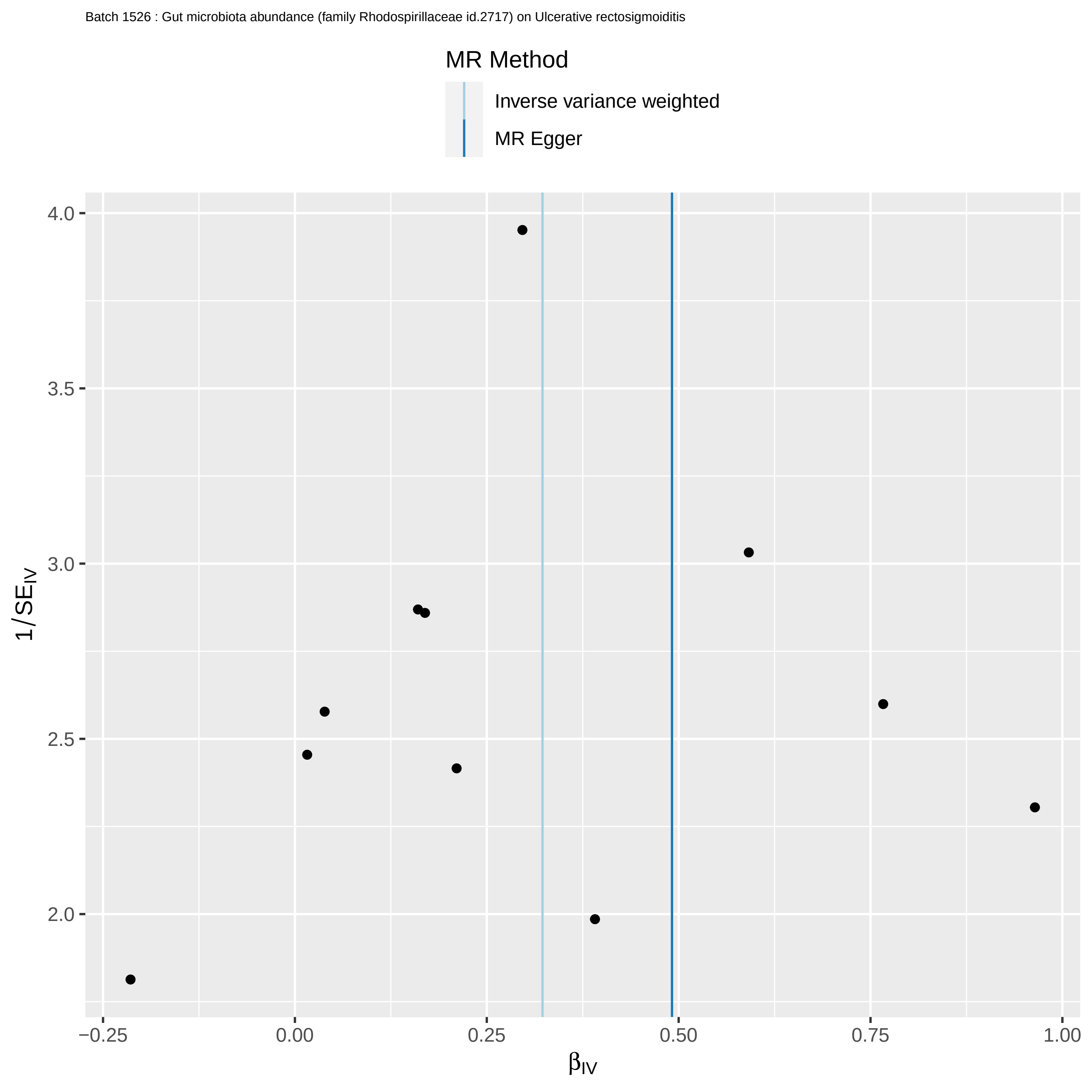

Batch 1526 : Gut microbiota abundance (family Rhodospirillaceae id.2717) on Ulcerative rectosigmoiditis
MR Method
Inverse variance weighted
MR Egger
4.0
3.5
3.0
2.5
2.0
1/SE_IV
−0.25
0.00
0.25
0.50
0.75
1.00
β_IV

Batch 1527 : Gut microbiota abundance (family Rikenellaceae id.967) on Ulcerative rectosigmoiditis

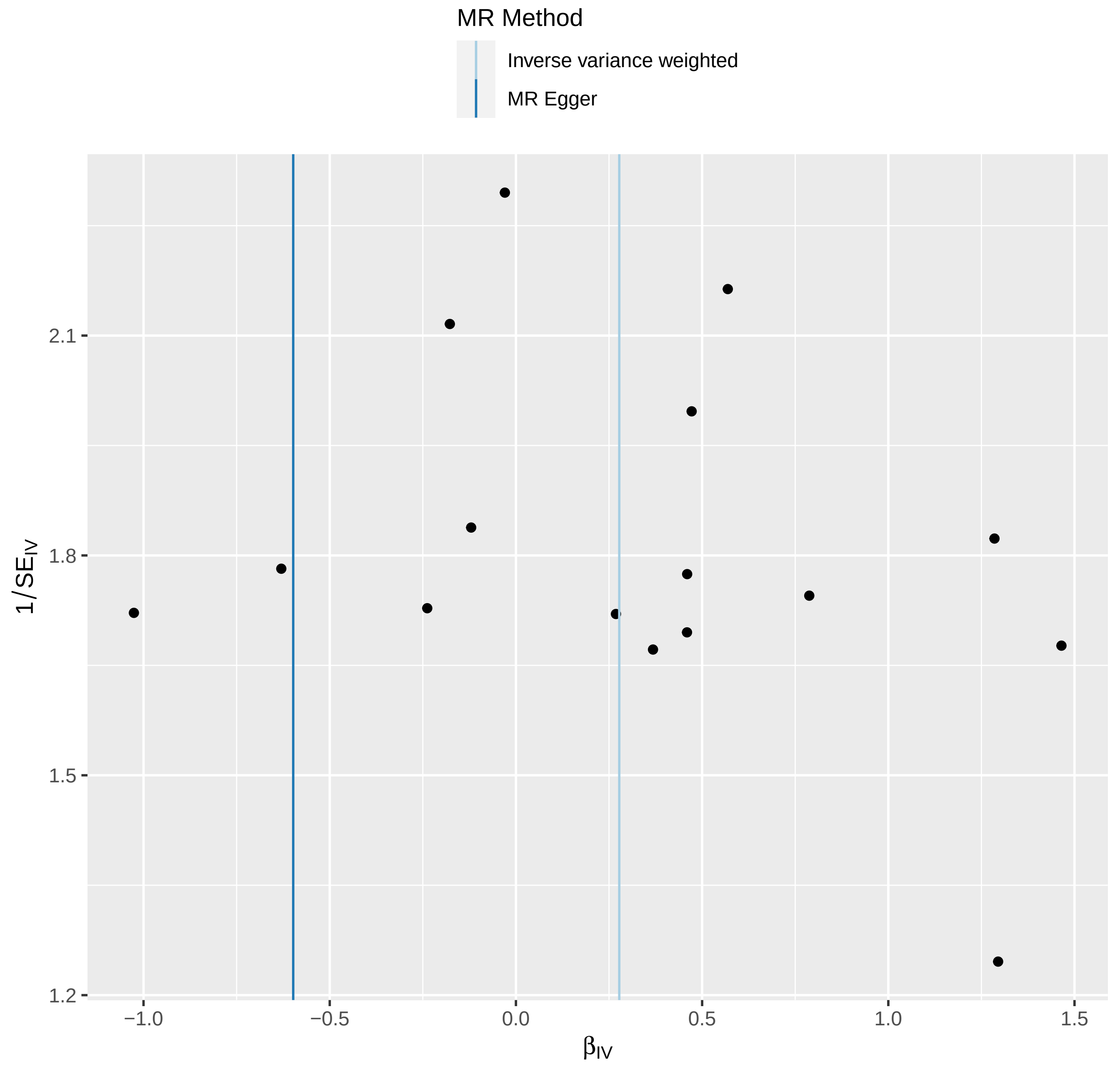

Batch 1528 : Gut microbiota abundance (family Ruminococcaceae id.2050) on Ulcerative rectosigmoiditis

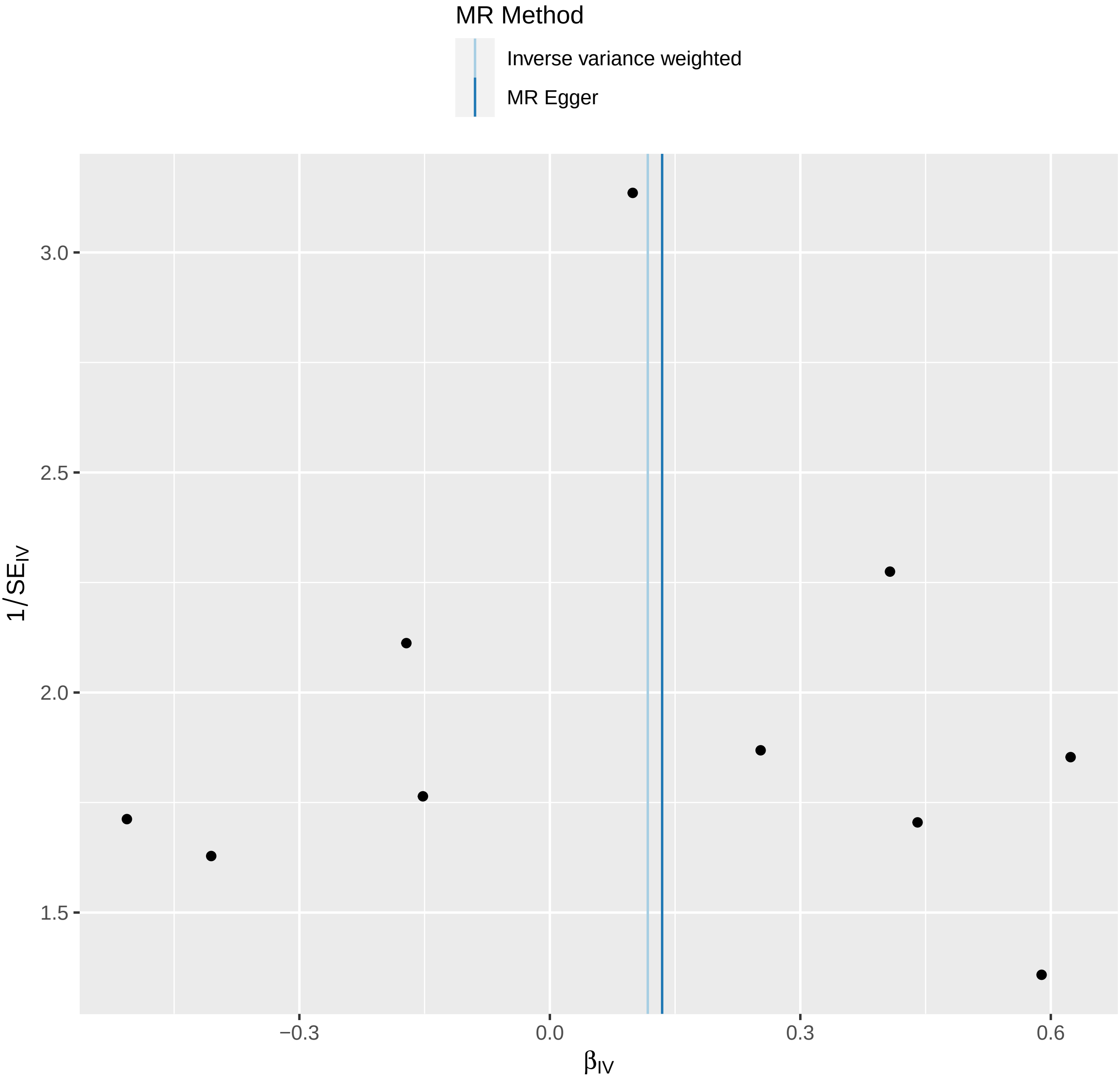

Batch 1529 : Gut microbiota abundance (family Streptococcaceae id.1850) on Ulcerative rectosigmoiditis

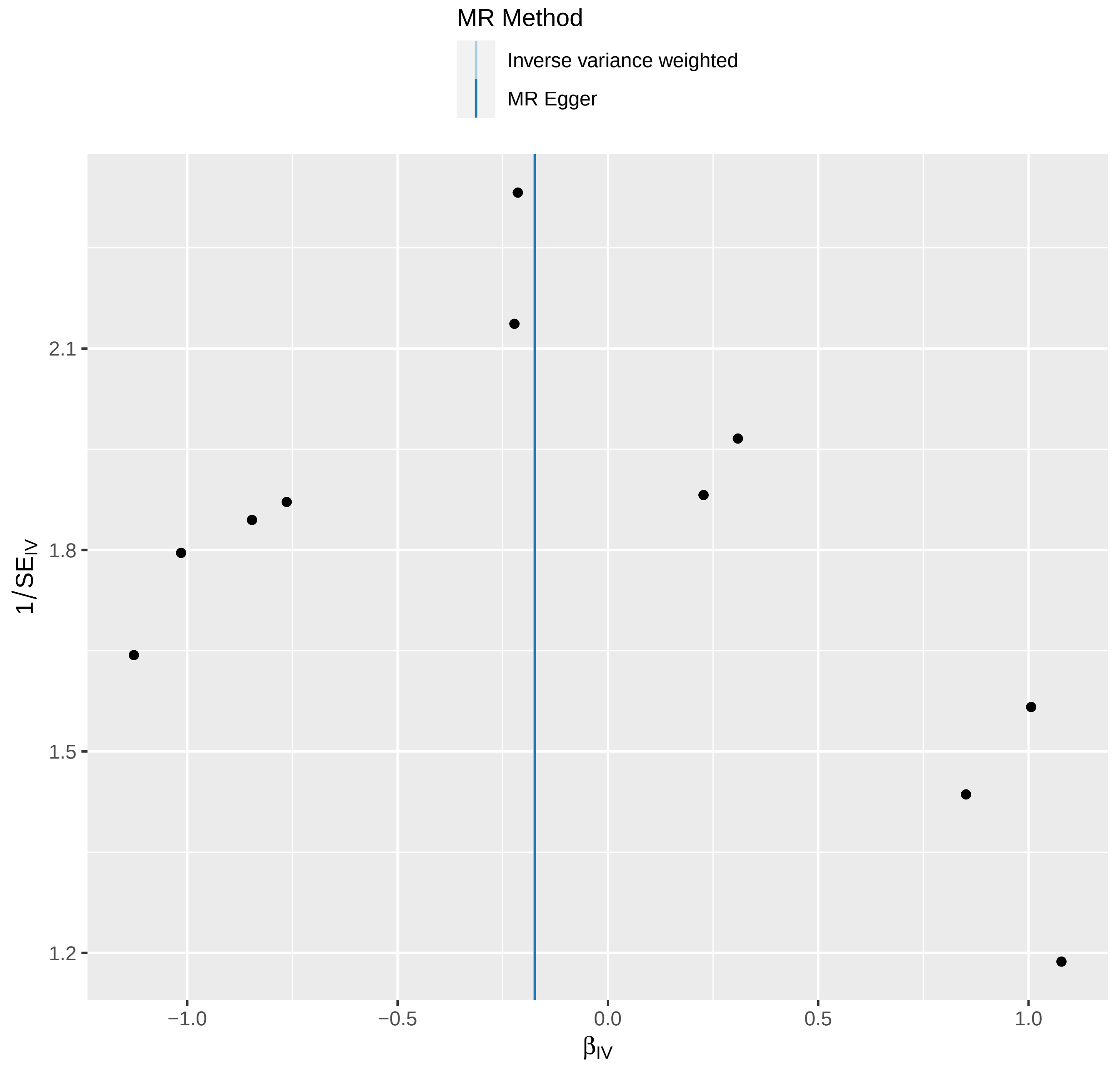

Batch 1530 : Gut microbiota abundance (family Veillonellaceae id.2172) on Ulcerative rectosigmoiditis

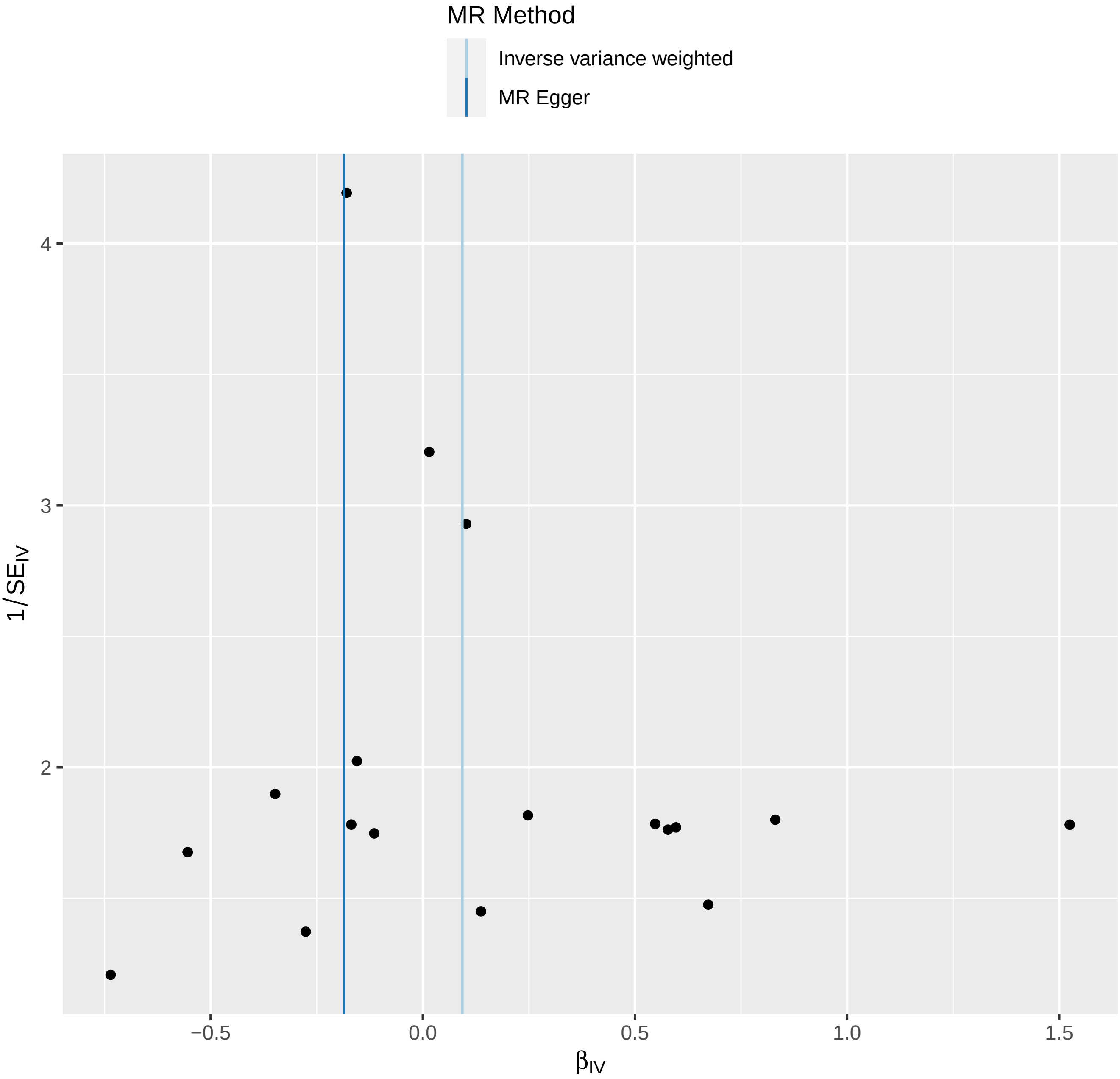

Batch 1531 : Gut microbiota abundance (family Verrucomicrobiaceae id.4036) on Ulcerative rectosigmoiditis

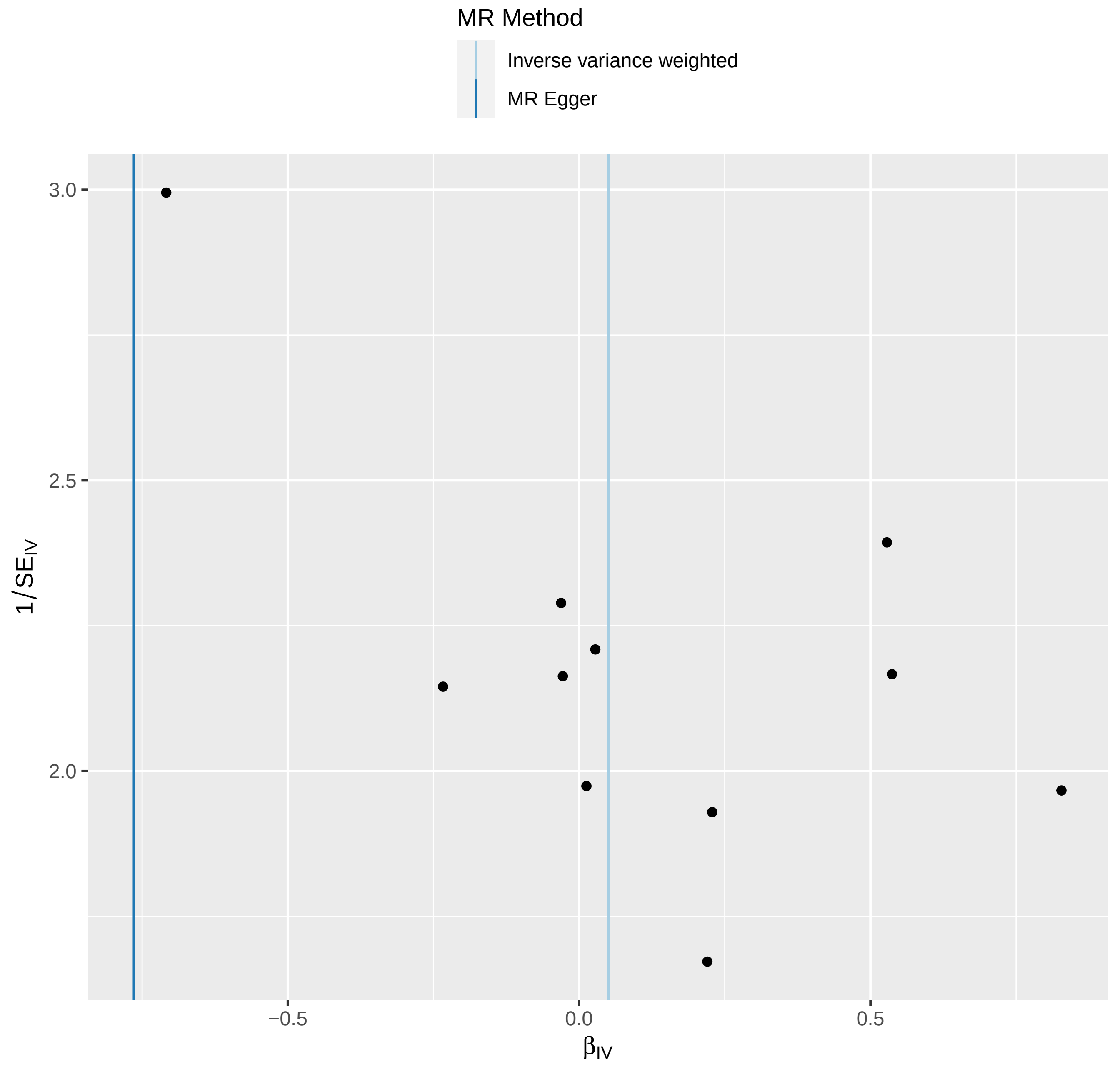

Batch 1532 : Gut microbiota abundance (family Victivallaceae id.2255) on Ulcerative rectosigmoiditis

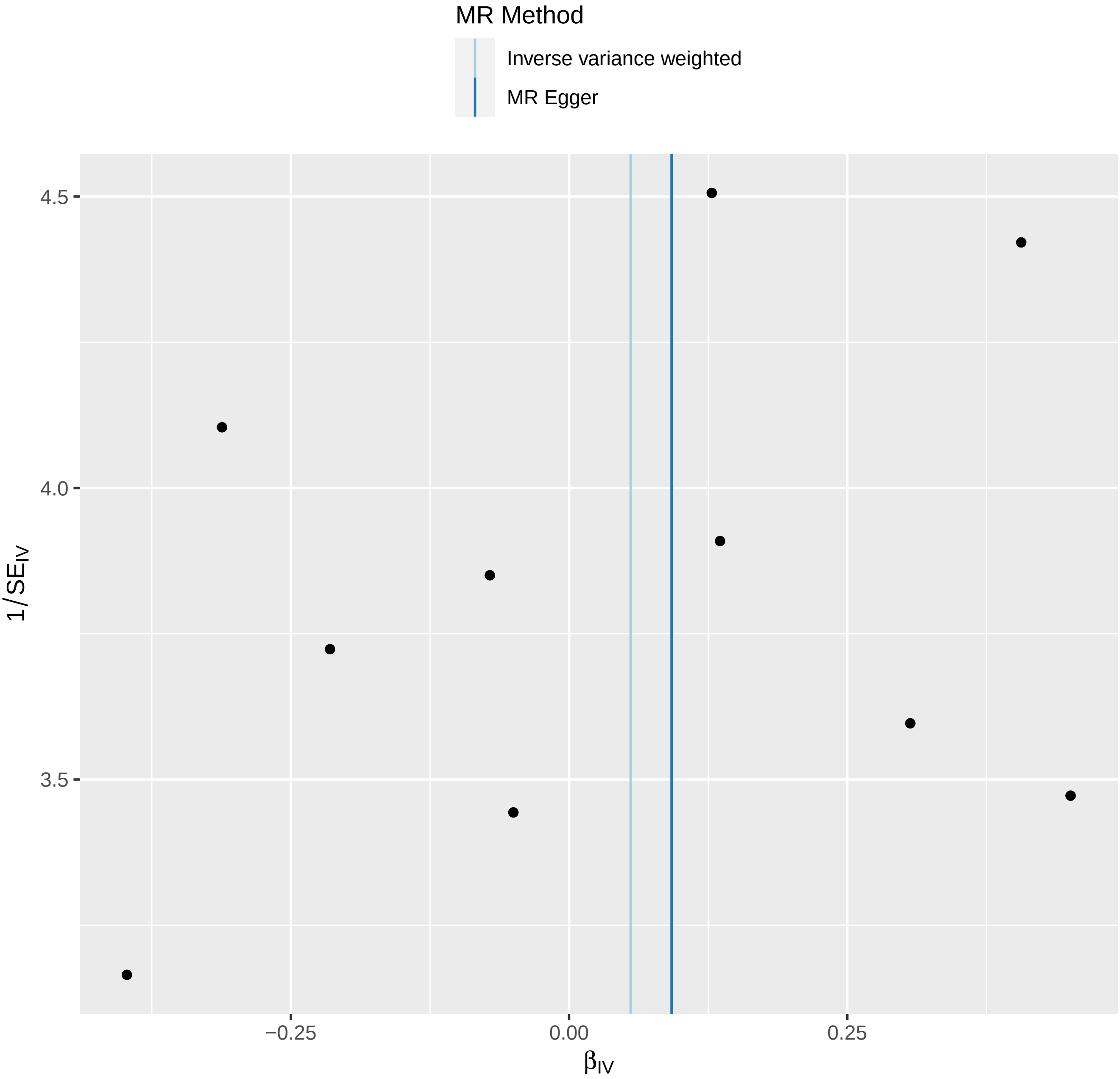

Batch 1533 : Gut microbiota abundance (genus Actinomyces id.423) on Ulcerative rectosigmoiditis

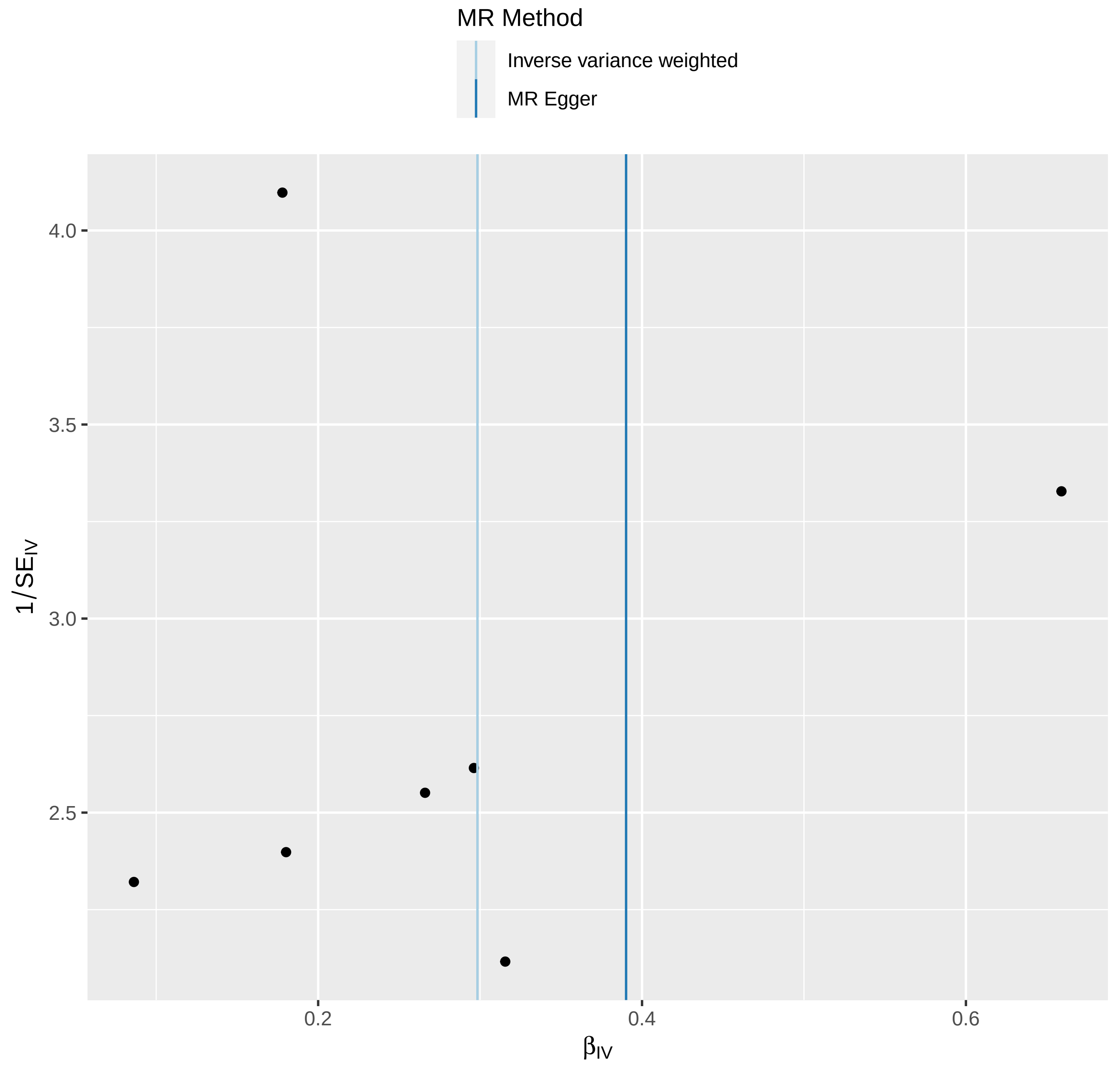

Batch 1534 : Gut microbiota abundance (genus Adlercreutzia id.812) on Ulcerative rectosigmoiditis

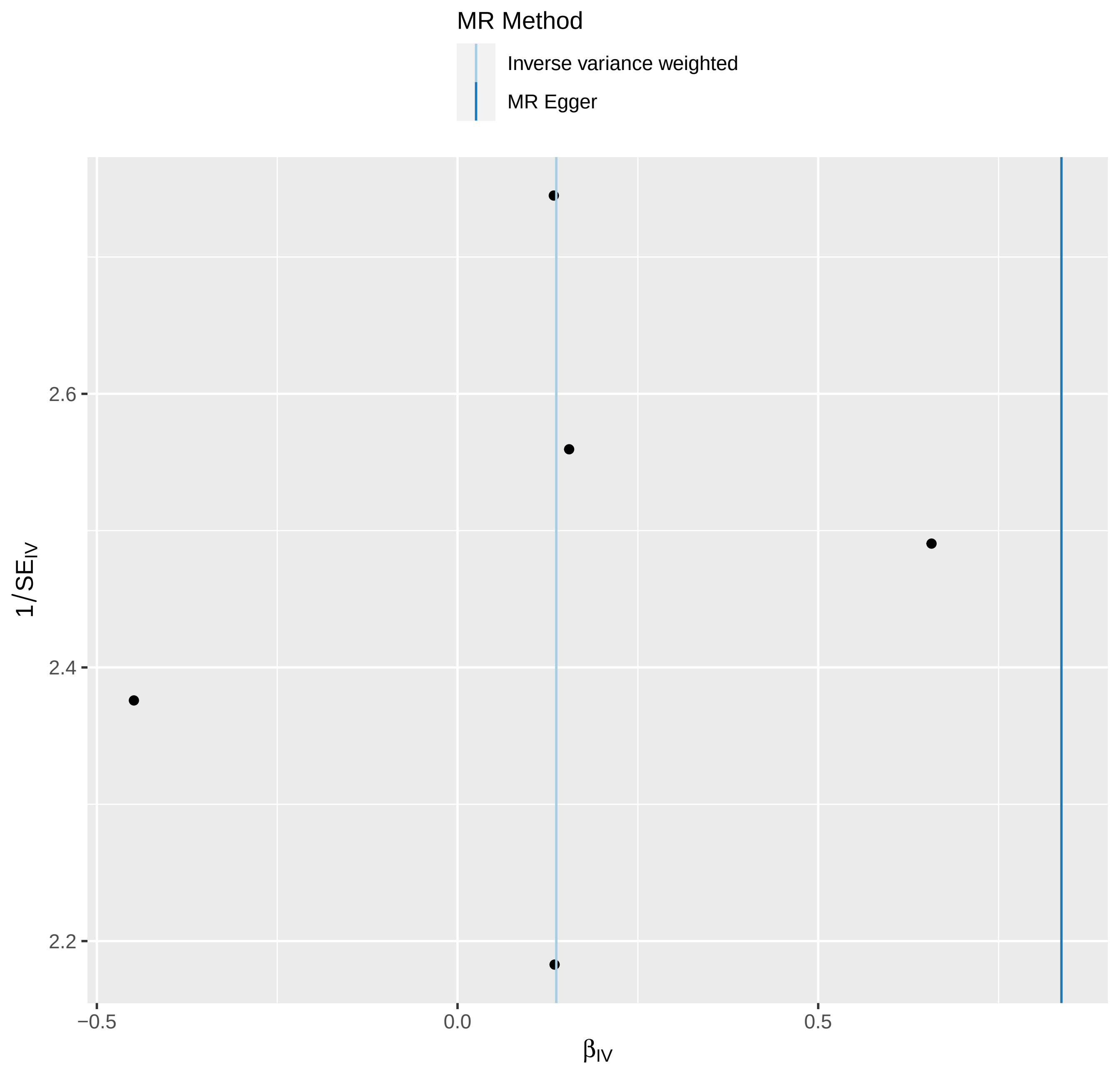

Batch 1535 : Gut microbiota abundance (genus Akkermansia id.4037) on Ulcerative rectosigmoiditis

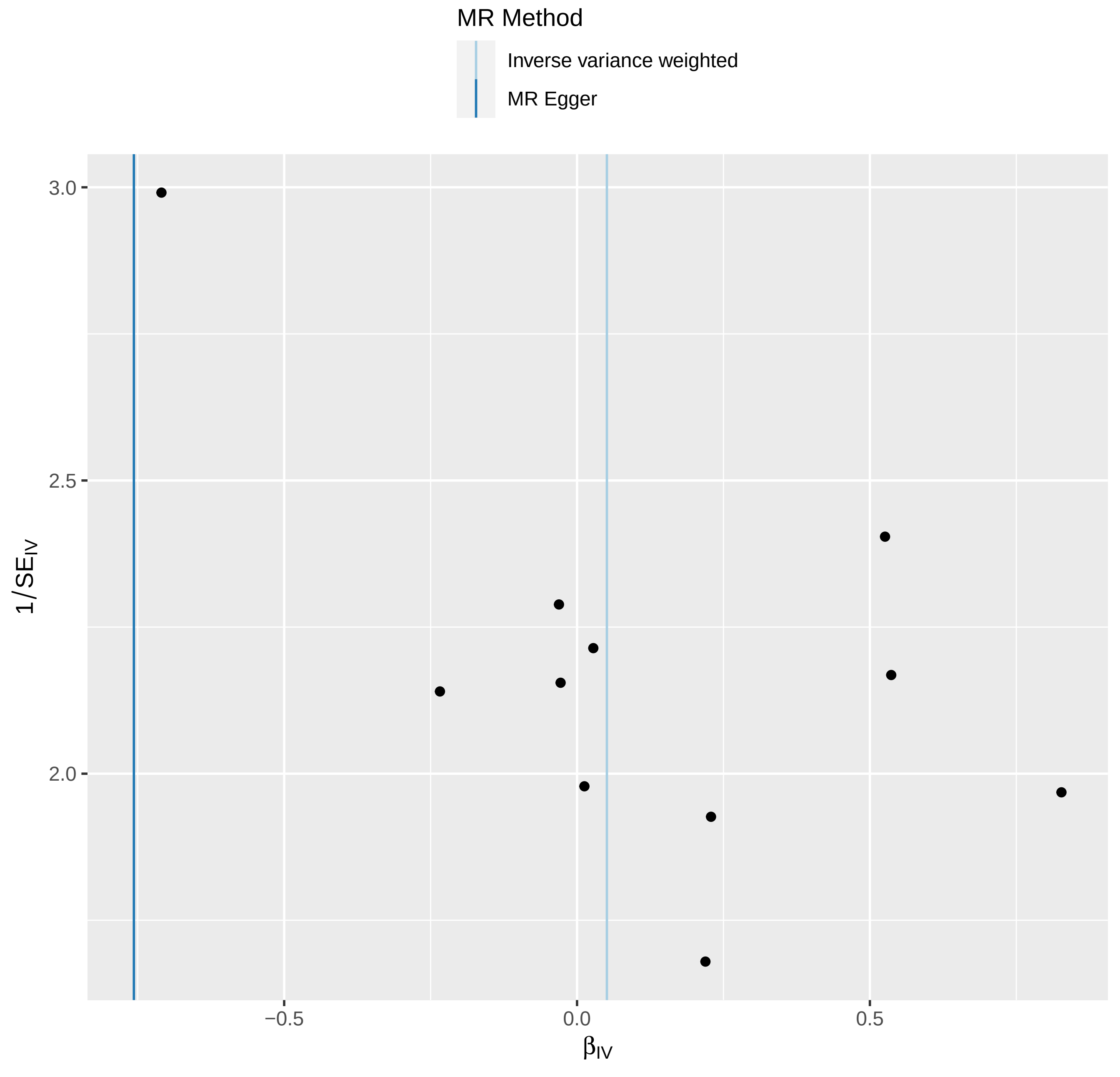

Batch 1536 : Gut microbiota abundance (genus Alistipes id.968) on Ulcerative rectosigmoiditis

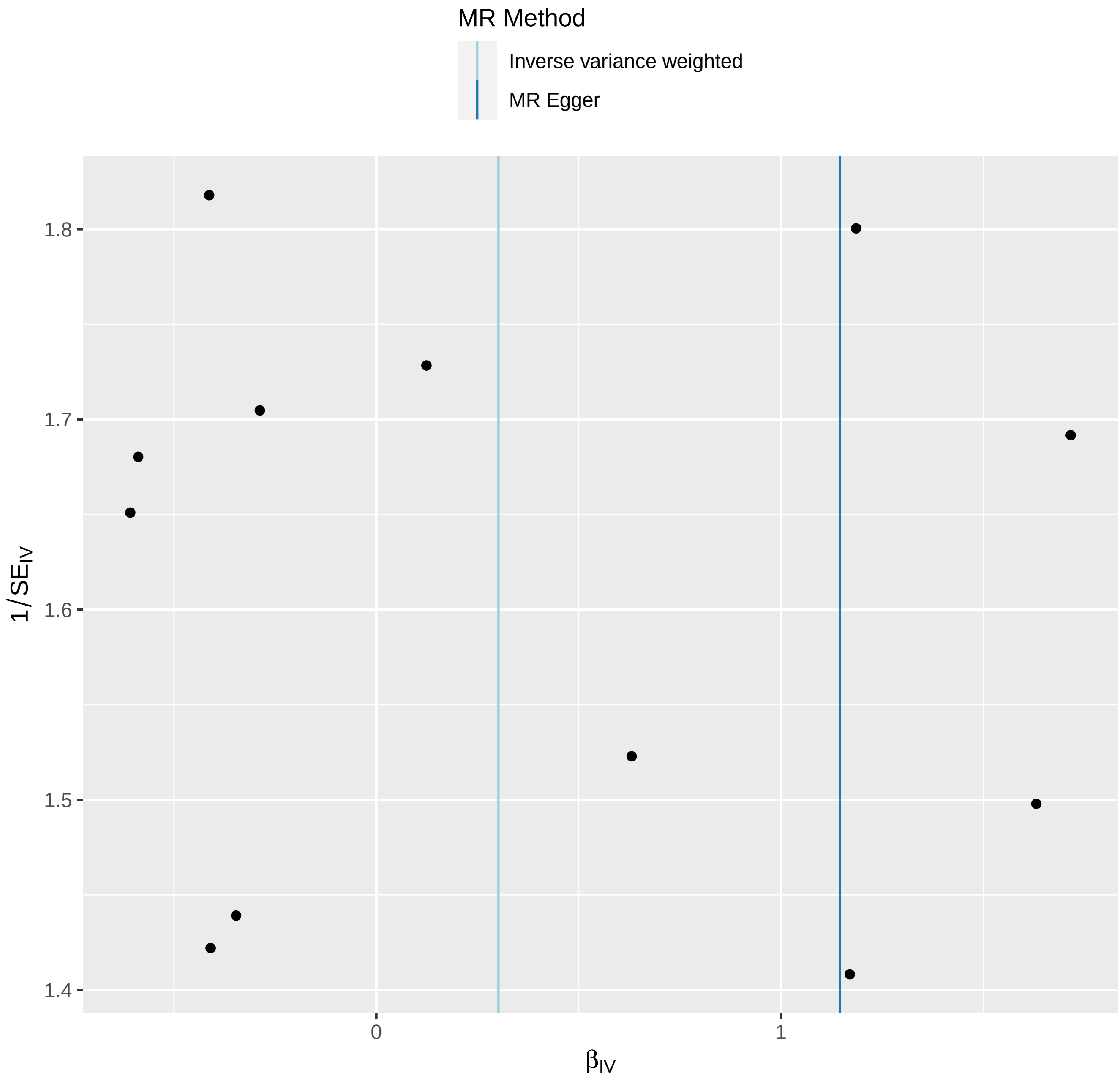

Batch 1537 : Gut microbiota abundance (genus Allisonella id.2174) on Ulcerative rectosigmoiditis

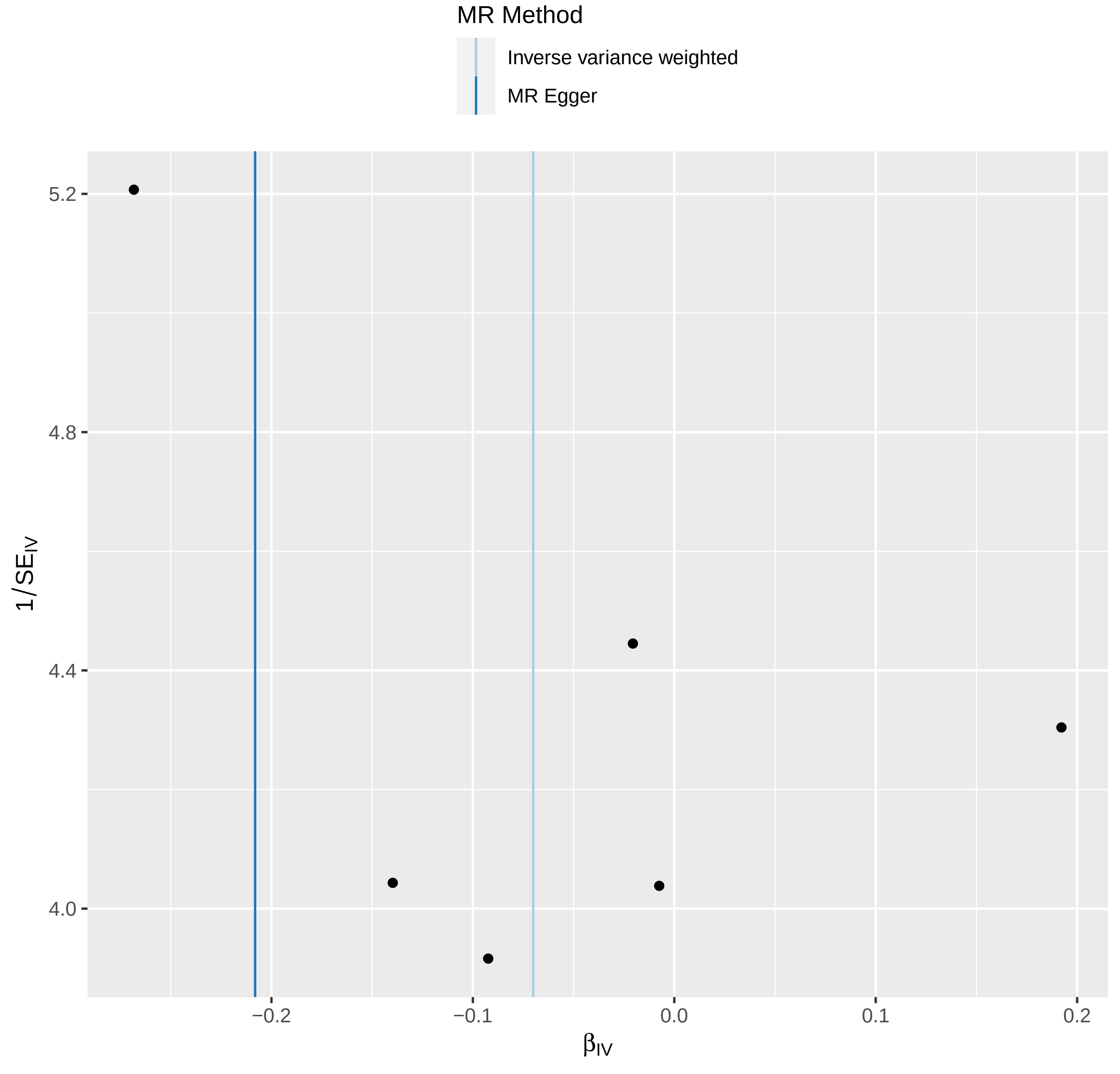

Batch 1538 : Gut microbiota abundance (genus Alloprevotella id.961) on Ulcerative rectosigmoiditis

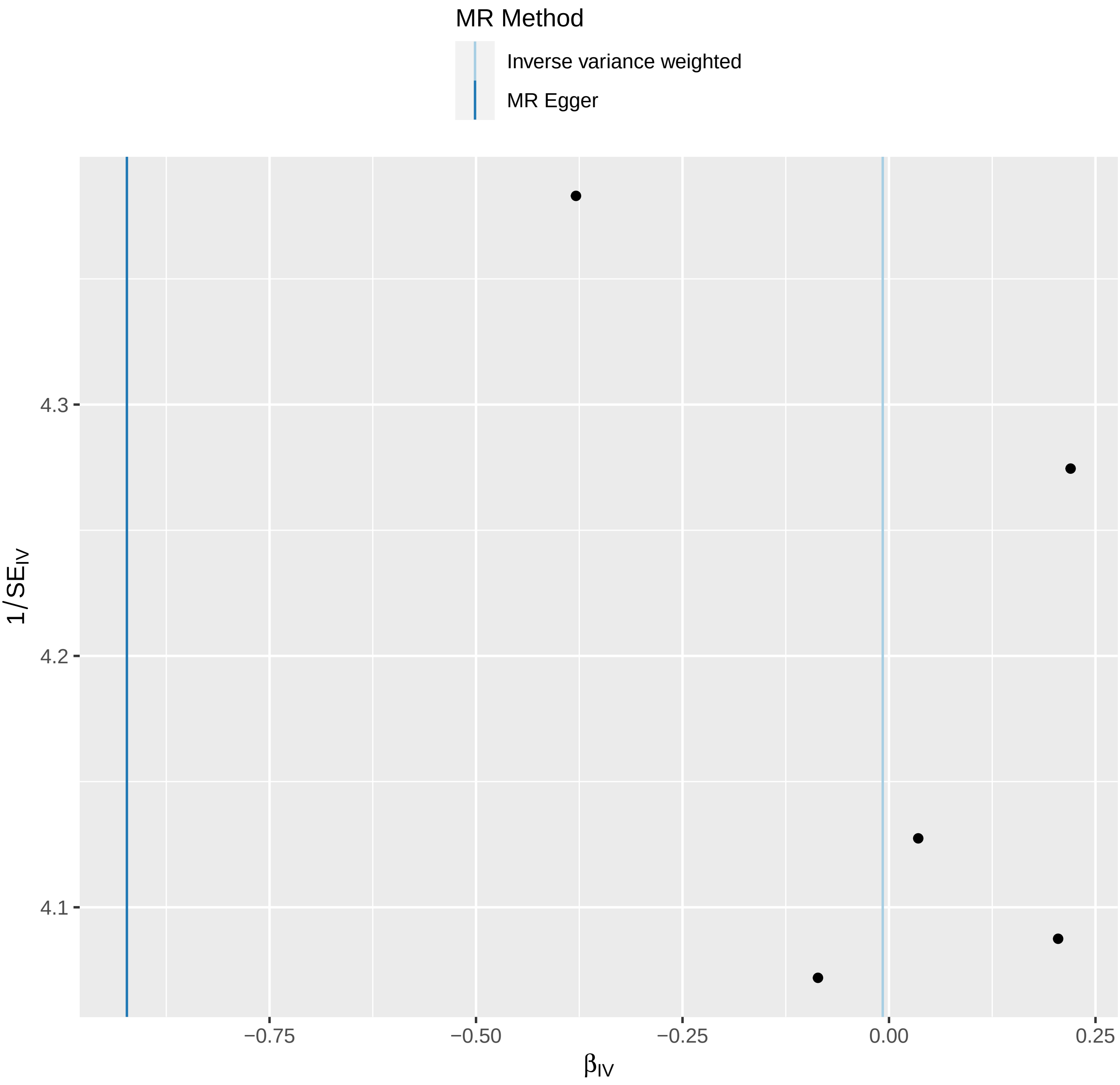

Batch 1539 : Gut microbiota abundance (genus Anaerofilum id.2053) on Ulcerative rectosigmoiditis

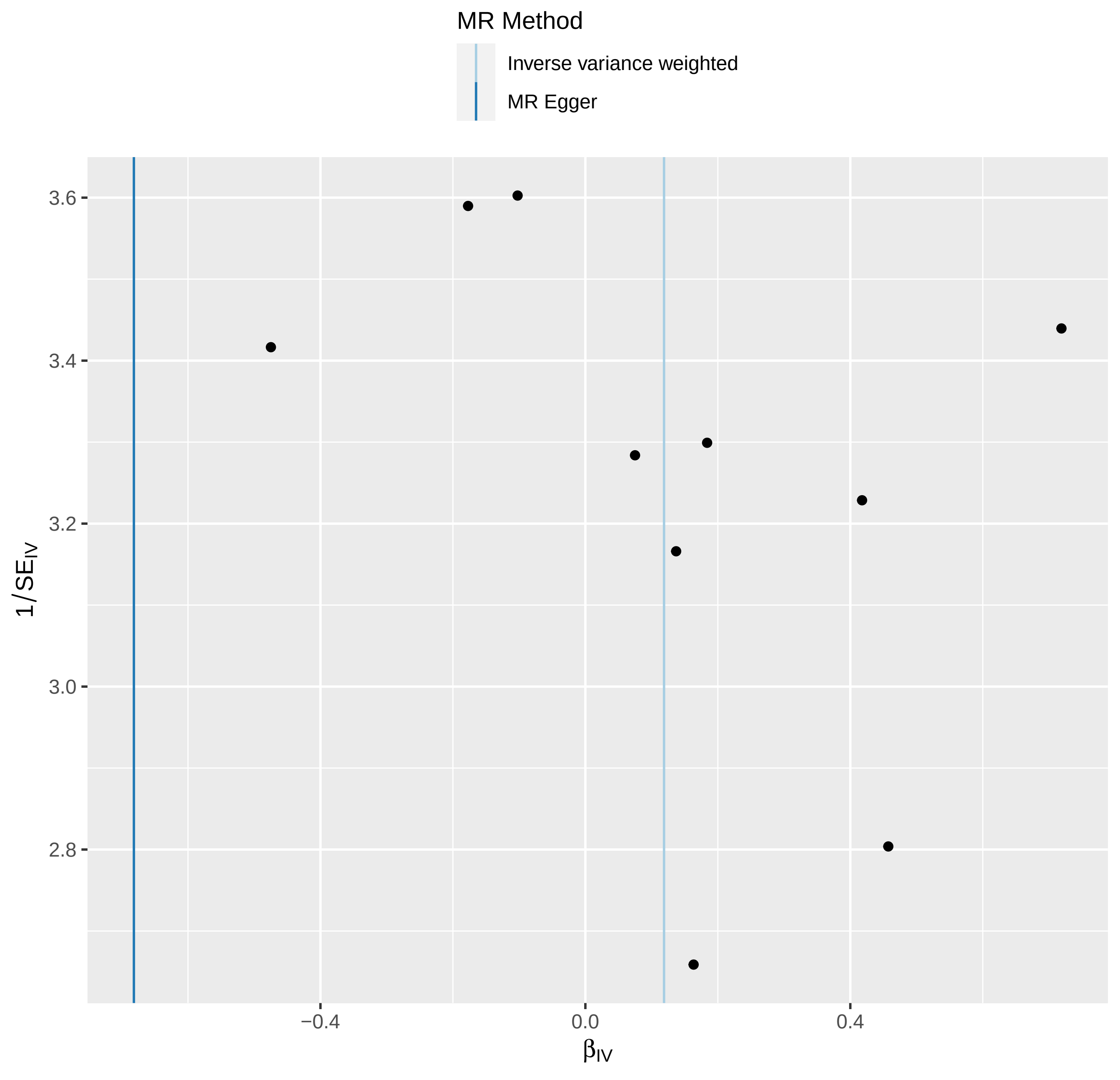

Batch 1540 : Gut microbiota abundance (genus Anaerostipes id.1991) on Ulcerative rectosigmoiditis

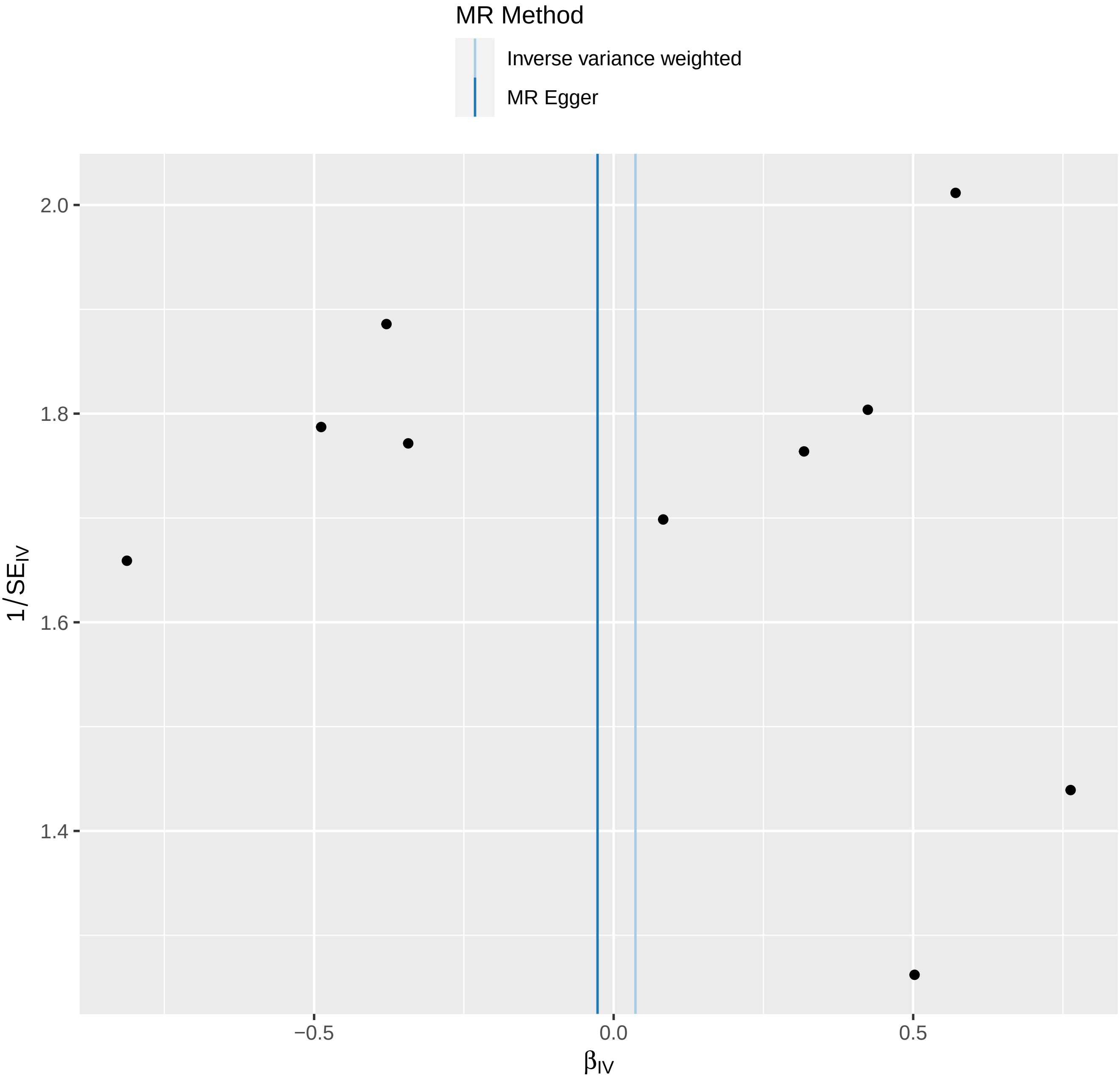

Batch 1541 : Gut microbiota abundance (genus Anaerotruncus id.2054) on Ulcerative rectosigmoiditis

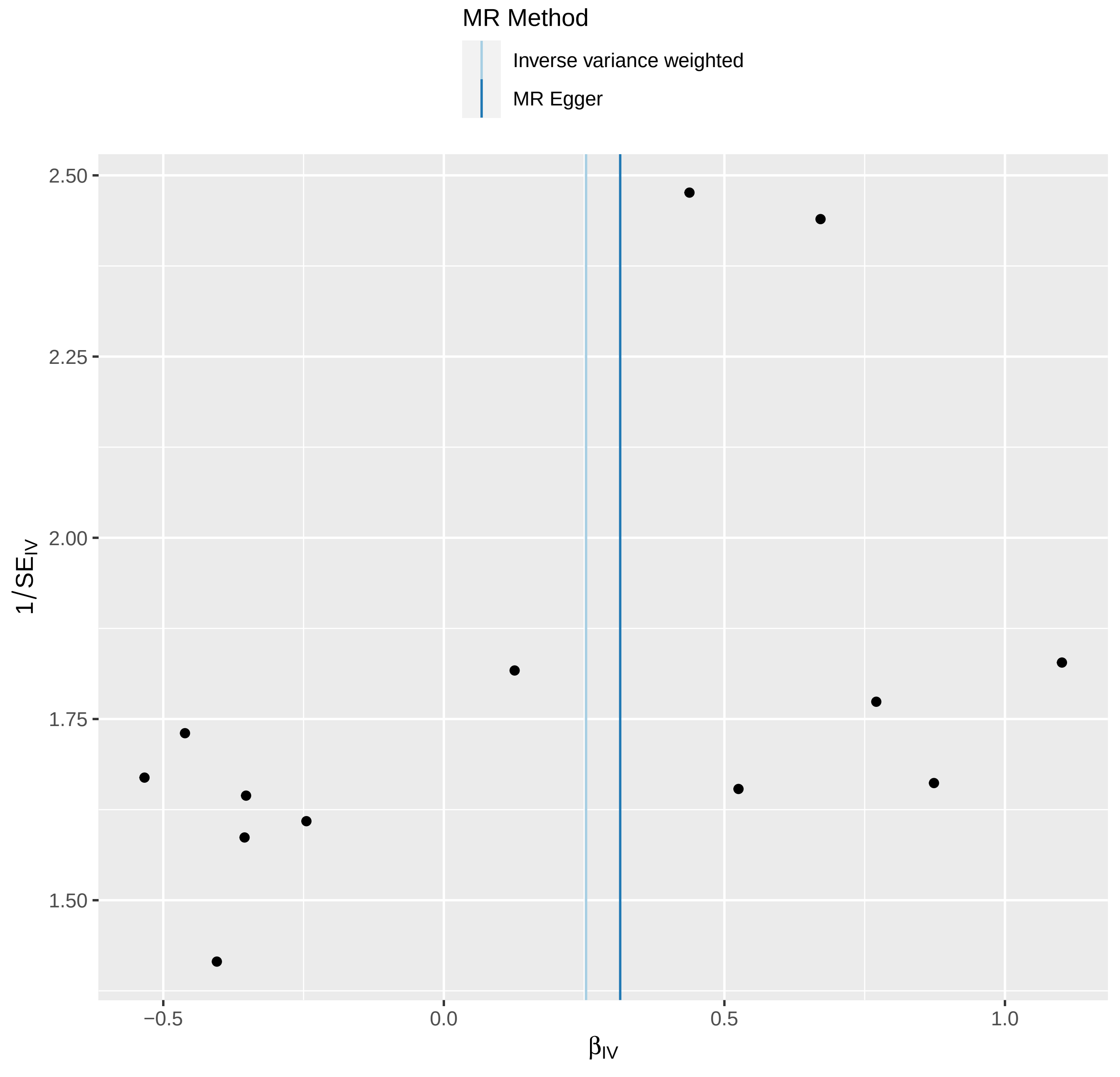

Batch 1542 : Gut microbiota abundance (genus Bacteroides id.918) on Ulcerative rectosigmoiditis

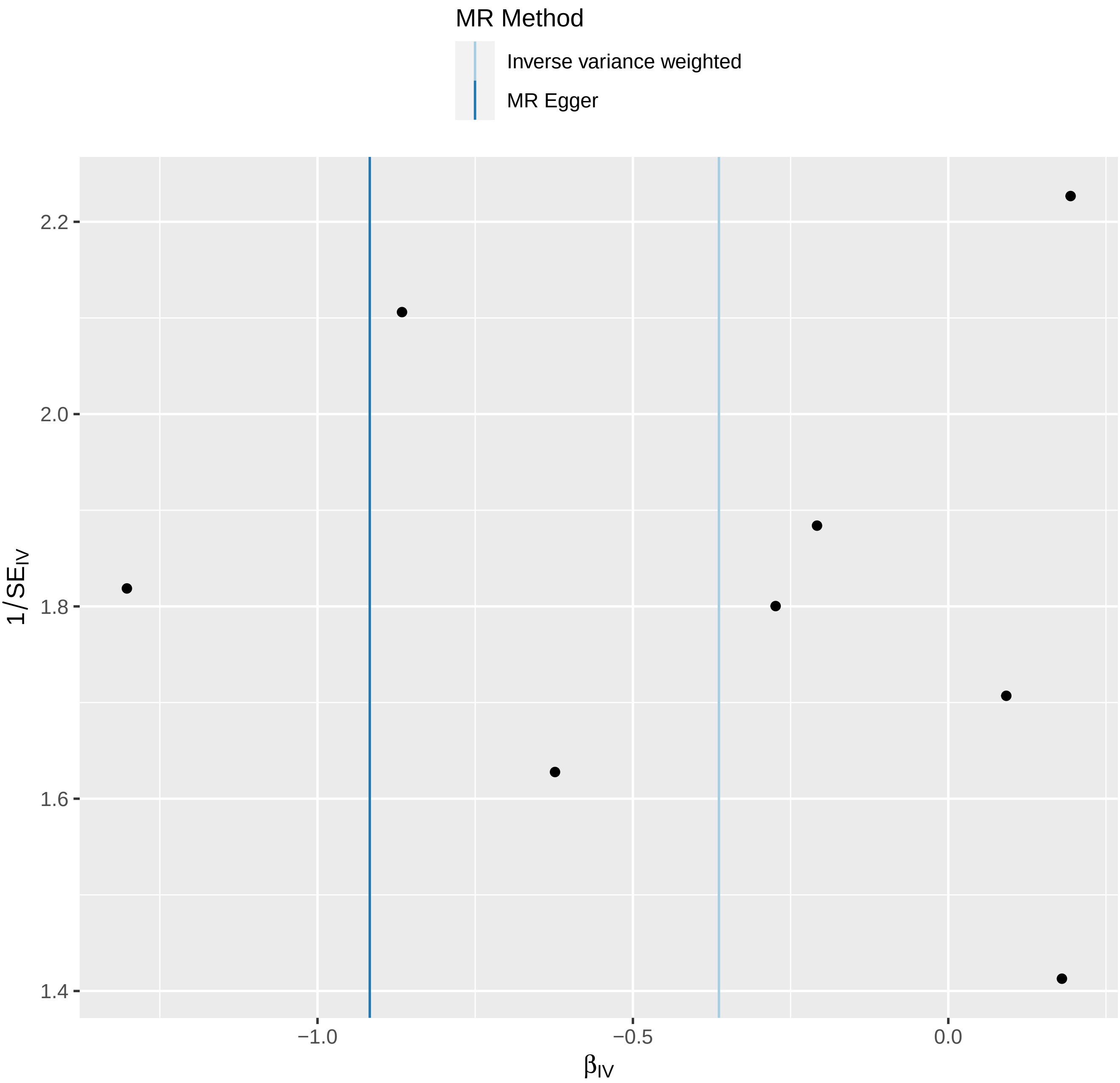

Batch 1543 : Gut microbiota abundance (genus Barnesiella id.944) on Ulcerative rectosigmoiditis

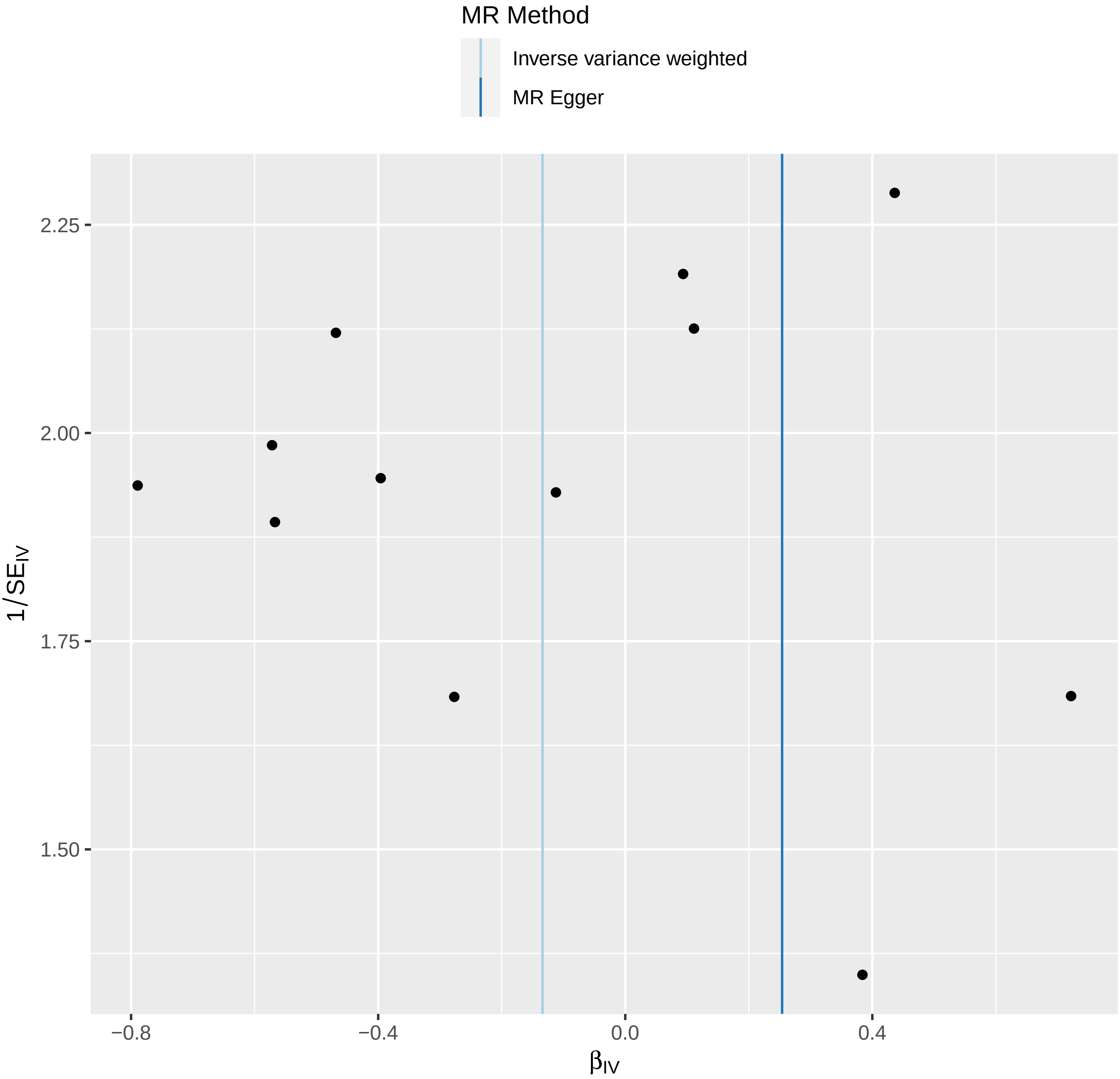

Batch 1544 : Gut microbiota abundance (genus Bifidobacterium id.436) on Ulcerative rectosigmoiditis

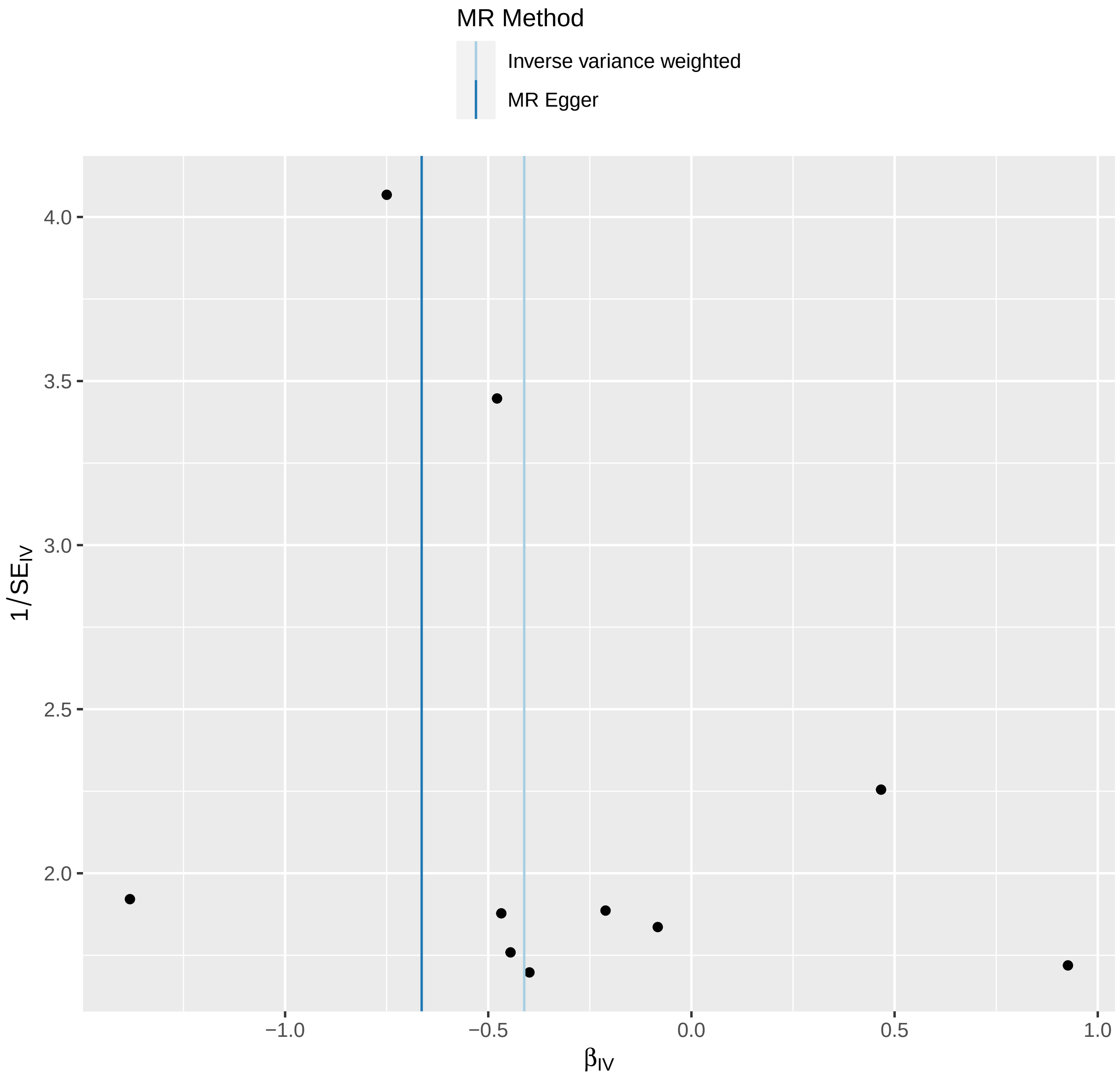

Batch 1545 : Gut microbiota abundance (genus Bilophila id.3170) on Ulcerative rectosigmoiditis

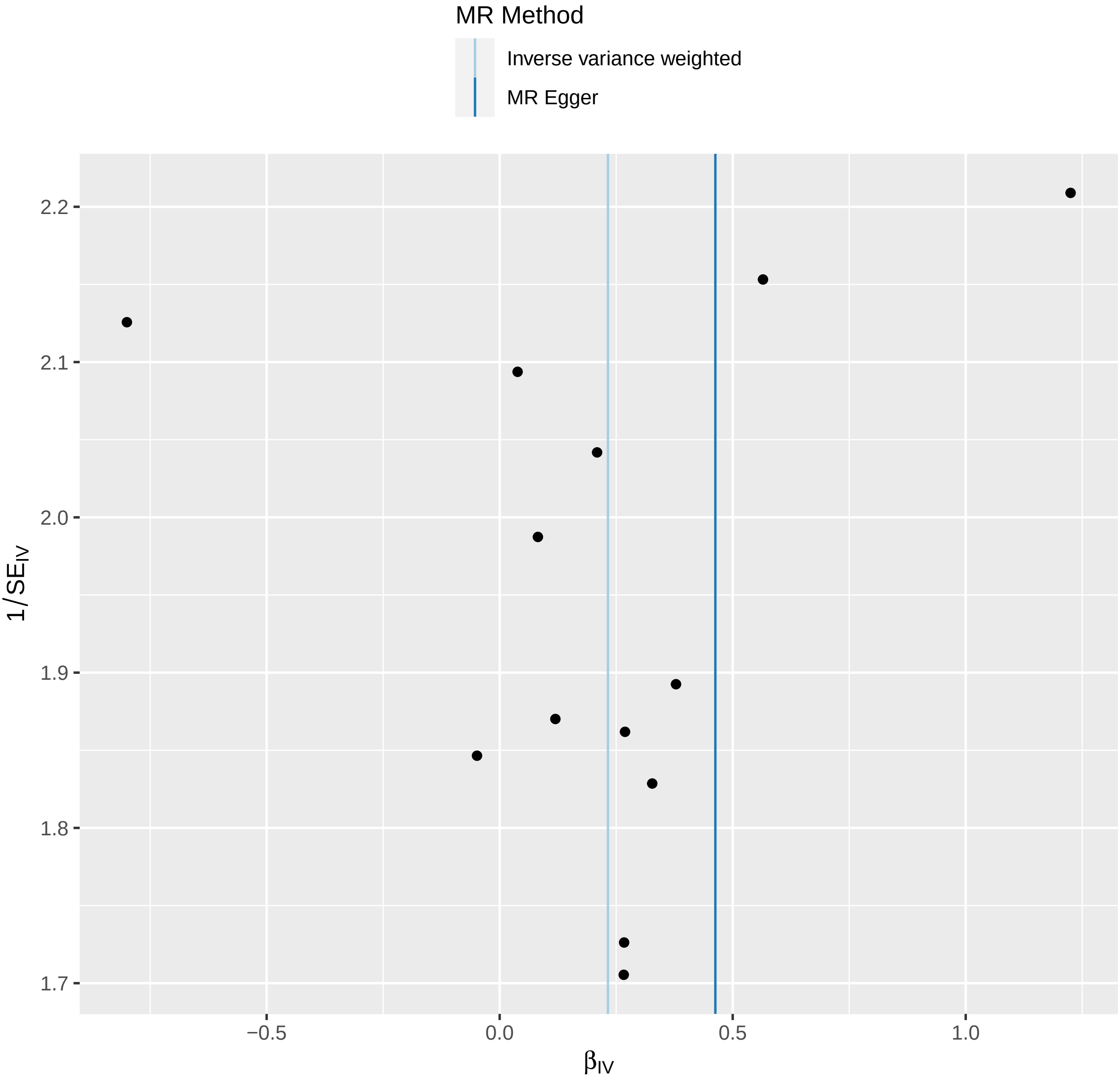

Batch 1546 : Gut microbiota abundance (genus Blautia id.1992) on Ulcerative rectosigmoiditis

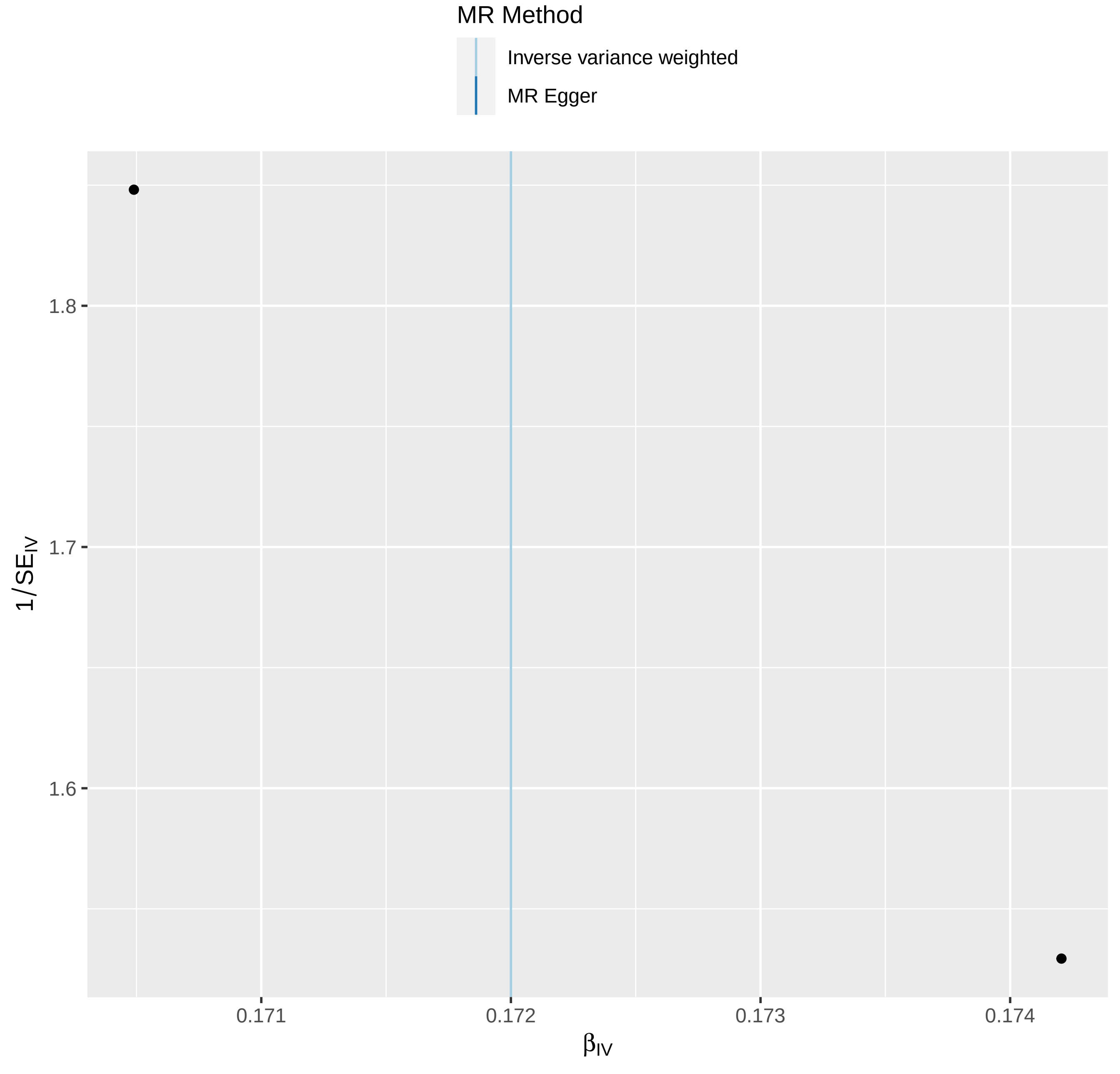

Batch 1547 : Gut microbiota abundance (genus Butyricicoccus id.2055) on Ulcerative rectosigmoiditis

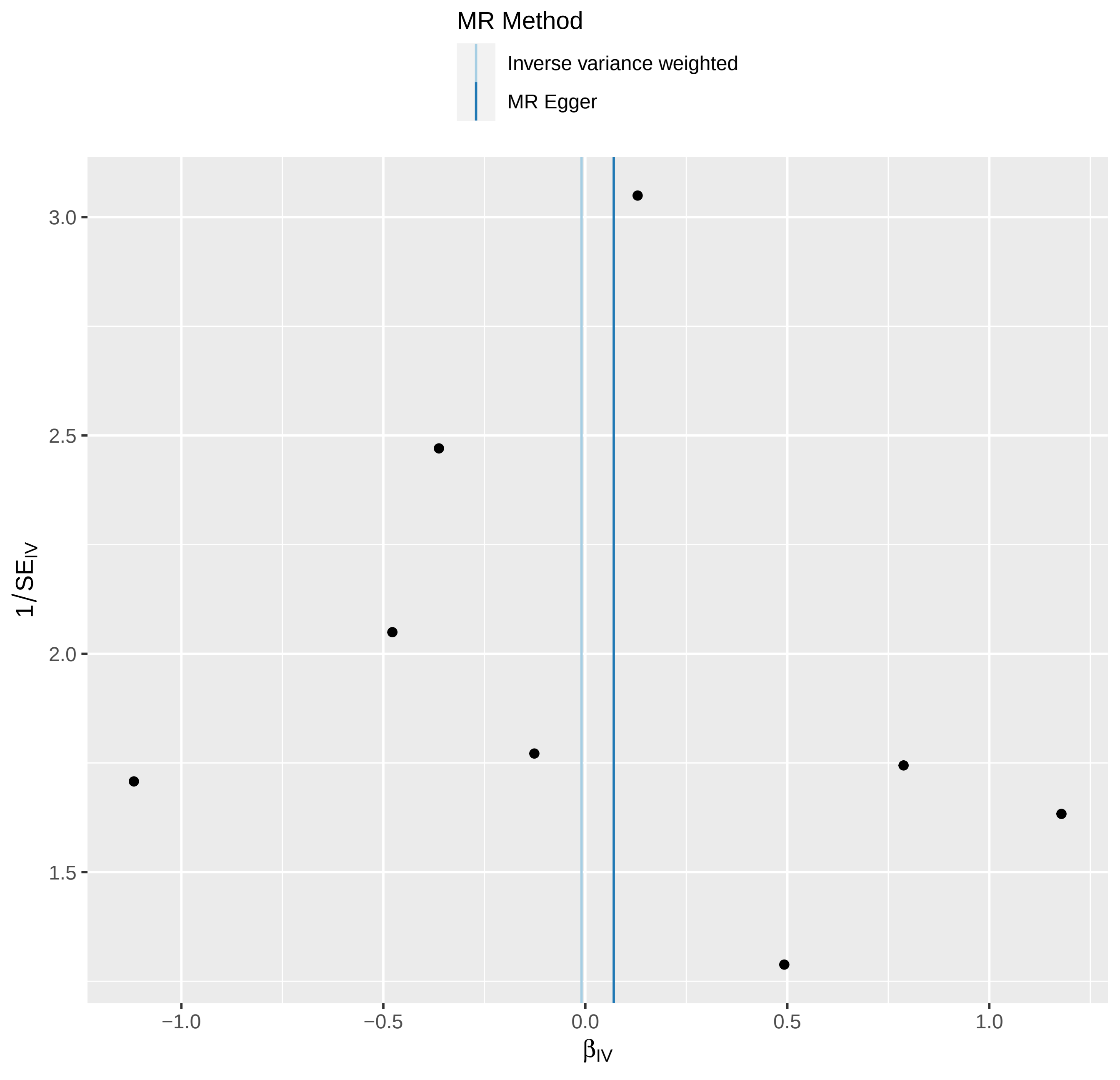

Batch 1548 : Gut microbiota abundance (genus Butyricimonas id.945) on Ulcerative rectosigmoiditis

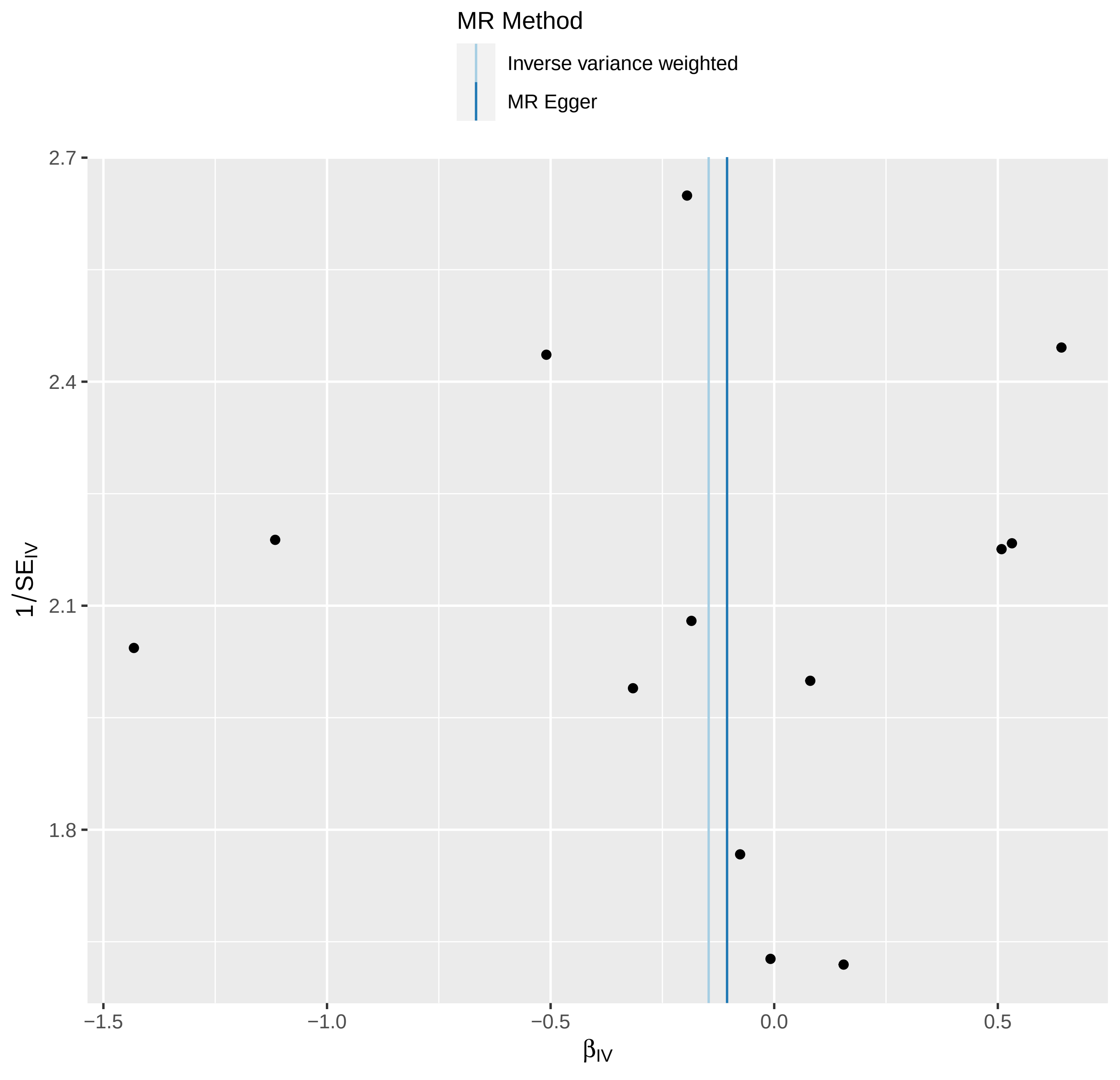

Batch 1549 : Gut microbiota abundance (genus Butyrivibrio id.1993) on Ulcerative rectosigmoiditis

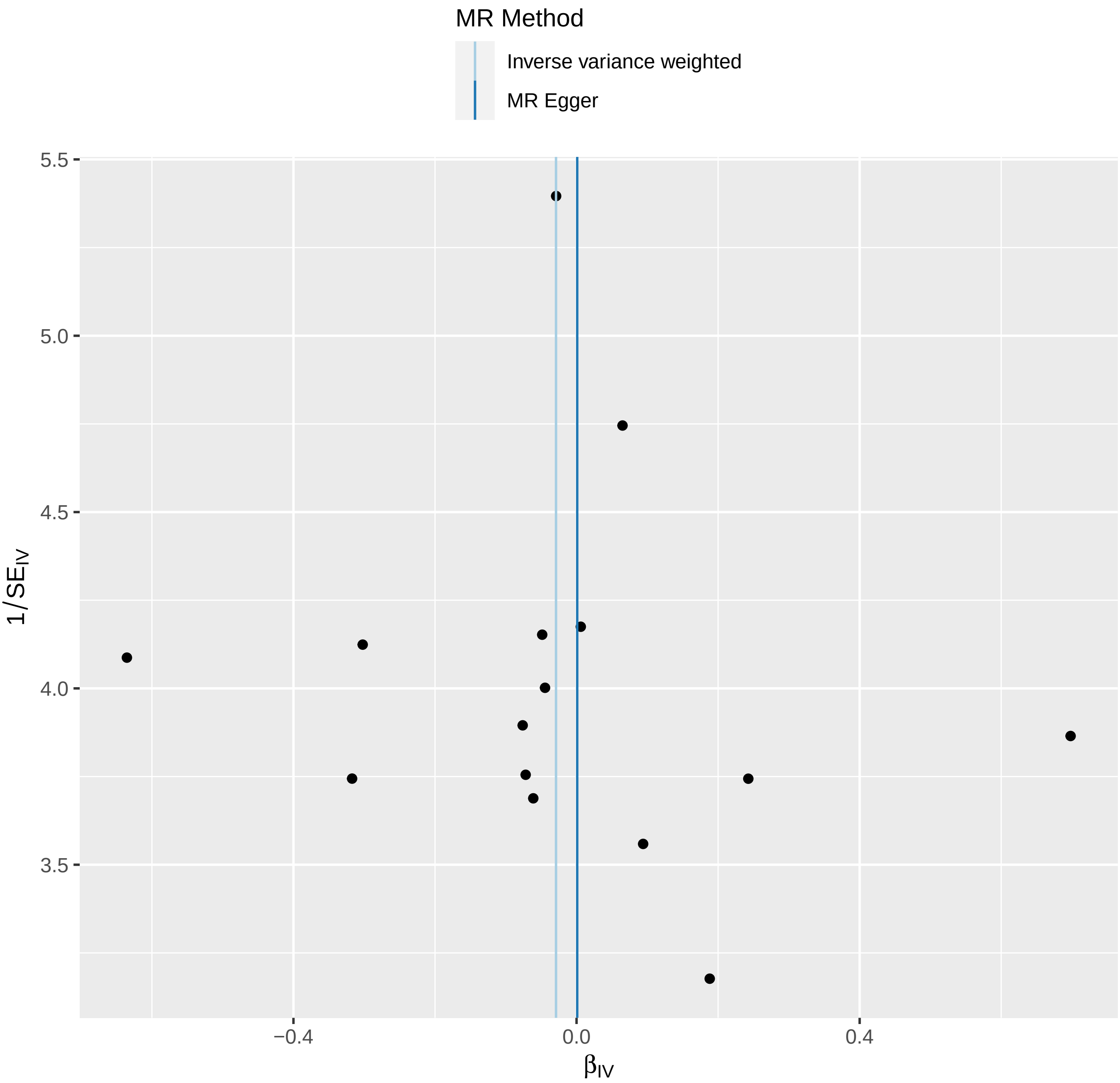

Batch 1550 : Gut microbiota abundance (genus Candidatus Soleaferrea id.11350) on Ulcerative rectosigmoiditis

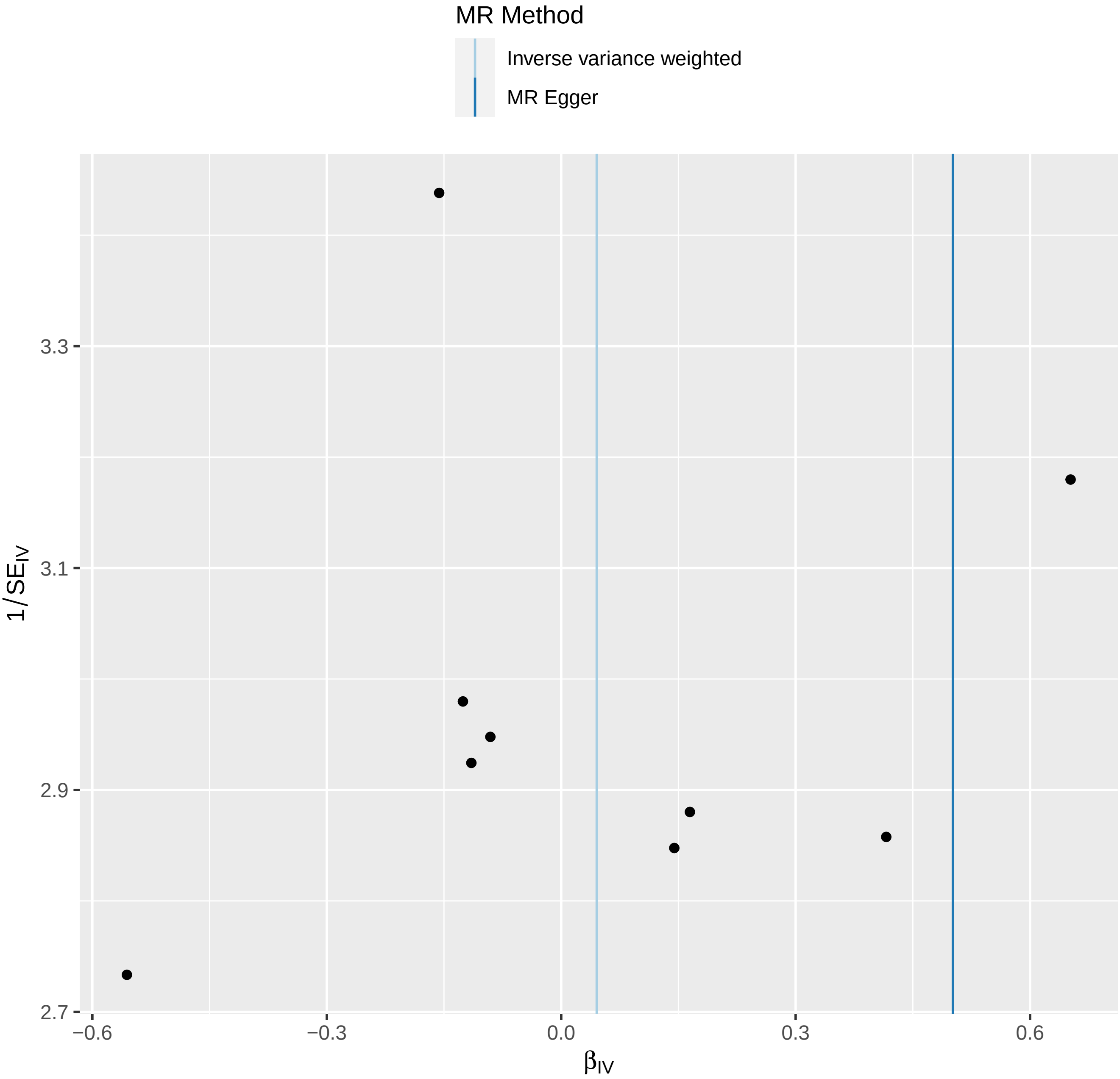

Batch 1551 : Gut microbiota abundance (genus Catenibacterium id.2153) on Ulcerative rectosigmoiditis

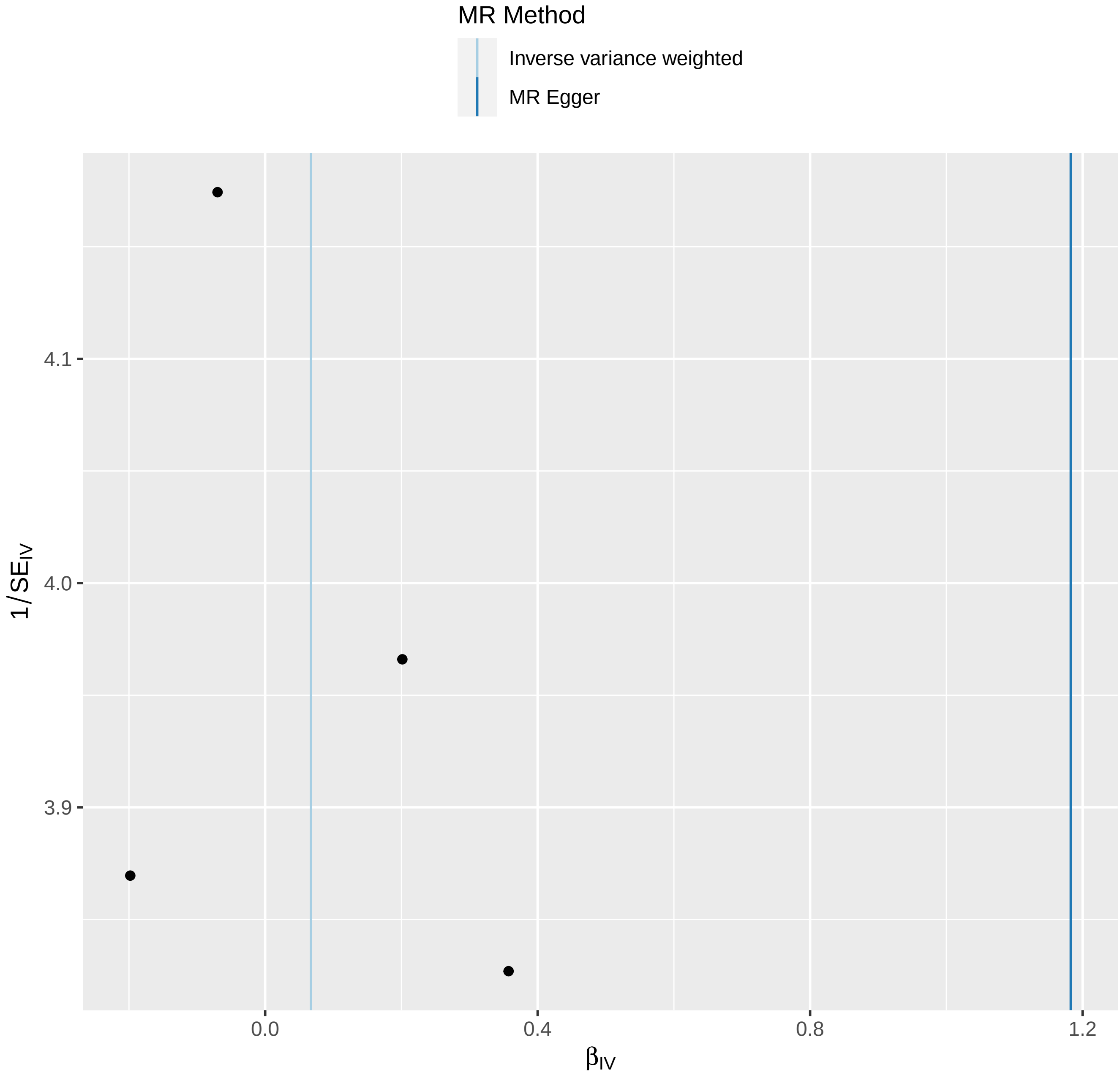

Batch 1552 : Gut microbiota abundance (genus Christensenellaceae R 7group id.11283) on Ulcerative rectosigmoiditis

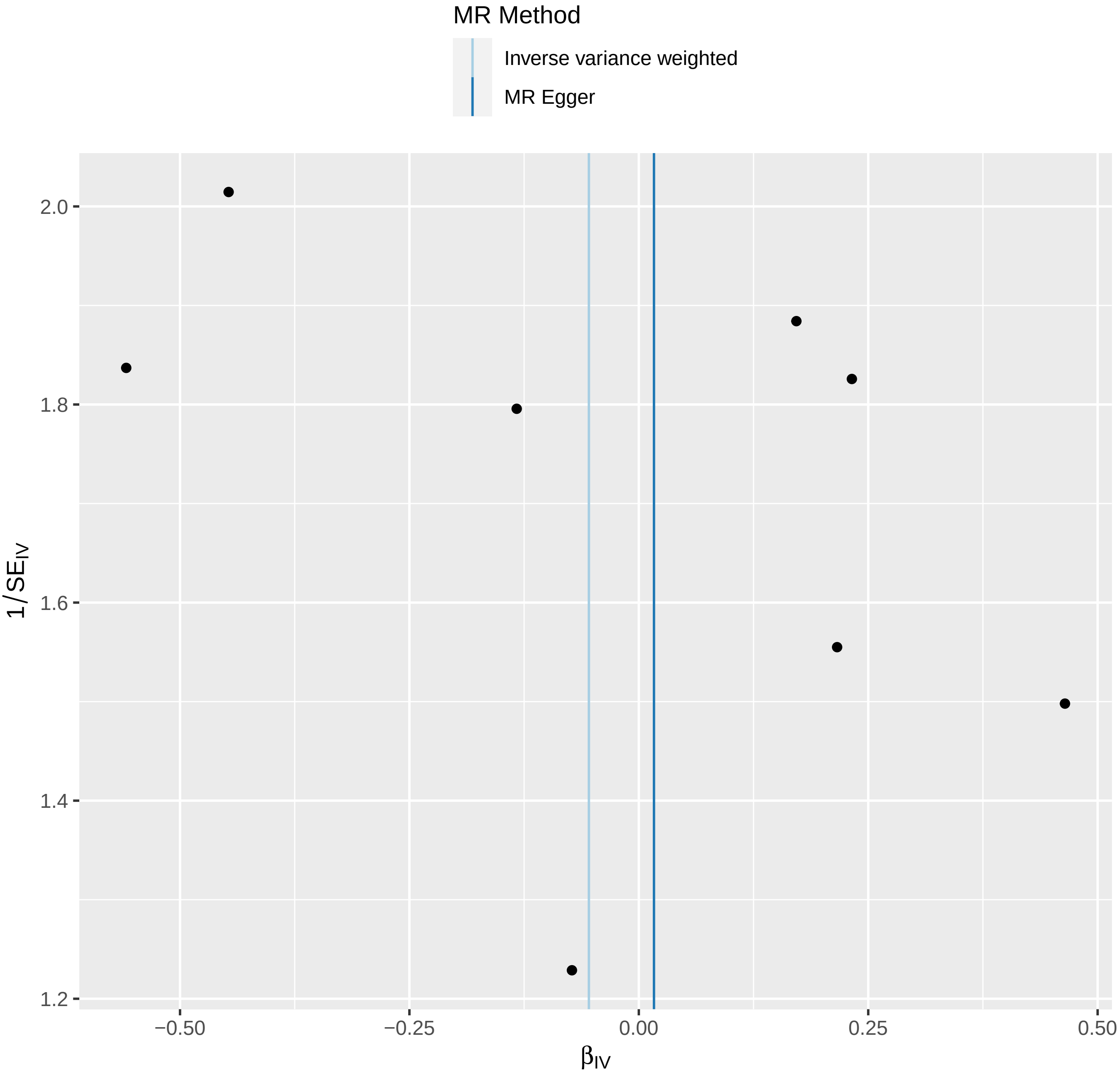

Batch 1553 : Gut microbiota abundance (genus Clostridium innocuum group id.14397) on Ulcerative rectosigmoiditis

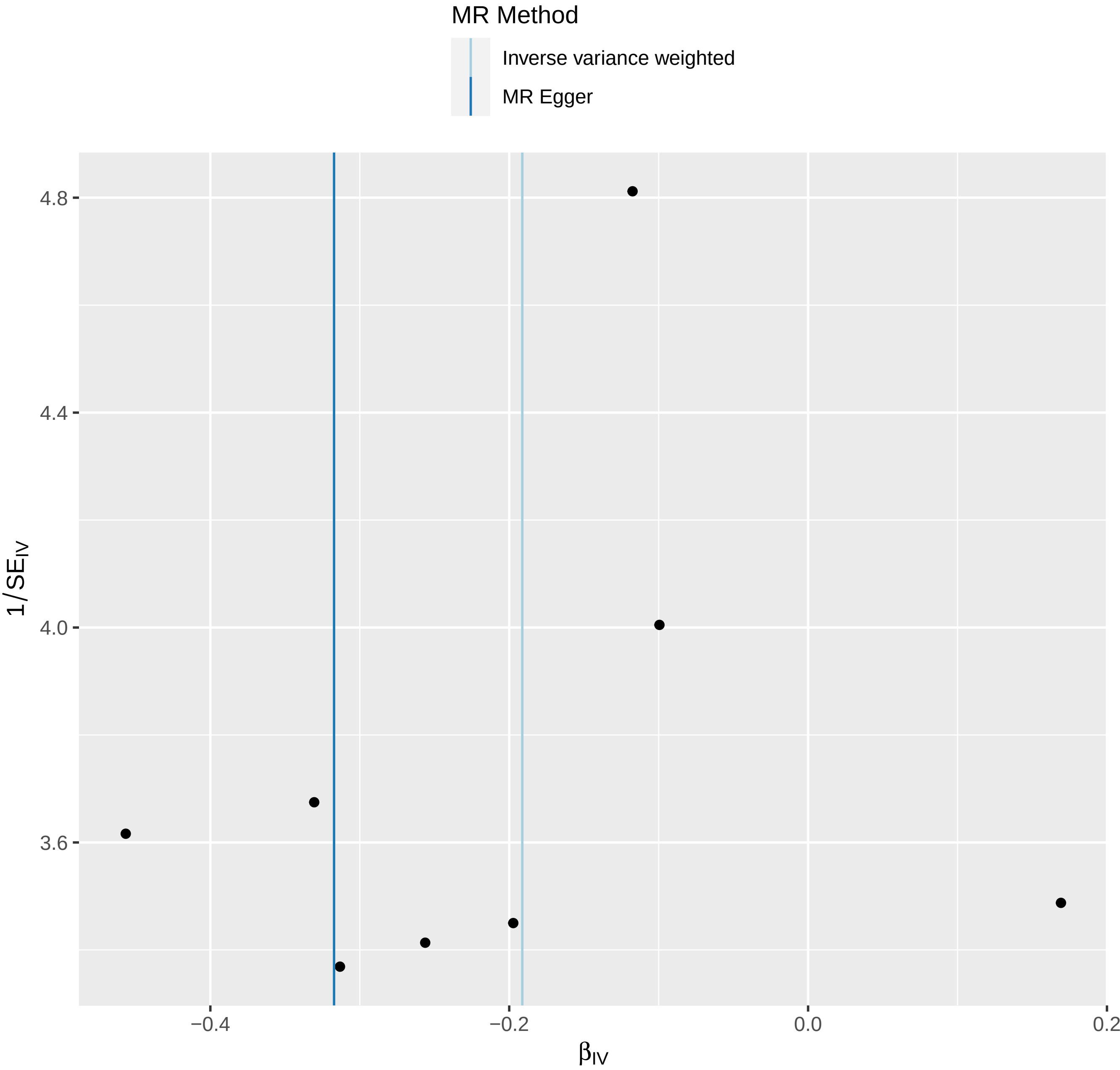

Batch 1554 : Gut microbiota abundance (genus Clostridium sensustricto1 id.1873) on Ulcerative rectosigmoiditis

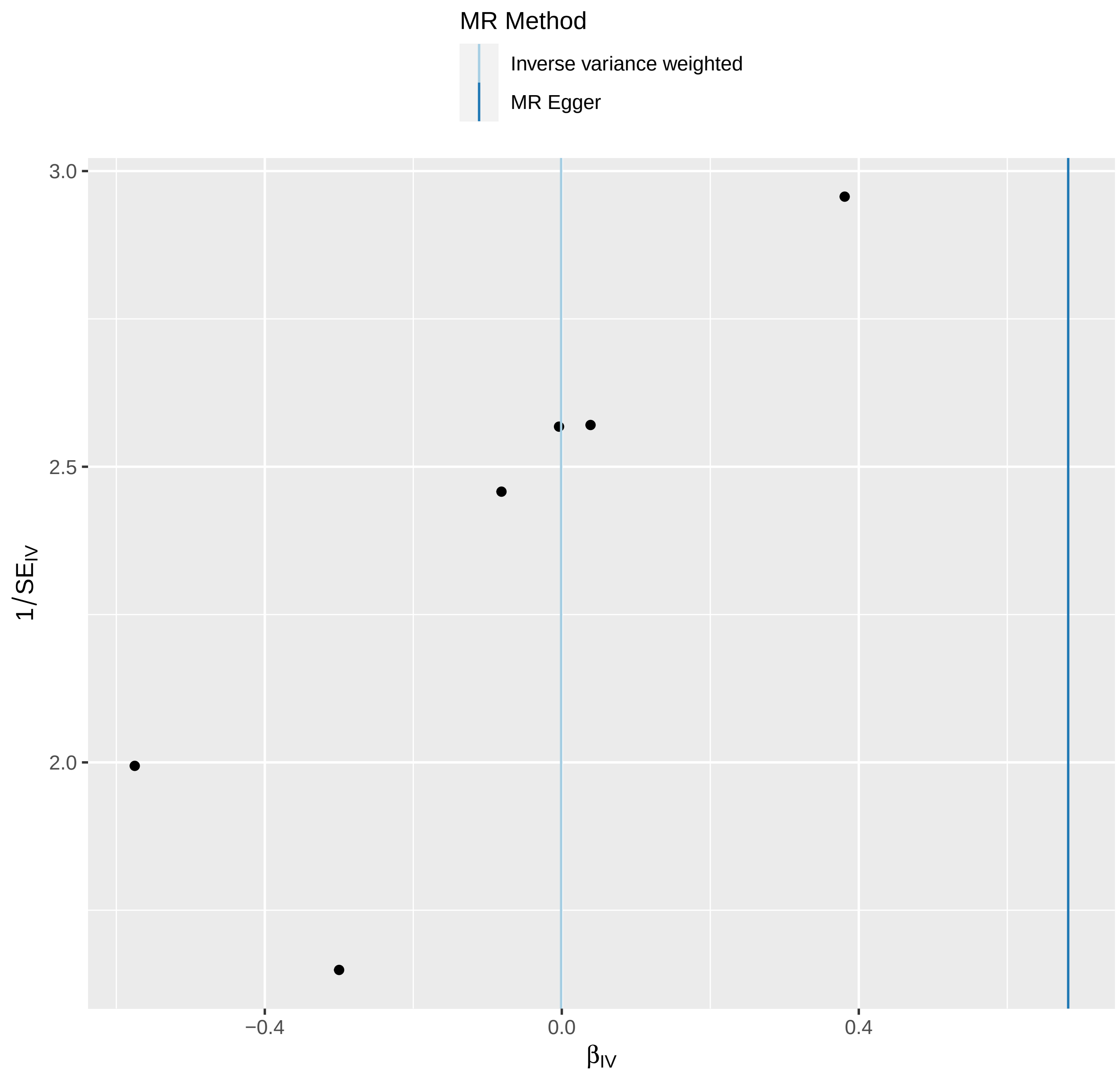

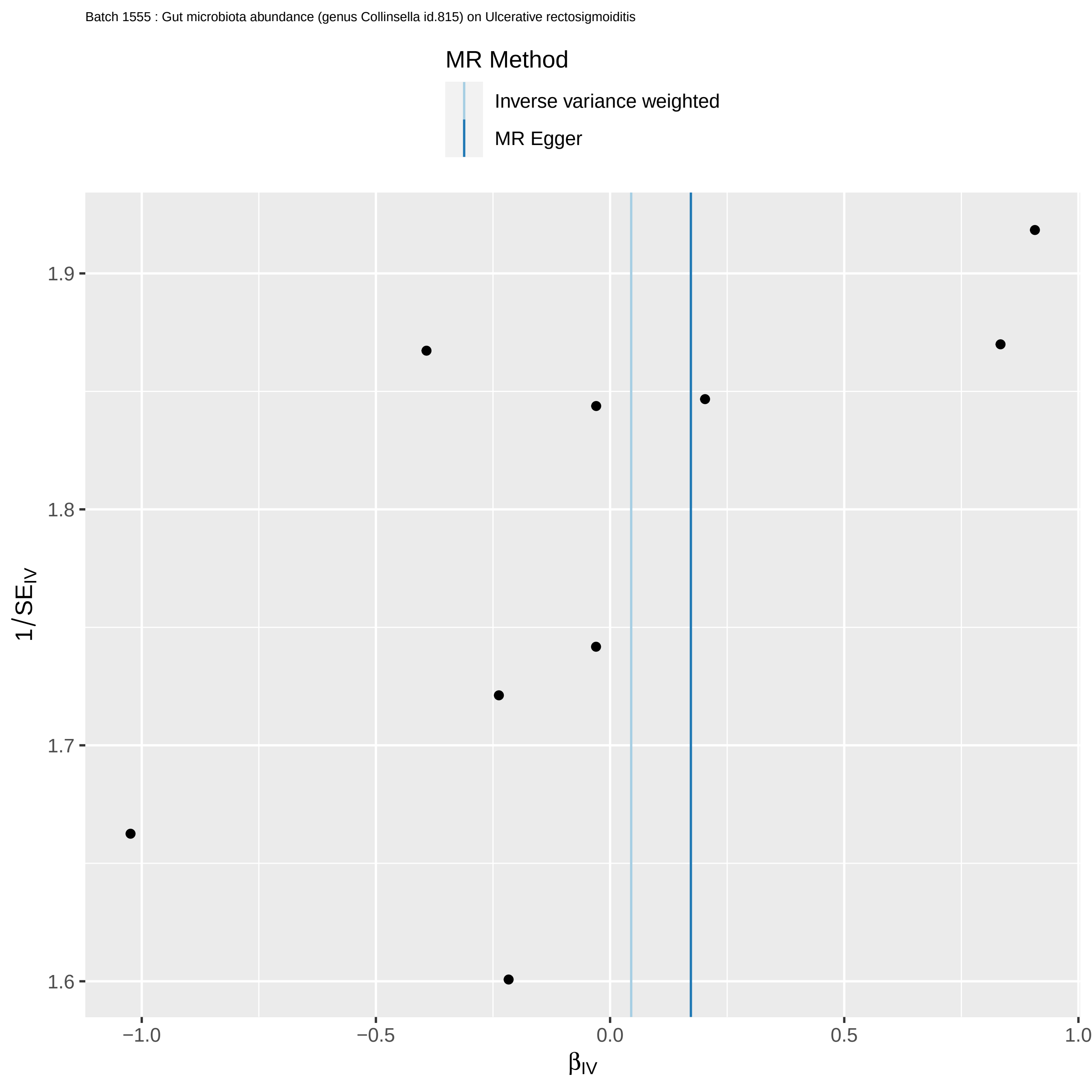
Batch 1555 : Gut microbiota abundance (genus Collinsella id.815) on Ulcerative rectosigmoiditis
MR Method
Inverse variance weighted
MR Egger
1.9
1.8
1.7
1.6
1/SE_IV
−1.0
−0.5
0.0
0.5
1.0
β_IV

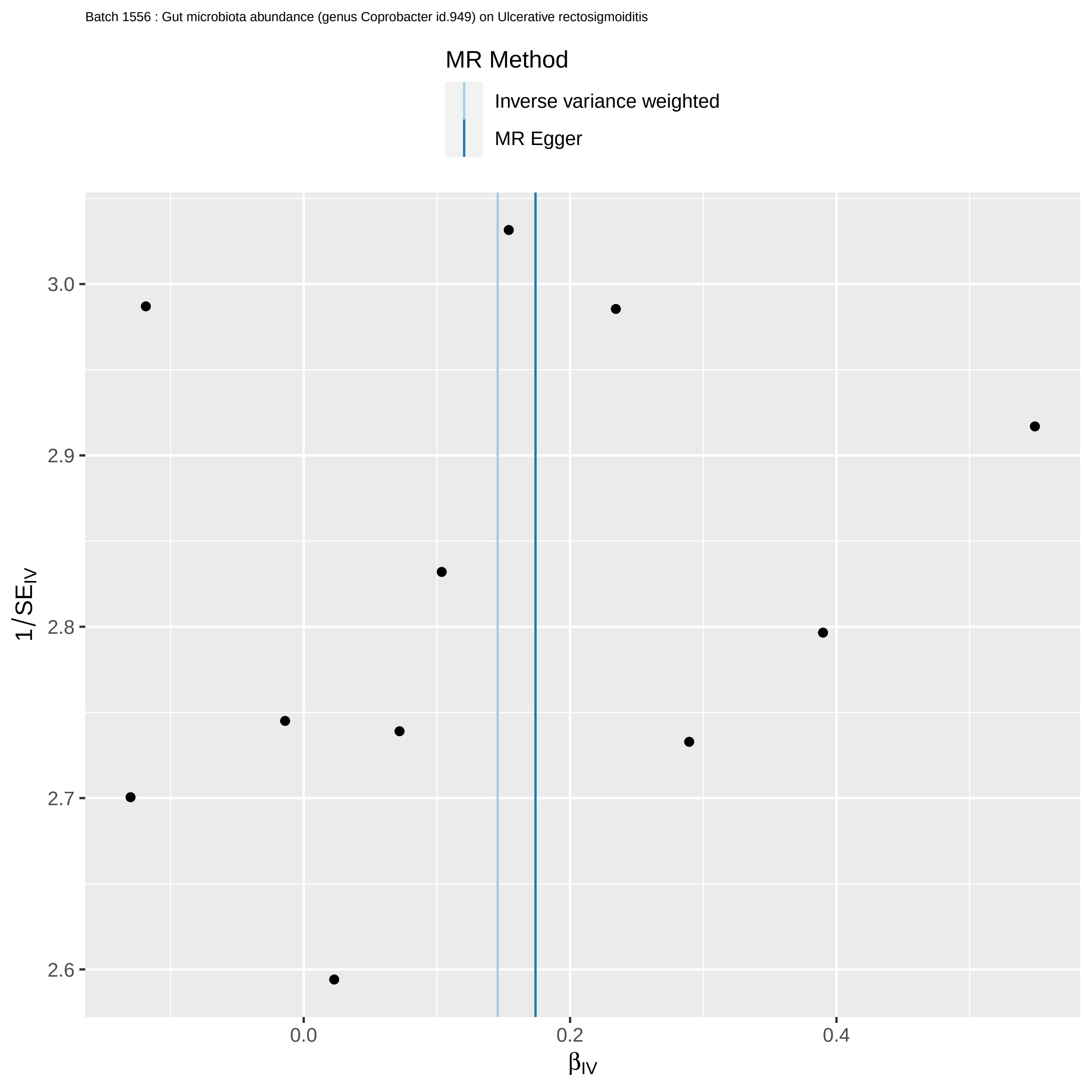
Batch 1556 : Gut microbiota abundance (genus Coprobacter id.949) on Ulcerative rectosigmoiditis
MR Method
Inverse variance weighted
MR Egger
3.0
2.9
2.8
2.7
2.6
1/SE_IV
0.0
0.2
0.4
β_IV

Batch 1557 : Gut microbiota abundance (genus Coprococcus1 id.11301) on Ulcerative rectosigmoiditis

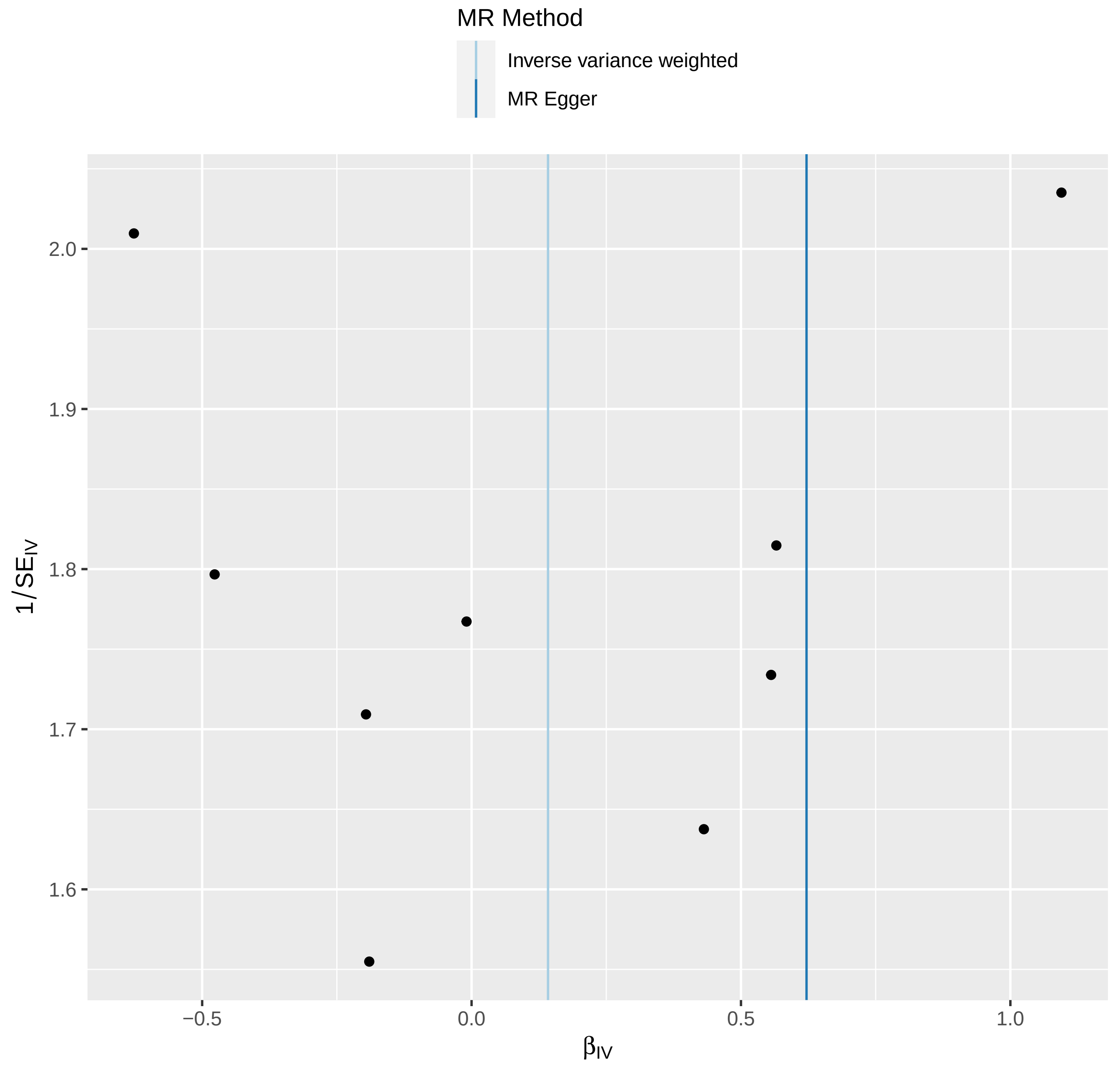

Batch 1558 : Gut microbiota abundance (genus Coprococcus2 id.11302) on Ulcerative rectosigmoiditis

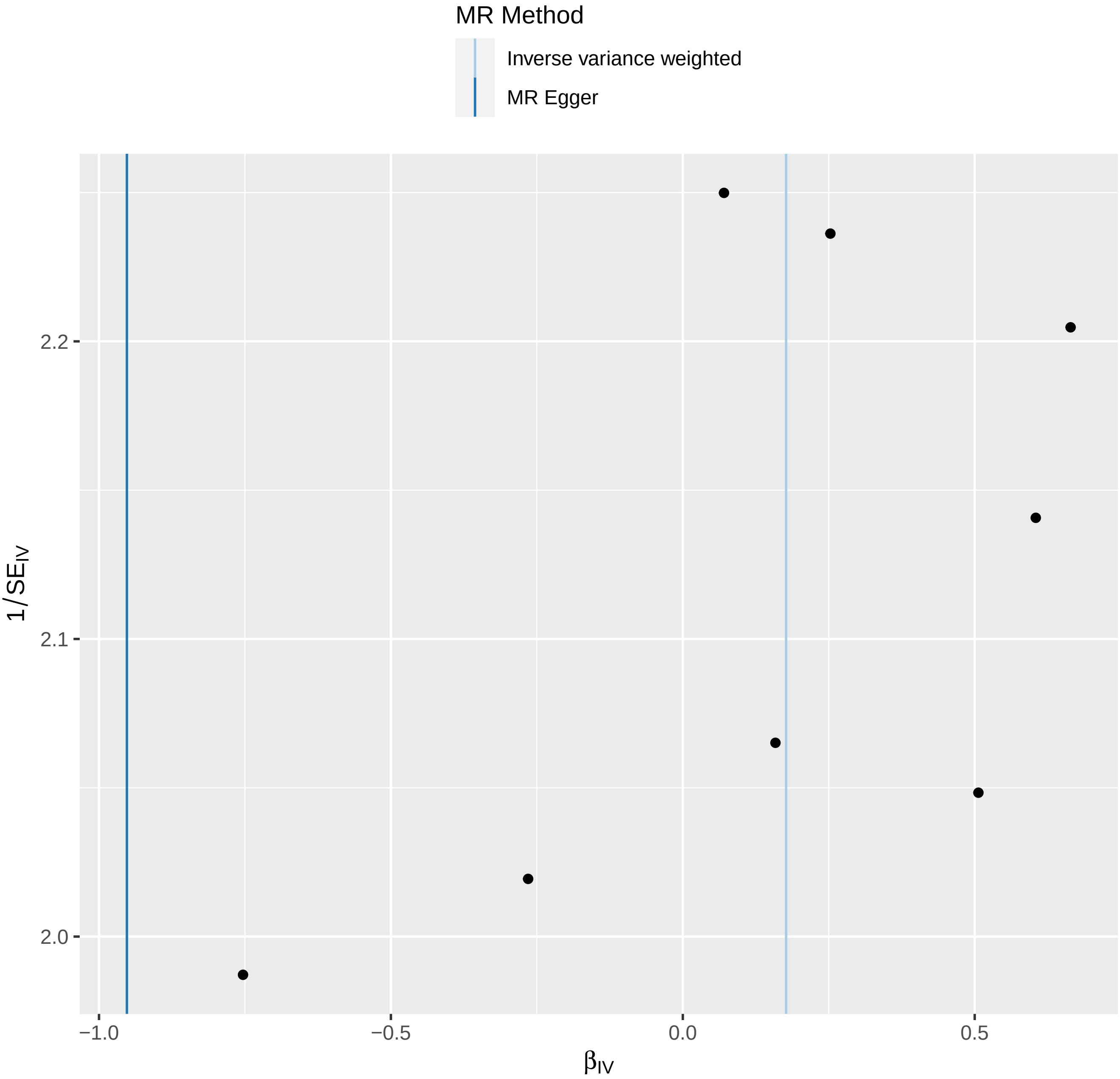

Batch 1559 : Gut microbiota abundance (genus Coprococcus3 id.11303) on Ulcerative rectosigmoiditis

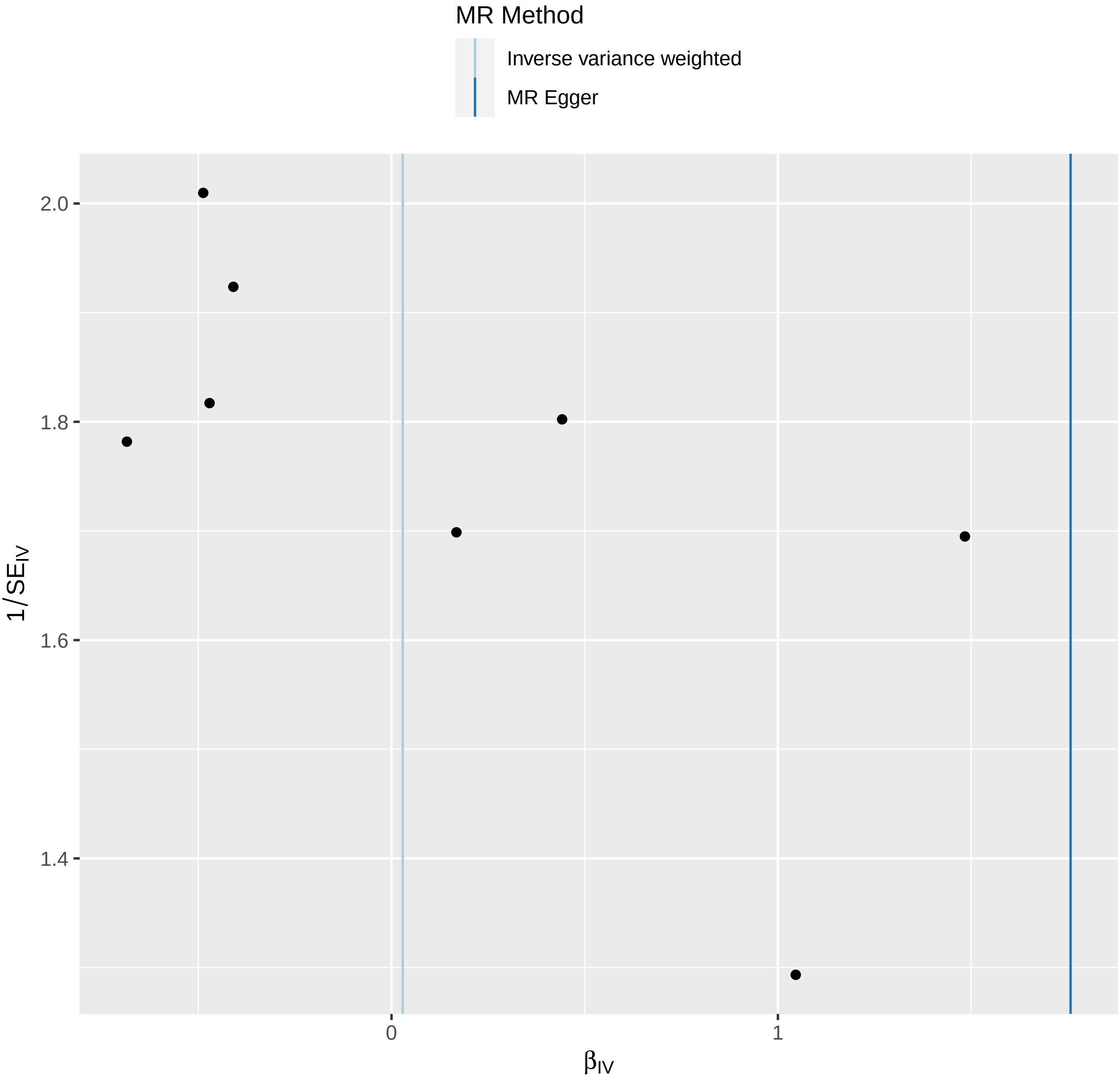

Batch 1560 : Gut microbiota abundance (genus Defluviitaleaceae UCG011 id.11287) on Ulcerative rectosigmoiditis

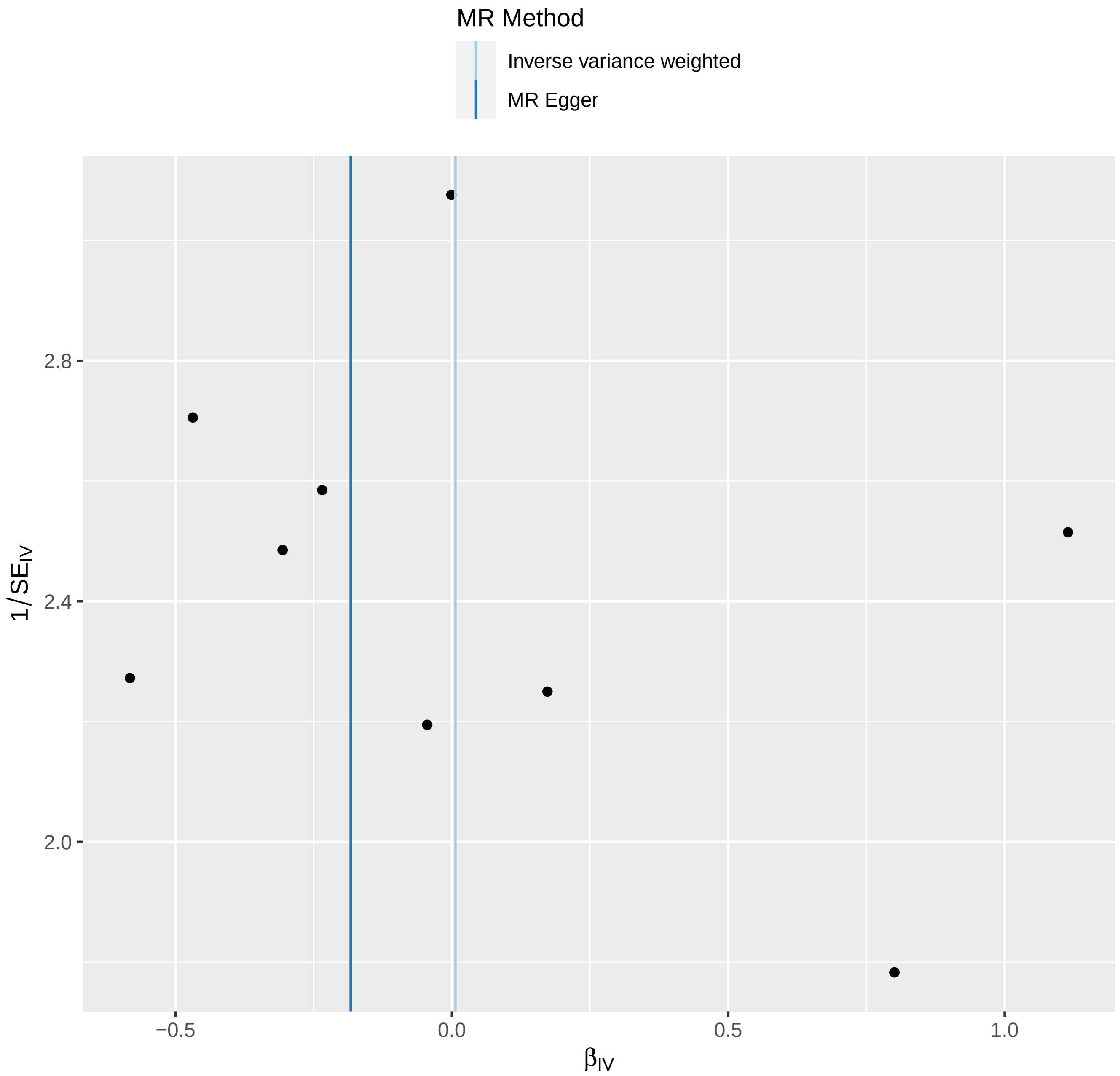

Batch 1561 : Gut microbiota abundance (genus Desulfovibrio id.3173) on Ulcerative rectosigmoiditis

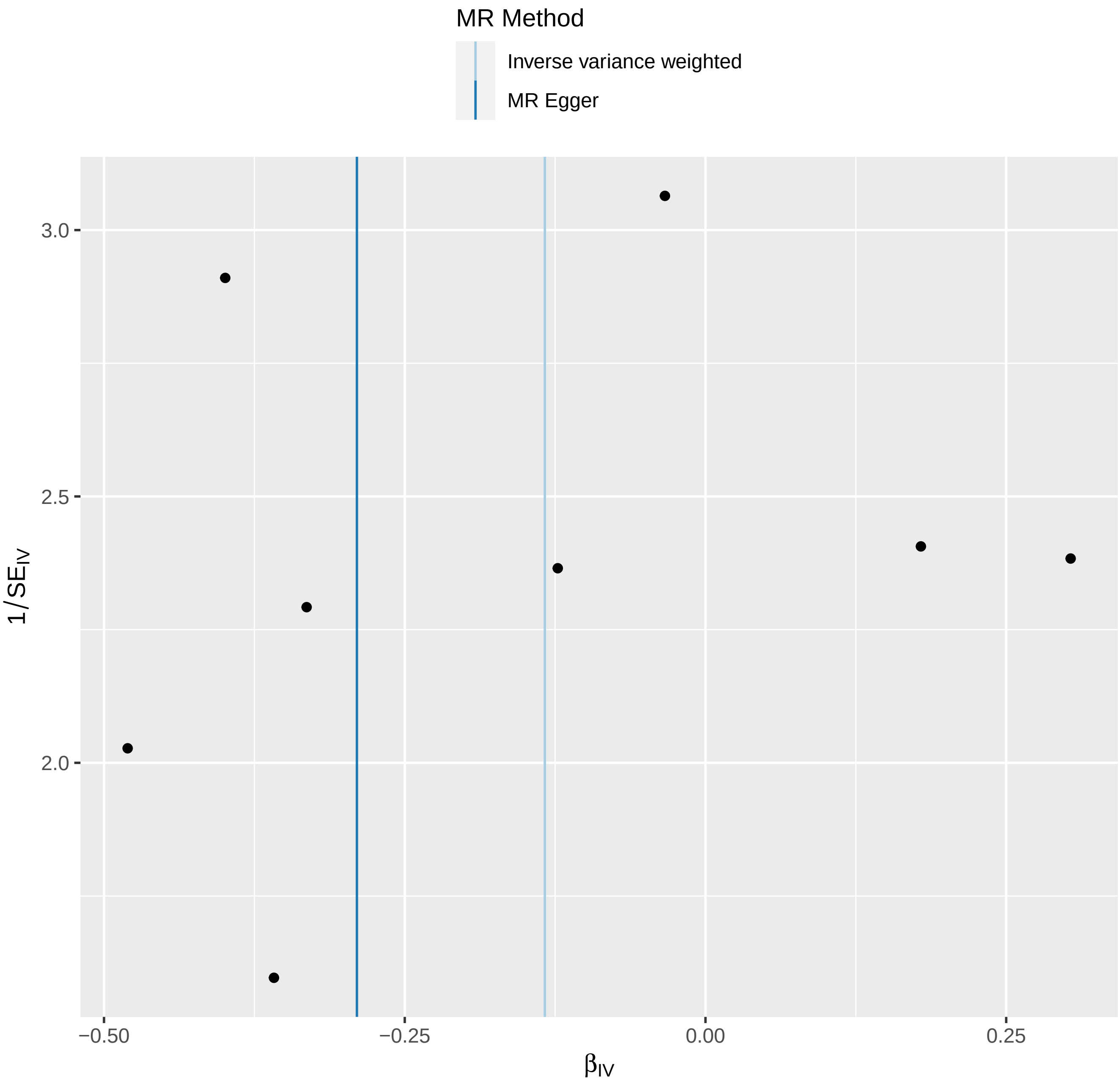

Batch 1562 : Gut microbiota abundance (genus Dialister id.2183) on Ulcerative rectosigmoiditis

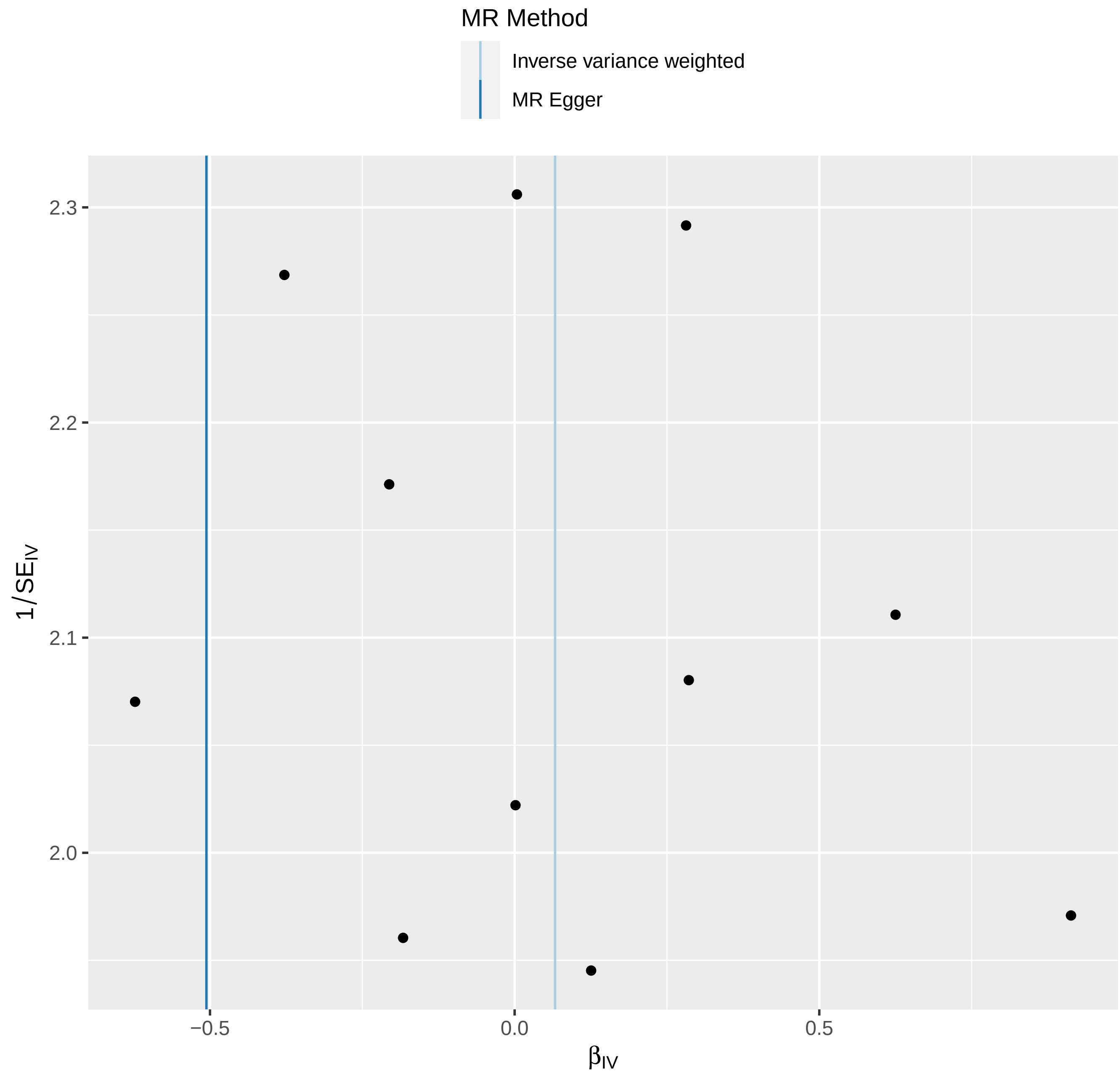

Batch 1563 : Gut microbiota abundance (genus Dorea id.1997) on Ulcerative rectosigmoiditis

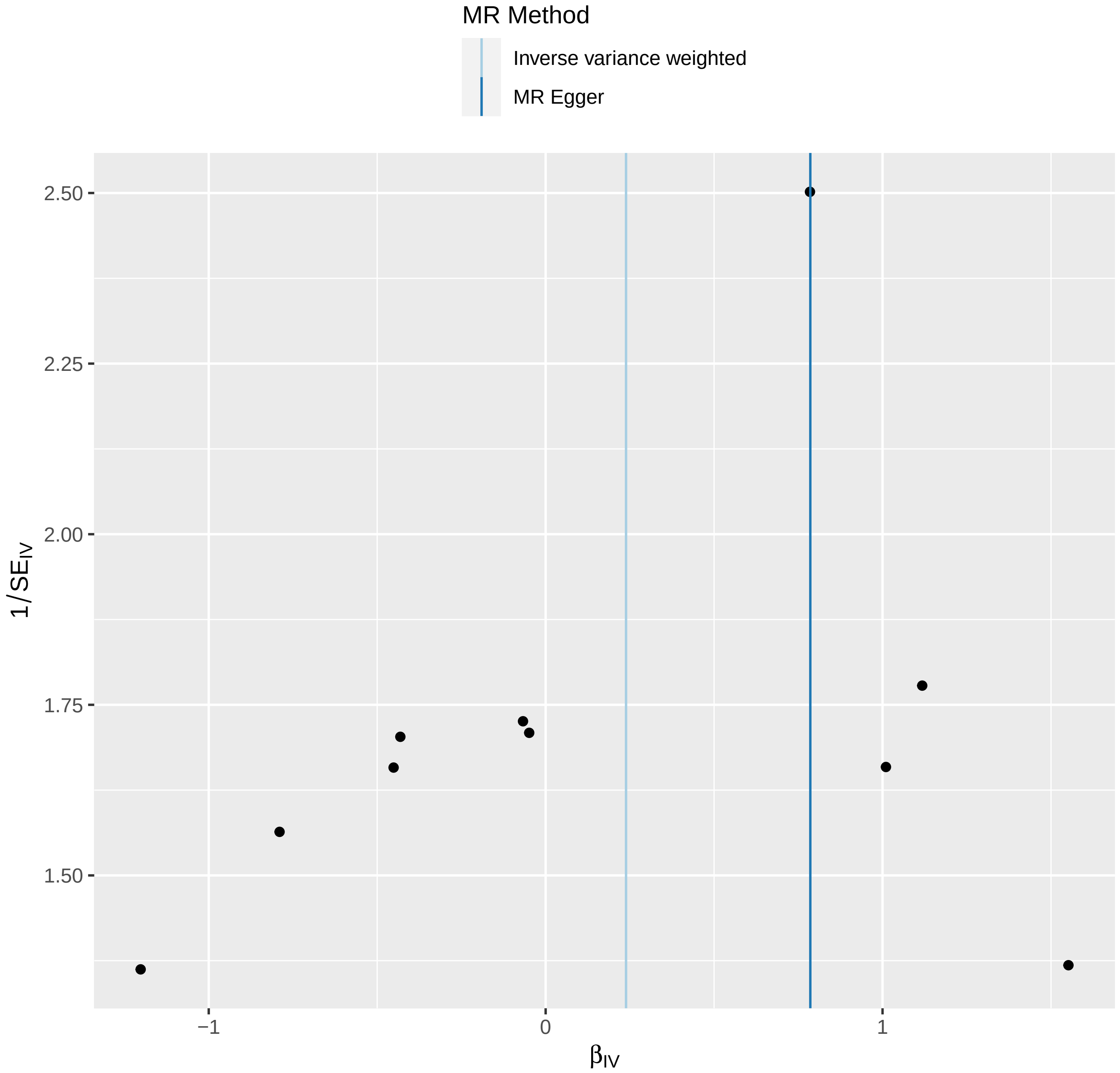

Batch 1564 : Gut microbiota abundance (genus Eggerthella id.819) on Ulcerative rectosigmoiditis

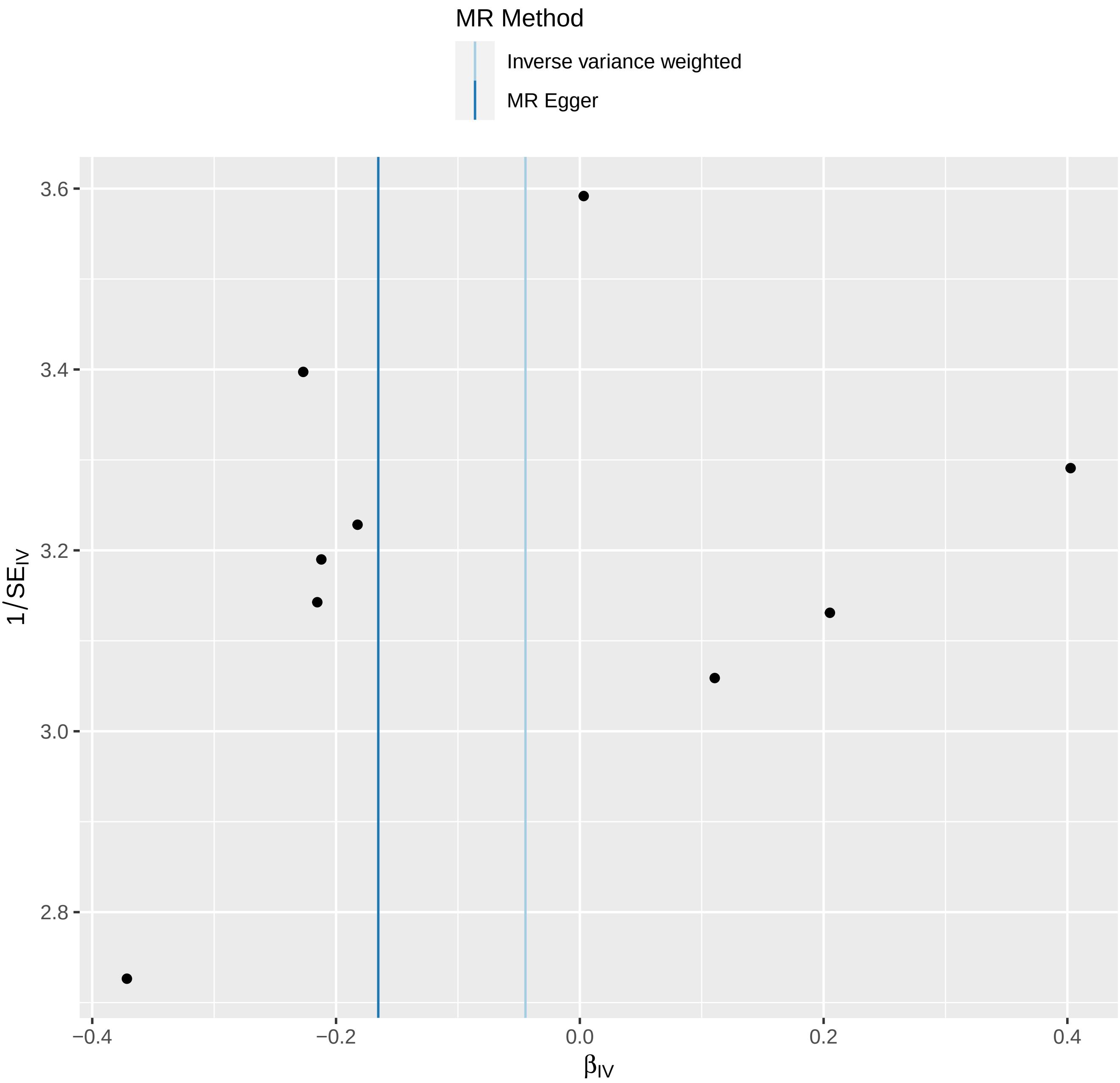

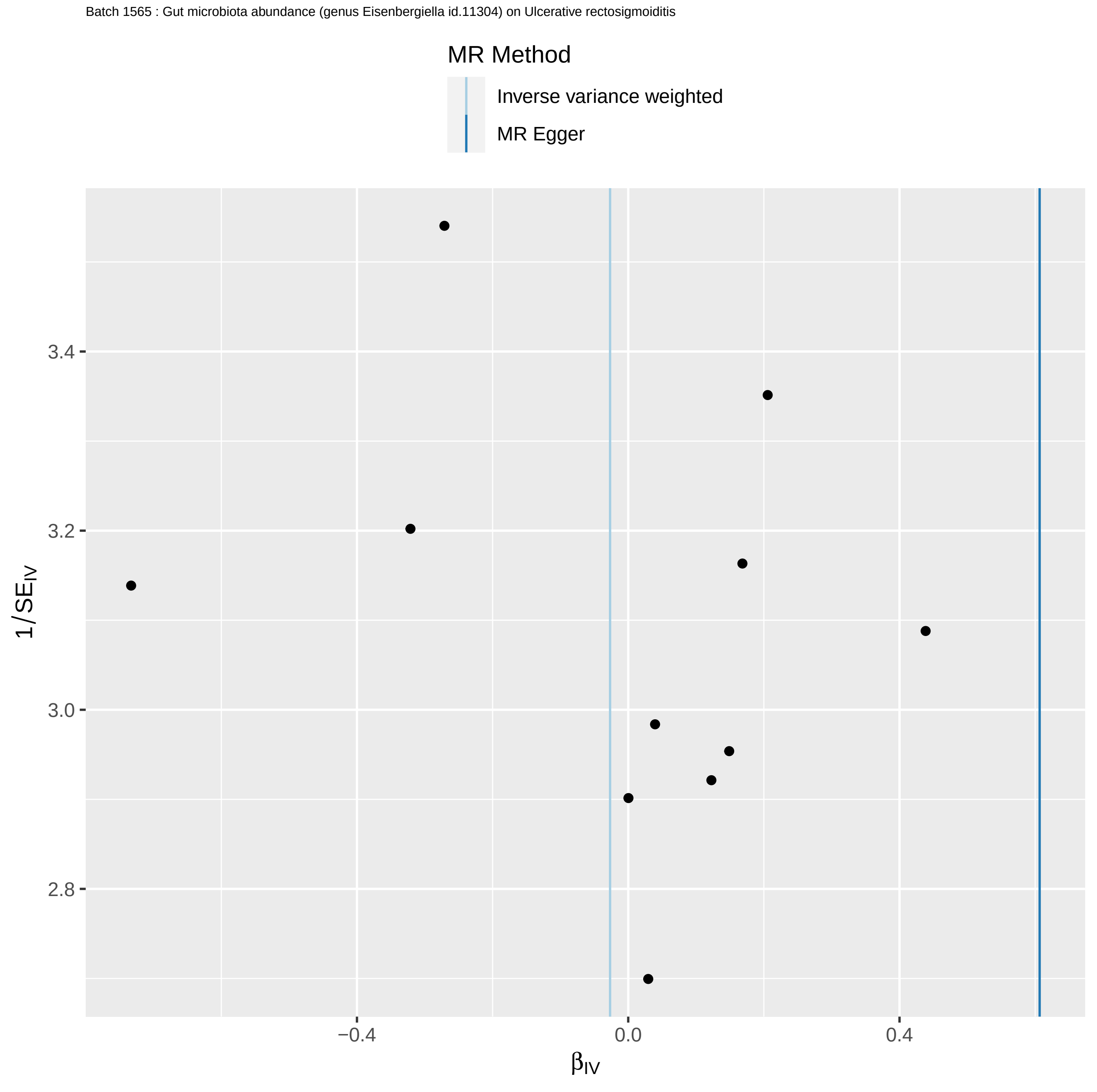
Batch 1565 : Gut microbiota abundance (genus Eisenbergiella id.11304) on Ulcerative rectosigmoiditis
MR Method
Inverse variance weighted
MR Egger
3.4
3.2
3.0
2.8
1/SE_IV
−0.4
0.0
0.4
β_IV

Batch 1566 : Gut microbiota abundance (genus Enterorhabdus id.820) on Ulcerative rectosigmoiditis

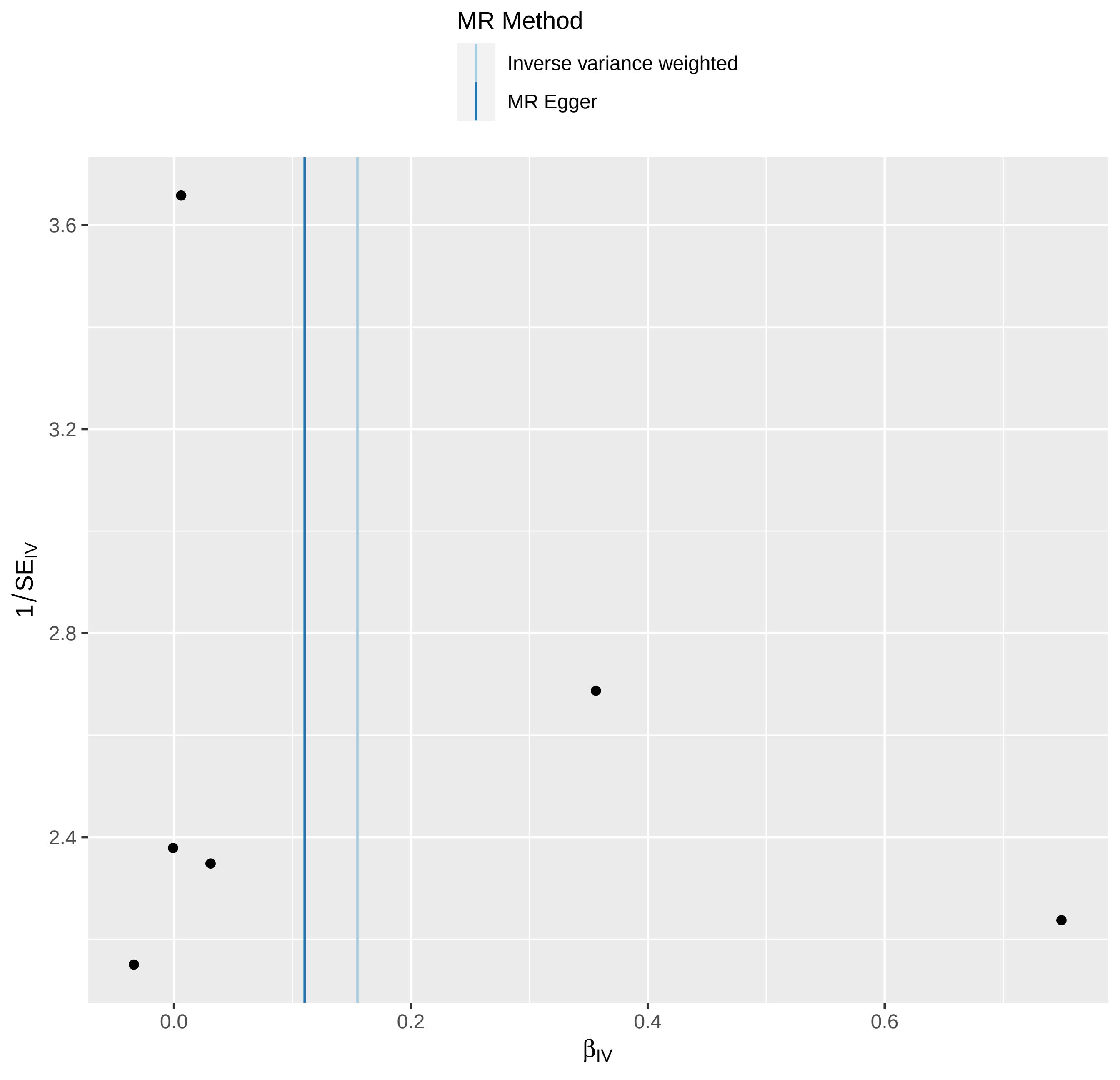

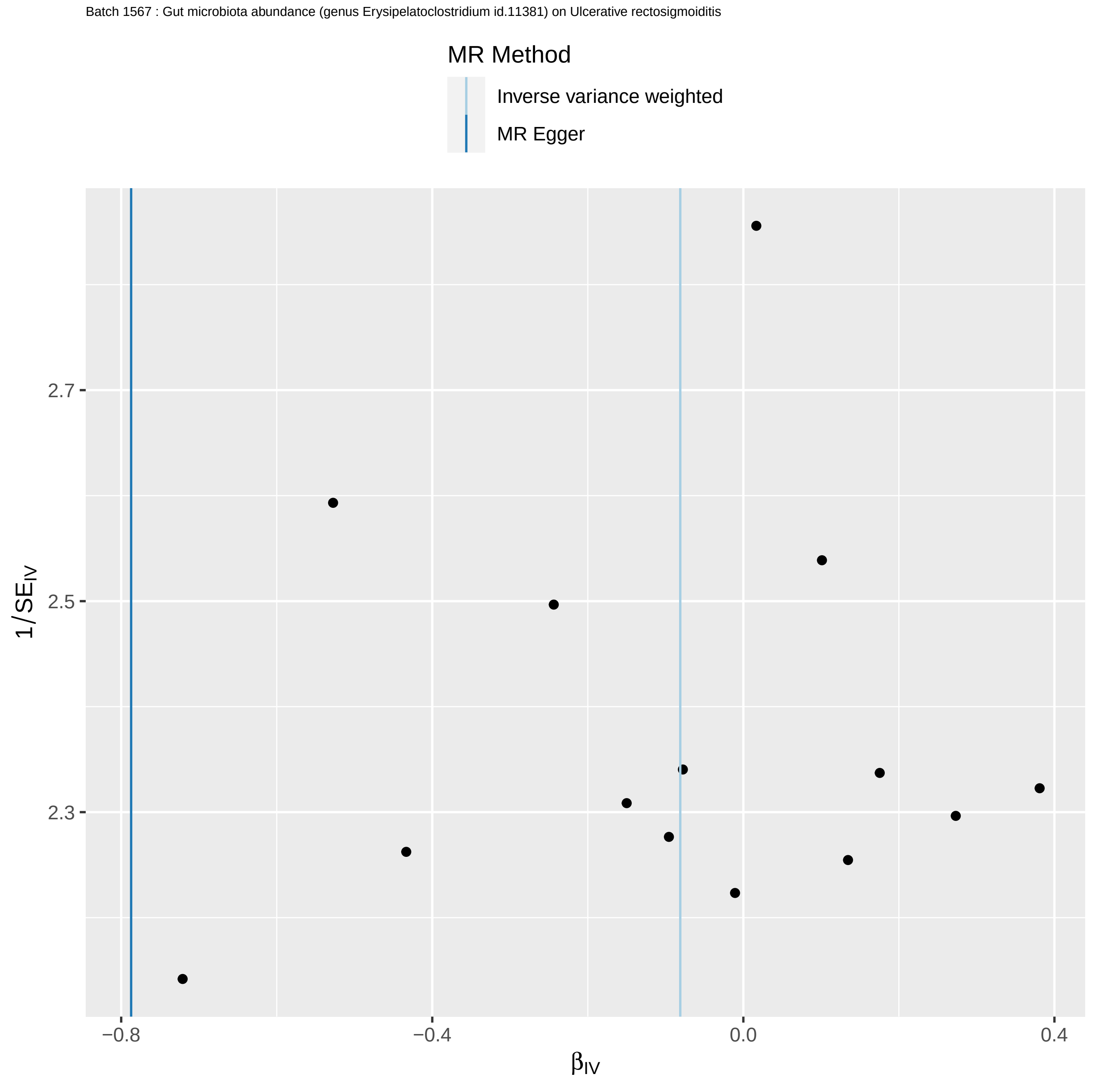

Batch 1567 : Gut microbiota abundance (genus Erysipelatoclostridium id.11381) on Ulcerative rectosigmoiditis
MR Method
Inverse variance weighted
MR Egger
2.7
2.5
2.3
1/SE_IV
−0.8
−0.4
0.0
0.4
β_IV

Insufficient number of SNPs

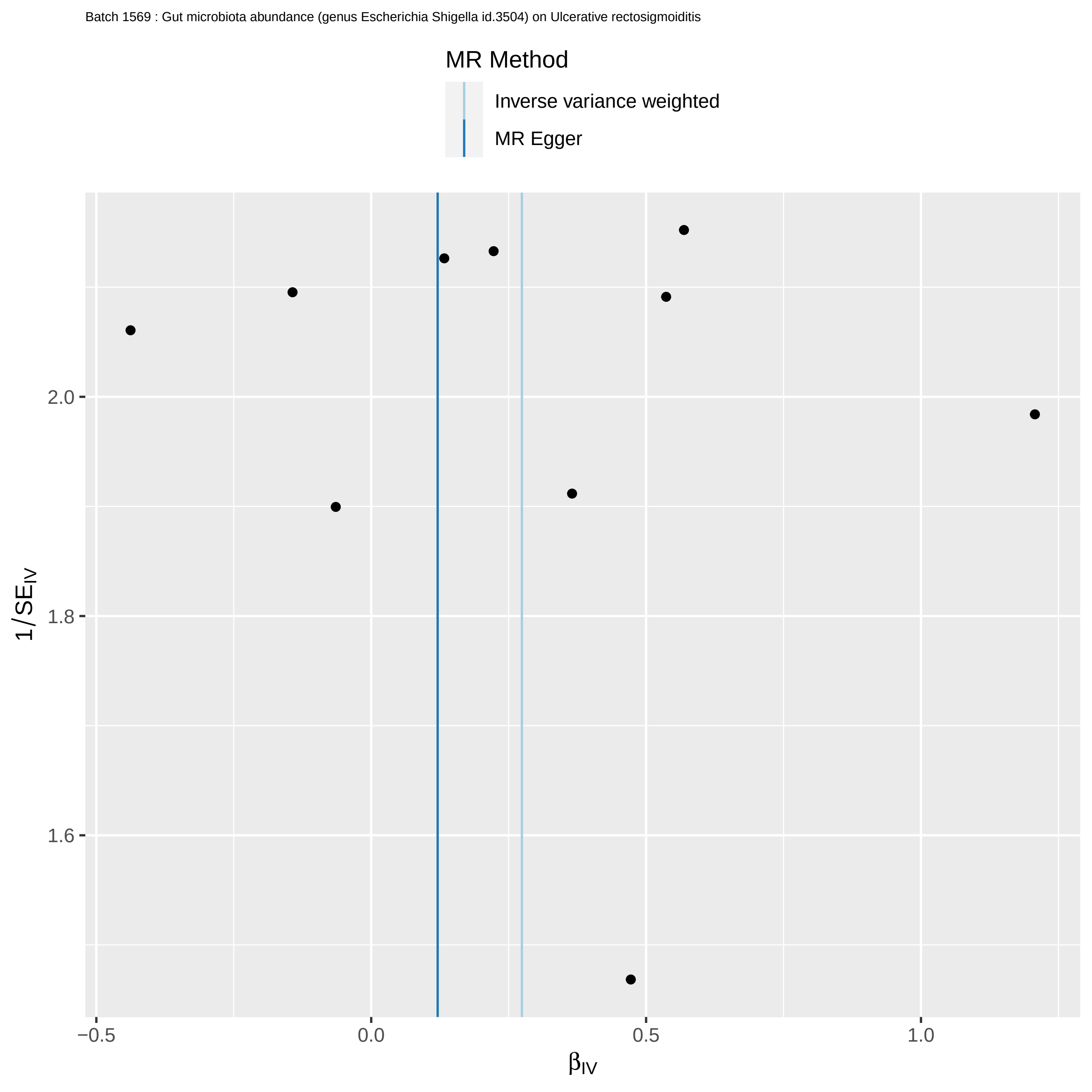

Batch 1569 : Gut microbiota abundance (genus Escherichia Shigella id.3504) on Ulcerative rectosigmoiditis
MR Method
Inverse variance weighted
MR Egger
2.0
1.8
1.6
1/SE$_{IV}$
−0.5
0.0
0.5
1.0
β$_{IV}$

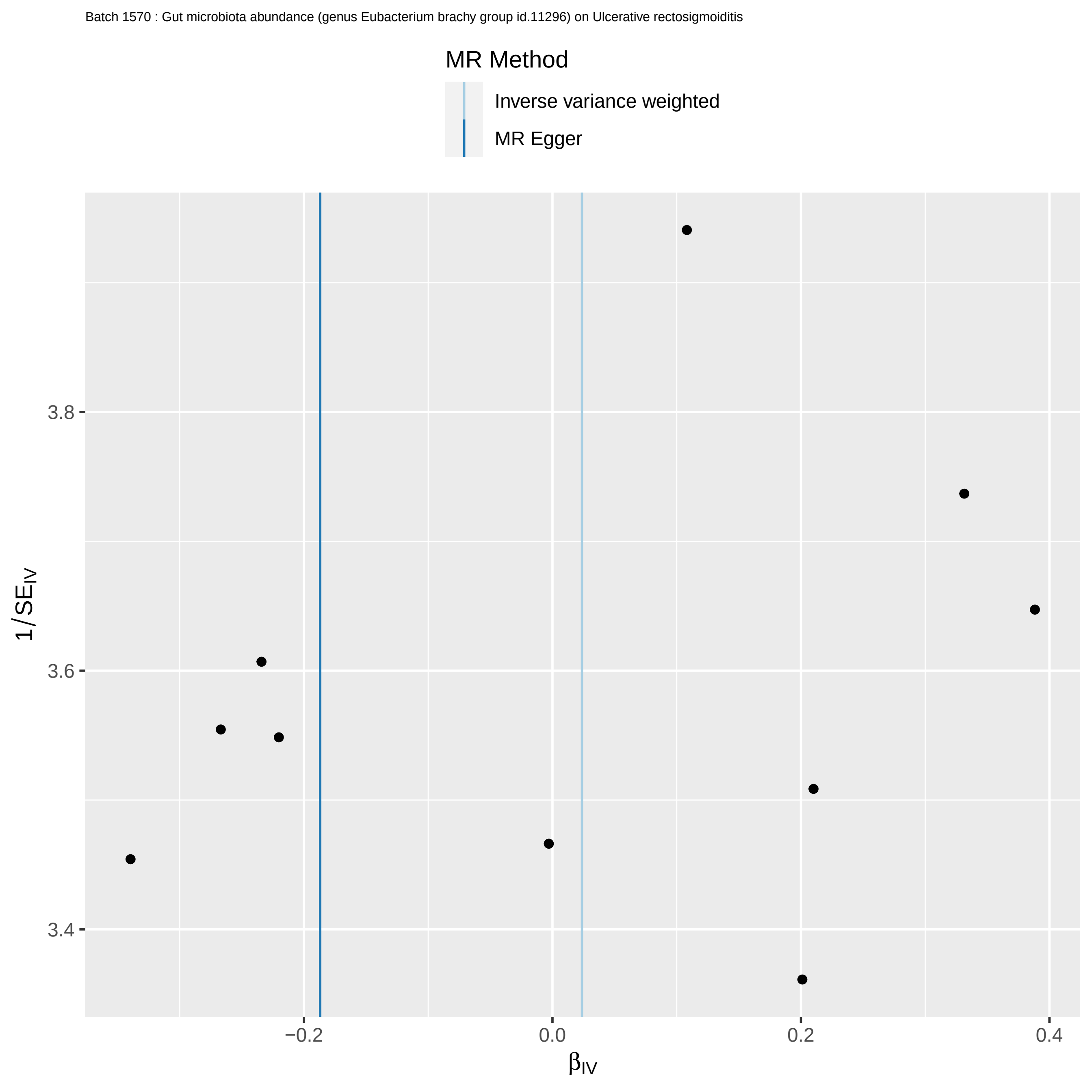
Batch 1570 : Gut microbiota abundance (genus Eubacterium brachy group id.11296) on Ulcerative rectosigmoiditis
MR Method
Inverse variance weighted
MR Egger
3.8
3.6
3.4
1/SE_IV
−0.2
0.0
0.2
0.4
β_IV

Batch 1571 : Gut microbiota abundance (genus Eubacterium coprostanoligenes group id.11375) on Ulcerative rectosigmoiditis

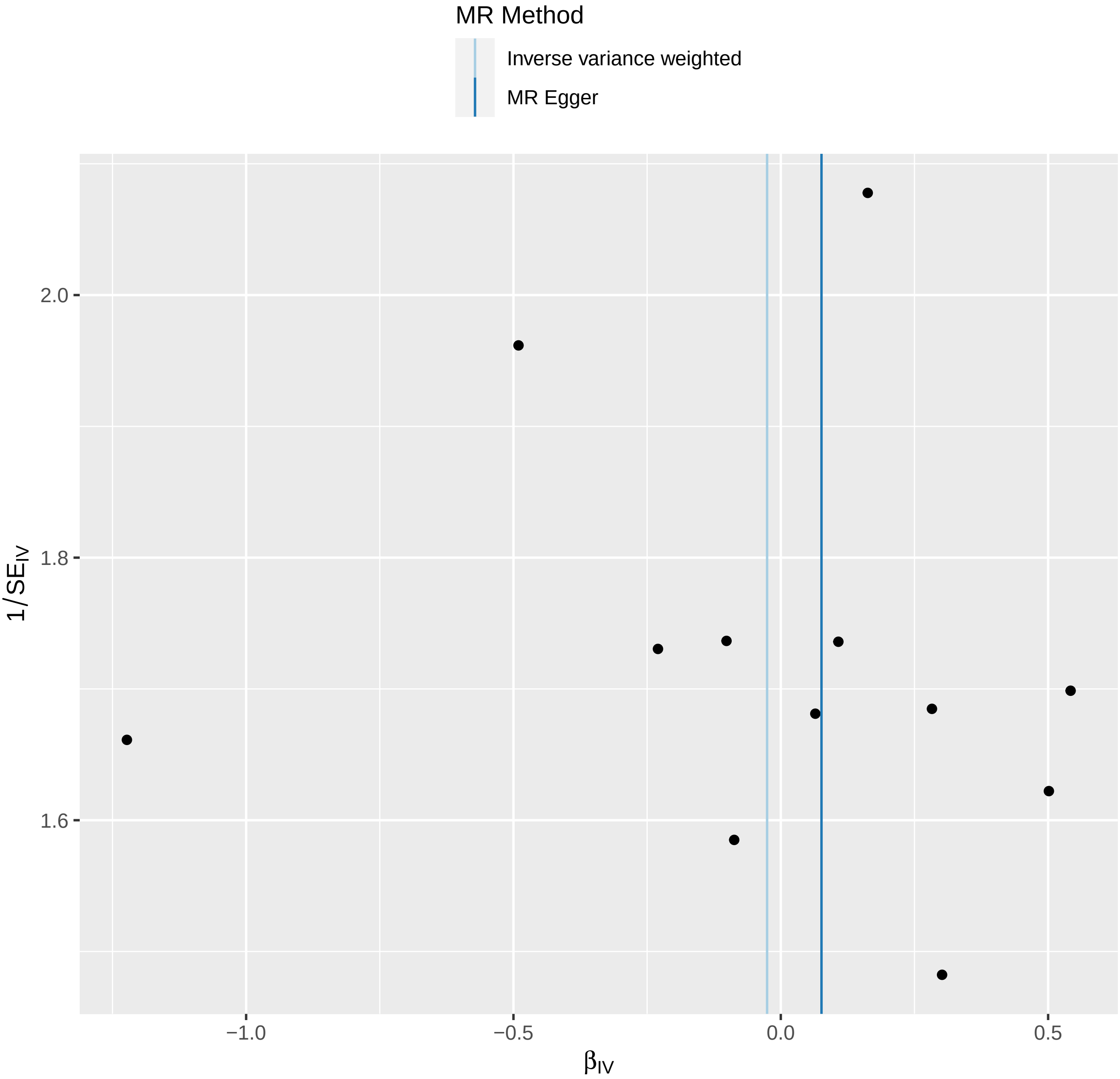

Batch 1572 : Gut microbiota abundance (genus Eubacterium eligens group id.14372) on Ulcerative rectosigmoiditis

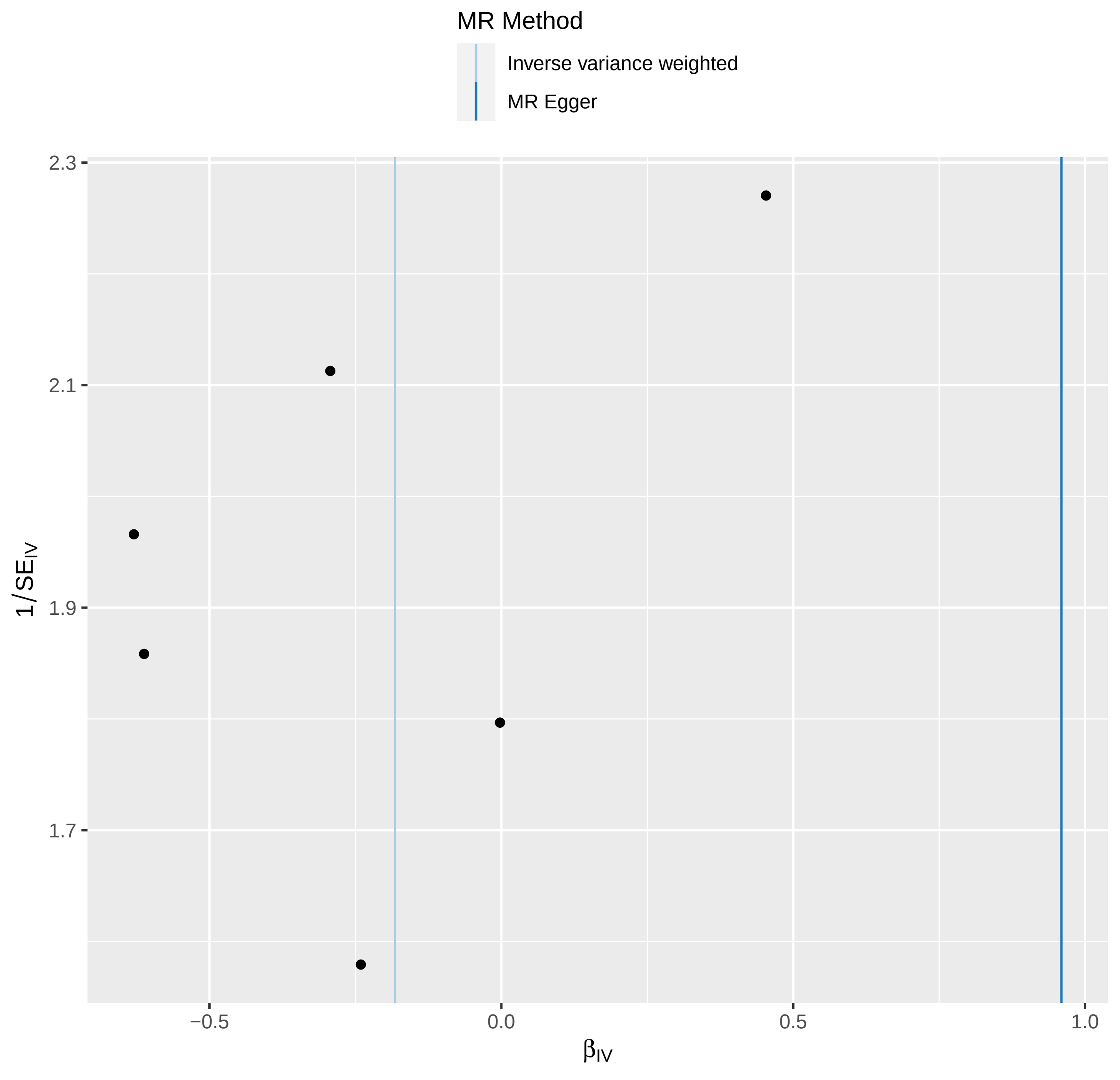

Batch 1573 : Gut microbiota abundance (genus Eubacterium fissicatena group id.14373) on Ulcerative rectosigmoiditis

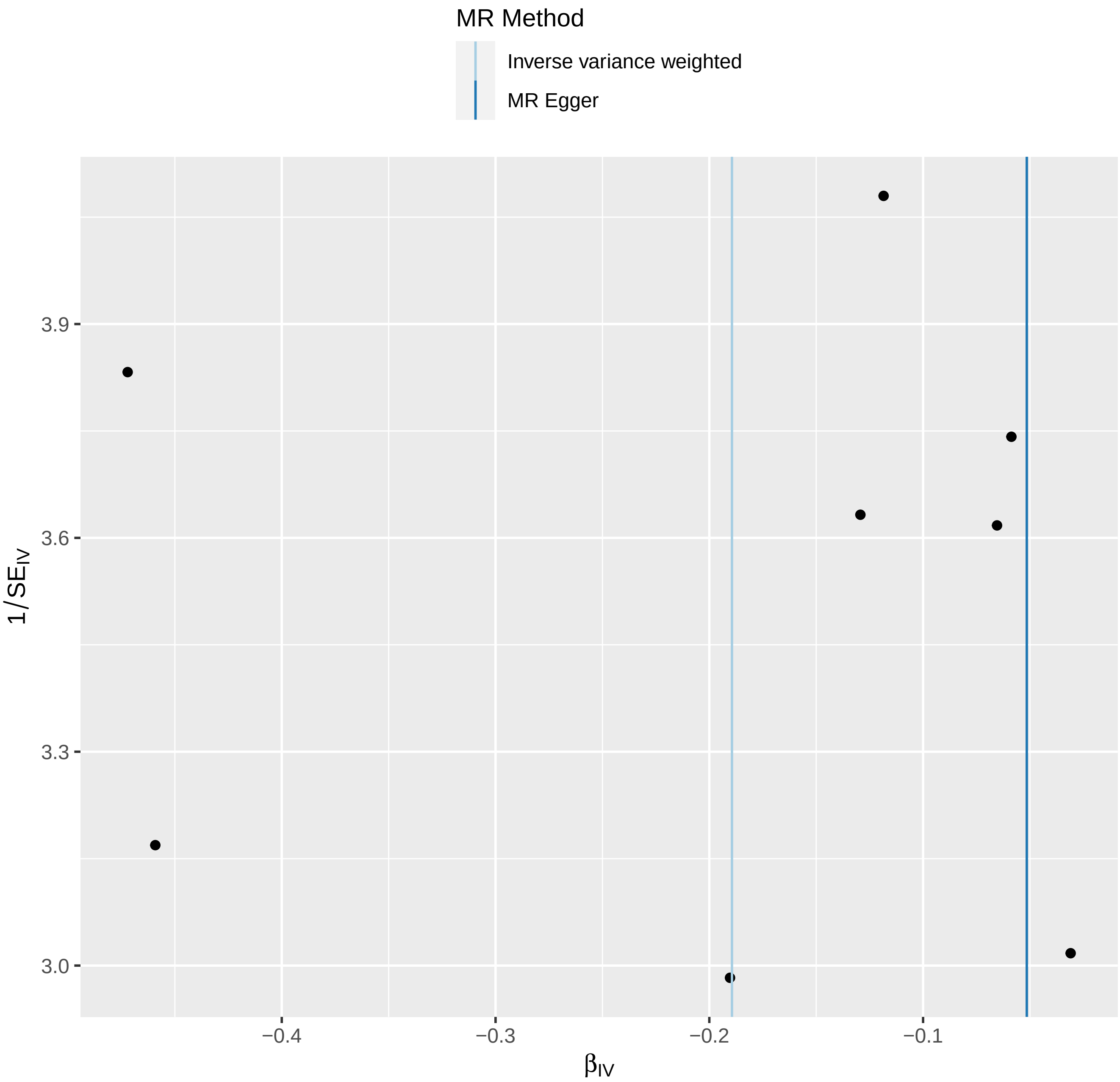

Batch 1574 : Gut microbiota abundance (genus Eubacterium hallii group id.11338) on Ulcerative rectosigmoiditis

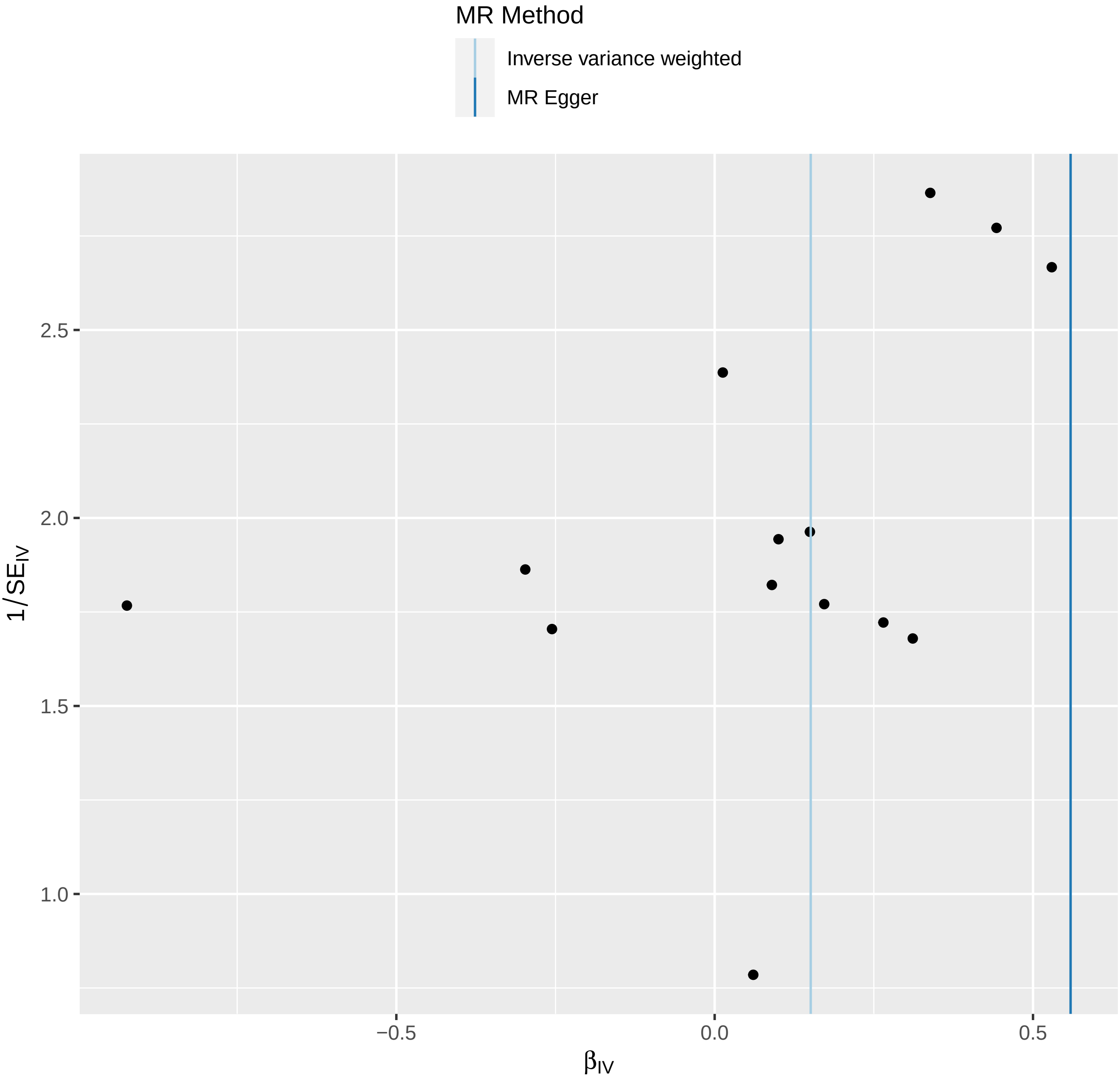

Batch 1575 : Gut microbiota abundance (genus Eubacterium nodatum group id.11297) on Ulcerative rectosigmoiditis

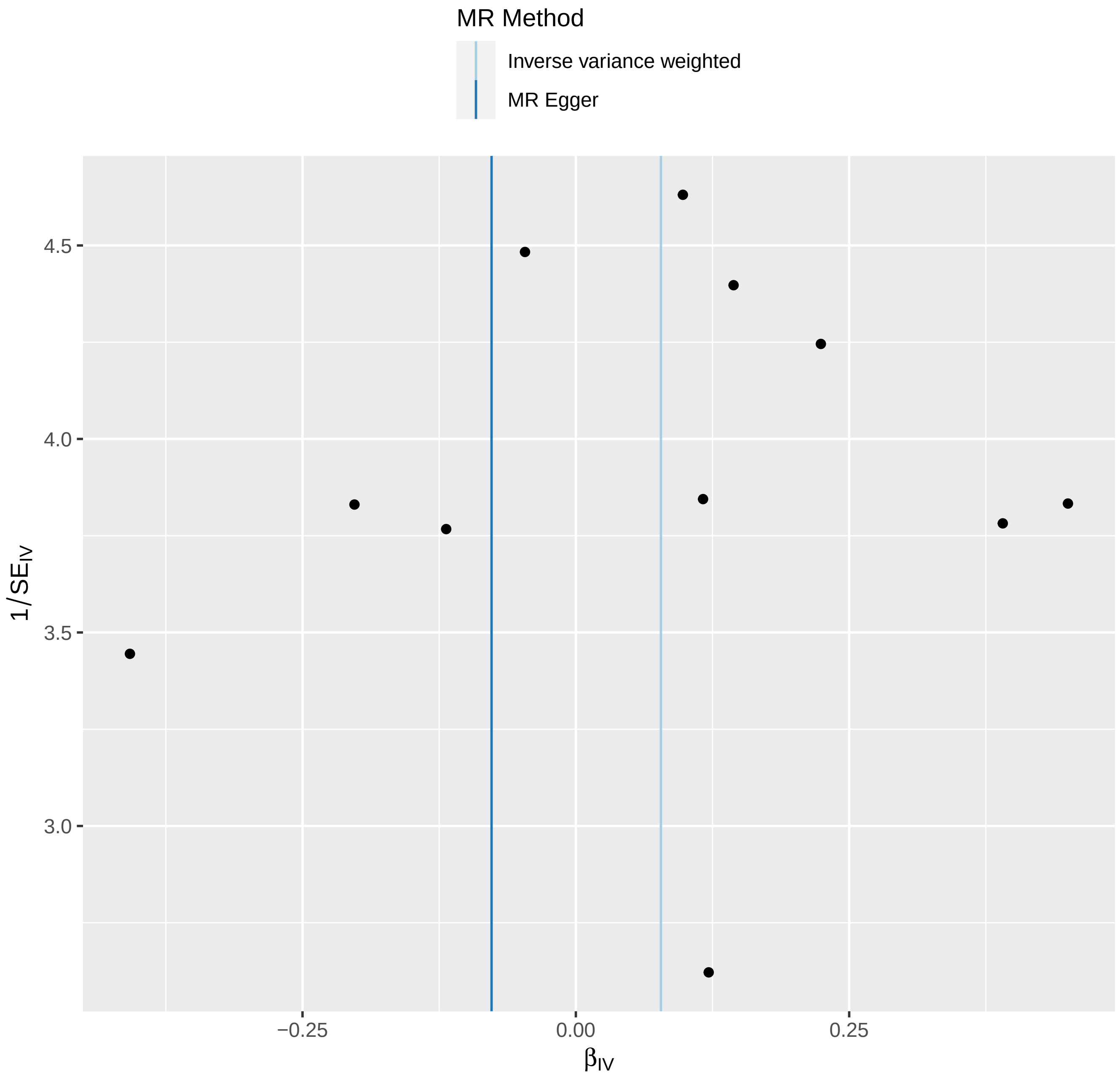

Batch 1576 : Gut microbiota abundance (genus Eubacterium oxidoreducens group id.11339) on Ulcerative rectosigmoiditis

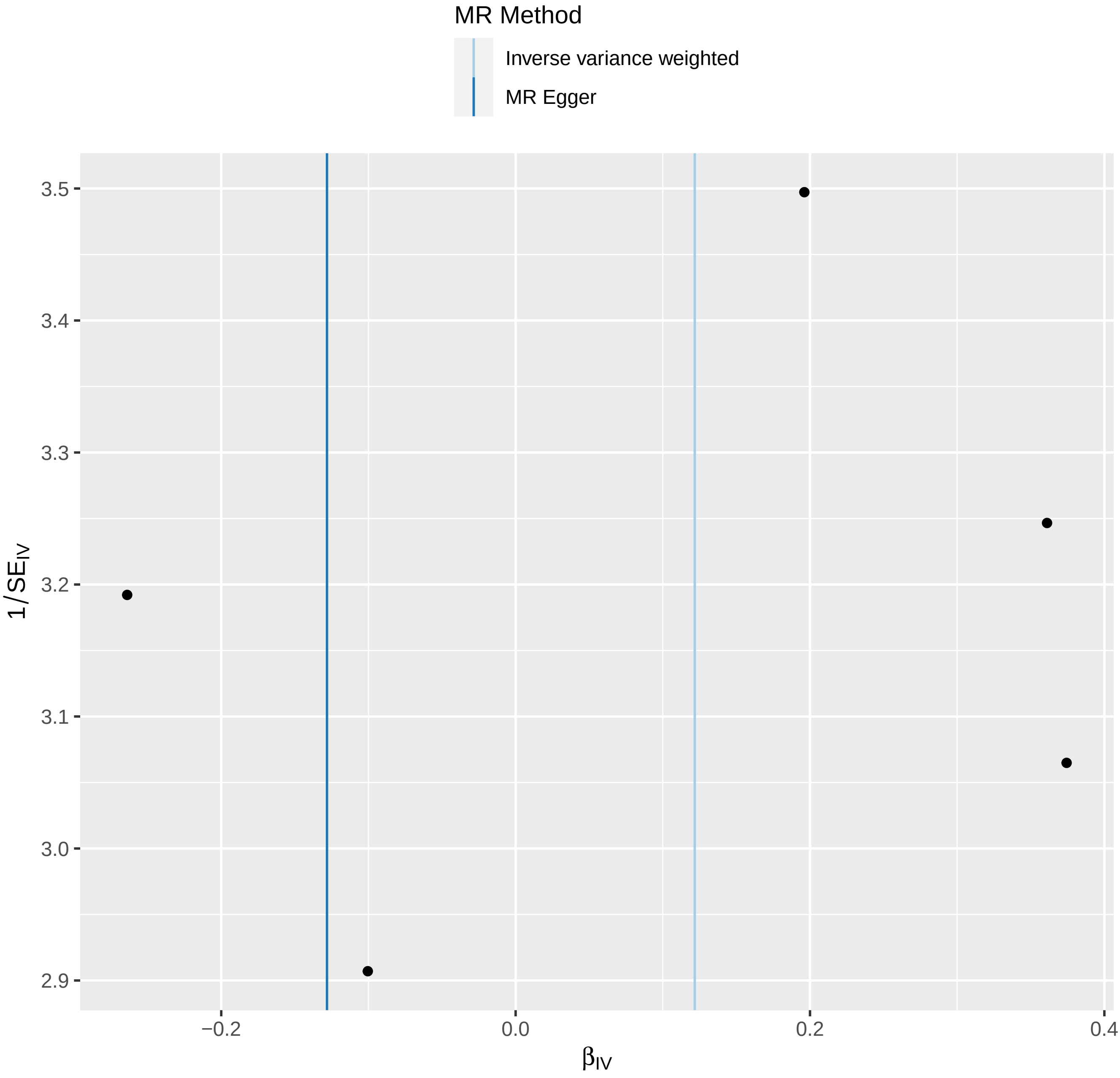

Batch 1577 : Gut microbiota abundance (genus Eubacterium rectale group id.14374) on Ulcerative rectosigmoiditis

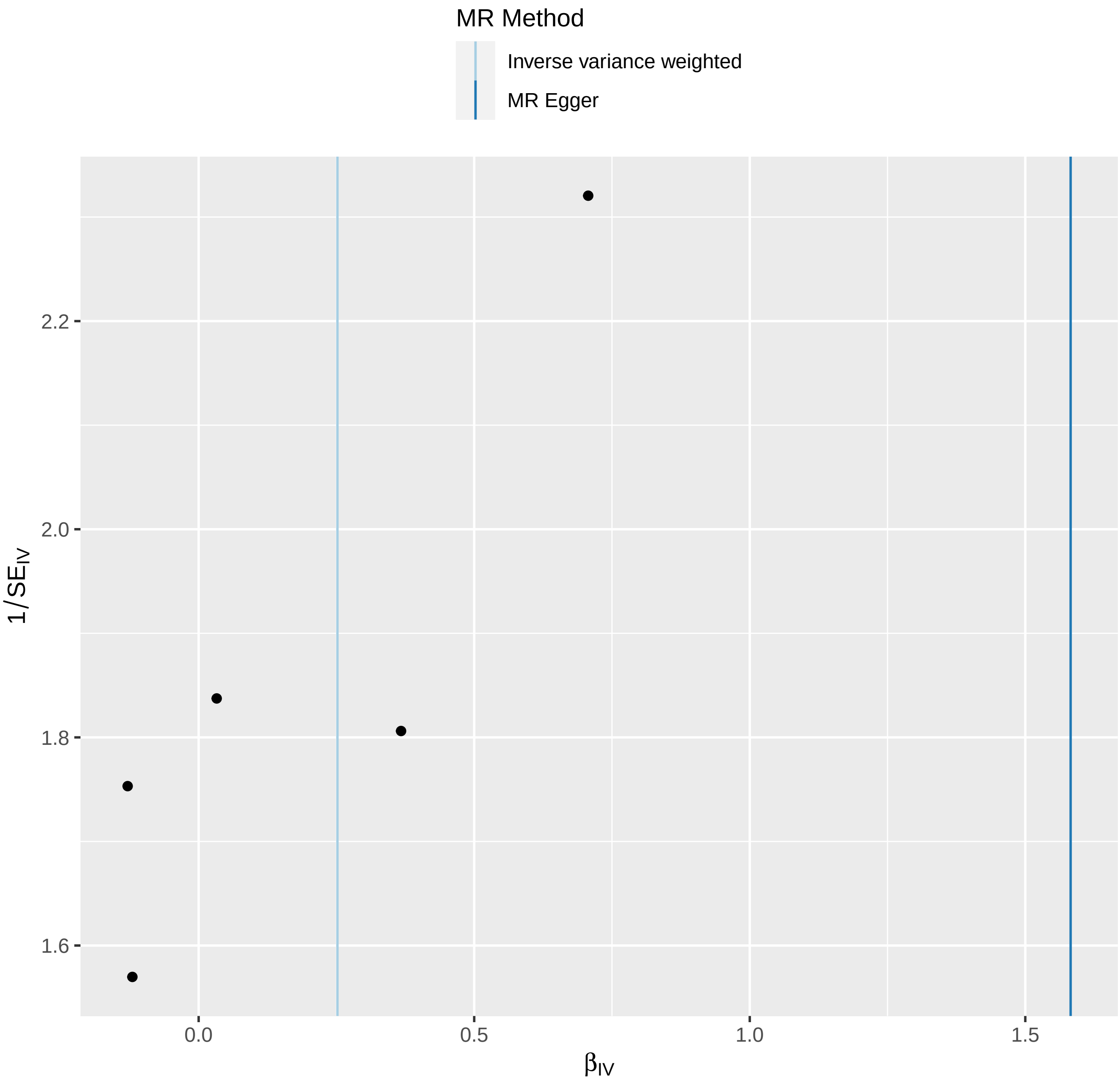

Batch 1578 : Gut microbiota abundance (genus Eubacterium ruminantium group id.11340) on Ulcerative rectosigmoiditis

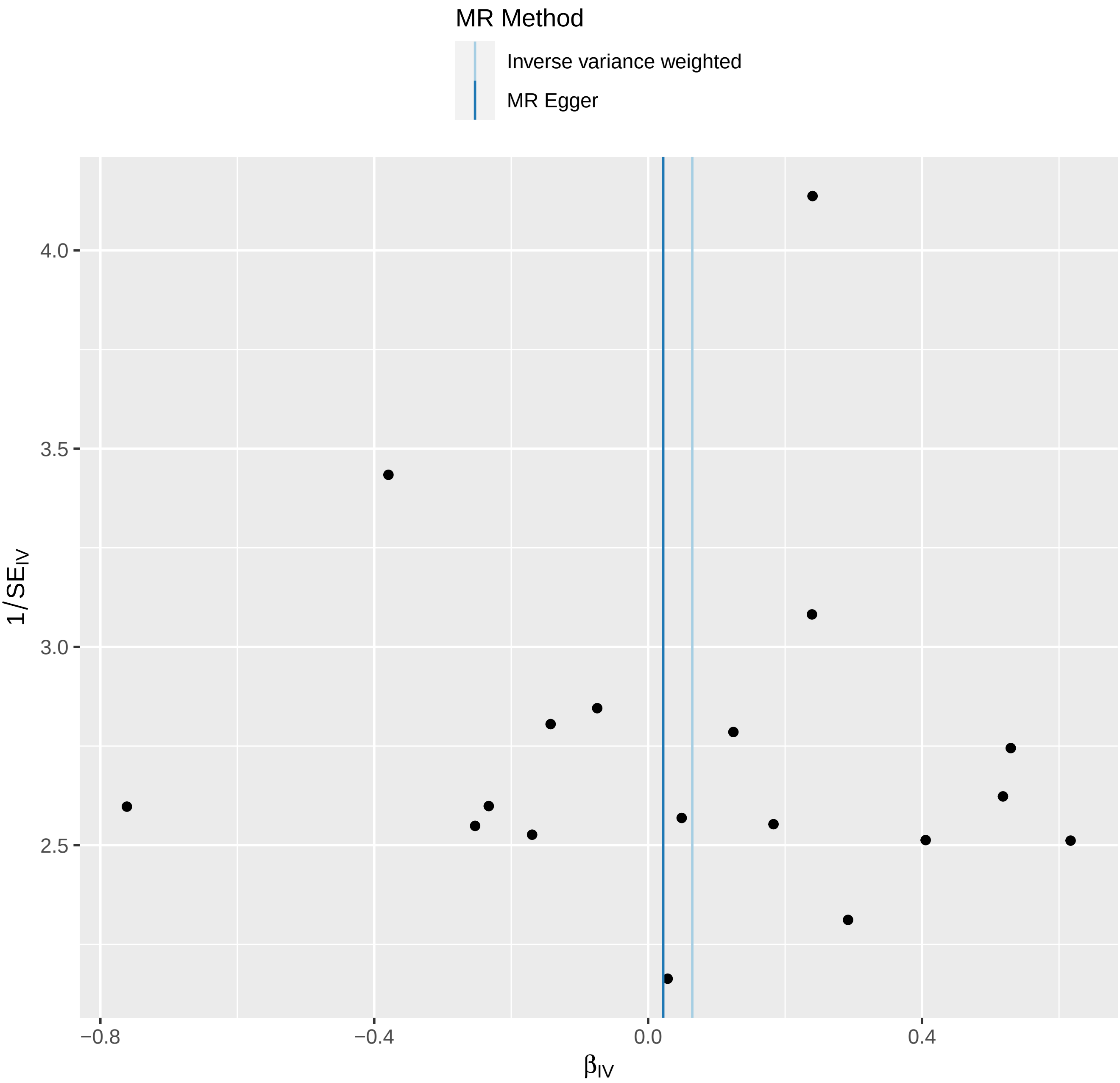

Batch 1579 : Gut microbiota abundance (genus Eubacterium ventriosum group id.11341) on Ulcerative rectosigmoiditis

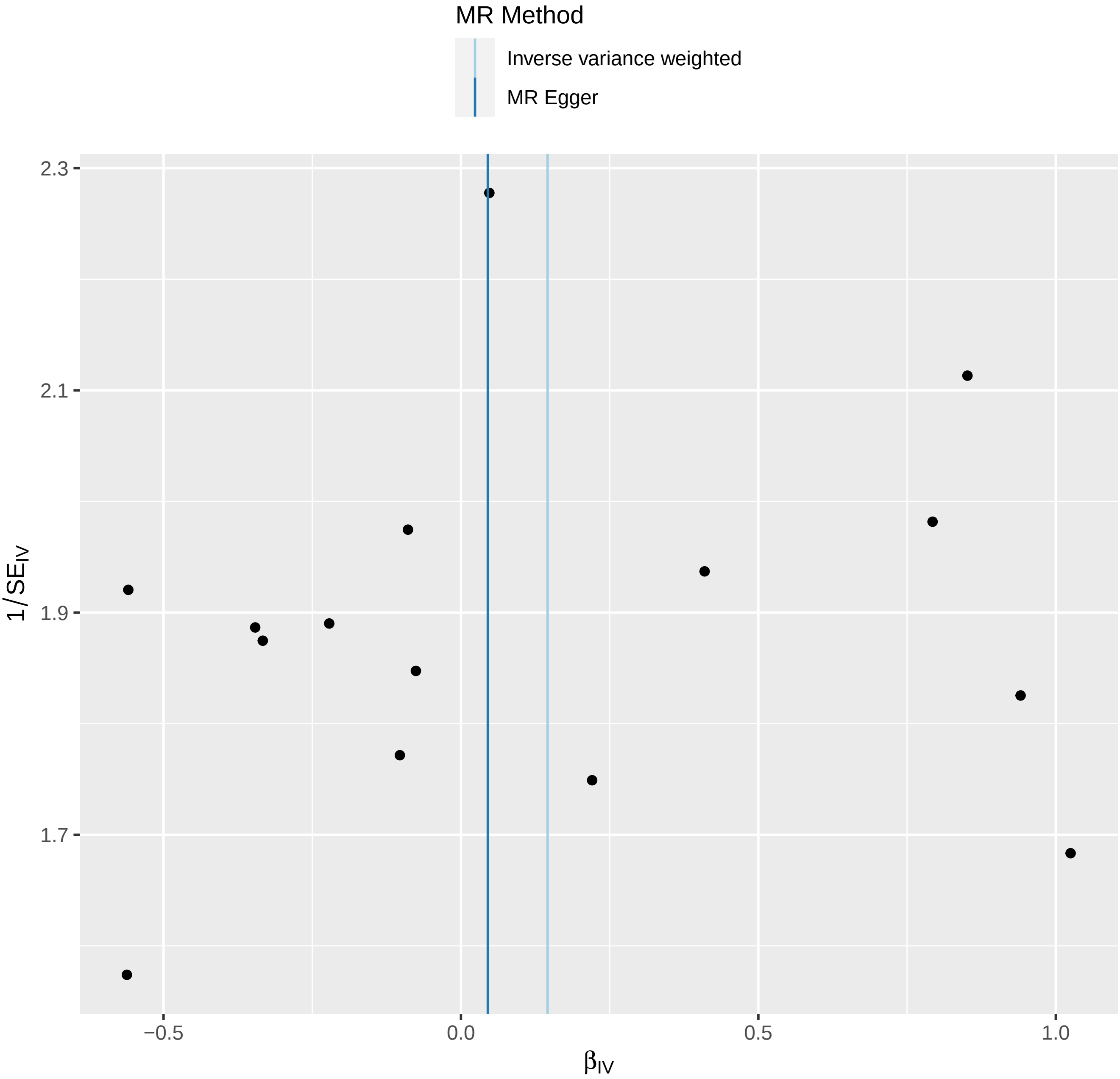

Batch 1580 : Gut microbiota abundance (genus Eubacterium xylanophilum group id.14375) on Ulcerative rectosigmoiditis

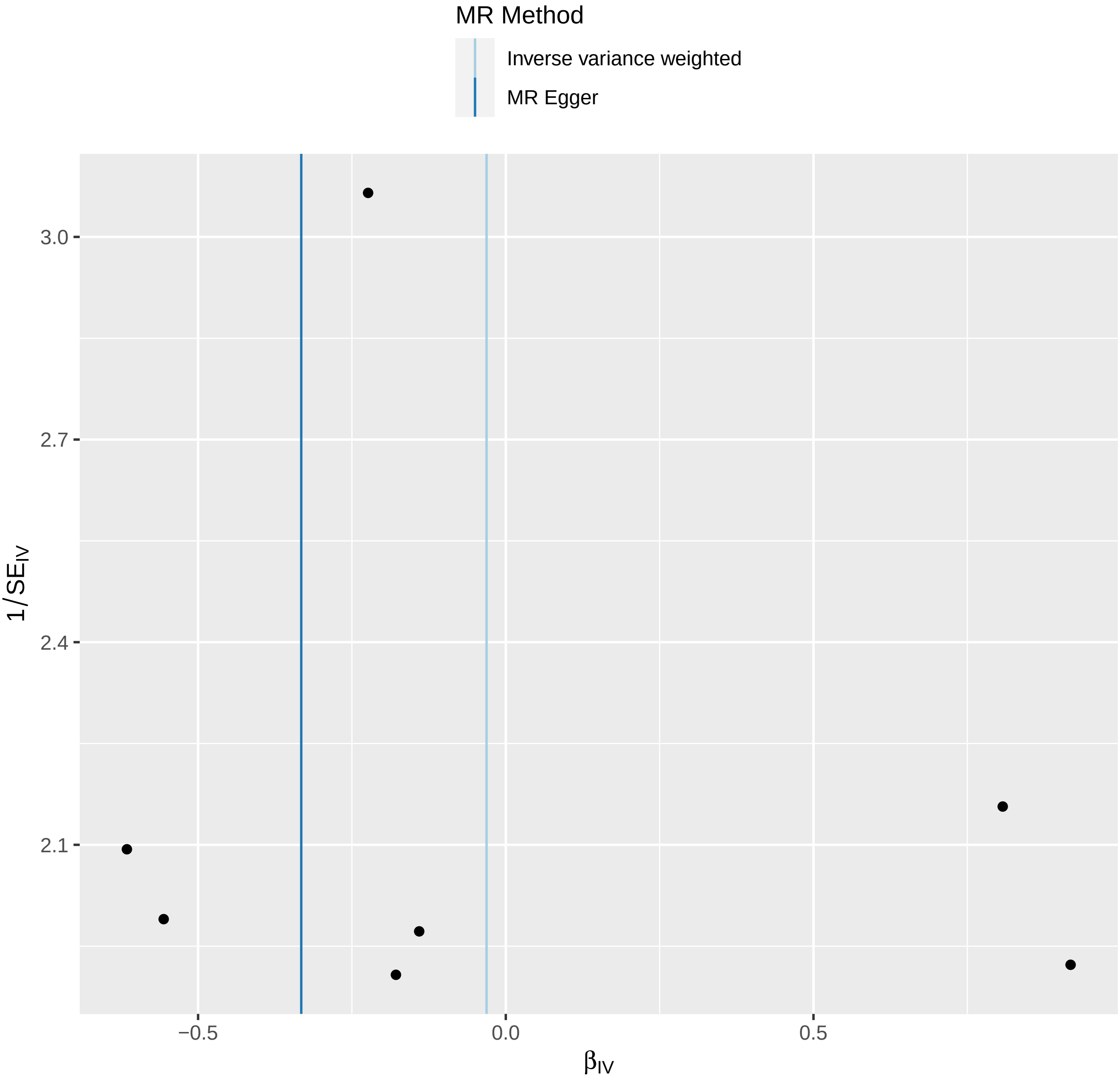

Batch 1581 : Gut microbiota abundance (genus Faecalibacterium id.2057) on Ulcerative rectosigmoiditis

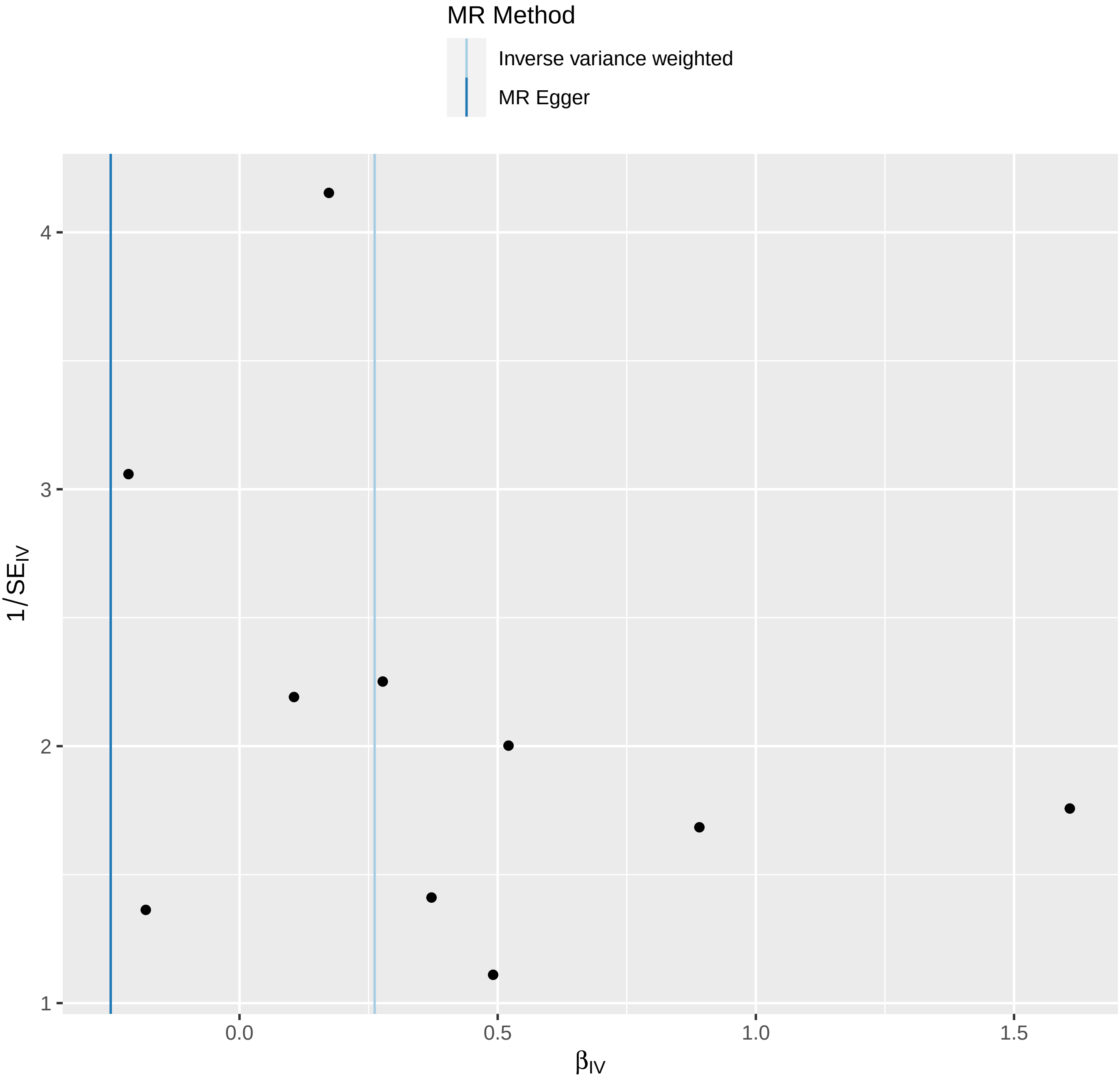

Batch 1582 : Gut microbiota abundance (genus Family XIII AD3011 group id.11293) on Ulcerative rectosigmoiditis

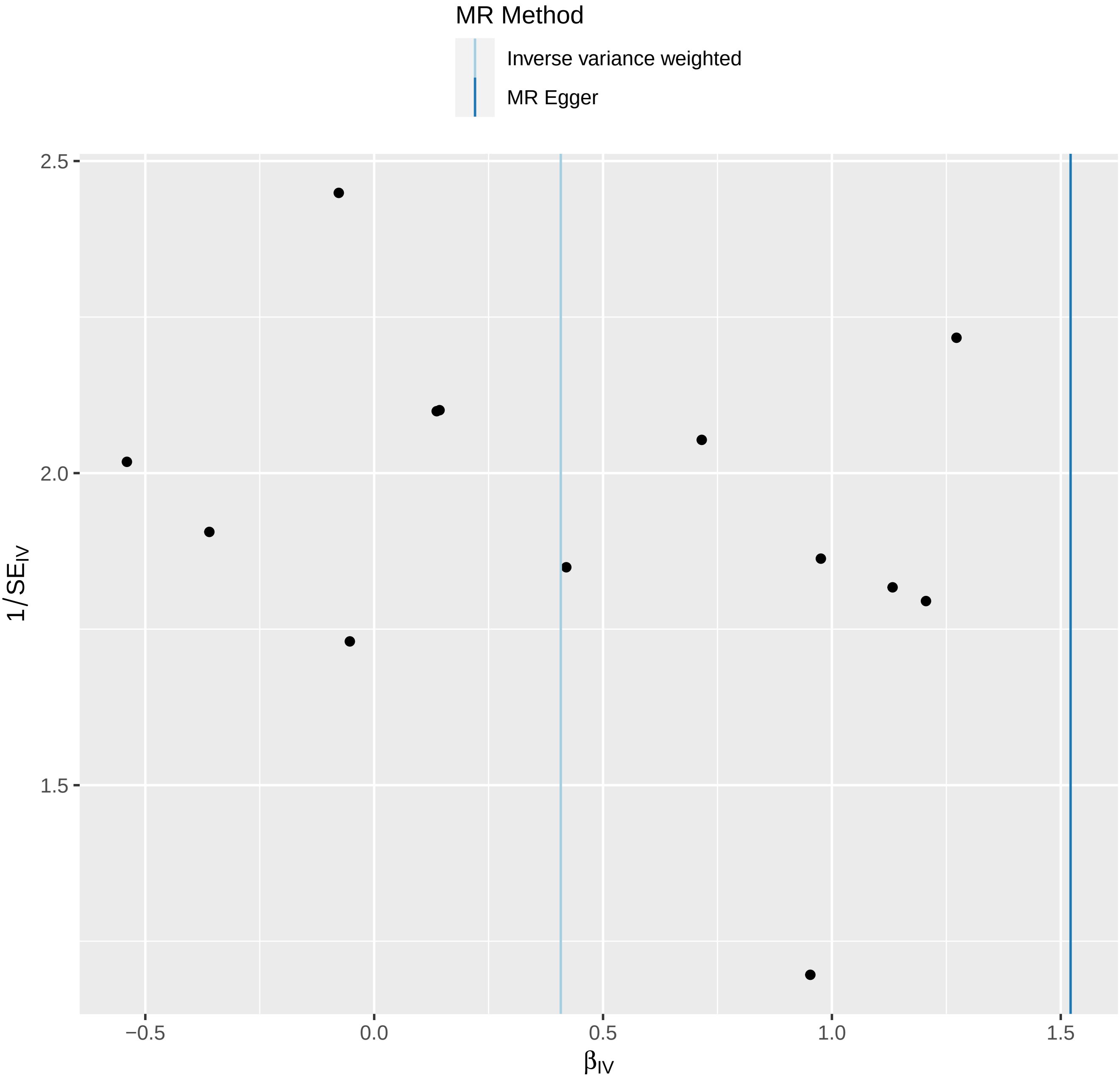

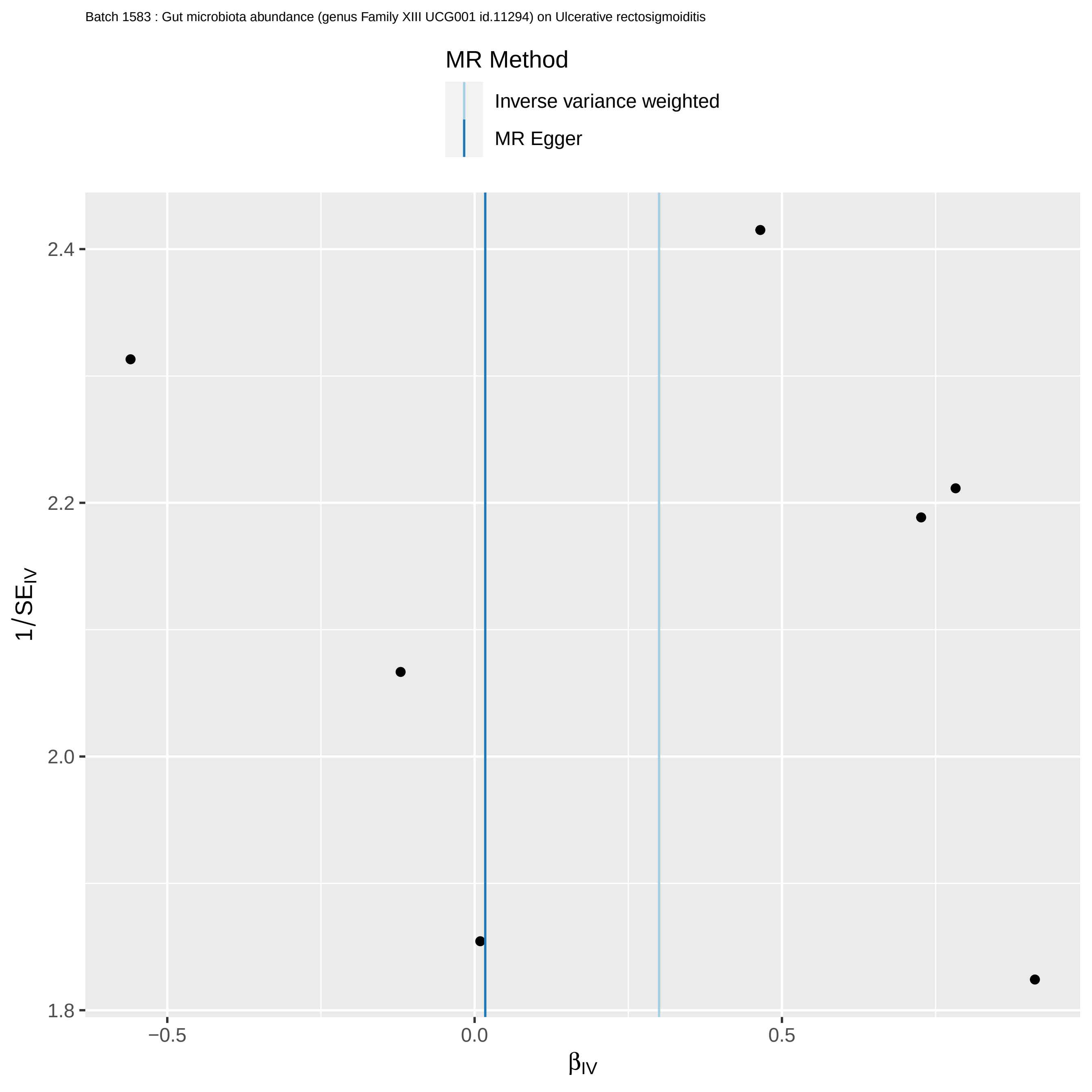
Batch 1583 : Gut microbiota abundance (genus Family XIII UCG001 id.11294) on Ulcerative rectosigmoiditis
MR Method
Inverse variance weighted
MR Egger
2.4
2.2
2.0
1.8
1/SE_IV
−0.5
0.0
0.5
β_IV

Batch 1584 : Gut microbiota abundance (genus Flavonifractor id.2059) on Ulcerative rectosigmoiditis

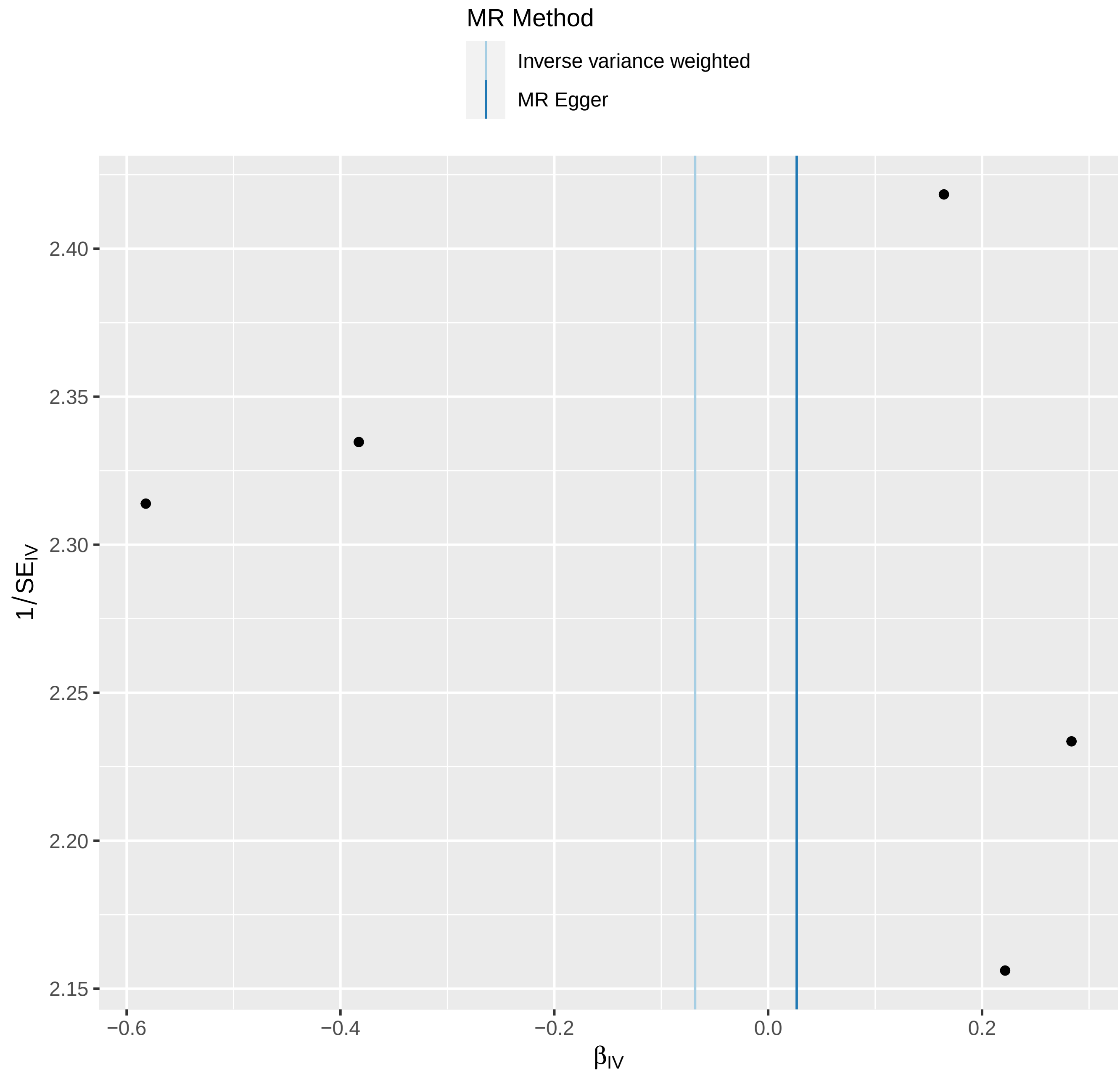

Batch 1585 : Gut microbiota abundance (genus Fusicatenibacter id.11305) on Ulcerative rectosigmoiditis

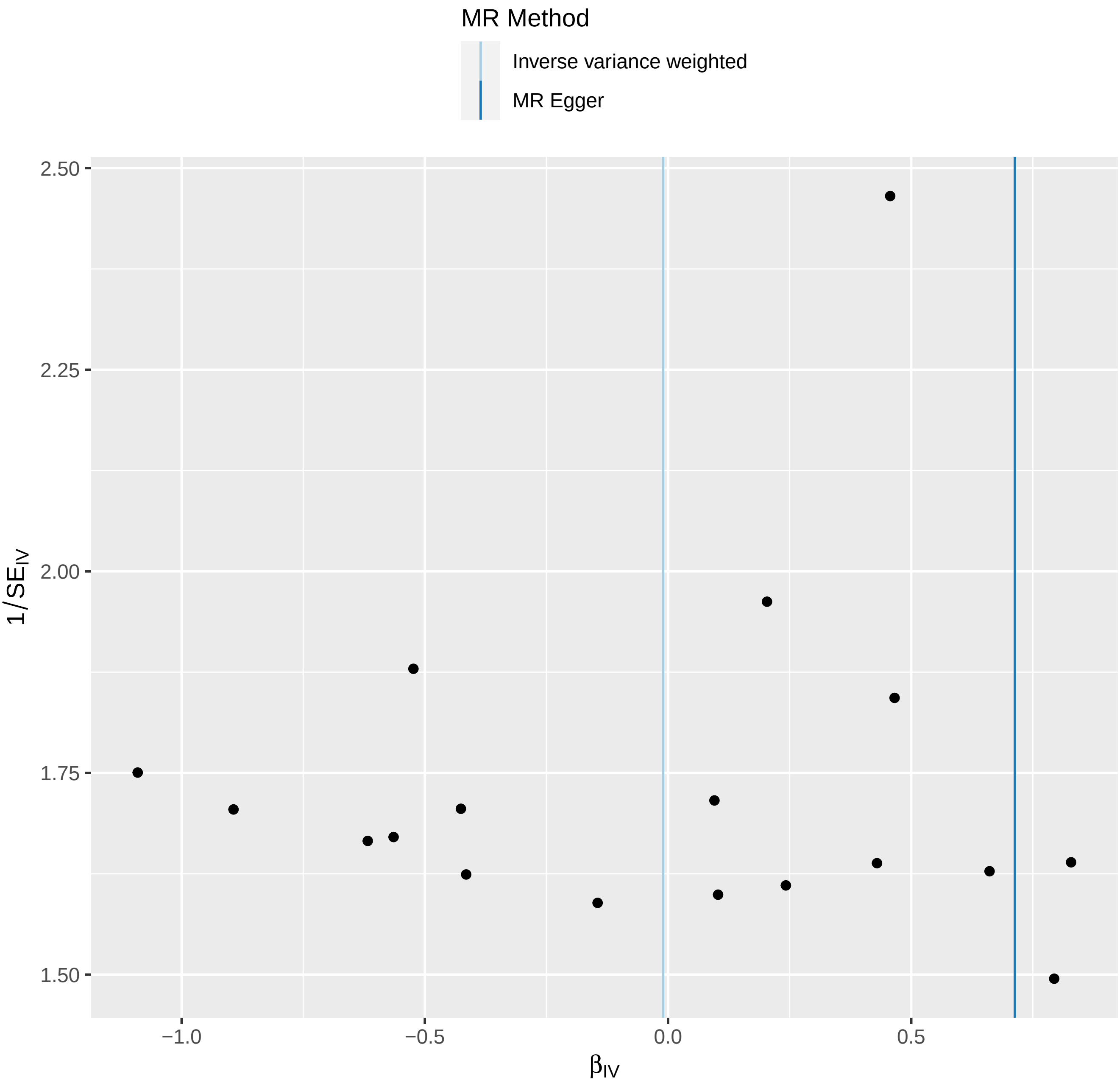

Batch 1586 : Gut microbiota abundance (genus Gordonibacter id.821) on Ulcerative rectosigmoiditis

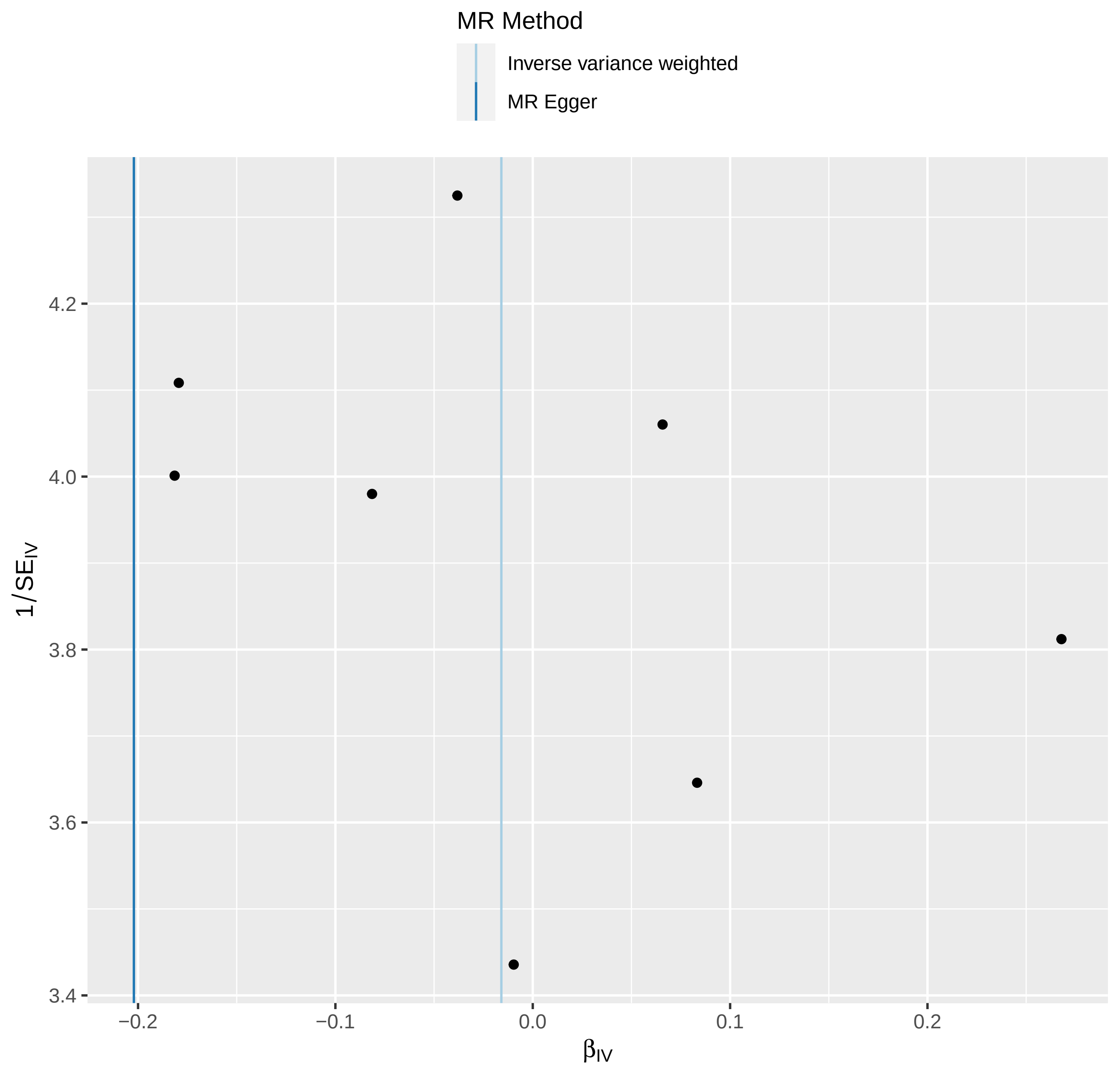

Batch 1587 : Gut microbiota abundance (genus Haemophilus id.3698) on Ulcerative rectosigmoiditis

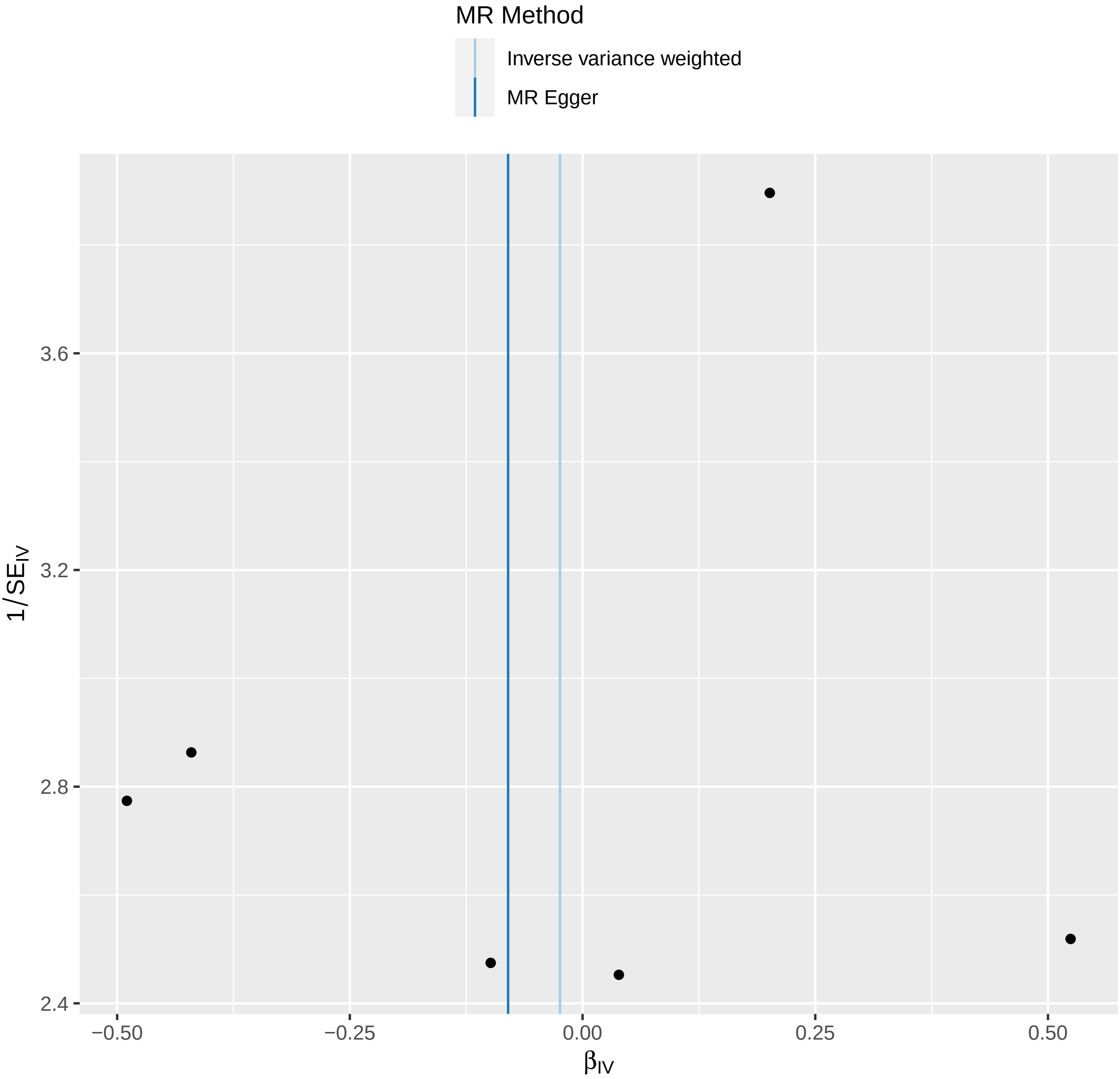

Batch 1588 : Gut microbiota abundance (genus Holdemanella id.11393) on Ulcerative rectosigmoiditis

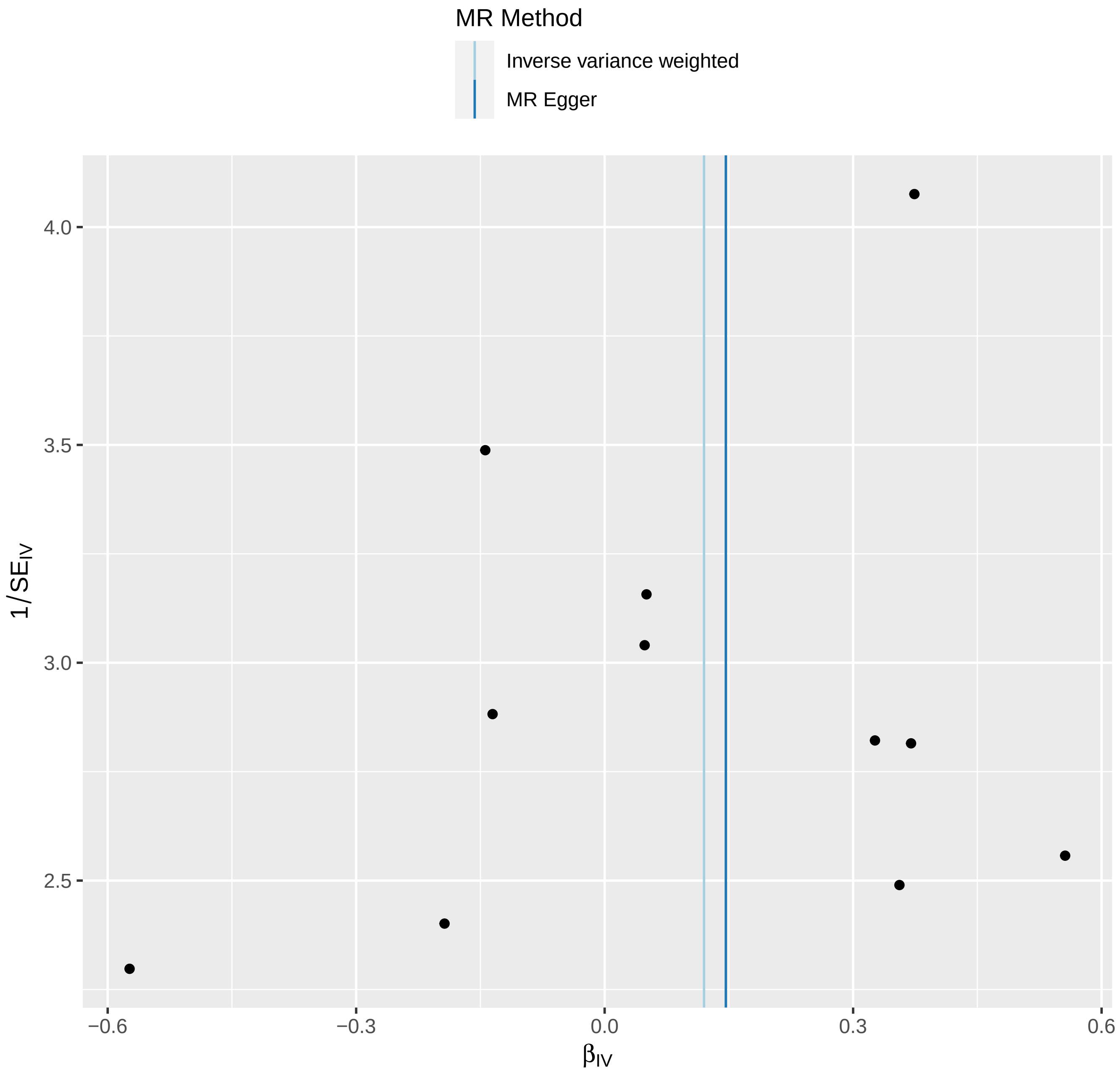

Batch 1589 : Gut microbiota abundance (genus Holdemania id.2157) on Ulcerative rectosigmoiditis

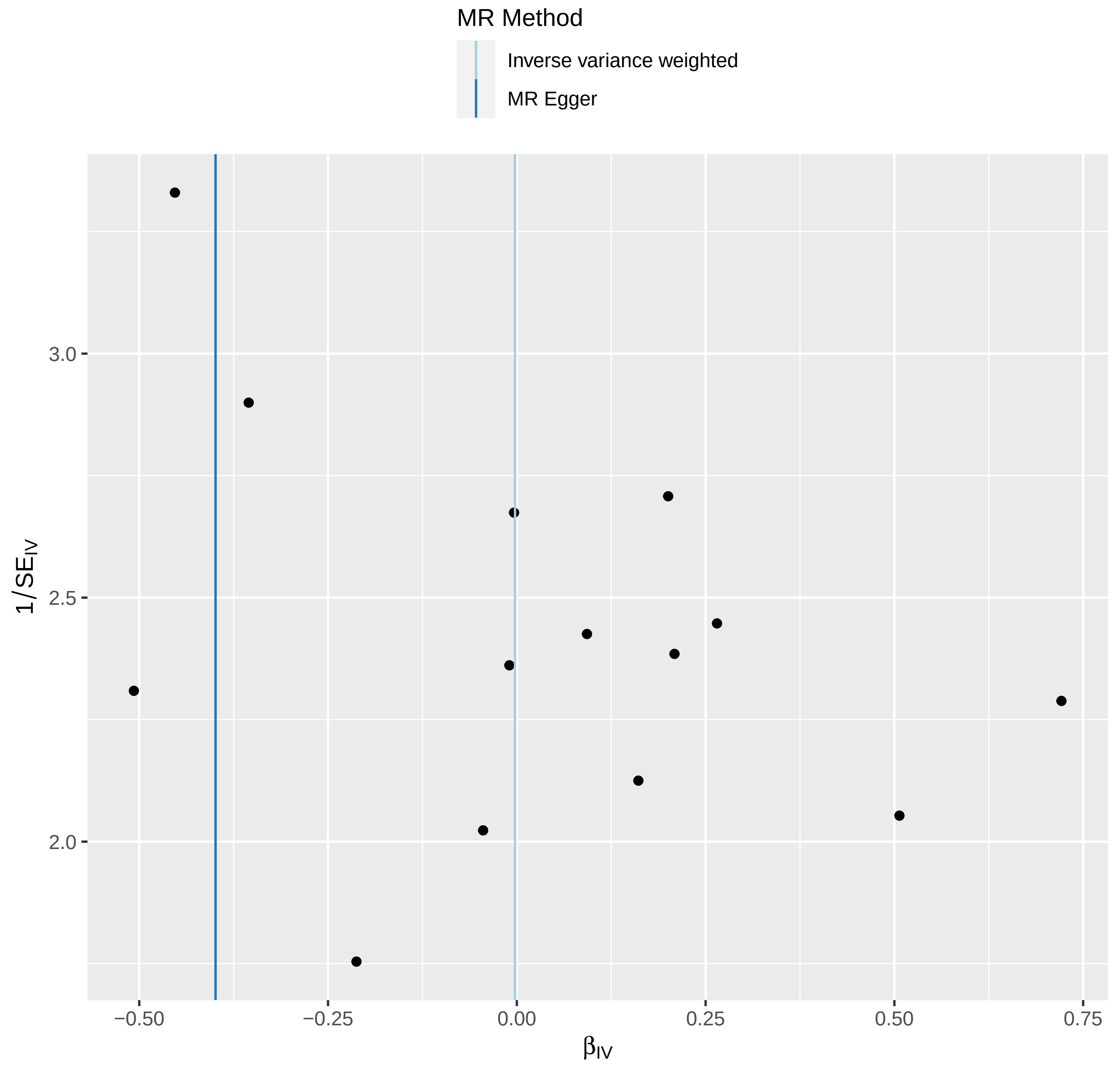

Batch 1590 : Gut microbiota abundance (genus Howardella id.2000) on Ulcerative rectosigmoiditis

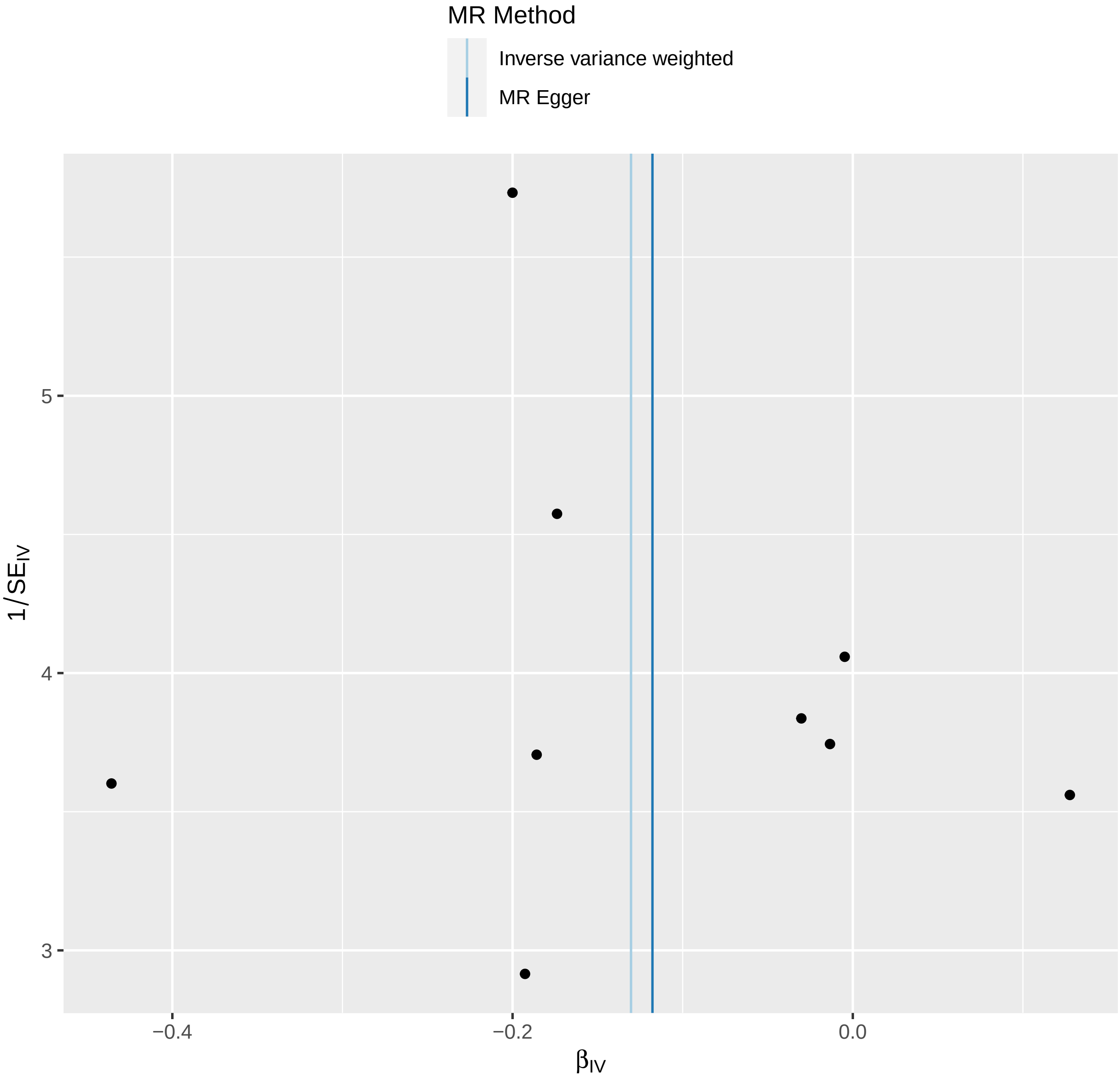

Batch 1591 : Gut microbiota abundance (genus Hungatella id.11306) on Ulcerative rectosigmoiditis

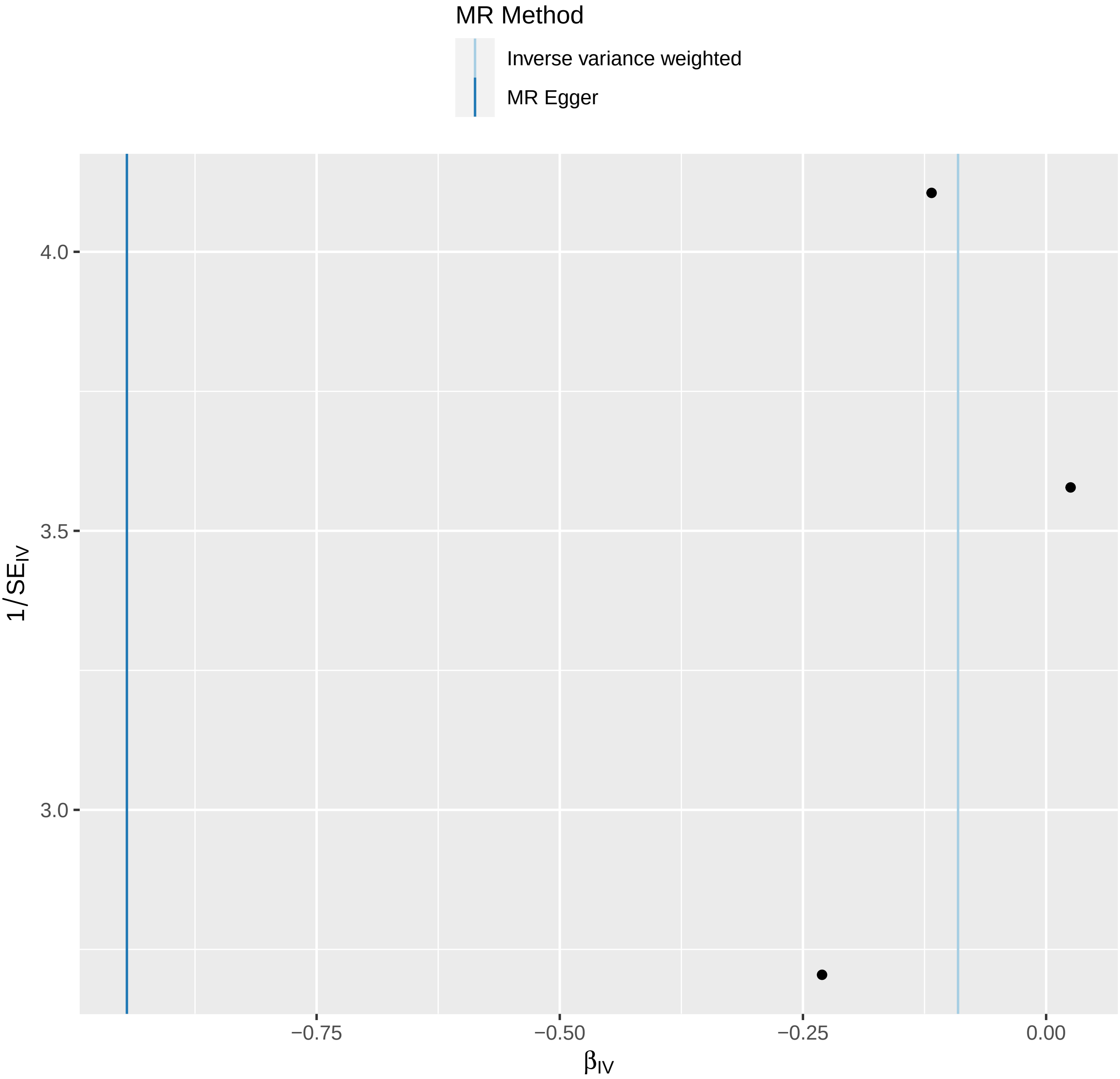

Batch 1592 : Gut microbiota abundance (genus Intestinibacter id.11345) on Ulcerative rectosigmoiditis

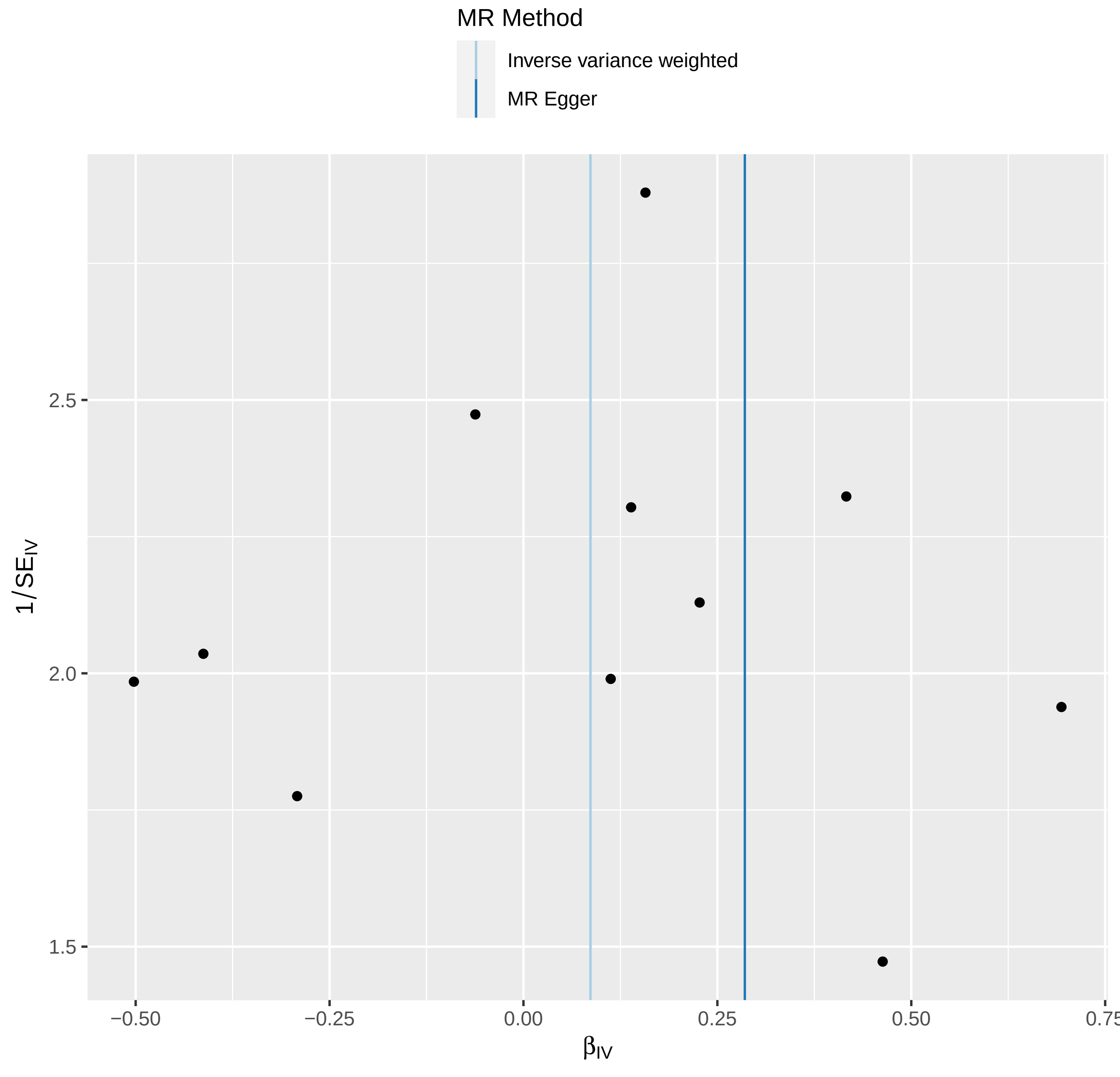

Batch 1593 : Gut microbiota abundance (genus Intestinimonas id.2062) on Ulcerative rectosigmoiditis

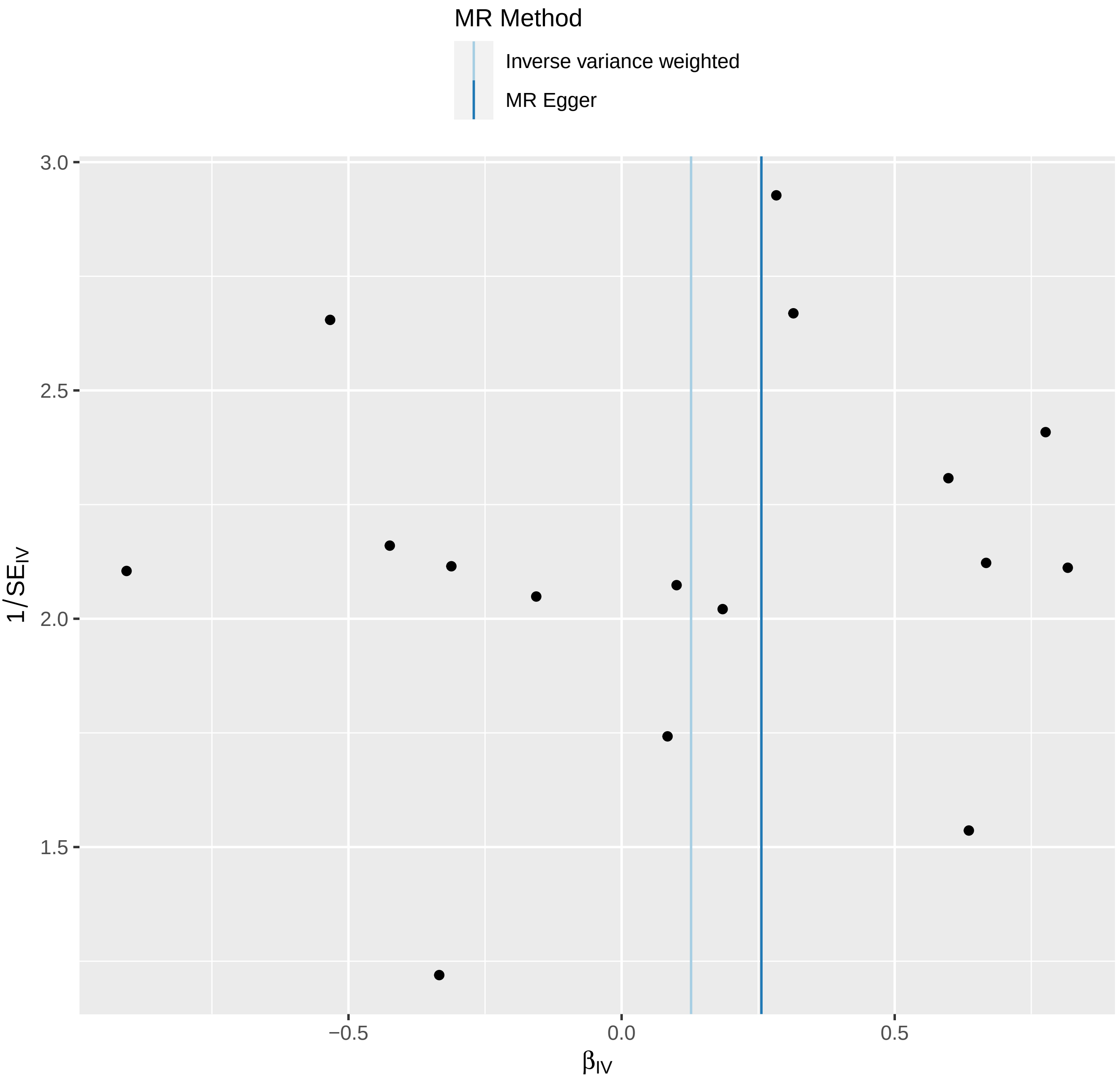

Batch 1594 : Gut microbiota abundance (genus Lachnoclostridium id.11308) on Ulcerative rectosigmoiditis

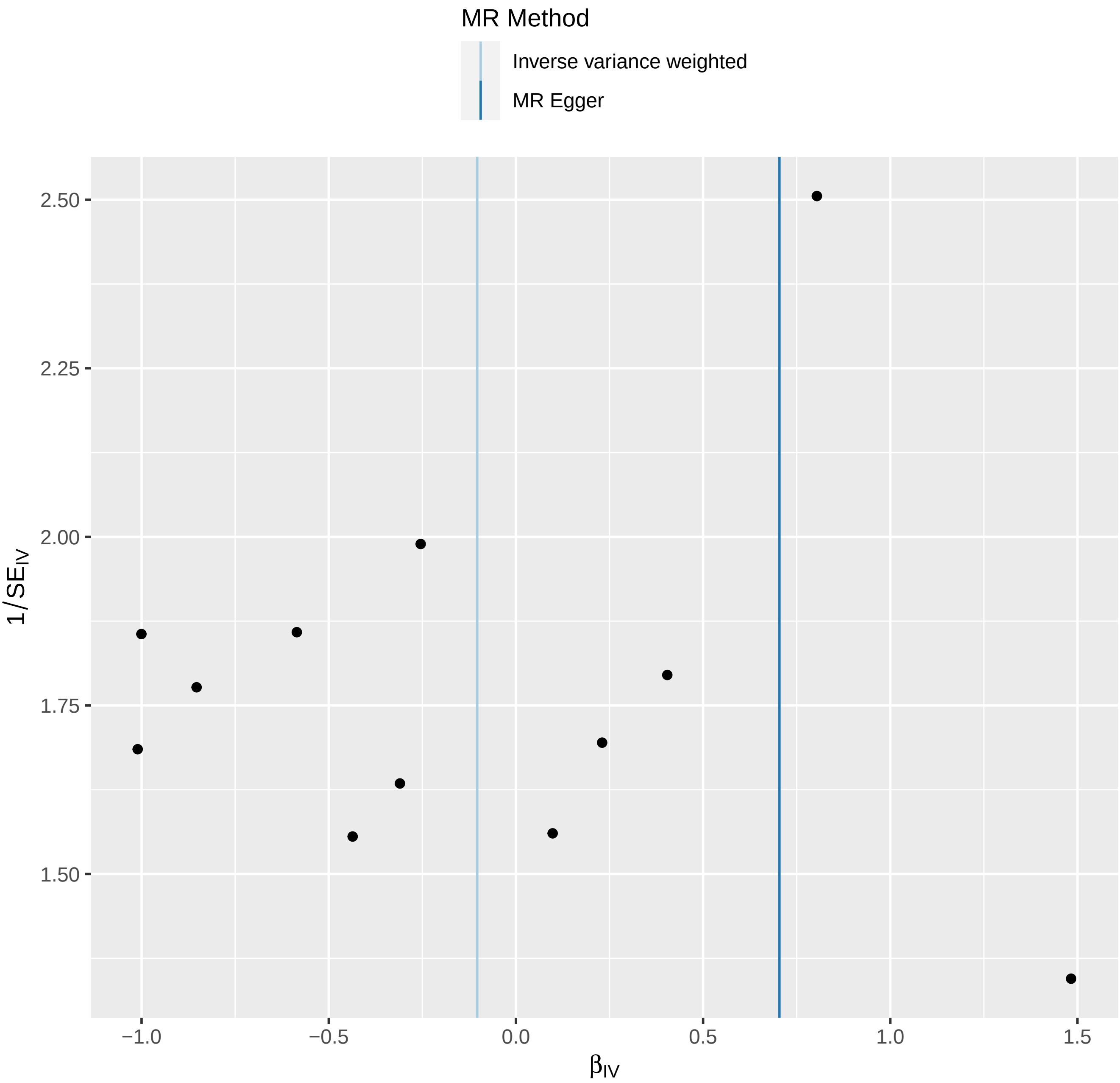

Batch 1595 : Gut microbiota abundance (genus Lachnospiraceae FCS020 group id.11314) on Ulcerative rectosigmoiditis

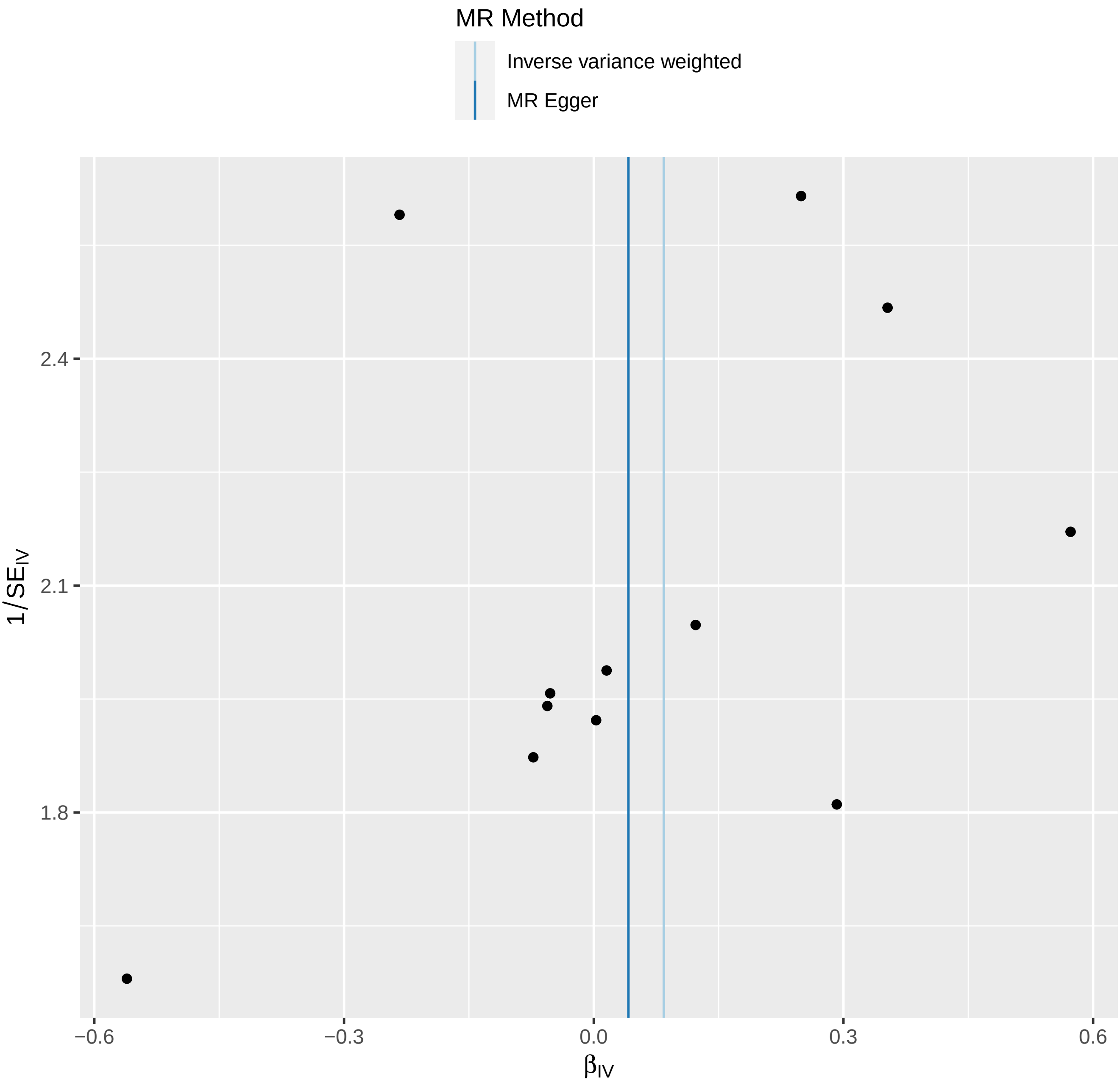

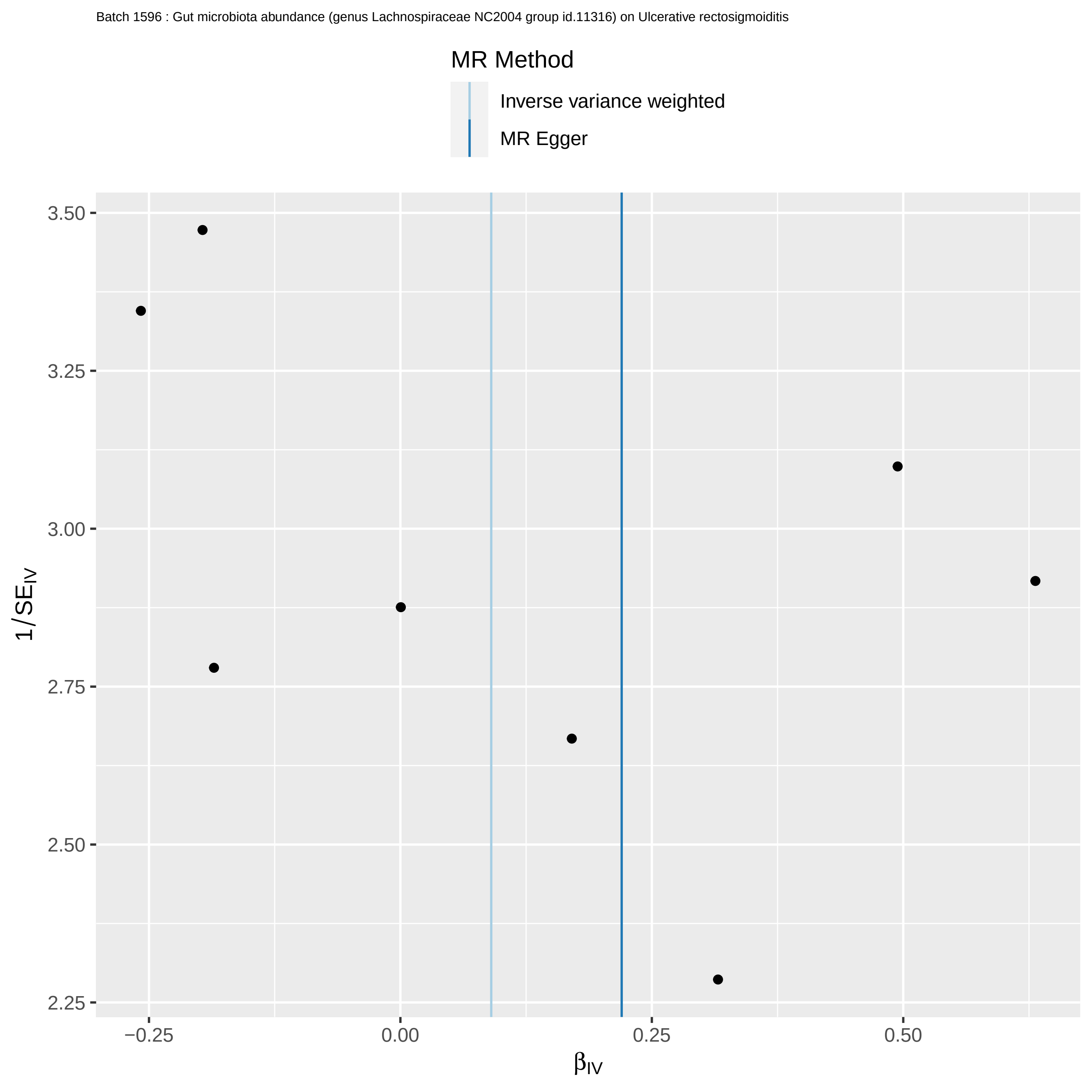
Batch 1596 : Gut microbiota abundance (genus Lachnospiraceae NC2004 group id.11316) on Ulcerative rectosigmoiditis
MR Method
Inverse variance weighted
MR Egger
3.50
3.25
3.00
2.75
2.50
2.25
1/SE$_{IV}$
−0.25
0.00
0.25
0.50
β_{IV}

Batch 1597 : Gut microbiota abundance (genus Lachnospiraceae ND3007 group id.11317) on Ulcerative rectosigmoiditis

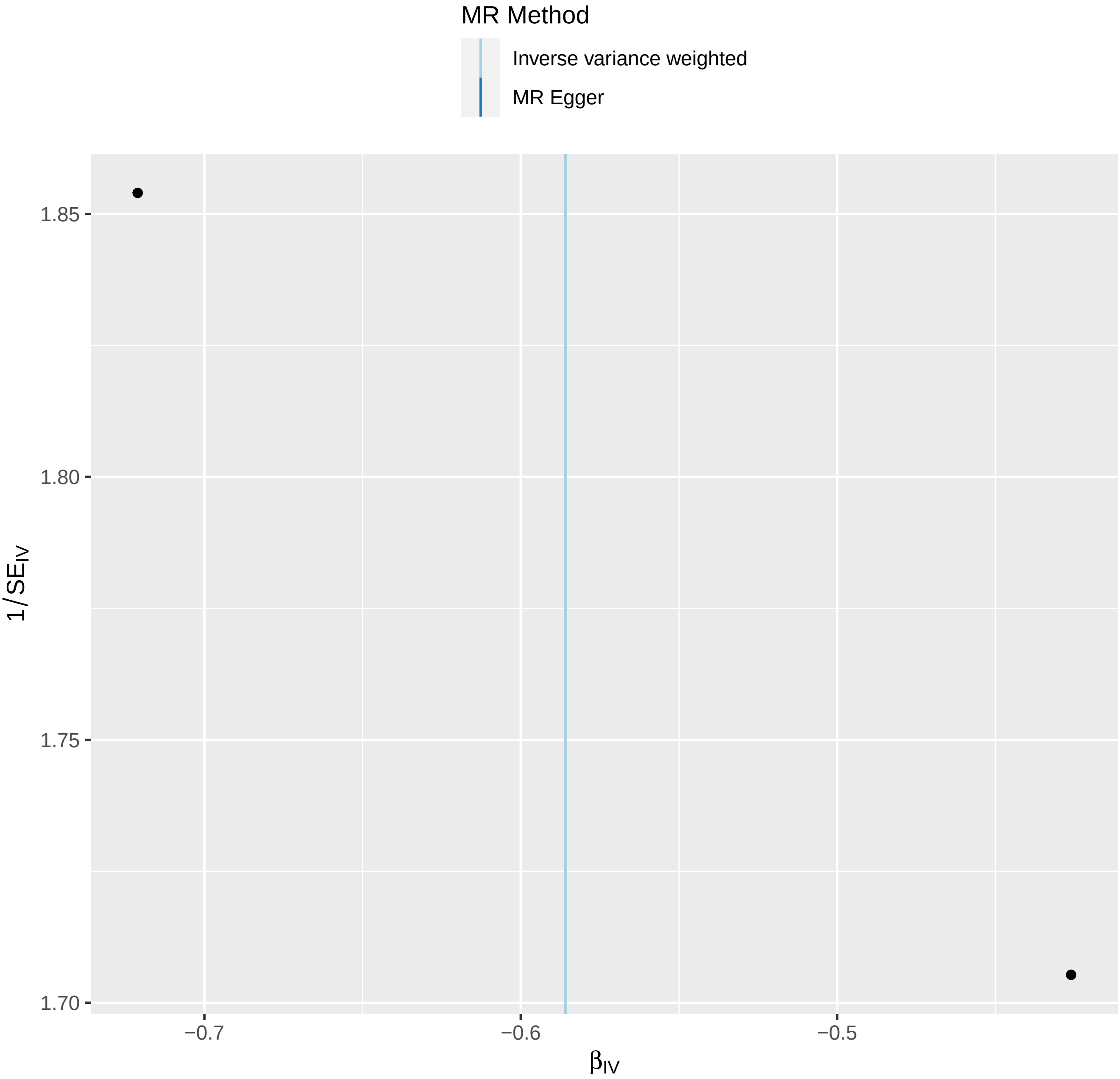

Batch 1598 : Gut microbiota abundance (genus Lachnospiraceae NK4A136 group id.11319) on Ulcerative rectosigmoiditis

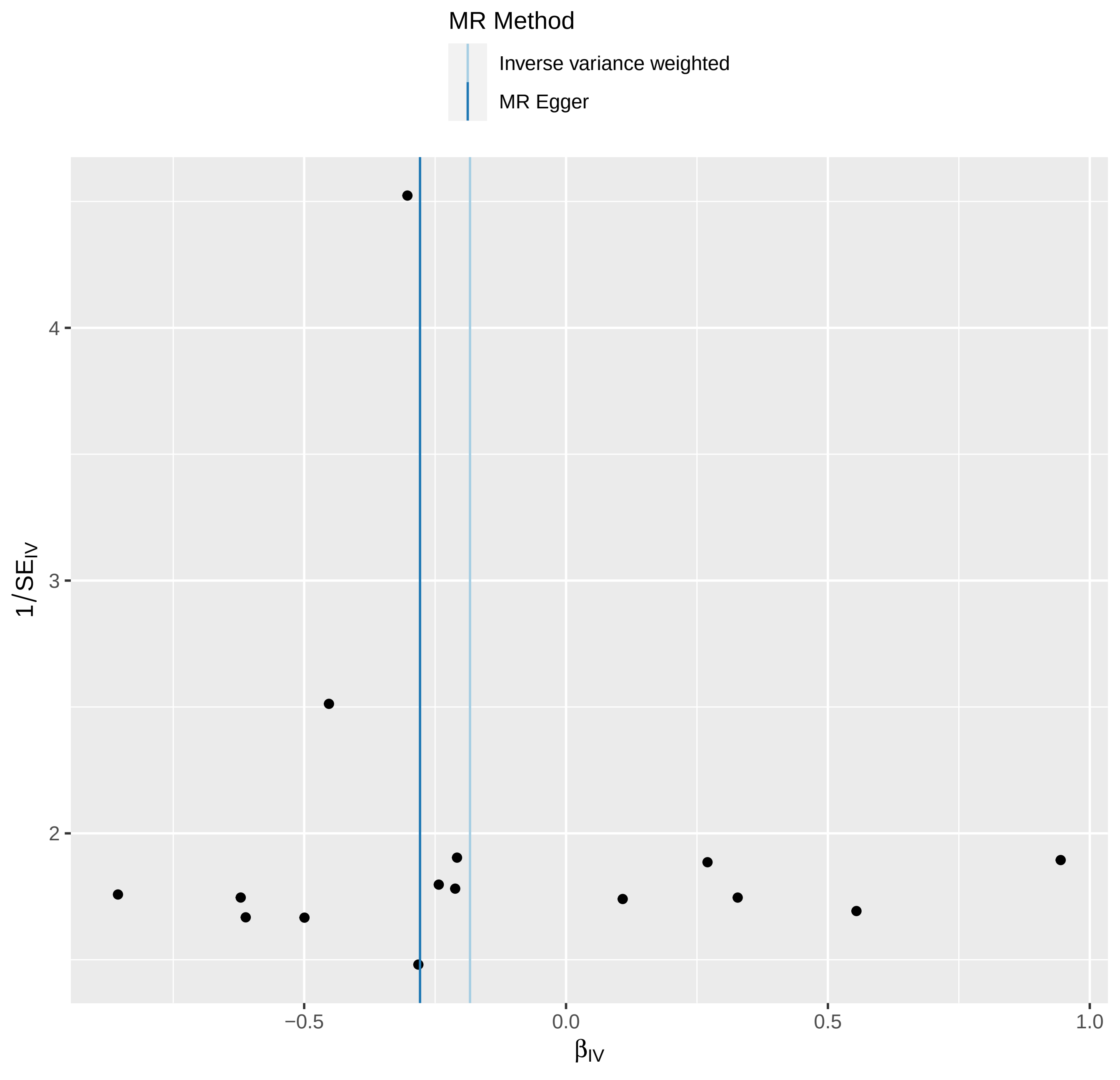

Batch 1599 : Gut microbiota abundance (genus Lachnospiraceae UCG001 id.11321) on Ulcerative rectosigmoiditis

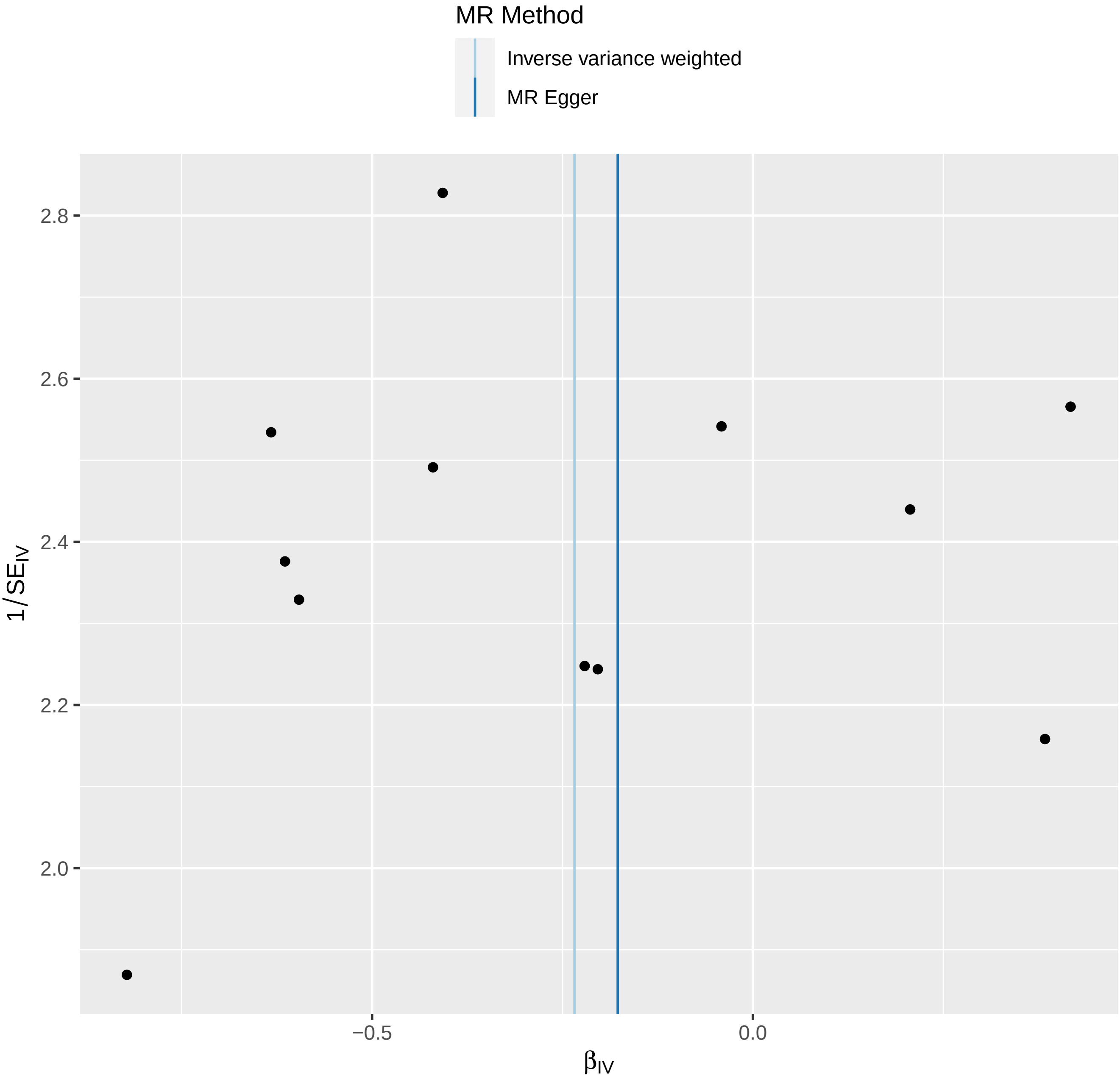

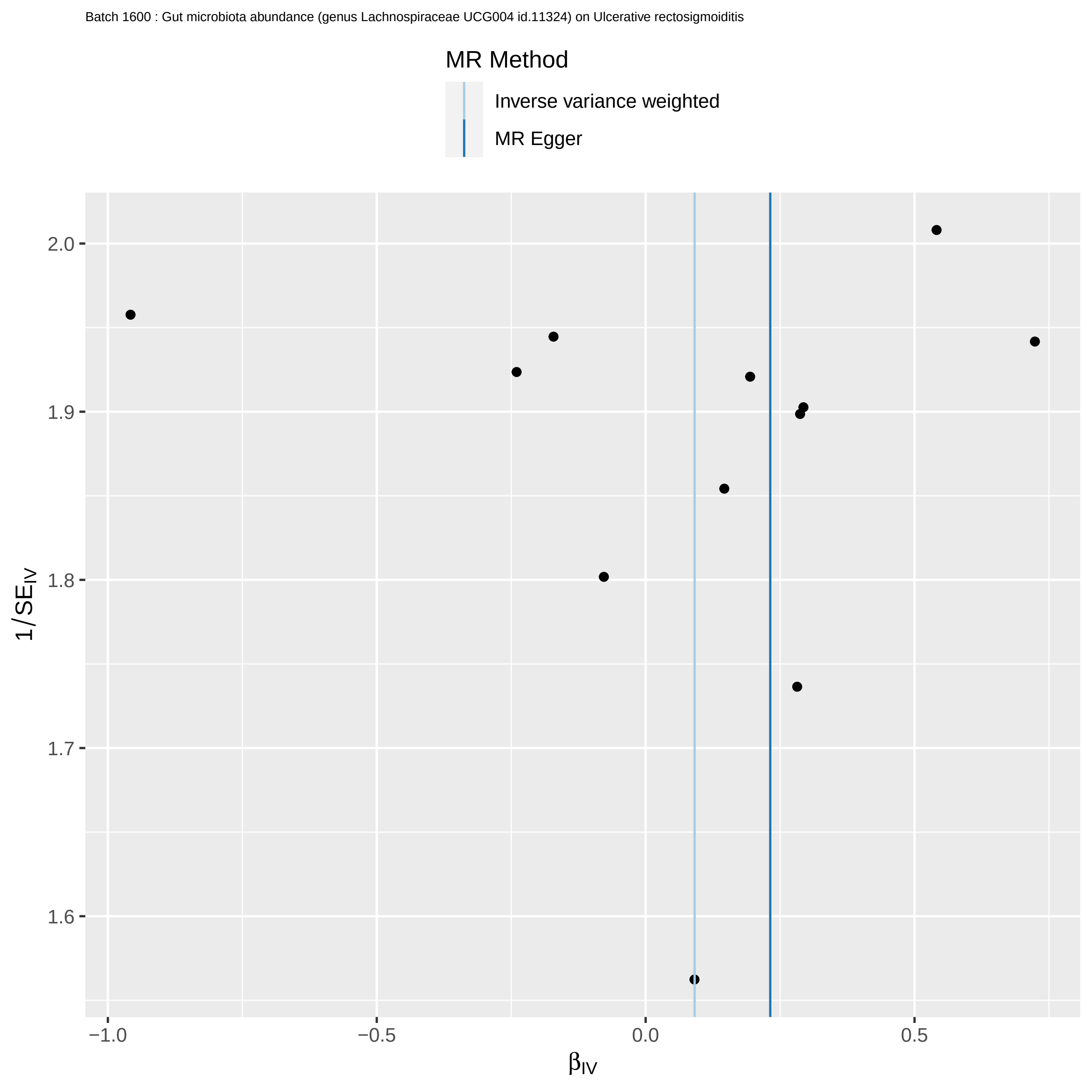
Batch 1600 : Gut microbiota abundance (genus Lachnospiraceae UCG004 id.11324) on Ulcerative rectosigmoiditis
MR Method
Inverse variance weighted
MR Egger
2.0
1.9
1.8
1.7
1.6
1/SE_IV
−1.0
−0.5
0.0
0.5
β_IV

Batch 1601 : Gut microbiota abundance (genus Lachnospiraceae UCG008 id.11328) on Ulcerative rectosigmoiditis

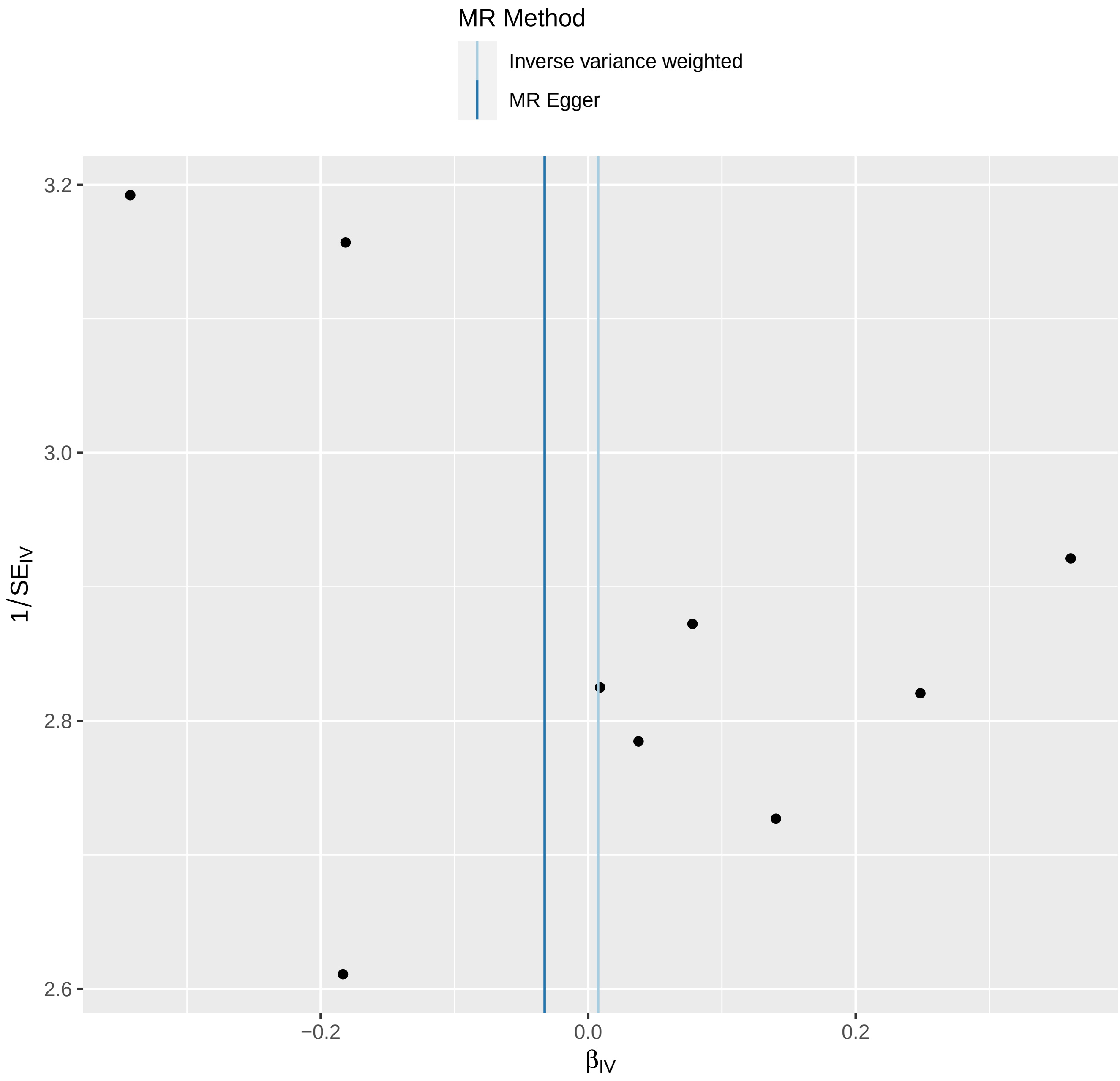

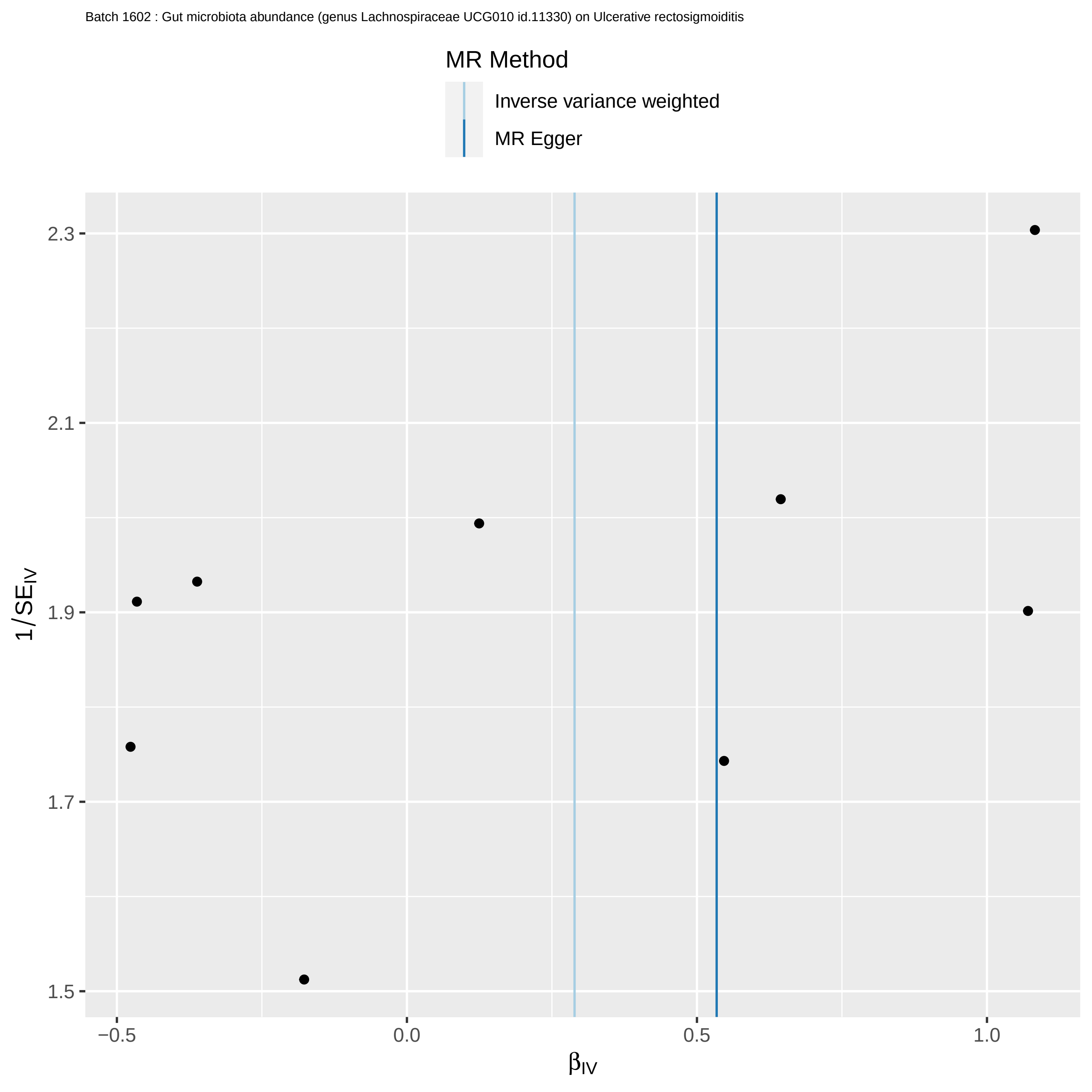
Batch 1602 : Gut microbiota abundance (genus Lachnospiraceae UCG010 id.11330) on Ulcerative rectosigmoiditis
MR Method
Inverse variance weighted
MR Egger
2.3
2.1
1.9
1.7
1.5
$1/SE_{IV}$
−0.5
0.0
0.5
1.0
β_{IV}

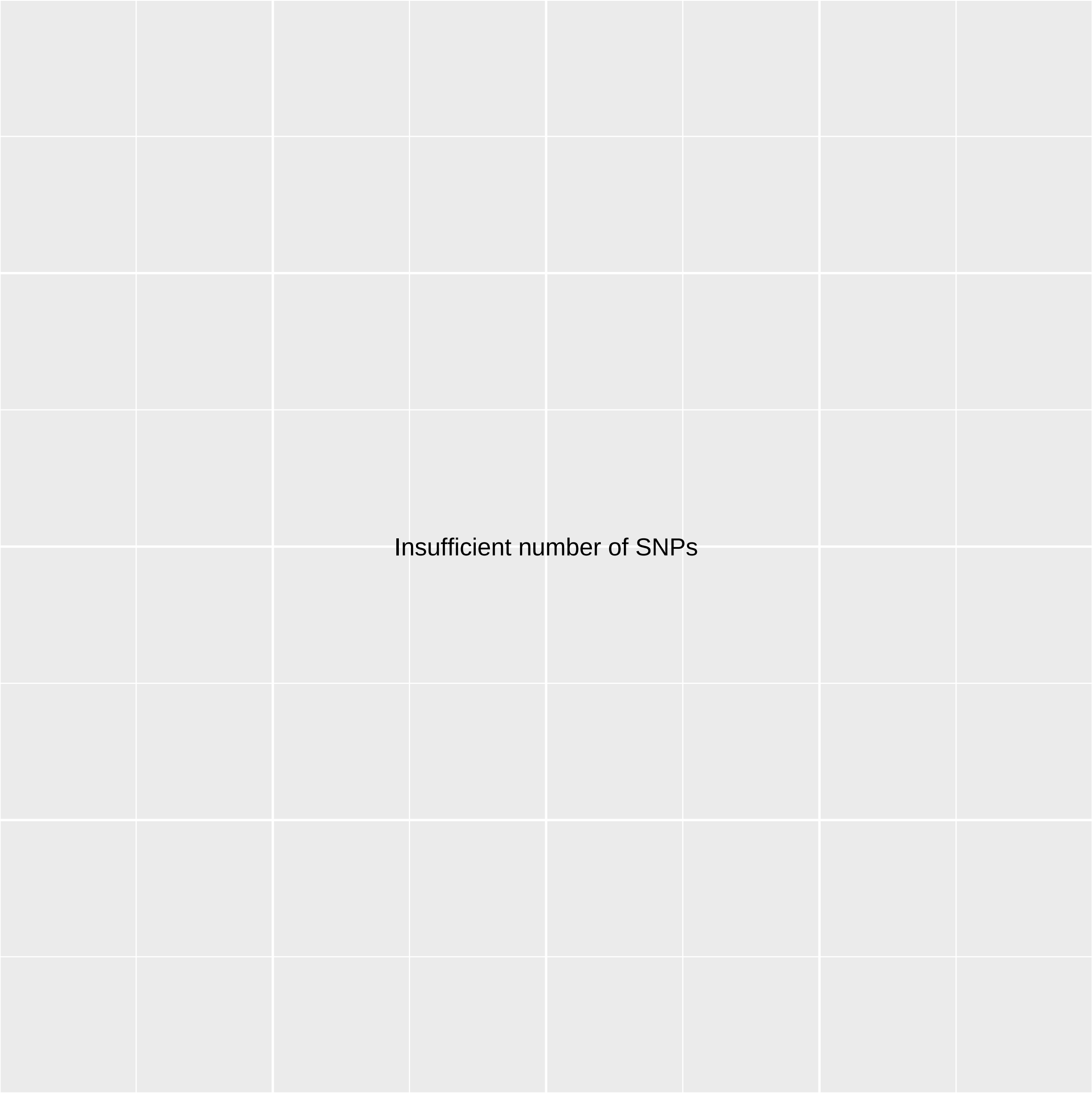
Insufficient number of SNPs

Batch 1604 : Gut microbiota abundance (genus Lactobacillus id.1837) on Ulcerative rectosigmoiditis

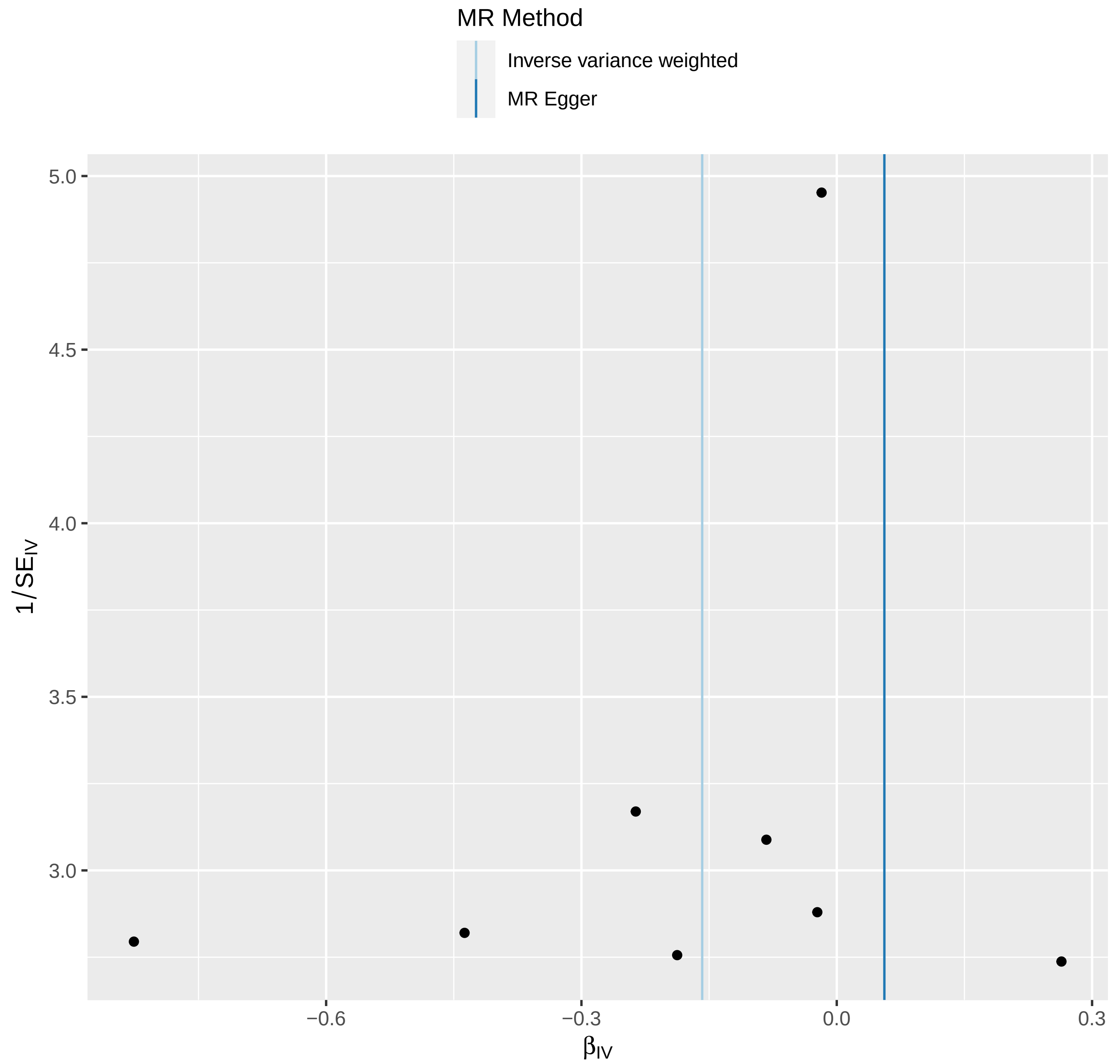

Batch 1605 : Gut microbiota abundance (genus Lactococcus id.1851) on Ulcerative rectosigmoiditis

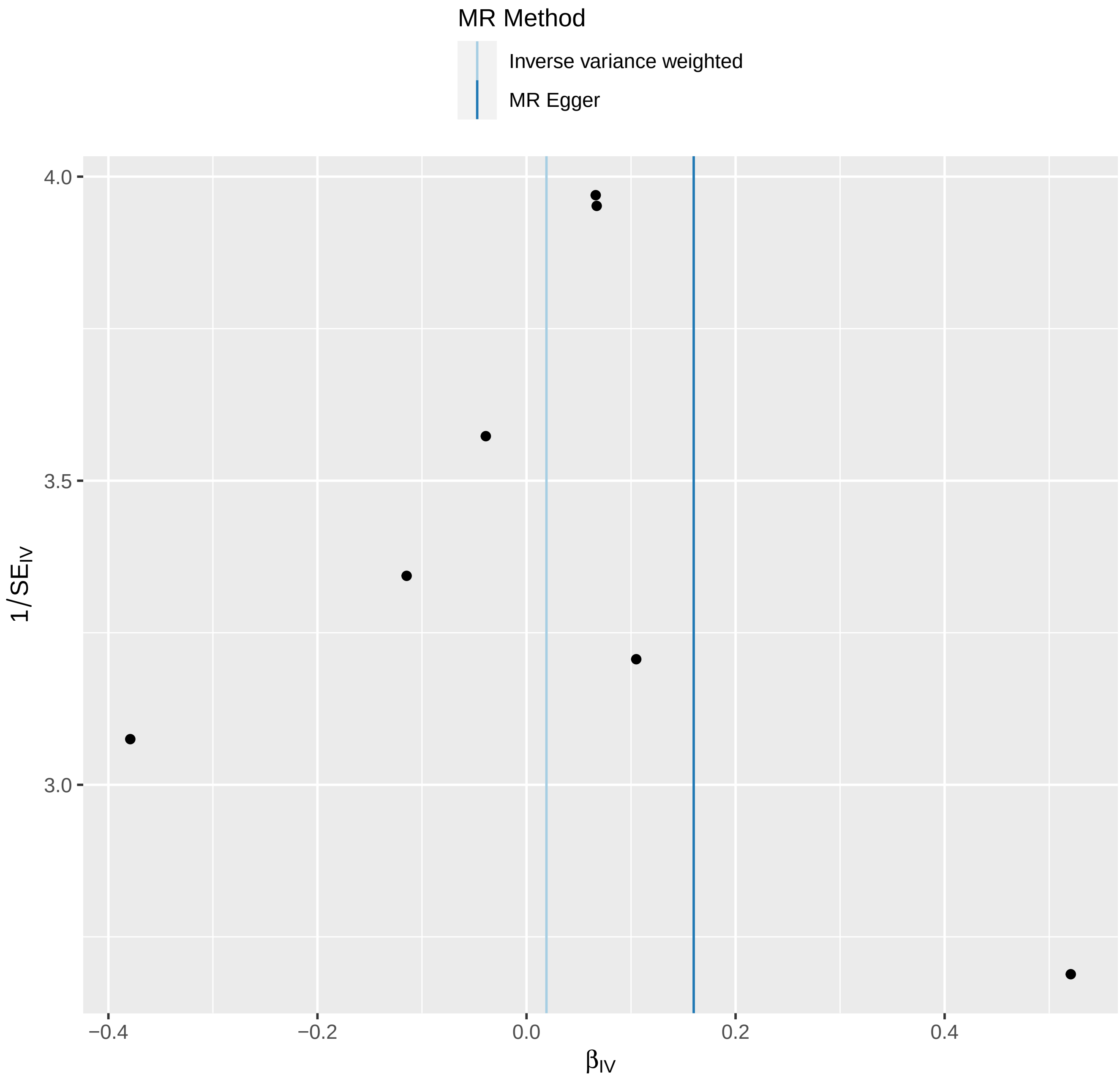

Batch 1606 : Gut microbiota abundance (genus Marvinbryantia id.2005) on Ulcerative rectosigmoiditis

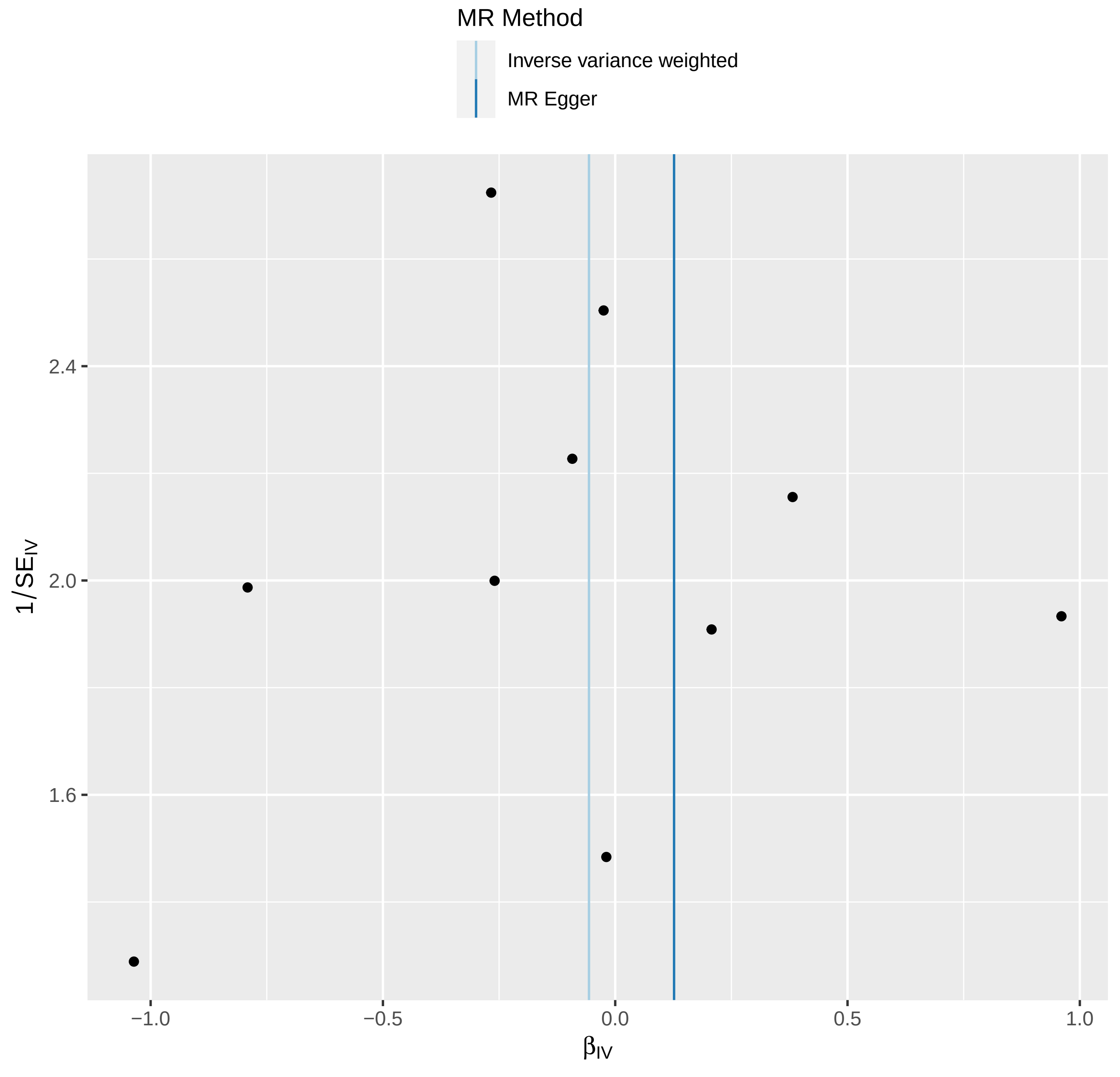

Batch 1607 : Gut microbiota abundance (genus Methanobrevibacter id.123) on Ulcerative rectosigmoiditis

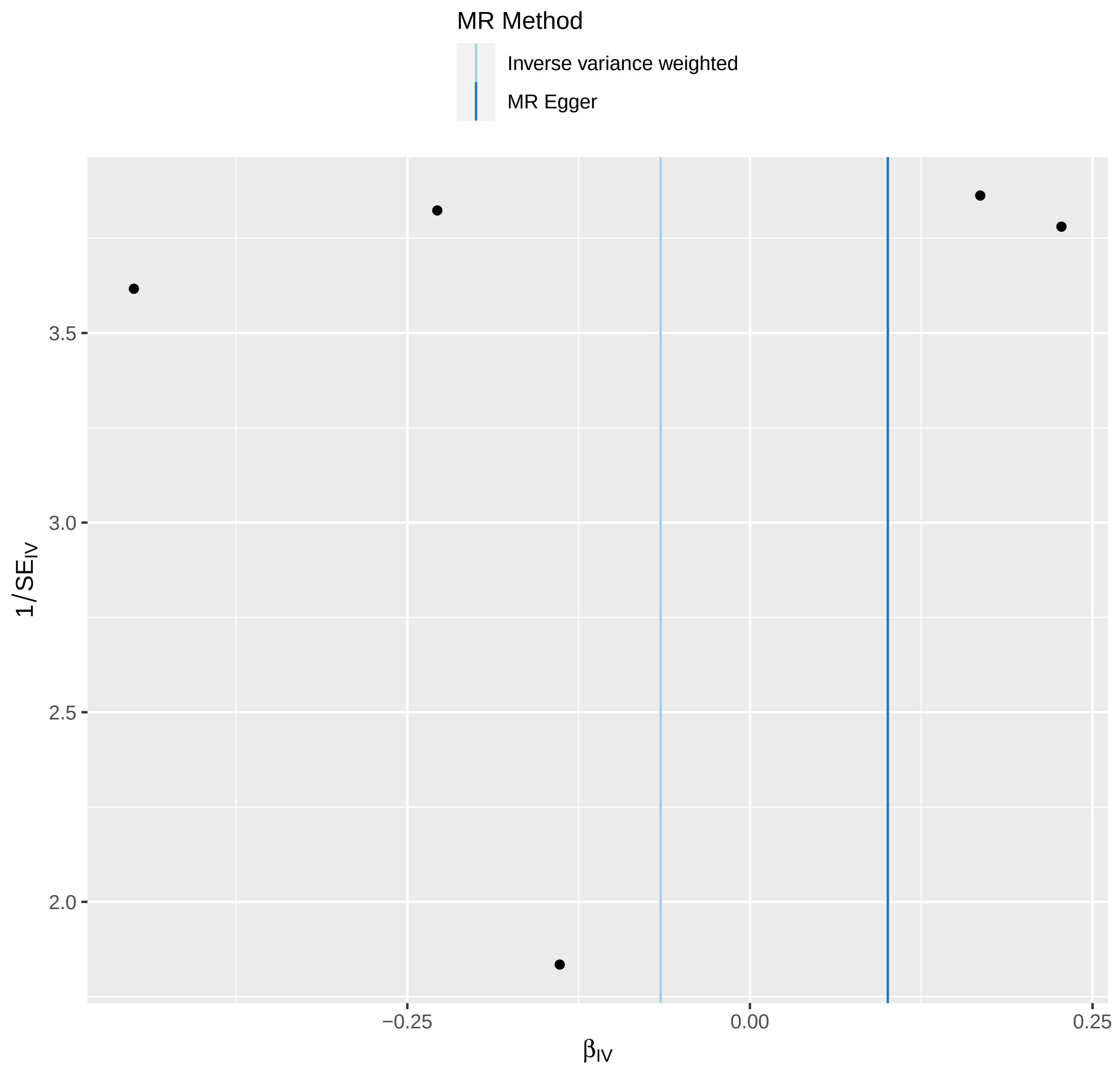

Batch 1608 : Gut microbiota abundance (genus Odoribacter id.952) on Ulcerative rectosigmoiditis

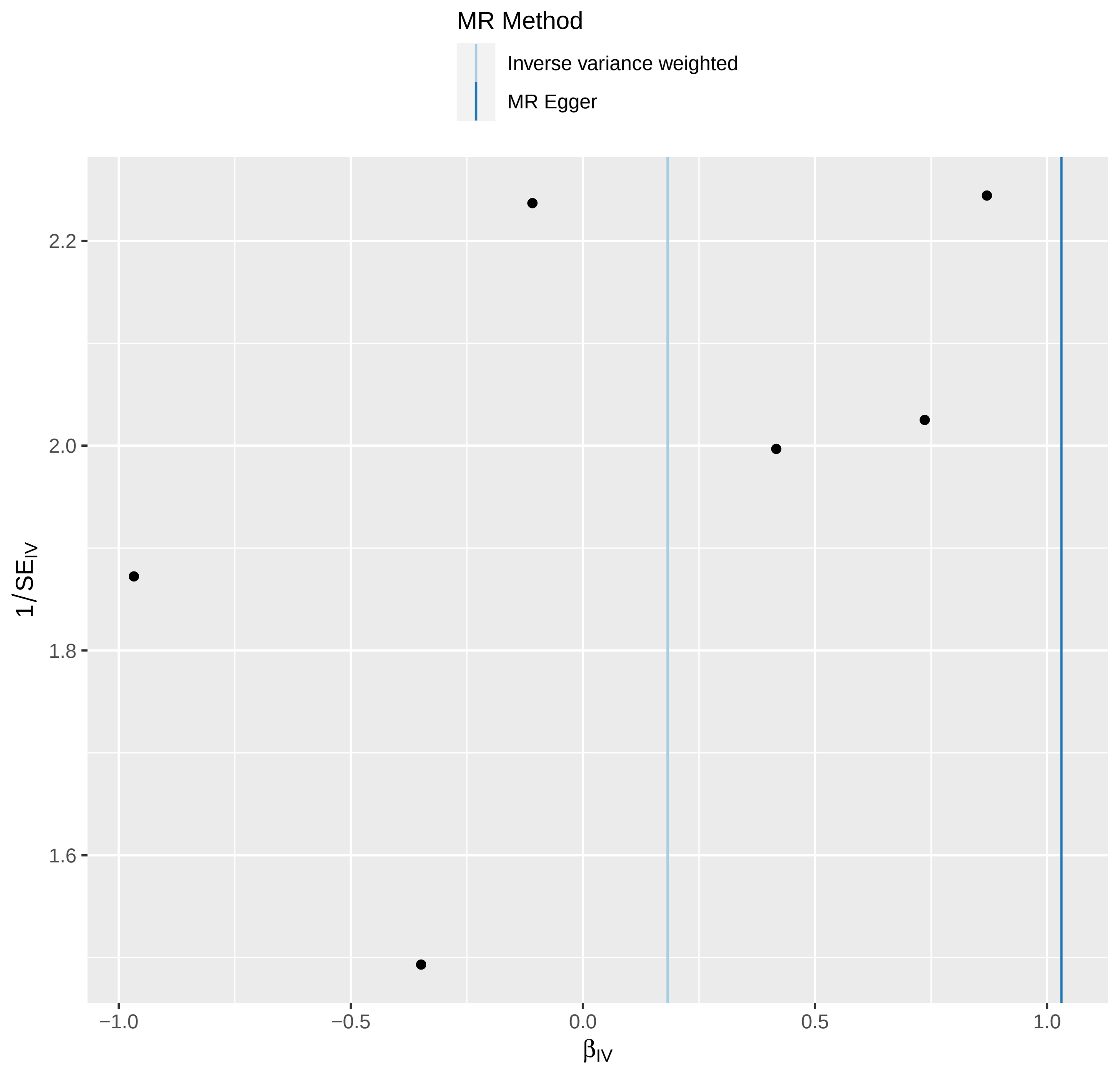

Batch 1609 : Gut microbiota abundance (genus Olsenella id.822) on Ulcerative rectosigmoiditis

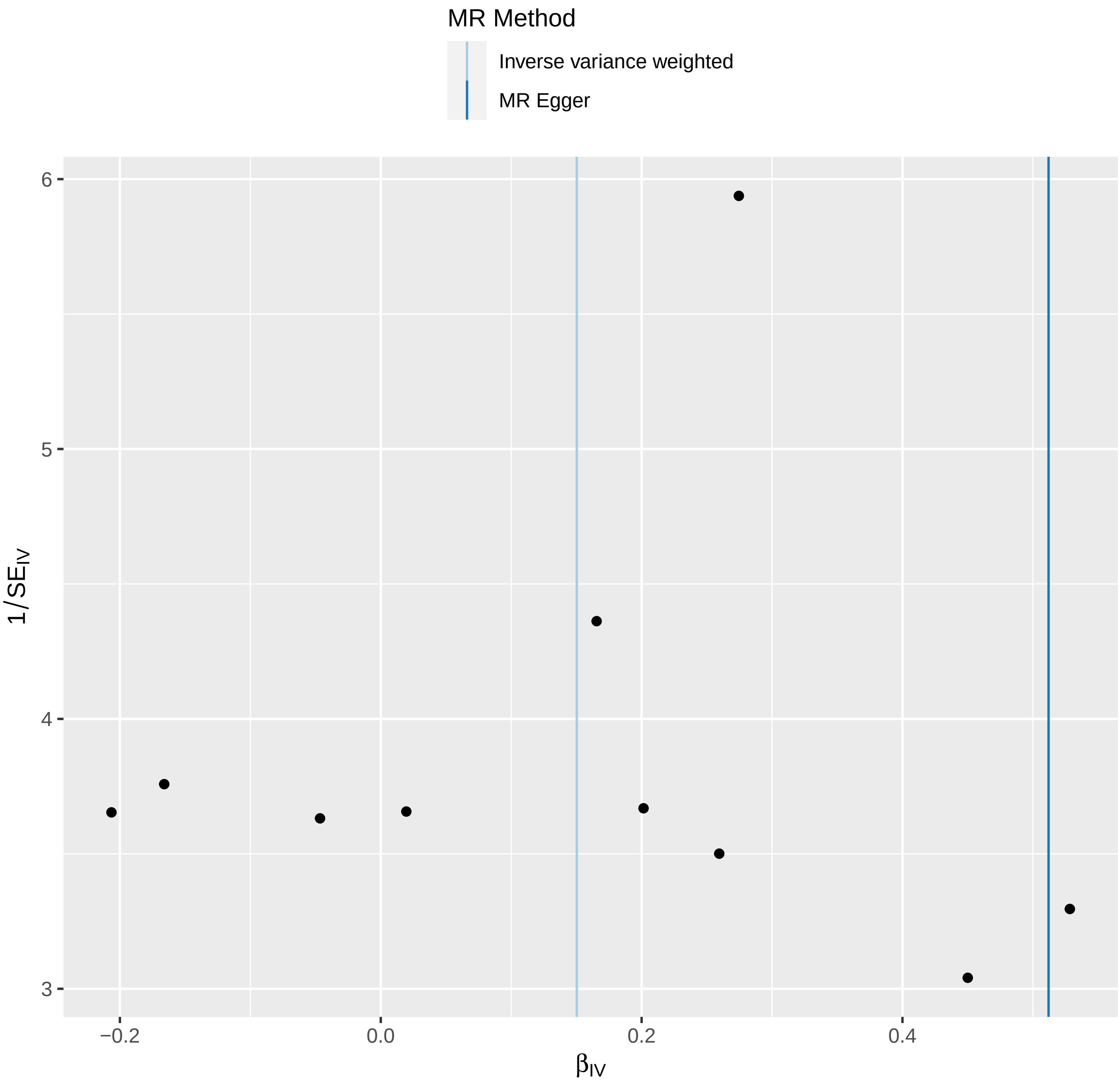

Batch 1610 : Gut microbiota abundance (genus Oscillibacter id.2063) on Ulcerative rectosigmoiditis

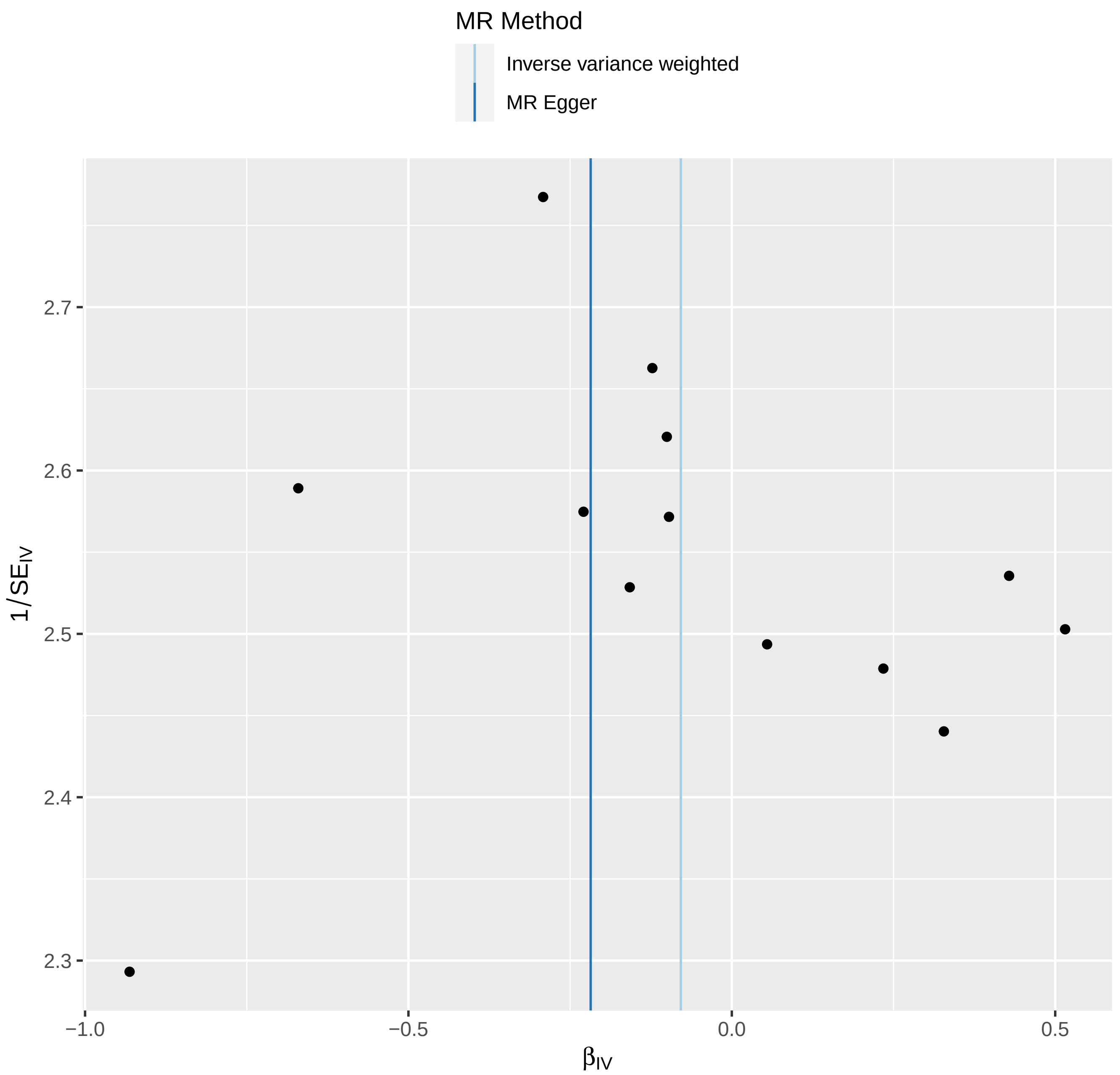

Batch 1611 : Gut microbiota abundance (genus Oscillospira id.2064) on Ulcerative rectosigmoiditis

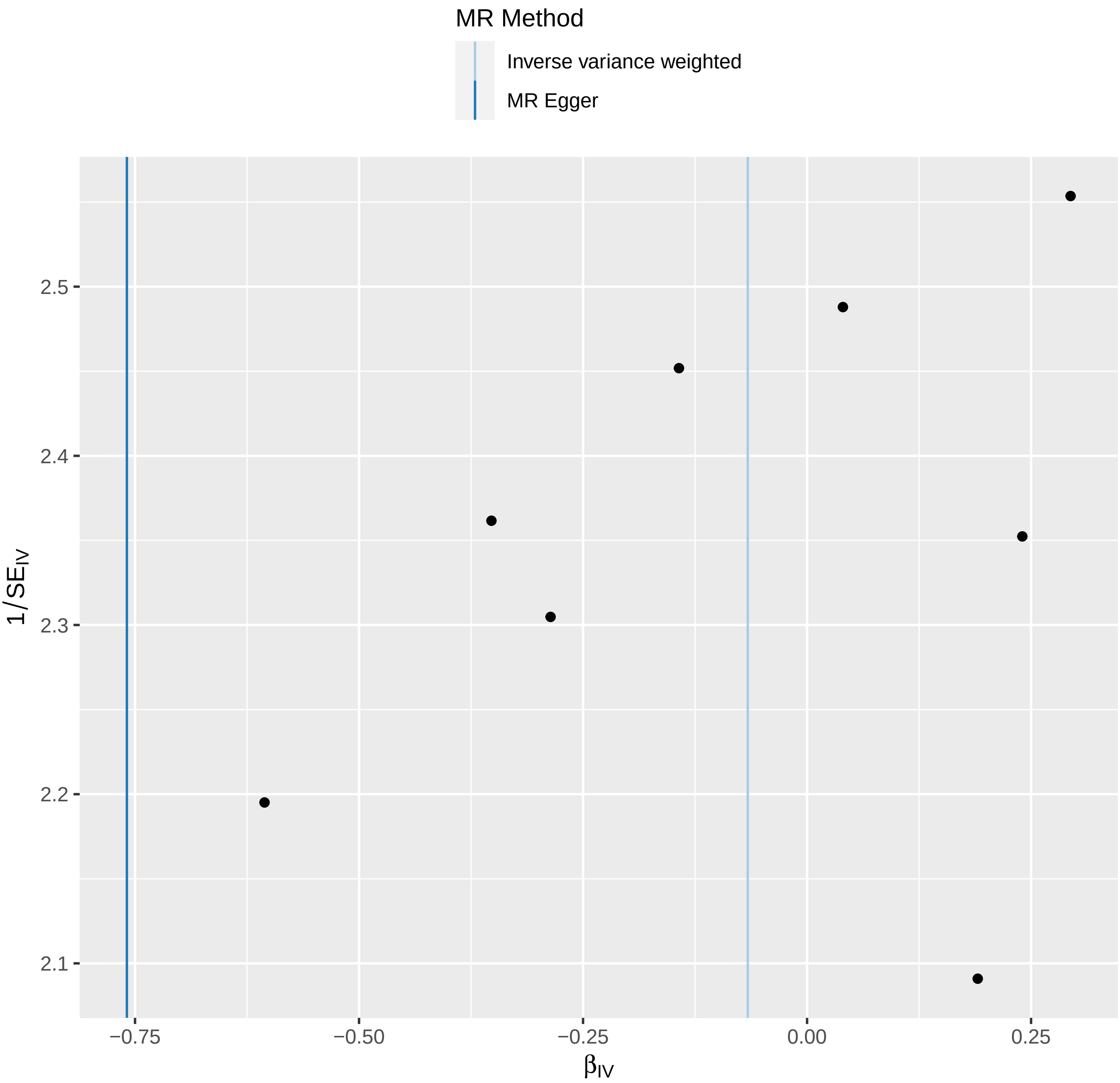

Batch 1612 : Gut microbiota abundance (genus Oxalobacter id.2978) on Ulcerative rectosigmoiditis

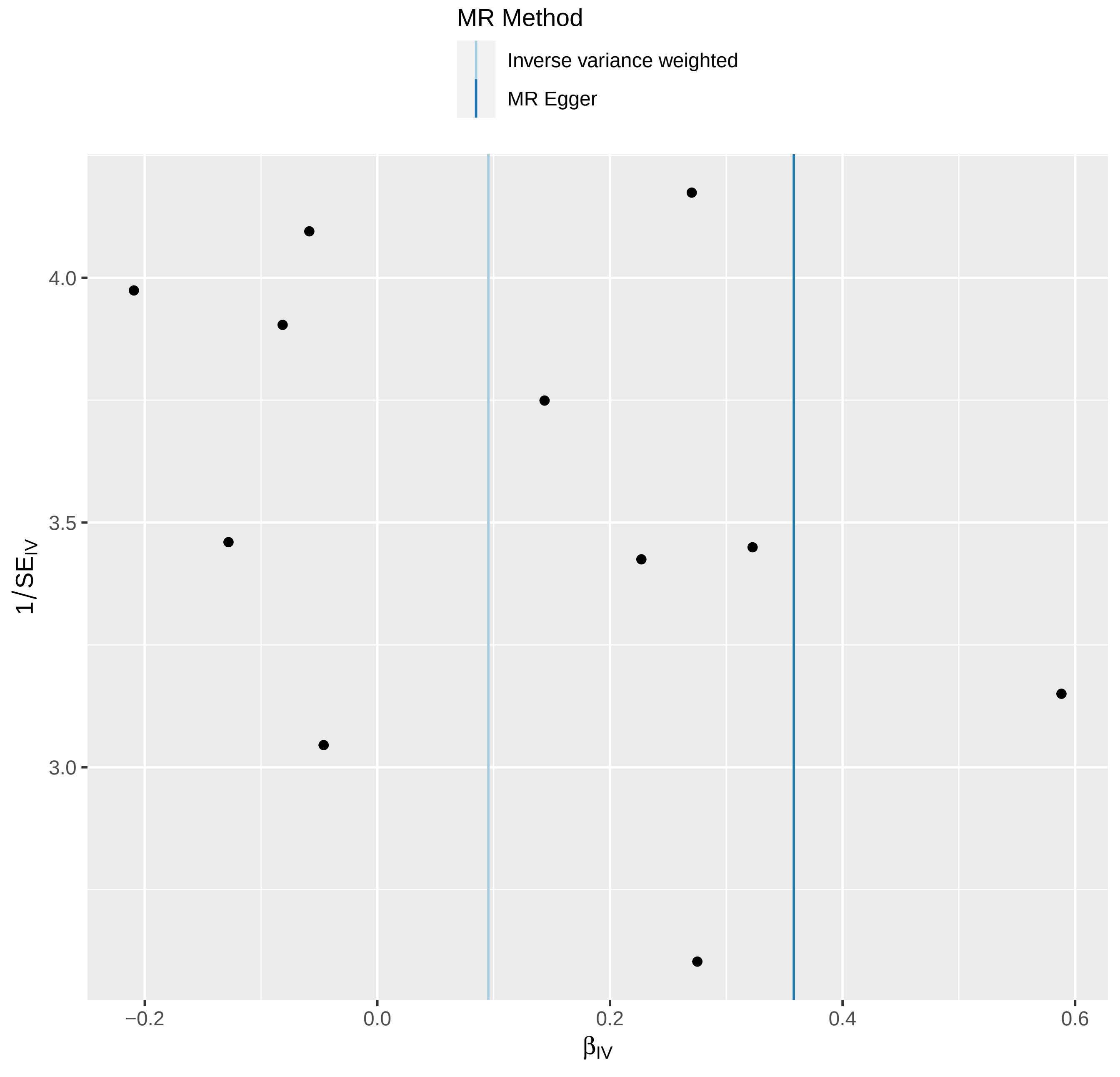

Batch 1613 : Gut microbiota abundance (genus Parabacteroides id.954) on Ulcerative rectosigmoiditis

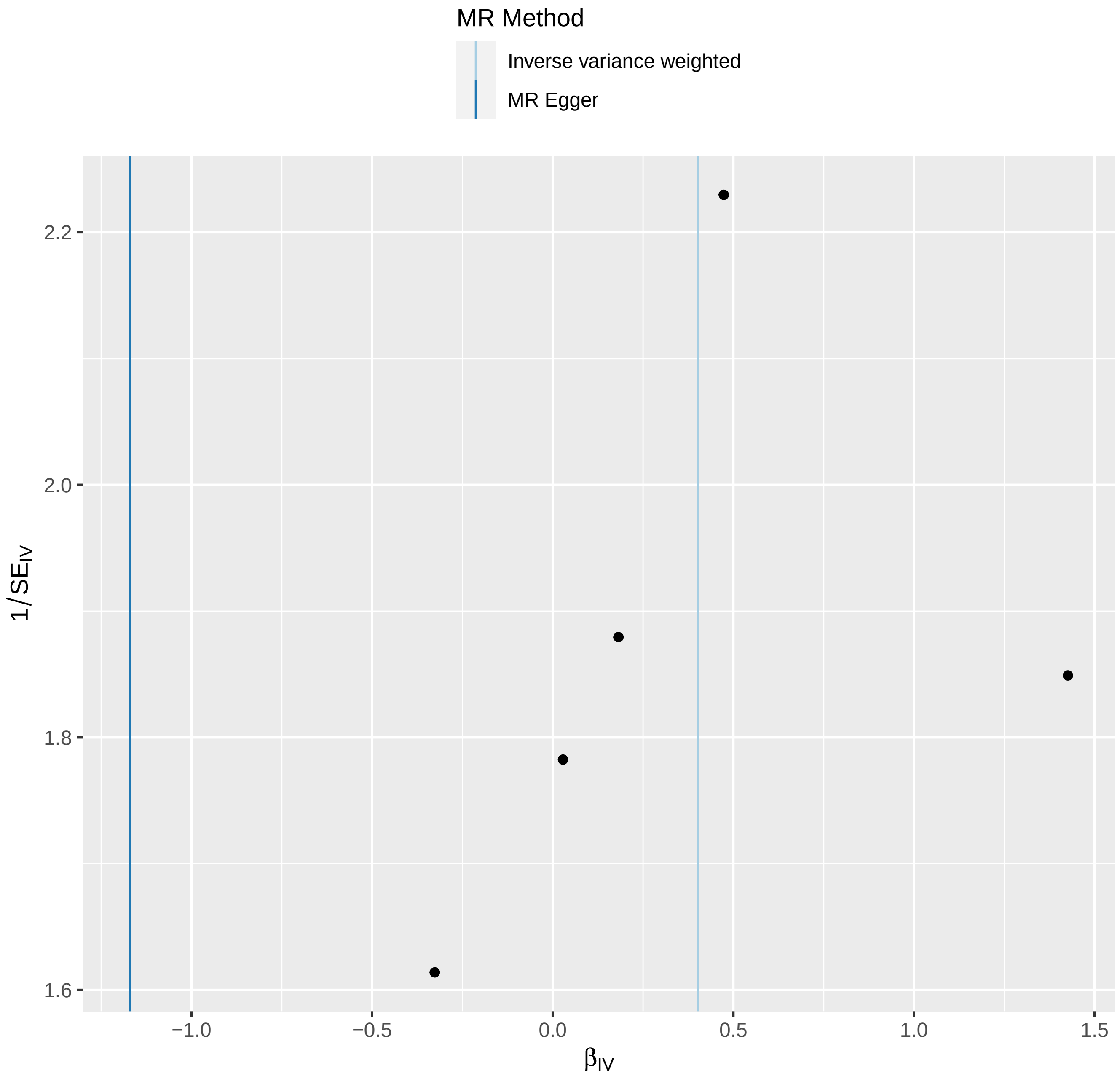

Batch 1614 : Gut microbiota abundance (genus Paraprevotella id.962) on Ulcerative rectosigmoiditis

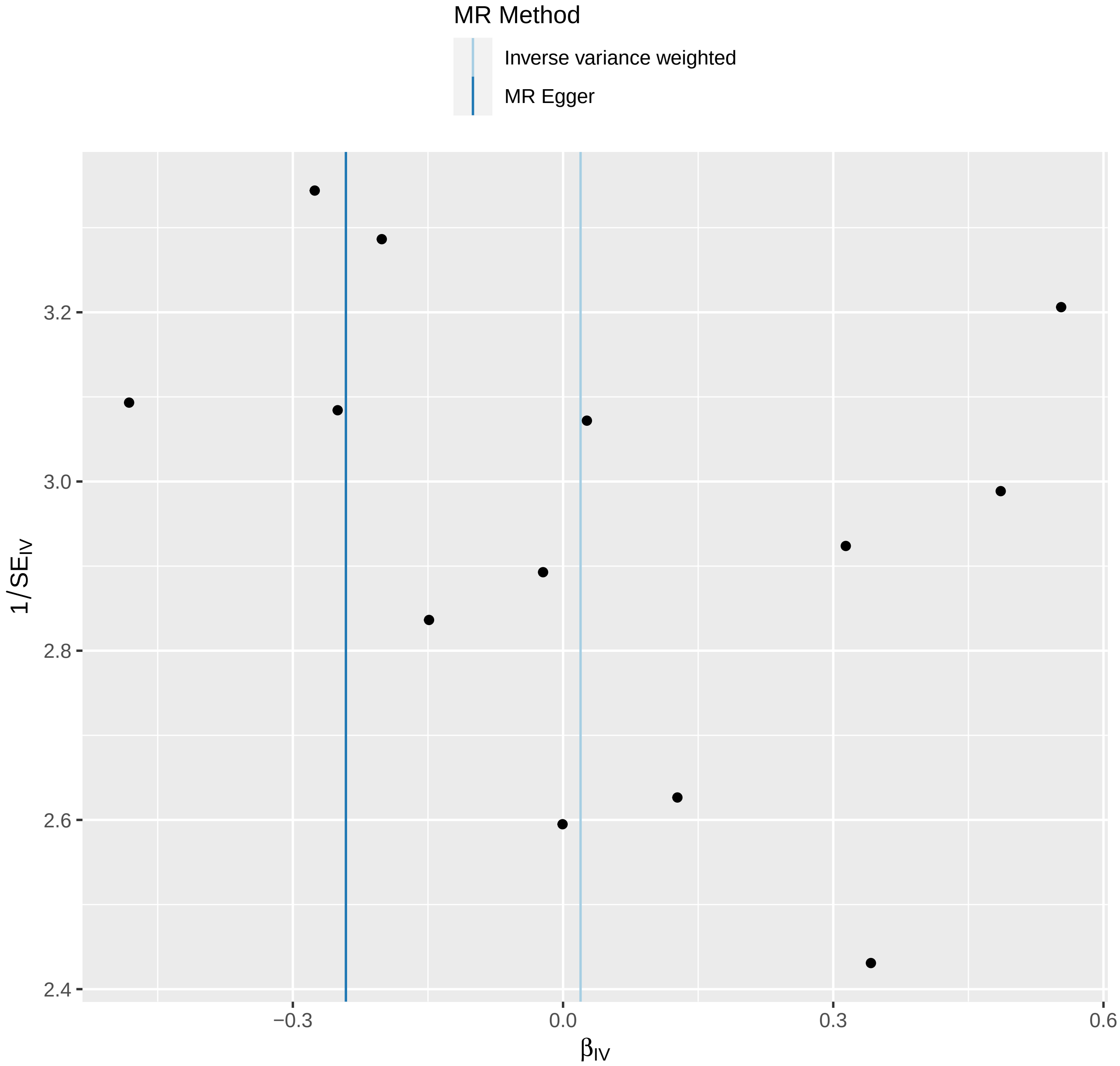

Batch 1615 : Gut microbiota abundance (genus Parasutterella id.2892) on Ulcerative rectosigmoiditis

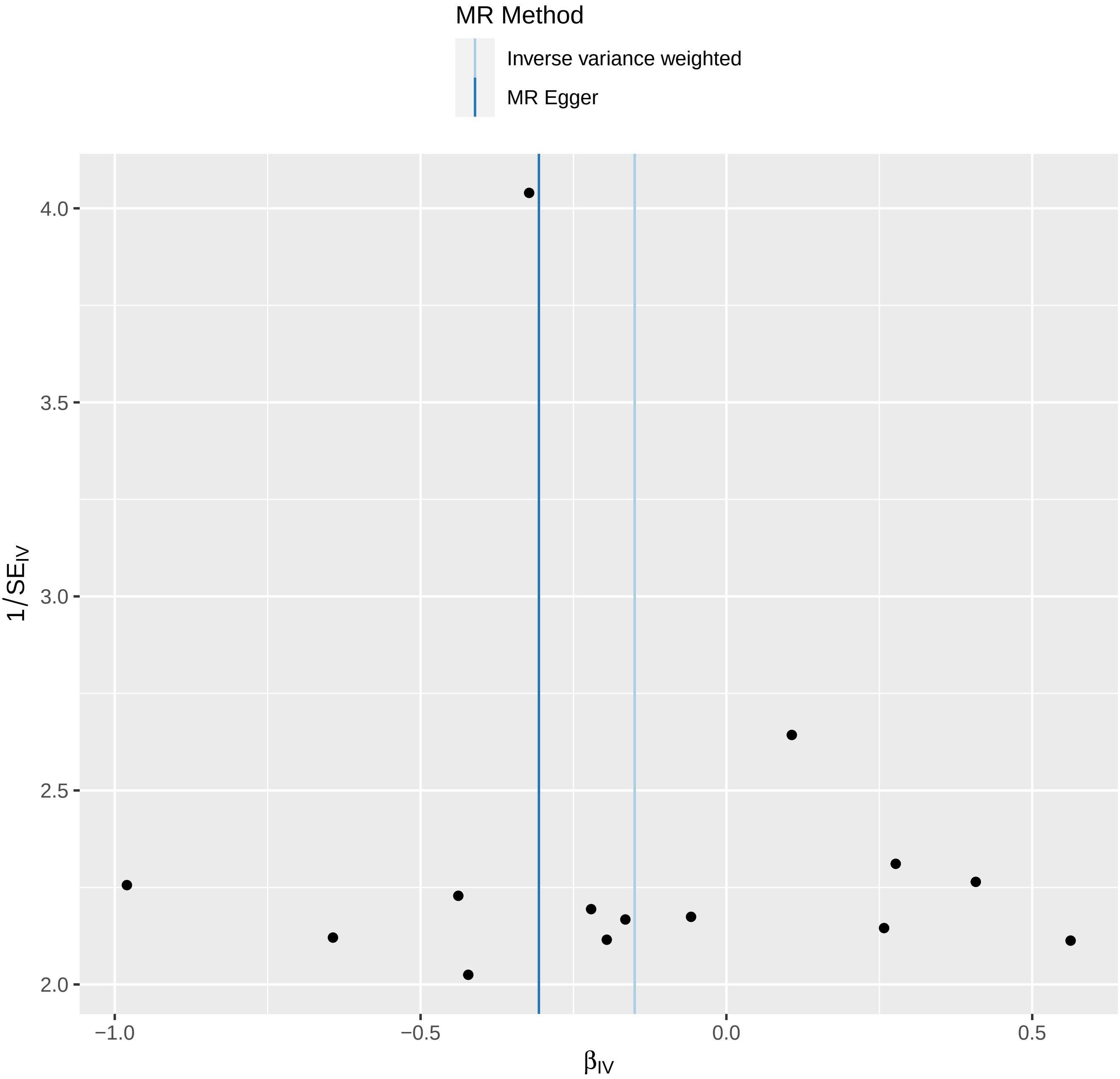

Batch 1616 : Gut microbiota abundance (genus Peptococcus id.2037) on Ulcerative rectosigmoiditis

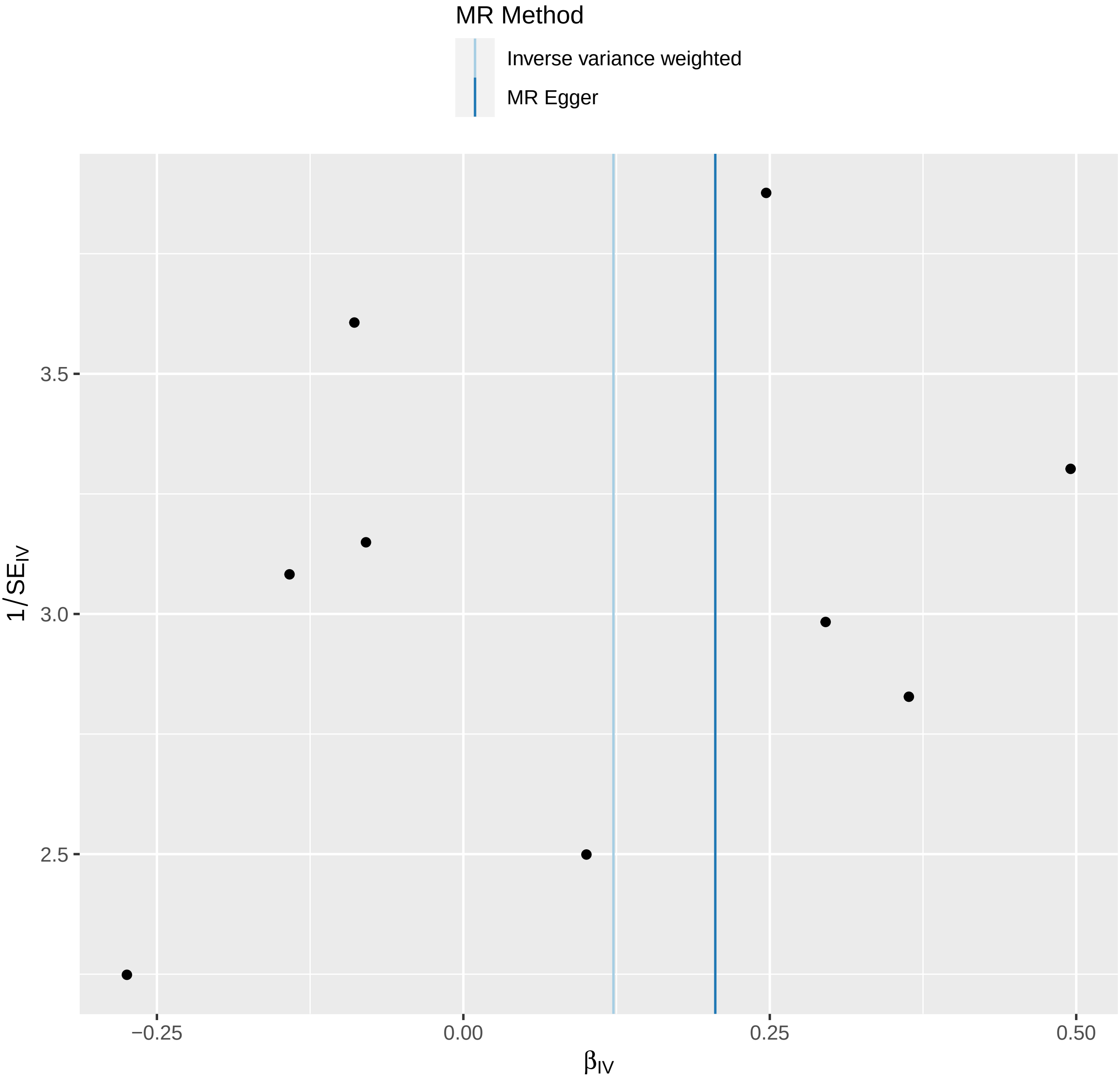

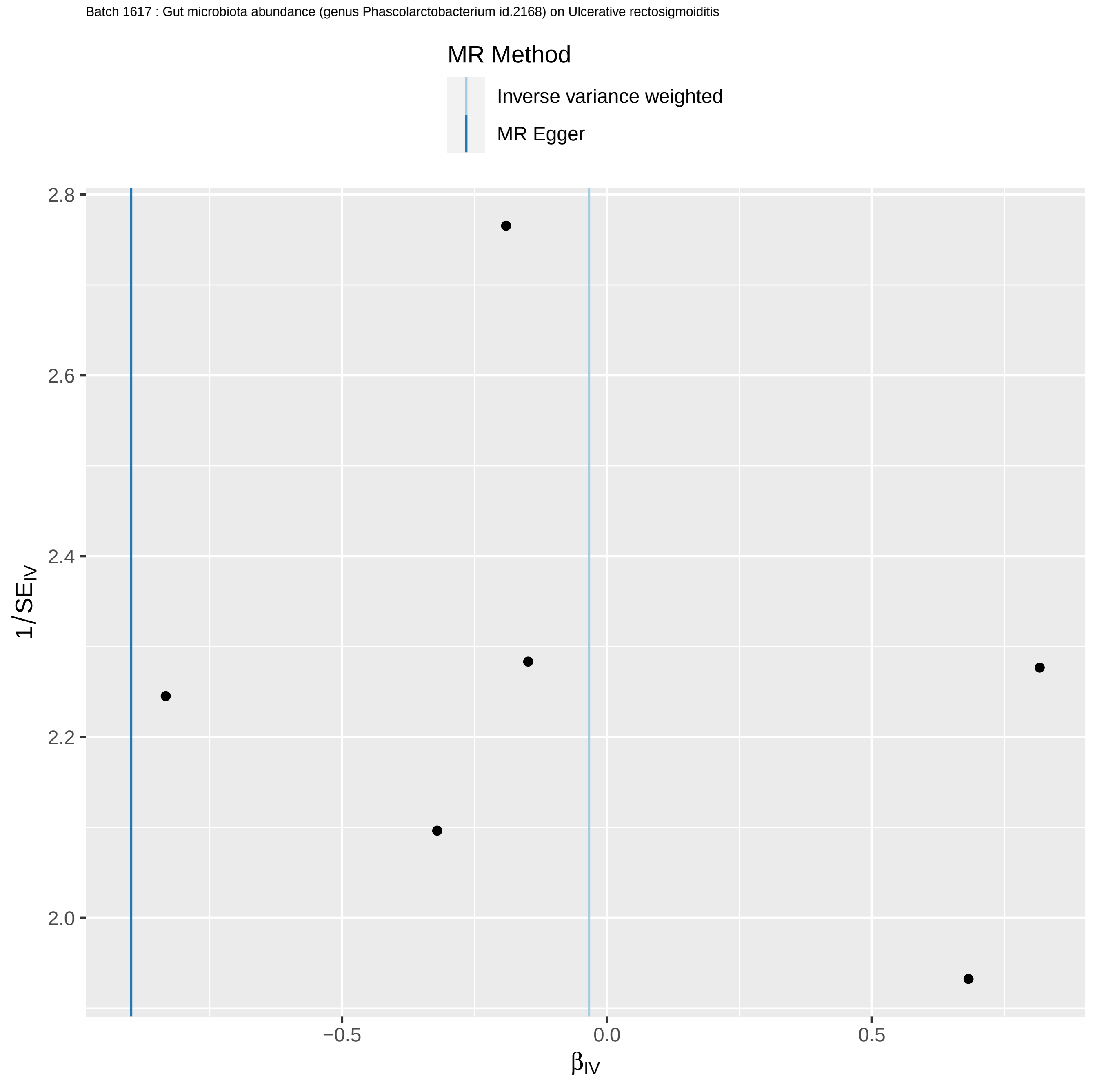
Batch 1617 : Gut microbiota abundance (genus Phascolarctobacterium id.2168) on Ulcerative rectosigmoiditis
MR Method
Inverse variance weighted
MR Egger
2.8
2.6
2.4
2.2
2.0
1/SE_IV
−0.5
0.0
0.5
β_IV

Batch 1618 : Gut microbiota abundance (genus Prevotella7 id.11182) on Ulcerative rectosigmoiditis

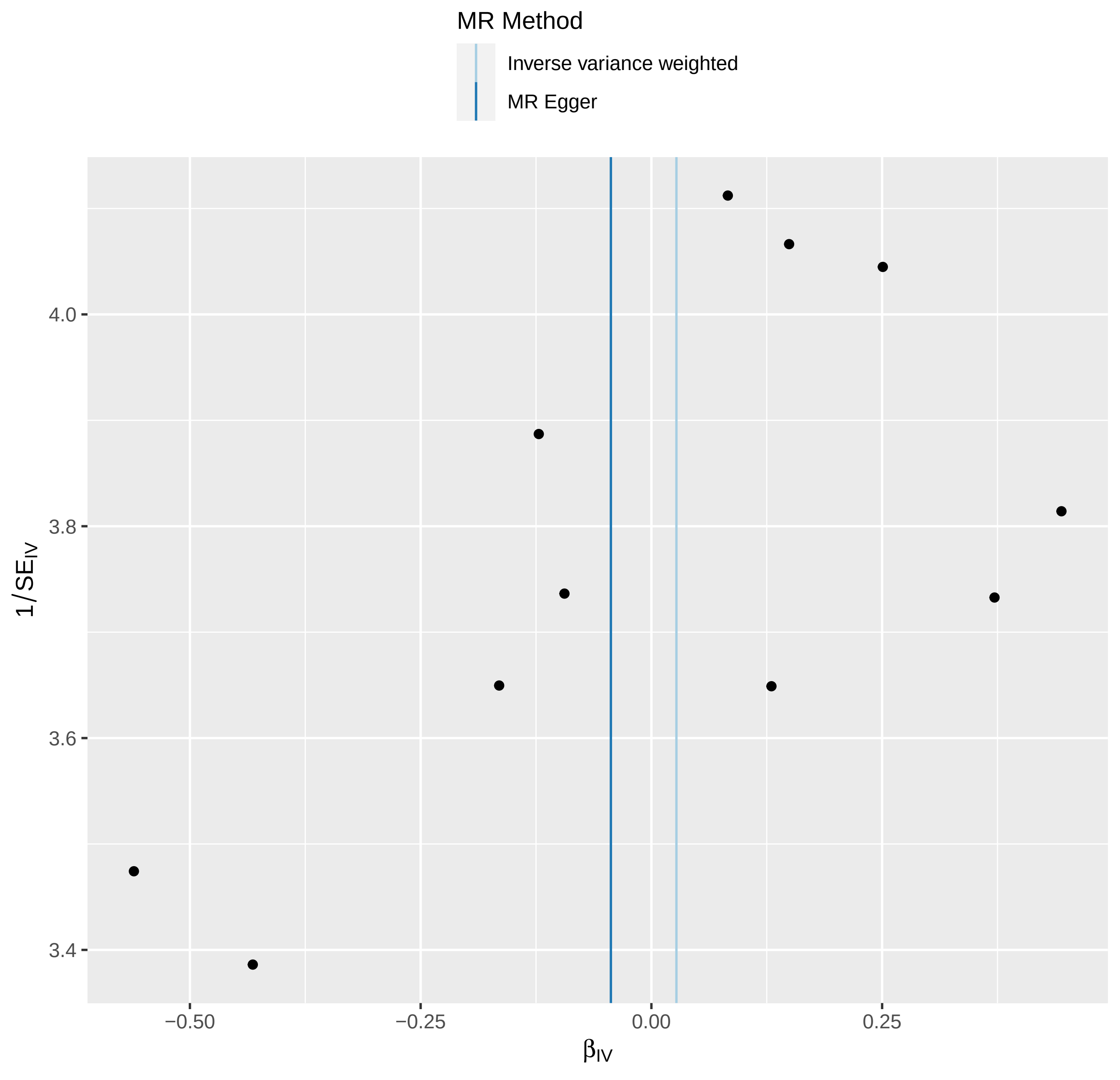

Batch 1619 : Gut microbiota abundance (genus Prevotella9 id.11183) on Ulcerative rectosigmoiditis

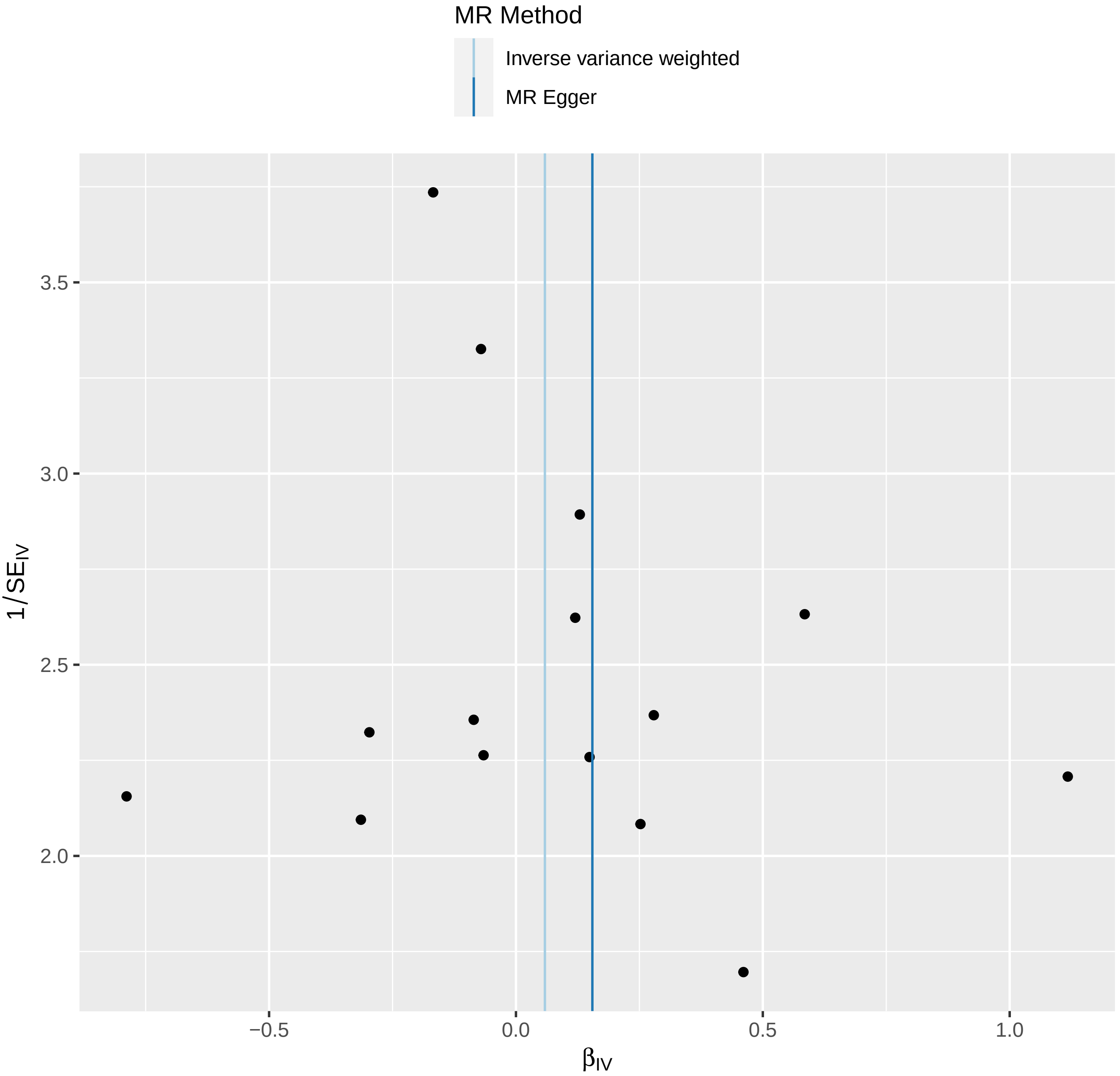

Batch 1620 : Gut microbiota abundance (genus Rikenellaceae RC9 gut group id.11191) on Ulcerative rectosigmoiditis

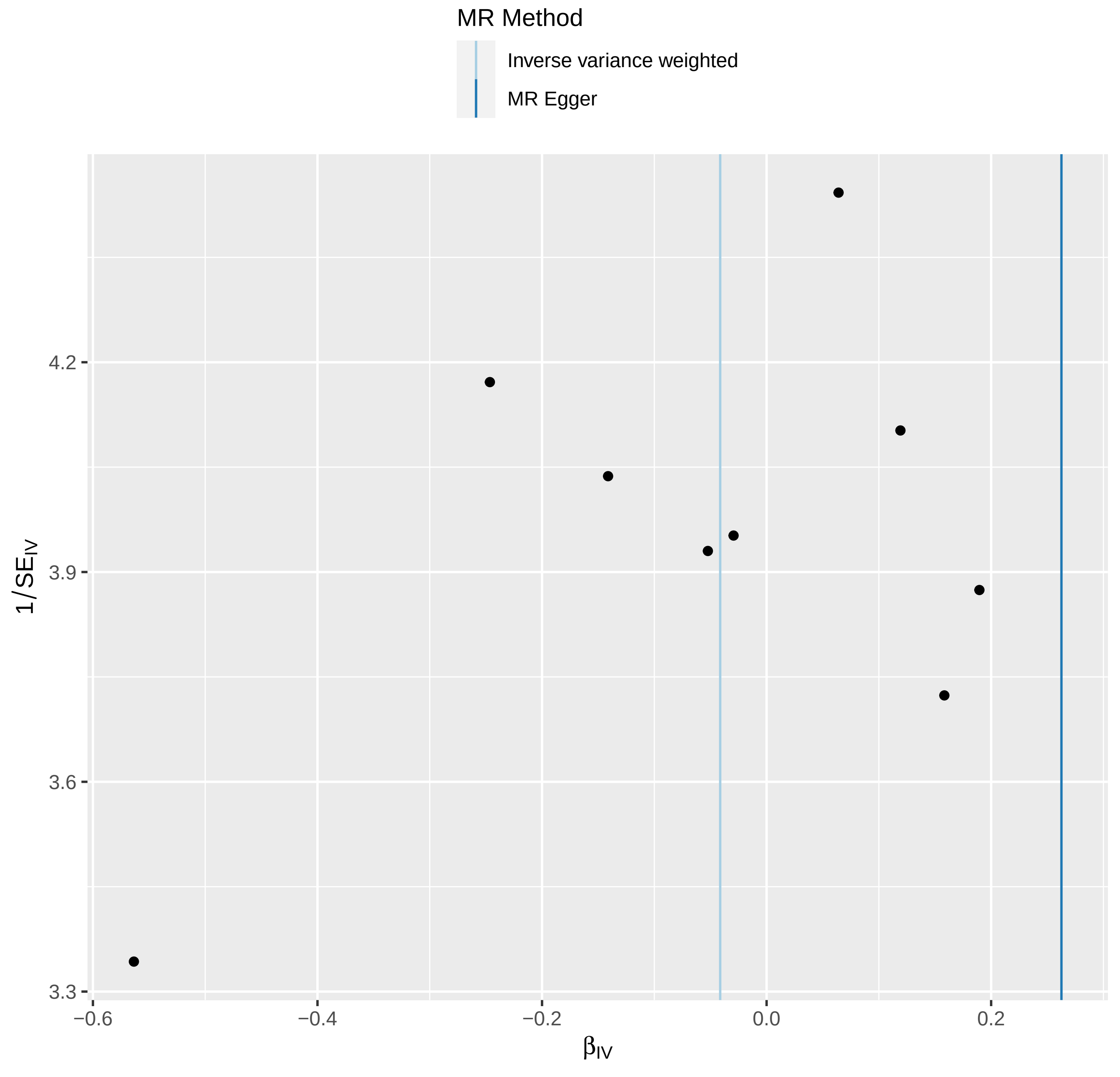

Batch 1621 : Gut microbiota abundance (genus Romboutsia id.11347) on Ulcerative rectosigmoiditis

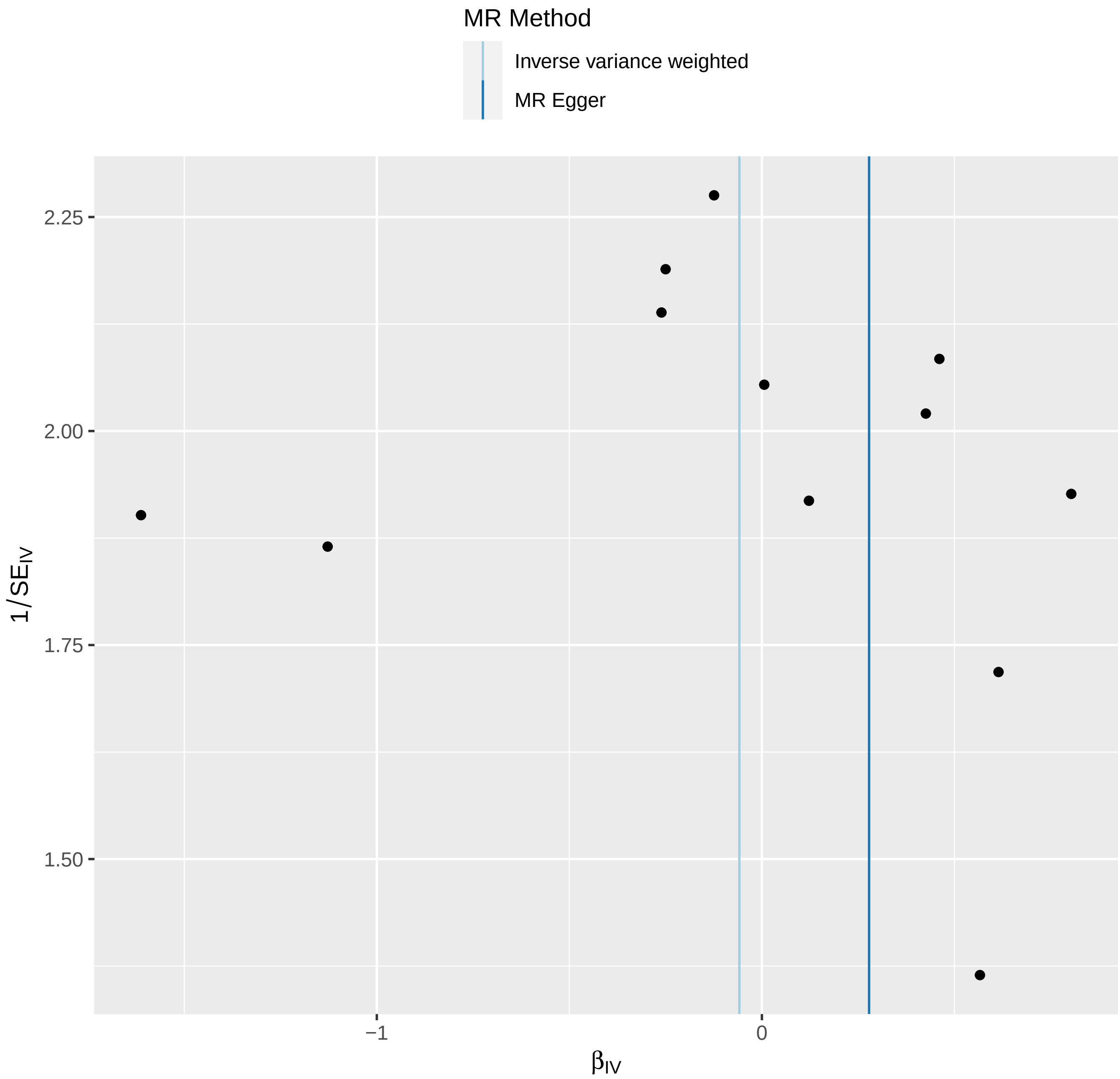

Batch 1622 : Gut microbiota abundance (genus Roseburia id.2012) on Ulcerative rectosigmoiditis

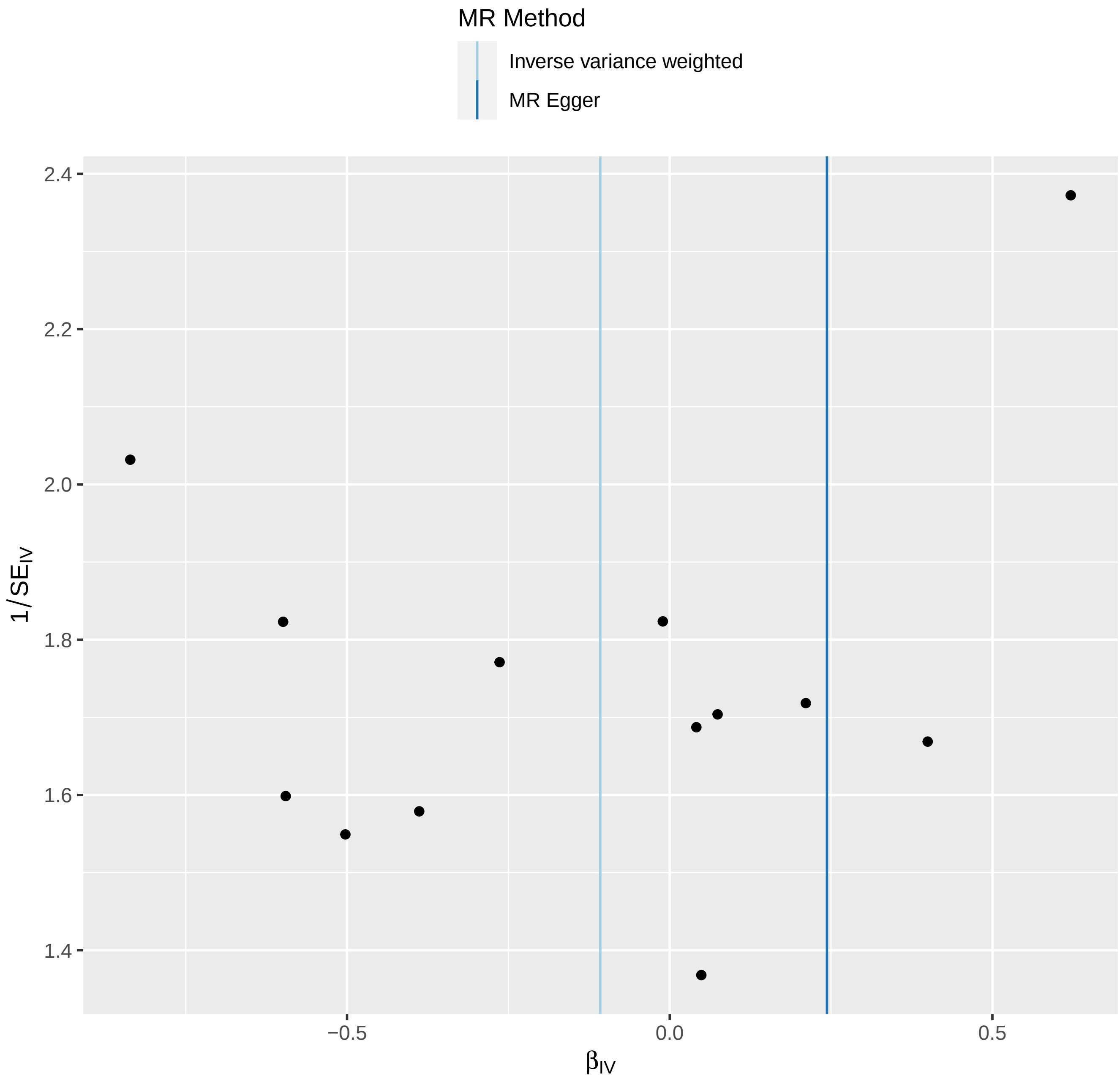

Batch 1623 : Gut microbiota abundance (genus Ruminiclostridium5 id.11355) on Ulcerative rectosigmoiditis

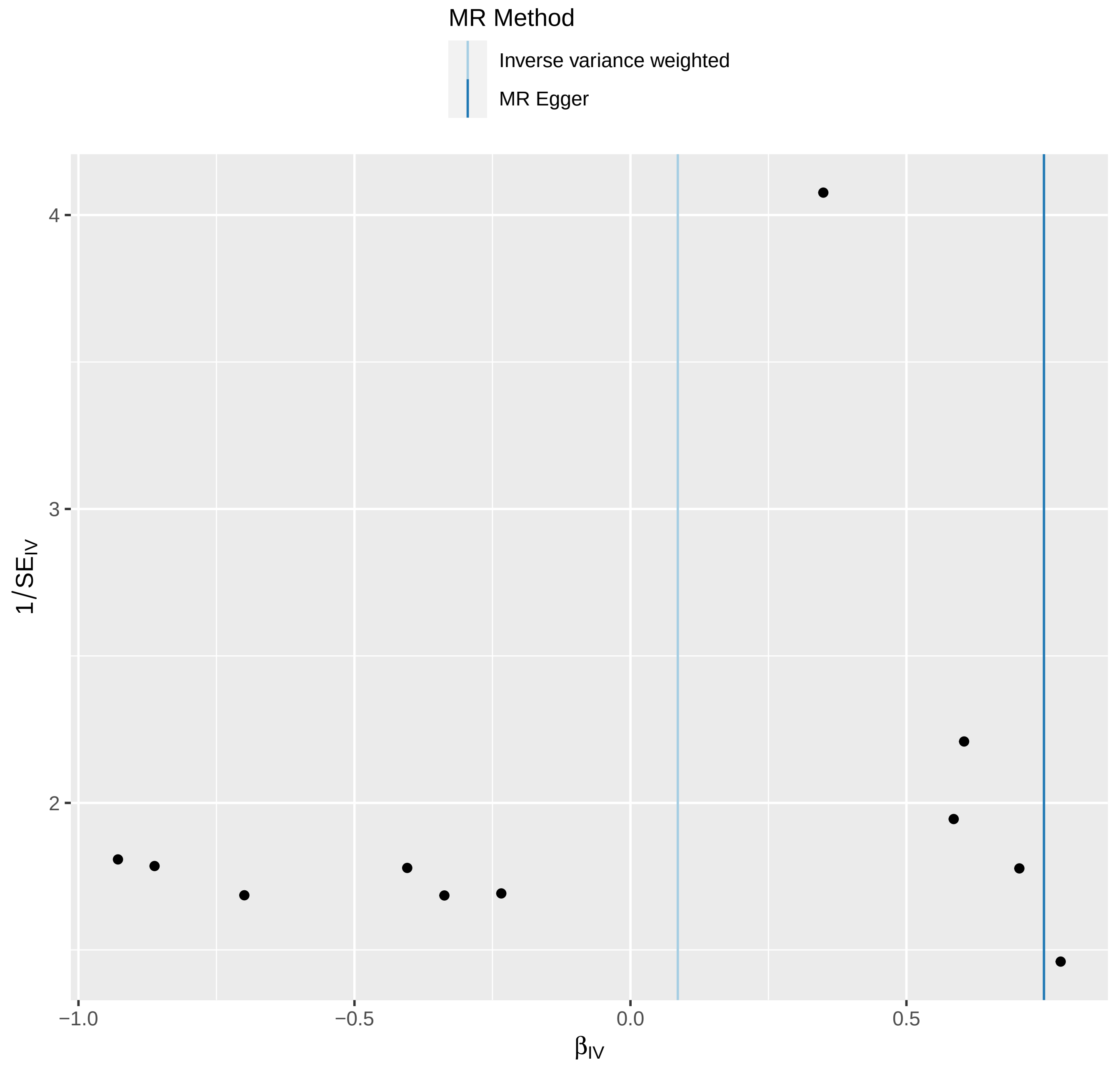

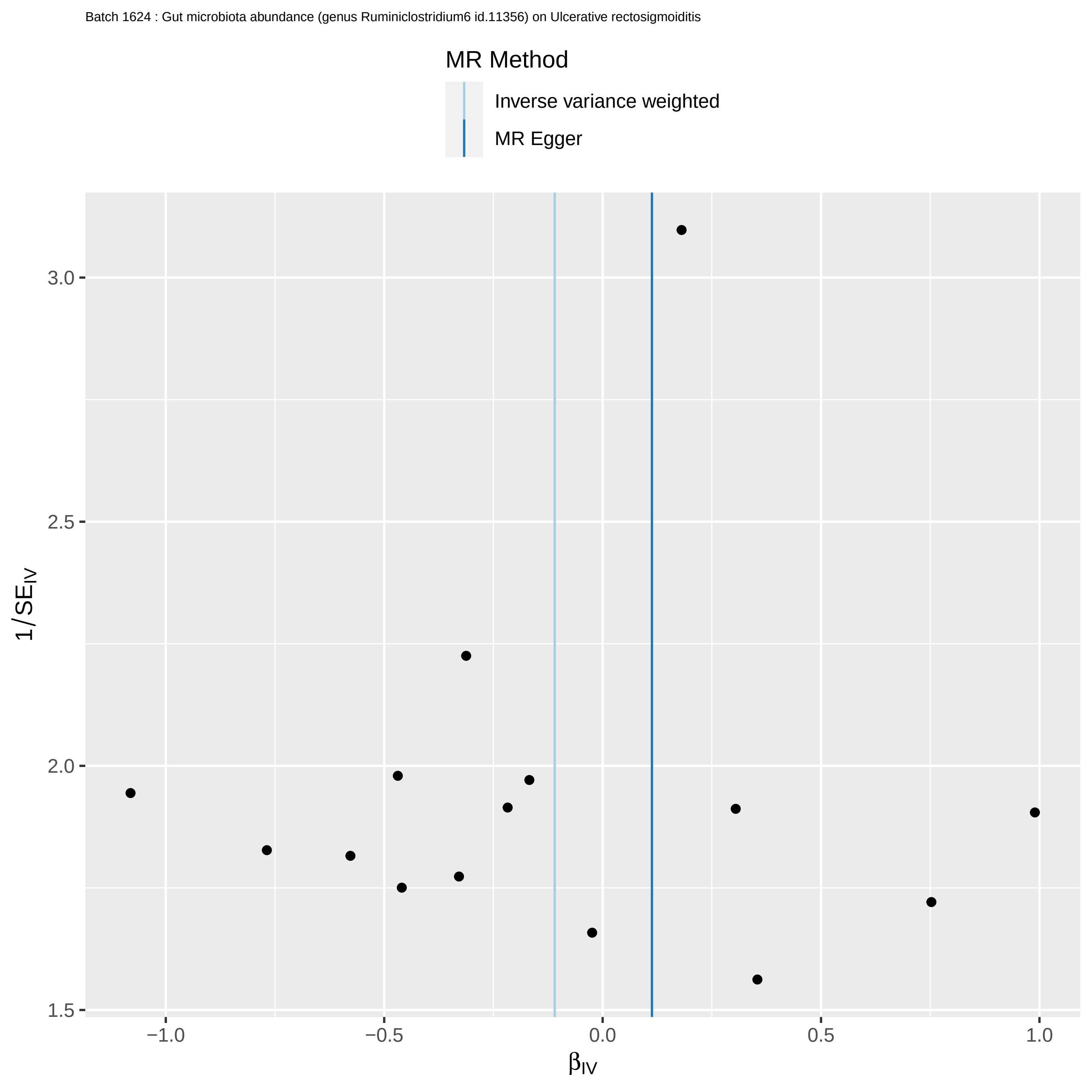
Batch 1624 : Gut microbiota abundance (genus Ruminiclostridium6 id.11356) on Ulcerative rectosigmoiditis
MR Method
Inverse variance weighted
MR Egger
3.0
2.5
2.0
1.5
1/SE_IV
−1.0
−0.5
0.0
0.5
1.0
β_IV

Batch 1625 : Gut microbiota abundance (genus Ruminiclostridium9 id.11357) on Ulcerative rectosigmoiditis

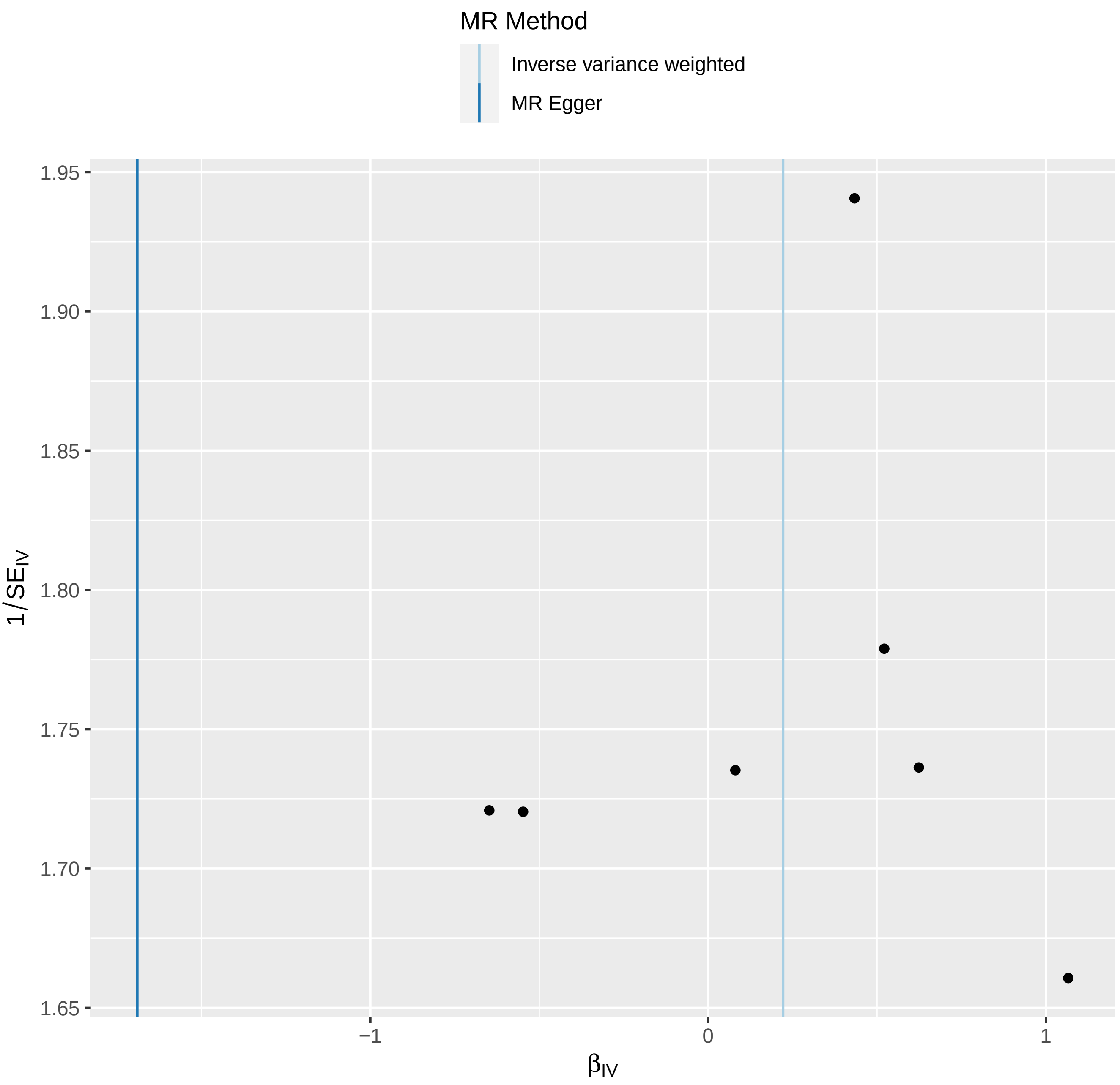

Batch 1626 : Gut microbiota abundance (genus Ruminococcaceae NK4A214 group id.11358) on Ulcerative rectosigmoiditis

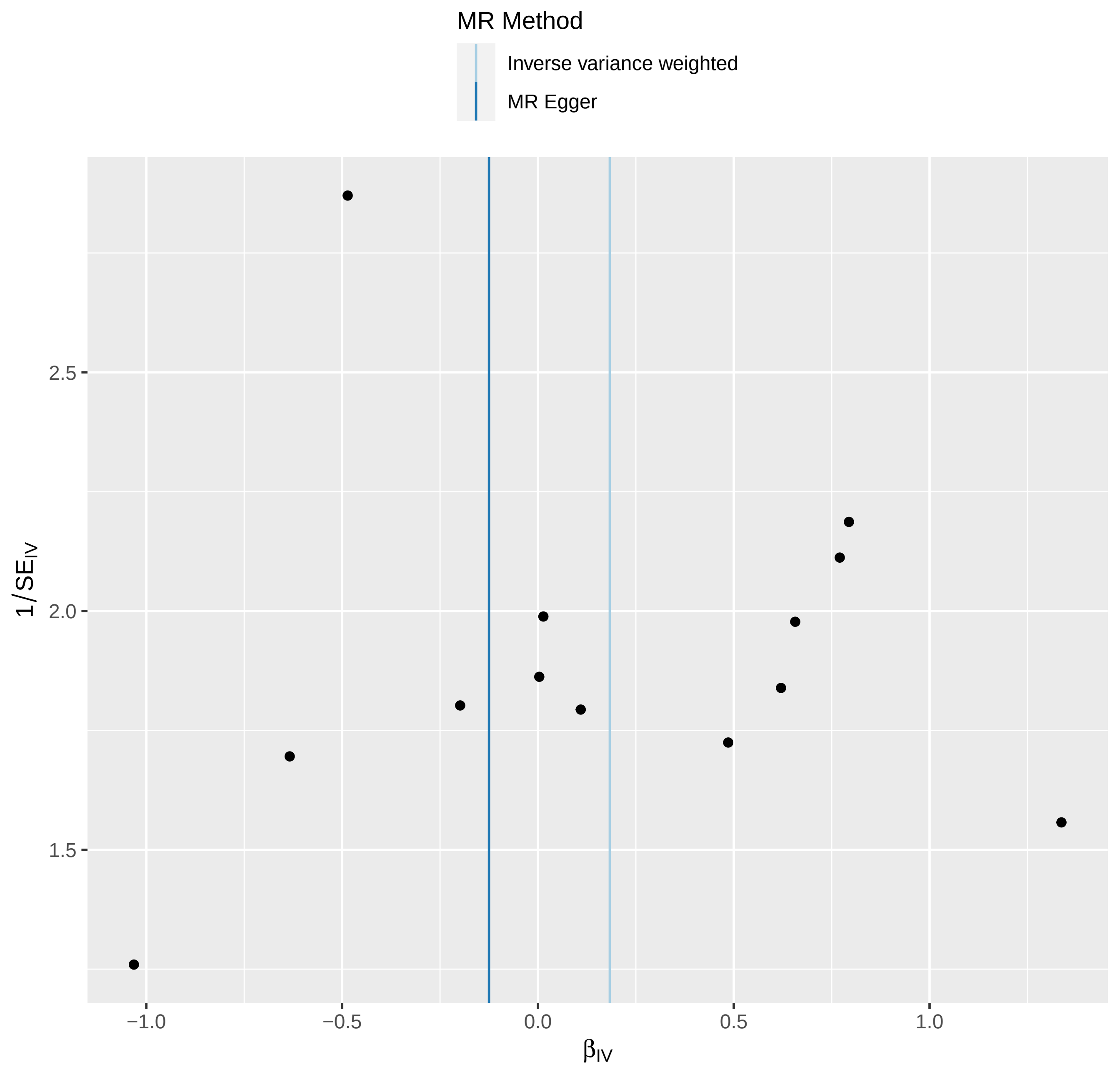

Batch 1627 : Gut microbiota abundance (genus Ruminococcaceae UCG002 id.11360) on Ulcerative rectosigmoiditis

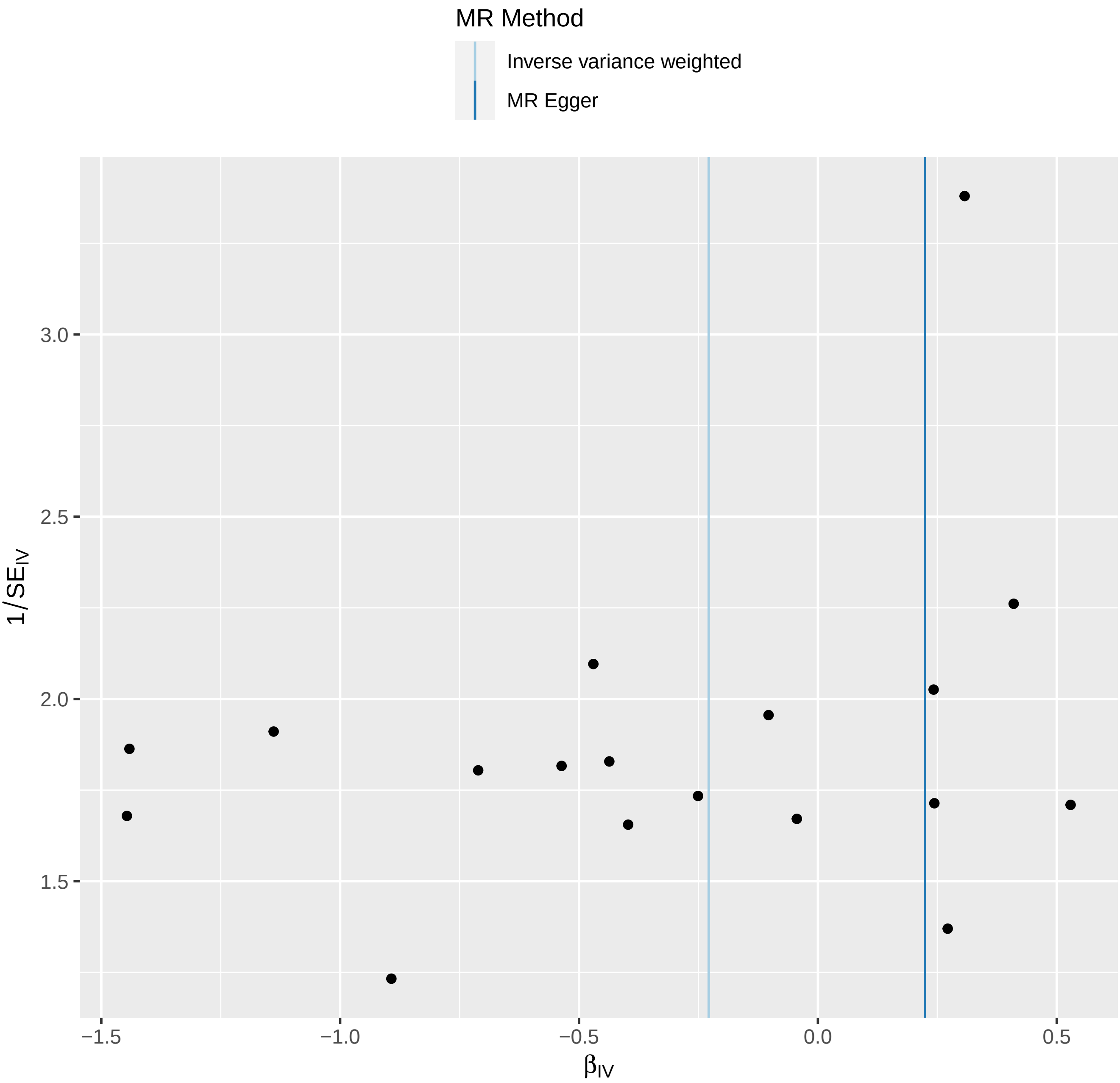

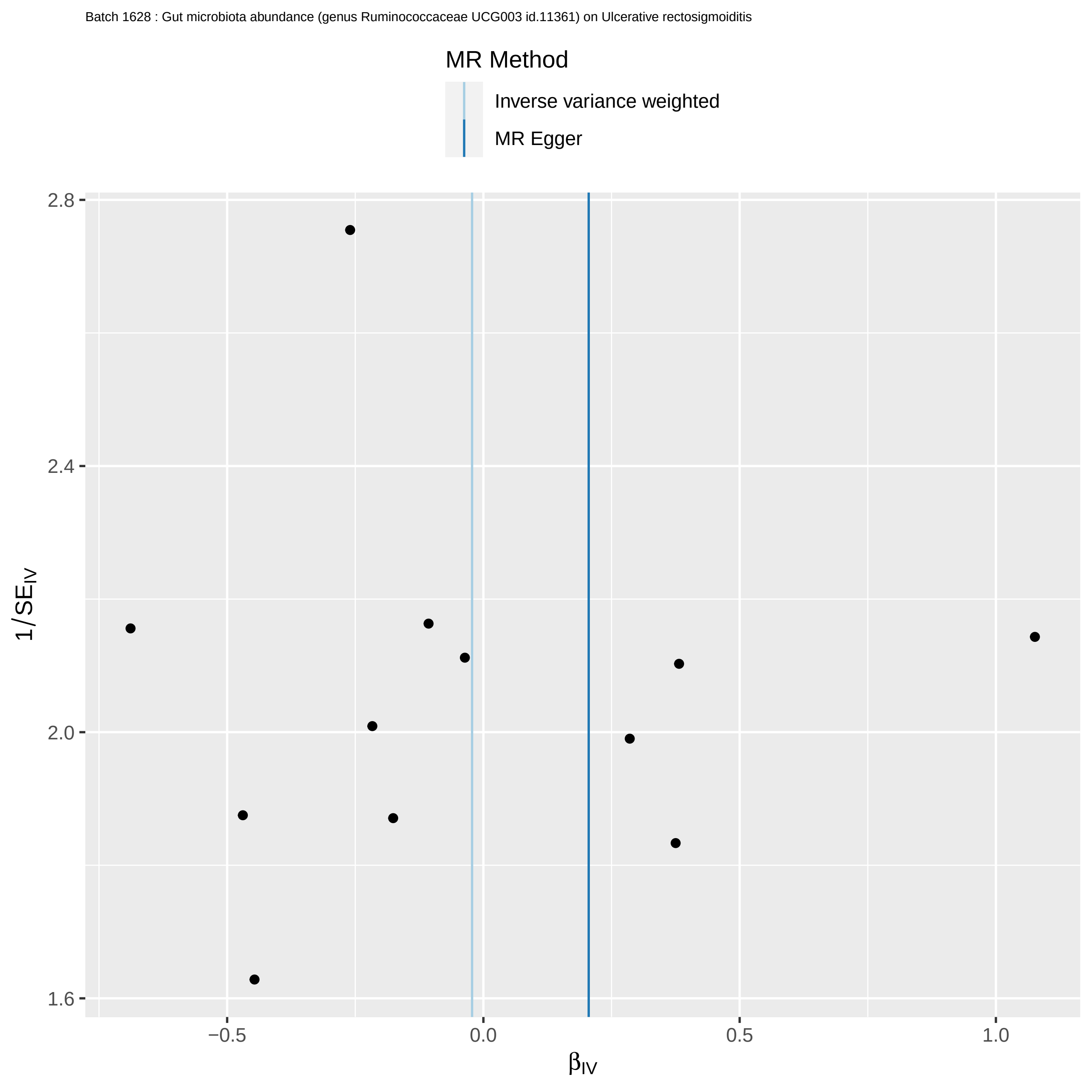

Batch 1628 : Gut microbiota abundance (genus Ruminococcaceae UCG003 id.11361) on Ulcerative rectosigmoiditis
MR Method
Inverse variance weighted
MR Egger
2.8
2.4
2.0
1.6
1/SE_IV
−0.5
0.0
0.5
1.0
β_IV

Batch 1629 : Gut microbiota abundance (genus Ruminococcaceae UCG004 id.11362) on Ulcerative rectosigmoiditis

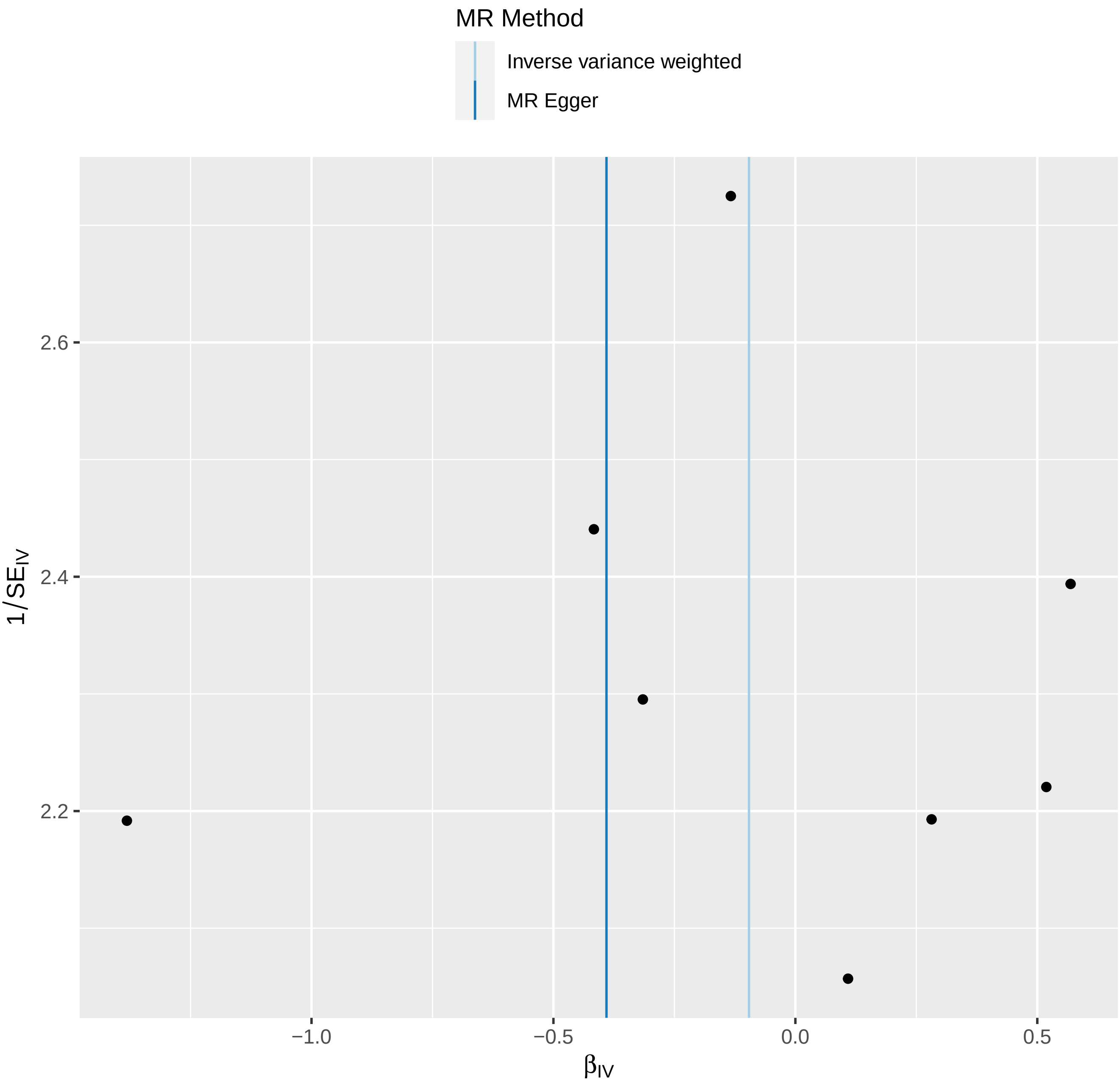

Batch 1630 : Gut microbiota abundance (genus Ruminococcaceae UCG005 id.11363) on Ulcerative rectosigmoiditis

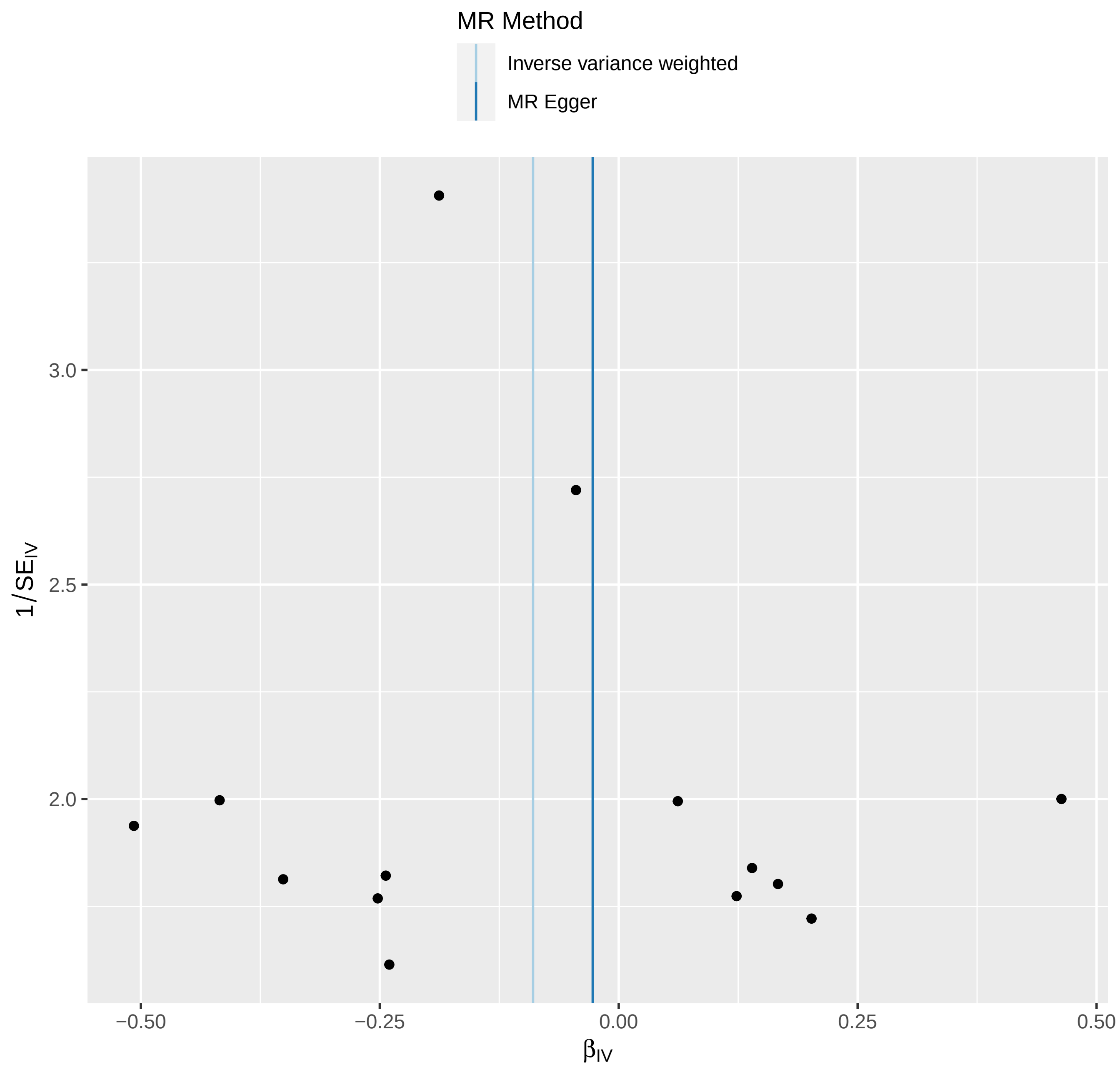

Batch 1631 : Gut microbiota abundance (genus Ruminococcaceae UCG009 id.11366) on Ulcerative rectosigmoiditis

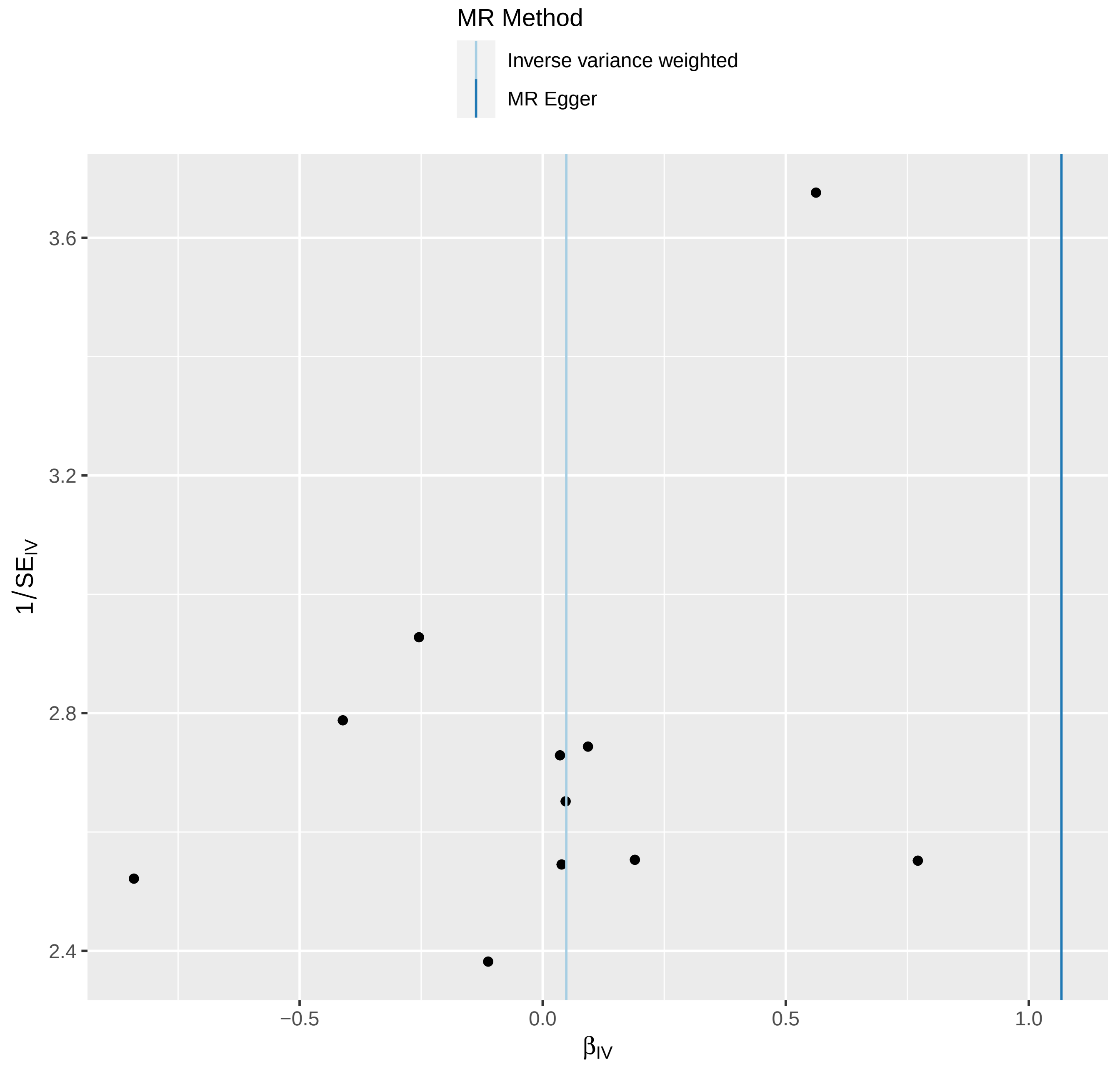

Batch 1632 : Gut microbiota abundance (genus Ruminococcaceae UCG010 id.11367) on Ulcerative rectosigmoiditis

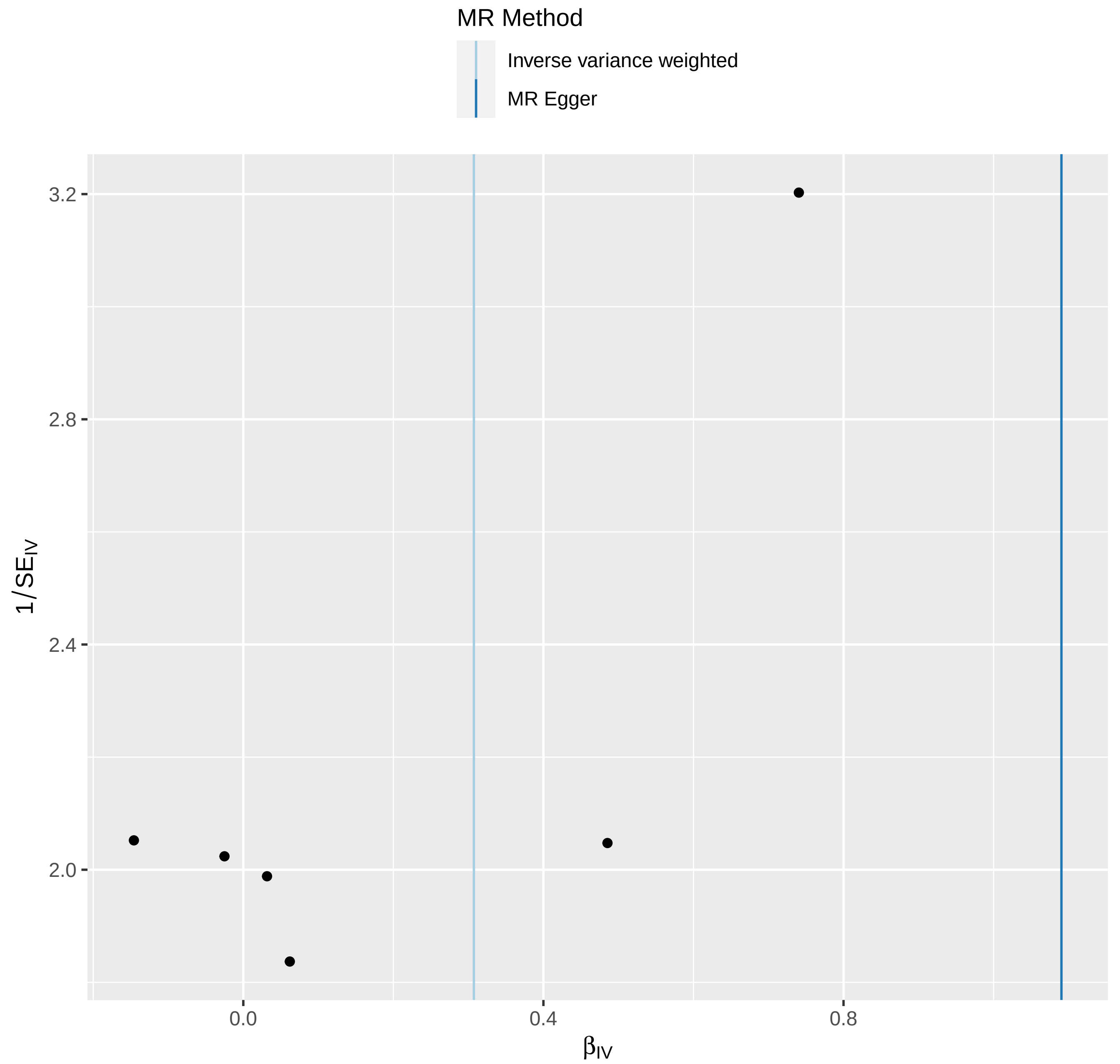

Batch 1633 : Gut microbiota abundance (genus Ruminococcaceae UCG011 id.11368) on Ulcerative rectosigmoiditis

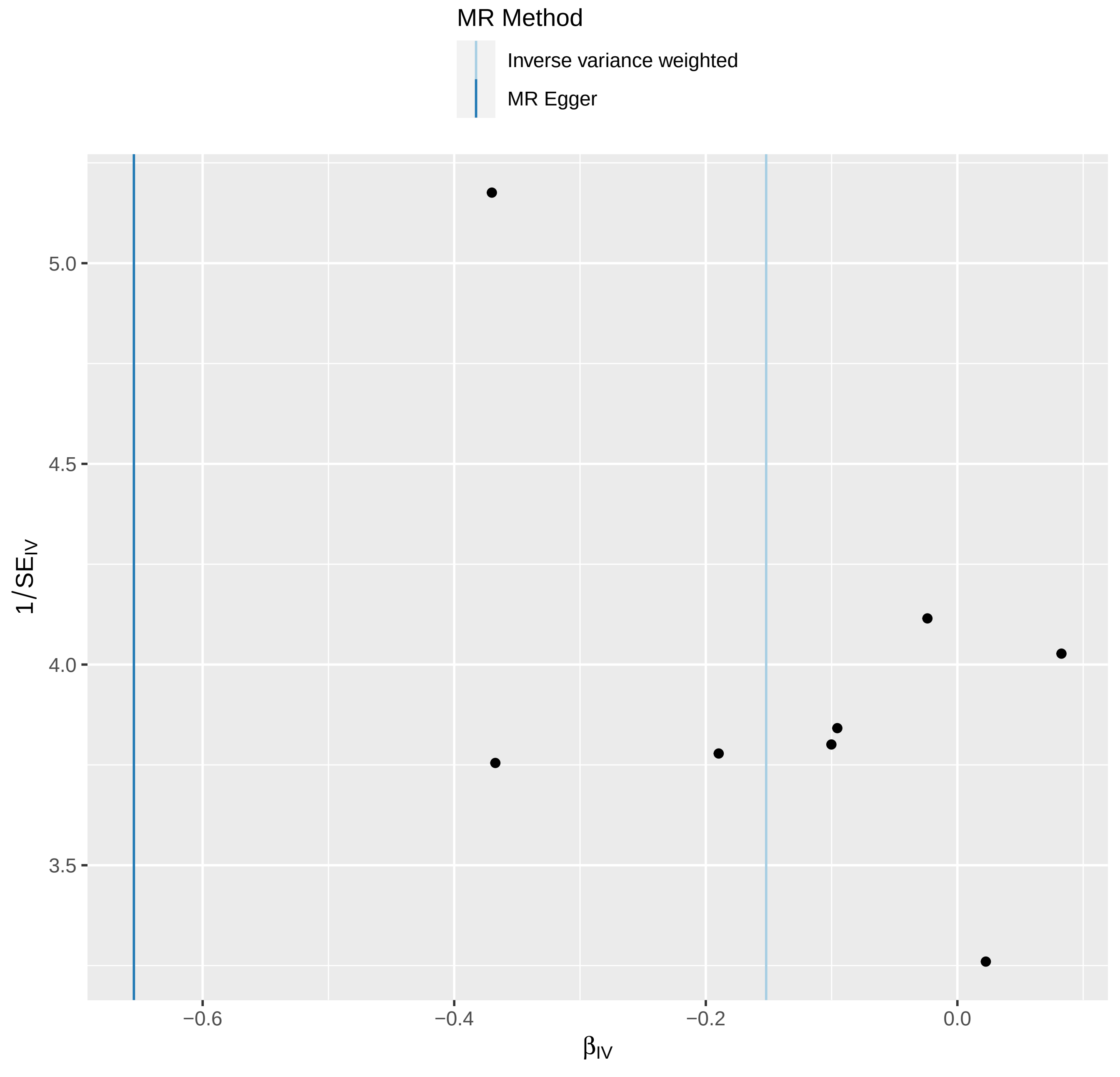

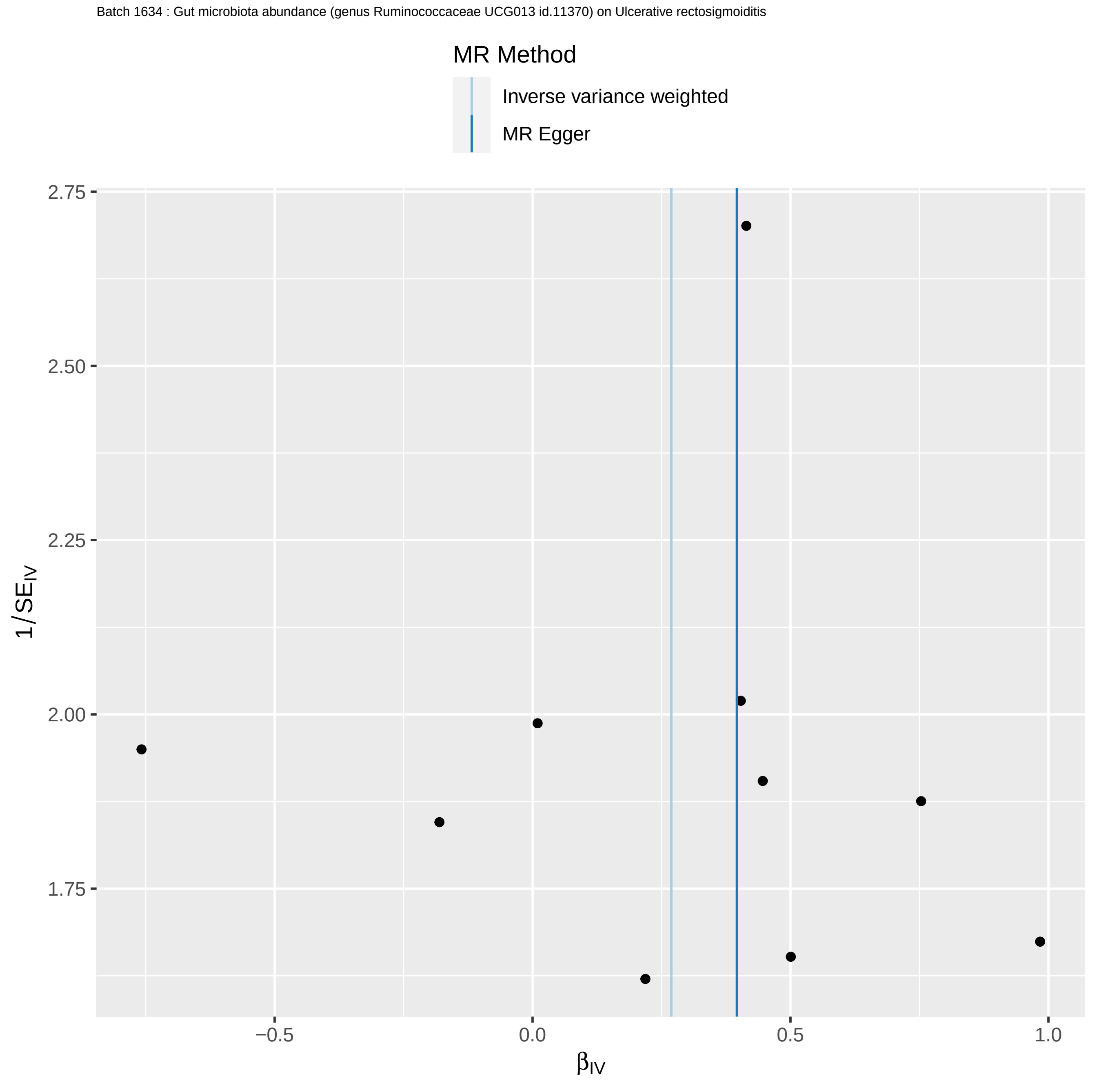
Batch 1634 : Gut microbiota abundance (genus Ruminococcaceae UCG013 id.11370) on Ulcerative rectosigmoiditis
MR Method
Inverse variance weighted
MR Egger
2.75
2.50
2.25
2.00
1.75
1/SE$_{IV}$
−0.5
0.0
0.5
1.0
β$_{IV}$

Batch 1635 : Gut microbiota abundance (genus Ruminococcaceae UCG014 id.11371) on Ulcerative rectosigmoiditis

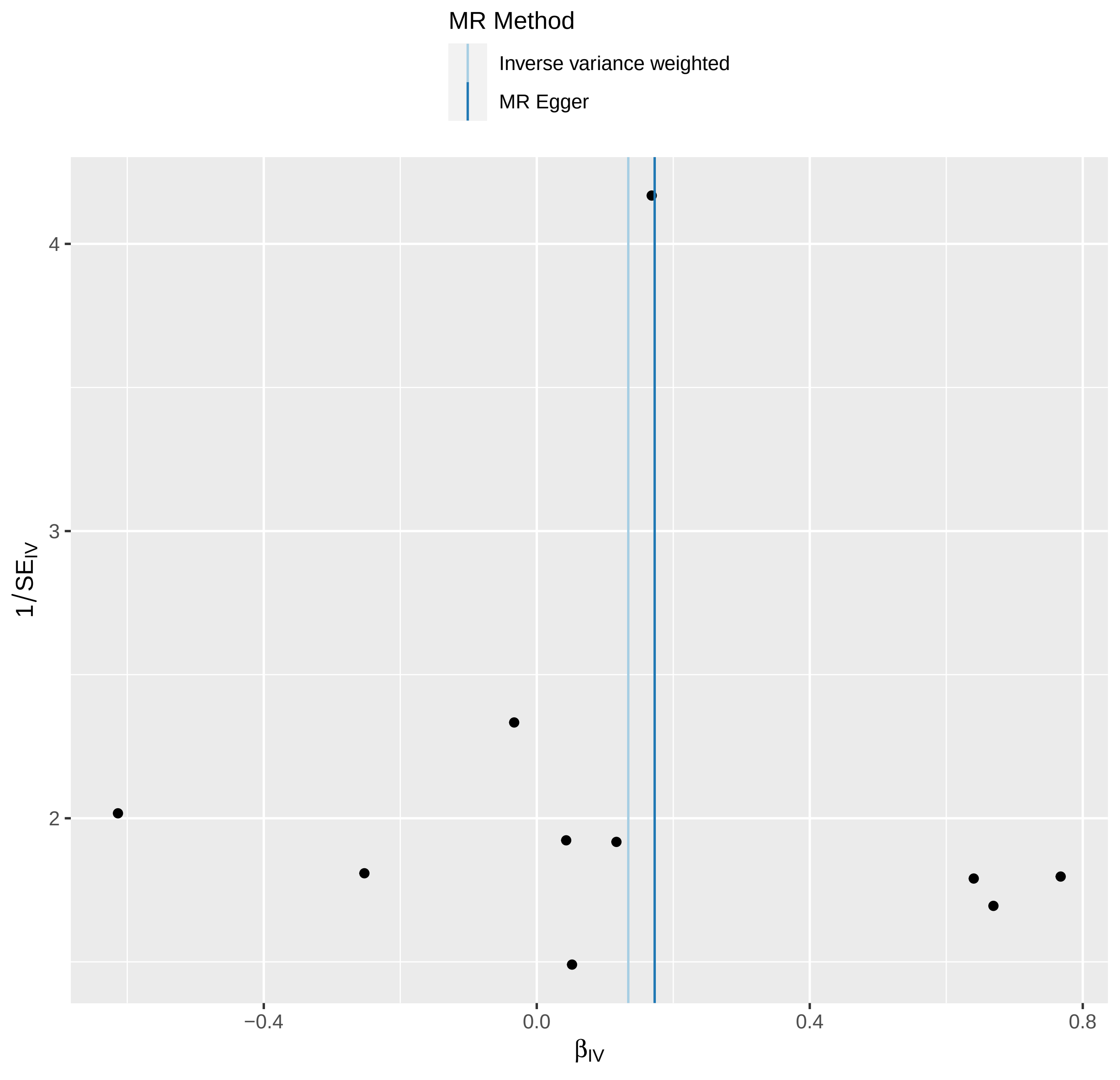

Batch 1636 : Gut microbiota abundance (genus Ruminococcus1 id.11373) on Ulcerative rectosigmoiditis

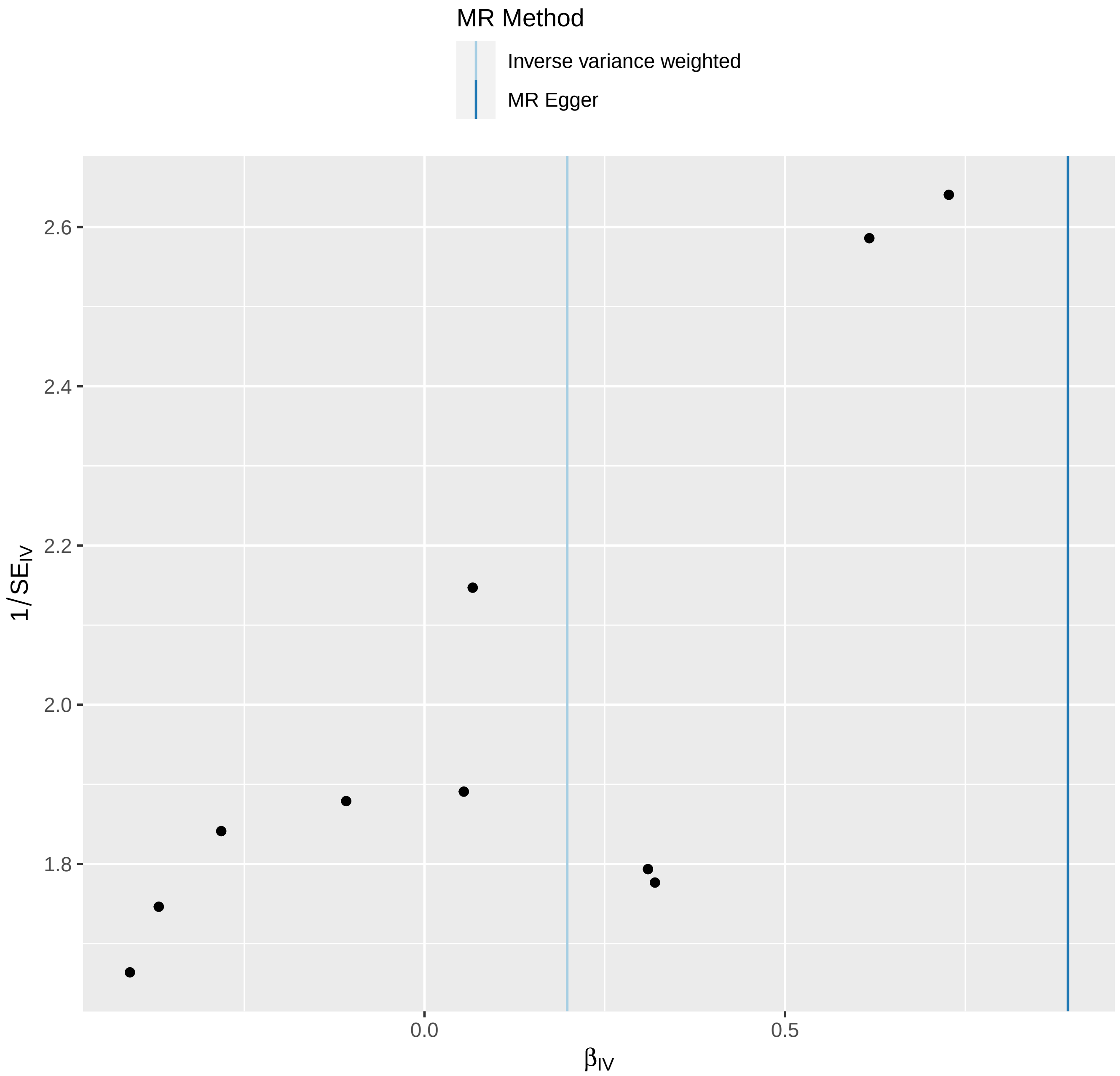

Batch 1637 : Gut microbiota abundance (genus Ruminococcus2 id.11374) on Ulcerative rectosigmoiditis

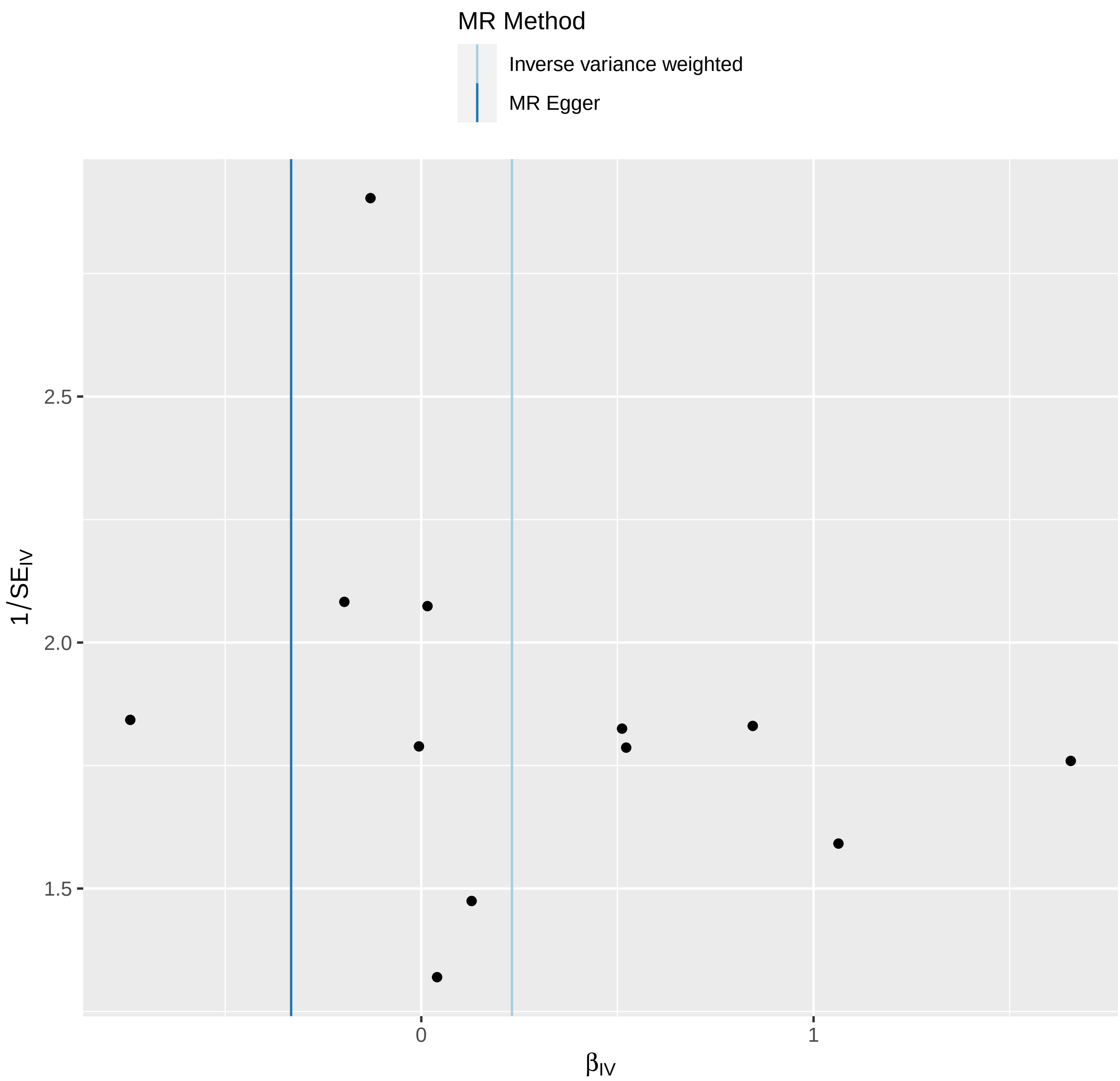

Batch 1638 : Gut microbiota abundance (genus Ruminococcus gauvreauii group id.11342) on Ulcerative rectosigmoiditis

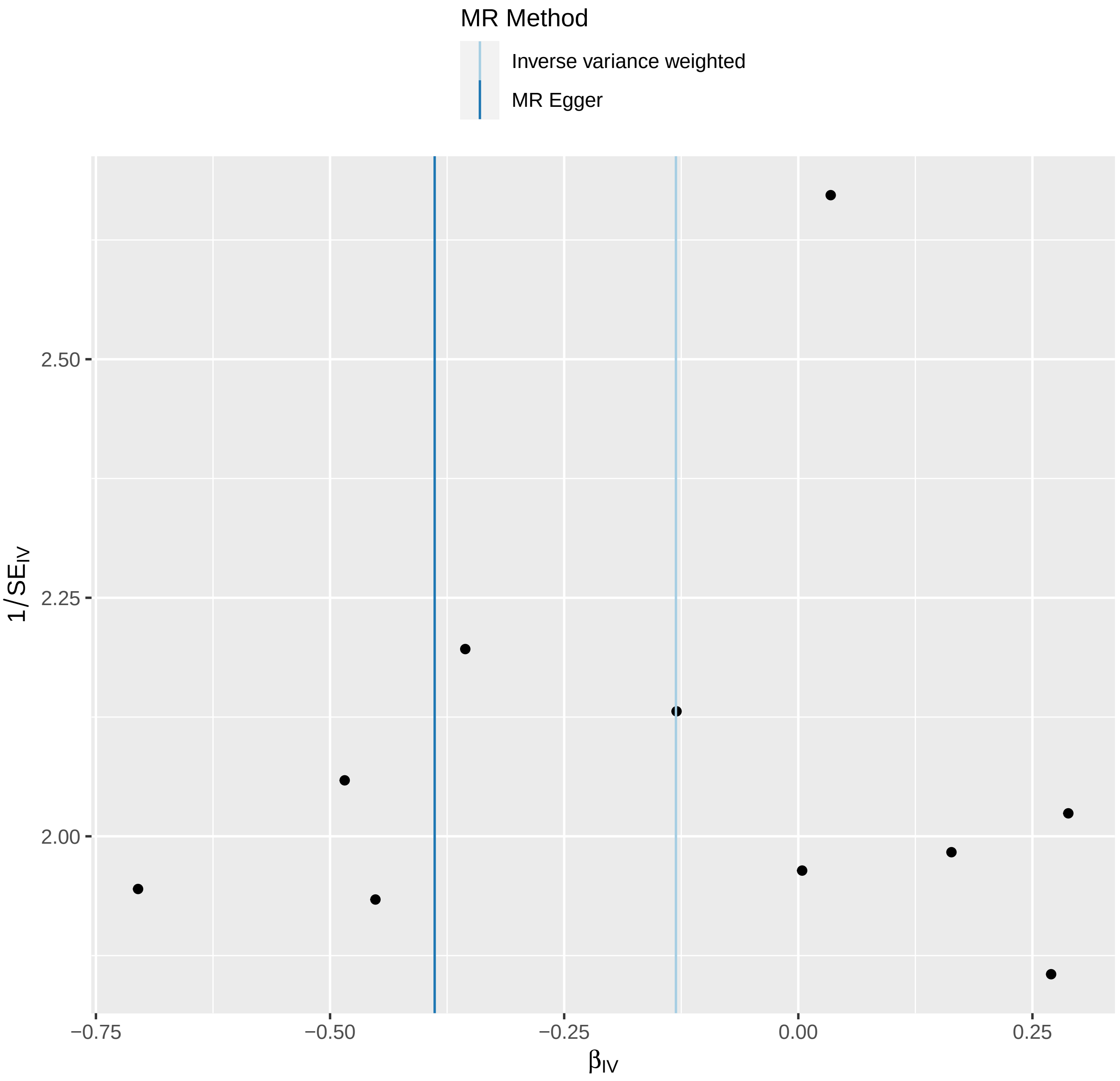

Batch 1639 : Gut microbiota abundance (genus Ruminococcus gnavus group id.14376) on Ulcerative rectosigmoiditis

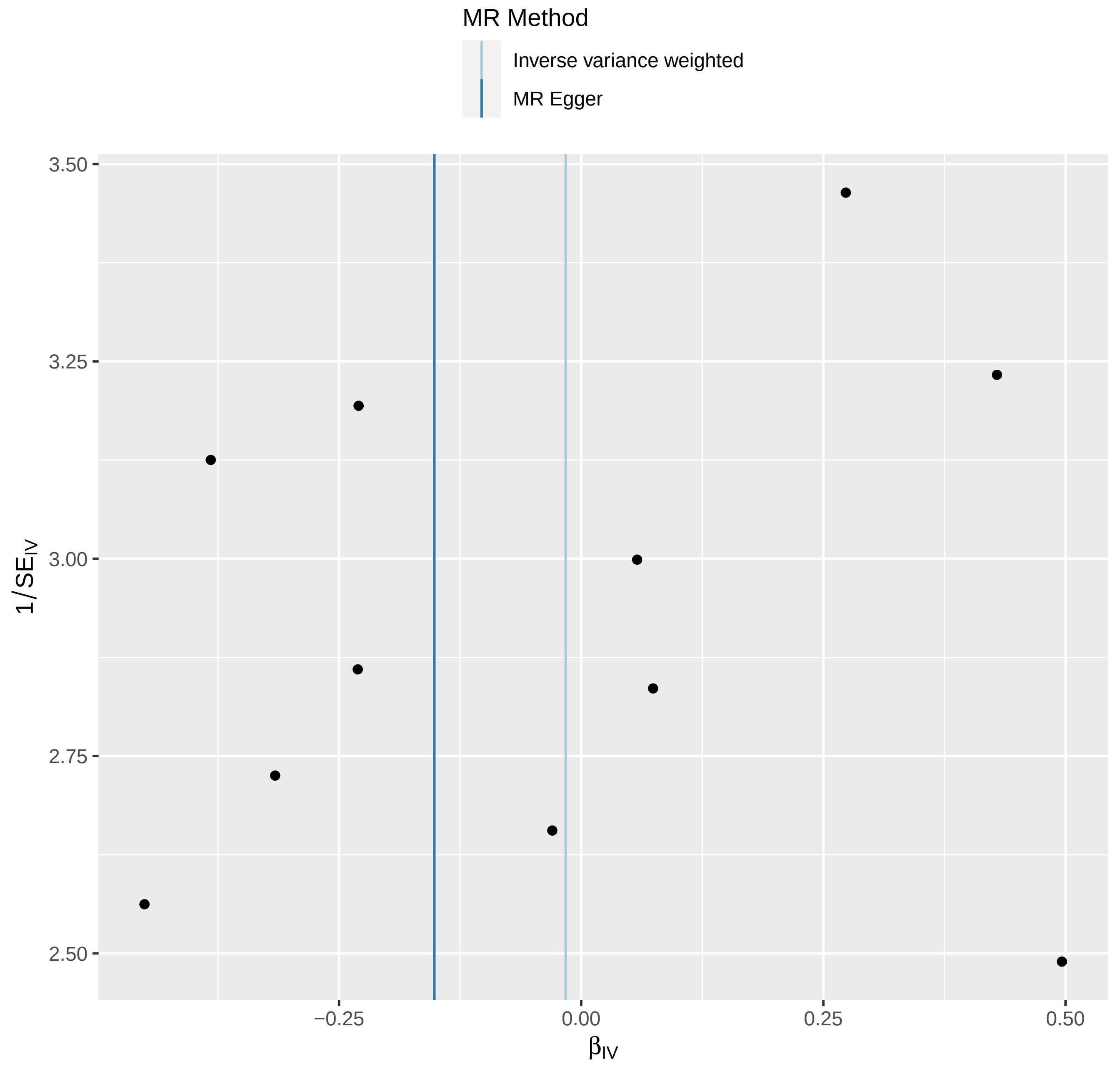

Batch 1640 : Gut microbiota abundance (genus Ruminococcus torques group id.14377) on Ulcerative rectosigmoiditis

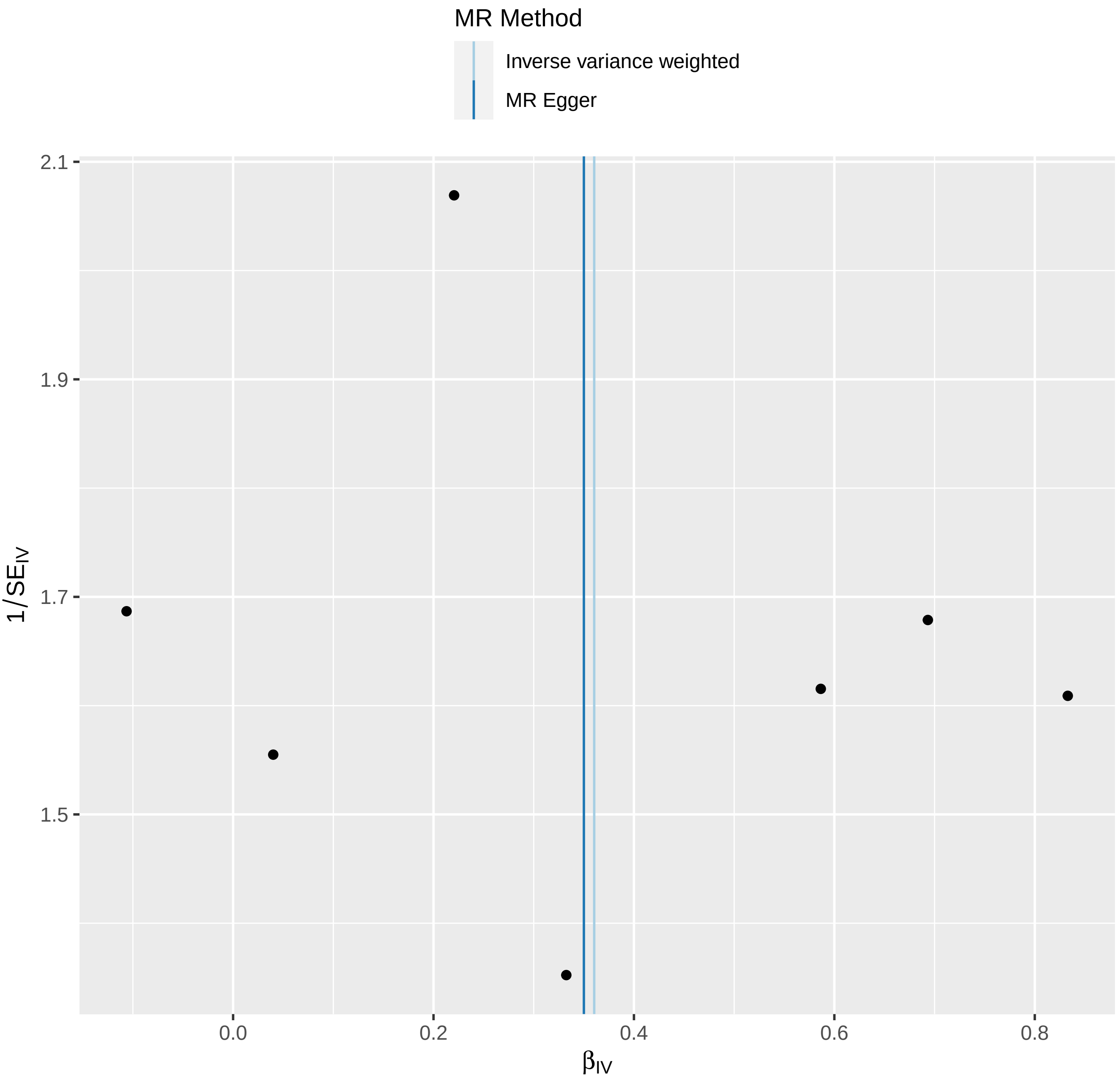

Batch 1641 : Gut microbiota abundance (genus Sellimonas id.14369) on Ulcerative rectosigmoiditis

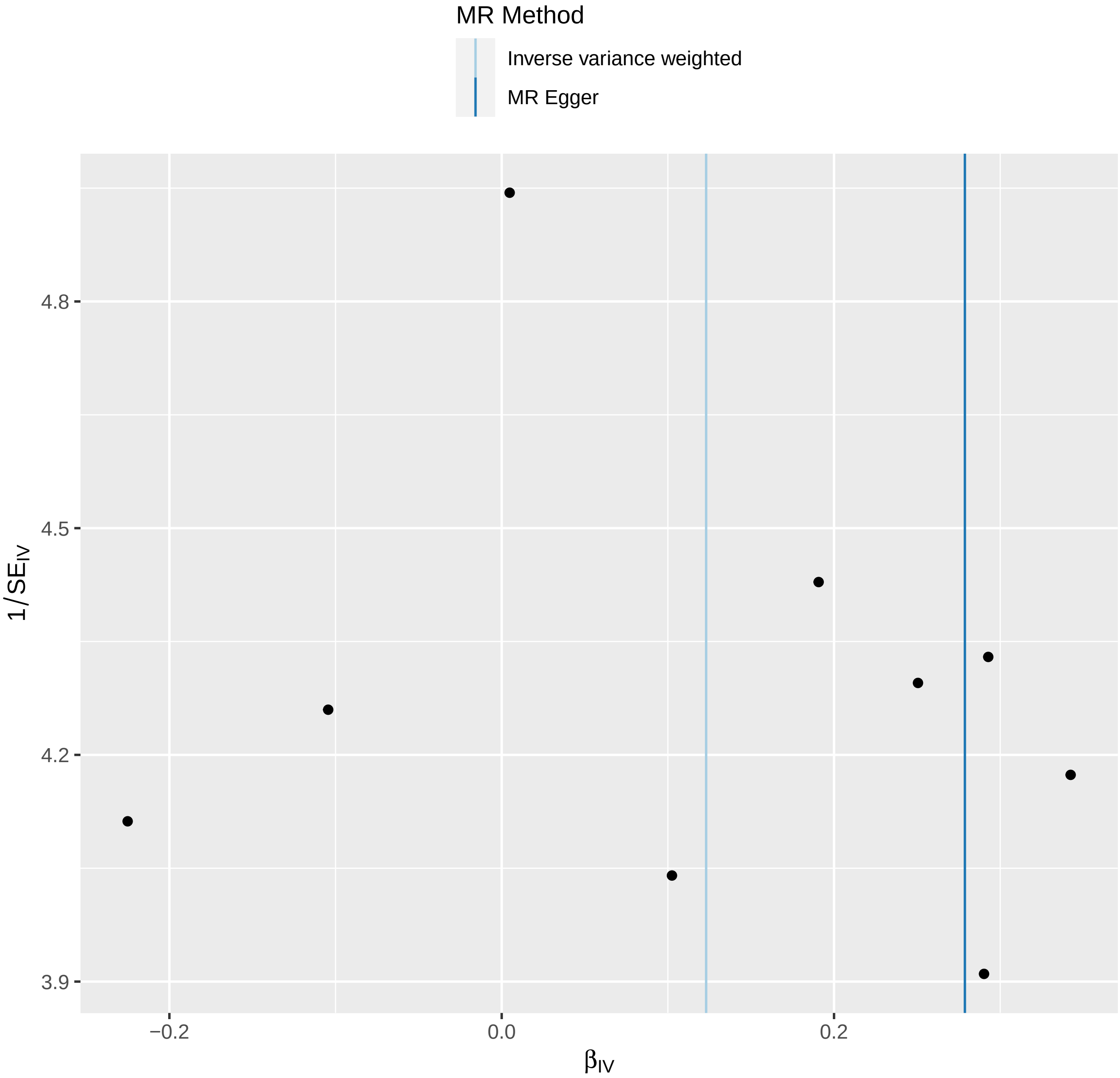

Batch 1642 : Gut microbiota abundance (genus Senegalimassilia id.11160) on Ulcerative rectosigmoiditis

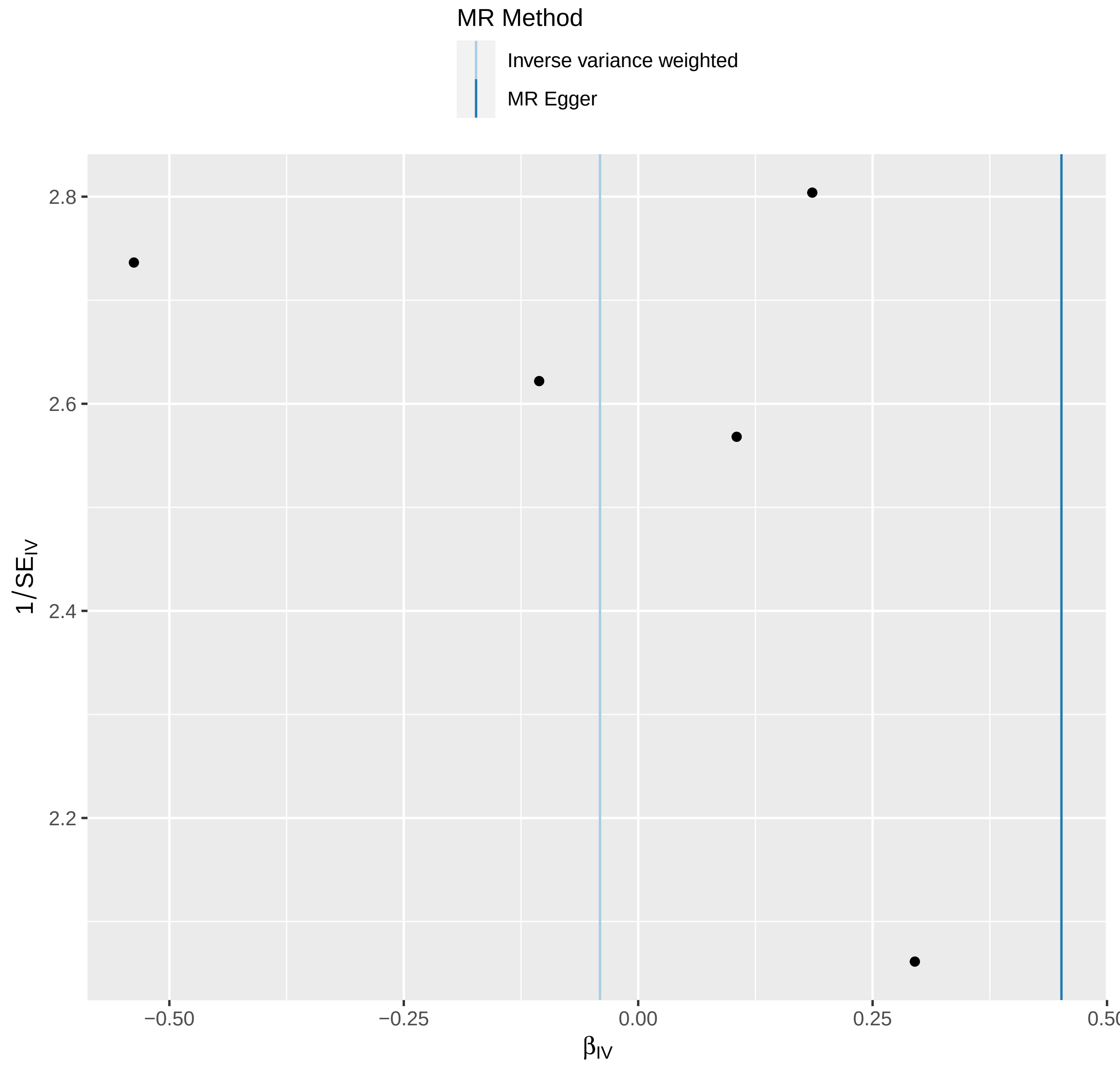

Batch 1643 : Gut microbiota abundance (genus Slackia id.825) on Ulcerative rectosigmoiditis

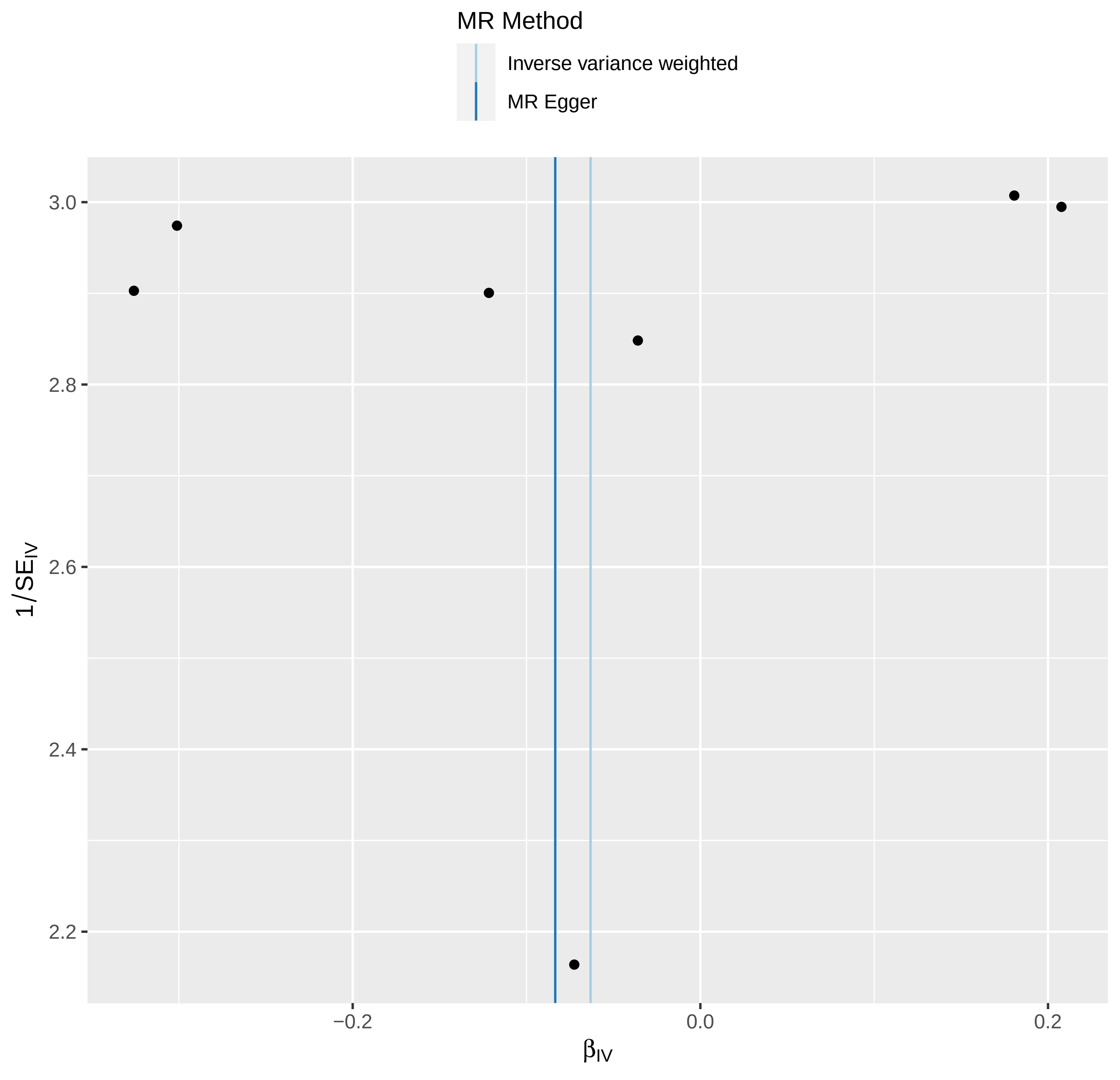

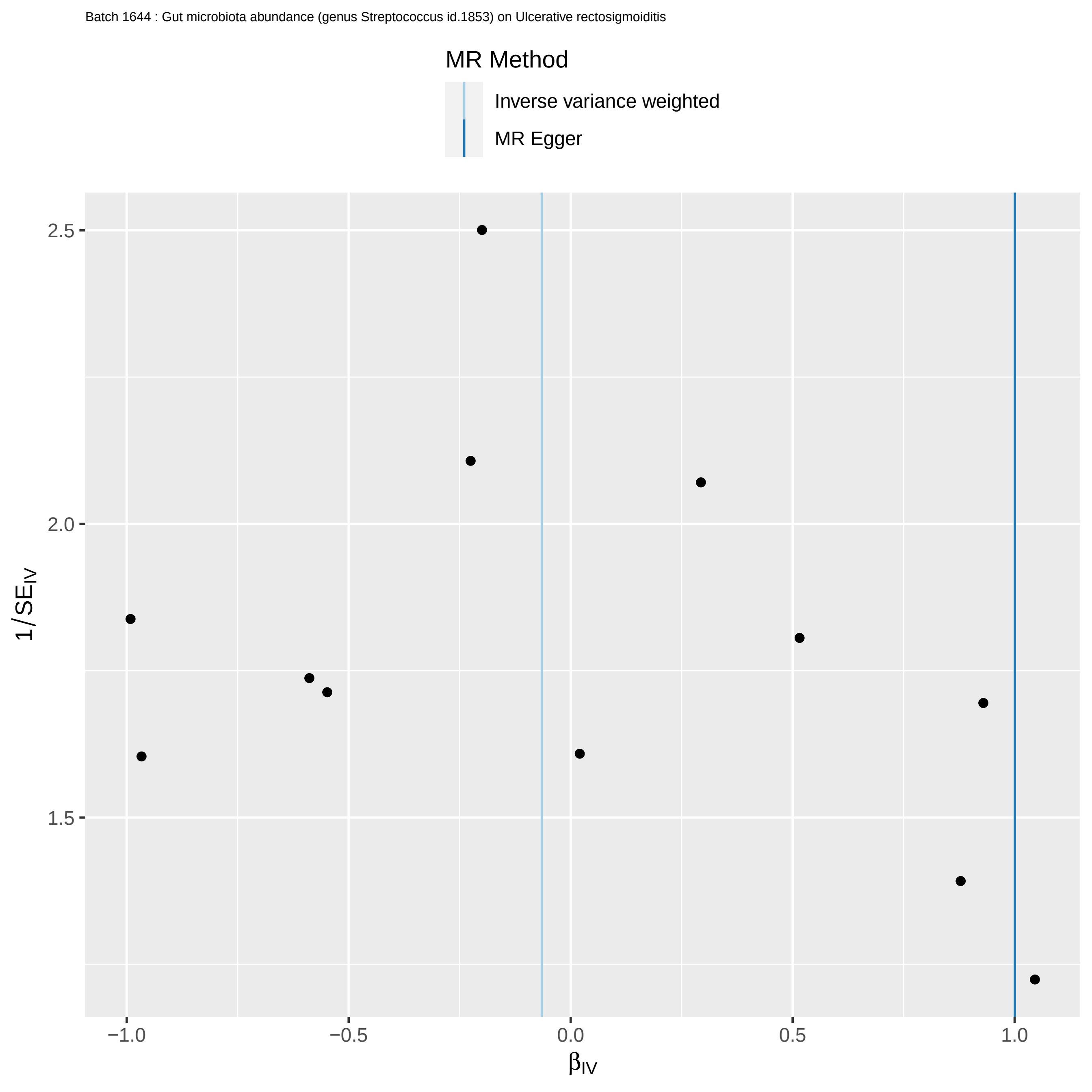
Batch 1644 : Gut microbiota abundance (genus Streptococcus id.1853) on Ulcerative rectosigmoiditis
MR Method
Inverse variance weighted
MR Egger
2.5
2.0
1.5
1/SE_IV
−1.0
−0.5
0.0
0.5
1.0
β_IV

Batch 1645 : Gut microbiota abundance (genus Subdoligranulum id.2070) on Ulcerative rectosigmoiditis

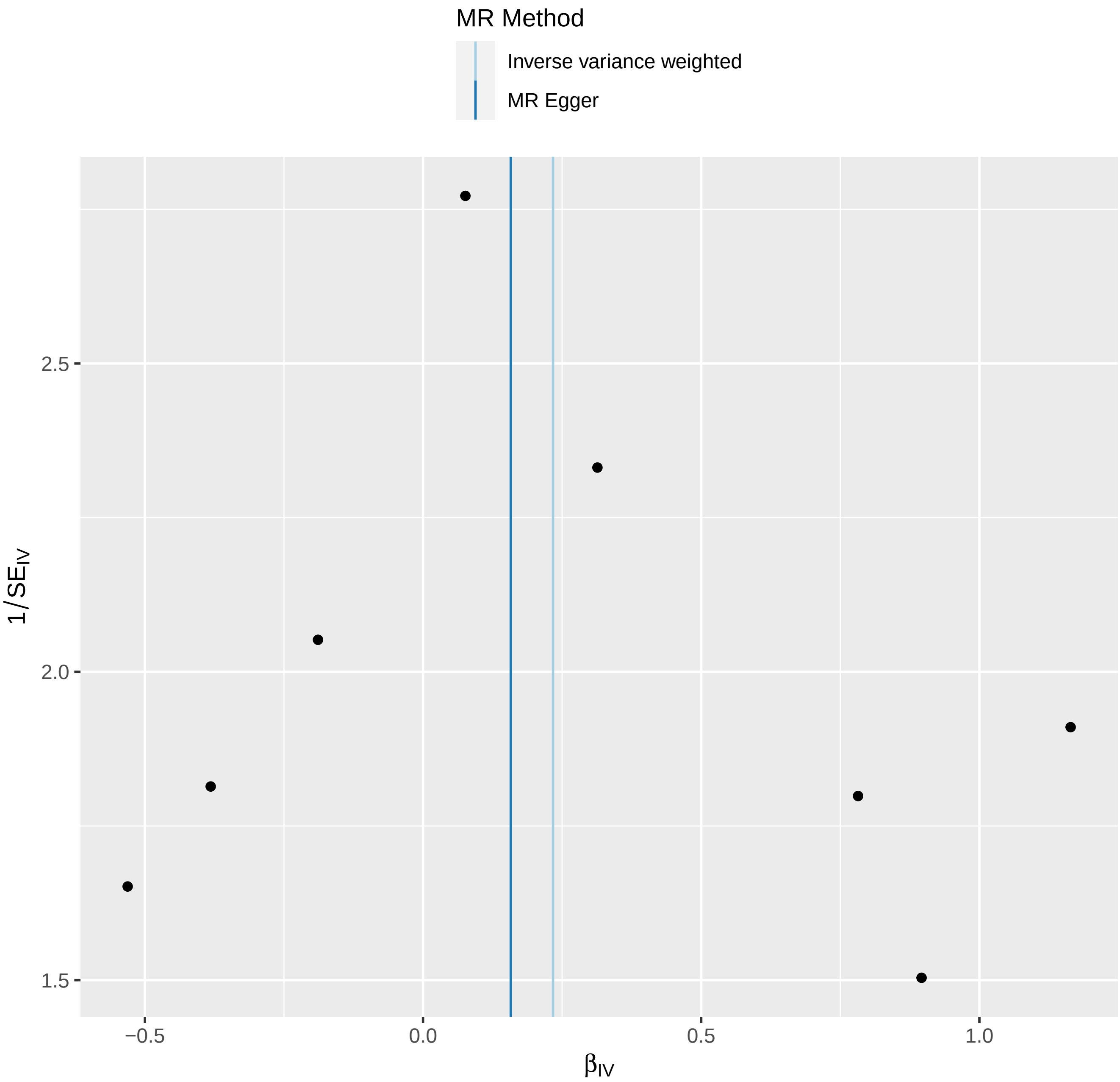

Batch 1646 : Gut microbiota abundance (genus Sutterella id.2896) on Ulcerative rectosigmoiditis

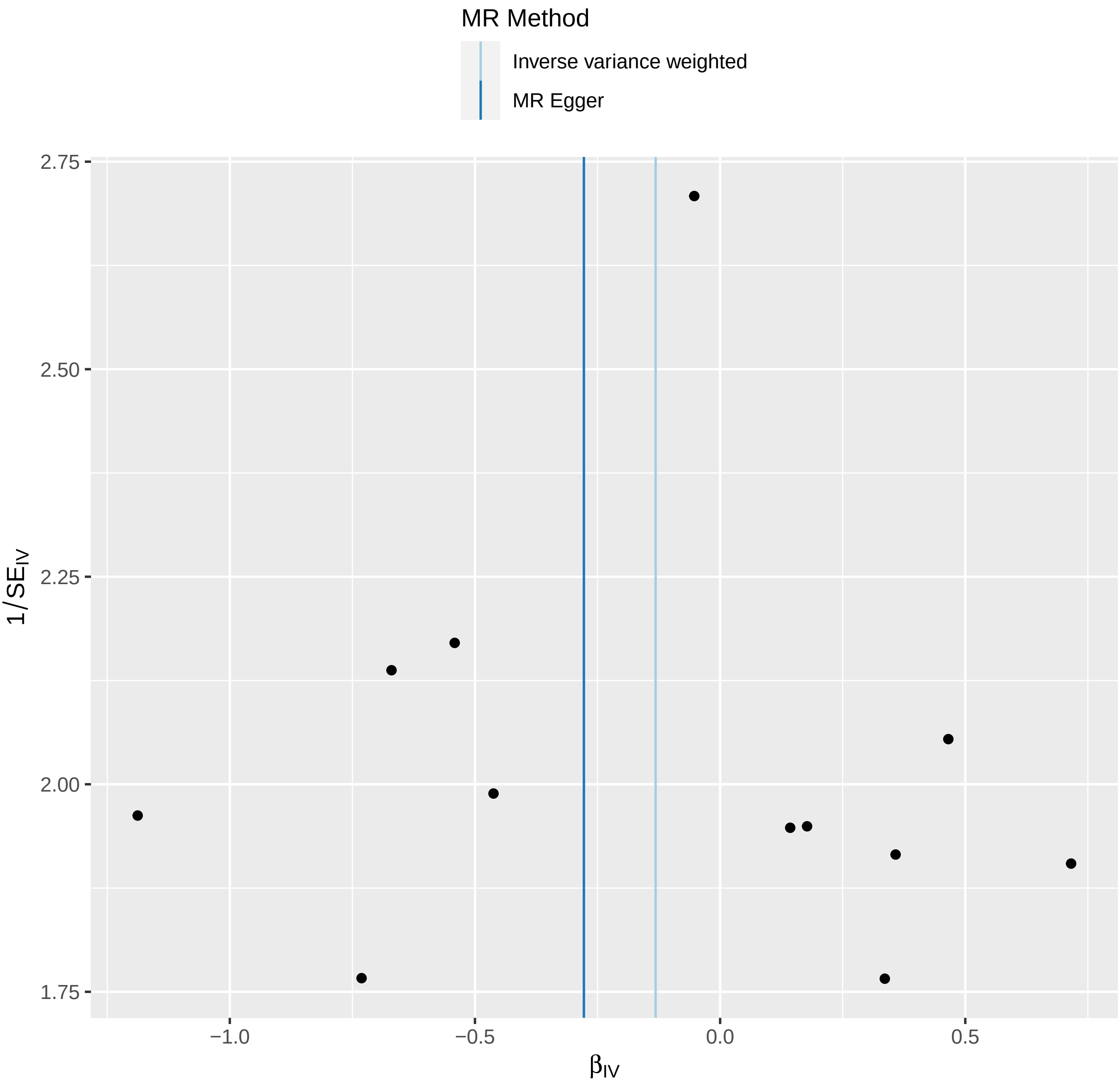

Batch 1647 : Gut microbiota abundance (genus Terrisporobacter id.11348) on Ulcerative rectosigmoiditis

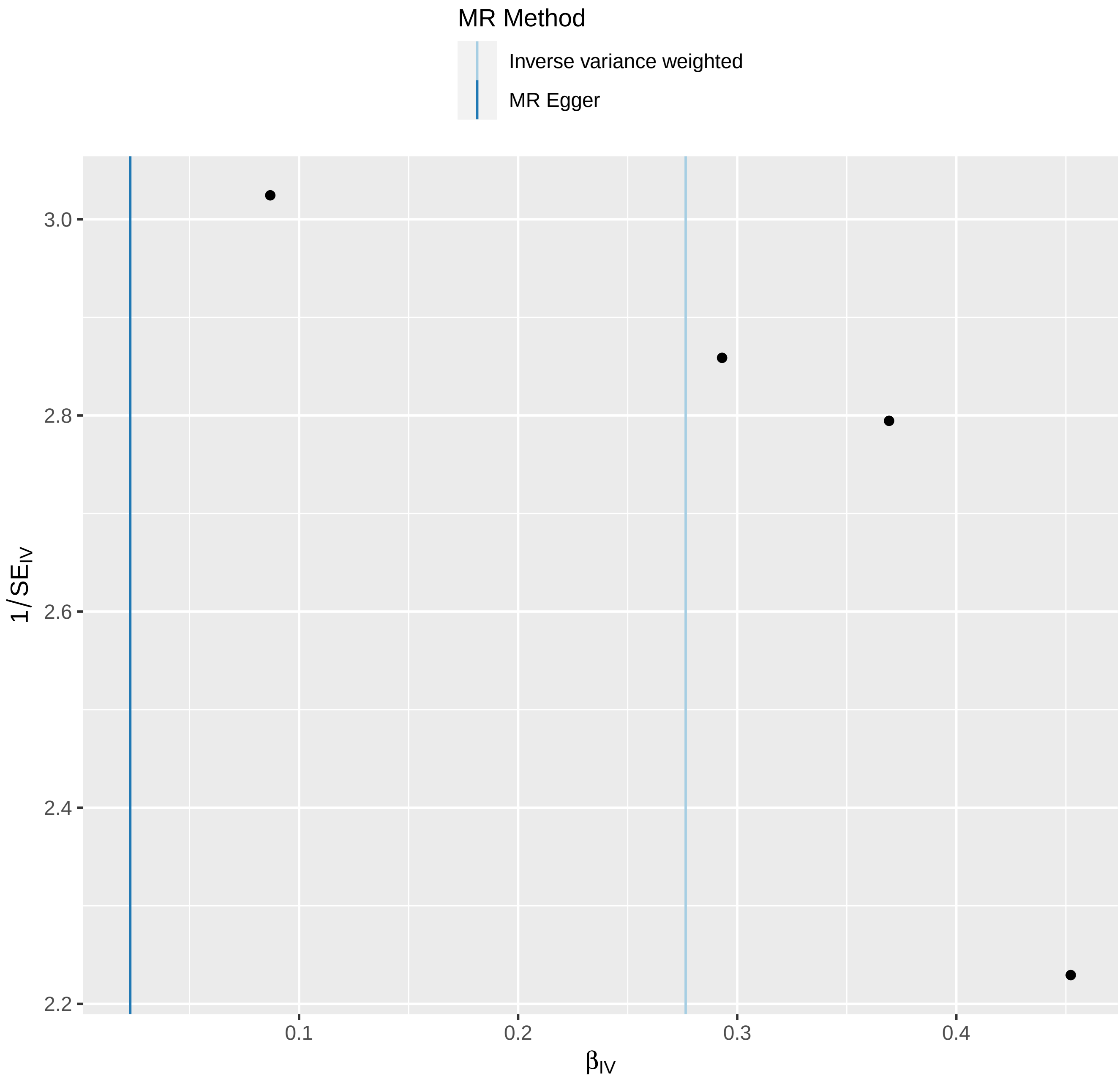

Batch 1648 : Gut microbiota abundance (genus Turicibacter id.2162) on Ulcerative rectosigmoiditis

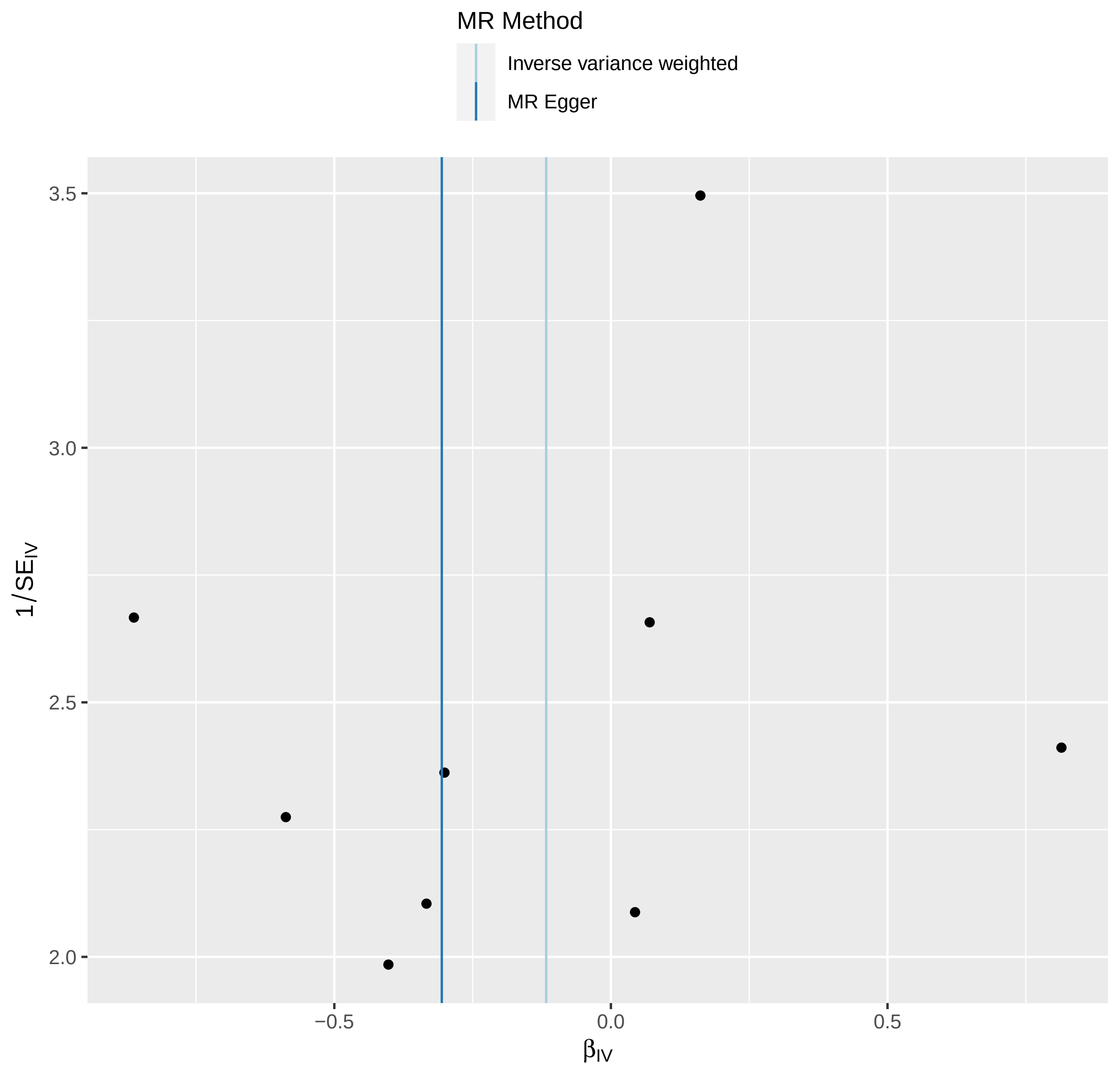

Batch 1649 : Gut microbiota abundance (genus Tyzzerella3 id.11335) on Ulcerative rectosigmoiditis

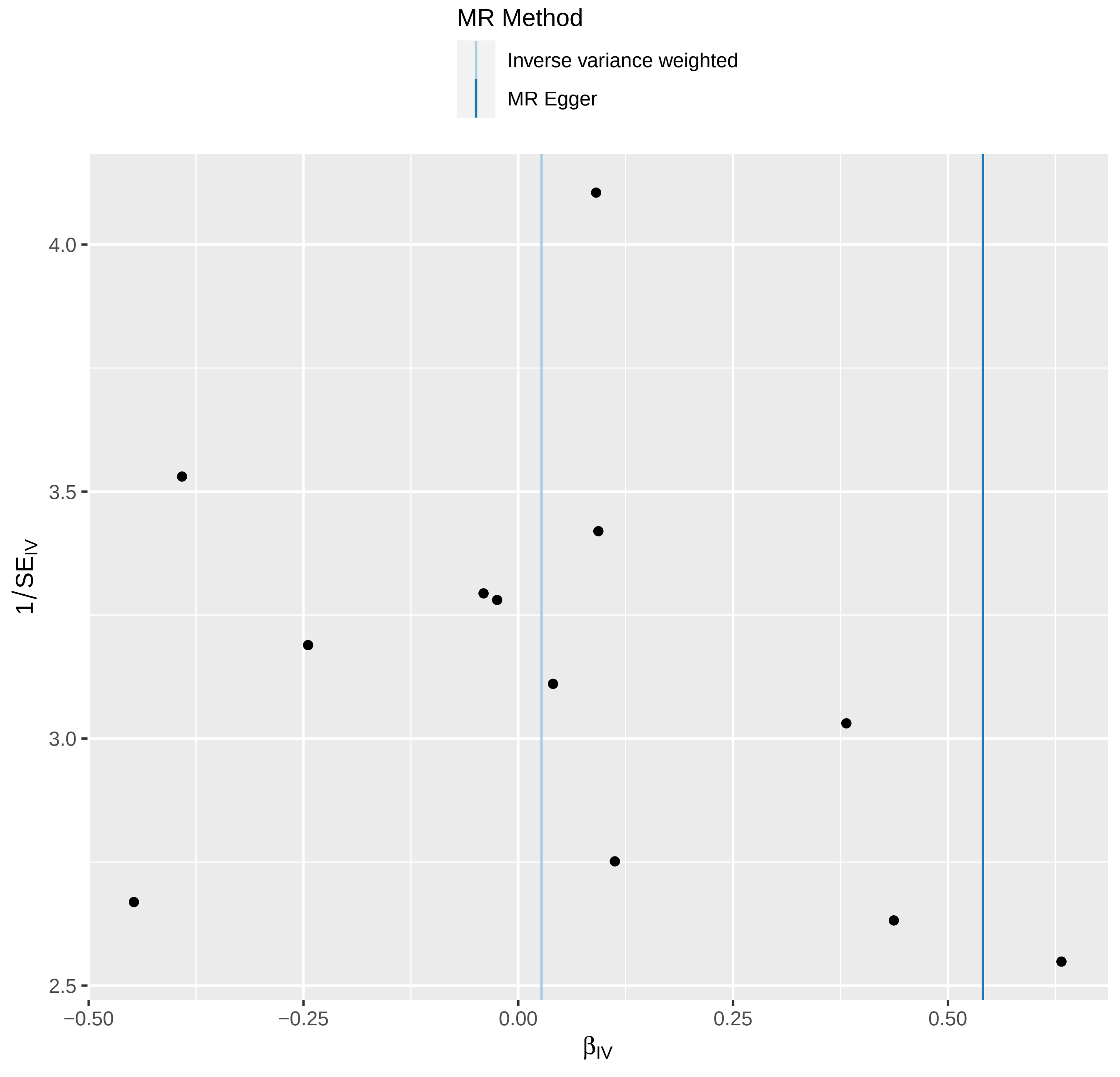

Batch 1650 : Gut microbiota abundance (genus Veillonella id.2198) on Ulcerative rectosigmoiditis

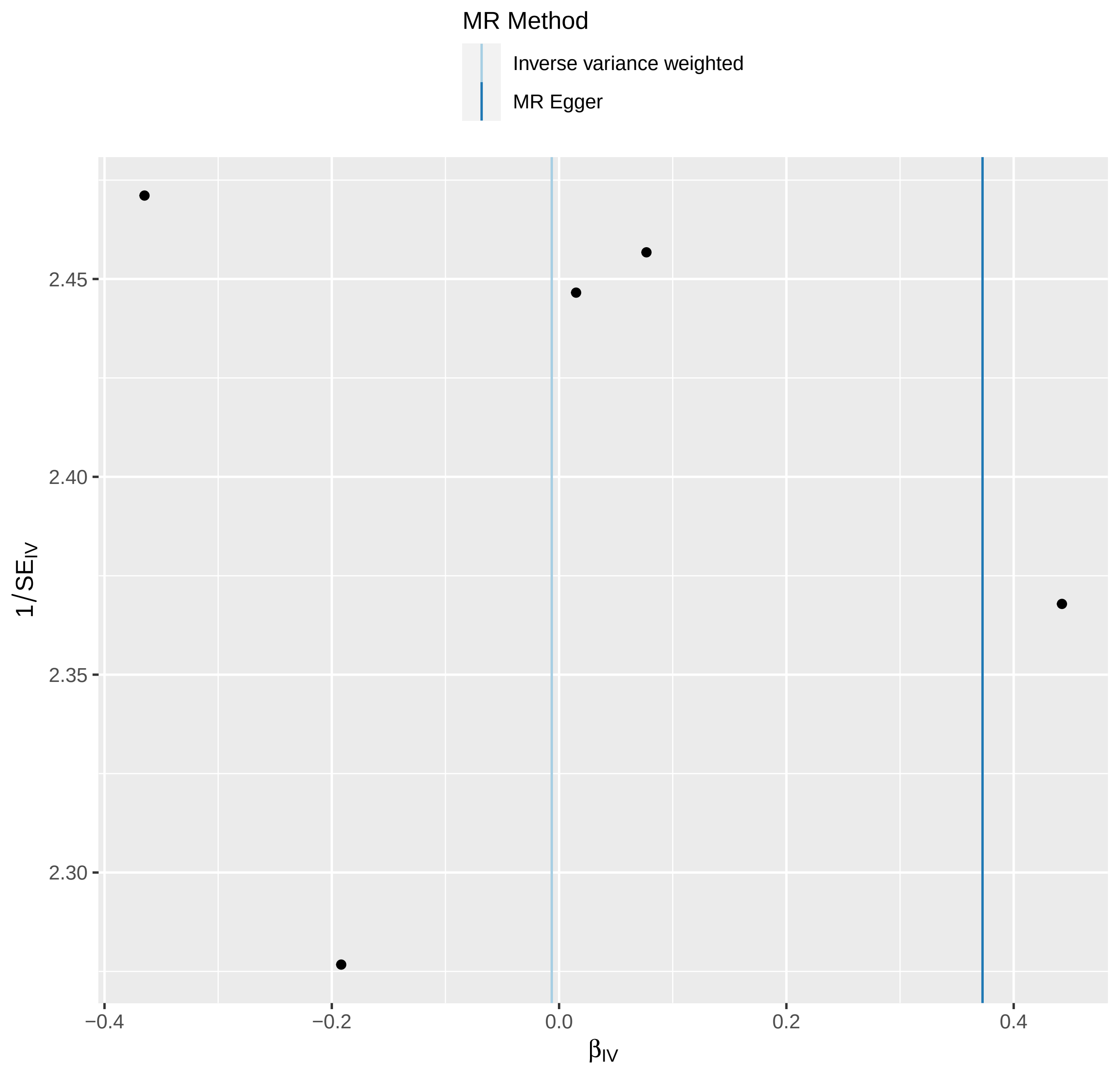

Supplement: Supplementary file 4 [file Image_4.pdf]
